# Pedology and Plant Provenance Can Improve Species Distribution Predictions of Australian Native Flora: A Calibrated and Validated Modeling Exercise on 5033 Species

**DOI:** 10.1002/ece3.71430

**Published:** 2025-06-24

**Authors:** Farzin Shabani, Mohsen Ahmadi, Niloufar Lorestani, Shazia Bibi, Atefeh Esmaeili, Tessa Lane, Martin F. Breed, John Llewelyn, Craig Liddicoat, Philip Kibet Langat, Bahareh Kalantar, Nadiezhda Ramírez‐Cabral, Pooja Singh, Ricardo Siqueira da Silva, Mohammed Abu‐Dieyeh, Masoud Nazarizadeh, Alessandro Ossola

**Affiliations:** ^1^ College of Arts and Sciences Qatar University Doha Qatar; ^2^ Department of Natural Resources Isfahan University of Technology Isfahan Iran; ^3^ College of Science and Engineering Flinders University Bedford Park South Australia Australia; ^4^ School of Engineering and Technology Central Queensland University Rockhampton Queensland Australia; ^5^ RIKEN Center for Advanced Intelligence Project Goal‐Oriented Technology Research Group, Disaster Resilience Science Team Tokyo Japan; ^6^ INIFAP National Research Institute of Agriculture, Forestry and Livestock Zacatecas México; ^7^ Department of Plant Sciences University of California Davis Davis California USA; ^8^ Department of Ecological Modelling Helmholtz Centre for Environmental Research—UFZ Leipzig Germany; ^9^ Postgraduate Program in Plant Science Diamantina Minas Gerais Brazil; ^10^ Faculty of Science University of South Bohemia České Budějovice Czech Republic; ^11^ Institute of Parasitology Biology Centre CAS České Budějovice Czech Republic; ^12^ School of Agriculture, Food and Ecosystem Sciences University of Melbourne Richmond Victoria Australia

**Keywords:** ecoregion‐specific data, ensemble models, habitat suitability, precipitation seasonality, predictor variables, species distributions

## Abstract

Species distribution models (SDMs) are valuable tools for assessing species' responses to environmental factors and identifying areas suitable for their survival. The careful selection of input variables is critical, as their interactions and correlations with other environmental factors can affect model performance. This study evaluates the influence of climate and soil variables on the performance of SDMs for 5033 Australian terrestrial vascular plant species, representing the largest phylogenetic diversity of native flora assessed in such an analysis. Using an ensemble of correlative models, we assessed the predictive performance of climate and soil variables, individually and in combination, across four distinct ecoregions: Desert (*n* = 640 species), Mediterranean (*n* = 1246), Temperate (*n* = 1936), and Tropical (*n* = 1211). Our results demonstrate that on a continental scale, climate variables have a greater influence on plant distributions than soil variables. Although incorporating soil and climate variables enhanced model performance in some ecoregions, our results indicate that relying solely on small‐scale variables such as soil may increase the likelihood of underfitting. The most influential predictor variables in the models varied across ecoregions and between specialist and generalist species. Mean annual rainfall (bio1) was consistently a strong climate predictor variable across ecoregions, but other climate variables became more important when analyses were restricted to ecoregion‐specific species (i.e., specialists). Soil organic carbon (SOC) was the most important soil variable in the Temperate and Tropical ecoregions across generalist and specialist species. In the Mediterranean ecoregion, clay content (CLY) became more important than SOC when analyses were restricted to ecoregion‐specific species, whereas nitrogen total organic (NTO) was consistently the strongest predictor soil variable for plants in the Desert. Our findings have significant implications for understanding the interplay between climate, soil, and plant distribution within diverse ecoregions. This study serves as a foundation for developing more accurate SDM predictions.

## Graphical abstract

Climate dominates species distribution models. Deserts depend more on soil factors. Higher thresholds improve model accuracy. Integrated models outperform single‐variable ones. Temperate regions show highest species richness.

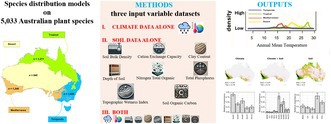

## 1 Introduction

Modeling the habitat requirements of species has become increasingly important in ecology, biogeography, and conservation (Veech 2021; Austin and Meyers 1996; Jarvis and Robertson 1999; Williams and Hero 2001). Species distribution models (SDMs) are numerical tools that integrate species occurrence and abundance records with environmental data to predict habitat suitability and species distributions (Guisan and Zimmermann 2000; Stauffer 2002; Franklin 2010; Kearney et al. 2010). By considering both abiotic and biotic factors, SDMs offer insights into the potential of species to survive across a landscape (Elith and Leathwick 2009). These models are often extrapolated across space and time to forecast species' responses to climate change (Brown et al. 2016; Hazen et al. 2013; Edwards and Richardson 2004; Ramirez‐Cabral et al. 2017; Shabani et al. 2017, 2019; Ramos et al. 2019; Wauchope‐Drumm et al. 2020; Ejaz et al. 2025; Lorestani et al. 2022). Thus, SDMs have become essential tools for conservation planning and environmental management because they can be used to assess habitat suitability and predict species' responses to environmental changes.

SDMs can be categorized into two main groups: mechanistic‐based and correlative‐based models (Kearney and Porter 2009; Dormann et al. 2012). Mechanistic models employ explicit functions to characterize relationships among different environmental variables and ecosystem components, which are typically defined a priori based on ecological theory or direct empirical evidence (Connolly et al. 2017; Dormann et al. 2012). However, mechanistic models require detailed and accurate physiological data for each species, making them less practical for large‐scale studies of entire floras (e.g., thousands of species spanning entire phylogenies). Although correlative models are conceptually simpler than mechanistic models, they can perform as well as (or better) than mechanistic models in predicting distributions when appropriate input variables are used (Robertson et al. 2003; Muhling et al. 2017). Additionally, their independence from explicit assumptions minimizes confirmation bias by relying on observed data to establish relationships between species distributions and environmental variables, rather than depending on predefined ecological or mechanistic hypotheses. This data‐driven approach improves the ability to detect emergent patterns and reduces the likelihood of misinterpretation stemming from incorrect or overly simplified assumptions about underlying processes (Connolly et al. 2017).

Significant attention has been devoted to addressing key challenges in correlative SDM, including the quality and quantity of species data, spatial resolution, and modeling methodologies (Wang et al. 2016; Datta et al. 2020; Valavi et al. 2022). However, it remains unclear which input variables will have the highest predictive power in such models. Often, the selection of input variables with low predictive power leads to underfitting, resulting in ecologically unreasonable and inaccurate model output (Shabani et al. 2016). Moreover, efforts to apply correlative models to continental‐wide species assemblages, such as entire floras, face challenges regarding input variable selection. Consequently, there is a growing need to comprehensively examine the performance and biases of SDMs with particular reference to the selection of input variables.

There is a large range of potential input variables available for SDMs. Currently, global databases such as WorldClim and CHELSA provide climatic data at a variety of spatiotemporal scales (Hijmans et al. 2004). Other datasets offer additional environmental layers relevant to species distributions, including topographic features, land use/land cover (e.g., Searle et al. (2022)), and soil properties (Hulshof and Spasojevic 2020). Given the array of available input data for SDMs, the question of “which input data are best to use?” is critical (Arenas‐Castro et al. 2022; Kearney and Porter 2009; Beck et al. 2014; Velazco et al. 2017). Additionally, there is growing concern regarding the assumptions and approaches used in SDM studies, especially when applied to broader spatial scales, such as entire biomes and ecoregions (Araújo and Guisan 2006; Svenning et al. 2008; Luoto et al. 2006). While some studies have included non‐climatic variables in SDMs, inconsistencies in the number and selection of these variables complicate comparisons (Iverson et al. 2008; Keith et al. 2008; Randin et al. 2009).

Pedology and the underlying soil properties are examples of non‐climate variables that could improve SDM accuracy, as they directly influence the physiology and ecology of terrestrial plants and thus their distribution patterns (Dubuis et al. 2013; Velazco et al. 2017). Soil is vital in the provision of water and nutrients (Aerts and Chapin 1999). It also physically supports root growth (Martre et al. 2002) and influences how the microbiome operates in the rhizosphere (Berg and Smalla 2009). While the use of soil properties in plant SDMs seems intuitive, empirical evidence of their broader impact is limited. Indeed, studies that have incorporated pedological factors have been limited to one or a few species (Fitzpatrick et al. 2008; Martinson et al. 2011; Hageer et al. 2017), and it remains unclear whether these findings apply to entire terrestrial floras.

In this study, we use ensemble correlative SDMs to model the entire Australian terrestrial vascular plant flora and address two questions: (1) How do predicted distribution maps change when using soil‐only data, climate‐only data, or a combination of both? (2) Do ecoregions differ in the relative importance of variables for predicting the distributions of their native species? Our modeling protocol was designed to find and utilize the most important environmental variables for each species (rather than use a consistent set of variables across all species). We applied this approach to model plant distributions across Australian terrestrial ecoregions. In Australia, diverse ecoregions—from arid Deserts to wetter Temperate and Tropical zones—present variable environmental conditions that influence species distribution patterns. Consequently, an ecoregion‐specific approach may be critical for accurately modeling the distribution of the continent's plants, as species' responses to climate, soil, and vegetation characteristics may vary significantly across ecoregions. By assessing the importance of climate and soil properties on SDM predictions across Australia's terrestrial ecoregions, we aim to provide a roadmap for input data selection concerning different ecoregions and whole continental floras.

## 2 Methods

### 2.1 Species Selection and Species Data Pre‐Processing

Native plant species' occurrences were obtained from various resources, including the Australian Native Plants Society (ANPSA 2024), Global Biodiversity Information Facility (Gbif 2024) (due to the use of multiple GBIF occurrence downloads, a full list of datasets and DOIs is provided in Table S5), and state‐specific flora databases (e.g., Electronic Flora of South Australia (Eflorasa 2024), Flora of Tasmania (Mf 2009), Flora of Victoria (Vicflora 2024), WetlandInfo (Wetlandinfo 2017), and Plants of the World Online (Powo 2017)). We used the Terrestrial Ecoregions of Australia map version 7 (Ibra 2024) to define ecoregions. These regions are distinct geographical units defined by a unique combination of species, natural communities, and environmental conditions; they are smaller and more localized than biomes (Olson et al. 2001). The terrestrial ecoregions of Australia map includes seven primary ecoregions, of which we focused on the four largest ones. The investigated ecoregions included (i) Desert and xeric shrublands (hereafter Desert), which accounted for around 49% of the total study area across Australia; (ii) Mediterranean forests, woodlands, and scrub (hereafter Mediterranean), covering around 11%; (iii) Temperate grasslands, savannas, and forests (hereafter Temperate), making up 12%; and (iv) Tropical and subtropical grasslands and savannas (hereafter Tropical), representing 28% of the total study area across Australia.

We downloaded the presence points of all Australian terrestrial plants with more than 100 presence points recorded in the four focal ecoregions in the Atlas of Living Australia (Ala 2024). This initial ALA dataset consisted of 11,322 plant species, which make up over 60% of the estimated flowering plant diversity on the continent, including terrestrial and aquatic species (Broadhurst and Coates 2017). We then matched the scientific names of species with their establishment status—whether native or alien—using the Australian Plant Census (Apc 2024). From this, we focused only on species identified as native, reducing the number of species to 9670. To achieve our main objective of performing an ecoregion‐specific SDM analysis, each species was associated with an ecoregion based on the proportion of its presence points across the four ecoregions. In this study, a species was assigned to a particular ecoregion if its frequency within that ecoregion exceeded thresholds of 0.6, 0.7, 0.8, 0.9, or 1 (i.e., 60%, 70%, 80%, 90%, or 100% of the species' records were in the focal ecoregion). All frequency thresholds were tested and documented; however, only the results for the 60% and 100% thresholds are presented in the main manuscript, while results for the other thresholds (70%, 80%, and 90%) are available in the Supporting Information.

The spatial resolution of our study was set to 2.5 arc‐minutes (~4.5 km). To ensure that each species had no more than one presence point per grid cell, we applied a geographical filtering approach (Kramer‐Schadt et al. 2013) and removed repeated presence points in each cell. We also excluded records that lacked precise coordinates and eliminated suspected outliers (i.e., records with coordinates falling outside the known range of the species) utilizing the ‘CoordinateCleaner’ package in R version 3.4.4 (Zizka et al. 2019). Ultimately, the number of acceptable species (after removal of aquatic plant species, for methodology refer to Supporting Information) with at least 60% frequency in any of the four ecoregions was reduced to 5033 (Table S2). Phylogenetic coverage for the 5033 angiosperm species studied was measured using the extended version of the Smith and Brown phylogeny implemented through the R package V.PhyloMaker2 (Jin and Qian 2022). This was performed to ensure species selection covered the breadth of phylogenetic diversity in our target flora. Details of the phylogenetic methods and the resulting tree are provided in Figure S1.

To mitigate the influence of potential biases in species location data used in modeling, in addition to the geographical filtering, we implemented two further strategies: environmental filtering (Varela et al. 2014) and background weighting (Elith et al. 2010). Environmental filtering was implemented using the *occfilt_env* function of the ‘flexsdm’ package in R, following the procedure by Varela et al. (2014). In this process, we created a multidimensional environmental grid based on selected environmental variables, where each dimension represents a variable. This grid divides the range of each variable into discrete intervals, or ‘bins,’ which are categories representing specific ranges of values. We then filtered the presence points by randomly retaining one point within each unique combination of these bins, thereby reducing the potential clustering of points in similar environmental conditions (Varela et al. 2014; Castellanos et al. 2019).

To address the issue of potential biases associated with selecting pseudo‐absences, we used background weighting. This method involved providing the models with environmental data that matched the spatial biases observed in the occurrence data (Renner and Warton 2013; Sequeira et al. 2012). To generate background weighting data, a weighting surface was created to emphasize areas that are geographically less dense in occurrence records. Following the methodology outlined by Elith et al. (2010), for each species a 2‐dimensional kernel density raster was first created from the presence points using the *kde2d* function of the ‘MASS’ package in R. Subsequently, a set of 10,000 background points was allocated based on the probability distribution of the density raster. This approach addresses the bias caused by spatially imbalanced or biased data, favoring pseudo‐absences in densely sampled areas over those in sparsely sampled areas (Ahmadi et al. 2023).

### 2.2 Explanatory Variables

We obtained 19 climate variables from the CHELSA database (Karger et al. 2017) in raster format at a resolution of 2.5 arc‐minutes. Climate variables can be highly correlated, which can cause problems in SDM (Naimi 2015). Thus, we selected nine of these climate variables for the modeling based on the variance inflation factor (Table S1): Annual Mean Temperature (bio1), Annual Mean Diurnal Temperature Range (bio2), Temperature Seasonality (bio4), Maximum Temperature of Warmest Month (bio5), Minimum Temperature of Coldest Month (bio6), Annual Precipitation (bio12), Precipitation of Wettest Month (bio13), Precipitation of Driest Month (bio14), and Precipitation Seasonality (bio15). In addition to climate variables, we considered eight soil variables: Bulk Density (BDW), Cation Exchange Capacity (CEC), Clay Content (CLY), Depth Of Soil (DES), Nitrogen Total Organic (NTO), Total Phosphorus (PTO), Topographic Wetness Index (TWI), and Soil Organic Carbon (SOC). These variables were incorporated using raster maps produced by the soil and landscape grid of Australia (SLGA; Grundy et al. 2015) at the same resolution as the climate data (i.e., at 2.5 arc‐minutes) (Table S1). The soil variables provide approximate measures of water and nutrient availability for plants (Duursma et al. 2013), and are likely impacted by soil parent material, climate, topography, and soil age (Delgado‐Baquerizo et al. 2020; Mcbratney et al. 2003).

Although we considered 17 environmental variables in total (nine climate variables and eight soil variables), we only used a subset of these variables in each species' SDM. For each species, we selected the most informative and predictive subset of variables out of the candidate variables, choosing five climate and five soil variables using the *covsel.embed* function from the ‘covsel’ package in R (Adde et al. 2023). This function is used to reduce the dimensionality of the predictor‐variable set and find highly informative covariates. It achieves this by integrating a collinearity‐filtering algorithm with three model‐specific embedded regularization techniques: a generalized linear model with elastic net regularization, a generalized additive model with null‐space penalization, and a guided regularized random forest. We then used the selected variables in the SDMs of each species with three arrangements: climate‐only variables (five variables), soil‐only variables (five variables), and climate + soil variables (ten variables).

### 2.3 Species Distribution Modeling

To predict suitable habitats and generate SDMs, we modeled each species across its entire distribution range in Australia. We utilized the biomod2 package ensemble platform in the R v. 3.3.4 environment (Thuiller et al. 2009). This platform employs multiple modeling techniques simultaneously, which can be used to build a consensus or “ensemble” model (Araújo and New 2007; Thuiller et al. 2009). We specifically selected four commonly used techniques known for their effectiveness in predicting species distribution: generalized linear model (GLM), generalized additive model (GAM), maximum entropy (MaxEnt), and random forest (RF). The choice of these techniques was based on various factors, including their ease of use, ability to handle uncertainty, and capacity to provide reliable error estimates (Merow et al. 2014). Furthermore, the combination of two regression‐based methods with two complex machine learning models allowed us to balance extrapolation (underfitting) and interpolation (overfitting) (Merow et al. 2014). Each technique was applied with 10 replicates for each species. We used the area under the ROC curve (AUC) and true‐skill statistic (TSS) to evaluate model performance. The AUC is a robust measure of discrimination ability, but its ecological realism in modeled distributions can be limited, especially when using presence and pseudo‐absence data rather than true absences (Lobo et al. 2008; Jiménez‐Valverde 2014; Booth 2017). Hence, we also calculated TSS as a classification accuracy measure since it is independent of prevalence (the ratio of presence versus absence records) and provides a more robust assessment of the predictive performance of SDMs when converted into presence/absence data (Hageer et al. 2017). While we assessed the predictive performance of each individual model, the final habitat suitability maps were generated using ensemble models to improve prediction accuracy and reduce model‐specific biases. To evaluate each model, the performance metrics were averaged across the 10‐fold validation subsets. We examined the relative importance of each explanatory variable to each model by analyzing the Pearson rank correlation between standard predictions and those based on five random permutations for each variable separately (Thuiller et al. 2009). For every variable, we then calculated the average variable importance across all implemented models.

### 2.4 Sensitivity Analysis of the Species Ecoregion Assignment

The thresholds used to assign species to ecoregions (i.e., the percentage of observations from an ecoregion required for assignment) can influence comparisons of SDM performance between ecoregions. To account for this, we conducted a sensitivity analysis across a range of thresholds (60%, 70%, 80%, 90%, and 100%) to refine species allocation within ecoregions. We evaluated SDM performance and the relative importance of variables across all thresholds using a two‐way ANOVA, followed by a post hoc Tukey HSD (Honestly Significant Difference) test to compare results across the five thresholds and four ecoregions. Due to space limitations and the consistency of results across thresholds, we present findings from the 60% and 100% thresholds in the main text, with results from other thresholds available in the Supporting Information.

### 2.5 Species Response to Individual Environmental Variables

We extracted raster values corresponding to specific geographic points in R, starting by loading the occurrence records for each species as spatial objects and the environmental variables as separate raster layers using the ‘sf’ (Pebesma 2018) and ‘raster’ packages (Hijmans 2023), respectively. The ‘extract()’ function from the ‘raster’ package was then used to retrieve raster values at each point location. These results enabled comparison of environmental ranges and suitability across ecoregions and facilitated the identification of overall species response patterns.

## 3 Results

### 3.1 Species Richness Patterns Under Different Density Thresholds

**FIGURE 1:**
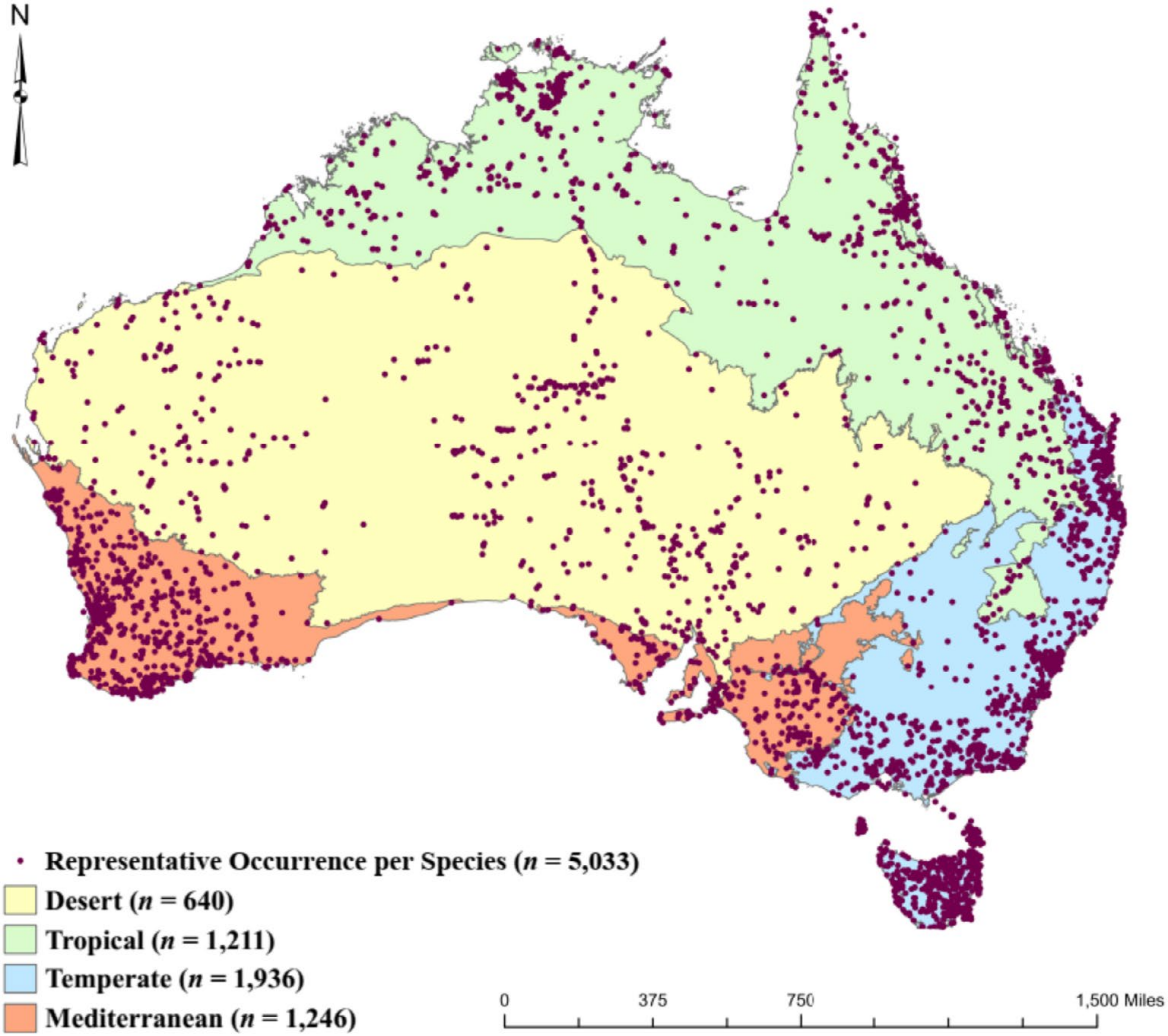
Spatial distribution of 5033 plant species across four major Australian ecoregions. Each purple dot represents a single species, based on one representative occurrence point per species. The background colors indicate the four ecoregions assessed: Desert (*n* = 640 species), Mediterranean (*n* = 1246), Temperate (*n* = 1936), and Tropical (*n* = 1211). Refer to Figure S1 for the phylogenetic tree.

Table 1 summarizes species richness in relation to occurrence thresholds (60%, 70%, 80%, 90%, and 100%) in each ecoregion. As the threshold increased from 60% to 100%, species richness decreased across all ecoregions. The Temperate ecoregion (Figure 1) consistently has the highest species richness across all thresholds. The Mediterranean and Tropical ecoregions also have a relatively high species richness, but they decrease more steeply as the threshold increases, particularly in the Mediterranean ecoregion. The Desert ecoregion has the lowest species richness at all thresholds.

**TABLE 1:** Species richness in relation to occurrence thresholds of 60%, 70%, 80%, 90%, and 100% within the investigated ecoregions (Desert, Mediterranean, Temperate, Tropical).

Threshold	Desert	Mediterranean	Temperate	Tropical
60% of total species occurrences	640	1246	1936	1211
70%	547	1132	1747	1110
80%	445	1042	1530	997
90%	289	921	1221	862
100%	38	476	502	453

### 3.2 Predictive Performance

#### 3.2.1 Density Occurrence Threshold Across Ecoregion

**FIGURE 2:**
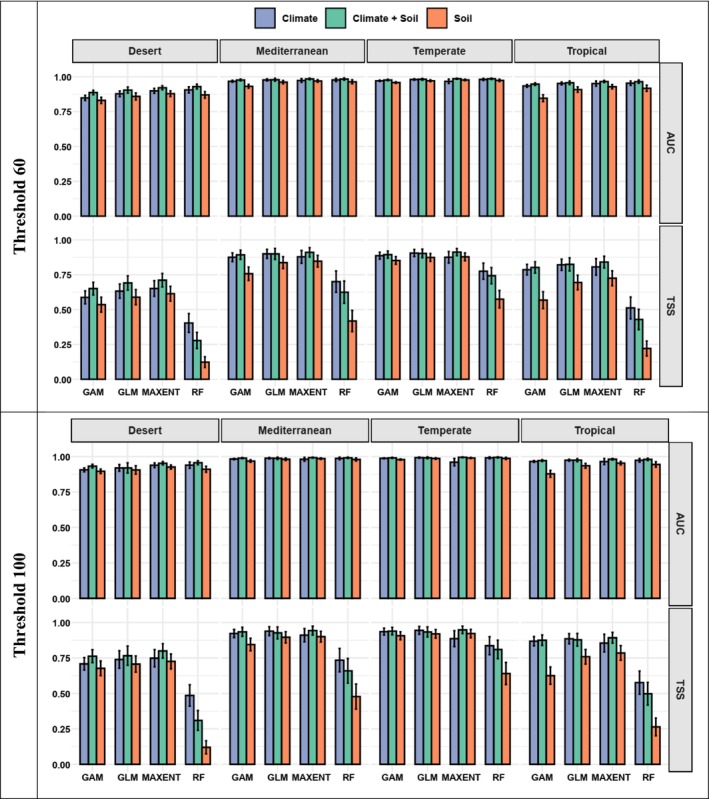
SDM performance results of generalized linear model (GLM), generalized additive model (GAM), random forest (RF), and maximum entropy (MaxEnt) for species across Australian ecoregions under 60% (*n =* 5033; top panel) and 100% (*n =* 1469; bottom panel) density occurrence ecoregion assignment thresholds. Refer to Figure S2 for details for 70%, 80%, and 90% density occurrence threshold. Continental‐scale performance metrics, averaged across all 5033 species, are provided in Table S4.

The highest AUC and TSS values were observed at the 100% occurrence density threshold across all four ecoregions, regardless of whether soil, climate, or both types of input variables were used in the SDMs (Figure 2). Conversely, the 60% threshold, which includes species with broader distributions, demonstrated the lowest model performance, particularly when only soil predictor variables were used.

#### 3.2.2 Ecoregions

Overall, the predictive performance was highest in the Temperate ecoregion, followed by the Mediterranean ecoregion (Figure 2). The Tropical ecoregion showed moderate performance, while the Desert exhibited the lowest predictive accuracy (Figure 2).

#### 3.2.3 Model Type

The comparison of predictive performance among the model types revealed that the MaxEnt model had the highest accuracy (TSS and AUC), particularly when both climate and soil variables were used together (Figure 2). Conversely, RF models using only soil data showed the lowest accuracy (TSS; Figure 2).

### 3.3 Variables Importance

When combining climate and soil variables, climate variables had a greater contribution to model predictions than soil variables, regardless of the threshold used (Table 2). To facilitate comparison, we calculated the average relative contribution of variables in climate + soil models across all species in each ecoregion (Table 2). The results showed that, at the 100% assignment thresholds (i.e., specialist plants), Annual Mean Temperature (bio1) was by far the most important variable in the Mediterranean ecoregion. For the Desert ecoregion, Temperature Seasonality (bio4) and Minimum Temperature Of Coldest Month (bio6) were the best predictors, whereas Precipitation Of Driest Month (bio14), Maximum Temperature Of Warmest Month (bio5), and Annual Mean Temperature (bio1) were most important in the Temperate ecoregion, and Annual Precipitation (bio12), Temperature Seasonality (bio4), and Annual Mean Temperature (bio1) were the most important variables for the Tropical ecoregion. The relative contribution of soil variables to specialist plants also varied between ecoregions (Table 2). The strongest soil predictor variables in the Desert ecoregion were Nitrogen Total Organic (NTO) and Exchange Capacity (CEC), whereas clay content (CLY) and Soil Organic Carbon (SOC) were the most important in the Mediterranean ecoregion, Soil Organic Carbon (SOC) and Bulk Density (BDW) were the most important in the Temperate ecoregion, and Soil Organic Carbon (SOC) and Nitrogen Total Organic (NTO) were the two most important soil variables for the Tropical ecoregion (Table 2). Interestingly, the mean relative contribution of climatic variables was highest in the Tropical ecoregion and lowest in the Desert ecoregion. Conversely, the mean relative contribution of soil variables was greatest in the Desert ecoregion and lowest in the Tropical ecoregion (Table 2). For both climatic and soil variables, these contrasts consistently became more pronounced as the assignment threshold increased from 60% (generalist species) to 100% (specialist species).

**TABLE 2:** Average relative contribution of variables in climate + soil models across all species in each ecoregion.

		Threshold 60				Threshold 100			
		Desert	Mediterranean	Temperate	Tropical	Desert	Mediterranean	Temperate	Tropical
Climatic variables	bio1	13.55	33.82	18.66	17.14	8.94	34.16	15.92	14.76
	bio2	3.59	1.49	3.22	2.48	5.8	1.14	3.32	1.9
	bio4	13.05	5.11	6.09	12.15	11.18	4.11	6.93	15.75
	bio5	9.09	6.65	14.76	4.55	5.52	4.64	16.32	2.46
	bio6	7.54	10.05	4.97	6.63	10.73	12.32	5.62	8.47
	bio12	9.88	6.27	6.88	16.17	10.28	4.95	5.43	17.15
	bio13	5.59	3.65	2.16	9.29	6.28	3.64	1.68	11.1
	bio14	0.82	2.08	16.28	1.61	1.76	1.21	17.96	1.07
	bio15	5.34	8.11	3.59	8.78	5.24	10.72	4.1	8.92
Mean		7.61	8.58	8.51	8.75	7.3	8.54	8.59	9.06
Soil variables	BDW	2.47	2.58	3.82	1.99	2.75	2.7	4.84	1.7
	CEC	5.66	3.49	2.13	2.38	5.89	2.7	1.74	2.49
	CLY	3.75	5.9	2.28	3.41	3.73	7.22	1.6	3.06
	DES	3.48	0.89	0.95	0.9	2.71	0.53	0.84	0.5
	NTO	7.7	3.1	3.21	3.27	10.74	2.74	4.67	3.39
	PTO	2.07	1.65	0.73	1.15	2.32	1.19	0.4	0.77
	SOC	3.8	4.97	8.98	6.55	4.13	5.83	7.59	5.38
	TWI	2.63	0.2	1.29	1.58	2.01	0.21	1.04	1.15
Mean		3.94	2.85	2.92	2.65	4.29	2.89	2.84	2.31

*Note:* A complete set of variable contributions across all assignment thresholds is provided in the Table S3.

### 3.4 Species Associations With Environmental Variables

**FIGURE 3:**
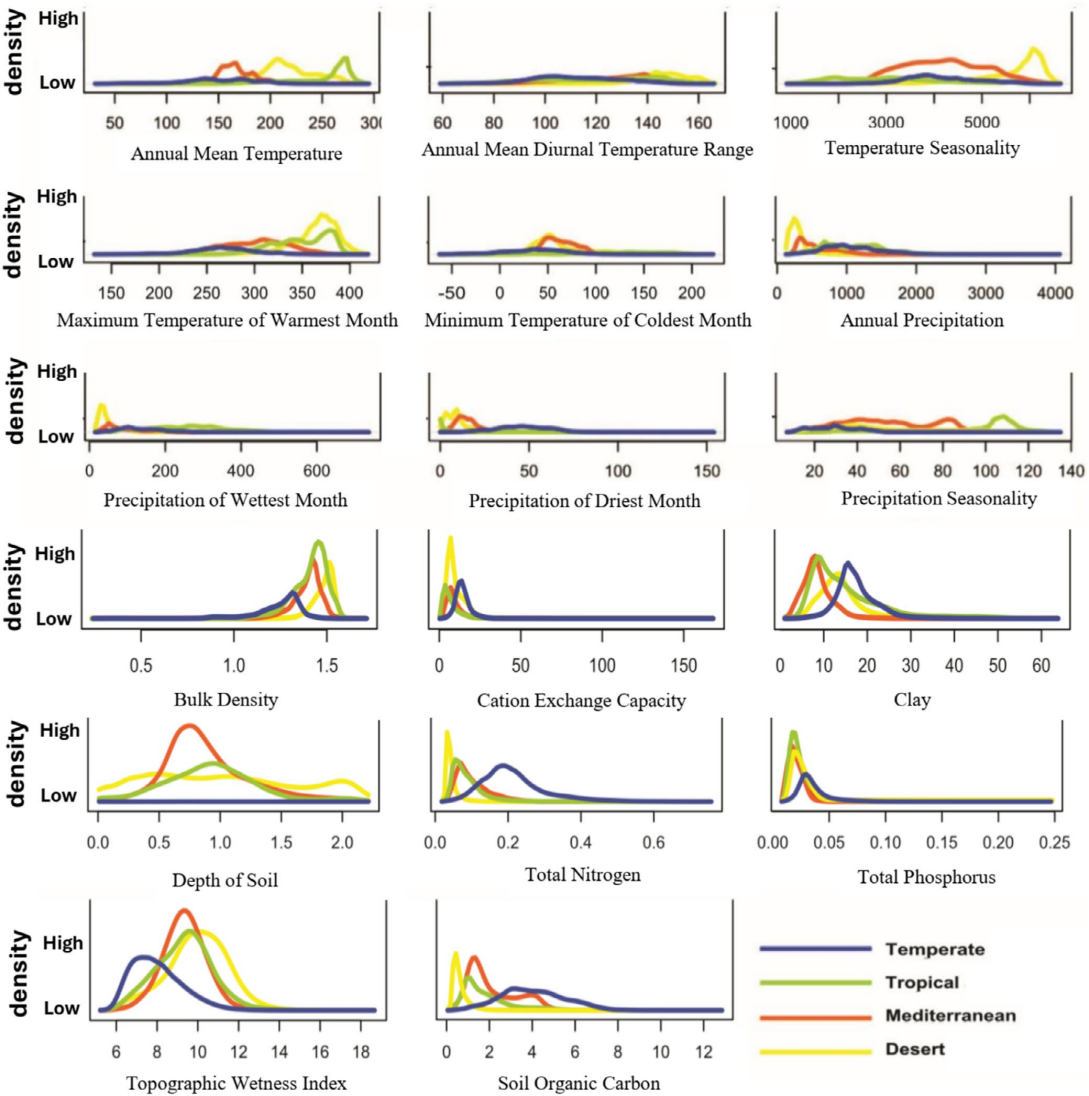
Density plots for different environmental variables across four different Australian ecoregions: Temperate, Tropical, Mediterranean, and Desert. In this study, we modeled 5033 native species, and the lines are the average of all species. The lines represent the average response of all species modeled within each ecoregion against the extracted raster values for each environmental variable. The x‐axis shows the range of each variable (see Table S1 for units, with temperature data presented in °C × 10 and precipitation data in millimeters), and the *y*‐axis represents density, indicating the frequency of values within that range in each ecoregion. For individual density plots of each species per ecoregion, please refer to Figure S3 in Supporting Information.

The response of modeled plants to bioclimatic and soil variables differs among the studied ecoregions, highlighting the unique conditions that characterize each ecoregion (Figure 3). Some ecoregions, like the Desert, show more extreme conditions with generally narrower ranges (see yellow line for Soil Organic Carbon (SOC), Exchange Capacity (CEC), Temperature Seasonality (bio4), or Annual Precipitation (bio12) in Figure 3), while others like the Tropical or Mediterranean ecoregions, exhibit broader ranges (see green and orange lines for bio4 and bio5).

### 3.5 ANOVA Analysis of Variable Importance Across Ecoregions

**FIGURE 4:**
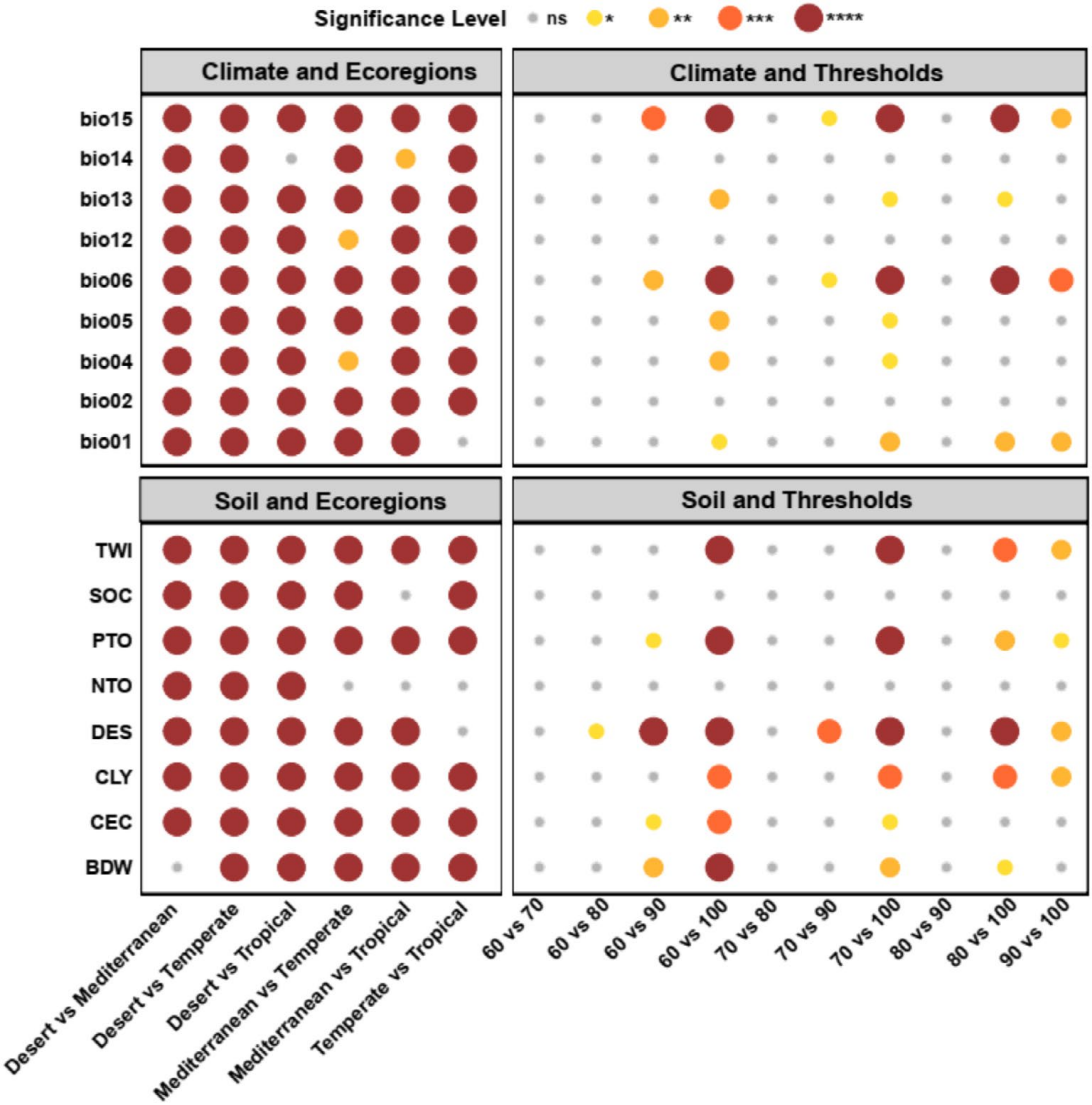
Statistical significance of environmental variables across ecoregions and thresholds using a dot plot representation. The figure consists of four facets: (1) Climate–Ecoregions, (2) Climate–Assignment Thresholds, (3) Soil–Ecoregions, and (4) Soil–Assignment Thresholds. Dot color and size correspond to significance levels, where larger dots indicate higher significance levels (****, ***, **, *), while “ns” represents non‐significant comparisons. Soil variables include: Cation Exchange Capacity (CEC), Depth of Soil (DES), Nitrogen Total Organic (NTO), Bulk Density (BDW), Soil Organic Carbon (SOC), Clay Content (CLY), Total Phosphorus (PTO), and Topographic Wetness Index (TWI). Climate variables include Annual Mean Temperature (bio1), Annual Mean Diurnal Temperature Range (bio2), Temperature Seasonality (bio4), Maximum Temperature Of Warmest Month (bio5), Minimum Temperature Of Coldest Month (bio6), Annual precipitation (bio12), Precipitation Of Wettest Month (bio13), Precipitation Of Driest Month (bio14), and Precipitation Seasonality (bio15).

The importance of predictor variables differed significantly between ecoregions (Figure 4). Most climate and soil variables showed very strong differences in between‐ecoregion comparisons. However, some variables did not show significant differences in certain ecoregion comparisons. Most importantly, the relative importance of Nitrogen Total Organic (NTO) was not significantly different between Mediterranean, Temperate, and Tropical ecoregions. Additionally, the importance of Bulk Density (BDW) and Precipitation Of The Driest Month (bio14) does not differ between some pairs, like Desert–Mediterranean and Desert–Tropical (Figure 4).

The post hoc tests also reveal varying significance levels across thresholds for certain environmental variables (Figure 4). For example, the importance of Precipitation Seasonality (bio15) and Minimum Temperature Of The Coldest Month (bio6) shows strong differences among ecoregion assignment thresholds (Figure 4). Similarly, Depth Of Soil (DES) and Total Phosphorus (PTO) are highly significant across the 60%–100% comparisons, suggesting that the importance of these variables differs more when the difference between assignment thresholds is larger. In contrast, Soil Organic Carbon (SOC), Nitrogen Total Organic (NTO), Annual Mean Diurnal Temperature Range (bio2), and Annual Precipitation (bio12) show mostly non‐significant (ns) results, indicating stable importance across thresholds and consistent influence in SDMs.

## 4 Discussion

We found that SDMs performed best when both climate and soil variables were integrated, with slightly higher AUC and TSS scores compared to climate‐only models and substantially higher than soil‐only models. This indicates that soil variables contribute important complementary information, particularly in combination with climate data, for modeling plant species distributions across Australian ecoregions. These findings support the idea that accuracy and model realism are largely dependent on the choice of environmental predictor variables used in SDMs (Crimmins et al. 2013; Mod et al. 2016). The integration of SDMs into environmental management can enhance decision‐making by identifying areas of high conservation value and predicting the impacts of environmental changes on species distributions.

### 4.1 The Effects of Pedology on Species Distribution Modeling of Floras

This study aimed to evaluate how environmental variables, both climatic and pedological (soil‐related), influence species distribution patterns of terrestrial, vascular plants across Australian ecoregions (Desert, Tropical, Temperate, and Mediterranean), using a range of SDMs and different occurrence density ecoregion assignment thresholds. Our study found that model performance, assessed using AUC and TSS metrics, was highest when climate and soil variables were integrated (Figure 2). To be more specific, models incorporating both climate and soil data exhibited marginally better performance than those utilizing climate data alone, whereas soil‐only models yielded the lowest accuracy. This is consistent with findings suggesting that adding soil variables can improve model accuracy alongside climate data in complex ecoregions (Fitzpatrick et al. 2008; Martinson et al. 2011; Dubuis et al. 2013; Hageer et al. 2017). For example, in another study on Australian shrub species, Hageer et al. (2017) demonstrated that utilizing soil variables enhances SDM performance. Similarly, Dubuis et al. (2013), studying 115 plant species in the Western Swiss Alps, showed that the inclusion of soil variables significantly improved model performance, particularly for species strongly associated with specific soil conditions. However, Zuquim et al. (2014) and Figueiredo et al. (2018), in studies with a similar spatial resolution to ours, reported contrasting findings for Amazonian plant species; they found that soil‐related variables had the greatest contribution for many of the species they modeled.

In this study, we found that when combining climate and soil variables, climate variables had a more pronounced influence on plant distributions across all Australian ecoregions compared to soil variables (Table 2, Table S3). This finding underscores the pivotal role of geographic scale in determining the relative importance of environmental factors. Indeed, the dominance of climate variables as determinants of plant distribution at a continental scale aligns with the concept of hierarchical environmental filters (Soberón 2007; Williams et al. 2009). Climate operates as a coarse‐scale filter, shaping the broad patterns of species distributions by delineating the physiological limits of plants (Körner 2012). In contrast, soil characteristics, such as nutrient availability, pH, and texture, often act as finer‐scale filters that mediate plant distributions within climatically suitable areas (Laliberté et al. 2013; John et al. 2007). More importantly, Australia's diverse biogeography, with its expansive arid interior, ropical north, and Temperate south, highlights climate as the primary driver of species distribution, likely overshadowing the localized effects of soil variables across its pronounced climatic gradients (Austin and Van Niel 2011).

While climate dominates at continental and global scales, the inclusion of soil variables enhances model performance by capturing additional nuances in species‐environment relationships. For example, in tropical rainforests, fine‐scale variations in soil properties are critical for explaining species diversity and distribution patterns (Zuquim et al. 2014; Paoli et al. 2008). Additionally, soil variables may become increasingly important in determining plant distributions under climate change scenarios, as species may need to shift their ranges to adapt to changing environmental conditions (Figueiredo et al. 2018). Nevertheless, a potential obstacle could arise from the limited availability of environmental variables used as predictors in the models. For instance, soil temperature and soil water capacity, which are crucial factors affecting plant growth (Dunne and Willmott 1996; Reddell et al. 1985), could be informative for SDMs, but the scarcity of data on these variables at a continental scale may pose a challenge for incorporating them into modeling studies.

### 4.2 Modeling Species Distributions of Floras Across Ecoregions

Species distribution patterns varied significantly across ecoregions (Figure S4), with the Temperate ecoregion consistently showing the highest species richness occurrences across all density thresholds, suggesting it supports a more diverse range of native angiosperm species compared to the other ecoregions studied (Table 1). In contrast, the Desert ecoregion had the lowest species richness, reflecting its harsher environmental conditions. In our study, lower ecoregion assignment thresholds (e.g., 60%) allowed for the inclusion of species that span multiple ecoregions and therefore likely had broader environmental tolerances (i.e., generalist species) (Stolar and Nielsen 2015; Ahmadi et al. 2023). In contrast, higher thresholds restrict the dataset to species with narrower, more defined distributions, characteristic of ecological specialists (Lay et al. 2010; Mccune 2016).

The highest AUC and TSS values were observed at the 100% density threshold across all four ecoregions, regardless of whether soil, climate, or a combination of both variable types were used in the SDMs (Figure 2). Generalist species, capable of thriving across a range of environmental conditions, often display lower predictive accuracy in SDMs due to their broad ecological niches, which could explain the lower performance of our models when lower ecoregion assignment thresholds were used (Mcpherson and Jetz 2007; Connor et al. 2017). These species can inhabit a variety of habitats, making it difficult for models to pinpoint precise environmental drivers. In contrast, specialists with narrow ecological niches exhibit stronger associations with a more limited range of environmental factors, which may help SDMs predict their distributions more accurately. Our findings highlight that the criteria used to include or exclude species when studying community responses to environmental variables are not merely a data‐handling decision but a significant factor that can influence a study's results.

More importantly, our findings revealed a clear contrast between the Deserts and Tropical ecoregions regarding the significance of climate and soil variables along the generalist to specialist species spectrum (i.e., across the assignment thresholds). The importance of climatic variables was highest in the Tropical ecoregion where the contributions of these variables increased with assignment threshold. Conversely, the contributions of soil variables were highest in the Desert ecoregion and the contribution of soil‐related variables escalated in this ecoregion at higher thresholds. In Tropical and Temperate regions, where rainfall is more abundant and climate conditions are generally milder, the role of soil in shaping plant distribution, while remaining essential in all ecosystems, becomes less pronounced (Brenes et al. 2008). In these enriched and ecologically diverse regions, other limiting factors such as temperature, sunlight, and competition with other species may play a more significant role in shaping plant distribution (Baltzer et al. 2008). Moreover, higher rainfall in Tropical regions results in increased leaching or lower mineralization rates, which decreases nutrient availability in the soils (Posada and Schuur 2011; Kurniawan et al. 2018). By contrast, in arid regions where water availability is more limited and the climate is harsh, soil characteristics become crucial determinants of plant survival and distribution (Martirosyan et al. 2016; Gamalero et al. 2020). The type and quality of soil directly influence water retention and drainage, which are critical for plant survival (Nielsen and Ball 2015). In line with this, Bui et al. (2014) discovered that in Australia, while climate plays a more significant role in influencing the distribution of Acacia species at a continental scale, the physical and chemical characteristics of soil proved to be more informative in explaining the distribution patterns of shrub species in xeric ecosystems. Altogether, understanding the specific mechanisms driving plant distribution at different spatial scales and various ecoregions is still challenging. In addition to the influence of the environment on species distributions, other factors such as dispersal limitations, neutral processes, and interactions with other species can play an important role (Franklin 2010; Chust et al. 2006).

The post hoc analysis (Figure 4) shows significant differences in the importance of climate (e.g., Annual Mean Diurnal Temperature Range (bio2), Precipitation Seasonality (bio15)), and soil variables (e.g., Exchange Capacity (CEC), Total Phosphorus (PTO), Clay Content (CLY)) to species distribution across ecoregions, indicating that species respond differently to environmental variables depending on which ecoregion they come from. These findings suggest that SDMs should be customized to each ecoregion, as key environmental drivers vary regionally. Additionally, analysis across thresholds reveals that the importance of some variables, such as Precipitation Seasonality (bio15), Depth Of Soil (DES), and Total Phosphorus (PTO), changes depending on the ecoregion assignment thresholds used (e.g., 60%–100%), suggesting the importance of variables differs between the generalist species included when a low threshold is used and the more specialized species that remain when a higher, more restrictive threshold is used. In contrast, variables like Annual Mean Diurnal Temperature Range (bio2) and Nitrogen Total Organic (NTO) remain stable, indicating their contributions as predictor variables are consistent across ecoregion assignment thresholds.

## 5 Conclusions

Our findings underscore the potential of SDMs to inform adaptive management practices, particularly in ecoregions where species distributions are strongly influenced by both climate and soil variables. By helping tailor environmental management strategies to the specific drivers of species distributions in each ecoregion, SDMs can contribute to the sustainable use of natural resources and the preservation of biodiversity under changing environmental conditions. Our study concludes that climate variables are the most important factors at the ecoregion scale across all assignment thresholds. Further, adding soil variables often improves SDMs, but soil‐only models are weak predictors compared to climate‐only or climate + soil models. Our study revealed that, among the various model types, MaxEnt stands out as one of the most effective models employed in this study. Overall, the 100%‐assignment threshold yielded the highest SDM performance, while the lowest performance was observed at the 60% threshold.

Future research should investigate the relationship between species' range sizes and the importance of different environmental variables. Species with narrow distributions, such as rare, endemic, or threatened species, may be disproportionately influenced by specific environmental factors, whereas widespread species may respond to a shared suite of variables. Understanding this relationship could yield valuable insights for conservation biology by identifying which environmental factors are most critical for the persistence of species with restricted ranges. While our study illustrates the complex interplay between climate and soil variables in shaping species distributions, other influential factors—such as dispersal limitations and neutral processes, including random dispersal, genetic drift, and stochastic demographic events—also play important roles (Chust et al. 2006; Franklin 2010). Additionally, correlated environmental variables complicate interpretations of model output (Davidar et al. 2007; Brenes et al. 2008). We recommend that future studies explore the potential benefits of incorporating additional variables, such as topographic and land use factors, to further enhance model accuracy and better capture the mechanisms driving plant distributions across spatial scales and ecoregions.

## Author Contributions

**Farzin Shabani:** conceptualization (equal), data curation (lead), formal analysis (lead), funding acquisition (lead), methodology (lead), project administration (lead), validation (lead), visualization (lead), writing – original draft (equal), writing – review and editing (lead). **Mohsen Ahmadi:** conceptualization (equal), data curation (lead), formal analysis (lead), methodology (lead), validation (supporting), visualization (lead), writing – original draft (lead), writing – review and editing (equal). **Niloufar Lorestani:** conceptualization (equal), data curation (lead), formal analysis (lead), methodology (equal), validation (supporting), visualization (lead), writing – original draft (lead), writing – review and editing (equal). **Shazia Bibi:** validation (supporting), writing – review and editing (equal). **Atefeh Esmaeili:** validation (supporting), writing – review and editing (equal). **Tessa Lane:** conceptualization (equal), validation (supporting), writing – review and editing (equal). **Martin F. Breed:** validation (supporting), writing – review and editing (equal). **John Llewelyn:** conceptualization (equal), validation (supporting), writing – review and editing (equal). **Craig Liddicoat:** data curation (supporting), validation (supporting), writing – review and editing (equal). **Philip Kibet Langat:** validation (supporting), writing – review and editing (equal). **Bahareh Kalantar:** data curation (supporting), methodology (supporting), validation (supporting), writing – review and editing (equal). **Nadiezhda Ramírez‐Cabral:** validation (supporting), writing – review and editing (equal). **Pooja Singh:** conceptualization (supporting), data curation (supporting), methodology (supporting), validation (supporting), writing – review and editing (equal). **Ricardo Siqueira da Silva:** data curation (supporting), validation (supporting), writing – review and editing (equal). **Mohammed Abu‐Dieyeh:** writing – review and editing (equal). **Masoud Nazarizadeh:** data curation (supporting), methodology (supporting), writing – review and editing (equal). **Alessandro Ossola:** conceptualization (supporting), data curation (supporting), methodology (supporting), validation (supporting), writing – review and editing (equal).

## Conflicts of Interest

The authors declare no conflicts of interest.

## Supporting information


Appendix S1.

## Data Availability

Data are available from the Dryad Digital Repository: https://doi.org/10.5061/dryad.9cnp5hqwn.
