# Supplementary material for: Pedology and Plant Provenance Can Improve Species Distribution Predictions of Australian Native Flora: A Calibrated and Validated Modeling Exercise on 5033 Species
Source: Ecol Evol. 2025 Jun 24;15(6):e71430. doi: 10.1002/ece3.71430 (PMC12185943; doi:10.1002/ece3.71430)
Supplement: Supplementary file 1 — Appendix S1. [file ECE3-15-e71430-s001.zip › SI/SI_Ecoregion.pdf]

Table S1. Environmental input variables (climate & soil) used in this study.

	Name	Abbreviation	Unit
**Climate**	Annual mean temperature	bio1	°C*10
	Annual mean diurnal temperature range	bio2	°C×10
	Temperature seasonality	bio4	%
	Maximum temperature of warmest month	bio5	°C×10
	Minimum temperature of coldest month	bio6	°C×10
	Annual precipitation	bio12	mm
	Precipitation of wettest month	bio13	mm
	Precipitation of driest month	bio14	mm
	Precipitation seasonality	bio15	%
**Soil**	Bulk Density	BDW	g cm^{-3} whole earth
	Cation Exchange Capacity	CEC	mEq per 100g
	Clay Content	CLY	%
	Depth of Soil	DES	cm
	Nitrogen Total Organic	NTO	%
	Total Phosphorus	PTO	%
	Topographic Wetness Index	TWI	adimensional
	Soil Organic Carbon	SOC	%

Table S2. List of all 5,033 native species covered in this study. It is obtained from various online databases including Australian Native Plants Society (ANPSA, 2024), Global Biodiversity Information Facility (GBIF, 2024), and state specific flora databases (e.g., Electronic Flora of South Australia (eFloraSA, 2024), Flora of Tasmania (MF, 2009), Flora of Victoria (VicFlora, 2024), WetlandInfo (WetlandInfo, 2017), Plants of the World Online (POWO, 2017))

Deserts	Mediterranean	Temperate	Tropical
Abutilon cryptopetalum	*Acacia acanthoclada*	*Abrophyllum ornans*	*Abelmoschus ficulneus*
Abutilon fraseri	*Acacia acuaria*	*Acacia acinacea*	*Abrus precatorius*
Abutilon halophilum	*Acacia acuminata*	*Acacia aculeatissima*	*Abutilon auritum*
Abutilon lepidum	*Acacia alata*	*Acacia aspera*	*Abutilon hannii*
Abutilon leucopetalum	*Acacia anceps*	*Acacia aulacocarpa*	*Acacia alleniana*
Abutilon macrum	*Acacia ancistrophylla*	*Acacia baueri*	*Acacia argyraea*
Abutilon malvifolium	*Acacia andrewsii*	*Acacia blakei*	*Acacia asperulacea*
Acacia abrupta	*Acacia applanata*	*Acacia brownii*	*Acacia bancroftiorum*
Acacia adoxa	*Acacia argyrophylla*	*Acacia buxifolia*	*Acacia burrowii*
Acacia adsurgens	*Acacia assimilis*	*Acacia caesiella*	*Acacia calligera*
Acacia ancistrocarpa	*Acacia bidentata*	*Acacia complanata*	*Acacia calyculata*
Acacia aneura	*Acacia blakelyi*	*Acacia concurrens*	*Acacia caroleae*
Acacia aptaneura	*Acacia brachybotrya*	*Acacia dawsonii*	*Acacia cheelii*
Acacia arida	*Acacia browniana*	*Acacia decora*	*Acacia chisholmii*

Acacia ayersiana	*Acacia camptoclada*	*Acacia difformis*	*Acacia colei*
Acacia beckleri	*Acacia celastrifolia*	*Acacia disparrima*	*Acacia conferta*
Acacia bivenosa	*Acacia chrysella*	*Acacia doratoxylon*	*Acacia conspersa*
Acacia brachystachya	*Acacia chrysocephala*	*Acacia echinula*	*Acacia crassa*
Acacia caesaneura	*Acacia cochlearis*	*Acacia falcata*	*Acacia crassicarpa*
Acacia calcicola	*Acacia colletioides*	*Acacia falciformis*	*Acacia difficilis*
Acacia carneorum	*Acacia coolgardiensis*	*Acacia filicifolia*	*Acacia dimidiata*
Acacia citrinoviridis	*Acacia divergens*	*Acacia flexifolia*	*Acacia drepanocarpa*
Acacia continua	*Acacia drummondii*	*Acacia genistifolia*	*Acacia eriopoda*
Acacia coriacea	*Acacia enervia*	*Acacia gunnii*	*Acacia fasciculifera*
Acacia craspedocarpa	*Acacia erinacea*	*Acacia hispidula*	*Acacia flavescens*
Acacia cuthbertsonii	*Acacia euthycarpa*	*Acacia homalophylla*	*Acacia galioides*
Acacia cyperophylla	*Acacia farinosa*	*Acacia implexa*	*Acacia gladiiformis*
Acacia dictyophleba	*Acacia fragilis*	*Acacia irrorata*	*Acacia gonocarpa*
Acacia estrophiolata	*Acacia glaucoptera*	*Acacia kettlewelliae*	*Acacia gonoclada*
Acacia fuscaneura	*Acacia gonophylla*	*Acacia lanigera*	*Acacia hammondii*
Acacia georginae	*Acacia halliana*	*Acacia leiocalyx*	*Acacia harpophylla*
Acacia grasbyi	*Acacia hemiteles*	*Acacia leprosa*	*Acacia hemignosta*
Acacia hilliana	*Acacia heteroneura*	*Acacia leucoclada*	*Acacia hemsleyi*
Acacia inaequilatera	*Acacia lasiocalyx*	*Acacia leucolobia*	*Acacia holosericea*
Acacia incurvaneura	*Acacia lasiocarpa*	*Acacia linearifolia*	*Acacia humifusa*
Acacia kempeana	*Acacia latipes*	*Acacia linifolia*	*Acacia julifera*
Acacia macdonnellensis	*Acacia leiophylla*	*Acacia longissima*	*Acacia laccata*
Acacia maitlandii	*Acacia leptopetala*	*Acacia mabellae*	*Acacia lamprocarpa*
Acacia melleodora	*Acacia leptospermoides*	*Acacia maidenii*	*Acacia latescens*
Acacia minyura	*Acacia lineolata*	*Acacia mitchellii*	*Acacia latifolia*
Acacia mulganeura	*Acacia loderi*	*Acacia montana*	*Acacia leptocarpa*
Acacia murrayana	*Acacia longispinea*	*Acacia mucronata*	*Acacia leptophleba*
Acacia pachyacra	*Acacia merrallii*	*Acacia myrtifolia*	*Acacia leptostachya*
Acacia papyrocarpa	*Acacia microbotrya*	*Acacia neriifolia*	*Acacia limbata*
Acacia paraneura	*Acacia microcarpa*	*Acacia obliquinervia*	*Acacia longispicata*
Acacia pruinocarpa	*Acacia multispicata*	*Acacia obtusata*	*Acacia lycopodiifolia*
Acacia pteraneura	*Acacia mutabilis*	*Acacia obtusifolia*	*Acacia lysiphloia*
Acacia pyrifolia	*Acacia nematophylla*	*Acacia oshanesii*	*Acacia mariae*
Acacia quadrimarginea	*Acacia nervosa*	*Acacia oxycedrus*	*Acacia mimula*
Acacia ramulosa	*Acacia neurophylla*	*Acacia parvipinnula*	*Acacia multisiliqua*
Acacia rhodophloia	*Acacia nigripilosa*	*Acacia penninervis*	*Acacia neurocarpa*
Acacia rivalis	*Acacia nyssophylla*	*Acacia quadrilateralis*	*Acacia nuperrima*
Acacia sclerosperma	*Acacia resinimarginea*	*Acacia rubida*	*Acacia oncinocarpa*
Acacia sericophylla	*Acacia restiacea*	*Acacia siculiformis*	*Acacia oraria*
Acacia sibirica	*Acacia rigens*	*Acacia silvestris*	*Acacia pellita*

Acacia spondylophylla	*Acacia sclerophylla*	*Acacia stenophylla*	*Acacia phlebocarpa*
Acacia stellaticeps	*Acacia spathulifolia*	*Acacia stricta*	*Acacia pilligaensis*
Acacia stipuligera	*Acacia sphacelata*	*Acacia suaveolens*	*Acacia platycarpa*
Acacia strongylophylla	*Acacia spinescens*	*Acacia ulicifolia*	*Acacia plectocarpa*
Acacia synchronicia	*Acacia stenoptera*	*Acacia venulosa*	*Acacia polybotrya*
Acacia tarculensis	*Acacia stereophylla*	*Acacia verniciflua*	*Acacia polystachya*
Acacia tetragonophylla	*Acacia subcaerulea*	*Acacia verticillata*	*Acacia producta*
Acacia trachycarpa	*Acacia sulcata*	*Acaena echinata*	*Acacia pustula*
Acacia validinervia	*Acacia triquetra*	*Acalypha capillipes*	*Acacia retivenea*
Acacia victoriae	*Acacia uncinella*	*Acalypha nemorum*	*Acacia rothii*
Acacia xiphophylla	*Acacia urophylla*	*Acianthus caudatus*	*Acacia sertiformis*
Afrohybanthus aurantiacus	*Acacia varia*	*Acianthus exsertus*	*Acacia shirleyi*
Allocasuarina decaisneana	*Acacia verriculum*	*Acianthus fornicatus*	*Acacia simsii*
Aluta maisonneuvei	*Acacia wilhelmiana*	*Aciphylla simplicifolia*	*Acacia sparsiflora*
Alyogyne pinoniana	*Acacia willdenowiana*	*Ackama paniculosa*	*Acacia stigmatophylla*
Amaranthus cuspidifolius	*Acacia yorkrakinensis*	*Acradenia euodiiformis*	*Acacia sublanata*
Amaranthus grandiflorus	*Acanthocarpus preissii*	*Acronychia imperforata*	*Acacia tephrina*
Amaranthus mitchellii	*Acrotriche affinis*	*Acronychia oblongifolia*	*Acacia torulosa*
Amphipogon sericeus	*Acrotriche cordata*	*Acronychia pauciflora*	*Acacia translucens*
Amyema fitzgeraldii	*Acrotriche depressa*	*Acronychia pubescens*	*Acacia tropica*
Amyema gibberula	*Acrotriche patula*	*Acronychia wilcoxiana*	*Acacia tumida*
Amyema hilliana	*Actinodium cunninghamii*	*Acrothamnus hookeri*	*Acacia umbellata*
Amyema maidenii	*Actinotus glomeratus*	*Acrothamnus maccraei*	*Acacia wickhamii*
Amyema nestor	*Adenanthos argyreus*	*Acrothamnus montanus*	*Acacia yirrkallensis*
Anacampseros australiana	*Adenanthos barbiger*	*Acrotriche aggregata*	*Acalypha eremorum*
Androcalva loxophylla	*Adenanthos cuneatus*	*Acrotriche divaricata*	*Acanthus ilicifolius*
Anemocarpa podolepidium	*Adenanthos cygnorum*	*Acrotriche prostrata*	*Achyranthes aspera*
Anemocarpa saxatilis	*Adenanthos obovatus*	*Acrotriche rigida*	*Acmella grandiflora*
Anthobolus leptomerioides	*Adenanthos terminalis*	*Acrotriche serrulata*	*Adansonia gregorii*
Apowollastonia stirlingii	*Adriana quadripartita*	*Actephila mooreana*	*Adenia heterophylla*
Arabidella eremigena	*Agonis flexuosa*	*Actinotus minor*	*Aegialitis annulata*
Arabidella glaucescens	*Agrostocrinum hirsutum*	*Actites megalocarpus*	*Aeschynomene brevifolia*
Arabidella nasturtium	*Agrostocrinum scabrum*	*Aegiceras corniculatum*	*Aeschynomene indica*
Arabidella trisecta	*Allocasuarina acutivalvis*	*Afrohybanthus stellarioides*	*Afrohybanthus enneaspermus*

Aristida arida	*Allocasuarina campestris*	*Agastachys odorata*	*Aglaia brownii*
Aristida biglandulosa	*Allocasuarina corniculata*	*Agrostis venusta*	*Aglaia elaeagnoidea*
Aristida nitidula	*Allocasuarina fraseriana*	*Ailanthus triphysa*	*Aglaia sapindina*
Aristida obscura	*Allocasuarina helmsii*	*Ajuga australis*	*Aidia racemosa*
Aristida strigosa	*Allocasuarina huegeliana*	*Akania bidwillii*	*Alectryon diversifolius*
Astrebla pectinata	*Allocasuarina humilis*	*Alangium villosum*	*Allocasuarina inophloia*
Atriplex angulata	*Allocasuarina lehmanniana*	*Alchornea ilicifolia*	*Allophylus cobbe*
Atriplex codonocarpa	*Allocasuarina mackliniana*	*Alectryon coriaceus*	*Allopterigeron filifolius*
Atriplex crassipes	*Allocasuarina microstachya*	*Alectryon subcinereus*	*Alloteropsis cimicina*
Atriplex elachophylla	*Allocasuarina muelleriana*	*Alectryon subdentatus*	*Alloteropsis semialata*
Atriplex fissivalvis	*Allocasuarina pusilla*	*Alectryon tomentosus*	*Alphitonia oblata*
Atriplex incrassata	*Allocasuarina spinosissima*	*Allittia cardiocarpa*	*Alphitonia pomaderroides*
Atriplex intermedia	*Allocasuarina striata*	*Allittia uliginosa*	*Alstonia actinophylla*
Atriplex limbata	*Allocasuarina thuyoides*	*Allocasuarina distyla*	*Alstonia scholaris*
Atriplex quinii	*Aluta aspera*	*Allocasuarina littoralis*	*Alstonia spectabilis*
Atriplex semilunaris	*Alyogyne hakeifolia*	*Allocasuarina misera*	*Alternanthera ficoidea*
Atriplex spongiosa	*Alyxia buxifolia*	*Allocasuarina monilifera*	*Alysicarpus muelleri*
Atriplex velutinella	*Amphipogon amphipogonoides*	*Allocasuarina nana*	*Alysicarpus schomburgkii*
Atriplex vesicaria	*Amyema melaleucae*	*Allocasuarina paludosa*	*Alyxia spicata*
Austrostipa pilata	*Anarthria gracilis*	*Allocasuarina torulosa*	*Ammannia baccifera*
Bergia henshallii	*Anarthria humilis*	*Almaleea subumbellata*	*Amorphophallus galbra*
Bergia perennis	*Anarthria laevis*	*Alphitonia excelsa*	*Ampelocissus acetosa*
Blennodia canescens	*Anarthria prolifera*	*Alpinia arundelliana*	*Ampelocissus frutescens*
Blennodia pterosperma	*Anarthria scabra*	*Alpinia caerulea*	*Amyema benthamii*
Boerhavia repleta	*Andersonia caerulea*	*Alternanthera denticulata*	*Amyema bifurcata*
Boerhavia schomburgkiana	*Andersonia lehmanniana*	*Alyxia magnifolia*	*Amyema conspicua*
Bonamia erecta	*Andersonia parvifolia*	*Alyxia ruscifolia*	*Amyema mackayensis*
Brachychiton gregorii	*Andersonia sprengelioides*	*Amaranthus macrocarpus*	*Amyema sanguinea*
Brachyscome dichromosomatica	*Angianthus preissianus*	*Amperea xiphoclada*	*Amyema villiflora*
Brachyscome gilesii	*Angianthus tomentosus*	*Amyema cambagei*	*Ancistrachne uncinulata*

Brachyscome tesquorum	*Anigozanthos bicolor*	*Amyema congener*	*Aneilema siliculosum*
Calandrinia balonensis	*Anigozanthos humilis*	*Amyema pendula*	*Anisomeles moschata*
Calandrinia polyandra	*Anigozanthos rufus*	*Amylotheca dictyophleba*	*Anthobolus filifolius*
Calandrinia ptychosperma	*Anthocercis littorea*	*Androcalva fraseri*	*Antidesma ghaesembilla*
Calandrinia pumila	*Aotus subspinescens*	*Aneilema acuminatum*	*Antidesma parvifolium*
Calandrinia remota	*Aphelia cyperoides*	*Aneilema biflorum*	*Aotus mollis*
Calandrinia reticulata	*Apium annuum*	*Angophora bakeri*	*Aphyllodium biarticulatum*
Calandrinia stagnensis	*Apodasmia brownii*	*Angophora floribunda*	*Aphyllodium schindleri*
Calocephalus knappii	*Argentipallium blandowskianum*	*Angophora hispida*	*Apowollastonia cylindrica*
Calocephalus platycephalus	*Argentipallium niveum*	*Angophora subvelutina*	*Apowollastonia spilanthoides*
Calotis cymbacantha	*Argentipallium obtusifolium*	*Angophora woodsiana*	*Archidendropsis basaltica*
Calotis latiuscula	*Arthropodium curvipes*	*Anisopogon avenaceus*	*Argusia argentea*
Calotis multicaulis	*Astartea scoparia*	*Anodopetalum biglandulosum*	*Argyrodendron polyandrum*
Calotis plumulifera	*Asteridea athrixioides*	*Anopterus glandulosus*	*Aristida exserta*
Calotis porphyroglossa	*Asteridea pulverulenta*	*Anopterus macleayanus*	*Aristida gracilipes*
Calytrix carinata	*Atriplex acutibractea*	*Anthocarapa nitidula*	*Aristida hygrometrica*
Capparis spinosa	*Atriplex paludosa*	*Anthosachne kingiana*	*Aristida ingrata*
Centipeda pleiocephala	*Atriplex stipitata*	*Anthosachne rectiseta*	*Aristida latifolia*
Centipeda thespidioides	*Austrostipa compressa*	*Aotus ericoides*	*Aristida lazaridis*
Centrolepis eremica	*Austrostipa drummondii*	*Aotus lanigera*	*Aristida leichhardtiana*
Chenopodium auricomum	*Austrostipa elegantissima*	*Aotus subglauca*	*Aristida leptopoda*
Chenopodium gaudichaudianum	*Austrostipa flavescens*	*Aphananthe philippinensis*	*Aristida macroclada*
Chloris pectinata	*Austrostipa hemipogon*	*Aphanes australiana*	*Aristida pruinosa*
Chrysocephalum eremaeum	*Austrostipa macalpinei*	*Aphanopetalum resinosum*	*Aristida queenslandica*
Chrysocephalum pterochaetum	*Austrostipa mundula*	*Aphelia gracilis*	*Aristida superpendens*
Codonocarpus cotinifolius	*Austrostipa puberula*	*Aphelia pumilio*	*Aristida utilis*
Codonocarpus pyramidalis	*Austrostipa tenuifolia*	*Apium prostratum*	*Aristolochia pubera*
Commicarpus australis	*Austrostipa variabilis*	*Archidendron grandiflorum*	*Arivela cleomoides*
Convolvulus clementii	*Baeckea crassifolia*	*Archirhodomyrtus beckleri*	*Arivela tetrandra*

Convolvulus eyreanus	*Baeckea elderiana*	*Archontophoenix cunninghamiana*	*Arivela viscosa*
Convolvulus microsepalus	*Baeckea preissiana*	*Argentipallium dealbatum*	*Arthrostylis aphylla*
Corchorus crozophorifolius	*Banksia armata*	*Argyrodendron actinophyllum*	*Arundinella nepalensis*
Corchorus lasiocarpus	*Banksia ashbyi*	*Argyrodendron trifoliolatum*	*Arundinella setosa*
Corchorus walcottii	*Banksia attenuata*	*Aristida vagans*	*Asparagus racemosus*
Corymbia aparrerinja	*Banksia baueri*	*Aristida warburgii*	*Asteromyrtus lysicephala*
Corymbia candida	*Banksia baxteri*	*Aristolochia praevenosa*	*Asteromyrtus symphyocarpa*
Corymbia chippendalei	*Banksia bipinnatifida*	*Aristotelia peduncularis*	*Astrebla squarrosa*
Corymbia deserticola	*Banksia cirsioides*	*Arrhenechthites mixtus*	*Atalaya variifolia*
Corymbia eremaea	*Banksia coccinea*	*Artanema fimbriatum*	*Atractocarpus fitzalanii*
Corymbia hamersleyana	*Banksia dallanneyi*	*Arthrochilus prolixus*	*Atractocarpus sessilis*
Corymbia opaca	*Banksia elderiana*	*Arthropodium milleflorum*	*Austrodolichos errabundus*
Crotalaria cunninghamii	*Banksia fraseri*	*Arthropodium minus*	*Avicennia marina*
Crotalaria eremaea	*Banksia gardneri*	*Arthropodium strictum*	*Bacopa floribunda*
Crotalaria smithiana	*Banksia grandis*	*Arytera distylis*	*Balanophora fungosa*
Cucumis argenteus	*Banksia ilicifolia*	*Arytera divaricata*	*Banksia dentata*
Cucumis variabilis	*Banksia leptophylla*	*Arytera foveolata*	*Barringtonia acutangula*
Cullen australasicum	*Banksia littoralis*	*Asperula cunninghamii*	*Basilicum polystachyon*
Cullen cinereum	*Banksia media*	*Asperula gunnii*	*Bergia pedicellaris*
Cullen discolor	*Banksia menziesii*	*Asperula pusilla*	*Blainvillea cunninghamii*
Cullen graveolens	*Banksia nivea*	*Asperula scoparia*	*Blepharocarya depauperata*
Cullen leucanthum	*Banksia nutans*	*Astelia alpina*	*Blepharocarya involucrigera*
Cullen martinii	*Banksia obovata*	*Asterolasia correifolia*	*Blumea axillaris*
Cullen pallidum	*Banksia ornata*	*Astrotricha asperifolia*	*Blumea diffusa*
Cullen patens	*Banksia prionotes*	*Astrotricha floccosa*	*Blumea integrifolia*
Cuphonotus andraeanus	*Banksia pulchella*	*Astrotricha latifolia*	*Blumea saxatilis*
Cuscuta victoriana	*Banksia quercifolia*	*Astrotricha ledifolia*	*Blumea tenella*
Cynanchum floribundum	*Banksia repens*	*Astrotricha longifolia*	*Boerhavia albiflora*
Cynodon simonii	*Banksia rufa*	*Atalaya salicifolia*	*Boerhavia gardneri*
Cyperus bulbosus	*Banksia seminuda*	*Atherosperma moschatum*	*Boerhavia paludosa*

Cyperus centralis	*Banksia sessilis*	*Atractocarpus benthamianus*	*Bombax ceiba*
Dampiera candicans	*Banksia speciosa*	*Atractocarpus chartaceus*	*Bonamia brevifolia*
Dampiera cinerea	*Banksia sphaerocarpa*	*Atriplex australasica*	*Bonamia media*
Dentella asperata	*Banksia squarrosa*	*Atriplex spinibractea*	*Bonamia multiflora*
Dentella pulvinata	*Banksia violacea*	*Australina pusilla*	*Bonamia pannosa*
Dichromochlamys dentatifolia	*Beaufortia bracteosa*	*Austromyrtus dulcis*	*Boronia glabra*
Dicrastylis beveridgei	*Beaufortia elegans*	*Austrosteenisia blackii*	*Boronia lanceolata*
Dicrastylis cordifolia	*Beaufortia empetrifolia*	*Austrosteenisia glabristyla*	*Boronia lanuginosa*
Dicrastylis costelloi	*Beaufortia micrantha*	*Austrostipa aristiglumis*	*Boronia occidentalis*
Dicrastylis exsuccosa	*Beaufortia puberula*	*Austrostipa bigeniculata*	*Bossiaea bossiaeoides*
Dicrastylis gilesii	*Beaufortia schaueri*	*Austrostipa densiflora*	*Bossiaea concolor*
Dicrastylis lewellinii	*Beaufortia sparsa*	*Austrostipa gibbosa*	*Brachychiton australis*
Diplatia grandibractea	*Bertya tasmanica*	*Austrostipa muelleri*	*Brachychiton diversifolius*
Diplopeltis stuartii	*Beyeria lechenaultii*	*Austrostipa oligostachya*	*Brachychiton fitzgeraldianus*
Dipteracanthus australasicus	*Beyeria opaca*	*Austrostipa pubescens*	*Brachychiton megaphyllus*
Dodonaea coriacea	*Beyeria sulcata*	*Austrostipa rudis*	*Brachychiton paradoxus*
Dodonaea lobulata	*Billardiera coriacea*	*Austrostipa semibarbata*	*Brachychiton rupestris*
Dodonaea microzyga	*Billardiera floribunda*	*Austrostipa stuposa*	*Brachychiton viscidulus*
Dodonaea petiolaris	*Billardiera sericophora*	*Austrostipa tuckeri*	*Breynia cernua*
Dodonaea rigida	*Billardiera variifolia*	*Austrostipa verticillata*	*Bridelia tomentosa*
Duperreya commixta	*Billardiera venusta*	*Backhousia leptopetala*	*Brucea javanica*
Dysphania kalpari	*Billardiera versicolor*	*Backhousia myrtifolia*	*Brunoniella acaulis*
Dysphania plantaginella	*Blennospora drummondii*	*Backhousia sciadophora*	*Buchanania arborescens*
Dysphania rhadinostachya	*Boronia coerulescens*	*Baeckea brevifolia*	*Buchanania obovata*
Dysphania truncata	*Boronia crassifolia*	*Baeckea diosmifolia*	*Buchnera asperata*
Enneapogon avenaceus	*Boronia crenulata*	*Baeckea frutescens*	*Buchnera gracilis*
Enneapogon caerulescens	*Boronia filifolia*	*Baeckea gunniana*	*Buchnera linearis*
Enneapogon cylindricus	*Boronia inornata*	*Baeckea imbricata*	*Buchnera ramosissima*
Enneapogon polyphyllus	*Boronia ramosa*	*Baeckea linifolia*	*Buchnera urticifolia*
Eragrostis basedowii	*Boronia scabra*	*Baeckea utilis*	*Burmannia juncea*
Eragrostis dielsii	*Boronia spathulata*	*Baloghia inophylla*	*Bursaria incana*
Eragrostis eriopoda	*Boronia ternata*	*Baloskion australe*	*Bursaria tenuifolia*
Eragrostis kennedyae	*Borya constricta*	*Baloskion fimbriatum*	*Byblis filifolia*

Eragrostis laniflora	*Borya sphaerocephala*	*Baloskion pallens*	*Byblis liniflora*
Eragrostis lanipes	*Bossiaea aquifolium*	*Baloskion tetraphyllum*	*Caesalpinia bonduc*
Eragrostis pergracilis	*Bossiaea eriocarpa*	*Banksia aemula*	*Cajanus acutifolius*
Eragrostis xerophila	*Bossiaea linophylla*	*Banksia marginata*	*Cajanus cinereus*
Eremophea spinosa	*Bossiaea ornata*	*Banksia oblongifolia*	*Cajanus geminatus*
Eremophila alternifolia	*Bossiaea preissii*	*Banksia paludosa*	*Cajanus latisepalus*
Eremophila bowmanii	*Bossiaea spinescens*	*Banksia robur*	*Cajanus marmoratus*
Eremophila christophori	*Brachyloma ericoides*	*Banksia serrata*	*Cajanus pubescens*
Eremophila cuneifolia	*Brachyscome exilis*	*Banksia spinulosa*	*Cajanus reticulatus*
Eremophila duttonii	*Brachyscome goniocarpa*	*Bauera capitata*	*Cajanus scarabaeoides*
Eremophila eriocalyx	*Brachyscome iberidifolia*	*Bauera rubioides*	*Calamus australis*
Eremophila exilifolia	*Burchardia congesta*	*Bedfordia arborescens*	*Calandrinia gracilis*
Eremophila forrestii	*Burchardia multiflora*	*Bedfordia salicina*	*Calandrinia quadrivalvis*
Eremophila fraseri	*Bursaria occidentalis*	*Beilschmiedia elliptica*	*Calandrinia uniflora*
Eremophila freelingii	*Caesia micrantha*	*Beilschmiedia obtusifolia*	*Callicarpa candicans*
Eremophila galeata	*Caladenia attingens*	*Bellendena montana*	*Calophyllum inophyllum*
Eremophila georgei	*Caladenia barbarossa*	*Berberidopsis beckleri*	*Calophyllum sil*
Eremophila gibsonii	*Caladenia bicalliata*	*Bertya cunninghamii*	*Calotis breviseta*
Eremophila gilesii	*Caladenia brownii*	*Beyeria lasiocarpa*	*Calytrix achaeta*
Eremophila glutinosa	*Caladenia cairnsiana*	*Beyeria viscosa*	*Calytrix brownii*
Eremophila goodwinii	*Caladenia capillata*	*Billardiera longiflora*	*Calytrix exstipulata*
Eremophila jucunda	*Caladenia cardiochila*	*Billardiera macrantha*	*Camptacra barbata*
Eremophila latrobei	*Caladenia denticulata*	*Billardiera mutabilis*	*Camptostemon schultzii*
Eremophila macdonnellii	*Caladenia dimidia*	*Billardiera scandens*	*Canarium australianum*
Eremophila margarethae	*Caladenia discoidea*	*Blandfordia grandiflora*	*Canavalia papuana*
Eremophila miniata	*Caladenia doutchiae*	*Blandfordia nobilis*	*Canscora diffusa*
Eremophila neglecta	*Caladenia falcata*	*Blandfordia punicea*	*Capillipedium parviflorum*
Eremophila obovata	*Caladenia filamentosa*	*Boronia algida*	*Capparis canescens*
Eremophila paisleyi	*Caladenia filifera*	*Boronia anemonifolia*	*Capparis lasiantha*
Eremophila pantonii	*Caladenia flava*	*Boronia anethifolia*	*Capparis lucida*
Eremophila platycalyx	*Caladenia hirta*	*Boronia citriodora*	*Capparis quiniflora*
Eremophila platythamnos	*Caladenia latifolia*	*Boronia falcifolia*	*Capparis sepiaria*
Eremophila punctata	*Caladenia longicauda*	*Boronia floribunda*	*Capparis umbonata*
Eremophila rotundifolia	*Caladenia longiclavata*	*Boronia ledifolia*	*Carissa lanceolata*

Eremophila serrulata	*Caladenia macrostylis*	*Boronia microphylla*	*Carpentaria acuminata*
Eremophila spectabilis	*Caladenia marginata*	*Boronia nana*	*Cartonema parviflorum*
Eremophila tietkensii	*Caladenia microchila*	*Boronia parviflora*	*Cartonema spicatum*
Eremophila willsii	*Caladenia nana*	*Boronia pilosa*	*Cassia brewsteri*
Eremophila youngii	*Caladenia pectinata*	*Boronia pinnata*	*Cassia tomentella*
Eriachne aristidea	*Caladenia polychroma*	*Boronia polygalifolia*	*Cassytha capillaris*
Eriachne benthamii	*Caladenia reptans*	*Boronia rosmarinifolia*	*Cassytha filiformis*
Eriachne flaccida	*Caladenia roei*	*Bossiaea buxifolia*	*Cathormion umbellatum*
Eriachne helmsii	*Caladenia stricta*	*Bossiaea cinerea*	*Causonis trifolia*
Eriachne ovata	*Caladenia tensa*	*Bossiaea cordigera*	*Cayratia maritima*
Eriachne pulchella	*Caladenia verrucosa*	*Bossiaea ensata*	*Celtis philippensis*
Eriochloa australiensis	*Caladenia vulgata*	*Bossiaea foliosa*	*Celtis strychnoides*
Erodium carolinianum	*Calandrinia brevipedata*	*Bossiaea heterophylla*	*Cenchrus elymoides*
Erodium janszii	*Calandrinia corrigioloides*	*Bossiaea neoanglica*	*Centipeda borealis*
Ethuliopsis cunninghamii	*Calandrinia granulifera*	*Bossiaea obcordata*	*Centranthera cochinchinensis*
Eucalyptus concinna	*Caleana nigrita*	*Bossiaea prostrata*	*Centrolepis banksii*
Eucalyptus eremicola	*Calectasia narragara*	*Bossiaea rhombifolia*	*Centrolepis exserta*
Eucalyptus gamophylla	*Calectasia valida*	*Bossiaea riparia*	*Chamaecrista absus*
Eucalyptus gillii	*Callistemon phoeniceus*	*Bossiaea scolopendria*	*Chamaecrista mimosoides*
Eucalyptus glomerosa	*Calocephalus multiflorus*	*Bossiaea scortechinii*	*Chamaecrista symonii*
Eucalyptus gongylocarpa	*Calothamnus gilesii*	*Bothriochloa decipiens*	*Chionachne cyathopoda*
Eucalyptus gypsophila	*Calothamnus gracilis*	*Bouchardatia neurococca*	*Chionachne hubbardiana*
Eucalyptus kingsmillii	*Calothamnus lateralis*	*Brachychiton discolor*	*Chionanthus ramiflorus*
Eucalyptus lucasii	*Calothamnus sanguineus*	*Brachyloma daphnoides*	*Chloris pumilio*
Eucalyptus mannensis	*Calytrix alpestris*	*Brachyscome aculeata*	*Chlorophytum laxum*
Eucalyptus normantonensis	*Calytrix angulata*	*Brachyscome curvicarpa*	*Choriceras tricorne*
Eucalyptus ochrophloia	*Calytrix breviseta*	*Brachyscome decipiens*	*Chrysopogon elongatus*
Eucalyptus odontocarpa	*Calytrix depressa*	*Brachyscome dentata*	*Chrysopogon fallax*
Eucalyptus oxymitra	*Calytrix flavescens*	*Brachyscome diversifolia*	*Chrysopogon filipes*
Eucalyptus pachyphylla	*Calytrix fraseri*	*Brachyscome gracilis*	*Chrysopogon latifolius*
Eucalyptus striaticalyx	*Calytrix involucrata*	*Brachyscome graminea*	*Chrysopogon oliganthus*

Eucalyptus trivalva	*Calytrix leschenaultii*	*Brachyscome melanocarpa*	*Chrysopogon pallidus*
Eucalyptus victrix	*Calytrix sapphirina*	*Brachyscome microcarpa*	*Chrysopogon setifolius*
Eucalyptus youngiana	*Calytrix strigosa*	*Brachyscome multifida*	*Cissus adnata*
Eucalyptus yumbarrana	*Carpobrotus rossii*	*Brachyscome paludicola*	*Cissus oblonga*
Euphorbia australis	*Carpobrotus virescens*	*Brachyscome radicans*	*Cissus reniformis*
Euphorbia boophthona	*Cassinia complanata*	*Brachyscome rigidula*	*Citrus glauca*
Euphorbia centralis	*Cassytha aurea*	*Brachyscome scapigera*	*Claoxylon hillii*
Euphorbia ferdinandi	*Cassytha flava*	*Brachyscome smithwhitei*	*Claoxylon tenerifolium*
Euphorbia multifaria	*Cassytha peninsularis*	*Brachyscome spathulata*	*Cleistochloa subjuncea*
Euphorbia porcata	*Cassytha pomiformis*	*Brachyscome stuartii*	*Clematis pickeringii*
Euphorbia stevenii	*Cassytha racemosa*	*Brachyscome triloba*	*Clerodendrum costatum*
Euphorbia thelephora	*Caustis dioica*	*Brachyscome willisii*	*Clerodendrum inerme*
Euphorbia vaccaria	*Centrolepis cephaloformis*	*Breynia oblongifolia*	*Clerodendrum longiflorum*
Euphorbia wheeleri	*Centrolepis drummondiana*	*Bridelia exaltata*	*Clerodendrum tatei*
Fimbristylis simulans	*Centrolepis glabra*	*Brunonia australis*	*Cochlospermum fraseri*
Frankenia cinerea	*Centrolepis polygyna*	*Brunoniella australis*	*Cochlospermum gillivraei*
Frankenia cordata	*Ceratogyne obionoides*	*Brunoniella pumilio*	*Cochlospermum gregorii*
Frankenia foliosa	*Chamaescilla spiralis*	*Bulbophyllum exiguum*	*Coelospermum decipiens*
Frankenia serpyllifolia	*Chamaexeros fimbriata*	*Bulbophyllum schillerianum*	*Coelospermum reticulatum*
Glycine rubiginosa	*Chamelaucium ciliatum*	*Bulbophyllum shepherdii*	*Coldenia procumbens*
Gnephosis arachnoidea	*Chamelaucium megalopetalum*	*Bulbostylis densa*	*Colubrina asiatica*
Gnephosis brevifolia	*Chamelaucium pauciflorum*	*Burchardia umbellata*	*Comesperma secundum*
Gnephosis eriocarpa	*Chenopodium curvispicatum*	*Bursaria longisepala*	*Commelina agrostophylla*
Gompholobium polyzygum	*Choretrum chrysanthum*	*Bursaria spinosa*	*Commelina ciliata*
Gomphrena cunninghamii	*Choretrum glomeratum*	*Caesia parviflora*	*Commelina ensifolia*
Goodenia albiflora	*Chorizema aciculare*	*Caladenia alata*	*Corchorus aestuans*
Goodenia azurea	*Chorizema cordatum*	*Caladenia alpina*	*Corchorus pumilio*
Goodenia calcarata	*Chorizema glycinifolium*	*Caladenia carnea*	*Corchorus sericeus*

Goodenia centralis	*Chorizema racemosum*	*Caladenia catenata*	*Corchorus sidoides*
Goodenia heterochila	*Chorizema rhombeum*	*Caladenia clavigera*	*Corchorus tridens*
Goodenia lunata	*Clematis pubescens*	*Caladenia congesta*	*Cordia dichotoma*
Goodenia microptera	*Comesperma calymega*	*Caladenia cucullata*	*Cordia subcordata*
Goodenia modesta	*Comesperma ciliatum*	*Caladenia dilatata*	*Cordyline cannifolia*
Goodenia mueckeana	*Comesperma confertum*	*Caladenia fuscata*	*Coronidium lanuginosum*
Goodenia ramelii	*Comesperma integerrimum*	*Caladenia gracilis*	*Coronidium rupicola*
Goodenia tenuiloba	*Comesperma polygaloides*	*Caladenia mentiens*	*Corymbia aspera*
Goodenia triodiophila	*Comesperma scoparium*	*Caladenia moschata*	*Corymbia bella*
Goodenia vilmoriniae	*Comesperma spinosum*	*Caladenia parva*	*Corymbia bleeseri*
Gossypium sturtianum	*Comesperma virgatum*	*Caladenia patersonii*	*Corymbia cadophora*
Grammosolen truncatus	*Conospermum caeruleum*	*Caladenia phaeoclavia*	*Corymbia clarksoniana*
Grevillea berryana	*Conospermum distichum*	*Caladenia picta*	*Corymbia confertiflora*
Grevillea deflexa	*Conospermum patens*	*Caladenia praecox*	*Corymbia dallachiana*
Grevillea juncifolia	*Conospermum stoechadis*	*Caladenia pusilla*	*Corymbia dichromophloia*
Grevillea nematophylla	*Conospermum teretifolium*	*Caladenia testacea*	*Corymbia disjuncta*
Grevillea stenobotrya	*Conostephium pendulum*	*Caladenia transitoria*	*Corymbia dunlopiana*
Gunniopsis zygophylloides	*Conostephium preissii*	*Caladenia vulgaris*	*Corymbia erythrophloia*
Gyrostemon ramulosus	*Conostylis aculeata*	*Calamus muelleri*	*Corymbia ferruginea*
Gyrostemon tepperi	*Conostylis aurea*	*Calandrinia pickeringii*	*Corymbia flavescens*
Hakea carinata	*Conostylis candicans*	*Calanthe triplicata*	*Corymbia foelscheana*
Hakea chordophylla	*Conostylis juncea*	*Caleana major*	*Corymbia grandifolia*
Hakea divaricata	*Conostylis prolifera*	*Caleana minor*	*Corymbia greeniana*
Hakea ednieana	*Conostylis pusilla*	*Calectasia intermedia*	*Corymbia kombolgiensis*
Hakea eyreana	*Conostylis seorsiflora*	*Callicarpa pedunculata*	*Corymbia latifolia*
Hakea macrocarpa	*Conostylis serrulata*	*Callicoma serratifolia*	*Corymbia leichhardtii*
Hakea minyma	*Conostylis setigera*	*Callistemon linearifolius*	*Corymbia nesophila*
Halgania erecta	*Coopernookia strophiolata*	*Callistemon pachyphyllus*	*Corymbia pocillum*
Halgania solanacea	*Correa backhouseana*	*Callistemon pallidus*	*Corymbia polycarpa*
Haloragis gossei	*Correa pulchella*	*Callistemon pinifolius*	*Corymbia polysciada*
Haloragis uncatipila	*Corunastylis nigricans*	*Callistemon pityoides*	*Corymbia porrecta*

Hannafordia bissillii	*Corybas despectans*	*Callistemon sieberi*	*Corymbia ptychocarpa*
Harmsiodoxa puberula	*Cotula cotuloides*	*Callistemon viminalis*	*Corymbia setosa*
Heliotropium ammophilum	*Cotula vulgaris*	*Callistemon viridiflorus*	*Corymbia stockeri*
Heliotropium asperrimum	*Crassula closiana*	*Callitriche muelleri*	*Corymbia terminalis*
Helipterum craspedioides	*Crassula colligata*	*Callitriche sonderi*	*Corymbia tessellaris*
Hibiscus burtonii	*Crassula exserta*	*Calocephalus citreus*	*Corymbia trachyphloia*
Hibiscus coatesii	*Cryptandra arbutiflora*	*Calocephalus lacteus*	*Corymbia watsoniana*
Hibiscus krichauffianus	*Cryptandra myriantha*	*Calocephalus sonderi*	*Crinum arenarium*
Hibiscus solanifolius	*Cryptandra nutans*	*Calochilus campestris*	*Crinum uniflorum*
Hydrocotyle trachycarpa	*Cryptandra pungens*	*Calochilus gracillimus*	*Crotalaria aridicola*
Hypertelis cerviana	*Cryptandra tomentosa*	*Calochilus herbaceus*	*Crotalaria brevis*
Indigofera cornuligera	*Cryptostylis ovata*	*Calochilus paludosus*	*Crotalaria calycina*
Indigofera georgei	*Cyanicula amplexans*	*Calochilus platychilus*	*Crotalaria crispata*
Indigofera helmsii	*Cyanicula gemmata*	*Calochilus robertsonii*	*Crotalaria dissitiflora*
Indigofera leucotricha	*Cyanicula sericea*	*Calomeria amaranthoides*	*Crotalaria medicaginea*
Indigofera monophylla	*Cyanostegia angustifolia*	*Calotis anthemoides*	*Crotalaria montana*
Indigofera psammophila	*Cyathochaeta avenacea*	*Calotis cuneifolia*	*Crotalaria ramosissima*
Ipomoea costata	*Cyphanthera myosotidea*	*Calotis dentex*	*Crotalaria verrucosa*
Ipomoea muelleri	*Cyrtostylis huegelii*	*Calotis lappulacea*	*Croton arnhemicus*
Ipomoea racemigera	*Cyrtostylis robusta*	*Calotis scabiosifolia*	*Croton habrophyllus*
Iseilema dolichotrichum	*Dampiera alata*	*Calotis scapigera*	*Croton phebalioides*
Iseilema eremaeum	*Dampiera dysantha*	*Calystegia marginata*	*Cryptocarya cunninghamii*
Isotoma petraea	*Dampiera eriocephala*	*Calystegia sepium*	*Cryptocarya exfoliata*
Isotropis atropurpurea	*Dampiera hederacea*	*Canarium australasicum*	*Cryptocarya hypospodia*
Isotropis wheeleri	*Dampiera incana*	*Capillipedium spicigerum*	*Cryptocarya vulgaris*
Ixiochlamys cuneifolia	*Dampiera juncea*	*Capparis arborea*	*Cucumis althaeoides*
Ixiochlamys filicifolia	*Dampiera lavandulacea*	*Capparis sarmentosa*	*Cucumis melo*
Jacksonia aculeata	*Dampiera lindleyi*	*Cardamine gunnii*	*Cucumis picrocarpus*
Jasminum calcareum	*Dampiera linearis*	*Cardamine lilacina*	*Cullen badocanum*
Kennedia prorepens	*Dampiera marifolia*	*Cardamine papillata*	*Cullen balsamicum*
Lawrencella davenportii	*Dampiera rosmarinifolia*	*Cardamine paucijuga*	*Cullen pustulatum*

Lawrencia glomerata	*Dampiera sacculata*	*Cardamine tenuifolia*	*Curculigo ensifolia*
Lechenaultia divaricata	*Dampiera spicigera*	*Carex brunnea*	*Curcuma australasica*
Leiocarpa semicalva	*Dampiera wellsiana*	*Carex declinata*	*Cyanotis axillaris*
Leiocarpa websteri	*Darwinia micropetala*	*Carex gunniana*	*Cyclophyllum schultzii*
Lepidium oxytrichum	*Darwinia vestita*	*Carex hebes*	*Cymbidium canaliculatum*
Lepidium phlebopetalum	*Dasymalla terminalis*	*Carex incomitata*	*Cymbopogon bombycinus*
Lepidium platypetalum	*Dasypogon bromeliifolius*	*Carex inversa*	*Cymbopogon procerus*
Leptosema chambersii	*Daviesia angulata*	*Carex longebrachiata*	*Cynanchum pedunculatum*
Leucochrysum stipitatum	*Daviesia aphylla*	*Carex maculata*	*Cynodon tenellus*
Lobelia darlingensis	*Daviesia arenaria*	*Carex polyantha*	*Cyperus breviculmis*
Lomandra densiflora	*Daviesia asperula*	*Carex pumila*	*Cyperus carinatus*
Lotus cruentus	*Daviesia benthamii*	*Carpobrotus glaucescens*	*Cyperus castaneus*
Lysiana murrayi	*Daviesia brevifolia*	*Casearia multinervosa*	*Cyperus conicus*
Lythrum wilsonii	*Daviesia cordata*	*Cassinia aculeata*	*Cyperus cuspidatus*
Macgregoria racemigera	*Daviesia decurrens*	*Cassinia aureonitens*	*Cyperus javanicus*
Maireana astrotricha	*Daviesia devito*	*Cassinia cunninghamii*	*Cyperus microcephalus*
Maireana campanulata	*Daviesia divaricata*	*Cassinia longifolia*	*Cyperus nervulosus*
Maireana ciliata	*Daviesia hakeoides*	*Cassinia quinquefaria*	*Cyperus pulchellus*
Maireana convexa	*Daviesia incrassata*	*Cassinia trinerva*	*Cyperus scaber*
Maireana eriantha	*Daviesia nematophylla*	*Cassinia uncata*	*Cyperus tenuispica*
Maireana integra	*Daviesia nudiflora*	*Cassytha glabella*	*Dampiera adpressa*
Maireana lobiflora	*Daviesia physodes*	*Cassytha phaeolasia*	*Dapsilanthus ramosus*
Maireana planifolia	*Daviesia preissii*	*Cassytha pubescens*	*Dapsilanthus spathaceus*
Maireana schistocarpa	*Desmocladus asper*	*Caustis blakei*	*Daviesia filipes*
Maireana scleroptera	*Desmocladus fasciculatus*	*Caustis flexuosa*	*Daviesia reclinata*
Maireana sedifolia	*Desmocladus flexuosus*	*Caustis pentandra*	*Decaisnina angustata*
Maireana spongiocarpa	*Desmocladus lateriflorus*	*Caustis recurvata*	*Decaisnina brittenii*
Maireana tomentosa	*Dianella brevicaulis*	*Cayratia clematidea*	*Decaisnina signata*
Maireana villosa	*Dichopogon capillipes*	*Cayratia eurynema*	*Dendrobium bigibbum*
Melaleuca dissitiflora	*Dicrastylis verticillata*	*Celastrus australis*	*Dendrobium canaliculatum*
Melaleuca glomerata	*Didymanthus roei*	*Celastrus subspicatus*	*Dendrobium dicuphum*
Melaleuca interioris	*Dillwynia laxiflora*	*Celmisia asteliifolia*	*Dendrobium discolor*
Melaleuca lasiandra	*Dillwynia uncinata*	*Celtis paniculata*	*Dendrobium johannis*

Melaleuca xerophila	*Dioscorea hastifolia*	*Cenarrhenes nitida*	*Dendrocnide moroides*
Menkea sphaerocarpa	*Diplopeltis huegelii*	*Cenchrus caliculatus*	*Dendrophthoe acacioides*
Menkea villosula	*Diuris brachyscapa*	*Centella cordifolia*	*Dendrophthoe curvata*
Micromyrtus flaviflora	*Diuris corymbosa*	*Centipeda cunninghamii*	*Dendrophthoe glabrescens*
Millotia greevesii	*Diuris decrementum*	*Centipeda elatinoides*	*Dendrophthoe odontocalyx*
Minuria cunninghamii	*Diuris laxiflora*	*Centipeda minima*	*Denhamia cunninghamii*
Minuria denticulata	*Diuris longifolia*	*Centratherum riparium*	*Denhamia disperma*
Minuria rigida	*Diuris palustris*	*Centrolepis fascicularis*	*Denhamia obscura*
Mirbelia viminalis	*Diuris porrifolia*	*Centrolepis strigosa*	*Denhamia oleaster*
Monachather paradoxus	*Diuris setacea*	*Cephalaralia cephalobotrys*	*Dentella repens*
Muelleranthus stipularis	*Dodonaea adenophora*	*Ceratopetalum apetalum*	*Derris trifoliata*
Myriocephalus oldfieldii	*Dodonaea baueri*	*Ceratopetalum gummiferum*	*Desmodiopsis campylocaulon*
Myriocephalus pluriflorus	*Dodonaea bursariifolia*	*Cheiranthera linearis*	*Dianella nervosa*
Myriocephalus rudallii	*Dodonaea caespitosa*	*Chenopodium nitrariaceum*	*Dianella odorata*
Neobassia astrocarpa	*Dodonaea ceratocarpa*	*Chiloglottis cornuta*	*Dichanthium fecundum*
Neobassia proceriflora	*Dodonaea hexandra*	*Chiloglottis curviclavia*	*Dichrostachys spicata*
Neurachne munroi	*Dodonaea humilis*	*Chiloglottis diphylla*	*Dicliptera armata*
Newcastelia cephalantha	*Dodonaea inaequifolia*	*Chiloglottis formicifera*	*Digitaria ctenantha*
Newcastelia cladotricha	*Dodonaea pinifolia*	*Chiloglottis reflexa*	*Digitaria diminuta*
Newcastelia spodiotricha	*Dodonaea stenozyga*	*Chiloglottis sylvestris*	*Digitaria gibbosa*
Nicotiana benthamiana	*Drakaea glyptodon*	*Chiloglottis trapeziformis*	*Digitaria longiflora*
Nicotiana occidentalis	*Drakaea livida*	*Chiloglottis trilabra*	*Digitaria porrecta*
Nicotiana rosulata	*Drosera bulbosa*	*Chiloglottis valida*	*Dillenia alata*
Nicotiana simulans	*Drosera collina*	*Chloanthes stoechadis*	*Dimeria ornithopoda*
Nicotiana velutina	*Drosera drummondii*	*Chloris ventricosa*	*Dinebra decipiens*
Oldenlandia pterospora	*Drosera erythrorhiza*	*Chordifex fastigiatus*	*Dinebra neesii*
Olearia stuartii	*Drosera gigantea*	*Choretrum candollei*	*Dioscorea bulbifera*
Osteocarpum dipterocarpum	*Drosera macrantha*	*Choretrum pauciflorum*	*Diospyros calycantha*
Owenia reticulata	*Drosera menziesii*	*Chorizema parviflorum*	*Diospyros compacta*
Pandorea doratoxylon	*Drosera neesii*	*Chrysocephalum apiculatum*	*Diospyros hebecarpa*
Paractaenum refractum	*Drosera pallida*	*Chrysocephalum semipapposum*	*Diospyros humilis*

Paraneurachne muelleri	*Drosera pulchella*	*Cinnamomum oliveri*	*Diospyros maritima*
Paspalidium basicladum	*Drosera rosulata*	*Cinnamomum virens*	*Diospyros rugosula*
Paspalidium clementii	*Drosera stolonifera*	*Cissus antarctica*	*Distimake quinatus*
Paspalidium reflexum	*Drosera subhirtella*	*Cissus hypoglauca*	*Dodonaea filifolia*
Peplidium aithocheilum	*Drosera whittakeri*	*Cissus sterculiifolia*	*Dodonaea heteromorpha*
Peplidium foecundum	*Drosera zonaria*	*Citronella moorei*	*Dodonaea hispidula*
Peplidium muelleri	*Drummondita hassellii*	*Claoxylon australe*	*Dodonaea lanceolata*
Petalostylis cassioides	*Ecdeiocolea monostachya*	*Cleistanthus cunninghamii*	*Dodonaea oxyptera*
Petalostylis labicheoides	*Elythranthera brunonis*	*Cleistochloa rigida*	*Dodonaea peduncularis*
Philotheca linearis	*Elythranthera emarginata*	*Clematicissus opaca*	*Dodonaea physocarpa*
Phlegmatospermum cochlearinum	*Enchylaena lanata*	*Clematis aristata*	*Dodonaea platyptera*
Phyllanthus erwinii	*Eremaea beaufortioides*	*Clematis glycinoides*	*Dodonaea polyandra*
Pimelea ammocharis	*Eremaea pauciflora*	*Clematis leptophylla*	*Dodonaea stenophylla*
Pimelea simplex	*Eremophila behriana*	*Clerodendrum tomentosum*	*Dolichandrone filiformis*
Pluchea dentex	*Eremophila clarkei*	*Coatesia paniculata*	*Dolichandrone heterophylla*
Pluchea dunlopii	*Eremophila crassifolia*	*Codonocarpus attenuatus*	*Dolichandrone occidentalis*
Pluchea rubelliflora	*Eremophila decipiens*	*Coelospermum paniculatum*	*Dracaena angustifolia*
Pluchea tetranthera	*Eremophila dempsteri*	*Comesperma defoliatum*	*Drosera burmanni*
Poa crassicaudex	*Eremophila drummondii*	*Comesperma ericinum*	*Drosera dilatatopetiolaris*
Podolepis eremaea	*Eremophila glabra*	*Comesperma retusum*	*Drosera lanata*
Polycarpaea arida	*Eremophila interstans*	*Comesperma sphaerocarpum*	*Drosera petiolaris*
Polygala glaucifolia	*Eremophila ionantha*	*Comesperma volubile*	*Drosera serpens*
Polygala isingii	*Eremophila lehmanniana*	*Commelina cyanea*	*Dysoxylum acutangulum*
Portulaca intraterranea	*Eremophila oldfieldii*	*Commersonia bartramia*	*Ectrosia agrostoides*
Prostanthera althoferi	*Eremophila subfloccosa*	*Commersonia dasyphylla*	*Ectrosia leporina*
Prostanthera sericea	*Eremophila weldii*	*Conospermum ericifolium*	*Ectrosia scabrida*
Prostanthera striatiflora	*Ericksonella saccharata*	*Conospermum longifolium*	*Ectrosia schultzii*
Prostanthera wilkieana	*Ericomyrtus serpyllifolia*	*Conospermum taxifolium*	*Ehretia saligna*

Psydrax latifolia	*Eriochilus dilatatus*	*Convolvulus angustissimus*	*Elaeagnus triflora*
Psydrax suaveolens	*Eriochilus scaber*	*Convolvulus erubescens*	*Elaeocarpus arnhemicus*
Ptilotus aervoides	*Erymophyllum tenellum*	*Coopernookia barbata*	*Elaeodendron melanocarpum*
Ptilotus arthrolasius	*Eryngium pinnatifidum*	*Coprosma hirtella*	*Emmenosperma cunninghamii*
Ptilotus astrolasius	*Eucalyptus accedens*	*Coprosma nitida*	*Endiandra glauca*
Ptilotus axillaris	*Eucalyptus albida*	*Cordyline petiolaris*	*Enneapogon pallidus*
Ptilotus calostachyus	*Eucalyptus albopurpurea*	*Cordyline rubra*	*Enneapogon purpurascens*
Ptilotus chamaecladus	*Eucalyptus angulosa*	*Coronidium elatum*	*Enneapogon truncatus*
Ptilotus clementii	*Eucalyptus arachnaea*	*Coronidium gunnianum*	*Enteropogon unispiceus*
Ptilotus decipiens	*Eucalyptus arenacea*	*Coronidium monticola*	*Eragrostis cumingii*
Ptilotus gaudichaudii	*Eucalyptus brachycalyx*	*Coronidium oxylepis*	*Eragrostis desertorum*
Ptilotus gomphrenoides	*Eucalyptus calcareana*	*Coronidium scorpioides*	*Eragrostis exigua*
Ptilotus helipteroides	*Eucalyptus calycogona*	*Coronidium waddelliae*	*Eragrostis fallax*
Ptilotus incanus	*Eucalyptus capillosa*	*Correa alba*	*Eragrostis megalosperma*
Ptilotus latifolius	*Eucalyptus celastroides*	*Correa lawrenceana*	*Eragrostis pubescens*
Ptilotus leucocoma	*Eucalyptus clelandiorum*	*Correa reflexa*	*Eragrostis schultzii*
Ptilotus murrayi	*Eucalyptus conglobata*	*Corunastylis archeri*	*Eragrostis sororia*
Ptilotus obovatus	*Eucalyptus cornuta*	*Corunastylis despectans*	*Eragrostis spartinoides*
Ptilotus roei	*Eucalyptus cosmophylla*	*Corunastylis fimbriata*	*Eragrostis tenellula*
Ptilotus rotundifolius	*Eucalyptus cyanophylla*	*Corunastylis morrisii*	*Eriachne armitii*
Ptilotus schwartzii	*Eucalyptus cylindrocarpa*	*Corunastylis nuda*	*Eriachne avenacea*
Ptilotus sessilifolius	*Eucalyptus decipiens*	*Corunastylis pumila*	*Eriachne basedowii*
Ptilotus whitei	*Eucalyptus densa*	*Corybas aconitiflorus*	*Eriachne burkittii*
Ptilotus xerophilus	*Eucalyptus dissimulata*	*Corybas barbarae*	*Eriachne capillaris*
Quoya loxocarpa	*Eucalyptus diversifolia*	*Corybas fimbriatus*	*Eriachne ciliata*
Rhagodia eremaea	*Eucalyptus drummondii*	*Corybas incurvus*	*Eriachne festucacea*
Rhodanthe charsleyae	*Eucalyptus dumosa*	*Corybas unguiculatus*	*Eriachne filiformis*
Rhodanthe floribunda	*Eucalyptus ebbanoensis*	*Corymbia eximia*	*Eriachne glauca*

Rhodanthe humboldtiana	*Eucalyptus eremophila*	*Corymbia gummifera*	*Eriachne melicacea*
Rhodanthe maryonii	*Eucalyptus eudesmioides*	*Corymbia henryi*	*Eriachne obtusa*
Rhodanthe microglossa	*Eucalyptus falcata*	*Corymbia intermedia*	*Eriachne pallescens*
Rhodanthe moschata	*Eucalyptus fasciculosa*	*Corymbia torelliana*	*Eriachne rara*
Rhodanthe stricta	*Eucalyptus flocktoniae*	*Cotula alpina*	*Eriachne schultziana*
Rhodanthe tietkensii	*Eucalyptus foecunda*	*Craspedia aurantia*	*Eriachne stipacea*
Rhodanthe uniflora	*Eucalyptus gracilis*	*Craspedia canens*	*Eriachne sulcata*
Roebuckiella similis	*Eucalyptus griffithsii*	*Craspedia glauca*	*Eriachne triseta*
Roepera compressa	*Eucalyptus horistes*	*Craspedia gracilis*	*Eriocaulon cinereum*
Roepera confluens	*Eucalyptus incrassata*	*Craspedia haplorrhiza*	*Eriocaulon depressum*
Roepera eichleri	*Eucalyptus kochii*	*Craspedia paludicola*	*Eriocaulon fistulosum*
Roepera emarginata	*Eucalyptus leptocalyx*	*Craspedia variabilis*	*Eriocaulon pygmaeum*
Roepera howittii	*Eucalyptus leptophylla*	*Crassula decumbens*	*Eriocaulon setaceum*
Roepera prismatotheca	*Eucalyptus leptopoda*	*Crassula helmsii*	*Eriocaulon spectabile*
Roepera similis	*Eucalyptus lesouefii*	*Crassula peduncularis*	*Eriocaulon tortuosum*
Roepera tesquorum	*Eucalyptus longicornis*	*Crassula sieberiana*	*Eriosema chinense*
Rutidosis helichrysoides	*Eucalyptus longissima*	*Crinum pedunculatum*	*Erythrina variegata*
Sauropus rigens	*Eucalyptus loxophleba*	*Croton acronychioides*	*Erythrophleum chlorostachys*
Scaevola amblyanthera	*Eucalyptus macrocarpa*	*Croton insularis*	*Erythroxylum australe*
Scaevola basedowii	*Eucalyptus marginata*	*Croton stigmatosus*	*Erythroxylum ellipticum*
Scaevola depauperata	*Eucalyptus melanoxylon*	*Croton verreauxii*	*Eucalyptus alba*
Scaevola glabrata	*Eucalyptus moderata*	*Cryptandra amara*	*Eucalyptus apodophylla*
Scaevola humilis	*Eucalyptus myriadena*	*Cryptandra spinescens*	*Eucalyptus bakeri*
Scaevola ovalifolia	*Eucalyptus obtusiflora*	*Cryptocarya bidwillii*	*Eucalyptus bigalerita*
Scaevola parvibarbata	*Eucalyptus oldfieldii*	*Cryptocarya erythroxylon*	*Eucalyptus brachyandra*
Scaevola parvifolia	*Eucalyptus oleosa*	*Cryptocarya foveolata*	*Eucalyptus brassiana*
Schoenia ayersii	*Eucalyptus olivina*	*Cryptocarya glaucescens*	*Eucalyptus brevifolia*
Schoenia ramosissima	*Eucalyptus patens*	*Cryptocarya laevigata*	*Eucalyptus brownii*
Scleranthus pungens	*Eucalyptus peninsularis*	*Cryptocarya macdonaldii*	*Eucalyptus cambageana*
Sclerolaena cornishiana	*Eucalyptus phaenophylla*	*Cryptocarya meisneriana*	*Eucalyptus chloroclada*
Sclerolaena costata	*Eucalyptus phenax*	*Cryptocarya microneura*	*Eucalyptus chlorophylla*
Sclerolaena cuneata	*Eucalyptus pileata*	*Cryptocarya obovata*	*Eucalyptus cloeziana*
Sclerolaena decurrens	*Eucalyptus platycorys*	*Cryptocarya rigida*	*Eucalyptus cullenii*

Sclerolaena densiflora	*Eucalyptus platypus*	*Cryptostylis erecta*	*Eucalyptus decorticans*
Sclerolaena deserticola	*Eucalyptus pleurocarpa*	*Cryptostylis hunteriana*	*Eucalyptus exserta*
Sclerolaena eriacantha	*Eucalyptus porosa*	*Cryptostylis leptochila*	*Eucalyptus granitica*
Sclerolaena glabra	*Eucalyptus preissiana*	*Cryptostylis subulata*	*Eucalyptus herbertiana*
Sclerolaena holtiana	*Eucalyptus ravida*	*Cullen microcephalum*	*Eucalyptus jensenii*
Sclerolaena intricata	*Eucalyptus rigidula*	*Cullen parvum*	*Eucalyptus leptophleba*
Sclerolaena johnsonii	*Eucalyptus rudis*	*Cullen tenax*	*Eucalyptus leucophloia*
Sclerolaena lanicuspis	*Eucalyptus rugosa*	*Cupaniopsis anacardioides*	*Eucalyptus longirostrata*
Sclerolaena limbata	*Eucalyptus salicola*	*Cupaniopsis parvifolia*	*Eucalyptus mediocris*
Sclerolaena uniflora	*Eucalyptus salmonophloia*	*Cuphonotus humistratus*	*Eucalyptus microneura*
Sclerolaena ventricosa	*Eucalyptus salubris*	*Cuttsia viburnea*	*Eucalyptus microtheca*
Senecio anethifolius	*Eucalyptus sheathiana*	*Cyanicula caerulea*	*Eucalyptus miniata*
Senecio depressicola	*Eucalyptus socialis*	*Cyathochaeta diandra*	*Eucalyptus oligantha*
Senecio gregorii	*Eucalyptus sporadica*	*Cyathodes glauca*	*Eucalyptus orgadophila*
Senecio lacustrinus	*Eucalyptus subangusta*	*Cyclophyllum coprosmoides*	*Eucalyptus patellaris*
Senecio lanibracteus	*Eucalyptus suggrandis*	*Cyclophyllum longipetalum*	*Eucalyptus persistens*
Senecio magnificus	*Eucalyptus tenera*	*Cymbidium madidum*	*Eucalyptus phoenicea*
Senna cardiosperma	*Eucalyptus thamnoides*	*Cymbidium suave*	*Eucalyptus platyphylla*
Senna glaucifolia	*Eucalyptus todtiana*	*Cymbonotus lawsonianus*	*Eucalyptus pruinosa*
Senna glutinosa	*Eucalyptus torquata*	*Cymbonotus preissianus*	*Eucalyptus shirleyi*
Senna notabilis	*Eucalyptus transcontinentalis*	*Cymbopogon refractus*	*Eucalyptus tectifica*
Senna phyllodinea	*Eucalyptus uncinata*	*Cynanchum elegans*	*Eucalyptus tenuipes*
Senna pleurocarpa	*Eucalyptus urna*	*Cyperus disjunctus*	*Eucalyptus tetrodonta*
Seringia exastia	*Eucalyptus vegrandis*	*Cyperus eglobosus*	*Eucalyptus tintinnans*
Seringia nephrosperma	*Eucalyptus wandoo*	*Cyperus enervis*	*Eucalyptus whitei*
Setaria dielsii	*Eucalyptus yalatensis*	*Cyperus imbecillis*	*Eucalyptus woollsiana*
Sida ammophila	*Eucalyptus yilgarnensis*	*Cyperus laevis*	*Eugenia reinwardtiana*
Sida arenicola	*Euryomyrtus maidenii*	*Cyperus sphaeroideus*	*Eulalia mackinlayi*
Sida argillacea	*Eutaxia diffusa*	*Cyperus tetraphyllus*	*Euphorbia bifida*
Sida calyxhymenia	*Eutaxia microphylla*	*Cyperus trinervis*	*Euphorbia coghlanii*
Sida cardiophylla	*Eutaxia myrtifolia*	*Cyphanthera albicans*	*Euphorbia hassallii*
Sida ectogama	*Eutaxia parvifolia*	*Cyrtostylis reniformis*	*Euphorbia mitchelliana*
Sida fibulifera	*Exocarpos aphyllus*	*Dampiera purpurea*	*Euphorbia muelleri*

Sida goniocarpa	*Exocarpos sparteus*	*Dampiera stricta*	*Euphorbia schizolepis*
Sida petrophila	*Exocarpos syrticola*	*Dampiera sylvestris*	*Euphorbia schultzii*
Sida phaeotricha	*Frankenia sessilis*	*Daphnandra apatela*	*Excoecaria ovalis*
Sida platycalyx	*Gahnia deusta*	*Daphnandra micrantha*	*Excoecaria parvifolia*
Sida spodochroma	*Gahnia lanigera*	*Daviesia acicularis*	*Exocarpos latifolius*
Solanum callosum	*Gastrolobium calycinum*	*Daviesia alata*	*Falcataria toona*
Solanum diversiflorum	*Gastrolobium capitatum*	*Daviesia arborea*	*Ficus aculeata*
Solanum ellipticum	*Gastrolobium parviflorum*	*Daviesia buxifolia*	*Ficus atricha*
Solanum lithophilum	*Gastrolobium spinosum*	*Daviesia corymbosa*	*Ficus cerasicarpa*
Solanum petrophilum	*Geijera linearifolia*	*Daviesia genistifolia*	*Ficus congesta*
Solanum phlomoides	*Geleznowia verrucosa*	*Daviesia latifolia*	*Ficus coronulata*
Solanum quadriloculatum	*Gilberta tenuifolia*	*Daviesia leptophylla*	*Ficus hispida*
Solanum sturtianum	*Gilruthia osbornei*	*Daviesia mimosoides*	*Ficus microcarpa*
Spermacoce hillii	*Glischrocaryon angustifolium*	*Daviesia squarrosa*	*Ficus opposita*
Sporobolus blakei	*Glischrocaryon aureum*	*Daviesia ulicifolia*	*Ficus platypoda*
Stackhousia clementii	*Glischrocaryon behrii*	*Daviesia umbellulata*	*Ficus racemosa*
Stemodia grossa	*Glischrocaryon flavescens*	*Daviesia villifera*	*Ficus virens*
Stemodia viscosa	*Glossostigma drummondii*	*Daviesia wyattiana*	*Fimbristylis acuminata*
Stenopetalum anfractum	*Gnephosis angianthoides*	*Decaspermum humile*	*Fimbristylis aestivalis*
Stenopetalum decipiens	*Gnephosis drummondii*	*Deeringia amaranthoides*	*Fimbristylis cephalophora*
Stenopetalum nutans	*Gompholobium capitatum*	*Deeringia arborescens*	*Fimbristylis cinnamometorum*
Stenopetalum velutinum	*Gompholobium confertum*	*Dendrobium aemulum*	*Fimbristylis cymosa*
Streptoglossa adscendens	*Gompholobium marginatum*	*Dendrobium gracilicaule*	*Fimbristylis densa*
Streptoglossa cylindriceps	*Gompholobium ovatum*	*Dendrobium monophyllum*	*Fimbristylis depauperata*
Streptoglossa decurrens	*Gompholobium polymorphum*	*Dendrobium speciosum*	*Fimbristylis furva*
Streptoglossa liatroides	*Gompholobium preissii*	*Dendrobium tetragonum*	*Fimbristylis lanceolata*
Streptoglossa macrocephala	*Gompholobium scabrum*	*Dendrocnide excelsa*	*Fimbristylis pterigosperma*
Stylidium desertorum	*Gompholobium tomentosum*	*Dendrocnide photinophylla*	*Fimbristylis punctata*

Stylobasium spathulatum	*Gonocarpus mezianus*	*Dendrophthoe vitellina*	*Fimbristylis recta*
Swainsona burkei	*Gonocarpus nodulosus*	*Denhamia bilocularis*	*Fimbristylis schultzii*
Swainsona campylantha	*Goodenia concinna*	*Denhamia celastroides*	*Fimbristylis simplex*
Swainsona canescens	*Goodenia incana*	*Denhamia pittosporoides*	*Fimbristylis sphaerocephala*
Swainsona flavicarinata	*Goodenia micrantha*	*Denhamia silvestris*	*Fimbristylis squarrulosa*
Swainsona formosa	*Goodenia mimuloides*	*Desmodium rhytidophyllum*	*Fimbristylis tetragona*
Swainsona kingii	*Goodenia pulchella*	*Desmodium varians*	*Fimbristylis trigastrocarya*
Swainsona laxa	*Goodenia robusta*	*Deyeuxia contracta*	*Fimbristylis xyridis*
Swainsona oligophylla	*Goodenia scapigera*	*Deyeuxia frigida*	*Flemingia lineata*
Swainsona phacoides	*Goodenia varia*	*Deyeuxia gunniana*	*Flemingia parviflora*
Swainsona stipularis	*Goodenia willisiana*	*Deyeuxia monticola*	*Flemingia pauciflora*
Swainsona tenuis	*Goodia medicaginea*	*Deyeuxia parviseta*	*Flindersia dissosperma*
Swainsona villosa	*Grevillea acacioides*	*Deyeuxia rodwayi*	*Flueggea leucopyrus*
Synostemon rhytidospermus	*Grevillea acuaria*	*Deyeuxia scaberula*	*Flueggea virosa*
Tecticornia disarticulata	*Grevillea anethifolia*	*Dianella amoena*	*Freycinetia excelsa*
Tecticornia medullosa	*Grevillea biformis*	*Dianella brevipedunculata*	*Fuirena ciliaris*
Tecticornia tenuis	*Grevillea bipinnatifida*	*Dianella caerulea*	*Galactia megalophylla*
Tephrosia sphaerospora	*Grevillea cagiana*	*Dianella congesta*	*Galactia muelleri*
Tephrosia supina	*Grevillea didymobotrya*	*Dianella longifolia*	*Galactia tenuiflora*
Teucrium teucriiflorum	*Grevillea excelsior*	*Dianella porracea*	*Ganophyllum falcatum*
Thryptomene decussata	*Grevillea hakeoides*	*Dianella prunina*	*Garcinia warrenii*
Thyridolepis multiculmis	*Grevillea hookeriana*	*Dianella rara*	*Gardenia ewartii*
Thysanotus exfimbriatus	*Grevillea huegelii*	*Dianella revoluta*	*Gardenia fucata*
Thysanotus exiliflorus	*Grevillea ilicifolia*	*Dianella tarda*	*Gardenia megasperma*
Trachymene glaucifolia	*Grevillea levis*	*Dianella tasmanica*	*Gardenia pyriformis*
Trachymene ochracea	*Grevillea nudiflora*	*Dichanthium setosum*	*Gardenia resinosa*
Trachymene oleracea	*Grevillea obliquistigma*	*Dichelachne hirtella*	*Gardenia schwarzii*
Trianthema pilosum	*Grevillea oligantha*	*Dichelachne inaequiglumis*	*Gastrolobium grandiflorum*
Trianthema triquetrum	*Grevillea paniculata*	*Dichelachne micrantha*	*Geodorum densiflorum*

Tribulus astrocarpus	*Grevillea paradoxa*	*Dichelachne rara*	*Germainia truncatiglumis*
Tribulus eichlerianus	*Grevillea pauciflora*	*Dichelachne sieberiana*	*Glochidion disparipes*
Tribulus hirsutus	*Grevillea pectinata*	*Dichondra repens*	*Glochidion harveyanum*
Tribulus hystrix	*Grevillea petrophiloides*	*Digitaria diffusa*	*Glochidion lobocarpum*
Tribulus macrocarpus	*Grevillea pilulifera*	*Digitaria parviflora*	*Glochidion xerocarpum*
Tribulus occidentalis	*Grevillea pterosperma*	*Digitaria ramularis*	*Glossocarya hemiderma*
Tribulus platypterus	*Grevillea pulchella*	*Dillwynia acicularis*	*Glycine falcata*
Tribulus suberosus	*Grevillea quercifolia*	*Dillwynia cinerascens*	*Glycine tomentella*
Trichanthodium skirrophorum	*Grevillea shuttleworthiana*	*Dillwynia elegans*	*Glycosmis trifoliata*
Trigastrotheca molluginea	*Grevillea synapheae*	*Dillwynia floribunda*	*Gompholobium nitidum*
Trigonella suavissima	*Grevillea teretifolia*	*Dillwynia glaberrima*	*Gompholobium subulatum*
Triodia brizoides	*Grevillea trifida*	*Dillwynia juniperina*	*Gomphrena breviflora*
Triodia epactia	*Grevillea vestita*	*Dillwynia phylicoides*	*Gomphrena canescens*
Triodia longiceps	*Guichenotia ledifolia*	*Dillwynia ramosissima*	*Gomphrena flaccida*
Triodia melvillei	*Guichenotia micrantha*	*Dillwynia retorta*	*Gomphrena lanata*
Triodia pungens	*Gyrostemon australasicus*	*Dillwynia rudis*	*Gomphrena leptoclada*
Triodia schinzii	*Gyrostemon racemiger*	*Dillwynia sericea*	*Gonocarpus acanthocarpus*
Triodia spicata	*Gyrostemon subnudus*	*Dillwynia sieberi*	*Gonocarpus leptothecus*
Triodia wiseana	*Gyrostemon thesioides*	*Dioscorea transversa*	*Goodenia armstrongiana*
Triraphis mollis	*Haemodorum discolor*	*Diospyros australis*	*Goodenia bicolor*
Urochloa praetervisa	*Haemodorum laxum*	*Diospyros fasciculosa*	*Goodenia byrnesii*
Vittadinia arida	*Haemodorum simplex*	*Diospyros pentamera*	*Goodenia coronopifolia*
Vittadinia blackii	*Haemodorum spicatum*	*Diplarrena moraea*	*Goodenia delicata*
Vittadinia eremaea	*Hakea amplexicaulis*	*Diploglottis australis*	*Goodenia heppleana*
Waltheria virgata	*Hakea ceratophylla*	*Dipodium pardalinum*	*Goodenia hispida*
Wurmbea australis	*Hakea corymbosa*	*Dipodium punctatum*	*Goodenia holtzeana*
Xanthorrhoea quadrangulata	*Hakea costata*	*Dipodium roseum*	*Goodenia janamba*
Xerochrysum interiore	*Hakea cycloptera*	*Dipodium variegatum*	*Goodenia leiosperma*
Yakirra australiensis	*Hakea cygnus*	*Discaria pubescens*	*Goodenia odonnellii*
Zornia albiflora	*Hakea erecta*	*Diuris abbreviata*	*Goodenia pilosa*
Zygochloa paradoxa	*Hakea francisiana*	*Diuris alba*	*Goodenia pumilio*

	Hakea lissocarpha	*Diuris aurea*	*Goodenia purpurascens*
	Hakea marginata	*Diuris behrii*	*Goodenia scaevolina*
	Hakea mitchellii	*Diuris chryseopsis*	*Goodenia sepalosa*
	Hakea multilineata	*Diuris dendrobioides*	*Goodenia strangfordii*
	Hakea nitida	*Diuris lanceolata*	*Grevillea agrifolia*
	Hakea obliqua	*Diuris maculata*	*Grevillea baileyana*
	Hakea preissii	*Diuris monticola*	*Grevillea decora*
	Hakea recurva	*Diuris pardina*	*Grevillea decurrens*
	Hakea rostrata	*Diuris punctata*	*Grevillea dimidiata*
	Hakea rugosa	*Diuris sulphurea*	*Grevillea dryandri*
	Hakea ruscifolia	*Diuris tricolor*	*Grevillea glauca*
	Hakea scoparia	*Dockrillia bowmanii*	*Grevillea goodii*
	Hakea sulcata	*Dockrillia fairfaxii*	*Grevillea heliosperma*
	Hakea trifurcata	*Dockrillia linguiformis*	*Grevillea mimosoides*
	Hakea varia	*Dockrillia pugioniformis*	*Grevillea parallela*
	Hakea vittata	*Dockrillia schoenina*	*Grevillea prasina*
	Halgania anagalloides	*Dockrillia striolata*	*Grevillea pteridifolia*
	Halgania andromedifolia	*Dockrillia teretifolia*	*Grevillea pungens*
	Halgania cyanea	*Dodonaea boroniifolia*	*Grevillea pyramidalis*
	Halgania integerrima	*Dodonaea multijuga*	*Grevillea refracta*
	Halgania lavandulacea	*Dodonaea triangularis*	*Grewia breviflora*
	Haloragis acutangula	*Dodonaea triquetra*	*Grewia mesomischa*
	Helichrysum leucopsideum	*Dodonaea truncatiales*	*Grewia oxyphylla*
	Hemiandra pungens	*Doryanthes excelsa*	*Grewia retusifolia*
	Hemigenia humilis	*Doryphora sassafras*	*Grewia savannicola*
	Hemigenia incana	*Dracophyllum secundum*	*Grona brownii*
	Hemigenia teretiuscula	*Drosera aberrans*	*Grona filiformis*
	Hibbertia acerosa	*Drosera arcturi*	*Grona glareosa*
	Hibbertia amplexicaulis	*Drosera auriculata*	*Grona heterocarpos*
	Hibbertia commutata	*Drosera hookeri*	*Grona muelleri*
	Hibbertia cuneiformis	*Drosera peltata*	*Grona pullenii*
	Hibbertia cunninghamii	*Drosera pygmaea*	*Grona pycnotricha*
	Hibbertia devitata	*Drosera spatulata*	*Grona trichostachya*
	Hibbertia exasperata	*Drymophila cyanocarpa*	*Guettarda speciosa*
	Hibbertia glomerosa	*Drymophila moorei*	*Guioa acutifolia*
	Hibbertia gracilipes	*Dryopoa dives*	*Gymnanthera oblonga*
	Hibbertia hemignosta	*Drypetes deplanchei*	*Gymnema erectum*
	Hibbertia huegelii	*Duboisia myoporoides*	*Gymnema geminatum*

	Hibbertia hypericoides	*Duma florulenta*	*Gyrocarpus americanus*
	Hibbertia lineata	*Duma horrida*	*Haemodorum brevicaule*
	Hibbertia pungens	*Dysoxylum fraserianum*	*Haemodorum coccineum*
	Hibbertia racemosa	*Dysoxylum mollissimum*	*Haemodorum ensifolium*
	Hibbertia spicata	*Dysoxylum rufum*	*Hakea arborescens*
	Hibbertia stellaris	*Dysphania carinata*	*Helicia australasica*
	Hibbertia striata	*Echinopogon caespitosus*	*Helicteres cana*
	Hibbertia subvaginata	*Echinopogon nutans*	*Helicteres integrifolia*
	Hibbertia virgata	*Echinostephia aculeata*	*Herissantia crispa*
	Homalocalyx thryptomenoides	*Eclipta platyglossa*	*Heritiera littoralis*
	Homalosciadium homalocarpum	*Ehretia acuminata*	*Heterachne abortiva*
	Homoranthus wilhelmii	*Einadia hastata*	*Heterachne gulliveri*
	Hovea chorizemifolia	*Einadia nutans*	*Heteropogon contortus*
	Hovea elliptica	*Elaeocarpus grandis*	*Heteropogon triticeus*
	Hovea pungens	*Elaeocarpus holopetalus*	*Hibbertia brownii*
	Hovea trisperma	*Elaeocarpus kirtonii*	*Hibbertia candicans*
	Hyalochlamys globifera	*Elaeocarpus obovatus*	*Hibbertia caudice*
	Hyalosperma cotula	*Elaeodendron australe*	*Hibbertia cistifolia*
	Hyalosperma glutinosum	*Elatine gratioloides*	*Hibbertia complanata*
	Hybanthus calycinus	*Elatostema reticulatum*	*Hibbertia dealbata*
	Hybanthus floribundus	*Elattostachys nervosa*	*Hibbertia dilatata*
	Hydrocotyle alata	*Elattostachys xylocarpa*	*Hibbertia echiifolia*
	Hydrocotyle capillaris	*Embelia australiana*	*Hibbertia juncea*
	Hydrocotyle diantha	*Emmenosperma alphitonioides*	*Hibbertia lepidota*
	Hydrocotyle intertexta	*Empodisma minus*	*Hibbertia oblongata*
	Hydrocotyle medicaginoides	*Endiandra discolor*	*Hibbertia sphenandra*
	Hydrocotyle rugulosa	*Endiandra muelleri*	*Hibiscus austrinus*
	Hypocalymma angustifolium	*Endiandra pubens*	*Hibiscus divaricatus*
	Hypocalymma robustum	*Endiandra sieberi*	*Hibiscus leptocladus*
	Hypolaena exsulca	*Entolasia marginata*	*Hibiscus meraukensis*
	Hysterobaeckea behrii	*Entolasia stricta*	*Hibiscus panduriformis*
	Isolepis stellata	*Entolasia whiteana*	*Hibiscus pentaphyllus*

	Isopogon divergens	*Enydra woollsii*	*Hibiscus verdcourtii*
	Isopogon dubius	*Epacris breviflora*	*Hibiscus zonatus*
	Isopogon formosus	*Epacris gunnii*	*Homalocalyx ericaeus*
	Isopogon scabriusculus	*Epacris impressa*	*Homoranthus flavescens*
	Isopogon spathulatus	*Epacris lanuginosa*	*Horsfieldia australiana*
	Isopogon teretifolius	*Epacris longiflora*	*Hovea longipes*
	Isopogon trilobus	*Epacris microphylla*	*Hovea parvicalyx*
	Isotoma hypocrateriformis	*Epacris obtusifolia*	*Hydriastele wendlandiana*
	Isotoma scapigera	*Epacris paludosa*	*Hygrophila angustifolia*
	Isotropis cuneifolia	*Epacris pulchella*	*Hypoestes floribunda*
	Jacksonia condensata	*Epacris serpyllifolia*	*Ichnocarpus frutescens*
	Jacksonia floribunda	*Epilobium billardiereanum*	*Ilex arnhemensis*
	Jacksonia furcellata	*Epilobium gunnianum*	*Indigastrum parviflorum*
	Jacksonia horrida	*Epilobium hirtigerum*	*Indigofera elachantha*
	Jacksonia racemosa	*Epilobium pallidiflorum*	*Indigofera ewartiana*
	Jacksonia sternbergiana	*Eragrostis interrupta*	*Indigofera haplophylla*
	Johnsonia lupulina	*Eragrostis trachycarpa*	*Indigofera linifolia*
	Kennedia coccinea	*Eremochloa bimaculata*	*Indigofera pratensis*
	Kingia australis	*Eremophila debilis*	*Indigofera trita*
	Kunzea micrantha	*Eremophila divaricata*	*Iphigenia indica*
	Kunzea pomifera	*Eriocaulon scariosum*	*Ipomoea abrupta*
	Kunzea preissiana	*Eriochilus cucullatus*	*Ipomoea argillicola*
	Kunzea recurva	*Eriostemon australasius*	*Ipomoea coptica*
	Labichea lanceolata	*Eryngium ovinum*	*Ipomoea diamantinensis*
	Labichea punctata	*Eryngium paludosum*	*Ipomoea diversifolia*
	Lachnostachys eriobotrya	*Eryngium vesiculosum*	*Ipomoea eriocarpa*
	Lagenophora huegelii	*Erythrorchis cassythoides*	*Ipomoea gracilis*
	Lambertia inermis	*Eucalyptus acaciiformis*	*Ipomoea graminea*
	Lambertia multiflora	*Eucalyptus acmenoides*	*Ipomoea lonchophylla*
	Lasiopetalum baueri	*Eucalyptus agglomerata*	*Ipomoea polymorpha*
	Lasiopetalum behrii	*Eucalyptus albens*	*Ipomoea violacea*
	Lasiopetalum discolor	*Eucalyptus amplifolia*	*Isachne confusa*
	Lasiopetalum floribundum	*Eucalyptus amygdalina*	*Ischaemum decumbens*

	Lasiopetalum rosmarinifolium	*Eucalyptus andrewsii*	*Ischaemum rugosum*
	Lasiopetalum schulzenii	*Eucalyptus angophoroides*	*Iseilema fragile*
	Lawrencella rosea	*Eucalyptus aromaphloia*	*Iseilema macratherum*
	Laxmannia grandiflora	*Eucalyptus bancroftii*	*Iseilema vaginiflorum*
	Laxmannia paleacea	*Eucalyptus baueriana*	*Ixora timorensis*
	Laxmannia ramosa	*Eucalyptus beyeriana*	*Jacksonia dilatata*
	Laxmannia sessiliflora	*Eucalyptus blakelyi*	*Jacksonia forrestii*
	Laxmannia squarrosa	*Eucalyptus blaxlandii*	*Jacksonia odontoclada*
	Lechenaultia biloba	*Eucalyptus bosistoana*	*Jacksonia thesioides*
	Lechenaultia floribunda	*Eucalyptus bridgesiana*	*Jacquemontia browniana*
	Lechenaultia formosa	*Eucalyptus brookeriana*	*Jacquemontia paniculata*
	Lechenaultia linarioides	*Eucalyptus caleyi*	*Jasminum elongatum*
	Lechenaultia tubiflora	*Eucalyptus caliginosa*	*Jasminum molle*
	Lepidium leptopetalum	*Eucalyptus cameronii*	*Lamprolobium fruticosum*
	Lepidium rotundum	*Eucalyptus campanulata*	*Larsenaikia ochreata*
	Lepidobolus chaetocephalus	*Eucalyptus camphora*	*Larsenaikia suffruticosa*
	Lepidobolus drapetocoleus	*Eucalyptus canaliculata*	*Lechenaultia filiformis*
	Lepidobolus preissianus	*Eucalyptus capitellata*	*Leea novoguineensis*
	Lepidosperma angustatum	*Eucalyptus carnea*	*Leea rubra*
	Lepidosperma canescens	*Eucalyptus cephalocarpa*	*Leichhardtia microlepis*
	Lepidosperma carphoides	*Eucalyptus cinerea*	*Leichhardtia viridiflora*
	Lepidosperma costale	*Eucalyptus conica*	*Leptospermum madidum*
	Lepidosperma leptostachyum	*Eucalyptus consideniana*	*Lepturus repens*
	Lepidosperma tenue	*Eucalyptus croajingolensis*	*Limnophila aromatica*
	Leporella fimbriata	*Eucalyptus cypellocarpa*	*Limnophila brownii*
	Leptocarpus coangustatus	*Eucalyptus dalrympleana*	*Limnophila fragrans*
	Leptocarpus scariosus	*Eucalyptus dealbata*	*Lindernia aplectra*
	Leptomeria aphylla	*Eucalyptus deanei*	*Lindernia clausa*
	Leptomeria cunninghamii	*Eucalyptus delegatensis*	*Lindernia lobelioides*

	Leptomeria pauciflora	*Eucalyptus dives*	*Lindernia scapigera*
	Leptomeria preissiana	*Eucalyptus dwyeri*	*Lithomyrtus obtusa*
	Leptomeria squarrulosa	*Eucalyptus elata*	*Lithomyrtus retusa*
	Leptorhynchos waitzia	*Eucalyptus eugenioides*	*Litsea breviumbellata*
	Leptospermum coriaceum	*Eucalyptus falciformis*	*Litsea fawcettiana*
	Leptospermum erubescens	*Eucalyptus fastigata*	*Litsea glutinosa*
	Leptospermum fastigiatum	*Eucalyptus glaucina*	*Livistona humilis*
	Leptospermum nitens	*Eucalyptus globoidea*	*Livistona inermis*
	Leptospermum oligandrum	*Eucalyptus goniocalyx*	*Lobelia dioica*
	Leucopogon capitellatus	*Eucalyptus grandis*	*Lobelia douglasiana*
	Leucopogon concurvus	*Eucalyptus haemastoma*	*Lomandra tropica*
	Leucopogon costatus	*Eucalyptus laevopinea*	*Lophostemon grandiflorus*
	Leucopogon fimbriatus	*Eucalyptus largiflorens*	*Lophostemon lactifluus*
	Leucopogon gibbosus	*Eucalyptus longifolia*	*Ludwigia adscendens*
	Leucopogon glabellus	*Eucalyptus macrorhyncha*	*Ludwigia octovalvis*
	Leucopogon obovatus	*Eucalyptus major*	*Ludwigia perennis*
	Leucopogon obtusatus	*Eucalyptus mannifera*	*Lumnitzera racemosa*
	Leucopogon polymorphus	*Eucalyptus melliodora*	*Luvunga monophylla*
	Leucopogon pulchellus	*Eucalyptus microcarpa*	*Lysiana spathulata*
	Leucopogon verticillatus	*Eucalyptus moluccana*	*Lysicarpus angustifolius*
	Levenhookia leptantha	*Eucalyptus muelleriana*	*Macaranga involucrata*
	Levenhookia pusilla	*Eucalyptus nicholii*	*Macarthuria vertex*
	Levenhookia stipitata	*Eucalyptus nitens*	*Mackinlaya macrosciadea*
	Lobelia rhombifolia	*Eucalyptus nitida*	*Macropteranthes kekwickii*
	Lobelia tenuior	*Eucalyptus nobilis*	*Mallotus nesophilus*
	Logania linifolia	*Eucalyptus nortonii*	*Mallotus polyadenos*
	Logania ovata	*Eucalyptus notabilis*	*Maranthes corymbosa*
	Logania vaginalis	*Eucalyptus obliqua*	*Melaleuca acacioides*
	Lomandra caespitosa	*Eucalyptus ovata*	*Melaleuca argentea*
	Lomandra collina	*Eucalyptus paniculata*	*Melaleuca cajuputi*
	Lomandra effusa	*Eucalyptus parramattensis*	*Melaleuca citrolens*

	Lomandra hermaphrodita	*Eucalyptus pauciflora*	*Melaleuca dealbata*
	Lomandra integra	*Eucalyptus pilularis*	*Melaleuca fluviatilis*
	Lomandra juncea	*Eucalyptus piperita*	*Melaleuca foliolosa*
	Lomandra leucocephala	*Eucalyptus planchoniana*	*Melaleuca leucadendra*
	Lomandra mucronata	*Eucalyptus polyanthemos*	*Melaleuca minutifolia*
	Lomandra nigricans	*Eucalyptus polybractea*	*Melaleuca nervosa*
	Lomandra preissii	*Eucalyptus prava*	*Melaleuca saligna*
	Lomandra purpurea	*Eucalyptus propinqua*	*Melaleuca stenostachya*
	Lomandra sericea	*Eucalyptus punctata*	*Melaleuca viridiflora*
	Lomandra sonderi	*Eucalyptus quadrangulata*	*Melastoma affine*
	Lomandra suaveolens	*Eucalyptus racemosa*	*Melochia corchorifolia*
	Loxocarya cinerea	*Eucalyptus radiata*	*Memecylon pauciflorum*
	Lyginia barbata	*Eucalyptus regnans*	*Merremia gemella*
	Lyginia imberbis	*Eucalyptus robusta*	*Merremia incisa*
	Lyperanthus serratus	*Eucalyptus rossii*	*Micromelum minutum*
	Lysinema ciliatum	*Eucalyptus rubida*	*Microstachys chamaelea*
	Lysinema pentapetalum	*Eucalyptus scias*	*Miliusa brahei*
	Maireana brevifolia	*Eucalyptus seeana*	*Miliusa traceyi*
	Maireana marginata	*Eucalyptus siderophloia*	*Mimusops elengi*
	Maireana oppositifolia	*Eucalyptus sieberi*	*Minuria macrorhiza*
	Maireana pentatropis	*Eucalyptus smithii*	*Mischocarpus exangulatus*
	Maireana radiata	*Eucalyptus sparsifolia*	*Mischocarpus lachnocarpus*
	Maireana rohrlachii	*Eucalyptus stellulata*	*Mitrasacme connata*
	Maireana trichoptera	*Eucalyptus stricta*	*Mitrasacme exserta*
	Malleostemon roseus	*Eucalyptus tereticornis*	*Mitrasacme laevis*
	Malleostemon tuberculatus	*Eucalyptus tindaliae*	*Mitrasacme multicaulis*
	Marianthus bicolor	*Eucalyptus umbra*	*Mitrasacme nudicaulis*
	Meionectes brownii	*Eucalyptus vicina*	*Mitrasacme nummularia*
	Melaleuca acuminata	*Eucalyptus viminalis*	*Mitrasacme pygmaea*
	Melaleuca adnata	*Eucalyptus yarraensis*	*Mitrasacme scrithicola*
	Melaleuca atroviridis	*Eucalyptus youmanii*	*Mitrasacme stellata*
	Melaleuca brevifolia	*Euchiton involucratus*	*Mitrasacme subvolubilis*

	Melaleuca cardiophylla	*Euchiton japonicus*	*Mnesithea formosa*
	Melaleuca carrii	*Euchiton sphaericus*	*Mnesithea rottboellioides*
	Melaleuca concreta	*Euchiton umbricola*	*Monoon australe*
	Melaleuca conothamnoides	*Eucryphia lucida*	*Morinda citrifolia*
	Melaleuca cordata	*Euphorbia planitiicola*	*Mucuna gigantea*
	Melaleuca cuticularis	*Euphrasia collina*	*Murdannia gigantea*
	Melaleuca elliptica	*Euphrasia gibbsiae*	*Myristica globosa*
	Melaleuca fulgens	*Eupomatia bennettii*	*Myristica insipida*
	Melaleuca gibbosa	*Eupomatia laurina*	*Myrsine porosa*
	Melaleuca glaberrima	*Euroschinus falcata*	*Nelsonia campestris*
	Melaleuca hamata	*Eurychorda complanata*	*Neofabricia myrtifolia*
	Melaleuca hamulosa	*Euryomyrtus ramosissima*	*Neolitsea brassii*
	Melaleuca huegelii	*Eustrephus latifolius*	*Neptunia dimorphantha*
	Melaleuca lateriflora	*Excoecaria dallachyana*	*Neptunia gracilis*
	Melaleuca lateritia	*Exocarpos cupressiformis*	*Neptunia monosperma*
	Melaleuca laxiflora	*Exocarpos strictus*	*Nervilia holochila*
	Melaleuca marginata	*Ficus coronata*	*Notoleptopus decaisnei*
	Melaleuca pauperiflora	*Ficus fraseri*	*Nymphoides aurantiaca*
	Melaleuca preissiana	*Ficus henneana*	*Nymphoides crenata*
	Melaleuca pungens	*Ficus obliqua*	*Nymphoides exiliflora*
	Melaleuca radula	*Ficus watkinsiana*	*Nymphoides indica*
	Melaleuca rhaphiophylla	*Fieldia australis*	*Nymphoides parvifolia*
	Melaleuca rigidifolia	*Fimbristylis velata*	*Nyssanthes erecta*
	Melaleuca scalena	*Flindersia australis*	*Ocimum caryophyllinum*
	Melaleuca stereophloia	*Flindersia bennettii*	*Olax aphylla*
	Melaleuca suberosa	*Flindersia collina*	*Oldenlandia argillacea*
	Melaleuca subtrigona	*Flindersia schottiana*	*Oldenlandia galioides*
	Melaleuca systena	*Flindersia xanthoxyla*	*Oldenlandia mitrasacmoides*
	Melaleuca thymoides	*Gahnia aspera*	*Operculina aequisepala*
	Melaleuca thyoides	*Gahnia grandis*	*Ophiuros exaltatus*
	Melaleuca trichophylla	*Gahnia melanocarpa*	*Opilia amentacea*

	Melaleuca tuberculata	*Gahnia microstachya*	*Oplismenus compositus*
	Melaleuca undulata	*Gahnia radula*	*Osbeckia australiana*
	Menkea australis	*Galium australe*	*Osbornia octodonta*
	Mesomelaena graciliceps	*Galium ciliare*	*Owenia vernicosa*
	Mesomelaena preissii	*Galium curvihirtum*	*Ozothamnus cassinioides*
	Mesomelaena pseudostygia	*Galium gaudichaudii*	*Pachygone ovata*
	Mesomelaena stygia	*Galium leiocarpum*	*Pandanus aquaticus*
	Mesomelaena tetragona	*Galium leptogonium*	*Pandanus cookii*
	Microcybe multiflora	*Galium liratum*	*Pandanus spiralis*
	Microcybe pauciflora	*Galium migrans*	*Panicum mindanaense*
	Micromyrtus obovata	*Galium propinquum*	*Panicum seminudum*
	Micromyrtus racemosa	*Gastrodia procera*	*Parinari nonda*
	Microtis alba	*Gastrodia sesamoides*	*Parsonsia plaesiophylla*
	Microtis atrata	*Gaultheria appressa*	*Paspalidium caespitosum*
	Microtis media	*Gaultheria hispida*	*Paspalidium globoideum*
	Microtis orbicularis	*Geitonoplesium cymosum*	*Paspalidium rarum*
	Millotia major	*Gentianella diemensis*	*Patersonia macrantha*
	Millotia muelleri	*Geranium antrorsum*	*Pavetta brownii*
	Millotia tenuifolia	*Geranium homeanum*	*Pemphis acidula*
	Mirbelia dilatata	*Geranium neglectum*	*Perotis rara*
	Mirbelia floribunda	*Geranium potentilloides*	*Persicaria barbata*
	Mirbelia microphylla	*Geranium retrorsum*	*Persoonia falcata*
	Mirbelia ramulosa	*Geranium solanderi*	*Petalostigma banksii*
	Mirbelia spinosa	*Geum urbanum*	*Petalostigma pubescens*
	Mirbelia trichocalyx	*Glochidion ferdinandi*	*Petalostigma quadriloculare*
	Monotaxis bracteata	*Glochidion sumatranum*	*Phacellothrix cladochaeta*
	Monotaxis grandiflora	*Glossocardia bidens*	*Phebalium nottii*
	Muehlenbeckia adpressa	*Glossodia major*	*Phyllanthus carpentariae*
	Muehlenbeckia diclina	*Glossodia minor*	*Phyllanthus ciccoides*
	Muehlenbeckia gunnii	*Glossostigma elatinoides*	*Phyllanthus exilis*
	Myoporum brevipes	*Glycine clandestina*	*Phyllanthus flagellaris*
	Myoporum platycarpum	*Glycine cyrtoloba*	*Phyllanthus maderaspatensis*

	Myoporum viscosum	*Glycine latrobeana*	*Phyllanthus minutiflorus*
	Myriocephalus guerinae	*Glycine tabacina*	*Phyllanthus urinaria*
	Myriocephalus pygmaeus	*Glycyrrhiza acanthocarpa*	*Pimelea cornucopiae*
	Nicotiana maritima	*Gmelina leichhardtii*	*Pimelea punicea*
	Nicotiana rotundifolia	*Gompholobium aspalathoides*	*Pimelea sanguinea*
	Nuytsia floribunda	*Gompholobium glabratum*	*Piper caninum*
	Olax benthamiana	*Gompholobium grandiflorum*	*Pisonia aculeata*
	Olax phyllanthi	*Gompholobium huegelii*	*Pisonia umbellifera*
	Olearia axillaris	*Gompholobium inconspicuum*	*Pittosporum ferrugineum*
	Olearia brachyphylla	*Gompholobium latifolium*	*Pittosporum rubiginosum*
	Olearia ciliata	*Gompholobium minus*	*Pittosporum spinescens*
	Olearia floribunda	*Gompholobium pinnatum*	*Pittosporum tinifolium*
	Olearia lanuginosa	*Gompholobium virgatum*	*Planchonella arnhemica*
	Olearia lepidophylla	*Gonocarpus humilis*	*Planchonella pubescens*
	Olearia magniflora	*Gonocarpus micranthus*	*Planchonia careya*
	Olearia minor	*Gonocarpus montanus*	*Platysace valida*
	Olearia muelleri	*Gonocarpus oreophilus*	*Pleiogynium timoriense*
	Olearia passerinoides	*Gonocarpus tetragynus*	*Pleurocarpaea denticulata*
	Olearia paucidentata	*Gonocarpus teucrioides*	*Pleurolobus gangeticus*
	Olearia picridifolia	*Goodenia bellidifolia*	*Pogostemon stellatus*
	Olearia pimeleoides	*Goodenia elongata*	*Pollia macrophylla*
	Olearia rudis	*Goodenia glauca*	*Polycarpaea breviflora*
	Opercularia apiciflora	*Goodenia gracilis*	*Polycarpaea corymbosa*
	Opercularia hispidula	*Goodenia hederacea*	*Polycarpaea holtzei*
	Opercularia scabrida	*Goodenia heteromera*	*Polycarpaea involucrata*
	Opercularia spermacocea	*Goodenia heterophylla*	*Polycarpaea longiflora*
	Opercularia turpis	*Goodenia humilis*	*Polycarpaea spirostylis*
	Orianthera campanulata	*Goodenia lanata*	*Polycarpaea violacea*

	Orianthera flaviflora	*Goodenia macbarronii*	*Polygala bifoliata*
	Orianthera nuda	*Goodenia ovata*	*Polygala coralliformis*
	Orianthera serpyllifolia	*Goodenia paniculata*	*Polygala eriocephala*
	Ornduffia parnassifolia	*Goodenia rotundifolia*	*Polygala exsquarrosa*
	Ornduffia umbricola	*Goodenia stelligera*	*Polygala longifolia*
	Orthrosanthus laxus	*Goodia lotifolia*	*Polygala orbicularis*
	Orthrosanthus multiflorus	*Gossia acmenoides*	*Polygala parviloba*
	Ozothamnus decurrens	*Gossia bidwillii*	*Polygala pterocarpa*
	Ozothamnus retusus	*Gossia hillii*	*Polygala triflora*
	Patersonia juncea	*Gratiola pedunculata*	*Polymeria ambigua*
	Patersonia umbrosa	*Gratiola peruviana*	*Polymeria longifolia*
	Pauridia occidentalis	*Gratiola pubescens*	*Polymeria pusilla*
	Pelargonium littorale	*Gratiola pumilo*	*Polyscias australiana*
	Pericalymma ellipticum	*Grevillea alpina*	*Portulaca australis*
	Persoonia coriacea	*Grevillea aquifolium*	*Portulaca bicolor*
	Persoonia longifolia	*Grevillea arenaria*	*Portulaca digyna*
	Persoonia quinquenervis	*Grevillea australis*	*Premna acuminata*
	Petrophile brevifolia	*Grevillea banksii*	*Premna herbacea*
	Petrophile diversifolia	*Grevillea buxifolia*	*Premna serratifolia*
	Petrophile ericifolia	*Grevillea hilliana*	*Prostanthera cryptandroides*
	Petrophile linearis	*Grevillea juniperina*	*Pseudopogonatherum contortum*
	Petrophile seminuda	*Grevillea lanigera*	*Pseudopogonatherum irritans*
	Petrophile serruriae	*Grevillea linearifolia*	*Psychotria nesophila*
	Petrophile squamata	*Grevillea mucronulata*	*Psychotria poliostemma*
	Petrophile striata	*Grevillea parviflora*	*Psydrax attenuata*
	Petrophile teretifolia	*Grevillea ramosissima*	*Pterocaulon ciliosum*
	Phebalium bullatum	*Grevillea sericea*	*Pterocaulon serrulatum*
	Phebalium canaliculatum	*Grevillea speciosa*	*Pterocaulon tricholobum*
	Phebalium filifolium	*Grevillea victoriae*	*Pterocaulon verbascifolium*
	Phebalium lepidotum	*Grona nemorosa*	*Ptilotus conicus*
	Phebalium tuberculosum	*Guilfoylia monostylis*	*Ptilotus corymbosus*
	Pheladenia deformis	*Guioa semiglauca*	*Ptilotus distans*
	Philotheca brucei	*Gymnema pleiadenium*	*Ptilotus fusiformis*

	Philotheca pungens	*Gymnostachys anceps*	*Ptilotus spicatus*
	Philotheca spicata	*Gynatrix pulchella*	*Pultenaea cinerascens*
	Philotheca tomentella	*Gynochthodes canthoides*	*Pycnospora lutescens*
	Philydrella pygmaea	*Gynochthodes jasminoides*	*Ranunculus meristus*
	Phlebocarya ciliata	*Hackelia latifolia*	*Rhodomyrtus macrocarpa*
	Phyllangium paradoxum	*Hackelia suaveolens*	*Rhodomyrtus trineura*
	Phyllangium sulcatum	*Haemodorum austroqueenslandicum*	*Rhynchosia australis*
	Phyllanthus calycinus	*Haemodorum corymbosum*	*Rhynchosia minima*
	Phyllota pleurandroides	*Haemodorum planifolium*	*Rhynchospora exserta*
	Phyllota remota	*Hakea actites*	*Rhynchospora longisetis*
	Pileanthus peduncularis	*Hakea epiglottis*	*Rhynchospora pterochaeta*
	Pimelea angustifolia	*Hakea eriantha*	*Rhynchospora subtenuifolia*
	Pimelea argentea	*Hakea florulenta*	*Ricinocarpos bowmanii*
	Pimelea brevifolia	*Hakea lissosperma*	*Rotala diandra*
	Pimelea ciliata	*Hakea microcarpa*	*Rotala mexicana*
	Pimelea ferruginea	*Hakea nodosa*	*Rotala occultiflora*
	Pimelea imbricata	*Hakea propinqua*	*Salomonia ciliata*
	Pimelea longiflora	*Hakea repullulans*	*Sauropus elachophyllus*
	Pimelea octophylla	*Hakea salicifolia*	*Sauropus stenocladus*
	Pimelea phylicoides	*Hakea teretifolia*	*Scaevola browniana*
	Pimelea rosea	*Hakea ulicina*	*Scaevola revoluta*
	Pimelea serpyllifolia	*Haloragis glauca*	*Scaevola taccada*
	Pimelea suaveolens	*Haloragis heterophylla*	*Schelhammera multiflora*
	Pimelea sulphurea	*Hardenbergia violacea*	*Schizachyrium crinizonatum*
	Pityrodia lepidota	*Harmogia densifolia*	*Schizachyrium fragile*
	Platysace compressa	*Harpullia hillii*	*Schizachyrium pachyarthron*
	Platysace filiformis	*Harpullia pendula*	*Schizachyrium pseudeulalia*
	Platysace trachymenioides	*Hedraianthera porphyropetala*	*Schoenoplectiella laevis*
	Poa drummondiana	*Hedycarya angustifolia*	*Schoenoplectiella lateriflora*
	Poa porphyroclados	*Helicia glabriflora*	*Schoenus calostachyus*
	Podolepis aristata	*Hibbertia acicularis*	*Schoenus kennyi*

	Podolepis capillaris	*Hibbertia aspera*	*Schoenus punctatus*
	Podolepis gracilis	*Hibbertia bracteata*	*Schoenus sparteus*
	Podolepis lessonii	*Hibbertia calycina*	*Scleria brownii*
	Podolepis rugata	*Hibbertia circumdans*	*Scleria ciliaris*
	Podolepis tepperi	*Hibbertia cistiflora*	*Scleria levis*
	Podotheca angustifolia	*Hibbertia dentata*	*Scleria lingulata*
	Podotheca gnaphalioides	*Hibbertia diffusa*	*Scleria polycarpa*
	Pogonolepis stricta	*Hibbertia empetrifolia*	*Scleria rugosa*
	Pomaderris paniculosa	*Hibbertia fasciculata*	*Scleria sphacelata*
	Poranthera huegelii	*Hibbertia linearis*	*Scleria tricuspidata*
	Poranthera triandra	*Hibbertia monogyna*	*Sclerolaena tetracuspis*
	Praecoxanthus aphyllus	*Hibbertia obtusifolia*	*Scyphiphora hydrophylacea*
	Prasophyllum cyphochilum	*Hibbertia pedunculata*	*Sehima nervosum*
	Prasophyllum fimbria	*Hibbertia procumbens*	*Senecio brigalowensis*
	Prasophyllum hians	*Hibbertia rufa*	*Senna coronilloides*
	Prasophyllum macrostachyum	*Hibbertia salicifolia*	*Senna costata*
	Prasophyllum occidentale	*Hibbertia scandens*	*Senna gaudichaudii*
	Prasophyllum parvifolium	*Hibbertia serpyllifolia*	*Senna oligoclada*
	Prasophyllum plumiforme	*Hibbertia stricta*	*Senna planitiicola*
	Prostanthera aspalathoides	*Hibbertia vestita*	*Seringia collina*
	Prostanthera serpyllifolia	*Hibiscus heterophyllus*	*Seringia corollata*
	Psammomoya choretroides	*Hibiscus splendens*	*Sersalisia sericea*
	Pterochaeta paniculata	*Hierochloe rariflora*	*Sesbania brachycarpa*
	Pterostylis arbuscula	*Hippocratea barbata*	*Sesbania simpliciuscula*
	Pterostylis barbata	*Hodgkinsonia ovatiflora*	*Setaria apiculata*
	Pterostylis excelsa	*Homalanthus stillingiifolius*	*Setaria surgens*
	Pterostylis pusilla	*Homoranthus virgatus*	*Sida atherophora*
	Pterostylis recurva	*Hovea acutifolia*	*Sida brachypoda*
	Pterostylis roensis	*Hovea asperifolia*	*Sida laevis*
	Pterostylis sanguinea	*Hovea heterophylla*	*Sida macropoda*
	Pterostylis sargentii	*Hovea lanceolata*	*Solanum dioicum*

	Pterostylis scabra	*Hovea linearis*	*Solanum echinatum*
	Pterostylis spathulata	*Hovea longifolia*	*Solanum jucundum*
	Pterostylis vittata	*Hovea montana*	*Solanum parvifolium*
	Ptilotus declinatus	*Hovea purpurea*	*Solanum succosum*
	Ptilotus drummondii	*Howittia trilocularis*	*Sonneratia alba*
	Ptilotus eremita	*Hyalosperma praecox*	*Sorghum bulbosum*
	Ptilotus holosericeus	*Hybanthus monopetalus*	*Sorghum plumosum*
	Ptilotus humilis	*Hybanthus vernonii*	*Sorghum stipoideum*
	Ptilotus manglesii	*Hydrocotyle acutiloba*	*Sorghum timorense*
	Ptilotus seminudus	*Hydrocotyle algida*	*Sowerbaea alliacea*
	Pultenaea acerosa	*Hydrocotyle foveolata*	*Spermacoce auriculata*
	Pultenaea canaliculata	*Hydrocotyle geraniifolia*	*Spermacoce brachystema*
	Pultenaea densifolia	*Hydrocotyle hirta*	*Spermacoce breviflora*
	Pultenaea elachista	*Hydrocotyle laxiflora*	*Spermacoce calliantha*
	Pultenaea prostrata	*Hydrocotyle pedicellosa*	*Spermacoce dolichosperma*
	Pultenaea reticulata	*Hydrocotyle pterocarpa*	*Spermacoce leptoloba*
	Pultenaea tenuifolia	*Hydrocotyle sibthorpioides*	*Spermacoce multicaulis*
	Pultenaea verruculosa	*Hydrocotyle tripartita*	*Spermacoce platyloba*
	Pyrorchis nigricans	*Hymenosporum flavum*	*Spermacoce stenophylla*
	Quinetia urvillei	*Hypericum gramineum*	*Sphaeranthus indicus*
	Rhagodia baccata	*Hypericum japonicum*	*Sporobolus pulchellus*
	Rhagodia crassifolia	*Hypoxis hygrometrica*	*Sporobolus scabridus*
	Rhagodia drummondii	*Hypoxis pratensis*	*Stackhousia intermedia*
	Rhagodia latifolia	*Indigofera adesmiifolia*	*Staurogyne spatulata*
	Rhagodia preissii	*Isolepis gaudichaudiana*	*Stemodia lythrifolia*
	Rhodanthe citrina	*Isolepis multicaulis*	*Stemodia tephropelina*
	Rhodanthe laevis	*Isolepis platycarpa*	*Stenocarpus acacioides*
	Rhodanthe manglesii	*Isolepis producta*	*Stenocarpus cunninghamii*
	Rhodanthe stuartiana	*Isopogon anemonifolius*	*Streptoglossa odora*
	Ricinocarpos velutinus	*Isopogon anethifolius*	*Striga curviflora*
	Rinzia ericaea	*Isotoma axillaris*	*Strychnos lucida*
	Roepera angustifolia	*Isotoma fluviatilis*	*Strychnos psilosperma*
	Roepera apiculata	*Ixora beckleri*	*Stylidium adenophorum*
	Roepera aurantiaca	*Jacksonia scoparia*	*Stylidium eglandulosum*
	Roepera billardierei	*Jagera pseudorhus*	*Stylidium eriorhizum*
	Roepera glauca	*Jasminum simplicifolium*	*Stylidium fissilobum*

	Roepera ovata	*Jasminum suavissimum*	*Stylidium pachyrrhizum*
	Rytidosperma occidentale	*Juncus filicaulis*	*Stylidium rotundifolium*
	Salicornia blackiana	*Juncus homalocaulis*	*Stylidium schizanthum*
	Samolus junceus	*Juncus pauciflorus*	*Stylidium semipartitum*
	Santalum acuminatum	*Juncus phaeanthus*	*Stylidium tenerum*
	Santalum murrayanum	*Juncus sandwithii*	*Synostemon glaucus*
	Scaevola bursariifolia	*Kunzea capitata*	*Synostemon lissocarpus*
	Scaevola calliptera	*Kunzea leptospermoides*	*Syzygium angophoroides*
	Scaevola canescens	*Lachnagrostis punicea*	*Syzygium armstrongii*
	Scaevola crassifolia	*Lachnagrostis robusta*	*Syzygium claviflorum*
	Scaevola glandulifera	*Lachnagrostis rudis*	*Syzygium cormiflorum*
	Scaevola linearis	*Lagenophora gracilis*	*Syzygium eucalyptoides*
	Scaevola phlebopetala	*Lagenophora montana*	*Syzygium forte*
	Scaevola repens	*Lagenophora stipitata*	*Syzygium hemilamprum*
	Scaevola striata	*Lagenophora sublyrata*	*Syzygium johnsonii*
	Scaevola thesioides	*Lambertia formosa*	*Syzygium nervosum*
	Schoenus breviculmis	*Lasiopetalum ferrugineum*	*Syzygium suborbiculare*
	Schoenus brevisetis	*Lasiopetalum macrophyllum*	*Tabernaemontana orientalis*
	Schoenus caespititius	*Laxmannia compacta*	*Tacca leontopetaloides*
	Schoenus clandestinus	*Laxmannia gracilis*	*Tarenna dallachiana*
	Schoenus deformis	*Legnephora moorei*	*Tarenna pentamera*
	Schoenus efoliatus	*Leichhardtia coronata*	*Tecticornia australasica*
	Schoenus laevigatus	*Leichhardtia flavescens*	*Templetonia hookeri*
	Schoenus pleiostemoneus	*Leichhardtia liisae*	*Tephrosia astragaloides*
	Schoenus racemosus	*Leichhardtia lloydii*	*Tephrosia brachyodon*
	Schoenus subaphyllus	*Leichhardtia longiloba*	*Tephrosia conspicua*
	Schoenus subfascicularis	*Leichhardtia rostrata*	*Tephrosia coriacea*
	Schoenus subflavus	*Leichhardtia suaveolens*	*Tephrosia filipes*
	Scleranthus minusculus	*Leiocarpa panaetioides*	*Tephrosia juncea*
	Senecio multicaulis	*Lepidium fasciculatum*	*Tephrosia lasiochlaena*
	Senecio spanomerus	*Lepidium foliosum*	*Tephrosia leptoclada*

	Seringia hermanniifolia	*Lepidium pseudohyssopifolium*	*Tephrosia oblongata*
	Seringia velutina	*Lepidium pseudotasmanicum*	*Tephrosia phaeosperma*
	Siloxerus humifusus	*Lepidosperma curtisiae*	*Tephrosia polyzyga*
	Solanum plicatile	*Lepidosperma filiforme*	*Tephrosia porrecta*
	Solanum simile	*Lepidosperma forsythii*	*Tephrosia purpurea*
	Solanum symonii	*Lepidosperma laterale*	*Tephrosia remotiflora*
	Sowerbaea laxiflora	*Lepidosperma limicola*	*Tephrosia simplicifolia*
	Spartochloa scirpoidea	*Lepidosperma tortuosum*	*Tephrosia spechtii*
	Sphaerolobium drummondii	*Leptecophylla pogonocalyx*	*Tephrosia subpectinata*
	Sphaerolobium medium	*Leptinella filicula*	*Tephrosia virens*
	Sphenotoma gracilis	*Leptinella longipes*	*Terminalia aridicola*
	Spiculaea ciliata	*Leptinella reptans*	*Terminalia arostrata*
	Spinifex hirsutus	*Leptocarpus tenax*	*Terminalia bursarina*
	Spyridium bifidum	*Leptomeria acida*	*Terminalia canescens*
	Spyridium eriocephalum	*Leptomeria drupacea*	*Terminalia carpentariae*
	Spyridium globulosum	*Leptorhynchos elongatus*	*Terminalia catappa*
	Spyridium phylicoides	*Leptorhynchos nitidulus*	*Terminalia ferdinandiana*
	Spyridium stenophyllum	*Leptorhynchos squamatus*	*Terminalia grandiflora*
	Spyridium subochreatum	*Leptorhynchos tenuifolius*	*Terminalia latipes*
	Stackhousia aspericocca	*Leptospermum arachnoides*	*Terminalia microcarpa*
	Stackhousia pubescens	*Leptospermum brachyandrum*	*Terminalia muelleri*
	Stellaria filiformis	*Leptospermum brevipes*	*Terminalia oblongata*
	Stenanthemum leucophractum	*Leptospermum continentale*	*Terminalia platyphylla*
	Stenanthemum notiale	*Leptospermum divaricatum*	*Terminalia platyptera*
	Stenanthera conostephioides	*Leptospermum glaucescens*	*Terminalia porphyrocarpa*
	Stenopetalum filifolium	*Leptospermum juniperinum*	*Terminalia pterocarya*
	Stenopetalum sphaerocarpum	*Leptospermum lanigerum*	*Terminalia subacroptera*
	Stirlingia latifolia	*Leptospermum liversidgei*	*Terminalia volucris*
	Stylidium adpressum	*Leptospermum microcarpum*	*Teucrium integrifolium*

	Stylidium amoenum	*Leptospermum morrisonii*	*Thaumastochloa brassii*
	Stylidium androsaceum	*Leptospermum multicaule*	*Thaumastochloa major*
	Stylidium brunonianum	*Leptospermum myrtifolium*	*Thaumastochloa pubescens*
	Stylidium calcaratum	*Leptospermum nitidum*	*Themeda arguens*
	Stylidium caricifolium	*Leptospermum obovatum*	*Thespesia populnea*
	Stylidium ciliatum	*Leptospermum parvifolium*	*Thespesia populneoides*
	Stylidium crassifolium	*Leptospermum polyanthum*	*Thryptomene oligandra*
	Stylidium crossocephalum	*Leptospermum polygalifolium*	*Thuarea involuta*
	Stylidium eriopodum	*Leptospermum scoparium*	*Thysanotus banksii*
	Stylidium guttatum	*Leptospermum squarrosum*	*Thysanotus chinensis*
	Stylidium junceum	*Leptospermum trinervium*	*Timonius timon*
	Stylidium petiolare	*Leptostigma reptans*	*Tinospora smilacina*
	Stylidium piliferum	*Lepyrodia anarthria*	*Trachymene didiscoides*
	Stylidium repens	*Lepyrodia muelleri*	*Trachymene microcephala*
	Stylidium rhynchocarpum	*Lepyrodia scariosa*	*Trianthema rhynchocalyptrum*
	Stylidium scandens	*Lespedeza juncea*	*Tribulopis angustifolia*
	Stylidium scariosum	*Leucochrysum albicans*	*Tribulopis pentandra*
	Stylidium schoenoides	*Leucochrysum molle*	*Tribulus cistoides*
	Stylidium spathulatum	*Leucopogon australis*	*Trichosanthes cucumerina*
	Stylidium tenue	*Leucopogon collinus*	*Tricoryne anceps*
	Stylidium uniflorum	*Leucopogon gelidus*	*Triodia bitextura*
	Stylidium zeicolor	*Leucopogon glacialis*	*Triodia burbidgeana*
	Stylobasium australe	*Leucopogon leptospermoides*	*Triodia bynoei*
	Styphelia exarrhena	*Leucopogon microphyllus*	*Triodia intermedia*
	Styphelia tenuiflora	*Leucopogon pimeleoides*	*Triodia microstachya*
	Synaphea gracillima	*Leucopogon virgatus*	*Triodia mitchellii*
	Synaphea interioris	*Libertia paniculata*	*Tristaniopsis exiliflora*
	Synaphea obtusata	*Libertia pulchella*	*Triumfetta albida*
	Synaphea petiolaris	*Lilaeopsis polyantha*	*Triumfetta micracantha*
	Synaphea spinulosa	*Limosella australis*	*Triumfetta plumigera*
	Taxandria linearifolia	*Linospadix monostachyos*	*Turraea pubescens*

	Taxandria spathulata	*Liparis reflexa*	*Uraria lagopodioides*
	Tecticornia lepidosperma	*Liparophyllum exaltatum*	*Urochloa holosericea*
	Tecticornia lylei	*Lissanthe strigosa*	*Urochloa polyphylla*
	Tecticornia pterygosperma	*Litsea australis*	*Urochloa pubigera*
	Tecticornia syncarpa	*Litsea reticulata*	*Utania racemosa*
	Templetonia retusa	*Livistona australis*	*Utricularia aurea*
	Templetonia rossii	*Lobelia anceps*	*Utricularia caerulea*
	Templetonia sulcata	*Lobelia andrewsii*	*Utricularia chrysantha*
	Tetraria octandra	*Lobelia browniana*	*Utricularia leptoplectra*
	Tetrarrhena laevis	*Lobelia concolor*	*Utricularia limosa*
	Tetratheca affinis	*Lobelia dentata*	*Utricularia odorata*
	Tetratheca hirsuta	*Lobelia gibbosa*	*Uvedalia linearis*
	Tetratheca setigera	*Lobelia irrigua*	*Vachellia bidwillii*
	Teucrium sessiliflorum	*Lobelia pedunculata*	*Vachellia pachyphloia*
	Thelymitra alcockiae	*Lobelia pratioides*	*Vachellia sutherlandii*
	Thelymitra antennifera	*Lobelia simplicicaulis*	*Vachellia valida*
	Thelymitra benthamiana	*Lobelia stenophylla*	*Vavaea amicorum*
	Thelymitra crinita	*Lobelia surrepens*	*Verticordia cunninghamii*
	Thelymitra fuscolutea	*Lobelia trigonocaulis*	*Verticordia verticillata*
	Thelymitra graminea	*Logania albiflora*	*Vigna lanceolata*
	Thelymitra macrophylla	*Lomandra bracteata*	*Vigna vexillata*
	Thelymitra petrophila	*Lomandra confertifolia*	*Vincetoxicum carnosum*
	Thelymitra villosa	*Lomandra cylindrica*	*Vincetoxicum cinerascens*
	Thelymitra vulgaris	*Lomandra filiformis*	*Vincetoxicum erectum*
	Thomasia foliosa	*Lomandra glauca*	*Vincetoxicum flexuosum*
	Thomasia grandiflora	*Lomandra gracilis*	*Vincetoxicum polyanthum*
	Thomasia petalocalyx	*Lomandra hystrix*	*Viscum articulatum*
	Threlkeldia diffusa	*Lomandra laxa*	*Vitex acuminata*
	Thryptomene australis	*Lomandra longifolia*	*Vitex glabrata*
	Thryptomene kochii	*Lomandra multiflora*	*Vitex rotundifolia*
	Thryptomene micrantha	*Lomandra obliqua*	*Vittadinia diffusa*
	Thysanotus baueri	*Lomandra patens*	*Wahlenbergia caryophylloides*

	Thysanotus dichotomus	*Lomandra spicata*	*Waltheria indica*
	Thysanotus manglesianus	*Lomatia arborescens*	*Westringia cheelii*
	Thysanotus multiflorus	*Lomatia fraseri*	*Whiteochloa airoides*
	Thysanotus racemoides	*Lomatia ilicifolia*	*Whiteochloa capillipes*
	Thysanotus sparteus	*Lomatia myricoides*	*Wrightia pubescens*
	Thysanotus speckii	*Lomatia polymorpha*	*Wrightia saligna*
	Thysanotus tenellus	*Lomatia silaifolia*	*Xanthophyllum octandrum*
	Thysanotus thyrsoideus	*Lomatia tinctoria*	*Xanthorrhoea acaulis*
	Thysanotus triandrus	*Lophostemon suaveolens*	*Xanthostemon chrysanthus*
	Trachymene cyanopetala	*Lordhowea amygdalifolia*	*Xanthostemon eucalyptoides*
	Trachymene ornata	*Lordhowea velleioides*	*Xanthostemon paradoxus*
	Trachymene pilosa	*Luzula flaccida*	*Xenostegia tridentata*
	Tremandra stelligera	*Luzula meridionalis*	*Xerochloa barbata*
	Tribonanthes australis	*Lycopus australis*	*Xerochloa imberbis*
	Tribonanthes longipetala	*Lyperanthus suaveolens*	*Xylocarpus granatum*
	Trichocline spathulata	*Macadamia integrifolia*	*Xylocarpus moluccensis*
	Tricoryne humilis	*Macaranga tanarius*	*Xylomelum cunninghamianum*
	Tricoryne tenella	*Macarthuria neocambrica*	*Xyris cheumatophila*
	Triodia irritans	*Machaerina acuta*	*Xyris complanata*
	Triodia scariosa	*Machaerina tetragona*	*Xyris indica*
	Tripterococcus brunonis	*Maclura cochinchinensis*	*Xyris oligantha*
	Trithuria submersa	*Maireana decalvans*	*Xyris pauciflora*
	Trymalium ledifolium	*Maireana microphylla*	*Yakirra majuscula*
	Trymalium odoratissimum	*Maireana pentagona*	*Yakirra nulla*
	Utricularia multifida	*Malacocera tricornis*	*Yakirra pauciflora*
	Veronica hillebrandii	*Malaisia scandens*	*Zanthoxylum rhetsa*
	Verticordia acerosa	*Mallotus claoxyloides*	*Ziziphus oenopolia*
	Verticordia chrysantha	*Mallotus discolor*	*Ziziphus quadrilocularis*
	Verticordia chrysanthella	*Mallotus philippensis*	*Zornia areolata*
	Verticordia densiflora	*Mazus pumilio*	*Zornia muriculata*
	Verticordia endlicheriana	*Melaleuca alternifolia*	*Zornia prostrata*

	Verticordia eriocephala	*Melaleuca decora*	
	Verticordia grandiflora	*Melaleuca diosmatifolia*	
	Verticordia grandis	*Melaleuca ericifolia*	
	Verticordia habrantha	*Melaleuca nodosa*	
	Verticordia huegelii	*Melaleuca sieberi*	
	Verticordia inclusa	*Melaleuca squamea*	
	Verticordia insignis	*Melaleuca squarrosa*	
	Verticordia nobilis	*Melaleuca thymifolia*	
	Verticordia pennigera	*Melichrus procumbens*	
	Verticordia picta	*Melichrus urceolatus*	
	Verticordia plumosa	*Melicope hayesii*	
	Verticordia roei	*Melicope micrococca*	
	Verticordia serrata	*Melicytus angustifolius*	
	Vittadinia australasica	*Melicytus dentatus*	
	Vittadinia megacephala	*Melodinus australis*	
	Wahlenbergia preissii	*Mentha australis*	
	Waitzia acuminata	*Mentha diemenica*	
	Waitzia nitida	*Mentha laxiflora*	
	Waitzia suaveolens	*Mentha satureioides*	
	Westringia cephalantha	*Mezoneuron brachycarpum*	
	Westringia dampieri	*Mezoneuron scortechinii*	
	Westringia rigida	*Micrantheum ericoides*	
	Wilsonia humilis	*Micrantheum hexandrum*	
	Wurmbea densiflora	*Micromyrtus ciliata*	
	Wurmbea tenella	*Micromyrtus striata*	
	Xanthorrhoea caespitosa	*Microseris lanceolata*	
	Xanthorrhoea drummondii	*Microseris scapigera*	
	Xanthorrhoea gracilis	*Microtis oblonga*	
	Xanthorrhoea preissii	*Microtis parviflora*	
	Xanthorrhoea semiplana	*Microtis rara*	
	Xanthosia candida	*Microtis unifolia*	
	Xanthosia huegelii	*Mirbelia oxylobioides*	
	Xanthosia leiophylla	*Mirbelia platylobioides*	
	Xanthosia rotundifolia	*Mirbelia rubiifolia*	
	Zieria veronicea	*Mirbelia speciosa*	
		Mischarytera lautereriana	
		Mischocarpus anodontus	

		Mischocarpus australis	
		Mischocarpus pyriformis	
		Mitrasacme alsinoides	
		Mitrasacme paludosa	
		Mitrasacme pilosa	
		Mitrasacme polymorpha	
		Mitrasacme serpyllifolia	
		Monotoca elliptica	
		Monotoca glauca	
		Monotoca scoparia	
		Montia australasica	
		Montia fontana	
		Muehlenbeckia gracillima	
		Muehlenbeckia rhyticarya	
		Muellerina celastroides	
		Muellerina eucalyptoides	
		Myoporum acuminatum	
		Myoporum boninense	
		Myosotis australis	
		Myriocephalus rhizocephalus	
		Myriophyllum crispatum	
		Myriophyllum papillosum	
		Myriophyllum pedunculatum	
		Myriophyllum simulans	
		Myriophyllum variifolium	
		Myrsine howittiana	
		Myrsine subsessilis	
		Myrsine variabilis	
		Nematolepis squamea	
		Neolitsea australiensis	
		Neolitsea dealbata	
		Nertera granadensis	
		Nicotiana forsteri	
		Nicotiana suaveolens	
		Notelaea ligustrina	
		Notelaea linearis	
		Notelaea longifolia	
		Notelaea microcarpa	
		Notelaea ovata	
		Notelaea punctata	
		Notelaea venosa	

		Nothofagus cunninghamii	
		Notothixos cornifolius	
		Notothixos incanus	
		Notothixos subaureus	
		Nymphoides geminata	
		Nyssanthes diffusa	
		Oberonia titania	
		Ochrosperma lineare	
		Olax stricta	
		Olea paniculata	
		Olearia algida	
		Olearia alpicola	
		Olearia argophylla	
		Olearia elliptica	
		Olearia erubescens	
		Olearia glandulosa	
		Olearia iodochroa	
		Olearia lirata	
		Olearia megalophylla	
		Olearia microphylla	
		Olearia myrsinoides	
		Olearia nernstii	
		Olearia phlogopappa	
		Olearia ramosissima	
		Olearia rugosa	
		Olearia stellulata	
		Olearia tenuifolia	
		Olearia tomentosa	
		Olearia viscidula	
		Olearia viscosa	
		Omphacomeria acerba	
		Opercularia aspera	
		Opercularia diphylla	
		Opercularia ovata	
		Opercularia varia	
		Oplismenus hirtellus	
		Oplismenus undulatifolius	
		Oreomyrrhis ciliata	
		Oreomyrrhis eriopoda	
		Orianthera pusilla	
		Orites excelsus	
		Orites revolutus	

		Ornduffia reniformis	
		Orthoceras strictum	
		Ottochloa gracillima	
		Owenia venosa	
		Oxalis exilis	
		Oxalis perennans	
		Oxalis rubens	
		Oxalis thompsoniae	
		Oxylobium arborescens	
		Oxylobium ellipticum	
		Oxytes brachypoda	
		Ozothamnus argophyllus	
		Ozothamnus conditus	
		Ozothamnus cuneifolius	
		Ozothamnus ferrugineus	
		Ozothamnus obcordatus	
		Ozothamnus rosmarinifolius	
		Ozothamnus rufescens	
		Ozothamnus secundiflorus	
		Ozothamnus thyrsoideus	
		Palmeria scandens	
		Pandanus tectorius	
		Pandorea floribunda	
		Pandorea jasminoides	
		Panicum effusum	
		Panicum pygmaeum	
		Panicum simile	
		Pararchidendron pruinosum	
		Parsonsia brisbanensis	
		Parsonsia brownii	
		Parsonsia induplicata	
		Parsonsia longipetiolata	
		Parsonsia purpurascens	
		Parsonsia rotata	
		Parsonsia straminea	
		Parsonsia velutina	
		Parsonsia ventricosa	
		Paspalidium criniforme	
		Paspalidium gausum	
		Passiflora aurantia	
		Passiflora herbertiana	
		Patersonia fragilis	

		Patersonia glabrata	
		Patersonia sericea	
		Pauridia vaginata	
		Pelargonium australe	
		Pelargonium inodorum	
		Pelargonium rodneyanum	
		Pennantia cunninghamii	
		Pentaceras australe	
		Pentachondra pumila	
		Peperomia blanda	
		Peperomia tetraphylla	
		Peristeranthus hillii	
		Persicaria decipiens	
		Persicaria dichotoma	
		Persicaria praetermissa	
		Persicaria prostrata	
		Persicaria strigosa	
		Persicaria subsessilis	
		Persoonia chamaepeuce	
		Persoonia confertiflora	
		Persoonia conjuncta	
		Persoonia cornifolia	
		Persoonia curvifolia	
		Persoonia juniperina	
		Persoonia lanceolata	
		Persoonia laurina	
		Persoonia levis	
		Persoonia linearis	
		Persoonia media	
		Persoonia mollis	
		Persoonia oleoides	
		Persoonia pinifolia	
		Persoonia rigida	
		Persoonia silvatica	
		Persoonia stradbrokensis	
		Persoonia tenuifolia	
		Persoonia virgata	
		Petalostigma triloculare	
		Petermannia cirrosa	
		Petrophile canescens	
		Petrophile pedunculata	
		Petrophile pulchella	

		Petrophile sessilis	
		Phebalium squamulosum	
		Phebalium woombye	
		Philotheca hispidula	
		Philotheca myoporoides	
		Philotheca salsolifolia	
		Philotheca trachyphylla	
		Philotheca verrucosa	
		Philydrum lanuginosum	
		Phyllanthus gunnii	
		Phyllanthus hirtellus	
		Phyllanthus microcladus	
		Phyllanthus occidentalis	
		Phyllanthus similis	
		Phyllanthus subcrenulatus	
		Phyllota phylicoides	
		Picris angustifolia	
		Picris squarrosa	
		Pilidiostigma glabrum	
		Pimelea alpina	
		Pimelea altior	
		Pimelea axiflora	
		Pimelea curviflora	
		Pimelea drupacea	
		Pimelea hirsuta	
		Pimelea humilis	
		Pimelea latifolia	
		Pimelea ligustrina	
		Pimelea linifolia	
		Pimelea neoanglica	
		Pimelea nivea	
		Pimelea pauciflora	
		Pimelea spinescens	
		Piper hederaceum	
		Pittosporum bicolor	
		Pittosporum multiflorum	
		Pittosporum revolutum	
		Pittosporum viscidum	
		Planchonella australis	
		Planchonella chartacea	
		Planchonella cotinifolia	

		Planchonella myrsinifolia	
		Plantago antarctica	
		Plantago cunninghamii	
		Plantago gaudichaudii	
		Plantago hispida	
		Plantago varia	
		Platylobium formosum	
		Platylobium montanum	
		Platylobium obtusangulum	
		Platylobium parviflorum	
		Platysace ericoides	
		Platysace heterophylla	
		Platysace lanceolata	
		Platysace linearifolia	
		Plectorrhiza tridentata	
		Pleioluma queenslandica	
		Pleogyne australis	
		Poa billardierei	
		Poa clivicola	
		Poa fawcettiae	
		Poa gunnii	
		Poa hiemata	
		Poa hothamensis	
		Poa meionectes	
		Poa morrisii	
		Poa phillipsiana	
		Poa rodwayi	
		Poa sieberiana	
		Podolepis arachnoidea	
		Podolepis decipiens	
		Podolepis hieracioides	
		Podolepis jaceoides	
		Podolepis muelleri	
		Podolepis neglecta	
		Podolepis robusta	
		Podolobium alpestre	
		Podolobium ilicifolium	
		Podolobium procumbens	
		Podolobium scandens	
		Pollia crispata	
		Polygala japonica	

		Polymeria calycina	
		Polyosma cunninghamii	
		Polyscias elegans	
		Polyscias murrayi	
		Pomaderris andromedifolia	
		Pomaderris angustifolia	
		Pomaderris apetala	
		Pomaderris argyrophylla	
		Pomaderris betulina	
		Pomaderris discolor	
		Pomaderris elachophylla	
		Pomaderris elliptica	
		Pomaderris eriocephala	
		Pomaderris ferruginea	
		Pomaderris intermedia	
		Pomaderris lanigera	
		Pomax umbellata	
		Poranthera corymbosa	
		Poranthera ericifolia	
		Poranthera microphylla	
		Pothos longipes	
		Prasophyllum australe	
		Prasophyllum brevilabre	
		Prasophyllum flavum	
		Prasophyllum patens	
		Prostanthera denticulata	
		Prostanthera hirtula	
		Prostanthera howelliae	
		Prostanthera incana	
		Prostanthera incisa	
		Prostanthera lasianthos	
		Prostanthera linearis	
		Prostanthera melissifolia	
		Prostanthera rotundifolia	
		Prostanthera saxicola	
		Prostanthera scutellarioides	
		Pseudanthus orientalis	
		Pseuderanthemum variabile	
		Pseudognaphalium luteoalbum	
		Pseudoweinmannia lachnocarpa	
		Psychotria daphnoides	
		Psychotria loniceroides	
		Psydrax lamprophylla	

		Psydrax odorata	
		Pterocaulon redolens	
		Pterostylis aciculiformis	
		Pterostylis acuminata	
		Pterostylis alpina	
		Pterostylis alveata	
		Pterostylis atrans	
		Pterostylis baptistii	
		Pterostylis bicolor	
		Pterostylis coccina	
		Pterostylis concinna	
		Pterostylis falcata	
		Pterostylis fischiorum	
		Pterostylis grandiflora	
		Pterostylis hamata	
		Pterostylis hispidula	
		Pterostylis longifolia	
		Pterostylis melagramma	
		Pterostylis monticola	
		Pterostylis nutans	
		Pterostylis obtusa	
		Pterostylis ophioglossa	
		Pterostylis parviflora	
		Pterostylis pedunculata	
		Pterostylis reflexa	
		Pterostylis revoluta	
		Pterostylis rubescens	
		Pterostylis rufa	
		Pterostylis smaragdyna	
		Pterostylis squamata	
		Pterostylis striata	
		Pterostylis truncata	
		Ptilothrix deusta	
		Pullenia gunnii	
		Pultenaea blakelyi	
		Pultenaea daphnoides	
		Pultenaea dentata	
		Pultenaea euchila	
		Pultenaea ferruginea	
		Pultenaea flexilis	
		Pultenaea foliolosa	
		Pultenaea gunnii	
		Pultenaea hispidula	

		Pultenaea humilis	
		Pultenaea juniperina	
		Pultenaea linophylla	
		Pultenaea microphylla	
		Pultenaea mollis	
		Pultenaea muelleri	
		Pultenaea myrtoides	
		Pultenaea paleacea	
		Pultenaea pedunculata	
		Pultenaea petiolaris	
		Pultenaea polifolia	
		Pultenaea procumbens	
		Pultenaea retusa	
		Pultenaea scabra	
		Pultenaea setulosa	
		Pultenaea spinosa	
		Pultenaea stricta	
		Pultenaea subspicata	
		Pultenaea tuberculata	
		Pultenaea villosa	
		Pycnosorus globosus	
		Pycnosorus thompsonianus	
		Quintinia sieberi	
		Quintinia verdonii	
		Ranunculus collinus	
		Ranunculus glabrifolius	
		Ranunculus graniticola	
		Ranunculus pachycarpus	
		Ranunculus plebeius	
		Ranunculus robertsonii	
		Ranunculus scapiger	
		Rhinerrhiza divitiflora	
		Rhodamnia argentea	
		Rhodamnia rubescens	
		Rhodanthe anthemoides	
		Rhodanthe corymbiflora	
		Rhodanthe diffusa	
		Rhodomyrtus psidioides	
		Rhodosphaera rhodanthema	
		Rhysotoechia bifoliolata	
		Rhytidosporum procumbens	
		Ricinocarpos pinifolius	
		Ripogonum album	

		Ripogonum brevifolium	
		Ripogonum discolor	
		Ripogonum elseyanum	
		Ripogonum fawcettianum	
		Rorippa dictyosperma	
		Rorippa eustylis	
		Rorippa gigantea	
		Rorippa laciniata	
		Rubus moluccanus	
		Rubus nebulosus	
		Rubus parvifolius	
		Rubus rosifolius	
		Rumex bidens	
		Rumex dumosus	
		Rumex tenax	
		Rytidosperma auriculatum	
		Rytidosperma bipartitum	
		Rytidosperma carphoides	
		Rytidosperma erianthum	
		Rytidosperma fulvum	
		Rytidosperma indutum	
		Rytidosperma laeve	
		Rytidosperma longifolium	
		Rytidosperma monticola	
		Rytidosperma nudiflorum	
		Rytidosperma pallidum	
		Rytidosperma penicillatum	
		Rytidosperma pilosum	
		Rytidosperma racemosum	
		Rytidosperma richardsonii	
		Rytidosperma tenuius	
		Salicornia quinqueflora	
		Sambucus australasica	
		Sannantha cunninghamii	
		Sannantha pluriflora	
		Santalum obtusifolium	
		Sarcochilus australis	
		Sarcochilus ceciliae	
		Sarcochilus falcatus	

		Sarcochilus hillii	
		Sarcochilus parviflorus	
		Sarcomelicope simplicifolia	
		Sarcopetalum harveyanum	
		Sarcopteryx stipata	
		Scaevola calendulacea	
		Scaevola hookeri	
		Scaevola ramosissima	
		Schelhammera undulata	
		Schizomeria ovata	
		Schoenus ericetorum	
		Schoenus imberbis	
		Schoenus maschalinus	
		Schoenus melanostachys	
		Schoenus moorei	
		Scleranthus biflorus	
		Scleranthus diander	
		Scleranthus fasciculatus	
		Scleria mackaviensis	
		Sclerolaena stelligera	
		Sclerolaena tricuspis	
		Scolopia braunii	
		Scutellaria humilis	
		Scutellaria mollis	
		Sebaea albidiflora	
		Sebaea ovata	
		Senecio bathurstianus	
		Senecio bipinnatisectus	
		Senecio biserratus	
		Senecio cunninghamii	
		Senecio diaschides	
		Senecio glomeratus	
		Senecio gunnii	
		Senecio hispidulus	
		Senecio lautus	
		Senecio linearifolius	
		Senecio minimus	
		Senecio phelleus	
		Senecio prenanthoides	
		Senecio quadridentatus	
		Senecio runcinifolius	
		Senecio spathulatus	

		Senecio tenuiflorus	
		Senecio vagus	
		Senna acclinis	
		Senna aciphylla	
		Senna barronfieldii	
		Senna sophera	
		Seringia arborescens	
		Sicyos australis	
		Sida corrugata	
		Sigesbeckia australiensis	
		Siphonodon australis	
		Sloanea australis	
		Sloanea woollsii	
		Smilax australis	
		Smilax glyciphylla	
		Solanum amblymerum	
		Solanum brownii	
		Solanum campanulatum	
		Solanum corifolium	
		Solanum densevestitum	
		Solanum esuriale	
		Solanum linearifolium	
		Solanum opacum	
		Solanum prinophyllum	
		Solanum pungetium	
		Solanum stelligerum	
		Solanum vescum	
		Solenogyne bellioides	
		Solenogyne dominii	
		Solenogyne gunnii	
		Sorghum leiocladum	
		Sowerbaea juncea	
		Spergularia brevifolia	
		Spergularia tasmanica	
		Sphaerolobium minus	
		Sphaerolobium vimineum	
		Sphaeromorphaea australis	
		Spiranthes australis	
		Sporadanthus interruptus	
		Sporadanthus tasmanicus	
		Sporobolus caroli	
		Sporobolus creber	
		Sporobolus elongatus	

		Sporobolus laxus	
		Sprengelia incarnata	
		Sprengelia sprengelioides	
		Spyridium parvifolium	
		Stackhousia monogyna	
		Stackhousia nuda	
		Stackhousia viminea	
		Stellaria angustifolia	
		Stellaria flaccida	
		Stellaria multiflora	
		Stellaria pungens	
		Stenanthera pinifolia	
		Stenocarpus salignus	
		Stenocarpus sinuatus	
		Stephania japonica	
		Streblus brunonianus	
		Stuartina muelleri	
		Stylidium armeria	
		Stylidium debile	
		Stylidium graminifolium	
		Stylidium montanum	
		Stylidium productum	
		Stypandra glauca	
		Styphelia laeta	
		Styphelia triflora	
		Styphelia tubiflora	
		Styphelia viridis	
		Suaeda australis	
		Swainsona behriana	
		Swainsona galegifolia	
		Swainsona greyana	
		Swainsona monticola	
		Swainsona murrayana	
		Swainsona procumbens	
		Sylvipoa queenslandica	
		Symplocos stawellii	
		Symplocos thwaitesii	
		Synoum glandulosum	
		Syzygium australe	
		Syzygium corynanthum	
		Syzygium crebrinerve	
		Syzygium floribundum	

		Syzygium francisii	
		Syzygium ingens	
		Syzygium luehmannii	
		Syzygium oleosum	
		Tabernaemontana pandacaqui	
		Tasmannia insipida	
		Tasmannia lanceolata	
		Tasmannia xerophila	
		Tecticornia triandra	
		Telopea speciosissima	
		Telopea truncata	
		Templetonia stenophylla	
		Tetragonia tetragonoides	
		Tetraria capillaris	
		Tetrarrhena acuminata	
		Tetrarrhena distichophylla	
		Tetrarrhena turfosa	
		Tetrastigma nitens	
		Tetratheca bauerifolia	
		Tetratheca ciliata	
		Tetratheca ericifolia	
		Tetratheca labillardierei	
		Tetratheca pilosa	
		Tetratheca thymifolia	
		Teucrium argutum	
		Teucrium corymbosum	
		Teucrium junceum	
		Thelionema caespitosum	
		Thelionema umbellatum	
		Thelymitra arenaria	
		Thelymitra aristata	
		Thelymitra brevifolia	
		Thelymitra carnea	
		Thelymitra cyanea	
		Thelymitra holmesii	
		Thelymitra ixioides	
		Thelymitra malvina	
		Thelymitra media	
		Thelymitra pauciflora	
		Thelymitra peniculata	
		Thesium australe	
		Thynninorchis huntiana	

		Thyridia repens	
		Thysanotus juncifolius	
		Thysanotus tuberosus	
		Toechima tenax	
		Trachymene composita	
		Trachymene humilis	
		Trachymene incisa	
		Trema tomentosa	
		Tricoryne elatior	
		Tricoryne simplex	
		Trimenia moorei	
		Tripladenia cunninghamii	
		Triptilodiscus pygmaeus	
		Trisetum spicatum	
		Tristaniopsis collina	
		Tristaniopsis laurina	
		Trochocarpa laurina	
		Typhonium brownii	
		Urtica incisa	
		Utricularia australis	
		Utricularia beaugleholei	
		Utricularia dichotoma	
		Utricularia lateriflora	
		Utricularia uniflora	
		Uvaria leichhardtii	
		Verbena gaudichaudii	
		Veronica calycina	
		Veronica derwentiana	
		Veronica gracilis	
		Veronica grosseserrata	
		Veronica notabilis	
		Veronica perfoliata	
		Veronica subtilis	
		Viminaria juncea	
		Vincetoxicum barbatum	
		Vincetoxicum ovatum	
		Vincetoxicum paniculatum	
		Viola betonicifolia	
		Viola caleyana	
		Viola cleistogamoides	
		Viola eminens	

		Viola fuscoviolacea	
		Viola hederacea	
		Viola silicestris	
		Vittadinia cuneata	
		Vittadinia muelleri	
		Vittadinia tenuissima	
		Wahlenbergia capillaris	
		Wahlenbergia ceracea	
		Wahlenbergia fluminalis	
		Wahlenbergia gloriosa	
		Wahlenbergia gracilis	
		Wahlenbergia graniticola	
		Wahlenbergia gymnoclada	
		Wahlenbergia luteola	
		Wahlenbergia multicaulis	
		Wahlenbergia planiflora	
		Wahlenbergia stricta	
		Wikstroemia indica	
		Wilkiea hugeliana	
		Wilkiea macrophylla	
		Wollastonia uniflora	
		Woollsia pungens	
		Wurmbea biglandulosa	
		Wurmbea dioica	
		Wurmbea latifolia	
		Xanthorrhoea arborea	
		Xanthorrhoea australis	
		Xanthorrhoea concava	
		Xanthorrhoea fulva	
		Xanthorrhoea glauca	
		Xanthorrhoea johnsonii	
		Xanthorrhoea latifolia	
		Xanthorrhoea macronema	
		Xanthorrhoea malacophylla	
		Xanthorrhoea media	
		Xanthorrhoea minor	
		Xanthorrhoea resinosa	
		Xanthosia atkinsoniana	
		Xanthosia dissecta	
		Xanthosia pilosa	
		Xanthosia tridentata	

		Xerochrysum bracteatum	
		Xerochrysum palustre	
		Xerochrysum subundulatum	
		Xylomelum pyriforme	
		Xyris gracilis	
		Xyris juncea	
		Xyris operculata	
		Youngia japonica	
		Zanthoxylum brachyacanthum	
		Zehneria cunninghamii	
		Zieria arborescens	
		Zieria compacta	
		Zieria cytisoides	
		Zieria laevigata	
		Zieria laxiflora	
		Zieria minutiflora	
		Zieria pilosa	
		Zieria smithii	
		Zornia dyctiocarpa	
		Zoysia macrantha	

Filtering Aquatic Plant Species

Aquatic species were excluded based on a literature review and online databases by first identifying families commonly associated with aquatic taxa (e.g., Alismataceae, Nymphaeaceae, Hydrocharitaceae, Cymodoceaceae, Pontederiaceae), followed by screening individual species within these families using published references such as (Ling et al., 2019, Lyons et al., 2004, Victoria and Rylah, 2012, Conservation;, 2012), and some online database such as WetlandInfo (2017).

Phylogenetic Tree

To reconstruct the phylogenetic relationships of the 5,033 angiosperm species analysed in this study, we employed the extended version of the Smith and Brown (2018)phylogeny, implemented through the R package V.PhyloMaker2 (Jin and Qian, 2022). We specifically utilized the megatree GBOTB.extended.WP.tre (Jin and Qian, 2022) and applied the functions build nodes.1 and Scenario 3 (Jin and Qian, 2022, Jin and Qian, 2023) to generate the phylogenetic tree. Because the megatree GBOTB.extended.WP.tre includes over three-quarters of the world's angiosperm genera, the resulting phylogenetic tree was well-resolved at the genus level (Jin and Qian, 2022). Previous studies have demonstrated that patterns of phylogenetic diversity and structure are consistent whether the phylogeny is resolved at the genus or species level (Qian and Jin, 2021, Qian et al., 2023b). Therefore, the phylogenetic tree generated in this study provides reliable values for the phylogenetic metrics applied. Phylogenetic trees created using V.PhyloMaker2 have also been

used in numerous studies examining phylogenetic diversity and structure across regional and global floras (Cai et al., 2023, Qian et al., 2023a, Zhou et al., 2023).

The phylogenetic tree revealed several basal lineages within the family Rubiaceae and the genus *Galium*. Additionally, numerous monophyletic families, such as Apocynaceae, Malvaceae, Poaceae, Ebenaceae, Fabaceae, Asteraceae, Orchidaceae, and Rosaceae, reflect clear evolutionary clades within these groups. In contrast, 13 families exhibit polyphyletic patterns, including Asparagaceae, Araliaceae, Amaranthaceae, Clusiaceae, and Apiaceae, where species are scattered across different branches of the phylogenetic tree, suggesting multiple evolutionary origins and a complex history of diversification. Furthermore, some families, like Asparagaceae, Amaranthaceae, and Clusiaceae, demonstrate paraphyletic characteristics. The results of the phylogenetic tree are shown in Figure S1. The phylogenetic analysis of angiosperms revealed considerable variability in branch lengths, with a median of 6.74 and a maximum of 138.97, suggesting a wide range of evolutionary divergence rates among lineages. Node ages ranged from 0.0038 to 138.97, with older nodes likely corresponding to basal divergences in the tree, providing insights into the deep evolutionary history of angiosperms. Species richness varied substantially across families, with Fabaceae (670 species), Myrtaceae (543 species), Asteraceae (342 species), Poaceae (310 species), Orchidaceae (264 species), and Proteaceae (243 species) emerging as the most diverse. These results highlight the evolutionary complexity and diversity within angiosperms, with certain families showing particularly high species richness, potentially due to adaptive radiation and ecological success.

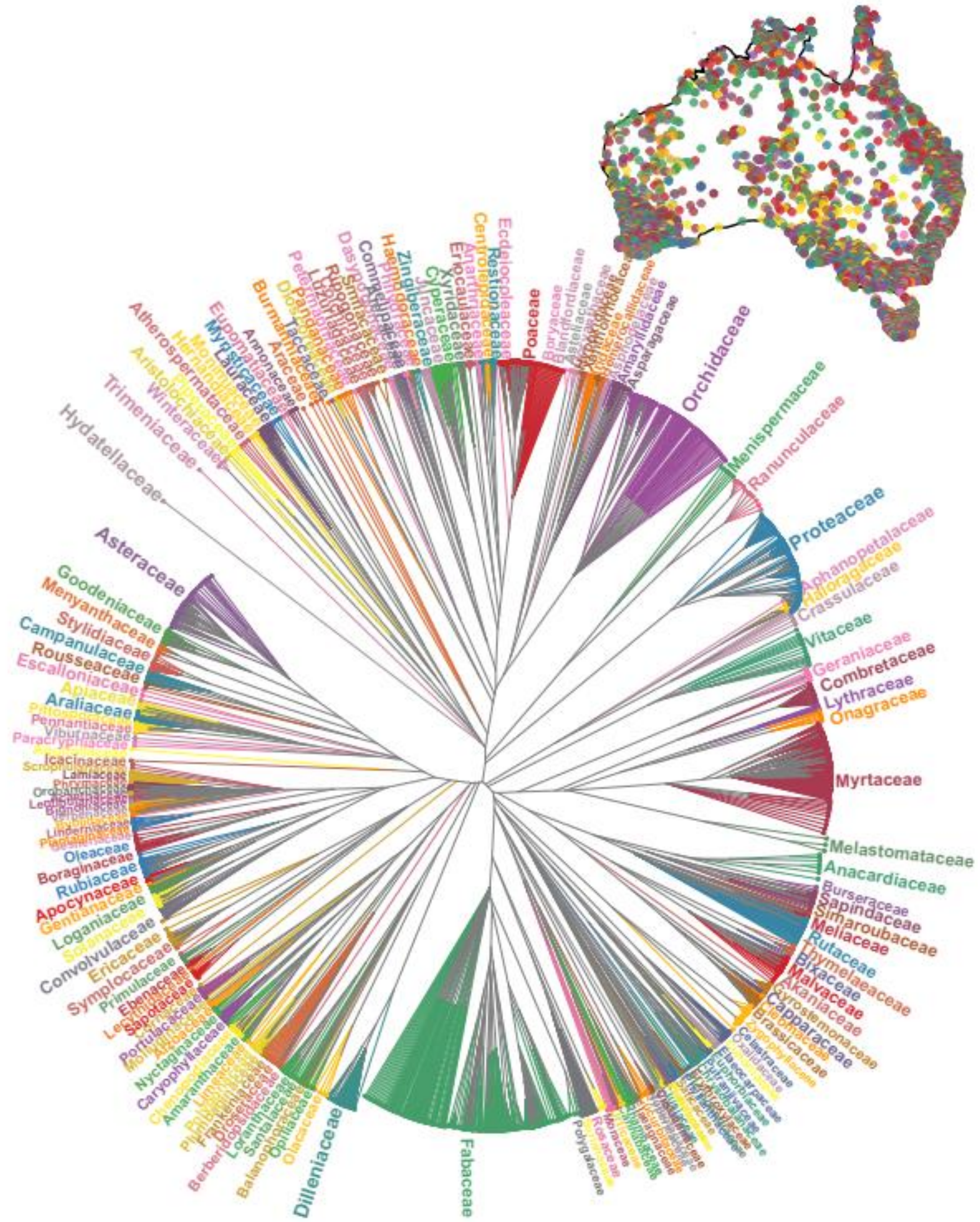

Figure S1. Phylogenetic tree of the 5,033 Australian plant species investigated in this study. The branching patterns reveal the relationships among taxa. Close branches suggest a closer evolutionary relationship, while longer branches typically indicate more significant evolutionary divergence or a longer time since the taxa split from a common ancestor. The phylogenetic analysis of angiosperms, based on V.PHYLOMAKER2, includes 166 families, 1,074 genera.

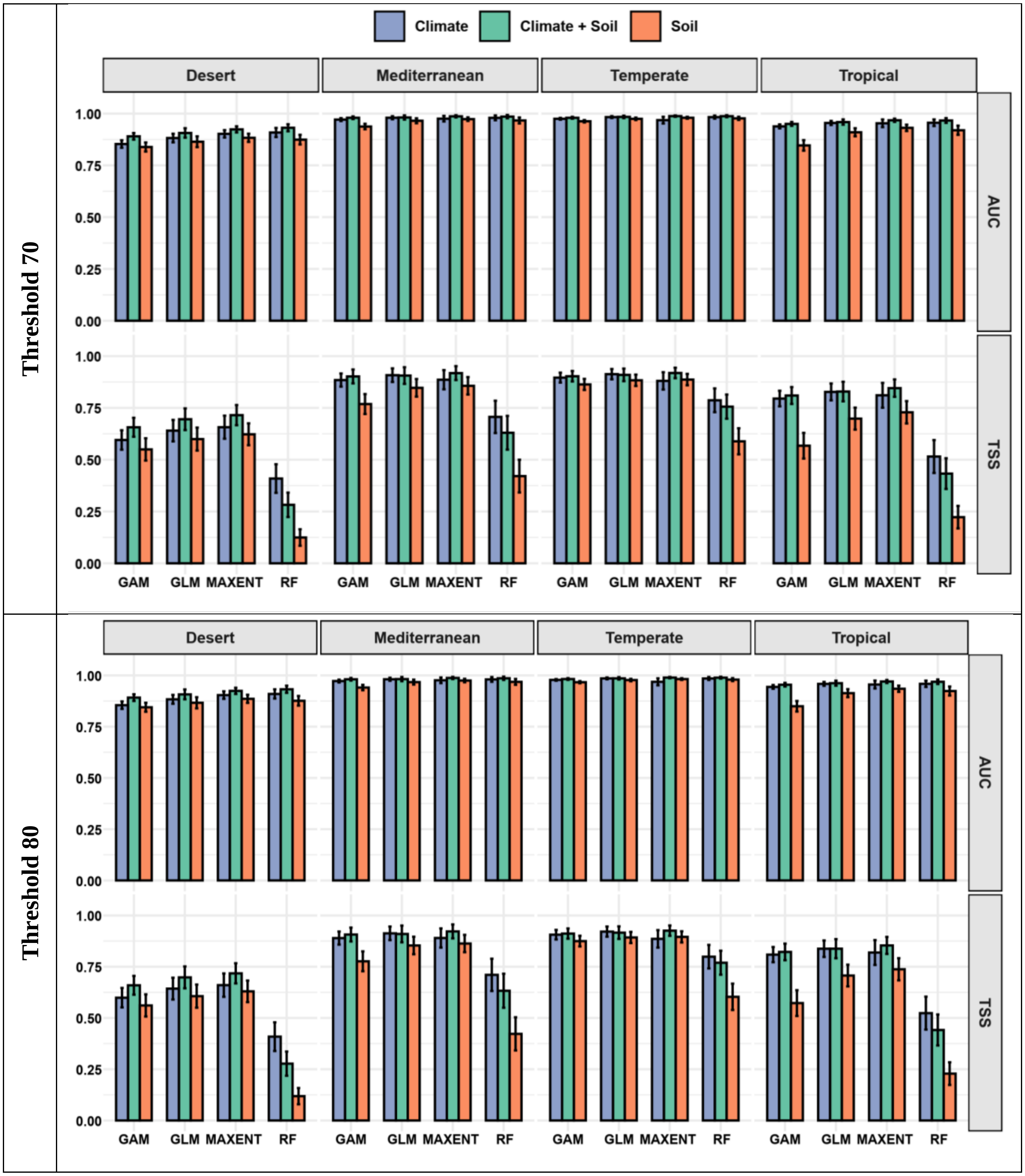
Threshold 70
Climate
Climate + Soil
Soil
Desert
Mediterranean
Temperate
Tropical
AUC
TSS
1.00
0.75
0.50
0.25
0.00
GAM
GLM
MAXENT
RF
Threshold 80

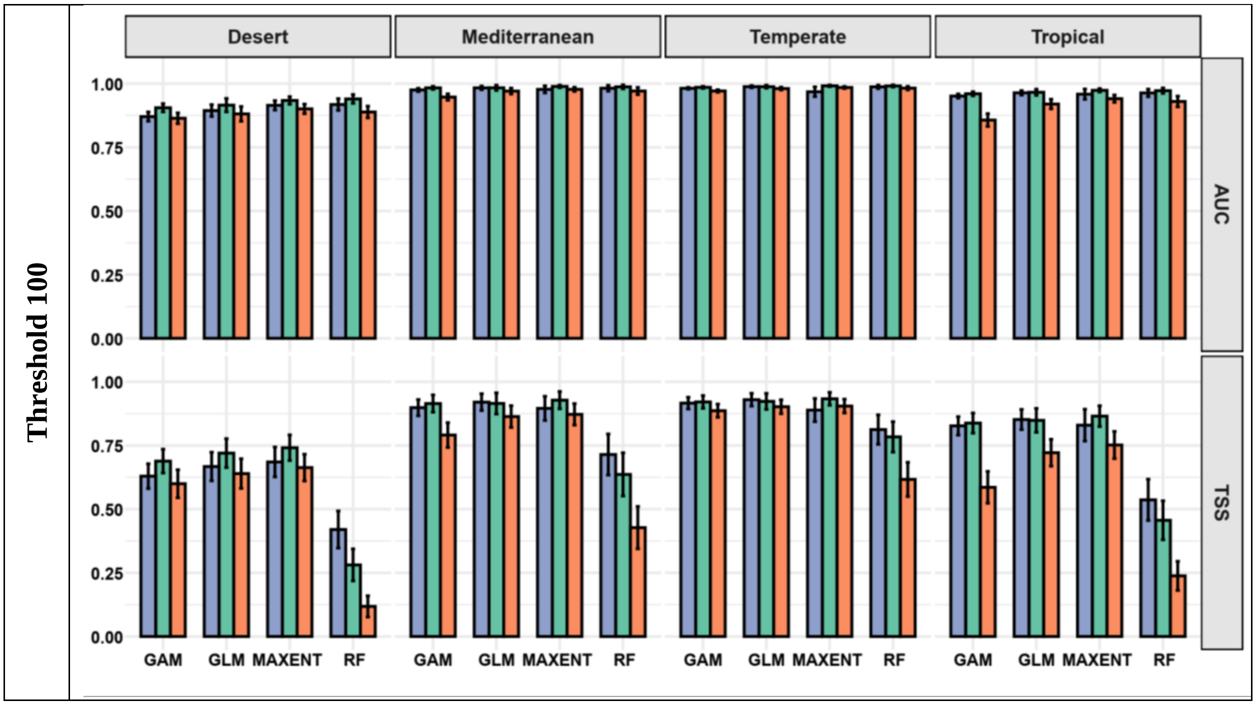

Figure S2. SDM assessment results of generalized linear model (GLM), generalized additive model (GAM), generalized boosting model (GBM), and maximum entropy (MaxEnt) for 5,033 species across Australian ecoregions under 70%, 80% and 90% density occurrence threshold

Table S3. Average relative contribution of variables in climate + soil models across all species in each ecoregion under 70%, 80%, and 90% density occurrence thresholds

		Threshold 70				Threshold 80				Threshold 90			
		Deserts	Mediterranean	Temperate	Tropical	Deserts	Mediterranean	Temperate	Tropical	Deserts	Mediterranean	Temperate	Tropical
**Climatic variables**	bio1	13.11	33.7	18.04	16.84	12.39	33.65	17.39	16.43	10.04	33.51	16	15.88
	bio2	3.64	1.3	3.17	2.5	3.83	1.22	3.1	2.44	4.15	1.13	3.11	2.47
	bio4	13.19	5.04	5.94	12.69	13.36	4.99	5.84	13.48	13.69	4.76	6.03	14.15
	bio5	8.81	6.39	15.35	3.93	8.46	6.13	15.73	3.56	8.15	5.82	16.5	3.11
	bio6	7.7	10.47	4.85	6.82	8.04	10.66	4.8	7.14	8.3	11.18	4.84	7.62
	bio12	10.03	6.15	6.74	16.28	10.2	6.06	6.62	16.29	10.4	5.89	6.37	16.34
	bio13	5.67	3.58	2.08	9.53	5.63	3.48	2.1	9.79	5.95	3.4	2.13	10.2
	bio14	0.85	2.02	16.83	1.46	0.73	1.92	17.63	1.3	0.67	1.77	18.37	1.08
	bio15	5.13	8.47	3.63	9.09	5.03	8.83	3.62	9.22	5.29	9.41	3.63	9.17
**Mean**		**7.57**	**8.57**	**8.51**	**8.79**	**7.52**	**8.55**	**8.54**	**8.85**	**7.41**	**8.54**	**8.55**	**8.89**
**Soil variables**	BDW	2.49	2.59	4.01	2	2.44	2.65	4.19	1.95	2.43	2.66	4.39	1.9
	CEC	5.54	3.36	1.97	2.41	5.41	3.3	1.8	2.44	5.56	3.17	1.67	2.47
	CLY	3.6	6.05	2.18	3.35	3.53	6.11	2.04	3.28	3.56	6.2	1.89	3.16
	DES	3.37	0.88	0.88	0.82	3.34	0.86	0.8	0.74	3.08	0.84	0.75	0.64
	NTO	8.24	3.05	3.29	3.3	8.86	3.06	3.36	3.35	9.75	3	3.61	3.34
	PTO	2.03	1.66	0.61	1.09	2.03	1.65	0.53	1.04	2	1.61	0.45	0.97
	SOC	3.83	5.11	9.12	6.38	3.89	5.25	9.18	6.13	4.12	5.48	9.01	6.15
	TWI	2.77	0.19	1.32	1.51	2.87	0.19	1.28	1.42	2.86	0.2	1.28	1.34
**Mean**		**3.98**	**2.86**	**2.92**	**2.61**	**4.04**	**2.88**	**2.9**	**2.54**	**4.17**	**2.89**	**2.88**	**2.5**

Table S4: Continental-scale performance metrics (AUC and TSS) for each modeling algorithm (GAM, GLM, MaxEnt, RF) under different thresholds and environmental variable combinations (climate only, soil only, climate + soil). Performance was calculated as the average across all 5,033 species. This summary provides an overall assessment of model accuracy at the continental level, complementing the regional-scale evaluations presented in the main text.

	Threshold	Model	AUC.mean	TSS.mean	AUC.sd	TSS.sd
soil	60	GAM	0.908	0.72	0.014	0.044
		GLM	0.939	0.784	0.013	0.041
		MAXENT	0.951	0.799	0.01	0.041
		RF	0.944	0.394	0.015	0.06
	70	GAM	0.913	0.729	0.014	0.044
		GLM	0.943	0.794	0.013	0.041
		MAXENT	0.954	0.808	0.01	0.041
		RF	0.947	0.402	0.015	0.061
	80	GAM	0.917	0.739	0.014	0.044
		GLM	0.946	0.804	0.013	0.041
		MAXENT	0.958	0.818	0.01	0.04
		RF	0.951	0.411	0.014	0.063
	90	GAM	0.925	0.756	0.014	0.044
		GLM	0.953	0.82	0.013	0.041
		MAXENT	0.964	0.834	0.009	0.04
		RF	0.957	0.422	0.014	0.066
	100	GAM	0.943	0.794	0.012	0.044
		GLM	0.967	0.857	0.01	0.04
		MAXENT	0.976	0.868	0.007	0.039
		RF	0.97	0.459	0.012	0.075
climate	60	GAM	0.946	0.821	0.008	0.032
		GLM	0.959	0.847	0.009	0.034
		MAXENT	0.956	0.83	0.016	0.048
		RF	0.963	0.645	0.012	0.068
	70	GAM	0.95	0.831	0.008	0.032
		GLM	0.963	0.856	0.008	0.034
		MAXENT	0.958	0.837	0.016	0.048
		RF	0.966	0.654	0.012	0.069
	80	GAM	0.954	0.843	0.007	0.032
		GLM	0.966	0.867	0.008	0.034
		MAXENT	0.96	0.845	0.016	0.049
		RF	0.969	0.663	0.012	0.07
	90	GAM	0.962	0.863	0.007	0.031
		GLM	0.972	0.883	0.008	0.034
		MAXENT	0.964	0.858	0.017	0.051
		RF	0.973	0.678	0.012	0.071
	100	GAM	0.977	0.905	0.005	0.029

		GLM	0.984	0.919	0.005	0.032
		MAXENT	0.968	0.881	0.02	0.055
		RF	0.983	0.714	0.009	0.075
**climate + soil**	60	GAM	0.958	0.839	0.008	0.034
		GLM	0.965	0.855	0.011	0.039
		MAXENT	0.972	0.868	0.006	0.034
		RF	0.973	0.578	0.009	0.067
	70	GAM	0.961	0.849	0.007	0.033
		GLM	0.967	0.862	0.011	0.039
		MAXENT	0.974	0.875	0.006	0.034
		RF	0.975	0.588	0.009	0.067
	80	GAM	0.965	0.859	0.007	0.033
		GLM	0.97	0.87	0.01	0.04
		MAXENT	0.977	0.883	0.006	0.034
		RF	0.977	0.598	0.008	0.069
	90	GAM	0.971	0.877	0.007	0.033
		GLM	0.975	0.883	0.01	0.04
		MAXENT	0.981	0.897	0.006	0.034
		RF	0.98	0.613	0.008	0.071
	100	GAM	0.983	0.914	0.004	0.032
		GLM	0.983	0.91	0.009	0.041
		MAXENT	0.989	0.926	0.004	0.032
		RF	0.988	0.653	0.006	0.077

	Density climatic variable
**Desert**	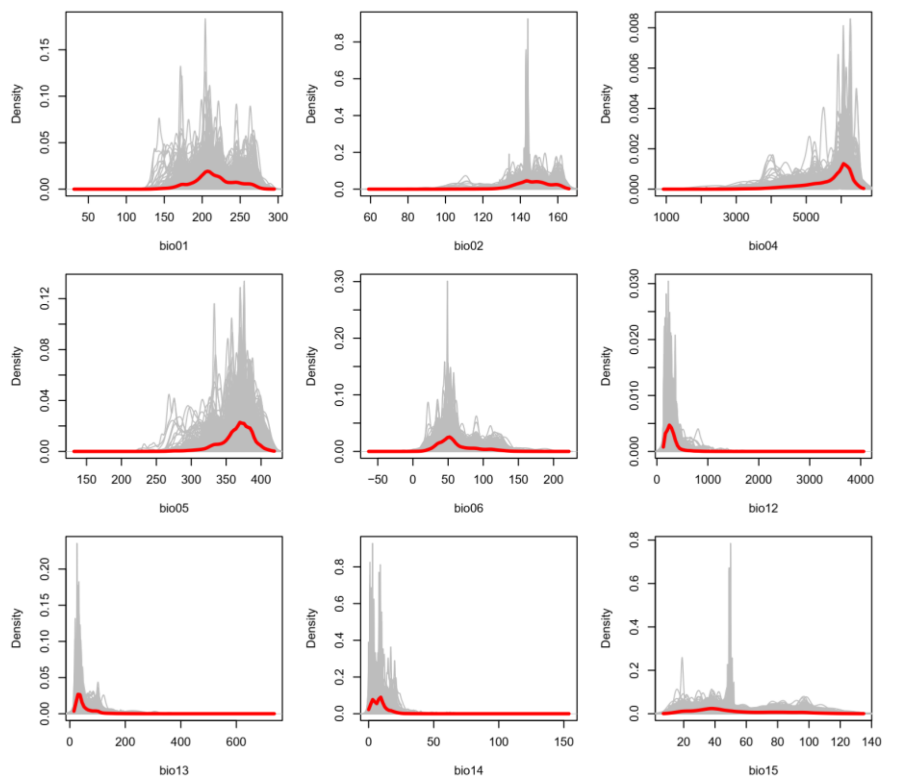
**Mediterranean**	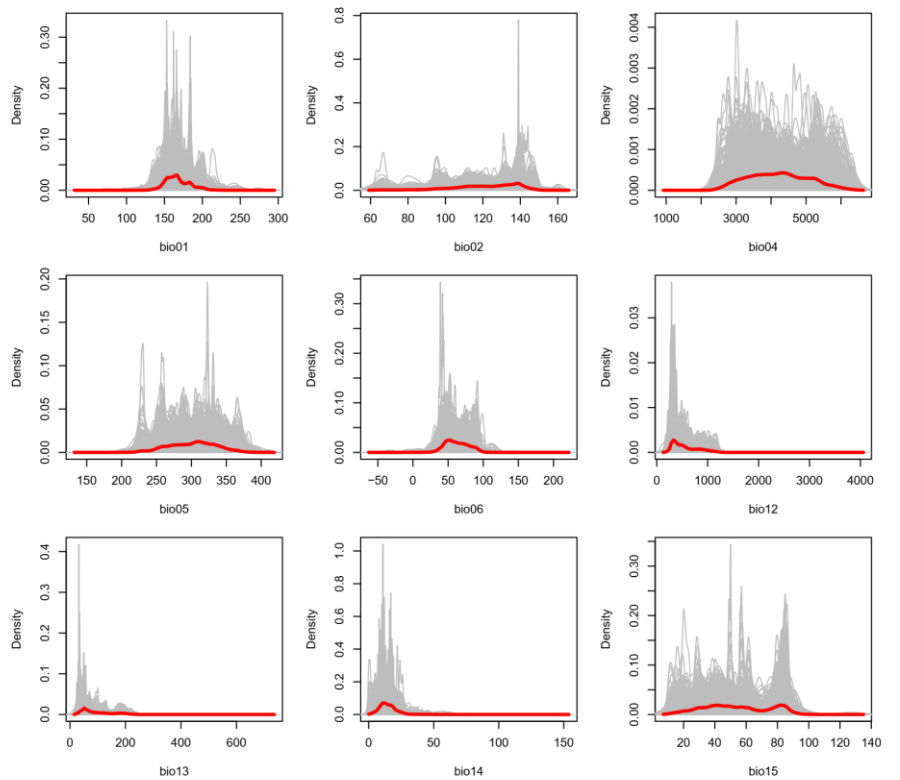

Temperate

Tropical

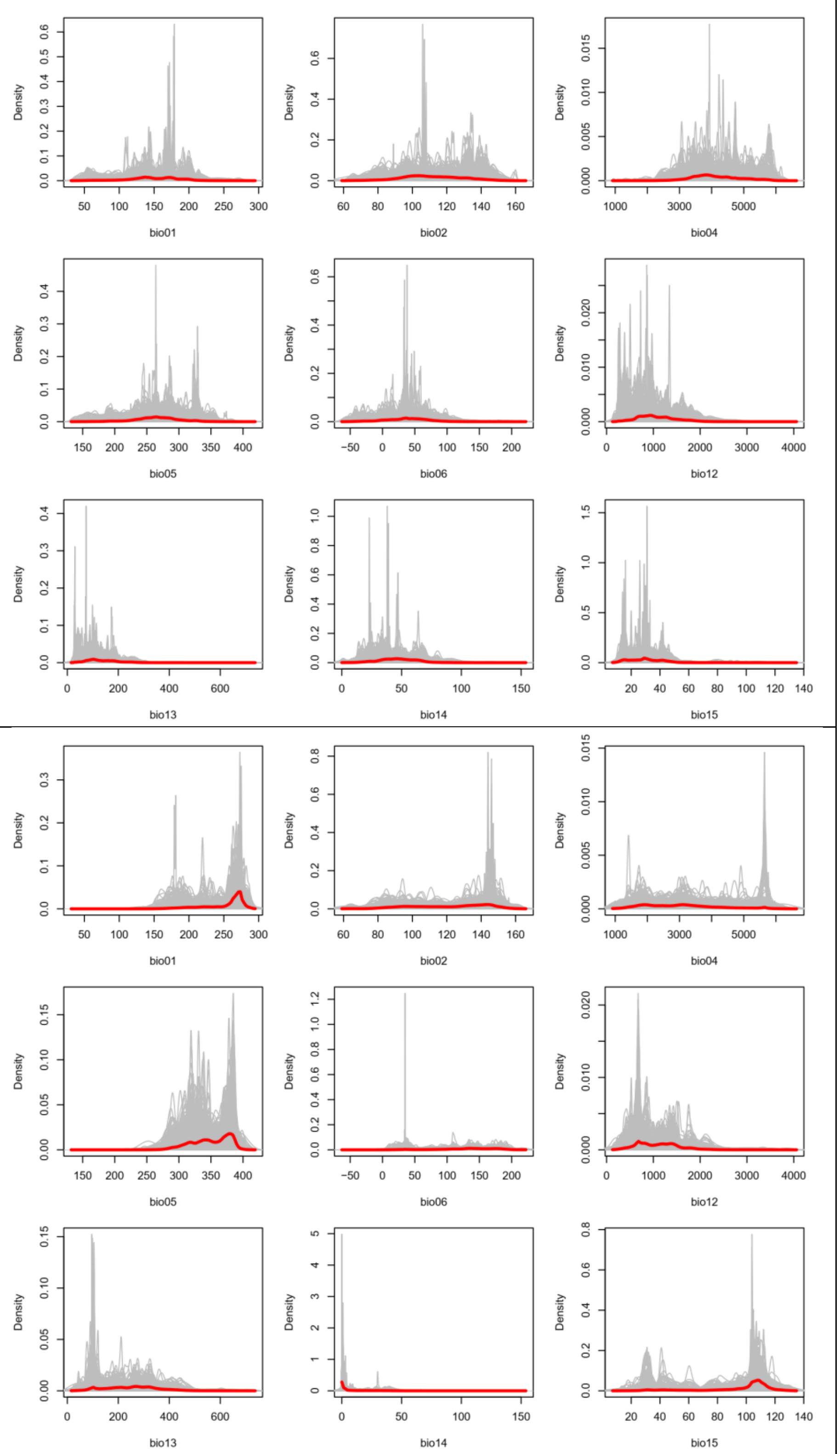

Density Soil variable

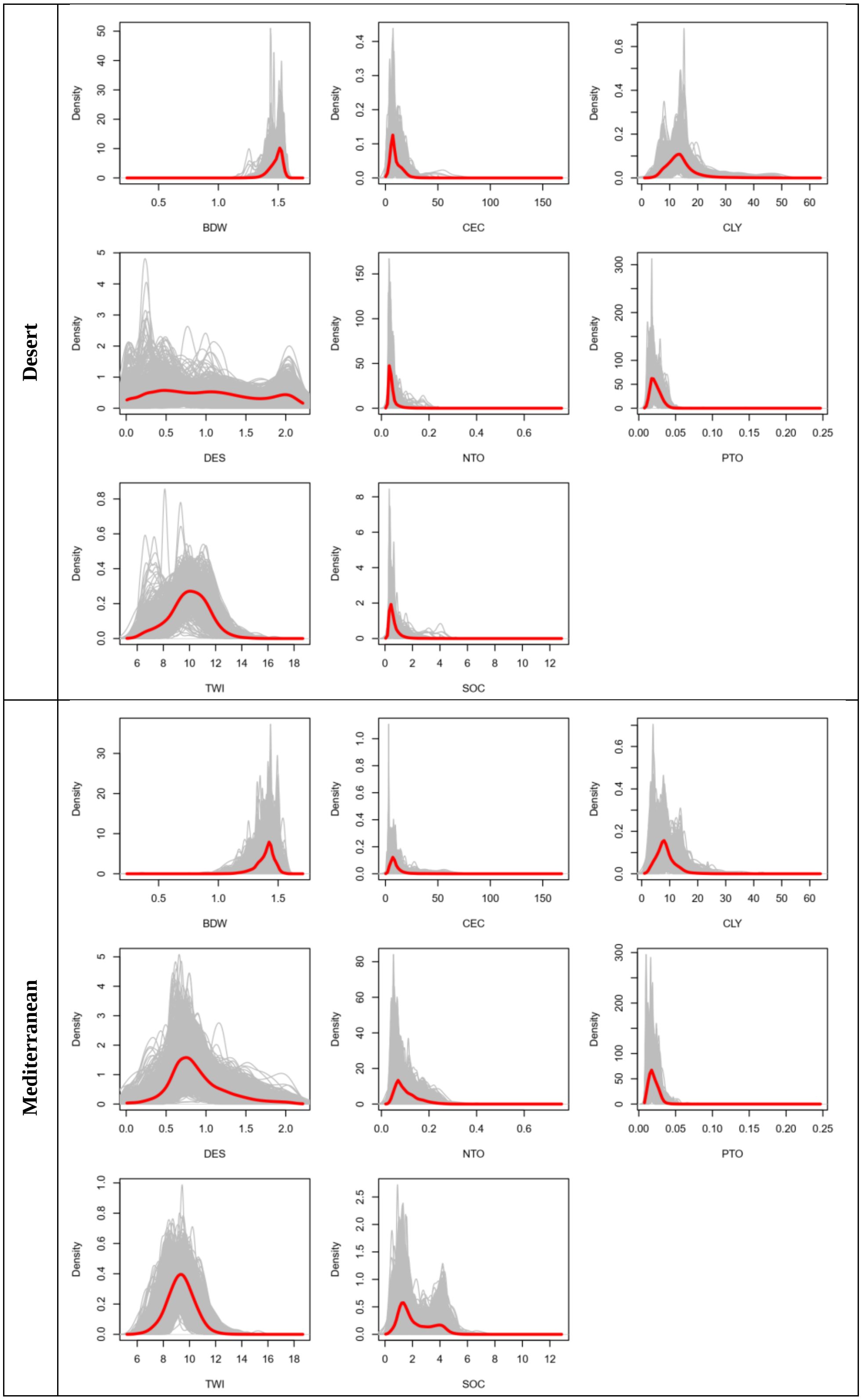
Desert
Mediterranean
Density
BDW
CEC
CLY
DES
NTO
PTO
TWI
SOC

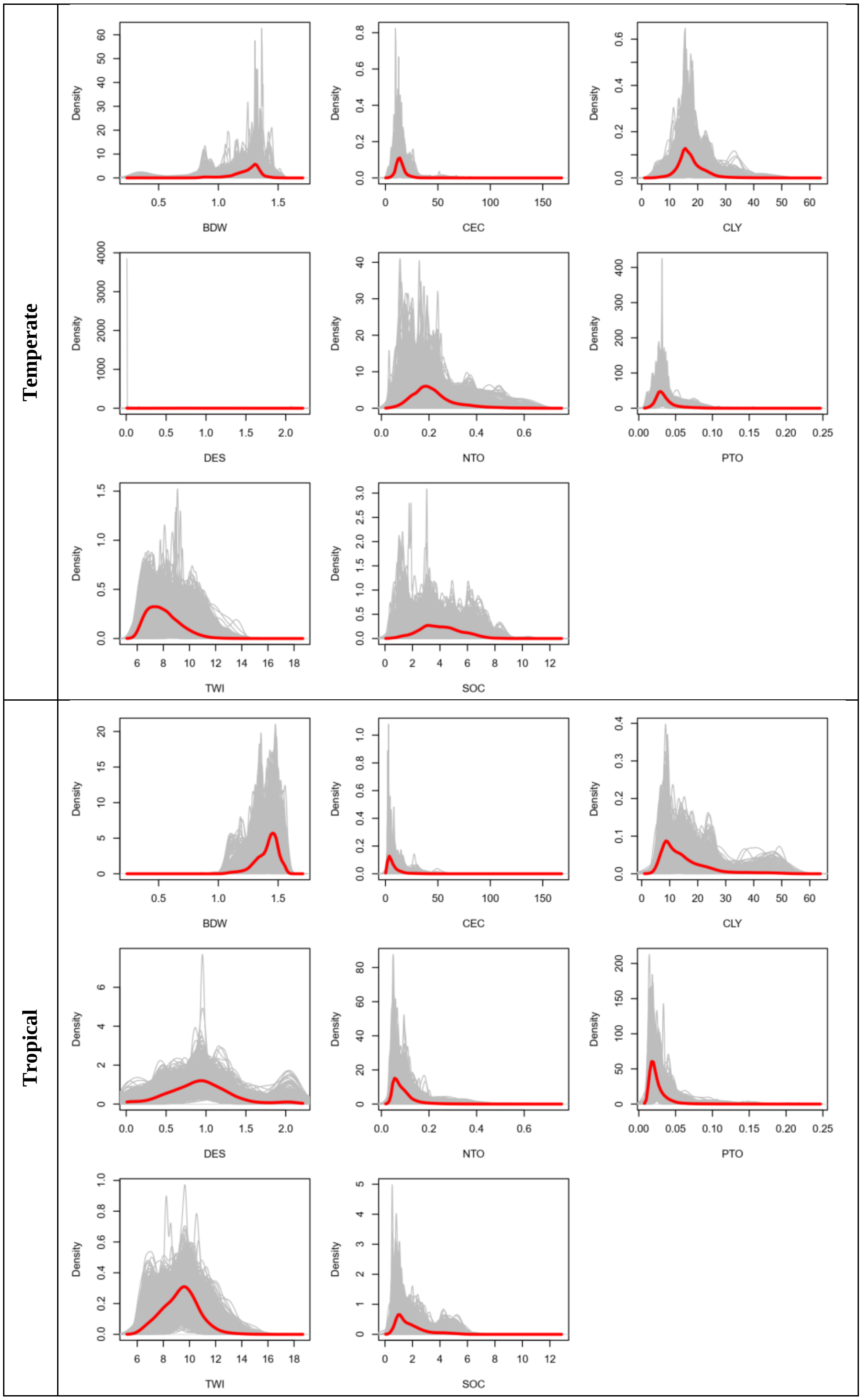
Temperate
Tropical
Density
BDW
CEC
CLY
DES
NTO
PTO
TWI
SOC

Figure S3. The individual density plots for each species within ecoregions in relation to environmental variables

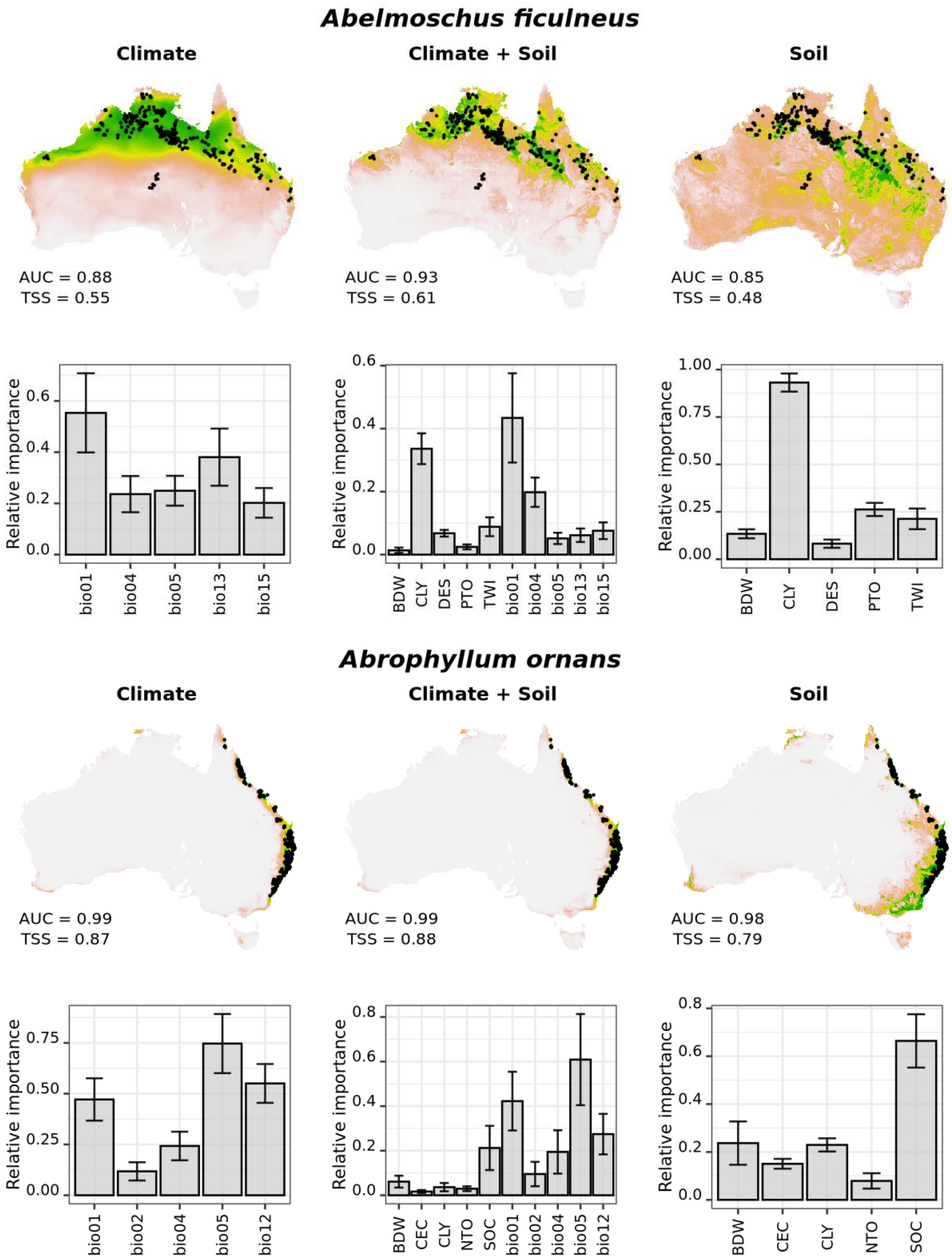
Abelmoschus ficulneus
Climate
Climate + Soil
Soil
AUC = 0.88
TSS = 0.55
AUC = 0.93
TSS = 0.61
AUC = 0.85
TSS = 0.48
Relative importance
0.0
0.2
0.4
0.6
bio01
bio04
bio05
bio13
bio15
Relative importance
0.0
0.2
0.4
0.6
BDW
CLY
DES
PTO
TWI
bio01
bio04
bio05
bio13
bio15
Relative importance
0.00
0.25
0.50
0.75
1.00
BDW
CLY
DES
PTO
TWI
Abrophyllum ornans
Climate
Climate + Soil
Soil
AUC = 0.99
TSS = 0.87
AUC = 0.99
TSS = 0.88
AUC = 0.98
TSS = 0.79
Relative importance
0.00
0.25
0.50
0.75
bio01
bio02
bio04
bio05
bio12
Relative importance
0.0
0.2
0.4
0.6
0.8
BDW
CEC
CLY
NTO
SOC
bio01
bio02
bio04
bio05
bio12
Relative importance
0.0
0.2
0.4
0.6
0.8
BDW
CEC
CLY
NTO
SOC

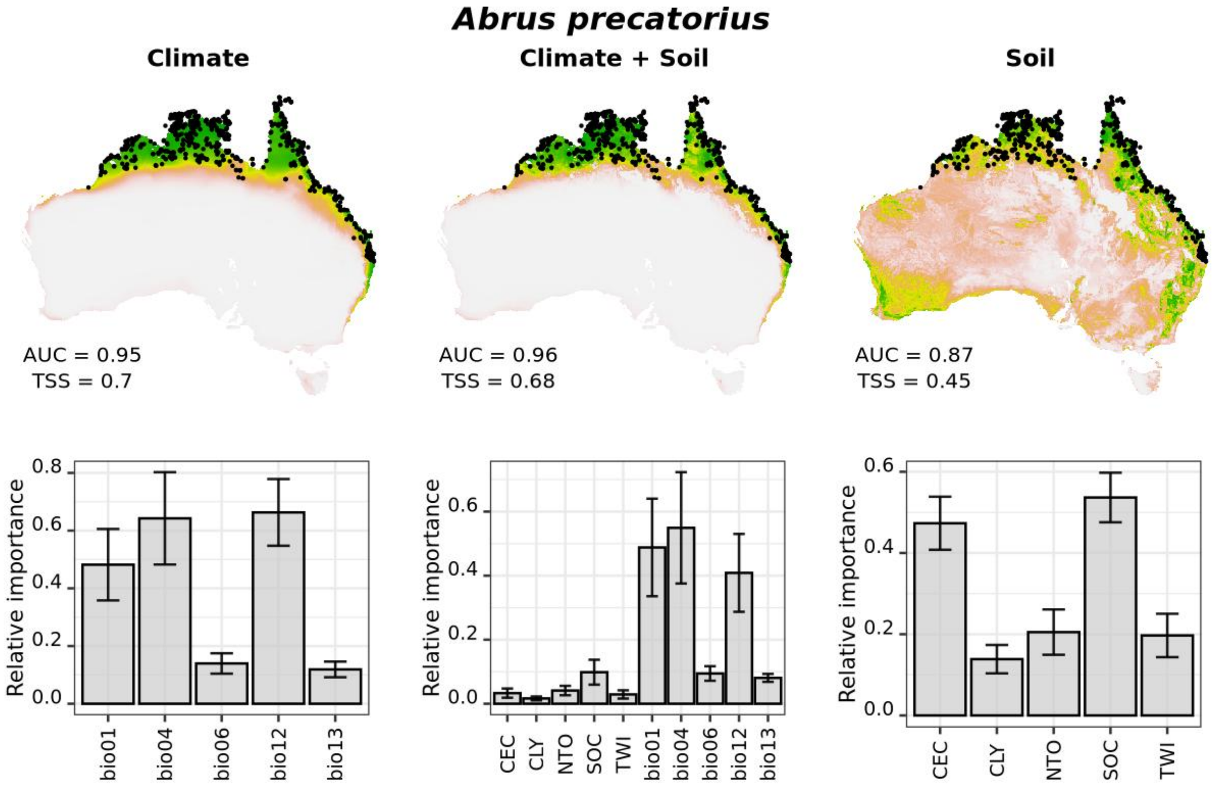

Abrus precatorius
Climate
Climate + Soil
Soil
AUC = 0.95
TSS = 0.7
AUC = 0.96
TSS = 0.68
AUC = 0.87
TSS = 0.45
Relative importance
bio01
bio04
bio06
bio12
bio13
CEC
CLY
NTO
SOC
TWI

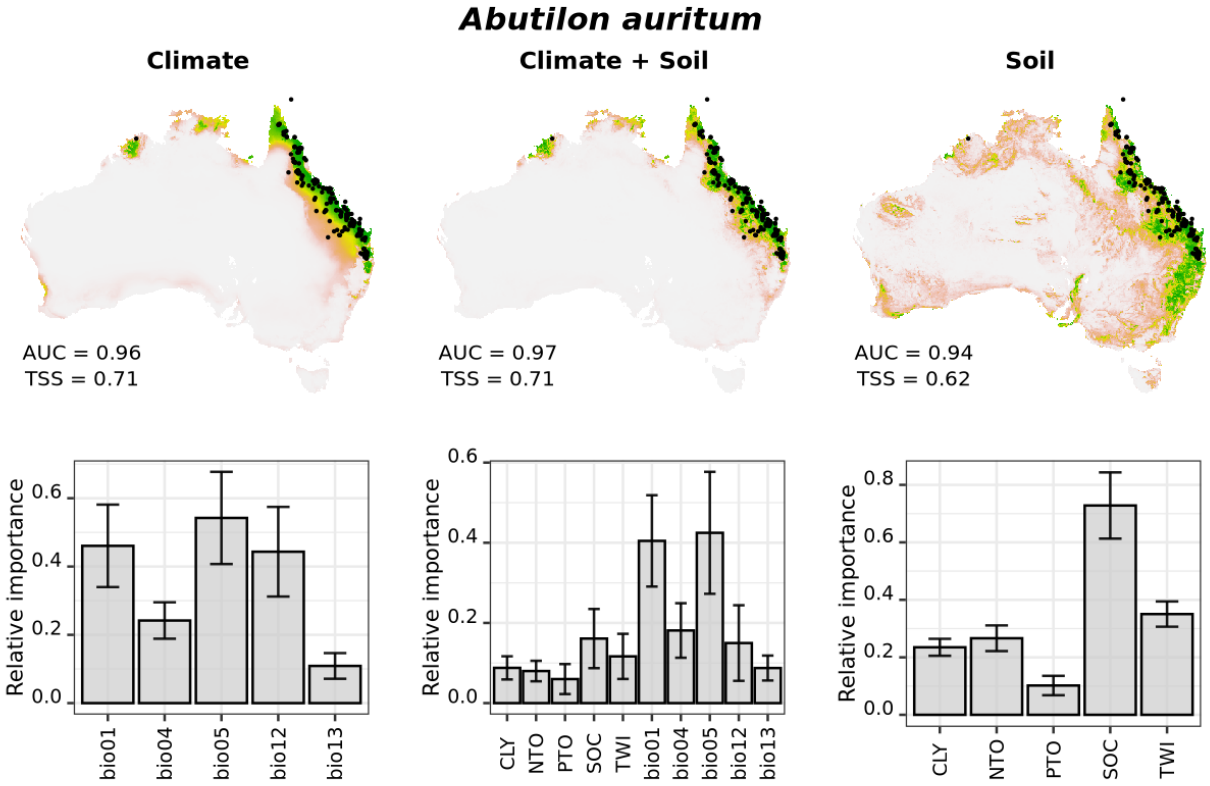

Abutilon auritum
Climate
Climate + Soil
Soil
AUC = 0.96
TSS = 0.71
AUC = 0.97
TSS = 0.71
AUC = 0.94
TSS = 0.62
Relative importance
bio01
bio04
bio05
bio12
bio13
CLY
NTO
PTO
SOC
TWI

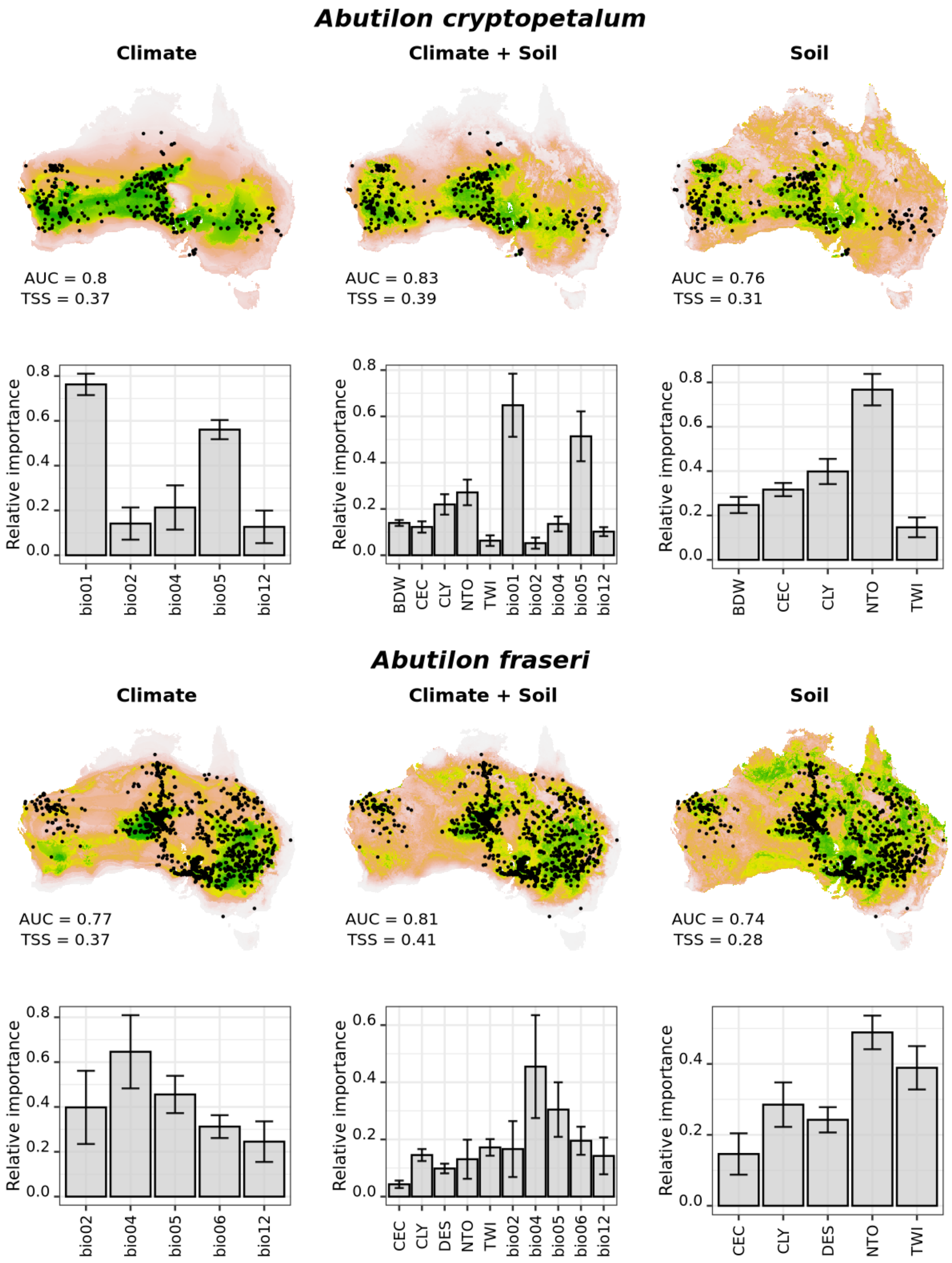
Abutilon cryptopetalum
Climate
Climate + Soil
Soil
AUC = 0.8
TSS = 0.37
AUC = 0.83
TSS = 0.39
AUC = 0.76
TSS = 0.31
Relative importance
bio01
bio02
bio04
bio05
bio12
BDW
CEC
CLY
NTO
TWI
Abutilon fraseri
AUC = 0.77
TSS = 0.37
AUC = 0.81
TSS = 0.41
AUC = 0.74
TSS = 0.28
bio06
DES

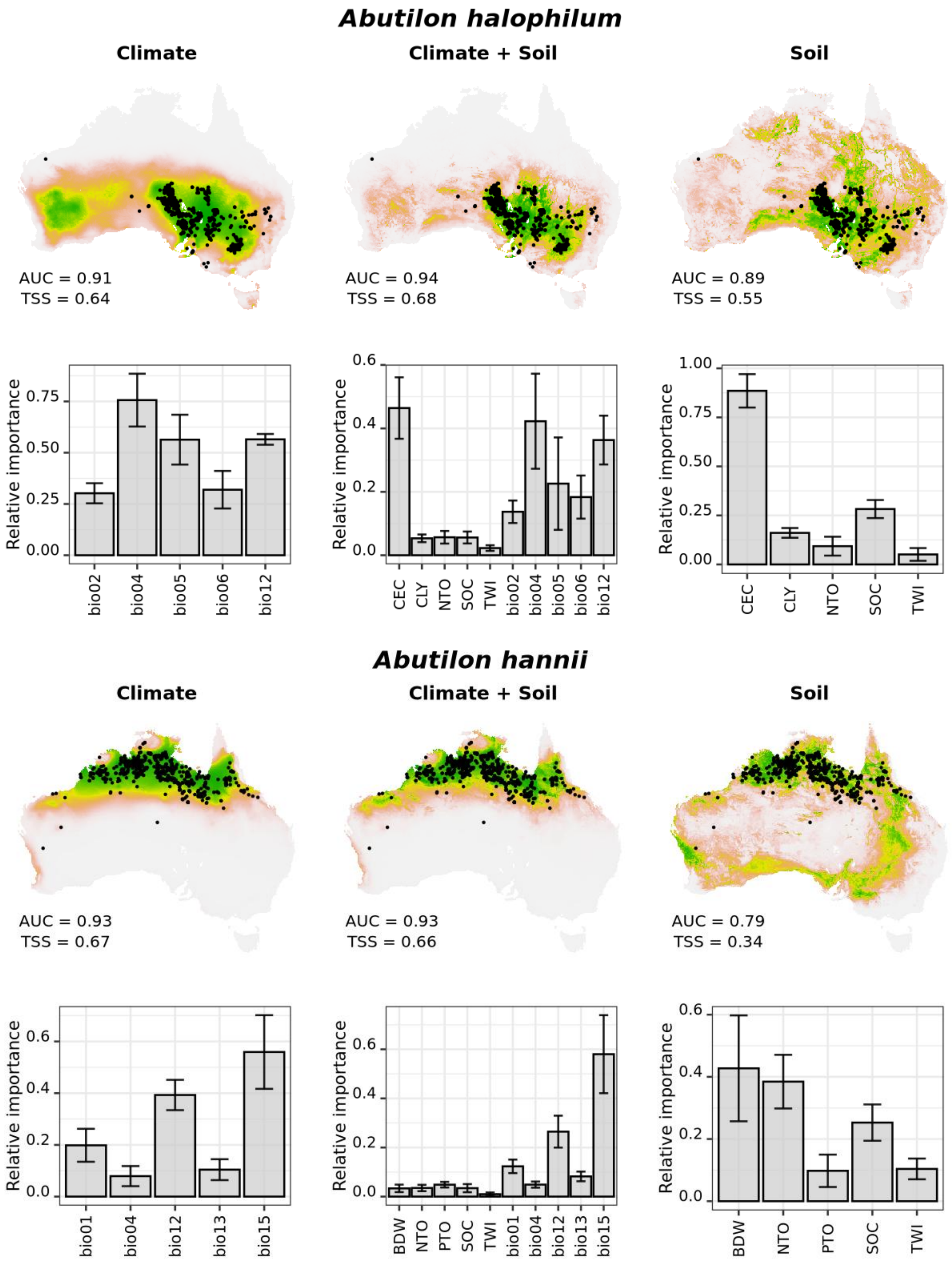
Abutilon halophilum
Climate
Climate + Soil
Soil
AUC = 0.91
TSS = 0.64
AUC = 0.94
TSS = 0.68
AUC = 0.89
TSS = 0.55
Relative importance
bio02 bio04 bio05 bio06 bio12
CEC CLY NTO SOC TWI bio02 bio04 bio05 bio06 bio12
CEC CLY NTO SOC TWI
Abutilon hannii
Climate
Climate + Soil
Soil
AUC = 0.93
TSS = 0.67
AUC = 0.93
TSS = 0.66
AUC = 0.79
TSS = 0.34
Relative importance
bio01 bio04 bio12 bio13 bio15
BDW NTO PTO SOC TWI bio01 bio04 bio12 bio13 bio15
BDW NTO PTO SOC TWI

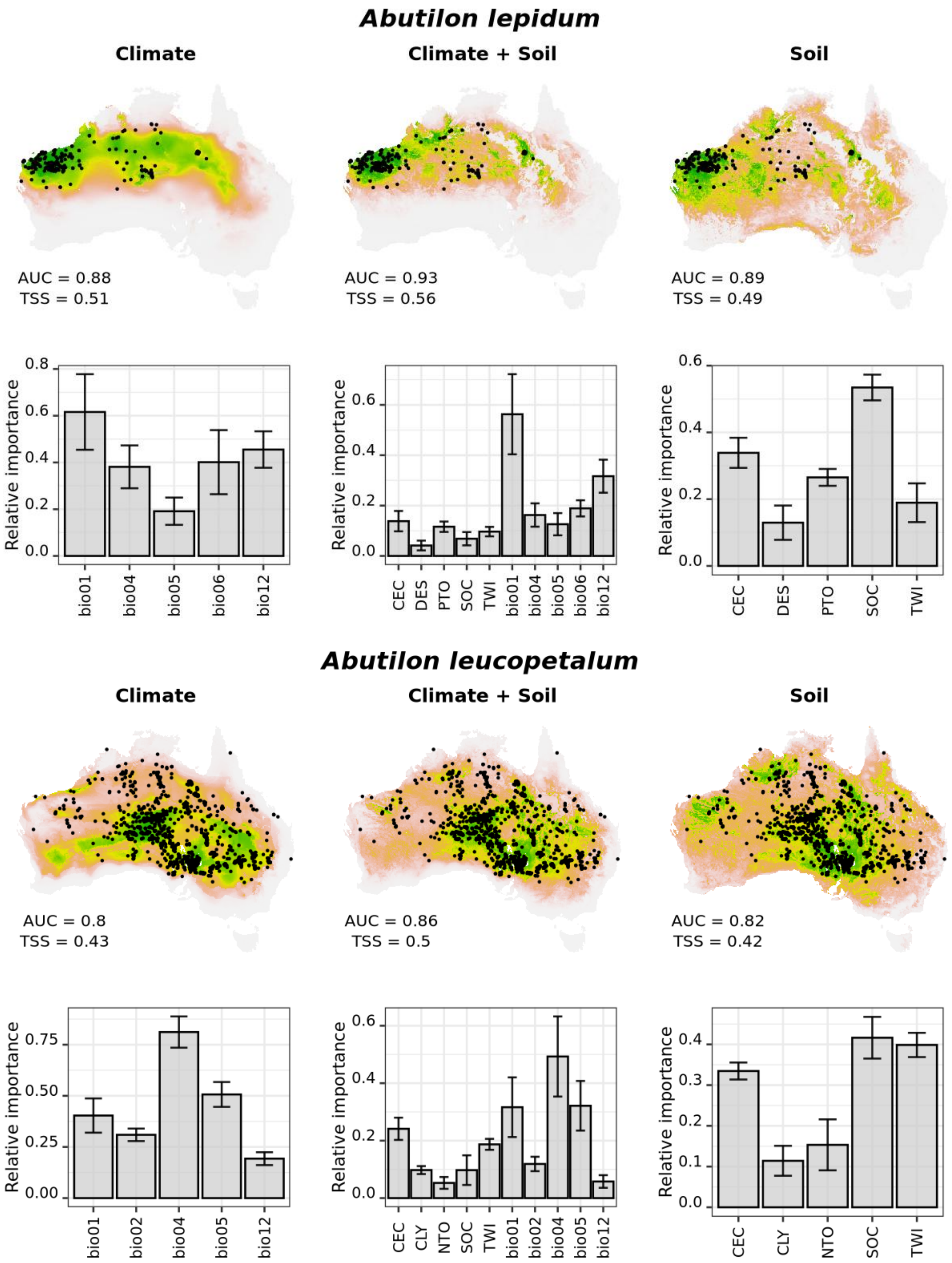
Abutilon lepidum
Climate
Climate + Soil
Soil
AUC = 0.88
TSS = 0.51
AUC = 0.93
TSS = 0.56
AUC = 0.89
TSS = 0.49
Relative importance
bio01
bio04
bio05
bio06
bio12
CEC
DES
PTO
SOC
TWI
Abutilon leucopetalum
Climate
Climate + Soil
Soil
AUC = 0.8
TSS = 0.43
AUC = 0.86
TSS = 0.5
AUC = 0.82
TSS = 0.42
Relative importance
bio01
bio02
bio04
bio05
bio12
CEC
CLY
NTO
SOC
TWI

Abutilon macrum

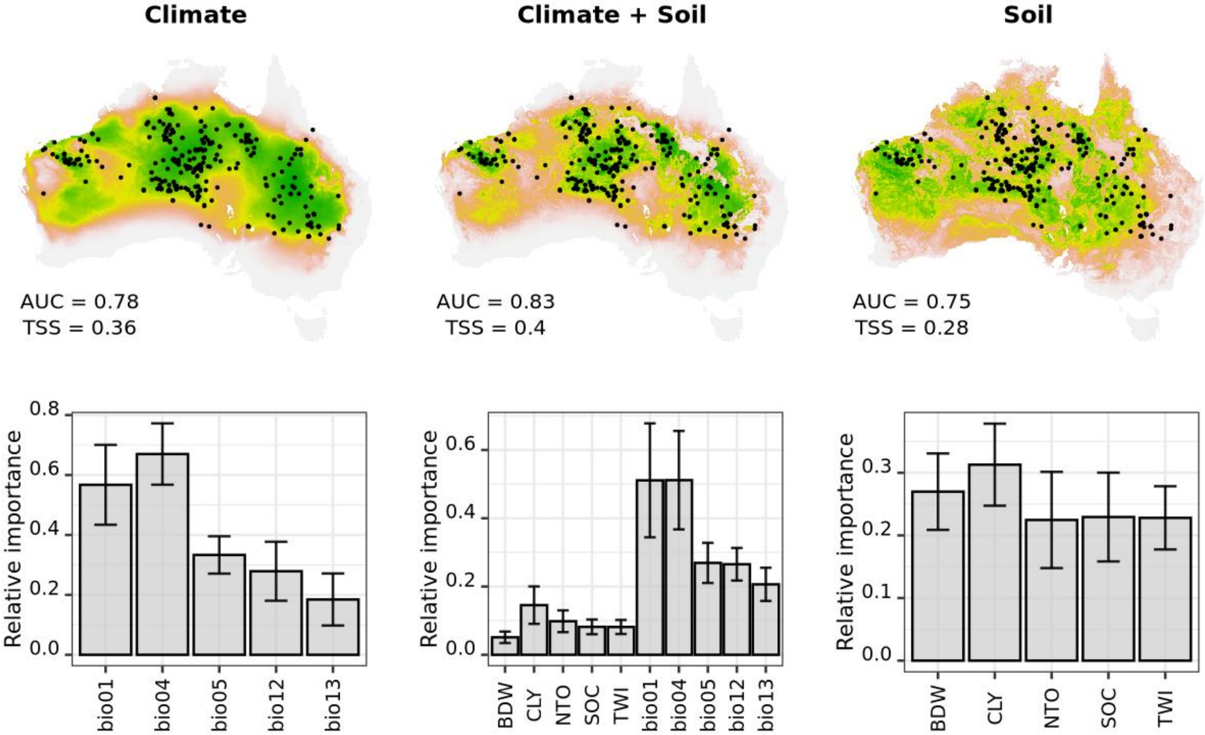

Abutilon malvifolium

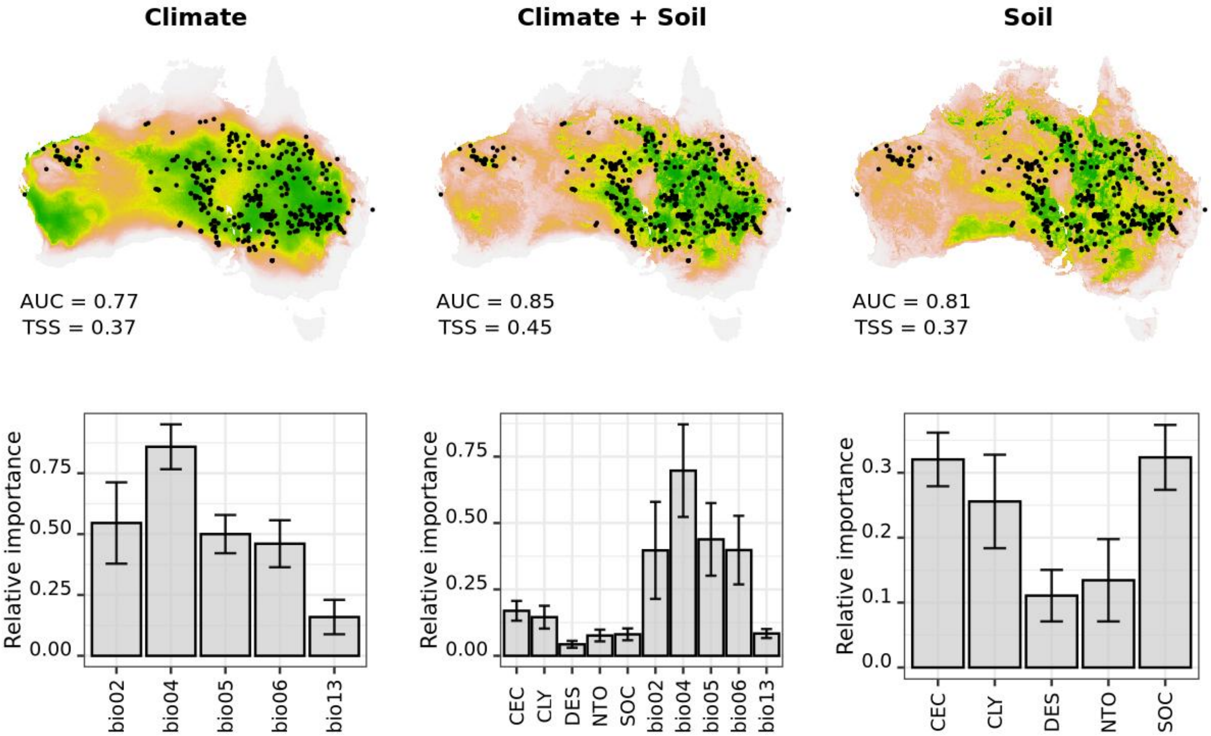

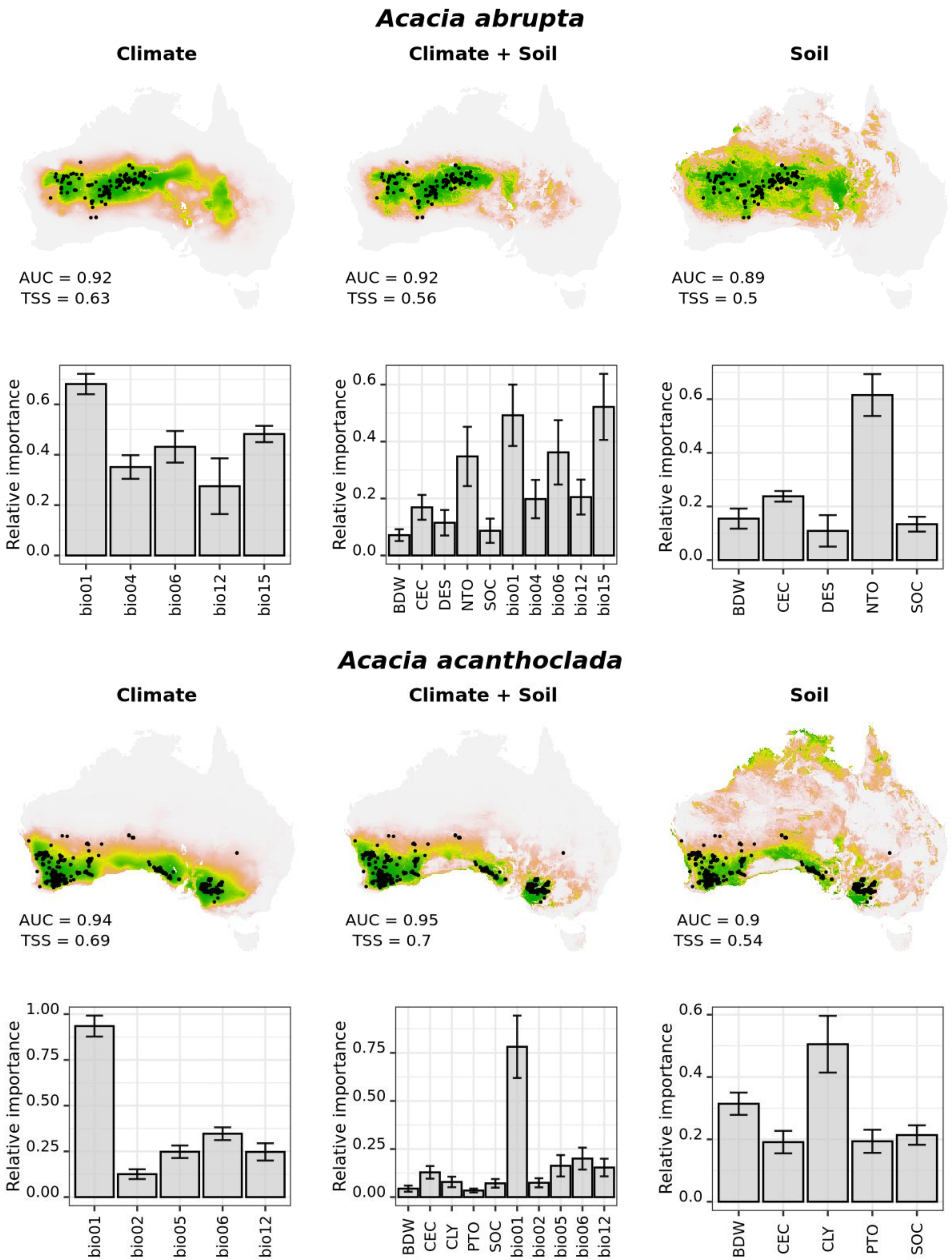
Acacia abrupta
Climate
Climate + Soil
Soil
AUC = 0.92
TSS = 0.63
AUC = 0.92
TSS = 0.56
AUC = 0.89
TSS = 0.5
Relative importance
bio01
bio04
bio06
bio12
bio15
BDW
CEC
DES
NTO
SOC
Acacia acanthoclada
Climate
Climate + Soil
Soil
AUC = 0.94
TSS = 0.69
AUC = 0.95
TSS = 0.7
AUC = 0.9
TSS = 0.54
Relative importance
bio01
bio02
bio05
bio06
bio12
BDW
CEC
CLY
PTO
SOC

Acacia acinacea

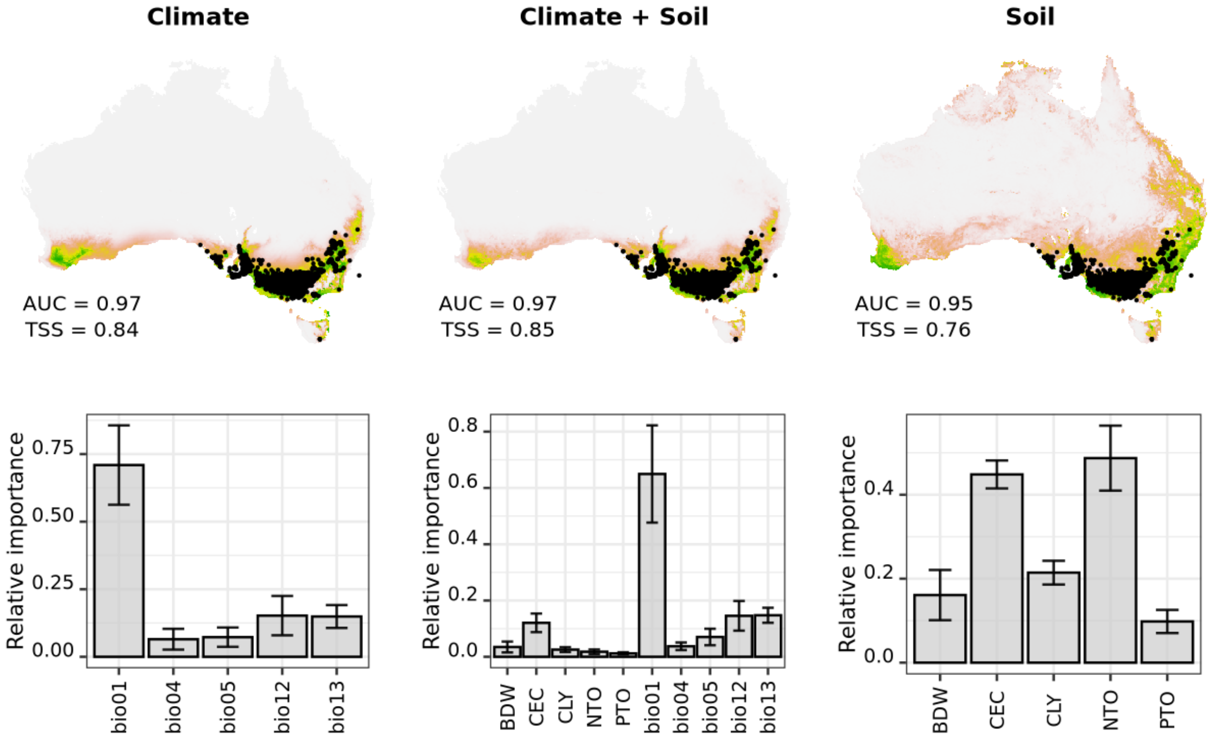

Acacia acuaria

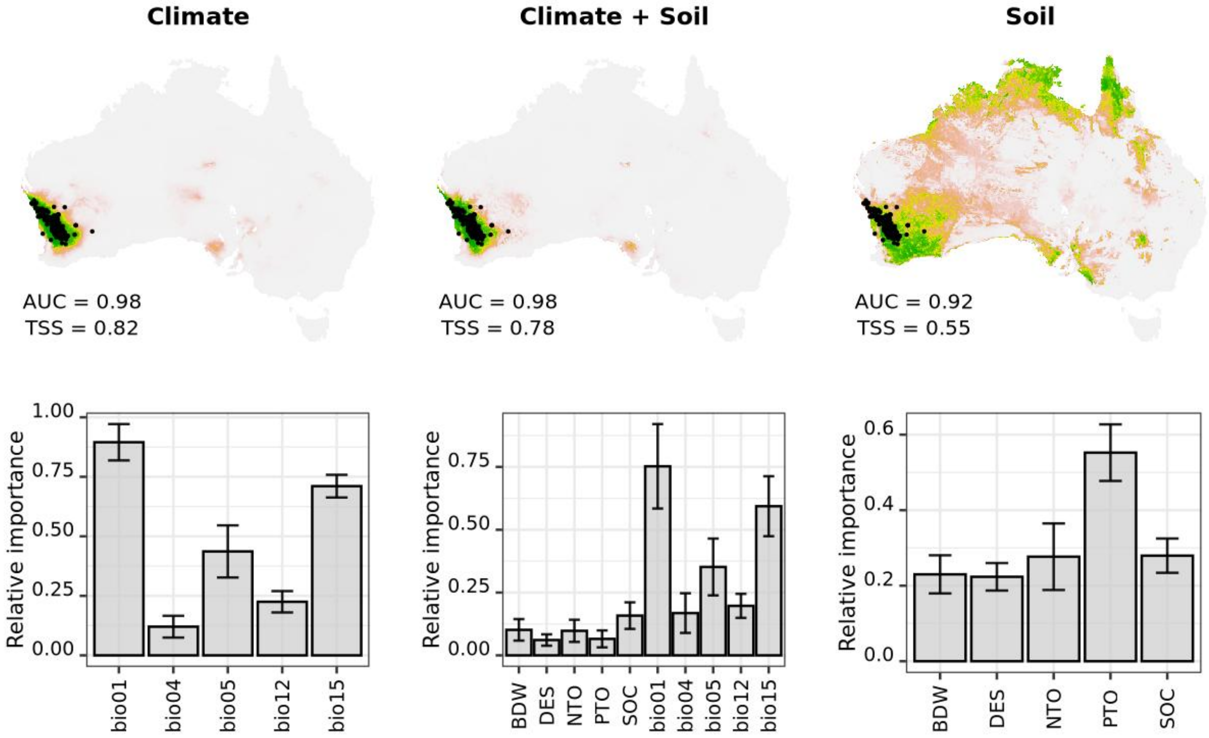

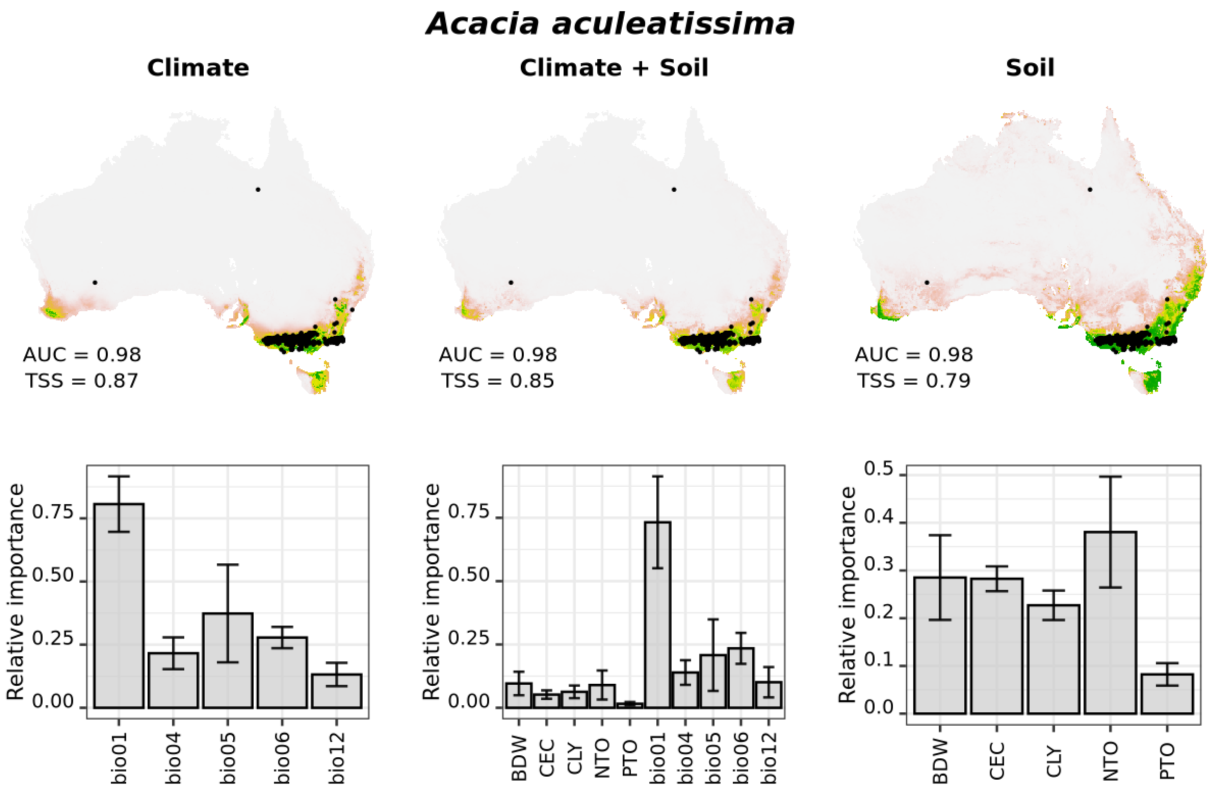
Acacia aculeatissima
Climate
Climate + Soil
Soil
AUC = 0.98
TSS = 0.87
AUC = 0.98
TSS = 0.85
AUC = 0.98
TSS = 0.79
Relative importance
bio01 bio04 bio05 bio06 bio12
BDW CEC CLY NTO PTO bio01 bio04 bio05 bio06 bio12
BDW CEC CLY NTO PTO

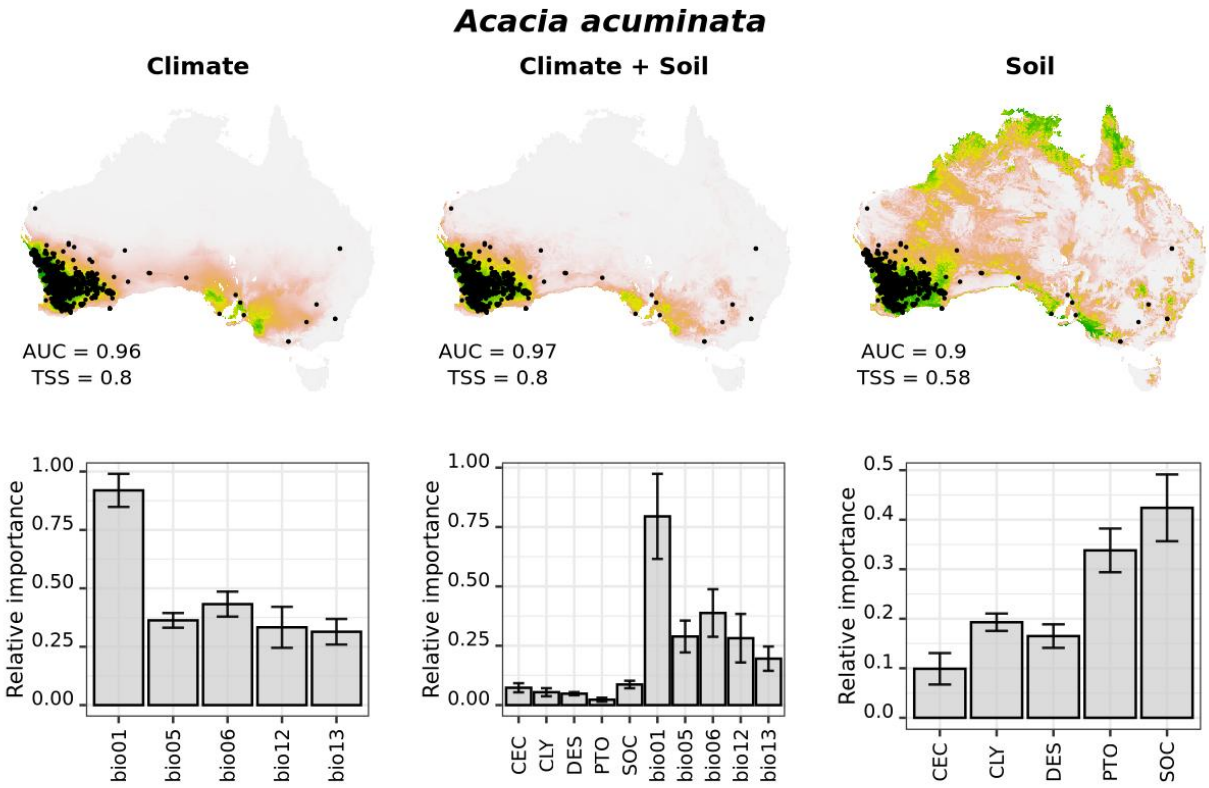
Acacia acuminata
Climate
Climate + Soil
Soil
AUC = 0.96
TSS = 0.8
AUC = 0.97
TSS = 0.8
AUC = 0.9
TSS = 0.58
Relative importance
bio01 bio05 bio06 bio12 bio13
CEC CLY DES PTO SOC bio01 bio05 bio06 bio12 bio13
CEC CLY DES PTO SOC

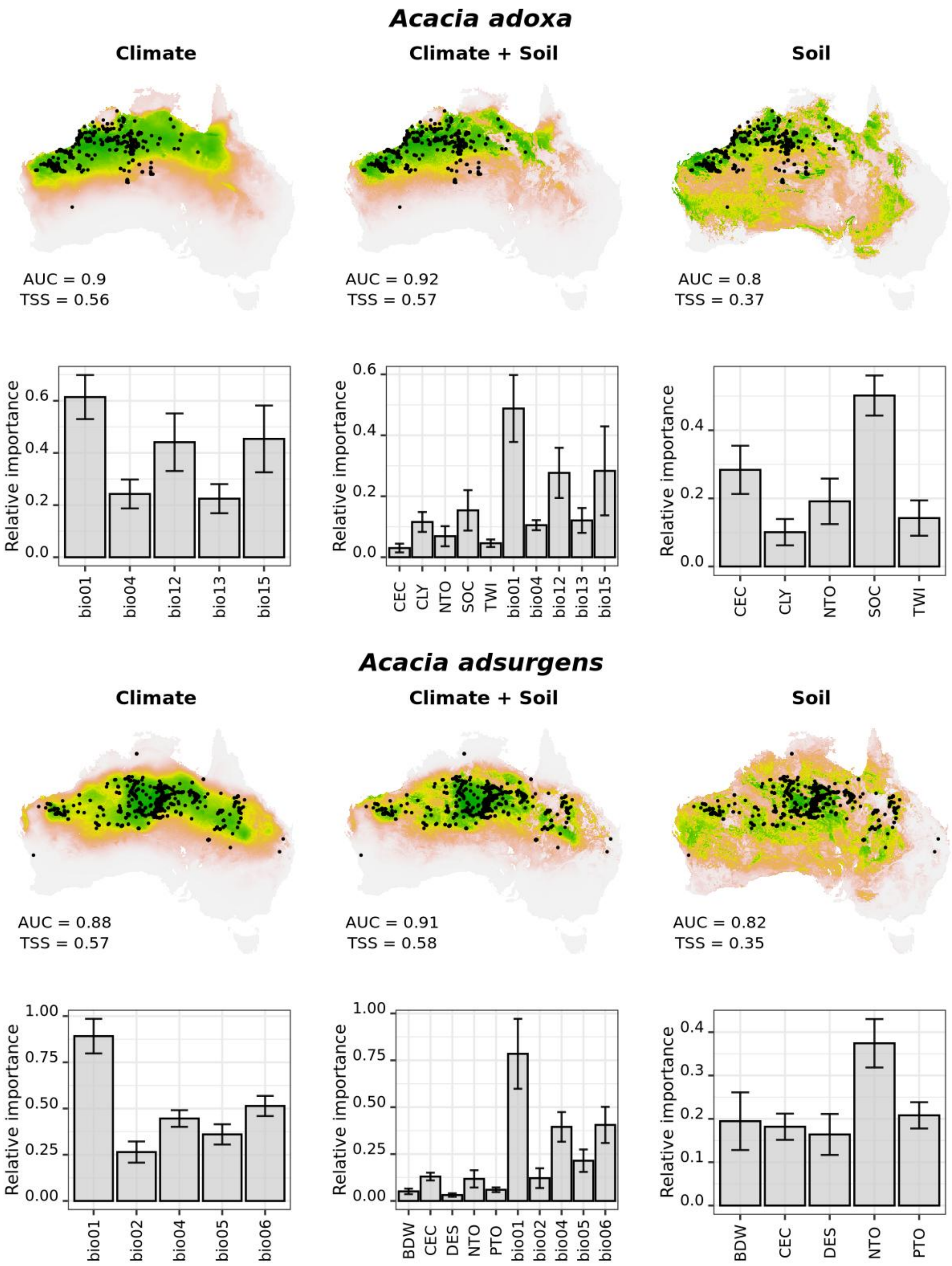

Acacia adoxa
Climate
Climate + Soil
Soil
AUC = 0.9
TSS = 0.56
AUC = 0.92
TSS = 0.57
AUC = 0.8
TSS = 0.37
Relative importance
bio01
bio04
bio12
bio13
bio15
CEC
CLY
NTO
SOC
TWI
Acacia adsurgens
Climate
Climate + Soil
Soil
AUC = 0.88
TSS = 0.57
AUC = 0.91
TSS = 0.58
AUC = 0.82
TSS = 0.35
Relative importance
bio01
bio02
bio04
bio05
bio06
BDW
CEC
DES
NTO
PTO

Acacia alata

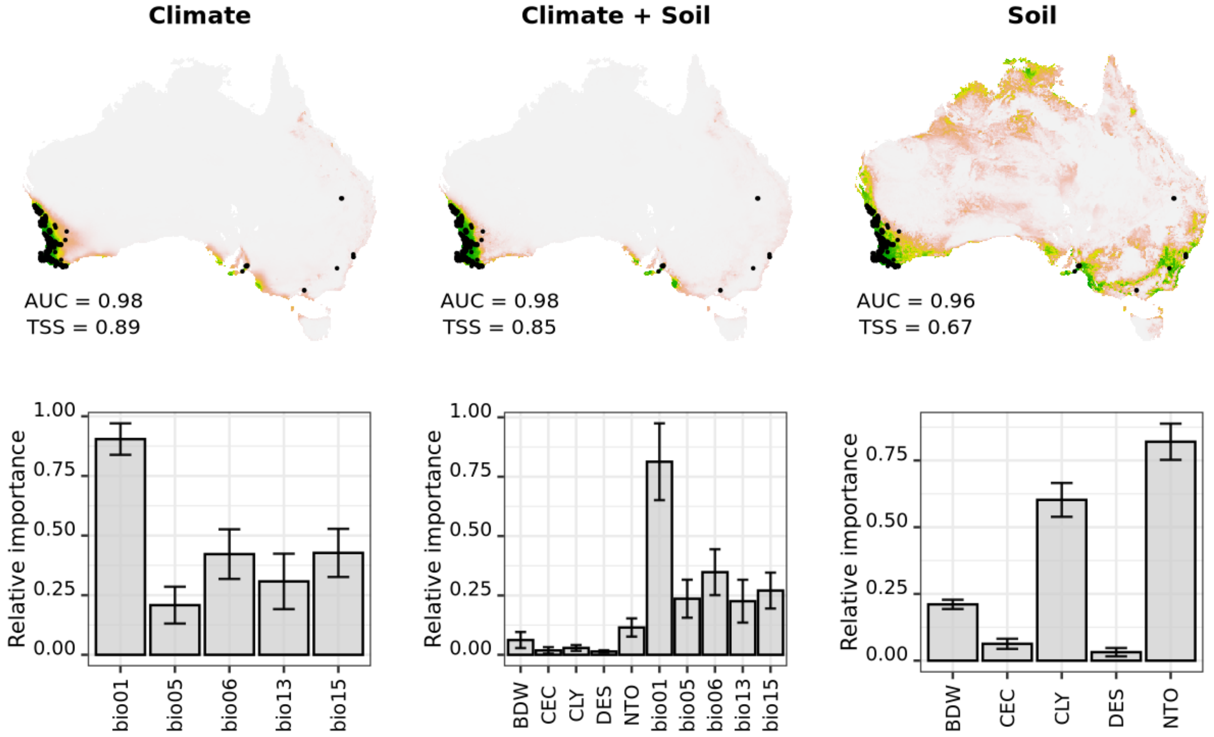

Acacia alleniana

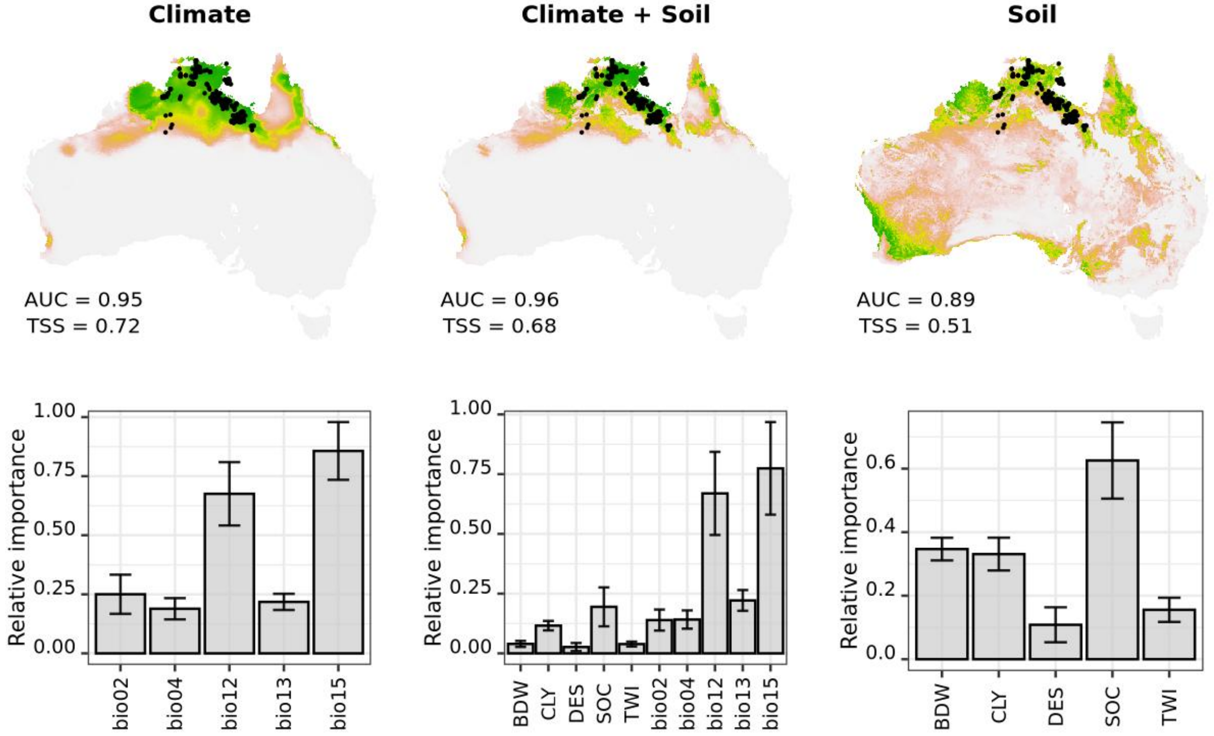

Acacia anceps

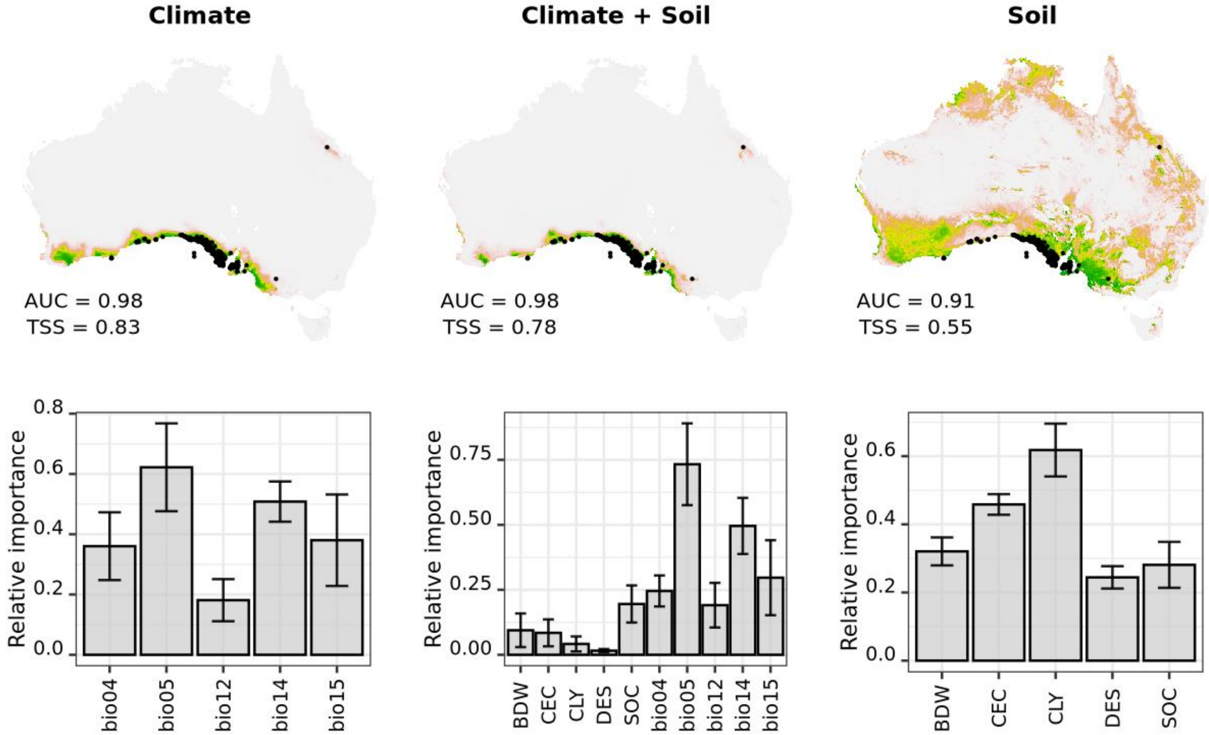

Acacia ancistrocarpa

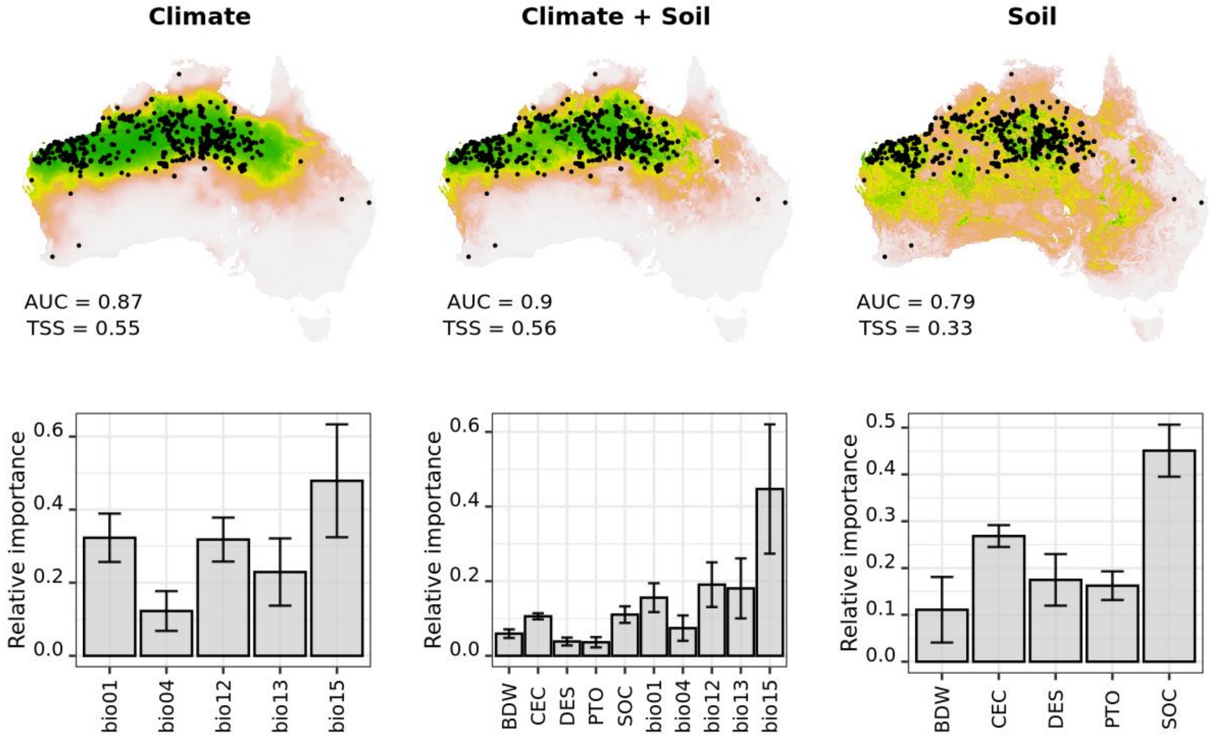

Acacia ancistrophylla

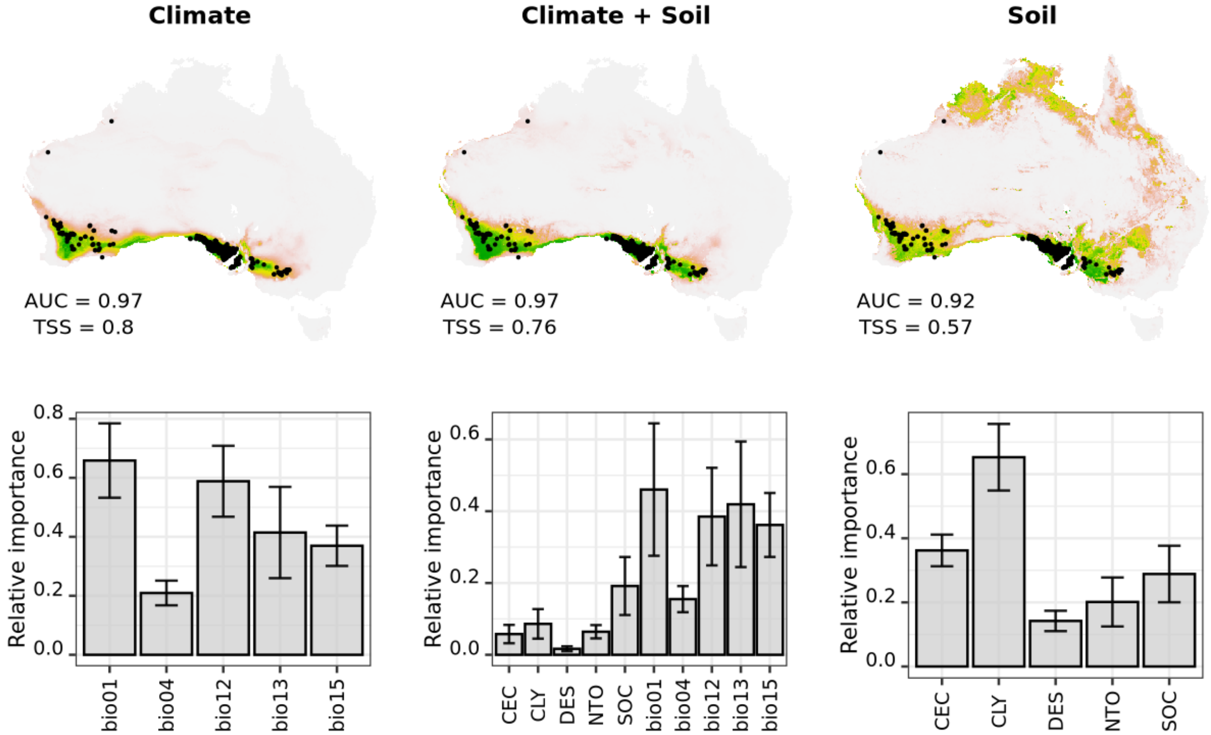

Acacia andrewsii

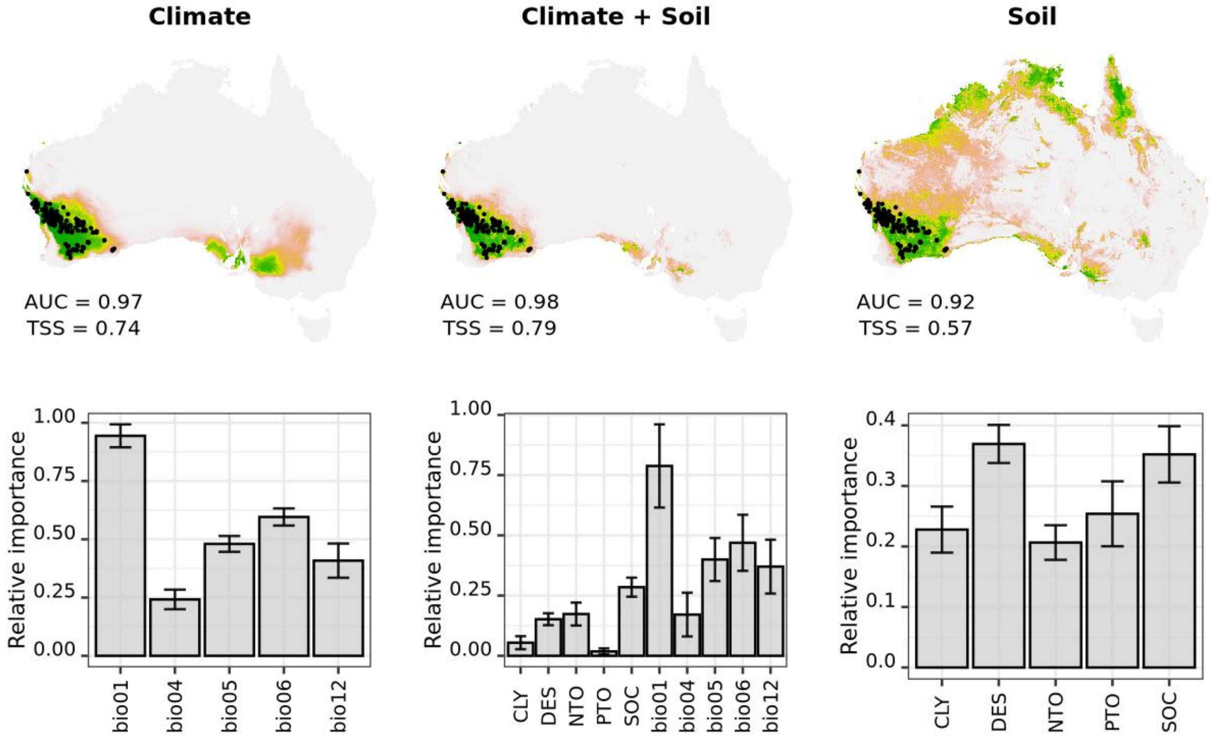

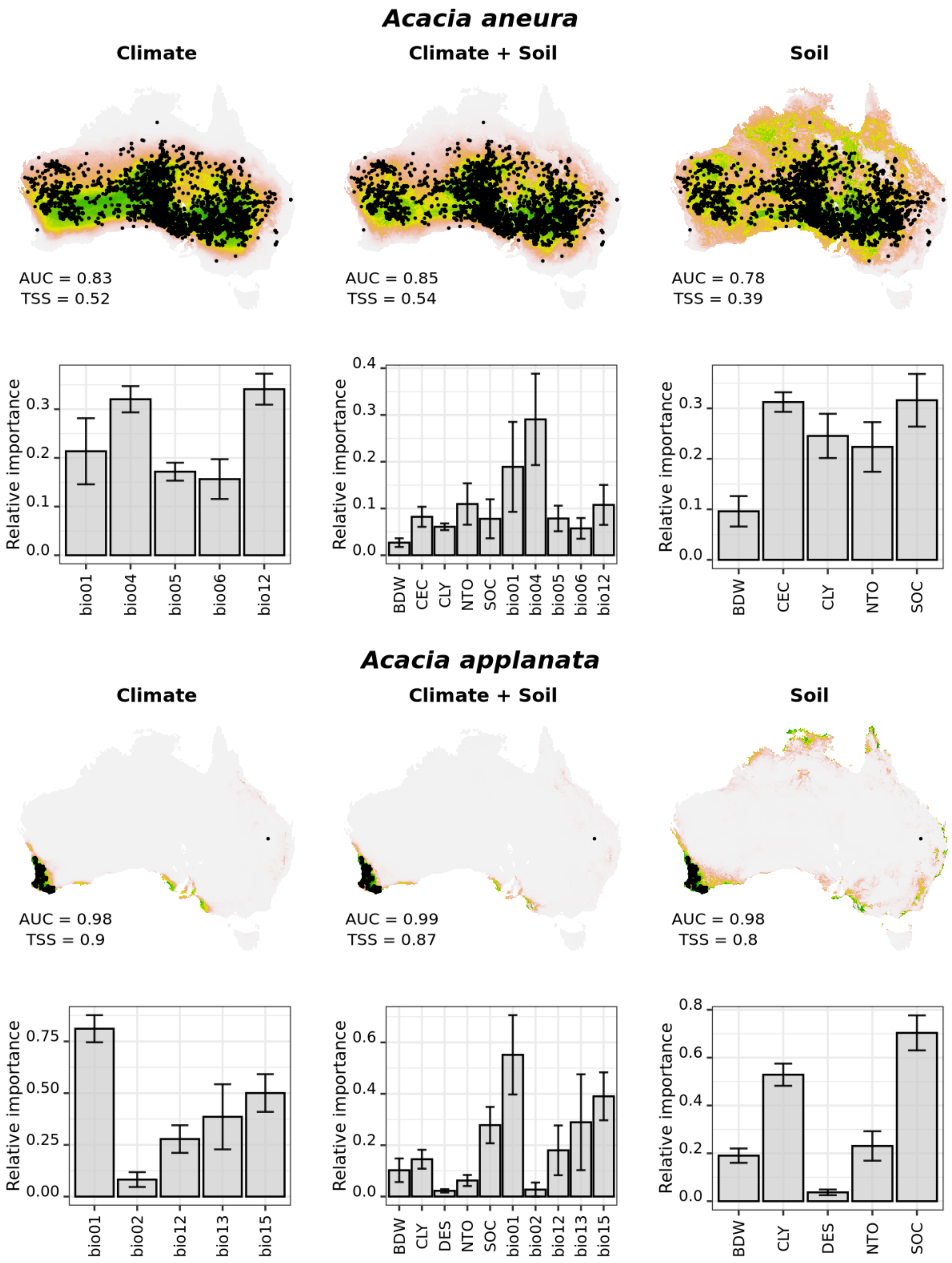
Acacia aneura
Climate
Climate + Soil
Soil
AUC = 0.83
TSS = 0.52
AUC = 0.85
TSS = 0.54
AUC = 0.78
TSS = 0.39
Relative importance
bio01
bio04
bio05
bio06
bio12
BDW
CEC
CLY
NTO
SOC
Acacia applanata
Climate
Climate + Soil
Soil
AUC = 0.98
TSS = 0.9
AUC = 0.99
TSS = 0.87
AUC = 0.98
TSS = 0.8
Relative importance
bio01
bio02
bio12
bio13
bio15
BDW
CLY
DES
NTO
SOC

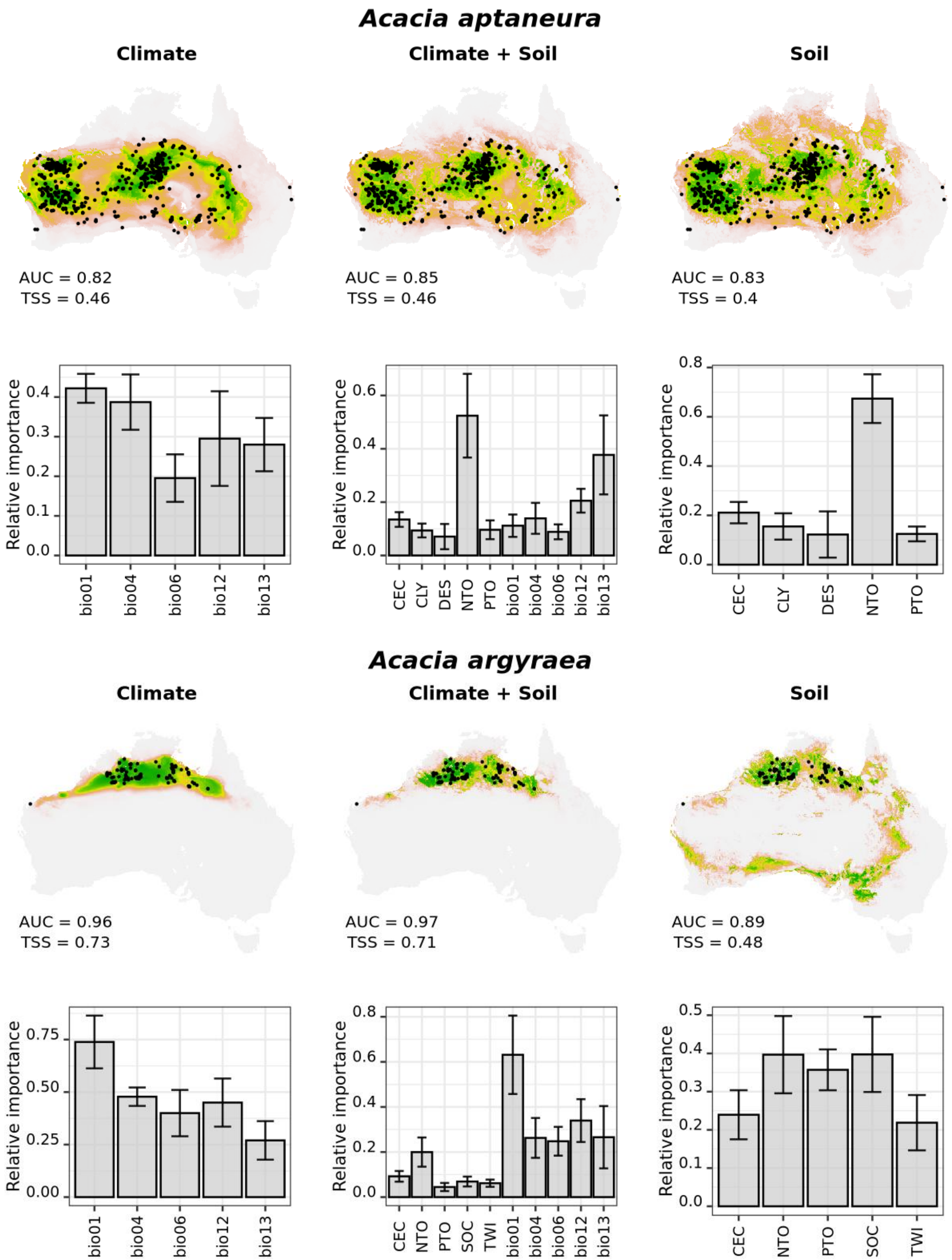

Acacia aptaneura
Climate
Climate + Soil
Soil
AUC = 0.82
TSS = 0.46
AUC = 0.85
TSS = 0.46
AUC = 0.83
TSS = 0.4
Relative importance
0.0
0.1
0.2
0.3
0.4
bio01
bio04
bio06
bio12
bio13
Relative importance
0.0
0.2
0.4
0.6
CEC
CLY
DES
NTO
PTO
bio01
bio04
bio06
bio12
bio13
Relative importance
0.0
0.2
0.4
0.6
0.8
CEC
CLY
DES
NTO
PTO
Acacia argyraea
Climate
Climate + Soil
Soil
AUC = 0.96
TSS = 0.73
AUC = 0.97
TSS = 0.71
AUC = 0.89
TSS = 0.48
Relative importance
0.00
0.25
0.50
0.75
bio01
bio04
bio06
bio12
bio13
Relative importance
0.0
0.2
0.4
0.6
0.8
CEC
NTO
PTO
SOC
TWI
bio01
bio04
bio06
bio12
bio13
Relative importance
0.0
0.1
0.2
0.3
0.4
0.5
CEC
NTO
PTO
SOC
TWI

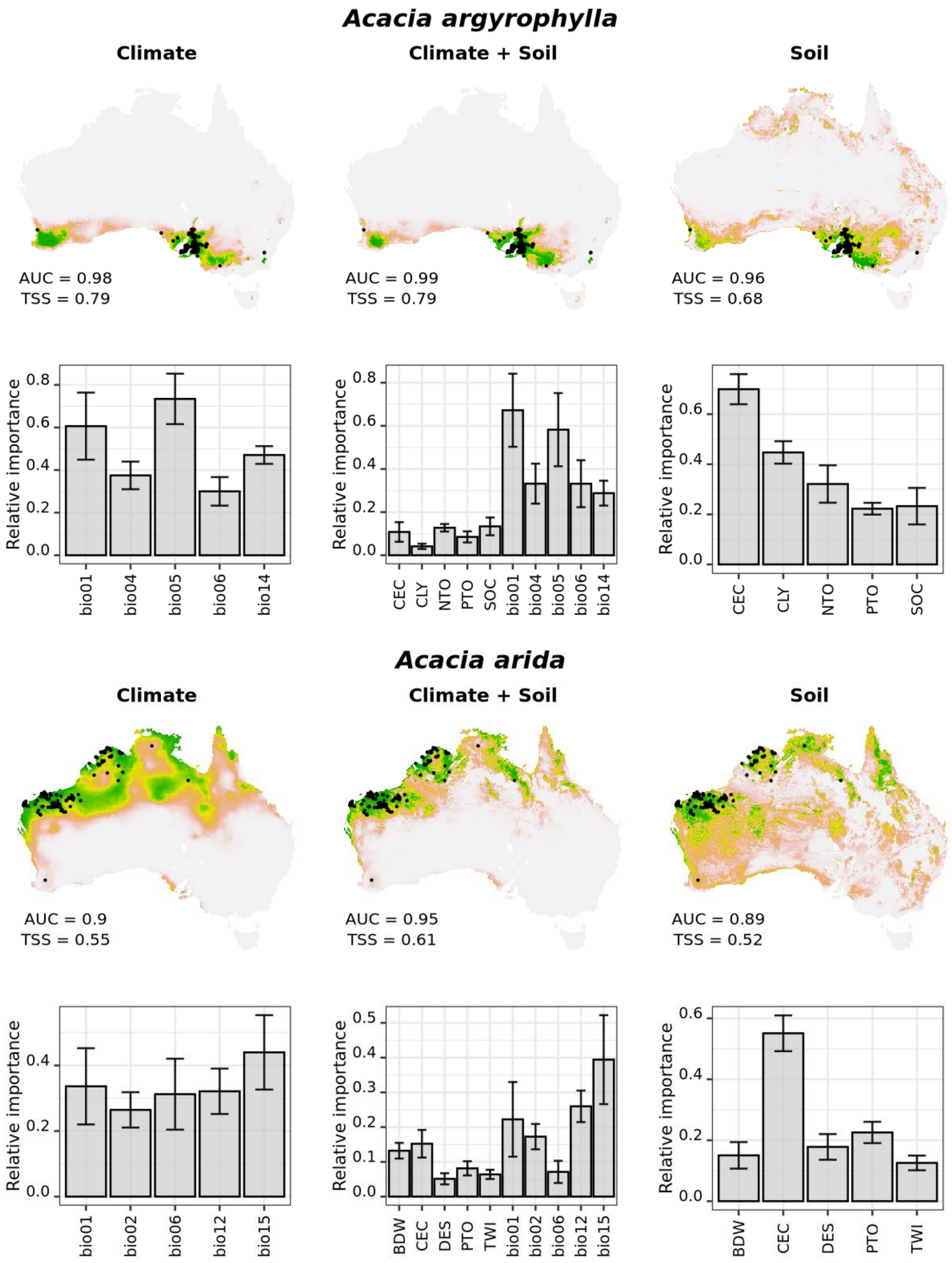
Acacia argyrophylla
Climate
Climate + Soil
Soil
AUC = 0.98
TSS = 0.79
AUC = 0.99
TSS = 0.79
AUC = 0.96
TSS = 0.68
Relative importance
bio01
bio04
bio05
bio06
bio14
CEC
CLY
NTO
PTO
SOC
Acacia arida
Climate
Climate + Soil
Soil
AUC = 0.9
TSS = 0.55
AUC = 0.95
TSS = 0.61
AUC = 0.89
TSS = 0.52
Relative importance
bio01
bio02
bio06
bio12
bio15
BDW
CEC
DES
PTO
TWI

Acacia aspera

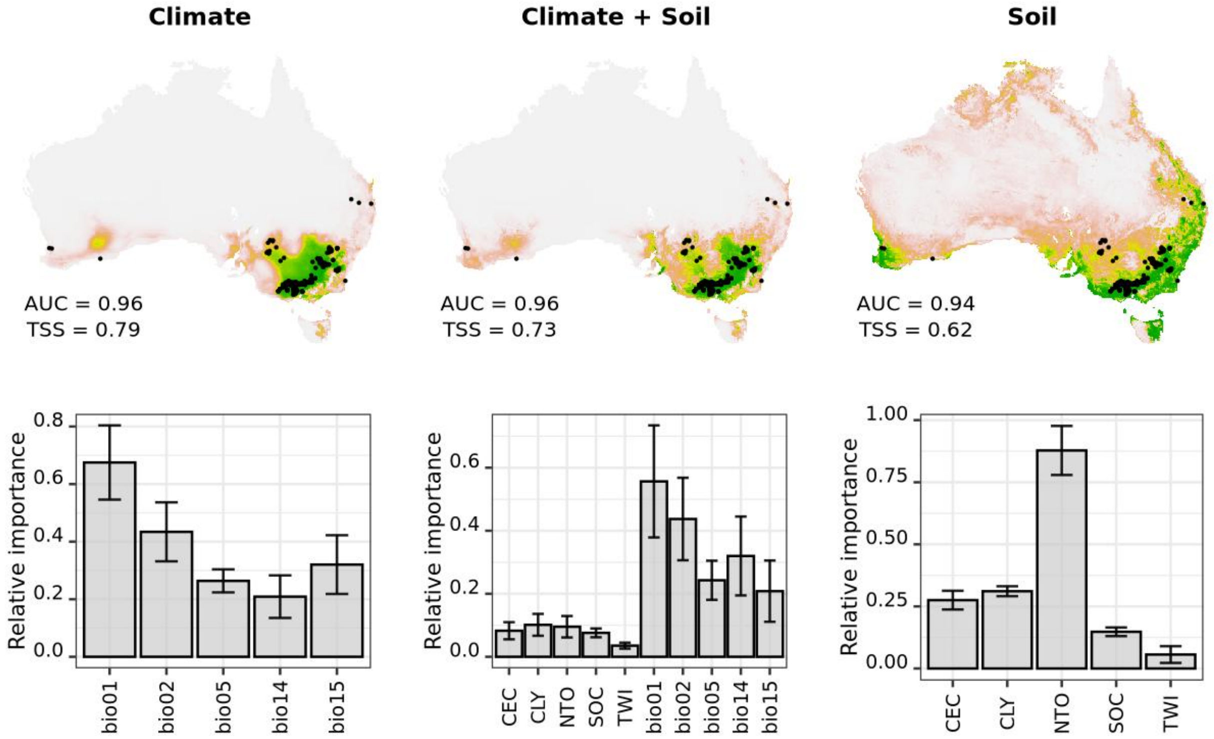

Acacia asperulacea

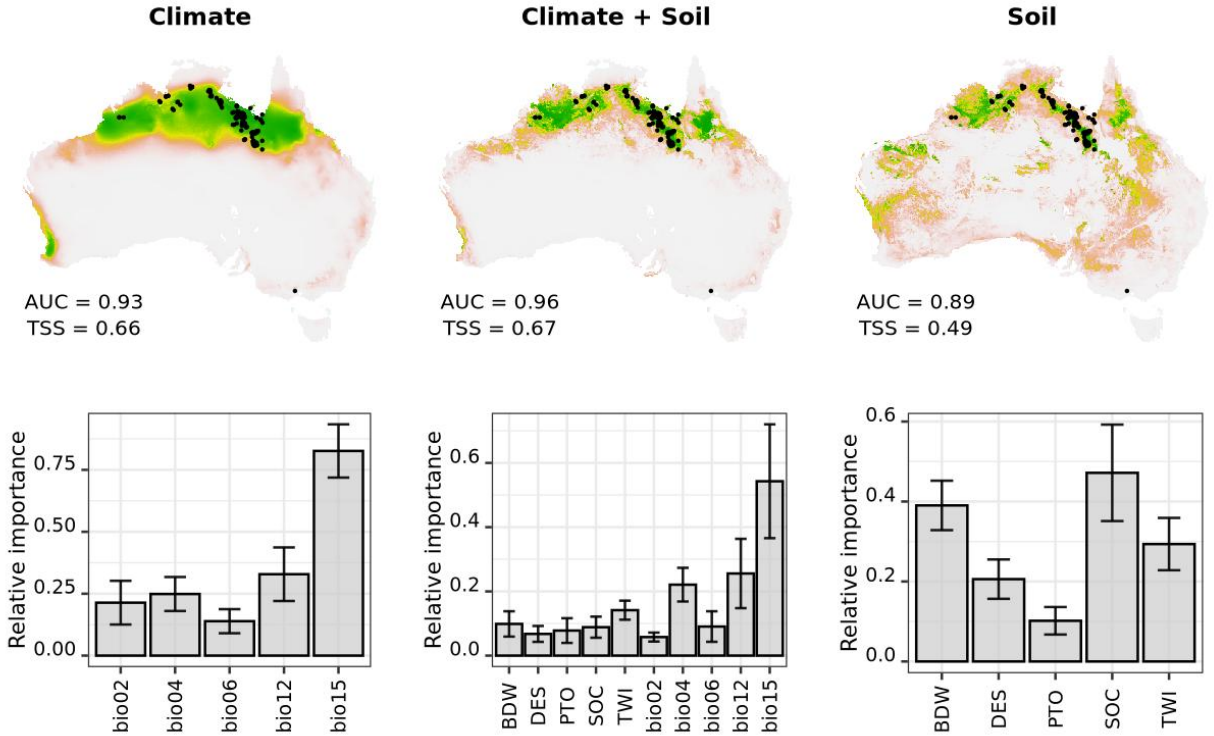

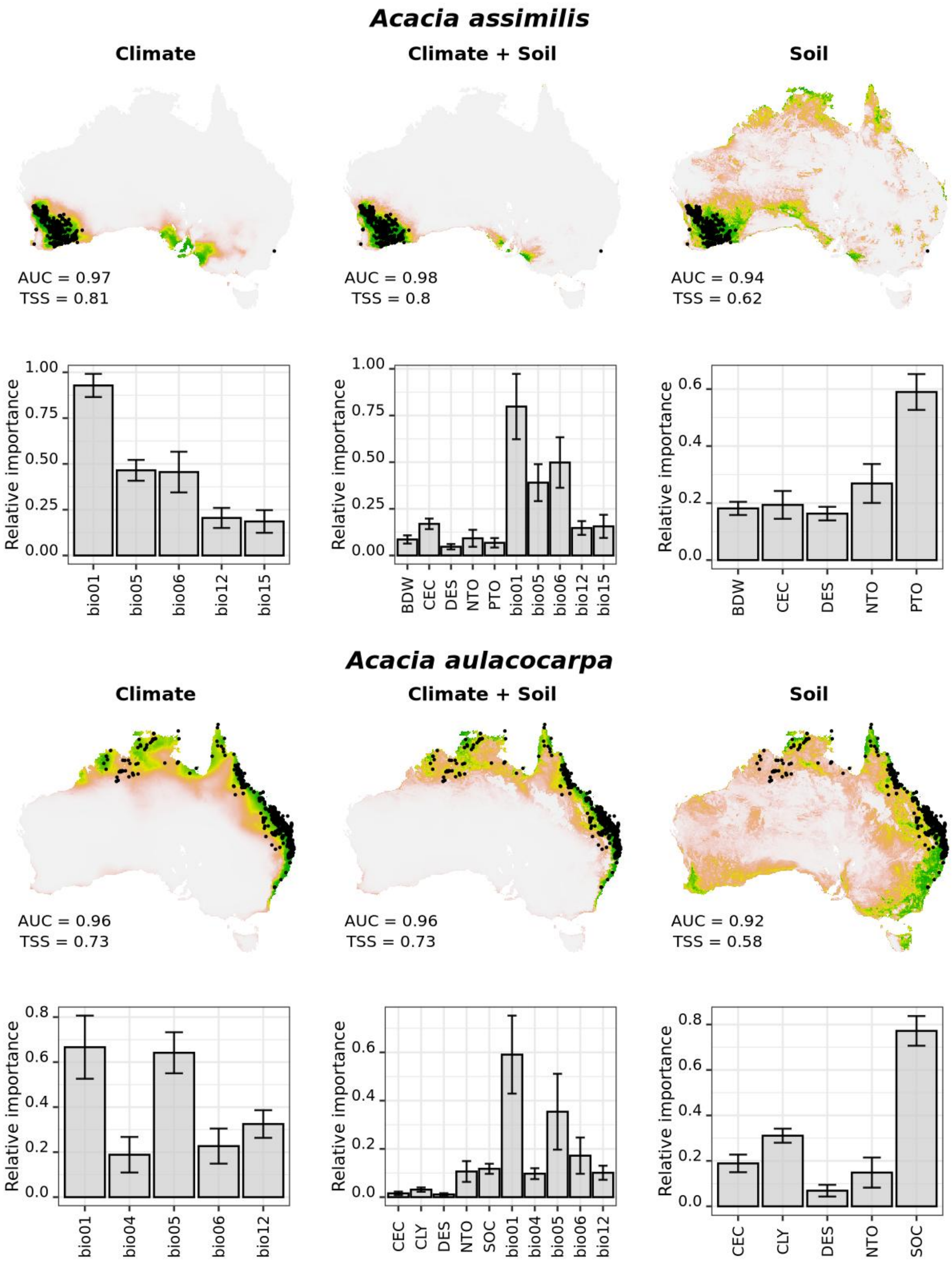

Acacia assimilis
Climate
Climate + Soil
Soil
AUC = 0.97
TSS = 0.81
AUC = 0.98
TSS = 0.8
AUC = 0.94
TSS = 0.62
Relative importance
1.00
0.75
0.50
0.25
0.00
bio01
bio05
bio06
bio12
bio15
BDW
CEC
DES
NTO
PTO
0.6
0.4
0.2
0.0
Acacia aulacocarpa
Climate
Climate + Soil
Soil
AUC = 0.96
TSS = 0.73
AUC = 0.96
TSS = 0.73
AUC = 0.92
TSS = 0.58
Relative importance
0.8
0.6
0.4
0.2
0.0
bio01
bio04
bio05
bio06
bio12
CEC
CLY
DES
NTO
SOC

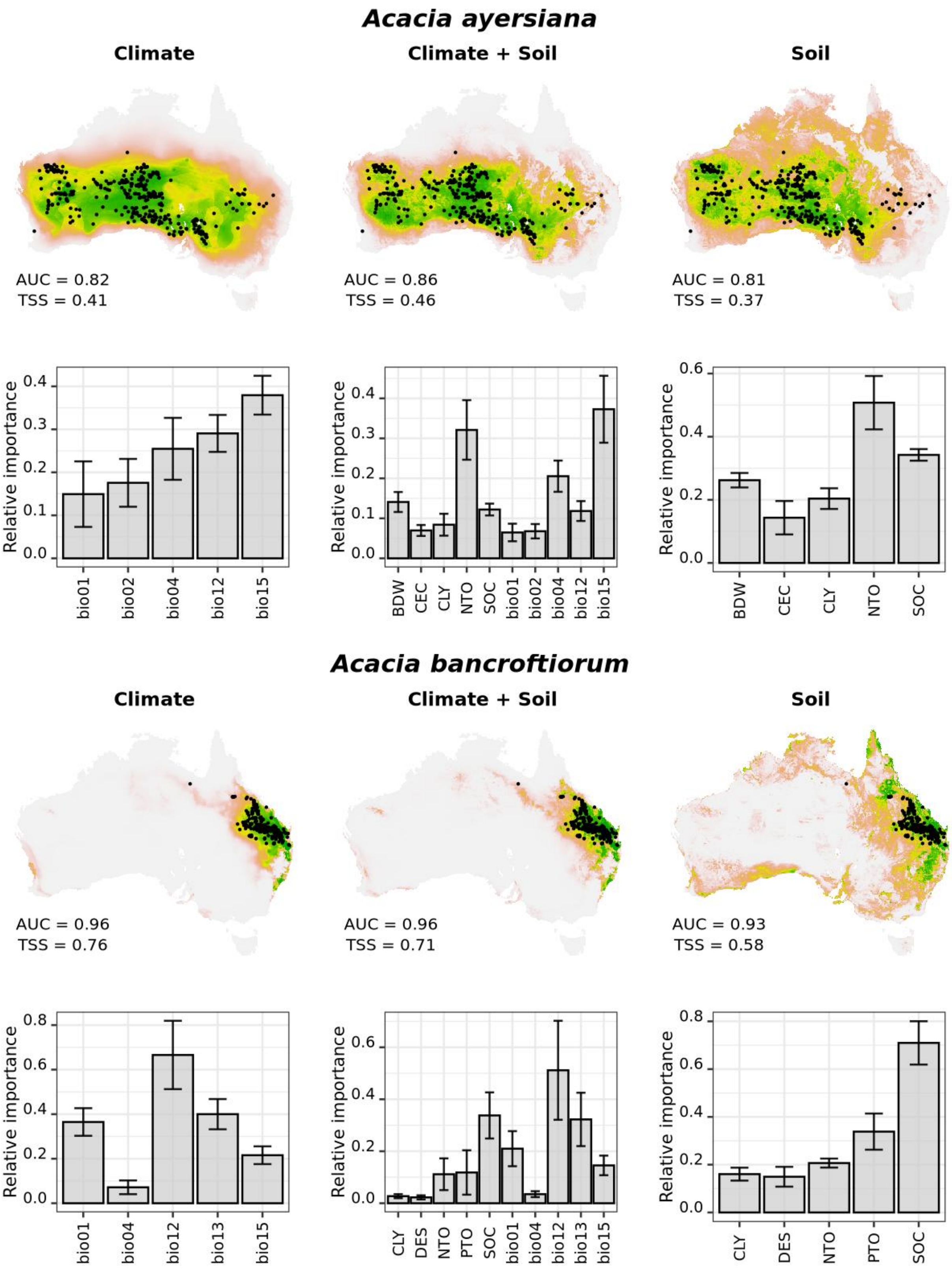
Acacia ayersiana
Climate
Climate + Soil
Soil
AUC = 0.82
TSS = 0.41
AUC = 0.86
TSS = 0.46
AUC = 0.81
TSS = 0.37
Relative importance
bio01
bio02
bio04
bio12
bio15
BDW
CEC
CLY
NTO
SOC
Acacia bancroftiorum
Climate
Climate + Soil
Soil
AUC = 0.96
TSS = 0.76
AUC = 0.96
TSS = 0.71
AUC = 0.93
TSS = 0.58
Relative importance
bio01
bio04
bio12
bio13
bio15
CLY
DES
NTO
PTO
SOC

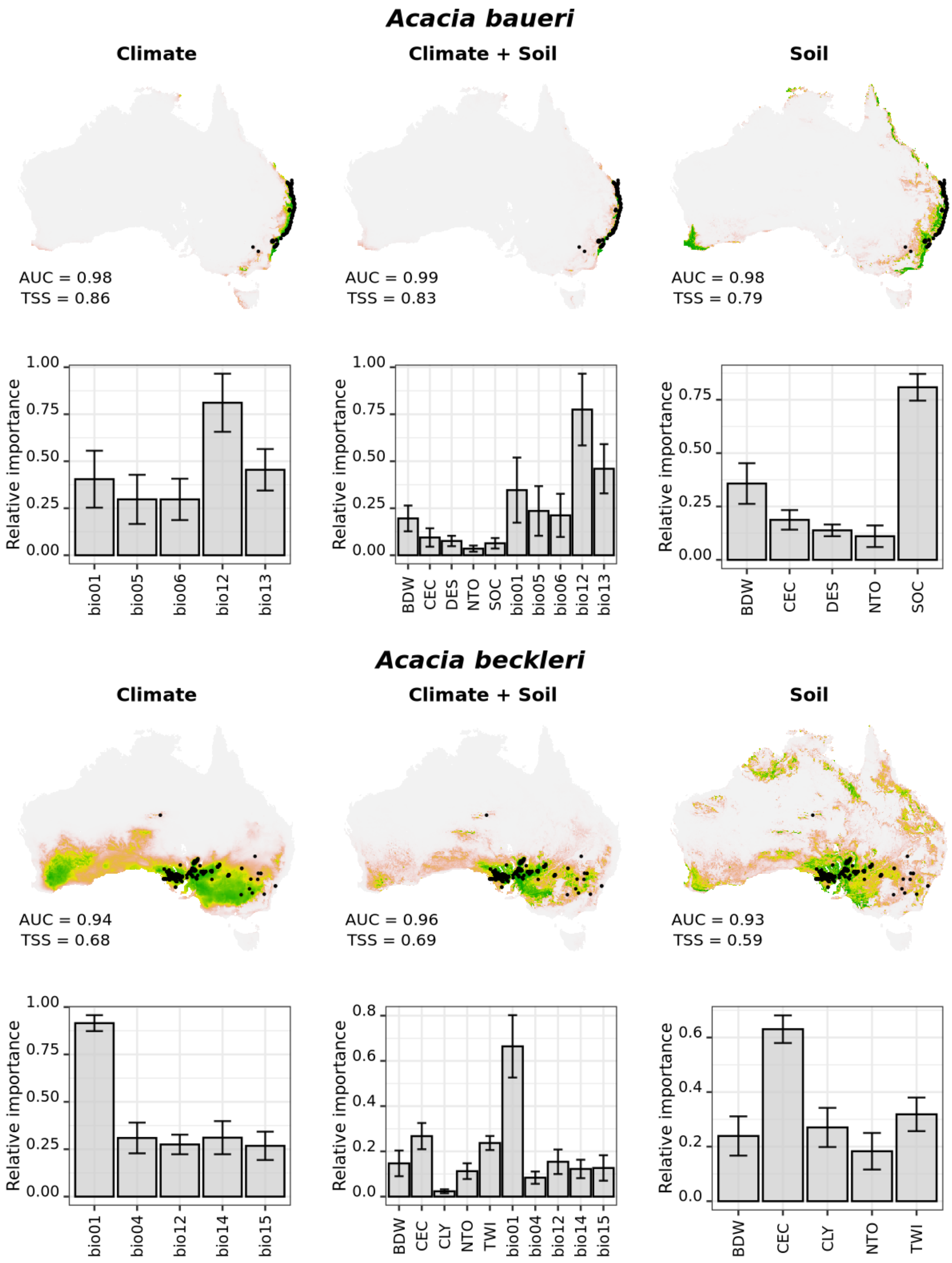
Acacia baueri
Climate
Climate + Soil
Soil
AUC = 0.98
TSS = 0.86
AUC = 0.99
TSS = 0.83
AUC = 0.98
TSS = 0.79
Relative importance
1.00
0.75
0.50
0.25
0.00
bio01
bio05
bio06
bio12
bio13
BDW
CEC
DES
NTO
SOC
Acacia beckleri
Climate
Climate + Soil
Soil
AUC = 0.94
TSS = 0.68
AUC = 0.96
TSS = 0.69
AUC = 0.93
TSS = 0.59
bio01
bio04
bio12
bio14
bio15
BDW
CEC
CLY
NTO
TWI
0.8
0.6
0.4
0.2
0.0

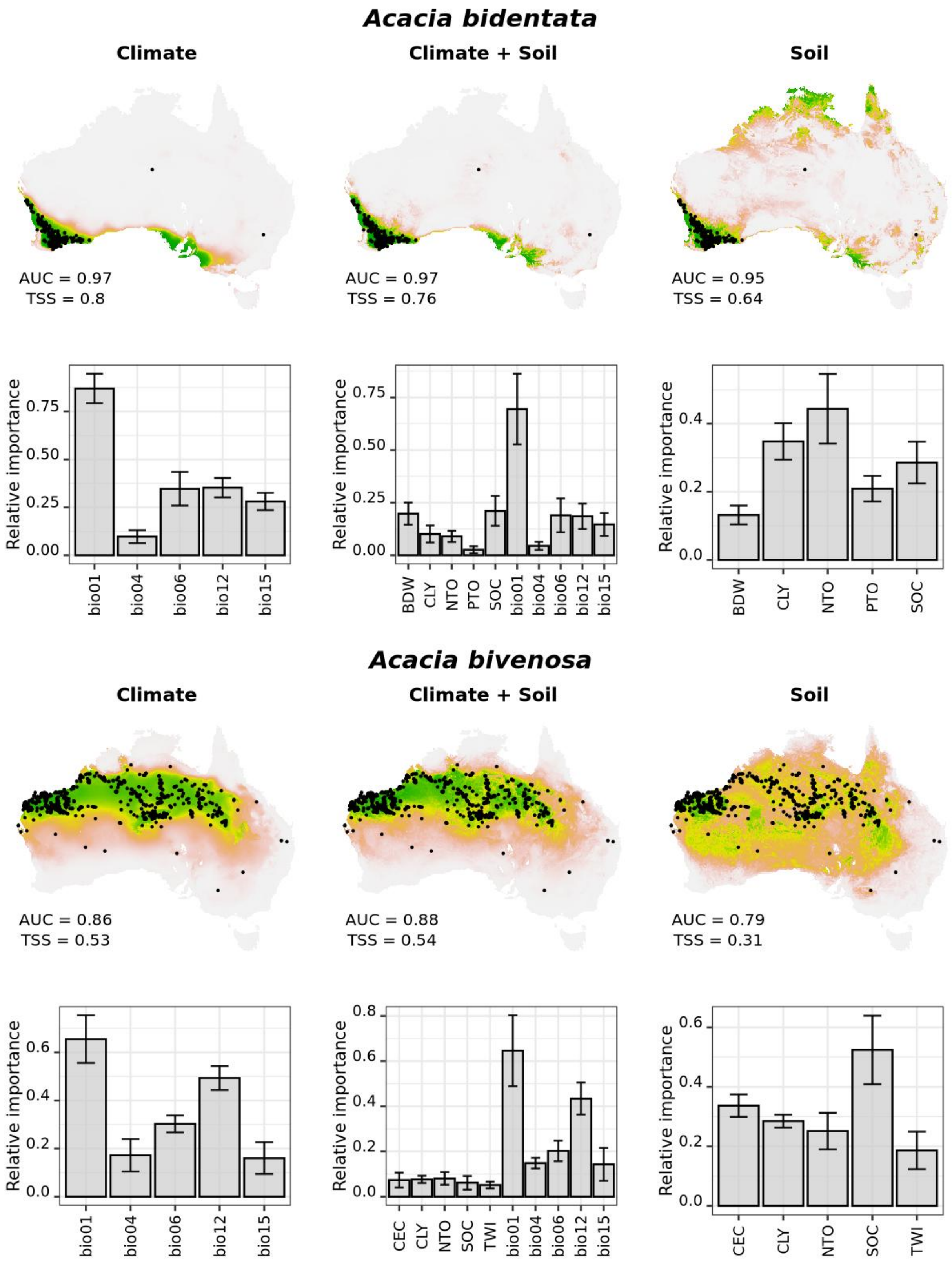
Acacia bidentata
Climate
Climate + Soil
Soil
AUC = 0.97
TSS = 0.8
AUC = 0.97
TSS = 0.76
AUC = 0.95
TSS = 0.64
Relative importance
0.00
0.25
0.50
0.75
bio01
bio04
bio06
bio12
bio15
BDW
CLY
NTO
PTO
SOC
0.0
0.2
0.4
Acacia bivenosa
Climate
Climate + Soil
Soil
AUC = 0.86
TSS = 0.53
AUC = 0.88
TSS = 0.54
AUC = 0.79
TSS = 0.31
Relative importance
0.0
0.2
0.4
0.6
0.8
bio01
bio04
bio06
bio12
bio15
CEC
CLY
NTO
SOC
TWI

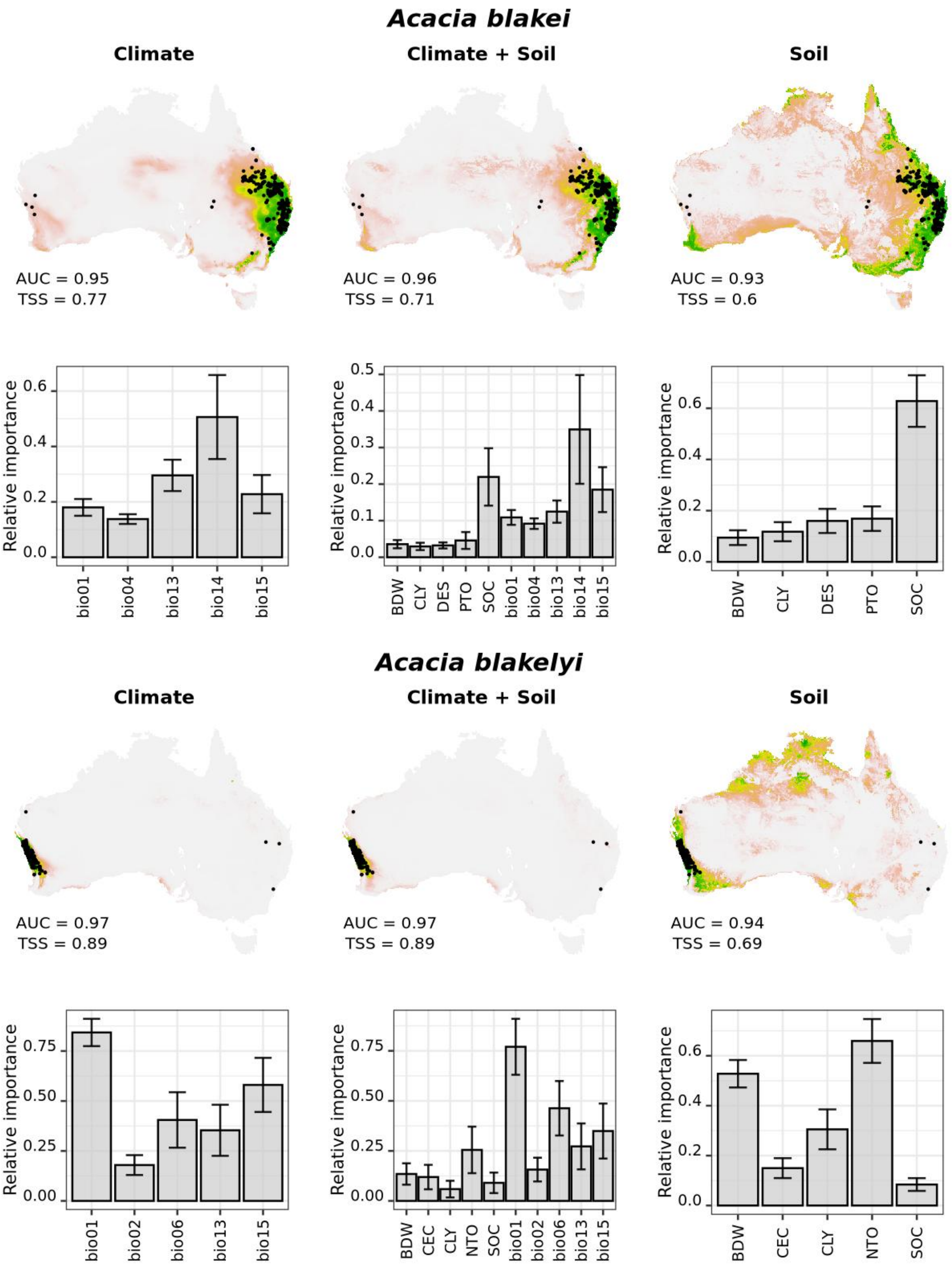
Acacia blakei
Climate
Climate + Soil
Soil
AUC = 0.95
TSS = 0.77
AUC = 0.96
TSS = 0.71
AUC = 0.93
TSS = 0.6
Relative importance
bio01
bio04
bio13
bio14
bio15
BDW
CLY
DES
PTO
SOC
Acacia blakelyi
Climate
Climate + Soil
Soil
AUC = 0.97
TSS = 0.89
AUC = 0.97
TSS = 0.89
AUC = 0.94
TSS = 0.69
Relative importance
bio01
bio02
bio06
bio13
bio15
BDW
CEC
CLY
NTO
SOC

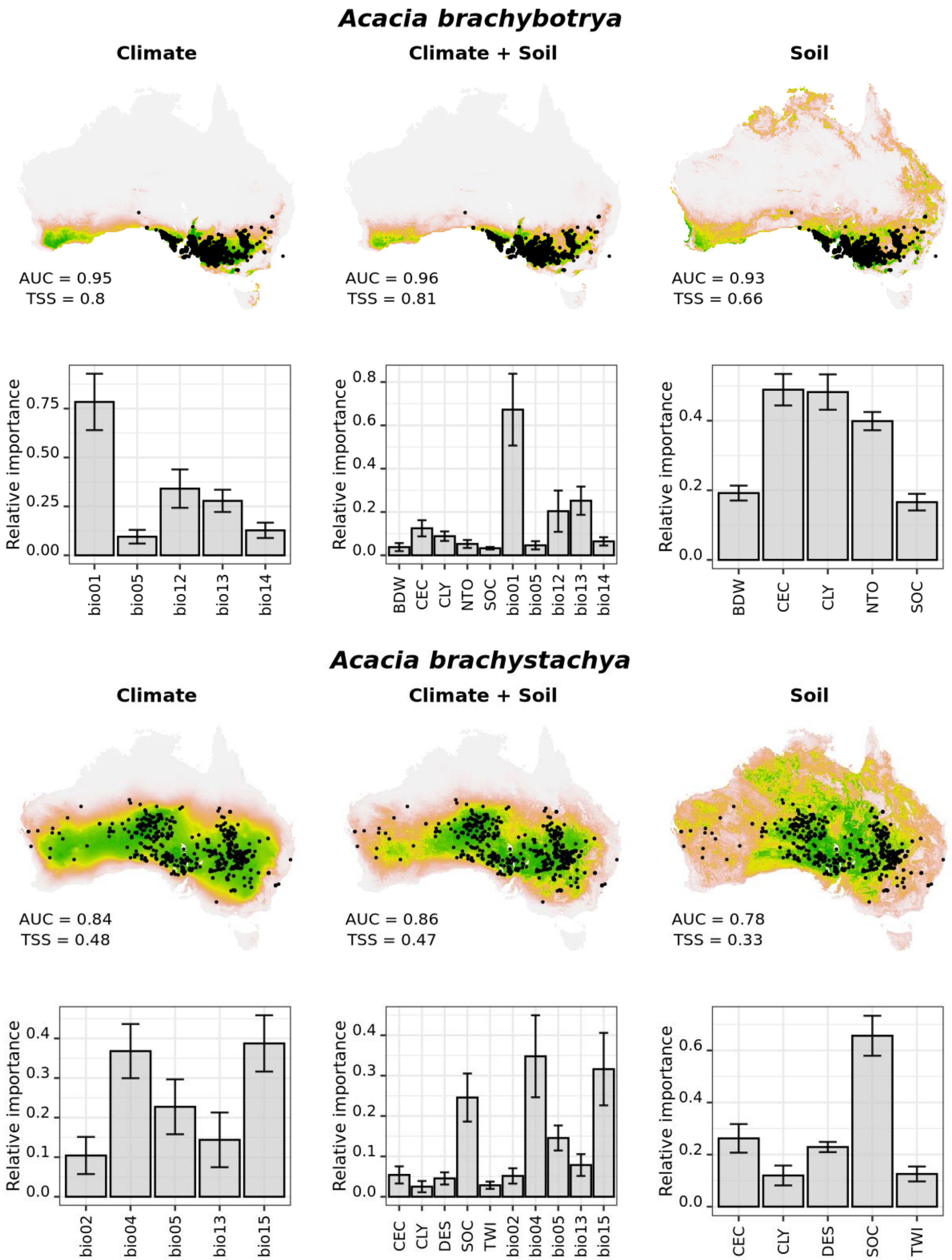

Acacia brachybotrya
Climate
Climate + Soil
Soil
AUC = 0.95
TSS = 0.8
AUC = 0.96
TSS = 0.81
AUC = 0.93
TSS = 0.66
Relative importance
bio01
bio05
bio12
bio13
bio14
BDW
CEC
CLY
NTO
SOC
Acacia brachystachya
Climate
Climate + Soil
Soil
AUC = 0.84
TSS = 0.48
AUC = 0.86
TSS = 0.47
AUC = 0.78
TSS = 0.33
Relative importance
bio02
bio04
bio05
bio13
bio15
CEC
CLY
DES
SOC
TWI

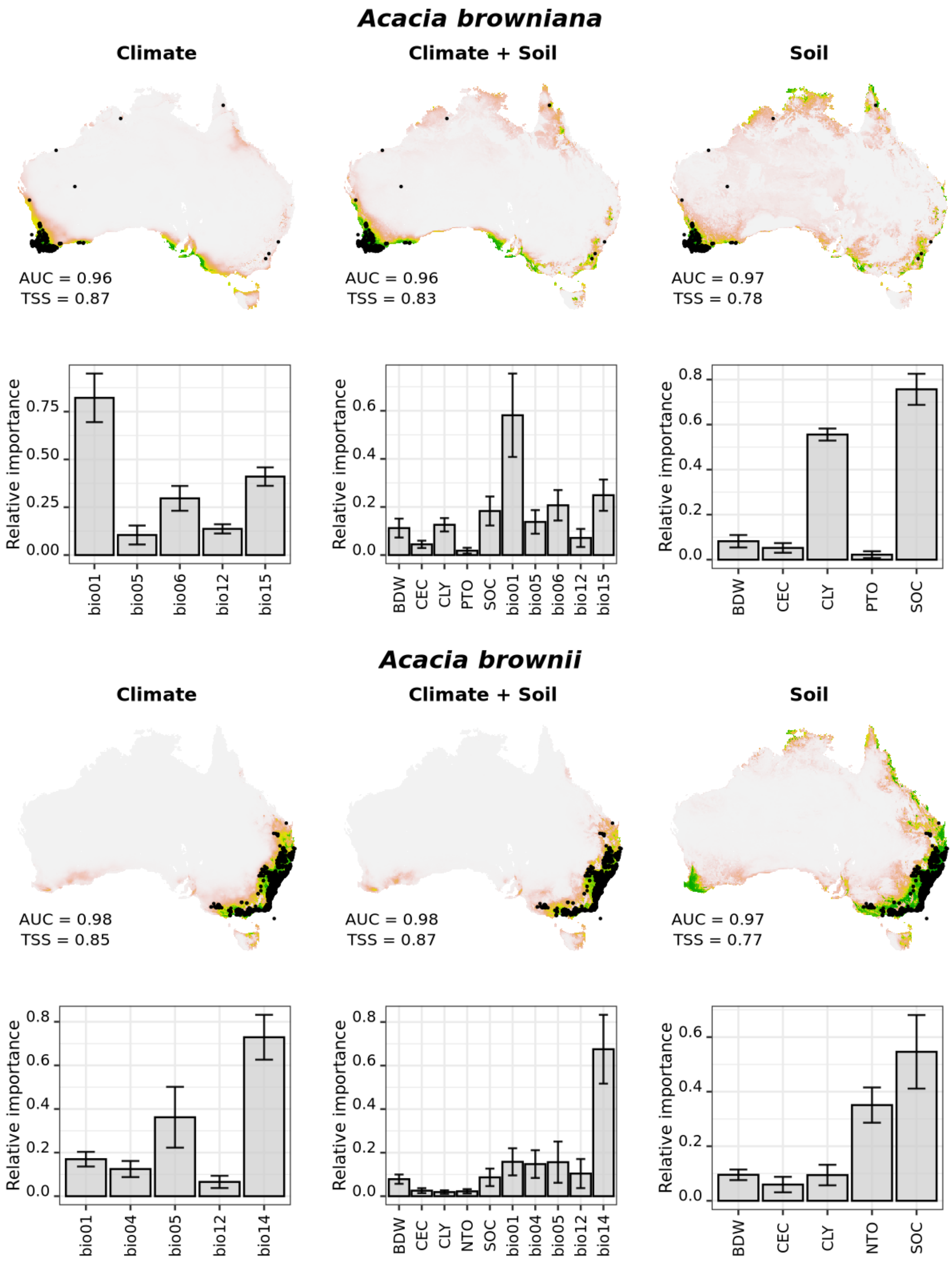
Acacia browniana
Climate
Climate + Soil
Soil
AUC = 0.96
TSS = 0.87
AUC = 0.96
TSS = 0.83
AUC = 0.97
TSS = 0.78
Relative importance
0.00
0.25
0.50
0.75
bio01
bio05
bio06
bio12
bio15
0.0
0.2
0.4
0.6
BDW
CEC
CLY
PTO
SOC
bio01
bio05
bio06
bio12
bio15
0.8
BDW
CEC
CLY
PTO
SOC
Acacia brownii
Climate
Climate + Soil
Soil
AUC = 0.98
TSS = 0.85
AUC = 0.98
TSS = 0.87
AUC = 0.97
TSS = 0.77
bio01
bio04
bio05
bio12
bio14
BDW
CEC
CLY
NTO
SOC
bio01
bio04
bio05
bio12
bio14
BDW
CEC
CLY
NTO
SOC

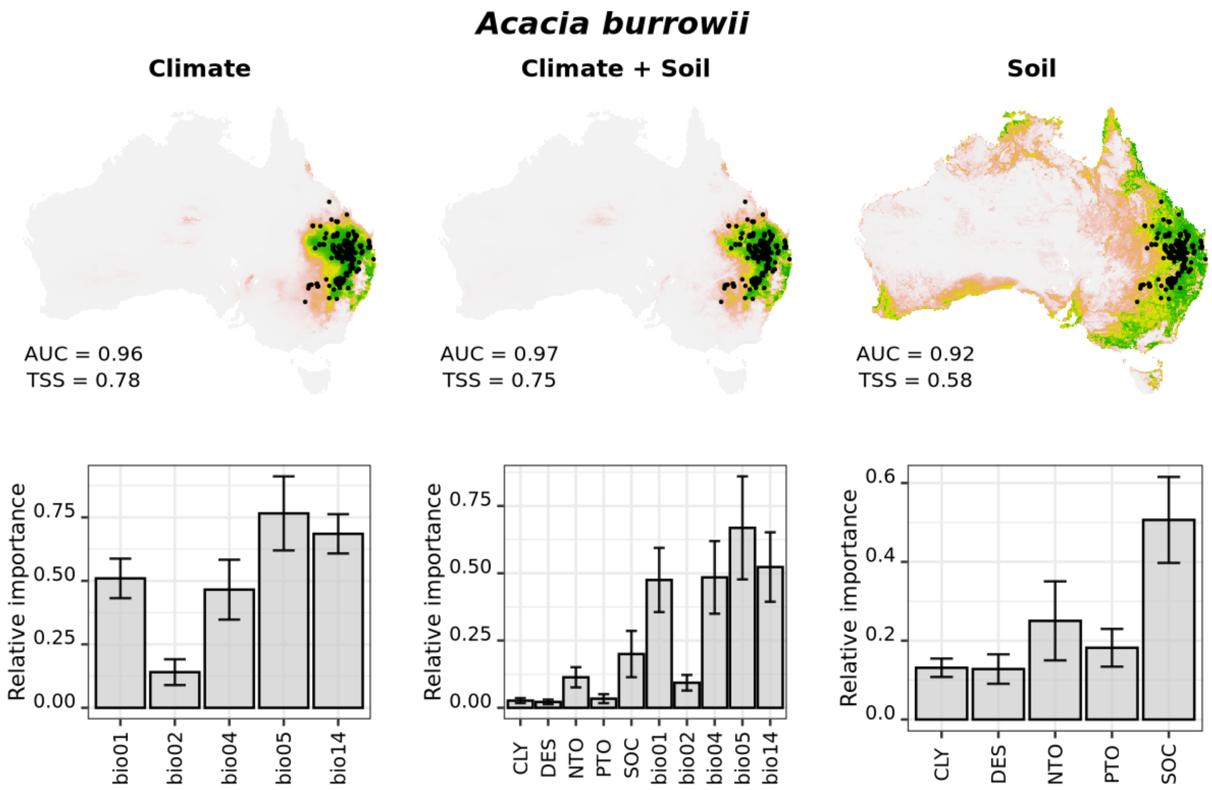
Acacia burrowii
Climate
Climate + Soil
Soil
AUC = 0.96
TSS = 0.78
AUC = 0.97
TSS = 0.75
AUC = 0.92
TSS = 0.58
Relative importance
0.00
0.25
0.50
0.75
bio01
bio02
bio04
bio05
bio14
CLY
DES
NTO
PTO
SOC
0.0
0.2
0.4
0.6

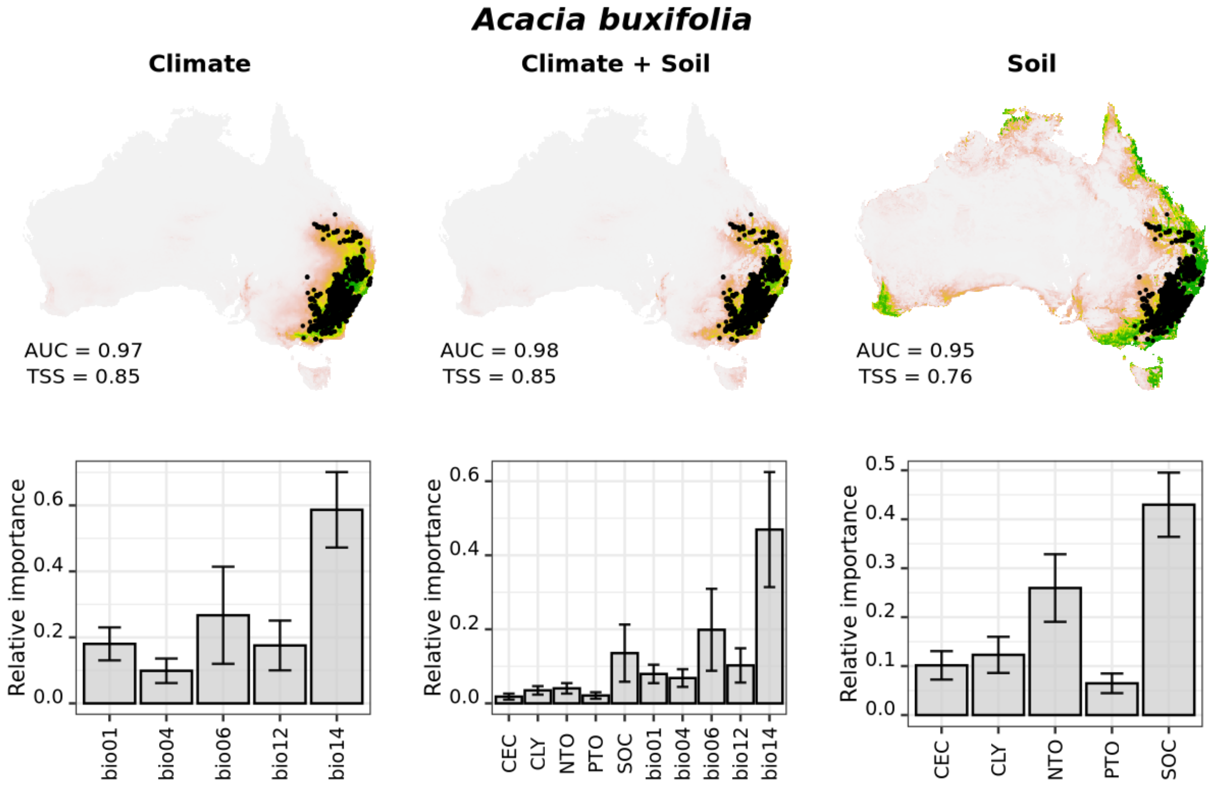
Acacia buxifolia
Climate
Climate + Soil
Soil
AUC = 0.97
TSS = 0.85
AUC = 0.98
TSS = 0.85
AUC = 0.95
TSS = 0.76
Relative importance
0.0
0.1
0.2
0.3
0.4
0.5
0.6
bio01
bio04
bio06
bio12
bio14
CEC
CLY
NTO
PTO
SOC

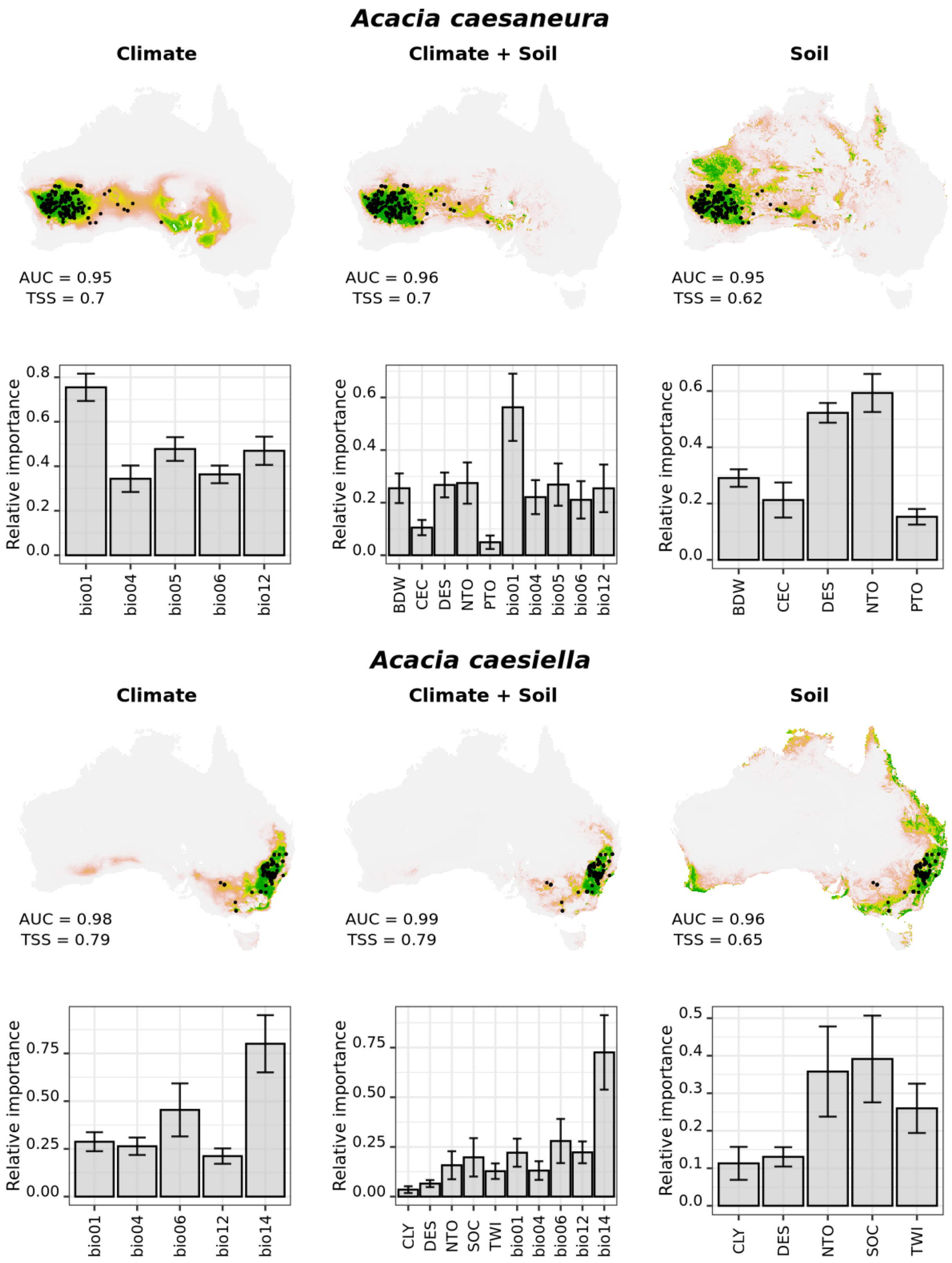
Acacia caesaneura
Climate
Climate + Soil
Soil
AUC = 0.95
TSS = 0.7
AUC = 0.96
TSS = 0.7
AUC = 0.95
TSS = 0.62
Relative importance
bio01
bio04
bio05
bio06
bio12
BDW
CEC
DES
NTO
PTO
Acacia caesiella
AUC = 0.98
TSS = 0.79
AUC = 0.99
TSS = 0.79
AUC = 0.96
TSS = 0.65
bio14
CLY
SOC
TWI

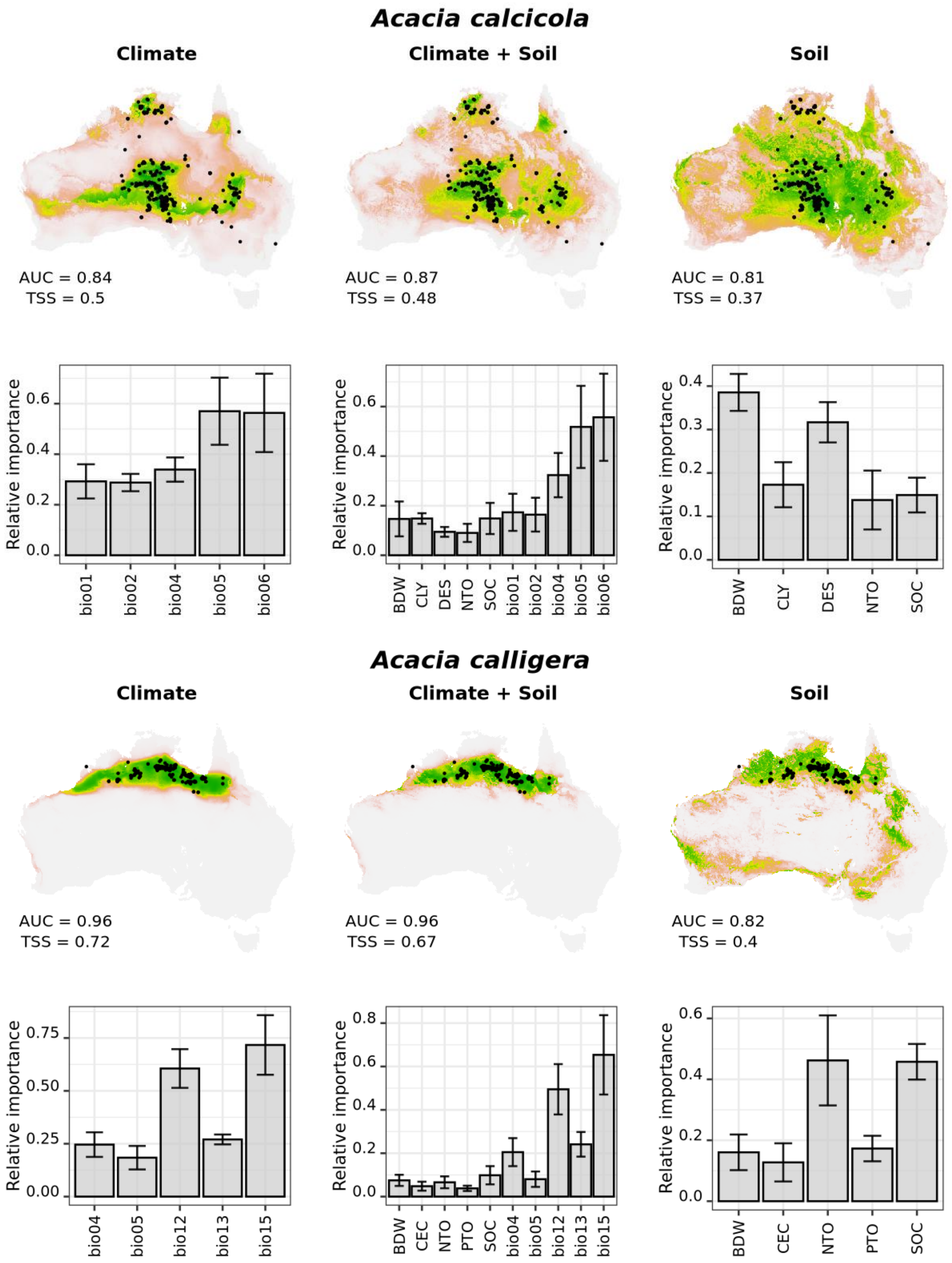

Acacia calcicola
Climate
Climate + Soil
Soil
AUC = 0.84
TSS = 0.5
AUC = 0.87
TSS = 0.48
AUC = 0.81
TSS = 0.37
Relative importance
bio01 bio02 bio04 bio05 bio06
BDW CLY DES NTO SOC bio01 bio02 bio04 bio05 bio06
BDW CLY DES NTO SOC
Acacia calligera
Climate
Climate + Soil
Soil
AUC = 0.96
TSS = 0.72
AUC = 0.96
TSS = 0.67
AUC = 0.82
TSS = 0.4
Relative importance
bio04 bio05 bio12 bio13 bio15
BDW CEC NTO PTO SOC bio04 bio05 bio12 bio13 bio15
BDW CEC NTO PTO SOC

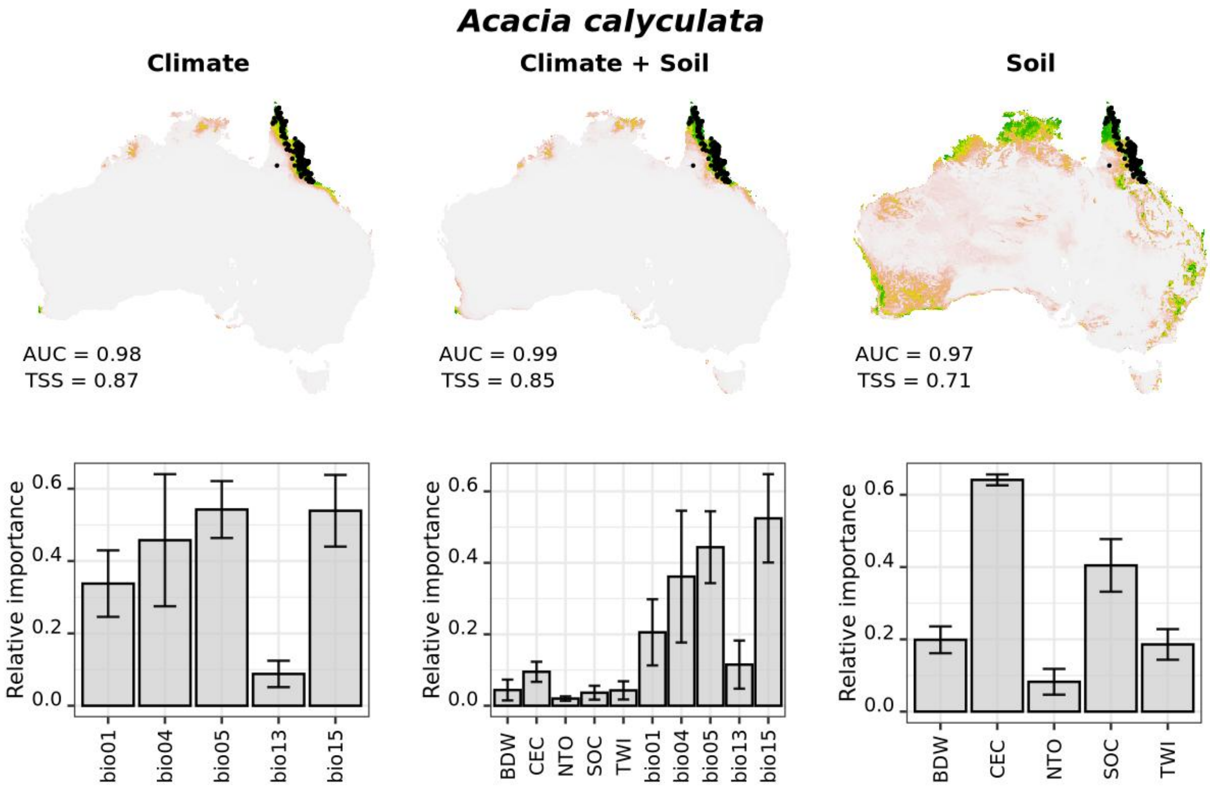
Acacia calyculata
Climate
Climate + Soil
Soil
AUC = 0.98
TSS = 0.87
AUC = 0.99
TSS = 0.85
AUC = 0.97
TSS = 0.71
Relative importance
0.0
0.2
0.4
0.6
bio01
bio04
bio05
bio13
bio15
BDW
CEC
NTO
SOC
TWI

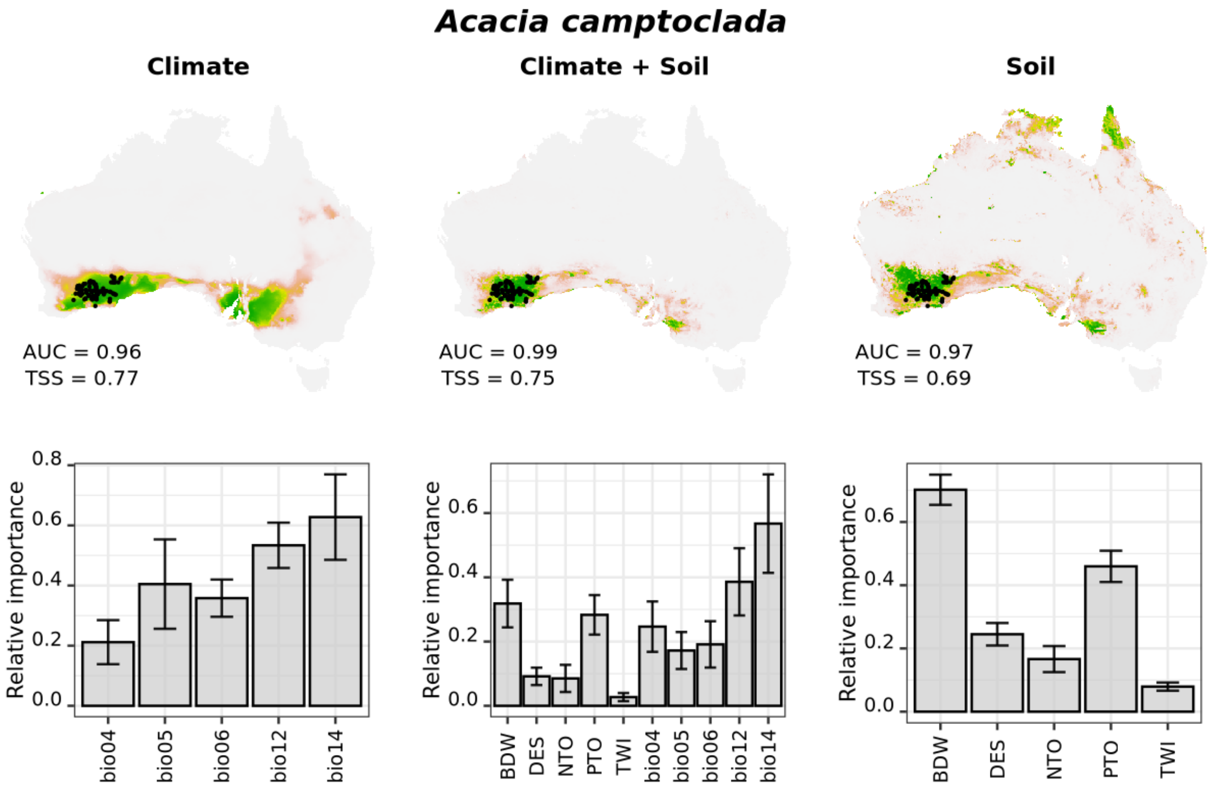
Acacia camptoclada
Climate
Climate + Soil
Soil
AUC = 0.96
TSS = 0.77
AUC = 0.99
TSS = 0.75
AUC = 0.97
TSS = 0.69
Relative importance
0.0
0.2
0.4
0.6
0.8
bio04
bio05
bio06
bio12
bio14
BDW
DES
NTO
PTO
TWI

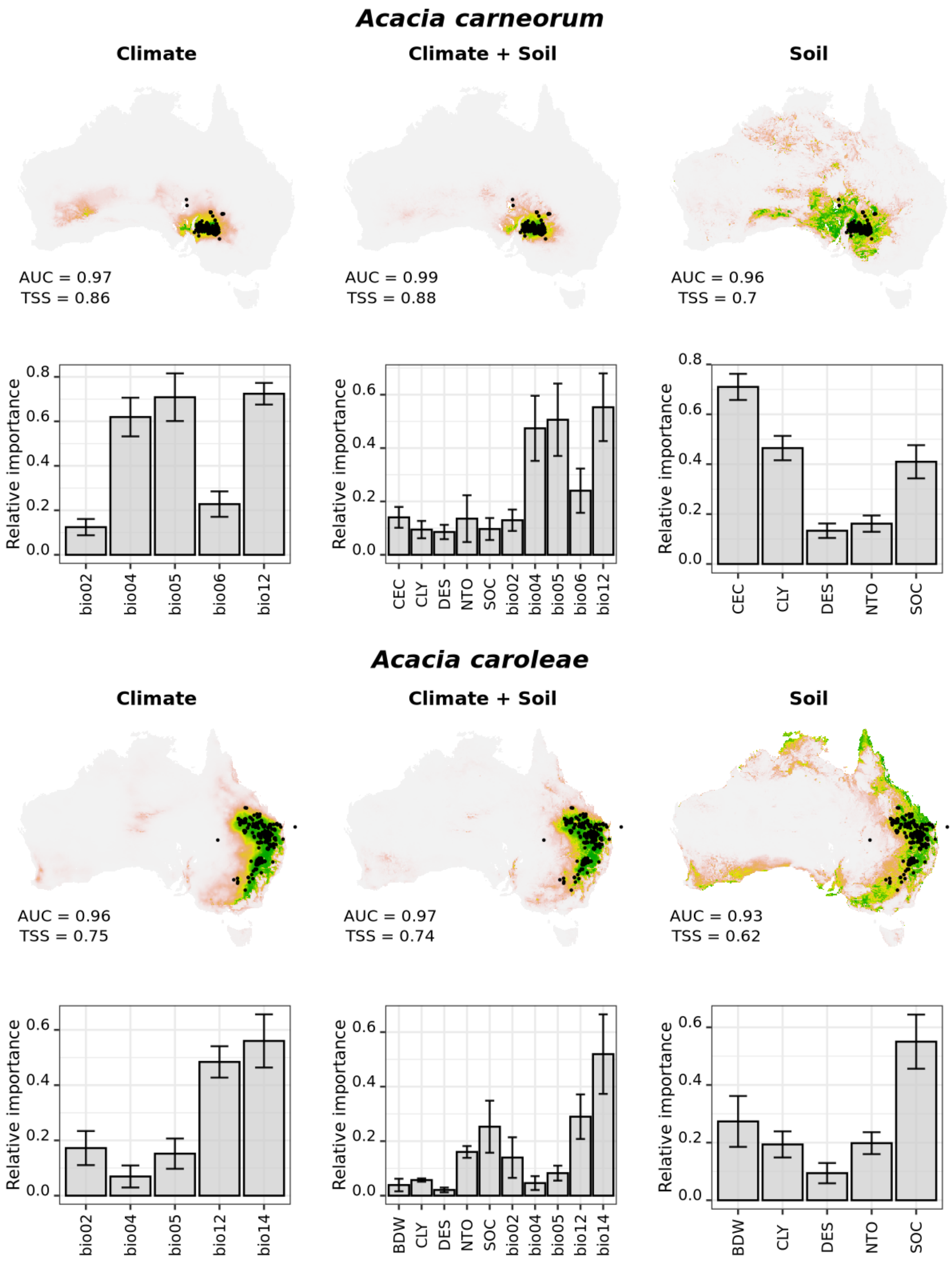
Acacia carneorum
Climate
Climate + Soil
Soil
AUC = 0.97
TSS = 0.86
AUC = 0.99
TSS = 0.88
AUC = 0.96
TSS = 0.7
Relative importance
bio02
bio04
bio05
bio06
bio12
CEC
CLY
DES
NTO
SOC
Acacia caroleae
AUC = 0.96
TSS = 0.75
AUC = 0.97
TSS = 0.74
AUC = 0.93
TSS = 0.62
bio14
BDW

Acacia celastrifolia

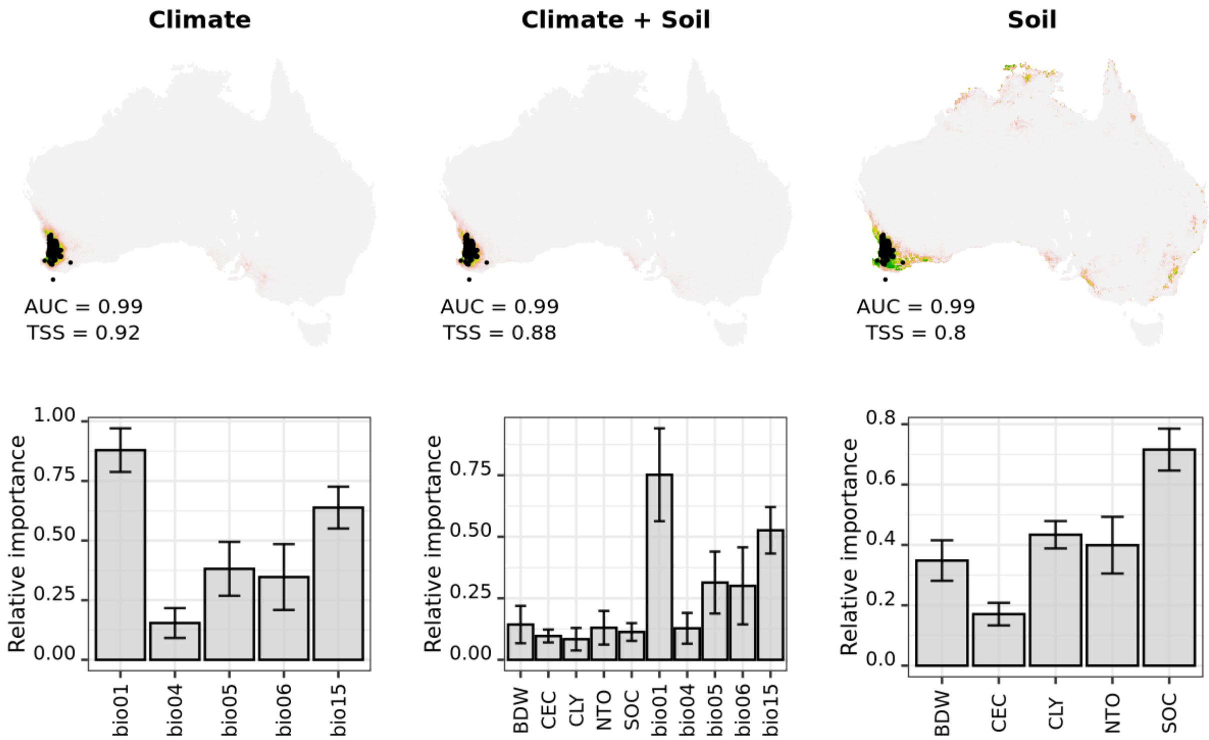

Acacia cheelii

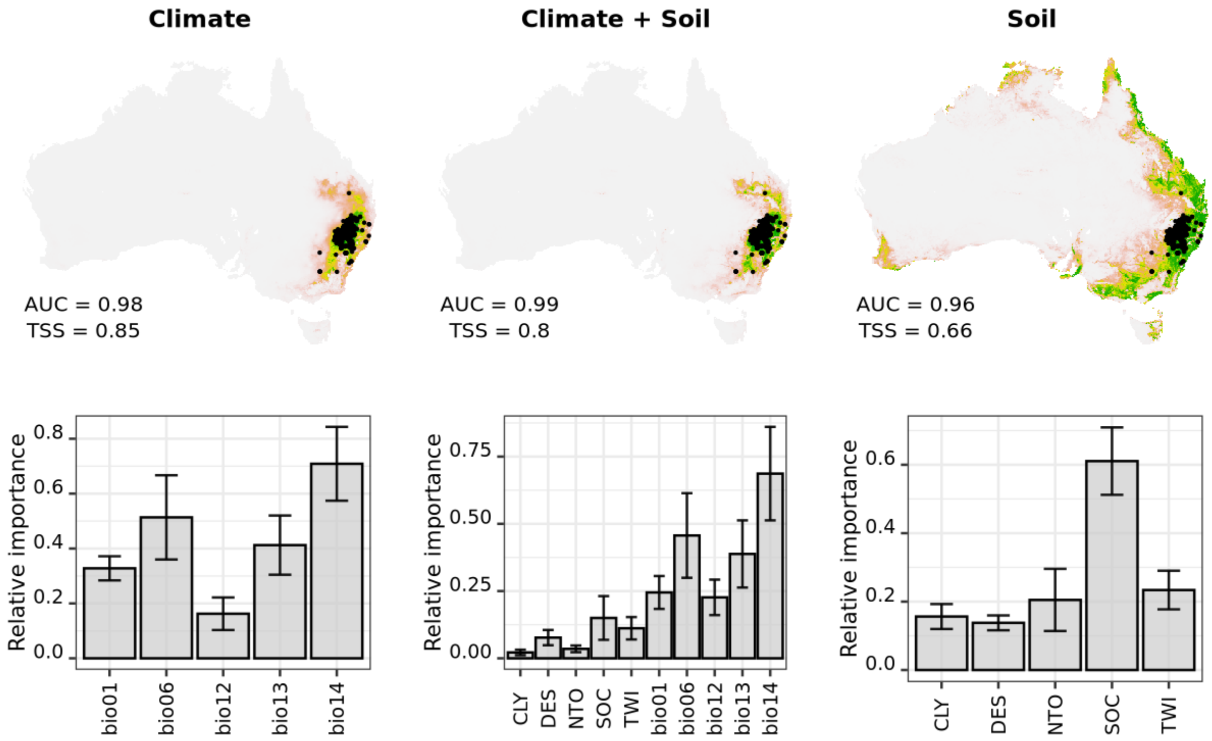

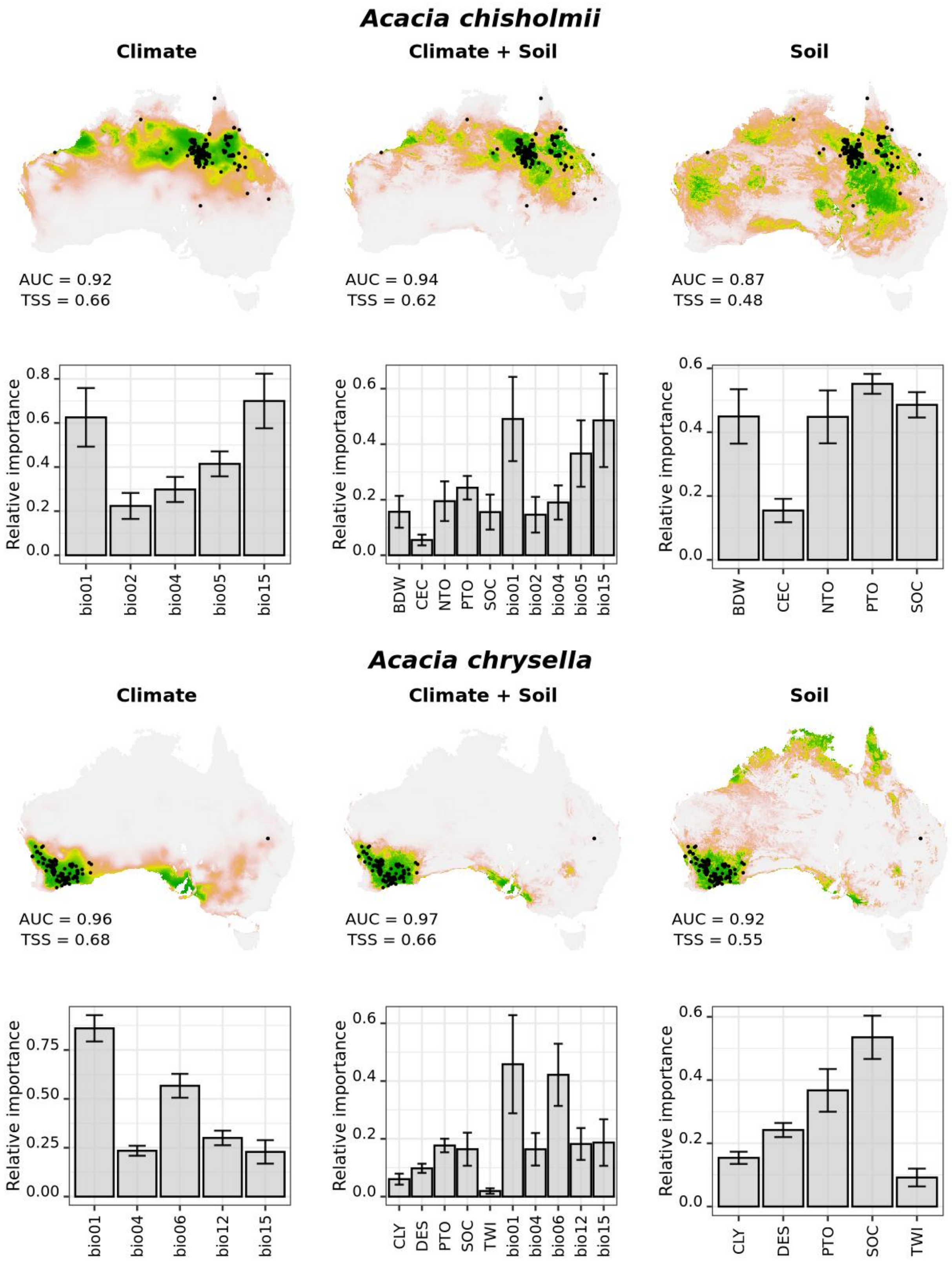
Acacia chisholmii
Climate
Climate + Soil
Soil
AUC = 0.92
TSS = 0.66
AUC = 0.94
TSS = 0.62
AUC = 0.87
TSS = 0.48
Relative importance
bio01
bio02
bio04
bio05
bio15
BDW
CEC
NTO
PTO
SOC
Acacia chrysella
Climate
Climate + Soil
Soil
AUC = 0.96
TSS = 0.68
AUC = 0.97
TSS = 0.66
AUC = 0.92
TSS = 0.55
Relative importance
bio01
bio04
bio06
bio12
bio15
CLY
DES
PTO
SOC
TWI

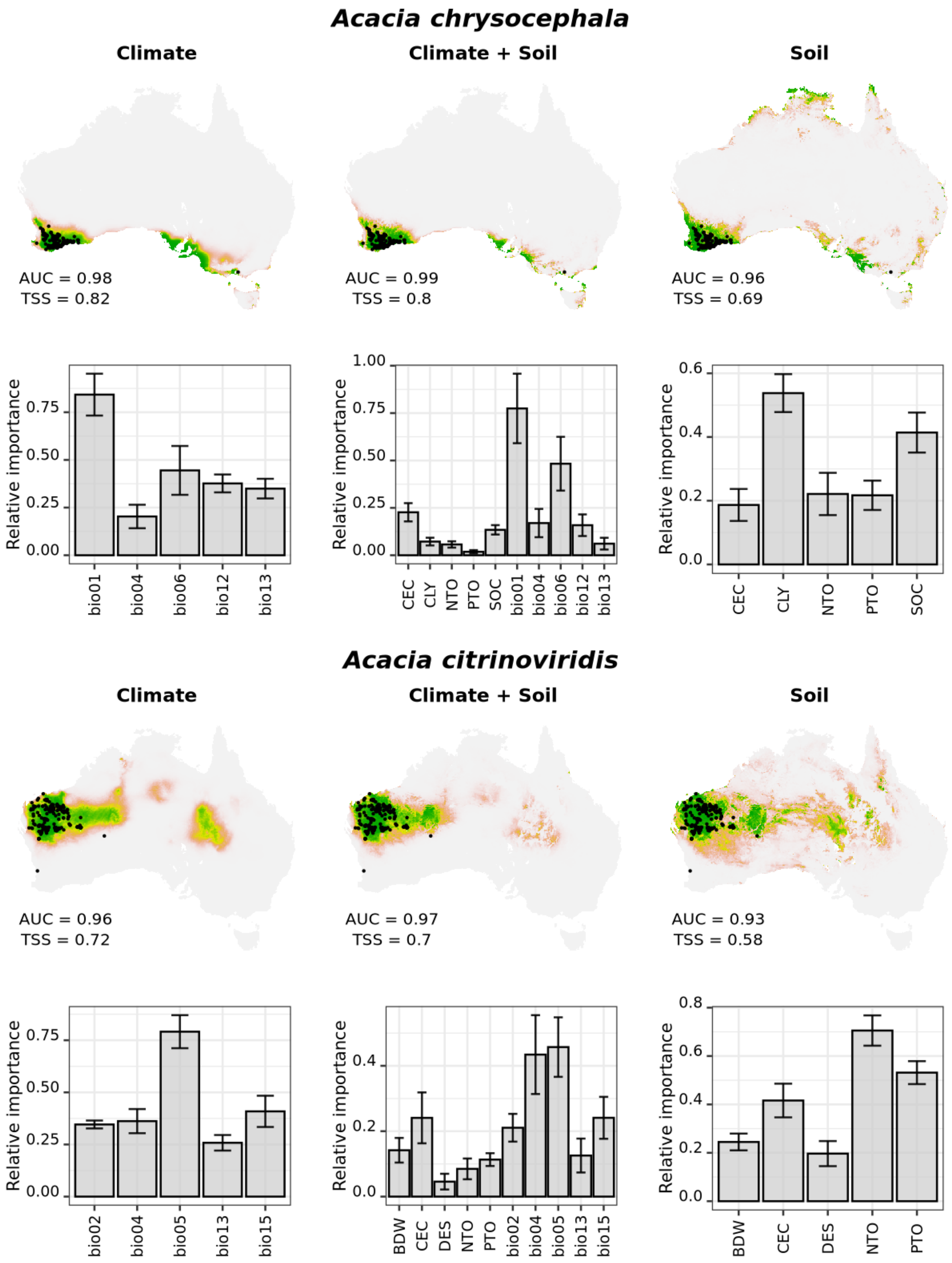

Acacia chrysocephala
Climate
Climate + Soil
Soil
AUC = 0.98
TSS = 0.82
AUC = 0.99
TSS = 0.8
AUC = 0.96
TSS = 0.69
Relative importance
0.00
0.25
0.50
0.75
1.00
0.0
0.2
0.4
0.6
bio01
bio04
bio06
bio12
bio13
CEC
CLY
NTO
PTO
SOC
Acacia citrinoviridis
AUC = 0.96
TSS = 0.72
AUC = 0.97
TSS = 0.7
AUC = 0.93
TSS = 0.58
0.8
bio02
bio05
bio15
BDW
DES

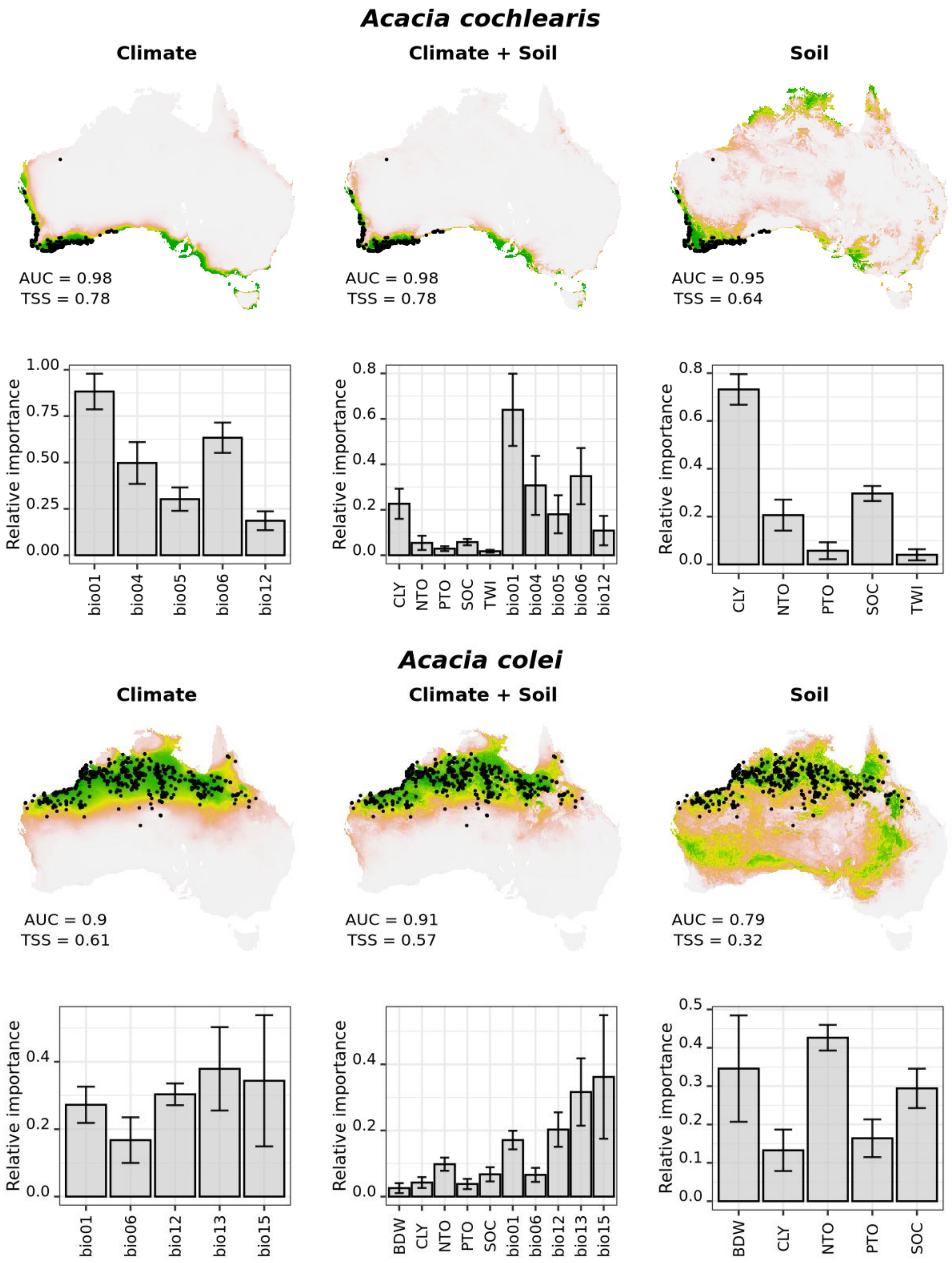

Acacia cochlearis
Climate
Climate + Soil
Soil
AUC = 0.98
TSS = 0.78
AUC = 0.98
TSS = 0.78
AUC = 0.95
TSS = 0.64
Relative importance
bio01
bio04
bio05
bio06
bio12
CLY
NTO
PTO
SOC
TWI
Acacia colei
Climate
Climate + Soil
Soil
AUC = 0.9
TSS = 0.61
AUC = 0.91
TSS = 0.57
AUC = 0.79
TSS = 0.32
bio01
bio06
bio12
bio13
bio15
BDW
CLY
NTO
PTO
SOC

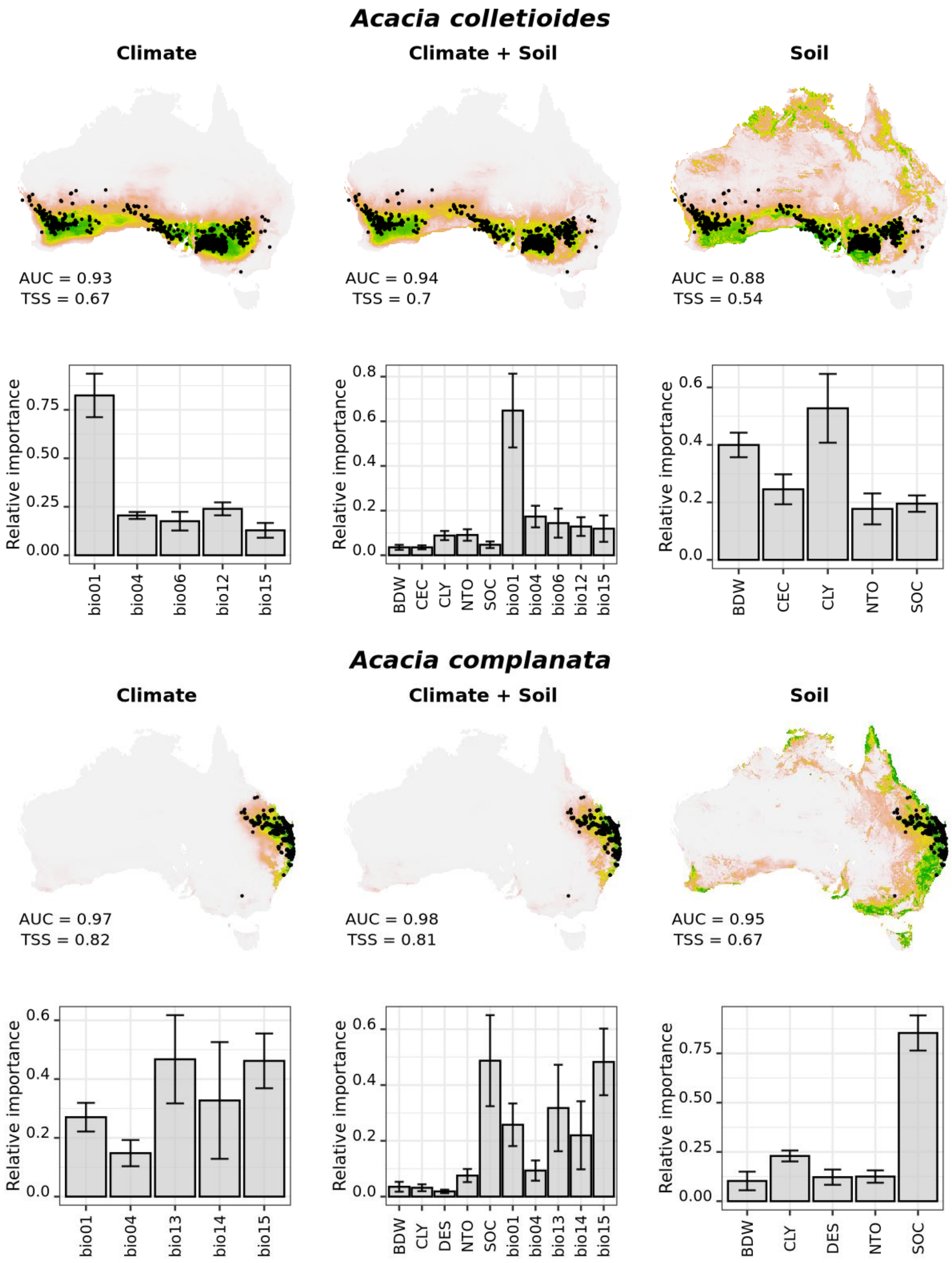

Acacia colletioides
Climate
Climate + Soil
Soil
AUC = 0.93
TSS = 0.67
AUC = 0.94
TSS = 0.7
AUC = 0.88
TSS = 0.54
Relative importance
0.00
0.25
0.50
0.75
bio01
bio04
bio06
bio12
bio15
Relative importance
0.0
0.2
0.4
0.6
0.8
BDW
CEC
CLY
NTO
SOC
bio01
bio04
bio06
bio12
bio15
Relative importance
0.0
0.2
0.4
0.6
BDW
CEC
CLY
NTO
SOC
Acacia complanata
Climate
Climate + Soil
Soil
AUC = 0.97
TSS = 0.82
AUC = 0.98
TSS = 0.81
AUC = 0.95
TSS = 0.67
Relative importance
0.0
0.2
0.4
0.6
bio01
bio04
bio13
bio14
bio15
Relative importance
0.0
0.2
0.4
0.6
BDW
CLY
DES
NTO
SOC
bio01
bio04
bio13
bio14
bio15
Relative importance
0.00
0.25
0.50
0.75
BDW
CLY
DES
NTO
SOC

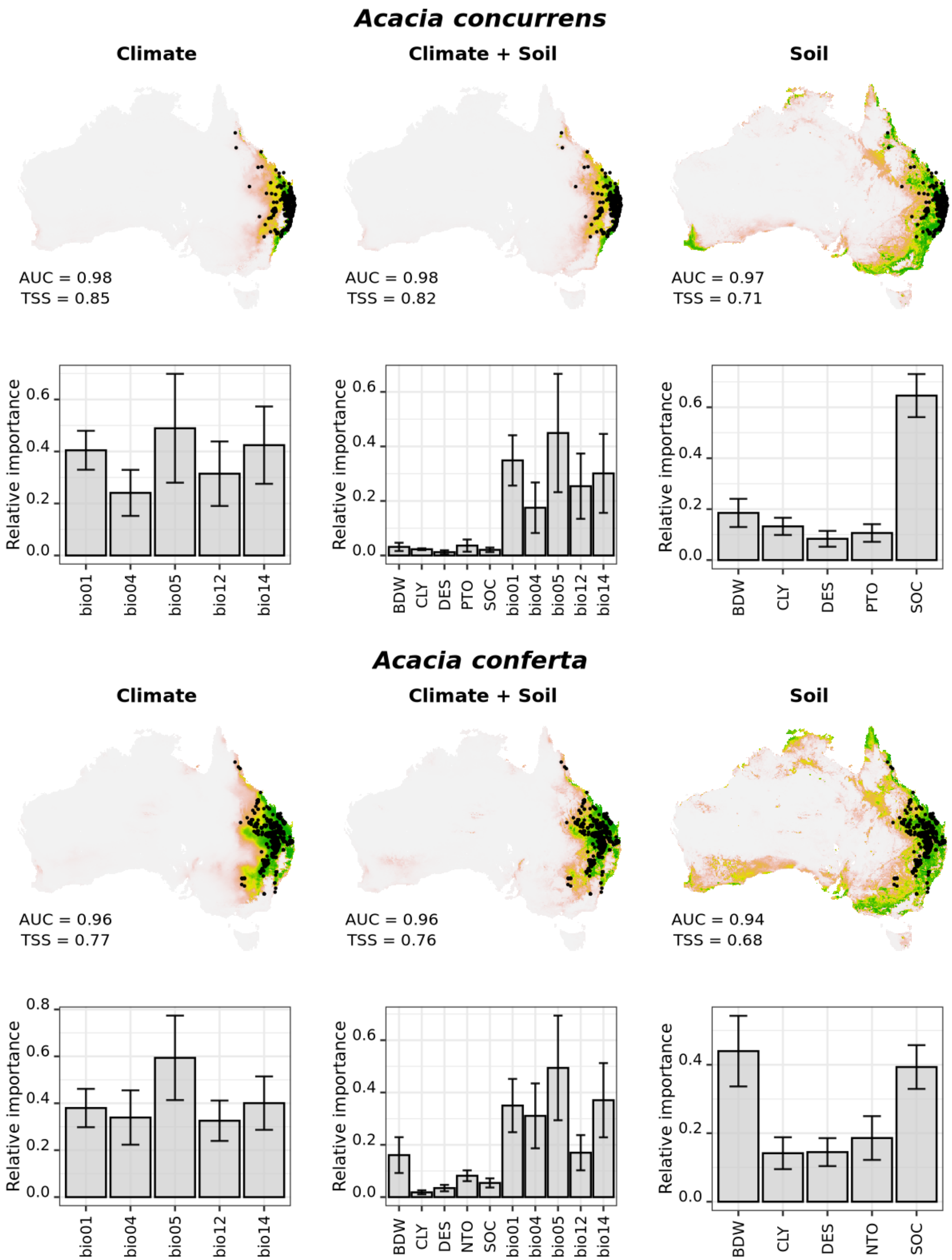
Acacia concurrens
Climate
Climate + Soil
Soil
AUC = 0.98
TSS = 0.85
AUC = 0.98
TSS = 0.82
AUC = 0.97
TSS = 0.71
Relative importance
bio01
bio04
bio05
bio12
bio14
BDW
CLY
DES
PTO
SOC
Acacia conferta
Climate
Climate + Soil
Soil
AUC = 0.96
TSS = 0.77
AUC = 0.96
TSS = 0.76
AUC = 0.94
TSS = 0.68
Relative importance
bio01
bio04
bio05
bio12
bio14
BDW
CLY
DES
NTO
SOC

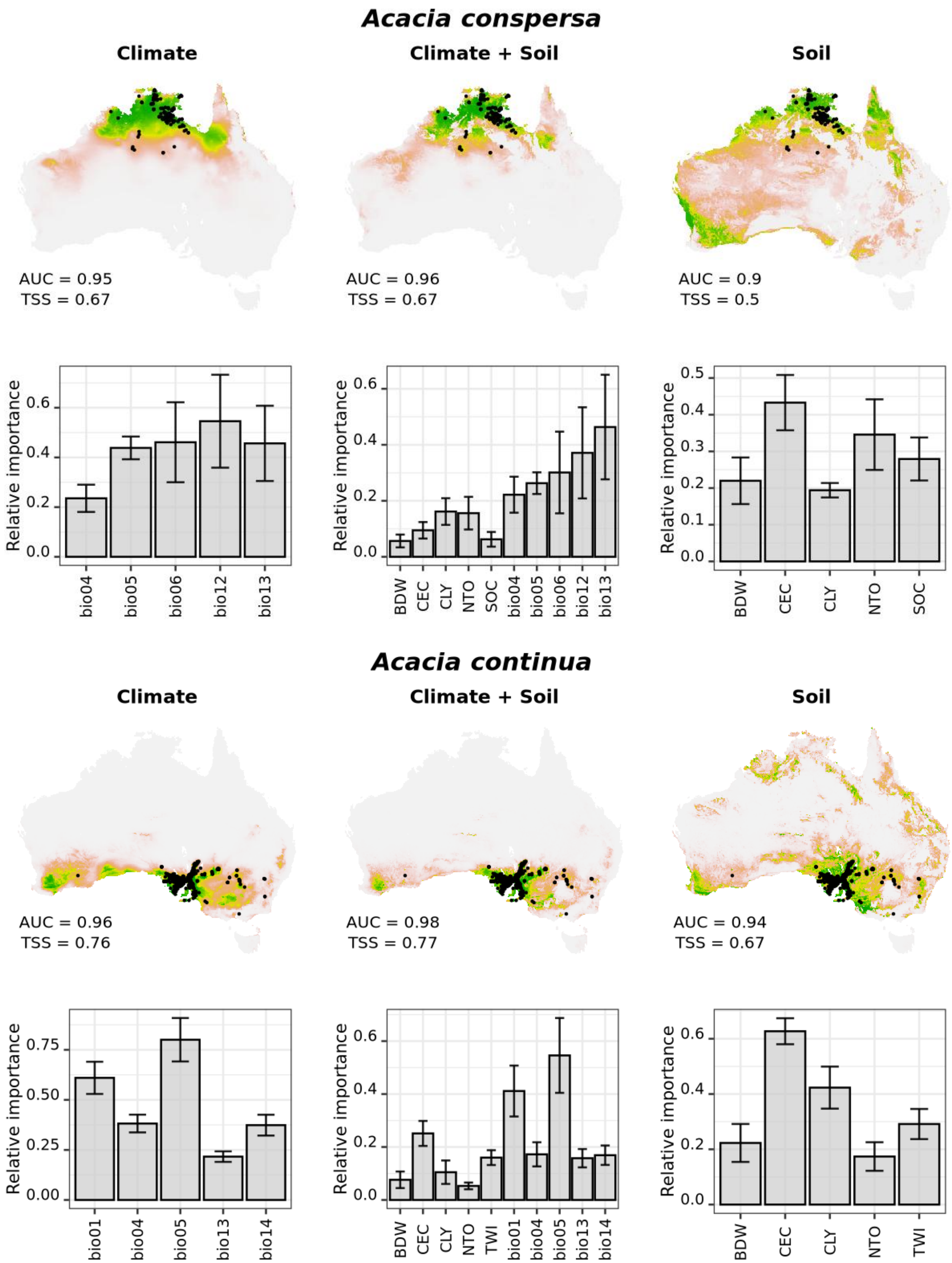

Acacia conspersa
Climate
Climate + Soil
Soil
AUC = 0.95
TSS = 0.67
AUC = 0.96
TSS = 0.67
AUC = 0.9
TSS = 0.5
Relative importance
bio04
bio05
bio06
bio12
bio13
BDW
CEC
CLY
NTO
SOC
Acacia continua
Climate
Climate + Soil
Soil
AUC = 0.96
TSS = 0.76
AUC = 0.98
TSS = 0.77
AUC = 0.94
TSS = 0.67
Relative importance
bio01
bio04
bio05
bio13
bio14
BDW
CEC
CLY
NTO
TWI

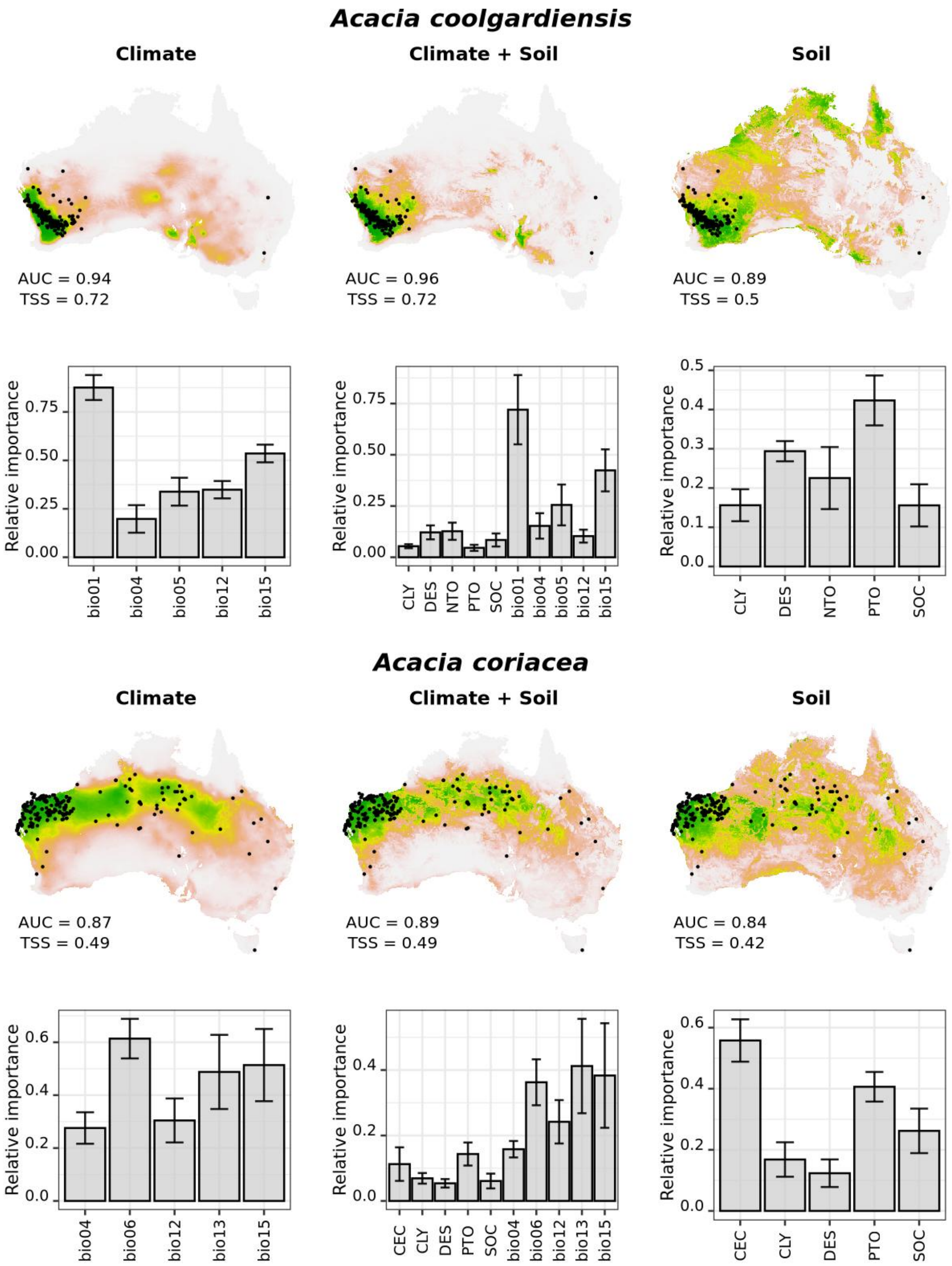
Acacia coolgardiensis
Climate
Climate + Soil
Soil
AUC = 0.94
TSS = 0.72
AUC = 0.96
TSS = 0.72
AUC = 0.89
TSS = 0.5
Relative importance
0.00
0.25
0.50
0.75
bio01
bio04
bio05
bio12
bio15
CLY
DES
NTO
PTO
SOC
0.0
0.1
0.2
0.3
0.4
0.5
Acacia coriacea
Climate
Climate + Soil
Soil
AUC = 0.87
TSS = 0.49
AUC = 0.89
TSS = 0.49
AUC = 0.84
TSS = 0.42
Relative importance
0.0
0.2
0.4
0.6
bio04
bio06
bio12
bio13
bio15
CEC
CLY
DES
PTO
SOC

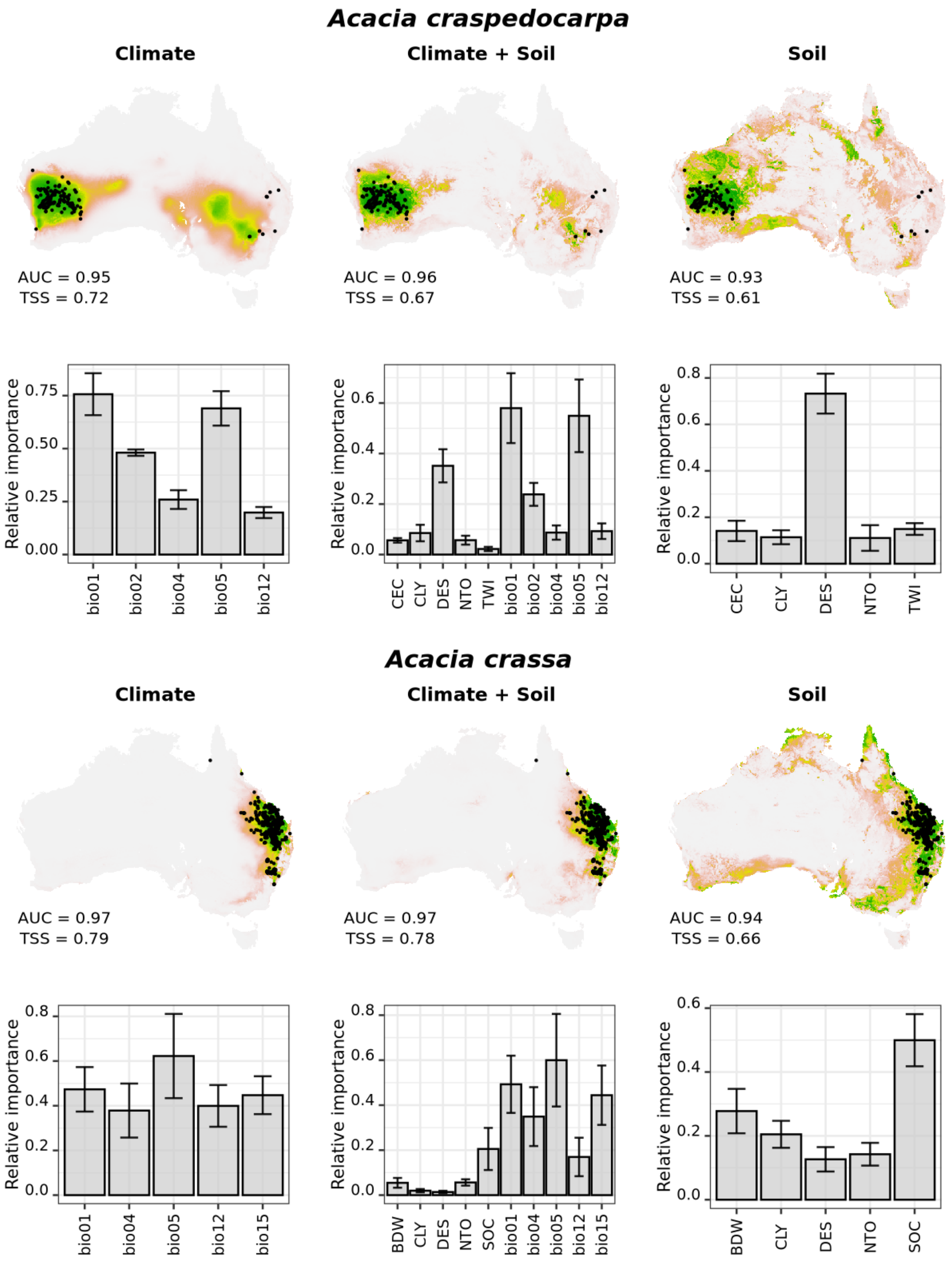
Acacia craspedocarpa
Climate
Climate + Soil
Soil
AUC = 0.95
TSS = 0.72
AUC = 0.96
TSS = 0.67
AUC = 0.93
TSS = 0.61
Relative importance
0.00
0.25
0.50
0.75
bio01
bio02
bio04
bio05
bio12
0.0
0.2
0.4
0.6
CEC
CLY
DES
NTO
TWI
bio01
bio02
bio04
bio05
bio12
0.8
CEC
CLY
DES
NTO
TWI
Acacia crassa
Climate
Climate + Soil
Soil
AUC = 0.97
TSS = 0.79
AUC = 0.97
TSS = 0.78
AUC = 0.94
TSS = 0.66
Relative importance
bio01
bio04
bio05
bio12
bio15
BDW
CLY
DES
NTO
SOC
bio01
bio04
bio05
bio12
bio15
BDW
CLY
DES
NTO
SOC

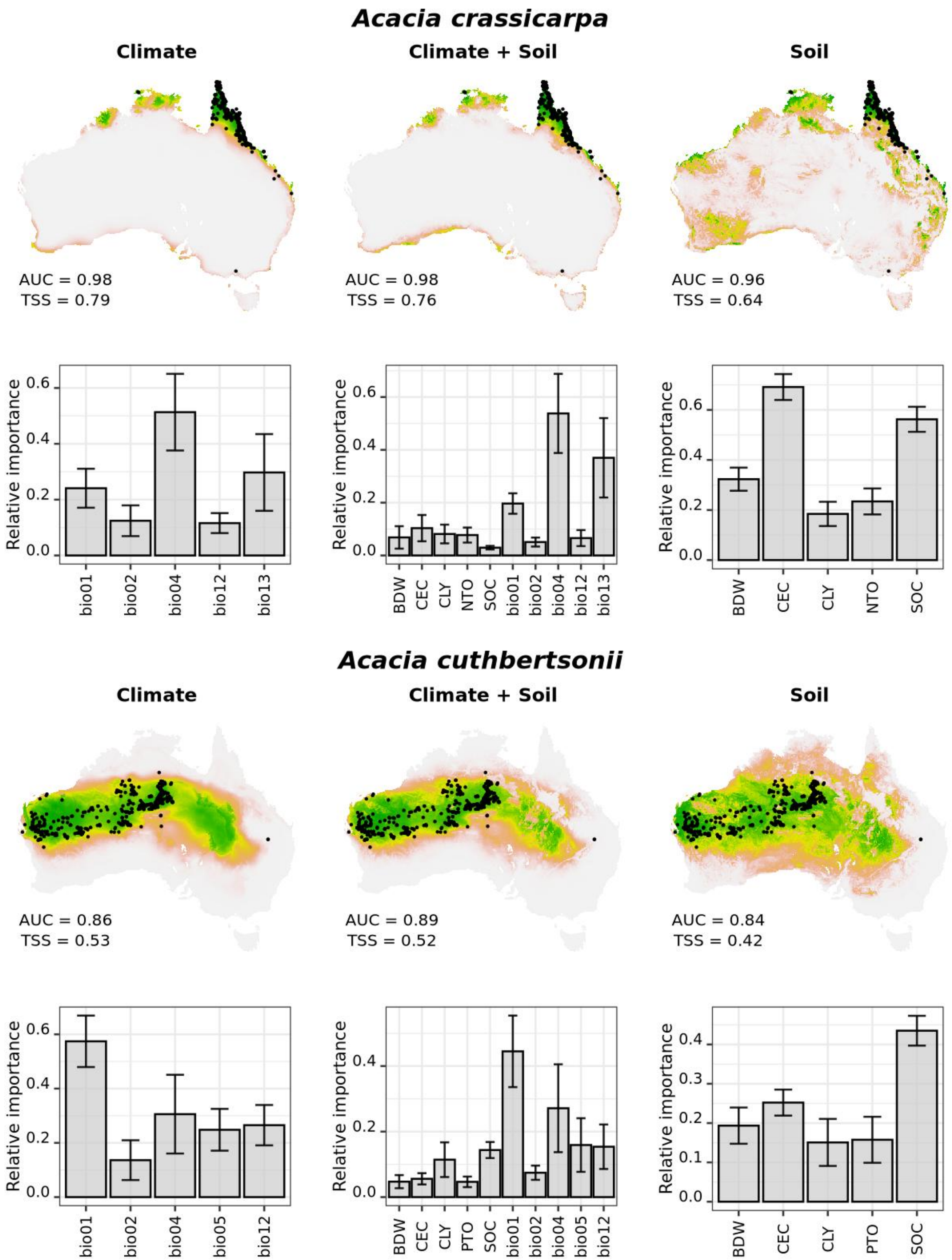
Acacia crassicarpa
Climate
Climate + Soil
Soil
AUC = 0.98
TSS = 0.79
AUC = 0.98
TSS = 0.76
AUC = 0.96
TSS = 0.64
Relative importance
0.0
0.2
0.4
0.6
bio01
bio02
bio04
bio12
bio13
BDW
CEC
CLY
NTO
SOC
Acacia cuthbertsonii
Climate
Climate + Soil
Soil
AUC = 0.86
TSS = 0.53
AUC = 0.89
TSS = 0.52
AUC = 0.84
TSS = 0.42
Relative importance
0.0
0.1
0.2
0.3
0.4
0.6
bio01
bio02
bio04
bio05
bio12
BDW
CEC
CLY
PTO
SOC

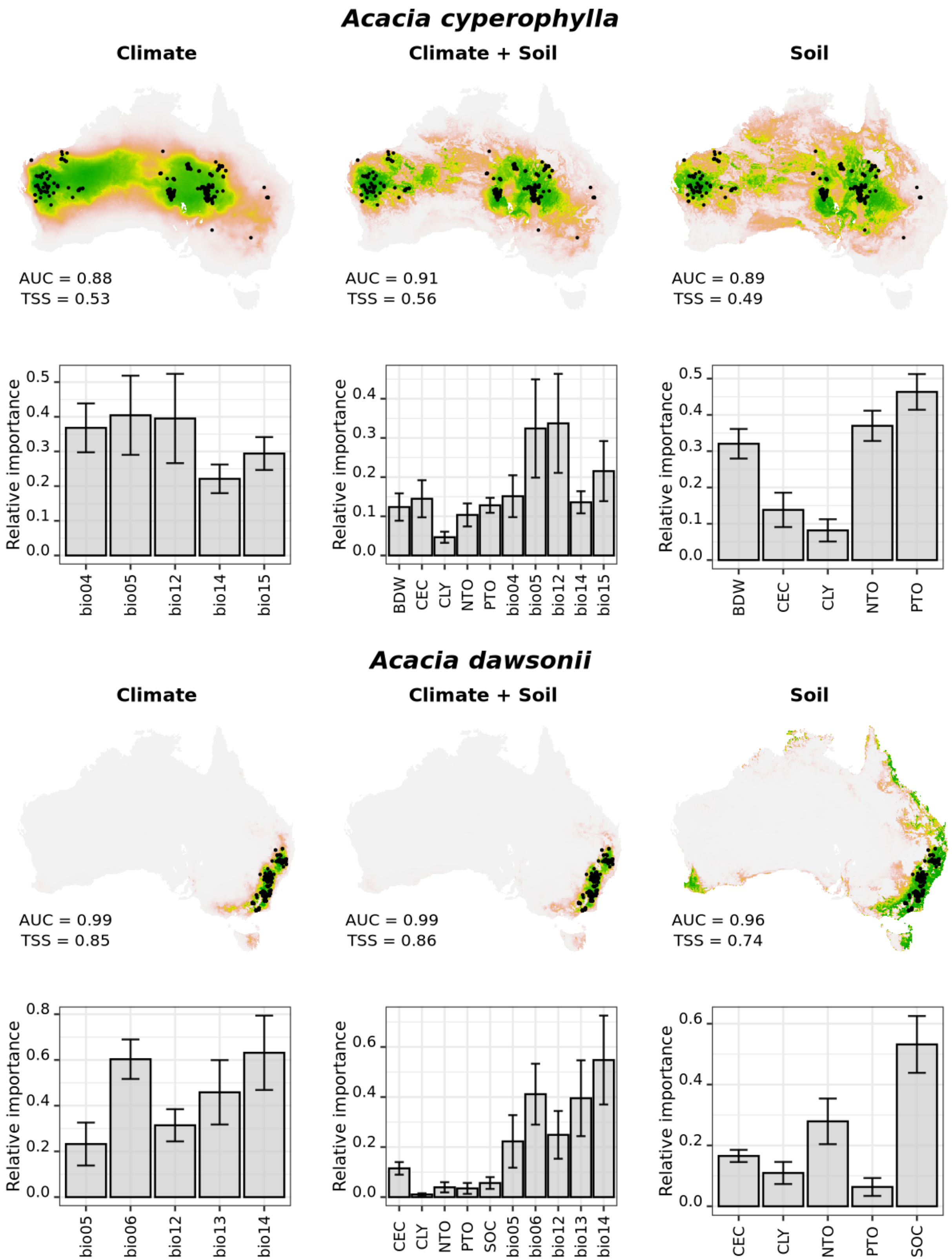
Acacia cyperophylla
Climate
Climate + Soil
Soil
AUC = 0.88
TSS = 0.53
AUC = 0.91
TSS = 0.56
AUC = 0.89
TSS = 0.49
Relative importance
bio04
bio05
bio12
bio14
bio15
BDW
CEC
CLY
NTO
PTO
Acacia dawsonii
AUC = 0.99
TSS = 0.85
AUC = 0.99
TSS = 0.86
AUC = 0.96
TSS = 0.74
bio06
bio13
SOC

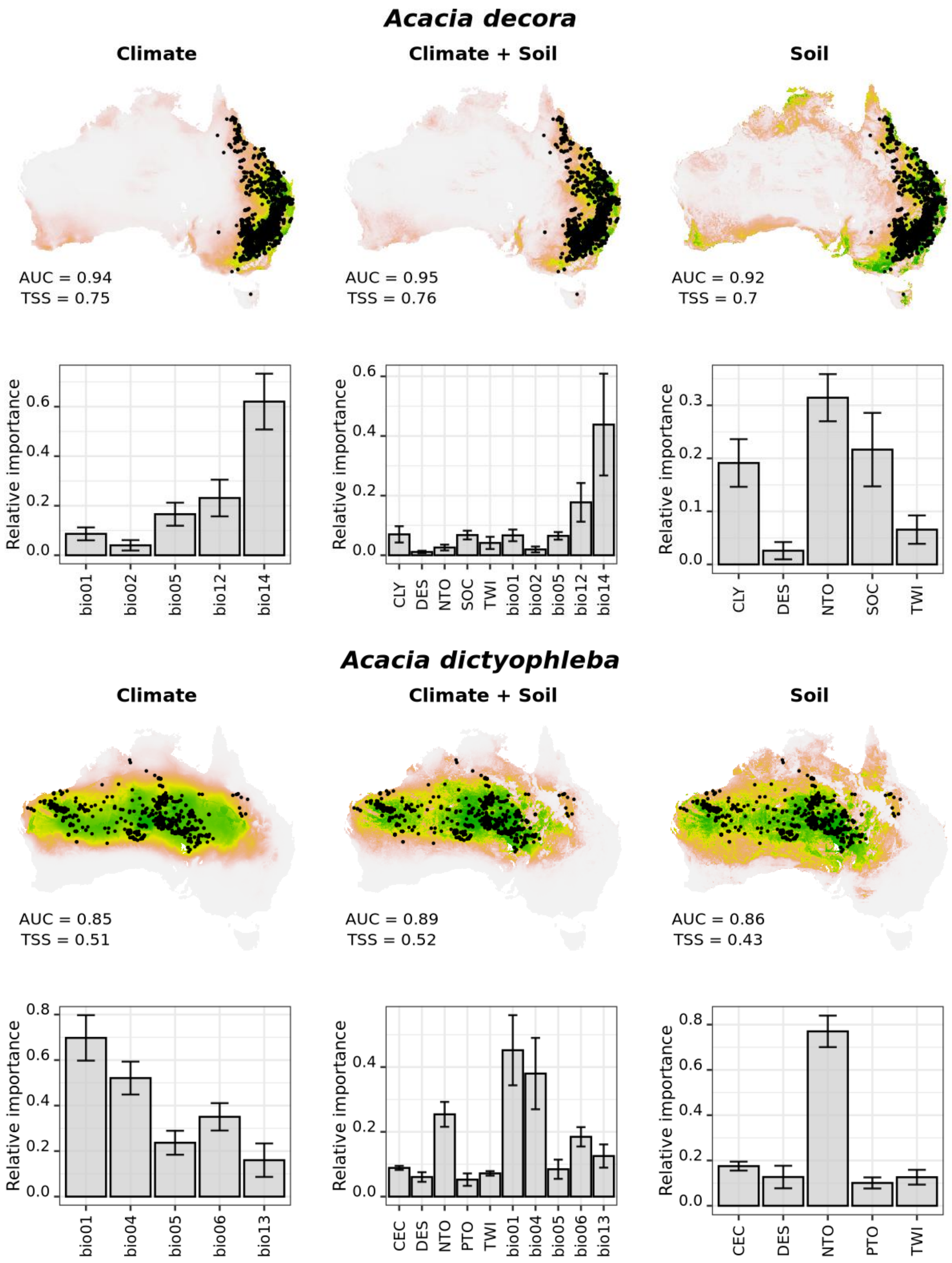

Acacia decora
Climate
Climate + Soil
Soil
AUC = 0.94
TSS = 0.75
AUC = 0.95
TSS = 0.76
AUC = 0.92
TSS = 0.7
Relative importance
bio01 bio02 bio05 bio12 bio14
CLY DES NTO SOC TWI bio01 bio02 bio05 bio12 bio14
CLY DES NTO SOC TWI
Acacia dictyophleba
Climate
Climate + Soil
Soil
AUC = 0.85
TSS = 0.51
AUC = 0.89
TSS = 0.52
AUC = 0.86
TSS = 0.43
Relative importance
bio01 bio04 bio05 bio06 bio13
CEC DES NTO PTO TWI bio01 bio04 bio05 bio06 bio13
CEC DES NTO PTO TWI

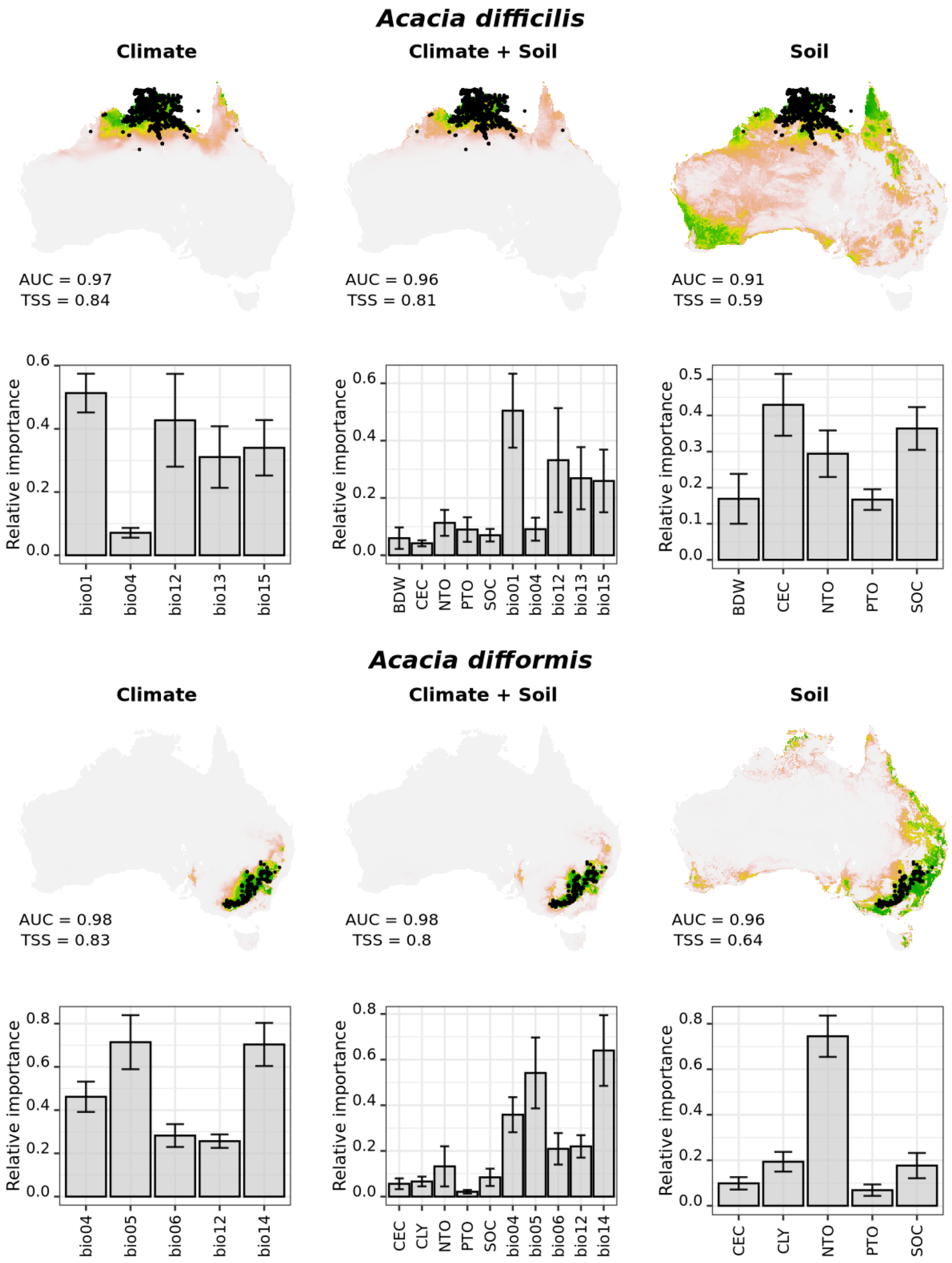
Acacia difficilis
Climate
Climate + Soil
Soil
AUC = 0.97
TSS = 0.84
AUC = 0.96
TSS = 0.81
AUC = 0.91
TSS = 0.59
Relative importance
bio01 bio04 bio12 bio13 bio15
BDW CEC NTO PTO SOC bio01 bio04 bio12 bio13 bio15
BDW CEC NTO PTO SOC
Acacia difformis
Climate
Climate + Soil
Soil
AUC = 0.98
TSS = 0.83
AUC = 0.98
TSS = 0.8
AUC = 0.96
TSS = 0.64
Relative importance
bio04 bio05 bio06 bio12 bio14
CEC CLY NTO PTO SOC bio04 bio05 bio06 bio12 bio14
CEC CLY NTO PTO SOC

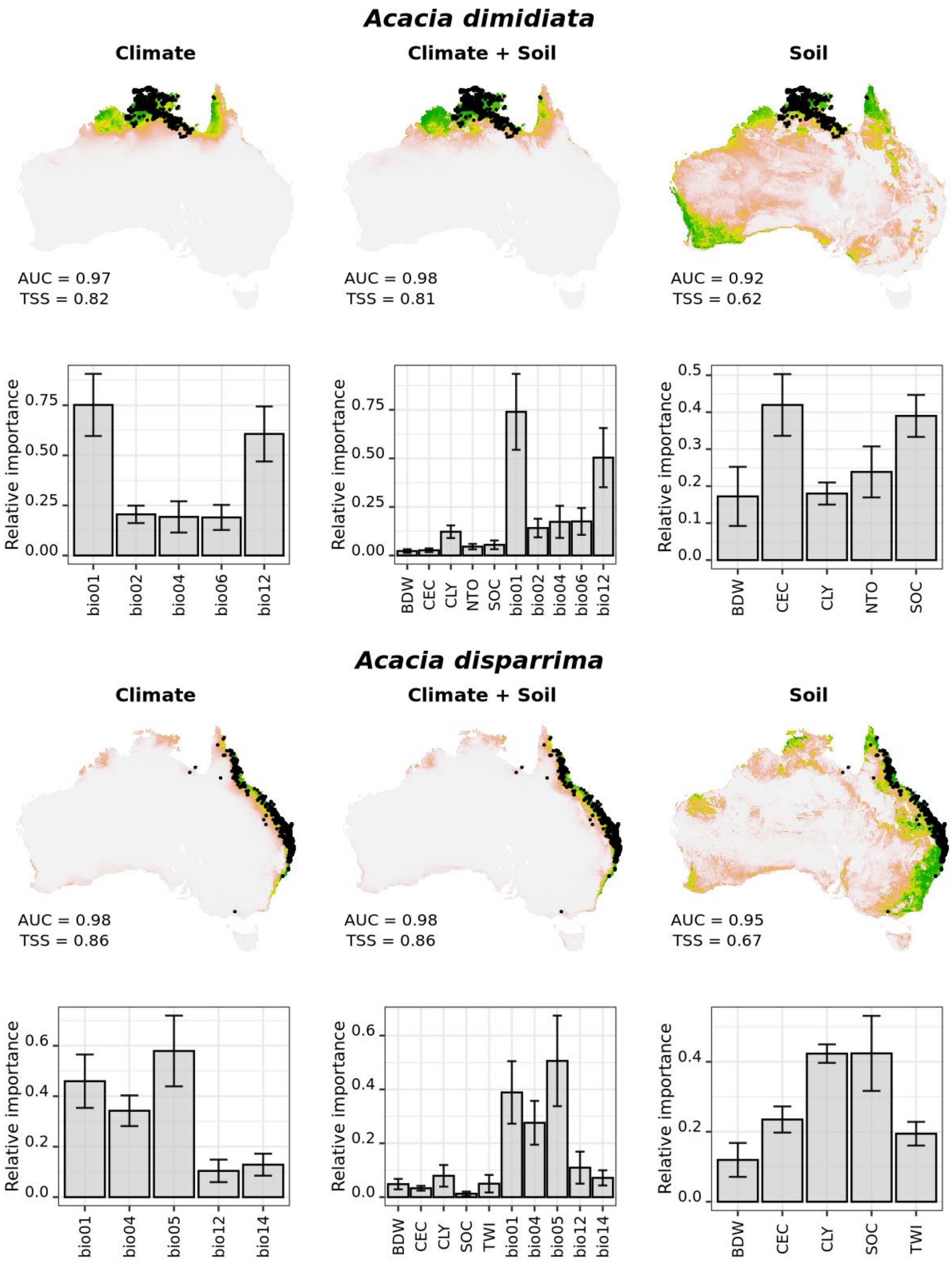
Acacia dimidiata
Climate
Climate + Soil
Soil
AUC = 0.97
TSS = 0.82
AUC = 0.98
TSS = 0.81
AUC = 0.92
TSS = 0.62
Relative importance
0.00
0.25
0.50
0.75
bio01
bio02
bio04
bio06
bio12
BDW
CEC
CLY
NTO
SOC
0.0
0.1
0.2
0.3
0.4
0.5
Acacia disparrima
Climate
Climate + Soil
Soil
AUC = 0.98
TSS = 0.86
AUC = 0.98
TSS = 0.86
AUC = 0.95
TSS = 0.67
Relative importance
0.0
0.2
0.4
0.6
bio01
bio04
bio05
bio12
bio14
BDW
CEC
CLY
SOC
TWI

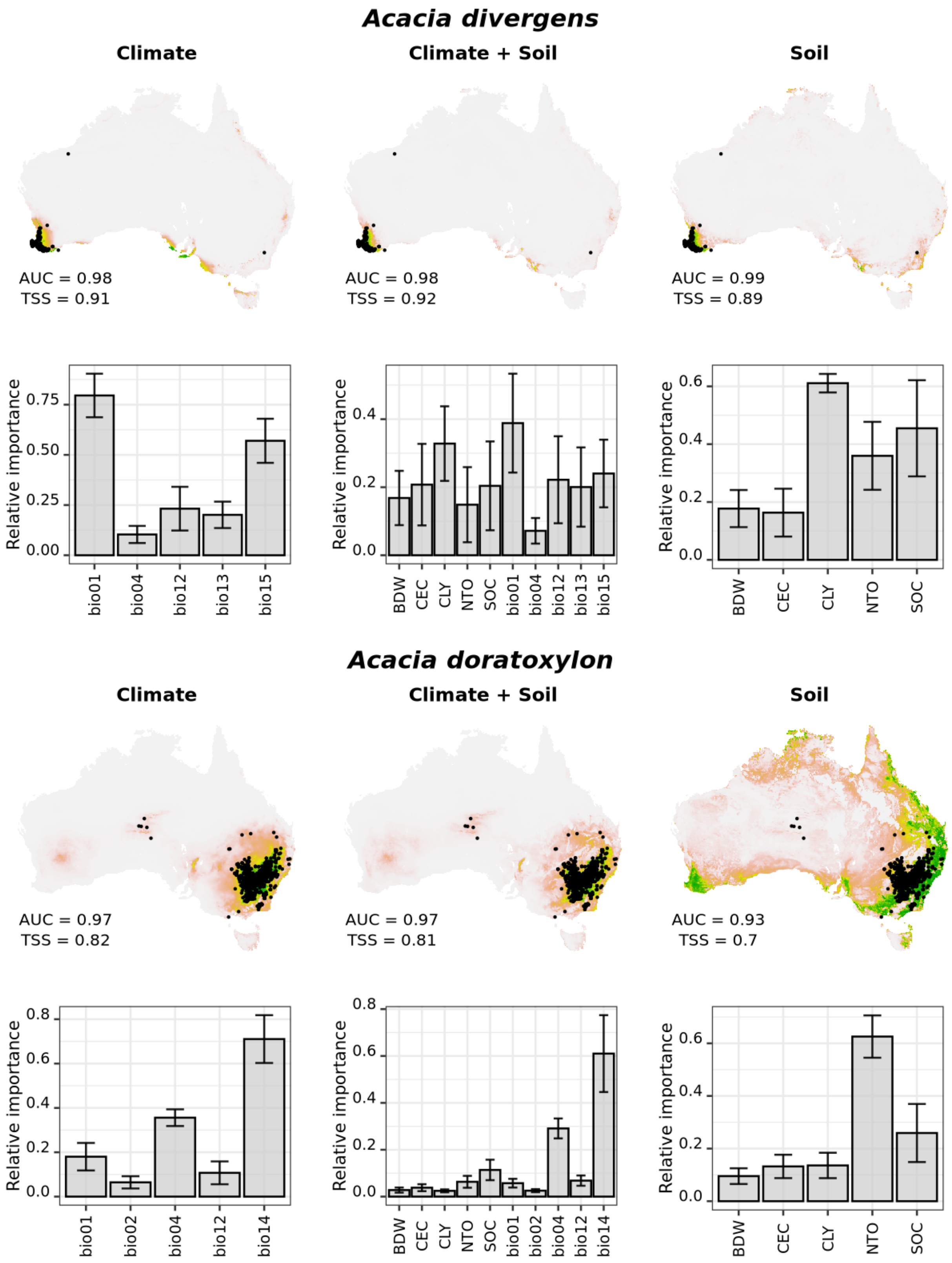
Acacia divergens
Climate
Climate + Soil
Soil
AUC = 0.98
TSS = 0.91
AUC = 0.98
TSS = 0.92
AUC = 0.99
TSS = 0.89
Relative importance
0.00
0.25
0.50
0.75
bio01
bio04
bio12
bio13
bio15
0.0
0.2
0.4
BDW
CEC
CLY
NTO
SOC
bio01
bio04
bio12
bio13
bio15
0.6
Acacia doratoxylon
AUC = 0.97
TSS = 0.82
AUC = 0.97
TSS = 0.81
AUC = 0.93
TSS = 0.7
0.8
bio02
bio14

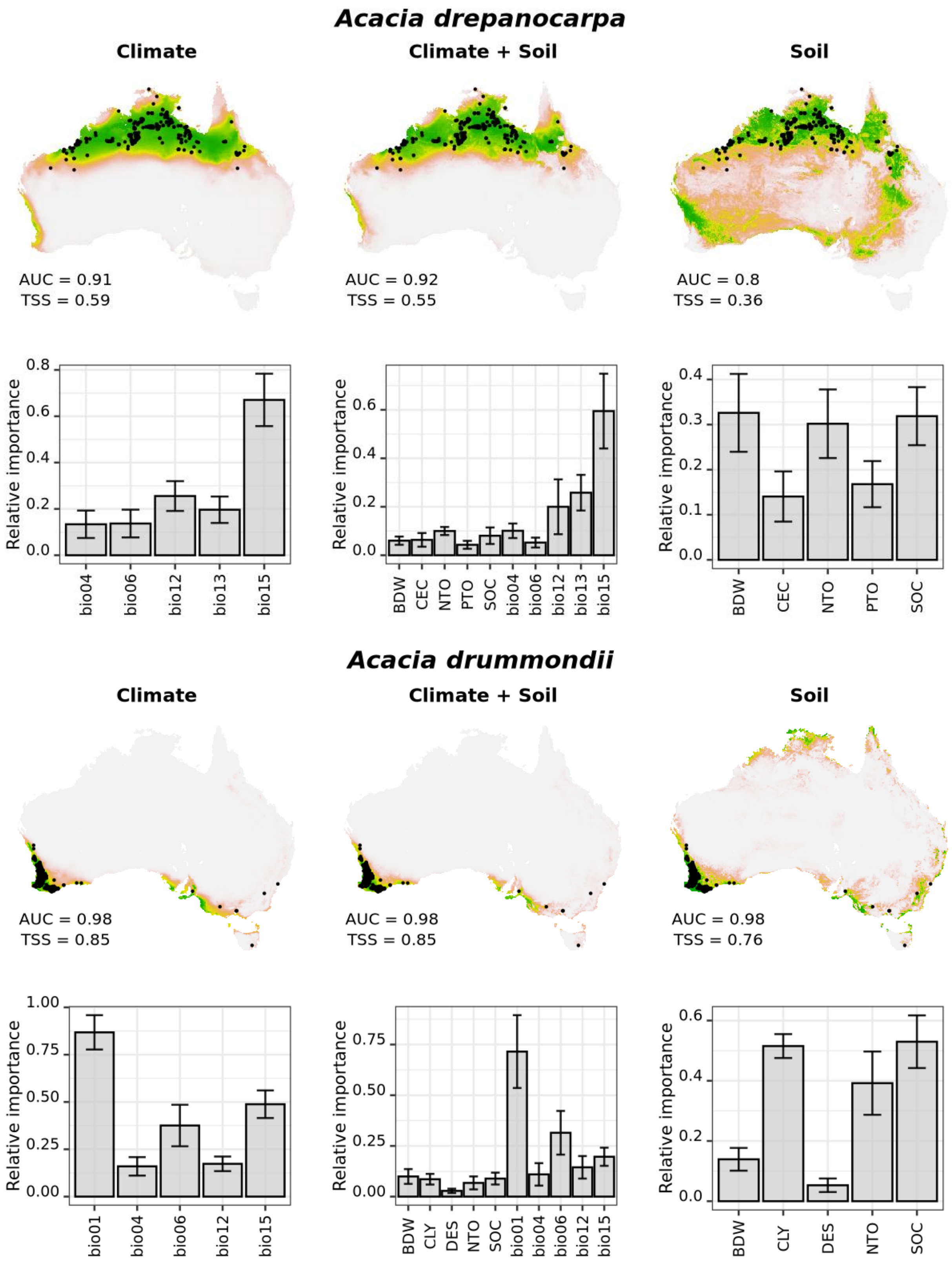
Acacia drepanocarpa
Climate
Climate + Soil
Soil
AUC = 0.91
TSS = 0.59
AUC = 0.92
TSS = 0.55
AUC = 0.8
TSS = 0.36
Relative importance
bio04 bio06 bio12 bio13 bio15
BDW CEC NTO PTO SOC bio04 bio06 bio12 bio13 bio15
BDW CEC NTO PTO SOC
Acacia drummondii
Climate
Climate + Soil
Soil
AUC = 0.98
TSS = 0.85
AUC = 0.98
TSS = 0.85
AUC = 0.98
TSS = 0.76
Relative importance
bio01 bio04 bio06 bio12 bio15
BDW CLY DES NTO SOC bio01 bio04 bio06 bio12 bio15
BDW CLY DES NTO SOC

Acacia echinula

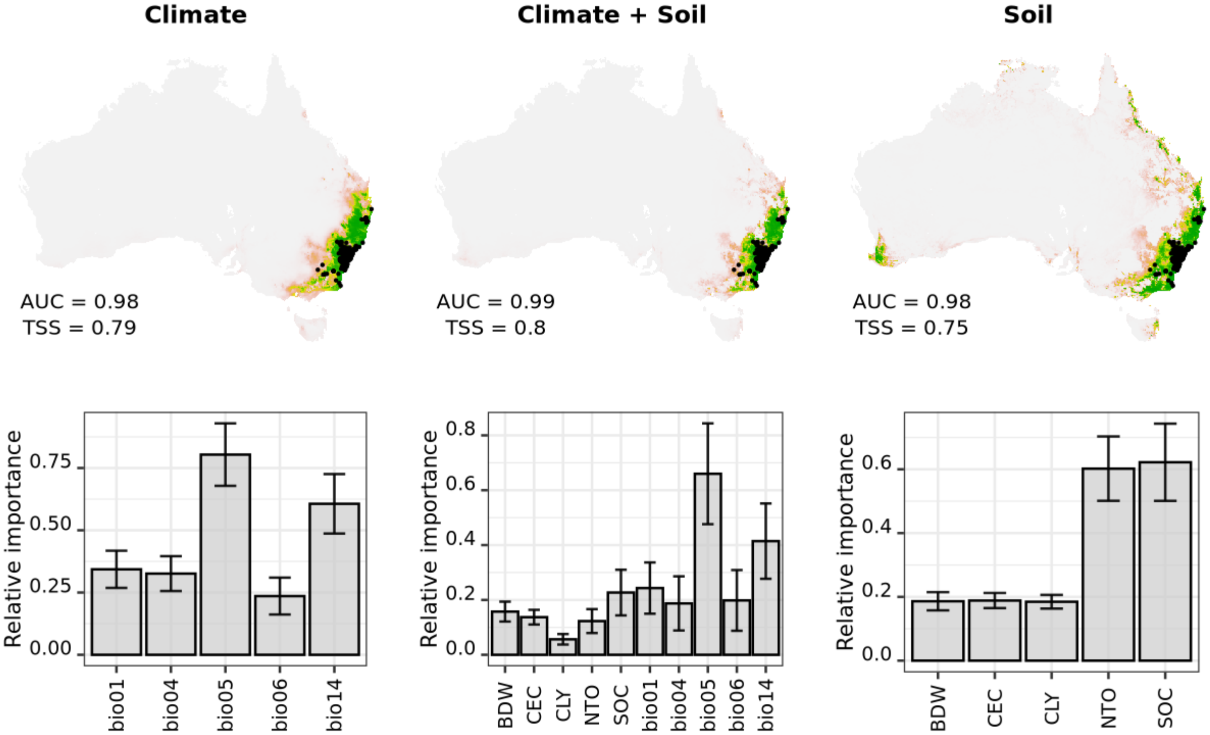

Acacia enervia

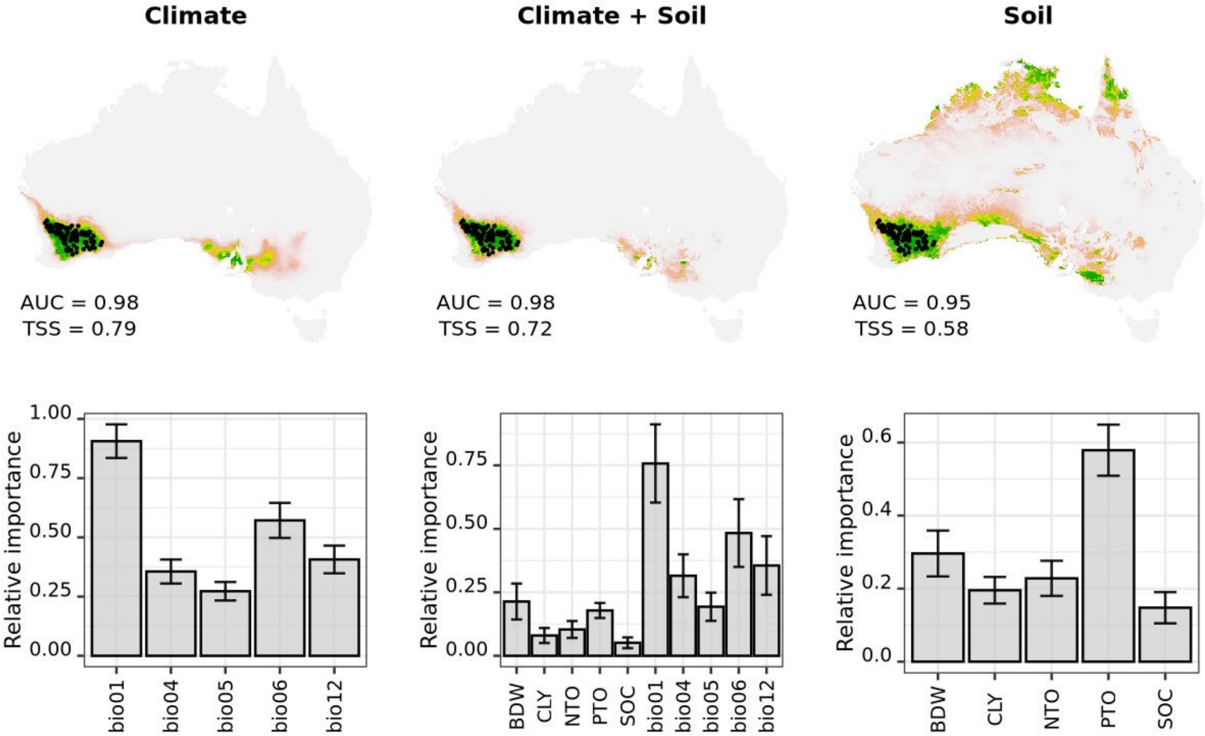

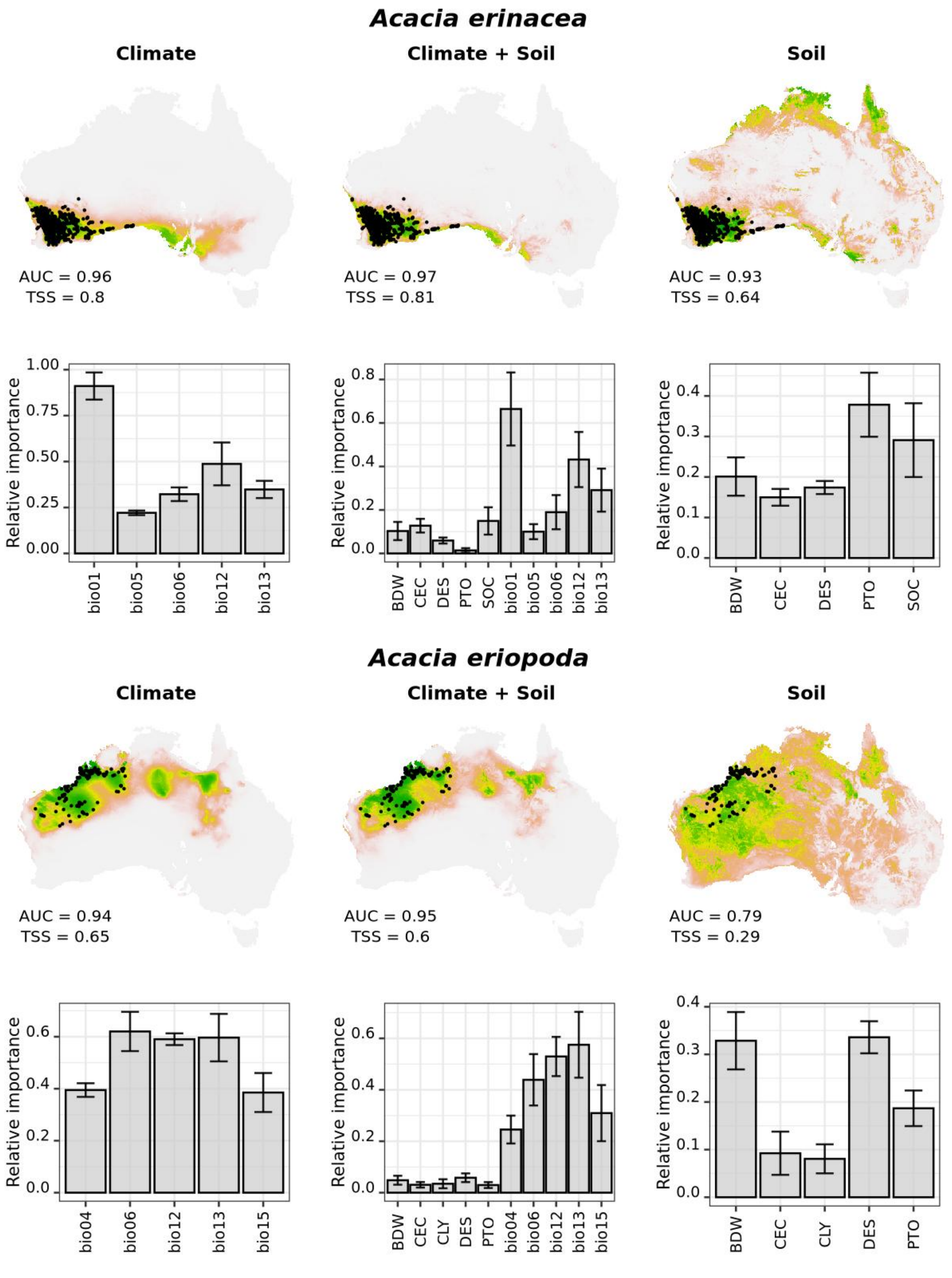
Acacia erinacea
Climate
Climate + Soil
Soil
AUC = 0.96
TSS = 0.8
AUC = 0.97
TSS = 0.81
AUC = 0.93
TSS = 0.64
Relative importance
bio01 bio05 bio06 bio12 bio13
BDW CEC DES PTO SOC bio01 bio05 bio06 bio12 bio13
BDW CEC DES PTO SOC
Acacia eriopoda
Climate
Climate + Soil
Soil
AUC = 0.94
TSS = 0.65
AUC = 0.95
TSS = 0.6
AUC = 0.79
TSS = 0.29
Relative importance
bio04 bio06 bio12 bio13 bio15
BDW CEC CLY DES PTO bio04 bio06 bio12 bio13 bio15
BDW CEC CLY DES PTO

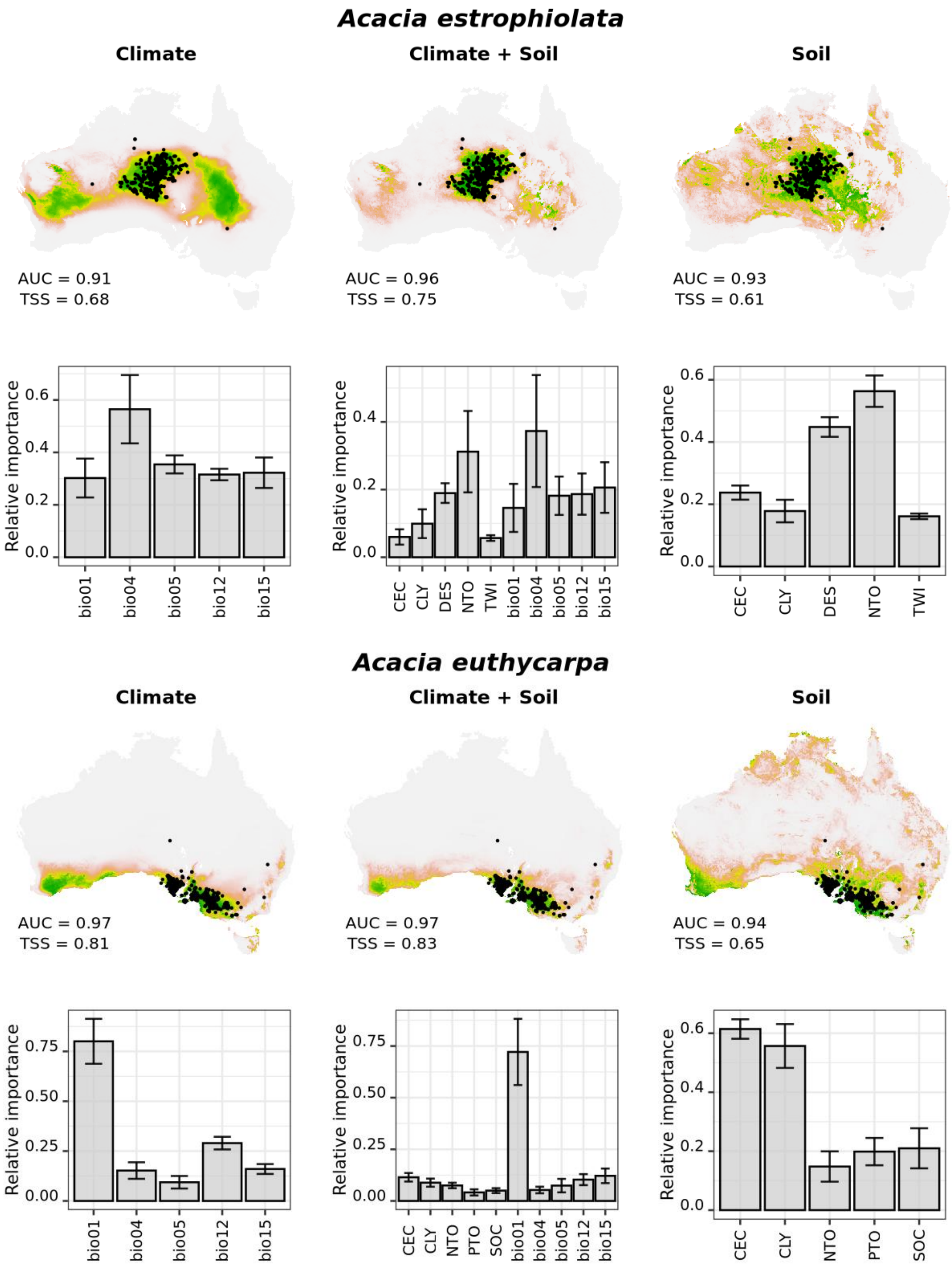
Acacia estrophiolata
Climate
Climate + Soil
Soil
AUC = 0.91
TSS = 0.68
AUC = 0.96
TSS = 0.75
AUC = 0.93
TSS = 0.61
Relative importance
bio01
bio04
bio05
bio12
bio15
CEC
CLY
DES
NTO
TWI
Acacia euthycarpa
Climate
Climate + Soil
Soil
AUC = 0.97
TSS = 0.81
AUC = 0.97
TSS = 0.83
AUC = 0.94
TSS = 0.65
Relative importance
PTO
SOC

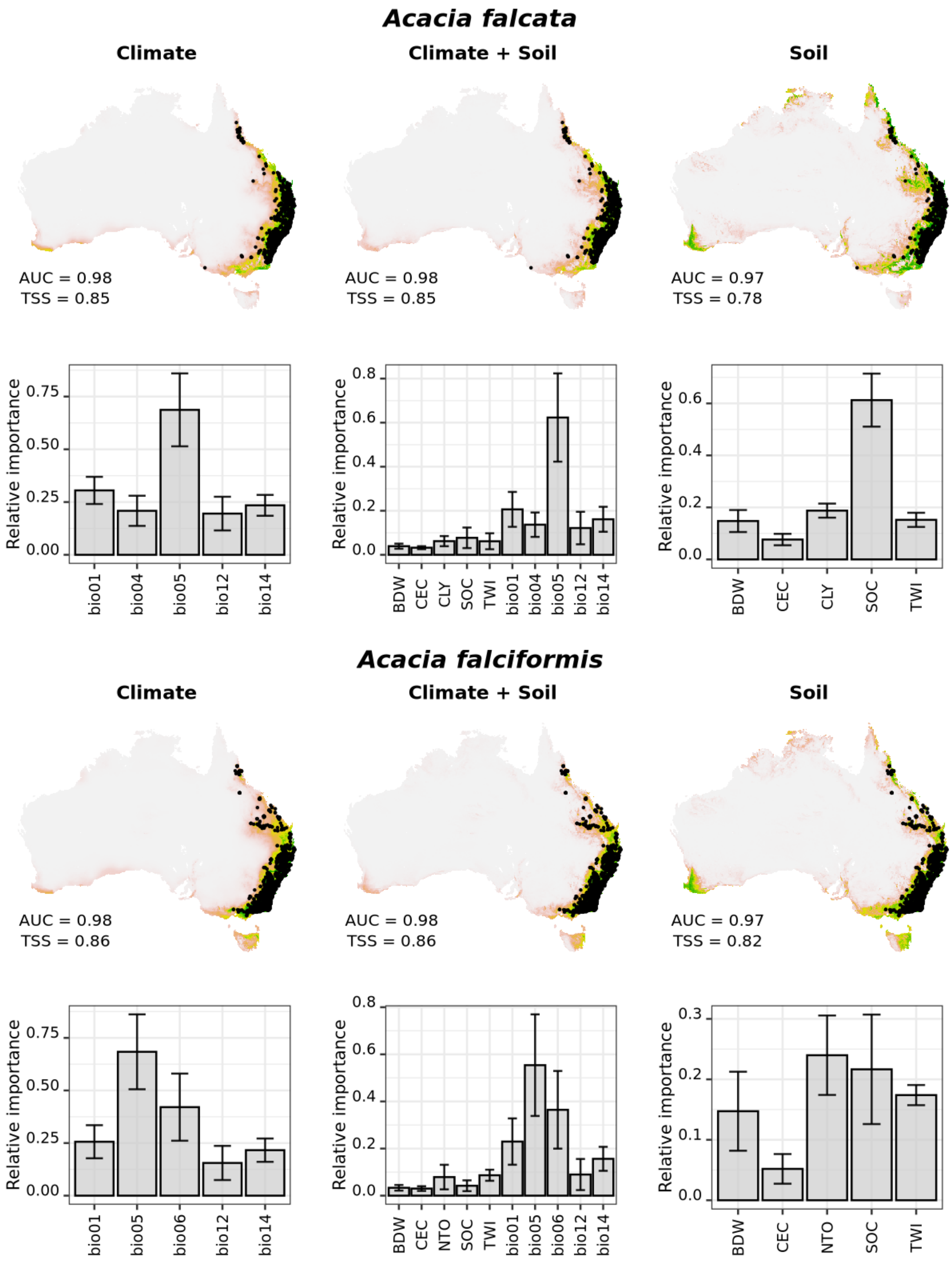
Acacia falcata
Climate
Climate + Soil
Soil
AUC = 0.98
TSS = 0.85
AUC = 0.98
TSS = 0.85
AUC = 0.97
TSS = 0.78
Relative importance
0.00
0.25
0.50
0.75
bio01
bio04
bio05
bio12
bio14
0.0
0.2
0.4
0.6
0.8
BDW
CEC
CLY
SOC
TWI
bio01
bio04
bio05
bio12
bio14
0.0
0.2
0.4
0.6
BDW
CEC
CLY
SOC
TWI
Acacia falciformis
Climate
Climate + Soil
Soil
AUC = 0.98
TSS = 0.86
AUC = 0.98
TSS = 0.86
AUC = 0.97
TSS = 0.82
Relative importance
0.00
0.25
0.50
0.75
bio01
bio05
bio06
bio12
bio14
0.0
0.2
0.4
0.6
0.8
BDW
CEC
NTO
SOC
TWI
bio01
bio05
bio06
bio12
bio14
0.0
0.1
0.2
0.3
BDW
CEC
NTO
SOC
TWI

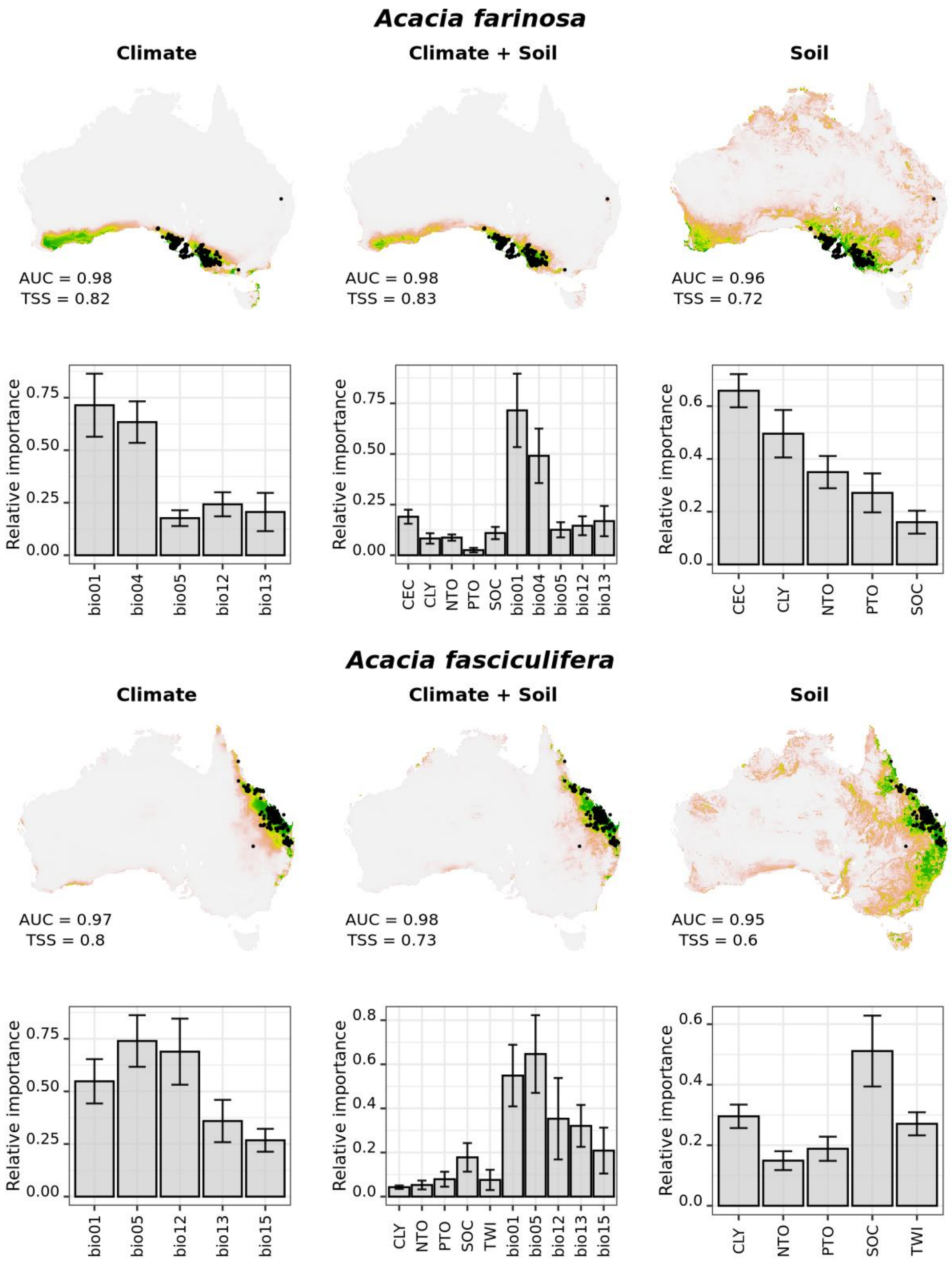

Acacia farinosa
Climate
Climate + Soil
Soil
AUC = 0.98
TSS = 0.82
AUC = 0.98
TSS = 0.83
AUC = 0.96
TSS = 0.72
Relative importance
bio01
bio04
bio05
bio12
bio13
CEC
CLY
NTO
PTO
SOC
Acacia fasciculifera
Climate
Climate + Soil
Soil
AUC = 0.97
TSS = 0.8
AUC = 0.98
TSS = 0.73
AUC = 0.95
TSS = 0.6
Relative importance
bio01
bio05
bio12
bio13
bio15
CLY
NTO
PTO
SOC
TWI

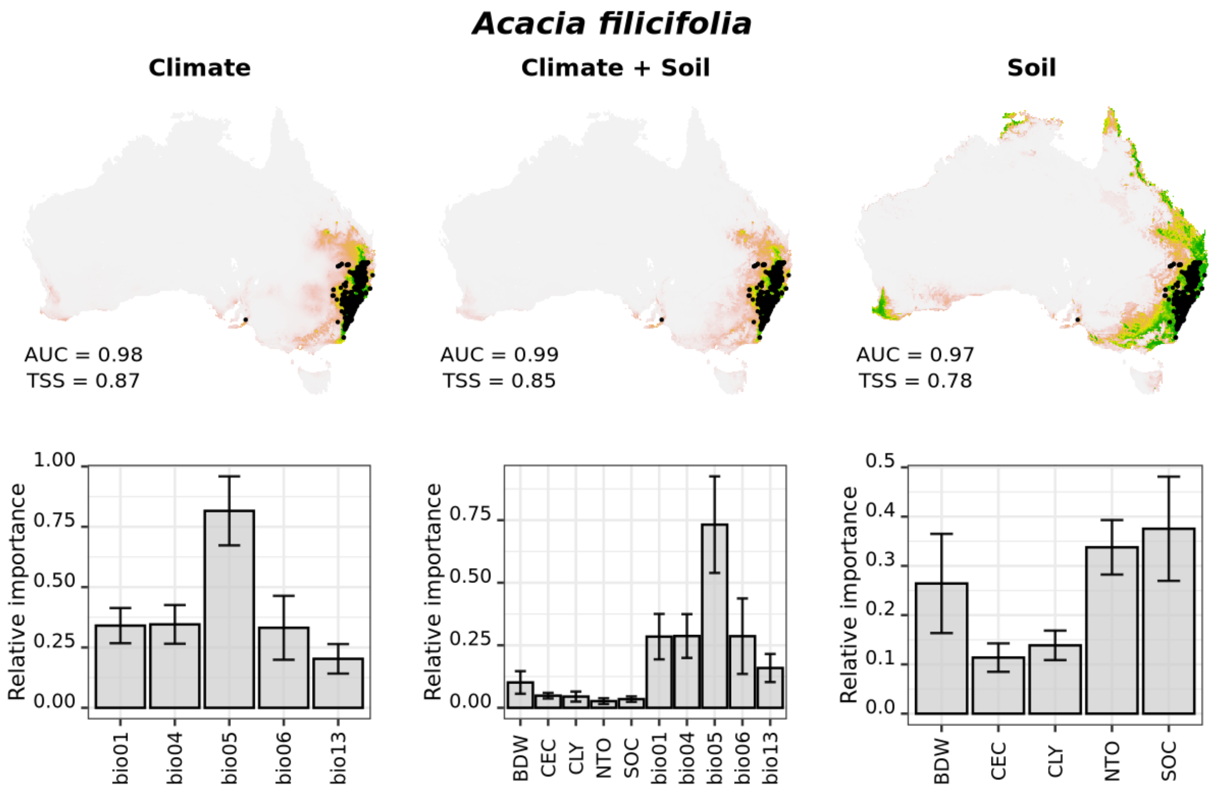
Acacia filicifolia
Climate
Climate + Soil
Soil
AUC = 0.98
TSS = 0.87
AUC = 0.99
TSS = 0.85
AUC = 0.97
TSS = 0.78
Relative importance
0.00
0.25
0.50
0.75
1.00
bio01
bio04
bio05
bio06
bio13
BDW
CEC
CLY
NTO
SOC
0.0
0.1
0.2
0.3
0.4
0.5

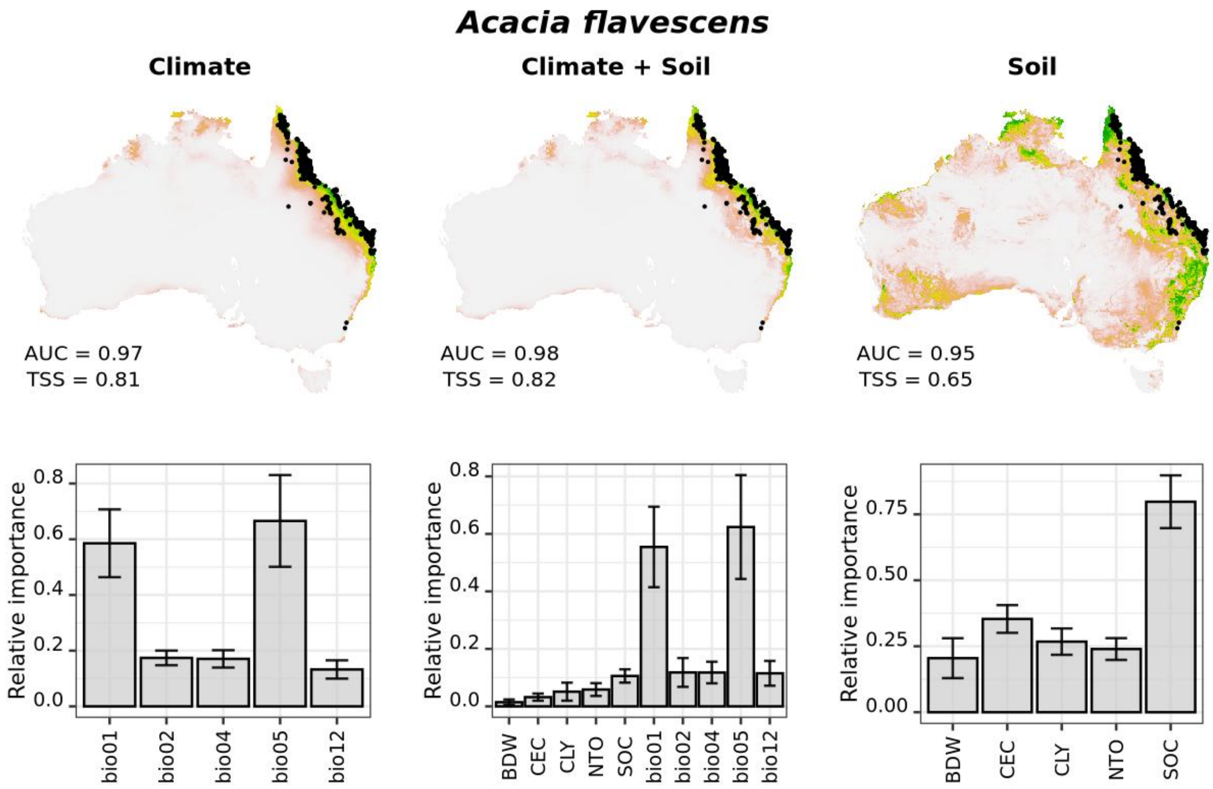
Acacia flavescens
Climate
Climate + Soil
Soil
AUC = 0.97
TSS = 0.81
AUC = 0.98
TSS = 0.82
AUC = 0.95
TSS = 0.65
Relative importance
0.0
0.2
0.4
0.6
0.8
bio01
bio02
bio04
bio05
bio12
BDW
CEC
CLY
NTO
SOC
0.00
0.25
0.50
0.75

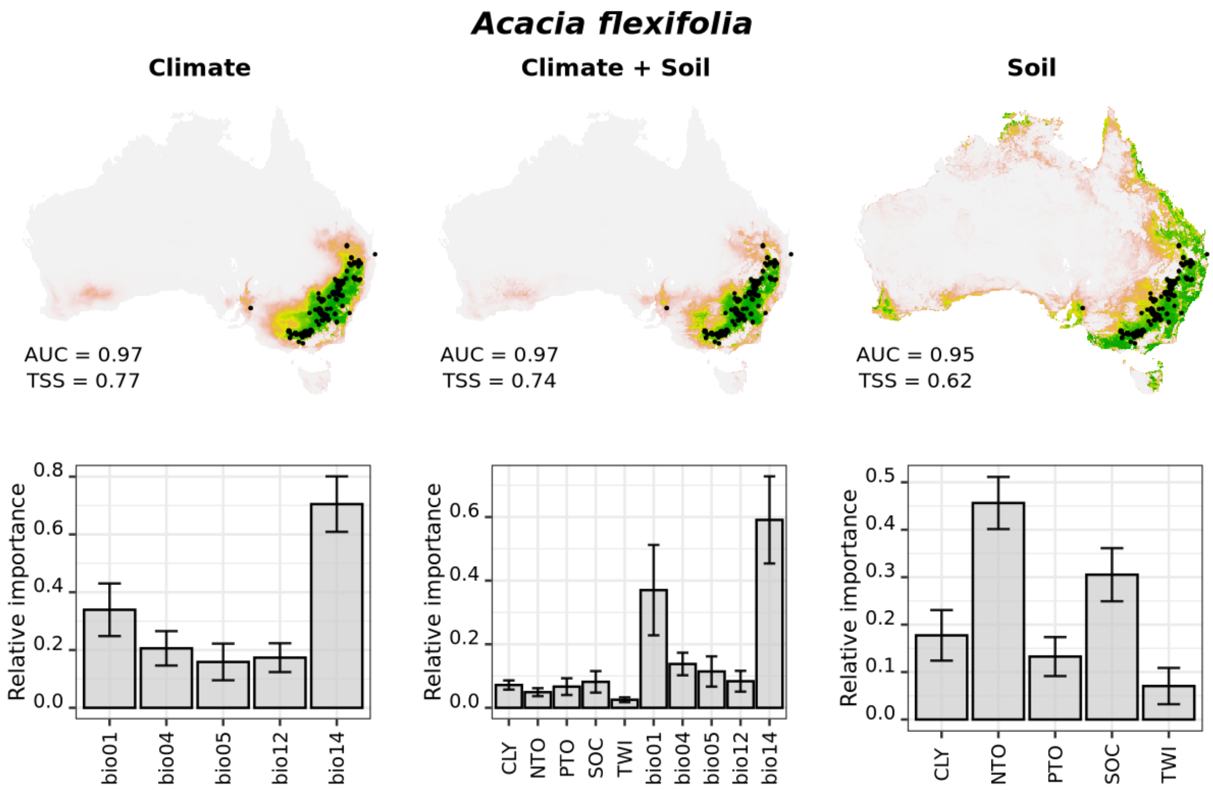

Acacia flexifolia
Climate
Climate + Soil
Soil
AUC = 0.97
TSS = 0.77
AUC = 0.97
TSS = 0.74
AUC = 0.95
TSS = 0.62
Relative importance
0.0
0.2
0.4
0.6
0.8
bio01
bio04
bio05
bio12
bio14
Relative importance
0.0
0.2
0.4
0.6
CLY
NTO
PTO
SOC
TWI
bio01
bio04
bio05
bio12
bio14
Relative importance
0.0
0.1
0.2
0.3
0.4
0.5
CLY
NTO
PTO
SOC
TWI

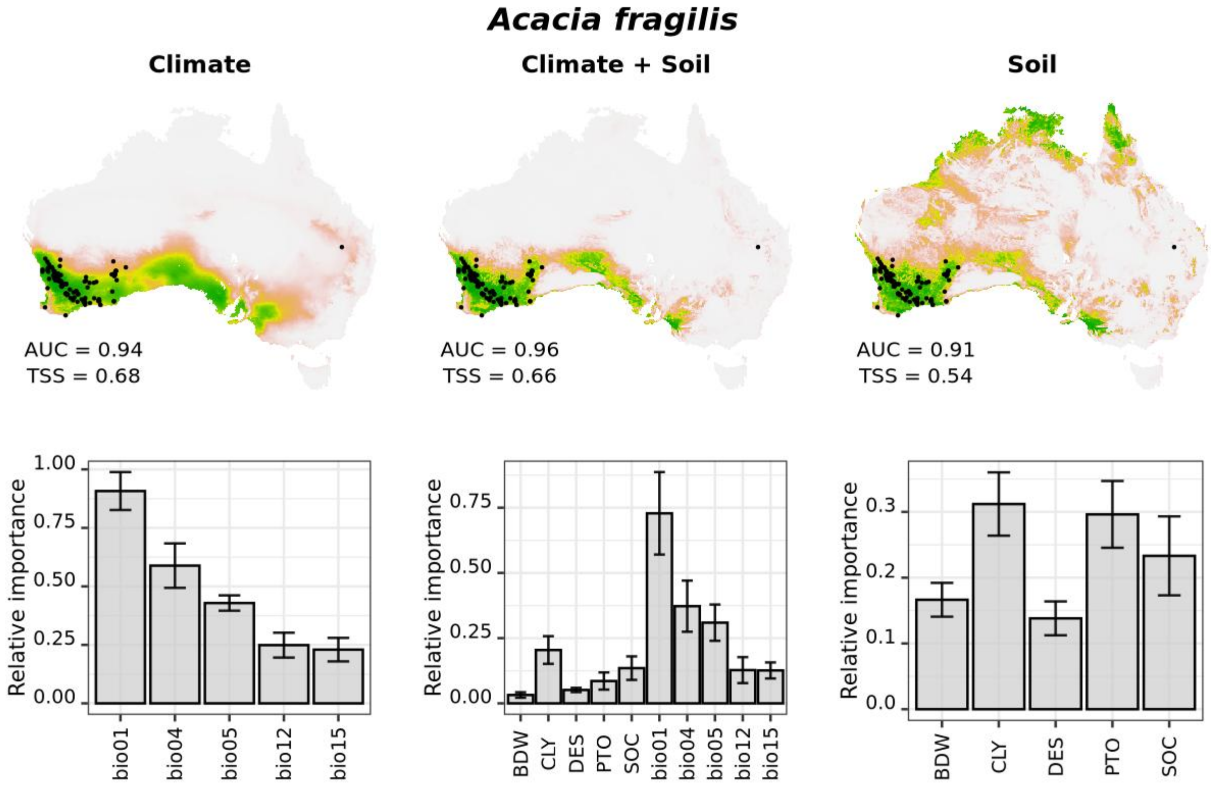

Acacia fragilis
Climate
Climate + Soil
Soil
AUC = 0.94
TSS = 0.68
AUC = 0.96
TSS = 0.66
AUC = 0.91
TSS = 0.54
Relative importance
0.00
0.25
0.50
0.75
1.00
bio01
bio04
bio05
bio12
bio15
Relative importance
0.00
0.25
0.50
0.75
BDW
CLY
DES
PTO
SOC
bio01
bio04
bio05
bio12
bio15
Relative importance
0.0
0.1
0.2
0.3
BDW
CLY
DES
PTO
SOC

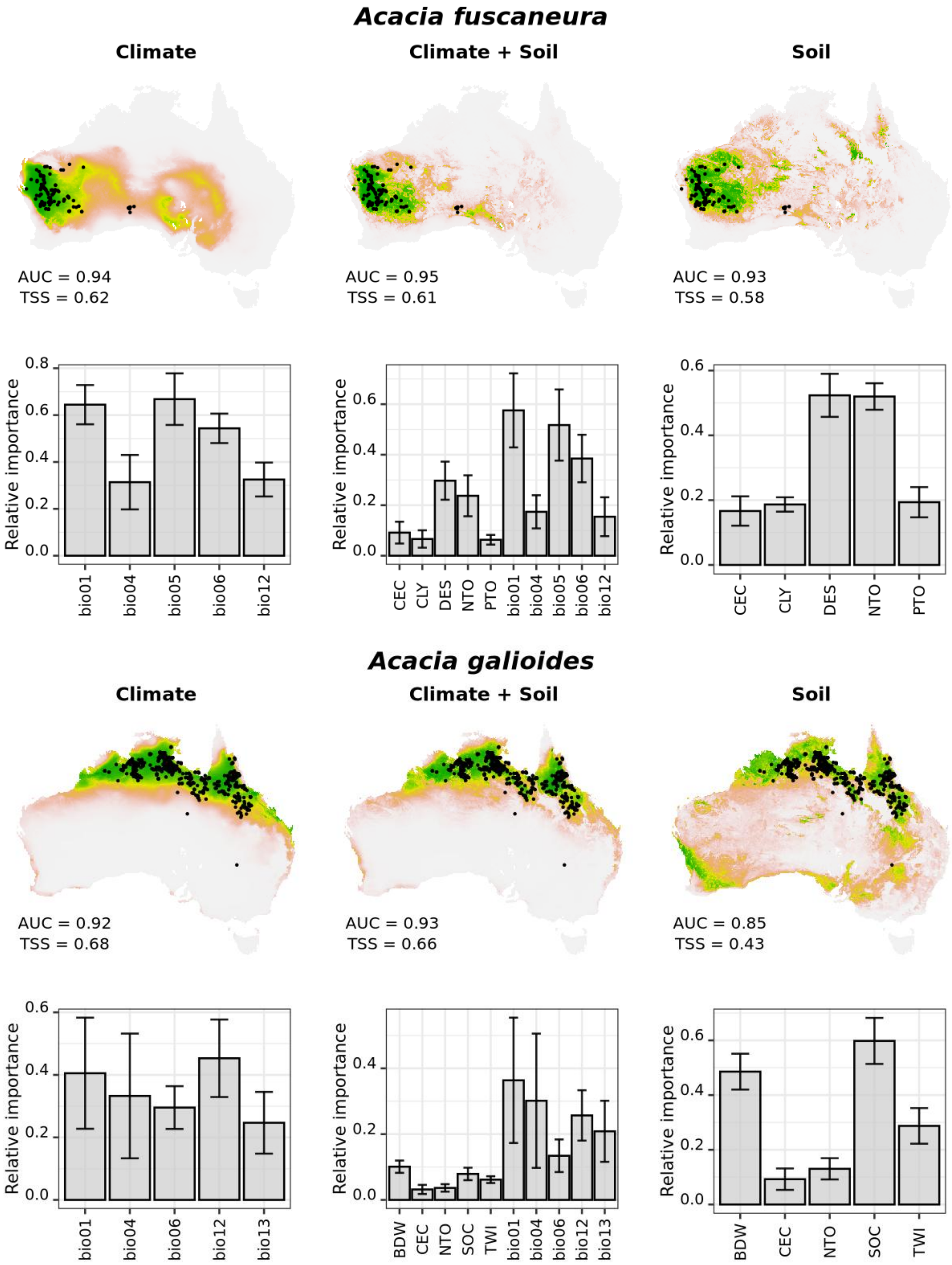

Acacia fuscaneura
Climate
Climate + Soil
Soil
AUC = 0.94
TSS = 0.62
AUC = 0.95
TSS = 0.61
AUC = 0.93
TSS = 0.58
Relative importance
bio01
bio04
bio05
bio06
bio12
CEC
CLY
DES
NTO
PTO
Acacia galioides
AUC = 0.92
TSS = 0.68
AUC = 0.93
TSS = 0.66
AUC = 0.85
TSS = 0.43
bio13
BDW
SOC
TWI

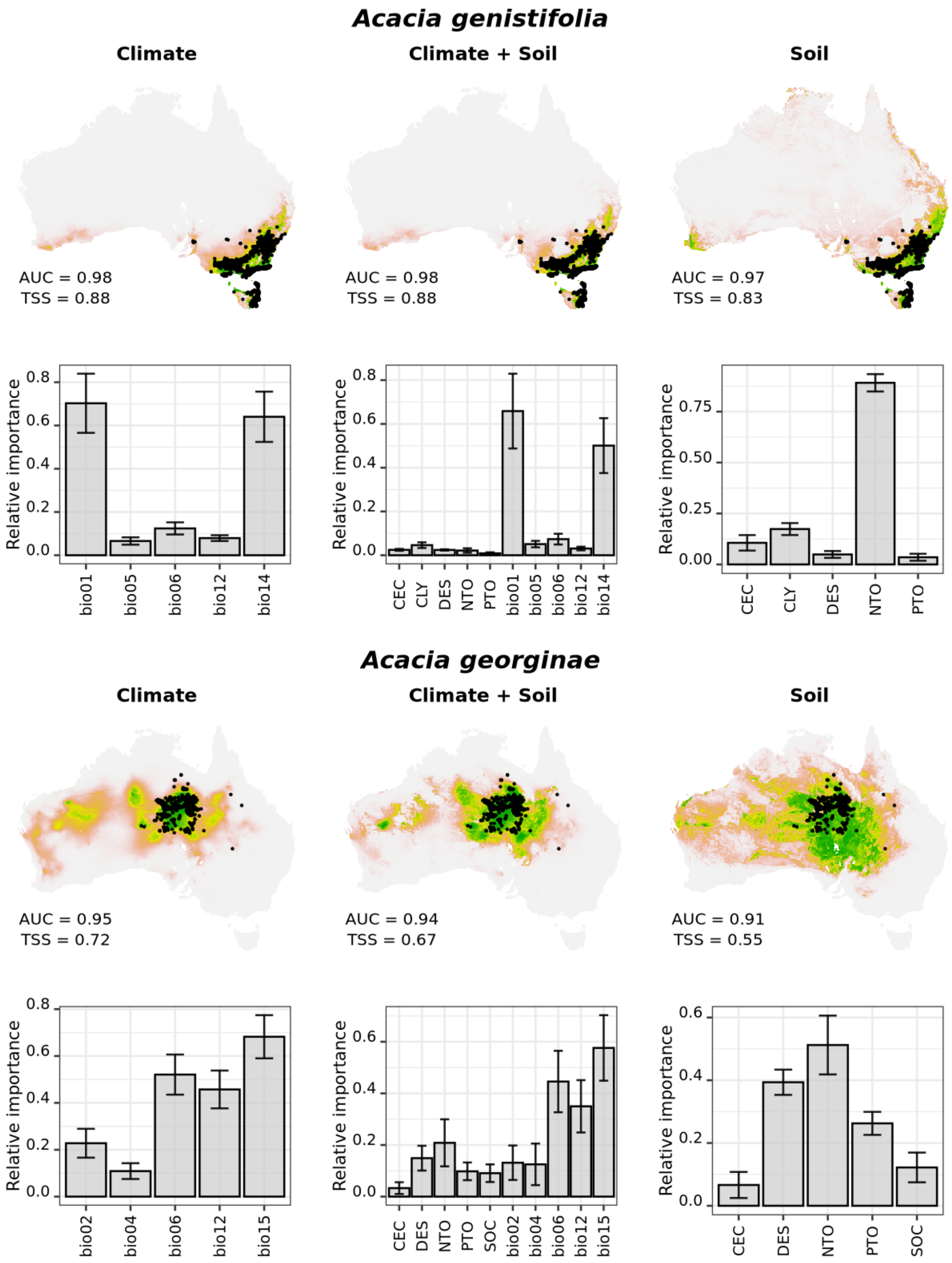
Acacia genistifolia
Climate
Climate + Soil
Soil
AUC = 0.98
TSS = 0.88
AUC = 0.98
TSS = 0.88
AUC = 0.97
TSS = 0.83
Relative importance
bio01
bio05
bio06
bio12
bio14
CEC
CLY
DES
NTO
PTO
Acacia georginae
Climate
Climate + Soil
Soil
AUC = 0.95
TSS = 0.72
AUC = 0.94
TSS = 0.67
AUC = 0.91
TSS = 0.55
Relative importance
bio02
bio04
bio06
bio12
bio15
CEC
DES
NTO
PTO
SOC

Acacia gladiiformis

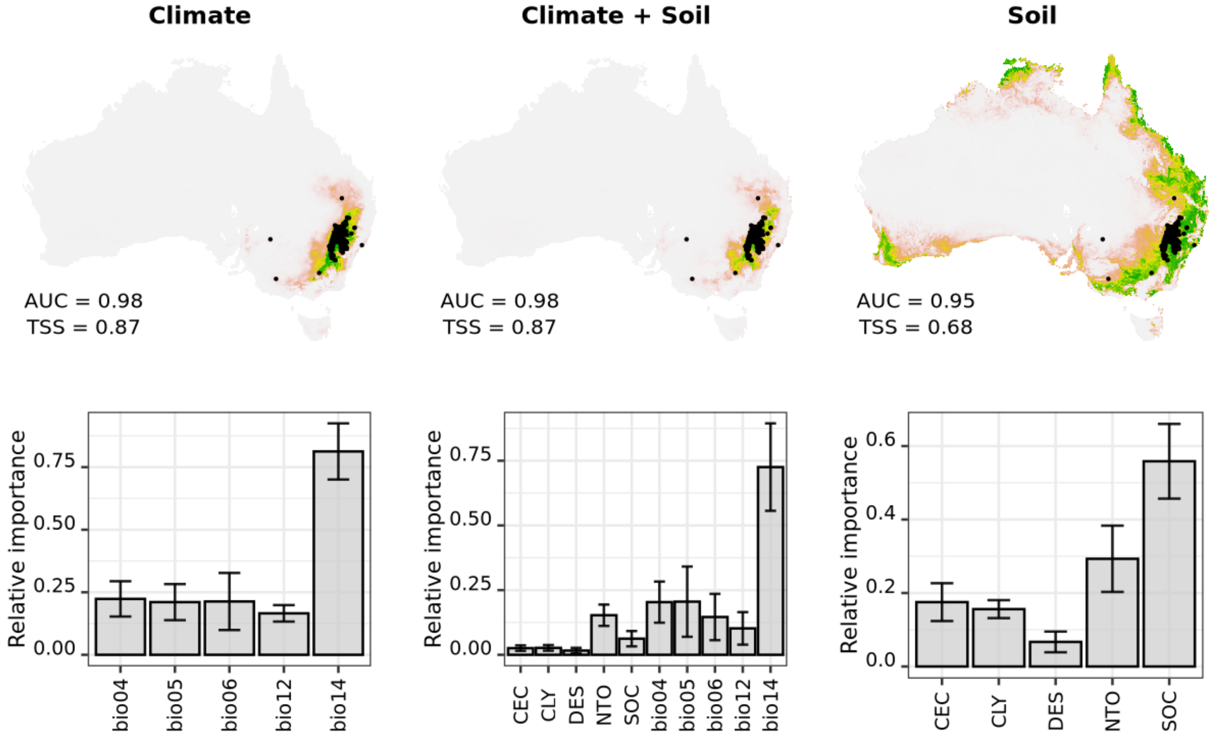

Acacia glaucoptera

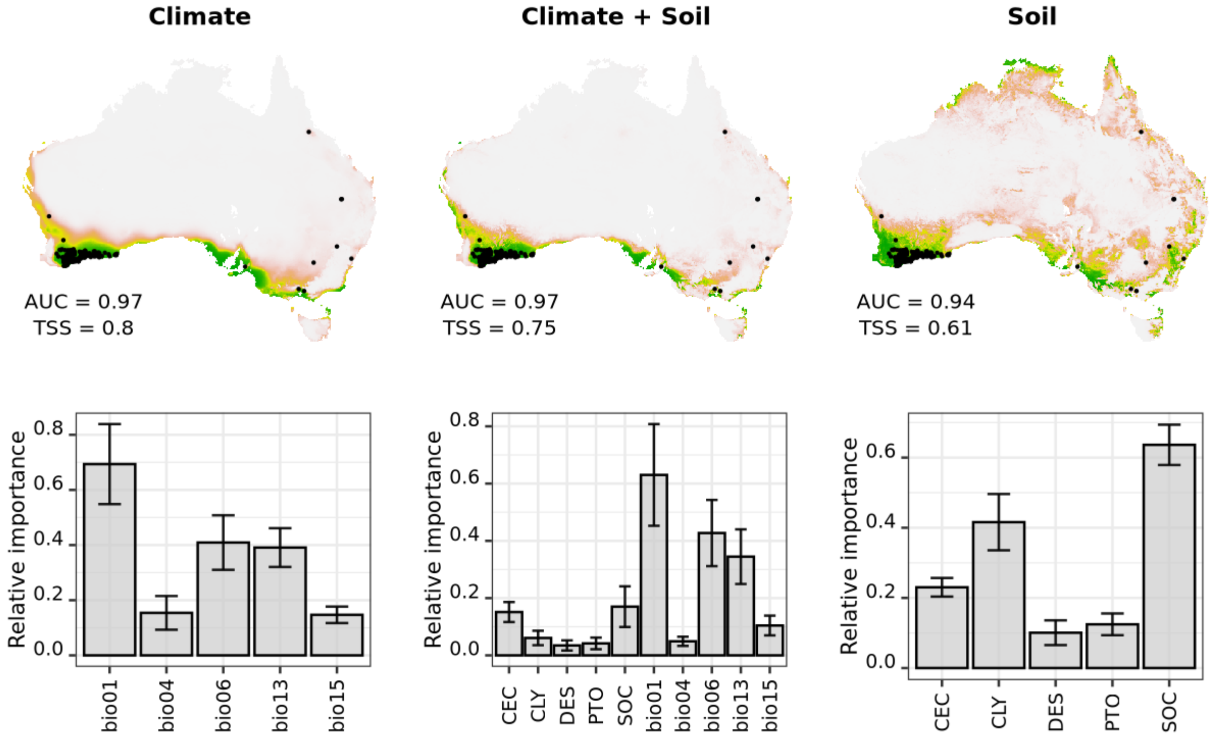

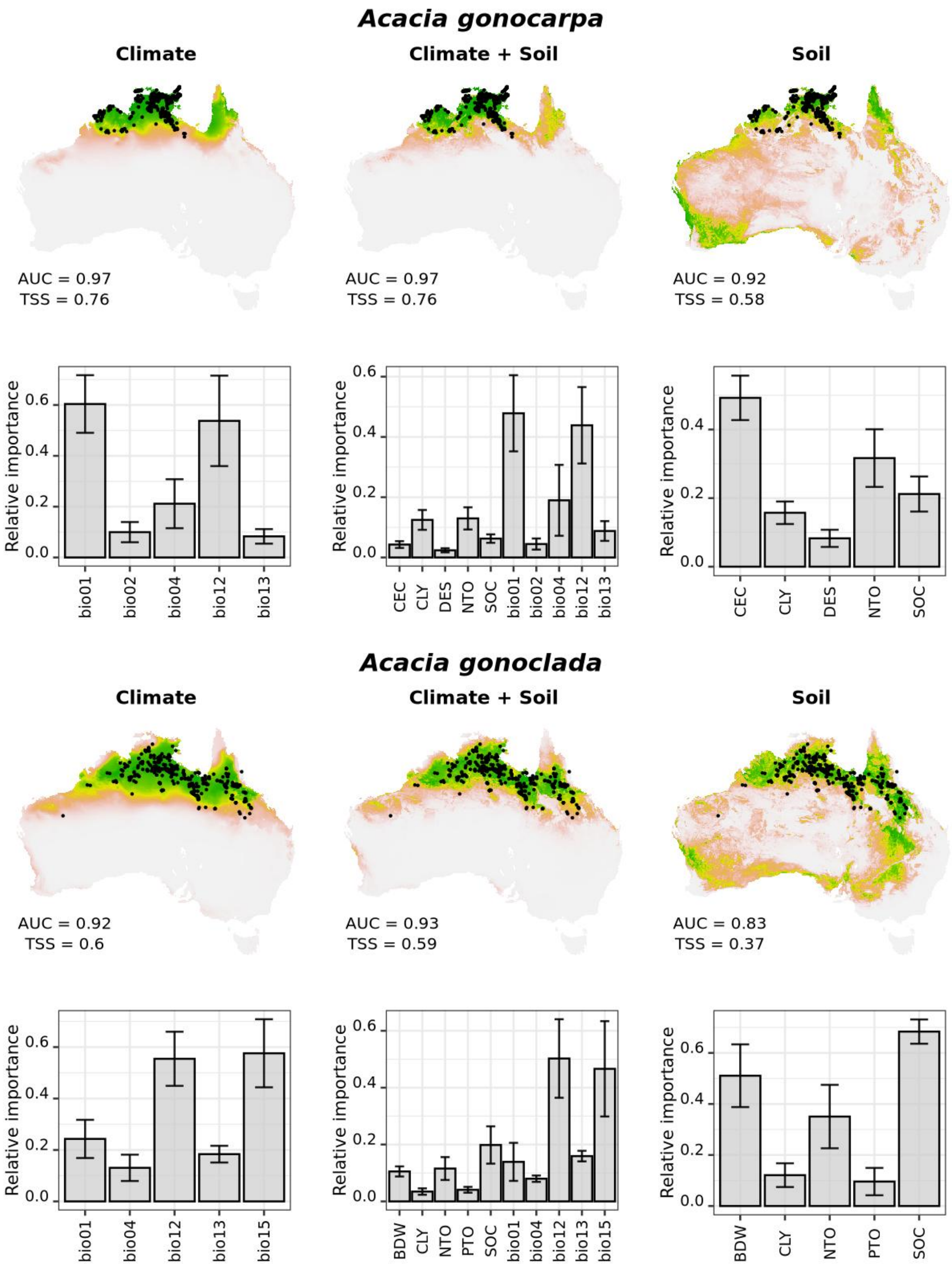
Acacia gonocarpa
Climate
Climate + Soil
Soil
AUC = 0.97
TSS = 0.76
AUC = 0.97
TSS = 0.76
AUC = 0.92
TSS = 0.58
Relative importance
bio01 bio02 bio04 bio12 bio13
CEC CLY DES NTO SOC bio01 bio02 bio04 bio12 bio13
CEC CLY DES NTO SOC
Acacia gonoclada
Climate
Climate + Soil
Soil
AUC = 0.92
TSS = 0.6
AUC = 0.93
TSS = 0.59
AUC = 0.83
TSS = 0.37
Relative importance
bio01 bio04 bio12 bio13 bio15
BDW CLY NTO PTO SOC bio01 bio04 bio12 bio13 bio15
BDW CLY NTO PTO SOC

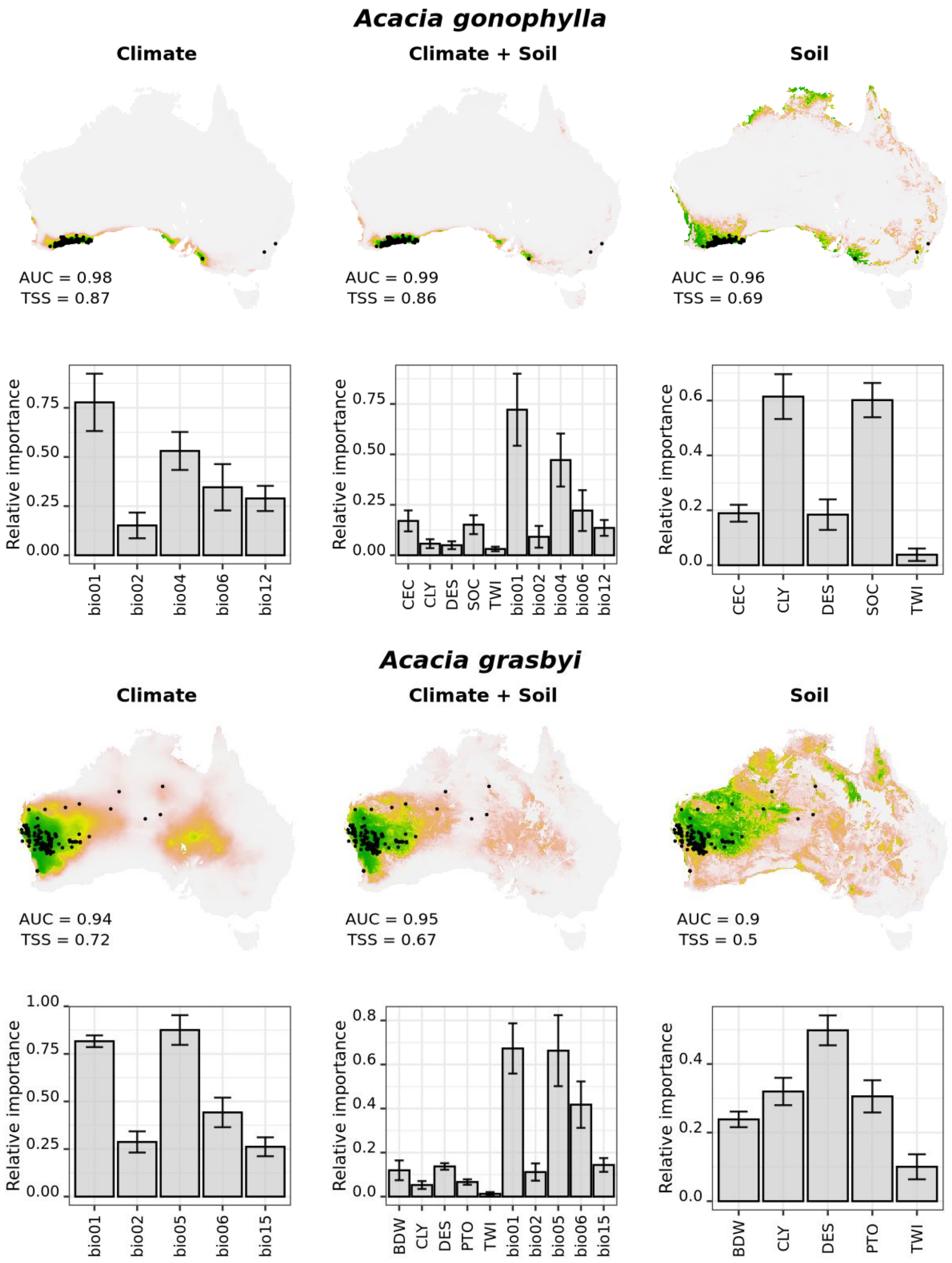

Acacia gonophylla
Climate
Climate + Soil
Soil
AUC = 0.98
TSS = 0.87
AUC = 0.99
TSS = 0.86
AUC = 0.96
TSS = 0.69
Relative importance
0.00
0.25
0.50
0.75
bio01
bio02
bio04
bio06
bio12
CEC
CLY
DES
SOC
TWI
0.0
0.2
0.4
0.6
Acacia grasbyi
Climate
Climate + Soil
Soil
AUC = 0.94
TSS = 0.72
AUC = 0.95
TSS = 0.67
AUC = 0.9
TSS = 0.5
1.00
bio05
bio15
BDW
PTO
0.8

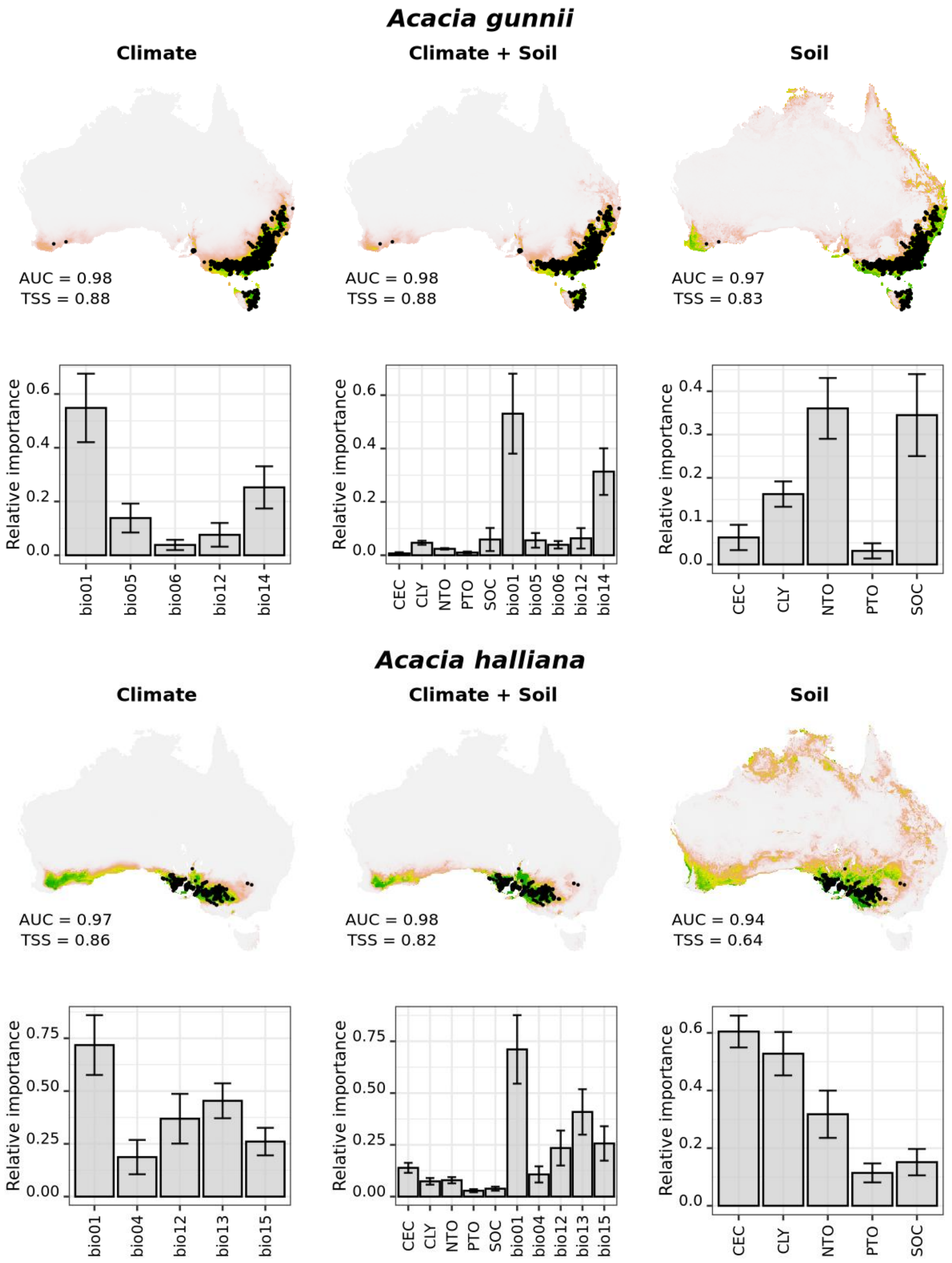
Acacia gunnii
Climate
Climate + Soil
Soil
AUC = 0.98
TSS = 0.88
AUC = 0.98
TSS = 0.88
AUC = 0.97
TSS = 0.83
Relative importance
bio01
bio05
bio06
bio12
bio14
CEC
CLY
NTO
PTO
SOC
Acacia halliana
AUC = 0.97
TSS = 0.86
AUC = 0.98
TSS = 0.82
AUC = 0.94
TSS = 0.64
bio04
bio13
bio15

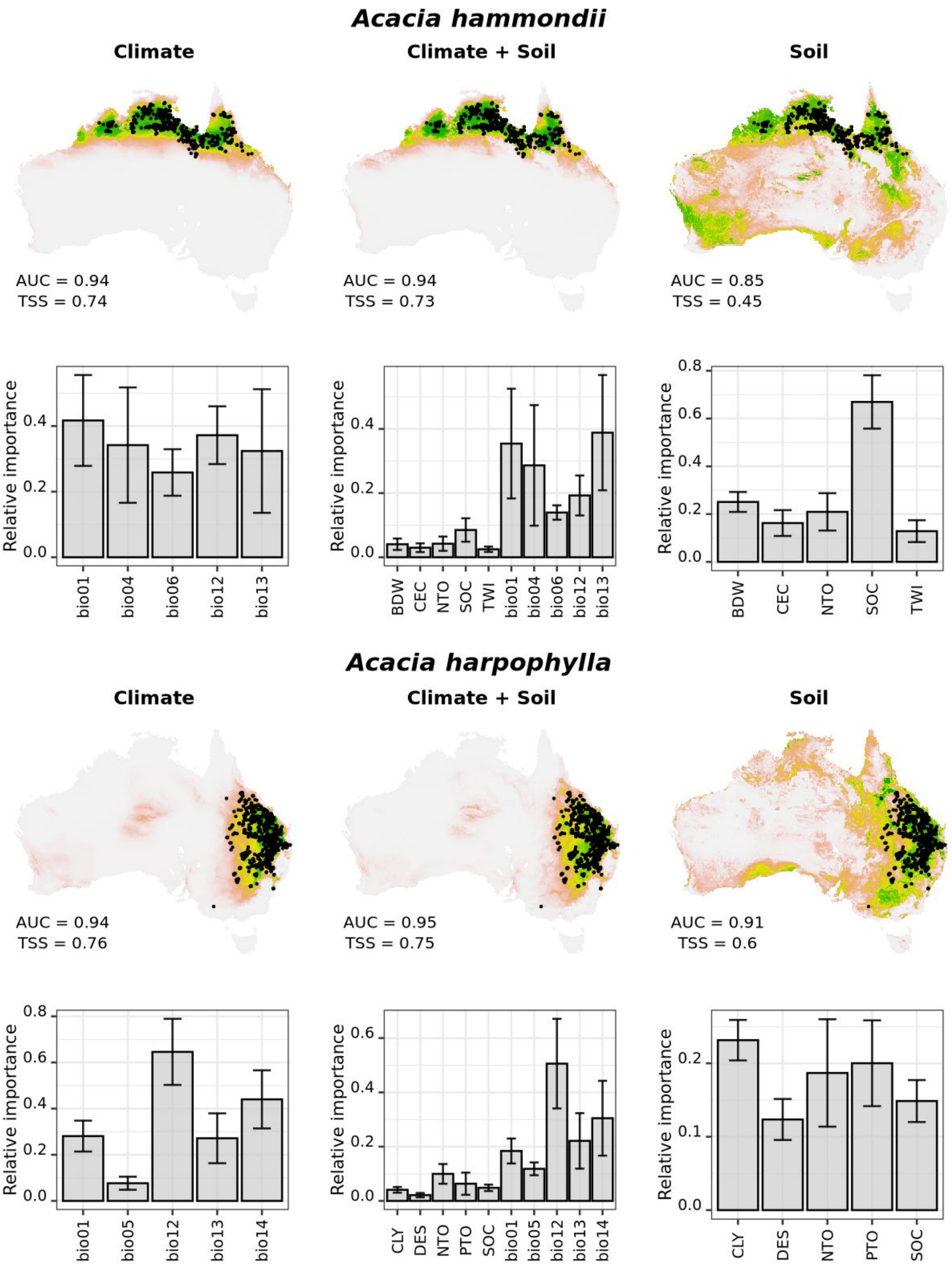
Acacia hammondii
Climate
Climate + Soil
Soil
AUC = 0.94
TSS = 0.74
AUC = 0.94
TSS = 0.73
AUC = 0.85
TSS = 0.45
Relative importance
bio01
bio04
bio06
bio12
bio13
BDW
CEC
NTO
SOC
TWI
Acacia harpophylla
Climate
Climate + Soil
Soil
AUC = 0.94
TSS = 0.76
AUC = 0.95
TSS = 0.75
AUC = 0.91
TSS = 0.6
Relative importance
bio01
bio05
bio12
bio13
bio14
CLY
DES
NTO
PTO
SOC

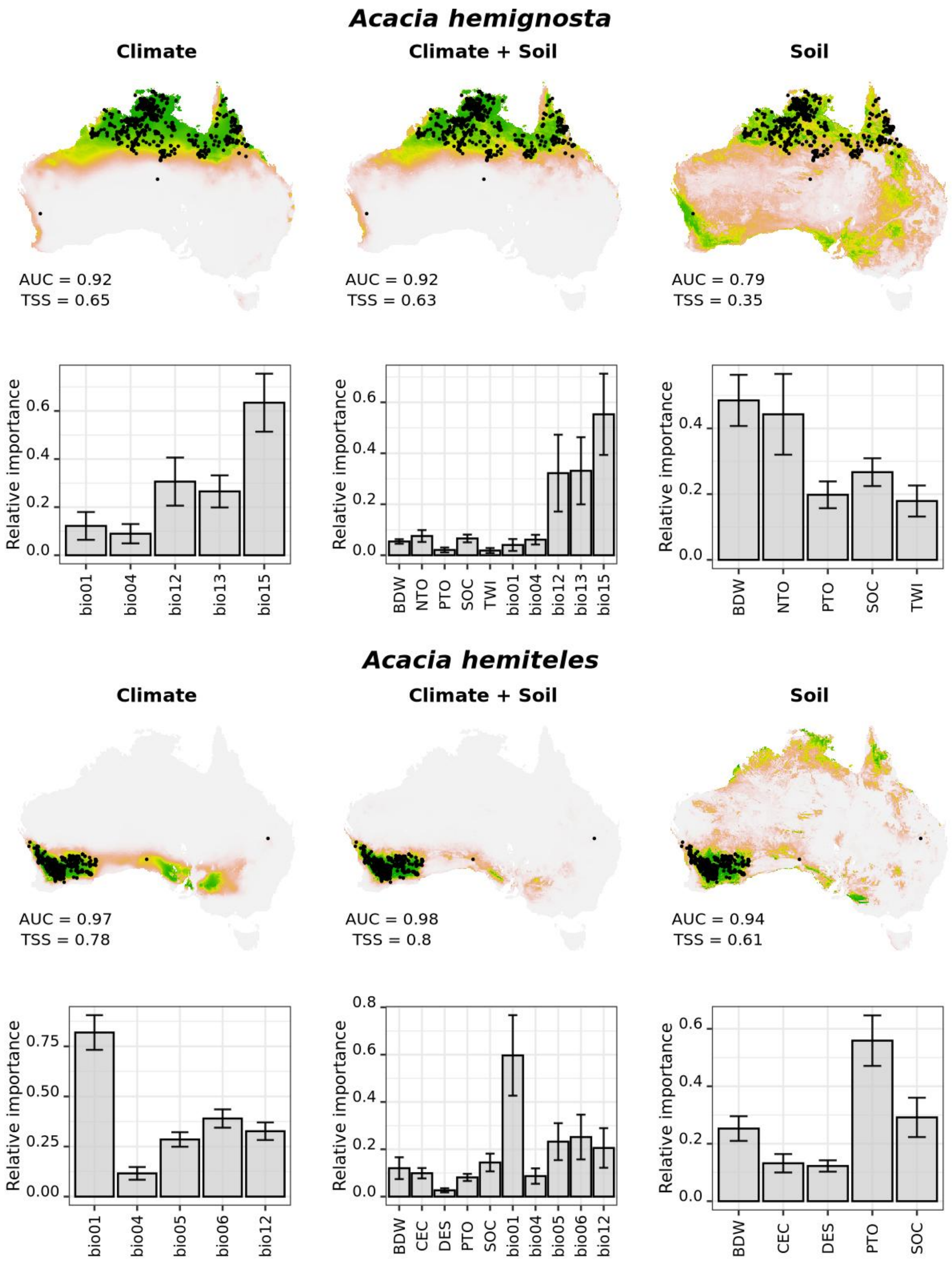
Acacia hemignosta
Climate
Climate + Soil
Soil
AUC = 0.92
TSS = 0.65
AUC = 0.92
TSS = 0.63
AUC = 0.79
TSS = 0.35
Relative importance
bio01 bio04 bio12 bio13 bio15
BDW NTO PTO SOC TWI bio01 bio04 bio12 bio13 bio15
BDW NTO PTO SOC TWI
Acacia hemiteles
Climate
Climate + Soil
Soil
AUC = 0.97
TSS = 0.78
AUC = 0.98
TSS = 0.8
AUC = 0.94
TSS = 0.61
Relative importance
bio01 bio04 bio05 bio06 bio12
BDW CEC DES PTO SOC bio01 bio04 bio05 bio06 bio12
BDW CEC DES PTO SOC

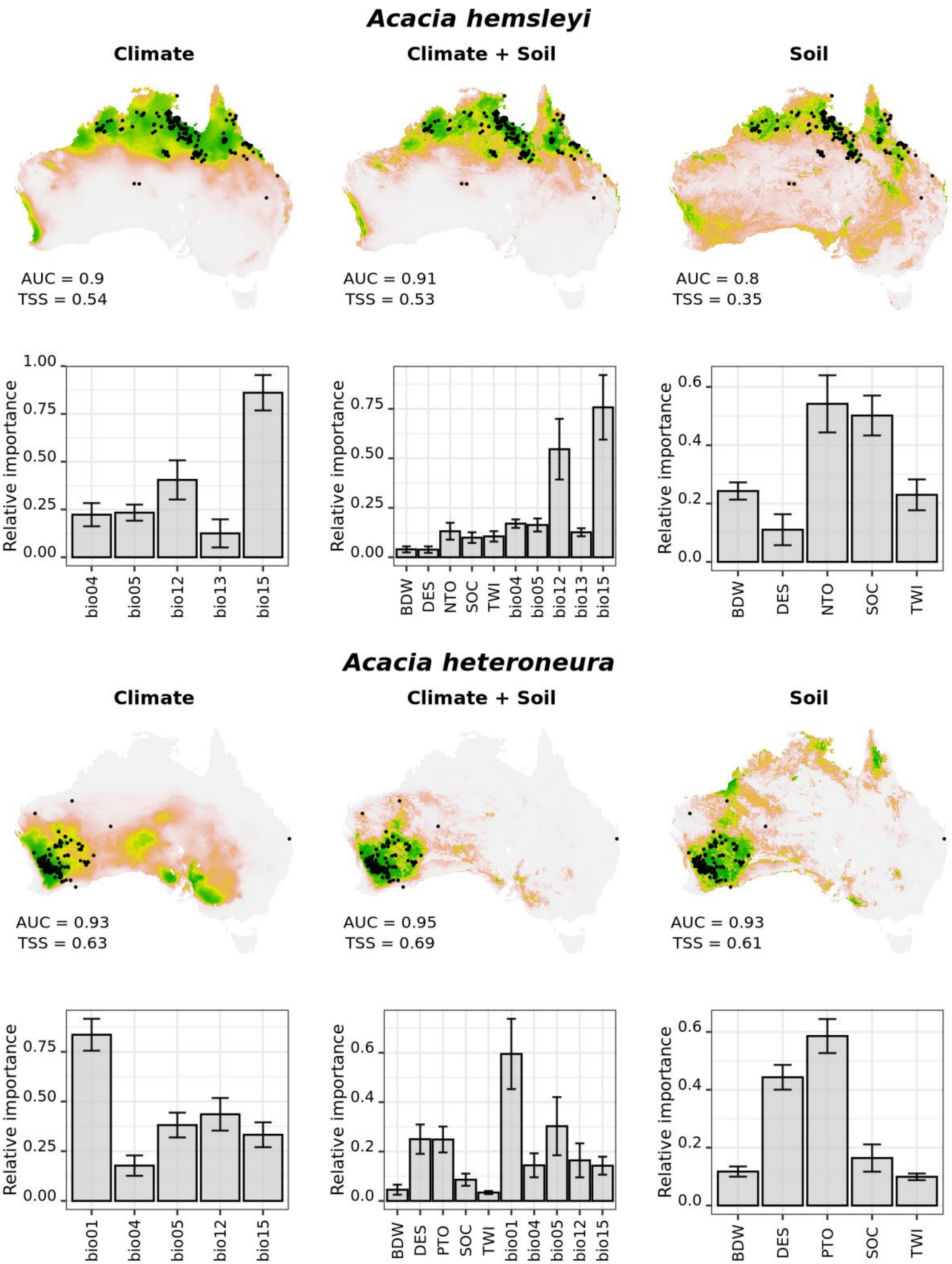
Acacia hemsleyi
Climate
Climate + Soil
Soil
AUC = 0.9
TSS = 0.54
AUC = 0.91
TSS = 0.53
AUC = 0.8
TSS = 0.35
Relative importance
1.00
0.75
0.50
0.25
0.00
bio04
bio05
bio12
bio13
bio15
BDW
DES
NTO
SOC
TWI
0.6
0.4
0.2
0.0
Acacia heteroneura
Climate
Climate + Soil
Soil
AUC = 0.93
TSS = 0.63
AUC = 0.95
TSS = 0.69
AUC = 0.93
TSS = 0.61
Relative importance
bio01
bio04
bio05
bio12
bio15
BDW
DES
PTO
SOC
TWI

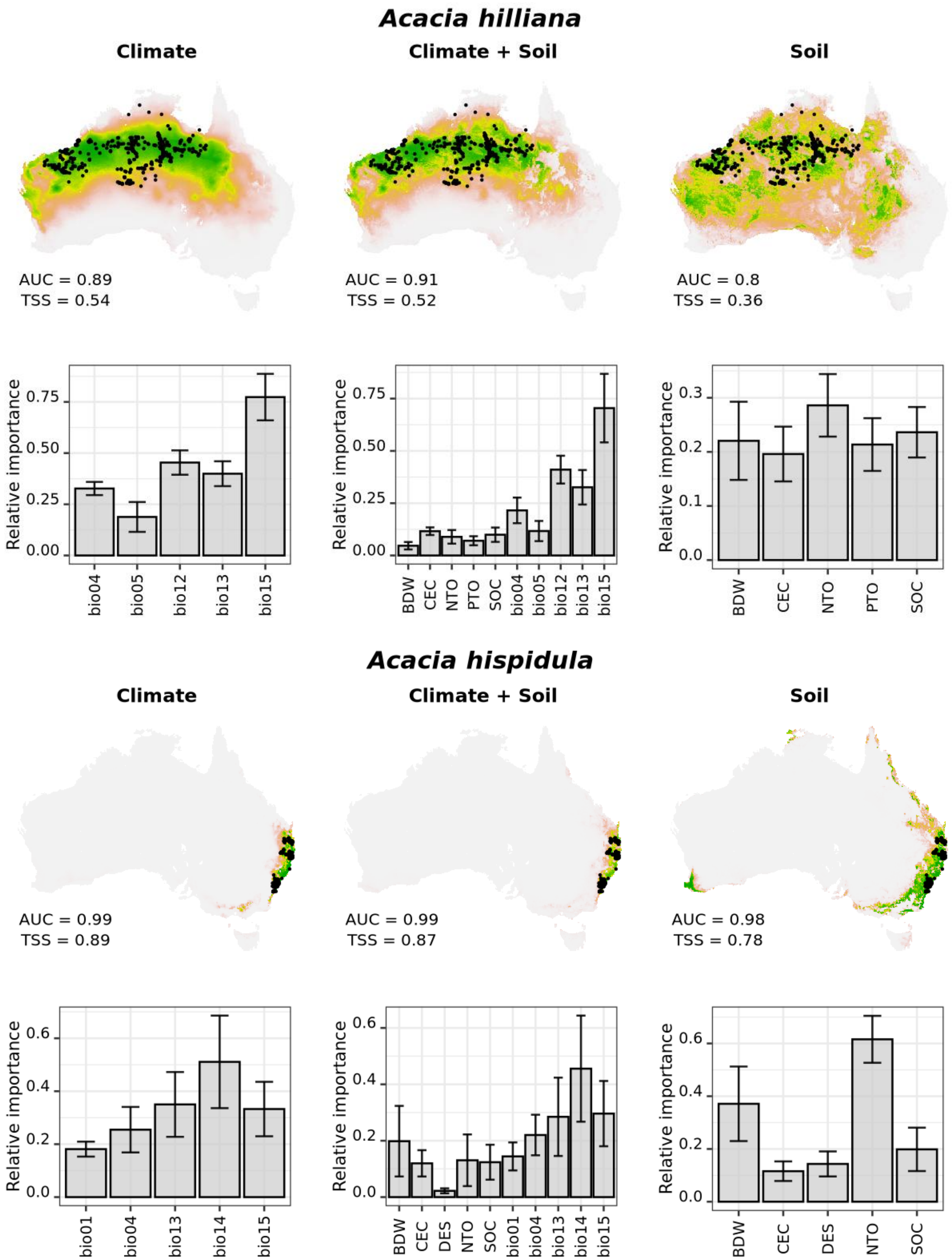
Acacia hilliana
Climate
Climate + Soil
Soil
AUC = 0.89
TSS = 0.54
AUC = 0.91
TSS = 0.52
AUC = 0.8
TSS = 0.36
Relative importance
bio04
bio05
bio12
bio13
bio15
BDW
CEC
NTO
PTO
SOC
Acacia hispidula
Climate
Climate + Soil
Soil
AUC = 0.99
TSS = 0.89
AUC = 0.99
TSS = 0.87
AUC = 0.98
TSS = 0.78
Relative importance
bio01
bio04
bio13
bio14
bio15
BDW
CEC
DES
NTO
SOC

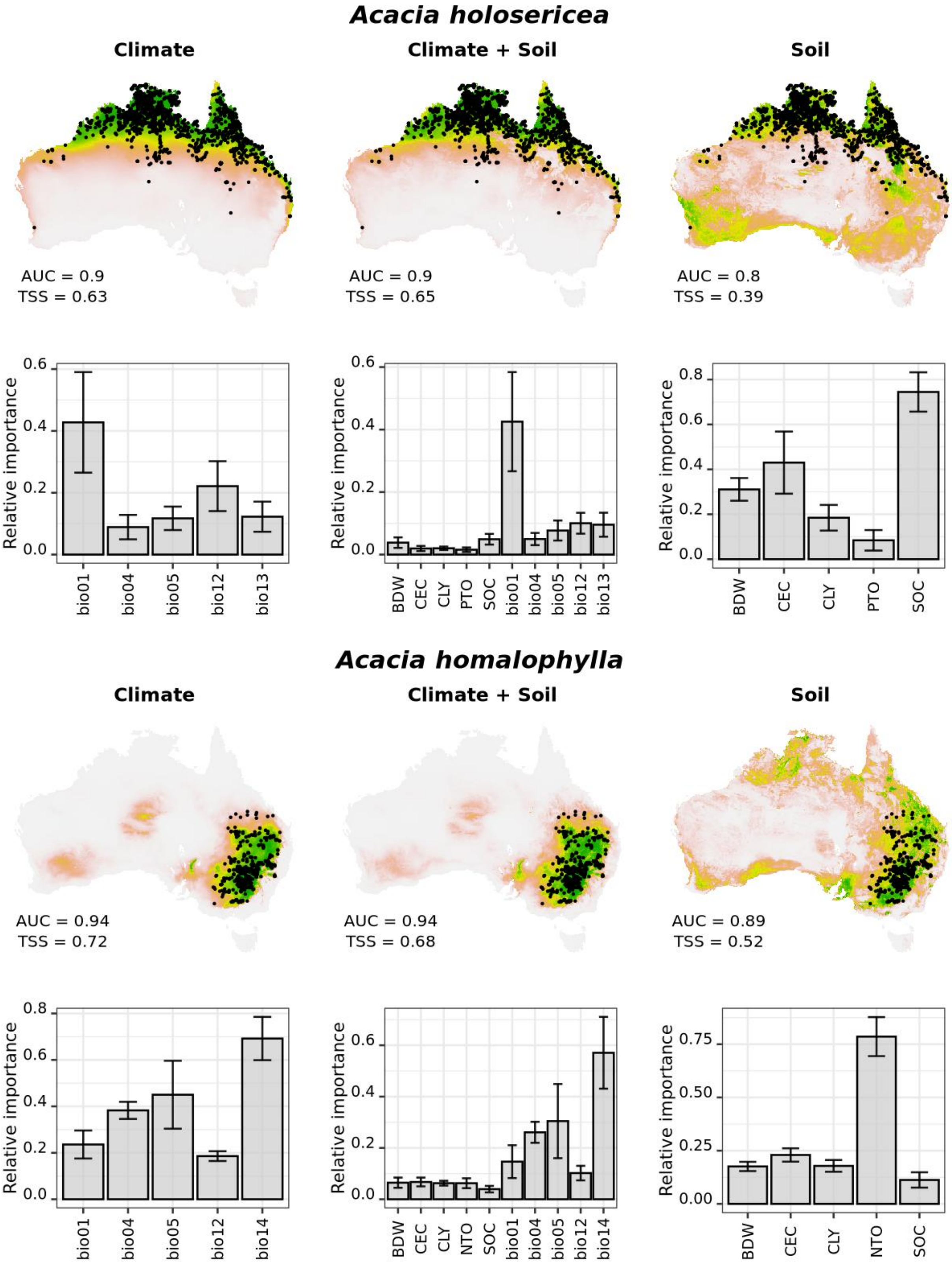
Acacia holosericea
Climate
Climate + Soil
Soil
AUC = 0.9
TSS = 0.63
AUC = 0.9
TSS = 0.65
AUC = 0.8
TSS = 0.39
Relative importance
bio01
bio04
bio05
bio12
bio13
BDW
CEC
CLY
PTO
SOC
Acacia homalophylla
Climate
Climate + Soil
Soil
AUC = 0.94
TSS = 0.72
AUC = 0.94
TSS = 0.68
AUC = 0.89
TSS = 0.52
Relative importance
bio01
bio04
bio05
bio12
bio14
BDW
CEC
CLY
NTO
SOC

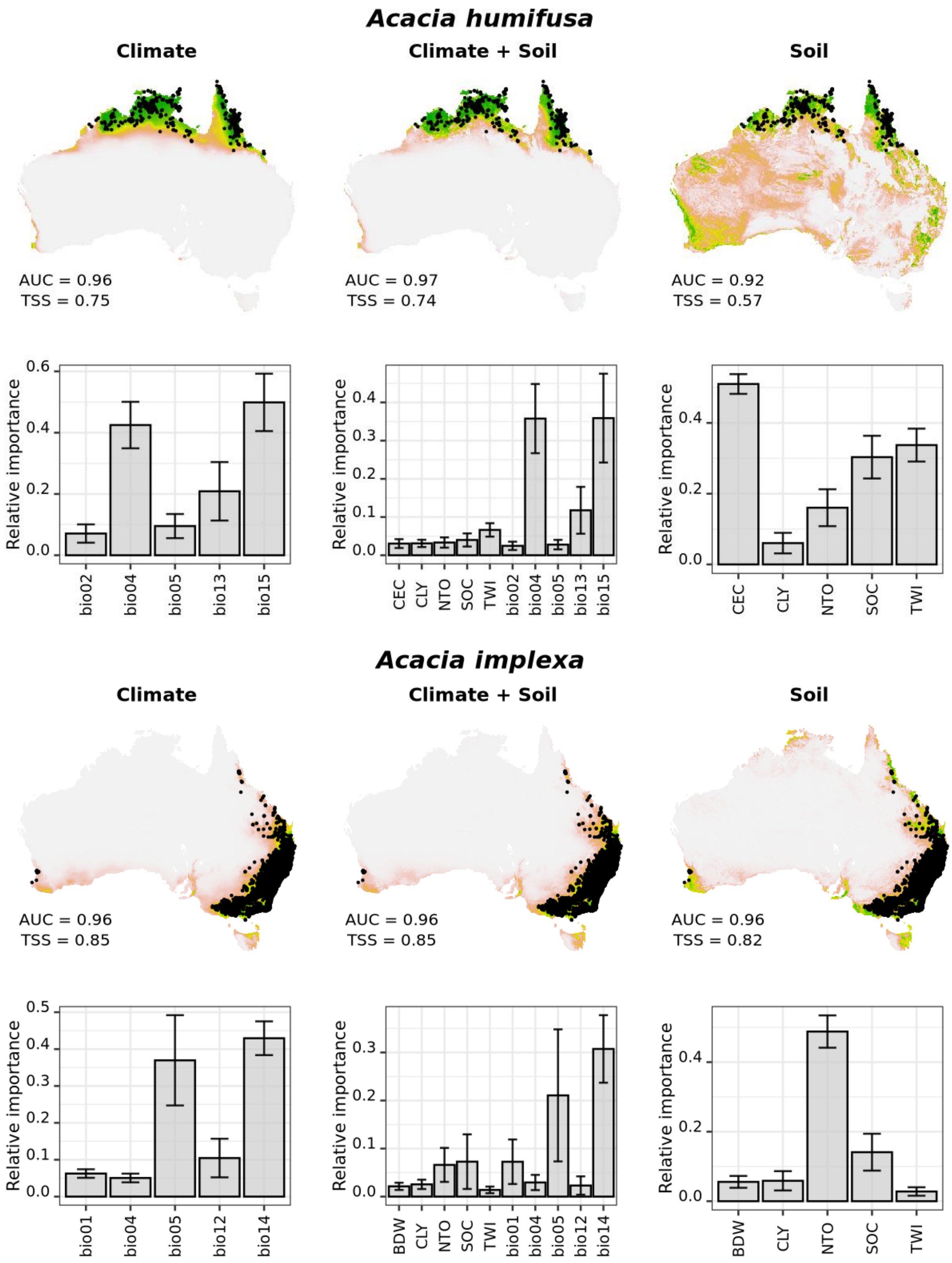

Acacia humifusa
Climate
Climate + Soil
Soil
AUC = 0.96
TSS = 0.75
AUC = 0.97
TSS = 0.74
AUC = 0.92
TSS = 0.57
Relative importance
bio02
bio04
bio05
bio13
bio15
CEC
CLY
NTO
SOC
TWI
bio02
bio04
bio05
bio13
bio15
CEC
CLY
NTO
SOC
TWI
Acacia implexa
Climate
Climate + Soil
Soil
AUC = 0.96
TSS = 0.85
AUC = 0.96
TSS = 0.85
AUC = 0.96
TSS = 0.82
Relative importance
bio01
bio04
bio05
bio12
bio14
BDW
CLY
NTO
SOC
TWI
bio01
bio04
bio05
bio12
bio14
BDW
CLY
NTO
SOC
TWI

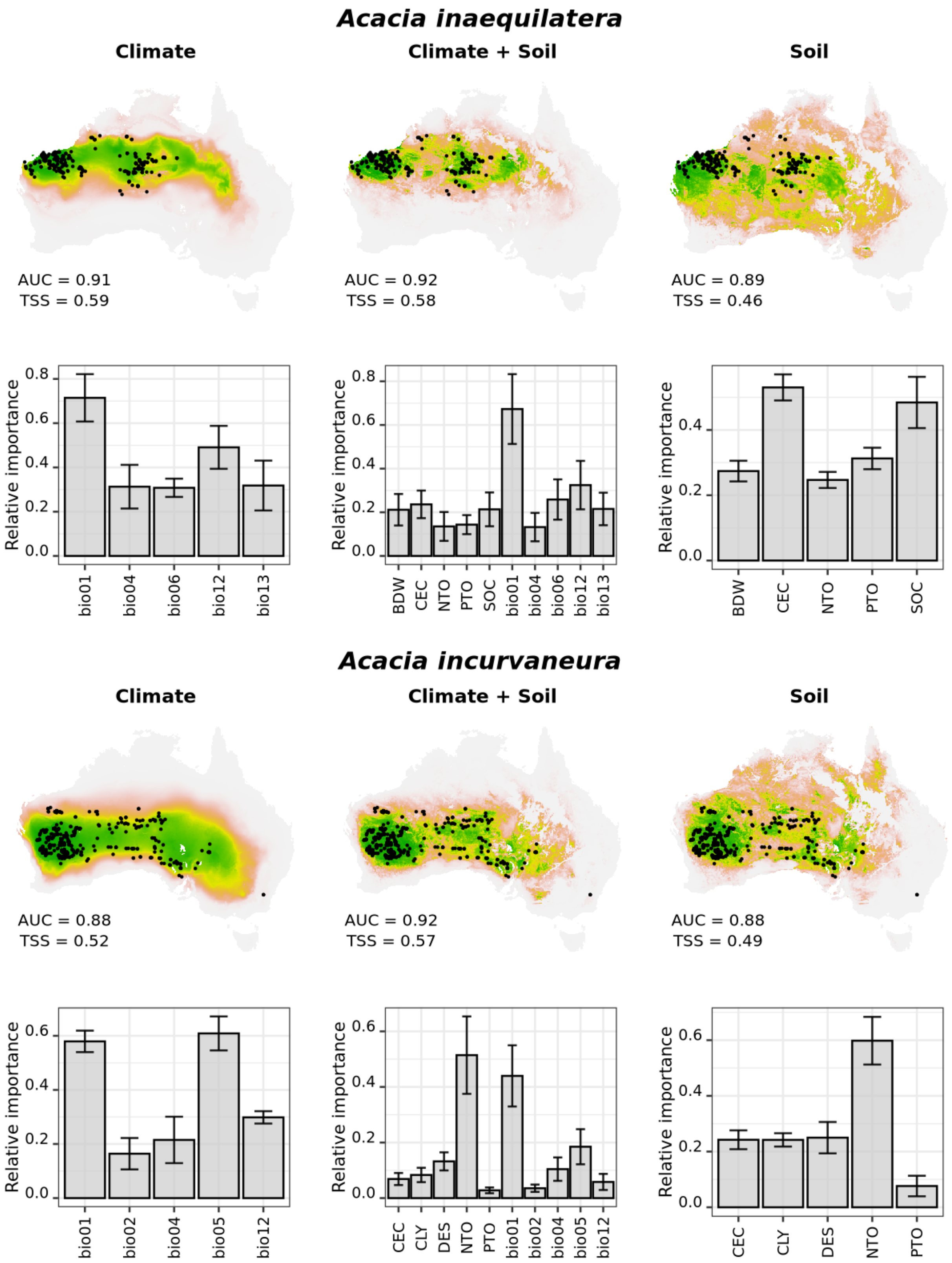
Acacia inaequilatera
Climate
Climate + Soil
Soil
AUC = 0.91
TSS = 0.59
AUC = 0.92
TSS = 0.58
AUC = 0.89
TSS = 0.46
Relative importance
bio01
bio04
bio06
bio12
bio13
BDW
CEC
NTO
PTO
SOC
Acacia incurvaneura
AUC = 0.88
TSS = 0.52
AUC = 0.92
TSS = 0.57
AUC = 0.88
TSS = 0.49
bio02
bio05
CLY
DES

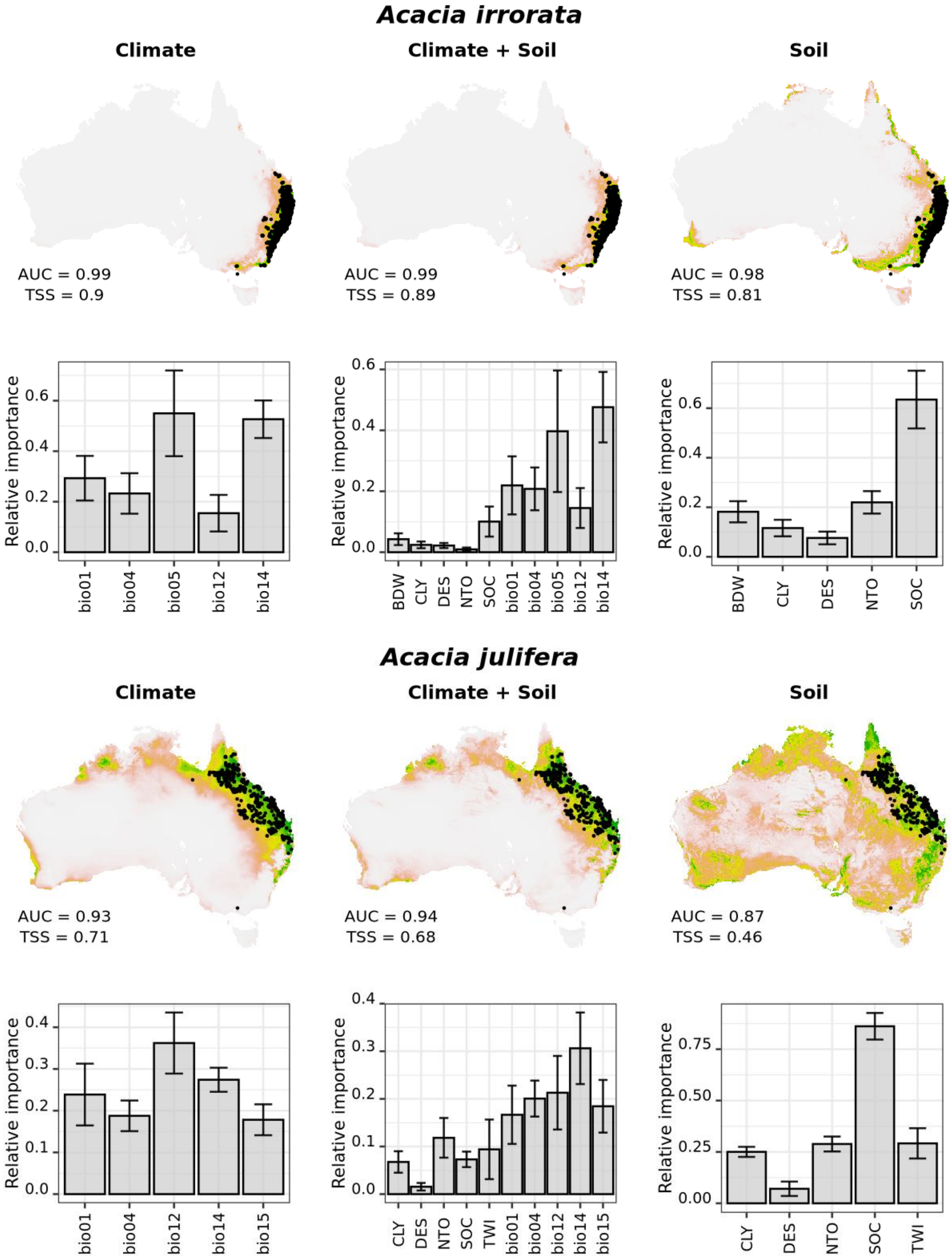

Acacia irrorata
Climate
Climate + Soil
Soil
AUC = 0.99
TSS = 0.9
AUC = 0.99
TSS = 0.89
AUC = 0.98
TSS = 0.81
Relative importance
bio01
bio04
bio05
bio12
bio14
BDW
CLY
DES
NTO
SOC
Acacia julifera
Climate
Climate + Soil
Soil
AUC = 0.93
TSS = 0.71
AUC = 0.94
TSS = 0.68
AUC = 0.87
TSS = 0.46
bio15
TWI

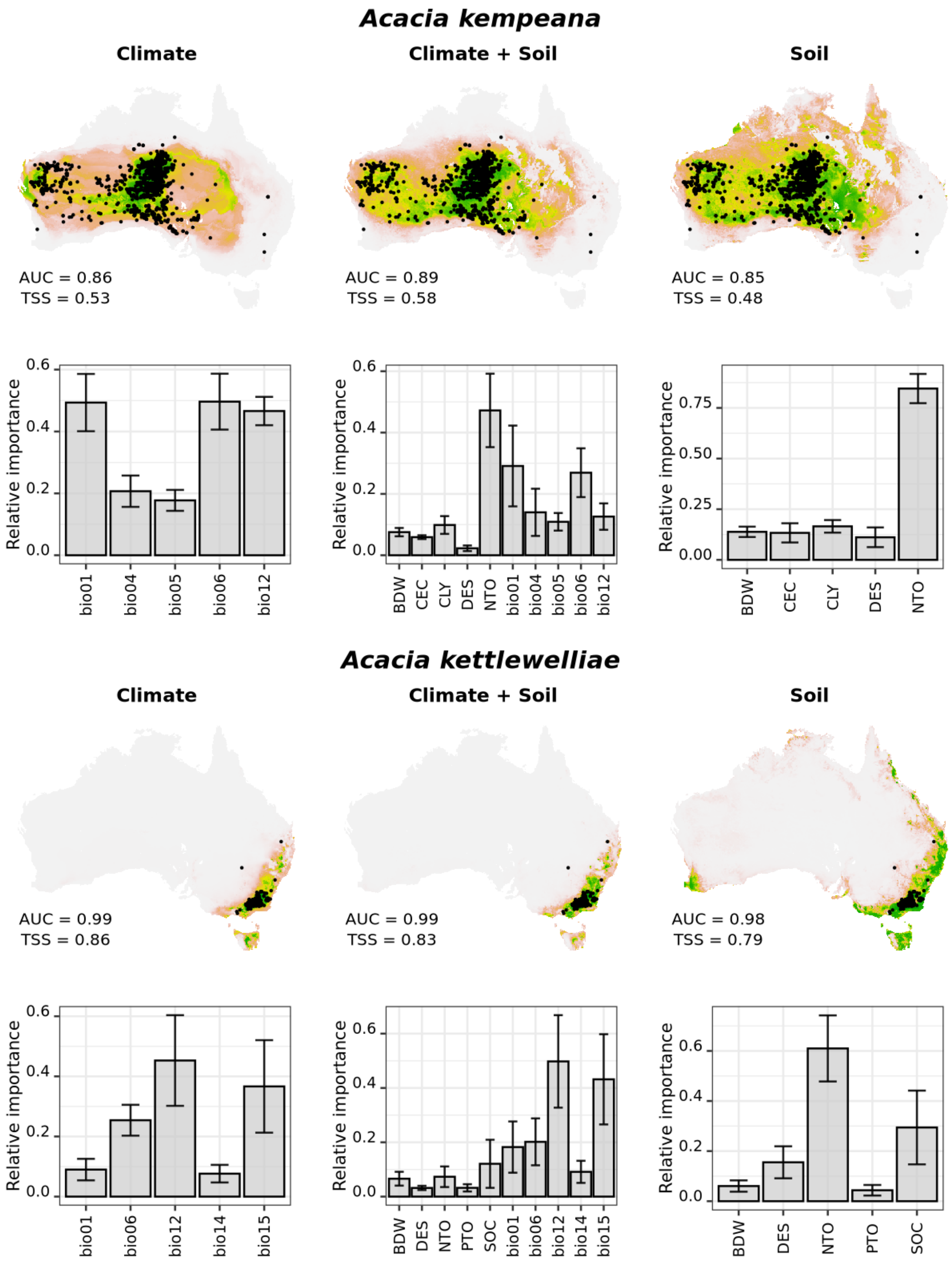
Acacia kempeana
Climate
Climate + Soil
Soil
AUC = 0.86
TSS = 0.53
AUC = 0.89
TSS = 0.58
AUC = 0.85
TSS = 0.48
Relative importance
0.0
0.2
0.4
0.6
0.00
0.25
0.50
0.75
bio01
bio04
bio05
bio06
bio12
BDW
CEC
CLY
DES
NTO
Acacia kettlewelliae
Climate
Climate + Soil
Soil
AUC = 0.99
TSS = 0.86
AUC = 0.99
TSS = 0.83
AUC = 0.98
TSS = 0.79
Relative importance
0.0
0.2
0.4
0.6
bio01
bio06
bio12
bio14
bio15
BDW
DES
NTO
PTO
SOC

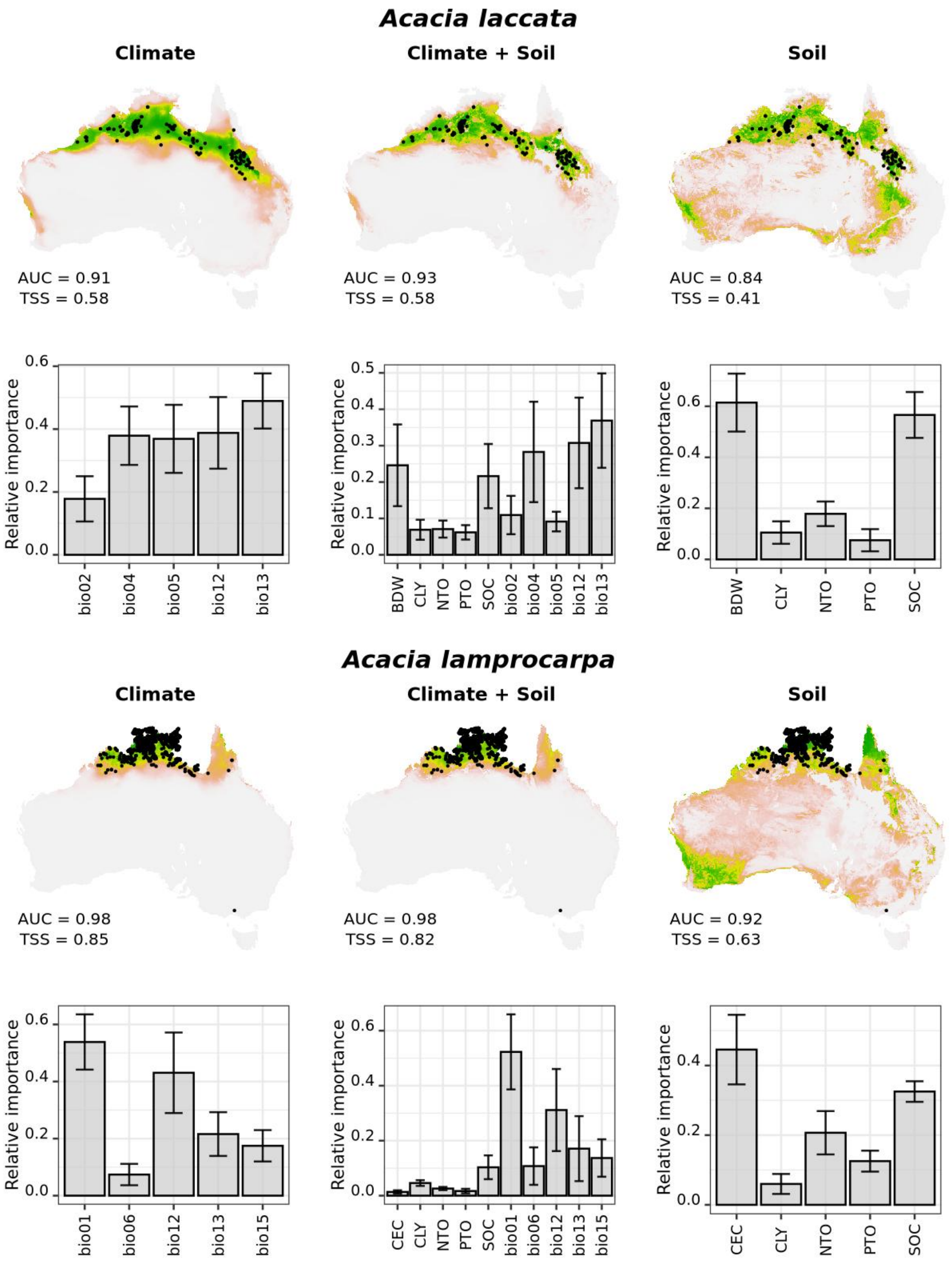

Acacia laccata
Climate
Climate + Soil
Soil
AUC = 0.91
TSS = 0.58
AUC = 0.93
TSS = 0.58
AUC = 0.84
TSS = 0.41
Relative importance
bio02
bio04
bio05
bio12
bio13
BDW
CLY
NTO
PTO
SOC
Acacia lamprocarpa
Climate
Climate + Soil
Soil
AUC = 0.98
TSS = 0.85
AUC = 0.98
TSS = 0.82
AUC = 0.92
TSS = 0.63
Relative importance
bio01
bio06
bio12
bio13
bio15
CEC
CLY
NTO
PTO
SOC

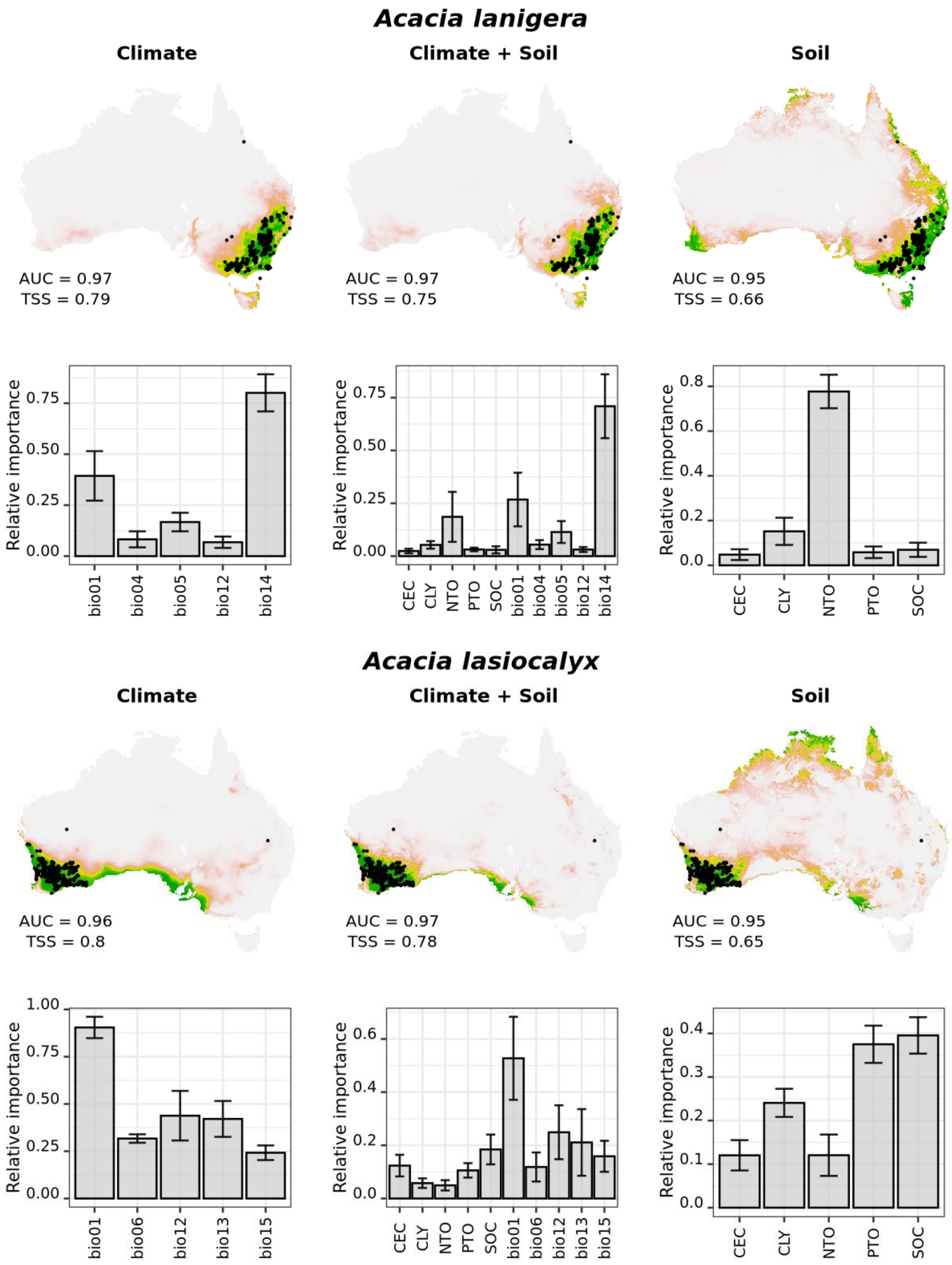
Acacia lanigera
Climate
Climate + Soil
Soil
AUC = 0.97
TSS = 0.79
AUC = 0.97
TSS = 0.75
AUC = 0.95
TSS = 0.66
Relative importance
bio01 bio04 bio05 bio12 bio14
CEC CLY NTO PTO SOC bio01 bio04 bio05 bio12 bio14
CEC CLY NTO PTO SOC
Acacia lasiocalyx
Climate
Climate + Soil
Soil
AUC = 0.96
TSS = 0.8
AUC = 0.97
TSS = 0.78
AUC = 0.95
TSS = 0.65
Relative importance
bio01 bio06 bio12 bio13 bio15
CEC CLY NTO PTO SOC bio01 bio06 bio12 bio13 bio15
CEC CLY NTO PTO SOC

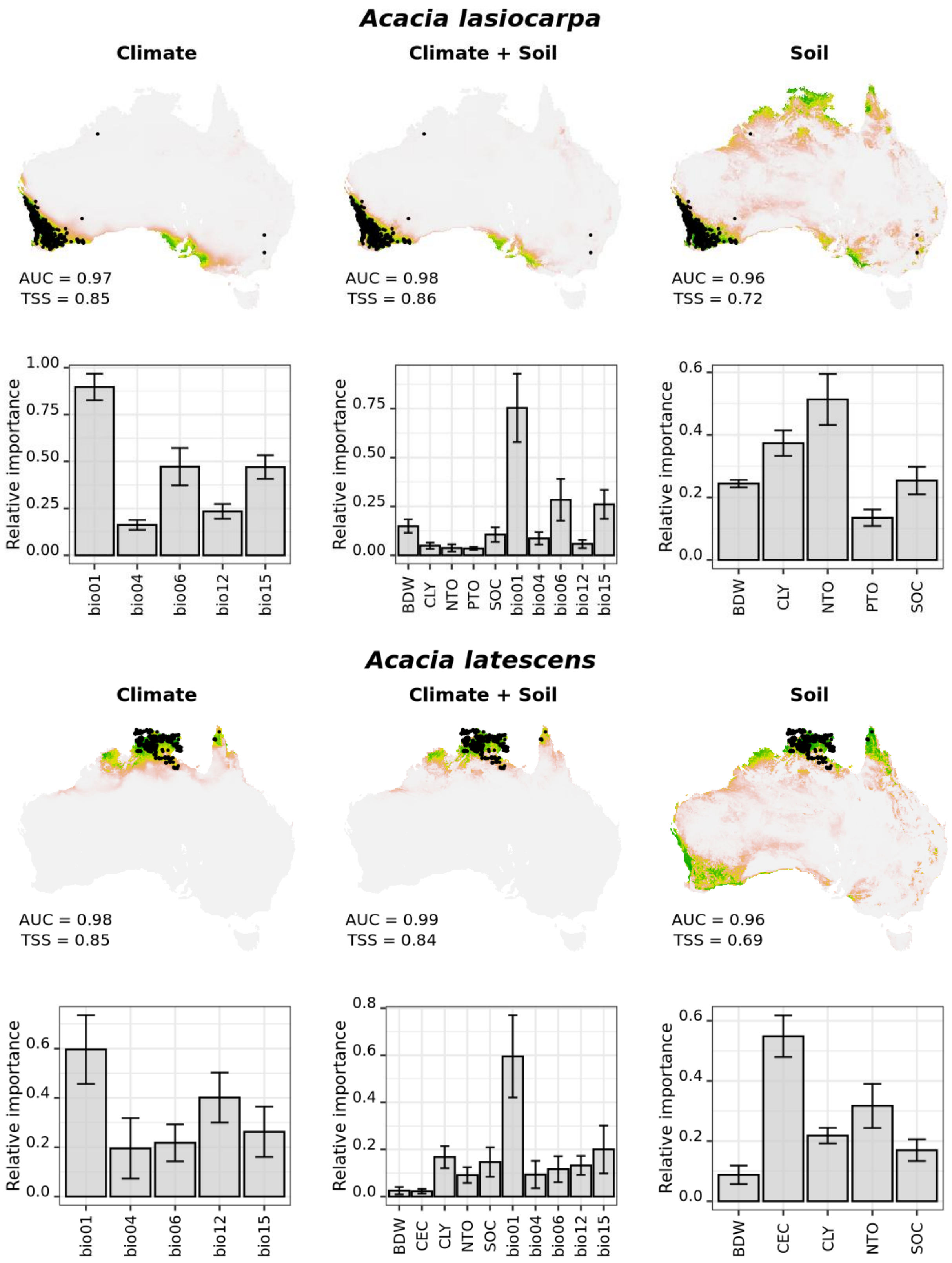
Acacia lasiocarpa
Climate
Climate + Soil
Soil
AUC = 0.97
TSS = 0.85
AUC = 0.98
TSS = 0.86
AUC = 0.96
TSS = 0.72
Relative importance
1.00
0.75
0.50
0.25
0.00
bio01
bio04
bio06
bio12
bio15
BDW
CLY
NTO
PTO
SOC
0.6
0.4
0.2
0.0
Acacia latescens
Climate
Climate + Soil
Soil
AUC = 0.98
TSS = 0.85
AUC = 0.99
TSS = 0.84
AUC = 0.96
TSS = 0.69
0.8
CEC

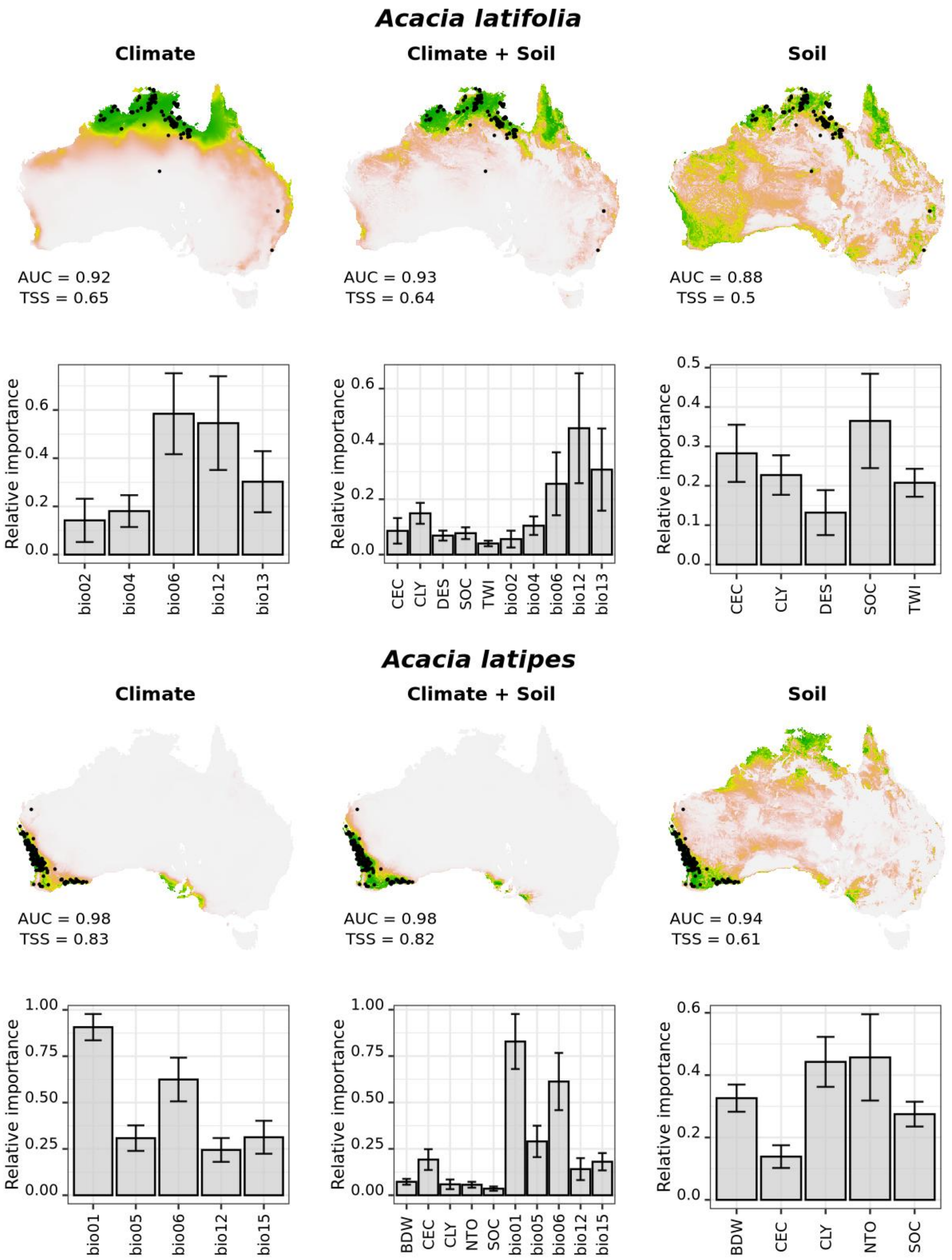

Acacia latifolia
Climate
Climate + Soil
Soil
AUC = 0.92
TSS = 0.65
AUC = 0.93
TSS = 0.64
AUC = 0.88
TSS = 0.5
Relative importance
bio02
bio04
bio06
bio12
bio13
CEC
CLY
DES
SOC
TWI
Acacia latipes
Climate
Climate + Soil
Soil
AUC = 0.98
TSS = 0.83
AUC = 0.98
TSS = 0.82
AUC = 0.94
TSS = 0.61
Relative importance
bio01
bio05
bio06
bio12
bio15
BDW
CEC
CLY
NTO
SOC

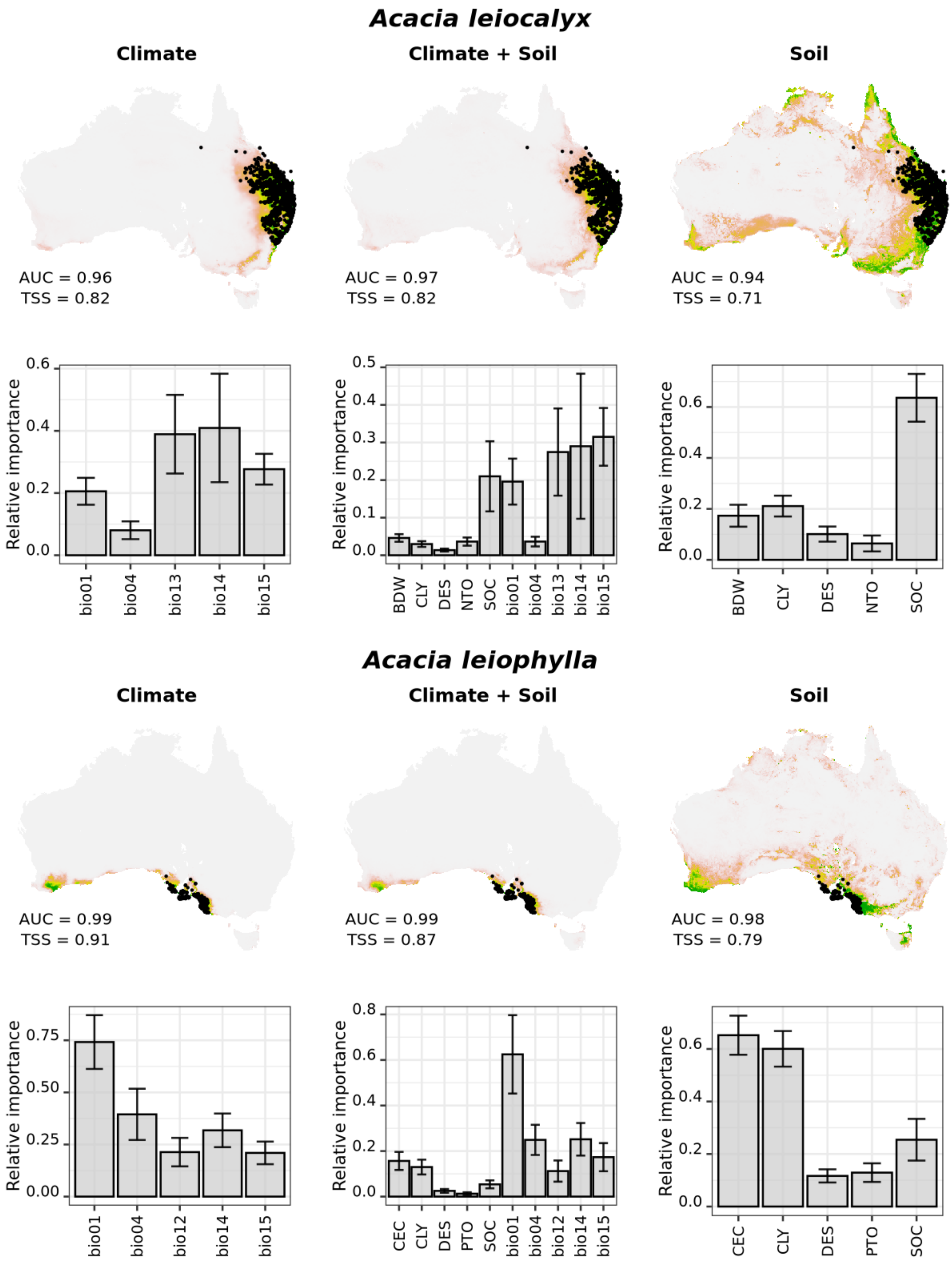
Acacia leiocalyx
Climate
Climate + Soil
Soil
AUC = 0.96
TSS = 0.82
AUC = 0.97
TSS = 0.82
AUC = 0.94
TSS = 0.71
Relative importance
bio01
bio04
bio13
bio14
bio15
BDW
CLY
DES
NTO
SOC
Acacia leiophylla
AUC = 0.99
TSS = 0.91
AUC = 0.99
TSS = 0.87
AUC = 0.98
TSS = 0.79
bio12
CEC
PTO

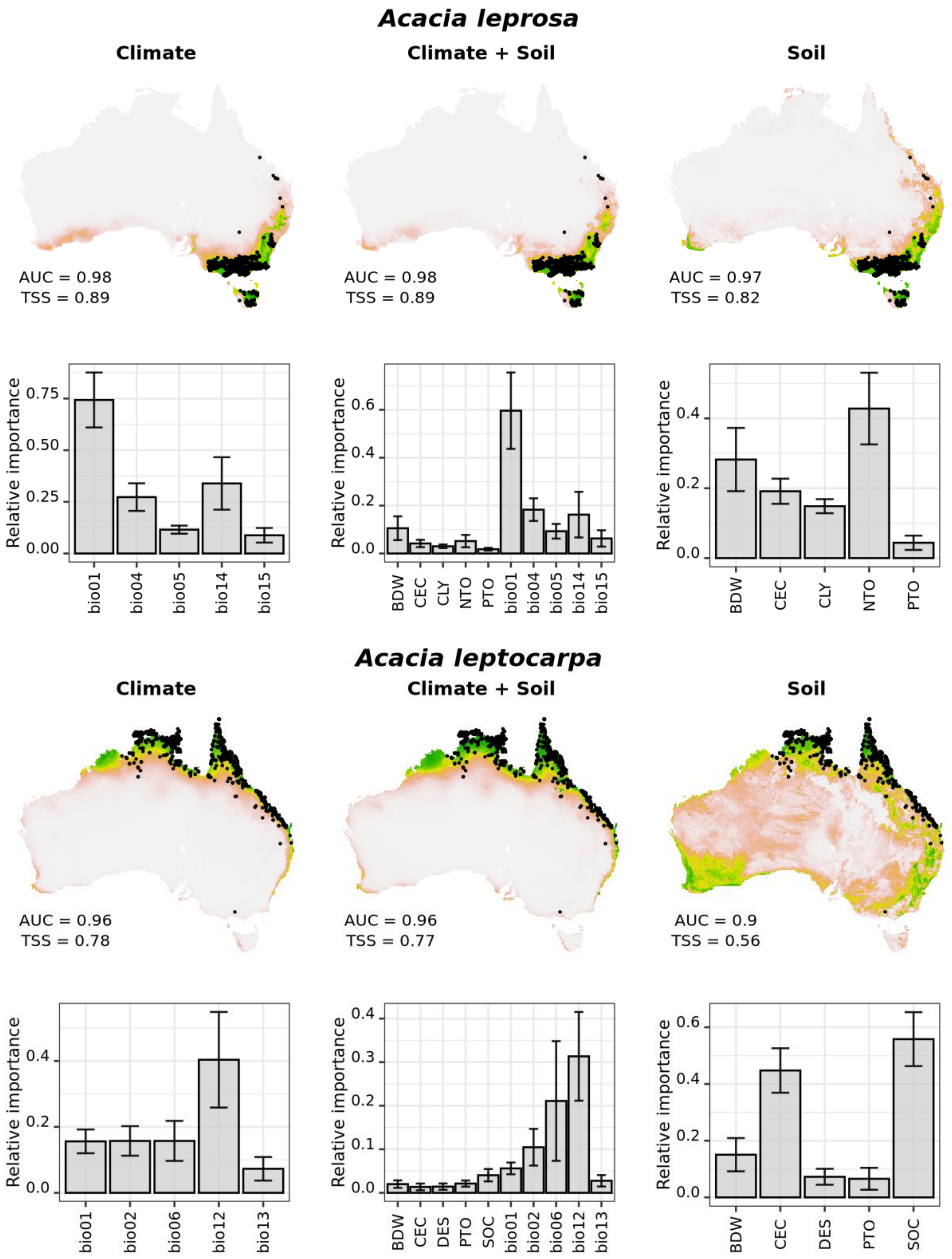
Acacia leprosa
Climate
Climate + Soil
Soil
AUC = 0.98
TSS = 0.89
AUC = 0.98
TSS = 0.89
AUC = 0.97
TSS = 0.82
Relative importance
0.00
0.25
0.50
0.75
bio01
bio04
bio05
bio14
bio15
0.0
0.2
0.4
0.6
BDW
CEC
CLY
NTO
PTO
bio01
bio04
bio05
bio14
bio15
0.0
0.2
0.4
BDW
CEC
CLY
NTO
PTO
Acacia leptocarpa
Climate
Climate + Soil
Soil
AUC = 0.96
TSS = 0.78
AUC = 0.96
TSS = 0.77
AUC = 0.9
TSS = 0.56
Relative importance
0.0
0.2
0.4
bio01
bio02
bio06
bio12
bio13
0.0
0.1
0.2
0.3
0.4
BDW
CEC
DES
PTO
SOC
bio01
bio02
bio06
bio12
bio13
0.0
0.2
0.4
0.6
BDW
CEC
DES
PTO
SOC

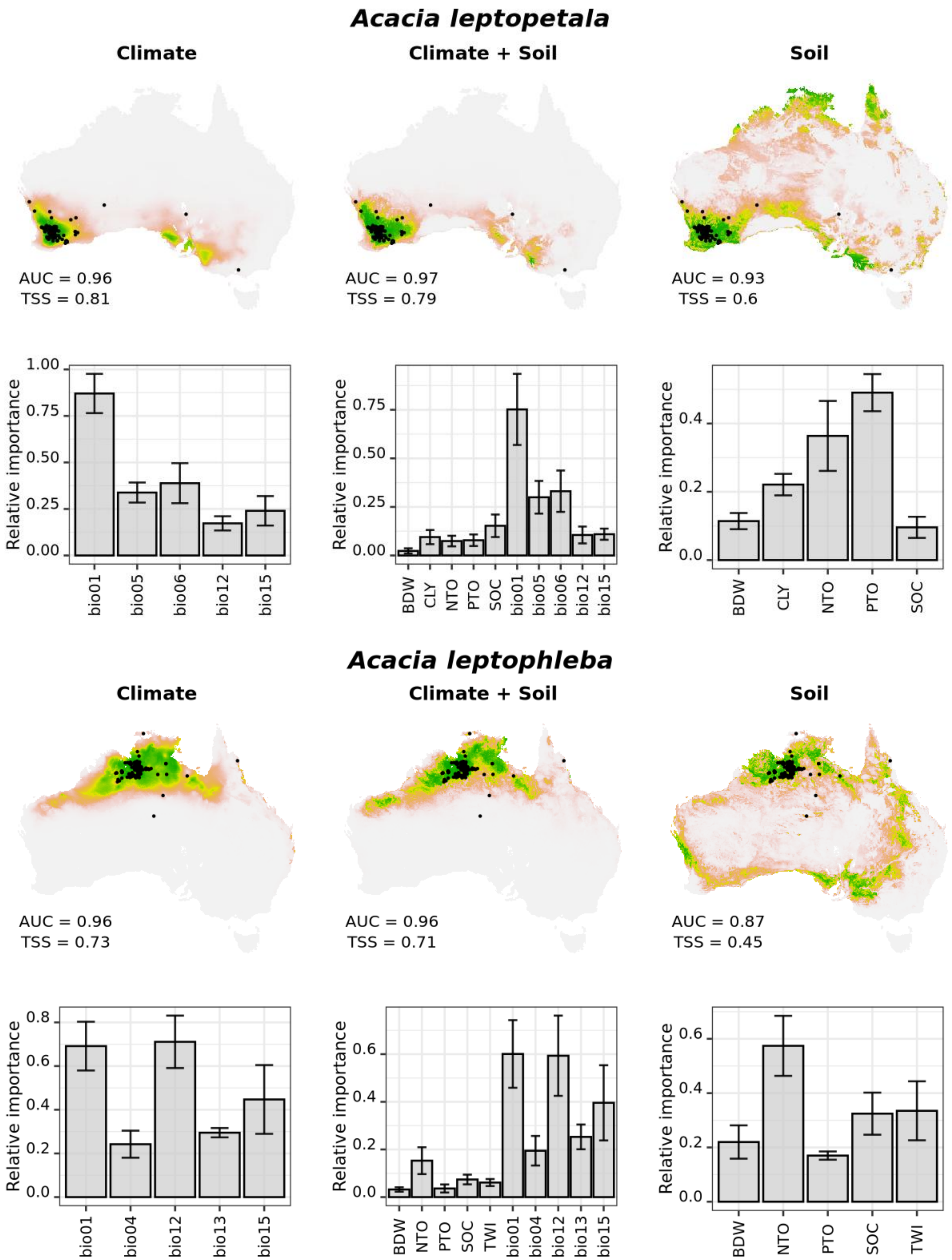
Acacia leptopetala
Climate
Climate + Soil
Soil
AUC = 0.96
TSS = 0.81
AUC = 0.97
TSS = 0.79
AUC = 0.93
TSS = 0.6
Relative importance
1.00
0.75
0.50
0.25
0.00
bio01
bio05
bio06
bio12
bio15
BDW
CLY
NTO
PTO
SOC
0.4
0.2
0.0
Acacia leptophleba
AUC = 0.96
TSS = 0.73
AUC = 0.96
TSS = 0.71
AUC = 0.87
TSS = 0.45
0.8
0.6
bio04
bio13
TWI

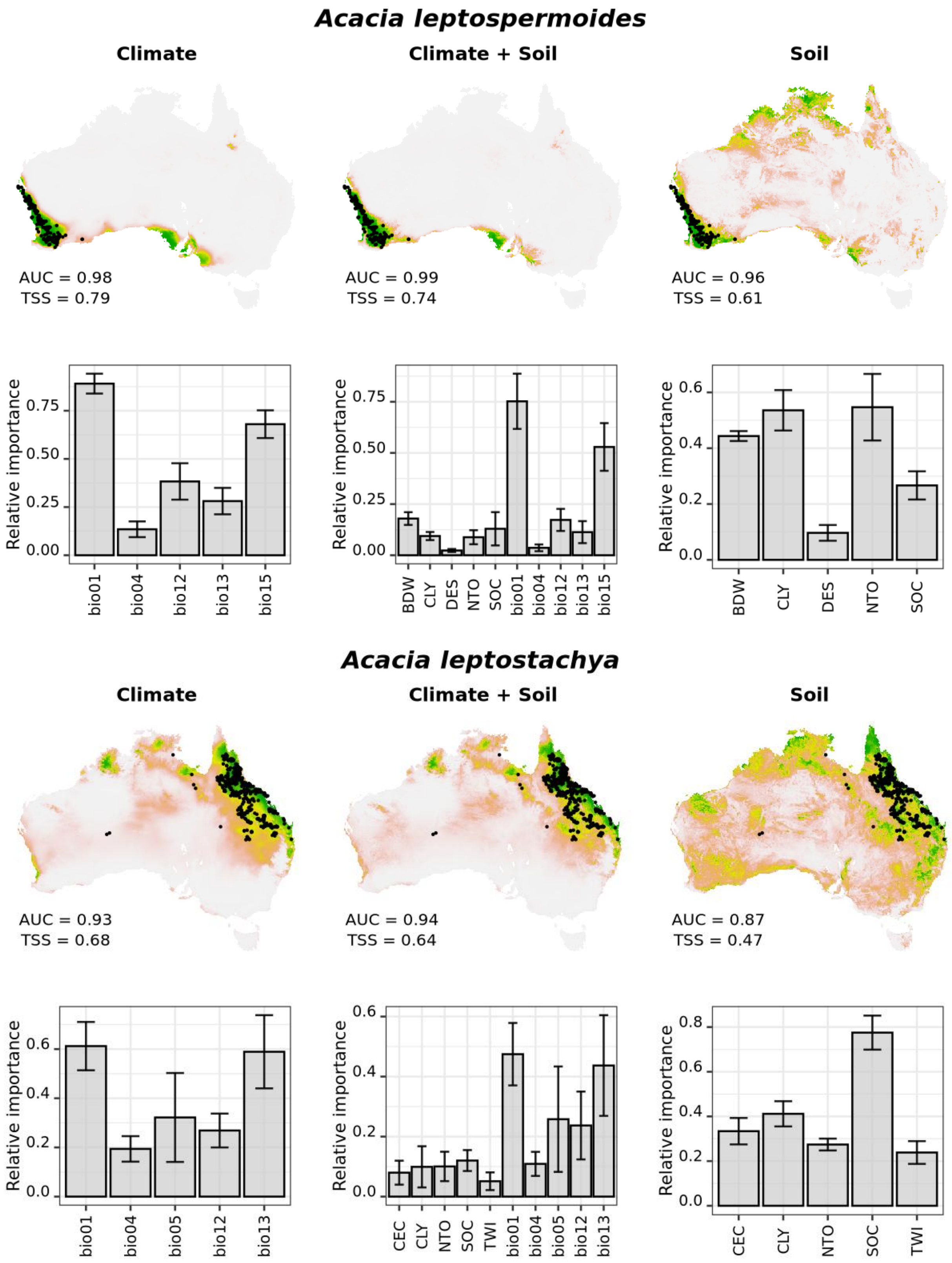
Acacia leptospermoides
Climate
Climate + Soil
Soil
AUC = 0.98
TSS = 0.79
AUC = 0.99
TSS = 0.74
AUC = 0.96
TSS = 0.61
Relative importance
0.00
0.25
0.50
0.75
bio01
bio04
bio12
bio13
bio15
BDW
CLY
DES
NTO
SOC
0.0
0.2
0.4
0.6
Acacia leptostachya
Climate
Climate + Soil
Soil
AUC = 0.93
TSS = 0.68
AUC = 0.94
TSS = 0.64
AUC = 0.87
TSS = 0.47
0.8
bio05
CEC
TWI

Acacia leucoclada

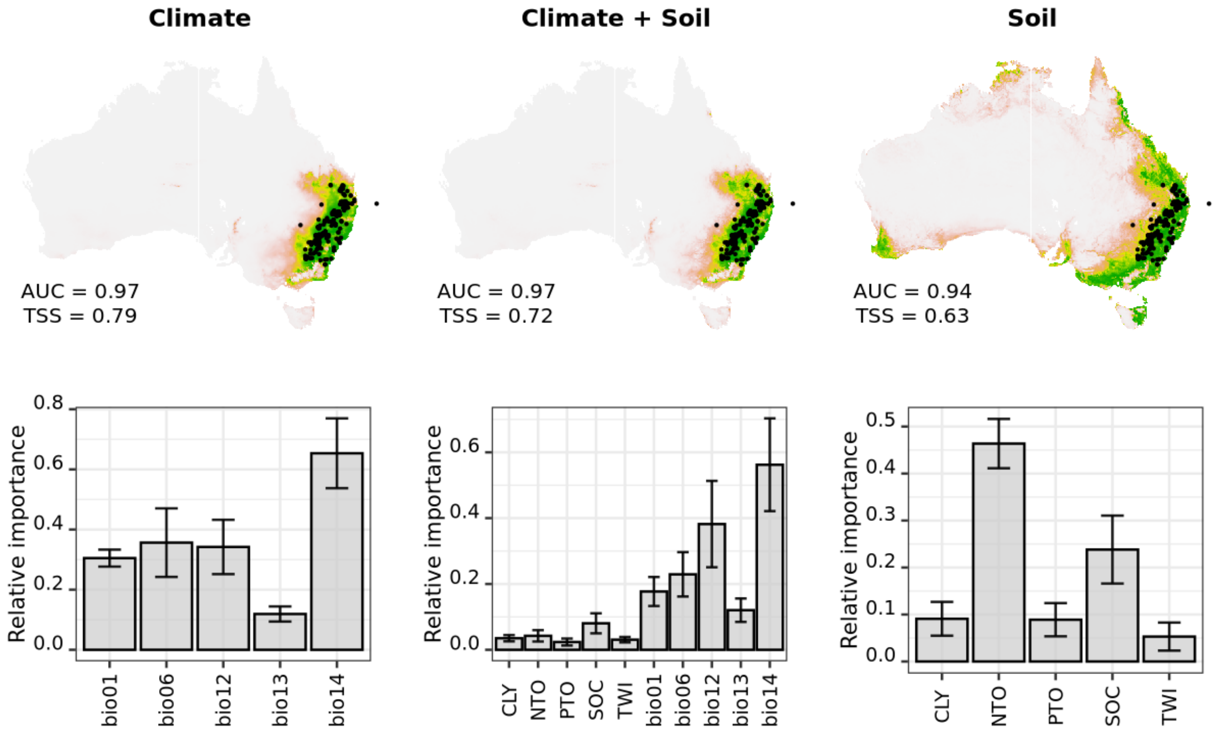

Acacia leucolobia

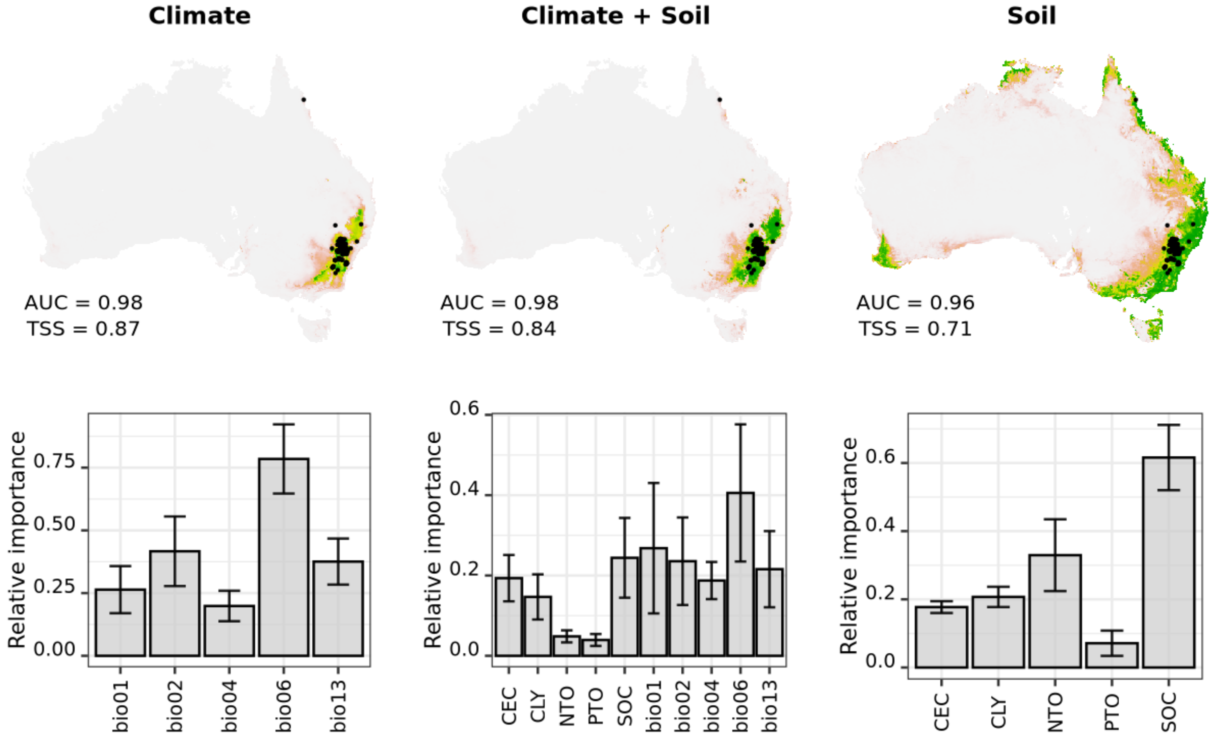

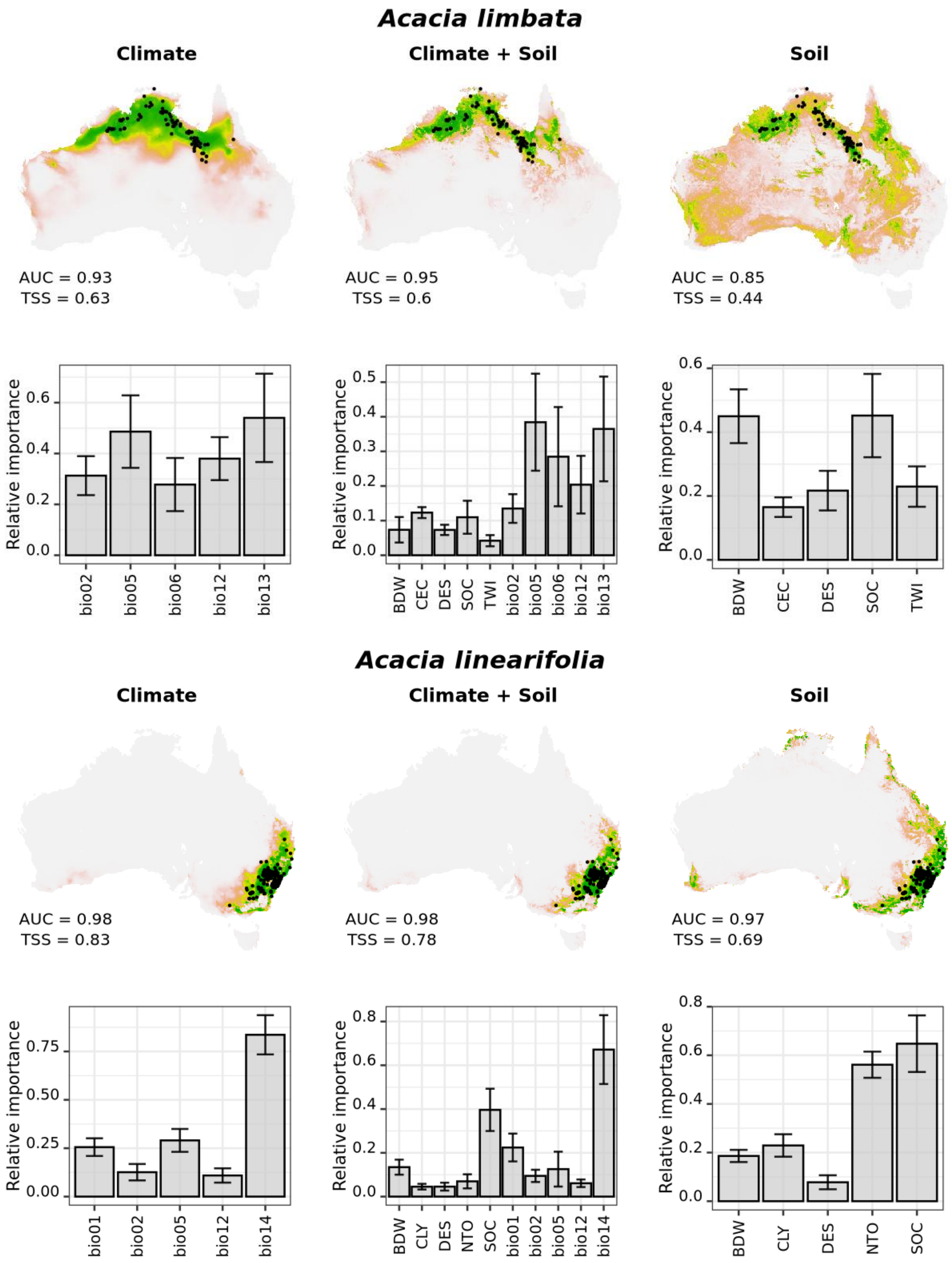
Acacia limbata
Climate
Climate + Soil
Soil
AUC = 0.93
TSS = 0.63
AUC = 0.95
TSS = 0.6
AUC = 0.85
TSS = 0.44
Relative importance
bio02
bio05
bio06
bio12
bio13
BDW
CEC
DES
SOC
TWI
bio02
bio05
bio06
bio12
bio13
BDW
CEC
DES
SOC
TWI
Acacia linearifolia
Climate
Climate + Soil
Soil
AUC = 0.98
TSS = 0.83
AUC = 0.98
TSS = 0.78
AUC = 0.97
TSS = 0.69
Relative importance
bio01
bio02
bio05
bio12
bio14
BDW
CLY
DES
NTO
SOC
bio01
bio02
bio05
bio12
bio14
BDW
CLY
DES
NTO
SOC

Acacia lineolata

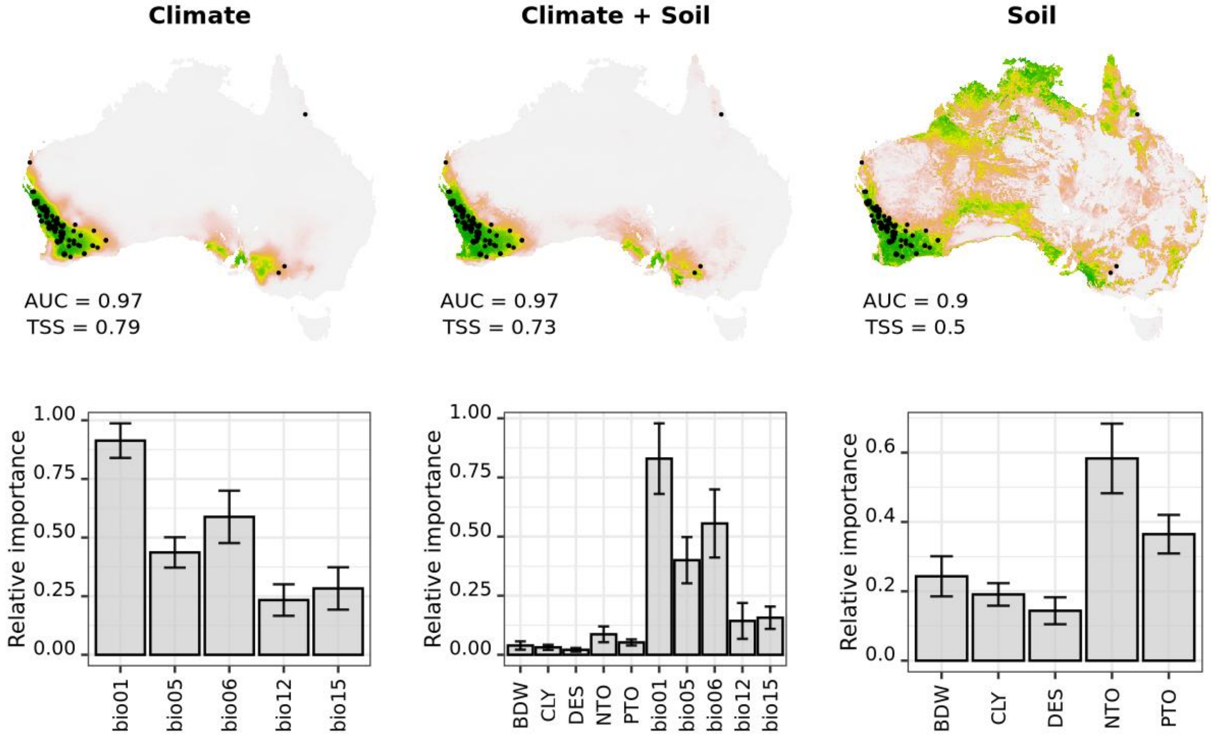

Acacia linifolia

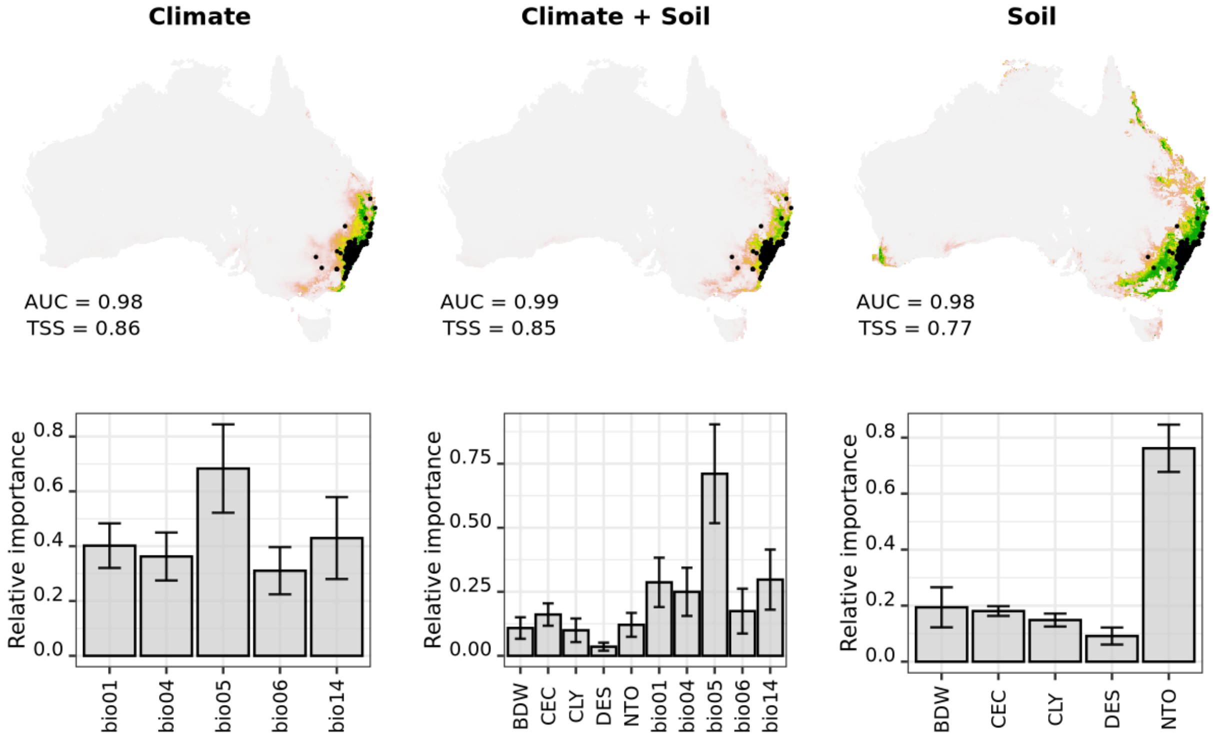

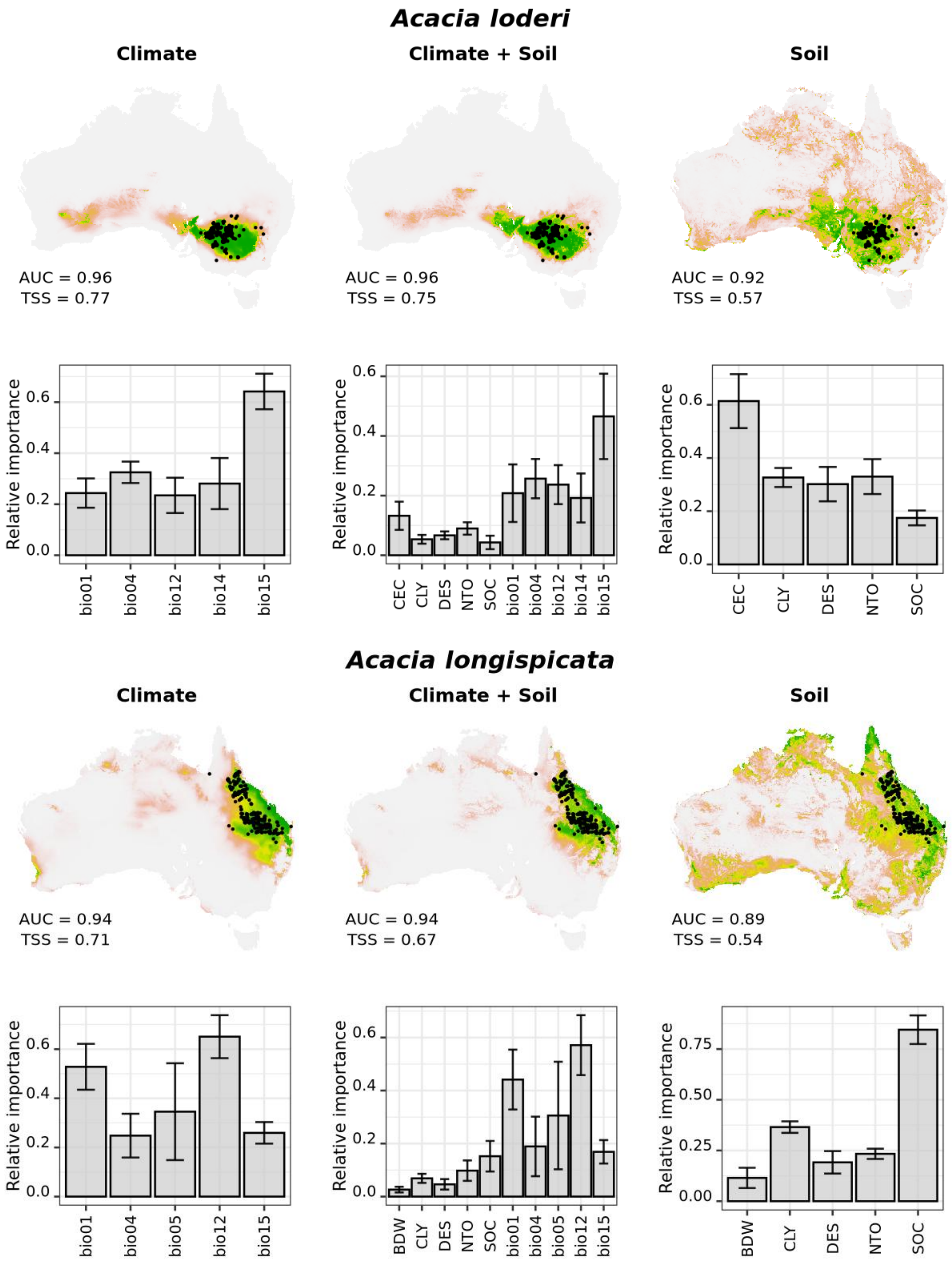

Acacia loderi
Climate
Climate + Soil
Soil
AUC = 0.96
TSS = 0.77
AUC = 0.96
TSS = 0.75
AUC = 0.92
TSS = 0.57
Relative importance
bio01
bio04
bio12
bio14
bio15
CEC
CLY
DES
NTO
SOC
Acacia longispicata
Climate
Climate + Soil
Soil
AUC = 0.94
TSS = 0.71
AUC = 0.94
TSS = 0.67
AUC = 0.89
TSS = 0.54
Relative importance
bio01
bio04
bio05
bio12
bio15
BDW
CLY
DES
NTO
SOC

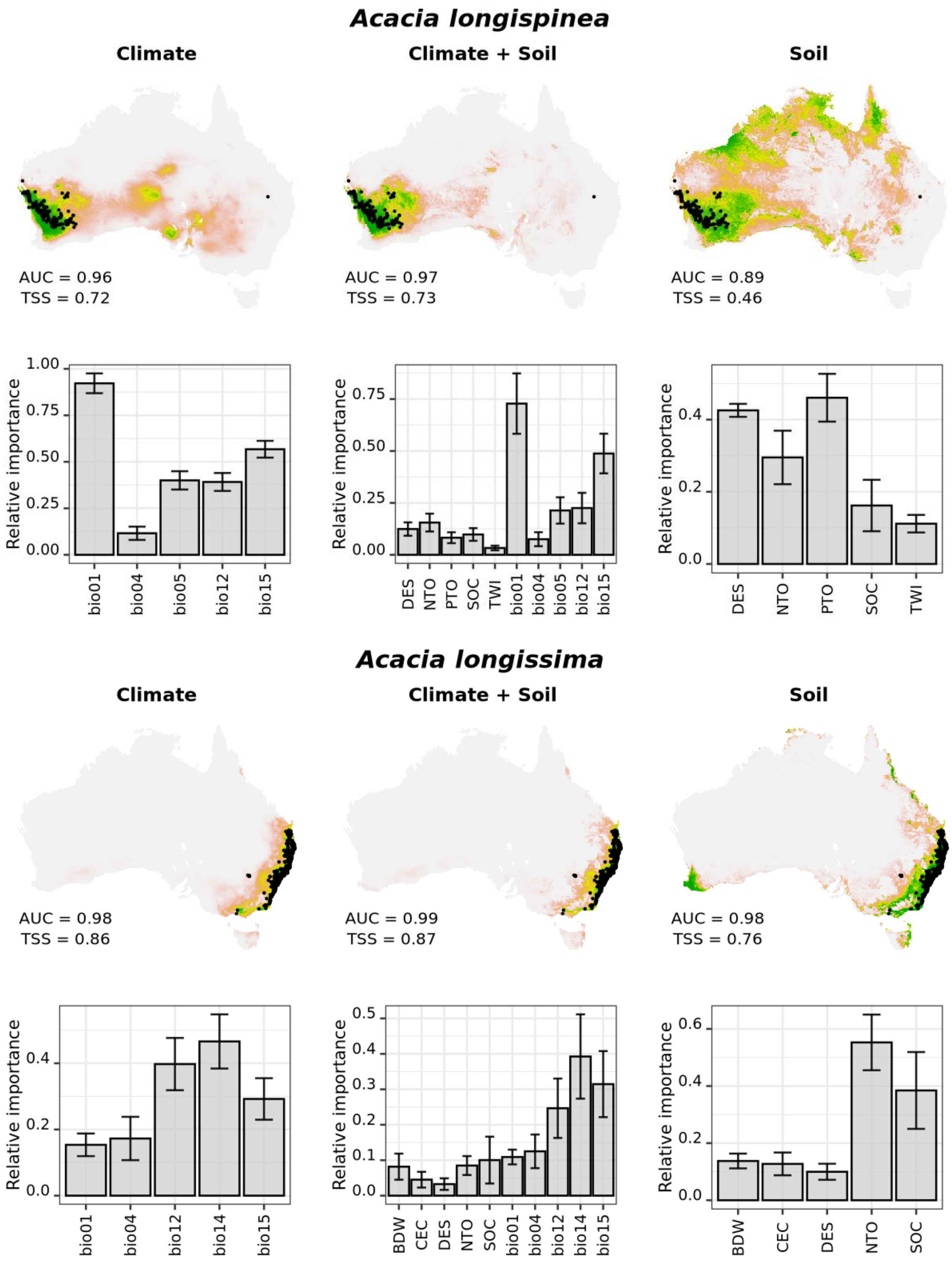
Acacia longispinea
Climate
Climate + Soil
Soil
AUC = 0.96
TSS = 0.72
AUC = 0.97
TSS = 0.73
AUC = 0.89
TSS = 0.46
Relative importance
bio01
bio04
bio05
bio12
bio15
DES
NTO
PTO
SOC
TWI
Acacia longissima
Climate
Climate + Soil
Soil
AUC = 0.98
TSS = 0.86
AUC = 0.99
TSS = 0.87
AUC = 0.98
TSS = 0.76
Relative importance
bio01
bio04
bio12
bio14
bio15
BDW
CEC
DES
NTO
SOC

Acacia lycopodiifolia

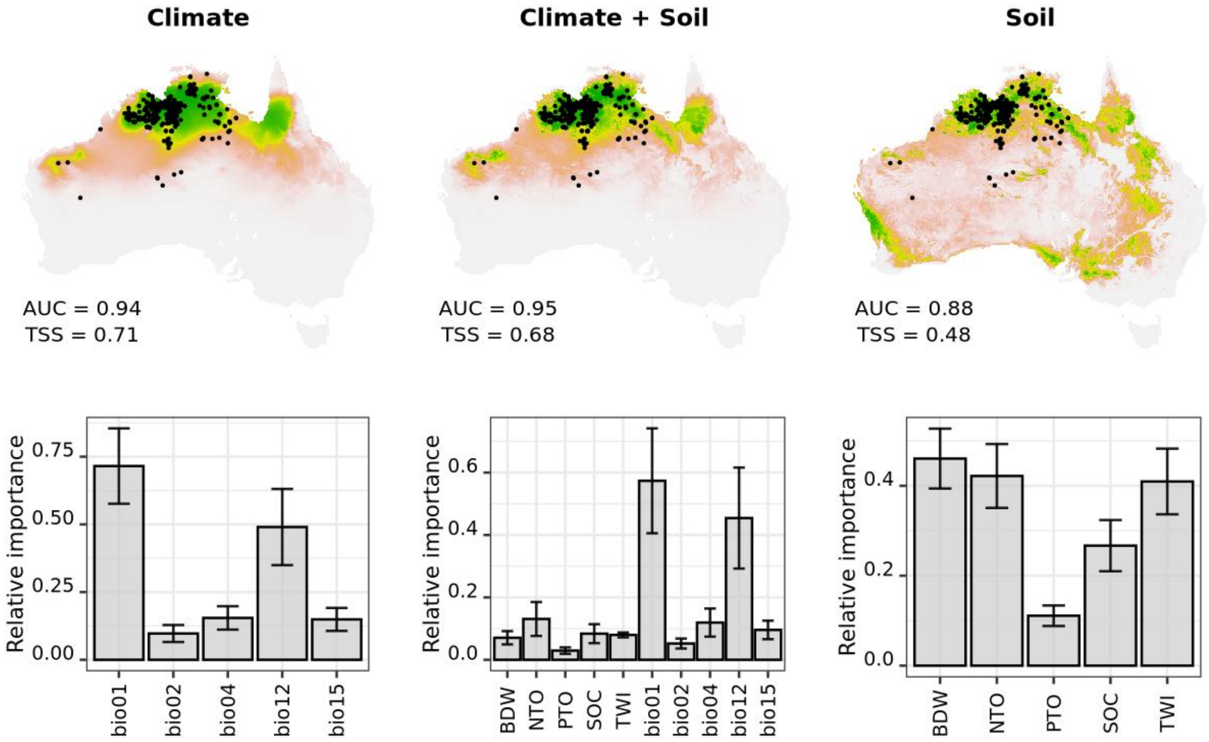

Acacia lysiphloia

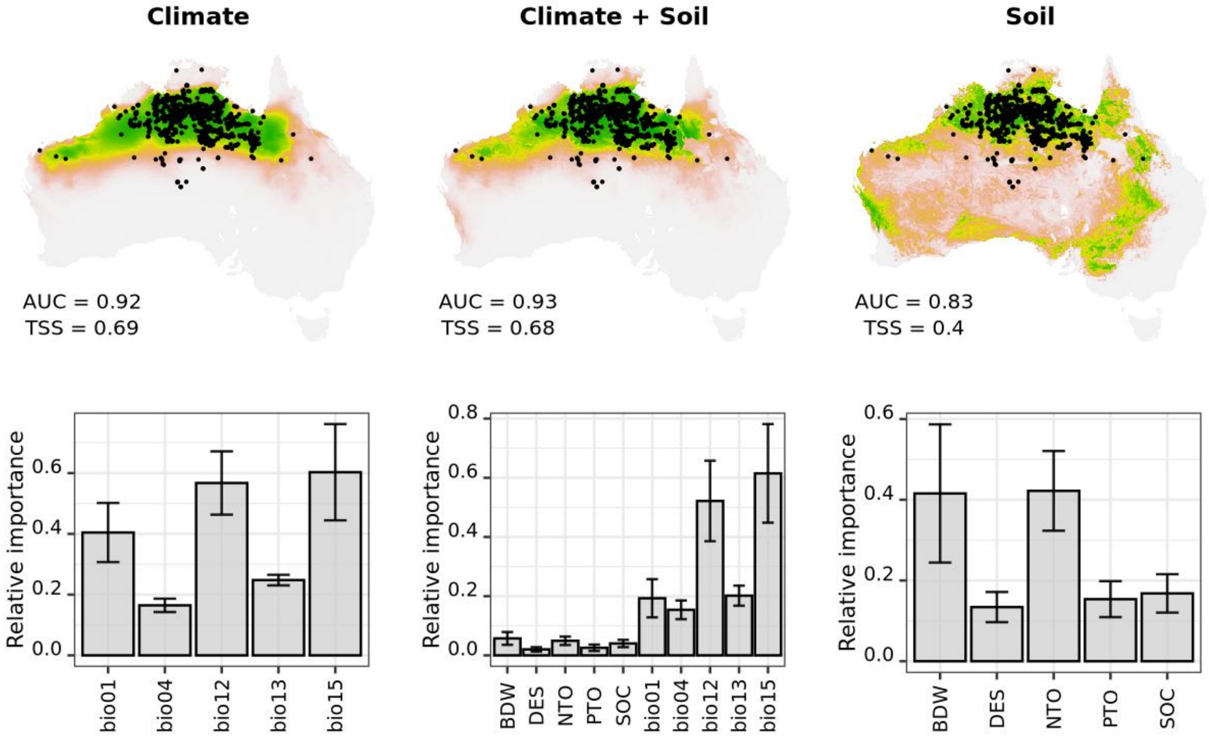

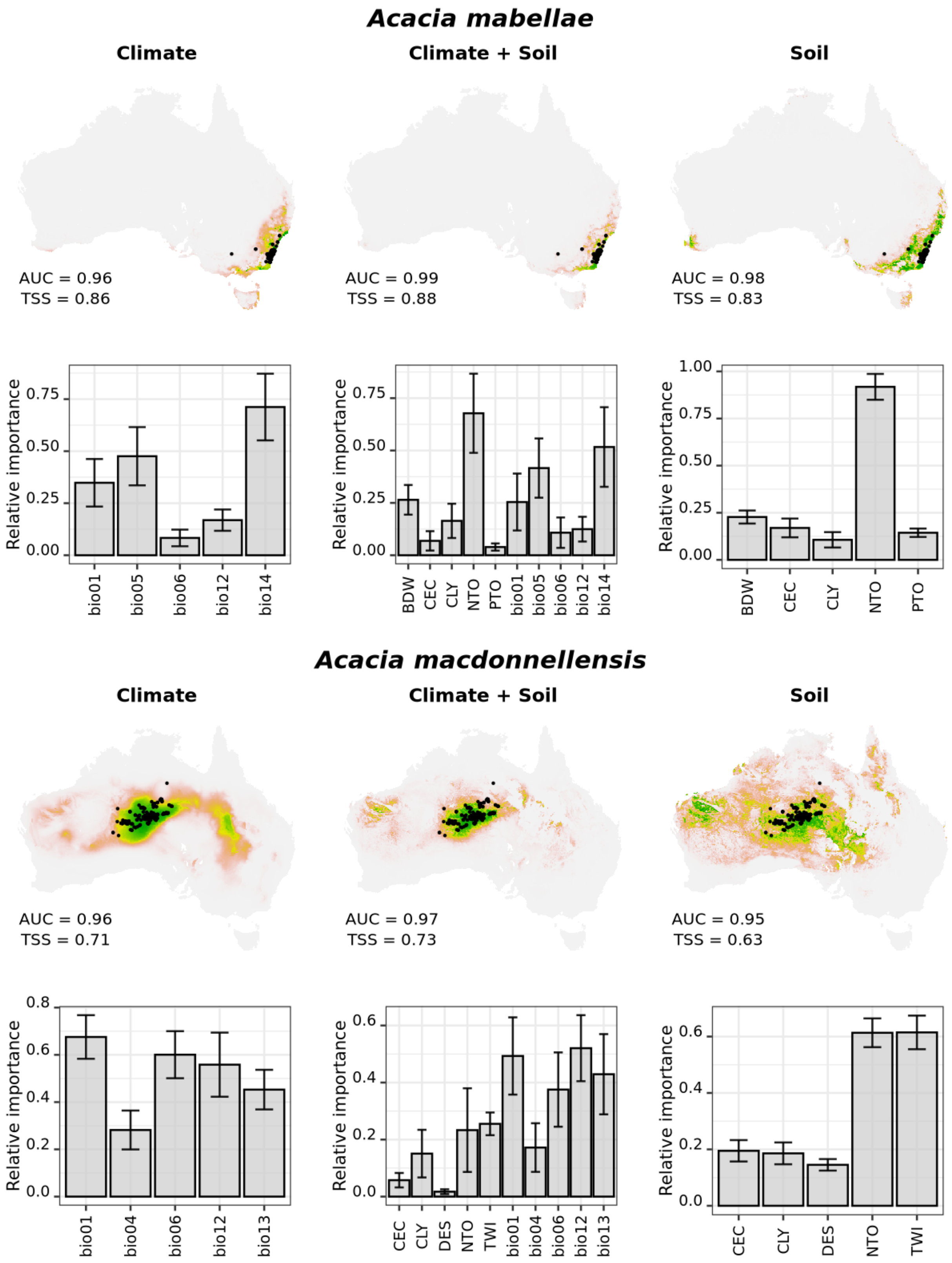
Acacia mabellae
Climate
Climate + Soil
Soil
AUC = 0.96
TSS = 0.86
AUC = 0.99
TSS = 0.88
AUC = 0.98
TSS = 0.83
Relative importance
0.00
0.25
0.50
0.75
1.00
bio01
bio05
bio06
bio12
bio14
BDW
CEC
CLY
NTO
PTO
Acacia macdonnellensis
Climate
Climate + Soil
Soil
AUC = 0.96
TSS = 0.71
AUC = 0.97
TSS = 0.73
AUC = 0.95
TSS = 0.63
Relative importance
0.0
0.2
0.4
0.6
0.8
bio01
bio04
bio06
bio12
bio13
CEC
CLY
DES
NTO
TWI

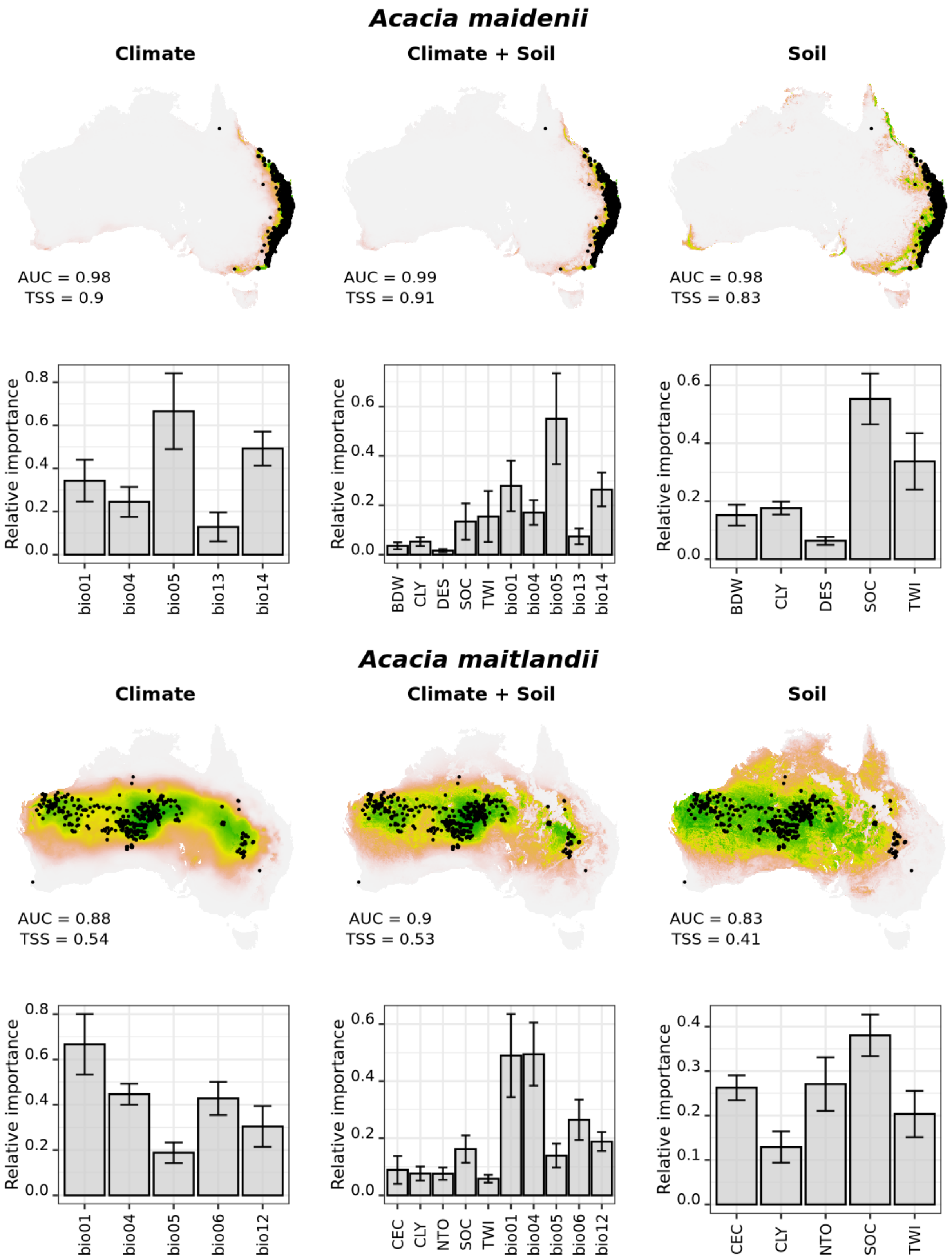
Acacia maidenii
Climate
Climate + Soil
Soil
AUC = 0.98
TSS = 0.9
AUC = 0.99
TSS = 0.91
AUC = 0.98
TSS = 0.83
Relative importance
0.0
0.2
0.4
0.6
0.8
bio01
bio04
bio05
bio13
bio14
BDW
CLY
DES
SOC
TWI
Acacia maitlandii
Climate
Climate + Soil
Soil
AUC = 0.88
TSS = 0.54
AUC = 0.9
TSS = 0.53
AUC = 0.83
TSS = 0.41
0.1
0.3
bio06
bio12
CEC
NTO

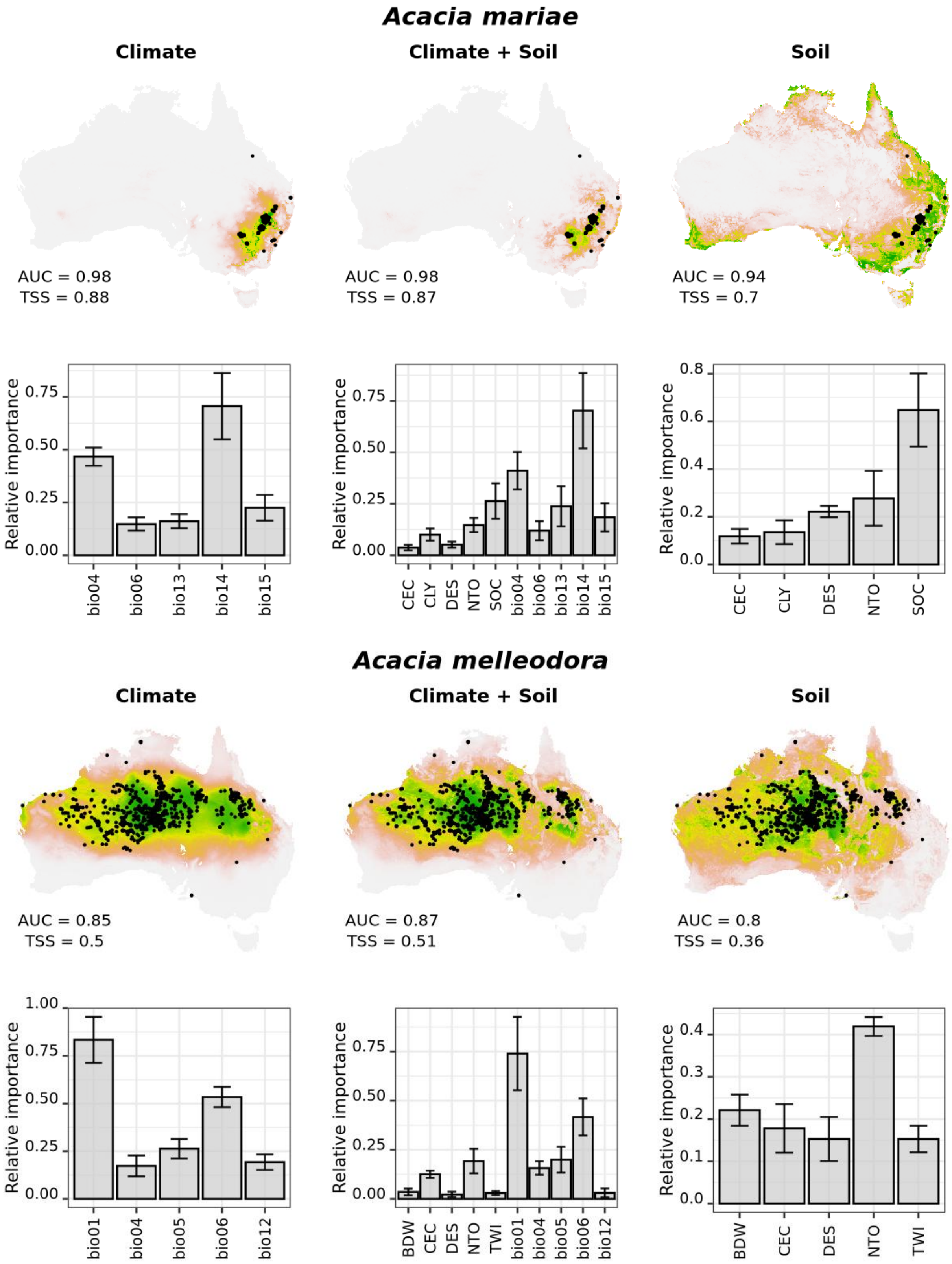

Acacia mariae
Climate
Climate + Soil
Soil
AUC = 0.98
TSS = 0.88
AUC = 0.98
TSS = 0.87
AUC = 0.94
TSS = 0.7
Relative importance
bio04
bio06
bio13
bio14
bio15
CEC
CLY
DES
NTO
SOC
Acacia melleodora
Climate
Climate + Soil
Soil
AUC = 0.85
TSS = 0.5
AUC = 0.87
TSS = 0.51
AUC = 0.8
TSS = 0.36
Relative importance
bio01
bio04
bio05
bio06
bio12
BDW
CEC
DES
NTO
TWI

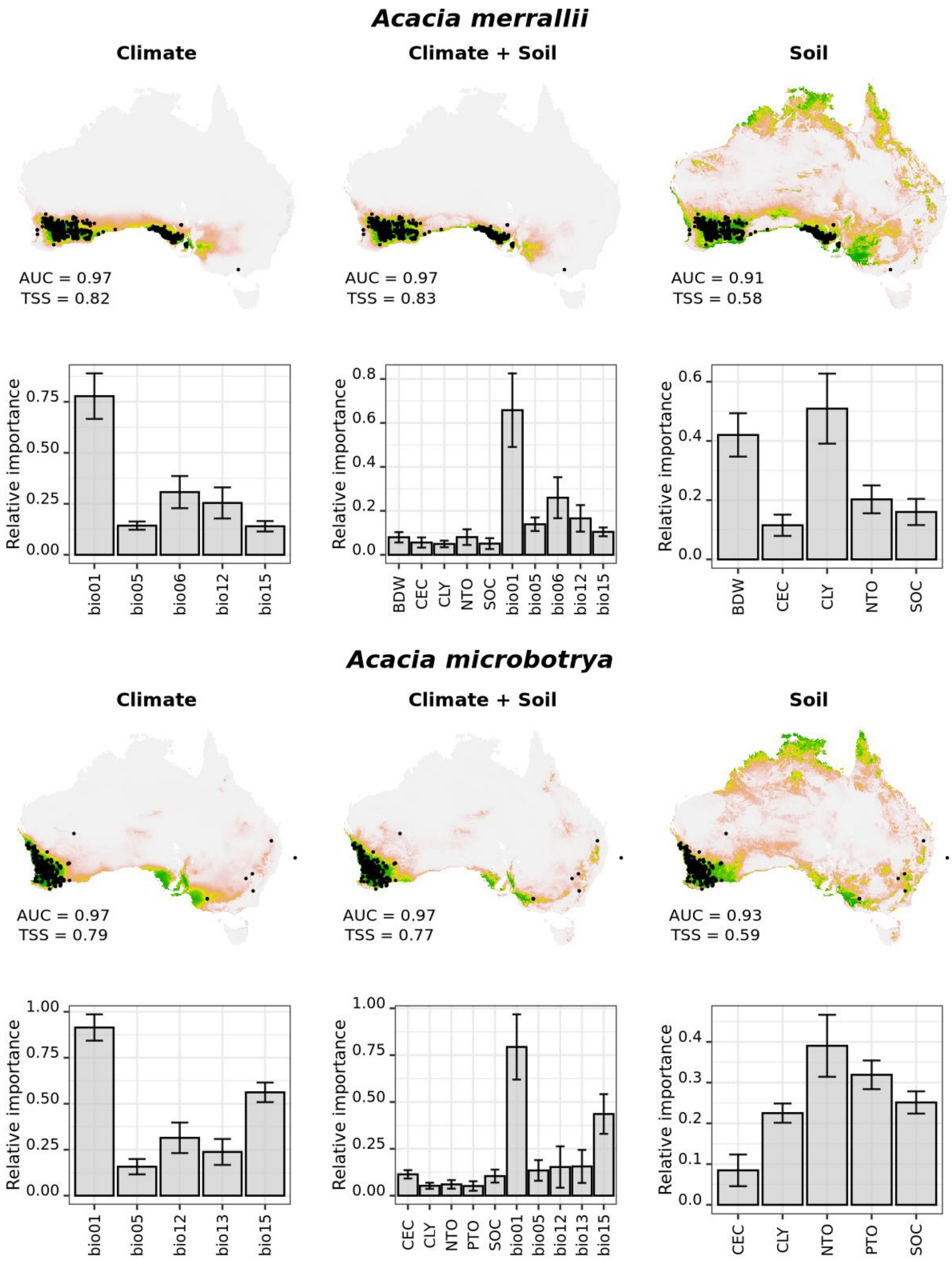
Acacia merrallii
Climate
Climate + Soil
Soil
AUC = 0.97
TSS = 0.82
AUC = 0.97
TSS = 0.83
AUC = 0.91
TSS = 0.58
Relative importance
0.00
0.25
0.50
0.75
bio01
bio05
bio06
bio12
bio15
0.0
0.2
0.4
0.6
0.8
BDW
CEC
CLY
NTO
SOC
bio01
bio05
bio06
bio12
bio15
0.0
0.2
0.4
0.6
BDW
CEC
CLY
NTO
SOC
Acacia microbotrya
Climate
Climate + Soil
Soil
AUC = 0.97
TSS = 0.79
AUC = 0.97
TSS = 0.77
AUC = 0.93
TSS = 0.59
Relative importance
0.00
0.25
0.50
0.75
1.00
bio01
bio05
bio12
bio13
bio15
CEC
CLY
NTO
PTO
SOC
bio01
bio05
bio12
bio13
bio15
0.0
0.1
0.2
0.3
0.4
CEC
CLY
NTO
PTO
SOC

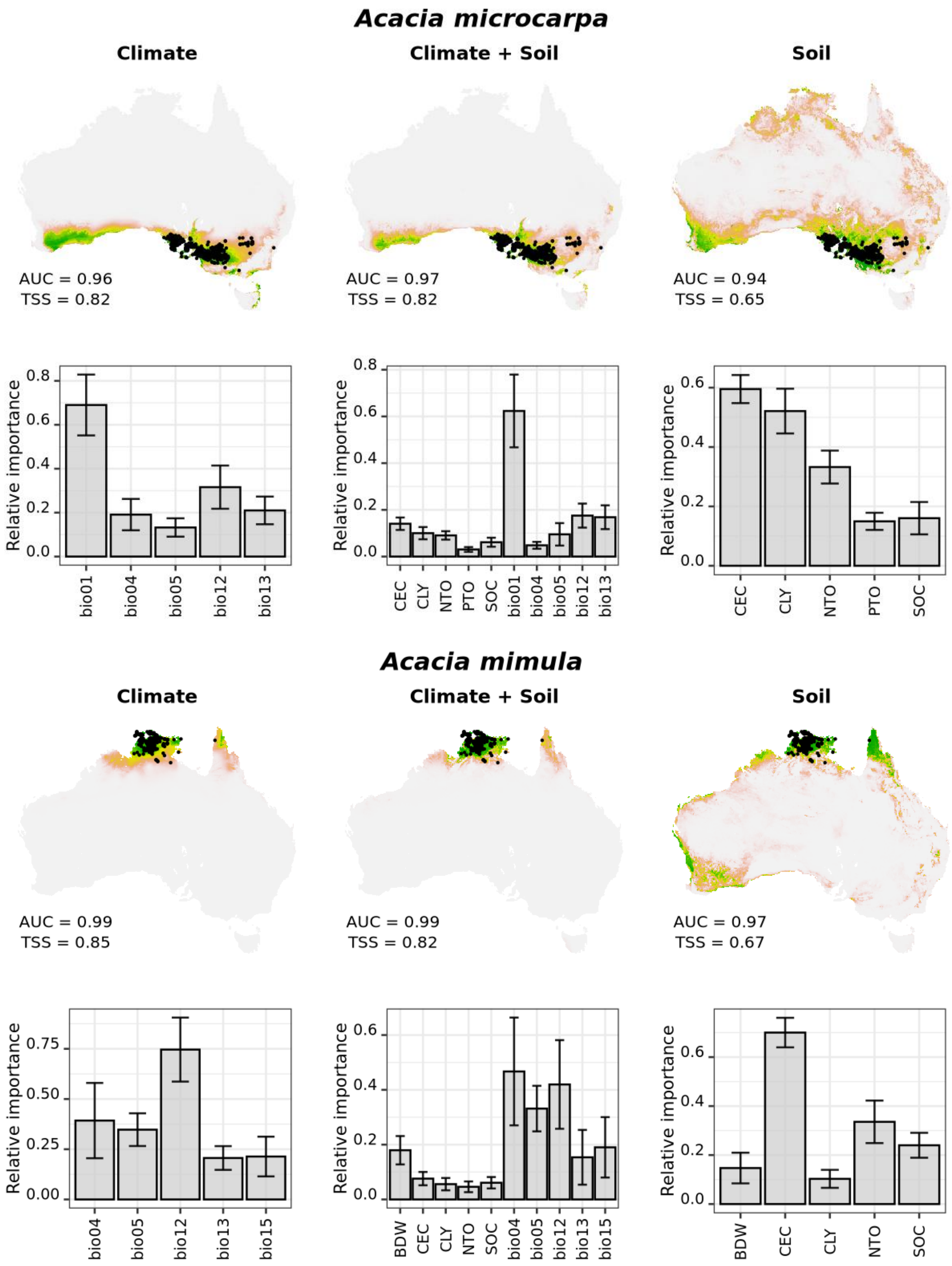
Acacia microcarpa
Climate
Climate + Soil
Soil
AUC = 0.96
TSS = 0.82
AUC = 0.97
TSS = 0.82
AUC = 0.94
TSS = 0.65
Relative importance
0.0
0.2
0.4
0.6
0.8
bio01
bio04
bio05
bio12
bio13
CEC
CLY
NTO
PTO
SOC
Acacia mimula
Climate
Climate + Soil
Soil
AUC = 0.99
TSS = 0.85
AUC = 0.99
TSS = 0.82
AUC = 0.97
TSS = 0.67
0.00
0.25
0.50
0.75
bio15
BDW

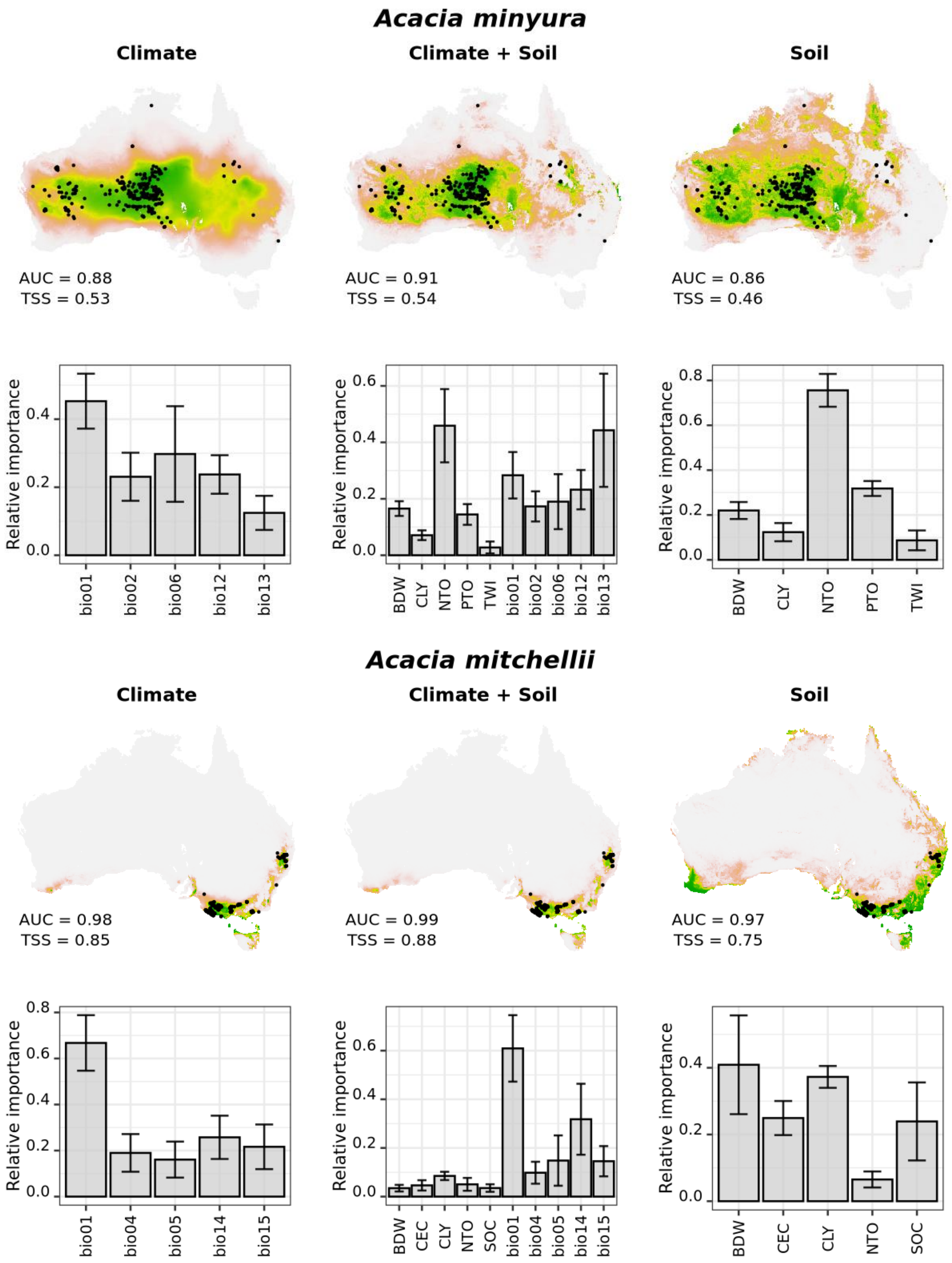

Acacia minyura
Climate
Climate + Soil
Soil
AUC = 0.88
TSS = 0.53
AUC = 0.91
TSS = 0.54
AUC = 0.86
TSS = 0.46
Relative importance
bio01
bio02
bio06
bio12
bio13
BDW
CLY
NTO
PTO
TWI
Acacia mitchellii
AUC = 0.98
TSS = 0.85
AUC = 0.99
TSS = 0.88
AUC = 0.97
TSS = 0.75
bio04
bio05
bio14
bio15
CEC
SOC

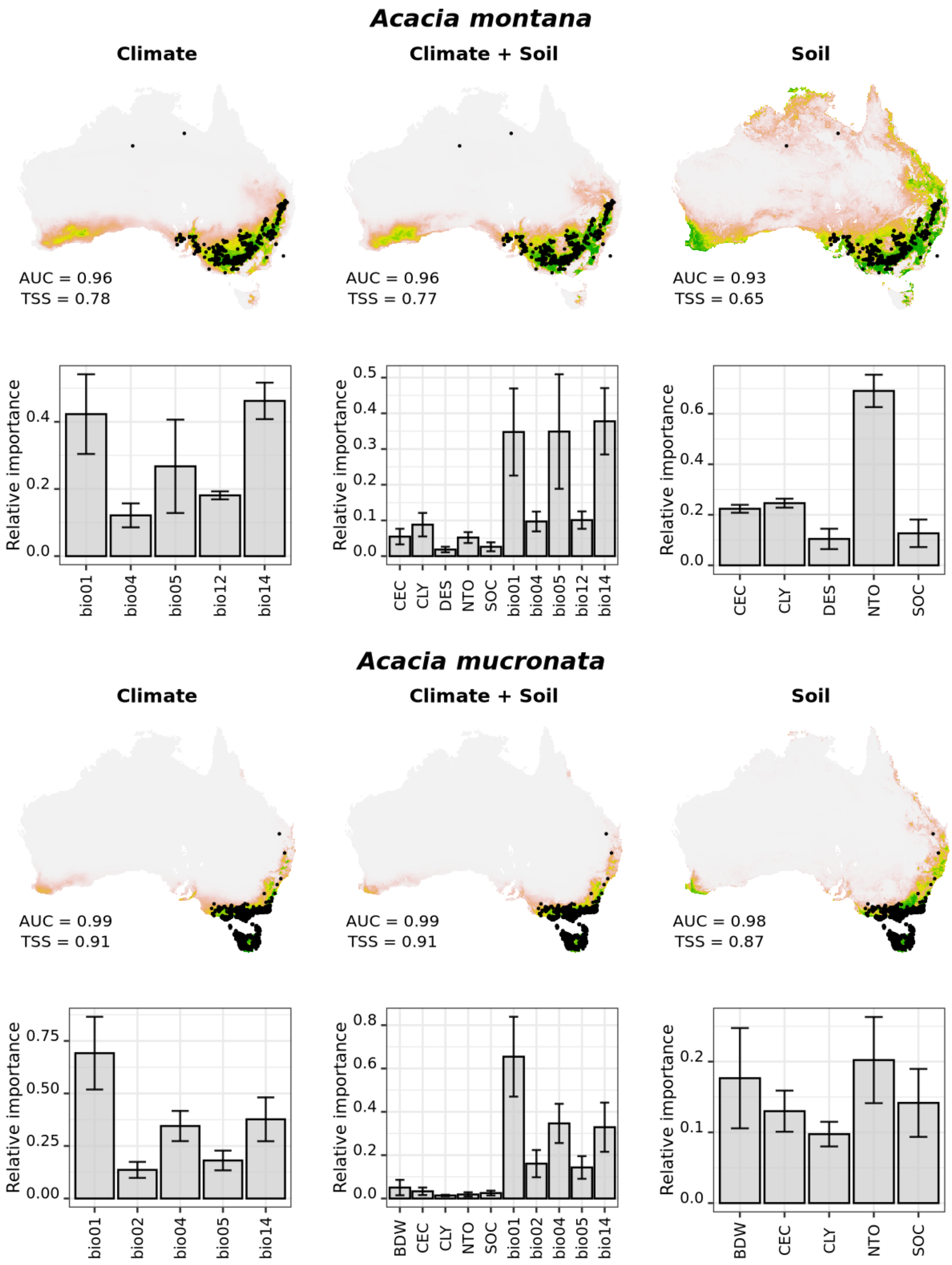

Acacia montana
Climate
Climate + Soil
Soil
AUC = 0.96
TSS = 0.78
AUC = 0.96
TSS = 0.77
AUC = 0.93
TSS = 0.65
Relative importance
bio01
bio04
bio05
bio12
bio14
CEC
CLY
DES
NTO
SOC
Acacia mucronata
Climate
Climate + Soil
Soil
AUC = 0.99
TSS = 0.91
AUC = 0.99
TSS = 0.91
AUC = 0.98
TSS = 0.87
Relative importance
bio01
bio02
bio04
bio05
bio14
BDW
CEC
CLY
NTO
SOC

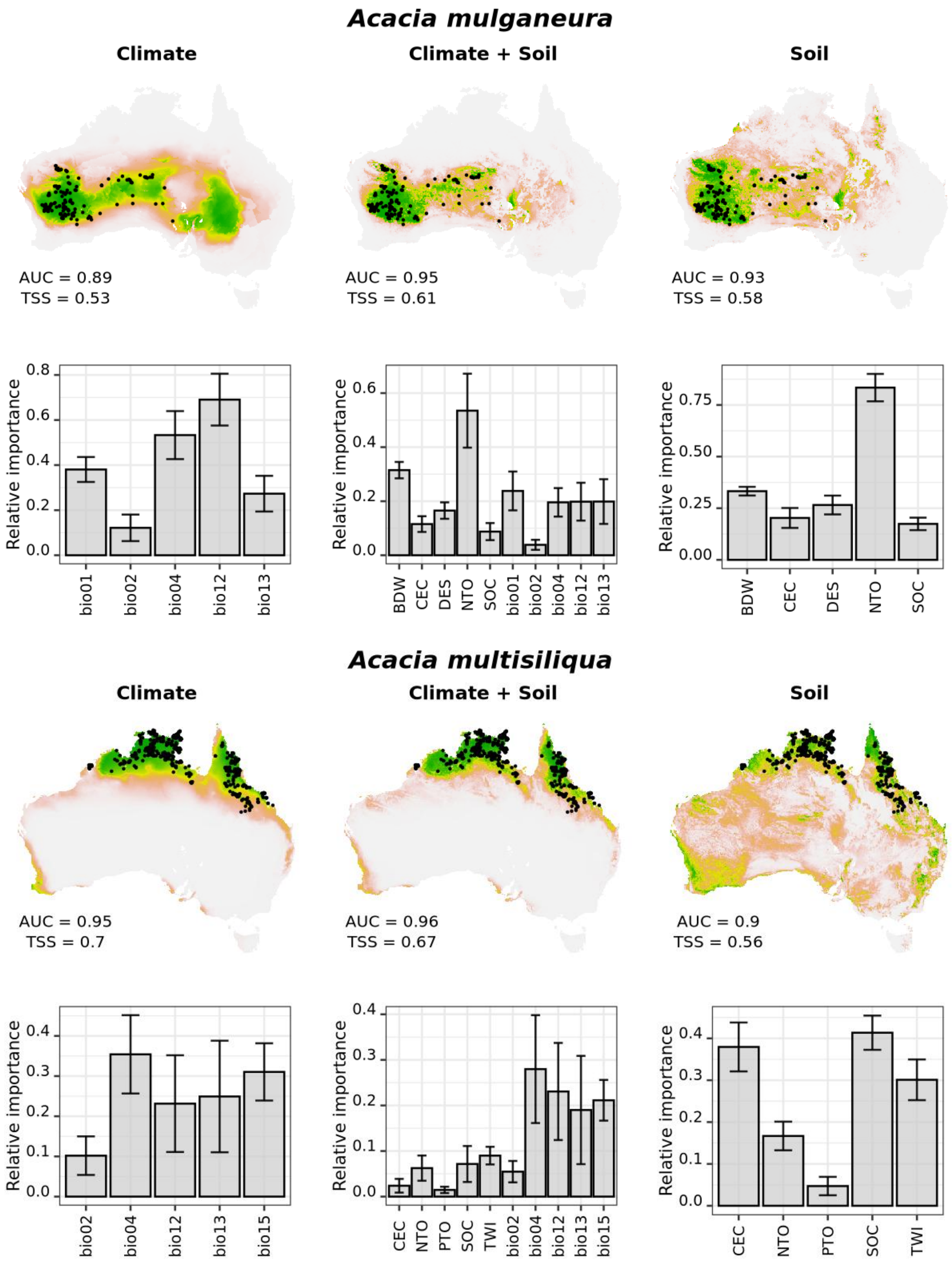

Acacia mulganeura
Climate
Climate + Soil
Soil
AUC = 0.89
TSS = 0.53
AUC = 0.95
TSS = 0.61
AUC = 0.93
TSS = 0.58
Relative importance
bio01
bio02
bio04
bio12
bio13
BDW
CEC
DES
NTO
SOC
Acacia multisiliqua
AUC = 0.95
TSS = 0.7
AUC = 0.96
TSS = 0.67
AUC = 0.9
TSS = 0.56
bio15
PTO
TWI

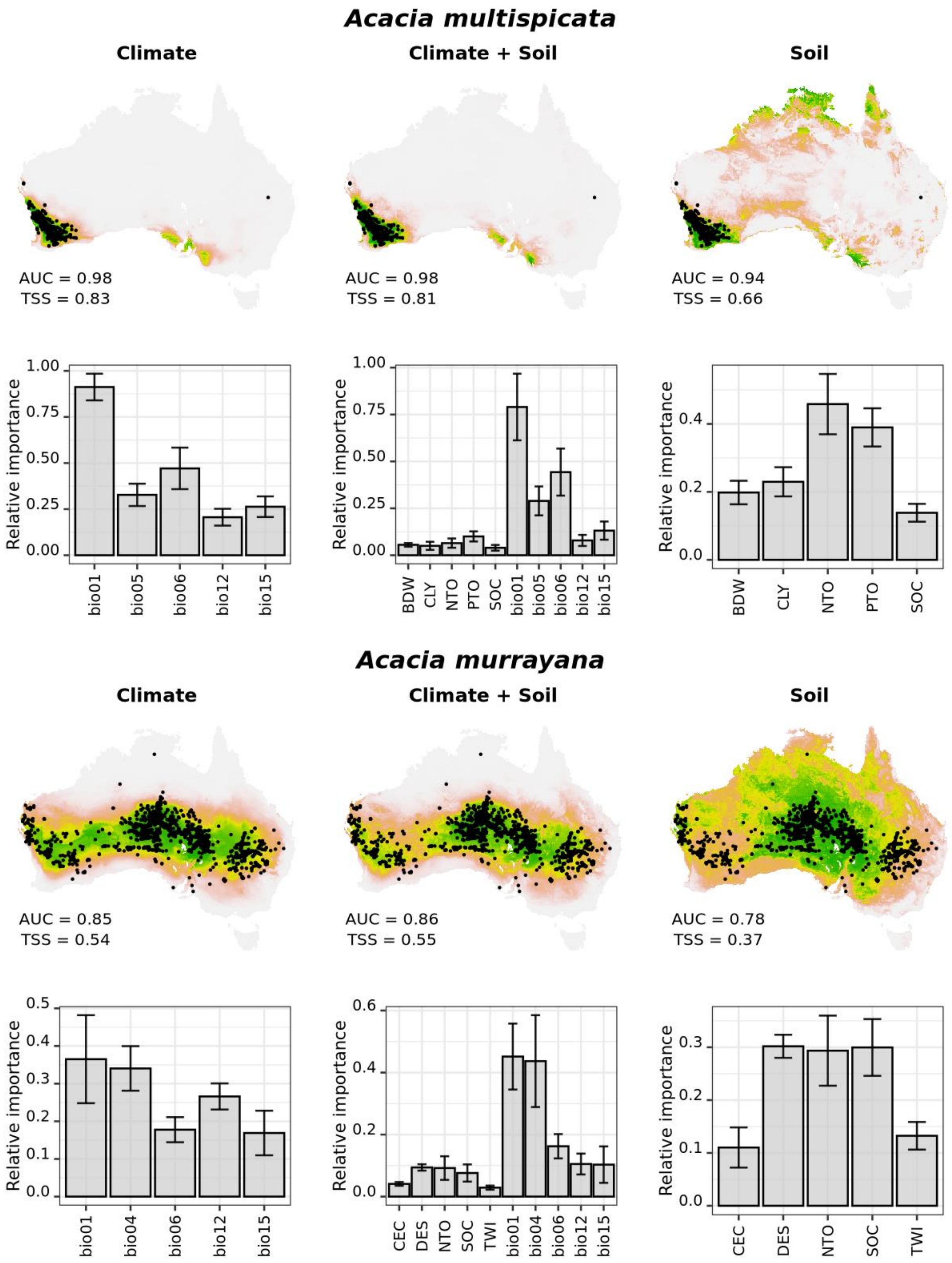

Acacia multispicata
Climate
Climate + Soil
Soil
AUC = 0.98
TSS = 0.83
AUC = 0.98
TSS = 0.81
AUC = 0.94
TSS = 0.66
Relative importance
1.00
0.75
0.50
0.25
0.00
bio01
bio05
bio06
bio12
bio15
BDW
CLY
NTO
PTO
SOC
0.4
0.2
0.0
Acacia murrayana
Climate
Climate + Soil
Soil
AUC = 0.85
TSS = 0.54
AUC = 0.86
TSS = 0.55
AUC = 0.78
TSS = 0.37
Relative importance
0.5
0.4
0.3
0.2
0.1
0.0
0.6
bio01
bio04
bio06
bio12
bio15
CEC
DES
NTO
SOC
TWI

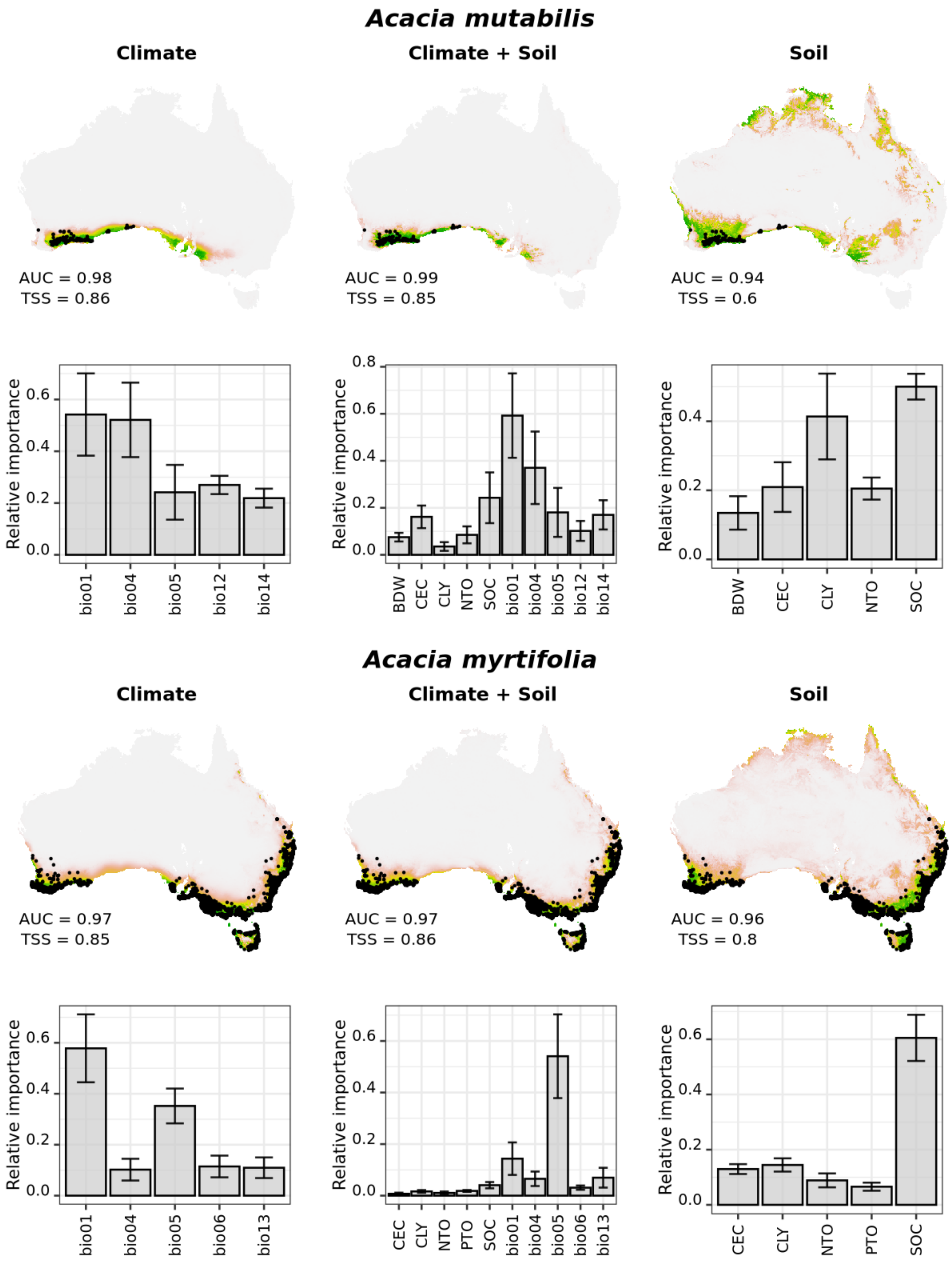
Acacia mutabilis
Climate
Climate + Soil
Soil
AUC = 0.98
TSS = 0.86
AUC = 0.99
TSS = 0.85
AUC = 0.94
TSS = 0.6
Relative importance
bio01
bio04
bio05
bio12
bio14
BDW
CEC
CLY
NTO
SOC
Acacia myrtifolia
Climate
Climate + Soil
Soil
AUC = 0.97
TSS = 0.85
AUC = 0.97
TSS = 0.86
AUC = 0.96
TSS = 0.8
Relative importance
bio01
bio04
bio05
bio06
bio13
CEC
CLY
NTO
PTO
SOC

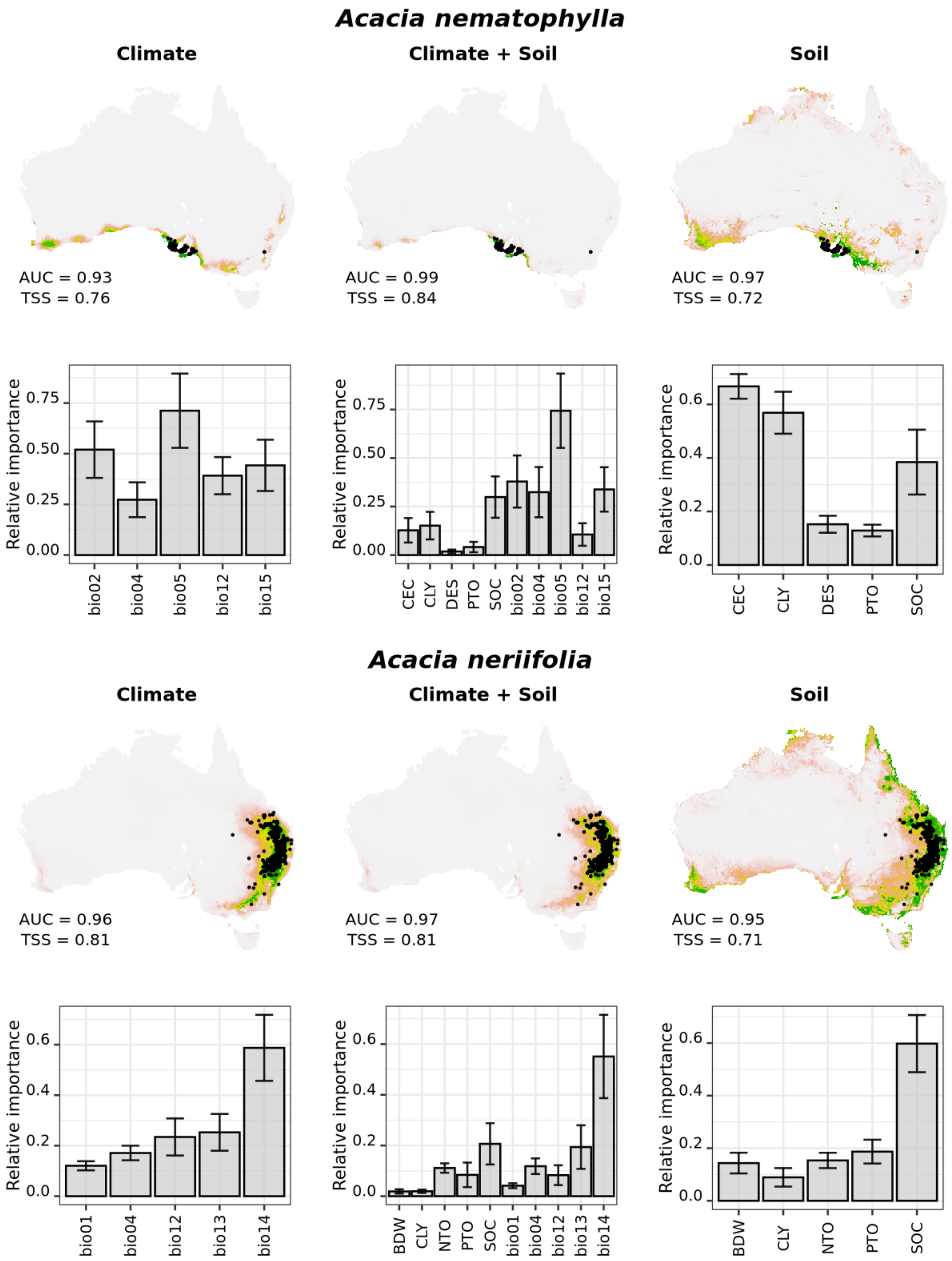
Acacia nematophylla
Climate
Climate + Soil
Soil
AUC = 0.93
TSS = 0.76
AUC = 0.99
TSS = 0.84
AUC = 0.97
TSS = 0.72
Relative importance
0.00
0.25
0.50
0.75
bio02
bio04
bio05
bio12
bio15
CEC
CLY
DES
PTO
SOC
0.0
0.2
0.4
0.6
Acacia neriifolia
AUC = 0.96
TSS = 0.81
AUC = 0.97
TSS = 0.81
AUC = 0.95
TSS = 0.71
bio01
bio13
bio14
BDW
NTO

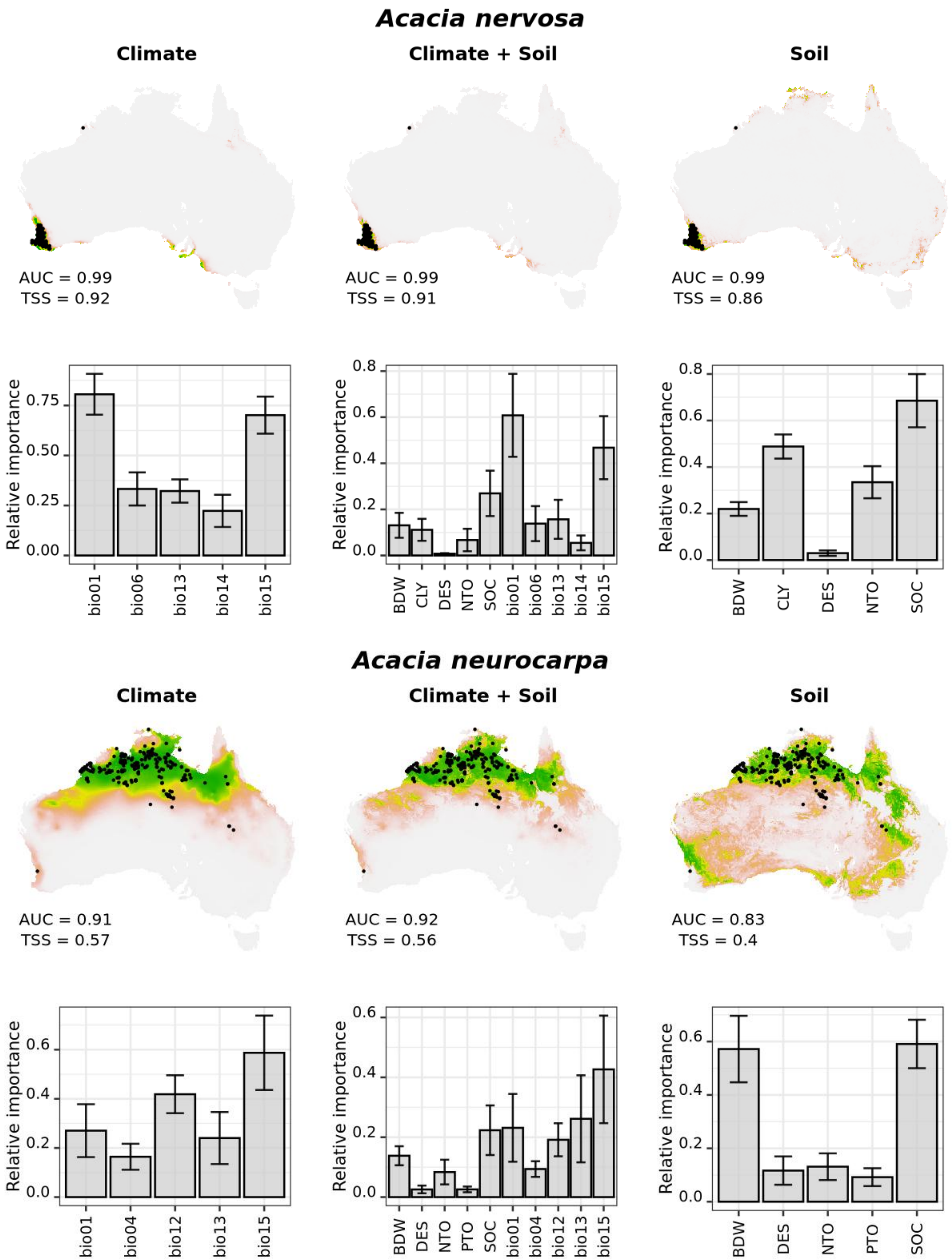
Acacia nervosa
Climate
Climate + Soil
Soil
AUC = 0.99
TSS = 0.92
AUC = 0.99
TSS = 0.91
AUC = 0.99
TSS = 0.86
Relative importance
bio01
bio06
bio13
bio14
bio15
BDW
CLY
DES
NTO
SOC
Acacia neurocarpa
Climate
Climate + Soil
Soil
AUC = 0.91
TSS = 0.57
AUC = 0.92
TSS = 0.56
AUC = 0.83
TSS = 0.4
Relative importance
bio01
bio04
bio12
bio13
bio15
BDW
DES
NTO
PTO
SOC

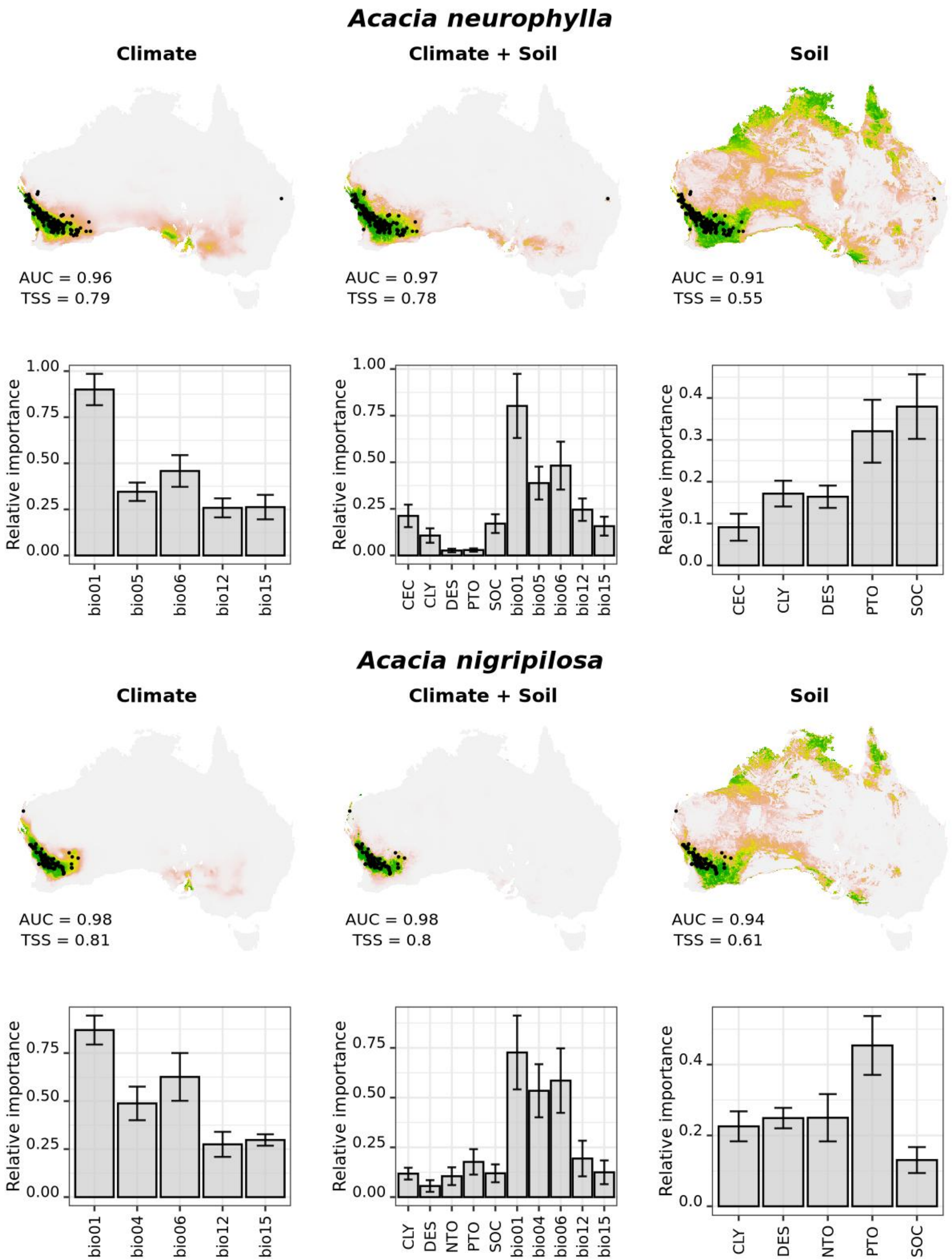

Acacia neurophylla
Climate
Climate + Soil
Soil
AUC = 0.96
TSS = 0.79
AUC = 0.97
TSS = 0.78
AUC = 0.91
TSS = 0.55
Relative importance
bio01
bio05
bio06
bio12
bio15
CEC
CLY
DES
PTO
SOC
Acacia nigripilosa
Climate
Climate + Soil
Soil
AUC = 0.98
TSS = 0.81
AUC = 0.98
TSS = 0.8
AUC = 0.94
TSS = 0.61
Relative importance
bio01
bio04
bio06
bio12
bio15
CLY
DES
NTO
PTO
SOC

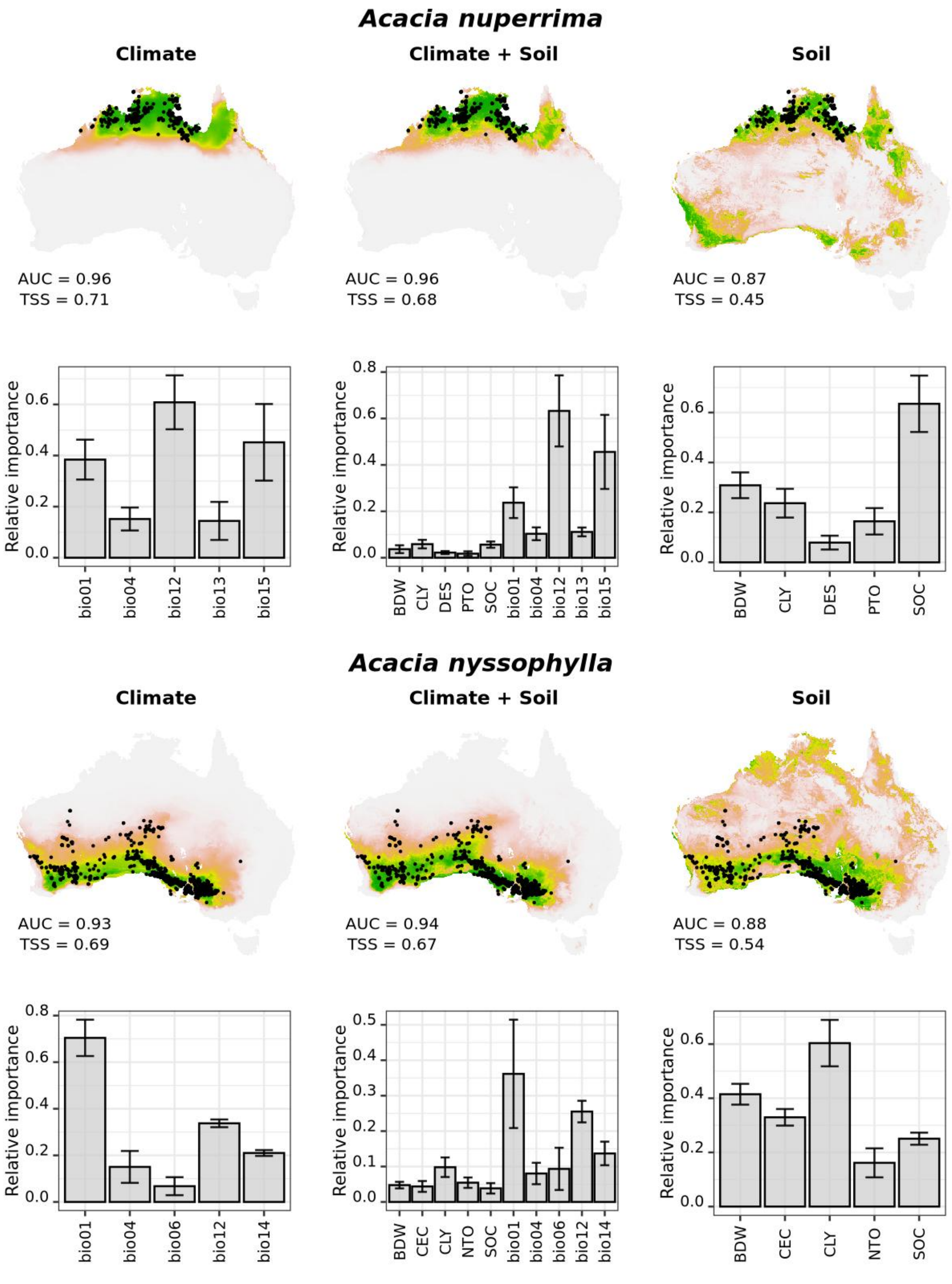
Acacia nuperrima
Climate
Climate + Soil
Soil
AUC = 0.96
TSS = 0.71
AUC = 0.96
TSS = 0.68
AUC = 0.87
TSS = 0.45
Relative importance
bio01 bio04 bio12 bio13 bio15
BDW CLY DES PTO SOC bio01 bio04 bio12 bio13 bio15
BDW CLY DES PTO SOC
Acacia nyssophylla
Climate
Climate + Soil
Soil
AUC = 0.93
TSS = 0.69
AUC = 0.94
TSS = 0.67
AUC = 0.88
TSS = 0.54
Relative importance
bio01 bio04 bio06 bio12 bio14
BDW CEC CLY NTO SOC bio01 bio04 bio06 bio12 bio14
BDW CEC CLY NTO SOC

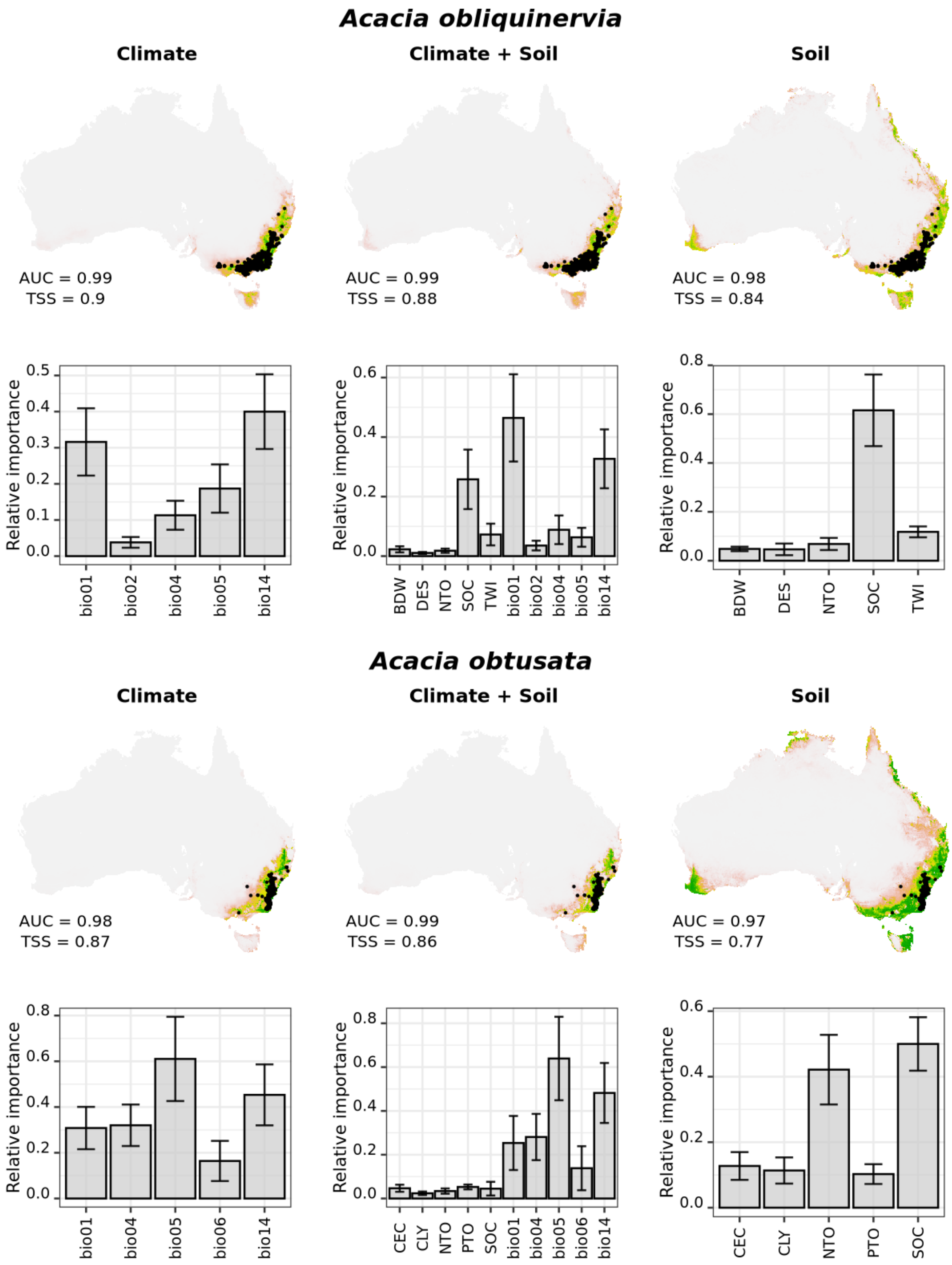
Acacia obliquinervia
Climate
Climate + Soil
Soil
AUC = 0.99
TSS = 0.9
AUC = 0.99
TSS = 0.88
AUC = 0.98
TSS = 0.84
Relative importance
bio01
bio02
bio04
bio05
bio14
BDW
DES
NTO
SOC
TWI
bio01
bio02
bio04
bio05
bio14
BDW
DES
NTO
SOC
TWI
Acacia obtusata
Climate
Climate + Soil
Soil
AUC = 0.98
TSS = 0.87
AUC = 0.99
TSS = 0.86
AUC = 0.97
TSS = 0.77
Relative importance
bio01
bio04
bio05
bio06
bio14
CEC
CLY
NTO
PTO
SOC
bio01
bio04
bio05
bio06
bio14
CEC
CLY
NTO
PTO
SOC

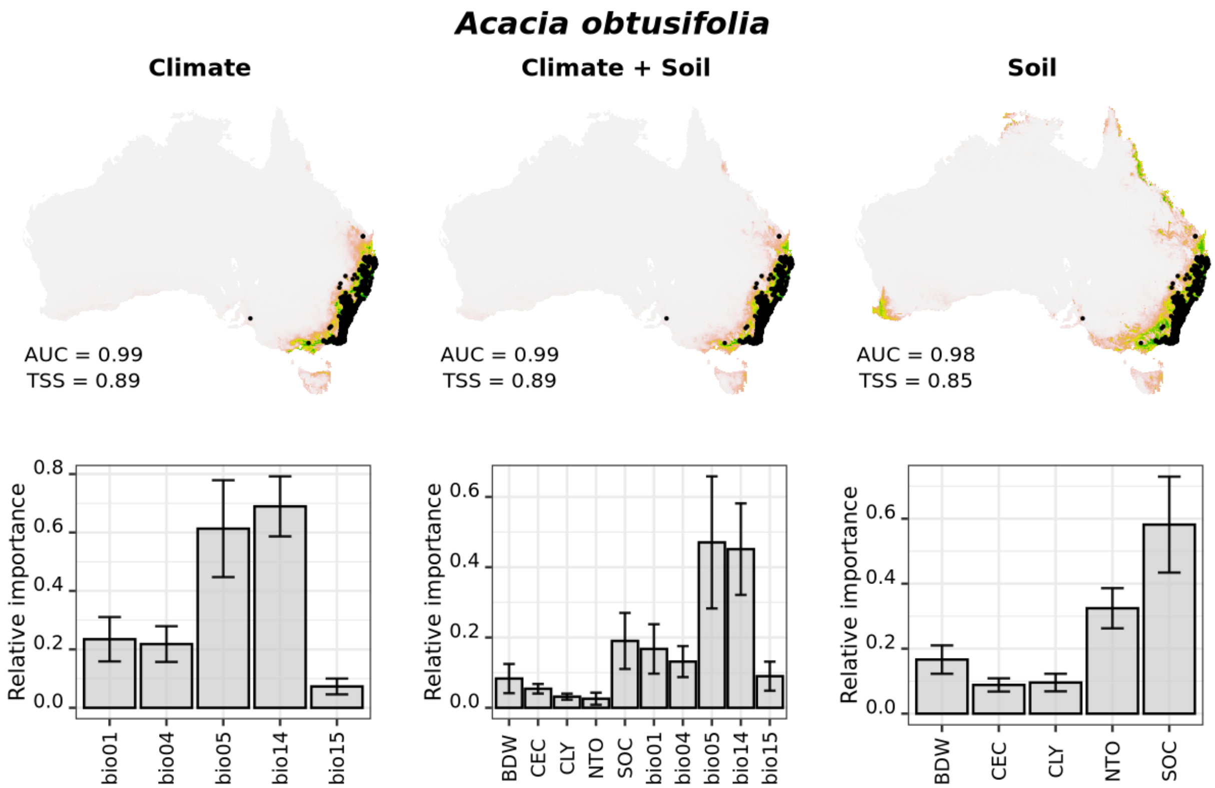

Acacia obtusifolia
Climate
Climate + Soil
Soil
AUC = 0.99
TSS = 0.89
AUC = 0.99
TSS = 0.89
AUC = 0.98
TSS = 0.85
Relative importance
0.0
0.2
0.4
0.6
0.8
bio01
bio04
bio05
bio14
bio15
BDW
CEC
CLY
NTO
SOC

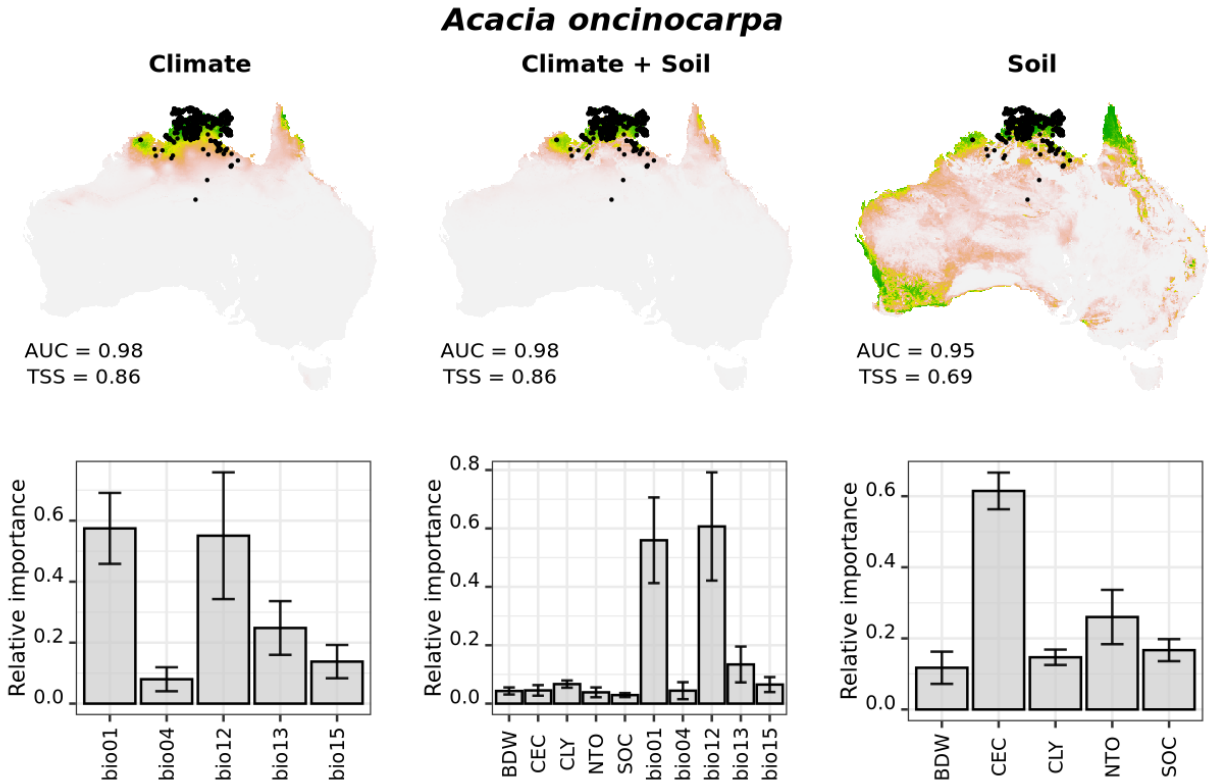

Acacia oncinocarpa
Climate
Climate + Soil
Soil
AUC = 0.98
TSS = 0.86
AUC = 0.98
TSS = 0.86
AUC = 0.95
TSS = 0.69
Relative importance
0.0
0.2
0.4
0.6
0.8
bio01
bio04
bio12
bio13
bio15
BDW
CEC
CLY
NTO
SOC

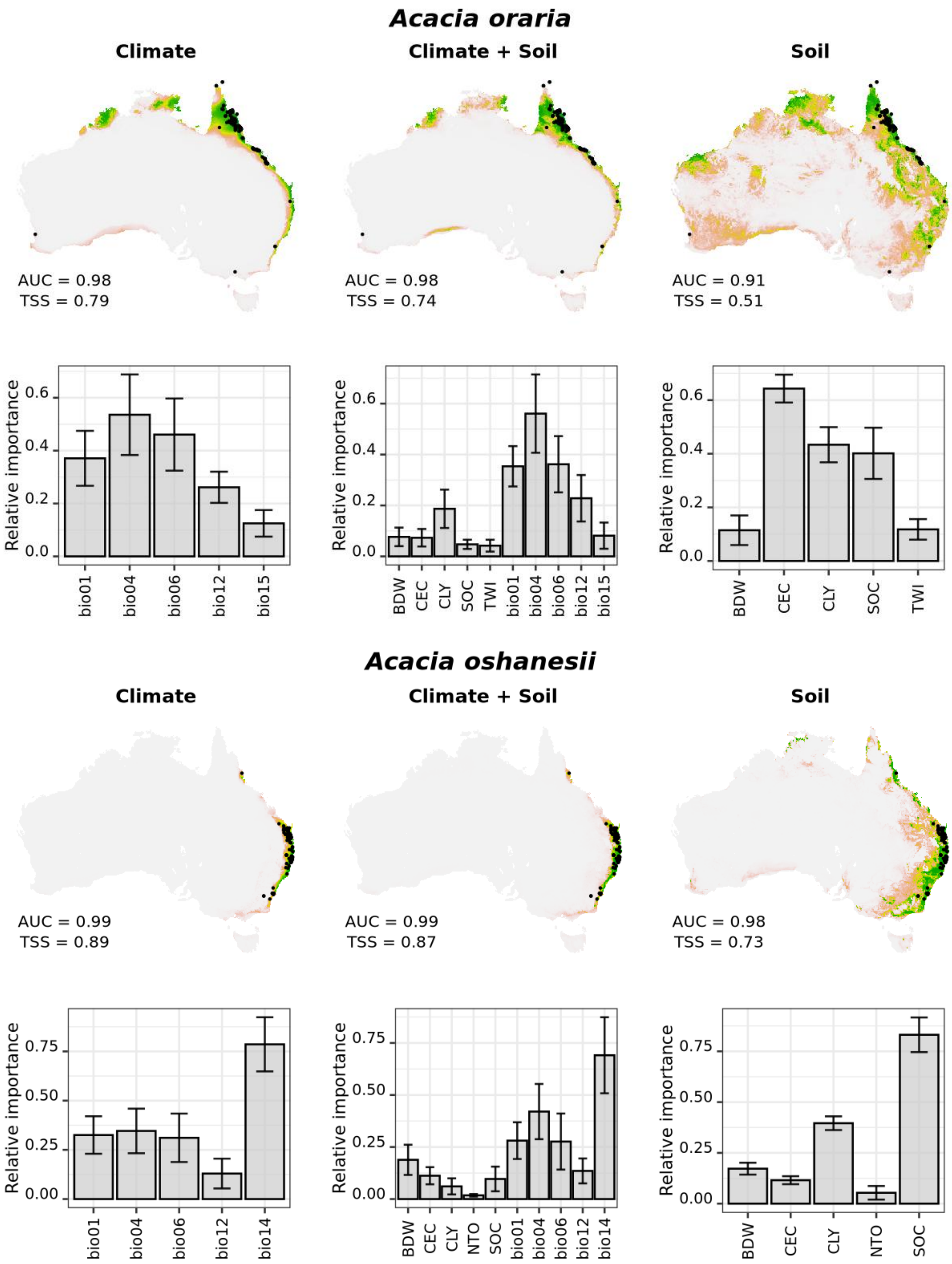

Acacia oraria
Climate
Climate + Soil
Soil
AUC = 0.98
TSS = 0.79
AUC = 0.98
TSS = 0.74
AUC = 0.91
TSS = 0.51
Relative importance
bio01
bio04
bio06
bio12
bio15
BDW
CEC
CLY
SOC
TWI
Acacia oshanesii
Climate
Climate + Soil
Soil
AUC = 0.99
TSS = 0.89
AUC = 0.99
TSS = 0.87
AUC = 0.98
TSS = 0.73
Relative importance
bio01
bio04
bio06
bio12
bio14
BDW
CEC
CLY
NTO
SOC

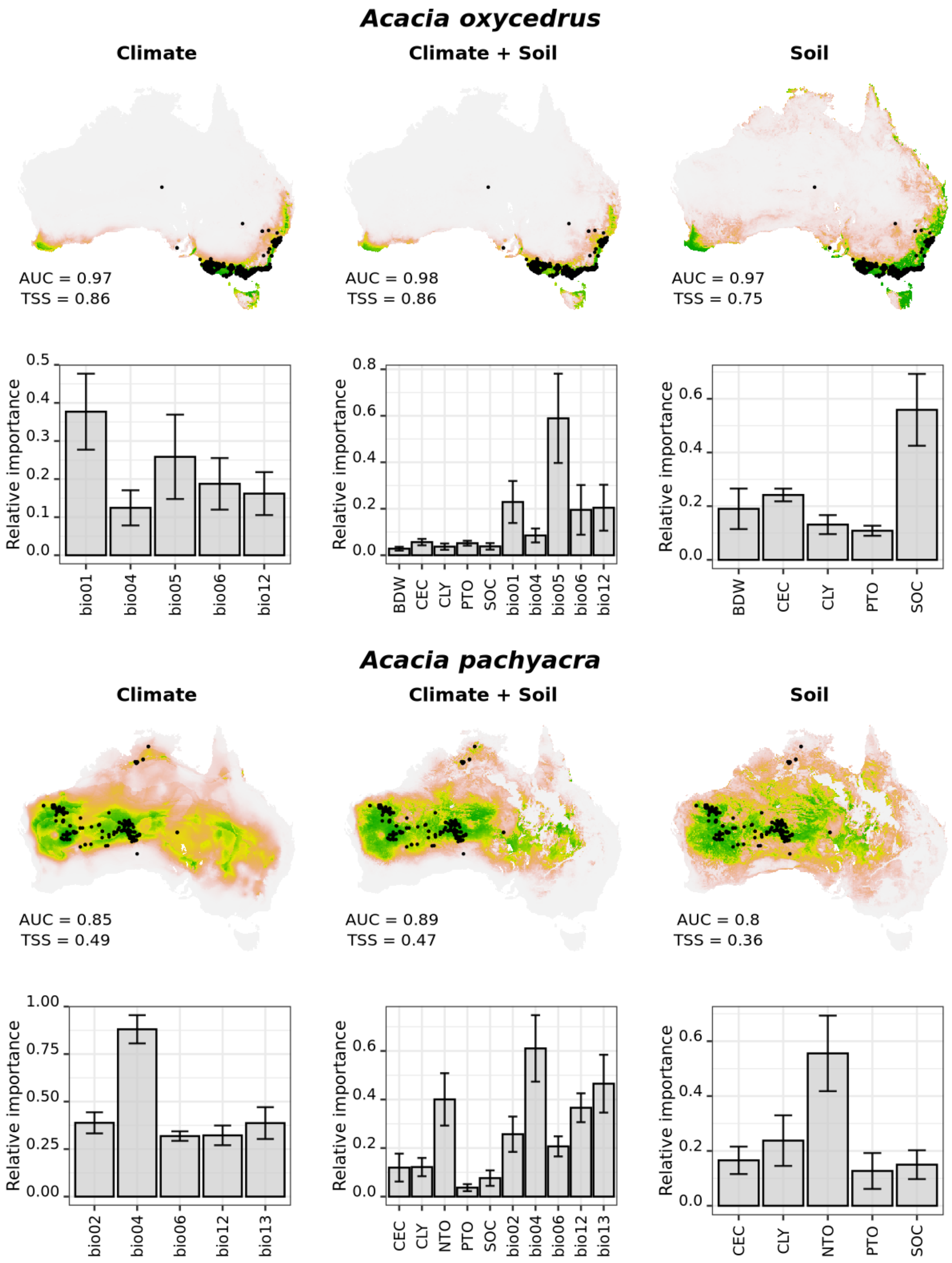
Acacia oxycedrus
Climate
Climate + Soil
Soil
AUC = 0.97
TSS = 0.86
AUC = 0.98
TSS = 0.86
AUC = 0.97
TSS = 0.75
Relative importance
bio01
bio04
bio05
bio06
bio12
BDW
CEC
CLY
PTO
SOC
Acacia pachyacra
Climate
Climate + Soil
Soil
AUC = 0.85
TSS = 0.49
AUC = 0.89
TSS = 0.47
AUC = 0.8
TSS = 0.36
Relative importance
bio02
bio04
bio06
bio12
bio13
CEC
CLY
NTO
PTO
SOC

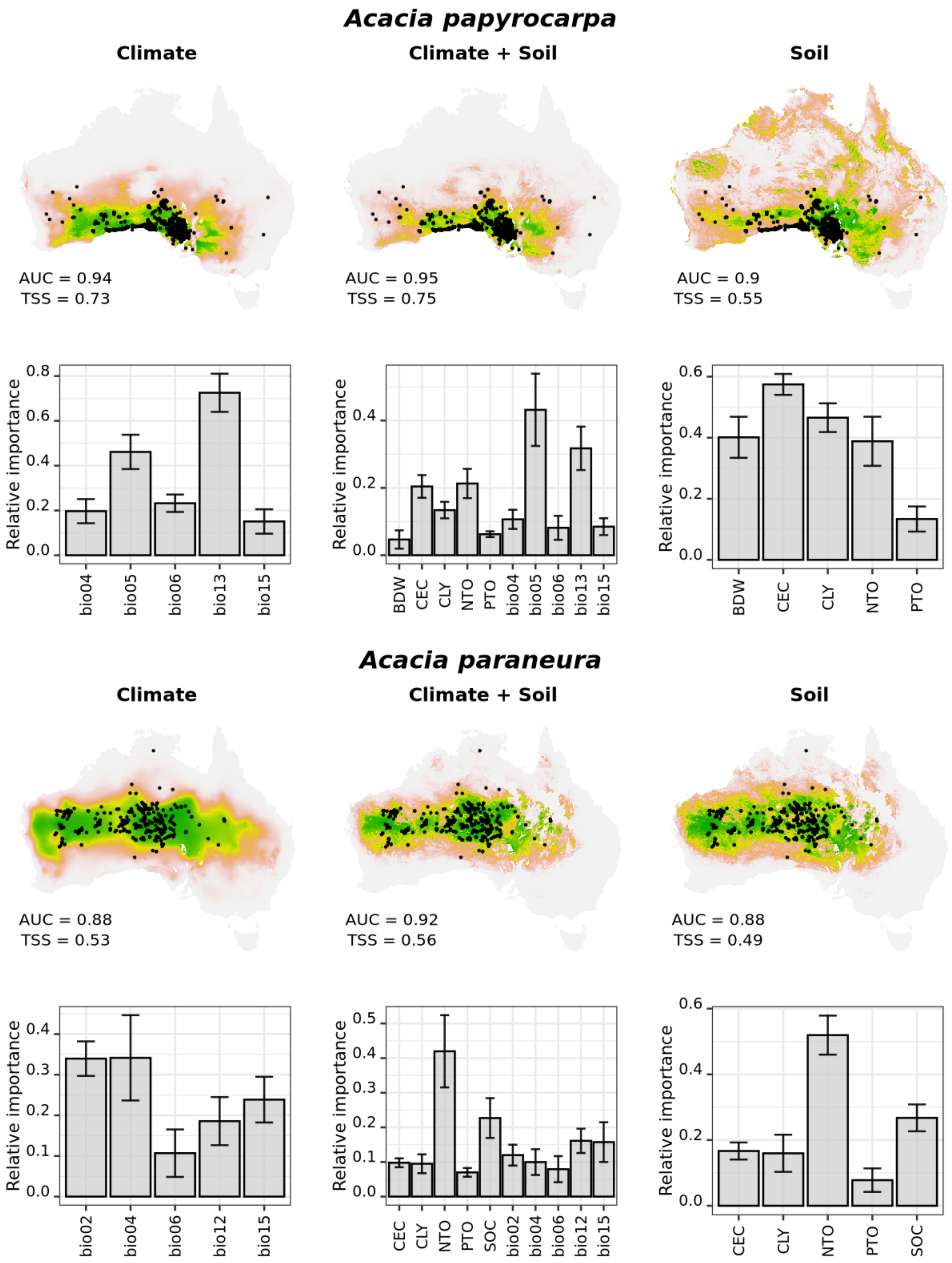
Acacia papyrocarpa
Climate
Climate + Soil
Soil
AUC = 0.94
TSS = 0.73
AUC = 0.95
TSS = 0.75
AUC = 0.9
TSS = 0.55
Relative importance
bio04
bio05
bio06
bio13
bio15
BDW
CEC
CLY
NTO
PTO
Acacia paraneura
AUC = 0.88
TSS = 0.53
AUC = 0.92
TSS = 0.56
AUC = 0.88
TSS = 0.49
bio02
bio12
SOC

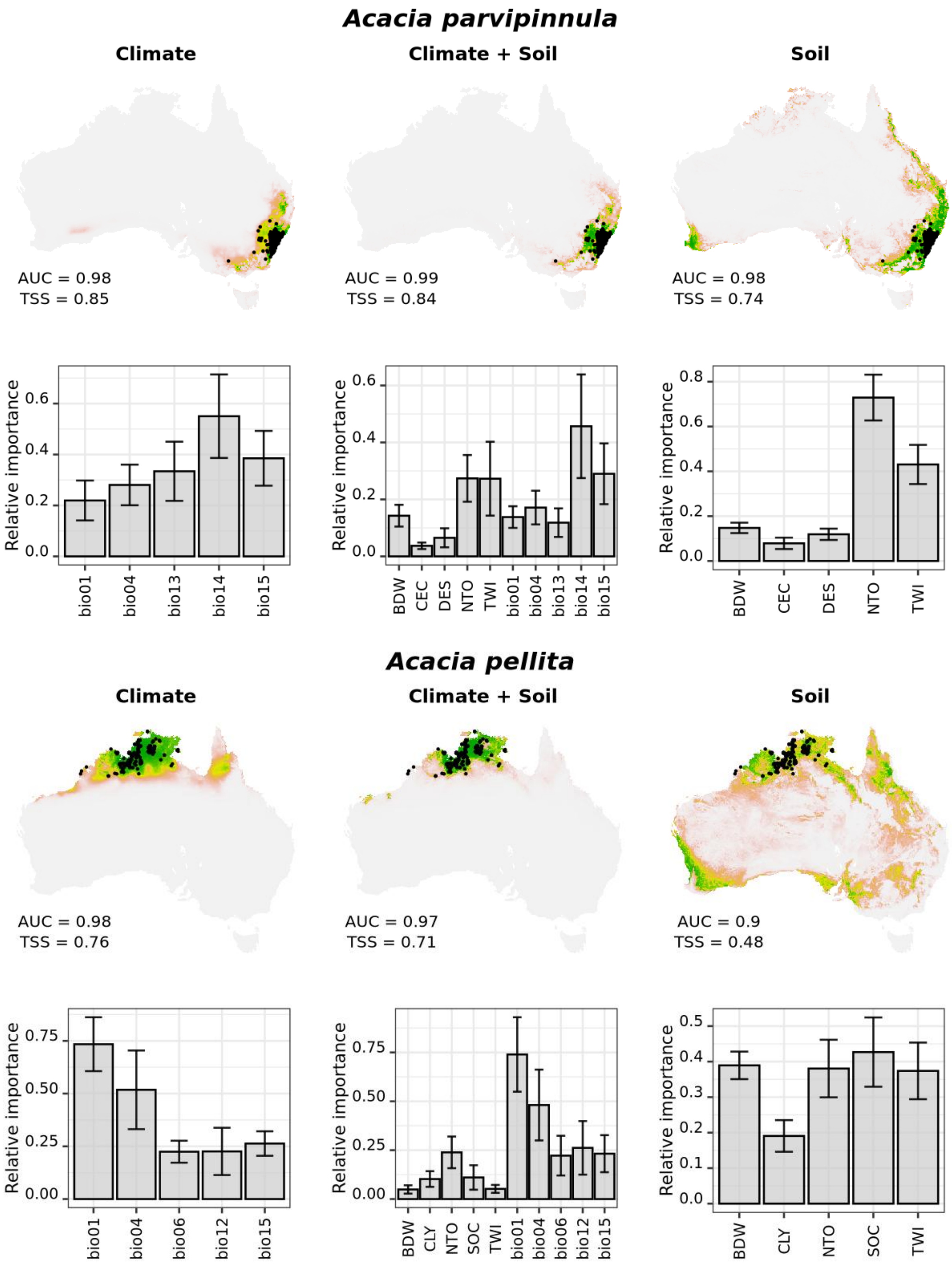
Acacia parvipinnula
Climate
Climate + Soil
Soil
AUC = 0.98
TSS = 0.85
AUC = 0.99
TSS = 0.84
AUC = 0.98
TSS = 0.74
Relative importance
0.0
0.2
0.4
0.6
0.8
bio01
bio04
bio13
bio14
bio15
BDW
CEC
DES
NTO
TWI
Acacia pellita
Climate
Climate + Soil
Soil
AUC = 0.98
TSS = 0.76
AUC = 0.97
TSS = 0.71
AUC = 0.9
TSS = 0.48
Relative importance
0.00
0.25
0.50
0.75
0.1
0.3
0.5
bio01
bio04
bio06
bio12
bio15
BDW
CLY
NTO
SOC
TWI

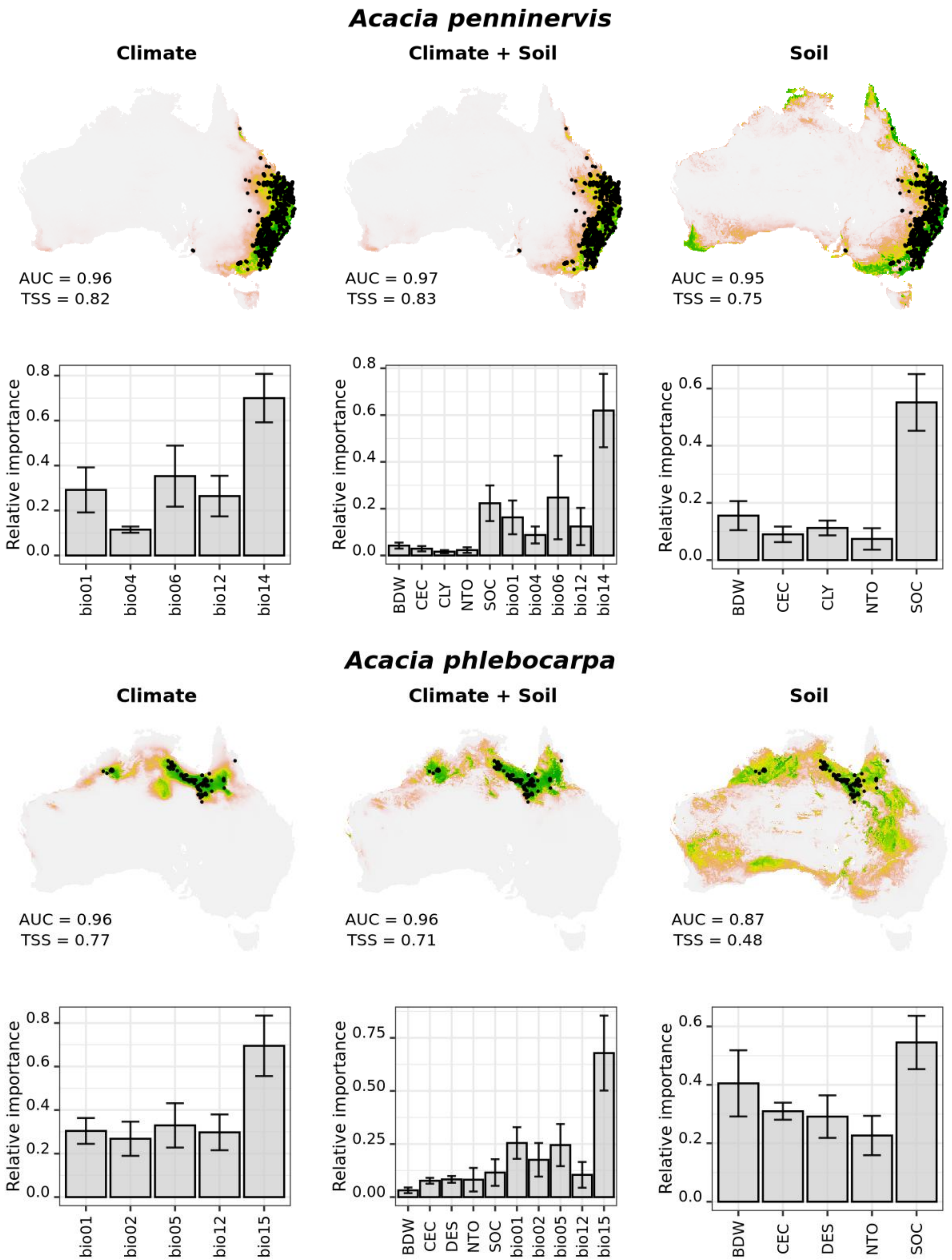
Acacia penninervis
Climate
Climate + Soil
Soil
AUC = 0.96
TSS = 0.82
AUC = 0.97
TSS = 0.83
AUC = 0.95
TSS = 0.75
Relative importance
0.0
0.2
0.4
0.6
0.8
bio01
bio04
bio06
bio12
bio14
BDW
CEC
CLY
NTO
SOC
Acacia phlebocarpa
Climate
Climate + Soil
Soil
AUC = 0.96
TSS = 0.77
AUC = 0.96
TSS = 0.71
AUC = 0.87
TSS = 0.48
0.00
0.25
0.50
0.75
bio01
bio02
bio05
bio12
bio15
BDW
CEC
DES
NTO
SOC

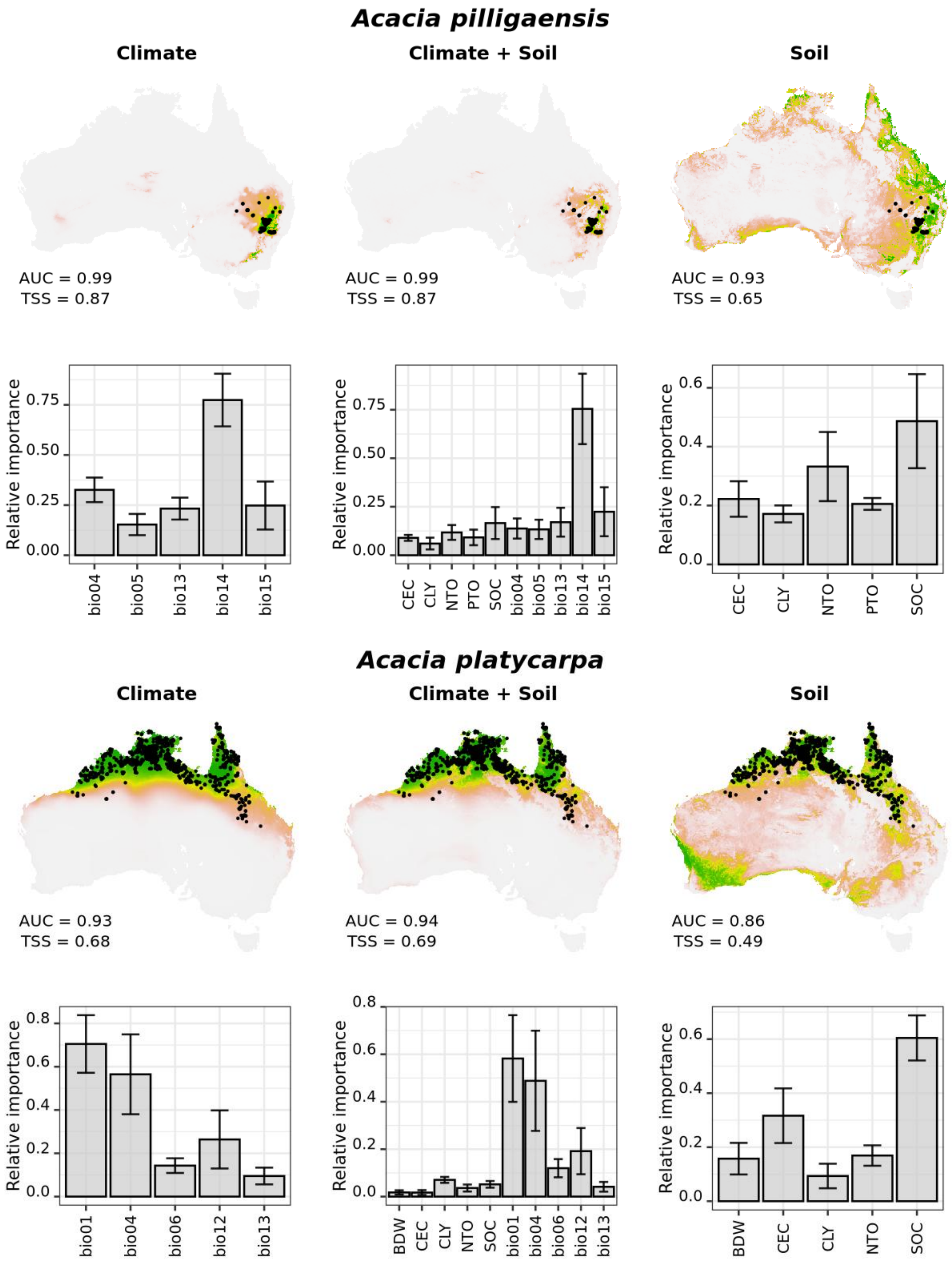
Acacia pilligaensis
Climate
Climate + Soil
Soil
AUC = 0.99
TSS = 0.87
AUC = 0.99
TSS = 0.87
AUC = 0.93
TSS = 0.65
Relative importance
bio04 bio05 bio13 bio14 bio15
CEC CLY NTO PTO SOC bio04 bio05 bio13 bio14 bio15
CEC CLY NTO PTO SOC
Acacia platycarpa
Climate
Climate + Soil
Soil
AUC = 0.93
TSS = 0.68
AUC = 0.94
TSS = 0.69
AUC = 0.86
TSS = 0.49
Relative importance
bio01 bio04 bio06 bio12 bio13
BDW CEC CLY NTO SOC bio01 bio04 bio06 bio12 bio13
BDW CEC CLY NTO SOC

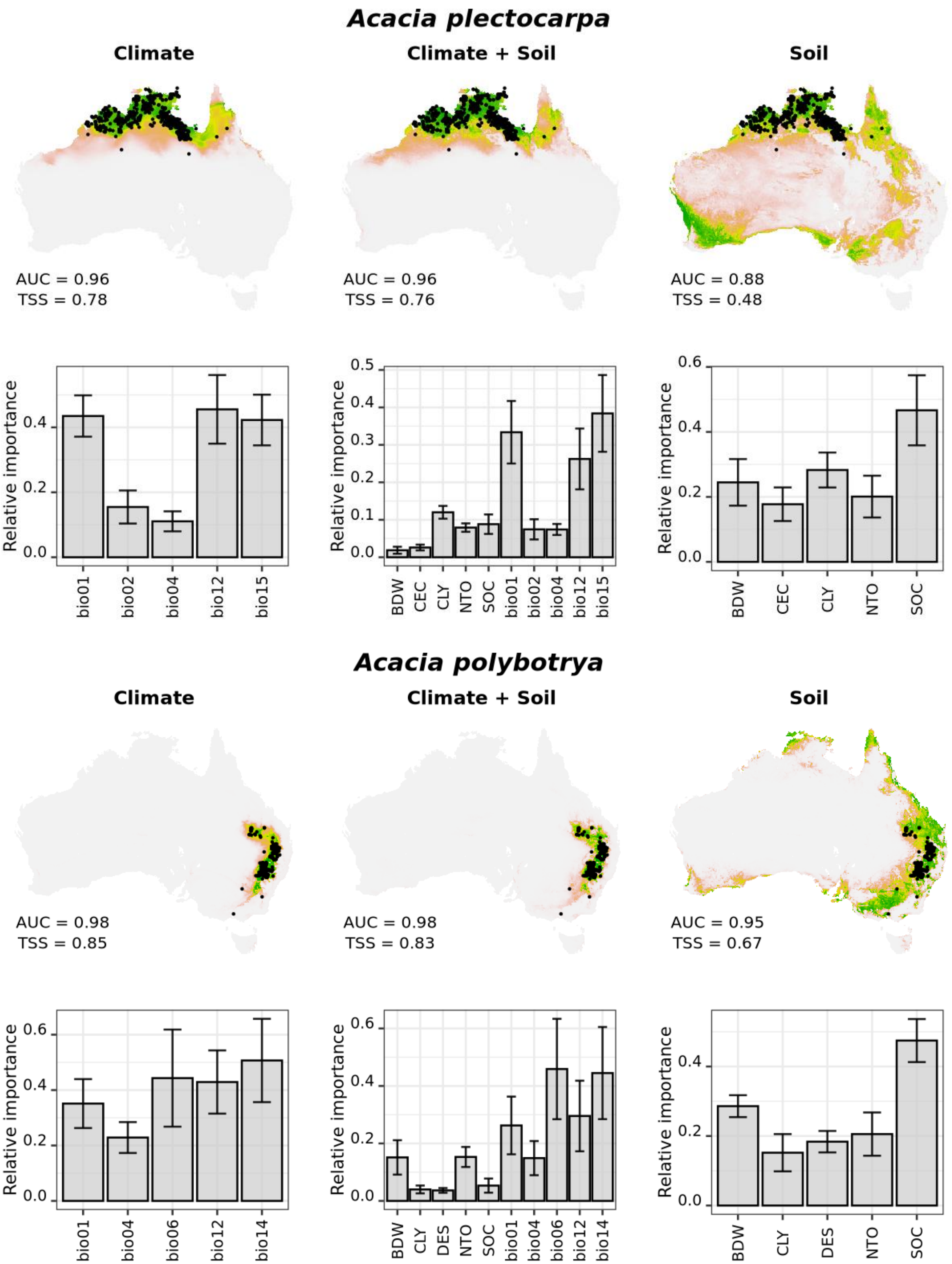

Acacia plectocarpa
Climate
Climate + Soil
Soil
AUC = 0.96
TSS = 0.78
AUC = 0.96
TSS = 0.76
AUC = 0.88
TSS = 0.48
Relative importance
bio01
bio02
bio04
bio12
bio15
BDW
CEC
CLY
NTO
SOC
Acacia polybotrya
AUC = 0.98
TSS = 0.85
AUC = 0.98
TSS = 0.83
AUC = 0.95
TSS = 0.67
bio06
bio14
DES

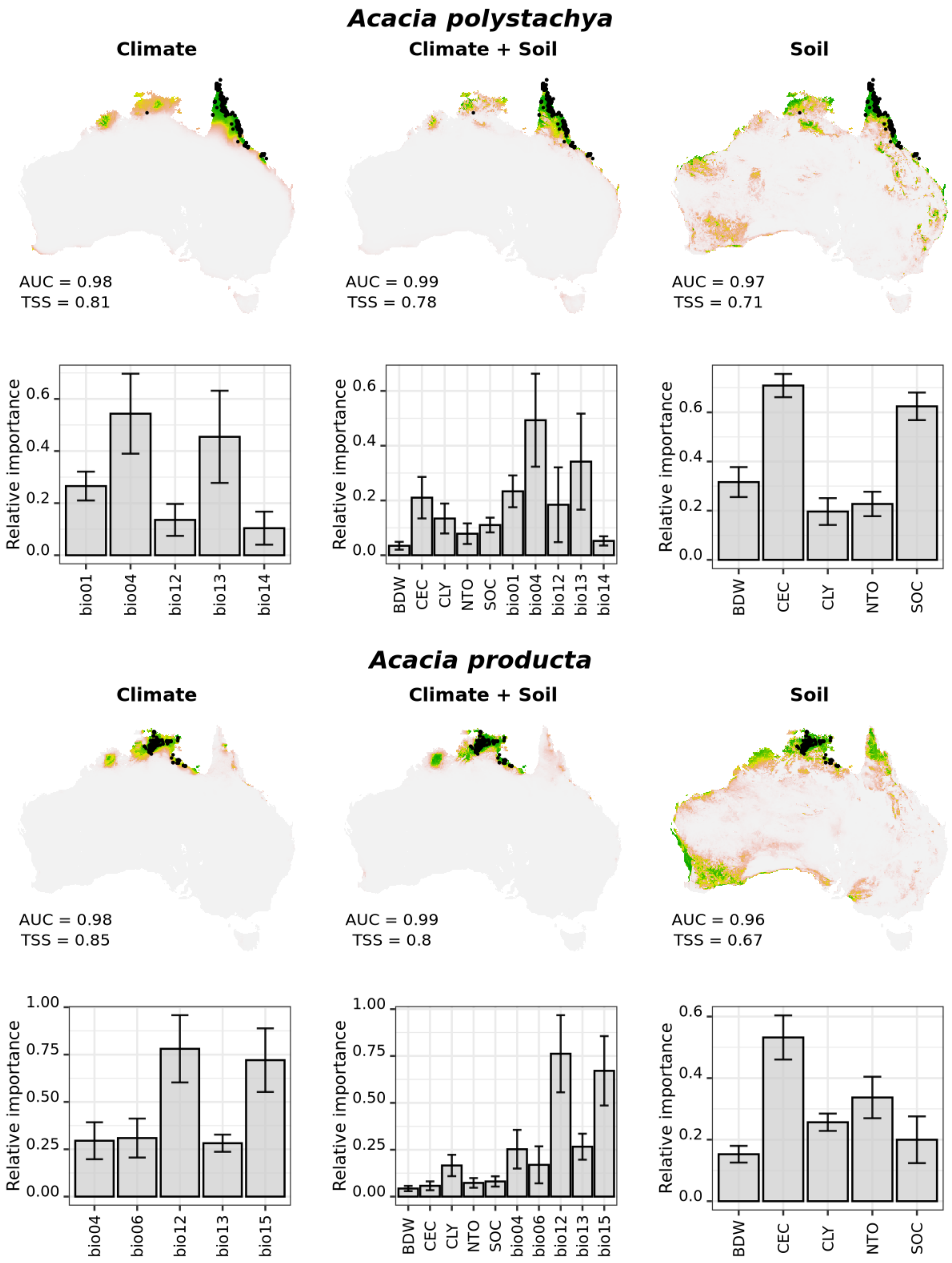

Acacia polystachya
Climate
Climate + Soil
Soil
AUC = 0.98
TSS = 0.81
AUC = 0.99
TSS = 0.78
AUC = 0.97
TSS = 0.71
Relative importance
bio01
bio04
bio12
bio13
bio14
BDW
CEC
CLY
NTO
SOC
Acacia producta
Climate
Climate + Soil
Soil
AUC = 0.98
TSS = 0.85
AUC = 0.99
TSS = 0.8
AUC = 0.96
TSS = 0.67
Relative importance
bio04
bio06
bio12
bio13
bio15
BDW
CEC
CLY
NTO
SOC

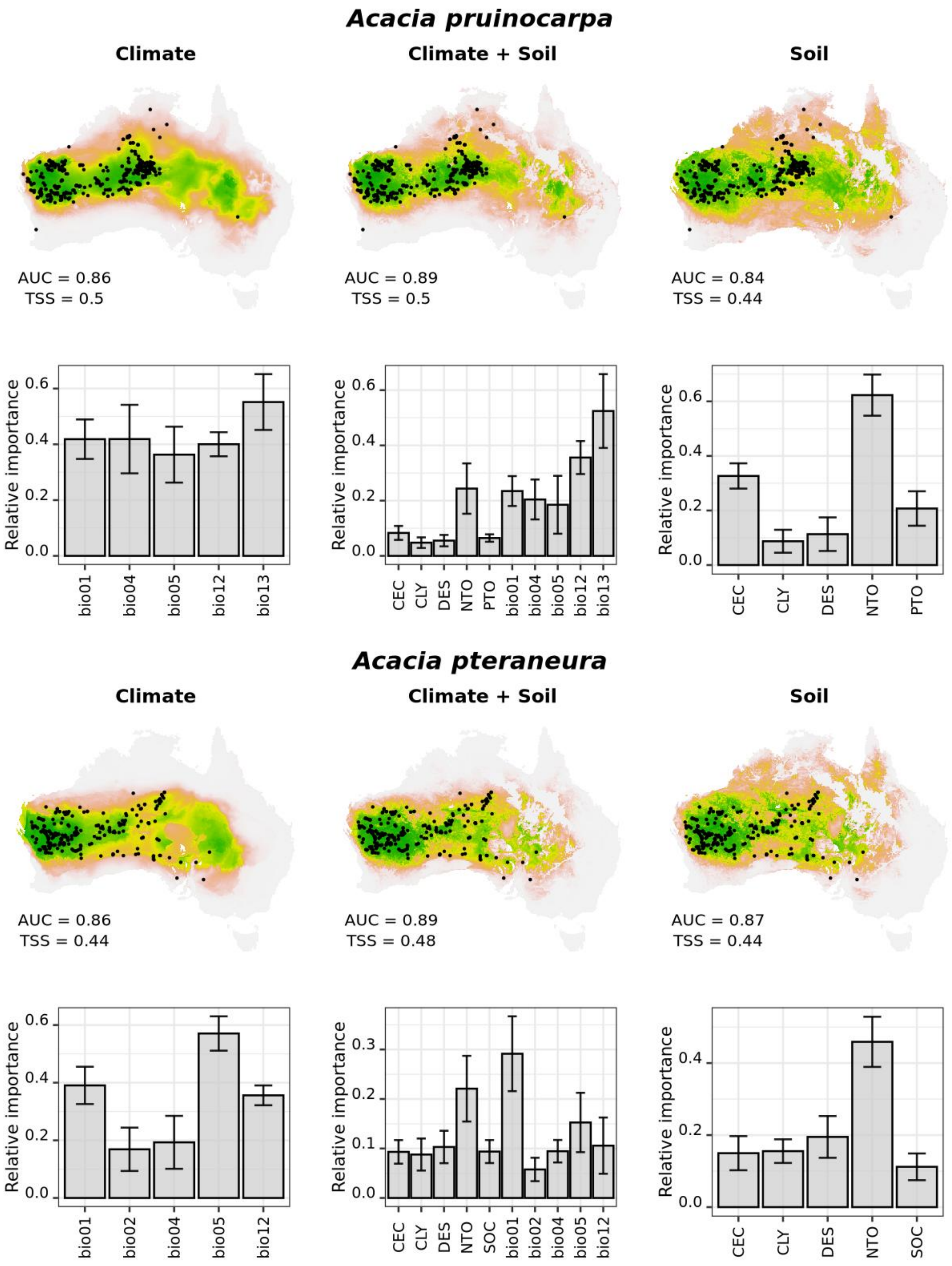
Acacia pruinocarpa
Climate
Climate + Soil
Soil
AUC = 0.86
TSS = 0.5
AUC = 0.89
TSS = 0.5
AUC = 0.84
TSS = 0.44
Relative importance
bio01
bio04
bio05
bio12
bio13
CEC
CLY
DES
NTO
PTO
Acacia pteraneura
Climate
Climate + Soil
Soil
AUC = 0.86
TSS = 0.44
AUC = 0.89
TSS = 0.48
AUC = 0.87
TSS = 0.44
Relative importance
bio01
bio02
bio04
bio05
bio12
CEC
CLY
DES
NTO
SOC

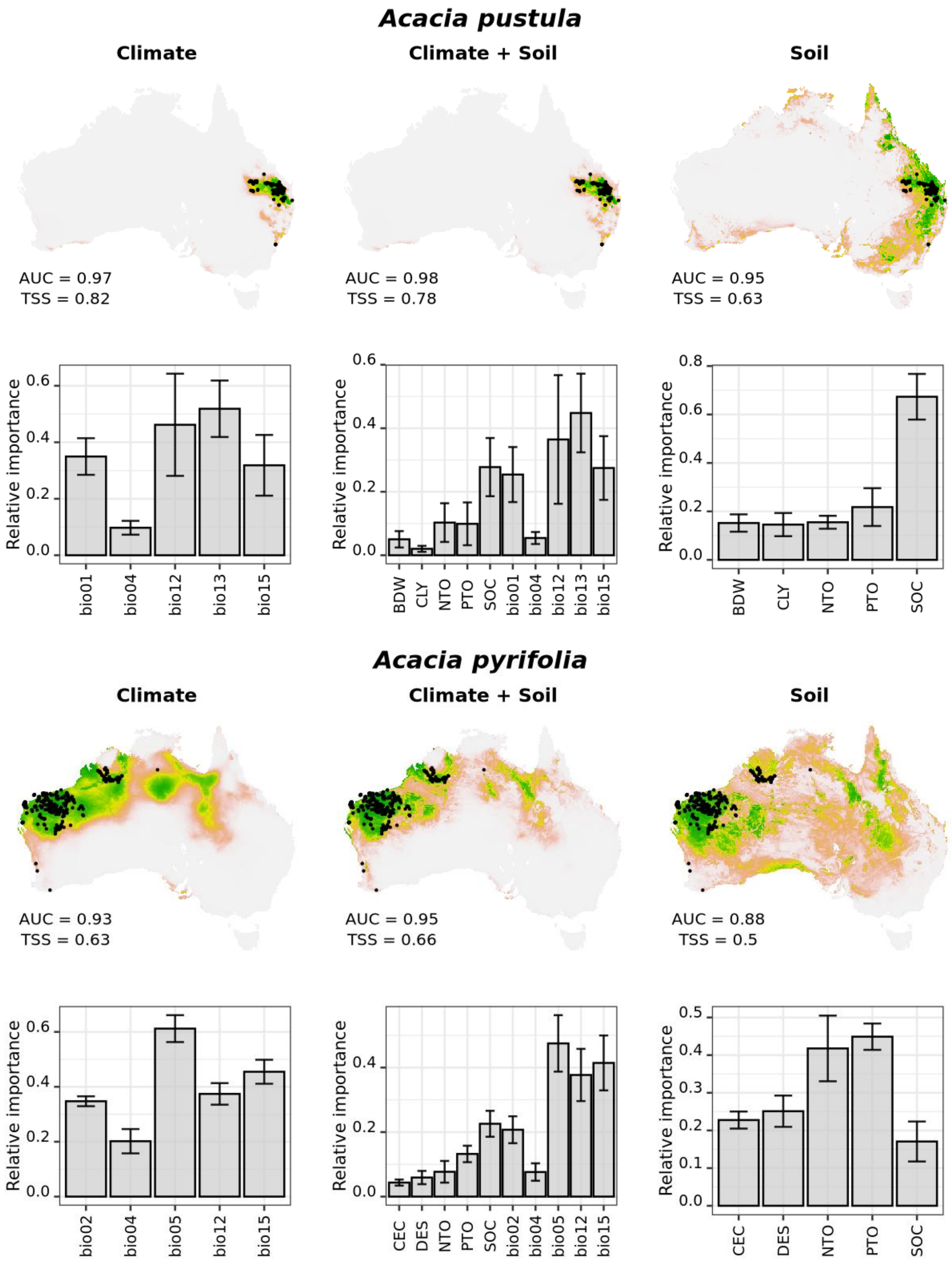
Acacia pustula
Climate
Climate + Soil
Soil
AUC = 0.97
TSS = 0.82
AUC = 0.98
TSS = 0.78
AUC = 0.95
TSS = 0.63
Relative importance
bio01
bio04
bio12
bio13
bio15
BDW
CLY
NTO
PTO
SOC
Acacia pyrifolia
AUC = 0.93
TSS = 0.63
AUC = 0.95
TSS = 0.66
AUC = 0.88
TSS = 0.5
bio02
bio05
CEC
DES

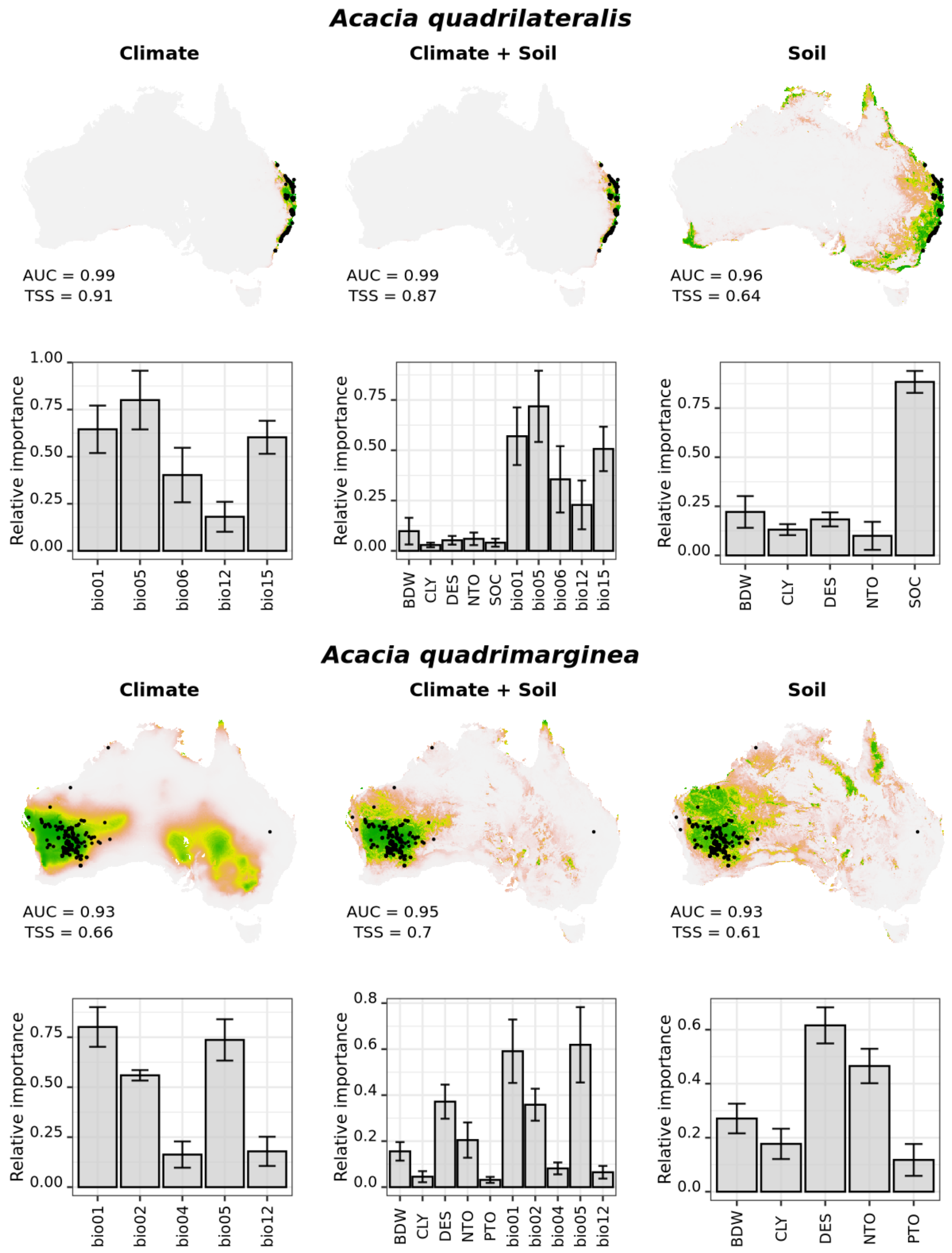

Acacia quadrilateralis
Climate
Climate + Soil
Soil
AUC = 0.99
TSS = 0.91
AUC = 0.99
TSS = 0.87
AUC = 0.96
TSS = 0.64
Relative importance
1.00
0.75
0.50
0.25
0.00
bio01
bio05
bio06
bio12
bio15
BDW
CLY
DES
NTO
SOC
Acacia quadrimarginea
Climate
Climate + Soil
Soil
AUC = 0.93
TSS = 0.66
AUC = 0.95
TSS = 0.7
AUC = 0.93
TSS = 0.61
0.8
0.6
0.4
0.2
0.0
bio02
bio04
PTO

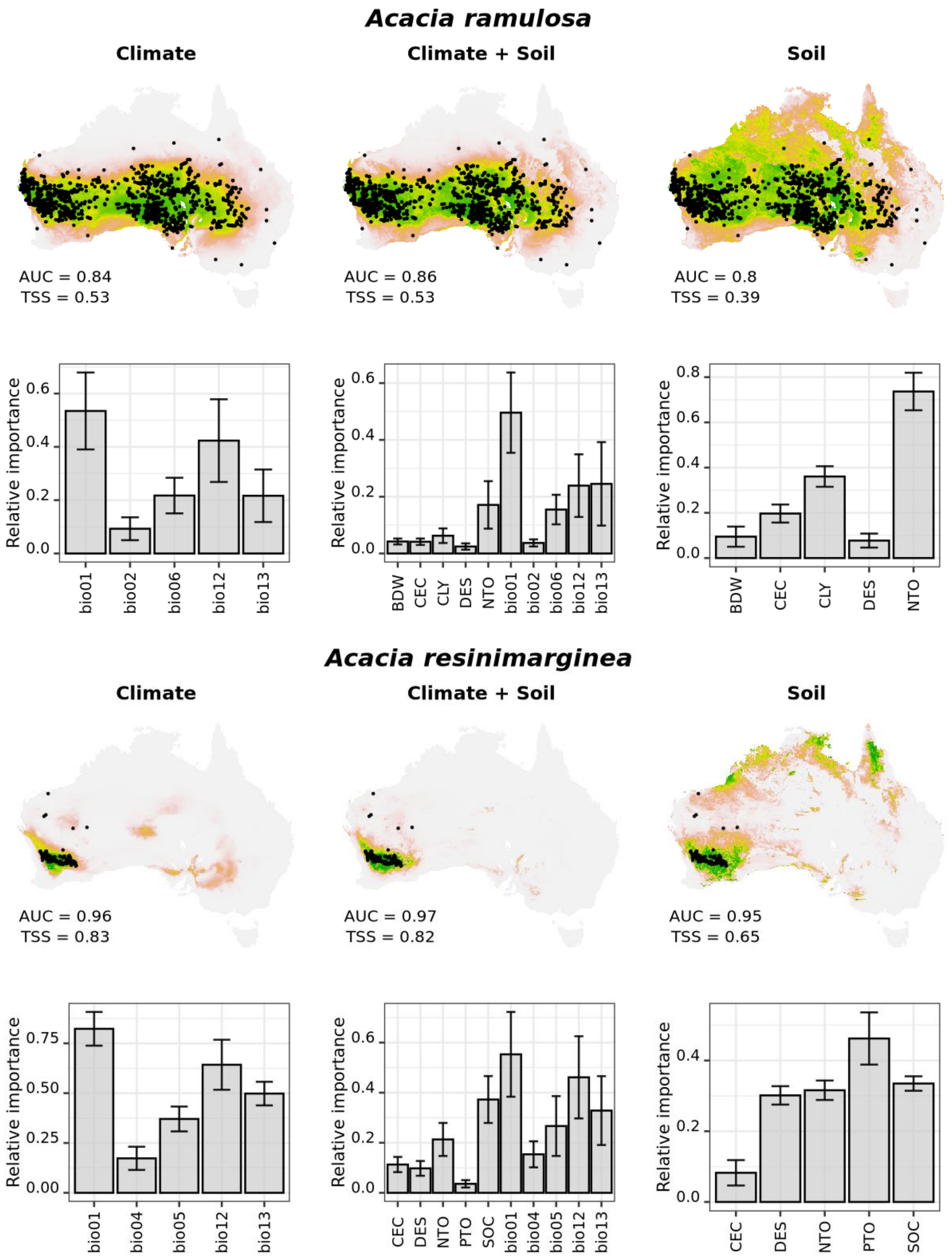

Acacia ramulosa
Climate
Climate + Soil
Soil
AUC = 0.84
TSS = 0.53
AUC = 0.86
TSS = 0.53
AUC = 0.8
TSS = 0.39
Relative importance
bio01
bio02
bio06
bio12
bio13
BDW
CEC
CLY
DES
NTO
Acacia resinimarginea
Climate
Climate + Soil
Soil
AUC = 0.96
TSS = 0.83
AUC = 0.97
TSS = 0.82
AUC = 0.95
TSS = 0.65
Relative importance
bio01
bio04
bio05
bio12
bio13
CEC
DES
NTO
PTO
SOC

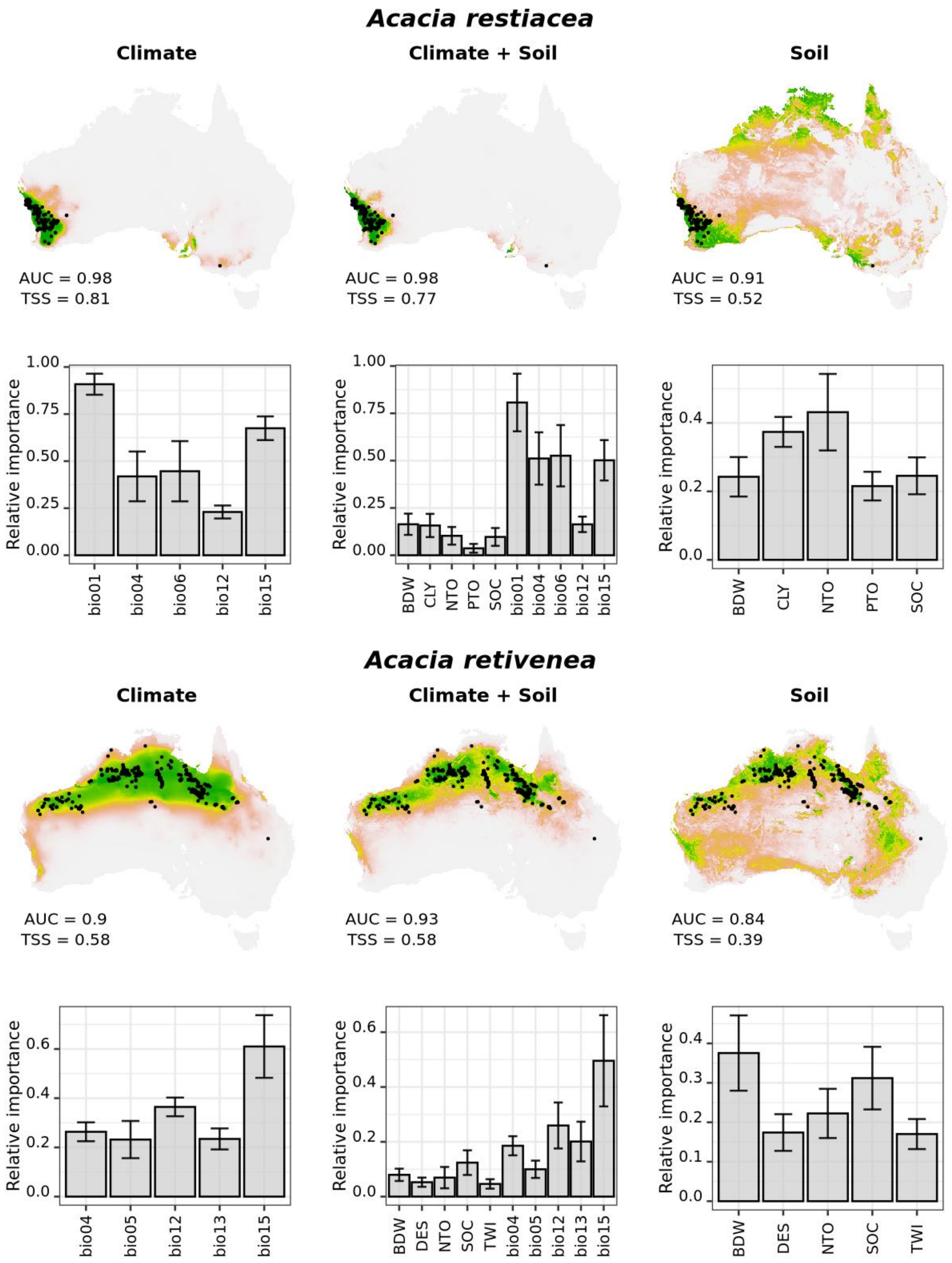

Acacia restiacea
Climate
Climate + Soil
Soil
AUC = 0.98
TSS = 0.81
AUC = 0.98
TSS = 0.77
AUC = 0.91
TSS = 0.52
Relative importance
bio01
bio04
bio06
bio12
bio15
BDW
CLY
NTO
PTO
SOC
Acacia retivenea
Climate
Climate + Soil
Soil
AUC = 0.9
TSS = 0.58
AUC = 0.93
TSS = 0.58
AUC = 0.84
TSS = 0.39
Relative importance
bio04
bio05
bio12
bio13
bio15
BDW
DES
NTO
SOC
TWI

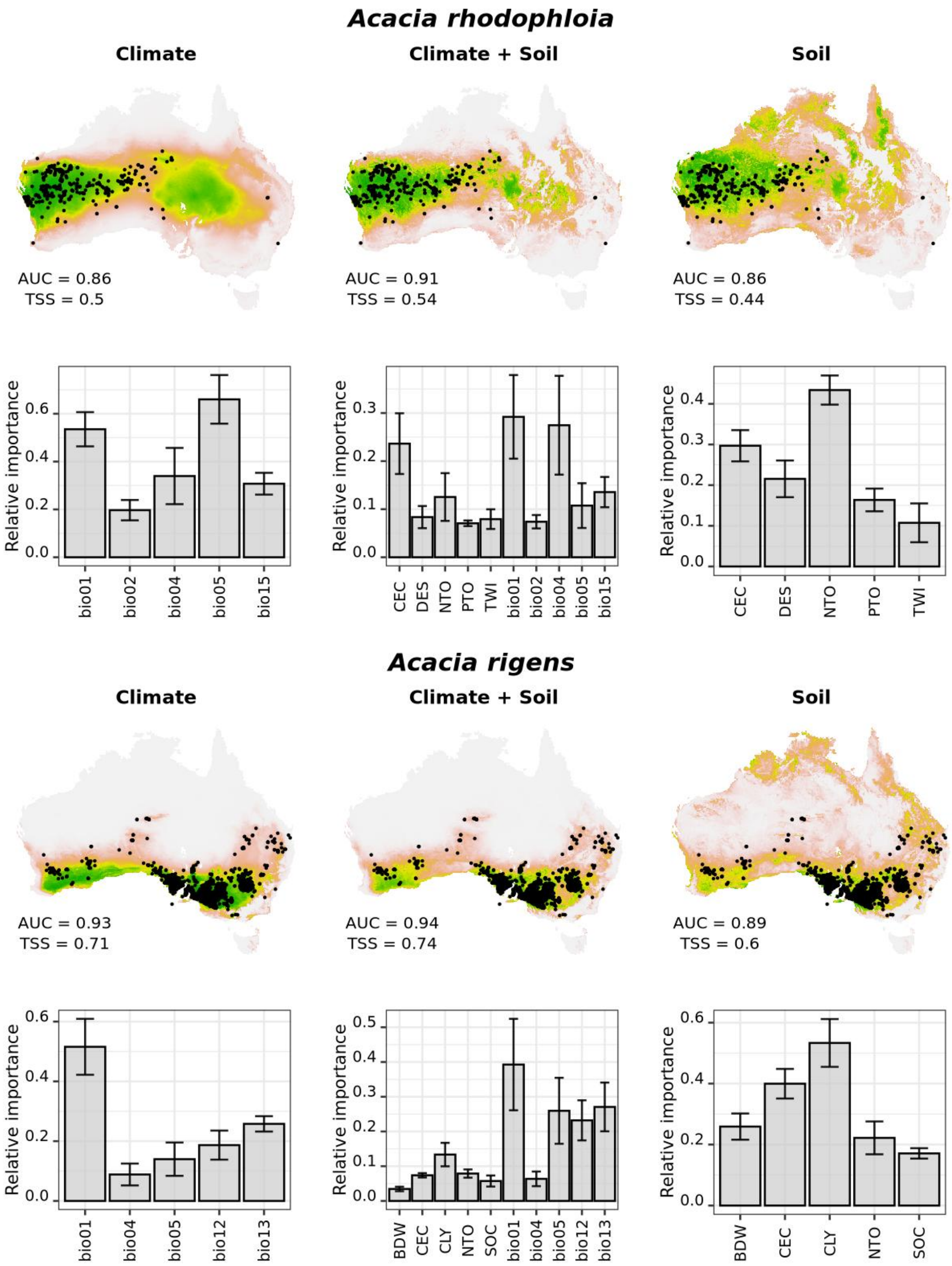
Acacia rhodophloia
Climate
Climate + Soil
Soil
AUC = 0.86
TSS = 0.5
AUC = 0.91
TSS = 0.54
AUC = 0.86
TSS = 0.44
Relative importance
bio01 bio02 bio04 bio05 bio15
CEC DES NTO PTO TWI bio01 bio02 bio04 bio05 bio15
CEC DES NTO PTO TWI
Acacia rigens
Climate
Climate + Soil
Soil
AUC = 0.93
TSS = 0.71
AUC = 0.94
TSS = 0.74
AUC = 0.89
TSS = 0.6
Relative importance
bio01 bio04 bio05 bio12 bio13
BDW CEC CLY NTO SOC bio01 bio04 bio05 bio12 bio13
BDW CEC CLY NTO SOC

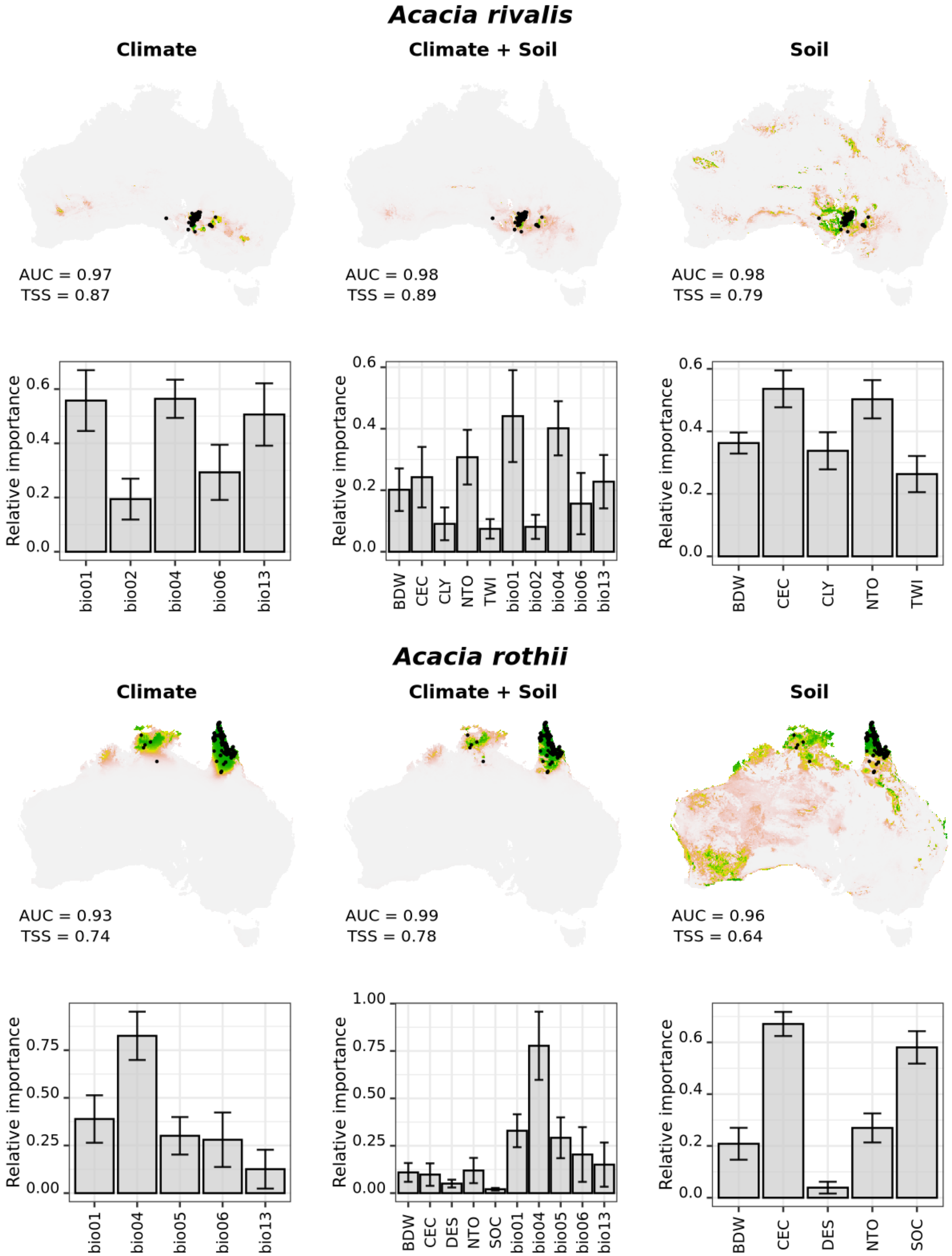
Acacia rivalis
Climate
Climate + Soil
Soil
AUC = 0.97
TSS = 0.87
AUC = 0.98
TSS = 0.89
AUC = 0.98
TSS = 0.79
Relative importance
bio01 bio02 bio04 bio06 bio13
BDW CEC CLY NTO TWI bio01 bio02 bio04 bio06 bio13
BDW CEC CLY NTO TWI
Acacia rothii
Climate
Climate + Soil
Soil
AUC = 0.93
TSS = 0.74
AUC = 0.99
TSS = 0.78
AUC = 0.96
TSS = 0.64
Relative importance
bio01 bio04 bio05 bio06 bio13
BDW CEC DES NTO SOC bio01 bio04 bio05 bio06 bio13
BDW CEC DES NTO SOC

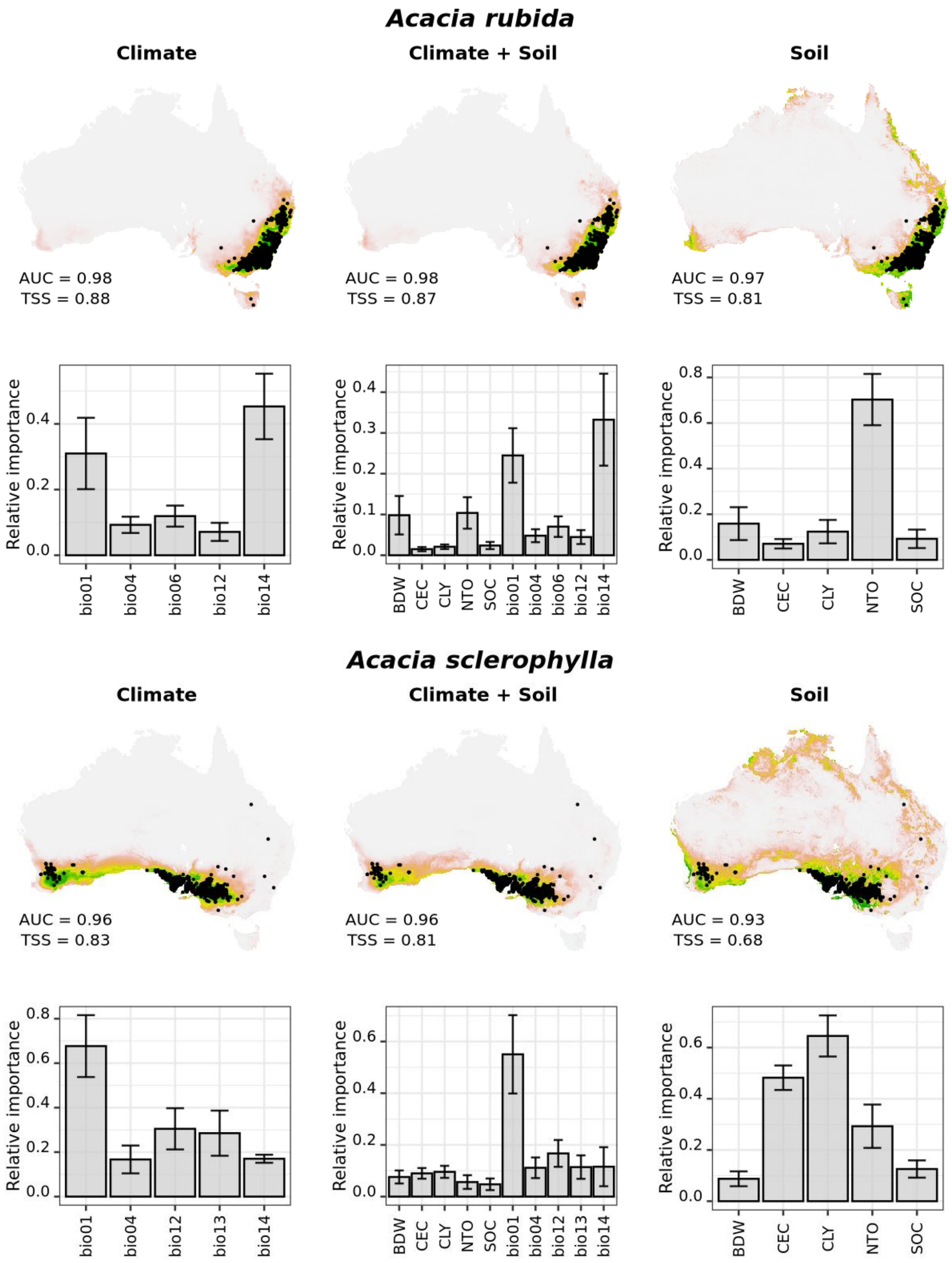

Acacia rubida
Climate
Climate + Soil
Soil
AUC = 0.98
TSS = 0.88
AUC = 0.98
TSS = 0.87
AUC = 0.97
TSS = 0.81
Relative importance
bio01 bio04 bio06 bio12 bio14
BDW CEC CLY NTO SOC bio01 bio04 bio06 bio12 bio14
BDW CEC CLY NTO SOC
Acacia sclerophylla
Climate
Climate + Soil
Soil
AUC = 0.96
TSS = 0.83
AUC = 0.96
TSS = 0.81
AUC = 0.93
TSS = 0.68
Relative importance
bio01 bio04 bio12 bio13 bio14
BDW CEC CLY NTO SOC bio01 bio04 bio12 bio13 bio14
BDW CEC CLY NTO SOC

Acacia sclerosperma

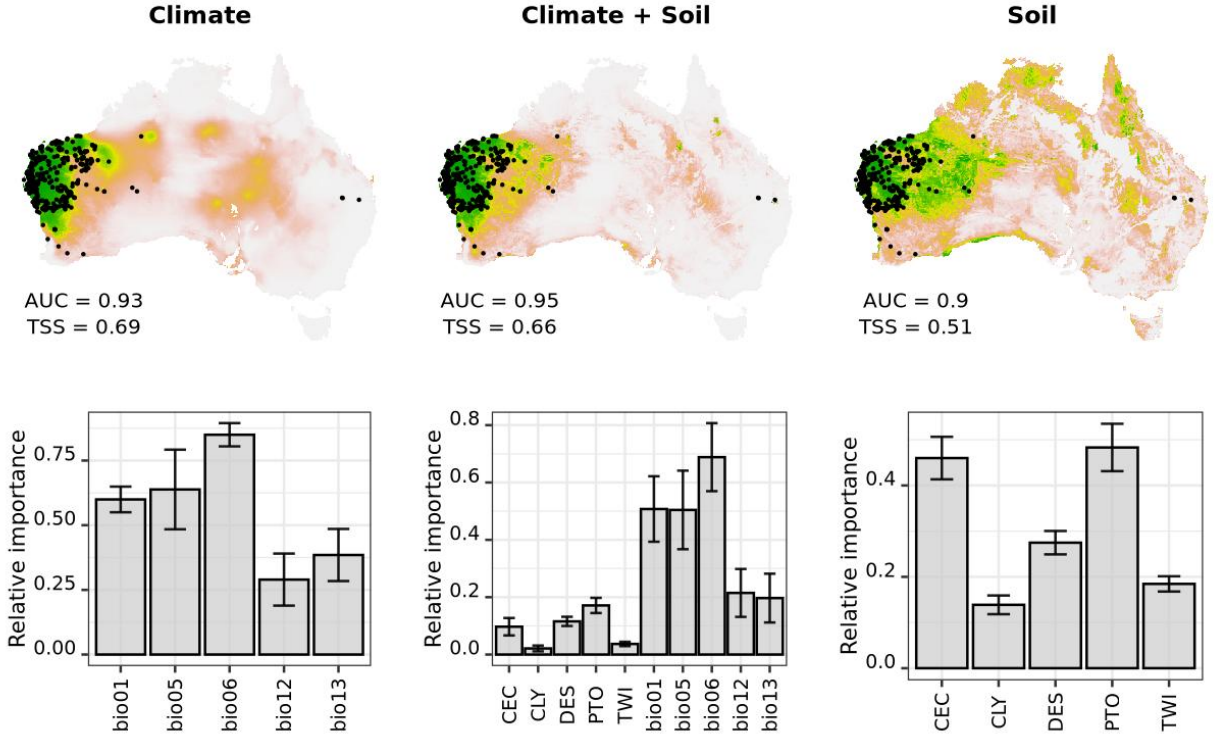

Acacia sericophylla

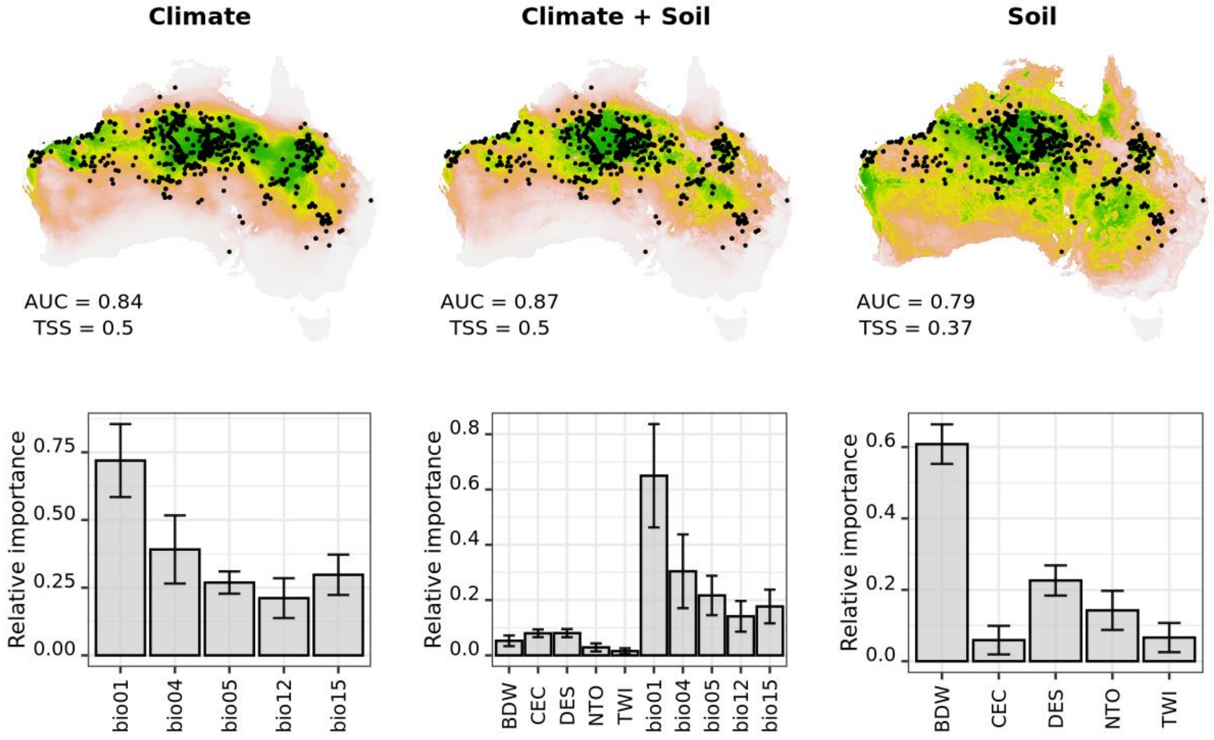

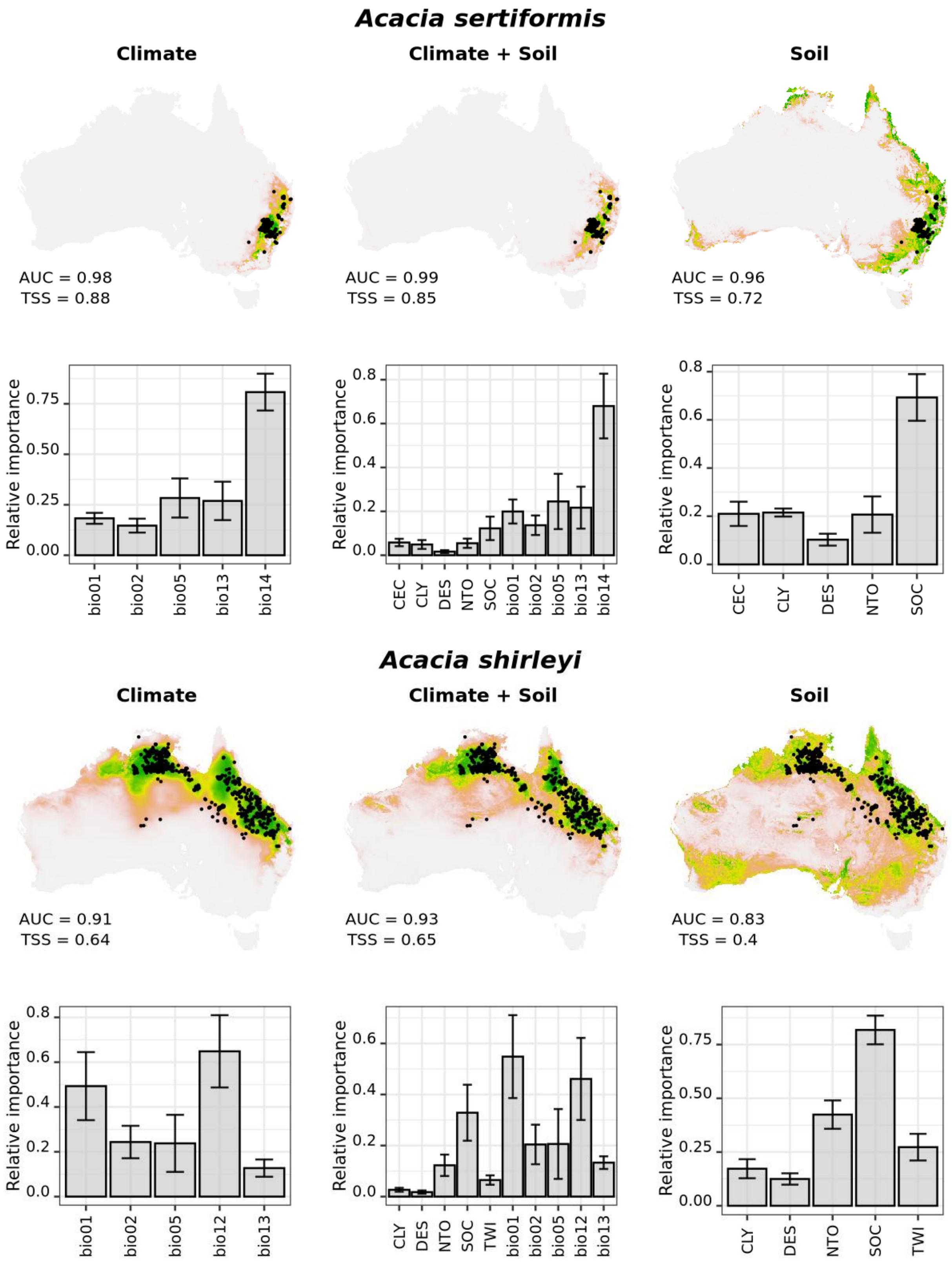

Acacia sertiformis
Climate
Climate + Soil
Soil
AUC = 0.98
TSS = 0.88
AUC = 0.99
TSS = 0.85
AUC = 0.96
TSS = 0.72
Relative importance
0.00
0.25
0.50
0.75
bio01
bio02
bio05
bio13
bio14
0.0
0.2
0.4
0.6
0.8
CEC
CLY
DES
NTO
SOC
bio01
bio02
bio05
bio13
bio14
CEC
CLY
DES
NTO
SOC
Acacia shirleyi
Climate
Climate + Soil
Soil
AUC = 0.91
TSS = 0.64
AUC = 0.93
TSS = 0.65
AUC = 0.83
TSS = 0.4
bio01
bio02
bio05
bio12
bio13
CLY
DES
NTO
SOC
TWI
bio01
bio02
bio05
bio12
bio13
CLY
DES
NTO
SOC
TWI

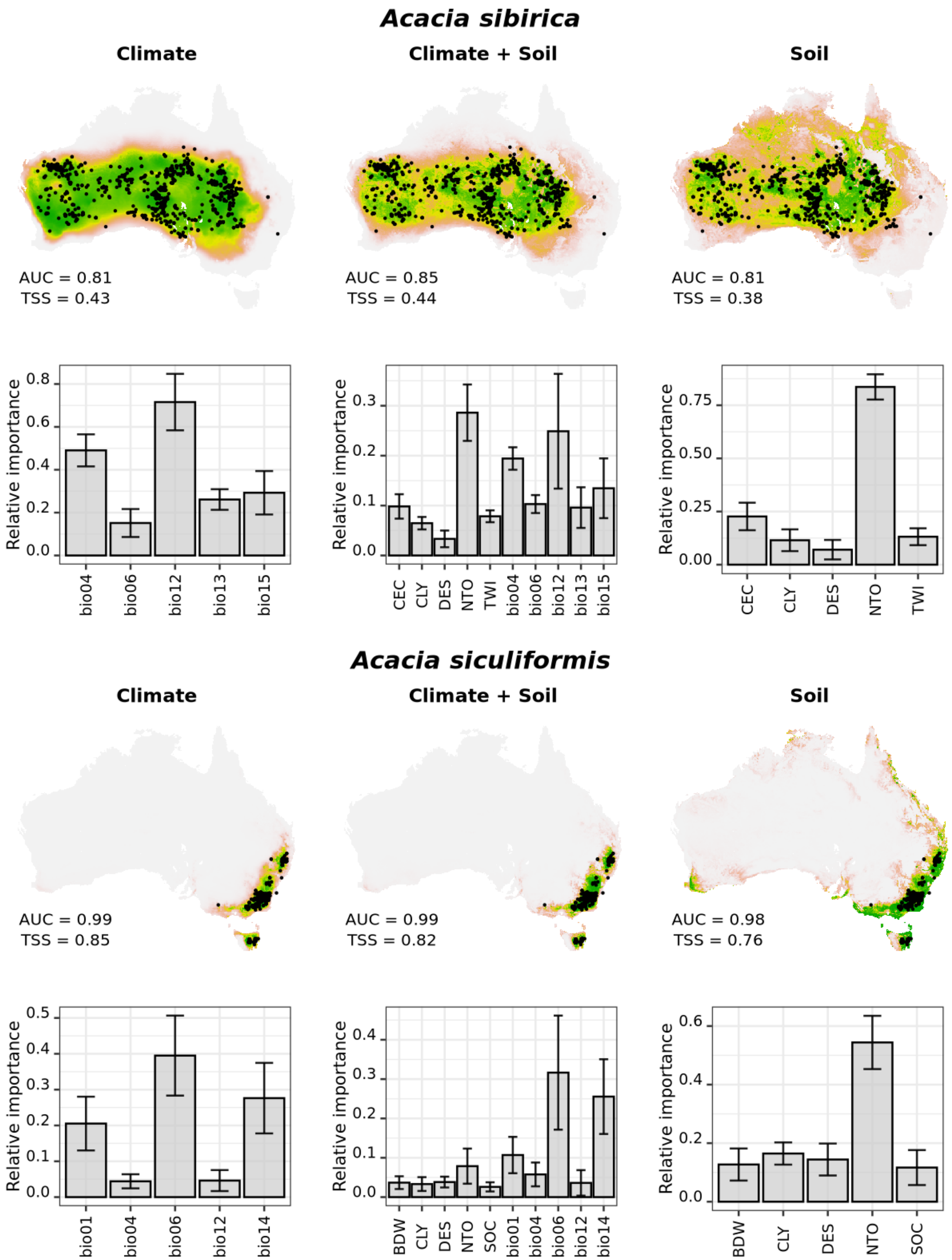
Acacia sibirica
Climate
Climate + Soil
Soil
AUC = 0.81
TSS = 0.43
AUC = 0.85
TSS = 0.44
AUC = 0.81
TSS = 0.38
Relative importance
bio04
bio06
bio12
bio13
bio15
CEC
CLY
DES
NTO
TWI
bio04
bio06
bio12
bio13
bio15
CEC
CLY
DES
NTO
TWI
Acacia siculiformis
Climate
Climate + Soil
Soil
AUC = 0.99
TSS = 0.85
AUC = 0.99
TSS = 0.82
AUC = 0.98
TSS = 0.76
Relative importance
bio01
bio04
bio06
bio12
bio14
BDW
CLY
DES
NTO
SOC
bio01
bio04
bio06
bio12
bio14
BDW
CLY
DES
NTO
SOC

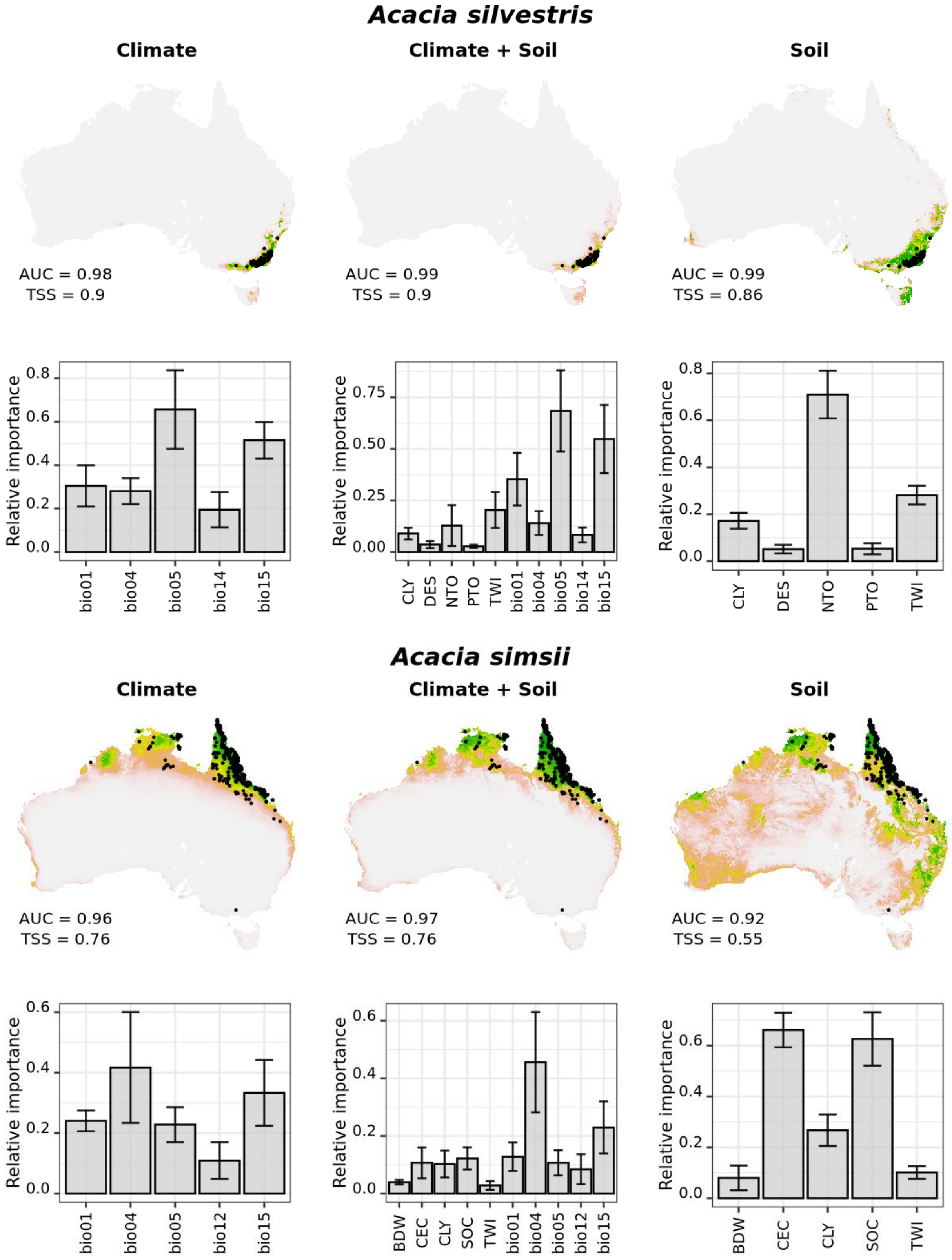
Acacia silvestris
Climate
Climate + Soil
Soil
AUC = 0.98
TSS = 0.9
AUC = 0.99
TSS = 0.9
AUC = 0.99
TSS = 0.86
Relative importance
0.0
0.2
0.4
0.6
0.8
bio01
bio04
bio05
bio14
bio15
0.00
0.25
0.50
0.75
CLY
DES
NTO
PTO
TWI
bio01
bio04
bio05
bio14
bio15
Acacia simsii
Climate
Climate + Soil
Soil
AUC = 0.96
TSS = 0.76
AUC = 0.97
TSS = 0.76
AUC = 0.92
TSS = 0.55
bio01
bio04
bio05
bio12
bio15
BDW
CEC
CLY
SOC
TWI

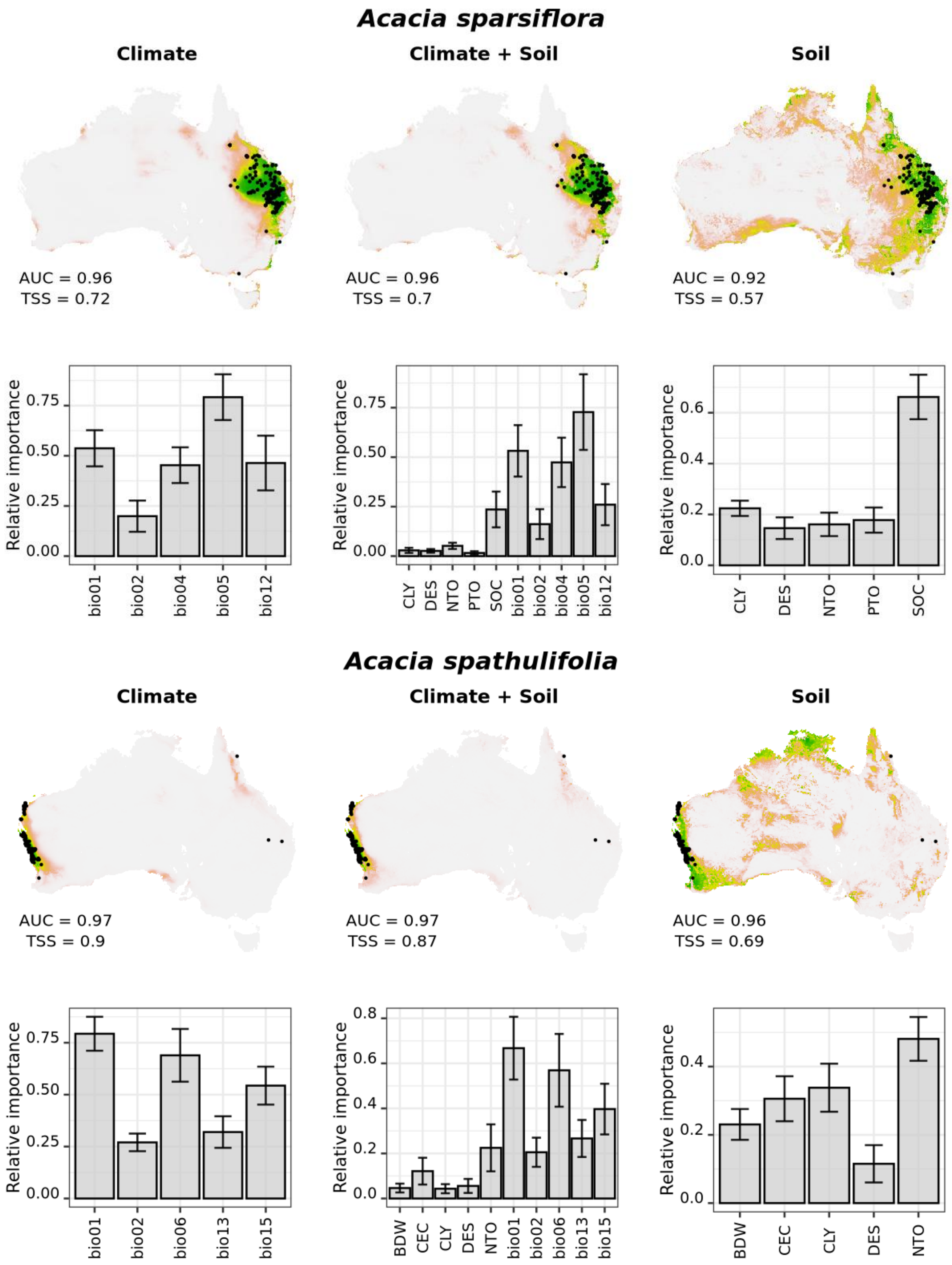
Acacia sparsiflora
Climate
Climate + Soil
Soil
AUC = 0.96
TSS = 0.72
AUC = 0.96
TSS = 0.7
AUC = 0.92
TSS = 0.57
Relative importance
0.00
0.25
0.50
0.75
bio01
bio02
bio04
bio05
bio12
CLY
DES
NTO
PTO
SOC
0.0
0.2
0.4
0.6
Acacia spathulifolia
Climate
Climate + Soil
Soil
AUC = 0.97
TSS = 0.9
AUC = 0.97
TSS = 0.87
AUC = 0.96
TSS = 0.69
bio06
bio13
bio15
BDW
CEC
0.8

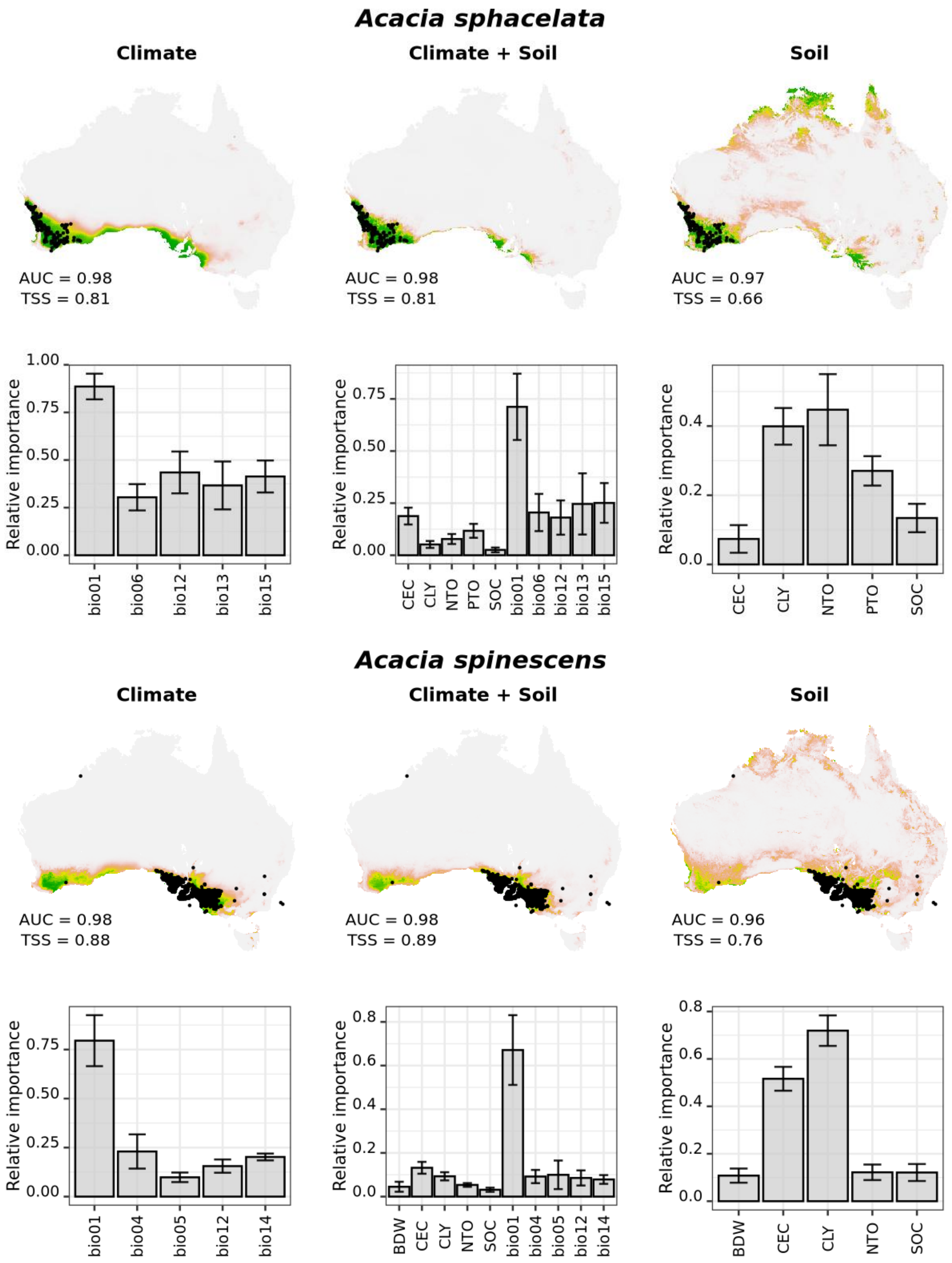
Acacia sphacelata
Climate
Climate + Soil
Soil
AUC = 0.98
TSS = 0.81
AUC = 0.98
TSS = 0.81
AUC = 0.97
TSS = 0.66
Relative importance
bio01
bio06
bio12
bio13
bio15
CEC
CLY
NTO
PTO
SOC
Acacia spinescens
Climate
Climate + Soil
Soil
AUC = 0.98
TSS = 0.88
AUC = 0.98
TSS = 0.89
AUC = 0.96
TSS = 0.76
Relative importance
bio01
bio04
bio05
bio12
bio14
BDW
CEC
CLY
NTO
SOC

Acacia spondylophylla

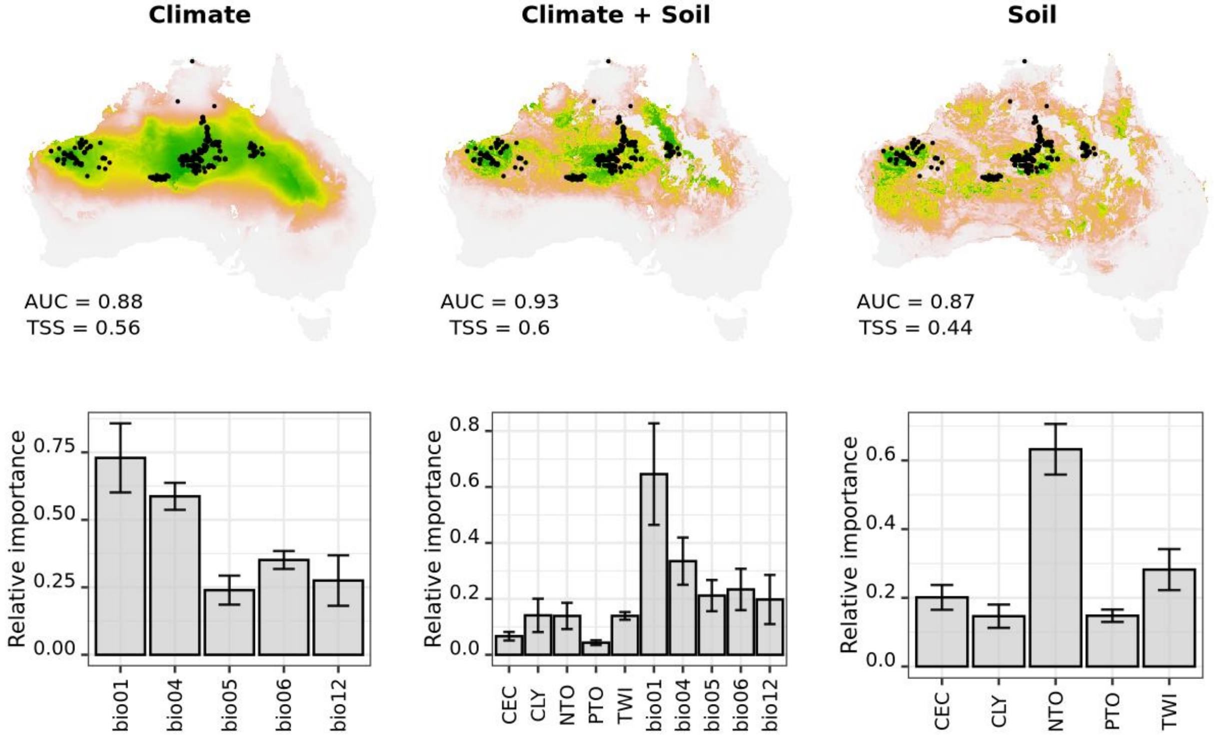

Acacia stellaticeps

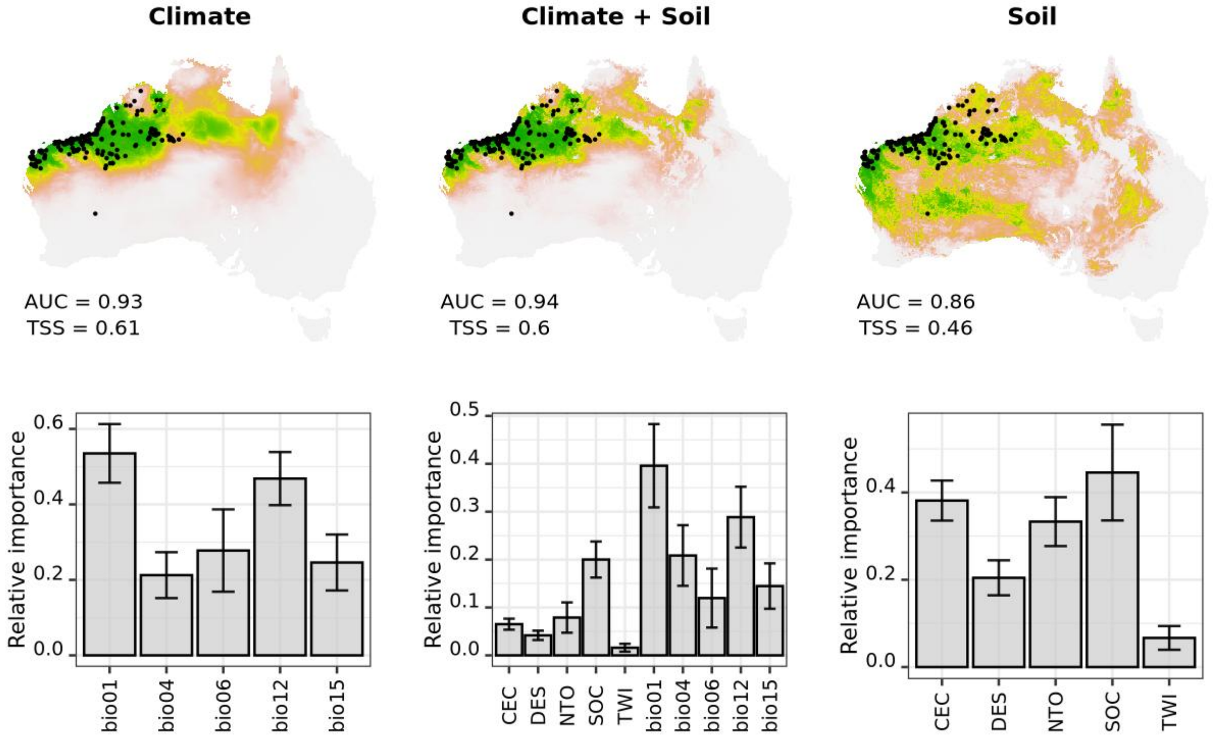

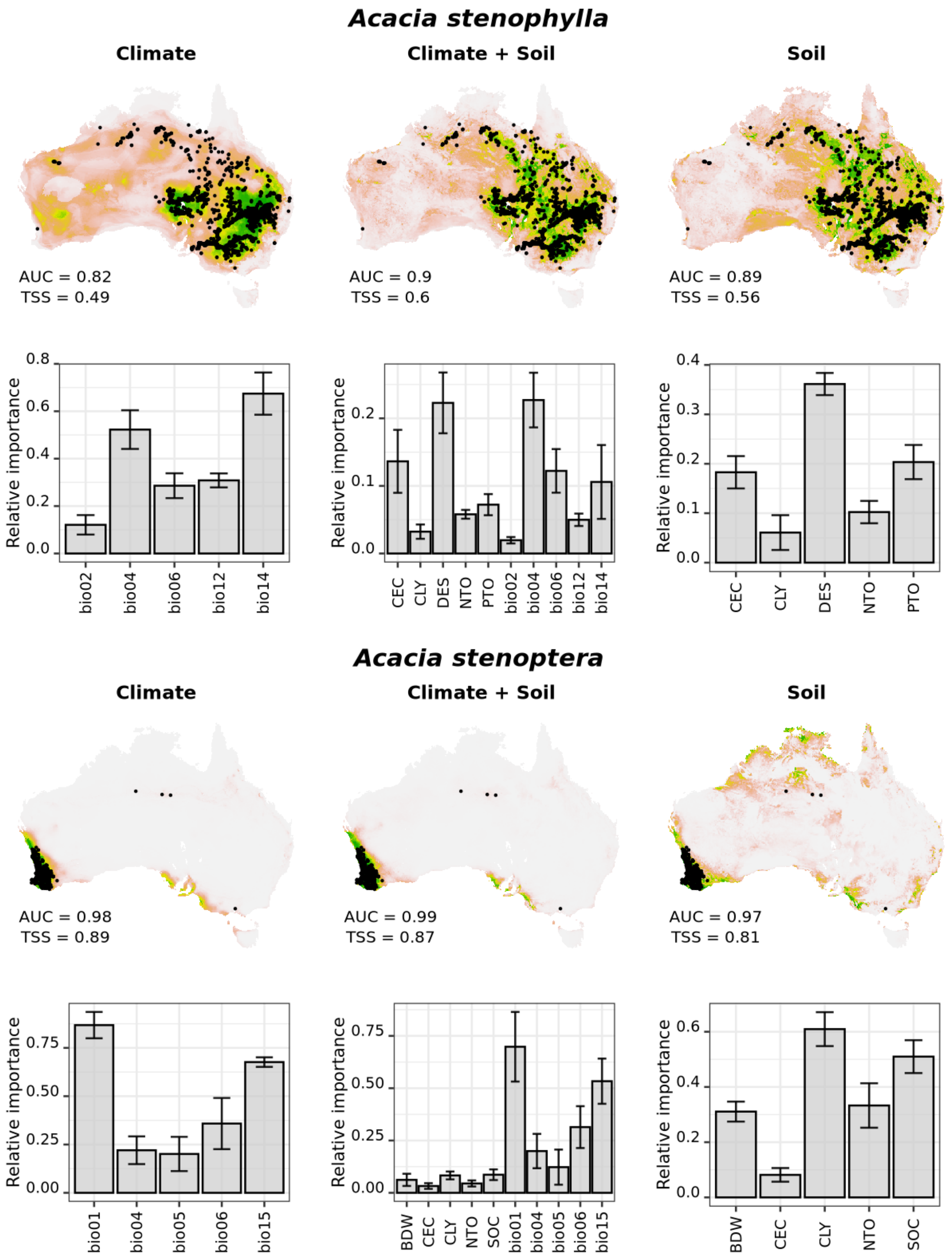

Acacia stenophylla
Climate
Climate + Soil
Soil
AUC = 0.82
TSS = 0.49
AUC = 0.9
TSS = 0.6
AUC = 0.89
TSS = 0.56
Relative importance
bio02
bio04
bio06
bio12
bio14
CEC
CLY
DES
NTO
PTO
Acacia stenoptera
AUC = 0.98
TSS = 0.89
AUC = 0.99
TSS = 0.87
AUC = 0.97
TSS = 0.81
bio01
bio05
bio15
BDW
SOC

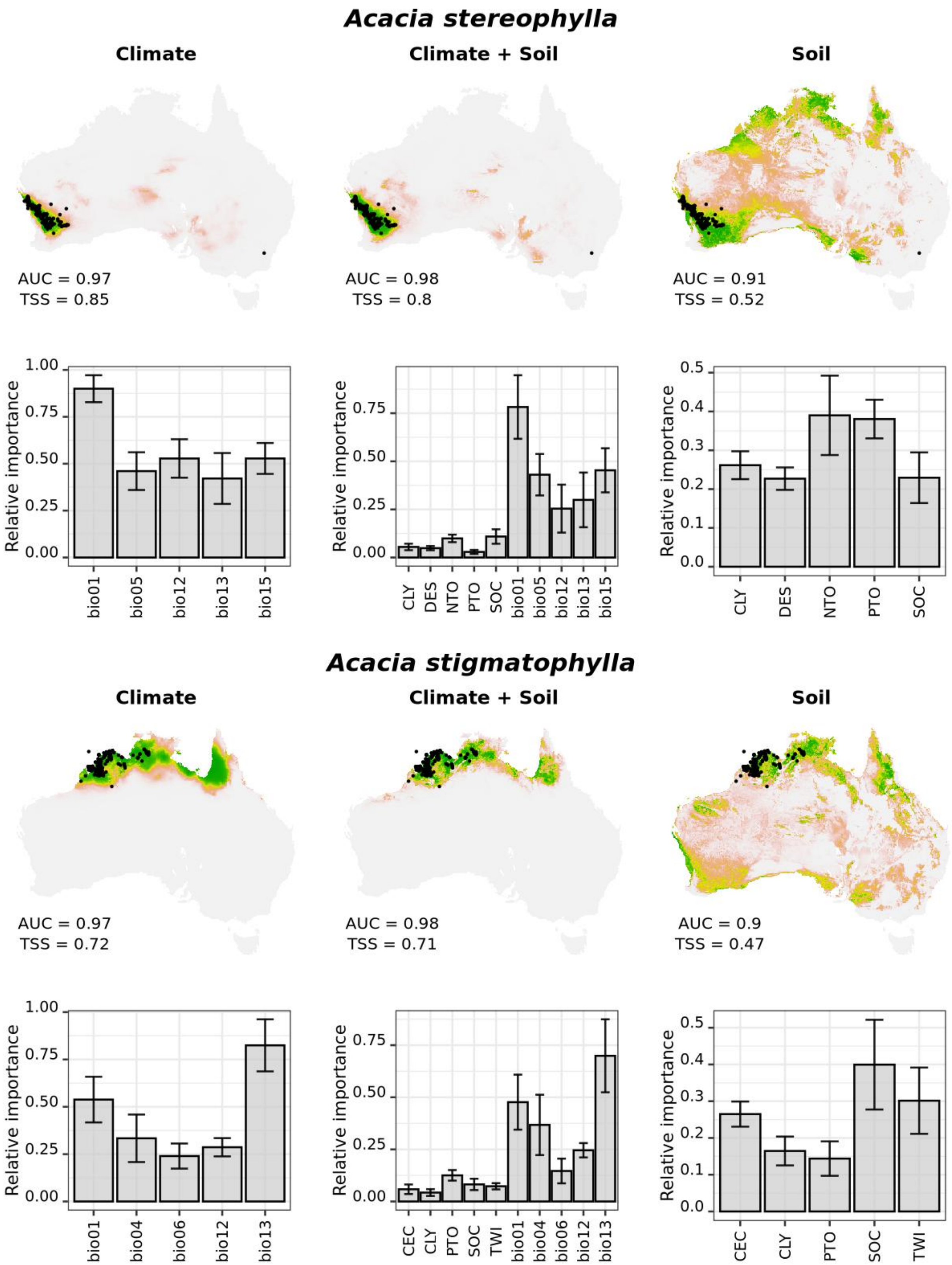

Acacia stereophylla
Climate
Climate + Soil
Soil
AUC = 0.97
TSS = 0.85
AUC = 0.98
TSS = 0.8
AUC = 0.91
TSS = 0.52
Relative importance
bio01
bio05
bio12
bio13
bio15
CLY
DES
NTO
PTO
SOC
Acacia stigmatophylla
Climate
Climate + Soil
Soil
AUC = 0.97
TSS = 0.72
AUC = 0.98
TSS = 0.71
AUC = 0.9
TSS = 0.47
Relative importance
bio01
bio04
bio06
bio12
bio13
CEC
CLY
PTO
SOC
TWI

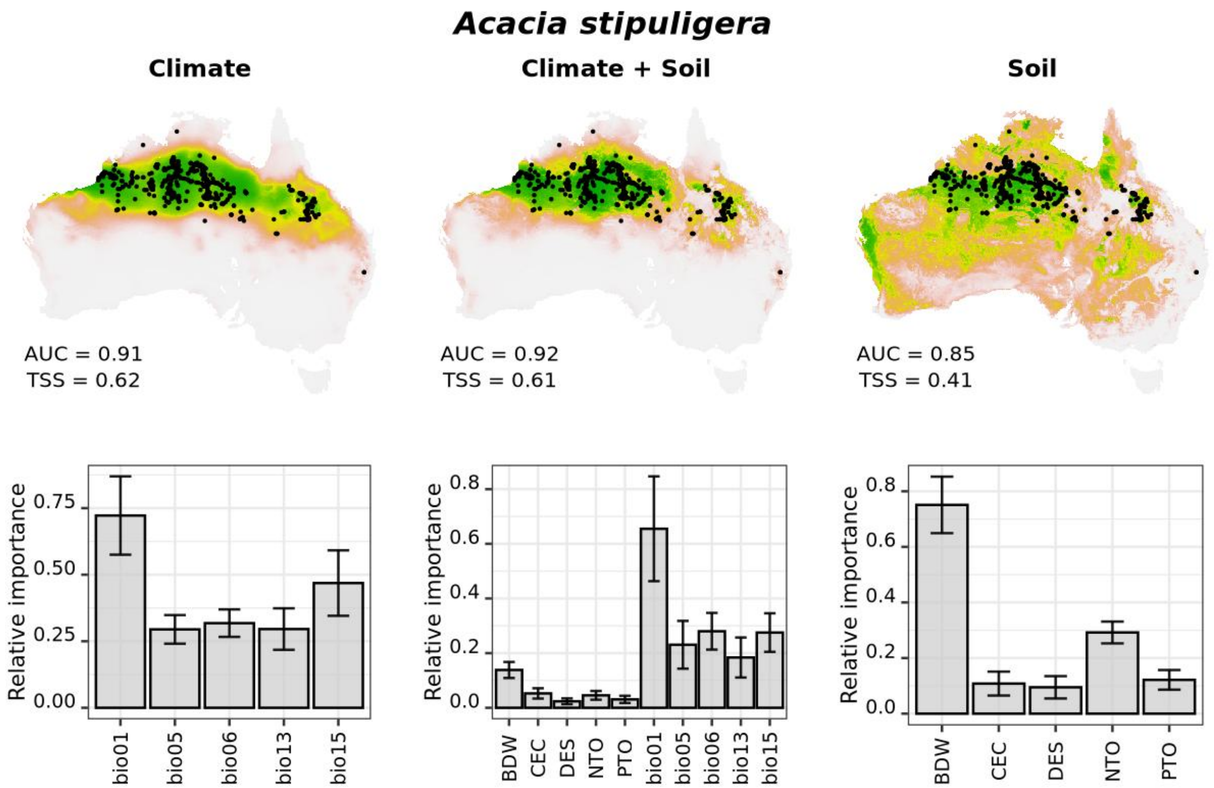
Acacia stipuligera
Climate
Climate + Soil
Soil
AUC = 0.91
TSS = 0.62
AUC = 0.92
TSS = 0.61
AUC = 0.85
TSS = 0.41
Relative importance
bio01
bio05
bio06
bio13
bio15
BDW
CEC
DES
NTO
PTO

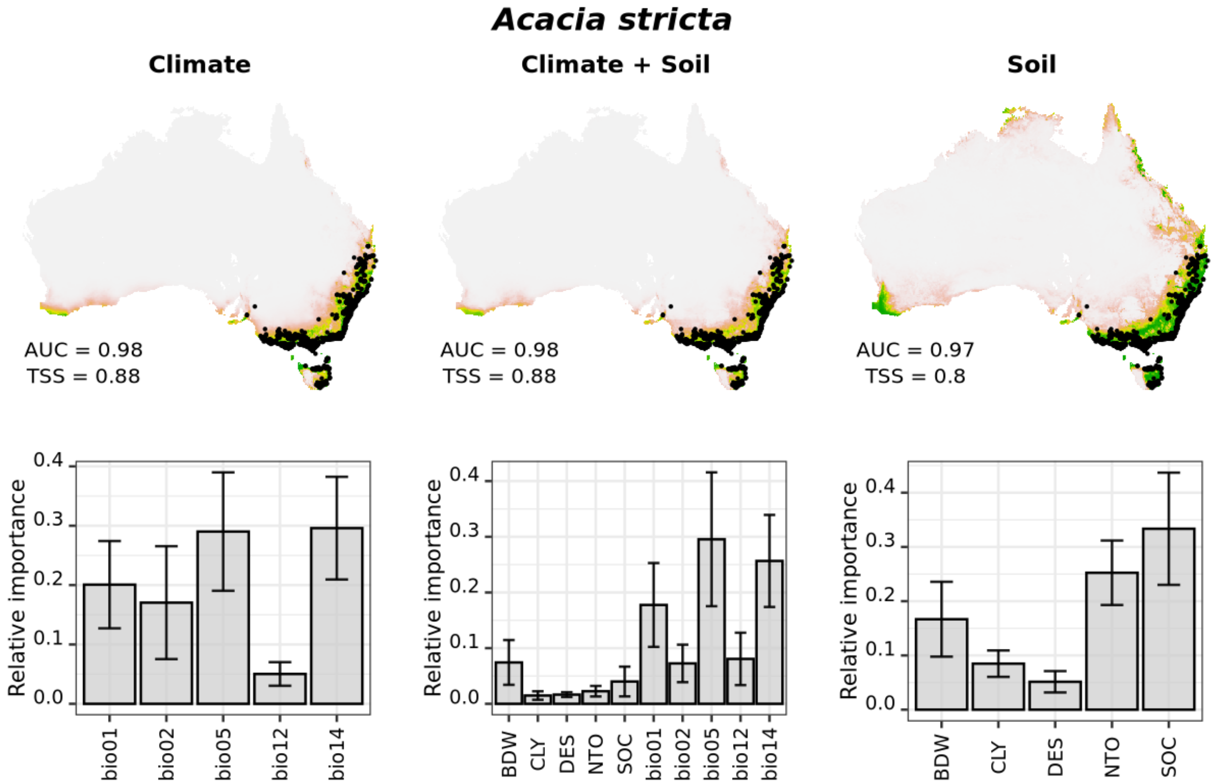
Acacia stricta
Climate
Climate + Soil
Soil
AUC = 0.98
TSS = 0.88
AUC = 0.98
TSS = 0.88
AUC = 0.97
TSS = 0.8
Relative importance
bio01
bio02
bio05
bio12
bio14
BDW
CLY
DES
NTO
SOC

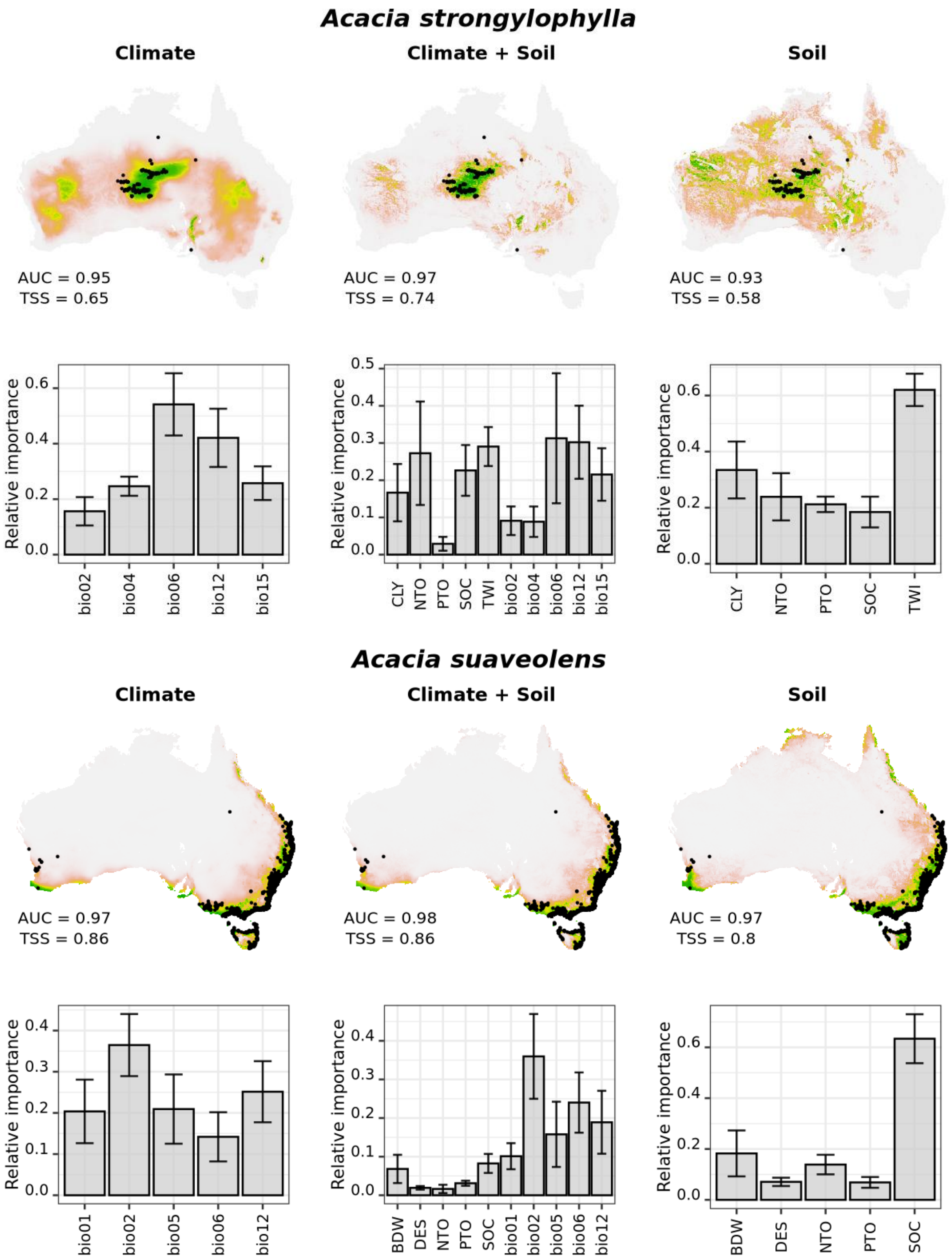

Acacia strongylophylla
Climate
Climate + Soil
Soil
AUC = 0.95
TSS = 0.65
AUC = 0.97
TSS = 0.74
AUC = 0.93
TSS = 0.58
Relative importance
bio02
bio04
bio06
bio12
bio15
CLY
NTO
PTO
SOC
TWI
bio02
bio04
bio06
bio12
bio15
CLY
NTO
PTO
SOC
TWI
Acacia suaveolens
Climate
Climate + Soil
Soil
AUC = 0.97
TSS = 0.86
AUC = 0.98
TSS = 0.86
AUC = 0.97
TSS = 0.8
Relative importance
bio01
bio02
bio05
bio06
bio12
BDW
DES
NTO
PTO
SOC
bio01
bio02
bio05
bio06
bio12
BDW
DES
NTO
PTO
SOC

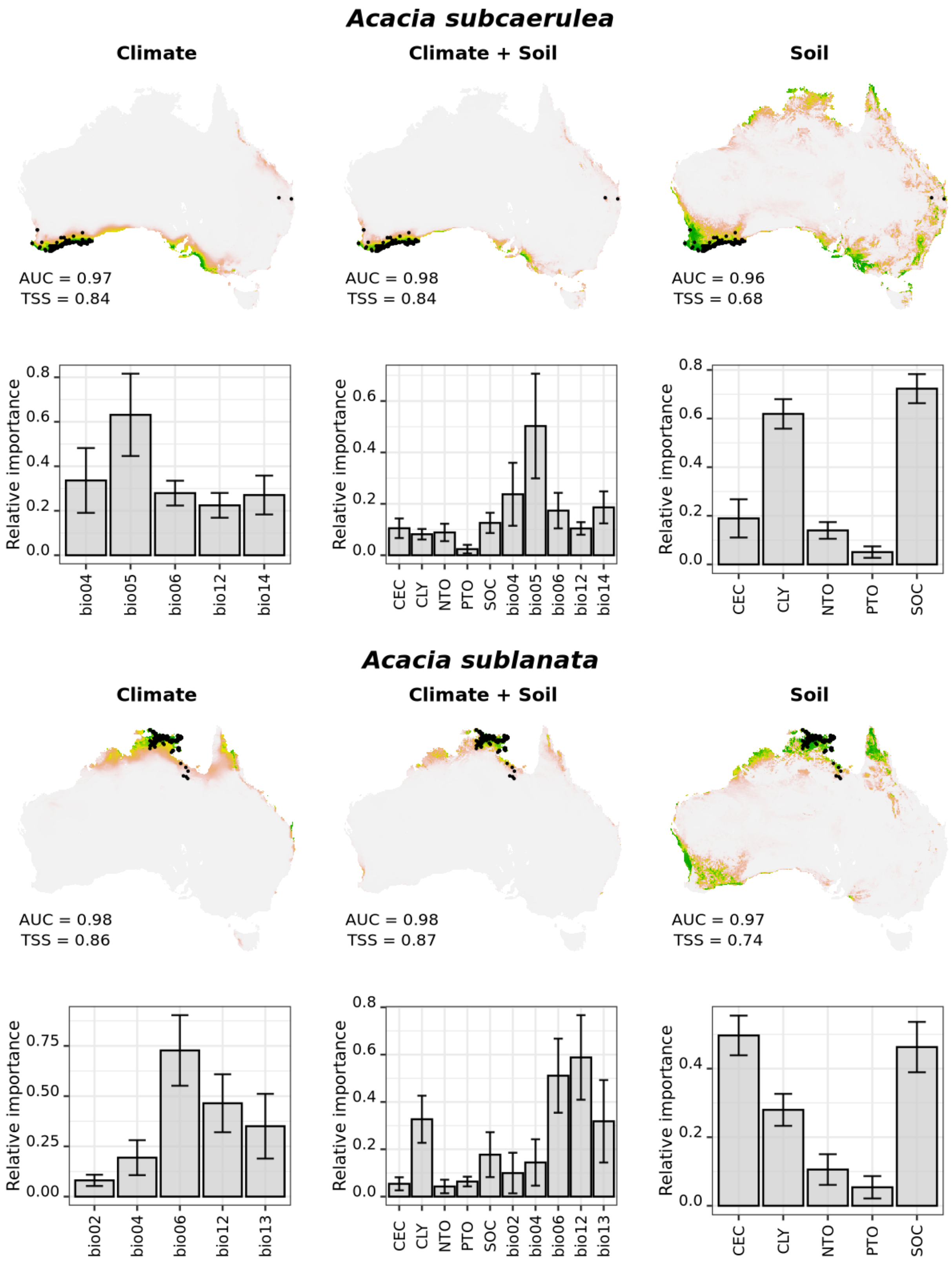
Acacia subcaerulea
Climate
Climate + Soil
Soil
AUC = 0.97
TSS = 0.84
AUC = 0.98
TSS = 0.84
AUC = 0.96
TSS = 0.68
Relative importance
bio04
bio05
bio06
bio12
bio14
CEC
CLY
NTO
PTO
SOC
Acacia sublanata
Climate
Climate + Soil
Soil
AUC = 0.98
TSS = 0.86
AUC = 0.98
TSS = 0.87
AUC = 0.97
TSS = 0.74
bio02
bio13

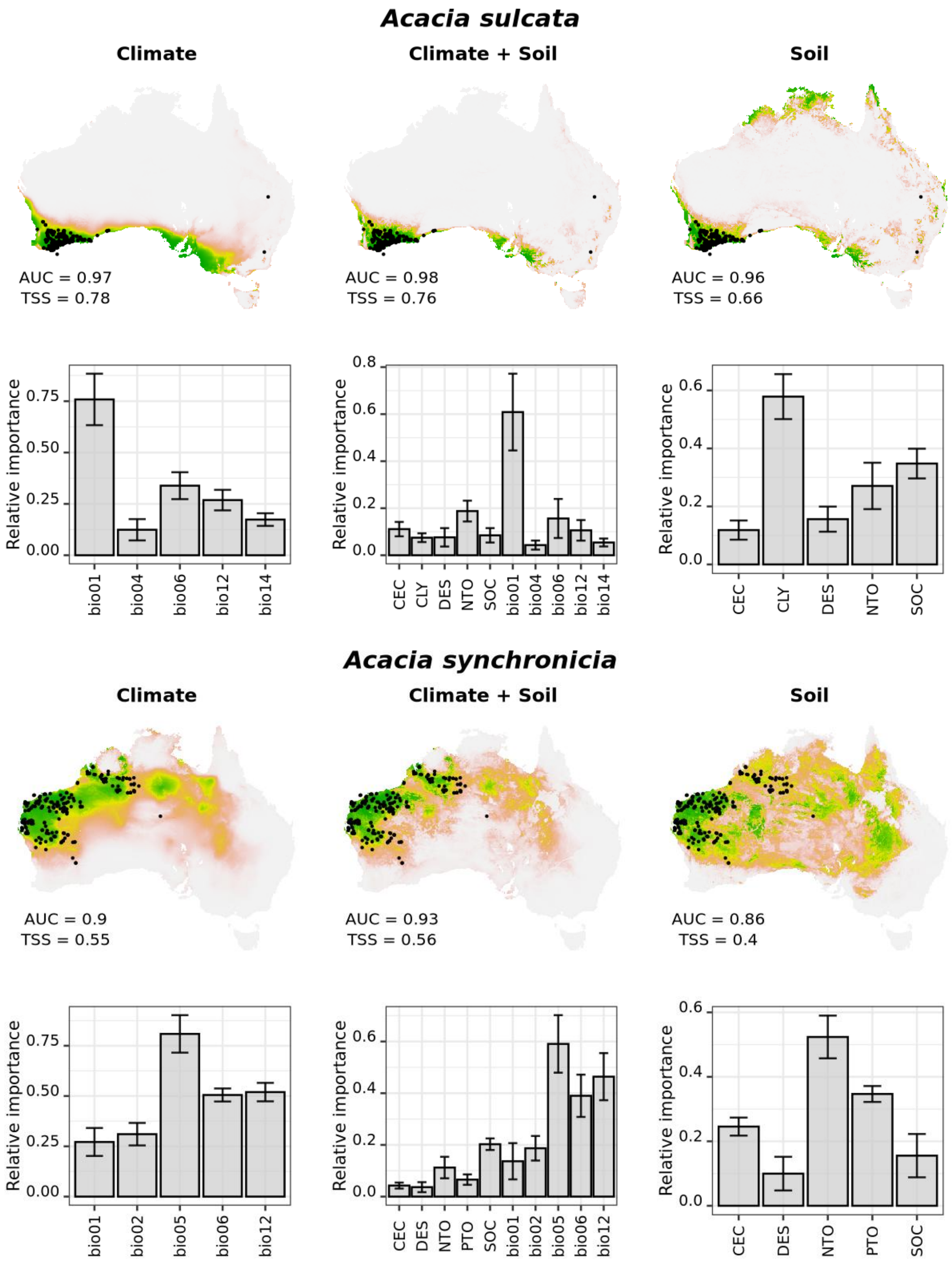
Acacia sulcata
Climate
Climate + Soil
Soil
AUC = 0.97
TSS = 0.78
AUC = 0.98
TSS = 0.76
AUC = 0.96
TSS = 0.66
Relative importance
bio01
bio04
bio06
bio12
bio14
CEC
CLY
DES
NTO
SOC
Acacia synchronicia
Climate
Climate + Soil
Soil
AUC = 0.9
TSS = 0.55
AUC = 0.93
TSS = 0.56
AUC = 0.86
TSS = 0.4
Relative importance
bio01
bio02
bio05
bio06
bio12
CEC
DES
NTO
PTO
SOC

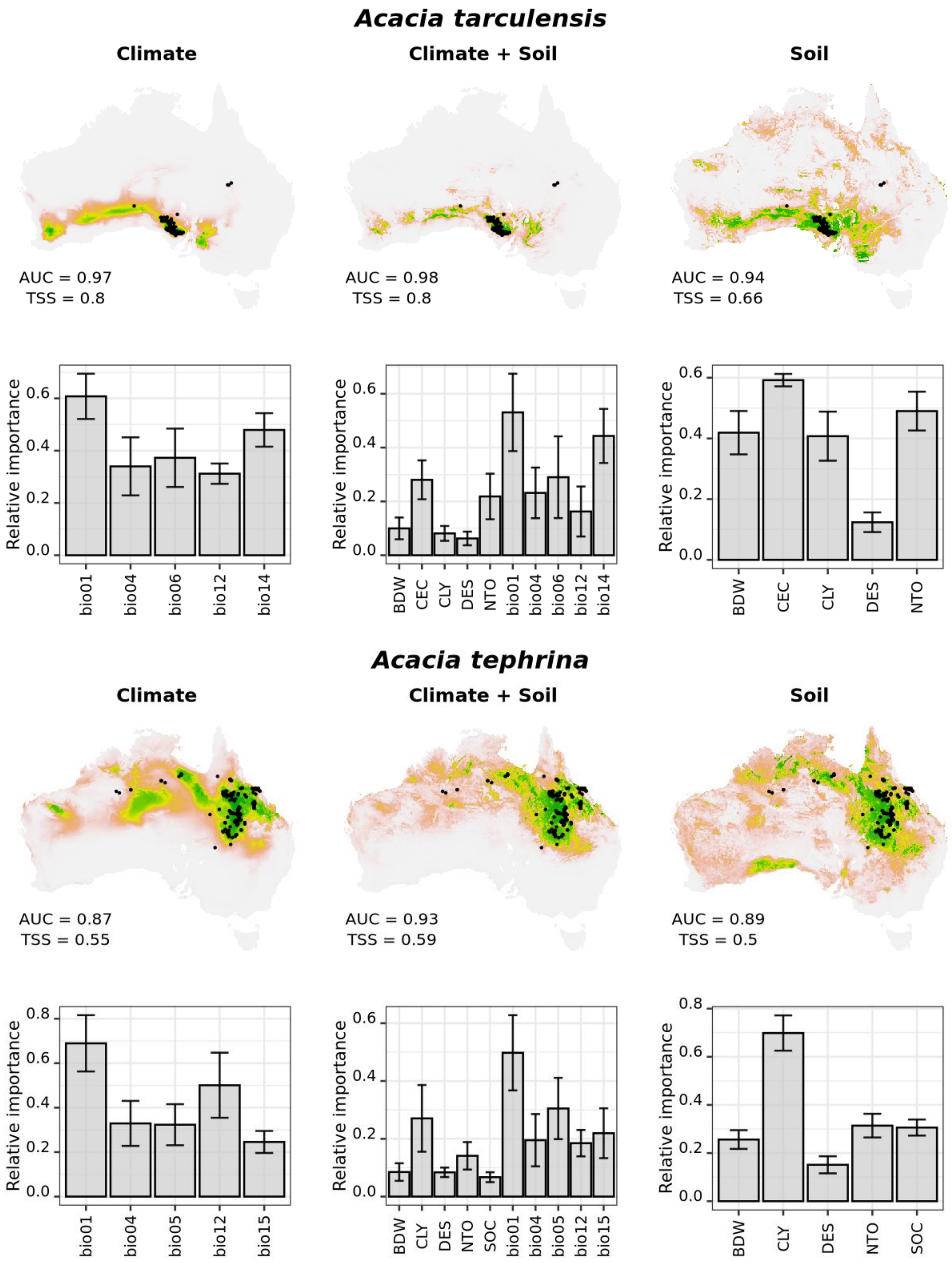

Acacia tarculensis
Climate
Climate + Soil
Soil
AUC = 0.97
TSS = 0.8
AUC = 0.98
TSS = 0.8
AUC = 0.94
TSS = 0.66
Relative importance
bio01 bio04 bio06 bio12 bio14
BDW CEC CLY DES NTO bio01 bio04 bio06 bio12 bio14
BDW CEC CLY DES NTO
Acacia tephrina
Climate
Climate + Soil
Soil
AUC = 0.87
TSS = 0.55
AUC = 0.93
TSS = 0.59
AUC = 0.89
TSS = 0.5
Relative importance
bio01 bio04 bio05 bio12 bio15
BDW CLY DES NTO SOC bio01 bio04 bio05 bio12 bio15
BDW CLY DES NTO SOC

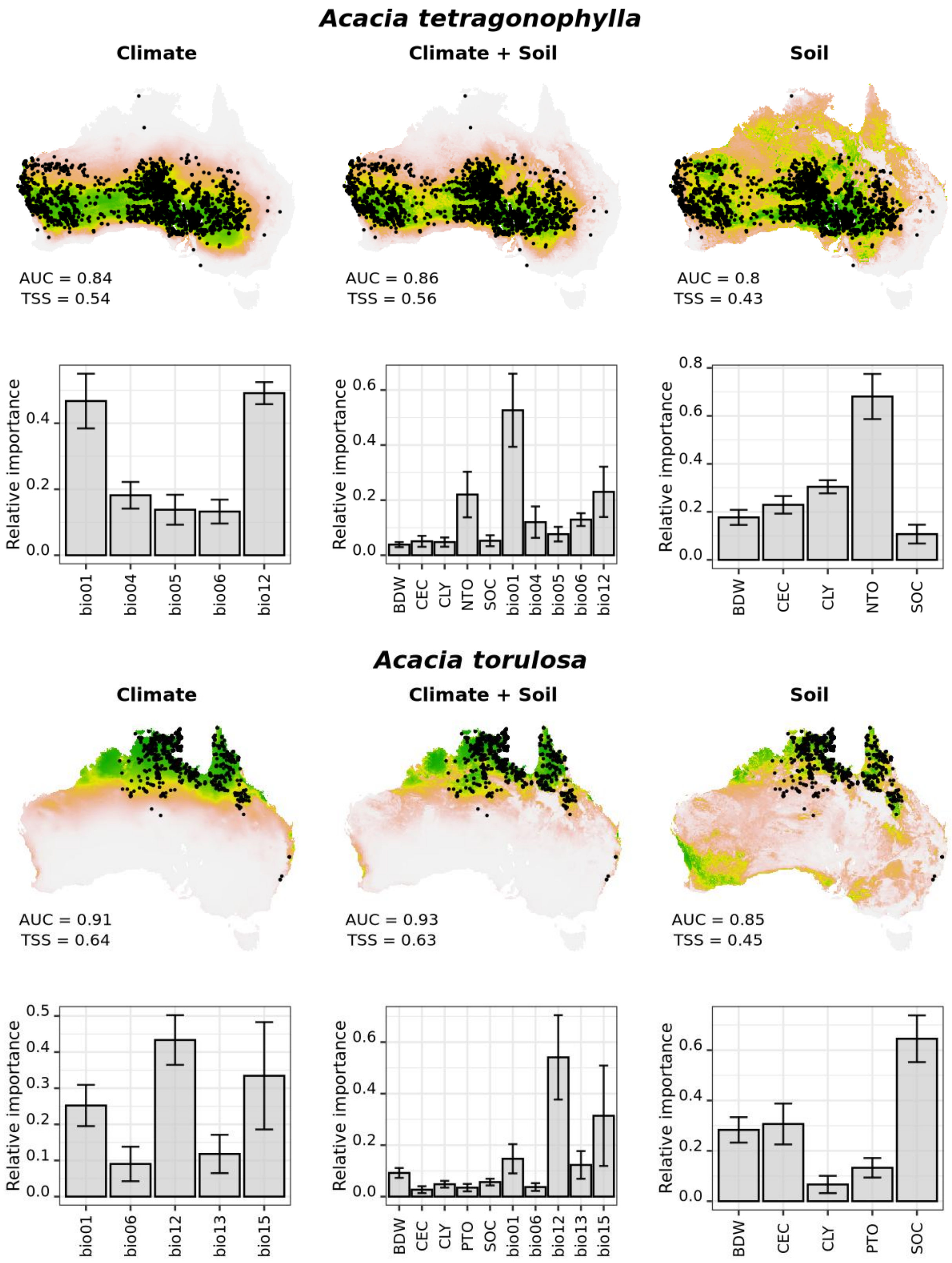
Acacia tetragonophylla
Climate
Climate + Soil
Soil
AUC = 0.84
TSS = 0.54
AUC = 0.86
TSS = 0.56
AUC = 0.8
TSS = 0.43
Relative importance
0.0
0.2
0.4
0.6
0.8
bio01
bio04
bio05
bio06
bio12
BDW
CEC
CLY
NTO
SOC
Acacia torulosa
Climate
Climate + Soil
Soil
AUC = 0.91
TSS = 0.64
AUC = 0.93
TSS = 0.63
AUC = 0.85
TSS = 0.45
Relative importance
0.0
0.1
0.2
0.3
0.4
0.5
0.6
bio01
bio06
bio12
bio13
bio15
BDW
CEC
CLY
PTO
SOC

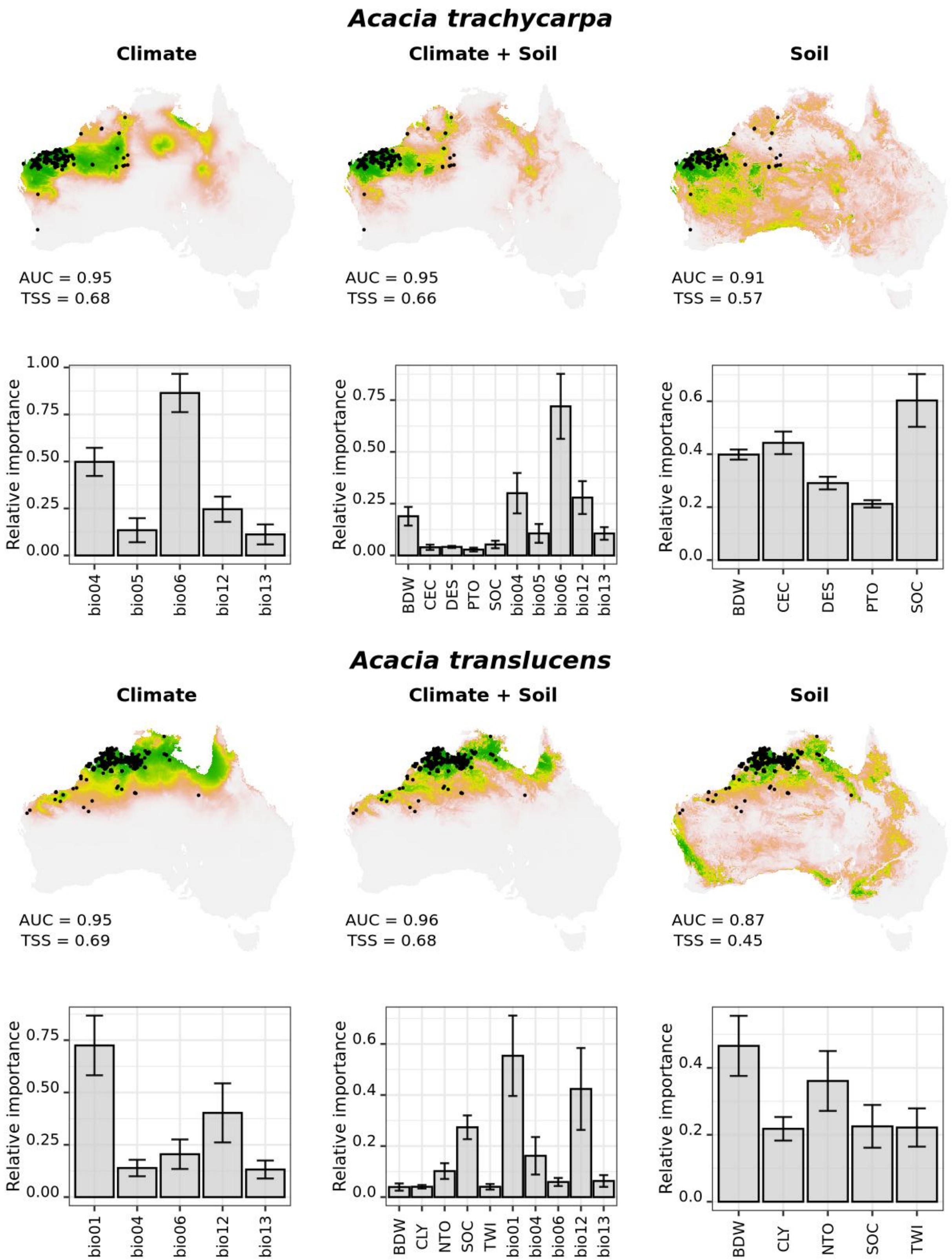
Acacia trachycarpa
Climate
Climate + Soil
Soil
AUC = 0.95
TSS = 0.68
AUC = 0.95
TSS = 0.66
AUC = 0.91
TSS = 0.57
Relative importance
bio04
bio05
bio06
bio12
bio13
BDW
CEC
DES
PTO
SOC
Acacia translucens
Climate
Climate + Soil
Soil
AUC = 0.95
TSS = 0.69
AUC = 0.96
TSS = 0.68
AUC = 0.87
TSS = 0.45
Relative importance
bio01
bio04
bio06
bio12
bio13
BDW
CLY
NTO
SOC
TWI

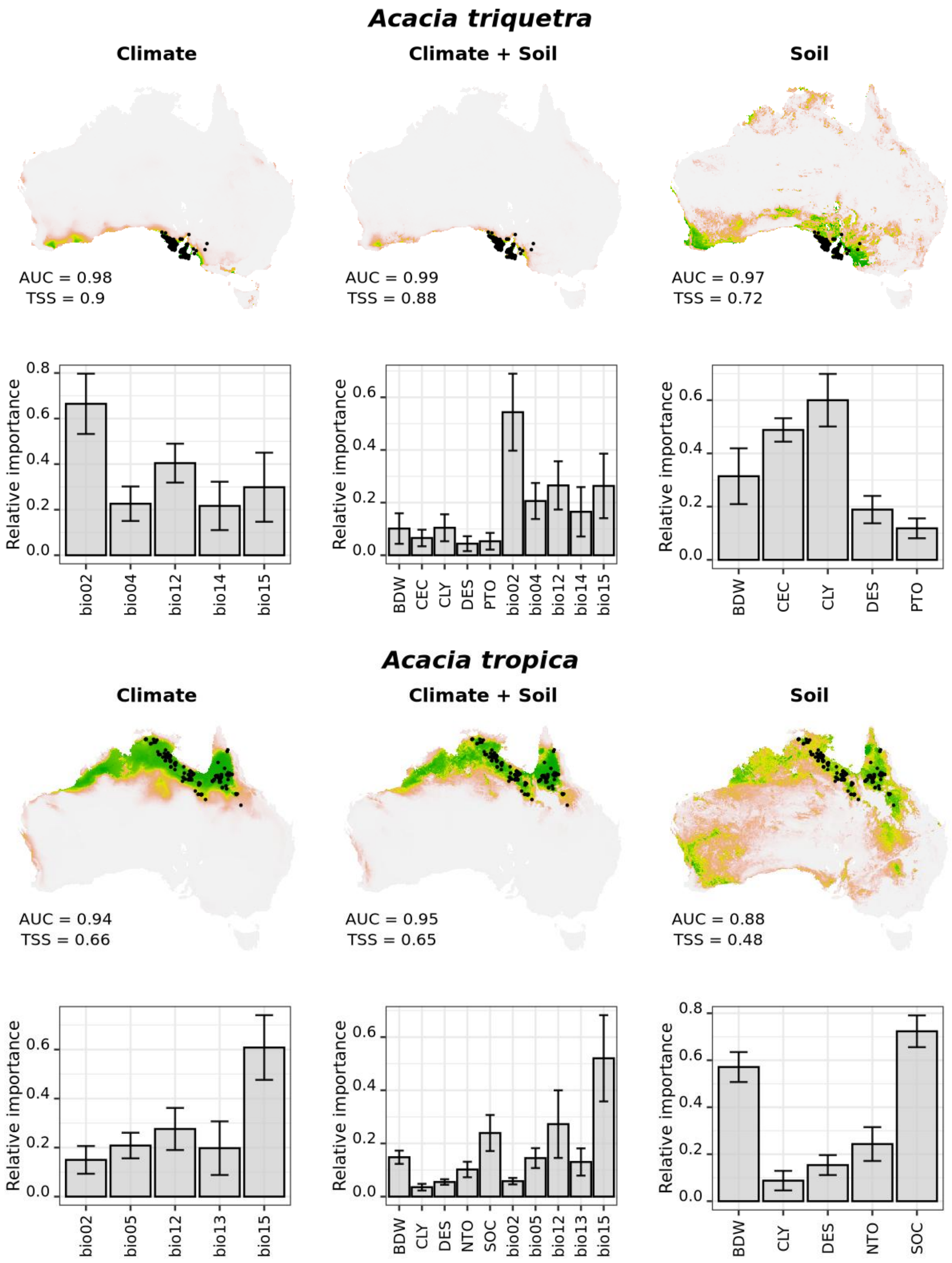
Acacia triquetra
Climate
Climate + Soil
Soil
AUC = 0.98
TSS = 0.9
AUC = 0.99
TSS = 0.88
AUC = 0.97
TSS = 0.72
Relative importance
bio02
bio04
bio12
bio14
bio15
BDW
CEC
CLY
DES
PTO
Acacia tropica
Climate
Climate + Soil
Soil
AUC = 0.94
TSS = 0.66
AUC = 0.95
TSS = 0.65
AUC = 0.88
TSS = 0.48
Relative importance
bio02
bio05
bio12
bio13
bio15
BDW
CLY
DES
NTO
SOC

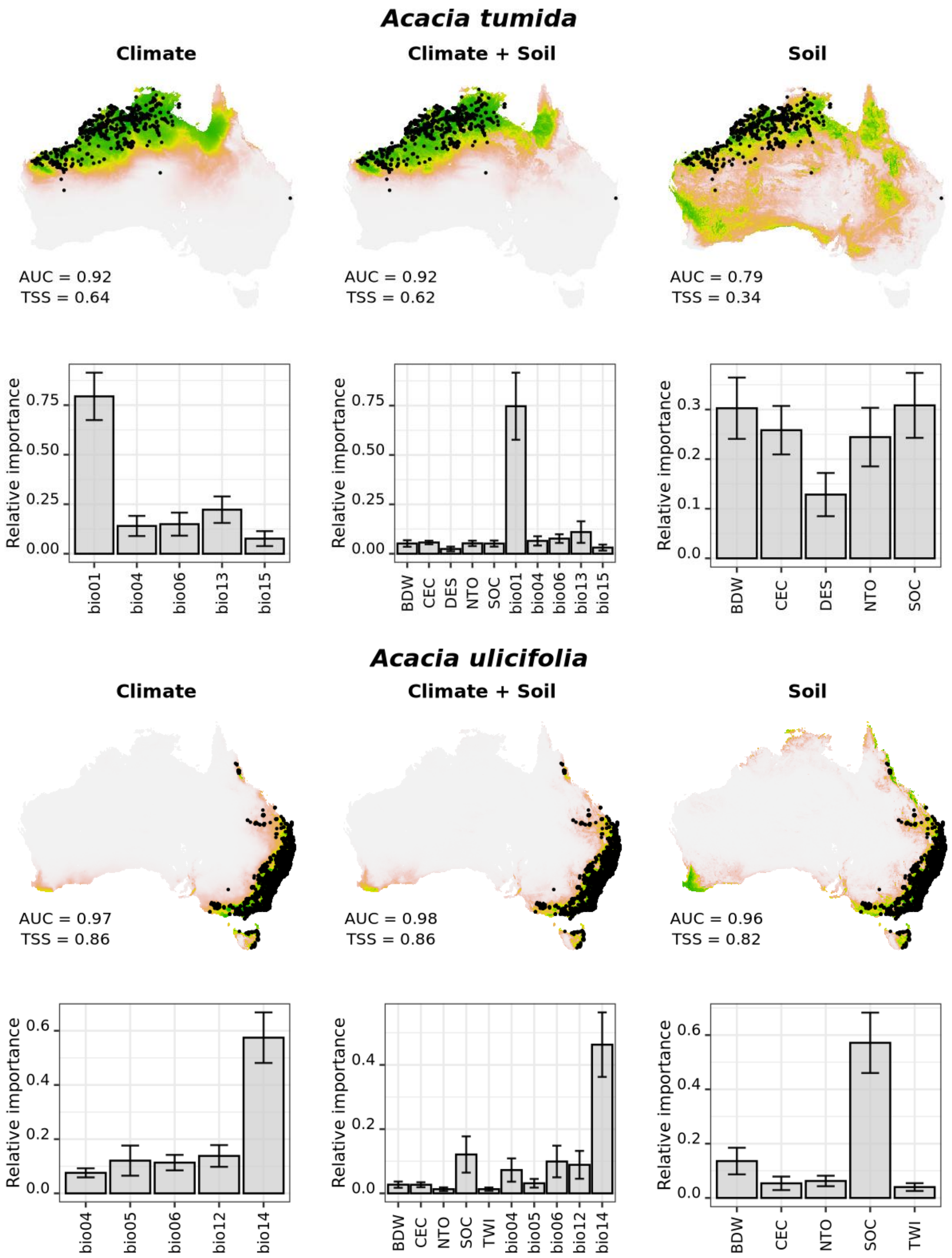

Acacia tumida
Climate
Climate + Soil
Soil
AUC = 0.92
TSS = 0.64
AUC = 0.92
TSS = 0.62
AUC = 0.79
TSS = 0.34
Relative importance
0.00
0.25
0.50
0.75
bio01
bio04
bio06
bio13
bio15
BDW
CEC
DES
NTO
SOC
0.0
0.1
0.2
0.3
Acacia ulicifolia
Climate
Climate + Soil
Soil
AUC = 0.97
TSS = 0.86
AUC = 0.98
TSS = 0.86
AUC = 0.96
TSS = 0.82
Relative importance
0.0
0.2
0.4
0.6
bio04
bio05
bio06
bio12
bio14
BDW
CEC
NTO
SOC
TWI

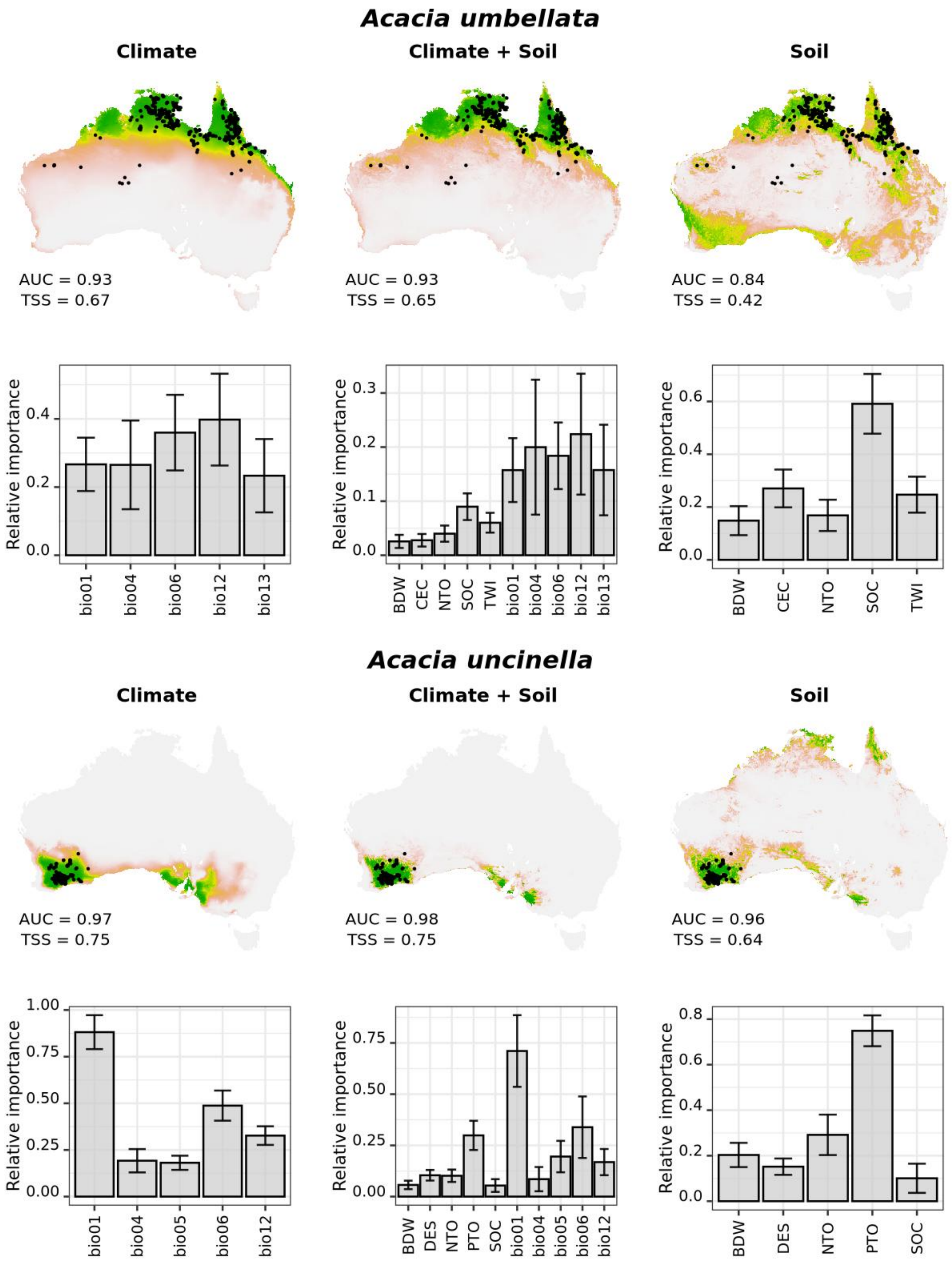

Acacia umbellata
Climate
Climate + Soil
Soil
AUC = 0.93
TSS = 0.67
AUC = 0.93
TSS = 0.65
AUC = 0.84
TSS = 0.42
Relative importance
bio01
bio04
bio06
bio12
bio13
BDW
CEC
NTO
SOC
TWI
Acacia uncinella
AUC = 0.97
TSS = 0.75
AUC = 0.98
TSS = 0.75
AUC = 0.96
TSS = 0.64
bio05
DES
PTO

Acacia urophylla

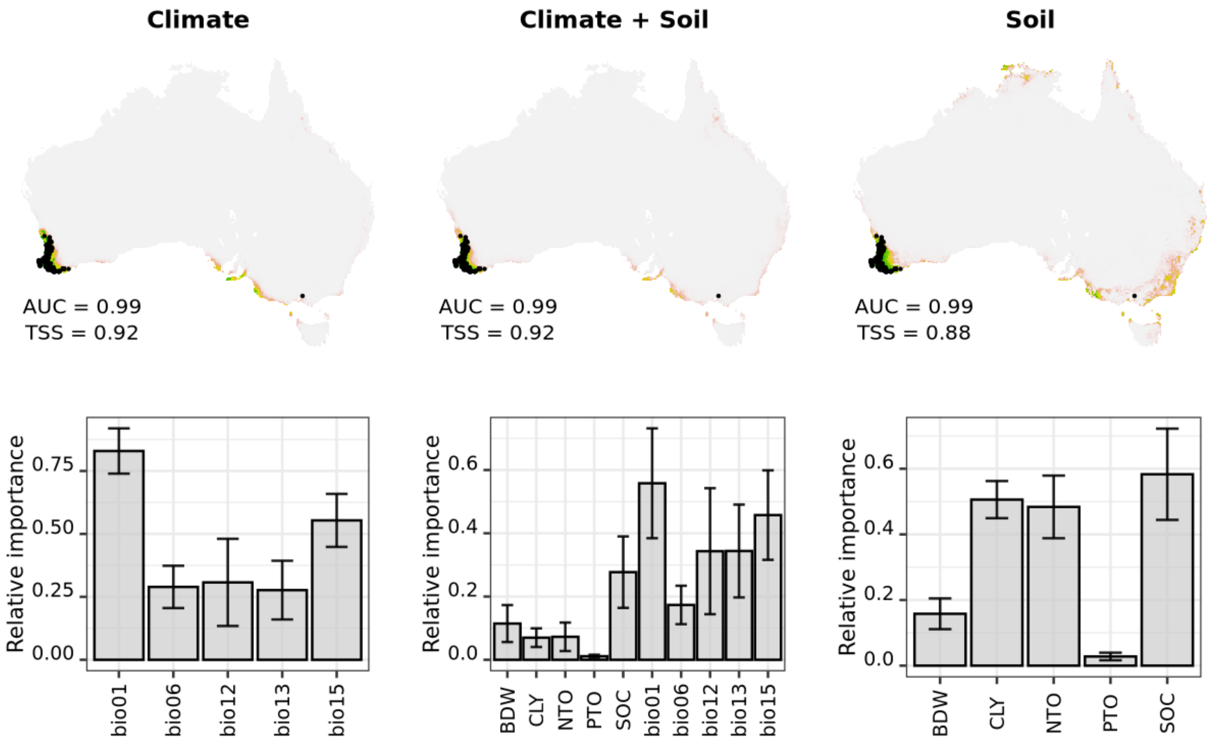

Acacia validinervia

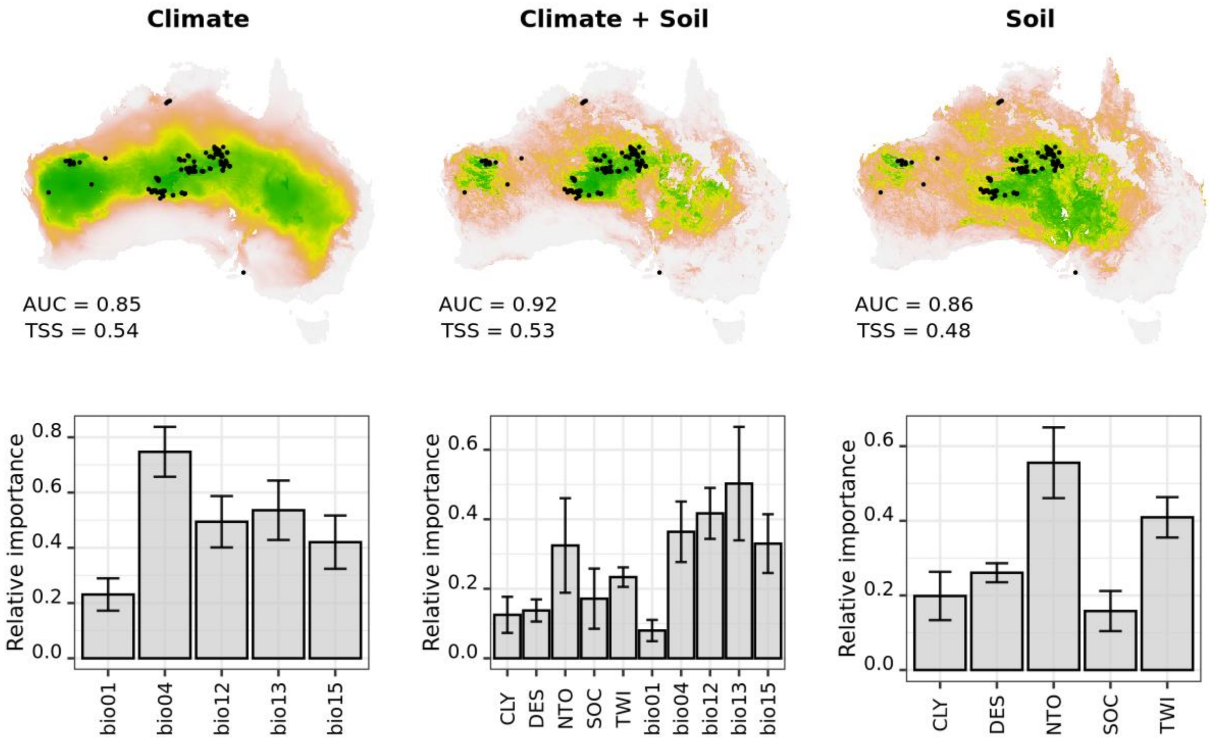

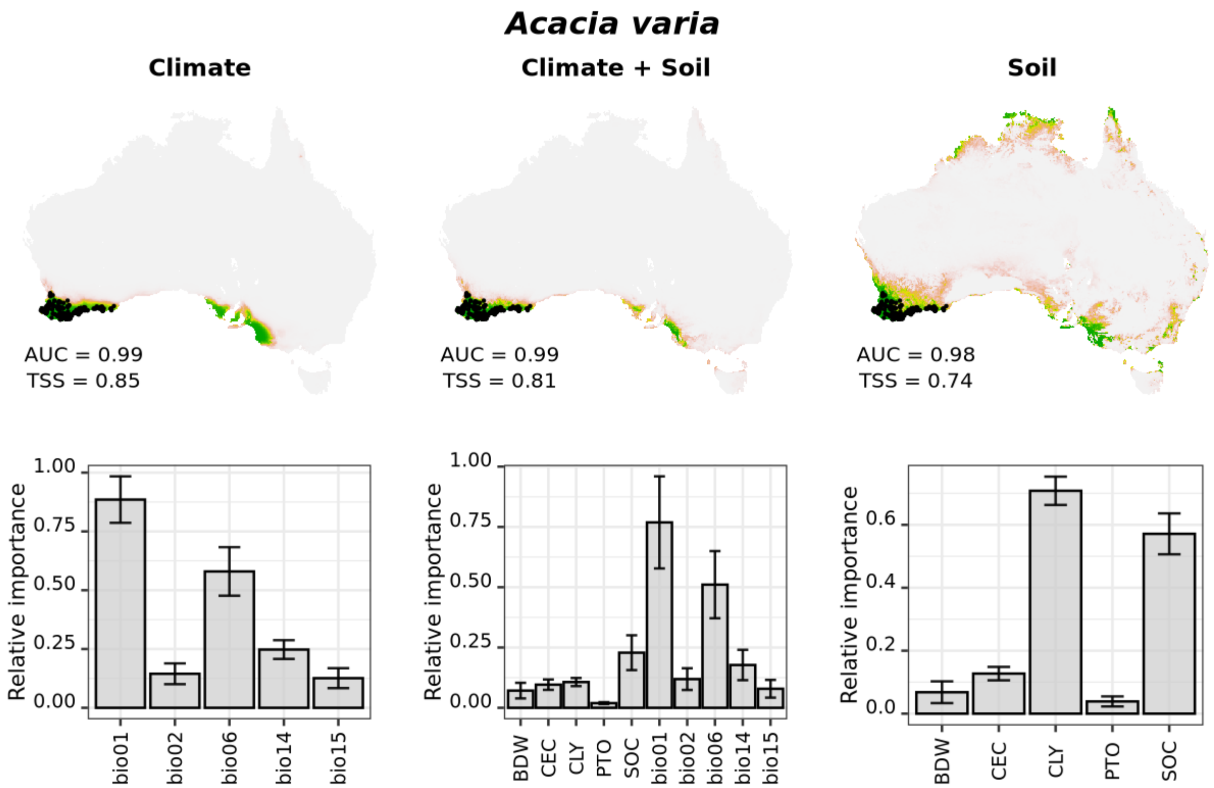
Acacia varia
Climate
Climate + Soil
Soil
AUC = 0.99
TSS = 0.85
AUC = 0.99
TSS = 0.81
AUC = 0.98
TSS = 0.74
Relative importance
1.00
0.75
0.50
0.25
0.00
bio01
bio02
bio06
bio14
bio15
BDW
CEC
CLY
PTO
SOC
0.6
0.4
0.2
0.0

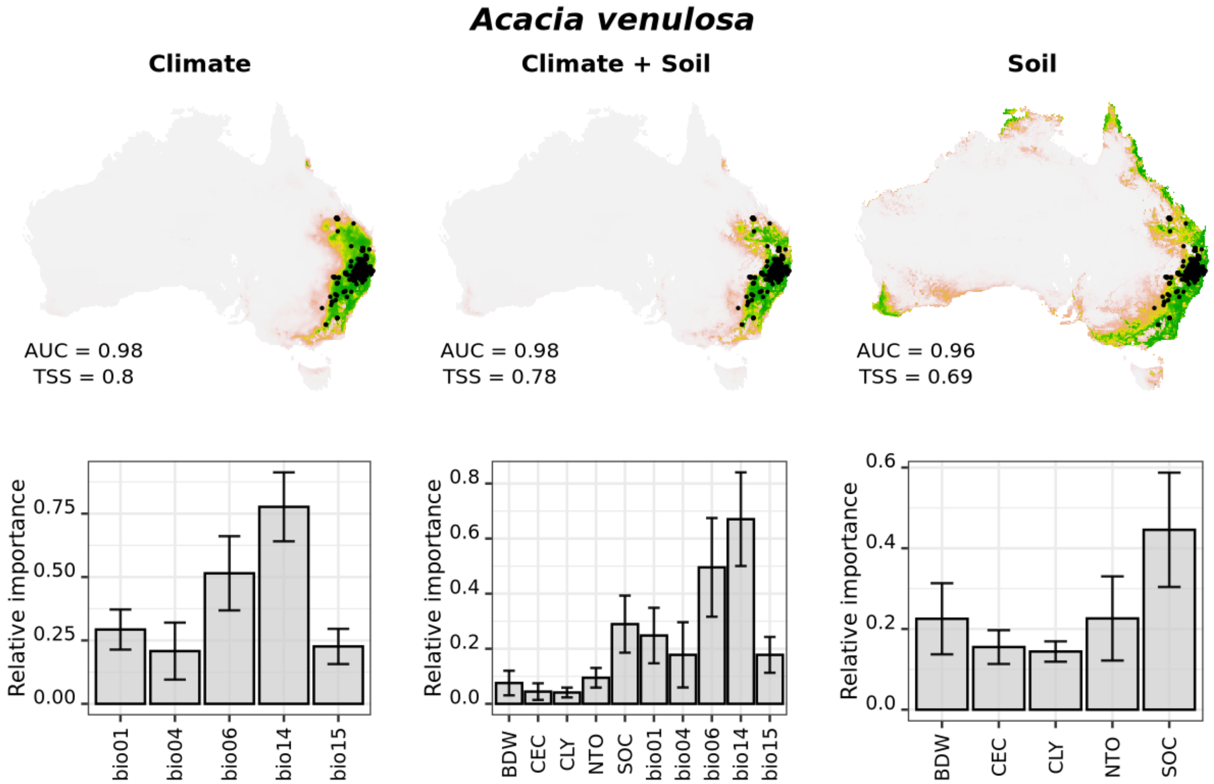
Acacia venulosa
Climate
Climate + Soil
Soil
AUC = 0.98
TSS = 0.8
AUC = 0.98
TSS = 0.78
AUC = 0.96
TSS = 0.69
Relative importance
0.75
0.50
0.25
0.00
bio01
bio04
bio06
bio14
bio15
0.8
0.6
0.4
0.2
0.0
BDW
CEC
CLY
NTO
SOC

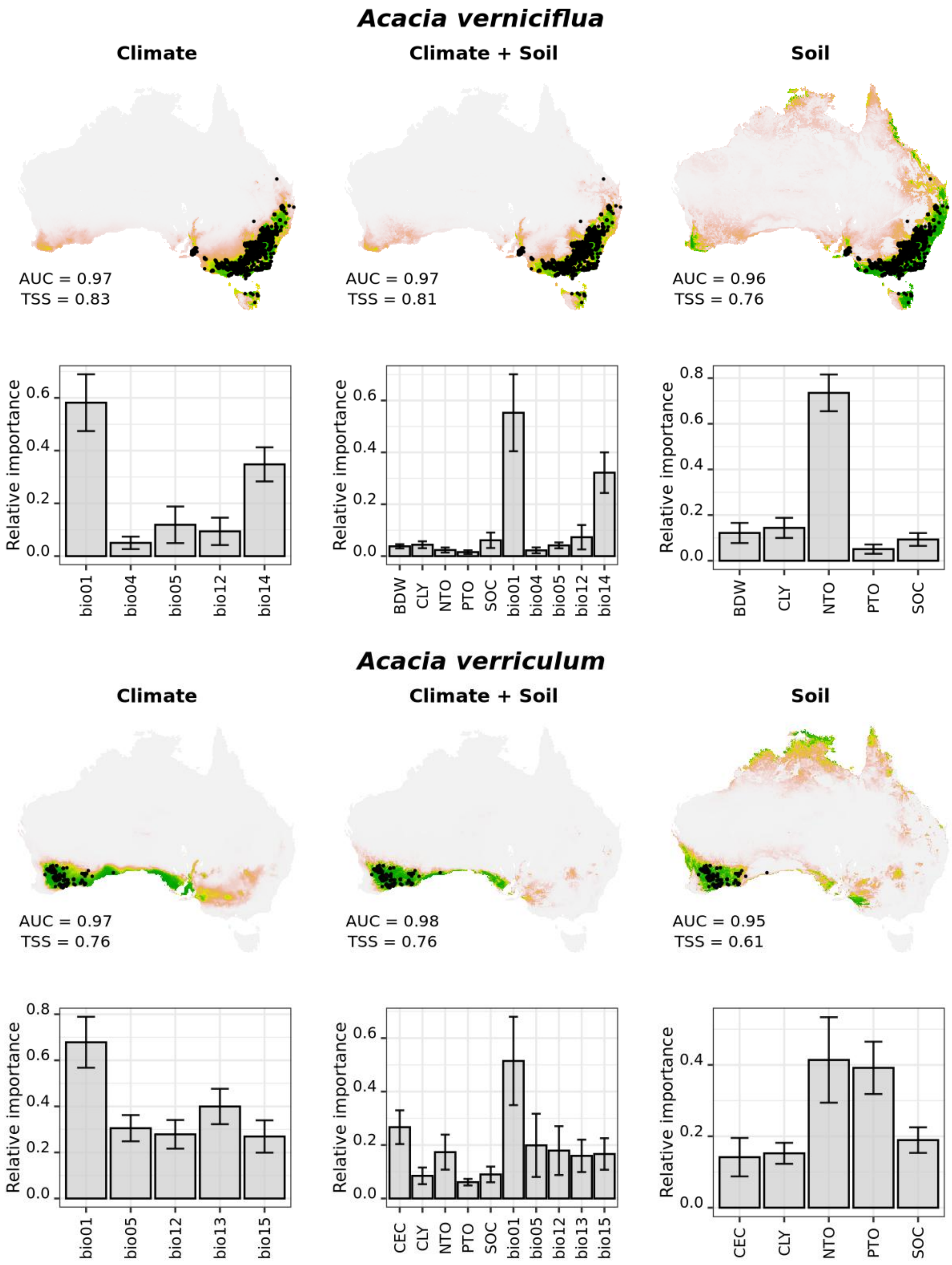
Acacia verniciflua
Climate
Climate + Soil
Soil
AUC = 0.97
TSS = 0.83
AUC = 0.97
TSS = 0.81
AUC = 0.96
TSS = 0.76
Relative importance
bio01 bio04 bio05 bio12 bio14
BDW CLY NTO PTO SOC bio01 bio04 bio05 bio12 bio14
BDW CLY NTO PTO SOC
Acacia verriculum
Climate
Climate + Soil
Soil
AUC = 0.97
TSS = 0.76
AUC = 0.98
TSS = 0.76
AUC = 0.95
TSS = 0.61
Relative importance
bio01 bio05 bio12 bio13 bio15
CEC CLY NTO PTO SOC bio01 bio05 bio12 bio13 bio15
CEC CLY NTO PTO SOC

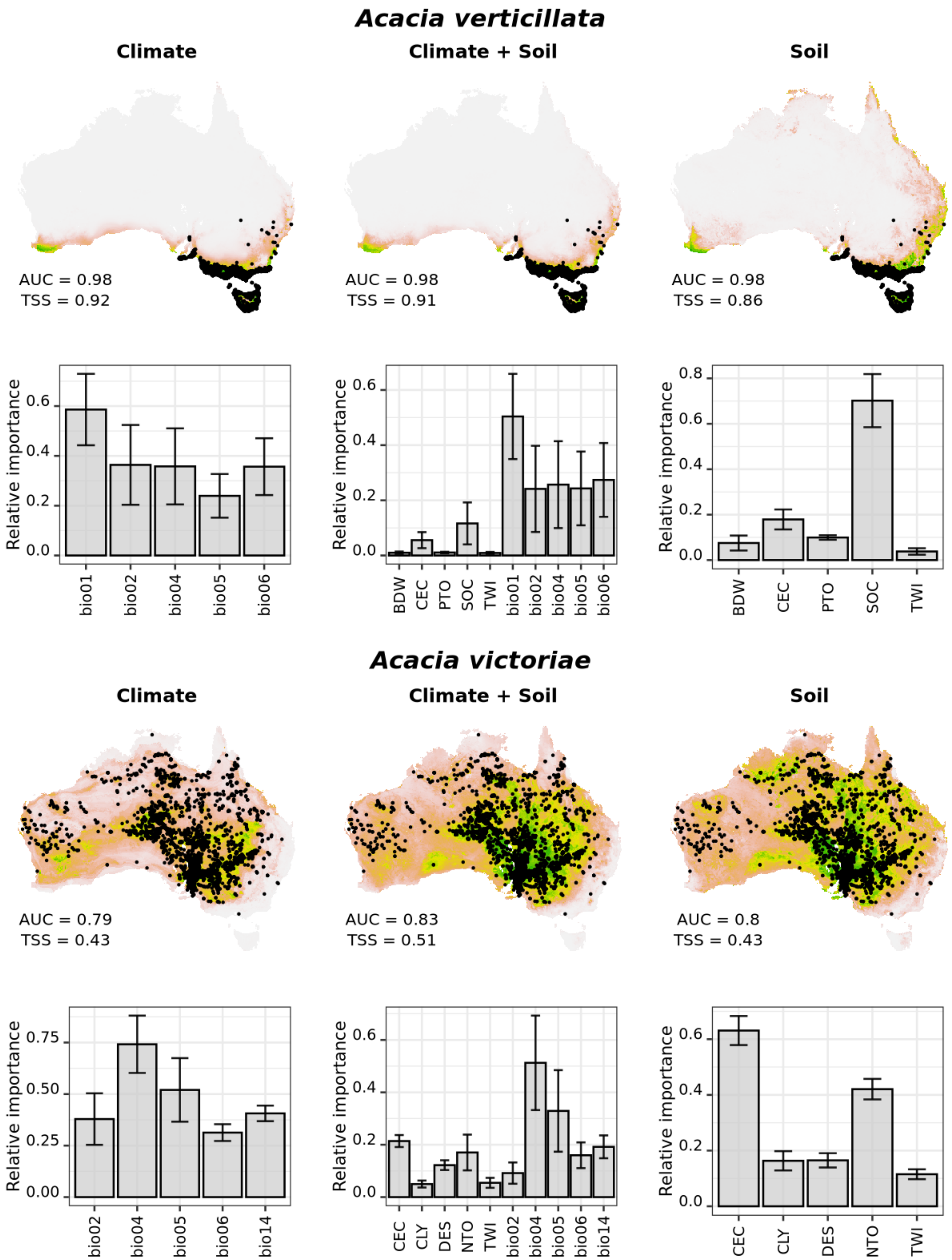
Acacia verticillata
Climate
Climate + Soil
Soil
AUC = 0.98
TSS = 0.92
AUC = 0.98
TSS = 0.91
AUC = 0.98
TSS = 0.86
Relative importance
0.0
0.2
0.4
0.6
0.8
bio01
bio02
bio04
bio05
bio06
BDW
CEC
PTO
SOC
TWI
Acacia victoriae
Climate
Climate + Soil
Soil
AUC = 0.79
TSS = 0.43
AUC = 0.83
TSS = 0.51
AUC = 0.8
TSS = 0.43
Relative importance
0.00
0.25
0.50
0.75
bio02
bio04
bio05
bio06
bio14
CEC
CLY
DES
NTO
TWI

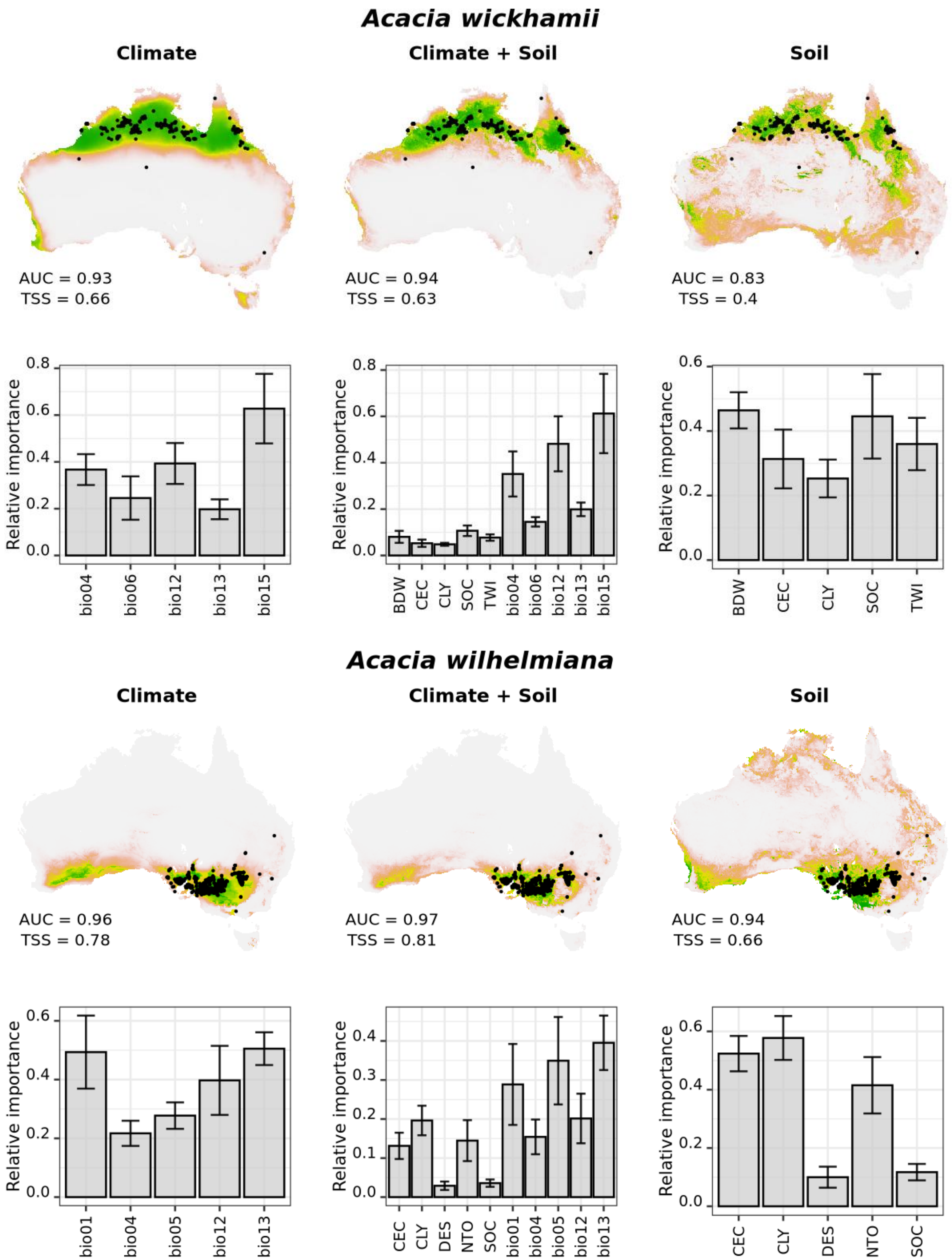
Acacia wickhamii
Climate
Climate + Soil
Soil
AUC = 0.93
TSS = 0.66
AUC = 0.94
TSS = 0.63
AUC = 0.83
TSS = 0.4
Relative importance
bio04
bio06
bio12
bio13
bio15
BDW
CEC
CLY
SOC
TWI
Acacia wilhelmiana
Climate
Climate + Soil
Soil
AUC = 0.96
TSS = 0.78
AUC = 0.97
TSS = 0.81
AUC = 0.94
TSS = 0.66
Relative importance
bio01
bio04
bio05
bio12
bio13
CEC
CLY
DES
NTO
SOC

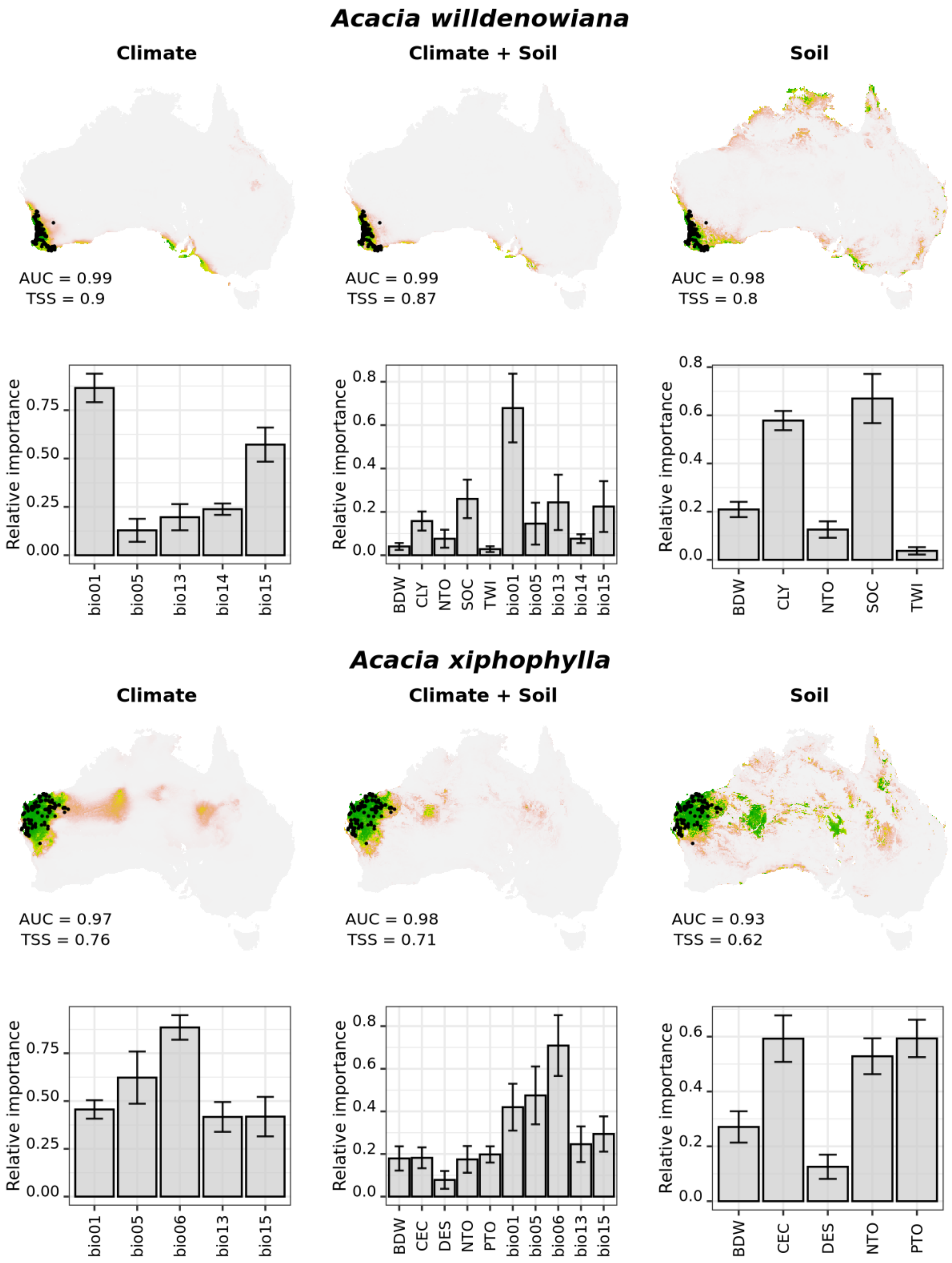
Acacia willdenowiana
Climate
Climate + Soil
Soil
AUC = 0.99
TSS = 0.9
AUC = 0.99
TSS = 0.87
AUC = 0.98
TSS = 0.8
Relative importance
0.00
0.25
0.50
0.75
bio01
bio05
bio13
bio14
bio15
0.0
0.2
0.4
0.6
0.8
BDW
CLY
NTO
SOC
TWI
bio01
bio05
bio13
bio14
bio15
Acacia xiphophylla
Climate
Climate + Soil
Soil
AUC = 0.97
TSS = 0.76
AUC = 0.98
TSS = 0.71
AUC = 0.93
TSS = 0.62
bio01
bio05
bio06
bio13
bio15
BDW
CEC
DES
NTO
PTO
bio01
bio05
bio06
bio13
bio15

Acacia yirrkallensis

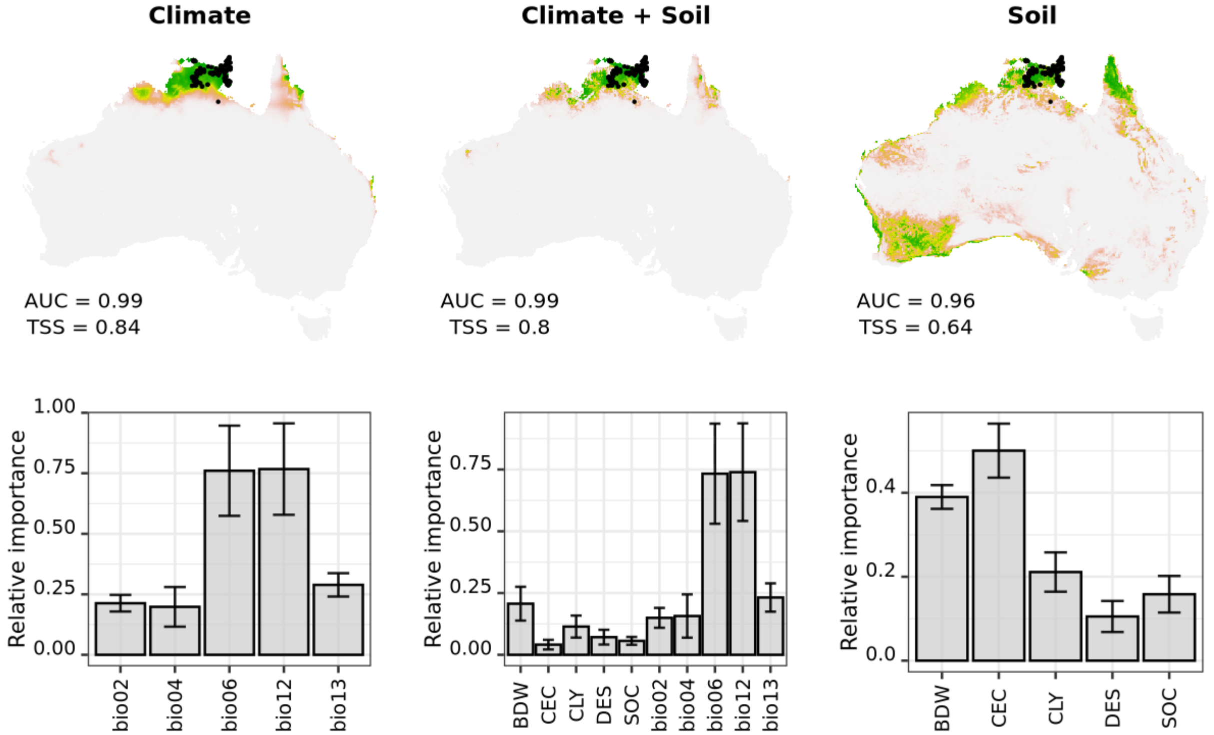

Acacia yorkrakinensis

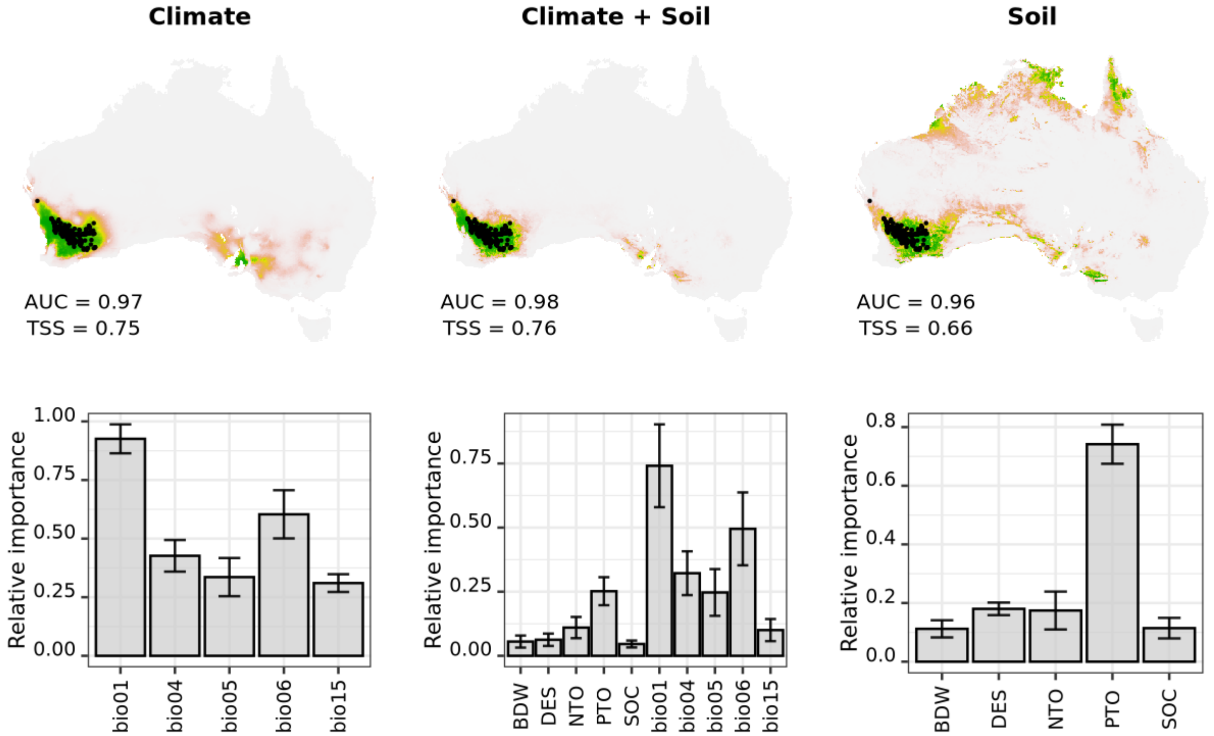

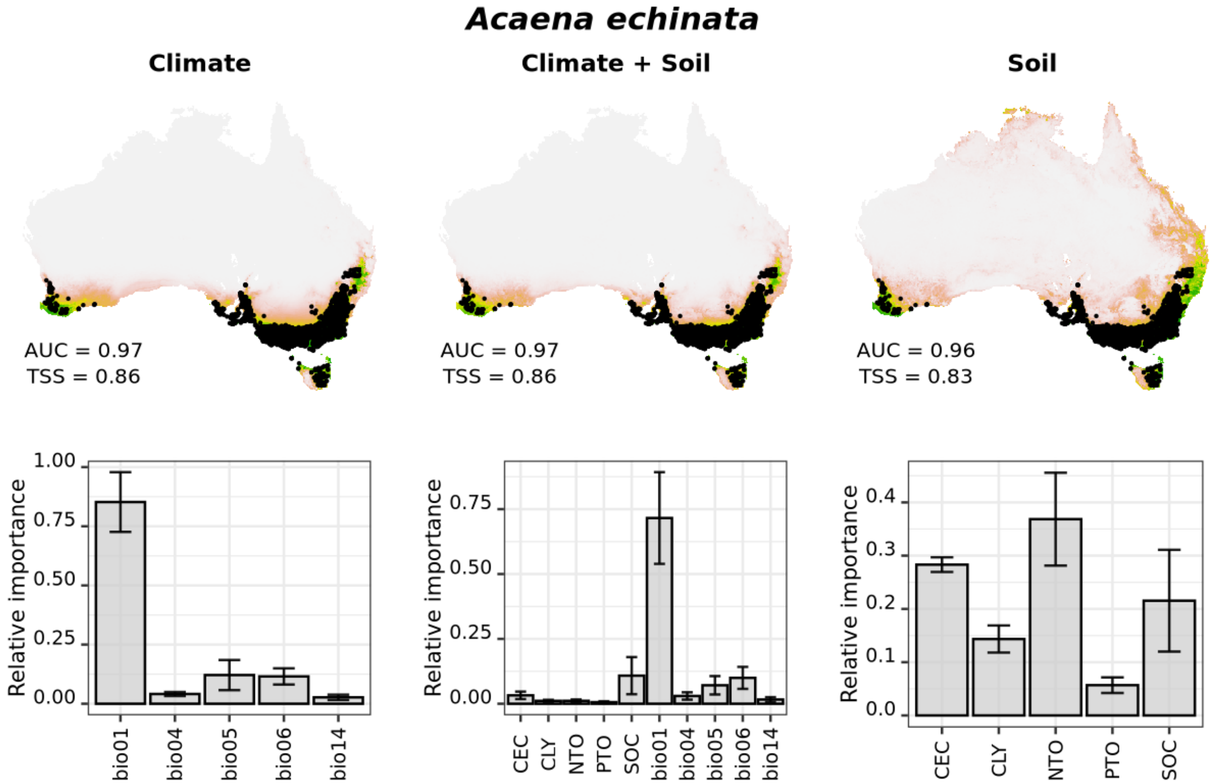
Acaena echinata
Climate
Climate + Soil
Soil
AUC = 0.97
TSS = 0.86
AUC = 0.97
TSS = 0.86
AUC = 0.96
TSS = 0.83
Relative importance
1.00
0.75
0.50
0.25
0.00
bio01
bio04
bio05
bio06
bio14
CEC
CLY
NTO
PTO
SOC
0.4
0.3
0.2
0.1
0.0

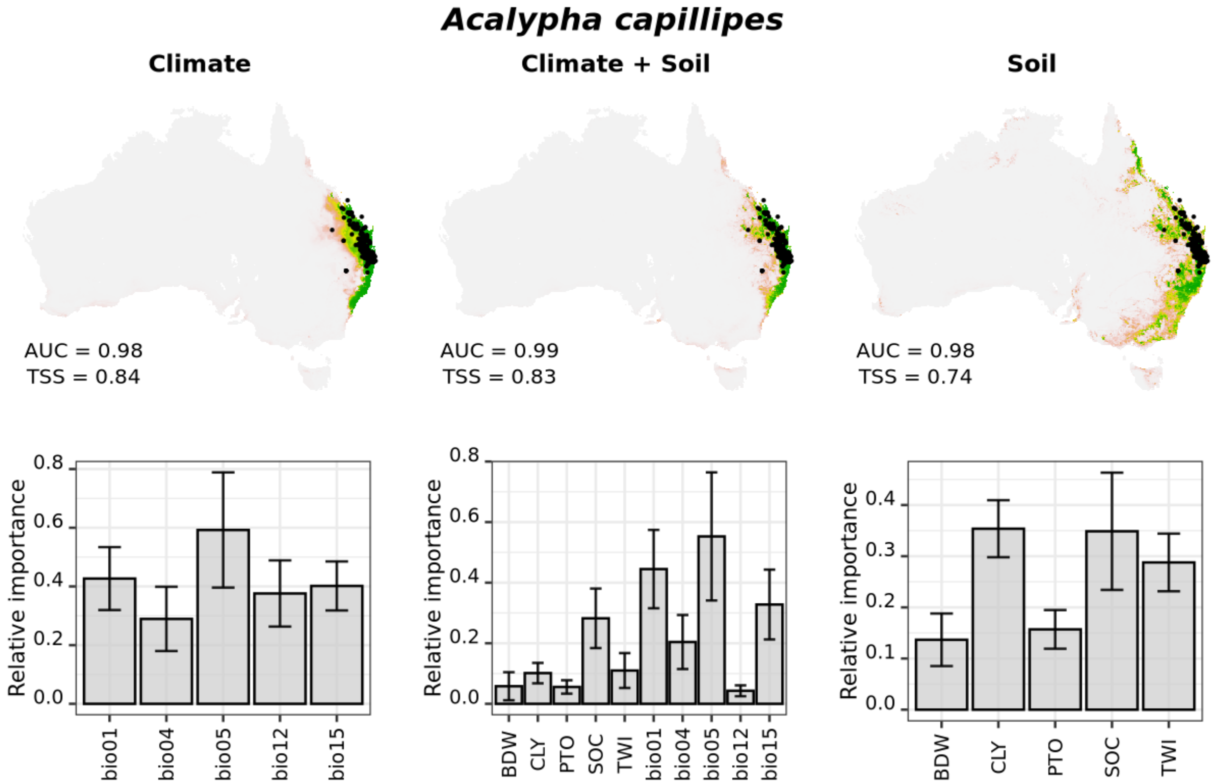
Acalypha capillipes
Climate
Climate + Soil
Soil
AUC = 0.98
TSS = 0.84
AUC = 0.99
TSS = 0.83
AUC = 0.98
TSS = 0.74
Relative importance
0.8
0.6
0.4
0.2
0.0
bio01
bio04
bio05
bio12
bio15
BDW
CLY
PTO
SOC
TWI

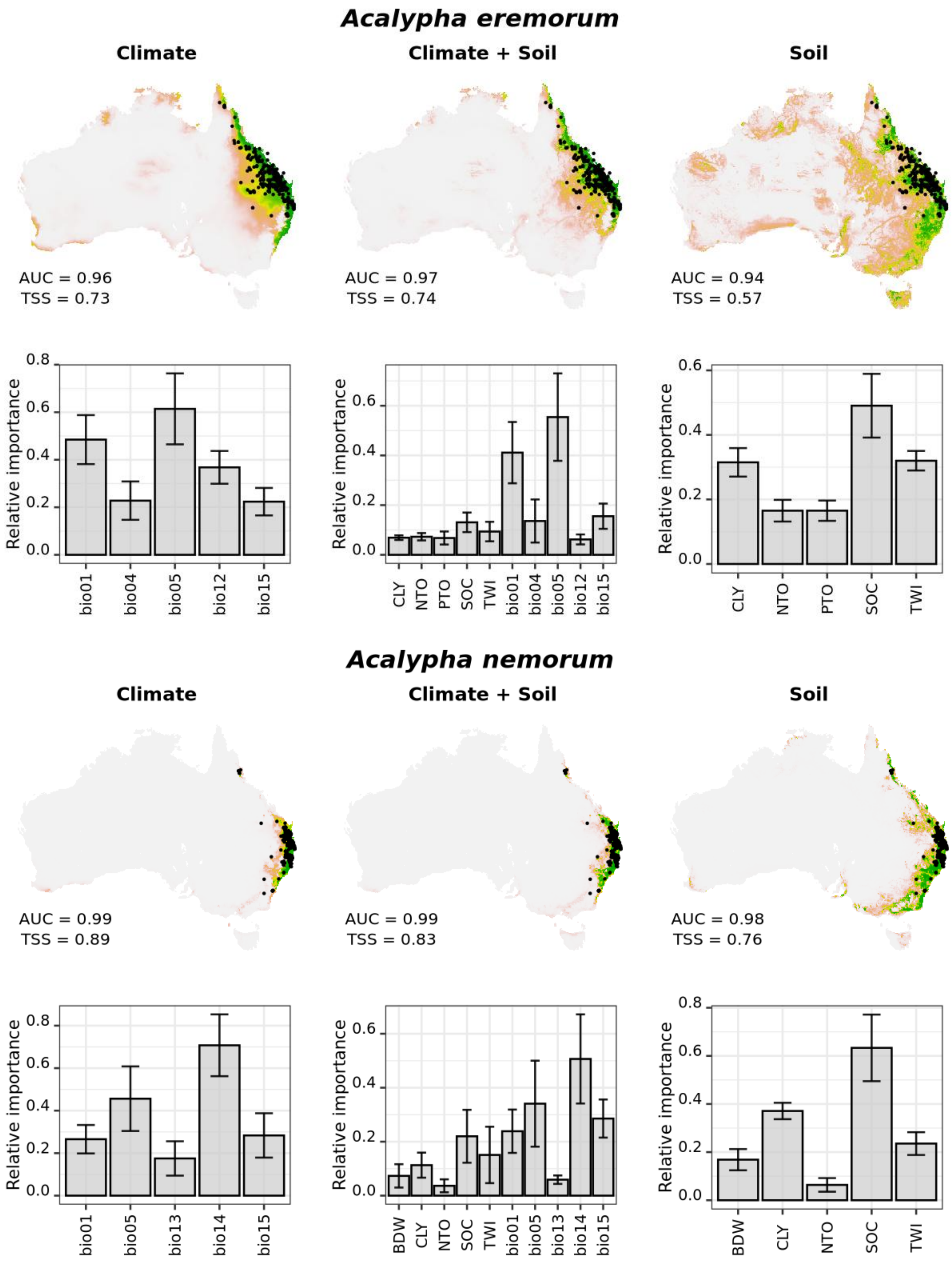
Acalypha eremorum
Climate
Climate + Soil
Soil
AUC = 0.96
TSS = 0.73
AUC = 0.97
TSS = 0.74
AUC = 0.94
TSS = 0.57
Relative importance
bio01
bio04
bio05
bio12
bio15
CLY
NTO
PTO
SOC
TWI
Acalypha nemorum
Climate
Climate + Soil
Soil
AUC = 0.99
TSS = 0.89
AUC = 0.99
TSS = 0.83
AUC = 0.98
TSS = 0.76
Relative importance
bio01
bio05
bio13
bio14
bio15
BDW
CLY
NTO
SOC
TWI

Acanthocarpus preissii

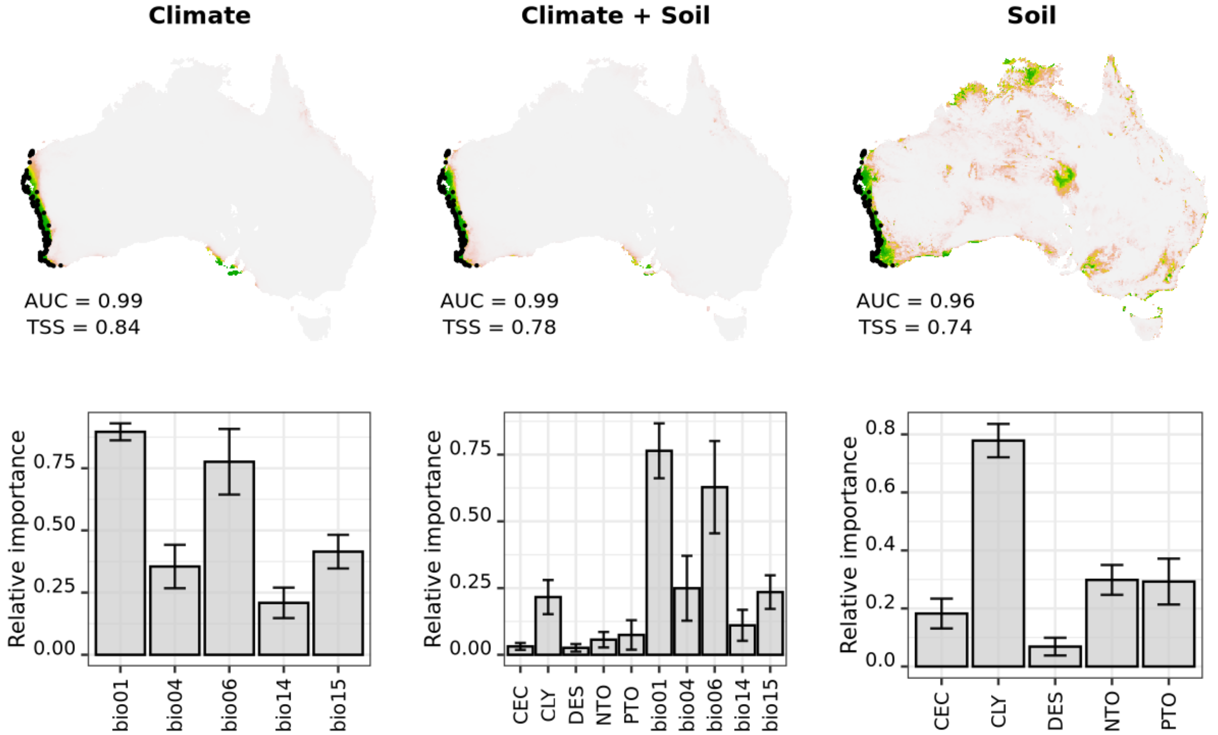

Acanthus ilicifolius

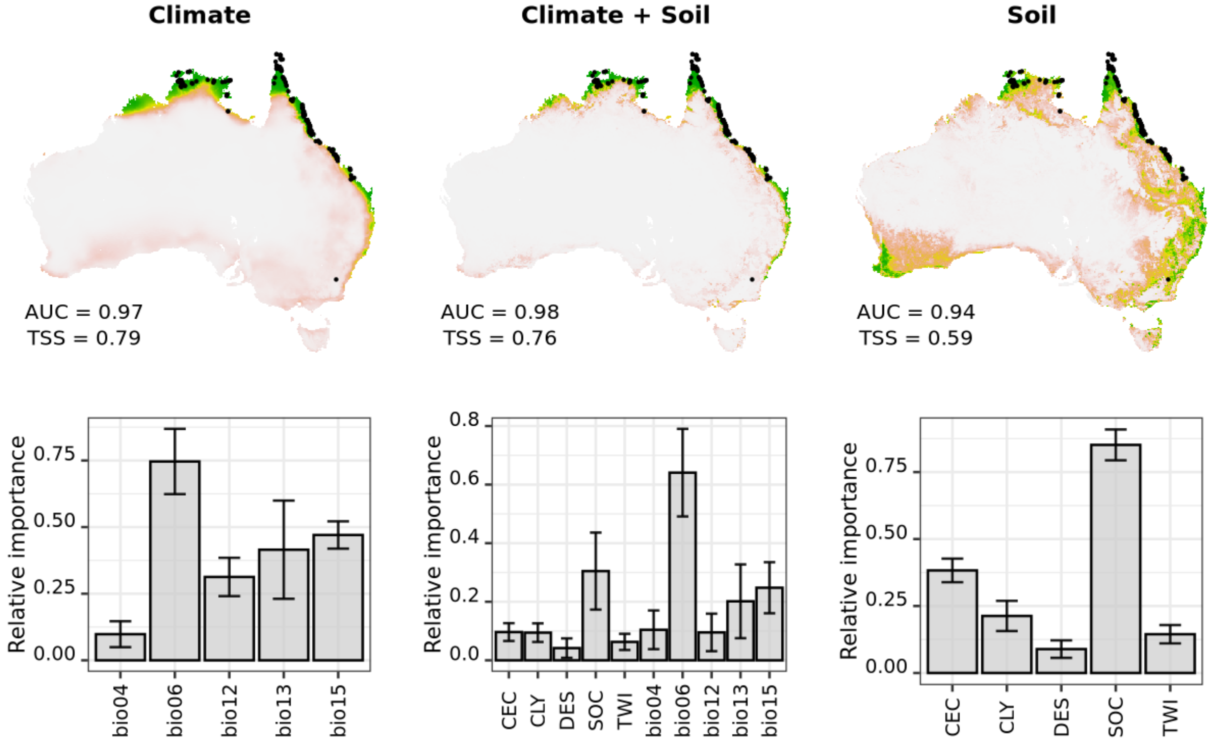

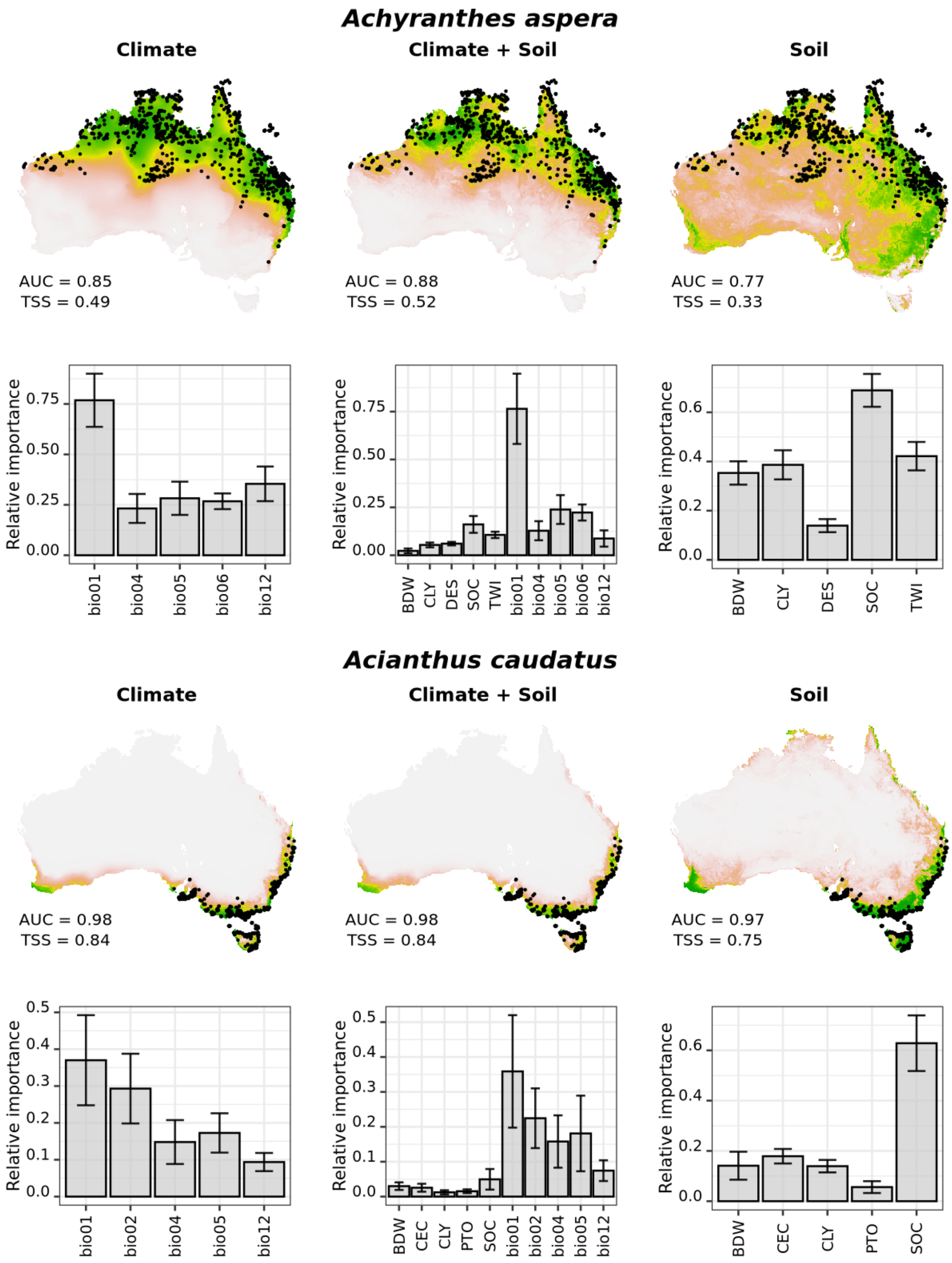
Achyranthes aspera
Climate
Climate + Soil
Soil
AUC = 0.85
TSS = 0.49
AUC = 0.88
TSS = 0.52
AUC = 0.77
TSS = 0.33
Relative importance
0.75
0.50
0.25
0.00
bio01
bio04
bio05
bio06
bio12
BDW
CLY
DES
SOC
TWI
0.6
0.4
0.2
0.0
Acianthus caudatus
Climate
Climate + Soil
Soil
AUC = 0.98
TSS = 0.84
AUC = 0.98
TSS = 0.84
AUC = 0.97
TSS = 0.75
Relative importance
0.5
0.4
0.3
0.2
0.1
0.0
bio01
bio02
bio04
bio05
bio12
BDW
CEC
CLY
PTO
SOC
0.6
0.4
0.2
0.0

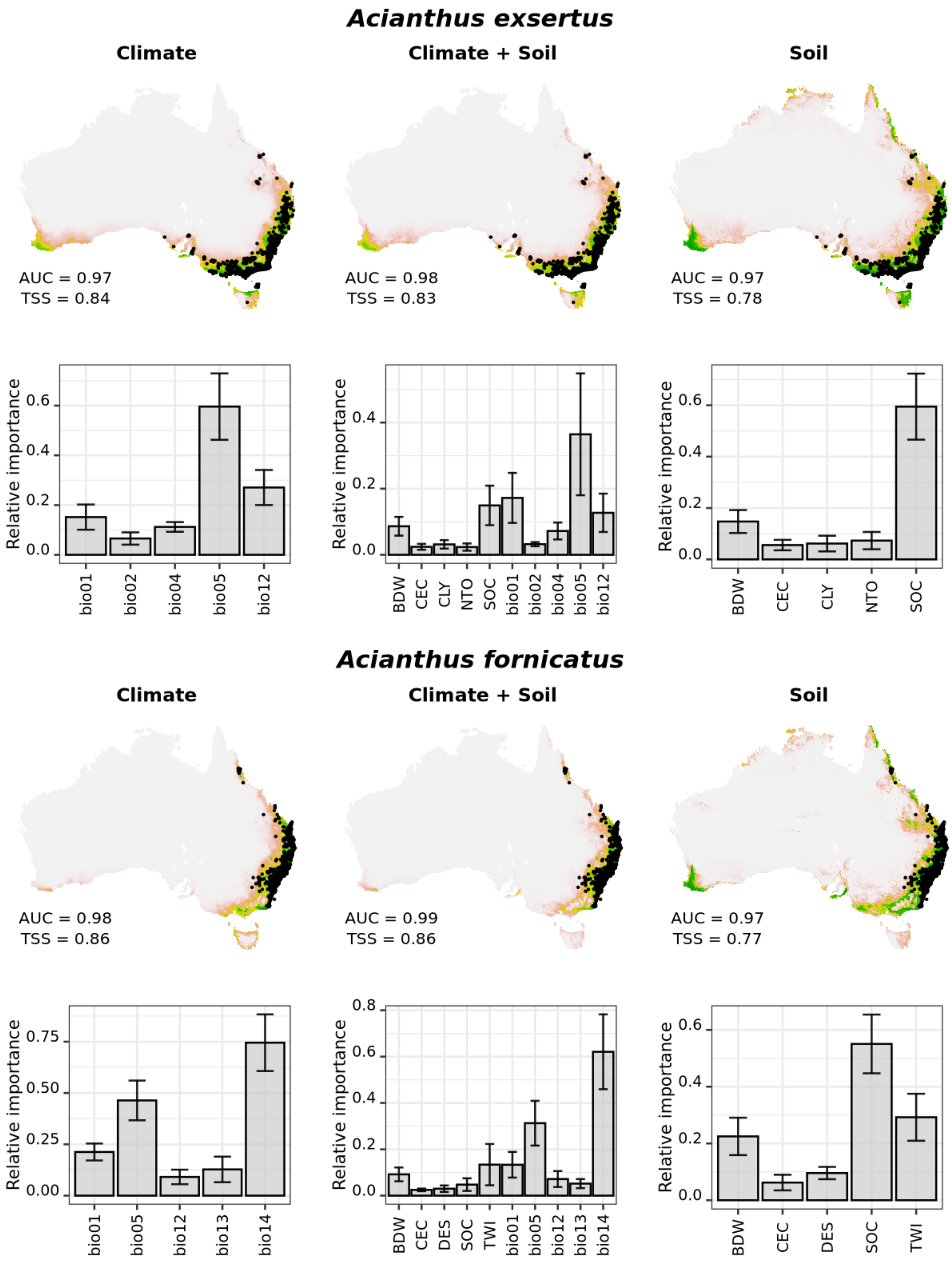
Acianthus exsertus
Climate
Climate + Soil
Soil
AUC = 0.97
TSS = 0.84
AUC = 0.98
TSS = 0.83
AUC = 0.97
TSS = 0.78
Relative importance
bio01
bio02
bio04
bio05
bio12
BDW
CEC
CLY
NTO
SOC
Acianthus fornicatus
Climate
Climate + Soil
Soil
AUC = 0.98
TSS = 0.86
AUC = 0.99
TSS = 0.86
AUC = 0.97
TSS = 0.77
Relative importance
bio01
bio05
bio12
bio13
bio14
BDW
CEC
DES
SOC
TWI

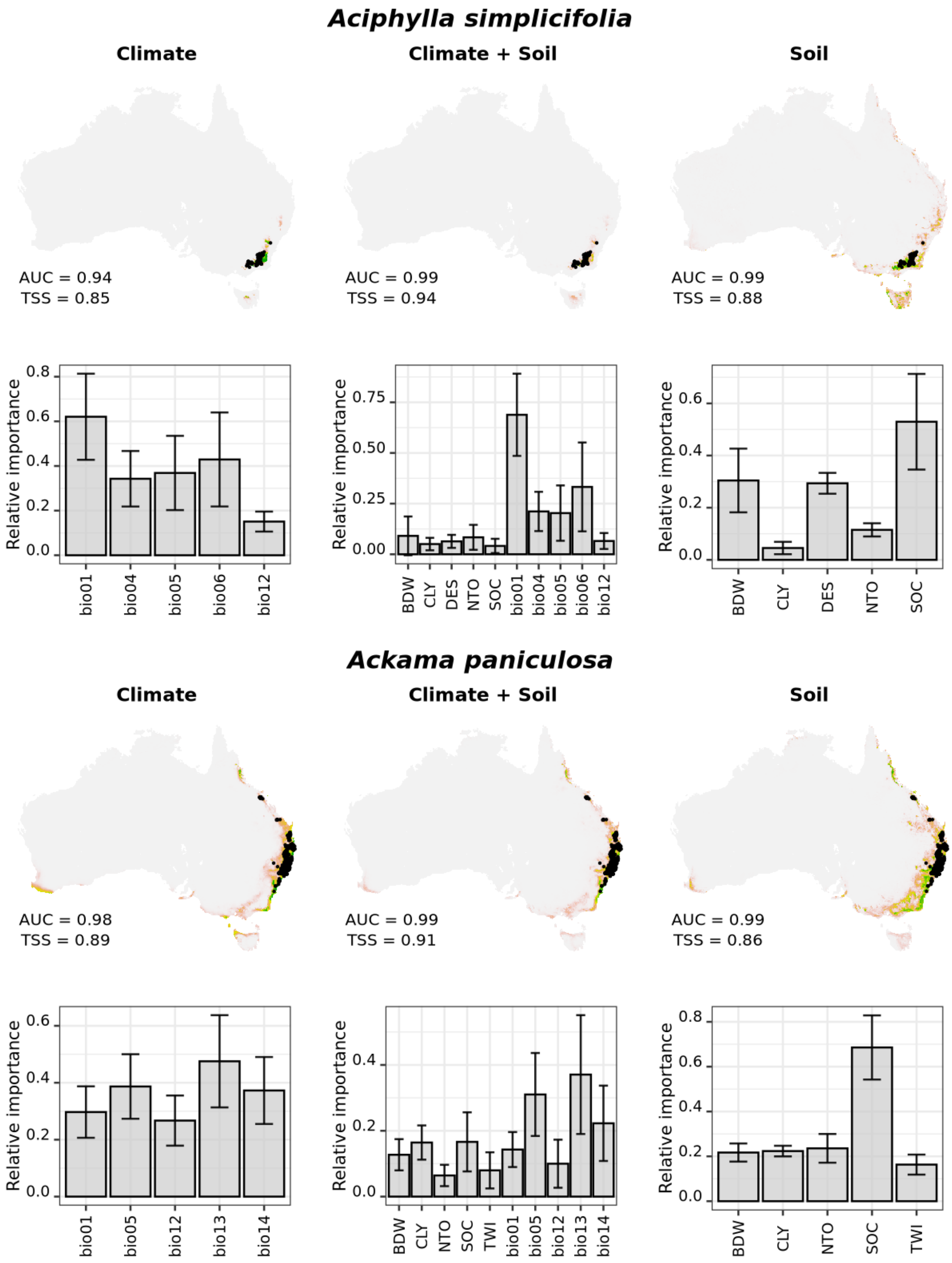

Aciphylla simplicifolia
Climate
Climate + Soil
Soil
AUC = 0.94
TSS = 0.85
AUC = 0.99
TSS = 0.94
AUC = 0.99
TSS = 0.88
Relative importance
0.0
0.2
0.4
0.6
0.8
bio01
bio04
bio05
bio06
bio12
Relative importance
0.00
0.25
0.50
0.75
BDW
CLY
DES
NTO
SOC
bio01
bio04
bio05
bio06
bio12
Relative importance
0.0
0.2
0.4
0.6
BDW
CLY
DES
NTO
SOC
Ackama paniculosa
Climate
Climate + Soil
Soil
AUC = 0.98
TSS = 0.89
AUC = 0.99
TSS = 0.91
AUC = 0.99
TSS = 0.86
Relative importance
0.0
0.2
0.4
0.6
bio01
bio05
bio12
bio13
bio14
Relative importance
0.0
0.2
0.4
BDW
CLY
NTO
SOC
TWI
bio01
bio05
bio12
bio13
bio14
Relative importance
0.0
0.2
0.4
0.6
0.8
BDW
CLY
NTO
SOC
TWI

Acmella grandiflora

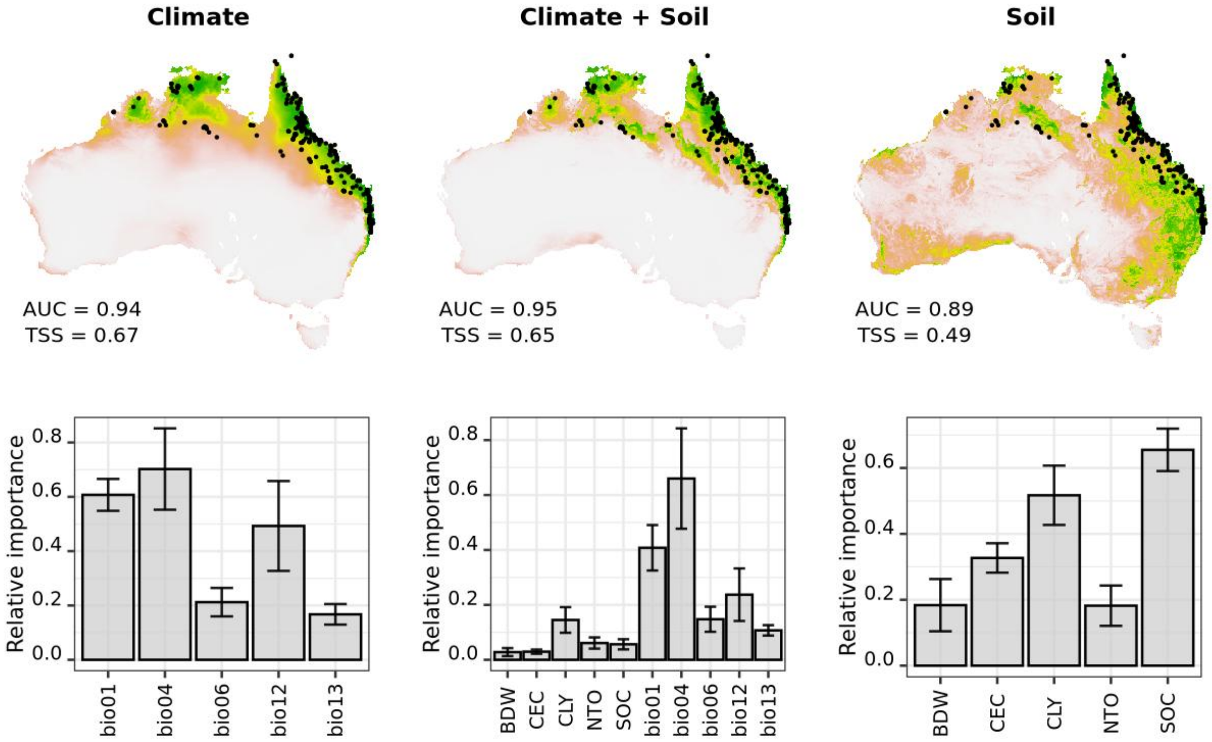

Acradenia euodiiformis

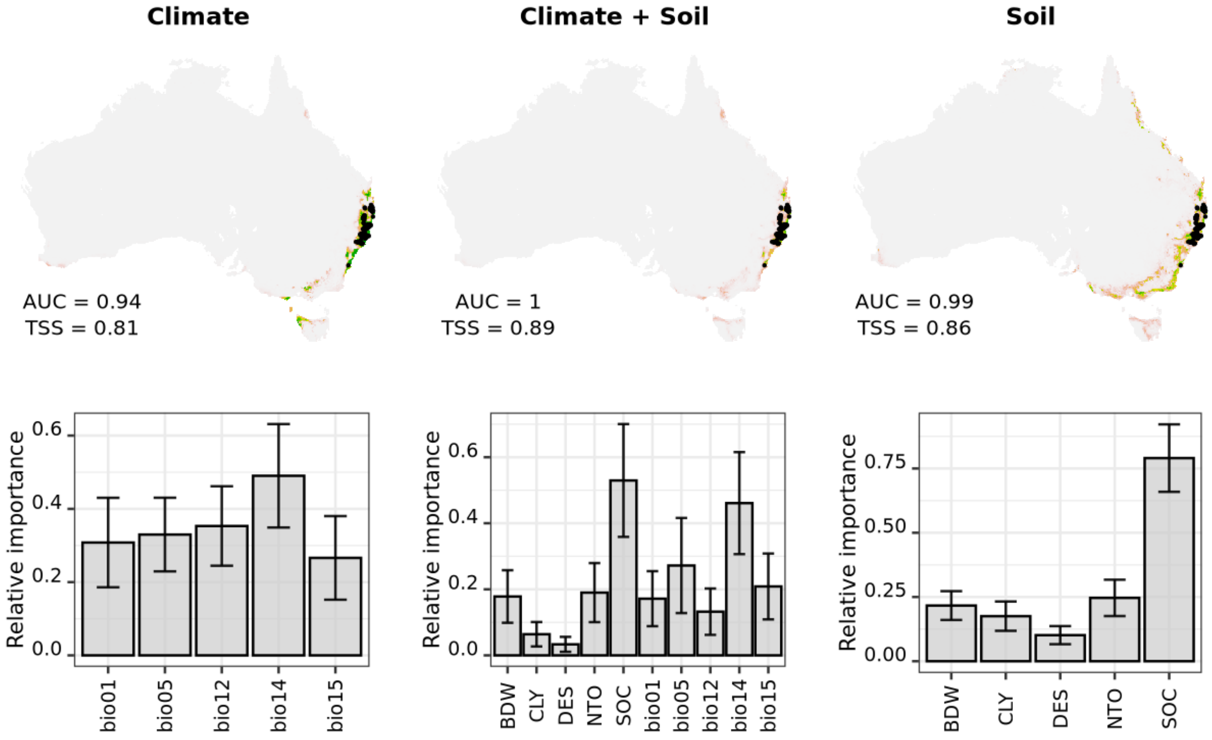

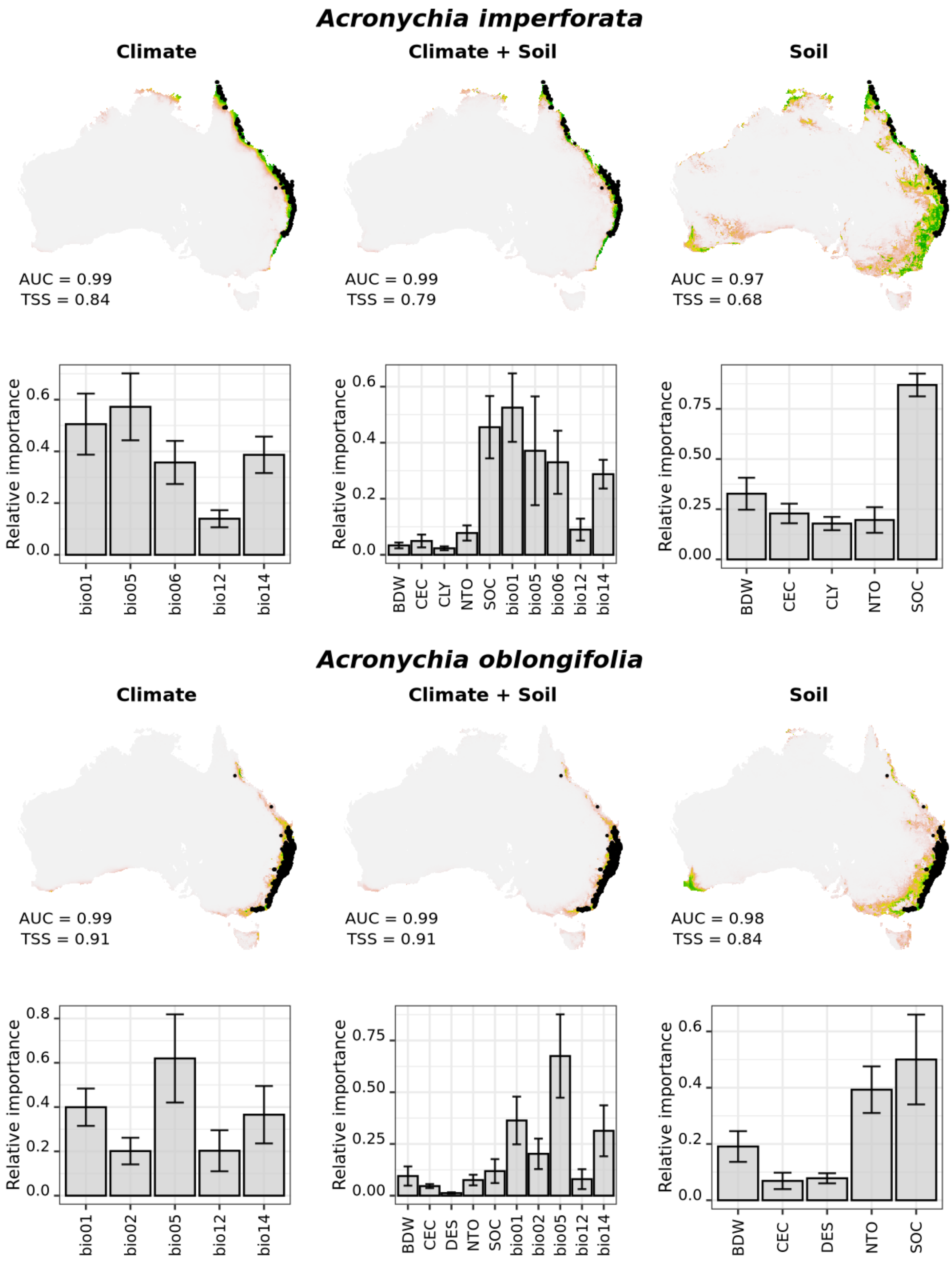
Acronychia imperforata
Climate
Climate + Soil
Soil
AUC = 0.99
TSS = 0.84
AUC = 0.99
TSS = 0.79
AUC = 0.97
TSS = 0.68
Relative importance
bio01
bio05
bio06
bio12
bio14
BDW
CEC
CLY
NTO
SOC
Acronychia oblongifolia
Climate
Climate + Soil
Soil
AUC = 0.99
TSS = 0.91
AUC = 0.99
TSS = 0.91
AUC = 0.98
TSS = 0.84
Relative importance
bio01
bio02
bio05
bio12
bio14
BDW
CEC
DES
NTO
SOC

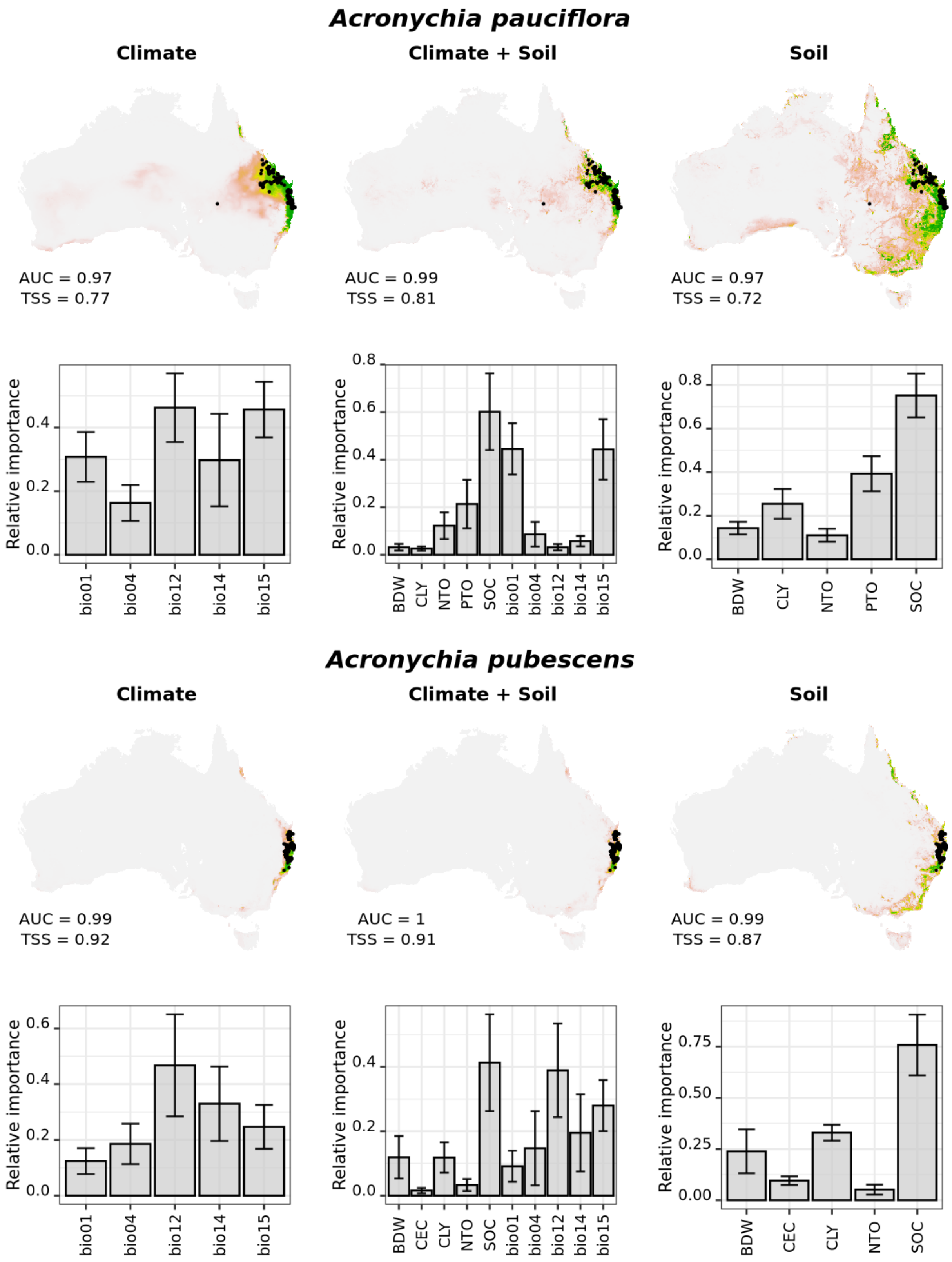

Acronychia pauciflora
Climate
Climate + Soil
Soil
AUC = 0.97
TSS = 0.77
AUC = 0.99
TSS = 0.81
AUC = 0.97
TSS = 0.72
Relative importance
bio01
bio04
bio12
bio14
bio15
BDW
CLY
NTO
PTO
SOC
Acronychia pubescens
Climate
Climate + Soil
Soil
AUC = 0.99
TSS = 0.92
AUC = 1
TSS = 0.91
AUC = 0.99
TSS = 0.87
Relative importance
CEC

Acronychia wilcoxiana

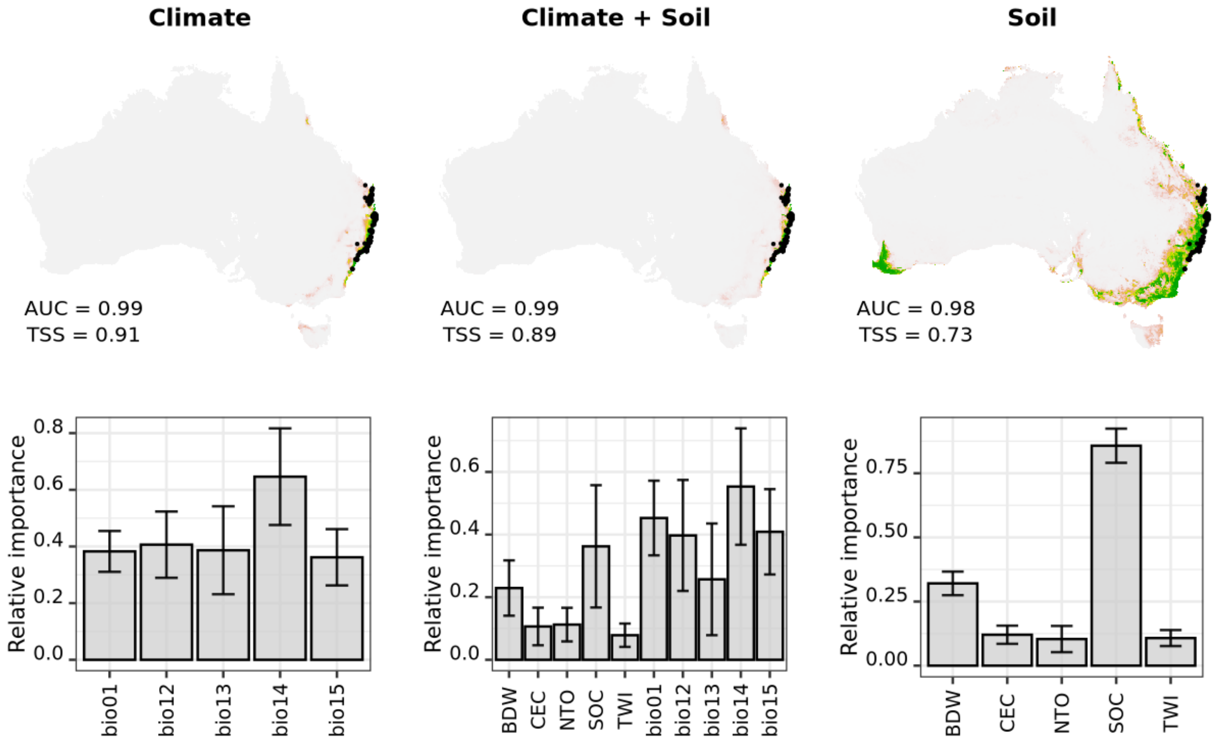

Acrothamnus hookeri

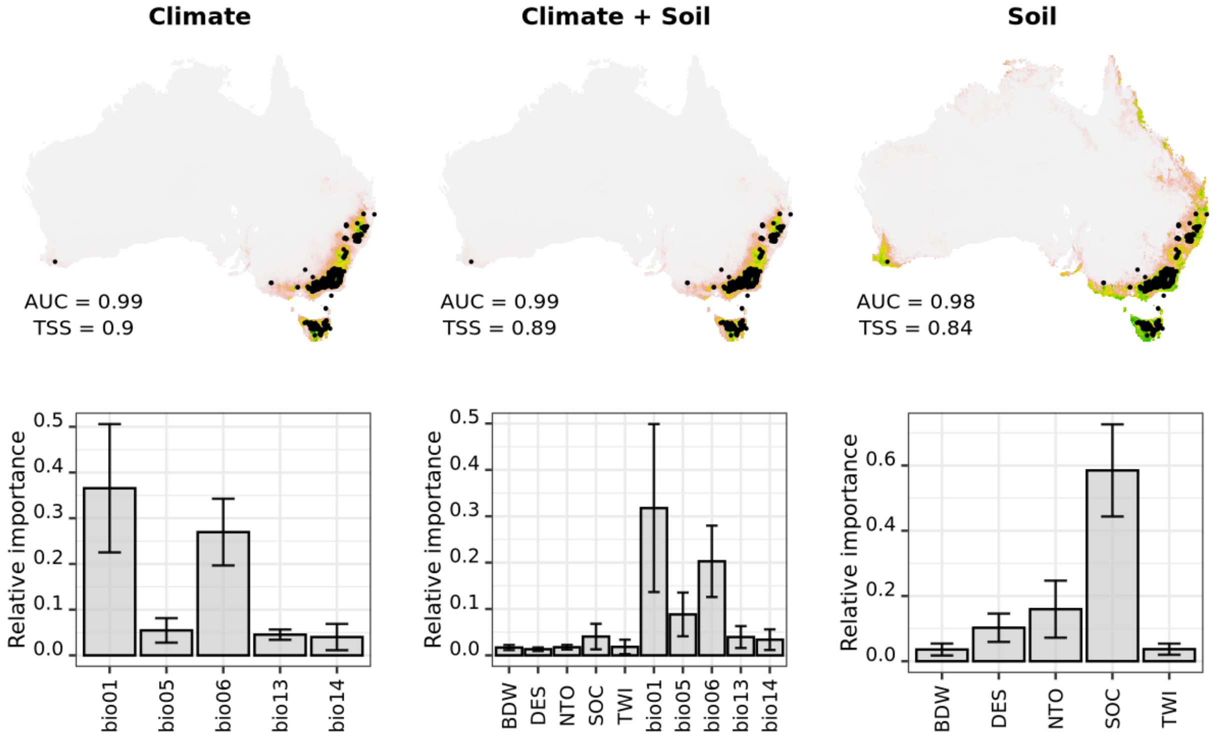

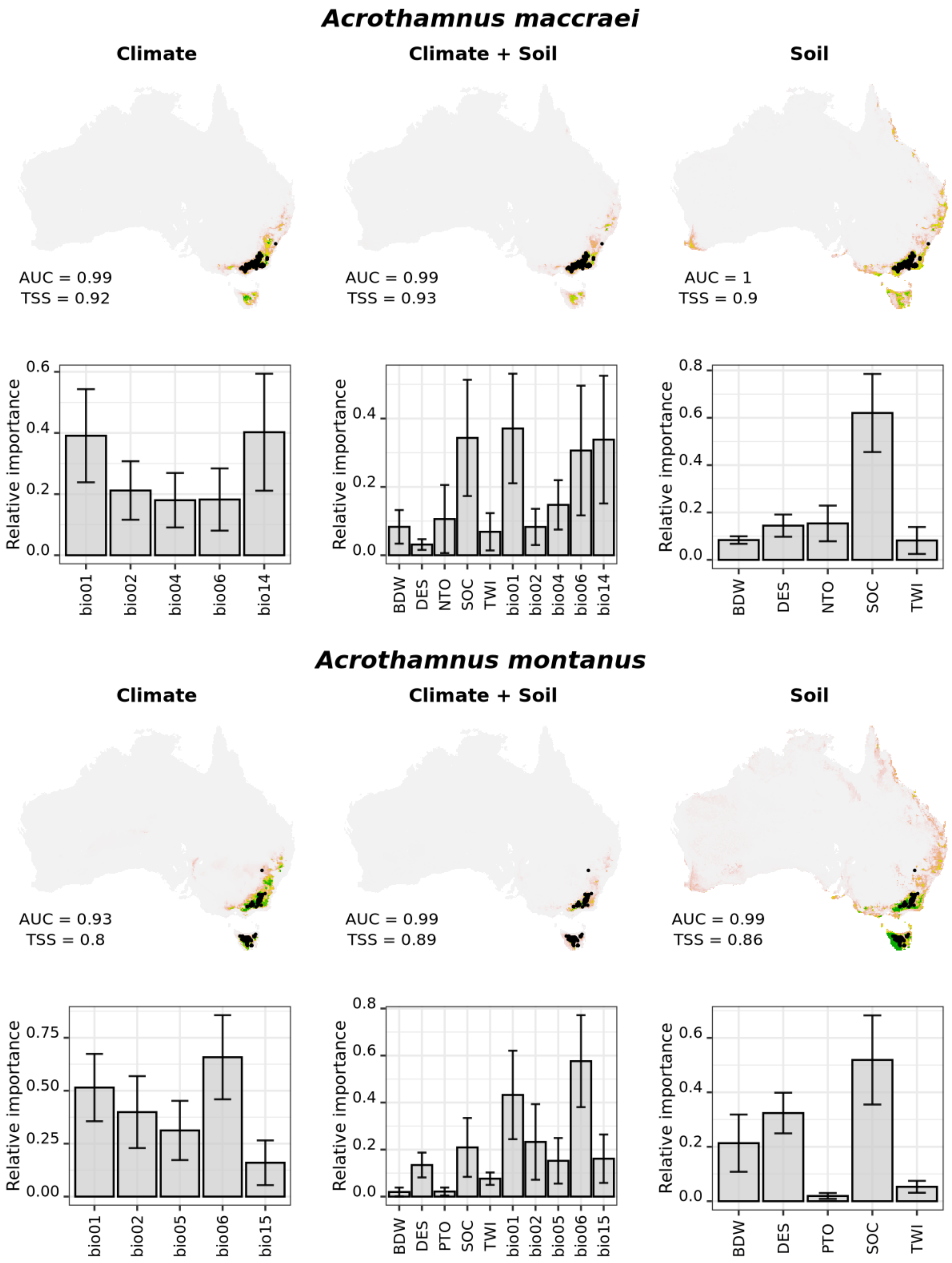

Acrothamnus maccraei
Climate
Climate + Soil
Soil
AUC = 0.99
TSS = 0.92
AUC = 0.99
TSS = 0.93
AUC = 1
TSS = 0.9
Relative importance
bio01
bio02
bio04
bio06
bio14
BDW
DES
NTO
SOC
TWI
Acrothamnus montanus
Climate
Climate + Soil
Soil
AUC = 0.93
TSS = 0.8
AUC = 0.99
TSS = 0.89
AUC = 0.99
TSS = 0.86
Relative importance
bio01
bio02
bio05
bio06
bio15
BDW
DES
PTO
SOC
TWI

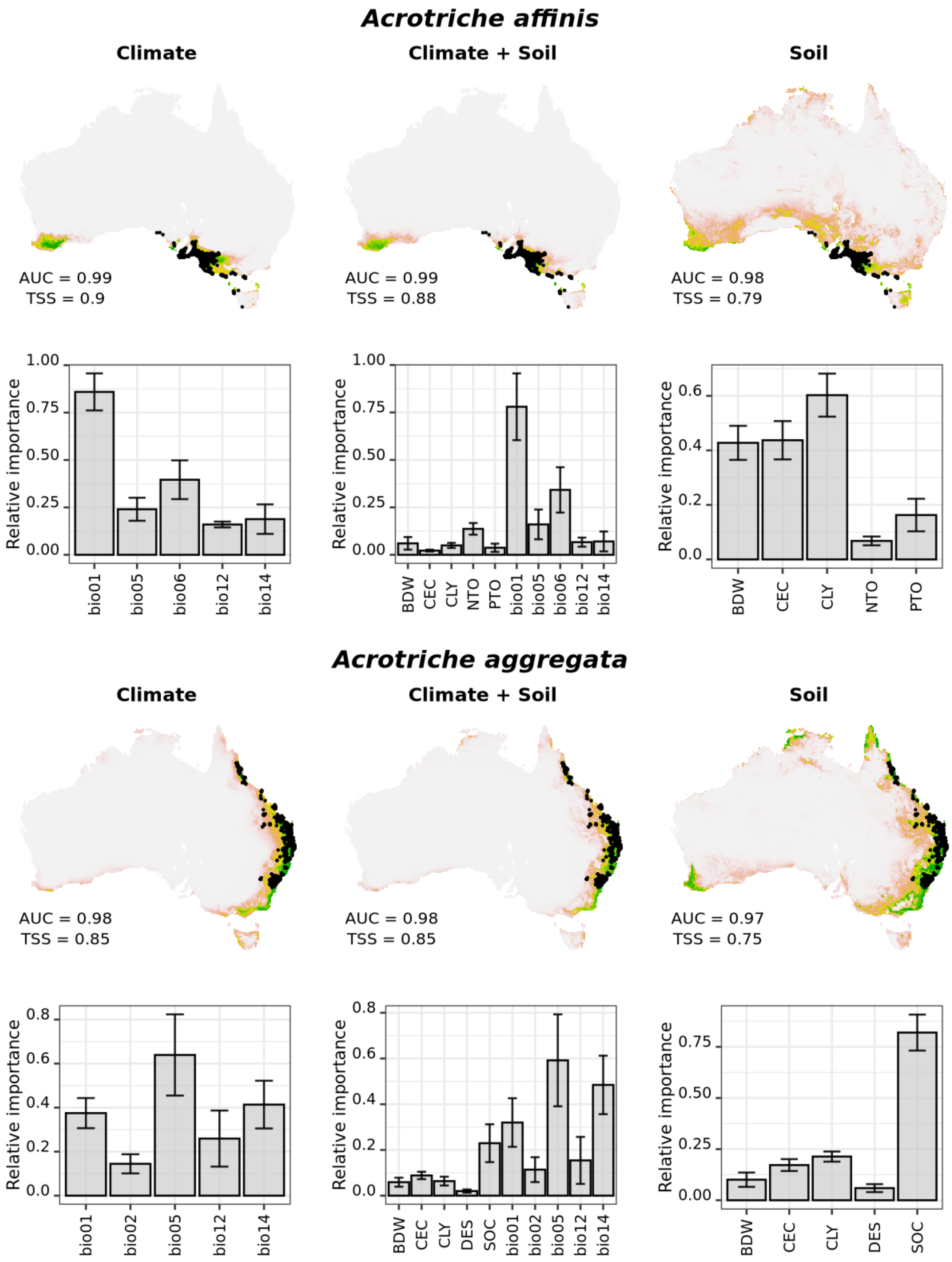

Acrotriche affinis
Climate
Climate + Soil
Soil
AUC = 0.99
TSS = 0.9
AUC = 0.99
TSS = 0.88
AUC = 0.98
TSS = 0.79
Relative importance
bio01
bio05
bio06
bio12
bio14
BDW
CEC
CLY
NTO
PTO
Acrotriche aggregata
AUC = 0.98
TSS = 0.85
AUC = 0.98
TSS = 0.85
AUC = 0.97
TSS = 0.75
bio02
DES
SOC

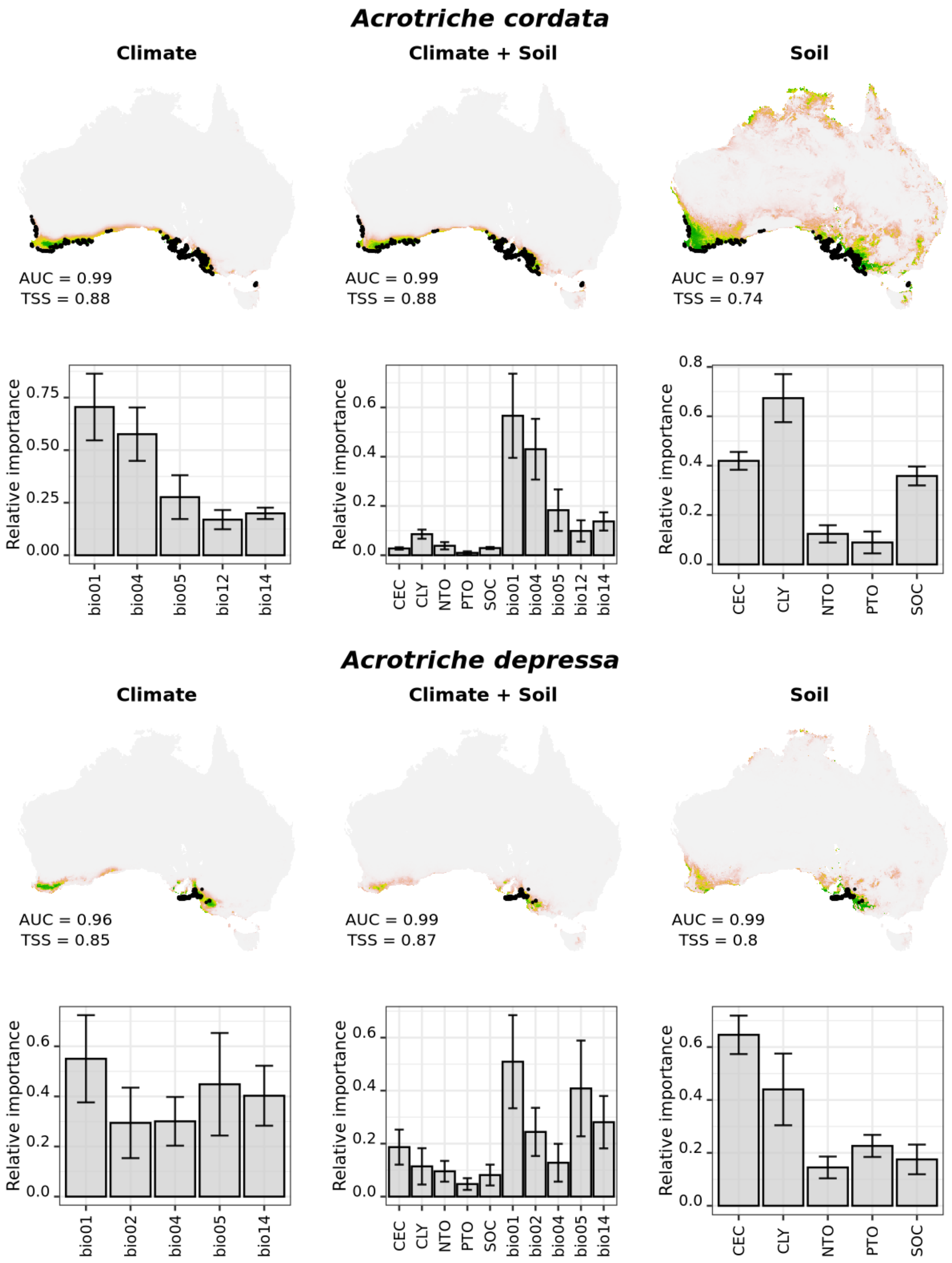
Acrotriche cordata
Climate
Climate + Soil
Soil
AUC = 0.99
TSS = 0.88
AUC = 0.99
TSS = 0.88
AUC = 0.97
TSS = 0.74
Relative importance
bio01
bio04
bio05
bio12
bio14
CEC
CLY
NTO
PTO
SOC
Acrotriche depressa
Climate
Climate + Soil
Soil
AUC = 0.96
TSS = 0.85
AUC = 0.99
TSS = 0.87
AUC = 0.99
TSS = 0.8
Relative importance
bio01
bio02
bio04
bio05
bio14
CEC
CLY
NTO
PTO
SOC

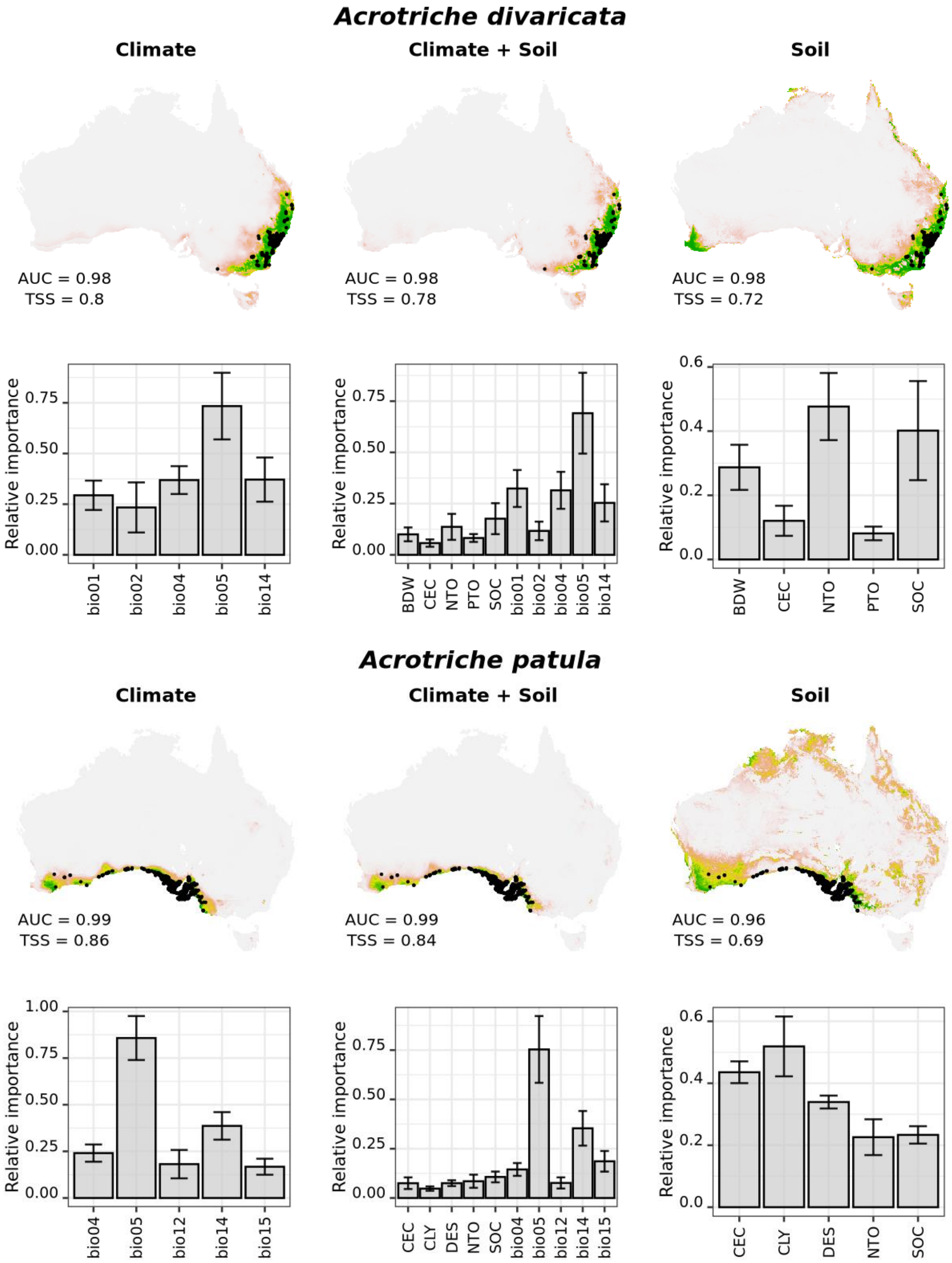

Acrotriche divaricata
Climate
Climate + Soil
Soil
AUC = 0.98
TSS = 0.8
AUC = 0.98
TSS = 0.78
AUC = 0.98
TSS = 0.72
Relative importance
0.00
0.25
0.50
0.75
bio01
bio02
bio04
bio05
bio14
BDW
CEC
NTO
PTO
SOC
bio01
bio02
bio04
bio05
bio14
0.0
0.2
0.4
0.6
BDW
CEC
NTO
PTO
SOC
Acrotriche patula
Climate
Climate + Soil
Soil
AUC = 0.99
TSS = 0.86
AUC = 0.99
TSS = 0.84
AUC = 0.96
TSS = 0.69
1.00
bio04
bio05
bio12
bio14
bio15
CEC
CLY
DES
NTO
SOC
bio04
bio05
bio12
bio14
bio15
CEC
CLY
DES
NTO
SOC

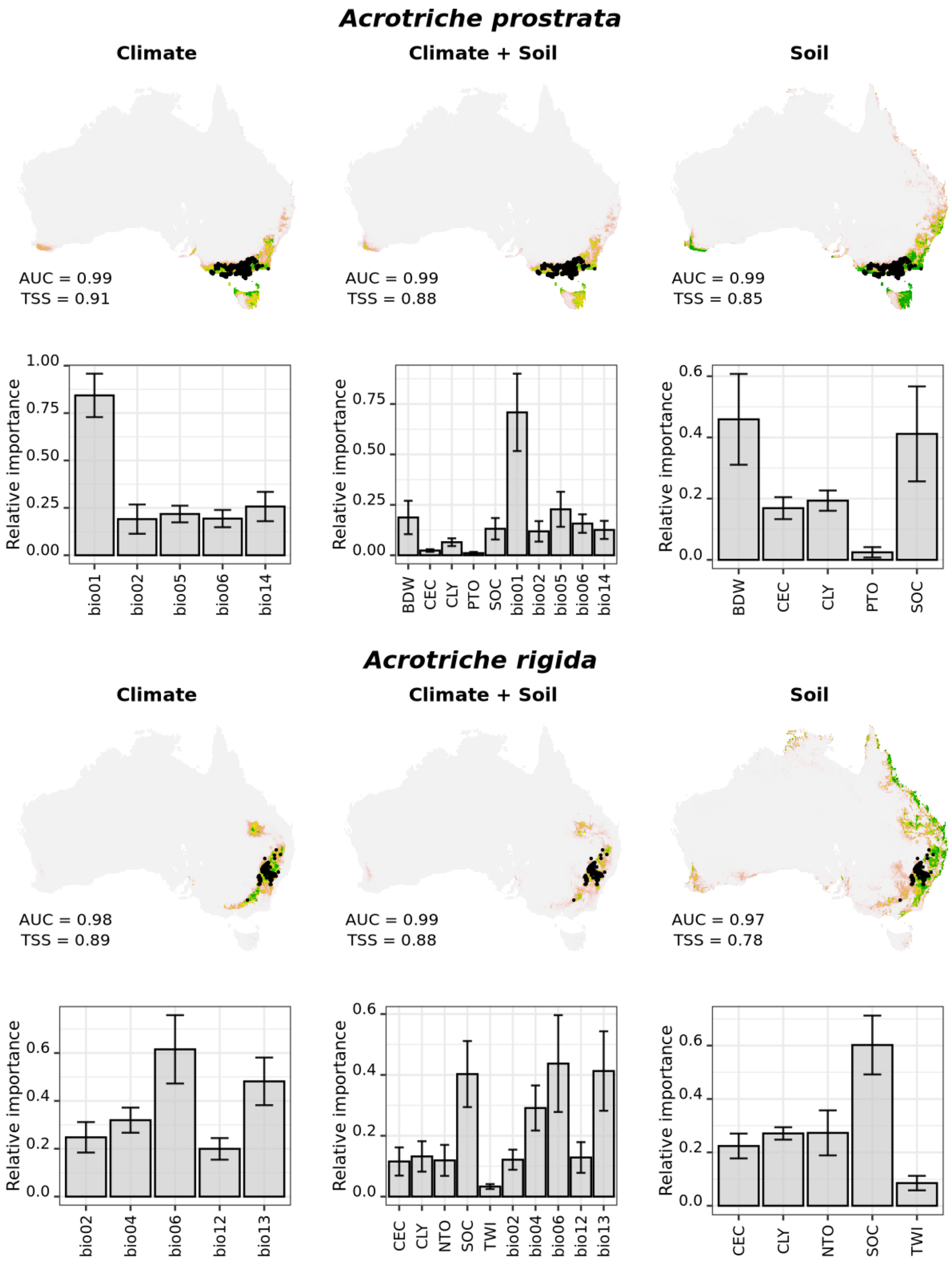

Acrotriche prostrata
Climate
Climate + Soil
Soil
AUC = 0.99
TSS = 0.91
AUC = 0.99
TSS = 0.88
AUC = 0.99
TSS = 0.85
Relative importance
bio01
bio02
bio05
bio06
bio14
BDW
CEC
CLY
PTO
SOC
Acrotriche rigida
AUC = 0.98
TSS = 0.89
AUC = 0.99
TSS = 0.88
AUC = 0.97
TSS = 0.78
bio04
bio12
bio13
NTO
TWI

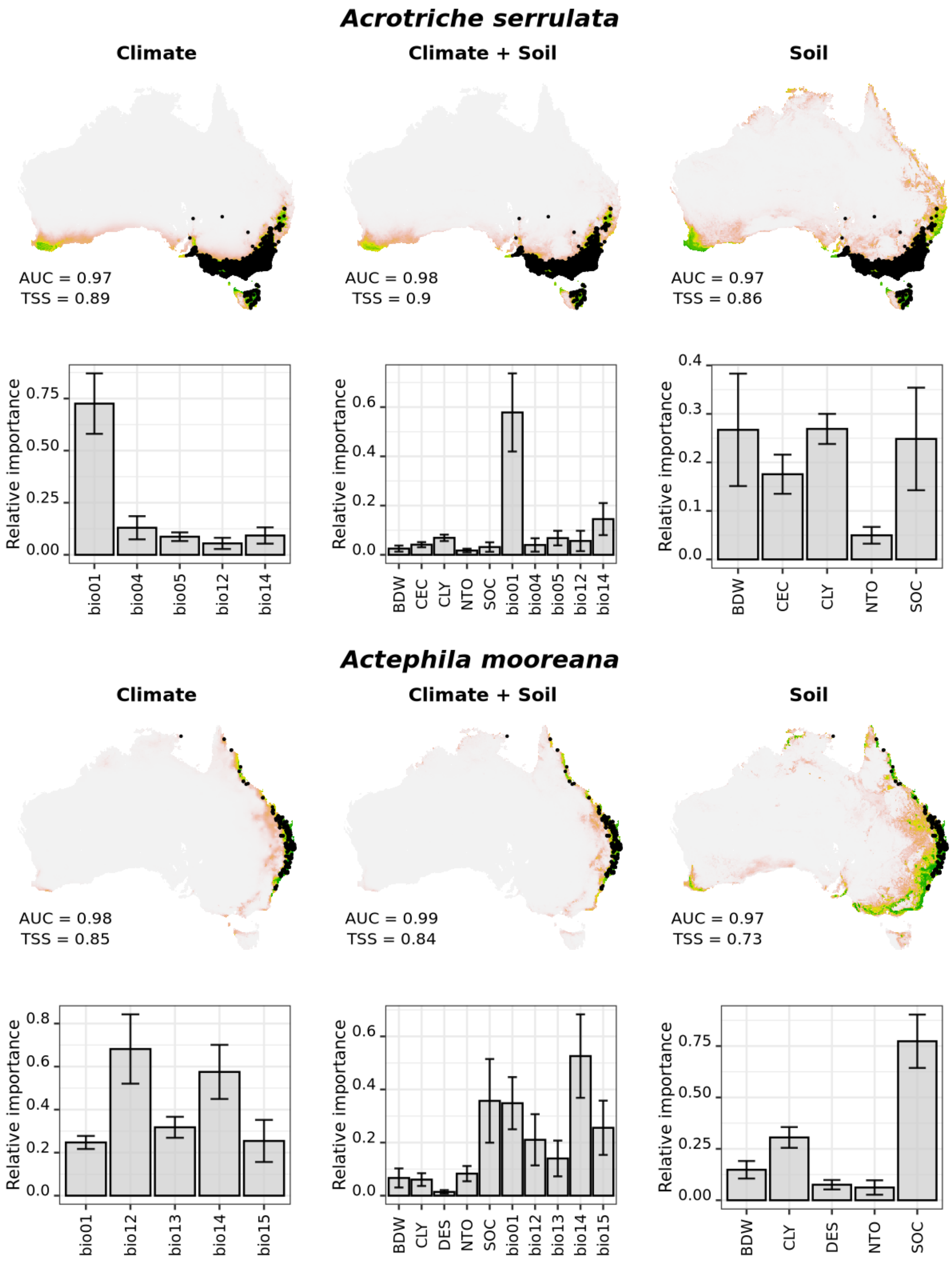

Acrotriche serrulata
Climate
Climate + Soil
Soil
AUC = 0.97
TSS = 0.89
AUC = 0.98
TSS = 0.9
AUC = 0.97
TSS = 0.86
Relative importance
bio01
bio04
bio05
bio12
bio14
BDW
CEC
CLY
NTO
SOC
Actephila mooreana
AUC = 0.98
TSS = 0.85
AUC = 0.99
TSS = 0.84
AUC = 0.97
TSS = 0.73
bio13
bio15
DES

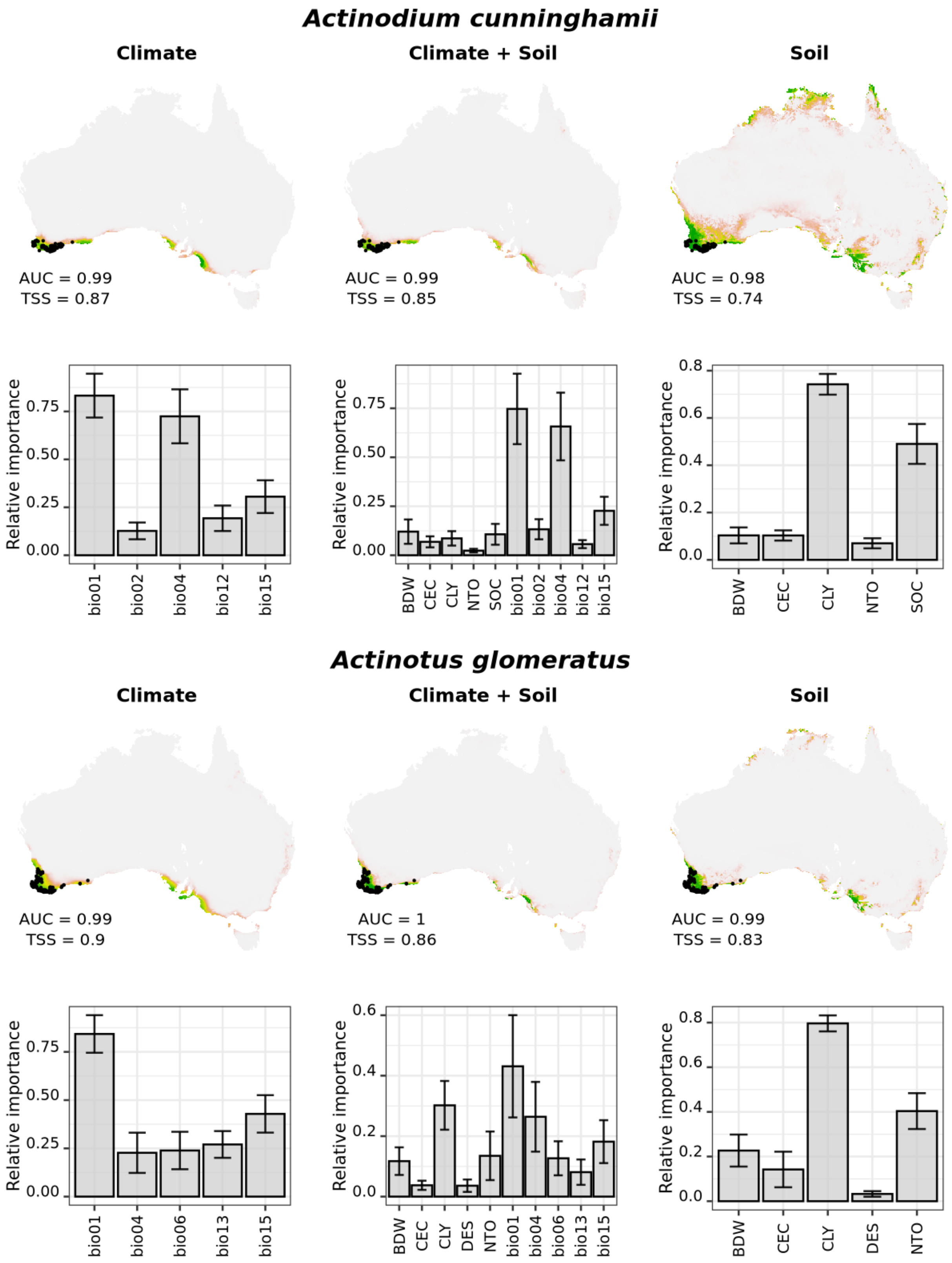
Actinodium cunninghamii
Climate
Climate + Soil
Soil
AUC = 0.99
TSS = 0.87
AUC = 0.99
TSS = 0.85
AUC = 0.98
TSS = 0.74
Relative importance
0.00
0.25
0.50
0.75
bio01
bio02
bio04
bio12
bio15
BDW
CEC
CLY
NTO
SOC
bio01
bio02
bio04
bio12
bio15
0.0
0.2
0.4
0.6
0.8
BDW
CEC
CLY
NTO
SOC
Actinotus glomeratus
Climate
Climate + Soil
Soil
AUC = 0.99
TSS = 0.9
AUC = 1
TSS = 0.86
AUC = 0.99
TSS = 0.83
Relative importance
0.00
0.25
0.50
0.75
bio01
bio04
bio06
bio13
bio15
0.0
0.2
0.4
0.6
BDW
CEC
CLY
DES
NTO
bio01
bio04
bio06
bio13
bio15
0.0
0.2
0.4
0.6
0.8
BDW
CEC
CLY
DES
NTO

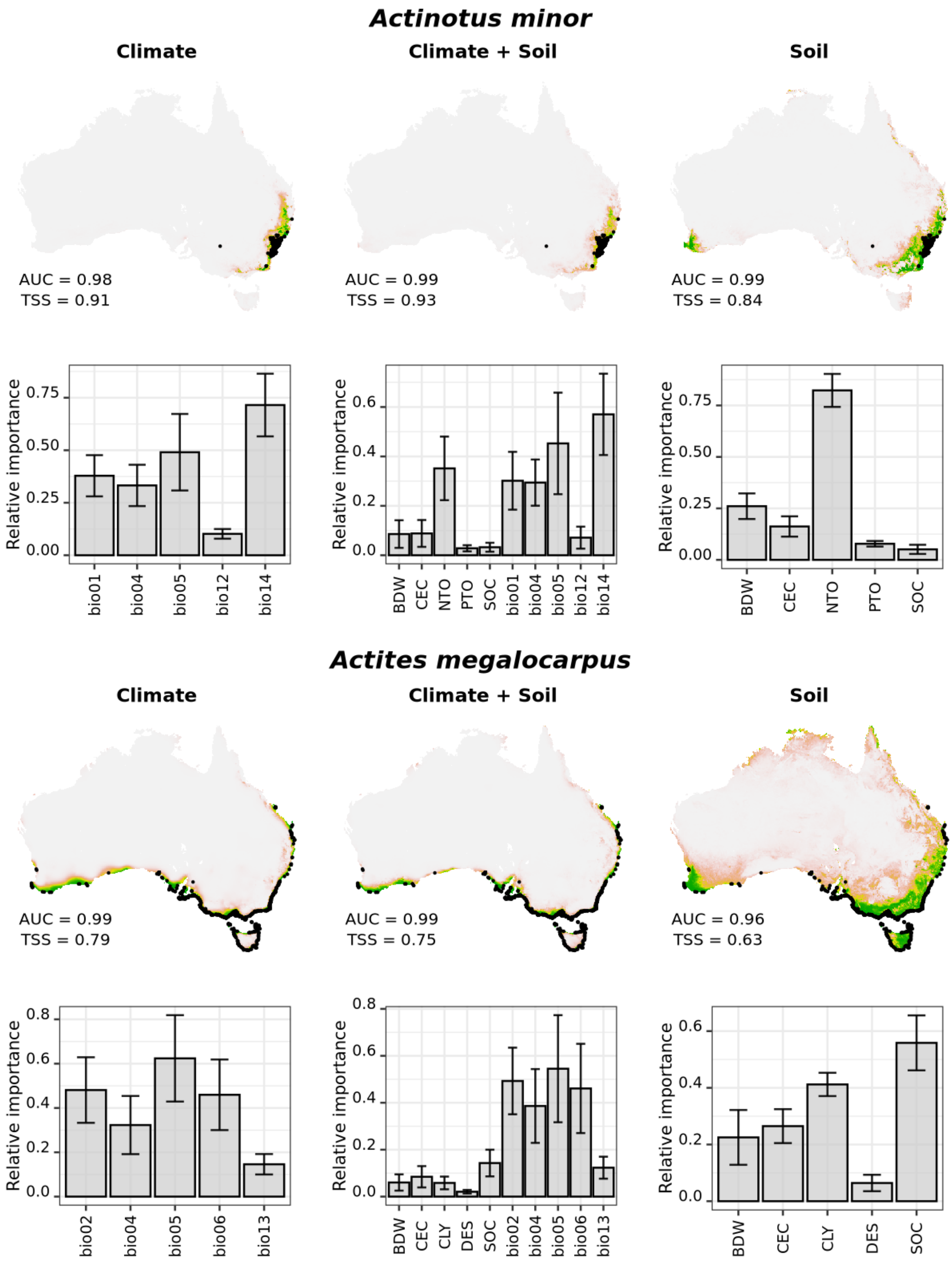

Actinotus minor
Climate
Climate + Soil
Soil
AUC = 0.98
TSS = 0.91
AUC = 0.99
TSS = 0.93
AUC = 0.99
TSS = 0.84
Relative importance
bio01
bio04
bio05
bio12
bio14
BDW
CEC
NTO
PTO
SOC
Actites megalocarpus
AUC = 0.99
TSS = 0.79
AUC = 0.99
TSS = 0.75
AUC = 0.96
TSS = 0.63
bio02
bio06
bio13
CLY
DES

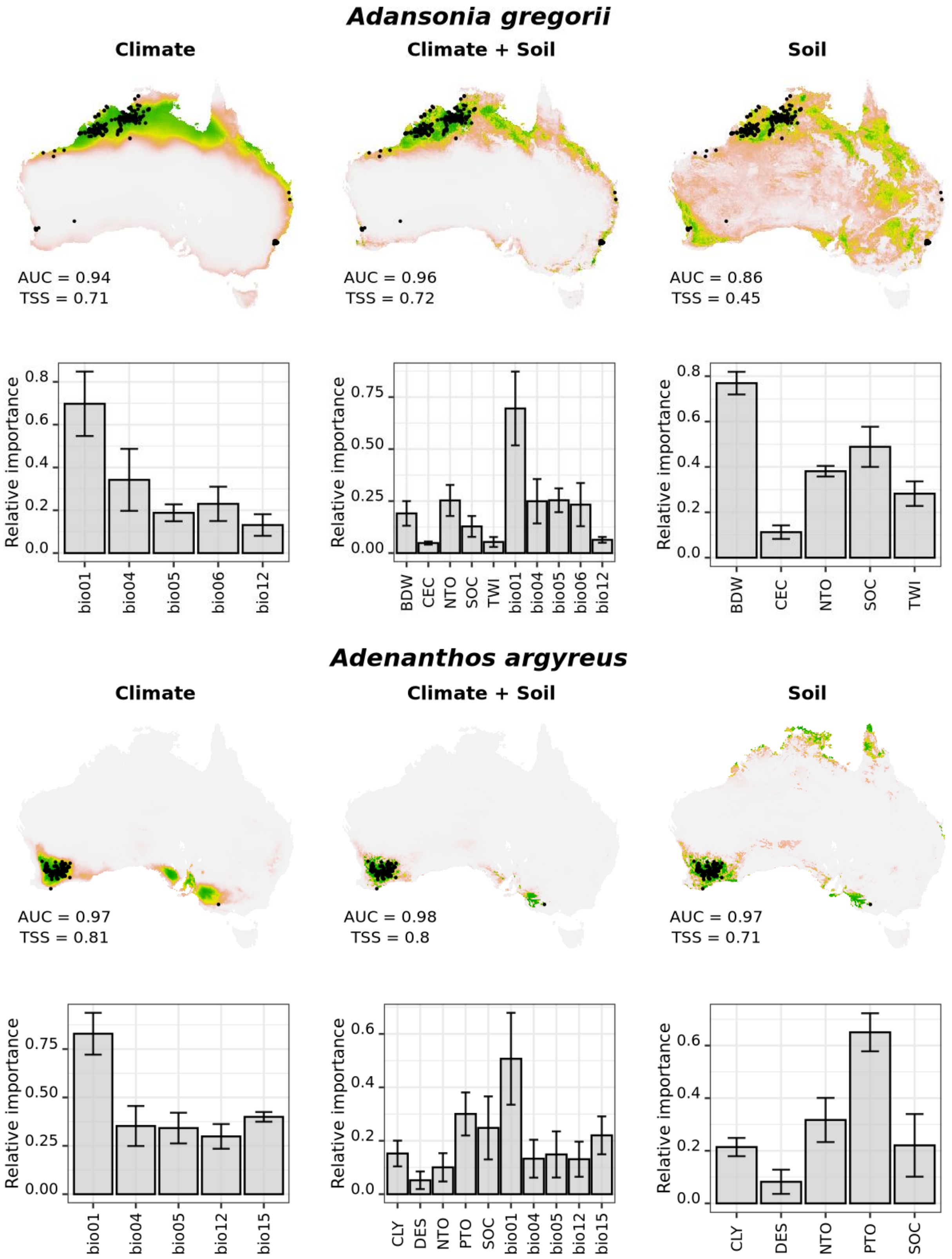
Adansonia gregorii
Climate
Climate + Soil
Soil
AUC = 0.94
TSS = 0.71
AUC = 0.96
TSS = 0.72
AUC = 0.86
TSS = 0.45
Relative importance
bio01
bio04
bio05
bio06
bio12
BDW
CEC
NTO
SOC
TWI
Adenanthos argyreus
Climate
Climate + Soil
Soil
AUC = 0.97
TSS = 0.81
AUC = 0.98
TSS = 0.8
AUC = 0.97
TSS = 0.71
Relative importance
bio01
bio04
bio05
bio12
bio15
CLY
DES
NTO
PTO
SOC

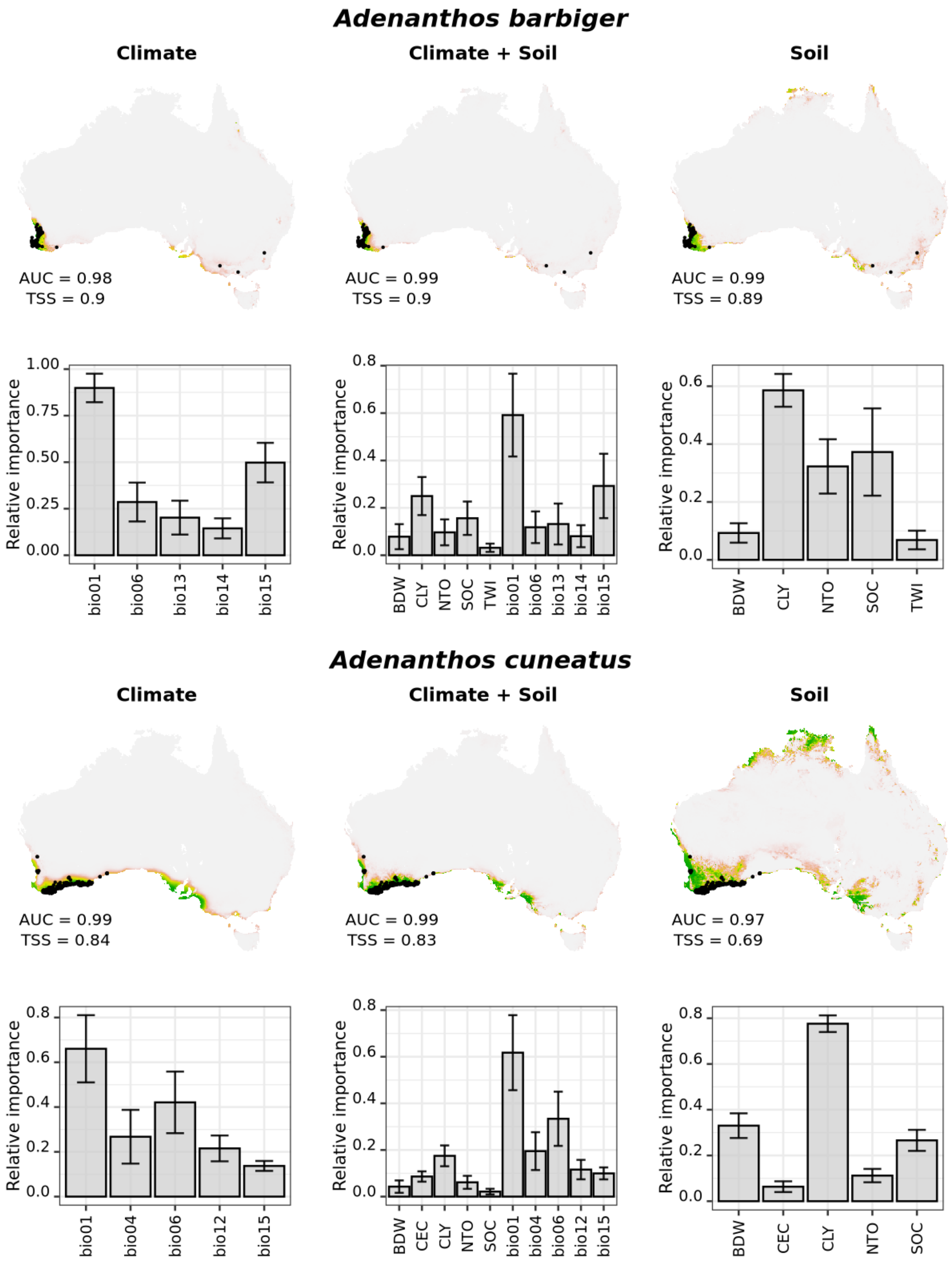
Adenanthos barbiger
Climate
Climate + Soil
Soil
AUC = 0.98
TSS = 0.9
AUC = 0.99
TSS = 0.9
AUC = 0.99
TSS = 0.89
Relative importance
bio01
bio06
bio13
bio14
bio15
BDW
CLY
NTO
SOC
TWI
Adenanthos cuneatus
Climate
Climate + Soil
Soil
AUC = 0.99
TSS = 0.84
AUC = 0.99
TSS = 0.83
AUC = 0.97
TSS = 0.69
Relative importance
bio01
bio04
bio06
bio12
bio15
BDW
CEC
CLY
NTO
SOC

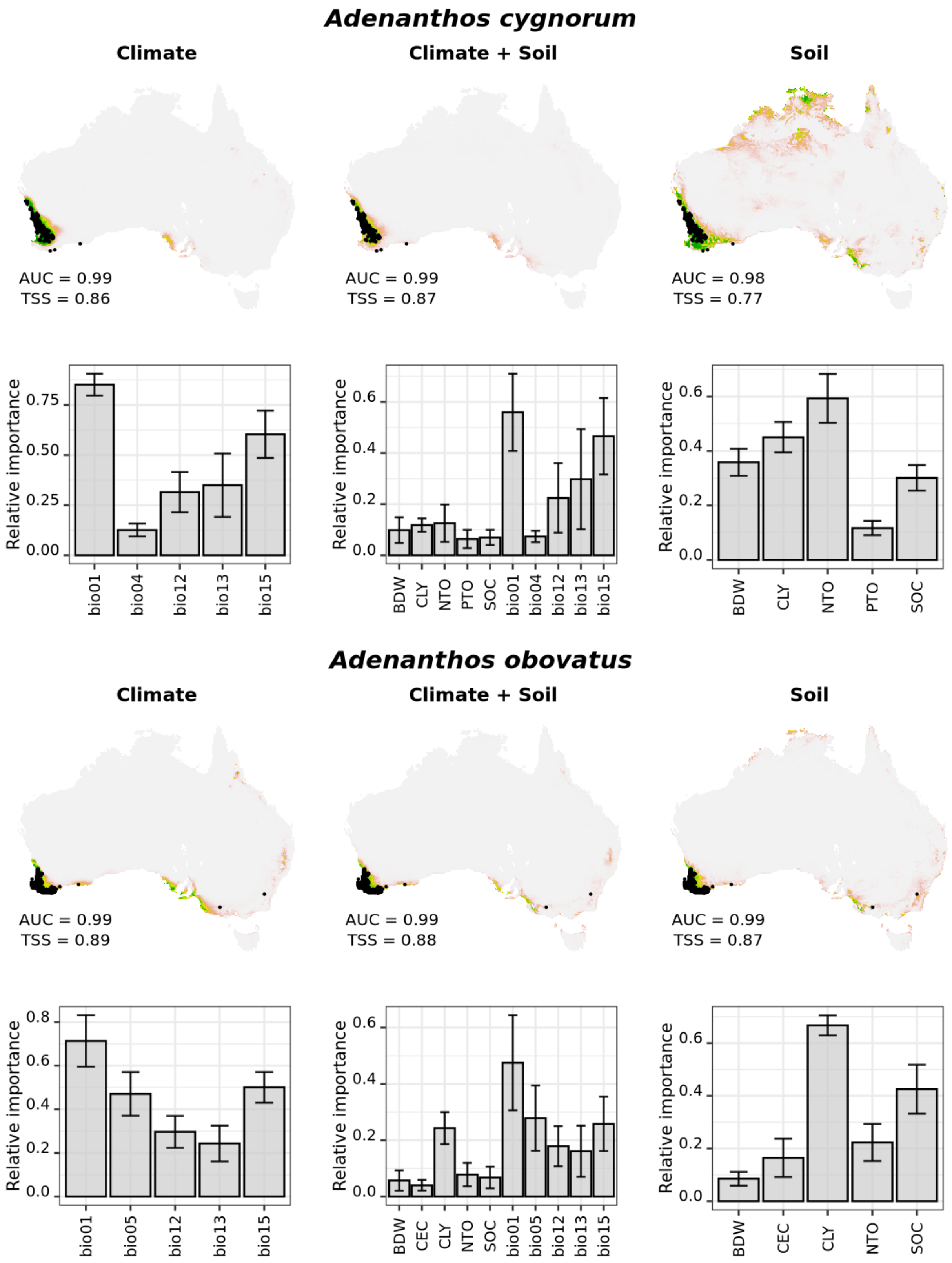
Adenanthos cygnorum
Climate
Climate + Soil
Soil
AUC = 0.99
TSS = 0.86
AUC = 0.99
TSS = 0.87
AUC = 0.98
TSS = 0.77
Relative importance
bio01
bio04
bio12
bio13
bio15
BDW
CLY
NTO
PTO
SOC
Adenanthos obovatus
Climate
Climate + Soil
Soil
AUC = 0.99
TSS = 0.89
AUC = 0.99
TSS = 0.88
AUC = 0.99
TSS = 0.87
Relative importance
bio01
bio05
bio12
bio13
bio15
BDW
CEC
CLY
NTO
SOC

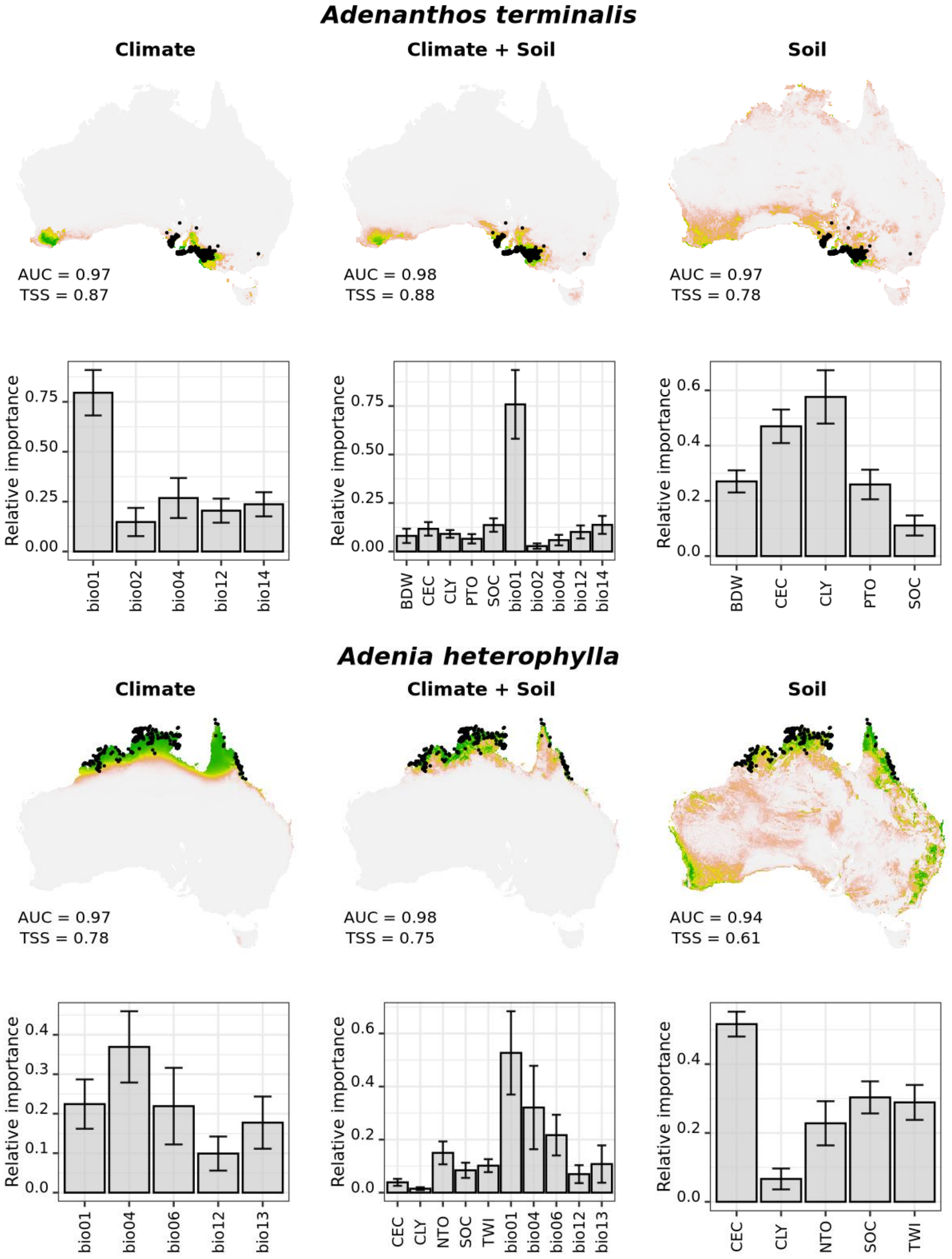
Adenanthos terminalis
Climate
Climate + Soil
Soil
AUC = 0.97
TSS = 0.87
AUC = 0.98
TSS = 0.88
AUC = 0.97
TSS = 0.78
Relative importance
0.00
0.25
0.50
0.75
bio01
bio02
bio04
bio12
bio14
BDW
CEC
CLY
PTO
SOC
bio01
bio02
bio04
bio12
bio14
0.0
0.2
0.4
0.6
BDW
CEC
CLY
PTO
SOC
Adenia heterophylla
Climate
Climate + Soil
Soil
AUC = 0.97
TSS = 0.78
AUC = 0.98
TSS = 0.75
AUC = 0.94
TSS = 0.61
Relative importance
0.0
0.1
0.2
0.3
0.4
bio01
bio04
bio06
bio12
bio13
0.0
0.2
0.4
0.6
CEC
CLY
NTO
SOC
TWI
bio01
bio04
bio06
bio12
bio13
0.0
0.2
0.4
CEC
CLY
NTO
SOC
TWI

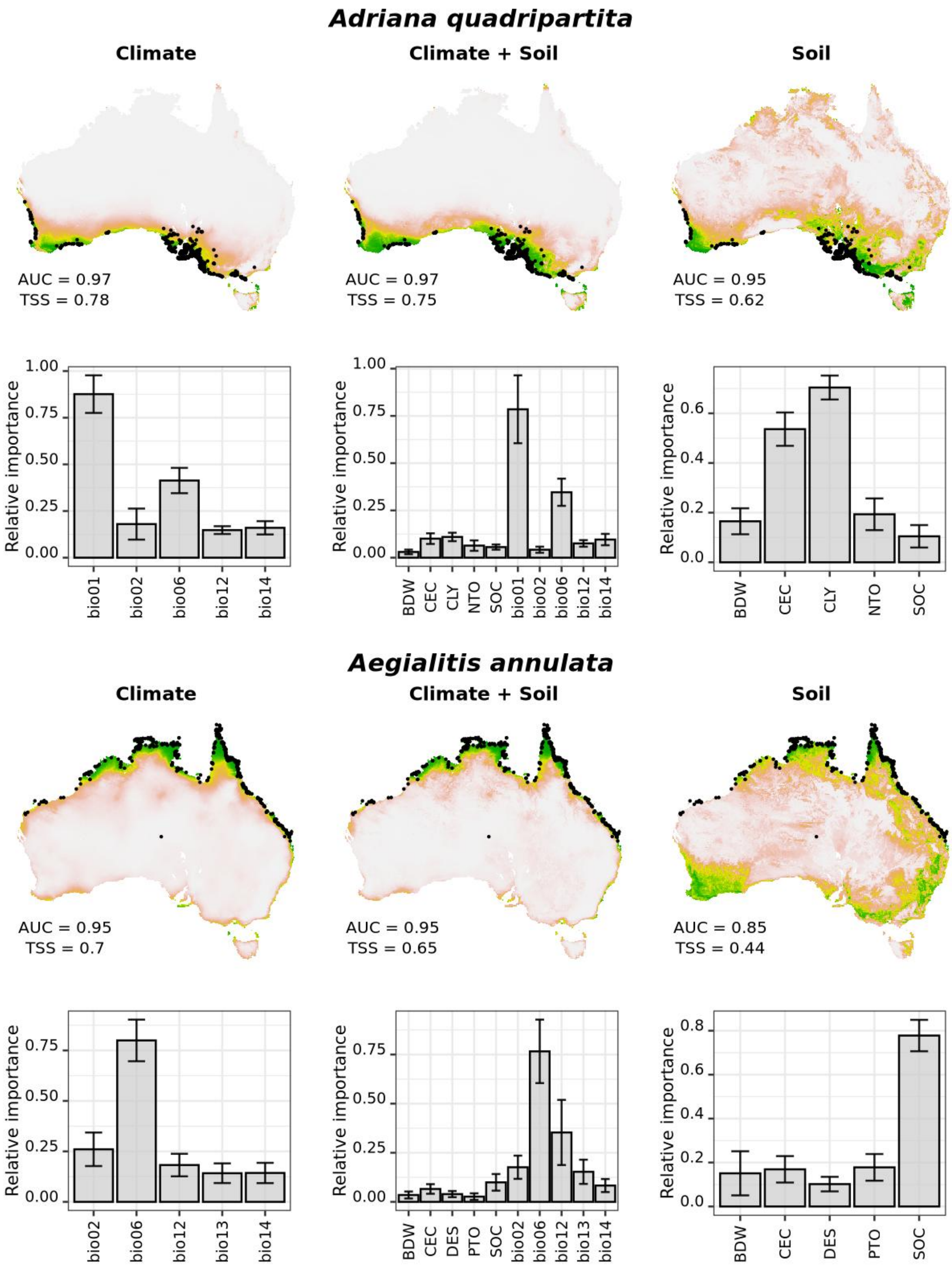
Adriana quadripartita
Climate
Climate + Soil
Soil
AUC = 0.97
TSS = 0.78
AUC = 0.97
TSS = 0.75
AUC = 0.95
TSS = 0.62
Relative importance
1.00
0.75
0.50
0.25
0.00
bio01
bio02
bio06
bio12
bio14
BDW
CEC
CLY
NTO
SOC
0.6
0.4
0.2
0.0
Aegialitis annulata
Climate
Climate + Soil
Soil
AUC = 0.95
TSS = 0.7
AUC = 0.95
TSS = 0.65
AUC = 0.85
TSS = 0.44
Relative importance
bio02
bio06
bio12
bio13
bio14
BDW
CEC
DES
PTO
SOC
0.8

Aegiceras corniculatum

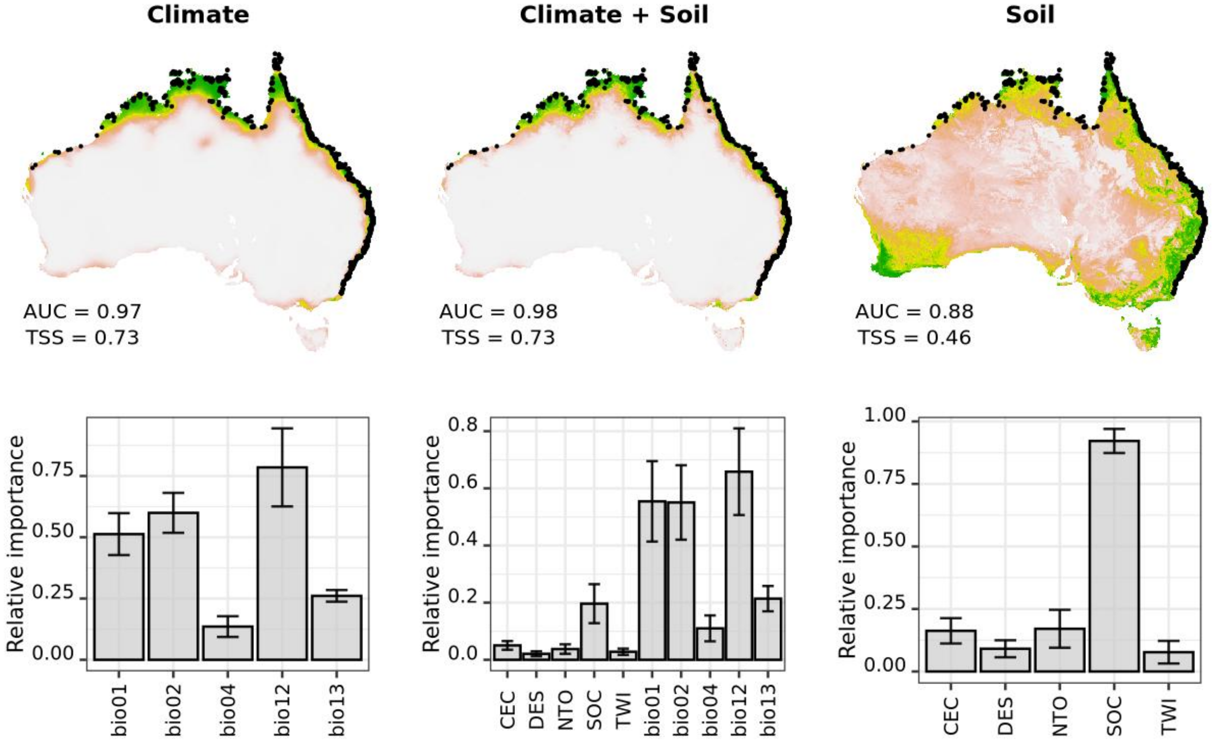

Aeschynomene brevifolia

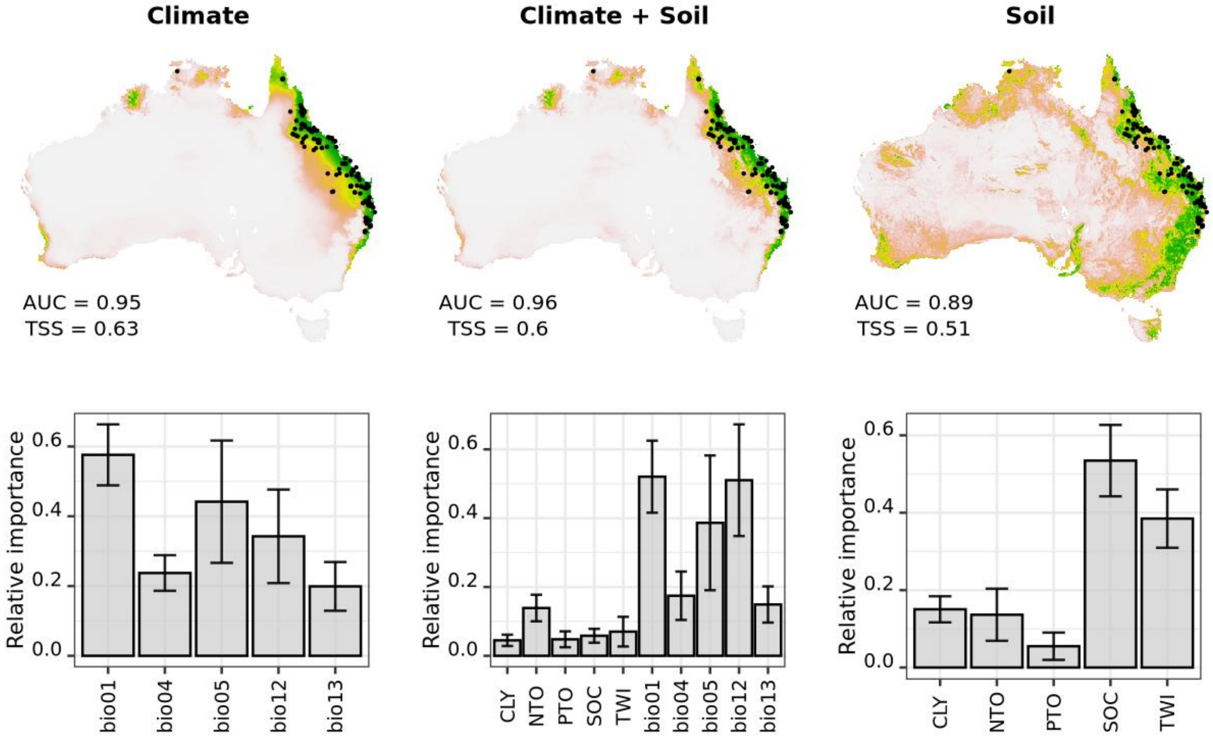

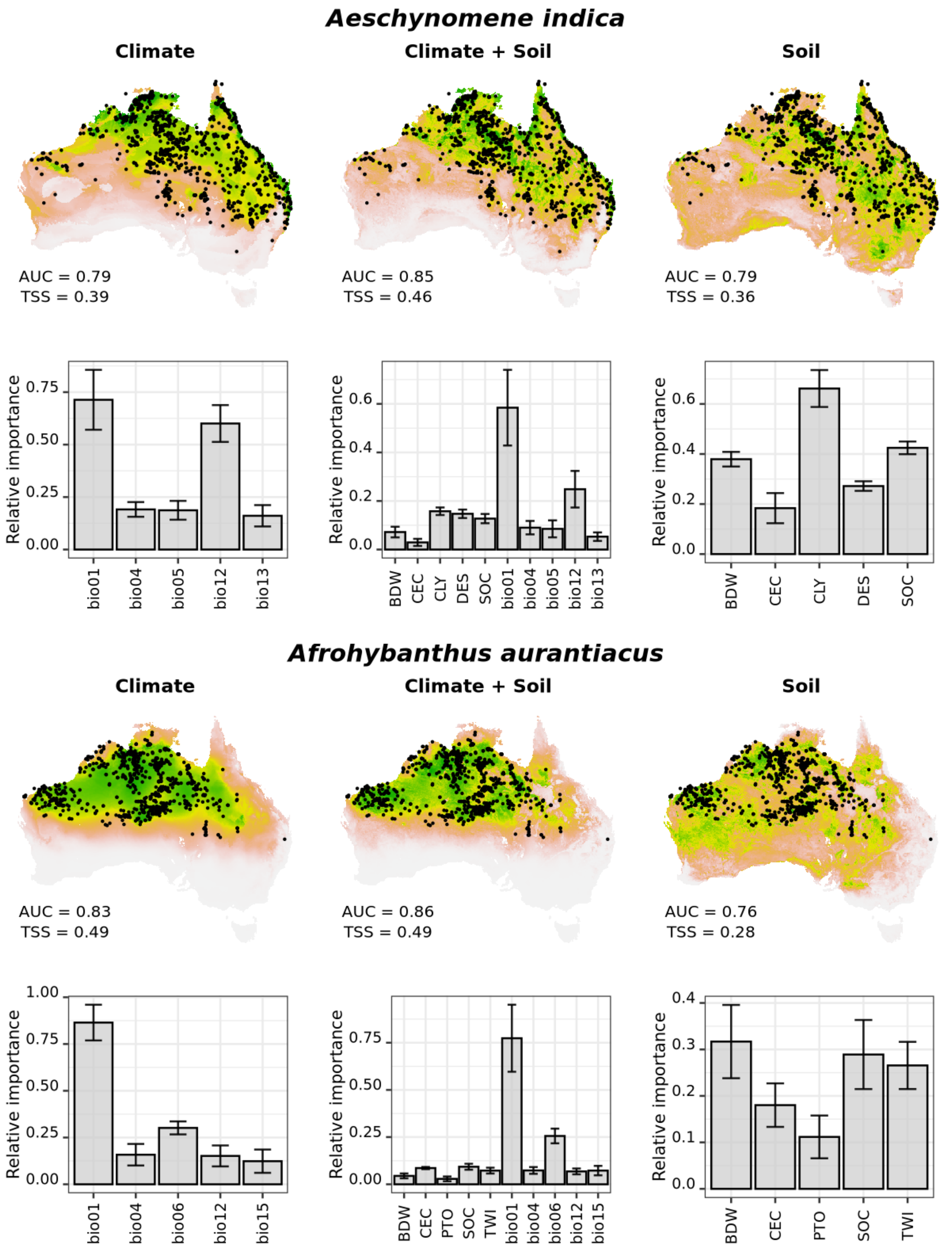
Aeschynomene indica
Climate
Climate + Soil
Soil
AUC = 0.79
TSS = 0.39
AUC = 0.85
TSS = 0.46
AUC = 0.79
TSS = 0.36
Relative importance
0.00
0.25
0.50
0.75
bio01
bio04
bio05
bio12
bio13
0.0
0.2
0.4
0.6
BDW
CEC
CLY
DES
SOC
bio01
bio04
bio05
bio12
bio13
BDW
CEC
CLY
DES
SOC
Afrohybanthus aurantiacus
Climate
Climate + Soil
Soil
AUC = 0.83
TSS = 0.49
AUC = 0.86
TSS = 0.49
AUC = 0.76
TSS = 0.28
1.00
bio01
bio04
bio06
bio12
bio15
BDW
CEC
PTO
SOC
TWI
bio01
bio04
bio06
bio12
bio15
0.1
0.3
BDW
CEC
PTO
SOC
TWI

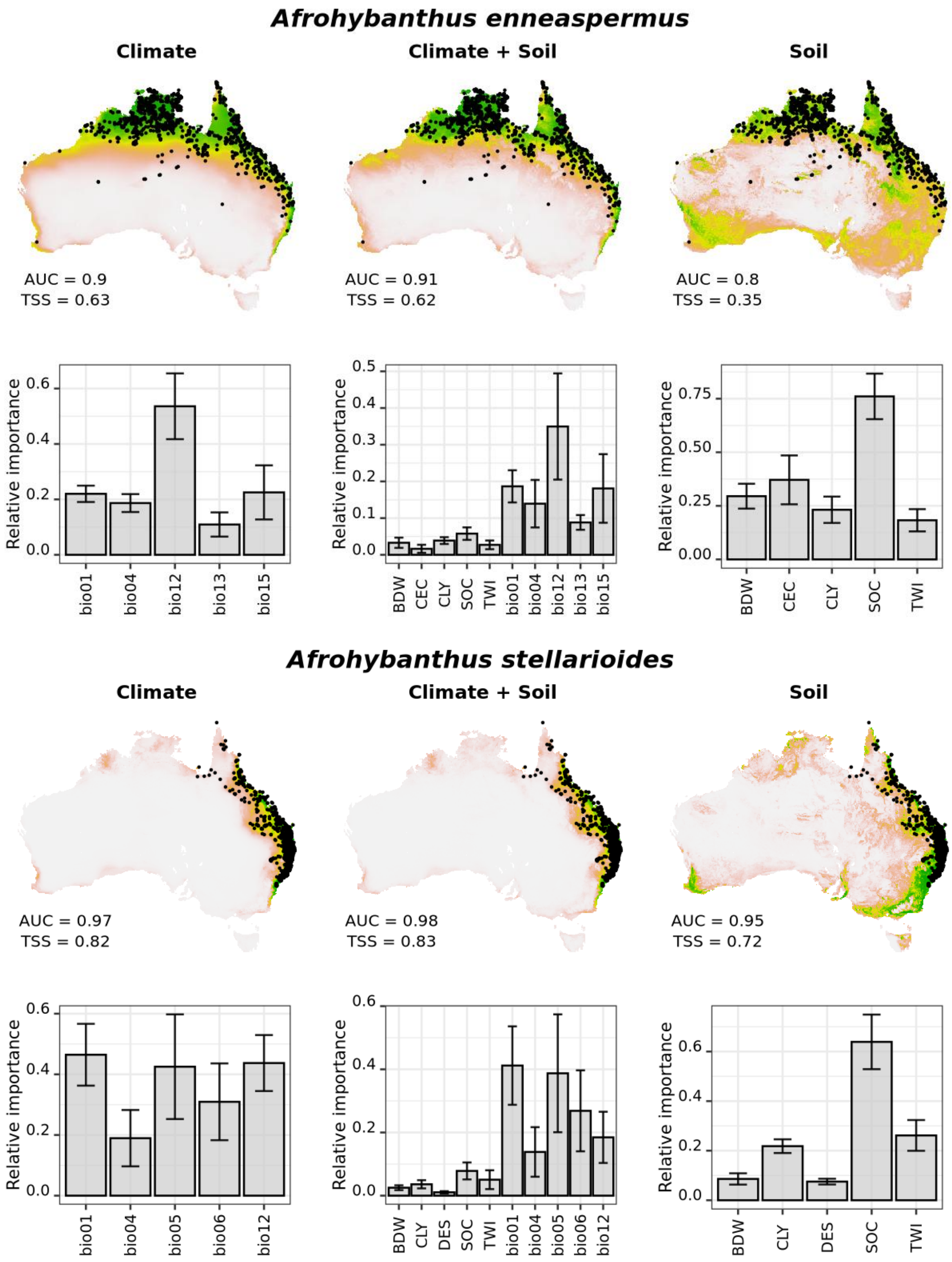

Afrohybanthus enneaspermus
Climate
Climate + Soil
Soil
AUC = 0.9
TSS = 0.63
AUC = 0.91
TSS = 0.62
AUC = 0.8
TSS = 0.35
Relative importance
bio01
bio04
bio12
bio13
bio15
BDW
CEC
CLY
SOC
TWI
Afrohybanthus stellarioides
Climate
Climate + Soil
Soil
AUC = 0.97
TSS = 0.82
AUC = 0.98
TSS = 0.83
AUC = 0.95
TSS = 0.72
Relative importance
bio01
bio04
bio05
bio06
bio12
BDW
CLY
DES
SOC
TWI

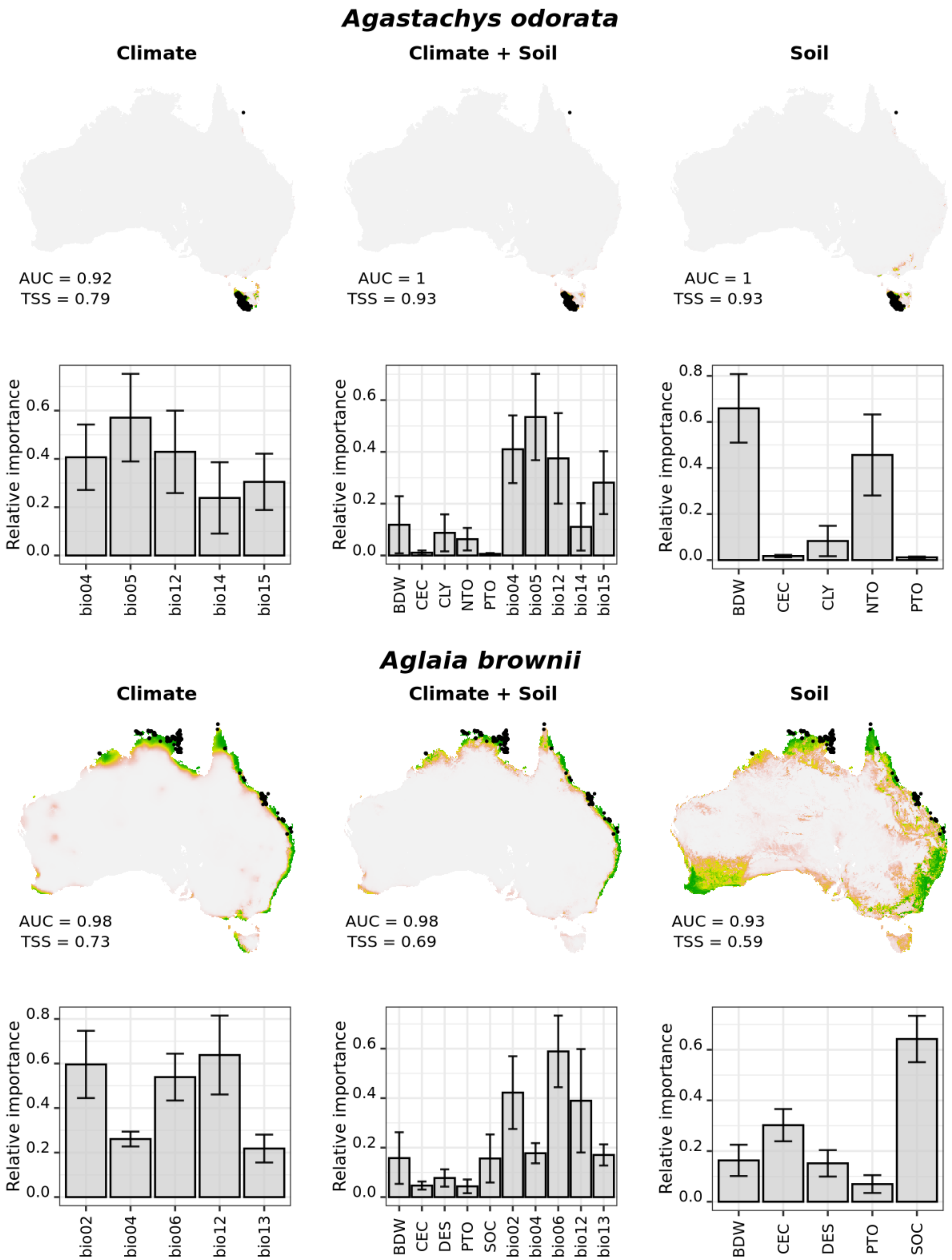
Agastachys odorata
Climate
Climate + Soil
Soil
AUC = 0.92
TSS = 0.79
AUC = 1
TSS = 0.93
AUC = 1
TSS = 0.93
Relative importance
bio04
bio05
bio12
bio14
bio15
BDW
CEC
CLY
NTO
PTO
Aglaia brownii
Climate
Climate + Soil
Soil
AUC = 0.98
TSS = 0.73
AUC = 0.98
TSS = 0.69
AUC = 0.93
TSS = 0.59
Relative importance
bio02
bio04
bio06
bio12
bio13
BDW
CEC
DES
PTO
SOC

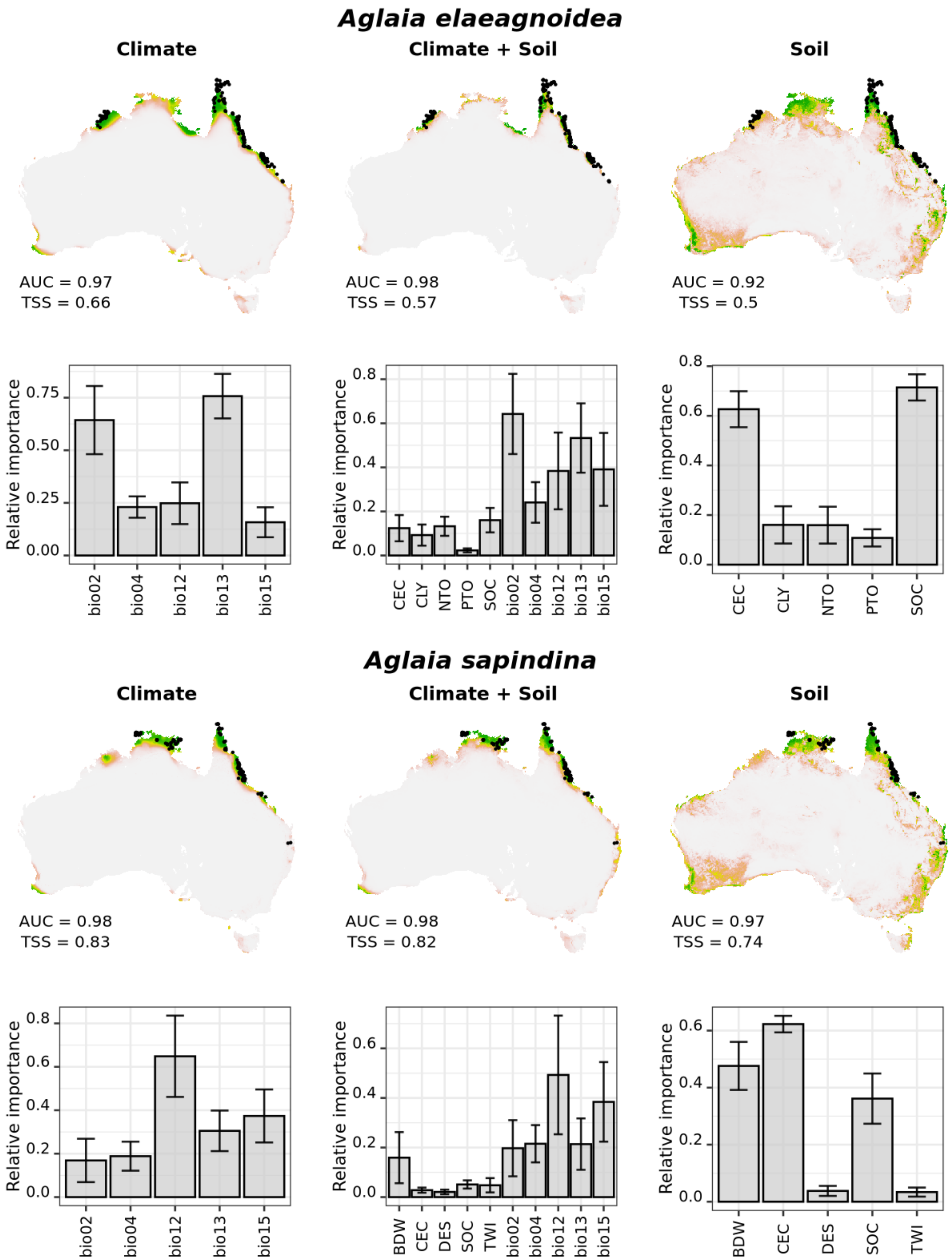
Aglaia elaeagnoidea
Climate
Climate + Soil
Soil
AUC = 0.97
TSS = 0.66
AUC = 0.98
TSS = 0.57
AUC = 0.92
TSS = 0.5
Relative importance
0.00
0.25
0.50
0.75
bio02
bio04
bio12
bio13
bio15
0.0
0.2
0.4
0.6
0.8
CEC
CLY
NTO
PTO
SOC
bio02
bio04
bio12
bio13
bio15
CEC
CLY
NTO
PTO
SOC
Aglaia sapindina
Climate
Climate + Soil
Soil
AUC = 0.98
TSS = 0.83
AUC = 0.98
TSS = 0.82
AUC = 0.97
TSS = 0.74
Relative importance
bio02
bio04
bio12
bio13
bio15
BDW
CEC
DES
SOC
TWI
bio02
bio04
bio12
bio13
bio15
BDW
CEC
DES
SOC
TWI

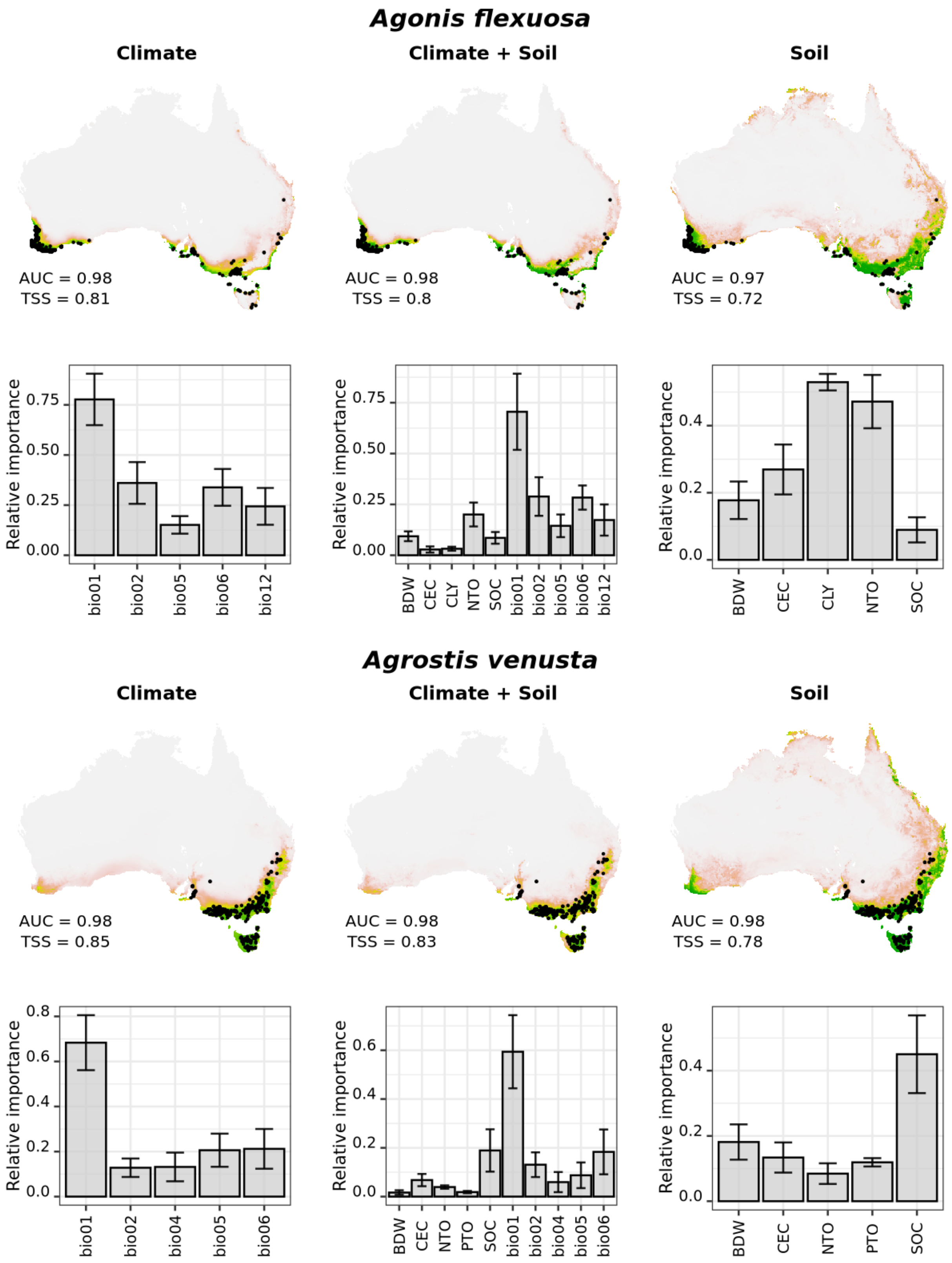

Agonis flexuosa
Climate
Climate + Soil
Soil
AUC = 0.98
TSS = 0.81
AUC = 0.98
TSS = 0.8
AUC = 0.97
TSS = 0.72
Relative importance
0.00
0.25
0.50
0.75
bio01
bio02
bio05
bio06
bio12
BDW
CEC
CLY
NTO
SOC
0.0
0.2
0.4
Agrostis venusta
AUC = 0.98
TSS = 0.85
AUC = 0.98
TSS = 0.83
AUC = 0.98
TSS = 0.78
0.6
0.8
bio04
PTO

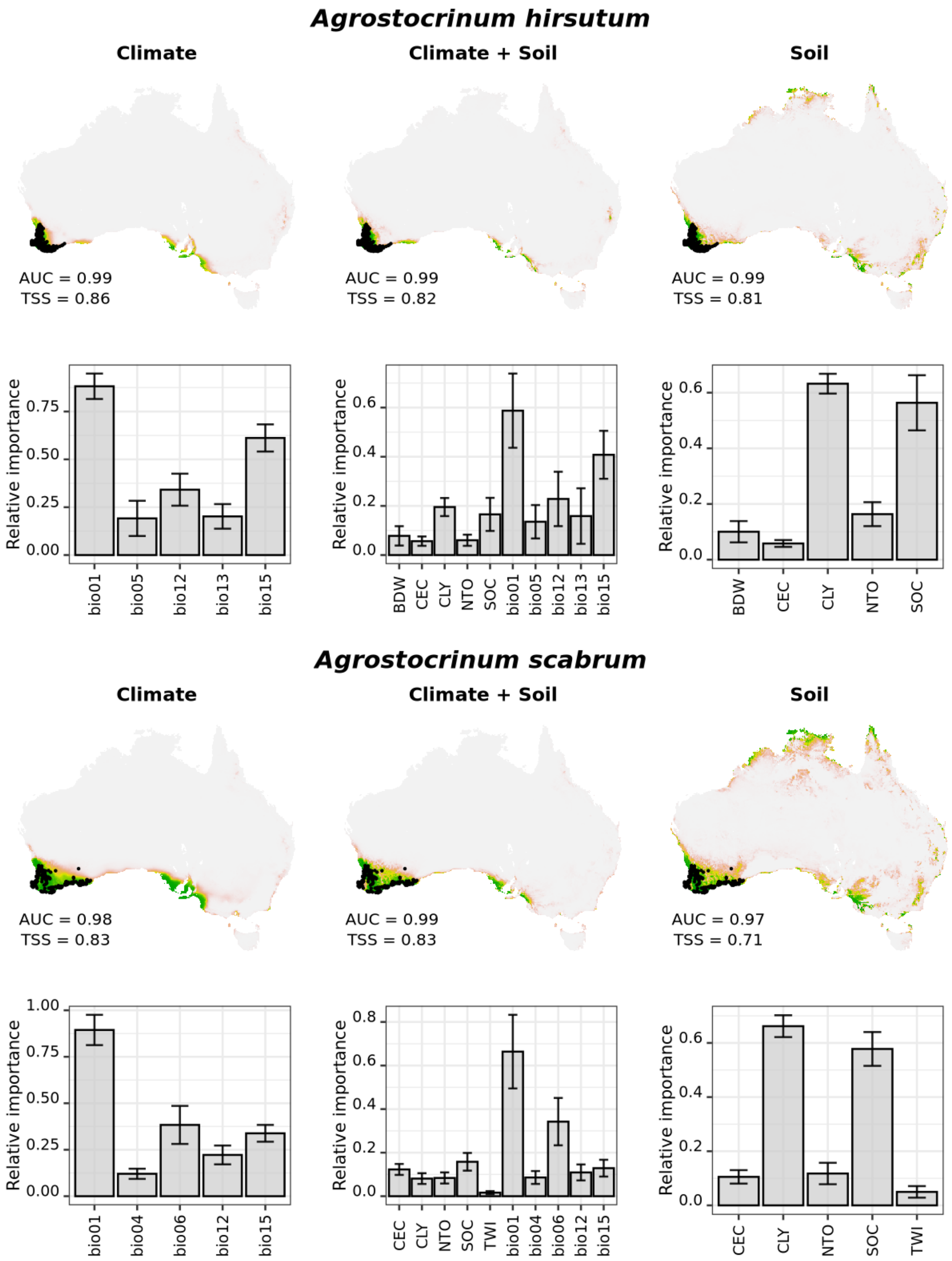
Agrostocrinum hirsutum
Climate
Climate + Soil
Soil
AUC = 0.99
TSS = 0.86
AUC = 0.99
TSS = 0.82
AUC = 0.99
TSS = 0.81
Relative importance
0.00
0.25
0.50
0.75
bio01
bio05
bio12
bio13
bio15
0.0
0.2
0.4
0.6
BDW
CEC
CLY
NTO
SOC
bio01
bio05
bio12
bio13
bio15
BDW
CEC
CLY
NTO
SOC
Agrostocrinum scabrum
Climate
Climate + Soil
Soil
AUC = 0.98
TSS = 0.83
AUC = 0.99
TSS = 0.83
AUC = 0.97
TSS = 0.71
Relative importance
1.00
0.75
0.50
0.25
0.00
bio01
bio04
bio06
bio12
bio15
0.8
CEC
CLY
NTO
SOC
TWI
bio01
bio04
bio06
bio12
bio15
CEC
CLY
NTO
SOC
TWI

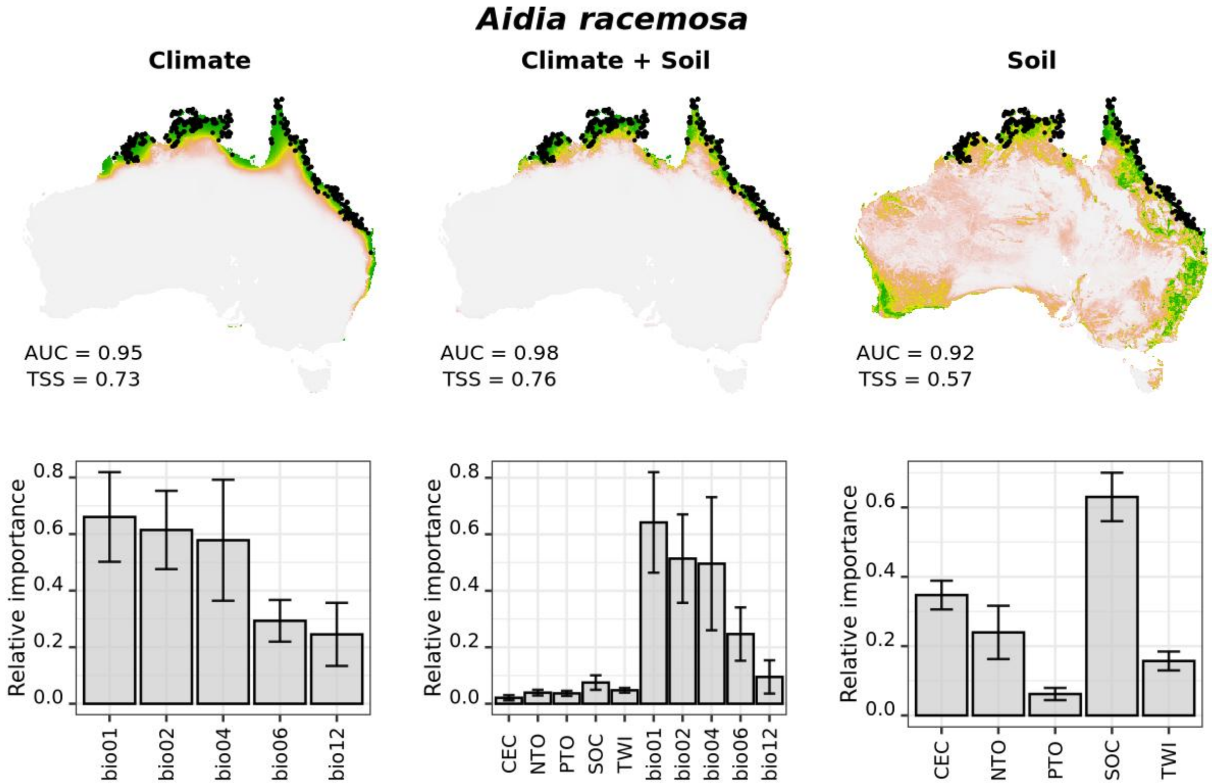
Aidia racemosa
Climate
Climate + Soil
Soil
AUC = 0.95
TSS = 0.73
AUC = 0.98
TSS = 0.76
AUC = 0.92
TSS = 0.57
Relative importance
0.0
0.2
0.4
0.6
0.8
bio01
bio02
bio04
bio06
bio12
CEC
NTO
PTO
SOC
TWI

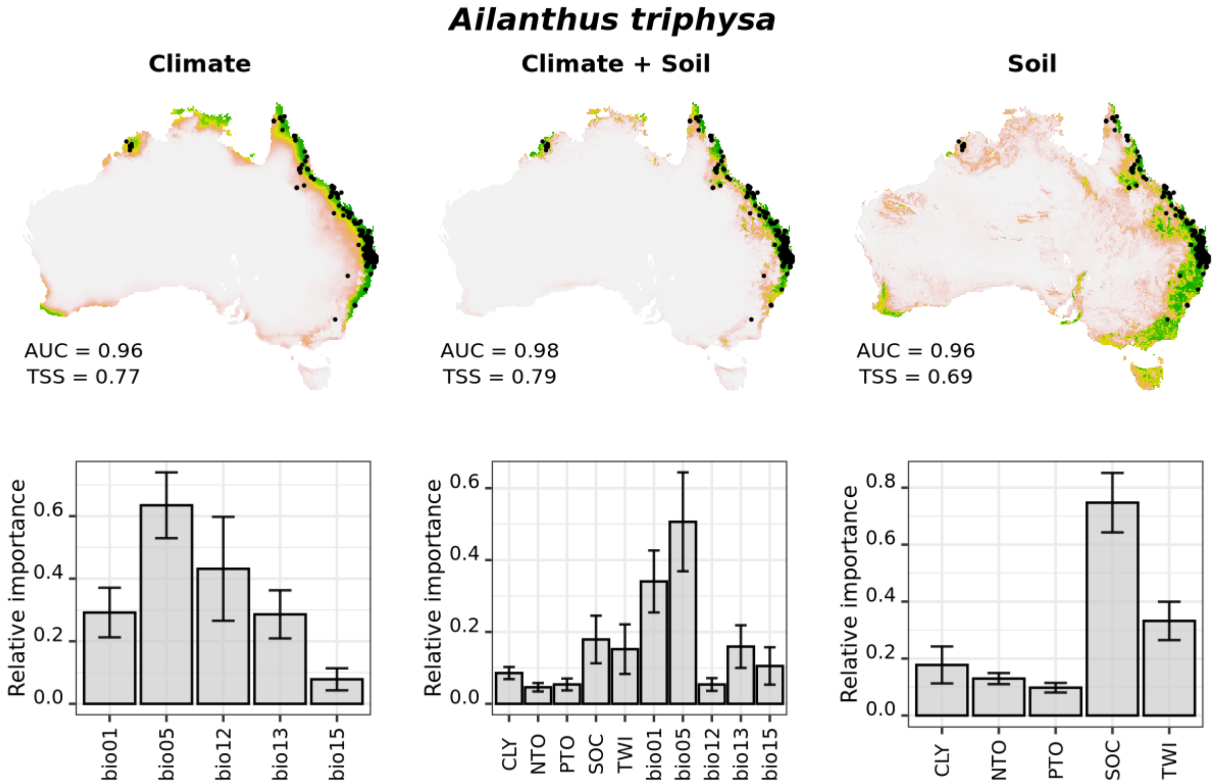
Ailanthus triphysa
Climate
Climate + Soil
Soil
AUC = 0.96
TSS = 0.77
AUC = 0.98
TSS = 0.79
AUC = 0.96
TSS = 0.69
Relative importance
0.0
0.2
0.4
0.6
0.8
bio01
bio05
bio12
bio13
bio15
CLY
NTO
PTO
SOC
TWI

Ajuga australis

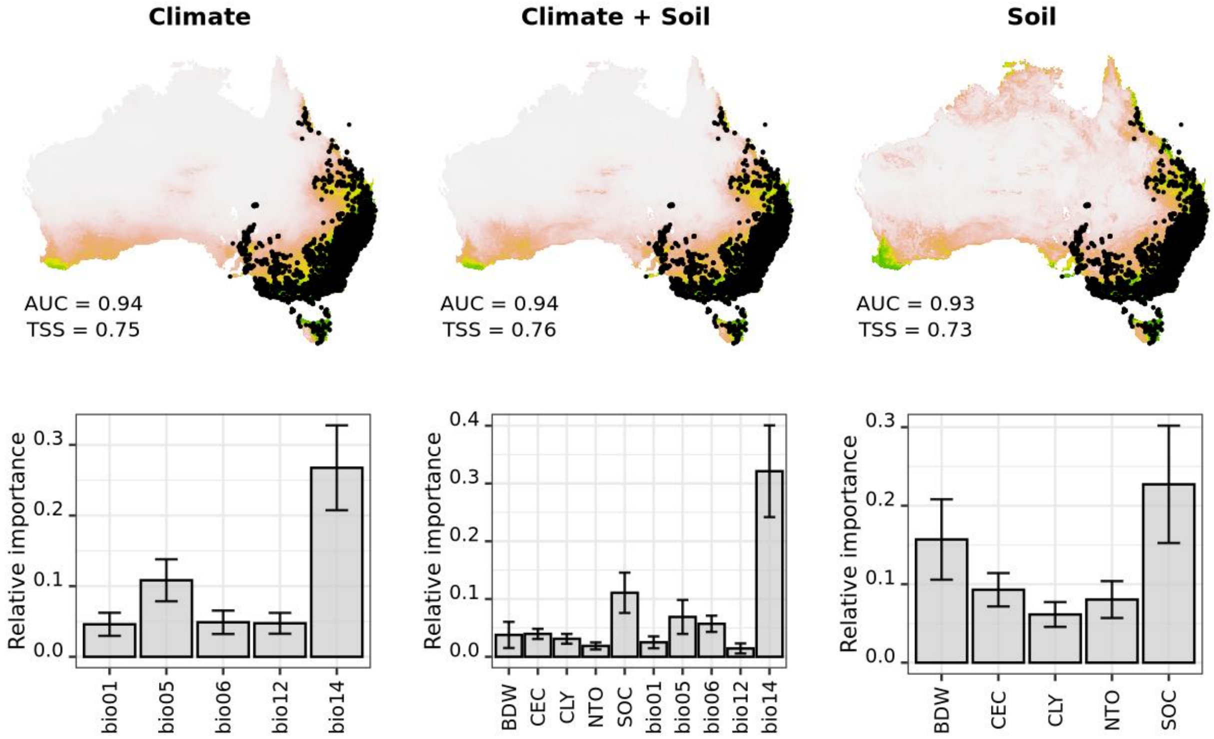

Akania bidwillii

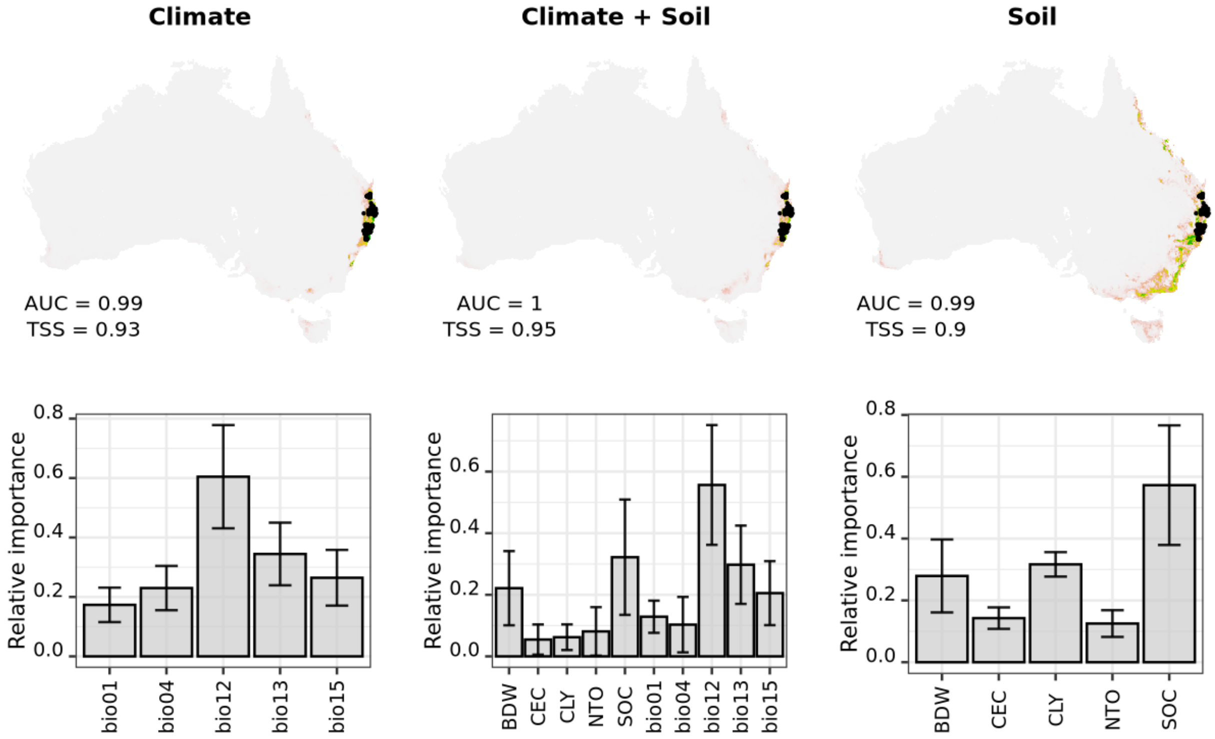

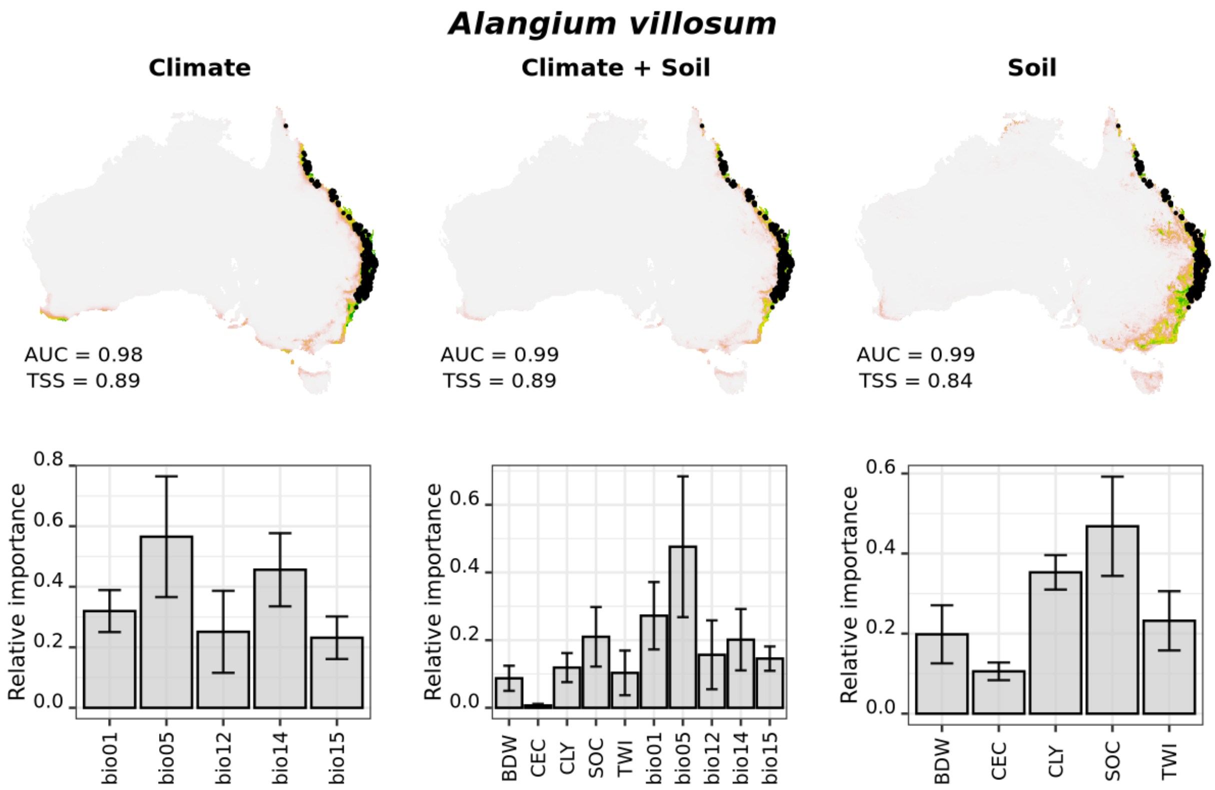
Alangium villosum
Climate
Climate + Soil
Soil
AUC = 0.98
TSS = 0.89
AUC = 0.99
TSS = 0.89
AUC = 0.99
TSS = 0.84
Relative importance
bio01 bio05 bio12 bio14 bio15
BDW CEC CLY SOC TWI bio01 bio05 bio12 bio14 bio15
BDW CEC CLY SOC TWI

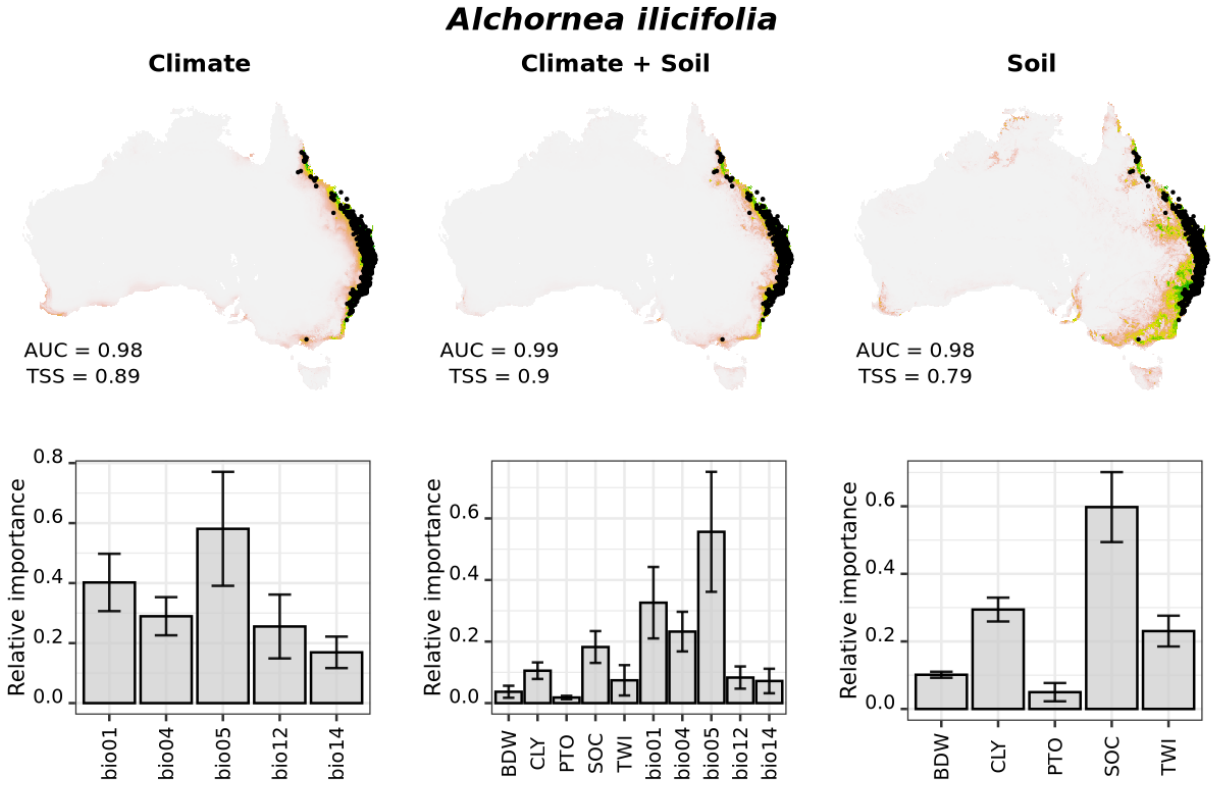
Alchornea ilicifolia
Climate
Climate + Soil
Soil
AUC = 0.98
TSS = 0.89
AUC = 0.99
TSS = 0.9
AUC = 0.98
TSS = 0.79
Relative importance
bio01 bio04 bio05 bio12 bio14
BDW CLY PTO SOC TWI bio01 bio04 bio05 bio12 bio14
BDW CLY PTO SOC TWI

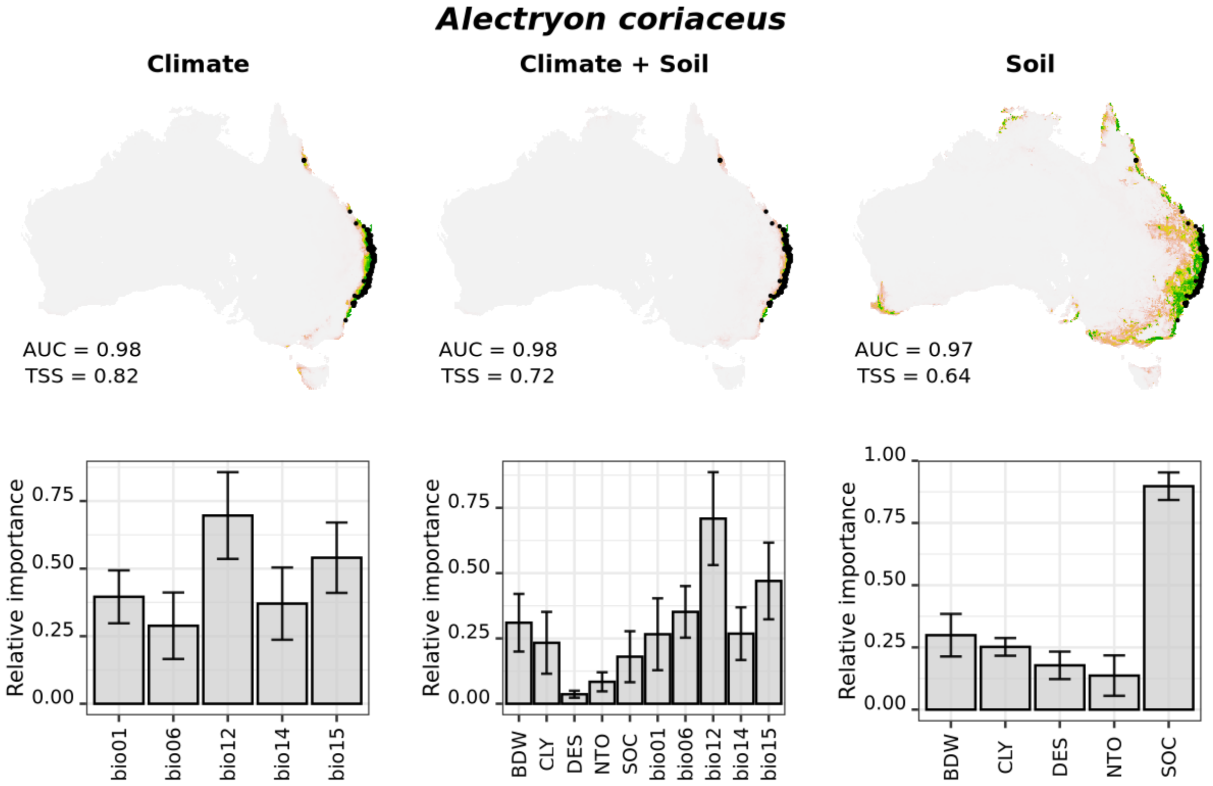
Alectryon coriaceus
Climate
Climate + Soil
Soil
AUC = 0.98
TSS = 0.82
AUC = 0.98
TSS = 0.72
AUC = 0.97
TSS = 0.64
Relative importance
0.00
0.25
0.50
0.75
1.00
bio01
bio06
bio12
bio14
bio15
BDW
CLY
DES
NTO
SOC

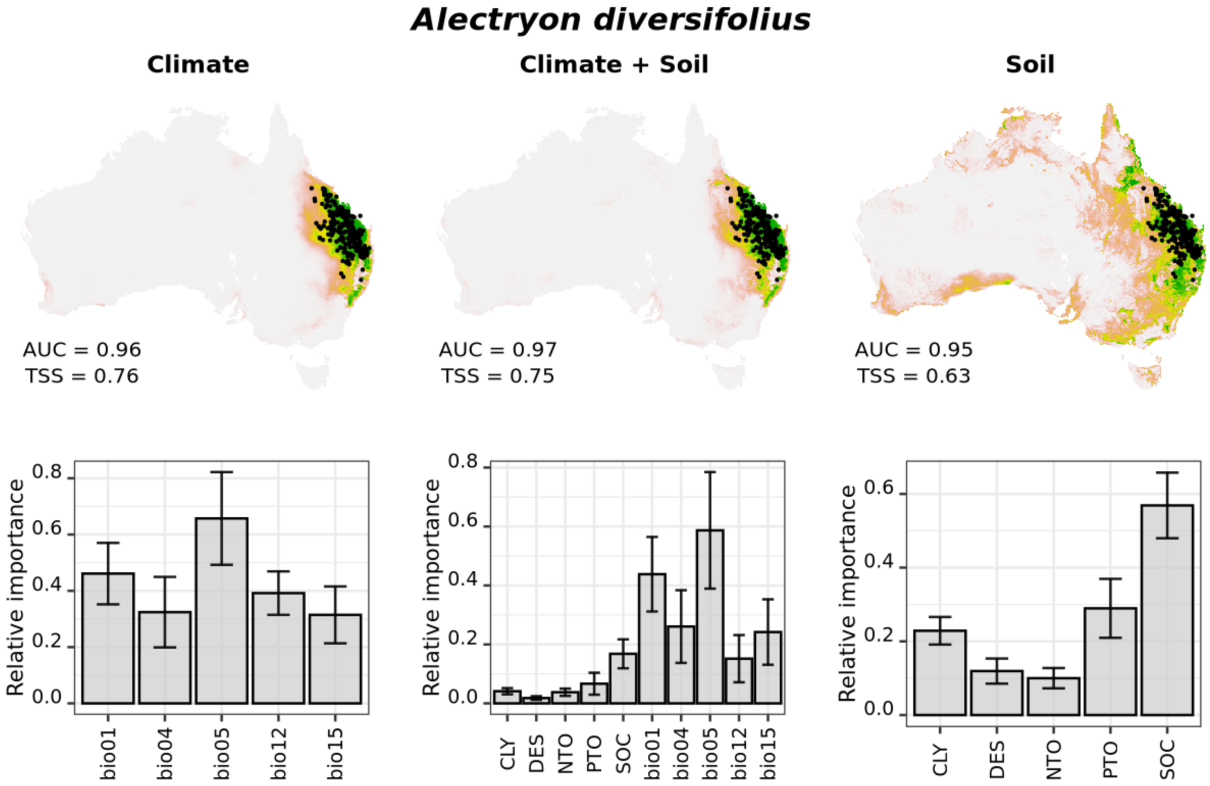
Alectryon diversifolius
Climate
Climate + Soil
Soil
AUC = 0.96
TSS = 0.76
AUC = 0.97
TSS = 0.75
AUC = 0.95
TSS = 0.63
Relative importance
0.0
0.2
0.4
0.6
0.8
bio01
bio04
bio05
bio12
bio15
CLY
DES
NTO
PTO
SOC

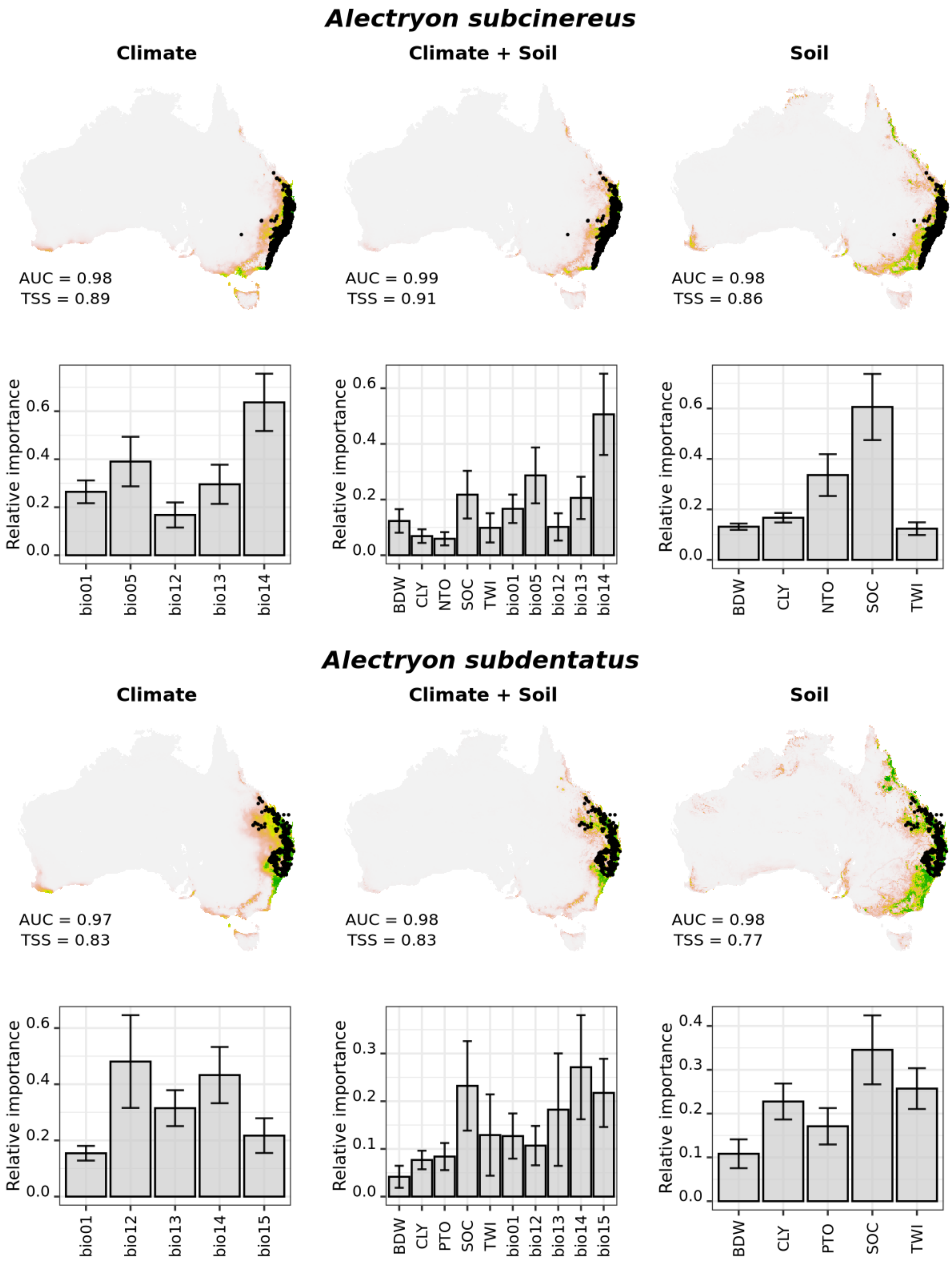
Alectryon subcinereus
Climate
Climate + Soil
Soil
AUC = 0.98
TSS = 0.89
AUC = 0.99
TSS = 0.91
AUC = 0.98
TSS = 0.86
Relative importance
bio01
bio05
bio12
bio13
bio14
BDW
CLY
NTO
SOC
TWI
Alectryon subdentatus
Climate
Climate + Soil
Soil
AUC = 0.97
TSS = 0.83
AUC = 0.98
TSS = 0.83
AUC = 0.98
TSS = 0.77
Relative importance
bio01
bio12
bio13
bio14
bio15
BDW
CLY
PTO
SOC
TWI

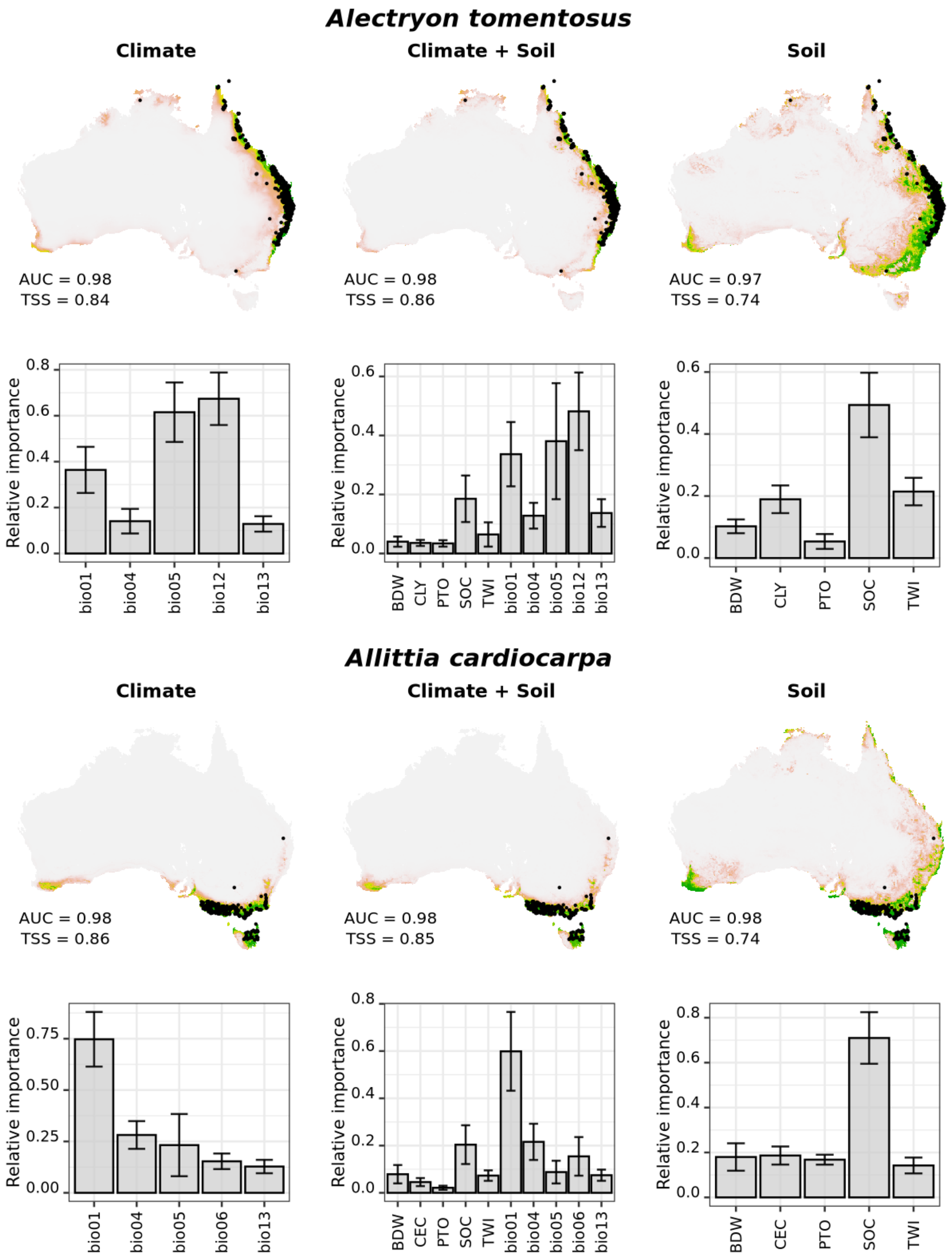
Alectryon tomentosus
Climate
Climate + Soil
Soil
AUC = 0.98
TSS = 0.84
AUC = 0.98
TSS = 0.86
AUC = 0.97
TSS = 0.74
Relative importance
0.0
0.2
0.4
0.6
0.8
bio01
bio04
bio05
bio12
bio13
BDW
CLY
PTO
SOC
TWI
Allittia cardiocarpa
AUC = 0.98
TSS = 0.86
AUC = 0.98
TSS = 0.85
AUC = 0.98
TSS = 0.74
0.00
0.25
0.50
0.75
bio06
CEC

Allittia uliginosa

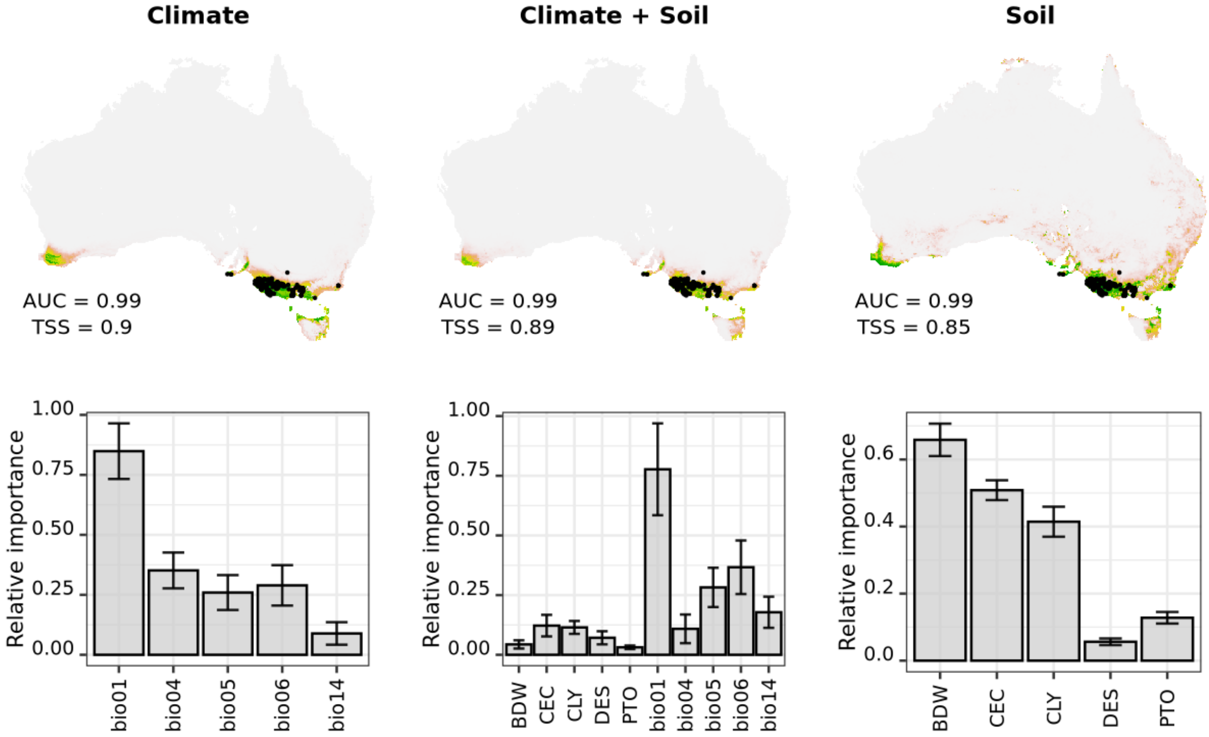

Allocasuarina acutivalvis

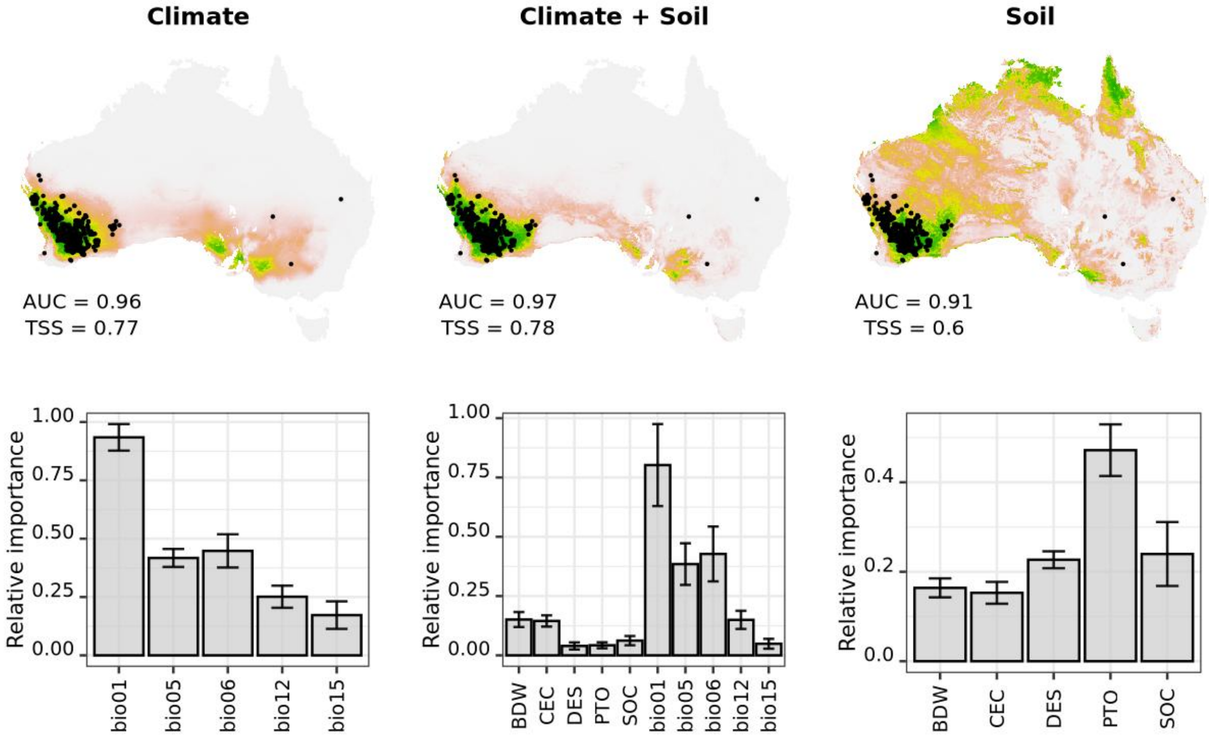

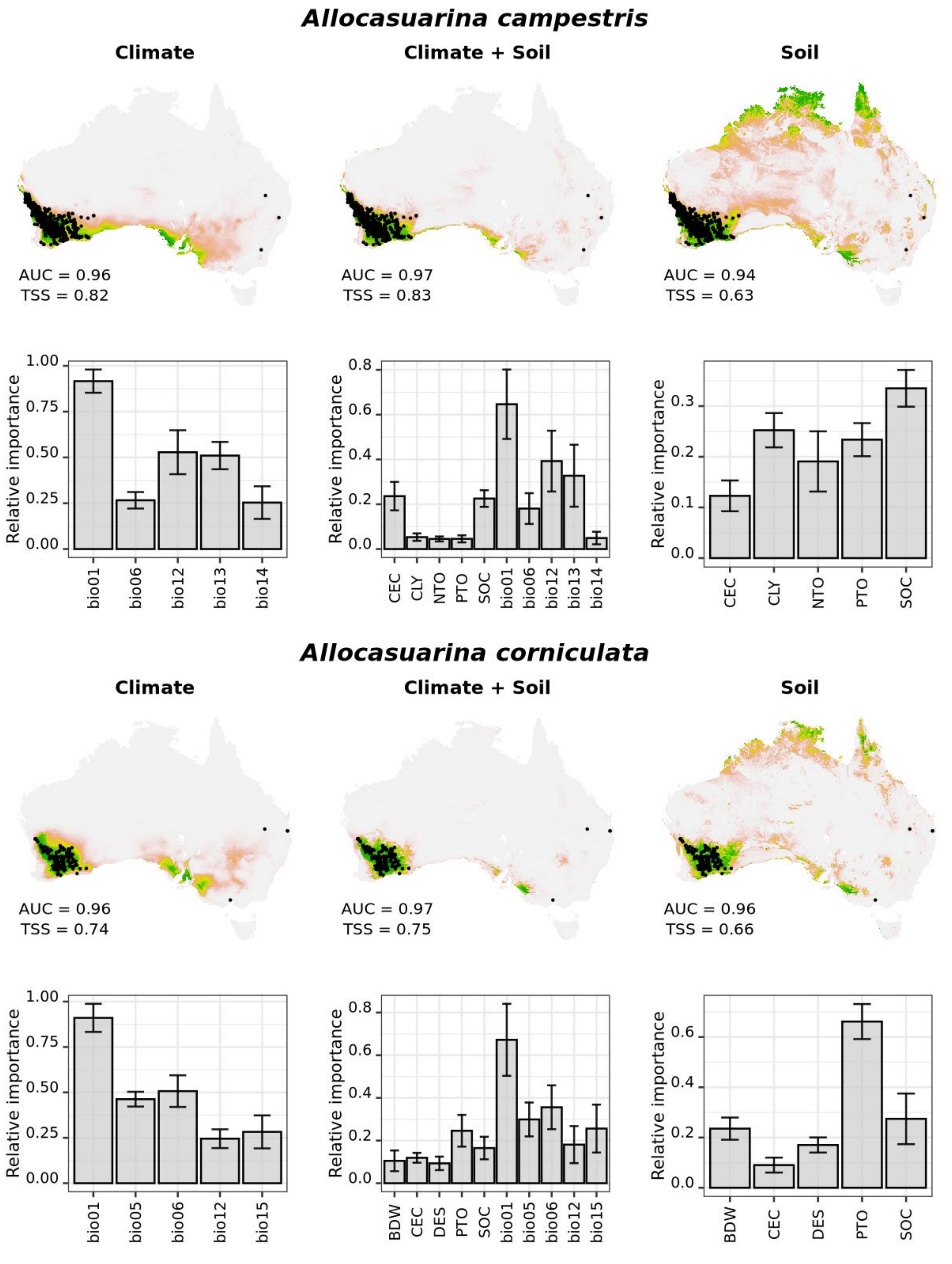

Allocasuarina campestris
Climate
Climate + Soil
Soil
AUC = 0.96
TSS = 0.82
AUC = 0.97
TSS = 0.83
AUC = 0.94
TSS = 0.63
Relative importance
1.00
0.75
0.50
0.25
0.00
bio01
bio06
bio12
bio13
bio14
0.8
0.6
0.4
0.2
0.0
CEC
CLY
NTO
PTO
SOC
bio01
bio06
bio12
bio13
bio14
0.3
0.2
0.1
0.0
CEC
CLY
NTO
PTO
SOC
Allocasuarina corniculata
Climate
Climate + Soil
Soil
AUC = 0.96
TSS = 0.74
AUC = 0.97
TSS = 0.75
AUC = 0.96
TSS = 0.66
Relative importance
1.00
0.75
0.50
0.25
0.00
bio01
bio05
bio06
bio12
bio15
0.8
0.6
0.4
0.2
0.0
BDW
CEC
DES
PTO
SOC
bio01
bio05
bio06
bio12
bio15
0.6
0.4
0.2
0.0
BDW
CEC
DES
PTO
SOC

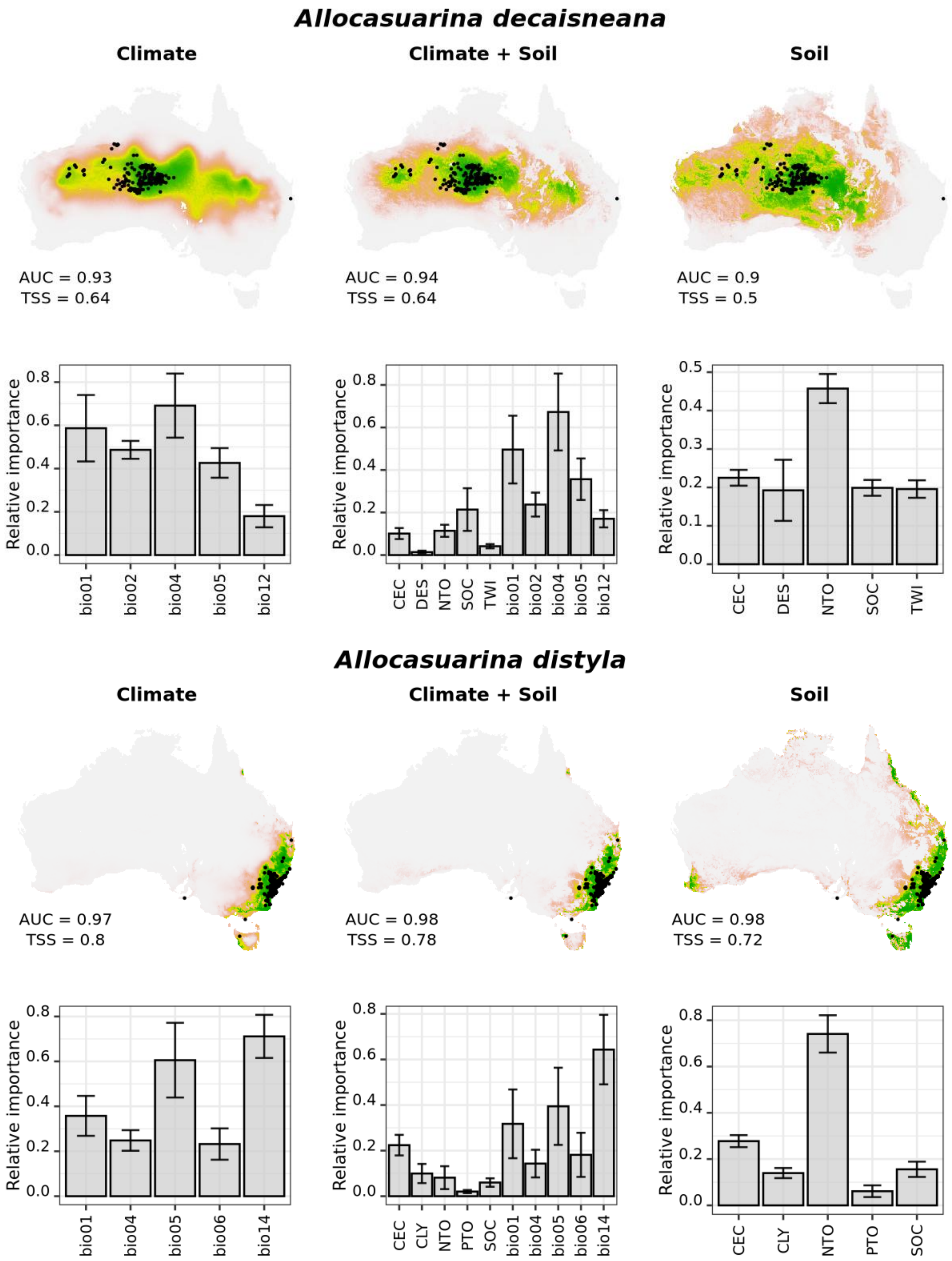

Allocasuarina decaisneana
Climate
Climate + Soil
Soil
AUC = 0.93
TSS = 0.64
AUC = 0.94
TSS = 0.64
AUC = 0.9
TSS = 0.5
Relative importance
bio01 bio02 bio04 bio05 bio12
CEC DES NTO SOC TWI bio01 bio02 bio04 bio05 bio12
CEC DES NTO SOC TWI
Allocasuarina distyla
Climate
Climate + Soil
Soil
AUC = 0.97
TSS = 0.8
AUC = 0.98
TSS = 0.78
AUC = 0.98
TSS = 0.72
Relative importance
bio01 bio04 bio05 bio06 bio14
CEC CLY NTO PTO SOC bio01 bio04 bio05 bio06 bio14
CEC CLY NTO PTO SOC

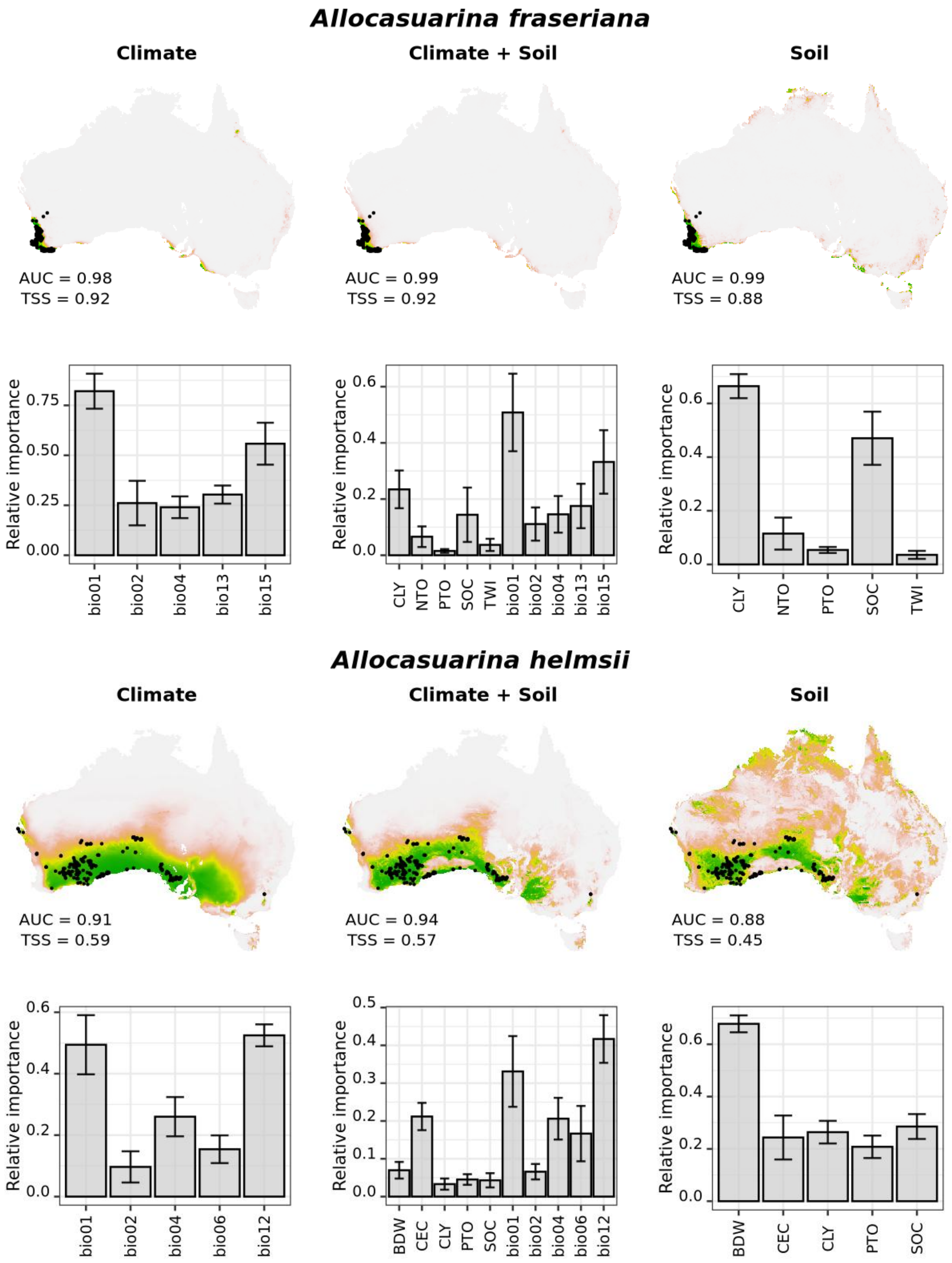

Allocasuarina fraseriana
Climate
Climate + Soil
Soil
AUC = 0.98
TSS = 0.92
AUC = 0.99
TSS = 0.92
AUC = 0.99
TSS = 0.88
Relative importance
bio01
bio02
bio04
bio13
bio15
CLY
NTO
PTO
SOC
TWI
Allocasuarina helmsii
Climate
Climate + Soil
Soil
AUC = 0.91
TSS = 0.59
AUC = 0.94
TSS = 0.57
AUC = 0.88
TSS = 0.45
Relative importance
bio01
bio02
bio04
bio06
bio12
BDW
CEC
CLY
PTO
SOC

Allocasuarina huegeliana

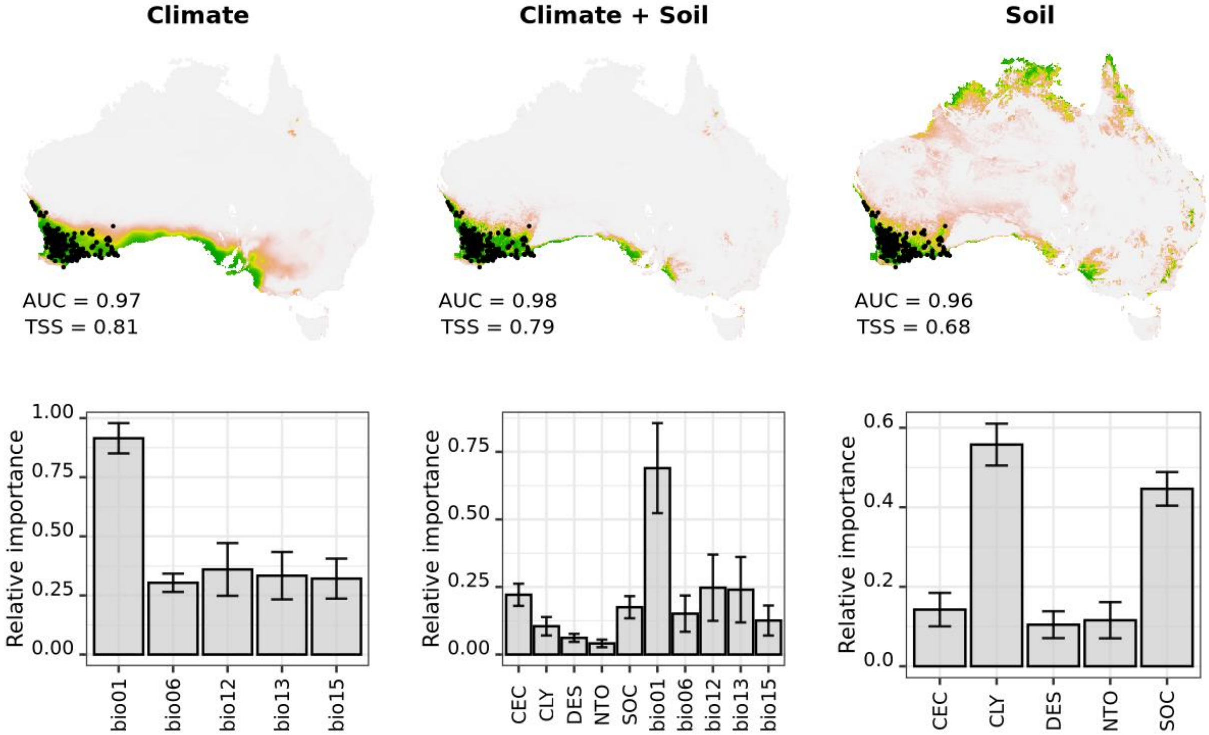

Allocasuarina humilis

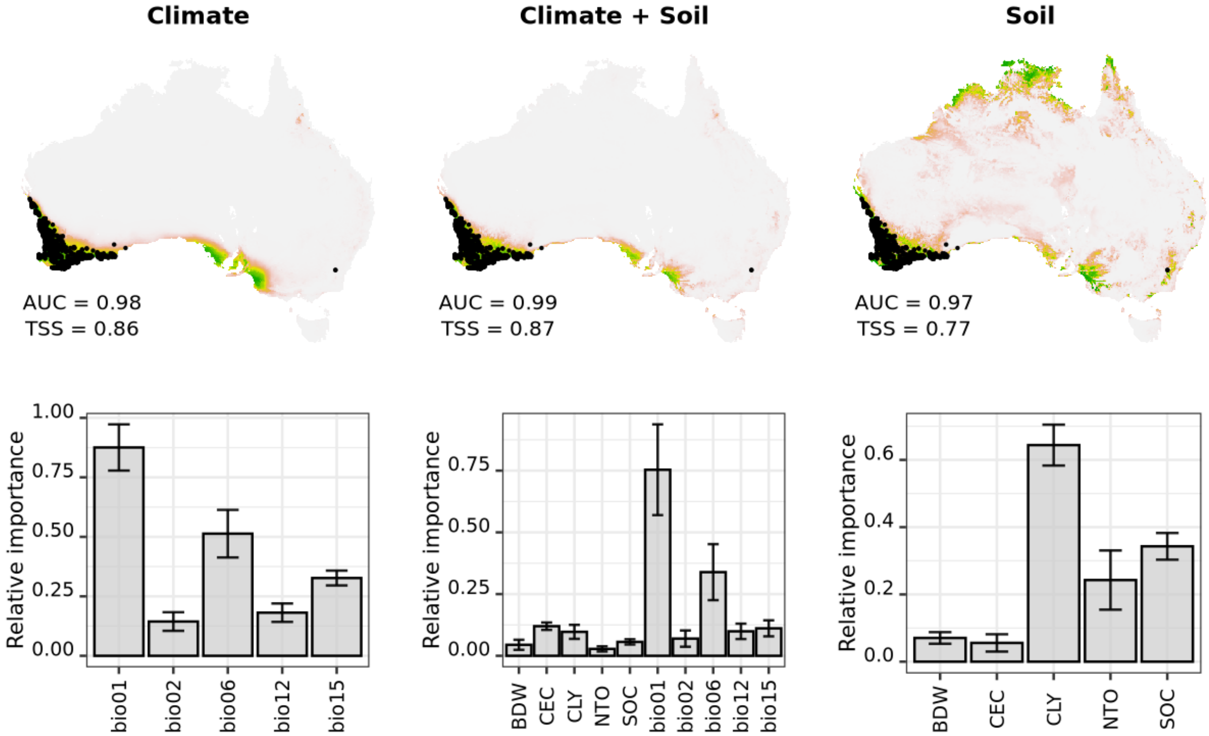

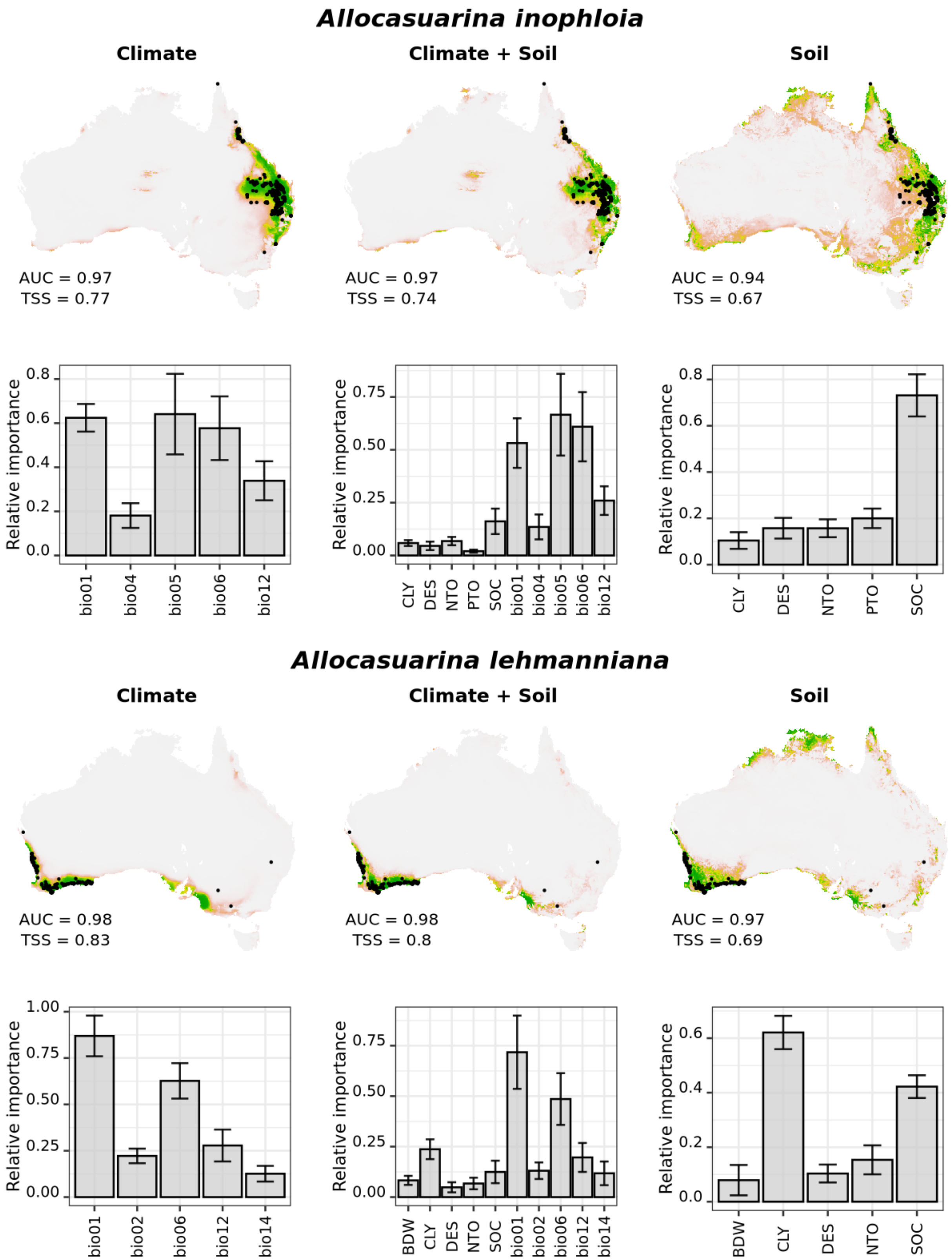
Allocasuarina inophloia
Climate
Climate + Soil
Soil
AUC = 0.97
TSS = 0.77
AUC = 0.97
TSS = 0.74
AUC = 0.94
TSS = 0.67
Relative importance
0.0
0.2
0.4
0.6
0.8
bio01
bio04
bio05
bio06
bio12
0.00
0.25
0.50
0.75
CLY
DES
NTO
PTO
SOC
bio01
bio04
bio05
bio06
bio12
CLY
DES
NTO
PTO
SOC
Allocasuarina lehmanniana
Climate
Climate + Soil
Soil
AUC = 0.98
TSS = 0.83
AUC = 0.98
TSS = 0.8
AUC = 0.97
TSS = 0.69
Relative importance
0.00
0.25
0.50
0.75
1.00
bio01
bio02
bio06
bio12
bio14
BDW
CLY
DES
NTO
SOC
bio01
bio02
bio06
bio12
bio14
0.0
0.2
0.4
0.6
BDW
CLY
DES
NTO
SOC

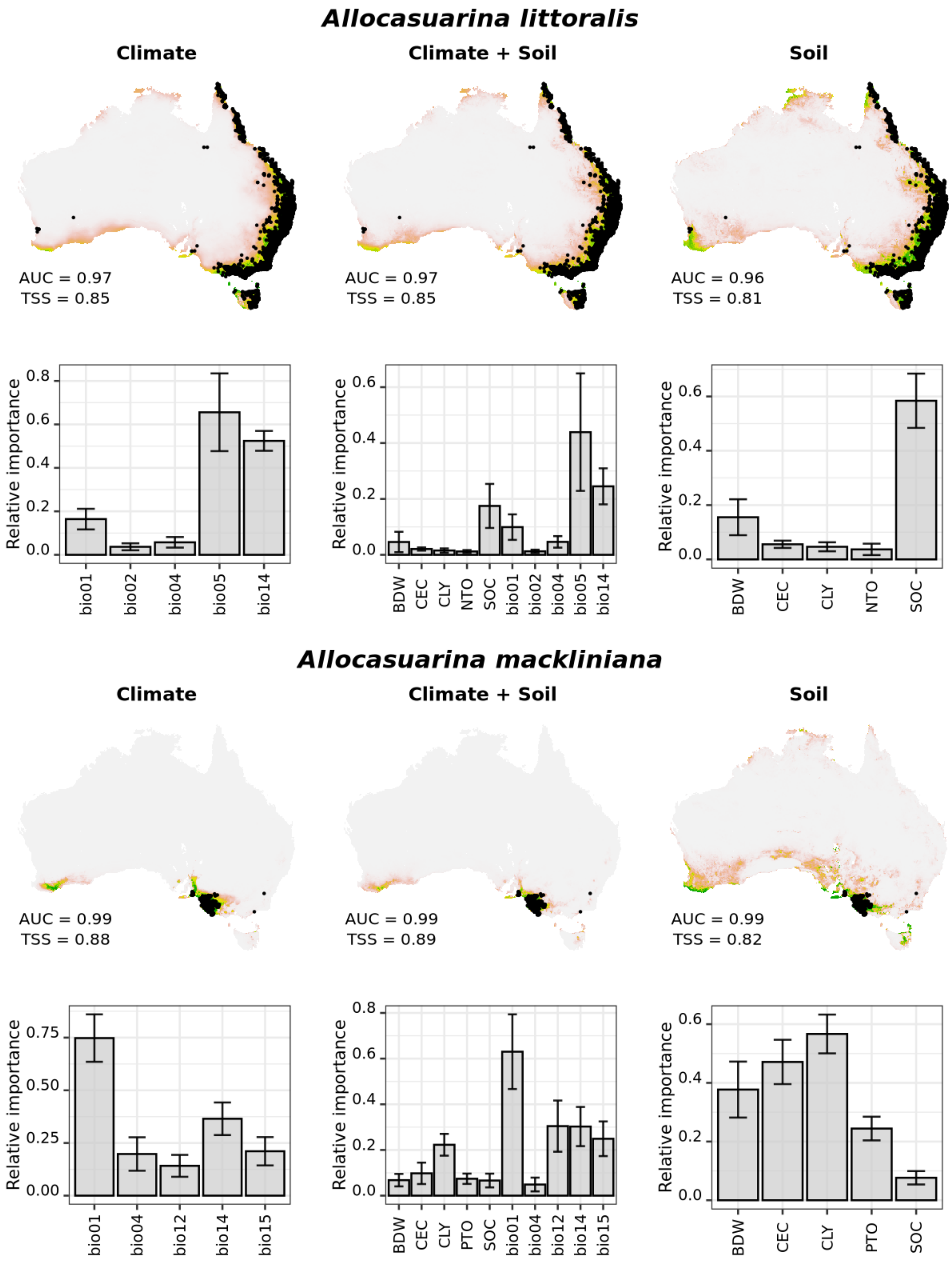

Allocasuarina littoralis
Climate
Climate + Soil
Soil
AUC = 0.97
TSS = 0.85
AUC = 0.97
TSS = 0.85
AUC = 0.96
TSS = 0.81
Relative importance
bio01
bio02
bio04
bio05
bio14
BDW
CEC
CLY
NTO
SOC
bio01
bio02
bio04
bio05
bio14
BDW
CEC
CLY
NTO
SOC
Allocasuarina mackliniana
Climate
Climate + Soil
Soil
AUC = 0.99
TSS = 0.88
AUC = 0.99
TSS = 0.89
AUC = 0.99
TSS = 0.82
Relative importance
bio01
bio04
bio12
bio14
bio15
BDW
CEC
CLY
PTO
SOC
bio01
bio04
bio12
bio14
bio15
BDW
CEC
CLY
PTO
SOC

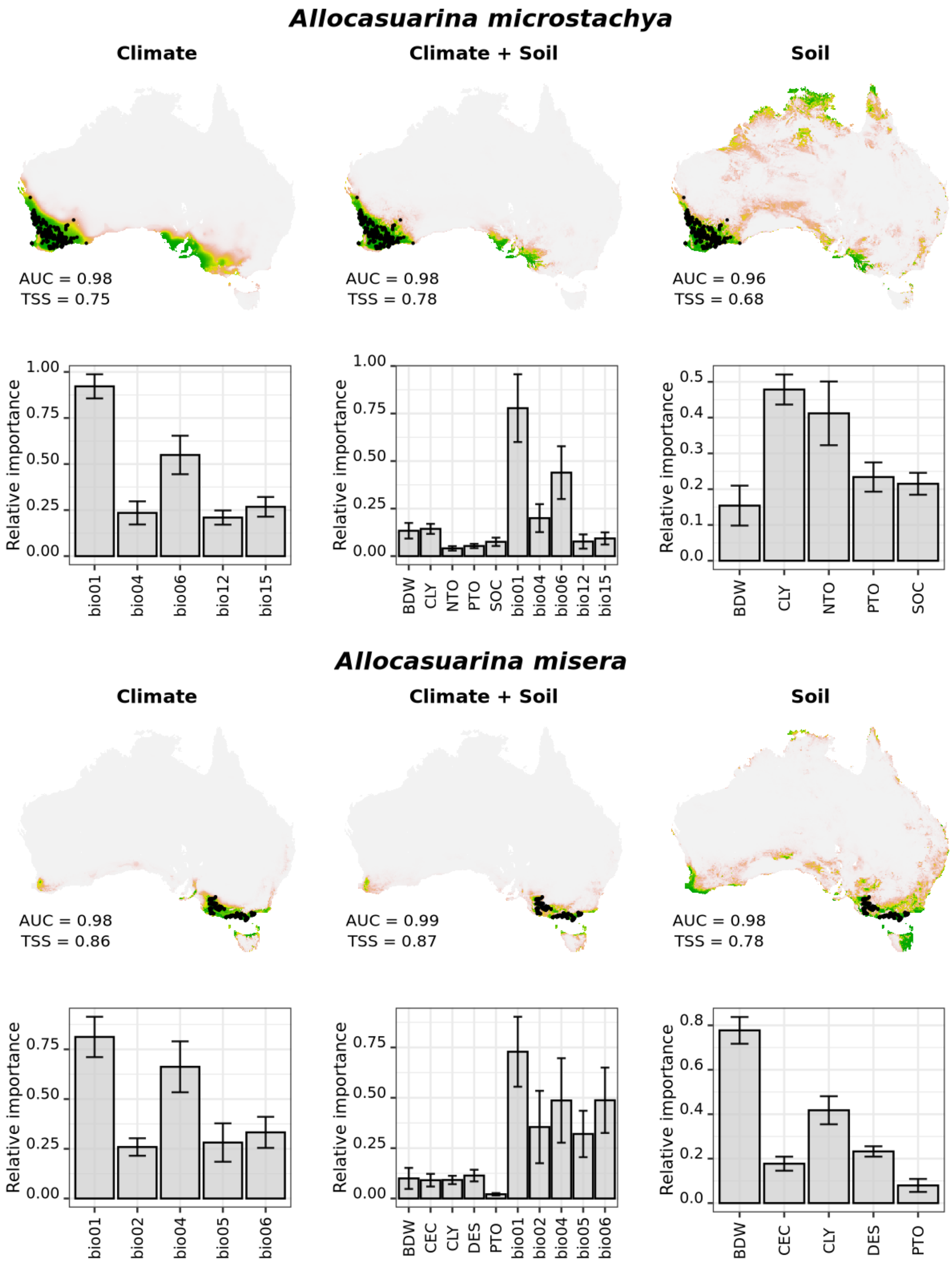

Allocasuarina microstachya
Climate
Climate + Soil
Soil
AUC = 0.98
TSS = 0.75
AUC = 0.98
TSS = 0.78
AUC = 0.96
TSS = 0.68
Relative importance
bio01
bio04
bio06
bio12
bio15
BDW
CLY
NTO
PTO
SOC
Allocasuarina misera
Climate
Climate + Soil
Soil
AUC = 0.98
TSS = 0.86
AUC = 0.99
TSS = 0.87
AUC = 0.98
TSS = 0.78
Relative importance
bio01
bio02
bio04
bio05
bio06
BDW
CEC
CLY
DES
PTO

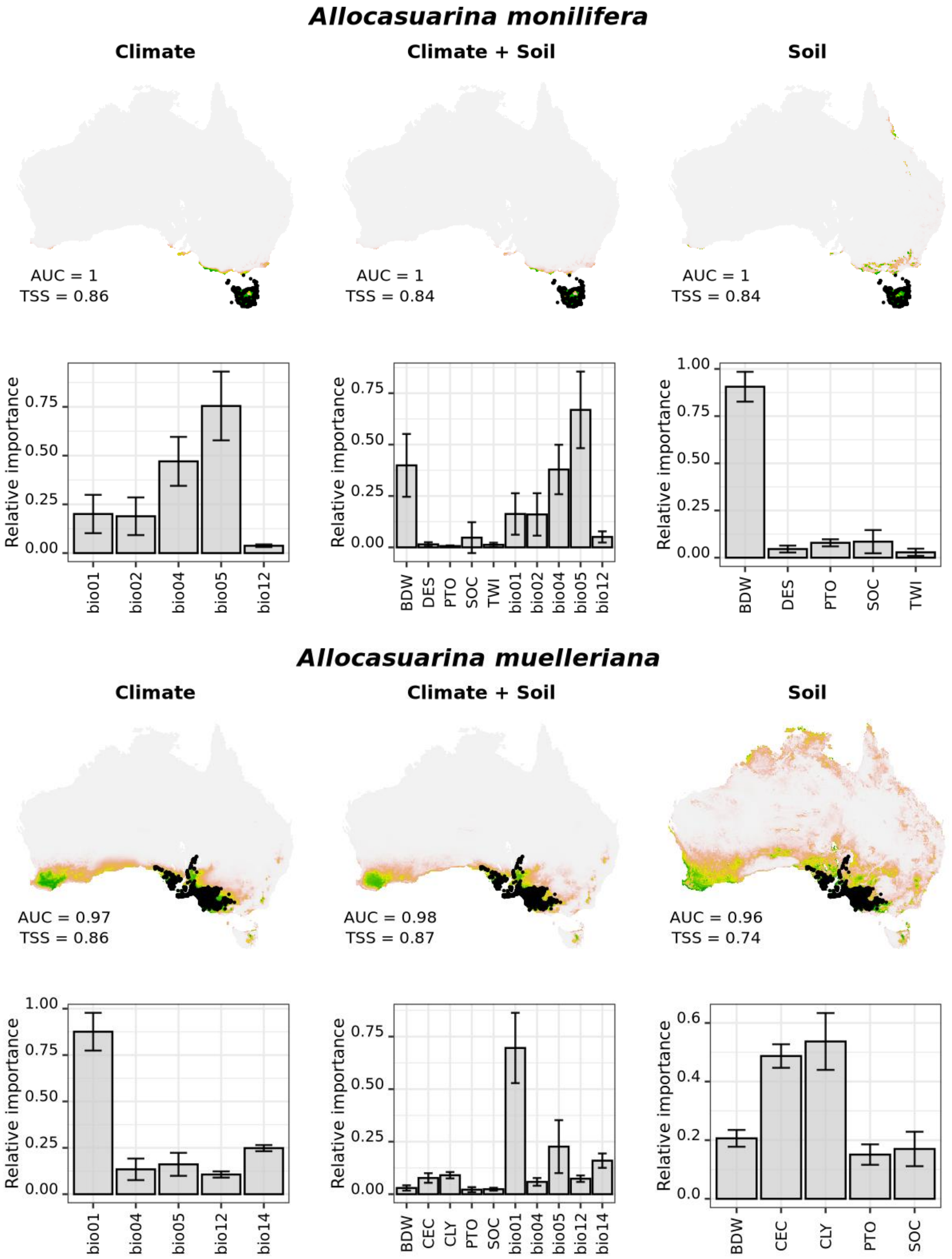
Allocasuarina monilifera
Climate
Climate + Soil
Soil
AUC = 1
TSS = 0.86
AUC = 1
TSS = 0.84
AUC = 1
TSS = 0.84
Relative importance
0.00
0.25
0.50
0.75
1.00
bio01
bio02
bio04
bio05
bio12
BDW
DES
PTO
SOC
TWI
Allocasuarina muelleriana
AUC = 0.97
TSS = 0.86
AUC = 0.98
TSS = 0.87
AUC = 0.96
TSS = 0.74
0.0
0.2
0.4
0.6
bio14
CEC
CLY

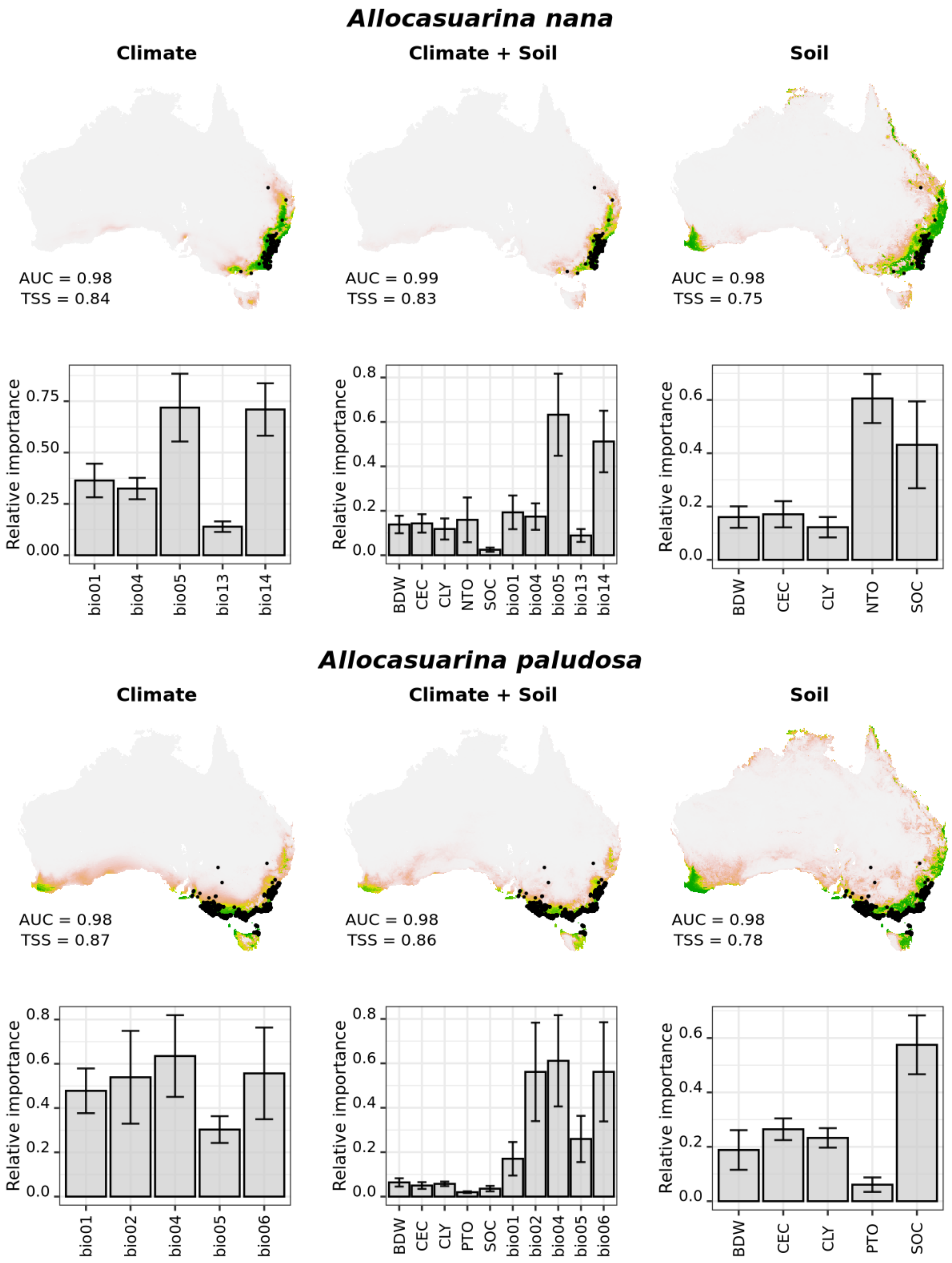

Allocasuarina nana
Climate
Climate + Soil
Soil
AUC = 0.98
TSS = 0.84
AUC = 0.99
TSS = 0.83
AUC = 0.98
TSS = 0.75
Relative importance
bio01 bio04 bio05 bio13 bio14
BDW CEC CLY NTO SOC bio01 bio04 bio05 bio13 bio14
BDW CEC CLY NTO SOC
Allocasuarina paludosa
Climate
Climate + Soil
Soil
AUC = 0.98
TSS = 0.87
AUC = 0.98
TSS = 0.86
AUC = 0.98
TSS = 0.78
Relative importance
bio01 bio02 bio04 bio05 bio06
BDW CEC CLY PTO SOC bio01 bio02 bio04 bio05 bio06
BDW CEC CLY PTO SOC

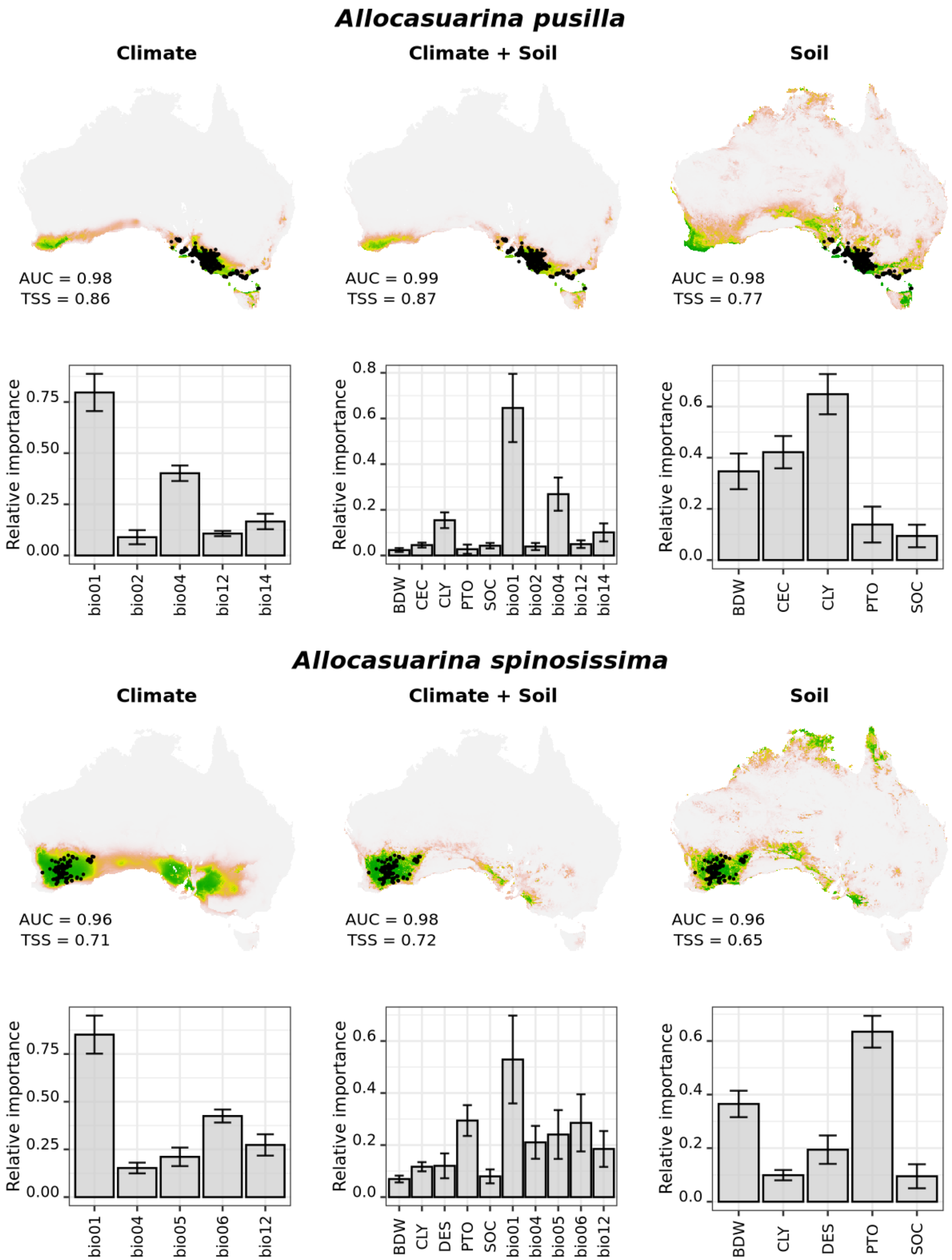

Allocasuarina pusilla
Climate
Climate + Soil
Soil
AUC = 0.98
TSS = 0.86
AUC = 0.99
TSS = 0.87
AUC = 0.98
TSS = 0.77
Relative importance
bio01
bio02
bio04
bio12
bio14
BDW
CEC
CLY
PTO
SOC
Allocasuarina spinosissima
AUC = 0.96
TSS = 0.71
AUC = 0.98
TSS = 0.72
AUC = 0.96
TSS = 0.65
DES
bio05
bio06

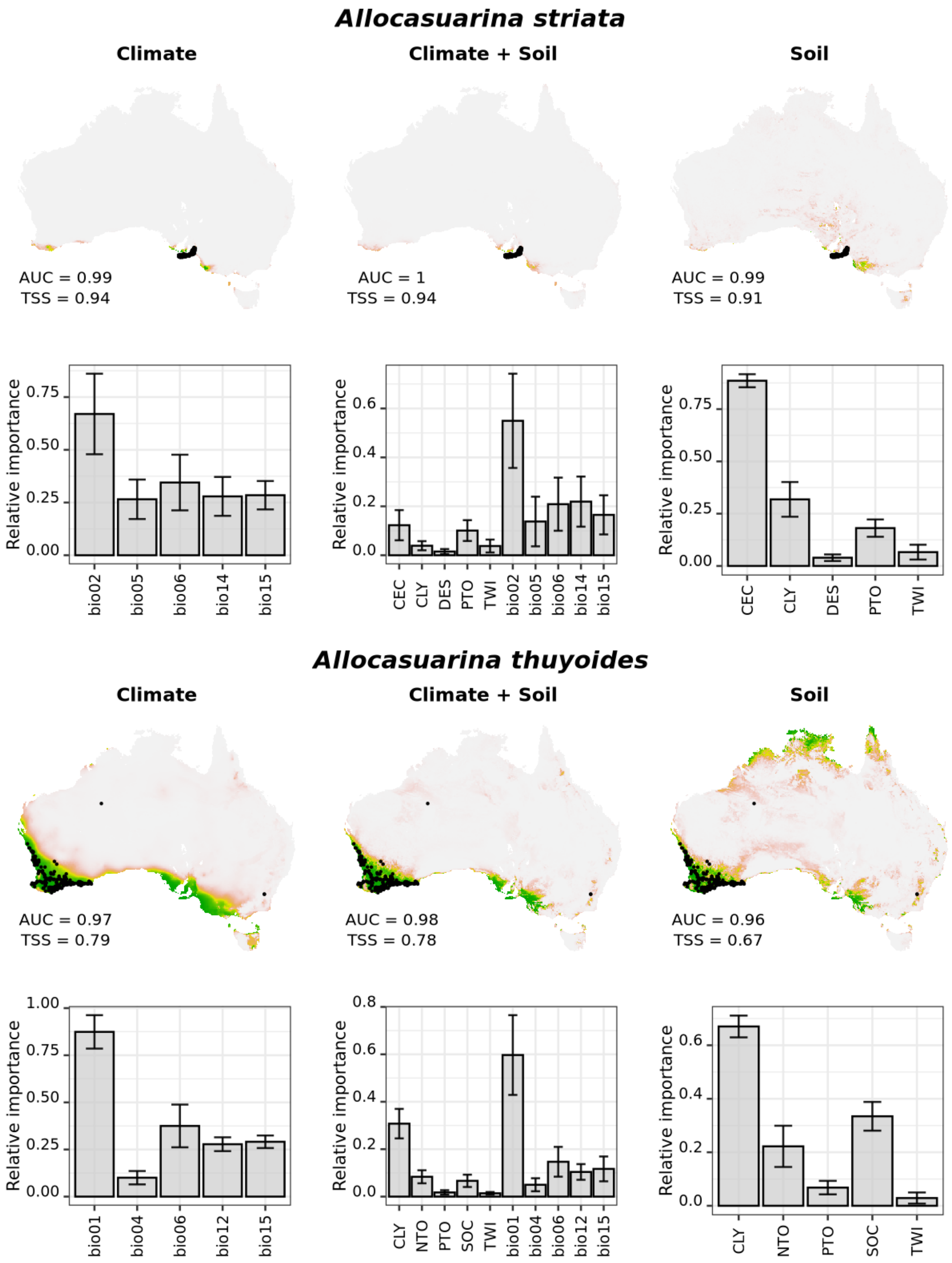

Allocasuarina striata
Climate
Climate + Soil
Soil
AUC = 0.99
TSS = 0.94
AUC = 1
TSS = 0.94
AUC = 0.99
TSS = 0.91
Relative importance
bio02
bio05
bio06
bio14
bio15
CEC
CLY
DES
PTO
TWI
Allocasuarina thuyoides
AUC = 0.97
TSS = 0.79
AUC = 0.98
TSS = 0.78
AUC = 0.96
TSS = 0.67
bio01
bio04
bio06
bio12
bio15
NTO
SOC

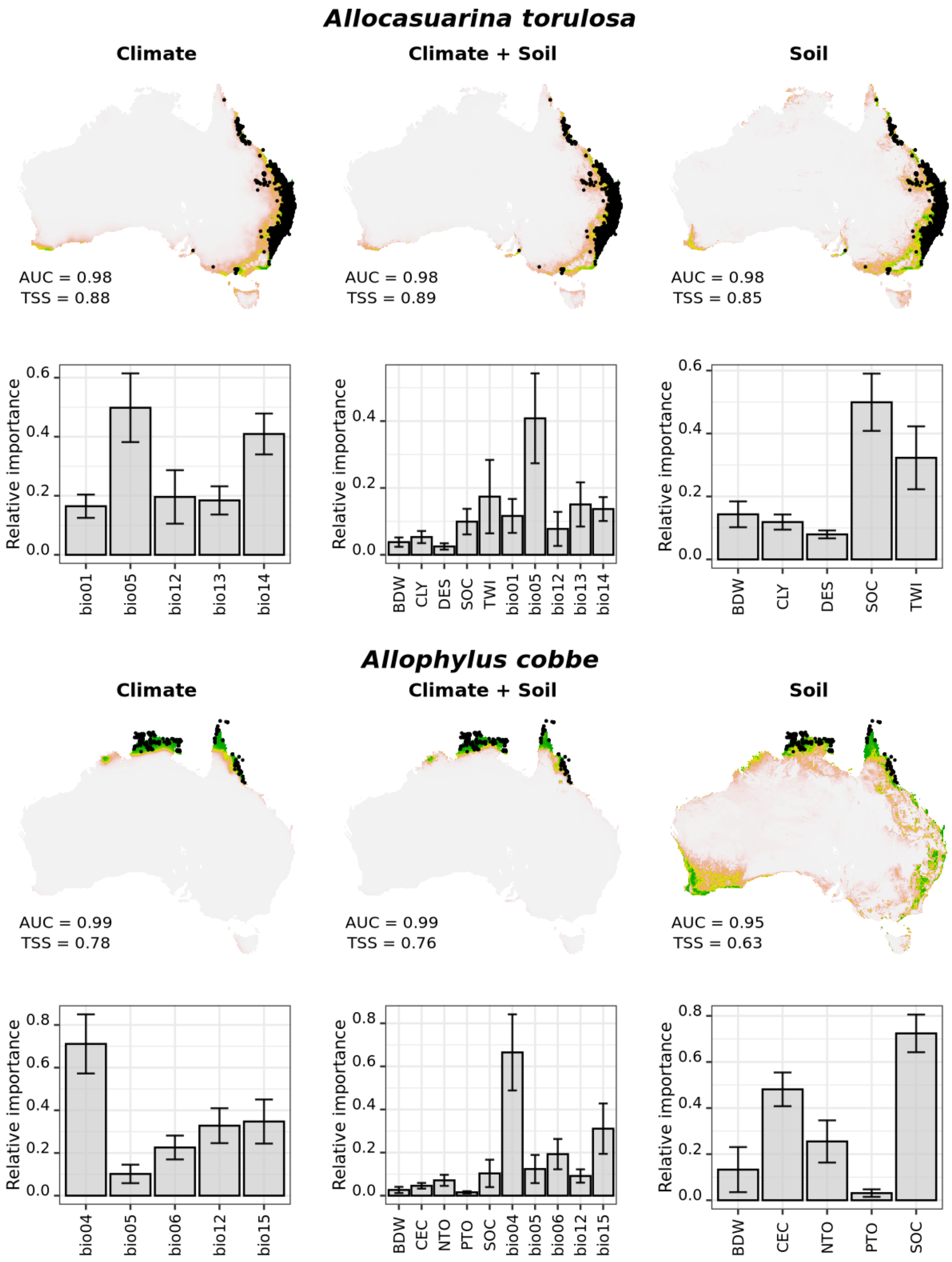

Allocasuarina torulosa
Climate
Climate + Soil
Soil
AUC = 0.98
TSS = 0.88
AUC = 0.98
TSS = 0.89
AUC = 0.98
TSS = 0.85
Relative importance
0.0
0.2
0.4
0.6
bio01
bio05
bio12
bio13
bio14
BDW
CLY
DES
SOC
TWI
bio01
bio05
bio12
bio13
bio14
BDW
CLY
DES
SOC
TWI
Allophylus cobbe
Climate
Climate + Soil
Soil
AUC = 0.99
TSS = 0.78
AUC = 0.99
TSS = 0.76
AUC = 0.95
TSS = 0.63
Relative importance
0.0
0.2
0.4
0.6
0.8
bio04
bio05
bio06
bio12
bio15
BDW
CEC
NTO
PTO
SOC
bio04
bio05
bio06
bio12
bio15
BDW
CEC
NTO
PTO
SOC

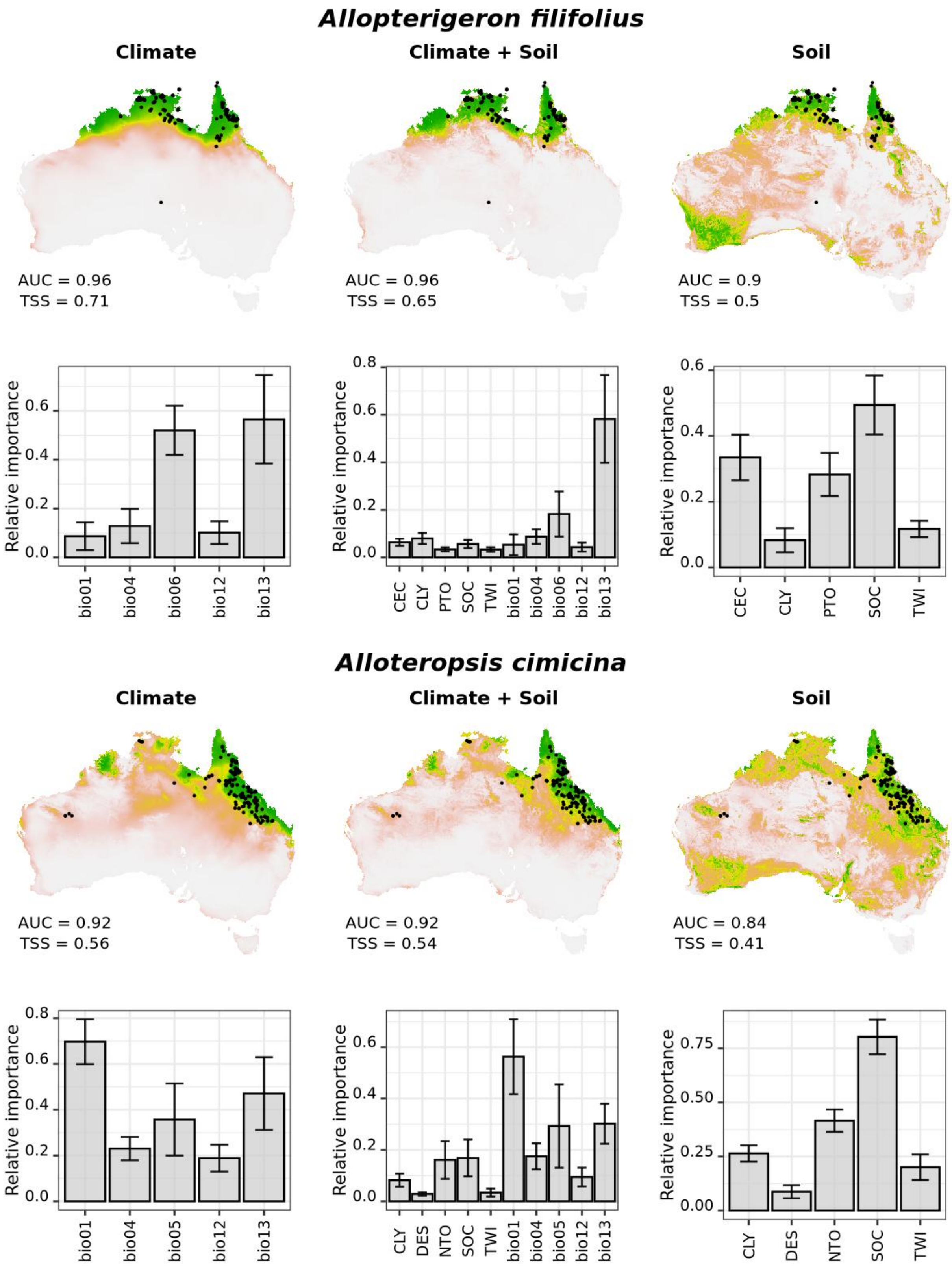
Allopterigeron filifolius
Climate
Climate + Soil
Soil
AUC = 0.96
TSS = 0.71
AUC = 0.96
TSS = 0.65
AUC = 0.9
TSS = 0.5
Relative importance
bio01 bio04 bio06 bio12 bio13
CEC CLY PTO SOC TWI bio01 bio04 bio06 bio12 bio13
CEC CLY PTO SOC TWI
Alloteropsis cimicina
Climate
Climate + Soil
Soil
AUC = 0.92
TSS = 0.56
AUC = 0.92
TSS = 0.54
AUC = 0.84
TSS = 0.41
Relative importance
bio01 bio04 bio05 bio12 bio13
CLY DES NTO SOC TWI bio01 bio04 bio05 bio12 bio13
CLY DES NTO SOC TWI

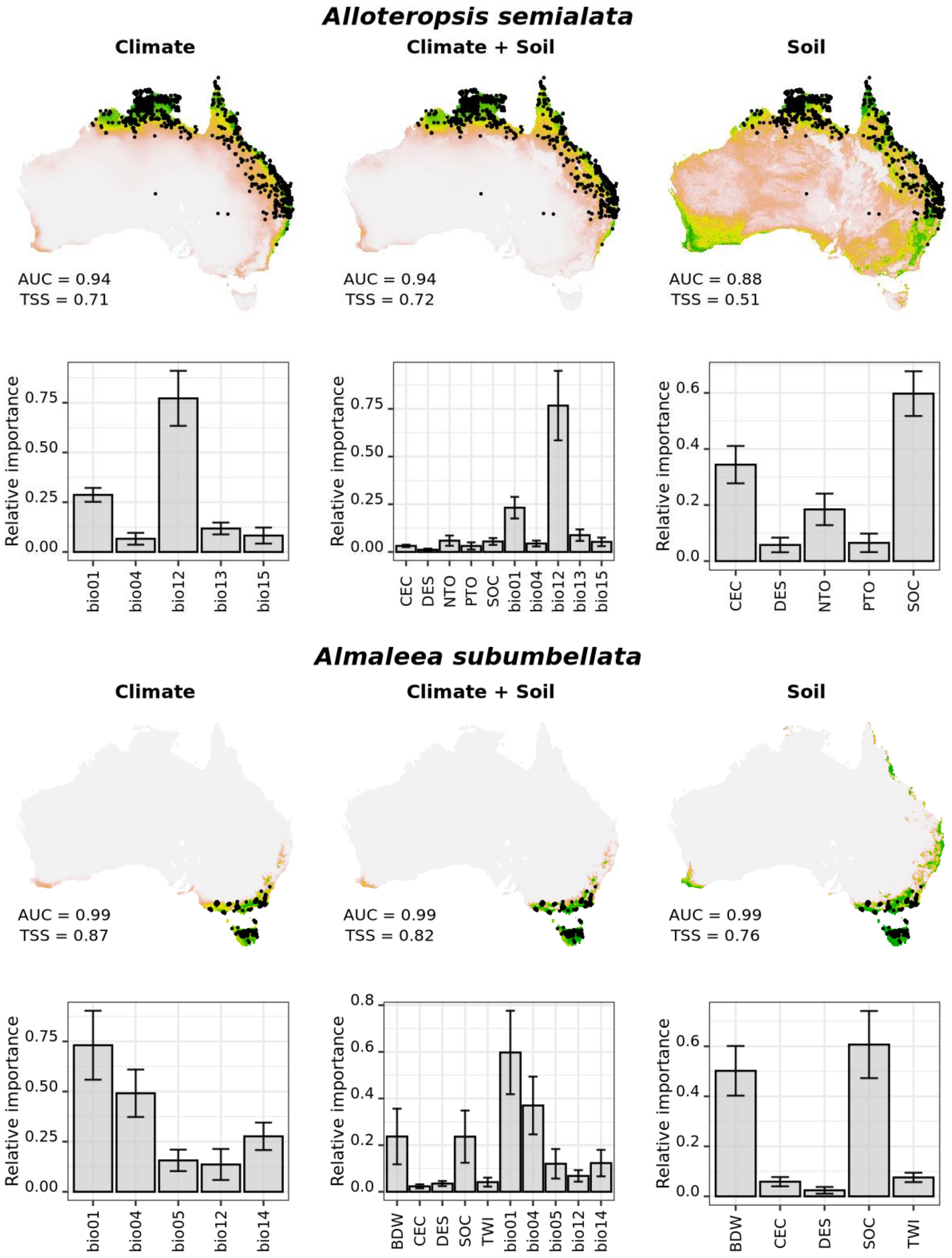

Alloteropsis semialata
Climate
Climate + Soil
Soil
AUC = 0.94
TSS = 0.71
AUC = 0.94
TSS = 0.72
AUC = 0.88
TSS = 0.51
Relative importance
0.00
0.25
0.50
0.75
bio01
bio04
bio12
bio13
bio15
Relative importance
0.00
0.25
0.50
0.75
CEC
DES
NTO
PTO
SOC
bio01
bio04
bio12
bio13
bio15
Relative importance
0.0
0.2
0.4
0.6
CEC
DES
NTO
PTO
SOC
Almaleea subumbellata
Climate
Climate + Soil
Soil
AUC = 0.99
TSS = 0.87
AUC = 0.99
TSS = 0.82
AUC = 0.99
TSS = 0.76
Relative importance
0.00
0.25
0.50
0.75
bio01
bio04
bio05
bio12
bio14
Relative importance
0.0
0.2
0.4
0.6
0.8
BDW
CEC
DES
SOC
TWI
bio01
bio04
bio05
bio12
bio14
Relative importance
0.0
0.2
0.4
0.6
BDW
CEC
DES
SOC
TWI

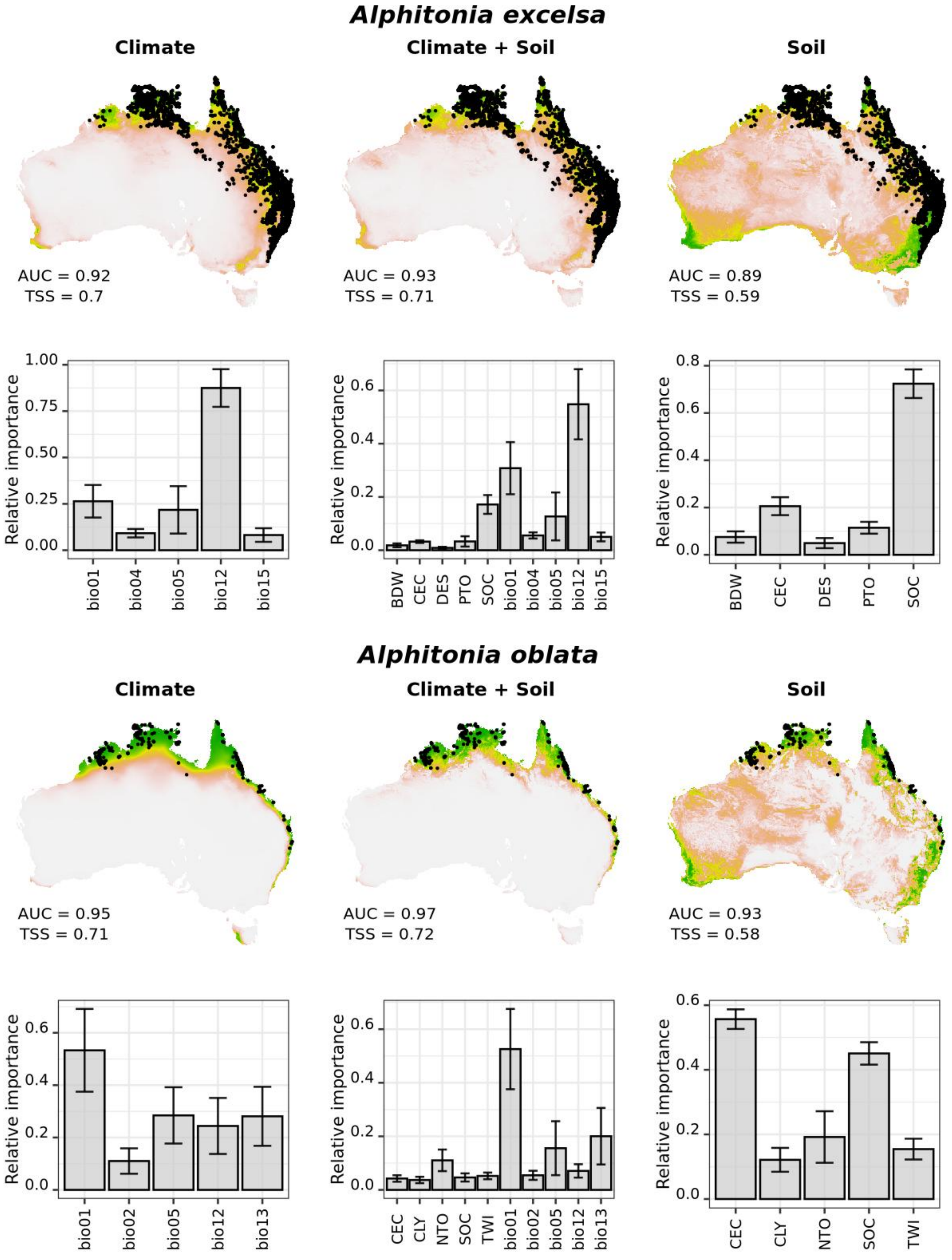

Alphitonia excelsa
Climate
Climate + Soil
Soil
AUC = 0.92
TSS = 0.7
AUC = 0.93
TSS = 0.71
AUC = 0.89
TSS = 0.59
Relative importance
bio01 bio04 bio05 bio12 bio15
BDW CEC DES PTO SOC bio01 bio04 bio05 bio12 bio15
BDW CEC DES PTO SOC
Alphitonia oblata
Climate
Climate + Soil
Soil
AUC = 0.95
TSS = 0.71
AUC = 0.97
TSS = 0.72
AUC = 0.93
TSS = 0.58
Relative importance
bio01 bio02 bio05 bio12 bio13
CEC CLY NTO SOC TWI bio01 bio02 bio05 bio12 bio13
CEC CLY NTO SOC TWI

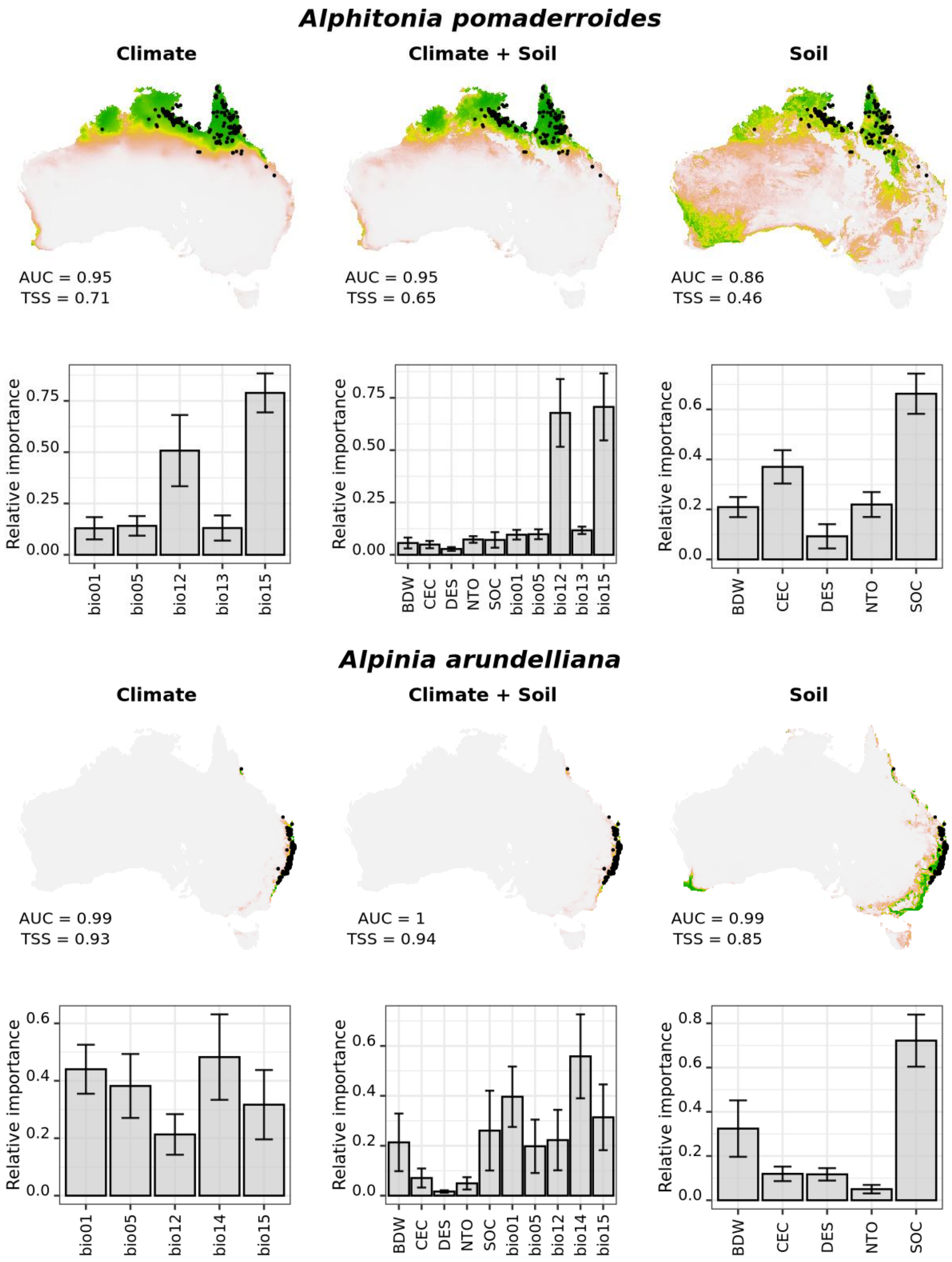

Alphitonia pomaderroides
Climate
Climate + Soil
Soil
AUC = 0.95
TSS = 0.71
AUC = 0.95
TSS = 0.65
AUC = 0.86
TSS = 0.46
Relative importance
0.00
0.25
0.50
0.75
bio01
bio05
bio12
bio13
bio15
BDW
CEC
DES
NTO
SOC
0.0
0.2
0.4
0.6
Alpinia arundelliana
Climate
Climate + Soil
Soil
AUC = 0.99
TSS = 0.93
AUC = 1
TSS = 0.94
AUC = 0.99
TSS = 0.85
Relative importance
0.8
bio14

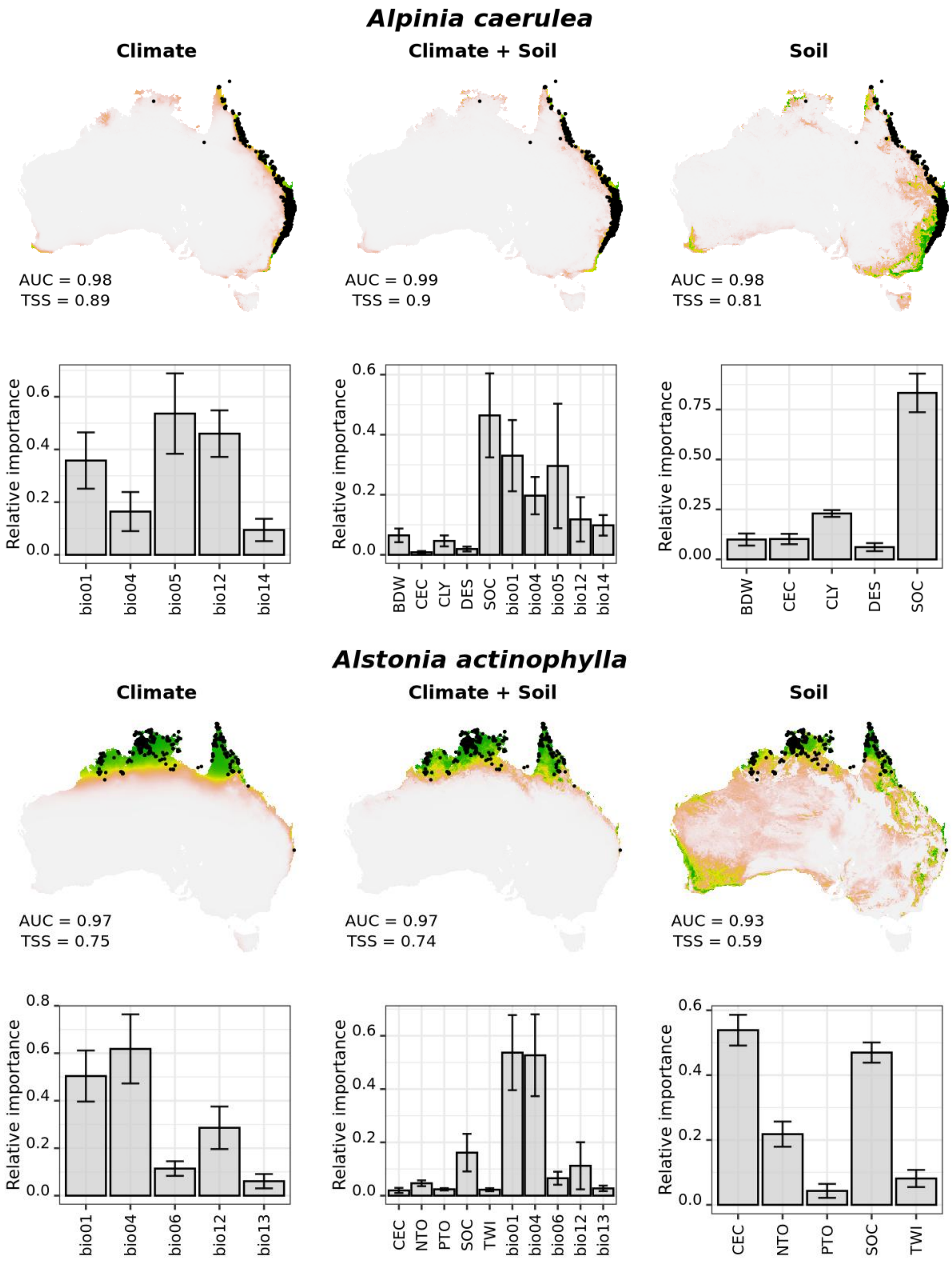
Alpinia caerulea
Climate
Climate + Soil
Soil
AUC = 0.98
TSS = 0.89
AUC = 0.99
TSS = 0.9
AUC = 0.98
TSS = 0.81
Relative importance
bio01 bio04 bio05 bio12 bio14
BDW CEC CLY DES SOC bio01 bio04 bio05 bio12 bio14
BDW CEC CLY DES SOC
Alstonia actinophylla
Climate
Climate + Soil
Soil
AUC = 0.97
TSS = 0.75
AUC = 0.97
TSS = 0.74
AUC = 0.93
TSS = 0.59
Relative importance
bio01 bio04 bio06 bio12 bio13
CEC NTO PTO SOC TWI bio01 bio04 bio06 bio12 bio13
CEC NTO PTO SOC TWI

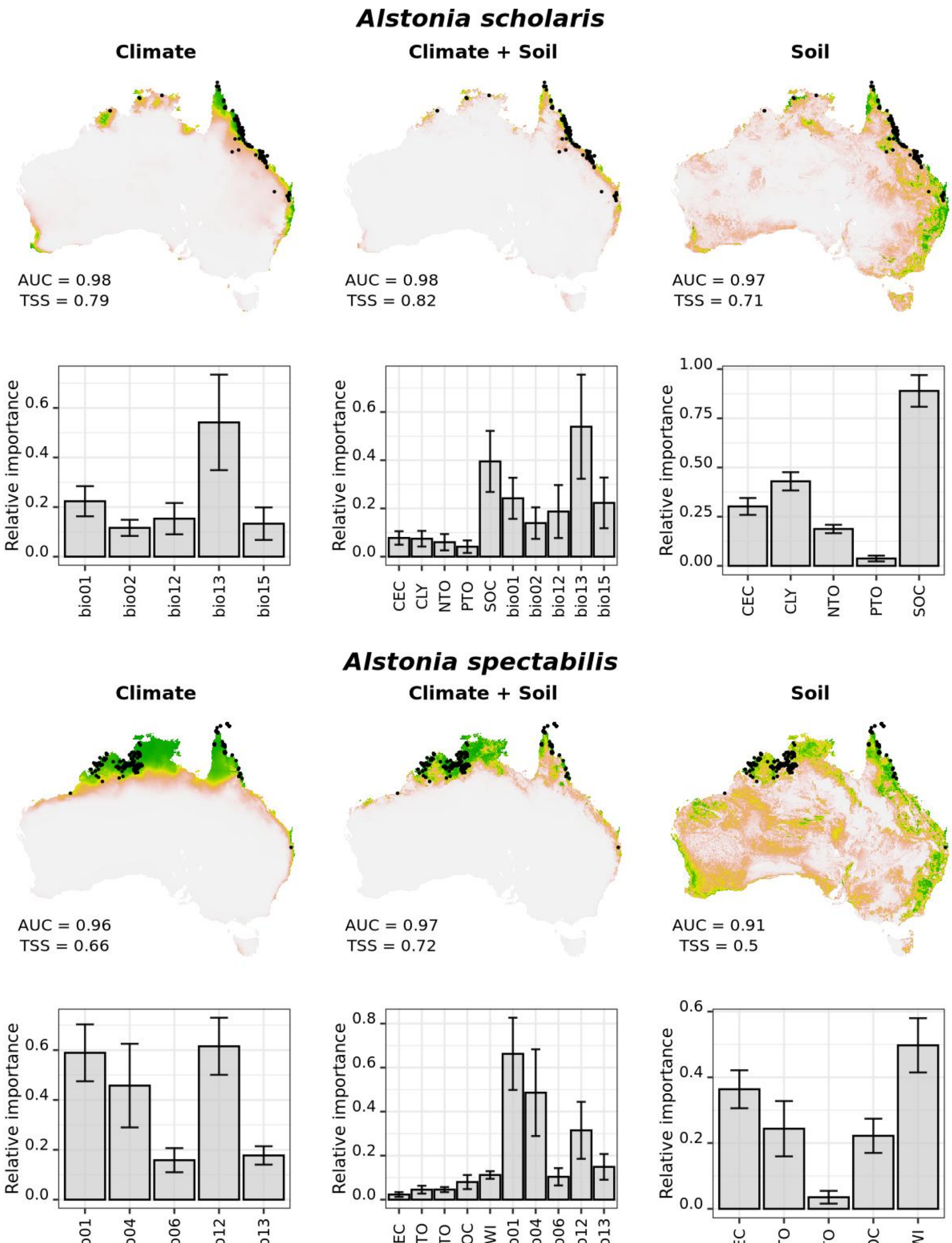
Alstonia scholaris
Climate
Climate + Soil
Soil
AUC = 0.98
TSS = 0.79
AUC = 0.98
TSS = 0.82
AUC = 0.97
TSS = 0.71
Relative importance
0.0 0.2 0.4 0.6
bio01 bio02 bio12 bio13 bio15
0.0 0.2 0.4 0.6
CEC CLY NTO PTO SOC bio01 bio02 bio12 bio13 bio15
0.00 0.25 0.50 0.75 1.00
CEC CLY NTO PTO SOC
Alstonia spectabilis
Climate
Climate + Soil
Soil
AUC = 0.96
TSS = 0.66
AUC = 0.97
TSS = 0.72
AUC = 0.91
TSS = 0.5
Relative importance
0.0 0.2 0.4 0.6
bio01 bio04 bio06 bio12 bio13
0.0 0.2 0.4 0.6 0.8
CEC NTO PTO SOC TWI bio01 bio04 bio06 bio12 bio13
0.0 0.2 0.4 0.6
CEC NTO PTO SOC TWI

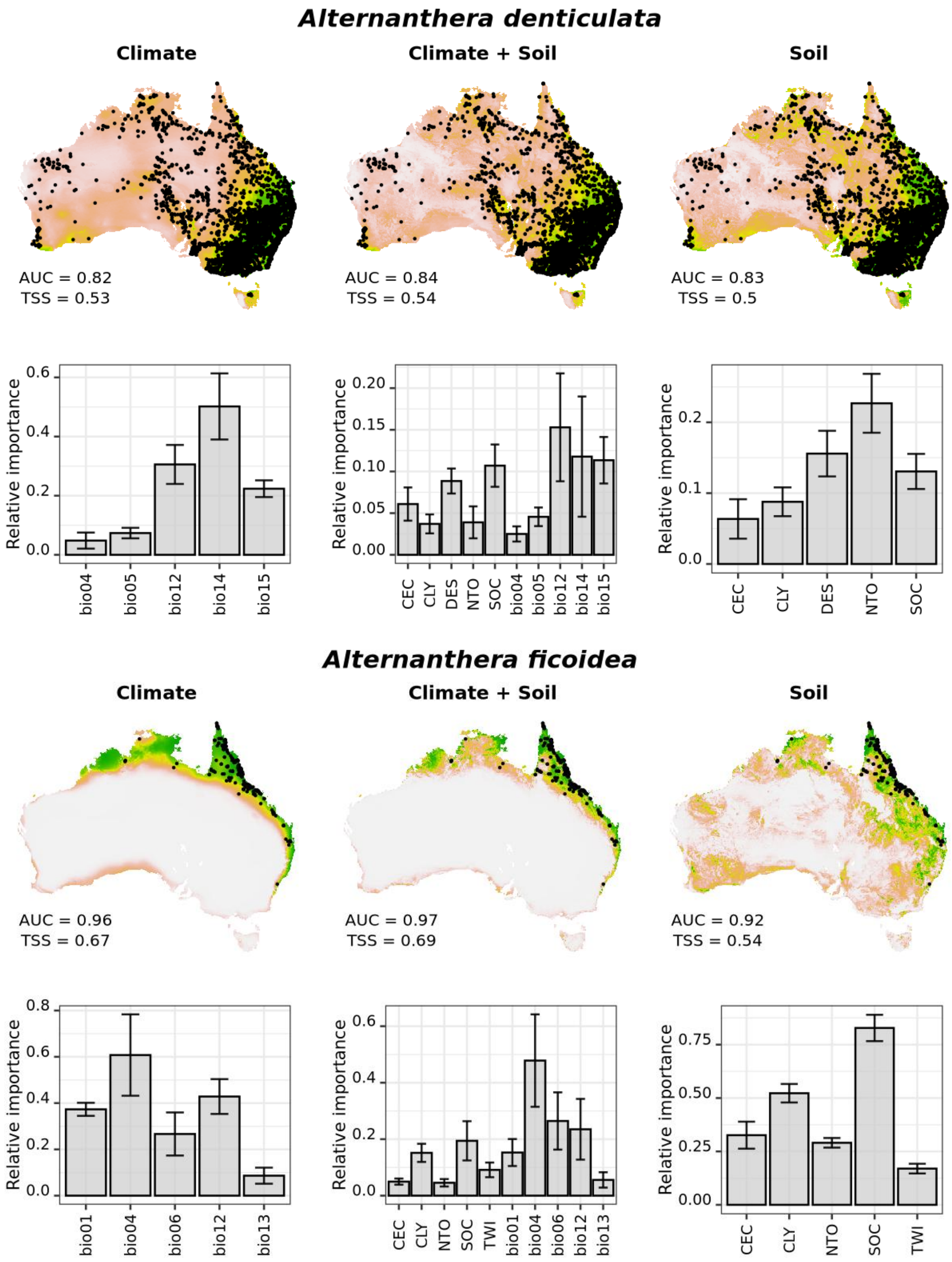
Alternanthera denticulata
Climate
Climate + Soil
Soil
AUC = 0.82
TSS = 0.53
AUC = 0.84
TSS = 0.54
AUC = 0.83
TSS = 0.5
Relative importance
bio04
bio05
bio12
bio14
bio15
CEC
CLY
DES
NTO
SOC
Alternanthera ficoidea
Climate
Climate + Soil
Soil
AUC = 0.96
TSS = 0.67
AUC = 0.97
TSS = 0.69
AUC = 0.92
TSS = 0.54
Relative importance
bio01
bio04
bio06
bio12
bio13
CEC
CLY
NTO
SOC
TWI

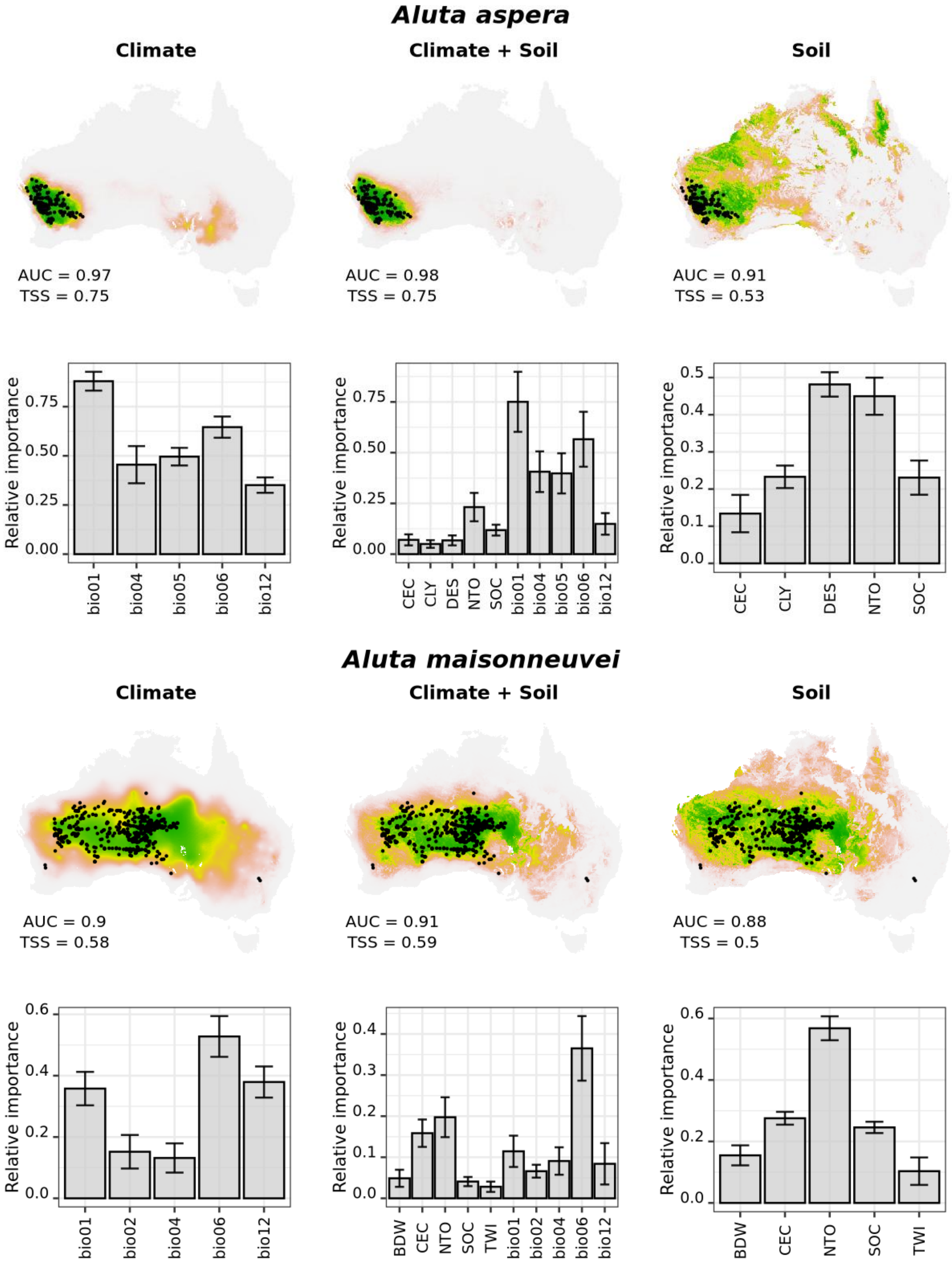

Aluta aspera
Climate
Climate + Soil
Soil
AUC = 0.97
TSS = 0.75
AUC = 0.98
TSS = 0.75
AUC = 0.91
TSS = 0.53
Relative importance
bio01
bio04
bio05
bio06
bio12
CEC
CLY
DES
NTO
SOC
Aluta maisonneuvei
Climate
Climate + Soil
Soil
AUC = 0.9
TSS = 0.58
AUC = 0.91
TSS = 0.59
AUC = 0.88
TSS = 0.5
Relative importance
bio01
bio02
bio04
bio06
bio12
BDW
CEC
NTO
SOC
TWI

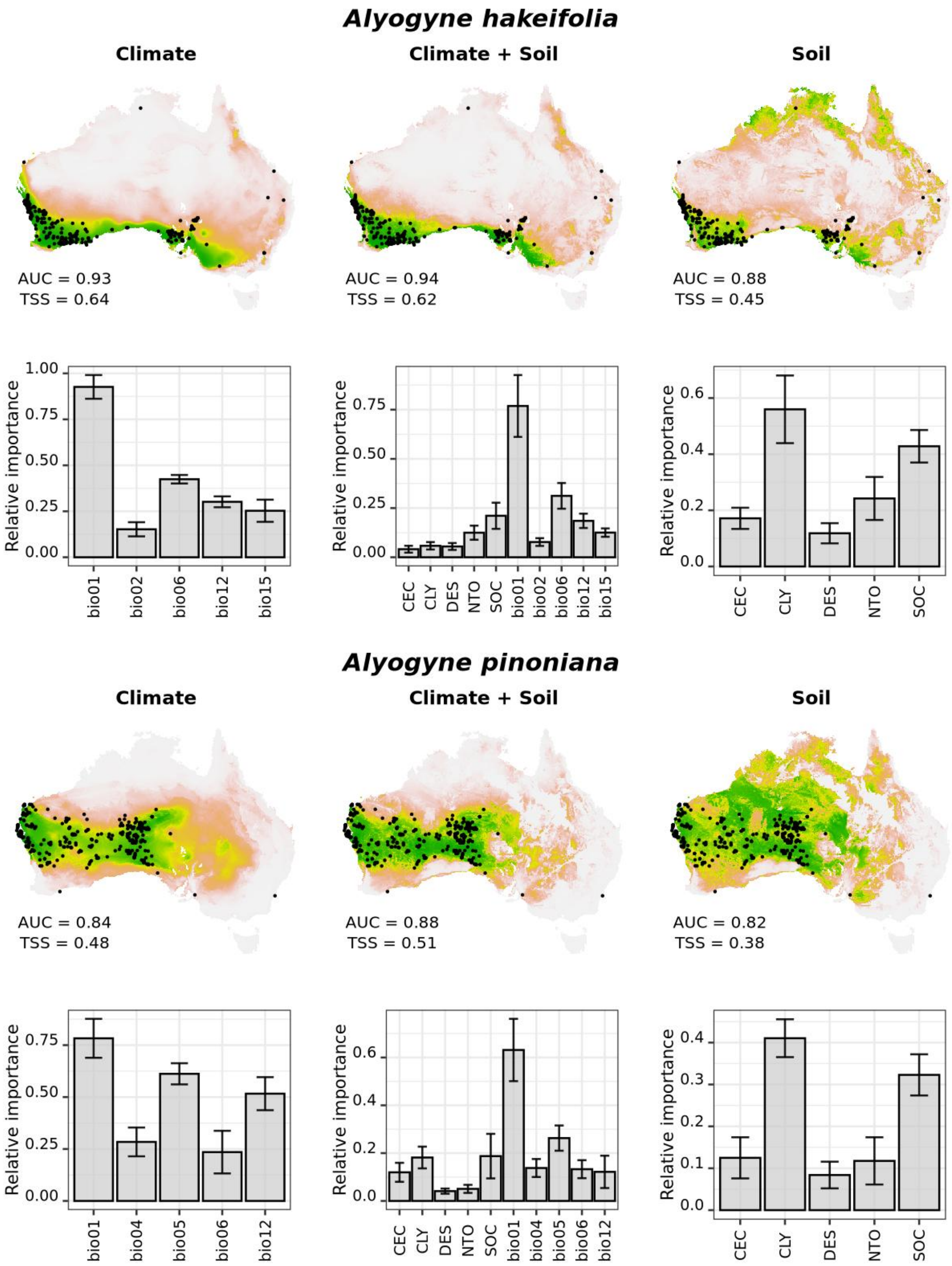

Alyogyne hakeifolia
Climate
Climate + Soil
Soil
AUC = 0.93
TSS = 0.64
AUC = 0.94
TSS = 0.62
AUC = 0.88
TSS = 0.45
Relative importance
1.00
0.75
0.50
0.25
0.00
bio01 bio02 bio06 bio12 bio15
CEC CLY DES NTO SOC bio01 bio02 bio06 bio12 bio15
0.6
0.4
0.2
0.0
CEC CLY DES NTO SOC
Alyogyne pinoniana
Climate
Climate + Soil
Soil
AUC = 0.84
TSS = 0.48
AUC = 0.88
TSS = 0.51
AUC = 0.82
TSS = 0.38
Relative importance
0.75
0.50
0.25
0.00
bio01 bio04 bio05 bio06 bio12
CEC CLY DES NTO SOC bio01 bio04 bio05 bio06 bio12
0.4
0.3
0.2
0.1
0.0
CEC CLY DES NTO SOC

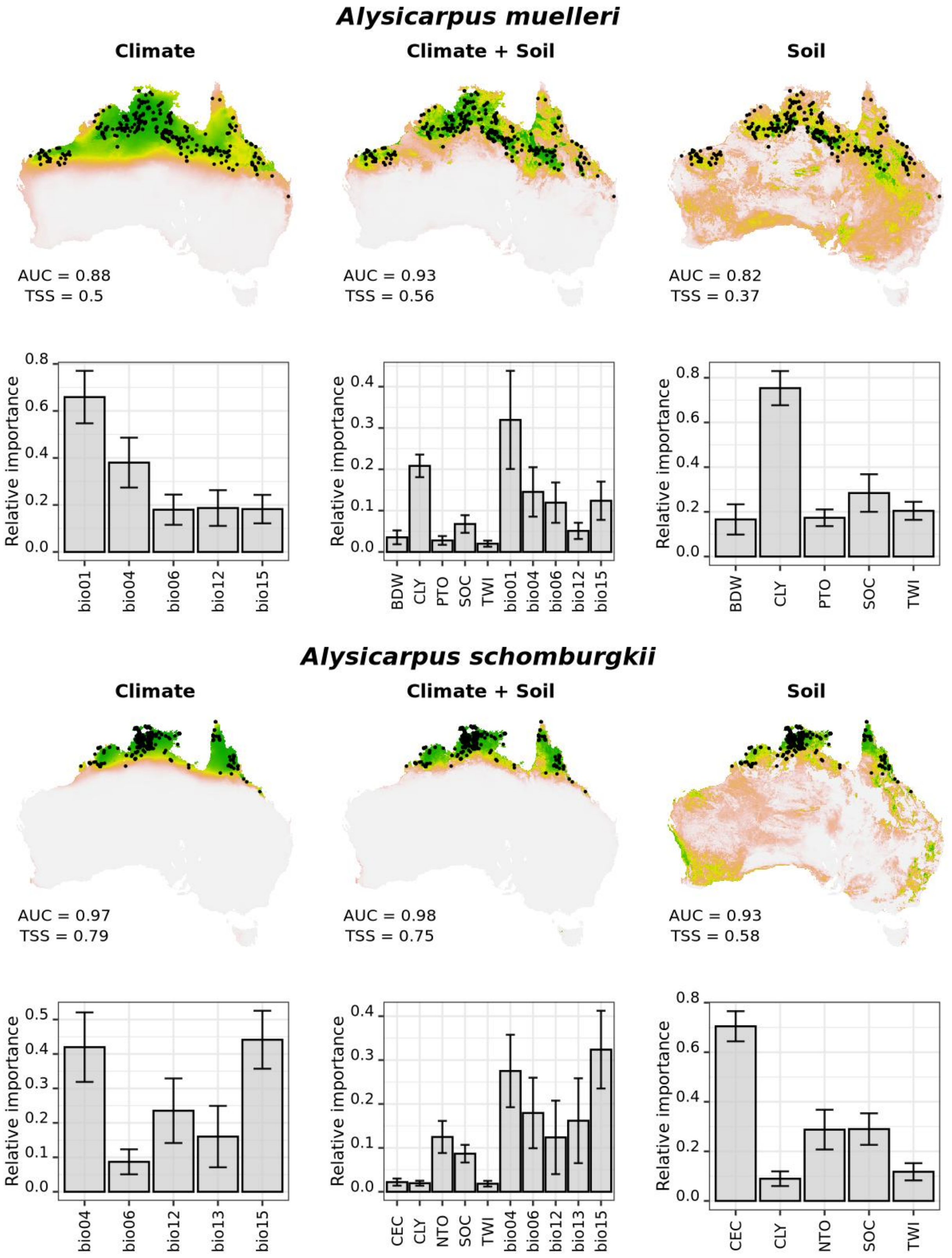
Alysicarpus muelleri
Climate
Climate + Soil
Soil
AUC = 0.88
TSS = 0.5
AUC = 0.93
TSS = 0.56
AUC = 0.82
TSS = 0.37
Relative importance
bio01
bio04
bio06
bio12
bio15
BDW
CLY
PTO
SOC
TWI
bio01
bio04
bio06
bio12
bio15
BDW
CLY
PTO
SOC
TWI
Alysicarpus schomburgkii
Climate
Climate + Soil
Soil
AUC = 0.97
TSS = 0.79
AUC = 0.98
TSS = 0.75
AUC = 0.93
TSS = 0.58
Relative importance
bio04
bio06
bio12
bio13
bio15
CEC
CLY
NTO
SOC
TWI
bio04
bio06
bio12
bio13
bio15
CEC
CLY
NTO
SOC
TWI

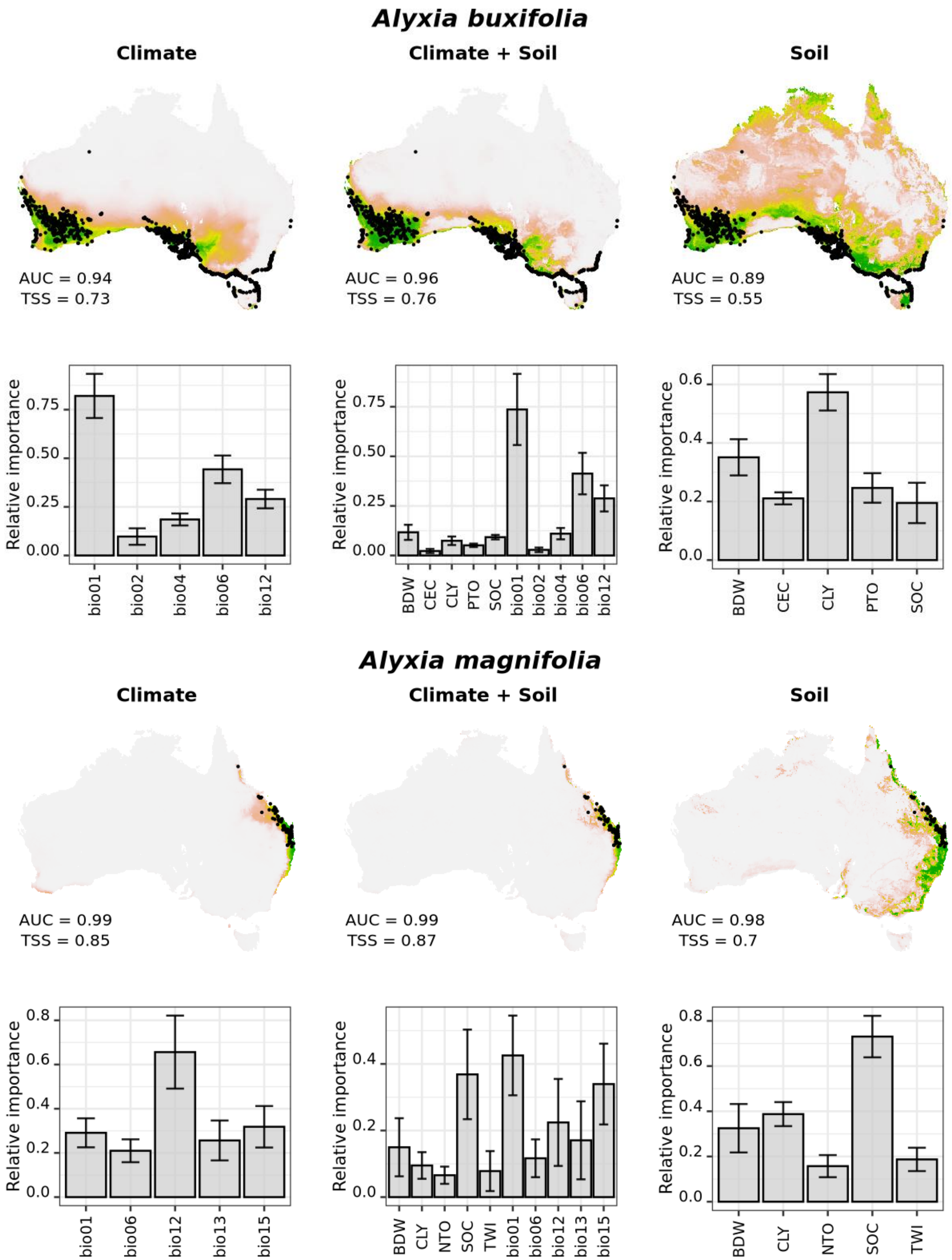

Alyxia buxifolia
Climate
Climate + Soil
Soil
AUC = 0.94
TSS = 0.73
AUC = 0.96
TSS = 0.76
AUC = 0.89
TSS = 0.55
Relative importance
0.00
0.25
0.50
0.75
bio01
bio02
bio04
bio06
bio12
BDW
CEC
CLY
PTO
SOC
0.0
0.2
0.4
0.6
Alyxia magnifolia
Climate
Climate + Soil
Soil
AUC = 0.99
TSS = 0.85
AUC = 0.99
TSS = 0.87
AUC = 0.98
TSS = 0.7
Relative importance
0.0
0.2
0.4
0.6
0.8
bio01
bio06
bio12
bio13
bio15
BDW
CLY
NTO
SOC
TWI

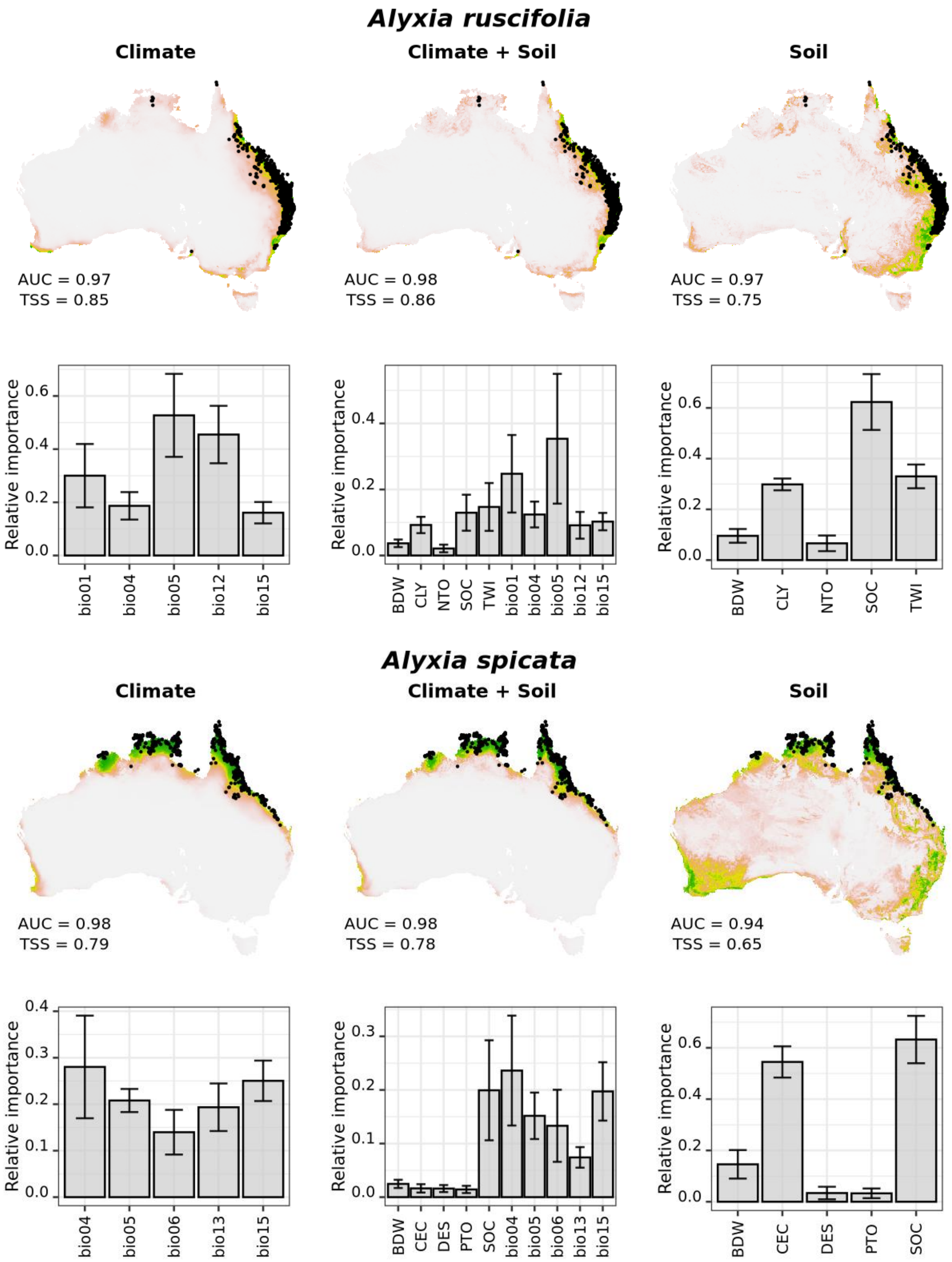

Alyxia ruscifolia
Climate
Climate + Soil
Soil
AUC = 0.97
TSS = 0.85
AUC = 0.98
TSS = 0.86
AUC = 0.97
TSS = 0.75
Relative importance
bio01
bio04
bio05
bio12
bio15
BDW
CLY
NTO
SOC
TWI
Alyxia spicata
AUC = 0.98
TSS = 0.79
AUC = 0.98
TSS = 0.78
AUC = 0.94
TSS = 0.65
bio06
bio13
CEC
DES
PTO

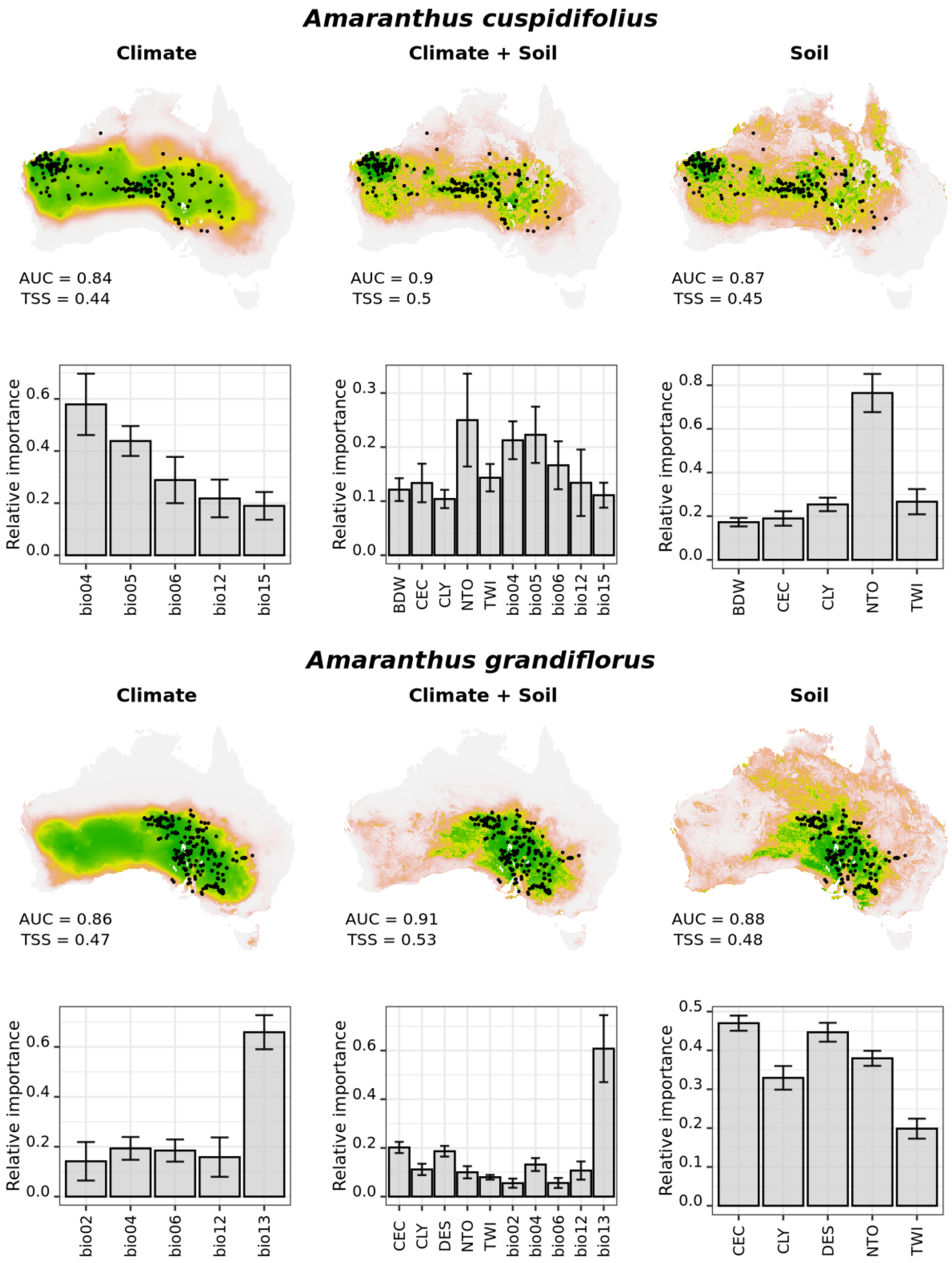

Amaranthus cuspidifolius
Climate
Climate + Soil
Soil
AUC = 0.84
TSS = 0.44
AUC = 0.9
TSS = 0.5
AUC = 0.87
TSS = 0.45
Relative importance
bio04
bio05
bio06
bio12
bio15
BDW
CEC
CLY
NTO
TWI
Amaranthus grandiflorus
Climate
Climate + Soil
Soil
AUC = 0.86
TSS = 0.47
AUC = 0.91
TSS = 0.53
AUC = 0.88
TSS = 0.48
Relative importance
bio02
bio04
bio06
bio12
bio13
CEC
CLY
DES
NTO
TWI

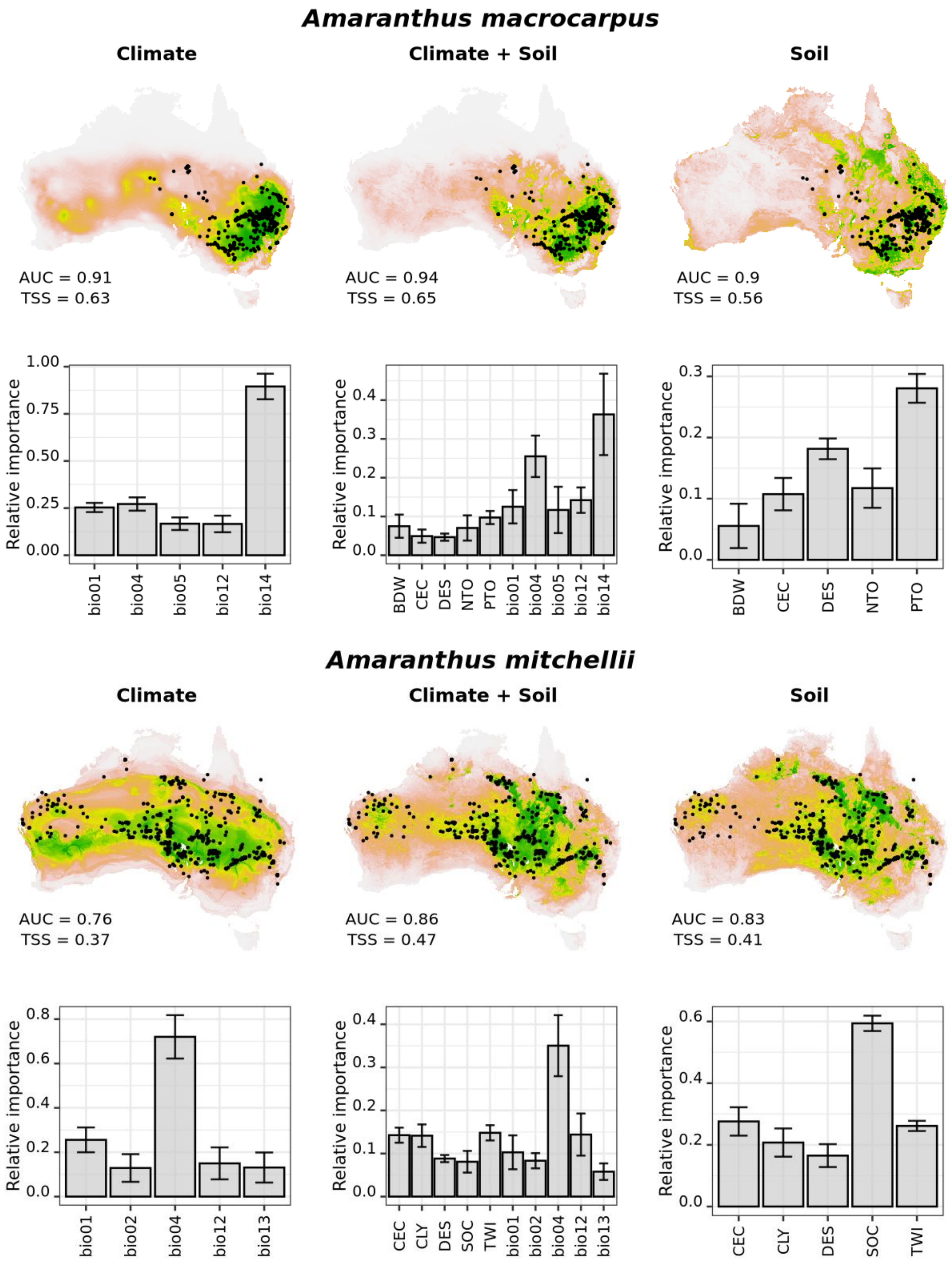
Amaranthus macrocarpus
Climate
Climate + Soil
Soil
AUC = 0.91
TSS = 0.63
AUC = 0.94
TSS = 0.65
AUC = 0.9
TSS = 0.56
Relative importance
bio01 bio04 bio05 bio12 bio14
BDW CEC DES NTO PTO bio01 bio04 bio05 bio12 bio14
BDW CEC DES NTO PTO
Amaranthus mitchellii
Climate
Climate + Soil
Soil
AUC = 0.76
TSS = 0.37
AUC = 0.86
TSS = 0.47
AUC = 0.83
TSS = 0.41
Relative importance
bio01 bio02 bio04 bio12 bio13
CEC CLY DES SOC TWI bio01 bio02 bio04 bio12 bio13
CEC CLY DES SOC TWI

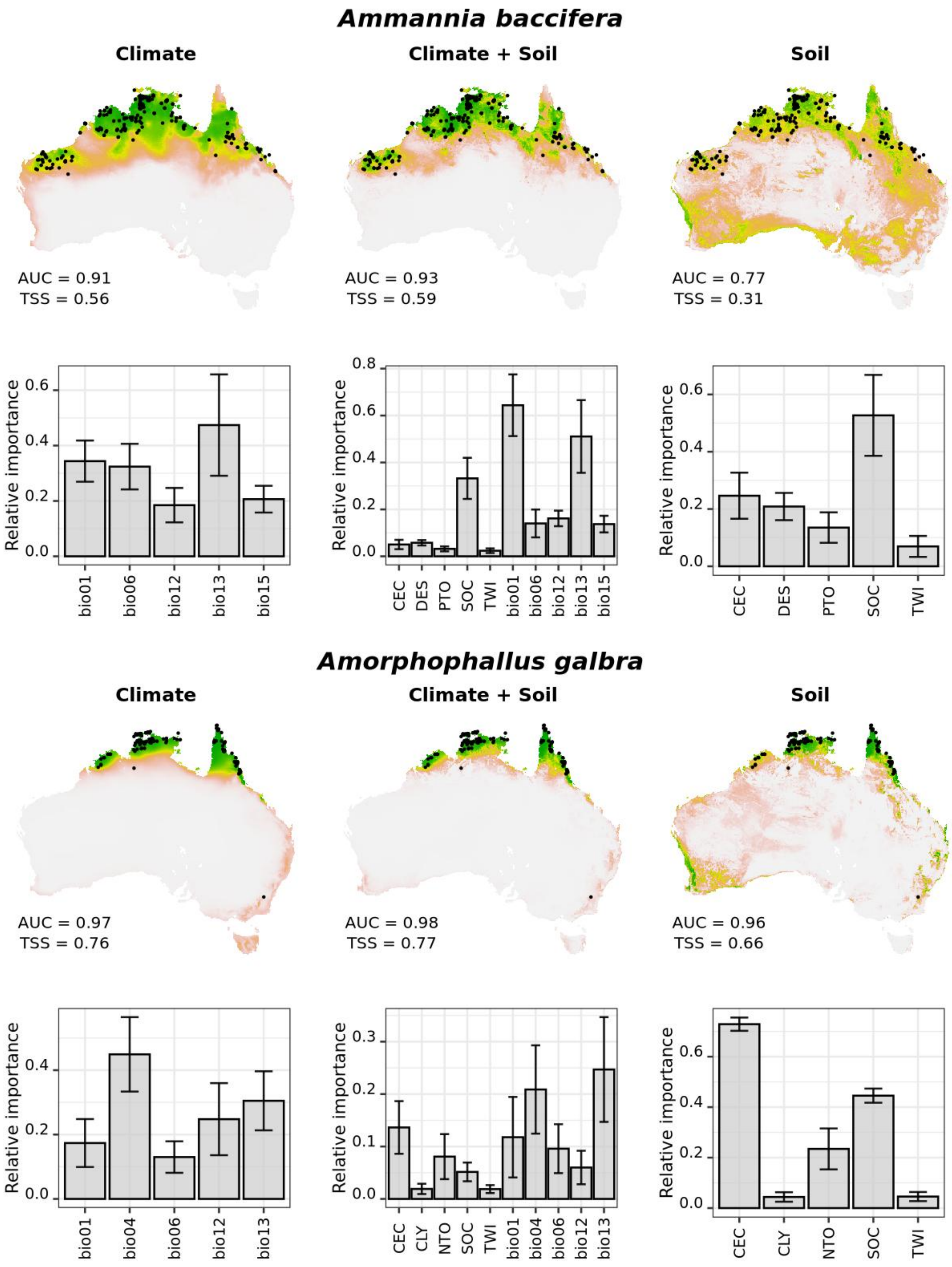

Ammannia baccifera
Climate
Climate + Soil
Soil
AUC = 0.91
TSS = 0.56
AUC = 0.93
TSS = 0.59
AUC = 0.77
TSS = 0.31
Relative importance
bio01 bio06 bio12 bio13 bio15
CEC DES PTO SOC TWI bio01 bio06 bio12 bio13 bio15
CEC DES PTO SOC TWI
Amorphophallus galbra
Climate
Climate + Soil
Soil
AUC = 0.97
TSS = 0.76
AUC = 0.98
TSS = 0.77
AUC = 0.96
TSS = 0.66
Relative importance
bio01 bio04 bio06 bio12 bio13
CEC CLY NTO SOC TWI bio01 bio04 bio06 bio12 bio13
CEC CLY NTO SOC TWI

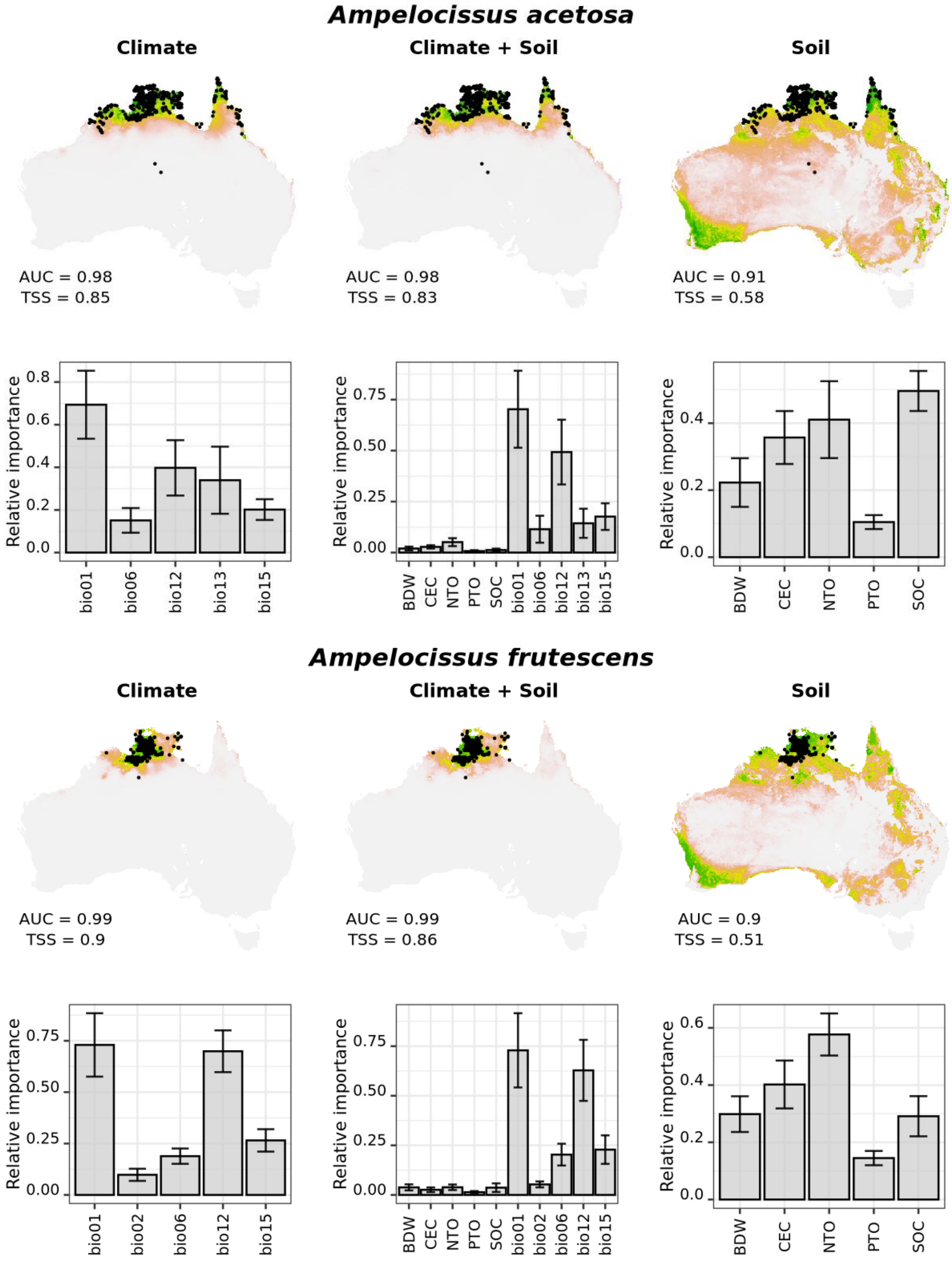

Ampelocissus acetosa
Climate
Climate + Soil
Soil
AUC = 0.98
TSS = 0.85
AUC = 0.98
TSS = 0.83
AUC = 0.91
TSS = 0.58
Relative importance
bio01
bio06
bio12
bio13
bio15
BDW
CEC
NTO
PTO
SOC
Ampelocissus frutescens
Climate
Climate + Soil
Soil
AUC = 0.99
TSS = 0.9
AUC = 0.99
TSS = 0.86
AUC = 0.9
TSS = 0.51
Relative importance
bio01
bio02
bio06
bio12
bio15
BDW
CEC
NTO
PTO
SOC

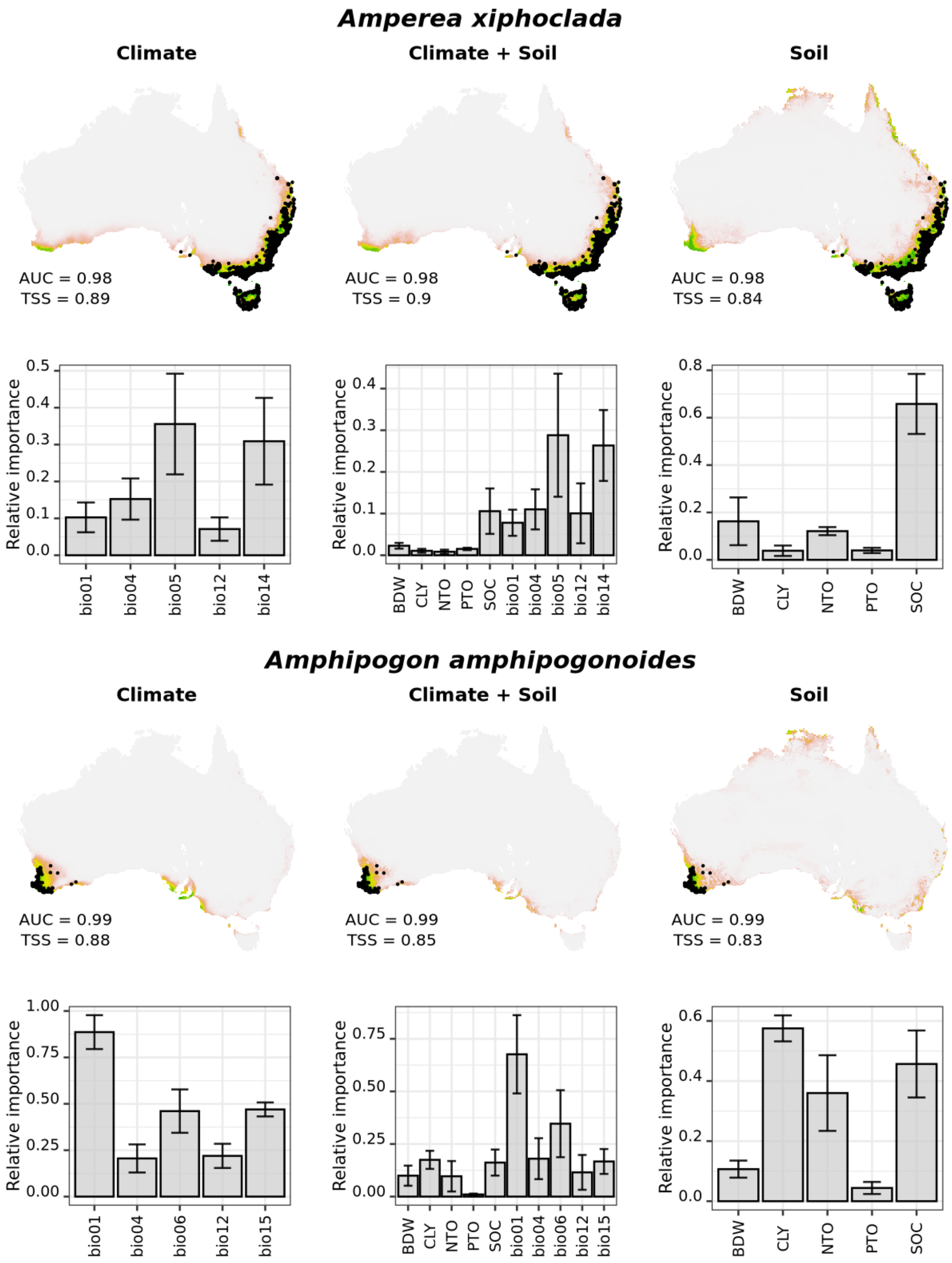
Amperea xiphoclada
Climate
Climate + Soil
Soil
AUC = 0.98
TSS = 0.89
AUC = 0.98
TSS = 0.9
AUC = 0.98
TSS = 0.84
Relative importance
bio01
bio04
bio05
bio12
bio14
BDW
CLY
NTO
PTO
SOC
Amphipogon amphipogonoides
Climate
Climate + Soil
Soil
AUC = 0.99
TSS = 0.88
AUC = 0.99
TSS = 0.85
AUC = 0.99
TSS = 0.83
Relative importance
bio01
bio04
bio06
bio12
bio15
BDW
CLY
NTO
PTO
SOC

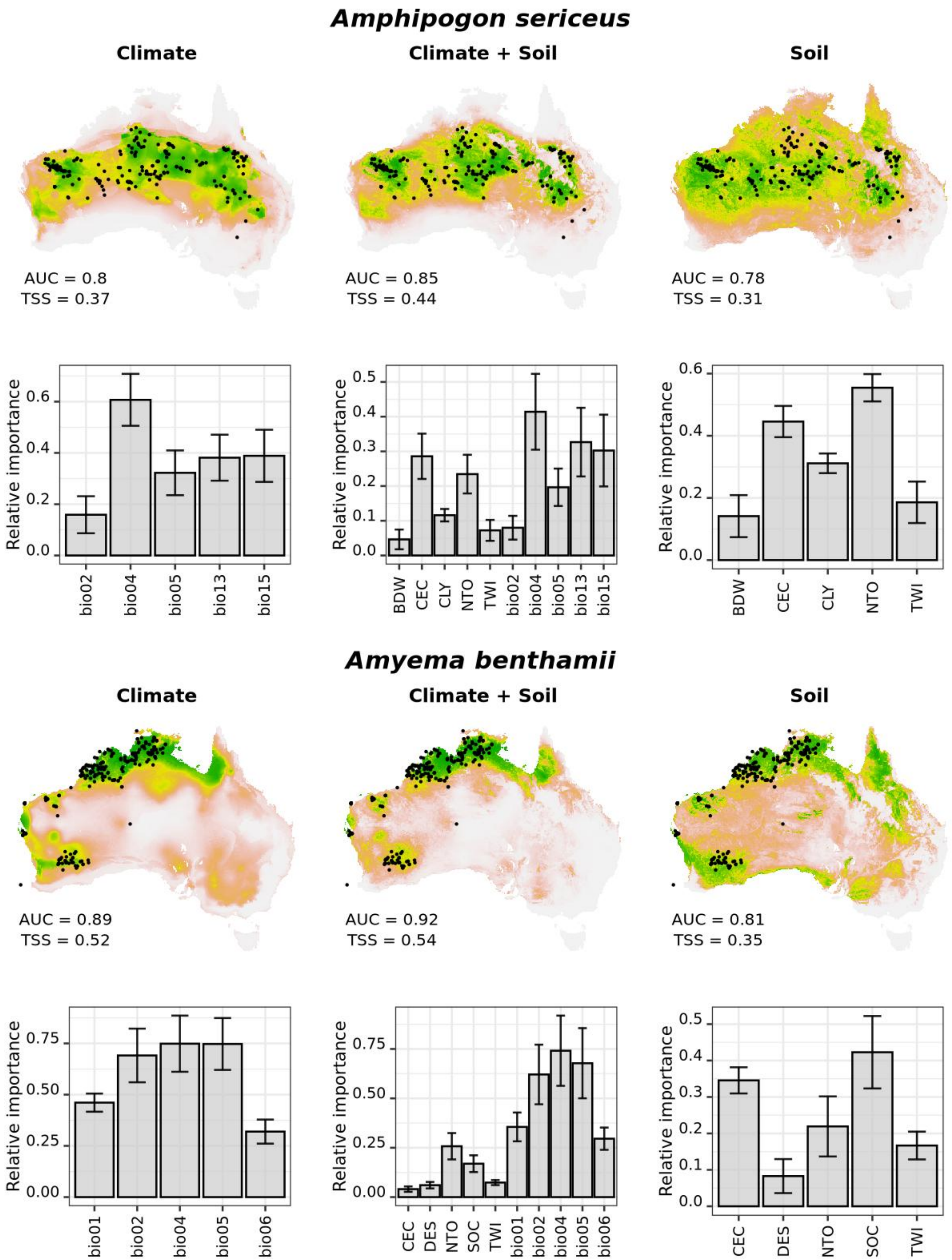

Amphipogon sericeus
Climate
Climate + Soil
Soil
AUC = 0.8
TSS = 0.37
AUC = 0.85
TSS = 0.44
AUC = 0.78
TSS = 0.31
Relative importance
bio02
bio04
bio05
bio13
bio15
BDW
CEC
CLY
NTO
TWI
Amyema benthamii
Climate
Climate + Soil
Soil
AUC = 0.89
TSS = 0.52
AUC = 0.92
TSS = 0.54
AUC = 0.81
TSS = 0.35
Relative importance
bio01
bio02
bio04
bio05
bio06
CEC
DES
NTO
SOC
TWI

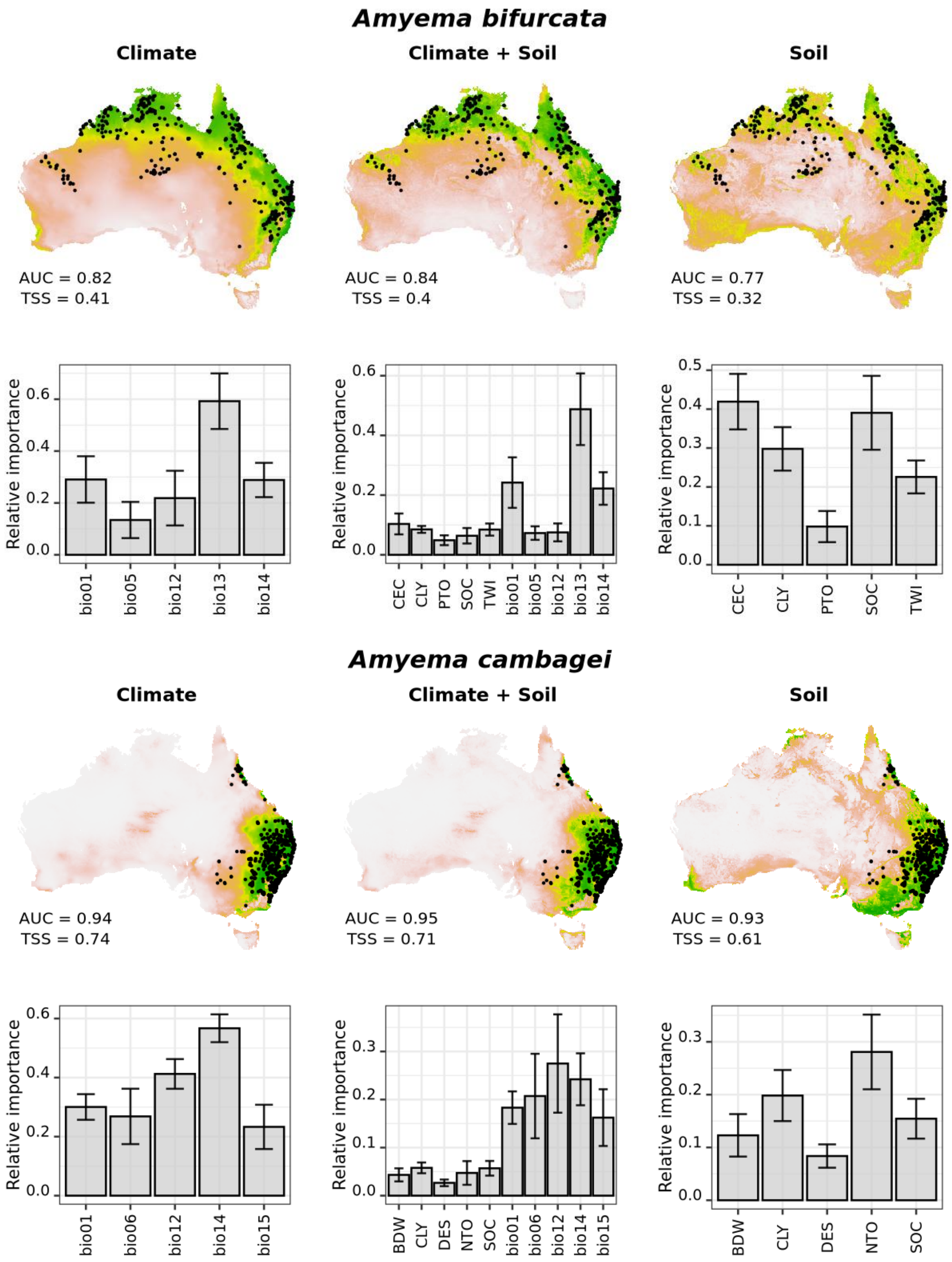
Amyema bifurcata
Climate
Climate + Soil
Soil
AUC = 0.82
TSS = 0.41
AUC = 0.84
TSS = 0.4
AUC = 0.77
TSS = 0.32
Relative importance
bio01
bio05
bio12
bio13
bio14
CEC
CLY
PTO
SOC
TWI
Amyema cambagei
Climate
Climate + Soil
Soil
AUC = 0.94
TSS = 0.74
AUC = 0.95
TSS = 0.71
AUC = 0.93
TSS = 0.61
Relative importance
bio01
bio06
bio12
bio14
bio15
BDW
CLY
DES
NTO
SOC

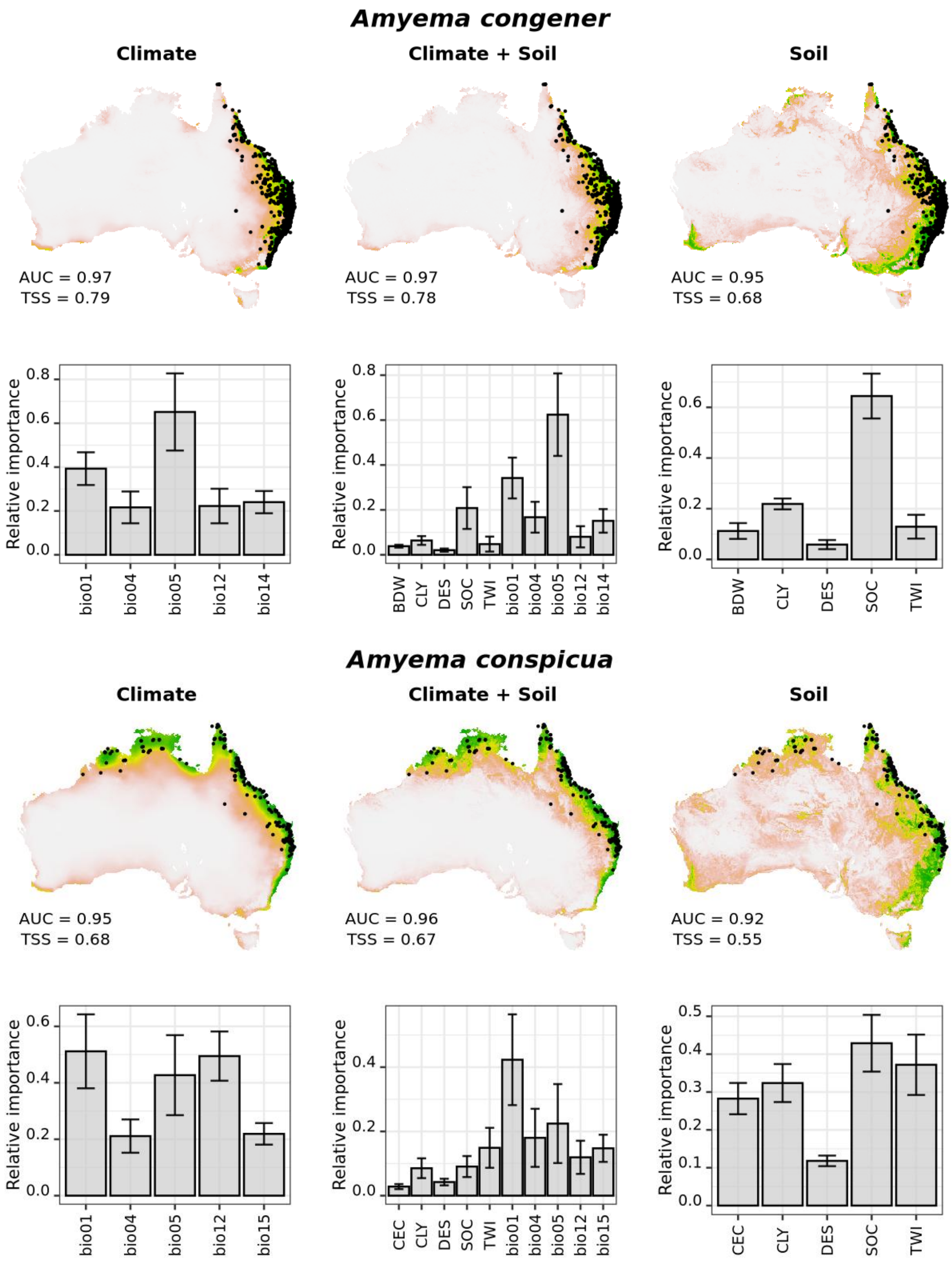
Amyema congener
Climate
Climate + Soil
Soil
AUC = 0.97
TSS = 0.79
AUC = 0.97
TSS = 0.78
AUC = 0.95
TSS = 0.68
Relative importance
bio01 bio04 bio05 bio12 bio14
BDW CLY DES SOC TWI bio01 bio04 bio05 bio12 bio14
BDW CLY DES SOC TWI
Amyema conspicua
Climate
Climate + Soil
Soil
AUC = 0.95
TSS = 0.68
AUC = 0.96
TSS = 0.67
AUC = 0.92
TSS = 0.55
Relative importance
bio01 bio04 bio05 bio12 bio15
CEC CLY DES SOC TWI bio01 bio04 bio05 bio12 bio15
CEC CLY DES SOC TWI

Amyema fitzgeraldii

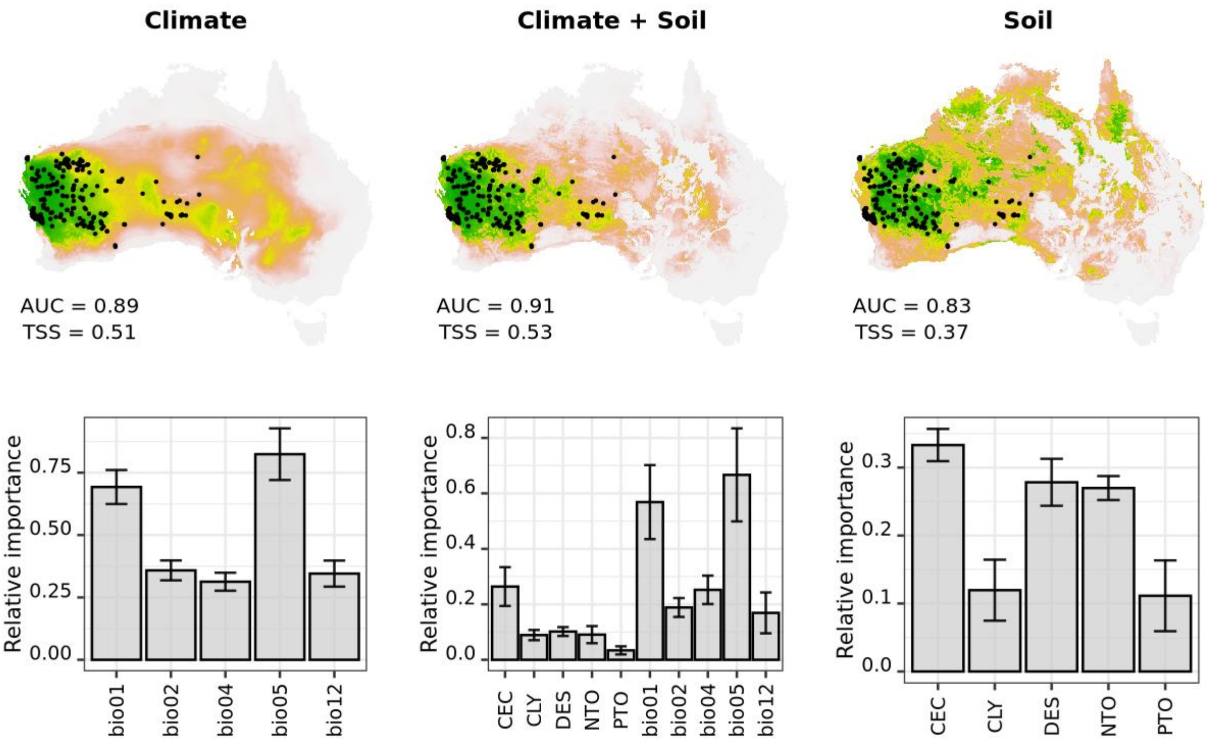

Amyema gibberula

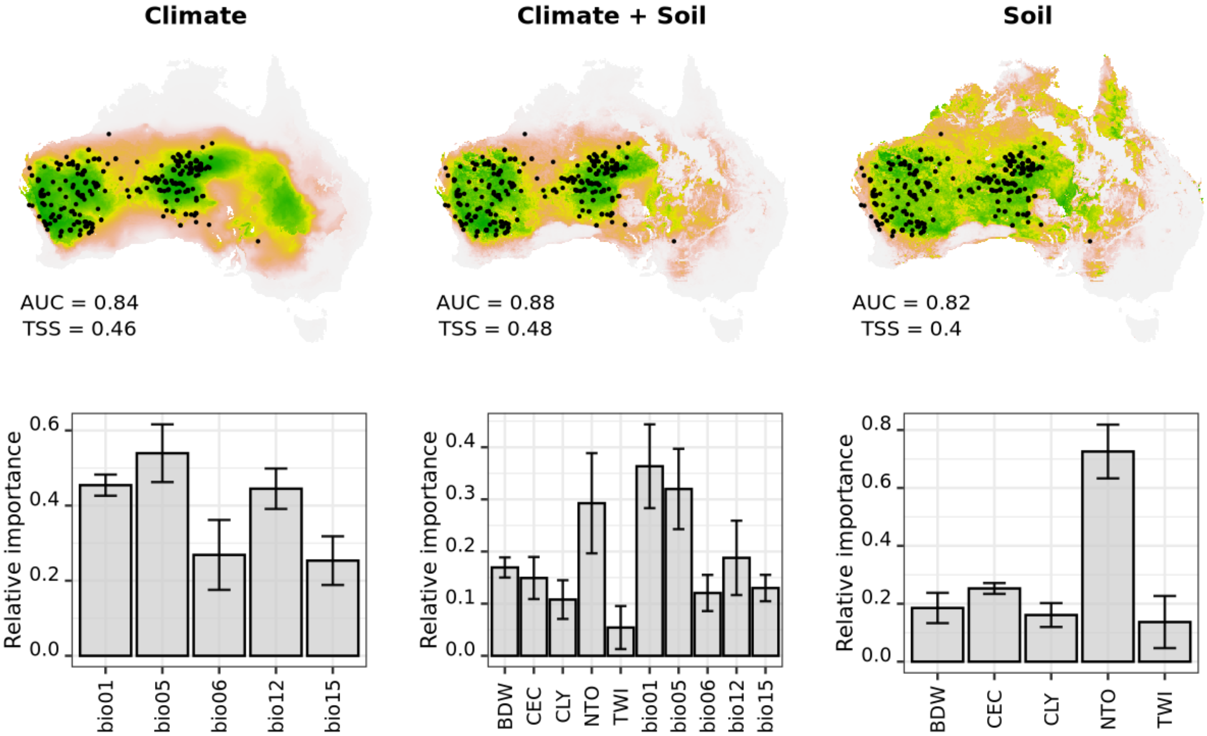

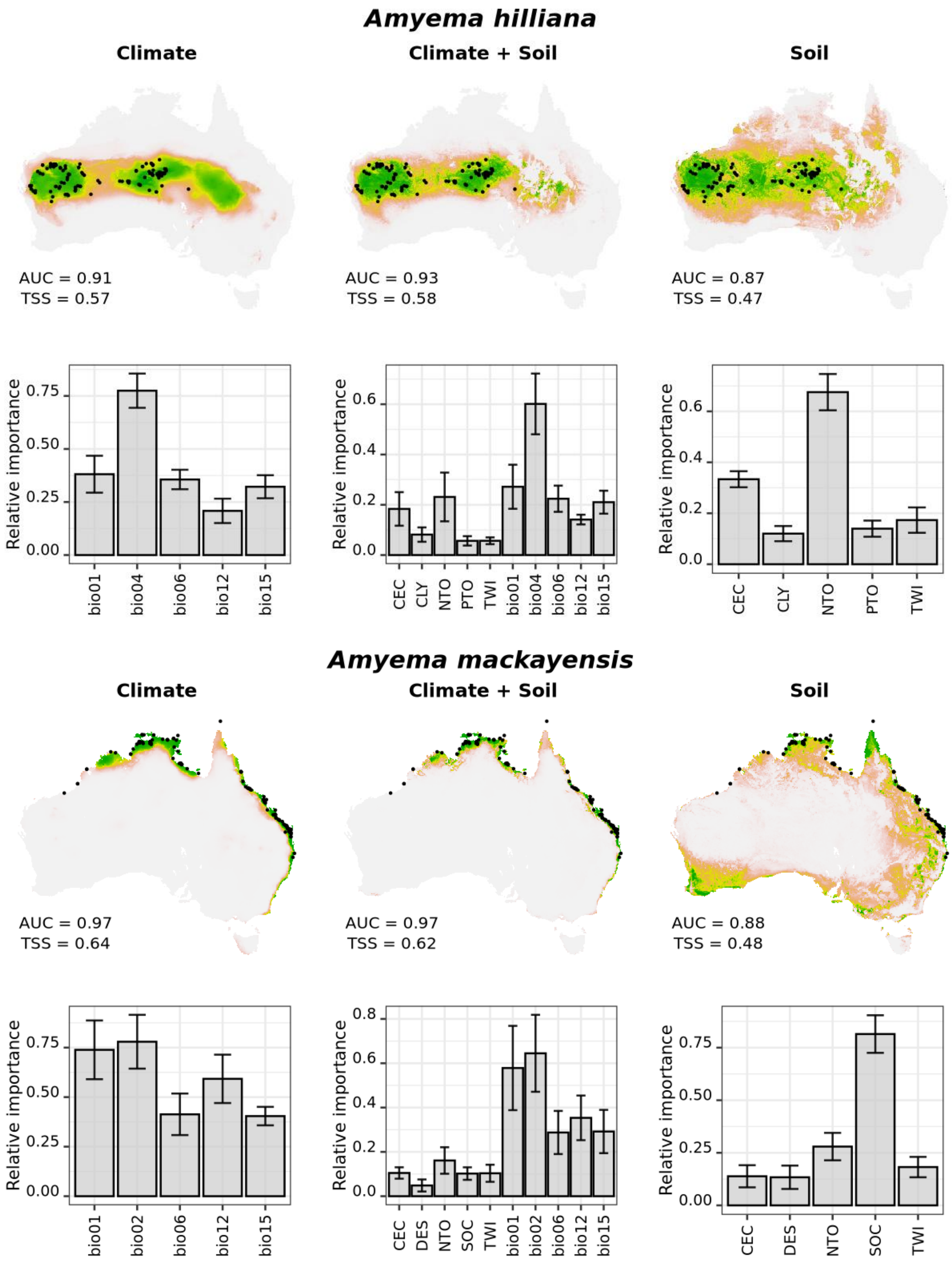

Amyema hilliana
Climate
Climate + Soil
Soil
AUC = 0.91
TSS = 0.57
AUC = 0.93
TSS = 0.58
AUC = 0.87
TSS = 0.47
Relative importance
0.00 0.25 0.50 0.75
bio01 bio04 bio06 bio12 bio15
0.0 0.2 0.4 0.6
CEC CLY NTO PTO TWI bio01 bio04 bio06 bio12 bio15
0.0 0.2 0.4 0.6
CEC CLY NTO PTO TWI
Amyema mackayensis
Climate
Climate + Soil
Soil
AUC = 0.97
TSS = 0.64
AUC = 0.97
TSS = 0.62
AUC = 0.88
TSS = 0.48
Relative importance
0.00 0.25 0.50 0.75
bio01 bio02 bio06 bio12 bio15
0.0 0.2 0.4 0.6 0.8
CEC DES NTO SOC TWI bio01 bio02 bio06 bio12 bio15
0.00 0.25 0.50 0.75
CEC DES NTO SOC TWI

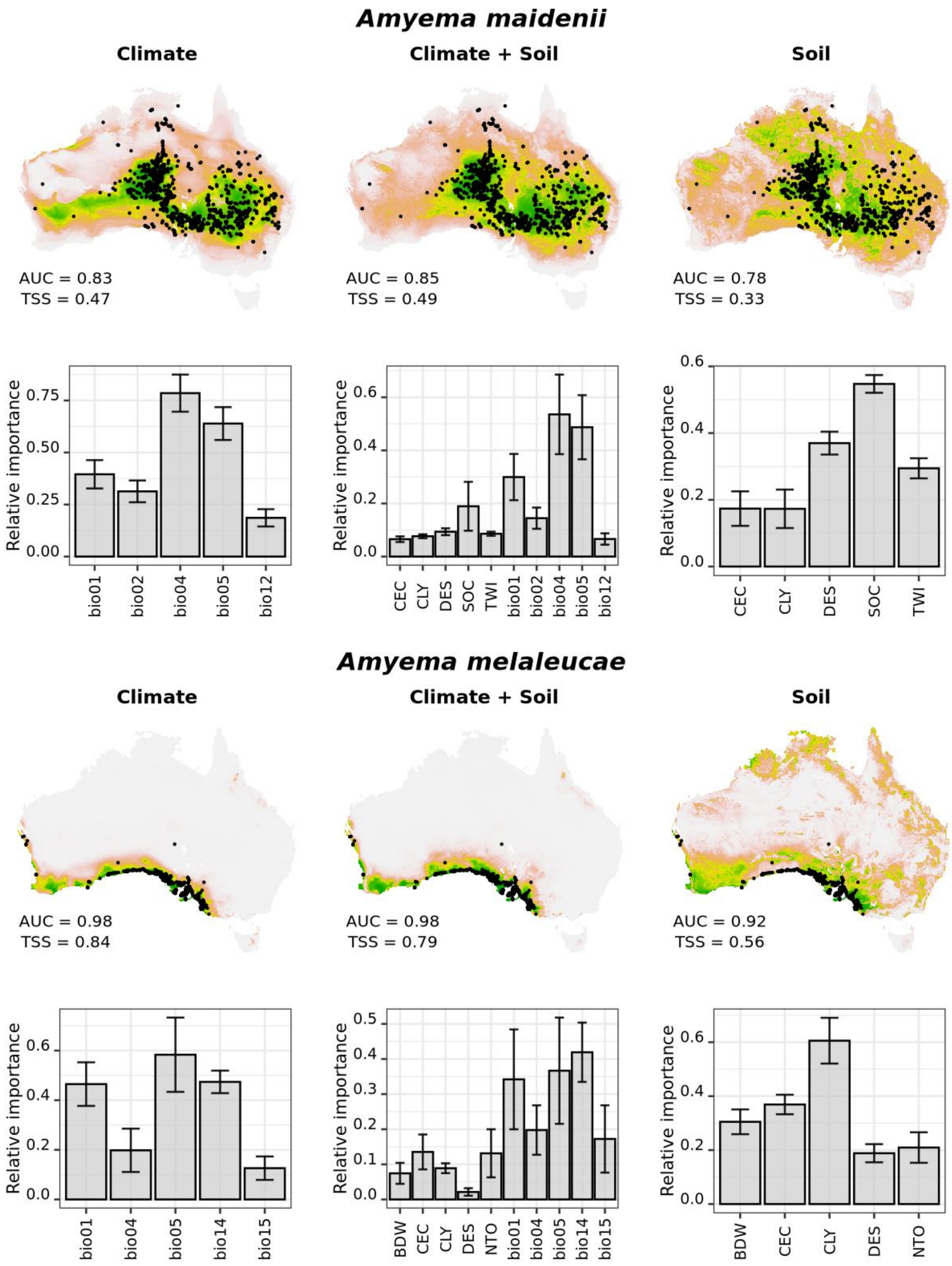

Amyema maidenii
Climate
Climate + Soil
Soil
AUC = 0.83
TSS = 0.47
AUC = 0.85
TSS = 0.49
AUC = 0.78
TSS = 0.33
Relative importance
bio01 bio02 bio04 bio05 bio12
CEC CLY DES SOC TWI bio01 bio02 bio04 bio05 bio12
CEC CLY DES SOC TWI
Amyema melaleucae
Climate
Climate + Soil
Soil
AUC = 0.98
TSS = 0.84
AUC = 0.98
TSS = 0.79
AUC = 0.92
TSS = 0.56
Relative importance
bio01 bio04 bio05 bio14 bio15
BDW CEC CLY DES NTO bio01 bio04 bio05 bio14 bio15
BDW CEC CLY DES NTO

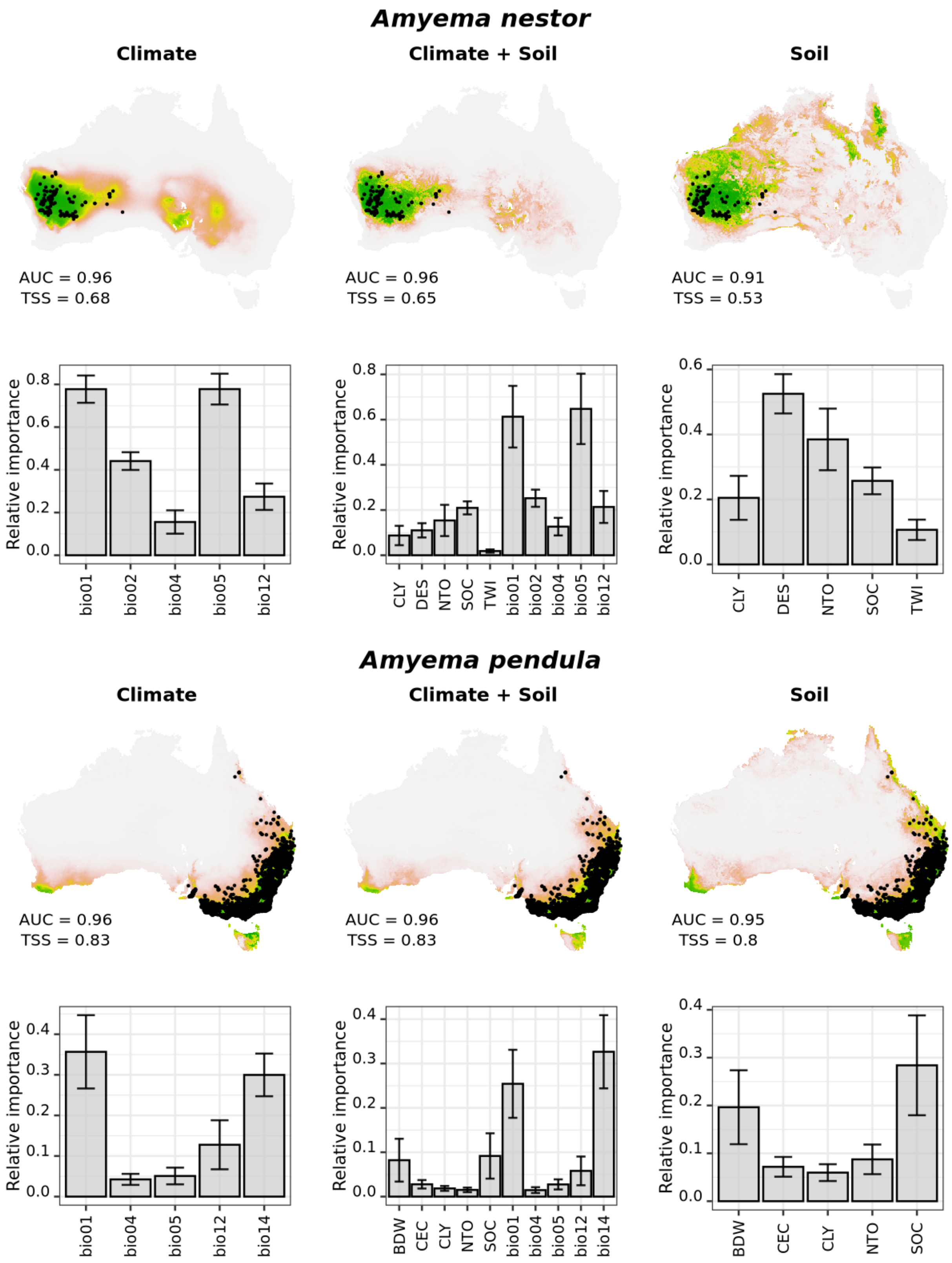
Amyema nestor
Climate
Climate + Soil
Soil
AUC = 0.96
TSS = 0.68
AUC = 0.96
TSS = 0.65
AUC = 0.91
TSS = 0.53
Relative importance
bio01
bio02
bio04
bio05
bio12
CLY
DES
NTO
SOC
TWI
Amyema pendula
Climate
Climate + Soil
Soil
AUC = 0.96
TSS = 0.83
AUC = 0.96
TSS = 0.83
AUC = 0.95
TSS = 0.8
Relative importance
bio01
bio04
bio05
bio12
bio14
BDW
CEC
CLY
NTO
SOC

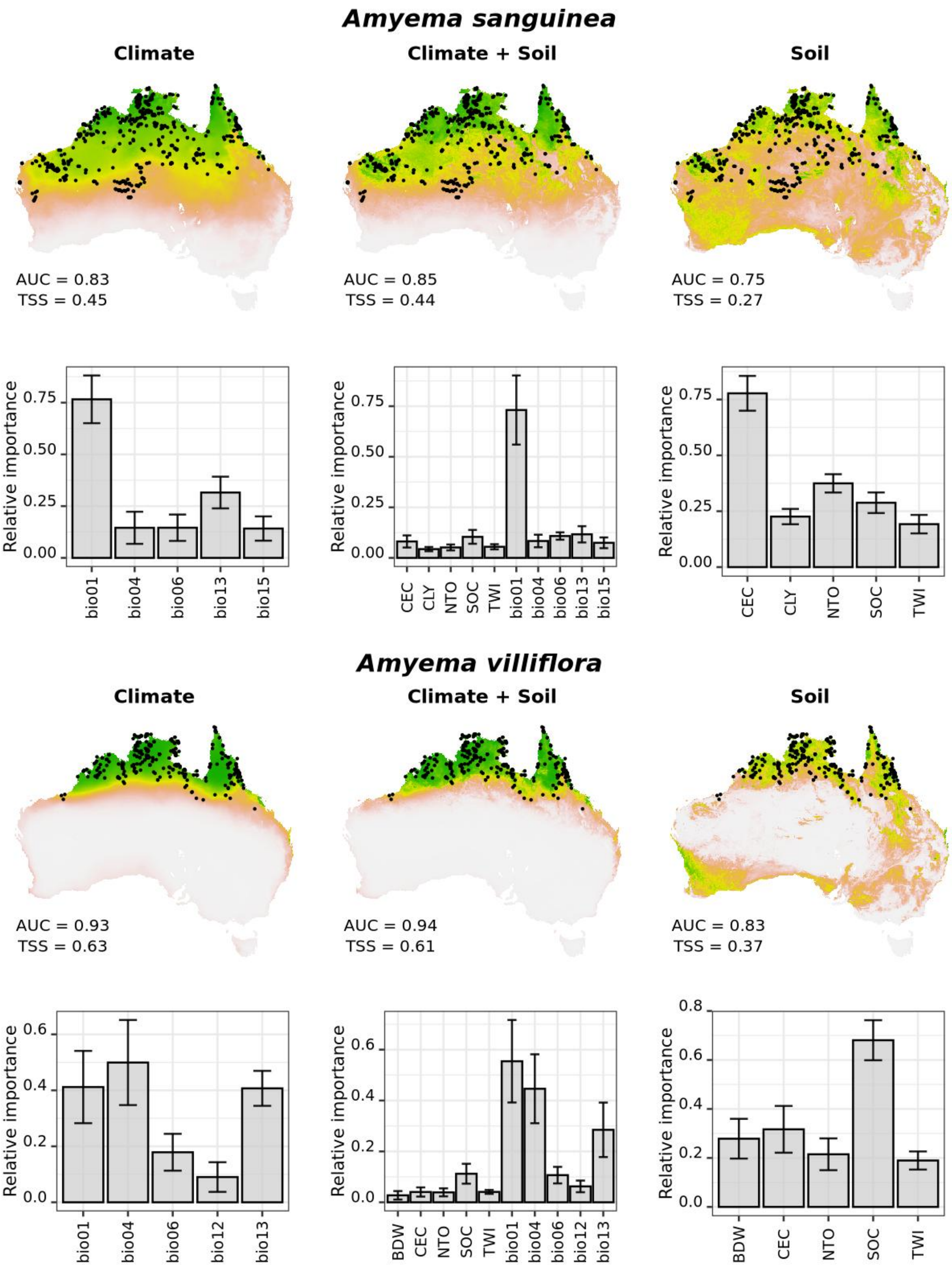

Amyema sanguinea
Climate
Climate + Soil
Soil
AUC = 0.83
TSS = 0.45
AUC = 0.85
TSS = 0.44
AUC = 0.75
TSS = 0.27
Relative importance
bio01 bio04 bio06 bio13 bio15
CEC CLY NTO SOC TWI bio01 bio04 bio06 bio13 bio15
CEC CLY NTO SOC TWI
Amyema villiflora
Climate
Climate + Soil
Soil
AUC = 0.93
TSS = 0.63
AUC = 0.94
TSS = 0.61
AUC = 0.83
TSS = 0.37
Relative importance
bio01 bio04 bio06 bio12 bio13
BDW CEC NTO SOC TWI bio01 bio04 bio06 bio12 bio13
BDW CEC NTO SOC TWI

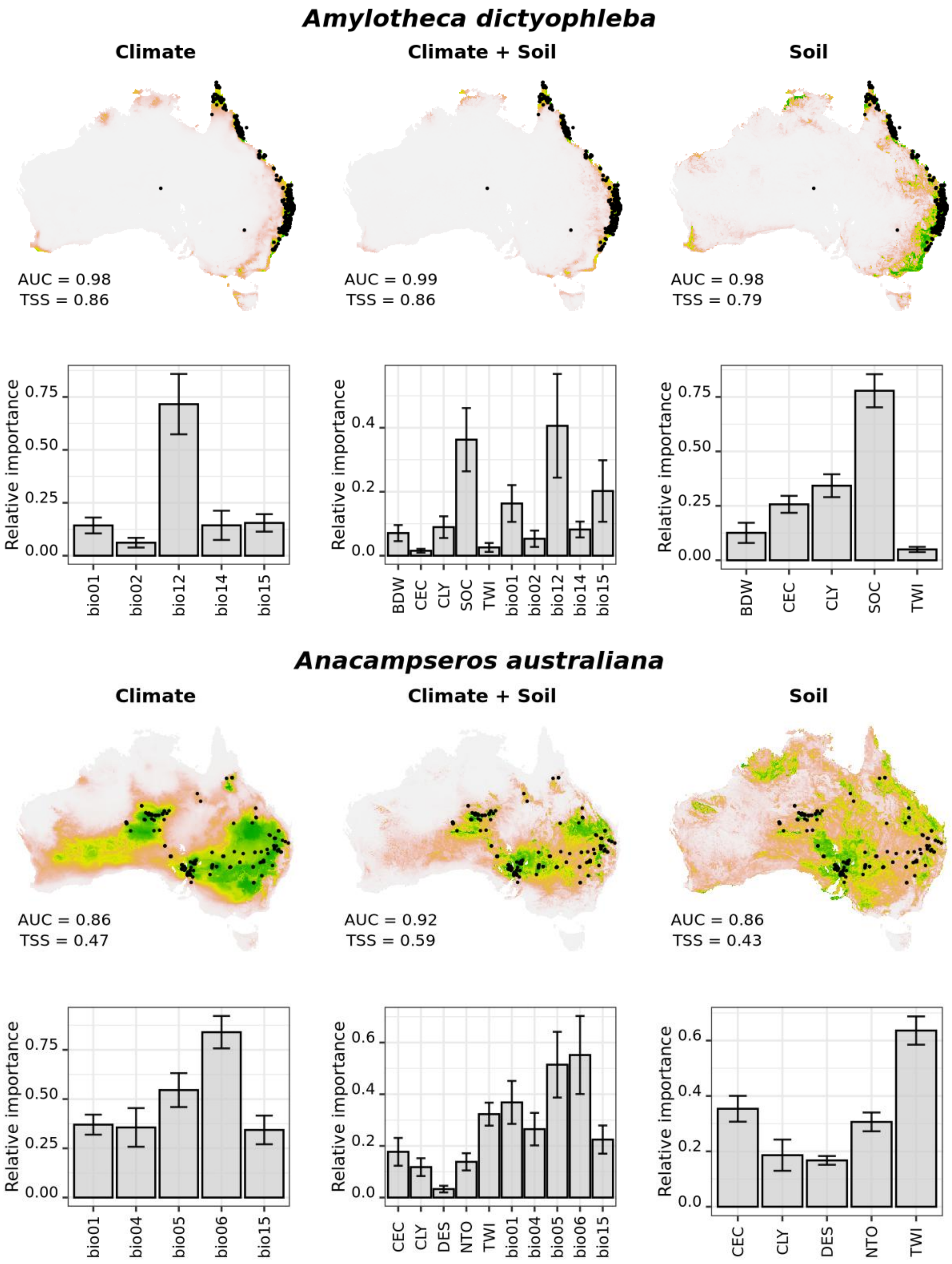
Amylotheca dictyophleba
Climate
Climate + Soil
Soil
AUC = 0.98
TSS = 0.86
AUC = 0.99
TSS = 0.86
AUC = 0.98
TSS = 0.79
Relative importance
bio01 bio02 bio12 bio14 bio15
BDW CEC CLY SOC TWI bio01 bio02 bio12 bio14 bio15
BDW CEC CLY SOC TWI
Anacampseros australiana
Climate
Climate + Soil
Soil
AUC = 0.86
TSS = 0.47
AUC = 0.92
TSS = 0.59
AUC = 0.86
TSS = 0.43
Relative importance
bio01 bio04 bio05 bio06 bio15
CEC CLY DES NTO TWI bio01 bio04 bio05 bio06 bio15
CEC CLY DES NTO TWI

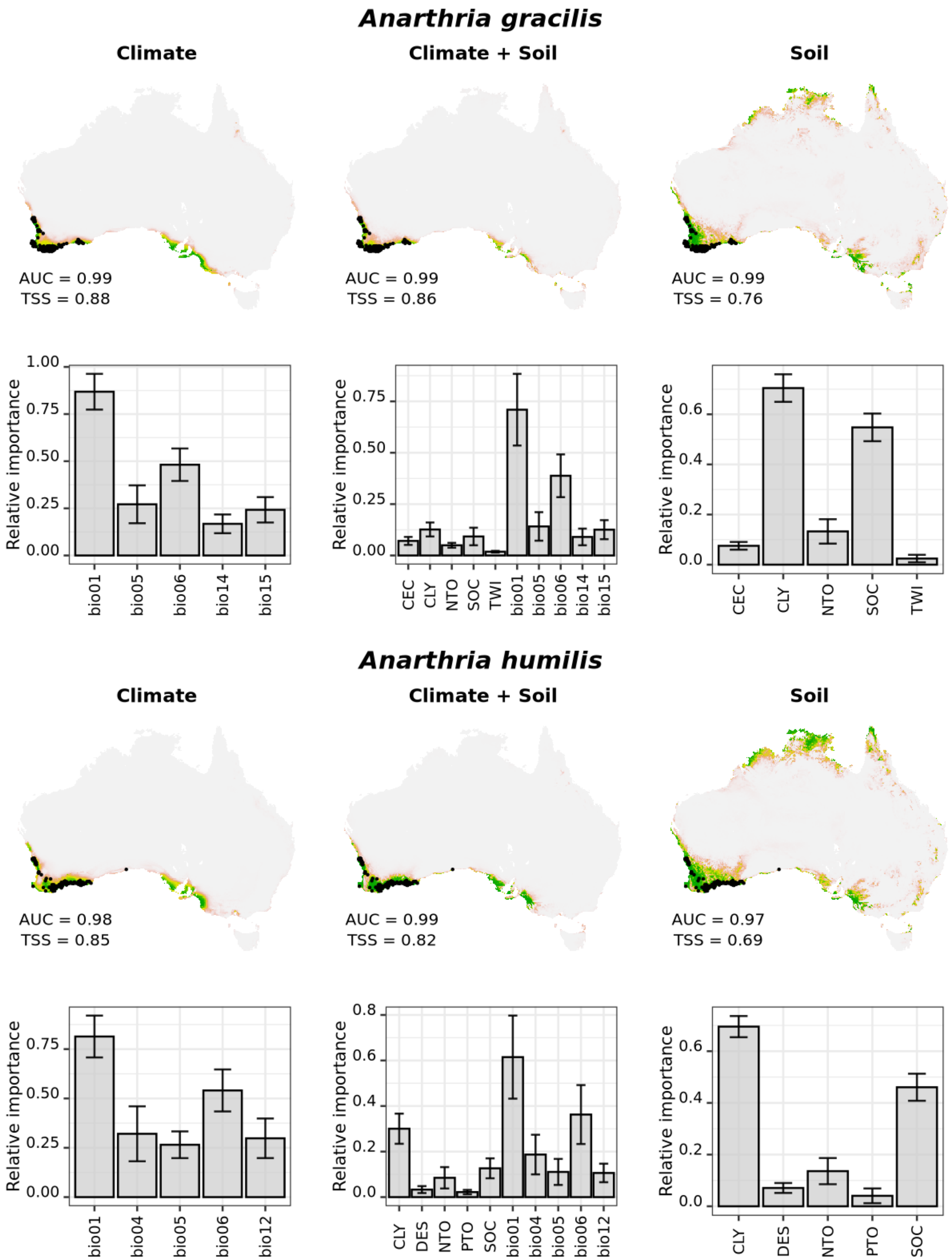

Anarthria gracilis
Climate
Climate + Soil
Soil
AUC = 0.99
TSS = 0.88
AUC = 0.99
TSS = 0.86
AUC = 0.99
TSS = 0.76
Relative importance
1.00
0.75
0.50
0.25
0.00
bio01
bio05
bio06
bio14
bio15
CEC
CLY
NTO
SOC
TWI
0.6
0.4
0.2
0.0
Anarthria humilis
Climate
Climate + Soil
Soil
AUC = 0.98
TSS = 0.85
AUC = 0.99
TSS = 0.82
AUC = 0.97
TSS = 0.69
bio01
bio04
bio05
bio06
bio12
CLY
DES
NTO
PTO
SOC
0.8

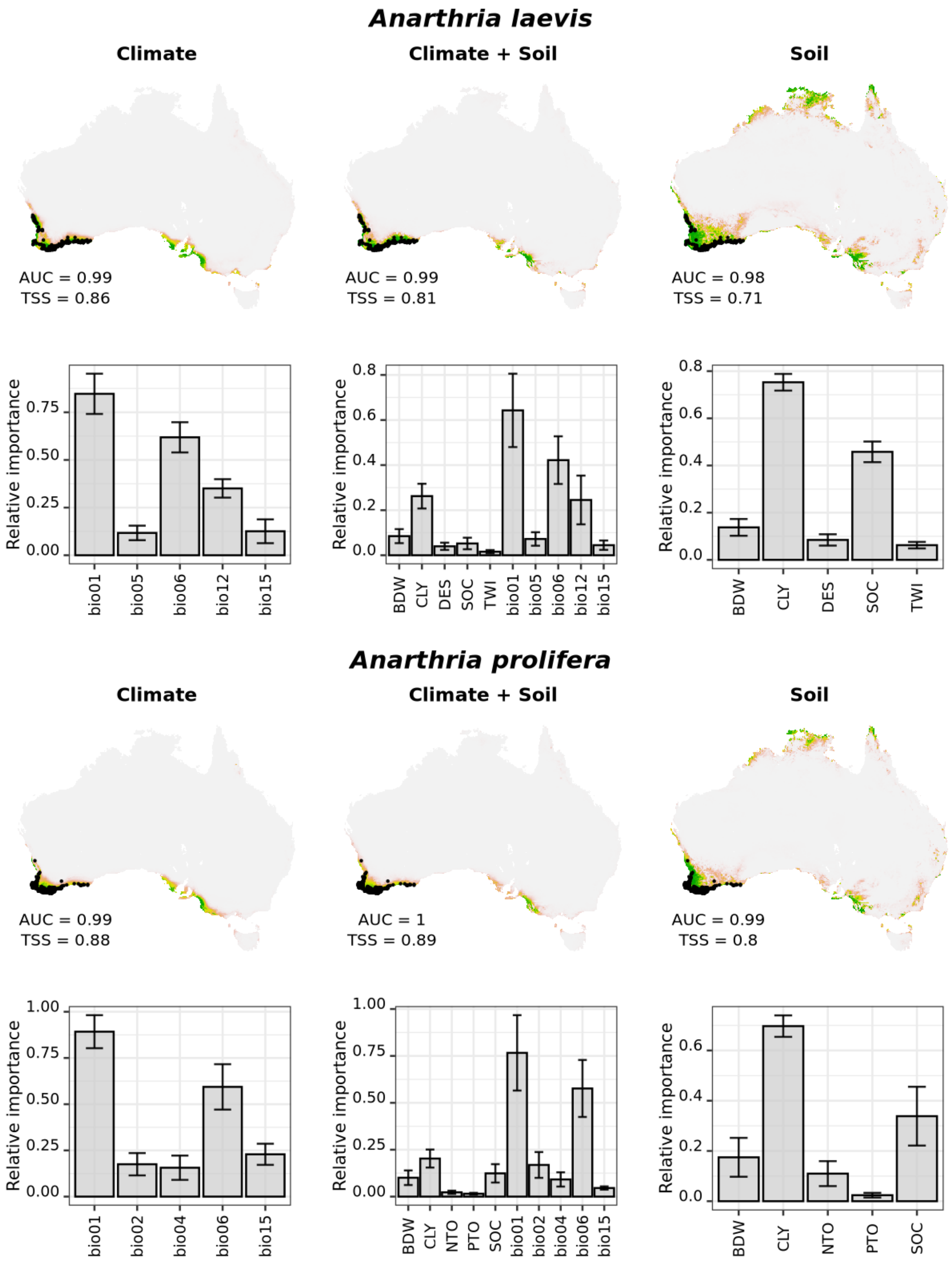

Anarthria laevis
Climate
Climate + Soil
Soil
AUC = 0.99
TSS = 0.86
AUC = 0.99
TSS = 0.81
AUC = 0.98
TSS = 0.71
Relative importance
0.00
0.25
0.50
0.75
bio01
bio05
bio06
bio12
bio15
Relative importance
0.0
0.2
0.4
0.6
0.8
BDW
CLY
DES
SOC
TWI
bio01
bio05
bio06
bio12
bio15
Relative importance
0.0
0.2
0.4
0.6
0.8
BDW
CLY
DES
SOC
TWI
Anarthria prolifera
Climate
Climate + Soil
Soil
AUC = 0.99
TSS = 0.88
AUC = 1
TSS = 0.89
AUC = 0.99
TSS = 0.8
Relative importance
0.00
0.25
0.50
0.75
1.00
bio01
bio02
bio04
bio06
bio15
Relative importance
0.00
0.25
0.50
0.75
1.00
BDW
CLY
NTO
PTO
SOC
bio01
bio02
bio04
bio06
bio15
Relative importance
0.0
0.2
0.4
0.6
BDW
CLY
NTO
PTO
SOC

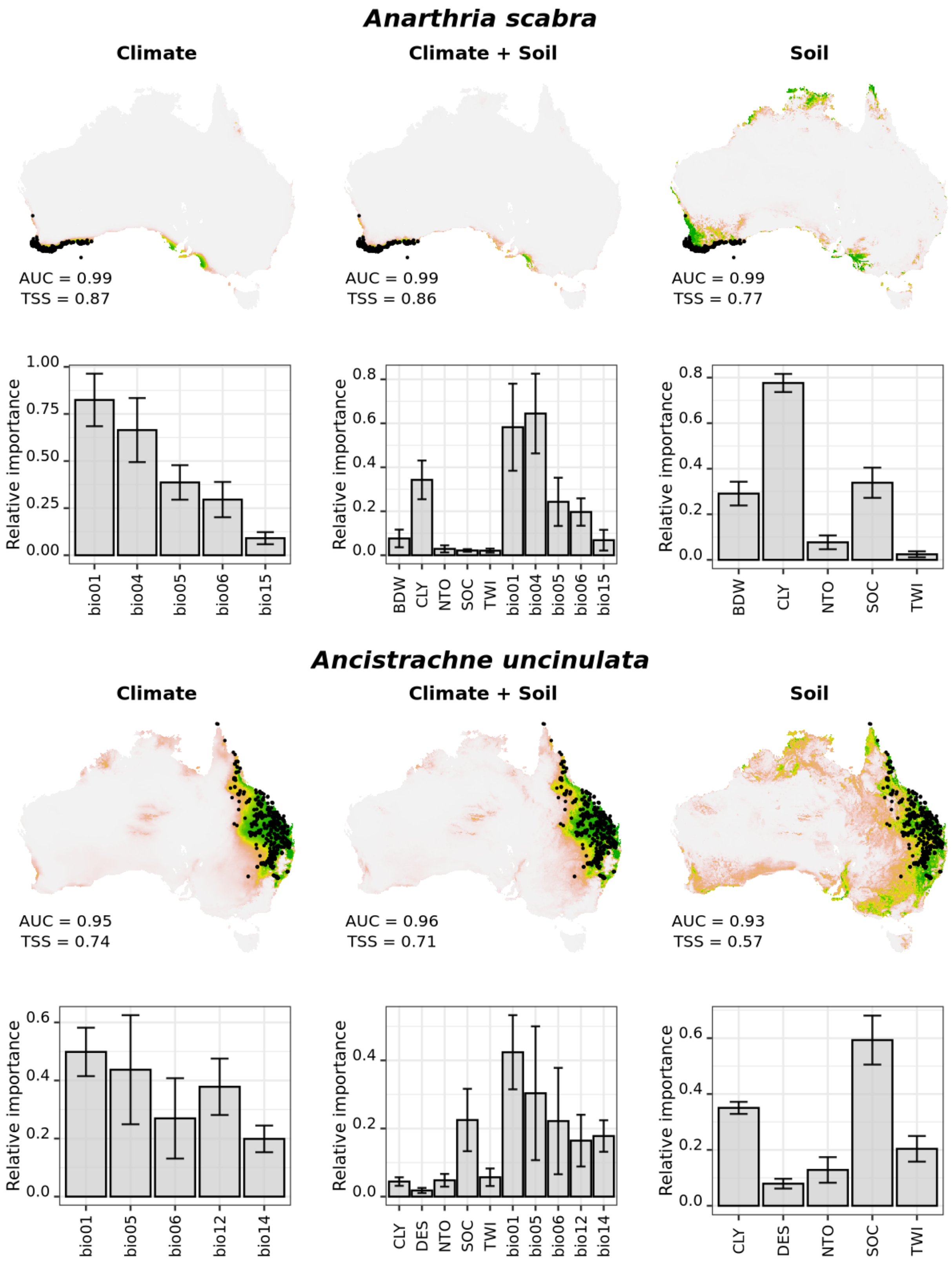
Anarthria scabra
Climate
Climate + Soil
Soil
AUC = 0.99
TSS = 0.87
AUC = 0.99
TSS = 0.86
AUC = 0.99
TSS = 0.77
Relative importance
bio01
bio04
bio05
bio06
bio15
BDW
CLY
NTO
SOC
TWI
Ancistrachne uncinulata
Climate
Climate + Soil
Soil
AUC = 0.95
TSS = 0.74
AUC = 0.96
TSS = 0.71
AUC = 0.93
TSS = 0.57
Relative importance
bio01
bio05
bio06
bio12
bio14
CLY
DES
NTO
SOC
TWI

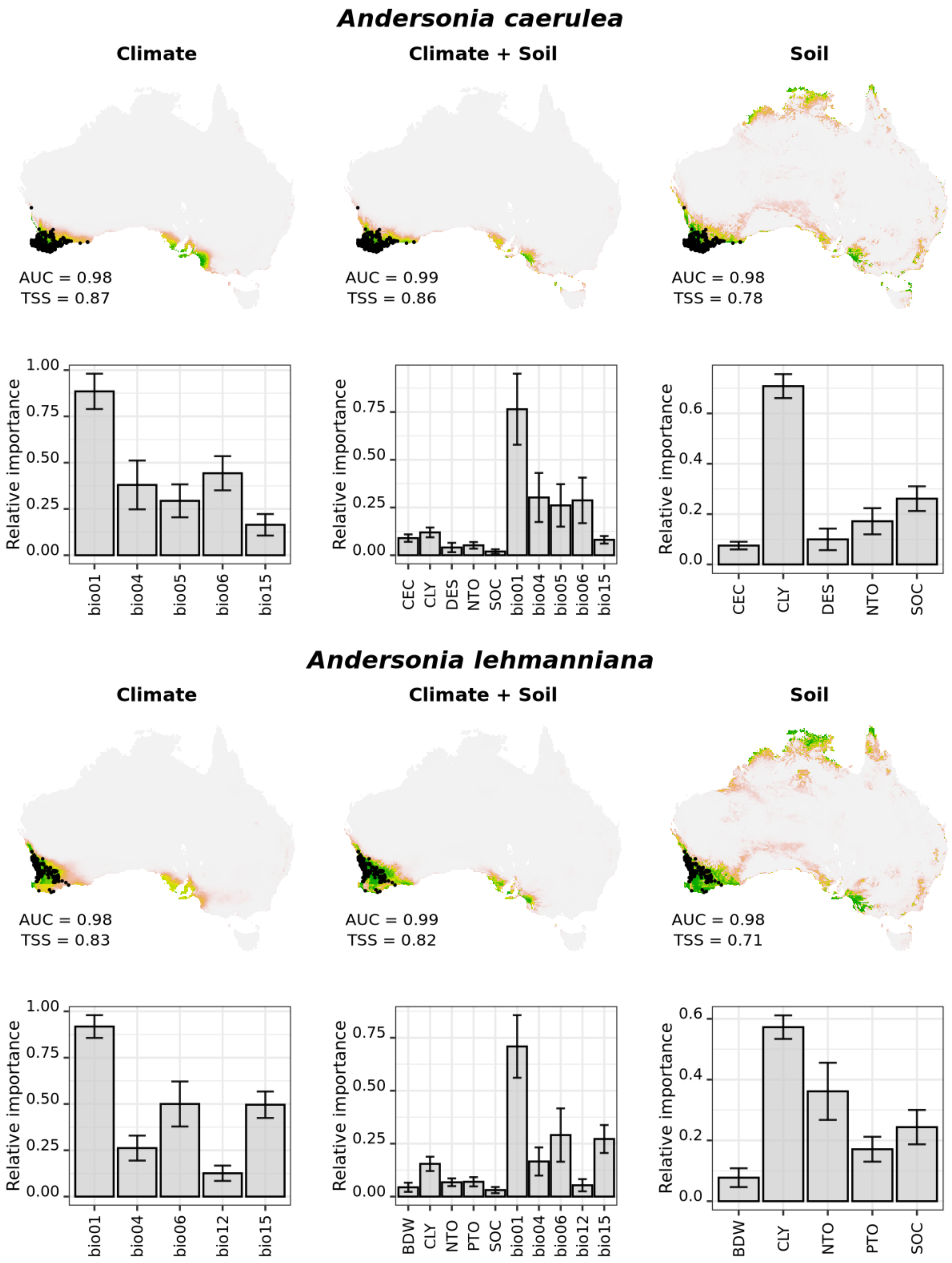

Andersonia caerulea
Climate
Climate + Soil
Soil
AUC = 0.98
TSS = 0.87
AUC = 0.99
TSS = 0.86
AUC = 0.98
TSS = 0.78
Relative importance
1.00
0.75
0.50
0.25
0.00
bio01
bio04
bio05
bio06
bio15
CEC
CLY
DES
NTO
SOC
0.6
0.4
0.2
0.0
Andersonia lehmanniana
Climate
Climate + Soil
Soil
AUC = 0.98
TSS = 0.83
AUC = 0.99
TSS = 0.82
AUC = 0.98
TSS = 0.71
bio12
BDW
PTO

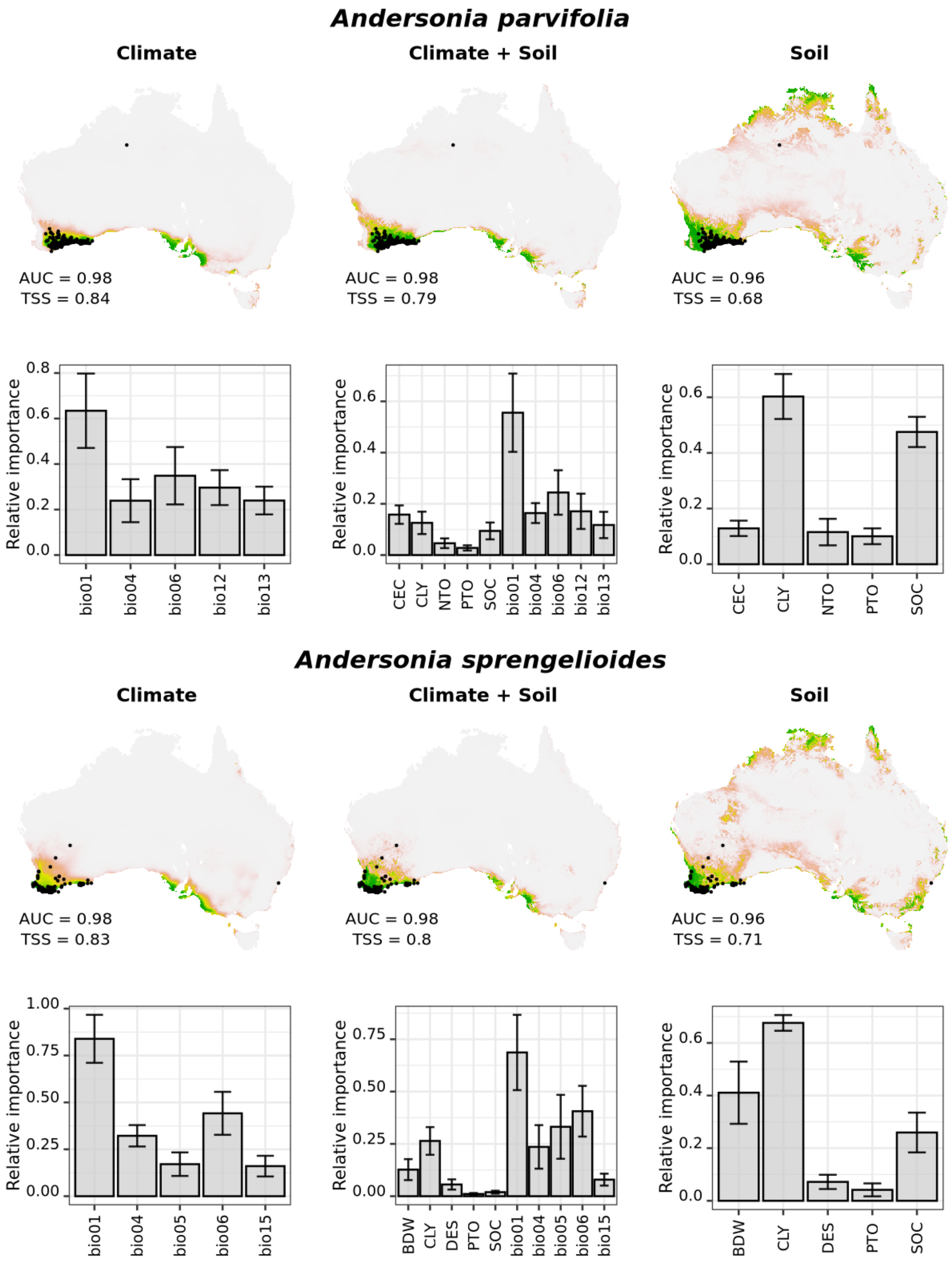

Andersonia parvifolia
Climate
Climate + Soil
Soil
AUC = 0.98
TSS = 0.84
AUC = 0.98
TSS = 0.79
AUC = 0.96
TSS = 0.68
Relative importance
bio01
bio04
bio06
bio12
bio13
CEC
CLY
NTO
PTO
SOC
Andersonia sprengelioides
Climate
Climate + Soil
Soil
AUC = 0.98
TSS = 0.83
AUC = 0.98
TSS = 0.8
AUC = 0.96
TSS = 0.71
Relative importance
bio01
bio04
bio05
bio06
bio15
BDW
CLY
DES
PTO
SOC

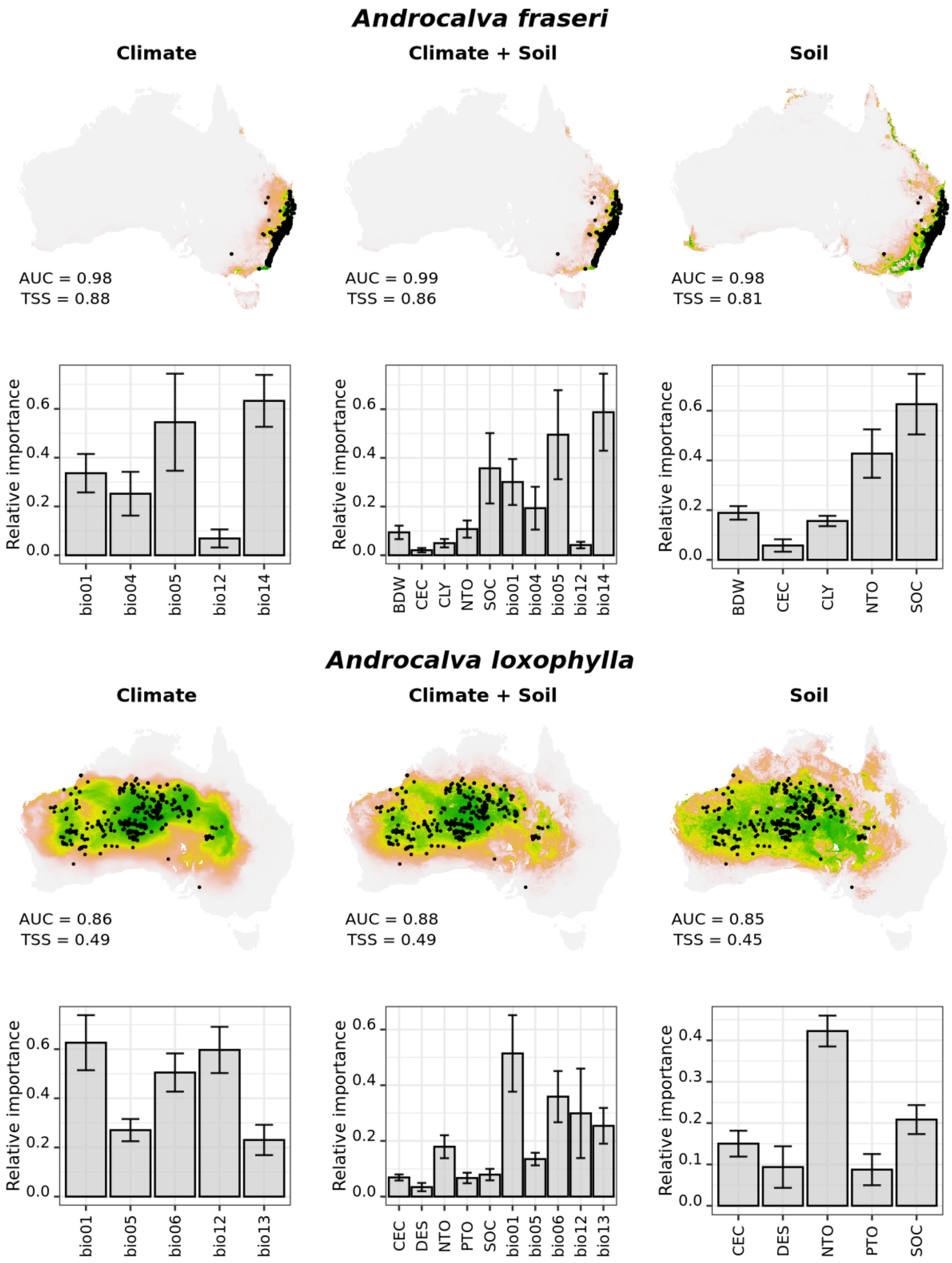

Androcalva fraseri
Climate
Climate + Soil
Soil
AUC = 0.98
TSS = 0.88
AUC = 0.99
TSS = 0.86
AUC = 0.98
TSS = 0.81
Relative importance
bio01
bio04
bio05
bio12
bio14
BDW
CEC
CLY
NTO
SOC
Androcalva loxophylla
Climate
Climate + Soil
Soil
AUC = 0.86
TSS = 0.49
AUC = 0.88
TSS = 0.49
AUC = 0.85
TSS = 0.45
bio06
bio13
DES
PTO

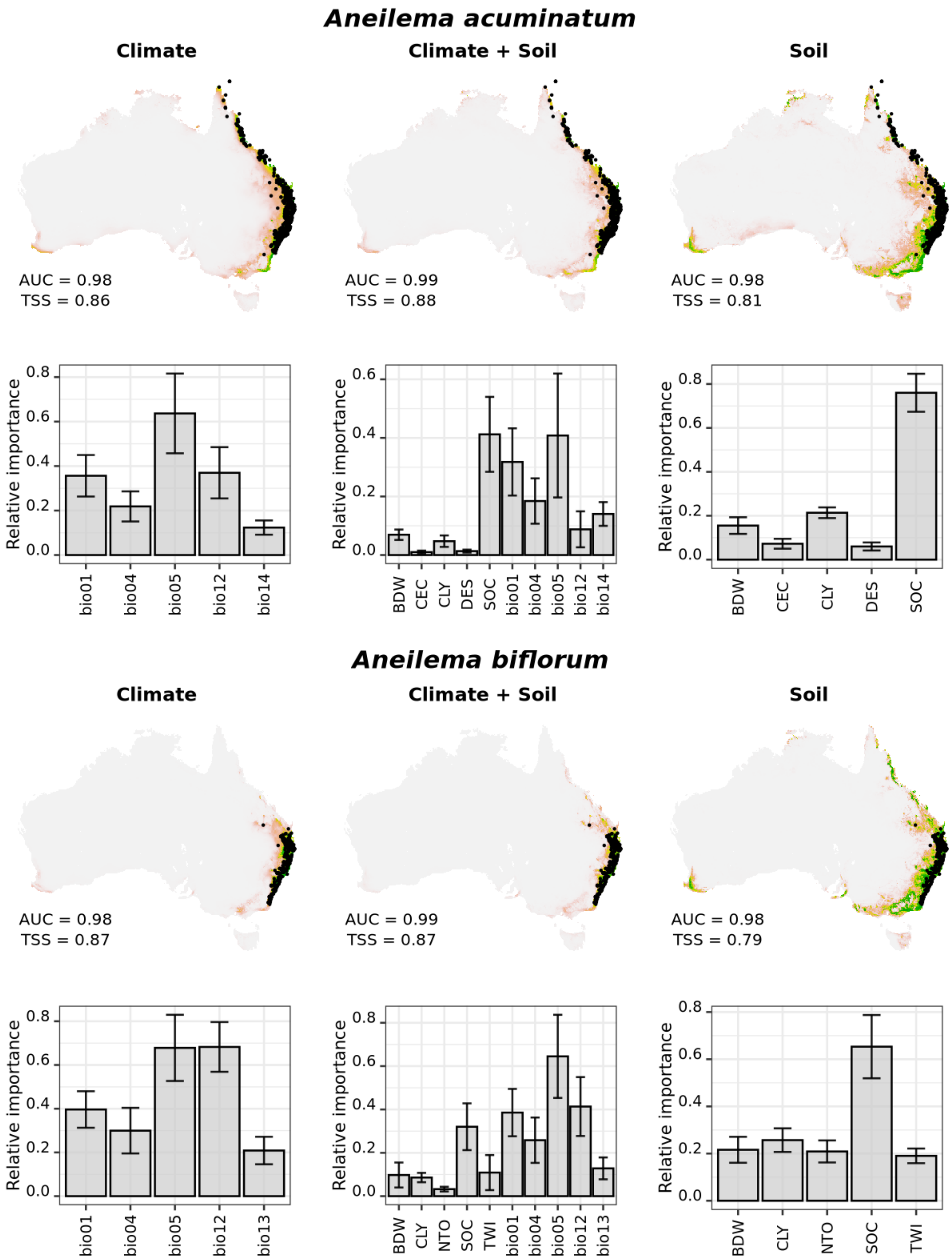
Aneilema acuminatum
Climate
Climate + Soil
Soil
AUC = 0.98
TSS = 0.86
AUC = 0.99
TSS = 0.88
AUC = 0.98
TSS = 0.81
Relative importance
bio01
bio04
bio05
bio12
bio14
BDW
CEC
CLY
DES
SOC
Aneilema biflorum
Climate
Climate + Soil
Soil
AUC = 0.98
TSS = 0.87
AUC = 0.99
TSS = 0.87
AUC = 0.98
TSS = 0.79
Relative importance
bio01
bio04
bio05
bio12
bio13
BDW
CLY
NTO
SOC
TWI

Aneilema siliculosum

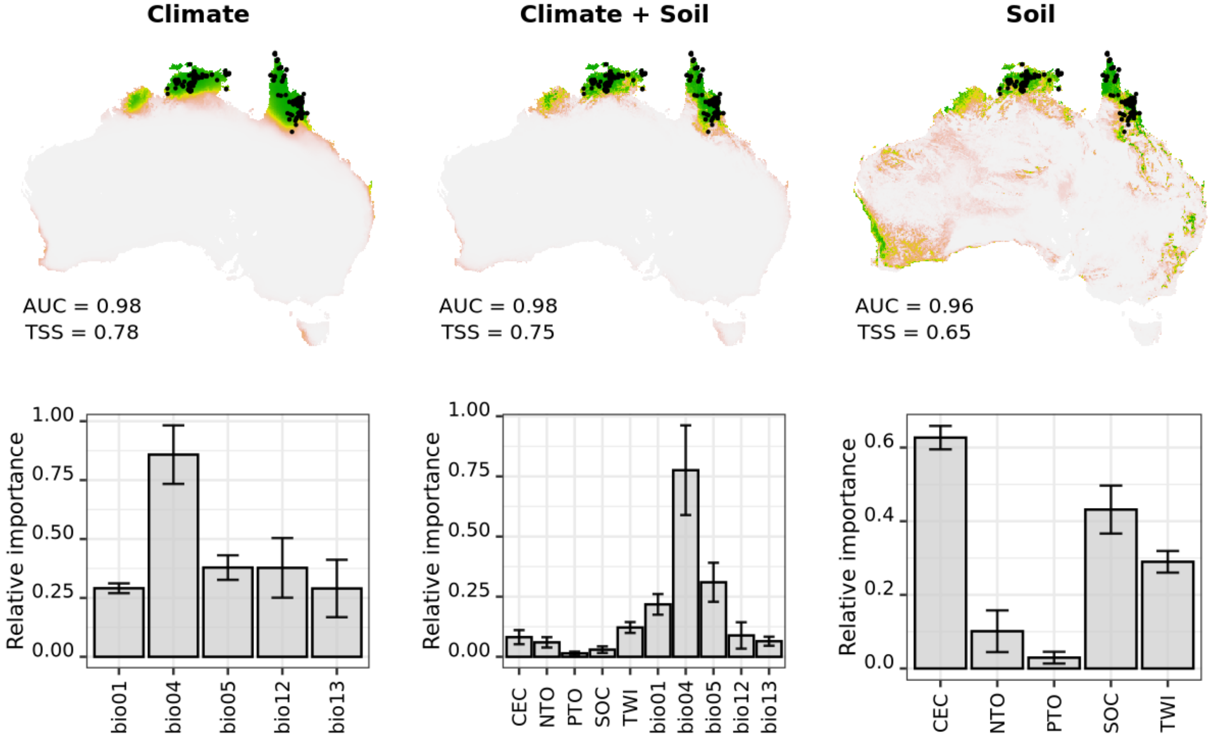

Anemocarpa podolepidium

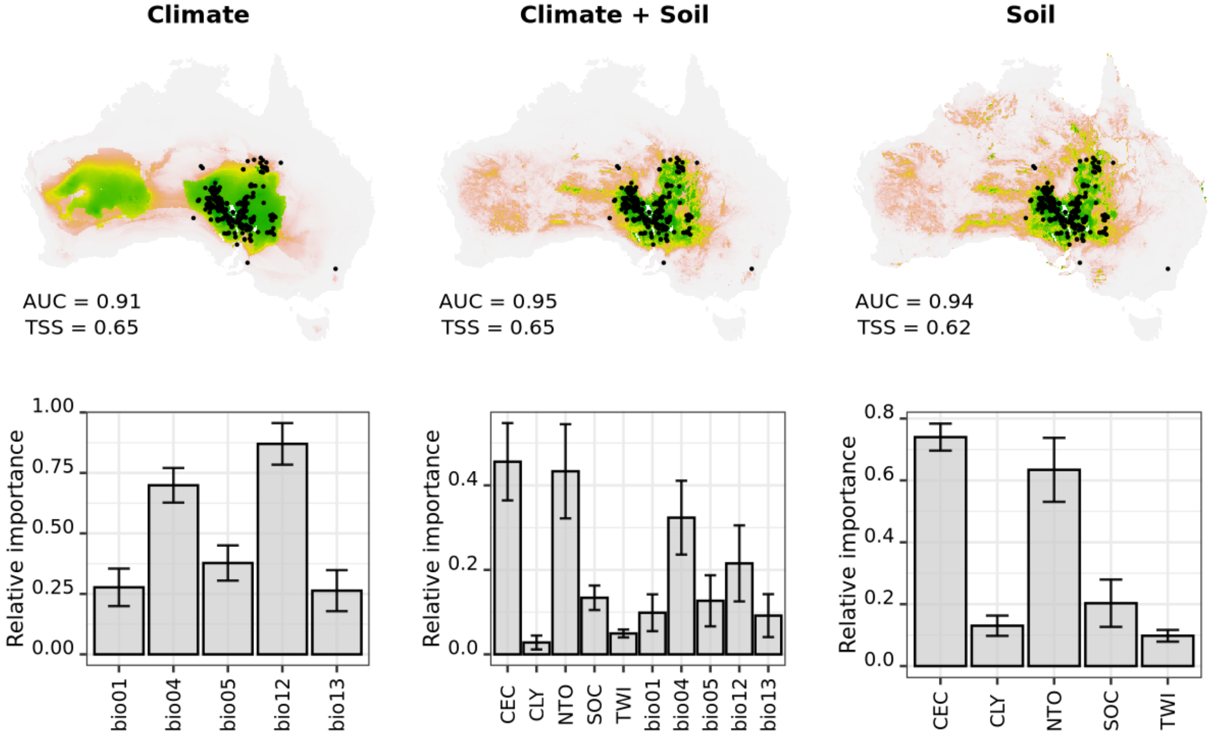

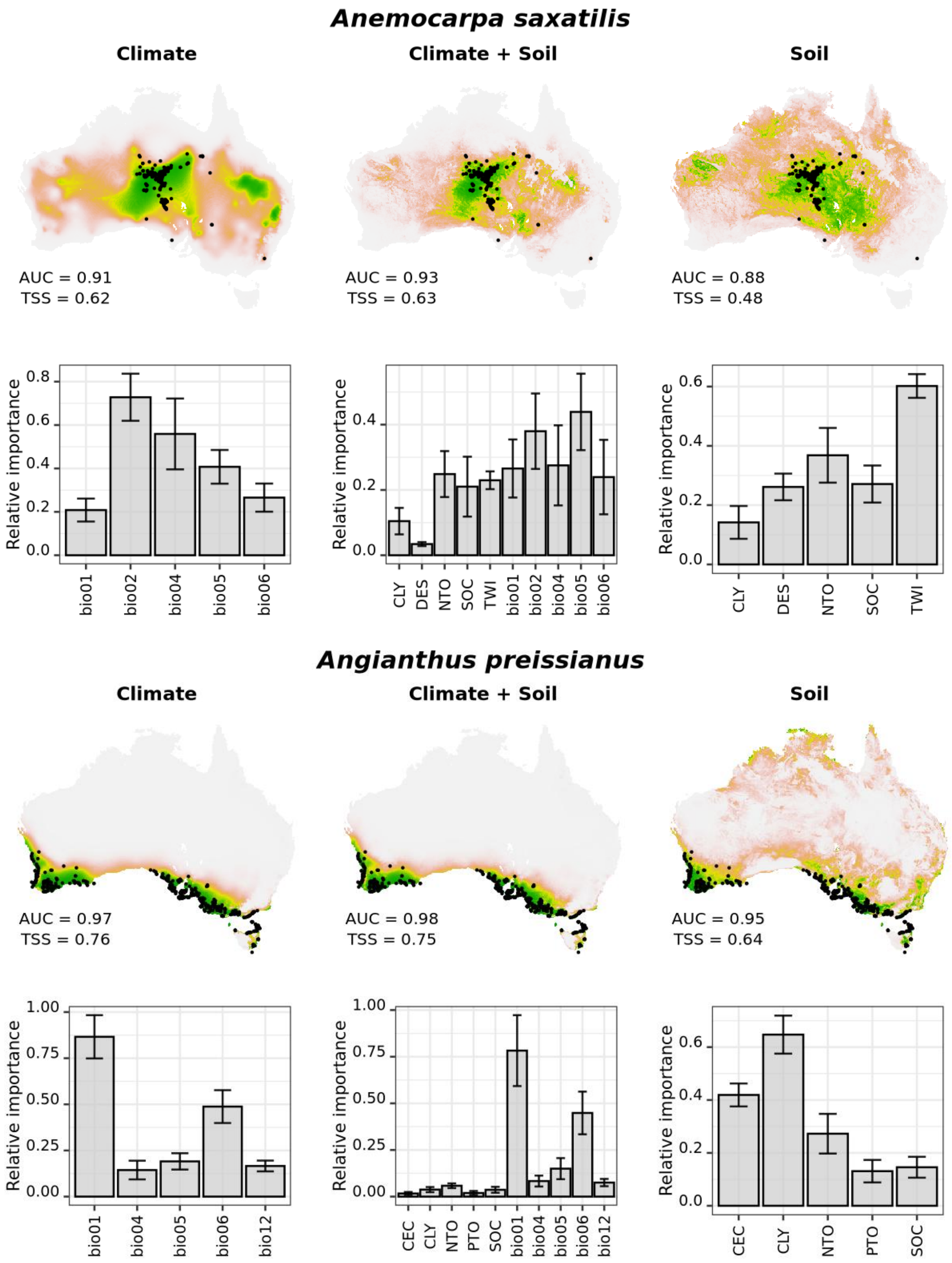

Anemocarpa saxatilis
Climate
Climate + Soil
Soil
AUC = 0.91
TSS = 0.62
AUC = 0.93
TSS = 0.63
AUC = 0.88
TSS = 0.48
Relative importance
bio01
bio02
bio04
bio05
bio06
CLY
DES
NTO
SOC
TWI
Angianthus preissianus
Climate
Climate + Soil
Soil
AUC = 0.97
TSS = 0.76
AUC = 0.98
TSS = 0.75
AUC = 0.95
TSS = 0.64
Relative importance
bio01
bio04
bio05
bio06
bio12
CEC
CLY
NTO
PTO
SOC

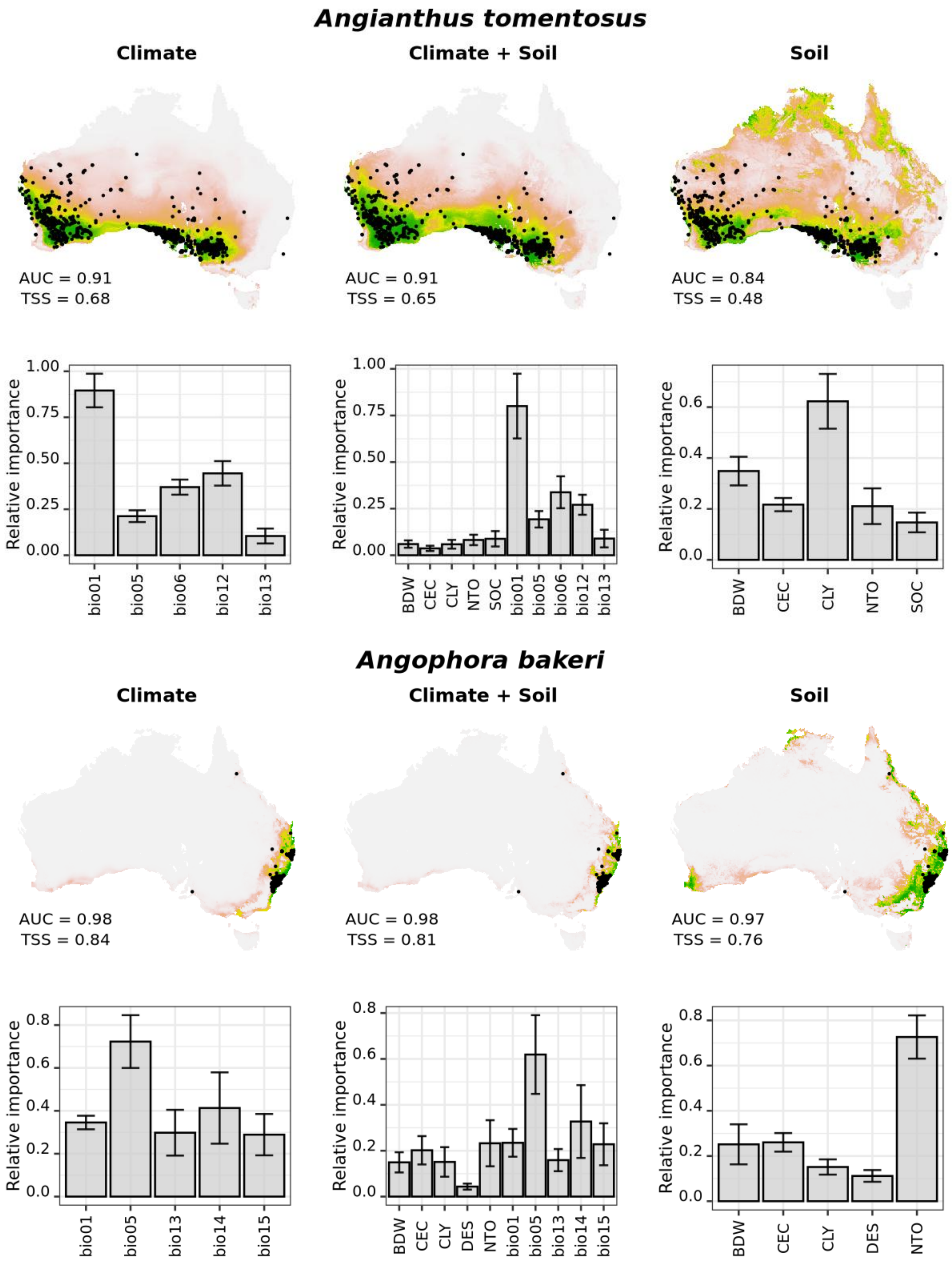
Angianthus tomentosus
Climate
Climate + Soil
Soil
AUC = 0.91
TSS = 0.68
AUC = 0.91
TSS = 0.65
AUC = 0.84
TSS = 0.48
Relative importance
bio01
bio05
bio06
bio12
bio13
BDW
CEC
CLY
NTO
SOC
Angophora bakeri
Climate
Climate + Soil
Soil
AUC = 0.98
TSS = 0.84
AUC = 0.98
TSS = 0.81
AUC = 0.97
TSS = 0.76
Relative importance
bio01
bio05
bio13
bio14
bio15
BDW
CEC
CLY
DES
NTO

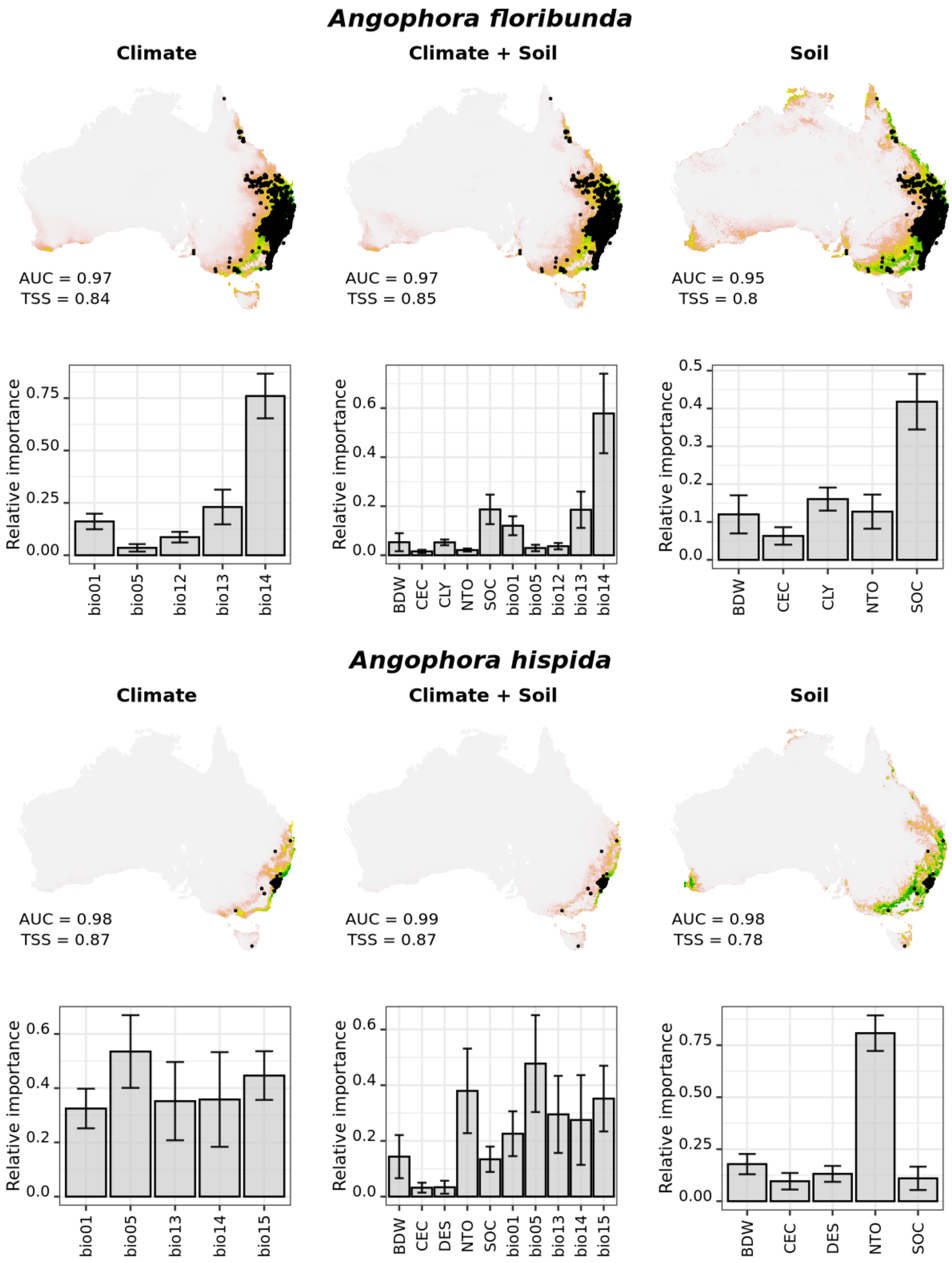

Angophora floribunda
Climate
Climate + Soil
Soil
AUC = 0.97
TSS = 0.84
AUC = 0.97
TSS = 0.85
AUC = 0.95
TSS = 0.8
Relative importance
0.00
0.25
0.50
0.75
bio01
bio05
bio12
bio13
bio14
0.0
0.2
0.4
0.6
BDW
CEC
CLY
NTO
SOC
bio01
bio05
bio12
bio13
bio14
0.0
0.1
0.2
0.3
0.4
0.5
BDW
CEC
CLY
NTO
SOC
Angophora hispida
Climate
Climate + Soil
Soil
AUC = 0.98
TSS = 0.87
AUC = 0.99
TSS = 0.87
AUC = 0.98
TSS = 0.78
Relative importance
0.0
0.2
0.4
0.6
bio01
bio05
bio13
bio14
bio15
0.0
0.2
0.4
0.6
BDW
CEC
DES
NTO
SOC
bio01
bio05
bio13
bio14
bio15
0.00
0.25
0.50
0.75
BDW
CEC
DES
NTO
SOC

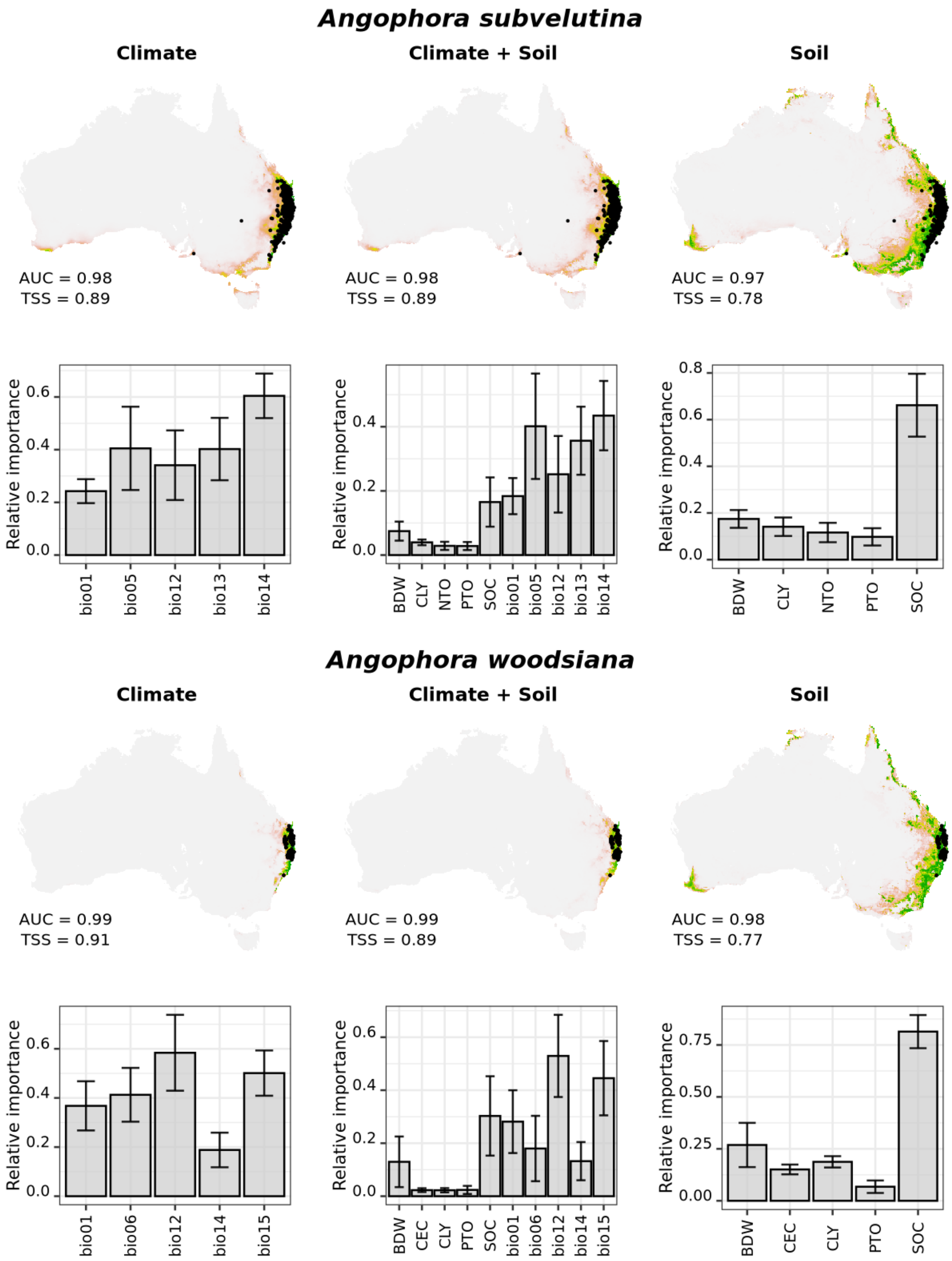

Angophora subvelutina
Climate
Climate + Soil
Soil
AUC = 0.98
TSS = 0.89
AUC = 0.98
TSS = 0.89
AUC = 0.97
TSS = 0.78
Relative importance
bio01
bio05
bio12
bio13
bio14
BDW
CLY
NTO
PTO
SOC
Angophora woodsiana
Climate
Climate + Soil
Soil
AUC = 0.99
TSS = 0.91
AUC = 0.99
TSS = 0.89
AUC = 0.98
TSS = 0.77
Relative importance
bio01
bio06
bio12
bio14
bio15
BDW
CEC
CLY
PTO
SOC

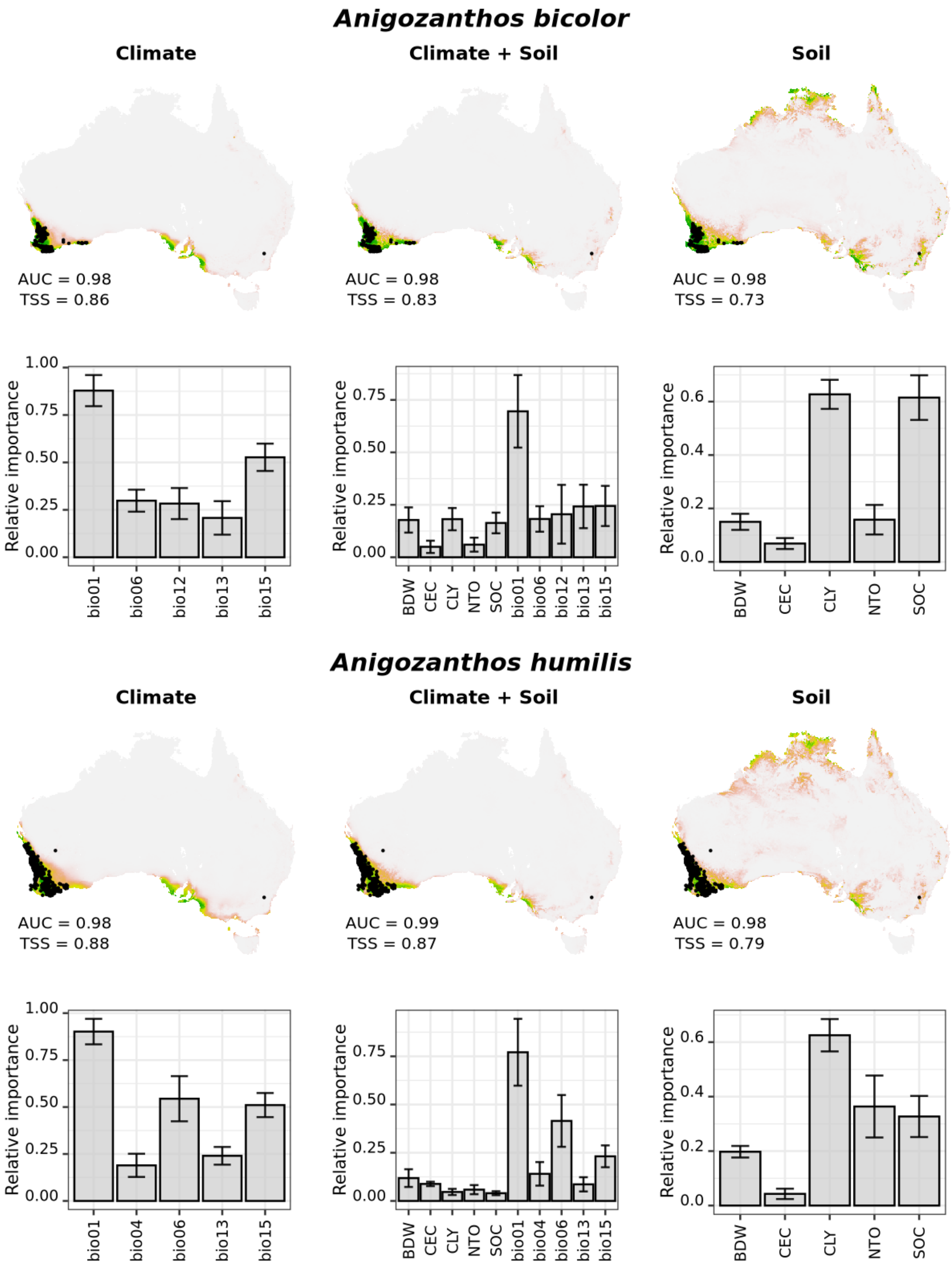
Anigozanthos bicolor
Climate
Climate + Soil
Soil
AUC = 0.98
TSS = 0.86
AUC = 0.98
TSS = 0.83
AUC = 0.98
TSS = 0.73
Relative importance
1.00
0.75
0.50
0.25
0.00
bio01
bio06
bio12
bio13
bio15
BDW
CEC
CLY
NTO
SOC
bio01
bio06
bio12
bio13
bio15
0.6
0.4
0.2
0.0
Anigozanthos humilis
Climate
Climate + Soil
Soil
AUC = 0.98
TSS = 0.88
AUC = 0.99
TSS = 0.87
AUC = 0.98
TSS = 0.79
bio01
bio04
bio06
bio13
bio15
BDW
CEC
CLY
NTO
SOC
bio01
bio04
bio06
bio13
bio15

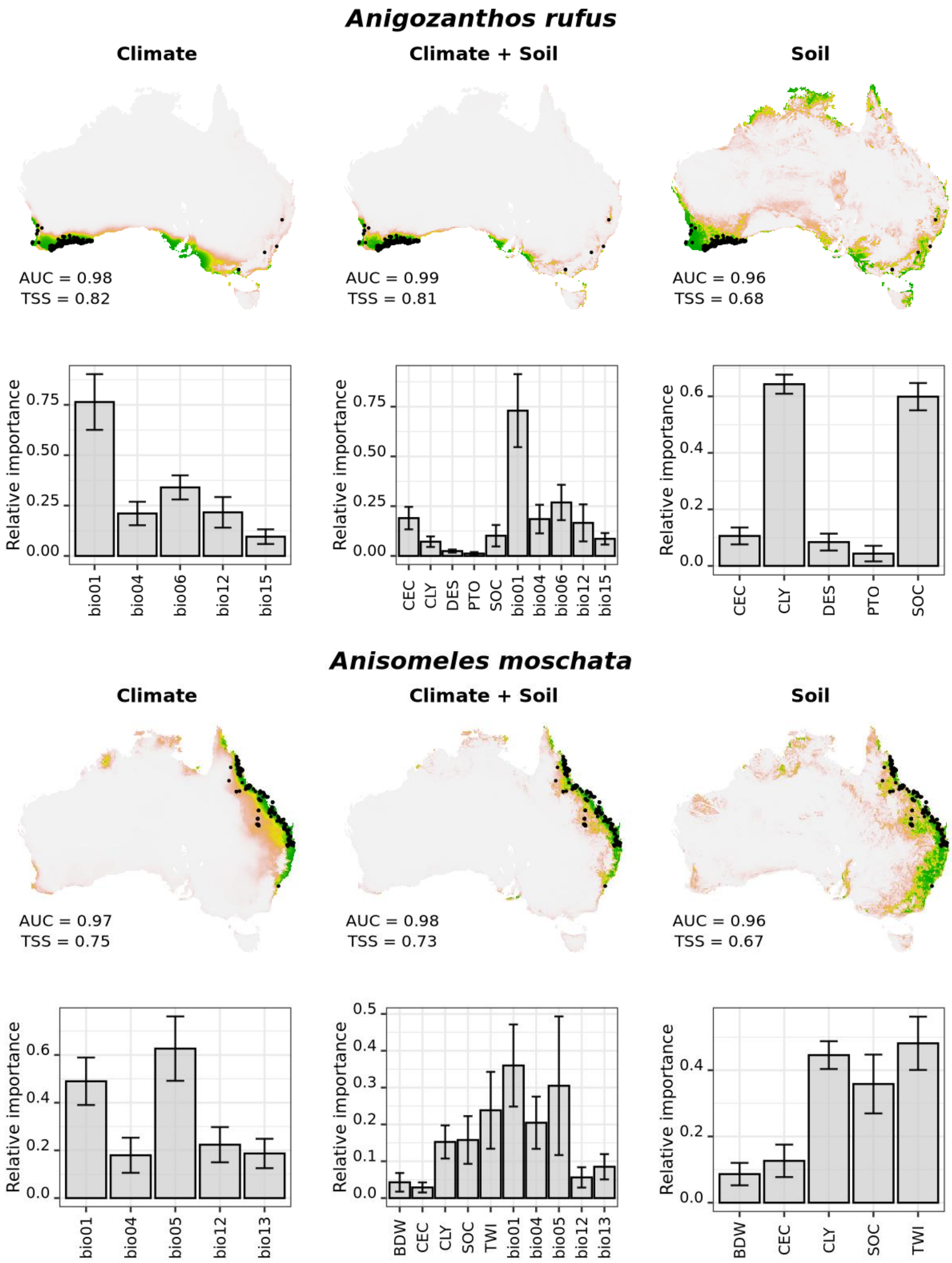
Anigozanthos rufus
Climate
Climate + Soil
Soil
AUC = 0.98
TSS = 0.82
AUC = 0.99
TSS = 0.81
AUC = 0.96
TSS = 0.68
Relative importance
0.00
0.25
0.50
0.75
bio01
bio04
bio06
bio12
bio15
CEC
CLY
DES
PTO
SOC
0.0
0.2
0.4
0.6
Anisomeles moschata
Climate
Climate + Soil
Soil
AUC = 0.97
TSS = 0.75
AUC = 0.98
TSS = 0.73
AUC = 0.96
TSS = 0.67
Relative importance
0.0
0.1
0.2
0.3
0.4
0.5
0.6
bio01
bio04
bio05
bio12
bio13
BDW
CEC
CLY
SOC
TWI

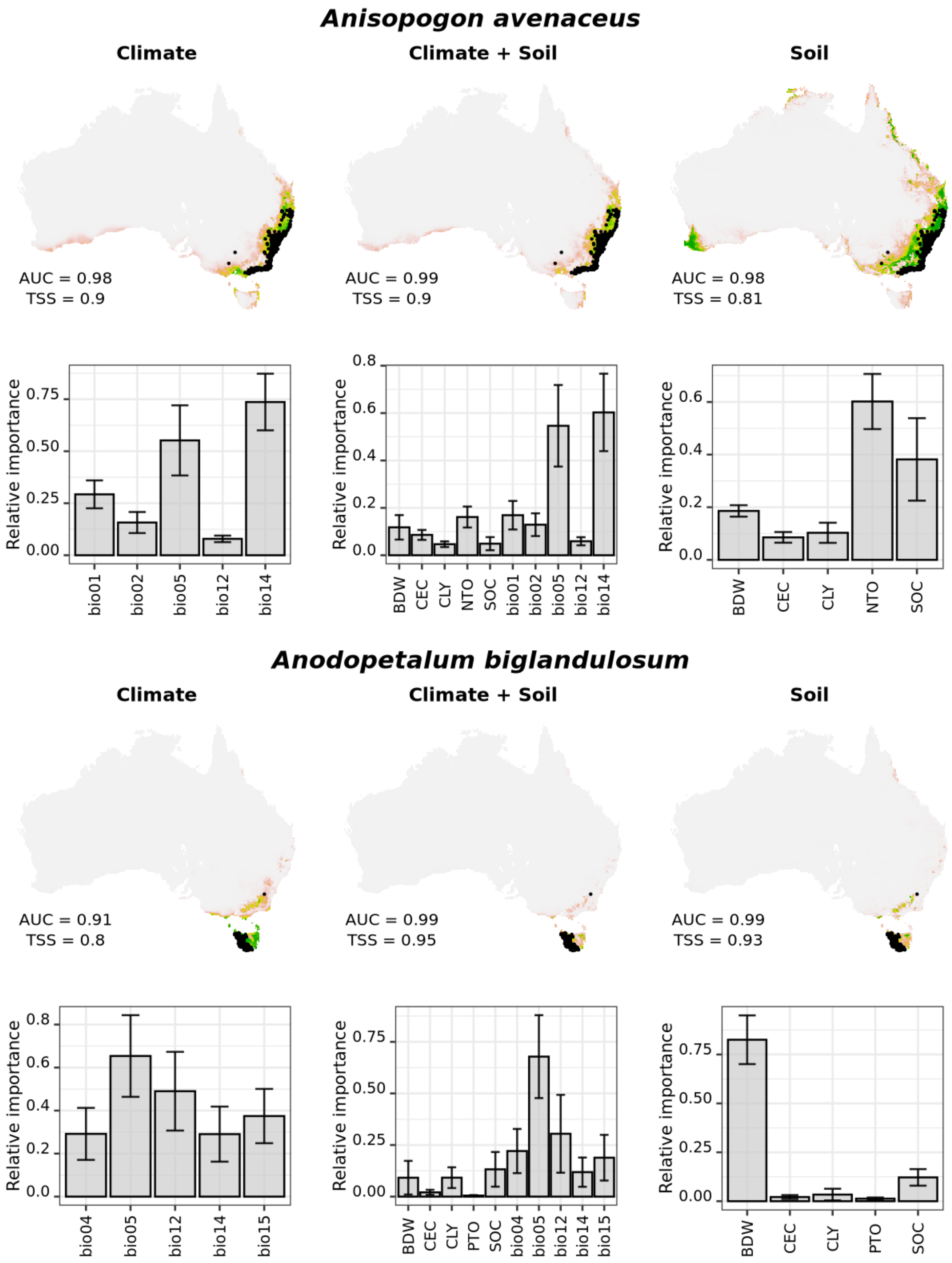

Anisopogon avenaceus
Climate
Climate + Soil
Soil
AUC = 0.98
TSS = 0.9
AUC = 0.99
TSS = 0.9
AUC = 0.98
TSS = 0.81
Relative importance
0.00
0.25
0.50
0.75
bio01
bio02
bio05
bio12
bio14
0.0
0.2
0.4
0.6
0.8
BDW
CEC
CLY
NTO
SOC
bio01
bio02
bio05
bio12
bio14
0.0
0.2
0.4
0.6
BDW
CEC
CLY
NTO
SOC
Anodopetalum biglandulosum
Climate
Climate + Soil
Soil
AUC = 0.91
TSS = 0.8
AUC = 0.99
TSS = 0.95
AUC = 0.99
TSS = 0.93
Relative importance
0.0
0.2
0.4
0.6
0.8
bio04
bio05
bio12
bio14
bio15
0.00
0.25
0.50
0.75
BDW
CEC
CLY
PTO
SOC
bio04
bio05
bio12
bio14
bio15
0.00
0.25
0.50
0.75
BDW
CEC
CLY
PTO
SOC

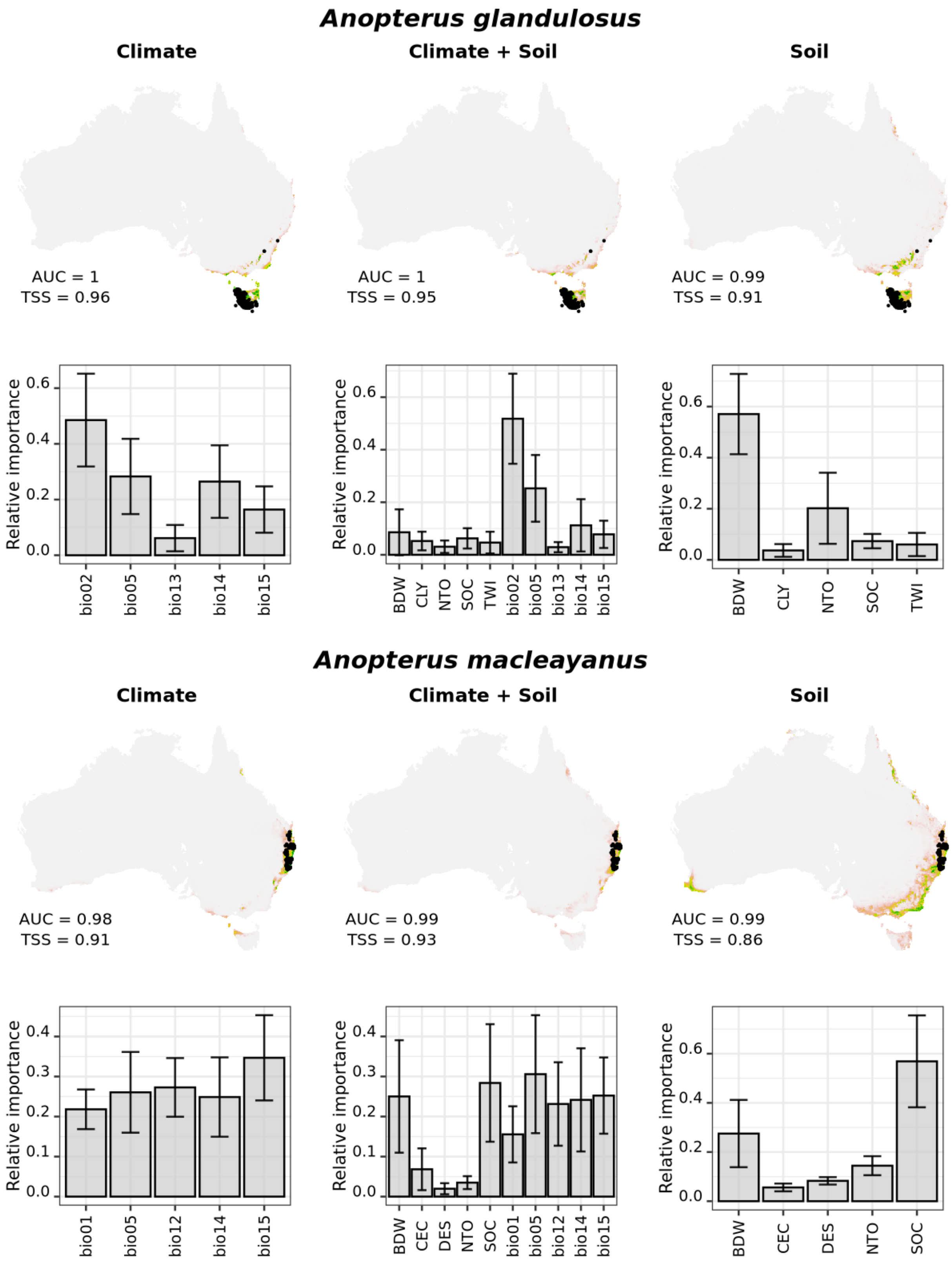
Anopterus glandulosus
Climate
Climate + Soil
Soil
AUC = 1
TSS = 0.96
AUC = 1
TSS = 0.95
AUC = 0.99
TSS = 0.91
Relative importance
bio02
bio05
bio13
bio14
bio15
BDW
CLY
NTO
SOC
TWI
Anopterus macleayanus
AUC = 0.98
TSS = 0.91
AUC = 0.99
TSS = 0.93
AUC = 0.99
TSS = 0.86
bio01
bio12
CEC
DES

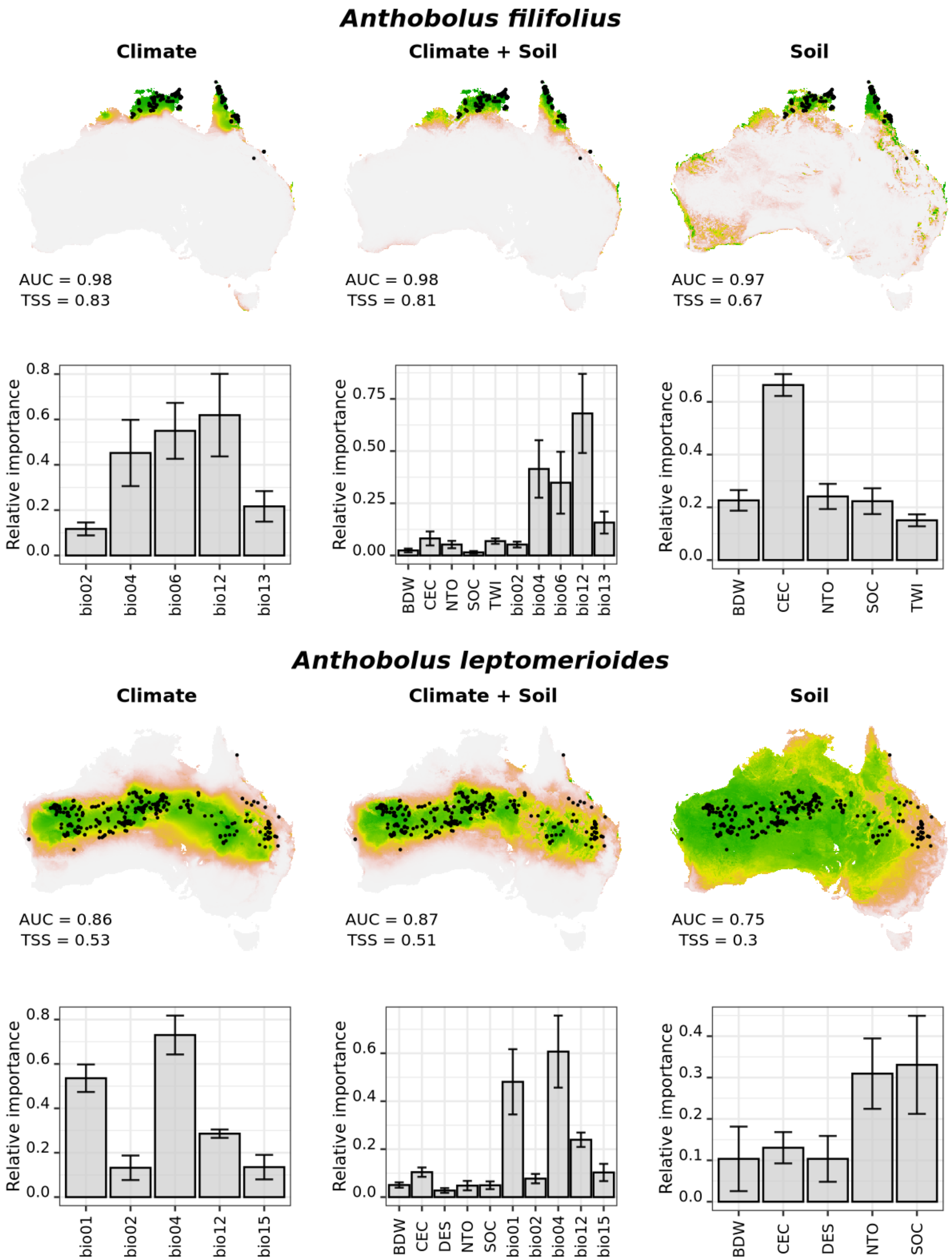
Anthobolus filifolius
Climate
Climate + Soil
Soil
AUC = 0.98
TSS = 0.83
AUC = 0.98
TSS = 0.81
AUC = 0.97
TSS = 0.67
Relative importance
bio02
bio04
bio06
bio12
bio13
BDW
CEC
NTO
SOC
TWI
Anthobolus leptomerioides
Climate
Climate + Soil
Soil
AUC = 0.86
TSS = 0.53
AUC = 0.87
TSS = 0.51
AUC = 0.75
TSS = 0.3
Relative importance
bio01
bio02
bio04
bio12
bio15
BDW
CEC
DES
NTO
SOC

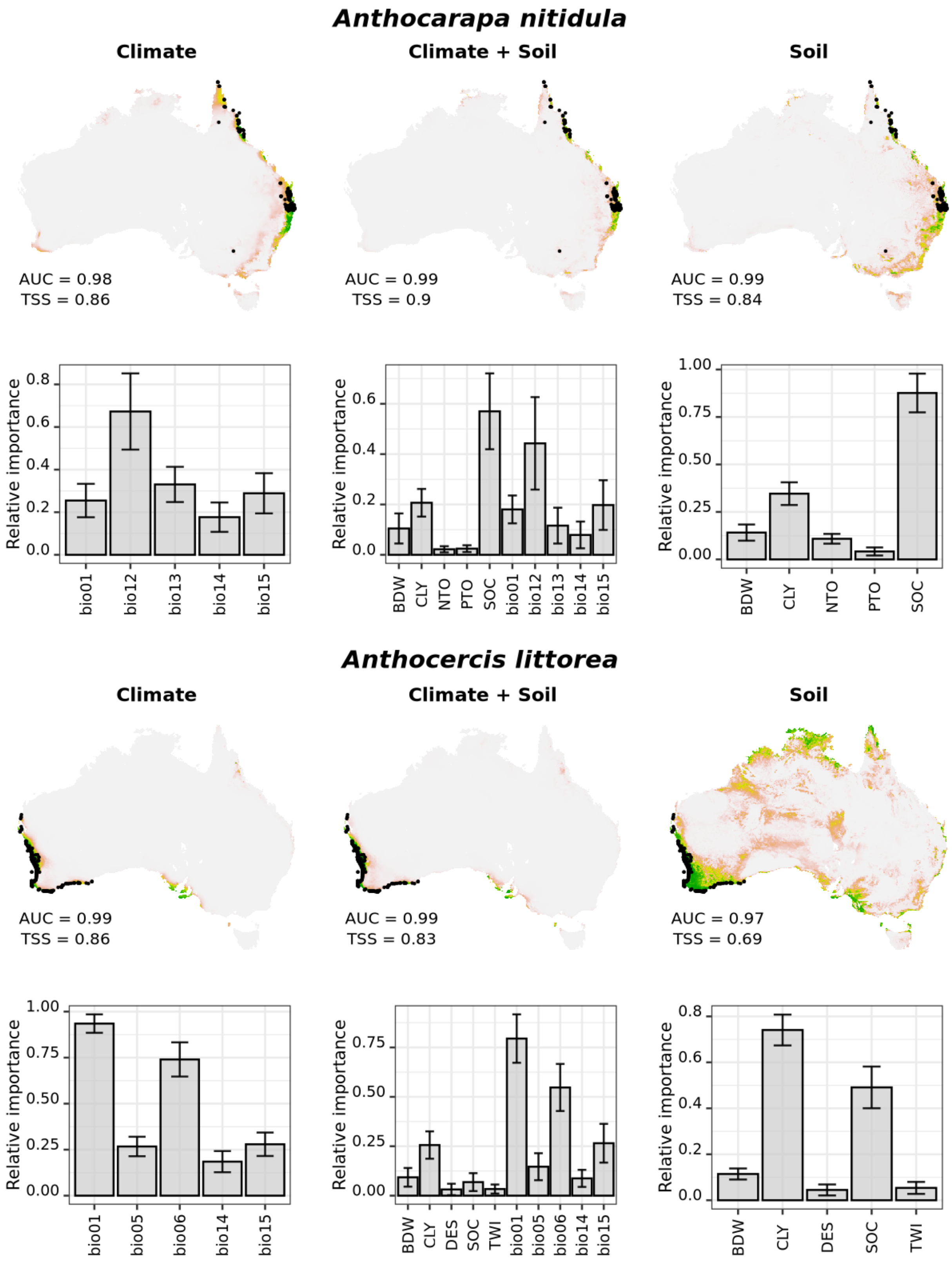
Anthocarapa nitidula
Climate
Climate + Soil
Soil
AUC = 0.98
TSS = 0.86
AUC = 0.99
TSS = 0.9
AUC = 0.99
TSS = 0.84
Relative importance
bio01
bio12
bio13
bio14
bio15
BDW
CLY
NTO
PTO
SOC
Anthocercis littorea
Climate
Climate + Soil
Soil
AUC = 0.99
TSS = 0.86
AUC = 0.99
TSS = 0.83
AUC = 0.97
TSS = 0.69
Relative importance
bio01
bio05
bio06
bio14
bio15
BDW
CLY
DES
SOC
TWI

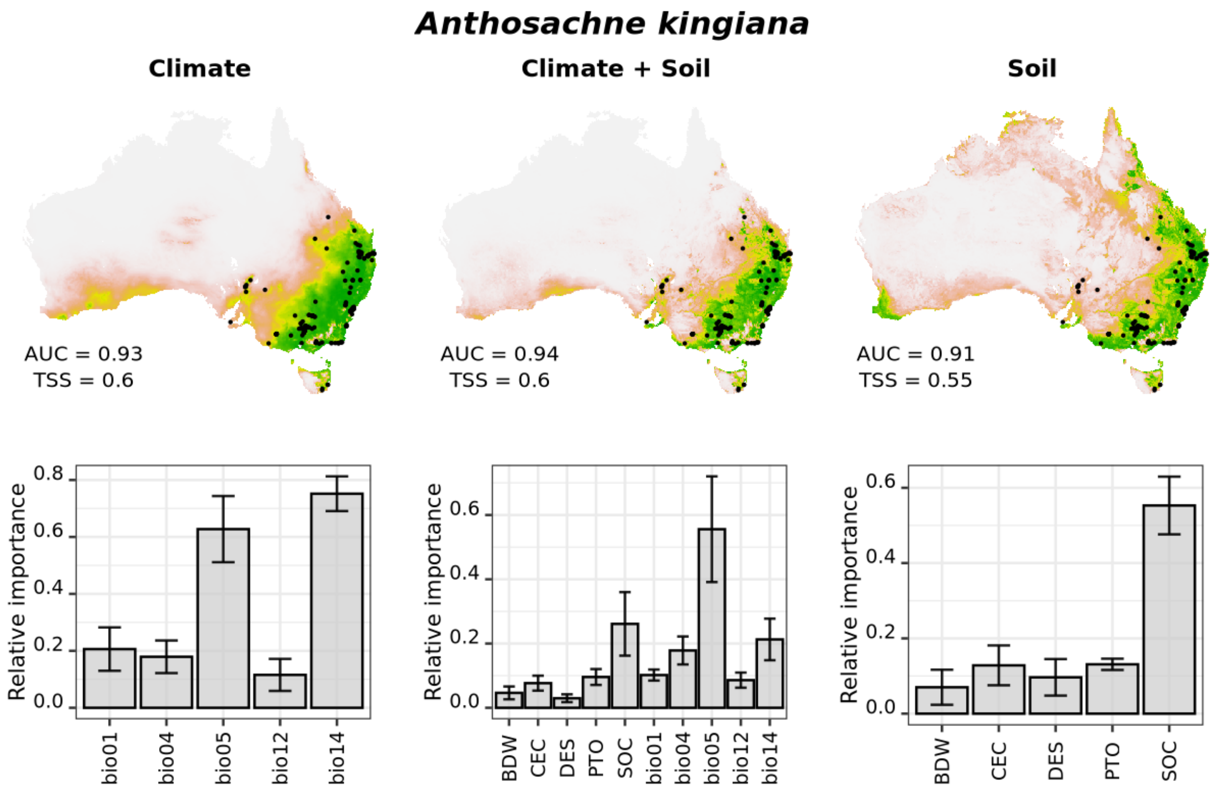
Anthosachne kingiana
Climate
Climate + Soil
Soil
AUC = 0.93
TSS = 0.6
AUC = 0.94
TSS = 0.6
AUC = 0.91
TSS = 0.55
Relative importance
0.0
0.2
0.4
0.6
0.8
bio01
bio04
bio05
bio12
bio14
BDW
CEC
DES
PTO
SOC

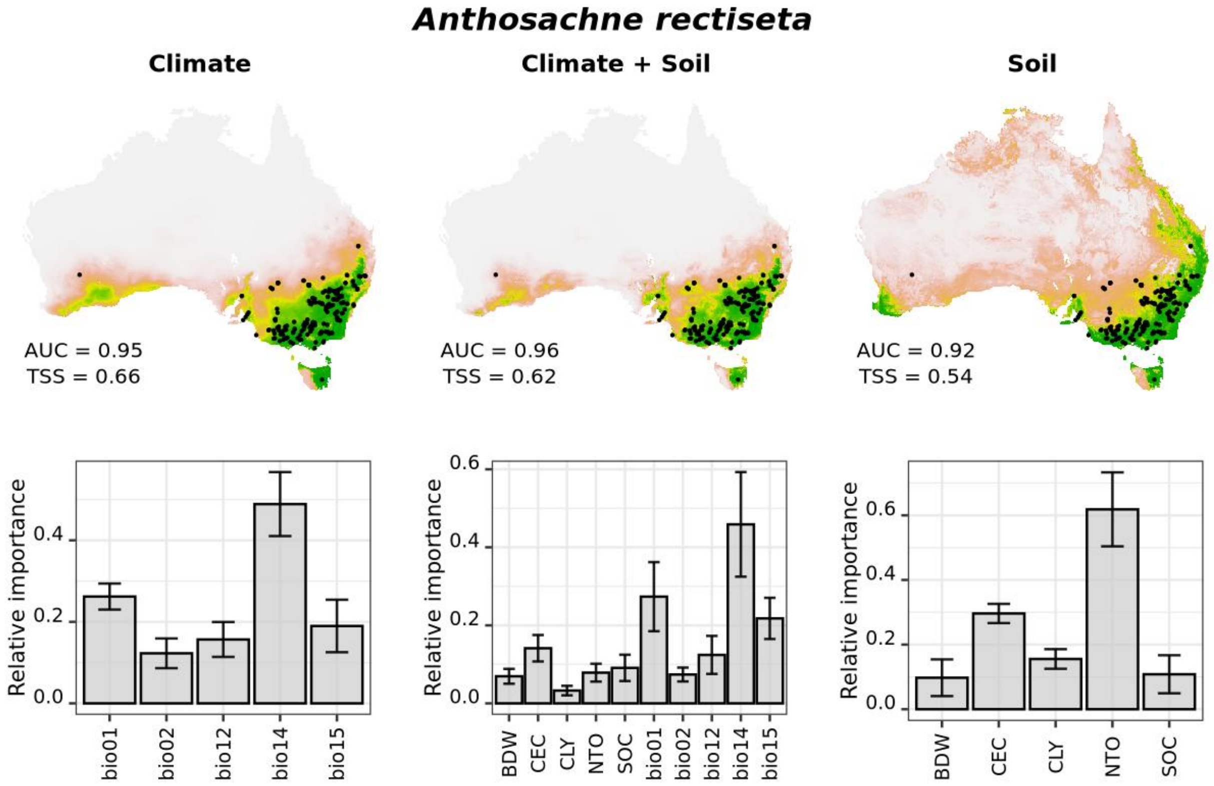
Anthosachne rectiseta
Climate
Climate + Soil
Soil
AUC = 0.95
TSS = 0.66
AUC = 0.96
TSS = 0.62
AUC = 0.92
TSS = 0.54
Relative importance
0.0
0.2
0.4
0.6
bio01
bio02
bio12
bio14
bio15
BDW
CEC
CLY
NTO
SOC

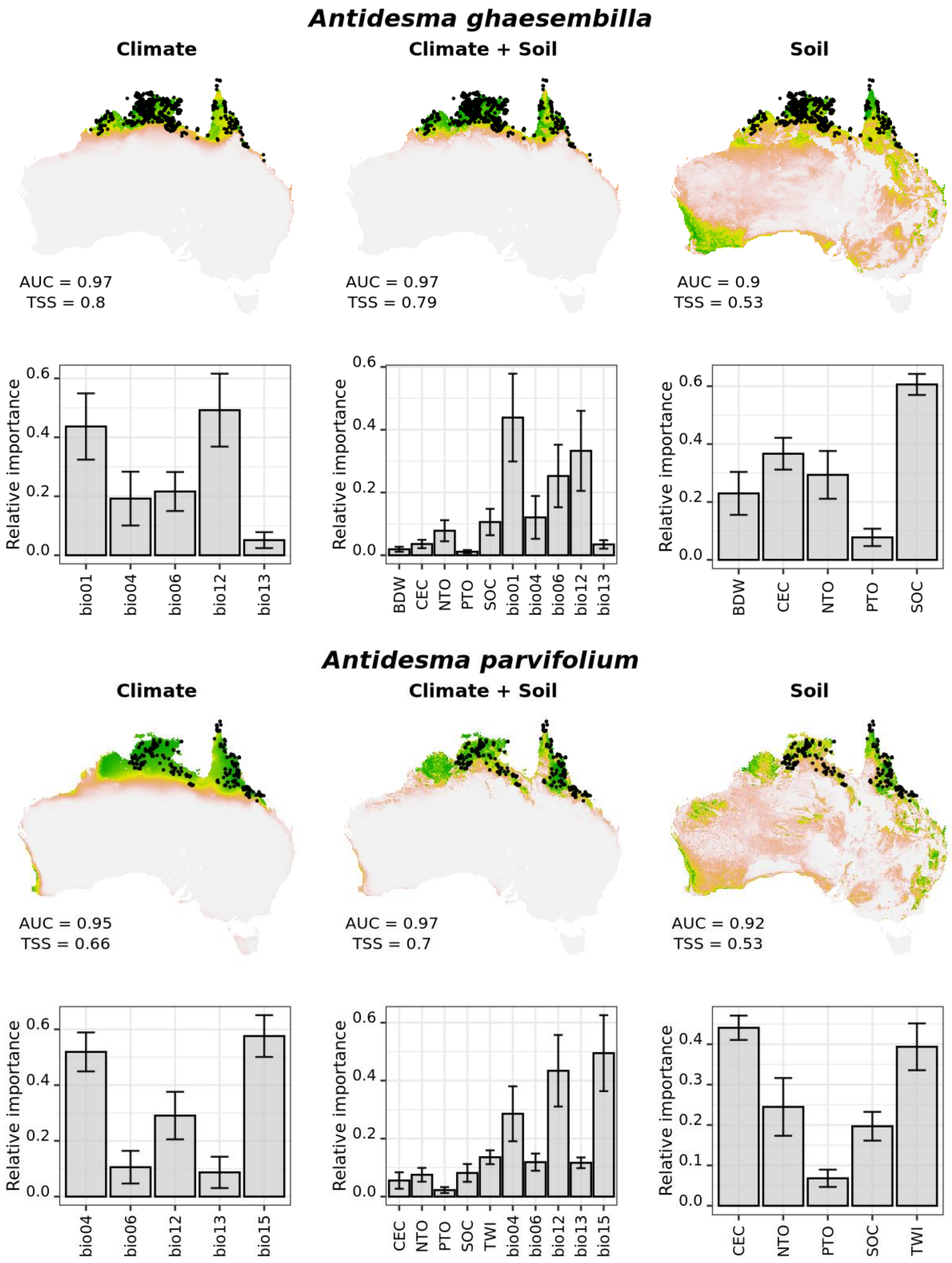

Antidesma ghaesembilla
Climate
Climate + Soil
Soil
AUC = 0.97
TSS = 0.8
AUC = 0.97
TSS = 0.79
AUC = 0.9
TSS = 0.53
Relative importance
0.0
0.2
0.4
0.6
bio01
bio04
bio06
bio12
bio13
BDW
CEC
NTO
PTO
SOC
Antidesma parvifolium
AUC = 0.95
TSS = 0.66
AUC = 0.97
TSS = 0.7
AUC = 0.92
TSS = 0.53
0.1
0.3
bio15
TWI

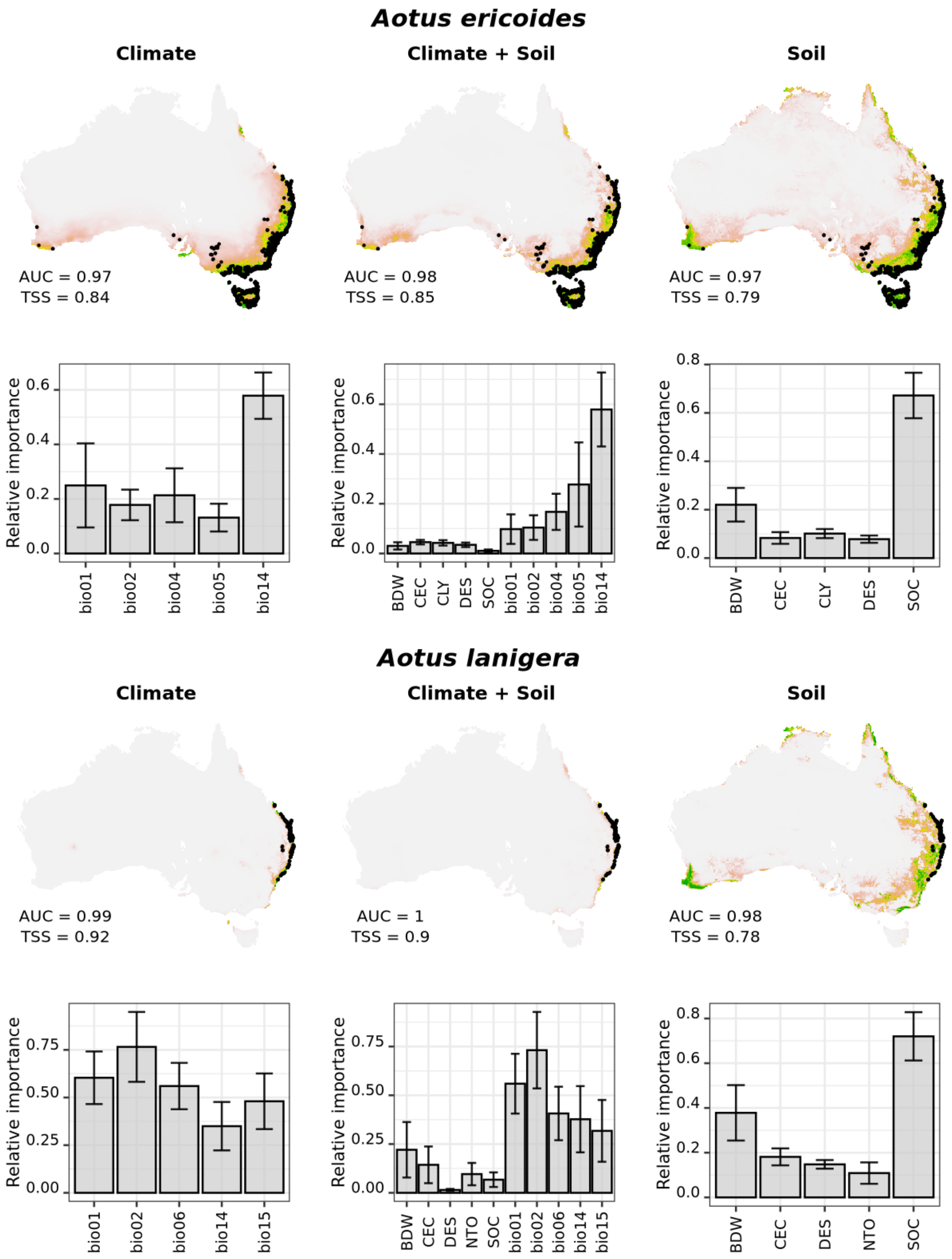
Aotus ericoides
Climate
Climate + Soil
Soil
AUC = 0.97
TSS = 0.84
AUC = 0.98
TSS = 0.85
AUC = 0.97
TSS = 0.79
Relative importance
bio01 bio02 bio04 bio05 bio14
BDW CEC CLY DES SOC bio01 bio02 bio04 bio05 bio14
BDW CEC CLY DES SOC
Aotus lanigera
Climate
Climate + Soil
Soil
AUC = 0.99
TSS = 0.92
AUC = 1
TSS = 0.9
AUC = 0.98
TSS = 0.78
Relative importance
bio01 bio02 bio06 bio14 bio15
BDW CEC DES NTO SOC bio01 bio02 bio06 bio14 bio15
BDW CEC DES NTO SOC

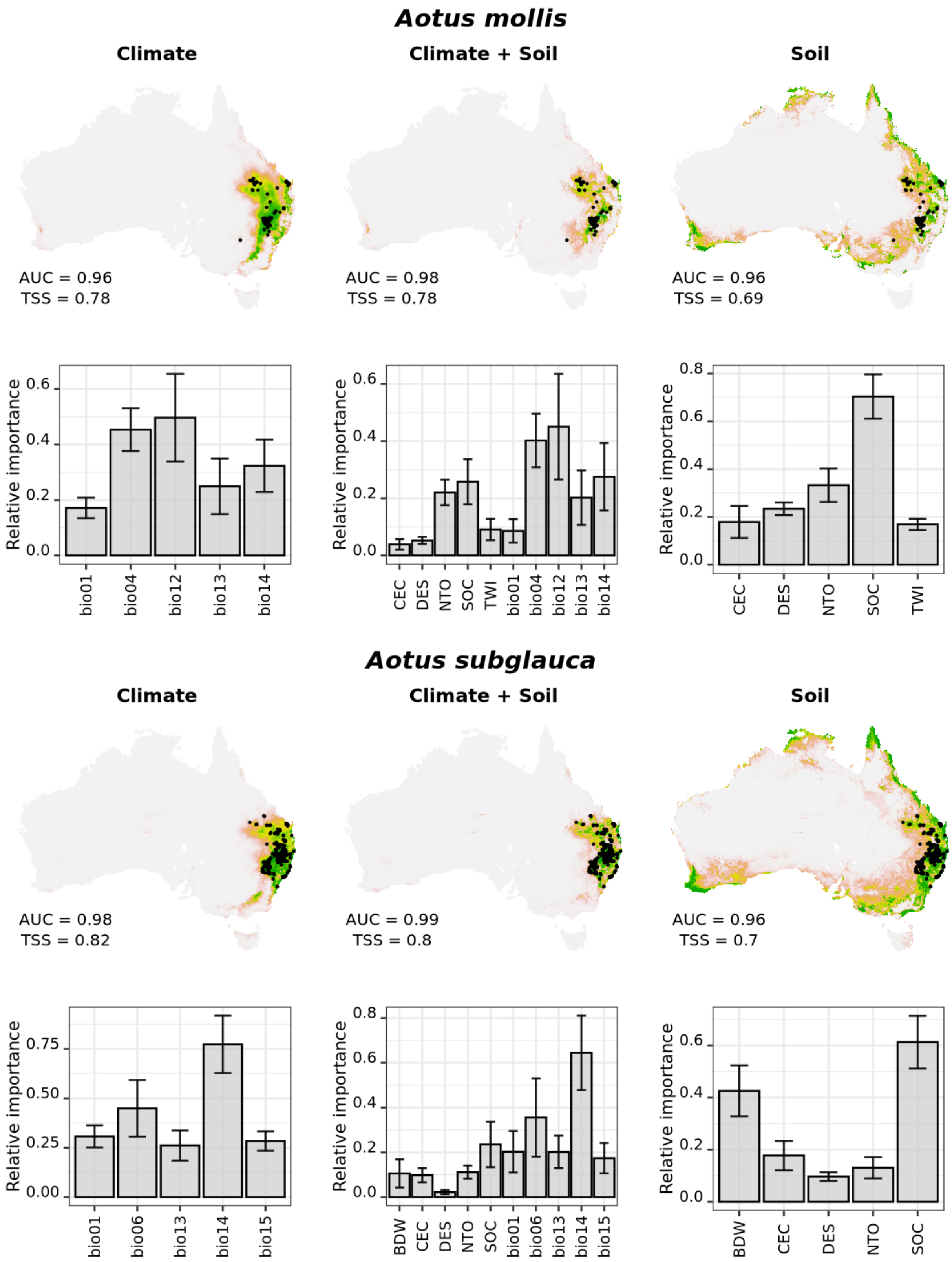
Aotus mollis
Climate
Climate + Soil
Soil
AUC = 0.96
TSS = 0.78
AUC = 0.98
TSS = 0.78
AUC = 0.96
TSS = 0.69
Relative importance
bio01
bio04
bio12
bio13
bio14
CEC
DES
NTO
SOC
TWI
Aotus subglauca
AUC = 0.98
TSS = 0.82
AUC = 0.99
TSS = 0.8
AUC = 0.96
TSS = 0.7
bio06
bio15
BDW

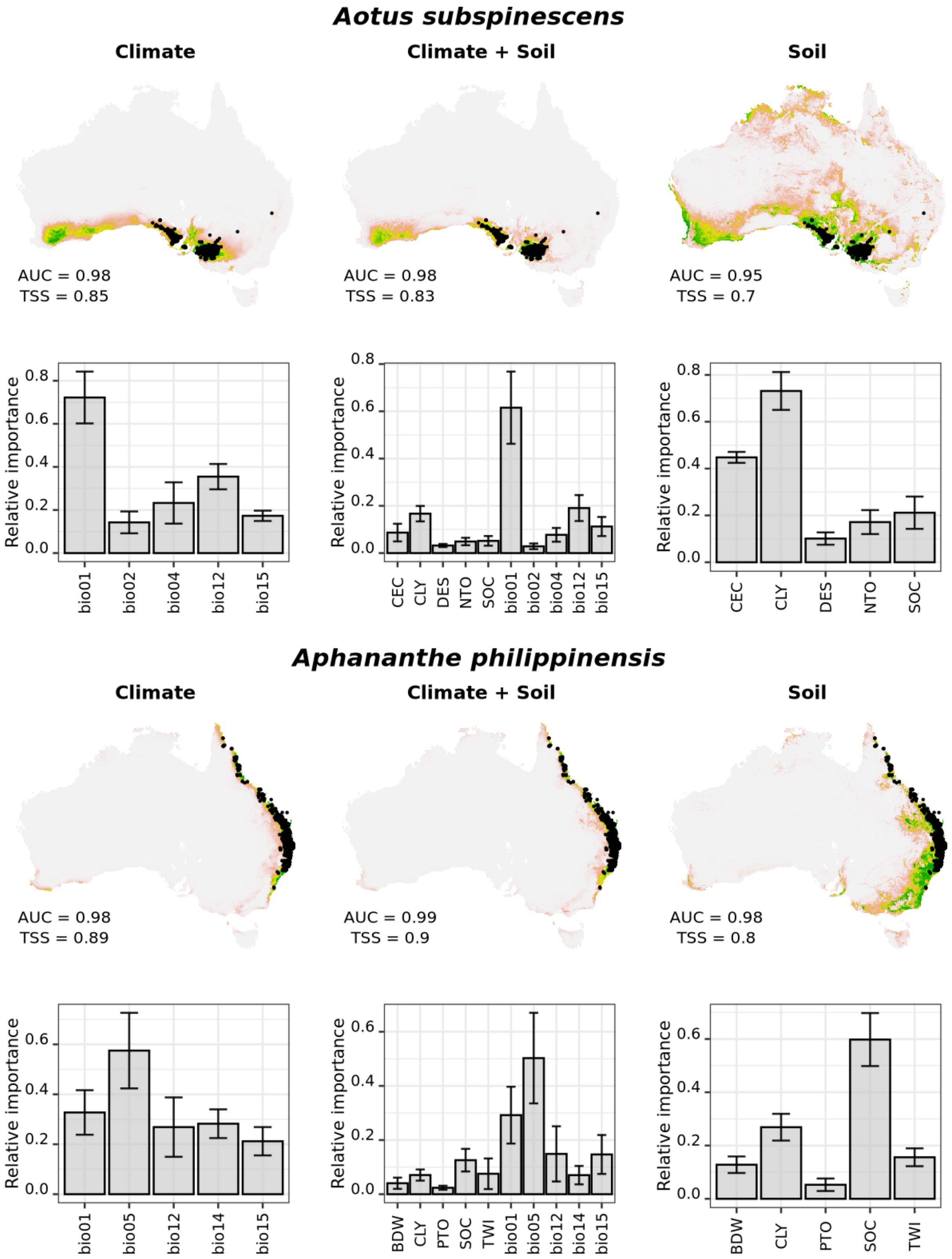
Aotus subspinescens
Climate
Climate + Soil
Soil
AUC = 0.98
TSS = 0.85
AUC = 0.98
TSS = 0.83
AUC = 0.95
TSS = 0.7
Relative importance
bio01 bio02 bio04 bio12 bio15
CEC CLY DES NTO SOC bio01 bio02 bio04 bio12 bio15
CEC CLY DES NTO SOC
Aphananthe philippinensis
Climate
Climate + Soil
Soil
AUC = 0.98
TSS = 0.89
AUC = 0.99
TSS = 0.9
AUC = 0.98
TSS = 0.8
Relative importance
bio01 bio05 bio12 bio14 bio15
BDW CLY PTO SOC TWI bio01 bio05 bio12 bio14 bio15
BDW CLY PTO SOC TWI

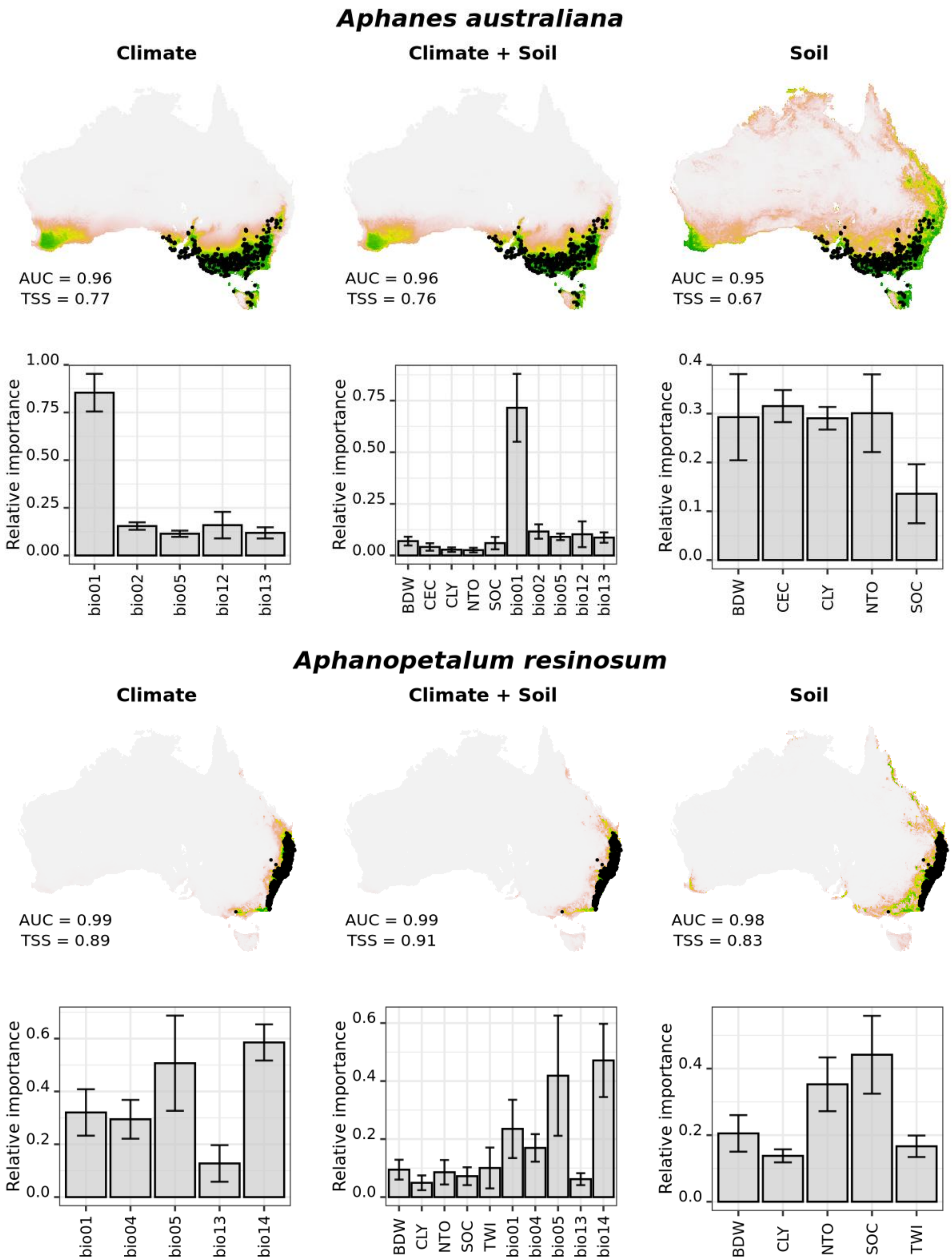

Aphanes australiana
Climate
Climate + Soil
Soil
AUC = 0.96
TSS = 0.77
AUC = 0.96
TSS = 0.76
AUC = 0.95
TSS = 0.67
Relative importance
bio01 bio02 bio05 bio12 bio13
BDW CEC CLY NTO SOC bio01 bio02 bio05 bio12 bio13
BDW CEC CLY NTO SOC
Aphanopetalum resinosum
Climate
Climate + Soil
Soil
AUC = 0.99
TSS = 0.89
AUC = 0.99
TSS = 0.91
AUC = 0.98
TSS = 0.83
Relative importance
bio01 bio04 bio05 bio13 bio14
BDW CLY NTO SOC TWI bio01 bio04 bio05 bio13 bio14
BDW CLY NTO SOC TWI

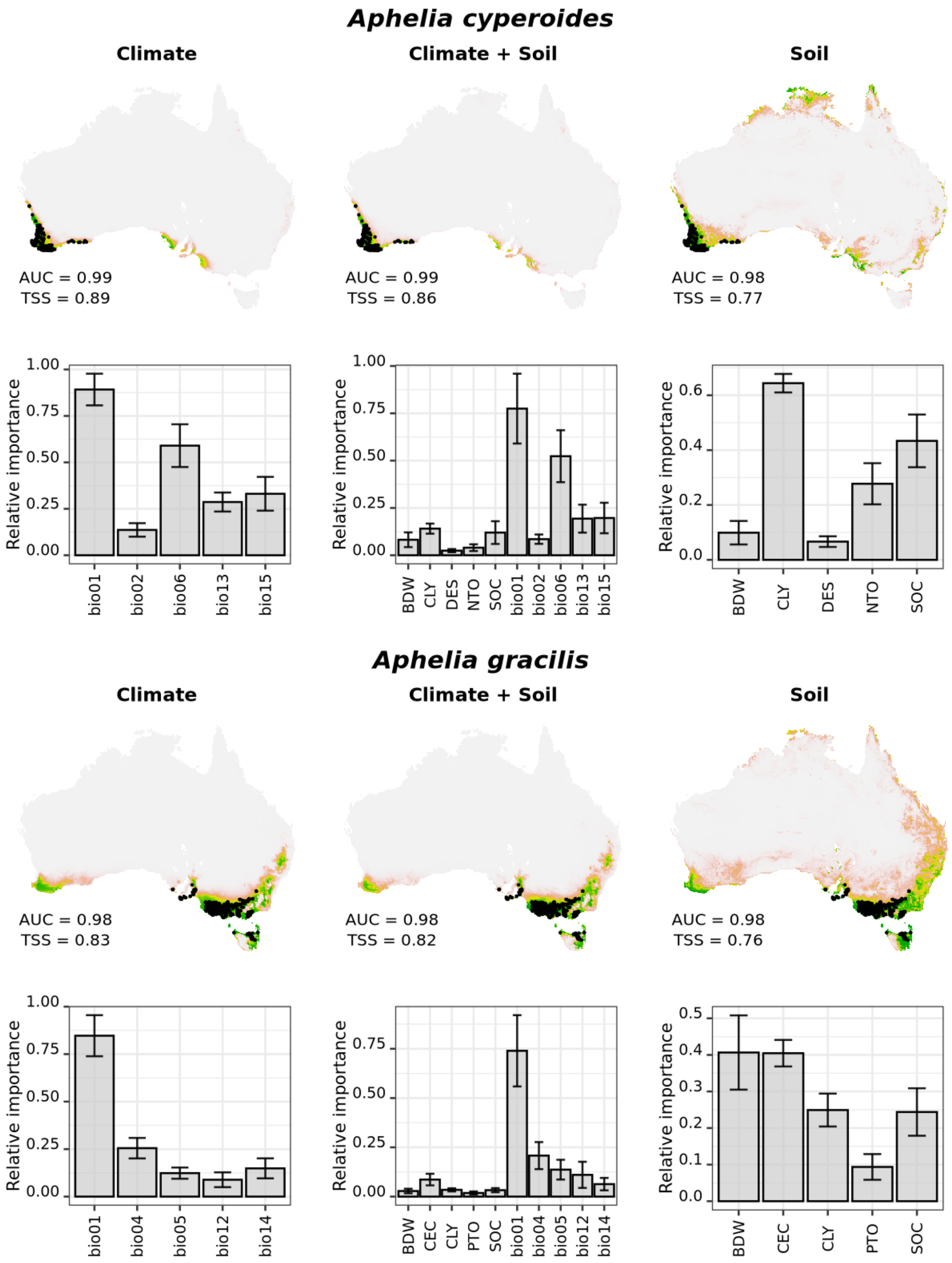

Aphelia cyperoides
Climate
Climate + Soil
Soil
AUC = 0.99
TSS = 0.89
AUC = 0.99
TSS = 0.86
AUC = 0.98
TSS = 0.77
Relative importance
bio01 bio02 bio06 bio13 bio15
BDW CLY DES NTO SOC bio01 bio02 bio06 bio13 bio15
BDW CLY DES NTO SOC
Aphelia gracilis
Climate
Climate + Soil
Soil
AUC = 0.98
TSS = 0.83
AUC = 0.98
TSS = 0.82
AUC = 0.98
TSS = 0.76
Relative importance
bio01 bio04 bio05 bio12 bio14
BDW CEC CLY PTO SOC bio01 bio04 bio05 bio12 bio14
BDW CEC CLY PTO SOC

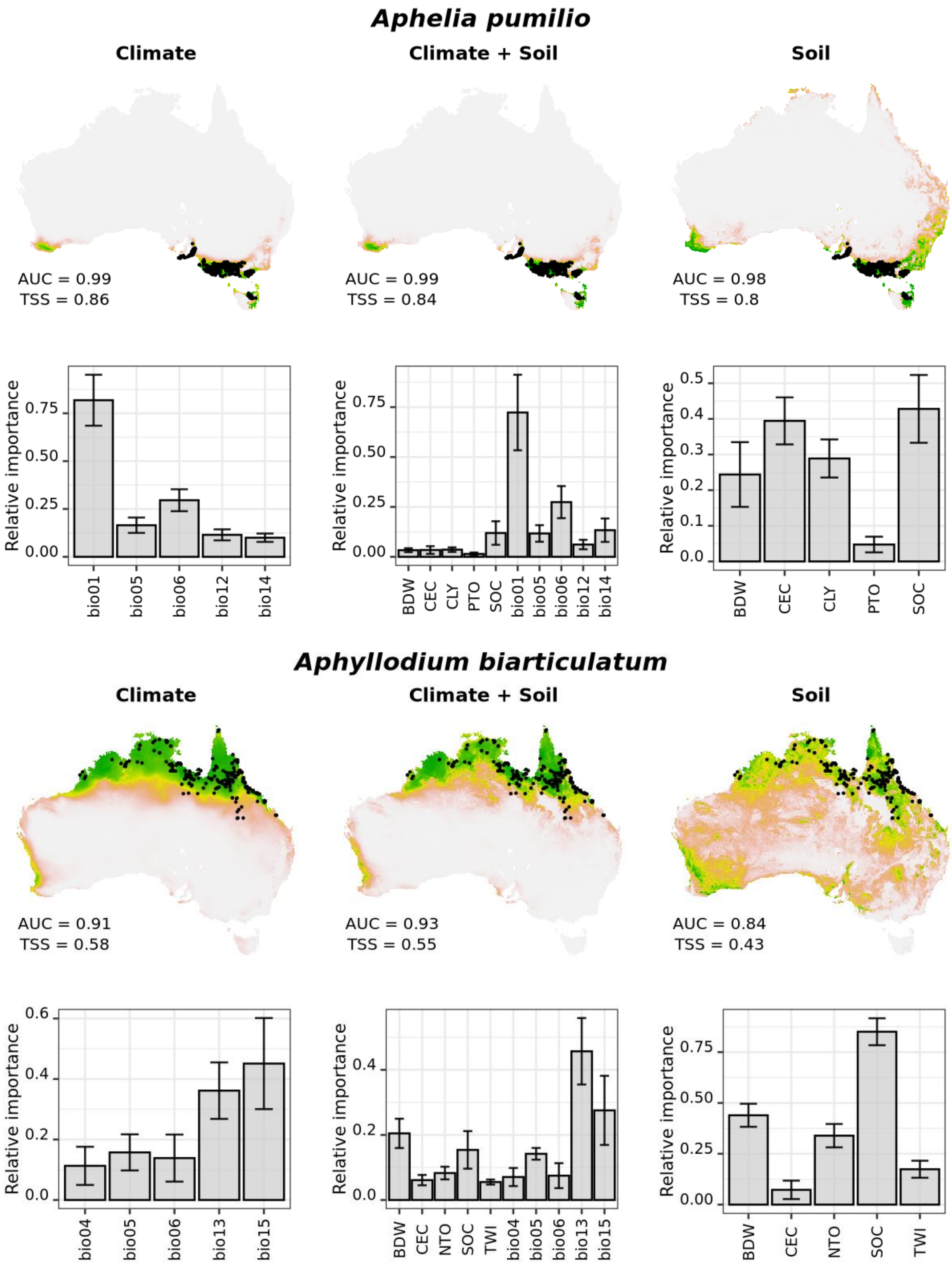
Aphelia pumilio
Climate
Climate + Soil
Soil
AUC = 0.99
TSS = 0.86
AUC = 0.99
TSS = 0.84
AUC = 0.98
TSS = 0.8
Relative importance
bio01
bio05
bio06
bio12
bio14
BDW
CEC
CLY
PTO
SOC
Aphyllodium biarticulatum
Climate
Climate + Soil
Soil
AUC = 0.91
TSS = 0.58
AUC = 0.93
TSS = 0.55
AUC = 0.84
TSS = 0.43
Relative importance
bio04
bio05
bio06
bio13
bio15
BDW
CEC
NTO
SOC
TWI

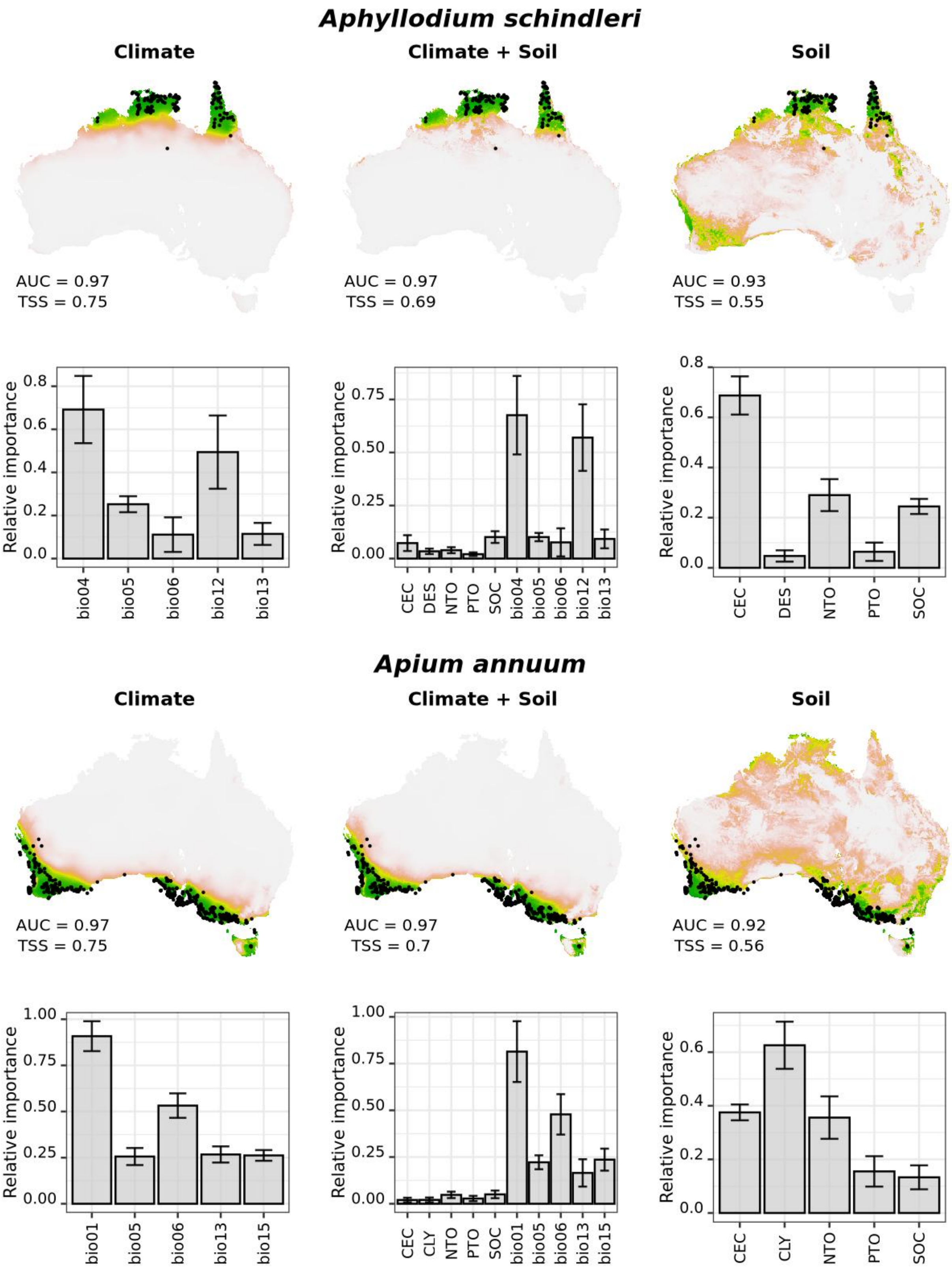
Aphyllodium schindleri
Climate
Climate + Soil
Soil
AUC = 0.97
TSS = 0.75
AUC = 0.97
TSS = 0.69
AUC = 0.93
TSS = 0.55
Relative importance
0.0
0.2
0.4
0.6
0.8
bio04
bio05
bio06
bio12
bio13
0.00
0.25
0.50
0.75
CEC
DES
NTO
PTO
SOC
bio04
bio05
bio06
bio12
bio13
0.0
0.2
0.4
0.6
0.8
CEC
DES
NTO
PTO
SOC
Apium annuum
Climate
Climate + Soil
Soil
AUC = 0.97
TSS = 0.75
AUC = 0.97
TSS = 0.7
AUC = 0.92
TSS = 0.56
Relative importance
0.00
0.25
0.50
0.75
1.00
bio01
bio05
bio06
bio13
bio15
CEC
CLY
NTO
PTO
SOC
bio01
bio05
bio06
bio13
bio15
0.0
0.2
0.4
0.6
CEC
CLY
NTO
PTO
SOC

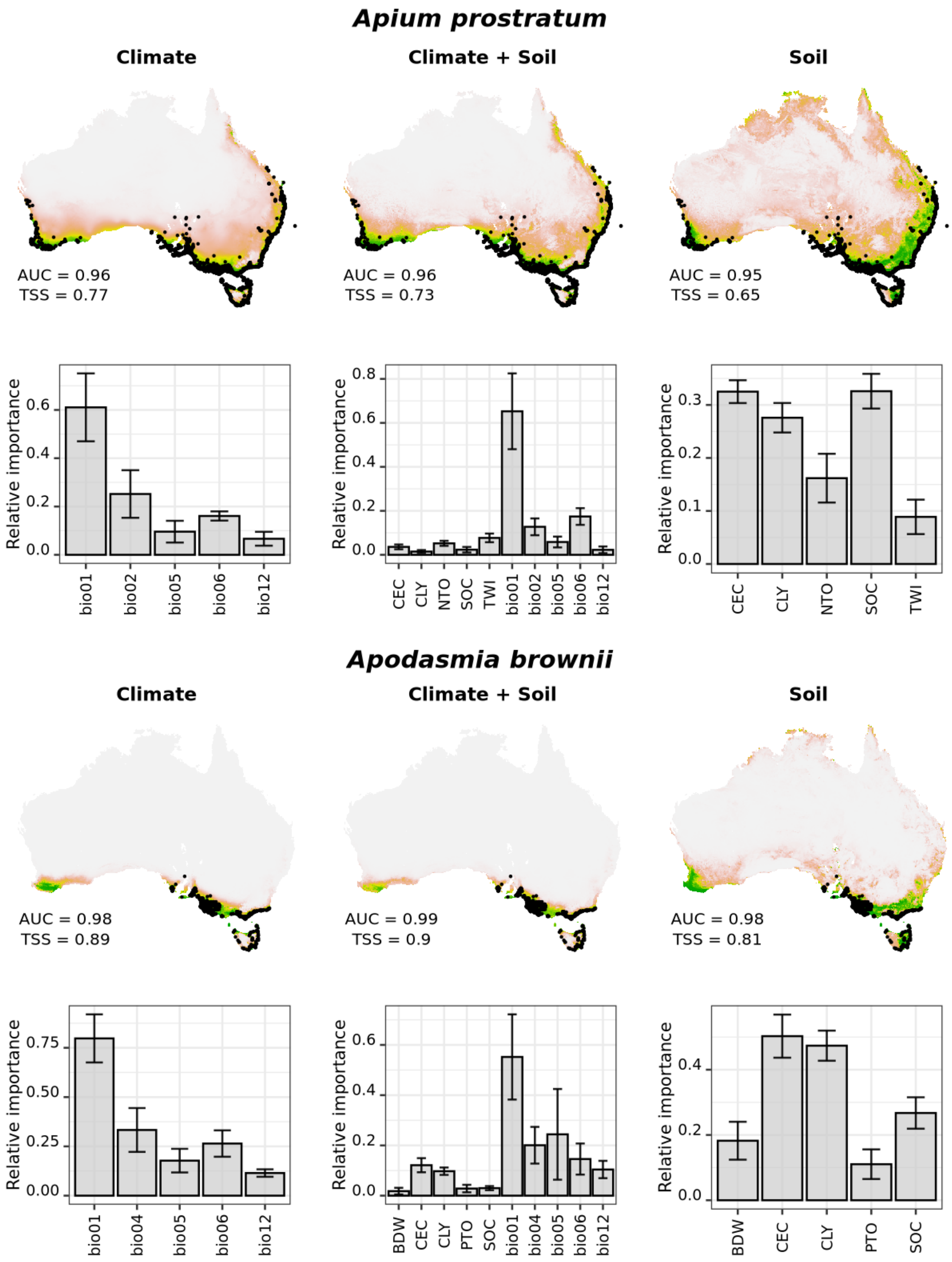
Apium prostratum
Climate
Climate + Soil
Soil
AUC = 0.96
TSS = 0.77
AUC = 0.96
TSS = 0.73
AUC = 0.95
TSS = 0.65
Relative importance
bio01
bio02
bio05
bio06
bio12
CEC
CLY
NTO
SOC
TWI
Apodasmia brownii
Climate
Climate + Soil
Soil
AUC = 0.98
TSS = 0.89
AUC = 0.99
TSS = 0.9
AUC = 0.98
TSS = 0.81
Relative importance
bio01
bio04
bio05
bio06
bio12
BDW
CEC
CLY
PTO
SOC

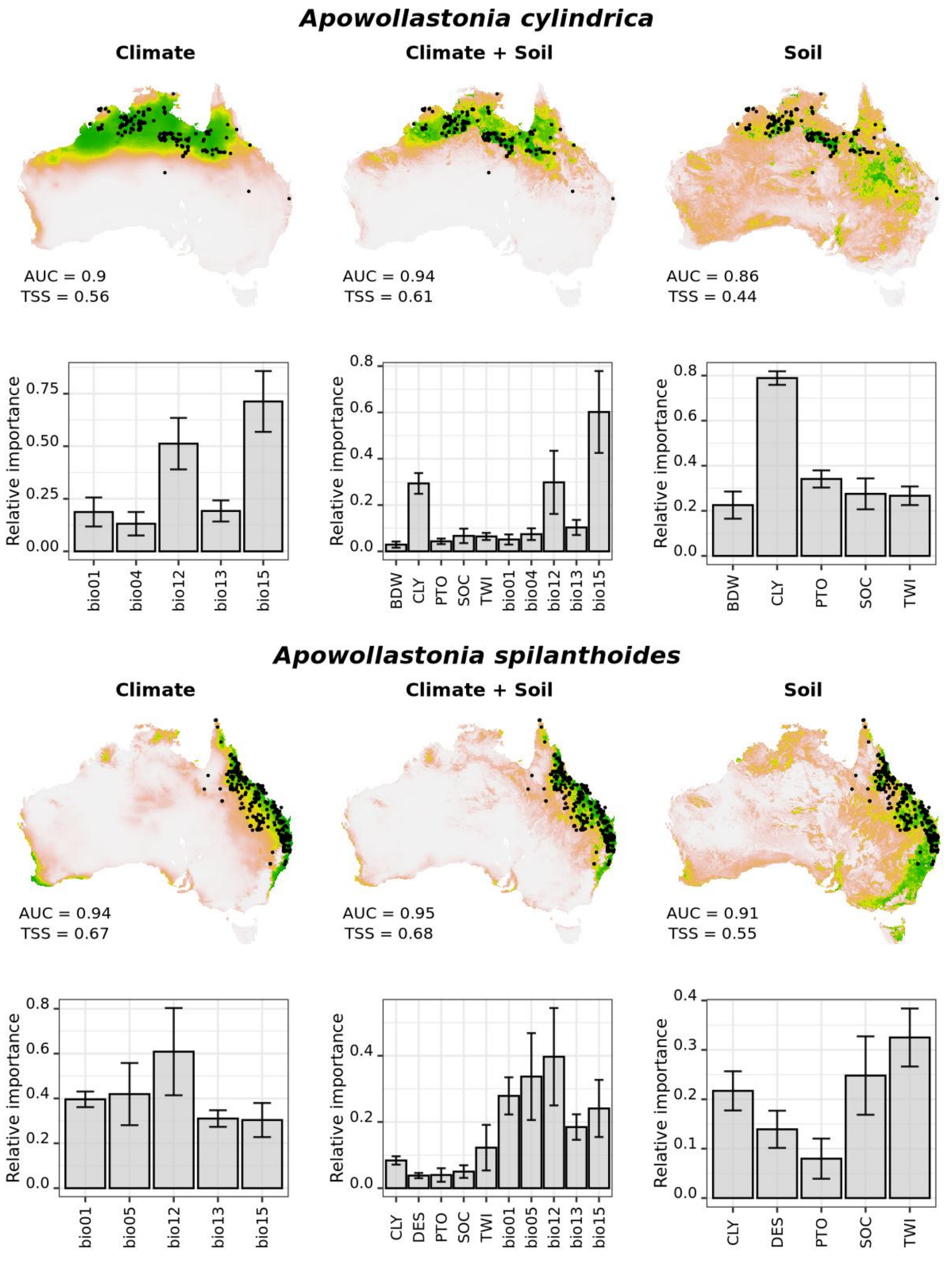

Apowollastonia cylindrica
Climate
Climate + Soil
Soil
AUC = 0.9
TSS = 0.56
AUC = 0.94
TSS = 0.61
AUC = 0.86
TSS = 0.44
Relative importance
bio01
bio04
bio12
bio13
bio15
BDW
CLY
PTO
SOC
TWI
Apowollastonia spilanthoides
Climate
Climate + Soil
Soil
AUC = 0.94
TSS = 0.67
AUC = 0.95
TSS = 0.68
AUC = 0.91
TSS = 0.55
Relative importance
bio01
bio05
bio12
bio13
bio15
CLY
DES
PTO
SOC
TWI

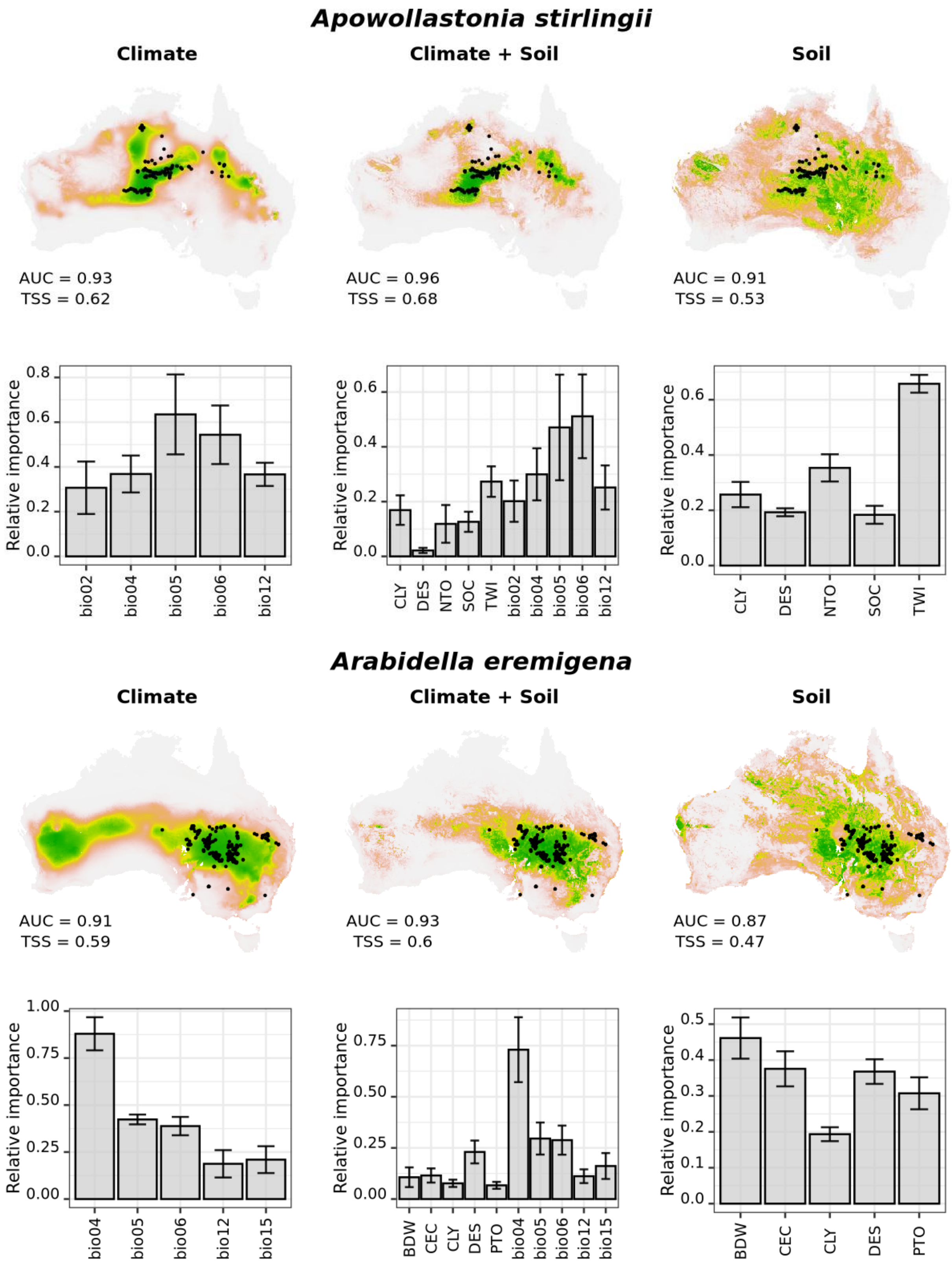

Apowollastonia stirlingii
Climate
Climate + Soil
Soil
AUC = 0.93
TSS = 0.62
AUC = 0.96
TSS = 0.68
AUC = 0.91
TSS = 0.53
Relative importance
bio02
bio04
bio05
bio06
bio12
CLY
DES
NTO
SOC
TWI
Arabidella eremigena
AUC = 0.91
TSS = 0.59
AUC = 0.93
TSS = 0.6
AUC = 0.87
TSS = 0.47
bio15
BDW
CEC
PTO

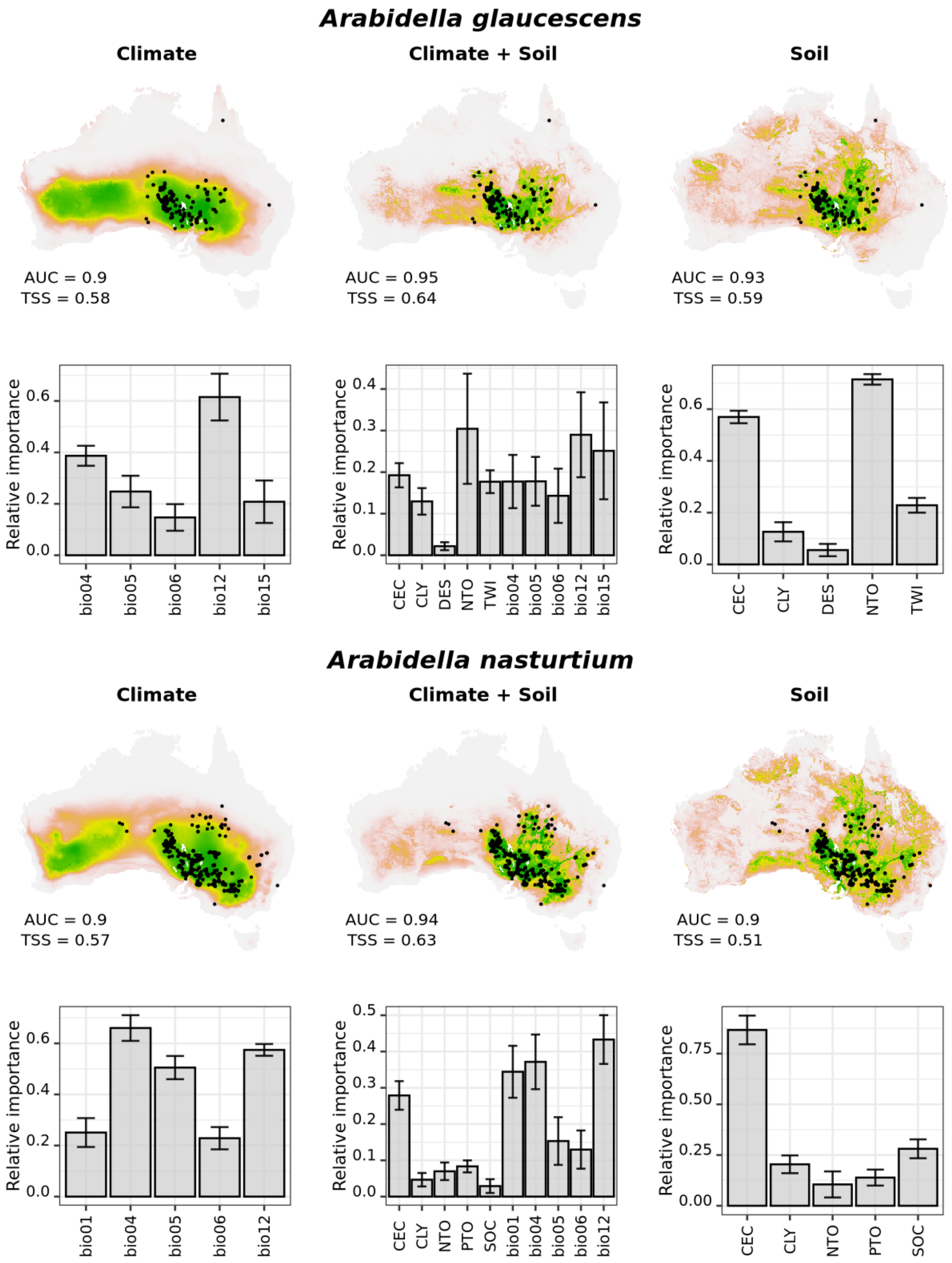
Arabidella glaucescens
Climate
Climate + Soil
Soil
AUC = 0.9
TSS = 0.58
AUC = 0.95
TSS = 0.64
AUC = 0.93
TSS = 0.59
Relative importance
0.0
0.2
0.4
0.6
bio04
bio05
bio06
bio12
bio15
Relative importance
0.0
0.1
0.2
0.3
0.4
CEC
CLY
DES
NTO
TWI
bio04
bio05
bio06
bio12
bio15
Relative importance
0.0
0.2
0.4
0.6
CEC
CLY
DES
NTO
TWI
Arabidella nasturtium
Climate
Climate + Soil
Soil
AUC = 0.9
TSS = 0.57
AUC = 0.94
TSS = 0.63
AUC = 0.9
TSS = 0.51
Relative importance
0.0
0.2
0.4
0.6
bio01
bio04
bio05
bio06
bio12
Relative importance
0.0
0.1
0.2
0.3
0.4
0.5
CEC
CLY
NTO
PTO
SOC
bio01
bio04
bio05
bio06
bio12
Relative importance
0.00
0.25
0.50
0.75
CEC
CLY
NTO
PTO
SOC

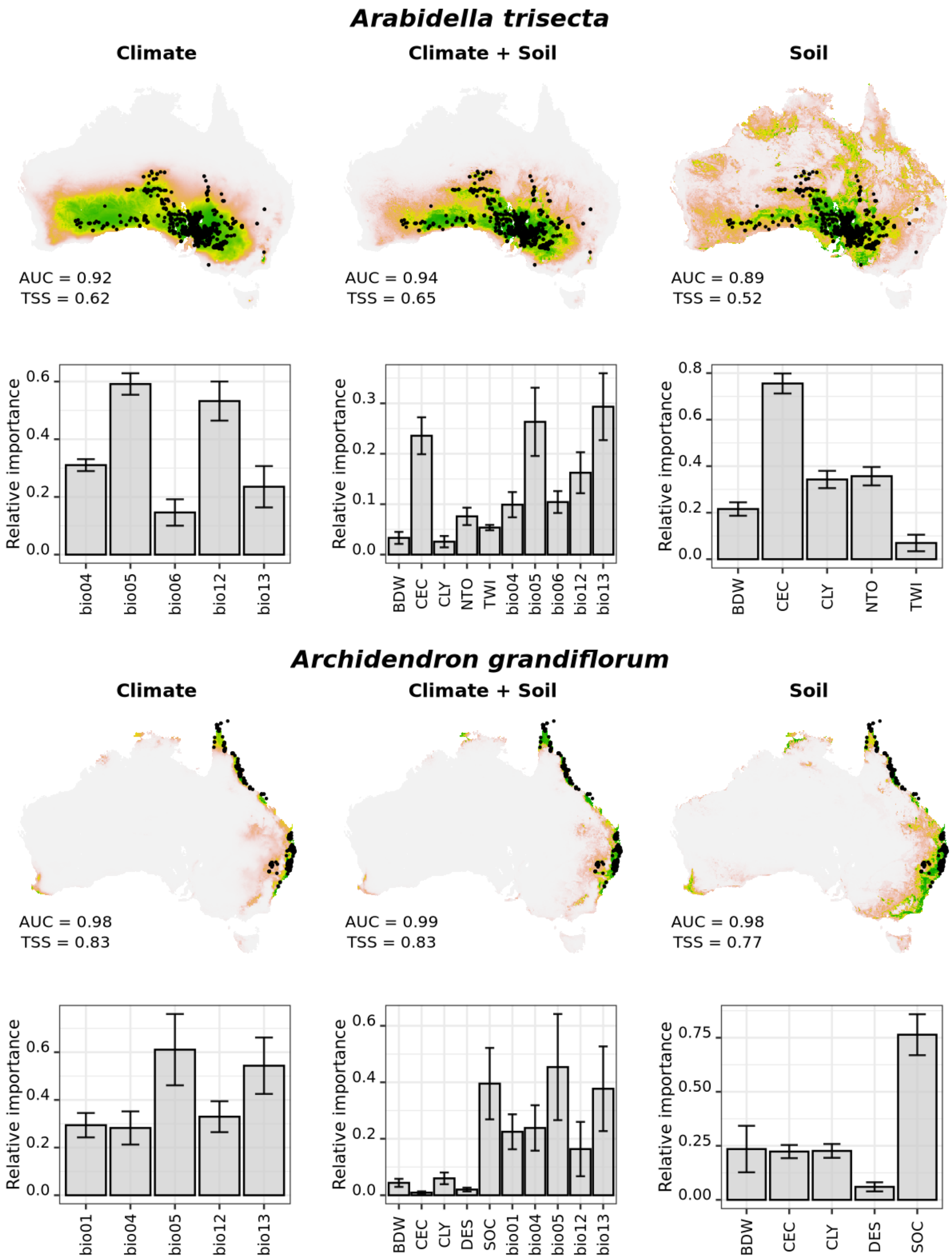
Arabidella trisecta
Climate
Climate + Soil
Soil
AUC = 0.92
TSS = 0.62
AUC = 0.94
TSS = 0.65
AUC = 0.89
TSS = 0.52
Relative importance
0.0
0.2
0.4
0.6
bio04
bio05
bio06
bio12
bio13
Relative importance
0.0
0.1
0.2
0.3
BDW
CEC
CLY
NTO
TWI
bio04
bio05
bio06
bio12
bio13
Relative importance
0.0
0.2
0.4
0.6
0.8
BDW
CEC
CLY
NTO
TWI
Archidendron grandiflorum
Climate
Climate + Soil
Soil
AUC = 0.98
TSS = 0.83
AUC = 0.99
TSS = 0.83
AUC = 0.98
TSS = 0.77
Relative importance
0.0
0.2
0.4
0.6
bio01
bio04
bio05
bio12
bio13
Relative importance
0.0
0.2
0.4
0.6
BDW
CEC
CLY
DES
SOC
bio01
bio04
bio05
bio12
bio13
Relative importance
0.00
0.25
0.50
0.75
BDW
CEC
CLY
DES
SOC

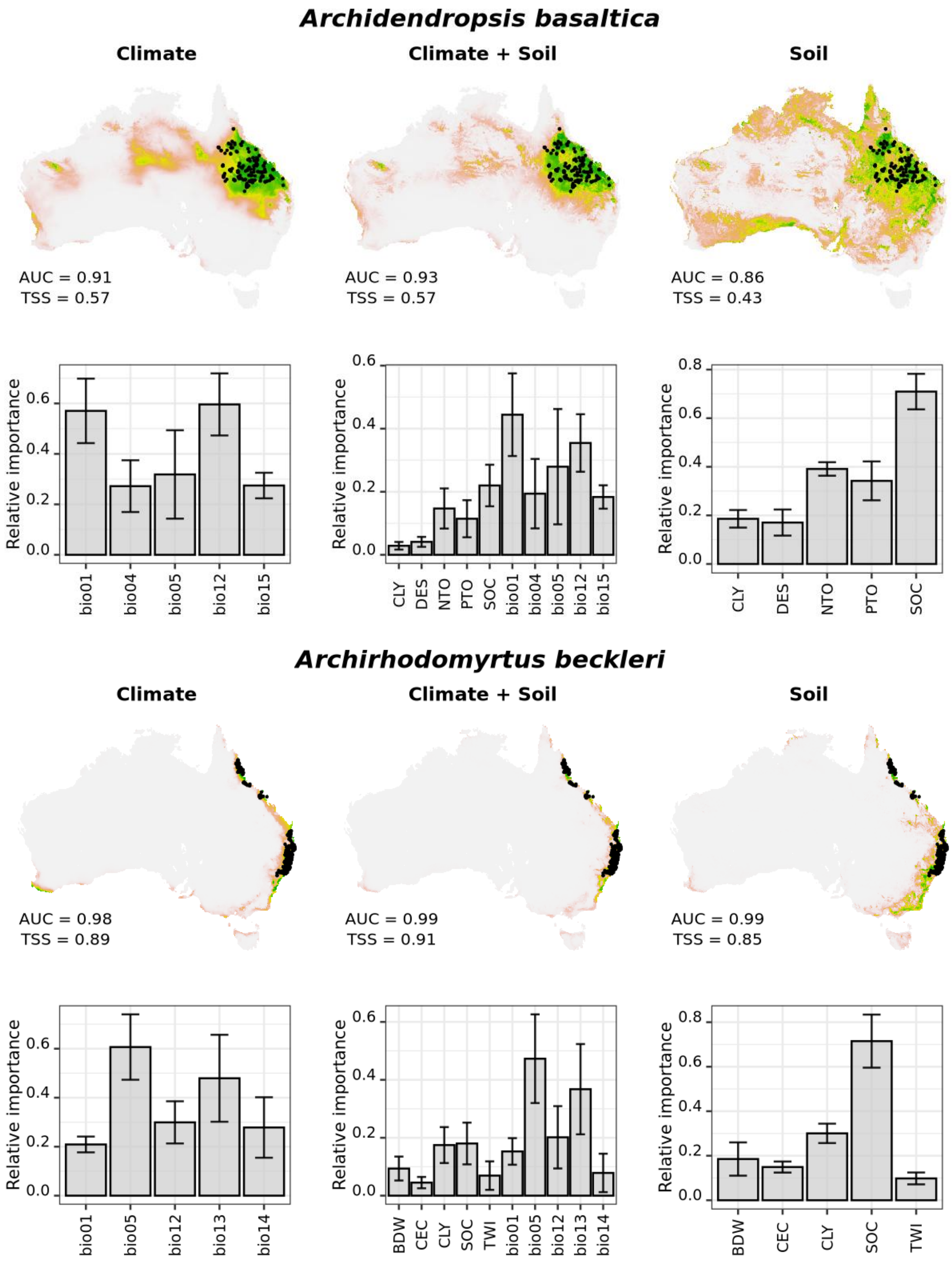

Archidendropsis basaltica
Climate
Climate + Soil
Soil
AUC = 0.91
TSS = 0.57
AUC = 0.93
TSS = 0.57
AUC = 0.86
TSS = 0.43
Relative importance
bio01 bio04 bio05 bio12 bio15
CLY DES NTO PTO SOC bio01 bio04 bio05 bio12 bio15
CLY DES NTO PTO SOC
Archirhodomyrtus beckleri
Climate
Climate + Soil
Soil
AUC = 0.98
TSS = 0.89
AUC = 0.99
TSS = 0.91
AUC = 0.99
TSS = 0.85
Relative importance
bio01 bio05 bio12 bio13 bio14
BDW CEC CLY SOC TWI bio01 bio05 bio12 bio13 bio14
BDW CEC CLY SOC TWI

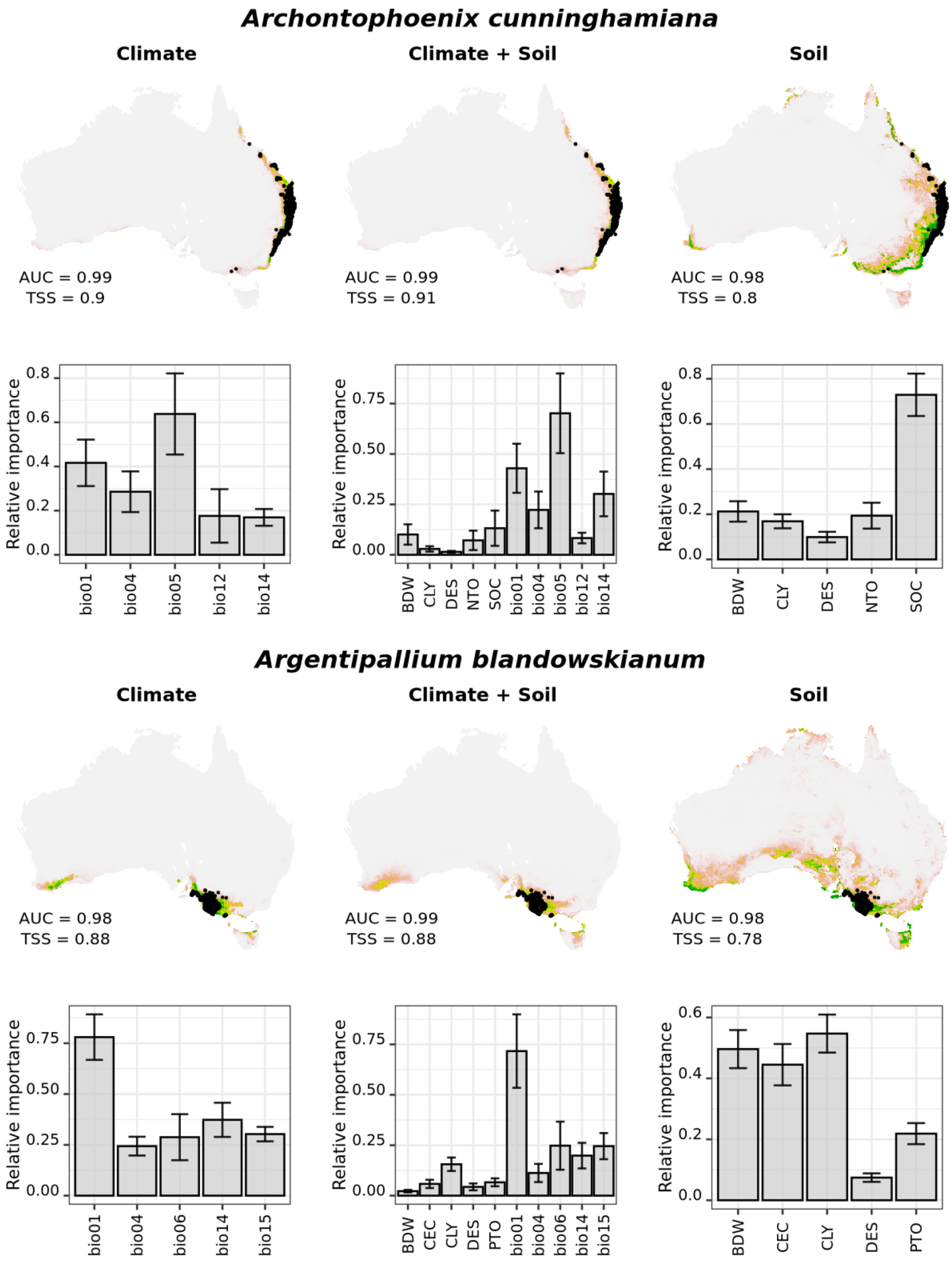

Archontophoenix cunninghamiana
Climate
Climate + Soil
Soil
AUC = 0.99
TSS = 0.9
AUC = 0.99
TSS = 0.91
AUC = 0.98
TSS = 0.8
Relative importance
bio01
bio04
bio05
bio12
bio14
BDW
CLY
DES
NTO
SOC
bio01
bio04
bio05
bio12
bio14
BDW
CLY
DES
NTO
SOC
Argentipallium blandowskianum
Climate
Climate + Soil
Soil
AUC = 0.98
TSS = 0.88
AUC = 0.99
TSS = 0.88
AUC = 0.98
TSS = 0.78
Relative importance
bio01
bio04
bio06
bio14
bio15
BDW
CEC
CLY
DES
PTO
bio01
bio04
bio06
bio14
bio15
BDW
CEC
CLY
DES
PTO

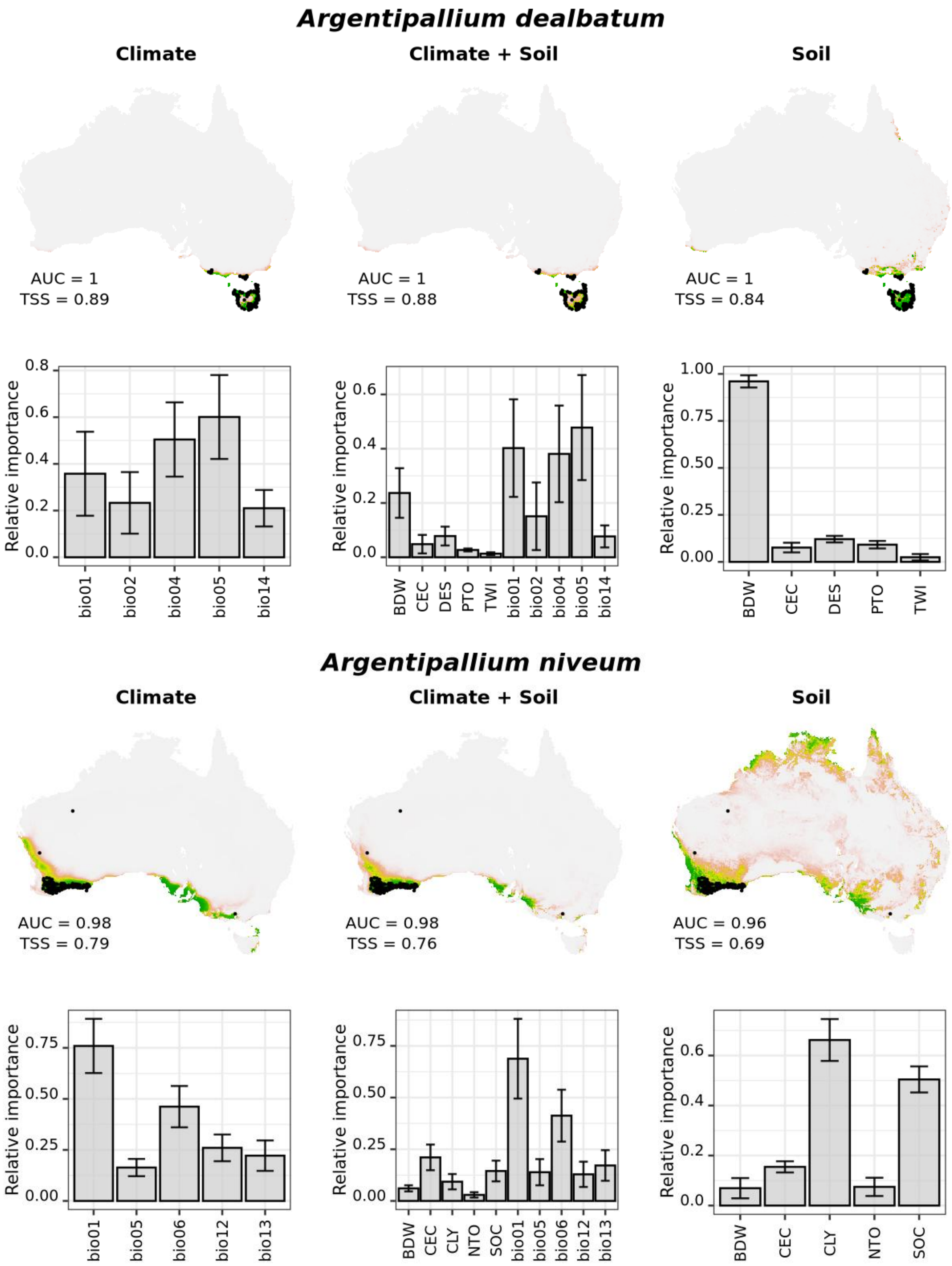

Argentipallium dealbatum
Climate
Climate + Soil
Soil
AUC = 1
TSS = 0.89
AUC = 1
TSS = 0.88
AUC = 1
TSS = 0.84
Relative importance
bio01 bio02 bio04 bio05 bio14
BDW CEC DES PTO TWI bio01 bio02 bio04 bio05 bio14
BDW CEC DES PTO TWI
Argentipallium niveum
Climate
Climate + Soil
Soil
AUC = 0.98
TSS = 0.79
AUC = 0.98
TSS = 0.76
AUC = 0.96
TSS = 0.69
Relative importance
bio01 bio05 bio06 bio12 bio13
BDW CEC CLY NTO SOC bio01 bio05 bio06 bio12 bio13
BDW CEC CLY NTO SOC

Argentipallium obtusifolium

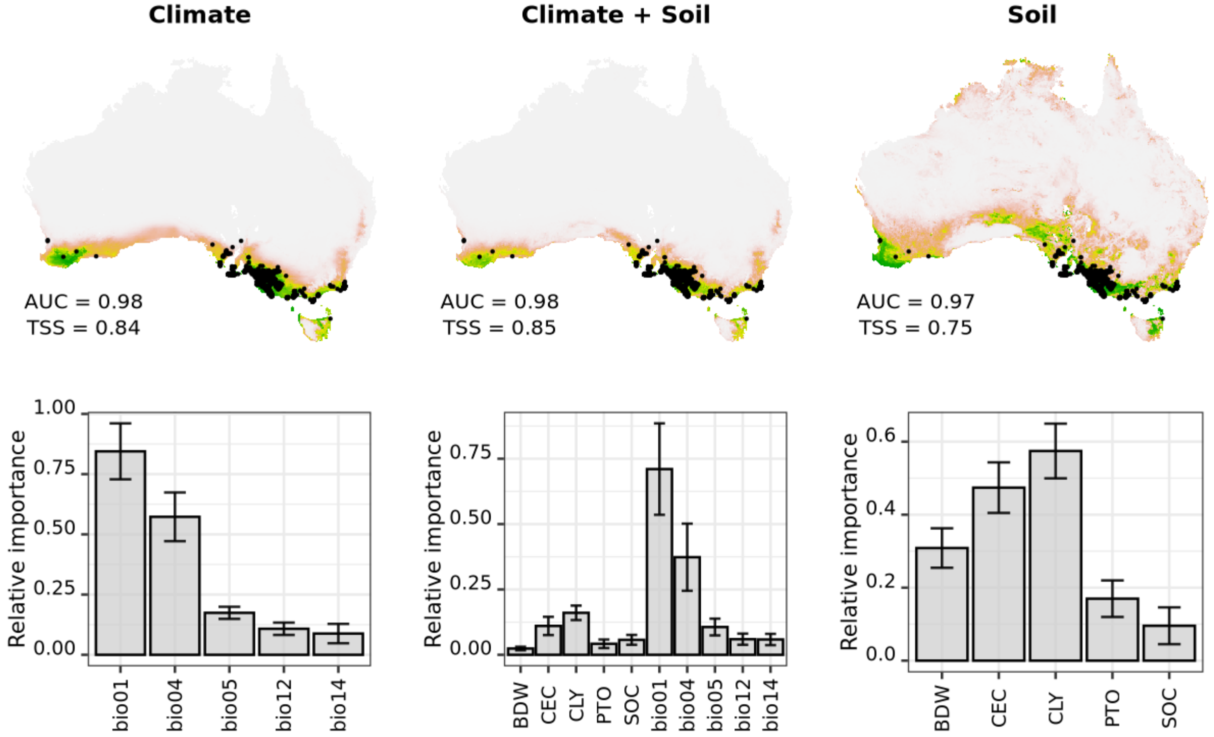

Argusia argentea

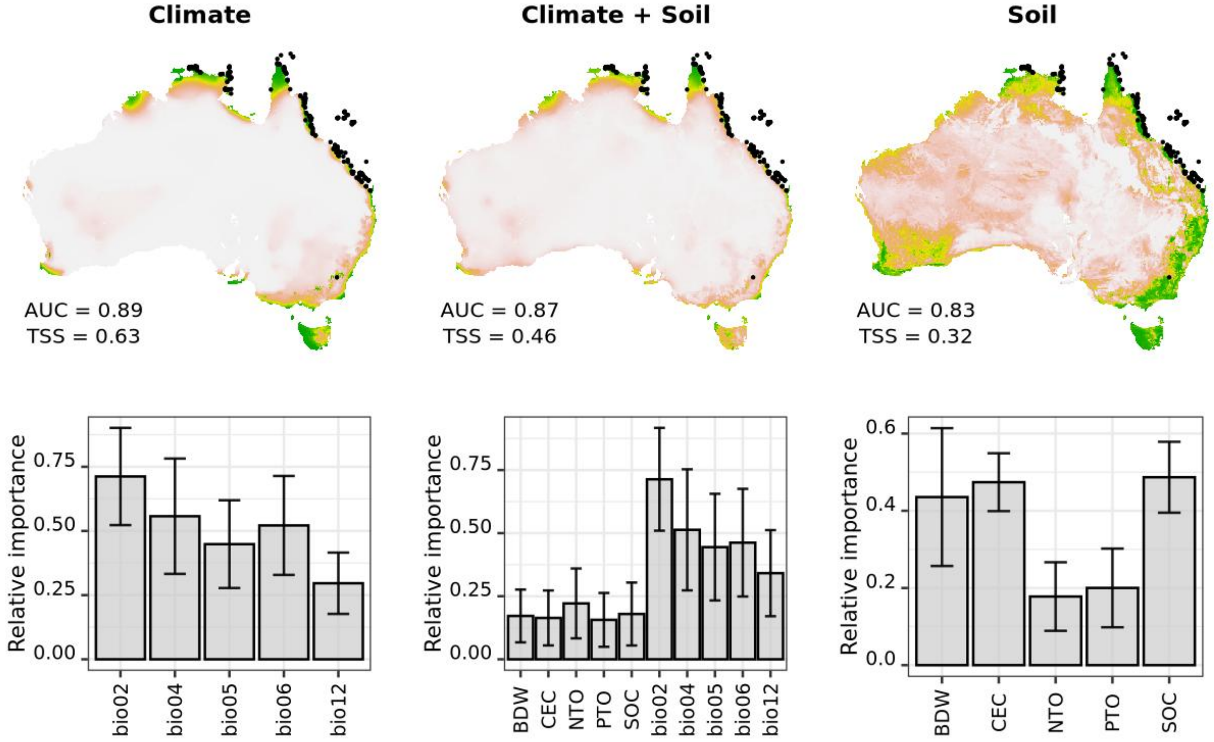

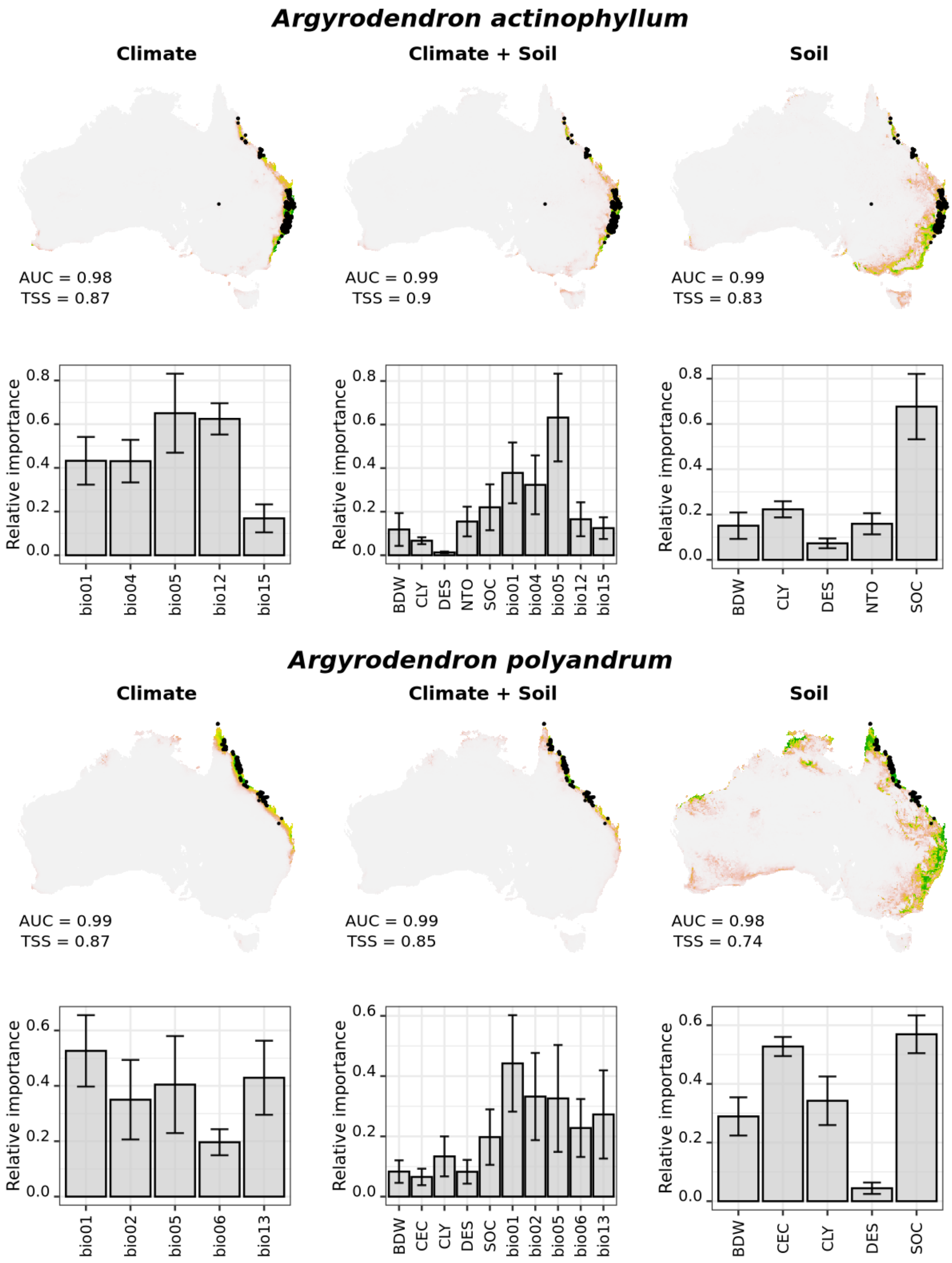

Argyrodendron actinophyllum
Climate
Climate + Soil
Soil
AUC = 0.98
TSS = 0.87
AUC = 0.99
TSS = 0.9
AUC = 0.99
TSS = 0.83
Relative importance
bio01
bio04
bio05
bio12
bio15
BDW
CLY
DES
NTO
SOC
Argyrodendron polyandrum
AUC = 0.99
TSS = 0.87
AUC = 0.99
TSS = 0.85
AUC = 0.98
TSS = 0.74
bio02
bio06
bio13
CEC

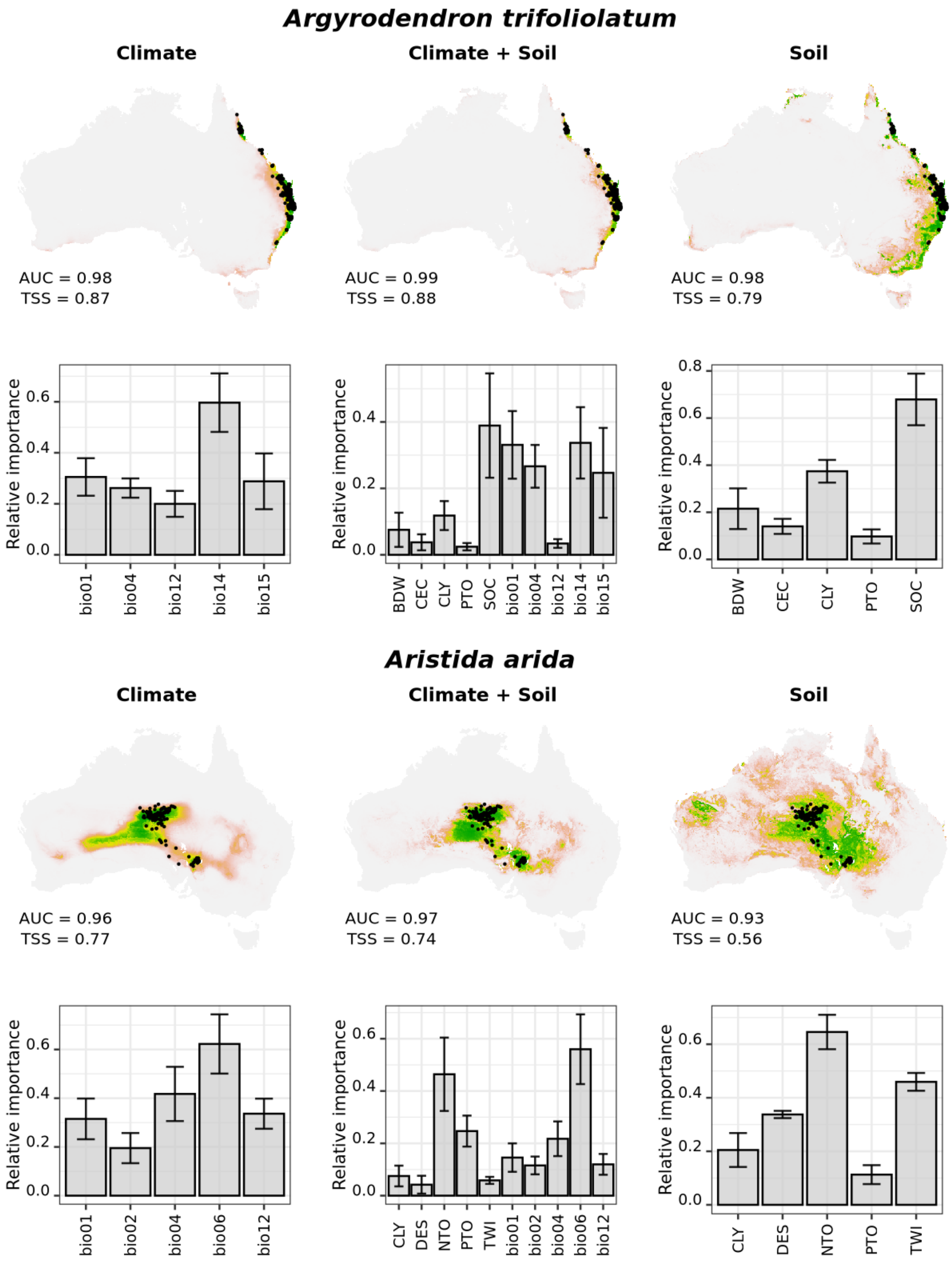

Argyrodendron trifoliolatum
Climate
Climate + Soil
Soil
AUC = 0.98
TSS = 0.87
AUC = 0.99
TSS = 0.88
AUC = 0.98
TSS = 0.79
Relative importance
bio01
bio04
bio12
bio14
bio15
BDW
CEC
CLY
PTO
SOC
Aristida arida
AUC = 0.96
TSS = 0.77
AUC = 0.97
TSS = 0.74
AUC = 0.93
TSS = 0.56
bio02
bio06
DES
NTO
TWI

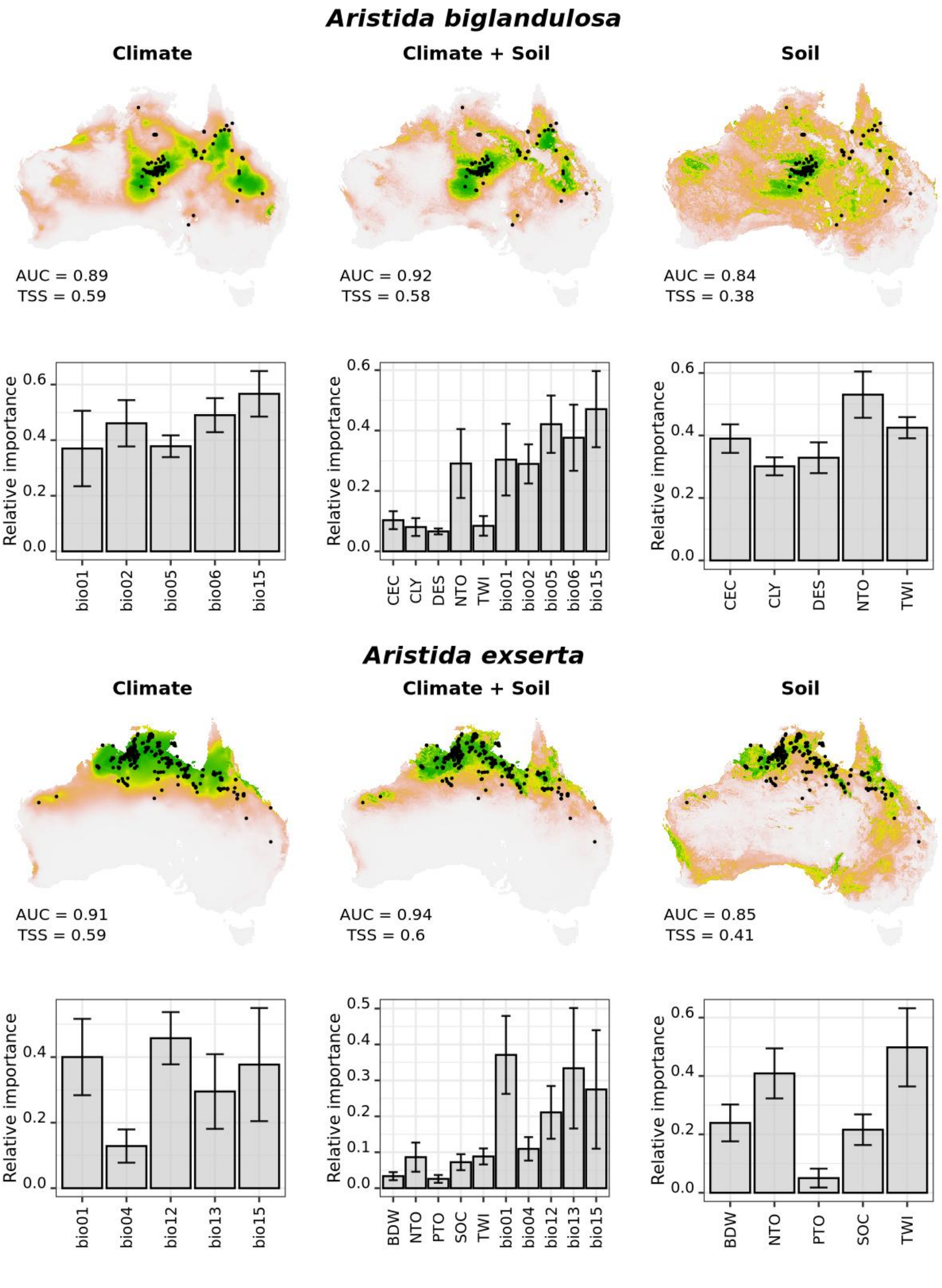
Aristida biglandulosa
Climate
Climate + Soil
Soil
AUC = 0.89
TSS = 0.59
AUC = 0.92
TSS = 0.58
AUC = 0.84
TSS = 0.38
Relative importance
bio01
bio02
bio05
bio06
bio15
CEC
CLY
DES
NTO
TWI
Aristida exserta
Climate
Climate + Soil
Soil
AUC = 0.91
TSS = 0.59
AUC = 0.94
TSS = 0.6
AUC = 0.85
TSS = 0.41
Relative importance
bio01
bio04
bio12
bio13
bio15
BDW
NTO
PTO
SOC
TWI

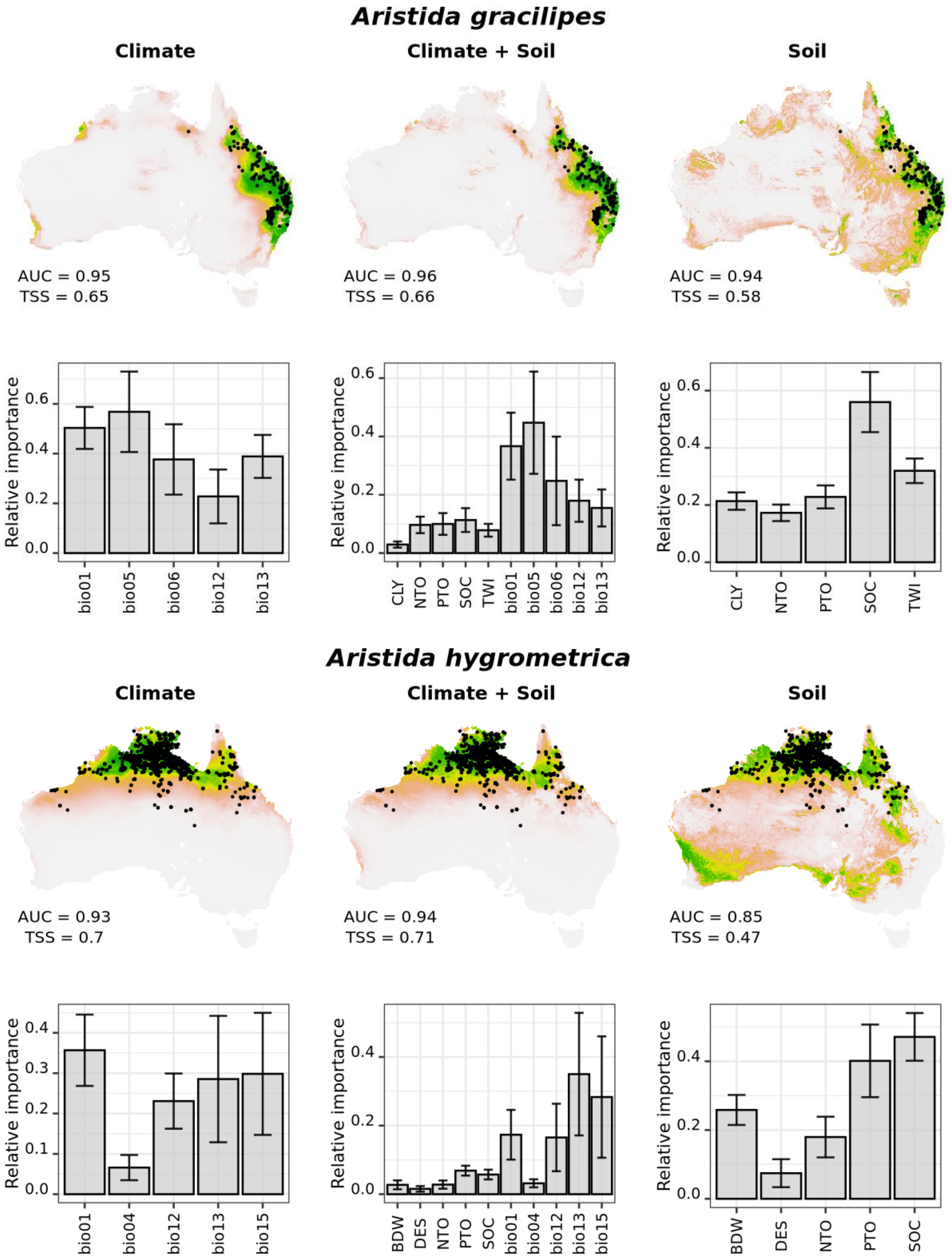

Aristida gracilipes
Climate
Climate + Soil
Soil
AUC = 0.95
TSS = 0.65
AUC = 0.96
TSS = 0.66
AUC = 0.94
TSS = 0.58
Relative importance
bio01 bio05 bio06 bio12 bio13
CLY NTO PTO SOC TWI bio01 bio05 bio06 bio12 bio13
CLY NTO PTO SOC TWI
Aristida hygrometrica
Climate
Climate + Soil
Soil
AUC = 0.93
TSS = 0.7
AUC = 0.94
TSS = 0.71
AUC = 0.85
TSS = 0.47
Relative importance
bio01 bio04 bio12 bio13 bio15
BDW DES NTO PTO SOC bio01 bio04 bio12 bio13 bio15
BDW DES NTO PTO SOC

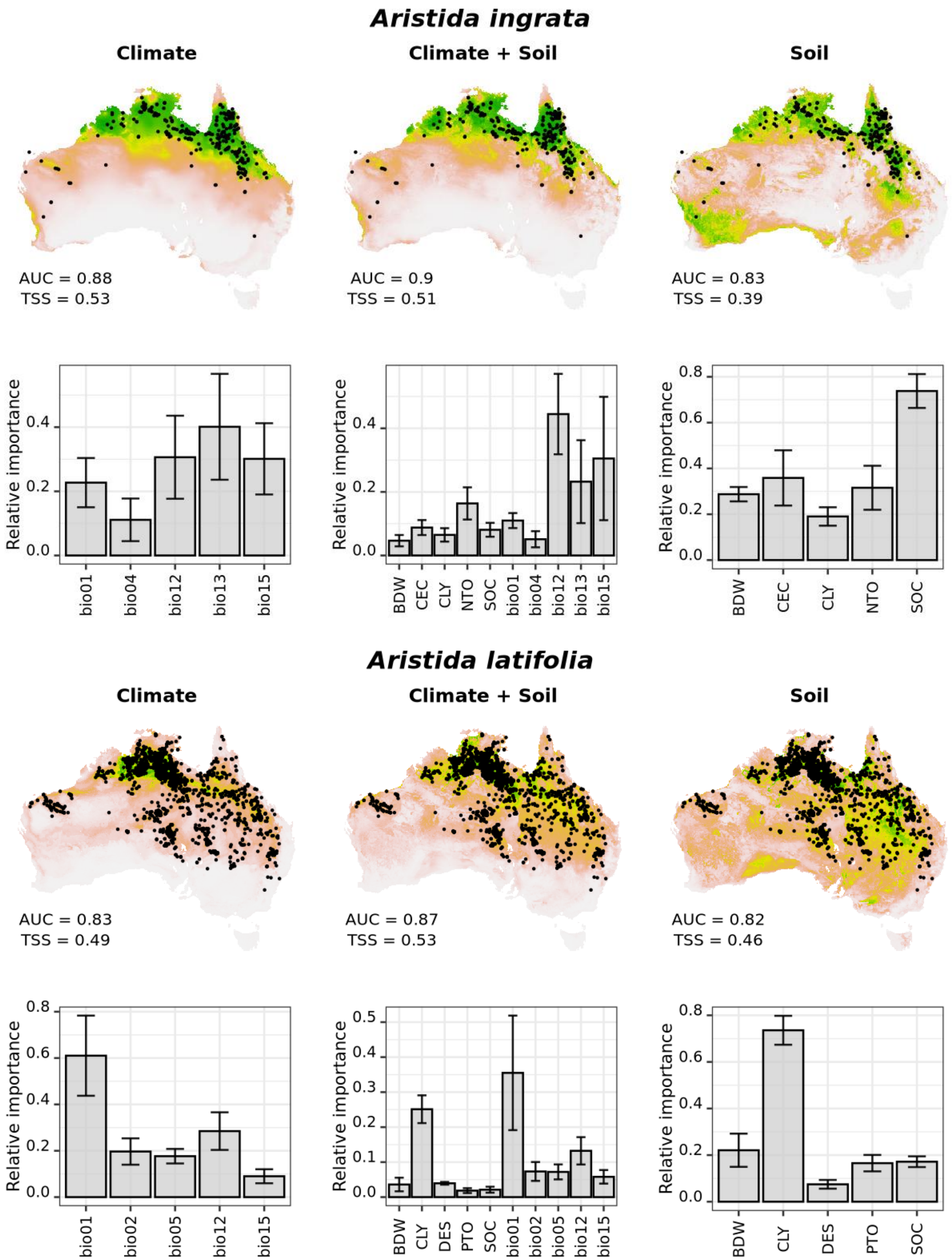

Aristida ingrata
Climate
Climate + Soil
Soil
AUC = 0.88
TSS = 0.53
AUC = 0.9
TSS = 0.51
AUC = 0.83
TSS = 0.39
Relative importance
bio01 bio04 bio12 bio13 bio15
BDW CEC CLY NTO SOC bio01 bio04 bio12 bio13 bio15
BDW CEC CLY NTO SOC
Aristida latifolia
Climate
Climate + Soil
Soil
AUC = 0.83
TSS = 0.49
AUC = 0.87
TSS = 0.53
AUC = 0.82
TSS = 0.46
Relative importance
bio01 bio02 bio05 bio12 bio15
BDW CLY DES PTO SOC bio01 bio02 bio05 bio12 bio15
BDW CLY DES PTO SOC

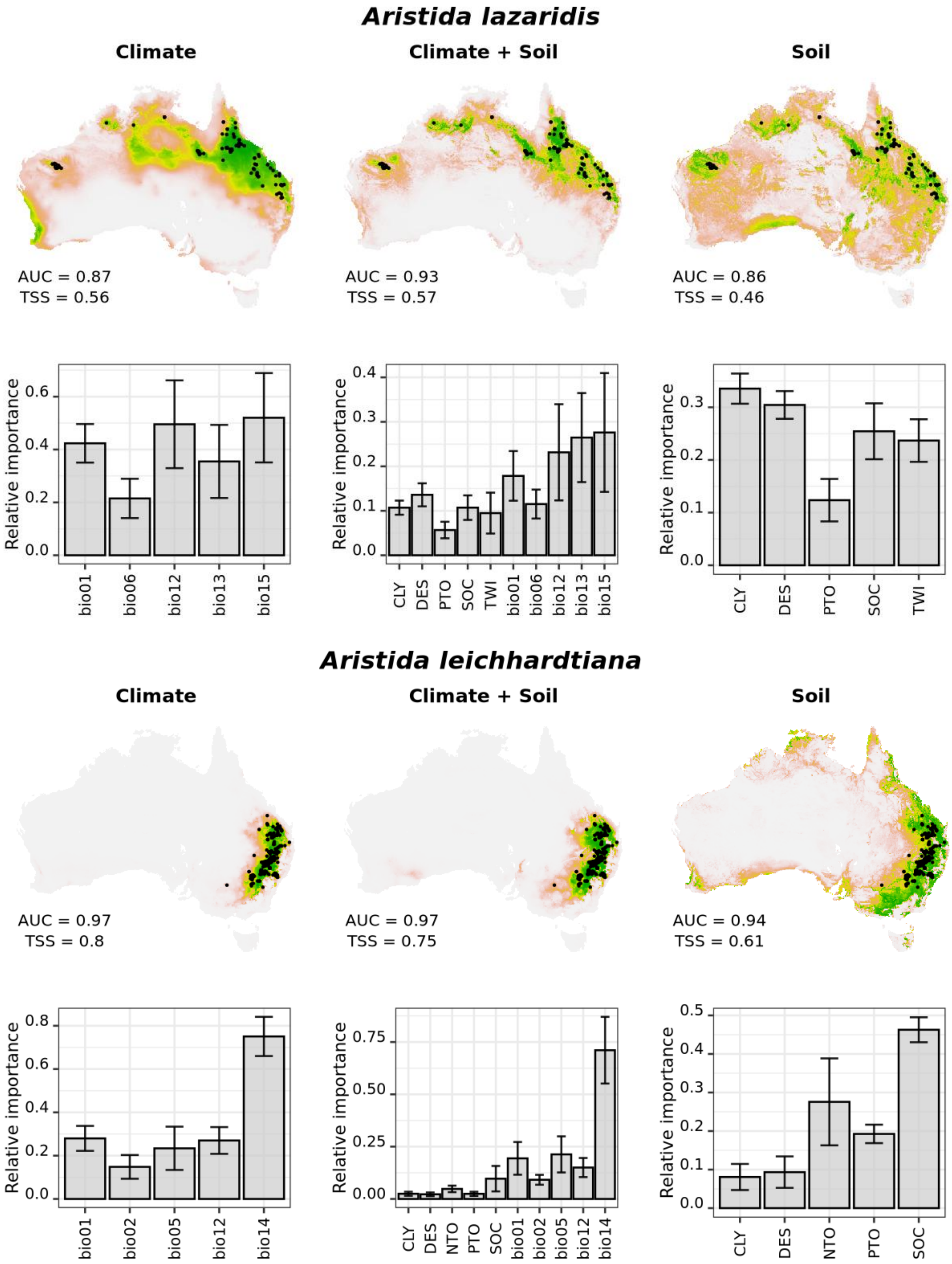
Aristida lazaridis
Climate
Climate + Soil
Soil
AUC = 0.87
TSS = 0.56
AUC = 0.93
TSS = 0.57
AUC = 0.86
TSS = 0.46
Relative importance
0.0
0.2
0.4
0.6
bio01
bio06
bio12
bio13
bio15
Relative importance
0.0
0.1
0.2
0.3
0.4
CLY
DES
PTO
SOC
TWI
bio01
bio06
bio12
bio13
bio15
Relative importance
0.0
0.1
0.2
0.3
CLY
DES
PTO
SOC
TWI
Aristida leichhardtiana
Climate
Climate + Soil
Soil
AUC = 0.97
TSS = 0.8
AUC = 0.97
TSS = 0.75
AUC = 0.94
TSS = 0.61
Relative importance
0.0
0.2
0.4
0.6
0.8
bio01
bio02
bio05
bio12
bio14
Relative importance
0.00
0.25
0.50
0.75
CLY
DES
NTO
PTO
SOC
bio01
bio02
bio05
bio12
bio14
Relative importance
0.0
0.1
0.2
0.3
0.4
0.5
CLY
DES
NTO
PTO
SOC

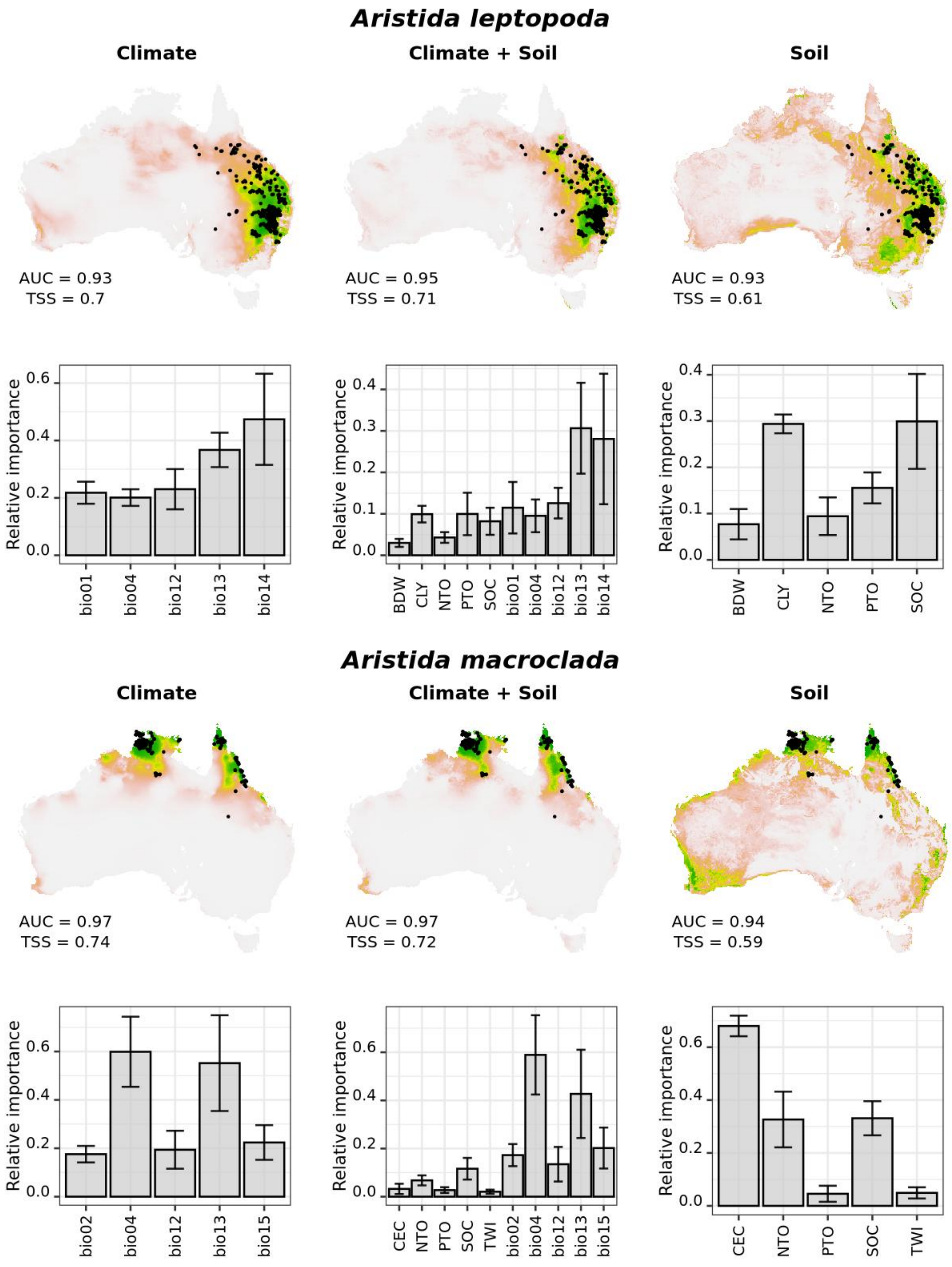
Aristida leptopoda
Climate
Climate + Soil
Soil
AUC = 0.93
TSS = 0.7
AUC = 0.95
TSS = 0.71
AUC = 0.93
TSS = 0.61
Relative importance
0.0
0.2
0.4
0.6
bio01
bio04
bio12
bio13
bio14
0.0
0.1
0.2
0.3
0.4
BDW
CLY
NTO
PTO
SOC
bio01
bio04
bio12
bio13
bio14
BDW
CLY
NTO
PTO
SOC
Aristida macroclada
Climate
Climate + Soil
Soil
AUC = 0.97
TSS = 0.74
AUC = 0.97
TSS = 0.72
AUC = 0.94
TSS = 0.59
bio02
bio04
bio12
bio13
bio15
CEC
NTO
PTO
SOC
TWI
bio02
bio04
bio12
bio13
bio15
CEC
NTO
PTO
SOC
TWI

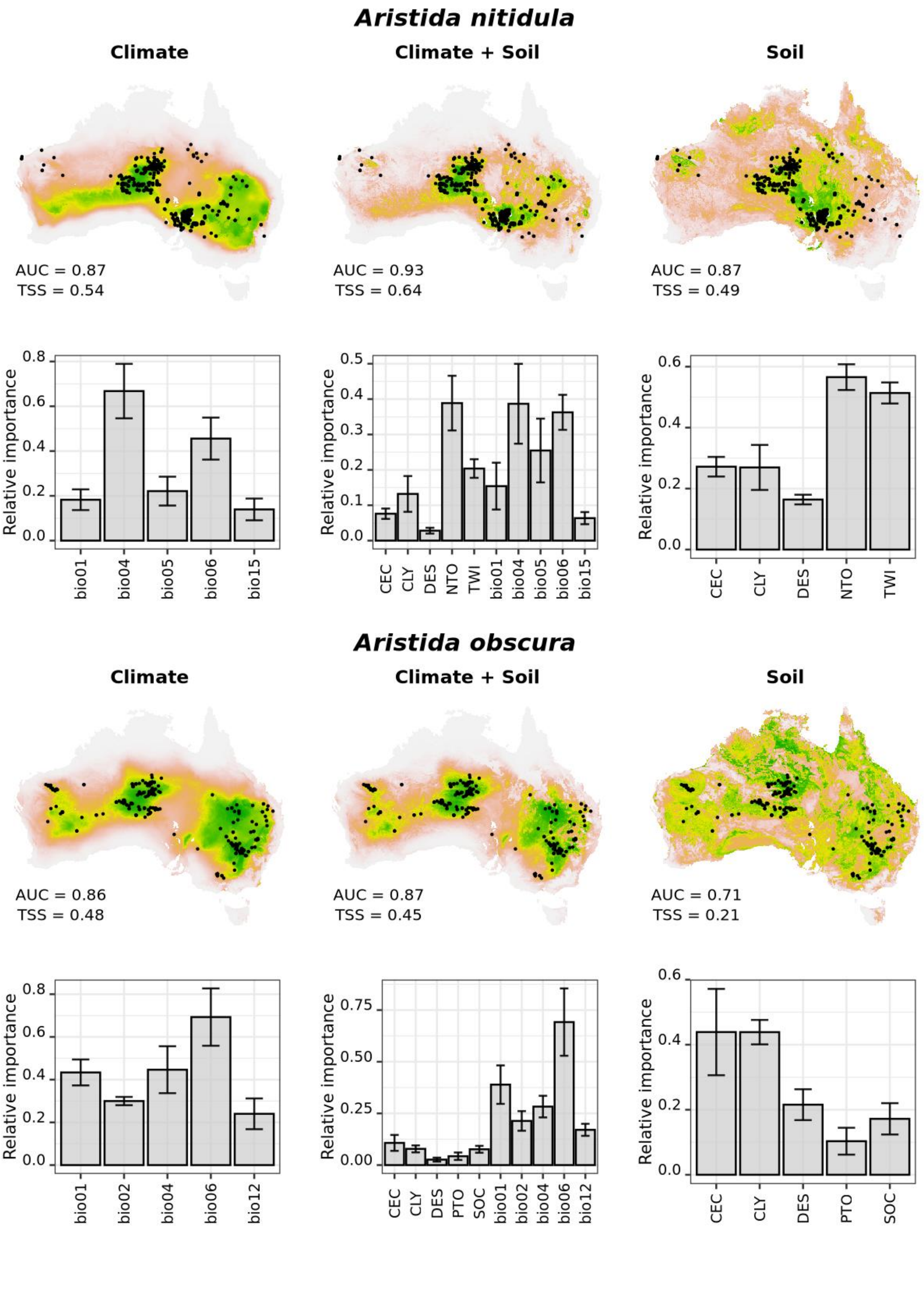
Aristida nitidula
Climate
Climate + Soil
Soil
AUC = 0.87
TSS = 0.54
AUC = 0.93
TSS = 0.64
AUC = 0.87
TSS = 0.49
Relative importance
bio01 bio04 bio05 bio06 bio15
CEC CLY DES NTO TWI bio01 bio04 bio05 bio06 bio15
CEC CLY DES NTO TWI
Aristida obscura
Climate
Climate + Soil
Soil
AUC = 0.86
TSS = 0.48
AUC = 0.87
TSS = 0.45
AUC = 0.71
TSS = 0.21
Relative importance
bio01 bio02 bio04 bio06 bio12
CEC CLY DES PTO SOC bio01 bio02 bio04 bio06 bio12
CEC CLY DES PTO SOC

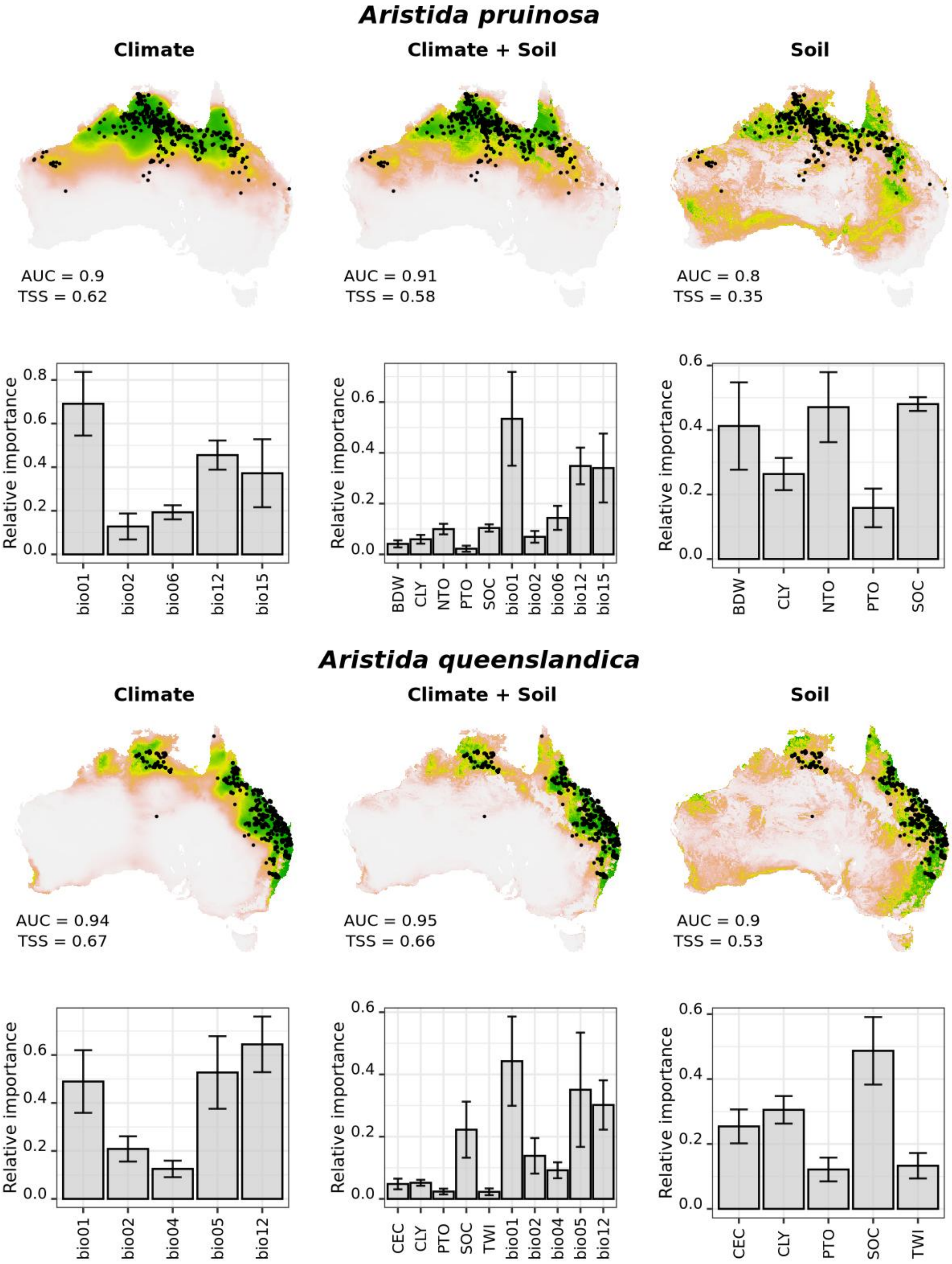
Aristida pruinosa
Climate
Climate + Soil
Soil
AUC = 0.9
TSS = 0.62
AUC = 0.91
TSS = 0.58
AUC = 0.8
TSS = 0.35
Relative importance
bio01 bio02 bio06 bio12 bio15
BDW CLY NTO PTO SOC bio01 bio02 bio06 bio12 bio15
BDW CLY NTO PTO SOC
Aristida queenslandica
Climate
Climate + Soil
Soil
AUC = 0.94
TSS = 0.67
AUC = 0.95
TSS = 0.66
AUC = 0.9
TSS = 0.53
Relative importance
bio01 bio02 bio04 bio05 bio12
CEC CLY PTO SOC TWI bio01 bio02 bio04 bio05 bio12
CEC CLY PTO SOC TWI

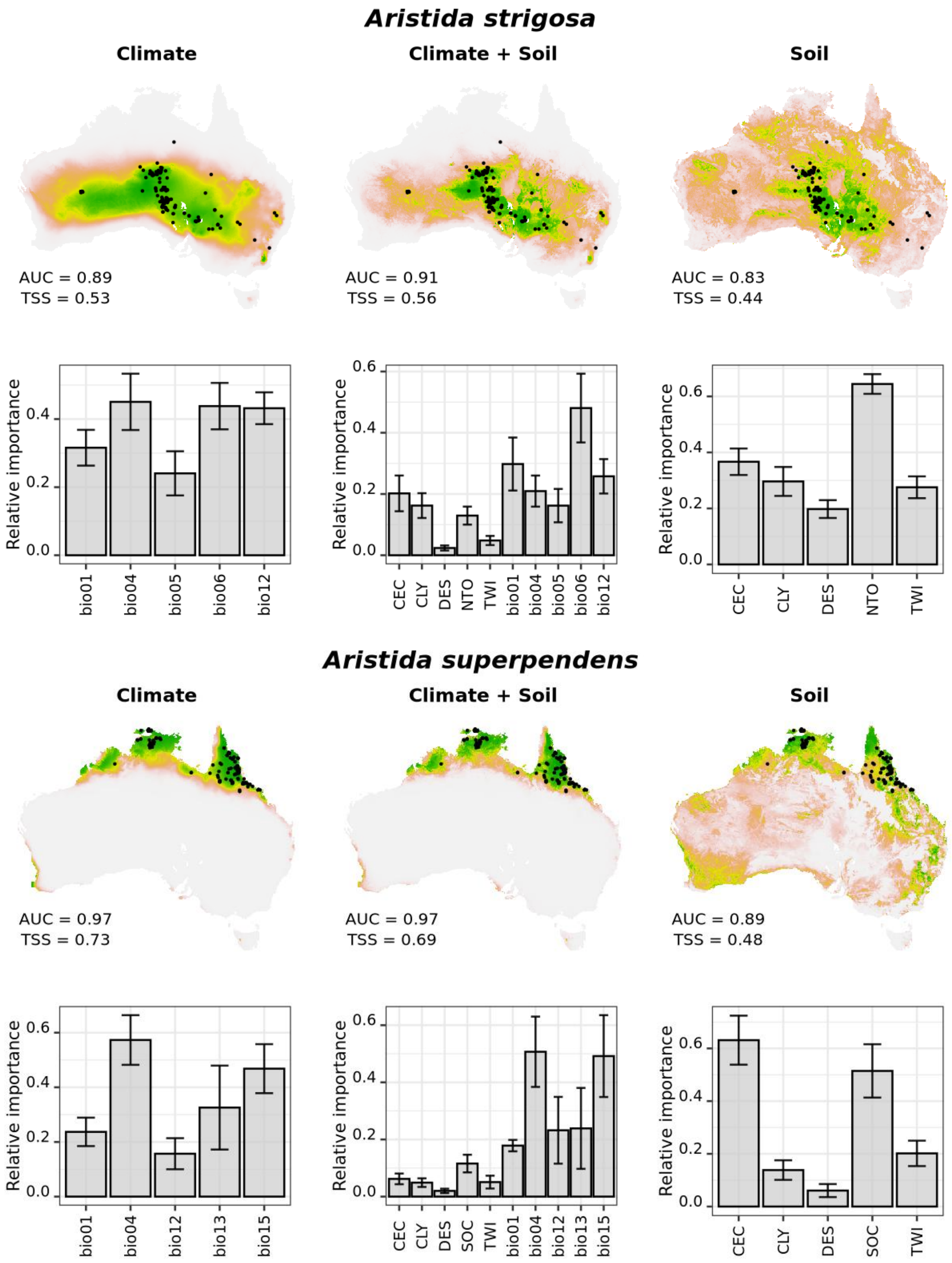
Aristida strigosa
Climate
Climate + Soil
Soil
AUC = 0.89
TSS = 0.53
AUC = 0.91
TSS = 0.56
AUC = 0.83
TSS = 0.44
Relative importance
bio01
bio04
bio05
bio06
bio12
CEC
CLY
DES
NTO
TWI
Aristida superpendens
Climate
Climate + Soil
Soil
AUC = 0.97
TSS = 0.73
AUC = 0.97
TSS = 0.69
AUC = 0.89
TSS = 0.48
Relative importance
bio01
bio04
bio12
bio13
bio15
CEC
CLY
DES
SOC
TWI

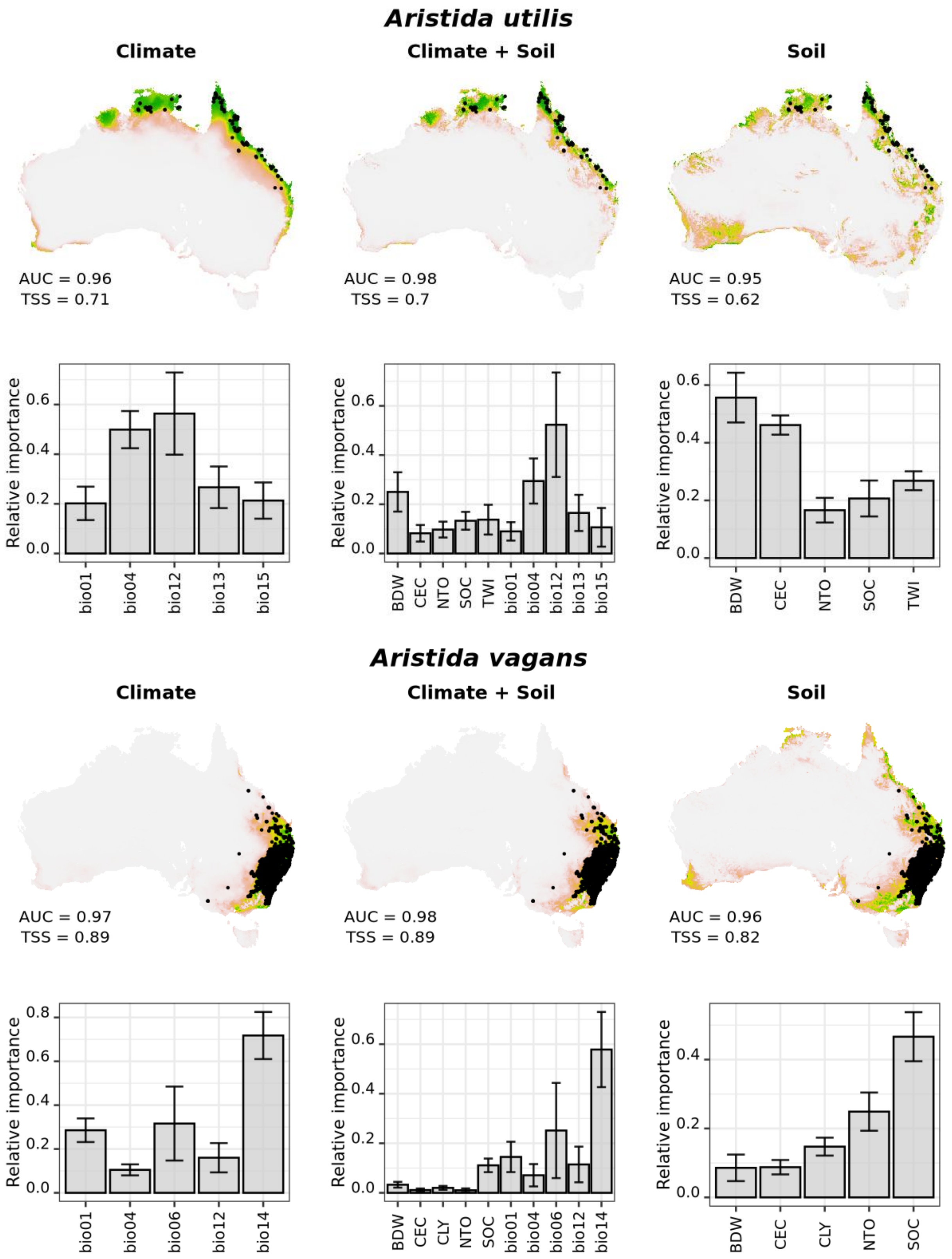
Aristida utilis
Climate
Climate + Soil
Soil
AUC = 0.96
TSS = 0.71
AUC = 0.98
TSS = 0.7
AUC = 0.95
TSS = 0.62
Relative importance
bio01
bio04
bio12
bio13
bio15
BDW
CEC
NTO
SOC
TWI
bio01
bio04
bio12
bio13
bio15
BDW
CEC
NTO
SOC
TWI
Aristida vagans
Climate
Climate + Soil
Soil
AUC = 0.97
TSS = 0.89
AUC = 0.98
TSS = 0.89
AUC = 0.96
TSS = 0.82
Relative importance
bio01
bio04
bio06
bio12
bio14
BDW
CEC
CLY
NTO
SOC
bio01
bio04
bio06
bio12
bio14
BDW
CEC
CLY
NTO
SOC

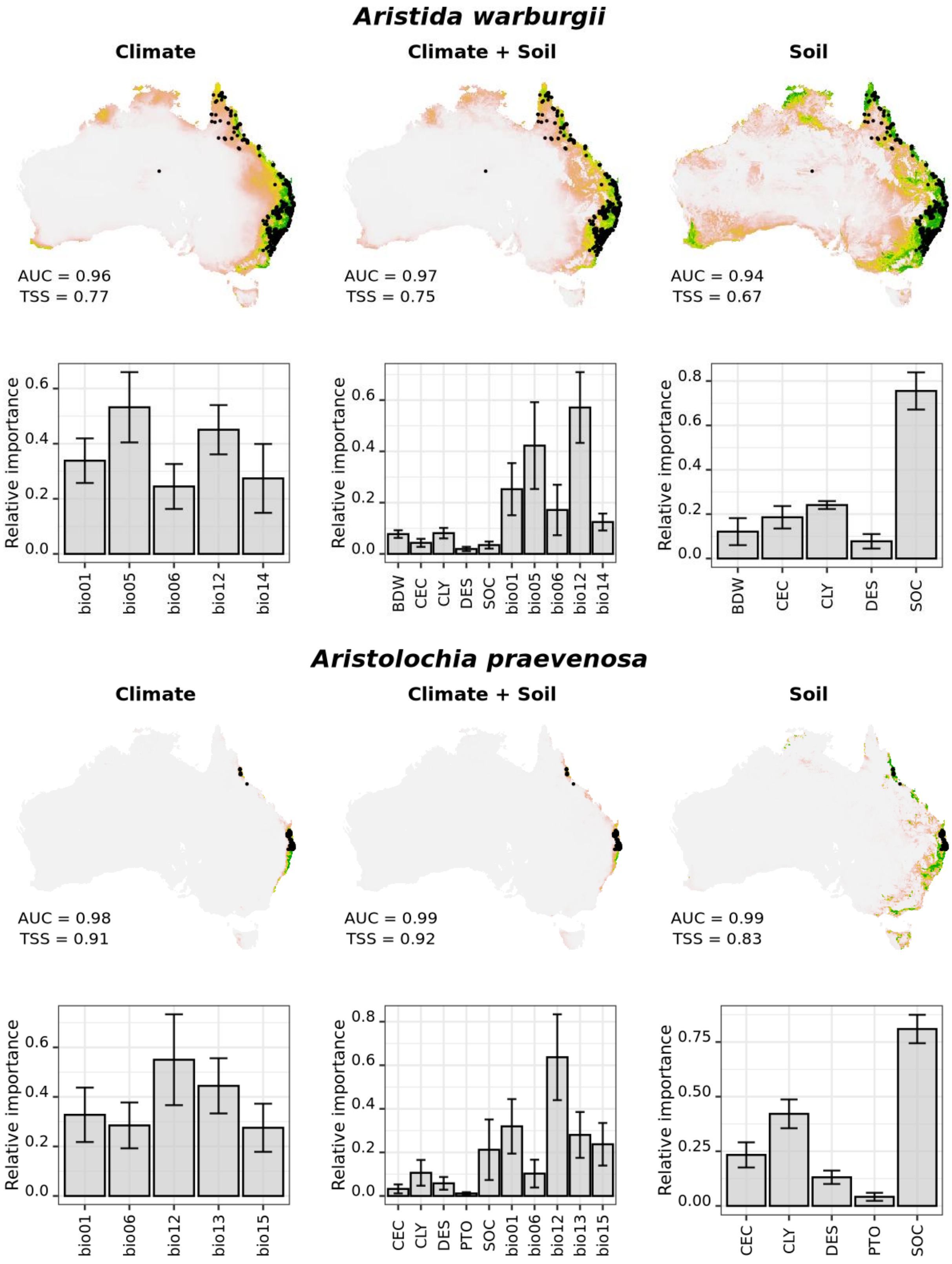

Aristida warburgii
Climate
Climate + Soil
Soil
AUC = 0.96
TSS = 0.77
AUC = 0.97
TSS = 0.75
AUC = 0.94
TSS = 0.67
Relative importance
bio01
bio05
bio06
bio12
bio14
BDW
CEC
CLY
DES
SOC
Aristolochia praevenosa
AUC = 0.98
TSS = 0.91
AUC = 0.99
TSS = 0.92
AUC = 0.99
TSS = 0.83
bio13
bio15
PTO

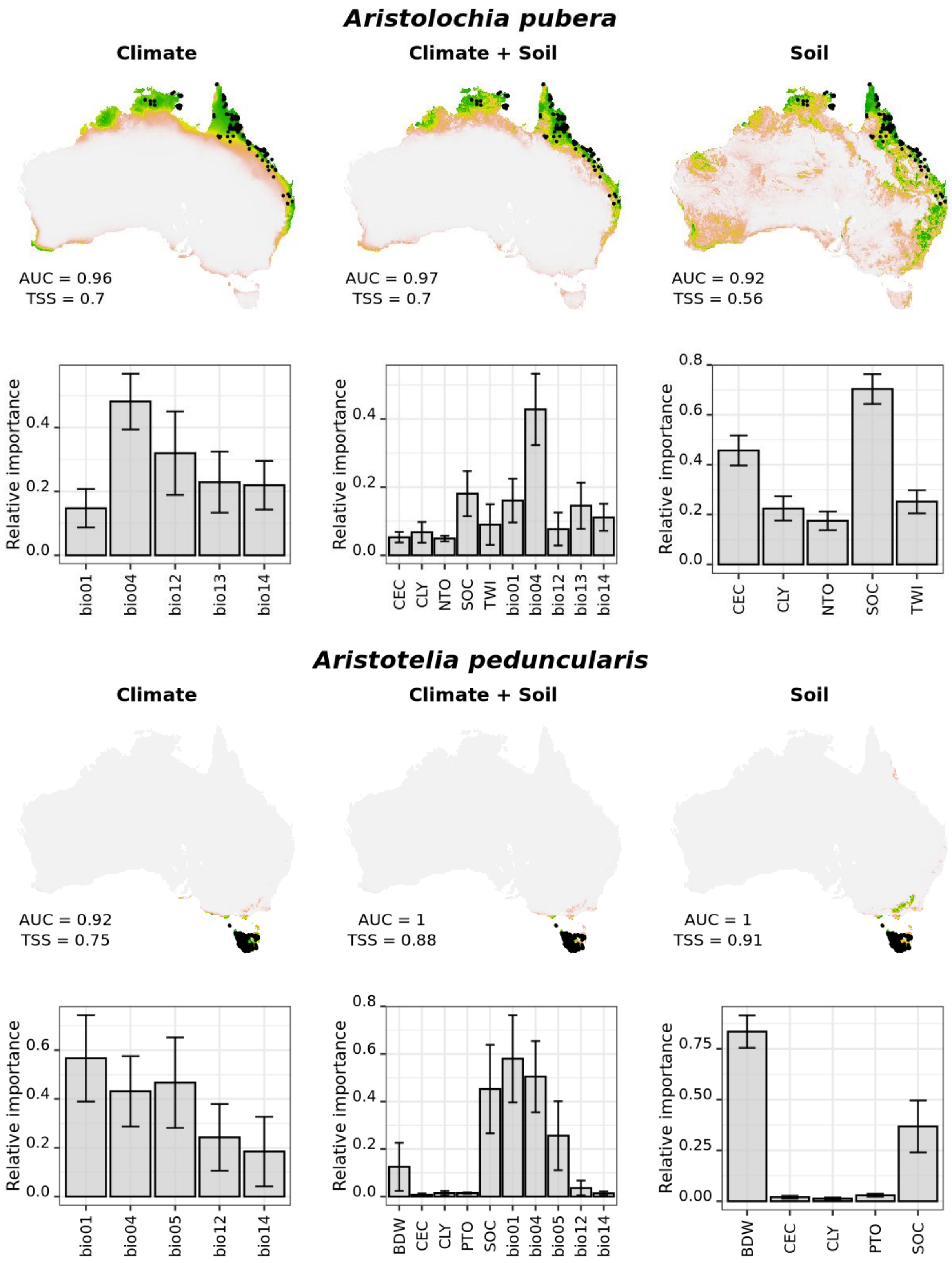
Aristolochia pubera
Climate
Climate + Soil
Soil
AUC = 0.96
TSS = 0.7
AUC = 0.97
TSS = 0.7
AUC = 0.92
TSS = 0.56
Relative importance
bio01
bio04
bio12
bio13
bio14
CEC
CLY
NTO
SOC
TWI
Aristotelia peduncularis
Climate
Climate + Soil
Soil
AUC = 0.92
TSS = 0.75
AUC = 1
TSS = 0.88
AUC = 1
TSS = 0.91
Relative importance
bio01
bio04
bio05
bio12
bio14
BDW
CEC
CLY
PTO
SOC

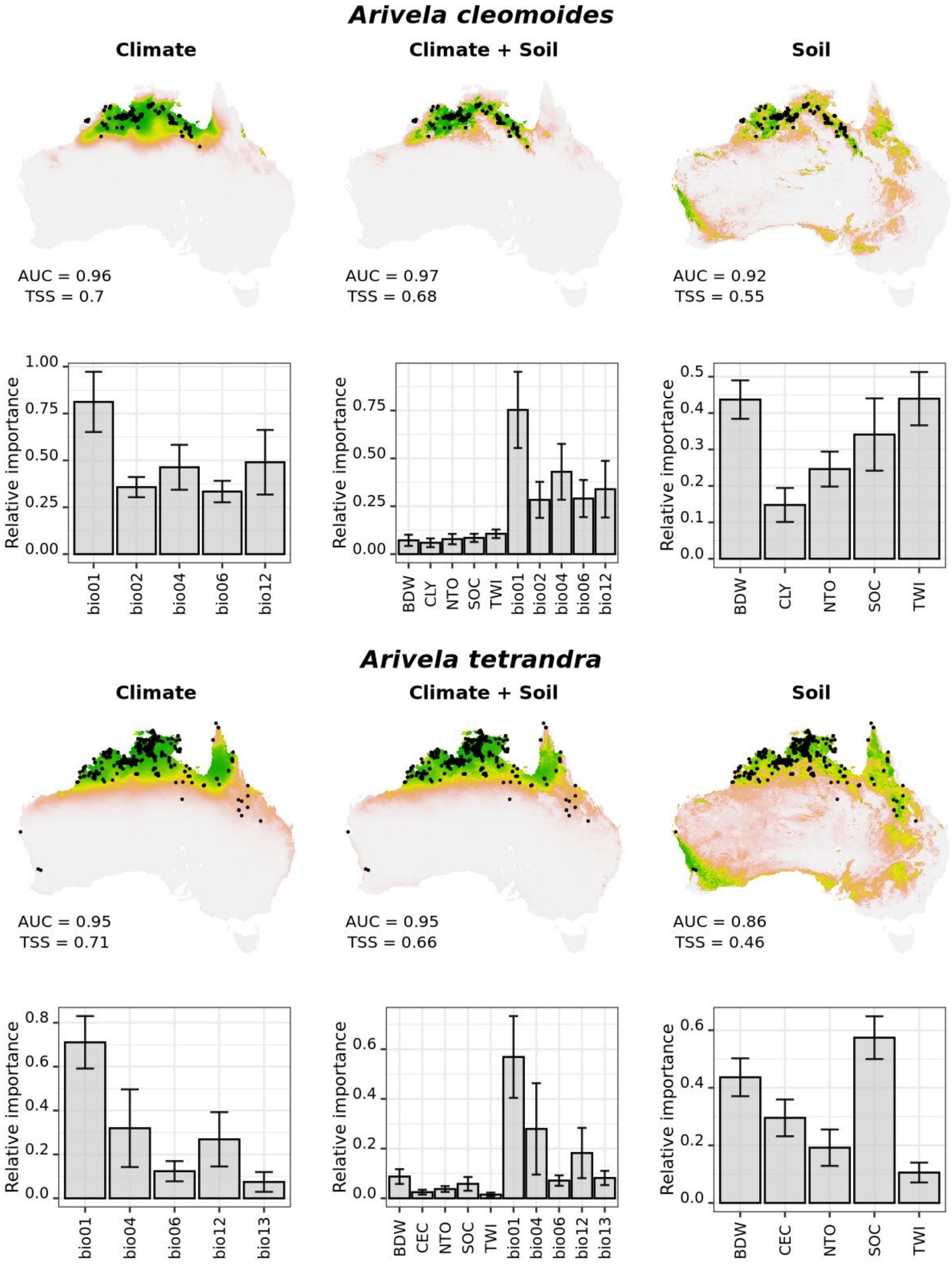

Arivela cleomoides
Climate
Climate + Soil
Soil
AUC = 0.96
TSS = 0.7
AUC = 0.97
TSS = 0.68
AUC = 0.92
TSS = 0.55
Relative importance
bio01
bio02
bio04
bio06
bio12
BDW
CLY
NTO
SOC
TWI
Arivela tetrandra
Climate
Climate + Soil
Soil
AUC = 0.95
TSS = 0.71
AUC = 0.95
TSS = 0.66
AUC = 0.86
TSS = 0.46
Relative importance
bio01
bio04
bio06
bio12
bio13
BDW
CEC
NTO
SOC
TWI

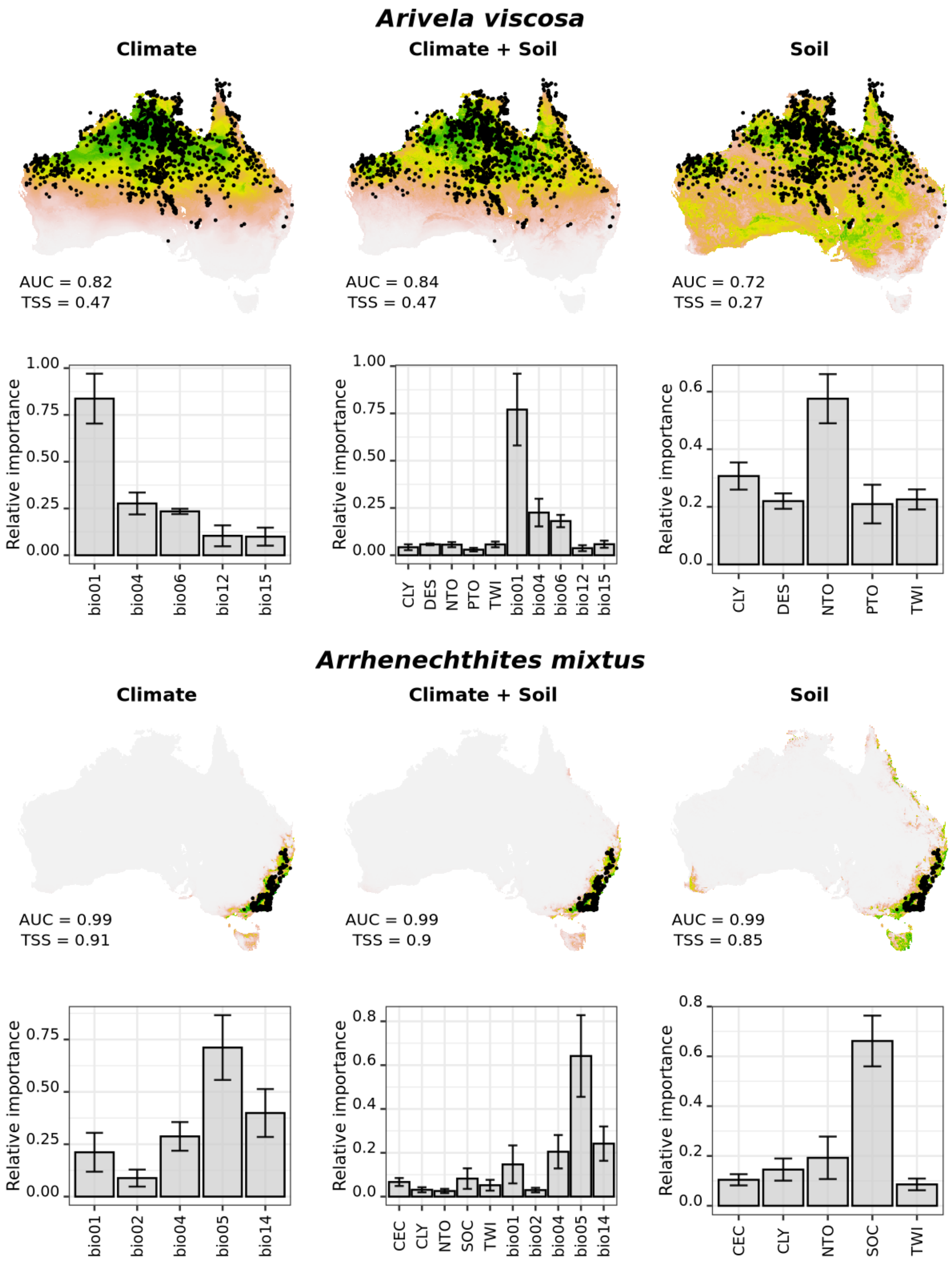

Arivela viscosa
Climate
Climate + Soil
Soil
AUC = 0.82
TSS = 0.47
AUC = 0.84
TSS = 0.47
AUC = 0.72
TSS = 0.27
Relative importance
1.00
0.75
0.50
0.25
0.00
bio01
bio04
bio06
bio12
bio15
Relative importance
1.00
0.75
0.50
0.25
0.00
CLY
DES
NTO
PTO
TWI
bio01
bio04
bio06
bio12
bio15
Relative importance
0.6
0.4
0.2
0.0
CLY
DES
NTO
PTO
TWI
Arrhenechthites mixtus
Climate
Climate + Soil
Soil
AUC = 0.99
TSS = 0.91
AUC = 0.99
TSS = 0.9
AUC = 0.99
TSS = 0.85
Relative importance
0.75
0.50
0.25
0.00
bio01
bio02
bio04
bio05
bio14
Relative importance
0.8
0.6
0.4
0.2
0.0
CEC
CLY
NTO
SOC
TWI
bio01
bio02
bio04
bio05
bio14
Relative importance
0.8
0.6
0.4
0.2
0.0
CEC
CLY
NTO
SOC
TWI

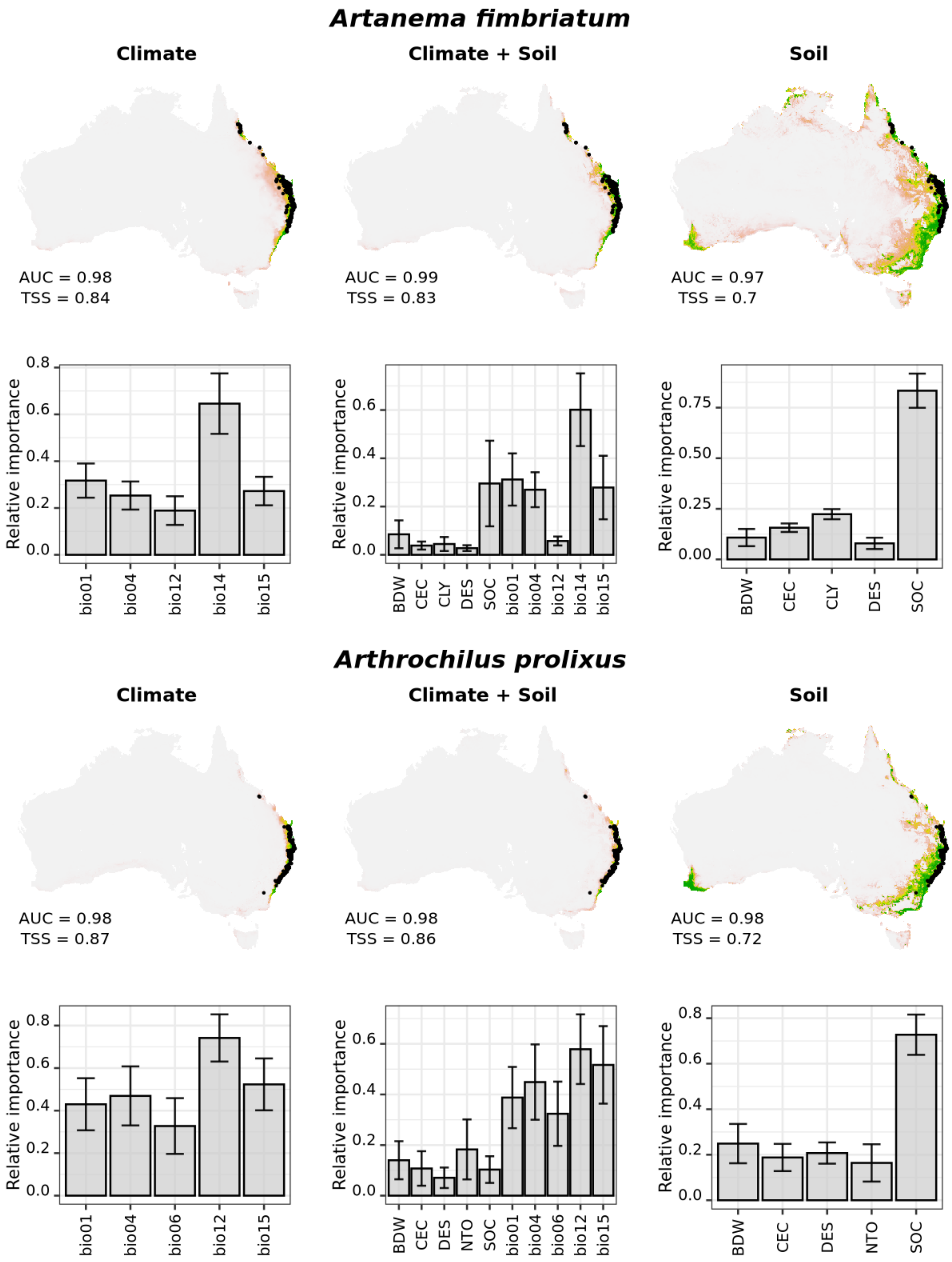
Artanema fimbriatum
Climate
Climate + Soil
Soil
AUC = 0.98
TSS = 0.84
AUC = 0.99
TSS = 0.83
AUC = 0.97
TSS = 0.7
Relative importance
0.0
0.2
0.4
0.6
0.8
bio01
bio04
bio12
bio14
bio15
BDW
CEC
CLY
DES
SOC
0.00
0.25
0.50
0.75
Arthrochilus prolixus
Climate
Climate + Soil
Soil
AUC = 0.98
TSS = 0.87
AUC = 0.98
TSS = 0.86
AUC = 0.98
TSS = 0.72
bio06
NTO

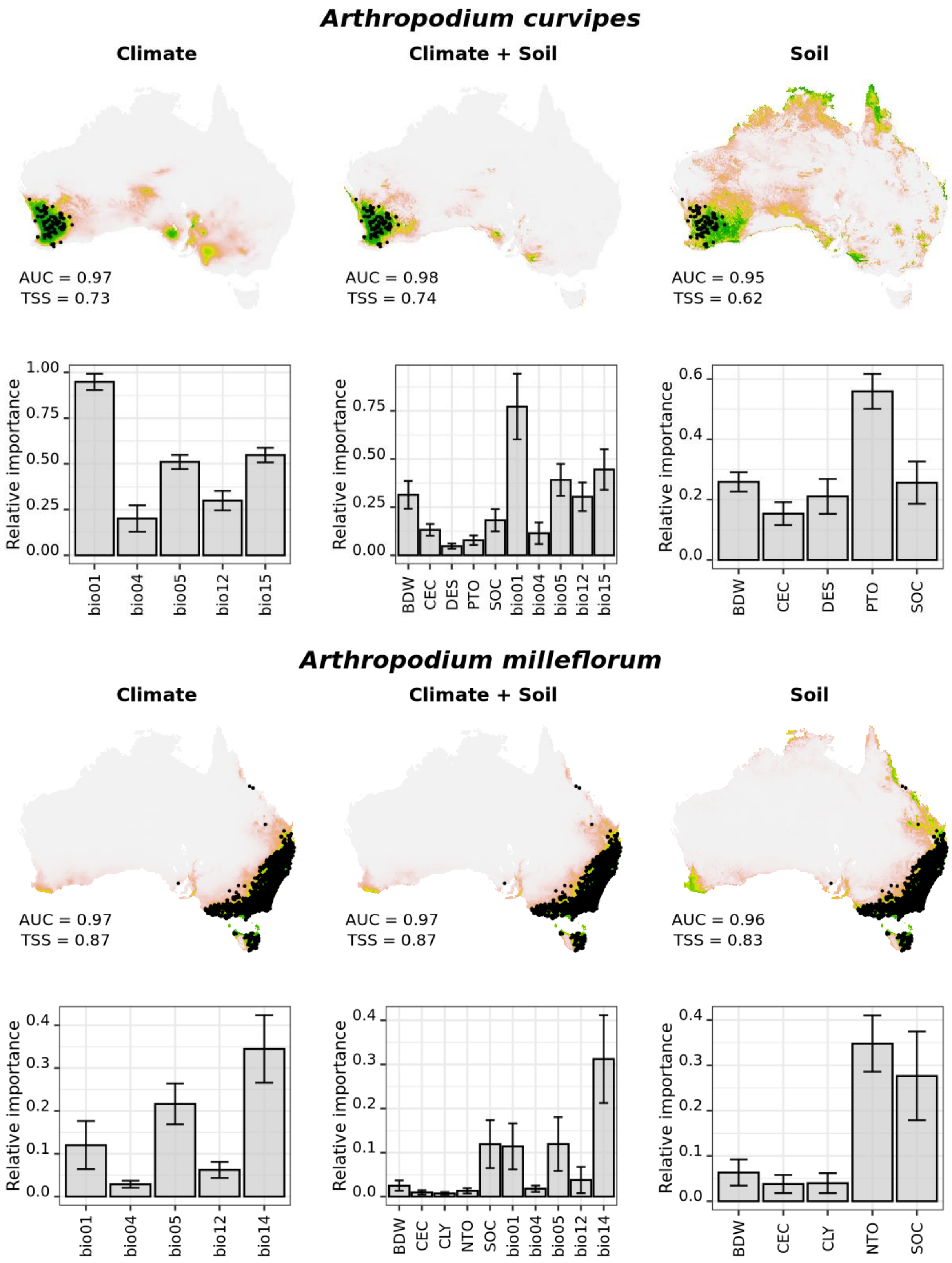

Arthropodium curvipes
Climate
Climate + Soil
Soil
AUC = 0.97
TSS = 0.73
AUC = 0.98
TSS = 0.74
AUC = 0.95
TSS = 0.62
Relative importance
bio01
bio04
bio05
bio12
bio15
BDW
CEC
DES
PTO
SOC
Arthropodium milleflorum
AUC = 0.97
TSS = 0.87
AUC = 0.97
TSS = 0.87
AUC = 0.96
TSS = 0.83
bio14
CLY
NTO

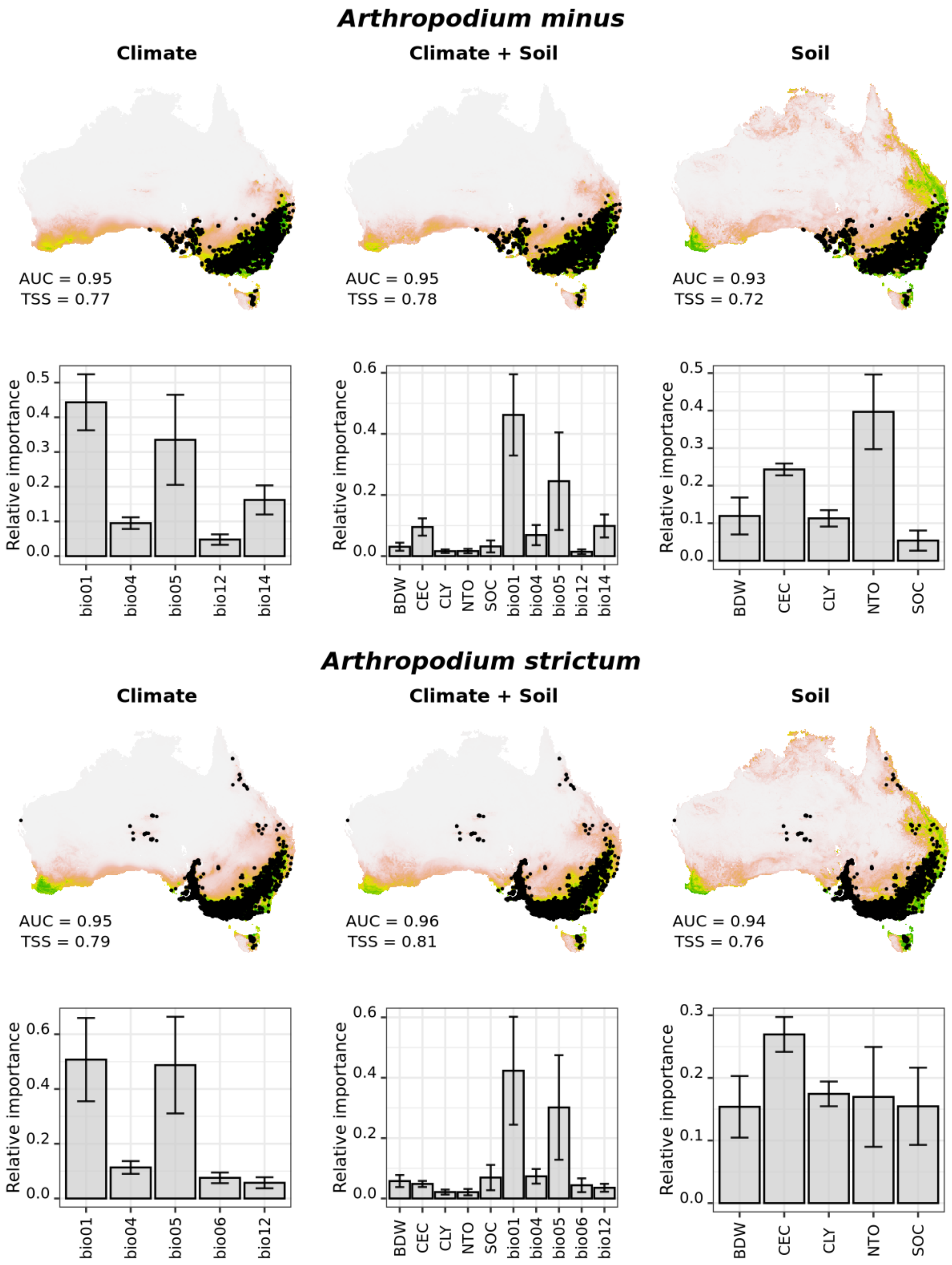
Arthropodium minus
Climate
Climate + Soil
Soil
AUC = 0.95
TSS = 0.77
AUC = 0.95
TSS = 0.78
AUC = 0.93
TSS = 0.72
Relative importance
bio01
bio04
bio05
bio12
bio14
BDW
CEC
CLY
NTO
SOC
Arthropodium strictum
Climate
Climate + Soil
Soil
AUC = 0.95
TSS = 0.79
AUC = 0.96
TSS = 0.81
AUC = 0.94
TSS = 0.76
Relative importance
bio01
bio04
bio05
bio06
bio12
BDW
CEC
CLY
NTO
SOC

Arthrostylis aphylla

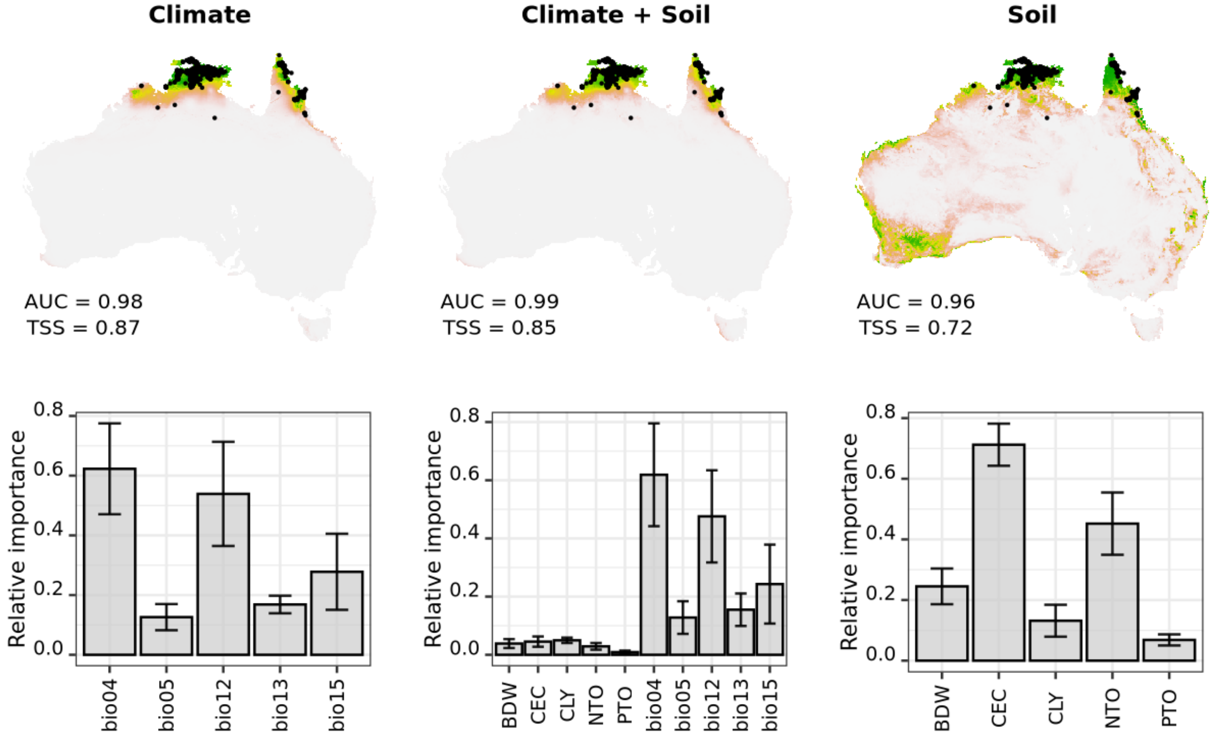

Arundinella nepalensis

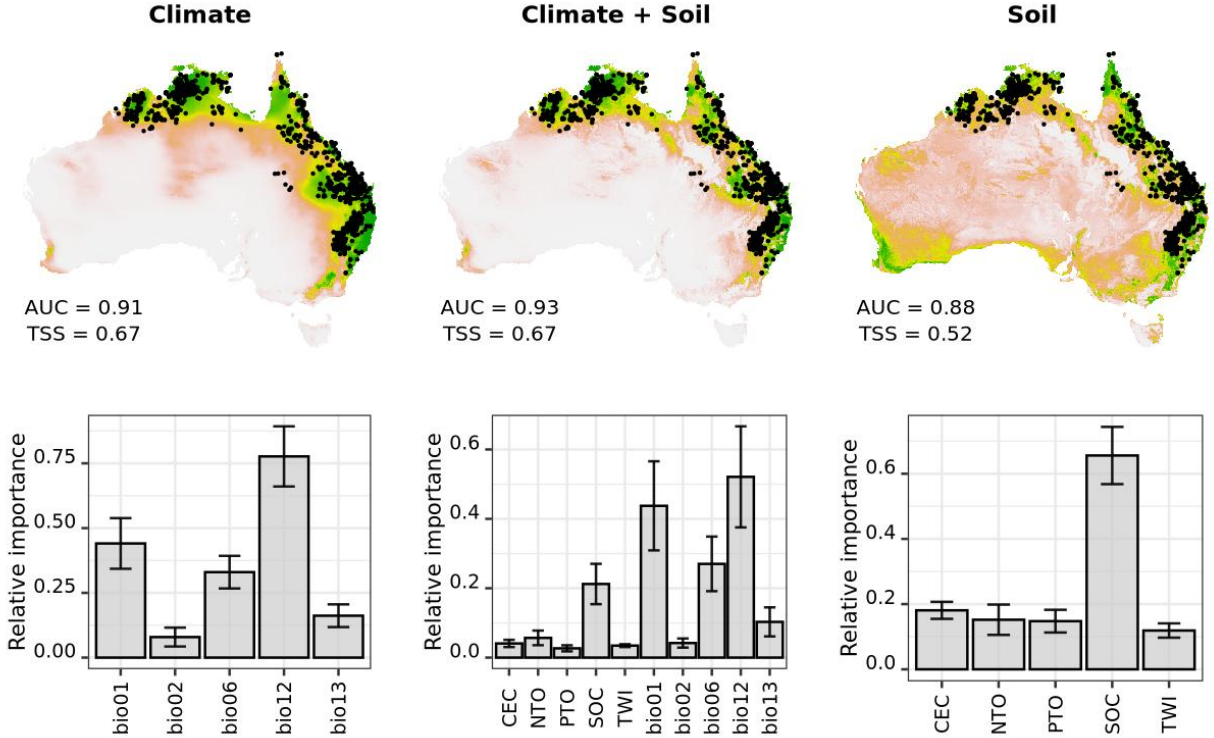

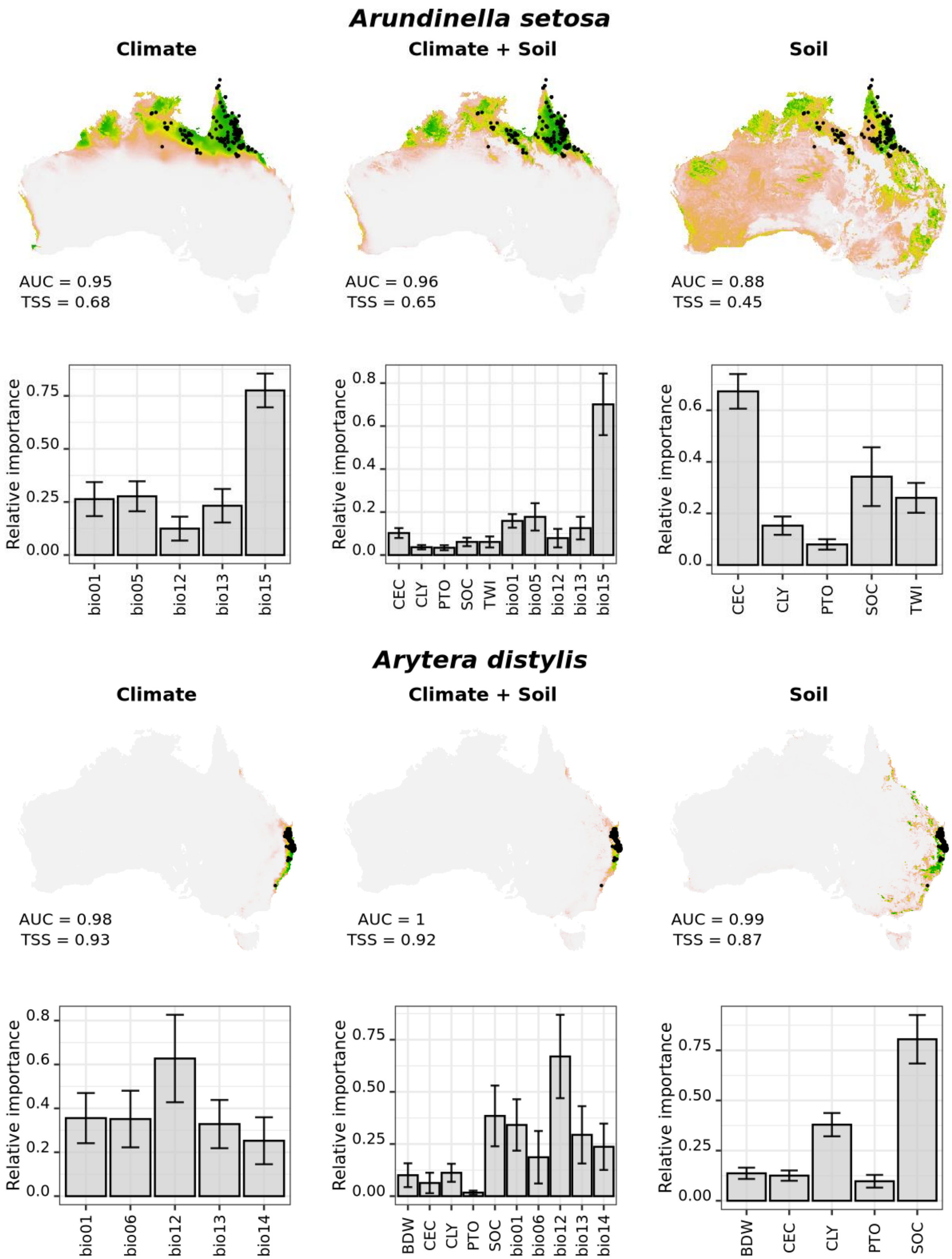

Arundinella setosa
Climate
Climate + Soil
Soil
AUC = 0.95
TSS = 0.68
AUC = 0.96
TSS = 0.65
AUC = 0.88
TSS = 0.45
Relative importance
0.00
0.25
0.50
0.75
bio01
bio05
bio12
bio13
bio15
0.0
0.2
0.4
0.6
0.8
CEC
CLY
PTO
SOC
TWI
bio01
bio05
bio12
bio13
bio15
0.0
0.2
0.4
0.6
CEC
CLY
PTO
SOC
TWI
Arytera distylis
Climate
Climate + Soil
Soil
AUC = 0.98
TSS = 0.93
AUC = 1
TSS = 0.92
AUC = 0.99
TSS = 0.87
Relative importance
0.0
0.2
0.4
0.6
0.8
bio01
bio06
bio12
bio13
bio14
0.00
0.25
0.50
0.75
BDW
CEC
CLY
PTO
SOC
bio01
bio06
bio12
bio13
bio14
0.00
0.25
0.50
0.75
BDW
CEC
CLY
PTO
SOC

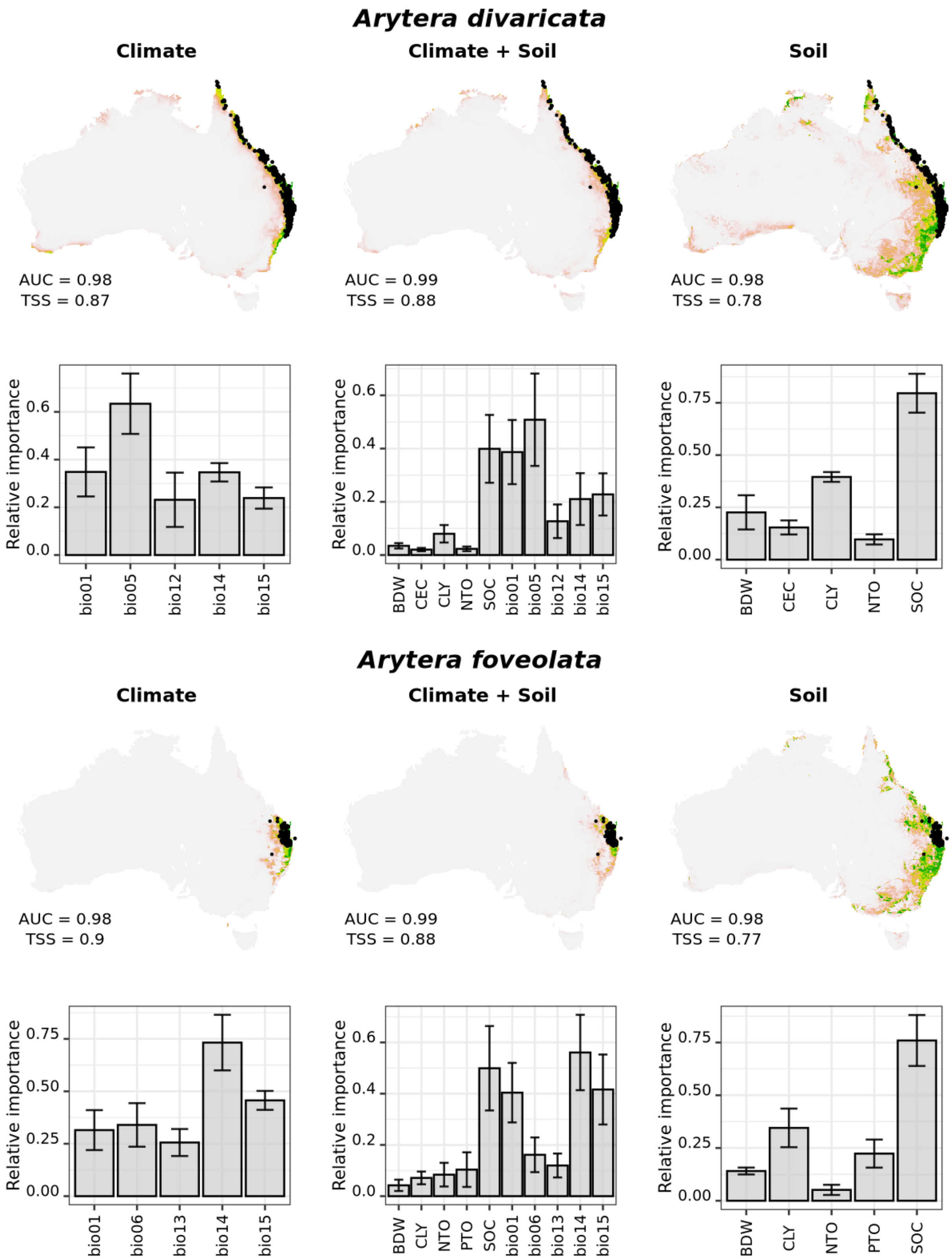
Arytera divaricata
Climate
Climate + Soil
Soil
AUC = 0.98
TSS = 0.87
AUC = 0.99
TSS = 0.88
AUC = 0.98
TSS = 0.78
Relative importance
bio01
bio05
bio12
bio14
bio15
BDW
CEC
CLY
NTO
SOC
Arytera foveolata
Climate
Climate + Soil
Soil
AUC = 0.98
TSS = 0.9
AUC = 0.99
TSS = 0.88
AUC = 0.98
TSS = 0.77
Relative importance
bio01
bio06
bio13
bio14
bio15
BDW
CLY
NTO
PTO
SOC

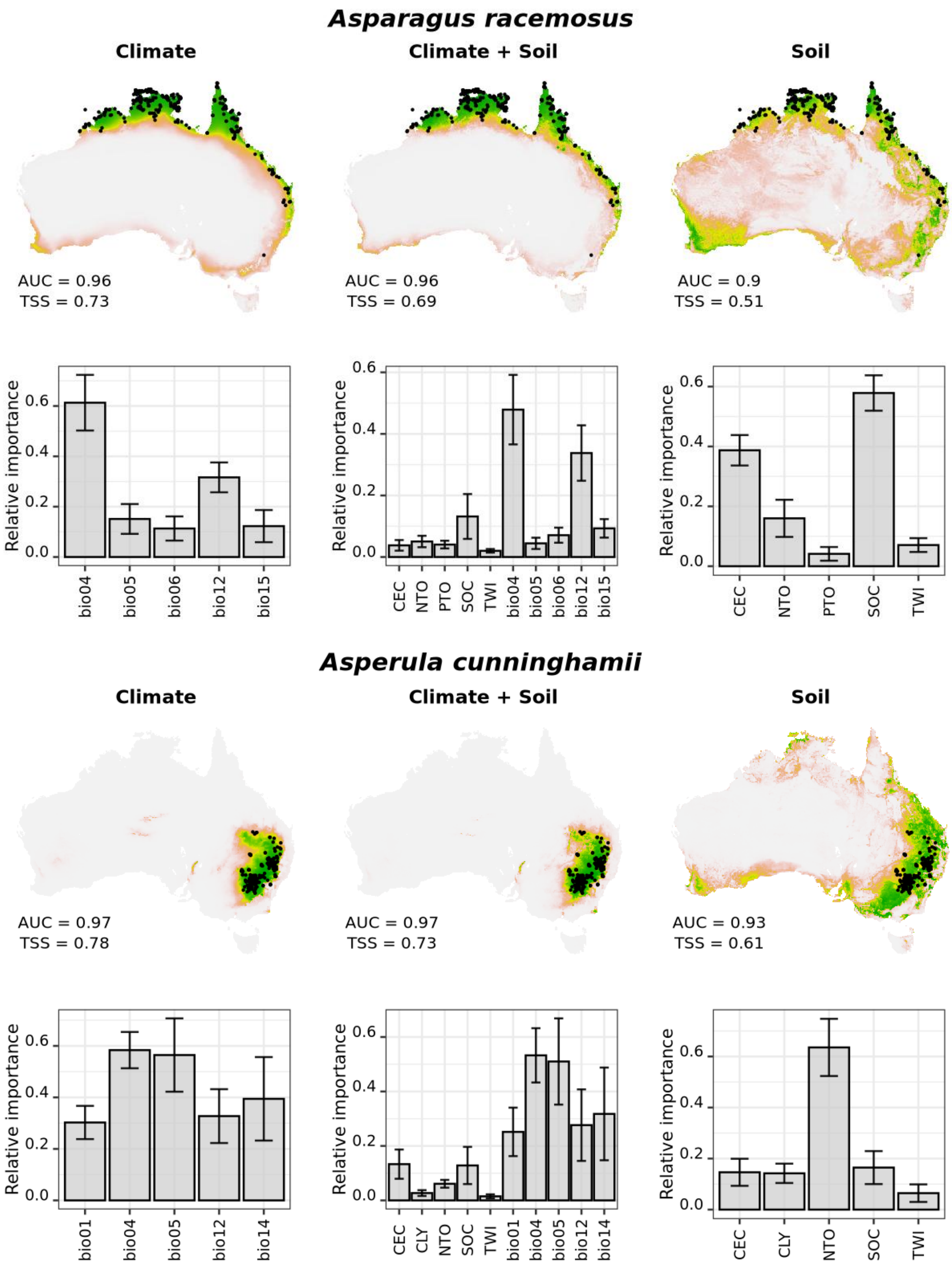
Asparagus racemosus
Climate
Climate + Soil
Soil
AUC = 0.96
TSS = 0.73
AUC = 0.96
TSS = 0.69
AUC = 0.9
TSS = 0.51
Relative importance
bio04
bio05
bio06
bio12
bio15
CEC
NTO
PTO
SOC
TWI
Asperula cunninghamii
Climate
Climate + Soil
Soil
AUC = 0.97
TSS = 0.78
AUC = 0.97
TSS = 0.73
AUC = 0.93
TSS = 0.61
Relative importance
bio01
bio04
bio05
bio12
bio14
CEC
CLY
NTO
SOC
TWI

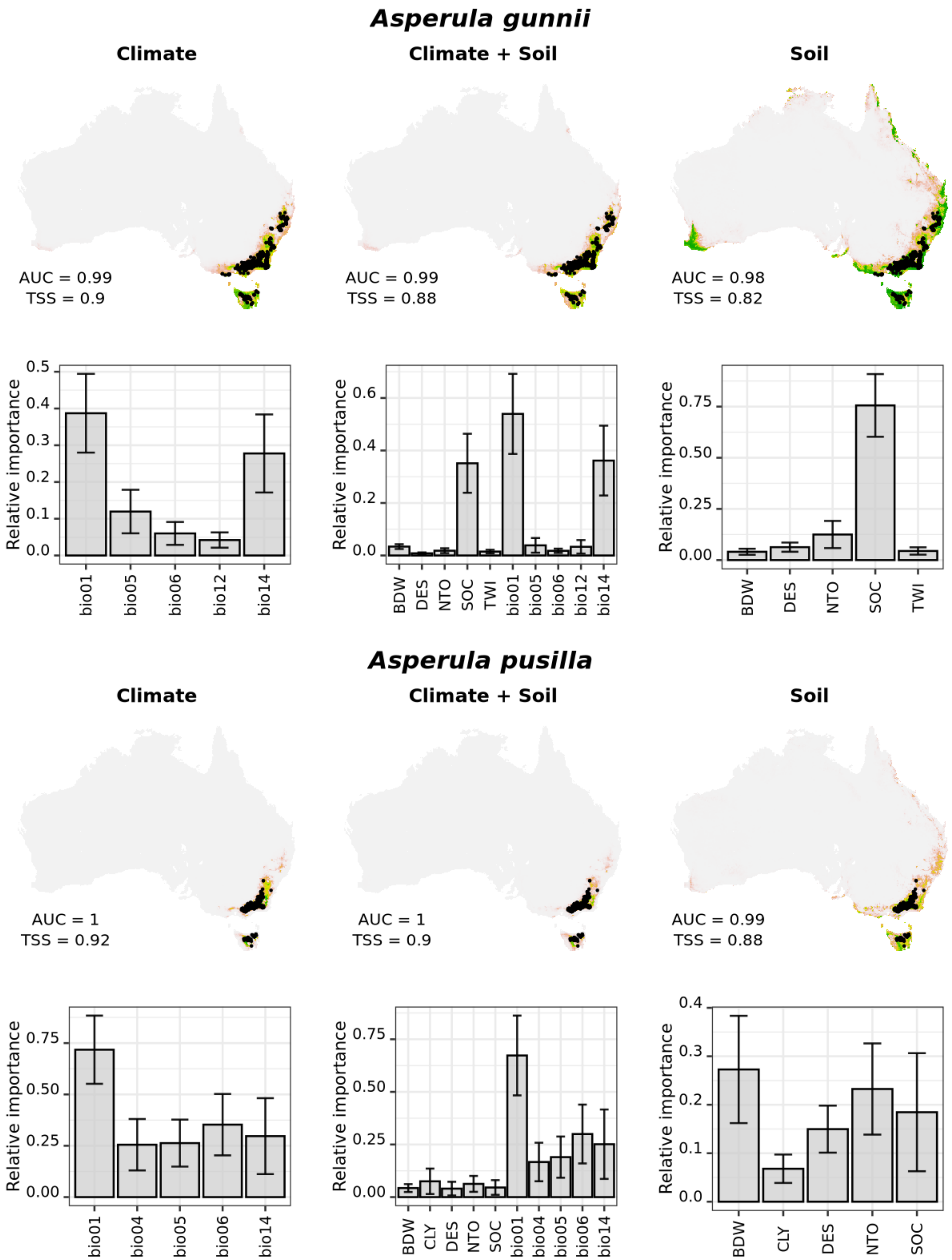
Asperula gunnii
Climate
Climate + Soil
Soil
AUC = 0.99
TSS = 0.9
AUC = 0.99
TSS = 0.88
AUC = 0.98
TSS = 0.82
Relative importance
bio01
bio05
bio06
bio12
bio14
BDW
DES
NTO
SOC
TWI
Asperula pusilla
AUC = 1
TSS = 0.92
AUC = 1
TSS = 0.9
AUC = 0.99
TSS = 0.88
bio04
CLY

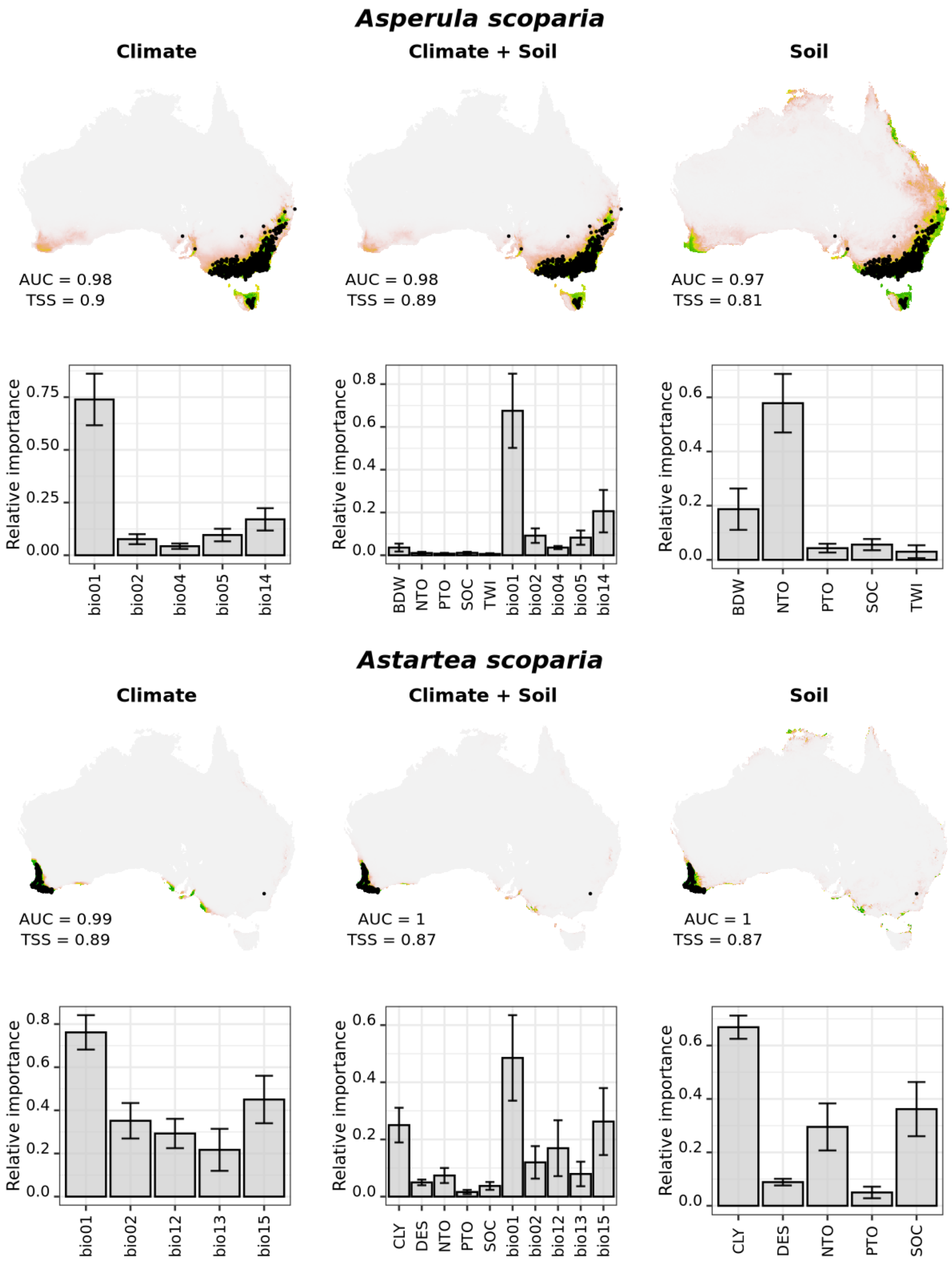

Asperula scoparia
Climate
Climate + Soil
Soil
AUC = 0.98
TSS = 0.9
AUC = 0.98
TSS = 0.89
AUC = 0.97
TSS = 0.81
Relative importance
bio01
bio02
bio04
bio05
bio14
BDW
NTO
PTO
SOC
TWI
Astartea scoparia
Climate
Climate + Soil
Soil
AUC = 0.99
TSS = 0.89
AUC = 1
TSS = 0.87
AUC = 1
TSS = 0.87
bio01
bio02
bio12
bio13
bio15
CLY
DES
NTO
PTO
SOC

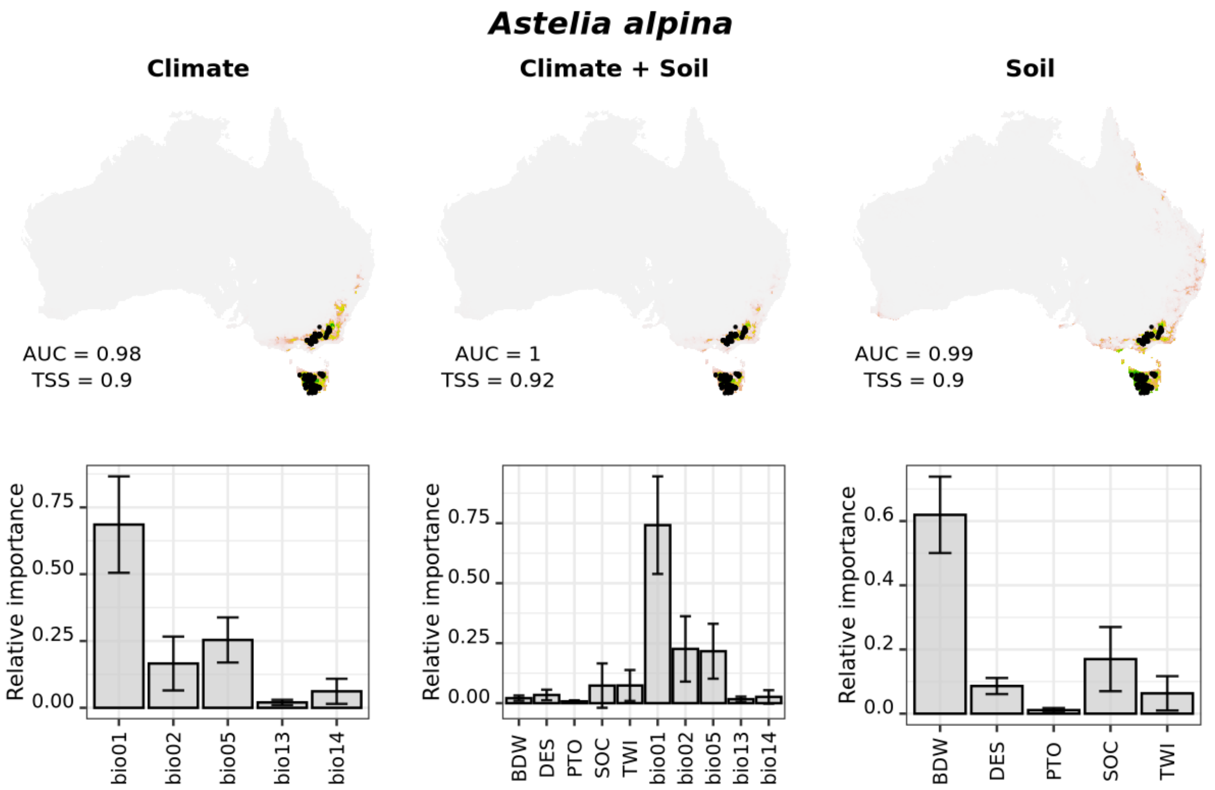
Astelia alpina
Climate
Climate + Soil
Soil
AUC = 0.98
TSS = 0.9
AUC = 1
TSS = 0.92
AUC = 0.99
TSS = 0.9
Relative importance
0.00
0.25
0.50
0.75
bio01
bio02
bio05
bio13
bio14
BDW
DES
PTO
SOC
TWI
0.0
0.2
0.4
0.6

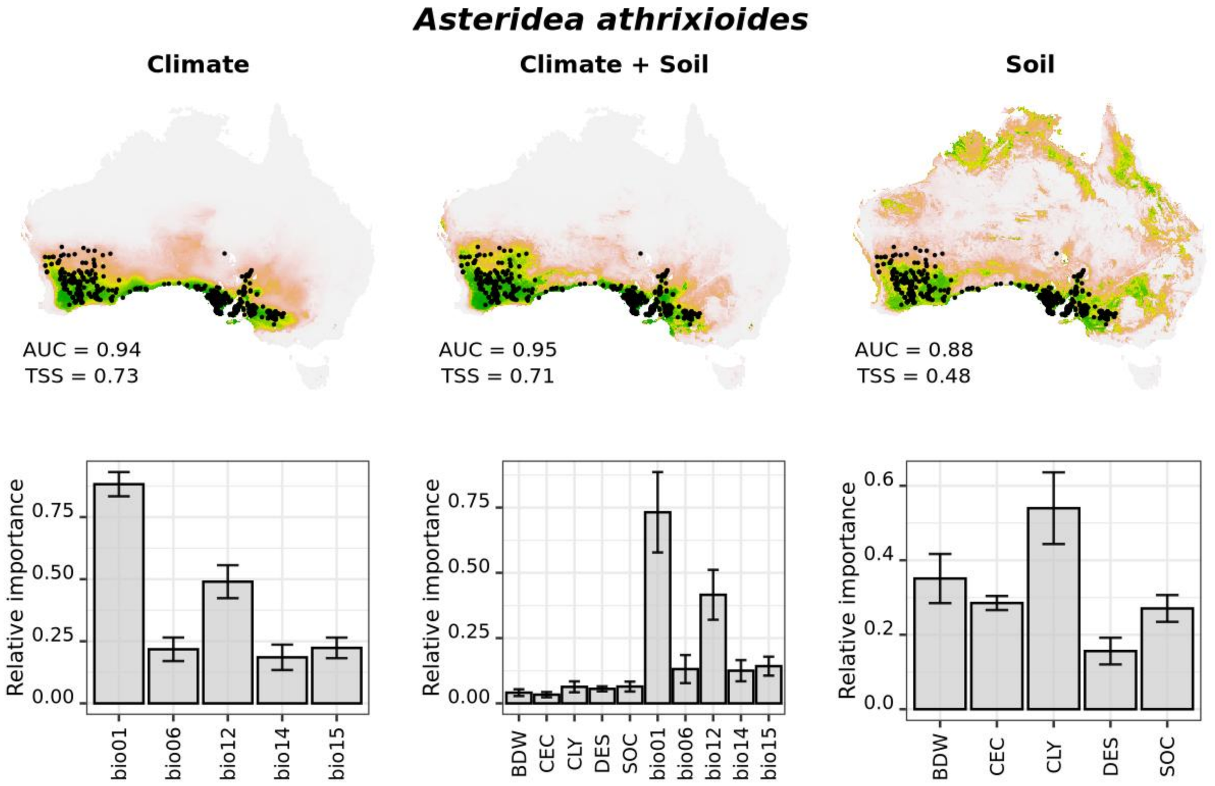
Asteridea athrixioides
Climate
Climate + Soil
Soil
AUC = 0.94
TSS = 0.73
AUC = 0.95
TSS = 0.71
AUC = 0.88
TSS = 0.48
Relative importance
0.00
0.25
0.50
0.75
bio01
bio06
bio12
bio14
bio15
BDW
CEC
CLY
DES
SOC
0.0
0.2
0.4
0.6

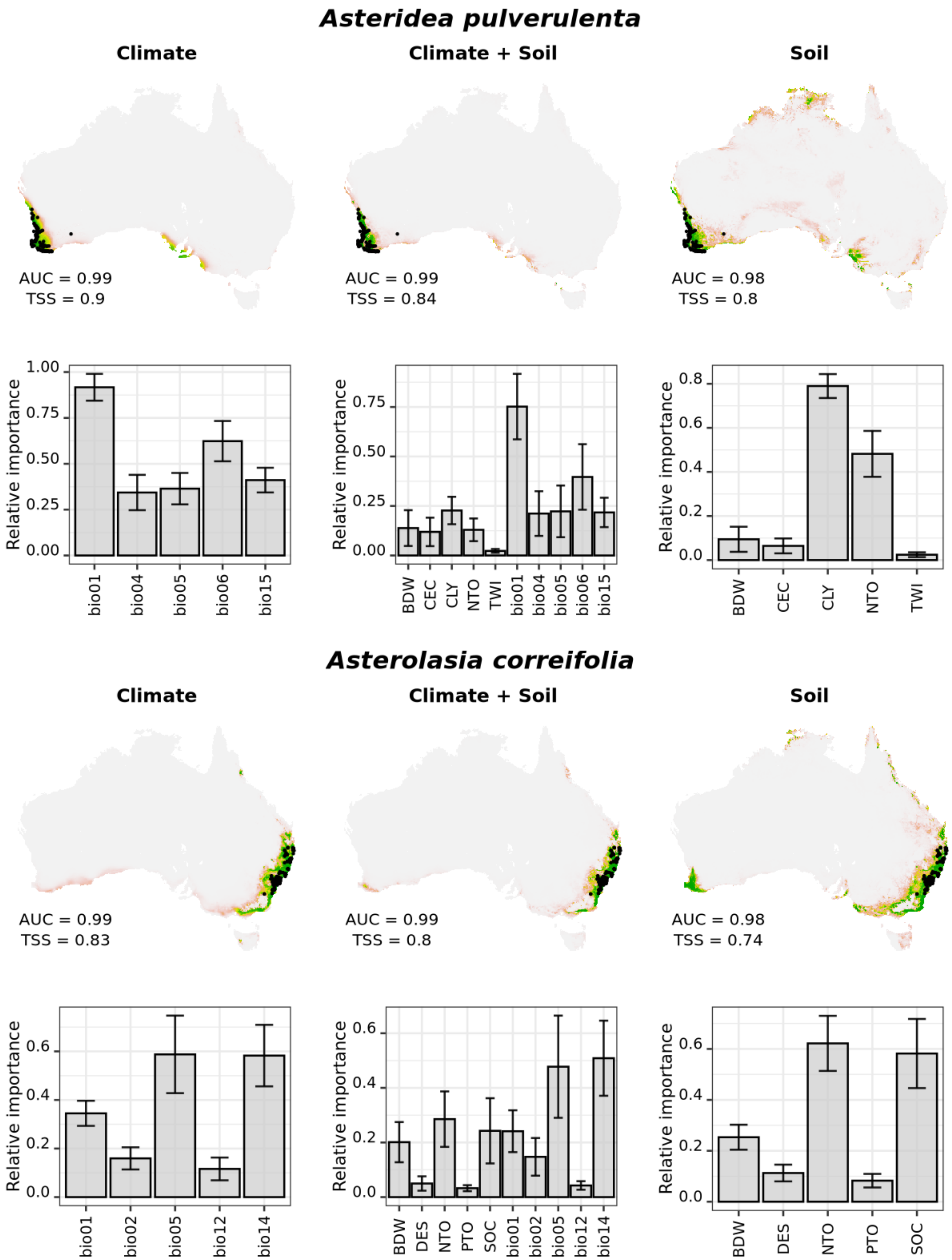
Asteridea pulverulenta
Climate
Climate + Soil
Soil
AUC = 0.99
TSS = 0.9
AUC = 0.99
TSS = 0.84
AUC = 0.98
TSS = 0.8
Relative importance
bio01
bio04
bio05
bio06
bio15
BDW
CEC
CLY
NTO
TWI
Asterolasia correifolia
AUC = 0.99
TSS = 0.83
AUC = 0.99
TSS = 0.8
AUC = 0.98
TSS = 0.74
bio02
bio12
bio14
DES
PTO
SOC

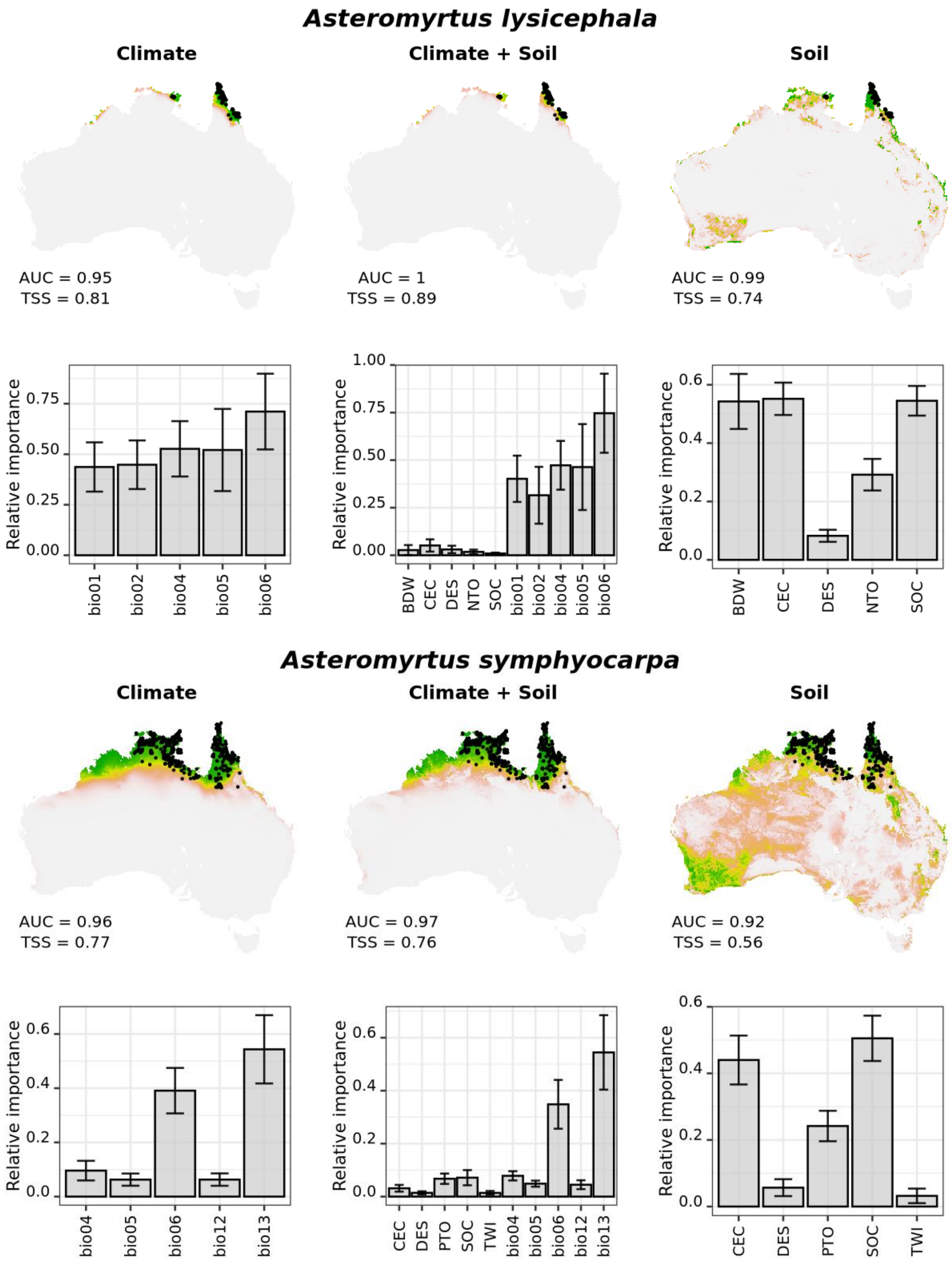
Asteromyrtus lysicephala
Climate
Climate + Soil
Soil
AUC = 0.95
TSS = 0.81
AUC = 1
TSS = 0.89
AUC = 0.99
TSS = 0.74
Relative importance
0.00
0.25
0.50
0.75
1.00
bio01
bio02
bio04
bio05
bio06
BDW
CEC
DES
NTO
SOC
0.0
0.2
0.4
0.6
Asteromyrtus symphyocarpa
Climate
Climate + Soil
Soil
AUC = 0.96
TSS = 0.77
AUC = 0.97
TSS = 0.76
AUC = 0.92
TSS = 0.56
Relative importance
bio04
bio05
bio06
bio12
bio13
CEC
DES
PTO
SOC
TWI

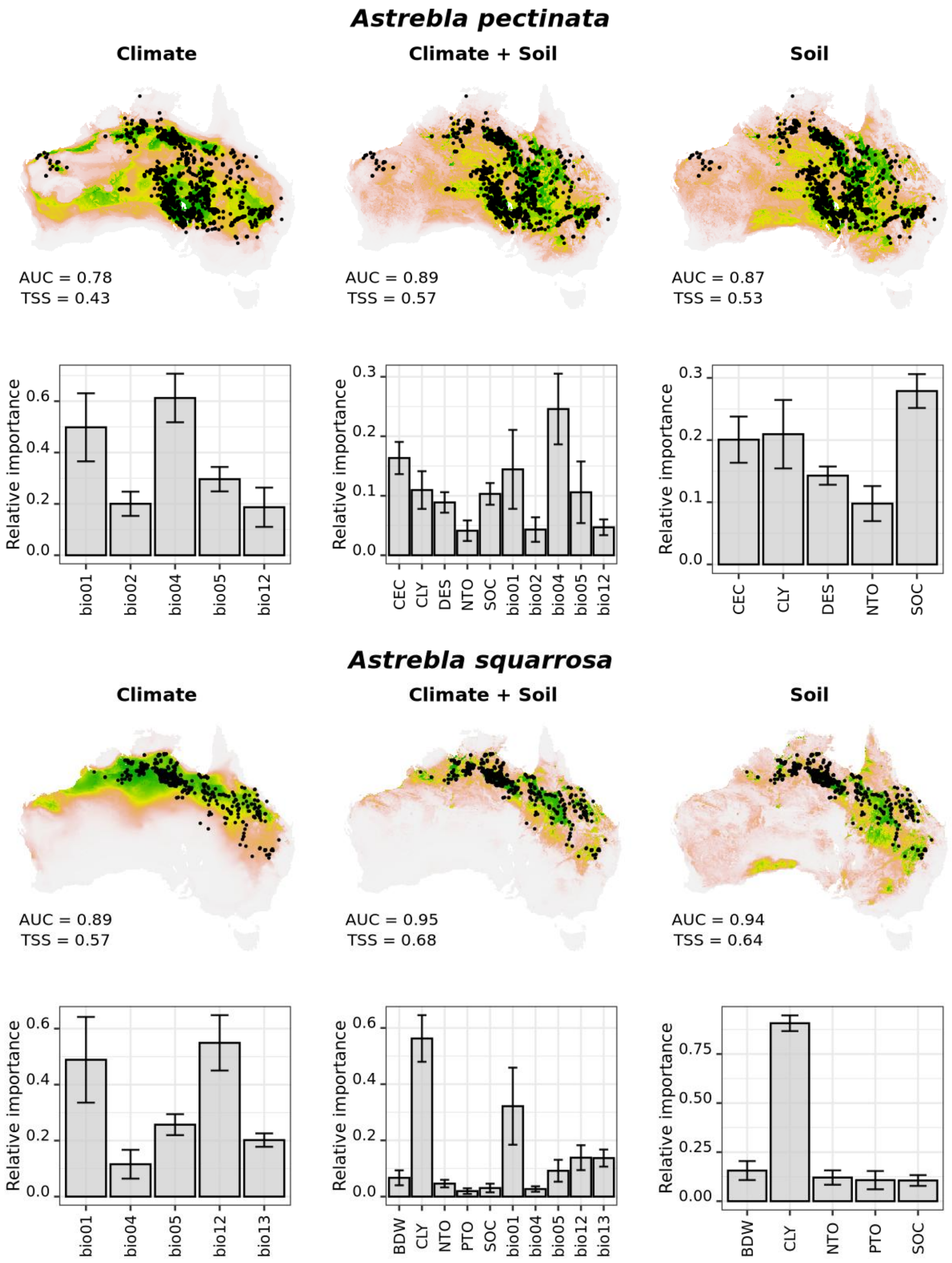
Astrebla pectinata
Climate
Climate + Soil
Soil
AUC = 0.78
TSS = 0.43
AUC = 0.89
TSS = 0.57
AUC = 0.87
TSS = 0.53
Relative importance
bio01
bio02
bio04
bio05
bio12
CEC
CLY
DES
NTO
SOC
Astrebla squarrosa
Climate
Climate + Soil
Soil
AUC = 0.89
TSS = 0.57
AUC = 0.95
TSS = 0.68
AUC = 0.94
TSS = 0.64
Relative importance
bio01
bio04
bio05
bio12
bio13
BDW
CLY
NTO
PTO
SOC

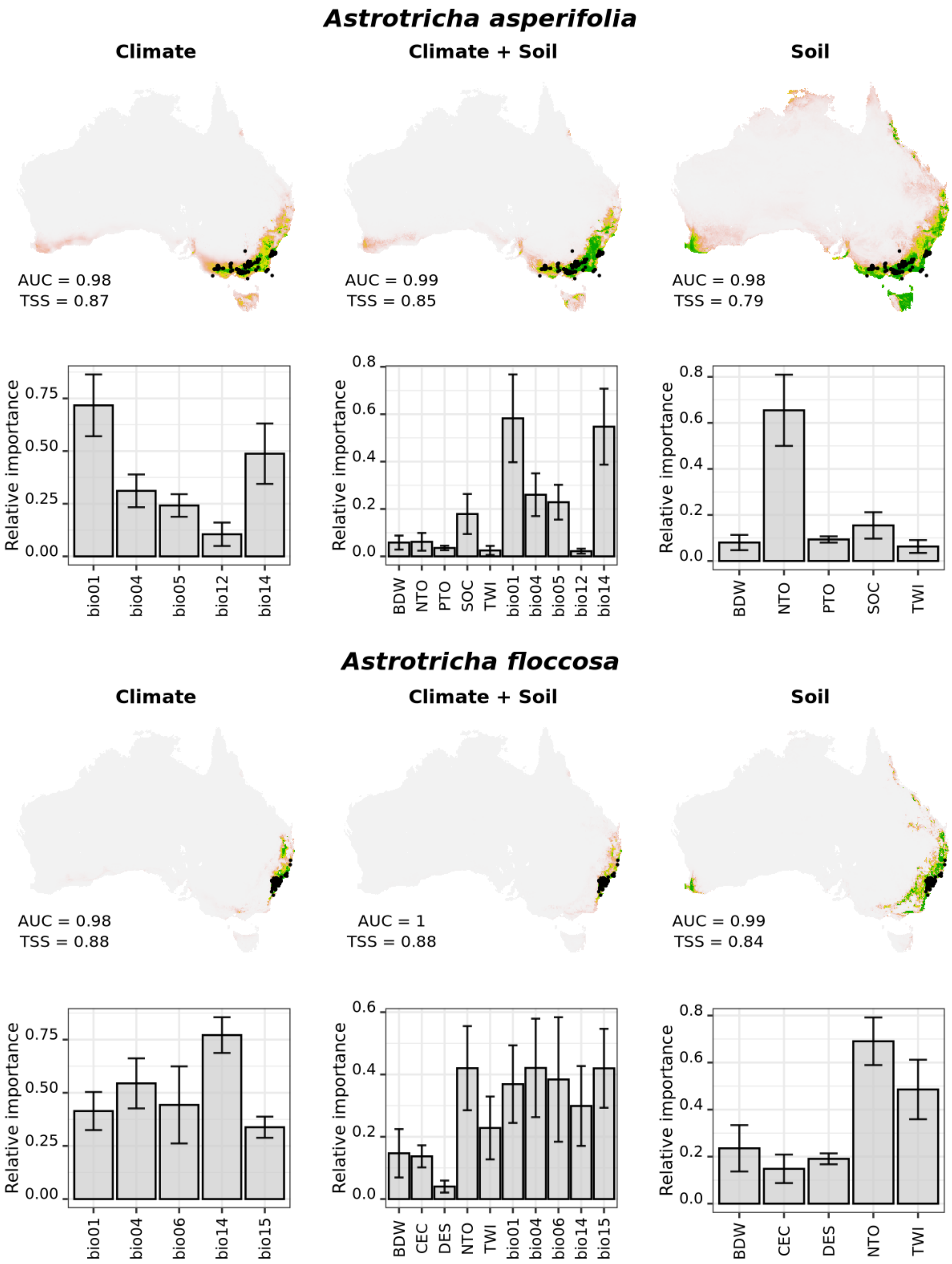

Astrotricha asperifolia
Climate
Climate + Soil
Soil
AUC = 0.98
TSS = 0.87
AUC = 0.99
TSS = 0.85
AUC = 0.98
TSS = 0.79
Relative importance
0.00
0.25
0.50
0.75
bio01
bio04
bio05
bio12
bio14
0.0
0.2
0.4
0.6
0.8
BDW
NTO
PTO
SOC
TWI
bio01
bio04
bio05
bio12
bio14
Astrotricha floccosa
Climate
Climate + Soil
Soil
AUC = 0.98
TSS = 0.88
AUC = 1
TSS = 0.88
AUC = 0.99
TSS = 0.84
bio01
bio04
bio06
bio14
bio15
BDW
CEC
DES
NTO
TWI
bio01
bio04
bio06
bio14
bio15

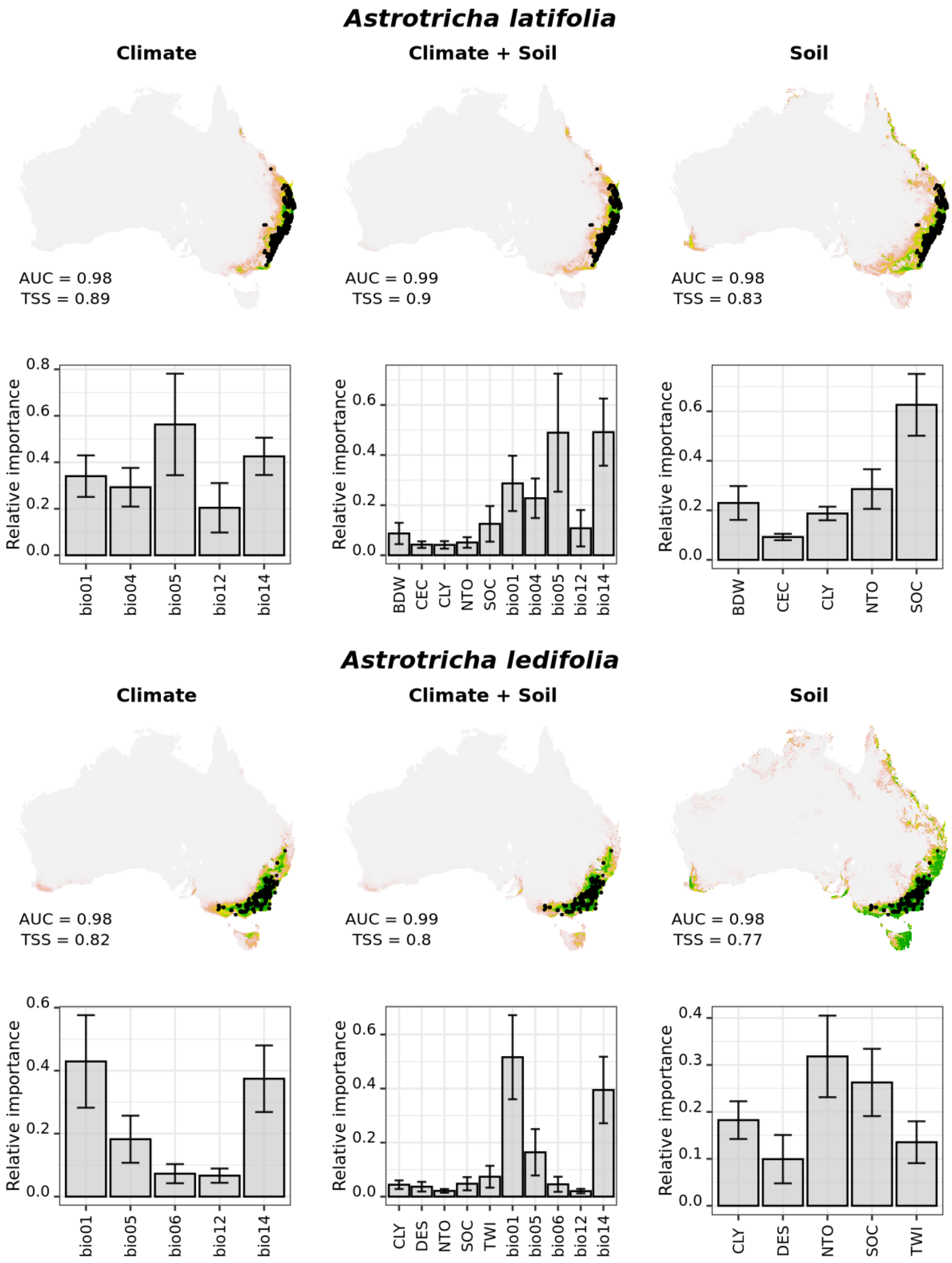
Astrotricha latifolia
Climate
Climate + Soil
Soil
AUC = 0.98
TSS = 0.89
AUC = 0.99
TSS = 0.9
AUC = 0.98
TSS = 0.83
Relative importance
bio01 bio04 bio05 bio12 bio14
BDW CEC CLY NTO SOC bio01 bio04 bio05 bio12 bio14
BDW CEC CLY NTO SOC
Astrotricha ledifolia
Climate
Climate + Soil
Soil
AUC = 0.98
TSS = 0.82
AUC = 0.99
TSS = 0.8
AUC = 0.98
TSS = 0.77
Relative importance
bio01 bio05 bio06 bio12 bio14
CLY DES NTO SOC TWI bio01 bio05 bio06 bio12 bio14
CLY DES NTO SOC TWI

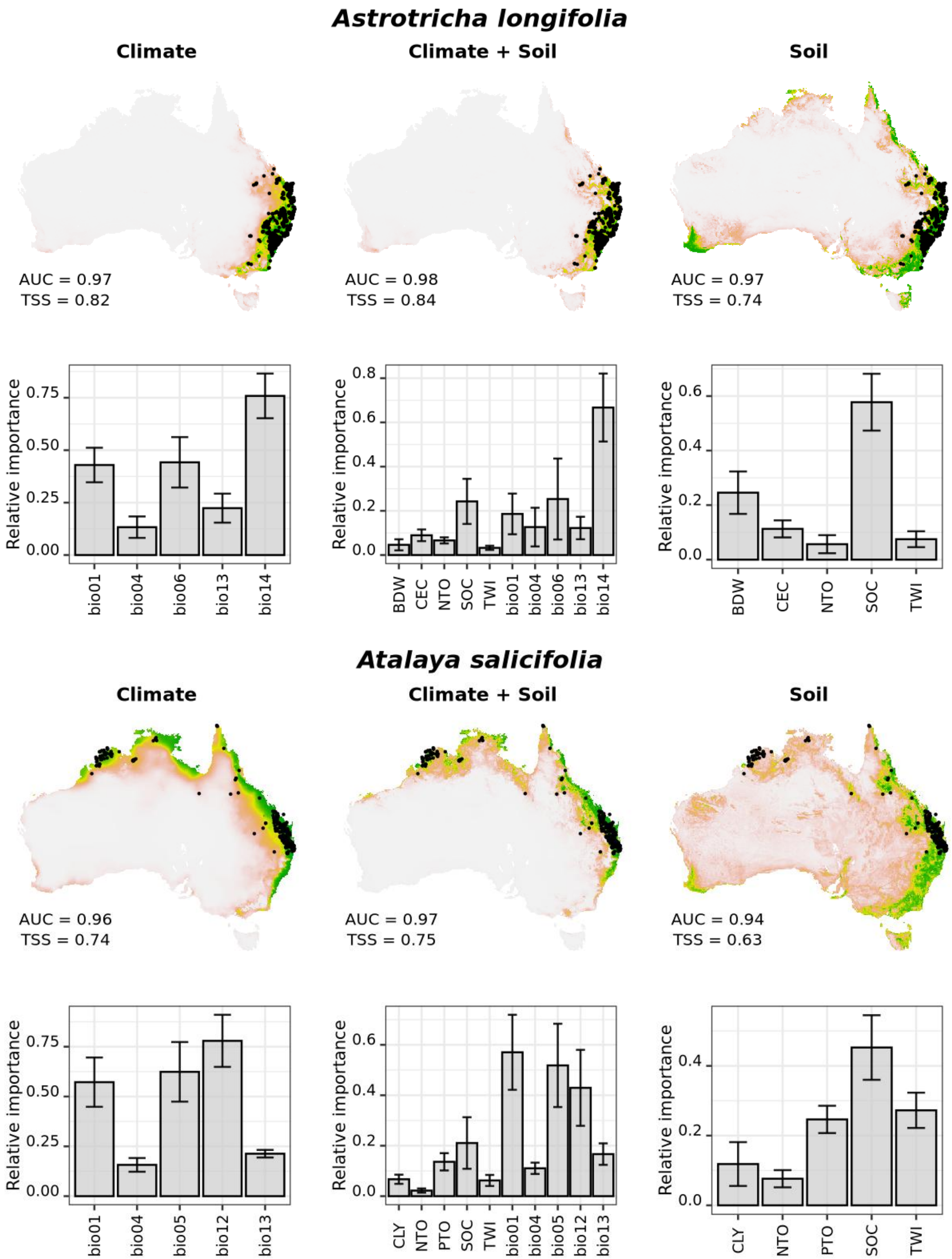
Astrotricha longifolia
Climate
Climate + Soil
Soil
AUC = 0.97
TSS = 0.82
AUC = 0.98
TSS = 0.84
AUC = 0.97
TSS = 0.74
Relative importance
0.00
0.25
0.50
0.75
bio01
bio04
bio06
bio13
bio14
0.0
0.2
0.4
0.6
0.8
BDW
CEC
NTO
SOC
TWI
bio01
bio04
bio06
bio13
bio14
0.0
0.2
0.4
0.6
BDW
CEC
NTO
SOC
TWI
Atalaya salicifolia
Climate
Climate + Soil
Soil
AUC = 0.96
TSS = 0.74
AUC = 0.97
TSS = 0.75
AUC = 0.94
TSS = 0.63
Relative importance
0.00
0.25
0.50
0.75
bio01
bio04
bio05
bio12
bio13
0.0
0.2
0.4
0.6
CLY
NTO
PTO
SOC
TWI
bio01
bio04
bio05
bio12
bio13
0.0
0.2
0.4
CLY
NTO
PTO
SOC
TWI

Atalaya variifolia

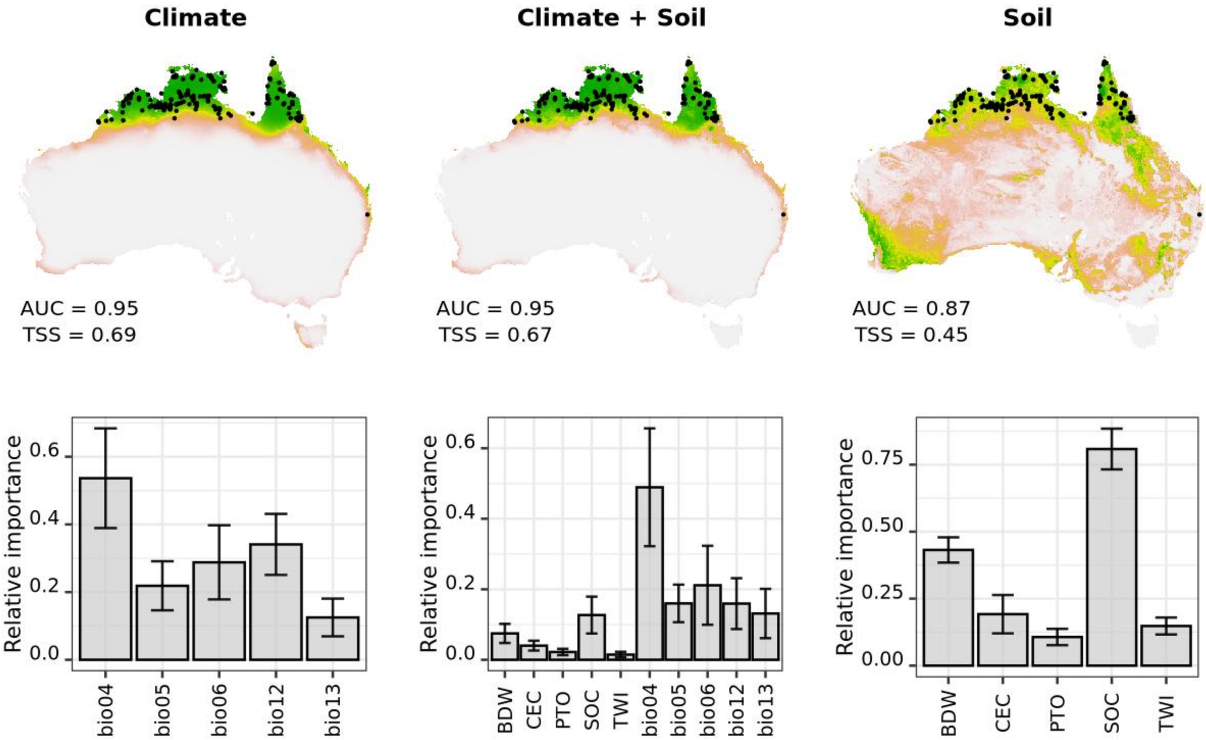

Atherosperma moschatum

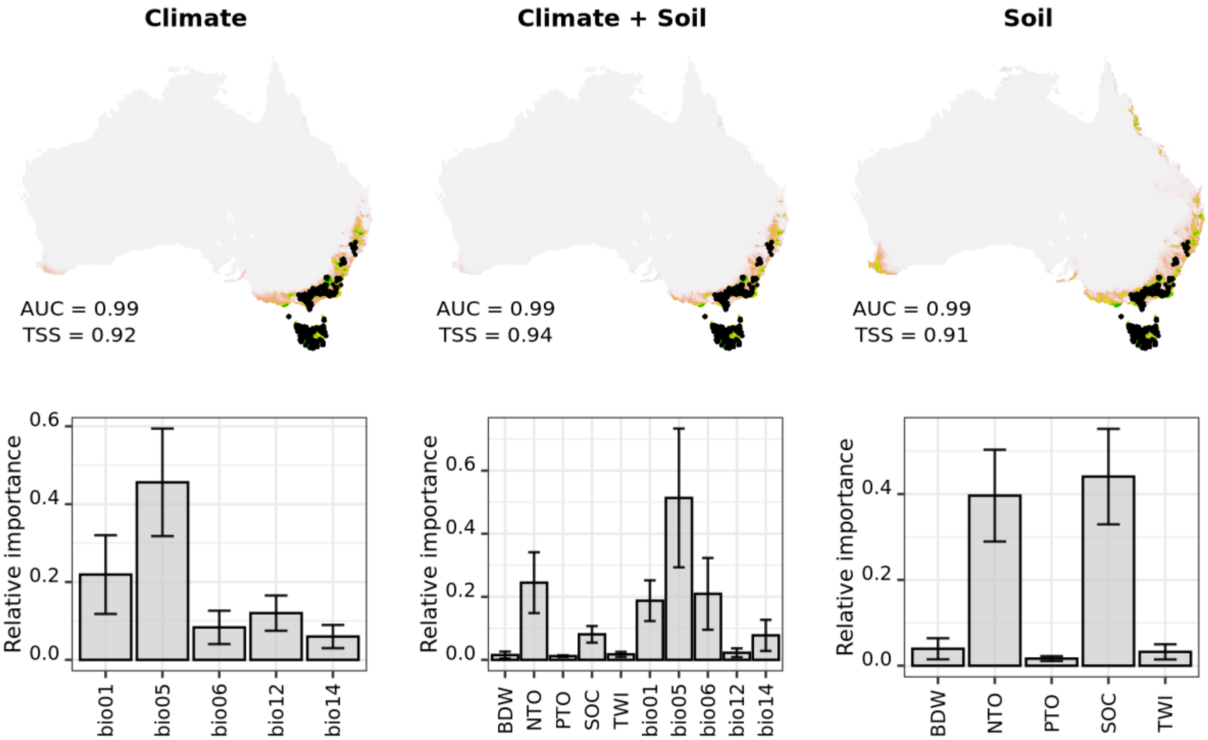

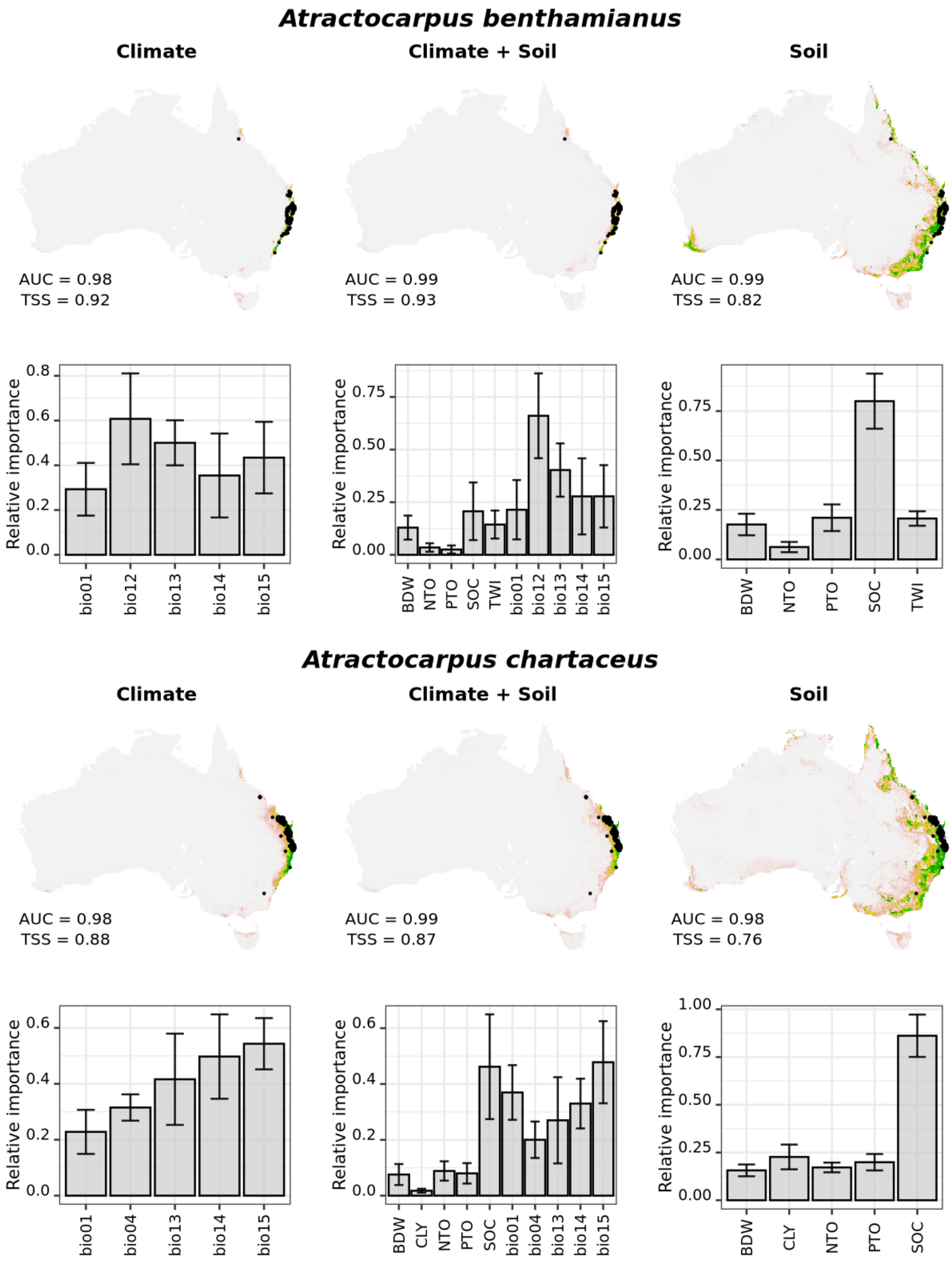
Atractocarpus benthamianus
Climate
Climate + Soil
Soil
AUC = 0.98
TSS = 0.92
AUC = 0.99
TSS = 0.93
AUC = 0.99
TSS = 0.82
Relative importance
0.0
0.2
0.4
0.6
0.8
bio01
bio12
bio13
bio14
bio15
Relative importance
0.00
0.25
0.50
0.75
BDW
NTO
PTO
SOC
TWI
bio01
bio12
bio13
bio14
bio15
Relative importance
0.00
0.25
0.50
0.75
BDW
NTO
PTO
SOC
TWI
Atractocarpus chartaceus
Climate
Climate + Soil
Soil
AUC = 0.98
TSS = 0.88
AUC = 0.99
TSS = 0.87
AUC = 0.98
TSS = 0.76
Relative importance
0.0
0.2
0.4
0.6
bio01
bio04
bio13
bio14
bio15
Relative importance
0.0
0.2
0.4
0.6
BDW
CLY
NTO
PTO
SOC
bio01
bio04
bio13
bio14
bio15
Relative importance
0.00
0.25
0.50
0.75
1.00
BDW
CLY
NTO
PTO
SOC

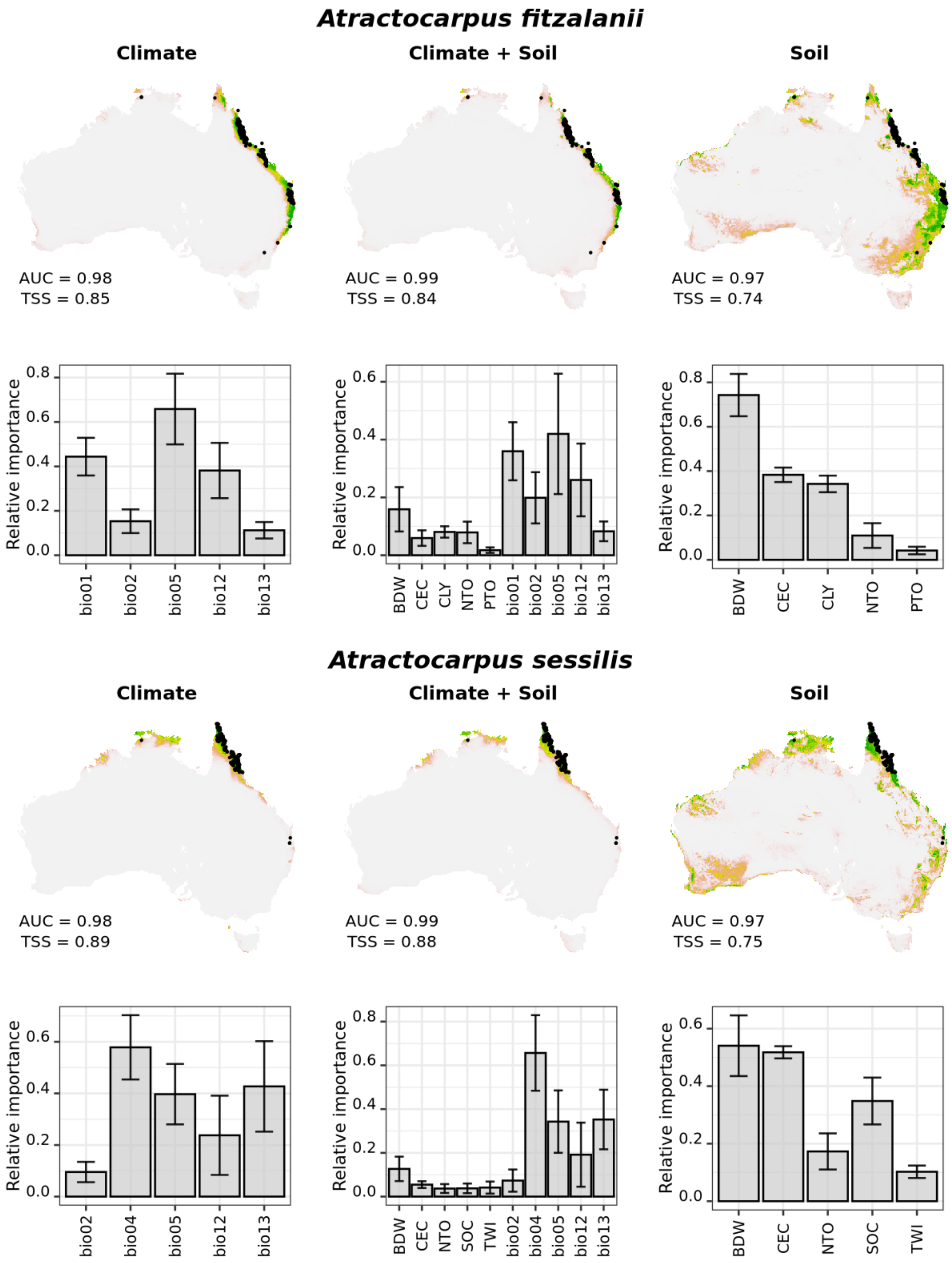

Atractocarpus fitzalanii
Climate
Climate + Soil
Soil
AUC = 0.98
TSS = 0.85
AUC = 0.99
TSS = 0.84
AUC = 0.97
TSS = 0.74
Relative importance
bio01
bio02
bio05
bio12
bio13
BDW
CEC
CLY
NTO
PTO
Atractocarpus sessilis
AUC = 0.98
TSS = 0.89
AUC = 0.99
TSS = 0.88
AUC = 0.97
TSS = 0.75
bio04
SOC
TWI

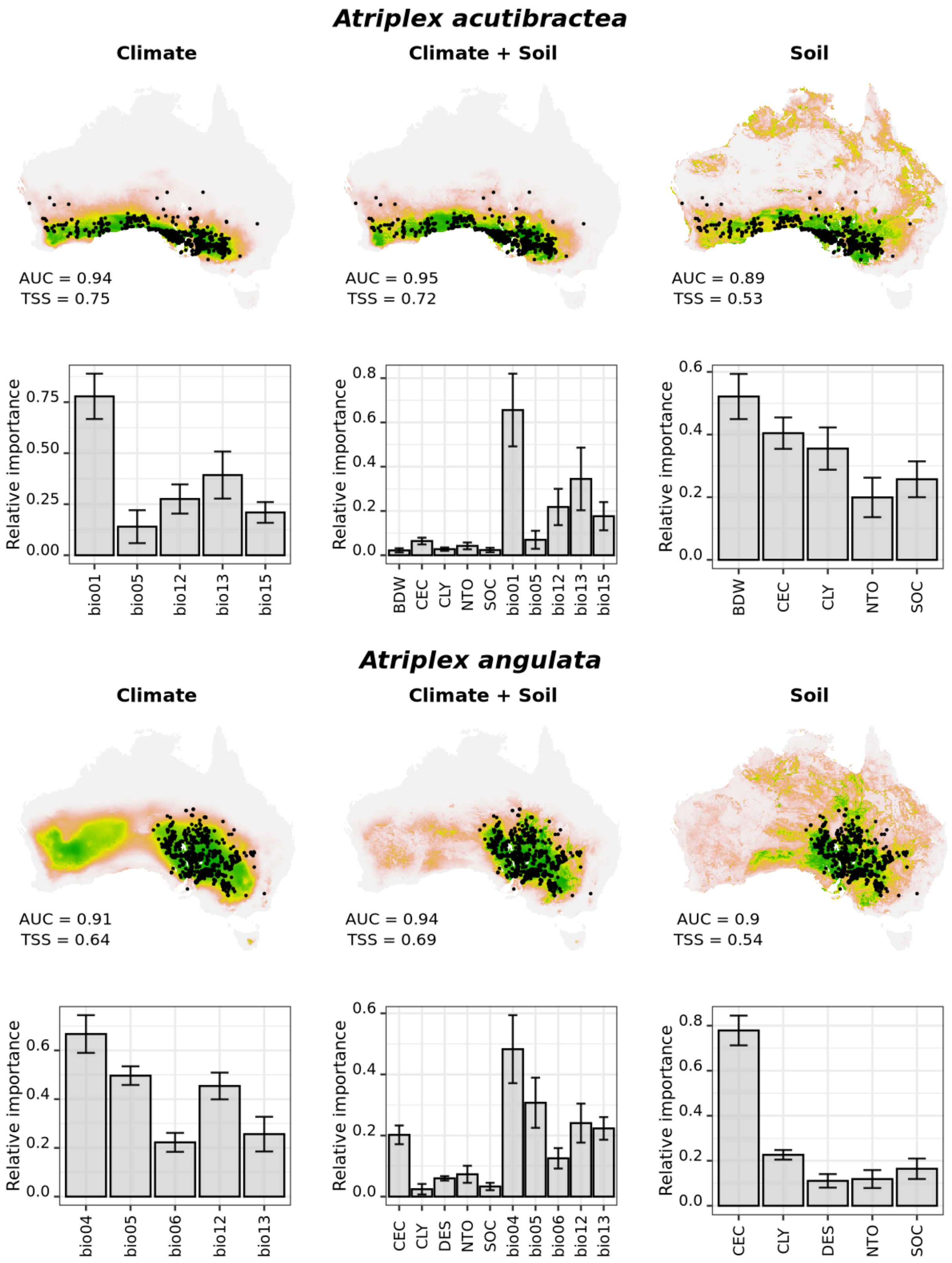

Atriplex acutibractea
Climate
Climate + Soil
Soil
AUC = 0.94
TSS = 0.75
AUC = 0.95
TSS = 0.72
AUC = 0.89
TSS = 0.53
Relative importance
bio01
bio05
bio12
bio13
bio15
BDW
CEC
CLY
NTO
SOC
Atriplex angulata
Climate
Climate + Soil
Soil
AUC = 0.91
TSS = 0.64
AUC = 0.94
TSS = 0.69
AUC = 0.9
TSS = 0.54
Relative importance
bio04
bio05
bio06
bio12
bio13
CEC
CLY
DES
NTO
SOC

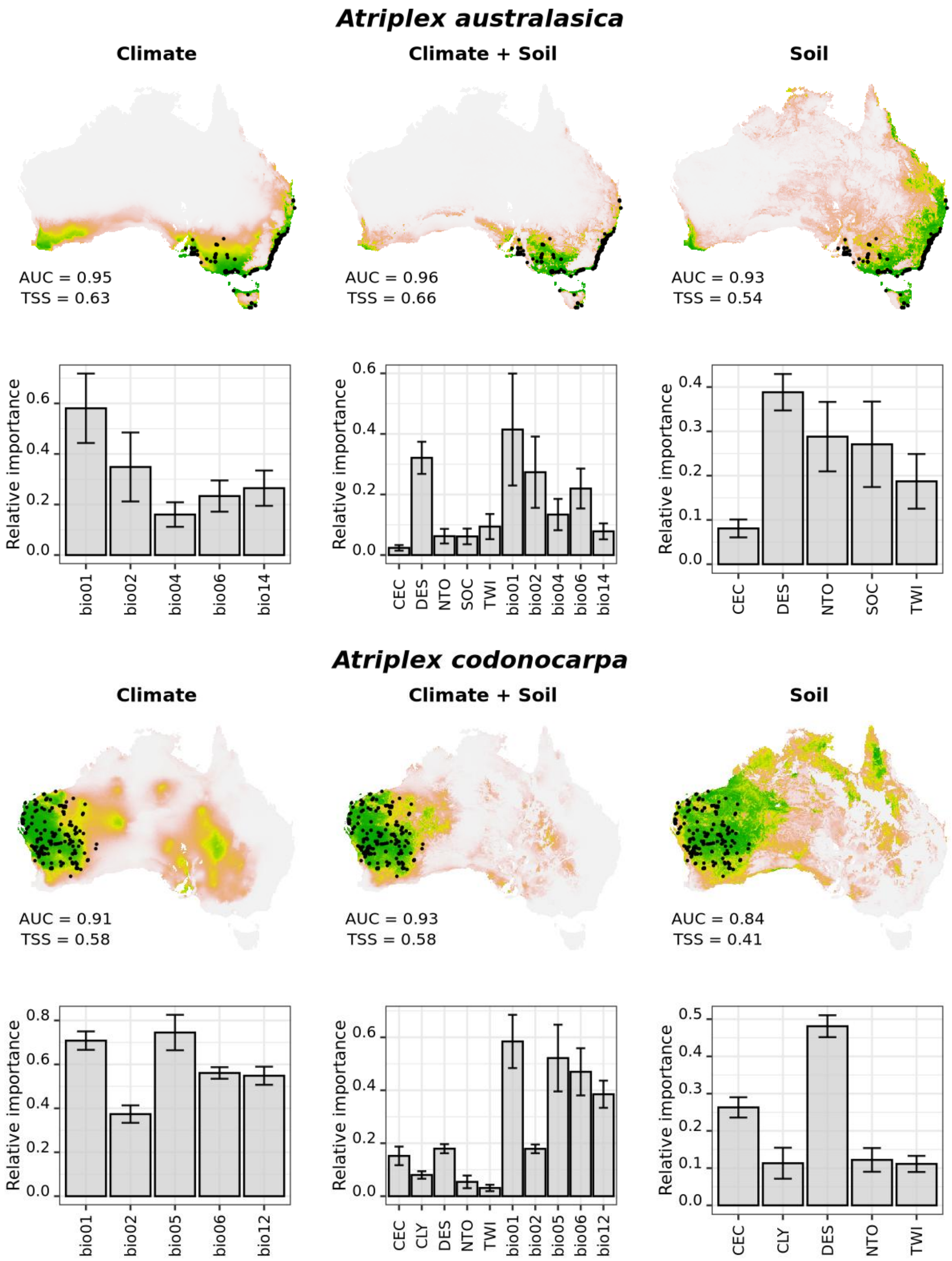
Atriplex australasica
Climate
Climate + Soil
Soil
AUC = 0.95
TSS = 0.63
AUC = 0.96
TSS = 0.66
AUC = 0.93
TSS = 0.54
Relative importance
bio01
bio02
bio04
bio06
bio14
CEC
DES
NTO
SOC
TWI
Atriplex codonocarpa
Climate
Climate + Soil
Soil
AUC = 0.91
TSS = 0.58
AUC = 0.93
TSS = 0.58
AUC = 0.84
TSS = 0.41
Relative importance
bio01
bio02
bio05
bio06
bio12
CEC
CLY
DES
NTO
TWI

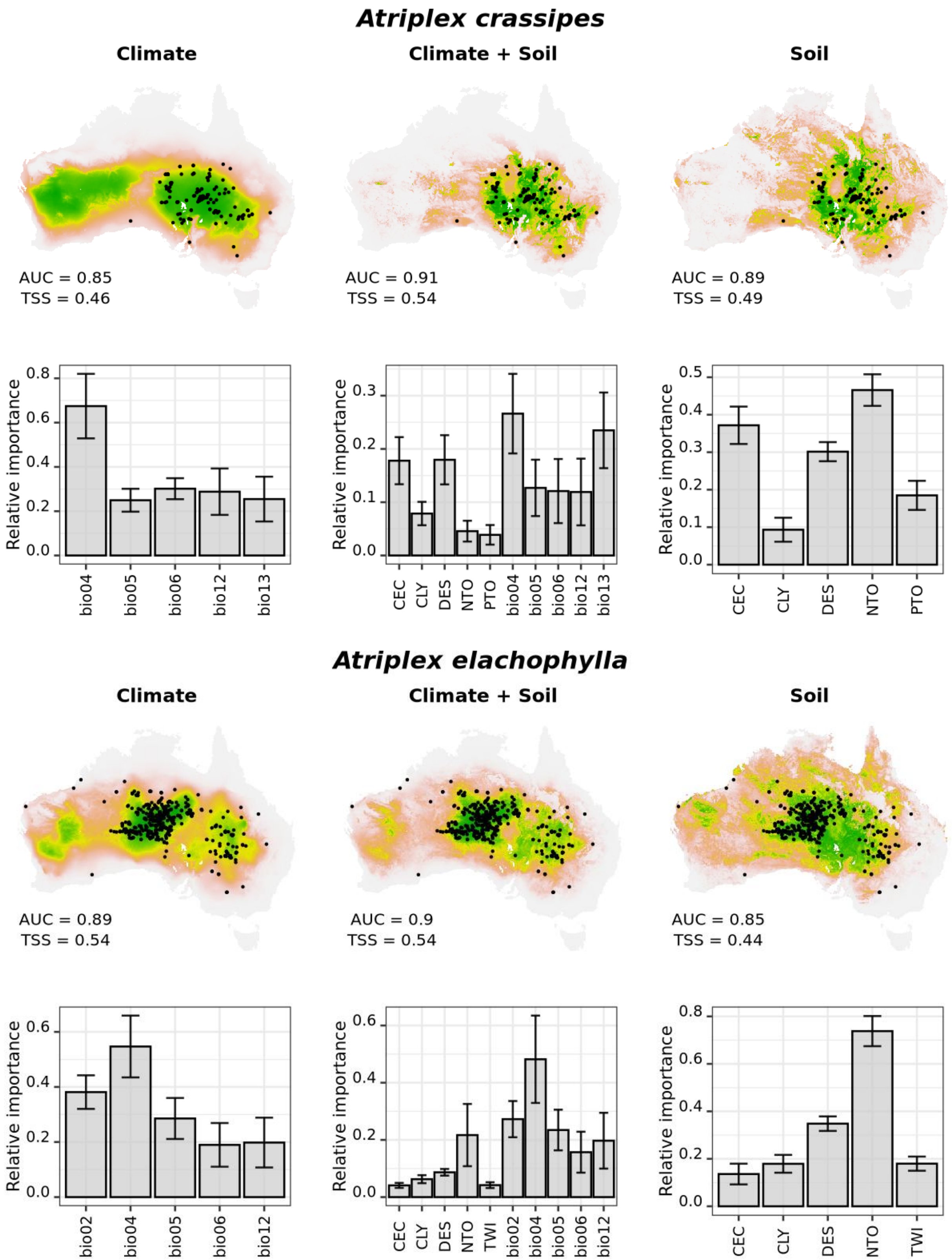

Atriplex crassipes
Climate
Climate + Soil
Soil
AUC = 0.85
TSS = 0.46
AUC = 0.91
TSS = 0.54
AUC = 0.89
TSS = 0.49
Relative importance
bio04
bio05
bio06
bio12
bio13
CEC
CLY
DES
NTO
PTO
Atriplex elachophylla
Climate
Climate + Soil
Soil
AUC = 0.89
TSS = 0.54
AUC = 0.9
TSS = 0.54
AUC = 0.85
TSS = 0.44
Relative importance
bio02
bio04
bio05
bio06
bio12
CEC
CLY
DES
NTO
TWI

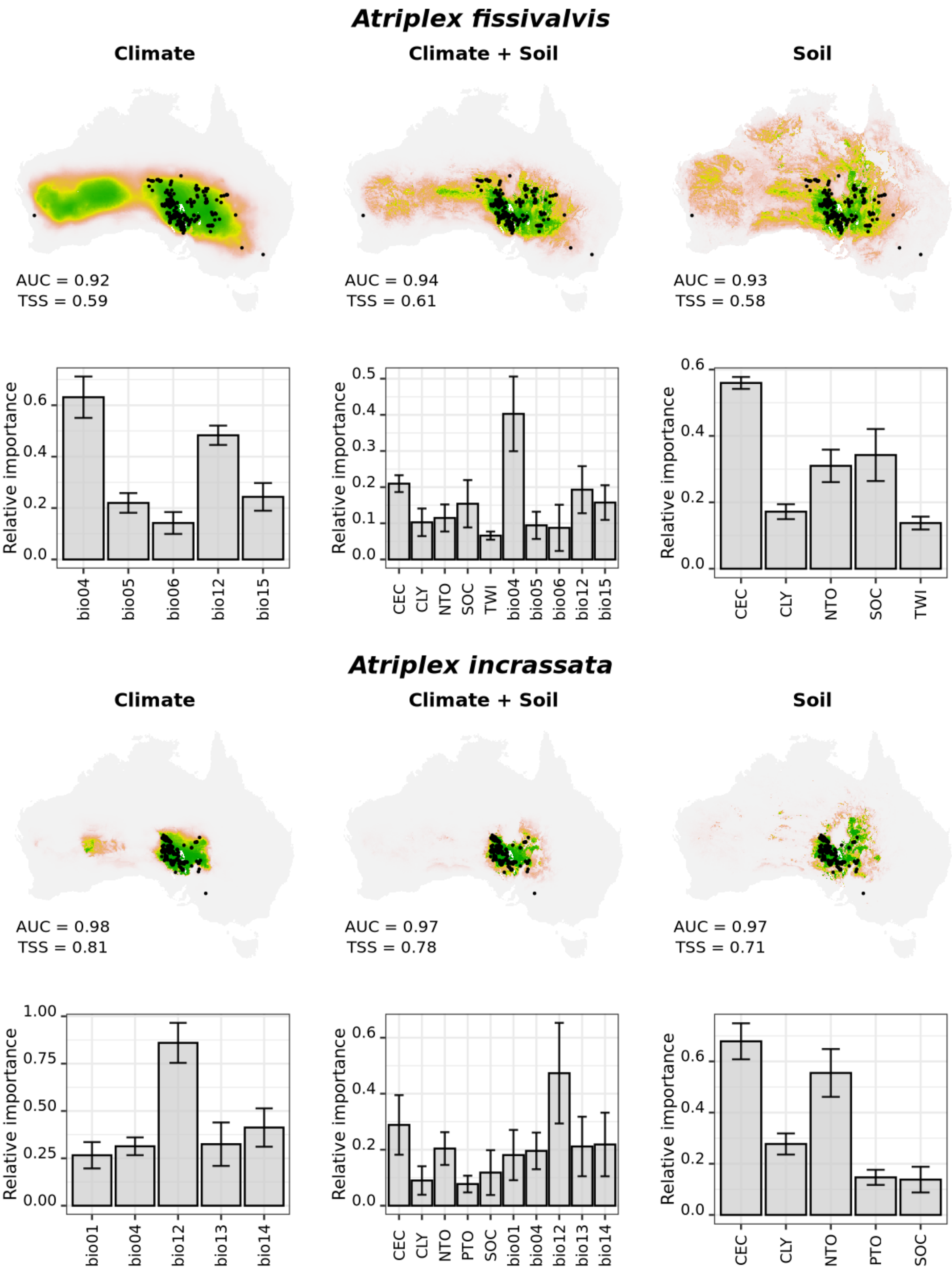
Atriplex fissivalvis
Climate
Climate + Soil
Soil
AUC = 0.92
TSS = 0.59
AUC = 0.94
TSS = 0.61
AUC = 0.93
TSS = 0.58
Relative importance
bio04
bio05
bio06
bio12
bio15
CEC
CLY
NTO
SOC
TWI
Atriplex incrassata
Climate
Climate + Soil
Soil
AUC = 0.98
TSS = 0.81
AUC = 0.97
TSS = 0.78
AUC = 0.97
TSS = 0.71
Relative importance
bio01
bio04
bio12
bio13
bio14
CEC
CLY
NTO
PTO
SOC

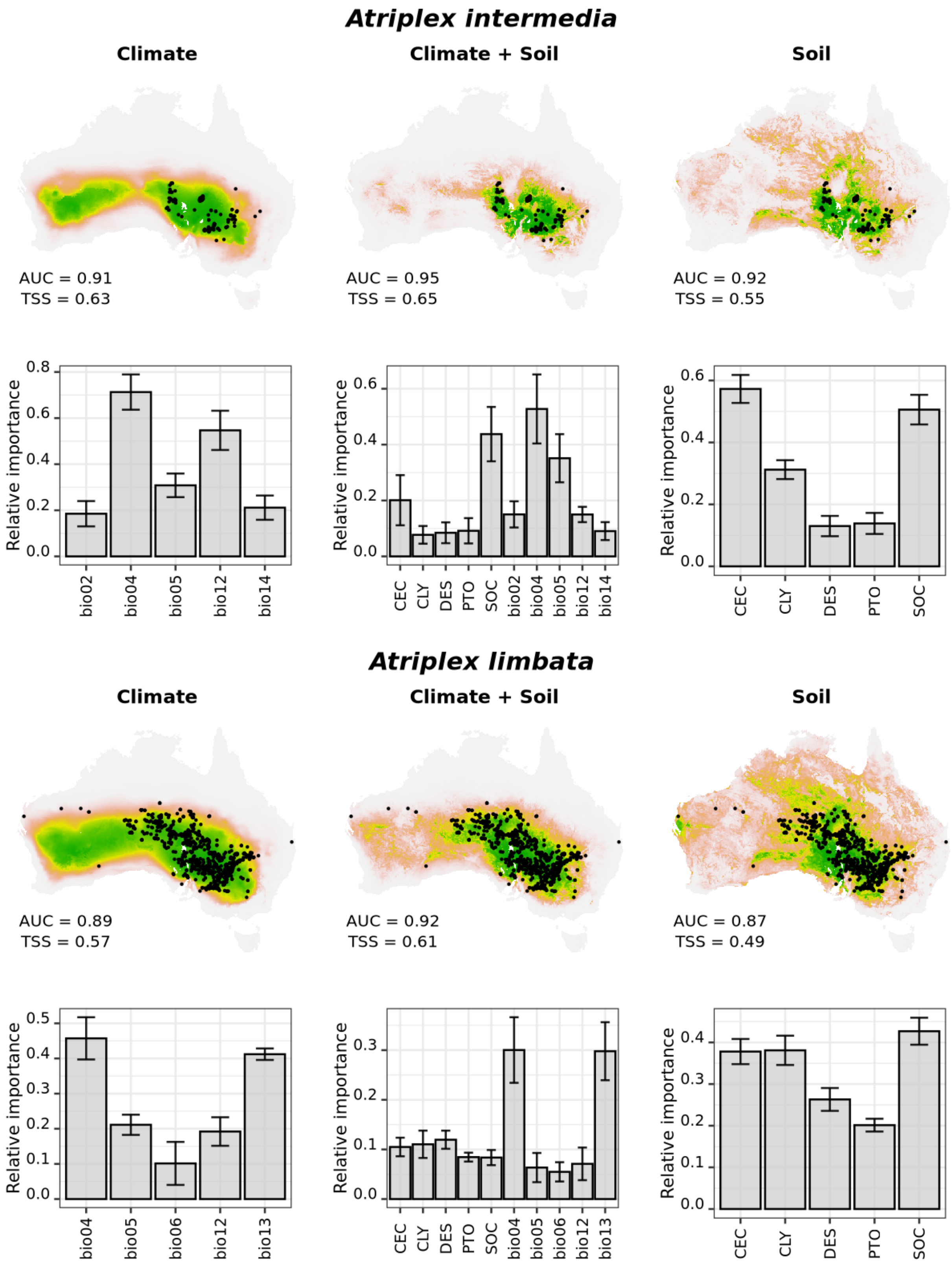
Atriplex intermedia
Climate
Climate + Soil
Soil
AUC = 0.91
TSS = 0.63
AUC = 0.95
TSS = 0.65
AUC = 0.92
TSS = 0.55
Relative importance
bio02
bio04
bio05
bio12
bio14
CEC
CLY
DES
PTO
SOC
Atriplex limbata
Climate
Climate + Soil
Soil
AUC = 0.89
TSS = 0.57
AUC = 0.92
TSS = 0.61
AUC = 0.87
TSS = 0.49
bio04
bio05
bio06
bio12
bio13

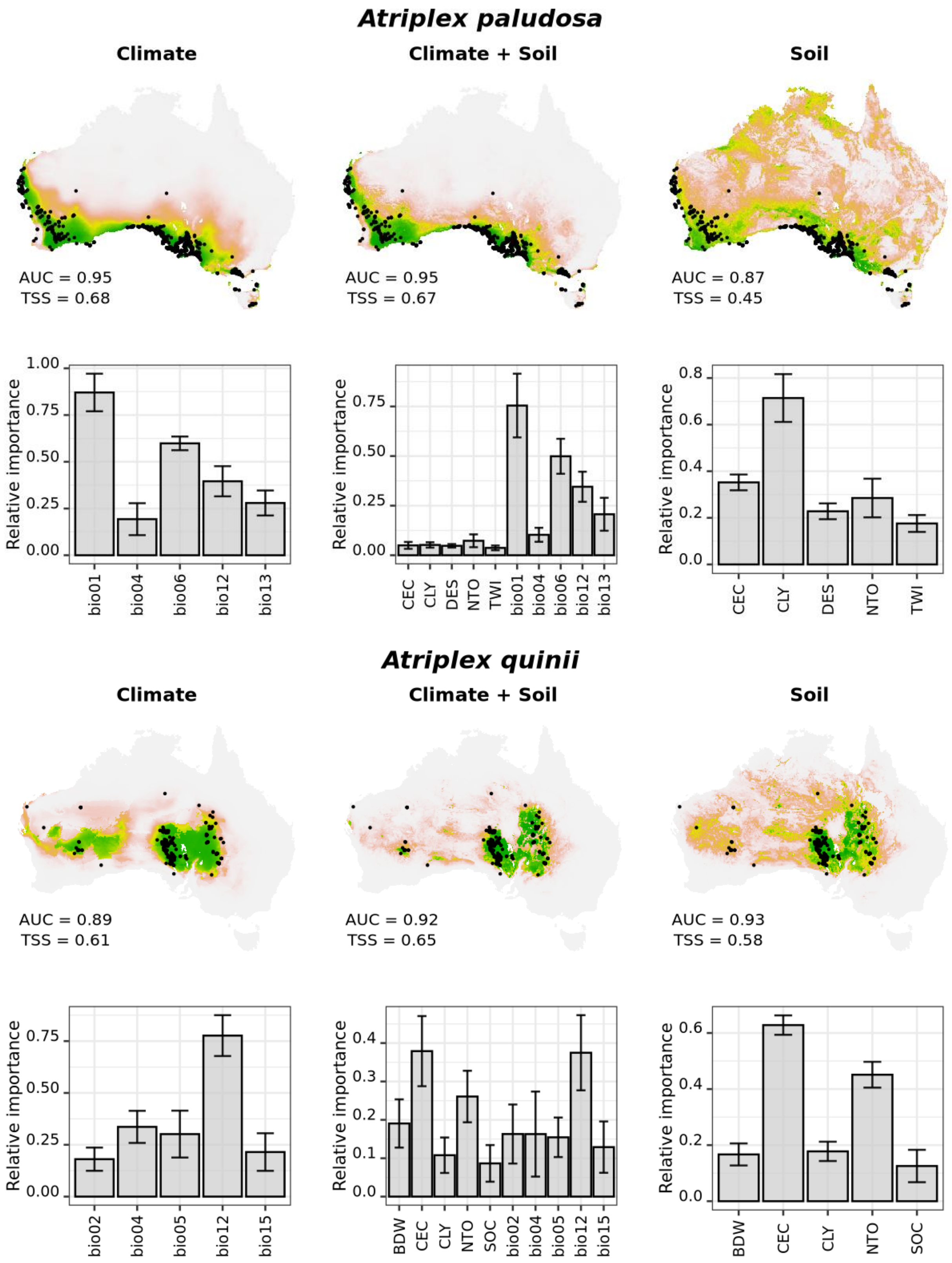

Atriplex paludosa
Climate
Climate + Soil
Soil
AUC = 0.95
TSS = 0.68
AUC = 0.95
TSS = 0.67
AUC = 0.87
TSS = 0.45
Relative importance
bio01
bio04
bio06
bio12
bio13
CEC
CLY
DES
NTO
TWI
Atriplex quinii
Climate
Climate + Soil
Soil
AUC = 0.89
TSS = 0.61
AUC = 0.92
TSS = 0.65
AUC = 0.93
TSS = 0.58
Relative importance
bio02
bio04
bio05
bio12
bio15
BDW
CEC
CLY
NTO
SOC

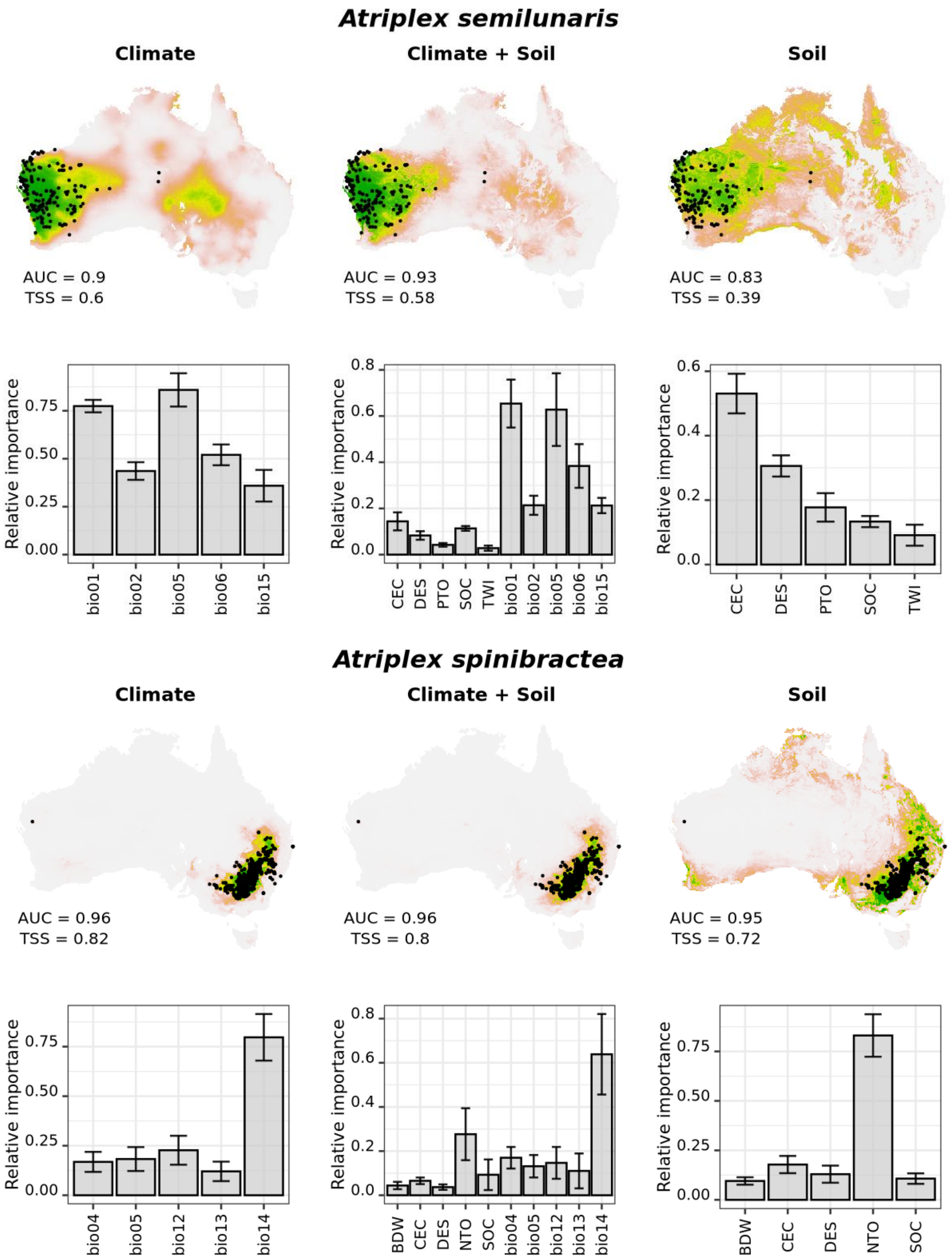
Atriplex semilunaris
Climate
Climate + Soil
Soil
AUC = 0.9
TSS = 0.6
AUC = 0.93
TSS = 0.58
AUC = 0.83
TSS = 0.39
Relative importance
0.00
0.25
0.50
0.75
bio01
bio02
bio05
bio06
bio15
0.0
0.2
0.4
0.6
0.8
CEC
DES
PTO
SOC
TWI
bio01
bio02
bio05
bio06
bio15
0.0
0.2
0.4
0.6
CEC
DES
PTO
SOC
TWI
Atriplex spinibractea
Climate
Climate + Soil
Soil
AUC = 0.96
TSS = 0.82
AUC = 0.96
TSS = 0.8
AUC = 0.95
TSS = 0.72
Relative importance
0.00
0.25
0.50
0.75
bio04
bio05
bio12
bio13
bio14
0.0
0.2
0.4
0.6
0.8
BDW
CEC
DES
NTO
SOC
bio04
bio05
bio12
bio13
bio14
0.00
0.25
0.50
0.75
BDW
CEC
DES
NTO
SOC

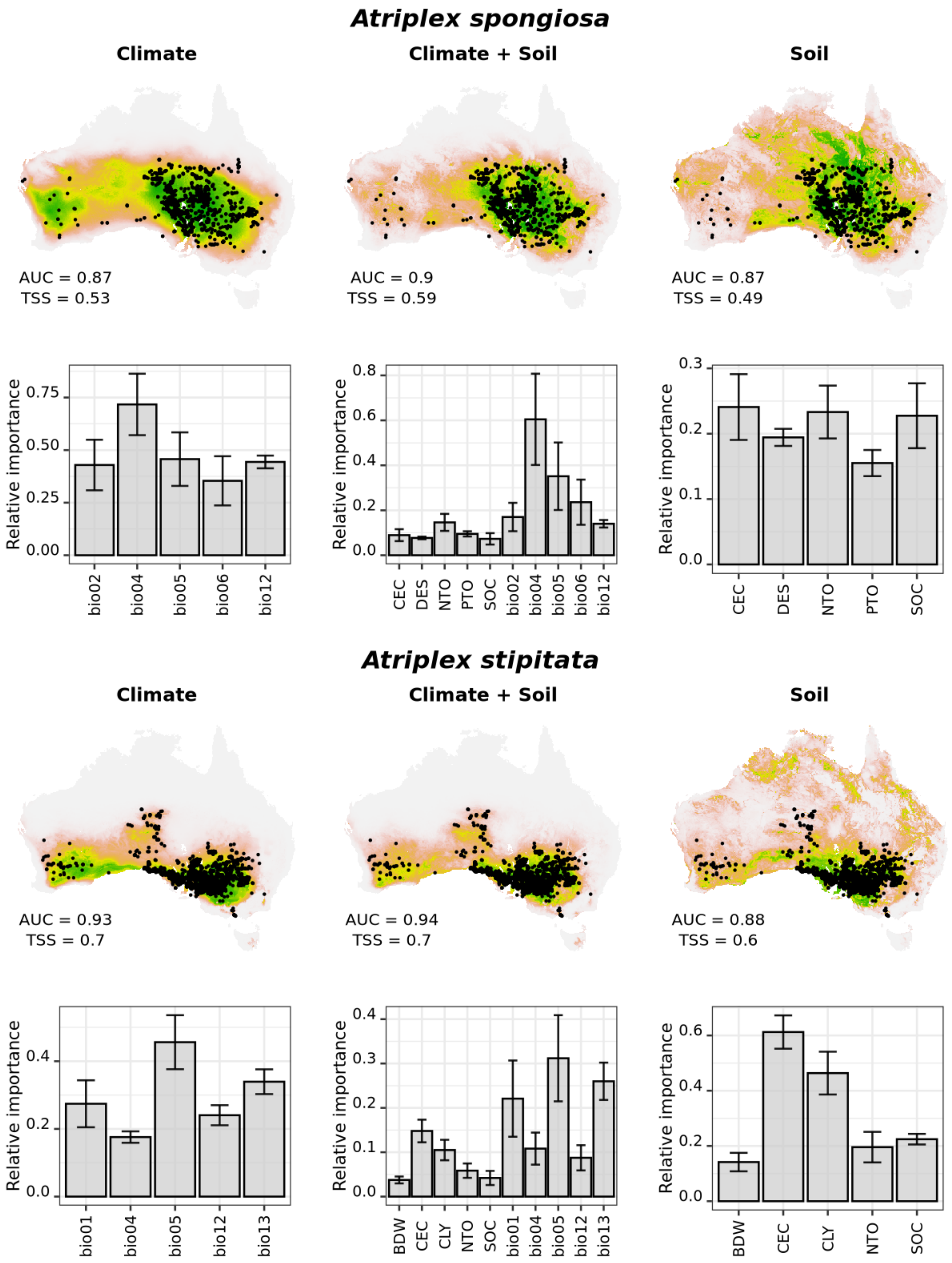
Atriplex spongiosa
Climate
Climate + Soil
Soil
AUC = 0.87
TSS = 0.53
AUC = 0.9
TSS = 0.59
AUC = 0.87
TSS = 0.49
Relative importance
bio02
bio04
bio05
bio06
bio12
CEC
DES
NTO
PTO
SOC
Atriplex stipitata
AUC = 0.93
TSS = 0.7
AUC = 0.94
TSS = 0.7
AUC = 0.88
TSS = 0.6
bio01
bio13
BDW
CLY

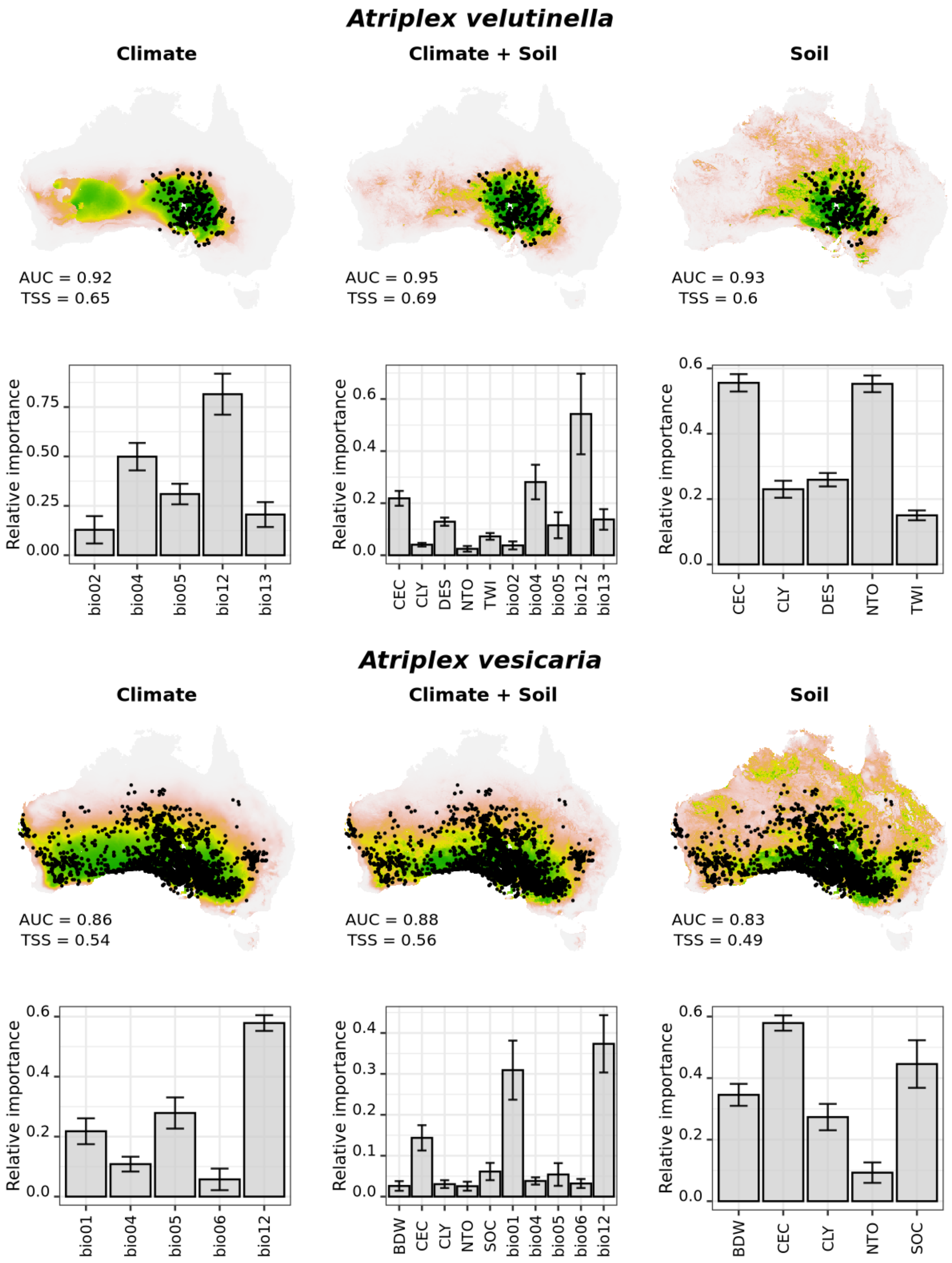

Atriplex velutinella
Climate
Climate + Soil
Soil
AUC = 0.92
TSS = 0.65
AUC = 0.95
TSS = 0.69
AUC = 0.93
TSS = 0.6
Relative importance
0.00
0.25
0.50
0.75
bio02
bio04
bio05
bio12
bio13
0.0
0.2
0.4
0.6
CEC
CLY
DES
NTO
TWI
bio02
bio04
bio05
bio12
bio13
CEC
CLY
DES
NTO
TWI
Atriplex vesicaria
Climate
Climate + Soil
Soil
AUC = 0.86
TSS = 0.54
AUC = 0.88
TSS = 0.56
AUC = 0.83
TSS = 0.49
Relative importance
0.0
0.2
0.4
0.6
bio01
bio04
bio05
bio06
bio12
0.0
0.1
0.2
0.3
0.4
BDW
CEC
CLY
NTO
SOC
bio01
bio04
bio05
bio06
bio12
BDW
CEC
CLY
NTO
SOC

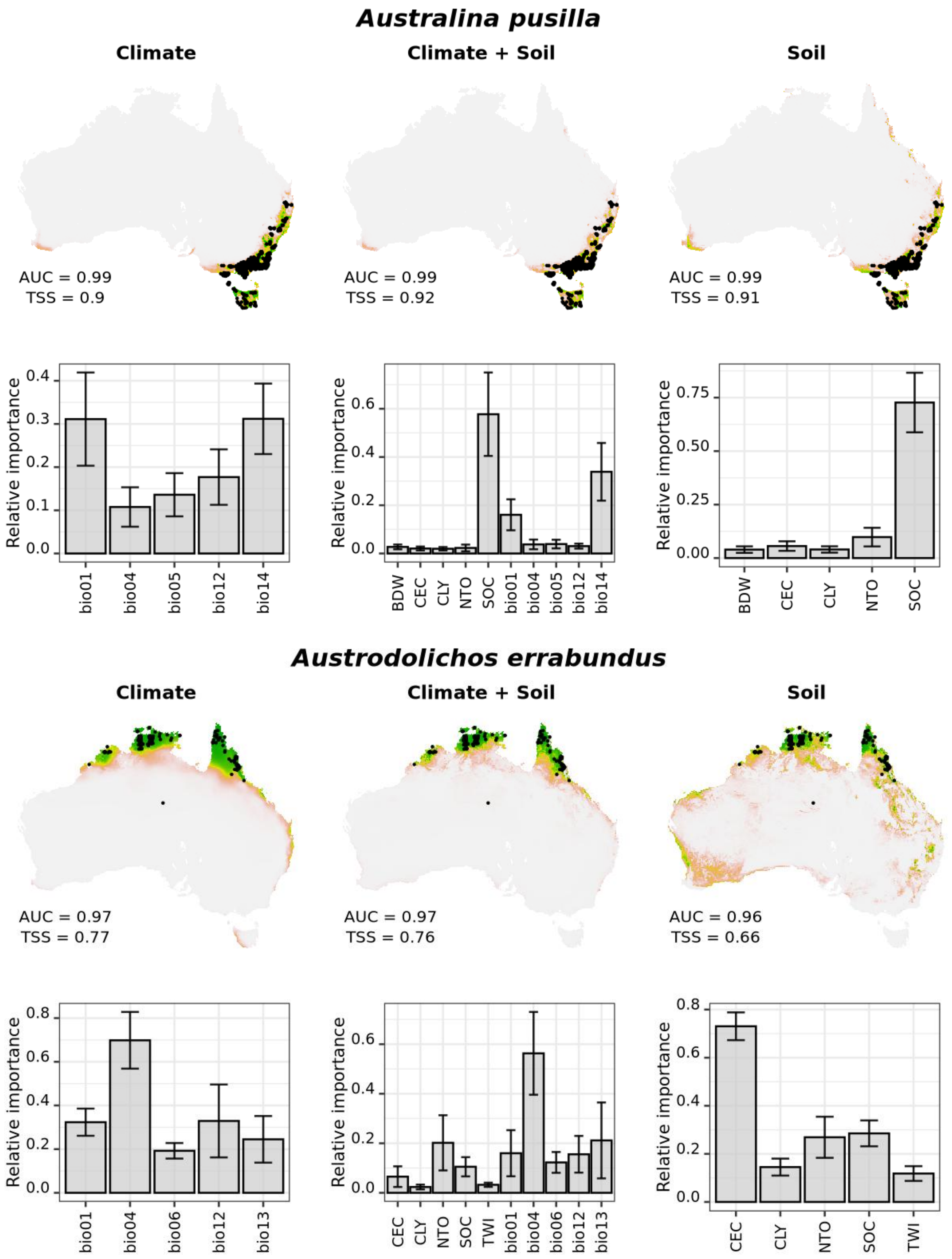
Australina pusilla
Climate
Climate + Soil
Soil
AUC = 0.99
TSS = 0.9
AUC = 0.99
TSS = 0.92
AUC = 0.99
TSS = 0.91
Relative importance
bio01
bio04
bio05
bio12
bio14
BDW
CEC
CLY
NTO
SOC
Austrodolichos errabundus
AUC = 0.97
TSS = 0.77
AUC = 0.97
TSS = 0.76
AUC = 0.96
TSS = 0.66
bio06
bio13
TWI

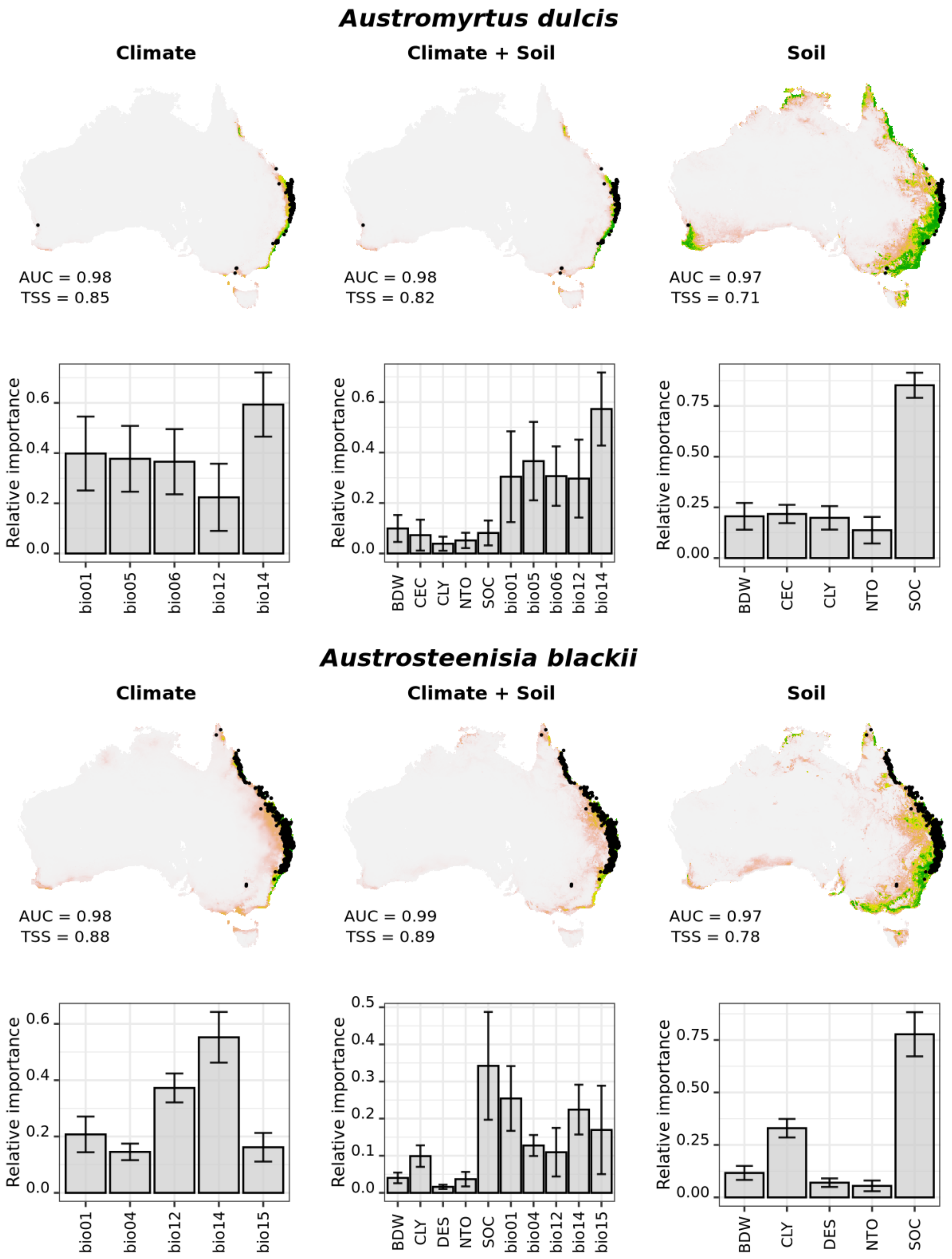
Austromyrtus dulcis
Climate
Climate + Soil
Soil
AUC = 0.98
TSS = 0.85
AUC = 0.98
TSS = 0.82
AUC = 0.97
TSS = 0.71
Relative importance
0.0
0.2
0.4
0.6
bio01
bio05
bio06
bio12
bio14
BDW
CEC
CLY
NTO
SOC
0.00
0.25
0.50
0.75
Austrosteenisia blackii
Climate
Climate + Soil
Soil
AUC = 0.98
TSS = 0.88
AUC = 0.99
TSS = 0.89
AUC = 0.97
TSS = 0.78
bio04
bio15
0.1
0.3
0.5
DES

Austrosteenisia glabristyla

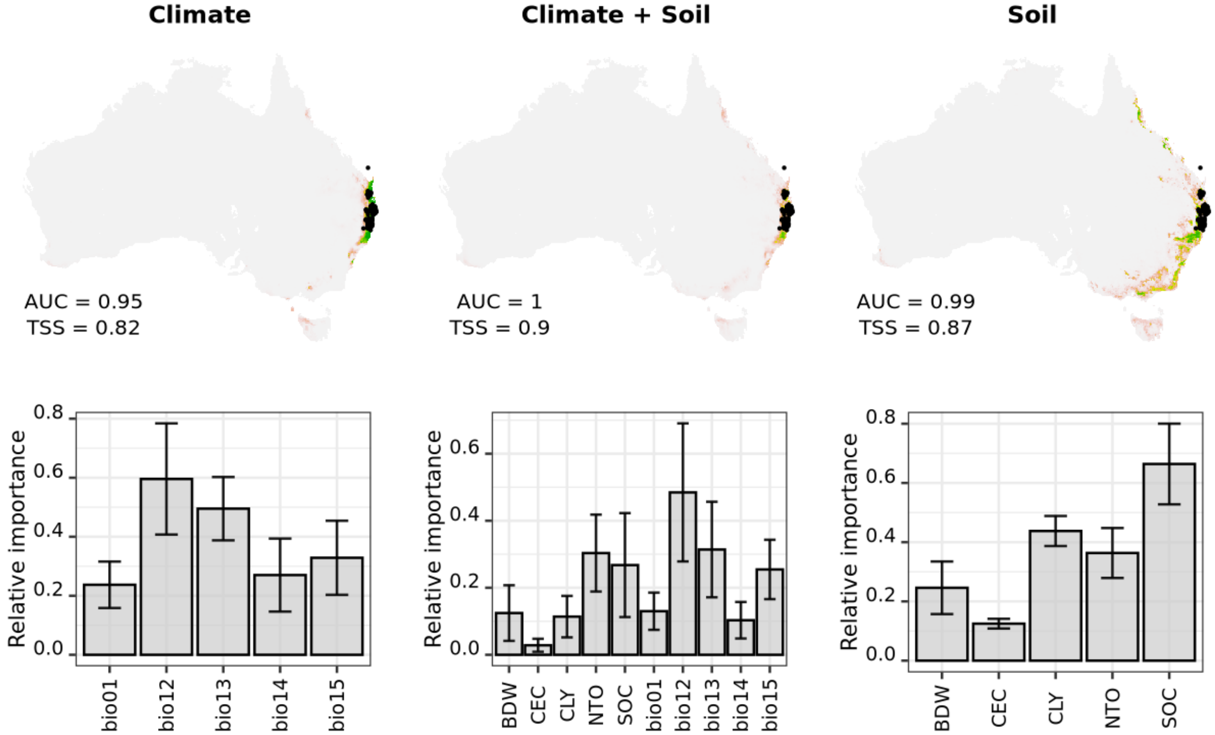

Austrostipa aristiglumis

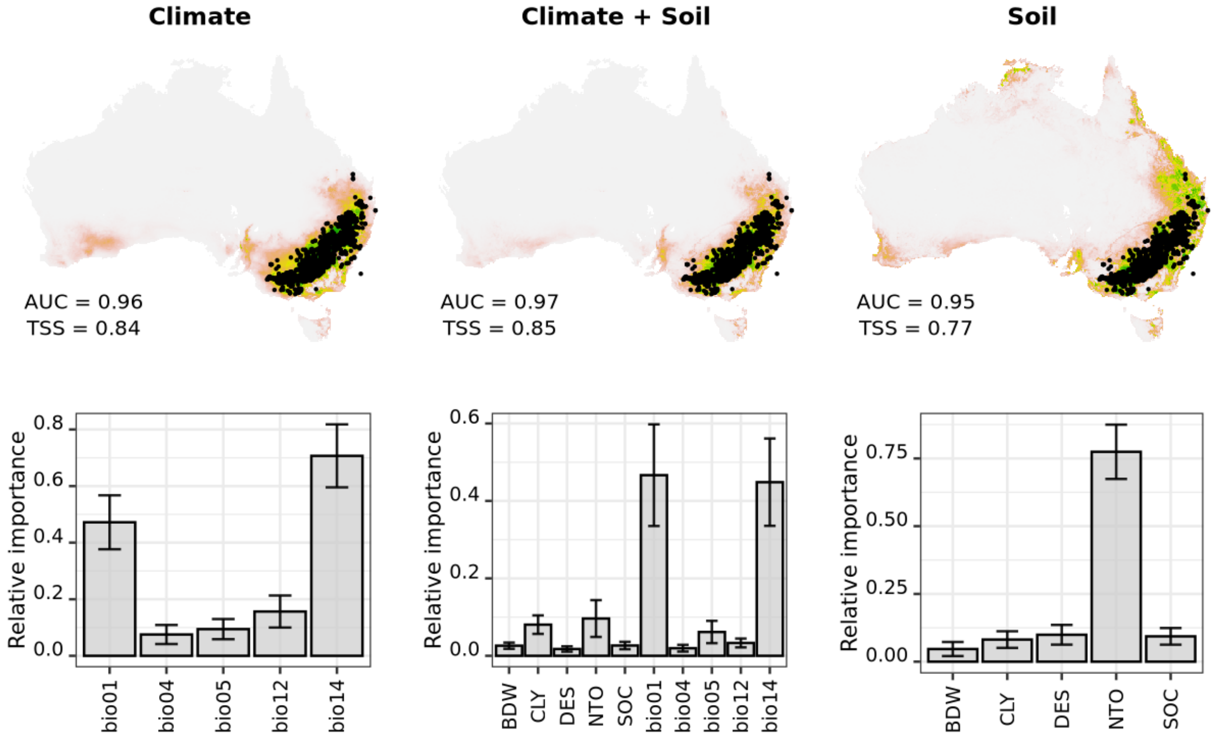

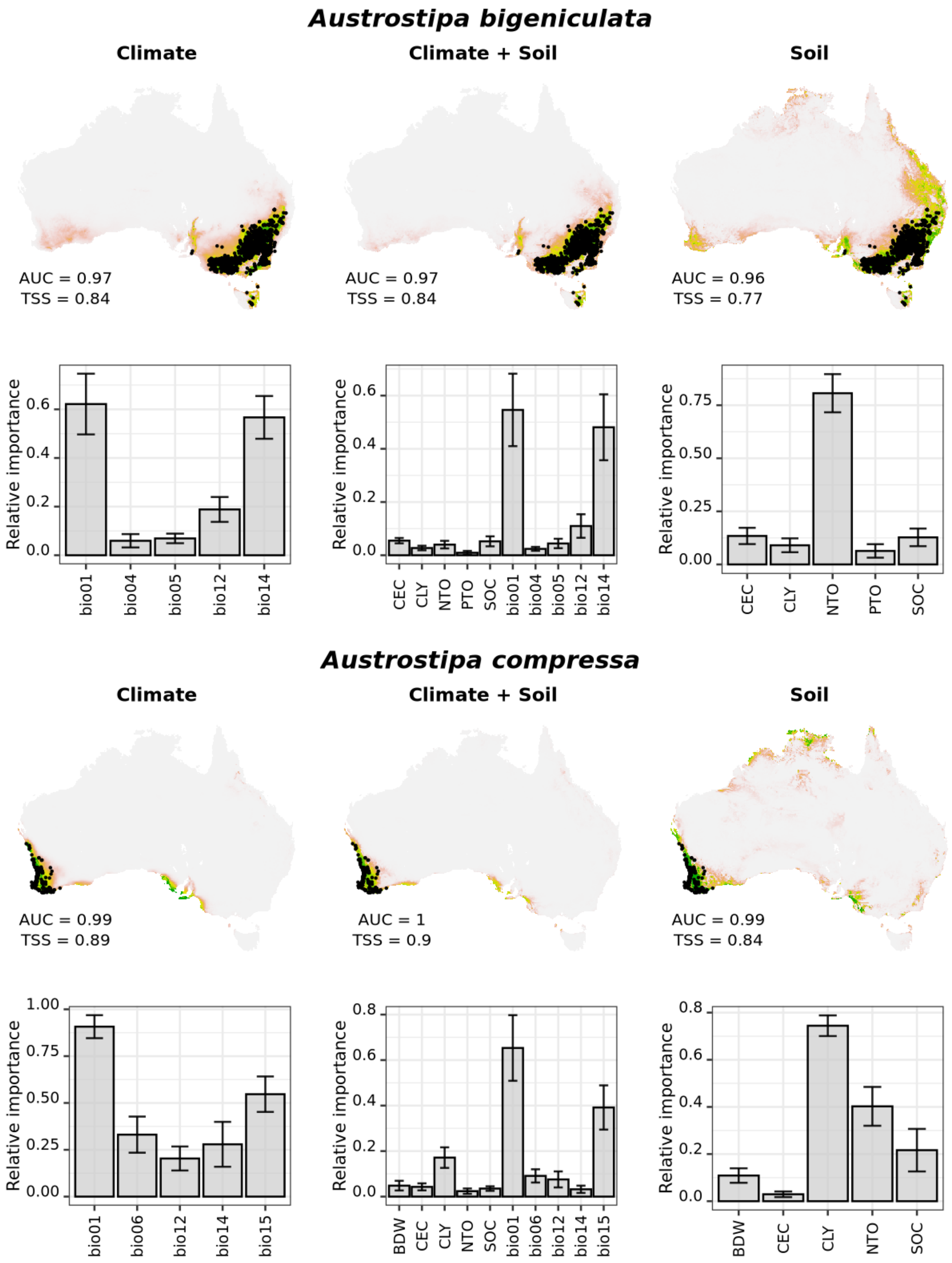
Austrostipa bigeniculata
Climate
Climate + Soil
Soil
AUC = 0.97
TSS = 0.84
AUC = 0.97
TSS = 0.84
AUC = 0.96
TSS = 0.77
Relative importance
bio01
bio04
bio05
bio12
bio14
CEC
CLY
NTO
PTO
SOC
Austrostipa compressa
Climate
Climate + Soil
Soil
AUC = 0.99
TSS = 0.89
AUC = 1
TSS = 0.9
AUC = 0.99
TSS = 0.84
Relative importance
bio01
bio06
bio12
bio14
bio15
BDW
CEC
CLY
NTO
SOC

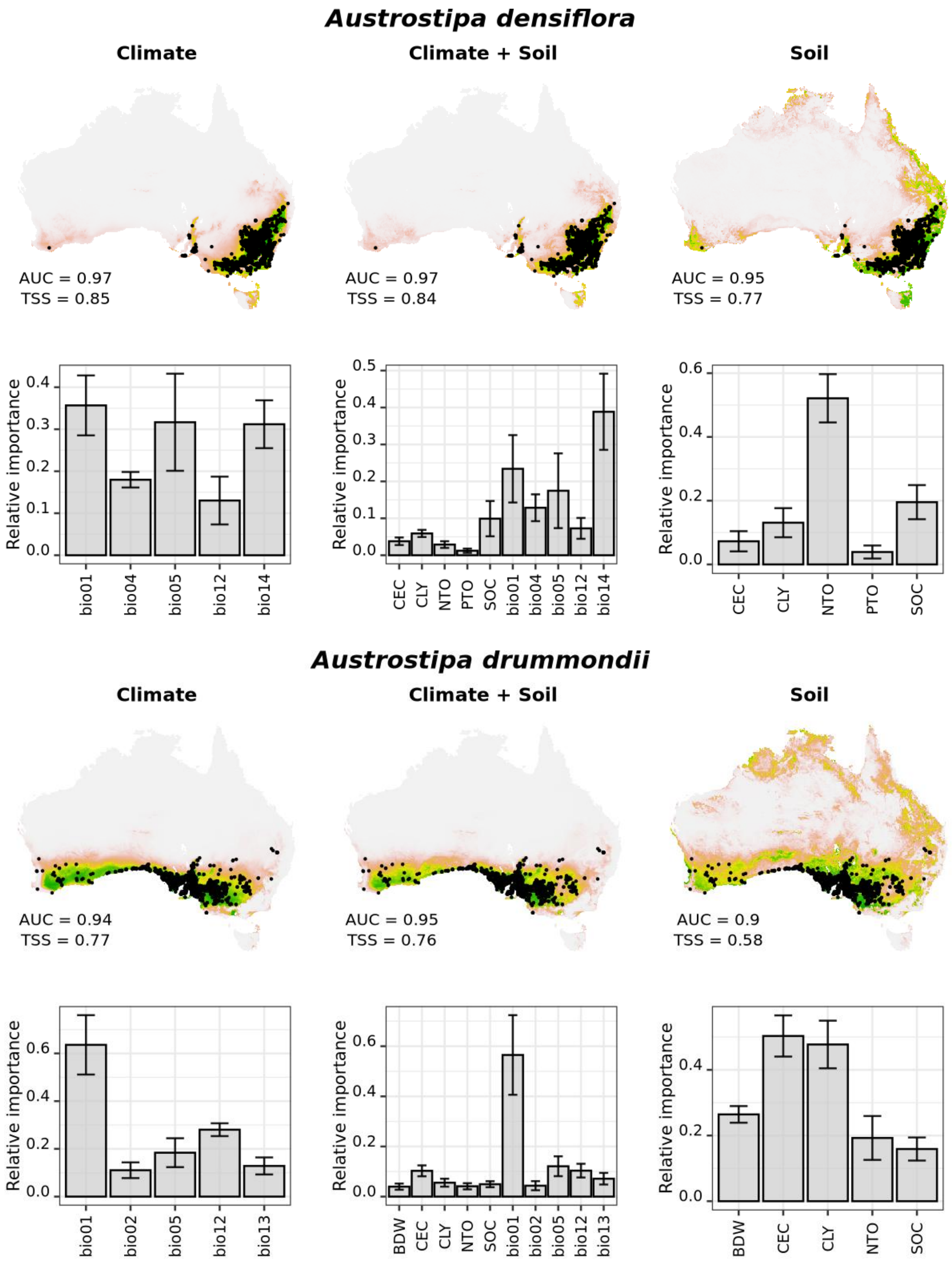

Austrostipa densiflora
Climate
Climate + Soil
Soil
AUC = 0.97
TSS = 0.85
AUC = 0.97
TSS = 0.84
AUC = 0.95
TSS = 0.77
Relative importance
bio01
bio04
bio05
bio12
bio14
CEC
CLY
NTO
PTO
SOC
Austrostipa drummondii
Climate
Climate + Soil
Soil
AUC = 0.94
TSS = 0.77
AUC = 0.95
TSS = 0.76
AUC = 0.9
TSS = 0.58
bio02
bio13
BDW

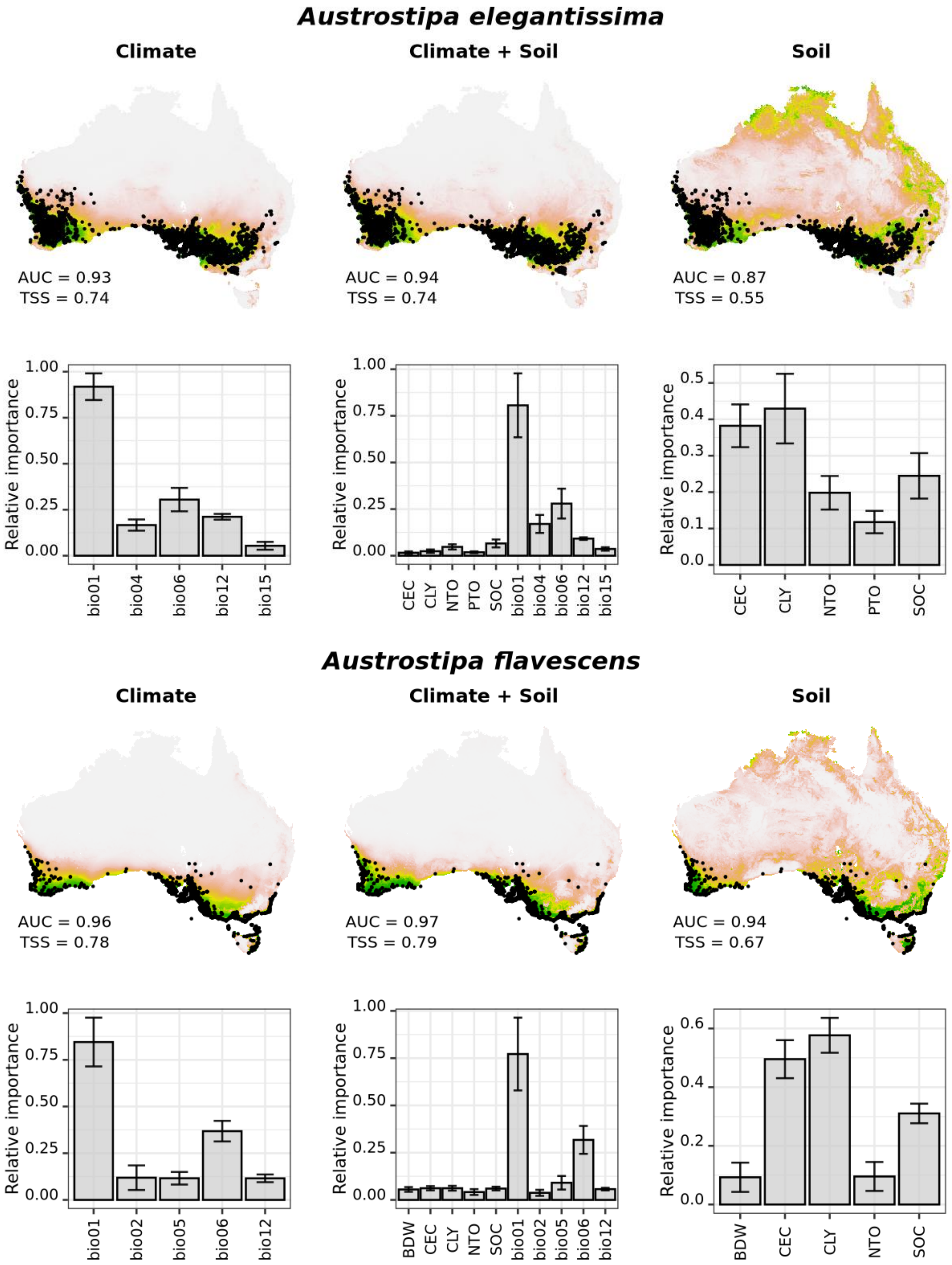
Austrostipa elegantissima
Climate
Climate + Soil
Soil
AUC = 0.93
TSS = 0.74
AUC = 0.94
TSS = 0.74
AUC = 0.87
TSS = 0.55
Relative importance
bio01 bio04 bio06 bio12 bio15
CEC CLY NTO PTO SOC bio01 bio04 bio06 bio12 bio15
CEC CLY NTO PTO SOC
Austrostipa flavescens
Climate
Climate + Soil
Soil
AUC = 0.96
TSS = 0.78
AUC = 0.97
TSS = 0.79
AUC = 0.94
TSS = 0.67
Relative importance
bio01 bio02 bio05 bio06 bio12
BDW CEC CLY NTO SOC bio01 bio02 bio05 bio06 bio12
BDW CEC CLY NTO SOC

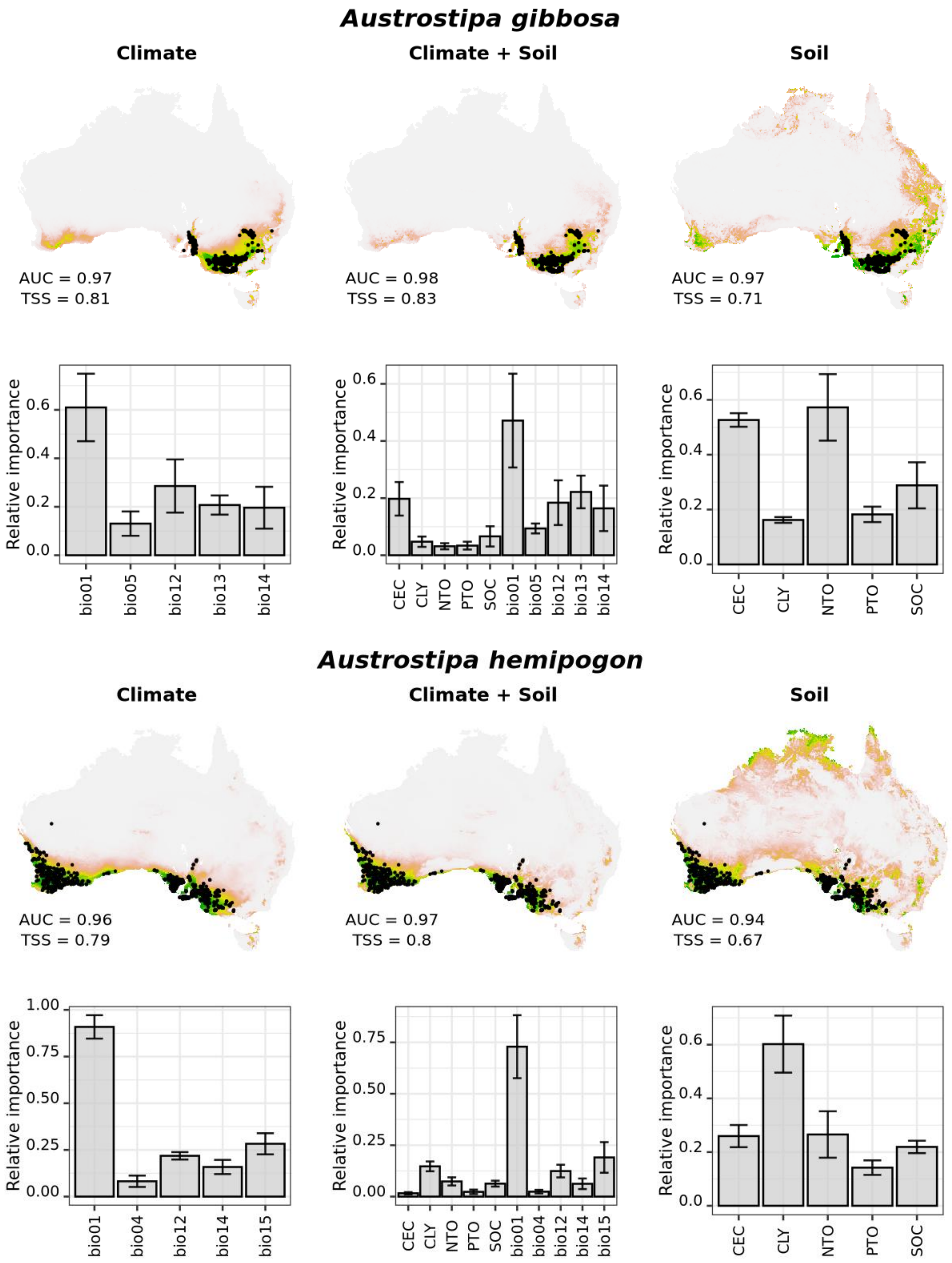

Austrostipa gibbosa
Climate
Climate + Soil
Soil
AUC = 0.97
TSS = 0.81
AUC = 0.98
TSS = 0.83
AUC = 0.97
TSS = 0.71
Relative importance
bio01
bio05
bio12
bio13
bio14
CEC
CLY
NTO
PTO
SOC
Austrostipa hemipogon
Climate
Climate + Soil
Soil
AUC = 0.96
TSS = 0.79
AUC = 0.97
TSS = 0.8
AUC = 0.94
TSS = 0.67
Relative importance
bio01
bio04
bio12
bio14
bio15
CEC
CLY
NTO
PTO
SOC

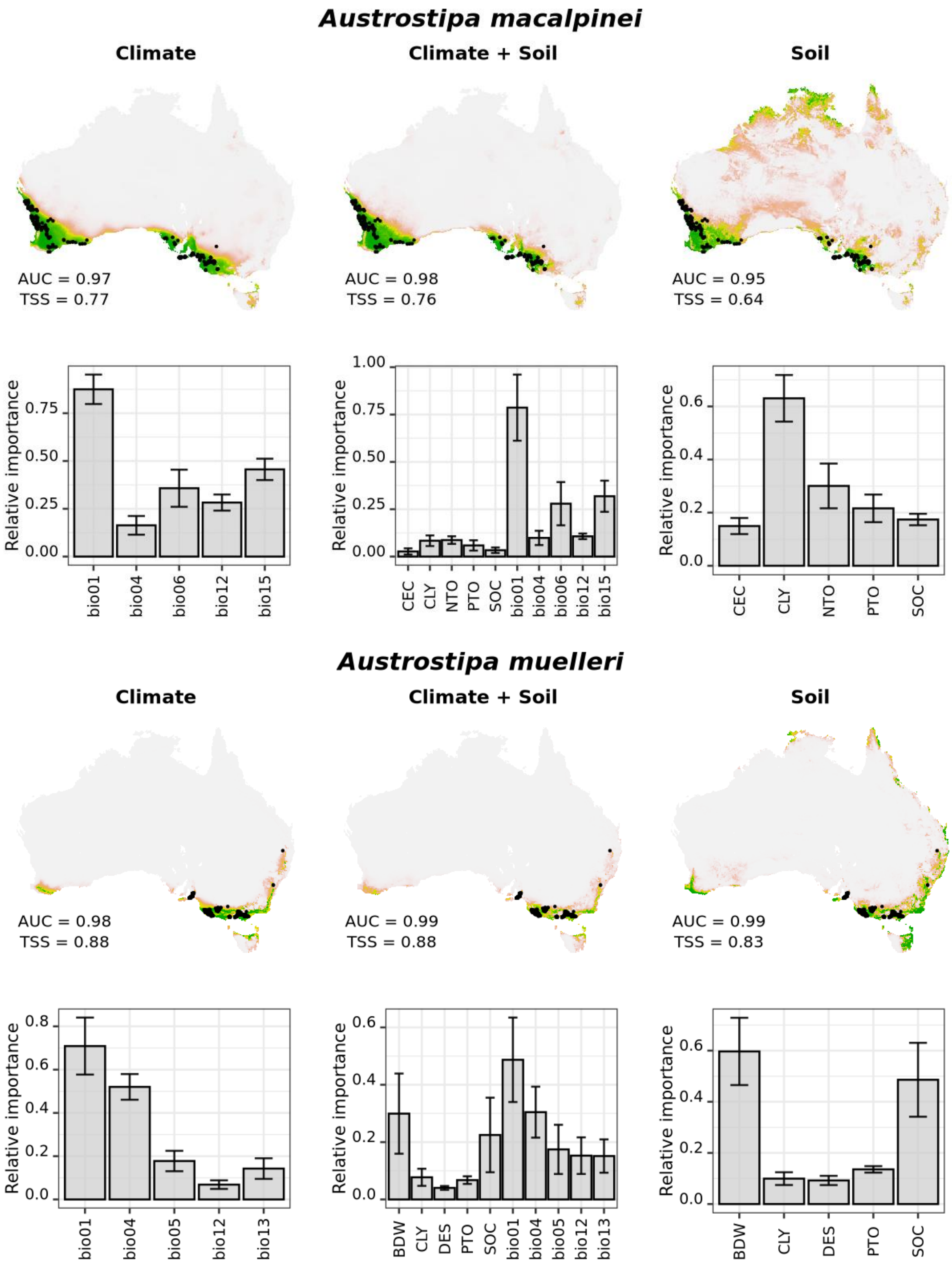

Austrostipa macalpinei
Climate
Climate + Soil
Soil
AUC = 0.97
TSS = 0.77
AUC = 0.98
TSS = 0.76
AUC = 0.95
TSS = 0.64
Relative importance
bio01
bio04
bio06
bio12
bio15
CEC
CLY
NTO
PTO
SOC
Austrostipa muelleri
Climate
Climate + Soil
Soil
AUC = 0.98
TSS = 0.88
AUC = 0.99
TSS = 0.88
AUC = 0.99
TSS = 0.83
Relative importance
bio01
bio04
bio05
bio12
bio13
BDW
CLY
DES
PTO
SOC

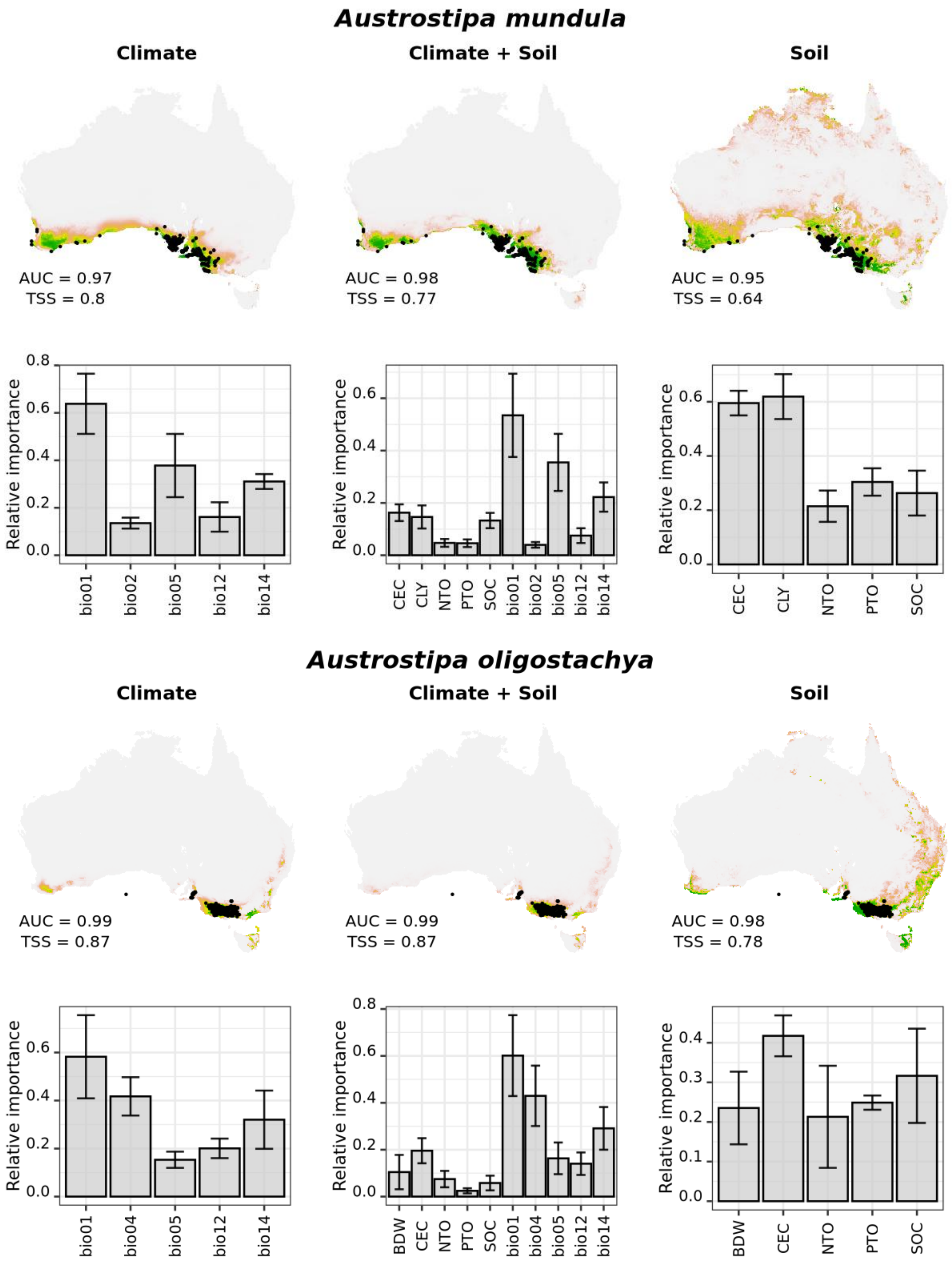

Austrostipa mundula
Climate
Climate + Soil
Soil
AUC = 0.97
TSS = 0.8
AUC = 0.98
TSS = 0.77
AUC = 0.95
TSS = 0.64
Relative importance
bio01 bio02 bio05 bio12 bio14
CEC CLY NTO PTO SOC bio01 bio02 bio05 bio12 bio14
CEC CLY NTO PTO SOC
Austrostipa oligostachya
Climate
Climate + Soil
Soil
AUC = 0.99
TSS = 0.87
AUC = 0.99
TSS = 0.87
AUC = 0.98
TSS = 0.78
Relative importance
bio01 bio04 bio05 bio12 bio14
BDW CEC NTO PTO SOC bio01 bio04 bio05 bio12 bio14
BDW CEC NTO PTO SOC

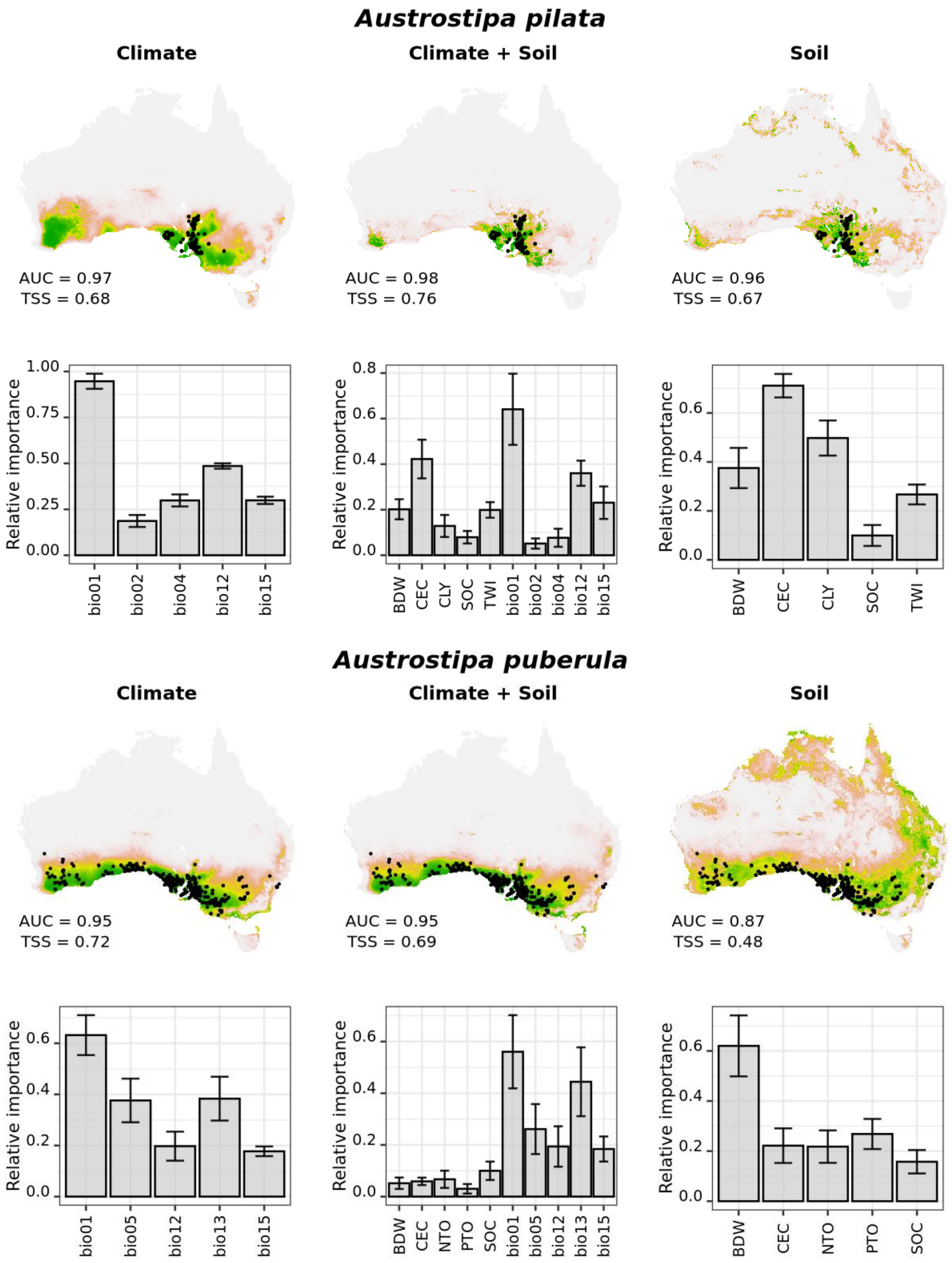
Austrostipa pilata
Climate
Climate + Soil
Soil
AUC = 0.97
TSS = 0.68
AUC = 0.98
TSS = 0.76
AUC = 0.96
TSS = 0.67
Relative importance
1.00
0.75
0.50
0.25
0.00
bio01
bio02
bio04
bio12
bio15
0.8
0.6
0.4
0.2
0.0
BDW
CEC
CLY
SOC
TWI
bio01
bio02
bio04
bio12
bio15
BDW
CEC
CLY
SOC
TWI
Austrostipa puberula
Climate
Climate + Soil
Soil
AUC = 0.95
TSS = 0.72
AUC = 0.95
TSS = 0.69
AUC = 0.87
TSS = 0.48
bio01
bio05
bio12
bio13
bio15
BDW
CEC
NTO
PTO
SOC
bio01
bio05
bio12
bio13
bio15
BDW
CEC
NTO
PTO
SOC

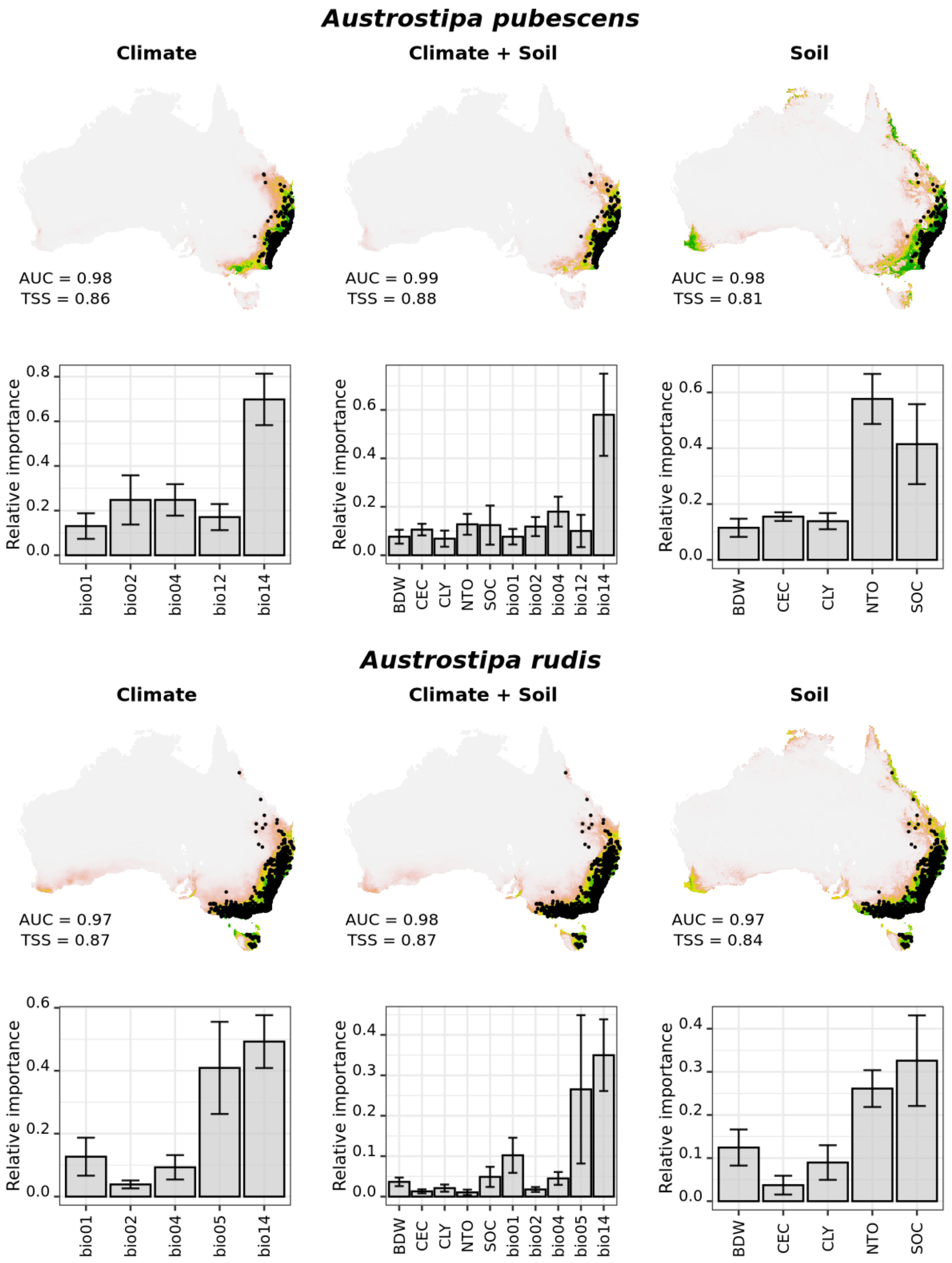

Austrostipa pubescens
Climate
Climate + Soil
Soil
AUC = 0.98
TSS = 0.86
AUC = 0.99
TSS = 0.88
AUC = 0.98
TSS = 0.81
Relative importance
bio01
bio02
bio04
bio12
bio14
BDW
CEC
CLY
NTO
SOC
Austrostipa rudis
Climate
Climate + Soil
Soil
AUC = 0.97
TSS = 0.87
AUC = 0.98
TSS = 0.87
AUC = 0.97
TSS = 0.84
Relative importance
bio01
bio02
bio04
bio05
bio14
BDW
CEC
CLY
NTO
SOC

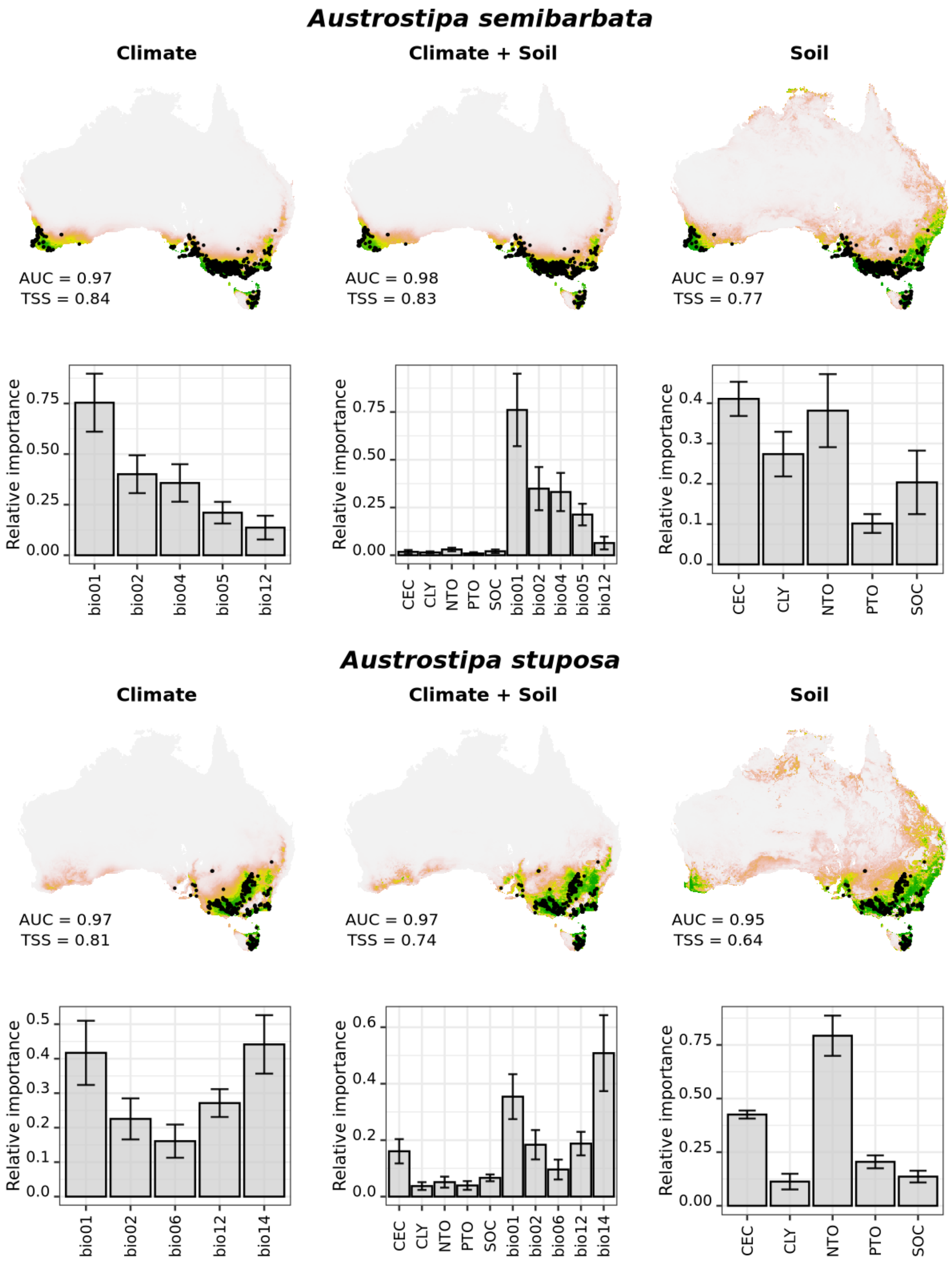
Austrostipa semibarbata
Climate
Climate + Soil
Soil
AUC = 0.97
TSS = 0.84
AUC = 0.98
TSS = 0.83
AUC = 0.97
TSS = 0.77
Relative importance
0.00
0.25
0.50
0.75
bio01
bio02
bio04
bio05
bio12
CEC
CLY
NTO
PTO
SOC
0.0
0.1
0.2
0.3
0.4
Austrostipa stupose
Climate
Climate + Soil
Soil
AUC = 0.97
TSS = 0.81
AUC = 0.97
TSS = 0.74
AUC = 0.95
TSS = 0.64
Relative importance
0.0
0.1
0.2
0.3
0.4
0.5
0.6
bio01
bio02
bio06
bio12
bio14
CEC
CLY
NTO
PTO
SOC
0.00
0.25
0.50
0.75

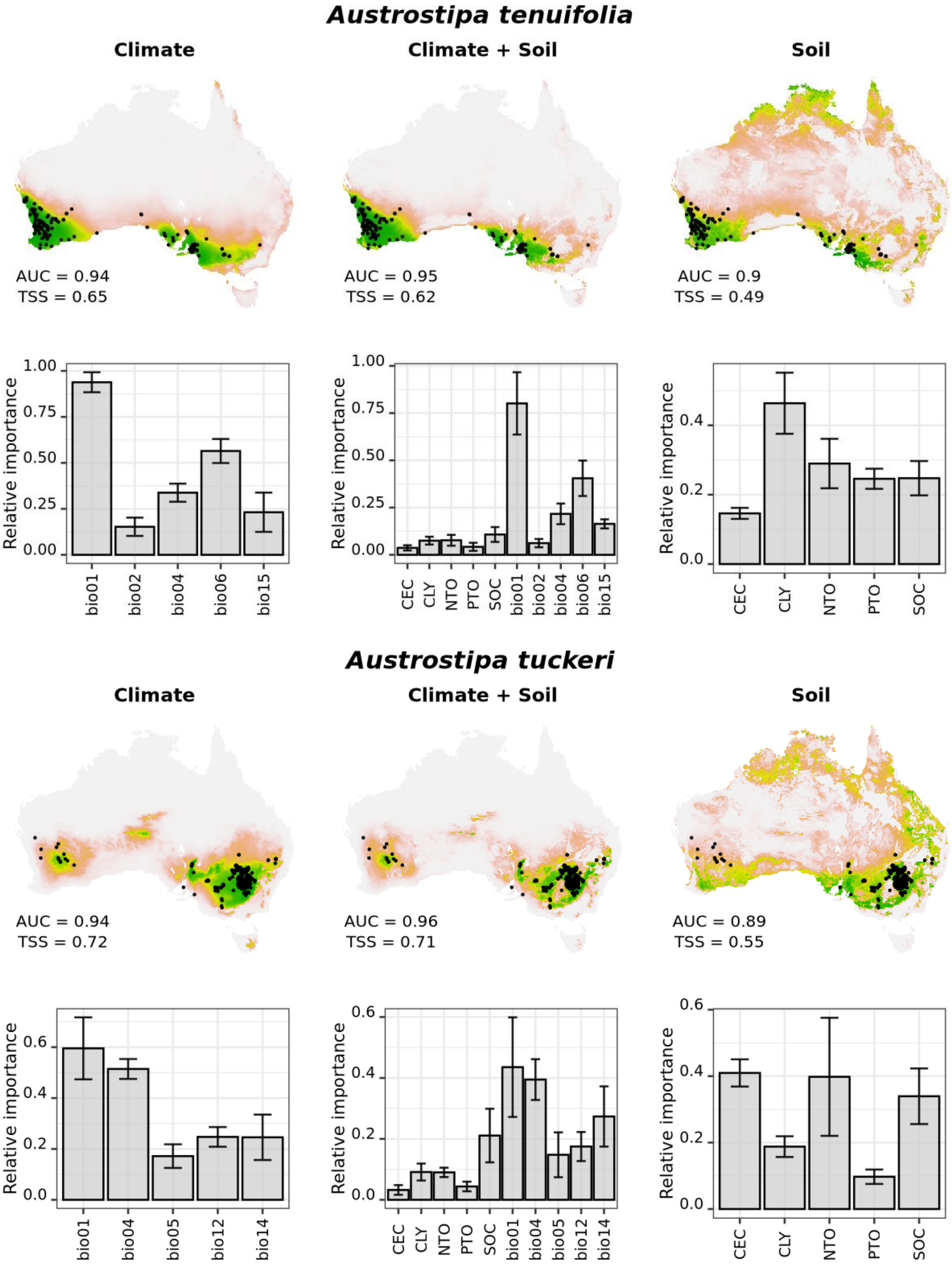

Austrostipa tenuifolia
Climate
Climate + Soil
Soil
AUC = 0.94
TSS = 0.65
AUC = 0.95
TSS = 0.62
AUC = 0.9
TSS = 0.49
Relative importance
1.00
0.75
0.50
0.25
0.00
bio01
bio02
bio04
bio06
bio15
CEC
CLY
NTO
PTO
SOC
0.4
0.2
0.0
Austrostipa tuckeri
Climate
Climate + Soil
Soil
AUC = 0.94
TSS = 0.72
AUC = 0.96
TSS = 0.71
AUC = 0.89
TSS = 0.55
0.6
bio05
bio12
bio14

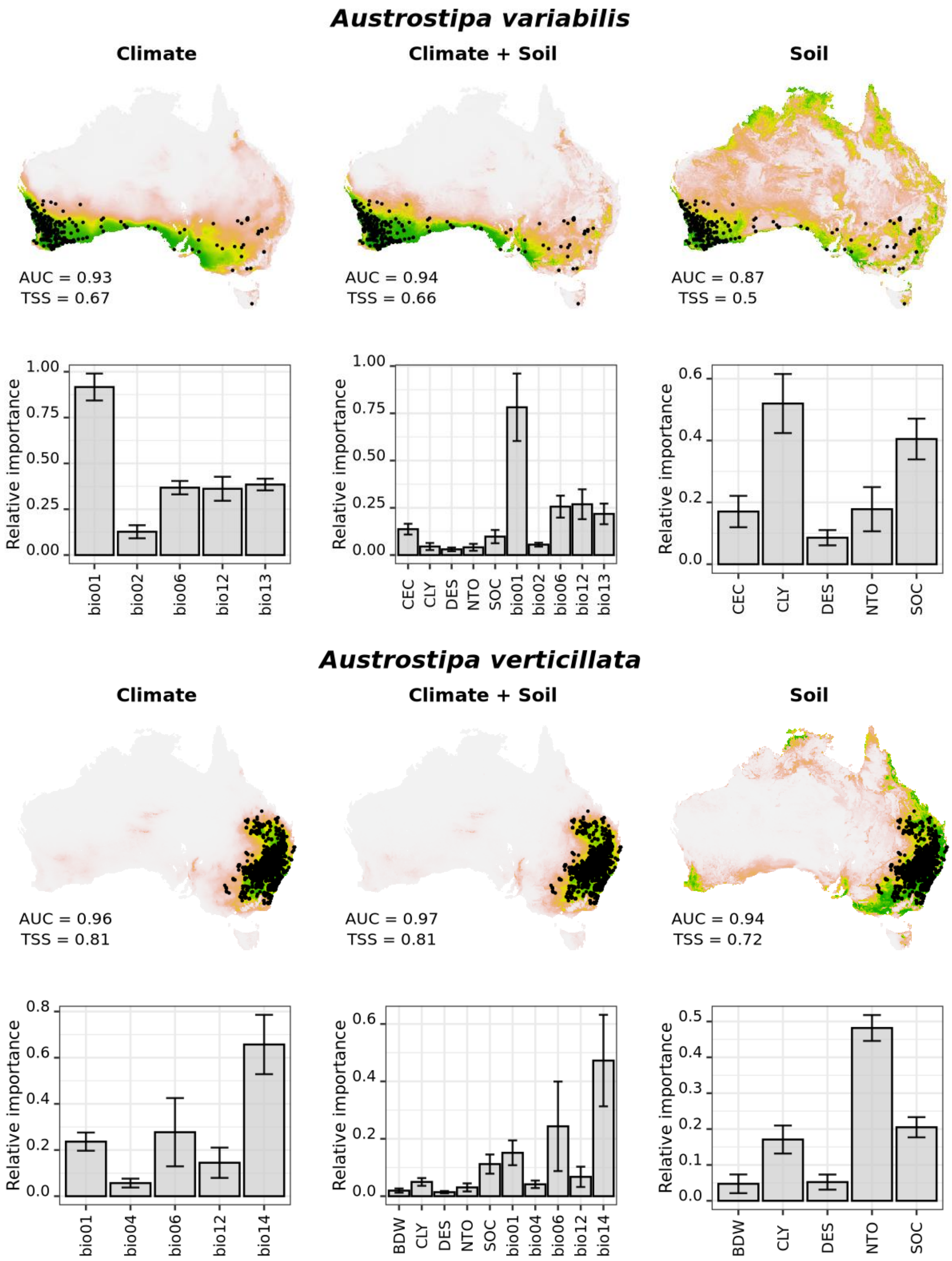
Austrostipa variabilis
Climate
Climate + Soil
Soil
AUC = 0.93
TSS = 0.67
AUC = 0.94
TSS = 0.66
AUC = 0.87
TSS = 0.5
Relative importance
bio01
bio02
bio06
bio12
bio13
CEC
CLY
DES
NTO
SOC
Austrostipa verticillata
Climate
Climate + Soil
Soil
AUC = 0.96
TSS = 0.81
AUC = 0.97
TSS = 0.81
AUC = 0.94
TSS = 0.72
Relative importance
bio01
bio04
bio06
bio12
bio14
BDW
CLY
DES
NTO
SOC

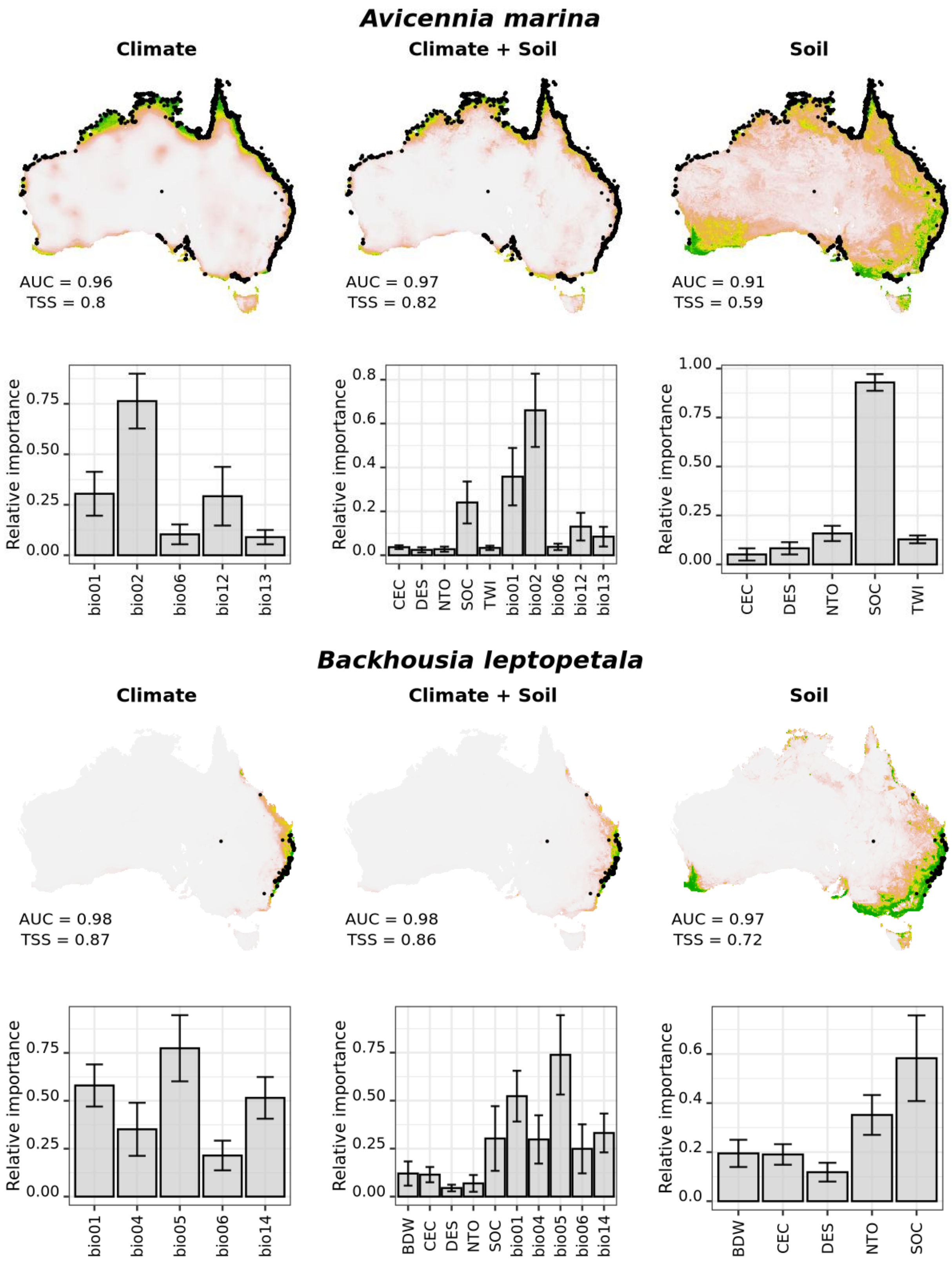
Avicennia marina
Climate
Climate + Soil
Soil
AUC = 0.96
TSS = 0.8
AUC = 0.97
TSS = 0.82
AUC = 0.91
TSS = 0.59
Relative importance
bio01 bio02 bio06 bio12 bio13
CEC DES NTO SOC TWI bio01 bio02 bio06 bio12 bio13
CEC DES NTO SOC TWI
Backhousia leptopetala
Climate
Climate + Soil
Soil
AUC = 0.98
TSS = 0.87
AUC = 0.98
TSS = 0.86
AUC = 0.97
TSS = 0.72
Relative importance
bio01 bio04 bio05 bio06 bio14
BDW CEC DES NTO SOC bio01 bio04 bio05 bio06 bio14
BDW CEC DES NTO SOC

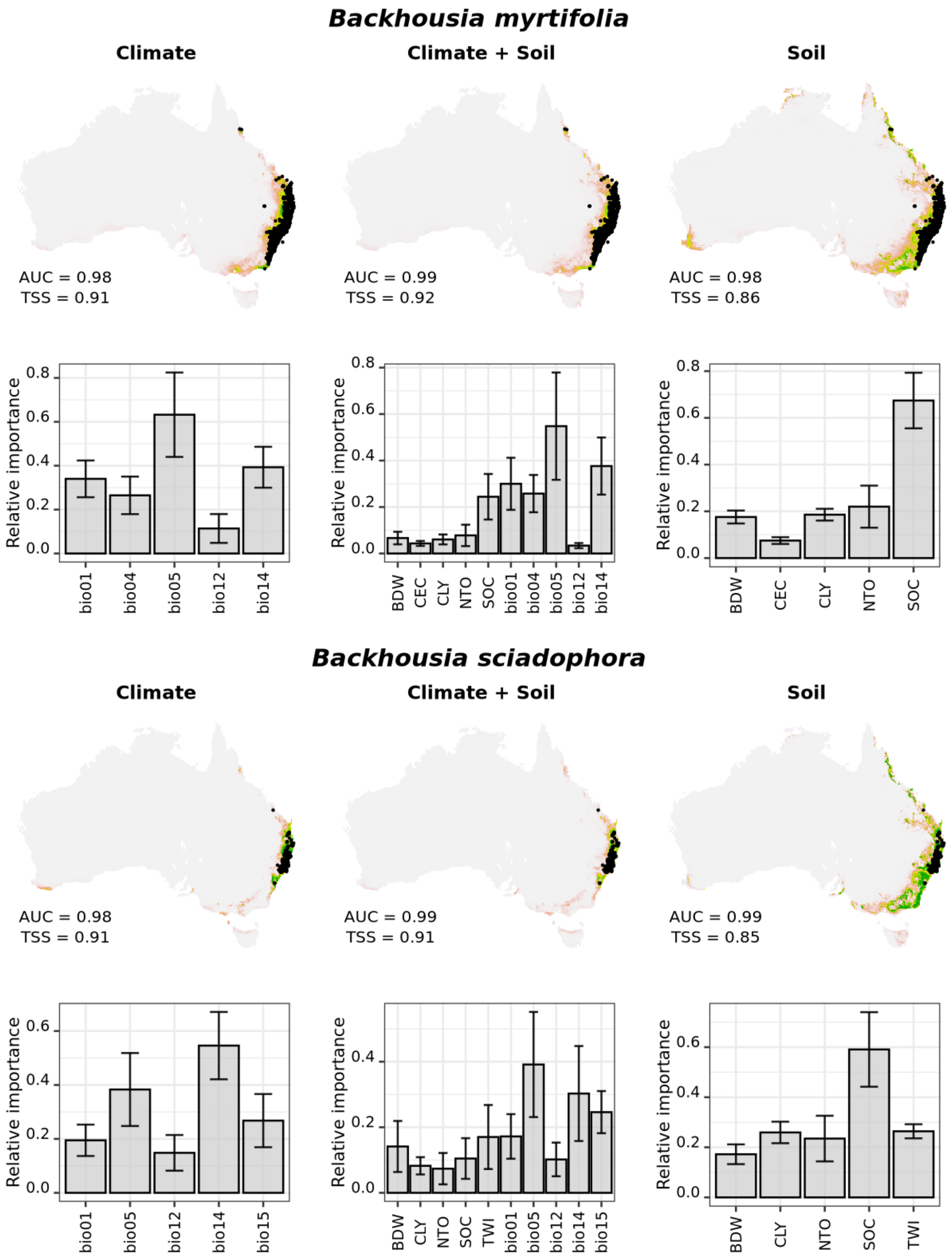

Backhousia myrtifolia
Climate
Climate + Soil
Soil
AUC = 0.98
TSS = 0.91
AUC = 0.99
TSS = 0.92
AUC = 0.98
TSS = 0.86
Relative importance
0.0
0.2
0.4
0.6
0.8
bio01
bio04
bio05
bio12
bio14
BDW
CEC
CLY
NTO
SOC
Backhousia sciadophora
AUC = 0.98
TSS = 0.91
AUC = 0.99
TSS = 0.91
AUC = 0.99
TSS = 0.85
bio15
TWI

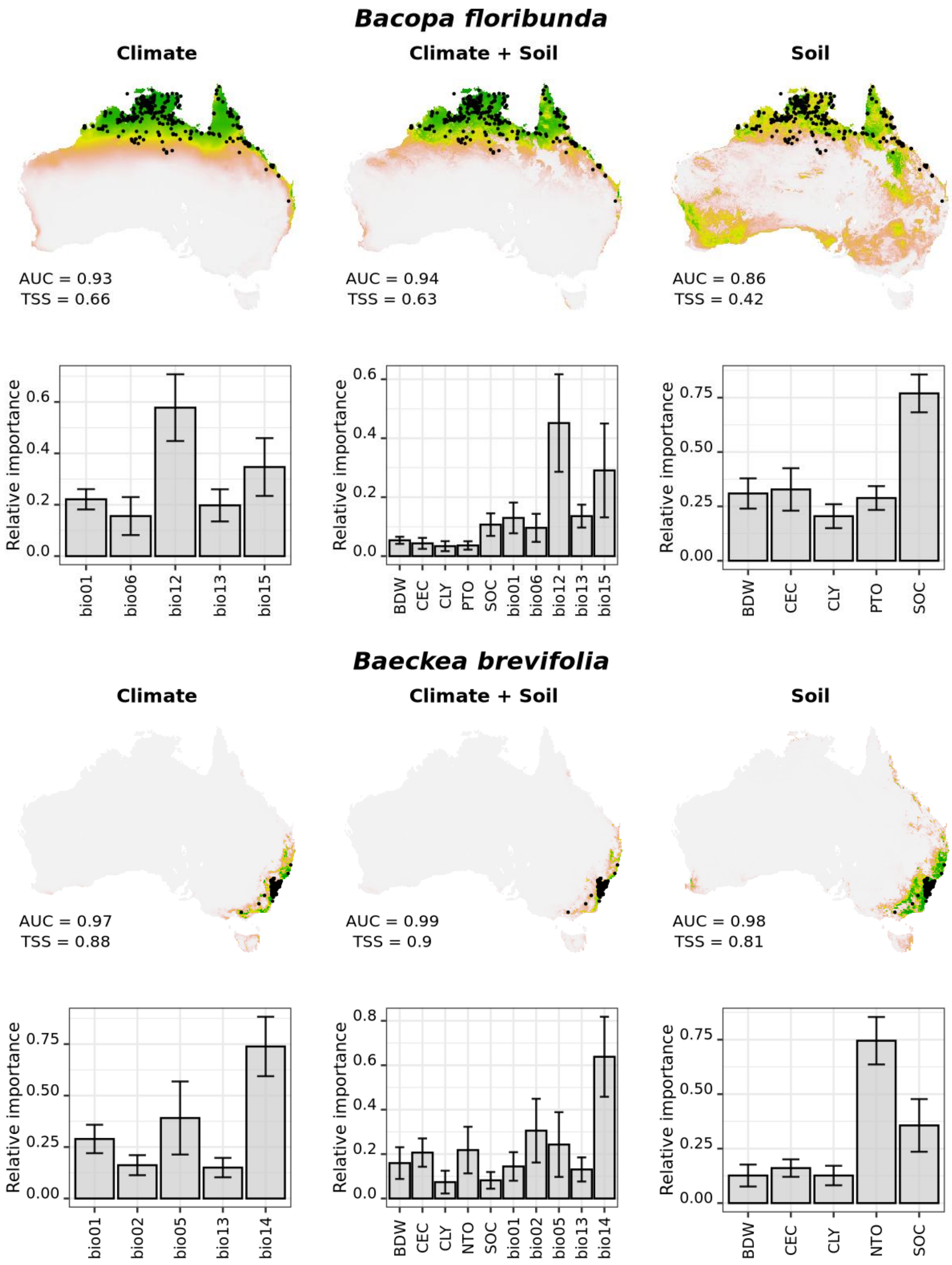
Bacopa floribunda
Climate
Climate + Soil
Soil
AUC = 0.93
TSS = 0.66
AUC = 0.94
TSS = 0.63
AUC = 0.86
TSS = 0.42
Relative importance
bio01
bio06
bio12
bio13
bio15
BDW
CEC
CLY
PTO
SOC
Baeckea brevifolia
Climate
Climate + Soil
Soil
AUC = 0.97
TSS = 0.88
AUC = 0.99
TSS = 0.9
AUC = 0.98
TSS = 0.81
Relative importance
bio01
bio02
bio05
bio13
bio14
BDW
CEC
CLY
NTO
SOC

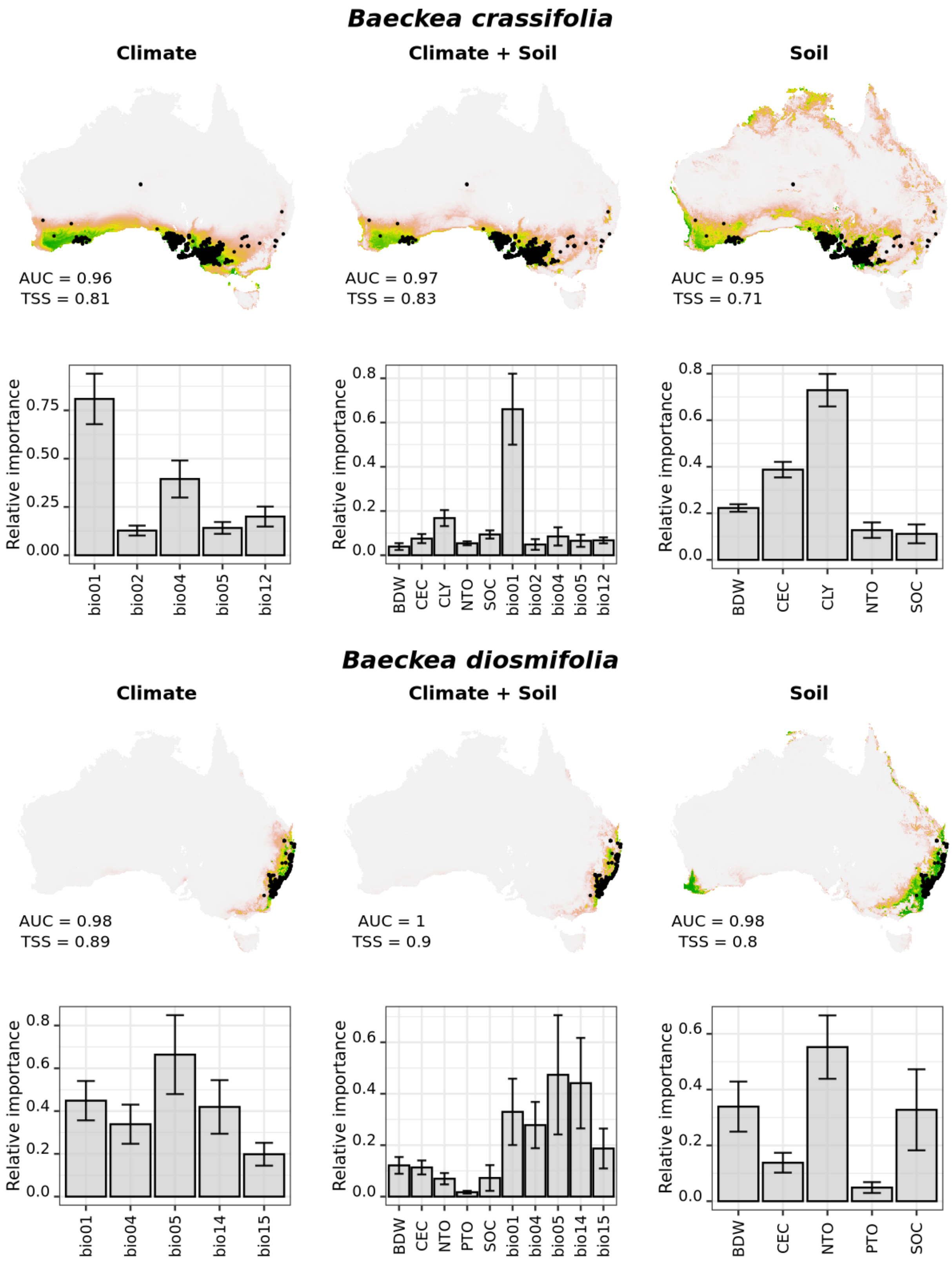
Baeckea crassifolia
Climate
Climate + Soil
Soil
AUC = 0.96
TSS = 0.81
AUC = 0.97
TSS = 0.83
AUC = 0.95
TSS = 0.71
Relative importance
bio01
bio02
bio04
bio05
bio12
BDW
CEC
CLY
NTO
SOC
Baeckea diosmifolia
Climate
Climate + Soil
Soil
AUC = 0.98
TSS = 0.89
AUC = 1
TSS = 0.9
AUC = 0.98
TSS = 0.8
Relative importance
bio01
bio04
bio05
bio14
bio15
BDW
CEC
NTO
PTO
SOC

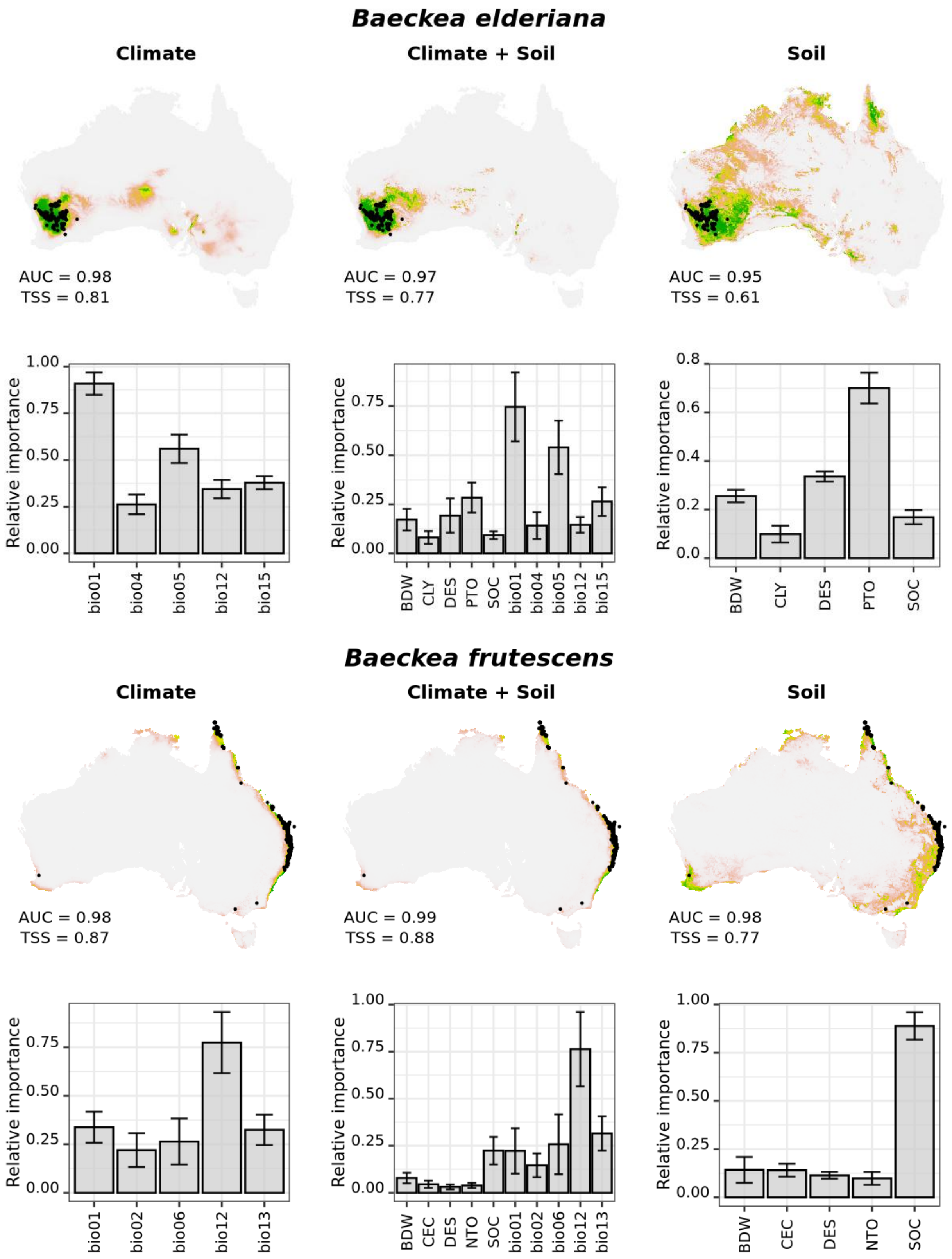

Baeckea elderiana
Climate
Climate + Soil
Soil
AUC = 0.98
TSS = 0.81
AUC = 0.97
TSS = 0.77
AUC = 0.95
TSS = 0.61
Relative importance
1.00
0.75
0.50
0.25
0.00
bio01
bio04
bio05
bio12
bio15
BDW
CLY
DES
PTO
SOC
0.8
0.6
0.4
0.2
0.0
Baeckea frutescens
AUC = 0.98
TSS = 0.87
AUC = 0.99
TSS = 0.88
AUC = 0.98
TSS = 0.77
bio02
bio06
bio13
CEC
NTO

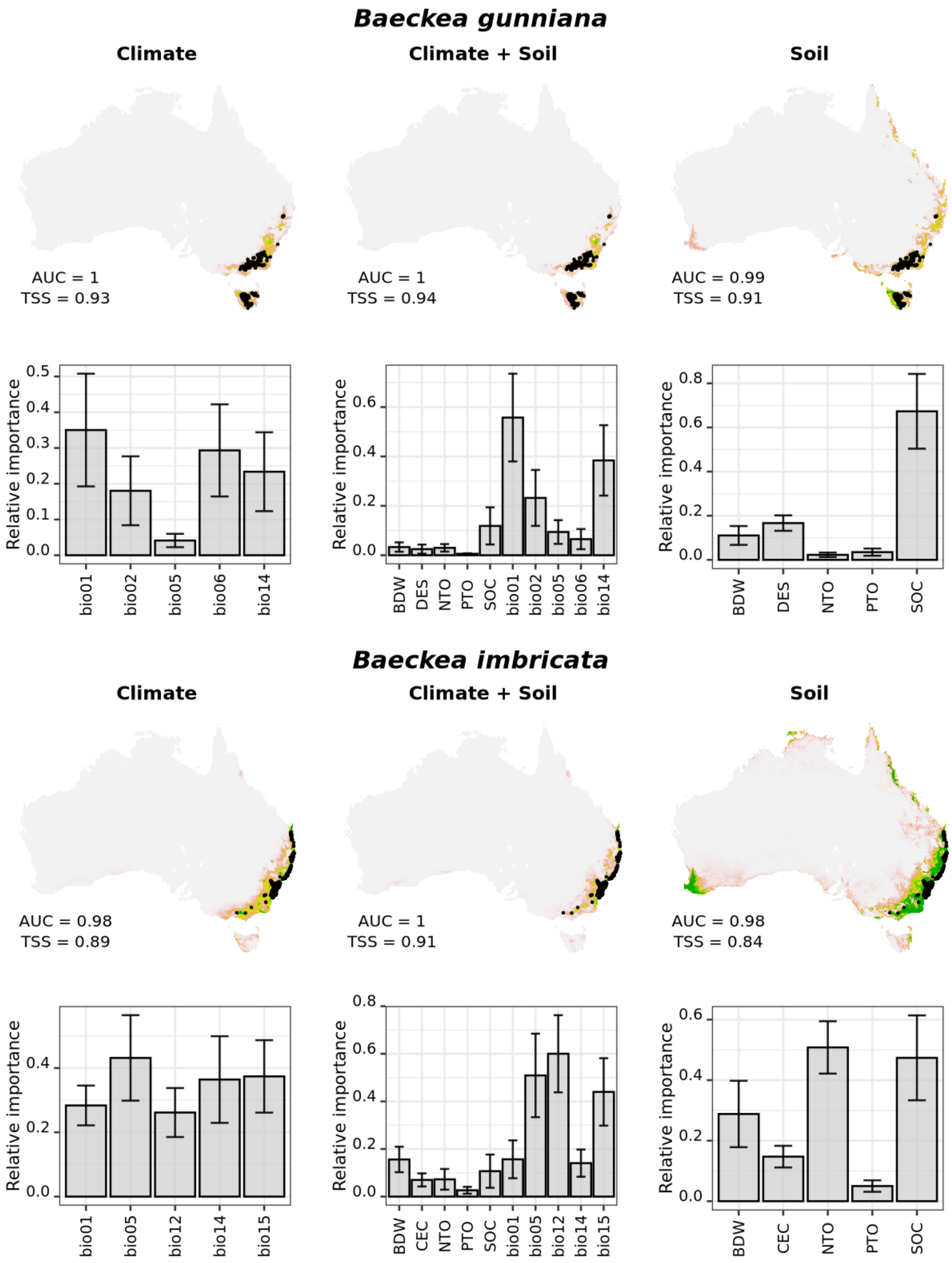
Baeckea gunniana
Climate
Climate + Soil
Soil
AUC = 1
TSS = 0.93
AUC = 1
TSS = 0.94
AUC = 0.99
TSS = 0.91
Relative importance
0.0
0.1
0.2
0.3
0.4
0.5
bio01
bio02
bio05
bio06
bio14
0.0
0.2
0.4
0.6
BDW
DES
NTO
PTO
SOC
bio01
bio02
bio05
bio06
bio14
0.0
0.2
0.4
0.6
0.8
BDW
DES
NTO
PTO
SOC
Baeckea imbricata
Climate
Climate + Soil
Soil
AUC = 0.98
TSS = 0.89
AUC = 1
TSS = 0.91
AUC = 0.98
TSS = 0.84
Relative importance
0.0
0.2
0.4
bio01
bio05
bio12
bio14
bio15
0.0
0.2
0.4
0.6
0.8
BDW
CEC
NTO
PTO
SOC
bio01
bio05
bio12
bio14
bio15
0.0
0.2
0.4
0.6
BDW
CEC
NTO
PTO
SOC

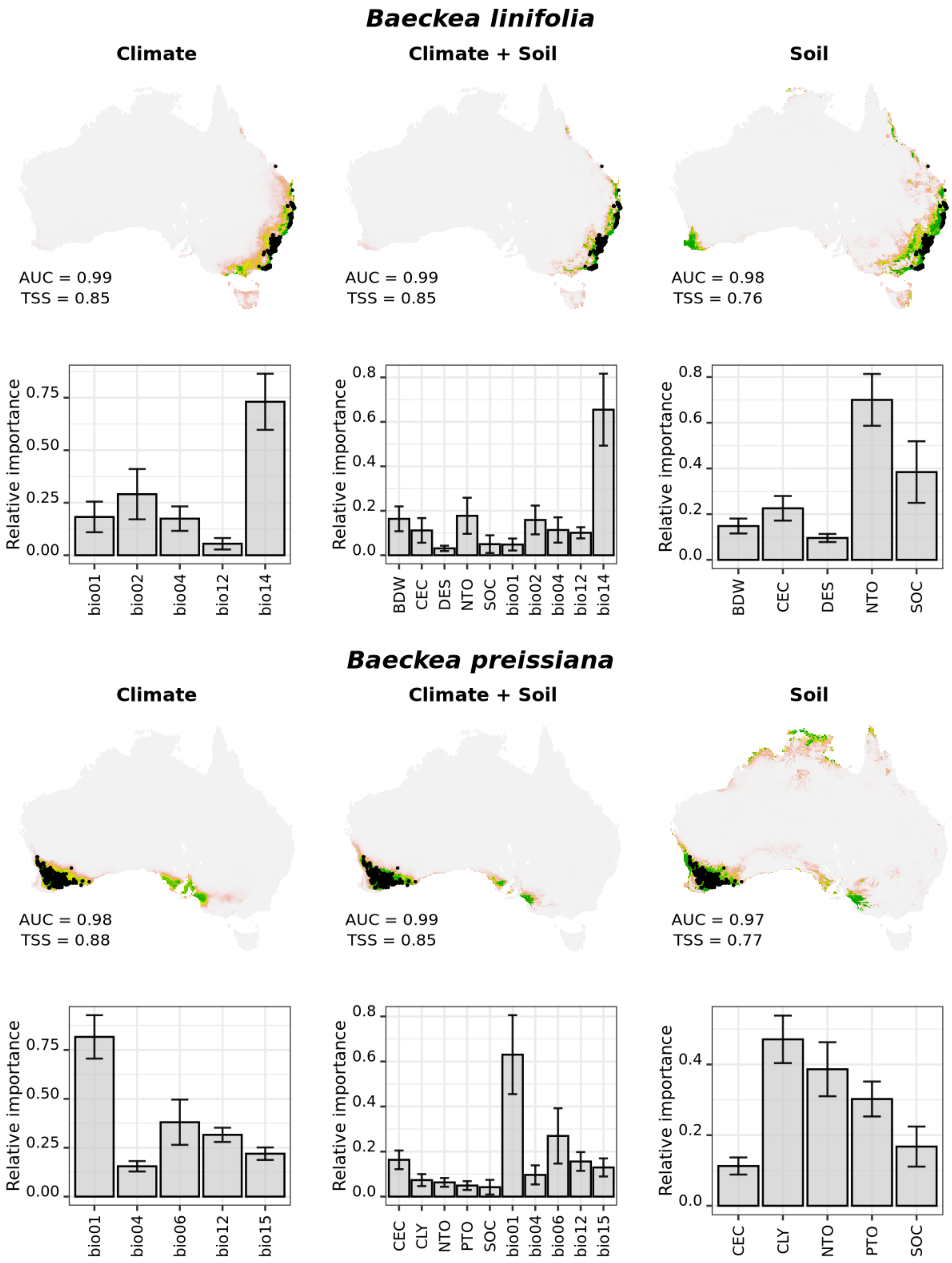
Baeckea linifolia
Climate
Climate + Soil
Soil
AUC = 0.99
TSS = 0.85
AUC = 0.99
TSS = 0.85
AUC = 0.98
TSS = 0.76
Relative importance
0.00
0.25
0.50
0.75
bio01
bio02
bio04
bio12
bio14
0.0
0.2
0.4
0.6
0.8
BDW
CEC
DES
NTO
SOC
bio01
bio02
bio04
bio12
bio14
BDW
CEC
DES
NTO
SOC
Baeckea preissiana
Climate
Climate + Soil
Soil
AUC = 0.98
TSS = 0.88
AUC = 0.99
TSS = 0.85
AUC = 0.97
TSS = 0.77
bio01
bio04
bio06
bio12
bio15
CEC
CLY
NTO
PTO
SOC
bio01
bio04
bio06
bio12
bio15
CEC
CLY
NTO
PTO
SOC

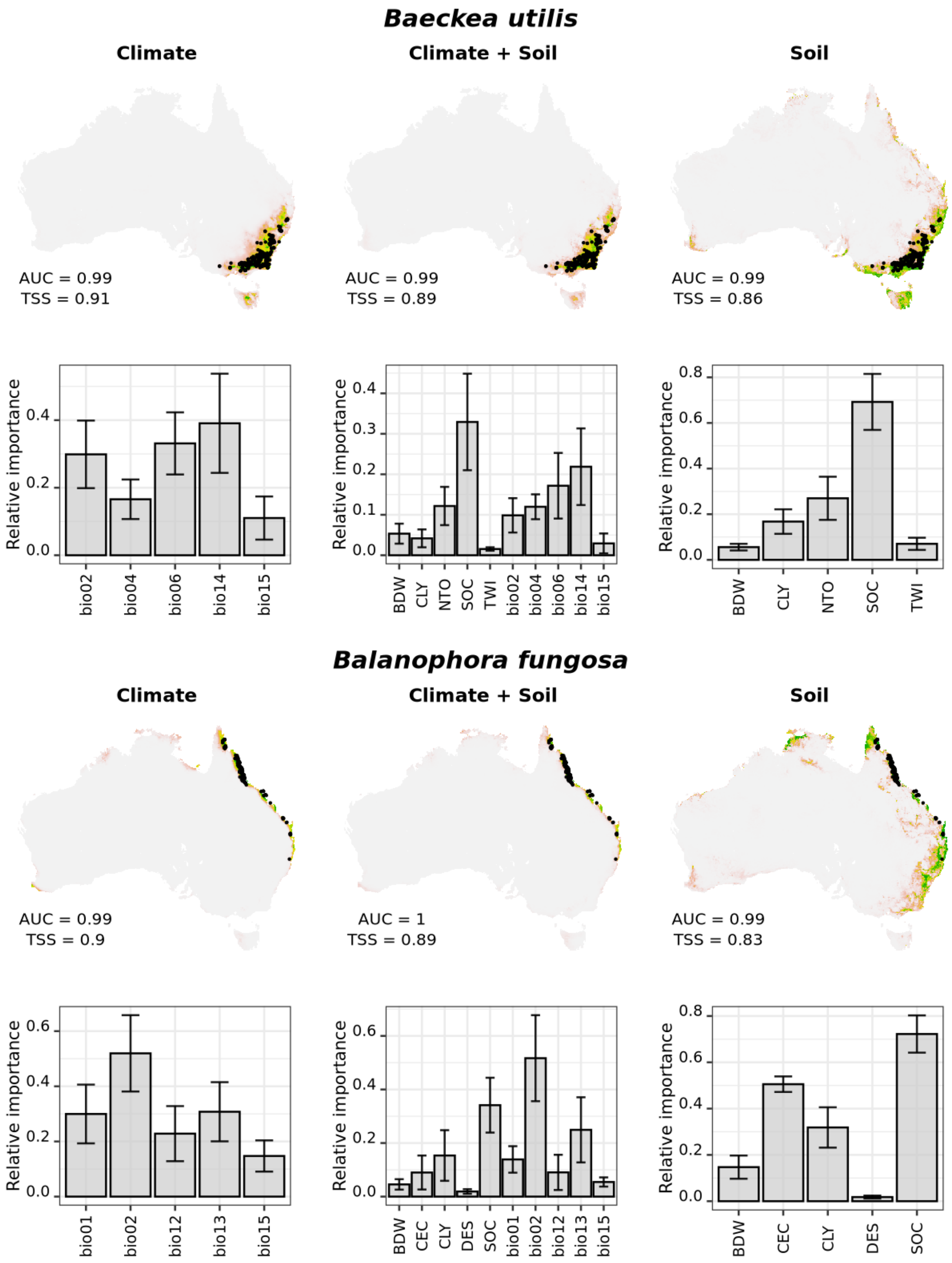

Baeckea utilis
Climate
Climate + Soil
Soil
AUC = 0.99
TSS = 0.91
AUC = 0.99
TSS = 0.89
AUC = 0.99
TSS = 0.86
Relative importance
bio02
bio04
bio06
bio14
bio15
BDW
CLY
NTO
SOC
TWI
bio02
bio04
bio06
bio14
bio15
BDW
CLY
NTO
SOC
TWI
Balanophora fungosa
Climate
Climate + Soil
Soil
AUC = 0.99
TSS = 0.9
AUC = 1
TSS = 0.89
AUC = 0.99
TSS = 0.83
Relative importance
bio01
bio02
bio12
bio13
bio15
BDW
CEC
CLY
DES
SOC
bio01
bio02
bio12
bio13
bio15
BDW
CEC
CLY
DES
SOC

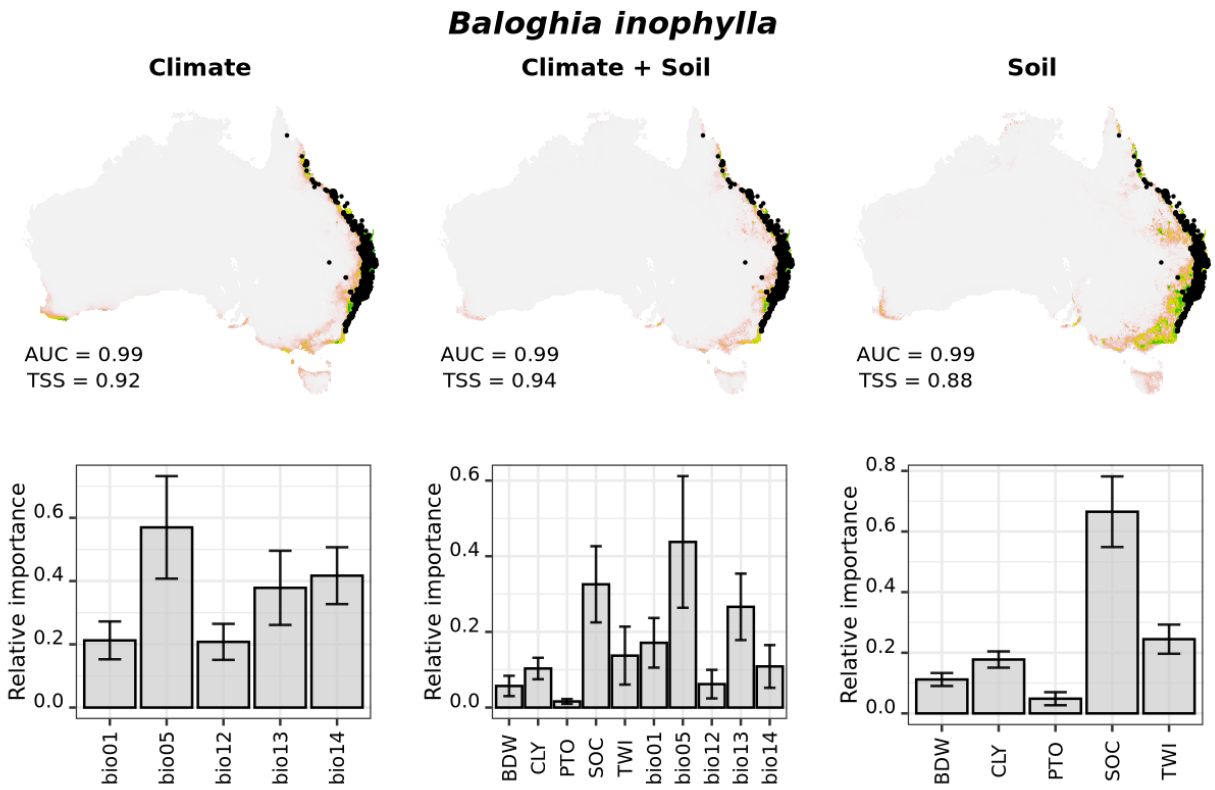
Baloghia inophylla
Climate
Climate + Soil
Soil
AUC = 0.99
TSS = 0.92
AUC = 0.99
TSS = 0.94
AUC = 0.99
TSS = 0.88
Relative importance
bio01 bio05 bio12 bio13 bio14
BDW CLY PTO SOC TWI bio01 bio05 bio12 bio13 bio14
BDW CLY PTO SOC TWI

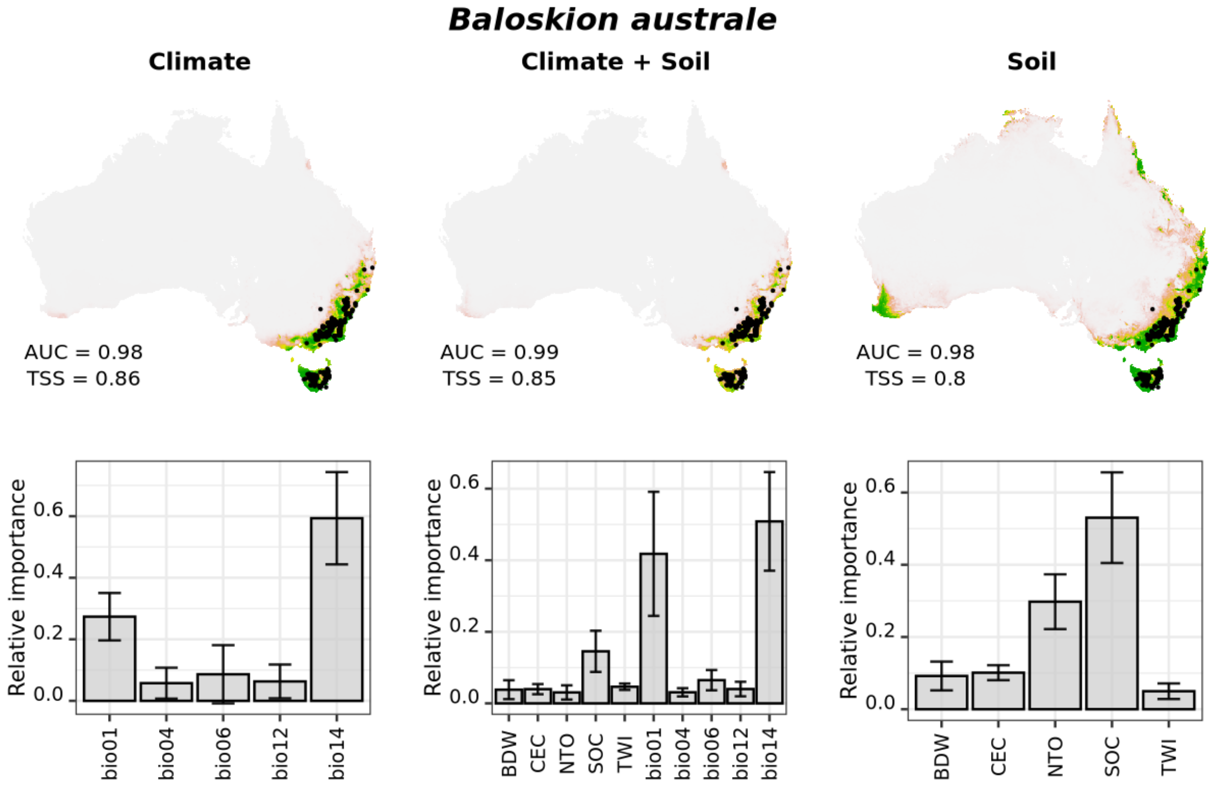
Baloskion australe
Climate
Climate + Soil
Soil
AUC = 0.98
TSS = 0.86
AUC = 0.99
TSS = 0.85
AUC = 0.98
TSS = 0.8
Relative importance
bio01 bio04 bio06 bio12 bio14
BDW CEC NTO SOC TWI bio01 bio04 bio06 bio12 bio14
BDW CEC NTO SOC TWI

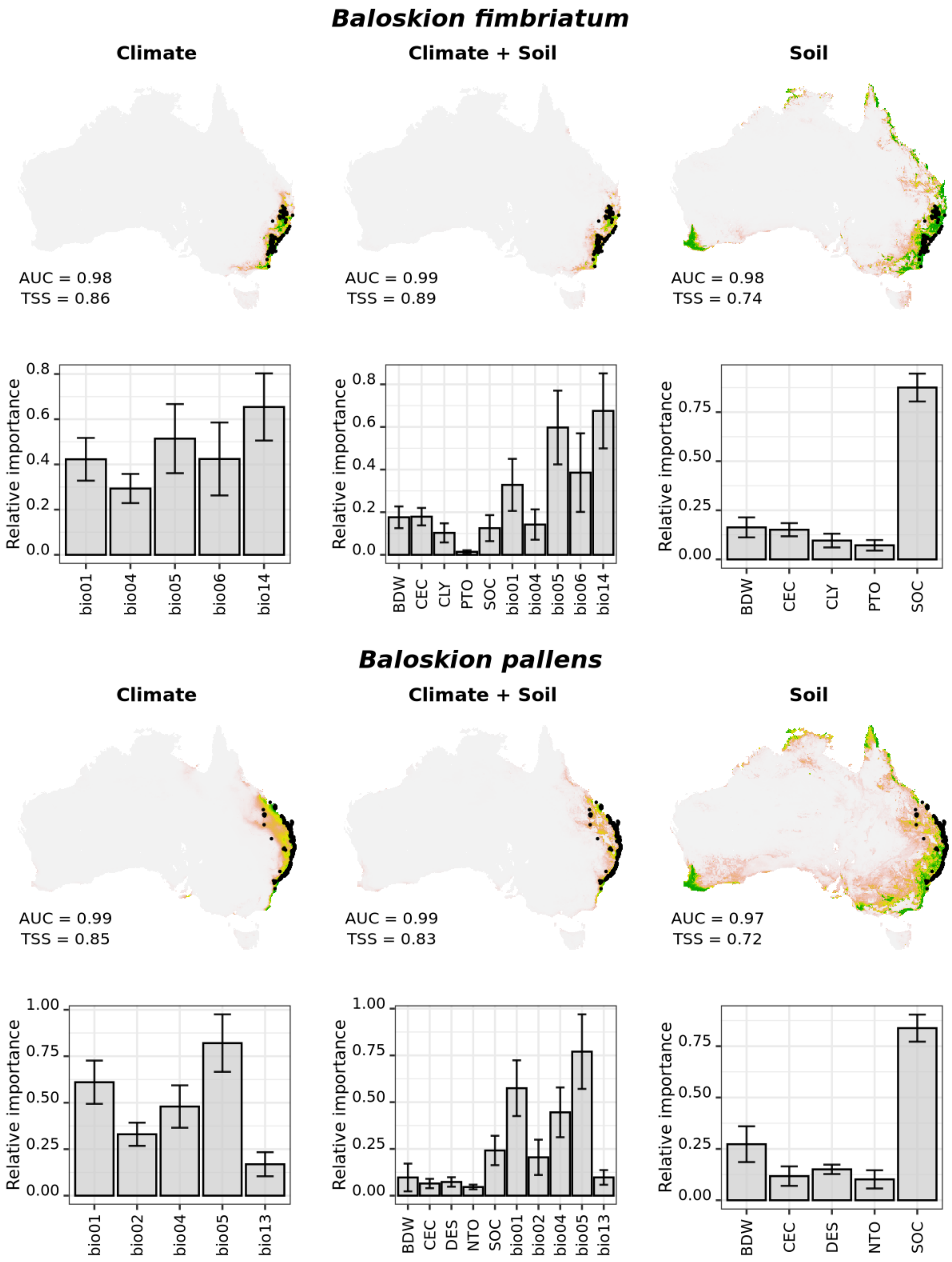

Baloskion fimbriatum
Climate
Climate + Soil
Soil
AUC = 0.98
TSS = 0.86
AUC = 0.99
TSS = 0.89
AUC = 0.98
TSS = 0.74
Relative importance
0.0
0.2
0.4
0.6
0.8
bio01
bio04
bio05
bio06
bio14
BDW
CEC
CLY
PTO
SOC
bio01
bio04
bio05
bio06
bio14
0.00
0.25
0.50
0.75
BDW
CEC
CLY
PTO
SOC
Baloskion pallens
Climate
Climate + Soil
Soil
AUC = 0.99
TSS = 0.85
AUC = 0.99
TSS = 0.83
AUC = 0.97
TSS = 0.72
Relative importance
0.00
0.25
0.50
0.75
1.00
bio01
bio02
bio04
bio05
bio13
BDW
CEC
DES
NTO
SOC
bio01
bio02
bio04
bio05
bio13
BDW
CEC
DES
NTO
SOC

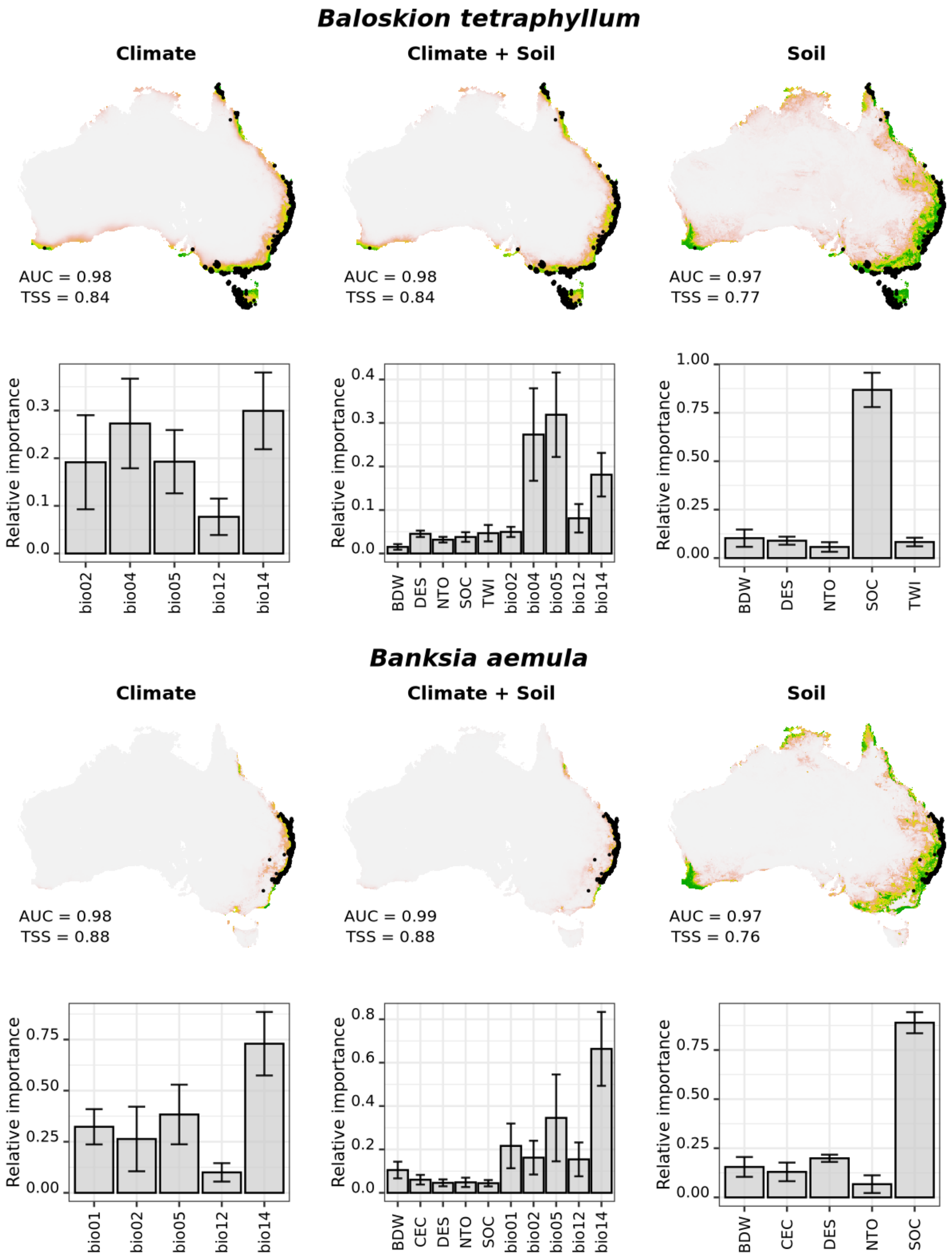
Baloskion tetraphyllum
Climate
Climate + Soil
Soil
AUC = 0.98
TSS = 0.84
AUC = 0.98
TSS = 0.84
AUC = 0.97
TSS = 0.77
Relative importance
bio02
bio04
bio05
bio12
bio14
BDW
DES
NTO
SOC
TWI
bio02
bio04
bio05
bio12
bio14
BDW
DES
NTO
SOC
TWI
Banksia aemula
Climate
Climate + Soil
Soil
AUC = 0.98
TSS = 0.88
AUC = 0.99
TSS = 0.88
AUC = 0.97
TSS = 0.76
Relative importance
bio01
bio02
bio05
bio12
bio14
BDW
CEC
DES
NTO
SOC
bio01
bio02
bio05
bio12
bio14
BDW
CEC
DES
NTO
SOC

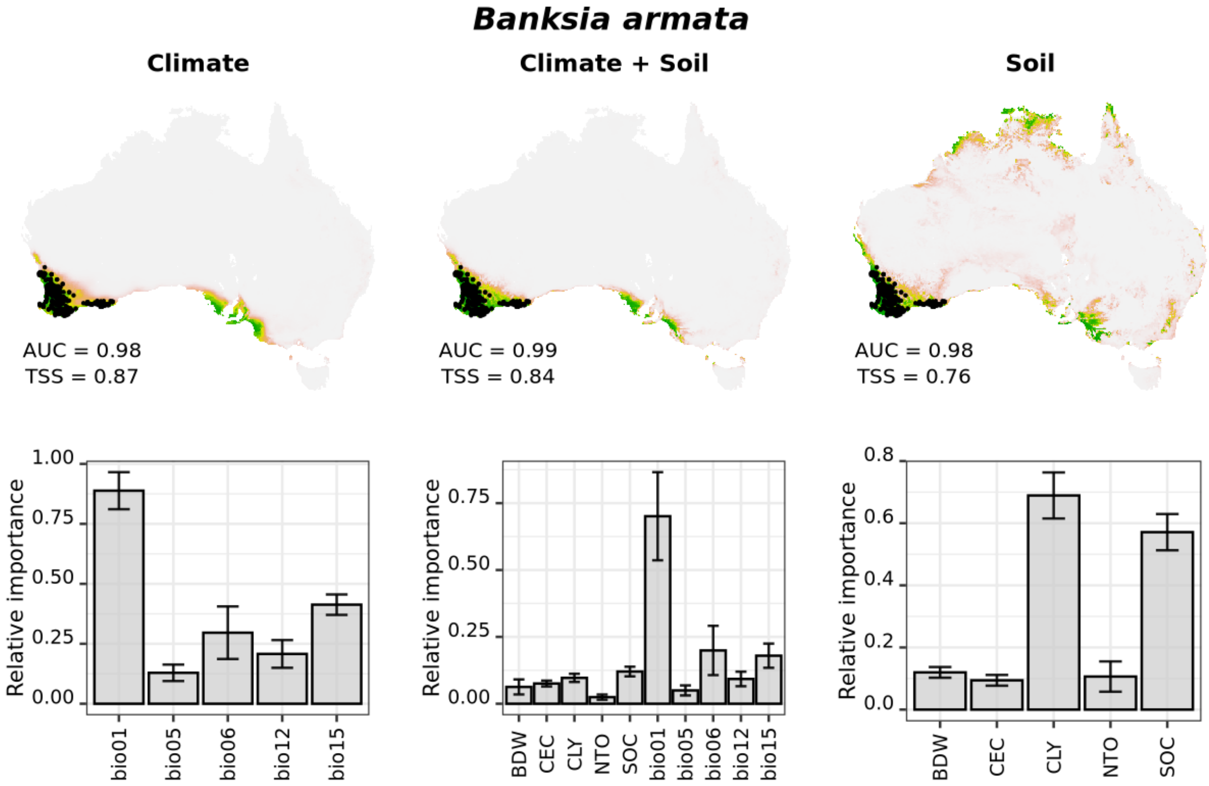
Banksia armata
Climate
Climate + Soil
Soil
AUC = 0.98
TSS = 0.87
AUC = 0.99
TSS = 0.84
AUC = 0.98
TSS = 0.76
Relative importance
bio01
bio05
bio06
bio12
bio15
BDW
CEC
CLY
NTO
SOC

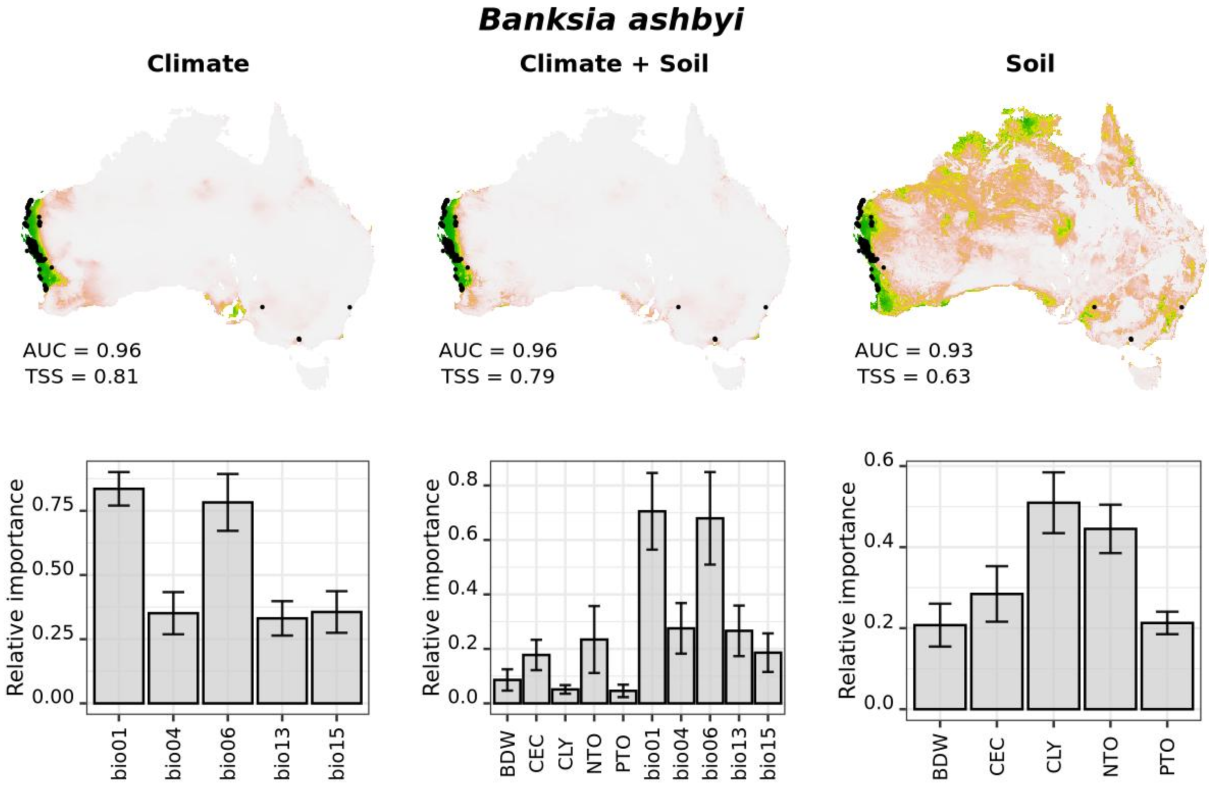
Banksia ashbyi
Climate
Climate + Soil
Soil
AUC = 0.96
TSS = 0.81
AUC = 0.96
TSS = 0.79
AUC = 0.93
TSS = 0.63
Relative importance
bio01
bio04
bio06
bio13
bio15
BDW
CEC
CLY
NTO
PTO

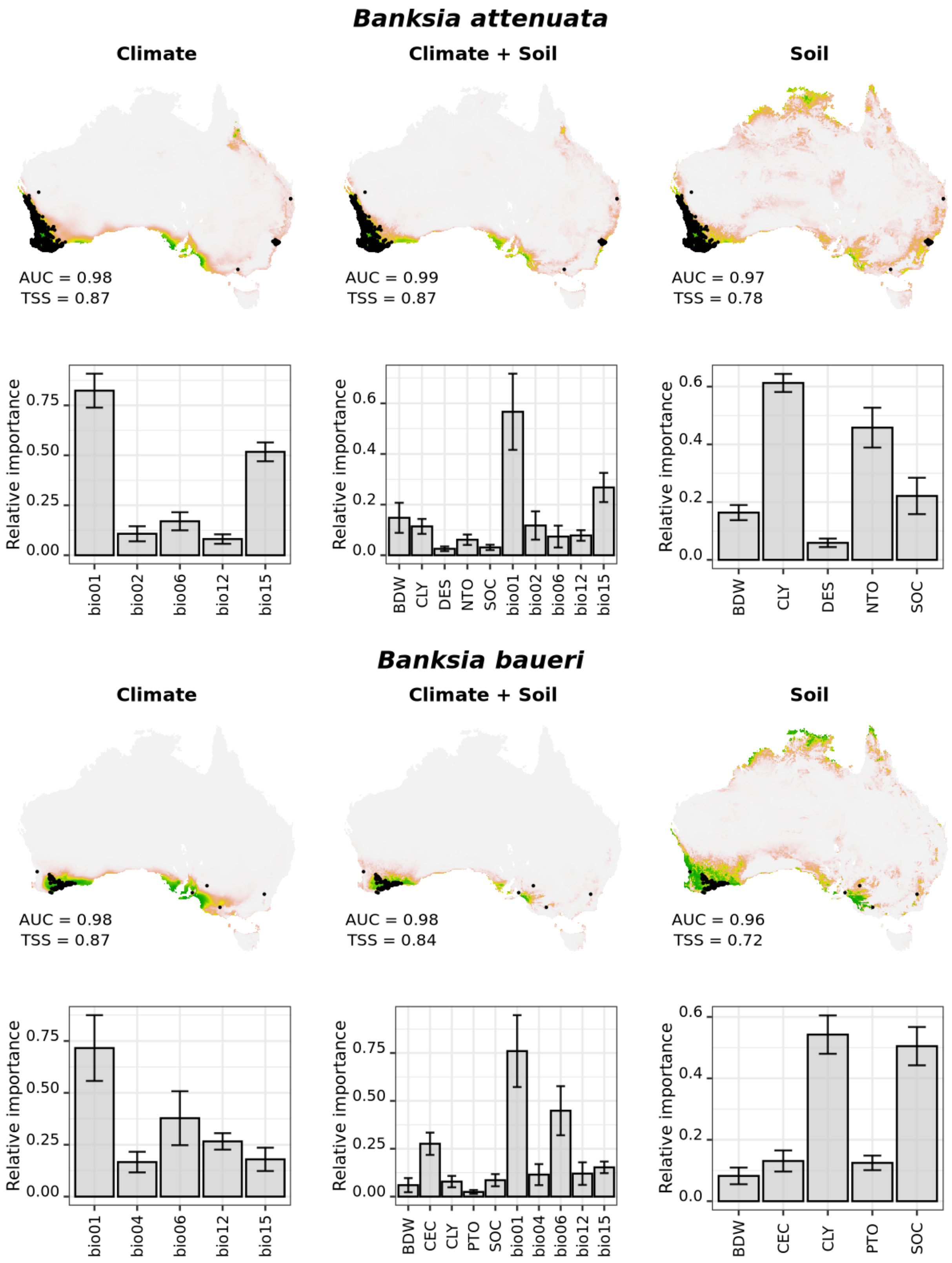
Banksia attenuata
Climate
Climate + Soil
Soil
AUC = 0.98
TSS = 0.87
AUC = 0.99
TSS = 0.87
AUC = 0.97
TSS = 0.78
Relative importance
bio01
bio02
bio06
bio12
bio15
BDW
CLY
DES
NTO
SOC
Banksia baueri
Climate
Climate + Soil
Soil
AUC = 0.98
TSS = 0.87
AUC = 0.98
TSS = 0.84
AUC = 0.96
TSS = 0.72
bio04
CEC
PTO

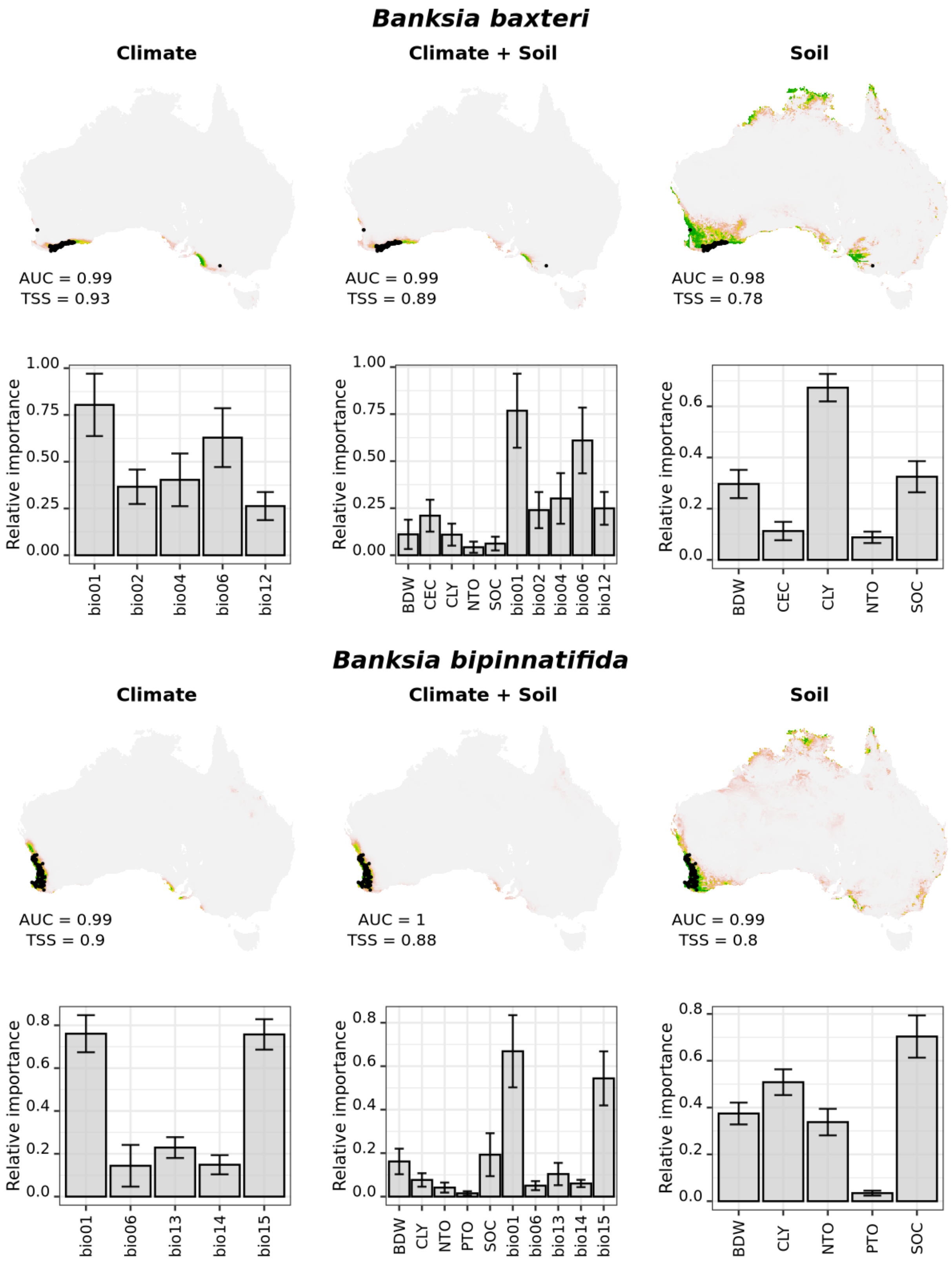
Banksia baxteri
Climate
Climate + Soil
Soil
AUC = 0.99
TSS = 0.93
AUC = 0.99
TSS = 0.89
AUC = 0.98
TSS = 0.78
Relative importance
0.00
0.25
0.50
0.75
1.00
bio01
bio02
bio04
bio06
bio12
BDW
CEC
CLY
NTO
SOC
0.0
0.2
0.4
0.6
Banksia bipinnatifida
Climate
Climate + Soil
Soil
AUC = 0.99
TSS = 0.9
AUC = 1
TSS = 0.88
AUC = 0.99
TSS = 0.8
Relative importance
0.0
0.2
0.4
0.6
0.8
bio01
bio06
bio13
bio14
bio15
BDW
CLY
NTO
PTO
SOC

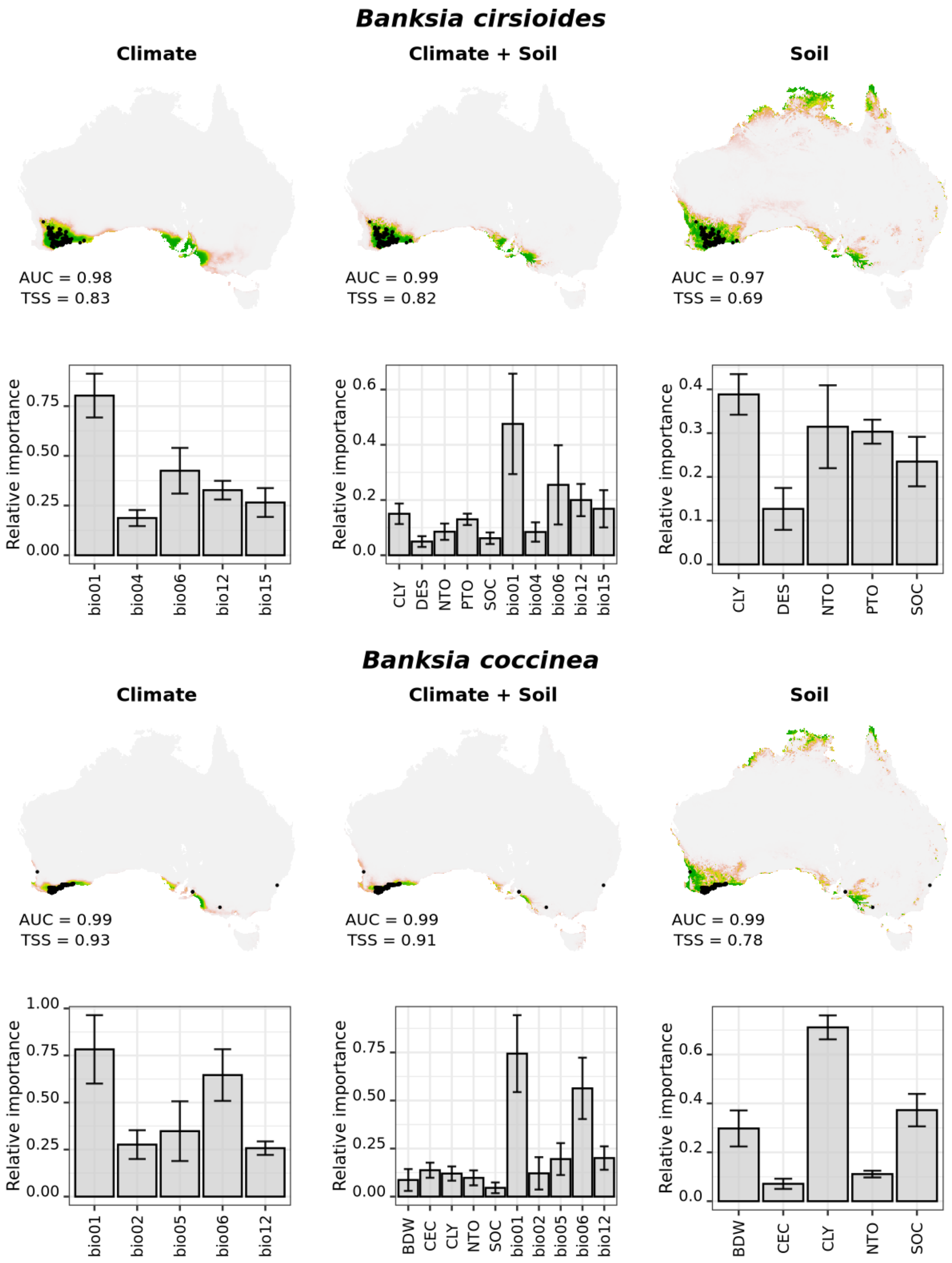
Banksia cirsioides
Climate
Climate + Soil
Soil
AUC = 0.98
TSS = 0.83
AUC = 0.99
TSS = 0.82
AUC = 0.97
TSS = 0.69
Relative importance
bio01
bio04
bio06
bio12
bio15
CLY
DES
NTO
PTO
SOC
Banksia coccinea
Climate
Climate + Soil
Soil
AUC = 0.99
TSS = 0.93
AUC = 0.99
TSS = 0.91
AUC = 0.99
TSS = 0.78
bio01
bio02
bio05
bio06
bio12
BDW
CEC
CLY
NTO
SOC

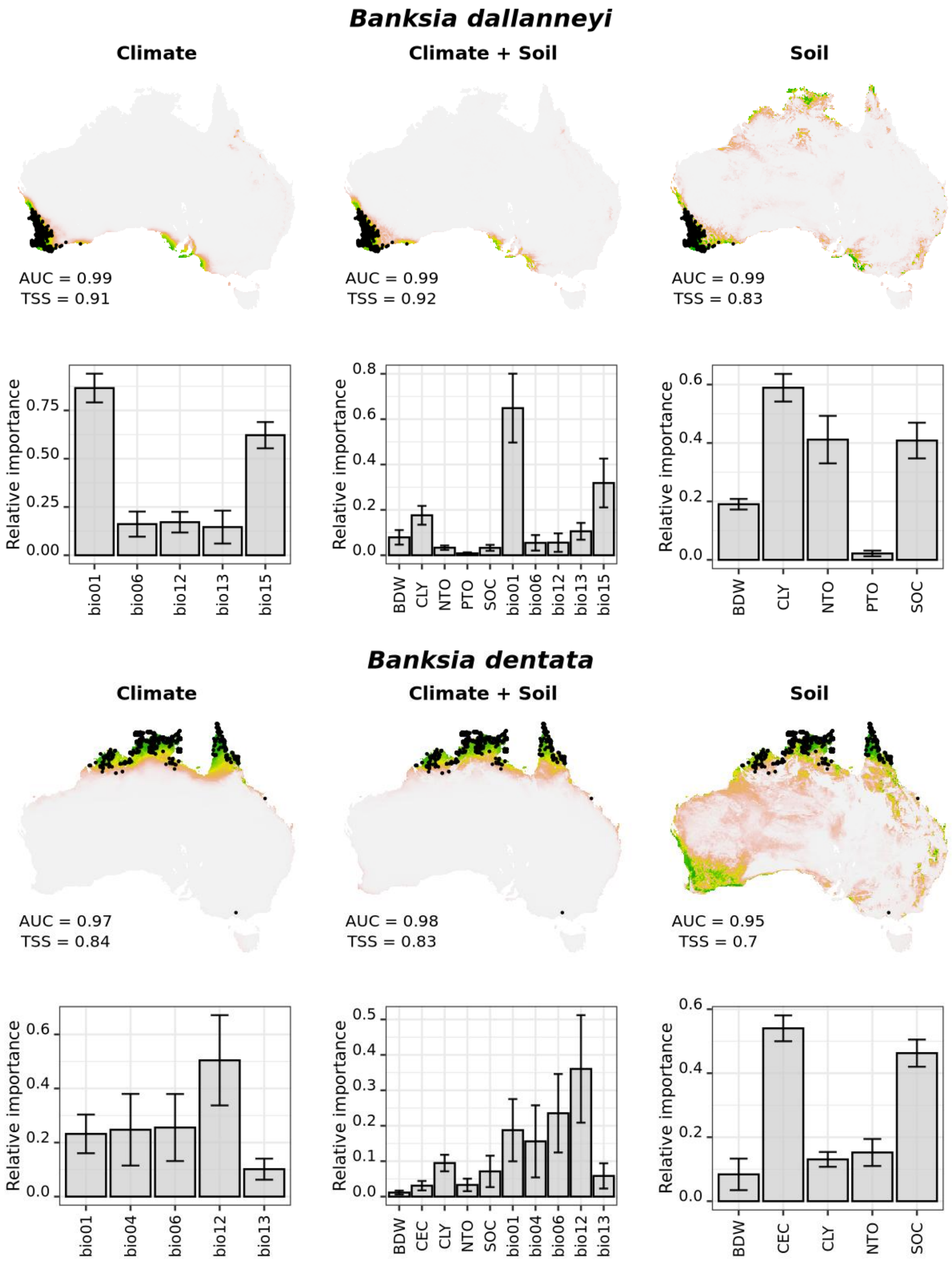
Banksia dallanneyi
Climate
Climate + Soil
Soil
AUC = 0.99
TSS = 0.91
AUC = 0.99
TSS = 0.92
AUC = 0.99
TSS = 0.83
Relative importance
bio01
bio06
bio12
bio13
bio15
BDW
CLY
NTO
PTO
SOC
Banksia dentata
Climate
Climate + Soil
Soil
AUC = 0.97
TSS = 0.84
AUC = 0.98
TSS = 0.83
AUC = 0.95
TSS = 0.7
Relative importance
bio01
bio04
bio06
bio12
bio13
BDW
CEC
CLY
NTO
SOC

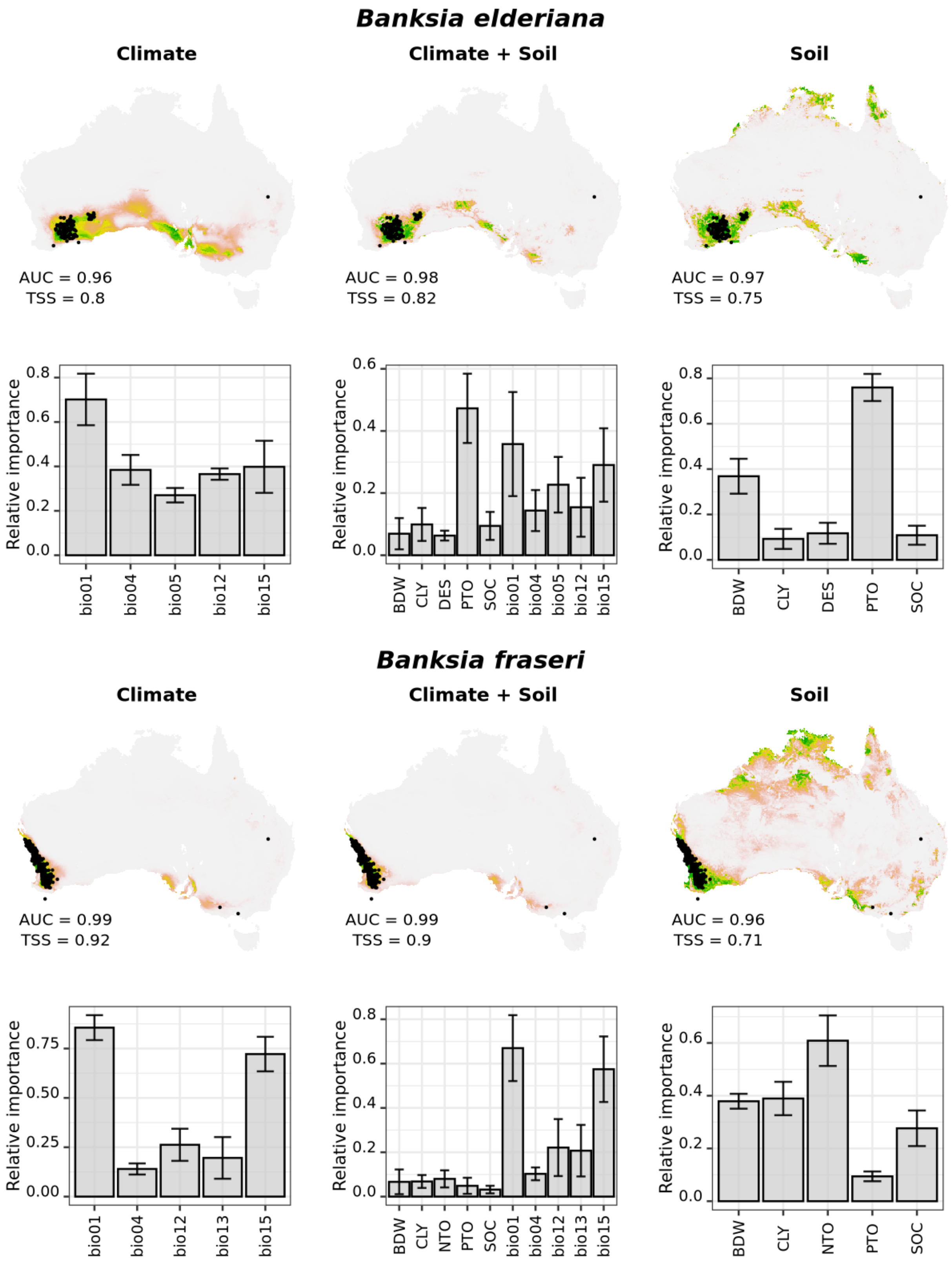
Banksia elderiana
Climate
Climate + Soil
Soil
AUC = 0.96
TSS = 0.8
AUC = 0.98
TSS = 0.82
AUC = 0.97
TSS = 0.75
Relative importance
bio01
bio04
bio05
bio12
bio15
BDW
CLY
DES
PTO
SOC
Banksia fraseri
Climate
Climate + Soil
Soil
AUC = 0.99
TSS = 0.92
AUC = 0.99
TSS = 0.9
AUC = 0.96
TSS = 0.71
Relative importance
bio01
bio04
bio12
bio13
bio15
BDW
CLY
NTO
PTO
SOC

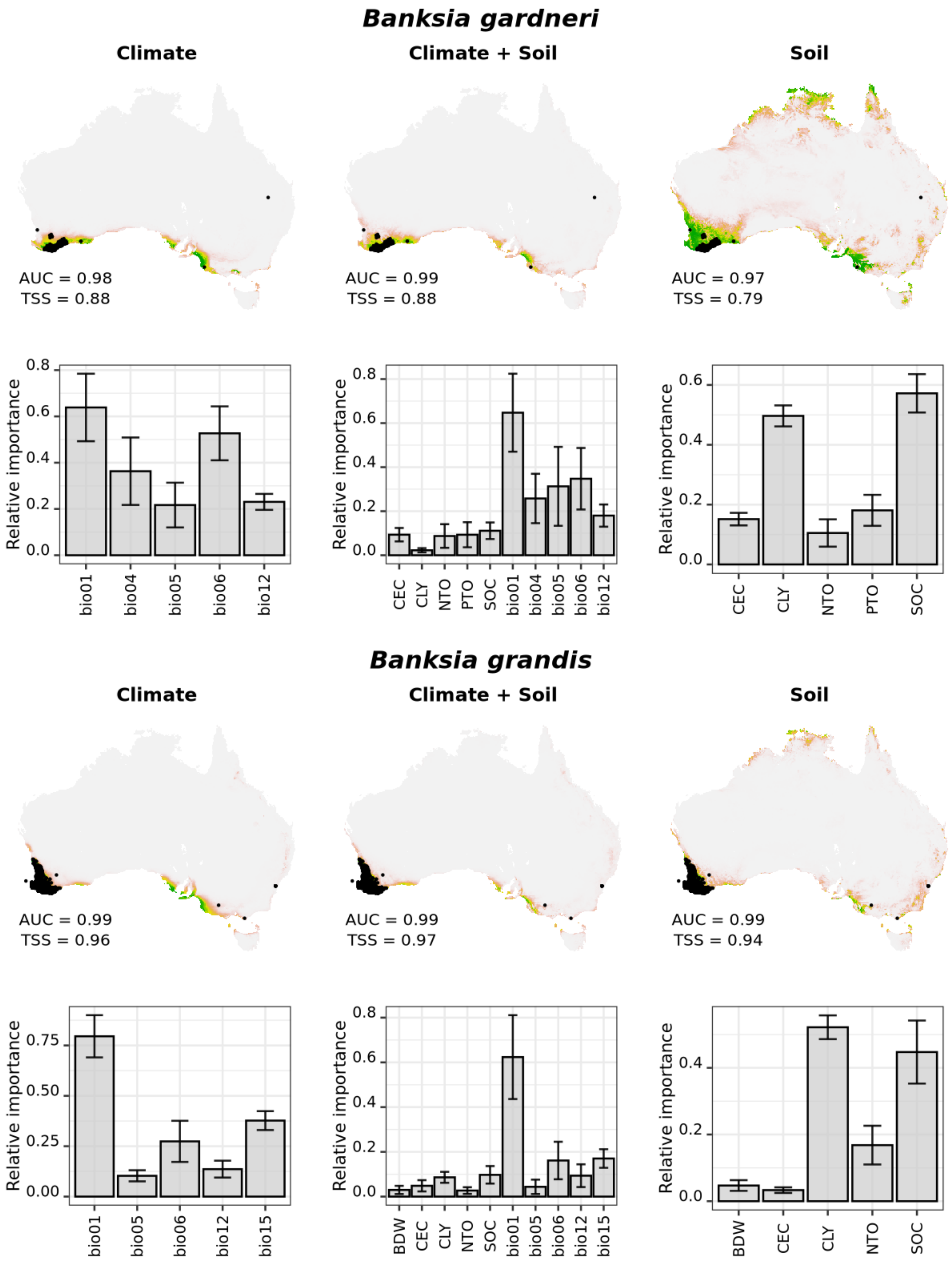
Banksia gardneri
Climate
Climate + Soil
Soil
AUC = 0.98
TSS = 0.88
AUC = 0.99
TSS = 0.88
AUC = 0.97
TSS = 0.79
Relative importance
0.0
0.2
0.4
0.6
0.8
bio01
bio04
bio05
bio06
bio12
CEC
CLY
NTO
PTO
SOC
Banksia grandis
Climate
Climate + Soil
Soil
AUC = 0.99
TSS = 0.96
AUC = 0.99
TSS = 0.97
AUC = 0.99
TSS = 0.94
0.00
0.25
0.50
0.75
bio15
BDW

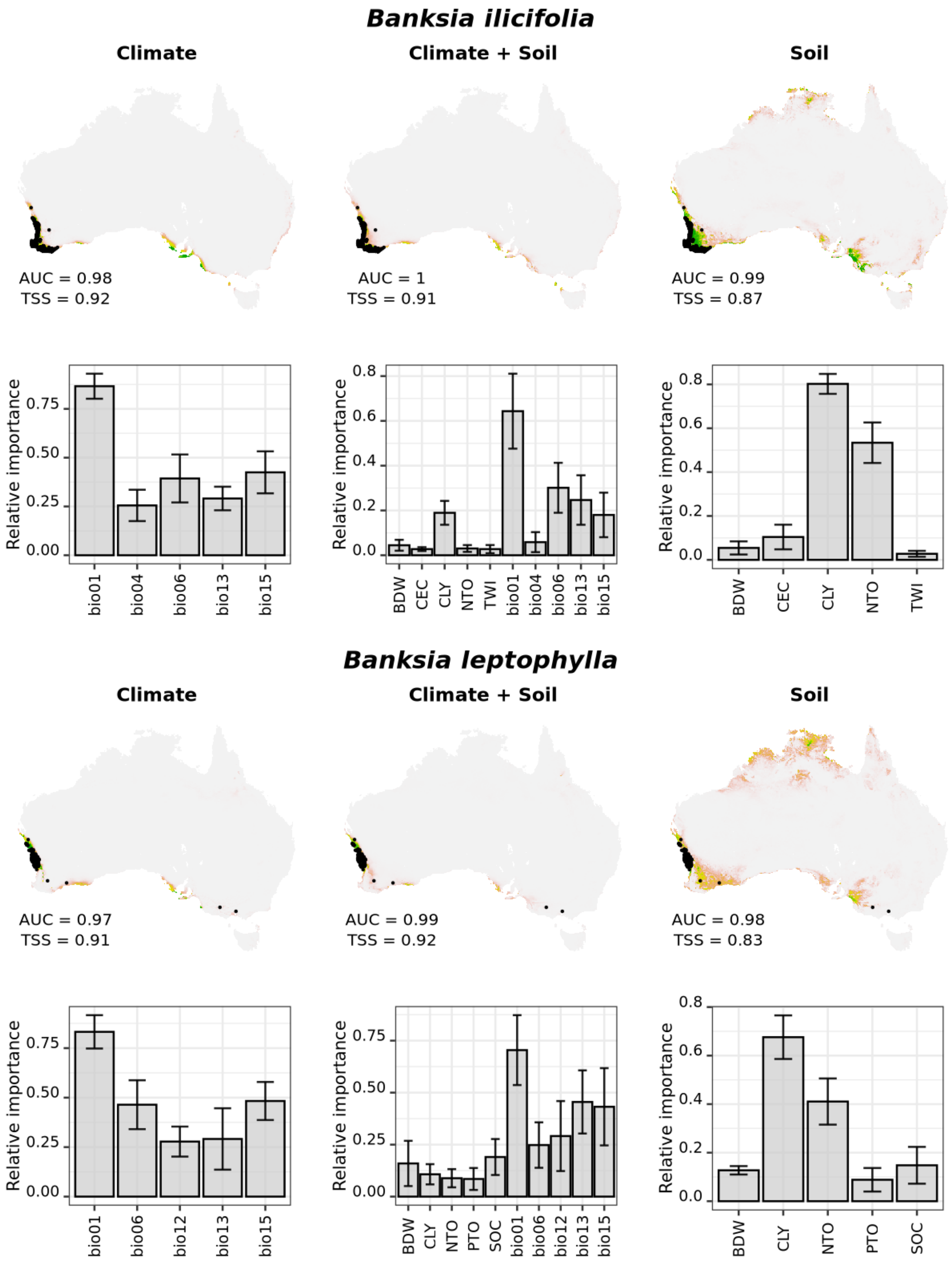

Banksia ilicifolia
Climate
Climate + Soil
Soil
AUC = 0.98
TSS = 0.92
AUC = 1
TSS = 0.91
AUC = 0.99
TSS = 0.87
Relative importance
0.00
0.25
0.50
0.75
bio01
bio04
bio06
bio13
bio15
0.0
0.2
0.4
0.6
0.8
BDW
CEC
CLY
NTO
TWI
bio01
bio04
bio06
bio13
bio15
BDW
CEC
CLY
NTO
TWI
Banksia leptophylla
Climate
Climate + Soil
Soil
AUC = 0.97
TSS = 0.91
AUC = 0.99
TSS = 0.92
AUC = 0.98
TSS = 0.83
Relative importance
bio01
bio06
bio12
bio13
bio15
BDW
CLY
NTO
PTO
SOC
bio01
bio06
bio12
bio13
bio15
BDW
CLY
NTO
PTO
SOC

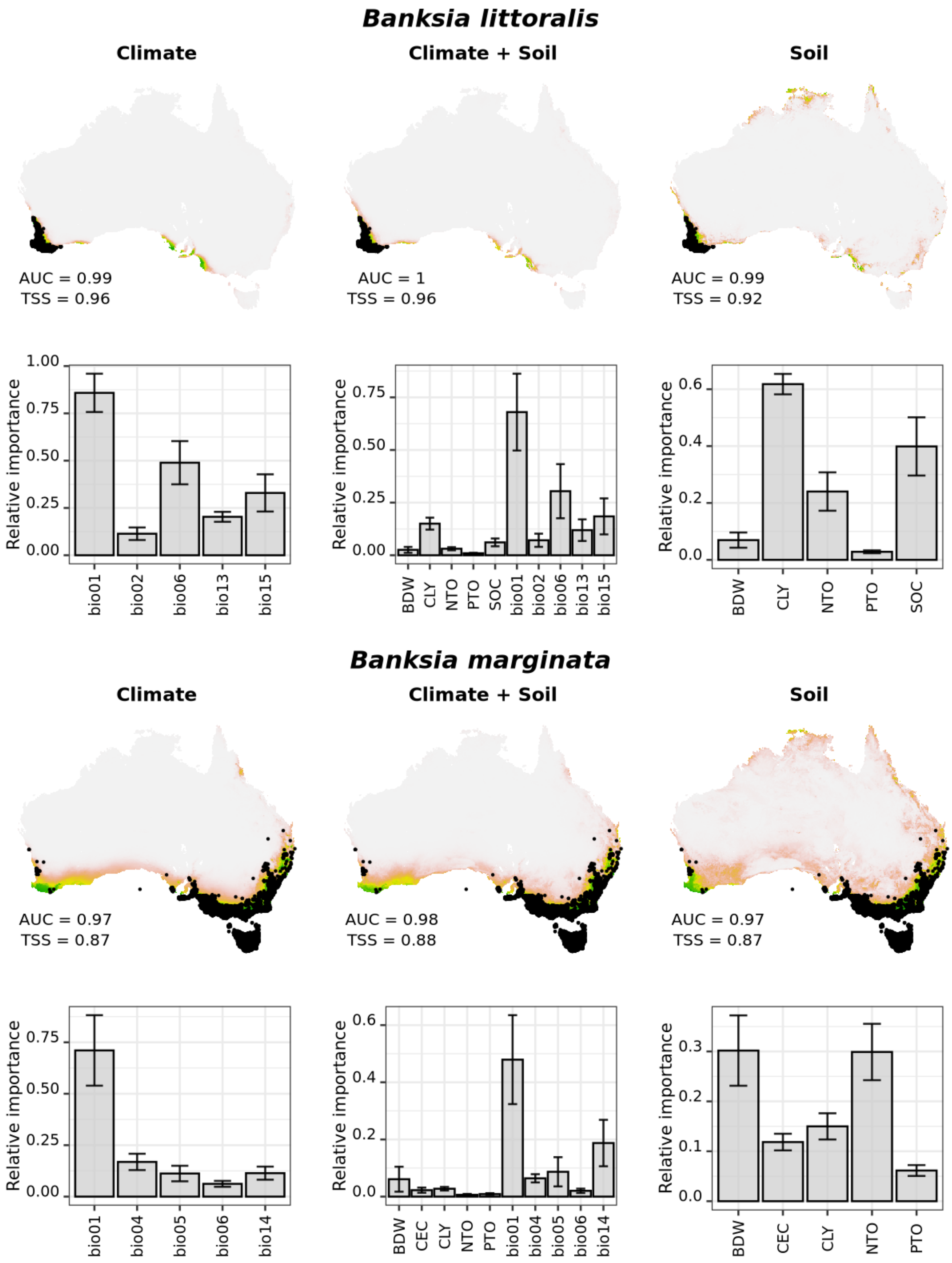
Banksia littoralis
Climate
Climate + Soil
Soil
AUC = 0.99
TSS = 0.96
AUC = 1
TSS = 0.96
AUC = 0.99
TSS = 0.92
Relative importance
bio01 bio02 bio06 bio13 bio15
BDW CLY NTO PTO SOC bio01 bio02 bio06 bio13 bio15
BDW CLY NTO PTO SOC
Banksia marginata
Climate
Climate + Soil
Soil
AUC = 0.97
TSS = 0.87
AUC = 0.98
TSS = 0.88
AUC = 0.97
TSS = 0.87
Relative importance
bio01 bio04 bio05 bio06 bio14
BDW CEC CLY NTO PTO bio01 bio04 bio05 bio06 bio14
BDW CEC CLY NTO PTO

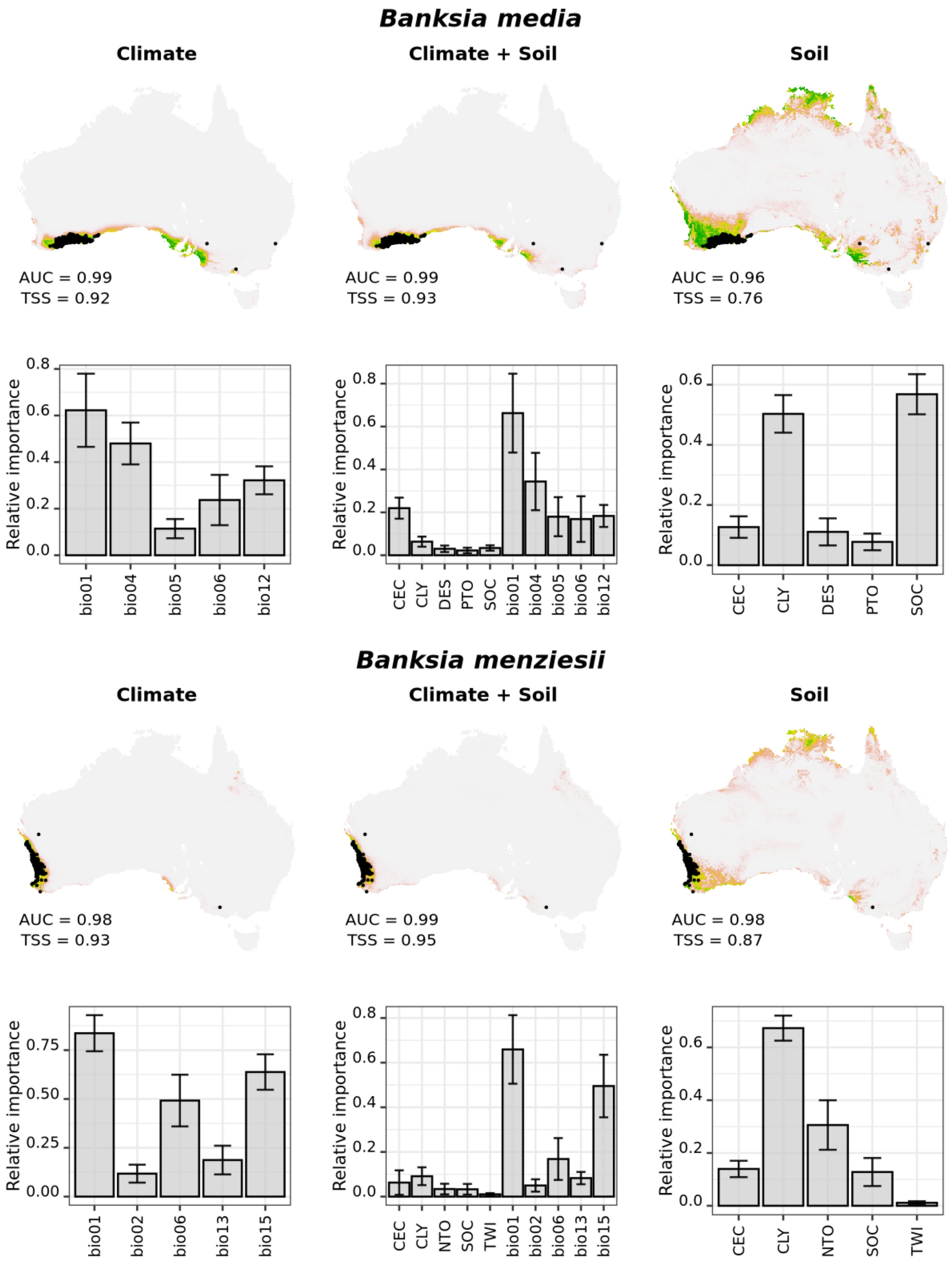

Banksia media
Climate
Climate + Soil
Soil
AUC = 0.99
TSS = 0.92
AUC = 0.99
TSS = 0.93
AUC = 0.96
TSS = 0.76
Relative importance
bio01 bio04 bio05 bio06 bio12
CEC CLY DES PTO SOC bio01 bio04 bio05 bio06 bio12
CEC CLY DES PTO SOC
Banksia menziesii
Climate
Climate + Soil
Soil
AUC = 0.98
TSS = 0.93
AUC = 0.99
TSS = 0.95
AUC = 0.98
TSS = 0.87
Relative importance
bio01 bio02 bio06 bio13 bio15
CEC CLY NTO SOC TWI bio01 bio02 bio06 bio13 bio15
CEC CLY NTO SOC TWI

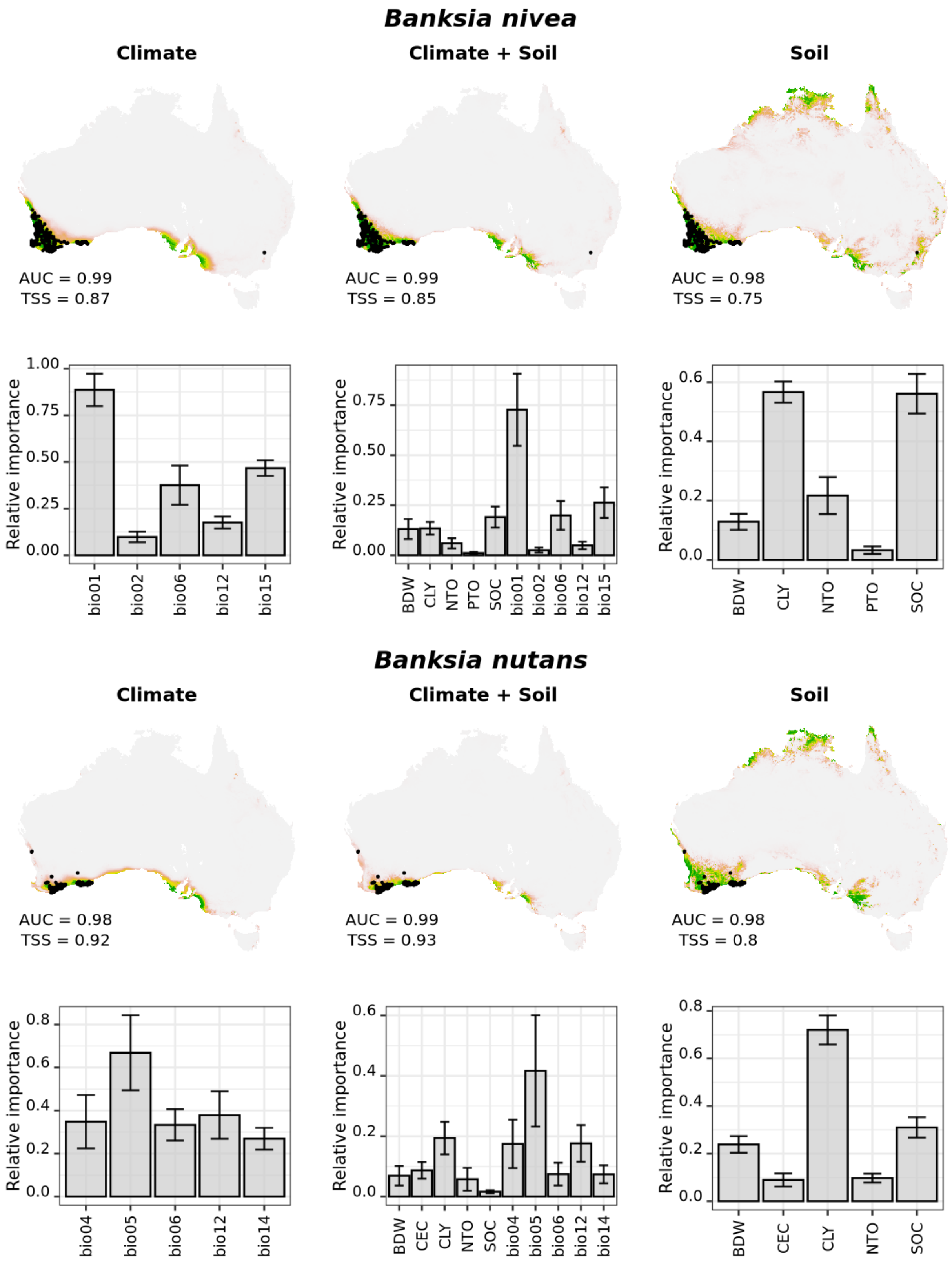
Banksia nivea
Climate
Climate + Soil
Soil
AUC = 0.99
TSS = 0.87
AUC = 0.99
TSS = 0.85
AUC = 0.98
TSS = 0.75
Relative importance
1.00
0.75
0.50
0.25
0.00
bio01
bio02
bio06
bio12
bio15
BDW
CLY
NTO
PTO
SOC
0.6
0.4
0.2
0.0
Banksia nutans
AUC = 0.98
TSS = 0.92
AUC = 0.99
TSS = 0.93
AUC = 0.98
TSS = 0.8
0.8
bio04
bio05
bio14
CEC

Banksia oblongifolia

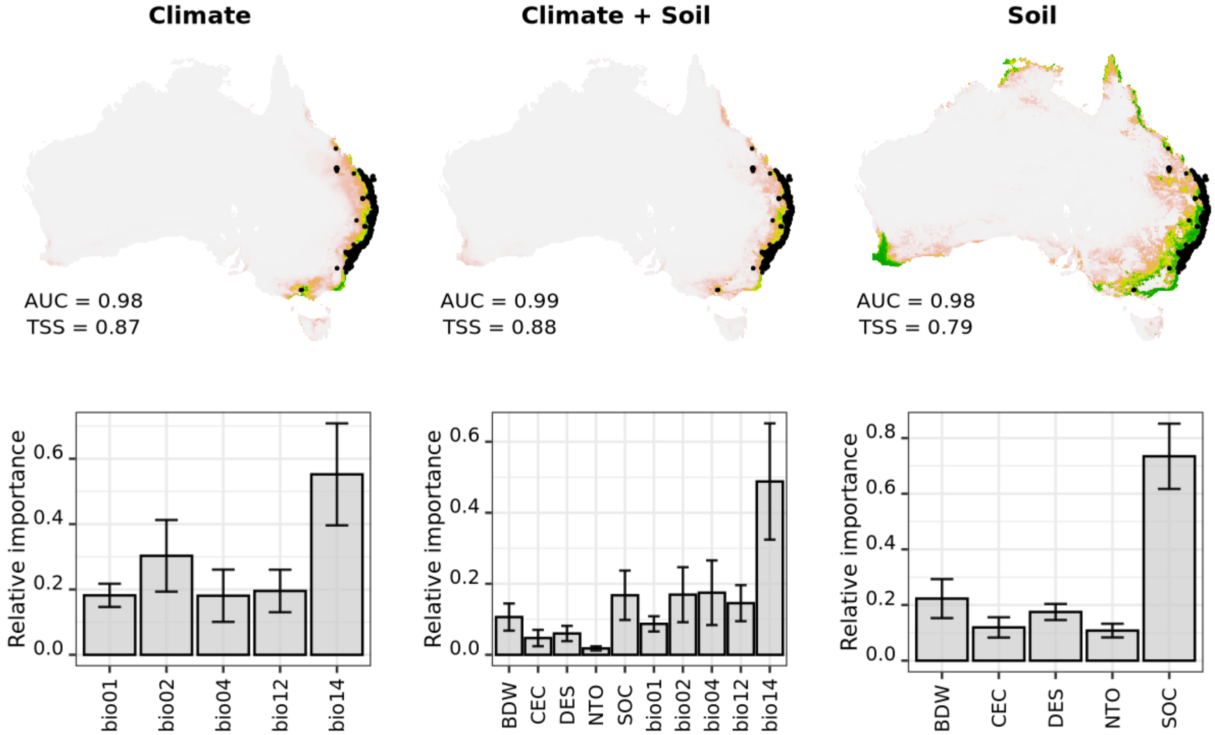

Banksia obovata

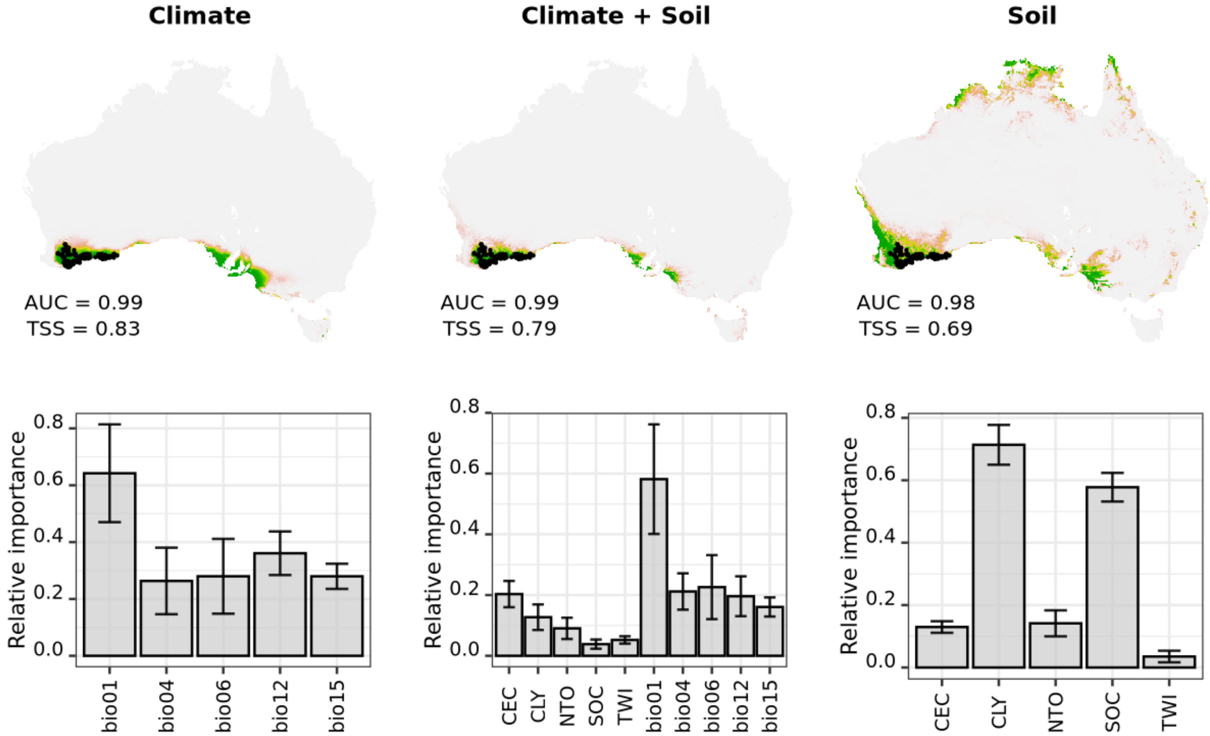

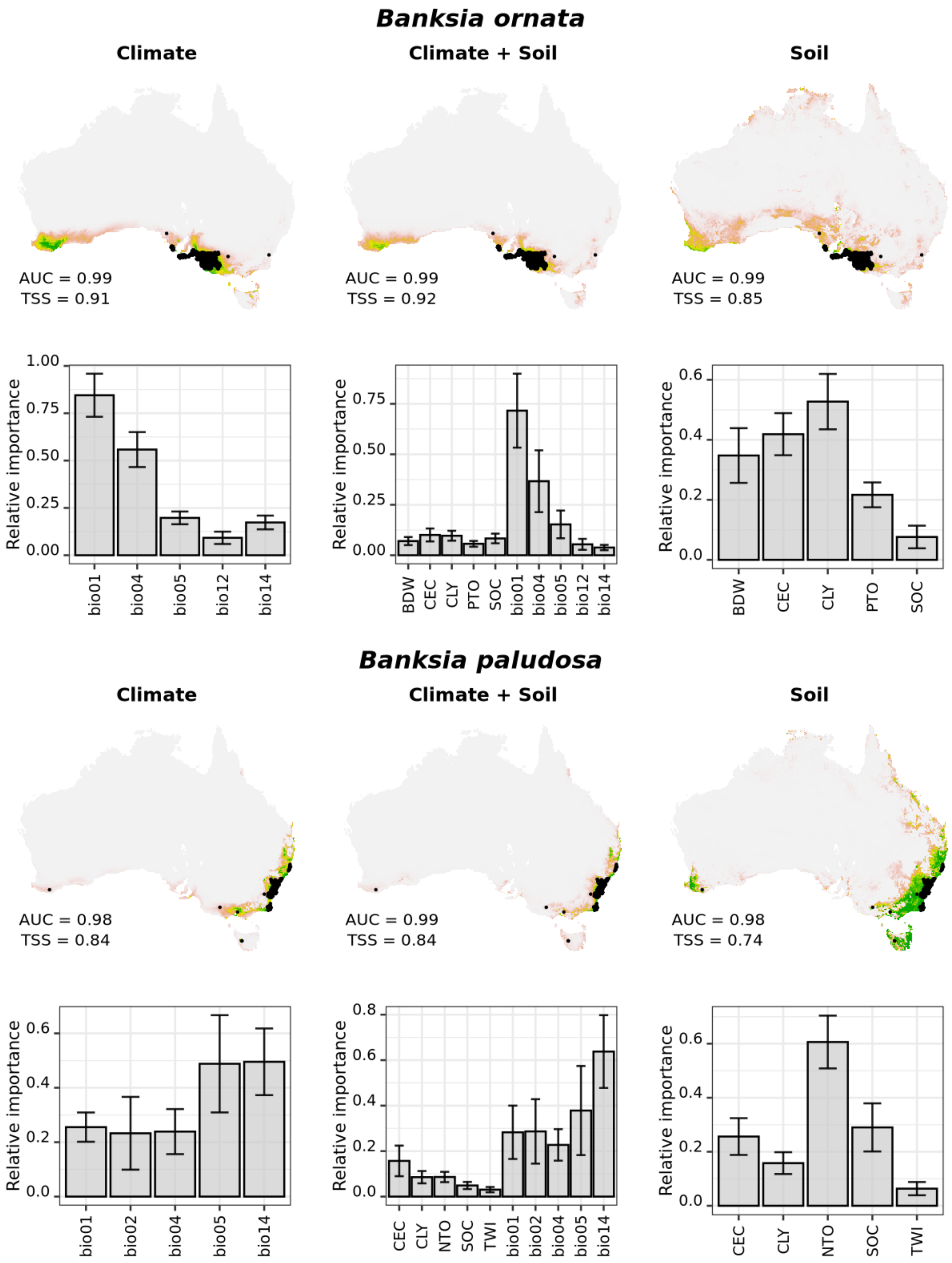
Banksia ornata
Climate
Climate + Soil
Soil
AUC = 0.99
TSS = 0.91
AUC = 0.99
TSS = 0.92
AUC = 0.99
TSS = 0.85
Relative importance
1.00
0.75
0.50
0.25
0.00
bio01
bio04
bio05
bio12
bio14
BDW
CEC
CLY
PTO
SOC
0.6
0.4
0.2
0.0
Banksia paludosa
AUC = 0.98
TSS = 0.84
AUC = 0.99
TSS = 0.84
AUC = 0.98
TSS = 0.74
bio02
NTO
TWI
0.8

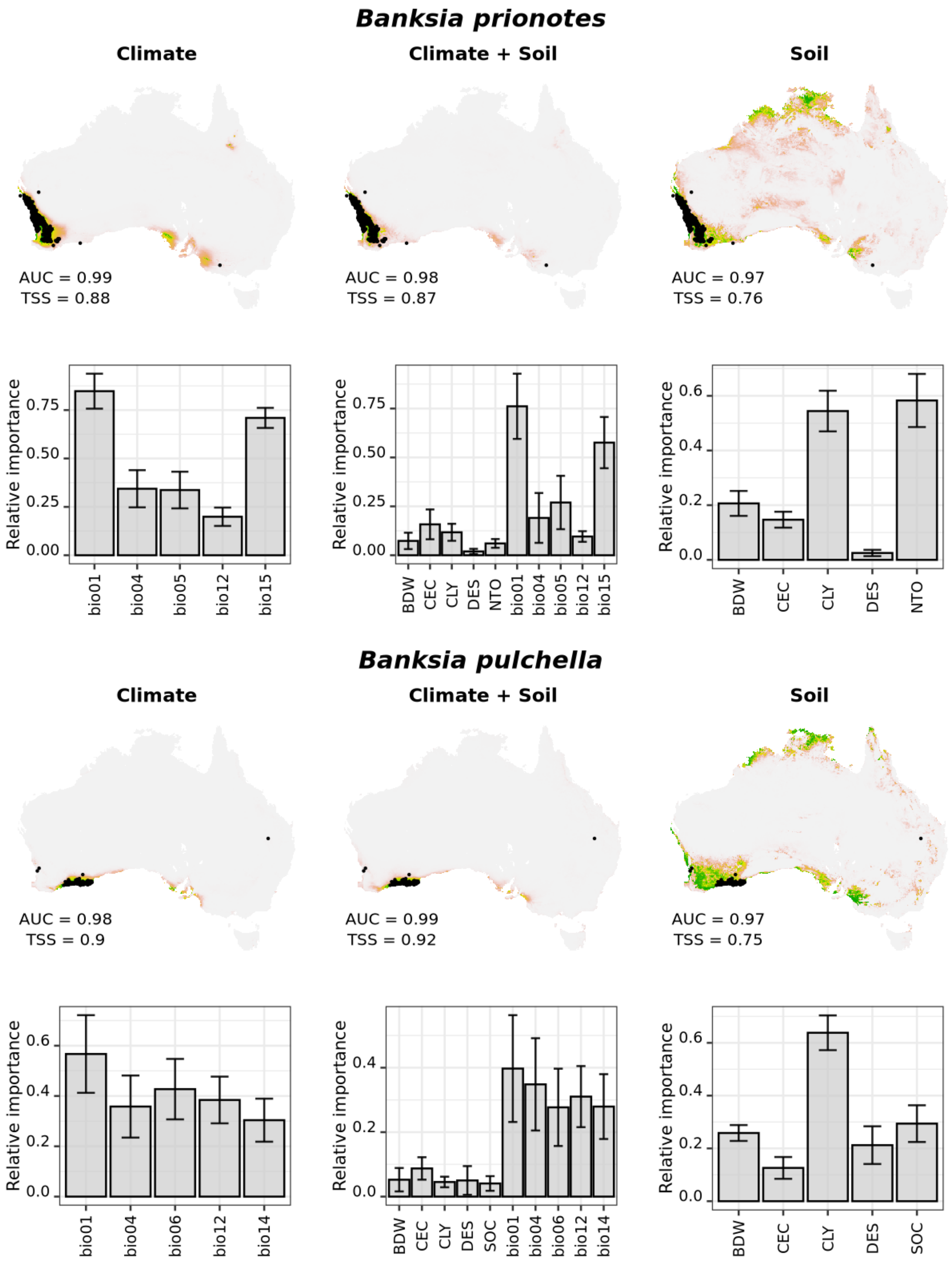

Banksia prionotes
Climate
Climate + Soil
Soil
AUC = 0.99
TSS = 0.88
AUC = 0.98
TSS = 0.87
AUC = 0.97
TSS = 0.76
Relative importance
0.00
0.25
0.50
0.75
bio01
bio04
bio05
bio12
bio15
BDW
CEC
CLY
DES
NTO
0.0
0.2
0.4
0.6
Banksia pulchella
Climate
Climate + Soil
Soil
AUC = 0.98
TSS = 0.9
AUC = 0.99
TSS = 0.92
AUC = 0.97
TSS = 0.75
bio06
bio14
SOC

Banksia quercifolia

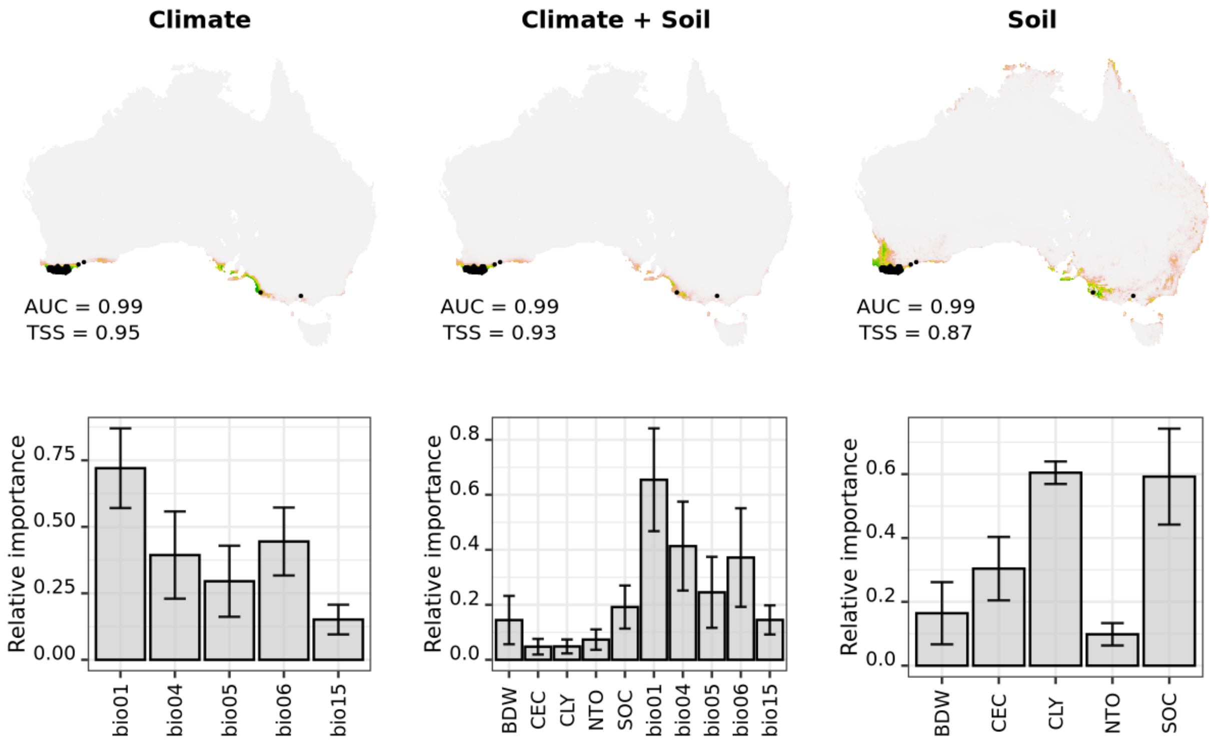

Banksia repens

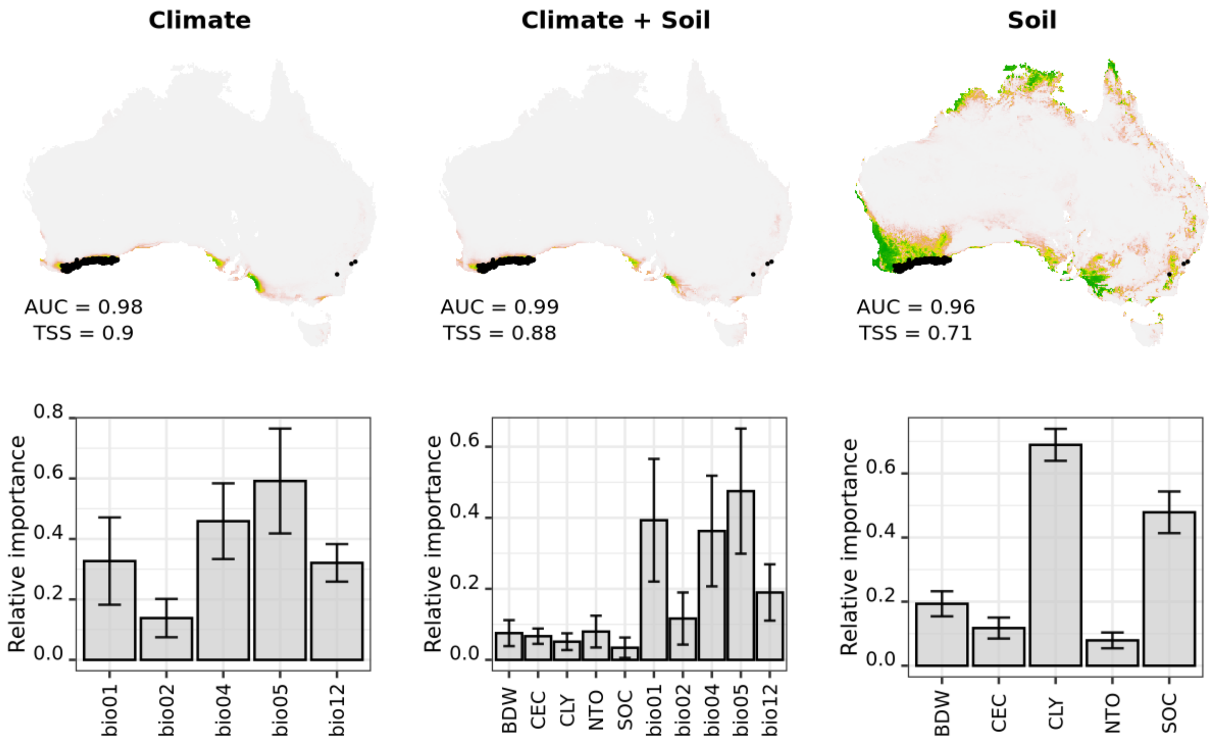

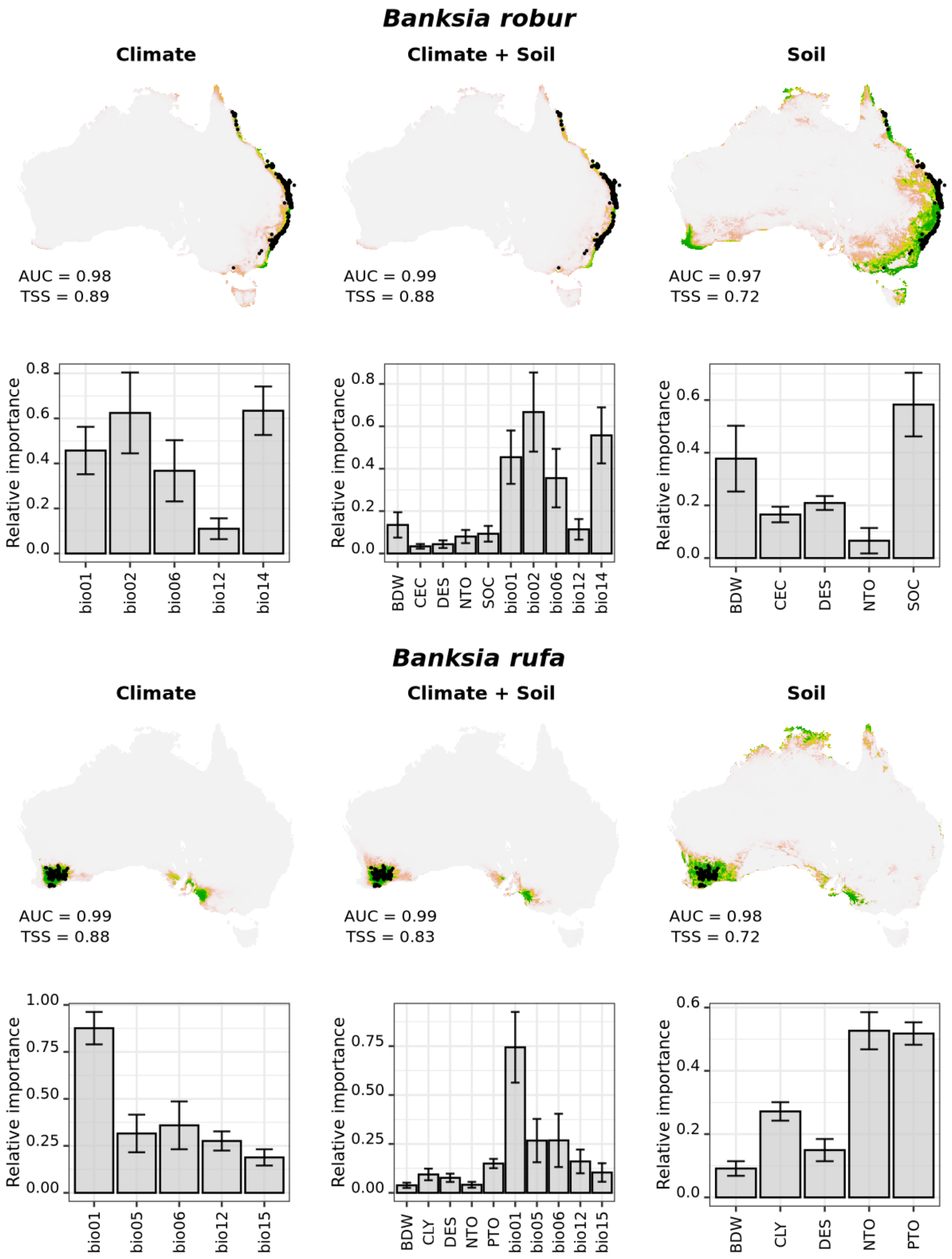
Banksia robur
Climate
Climate + Soil
Soil
AUC = 0.98
TSS = 0.89
AUC = 0.99
TSS = 0.88
AUC = 0.97
TSS = 0.72
Relative importance
bio01
bio02
bio06
bio12
bio14
BDW
CEC
DES
NTO
SOC
Banksia rufa
Climate
Climate + Soil
Soil
AUC = 0.99
TSS = 0.88
AUC = 0.99
TSS = 0.83
AUC = 0.98
TSS = 0.72
Relative importance
bio01
bio05
bio06
bio12
bio15
BDW
CLY
DES
NTO
PTO

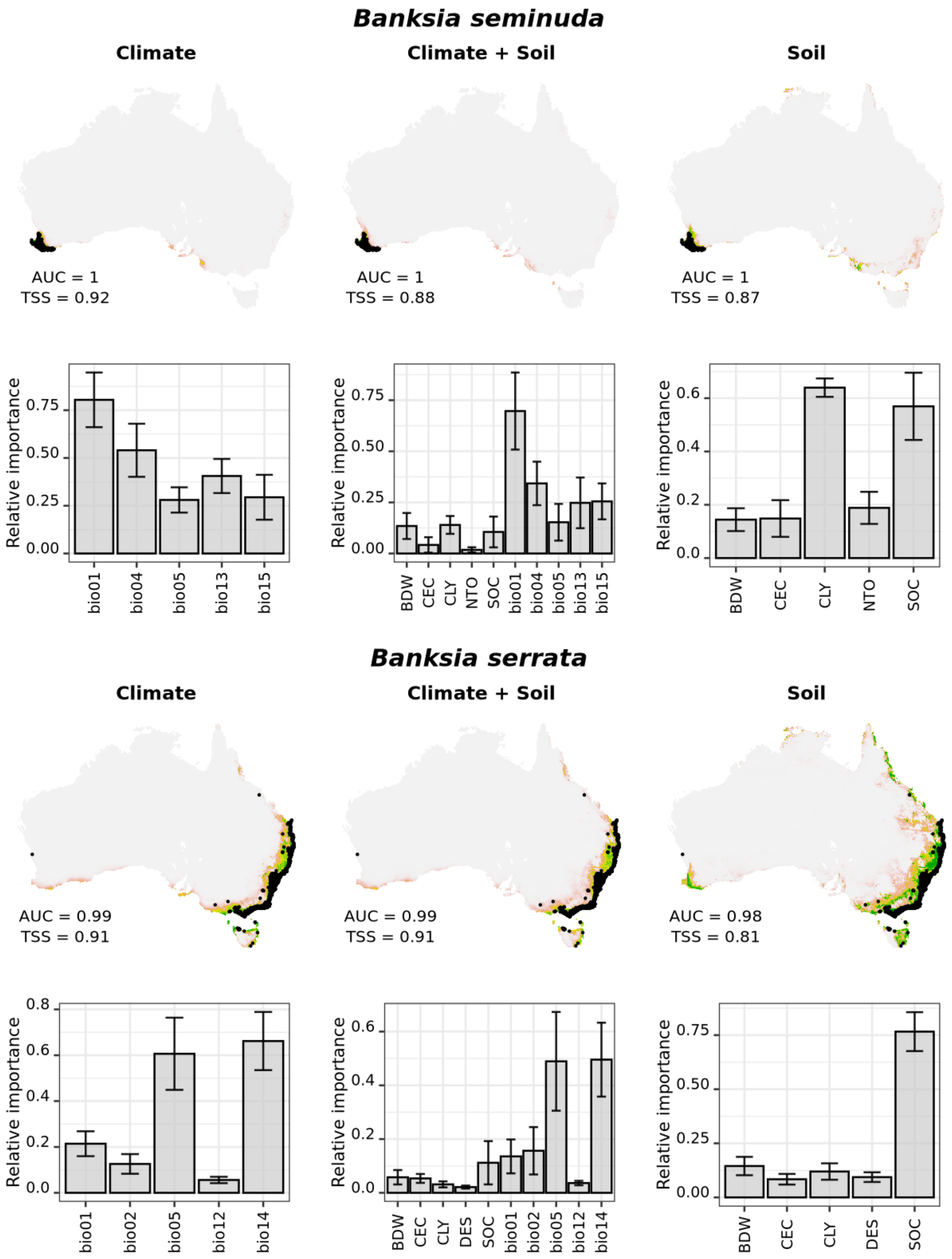
Banksia seminuda
Climate
Climate + Soil
Soil
AUC = 1
TSS = 0.92
AUC = 1
TSS = 0.88
AUC = 1
TSS = 0.87
Relative importance
bio01
bio04
bio05
bio13
bio15
BDW
CEC
CLY
NTO
SOC
Banksia serrata
Climate
Climate + Soil
Soil
AUC = 0.99
TSS = 0.91
AUC = 0.99
TSS = 0.91
AUC = 0.98
TSS = 0.81
Relative importance
bio01
bio02
bio05
bio12
bio14
BDW
CEC
CLY
DES
SOC

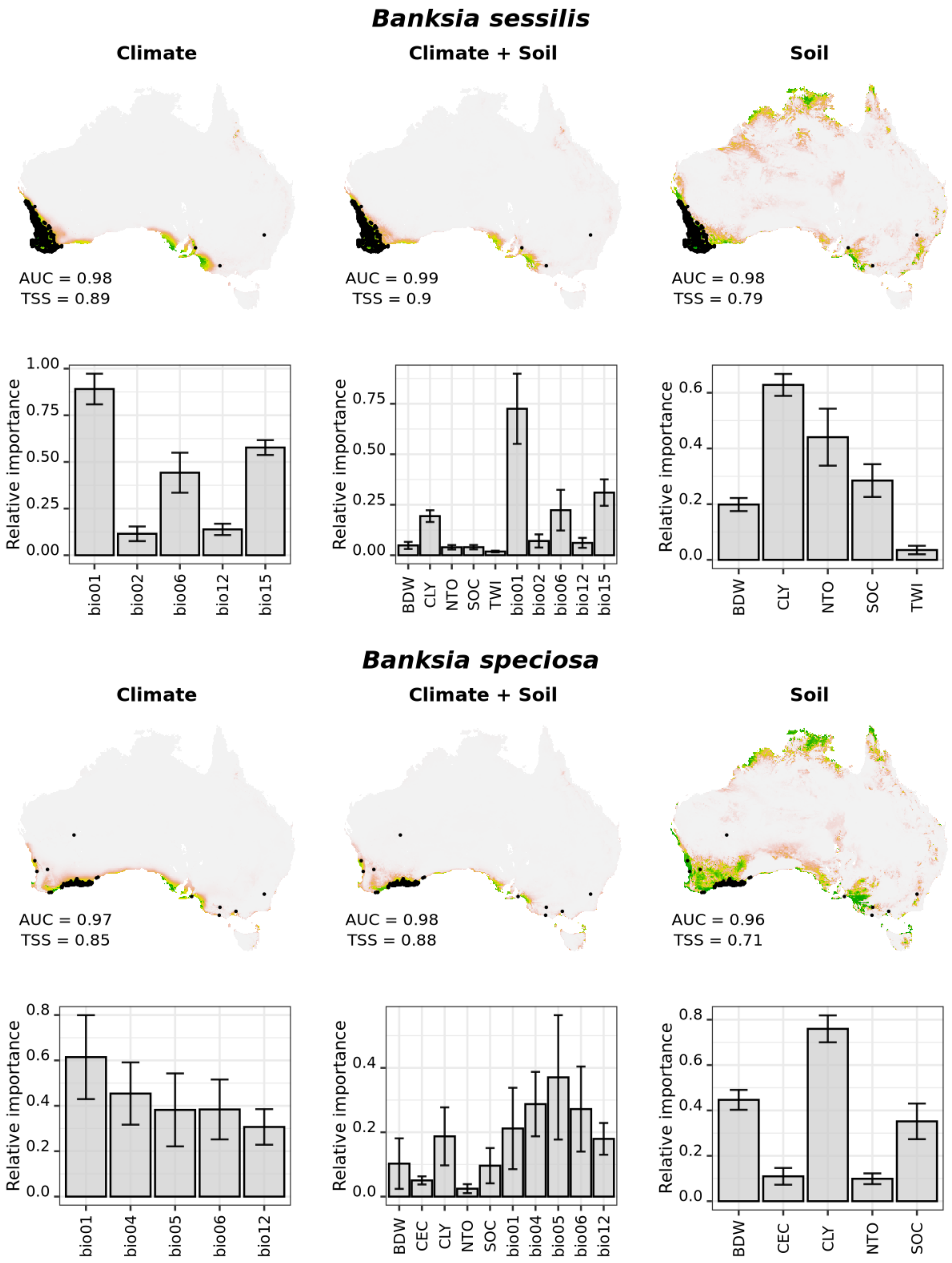
Banksia sessilis
Climate
Climate + Soil
Soil
AUC = 0.98
TSS = 0.89
AUC = 0.99
TSS = 0.9
AUC = 0.98
TSS = 0.79
Relative importance
bio01
bio02
bio06
bio12
bio15
BDW
CLY
NTO
SOC
TWI
Banksia speciosa
Climate
Climate + Soil
Soil
AUC = 0.97
TSS = 0.85
AUC = 0.98
TSS = 0.88
AUC = 0.96
TSS = 0.71
Relative importance
bio01
bio04
bio05
bio06
bio12
BDW
CEC
CLY
NTO
SOC

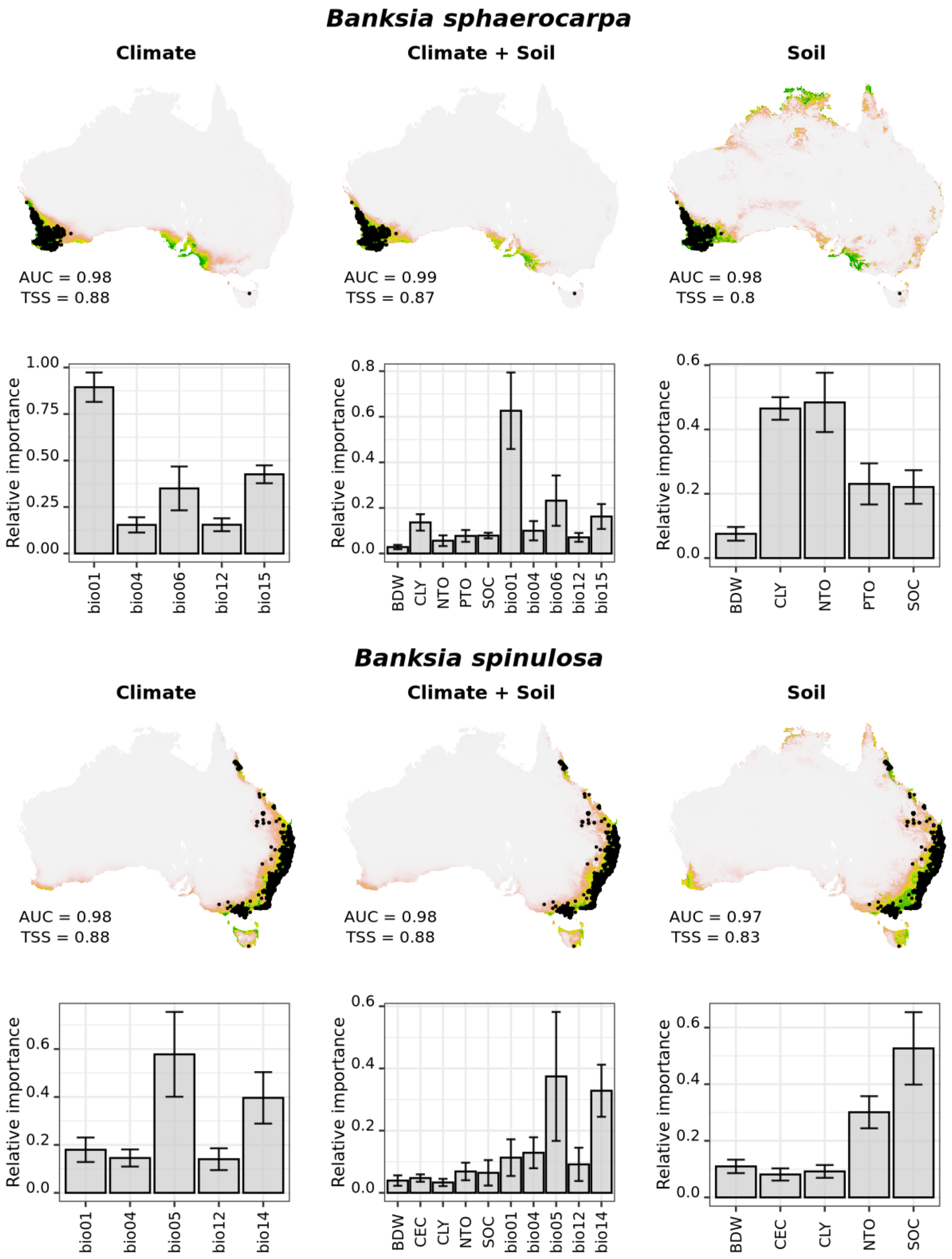
Banksia sphaerocarpa
Climate
Climate + Soil
Soil
AUC = 0.98
TSS = 0.88
AUC = 0.99
TSS = 0.87
AUC = 0.98
TSS = 0.8
Relative importance
1.00
0.75
0.50
0.25
0.00
bio01
bio04
bio06
bio12
bio15
0.8
0.6
0.4
0.2
0.0
BDW
CLY
NTO
PTO
SOC
bio01
bio04
bio06
bio12
bio15
0.6
0.4
0.2
0.0
BDW
CLY
NTO
PTO
SOC
Banksia spinulosa
Climate
Climate + Soil
Soil
AUC = 0.98
TSS = 0.88
AUC = 0.98
TSS = 0.88
AUC = 0.97
TSS = 0.83
Relative importance
0.6
0.4
0.2
0.0
bio01
bio04
bio05
bio12
bio14
0.6
0.4
0.2
0.0
BDW
CEC
CLY
NTO
SOC
bio01
bio04
bio05
bio12
bio14
0.6
0.4
0.2
0.0
BDW
CEC
CLY
NTO
SOC

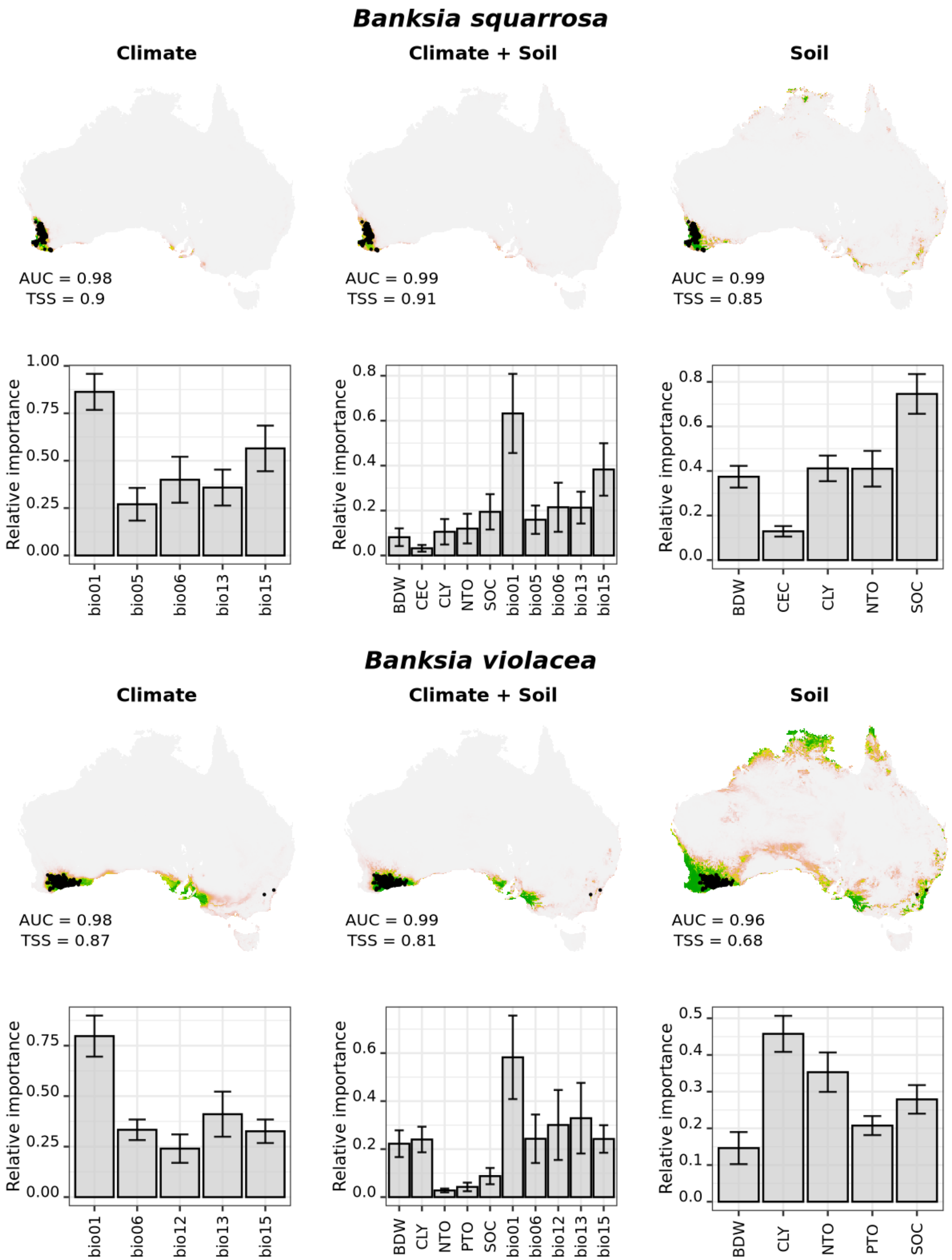

Banksia squarrosa
Climate
Climate + Soil
Soil
AUC = 0.98
TSS = 0.9
AUC = 0.99
TSS = 0.91
AUC = 0.99
TSS = 0.85
Relative importance
1.00
0.75
0.50
0.25
0.00
bio01
bio05
bio06
bio13
bio15
0.8
0.6
0.4
0.2
0.0
BDW
CEC
CLY
NTO
SOC
bio01
bio05
bio06
bio13
bio15
BDW
CEC
CLY
NTO
SOC
Banksia violacea
Climate
Climate + Soil
Soil
AUC = 0.98
TSS = 0.87
AUC = 0.99
TSS = 0.81
AUC = 0.96
TSS = 0.68
bio01
bio06
bio12
bio13
bio15
BDW
CLY
NTO
PTO
SOC
bio01
bio06
bio12
bio13
bio15
BDW
CLY
NTO
PTO
SOC
0.5
0.4
0.3
0.2
0.1
0.0

Barringtonia acutangula

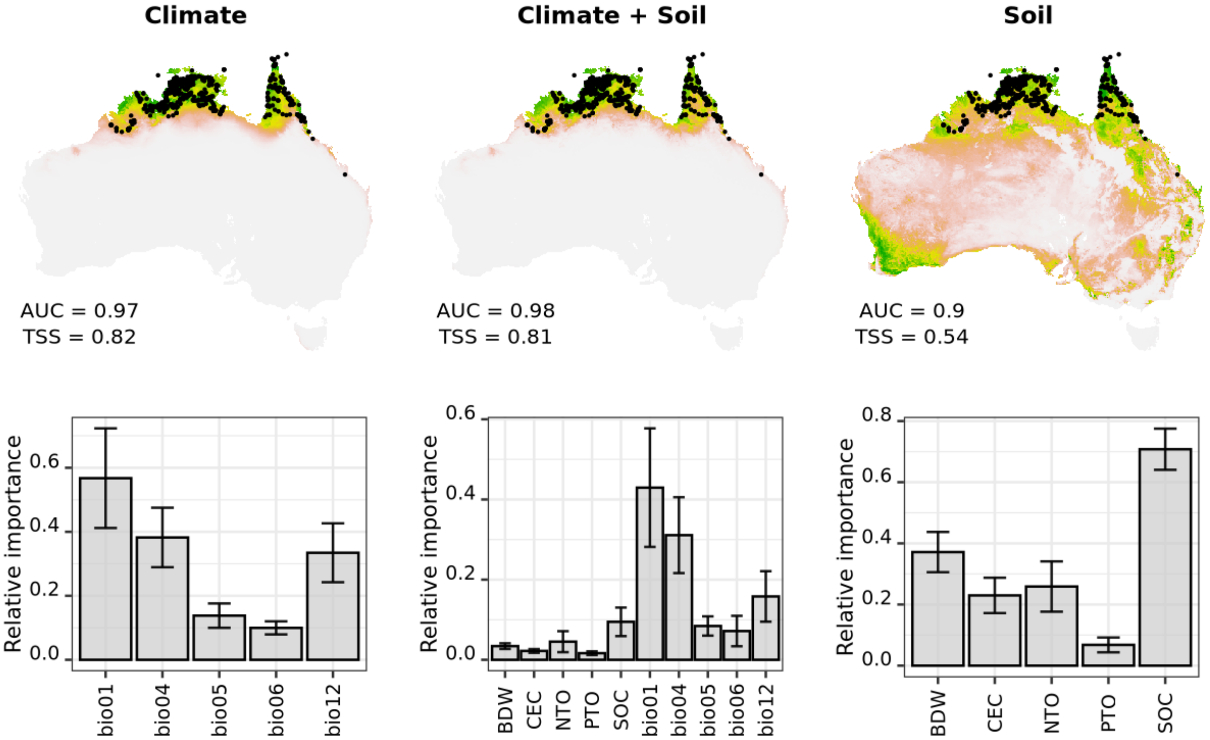

Basilicum polystachyon

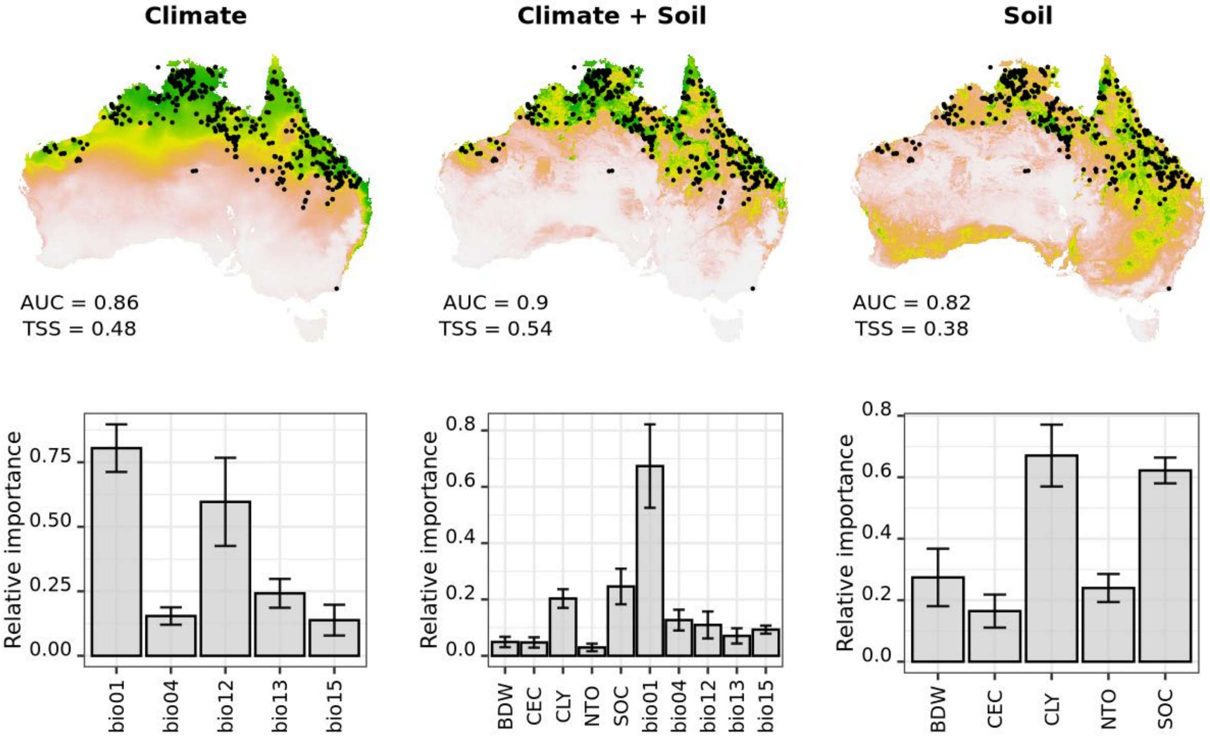

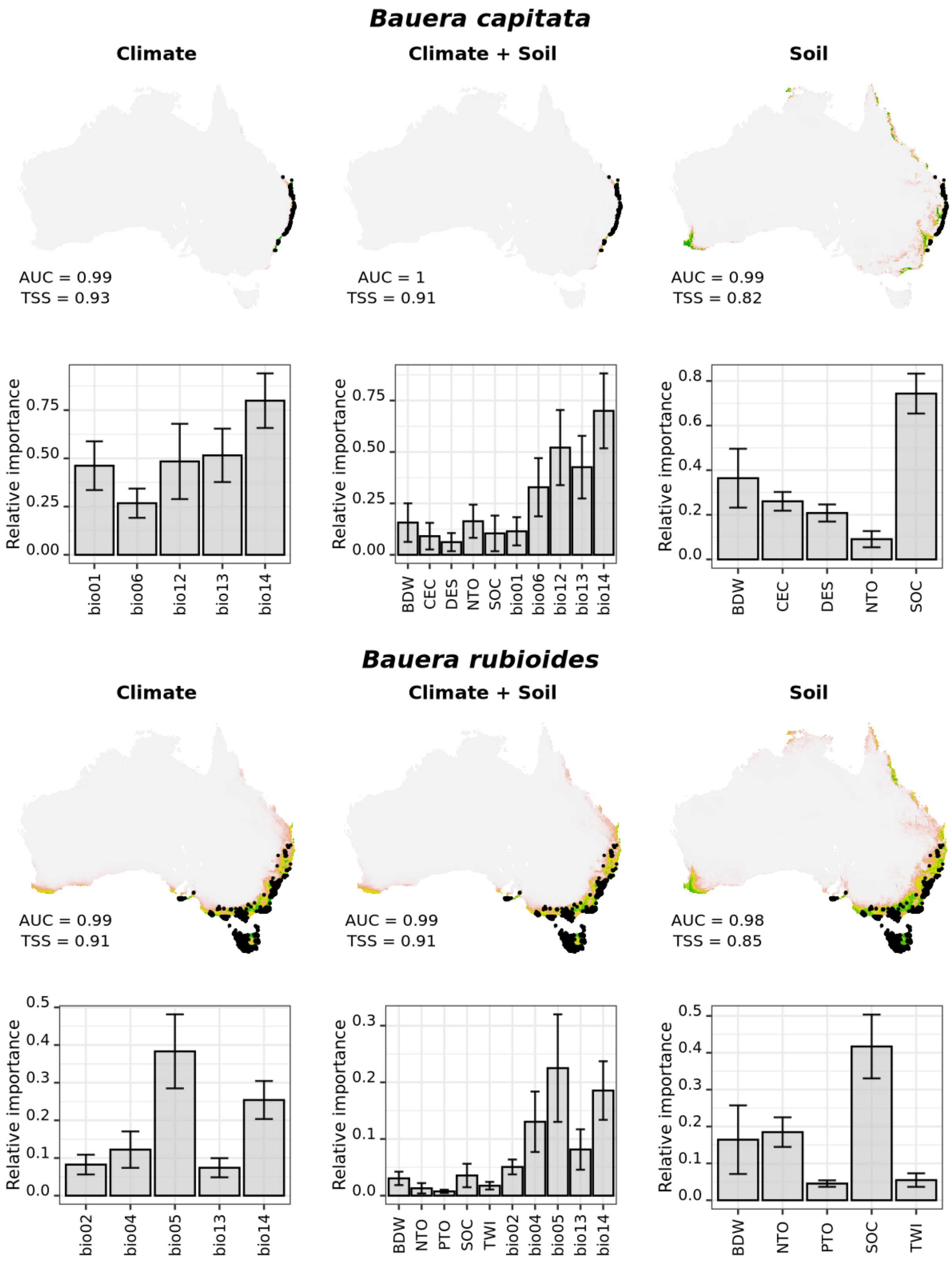

Bauera capitata
Climate
Climate + Soil
Soil
AUC = 0.99
TSS = 0.93
AUC = 1
TSS = 0.91
AUC = 0.99
TSS = 0.82
Relative importance
bio01 bio06 bio12 bio13 bio14
BDW CEC DES NTO SOC bio01 bio06 bio12 bio13 bio14
BDW CEC DES NTO SOC
Bauera rubioides
Climate
Climate + Soil
Soil
AUC = 0.99
TSS = 0.91
AUC = 0.99
TSS = 0.91
AUC = 0.98
TSS = 0.85
Relative importance
bio02 bio04 bio05 bio13 bio14
BDW NTO PTO SOC TWI bio02 bio04 bio05 bio13 bio14
BDW NTO PTO SOC TWI

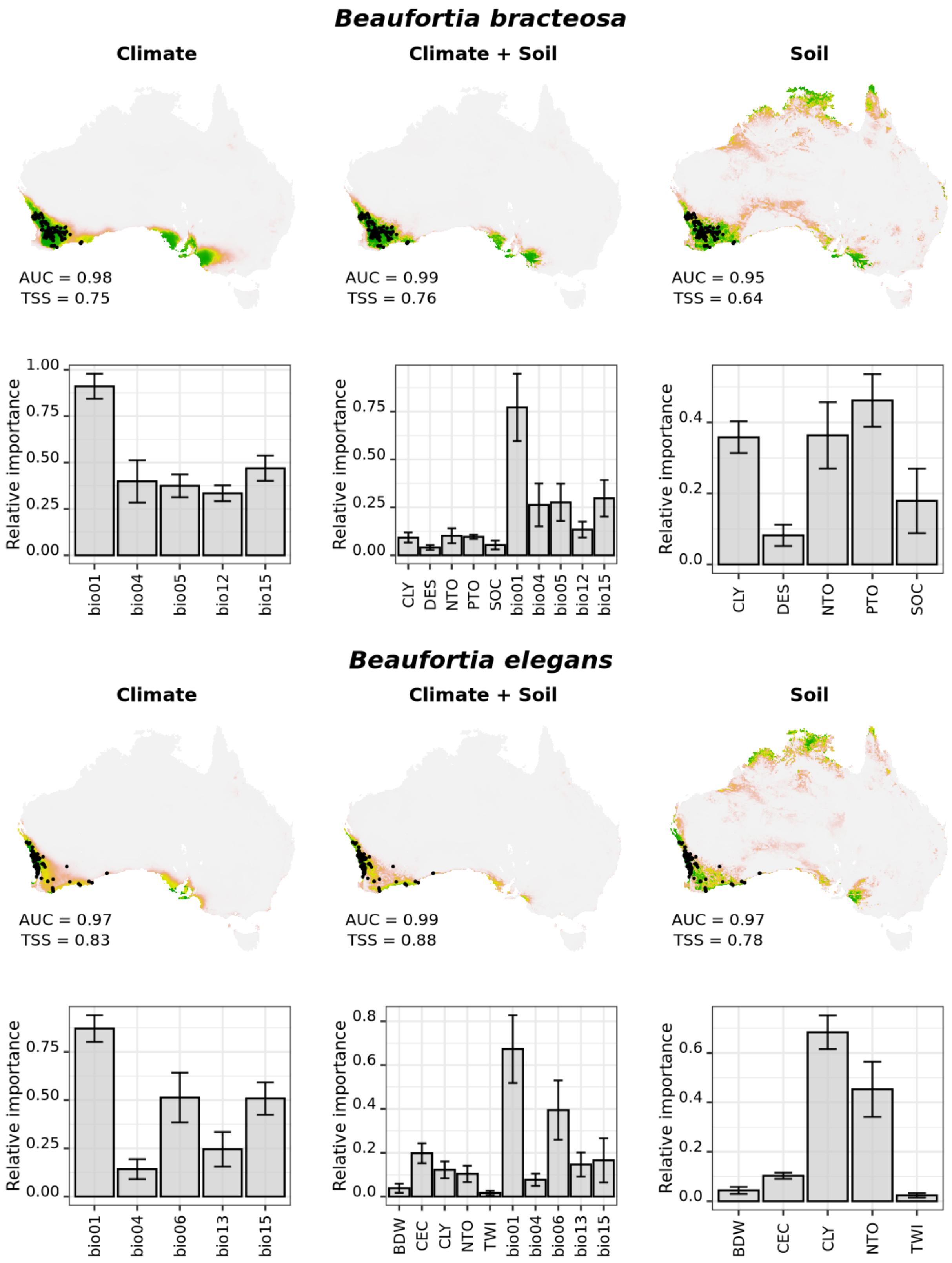

Beaufortia bracteosa
Climate
Climate + Soil
Soil
AUC = 0.98
TSS = 0.75
AUC = 0.99
TSS = 0.76
AUC = 0.95
TSS = 0.64
Relative importance
bio01
bio04
bio05
bio12
bio15
CLY
DES
NTO
PTO
SOC
Beaufortia elegans
Climate
Climate + Soil
Soil
AUC = 0.97
TSS = 0.83
AUC = 0.99
TSS = 0.88
AUC = 0.97
TSS = 0.78
Relative importance
bio01
bio04
bio06
bio13
bio15
BDW
CEC
CLY
NTO
TWI

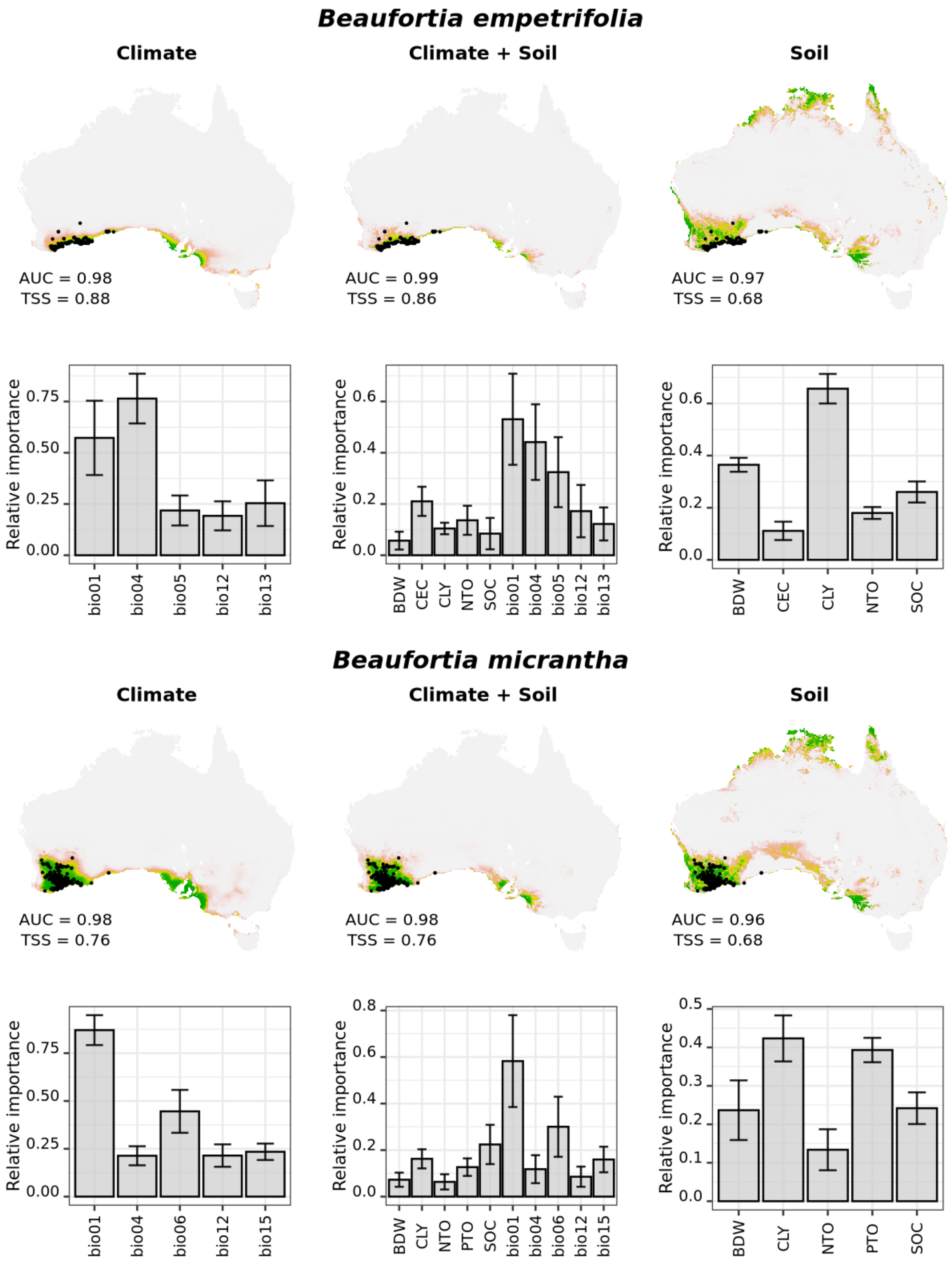

Beaufortia empetrifolia
Climate
Climate + Soil
Soil
AUC = 0.98
TSS = 0.88
AUC = 0.99
TSS = 0.86
AUC = 0.97
TSS = 0.68
Relative importance
0.00
0.25
0.50
0.75
bio01
bio04
bio05
bio12
bio13
0.0
0.2
0.4
0.6
BDW
CEC
CLY
NTO
SOC
bio01
bio04
bio05
bio12
bio13
BDW
CEC
CLY
NTO
SOC
Beaufortia micrantha
Climate
Climate + Soil
Soil
AUC = 0.98
TSS = 0.76
AUC = 0.98
TSS = 0.76
AUC = 0.96
TSS = 0.68
Relative importance
bio01
bio04
bio06
bio12
bio15
0.8
BDW
CLY
NTO
PTO
SOC
bio01
bio04
bio06
bio12
bio15
0.1
0.3
0.5
BDW
CLY
NTO
PTO
SOC

Beaufortia puberula

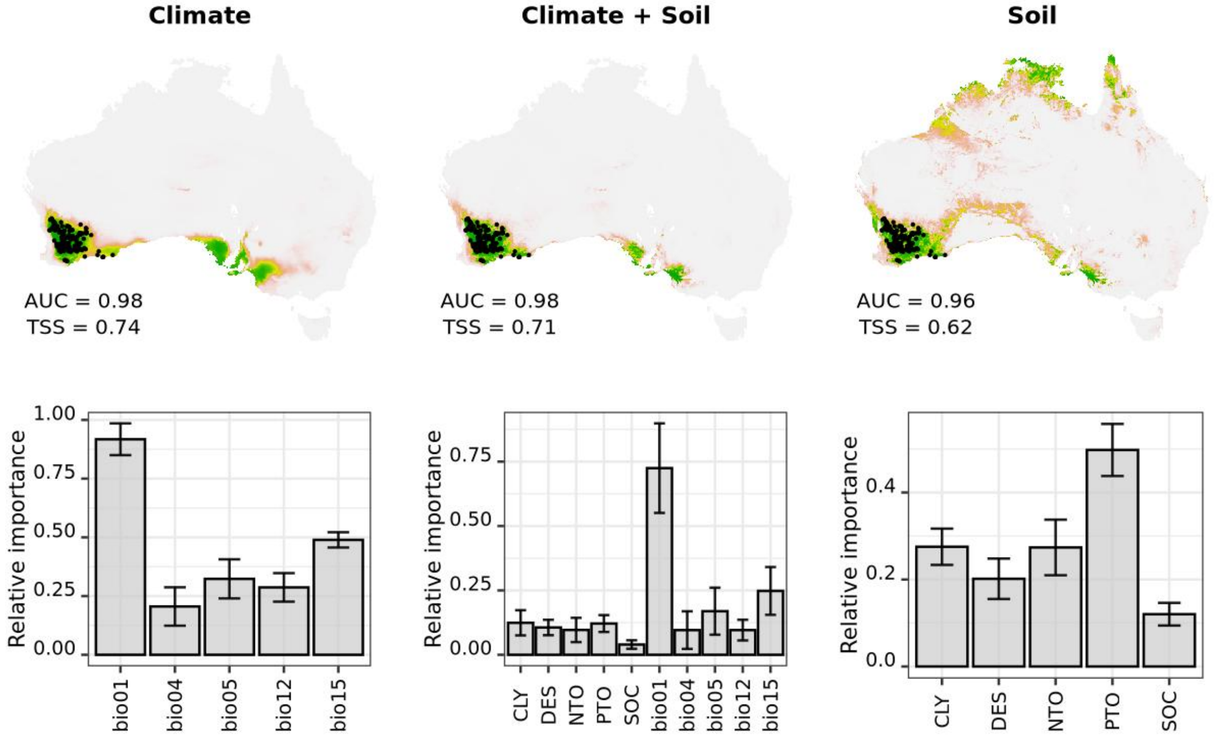

Beaufortia schaueri

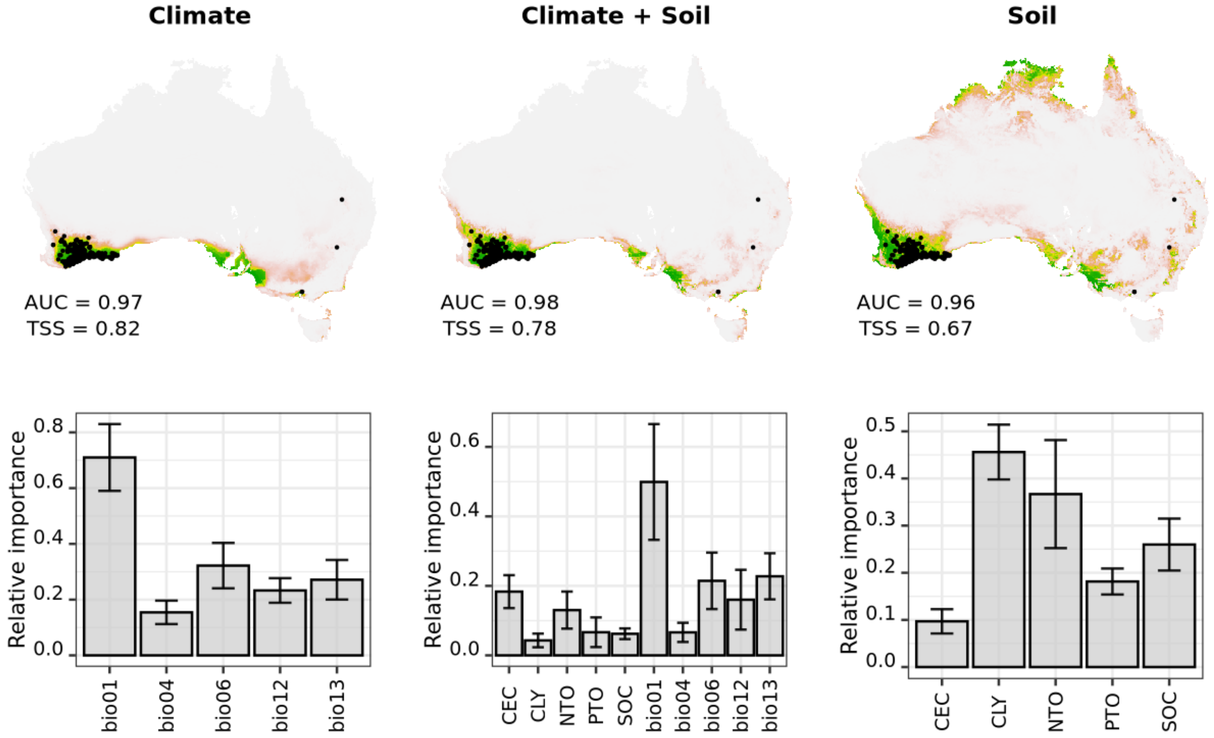

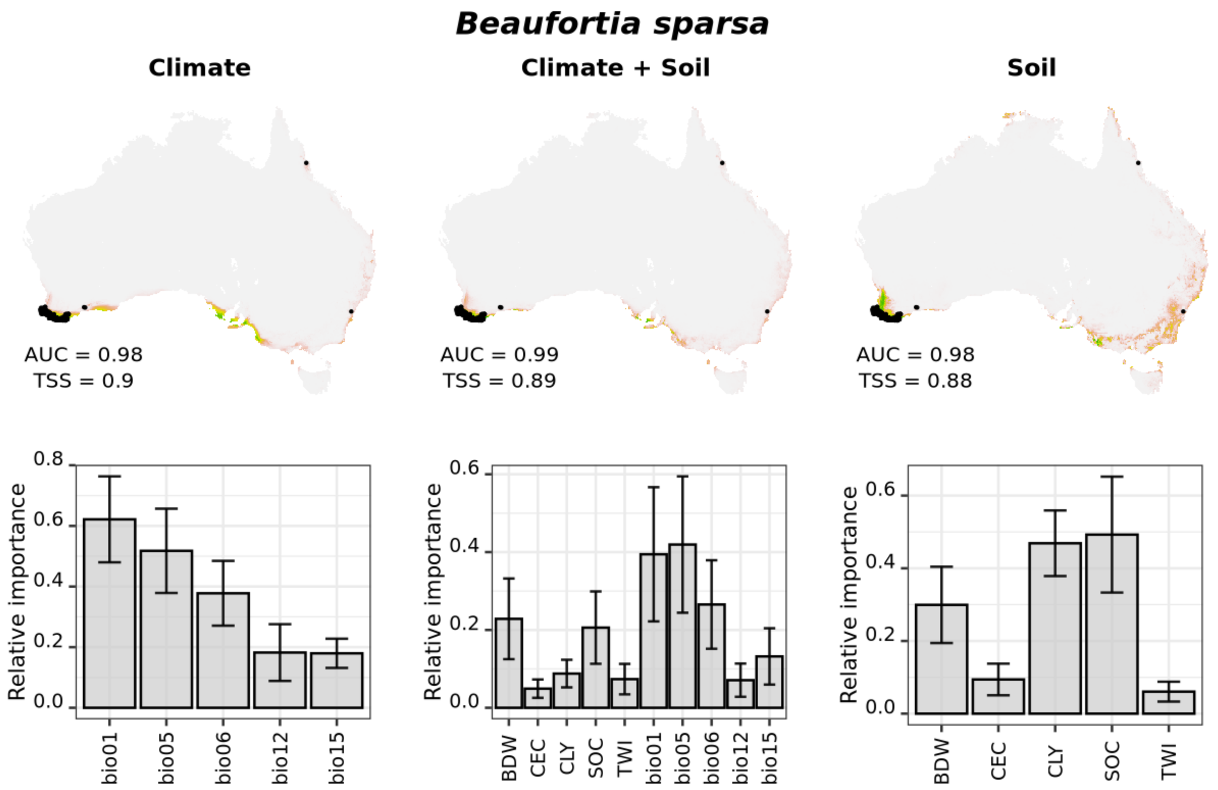
Beaufortia sparsa
Climate
Climate + Soil
Soil
AUC = 0.98
TSS = 0.9
AUC = 0.99
TSS = 0.89
AUC = 0.98
TSS = 0.88
Relative importance
0.0
0.2
0.4
0.6
0.8
bio01
bio05
bio06
bio12
bio15
BDW
CEC
CLY
SOC
TWI
bio01
bio05
bio06
bio12
bio15
BDW
CEC
CLY
SOC
TWI

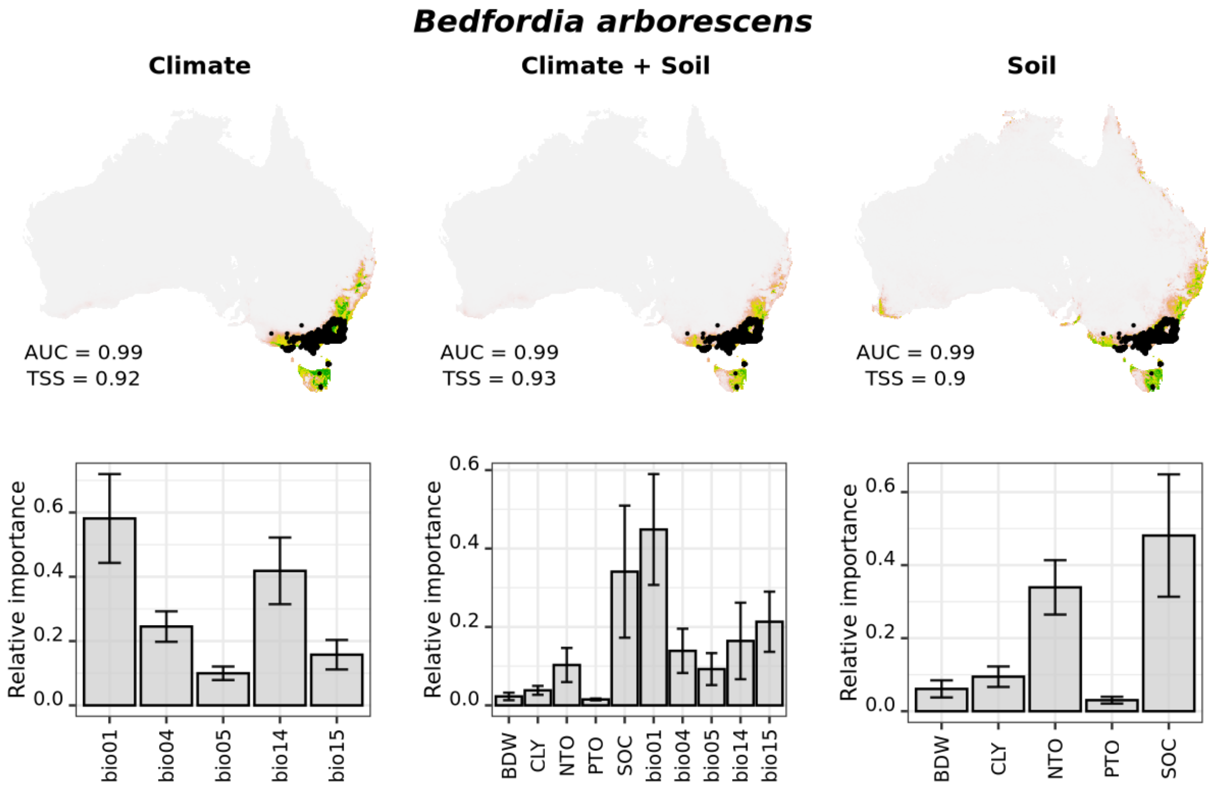
Bedfordia arborescens
Climate
Climate + Soil
Soil
AUC = 0.99
TSS = 0.92
AUC = 0.99
TSS = 0.93
AUC = 0.99
TSS = 0.9
Relative importance
0.0
0.2
0.4
0.6
bio01
bio04
bio05
bio14
bio15
BDW
CLY
NTO
PTO
SOC
bio01
bio04
bio05
bio14
bio15
BDW
CLY
NTO
PTO
SOC

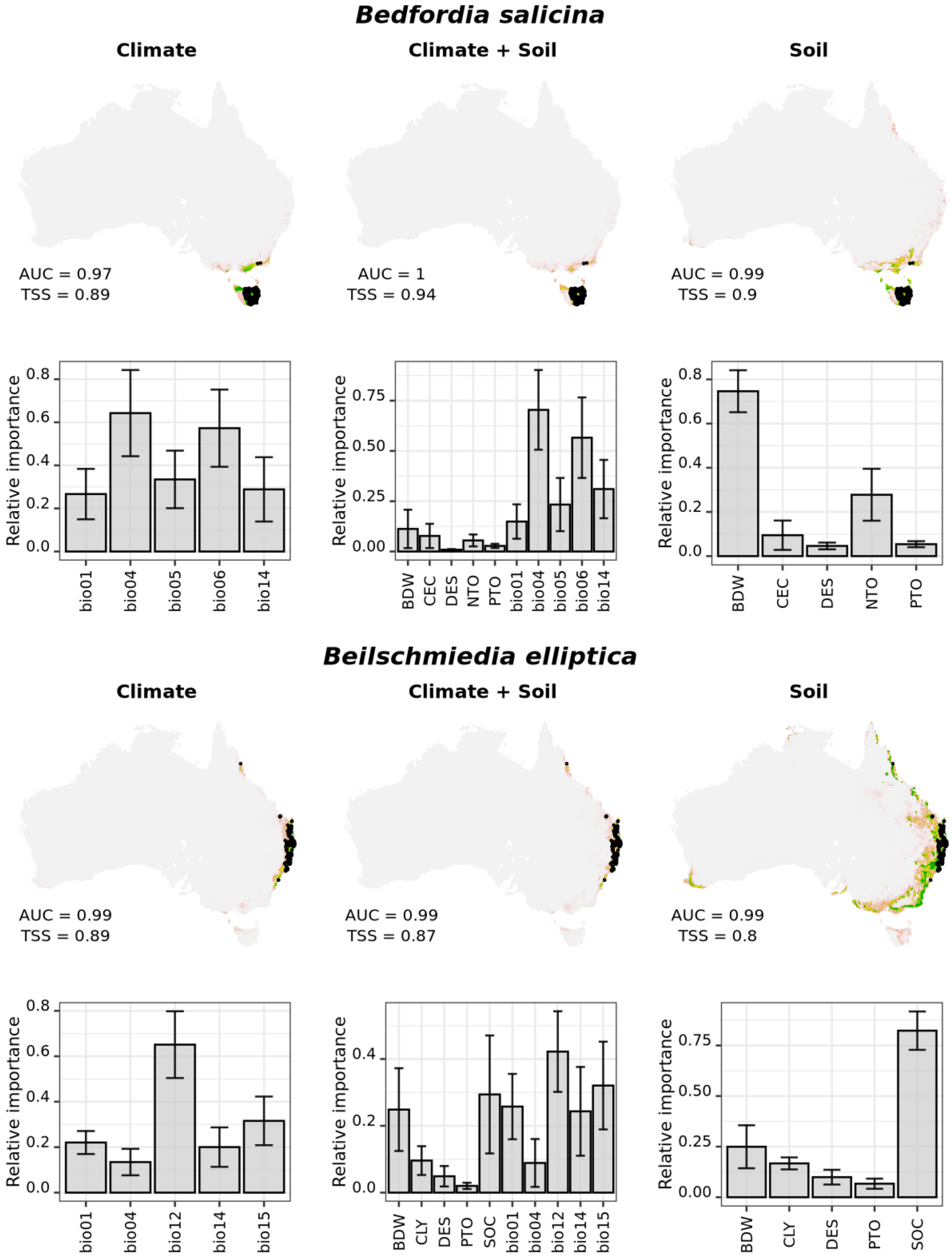

Bedfordia salicina
Climate
Climate + Soil
Soil
AUC = 0.97
TSS = 0.89
AUC = 1
TSS = 0.94
AUC = 0.99
TSS = 0.9
Relative importance
bio01
bio04
bio05
bio06
bio14
BDW
CEC
DES
NTO
PTO
Beilschmiedia elliptica
Climate
Climate + Soil
Soil
AUC = 0.99
TSS = 0.89
AUC = 0.99
TSS = 0.87
AUC = 0.99
TSS = 0.8
Relative importance
bio01
bio04
bio12
bio14
bio15
BDW
CLY
DES
PTO
SOC

Beilschmiedia obtusifolia

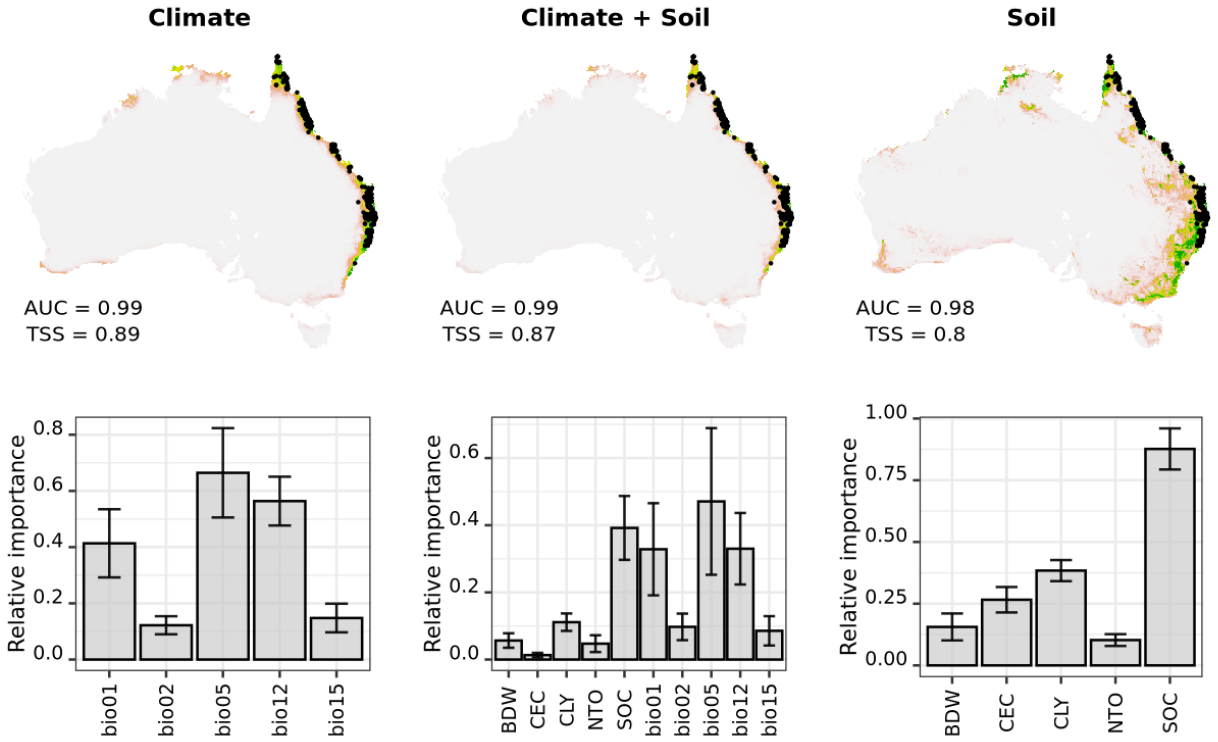

Bellendena montana

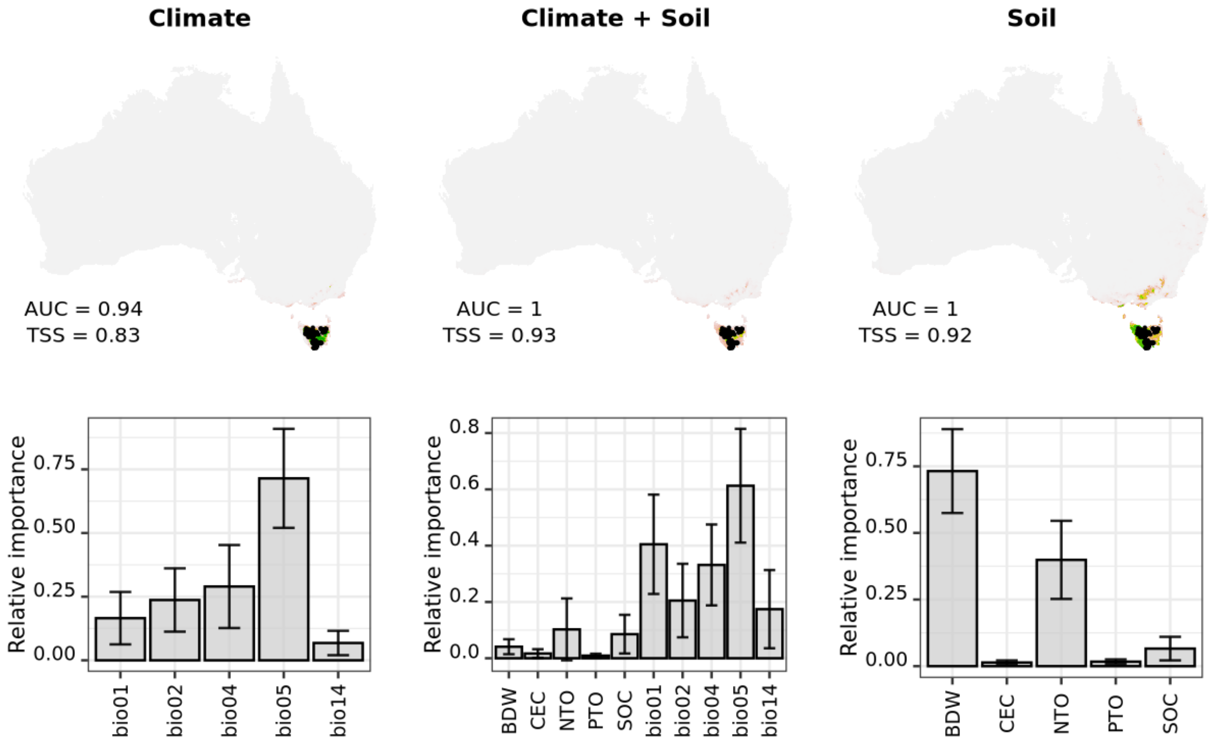

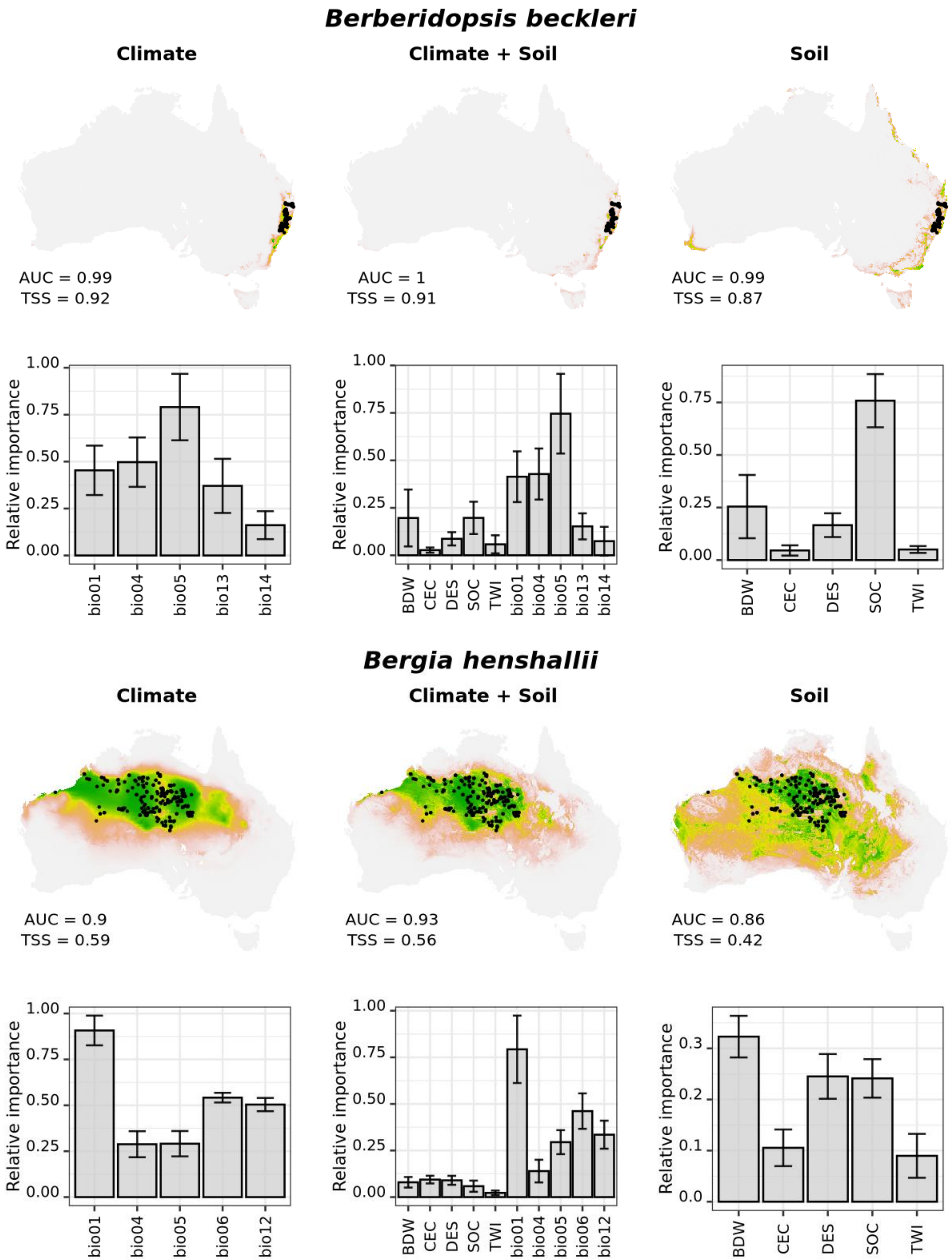

Berberidopsis beckleri
Climate
Climate + Soil
Soil
AUC = 0.99
TSS = 0.92
AUC = 1
TSS = 0.91
AUC = 0.99
TSS = 0.87
Relative importance
1.00
0.75
0.50
0.25
0.00
bio01
bio04
bio05
bio13
bio14
BDW
CEC
DES
SOC
TWI
Bergia henshallii
Climate
Climate + Soil
Soil
AUC = 0.9
TSS = 0.59
AUC = 0.93
TSS = 0.56
AUC = 0.86
TSS = 0.42
bio06
bio12
0.3
0.2
0.1
0.0

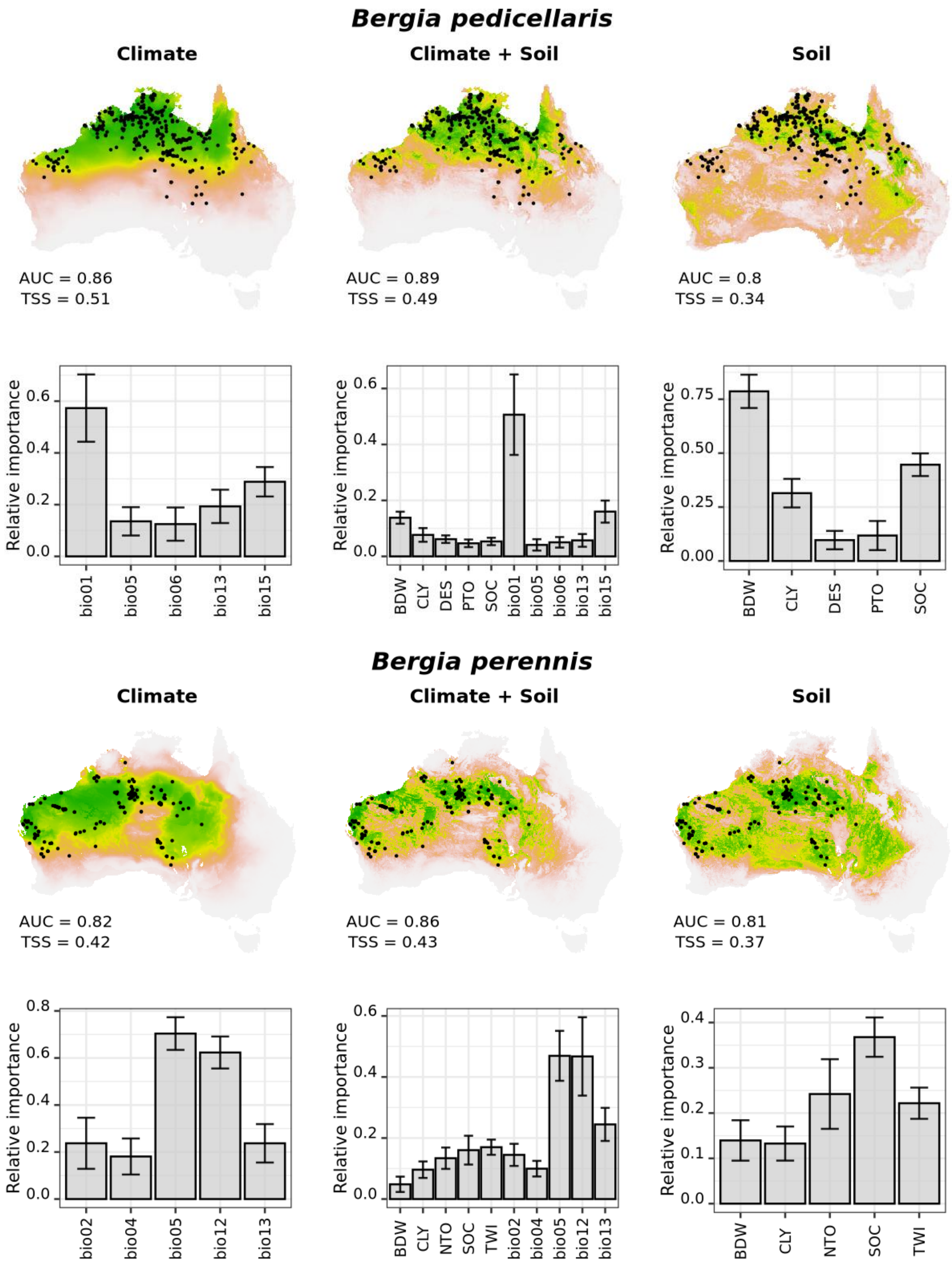
Bergia pedicellaris
Climate
Climate + Soil
Soil
AUC = 0.86
TSS = 0.51
AUC = 0.89
TSS = 0.49
AUC = 0.8
TSS = 0.34
Relative importance
bio01
bio05
bio06
bio13
bio15
BDW
CLY
DES
PTO
SOC
Bergia perennis
Climate
Climate + Soil
Soil
AUC = 0.82
TSS = 0.42
AUC = 0.86
TSS = 0.43
AUC = 0.81
TSS = 0.37
Relative importance
bio02
bio04
bio05
bio12
bio13
BDW
CLY
NTO
SOC
TWI

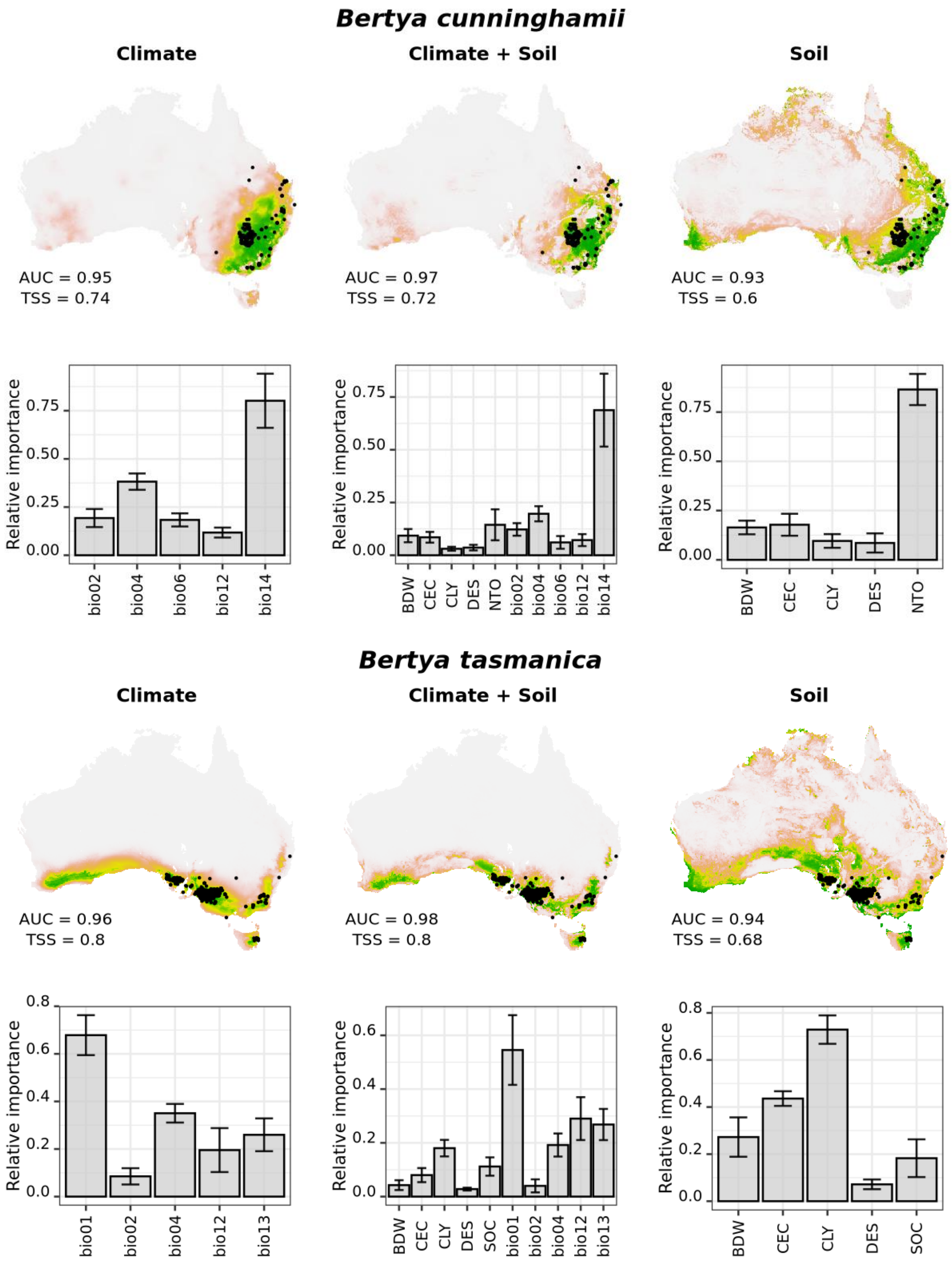
Bertya cunninghamii
Climate
Climate + Soil
Soil
AUC = 0.95
TSS = 0.74
AUC = 0.97
TSS = 0.72
AUC = 0.93
TSS = 0.6
Relative importance
0.00
0.25
0.50
0.75
bio02
bio04
bio06
bio12
bio14
BDW
CEC
CLY
DES
NTO
Bertya tasmanica
Climate
Climate + Soil
Soil
AUC = 0.96
TSS = 0.8
AUC = 0.98
TSS = 0.8
AUC = 0.94
TSS = 0.68
Relative importance
0.0
0.2
0.4
0.6
0.8
bio01
bio02
bio04
bio12
bio13
BDW
CEC
CLY
DES
SOC

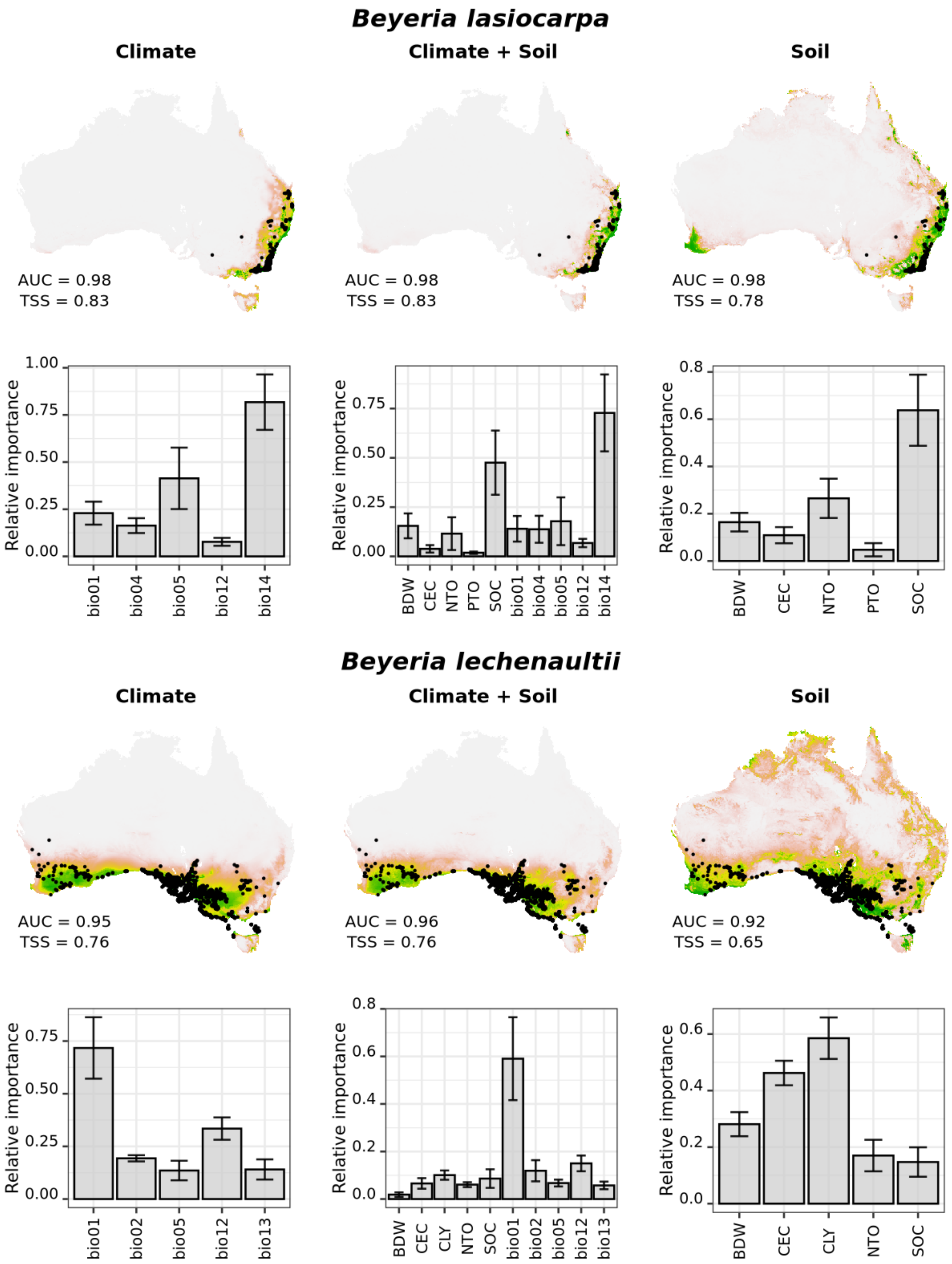
Beyeria lasiocarpa
Climate
Climate + Soil
Soil
AUC = 0.98
TSS = 0.83
AUC = 0.98
TSS = 0.83
AUC = 0.98
TSS = 0.78
Relative importance
1.00
0.75
0.50
0.25
0.00
bio01
bio04
bio05
bio12
bio14
Relative importance
0.75
0.50
0.25
0.00
BDW
CEC
NTO
PTO
SOC
bio01
bio04
bio05
bio12
bio14
Relative importance
0.8
0.6
0.4
0.2
0.0
BDW
CEC
NTO
PTO
SOC
Beyeria lechenaultii
Climate
Climate + Soil
Soil
AUC = 0.95
TSS = 0.76
AUC = 0.96
TSS = 0.76
AUC = 0.92
TSS = 0.65
Relative importance
0.75
0.50
0.25
0.00
bio01
bio02
bio05
bio12
bio13
Relative importance
0.8
0.6
0.4
0.2
0.0
BDW
CEC
CLY
NTO
SOC
bio01
bio02
bio05
bio12
bio13
Relative importance
0.6
0.4
0.2
0.0
BDW
CEC
CLY
NTO
SOC

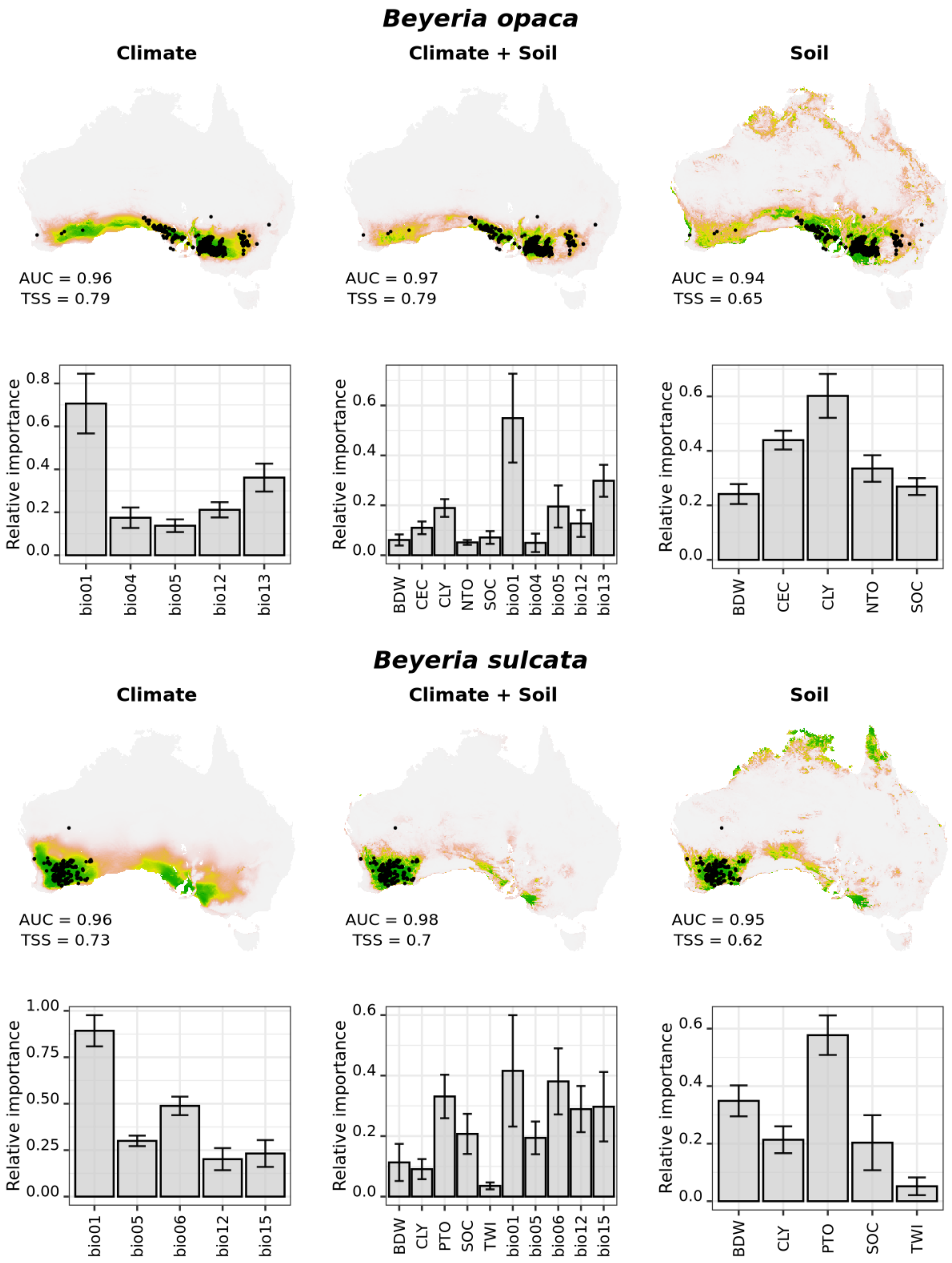
Beyeria opaca
Climate
Climate + Soil
Soil
AUC = 0.96
TSS = 0.79
AUC = 0.97
TSS = 0.79
AUC = 0.94
TSS = 0.65
Relative importance
bio01
bio04
bio05
bio12
bio13
BDW
CEC
CLY
NTO
SOC
Beyeria sulcata
AUC = 0.96
TSS = 0.73
AUC = 0.98
TSS = 0.7
AUC = 0.95
TSS = 0.62
bio06
bio15
PTO
TWI

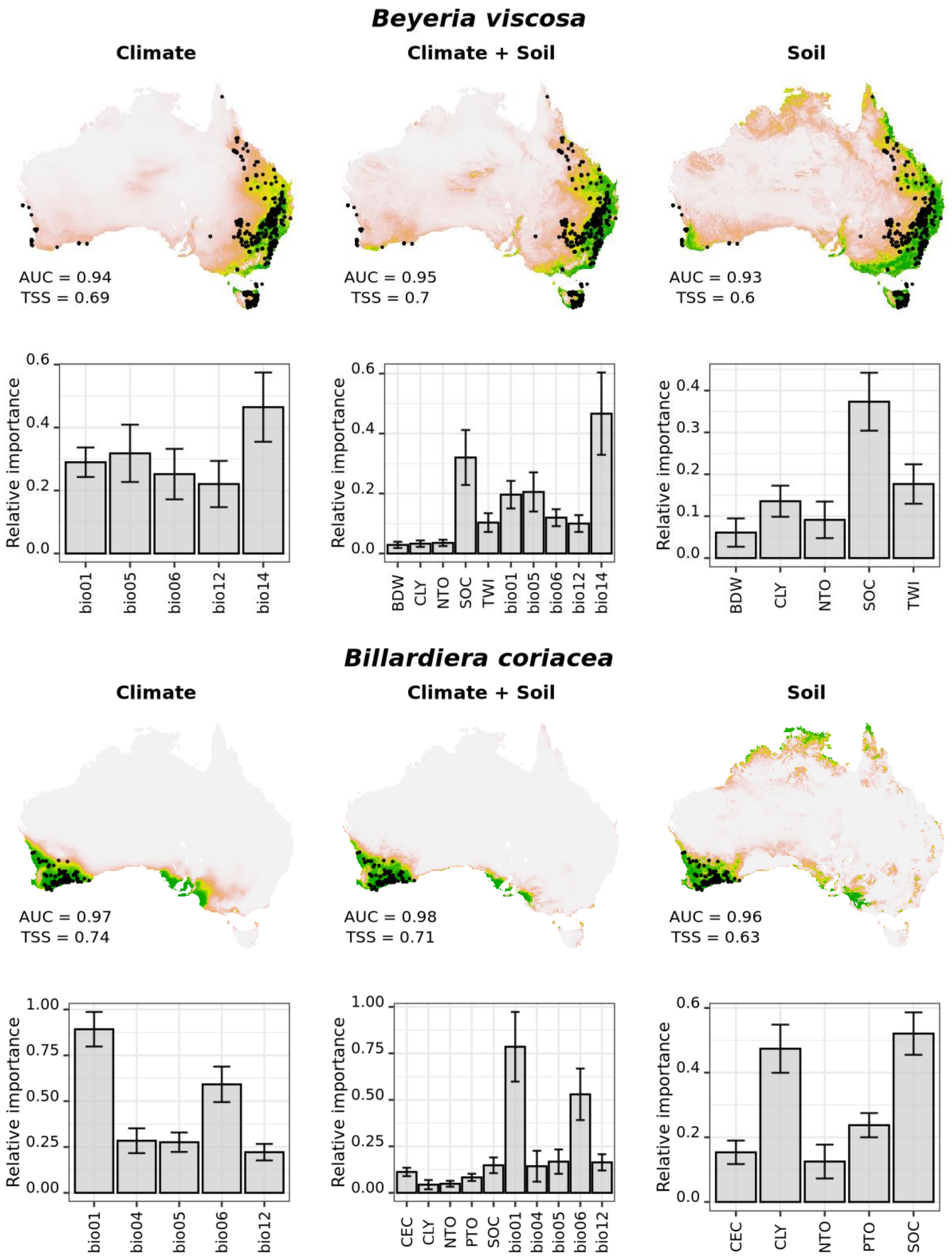

Beyeria viscosa
Climate
Climate + Soil
Soil
AUC = 0.94
TSS = 0.69
AUC = 0.95
TSS = 0.7
AUC = 0.93
TSS = 0.6
Relative importance
bio01
bio05
bio06
bio12
bio14
BDW
CLY
NTO
SOC
TWI
Billardiera coriacea
Climate
Climate + Soil
Soil
AUC = 0.97
TSS = 0.74
AUC = 0.98
TSS = 0.71
AUC = 0.96
TSS = 0.63
Relative importance
bio01
bio04
bio05
bio06
bio12
CEC
CLY
NTO
PTO
SOC

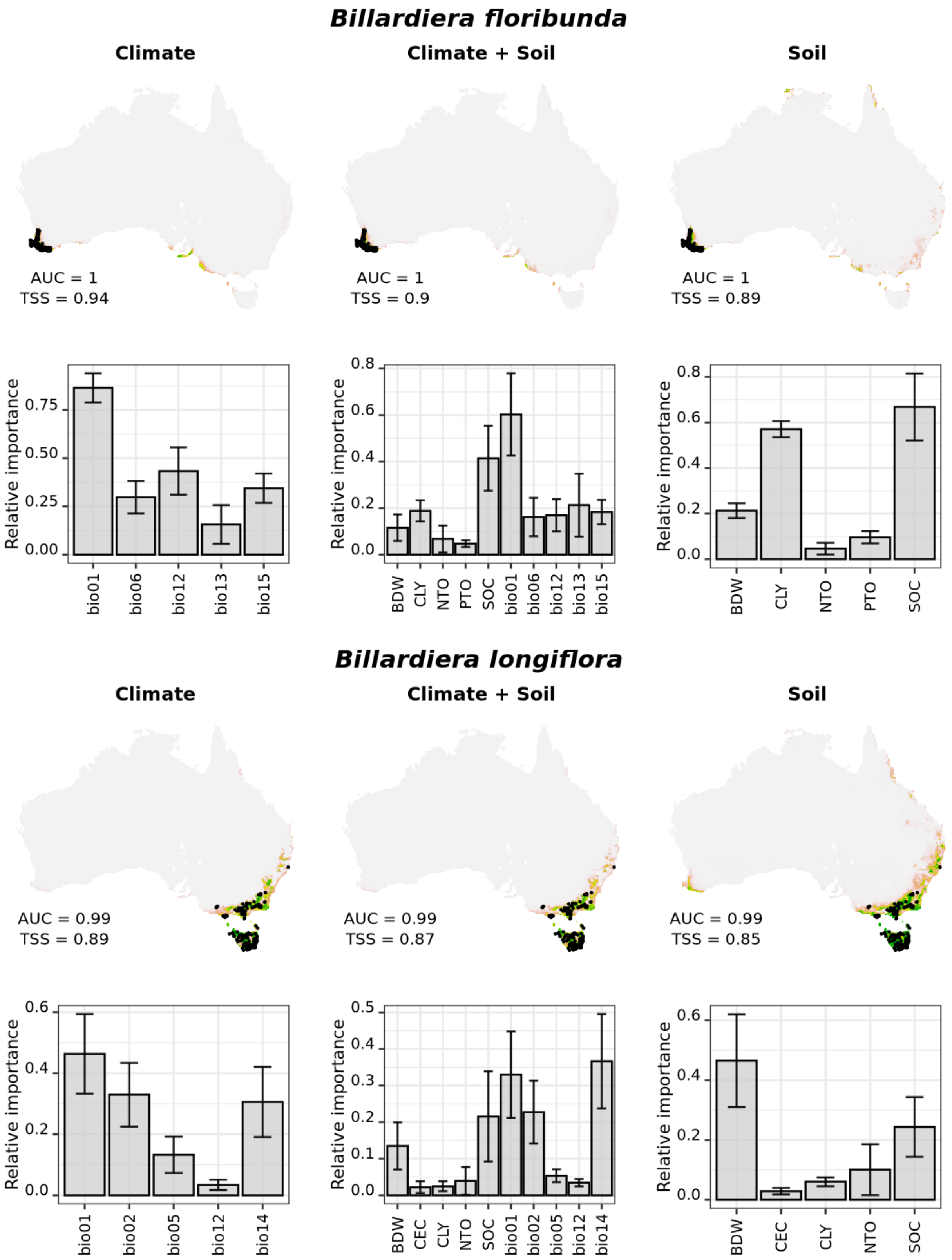
Billardiera floribunda
Climate
Climate + Soil
Soil
AUC = 1
TSS = 0.94
AUC = 1
TSS = 0.9
AUC = 1
TSS = 0.89
Relative importance
0.00
0.25
0.50
0.75
bio01
bio06
bio12
bio13
bio15
0.0
0.2
0.4
0.6
0.8
BDW
CLY
NTO
PTO
SOC
bio01
bio06
bio12
bio13
bio15
BDW
CLY
NTO
PTO
SOC
Billardiera longiflora
Climate
Climate + Soil
Soil
AUC = 0.99
TSS = 0.89
AUC = 0.99
TSS = 0.87
AUC = 0.99
TSS = 0.85
0.1
0.3
0.5
bio01
bio02
bio05
bio12
bio14
BDW
CEC
CLY
NTO
SOC
bio01
bio02
bio05
bio12
bio14
BDW
CEC
CLY
NTO
SOC

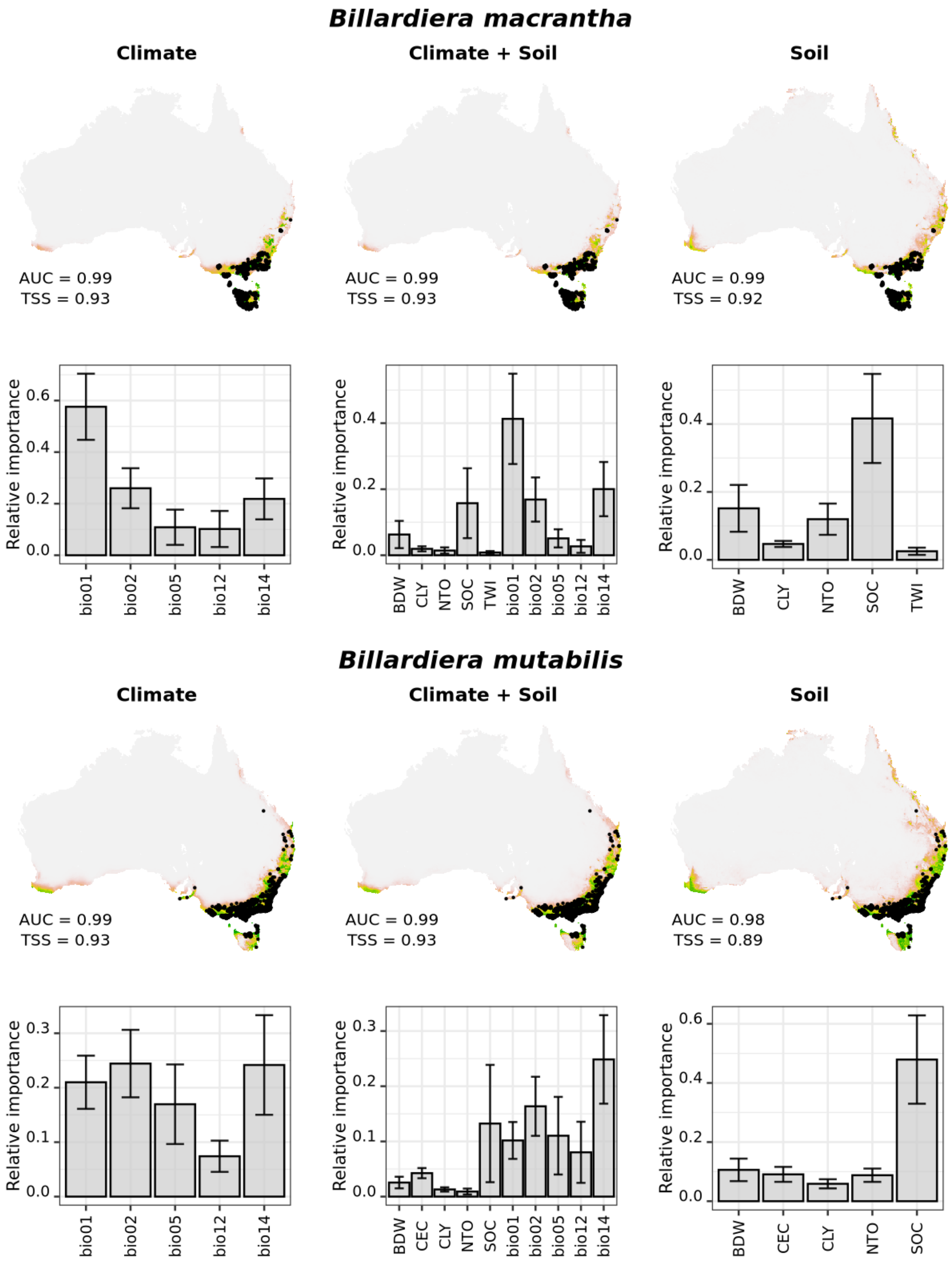
Billardiera macrantha
Climate
Climate + Soil
Soil
AUC = 0.99
TSS = 0.93
AUC = 0.99
TSS = 0.93
AUC = 0.99
TSS = 0.92
Relative importance
bio01
bio02
bio05
bio12
bio14
BDW
CLY
NTO
SOC
TWI
Billardiera mutabilis
Climate
Climate + Soil
Soil
AUC = 0.99
TSS = 0.93
AUC = 0.99
TSS = 0.93
AUC = 0.98
TSS = 0.89
Relative importance
bio01
bio02
bio05
bio12
bio14
BDW
CEC
CLY
NTO
SOC

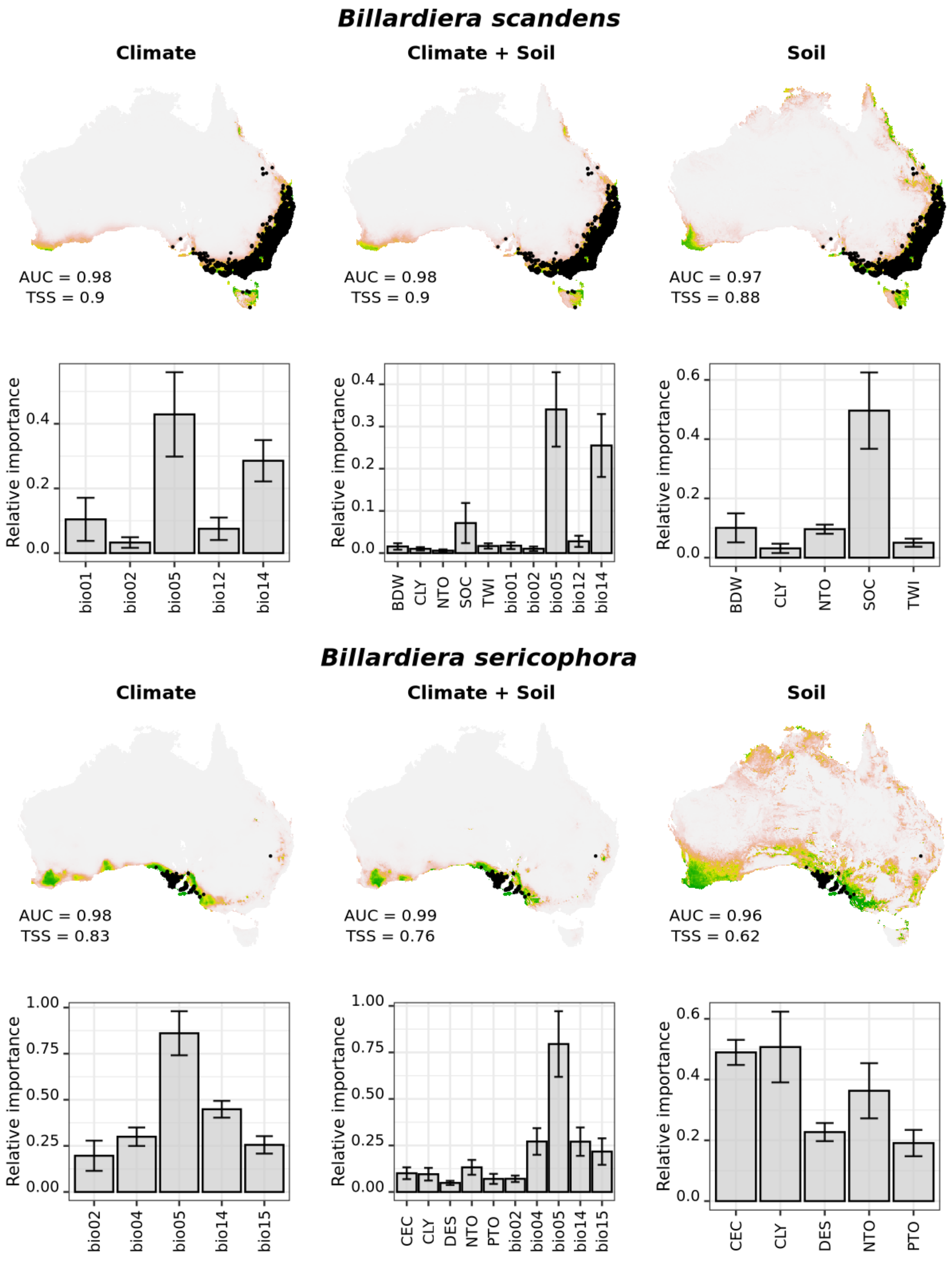

Billardiera scandens
Climate
Climate + Soil
Soil
AUC = 0.98
TSS = 0.9
AUC = 0.98
TSS = 0.9
AUC = 0.97
TSS = 0.88
Relative importance
bio01
bio02
bio05
bio12
bio14
BDW
CLY
NTO
SOC
TWI
Billardiera sericophora
Climate
Climate + Soil
Soil
AUC = 0.98
TSS = 0.83
AUC = 0.99
TSS = 0.76
AUC = 0.96
TSS = 0.62
bio02
bio04
bio05
bio14
bio15
CEC
CLY
DES
NTO
PTO

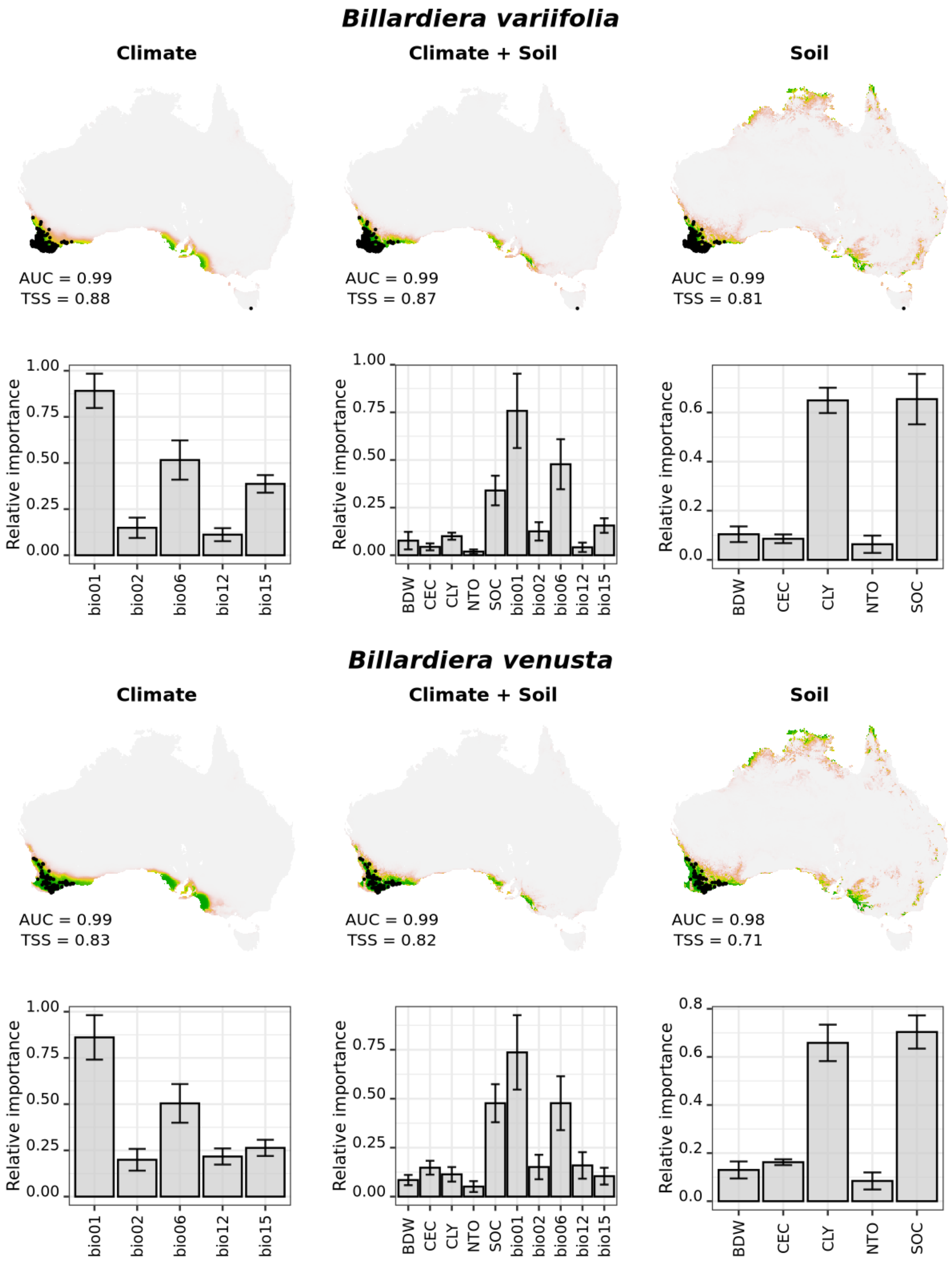

Billardiera variifolia
Climate
Climate + Soil
Soil
AUC = 0.99
TSS = 0.88
AUC = 0.99
TSS = 0.87
AUC = 0.99
TSS = 0.81
Relative importance
bio01
bio02
bio06
bio12
bio15
BDW
CEC
CLY
NTO
SOC
Billardiera venusta
Climate
Climate + Soil
Soil
AUC = 0.99
TSS = 0.83
AUC = 0.99
TSS = 0.82
AUC = 0.98
TSS = 0.71
Relative importance

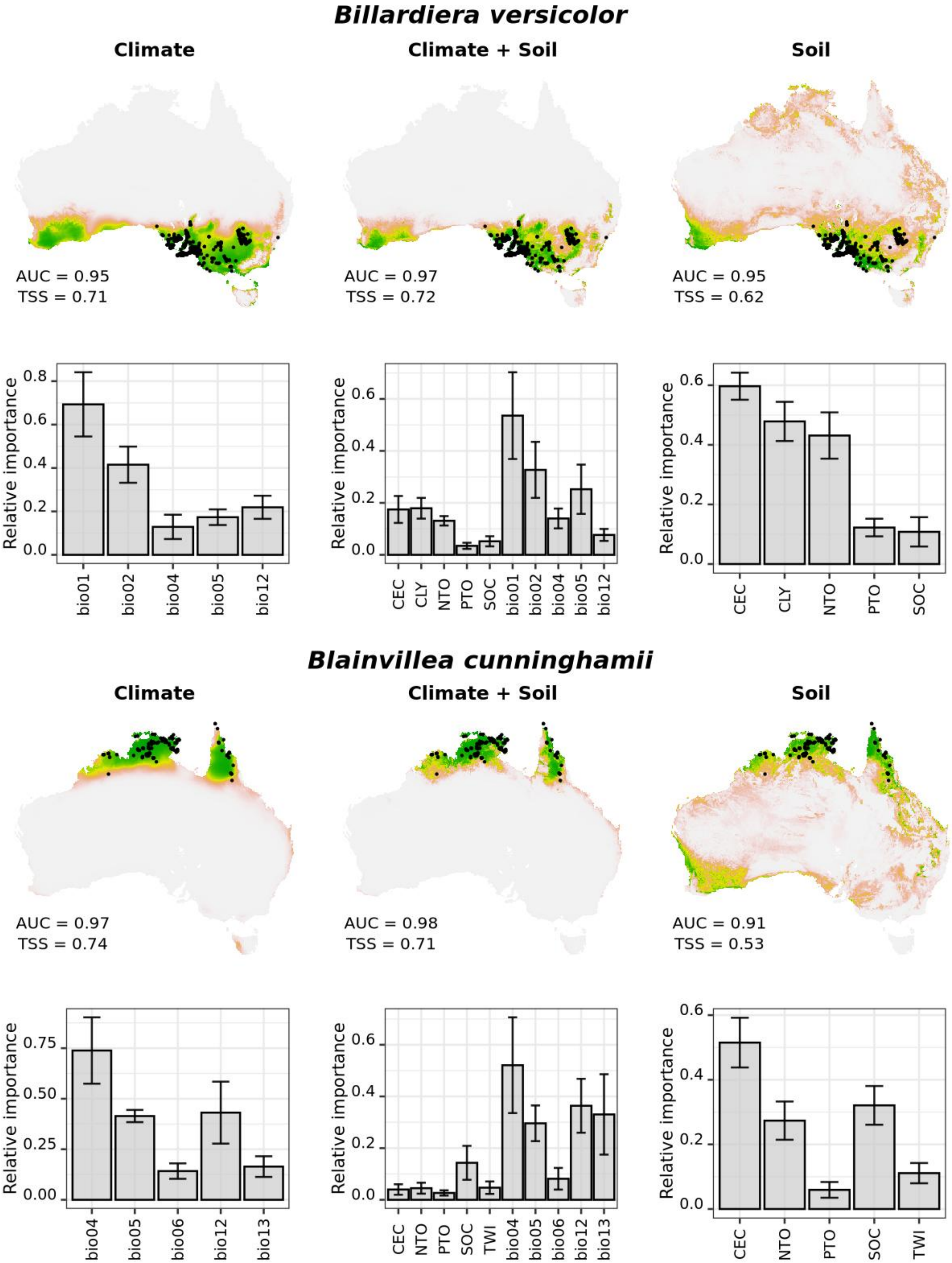
Billardiera versicolor
Climate
Climate + Soil
Soil
AUC = 0.95
TSS = 0.71
AUC = 0.97
TSS = 0.72
AUC = 0.95
TSS = 0.62
Relative importance
bio01
bio02
bio04
bio05
bio12
CEC
CLY
NTO
PTO
SOC
Blainvillea cunninghamii
Climate
Climate + Soil
Soil
AUC = 0.97
TSS = 0.74
AUC = 0.98
TSS = 0.71
AUC = 0.91
TSS = 0.53
Relative importance
bio04
bio05
bio06
bio12
bio13
CEC
NTO
PTO
SOC
TWI

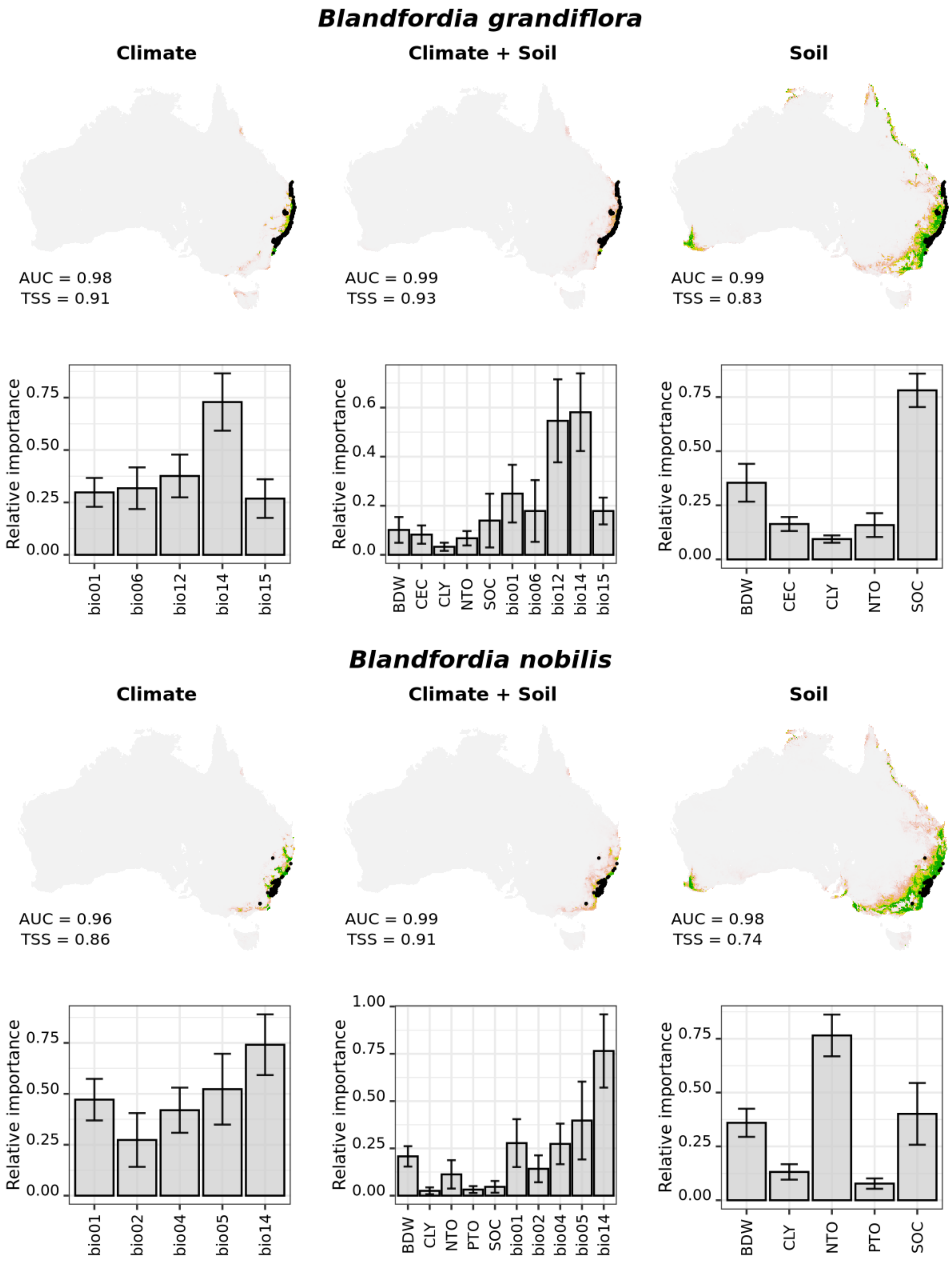

Blandfordia grandiflora
Climate
Climate + Soil
Soil
AUC = 0.98
TSS = 0.91
AUC = 0.99
TSS = 0.93
AUC = 0.99
TSS = 0.83
Relative importance
bio01
bio06
bio12
bio14
bio15
BDW
CEC
CLY
NTO
SOC
Blandfordia nobilis
Climate
Climate + Soil
Soil
AUC = 0.96
TSS = 0.86
AUC = 0.99
TSS = 0.91
AUC = 0.98
TSS = 0.74
Relative importance
bio01
bio02
bio04
bio05
bio14
BDW
CLY
NTO
PTO
SOC

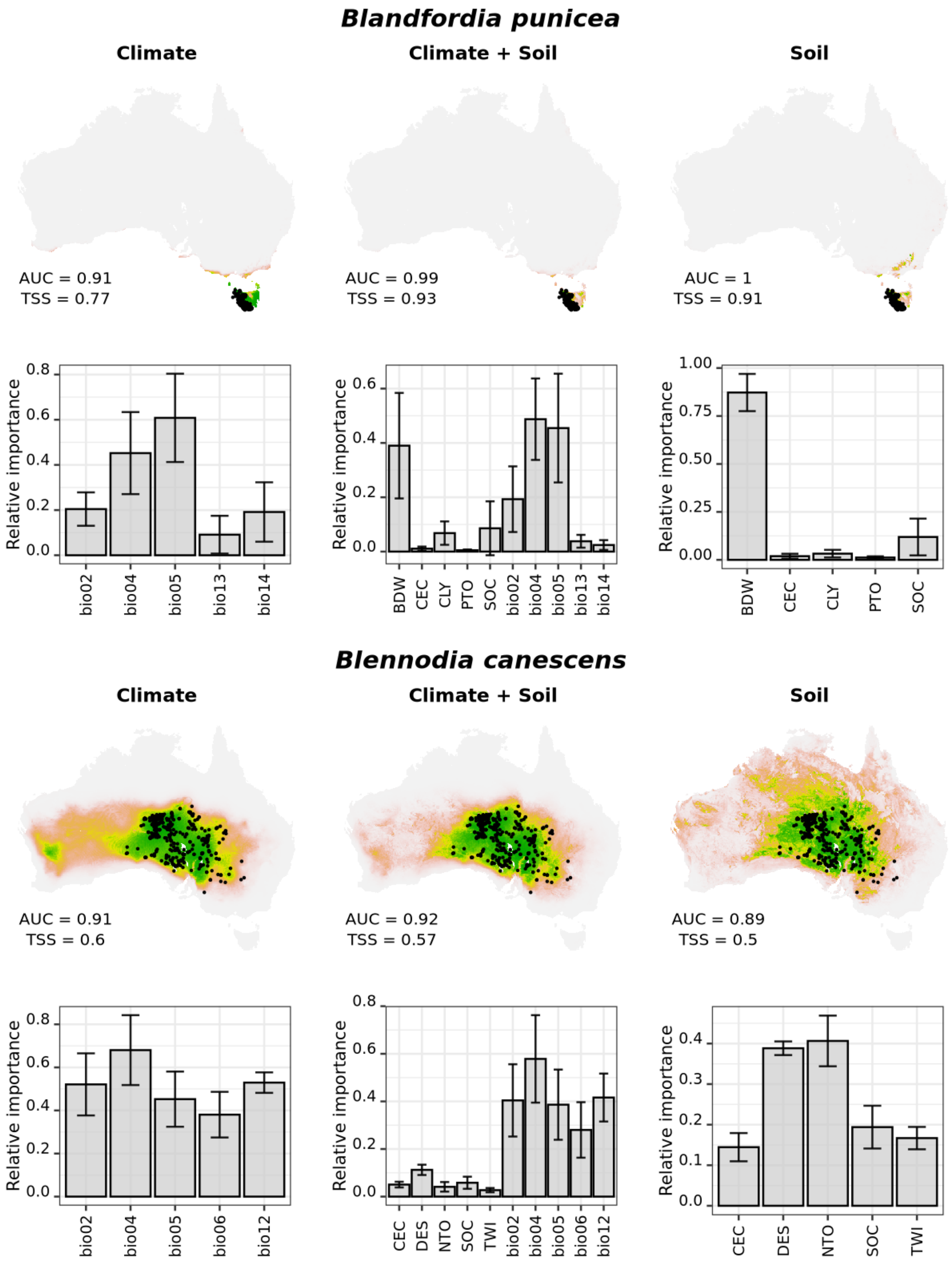
Blandfordia punicea
Climate
Climate + Soil
Soil
AUC = 0.91
TSS = 0.77
AUC = 0.99
TSS = 0.93
AUC = 1
TSS = 0.91
Relative importance
bio02
bio04
bio05
bio13
bio14
BDW
CEC
CLY
PTO
SOC
Blennodia canescens
AUC = 0.91
TSS = 0.6
AUC = 0.92
TSS = 0.57
AUC = 0.89
TSS = 0.5
bio06
bio12
DES
NTO
TWI

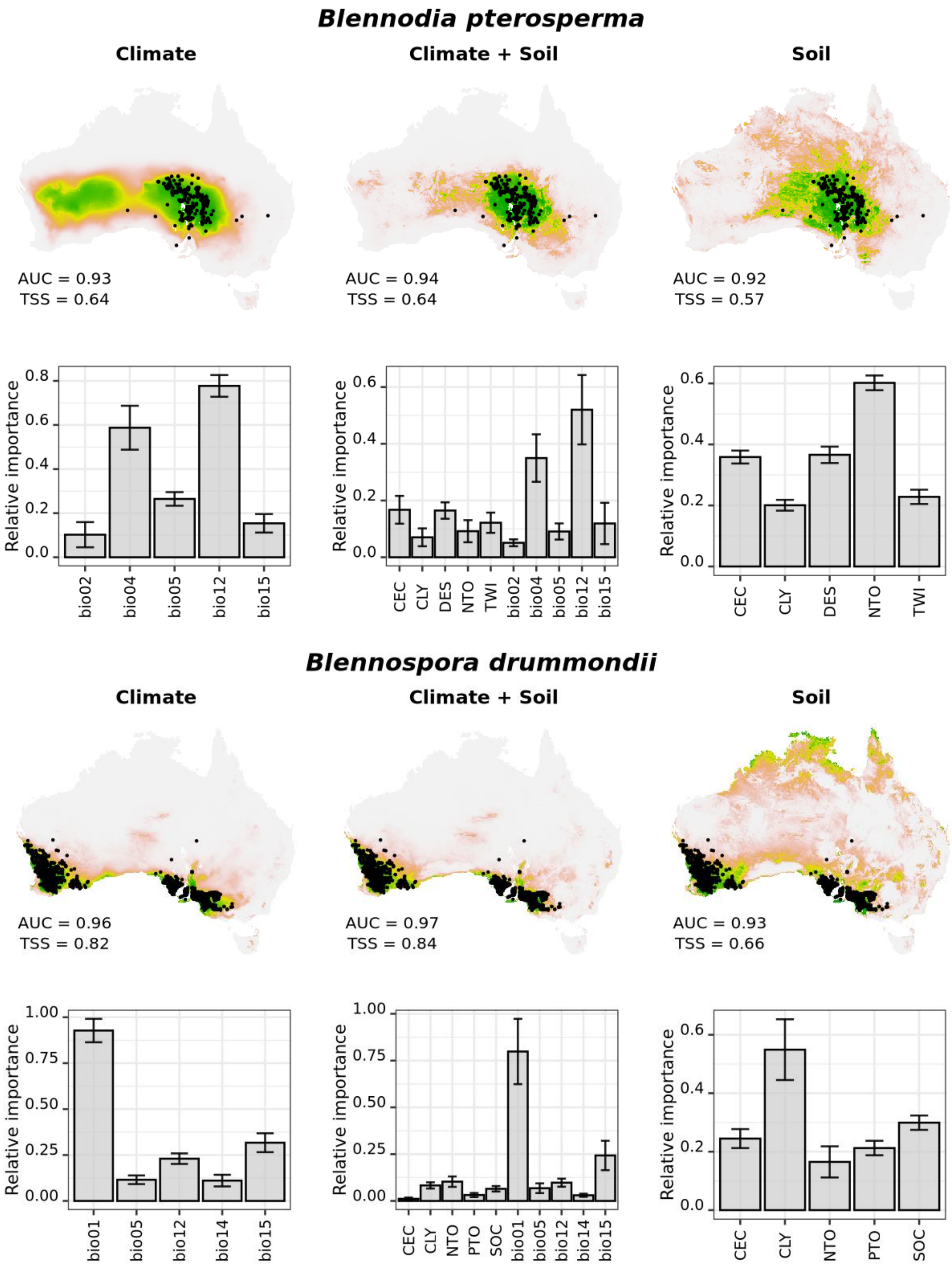
Blennodia pterosperma
Climate
Climate + Soil
Soil
AUC = 0.93
TSS = 0.64
AUC = 0.94
TSS = 0.64
AUC = 0.92
TSS = 0.57
Relative importance
bio02
bio04
bio05
bio12
bio15
CEC
CLY
DES
NTO
TWI
Blennospora drummondii
AUC = 0.96
TSS = 0.82
AUC = 0.97
TSS = 0.84
AUC = 0.93
TSS = 0.66
bio01
bio14
PTO
SOC

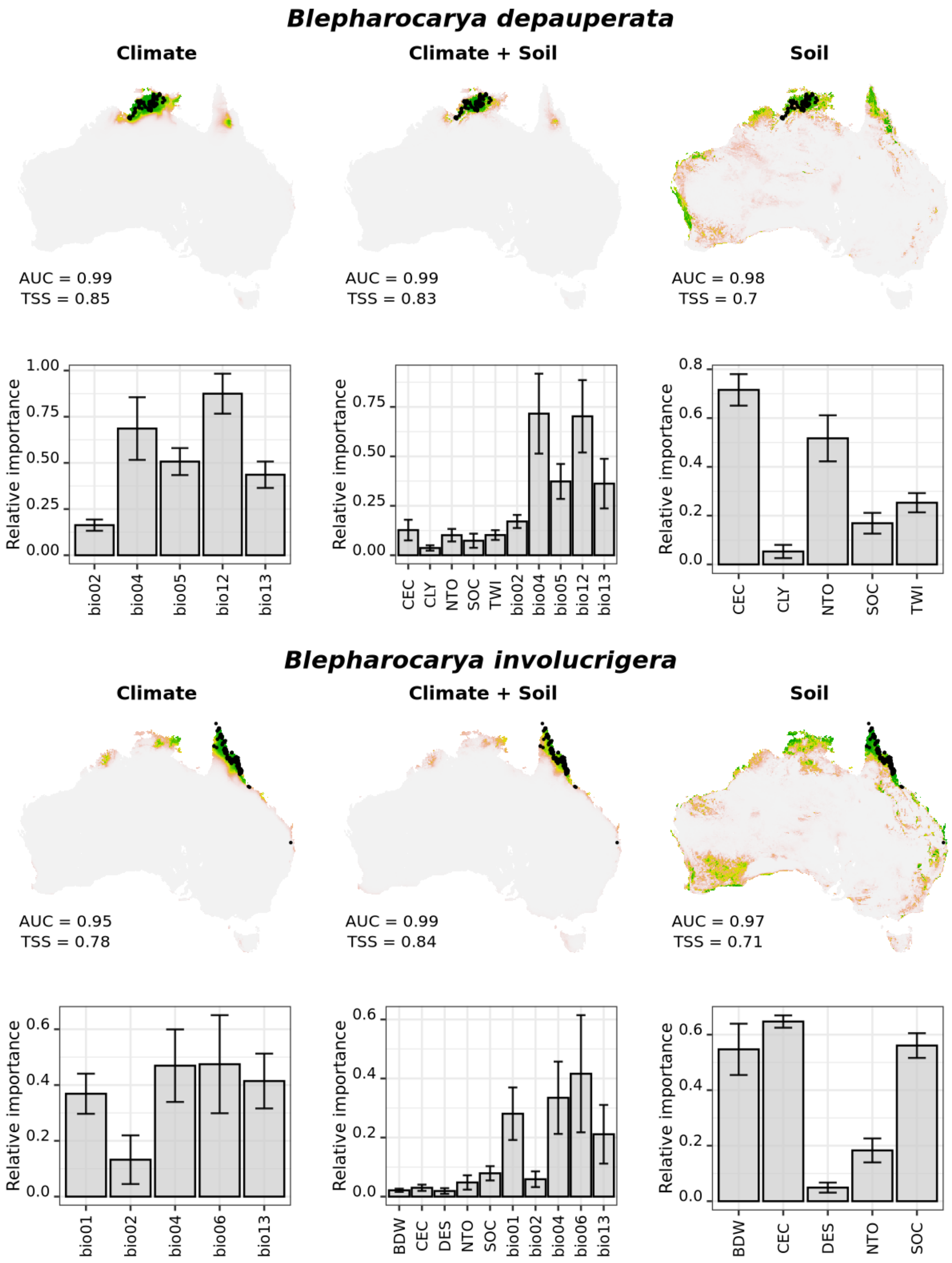

Blepharocarya depauperata
Climate
Climate + Soil
Soil
AUC = 0.99
TSS = 0.85
AUC = 0.99
TSS = 0.83
AUC = 0.98
TSS = 0.7
Relative importance
bio02
bio04
bio05
bio12
bio13
CEC
CLY
NTO
SOC
TWI
Blepharocarya involucrigera
Climate
Climate + Soil
Soil
AUC = 0.95
TSS = 0.78
AUC = 0.99
TSS = 0.84
AUC = 0.97
TSS = 0.71
bio01
bio06
BDW
DES

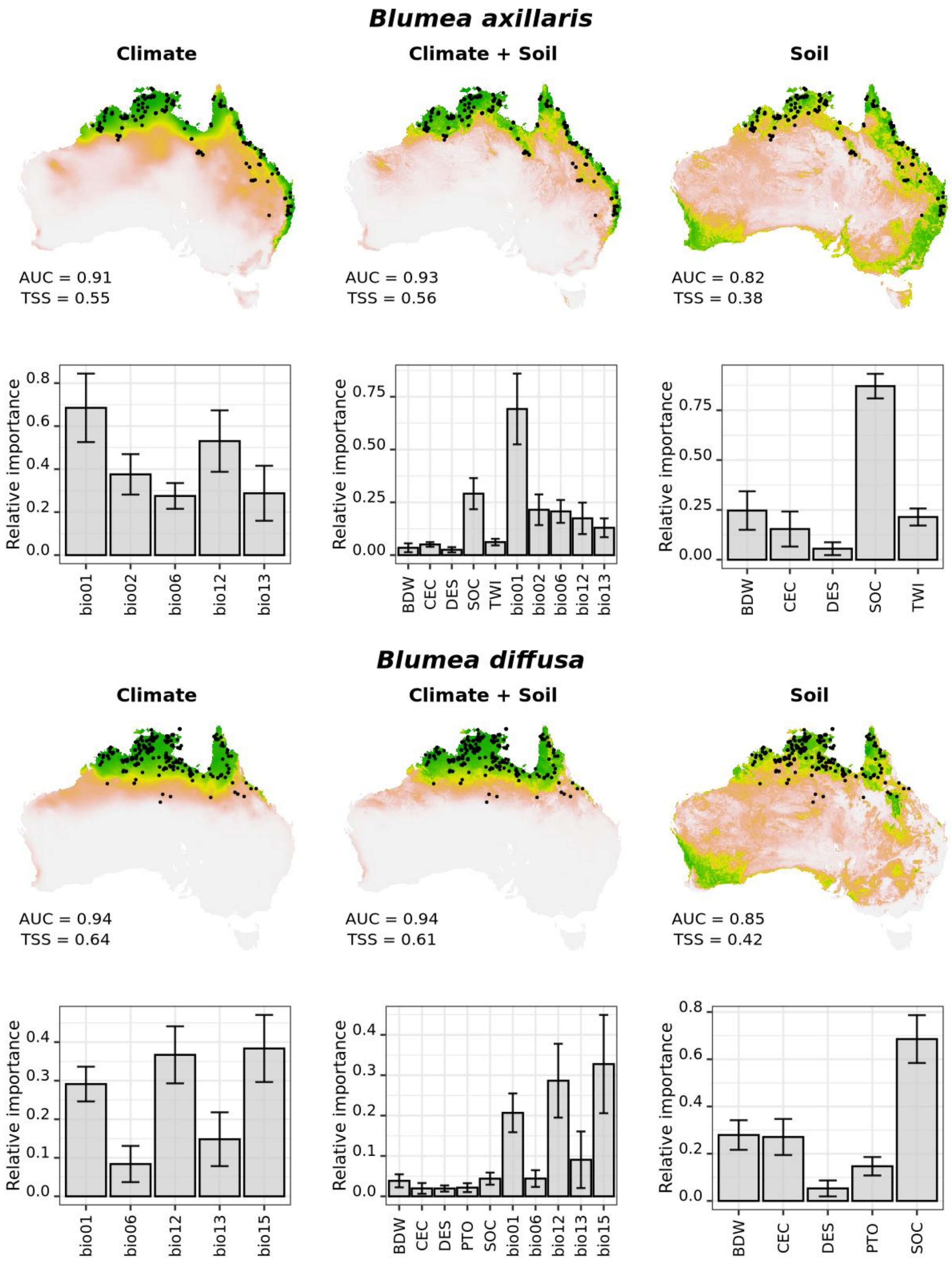

Blumea axillaris
Climate
Climate + Soil
Soil
AUC = 0.91
TSS = 0.55
AUC = 0.93
TSS = 0.56
AUC = 0.82
TSS = 0.38
Relative importance
bio01
bio02
bio06
bio12
bio13
BDW
CEC
DES
SOC
TWI
Blumea diffusa
Climate
Climate + Soil
Soil
AUC = 0.94
TSS = 0.64
AUC = 0.94
TSS = 0.61
AUC = 0.85
TSS = 0.42
Relative importance
bio01
bio06
bio12
bio13
bio15
BDW
CEC
DES
PTO
SOC

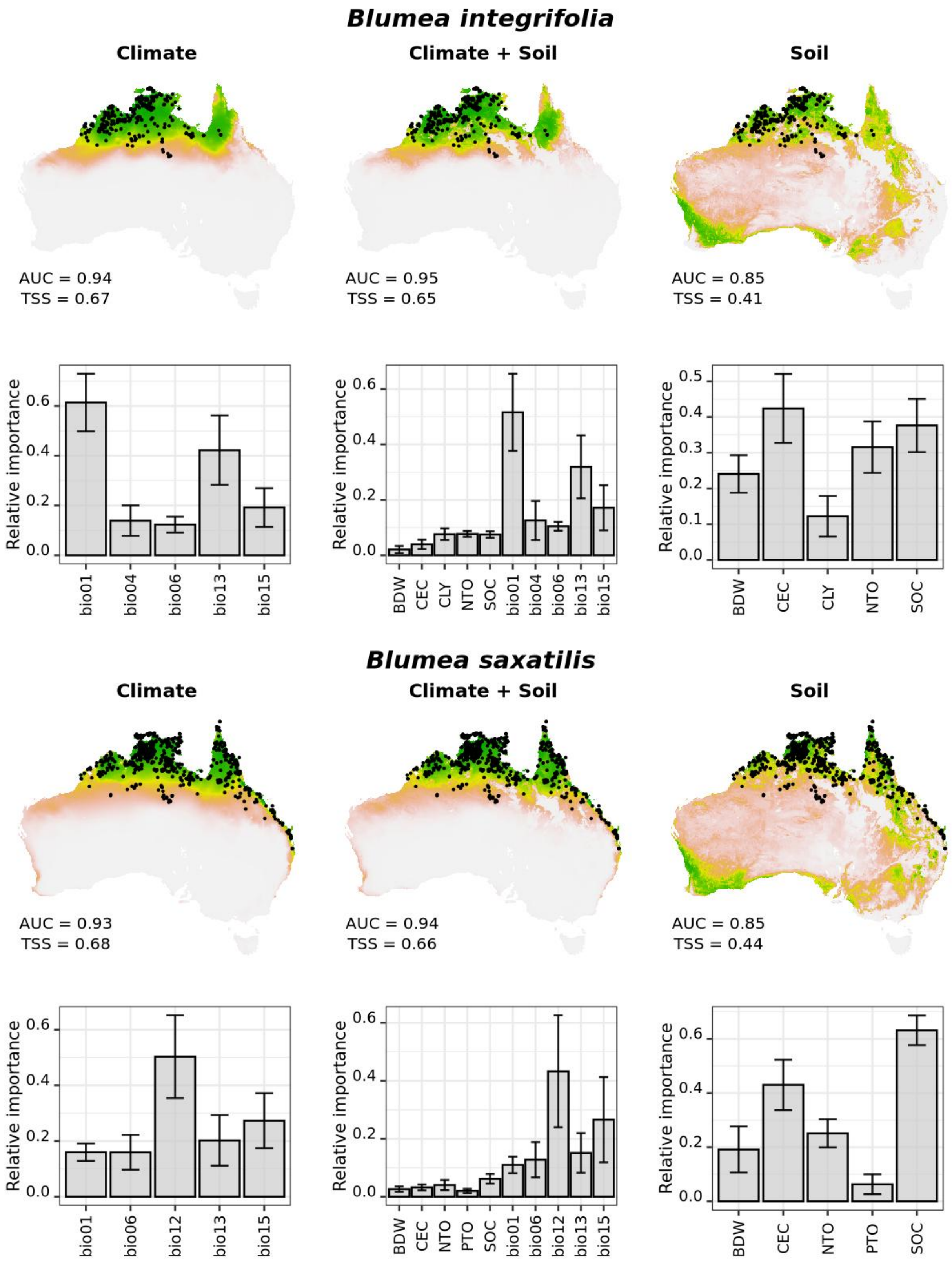

Blumea integrifolia
Climate
Climate + Soil
Soil
AUC = 0.94
TSS = 0.67
AUC = 0.95
TSS = 0.65
AUC = 0.85
TSS = 0.41
Relative importance
bio01
bio04
bio06
bio13
bio15
BDW
CEC
CLY
NTO
SOC
Blumea saxatilis
Climate
Climate + Soil
Soil
AUC = 0.93
TSS = 0.68
AUC = 0.94
TSS = 0.66
AUC = 0.85
TSS = 0.44
Relative importance
bio01
bio06
bio12
bio13
bio15
BDW
CEC
NTO
PTO
SOC

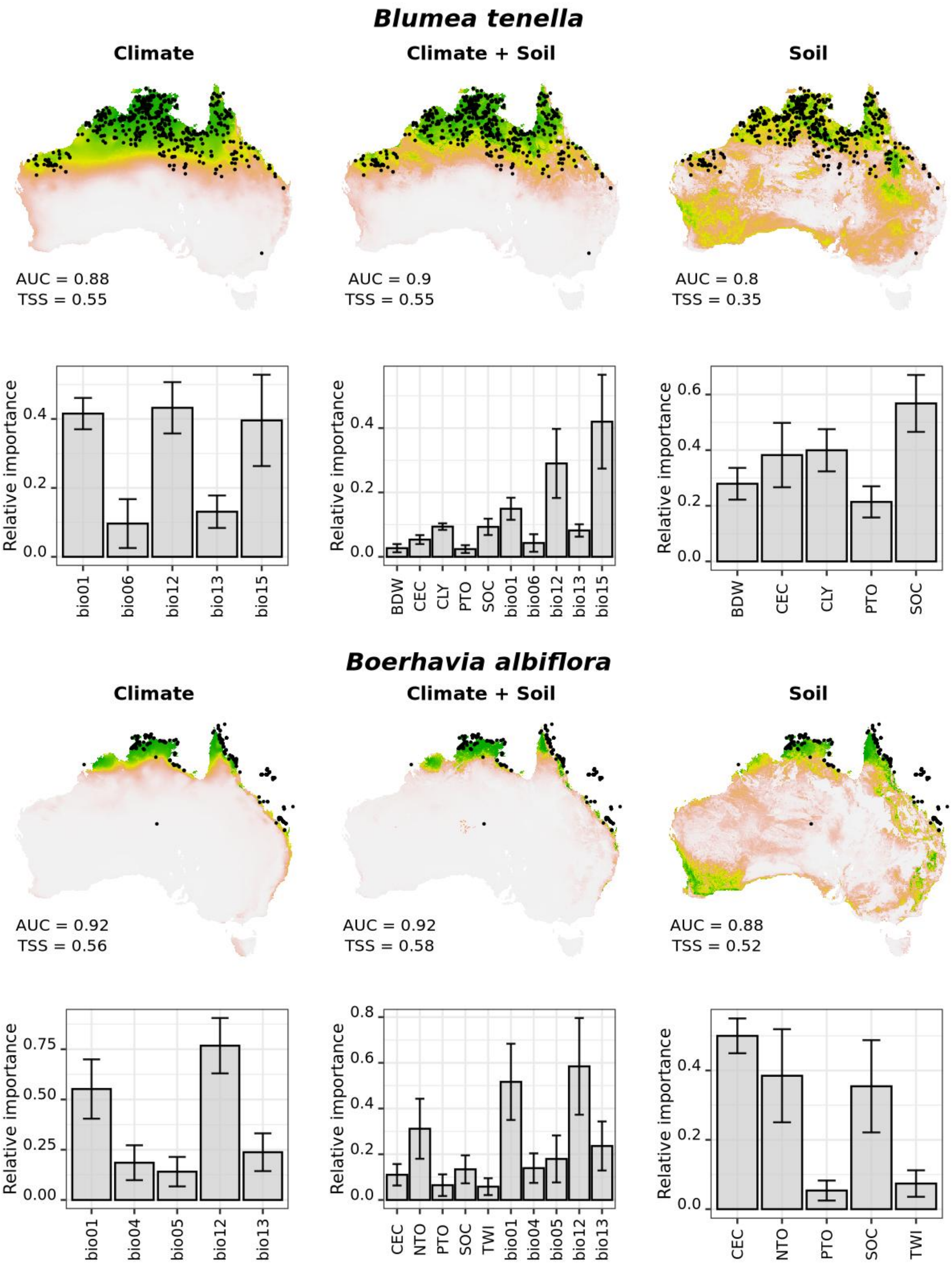
Blumea tenella
Climate
Climate + Soil
Soil
AUC = 0.88
TSS = 0.55
AUC = 0.9
TSS = 0.55
AUC = 0.8
TSS = 0.35
Relative importance
bio01
bio06
bio12
bio13
bio15
BDW
CEC
CLY
PTO
SOC
Boerhavia albiflora
Climate
Climate + Soil
Soil
AUC = 0.92
TSS = 0.56
AUC = 0.92
TSS = 0.58
AUC = 0.88
TSS = 0.52
Relative importance
bio01
bio04
bio05
bio12
bio13
CEC
NTO
PTO
SOC
TWI

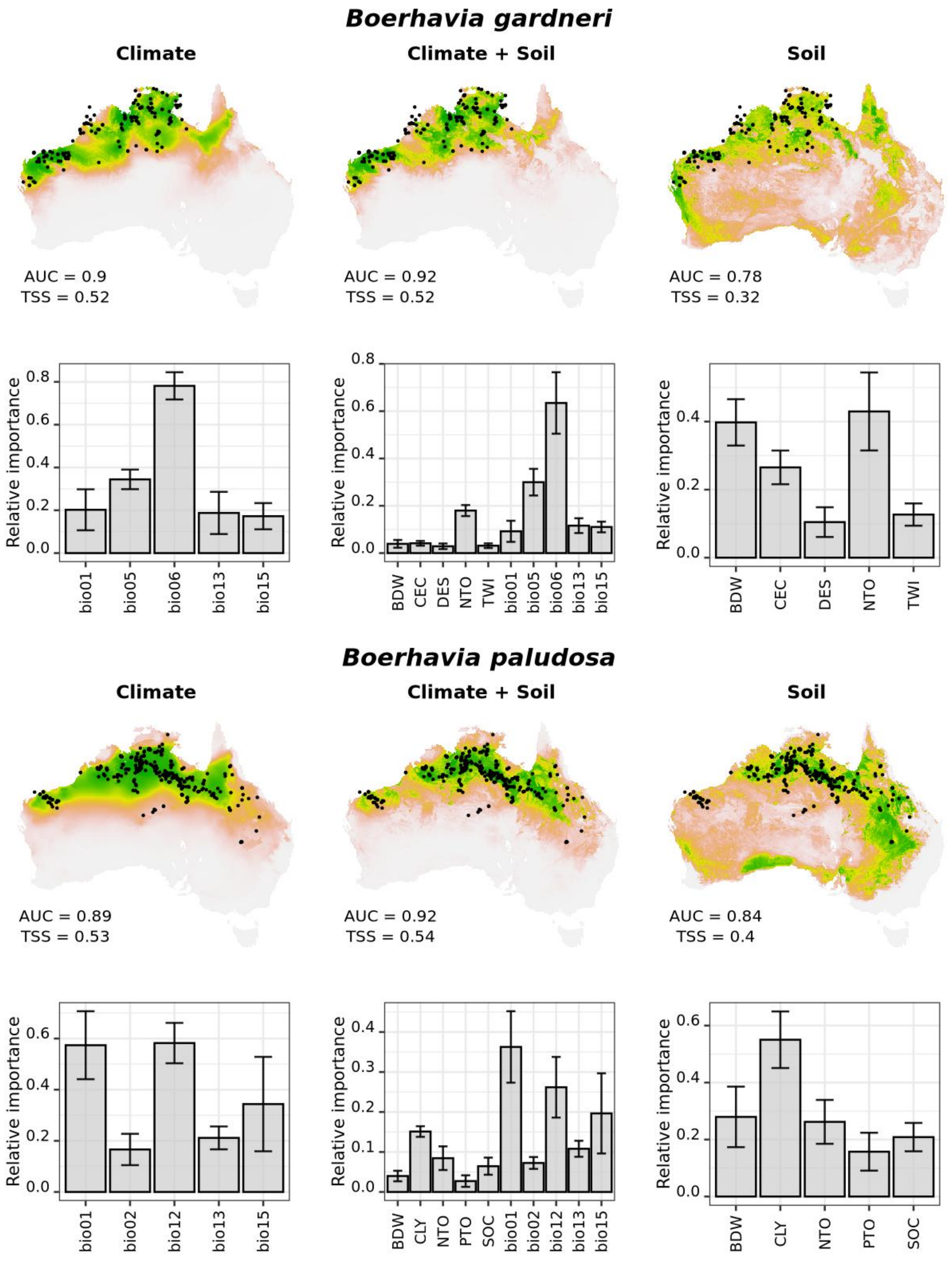

Boerhavia gardneri
Climate
Climate + Soil
Soil
AUC = 0.9
TSS = 0.52
AUC = 0.92
TSS = 0.52
AUC = 0.78
TSS = 0.32
Relative importance
bio01
bio05
bio06
bio13
bio15
BDW
CEC
DES
NTO
TWI
Boerhavia paludosa
Climate
Climate + Soil
Soil
AUC = 0.89
TSS = 0.53
AUC = 0.92
TSS = 0.54
AUC = 0.84
TSS = 0.4
Relative importance
bio01
bio02
bio12
bio13
bio15
BDW
CLY
NTO
PTO
SOC

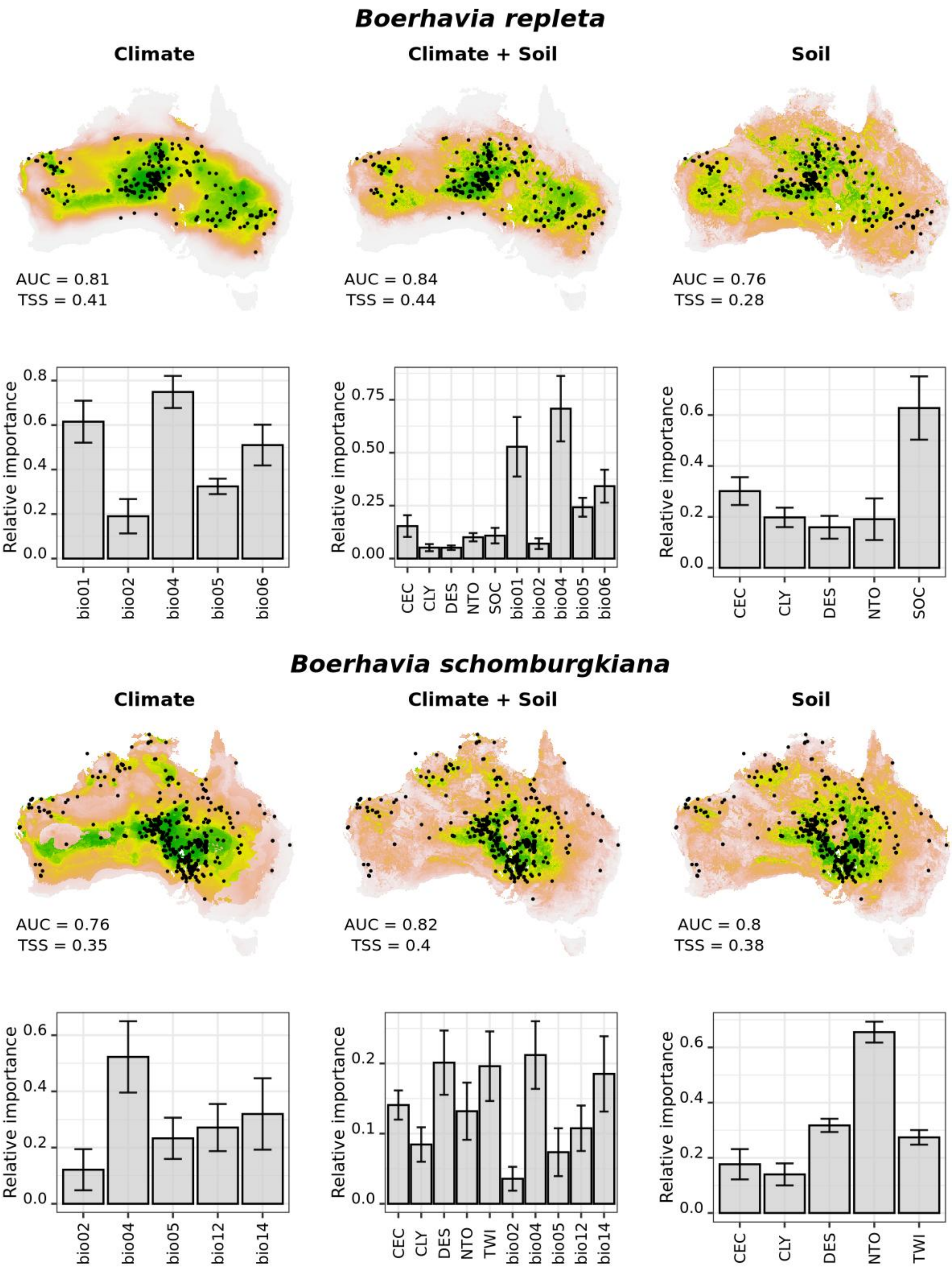
Boerhavia repleta
Climate
Climate + Soil
Soil
AUC = 0.81
TSS = 0.41
AUC = 0.84
TSS = 0.44
AUC = 0.76
TSS = 0.28
Relative importance
bio01 bio02 bio04 bio05 bio06
CEC CLY DES NTO SOC bio01 bio02 bio04 bio05 bio06
CEC CLY DES NTO SOC
Boerhavia schomburgkiana
Climate
Climate + Soil
Soil
AUC = 0.76
TSS = 0.35
AUC = 0.82
TSS = 0.4
AUC = 0.8
TSS = 0.38
Relative importance
bio02 bio04 bio05 bio12 bio14
CEC CLY DES NTO TWI bio02 bio04 bio05 bio12 bio14
CEC CLY DES NTO TWI

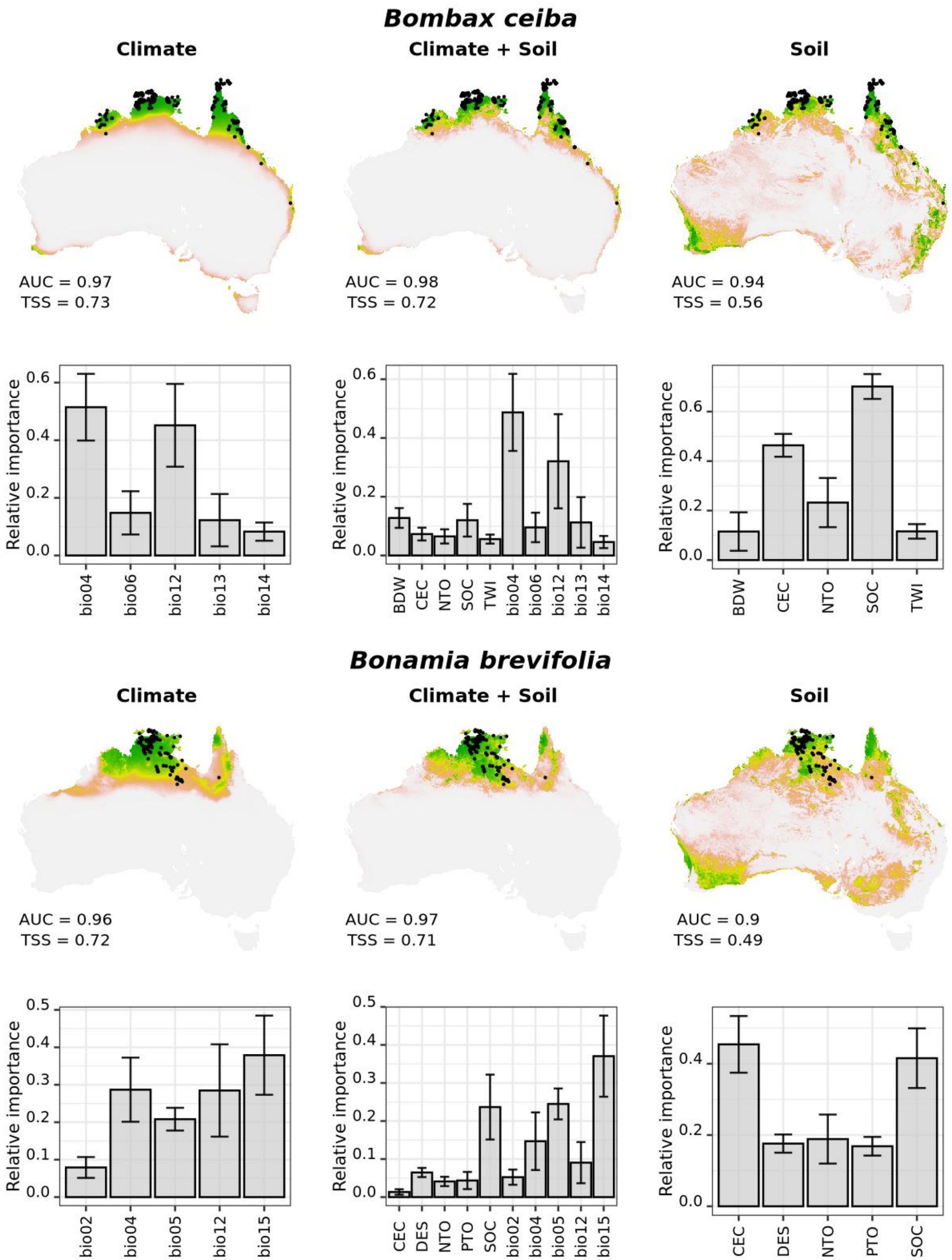

Bombax ceiba
Climate
Climate + Soil
Soil
AUC = 0.97
TSS = 0.73
AUC = 0.98
TSS = 0.72
AUC = 0.94
TSS = 0.56
Relative importance
0.0
0.2
0.4
0.6
bio04
bio06
bio12
bio13
bio14
BDW
CEC
NTO
SOC
TWI
Bonamia brevifolia
Climate
Climate + Soil
Soil
AUC = 0.96
TSS = 0.72
AUC = 0.97
TSS = 0.71
AUC = 0.9
TSS = 0.49
Relative importance
0.1
0.3
0.5
bio02
bio05
bio15
DES
PTO

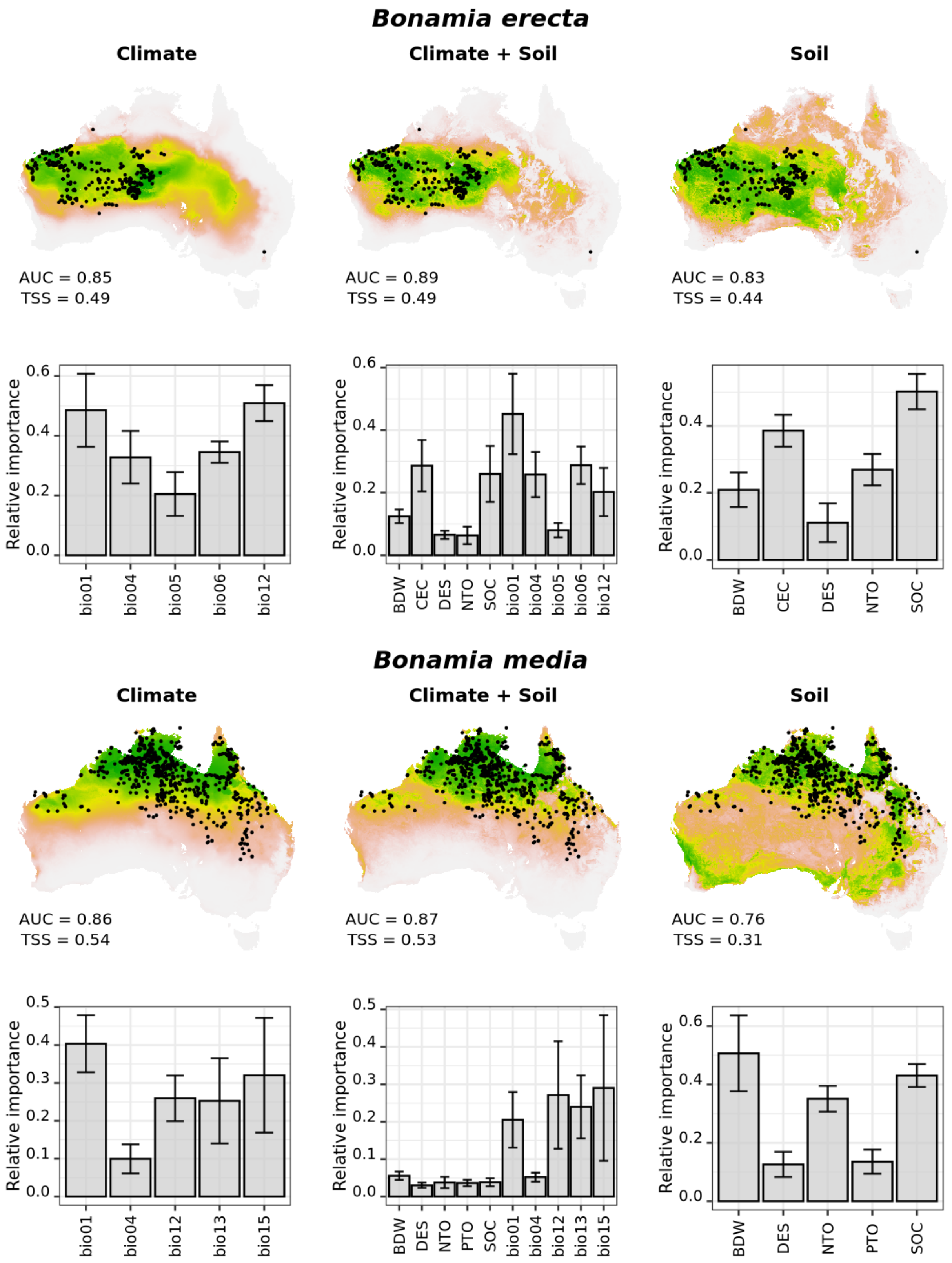

Bonamia erecta
Climate
Climate + Soil
Soil
AUC = 0.85
TSS = 0.49
AUC = 0.89
TSS = 0.49
AUC = 0.83
TSS = 0.44
Relative importance
bio01 bio04 bio05 bio06 bio12
BDW CEC DES NTO SOC bio01 bio04 bio05 bio06 bio12
BDW CEC DES NTO SOC
Bonamia media
Climate
Climate + Soil
Soil
AUC = 0.86
TSS = 0.54
AUC = 0.87
TSS = 0.53
AUC = 0.76
TSS = 0.31
Relative importance
bio01 bio04 bio12 bio13 bio15
BDW DES NTO PTO SOC bio01 bio04 bio12 bio13 bio15
BDW DES NTO PTO SOC

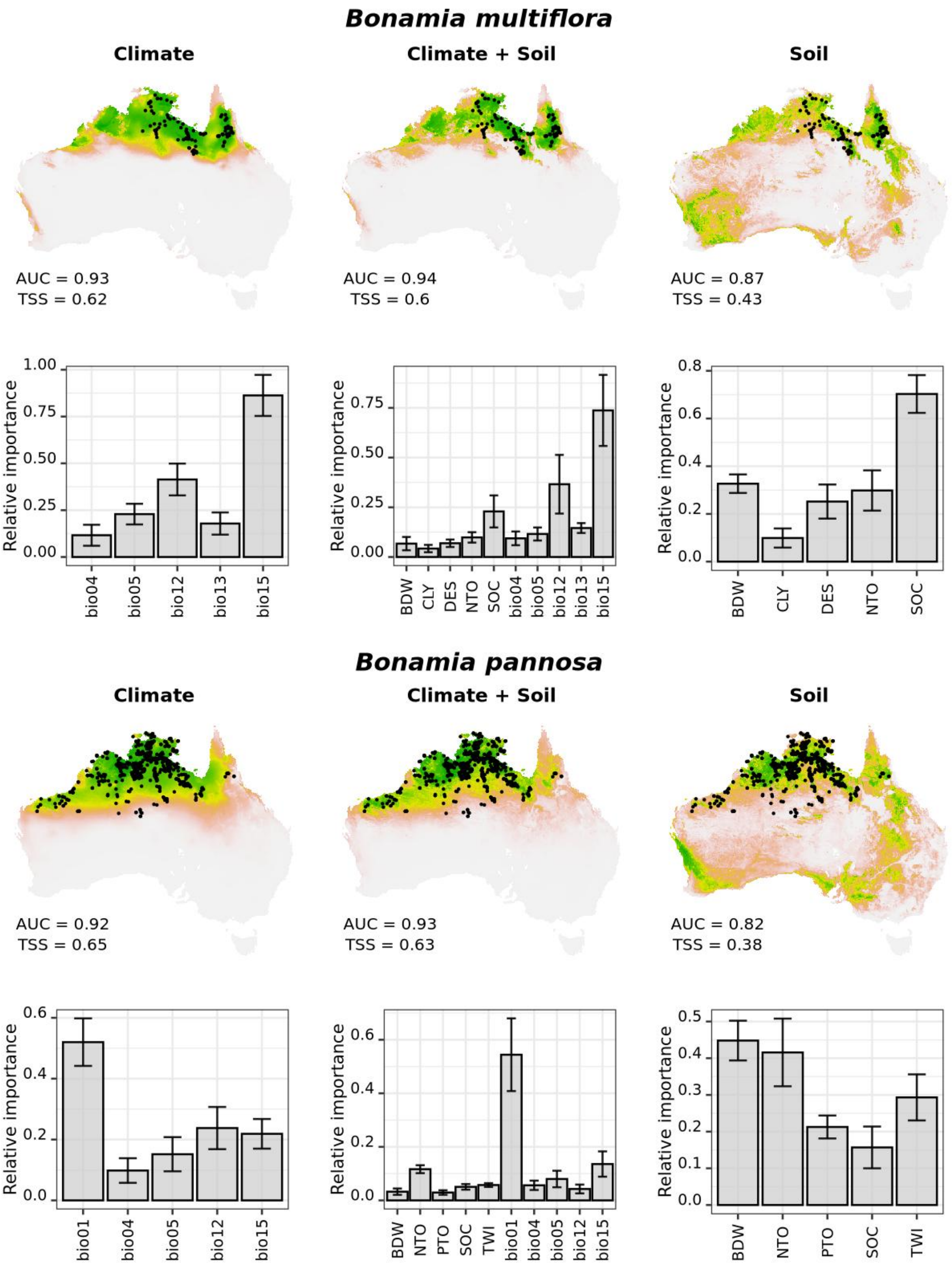
Bonamia multiflora
Climate
Climate + Soil
Soil
AUC = 0.93
TSS = 0.62
AUC = 0.94
TSS = 0.6
AUC = 0.87
TSS = 0.43
Relative importance
1.00
0.75
0.50
0.25
0.00
bio04
bio05
bio12
bio13
bio15
BDW
CLY
DES
NTO
SOC
0.8
0.6
0.4
0.2
0.0
Bonamia pannosa
Climate
Climate + Soil
Soil
AUC = 0.92
TSS = 0.65
AUC = 0.93
TSS = 0.63
AUC = 0.82
TSS = 0.38
bio01
PTO
TWI
0.5
0.3
0.1

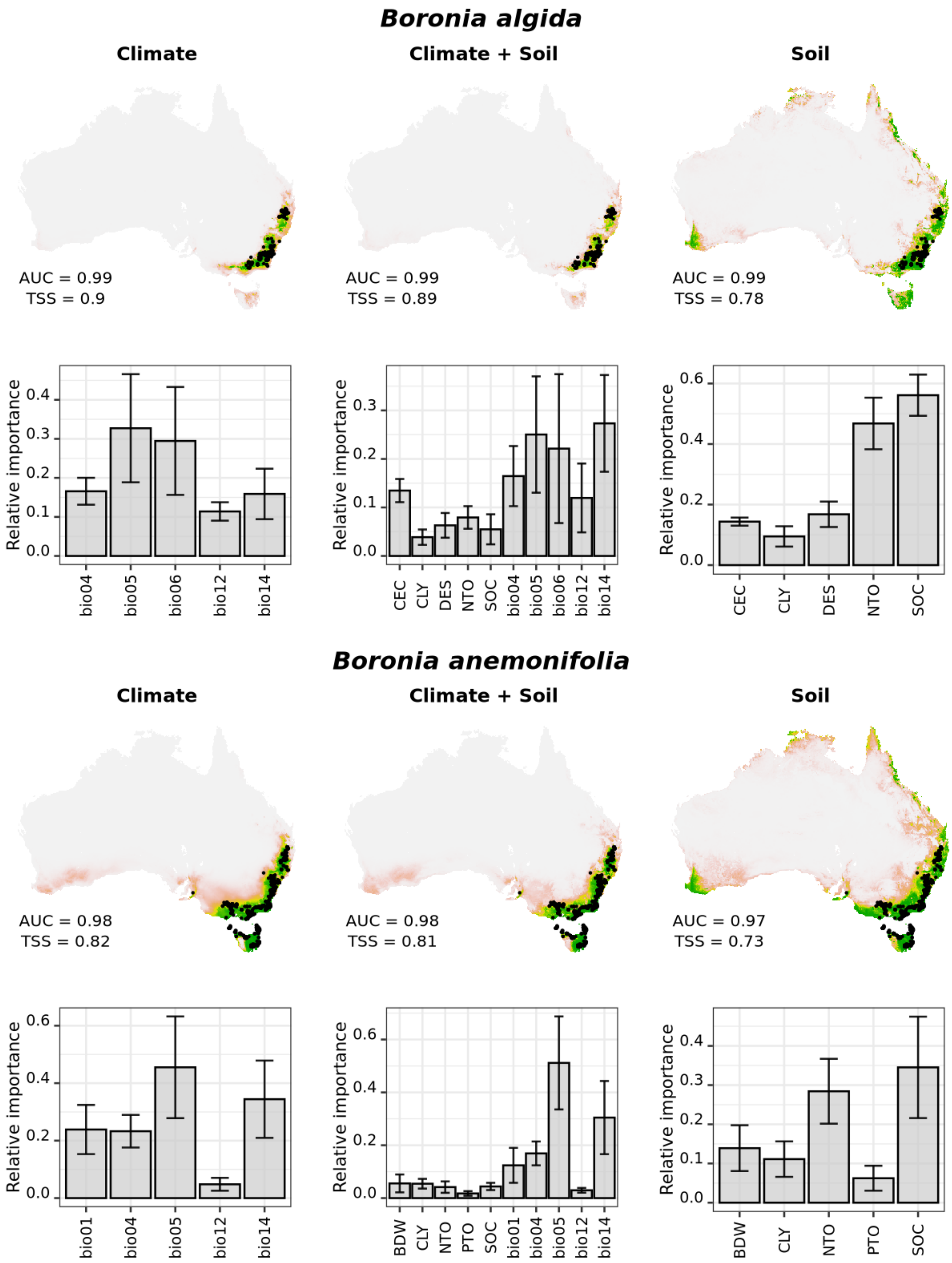
Boronia algida
Climate
Climate + Soil
Soil
AUC = 0.99
TSS = 0.9
AUC = 0.99
TSS = 0.89
AUC = 0.99
TSS = 0.78
Relative importance
bio04
bio05
bio06
bio12
bio14
CEC
CLY
DES
NTO
SOC
bio04
bio05
bio06
bio12
bio14
CEC
CLY
DES
NTO
SOC
Boronia anemonifolia
Climate
Climate + Soil
Soil
AUC = 0.98
TSS = 0.82
AUC = 0.98
TSS = 0.81
AUC = 0.97
TSS = 0.73
Relative importance
bio01
bio04
bio05
bio12
bio14
BDW
CLY
NTO
PTO
SOC
bio01
bio04
bio05
bio12
bio14
BDW
CLY
NTO
PTO
SOC

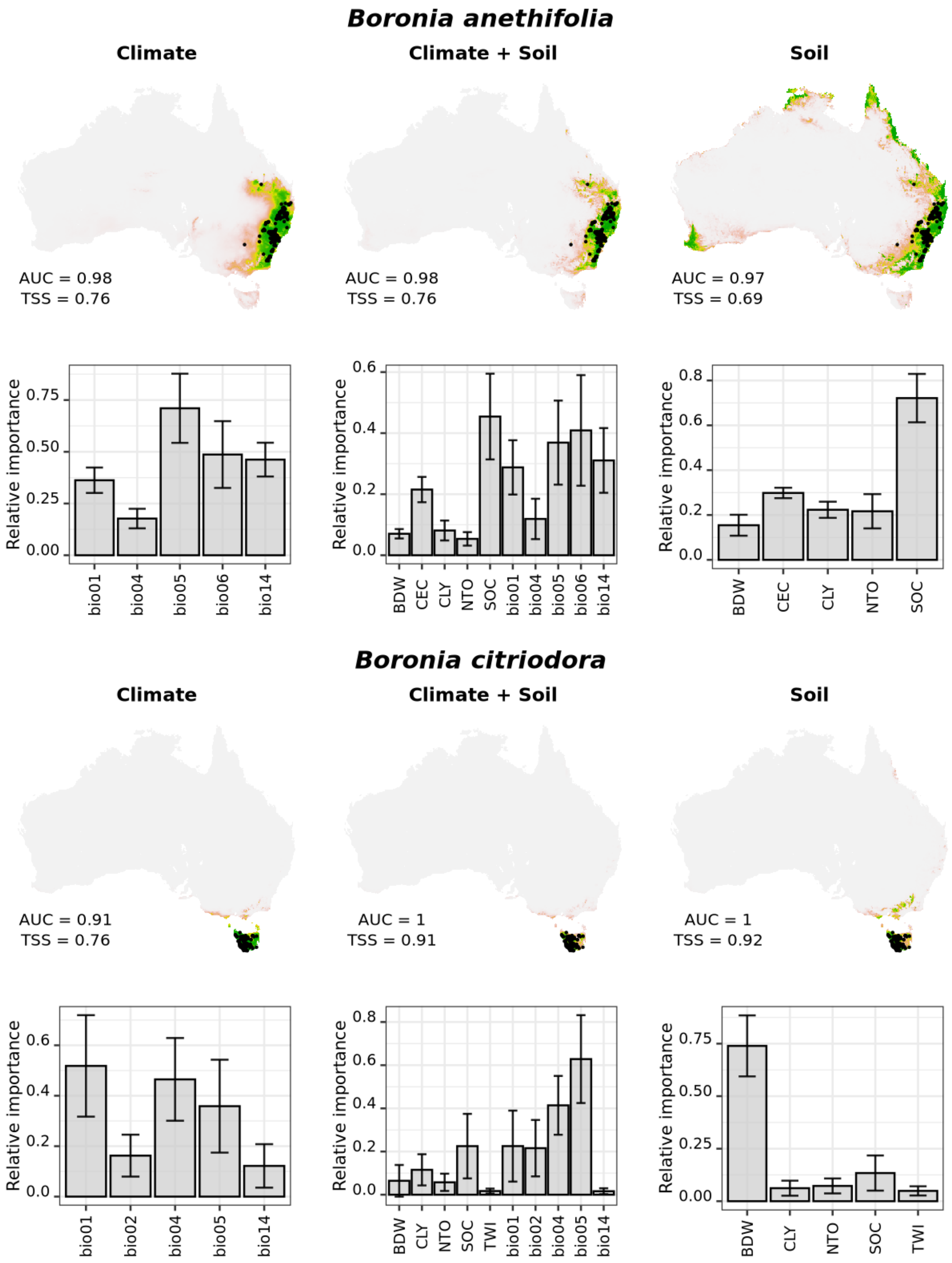
Boronia anethifolia
Climate
Climate + Soil
Soil
AUC = 0.98
TSS = 0.76
AUC = 0.98
TSS = 0.76
AUC = 0.97
TSS = 0.69
Relative importance
0.00
0.25
0.50
0.75
bio01
bio04
bio05
bio06
bio14
0.0
0.2
0.4
0.6
BDW
CEC
CLY
NTO
SOC
bio01
bio04
bio05
bio06
bio14
0.0
0.2
0.4
0.6
0.8
BDW
CEC
CLY
NTO
SOC
Boronia citriodora
Climate
Climate + Soil
Soil
AUC = 0.91
TSS = 0.76
AUC = 1
TSS = 0.91
AUC = 1
TSS = 0.92
Relative importance
0.0
0.2
0.4
0.6
bio01
bio02
bio04
bio05
bio14
0.0
0.2
0.4
0.6
0.8
BDW
CLY
NTO
SOC
TWI
bio01
bio02
bio04
bio05
bio14
0.00
0.25
0.50
0.75
BDW
CLY
NTO
SOC
TWI

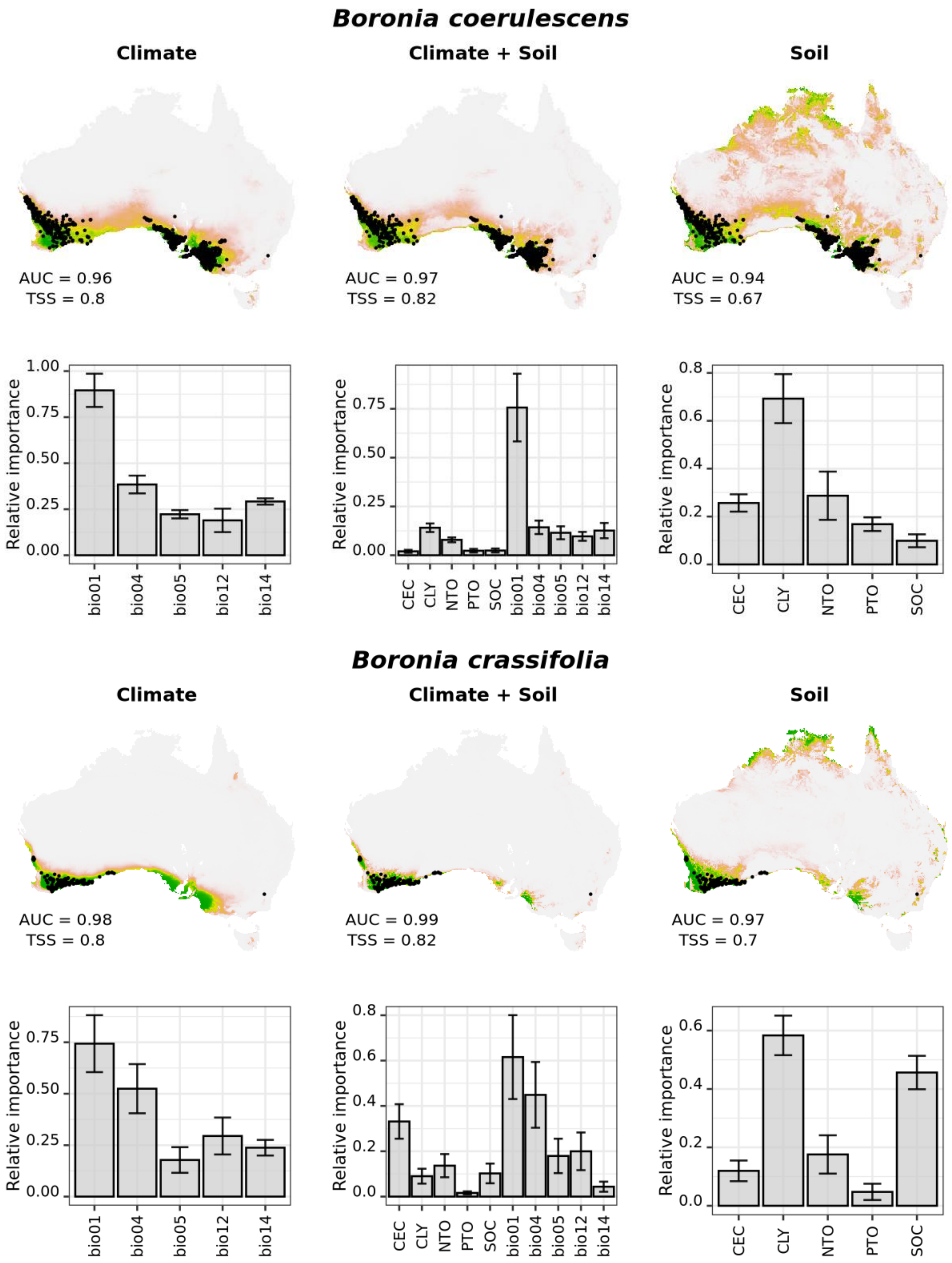
Boronia coerulescens
Climate
Climate + Soil
Soil
AUC = 0.96
TSS = 0.8
AUC = 0.97
TSS = 0.82
AUC = 0.94
TSS = 0.67
Relative importance
1.00
0.75
0.50
0.25
0.00
bio01
bio04
bio05
bio12
bio14
CEC
CLY
NTO
PTO
SOC
0.8
0.6
0.4
0.2
0.0
Boronia crassifolia
Climate
Climate + Soil
Soil
AUC = 0.98
TSS = 0.8
AUC = 0.99
TSS = 0.82
AUC = 0.97
TSS = 0.7

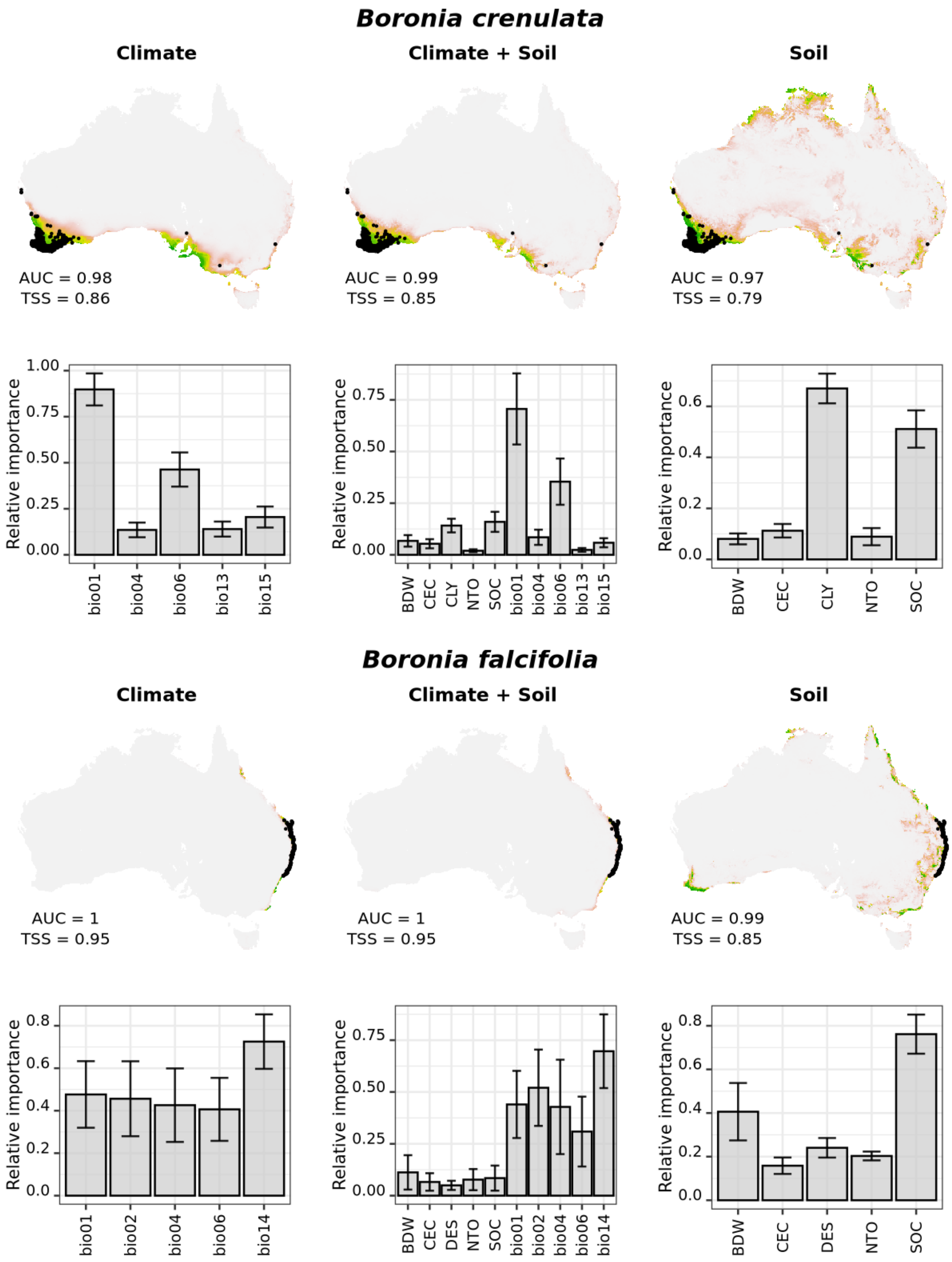

Boronia crenulata
Climate
Climate + Soil
Soil
AUC = 0.98
TSS = 0.86
AUC = 0.99
TSS = 0.85
AUC = 0.97
TSS = 0.79
Relative importance
bio01
bio04
bio06
bio13
bio15
BDW
CEC
CLY
NTO
SOC
Boronia falcifolia
AUC = 1
TSS = 0.95
AUC = 1
TSS = 0.95
AUC = 0.99
TSS = 0.85
bio02
bio14
DES

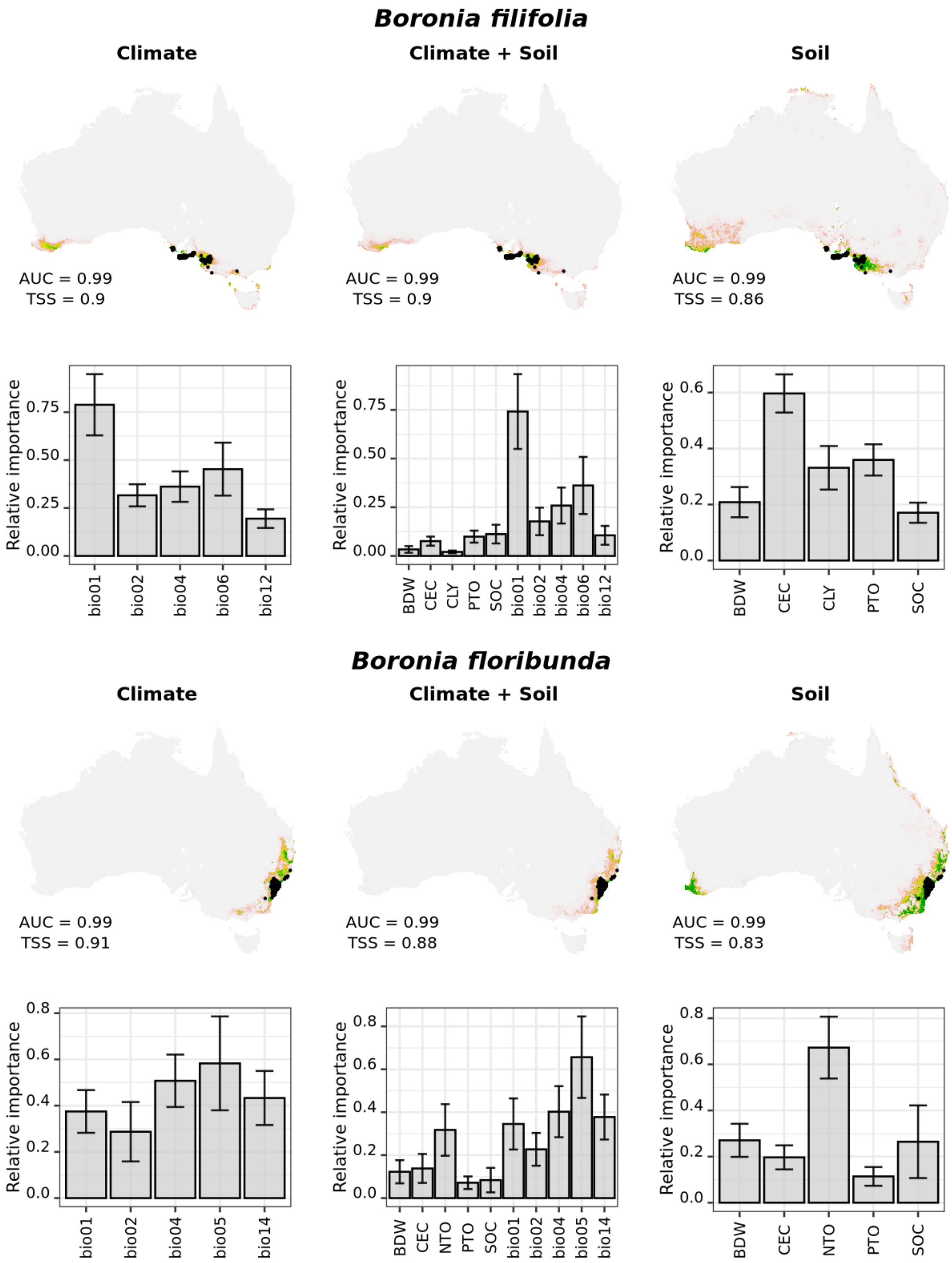
Boronia filifolia
Climate
Climate + Soil
Soil
AUC = 0.99
TSS = 0.9
AUC = 0.99
TSS = 0.9
AUC = 0.99
TSS = 0.86
Relative importance
0.00
0.25
0.50
0.75
bio01
bio02
bio04
bio06
bio12
Relative importance
0.00
0.25
0.50
0.75
BDW
CEC
CLY
PTO
SOC
bio01
bio02
bio04
bio06
bio12
Relative importance
0.0
0.2
0.4
0.6
BDW
CEC
CLY
PTO
SOC
Boronia floribunda
Climate
Climate + Soil
Soil
AUC = 0.99
TSS = 0.91
AUC = 0.99
TSS = 0.88
AUC = 0.99
TSS = 0.83
Relative importance
0.0
0.2
0.4
0.6
0.8
bio01
bio02
bio04
bio05
bio14
Relative importance
0.0
0.2
0.4
0.6
0.8
BDW
CEC
NTO
PTO
SOC
bio01
bio02
bio04
bio05
bio14
Relative importance
0.0
0.2
0.4
0.6
0.8
BDW
CEC
NTO
PTO
SOC

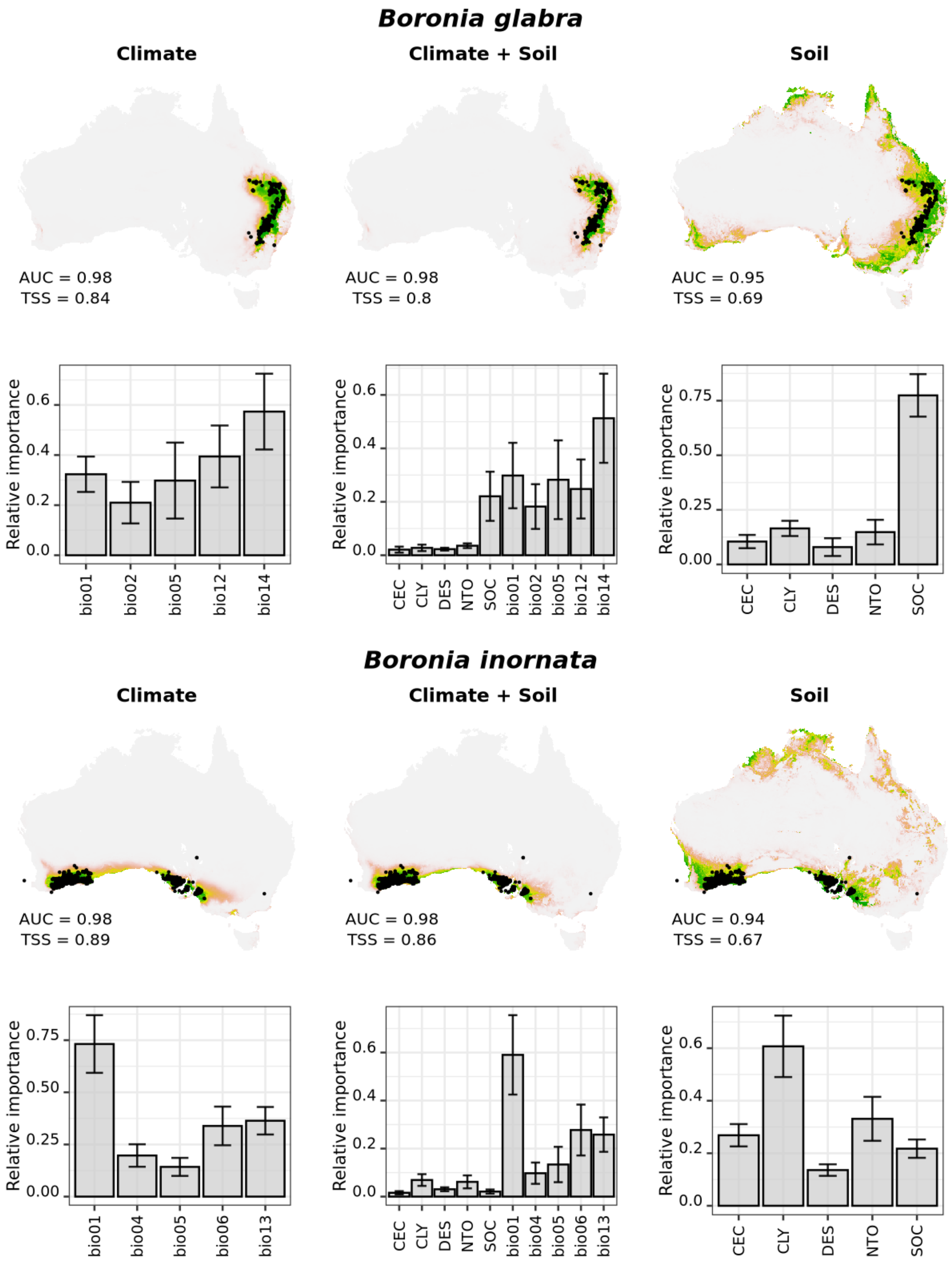
Boronia glabra
Climate
Climate + Soil
Soil
AUC = 0.98
TSS = 0.84
AUC = 0.98
TSS = 0.8
AUC = 0.95
TSS = 0.69
Relative importance
0.0
0.2
0.4
0.6
bio01
bio02
bio05
bio12
bio14
Relative importance
0.0
0.2
0.4
0.6
CEC
CLY
DES
NTO
SOC
bio01
bio02
bio05
bio12
bio14
Relative importance
0.00
0.25
0.50
0.75
CEC
CLY
DES
NTO
SOC
Boronia inornata
Climate
Climate + Soil
Soil
AUC = 0.98
TSS = 0.89
AUC = 0.98
TSS = 0.86
AUC = 0.94
TSS = 0.67
Relative importance
0.00
0.25
0.50
0.75
bio01
bio04
bio05
bio06
bio13
Relative importance
0.0
0.2
0.4
0.6
CEC
CLY
DES
NTO
SOC
bio01
bio04
bio05
bio06
bio13
Relative importance
0.0
0.2
0.4
0.6
CEC
CLY
DES
NTO
SOC

Boronia lanceolata

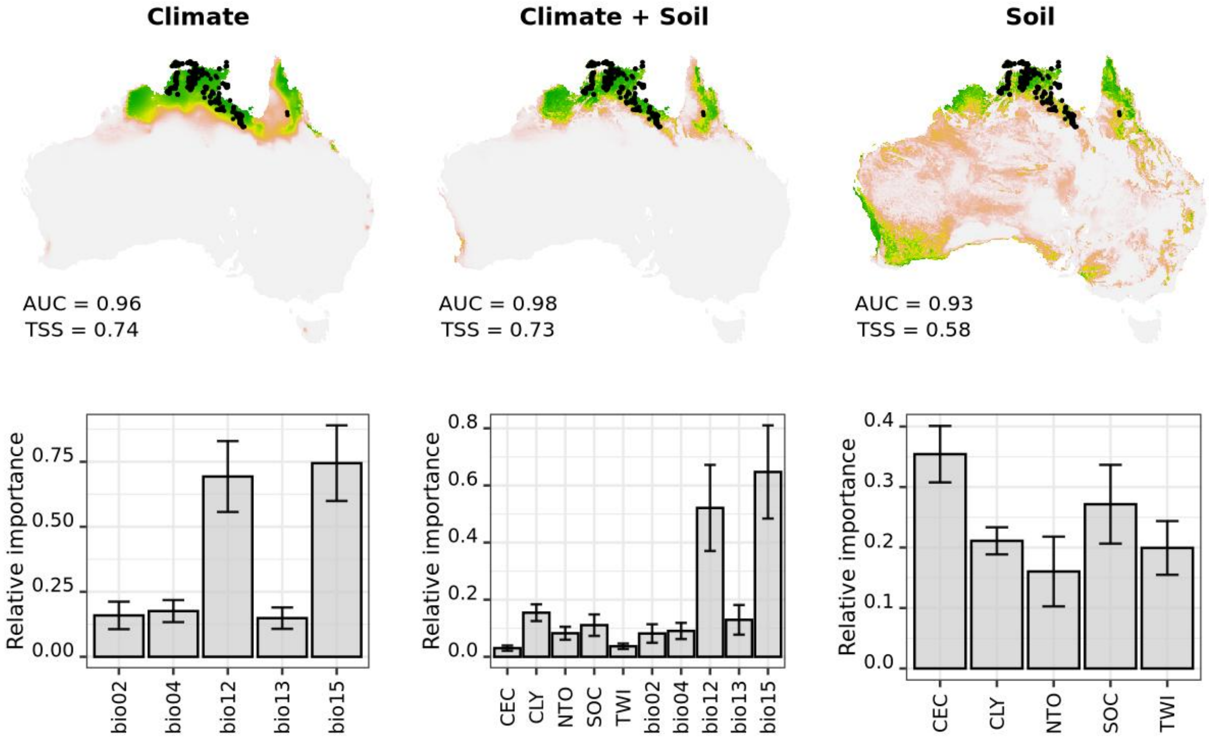

Boronia lanuginosa

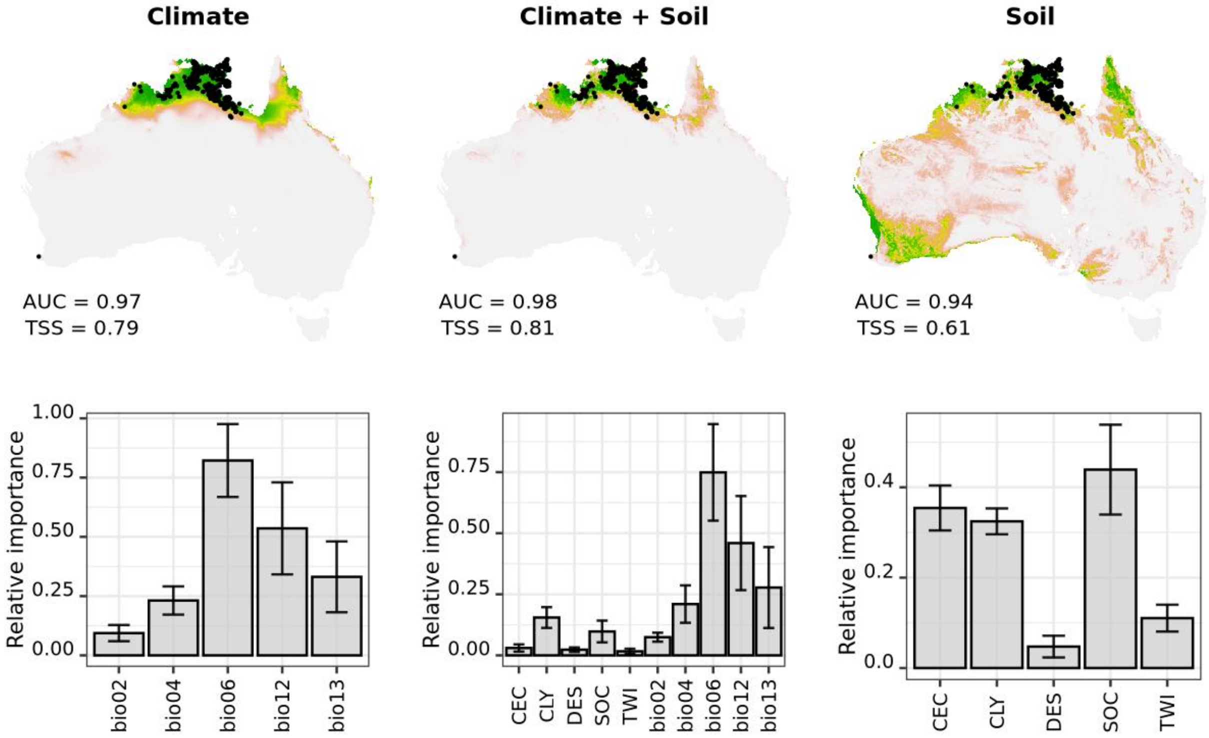

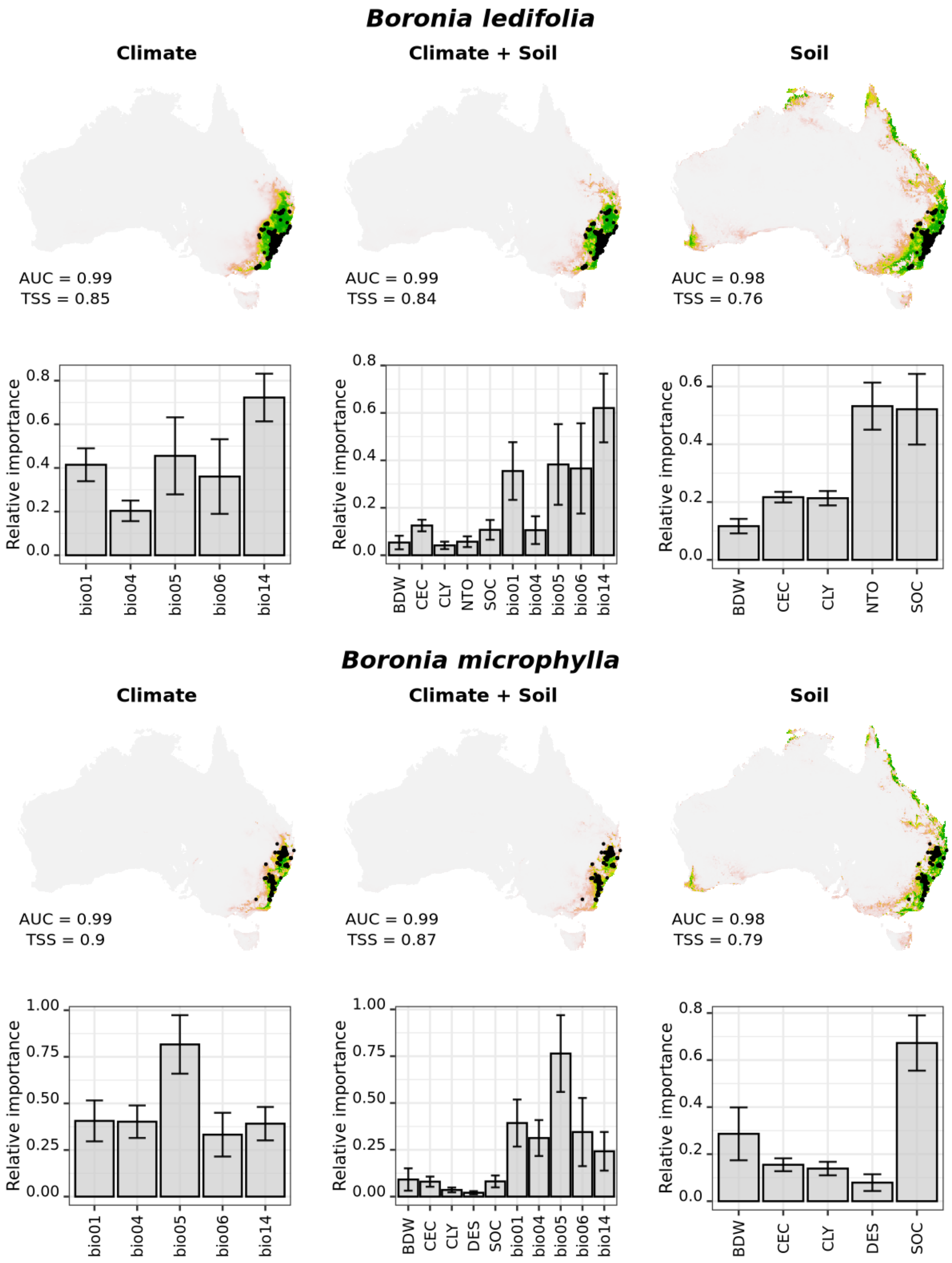

Boronia ledifolia
Climate
Climate + Soil
Soil
AUC = 0.99
TSS = 0.85
AUC = 0.99
TSS = 0.84
AUC = 0.98
TSS = 0.76
Relative importance
0.0
0.2
0.4
0.6
0.8
bio01
bio04
bio05
bio06
bio14
BDW
CEC
CLY
NTO
SOC
Boronia microphylla
Climate
Climate + Soil
Soil
AUC = 0.99
TSS = 0.9
AUC = 0.99
TSS = 0.87
AUC = 0.98
TSS = 0.79
Relative importance
0.00
0.25
0.50
0.75
1.00
bio01
bio04
bio05
bio06
bio14
BDW
CEC
CLY
DES
SOC

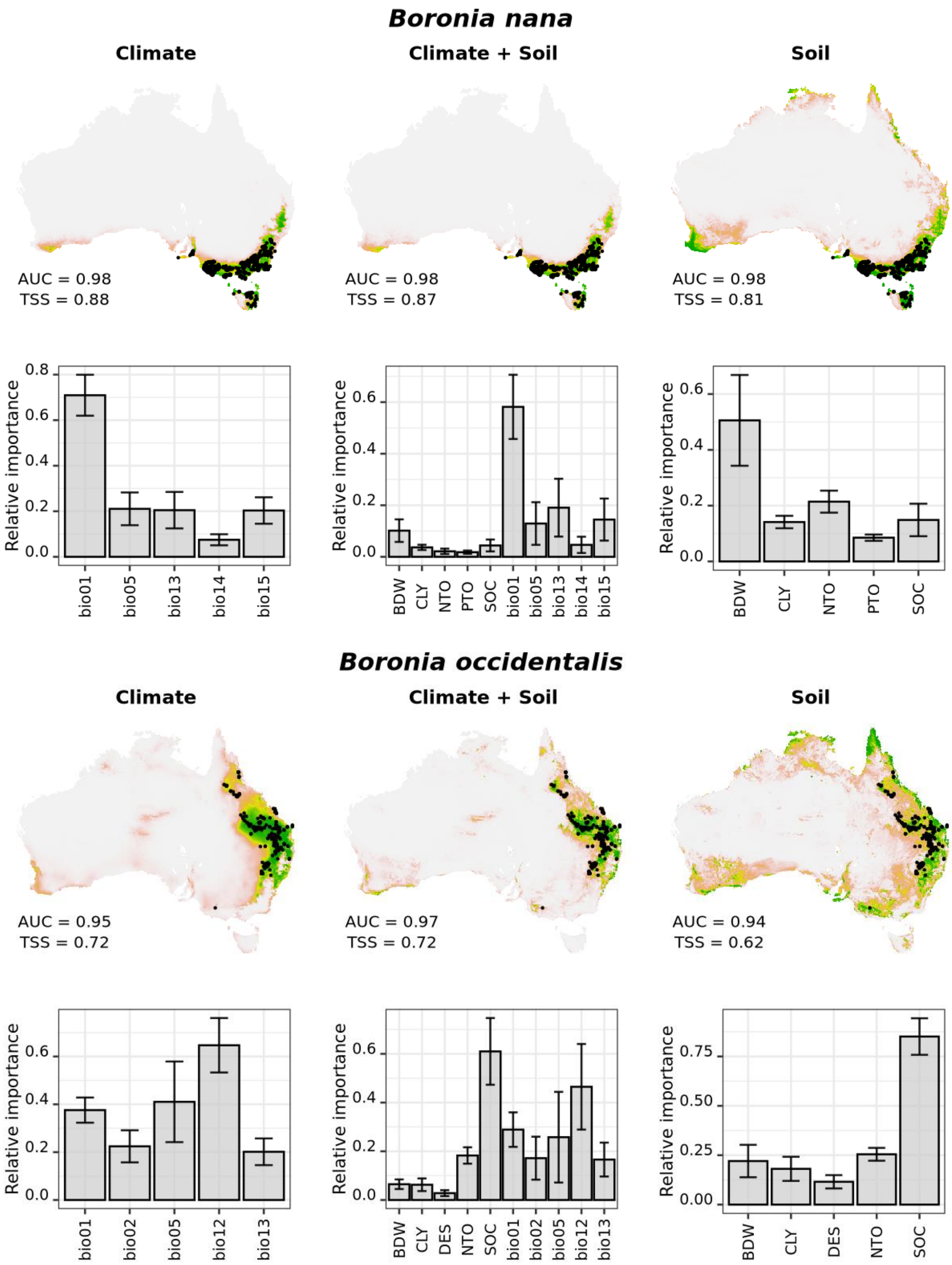
Boronia nana
Climate
Climate + Soil
Soil
AUC = 0.98
TSS = 0.88
AUC = 0.98
TSS = 0.87
AUC = 0.98
TSS = 0.81
Relative importance
bio01
bio05
bio13
bio14
bio15
BDW
CLY
NTO
PTO
SOC
Boronia occidentalis
Climate
Climate + Soil
Soil
AUC = 0.95
TSS = 0.72
AUC = 0.97
TSS = 0.72
AUC = 0.94
TSS = 0.62
Relative importance
bio01
bio02
bio05
bio12
bio13
BDW
CLY
DES
NTO
SOC

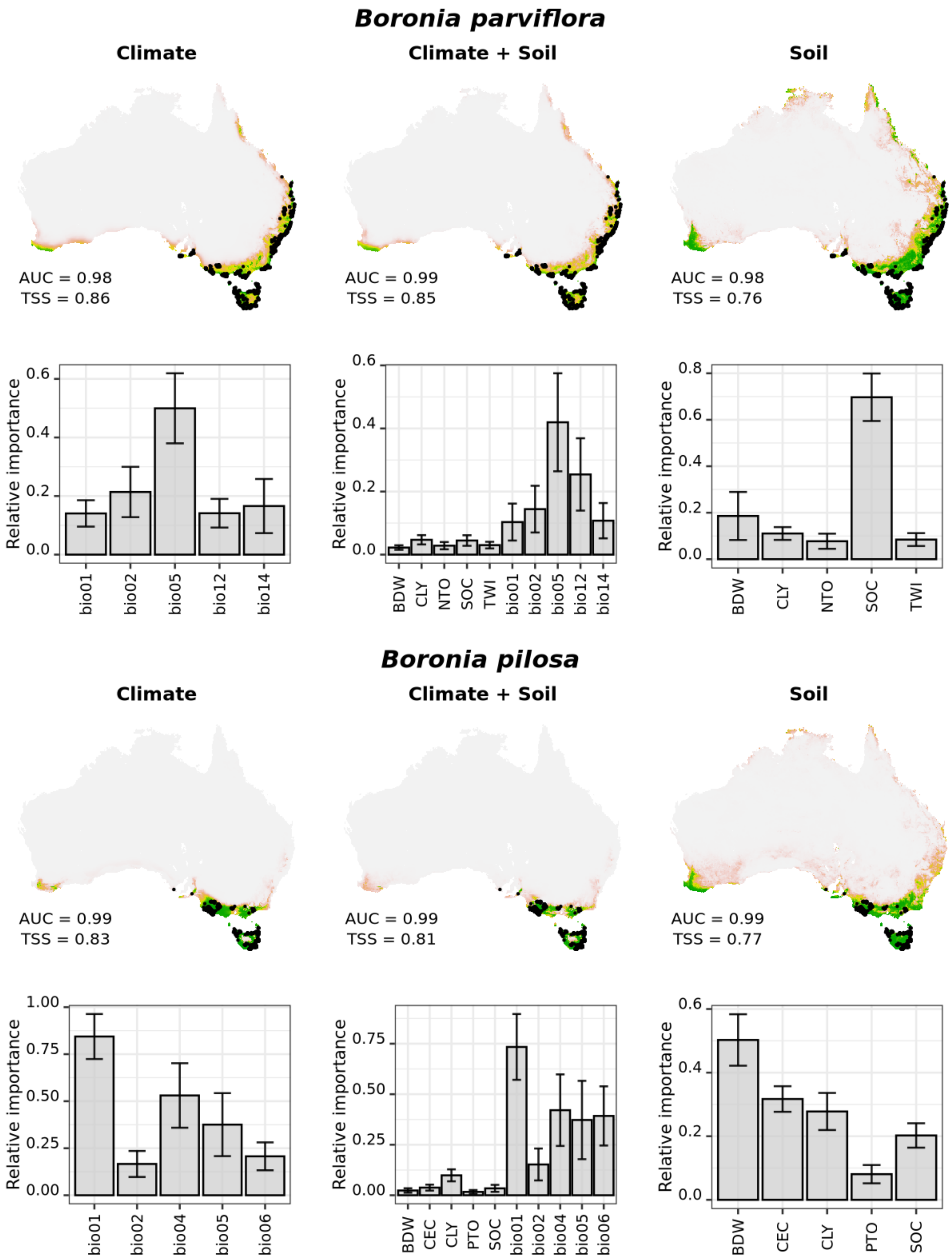

Boronia parviflora
Climate
Climate + Soil
Soil
AUC = 0.98
TSS = 0.86
AUC = 0.99
TSS = 0.85
AUC = 0.98
TSS = 0.76
Relative importance
bio01
bio02
bio05
bio12
bio14
BDW
CLY
NTO
SOC
TWI
Boronia pilosa
Climate
Climate + Soil
Soil
AUC = 0.99
TSS = 0.83
AUC = 0.99
TSS = 0.81
AUC = 0.99
TSS = 0.77
Relative importance
bio01
bio02
bio04
bio05
bio06
BDW
CEC
CLY
PTO
SOC

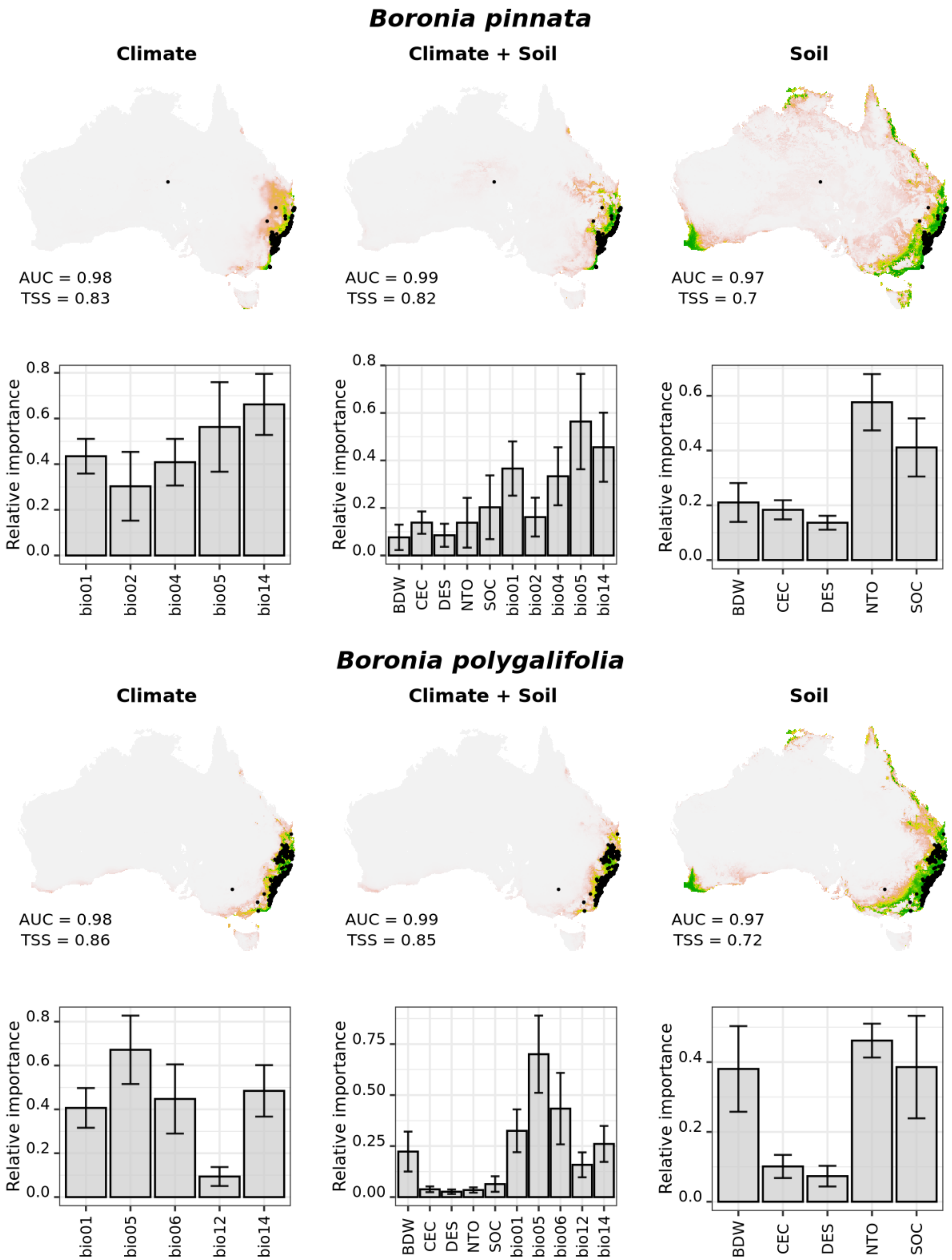
Boronia pinnata
Climate
Climate + Soil
Soil
AUC = 0.98
TSS = 0.83
AUC = 0.99
TSS = 0.82
AUC = 0.97
TSS = 0.7
Relative importance
bio01 bio02 bio04 bio05 bio14
BDW CEC DES NTO SOC bio01 bio02 bio04 bio05 bio14
BDW CEC DES NTO SOC
Boronia polygalifolia
Climate
Climate + Soil
Soil
AUC = 0.98
TSS = 0.86
AUC = 0.99
TSS = 0.85
AUC = 0.97
TSS = 0.72
Relative importance
bio01 bio05 bio06 bio12 bio14
BDW CEC DES NTO SOC bio01 bio05 bio06 bio12 bio14
BDW CEC DES NTO SOC

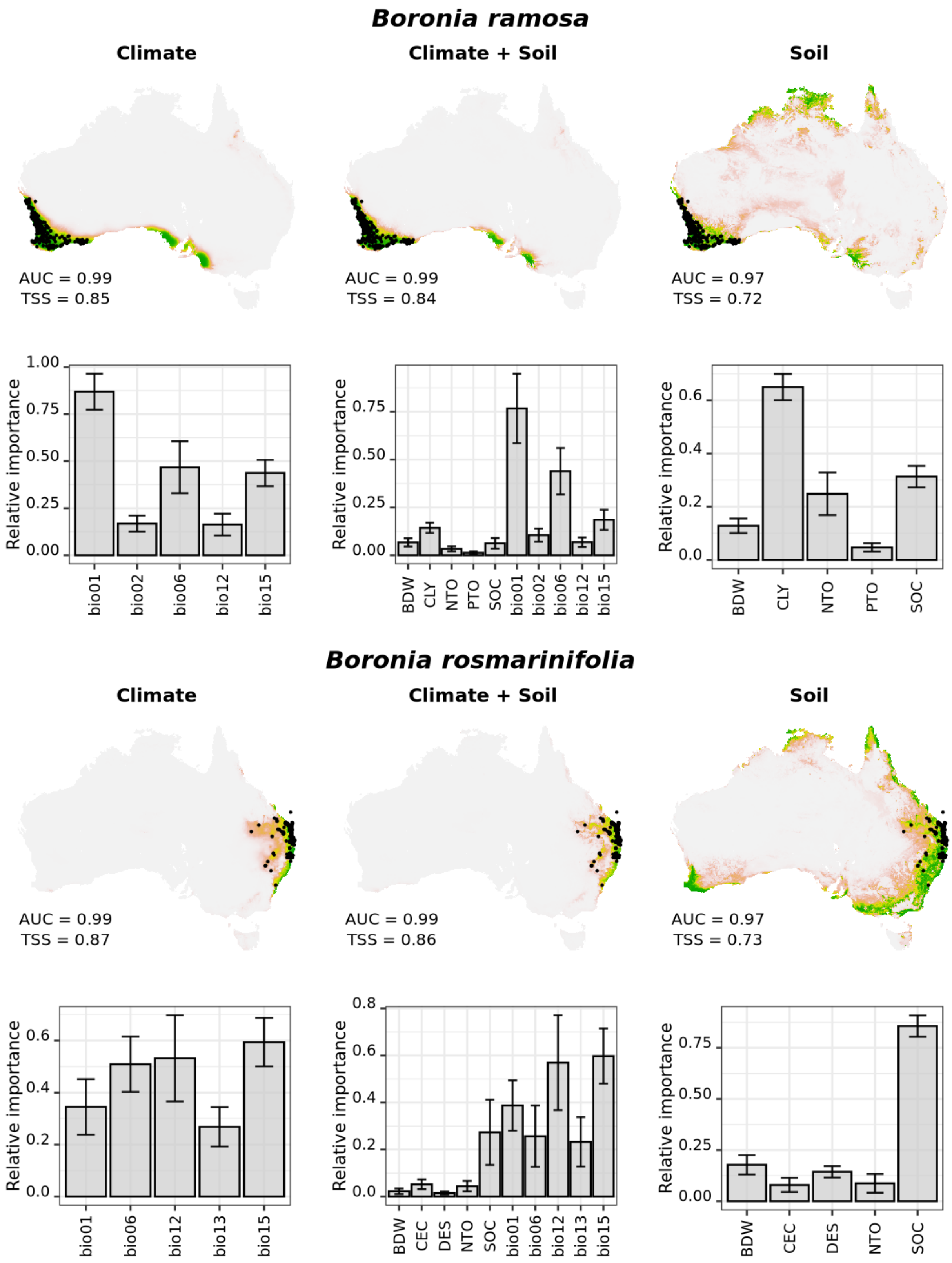

Boronia ramosa
Climate
Climate + Soil
Soil
AUC = 0.99
TSS = 0.85
AUC = 0.99
TSS = 0.84
AUC = 0.97
TSS = 0.72
Relative importance
bio01
bio02
bio06
bio12
bio15
BDW
CLY
NTO
PTO
SOC
Boronia rosmarinifolia
Climate
Climate + Soil
Soil
AUC = 0.99
TSS = 0.87
AUC = 0.99
TSS = 0.86
AUC = 0.97
TSS = 0.73
Relative importance
bio01
bio06
bio12
bio13
bio15
BDW
CEC
DES
NTO
SOC

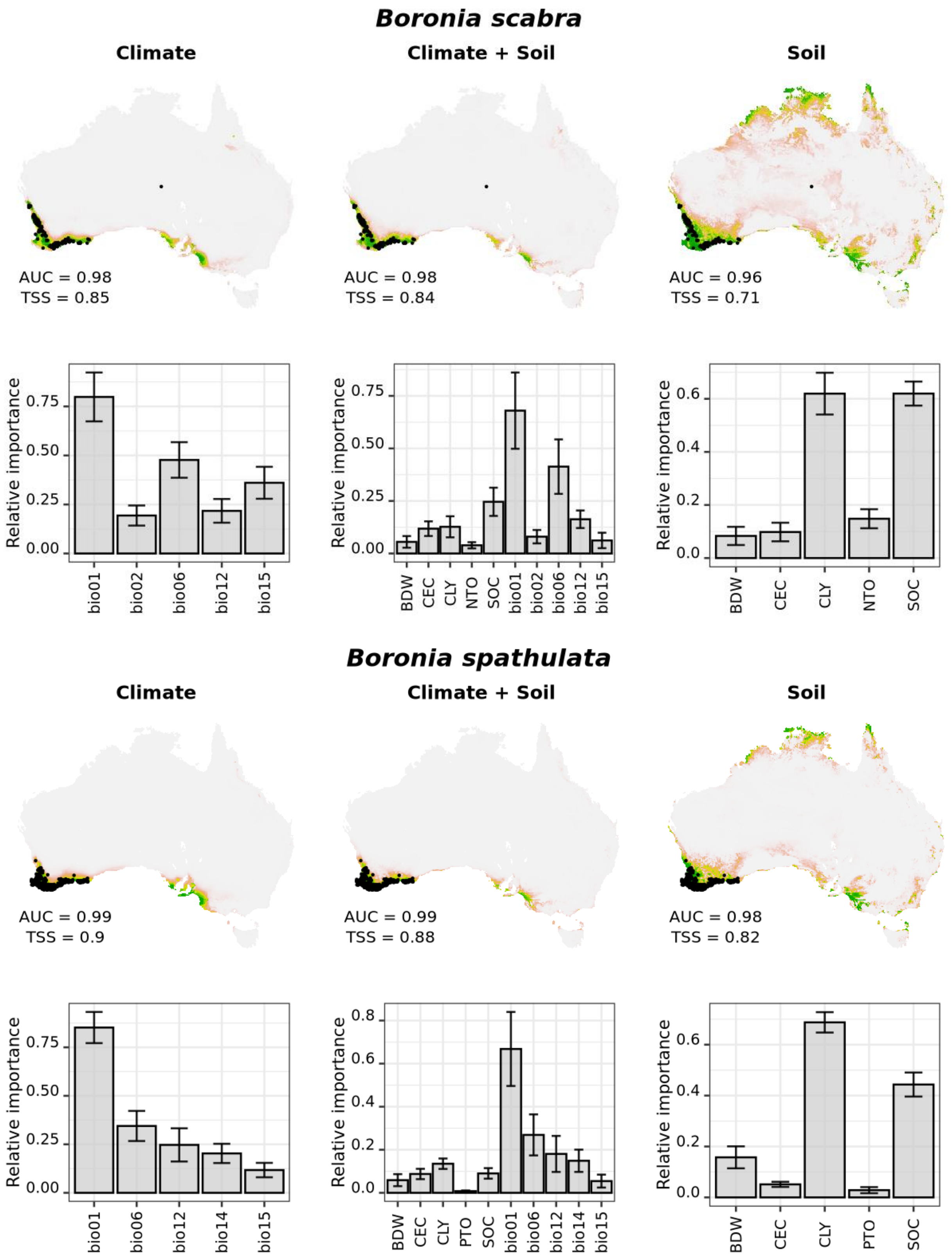

Boronia scabra
Climate
Climate + Soil
Soil
AUC = 0.98
TSS = 0.85
AUC = 0.98
TSS = 0.84
AUC = 0.96
TSS = 0.71
Relative importance
bio01
bio02
bio06
bio12
bio15
BDW
CEC
CLY
NTO
SOC
Boronia spathulata
Climate
Climate + Soil
Soil
AUC = 0.99
TSS = 0.9
AUC = 0.99
TSS = 0.88
AUC = 0.98
TSS = 0.82
Relative importance
bio01
bio06
bio12
bio14
bio15
BDW
CEC
CLY
PTO
SOC

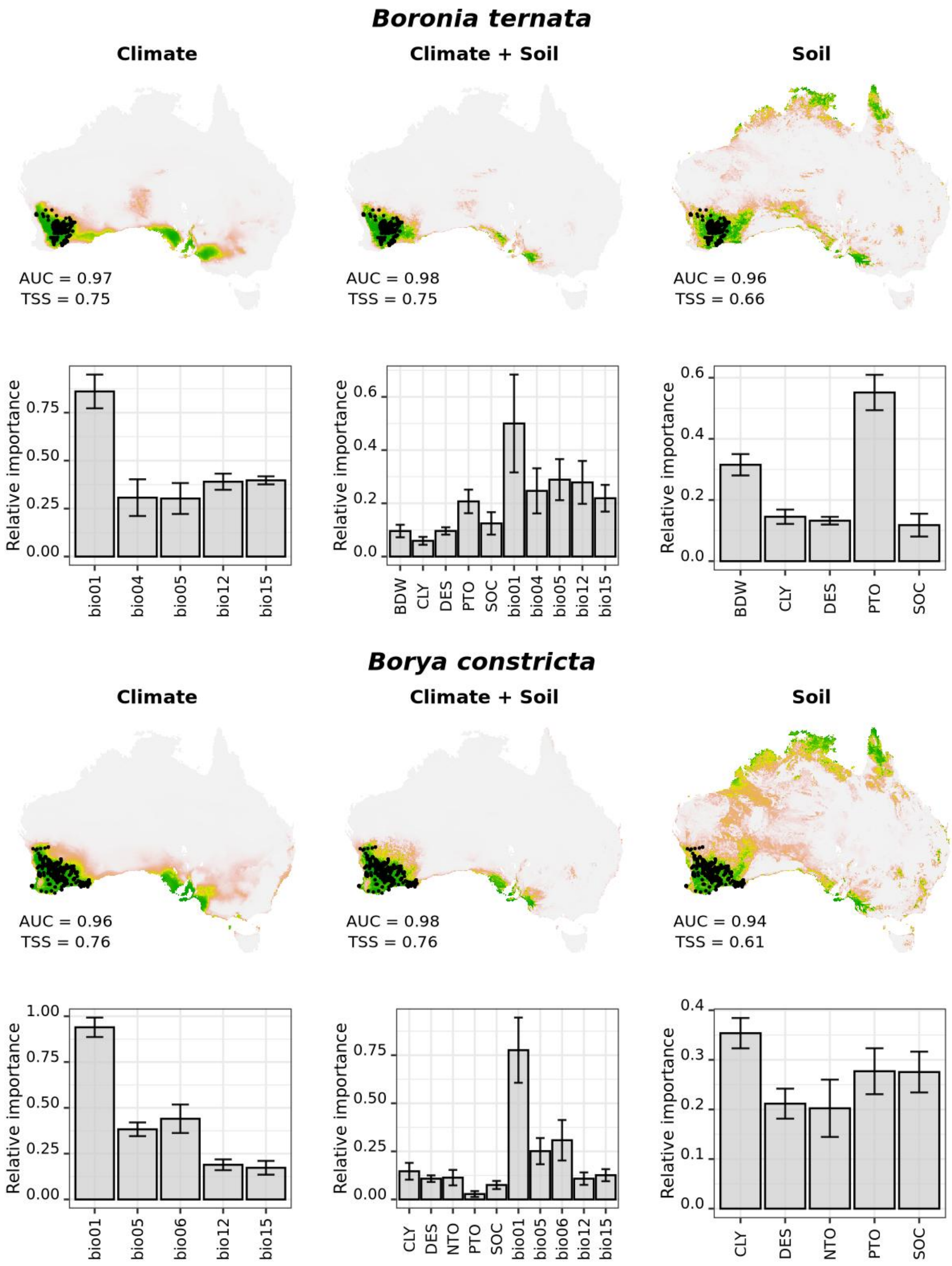
Boronia ternata
Climate
Climate + Soil
Soil
AUC = 0.97
TSS = 0.75
AUC = 0.98
TSS = 0.75
AUC = 0.96
TSS = 0.66
Relative importance
0.00 0.25 0.50 0.75
bio01 bio04 bio05 bio12 bio15
0.0 0.2 0.4 0.6
BDW CLY DES PTO SOC bio01 bio04 bio05 bio12 bio15
0.0 0.2 0.4 0.6
BDW CLY DES PTO SOC
Borya constricta
Climate
Climate + Soil
Soil
AUC = 0.96
TSS = 0.76
AUC = 0.98
TSS = 0.76
AUC = 0.94
TSS = 0.61
0.00 0.25 0.50 0.75 1.00
bio01 bio05 bio06 bio12 bio15
0.00 0.25 0.50 0.75
CLY DES NTO PTO SOC bio01 bio05 bio06 bio12 bio15
0.0 0.1 0.2 0.3 0.4
CLY DES NTO PTO SOC

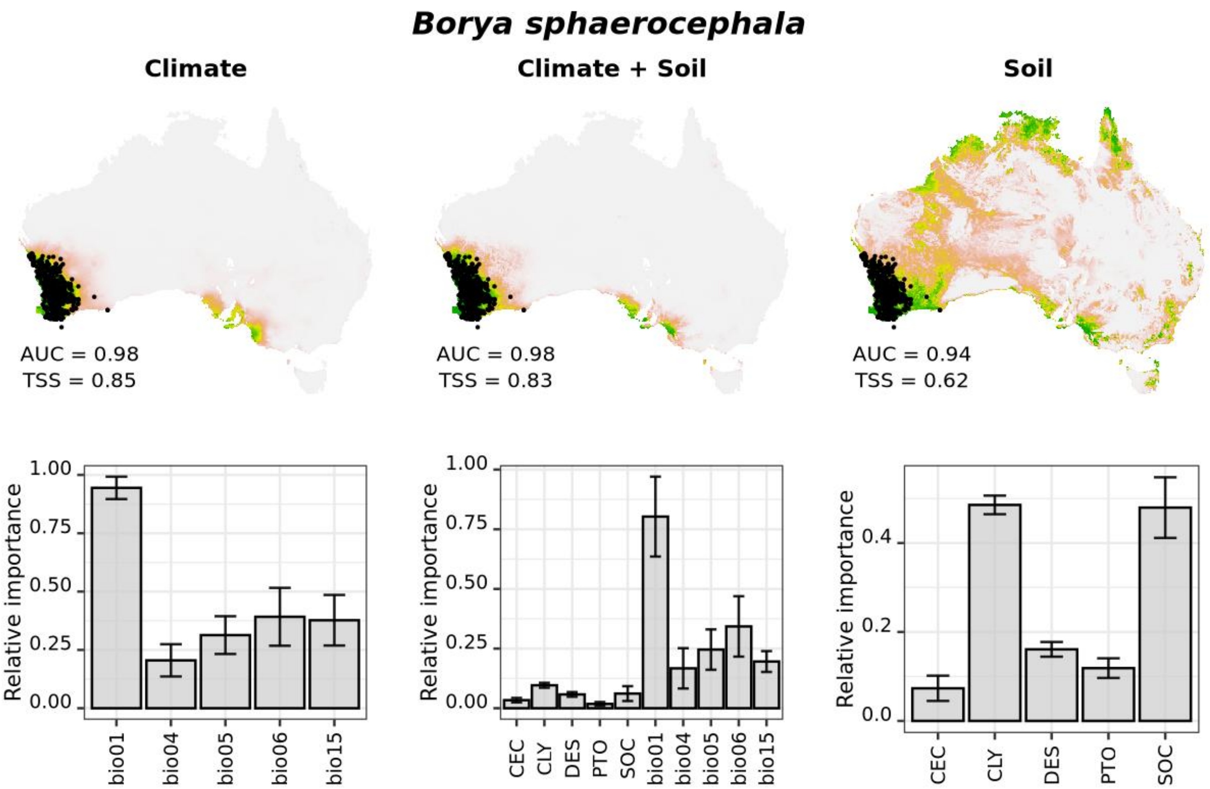
Borya sphaerocephala
Climate
Climate + Soil
Soil
AUC = 0.98
TSS = 0.85
AUC = 0.98
TSS = 0.83
AUC = 0.94
TSS = 0.62
Relative importance
1.00
0.75
0.50
0.25
0.00
bio01
bio04
bio05
bio06
bio15
CEC
CLY
DES
PTO
SOC
0.4
0.2
0.0

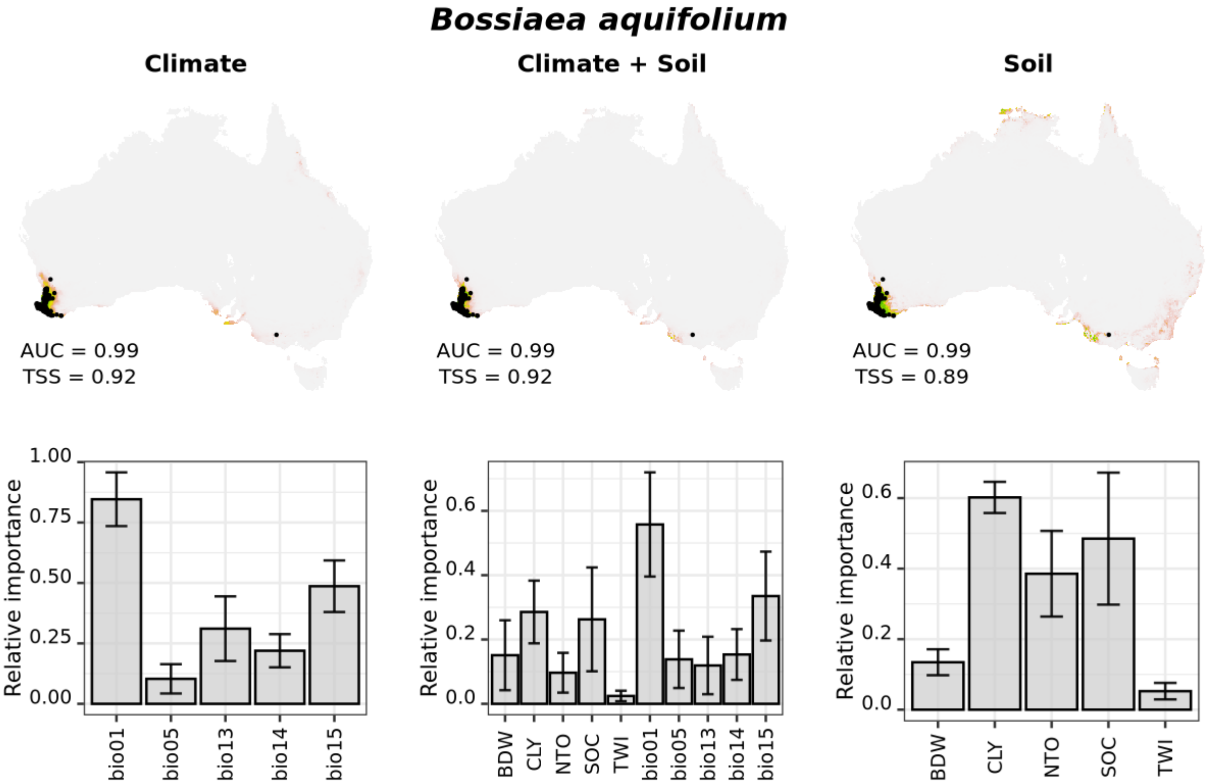
Bossiaea aquifolium
Climate
Climate + Soil
Soil
AUC = 0.99
TSS = 0.92
AUC = 0.99
TSS = 0.92
AUC = 0.99
TSS = 0.89
Relative importance
1.00
0.75
0.50
0.25
0.00
bio01
bio05
bio13
bio14
bio15
0.6
0.4
0.2
0.0
BDW
CLY
NTO
SOC
TWI

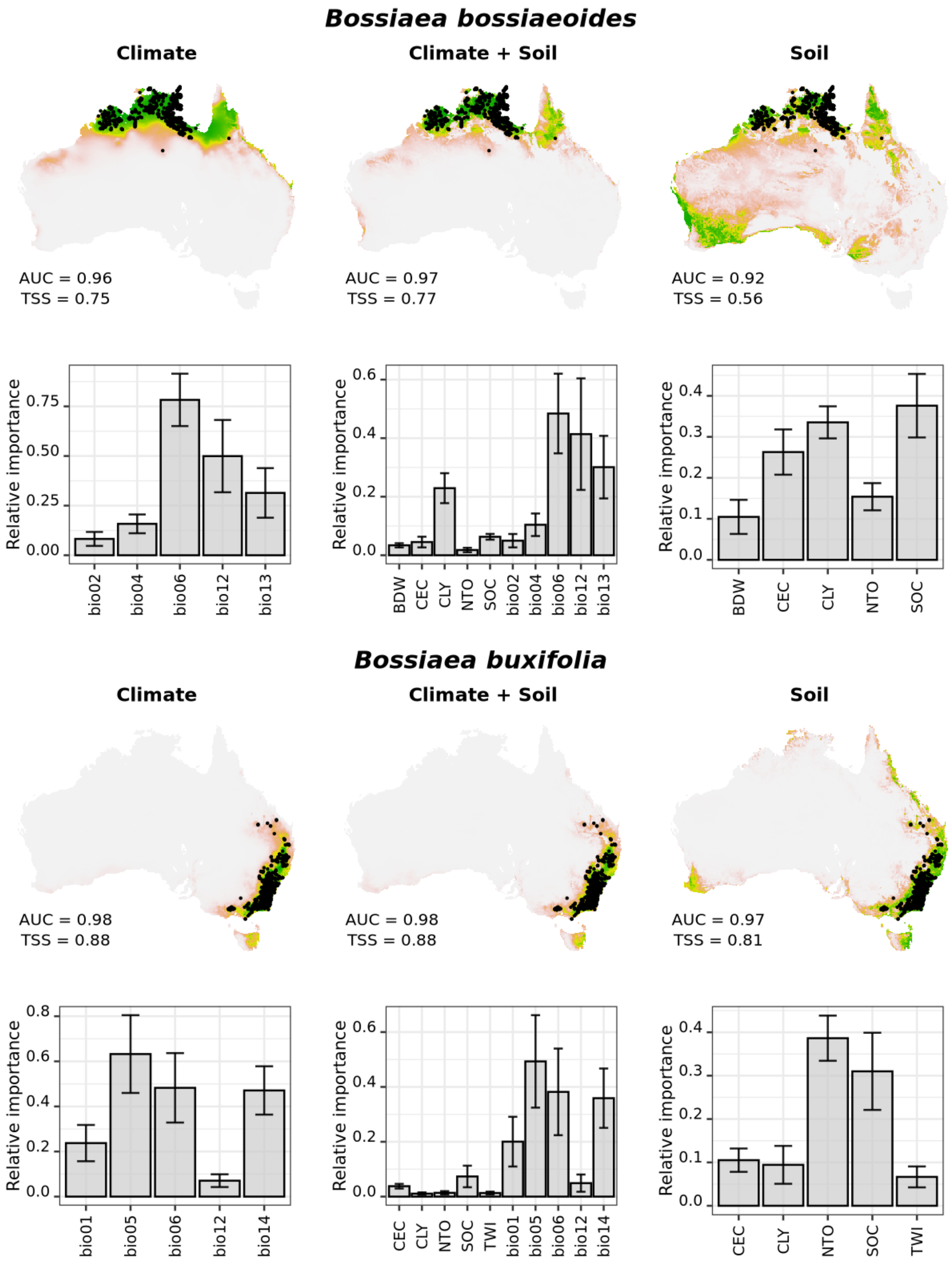

Bossiaea bossiaeoides
Climate
Climate + Soil
Soil
AUC = 0.96
TSS = 0.75
AUC = 0.97
TSS = 0.77
AUC = 0.92
TSS = 0.56
Relative importance
bio02
bio04
bio06
bio12
bio13
BDW
CEC
CLY
NTO
SOC
Bossiaea buxifolia
Climate
Climate + Soil
Soil
AUC = 0.98
TSS = 0.88
AUC = 0.98
TSS = 0.88
AUC = 0.97
TSS = 0.81
Relative importance
bio01
bio05
bio06
bio12
bio14
CEC
CLY
NTO
SOC
TWI

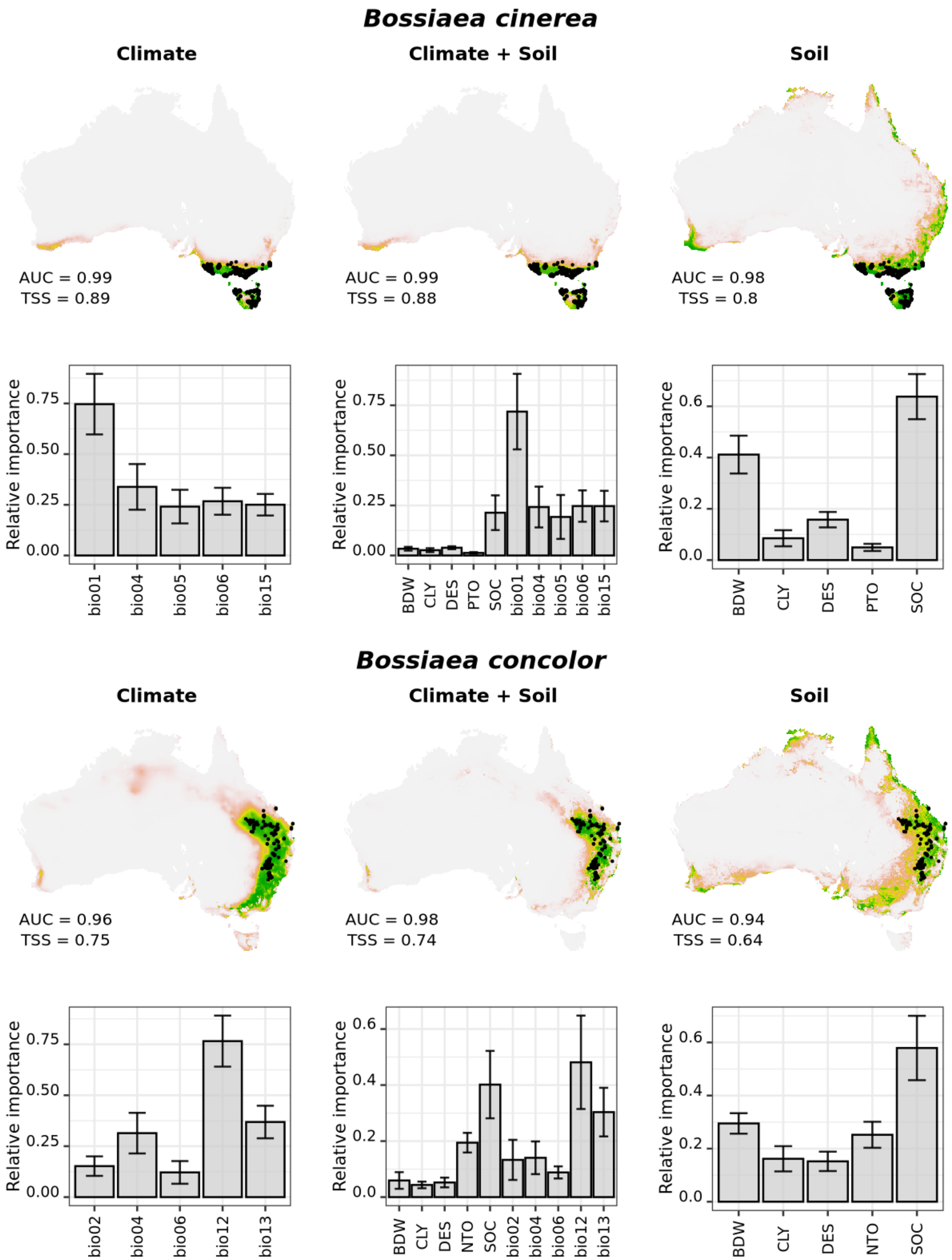
Bossiaea cinerea
Climate
Climate + Soil
Soil
AUC = 0.99
TSS = 0.89
AUC = 0.99
TSS = 0.88
AUC = 0.98
TSS = 0.8
Relative importance
bio01
bio04
bio05
bio06
bio15
BDW
CLY
DES
PTO
SOC
Bossiaea concolor
Climate
Climate + Soil
Soil
AUC = 0.96
TSS = 0.75
AUC = 0.98
TSS = 0.74
AUC = 0.94
TSS = 0.64
Relative importance
bio02
bio04
bio06
bio12
bio13
BDW
CLY
DES
NTO
SOC

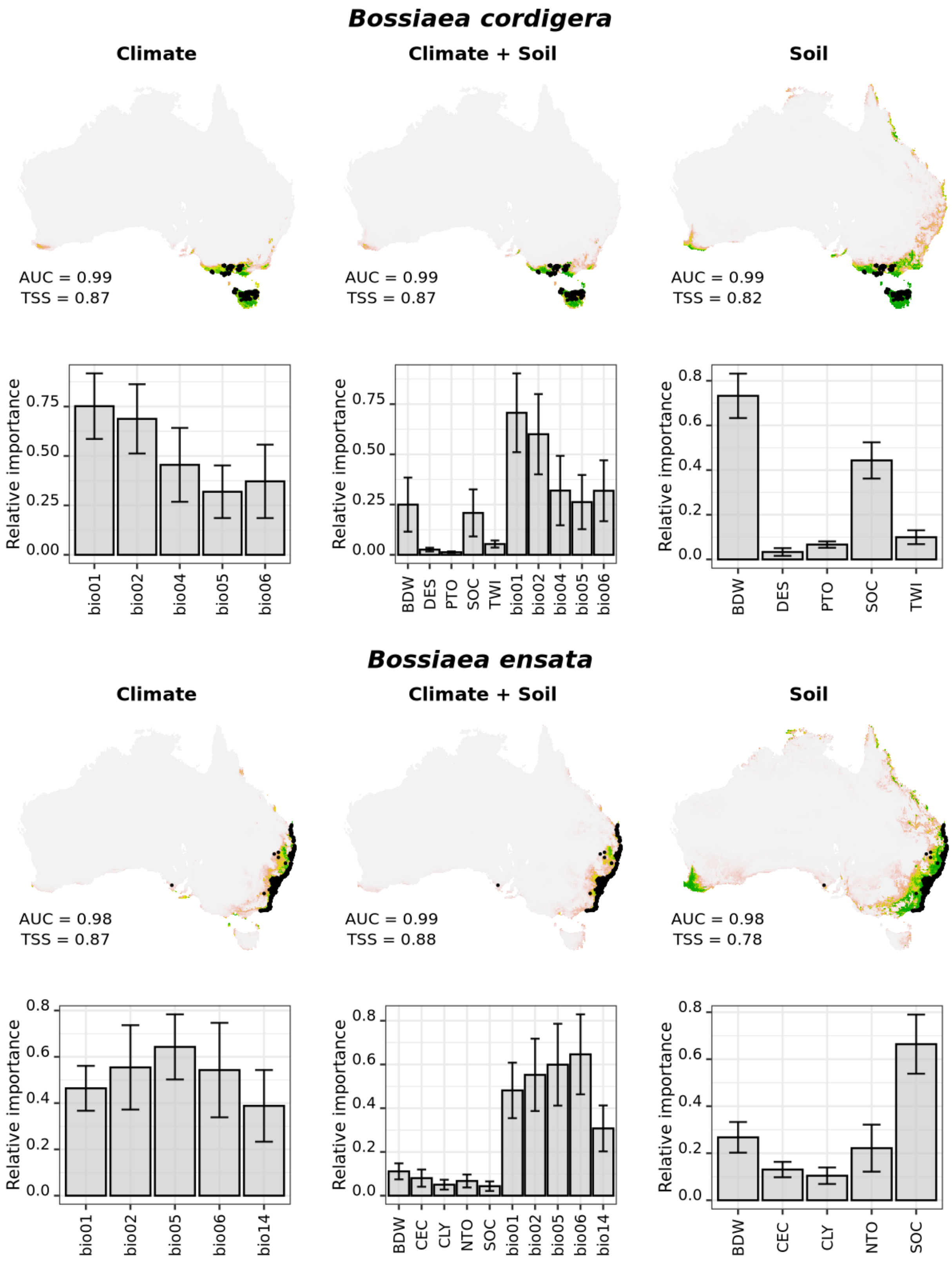

Bossiaea cordigera
Climate
Climate + Soil
Soil
AUC = 0.99
TSS = 0.87
AUC = 0.99
TSS = 0.87
AUC = 0.99
TSS = 0.82
Relative importance
0.00
0.25
0.50
0.75
bio01
bio02
bio04
bio05
bio06
BDW
DES
PTO
SOC
TWI
0.0
0.2
0.4
0.6
0.8
Bossiaea ensata
Climate
Climate + Soil
Soil
AUC = 0.98
TSS = 0.87
AUC = 0.99
TSS = 0.88
AUC = 0.98
TSS = 0.78
Relative importance
bio01
bio02
bio05
bio06
bio14
BDW
CEC
CLY
NTO
SOC

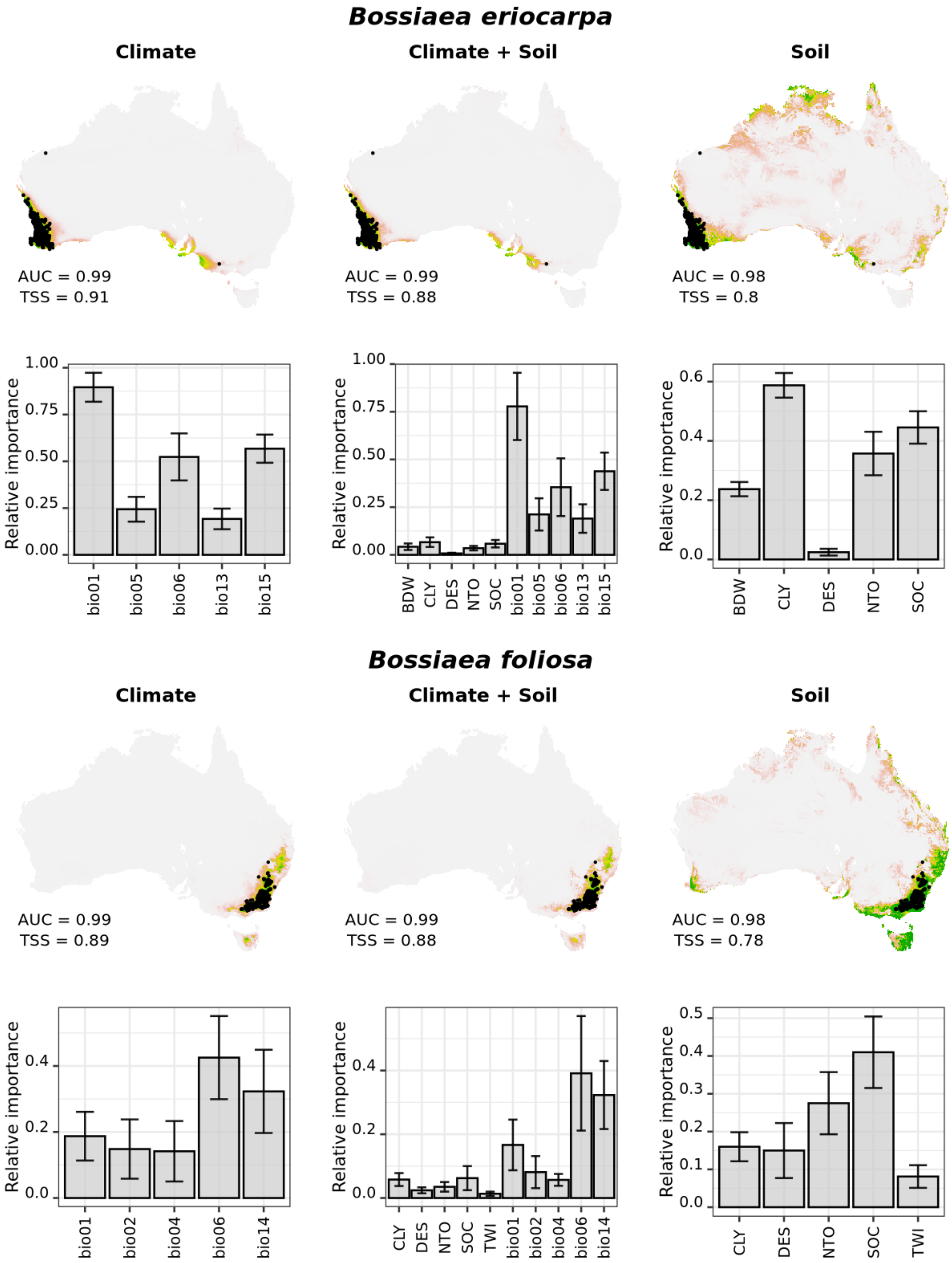
Bossiaea eriocarpa
Climate
Climate + Soil
Soil
AUC = 0.99
TSS = 0.91
AUC = 0.99
TSS = 0.88
AUC = 0.98
TSS = 0.8
Relative importance
bio01
bio05
bio06
bio13
bio15
BDW
CLY
DES
NTO
SOC
Bossiaea foliosa
Climate
Climate + Soil
Soil
AUC = 0.99
TSS = 0.89
AUC = 0.99
TSS = 0.88
AUC = 0.98
TSS = 0.78
Relative importance
bio01
bio02
bio04
bio06
bio14
CLY
DES
NTO
SOC
TWI

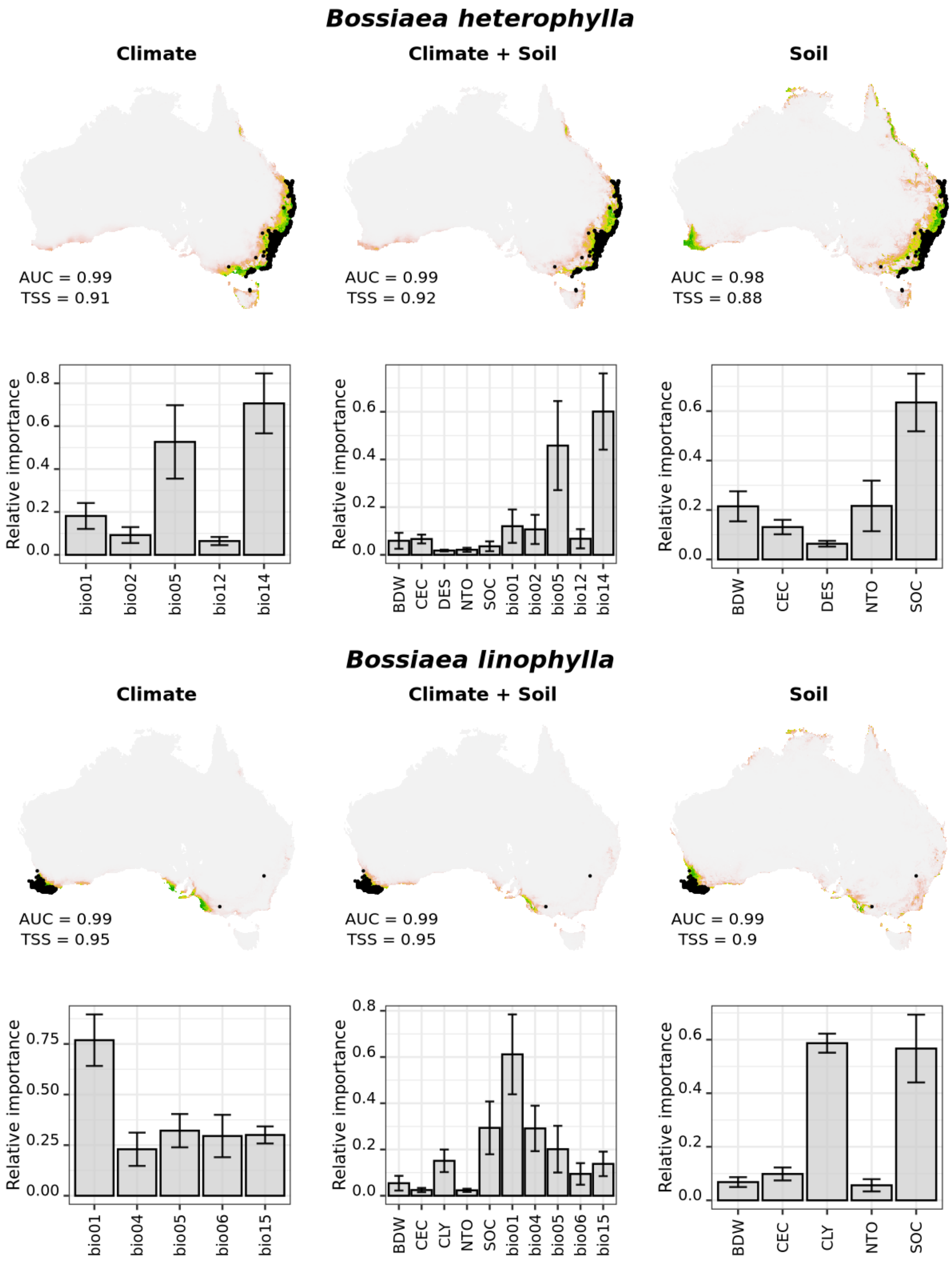
Bossiaea heterophylla
Climate
Climate + Soil
Soil
AUC = 0.99
TSS = 0.91
AUC = 0.99
TSS = 0.92
AUC = 0.98
TSS = 0.88
Relative importance
0.0
0.2
0.4
0.6
0.8
bio01
bio02
bio05
bio12
bio14
BDW
CEC
DES
NTO
SOC
Bossiaea linophylla
AUC = 0.99
TSS = 0.95
AUC = 0.99
TSS = 0.95
AUC = 0.99
TSS = 0.9
0.00
0.25
0.50
0.75
bio04
bio06
bio15
CLY

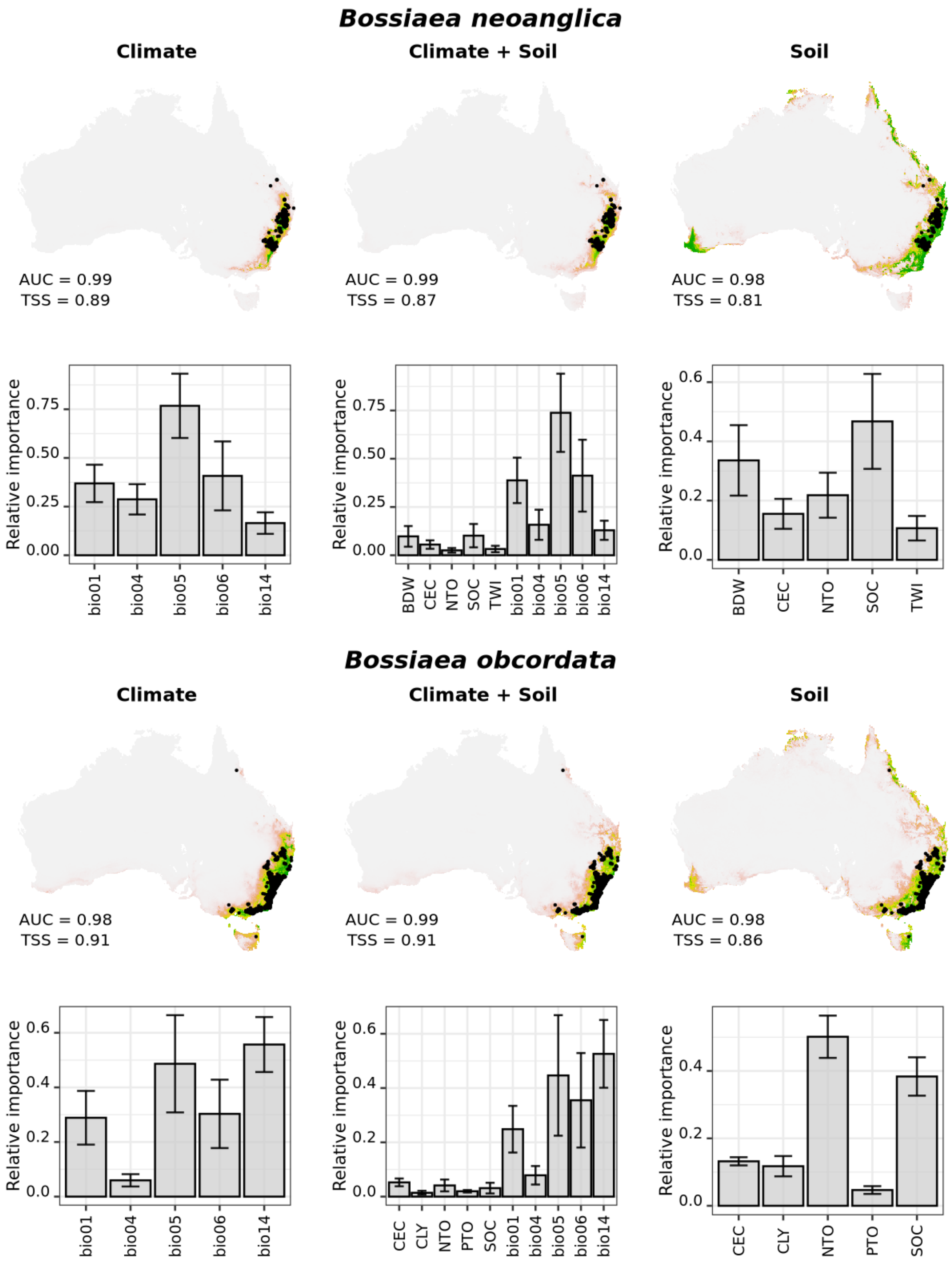
Bossiaea neoanglica
Climate
Climate + Soil
Soil
AUC = 0.99
TSS = 0.89
AUC = 0.99
TSS = 0.87
AUC = 0.98
TSS = 0.81
Relative importance
0.00
0.25
0.50
0.75
bio01
bio04
bio05
bio06
bio14
BDW
CEC
NTO
SOC
TWI
0.0
0.2
0.4
0.6
Bossiaea obcordata
AUC = 0.98
TSS = 0.91
AUC = 0.99
TSS = 0.91
AUC = 0.98
TSS = 0.86
CLY
PTO

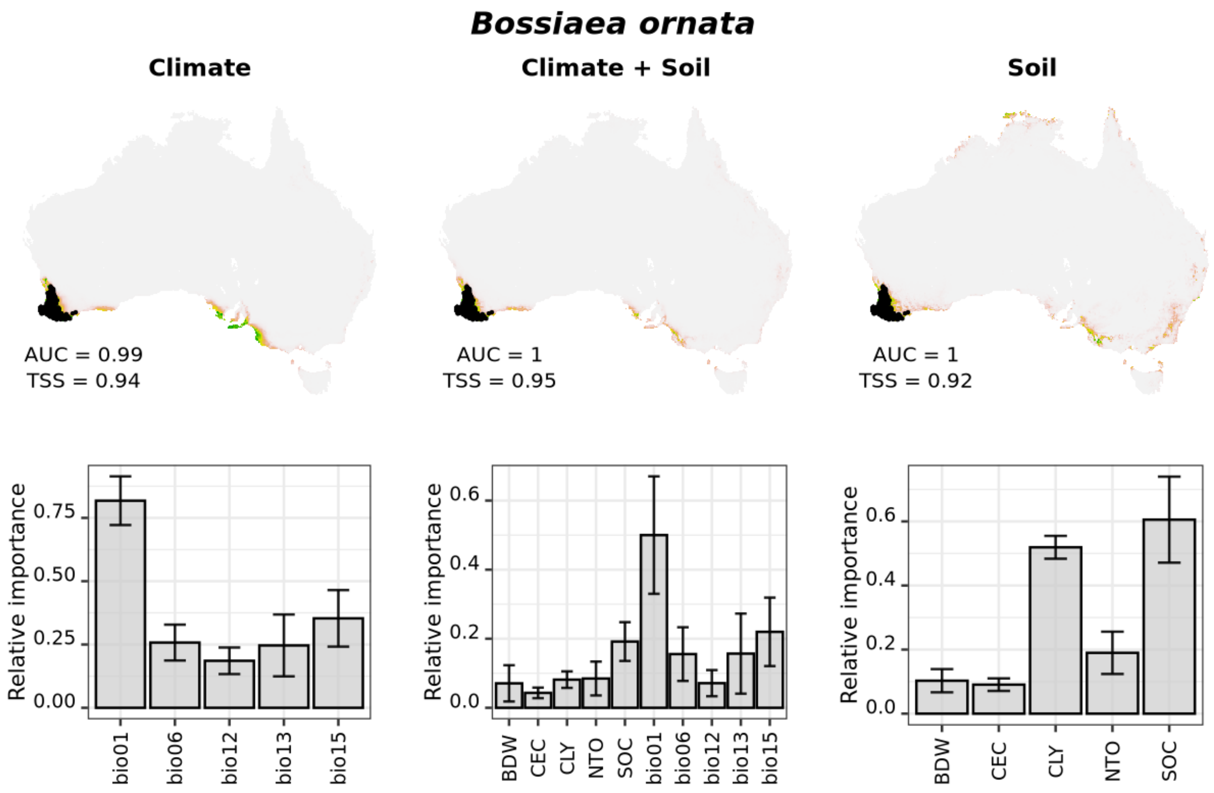
Bossiaea ornata
Climate
Climate + Soil
Soil
AUC = 0.99
TSS = 0.94
AUC = 1
TSS = 0.95
AUC = 1
TSS = 0.92
Relative importance
0.00
0.25
0.50
0.75
bio01
bio06
bio12
bio13
bio15
0.0
0.2
0.4
0.6
BDW
CEC
CLY
NTO
SOC
bio01
bio06
bio12
bio13
bio15
BDW
CEC
CLY
NTO
SOC

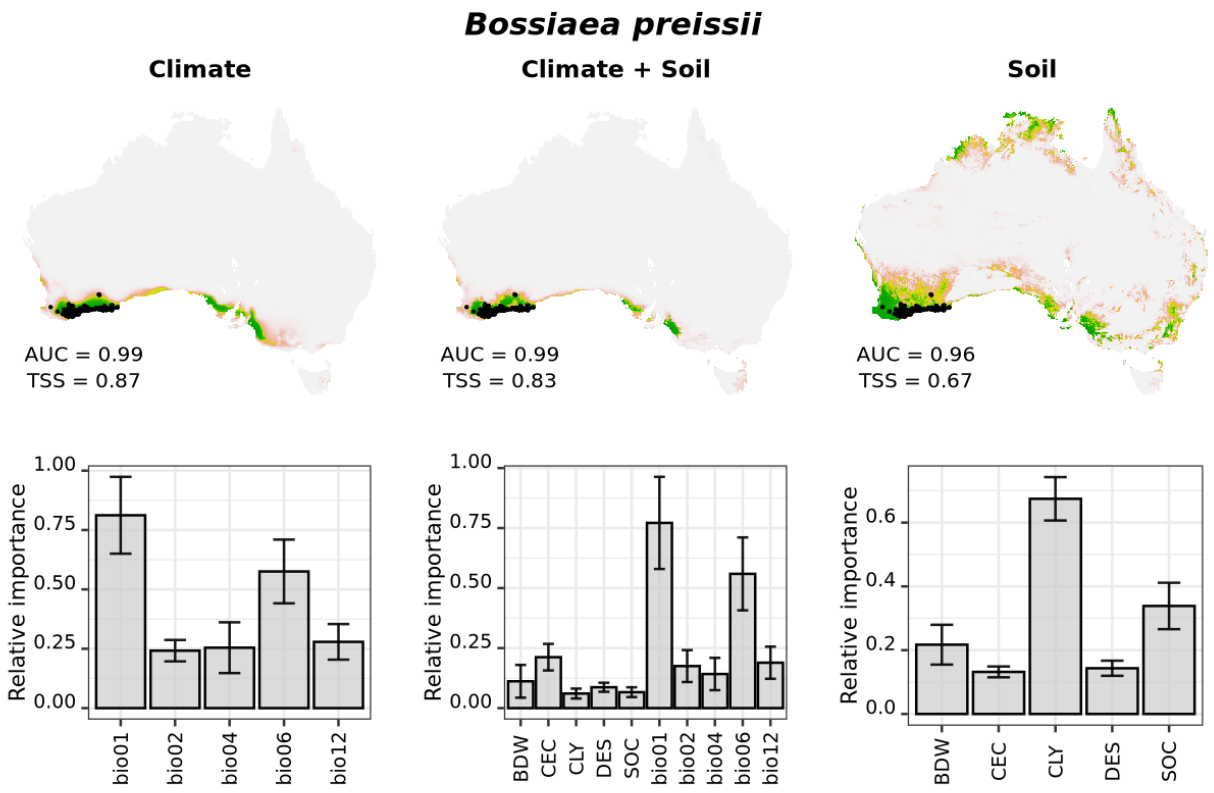
Bossiaea preissii
Climate
Climate + Soil
Soil
AUC = 0.99
TSS = 0.87
AUC = 0.99
TSS = 0.83
AUC = 0.96
TSS = 0.67
Relative importance
0.00
0.25
0.50
0.75
1.00
bio01
bio02
bio04
bio06
bio12
BDW
CEC
CLY
DES
SOC
bio01
bio02
bio04
bio06
bio12
0.0
0.2
0.4
0.6
BDW
CEC
CLY
DES
SOC

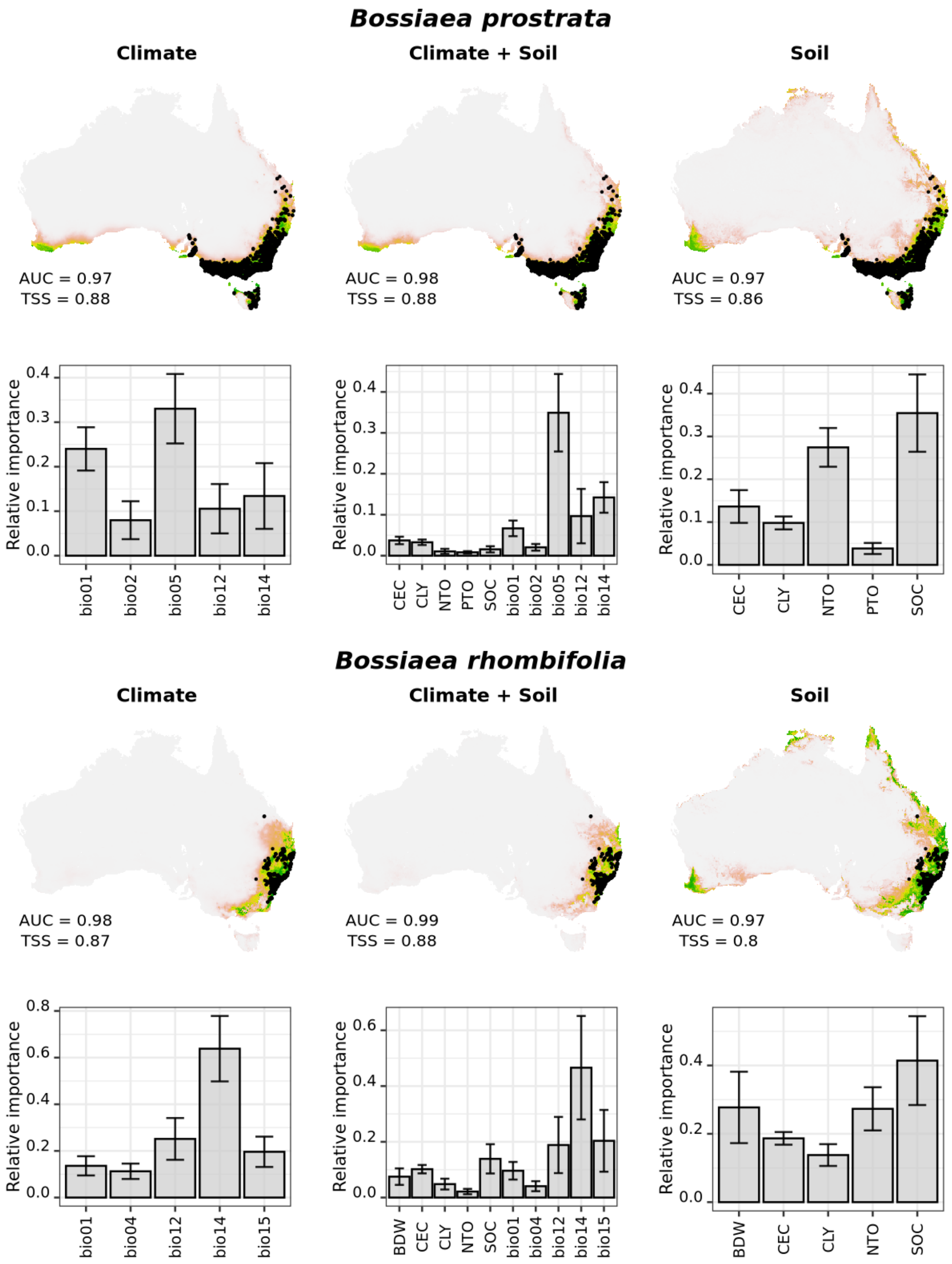

Bossiaea prostrata
Climate
Climate + Soil
Soil
AUC = 0.97
TSS = 0.88
AUC = 0.98
TSS = 0.88
AUC = 0.97
TSS = 0.86
Relative importance
0.0
0.1
0.2
0.3
0.4
bio01
bio02
bio05
bio12
bio14
CEC
CLY
NTO
PTO
SOC
Bossiaea rhombifolia
AUC = 0.98
TSS = 0.87
AUC = 0.99
TSS = 0.88
AUC = 0.97
TSS = 0.8
0.6
0.8
bio04
bio15
BDW

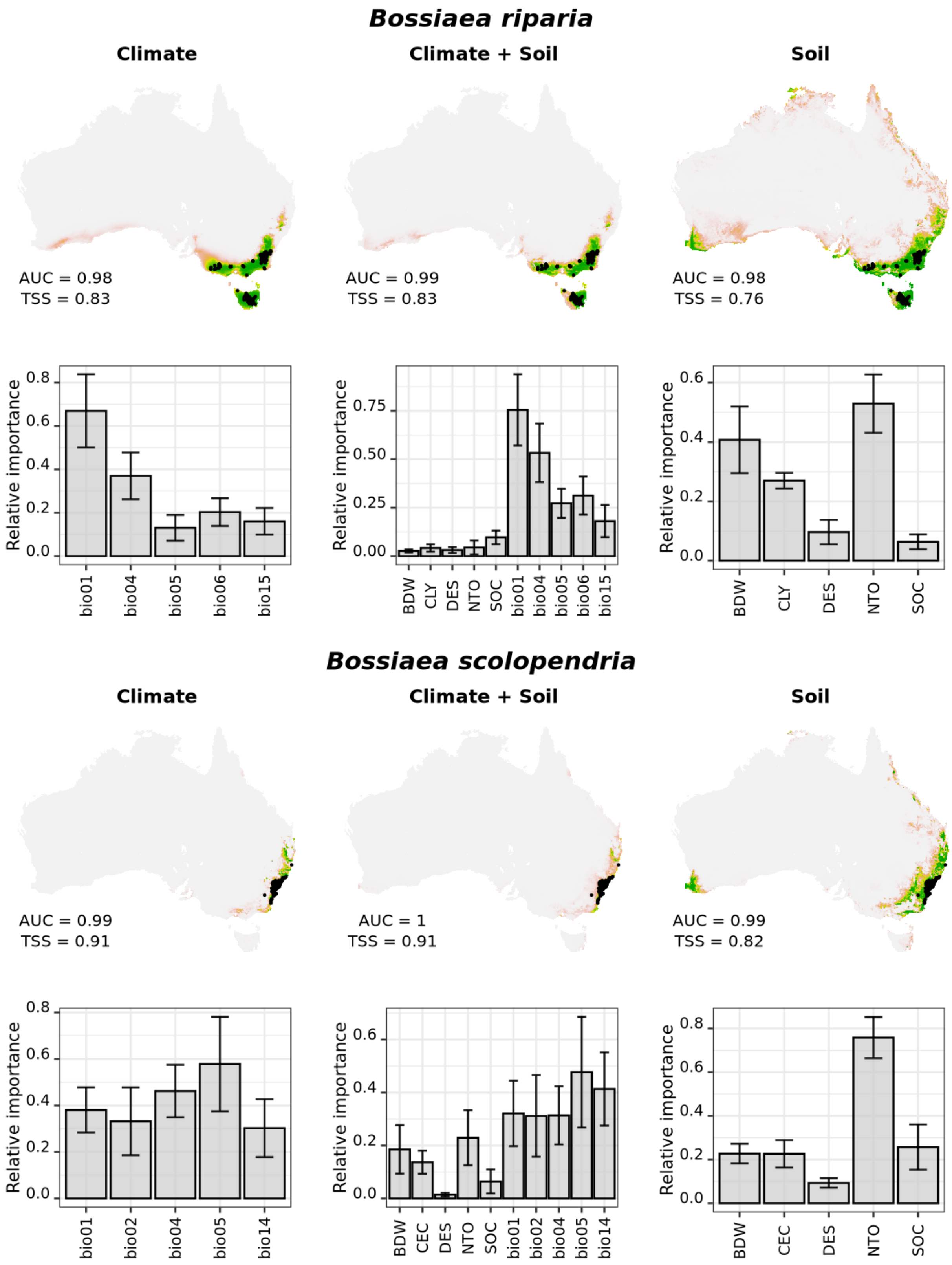
Bossiaea riparia
Climate
Climate + Soil
Soil
AUC = 0.98
TSS = 0.83
AUC = 0.99
TSS = 0.83
AUC = 0.98
TSS = 0.76
Relative importance
bio01
bio04
bio05
bio06
bio15
BDW
CLY
DES
NTO
SOC
Bossiaea scolopendria
Climate
Climate + Soil
Soil
AUC = 0.99
TSS = 0.91
AUC = 1
TSS = 0.91
AUC = 0.99
TSS = 0.82
Relative importance
bio01
bio02
bio04
bio05
bio14
BDW
CEC
DES
NTO
SOC

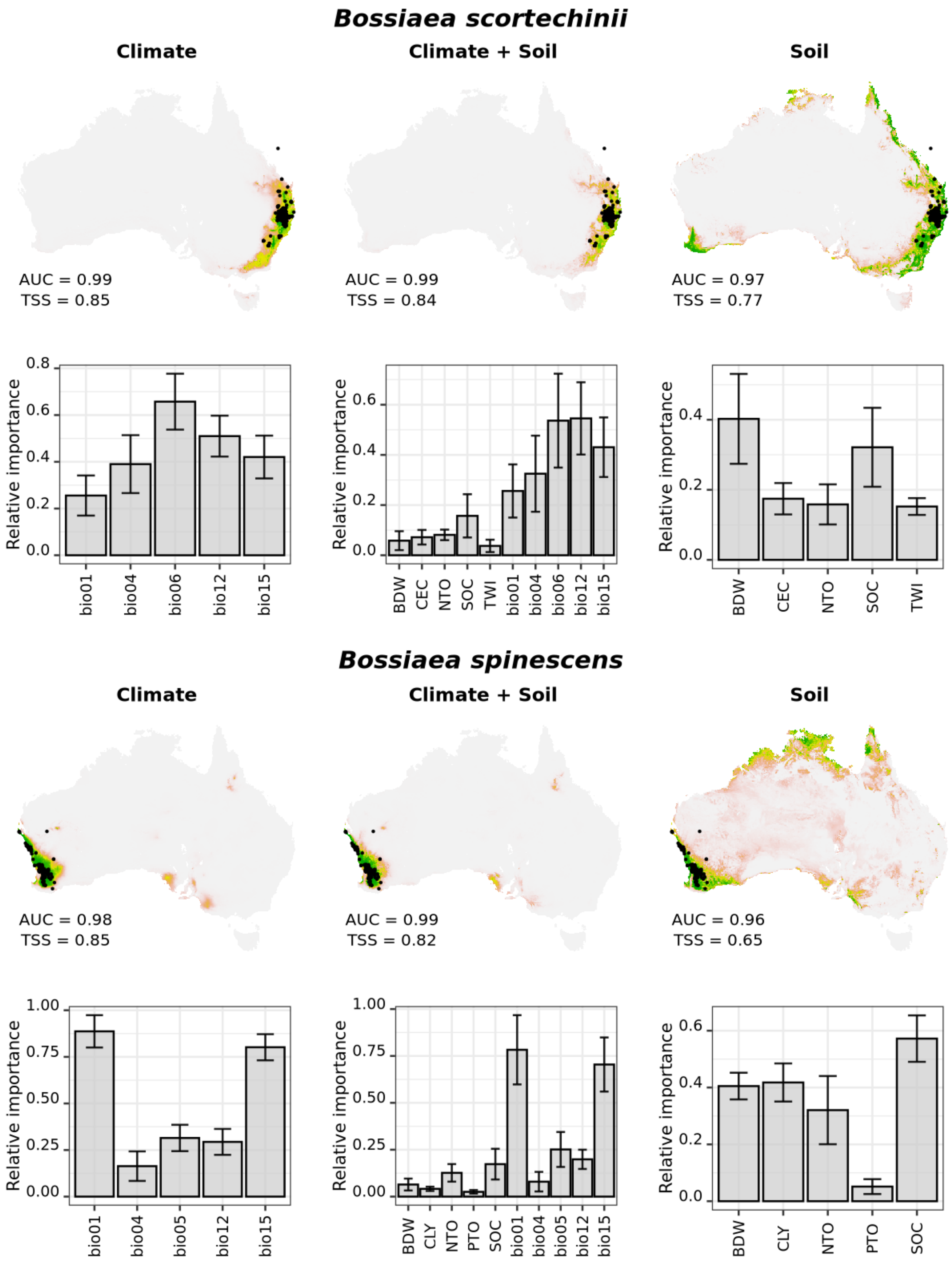

Bossiaea scortechinii
Climate
Climate + Soil
Soil
AUC = 0.99
TSS = 0.85
AUC = 0.99
TSS = 0.84
AUC = 0.97
TSS = 0.77
Relative importance
bio01
bio04
bio06
bio12
bio15
BDW
CEC
NTO
SOC
TWI
Bossiaea spinescens
Climate
Climate + Soil
Soil
AUC = 0.98
TSS = 0.85
AUC = 0.99
TSS = 0.82
AUC = 0.96
TSS = 0.65
Relative importance
bio01
bio04
bio05
bio12
bio15
BDW
CLY
NTO
PTO
SOC

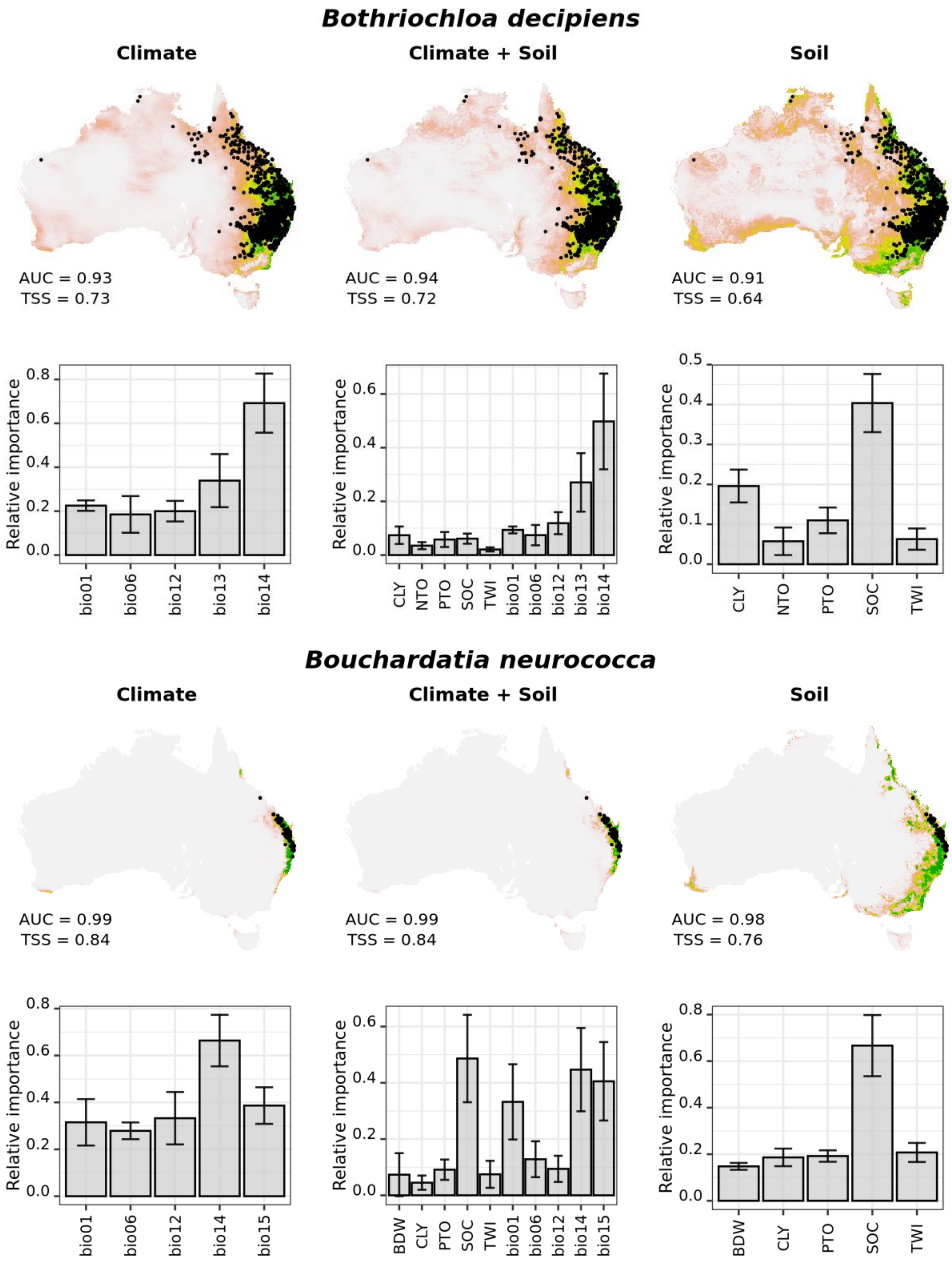
Bothriochloa decipiens
Climate
Climate + Soil
Soil
AUC = 0.93
TSS = 0.73
AUC = 0.94
TSS = 0.72
AUC = 0.91
TSS = 0.64
Relative importance
bio01
bio06
bio12
bio13
bio14
CLY
NTO
PTO
SOC
TWI
Bouchardatia neurococca
Climate
Climate + Soil
Soil
AUC = 0.99
TSS = 0.84
AUC = 0.99
TSS = 0.84
AUC = 0.98
TSS = 0.76
Relative importance
bio01
bio06
bio12
bio14
bio15
BDW
CLY
PTO
SOC
TWI

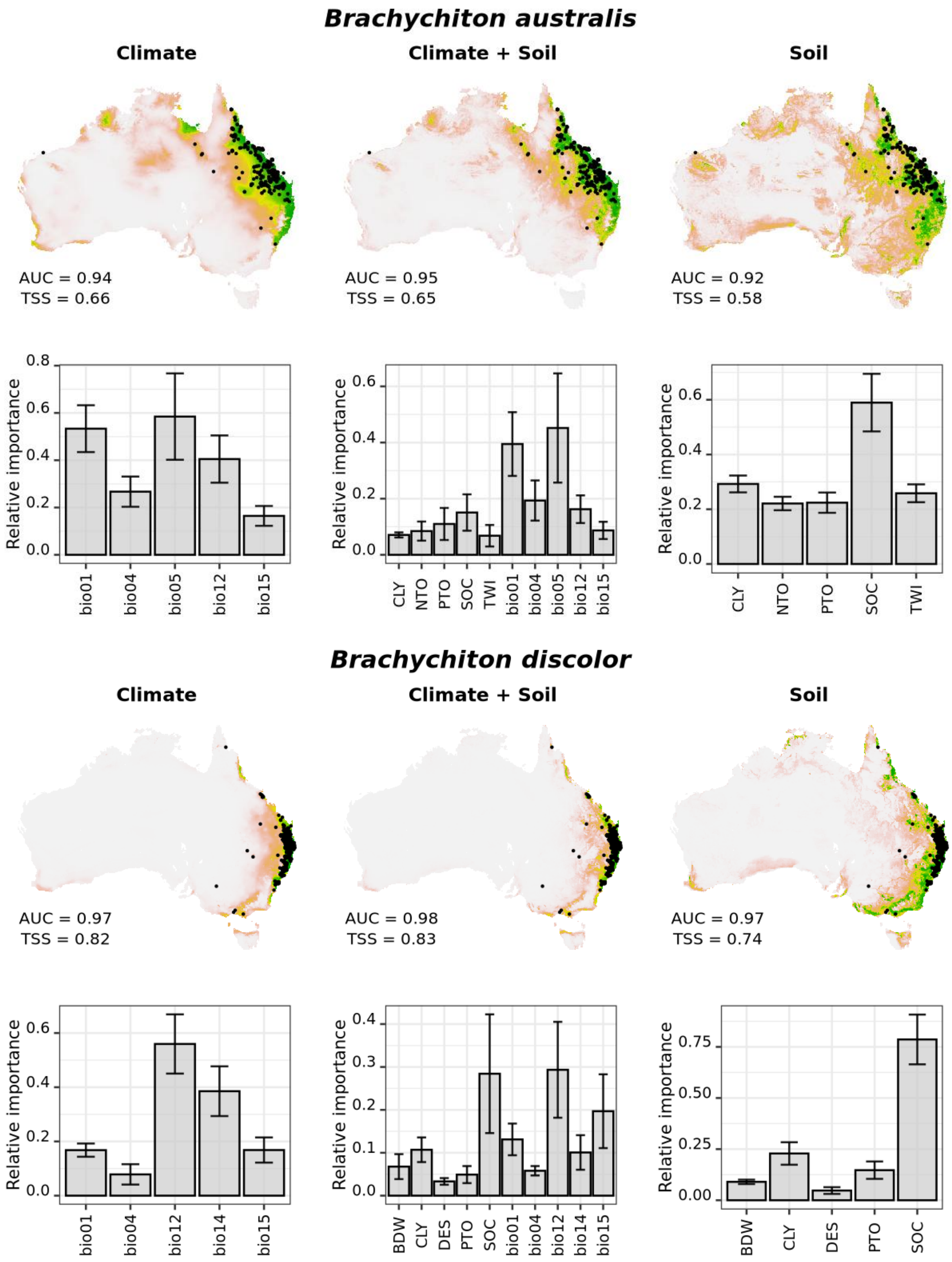
Brachychiton australis
Climate
Climate + Soil
Soil
AUC = 0.94
TSS = 0.66
AUC = 0.95
TSS = 0.65
AUC = 0.92
TSS = 0.58
Relative importance
bio01
bio04
bio05
bio12
bio15
CLY
NTO
PTO
SOC
TWI
Brachychiton discolor
AUC = 0.97
TSS = 0.82
AUC = 0.98
TSS = 0.83
AUC = 0.97
TSS = 0.74
bio14
BDW
DES

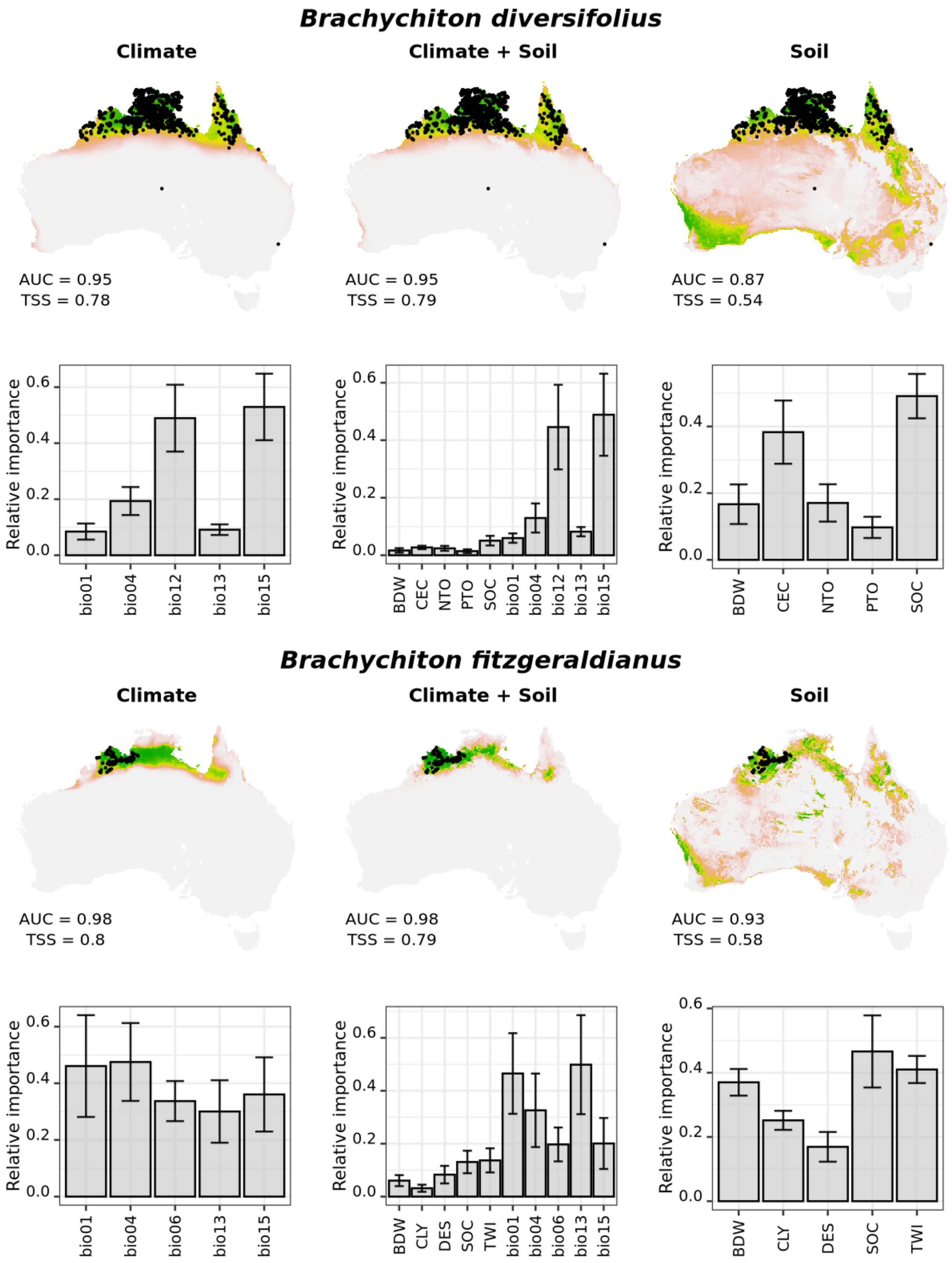

Brachychiton diversifolius
Climate
Climate + Soil
Soil
AUC = 0.95
TSS = 0.78
AUC = 0.95
TSS = 0.79
AUC = 0.87
TSS = 0.54
Relative importance
0.0
0.2
0.4
0.6
bio01
bio04
bio12
bio13
bio15
BDW
CEC
NTO
PTO
SOC
Brachychiton fitzgeraldianus
AUC = 0.98
TSS = 0.8
AUC = 0.98
TSS = 0.79
AUC = 0.93
TSS = 0.58
bio06
CLY
DES
TWI

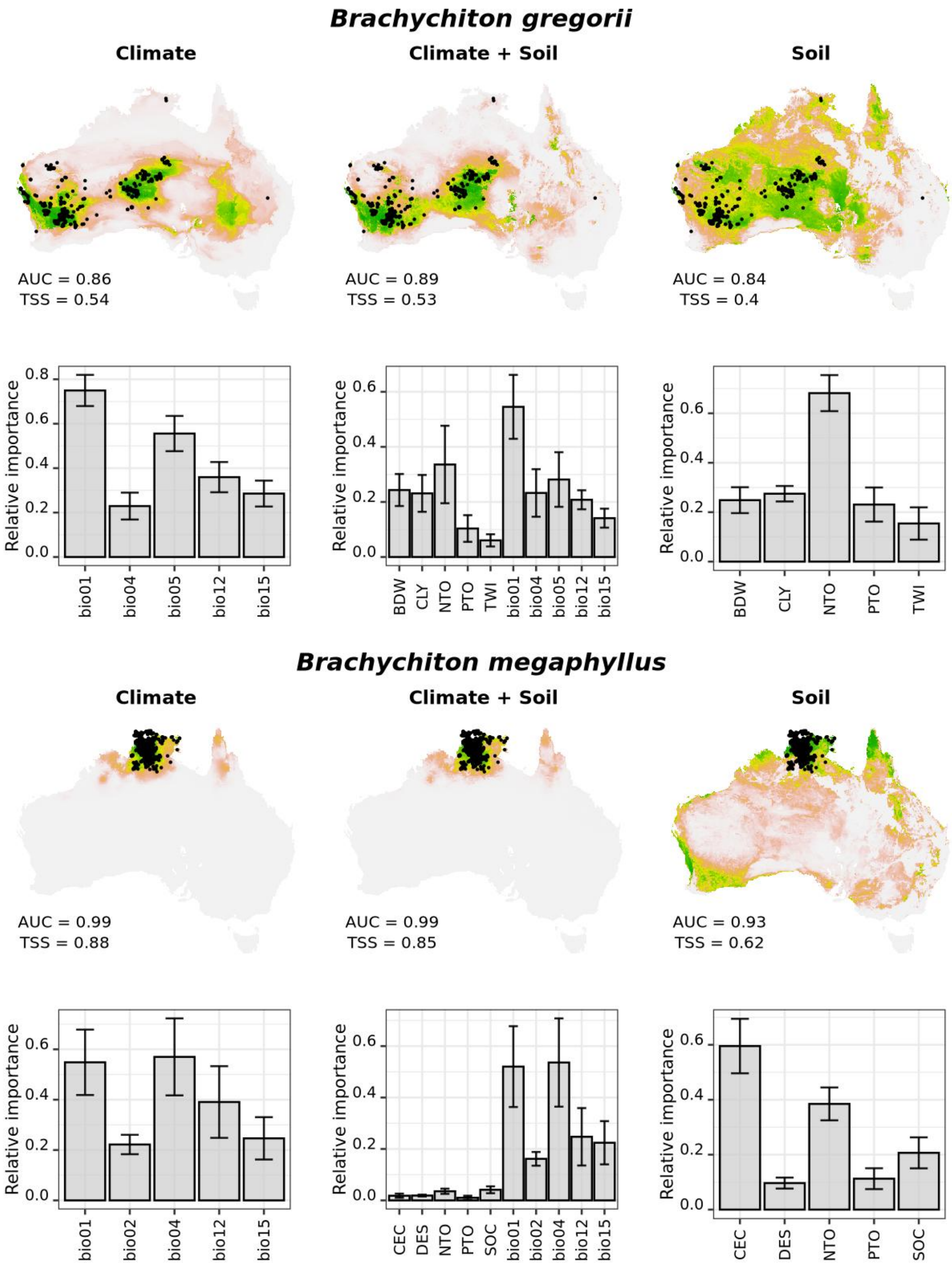
Brachychiton gregorii
Climate
Climate + Soil
Soil
AUC = 0.86
TSS = 0.54
AUC = 0.89
TSS = 0.53
AUC = 0.84
TSS = 0.4
Relative importance
0.0
0.2
0.4
0.6
0.8
bio01
bio04
bio05
bio12
bio15
BDW
CLY
NTO
PTO
TWI
Brachychiton megaphyllus
Climate
Climate + Soil
Soil
AUC = 0.99
TSS = 0.88
AUC = 0.99
TSS = 0.85
AUC = 0.93
TSS = 0.62
bio01
bio02
bio04
bio12
bio15
CEC
DES
NTO
PTO
SOC

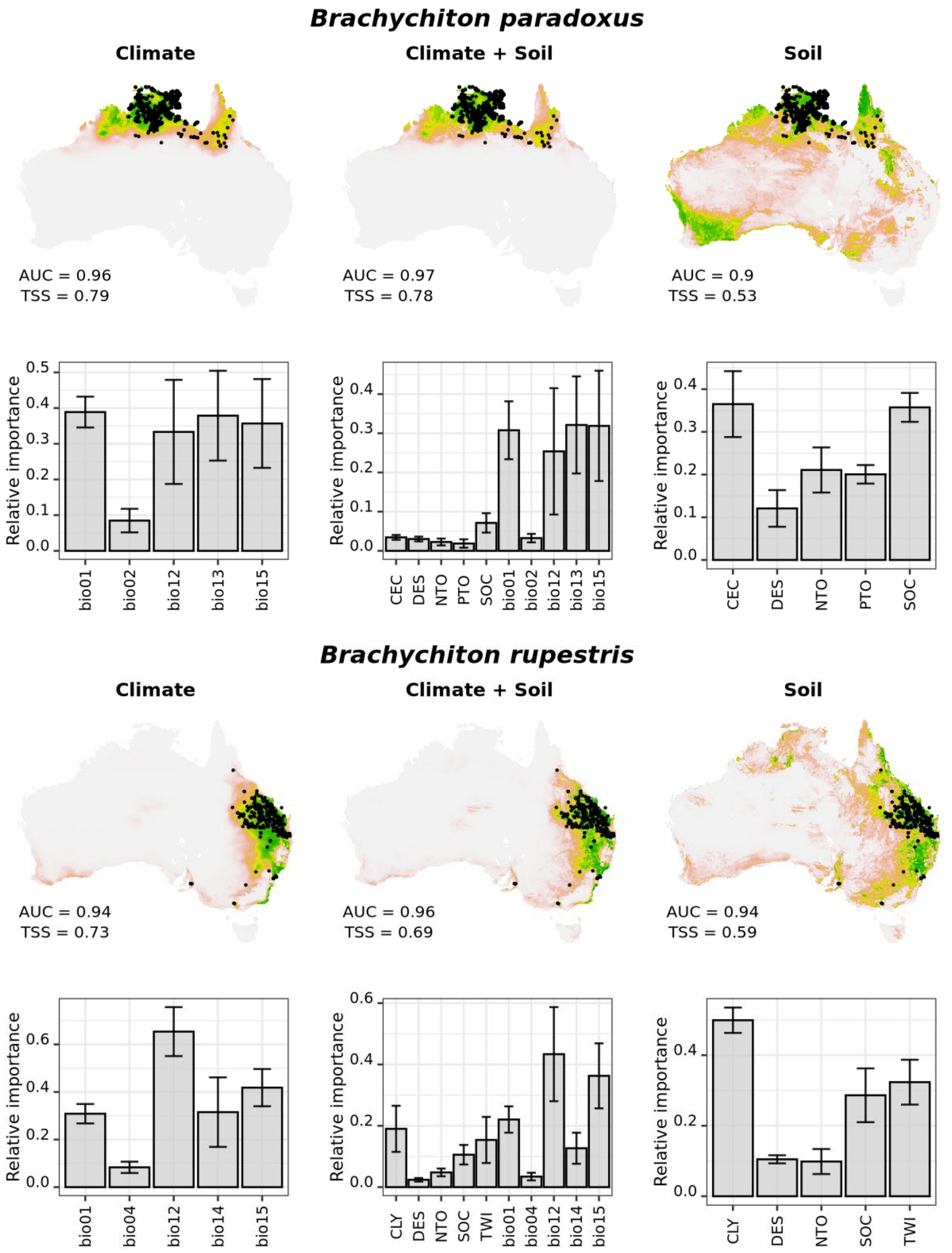

Brachychiton paradoxus
Climate
Climate + Soil
Soil
AUC = 0.96
TSS = 0.79
AUC = 0.97
TSS = 0.78
AUC = 0.9
TSS = 0.53
Relative importance
bio01
bio02
bio12
bio13
bio15
CEC
DES
NTO
PTO
SOC
Brachychiton rupestris
Climate
Climate + Soil
Soil
AUC = 0.94
TSS = 0.73
AUC = 0.96
TSS = 0.69
AUC = 0.94
TSS = 0.59
Relative importance
bio01
bio04
bio12
bio14
bio15
CLY
DES
NTO
SOC
TWI

Brachychiton viscidulus

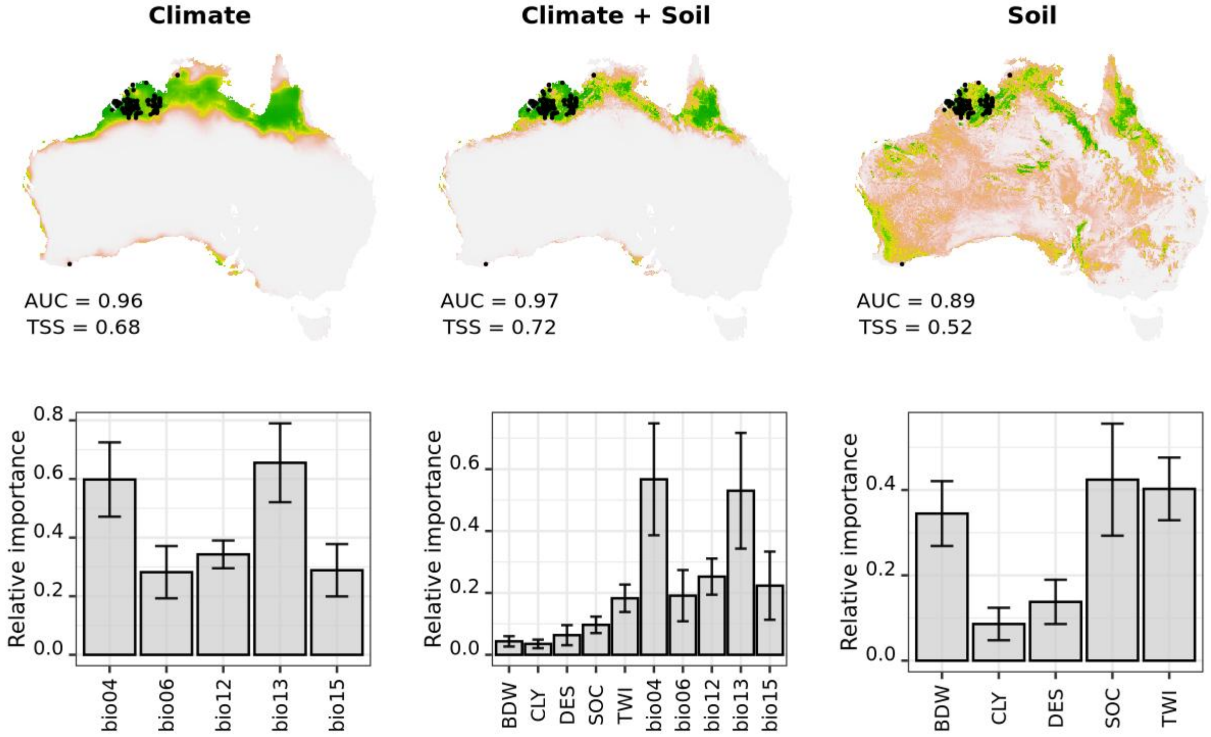

Brachyloma daphnoides

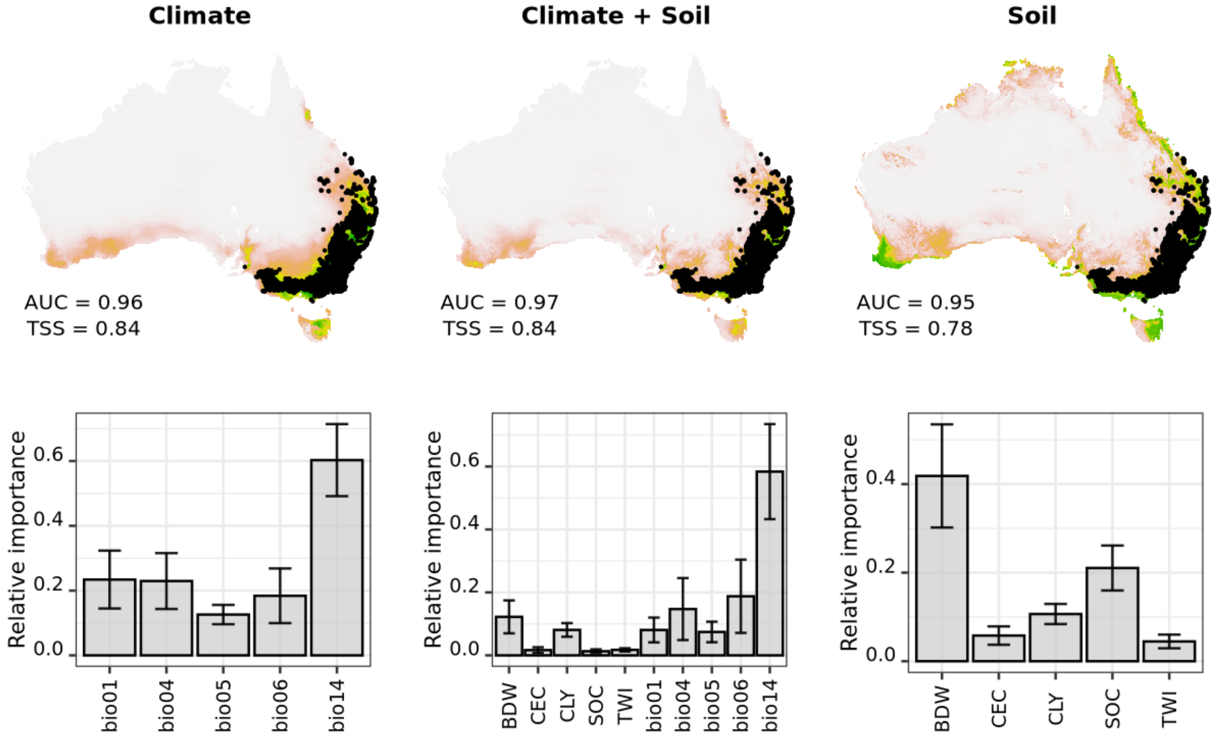

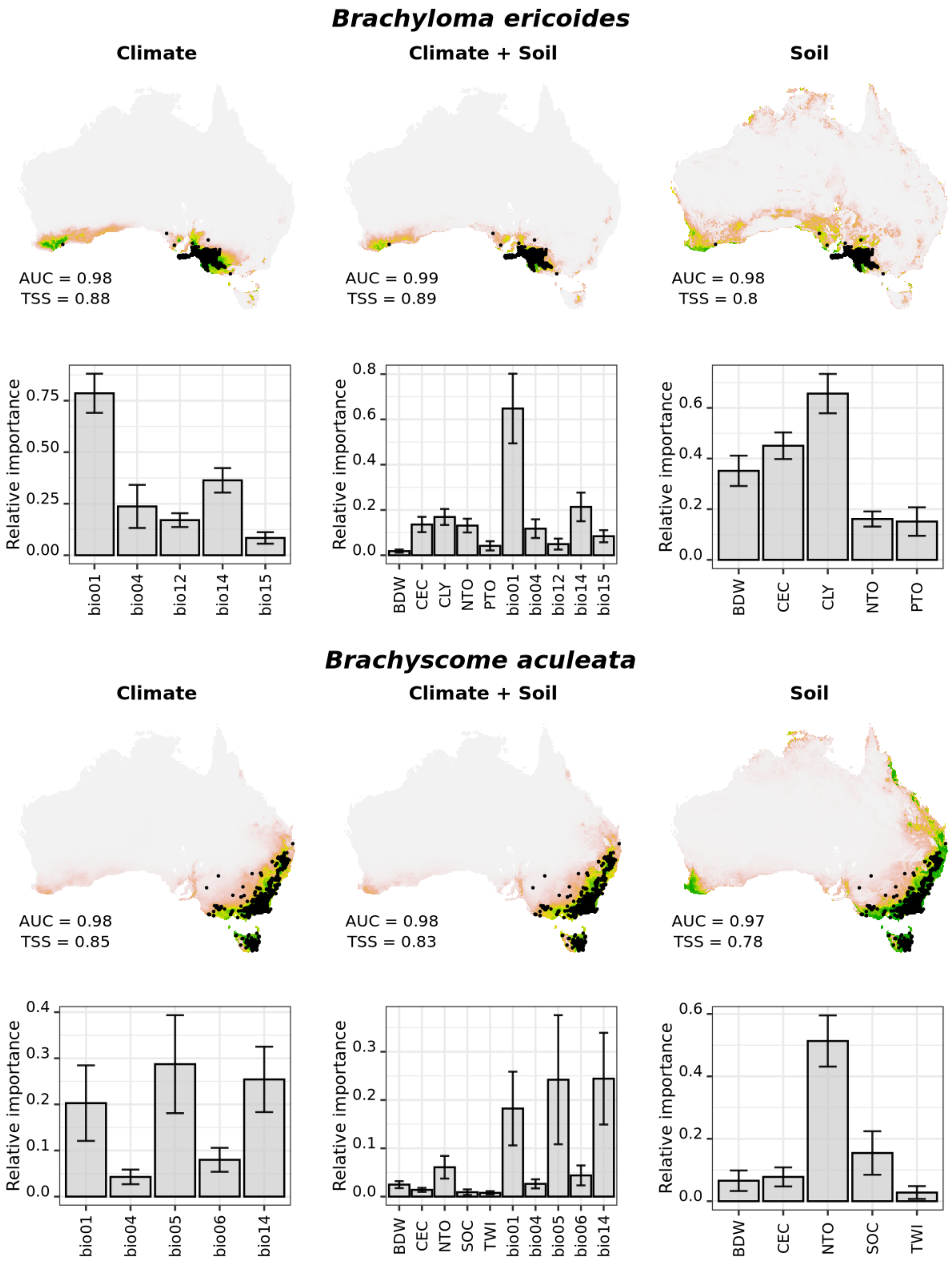
Brachyloma ericoides
Climate
Climate + Soil
Soil
AUC = 0.98
TSS = 0.88
AUC = 0.99
TSS = 0.89
AUC = 0.98
TSS = 0.8
Relative importance
bio01
bio04
bio12
bio14
bio15
BDW
CEC
CLY
NTO
PTO
Brachyscome aculeata
Climate
Climate + Soil
Soil
AUC = 0.98
TSS = 0.85
AUC = 0.98
TSS = 0.83
AUC = 0.97
TSS = 0.78
Relative importance
bio01
bio04
bio05
bio06
bio14
BDW
CEC
NTO
SOC
TWI

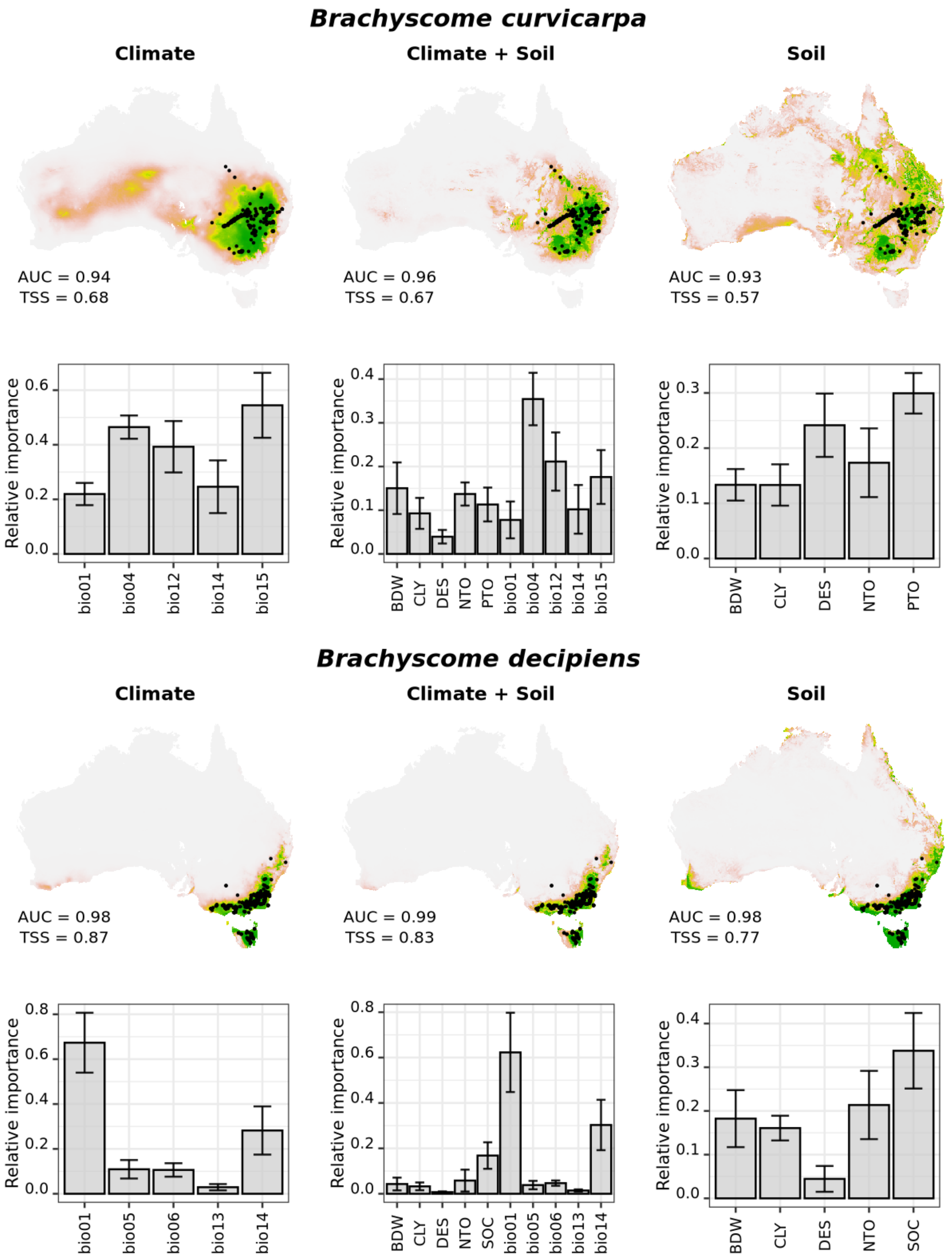
Brachyscome curvicarpa
Climate
Climate + Soil
Soil
AUC = 0.94
TSS = 0.68
AUC = 0.96
TSS = 0.67
AUC = 0.93
TSS = 0.57
Relative importance
bio01
bio04
bio12
bio14
bio15
BDW
CLY
DES
NTO
PTO
Brachyscome decipiens
Climate
Climate + Soil
Soil
AUC = 0.98
TSS = 0.87
AUC = 0.99
TSS = 0.83
AUC = 0.98
TSS = 0.77
Relative importance
bio01
bio05
bio06
bio13
bio14
BDW
CLY
DES
NTO
SOC

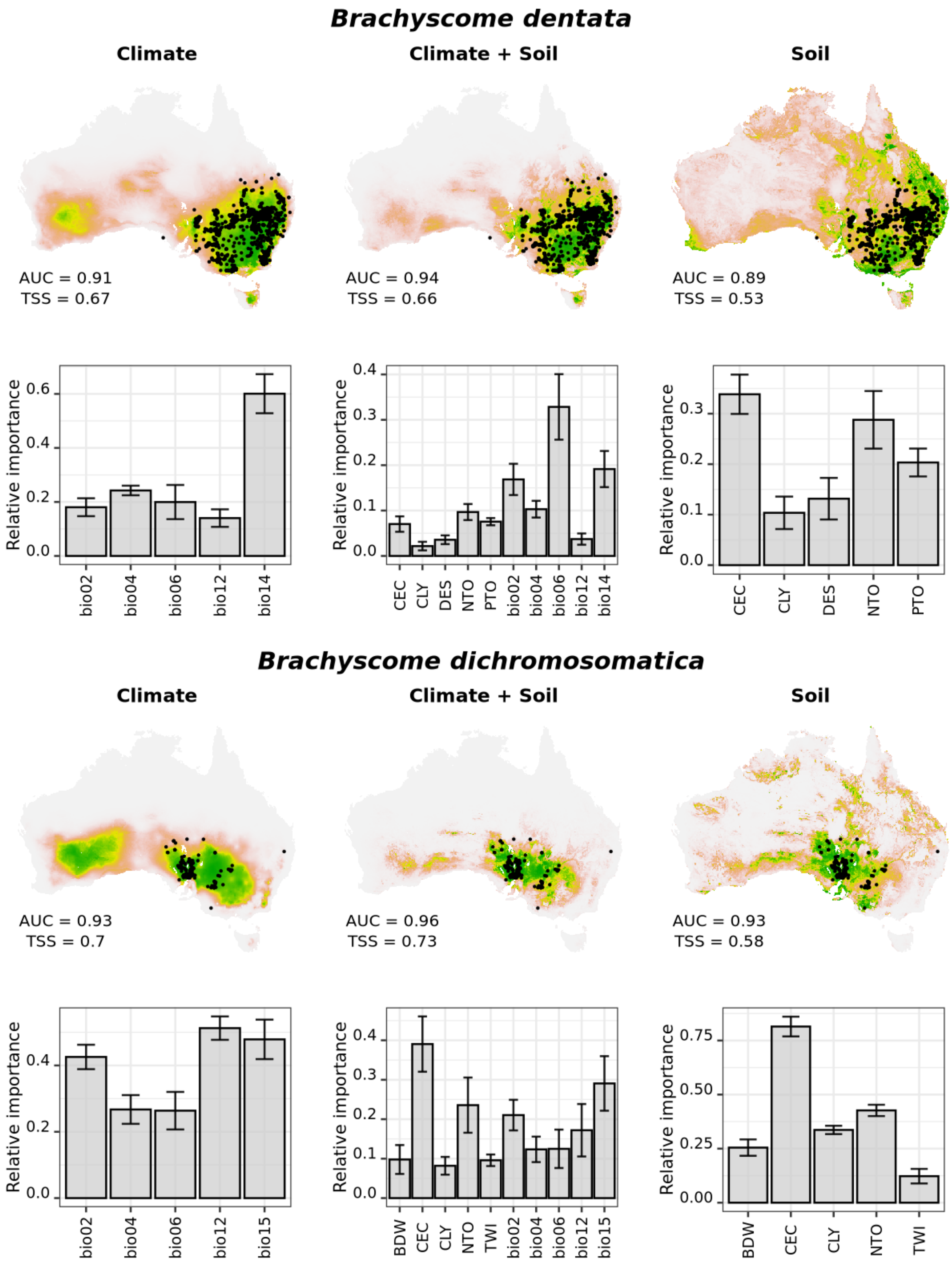
Brachyscome dentata
Climate
Climate + Soil
Soil
AUC = 0.91
TSS = 0.67
AUC = 0.94
TSS = 0.66
AUC = 0.89
TSS = 0.53
Relative importance
bio02
bio04
bio06
bio12
bio14
CEC
CLY
DES
NTO
PTO
Brachyscome dichromosomatica
Climate
Climate + Soil
Soil
AUC = 0.93
TSS = 0.7
AUC = 0.96
TSS = 0.73
AUC = 0.93
TSS = 0.58
Relative importance
bio02
bio04
bio06
bio12
bio15
BDW
CEC
CLY
NTO
TWI

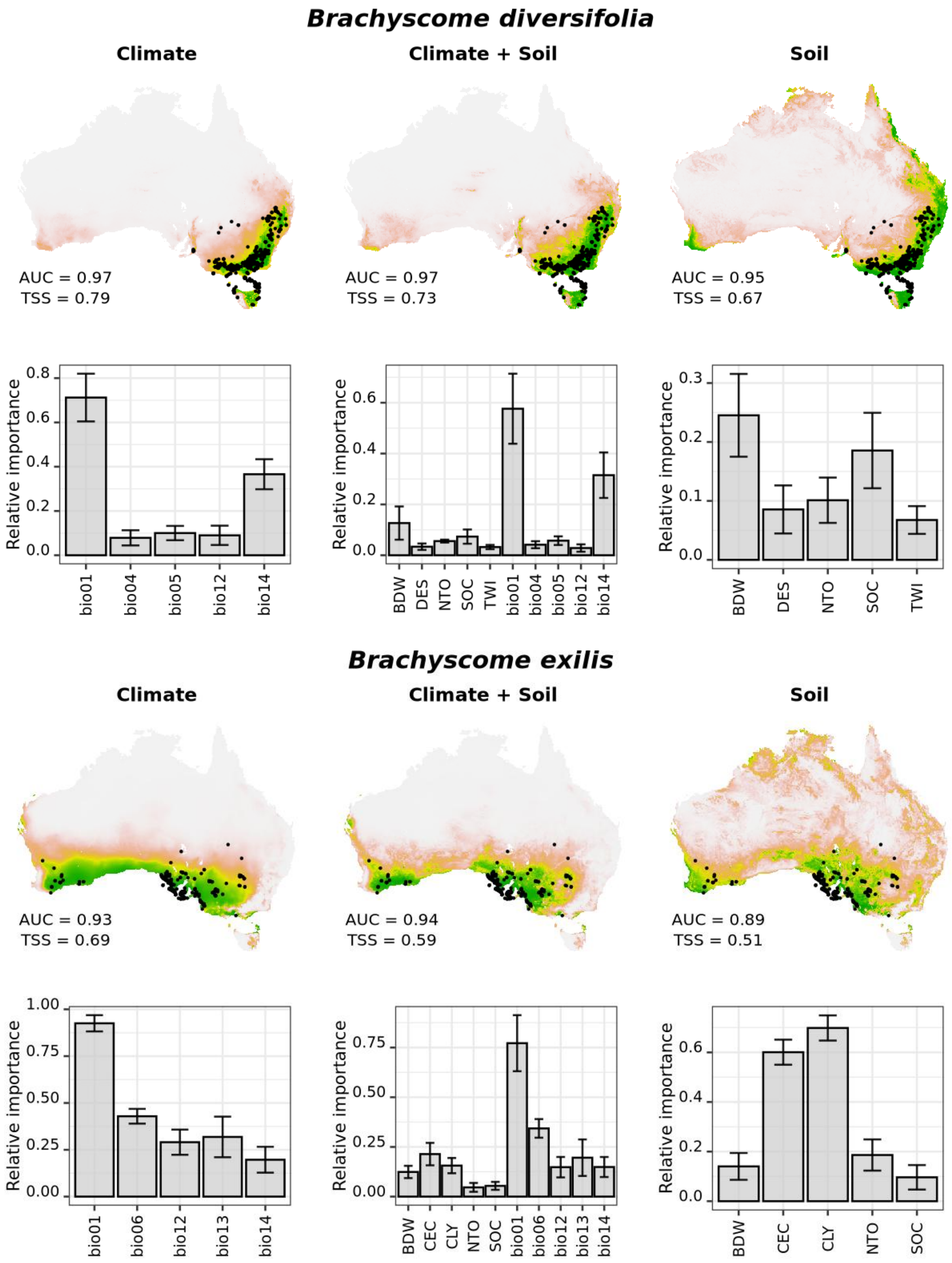
Brachyscome diversifolia
Climate
Climate + Soil
Soil
AUC = 0.97
TSS = 0.79
AUC = 0.97
TSS = 0.73
AUC = 0.95
TSS = 0.67
Relative importance
bio01 bio04 bio05 bio12 bio14
BDW DES NTO SOC TWI bio01 bio04 bio05 bio12 bio14
BDW DES NTO SOC TWI
Brachyscome exilis
Climate
Climate + Soil
Soil
AUC = 0.93
TSS = 0.69
AUC = 0.94
TSS = 0.59
AUC = 0.89
TSS = 0.51
Relative importance
bio01 bio06 bio12 bio13 bio14
BDW CEC CLY NTO SOC bio01 bio06 bio12 bio13 bio14
BDW CEC CLY NTO SOC

Brachyscome gilesii

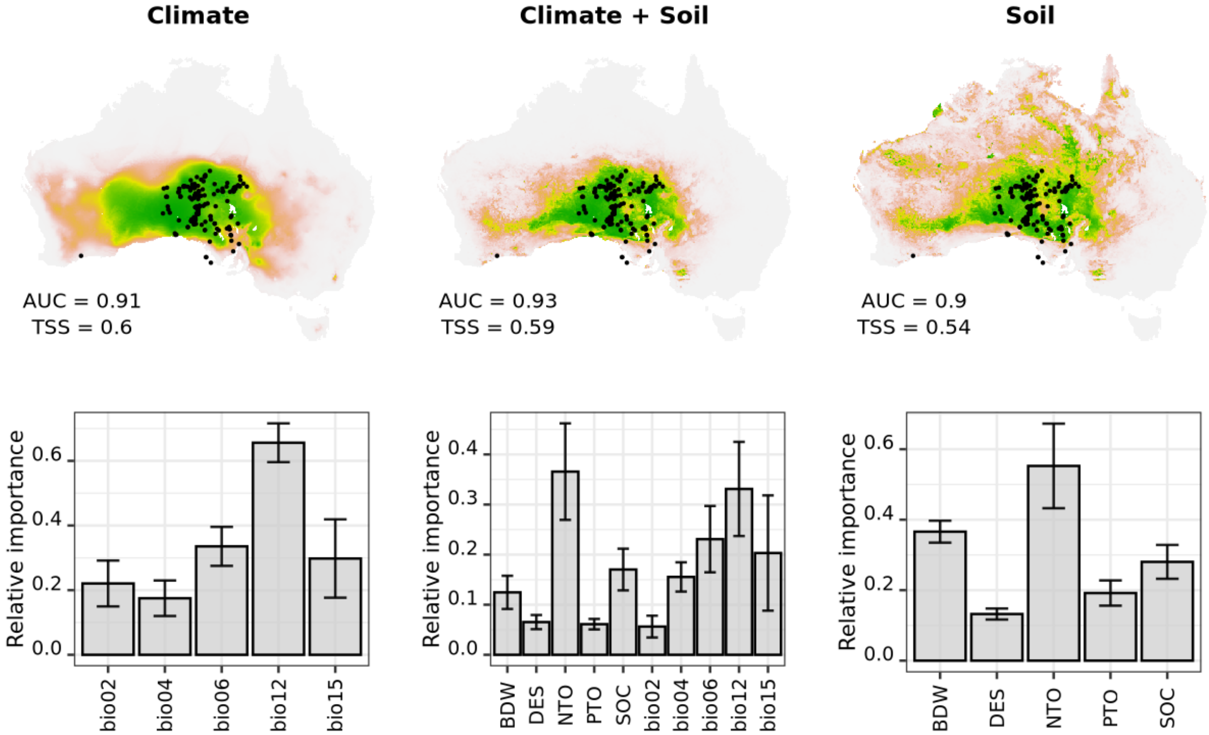

Brachyscome goniocarpa

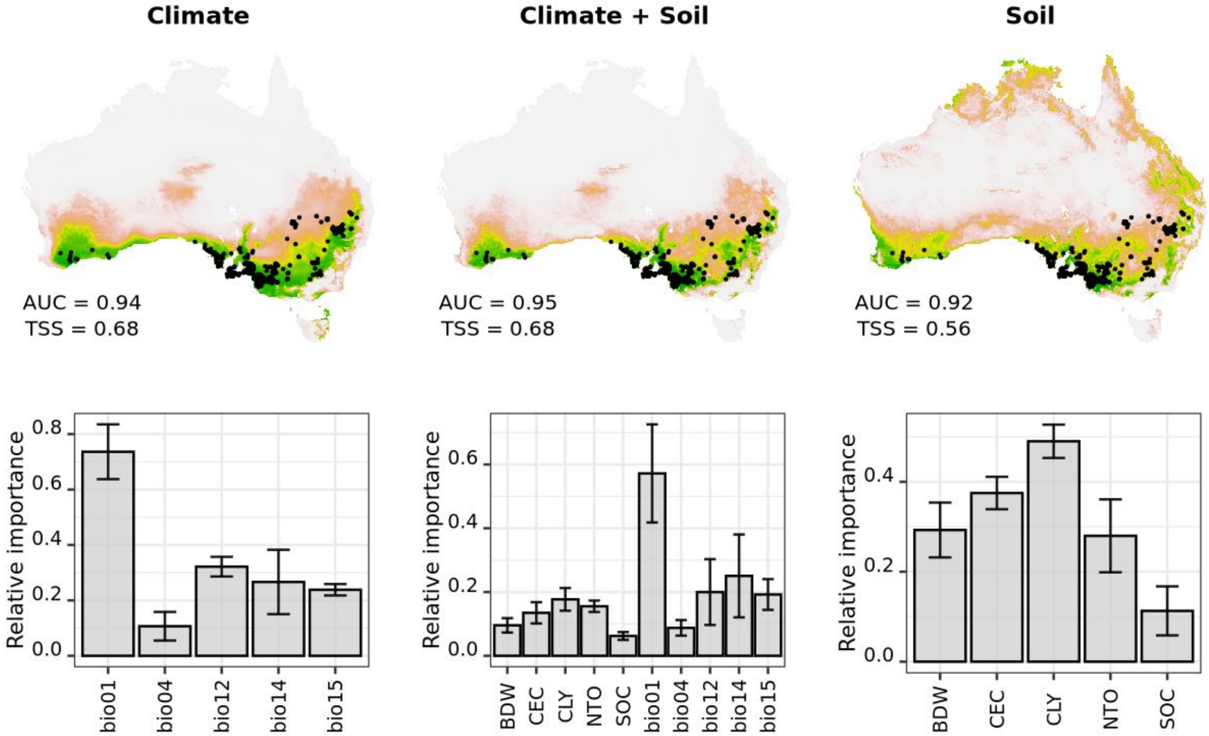

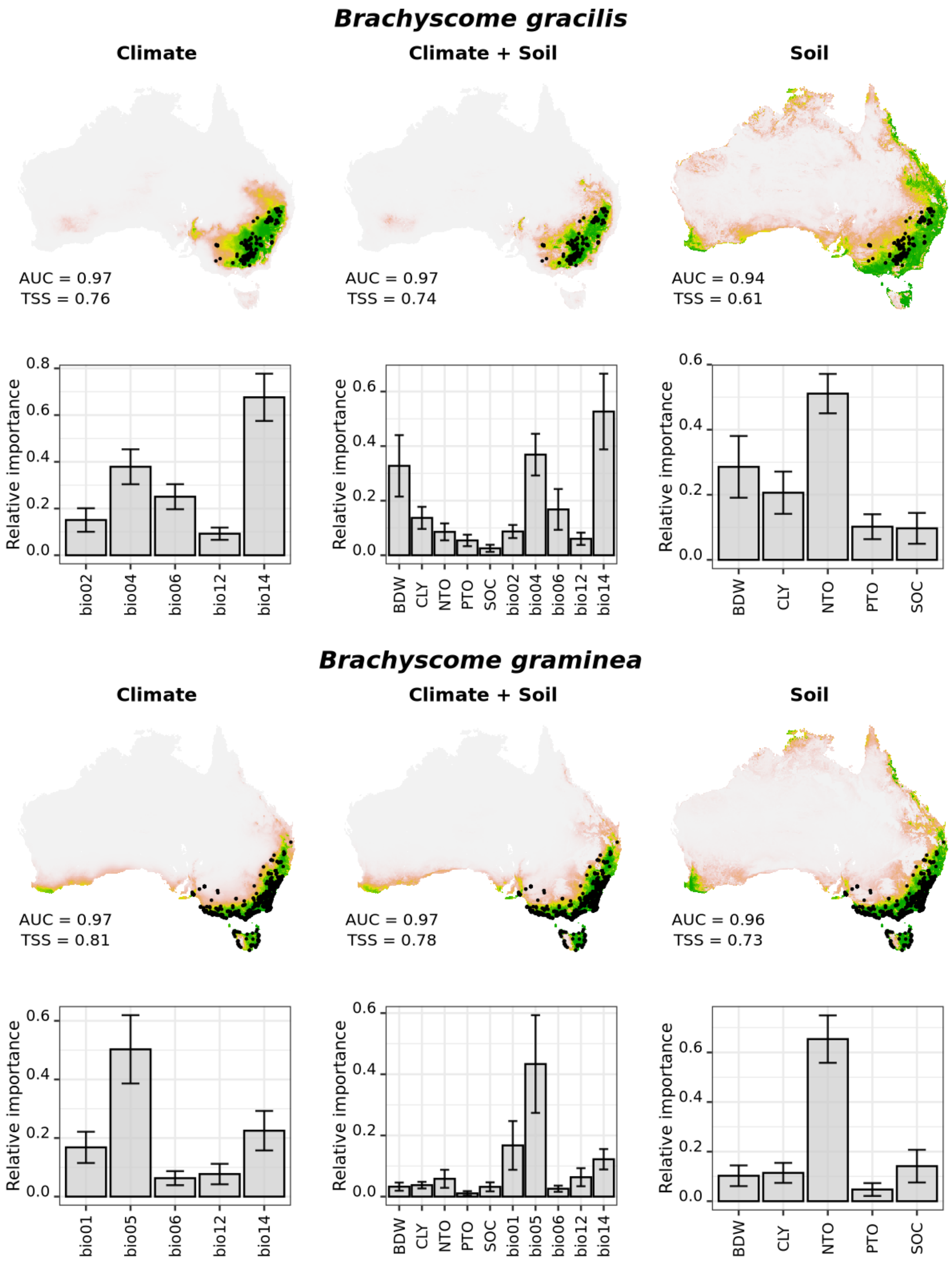
Brachyscome gracilis
Climate
Climate + Soil
Soil
AUC = 0.97
TSS = 0.76
AUC = 0.97
TSS = 0.74
AUC = 0.94
TSS = 0.61
Relative importance
bio02
bio04
bio06
bio12
bio14
BDW
CLY
NTO
PTO
SOC
Brachyscome graminea
Climate
Climate + Soil
Soil
AUC = 0.97
TSS = 0.81
AUC = 0.97
TSS = 0.78
AUC = 0.96
TSS = 0.73
Relative importance
bio01
bio05
bio06
bio12
bio14
BDW
CLY
NTO
PTO
SOC

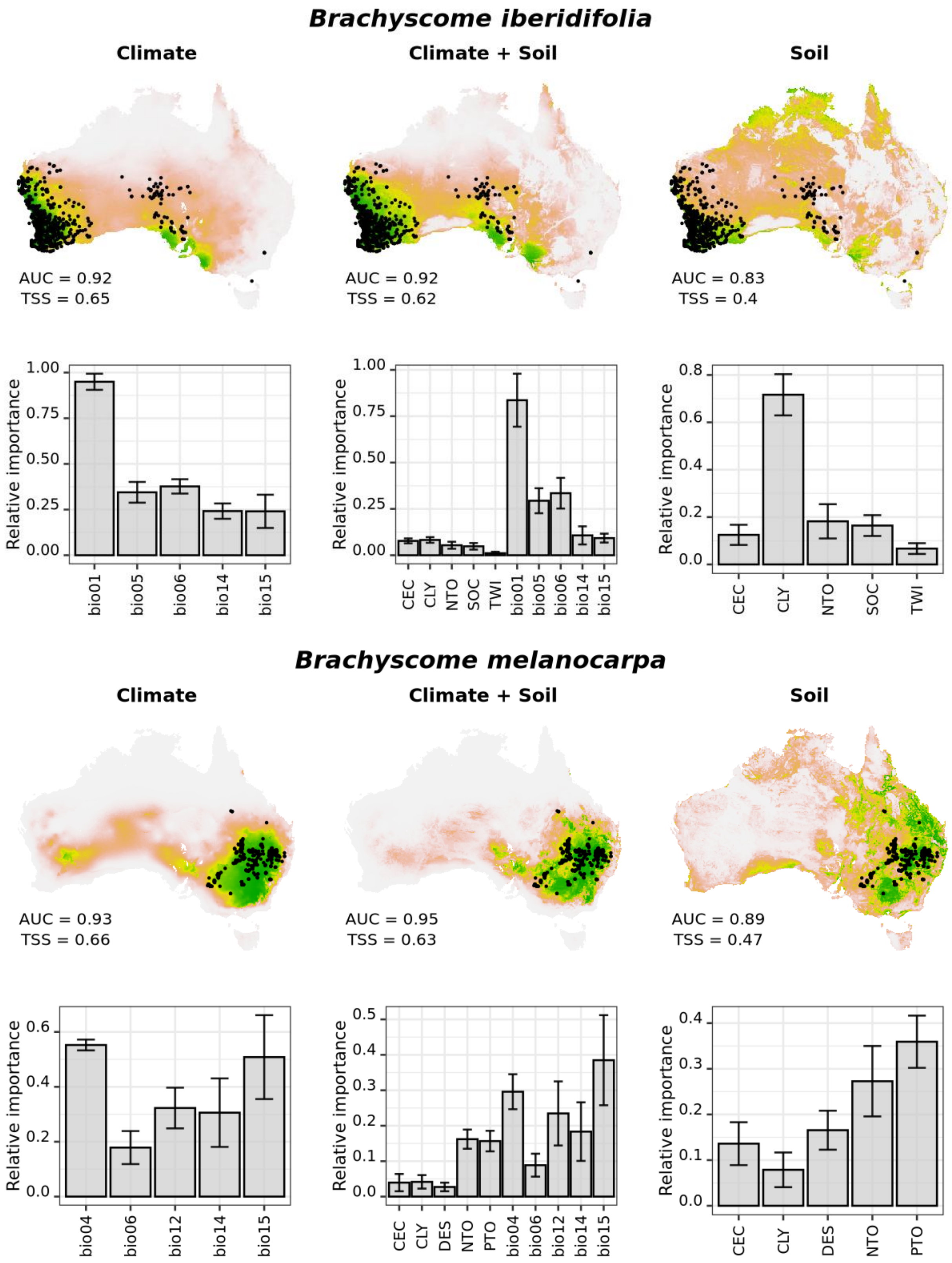

Brachyscome iberidifolia
Climate
Climate + Soil
Soil
AUC = 0.92
TSS = 0.65
AUC = 0.92
TSS = 0.62
AUC = 0.83
TSS = 0.4
Relative importance
bio01
bio05
bio06
bio14
bio15
CEC
CLY
NTO
SOC
TWI
Brachyscome melanocarpa
Climate
Climate + Soil
Soil
AUC = 0.93
TSS = 0.66
AUC = 0.95
TSS = 0.63
AUC = 0.89
TSS = 0.47
Relative importance
bio04
bio06
bio12
bio14
bio15
CEC
CLY
DES
NTO
PTO

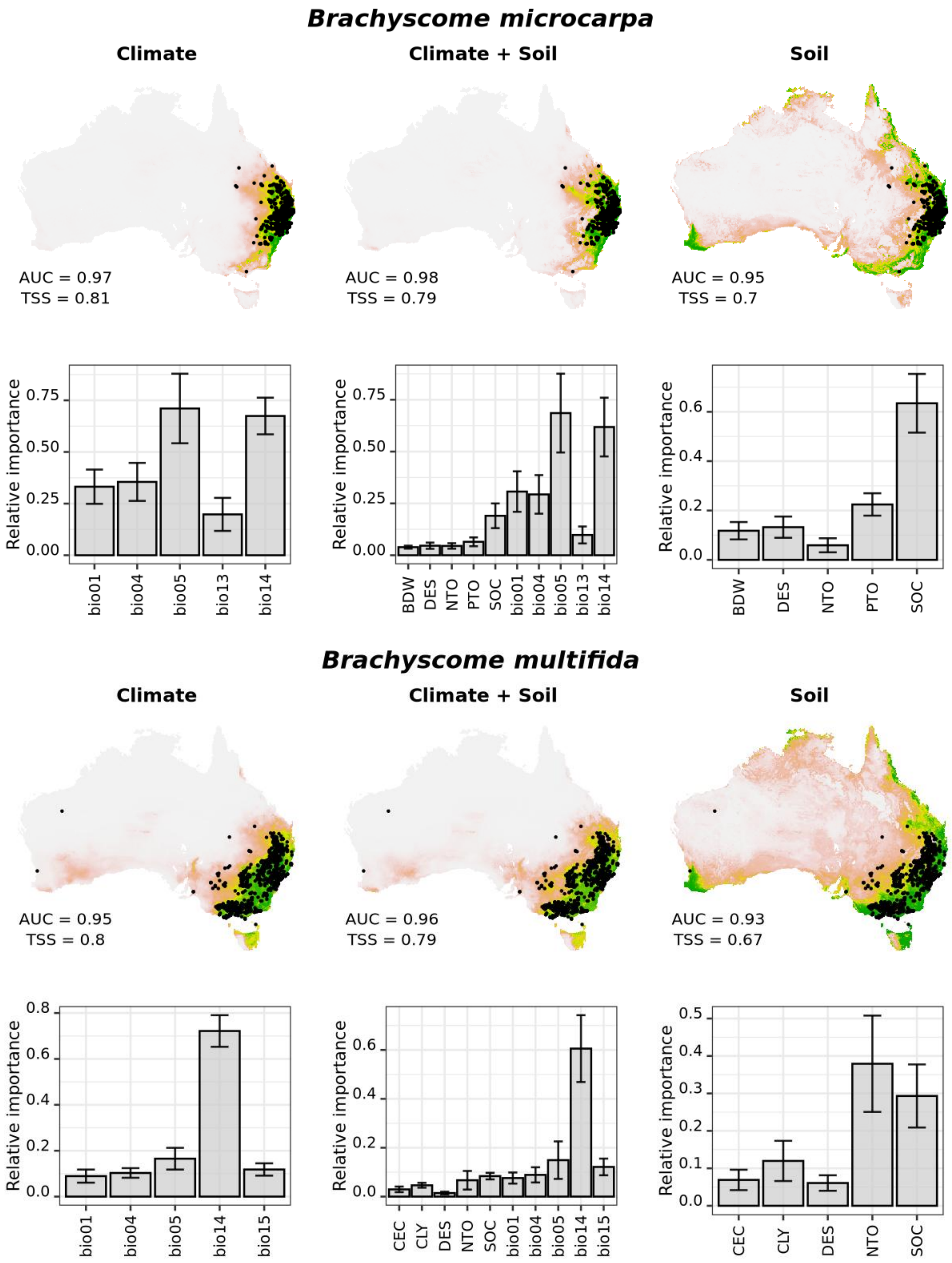
Brachyscome microcarpa
Climate
Climate + Soil
Soil
AUC = 0.97
TSS = 0.81
AUC = 0.98
TSS = 0.79
AUC = 0.95
TSS = 0.7
Relative importance
0.00
0.25
0.50
0.75
bio01
bio04
bio05
bio13
bio14
BDW
DES
NTO
PTO
SOC
0.0
0.2
0.4
0.6
Brachyscome multifida
Climate
Climate + Soil
Soil
AUC = 0.95
TSS = 0.8
AUC = 0.96
TSS = 0.79
AUC = 0.93
TSS = 0.67
Relative importance
0.0
0.2
0.4
0.6
0.8
bio01
bio04
bio05
bio14
bio15
CEC
CLY
DES
NTO
SOC
0.1
0.3
0.5

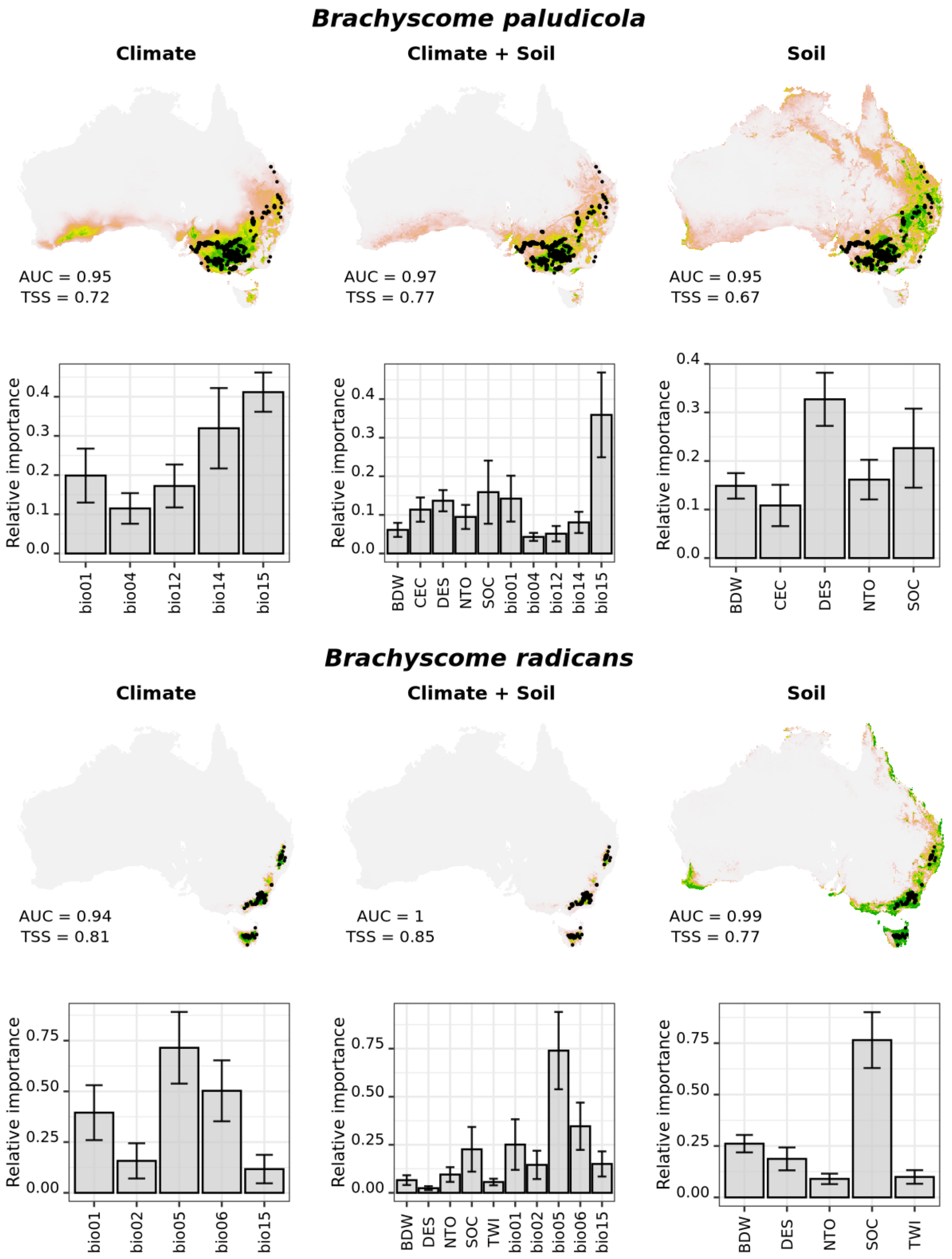
Brachyscome paludicola
Climate
Climate + Soil
Soil
AUC = 0.95
TSS = 0.72
AUC = 0.97
TSS = 0.77
AUC = 0.95
TSS = 0.67
Relative importance
bio01
bio04
bio12
bio14
bio15
BDW
CEC
DES
NTO
SOC
Brachyscome radicans
Climate
Climate + Soil
Soil
AUC = 0.94
TSS = 0.81
AUC = 1
TSS = 0.85
AUC = 0.99
TSS = 0.77
Relative importance
bio01
bio02
bio05
bio06
bio15
BDW
DES
NTO
SOC
TWI

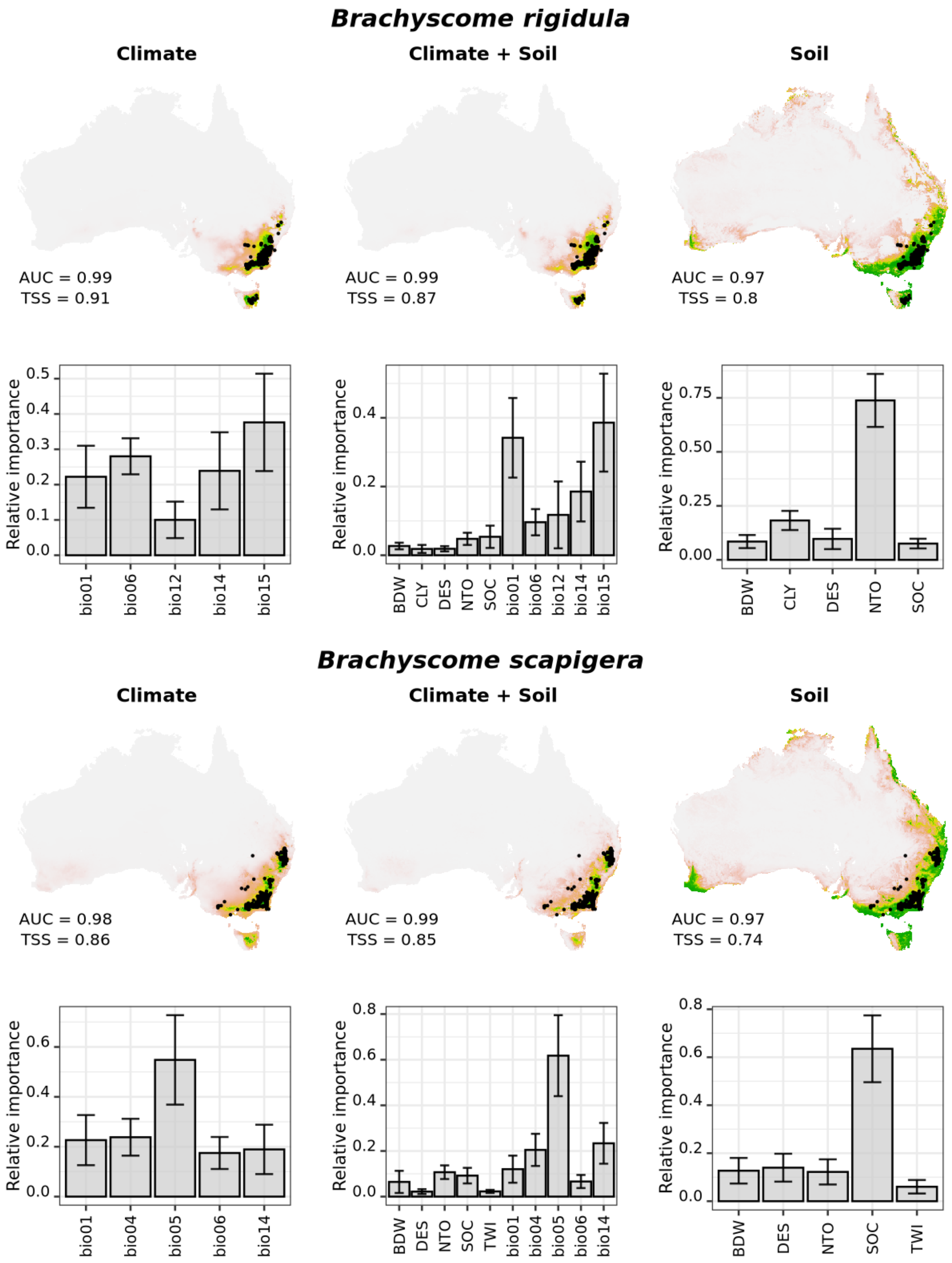
Brachyscome rigidula
Climate
Climate + Soil
Soil
AUC = 0.99
TSS = 0.91
AUC = 0.99
TSS = 0.87
AUC = 0.97
TSS = 0.8
Relative importance
bio01 bio06 bio12 bio14 bio15
BDW CLY DES NTO SOC bio01 bio06 bio12 bio14 bio15
BDW CLY DES NTO SOC
Brachyscome scapigera
Climate
Climate + Soil
Soil
AUC = 0.98
TSS = 0.86
AUC = 0.99
TSS = 0.85
AUC = 0.97
TSS = 0.74
Relative importance
bio01 bio04 bio05 bio06 bio14
BDW DES NTO SOC TWI bio01 bio04 bio05 bio06 bio14
BDW DES NTO SOC TWI

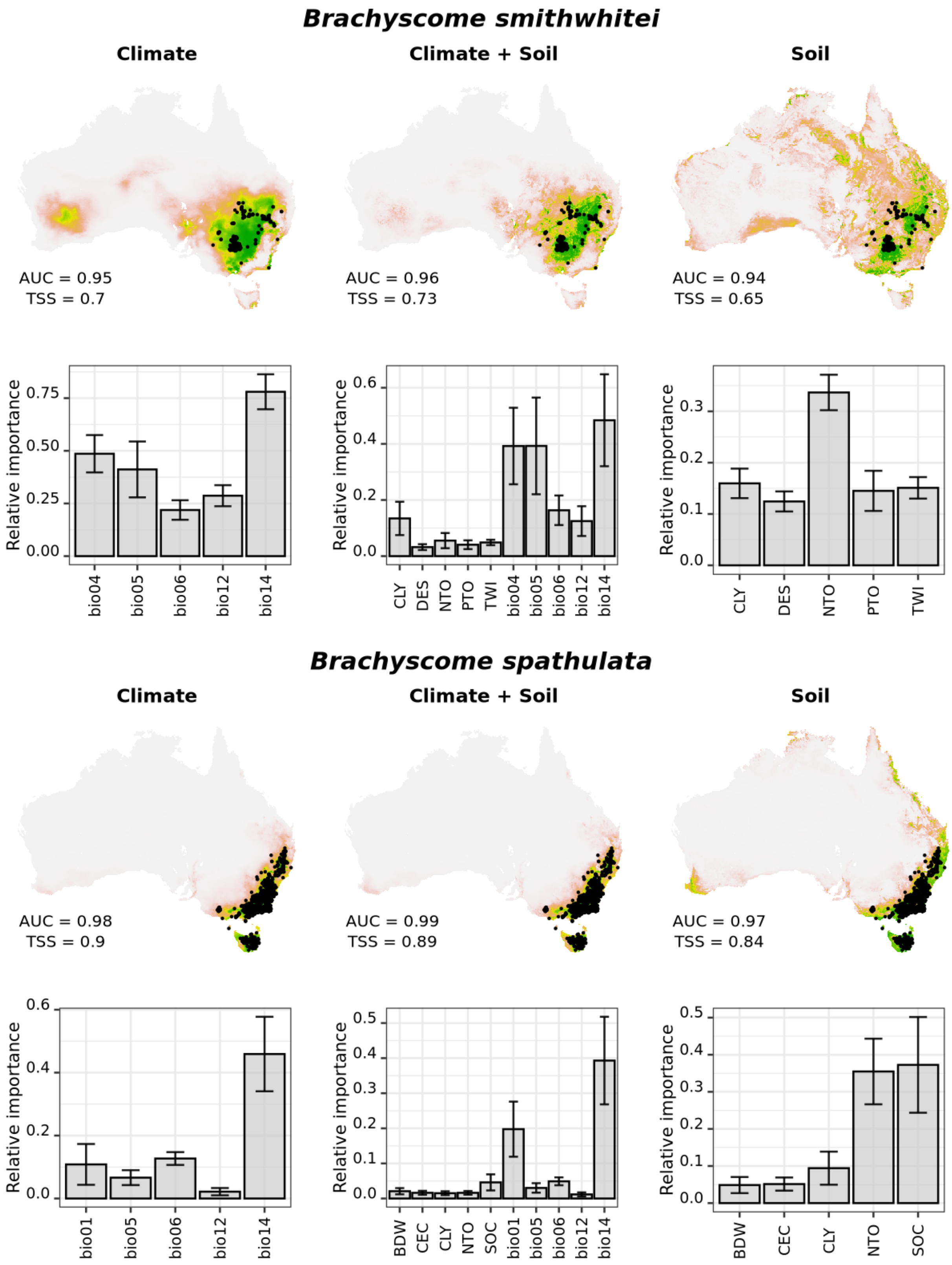

Brachyscome smithwhitei
Climate
Climate + Soil
Soil
AUC = 0.95
TSS = 0.7
AUC = 0.96
TSS = 0.73
AUC = 0.94
TSS = 0.65
Relative importance
0.00
0.25
0.50
0.75
bio04
bio05
bio06
bio12
bio14
0.0
0.2
0.4
0.6
CLY
DES
NTO
PTO
TWI
0.1
0.3
Brachyscome spathulata
AUC = 0.98
TSS = 0.9
AUC = 0.99
TSS = 0.89
AUC = 0.97
TSS = 0.84
bio01
BDW
CEC
SOC
0.5

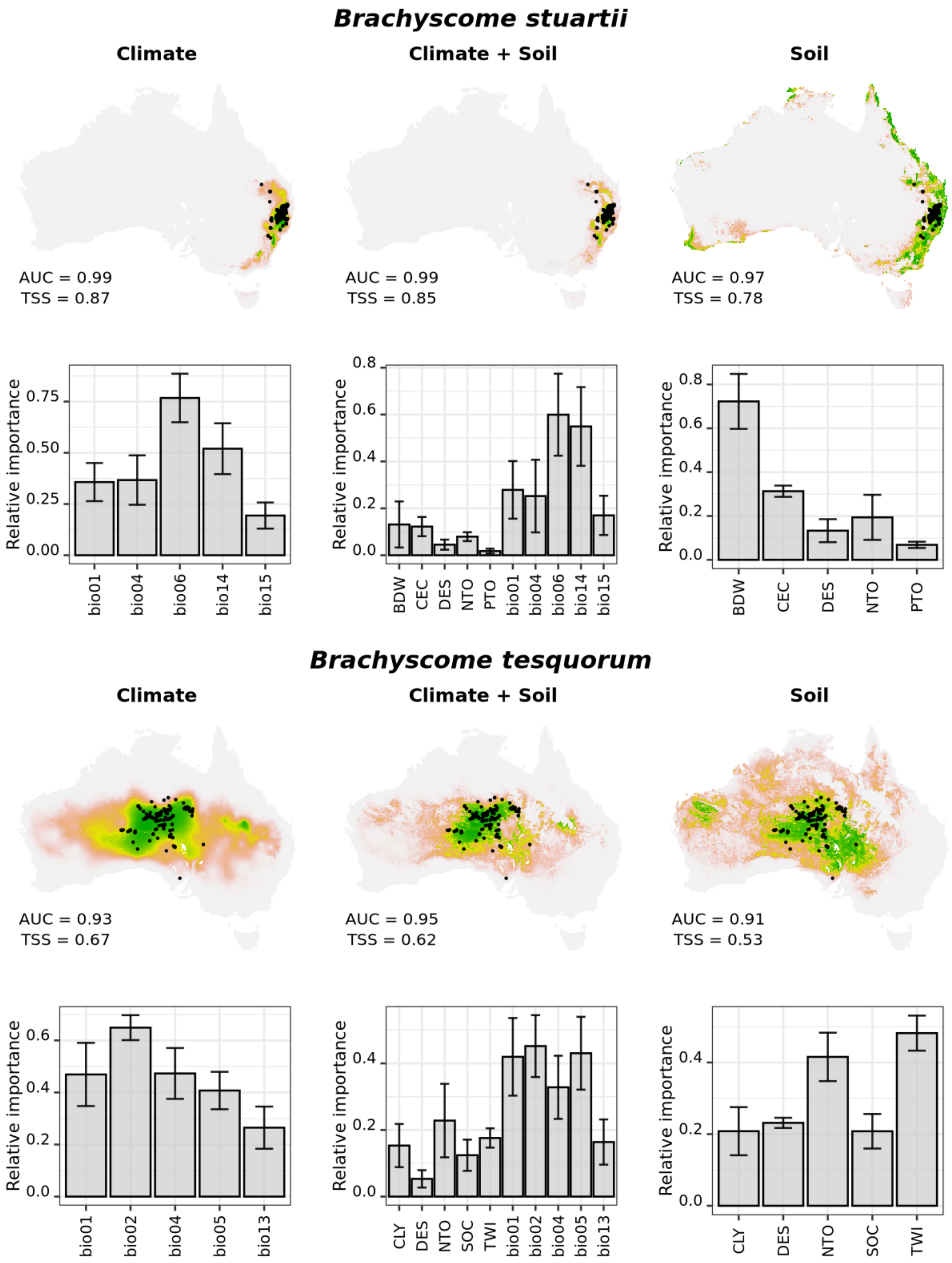

Brachyscome stuartii
Climate
Climate + Soil
Soil
AUC = 0.99
TSS = 0.87
AUC = 0.99
TSS = 0.85
AUC = 0.97
TSS = 0.78
Relative importance
bio01 bio04 bio06 bio14 bio15
BDW CEC DES NTO PTO bio01 bio04 bio06 bio14 bio15
BDW CEC DES NTO PTO
Brachyscome tesquorum
Climate
Climate + Soil
Soil
AUC = 0.93
TSS = 0.67
AUC = 0.95
TSS = 0.62
AUC = 0.91
TSS = 0.53
Relative importance
bio01 bio02 bio04 bio05 bio13
CLY DES NTO SOC TWI bio01 bio02 bio04 bio05 bio13
CLY DES NTO SOC TWI

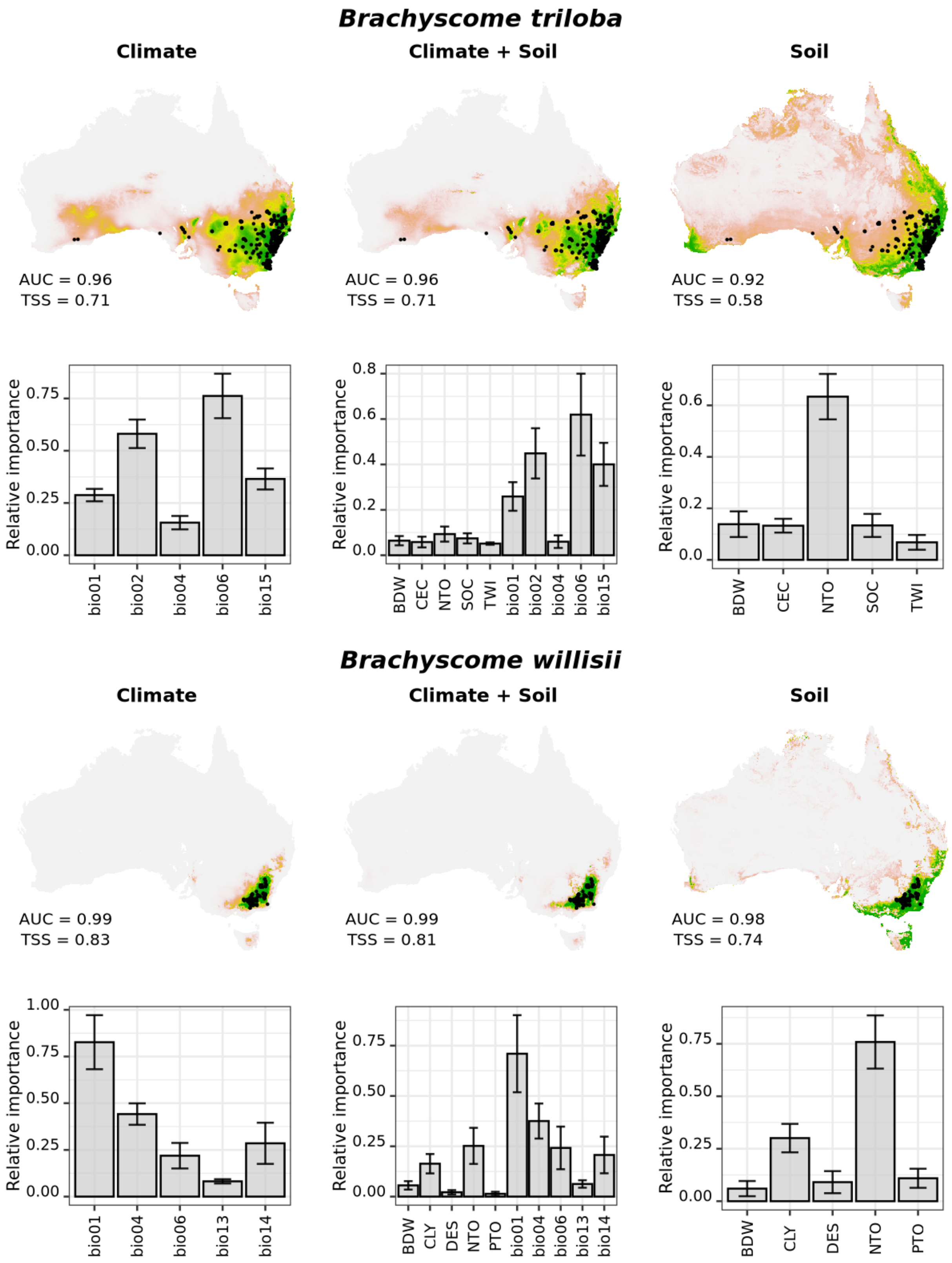

Brachyscome triloba
Climate
Climate + Soil
Soil
AUC = 0.96
TSS = 0.71
AUC = 0.96
TSS = 0.71
AUC = 0.92
TSS = 0.58
Relative importance
bio01
bio02
bio04
bio06
bio15
BDW
CEC
NTO
SOC
TWI
Brachyscome willisii
Climate
Climate + Soil
Soil
AUC = 0.99
TSS = 0.83
AUC = 0.99
TSS = 0.81
AUC = 0.98
TSS = 0.74
Relative importance
bio01
bio04
bio06
bio13
bio14
BDW
CLY
DES
NTO
PTO

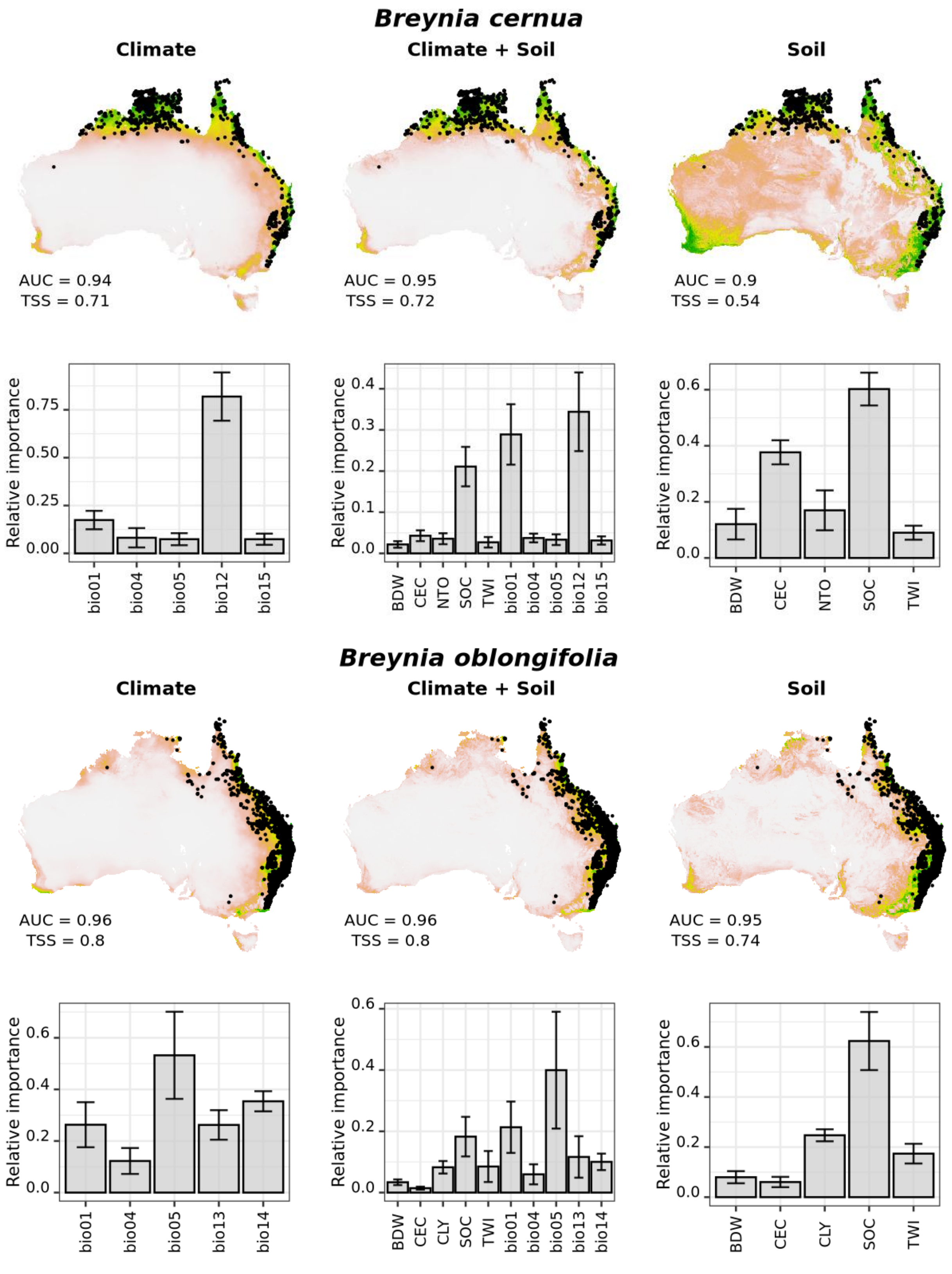
Breynia cernua
Climate
Climate + Soil
Soil
AUC = 0.94
TSS = 0.71
AUC = 0.95
TSS = 0.72
AUC = 0.9
TSS = 0.54
Relative importance
bio01
bio04
bio05
bio12
bio15
BDW
CEC
NTO
SOC
TWI
Breynia oblongifolia
Climate
Climate + Soil
Soil
AUC = 0.96
TSS = 0.8
AUC = 0.96
TSS = 0.8
AUC = 0.95
TSS = 0.74
Relative importance
bio01
bio04
bio05
bio13
bio14
BDW
CEC
CLY
SOC
TWI

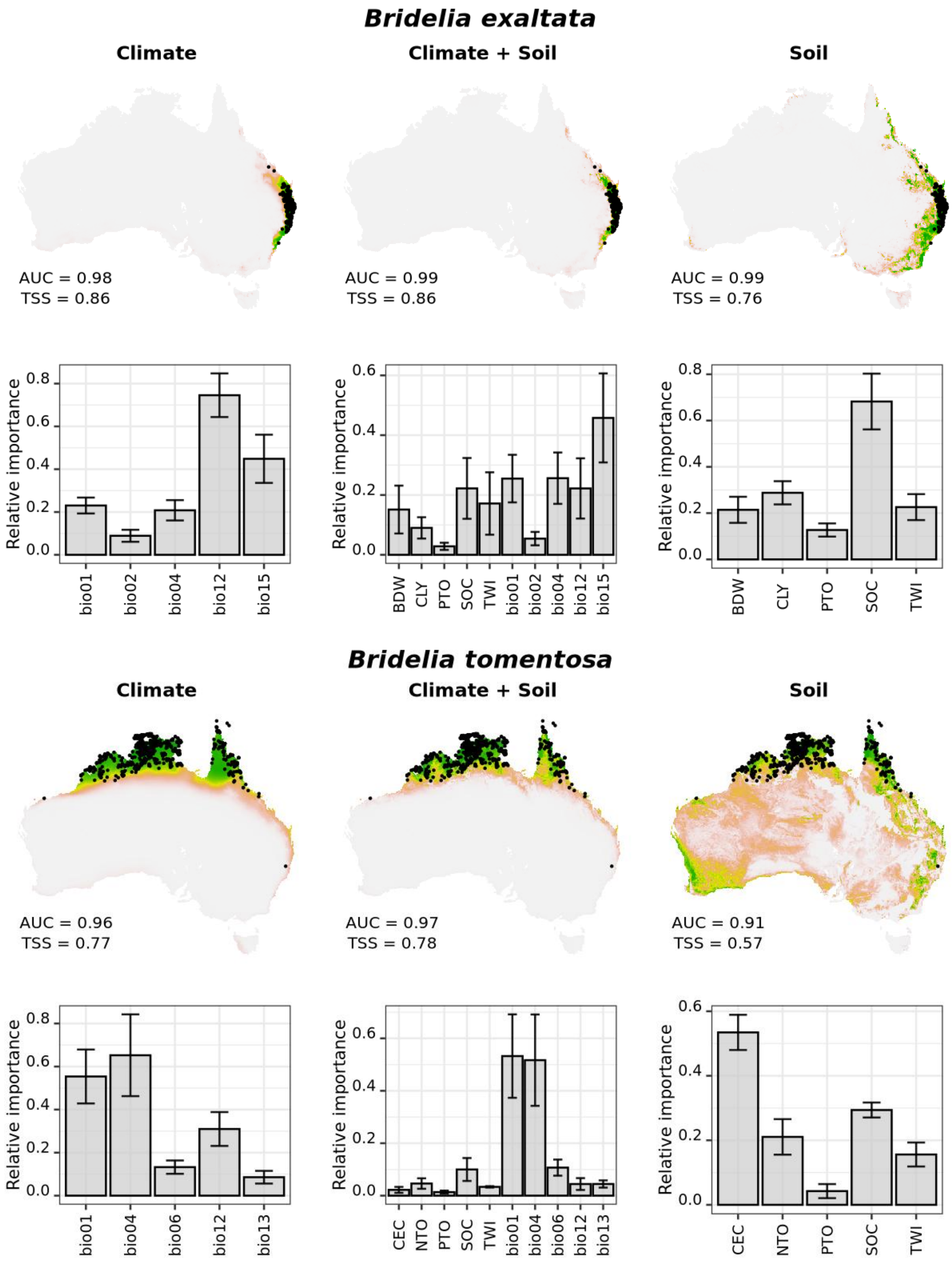

Bridelia exaltata
Climate
Climate + Soil
Soil
AUC = 0.98
TSS = 0.86
AUC = 0.99
TSS = 0.86
AUC = 0.99
TSS = 0.76
Relative importance
bio01
bio02
bio04
bio12
bio15
BDW
CLY
PTO
SOC
TWI
bio01
bio02
bio04
bio12
bio15
BDW
CLY
PTO
SOC
TWI
Bridelia tomentosa
Climate
Climate + Soil
Soil
AUC = 0.96
TSS = 0.77
AUC = 0.97
TSS = 0.78
AUC = 0.91
TSS = 0.57
Relative importance
bio01
bio04
bio06
bio12
bio13
CEC
NTO
PTO
SOC
TWI
bio01
bio04
bio06
bio12
bio13
CEC
NTO
PTO
SOC
TWI

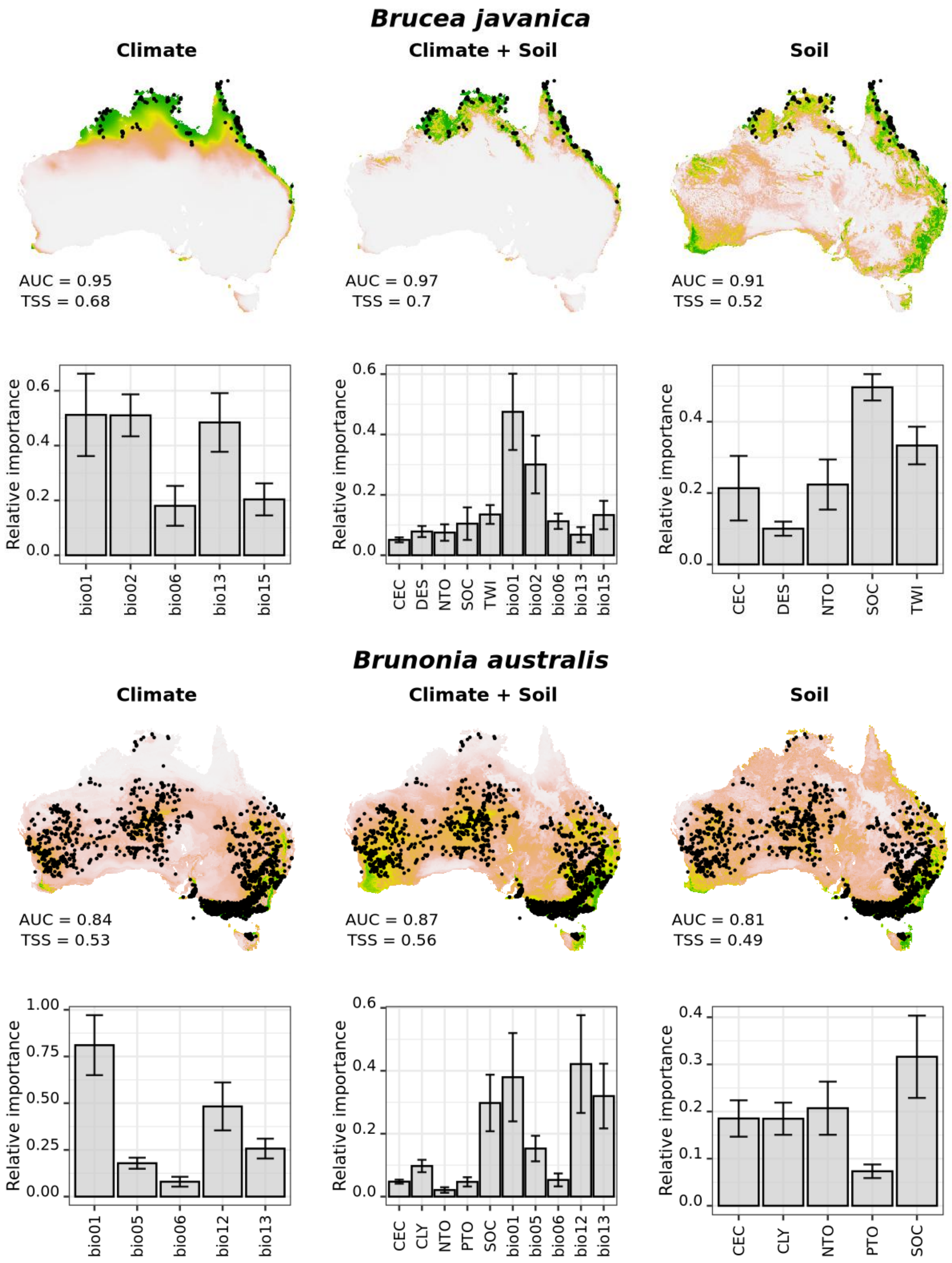

Brucea javanica
Climate
Climate + Soil
Soil
AUC = 0.95
TSS = 0.68
AUC = 0.97
TSS = 0.7
AUC = 0.91
TSS = 0.52
Relative importance
bio01 bio02 bio06 bio13 bio15
CEC DES NTO SOC TWI bio01 bio02 bio06 bio13 bio15
CEC DES NTO SOC TWI
Brunonia australis
Climate
Climate + Soil
Soil
AUC = 0.84
TSS = 0.53
AUC = 0.87
TSS = 0.56
AUC = 0.81
TSS = 0.49
Relative importance
bio01 bio05 bio06 bio12 bio13
CEC CLY NTO PTO SOC bio01 bio05 bio06 bio12 bio13
CEC CLY NTO PTO SOC

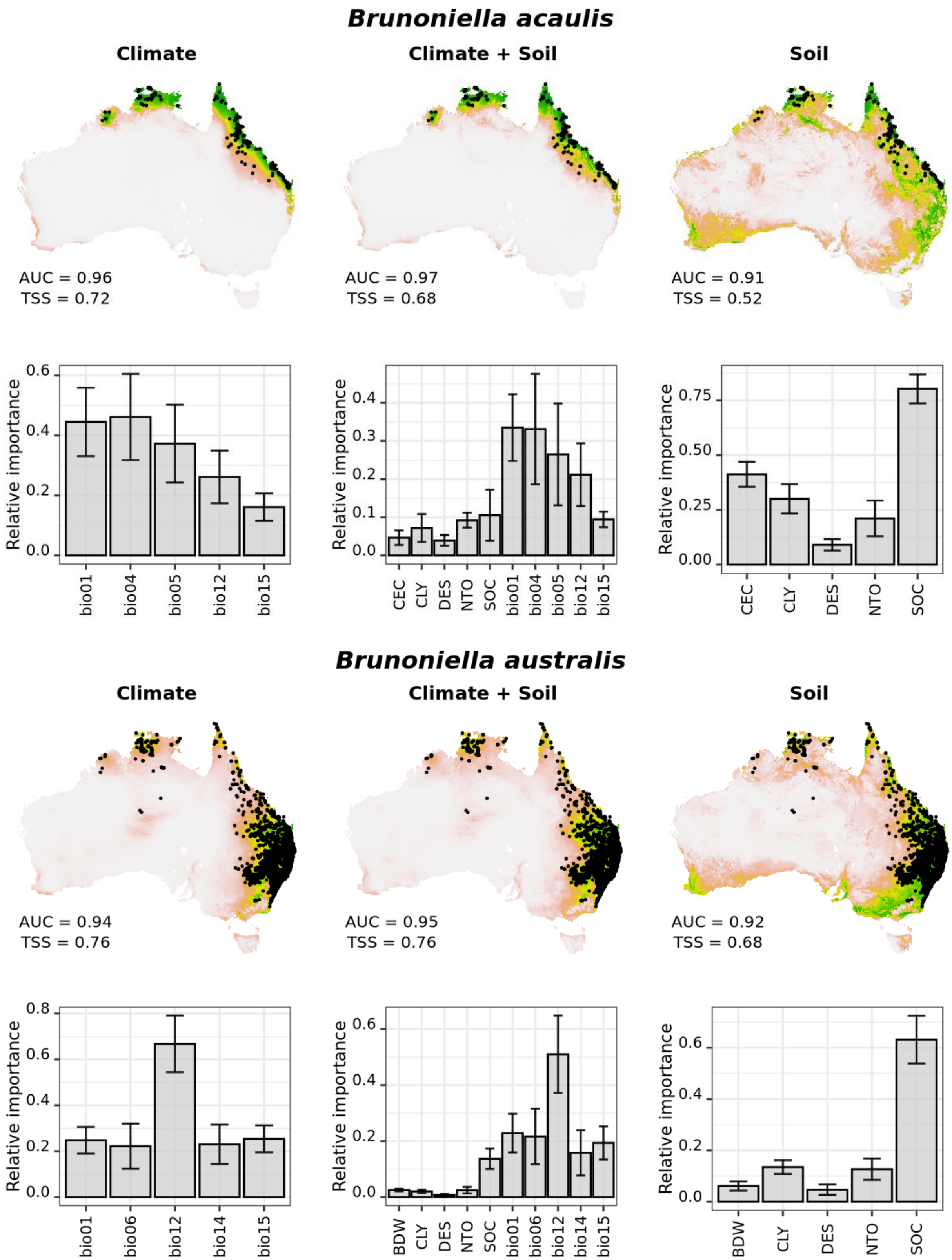

Brunoniella acaulis
Climate
Climate + Soil
Soil
AUC = 0.96
TSS = 0.72
AUC = 0.97
TSS = 0.68
AUC = 0.91
TSS = 0.52
Relative importance
bio01
bio04
bio05
bio12
bio15
CEC
CLY
DES
NTO
SOC
Brunoniella australis
Climate
Climate + Soil
Soil
AUC = 0.94
TSS = 0.76
AUC = 0.95
TSS = 0.76
AUC = 0.92
TSS = 0.68
Relative importance
bio01
bio06
bio12
bio14
bio15
BDW
CLY
DES
NTO
SOC

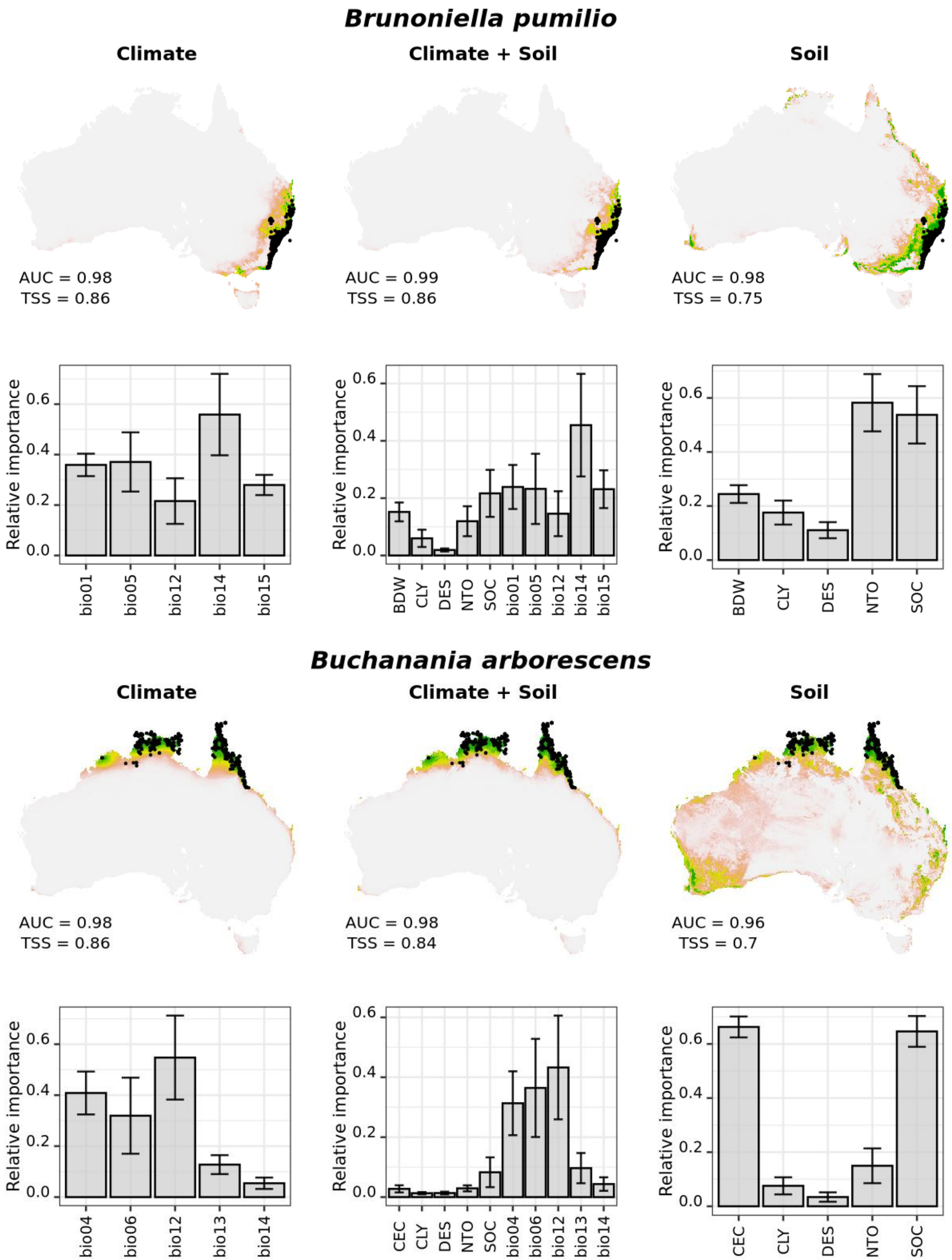
Brunoniella pumilio
Climate
Climate + Soil
Soil
AUC = 0.98
TSS = 0.86
AUC = 0.99
TSS = 0.86
AUC = 0.98
TSS = 0.75
Relative importance
0.0
0.2
0.4
0.6
bio01
bio05
bio12
bio14
bio15
BDW
CLY
DES
NTO
SOC
Buchanania arborescens
Climate
Climate + Soil
Soil
AUC = 0.98
TSS = 0.86
AUC = 0.98
TSS = 0.84
AUC = 0.96
TSS = 0.7
bio04
bio06
bio13
CEC

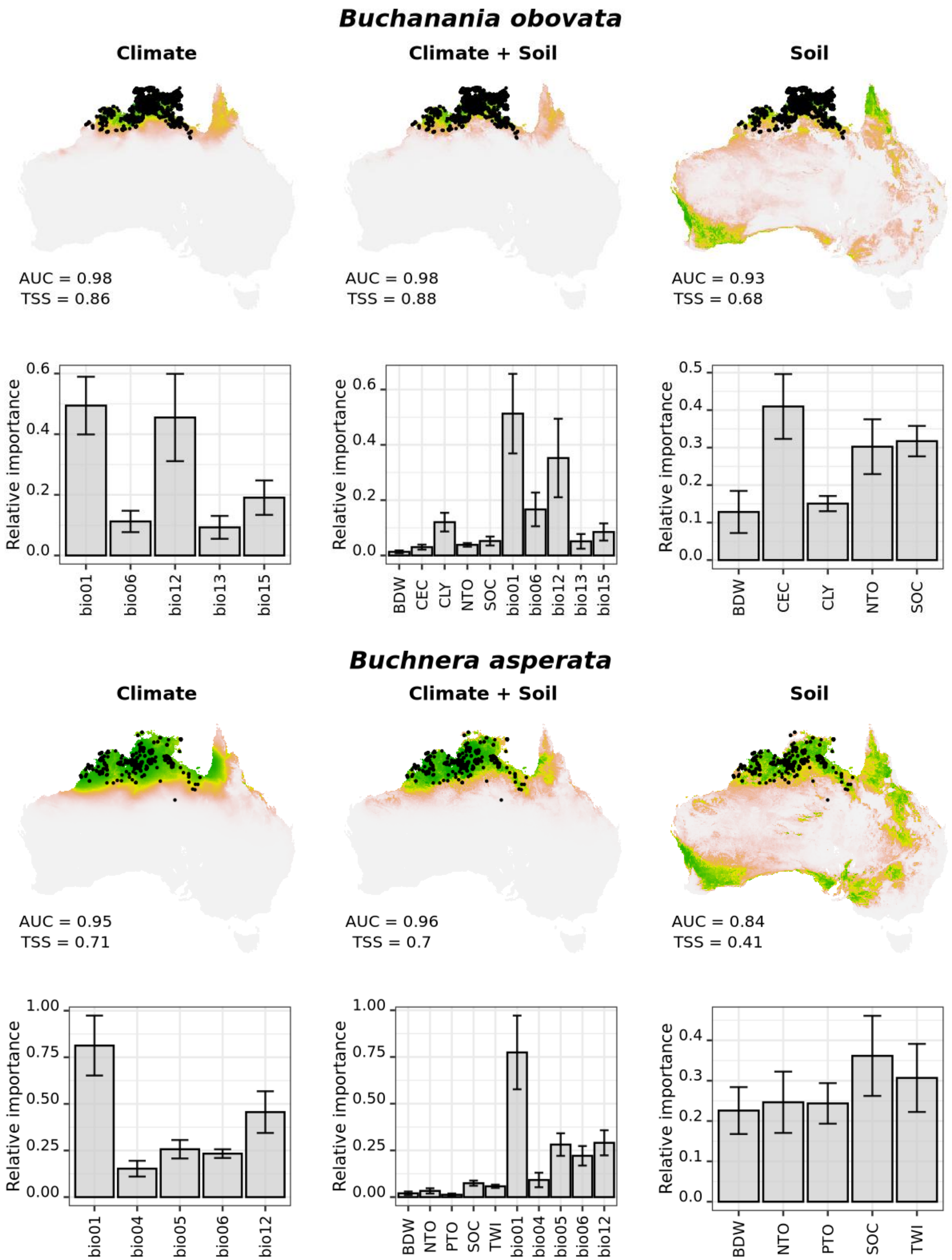

Buchanania obovata
Climate
Climate + Soil
Soil
AUC = 0.98
TSS = 0.86
AUC = 0.98
TSS = 0.88
AUC = 0.93
TSS = 0.68
Relative importance
0.0
0.2
0.4
0.6
bio01
bio06
bio12
bio13
bio15
BDW
CEC
CLY
NTO
SOC
bio01
bio06
bio12
bio13
bio15
0.1
0.3
0.5
BDW
CEC
CLY
NTO
SOC
Buchnera asperata
Climate
Climate + Soil
Soil
AUC = 0.95
TSS = 0.71
AUC = 0.96
TSS = 0.7
AUC = 0.84
TSS = 0.41
Relative importance
0.00
0.25
0.50
0.75
1.00
bio01
bio04
bio05
bio06
bio12
BDW
NTO
PTO
SOC
TWI
bio01
bio04
bio05
bio06
bio12
0.0
0.1
0.2
0.3
0.4
BDW
NTO
PTO
SOC
TWI

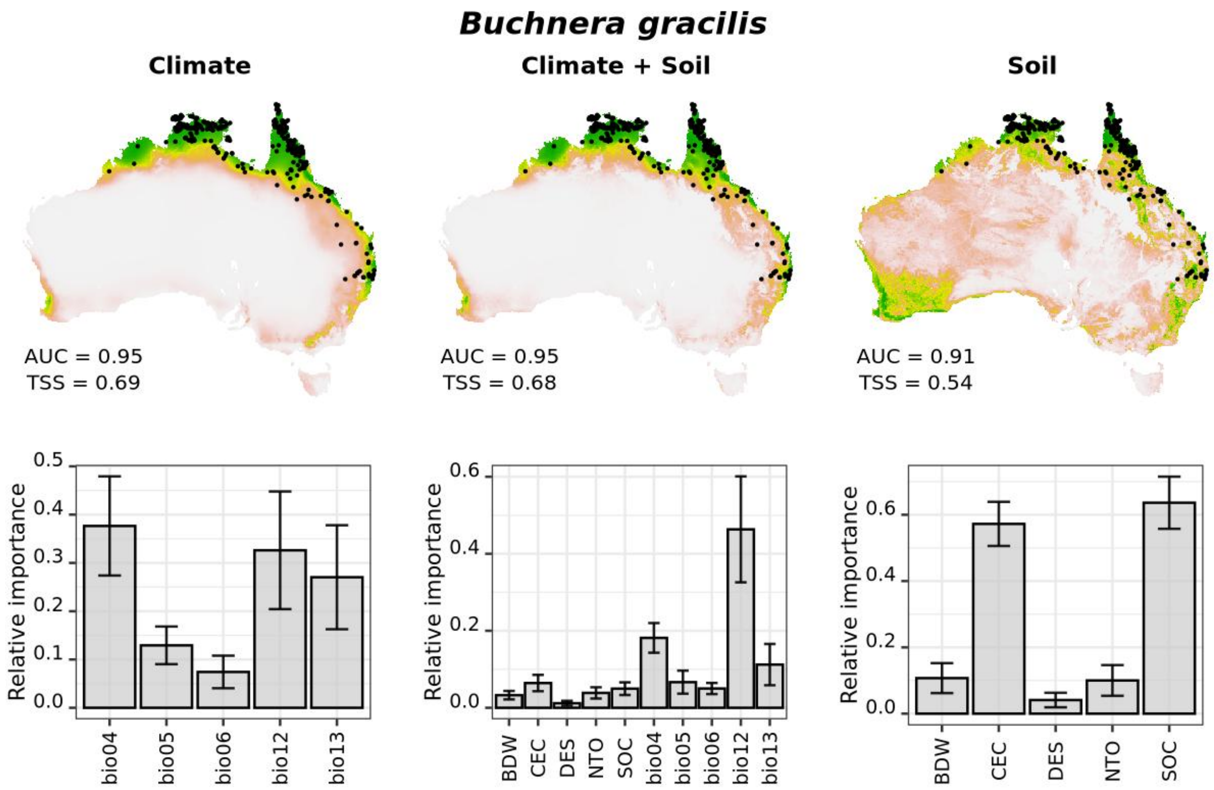
Buchnera gracilis
Climate
Climate + Soil
Soil
AUC = 0.95
TSS = 0.69
AUC = 0.95
TSS = 0.68
AUC = 0.91
TSS = 0.54
Relative importance
bio04
bio05
bio06
bio12
bio13
BDW
CEC
DES
NTO
SOC
bio04
bio05
bio06
bio12
bio13
BDW
CEC
DES
NTO
SOC

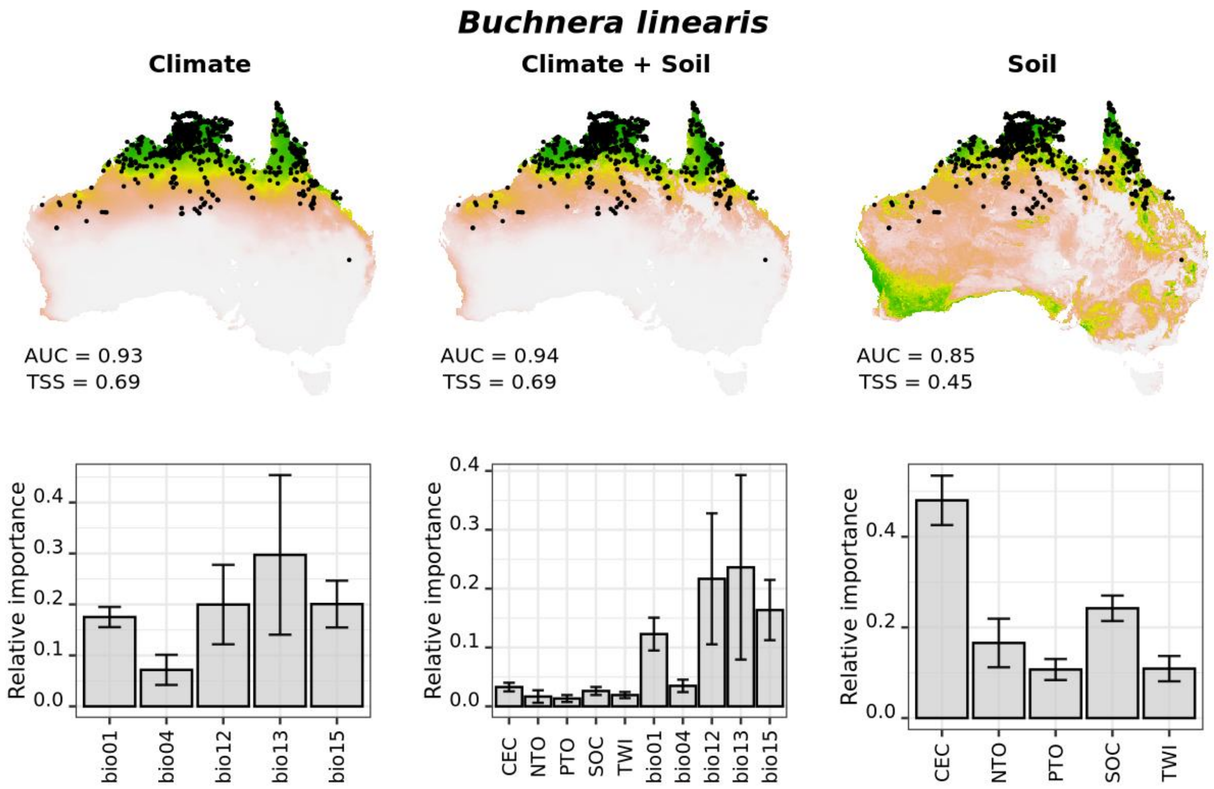
Buchnera linearis
Climate
Climate + Soil
Soil
AUC = 0.93
TSS = 0.69
AUC = 0.94
TSS = 0.69
AUC = 0.85
TSS = 0.45
Relative importance
bio01
bio04
bio12
bio13
bio15
CEC
NTO
PTO
SOC
TWI
bio01
bio04
bio12
bio13
bio15
CEC
NTO
PTO
SOC
TWI

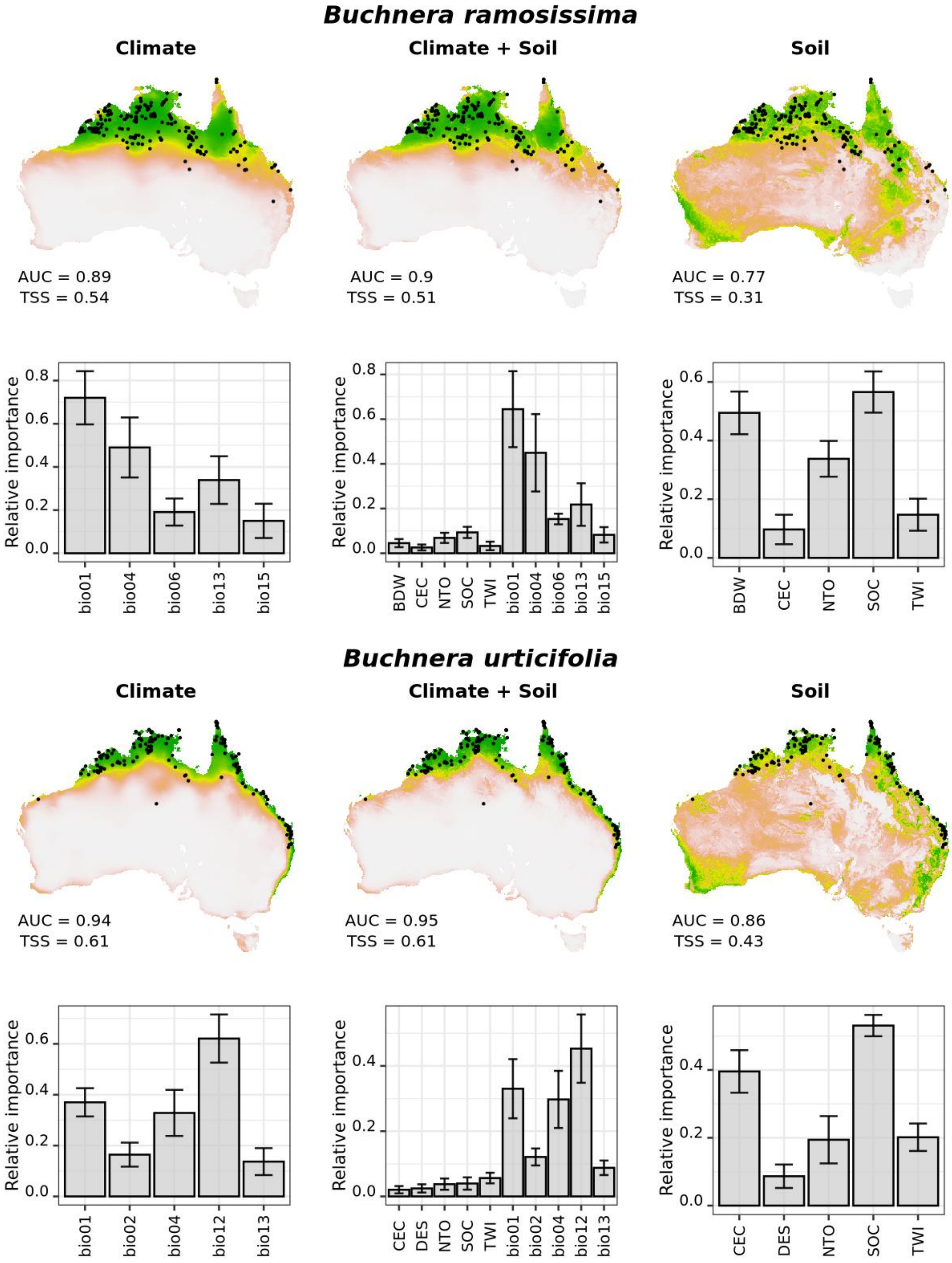

Buchnera ramosissima
Climate
Climate + Soil
Soil
AUC = 0.89
TSS = 0.54
AUC = 0.9
TSS = 0.51
AUC = 0.77
TSS = 0.31
Relative importance
bio01
bio04
bio06
bio13
bio15
BDW
CEC
NTO
SOC
TWI
Buchnera urticifolia
Climate
Climate + Soil
Soil
AUC = 0.94
TSS = 0.61
AUC = 0.95
TSS = 0.61
AUC = 0.86
TSS = 0.43
Relative importance
bio01
bio02
bio04
bio12
bio13
CEC
DES
NTO
SOC
TWI

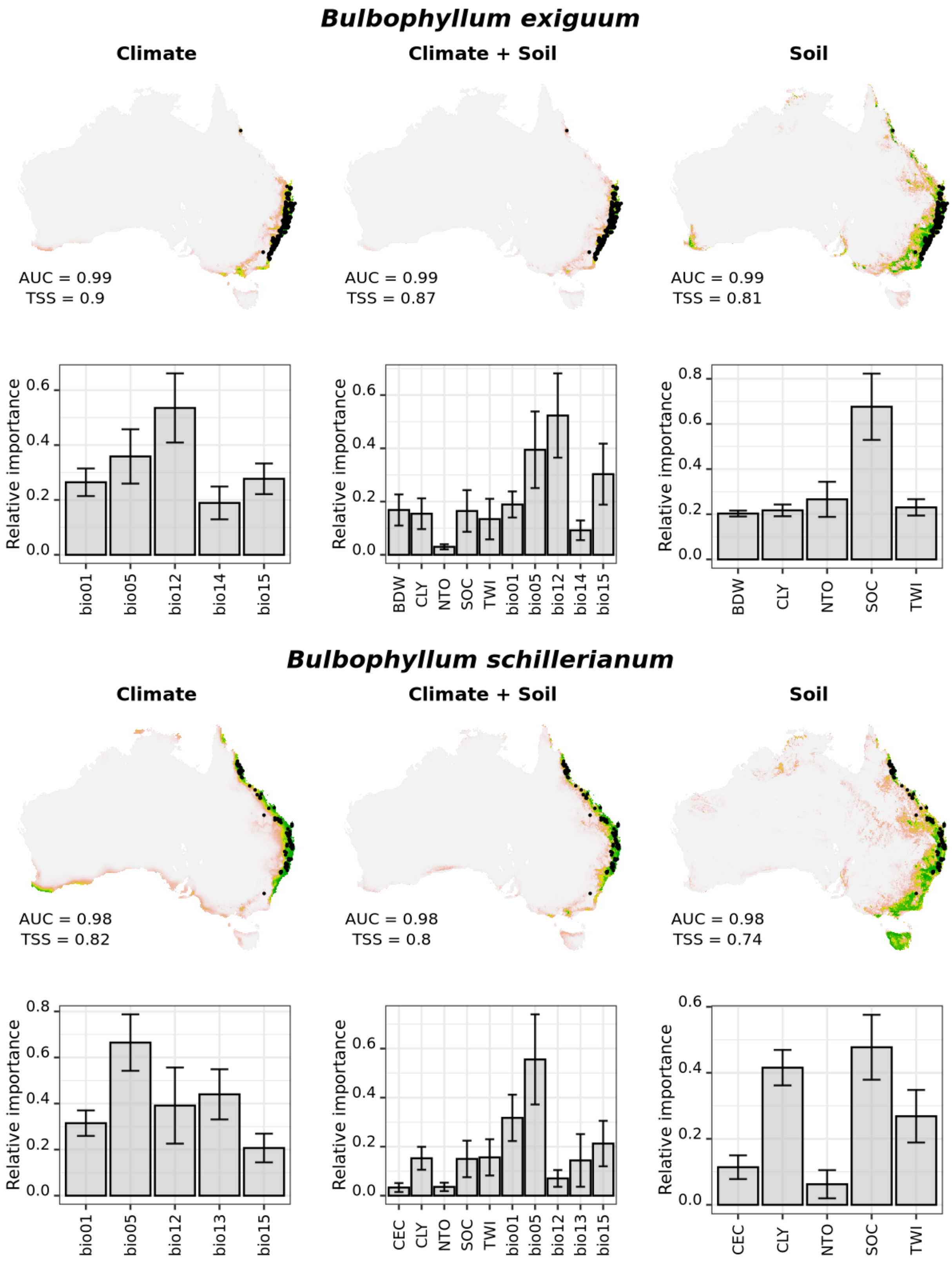
Bulbophyllum exiguum
Climate
Climate + Soil
Soil
AUC = 0.99
TSS = 0.9
AUC = 0.99
TSS = 0.87
AUC = 0.99
TSS = 0.81
Relative importance
0.0
0.2
0.4
0.6
0.8
bio01
bio05
bio12
bio14
bio15
BDW
CLY
NTO
SOC
TWI
Bulbophyllum schillerianum
Climate
Climate + Soil
Soil
AUC = 0.98
TSS = 0.82
AUC = 0.98
TSS = 0.8
AUC = 0.98
TSS = 0.74
Relative importance
0.0
0.2
0.4
0.6
0.8
bio01
bio05
bio12
bio13
bio15
CEC
CLY
NTO
SOC
TWI

Bulbophyllum shepherdii

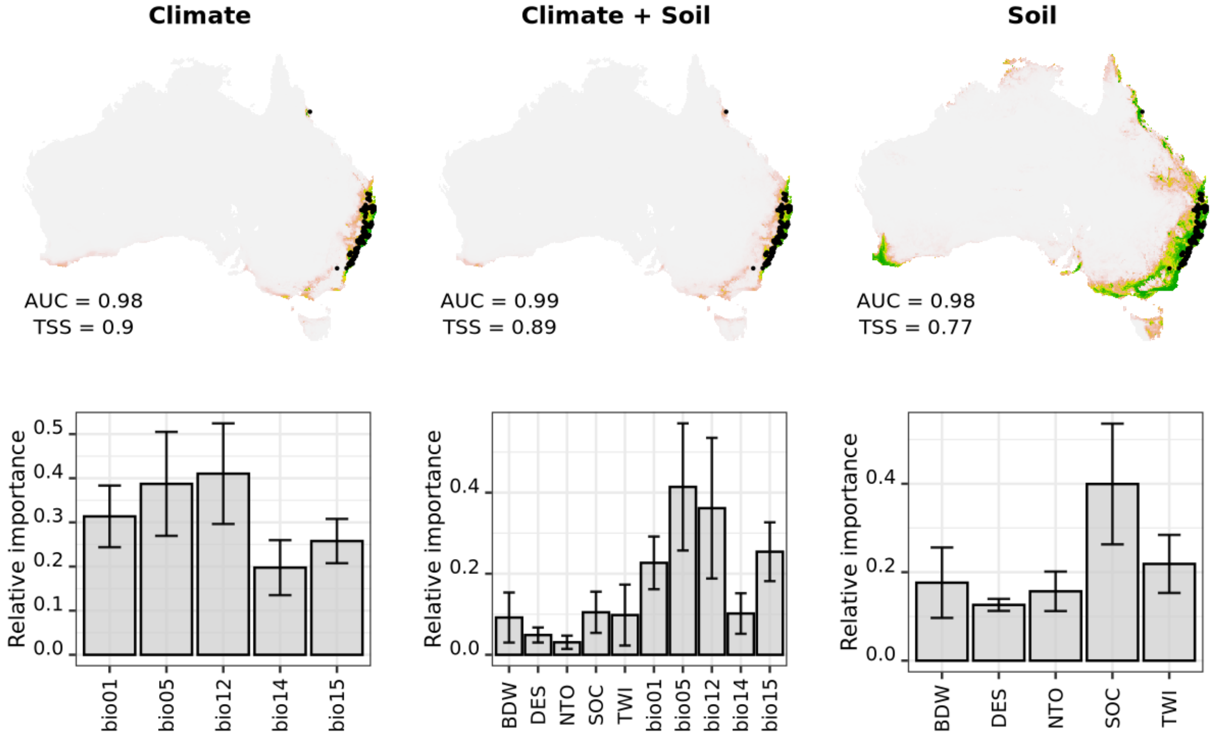

Bulbostylis densa

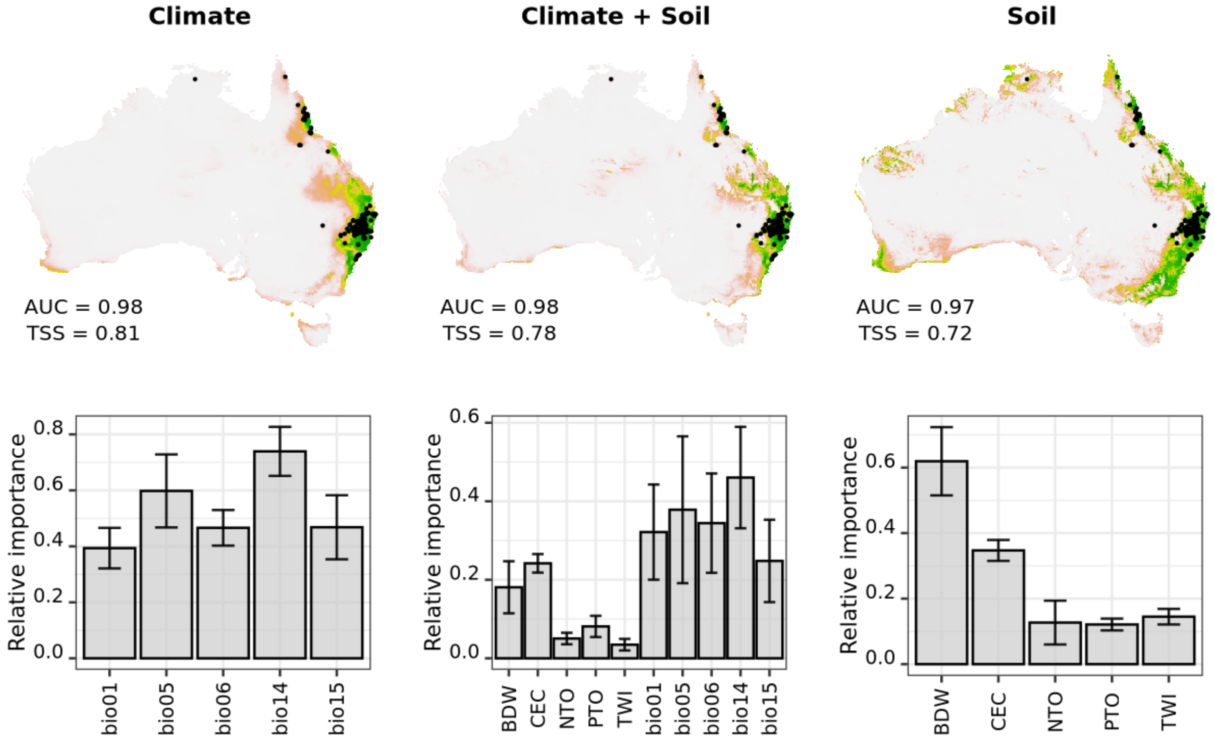

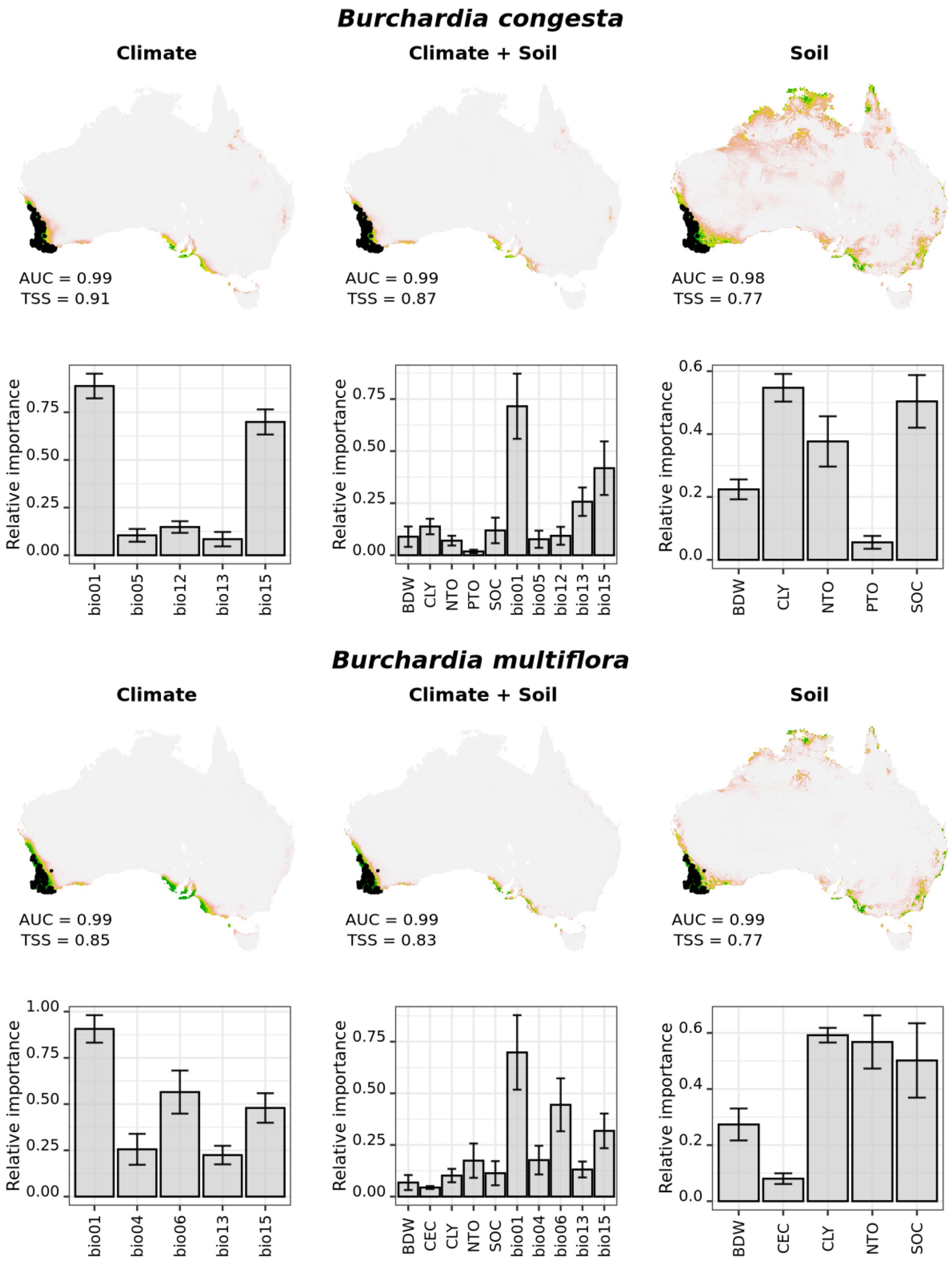
Burchardia congesta
Climate
Climate + Soil
Soil
AUC = 0.99
TSS = 0.91
AUC = 0.99
TSS = 0.87
AUC = 0.98
TSS = 0.77
Relative importance
0.00
0.25
0.50
0.75
bio01
bio05
bio12
bio13
bio15
BDW
CLY
NTO
PTO
SOC
0.0
0.2
0.4
0.6
Burchardia multiflora
Climate
Climate + Soil
Soil
AUC = 0.99
TSS = 0.85
AUC = 0.99
TSS = 0.83
AUC = 0.99
TSS = 0.77
1.00
bio04
bio06
CEC

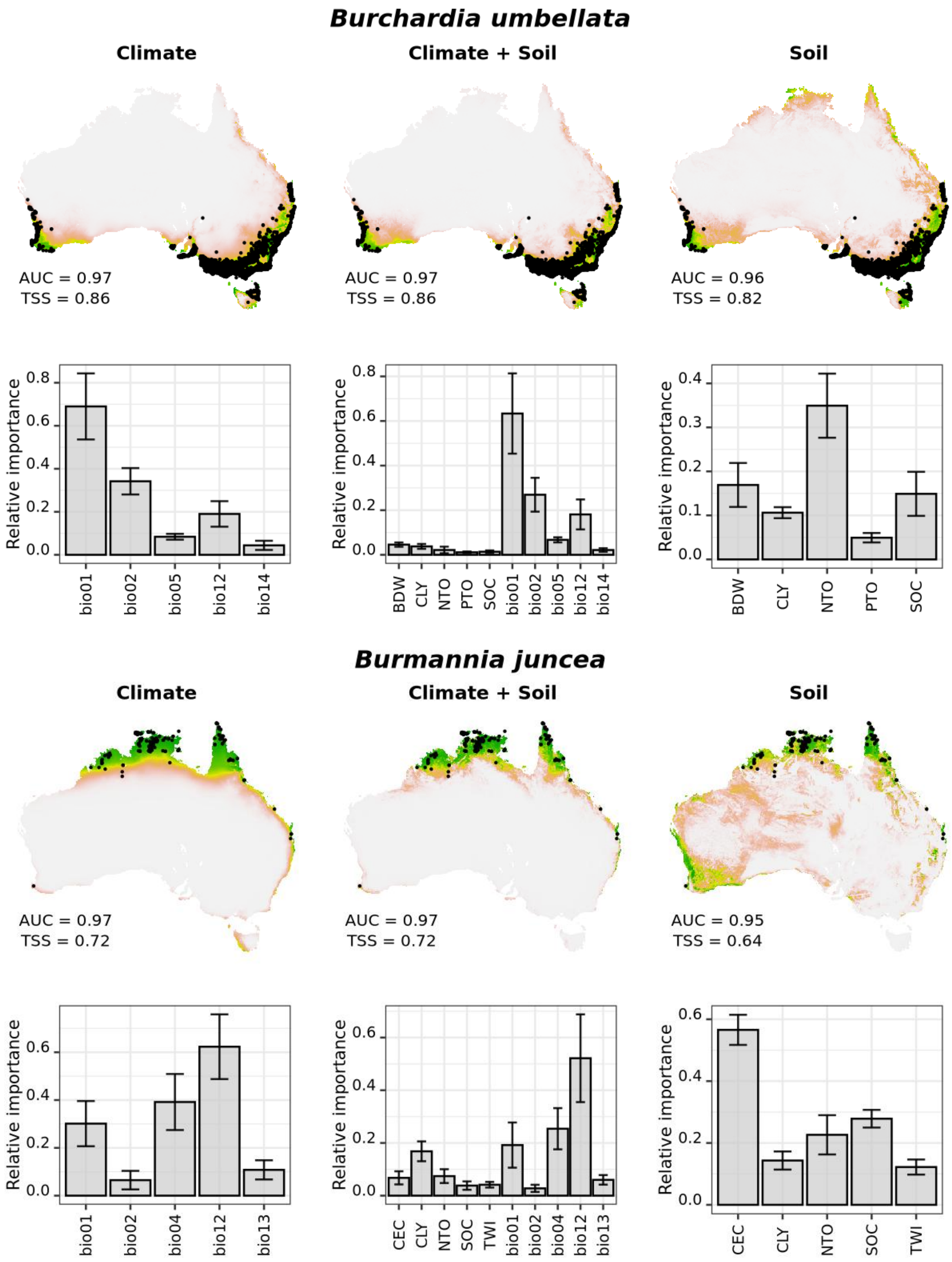

Burchardia umbellata
Climate
Climate + Soil
Soil
AUC = 0.97
TSS = 0.86
AUC = 0.97
TSS = 0.86
AUC = 0.96
TSS = 0.82
Relative importance
bio01
bio02
bio05
bio12
bio14
BDW
CLY
NTO
PTO
SOC
Burmannia juncea
Climate
Climate + Soil
Soil
AUC = 0.97
TSS = 0.72
AUC = 0.97
TSS = 0.72
AUC = 0.95
TSS = 0.64
Relative importance
bio01
bio02
bio04
bio12
bio13
CEC
CLY
NTO
SOC
TWI

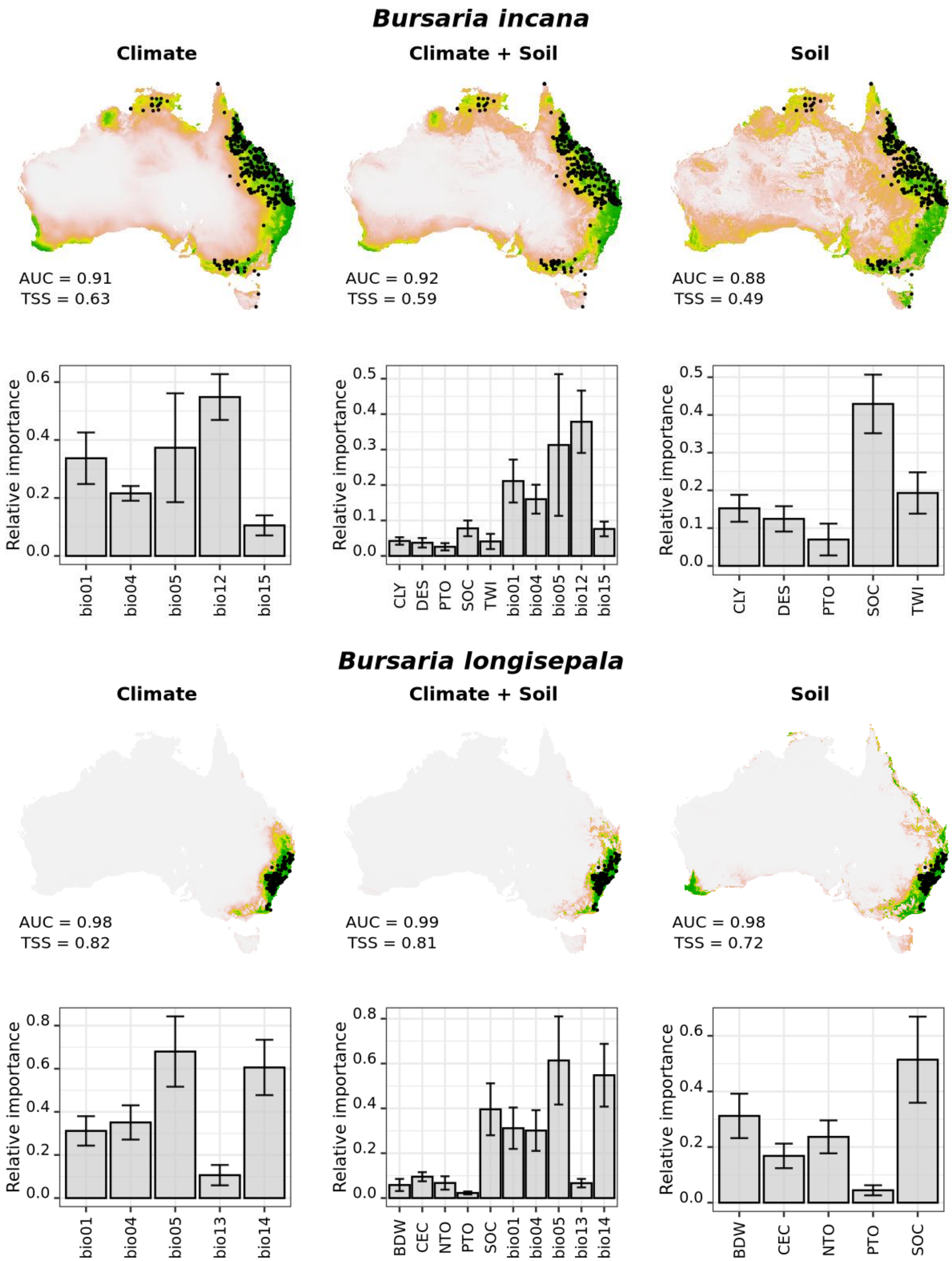

Bursaria incana
Climate
Climate + Soil
Soil
AUC = 0.91
TSS = 0.63
AUC = 0.92
TSS = 0.59
AUC = 0.88
TSS = 0.49
Relative importance
bio01
bio04
bio05
bio12
bio15
CLY
DES
PTO
SOC
TWI
Bursaria longisepala
Climate
Climate + Soil
Soil
AUC = 0.98
TSS = 0.82
AUC = 0.99
TSS = 0.81
AUC = 0.98
TSS = 0.72
Relative importance
bio01
bio04
bio05
bio13
bio14
BDW
CEC
NTO
PTO
SOC

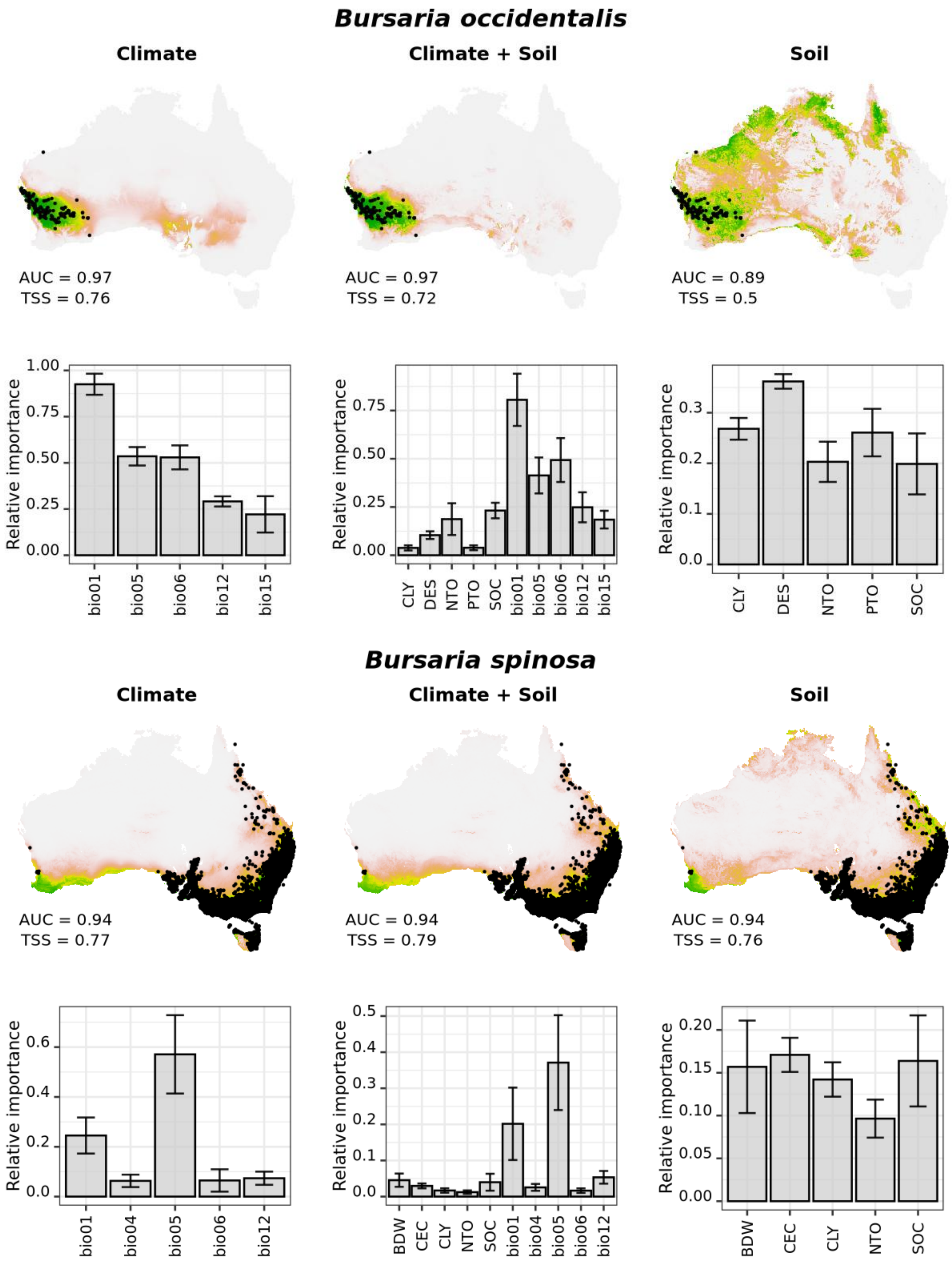
Bursaria occidentalis
Climate
Climate + Soil
Soil
AUC = 0.97
TSS = 0.76
AUC = 0.97
TSS = 0.72
AUC = 0.89
TSS = 0.5
Relative importance
bio01
bio05
bio06
bio12
bio15
CLY
DES
NTO
PTO
SOC
Bursaria spinosa
Climate
Climate + Soil
Soil
AUC = 0.94
TSS = 0.77
AUC = 0.94
TSS = 0.79
AUC = 0.94
TSS = 0.76
Relative importance
bio01
bio04
bio05
bio06
bio12
BDW
CEC
CLY
NTO
SOC

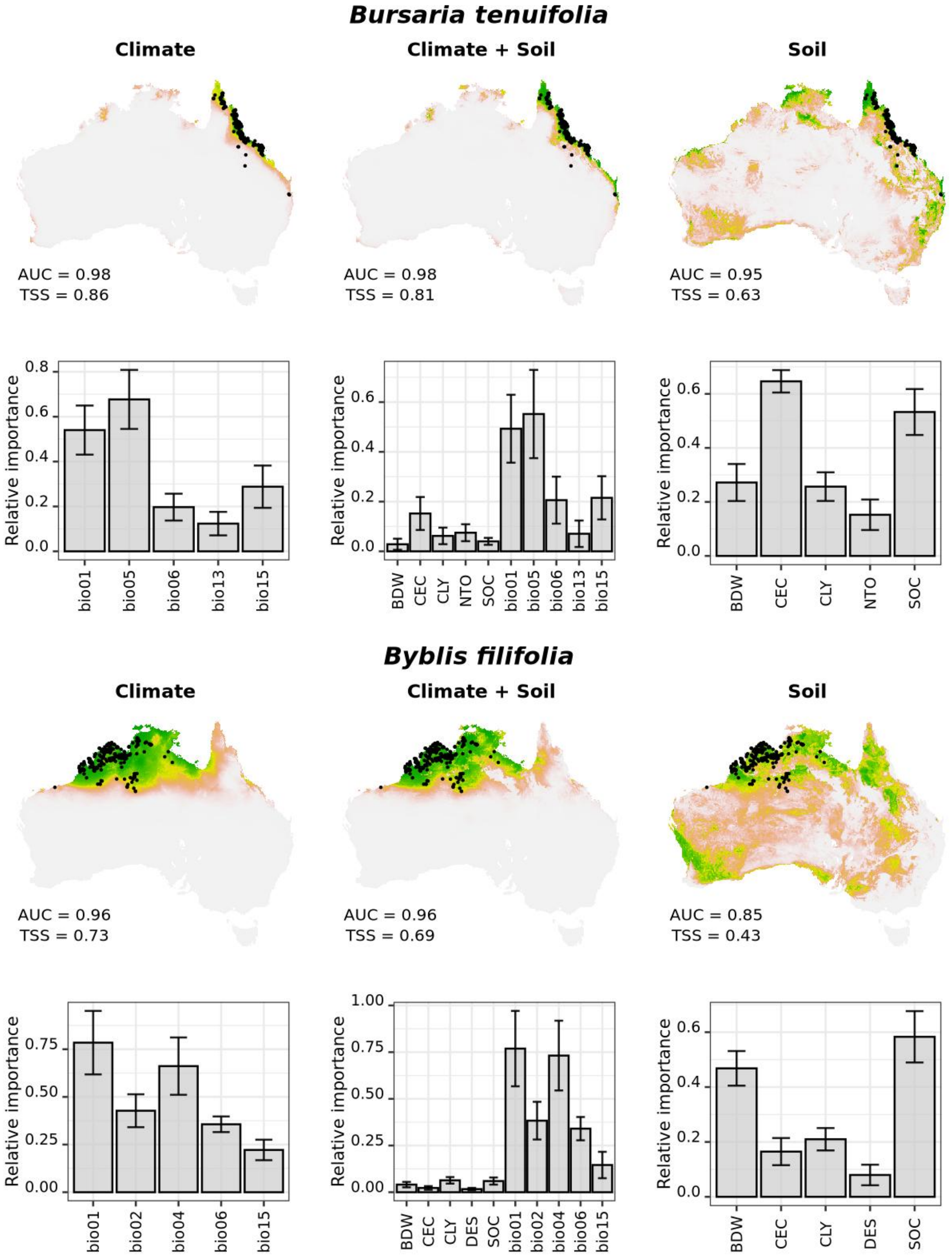

Bursaria tenuifolia
Climate
Climate + Soil
Soil
AUC = 0.98
TSS = 0.86
AUC = 0.98
TSS = 0.81
AUC = 0.95
TSS = 0.63
Relative importance
bio01
bio05
bio06
bio13
bio15
BDW
CEC
CLY
NTO
SOC
Byblis filifolia
Climate
Climate + Soil
Soil
AUC = 0.96
TSS = 0.73
AUC = 0.96
TSS = 0.69
AUC = 0.85
TSS = 0.43
Relative importance
bio01
bio02
bio04
bio06
bio15
BDW
CEC
CLY
DES
SOC

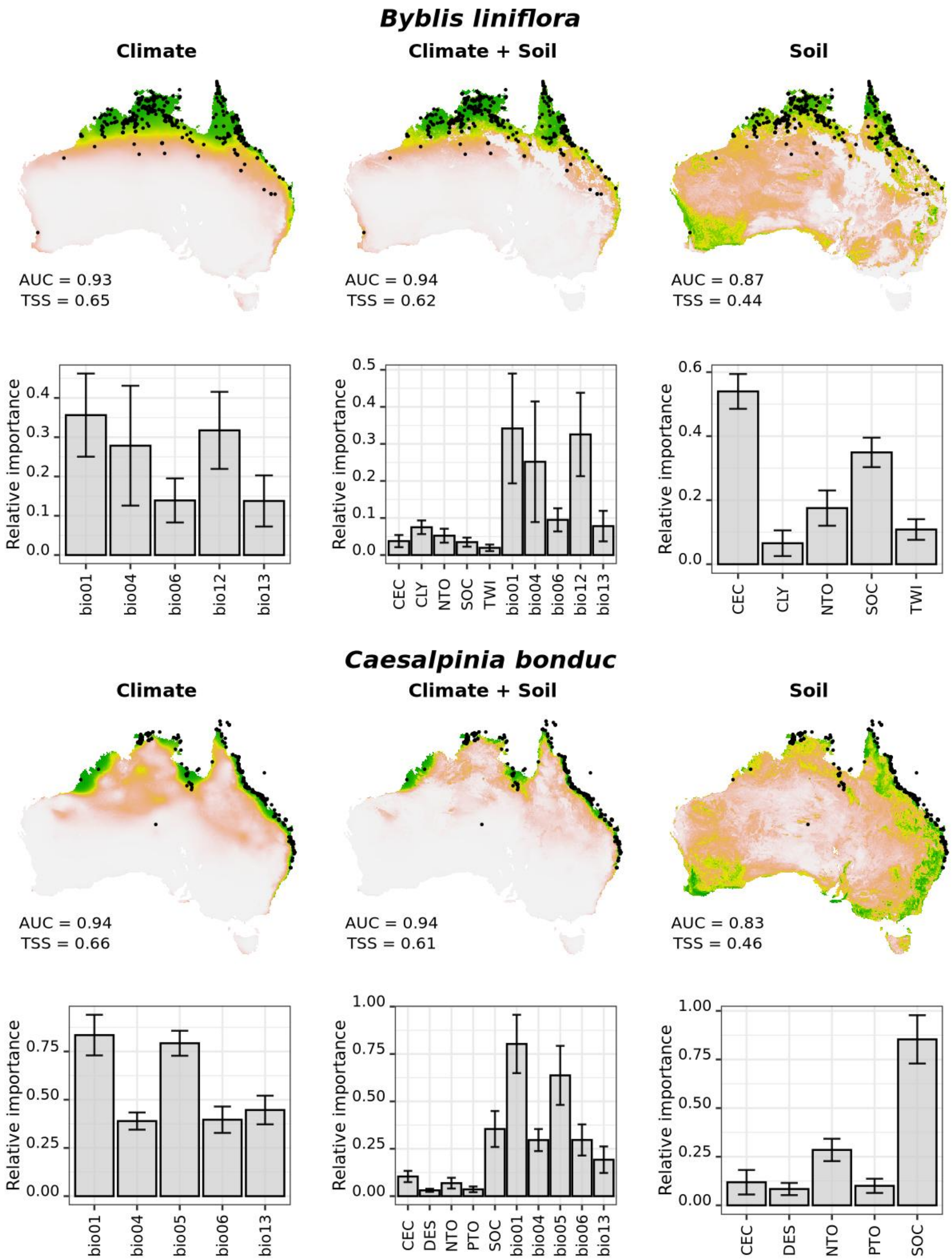

Byblis liniflora
Climate
Climate + Soil
Soil
AUC = 0.93
TSS = 0.65
AUC = 0.94
TSS = 0.62
AUC = 0.87
TSS = 0.44
Relative importance
bio01
bio04
bio06
bio12
bio13
CEC
CLY
NTO
SOC
TWI
Caesalpinia bonduc
Climate
Climate + Soil
Soil
AUC = 0.94
TSS = 0.66
AUC = 0.94
TSS = 0.61
AUC = 0.83
TSS = 0.46
Relative importance
bio01
bio04
bio05
bio06
bio13
CEC
DES
NTO
PTO
SOC

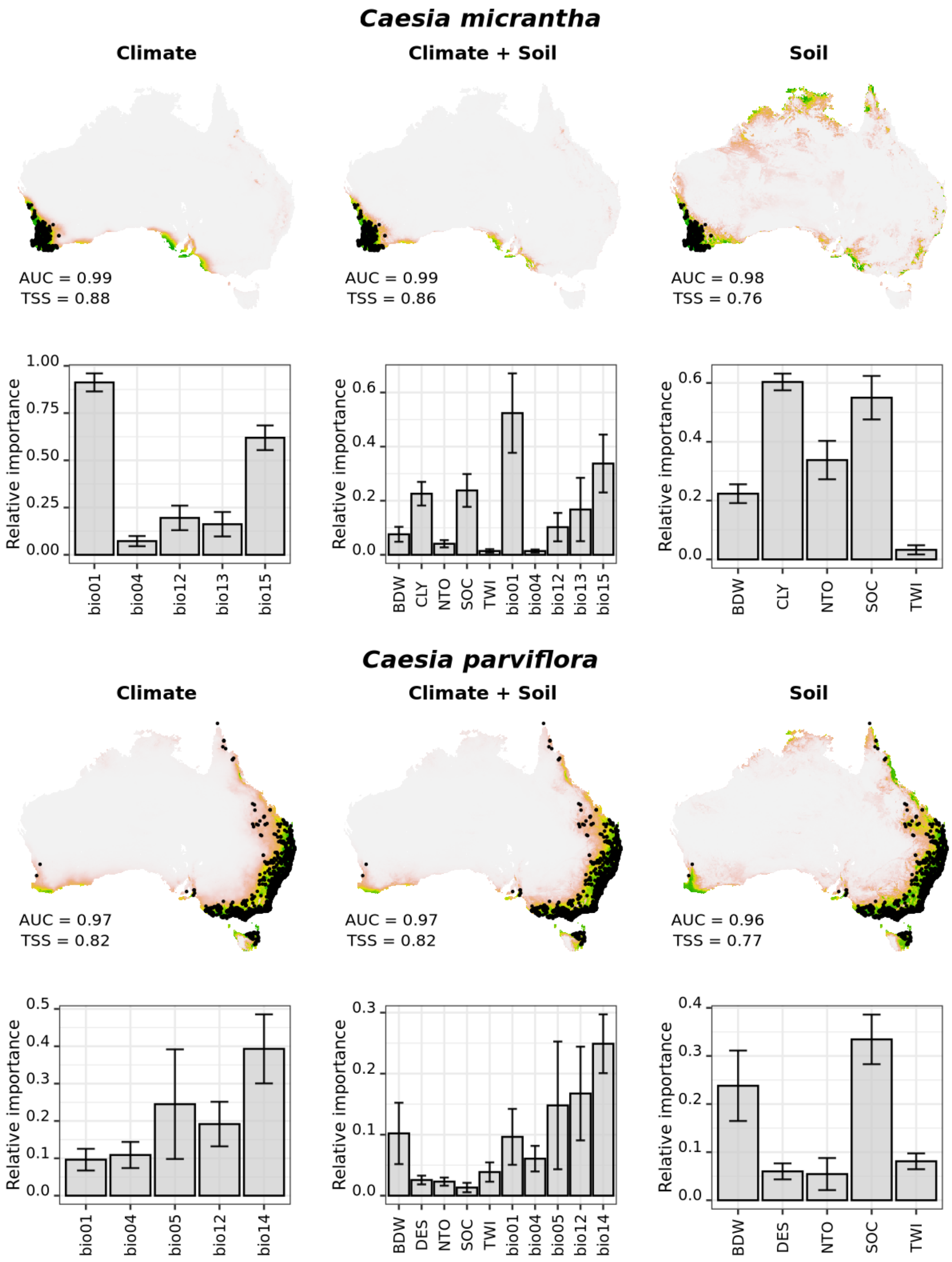

Caesia micrantha
Climate
Climate + Soil
Soil
AUC = 0.99
TSS = 0.88
AUC = 0.99
TSS = 0.86
AUC = 0.98
TSS = 0.76
Relative importance
bio01
bio04
bio12
bio13
bio15
BDW
CLY
NTO
SOC
TWI
Caesia parviflora
Climate
Climate + Soil
Soil
AUC = 0.97
TSS = 0.82
AUC = 0.97
TSS = 0.82
AUC = 0.96
TSS = 0.77
Relative importance
bio01
bio04
bio05
bio12
bio14
BDW
DES
NTO
SOC
TWI

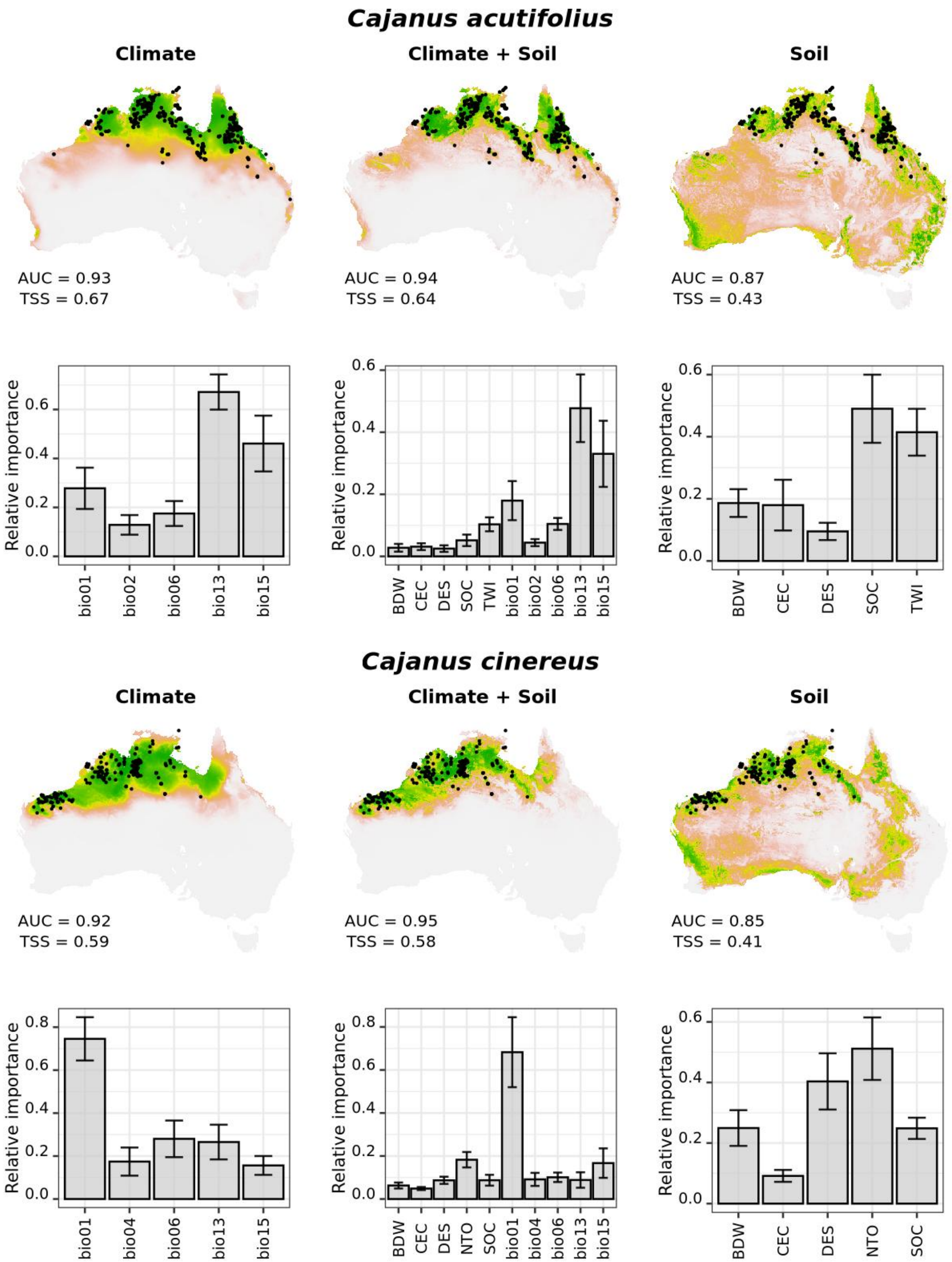

Cajanus acutifolius
Climate
Climate + Soil
Soil
AUC = 0.93
TSS = 0.67
AUC = 0.94
TSS = 0.64
AUC = 0.87
TSS = 0.43
Relative importance
bio01
bio02
bio06
bio13
bio15
BDW
CEC
DES
SOC
TWI
Cajanus cinereus
Climate
Climate + Soil
Soil
AUC = 0.92
TSS = 0.59
AUC = 0.95
TSS = 0.58
AUC = 0.85
TSS = 0.41
Relative importance
bio01
bio04
bio06
bio13
bio15
BDW
CEC
DES
NTO
SOC

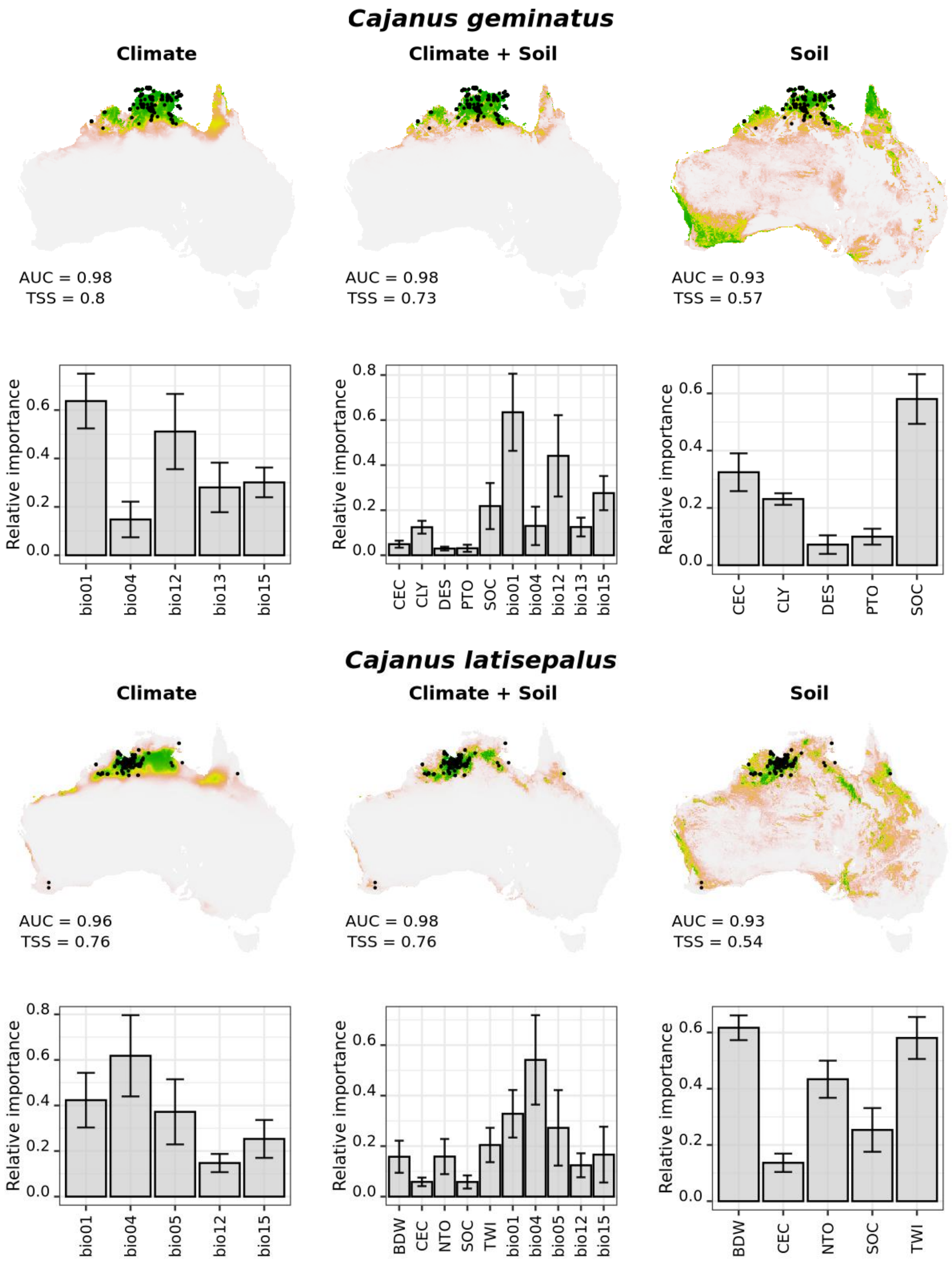

Cajanus geminatus
Climate
Climate + Soil
Soil
AUC = 0.98
TSS = 0.8
AUC = 0.98
TSS = 0.73
AUC = 0.93
TSS = 0.57
Relative importance
bio01
bio04
bio12
bio13
bio15
CEC
CLY
DES
PTO
SOC
Cajanus latisepalus
AUC = 0.96
TSS = 0.76
AUC = 0.98
TSS = 0.76
AUC = 0.93
TSS = 0.54
bio05
BDW
NTO
TWI

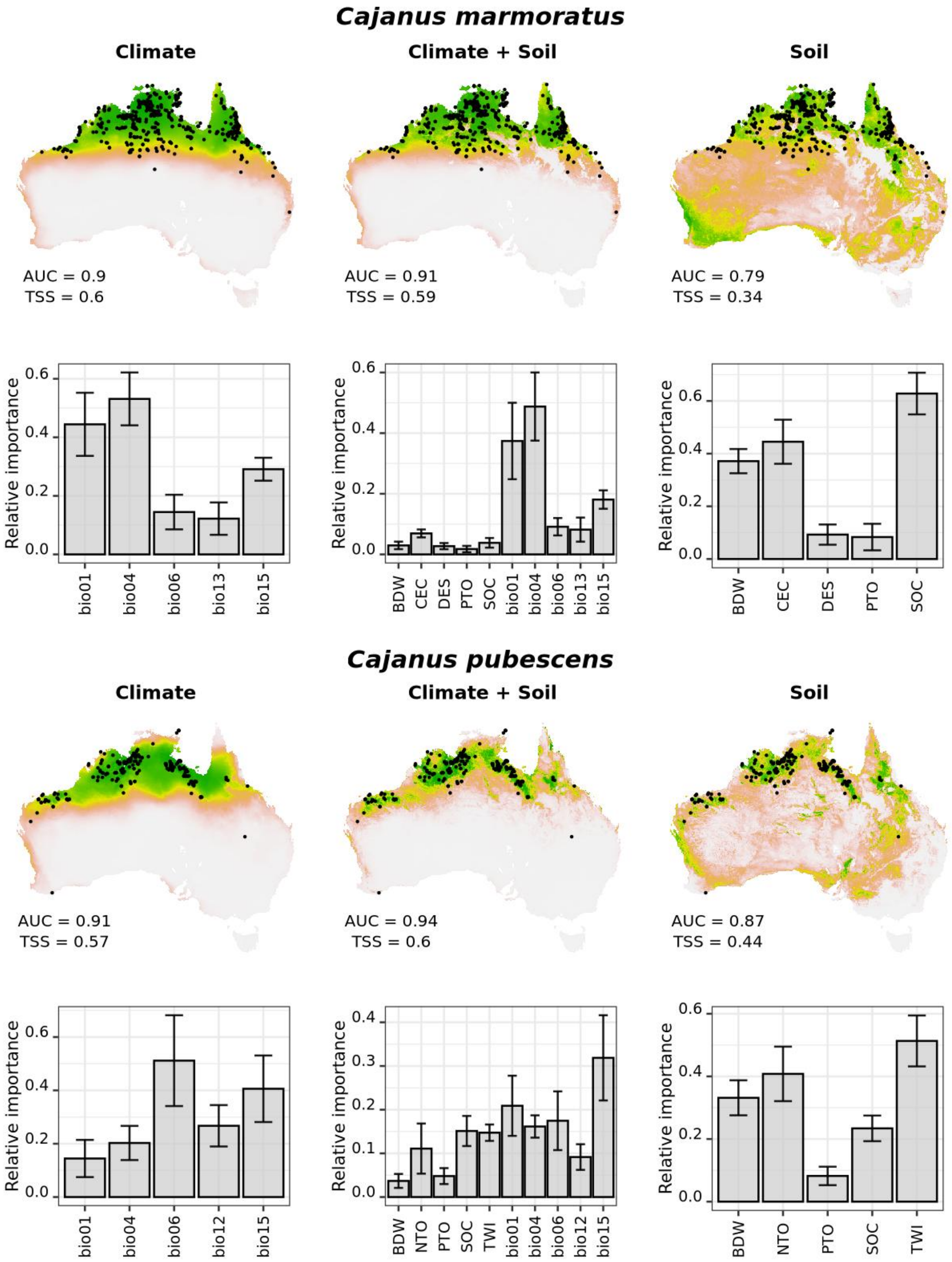
Cajanus marmoratus
Climate
Climate + Soil
Soil
AUC = 0.9
TSS = 0.6
AUC = 0.91
TSS = 0.59
AUC = 0.79
TSS = 0.34
Relative importance
bio01 bio04 bio06 bio13 bio15
BDW CEC DES PTO SOC bio01 bio04 bio06 bio13 bio15
BDW CEC DES PTO SOC
Cajanus pubescens
Climate
Climate + Soil
Soil
AUC = 0.91
TSS = 0.57
AUC = 0.94
TSS = 0.6
AUC = 0.87
TSS = 0.44
Relative importance
bio01 bio04 bio06 bio12 bio15
BDW NTO PTO SOC TWI bio01 bio04 bio06 bio12 bio15
BDW NTO PTO SOC TWI

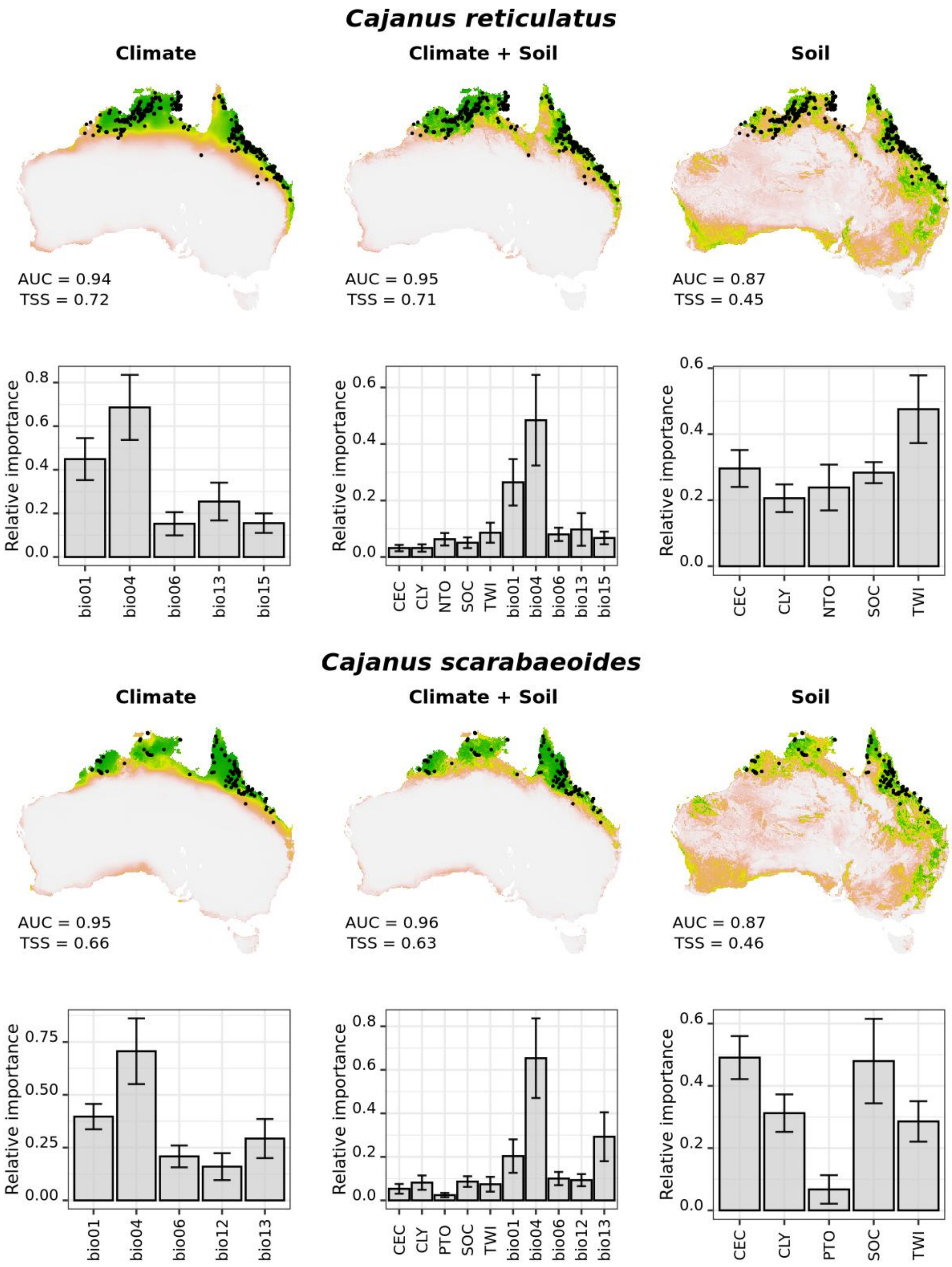
Cajanus reticulatus
Climate
Climate + Soil
Soil
AUC = 0.94
TSS = 0.72
AUC = 0.95
TSS = 0.71
AUC = 0.87
TSS = 0.45
Relative importance
bio01
bio04
bio06
bio13
bio15
CEC
CLY
NTO
SOC
TWI
Cajanus scarabaeoides
AUC = 0.95
TSS = 0.66
AUC = 0.96
TSS = 0.63
AUC = 0.87
TSS = 0.46
bio12
PTO

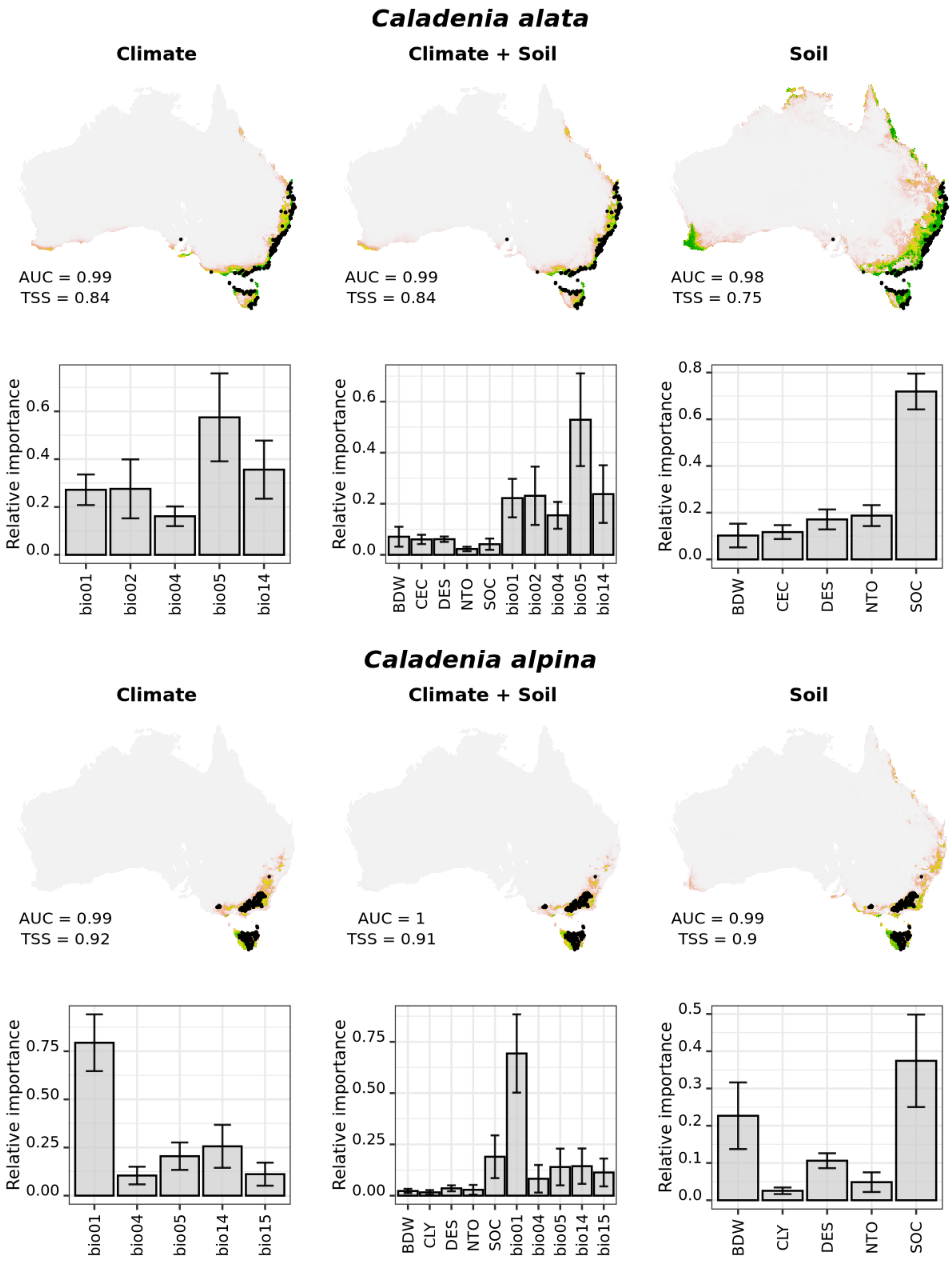

Caladenia alata
Climate
Climate + Soil
Soil
AUC = 0.99
TSS = 0.84
AUC = 0.99
TSS = 0.84
AUC = 0.98
TSS = 0.75
Relative importance
bio01 bio02 bio04 bio05 bio14
BDW CEC DES NTO SOC bio01 bio02 bio04 bio05 bio14
BDW CEC DES NTO SOC
Caladenia alpina
Climate
Climate + Soil
Soil
AUC = 0.99
TSS = 0.92
AUC = 1
TSS = 0.91
AUC = 0.99
TSS = 0.9
Relative importance
bio01 bio04 bio05 bio14 bio15
BDW CLY DES NTO SOC bio01 bio04 bio05 bio14 bio15
BDW CLY DES NTO SOC

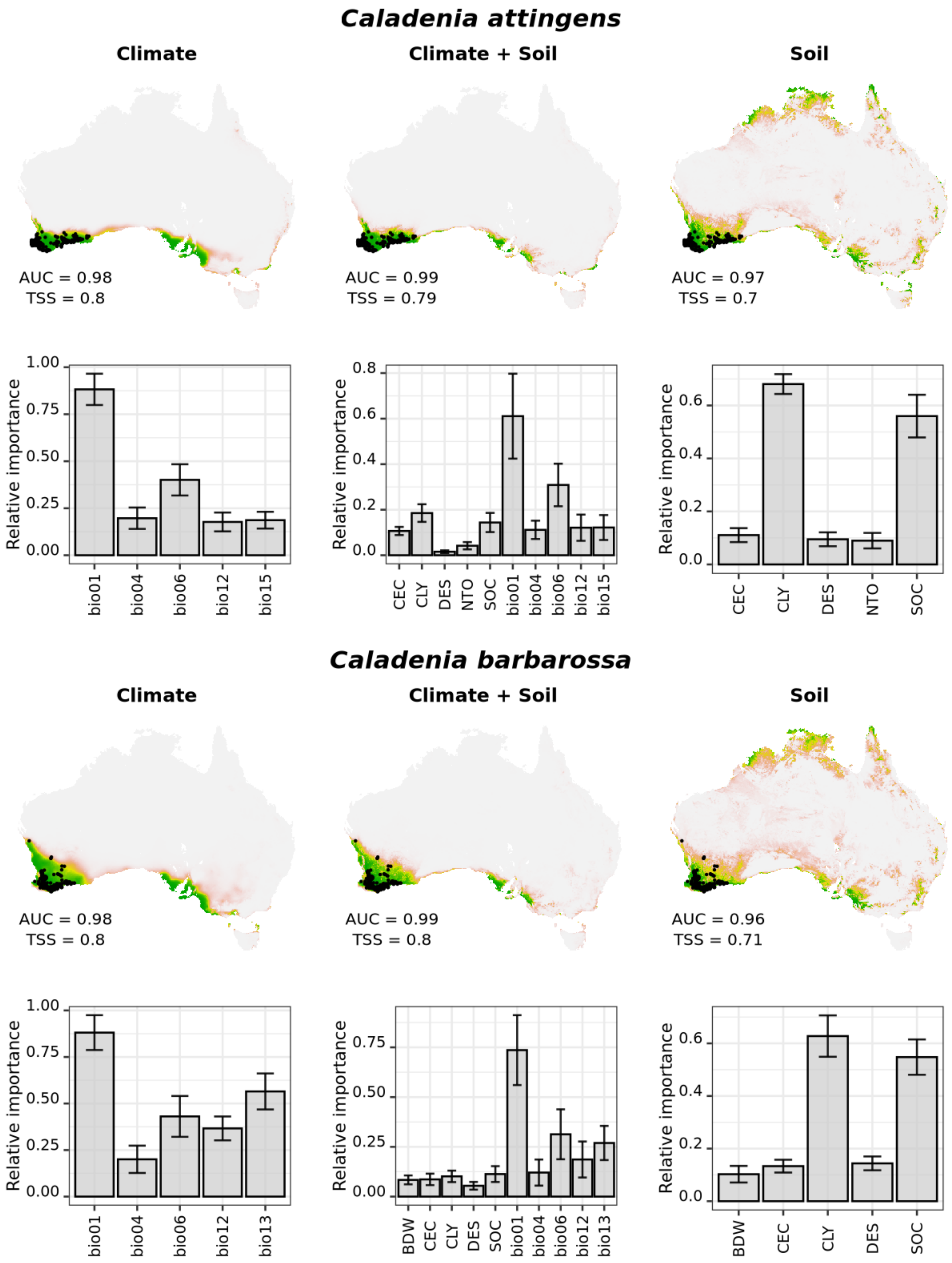
Caladenia attingens
Climate
Climate + Soil
Soil
AUC = 0.98
TSS = 0.8
AUC = 0.99
TSS = 0.79
AUC = 0.97
TSS = 0.7
Relative importance
1.00
0.75
0.50
0.25
0.00
bio01
bio04
bio06
bio12
bio15
0.8
0.6
0.4
0.2
0.0
CEC
CLY
DES
NTO
SOC
bio01
bio04
bio06
bio12
bio15
CEC
CLY
DES
NTO
SOC
Caladenia barbarossa
Climate
Climate + Soil
Soil
AUC = 0.98
TSS = 0.8
AUC = 0.99
TSS = 0.8
AUC = 0.96
TSS = 0.71
bio01
bio04
bio06
bio12
bio13
BDW
CEC
CLY
DES
SOC
bio01
bio04
bio06
bio12
bio13
BDW
CEC
CLY
DES
SOC

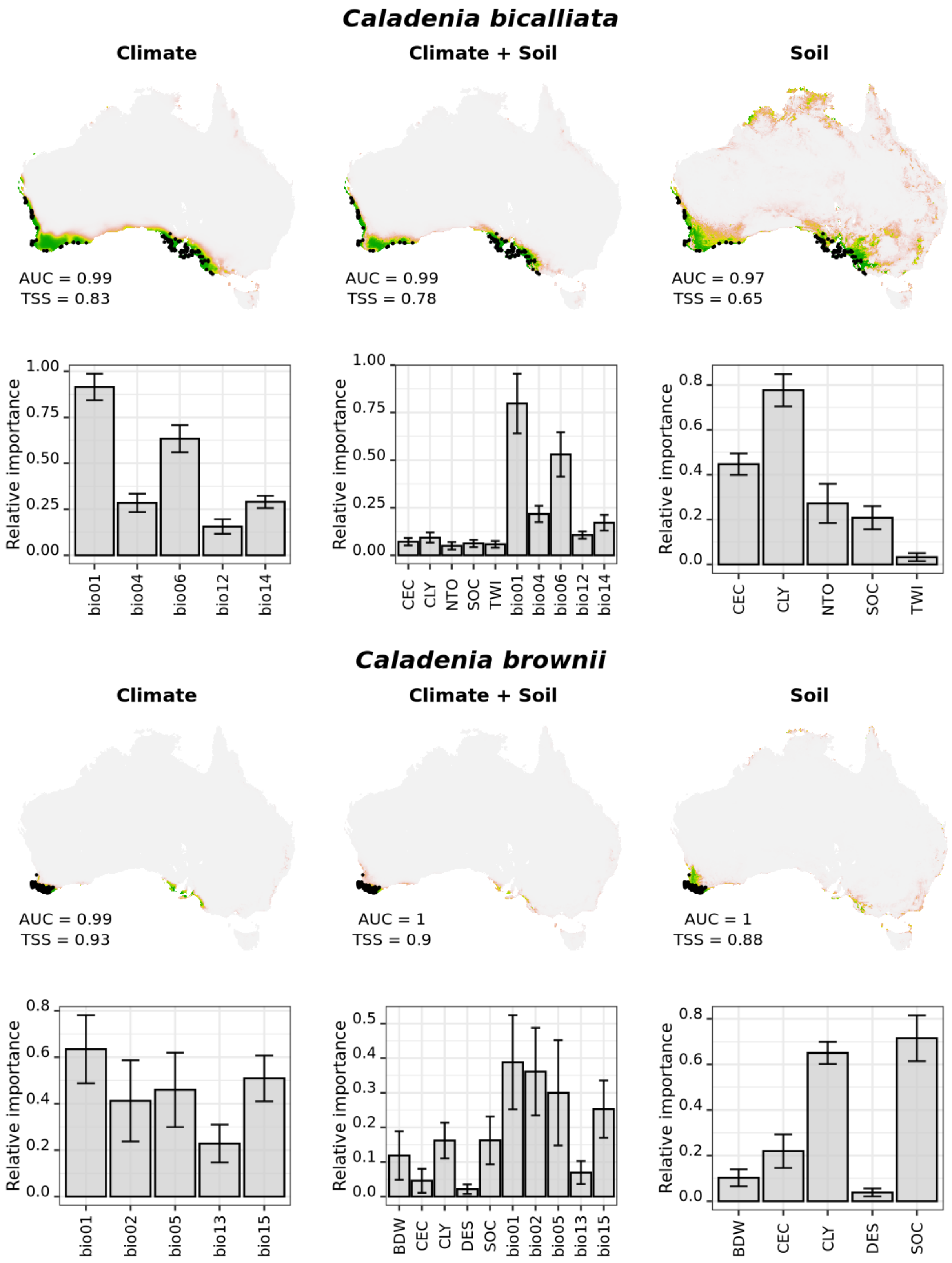
Caladenia bicalliata
Climate
Climate + Soil
Soil
AUC = 0.99
TSS = 0.83
AUC = 0.99
TSS = 0.78
AUC = 0.97
TSS = 0.65
Relative importance
1.00
0.75
0.50
0.25
0.00
bio01
bio04
bio06
bio12
bio14
CEC
CLY
NTO
SOC
TWI
0.8
0.6
0.4
0.2
0.0
Caladenia brownii
Climate
Climate + Soil
Soil
AUC = 0.99
TSS = 0.93
AUC = 1
TSS = 0.9
AUC = 1
TSS = 0.88
Relative importance
0.5
0.4
0.3
0.2
0.1
bio01
bio02
bio05
bio13
bio15
BDW
CEC
CLY
DES
SOC

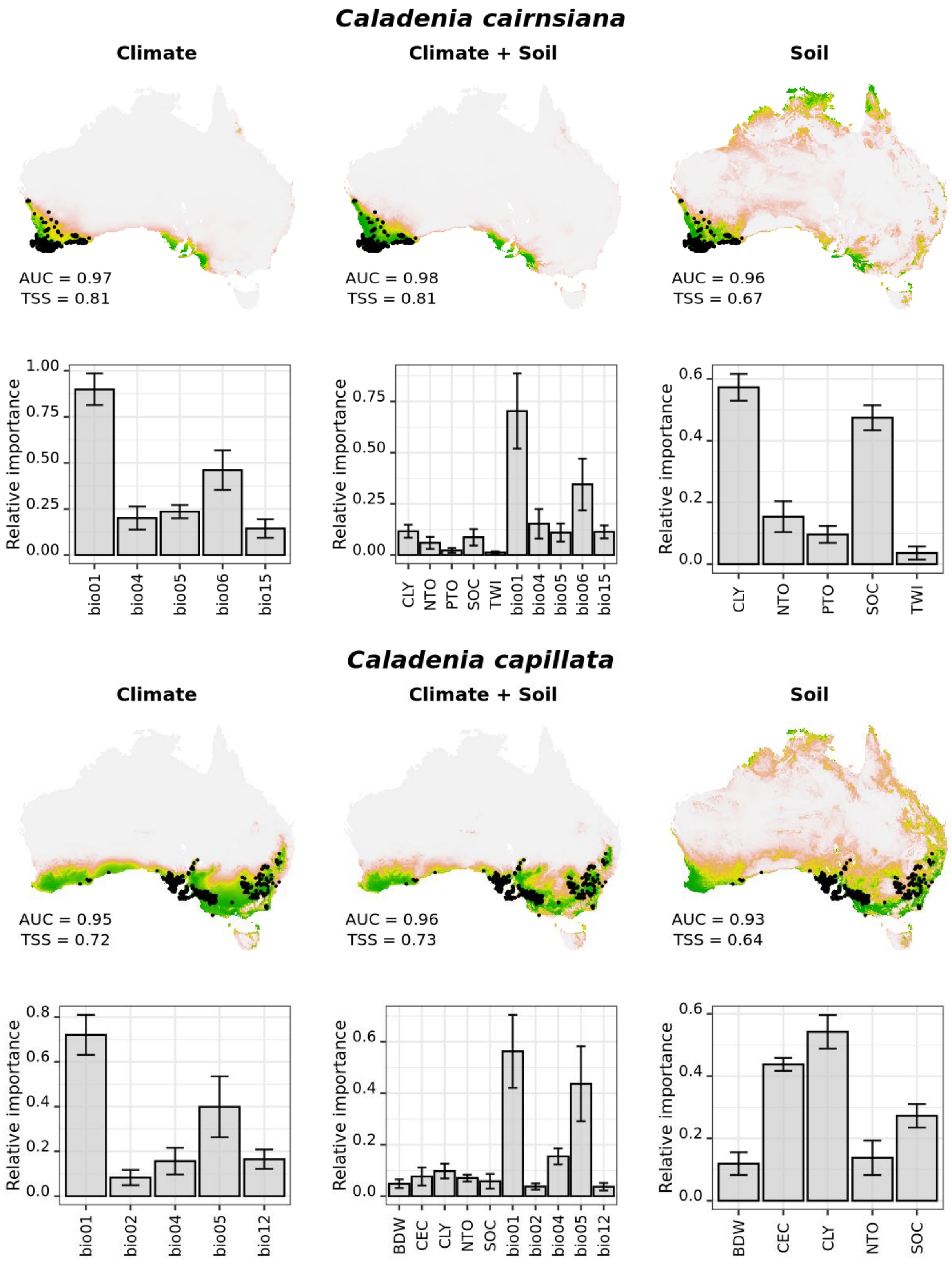

Caladenia cairnsiana
Climate
Climate + Soil
Soil
AUC = 0.97
TSS = 0.81
AUC = 0.98
TSS = 0.81
AUC = 0.96
TSS = 0.67
Relative importance
1.00
0.75
0.50
0.25
0.00
bio01
bio04
bio05
bio06
bio15
CLY
NTO
PTO
SOC
TWI
0.6
0.4
0.2
0.0
Caladenia capillata
AUC = 0.95
TSS = 0.72
AUC = 0.96
TSS = 0.73
AUC = 0.93
TSS = 0.64
0.8
bio02
bio12
BDW
CEC

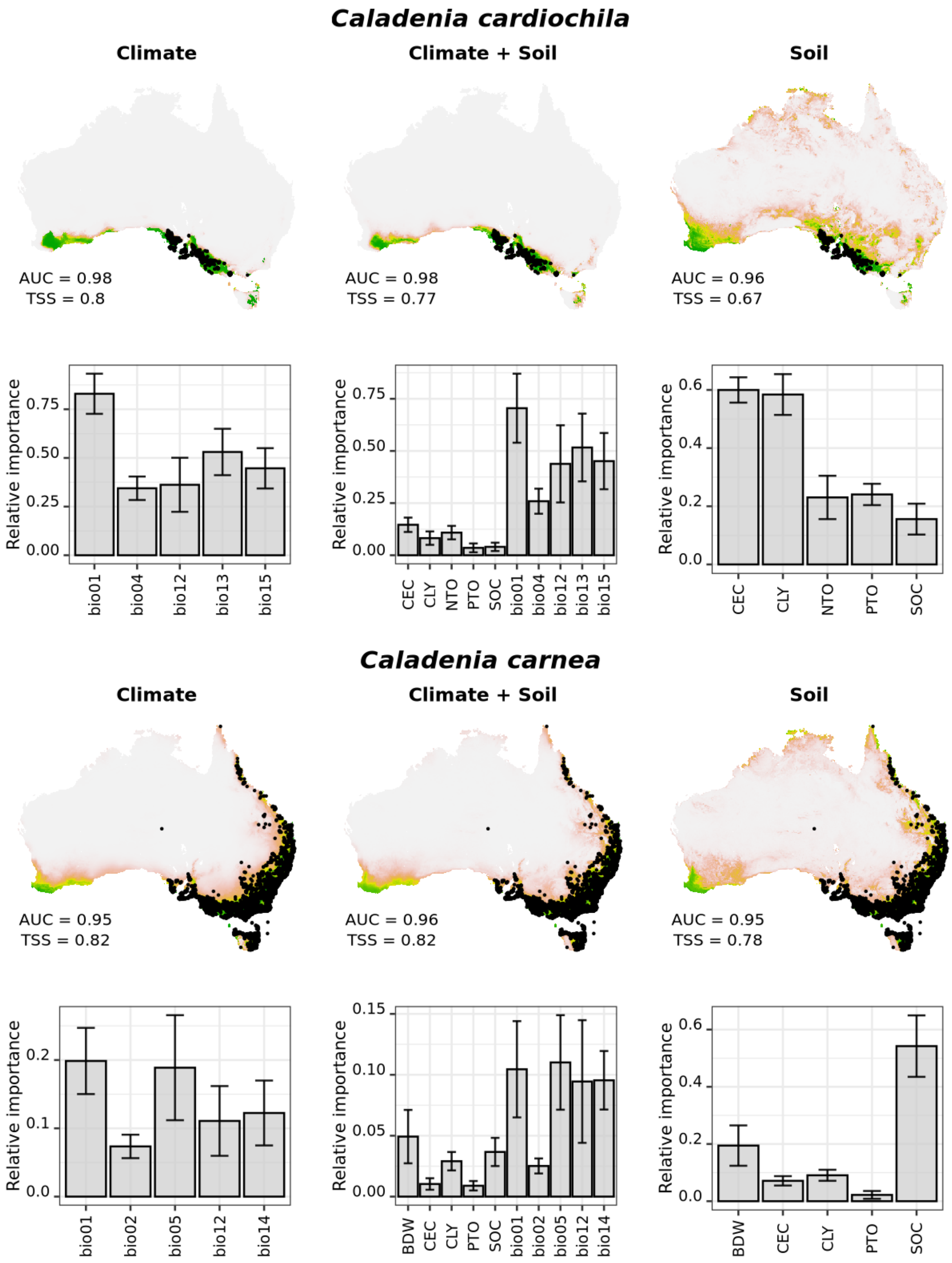
Caladenia cardiochila
Climate
Climate + Soil
Soil
AUC = 0.98
TSS = 0.8
AUC = 0.98
TSS = 0.77
AUC = 0.96
TSS = 0.67
Relative importance
0.00
0.25
0.50
0.75
bio01
bio04
bio12
bio13
bio15
CEC
CLY
NTO
PTO
SOC
0.0
0.2
0.4
0.6
Caladenia carnea
Climate
Climate + Soil
Soil
AUC = 0.95
TSS = 0.82
AUC = 0.96
TSS = 0.82
AUC = 0.95
TSS = 0.78
0.0
0.1
0.2
bio01
bio02
bio05
bio12
bio14
0.00
0.05
0.10
0.15
BDW
CEC
CLY
PTO
SOC

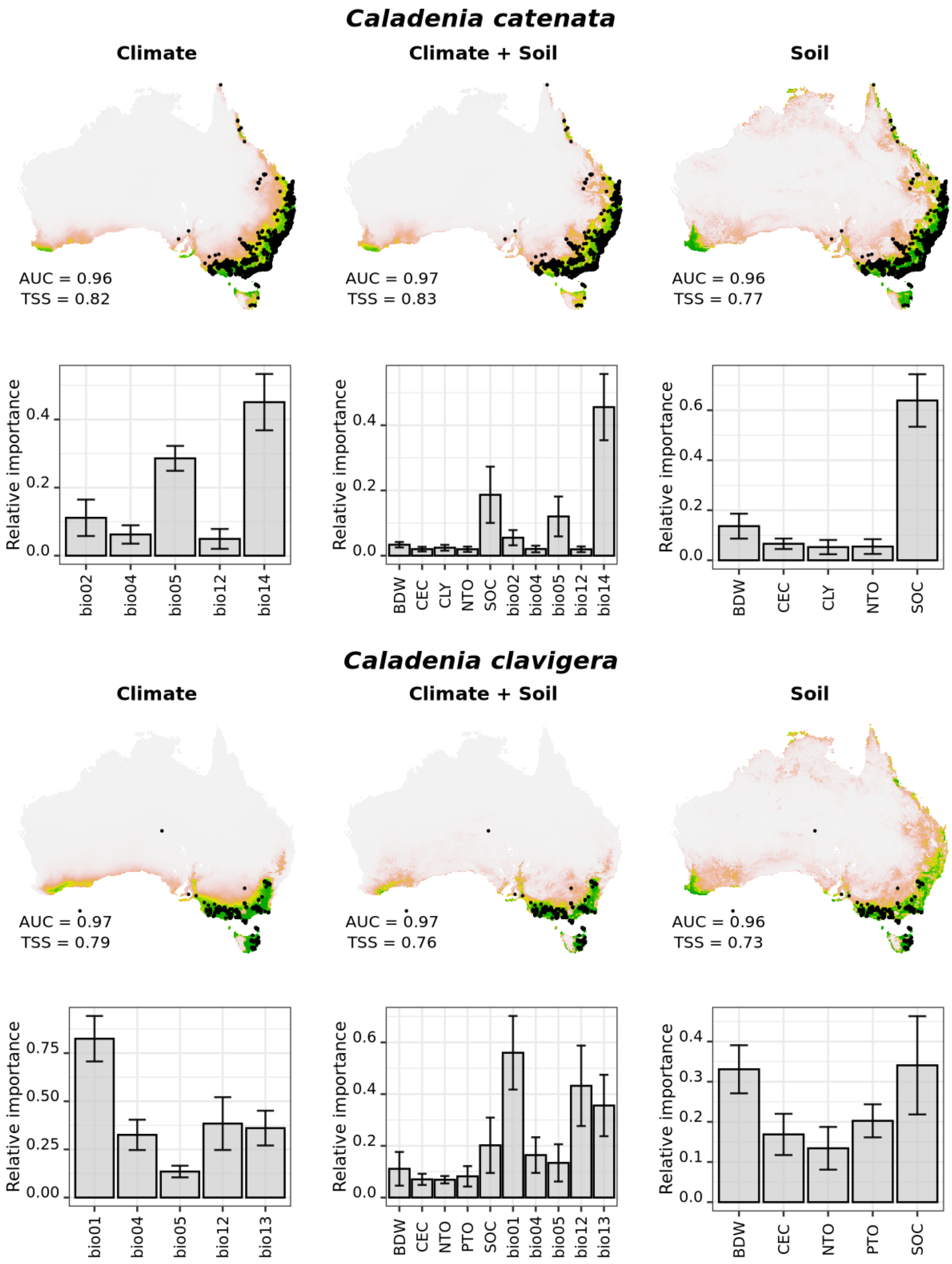

Caladenia catenata
Climate
Climate + Soil
Soil
AUC = 0.96
TSS = 0.82
AUC = 0.97
TSS = 0.83
AUC = 0.96
TSS = 0.77
Relative importance
bio02
bio04
bio05
bio12
bio14
BDW
CEC
CLY
NTO
SOC
Caladenia clavigera
Climate
Climate + Soil
Soil
AUC = 0.97
TSS = 0.79
AUC = 0.97
TSS = 0.76
AUC = 0.96
TSS = 0.73
Relative importance
bio01
bio04
bio05
bio12
bio13
BDW
CEC
NTO
PTO
SOC

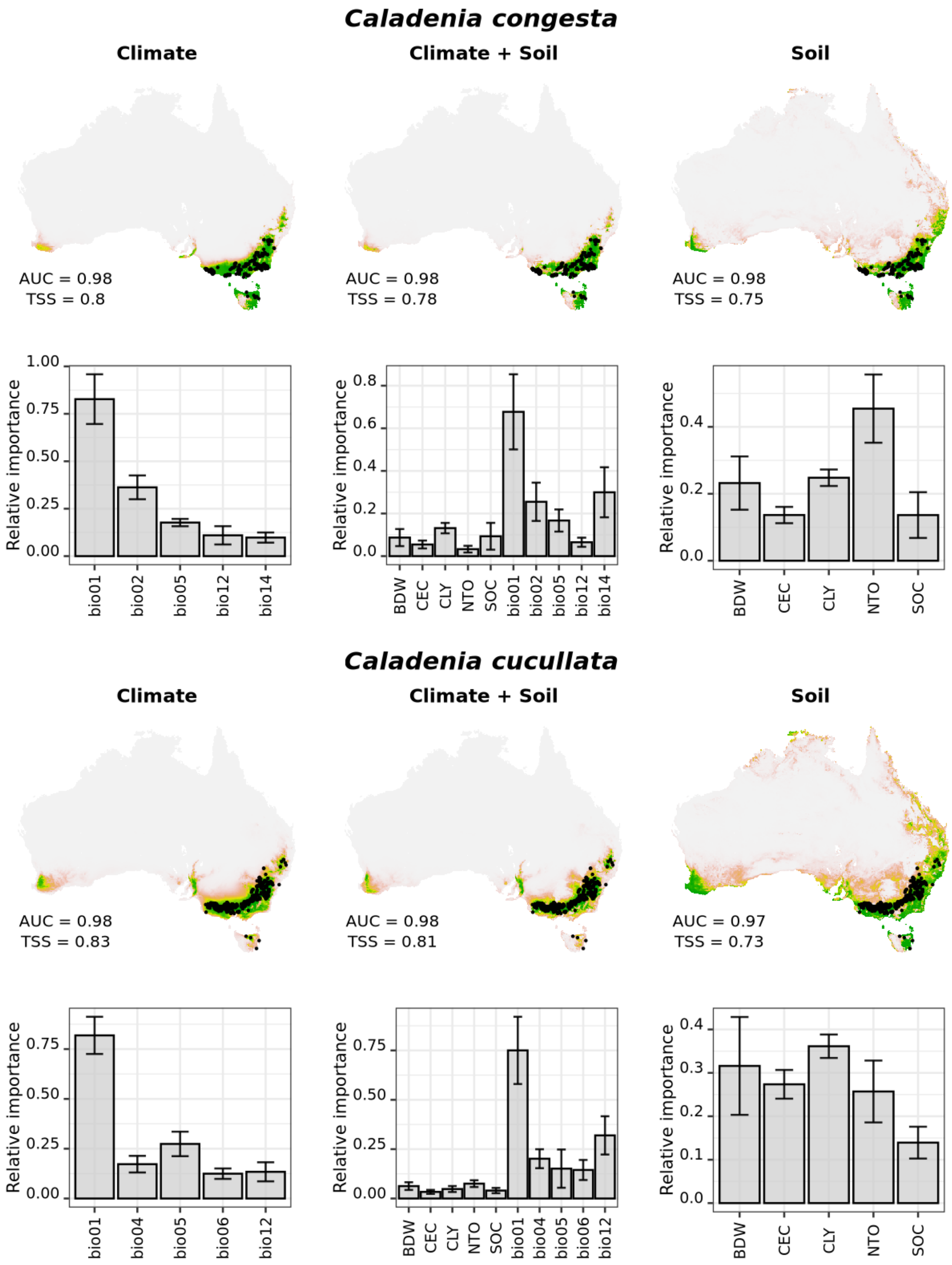
Caladenia congesta
Climate
Climate + Soil
Soil
AUC = 0.98
TSS = 0.8
AUC = 0.98
TSS = 0.78
AUC = 0.98
TSS = 0.75
Relative importance
bio01
bio02
bio05
bio12
bio14
BDW
CEC
CLY
NTO
SOC
Caladenia cucullata
Climate
Climate + Soil
Soil
AUC = 0.98
TSS = 0.83
AUC = 0.98
TSS = 0.81
AUC = 0.97
TSS = 0.73
Relative importance
bio01
bio04
bio05
bio06
bio12
BDW
CEC
CLY
NTO
SOC

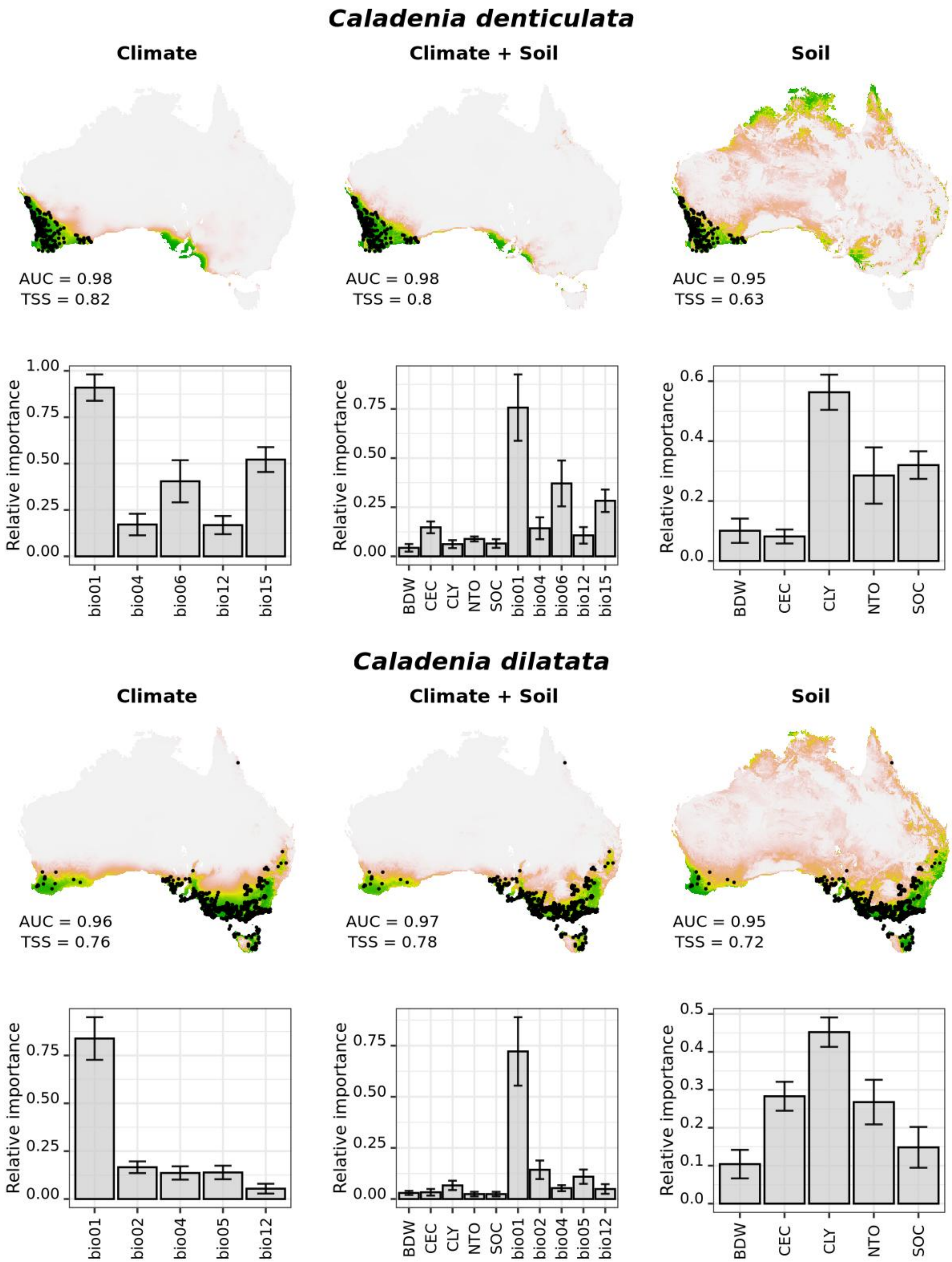
Caladenia denticulata
Climate
Climate + Soil
Soil
AUC = 0.98
TSS = 0.82
AUC = 0.98
TSS = 0.8
AUC = 0.95
TSS = 0.63
Relative importance
bio01
bio04
bio06
bio12
bio15
BDW
CEC
CLY
NTO
SOC
Caladenia dilatata
Climate
Climate + Soil
Soil
AUC = 0.96
TSS = 0.76
AUC = 0.97
TSS = 0.78
AUC = 0.95
TSS = 0.72
Relative importance
bio01
bio02
bio04
bio05
bio12
BDW
CEC
CLY
NTO
SOC

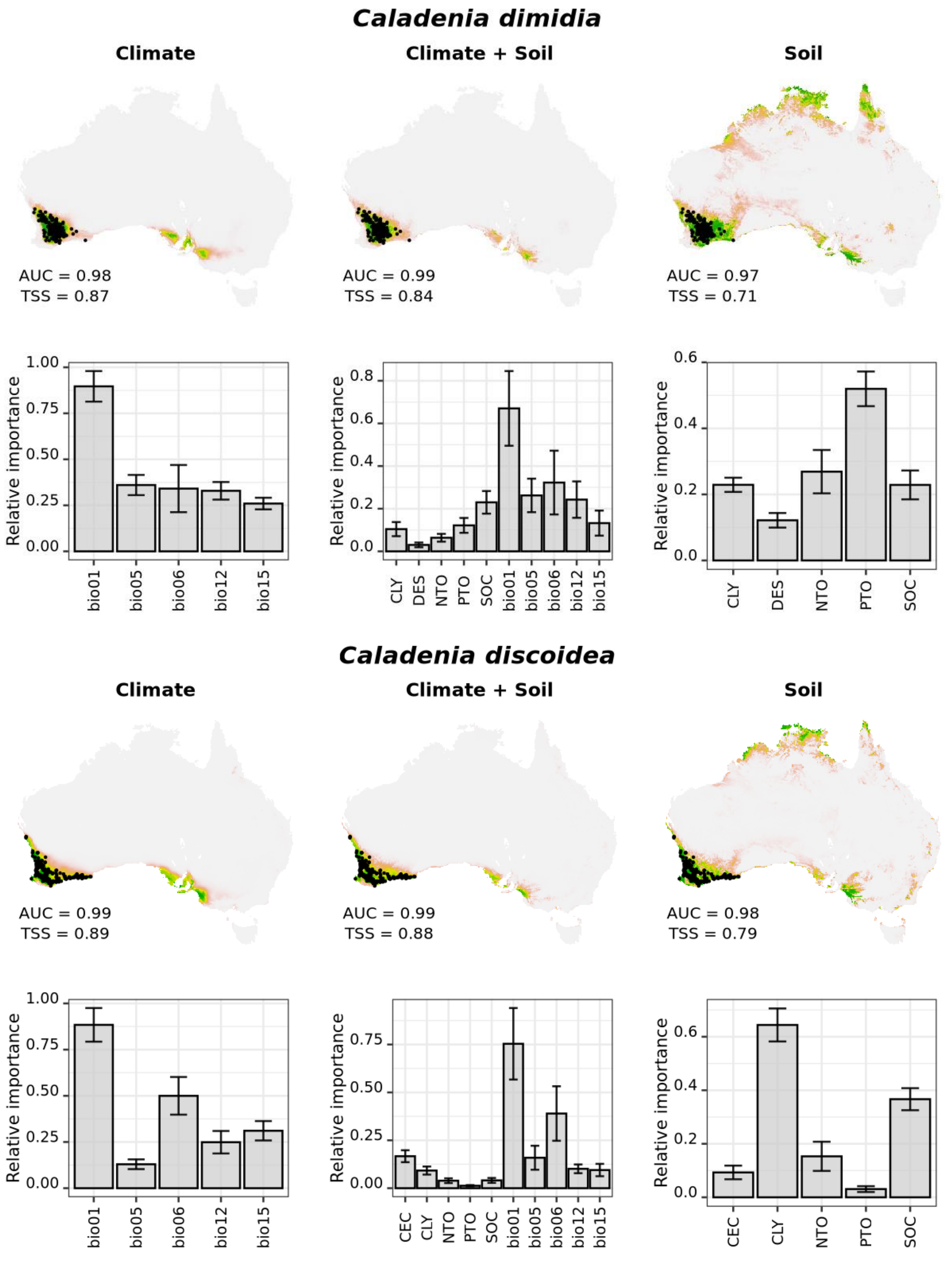
Caladenia dimidia
Climate
Climate + Soil
Soil
AUC = 0.98
TSS = 0.87
AUC = 0.99
TSS = 0.84
AUC = 0.97
TSS = 0.71
Relative importance
bio01
bio05
bio06
bio12
bio15
CLY
DES
NTO
PTO
SOC
Caladenia discoidea
Climate
Climate + Soil
Soil
AUC = 0.99
TSS = 0.89
AUC = 0.99
TSS = 0.88
AUC = 0.98
TSS = 0.79
Relative importance
bio01
bio05
bio06
bio12
bio15
CEC
CLY
NTO
PTO
SOC

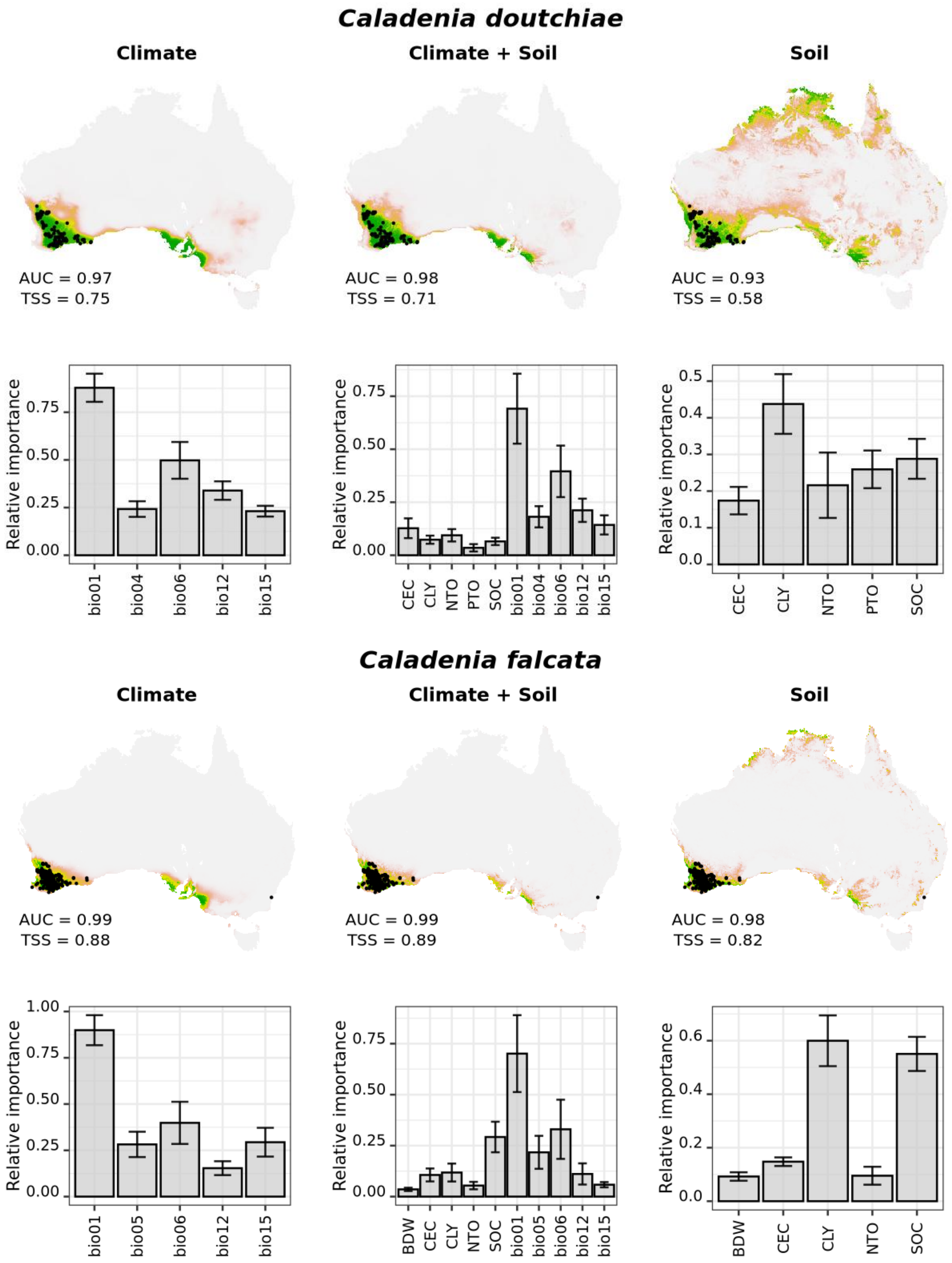
Caladenia doutchiae
Climate
Climate + Soil
Soil
AUC = 0.97
TSS = 0.75
AUC = 0.98
TSS = 0.71
AUC = 0.93
TSS = 0.58
Relative importance
bio01
bio04
bio06
bio12
bio15
CEC
CLY
NTO
PTO
SOC
Caladenia falcata
Climate
Climate + Soil
Soil
AUC = 0.99
TSS = 0.88
AUC = 0.99
TSS = 0.89
AUC = 0.98
TSS = 0.82
Relative importance
bio01
bio05
bio06
bio12
bio15
BDW
CEC
CLY
NTO
SOC

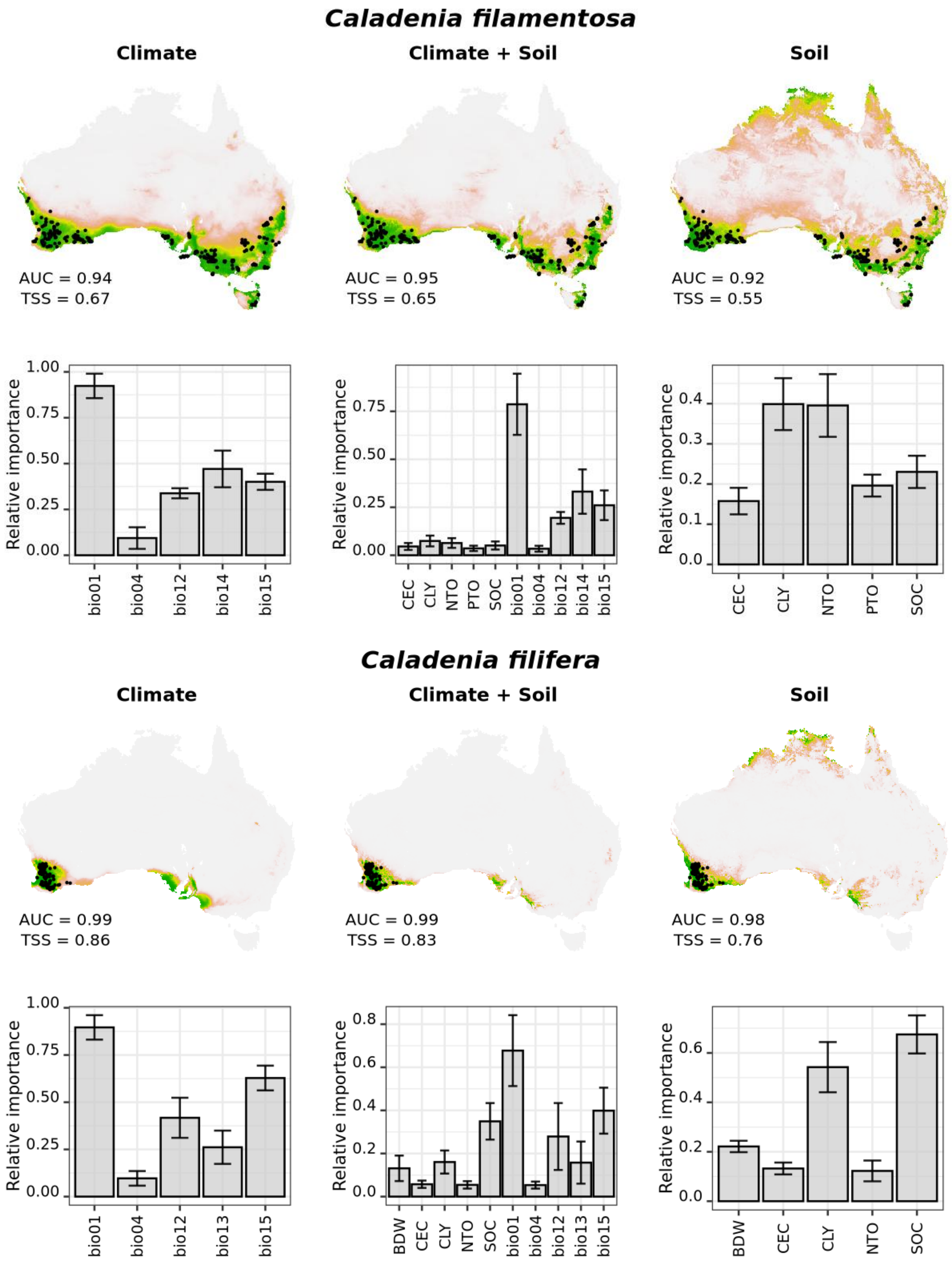

Caladenia filamentosa
Climate
Climate + Soil
Soil
AUC = 0.94
TSS = 0.67
AUC = 0.95
TSS = 0.65
AUC = 0.92
TSS = 0.55
Relative importance
bio01
bio04
bio12
bio14
bio15
CEC
CLY
NTO
PTO
SOC
Caladenia filifera
Climate
Climate + Soil
Soil
AUC = 0.99
TSS = 0.86
AUC = 0.99
TSS = 0.83
AUC = 0.98
TSS = 0.76
Relative importance
bio01
bio04
bio12
bio13
bio15
BDW
CEC
CLY
NTO
SOC

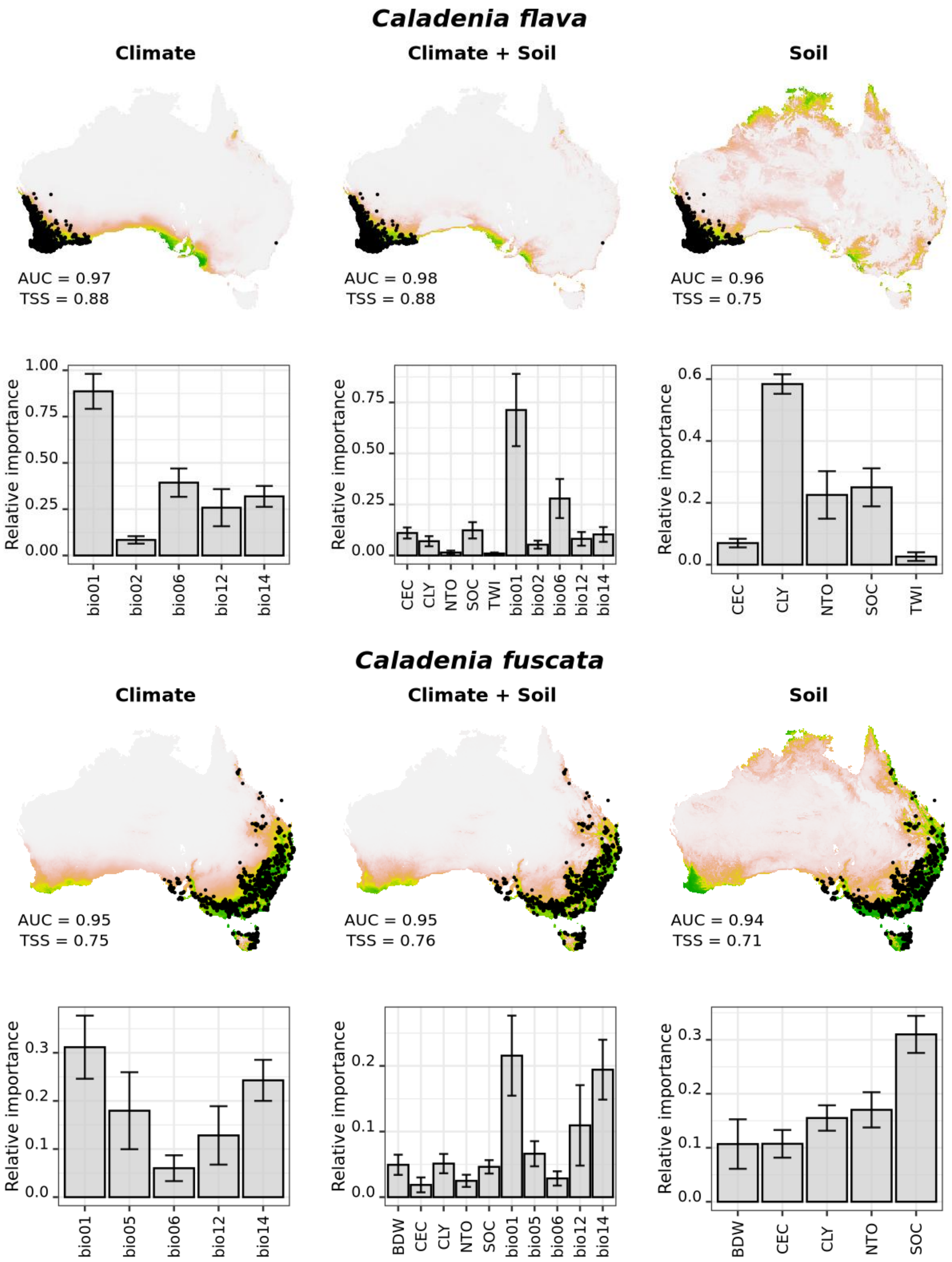
Caladenia flava
Climate
Climate + Soil
Soil
AUC = 0.97
TSS = 0.88
AUC = 0.98
TSS = 0.88
AUC = 0.96
TSS = 0.75
Relative importance
bio01
bio02
bio06
bio12
bio14
CEC
CLY
NTO
SOC
TWI
Caladenia fuscata
Climate
Climate + Soil
Soil
AUC = 0.95
TSS = 0.75
AUC = 0.95
TSS = 0.76
AUC = 0.94
TSS = 0.71
Relative importance
bio01
bio05
bio06
bio12
bio14
BDW
CEC
CLY
NTO
SOC

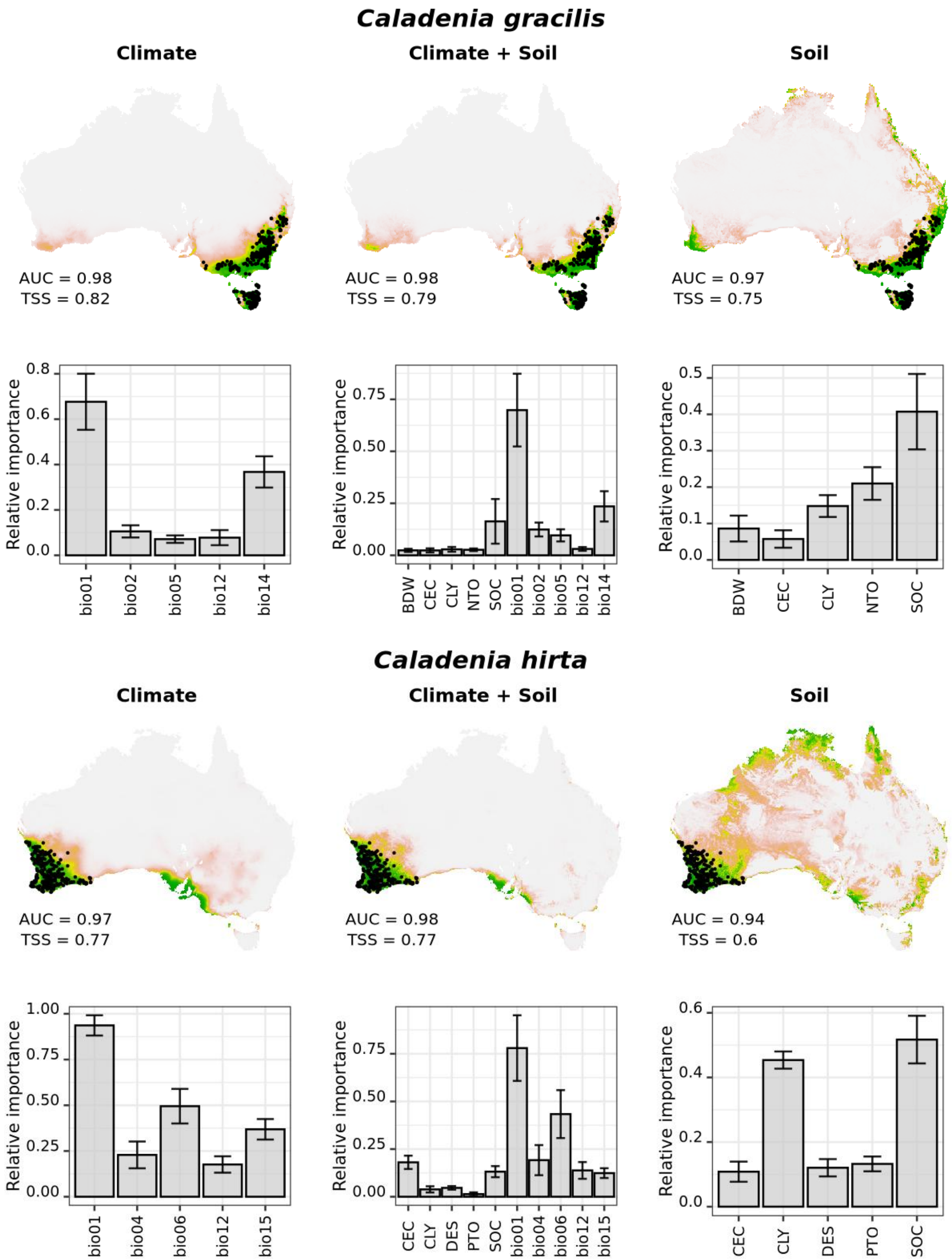
Caladenia gracilis
Climate
Climate + Soil
Soil
AUC = 0.98
TSS = 0.82
AUC = 0.98
TSS = 0.79
AUC = 0.97
TSS = 0.75
Relative importance
bio01 bio02 bio05 bio12 bio14
BDW CEC CLY NTO SOC bio01 bio02 bio05 bio12 bio14
BDW CEC CLY NTO SOC
Caladenia hirta
Climate
Climate + Soil
Soil
AUC = 0.97
TSS = 0.77
AUC = 0.98
TSS = 0.77
AUC = 0.94
TSS = 0.6
Relative importance
bio01 bio04 bio06 bio12 bio15
CEC CLY DES PTO SOC bio01 bio04 bio06 bio12 bio15
CEC CLY DES PTO SOC

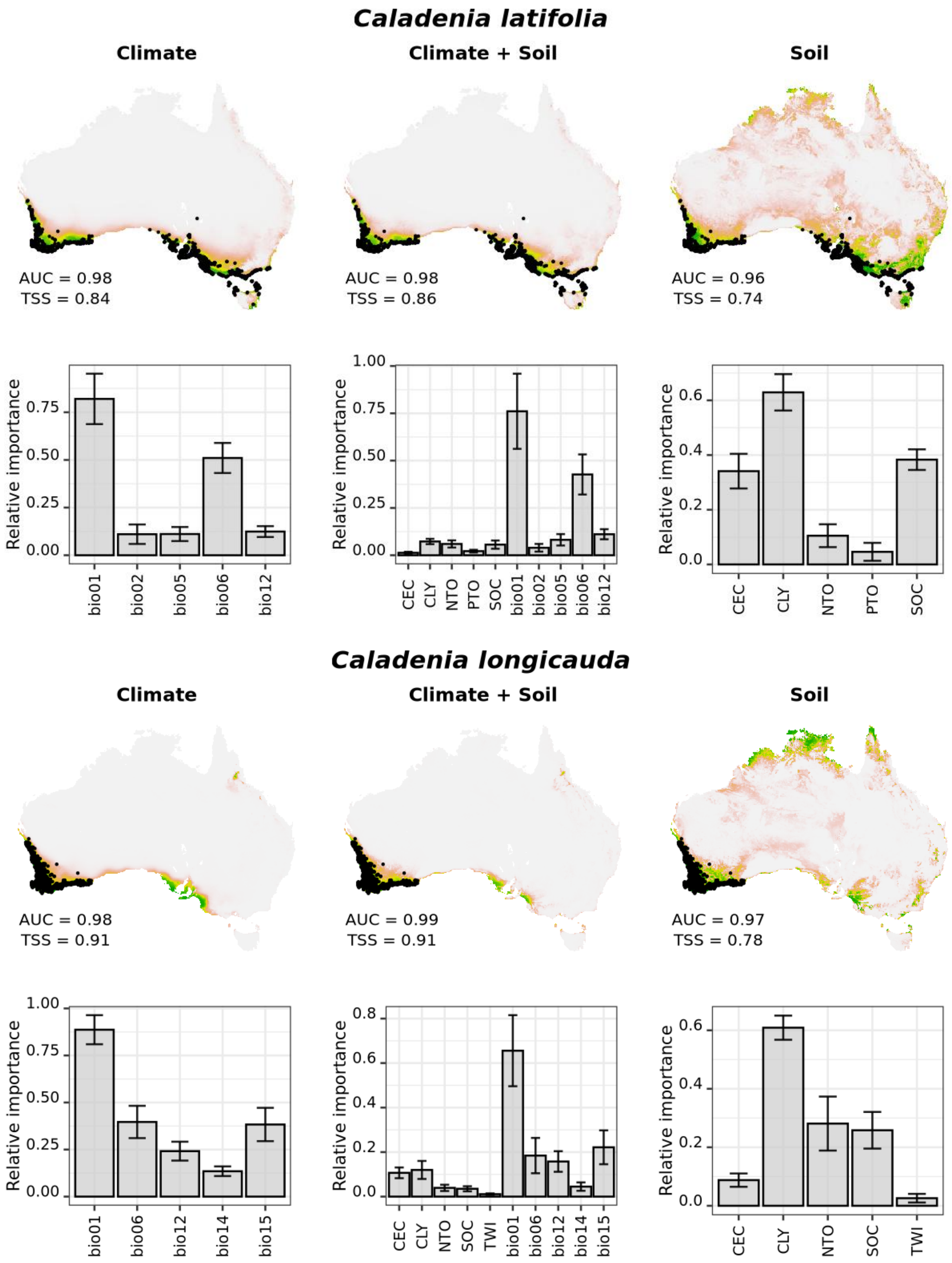
Caladenia latifolia
Climate
Climate + Soil
Soil
AUC = 0.98
TSS = 0.84
AUC = 0.98
TSS = 0.86
AUC = 0.96
TSS = 0.74
Relative importance
0.00
0.25
0.50
0.75
1.00
bio01
bio02
bio05
bio06
bio12
CEC
CLY
NTO
PTO
SOC
0.0
0.2
0.4
0.6
Caladenia longicauda
Climate
Climate + Soil
Soil
AUC = 0.98
TSS = 0.91
AUC = 0.99
TSS = 0.91
AUC = 0.97
TSS = 0.78
Relative importance
0.8
bio14
bio15
TWI

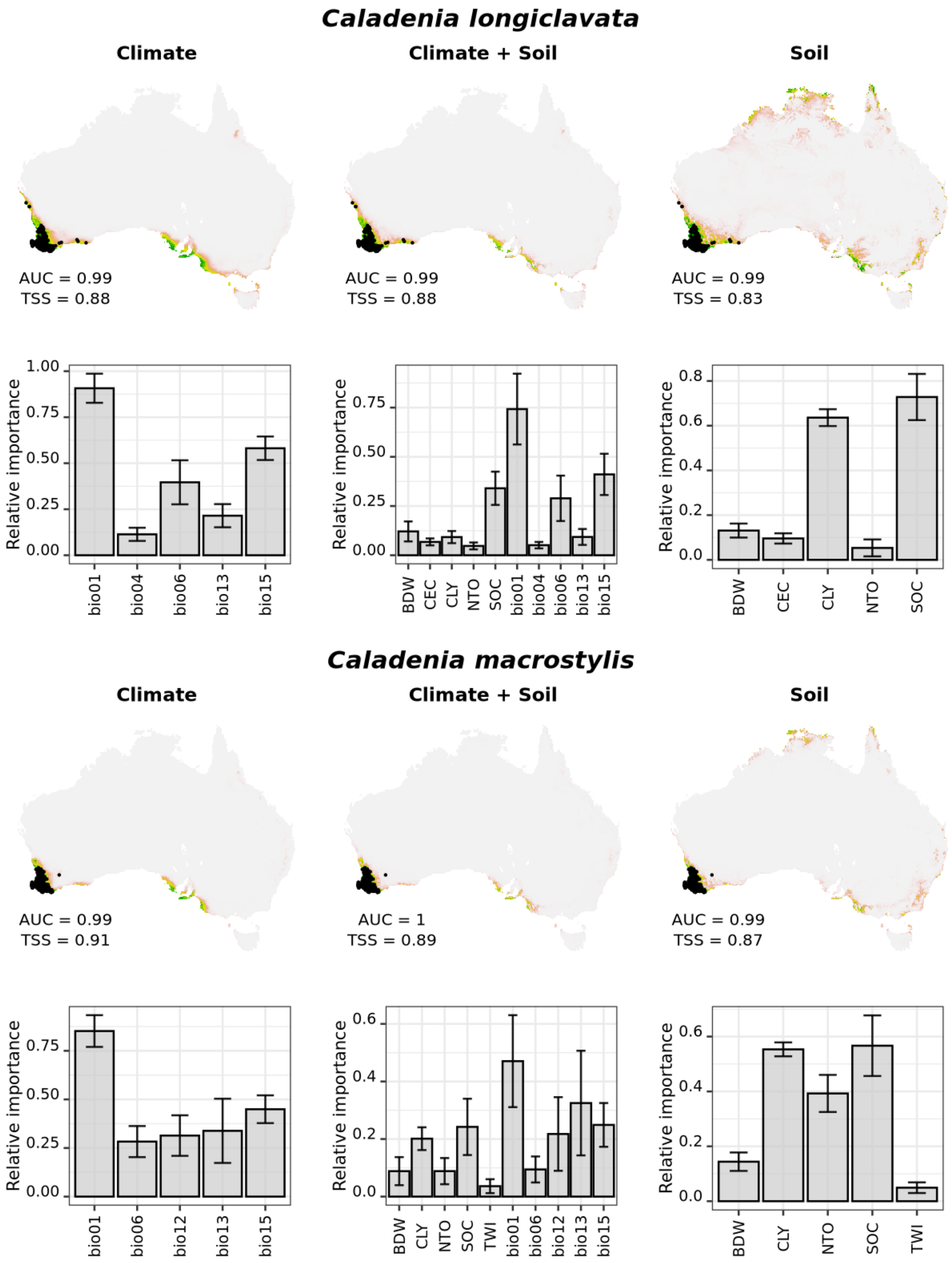
Caladenia longiclavata
Climate
Climate + Soil
Soil
AUC = 0.99
TSS = 0.88
AUC = 0.99
TSS = 0.88
AUC = 0.99
TSS = 0.83
Relative importance
1.00
0.75
0.50
0.25
0.00
bio01
bio04
bio06
bio13
bio15
Relative importance
0.75
0.50
0.25
0.00
BDW
CEC
CLY
NTO
SOC
bio01
bio04
bio06
bio13
bio15
Relative importance
0.8
0.6
0.4
0.2
0.0
BDW
CEC
CLY
NTO
SOC
Caladenia macrostylis
Climate
Climate + Soil
Soil
AUC = 0.99
TSS = 0.91
AUC = 1
TSS = 0.89
AUC = 0.99
TSS = 0.87
Relative importance
0.75
0.50
0.25
0.00
bio01
bio06
bio12
bio13
bio15
Relative importance
0.6
0.4
0.2
0.0
BDW
CLY
NTO
SOC
TWI
bio01
bio06
bio12
bio13
bio15
Relative importance
0.6
0.4
0.2
0.0
BDW
CLY
NTO
SOC
TWI

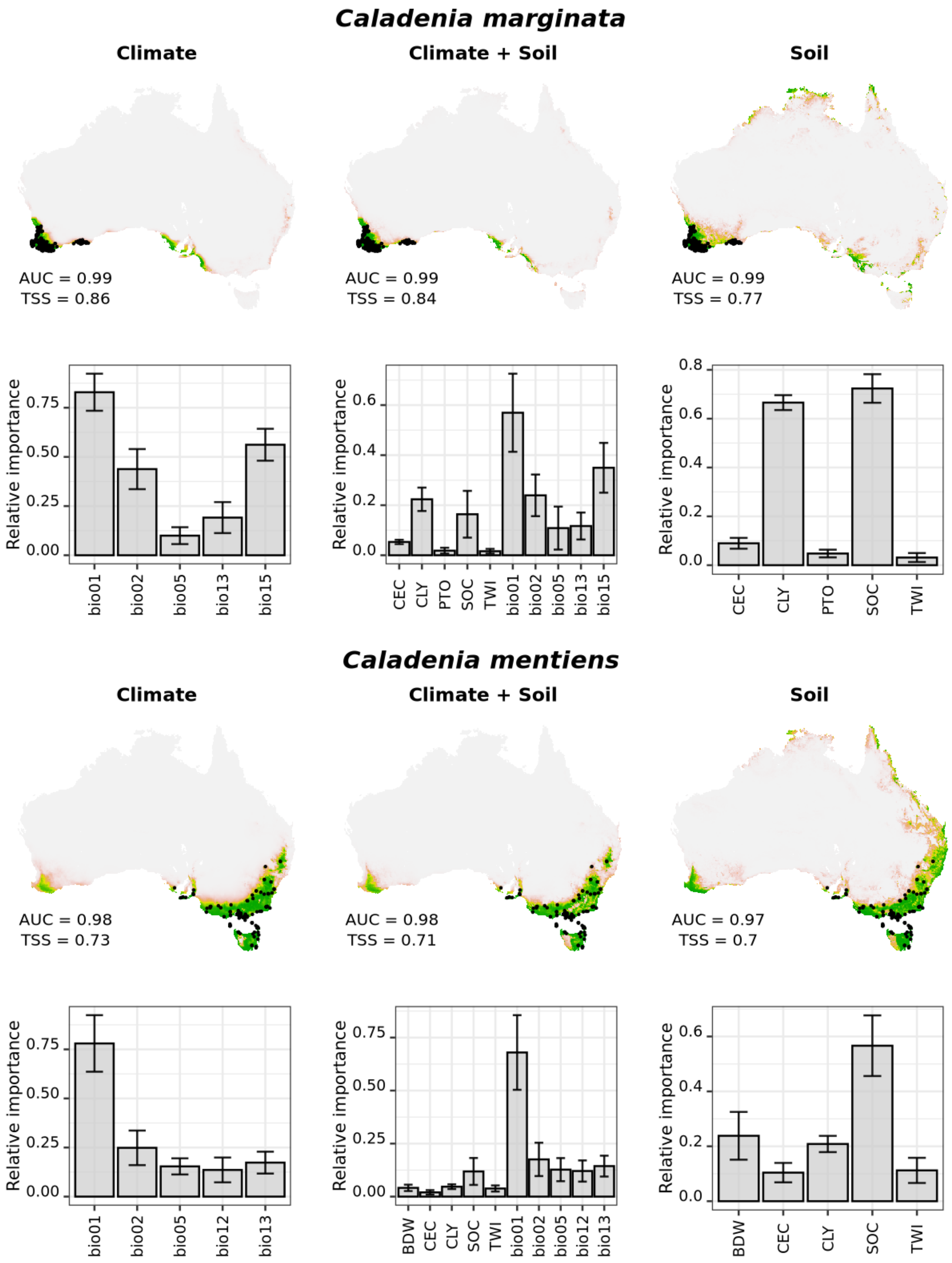

Caladenia marginata
Climate
Climate + Soil
Soil
AUC = 0.99
TSS = 0.86
AUC = 0.99
TSS = 0.84
AUC = 0.99
TSS = 0.77
Relative importance
0.00
0.25
0.50
0.75
bio01
bio02
bio05
bio13
bio15
0.0
0.2
0.4
0.6
CEC
CLY
PTO
SOC
TWI
bio01
bio02
bio05
bio13
bio15
0.0
0.2
0.4
0.6
0.8
CEC
CLY
PTO
SOC
TWI
Caladenia mentiens
Climate
Climate + Soil
Soil
AUC = 0.98
TSS = 0.73
AUC = 0.98
TSS = 0.71
AUC = 0.97
TSS = 0.7
Relative importance
0.00
0.25
0.50
0.75
bio01
bio02
bio05
bio12
bio13
BDW
CEC
CLY
SOC
TWI
bio01
bio02
bio05
bio12
bio13
0.0
0.2
0.4
0.6
BDW
CEC
CLY
SOC
TWI

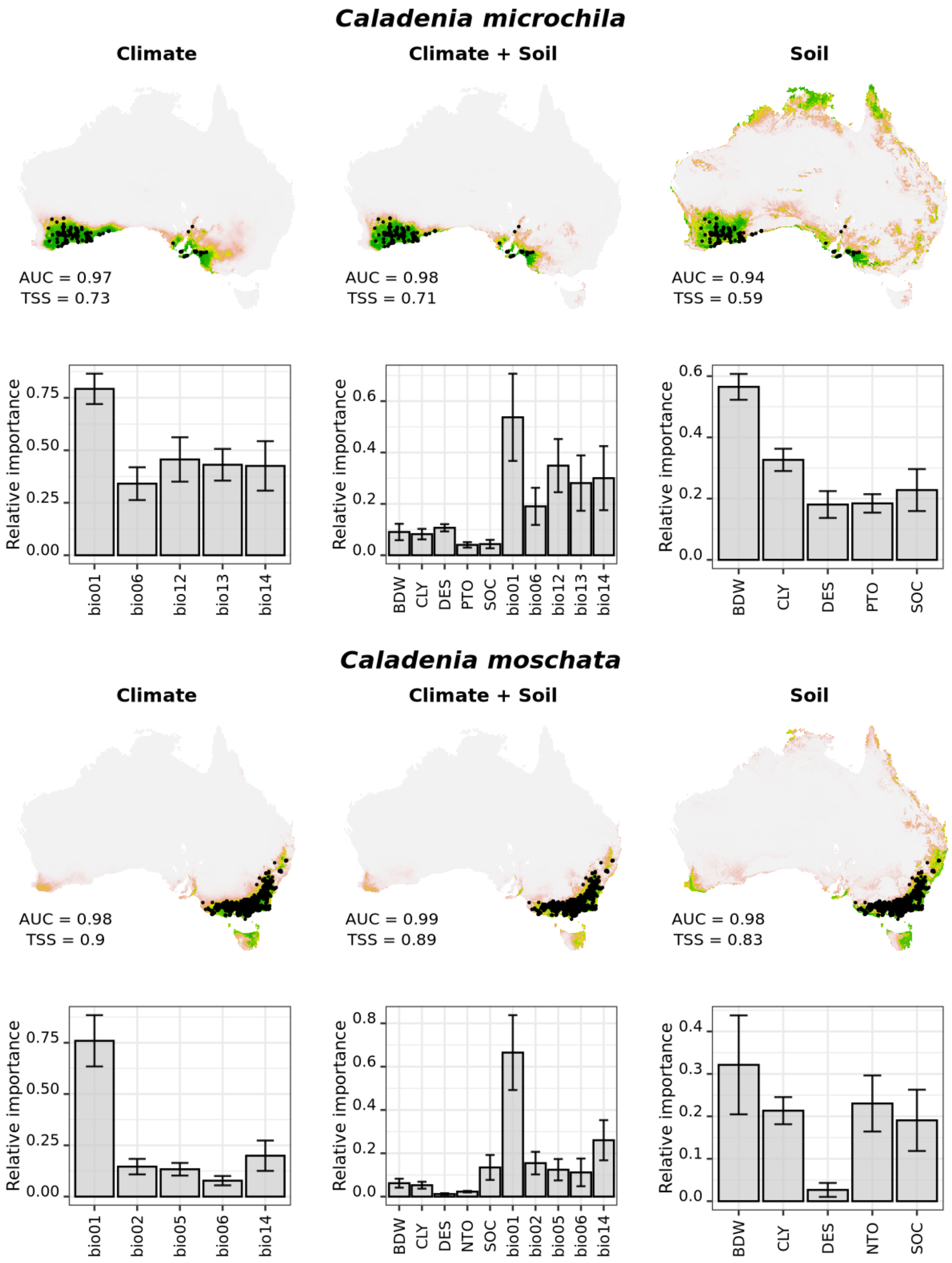

Caladenia microchila
Climate
Climate + Soil
Soil
AUC = 0.97
TSS = 0.73
AUC = 0.98
TSS = 0.71
AUC = 0.94
TSS = 0.59
Relative importance
0.00
0.25
0.50
0.75
bio01
bio06
bio12
bio13
bio14
0.0
0.2
0.4
0.6
BDW
CLY
DES
PTO
SOC
bio01
bio06
bio12
bio13
bio14
BDW
CLY
DES
PTO
SOC
Caladenia moschata
Climate
Climate + Soil
Soil
AUC = 0.98
TSS = 0.9
AUC = 0.99
TSS = 0.89
AUC = 0.98
TSS = 0.83
Relative importance
bio01
bio02
bio05
bio06
bio14
0.8
BDW
CLY
DES
NTO
SOC
bio01
bio02
bio05
bio06
bio14
0.1
0.3
BDW
CLY
DES
NTO
SOC

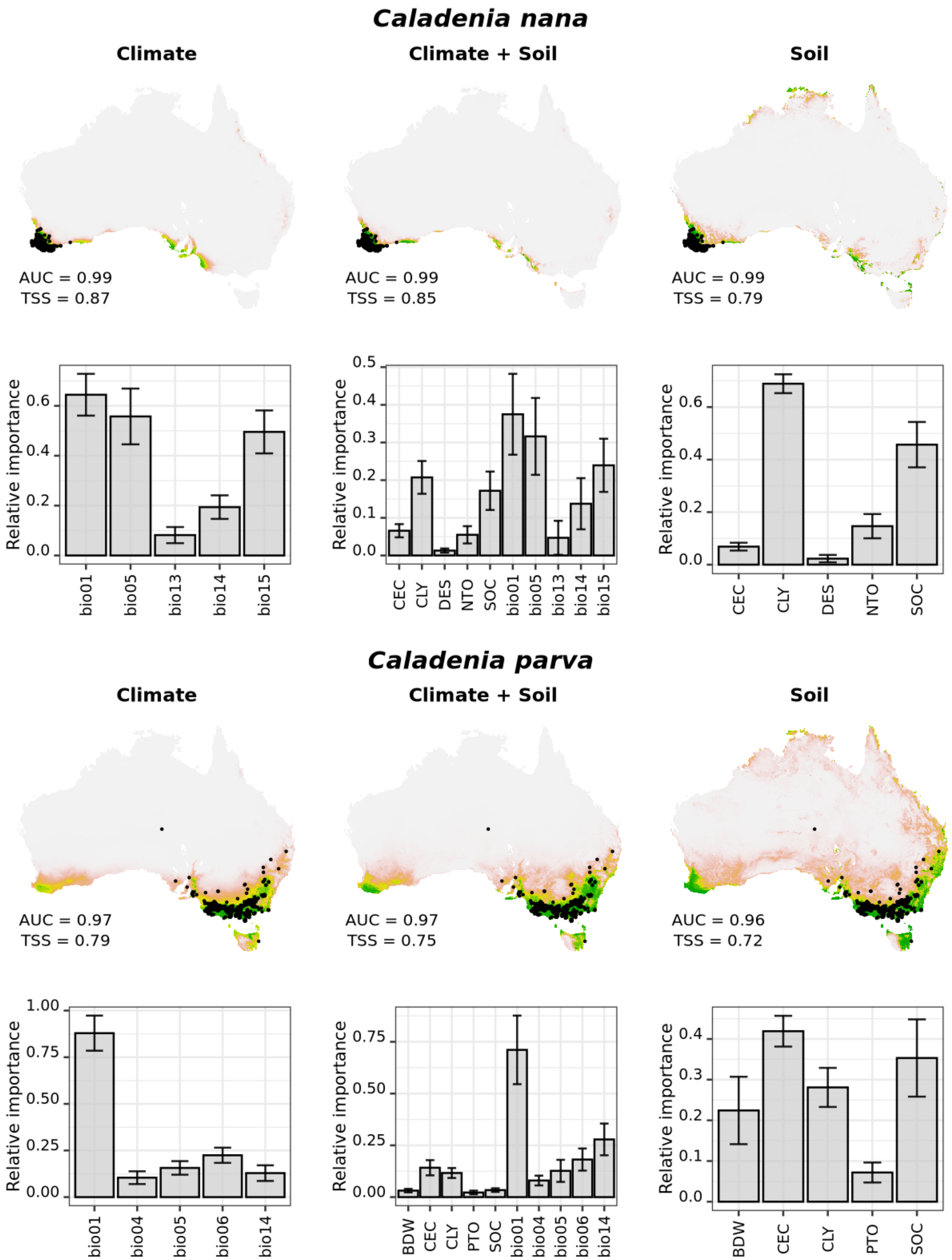
Caladenia nana
Climate
Climate + Soil
Soil
AUC = 0.99
TSS = 0.87
AUC = 0.99
TSS = 0.85
AUC = 0.99
TSS = 0.79
Relative importance
bio01
bio05
bio13
bio14
bio15
CEC
CLY
DES
NTO
SOC
Caladenia parva
AUC = 0.97
TSS = 0.79
AUC = 0.97
TSS = 0.75
AUC = 0.96
TSS = 0.72
bio04
bio06
BDW
PTO

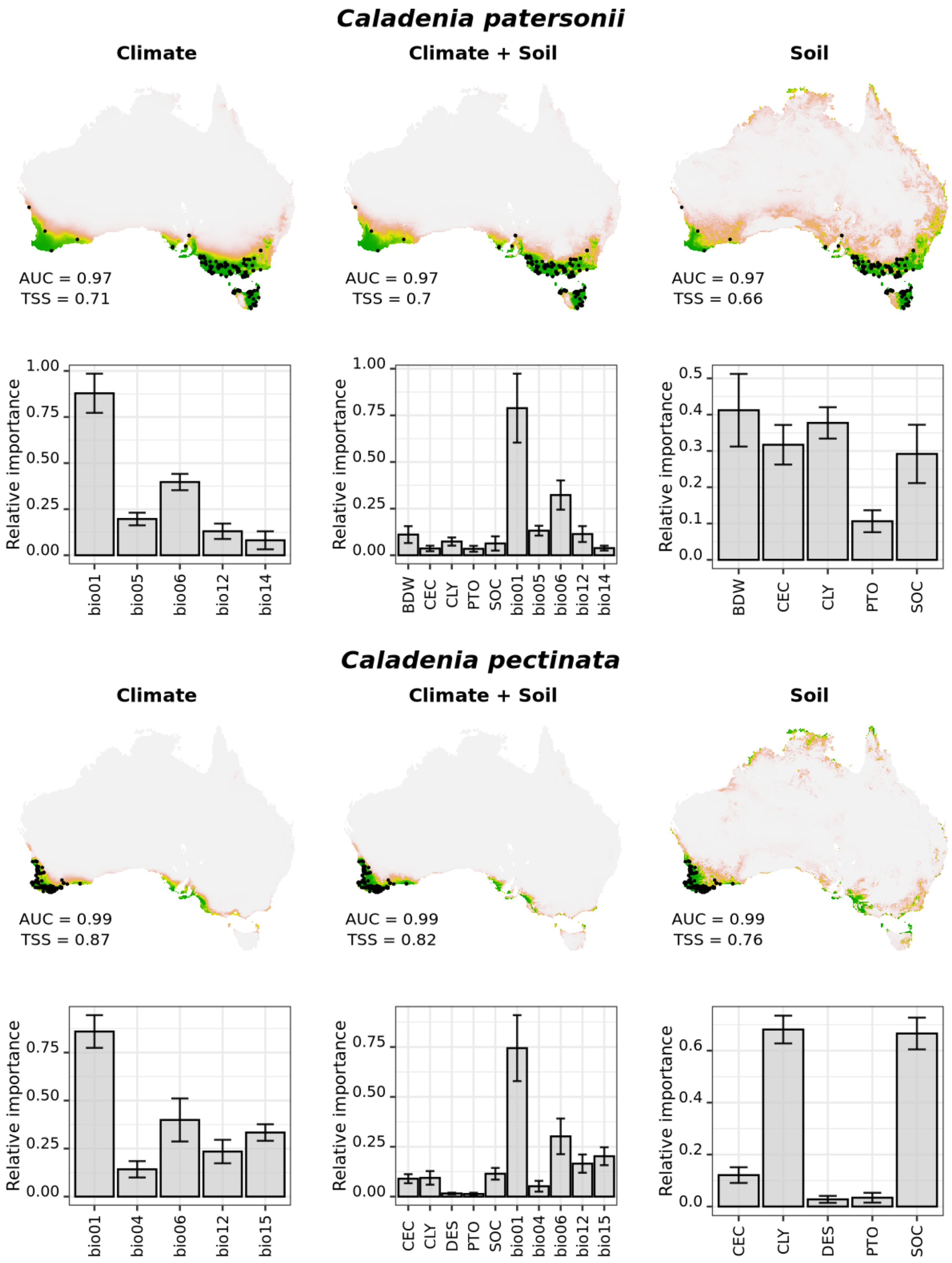
Caladenia patersonii
Climate
Climate + Soil
Soil
AUC = 0.97
TSS = 0.71
AUC = 0.97
TSS = 0.7
AUC = 0.97
TSS = 0.66
Relative importance
1.00
0.75
0.50
0.25
0.00
bio01
bio05
bio06
bio12
bio14
BDW
CEC
CLY
PTO
SOC
0.5
0.4
0.3
0.2
0.1
0.0
Caladenia pectinata
Climate
Climate + Soil
Soil
AUC = 0.99
TSS = 0.87
AUC = 0.99
TSS = 0.82
AUC = 0.99
TSS = 0.76
bio04
bio15
DES
0.6

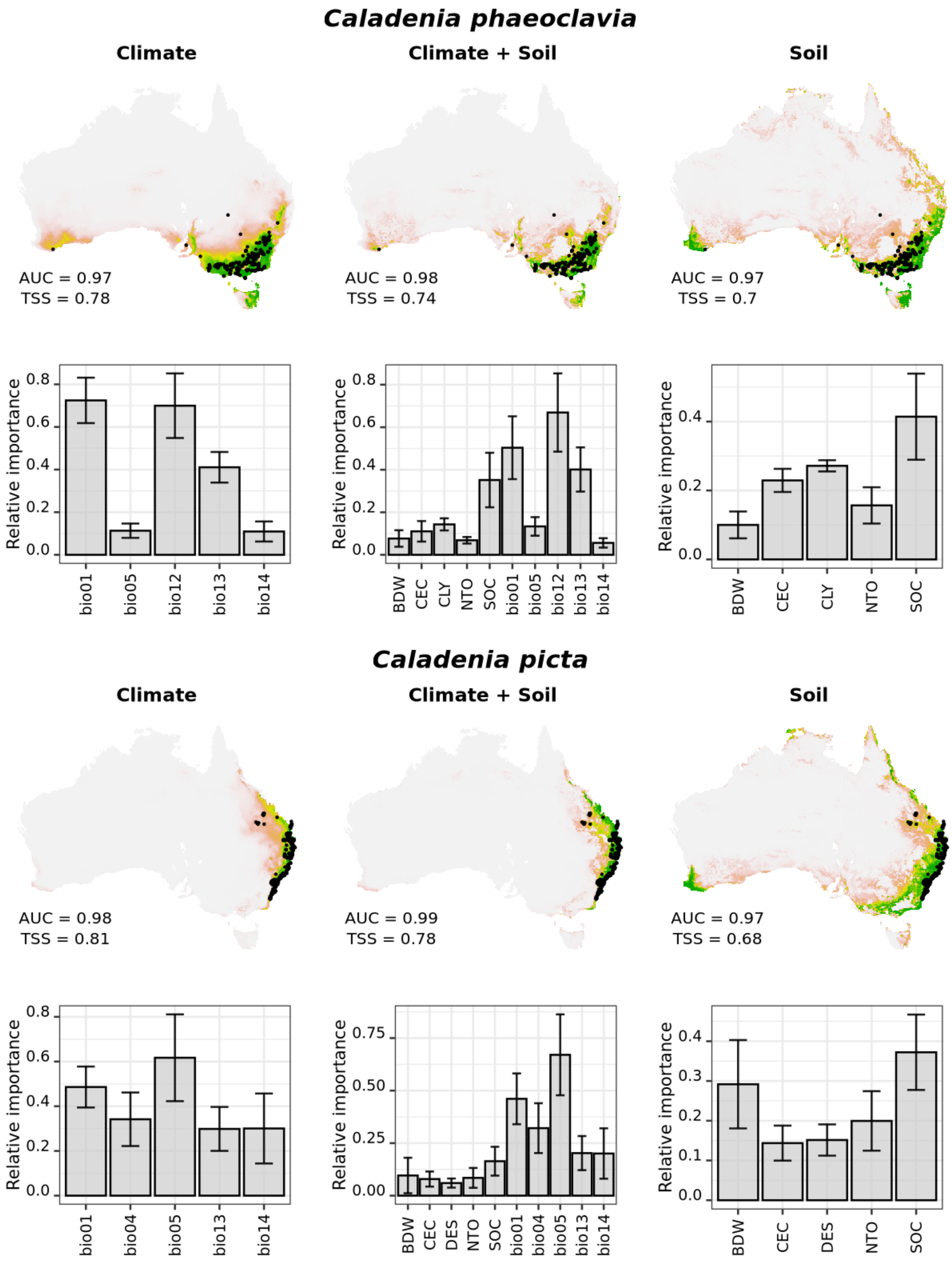
Caladenia phaeoclavia
Climate
Climate + Soil
Soil
AUC = 0.97
TSS = 0.78
AUC = 0.98
TSS = 0.74
AUC = 0.97
TSS = 0.7
Relative importance
bio01
bio05
bio12
bio13
bio14
BDW
CEC
CLY
NTO
SOC
Caladenia picta
Climate
Climate + Soil
Soil
AUC = 0.98
TSS = 0.81
AUC = 0.99
TSS = 0.78
AUC = 0.97
TSS = 0.68
bio04
DES

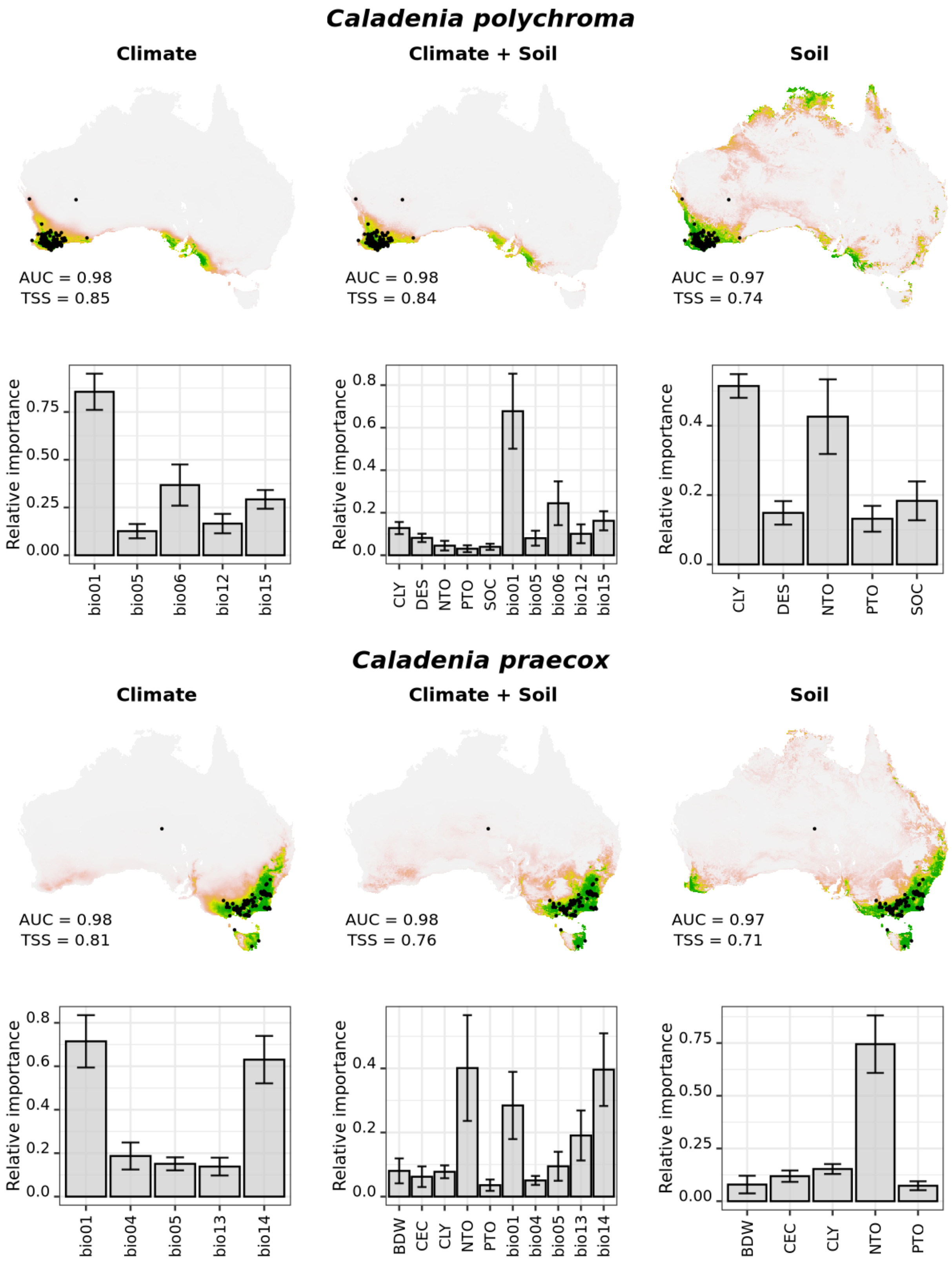
Caladenia polychroma
Climate
Climate + Soil
Soil
AUC = 0.98
TSS = 0.85
AUC = 0.98
TSS = 0.84
AUC = 0.97
TSS = 0.74
Relative importance
bio01
bio05
bio06
bio12
bio15
CLY
DES
NTO
PTO
SOC
Caladenia praecox
Climate
Climate + Soil
Soil
AUC = 0.98
TSS = 0.81
AUC = 0.98
TSS = 0.76
AUC = 0.97
TSS = 0.71
Relative importance
bio01
bio04
bio05
bio13
bio14
BDW
CEC
CLY
NTO
PTO

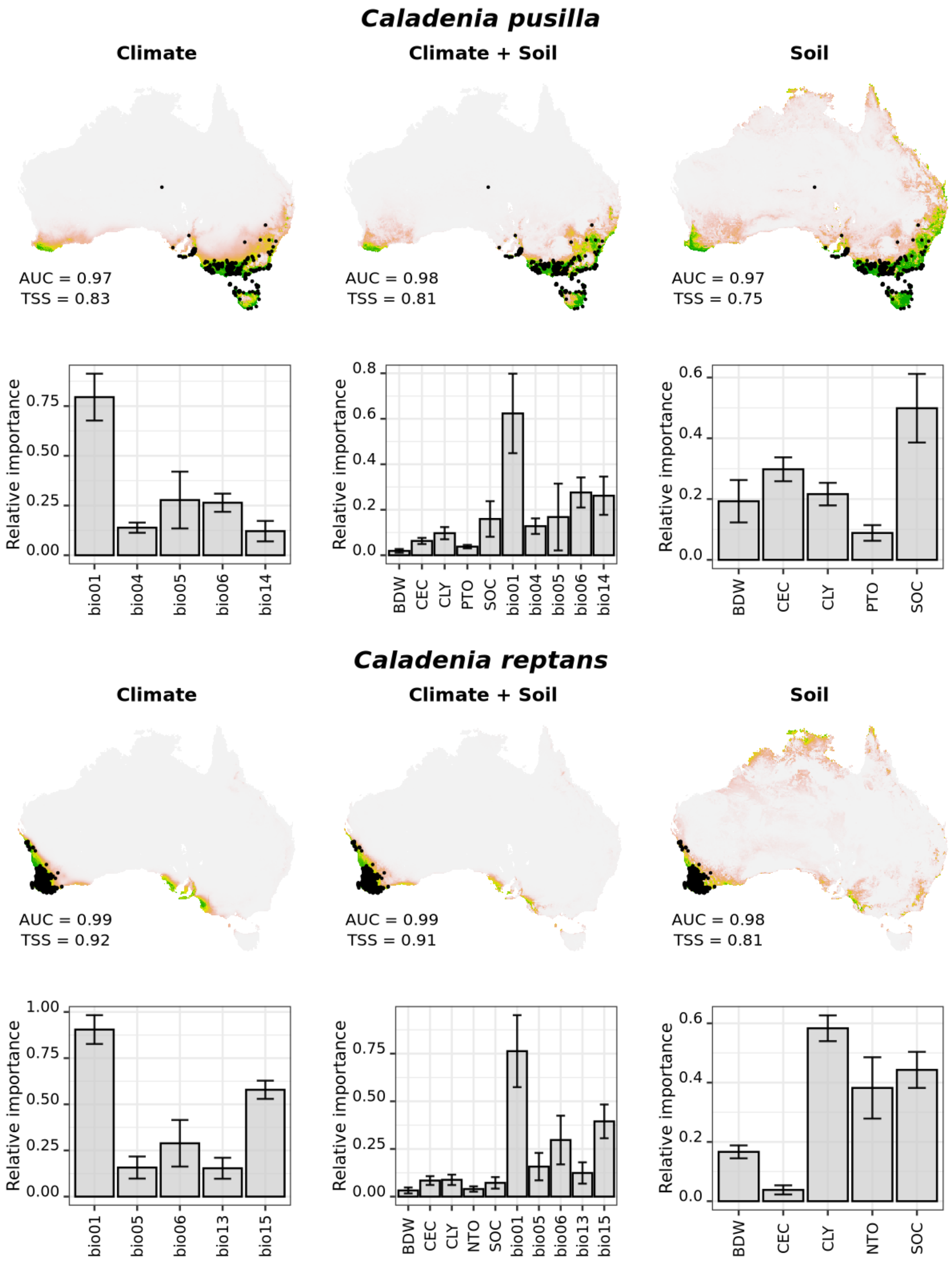
Caladenia pusilla
Climate
Climate + Soil
Soil
AUC = 0.97
TSS = 0.83
AUC = 0.98
TSS = 0.81
AUC = 0.97
TSS = 0.75
Relative importance
0.00
0.25
0.50
0.75
bio01
bio04
bio05
bio06
bio14
0.0
0.2
0.4
0.6
0.8
BDW
CEC
CLY
PTO
SOC
bio01
bio04
bio05
bio06
bio14
0.0
0.2
0.4
0.6
BDW
CEC
CLY
PTO
SOC
Caladenia reptans
Climate
Climate + Soil
Soil
AUC = 0.99
TSS = 0.92
AUC = 0.99
TSS = 0.91
AUC = 0.98
TSS = 0.81
Relative importance
0.00
0.25
0.50
0.75
1.00
bio01
bio05
bio06
bio13
bio15
0.00
0.25
0.50
0.75
BDW
CEC
CLY
NTO
SOC
bio01
bio05
bio06
bio13
bio15
0.0
0.2
0.4
0.6
BDW
CEC
CLY
NTO
SOC

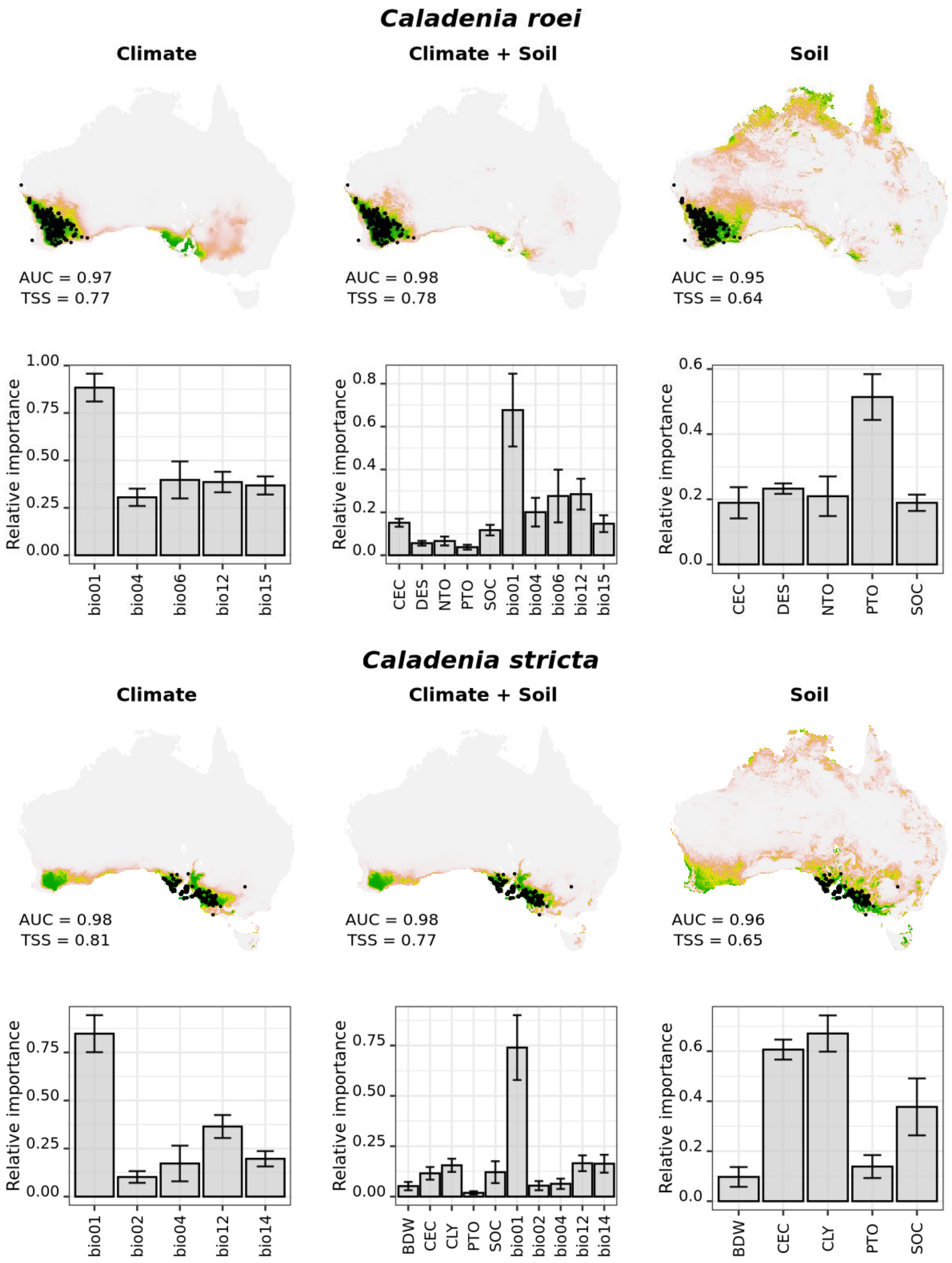
Caladenia roei
Climate
Climate + Soil
Soil
AUC = 0.97
TSS = 0.77
AUC = 0.98
TSS = 0.78
AUC = 0.95
TSS = 0.64
Relative importance
bio01
bio04
bio06
bio12
bio15
CEC
DES
NTO
PTO
SOC
Caladenia stricta
Climate
Climate + Soil
Soil
AUC = 0.98
TSS = 0.81
AUC = 0.98
TSS = 0.77
AUC = 0.96
TSS = 0.65
Relative importance
bio01
bio02
bio04
bio12
bio14
BDW
CEC
CLY
PTO
SOC

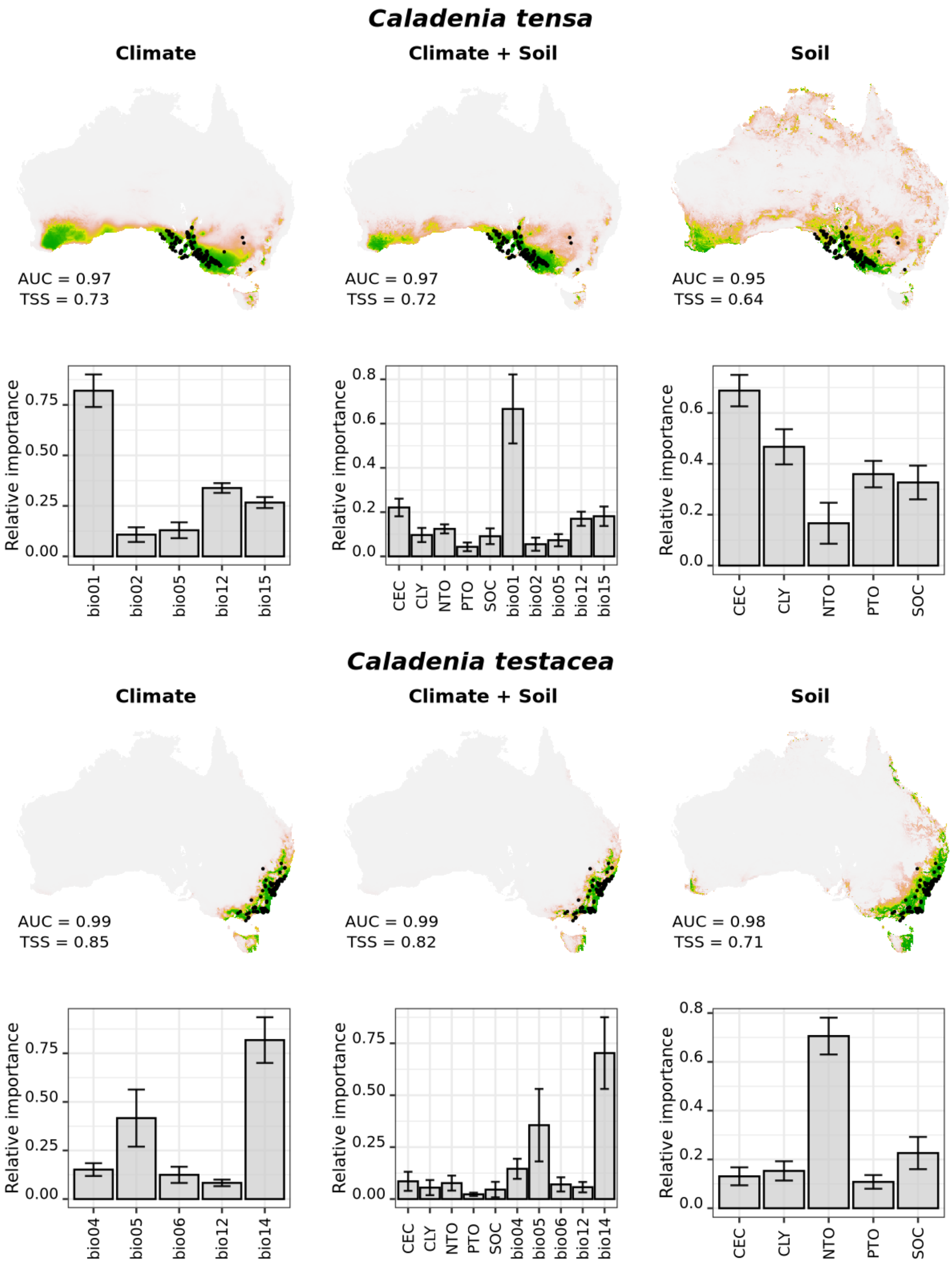
Caladenia tensa
Climate
Climate + Soil
Soil
AUC = 0.97
TSS = 0.73
AUC = 0.97
TSS = 0.72
AUC = 0.95
TSS = 0.64
Relative importance
bio01
bio02
bio05
bio12
bio15
CEC
CLY
NTO
PTO
SOC
Caladenia testacea
Climate
Climate + Soil
Soil
AUC = 0.99
TSS = 0.85
AUC = 0.99
TSS = 0.82
AUC = 0.98
TSS = 0.71
Relative importance
bio04
bio05
bio06
bio12
bio14
CEC
CLY
NTO
PTO
SOC

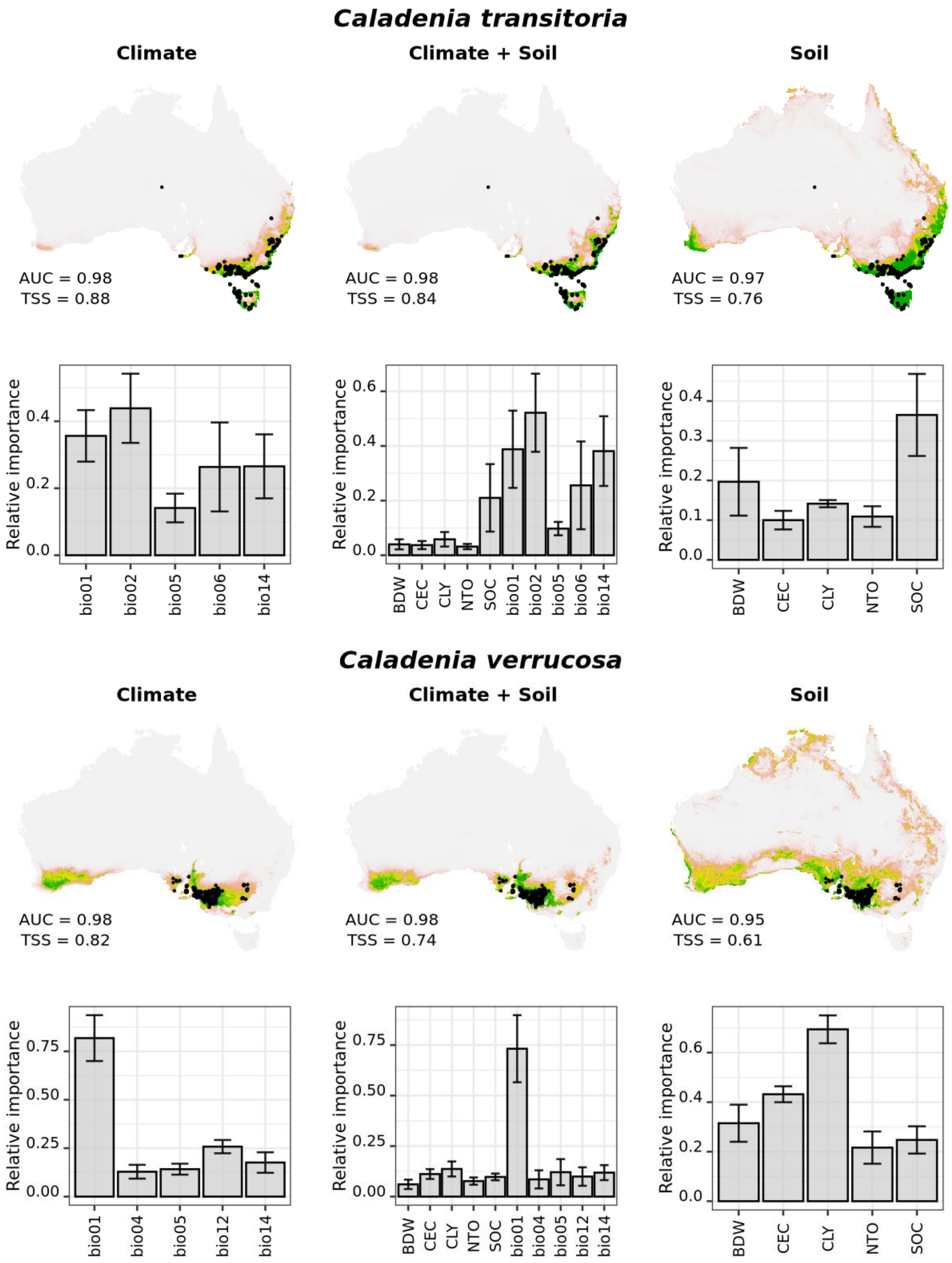

Caladenia transitoria
Climate
Climate + Soil
Soil
AUC = 0.98
TSS = 0.88
AUC = 0.98
TSS = 0.84
AUC = 0.97
TSS = 0.76
Relative importance
bio01
bio02
bio05
bio06
bio14
BDW
CEC
CLY
NTO
SOC
Caladenia verrucosa
Climate
Climate + Soil
Soil
AUC = 0.98
TSS = 0.82
AUC = 0.98
TSS = 0.74
AUC = 0.95
TSS = 0.61
Relative importance
bio01
bio04
bio05
bio12
bio14
BDW
CEC
CLY
NTO
SOC

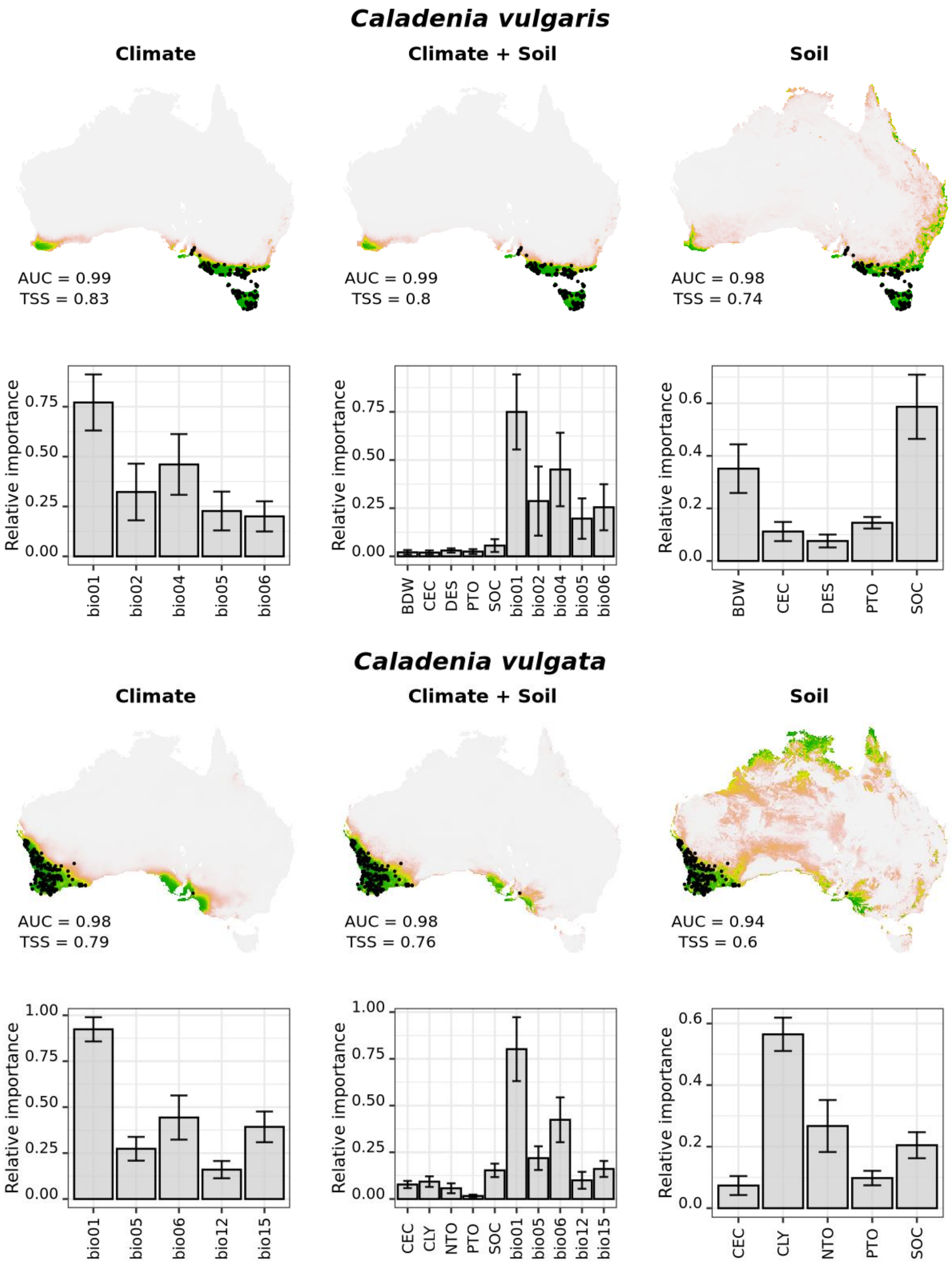
Caladenia vulgaris
Climate
Climate + Soil
Soil
AUC = 0.99
TSS = 0.83
AUC = 0.99
TSS = 0.8
AUC = 0.98
TSS = 0.74
Relative importance
0.00
0.25
0.50
0.75
bio01
bio02
bio04
bio05
bio06
BDW
CEC
DES
PTO
SOC
0.0
0.2
0.4
0.6
Caladenia vulgata
Climate
Climate + Soil
Soil
AUC = 0.98
TSS = 0.79
AUC = 0.98
TSS = 0.76
AUC = 0.94
TSS = 0.6
1.00
bio12
bio15
CLY
NTO

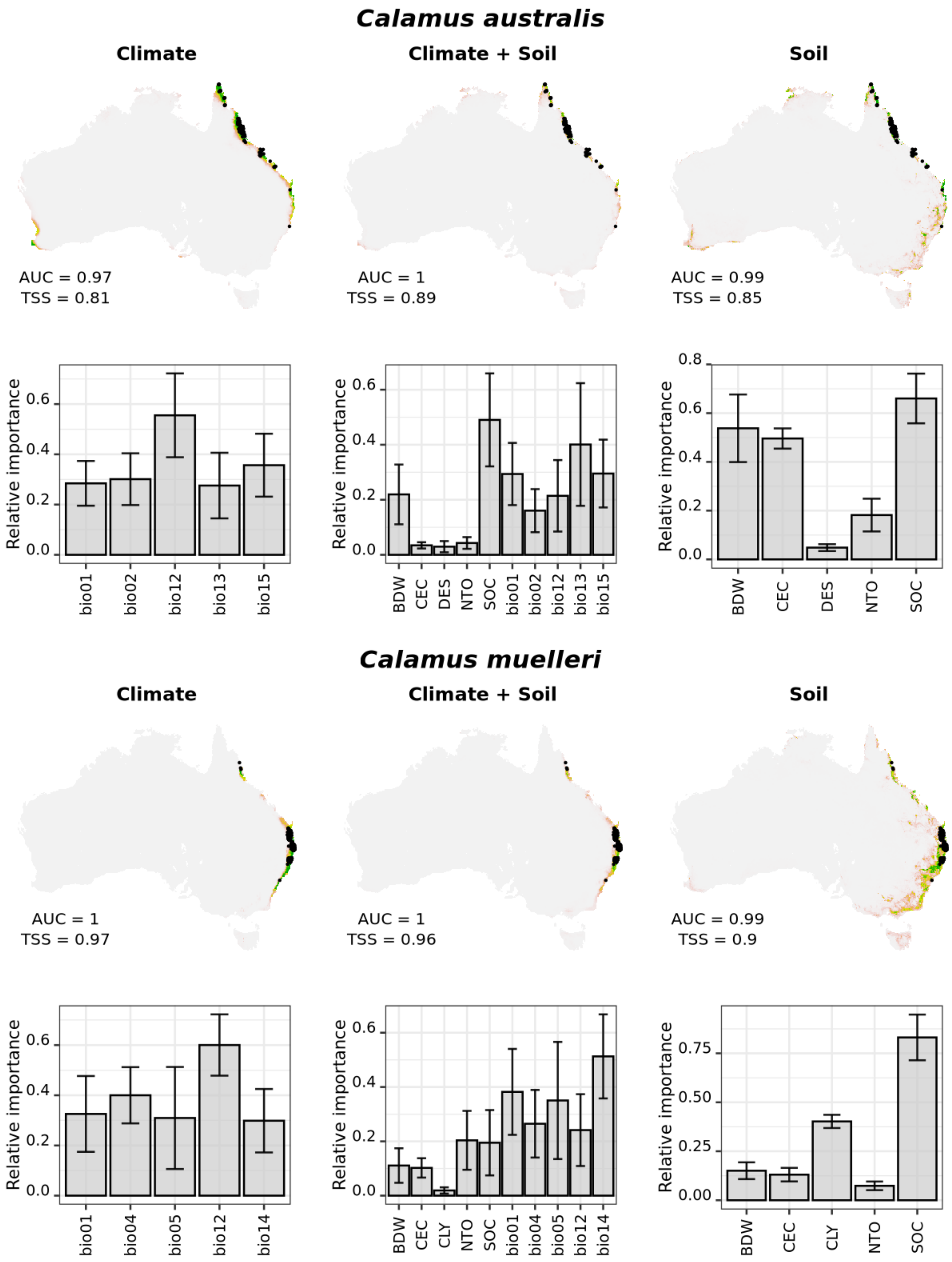
Calamus australis
Climate
Climate + Soil
Soil
AUC = 0.97
TSS = 0.81
AUC = 1
TSS = 0.89
AUC = 0.99
TSS = 0.85
Relative importance
bio01
bio02
bio12
bio13
bio15
BDW
CEC
DES
NTO
SOC
Calamus muelleri
Climate
Climate + Soil
Soil
AUC = 1
TSS = 0.97
AUC = 1
TSS = 0.96
AUC = 0.99
TSS = 0.9
Relative importance
bio01
bio04
bio05
bio12
bio14
BDW
CEC
CLY
NTO
SOC

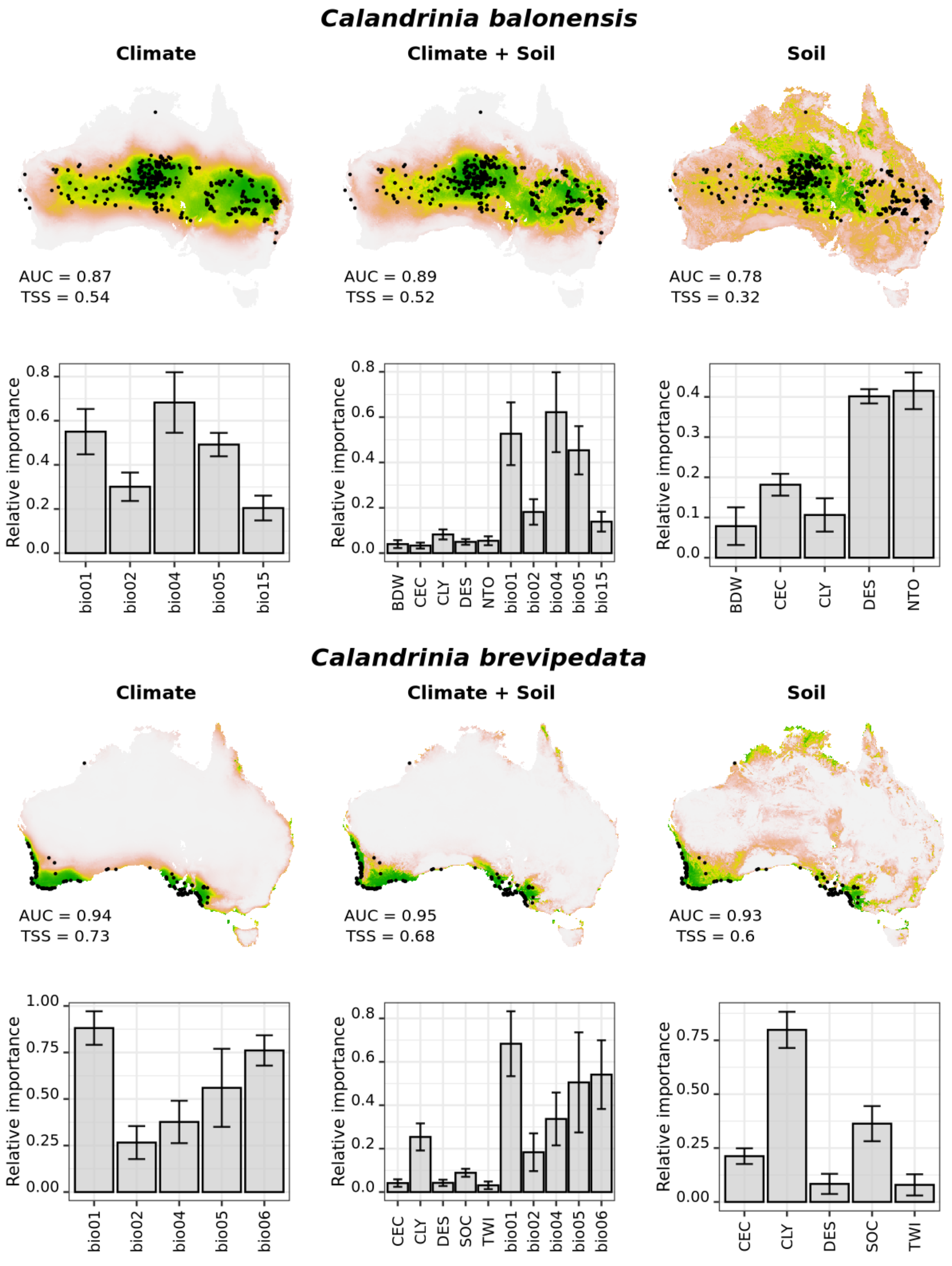
Calandrinia balonensis
Climate
Climate + Soil
Soil
AUC = 0.87
TSS = 0.54
AUC = 0.89
TSS = 0.52
AUC = 0.78
TSS = 0.32
Relative importance
bio01
bio02
bio04
bio05
bio15
BDW
CEC
CLY
DES
NTO
Calandrinia brevipedata
Climate
Climate + Soil
Soil
AUC = 0.94
TSS = 0.73
AUC = 0.95
TSS = 0.68
AUC = 0.93
TSS = 0.6
Relative importance
bio01
bio02
bio04
bio05
bio06
CEC
CLY
DES
SOC
TWI

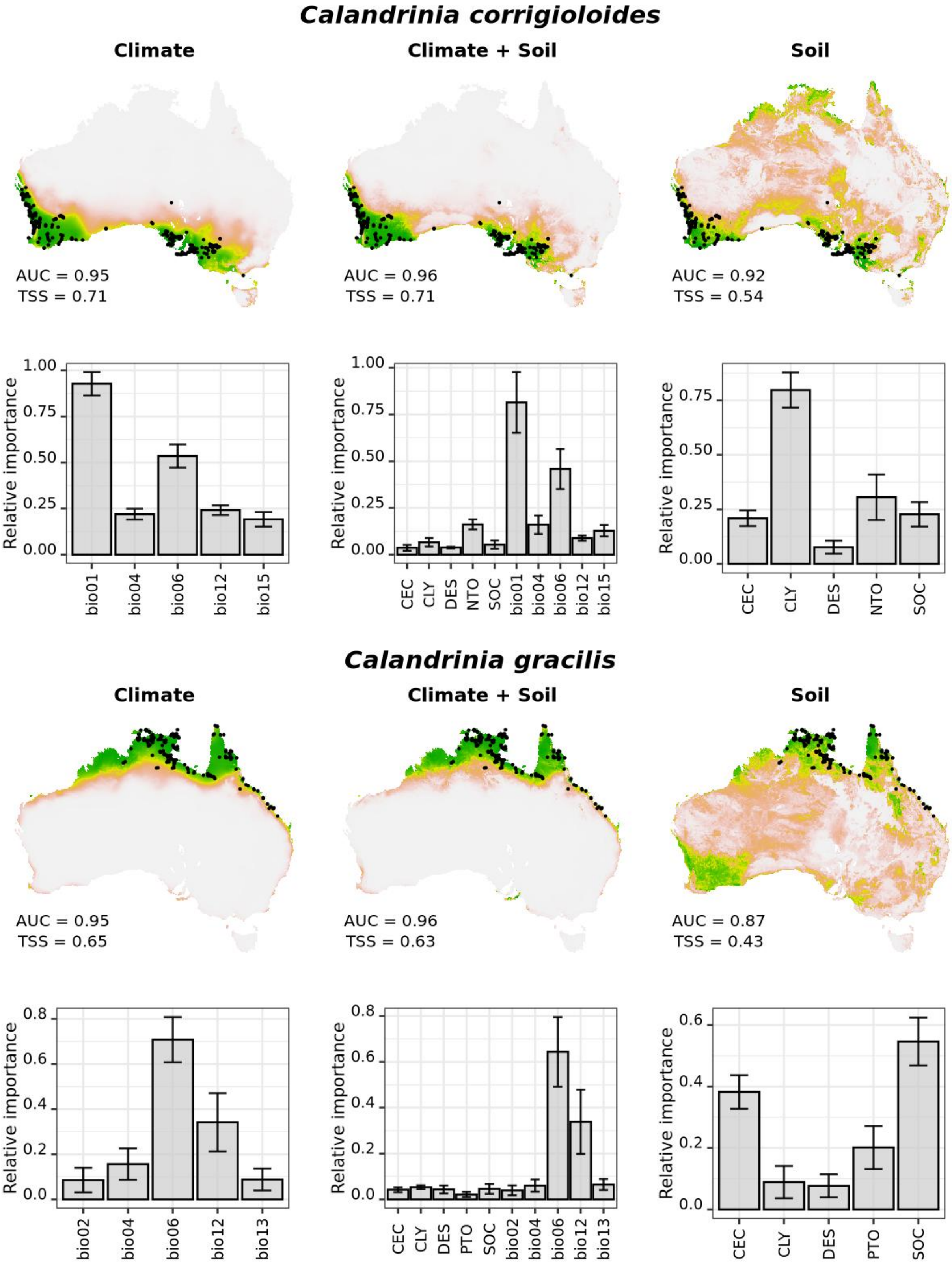
Calandrinia corrigioloides
Climate
Climate + Soil
Soil
AUC = 0.95
TSS = 0.71
AUC = 0.96
TSS = 0.71
AUC = 0.92
TSS = 0.54
Relative importance
1.00
0.75
0.50
0.25
0.00
bio01
bio04
bio06
bio12
bio15
CEC
CLY
DES
NTO
SOC
Calandrinia gracilis
Climate
Climate + Soil
Soil
AUC = 0.95
TSS = 0.65
AUC = 0.96
TSS = 0.63
AUC = 0.87
TSS = 0.43
Relative importance
0.8
0.6
0.4
0.2
0.0
bio02
bio04
bio06
bio12
bio13
CEC
CLY
DES
PTO
SOC

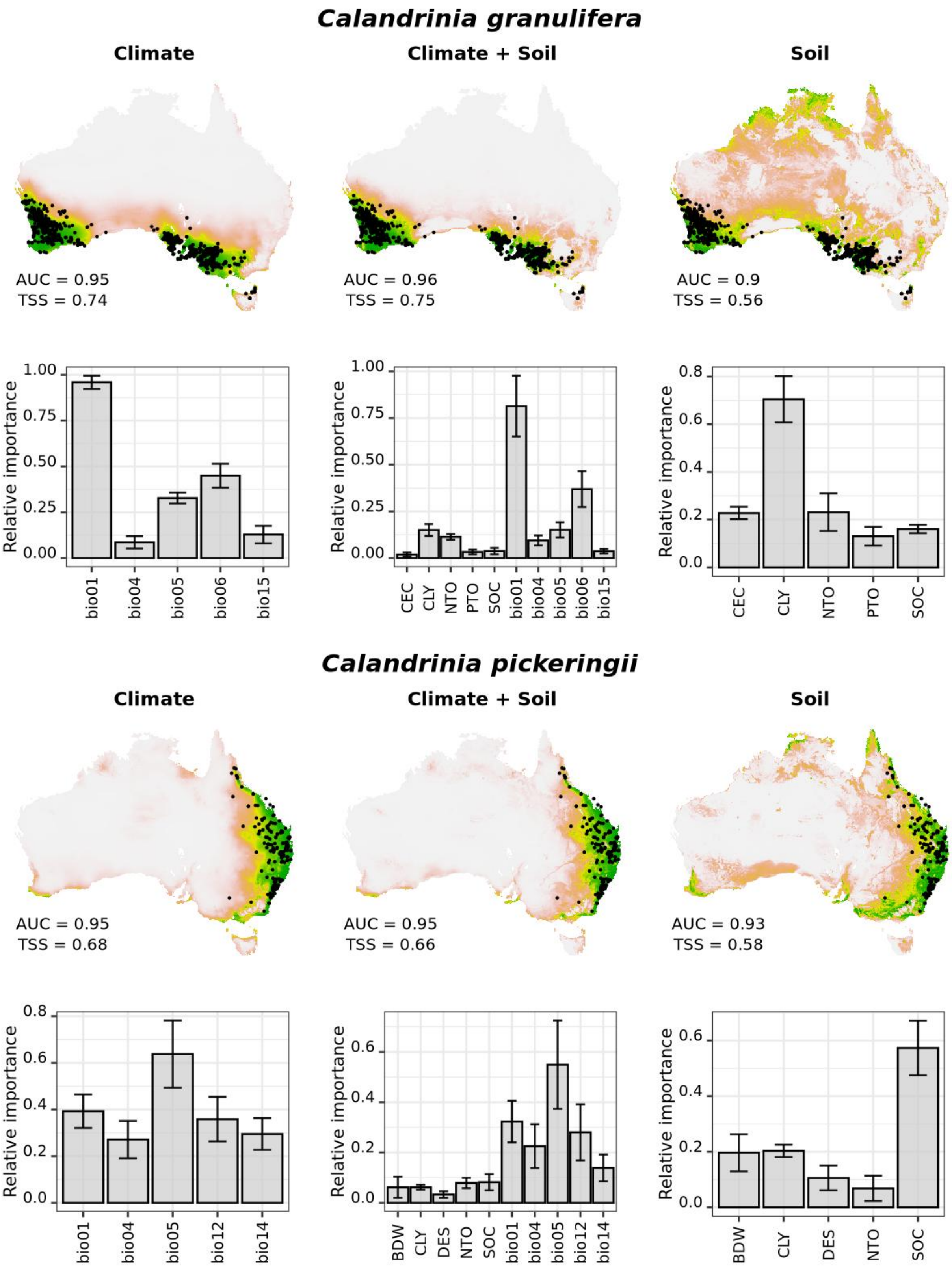
Calandrinia granulifera
Climate
Climate + Soil
Soil
AUC = 0.95
TSS = 0.74
AUC = 0.96
TSS = 0.75
AUC = 0.9
TSS = 0.56
Relative importance
bio01
bio04
bio05
bio06
bio15
CEC
CLY
NTO
PTO
SOC
Calandrinia pickeringii
AUC = 0.95
TSS = 0.68
AUC = 0.95
TSS = 0.66
AUC = 0.93
TSS = 0.58
bio12
bio14
BDW
DES

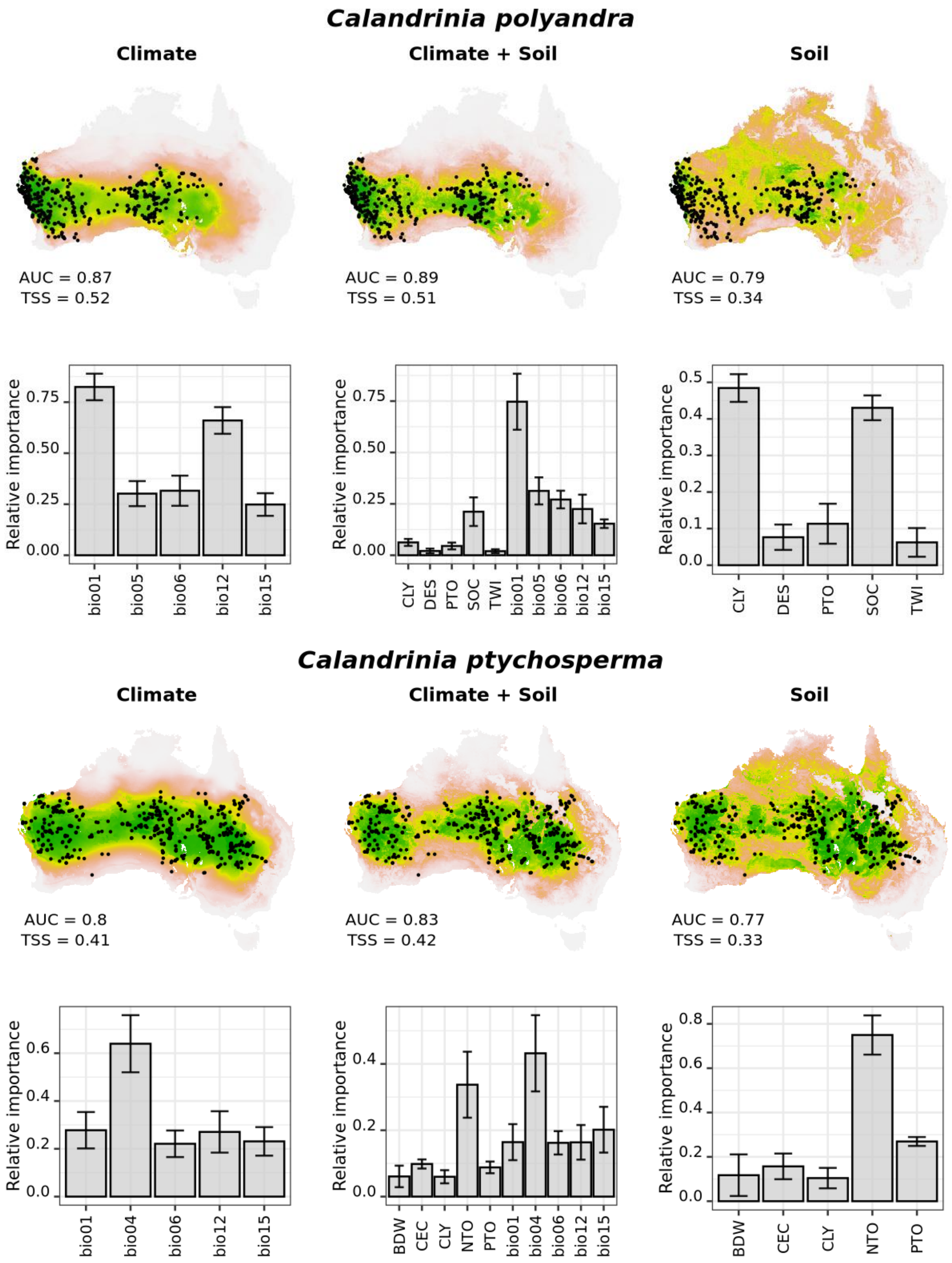
Calandrinia polyandra
Climate
Climate + Soil
Soil
AUC = 0.87
TSS = 0.52
AUC = 0.89
TSS = 0.51
AUC = 0.79
TSS = 0.34
Relative importance
bio01 bio05 bio06 bio12 bio15
CLY DES PTO SOC TWI bio01 bio05 bio06 bio12 bio15
CLY DES PTO SOC TWI
Calandrinia ptychosperma
Climate
Climate + Soil
Soil
AUC = 0.8
TSS = 0.41
AUC = 0.83
TSS = 0.42
AUC = 0.77
TSS = 0.33
Relative importance
bio01 bio04 bio06 bio12 bio15
BDW CEC CLY NTO PTO bio01 bio04 bio06 bio12 bio15
BDW CEC CLY NTO PTO

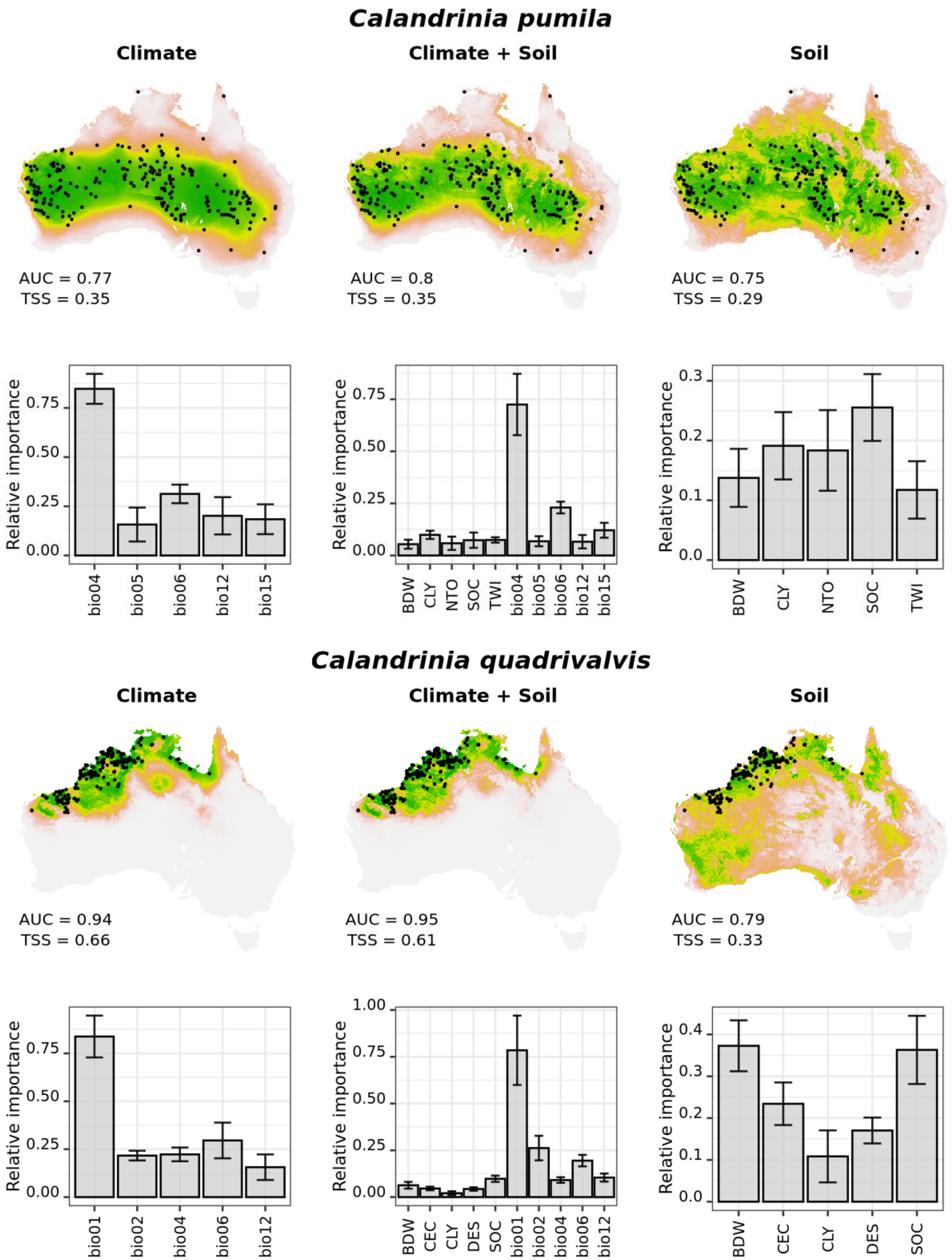
Calandrinia pumila
Climate
Climate + Soil
Soil
AUC = 0.77
TSS = 0.35
AUC = 0.8
TSS = 0.35
AUC = 0.75
TSS = 0.29
Relative importance
0.00
0.25
0.50
0.75
bio04
bio05
bio06
bio12
bio15
BDW
CLY
NTO
SOC
TWI
bio04
bio05
bio06
bio12
bio15
0.0
0.1
0.2
0.3
BDW
CLY
NTO
SOC
TWI
Calandrinia quadrivalvis
Climate
Climate + Soil
Soil
AUC = 0.94
TSS = 0.66
AUC = 0.95
TSS = 0.61
AUC = 0.79
TSS = 0.33
Relative importance
0.00
0.25
0.50
0.75
1.00
bio01
bio02
bio04
bio06
bio12
BDW
CEC
CLY
DES
SOC
bio01
bio02
bio04
bio06
bio12
0.0
0.1
0.2
0.3
0.4
BDW
CEC
CLY
DES
SOC

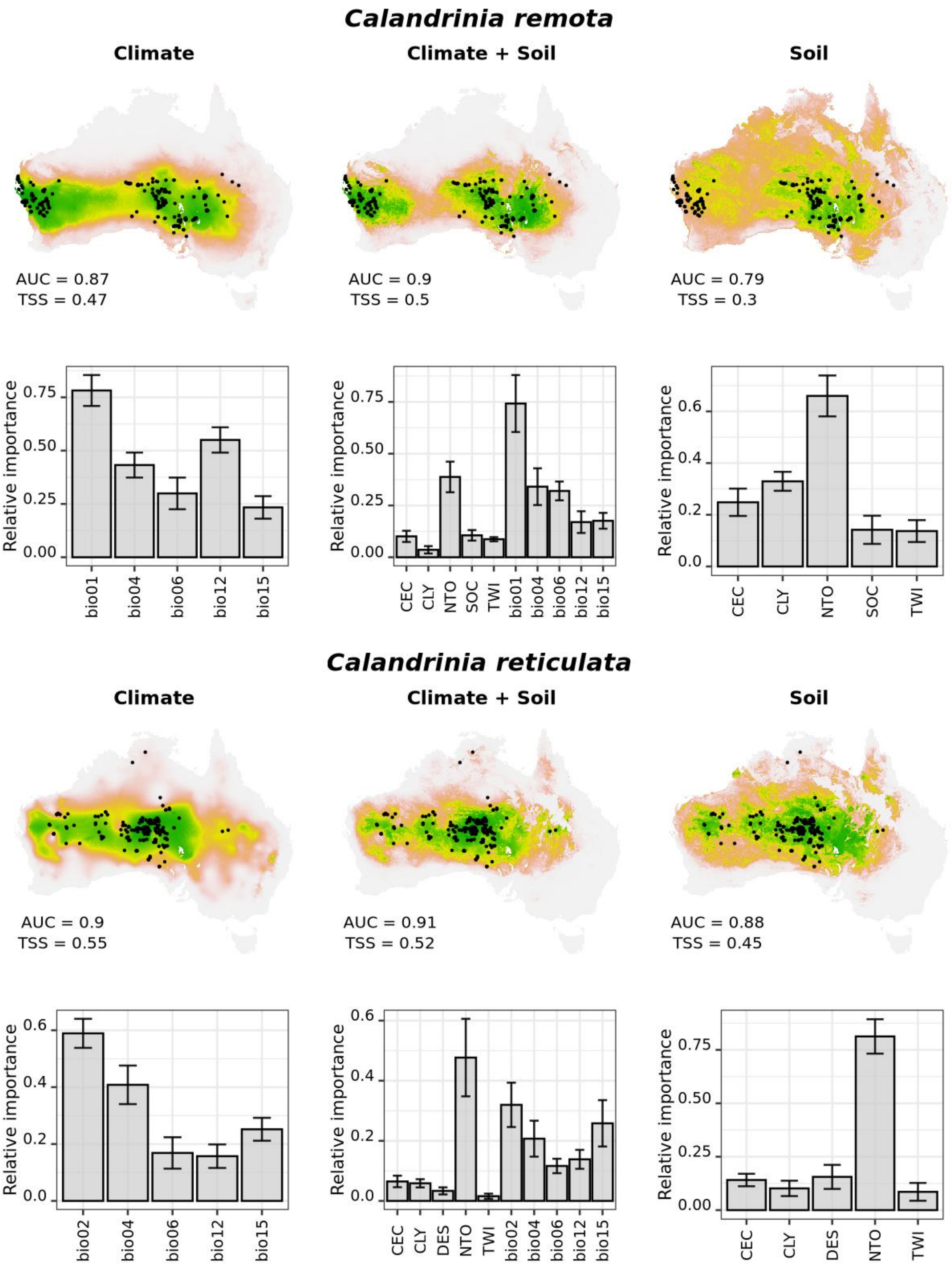

Calandrinia remota
Climate
Climate + Soil
Soil
AUC = 0.87
TSS = 0.47
AUC = 0.9
TSS = 0.5
AUC = 0.79
TSS = 0.3
Relative importance
bio01
bio04
bio06
bio12
bio15
CEC
CLY
NTO
SOC
TWI
Calandrinia reticulata
Climate
Climate + Soil
Soil
AUC = 0.9
TSS = 0.55
AUC = 0.91
TSS = 0.52
AUC = 0.88
TSS = 0.45
Relative importance
bio02
bio04
bio06
bio12
bio15
CEC
CLY
DES
NTO
TWI

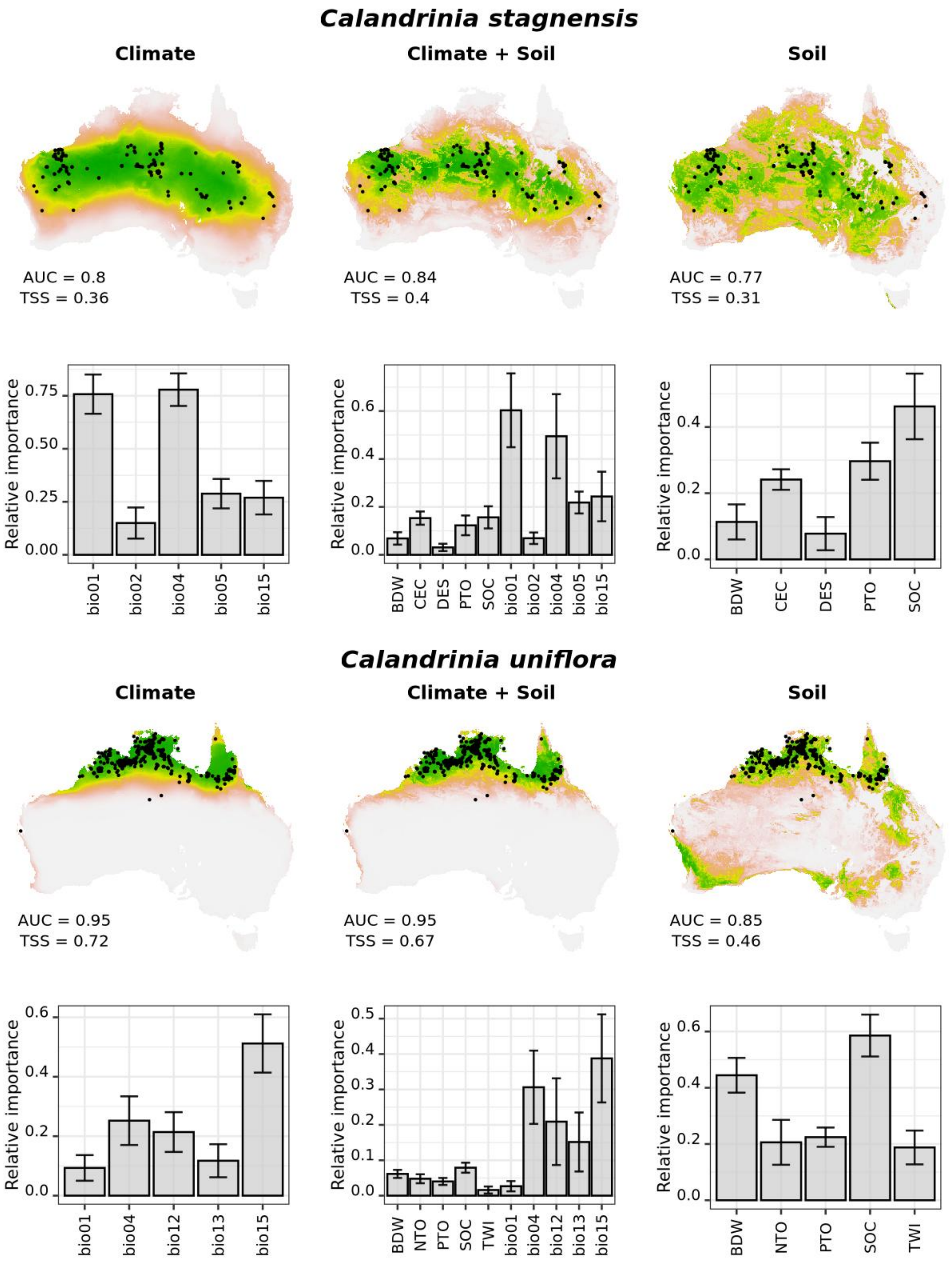
Calandrinia stagnensis
Climate
Climate + Soil
Soil
AUC = 0.8
TSS = 0.36
AUC = 0.84
TSS = 0.4
AUC = 0.77
TSS = 0.31
Relative importance
0.00
0.25
0.50
0.75
bio01
bio02
bio04
bio05
bio15
0.0
0.2
0.4
0.6
BDW
CEC
DES
PTO
SOC
bio01
bio02
bio04
bio05
bio15
0.0
0.2
0.4
BDW
CEC
DES
PTO
SOC
Calandrinia uniflora
Climate
Climate + Soil
Soil
AUC = 0.95
TSS = 0.72
AUC = 0.95
TSS = 0.67
AUC = 0.85
TSS = 0.46
Relative importance
0.0
0.2
0.4
0.6
bio01
bio04
bio12
bio13
bio15
0.0
0.1
0.2
0.3
0.4
0.5
BDW
NTO
PTO
SOC
TWI
bio01
bio04
bio12
bio13
bio15
0.0
0.2
0.4
0.6
BDW
NTO
PTO
SOC
TWI

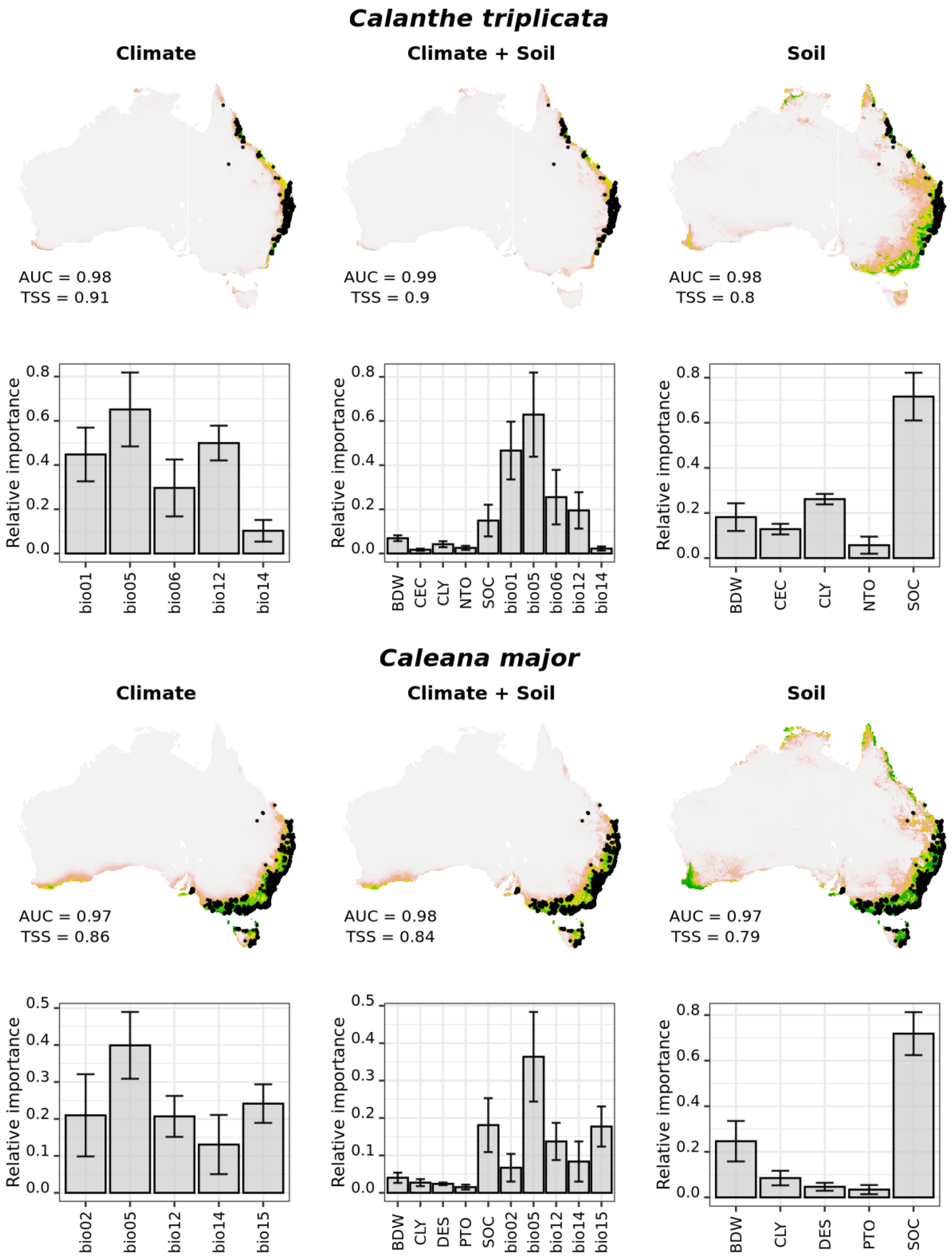
Calanthe triplicata
Climate
Climate + Soil
Soil
AUC = 0.98
TSS = 0.91
AUC = 0.99
TSS = 0.9
AUC = 0.98
TSS = 0.8
Relative importance
0.0
0.2
0.4
0.6
0.8
bio01
bio05
bio06
bio12
bio14
BDW
CEC
CLY
NTO
SOC
Caleana major
Climate
Climate + Soil
Soil
AUC = 0.97
TSS = 0.86
AUC = 0.98
TSS = 0.84
AUC = 0.97
TSS = 0.79
Relative importance
0.0
0.1
0.2
0.3
0.4
0.5
bio02
bio05
bio12
bio14
bio15
DES
PTO

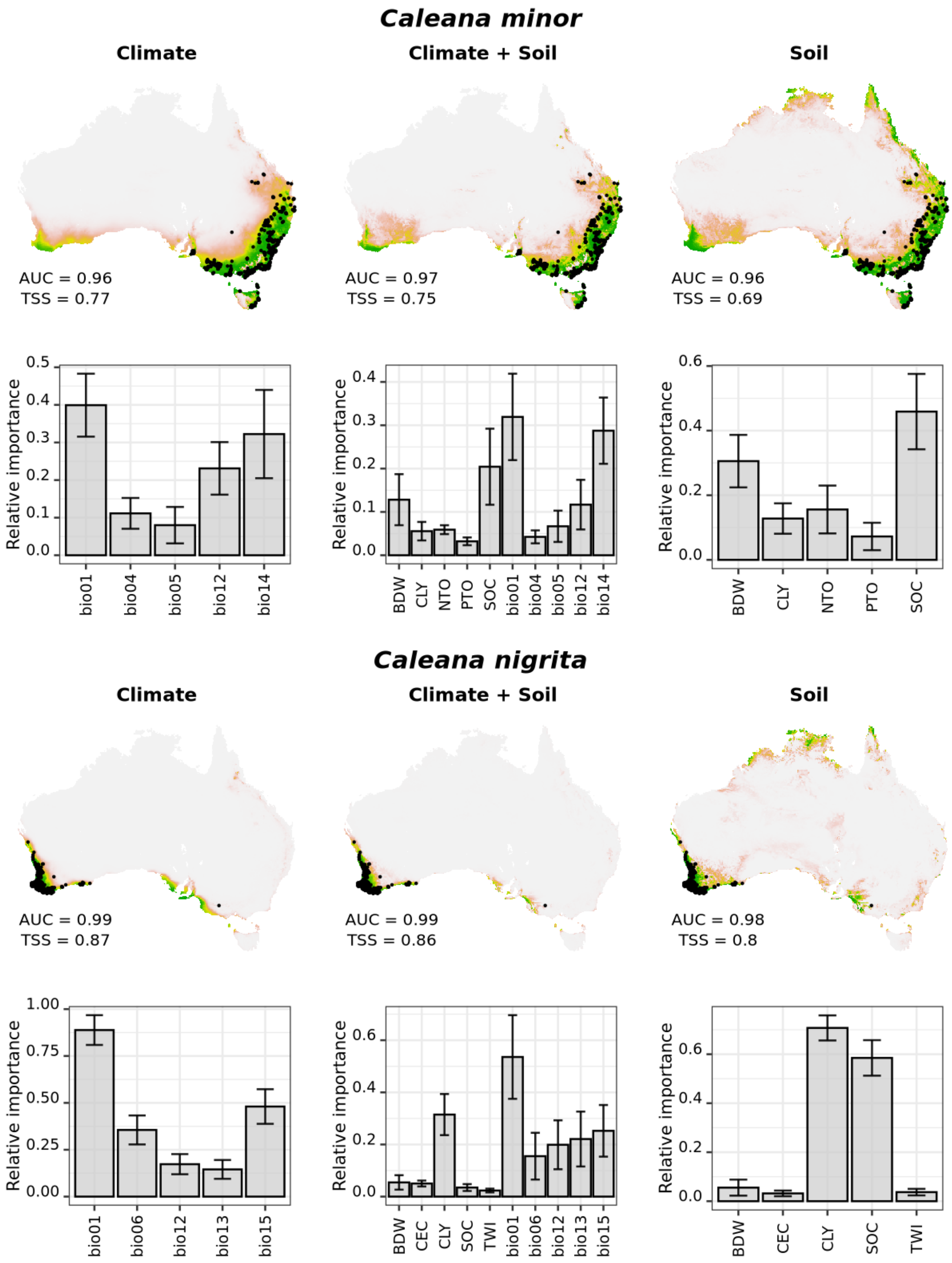

Caleana minor
Climate
Climate + Soil
Soil
AUC = 0.96
TSS = 0.77
AUC = 0.97
TSS = 0.75
AUC = 0.96
TSS = 0.69
Relative importance
bio01
bio04
bio05
bio12
bio14
BDW
CLY
NTO
PTO
SOC
Caleana nigrita
Climate
Climate + Soil
Soil
AUC = 0.99
TSS = 0.87
AUC = 0.99
TSS = 0.86
AUC = 0.98
TSS = 0.8
Relative importance
bio01
bio06
bio12
bio13
bio15
BDW
CEC
CLY
SOC
TWI

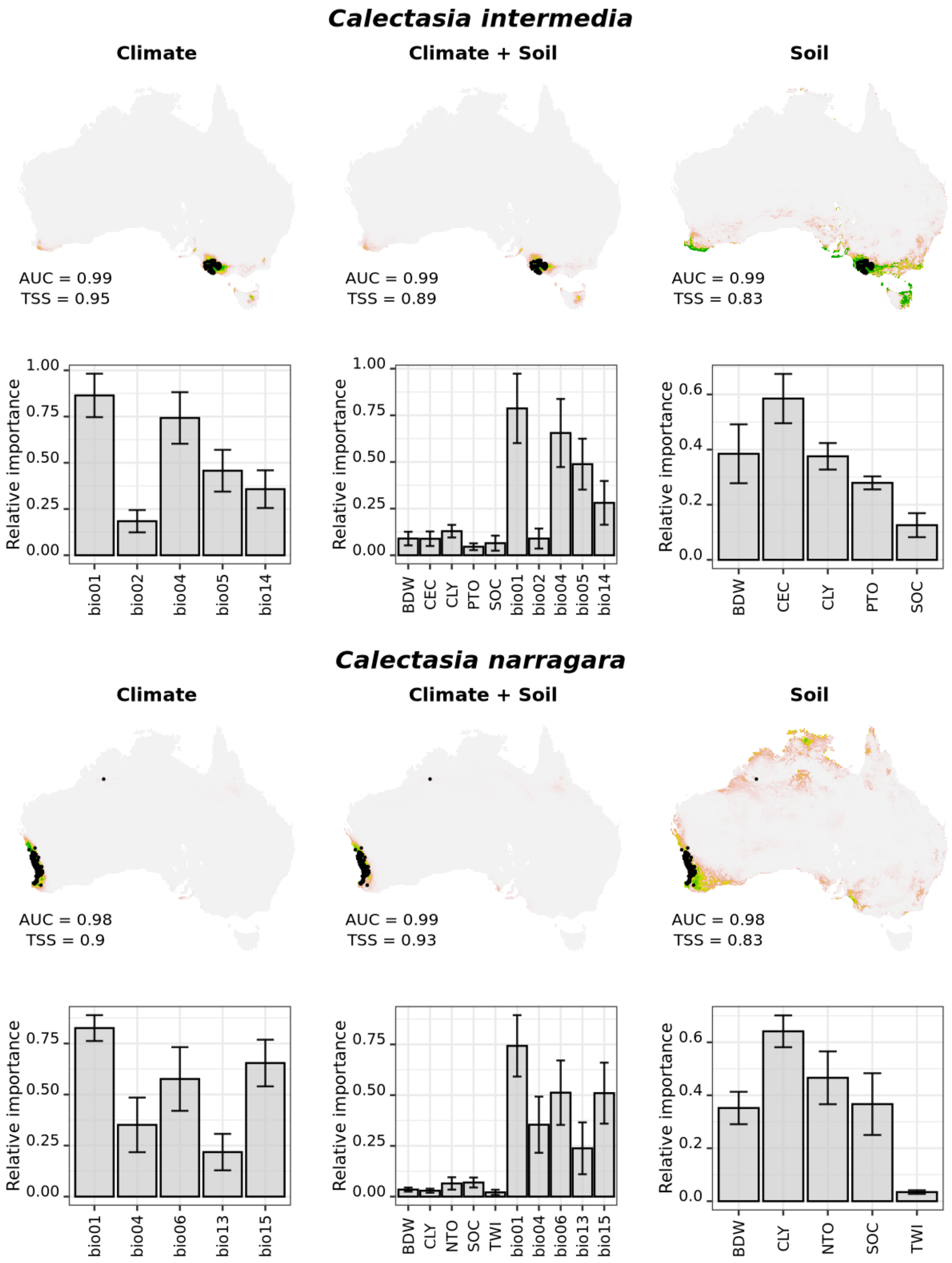
Calectasia intermedia
Climate
Climate + Soil
Soil
AUC = 0.99
TSS = 0.95
AUC = 0.99
TSS = 0.89
AUC = 0.99
TSS = 0.83
Relative importance
1.00
0.75
0.50
0.25
0.00
bio01
bio02
bio04
bio05
bio14
BDW
CEC
CLY
PTO
SOC
0.6
0.4
0.2
0.0
Calectasia narragara
AUC = 0.98
TSS = 0.9
AUC = 0.99
TSS = 0.93
AUC = 0.98
TSS = 0.83
bio06
bio13
bio15
NTO
TWI

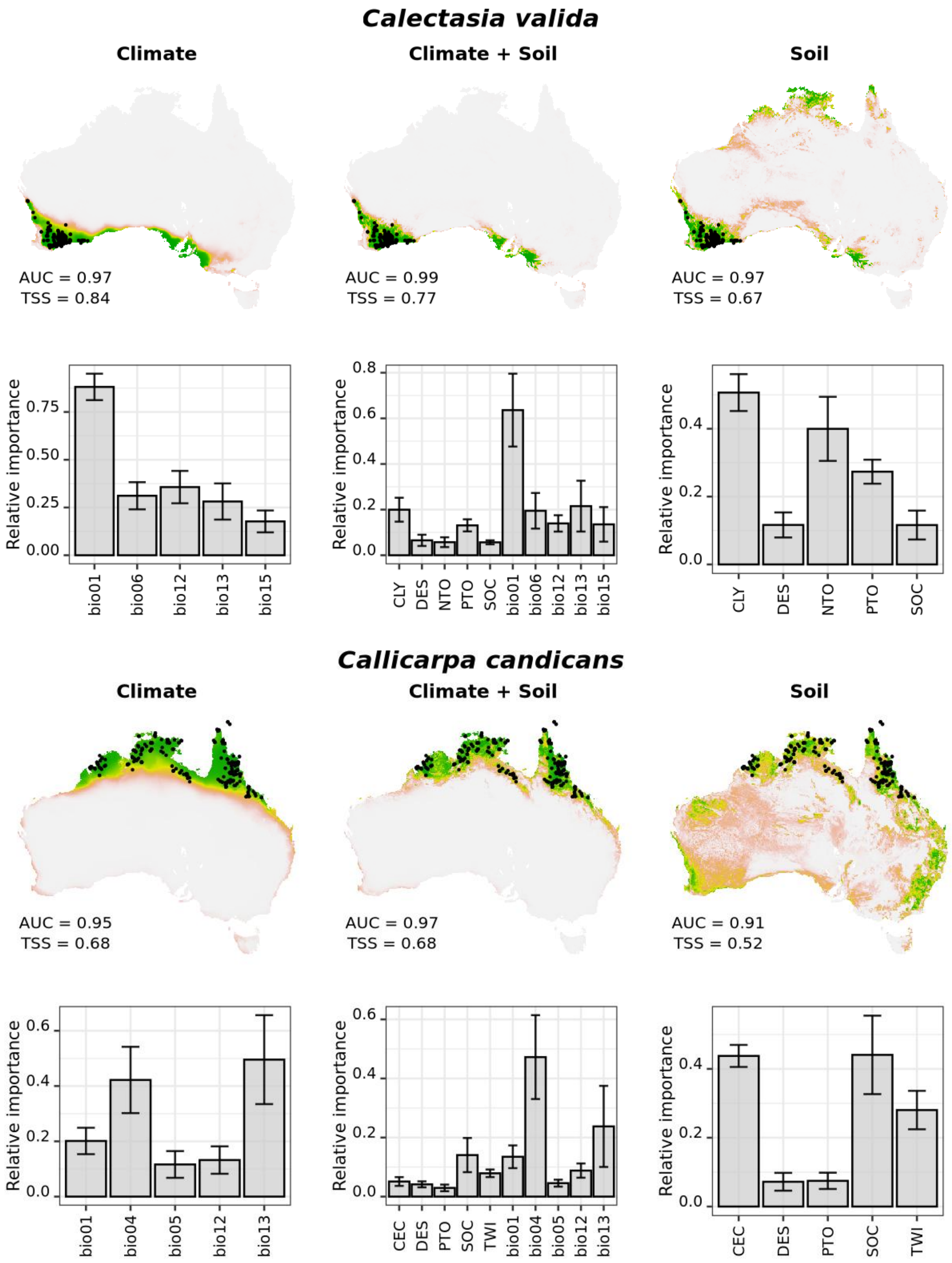
Calectasia valida
Climate
Climate + Soil
Soil
AUC = 0.97
TSS = 0.84
AUC = 0.99
TSS = 0.77
AUC = 0.97
TSS = 0.67
Relative importance
0.00
0.25
0.50
0.75
bio01
bio06
bio12
bio13
bio15
0.0
0.2
0.4
0.6
0.8
CLY
DES
NTO
PTO
SOC
bio01
bio06
bio12
bio13
bio15
0.0
0.2
0.4
CLY
DES
NTO
PTO
SOC
Callicarpa candicans
Climate
Climate + Soil
Soil
AUC = 0.95
TSS = 0.68
AUC = 0.97
TSS = 0.68
AUC = 0.91
TSS = 0.52
Relative importance
0.0
0.2
0.4
0.6
bio01
bio04
bio05
bio12
bio13
CEC
DES
PTO
SOC
TWI
bio01
bio04
bio05
bio12
bio13
CEC
DES
PTO
SOC
TWI

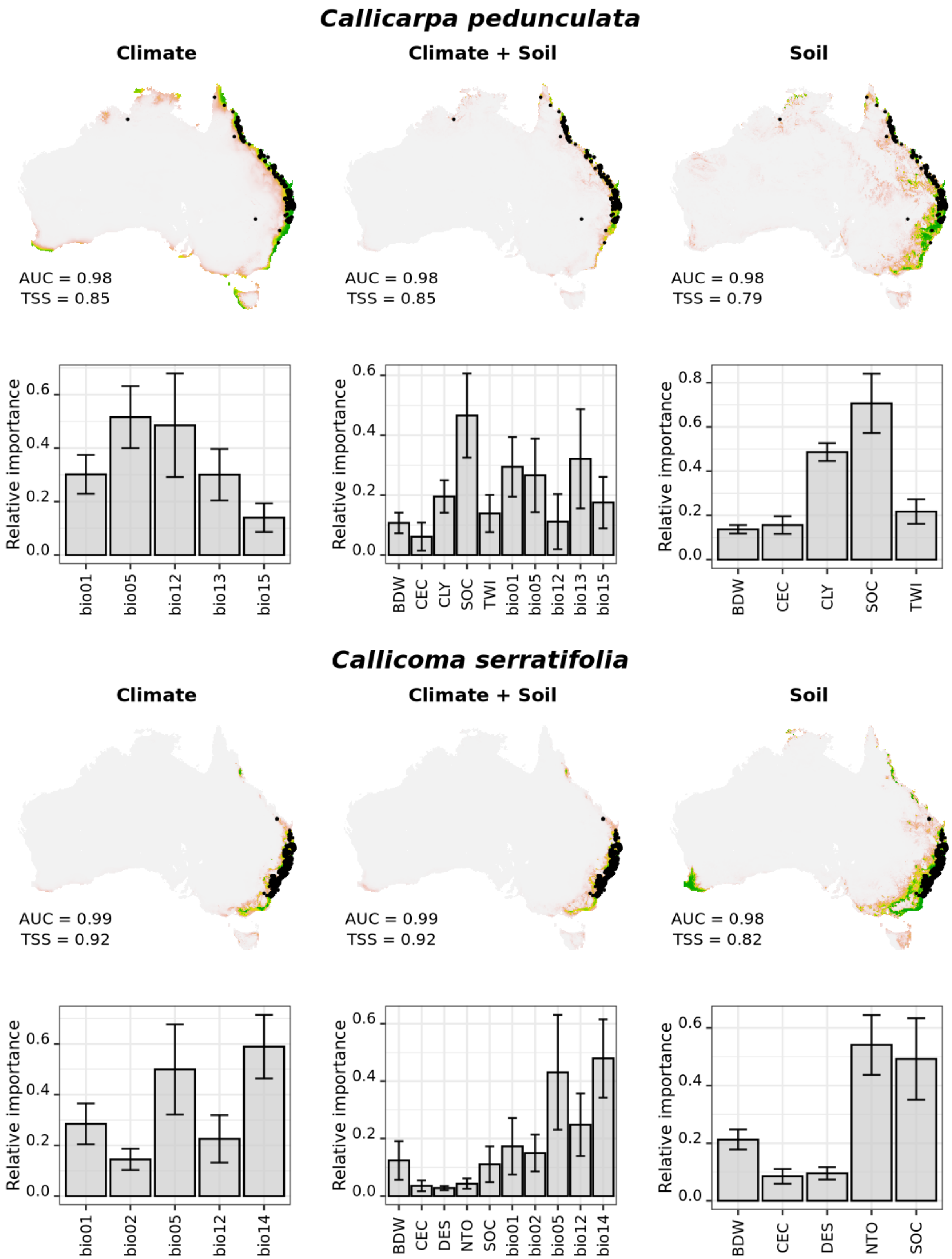
Callicarpa pedunculata
Climate
Climate + Soil
Soil
AUC = 0.98
TSS = 0.85
AUC = 0.98
TSS = 0.85
AUC = 0.98
TSS = 0.79
Relative importance
bio01 bio05 bio12 bio13 bio15
BDW CEC CLY SOC TWI bio01 bio05 bio12 bio13 bio15
BDW CEC CLY SOC TWI
Callicoma serratifolia
Climate
Climate + Soil
Soil
AUC = 0.99
TSS = 0.92
AUC = 0.99
TSS = 0.92
AUC = 0.98
TSS = 0.82
Relative importance
bio01 bio02 bio05 bio12 bio14
BDW CEC DES NTO SOC bio01 bio02 bio05 bio12 bio14
BDW CEC DES NTO SOC

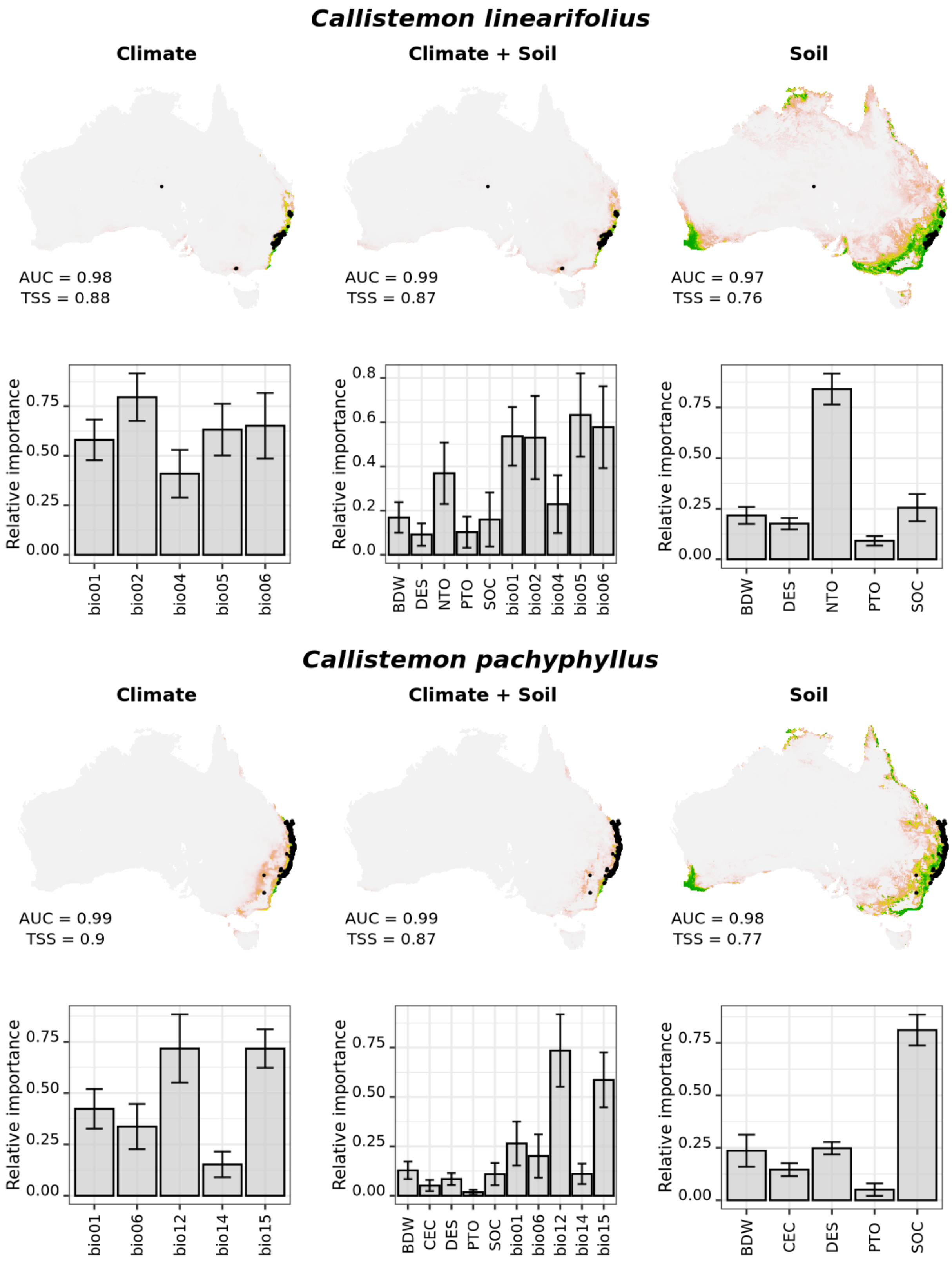

Callistemon linearifolius
Climate
Climate + Soil
Soil
AUC = 0.98
TSS = 0.88
AUC = 0.99
TSS = 0.87
AUC = 0.97
TSS = 0.76
Relative importance
0.00
0.25
0.50
0.75
bio01
bio02
bio04
bio05
bio06
0.0
0.2
0.4
0.6
0.8
BDW
DES
NTO
PTO
SOC
bio01
bio02
bio04
bio05
bio06
BDW
DES
NTO
PTO
SOC
Callistemon pachyphyllus
Climate
Climate + Soil
Soil
AUC = 0.99
TSS = 0.9
AUC = 0.99
TSS = 0.87
AUC = 0.98
TSS = 0.77
Relative importance
0.00
0.25
0.50
0.75
bio01
bio06
bio12
bio14
bio15
BDW
CEC
DES
PTO
SOC
bio01
bio06
bio12
bio14
bio15
BDW
CEC
DES
PTO
SOC

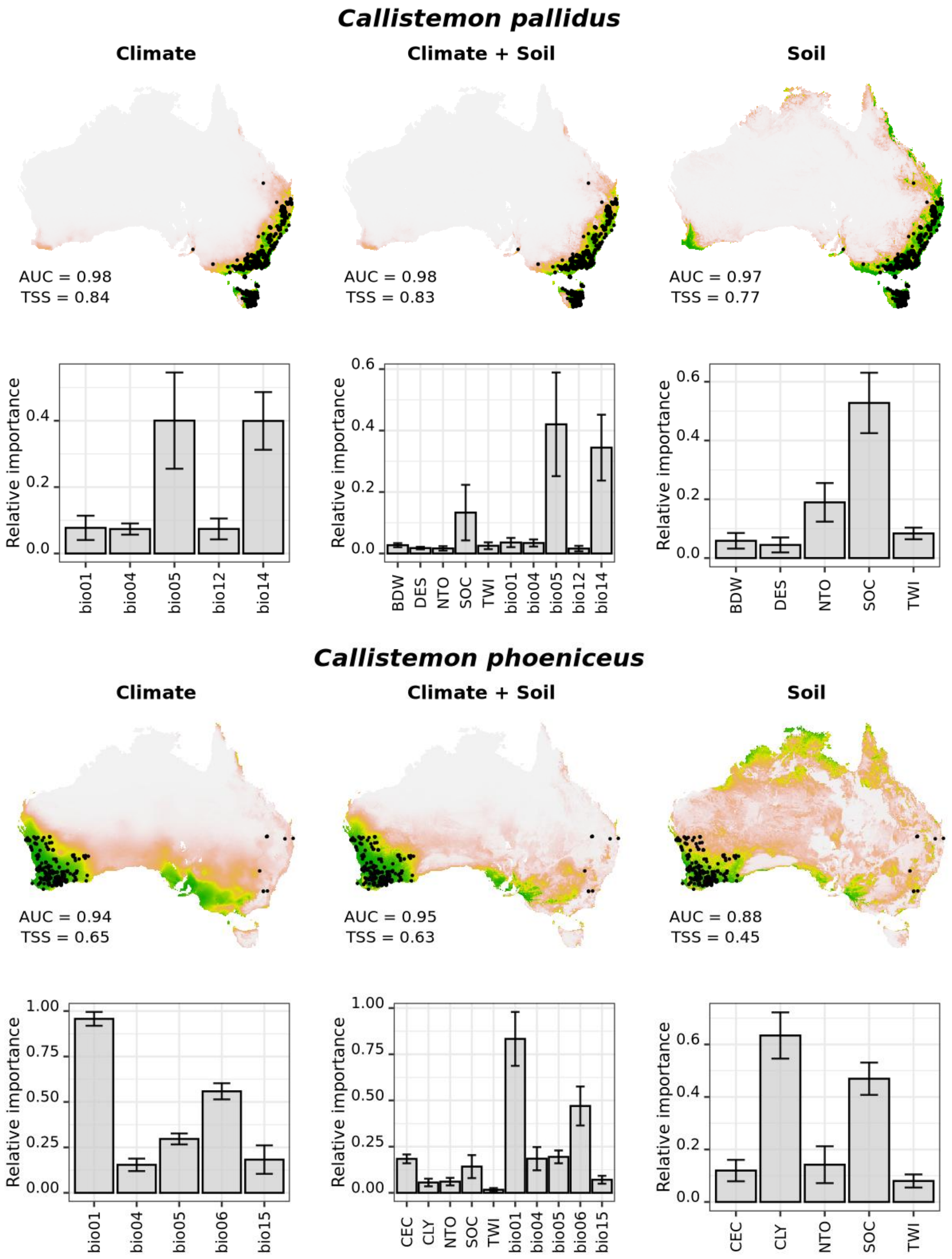
Callistemon pallidus
Climate
Climate + Soil
Soil
AUC = 0.98
TSS = 0.84
AUC = 0.98
TSS = 0.83
AUC = 0.97
TSS = 0.77
Relative importance
bio01 bio04 bio05 bio12 bio14
BDW DES NTO SOC TWI bio01 bio04 bio05 bio12 bio14
BDW DES NTO SOC TWI
Callistemon phoeniceus
Climate
Climate + Soil
Soil
AUC = 0.94
TSS = 0.65
AUC = 0.95
TSS = 0.63
AUC = 0.88
TSS = 0.45
Relative importance
bio01 bio04 bio05 bio06 bio15
CEC CLY NTO SOC TWI bio01 bio04 bio05 bio06 bio15
CEC CLY NTO SOC TWI

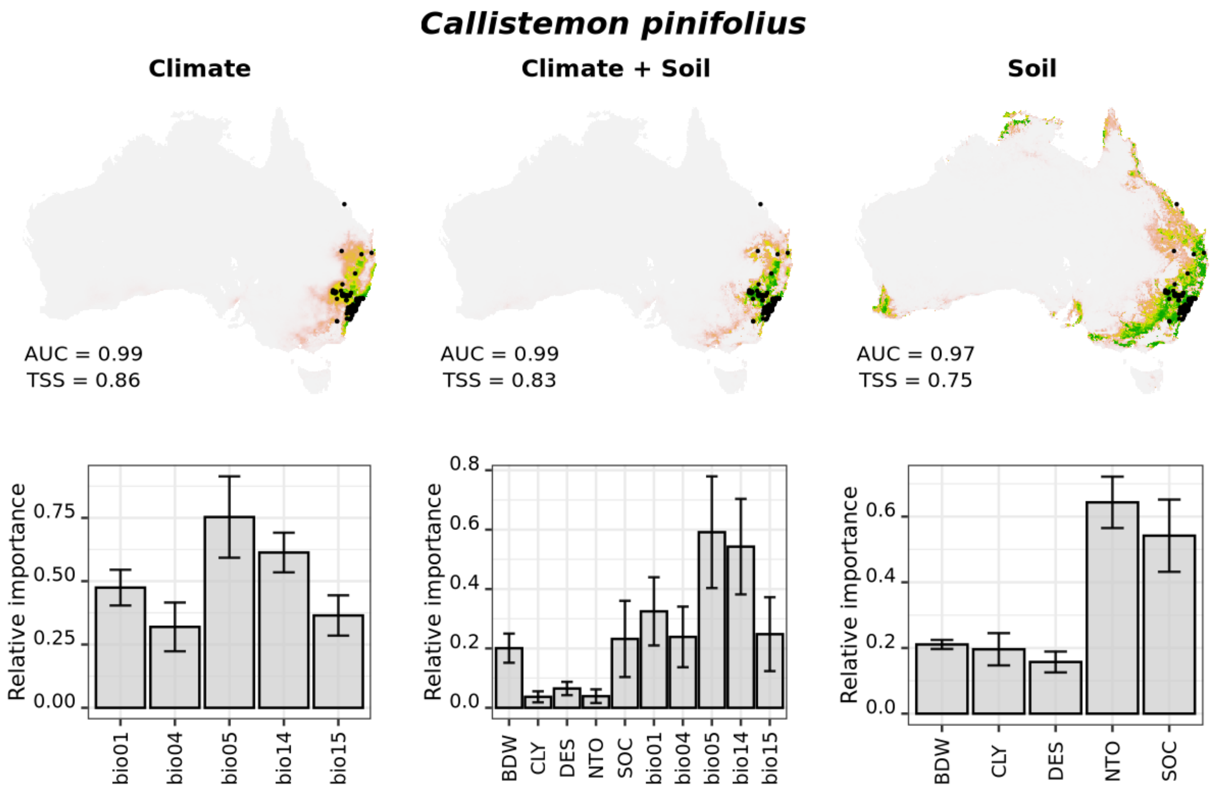

Callistemon pinifolius
Climate
Climate + Soil
Soil
AUC = 0.99
TSS = 0.86
AUC = 0.99
TSS = 0.83
AUC = 0.97
TSS = 0.75
Relative importance
0.00
0.25
0.50
0.75
bio01
bio04
bio05
bio14
bio15
0.0
0.2
0.4
0.6
0.8
BDW
CLY
DES
NTO
SOC
bio01
bio04
bio05
bio14
bio15
0.0
0.2
0.4
0.6
BDW
CLY
DES
NTO
SOC

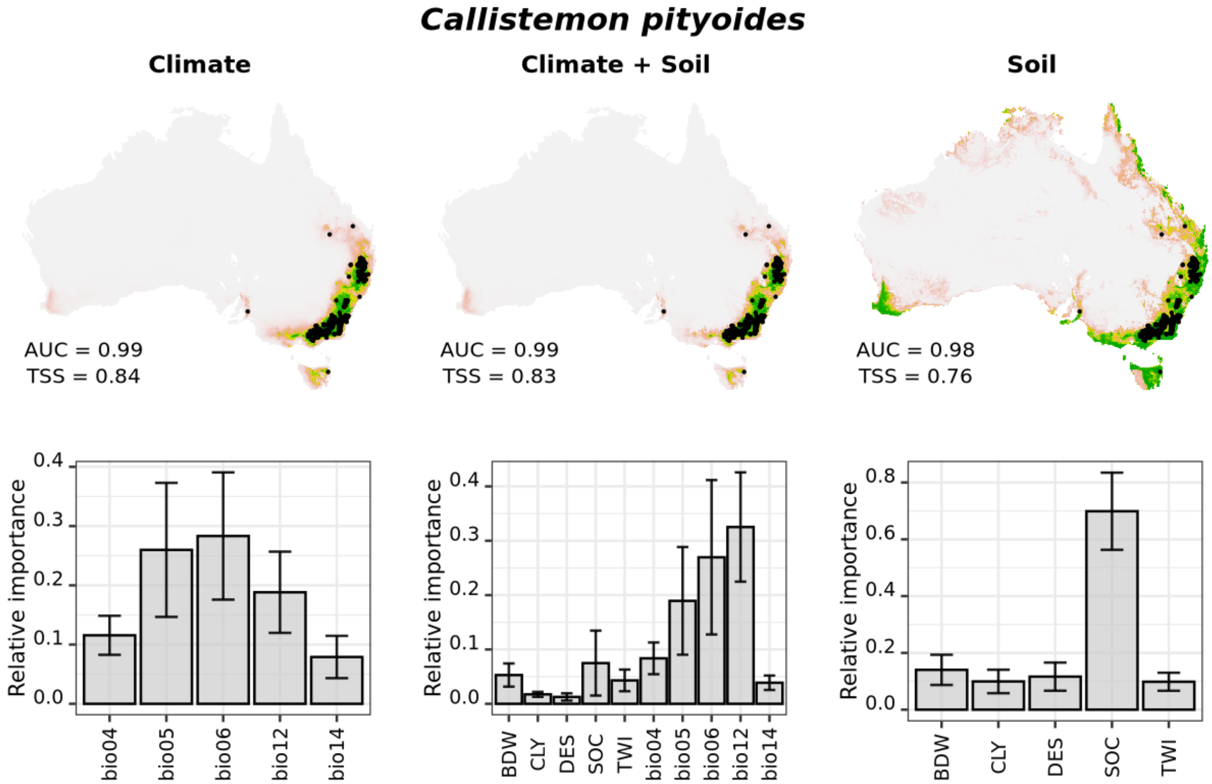

Callistemon pityoides
Climate
Climate + Soil
Soil
AUC = 0.99
TSS = 0.84
AUC = 0.99
TSS = 0.83
AUC = 0.98
TSS = 0.76
Relative importance
0.0
0.1
0.2
0.3
0.4
bio04
bio05
bio06
bio12
bio14
BDW
CLY
DES
SOC
TWI
bio04
bio05
bio06
bio12
bio14
0.0
0.2
0.4
0.6
0.8
BDW
CLY
DES
SOC
TWI

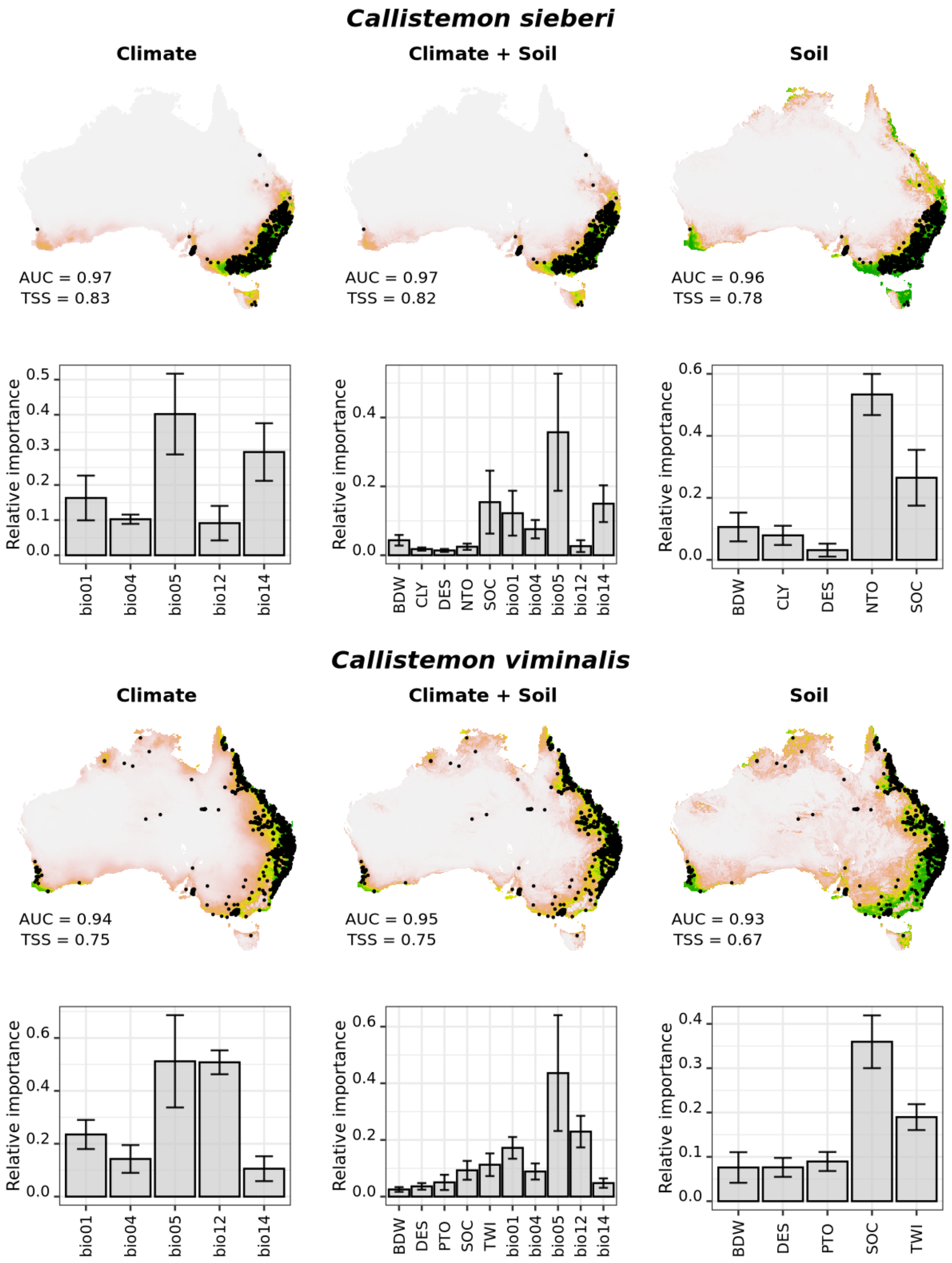

Callistemon sieberi
Climate
Climate + Soil
Soil
AUC = 0.97
TSS = 0.83
AUC = 0.97
TSS = 0.82
AUC = 0.96
TSS = 0.78
Relative importance
bio01
bio04
bio05
bio12
bio14
BDW
CLY
DES
NTO
SOC
Callistemon viminalis
Climate
Climate + Soil
Soil
AUC = 0.94
TSS = 0.75
AUC = 0.95
TSS = 0.75
AUC = 0.93
TSS = 0.67
Relative importance
bio01
bio04
bio05
bio12
bio14
BDW
DES
PTO
SOC
TWI

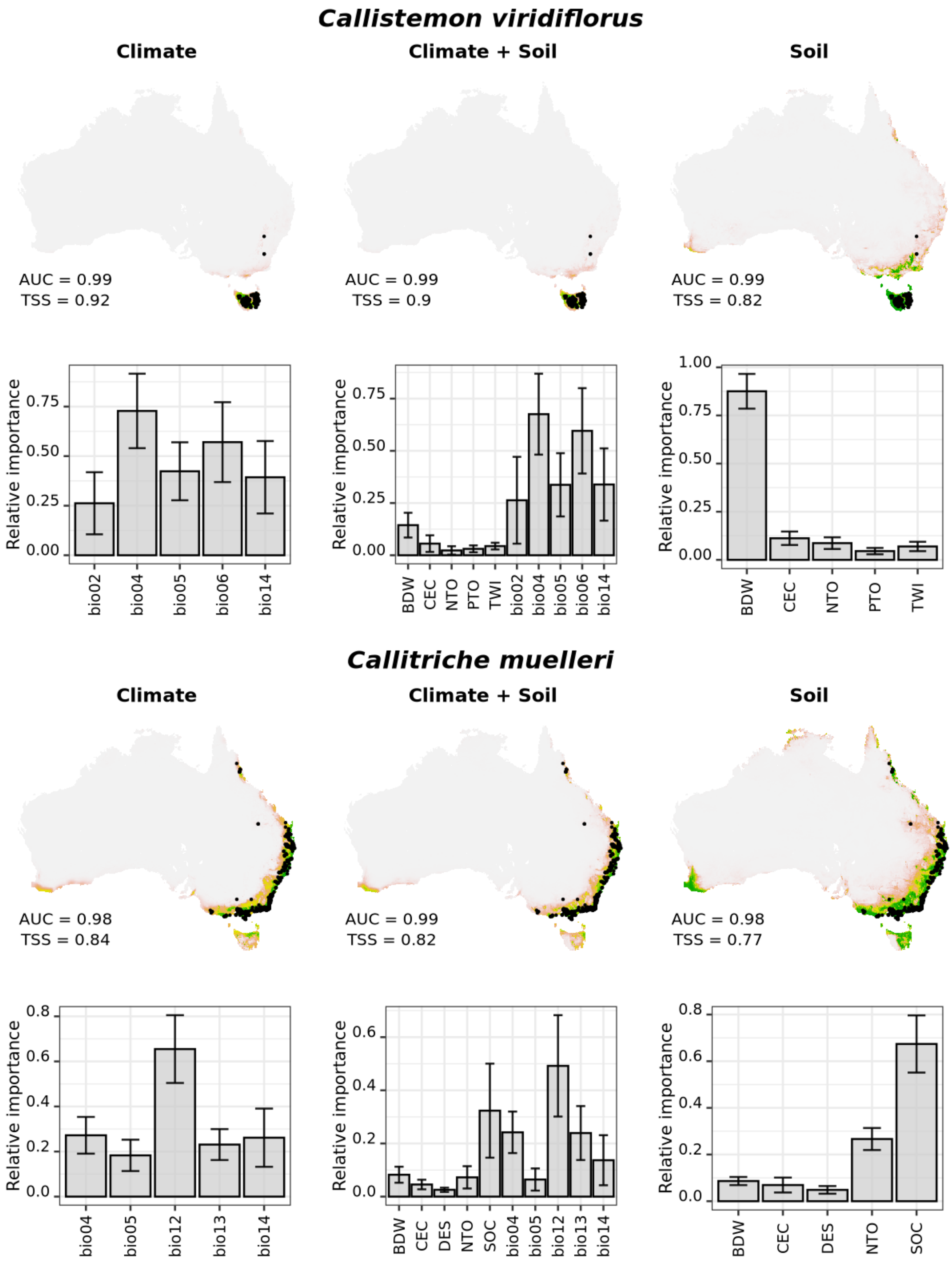
Callistemon viridiflorus
Climate
Climate + Soil
Soil
AUC = 0.99
TSS = 0.92
AUC = 0.99
TSS = 0.9
AUC = 0.99
TSS = 0.82
Relative importance
bio02
bio04
bio05
bio06
bio14
BDW
CEC
NTO
PTO
TWI
Callitriche muelleri
Climate
Climate + Soil
Soil
AUC = 0.98
TSS = 0.84
AUC = 0.99
TSS = 0.82
AUC = 0.98
TSS = 0.77
Relative importance
bio04
bio05
bio12
bio13
bio14
BDW
CEC
DES
NTO
SOC

Callitriche sonderi

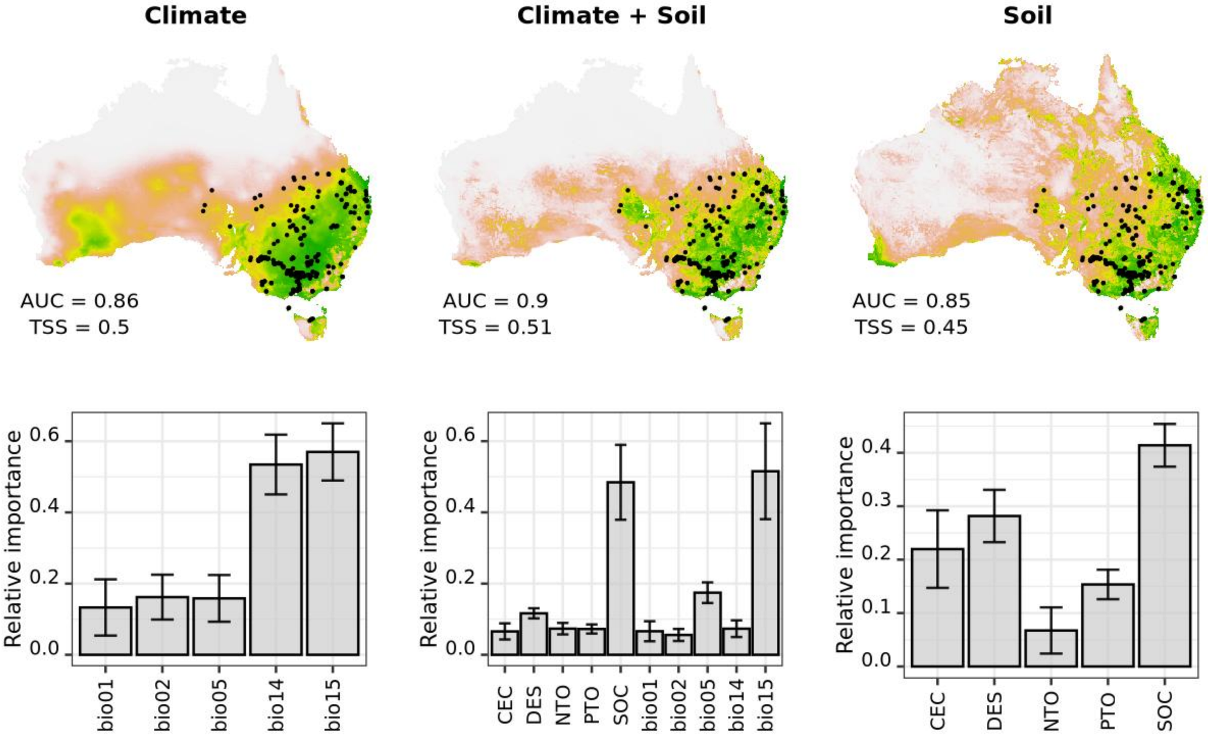

Calocephalus citreus

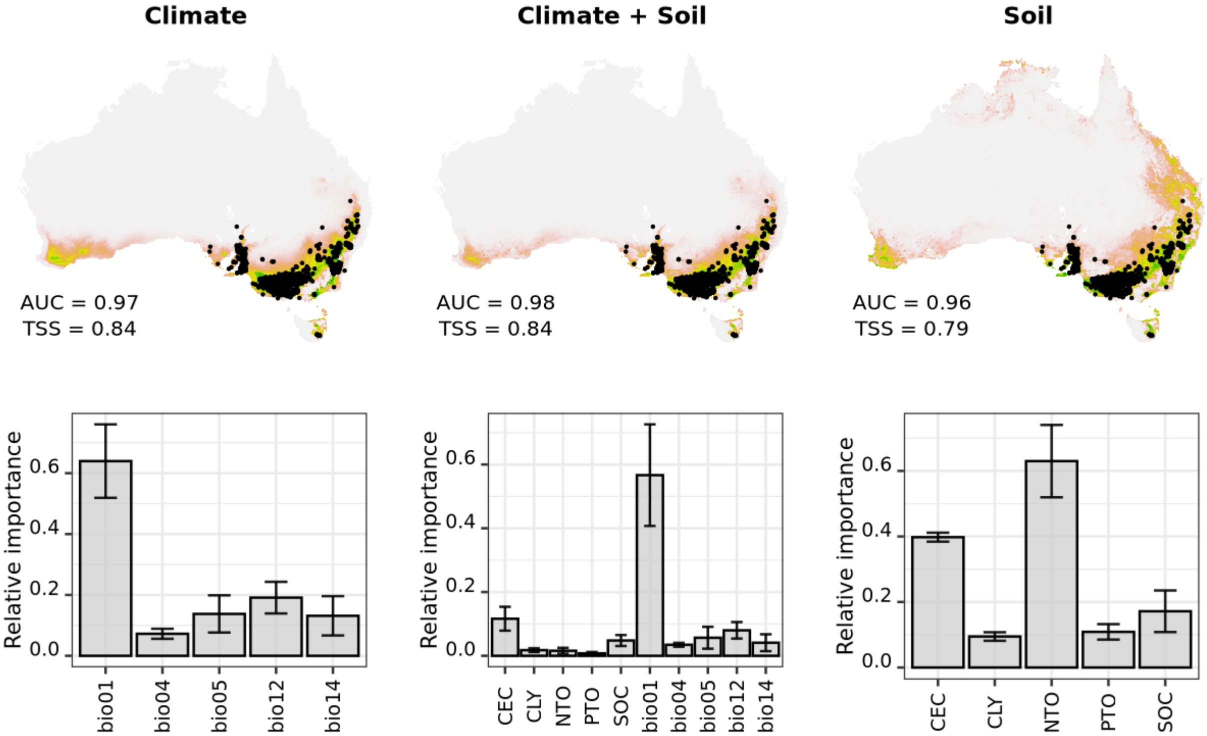

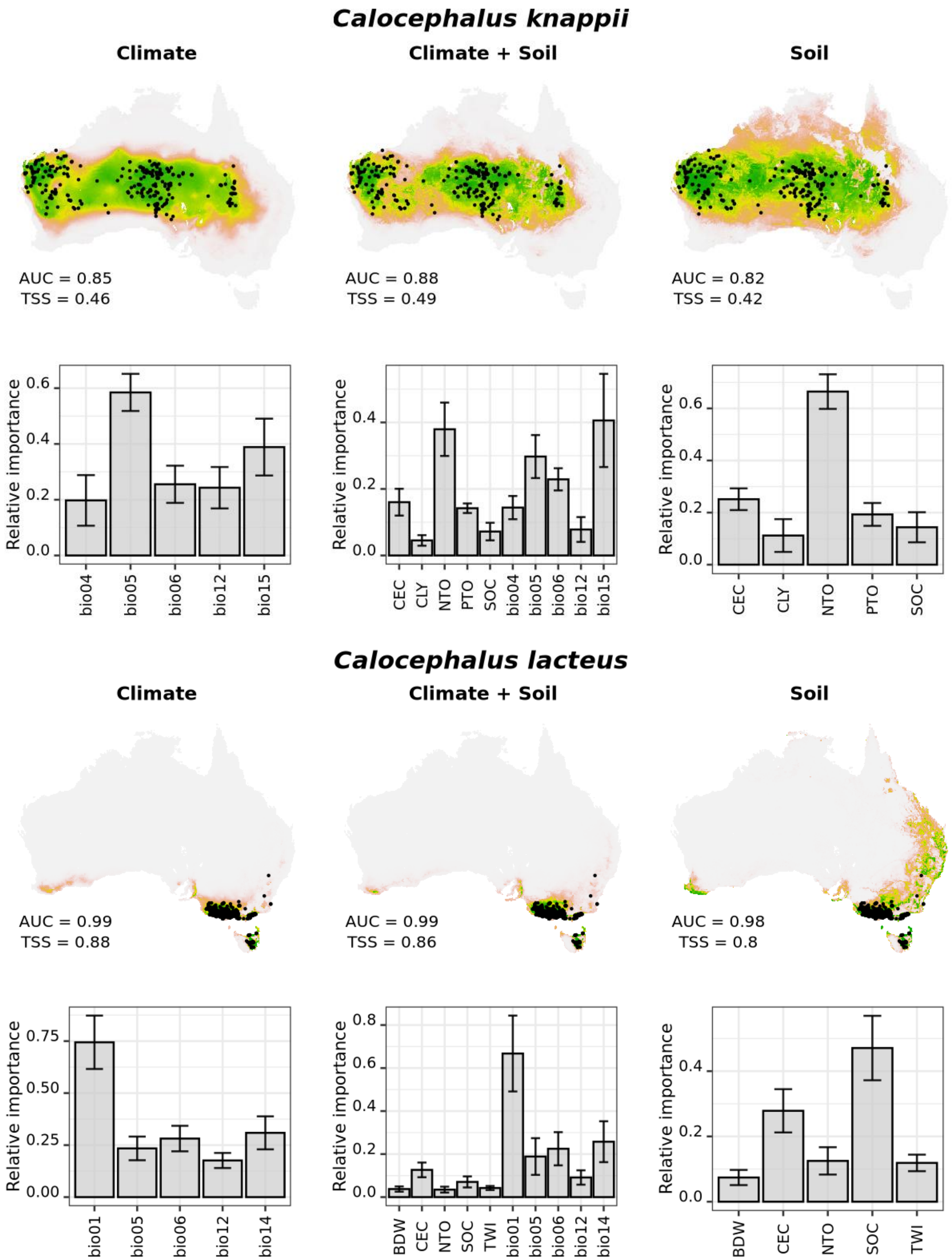
Calocephalus knappii
Climate
Climate + Soil
Soil
AUC = 0.85
TSS = 0.46
AUC = 0.88
TSS = 0.49
AUC = 0.82
TSS = 0.42
Relative importance
bio04
bio05
bio06
bio12
bio15
CEC
CLY
NTO
PTO
SOC
Calocephalus lacteus
AUC = 0.99
TSS = 0.88
AUC = 0.99
TSS = 0.86
AUC = 0.98
TSS = 0.8
bio01
bio14
BDW
TWI

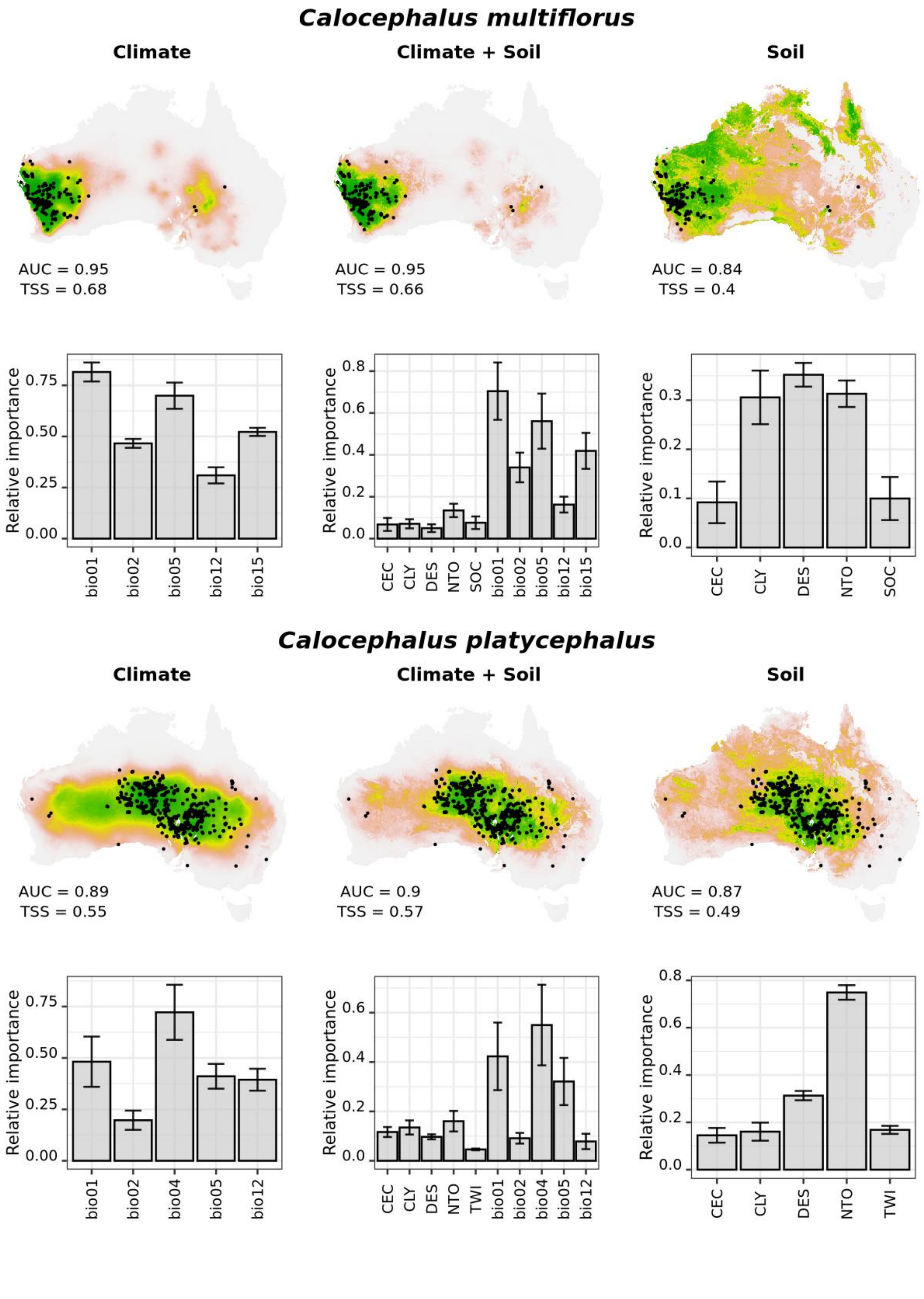
Calocephalus multiflorus
Climate
Climate + Soil
Soil
AUC = 0.95
TSS = 0.68
AUC = 0.95
TSS = 0.66
AUC = 0.84
TSS = 0.4
Relative importance
bio01
bio02
bio05
bio12
bio15
CEC
CLY
DES
NTO
SOC
Calocephalus platycephalus
Climate
Climate + Soil
Soil
AUC = 0.89
TSS = 0.55
AUC = 0.9
TSS = 0.57
AUC = 0.87
TSS = 0.49
Relative importance
bio01
bio02
bio04
bio05
bio12
CEC
CLY
DES
NTO
TWI

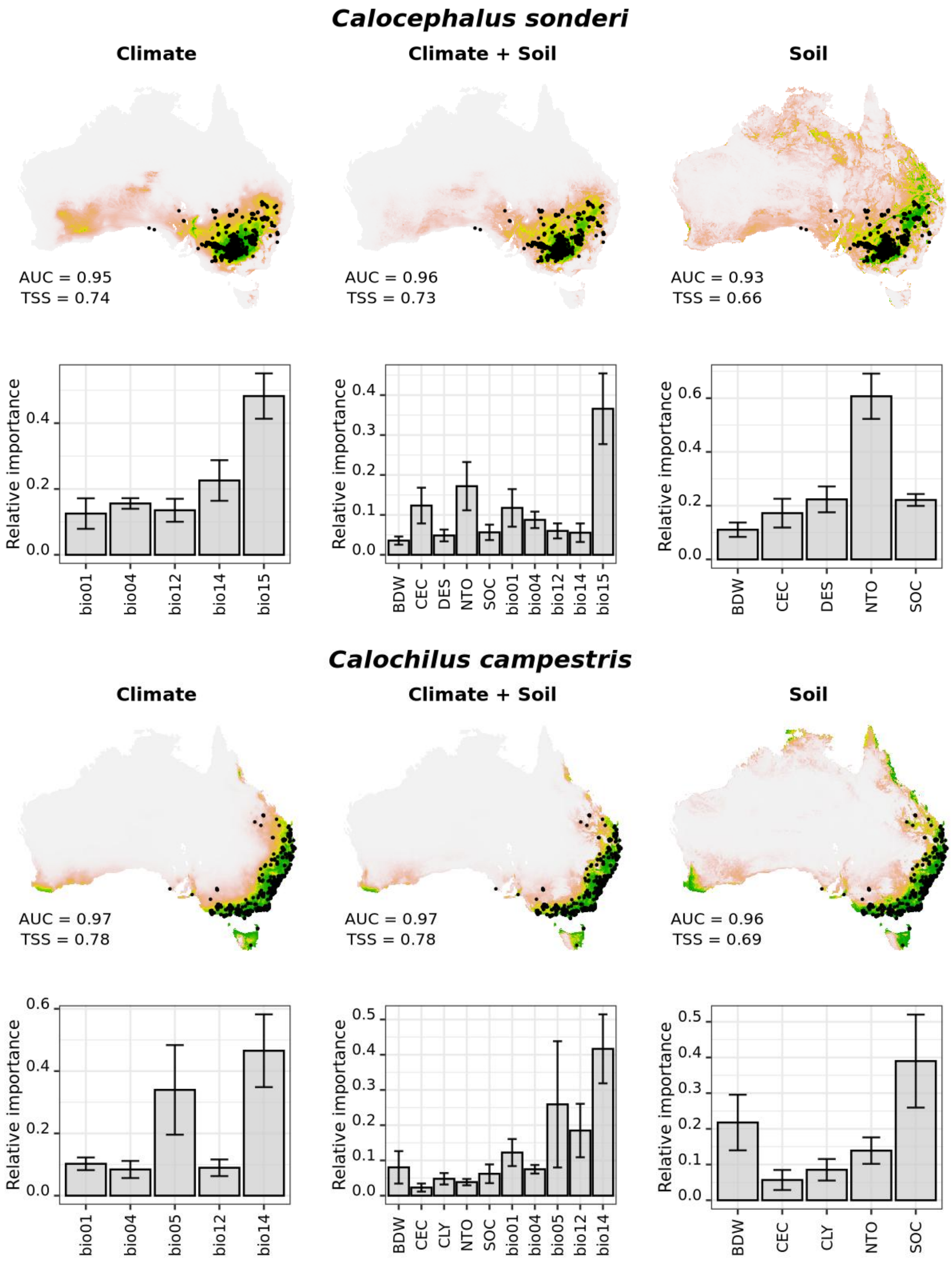
Calocephalus sonderi
Climate
Climate + Soil
Soil
AUC = 0.95
TSS = 0.74
AUC = 0.96
TSS = 0.73
AUC = 0.93
TSS = 0.66
Relative importance
bio01 bio04 bio12 bio14 bio15
BDW CEC DES NTO SOC bio01 bio04 bio12 bio14 bio15
BDW CEC DES NTO SOC
Calochilus campestris
Climate
Climate + Soil
Soil
AUC = 0.97
TSS = 0.78
AUC = 0.97
TSS = 0.78
AUC = 0.96
TSS = 0.69
Relative importance
bio01 bio04 bio05 bio12 bio14
BDW CEC CLY NTO SOC bio01 bio04 bio05 bio12 bio14
BDW CEC CLY NTO SOC

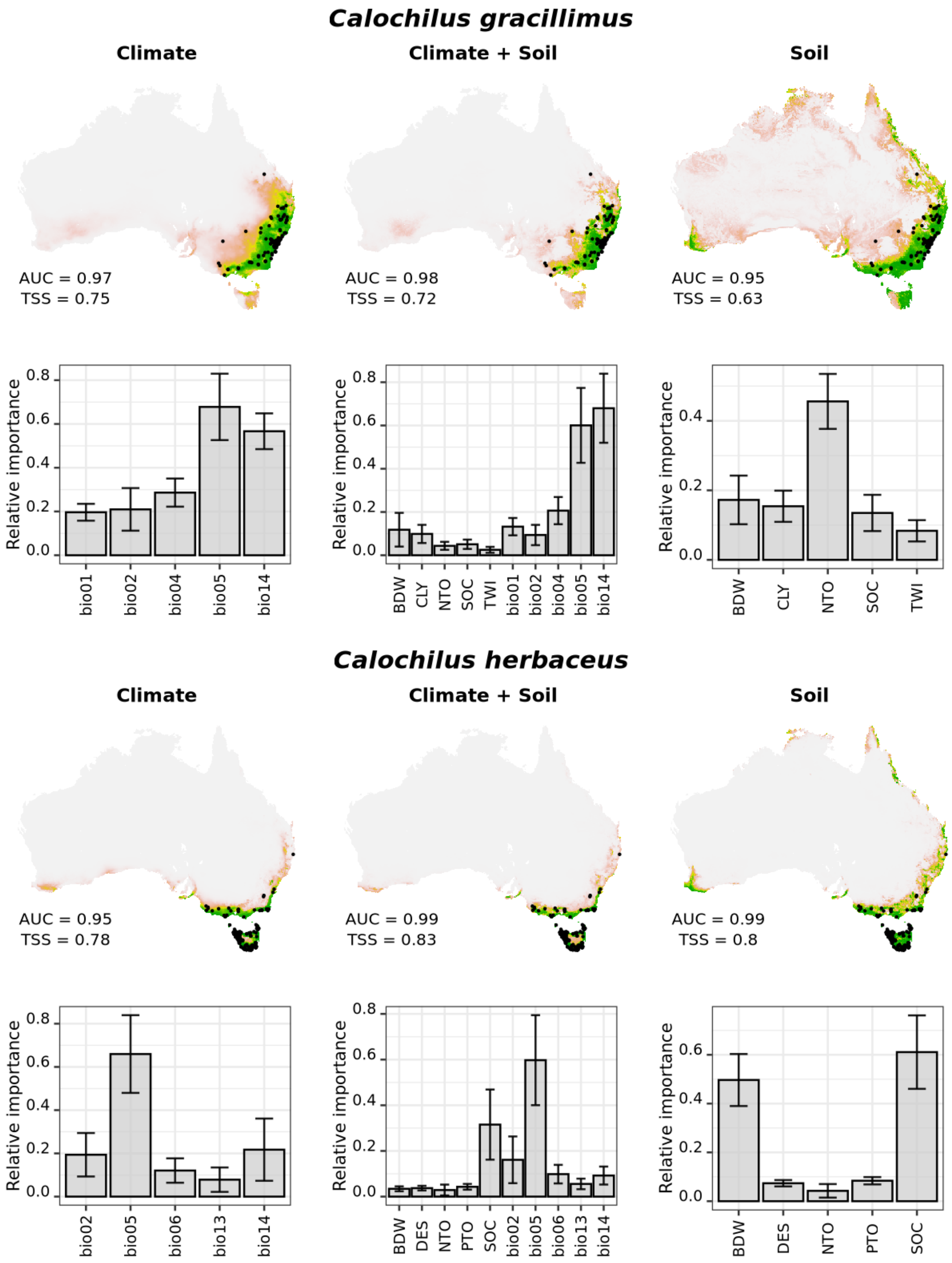

Calochilus gracillimus
Climate
Climate + Soil
Soil
AUC = 0.97
TSS = 0.75
AUC = 0.98
TSS = 0.72
AUC = 0.95
TSS = 0.63
Relative importance
0.0
0.2
0.4
0.6
0.8
bio01
bio02
bio04
bio05
bio14
BDW
CLY
NTO
SOC
TWI
bio01
bio02
bio04
bio05
bio14
BDW
CLY
NTO
SOC
TWI
Calochilus herbaceus
Climate
Climate + Soil
Soil
AUC = 0.95
TSS = 0.78
AUC = 0.99
TSS = 0.83
AUC = 0.99
TSS = 0.8
Relative importance
bio02
bio05
bio06
bio13
bio14
BDW
DES
NTO
PTO
SOC
bio02
bio05
bio06
bio13
bio14
BDW
DES
NTO
PTO
SOC

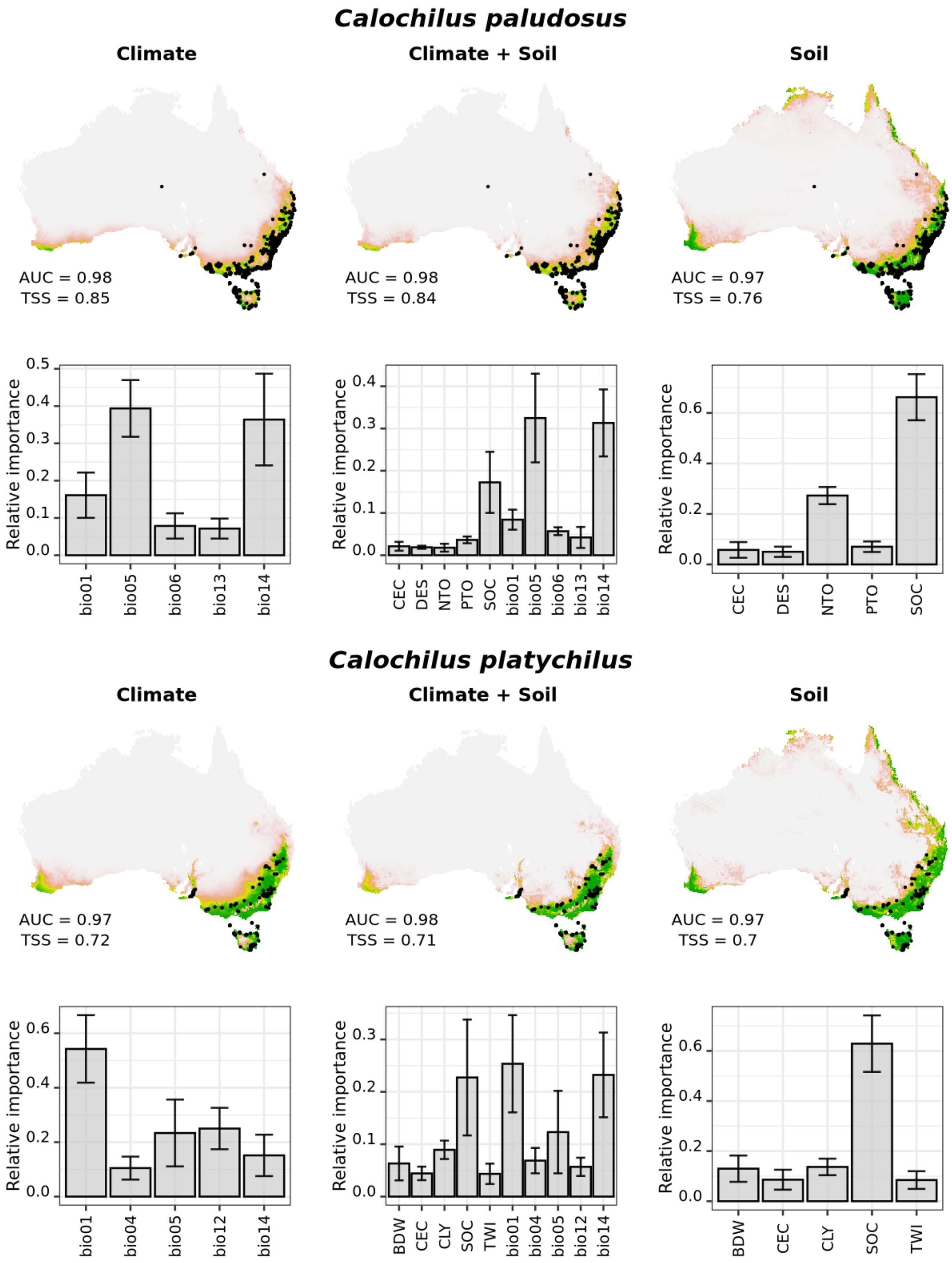

Calochilus paludosus
Climate
Climate + Soil
Soil
AUC = 0.98
TSS = 0.85
AUC = 0.98
TSS = 0.84
AUC = 0.97
TSS = 0.76
Relative importance
bio01 bio05 bio06 bio13 bio14
CEC DES NTO PTO SOC bio01 bio05 bio06 bio13 bio14
CEC DES NTO PTO SOC
Calochilus platychilus
Climate
Climate + Soil
Soil
AUC = 0.97
TSS = 0.72
AUC = 0.98
TSS = 0.71
AUC = 0.97
TSS = 0.7
Relative importance
bio01 bio04 bio05 bio12 bio14
BDW CEC CLY SOC TWI bio01 bio04 bio05 bio12 bio14
BDW CEC CLY SOC TWI

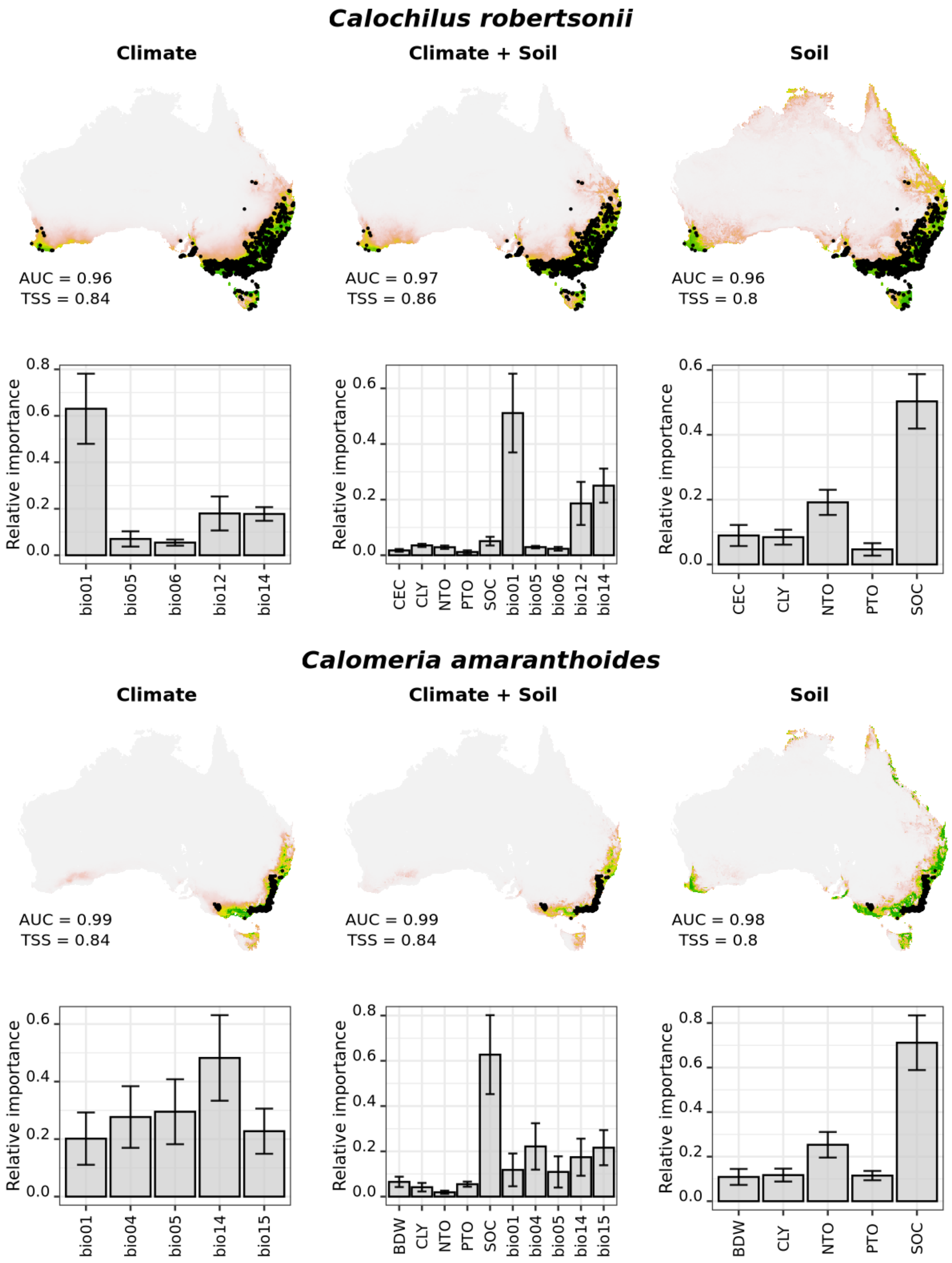

Calochilus robertsonii
Climate
Climate + Soil
Soil
AUC = 0.96
TSS = 0.84
AUC = 0.97
TSS = 0.86
AUC = 0.96
TSS = 0.8
Relative importance
bio01
bio05
bio06
bio12
bio14
CEC
CLY
NTO
PTO
SOC
Calomeria amaranthoides
AUC = 0.99
TSS = 0.84
AUC = 0.99
TSS = 0.84
AUC = 0.98
TSS = 0.8
bio04
bio15
BDW

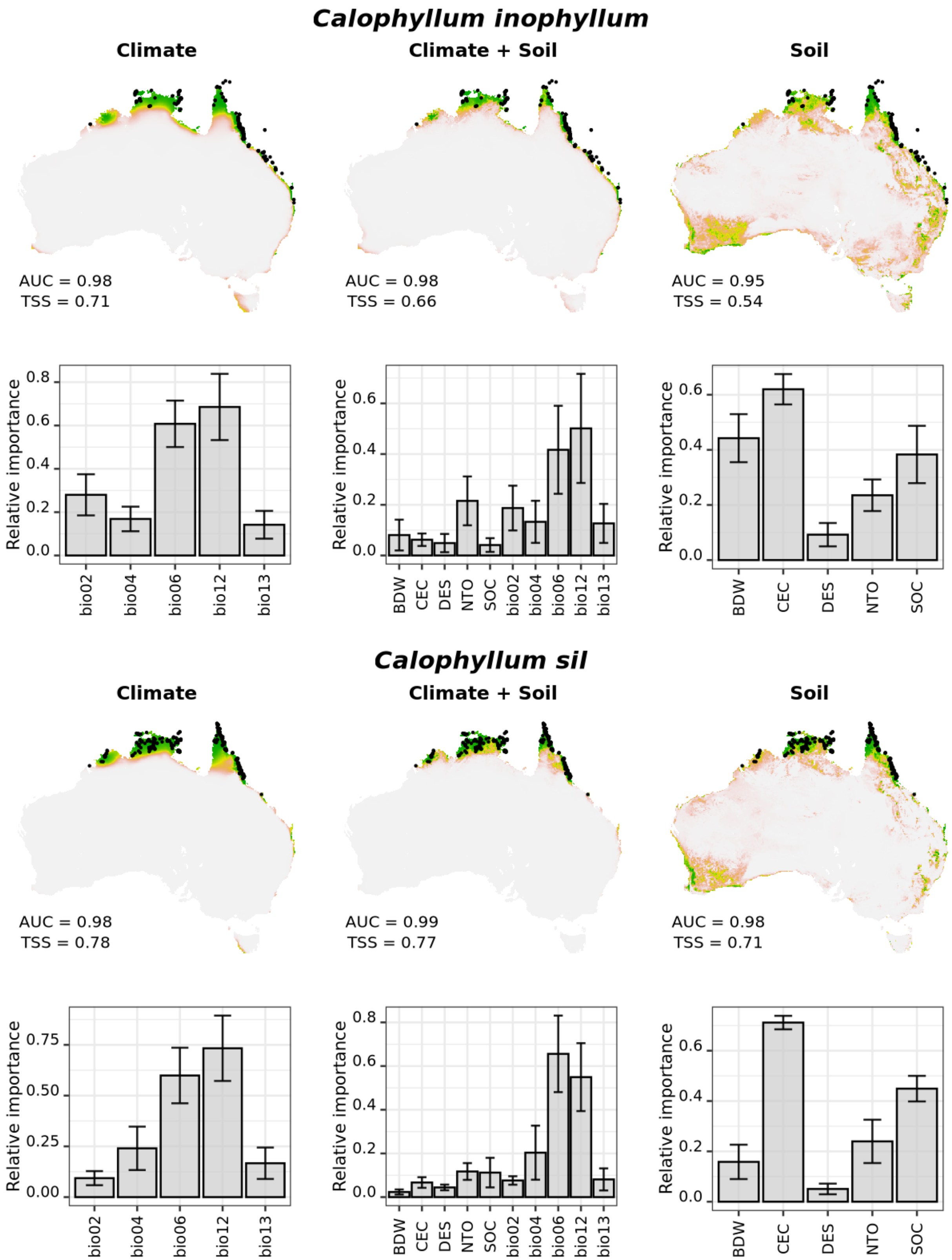
Calophyllum inophyllum
Climate
Climate + Soil
Soil
AUC = 0.98
TSS = 0.71
AUC = 0.98
TSS = 0.66
AUC = 0.95
TSS = 0.54
Relative importance
bio02 bio04 bio06 bio12 bio13
BDW CEC DES NTO SOC bio02 bio04 bio06 bio12 bio13
BDW CEC DES NTO SOC
Calophyllum sil
Climate
Climate + Soil
Soil
AUC = 0.98
TSS = 0.78
AUC = 0.99
TSS = 0.77
AUC = 0.98
TSS = 0.71
Relative importance
bio02 bio04 bio06 bio12 bio13
BDW CEC DES NTO SOC bio02 bio04 bio06 bio12 bio13
BDW CEC DES NTO SOC

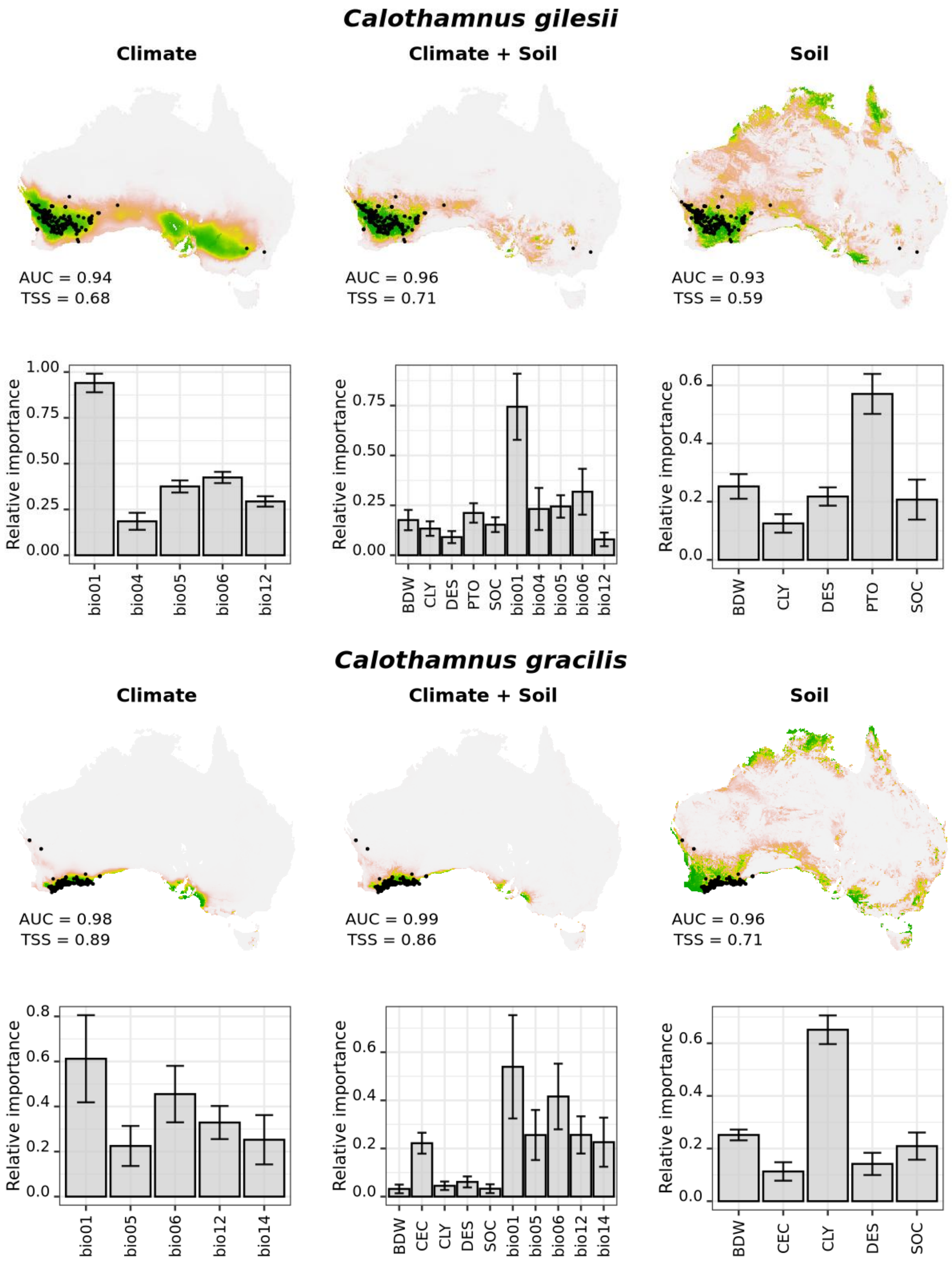
Calothamnus gilesii
Climate
Climate + Soil
Soil
AUC = 0.94
TSS = 0.68
AUC = 0.96
TSS = 0.71
AUC = 0.93
TSS = 0.59
Relative importance
1.00
0.75
0.50
0.25
0.00
bio01
bio04
bio05
bio06
bio12
BDW
CLY
DES
PTO
SOC
0.6
0.4
0.2
0.0
Calothamnus gracilis
Climate
Climate + Soil
Soil
AUC = 0.98
TSS = 0.89
AUC = 0.99
TSS = 0.86
AUC = 0.96
TSS = 0.71
0.8
bio14
CEC

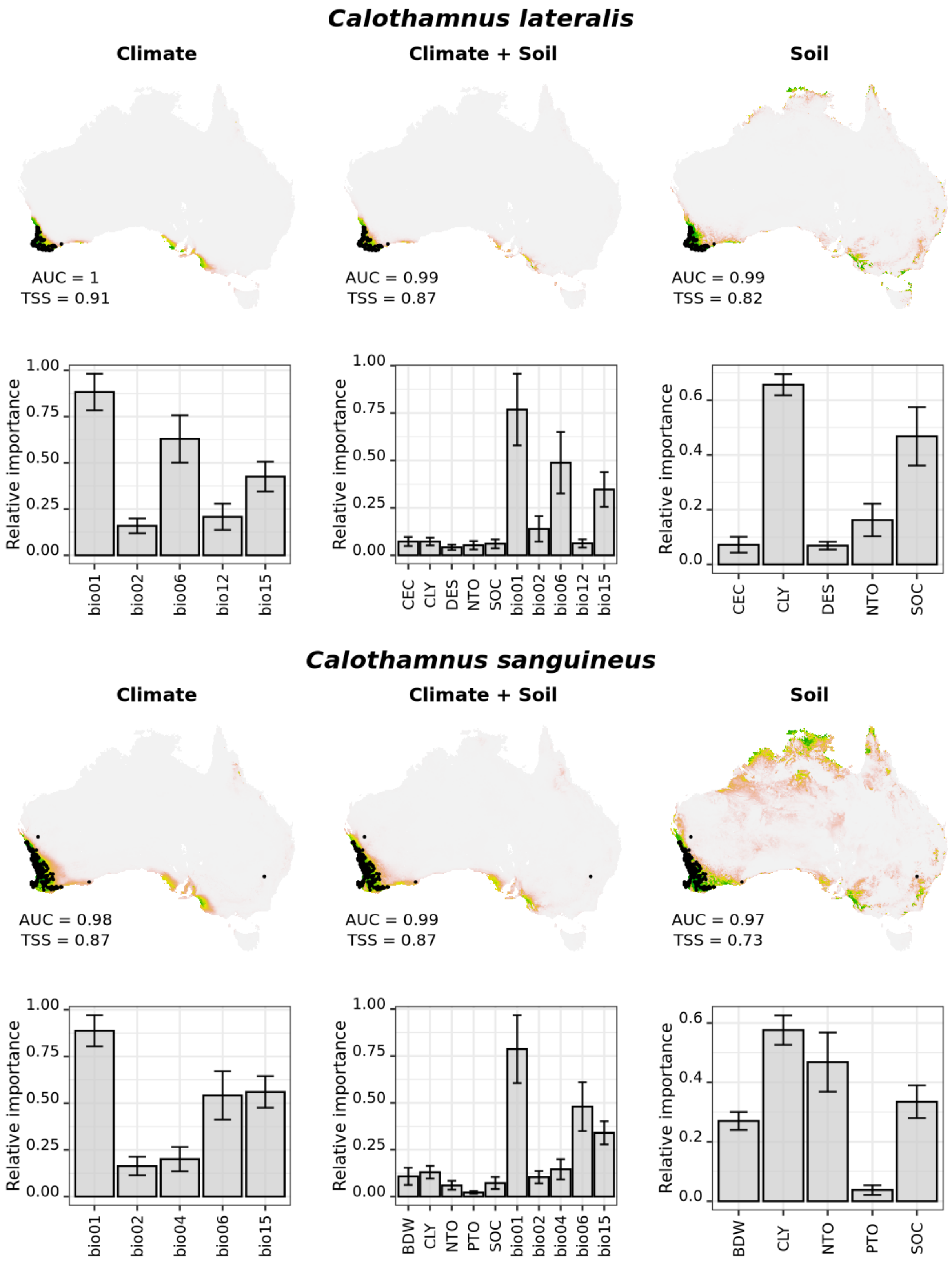
Calothamnus lateralis
Climate
Climate + Soil
Soil
AUC = 1
TSS = 0.91
AUC = 0.99
TSS = 0.87
AUC = 0.99
TSS = 0.82
Relative importance
bio01
bio02
bio06
bio12
bio15
CEC
CLY
DES
NTO
SOC
Calothamnus sanguineus
AUC = 0.98
TSS = 0.87
AUC = 0.99
TSS = 0.87
AUC = 0.97
TSS = 0.73
bio04
BDW
PTO

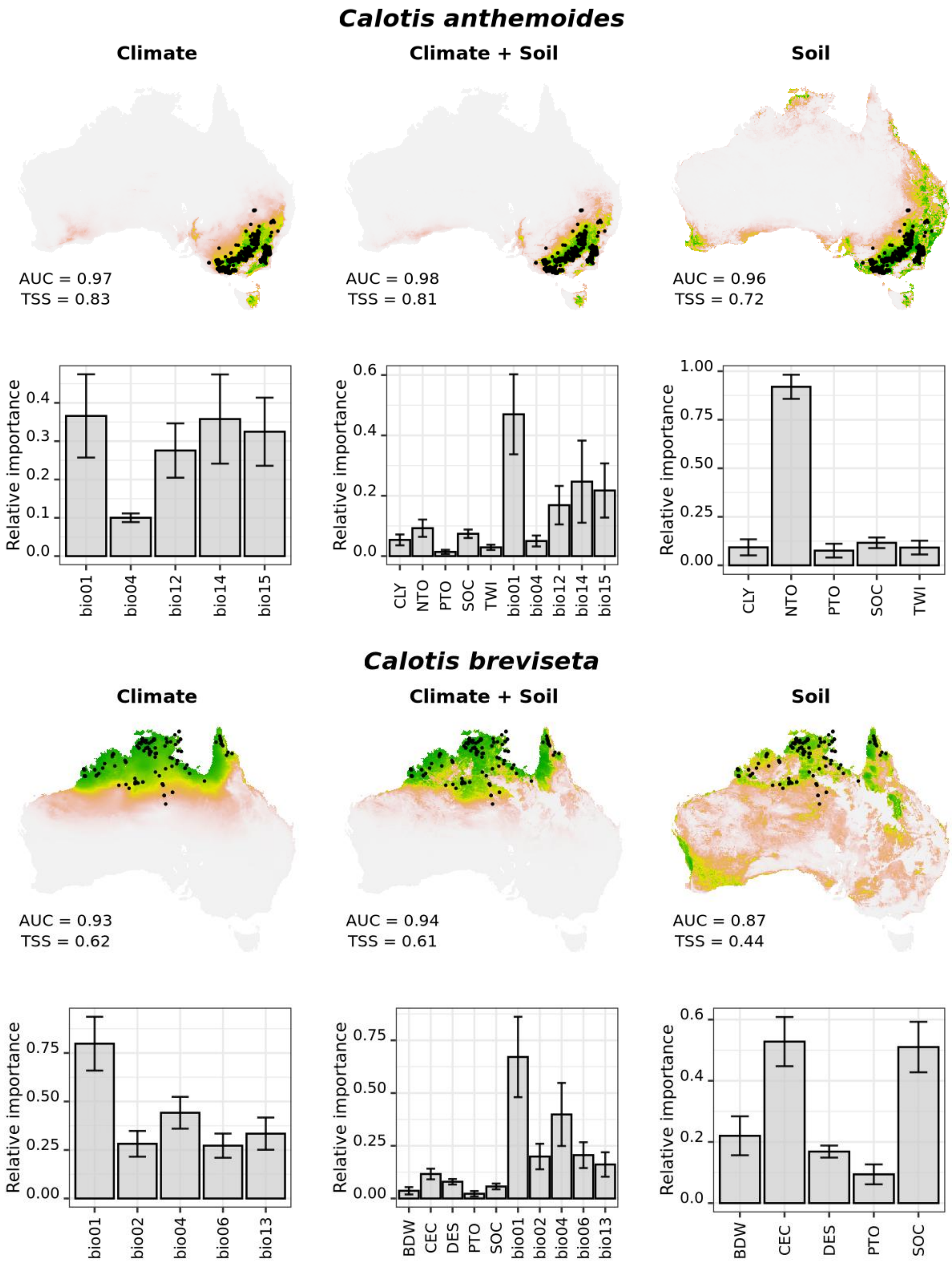

Calotis anthemoides
Climate
Climate + Soil
Soil
AUC = 0.97
TSS = 0.83
AUC = 0.98
TSS = 0.81
AUC = 0.96
TSS = 0.72
Relative importance
bio01
bio04
bio12
bio14
bio15
CLY
NTO
PTO
SOC
TWI
Calotis breviseta
Climate
Climate + Soil
Soil
AUC = 0.93
TSS = 0.62
AUC = 0.94
TSS = 0.61
AUC = 0.87
TSS = 0.44
Relative importance
bio01
bio02
bio04
bio06
bio13
BDW
CEC
DES
PTO
SOC

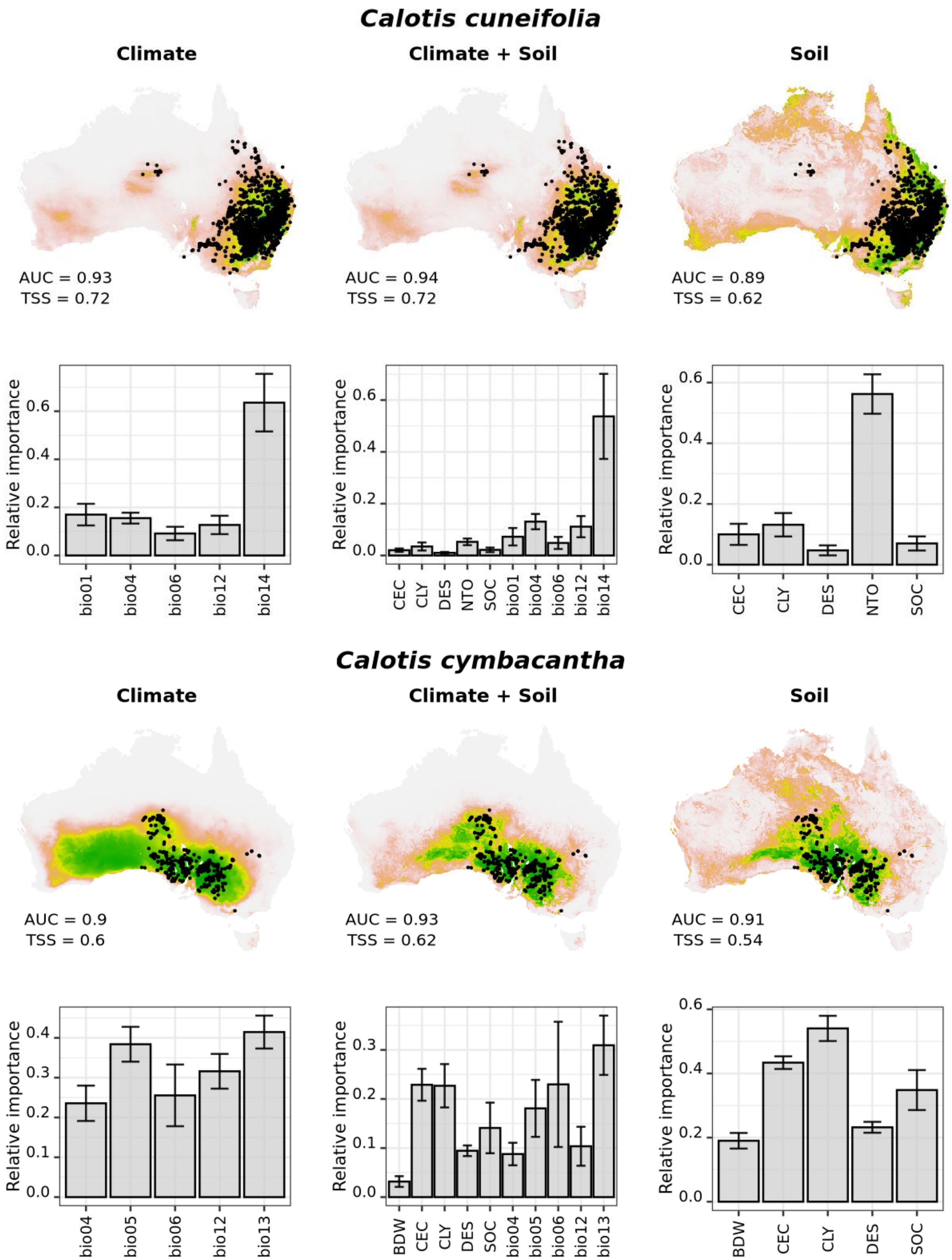
Calotis cuneifolia
Climate
Climate + Soil
Soil
AUC = 0.93
TSS = 0.72
AUC = 0.94
TSS = 0.72
AUC = 0.89
TSS = 0.62
Relative importance
bio01
bio04
bio06
bio12
bio14
CEC
CLY
DES
NTO
SOC
Calotis cymbacantha
AUC = 0.9
TSS = 0.6
AUC = 0.93
TSS = 0.62
AUC = 0.91
TSS = 0.54
BDW
bio05
bio13

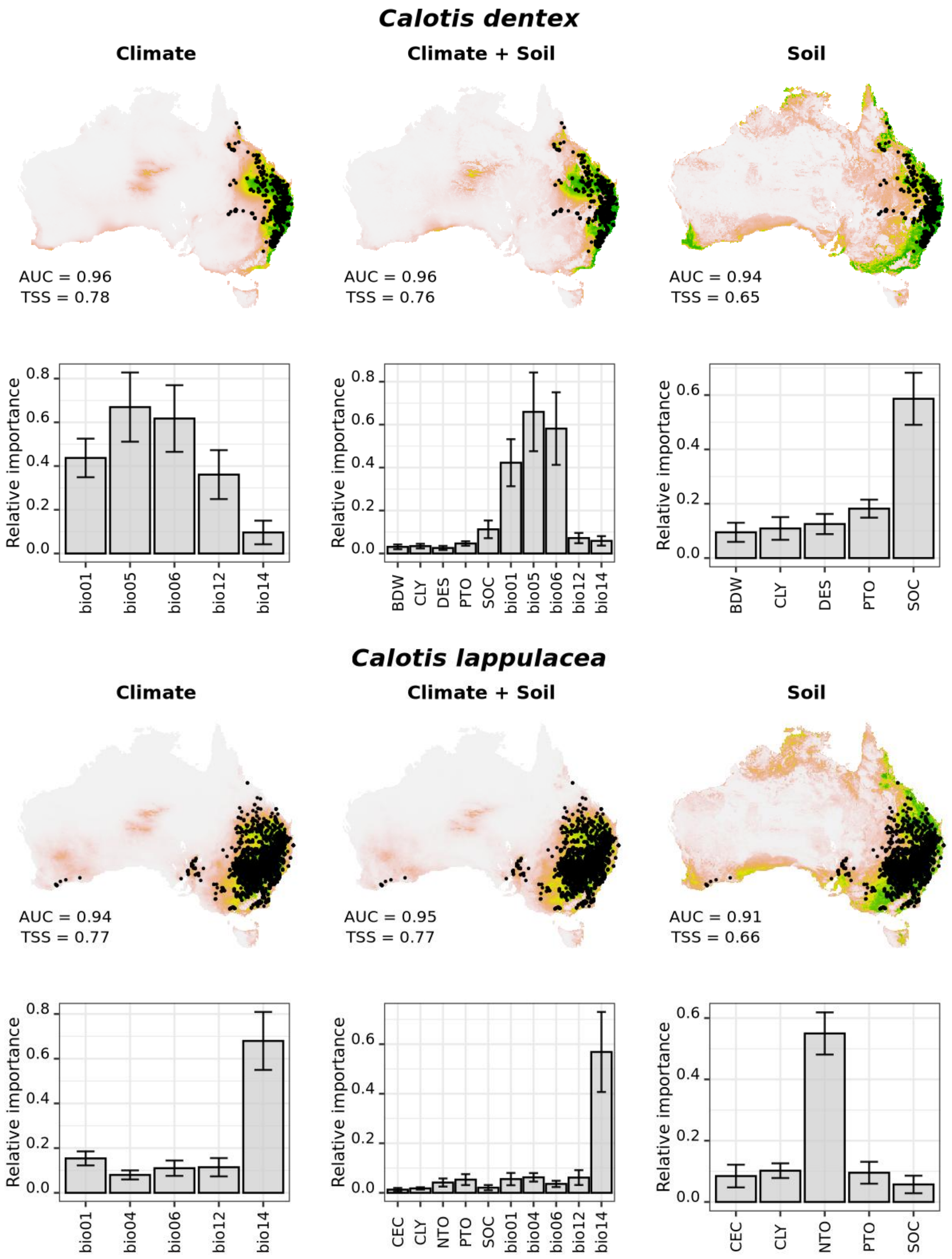
Calotis dentex
Climate
Climate + Soil
Soil
AUC = 0.96
TSS = 0.78
AUC = 0.96
TSS = 0.76
AUC = 0.94
TSS = 0.65
Relative importance
bio01
bio05
bio06
bio12
bio14
BDW
CLY
DES
PTO
SOC
Calotis lappulacea
Climate
Climate + Soil
Soil
AUC = 0.94
TSS = 0.77
AUC = 0.95
TSS = 0.77
AUC = 0.91
TSS = 0.66
Relative importance
bio01
bio04
bio06
bio12
bio14
CEC
CLY
NTO
PTO
SOC

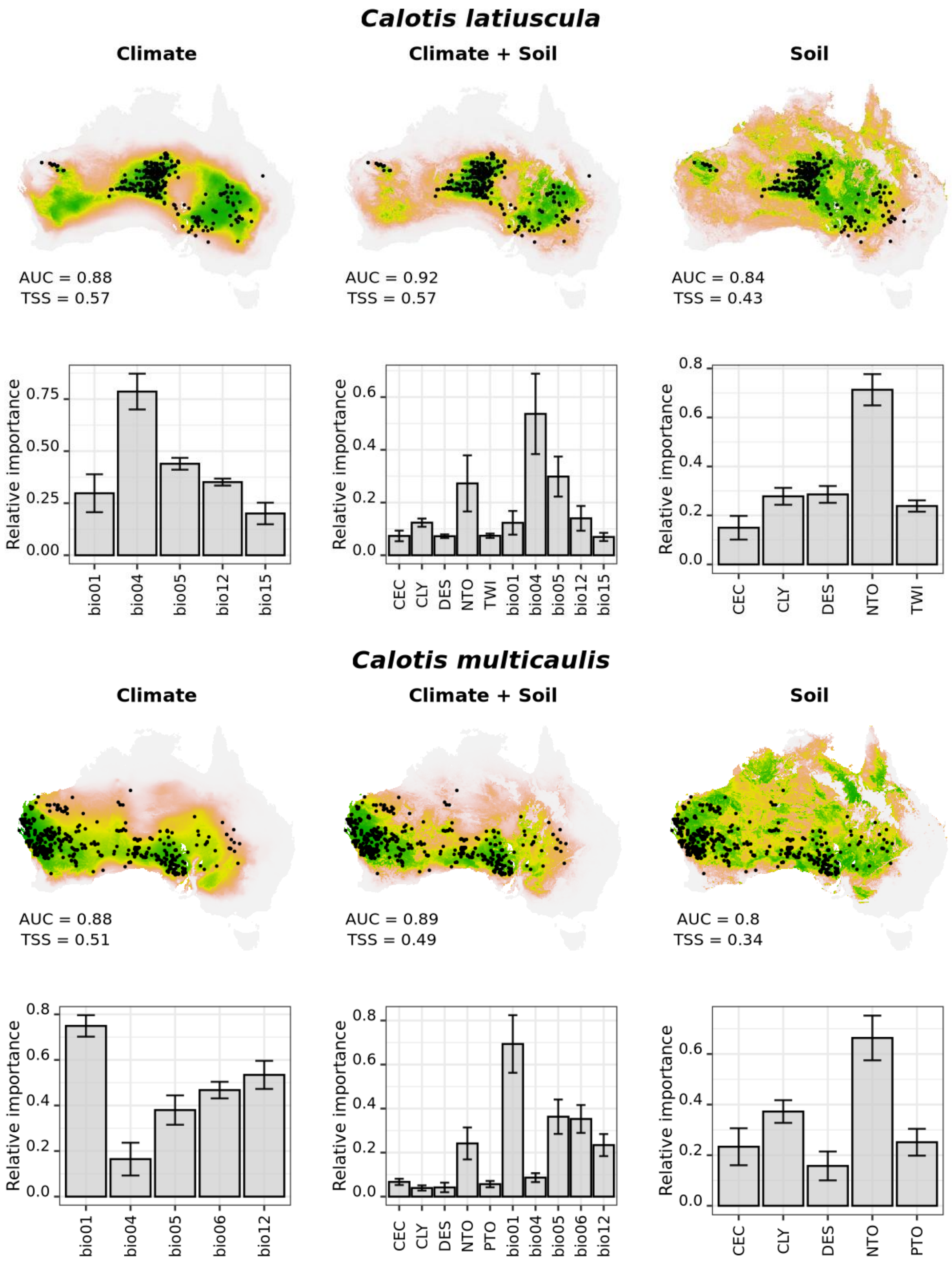
Calotis latiuscula
Climate
Climate + Soil
Soil
AUC = 0.88
TSS = 0.57
AUC = 0.92
TSS = 0.57
AUC = 0.84
TSS = 0.43
Relative importance
0.00
0.25
0.50
0.75
bio01
bio04
bio05
bio12
bio15
Relative importance
0.0
0.2
0.4
0.6
CEC
CLY
DES
NTO
TWI
bio01
bio04
bio05
bio12
bio15
Relative importance
0.0
0.2
0.4
0.6
0.8
CEC
CLY
DES
NTO
TWI
Calotis multicaulis
Climate
Climate + Soil
Soil
AUC = 0.88
TSS = 0.51
AUC = 0.89
TSS = 0.49
AUC = 0.8
TSS = 0.34
Relative importance
0.0
0.2
0.4
0.6
0.8
bio01
bio04
bio05
bio06
bio12
Relative importance
0.0
0.2
0.4
0.6
0.8
CEC
CLY
DES
NTO
PTO
bio01
bio04
bio05
bio06
bio12
Relative importance
0.0
0.2
0.4
0.6
CEC
CLY
DES
NTO
PTO

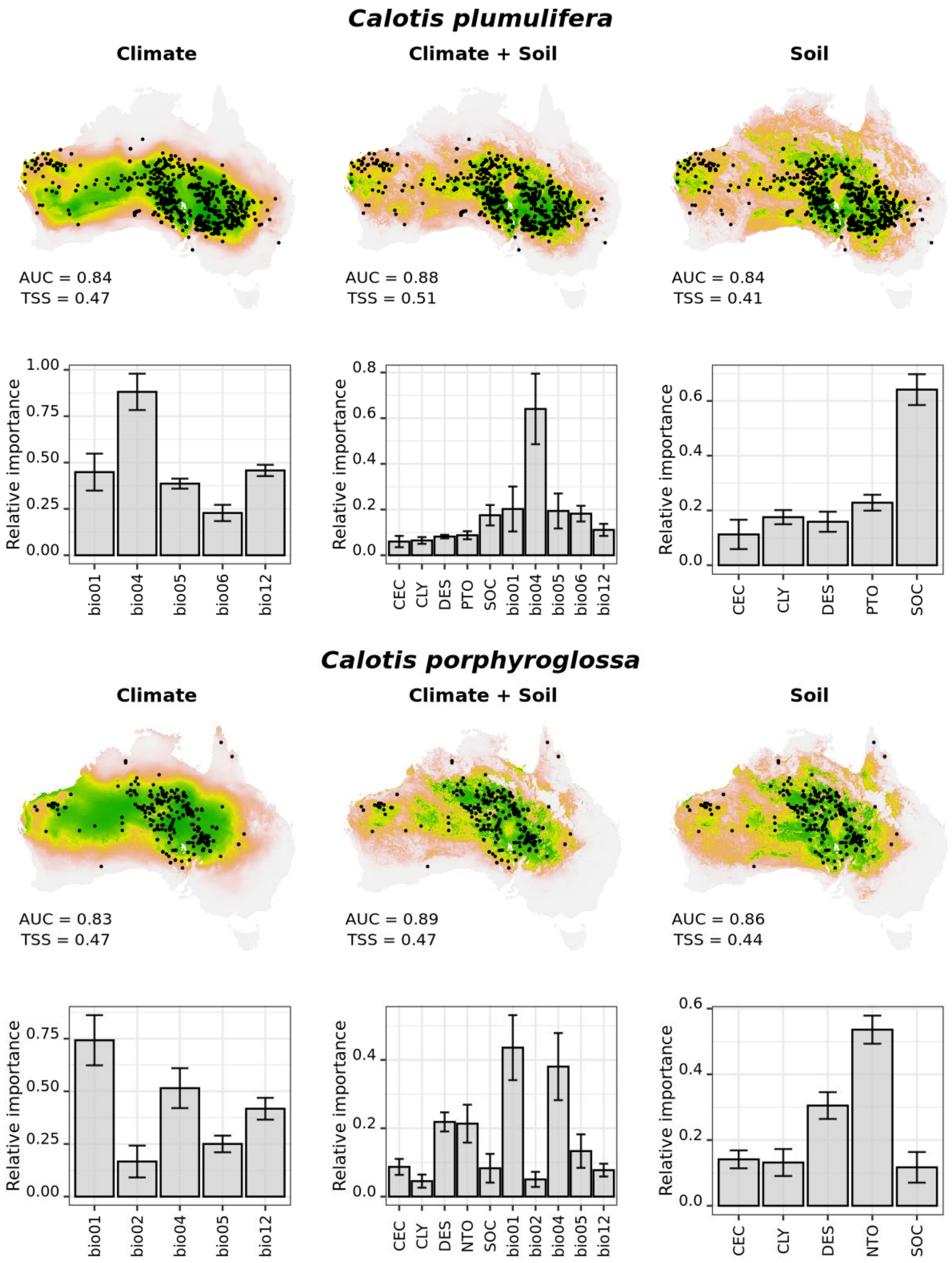
Calotis plumulifera
Climate
Climate + Soil
Soil
AUC = 0.84
TSS = 0.47
AUC = 0.88
TSS = 0.51
AUC = 0.84
TSS = 0.41
Relative importance
1.00
0.75
0.50
0.25
0.00
bio01
bio04
bio05
bio06
bio12
0.8
0.6
0.4
0.2
0.0
CEC
CLY
DES
PTO
SOC
bio01
bio04
bio05
bio06
bio12
0.6
0.4
0.2
0.0
CEC
CLY
DES
PTO
SOC
Calotis porphyroglossa
Climate
Climate + Soil
Soil
AUC = 0.83
TSS = 0.47
AUC = 0.89
TSS = 0.47
AUC = 0.86
TSS = 0.44
Relative importance
0.75
0.50
0.25
0.00
bio01
bio02
bio04
bio05
bio12
0.4
0.2
0.0
CEC
CLY
DES
NTO
SOC
bio01
bio02
bio04
bio05
bio12
0.6
0.4
0.2
0.0
CEC
CLY
DES
NTO
SOC

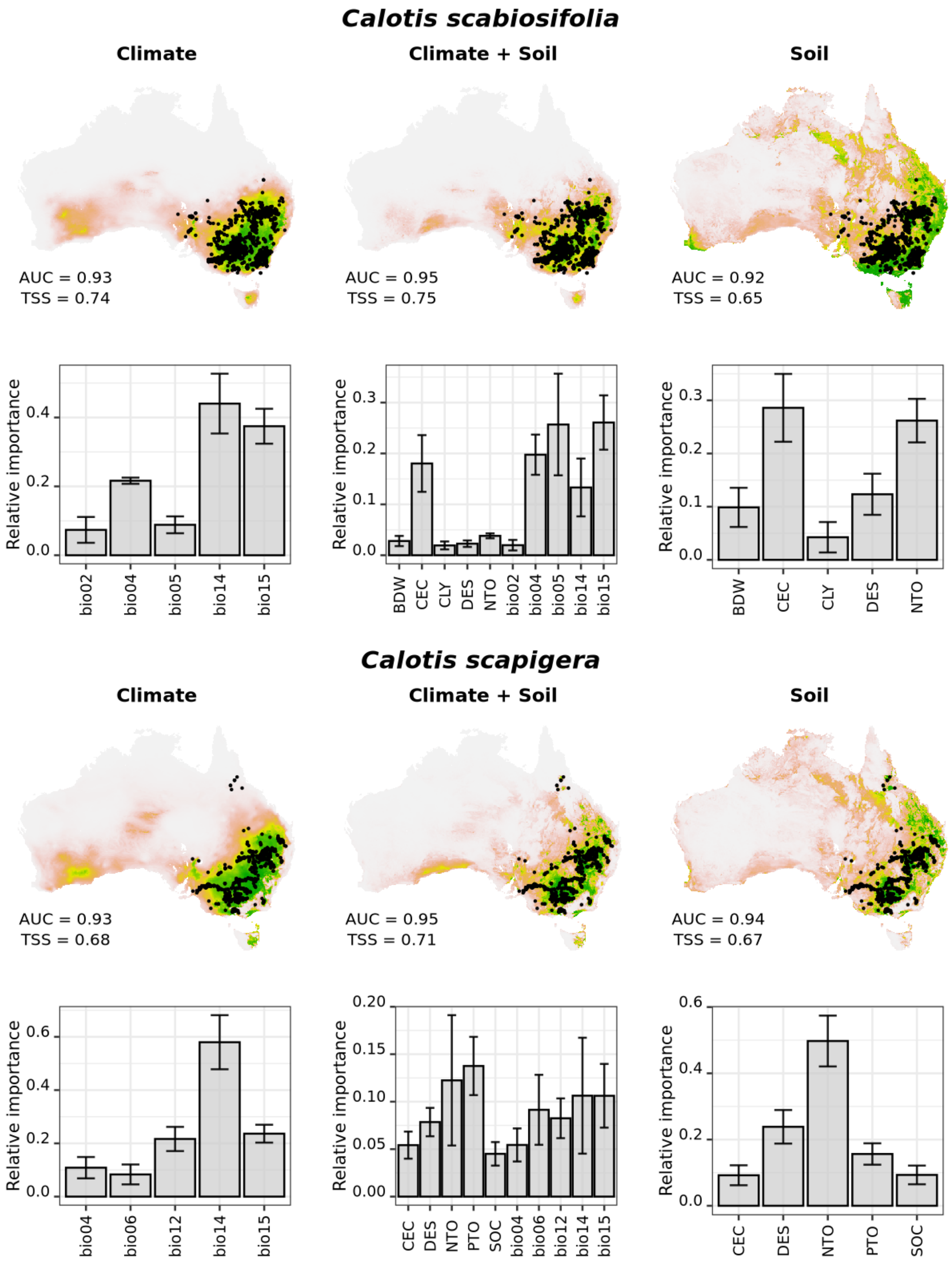

Calotis scabiosifolia
Climate
Climate + Soil
Soil
AUC = 0.93
TSS = 0.74
AUC = 0.95
TSS = 0.75
AUC = 0.92
TSS = 0.65
Relative importance
bio02
bio04
bio05
bio14
bio15
BDW
CEC
CLY
DES
NTO
bio02
bio04
bio05
bio14
bio15
BDW
CEC
CLY
DES
NTO
Calotis scapigera
Climate
Climate + Soil
Soil
AUC = 0.93
TSS = 0.68
AUC = 0.95
TSS = 0.71
AUC = 0.94
TSS = 0.67
Relative importance
bio04
bio06
bio12
bio14
bio15
CEC
DES
NTO
PTO
SOC
bio04
bio06
bio12
bio14
bio15
CEC
DES
NTO
PTO
SOC

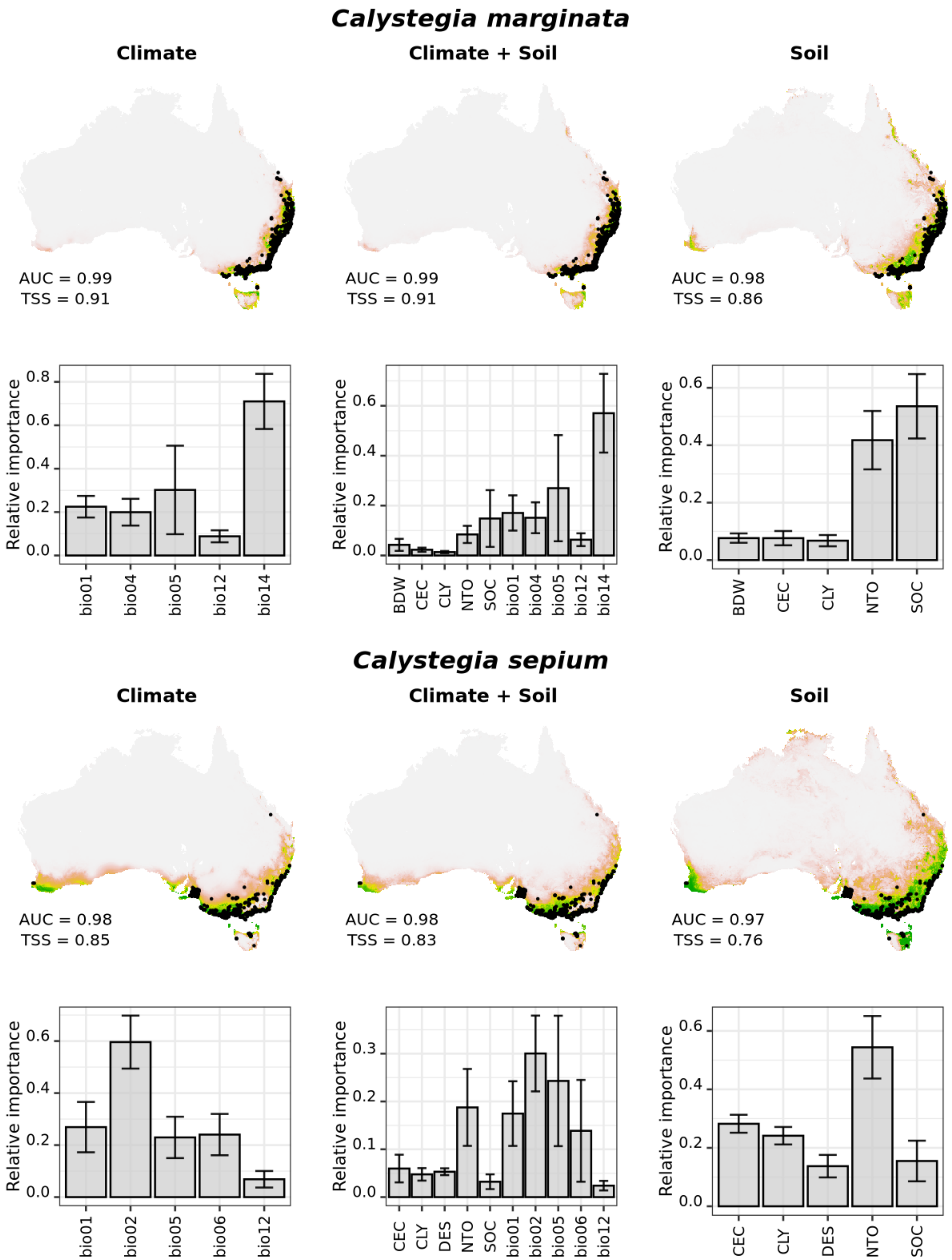

Calystegia marginata
Climate
Climate + Soil
Soil
AUC = 0.99
TSS = 0.91
AUC = 0.99
TSS = 0.91
AUC = 0.98
TSS = 0.86
Relative importance
bio01 bio04 bio05 bio12 bio14
BDW CEC CLY NTO SOC bio01 bio04 bio05 bio12 bio14
BDW CEC CLY NTO SOC
Calystegia sepium
Climate
Climate + Soil
Soil
AUC = 0.98
TSS = 0.85
AUC = 0.98
TSS = 0.83
AUC = 0.97
TSS = 0.76
Relative importance
bio01 bio02 bio05 bio06 bio12
CEC CLY DES NTO SOC bio01 bio02 bio05 bio06 bio12
CEC CLY DES NTO SOC

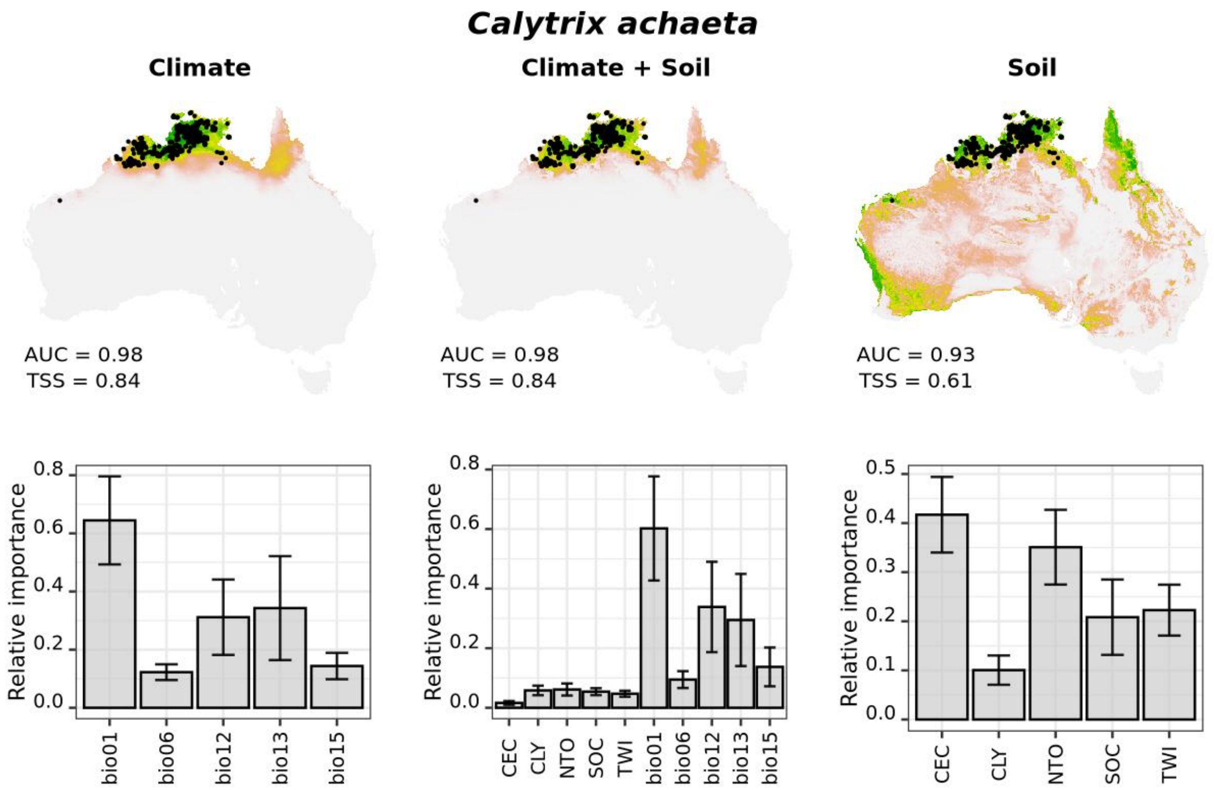
Calytrix achaeta
Climate
Climate + Soil
Soil
AUC = 0.98
TSS = 0.84
AUC = 0.98
TSS = 0.84
AUC = 0.93
TSS = 0.61
Relative importance
0.0
0.2
0.4
0.6
0.8
bio01
bio06
bio12
bio13
bio15
CEC
CLY
NTO
SOC
TWI
bio01
bio06
bio12
bio13
bio15
0.5
0.1
0.3
CEC
CLY
NTO
SOC
TWI

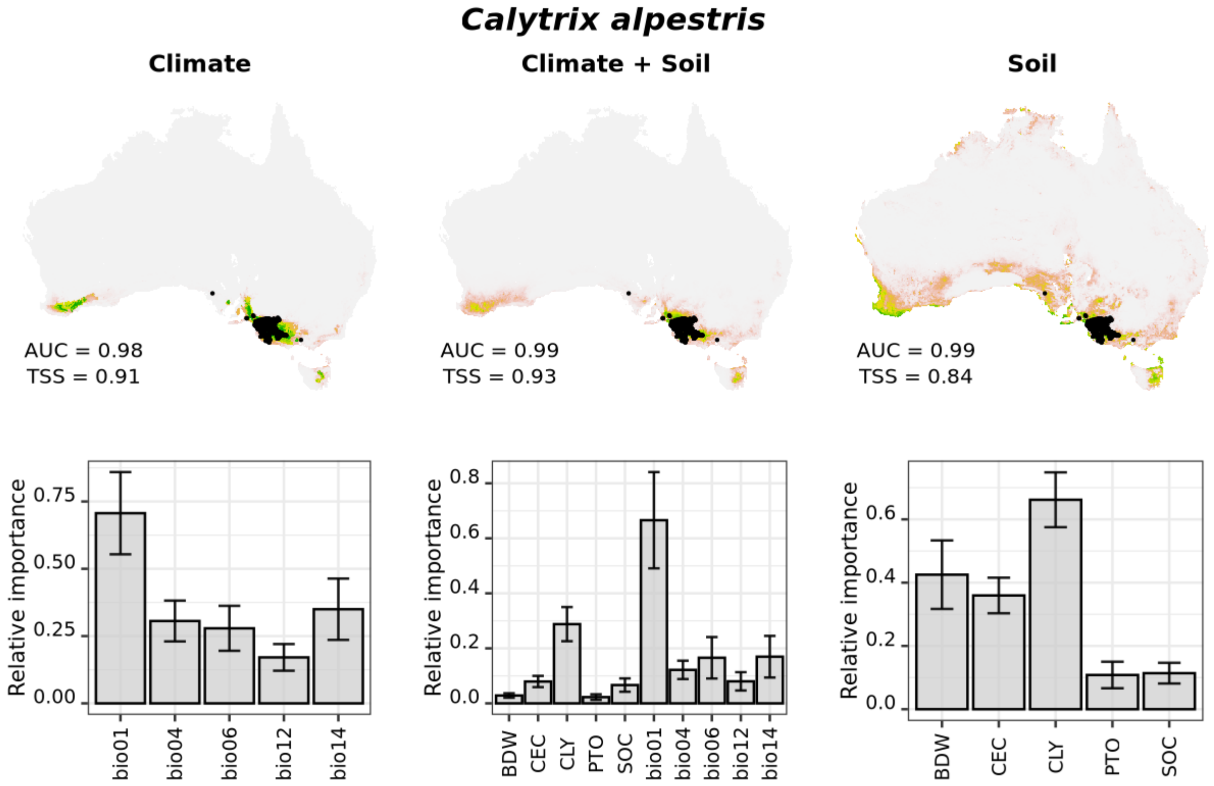
Calytrix alpestris
Climate
Climate + Soil
Soil
AUC = 0.98
TSS = 0.91
AUC = 0.99
TSS = 0.93
AUC = 0.99
TSS = 0.84
Relative importance
0.00
0.25
0.50
0.75
bio01
bio04
bio06
bio12
bio14
0.0
0.2
0.4
0.6
0.8
BDW
CEC
CLY
PTO
SOC
bio01
bio04
bio06
bio12
bio14
BDW
CEC
CLY
PTO
SOC

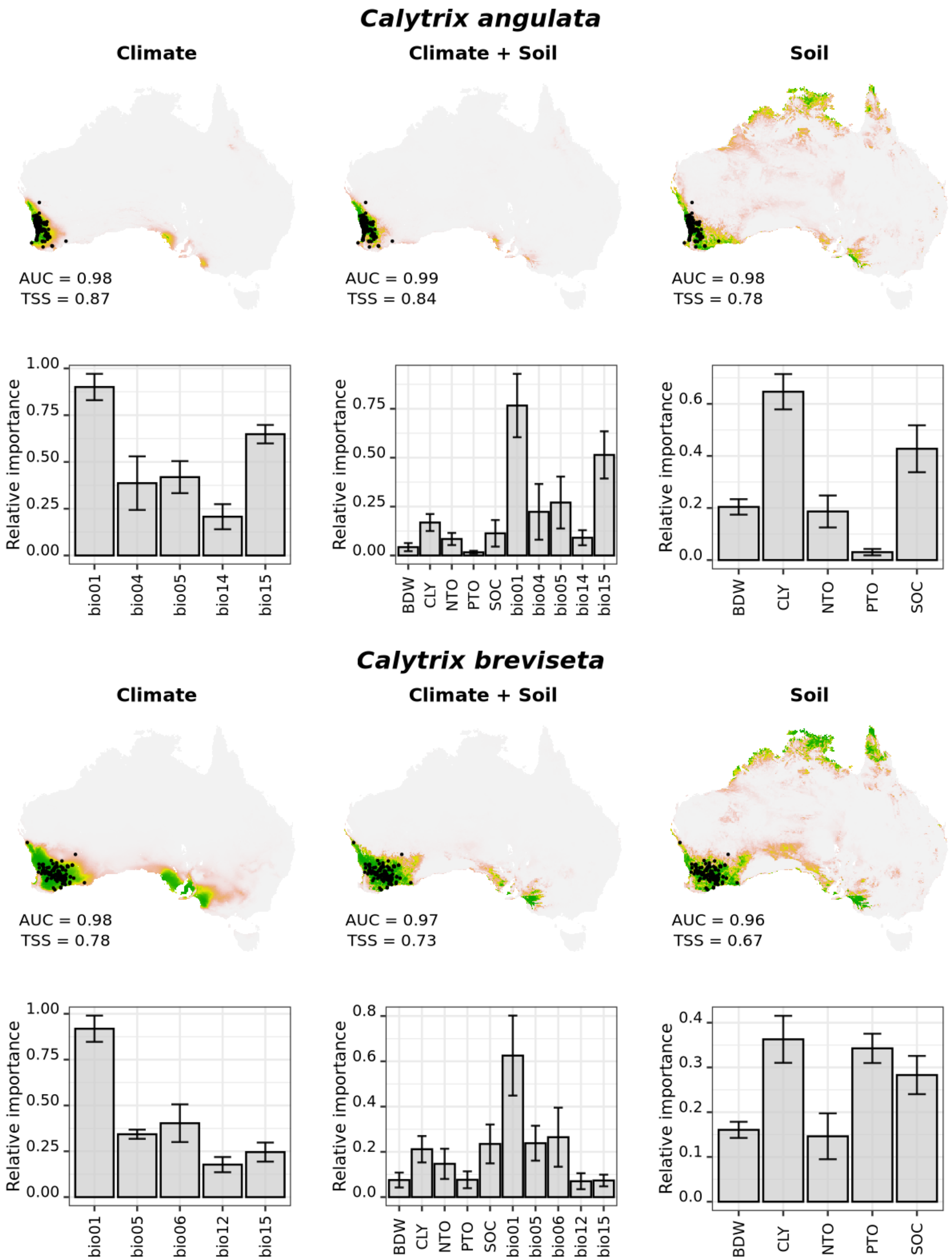
Calytrix angulata
Climate
Climate + Soil
Soil
AUC = 0.98
TSS = 0.87
AUC = 0.99
TSS = 0.84
AUC = 0.98
TSS = 0.78
Relative importance
1.00
0.75
0.50
0.25
0.00
bio01
bio04
bio05
bio14
bio15
BDW
CLY
NTO
PTO
SOC
0.6
0.4
0.2
0.0
Calytrix breviseta
AUC = 0.98
TSS = 0.78
AUC = 0.97
TSS = 0.73
AUC = 0.96
TSS = 0.67
bio06
bio12
0.8
0.3
0.1

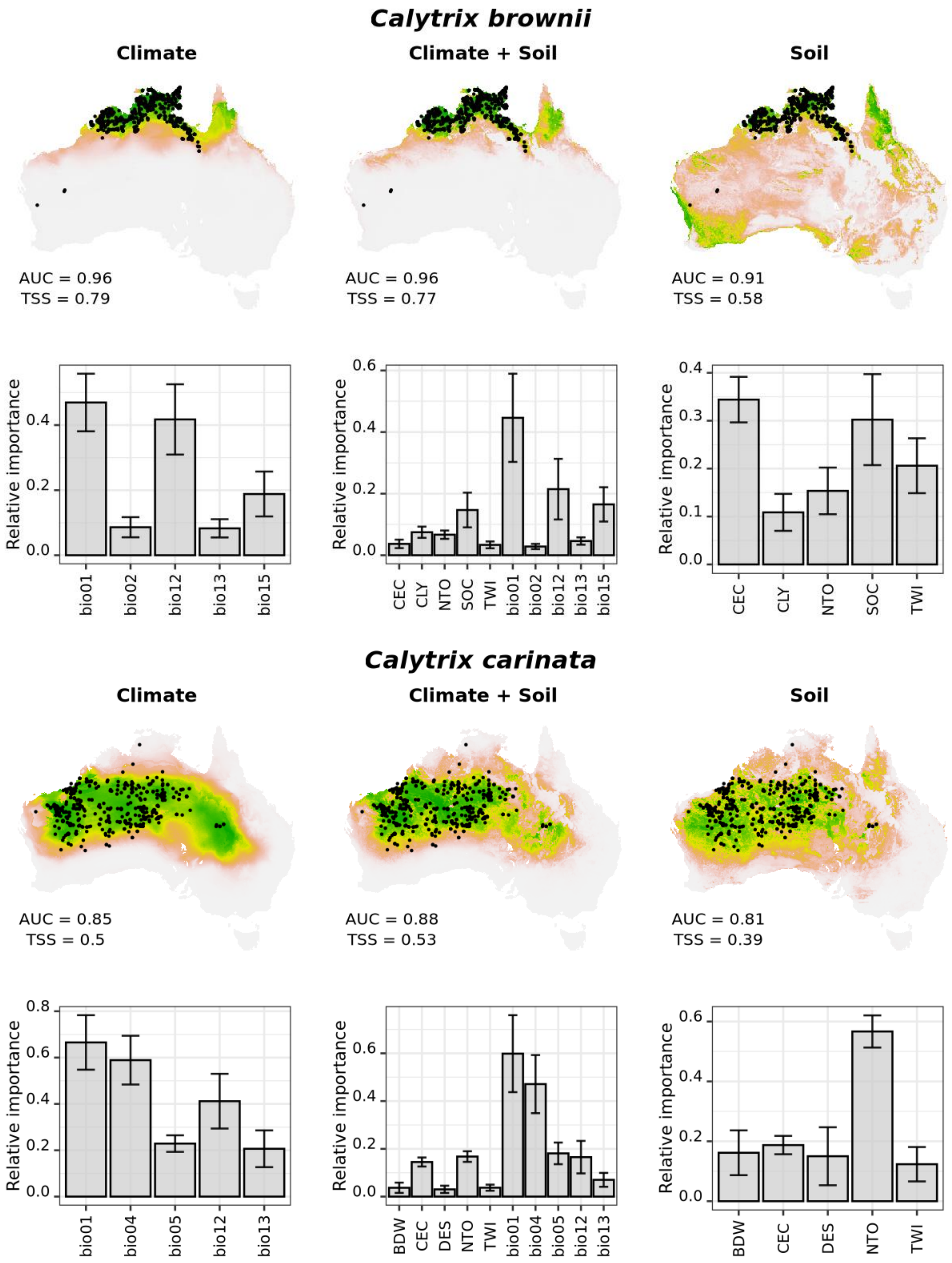
Calytrix brownii
Climate
Climate + Soil
Soil
AUC = 0.96
TSS = 0.79
AUC = 0.96
TSS = 0.77
AUC = 0.91
TSS = 0.58
Relative importance
bio01
bio02
bio12
bio13
bio15
CEC
CLY
NTO
SOC
TWI
Calytrix carinata
Climate
Climate + Soil
Soil
AUC = 0.85
TSS = 0.5
AUC = 0.88
TSS = 0.53
AUC = 0.81
TSS = 0.39
Relative importance
bio01
bio04
bio05
bio12
bio13
BDW
CEC
DES
NTO
TWI

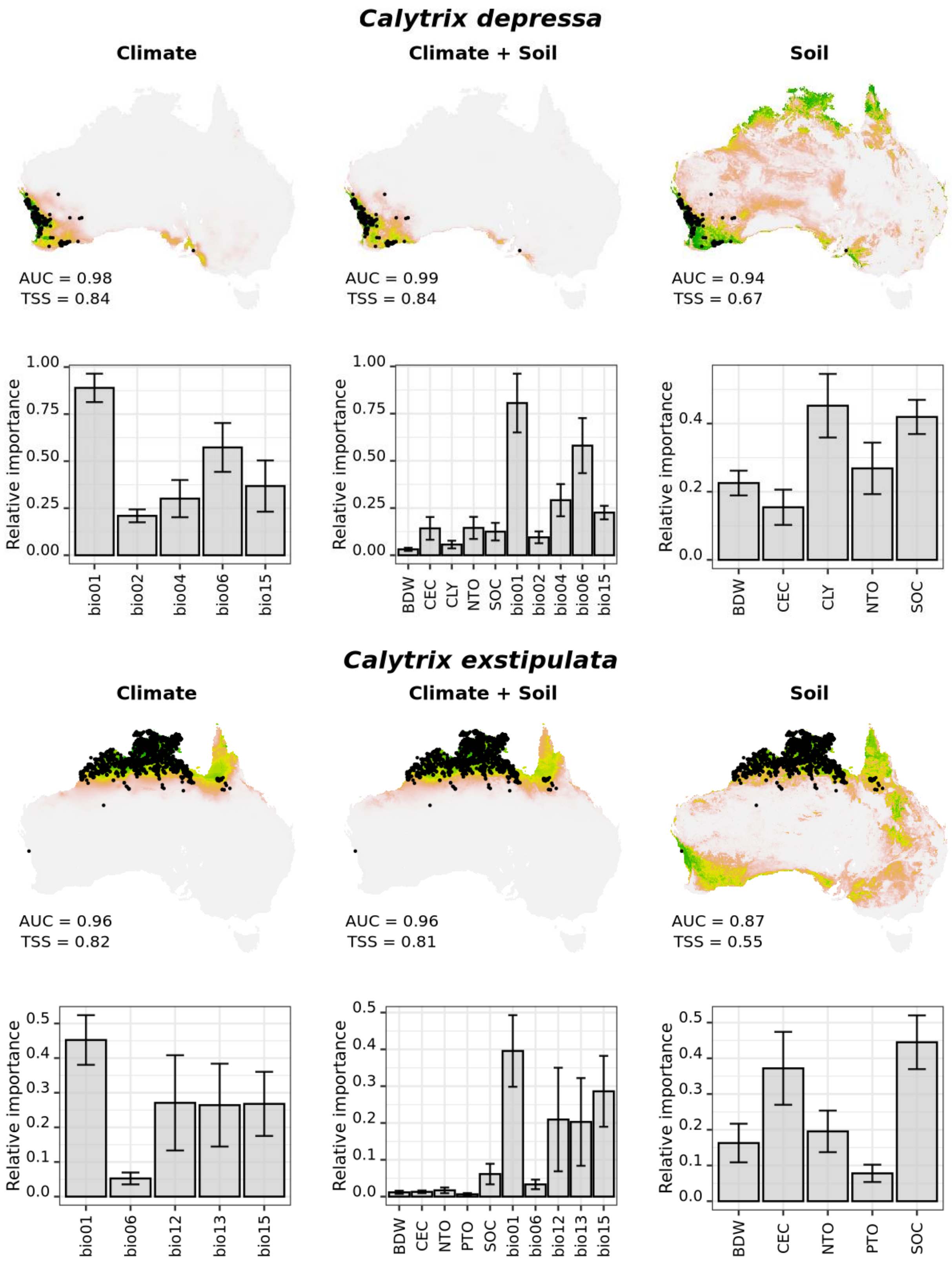
Calytrix depressa
Climate
Climate + Soil
Soil
AUC = 0.98
TSS = 0.84
AUC = 0.99
TSS = 0.84
AUC = 0.94
TSS = 0.67
Relative importance
0.00
0.25
0.50
0.75
1.00
bio01
bio02
bio04
bio06
bio15
BDW
CEC
CLY
NTO
SOC
0.0
0.2
0.4
Calytrix exstipulata
AUC = 0.96
TSS = 0.82
AUC = 0.96
TSS = 0.81
AUC = 0.87
TSS = 0.55
0.0
0.1
0.2
0.3
0.4
0.5
bio01
bio06
bio12
bio13
bio15
BDW
CEC
NTO
PTO
SOC

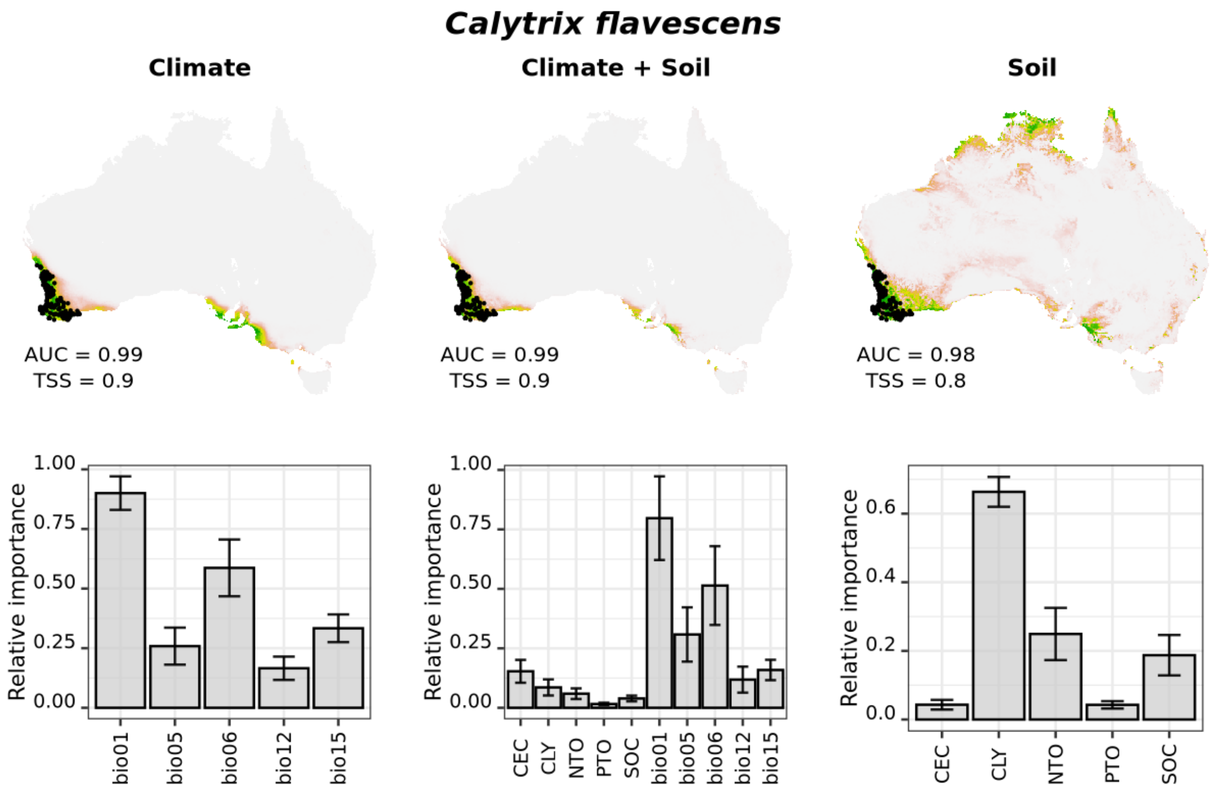

Calytrix flavescens
Climate
Climate + Soil
Soil
AUC = 0.99
TSS = 0.9
AUC = 0.99
TSS = 0.9
AUC = 0.98
TSS = 0.8
Relative importance
bio01
bio05
bio06
bio12
bio15
CEC
CLY
NTO
PTO
SOC

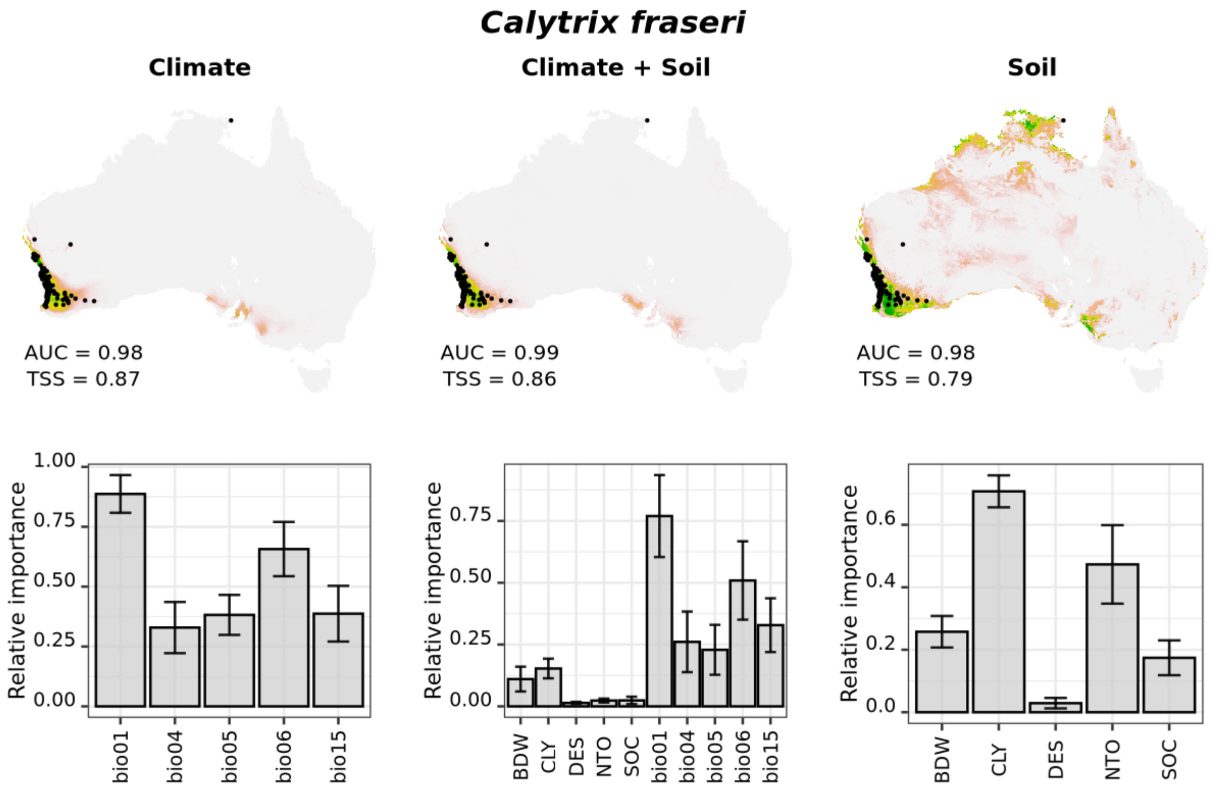

Calytrix fraseri
Climate
Climate + Soil
Soil
AUC = 0.98
TSS = 0.87
AUC = 0.99
TSS = 0.86
AUC = 0.98
TSS = 0.79
Relative importance
bio01
bio04
bio05
bio06
bio15
BDW
CLY
DES
NTO
SOC

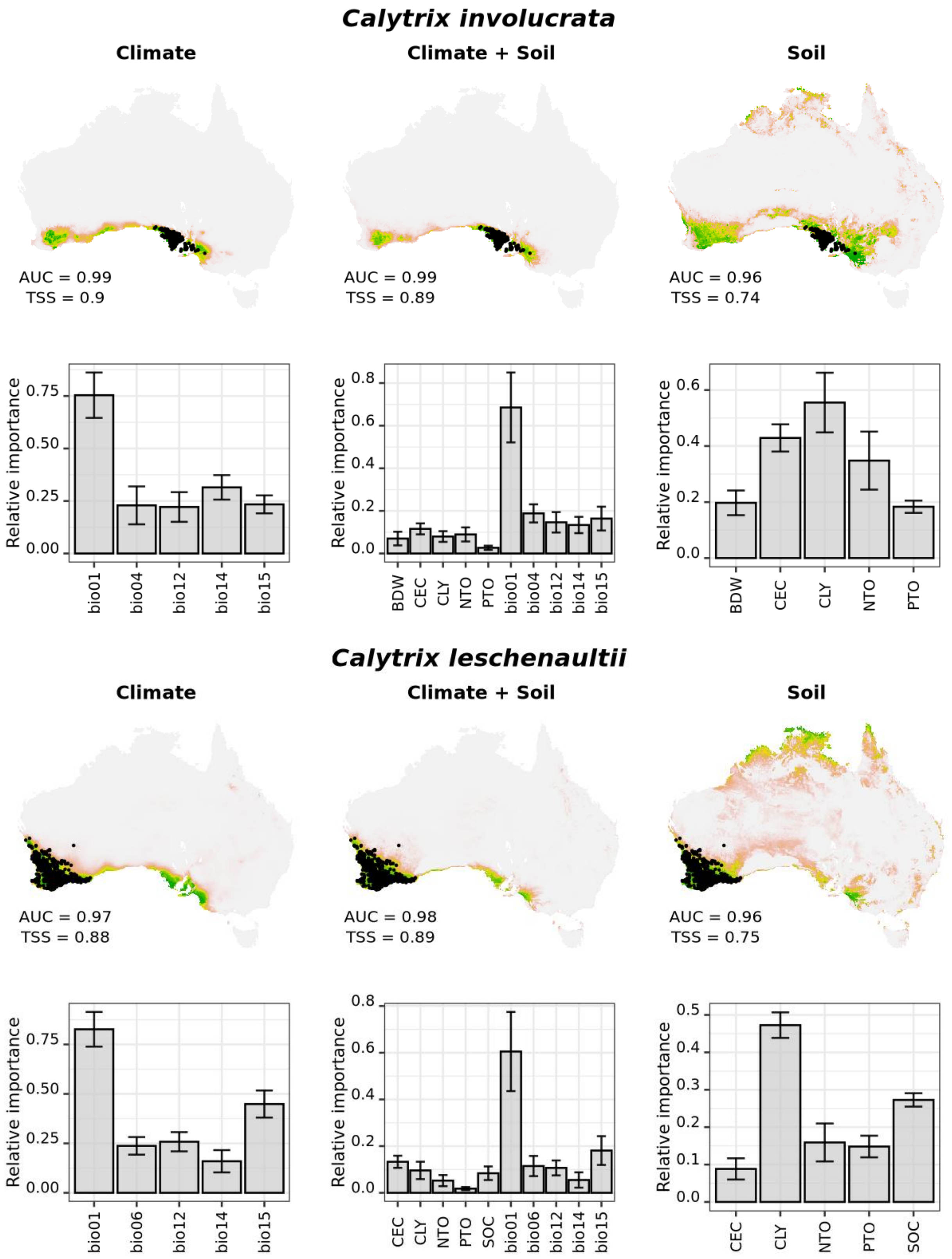
Calytrix involucrata
Climate
Climate + Soil
Soil
AUC = 0.99
TSS = 0.9
AUC = 0.99
TSS = 0.89
AUC = 0.96
TSS = 0.74
Relative importance
0.00
0.25
0.50
0.75
bio01
bio04
bio12
bio14
bio15
0.0
0.2
0.4
0.6
0.8
BDW
CEC
CLY
NTO
PTO
bio01
bio04
bio12
bio14
bio15
0.0
0.2
0.4
0.6
BDW
CEC
CLY
NTO
PTO
Calytrix leschenaultii
Climate
Climate + Soil
Soil
AUC = 0.97
TSS = 0.88
AUC = 0.98
TSS = 0.89
AUC = 0.96
TSS = 0.75
Relative importance
0.00
0.25
0.50
0.75
bio01
bio06
bio12
bio14
bio15
0.0
0.2
0.4
0.6
0.8
CEC
CLY
NTO
PTO
SOC
bio01
bio06
bio12
bio14
bio15
0.0
0.1
0.2
0.3
0.4
0.5
CEC
CLY
NTO
PTO
SOC

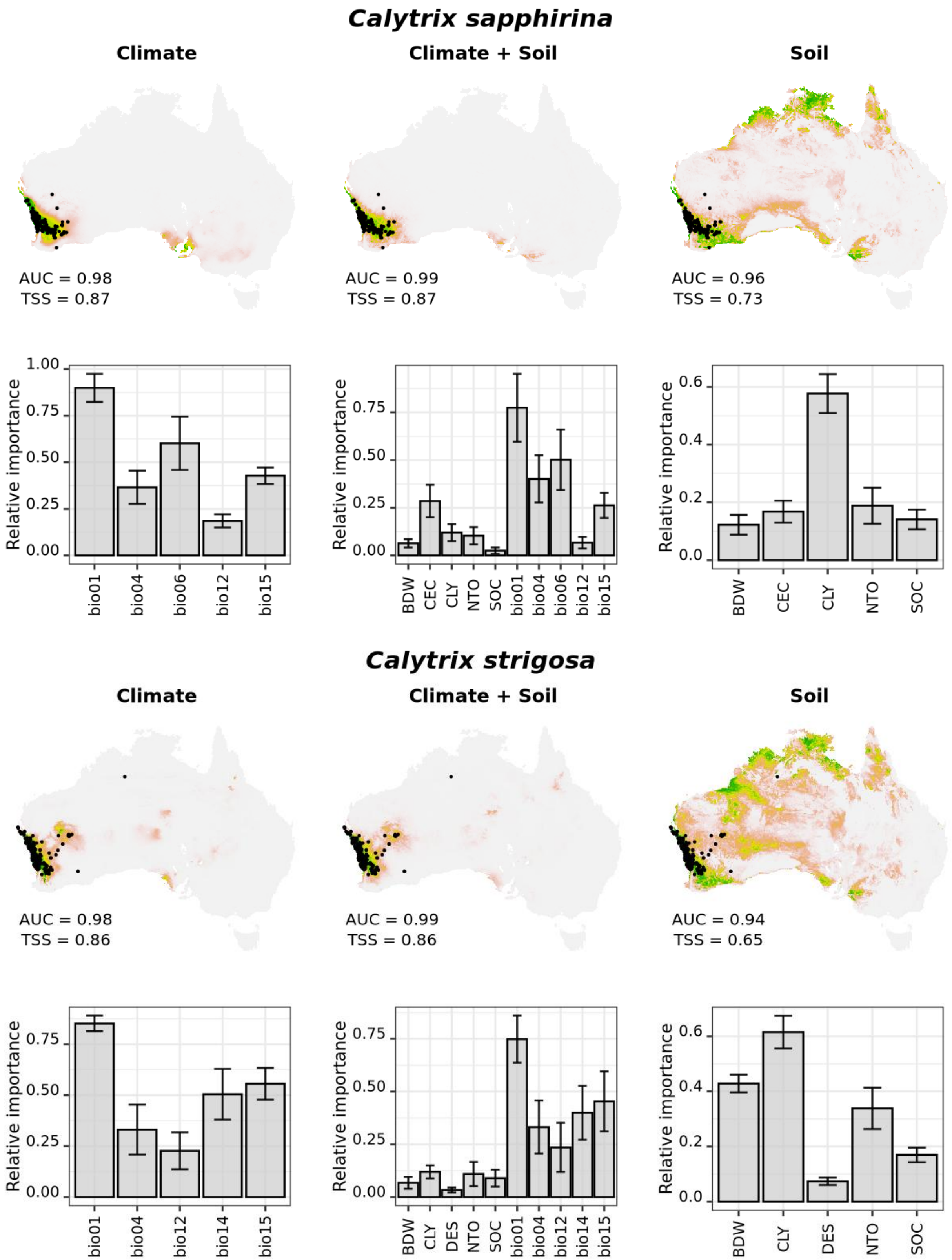
Calytrix sapphirina
Climate
Climate + Soil
Soil
AUC = 0.98
TSS = 0.87
AUC = 0.99
TSS = 0.87
AUC = 0.96
TSS = 0.73
Relative importance
1.00
0.75
0.50
0.25
0.00
bio01
bio04
bio06
bio12
bio15
BDW
CEC
CLY
NTO
SOC
0.6
0.4
0.2
0.0
Calytrix strigosa
Climate
Climate + Soil
Soil
AUC = 0.98
TSS = 0.86
AUC = 0.99
TSS = 0.86
AUC = 0.94
TSS = 0.65
bio14
DES

Camptacra barbata

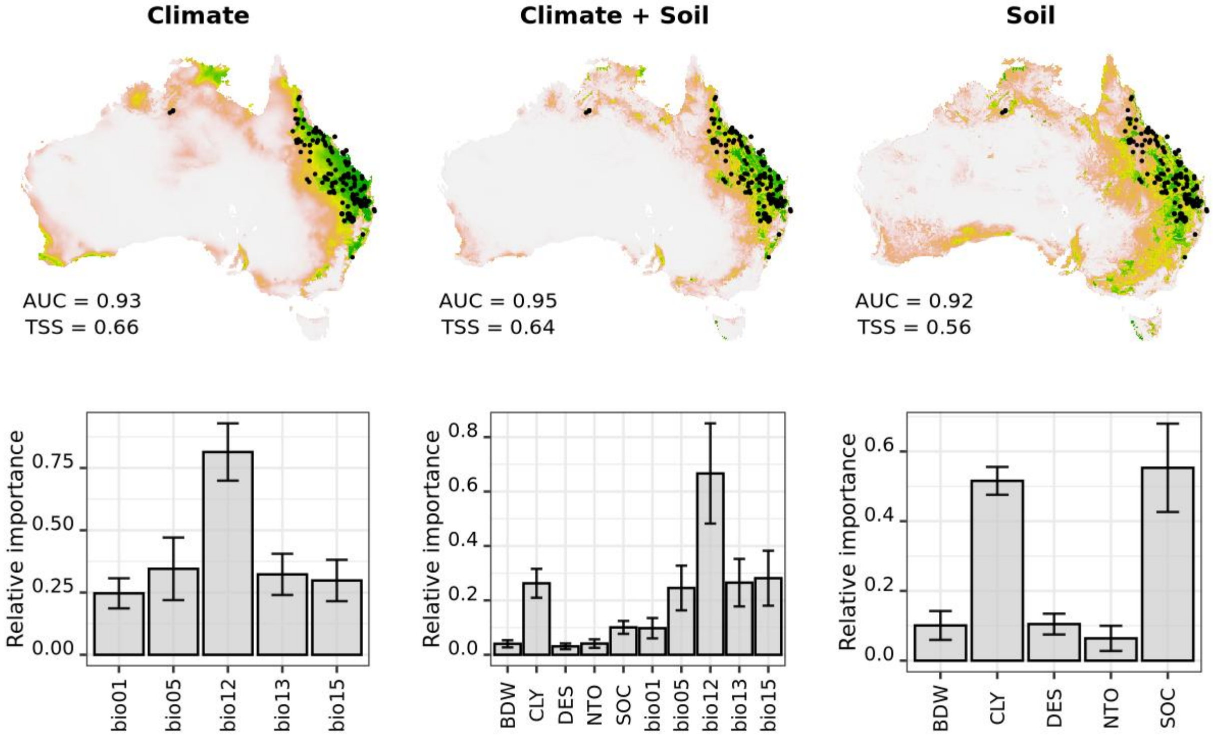

Camptostemon schultzii

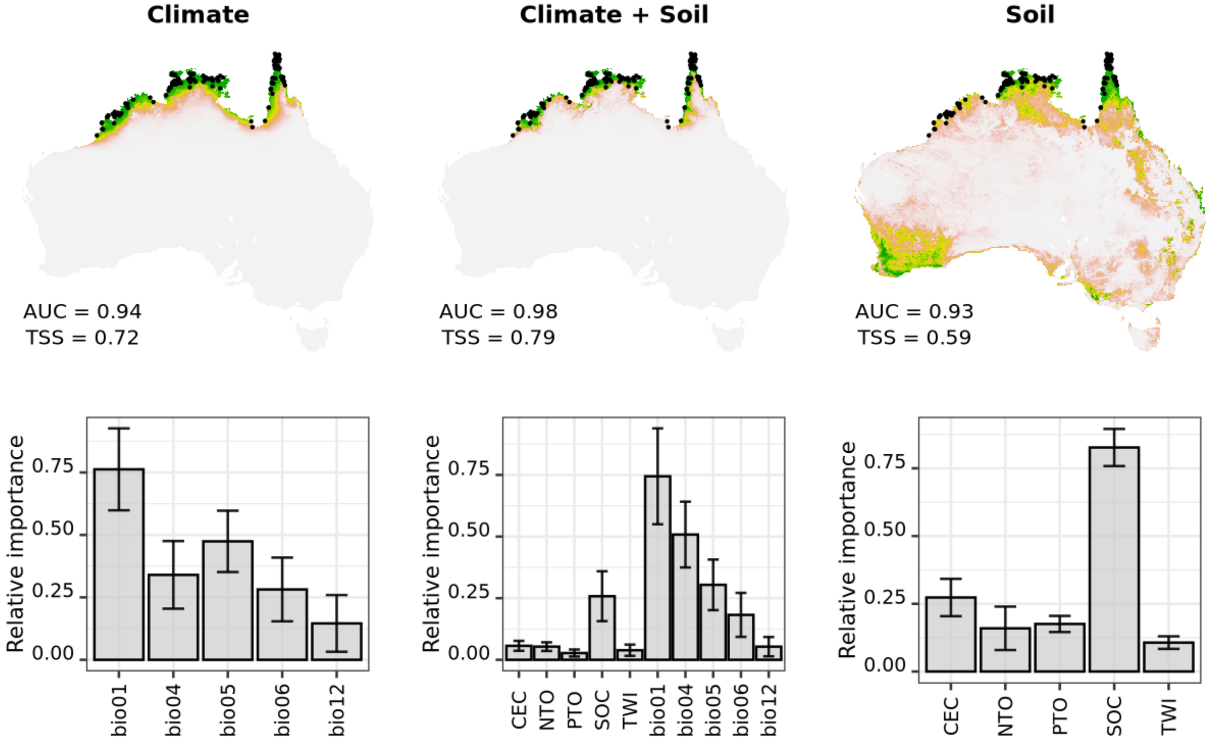

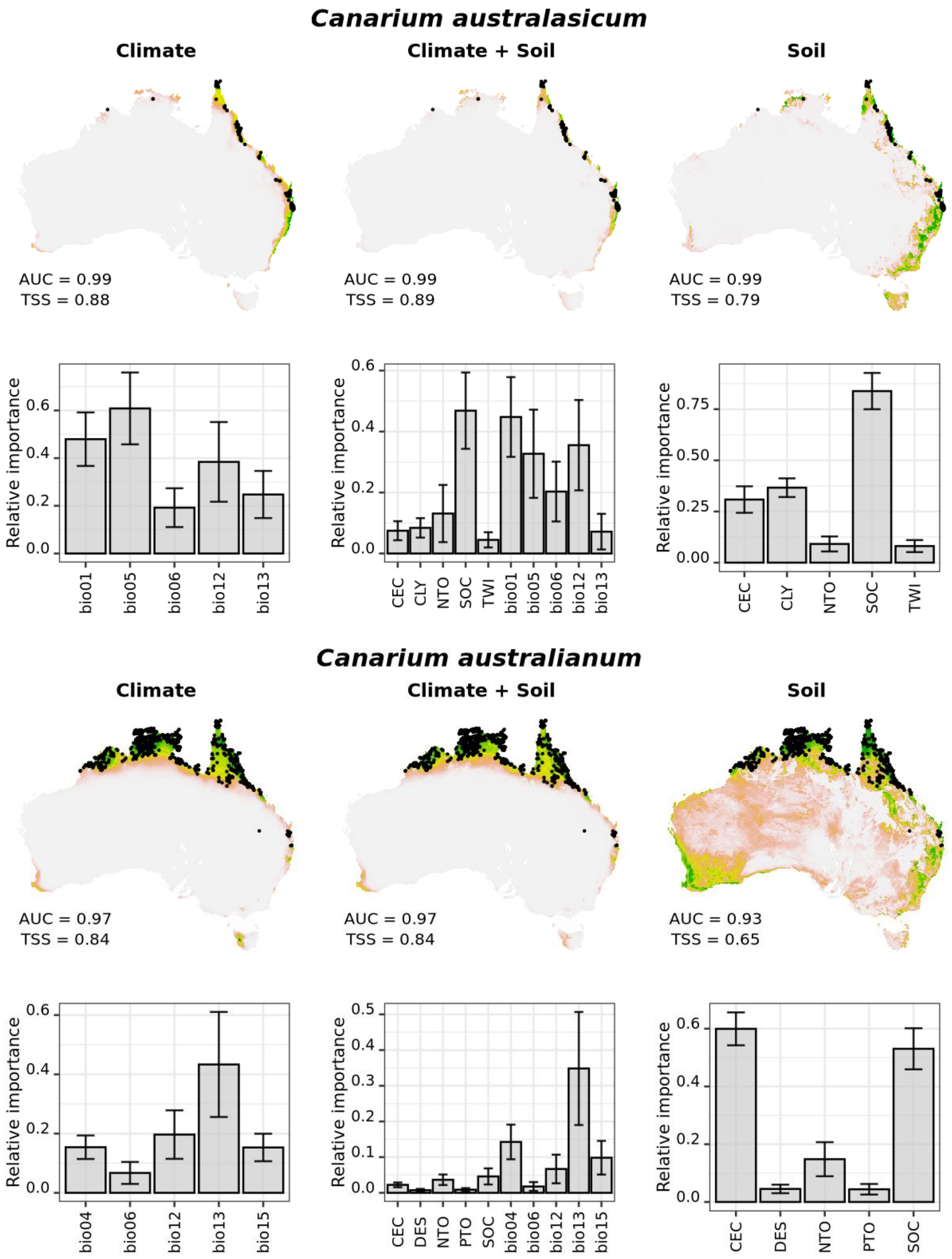

Canarium australasicum
Climate
Climate + Soil
Soil
AUC = 0.99
TSS = 0.88
AUC = 0.99
TSS = 0.89
AUC = 0.99
TSS = 0.79
Relative importance
0.0
0.2
0.4
0.6
bio01
bio05
bio06
bio12
bio13
CEC
CLY
NTO
SOC
TWI
bio01
bio05
bio06
bio12
bio13
0.00
0.25
0.50
0.75
Canarium australianum
Climate
Climate + Soil
Soil
AUC = 0.97
TSS = 0.84
AUC = 0.97
TSS = 0.84
AUC = 0.93
TSS = 0.65
bio04
bio06
bio12
bio13
bio15
0.1
0.3
0.5
CEC
DES
NTO
PTO
SOC
bio04
bio06
bio12
bio13
bio15

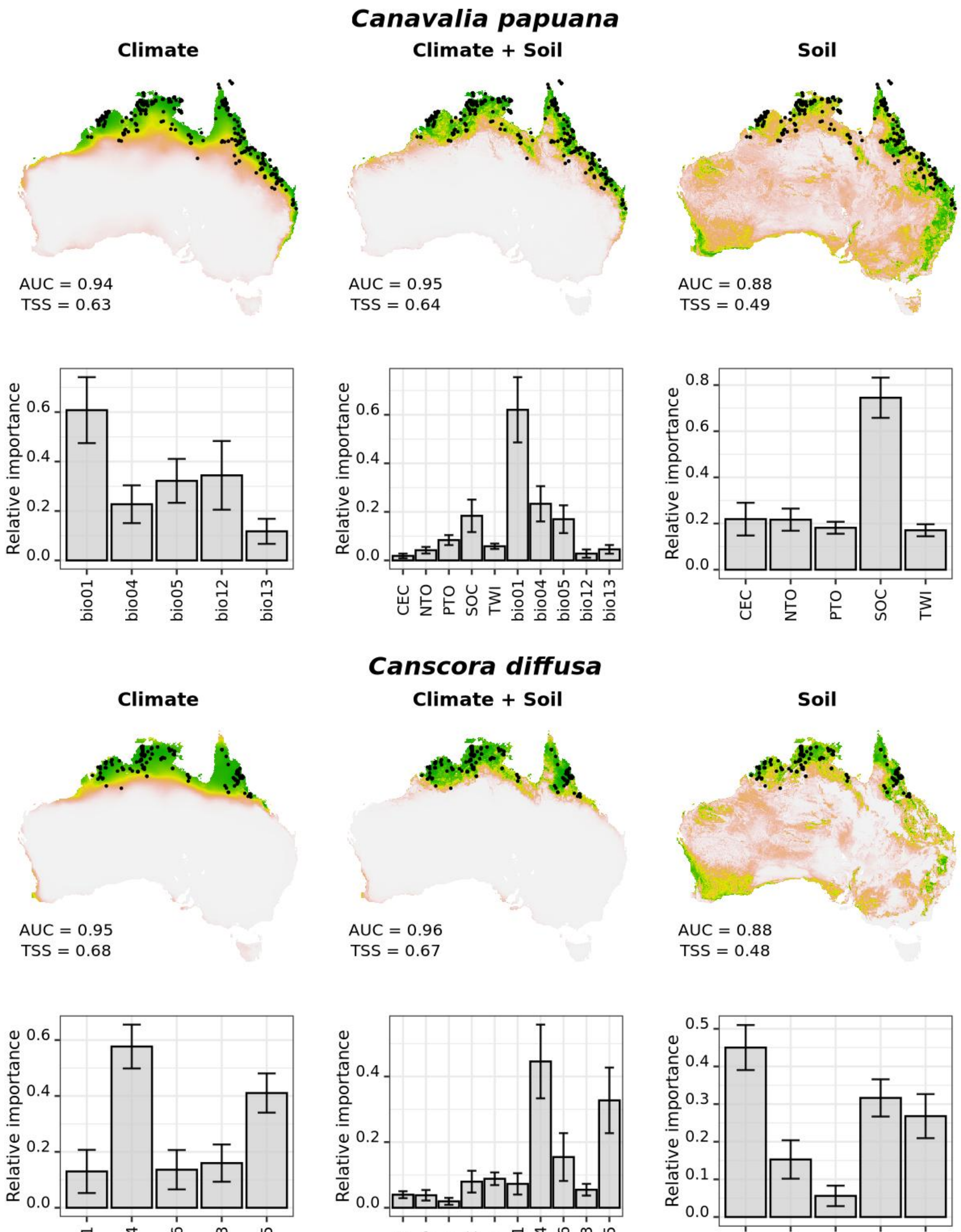

Canavalia papuana
Climate
Climate + Soil
Soil
AUC = 0.94
TSS = 0.63
AUC = 0.95
TSS = 0.64
AUC = 0.88
TSS = 0.49
Relative importance
bio01
bio04
bio05
bio12
bio13
CEC
NTO
PTO
SOC
TWI
Canscora diffusa
Climate
Climate + Soil
Soil
AUC = 0.95
TSS = 0.68
AUC = 0.96
TSS = 0.67
AUC = 0.88
TSS = 0.48
Relative importance
bio01
bio04
bio06
bio13
bio15
CEC
NTO
PTO
SOC
TWI

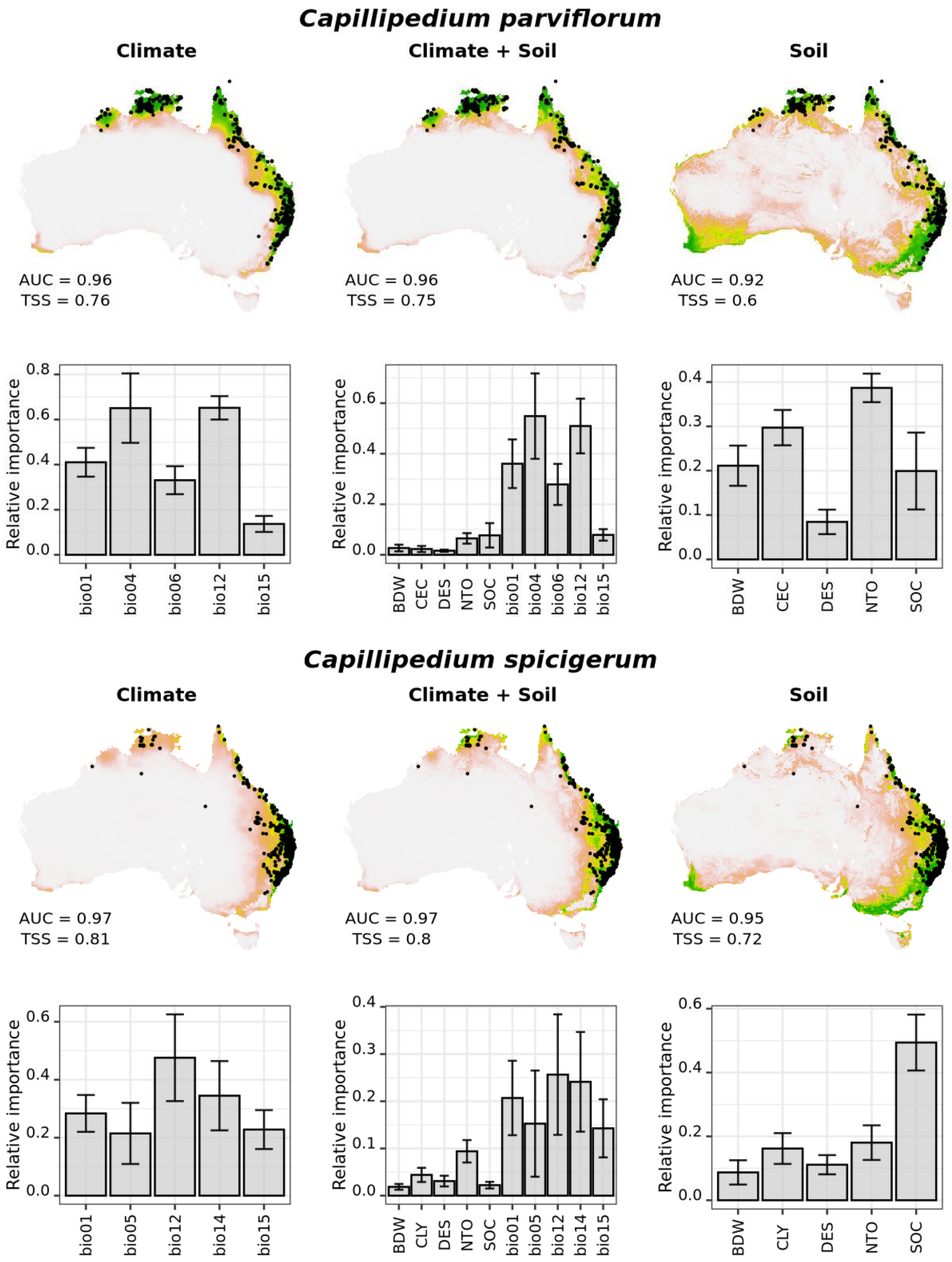

Capillipedium parviflorum
Climate
Climate + Soil
Soil
AUC = 0.96
TSS = 0.76
AUC = 0.96
TSS = 0.75
AUC = 0.92
TSS = 0.6
Relative importance
bio01 bio04 bio06 bio12 bio15
BDW CEC DES NTO SOC bio01 bio04 bio06 bio12 bio15
BDW CEC DES NTO SOC
Capillipedium spicigerum
Climate
Climate + Soil
Soil
AUC = 0.97
TSS = 0.81
AUC = 0.97
TSS = 0.8
AUC = 0.95
TSS = 0.72
Relative importance
bio01 bio05 bio12 bio14 bio15
BDW CLY DES NTO SOC bio01 bio05 bio12 bio14 bio15
BDW CLY DES NTO SOC

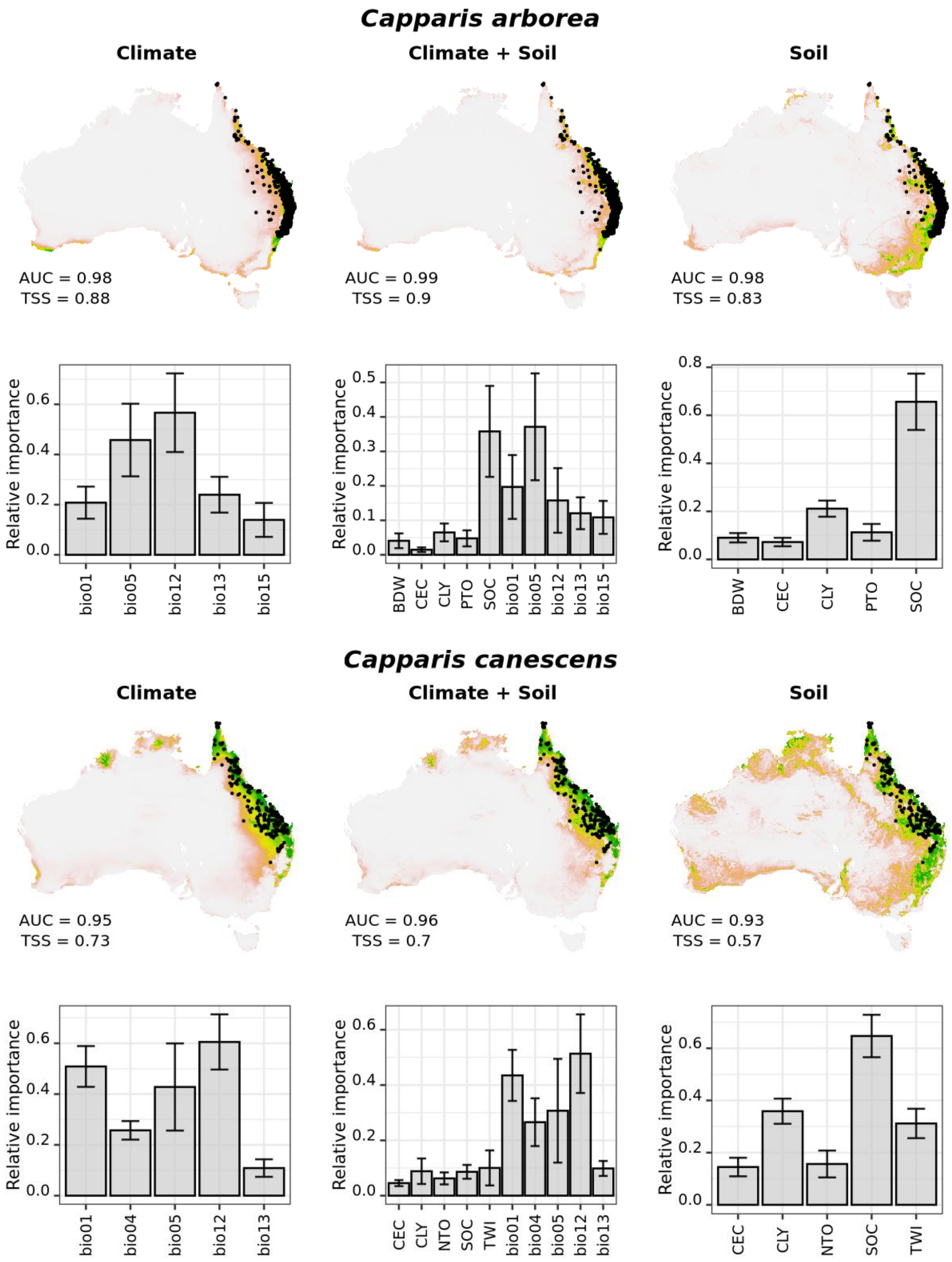
Capparis arborea
Climate
Climate + Soil
Soil
AUC = 0.98
TSS = 0.88
AUC = 0.99
TSS = 0.9
AUC = 0.98
TSS = 0.83
Relative importance
bio01 bio05 bio12 bio13 bio15
BDW CEC CLY PTO SOC bio01 bio05 bio12 bio13 bio15
BDW CEC CLY PTO SOC
Capparis canescens
Climate
Climate + Soil
Soil
AUC = 0.95
TSS = 0.73
AUC = 0.96
TSS = 0.7
AUC = 0.93
TSS = 0.57
Relative importance
bio01 bio04 bio05 bio12 bio13
CEC CLY NTO SOC TWI bio01 bio04 bio05 bio12 bio13
CEC CLY NTO SOC TWI

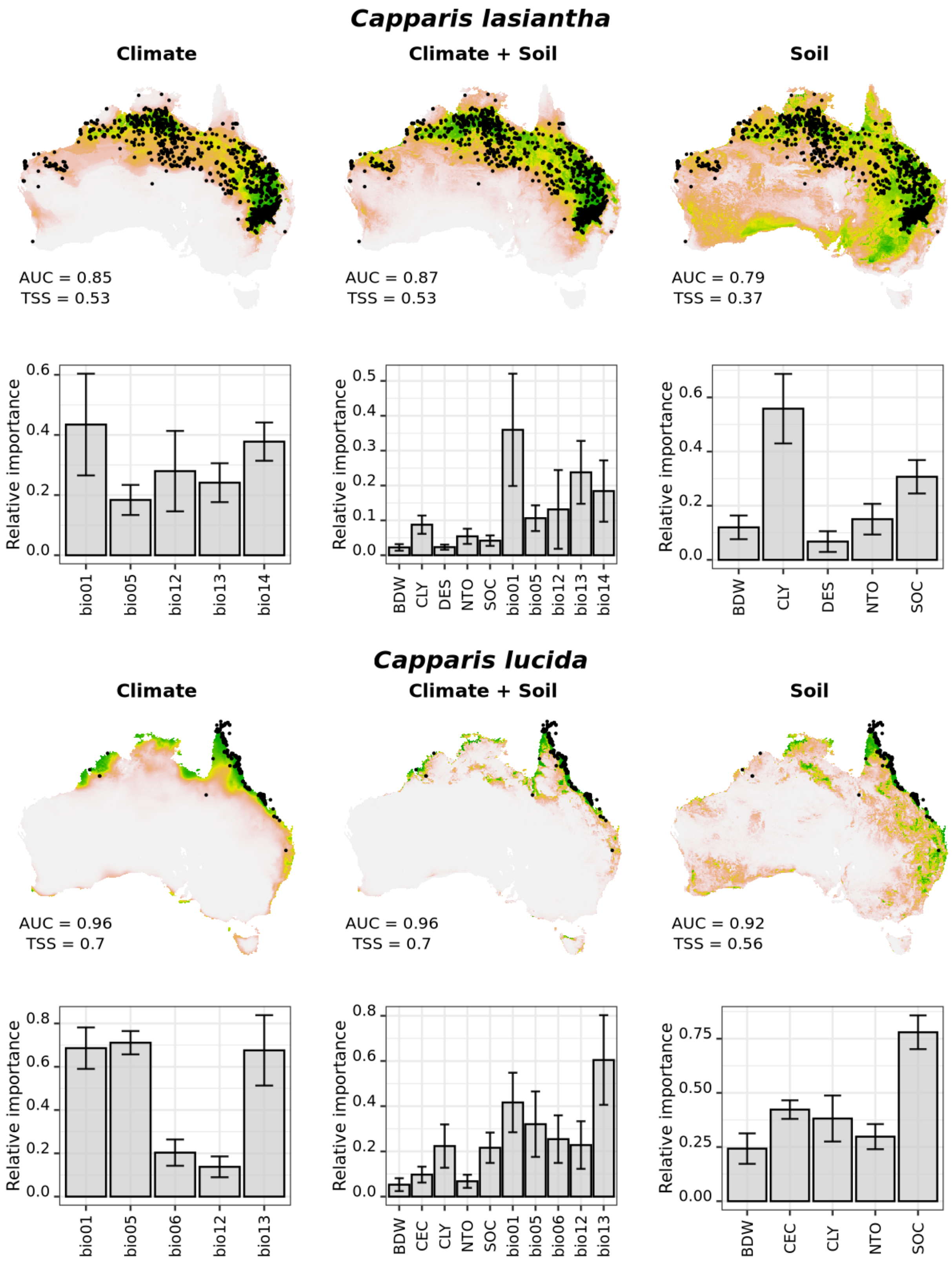
Capparis lasiantha
Climate
Climate + Soil
Soil
AUC = 0.85
TSS = 0.53
AUC = 0.87
TSS = 0.53
AUC = 0.79
TSS = 0.37
Relative importance
0.0
0.2
0.4
0.6
bio01
bio05
bio12
bio13
bio14
0.0
0.1
0.2
0.3
0.4
0.5
BDW
CLY
DES
NTO
SOC
bio01
bio05
bio12
bio13
bio14
BDW
CLY
DES
NTO
SOC
Capparis lucida
Climate
Climate + Soil
Soil
AUC = 0.96
TSS = 0.7
AUC = 0.96
TSS = 0.7
AUC = 0.92
TSS = 0.56
0.8
bio01
bio05
bio06
bio12
bio13
BDW
CEC
CLY
NTO
SOC
bio01
bio05
bio06
bio12
bio13
0.00
0.25
0.50
0.75
BDW
CEC
CLY
NTO
SOC

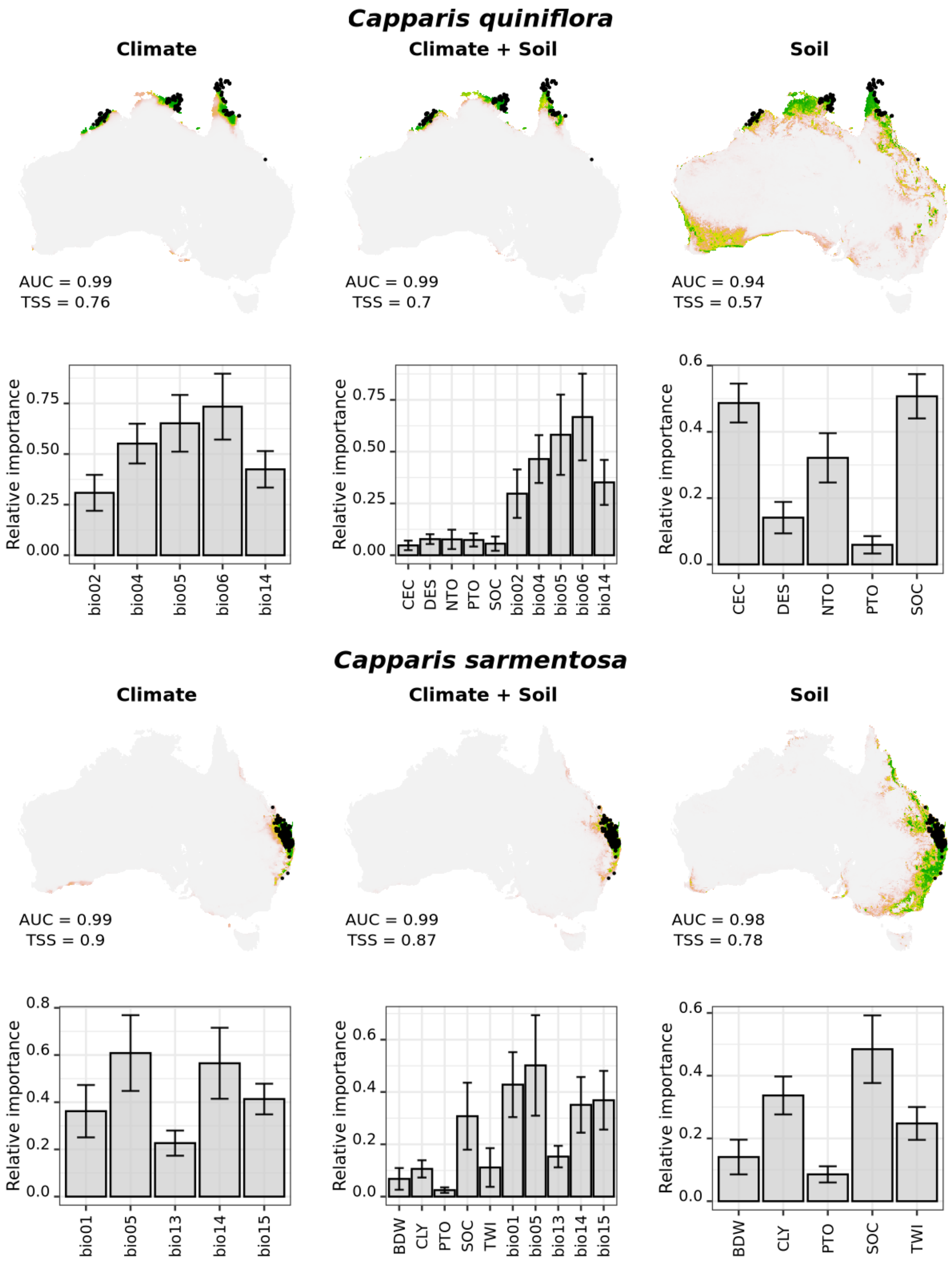

Capparis quiniflora
Climate
Climate + Soil
Soil
AUC = 0.99
TSS = 0.76
AUC = 0.99
TSS = 0.7
AUC = 0.94
TSS = 0.57
Relative importance
bio02
bio04
bio05
bio06
bio14
CEC
DES
NTO
PTO
SOC
Capparis sarmentosa
AUC = 0.99
TSS = 0.9
AUC = 0.99
TSS = 0.87
AUC = 0.98
TSS = 0.78
bio01
bio13
bio15
BDW
CLY
TWI

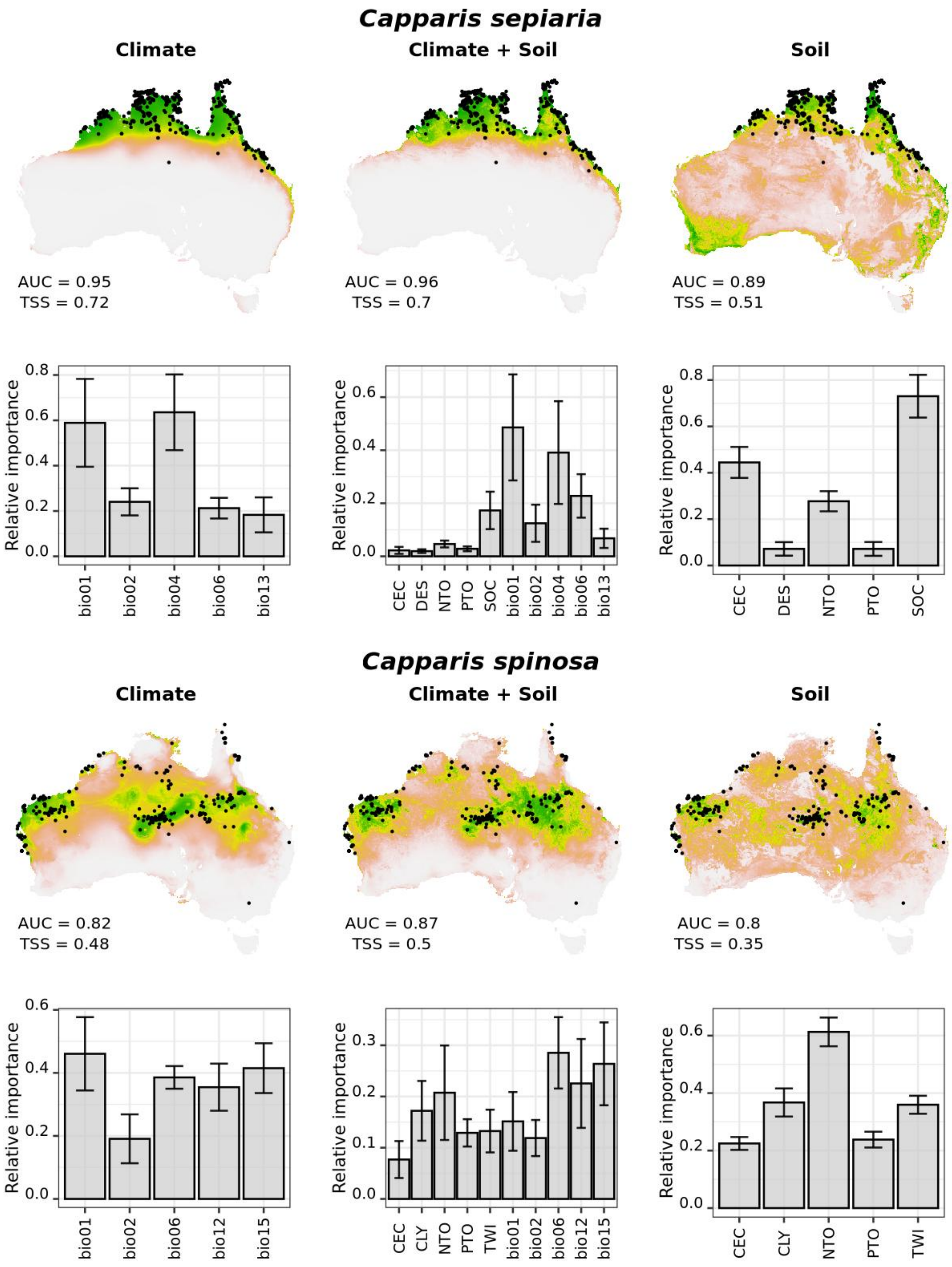

Capparis sepiaria
Climate
Climate + Soil
Soil
AUC = 0.95
TSS = 0.72
AUC = 0.96
TSS = 0.7
AUC = 0.89
TSS = 0.51
Relative importance
bio01
bio02
bio04
bio06
bio13
CEC
DES
NTO
PTO
SOC
Capparis spinosa
Climate
Climate + Soil
Soil
AUC = 0.82
TSS = 0.48
AUC = 0.87
TSS = 0.5
AUC = 0.8
TSS = 0.35
Relative importance
bio01
bio02
bio06
bio12
bio15
CEC
CLY
NTO
PTO
TWI

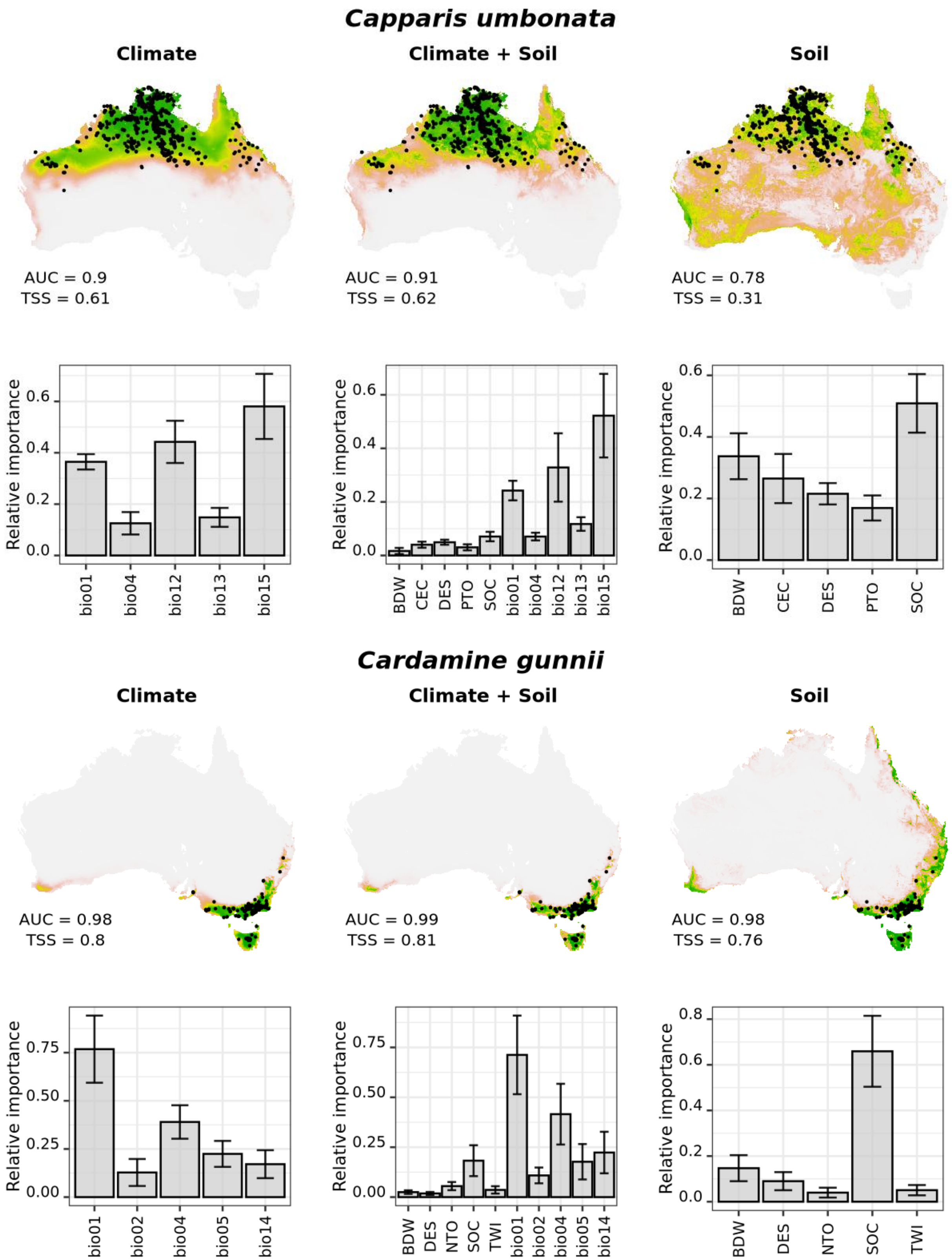

Capparis umbonata
Climate
Climate + Soil
Soil
AUC = 0.9
TSS = 0.61
AUC = 0.91
TSS = 0.62
AUC = 0.78
TSS = 0.31
Relative importance
0.0
0.2
0.4
0.6
bio01
bio04
bio12
bio13
bio15
BDW
CEC
DES
PTO
SOC
Cardamine gunnii
Climate
Climate + Soil
Soil
AUC = 0.98
TSS = 0.8
AUC = 0.99
TSS = 0.81
AUC = 0.98
TSS = 0.76
Relative importance
0.00
0.25
0.50
0.75
0.8
bio01
bio02
bio04
bio05
bio14
BDW
DES
NTO
SOC
TWI

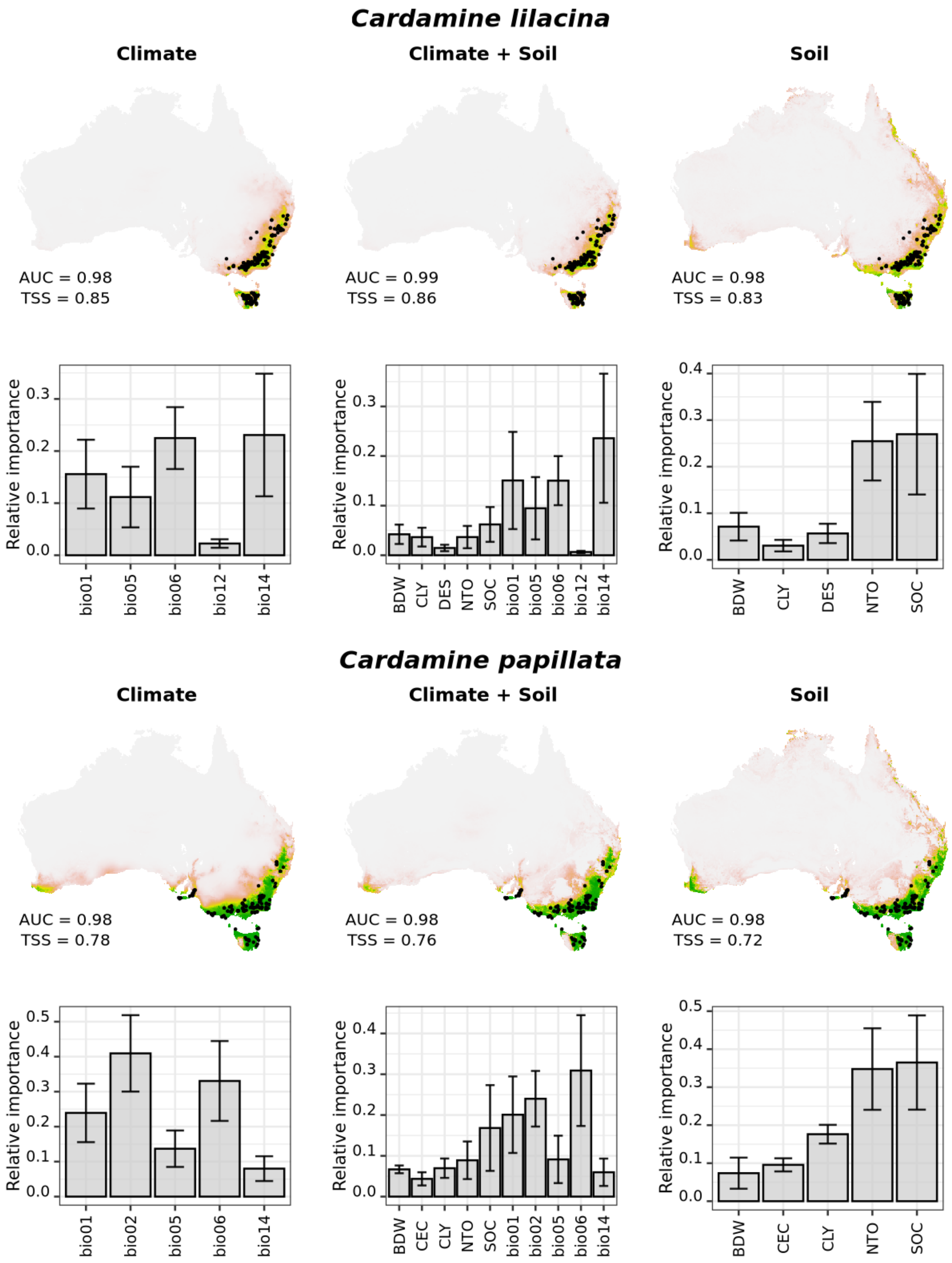
Cardamine lilacina
Climate
Climate + Soil
Soil
AUC = 0.98
TSS = 0.85
AUC = 0.99
TSS = 0.86
AUC = 0.98
TSS = 0.83
Relative importance
bio01
bio05
bio06
bio12
bio14
BDW
CLY
DES
NTO
SOC
Cardamine papillata
Climate
Climate + Soil
Soil
AUC = 0.98
TSS = 0.78
AUC = 0.98
TSS = 0.76
AUC = 0.98
TSS = 0.72
Relative importance
bio01
bio02
bio05
bio06
bio14
BDW
CEC
CLY
NTO
SOC

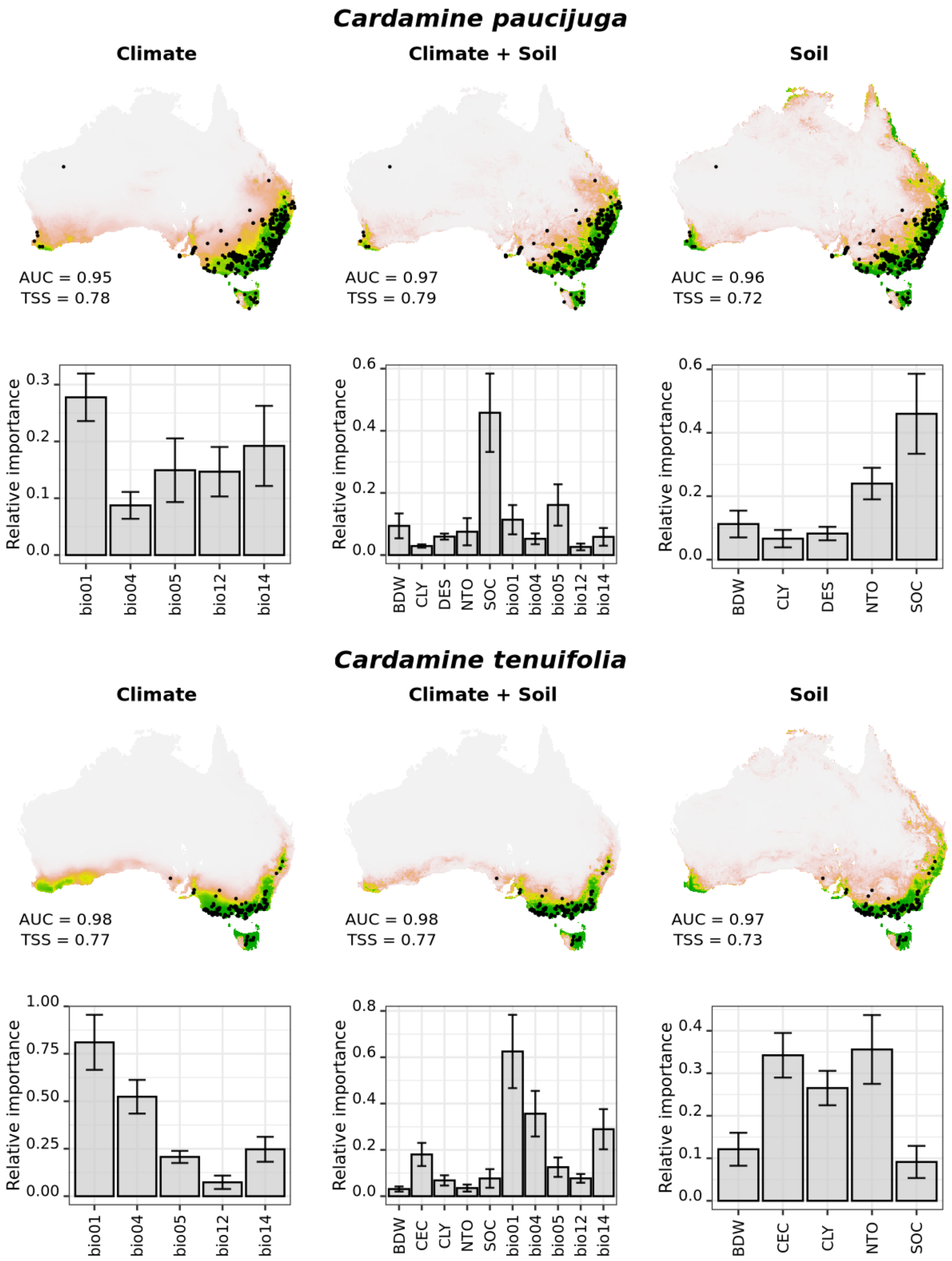
Cardamine paucijuga
Climate
Climate + Soil
Soil
AUC = 0.95
TSS = 0.78
AUC = 0.97
TSS = 0.79
AUC = 0.96
TSS = 0.72
Relative importance
0.0
0.1
0.2
0.3
bio01
bio04
bio05
bio12
bio14
Relative importance
0.0
0.2
0.4
0.6
BDW
CLY
DES
NTO
SOC
bio01
bio04
bio05
bio12
bio14
Relative importance
0.0
0.2
0.4
0.6
BDW
CLY
DES
NTO
SOC
Cardamine tenuifolia
Climate
Climate + Soil
Soil
AUC = 0.98
TSS = 0.77
AUC = 0.98
TSS = 0.77
AUC = 0.97
TSS = 0.73
Relative importance
0.00
0.25
0.50
0.75
1.00
bio01
bio04
bio05
bio12
bio14
Relative importance
0.0
0.2
0.4
0.6
0.8
BDW
CEC
CLY
NTO
SOC
bio01
bio04
bio05
bio12
bio14
Relative importance
0.0
0.1
0.2
0.3
0.4
BDW
CEC
CLY
NTO
SOC

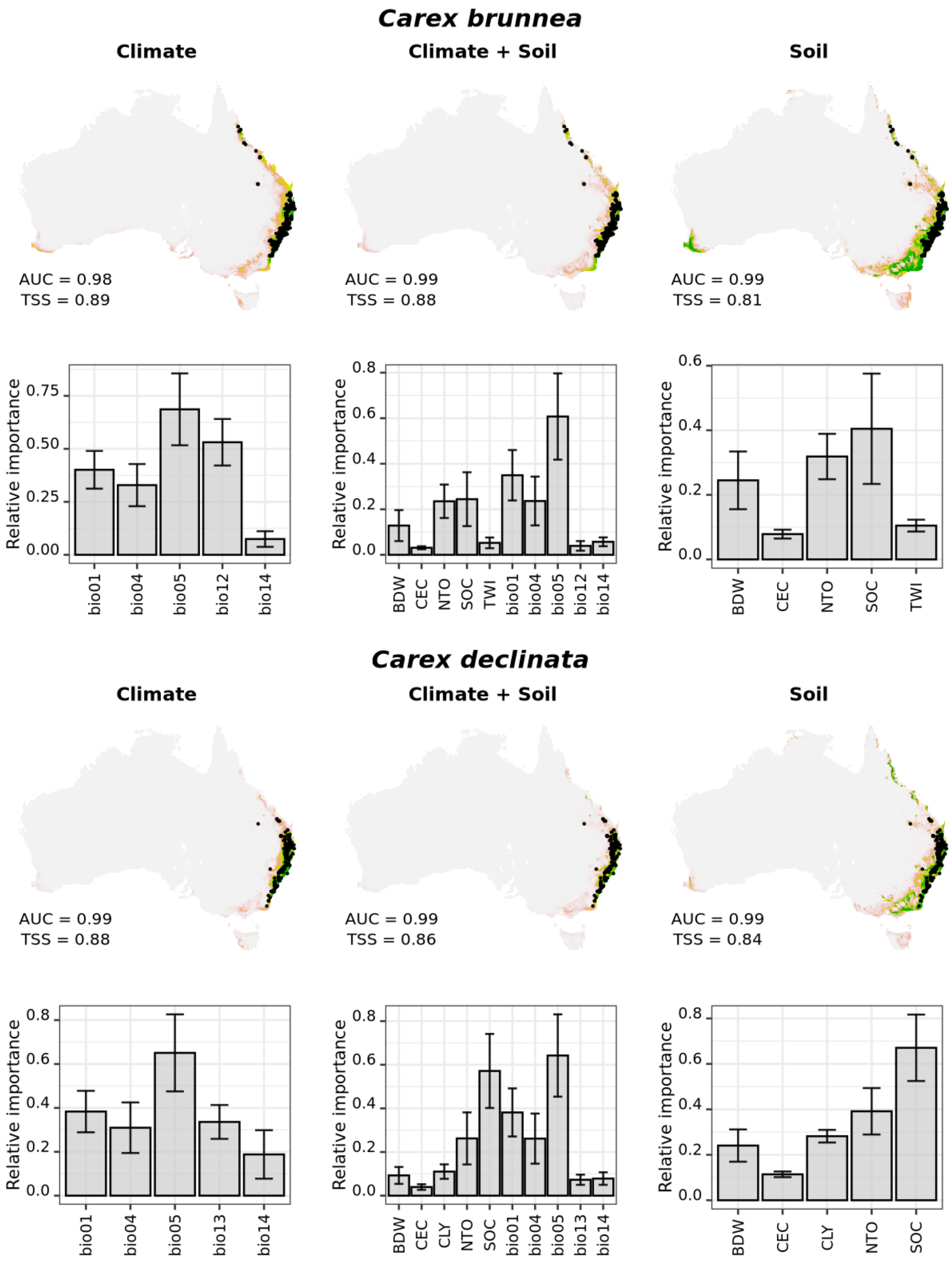
Carex brunnea
Climate
Climate + Soil
Soil
AUC = 0.98
TSS = 0.89
AUC = 0.99
TSS = 0.88
AUC = 0.99
TSS = 0.81
Relative importance
0.00
0.25
0.50
0.75
bio01
bio04
bio05
bio12
bio14
0.0
0.2
0.4
0.6
0.8
BDW
CEC
NTO
SOC
TWI
bio01
bio04
bio05
bio12
bio14
0.0
0.2
0.4
0.6
BDW
CEC
NTO
SOC
TWI
Carex declinata
Climate
Climate + Soil
Soil
AUC = 0.99
TSS = 0.88
AUC = 0.99
TSS = 0.86
AUC = 0.99
TSS = 0.84
Relative importance
0.0
0.2
0.4
0.6
0.8
bio01
bio04
bio05
bio13
bio14
BDW
CEC
CLY
NTO
SOC
bio01
bio04
bio05
bio13
bio14
BDW
CEC
CLY
NTO
SOC

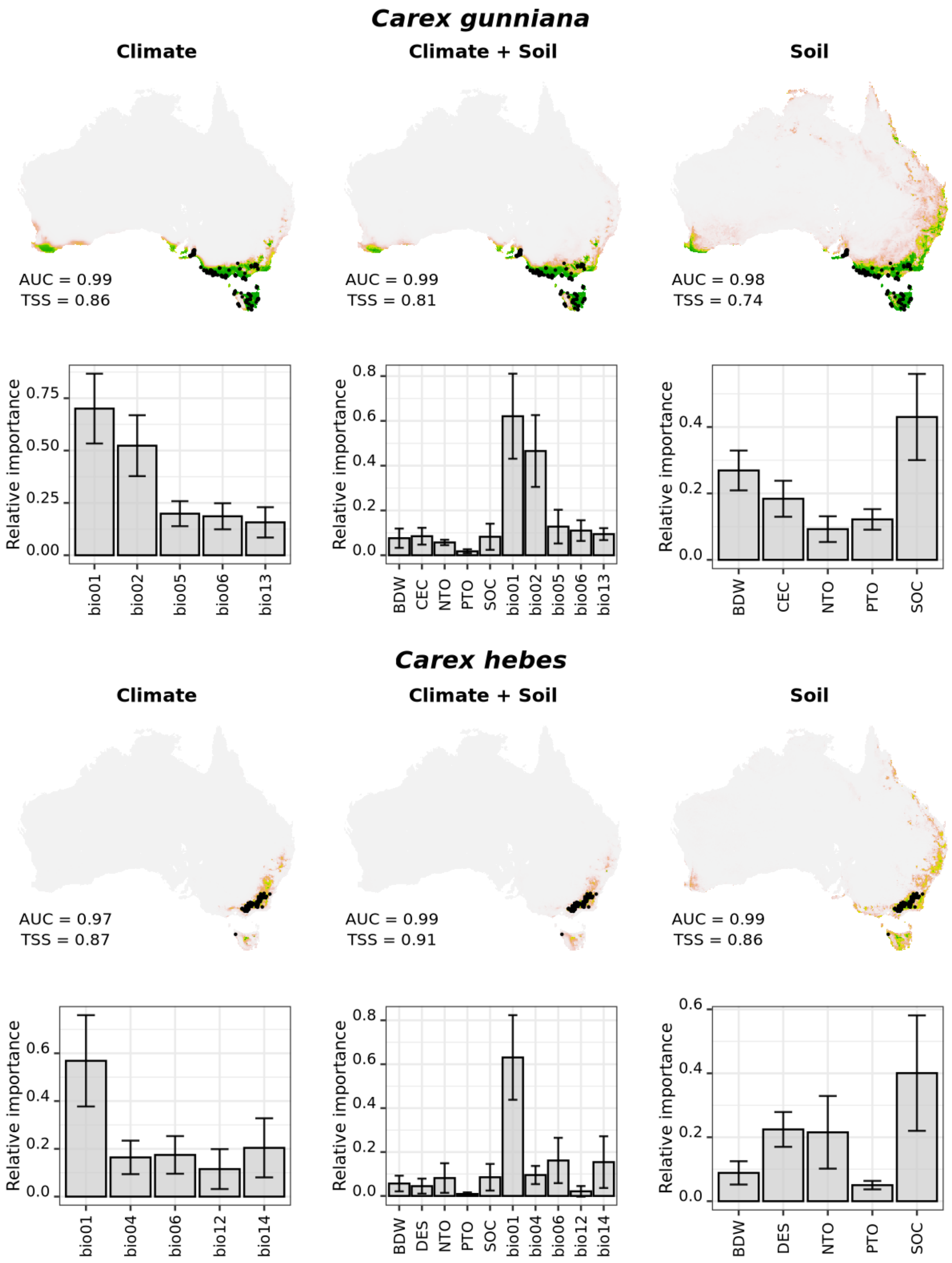

Carex gunniana
Climate
Climate + Soil
Soil
AUC = 0.99
TSS = 0.86
AUC = 0.99
TSS = 0.81
AUC = 0.98
TSS = 0.74
Relative importance
0.00
0.25
0.50
0.75
bio01
bio02
bio05
bio06
bio13
Relative importance
0.0
0.2
0.4
0.6
0.8
BDW
CEC
NTO
PTO
SOC
bio01
bio02
bio05
bio06
bio13
Relative importance
0.0
0.2
0.4
BDW
CEC
NTO
PTO
SOC
Carex hebes
Climate
Climate + Soil
Soil
AUC = 0.97
TSS = 0.87
AUC = 0.99
TSS = 0.91
AUC = 0.99
TSS = 0.86
Relative importance
0.0
0.2
0.4
0.6
bio01
bio04
bio06
bio12
bio14
Relative importance
0.0
0.2
0.4
0.6
0.8
BDW
DES
NTO
PTO
SOC
bio01
bio04
bio06
bio12
bio14
Relative importance
0.0
0.2
0.4
0.6
BDW
DES
NTO
PTO
SOC

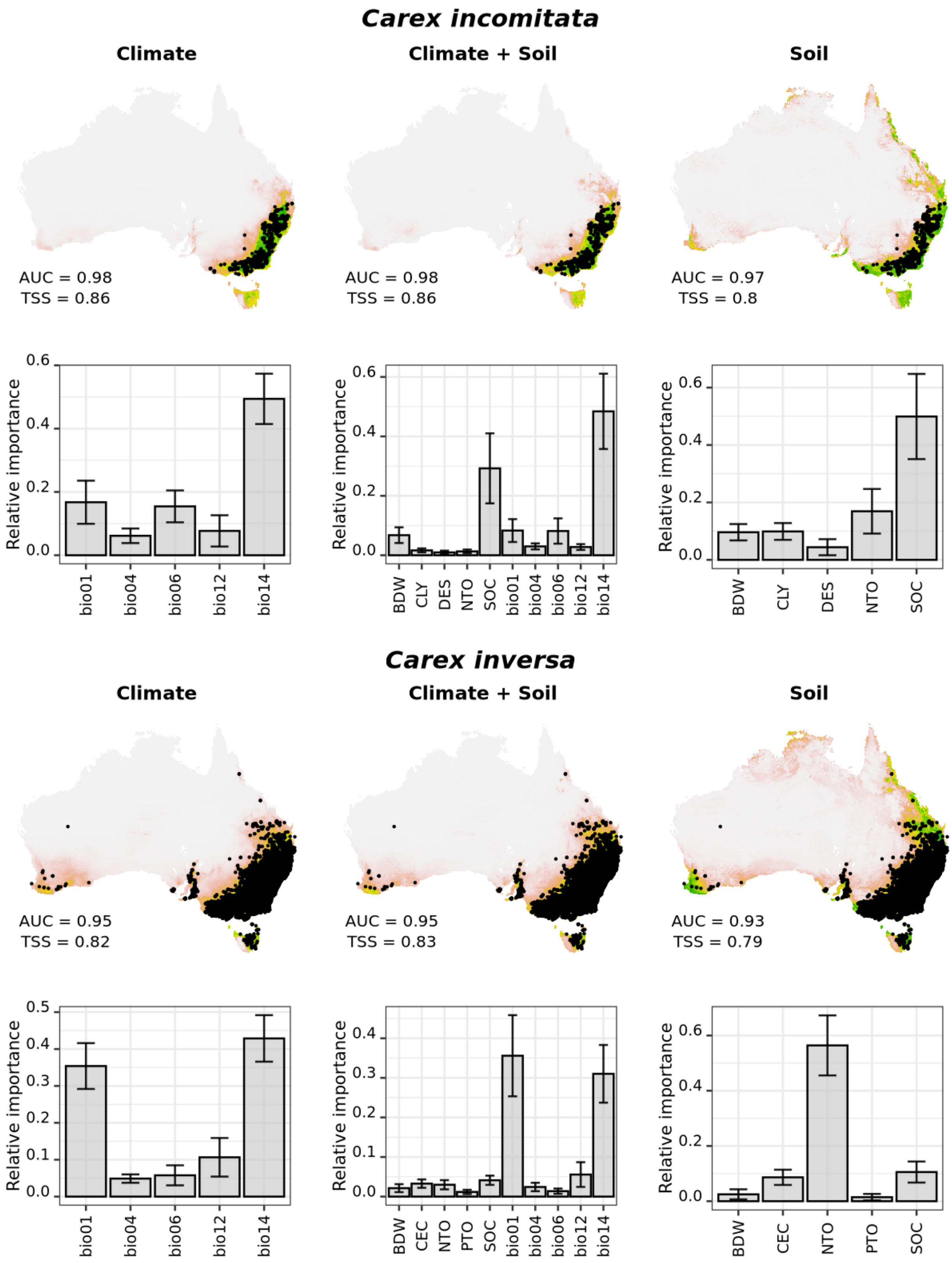
Carex incomitata
Climate
Climate + Soil
Soil
AUC = 0.98
TSS = 0.86
AUC = 0.98
TSS = 0.86
AUC = 0.97
TSS = 0.8
Relative importance
0.0
0.2
0.4
0.6
bio01
bio04
bio06
bio12
bio14
BDW
CLY
DES
NTO
SOC
Carex inversa
Climate
Climate + Soil
Soil
AUC = 0.95
TSS = 0.82
AUC = 0.95
TSS = 0.83
AUC = 0.93
TSS = 0.79
Relative importance
0.0
0.1
0.2
0.3
0.4
0.5
0.6
bio01
bio04
bio06
bio12
bio14
BDW
CEC
NTO
PTO
SOC

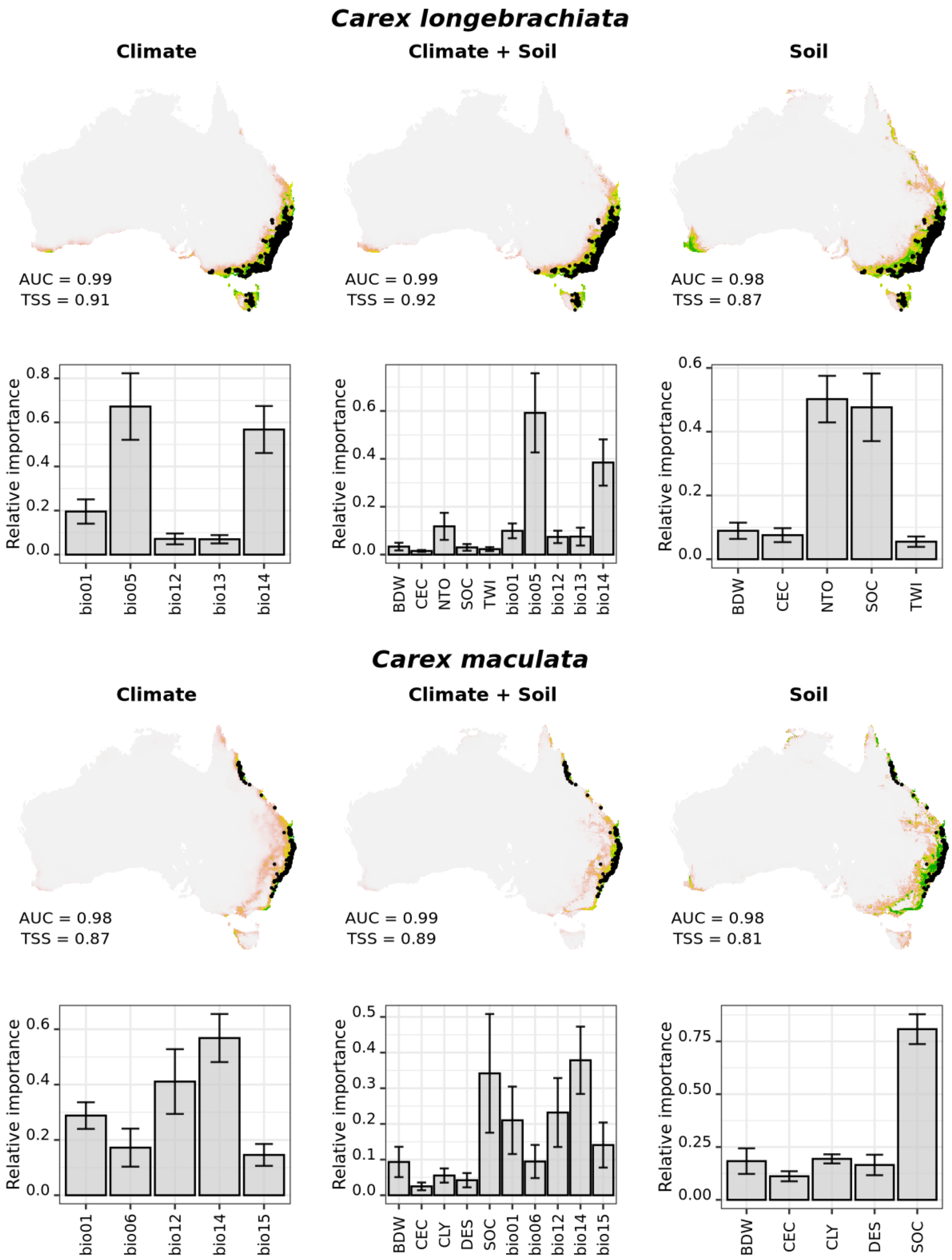

Carex longebrachiata
Climate
Climate + Soil
Soil
AUC = 0.99
TSS = 0.91
AUC = 0.99
TSS = 0.92
AUC = 0.98
TSS = 0.87
Relative importance
bio01
bio05
bio12
bio13
bio14
BDW
CEC
NTO
SOC
TWI
Carex maculata
Climate
Climate + Soil
Soil
AUC = 0.98
TSS = 0.87
AUC = 0.99
TSS = 0.89
AUC = 0.98
TSS = 0.81
Relative importance
bio01
bio06
bio12
bio14
bio15
BDW
CEC
CLY
DES
SOC

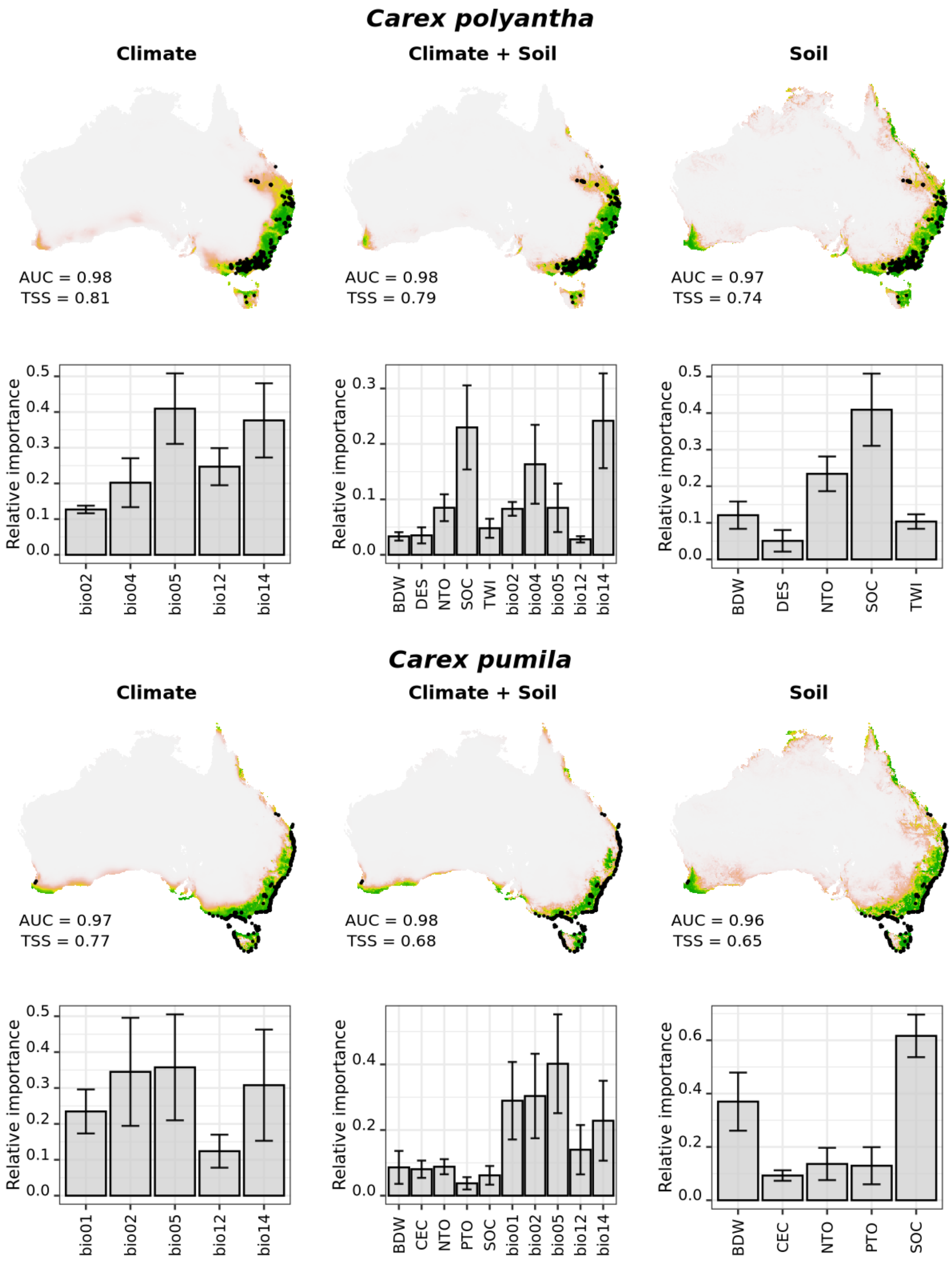
Carex polyantha
Climate
Climate + Soil
Soil
AUC = 0.98
TSS = 0.81
AUC = 0.98
TSS = 0.79
AUC = 0.97
TSS = 0.74
Relative importance
bio02
bio04
bio05
bio12
bio14
BDW
DES
NTO
SOC
TWI
Carex pumila
Climate
Climate + Soil
Soil
AUC = 0.97
TSS = 0.77
AUC = 0.98
TSS = 0.68
AUC = 0.96
TSS = 0.65
Relative importance
bio01
bio02
bio05
bio12
bio14
BDW
CEC
NTO
PTO
SOC

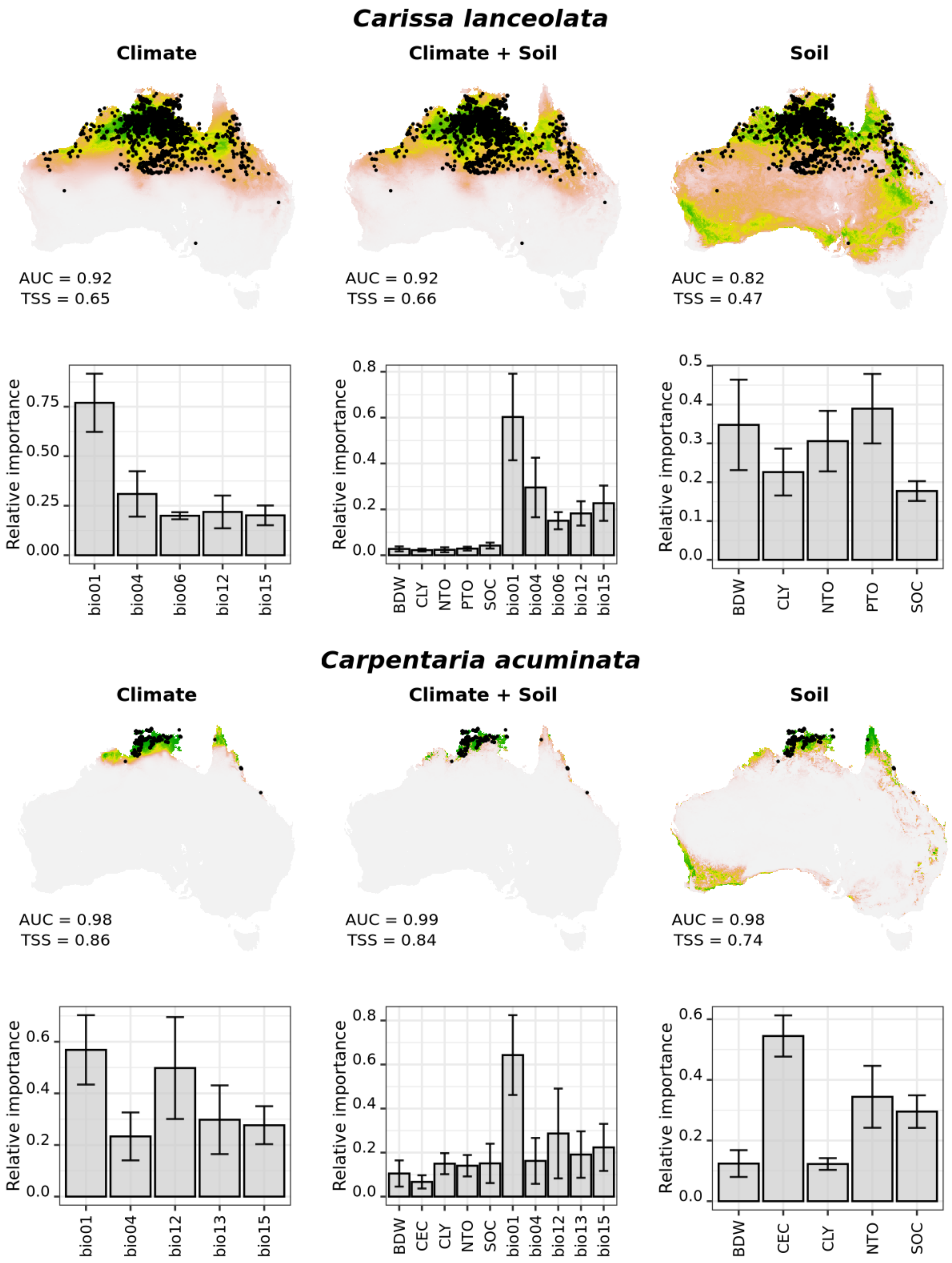
Carissa lanceolata
Climate
Climate + Soil
Soil
AUC = 0.92
TSS = 0.65
AUC = 0.92
TSS = 0.66
AUC = 0.82
TSS = 0.47
Relative importance
0.00
0.25
0.50
0.75
bio01
bio04
bio06
bio12
bio15
0.0
0.2
0.4
0.6
0.8
BDW
CLY
NTO
PTO
SOC
bio01
bio04
bio06
bio12
bio15
0.0
0.1
0.2
0.3
0.4
0.5
BDW
CLY
NTO
PTO
SOC
Carpentaria acuminata
Climate
Climate + Soil
Soil
AUC = 0.98
TSS = 0.86
AUC = 0.99
TSS = 0.84
AUC = 0.98
TSS = 0.74
Relative importance
0.0
0.2
0.4
0.6
bio01
bio04
bio12
bio13
bio15
0.0
0.2
0.4
0.6
0.8
BDW
CEC
CLY
NTO
SOC
bio01
bio04
bio12
bio13
bio15
0.0
0.2
0.4
0.6
BDW
CEC
CLY
NTO
SOC

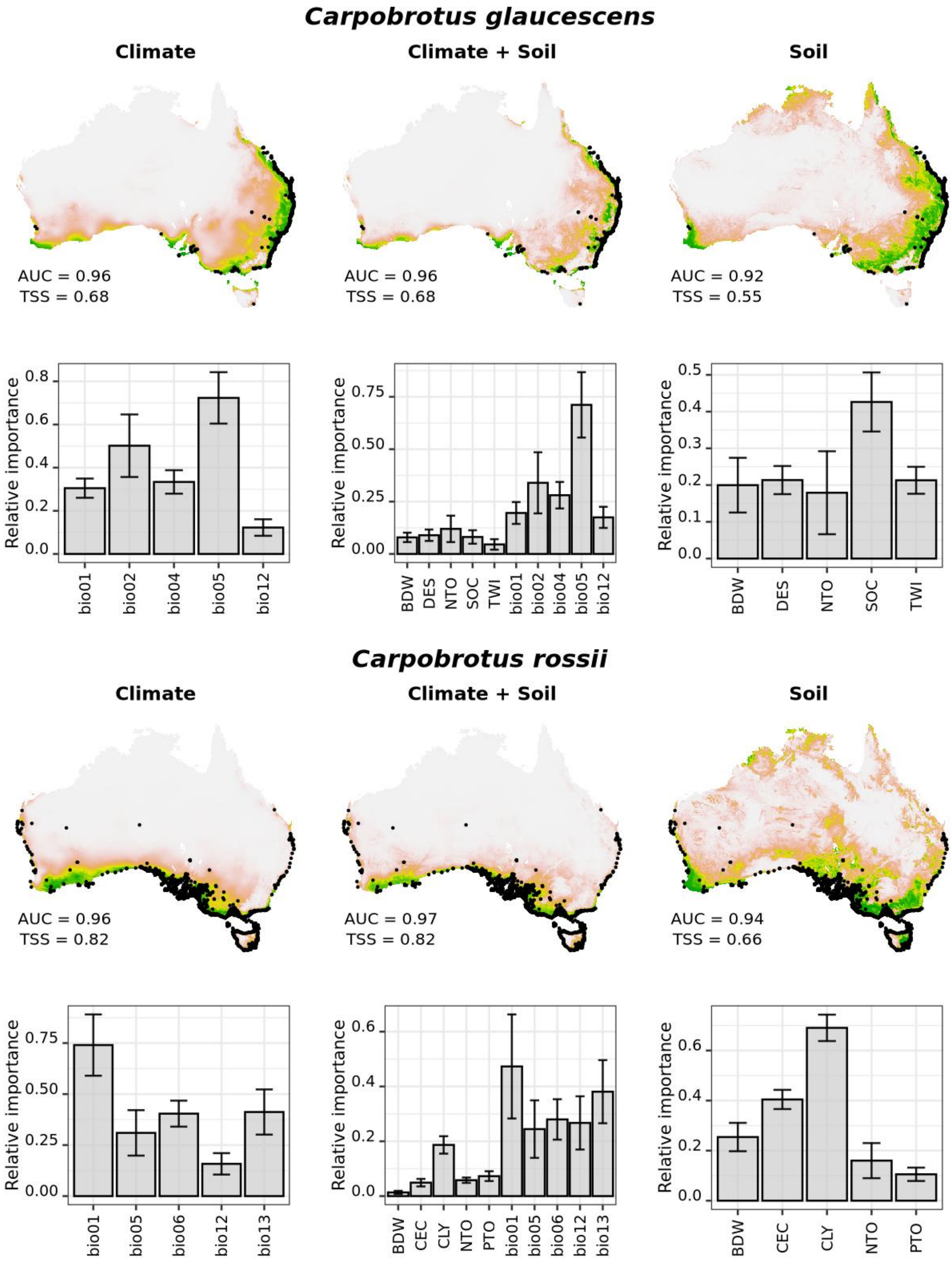
Carpobrotus glaucescens
Climate
Climate + Soil
Soil
AUC = 0.96
TSS = 0.68
AUC = 0.96
TSS = 0.68
AUC = 0.92
TSS = 0.55
Relative importance
bio01 bio02 bio04 bio05 bio12
BDW DES NTO SOC TWI bio01 bio02 bio04 bio05 bio12
BDW DES NTO SOC TWI
Carpobrotus rossii
Climate
Climate + Soil
Soil
AUC = 0.96
TSS = 0.82
AUC = 0.97
TSS = 0.82
AUC = 0.94
TSS = 0.66
Relative importance
bio01 bio05 bio06 bio12 bio13
BDW CEC CLY NTO PTO bio01 bio05 bio06 bio12 bio13
BDW CEC CLY NTO PTO

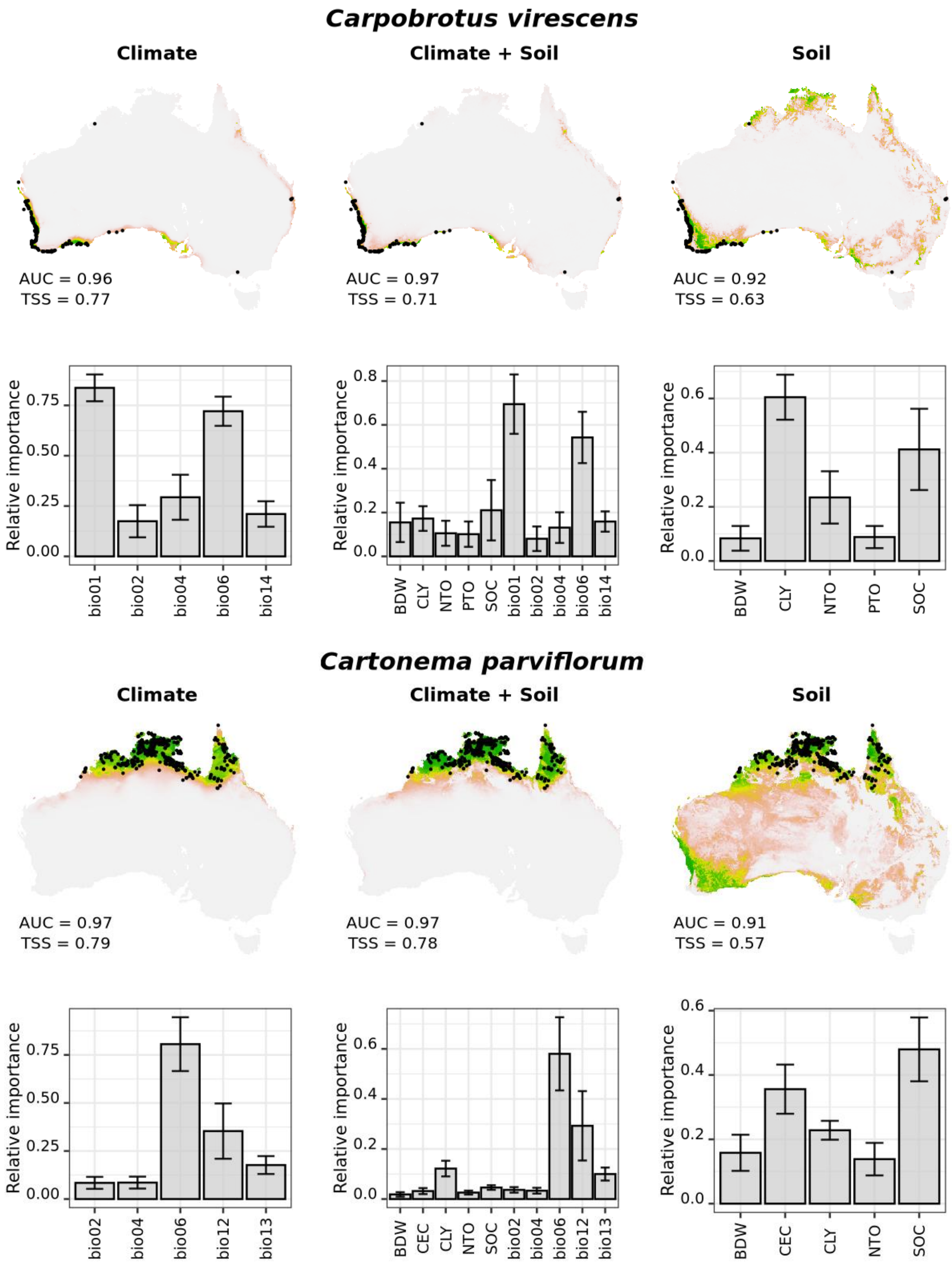

Carpobrotus virescens
Climate
Climate + Soil
Soil
AUC = 0.96
TSS = 0.77
AUC = 0.97
TSS = 0.71
AUC = 0.92
TSS = 0.63
Relative importance
0.00
0.25
0.50
0.75
bio01
bio02
bio04
bio06
bio14
0.0
0.2
0.4
0.6
0.8
BDW
CLY
NTO
PTO
SOC
bio01
bio02
bio04
bio06
bio14
BDW
CLY
NTO
PTO
SOC
Cartonema parviflorum
Climate
Climate + Soil
Soil
AUC = 0.97
TSS = 0.79
AUC = 0.97
TSS = 0.78
AUC = 0.91
TSS = 0.57
bio02
bio04
bio06
bio12
bio13
BDW
CEC
CLY
NTO
SOC
bio02
bio04
bio06
bio12
bio13
BDW
CEC
CLY
NTO
SOC

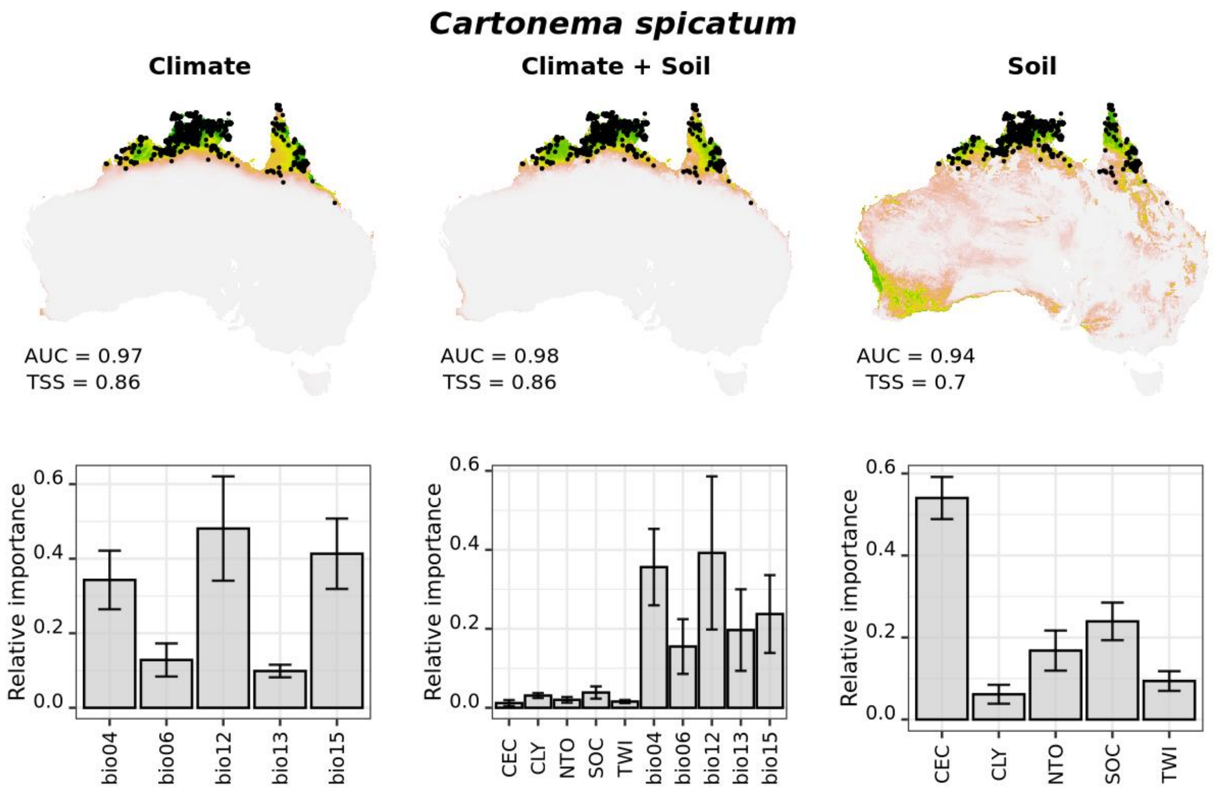

Cartonema spicatum
Climate
Climate + Soil
Soil
AUC = 0.97
TSS = 0.86
AUC = 0.98
TSS = 0.86
AUC = 0.94
TSS = 0.7
Relative importance
bio04
bio06
bio12
bio13
bio15
CEC
CLY
NTO
SOC
TWI

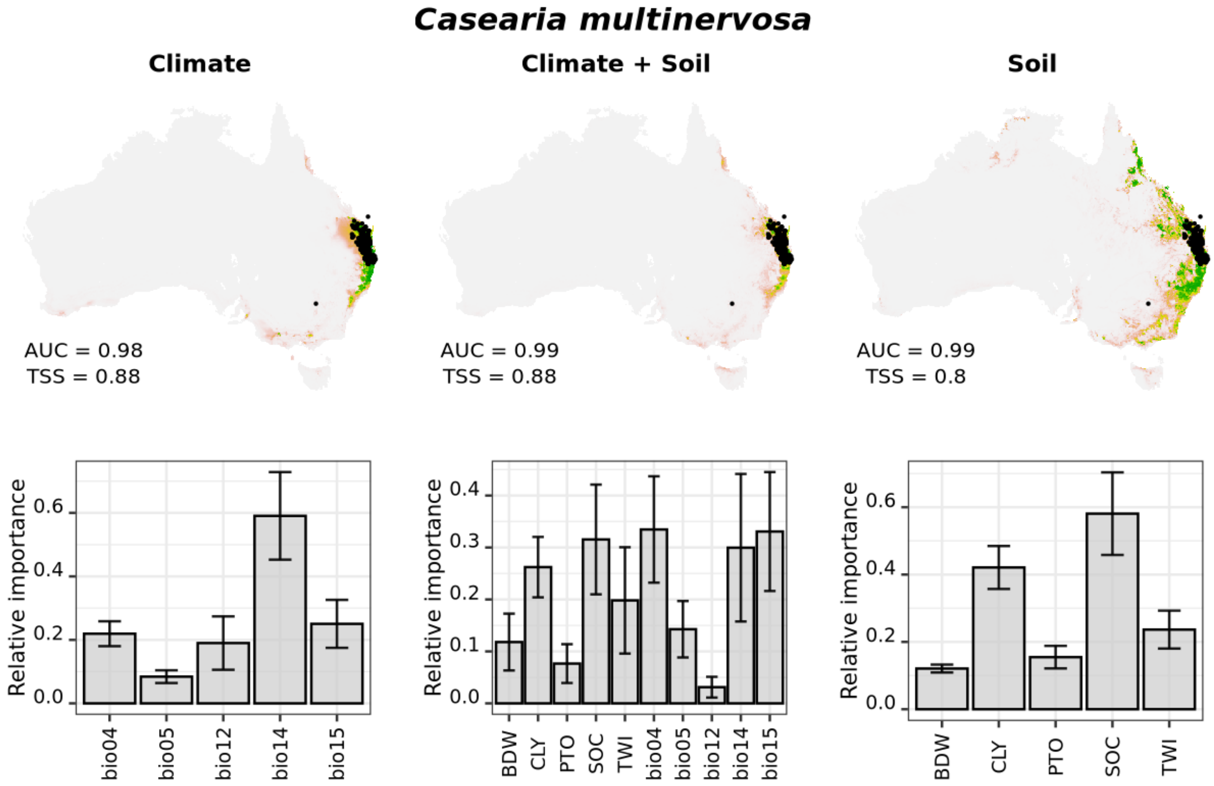

Casearia multinervosa
Climate
Climate + Soil
Soil
AUC = 0.98
TSS = 0.88
AUC = 0.99
TSS = 0.88
AUC = 0.99
TSS = 0.8
Relative importance
bio04
bio05
bio12
bio14
bio15
BDW
CLY
PTO
SOC
TWI

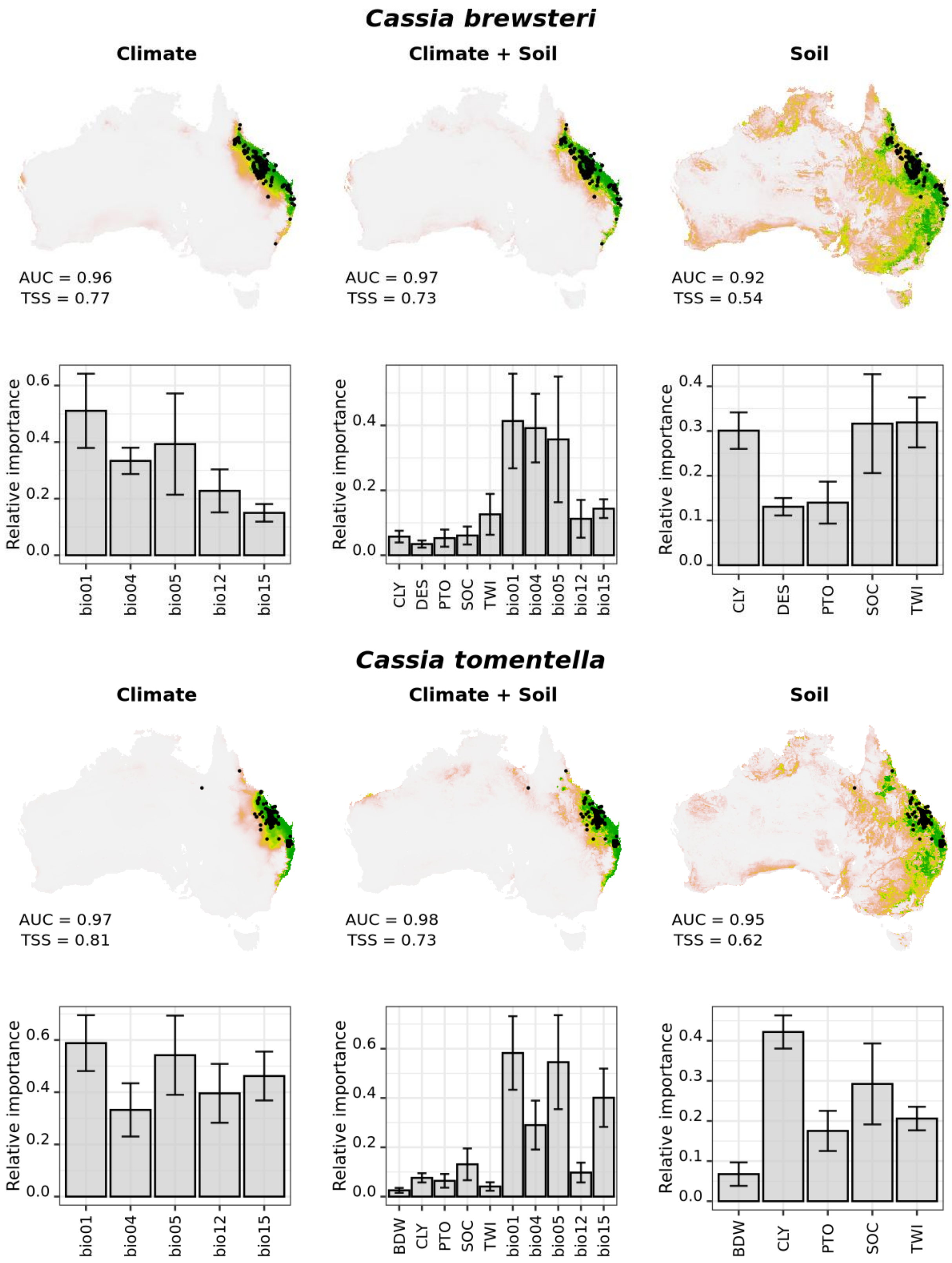
Cassia brewsteri
Climate
Climate + Soil
Soil
AUC = 0.96
TSS = 0.77
AUC = 0.97
TSS = 0.73
AUC = 0.92
TSS = 0.54
Relative importance
bio01 bio04 bio05 bio12 bio15
CLY DES PTO SOC TWI bio01 bio04 bio05 bio12 bio15
CLY DES PTO SOC TWI
Cassia tomentella
Climate
Climate + Soil
Soil
AUC = 0.97
TSS = 0.81
AUC = 0.98
TSS = 0.73
AUC = 0.95
TSS = 0.62
Relative importance
bio01 bio04 bio05 bio12 bio15
BDW CLY PTO SOC TWI bio01 bio04 bio05 bio12 bio15
BDW CLY PTO SOC TWI

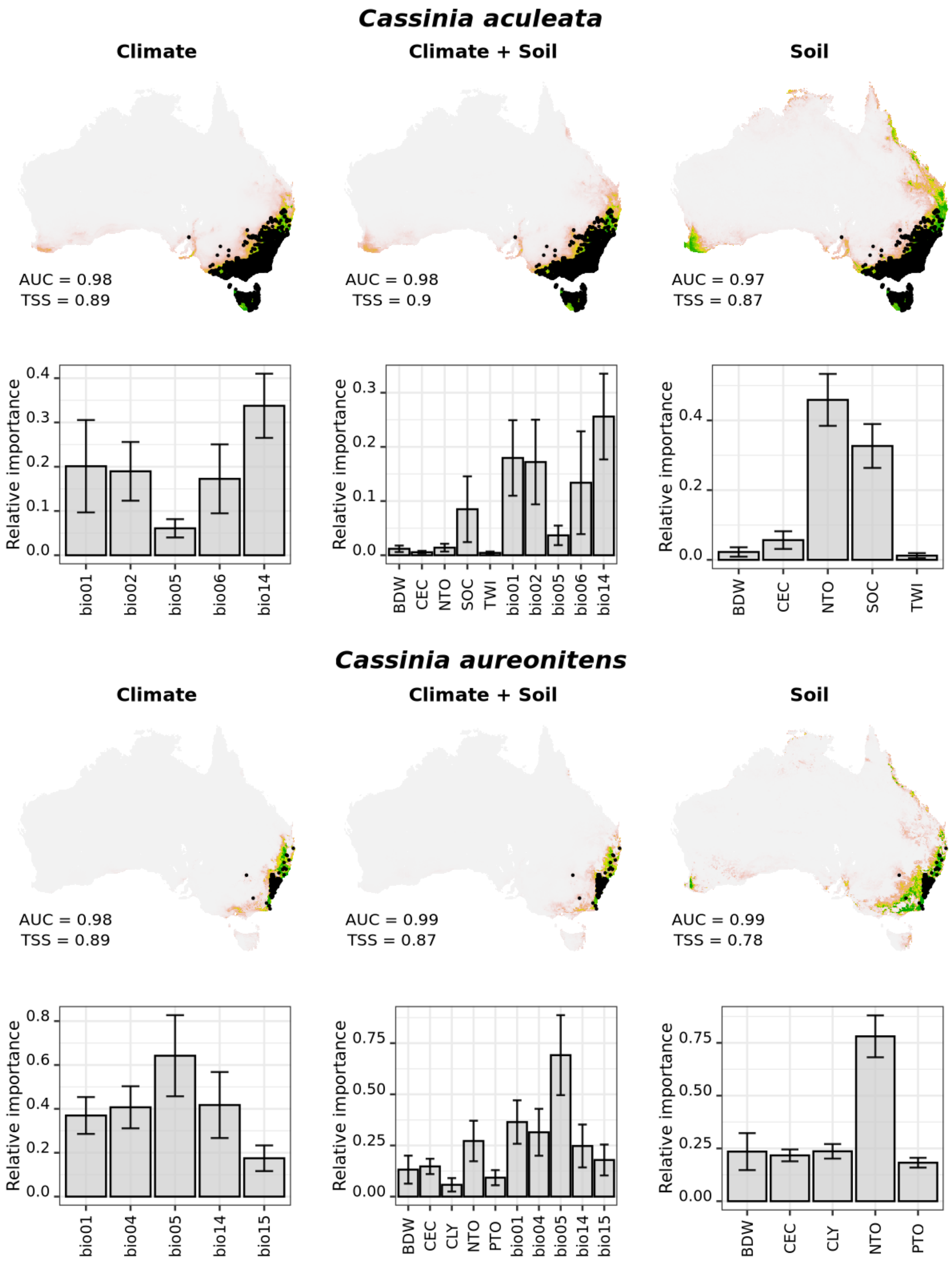
Cassinia aculeata
Climate
Climate + Soil
Soil
AUC = 0.98
TSS = 0.89
AUC = 0.98
TSS = 0.9
AUC = 0.97
TSS = 0.87
Relative importance
0.0
0.1
0.2
0.3
0.4
bio01
bio02
bio05
bio06
bio14
BDW
CEC
NTO
SOC
TWI
bio01
bio02
bio05
bio06
bio14
BDW
CEC
NTO
SOC
TWI
Cassinia aureonitens
Climate
Climate + Soil
Soil
AUC = 0.98
TSS = 0.89
AUC = 0.99
TSS = 0.87
AUC = 0.99
TSS = 0.78
Relative importance
0.0
0.2
0.4
0.6
0.8
0.00
0.25
0.50
0.75
bio01
bio04
bio05
bio14
bio15
BDW
CEC
CLY
NTO
PTO
bio01
bio04
bio05
bio14
bio15
BDW
CEC
CLY
NTO
PTO

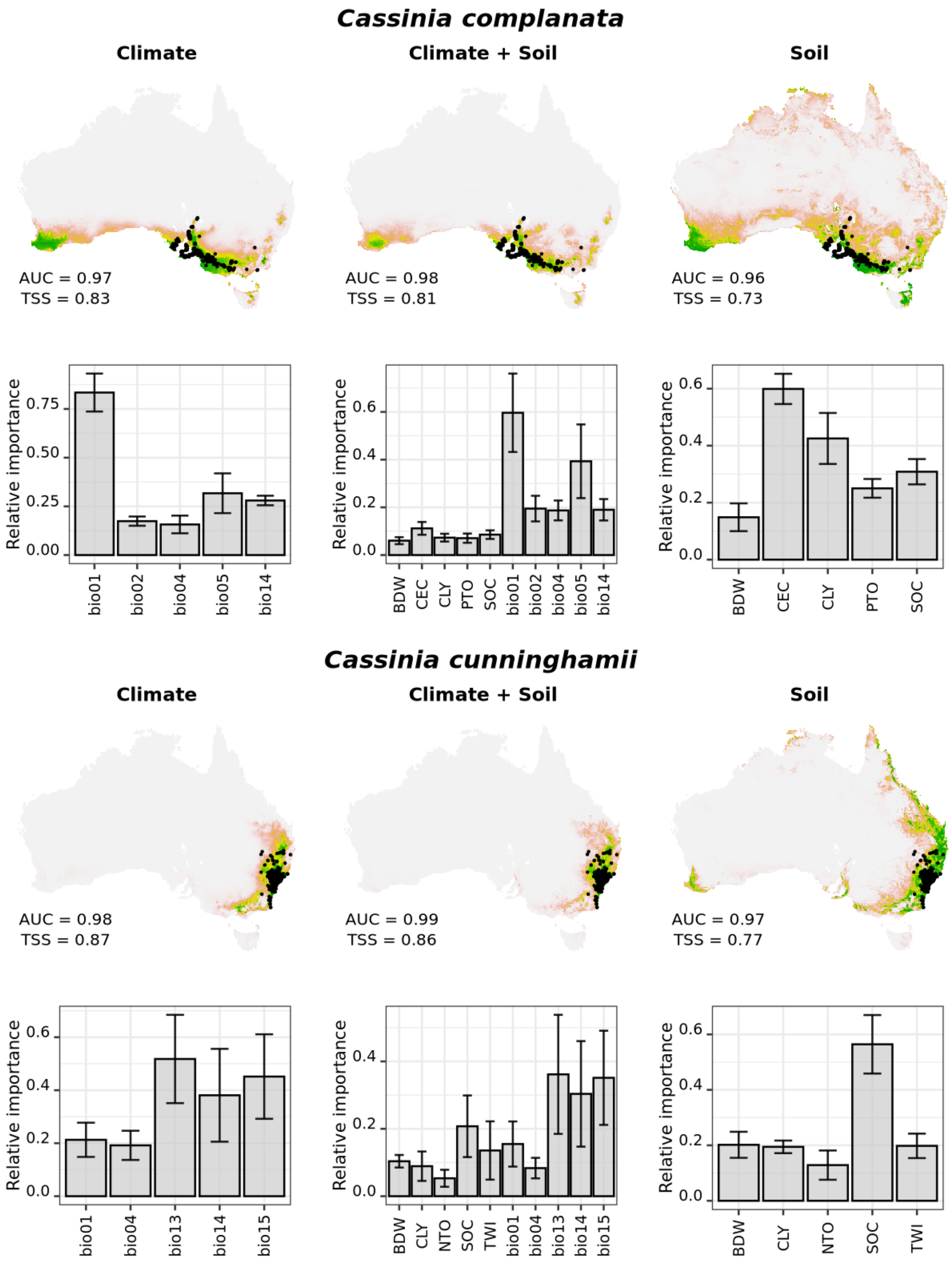
Cassinia complanata
Climate
Climate + Soil
Soil
AUC = 0.97
TSS = 0.83
AUC = 0.98
TSS = 0.81
AUC = 0.96
TSS = 0.73
Relative importance
0.00
0.25
0.50
0.75
bio01
bio02
bio04
bio05
bio14
0.0
0.2
0.4
0.6
BDW
CEC
CLY
PTO
SOC
Cassinia cunninghamii
AUC = 0.98
TSS = 0.87
AUC = 0.99
TSS = 0.86
AUC = 0.97
TSS = 0.77
bio13
bio15
NTO
TWI

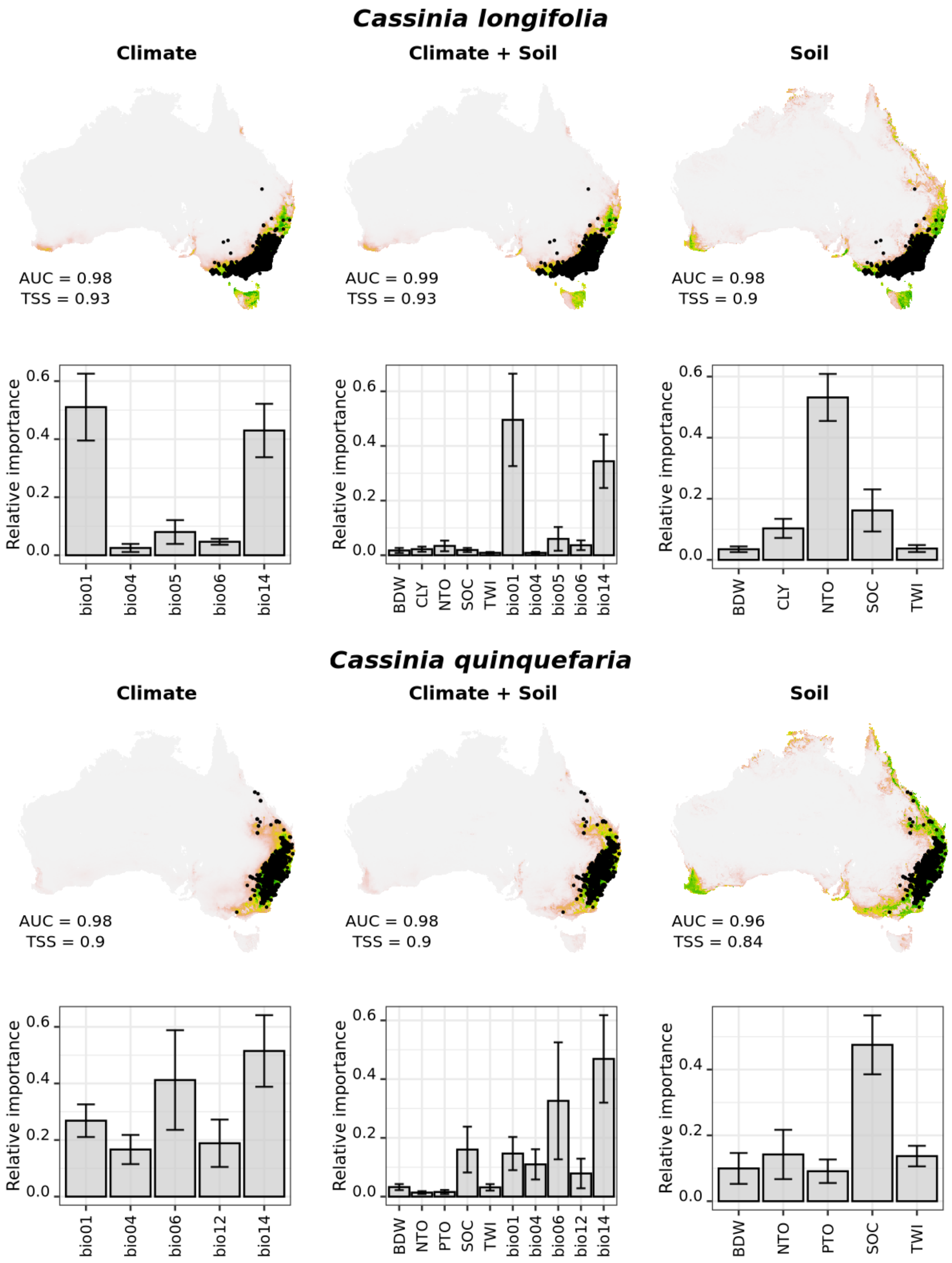
Cassinia longifolia
Climate
Climate + Soil
Soil
AUC = 0.98
TSS = 0.93
AUC = 0.99
TSS = 0.93
AUC = 0.98
TSS = 0.9
Relative importance
0.0
0.2
0.4
0.6
bio01
bio04
bio05
bio06
bio14
BDW
CLY
NTO
SOC
TWI
Cassinia quinquefaria
Climate
Climate + Soil
Soil
AUC = 0.98
TSS = 0.9
AUC = 0.98
TSS = 0.9
AUC = 0.96
TSS = 0.84
Relative importance
bio01
bio04
bio06
bio12
bio14
BDW
NTO
PTO
SOC
TWI

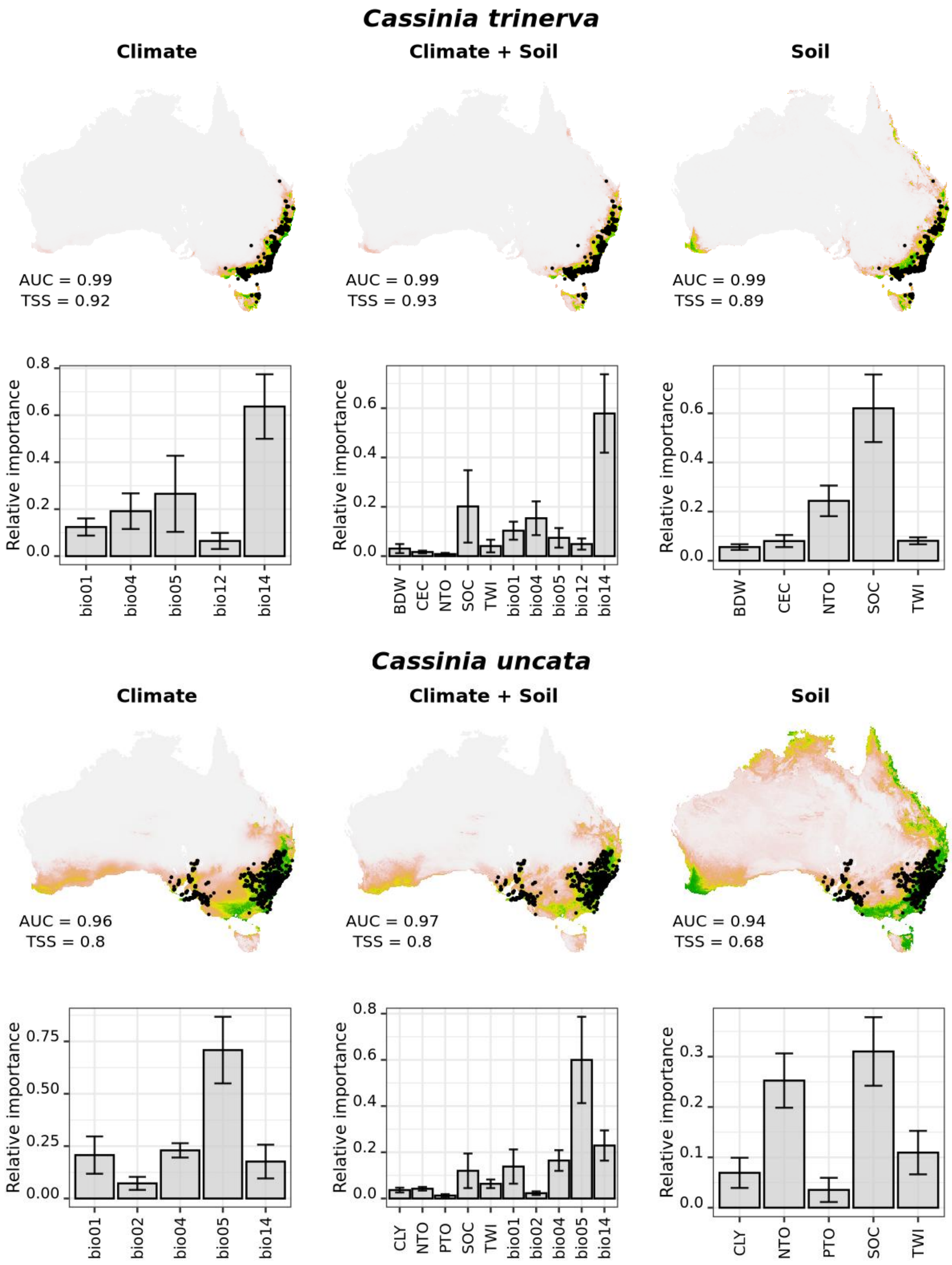
Cassinia trinerva
Climate
Climate + Soil
Soil
AUC = 0.99
TSS = 0.92
AUC = 0.99
TSS = 0.93
AUC = 0.99
TSS = 0.89
Relative importance
bio01
bio04
bio05
bio12
bio14
BDW
CEC
NTO
SOC
TWI
Cassinia uncata
AUC = 0.96
TSS = 0.8
AUC = 0.97
TSS = 0.8
AUC = 0.94
TSS = 0.68
bio02
CLY
PTO

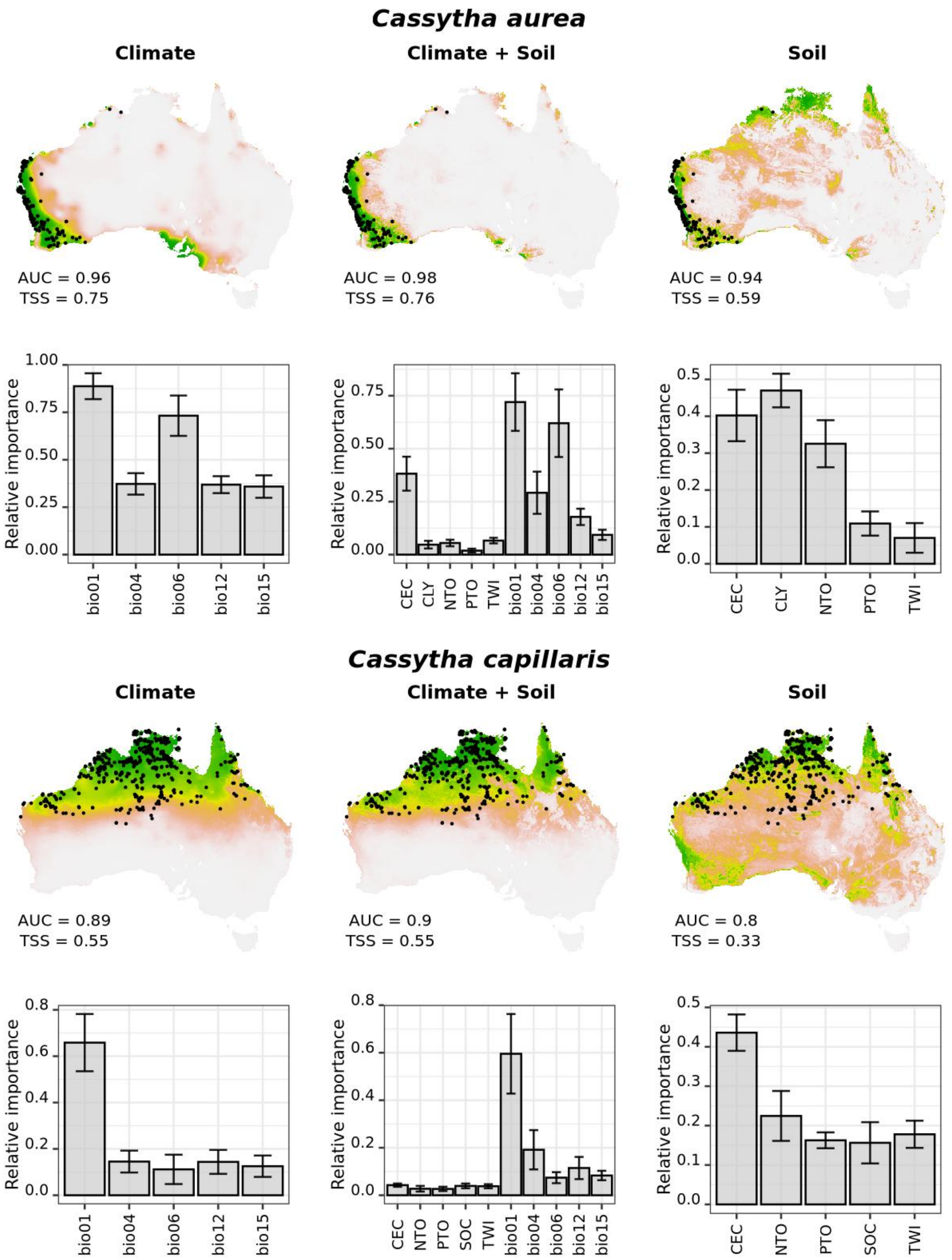

Cassytha aurea
Climate
Climate + Soil
Soil
AUC = 0.96
TSS = 0.75
AUC = 0.98
TSS = 0.76
AUC = 0.94
TSS = 0.59
Relative importance
bio01 bio04 bio06 bio12 bio15
CEC CLY NTO PTO TWI bio01 bio04 bio06 bio12 bio15
CEC CLY NTO PTO TWI
Cassytha capillaris
Climate
Climate + Soil
Soil
AUC = 0.89
TSS = 0.55
AUC = 0.9
TSS = 0.55
AUC = 0.8
TSS = 0.33
Relative importance
bio01 bio04 bio06 bio12 bio15
CEC NTO PTO SOC TWI bio01 bio04 bio06 bio12 bio15
CEC NTO PTO SOC TWI

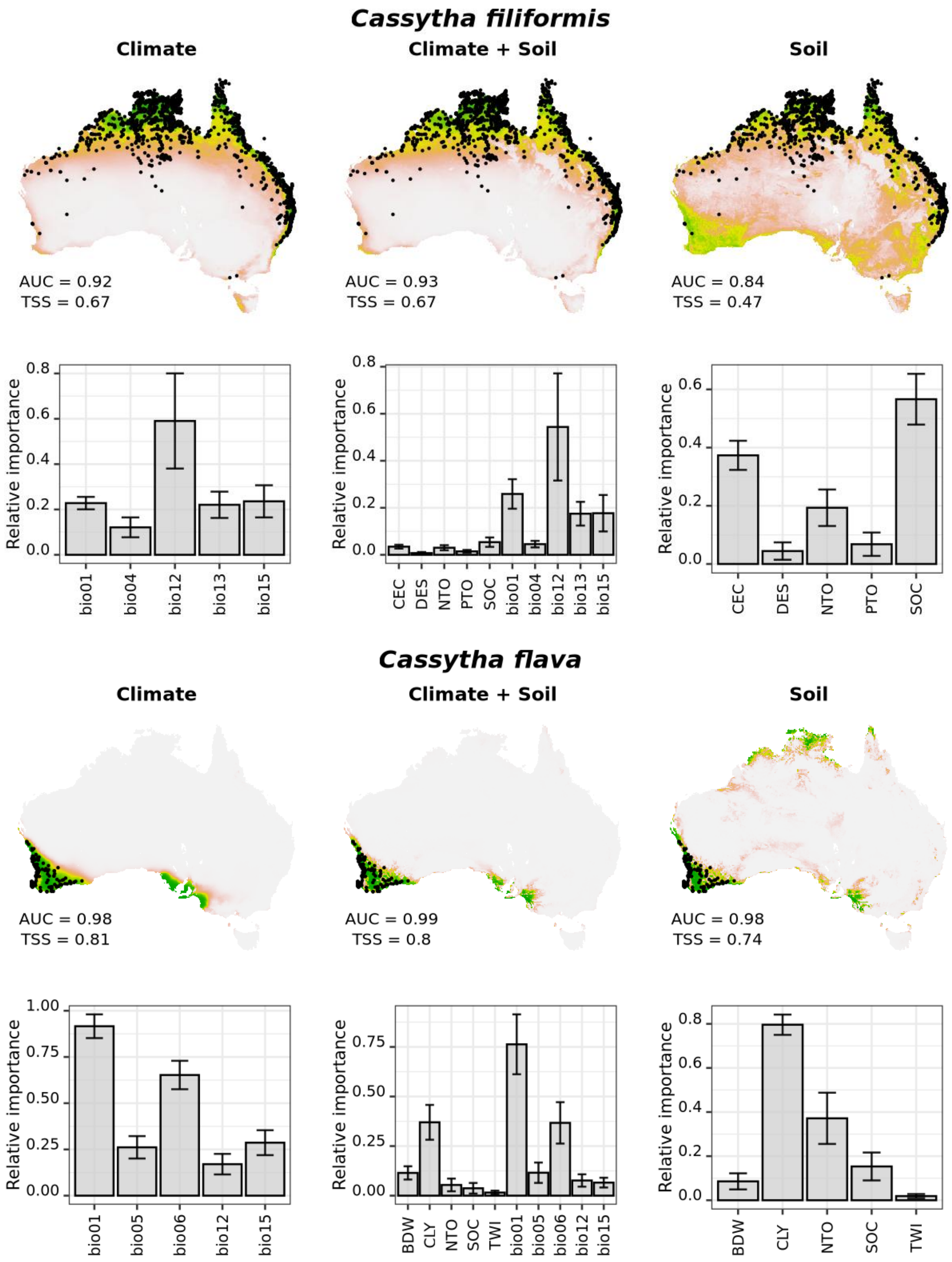

Cassytha filiformis
Climate
Climate + Soil
Soil
AUC = 0.92
TSS = 0.67
AUC = 0.93
TSS = 0.67
AUC = 0.84
TSS = 0.47
Relative importance
bio01
bio04
bio12
bio13
bio15
CEC
DES
NTO
PTO
SOC
Cassytha flava
AUC = 0.98
TSS = 0.81
AUC = 0.99
TSS = 0.8
AUC = 0.98
TSS = 0.74
bio05
bio06
BDW
CLY
TWI

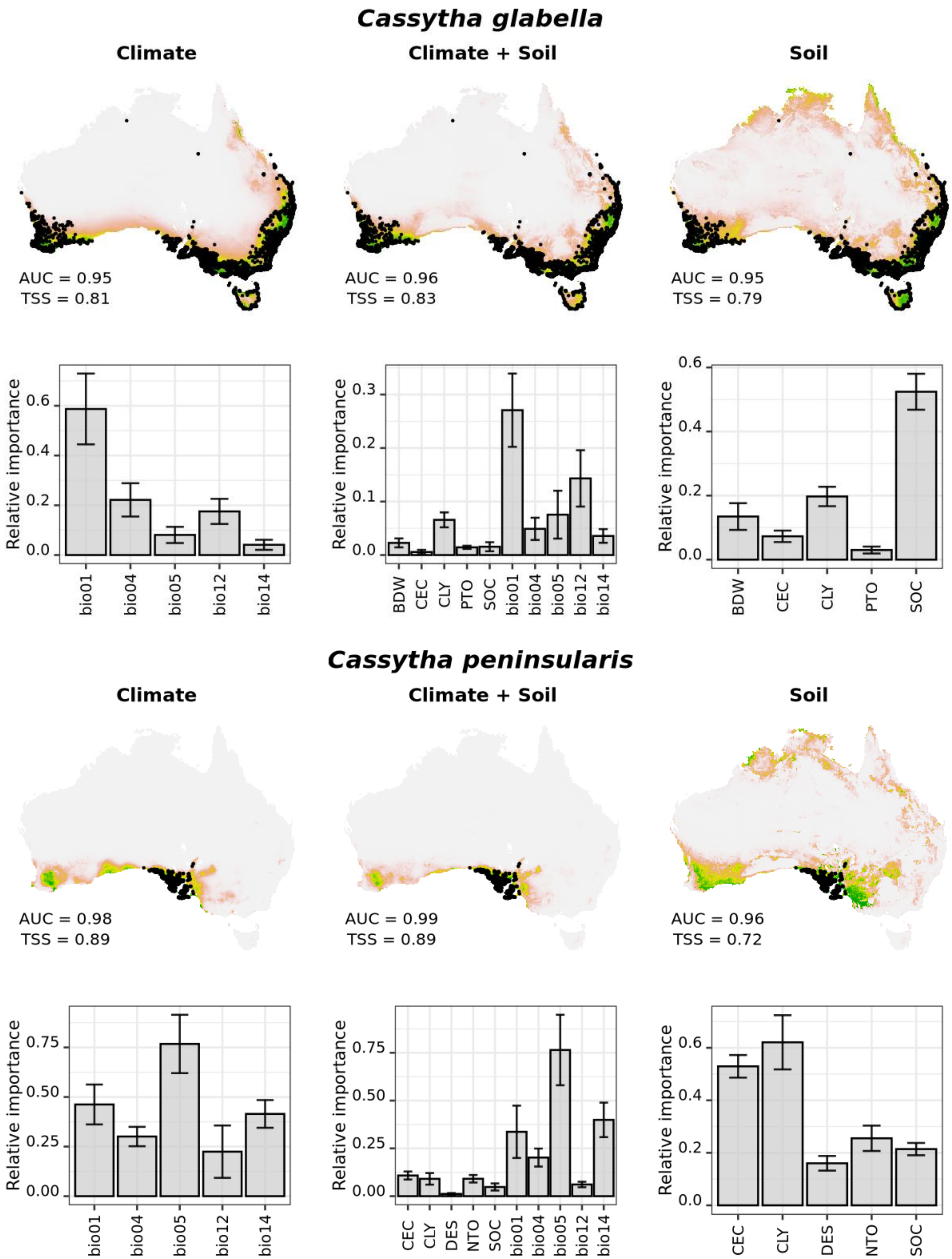
Cassytha glabella
Climate
Climate + Soil
Soil
AUC = 0.95
TSS = 0.81
AUC = 0.96
TSS = 0.83
AUC = 0.95
TSS = 0.79
Relative importance
bio01
bio04
bio05
bio12
bio14
BDW
CEC
CLY
PTO
SOC
Cassytha peninsularis
AUC = 0.98
TSS = 0.89
AUC = 0.99
TSS = 0.89
AUC = 0.96
TSS = 0.72
DES
NTO

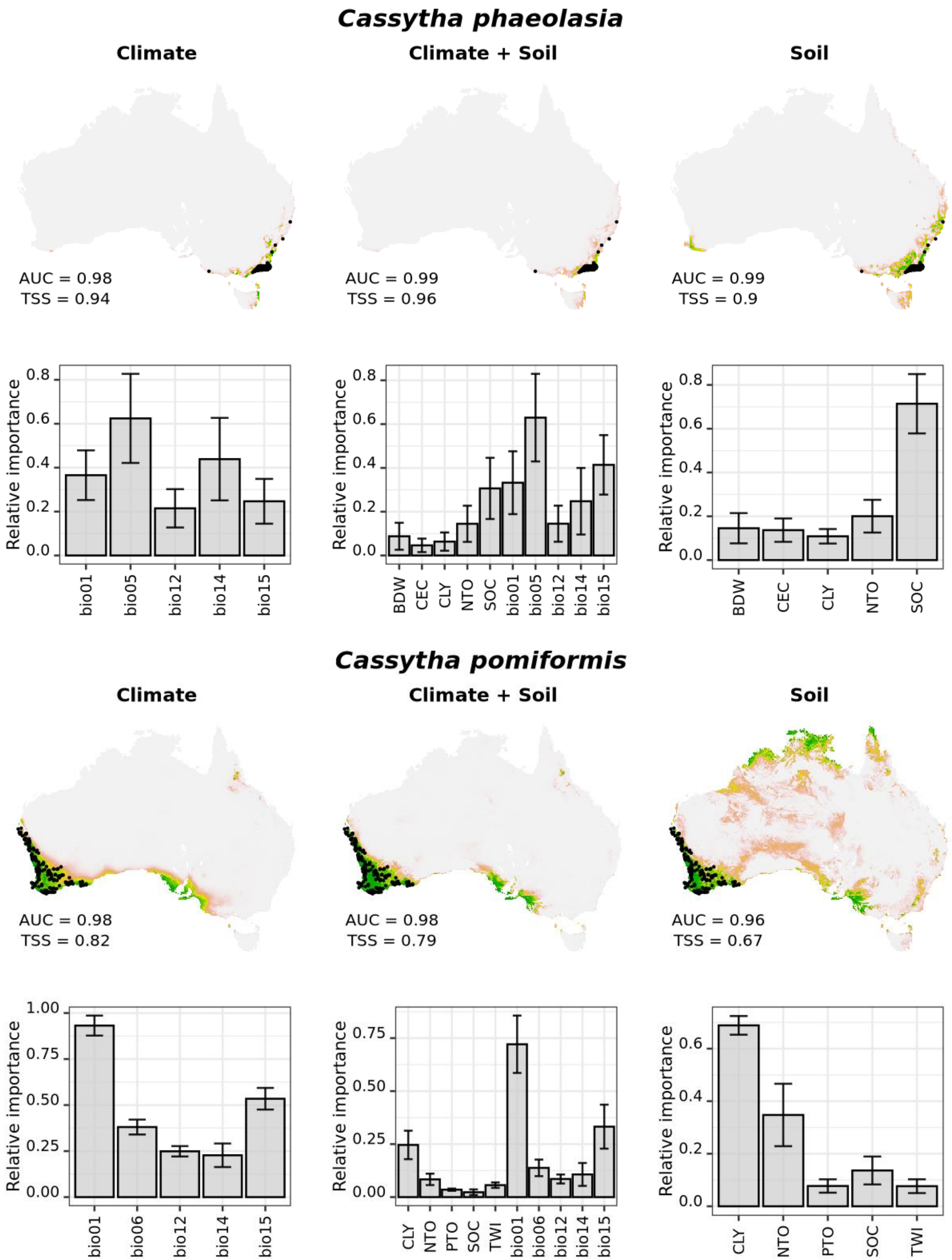
Cassytha phaeolasia
Climate
Climate + Soil
Soil
AUC = 0.98
TSS = 0.94
AUC = 0.99
TSS = 0.96
AUC = 0.99
TSS = 0.9
Relative importance
0.0
0.2
0.4
0.6
0.8
bio01
bio05
bio12
bio14
bio15
BDW
CEC
CLY
NTO
SOC
Cassytha pomiformis
AUC = 0.98
TSS = 0.82
AUC = 0.98
TSS = 0.79
AUC = 0.96
TSS = 0.67
0.00
0.25
0.50
0.75
1.00
bio06
PTO
TWI

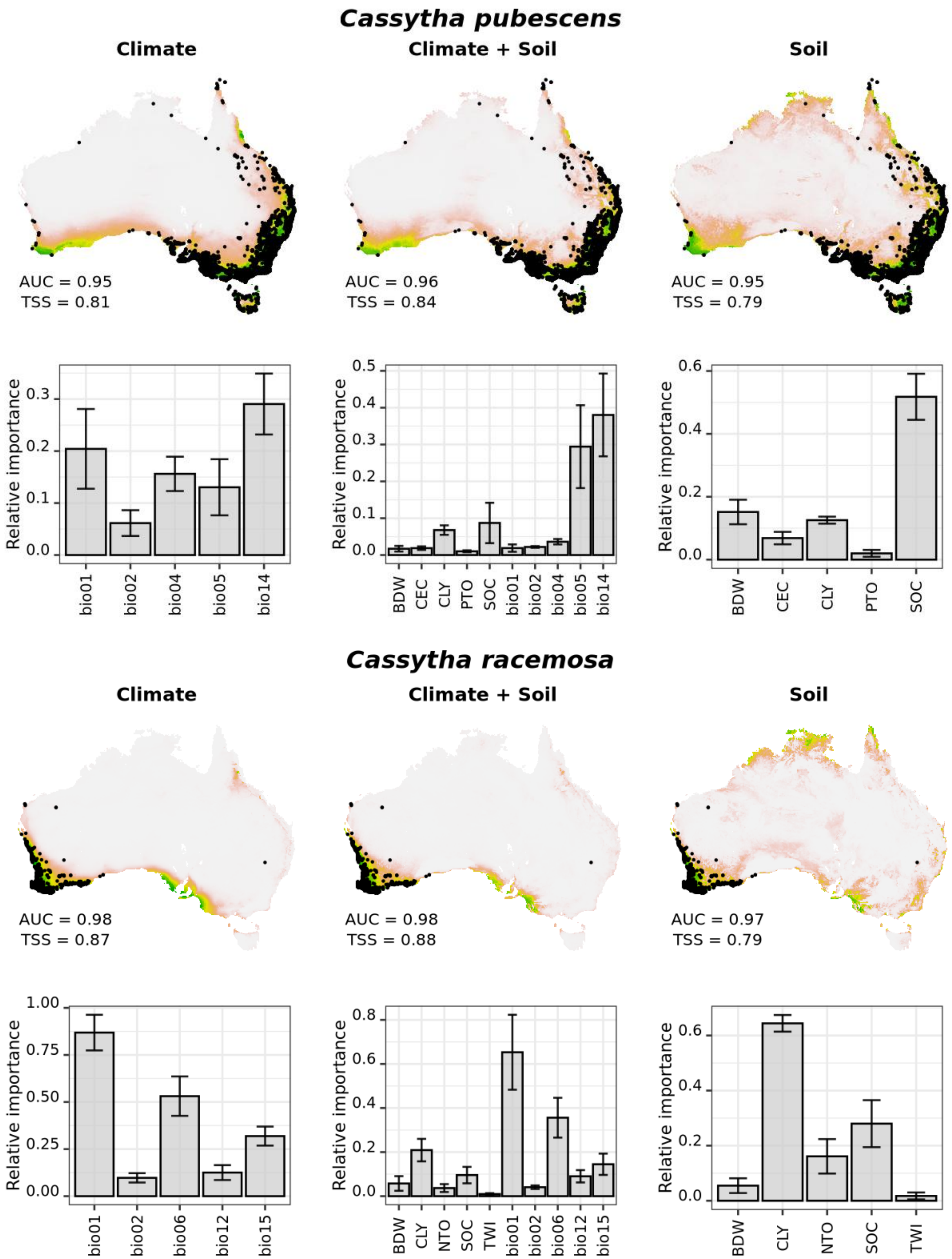

Cassytha pubescens
Climate
Climate + Soil
Soil
AUC = 0.95
TSS = 0.81
AUC = 0.96
TSS = 0.84
AUC = 0.95
TSS = 0.79
Relative importance
bio01
bio02
bio04
bio05
bio14
BDW
CEC
CLY
PTO
SOC
bio01
bio02
bio04
bio05
bio14
BDW
CEC
CLY
PTO
SOC
Cassytha racemosa
Climate
Climate + Soil
Soil
AUC = 0.98
TSS = 0.87
AUC = 0.98
TSS = 0.88
AUC = 0.97
TSS = 0.79
Relative importance
bio01
bio02
bio06
bio12
bio15
BDW
CLY
NTO
SOC
TWI
bio01
bio02
bio06
bio12
bio15
BDW
CLY
NTO
SOC
TWI

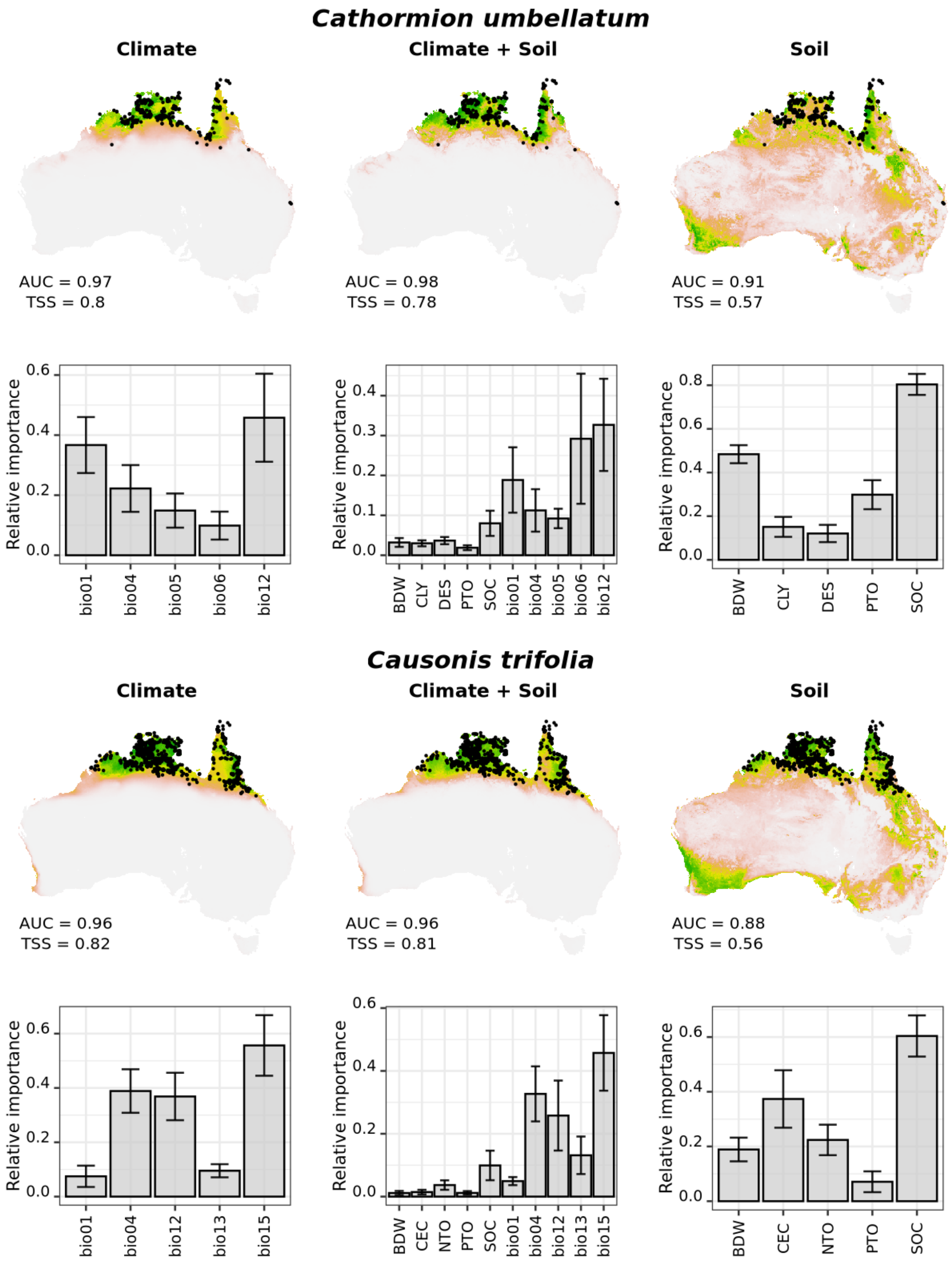

Cathormion umbellatum
Climate
Climate + Soil
Soil
AUC = 0.97
TSS = 0.8
AUC = 0.98
TSS = 0.78
AUC = 0.91
TSS = 0.57
Relative importance
bio01 bio04 bio05 bio06 bio12
BDW CLY DES PTO SOC bio01 bio04 bio05 bio06 bio12
BDW CLY DES PTO SOC
Causonis trifolia
Climate
Climate + Soil
Soil
AUC = 0.96
TSS = 0.82
AUC = 0.96
TSS = 0.81
AUC = 0.88
TSS = 0.56
Relative importance
bio01 bio04 bio12 bio13 bio15
BDW CEC NTO PTO SOC bio01 bio04 bio12 bio13 bio15
BDW CEC NTO PTO SOC

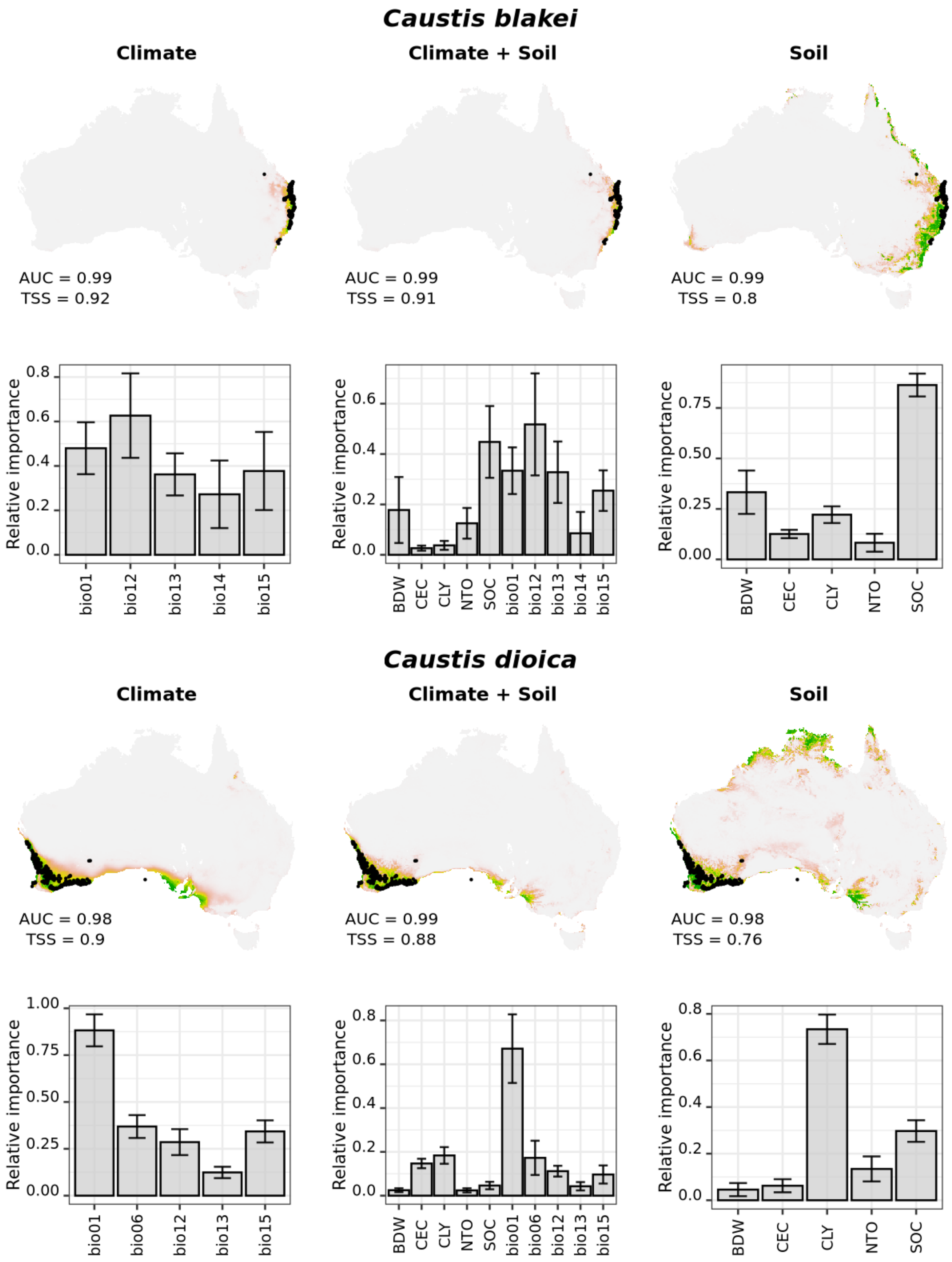

Caustis blakei
Climate
Climate + Soil
Soil
AUC = 0.99
TSS = 0.92
AUC = 0.99
TSS = 0.91
AUC = 0.99
TSS = 0.8
Relative importance
bio01
bio12
bio13
bio14
bio15
BDW
CEC
CLY
NTO
SOC
Caustis dioica
Climate
Climate + Soil
Soil
AUC = 0.98
TSS = 0.9
AUC = 0.99
TSS = 0.88
AUC = 0.98
TSS = 0.76
Relative importance
bio01
bio06
bio12
bio13
bio15
BDW
CEC
CLY
NTO
SOC

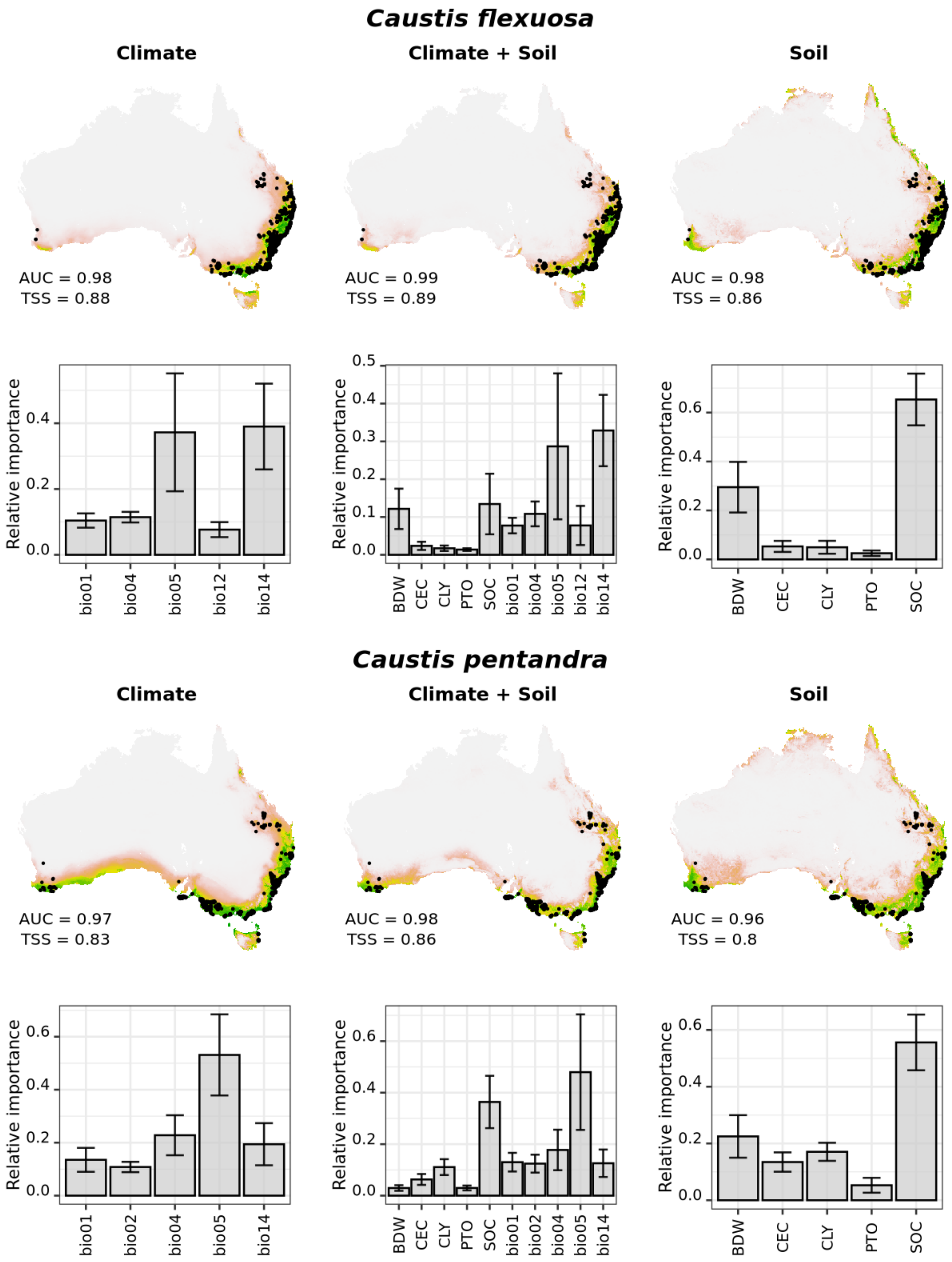

Caustis flexuosa
Climate
Climate + Soil
Soil
AUC = 0.98
TSS = 0.88
AUC = 0.99
TSS = 0.89
AUC = 0.98
TSS = 0.86
Relative importance
bio01
bio04
bio05
bio12
bio14
BDW
CEC
CLY
PTO
SOC
Caustis pentandra
Climate
Climate + Soil
Soil
AUC = 0.97
TSS = 0.83
AUC = 0.98
TSS = 0.86
AUC = 0.96
TSS = 0.8
Relative importance
bio01
bio02
bio04
bio05
bio14
BDW
CEC
CLY
PTO
SOC

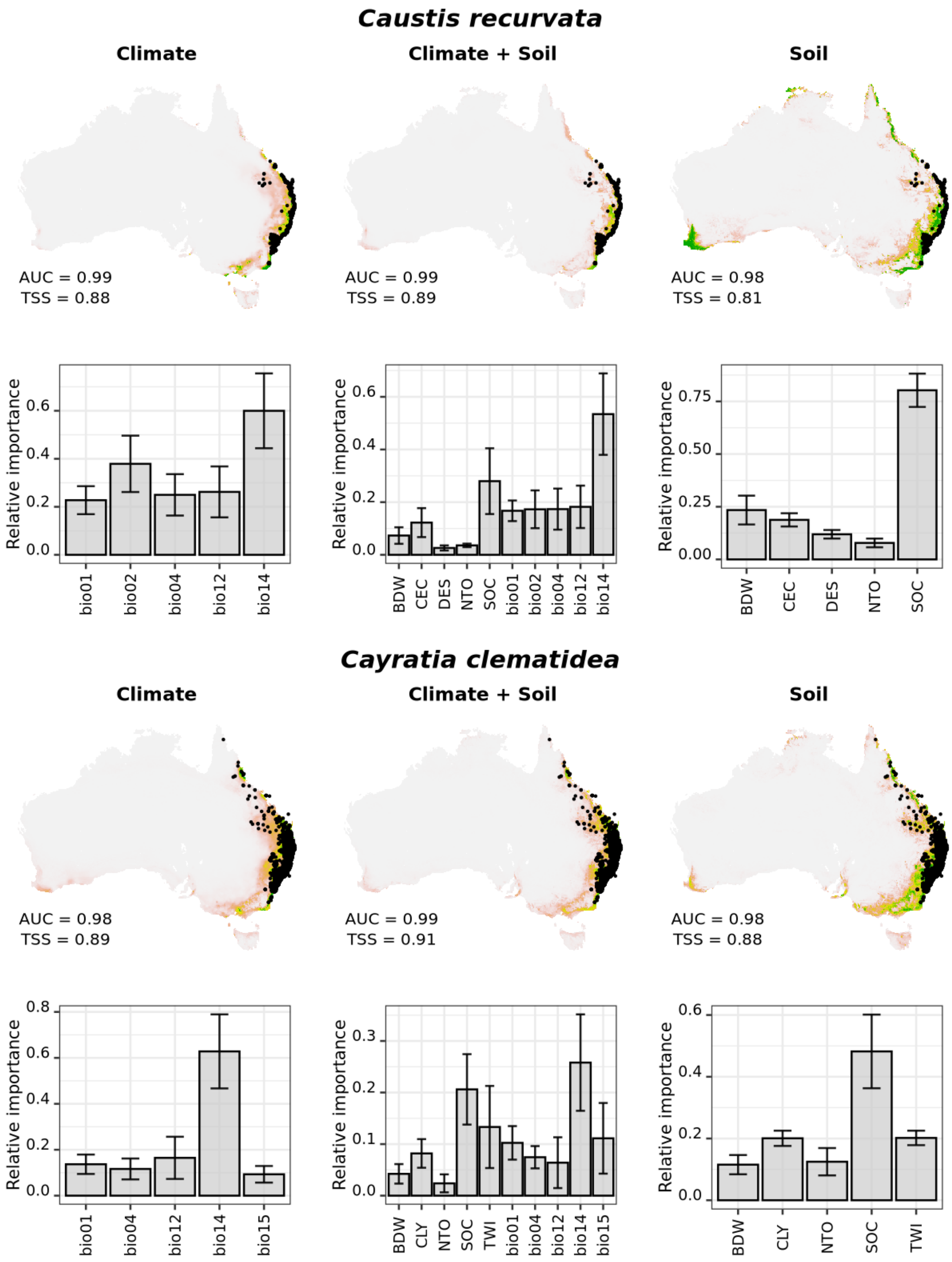
Caustis recurvata
Climate
Climate + Soil
Soil
AUC = 0.99
TSS = 0.88
AUC = 0.99
TSS = 0.89
AUC = 0.98
TSS = 0.81
Relative importance
bio01 bio02 bio04 bio12 bio14
BDW CEC DES NTO SOC bio01 bio02 bio04 bio12 bio14
BDW CEC DES NTO SOC
Cayratia clematidea
Climate
Climate + Soil
Soil
AUC = 0.98
TSS = 0.89
AUC = 0.99
TSS = 0.91
AUC = 0.98
TSS = 0.88
Relative importance
bio01 bio04 bio12 bio14 bio15
BDW CLY NTO SOC TWI bio01 bio04 bio12 bio14 bio15
BDW CLY NTO SOC TWI

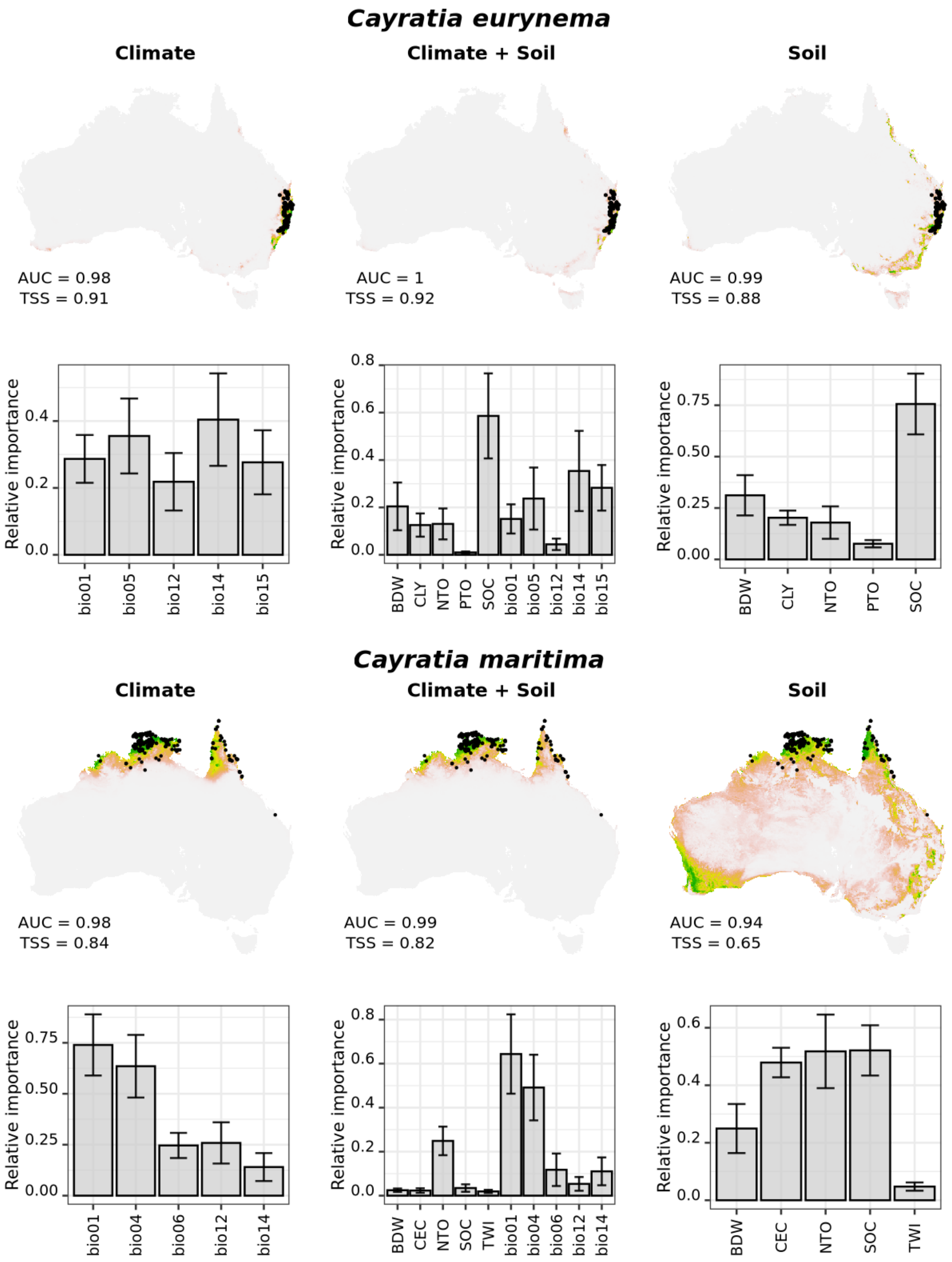

Cayratia eurynema
Climate
Climate + Soil
Soil
AUC = 0.98
TSS = 0.91
AUC = 1
TSS = 0.92
AUC = 0.99
TSS = 0.88
Relative importance
0.0
0.2
0.4
0.6
0.8
0.00
0.25
0.50
0.75
bio01
bio05
bio12
bio14
bio15
BDW
CLY
NTO
PTO
SOC
Cayratia maritima
Climate
Climate + Soil
Soil
AUC = 0.98
TSS = 0.84
AUC = 0.99
TSS = 0.82
AUC = 0.94
TSS = 0.65
Relative importance
bio01
bio04
bio06
bio12
bio14
BDW
CEC
NTO
SOC
TWI

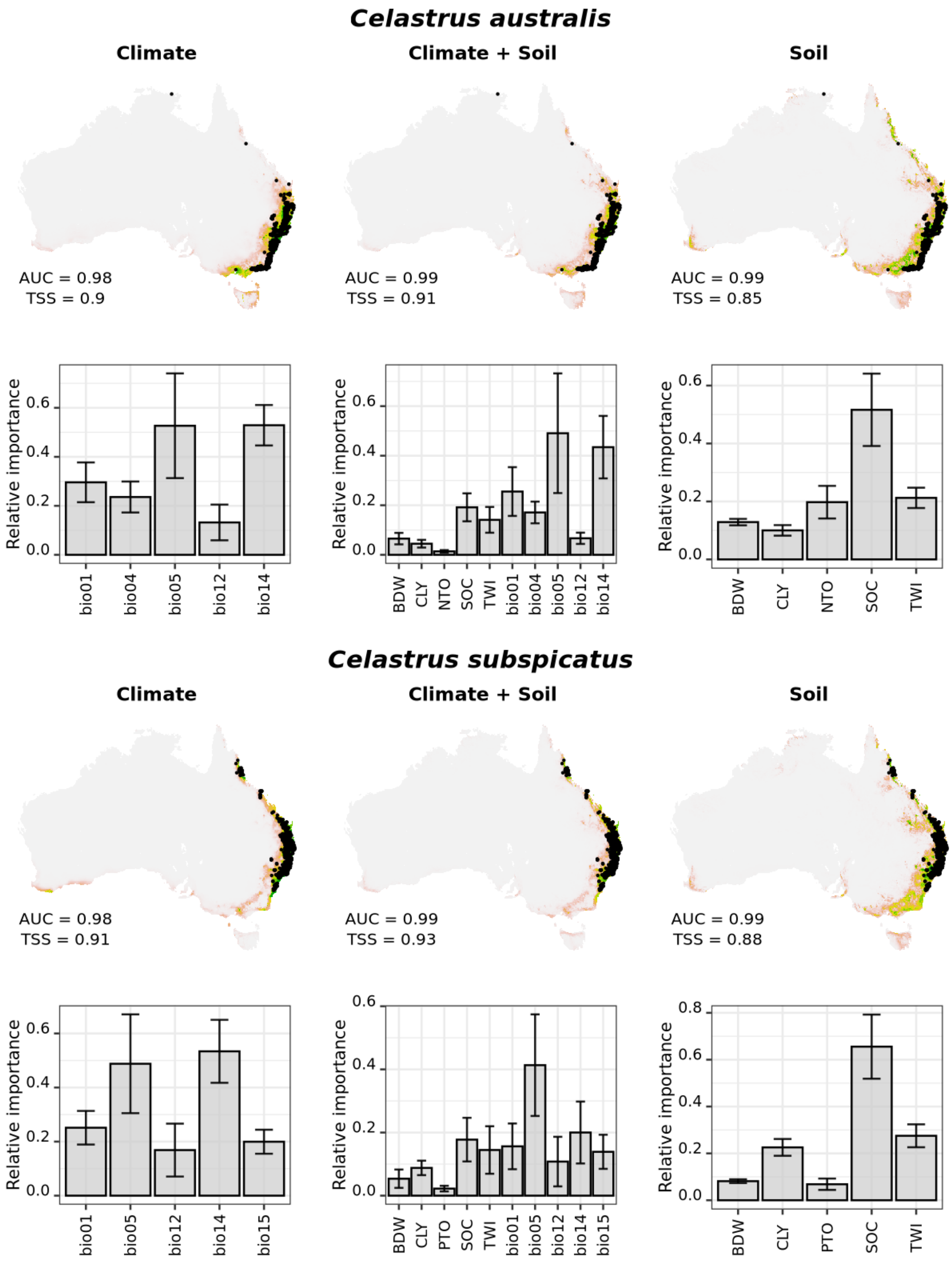

Celastrus australis
Climate
Climate + Soil
Soil
AUC = 0.98
TSS = 0.9
AUC = 0.99
TSS = 0.91
AUC = 0.99
TSS = 0.85
Relative importance
0.0
0.2
0.4
0.6
bio01
bio04
bio05
bio12
bio14
BDW
CLY
NTO
SOC
TWI
Celastrus subspicatus
AUC = 0.98
TSS = 0.91
AUC = 0.99
TSS = 0.93
AUC = 0.99
TSS = 0.88
0.8
bio15
PTO

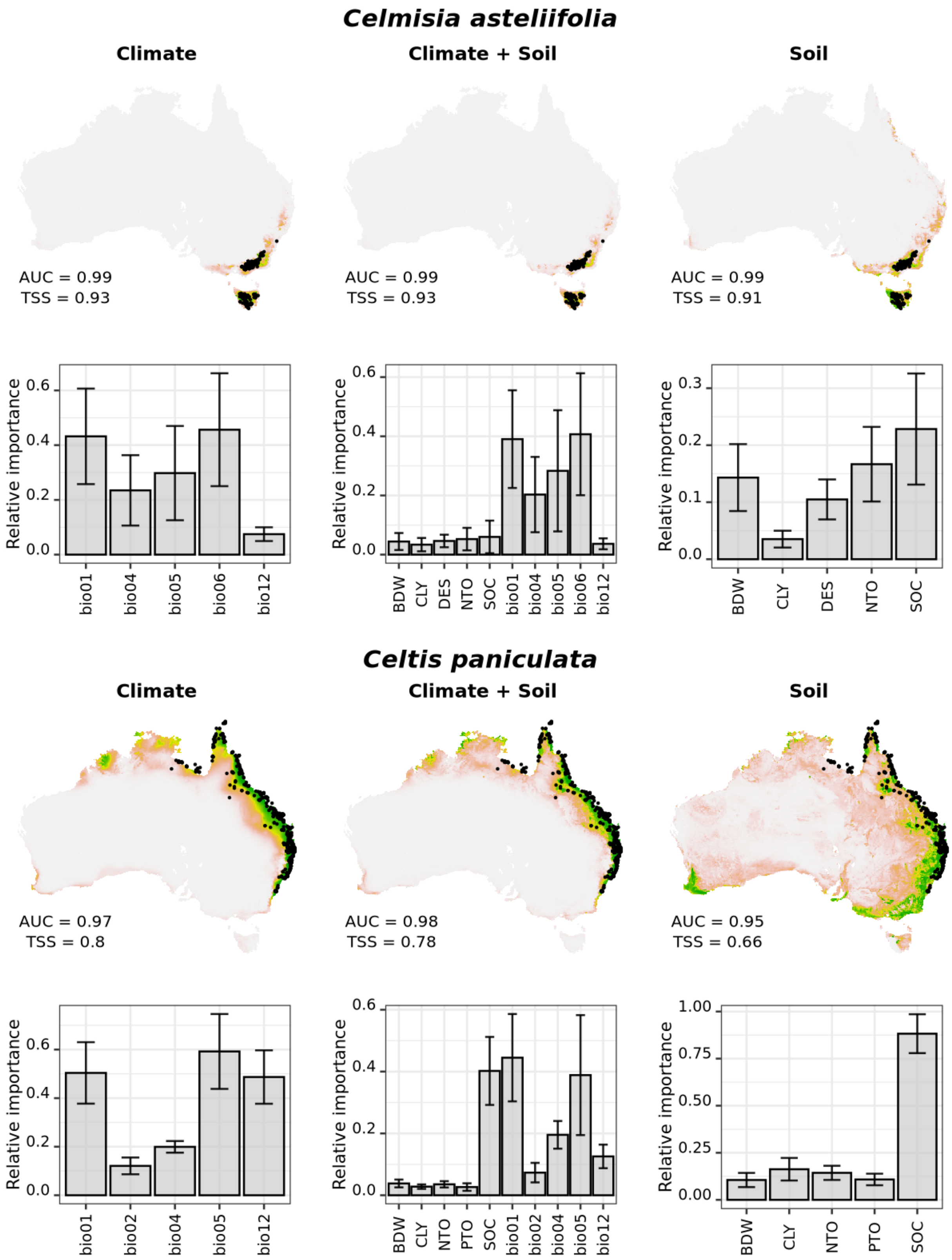
Celmisia asteliifolia
Climate
Climate + Soil
Soil
AUC = 0.99
TSS = 0.93
AUC = 0.99
TSS = 0.93
AUC = 0.99
TSS = 0.91
Relative importance
bio01 bio04 bio05 bio06 bio12
BDW CLY DES NTO SOC bio01 bio04 bio05 bio06 bio12
BDW CLY DES NTO SOC
Celtis paniculata
Climate
Climate + Soil
Soil
AUC = 0.97
TSS = 0.8
AUC = 0.98
TSS = 0.78
AUC = 0.95
TSS = 0.66
Relative importance
bio01 bio02 bio04 bio05 bio12
BDW CLY NTO PTO SOC bio01 bio02 bio04 bio05 bio12
BDW CLY NTO PTO SOC

Celtis philippensis

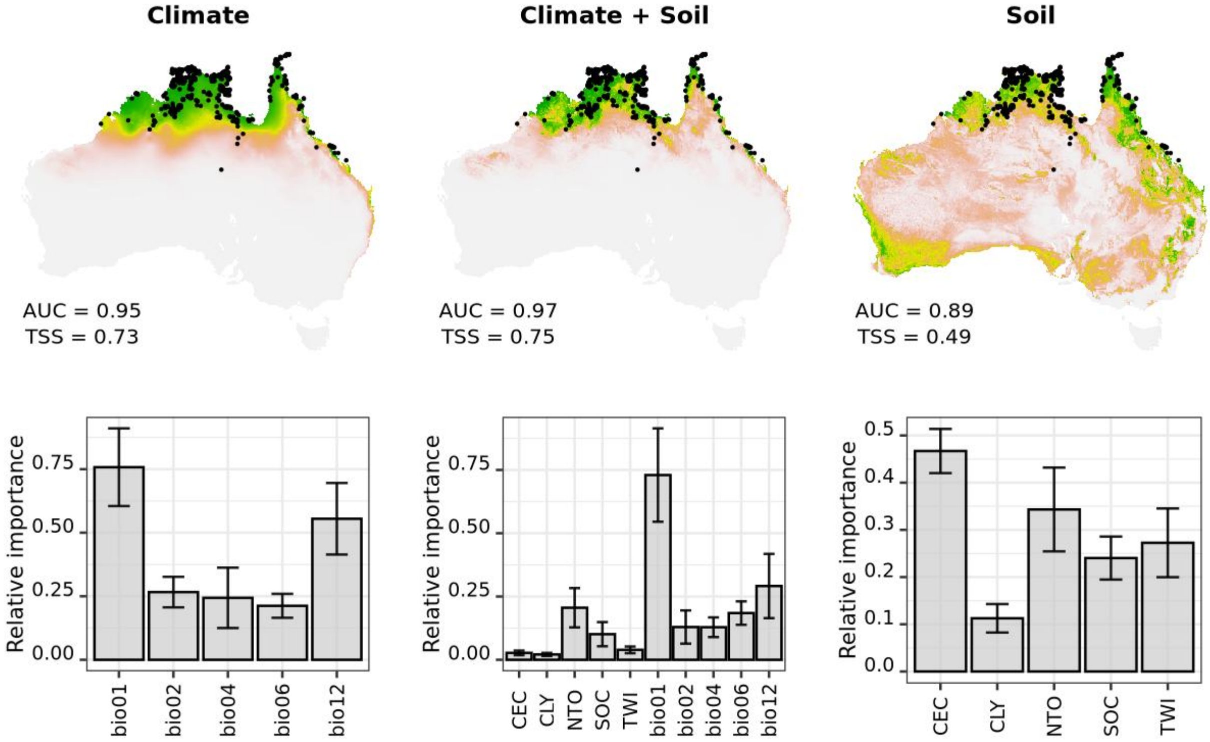

Celtis strychnoides

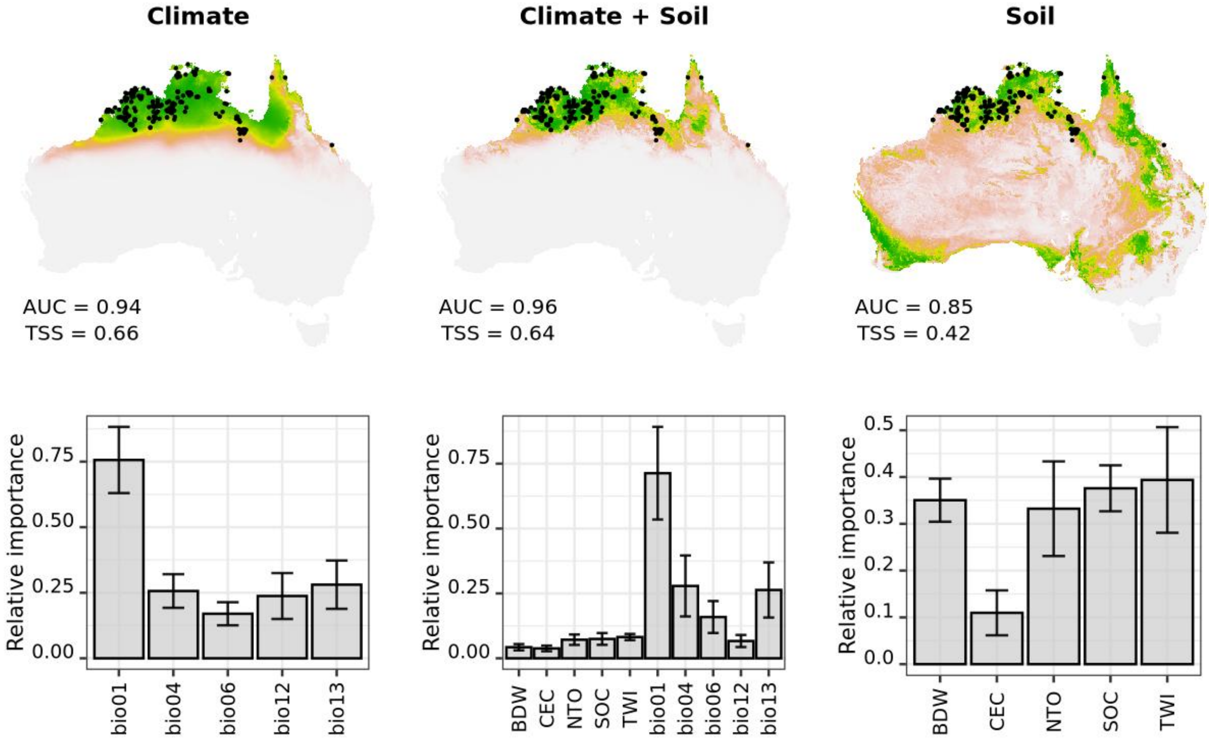

Cenarrhenes nitida

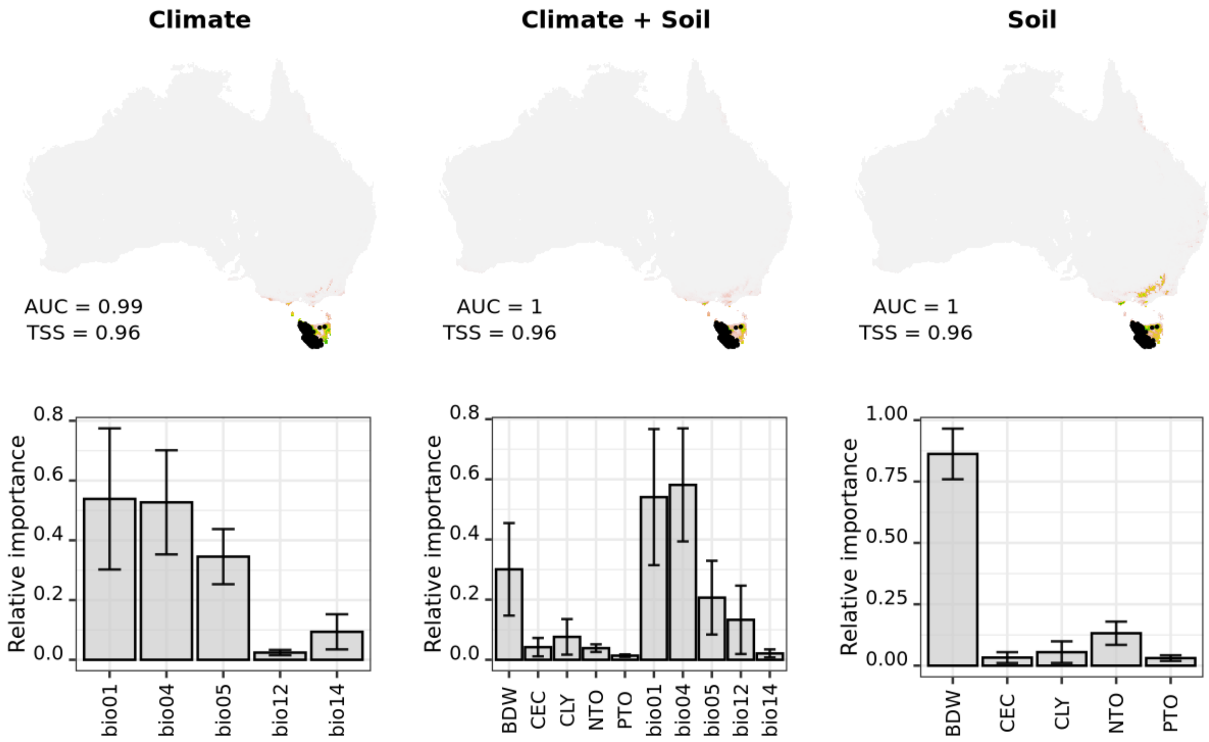

Cenchrus caliculatus

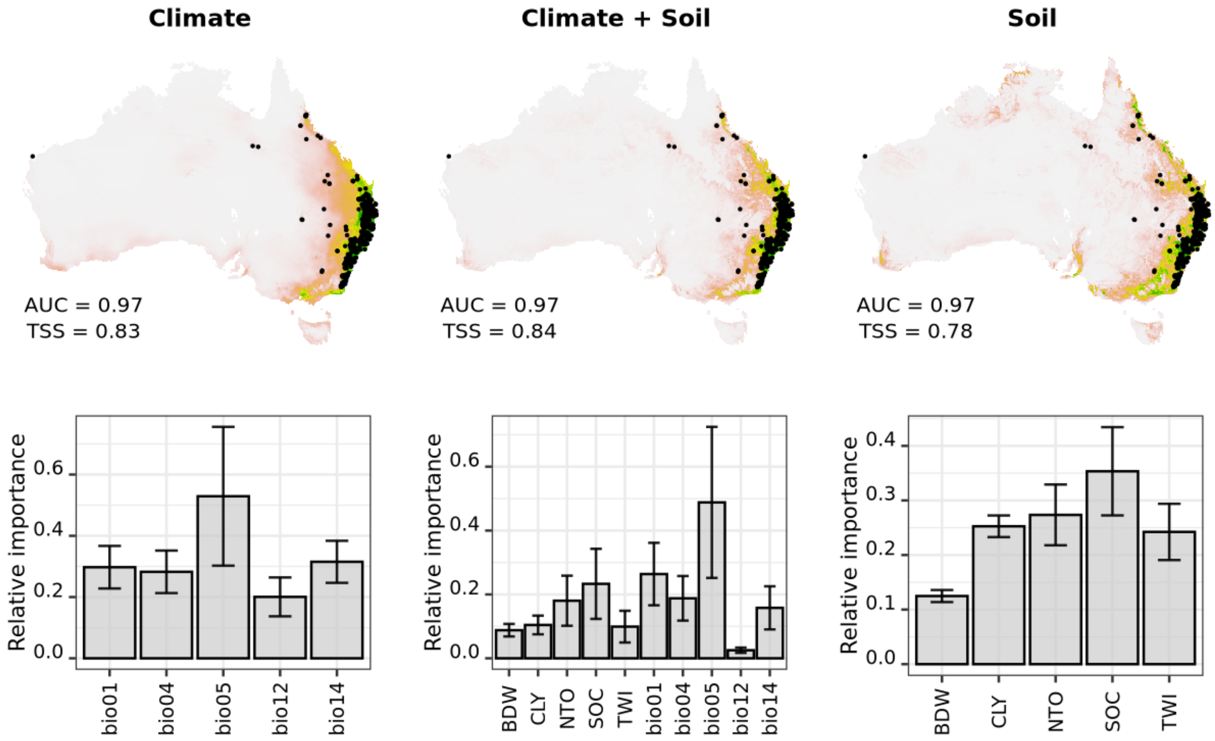

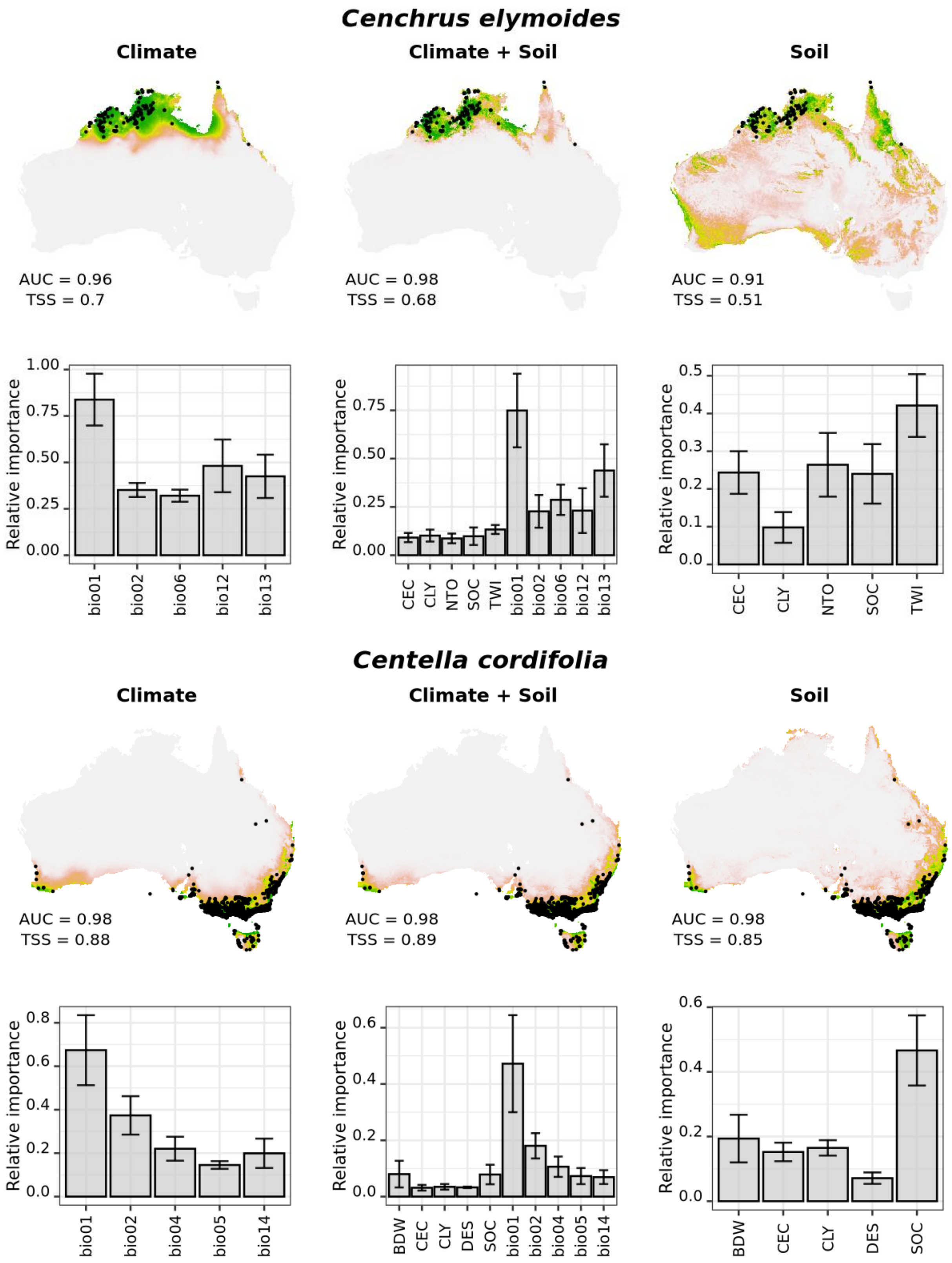
Cenchrus elymoides
Climate
Climate + Soil
Soil
AUC = 0.96
TSS = 0.7
AUC = 0.98
TSS = 0.68
AUC = 0.91
TSS = 0.51
Relative importance
bio01
bio02
bio06
bio12
bio13
CEC
CLY
NTO
SOC
TWI
Centella cordifolia
Climate
Climate + Soil
Soil
AUC = 0.98
TSS = 0.88
AUC = 0.98
TSS = 0.89
AUC = 0.98
TSS = 0.85
Relative importance
bio01
bio02
bio04
bio05
bio14
BDW
CEC
CLY
DES
SOC

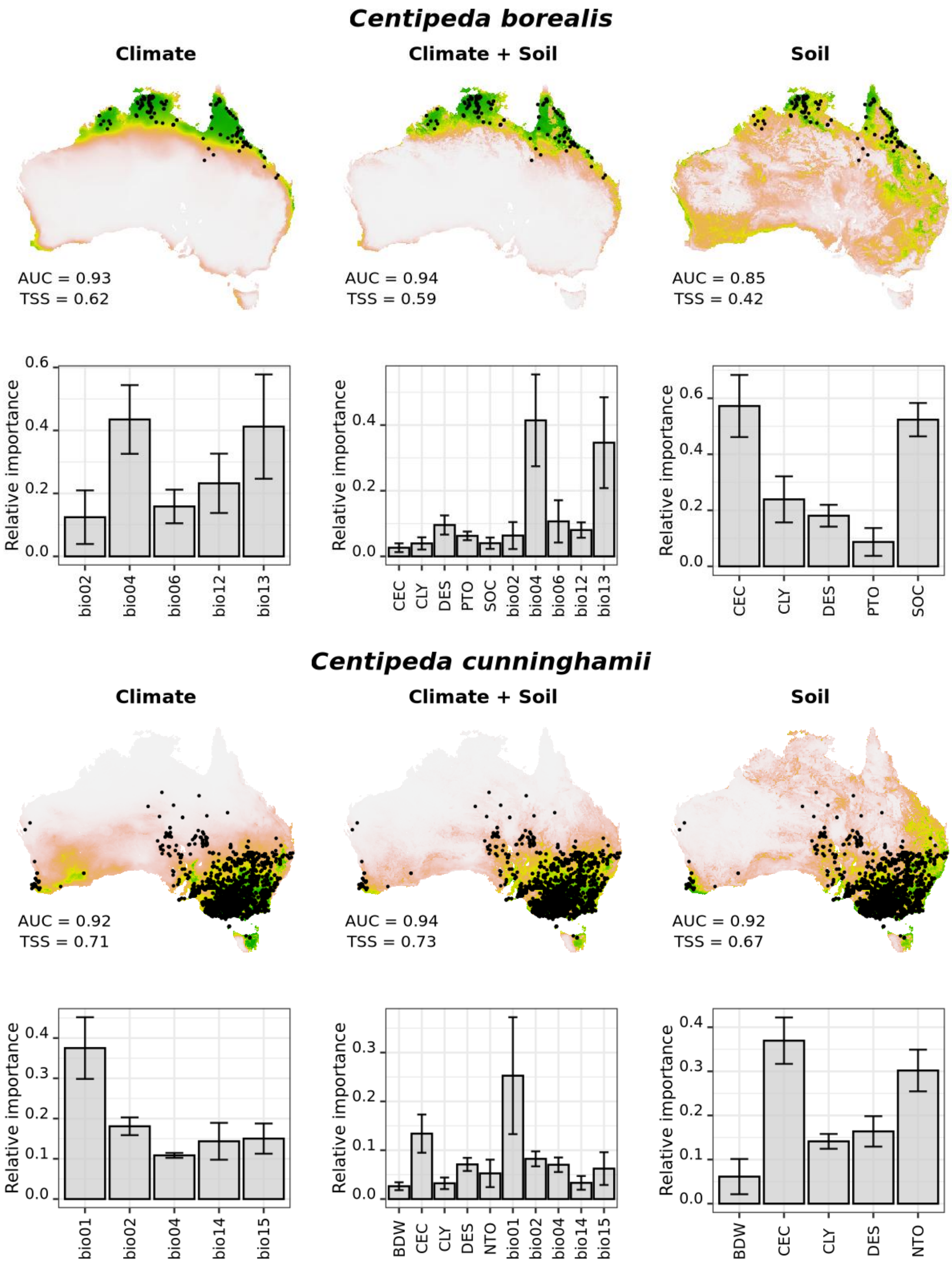
Centipeda borealis
Climate
Climate + Soil
Soil
AUC = 0.93
TSS = 0.62
AUC = 0.94
TSS = 0.59
AUC = 0.85
TSS = 0.42
Relative importance
bio02
bio04
bio06
bio12
bio13
CEC
CLY
DES
PTO
SOC
Centipeda cunninghamii
AUC = 0.92
TSS = 0.71
AUC = 0.94
TSS = 0.73
AUC = 0.92
TSS = 0.67
bio01
bio14
bio15
BDW
NTO

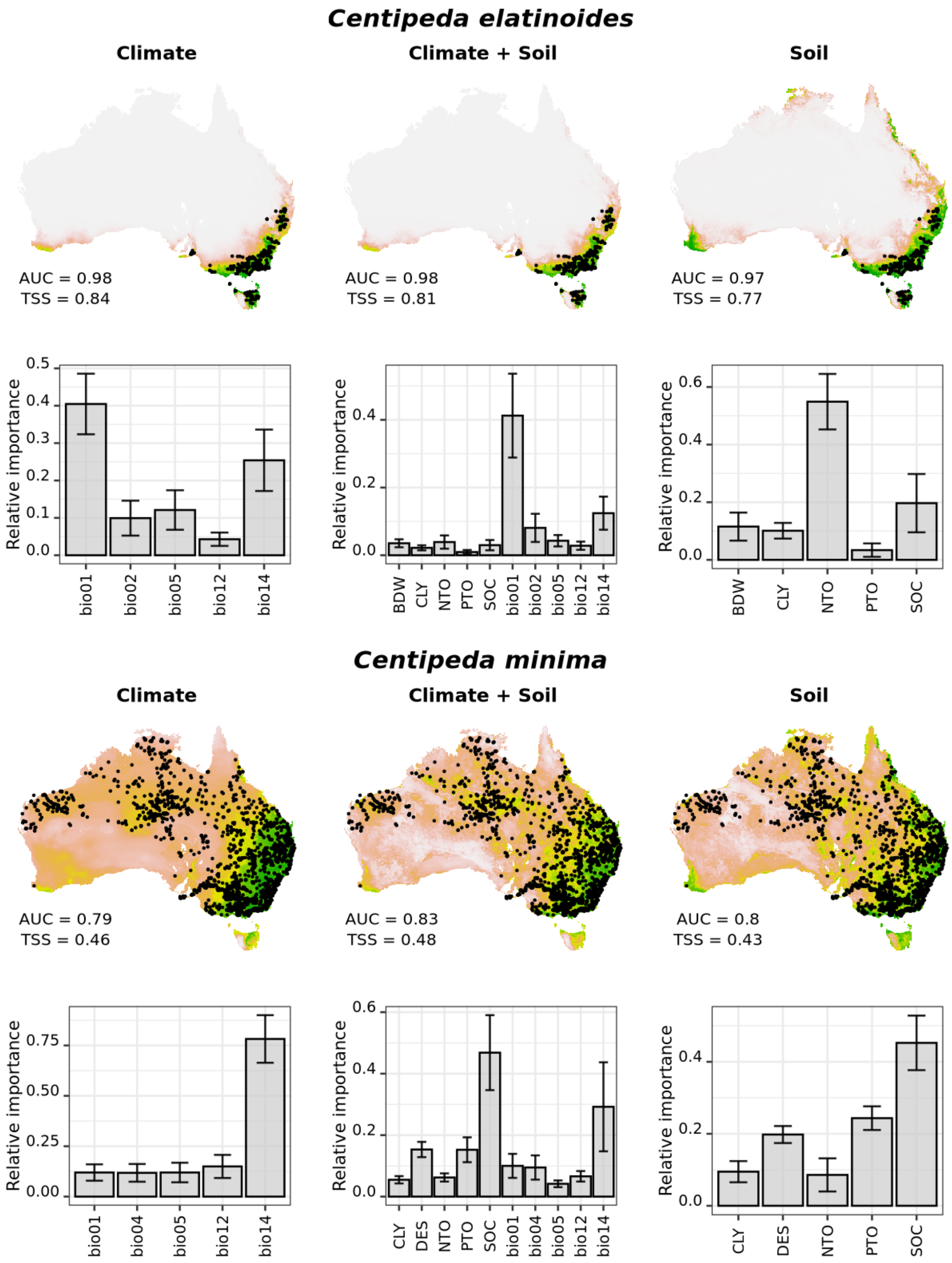

Centipeda elatinoides
Climate
Climate + Soil
Soil
AUC = 0.98
TSS = 0.84
AUC = 0.98
TSS = 0.81
AUC = 0.97
TSS = 0.77
Relative importance
bio01
bio02
bio05
bio12
bio14
BDW
CLY
NTO
PTO
SOC
Centipeda minima
AUC = 0.79
TSS = 0.46
AUC = 0.83
TSS = 0.48
AUC = 0.8
TSS = 0.43
bio04
DES

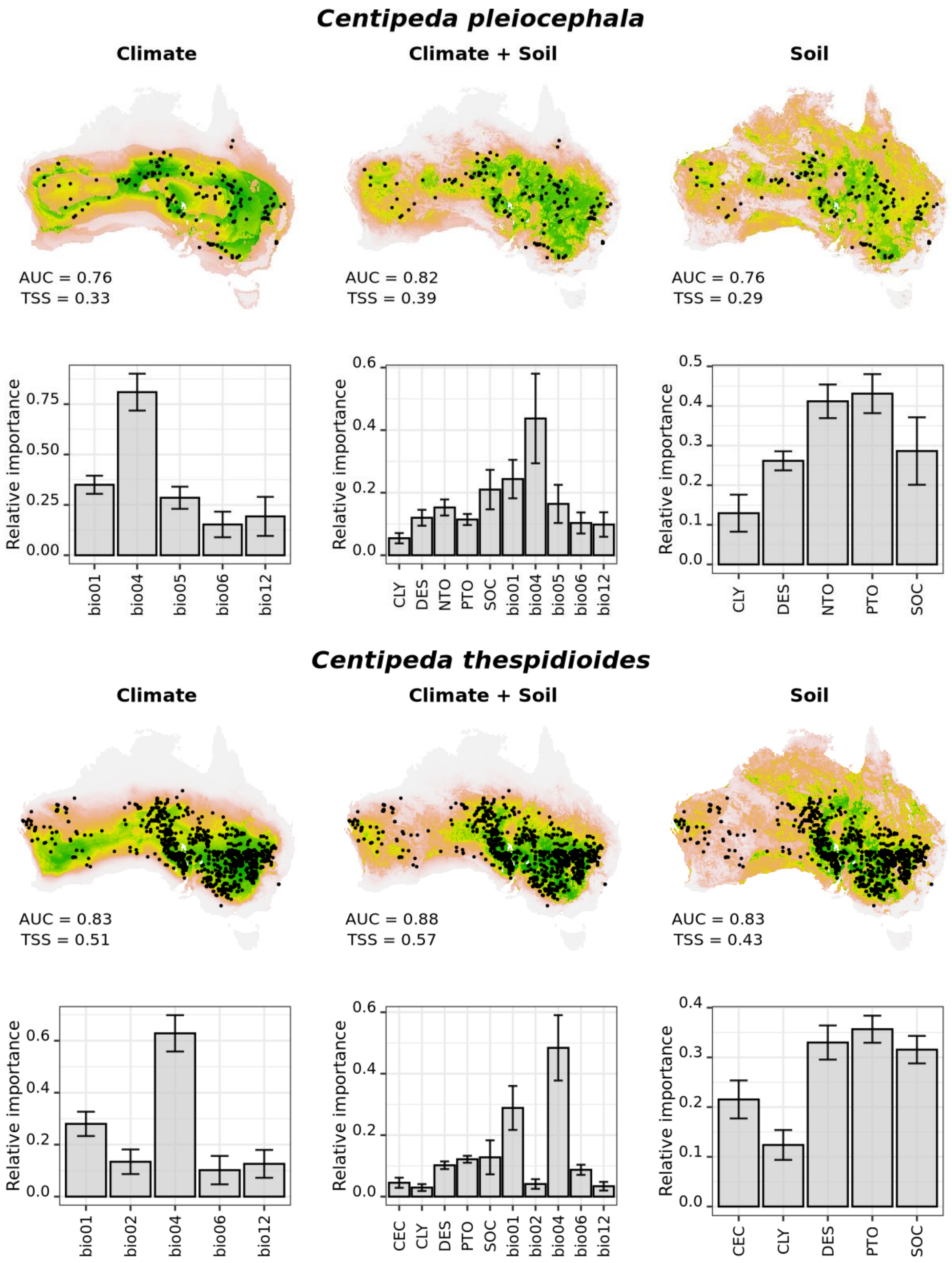

Centipeda pleiocephala
Climate
Climate + Soil
Soil
AUC = 0.76
TSS = 0.33
AUC = 0.82
TSS = 0.39
AUC = 0.76
TSS = 0.29
Relative importance
bio01
bio04
bio05
bio06
bio12
CLY
DES
NTO
PTO
SOC
Centipeda thespidioides
Climate
Climate + Soil
Soil
AUC = 0.83
TSS = 0.51
AUC = 0.88
TSS = 0.57
AUC = 0.83
TSS = 0.43
Relative importance
bio01
bio02
bio04
bio06
bio12
CEC
CLY
DES
PTO
SOC

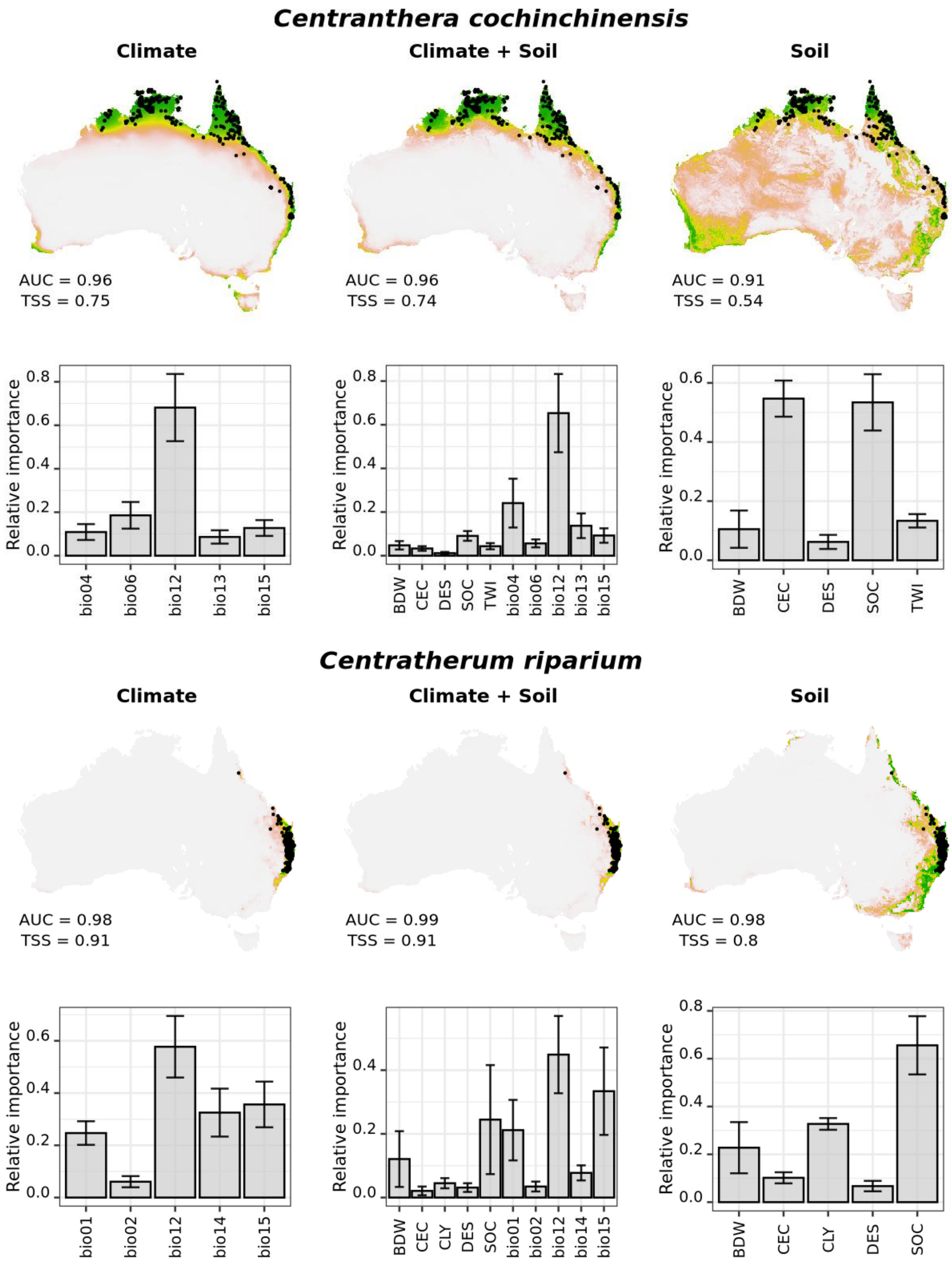
Centranthera cochinchinensis
Climate
Climate + Soil
Soil
AUC = 0.96
TSS = 0.75
AUC = 0.96
TSS = 0.74
AUC = 0.91
TSS = 0.54
Relative importance
bio04
bio06
bio12
bio13
bio15
BDW
CEC
DES
SOC
TWI
Centratherum riparium
Climate
Climate + Soil
Soil
AUC = 0.98
TSS = 0.91
AUC = 0.99
TSS = 0.91
AUC = 0.98
TSS = 0.8
bio01
bio02
bio12
bio14
bio15
BDW
CEC
CLY
DES
SOC

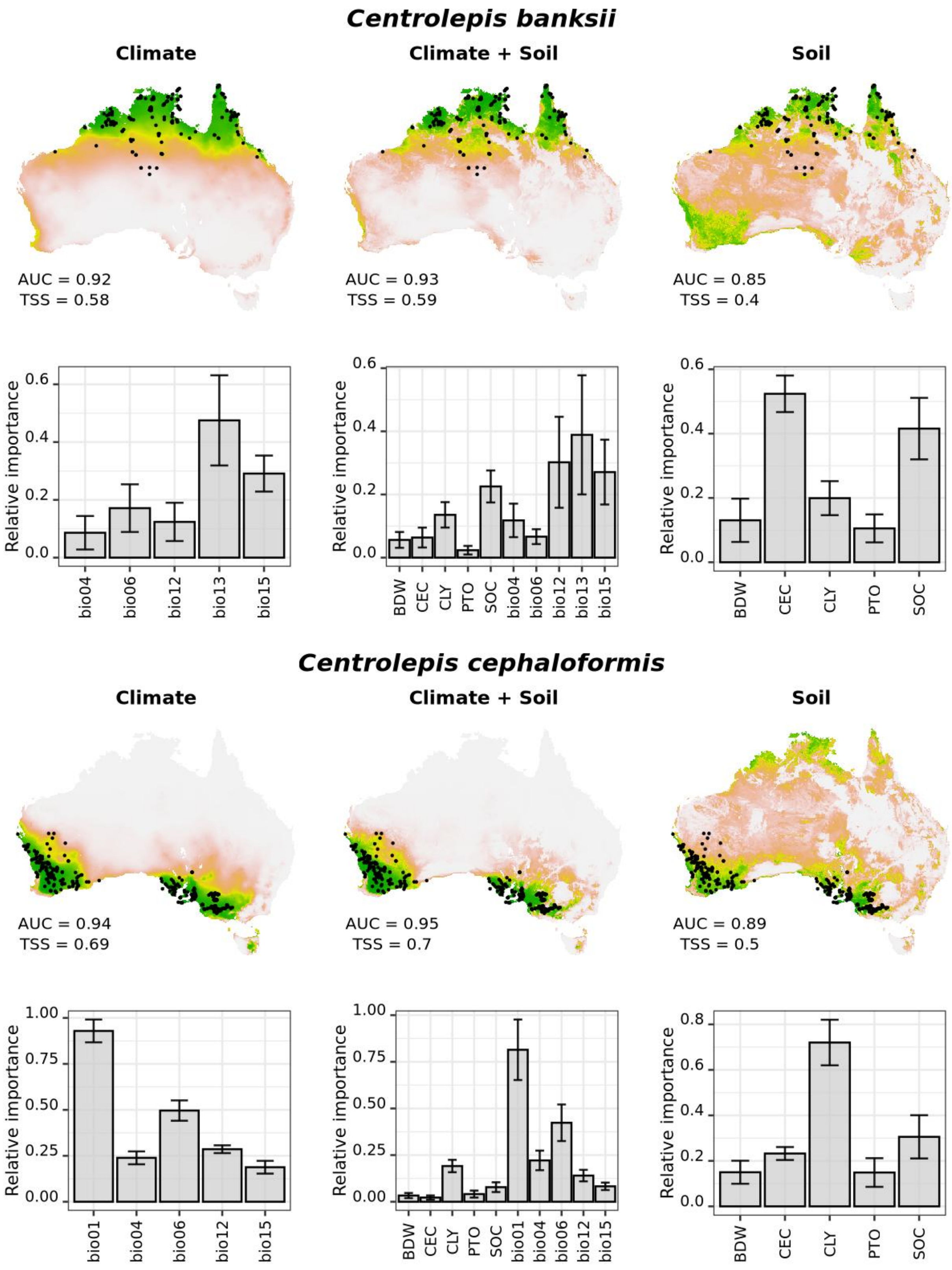

Centrolepis banksii
Climate
Climate + Soil
Soil
AUC = 0.92
TSS = 0.58
AUC = 0.93
TSS = 0.59
AUC = 0.85
TSS = 0.4
Relative importance
bio04
bio06
bio12
bio13
bio15
BDW
CEC
CLY
PTO
SOC
Centrolepis cephaloformis
Climate
Climate + Soil
Soil
AUC = 0.94
TSS = 0.69
AUC = 0.95
TSS = 0.7
AUC = 0.89
TSS = 0.5
Relative importance
bio01
bio04
bio06
bio12
bio15
BDW
CEC
CLY
PTO
SOC

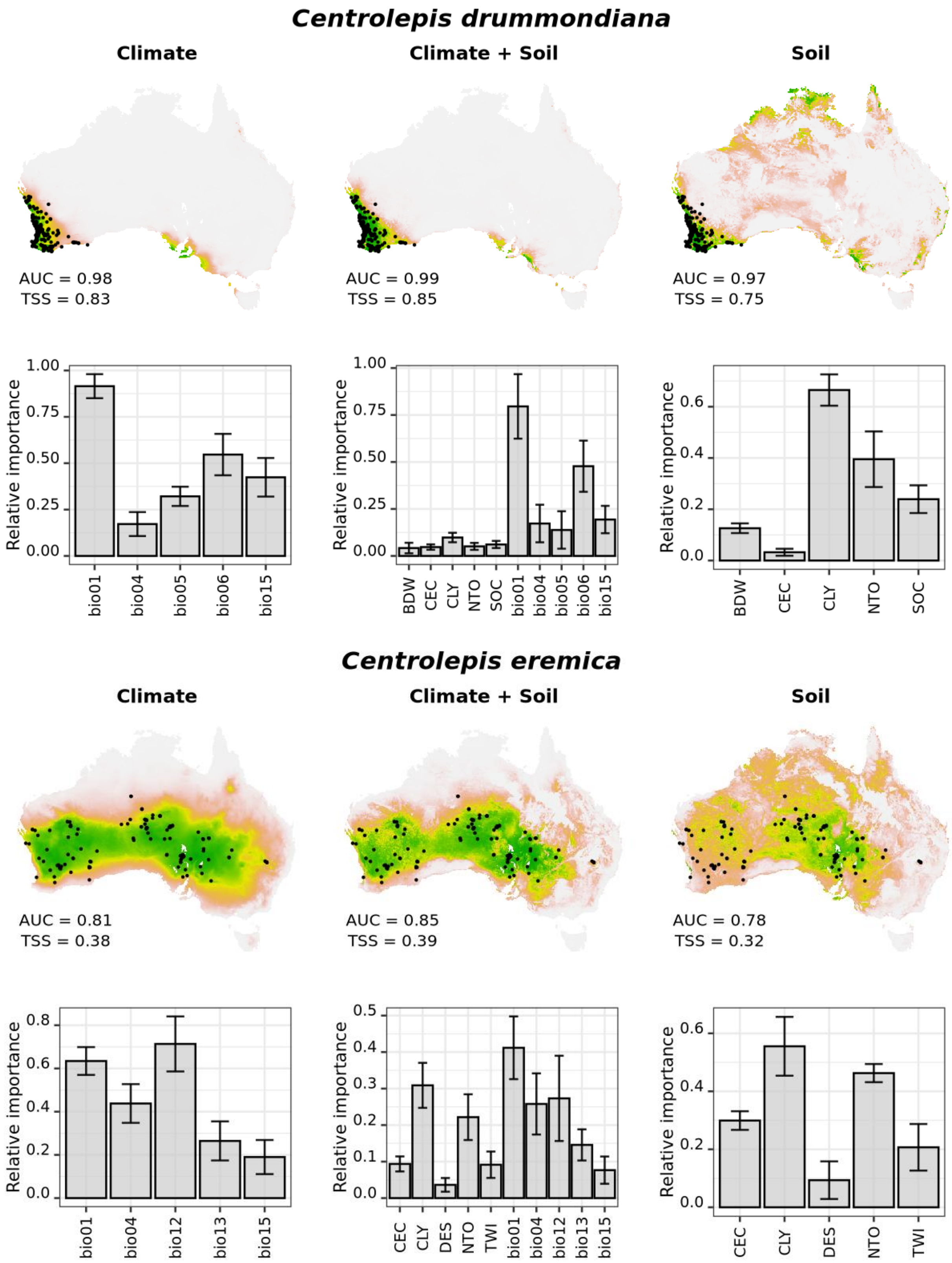

Centrolepis drummondiana
Climate
Climate + Soil
Soil
AUC = 0.98
TSS = 0.83
AUC = 0.99
TSS = 0.85
AUC = 0.97
TSS = 0.75
Relative importance
bio01
bio04
bio05
bio06
bio15
BDW
CEC
CLY
NTO
SOC
Centrolepis eremica
Climate
Climate + Soil
Soil
AUC = 0.81
TSS = 0.38
AUC = 0.85
TSS = 0.39
AUC = 0.78
TSS = 0.32
Relative importance
bio01
bio04
bio12
bio13
bio15
CEC
CLY
DES
NTO
TWI

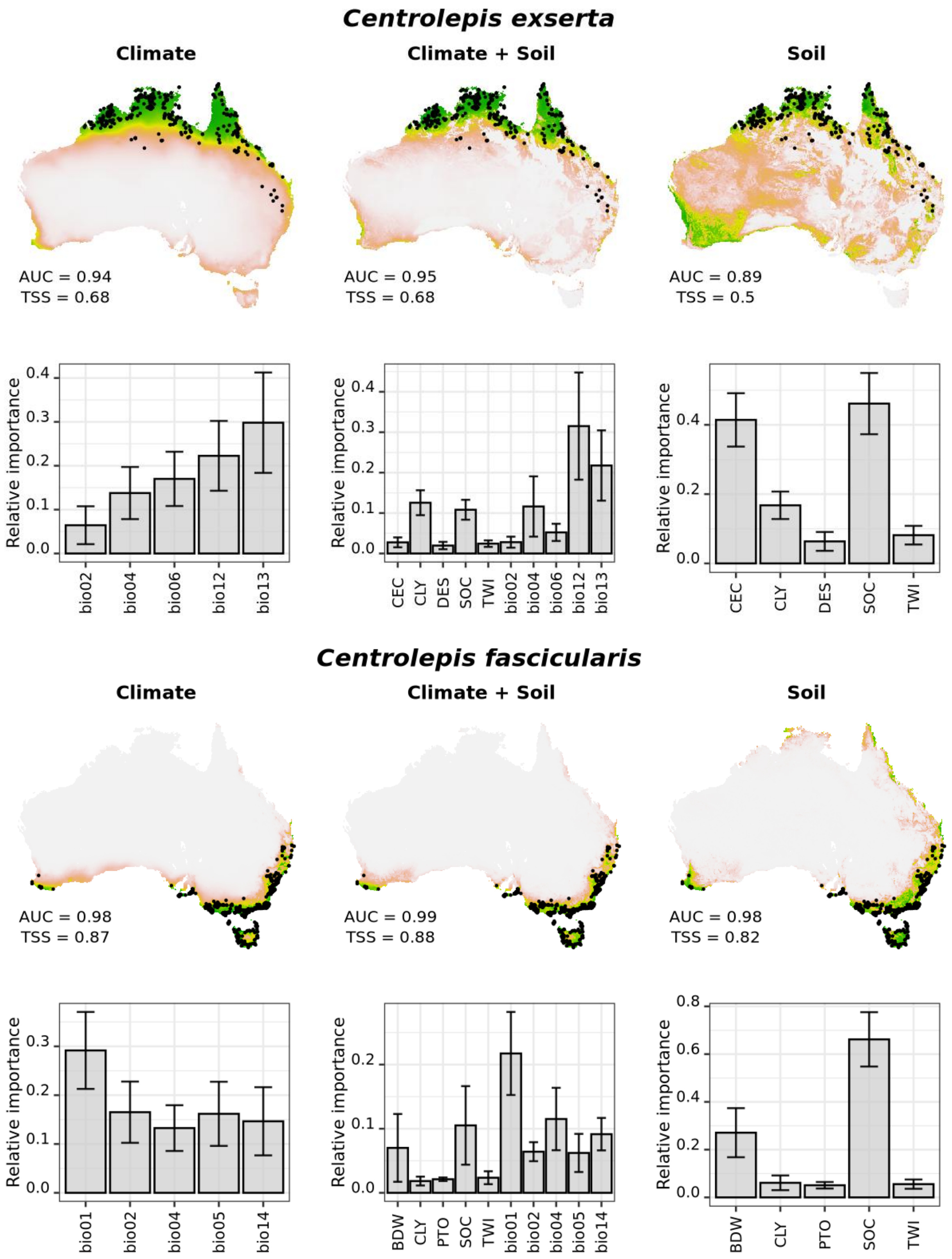

Centrolepis exserta
Climate
Climate + Soil
Soil
AUC = 0.94
TSS = 0.68
AUC = 0.95
TSS = 0.68
AUC = 0.89
TSS = 0.5
Relative importance
0.0
0.1
0.2
0.3
0.4
bio02
bio04
bio06
bio12
bio13
CEC
CLY
DES
SOC
TWI
bio02
bio04
bio06
bio12
bio13
CEC
CLY
DES
SOC
TWI
Centrolepis fascicularis
Climate
Climate + Soil
Soil
AUC = 0.98
TSS = 0.87
AUC = 0.99
TSS = 0.88
AUC = 0.98
TSS = 0.82
Relative importance
0.0
0.1
0.2
0.3
0.4
0.6
0.8
bio01
bio02
bio04
bio05
bio14
BDW
CLY
PTO
SOC
TWI
bio01
bio02
bio04
bio05
bio14
BDW
CLY
PTO
SOC
TWI

Centrolepis glabra

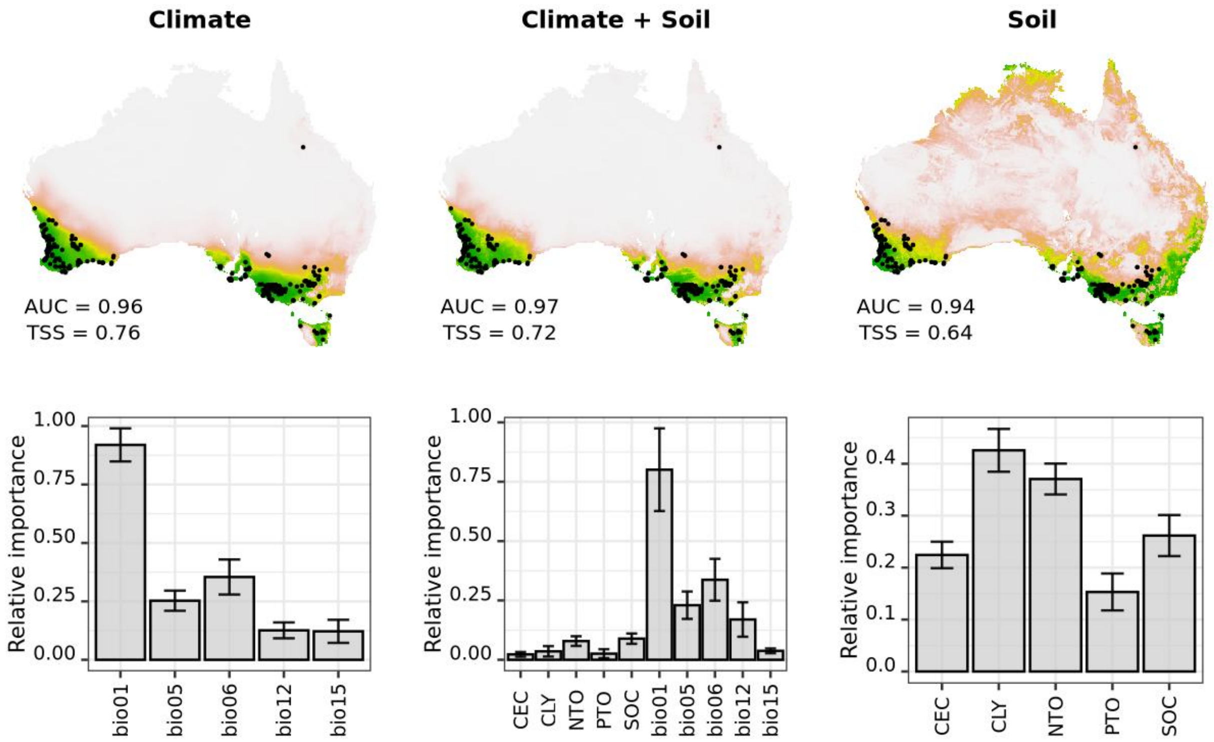

Centrolepis polygyna

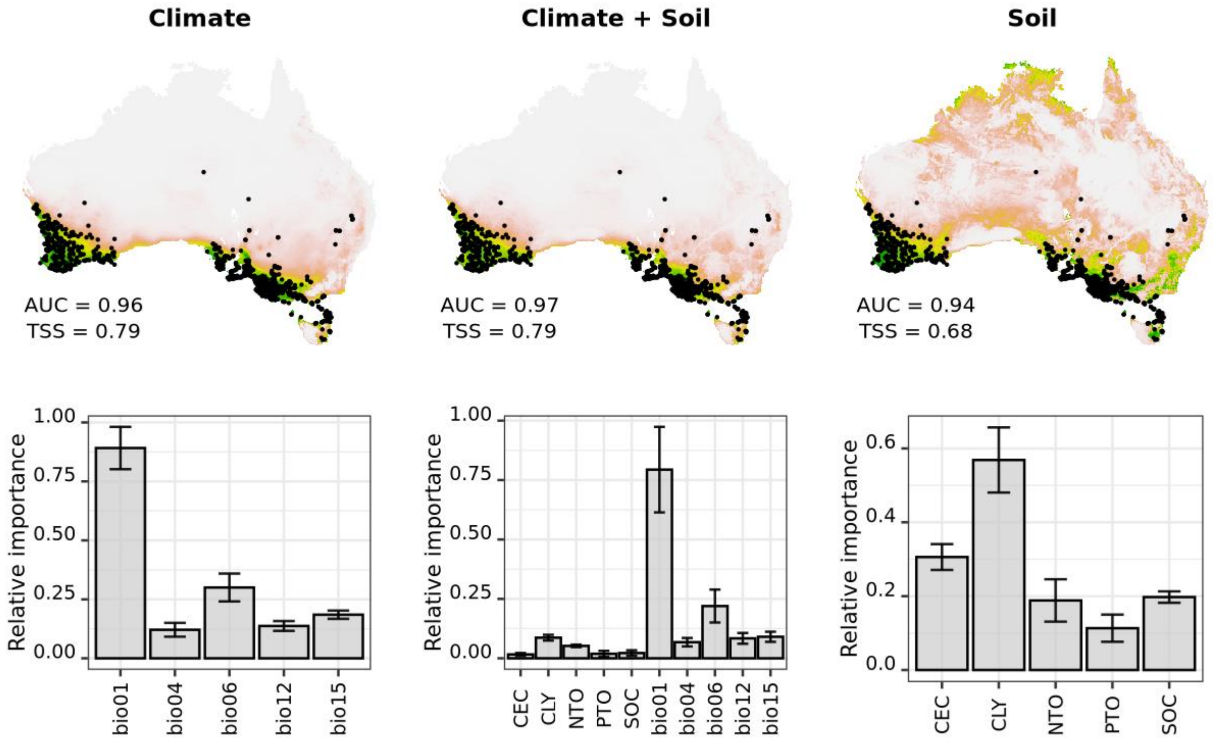

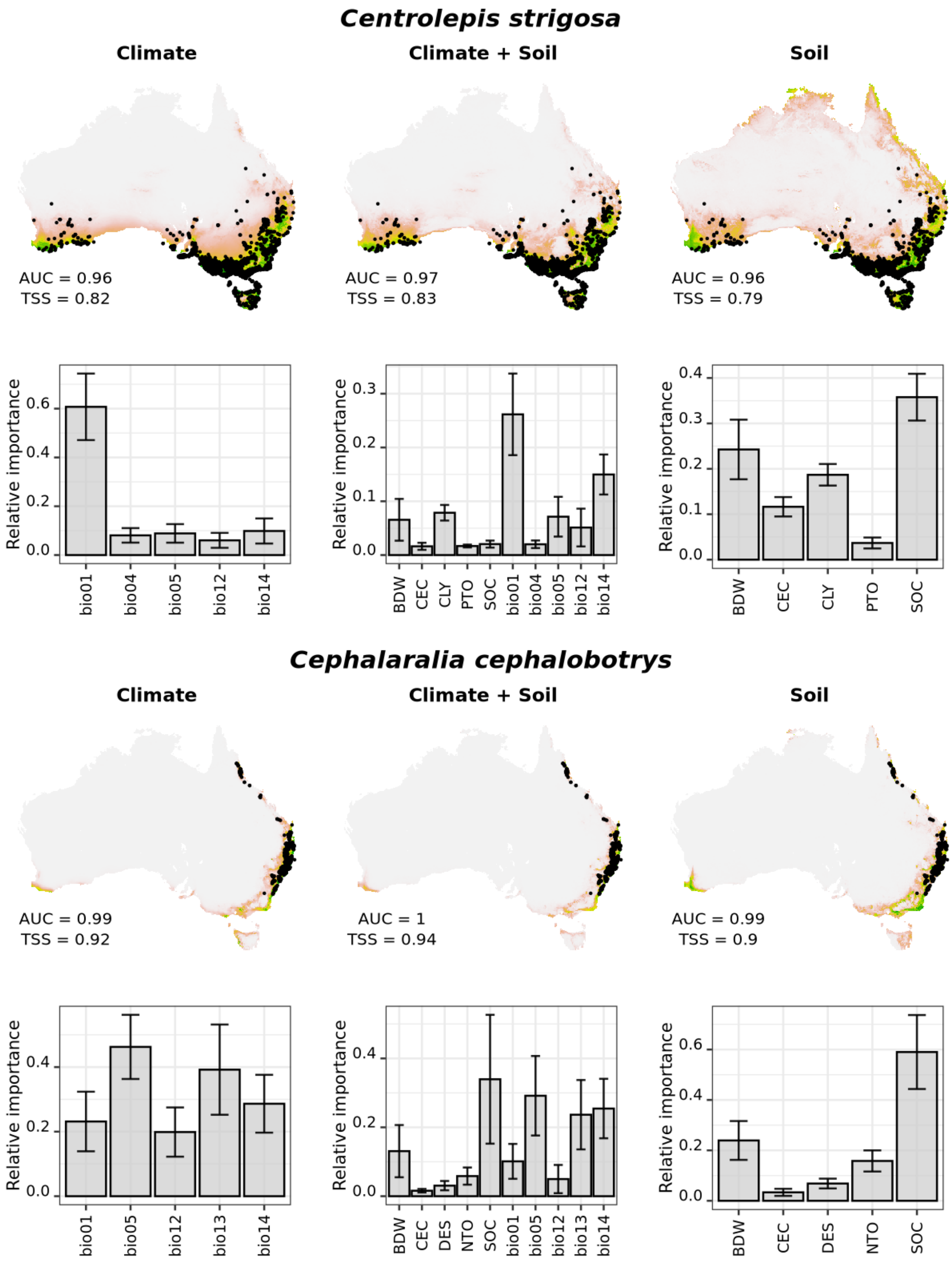
Centrolepis strigosa
Climate
Climate + Soil
Soil
AUC = 0.96
TSS = 0.82
AUC = 0.97
TSS = 0.83
AUC = 0.96
TSS = 0.79
Relative importance
bio01
bio04
bio05
bio12
bio14
BDW
CEC
CLY
PTO
SOC
Cephalaralia cephalobotrys
Climate
Climate + Soil
Soil
AUC = 0.99
TSS = 0.92
AUC = 1
TSS = 0.94
AUC = 0.99
TSS = 0.9
Relative importance
bio01
bio05
bio12
bio13
bio14
BDW
CEC
DES
NTO
SOC

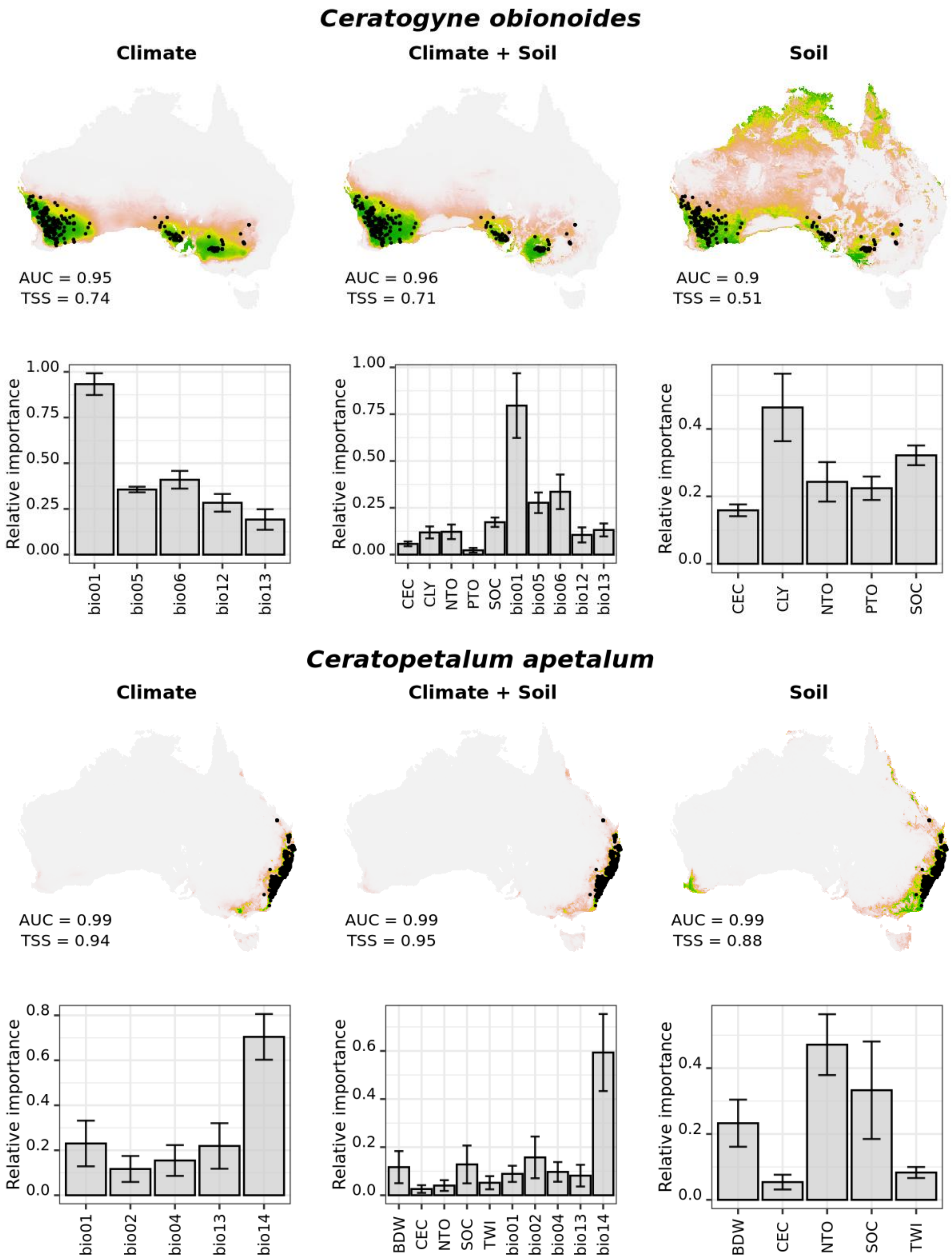
Ceratogyne obionoides
Climate
Climate + Soil
Soil
AUC = 0.95
TSS = 0.74
AUC = 0.96
TSS = 0.71
AUC = 0.9
TSS = 0.51
Relative importance
bio01 bio05 bio06 bio12 bio13
CEC CLY NTO PTO SOC bio01 bio05 bio06 bio12 bio13
CEC CLY NTO PTO SOC
Ceratopetalum apetalum
Climate
Climate + Soil
Soil
AUC = 0.99
TSS = 0.94
AUC = 0.99
TSS = 0.95
AUC = 0.99
TSS = 0.88
Relative importance
bio01 bio02 bio04 bio13 bio14
BDW CEC NTO SOC TWI bio01 bio02 bio04 bio13 bio14
BDW CEC NTO SOC TWI

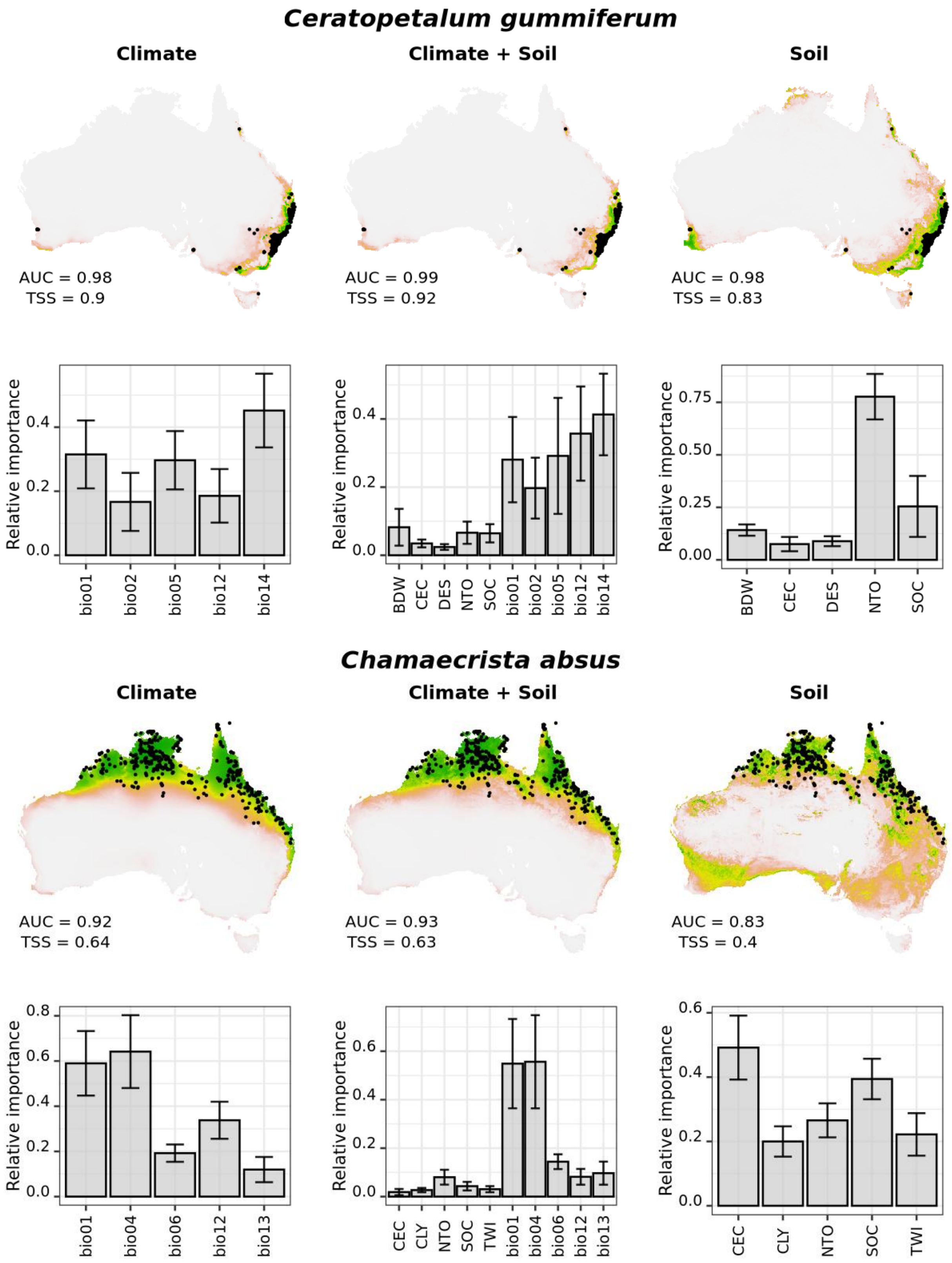
Ceratopetalum gummiferum
Climate
Climate + Soil
Soil
AUC = 0.98
TSS = 0.9
AUC = 0.99
TSS = 0.92
AUC = 0.98
TSS = 0.83
Relative importance
0.0
0.2
0.4
bio01
bio02
bio05
bio12
bio14
BDW
CEC
DES
NTO
SOC
0.00
0.25
0.50
0.75
Chamaecrista absus
Climate
Climate + Soil
Soil
AUC = 0.92
TSS = 0.64
AUC = 0.93
TSS = 0.63
AUC = 0.83
TSS = 0.4
0.6
0.8
bio04
bio06
bio13
CLY
TWI

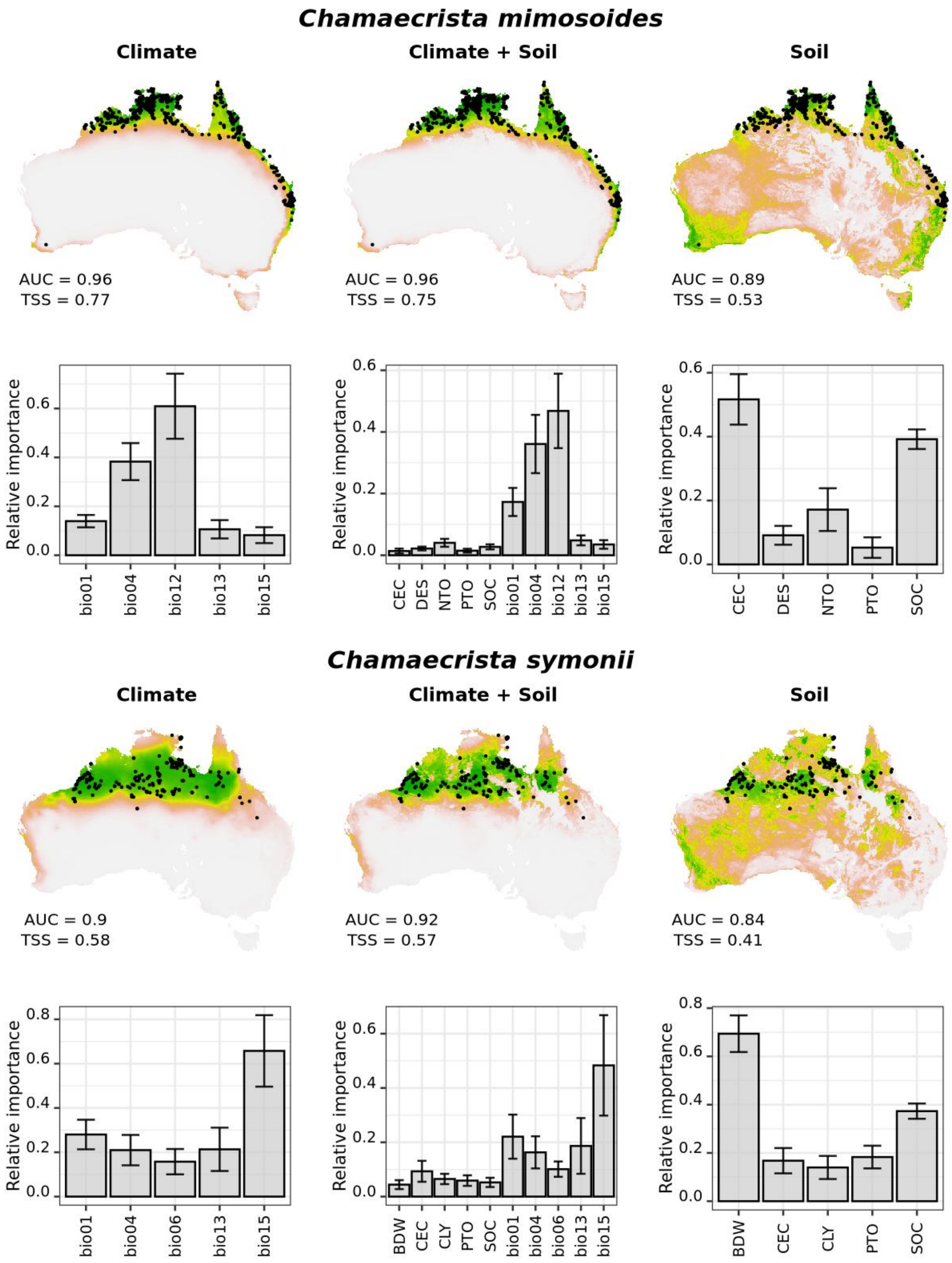

Chamaecrista mimosoides
Climate
Climate + Soil
Soil
AUC = 0.96
TSS = 0.77
AUC = 0.96
TSS = 0.75
AUC = 0.89
TSS = 0.53
Relative importance
bio01
bio04
bio12
bio13
bio15
CEC
DES
NTO
PTO
SOC
Chamaecrista symonii
Climate
Climate + Soil
Soil
AUC = 0.9
TSS = 0.58
AUC = 0.92
TSS = 0.57
AUC = 0.84
TSS = 0.41
Relative importance
bio01
bio04
bio06
bio13
bio15
BDW
CEC
CLY
PTO
SOC

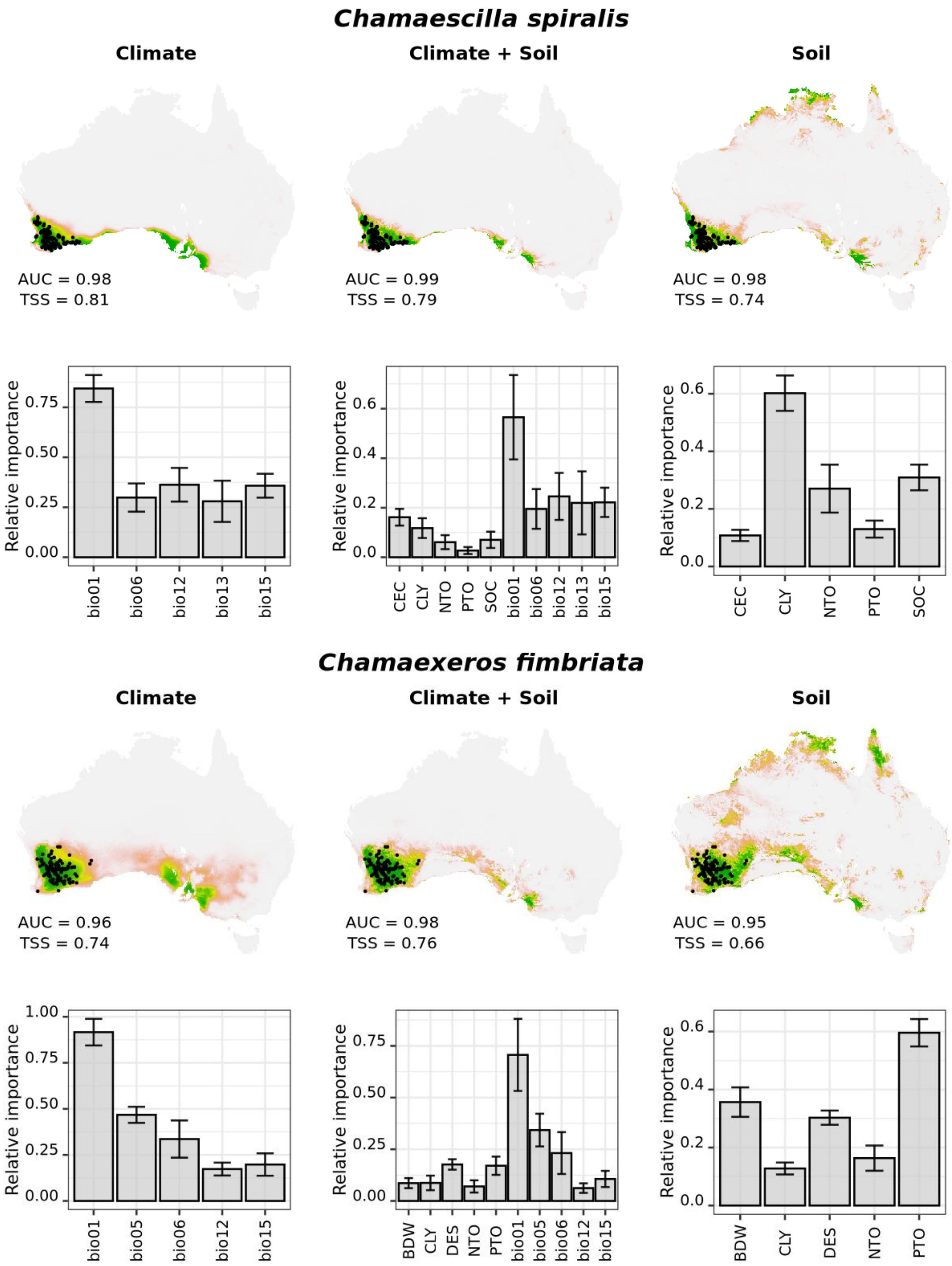
Chamaescilla spiralis
Climate
Climate + Soil
Soil
AUC = 0.98
TSS = 0.81
AUC = 0.99
TSS = 0.79
AUC = 0.98
TSS = 0.74
Relative importance
0.00
0.25
0.50
0.75
bio01
bio06
bio12
bio13
bio15
0.0
0.2
0.4
0.6
CEC
CLY
NTO
PTO
SOC
bio01
bio06
bio12
bio13
bio15
CEC
CLY
NTO
PTO
SOC
Chamaexeros fimbriata
Climate
Climate + Soil
Soil
AUC = 0.96
TSS = 0.74
AUC = 0.98
TSS = 0.76
AUC = 0.95
TSS = 0.66
Relative importance
0.00
0.25
0.50
0.75
1.00
bio01
bio05
bio06
bio12
bio15
BDW
CLY
DES
NTO
PTO
bio01
bio05
bio06
bio12
bio15
0.0
0.2
0.4
0.6
BDW
CLY
DES
NTO
PTO

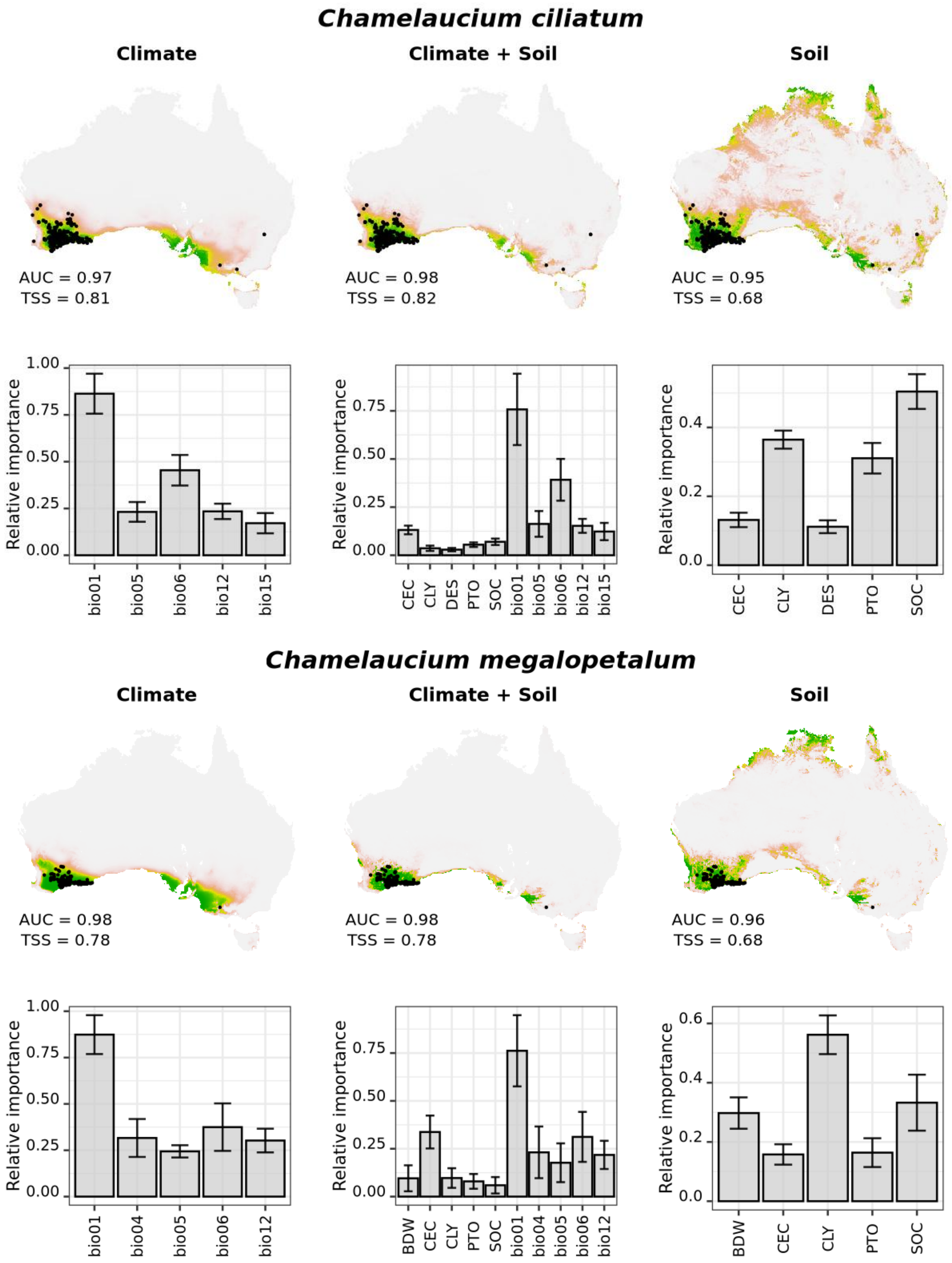

Chamelaucium ciliatum
Climate
Climate + Soil
Soil
AUC = 0.97
TSS = 0.81
AUC = 0.98
TSS = 0.82
AUC = 0.95
TSS = 0.68
Relative importance
1.00
0.75
0.50
0.25
0.00
bio01
bio05
bio06
bio12
bio15
CEC
CLY
DES
PTO
SOC
0.4
0.2
0.0
Chamelaucium megalopetalum
Climate
Climate + Soil
Soil
AUC = 0.98
TSS = 0.78
AUC = 0.98
TSS = 0.78
AUC = 0.96
TSS = 0.68
bio04
BDW
0.6

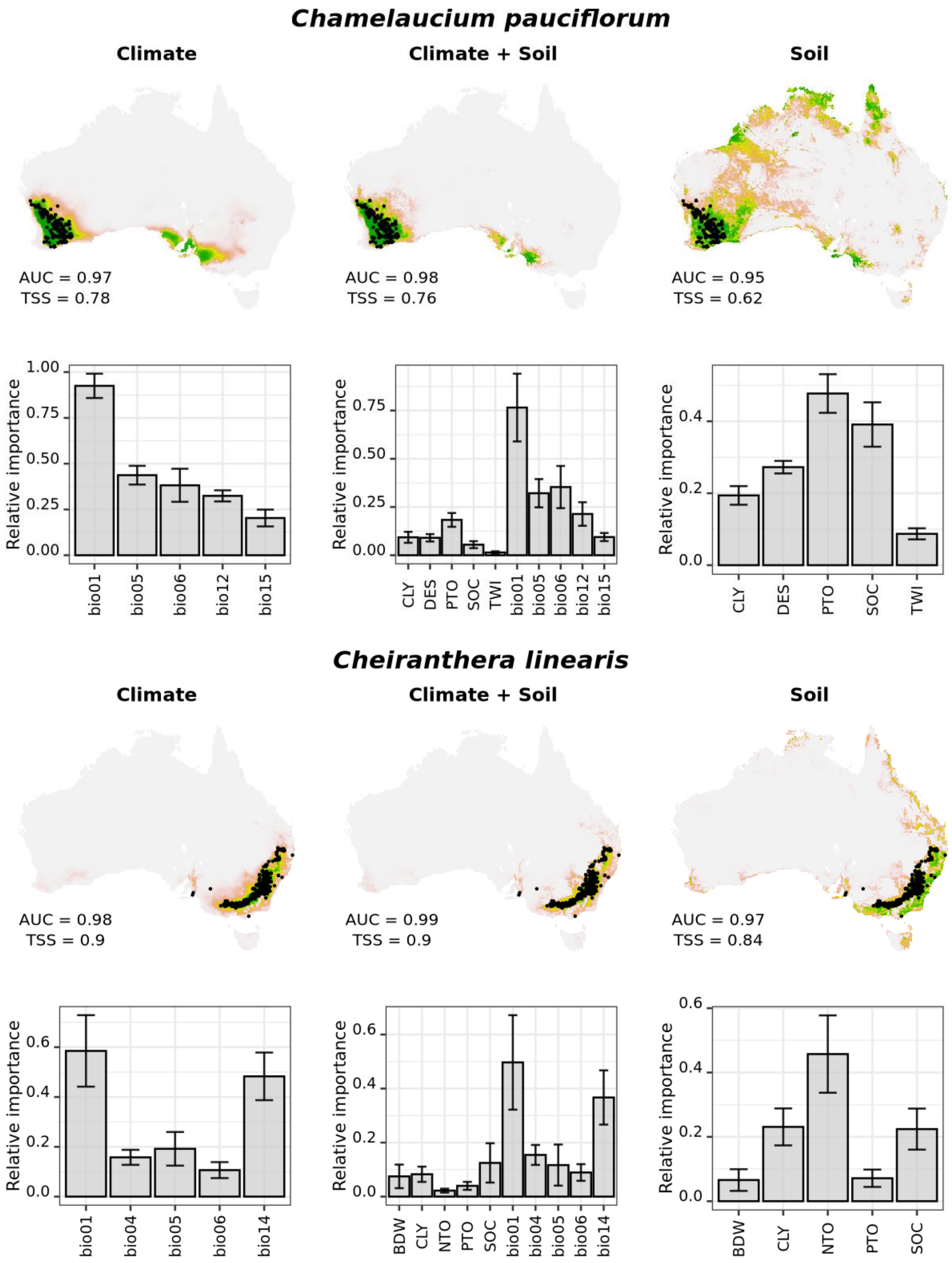

Chamelaucium pauciflorum
Climate
Climate + Soil
Soil
AUC = 0.97
TSS = 0.78
AUC = 0.98
TSS = 0.76
AUC = 0.95
TSS = 0.62
Relative importance
bio01
bio05
bio06
bio12
bio15
CLY
DES
PTO
SOC
TWI
Cheiranthera linearis
AUC = 0.98
TSS = 0.9
AUC = 0.99
TSS = 0.9
AUC = 0.97
TSS = 0.84
bio04
bio14
BDW
NTO

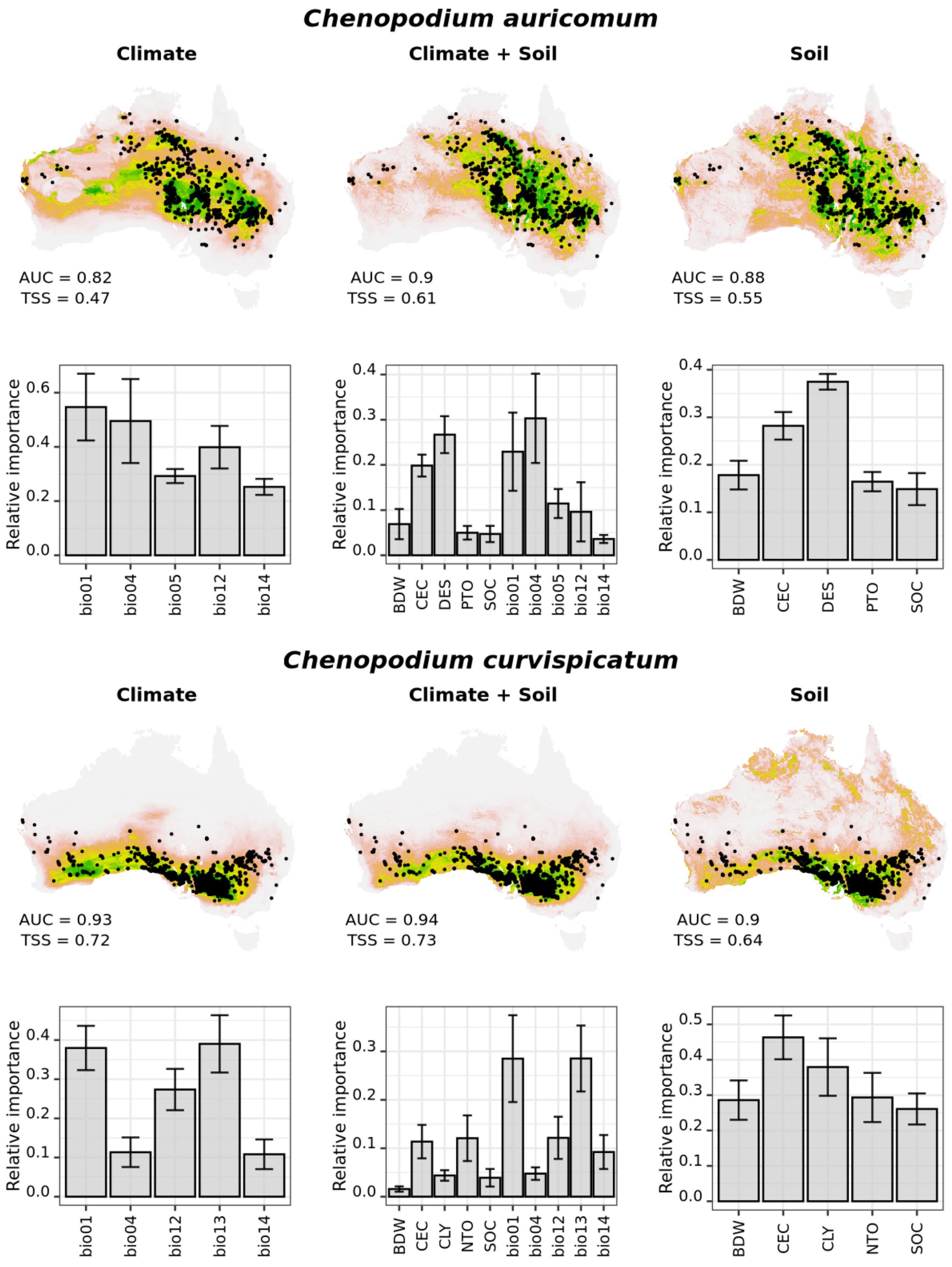
Chenopodium auricomum
Climate
Climate + Soil
Soil
AUC = 0.82
TSS = 0.47
AUC = 0.9
TSS = 0.61
AUC = 0.88
TSS = 0.55
Relative importance
bio01 bio04 bio05 bio12 bio14
BDW CEC DES PTO SOC bio01 bio04 bio05 bio12 bio14
BDW CEC DES PTO SOC
Chenopodium curvispicatum
Climate
Climate + Soil
Soil
AUC = 0.93
TSS = 0.72
AUC = 0.94
TSS = 0.73
AUC = 0.9
TSS = 0.64
Relative importance
bio01 bio04 bio12 bio13 bio14
BDW CEC CLY NTO SOC bio01 bio04 bio12 bio13 bio14
BDW CEC CLY NTO SOC

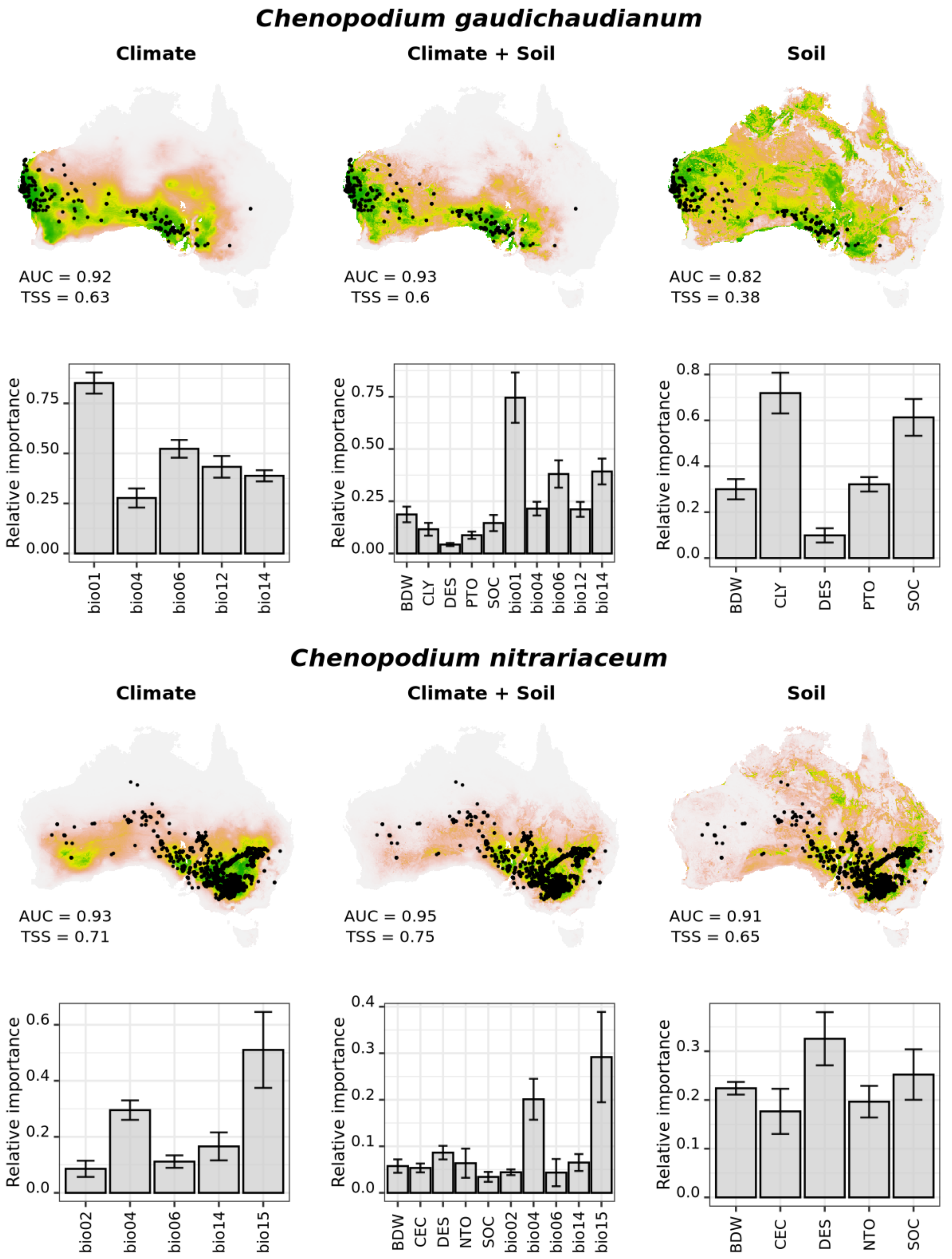
Chenopodium gaudichaudianum
Climate
Climate + Soil
Soil
AUC = 0.92
TSS = 0.63
AUC = 0.93
TSS = 0.6
AUC = 0.82
TSS = 0.38
Relative importance
bio01
bio04
bio06
bio12
bio14
BDW
CLY
DES
PTO
SOC
Chenopodium nitrariaceum
Climate
Climate + Soil
Soil
AUC = 0.93
TSS = 0.71
AUC = 0.95
TSS = 0.75
AUC = 0.91
TSS = 0.65
Relative importance
bio02
bio04
bio06
bio14
bio15
BDW
CEC
DES
NTO
SOC

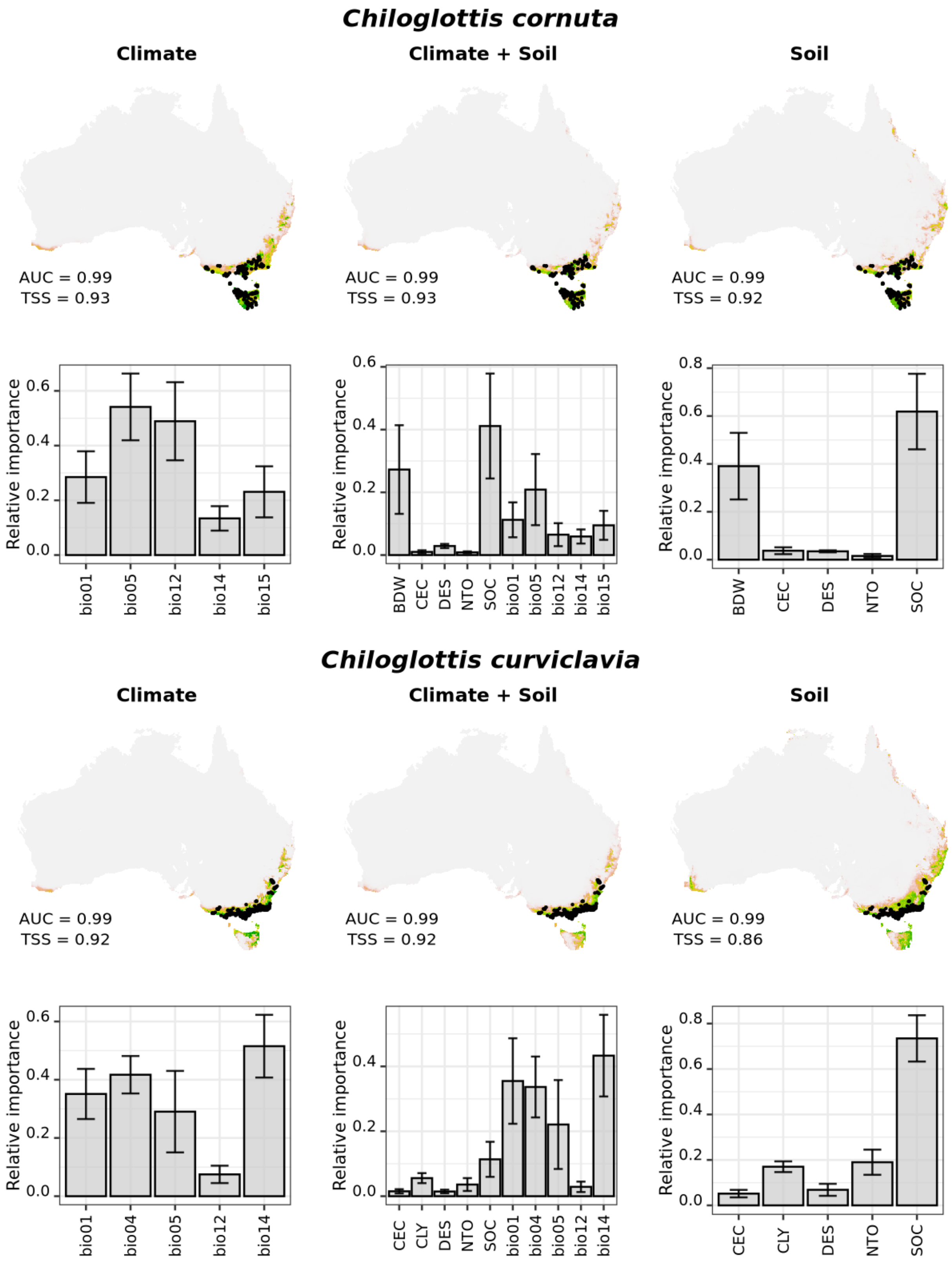
Chiloglottis cornuta
Climate
Climate + Soil
Soil
AUC = 0.99
TSS = 0.93
AUC = 0.99
TSS = 0.93
AUC = 0.99
TSS = 0.92
Relative importance
bio01
bio05
bio12
bio14
bio15
BDW
CEC
DES
NTO
SOC
Chiloglottis curviclavia
Climate
Climate + Soil
Soil
AUC = 0.99
TSS = 0.92
AUC = 0.99
TSS = 0.92
AUC = 0.99
TSS = 0.86
Relative importance
bio01
bio04
bio05
bio12
bio14
CEC
CLY
DES
NTO
SOC

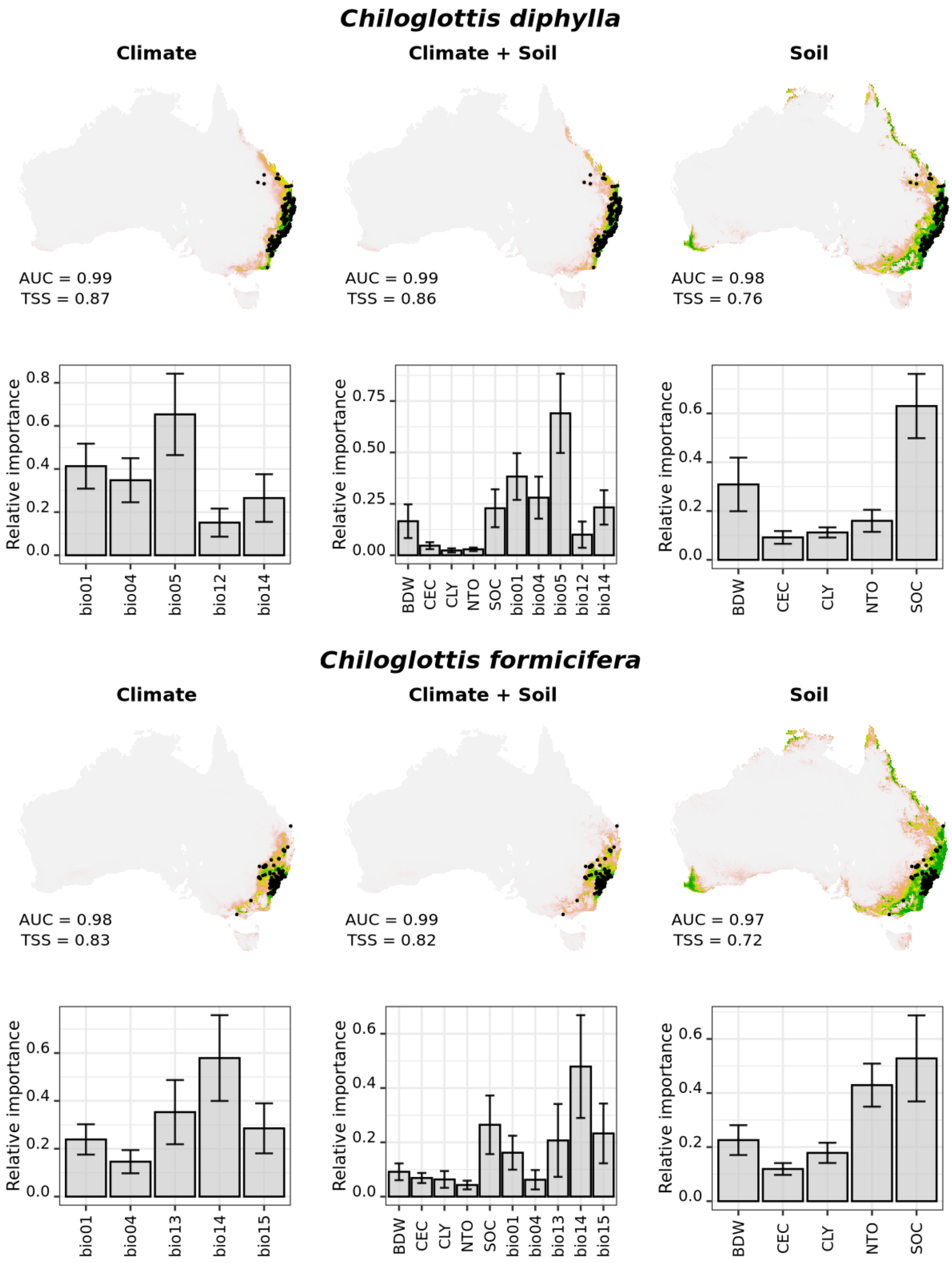
Chiloglottis diphylla
Climate
Climate + Soil
Soil
AUC = 0.99
TSS = 0.87
AUC = 0.99
TSS = 0.86
AUC = 0.98
TSS = 0.76
Relative importance
0.0
0.2
0.4
0.6
0.8
bio01
bio04
bio05
bio12
bio14
0.00
0.25
0.50
0.75
BDW
CEC
CLY
NTO
SOC
bio01
bio04
bio05
bio12
bio14
0.0
0.2
0.4
0.6
BDW
CEC
CLY
NTO
SOC
Chiloglottis formicifera
Climate
Climate + Soil
Soil
AUC = 0.98
TSS = 0.83
AUC = 0.99
TSS = 0.82
AUC = 0.97
TSS = 0.72
Relative importance
0.0
0.2
0.4
0.6
bio01
bio04
bio13
bio14
bio15
0.0
0.2
0.4
0.6
BDW
CEC
CLY
NTO
SOC
bio01
bio04
bio13
bio14
bio15
0.0
0.2
0.4
0.6
BDW
CEC
CLY
NTO
SOC

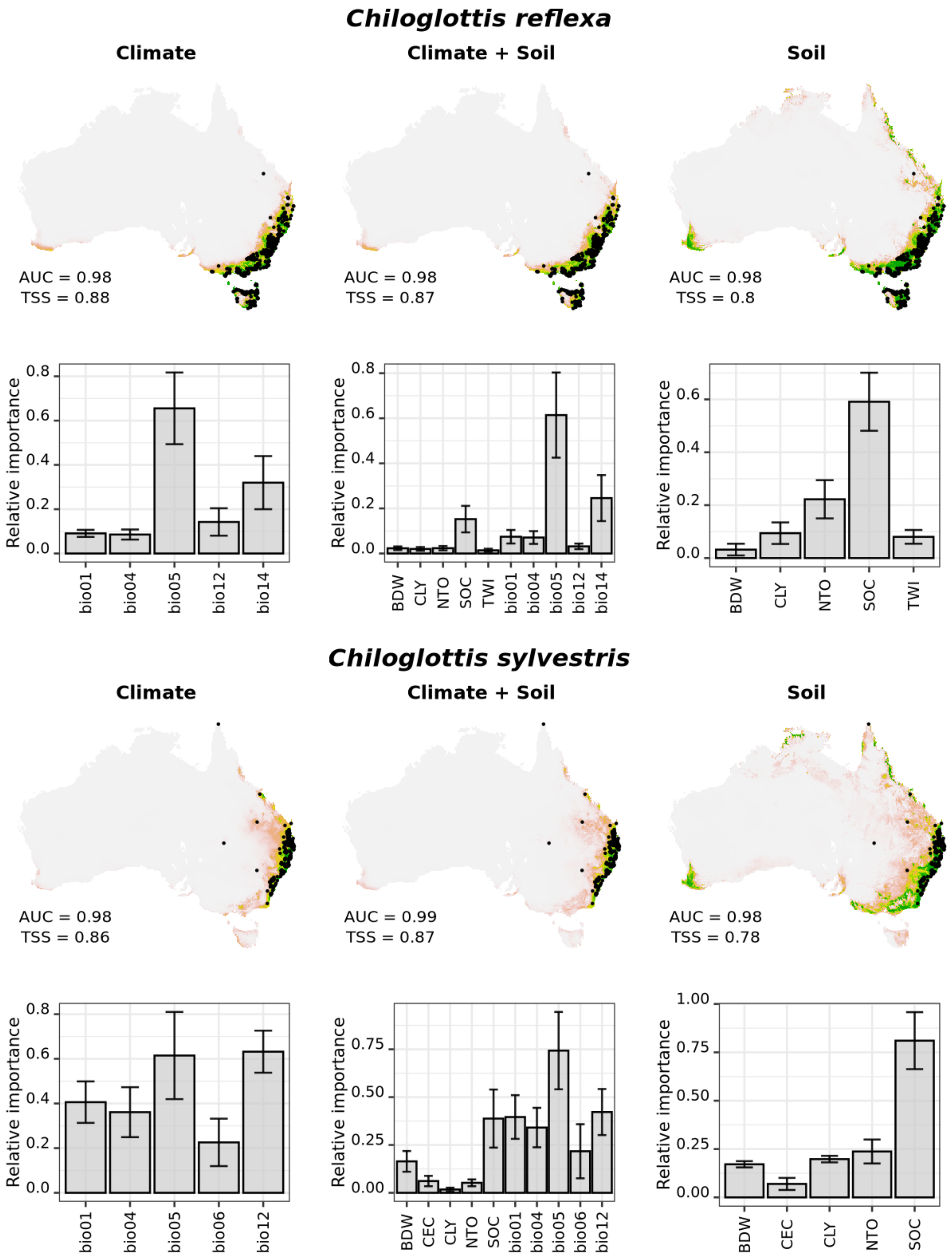

Chiloglottis reflexa
Climate
Climate + Soil
Soil
AUC = 0.98
TSS = 0.88
AUC = 0.98
TSS = 0.87
AUC = 0.98
TSS = 0.8
Relative importance
bio01
bio04
bio05
bio12
bio14
BDW
CLY
NTO
SOC
TWI
Chiloglottis sylvestris
AUC = 0.98
TSS = 0.86
AUC = 0.99
TSS = 0.87
AUC = 0.98
TSS = 0.78
bio06
CEC

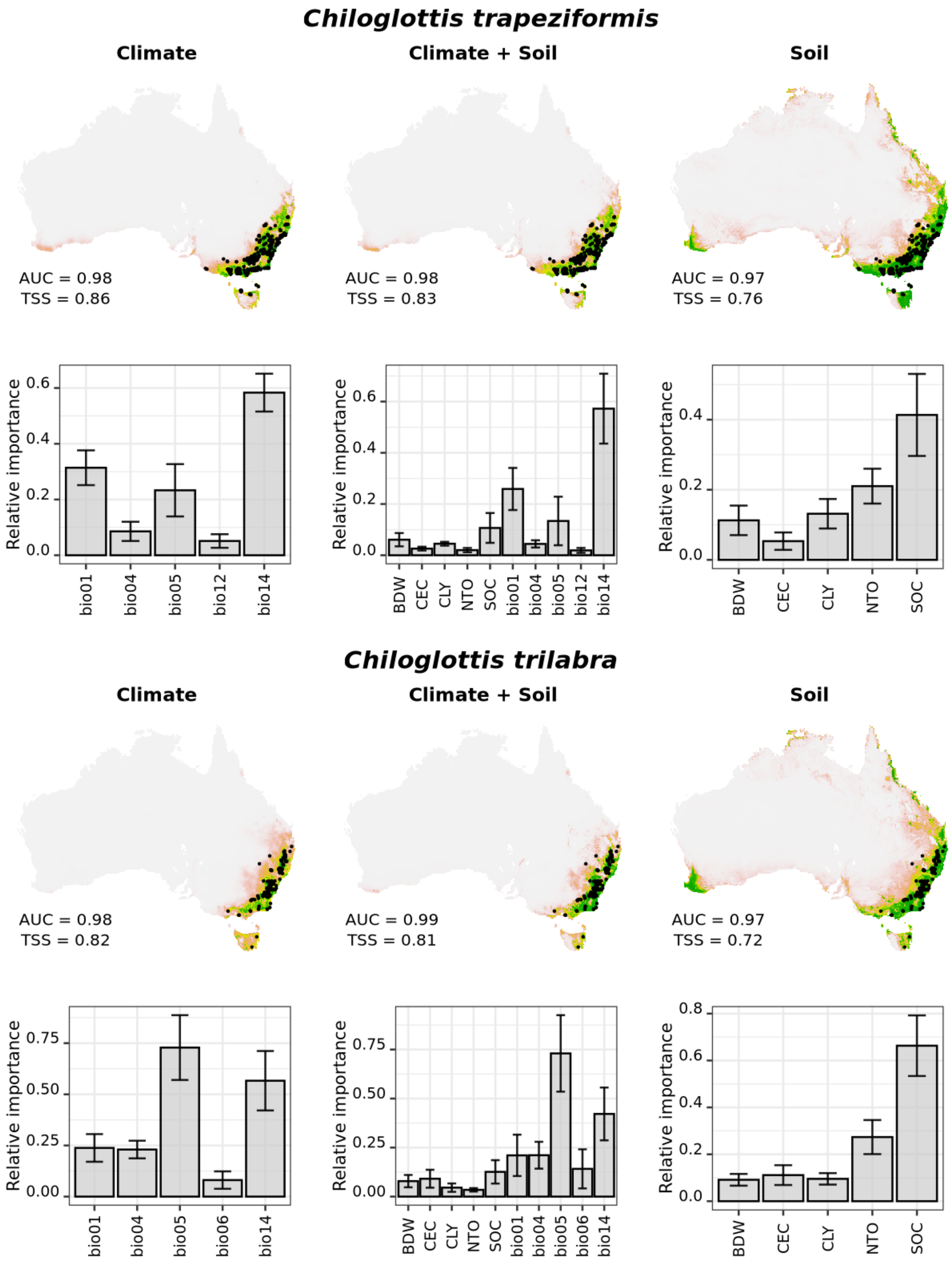
Chiloglottis trapeziformis
Climate
Climate + Soil
Soil
AUC = 0.98
TSS = 0.86
AUC = 0.98
TSS = 0.83
AUC = 0.97
TSS = 0.76
Relative importance
0.0
0.2
0.4
0.6
bio01
bio04
bio05
bio12
bio14
BDW
CEC
CLY
NTO
SOC
Chiloglottis trilabra
AUC = 0.98
TSS = 0.82
AUC = 0.99
TSS = 0.81
AUC = 0.97
TSS = 0.72
0.00
0.25
0.50
0.75
0.8
bio06

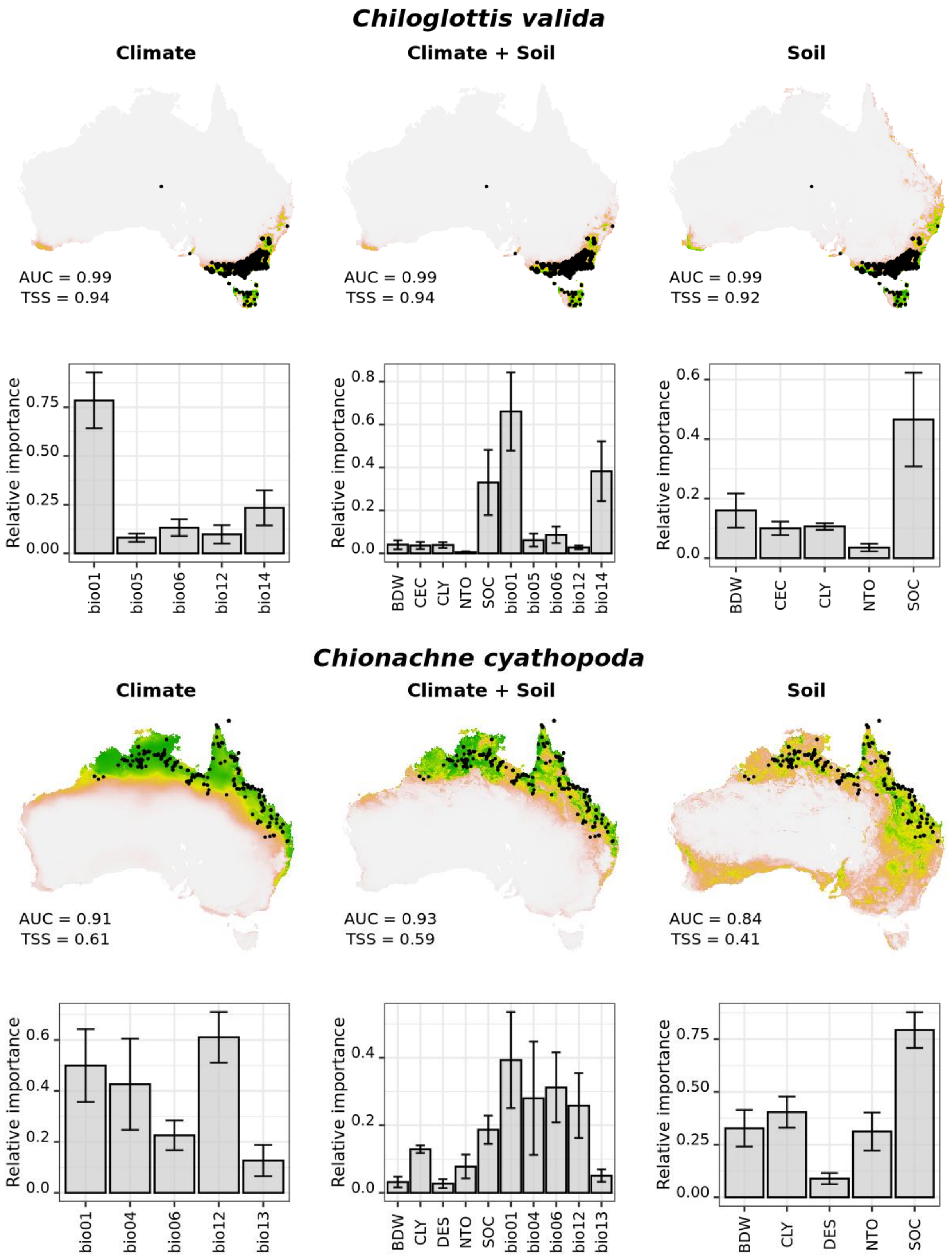

Chiloglottis valida
Climate
Climate + Soil
Soil
AUC = 0.99
TSS = 0.94
AUC = 0.99
TSS = 0.94
AUC = 0.99
TSS = 0.92
Relative importance
bio01
bio05
bio06
bio12
bio14
BDW
CEC
CLY
NTO
SOC
Chionachne cyathopoda
Climate
Climate + Soil
Soil
AUC = 0.91
TSS = 0.61
AUC = 0.93
TSS = 0.59
AUC = 0.84
TSS = 0.41
bio04
bio13
DES

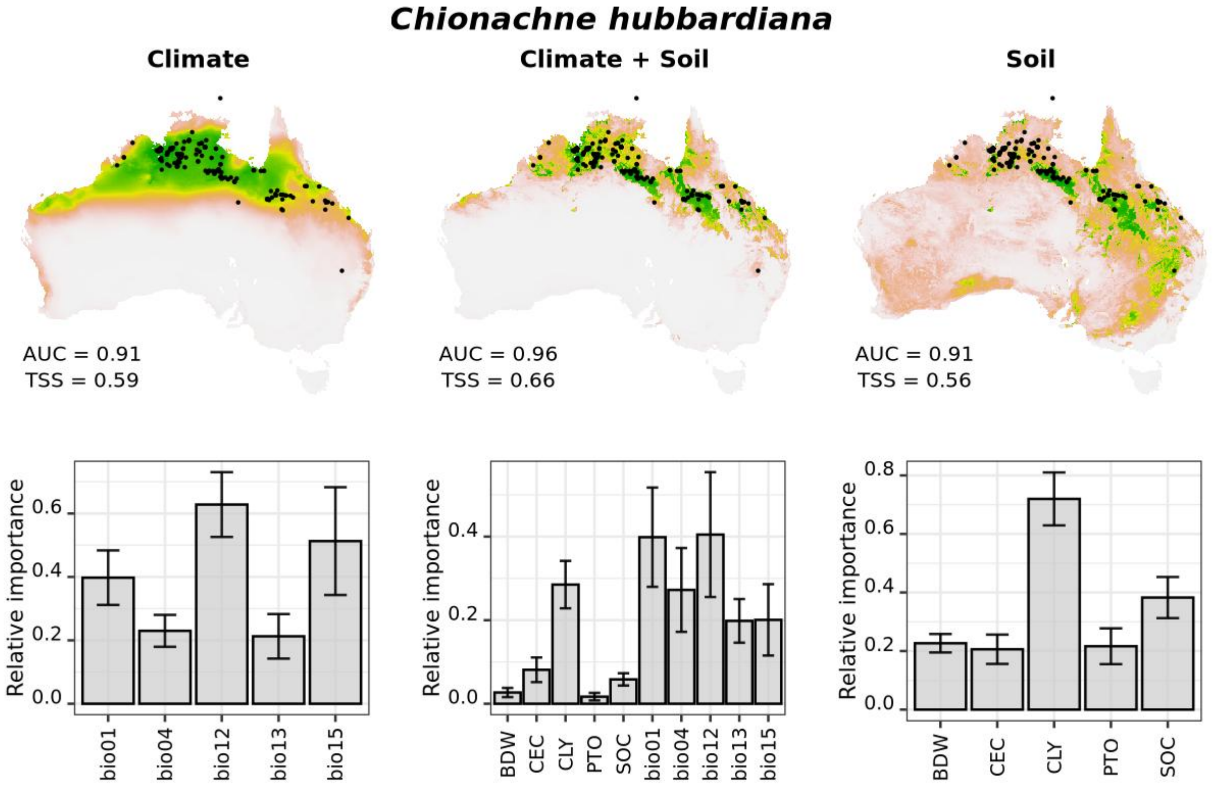

Chionachne hubbardiana
Climate
Climate + Soil
Soil
AUC = 0.91
TSS = 0.59
AUC = 0.96
TSS = 0.66
AUC = 0.91
TSS = 0.56
Relative importance
bio01
bio04
bio12
bio13
bio15
BDW
CEC
CLY
PTO
SOC

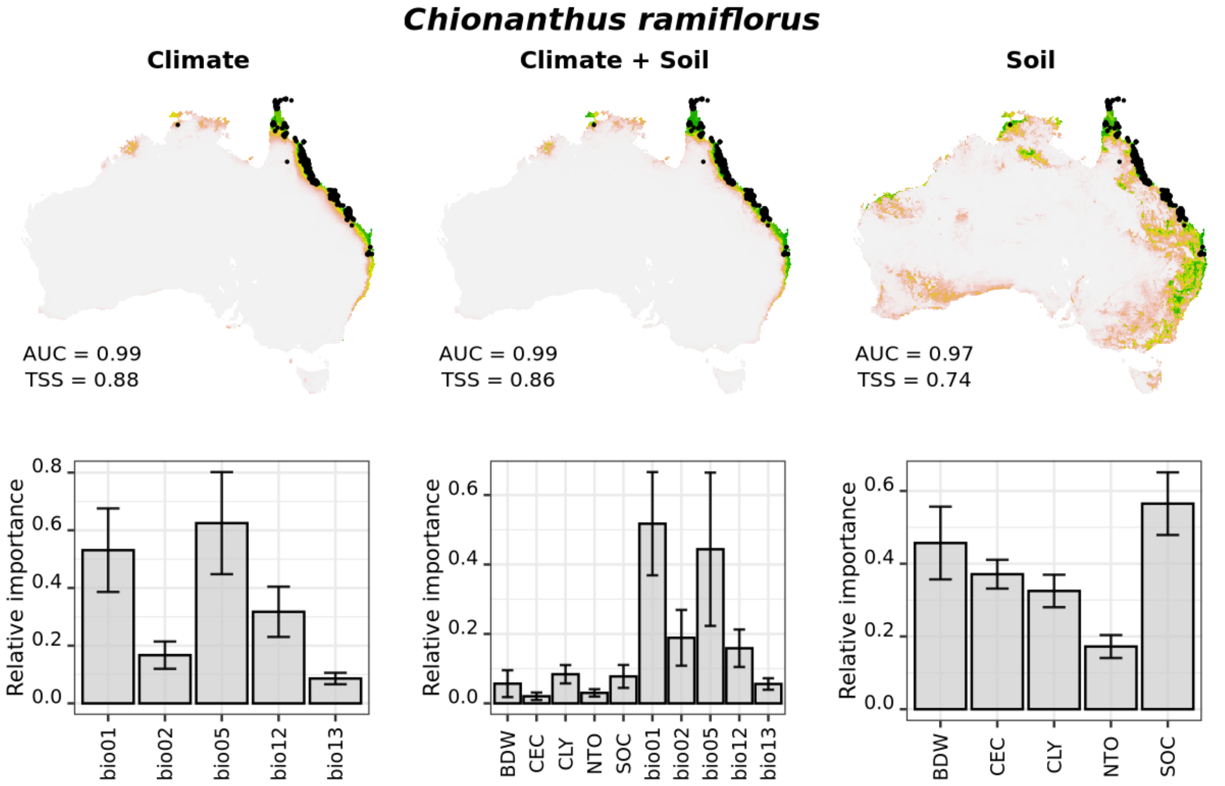

Chionanthus ramiflorus
Climate
Climate + Soil
Soil
AUC = 0.99
TSS = 0.88
AUC = 0.99
TSS = 0.86
AUC = 0.97
TSS = 0.74
Relative importance
bio01
bio02
bio05
bio12
bio13
BDW
CEC
CLY
NTO
SOC

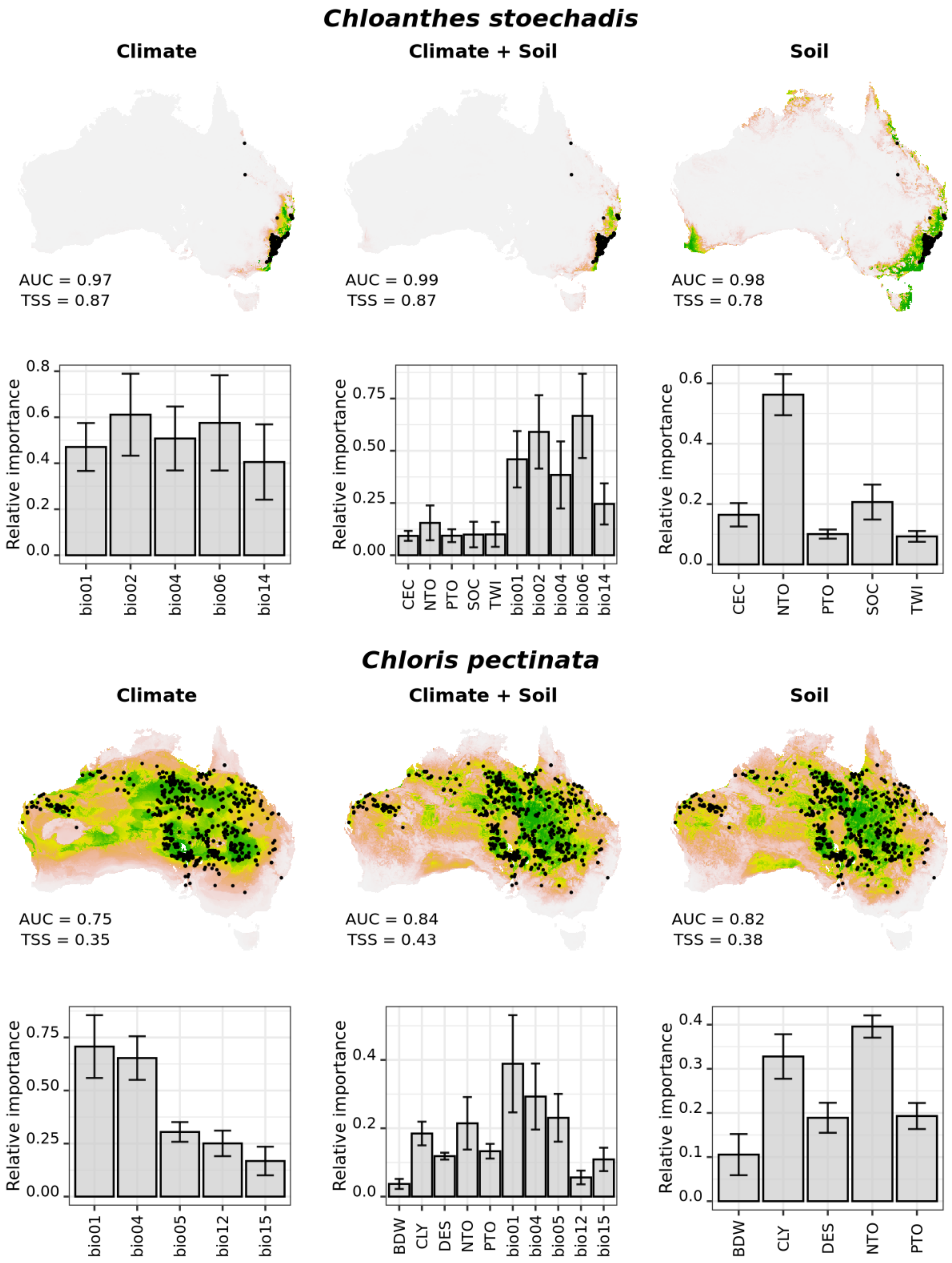
Chloanthes stoechadis
Climate
Climate + Soil
Soil
AUC = 0.97
TSS = 0.87
AUC = 0.99
TSS = 0.87
AUC = 0.98
TSS = 0.78
Relative importance
bio01
bio02
bio04
bio06
bio14
CEC
NTO
PTO
SOC
TWI
Chloris pectinata
Climate
Climate + Soil
Soil
AUC = 0.75
TSS = 0.35
AUC = 0.84
TSS = 0.43
AUC = 0.82
TSS = 0.38
Relative importance
bio01
bio04
bio05
bio12
bio15
BDW
CLY
DES
NTO
PTO

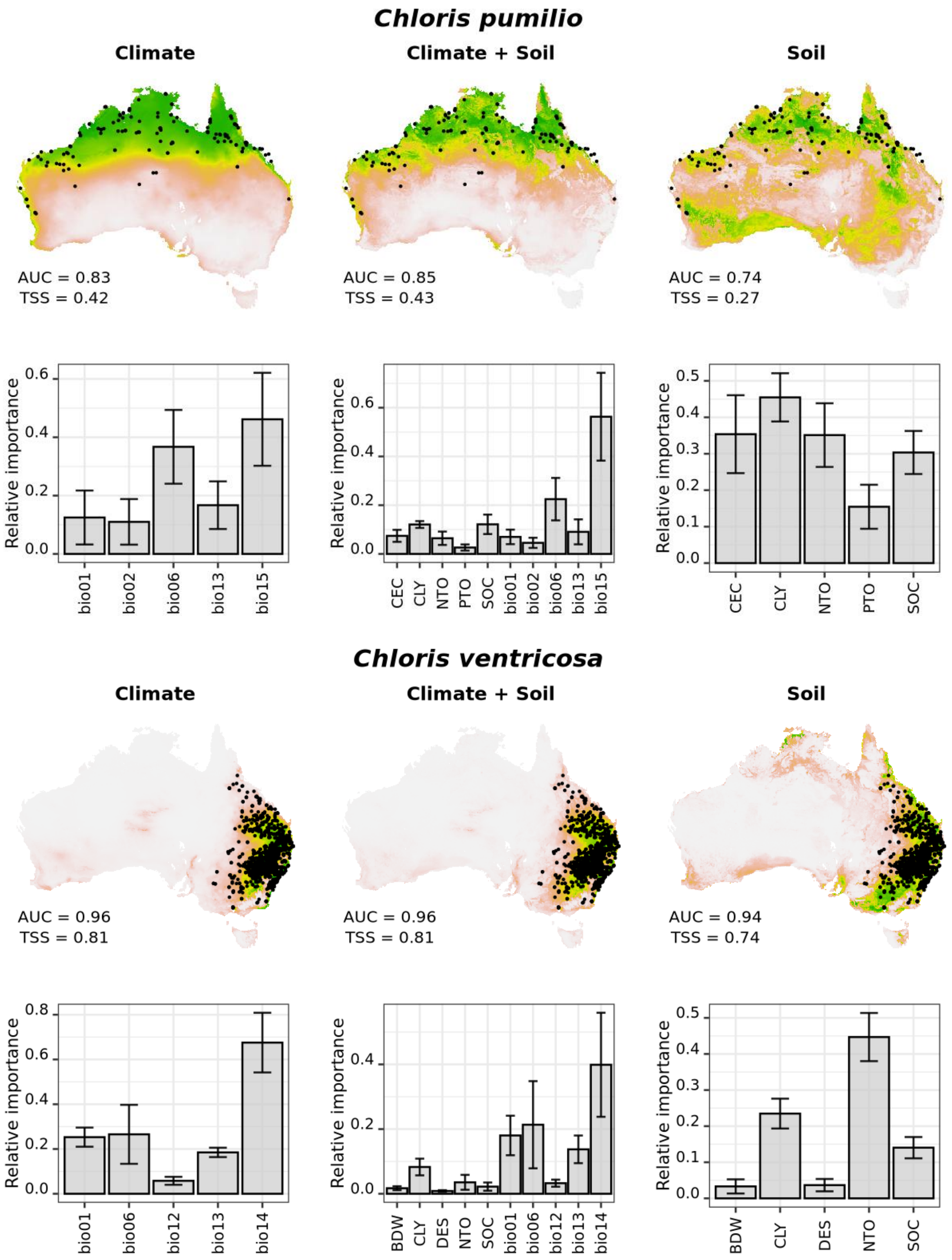

Chloris pumilio
Climate
Climate + Soil
Soil
AUC = 0.83
TSS = 0.42
AUC = 0.85
TSS = 0.43
AUC = 0.74
TSS = 0.27
Relative importance
bio01
bio02
bio06
bio13
bio15
CEC
CLY
NTO
PTO
SOC
bio01
bio02
bio06
bio13
bio15
CEC
CLY
NTO
PTO
SOC
Chloris ventricosa
Climate
Climate + Soil
Soil
AUC = 0.96
TSS = 0.81
AUC = 0.96
TSS = 0.81
AUC = 0.94
TSS = 0.74
Relative importance
bio01
bio06
bio12
bio13
bio14
BDW
CLY
DES
NTO
SOC
bio01
bio06
bio12
bio13
bio14
BDW
CLY
DES
NTO
SOC

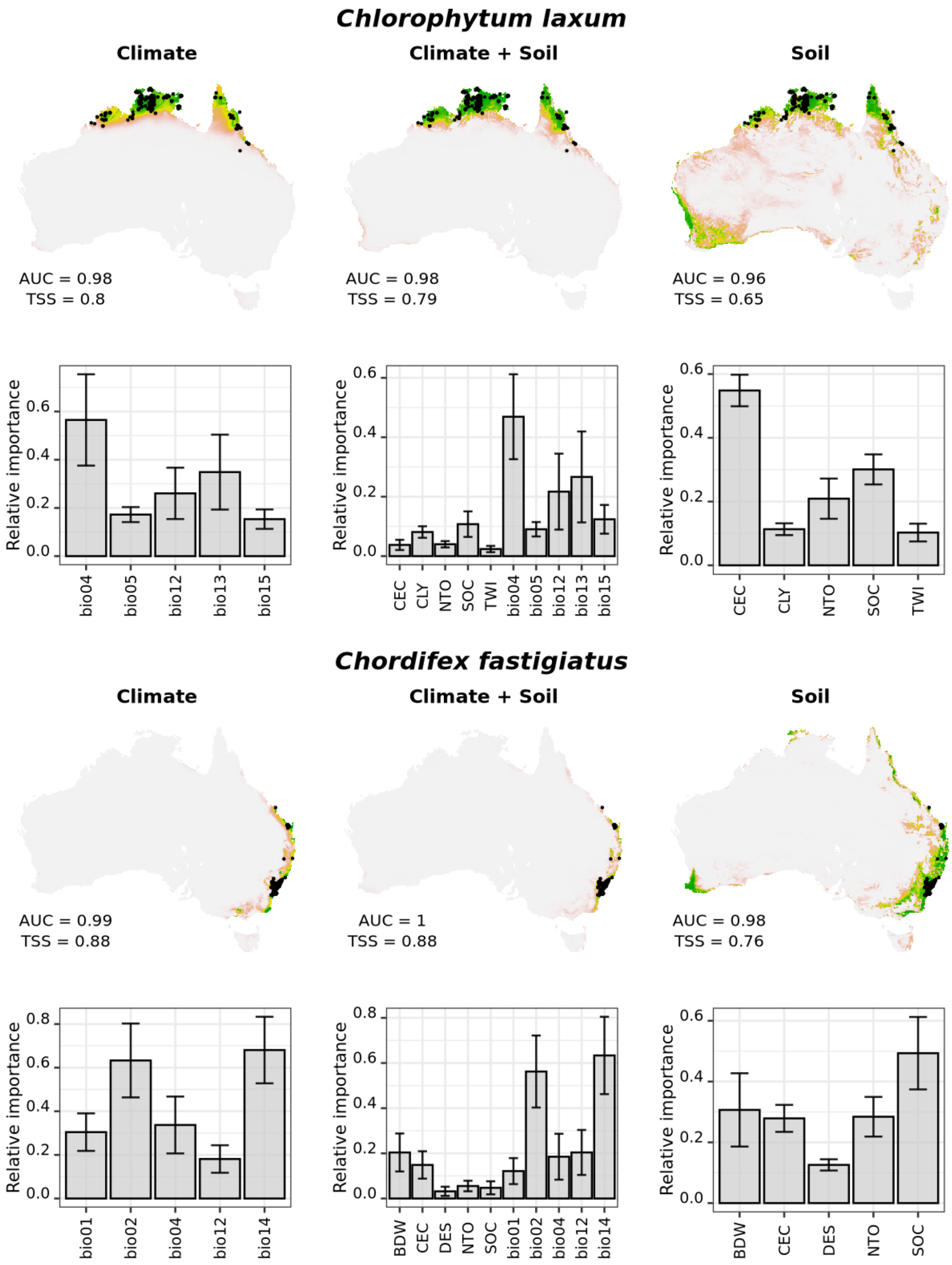
Chlorophytum laxum
Climate
Climate + Soil
Soil
AUC = 0.98
TSS = 0.8
AUC = 0.98
TSS = 0.79
AUC = 0.96
TSS = 0.65
Relative importance
0.0
0.2
0.4
0.6
bio04
bio05
bio12
bio13
bio15
CEC
CLY
NTO
SOC
TWI
Chordifex fastigiatus
Climate
Climate + Soil
Soil
AUC = 0.99
TSS = 0.88
AUC = 1
TSS = 0.88
AUC = 0.98
TSS = 0.76
Relative importance
0.8
bio01
bio02
bio14
BDW
DES

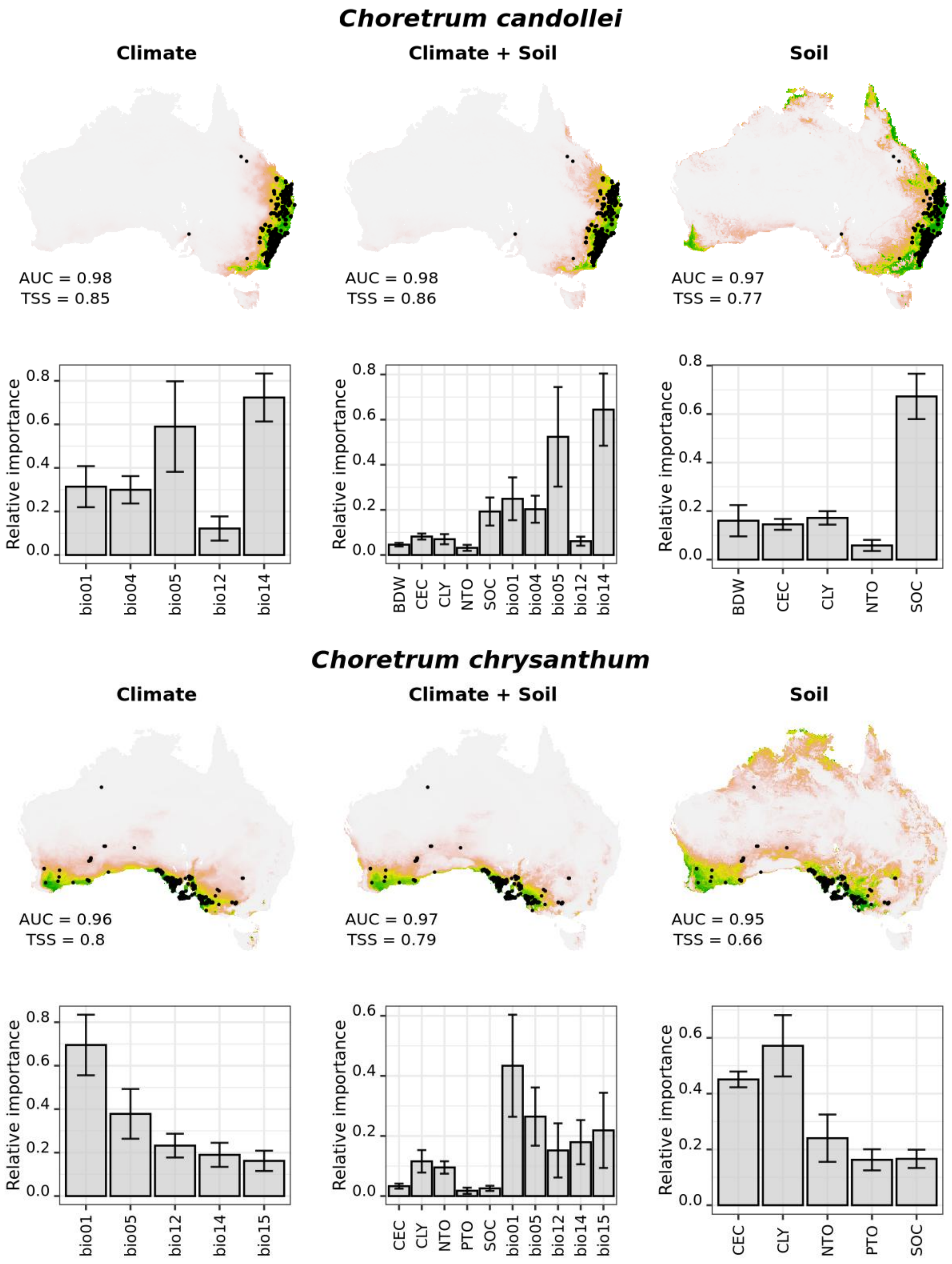
Choretrum candollei
Climate
Climate + Soil
Soil
AUC = 0.98
TSS = 0.85
AUC = 0.98
TSS = 0.86
AUC = 0.97
TSS = 0.77
Relative importance
0.0
0.2
0.4
0.6
0.8
bio01
bio04
bio05
bio12
bio14
BDW
CEC
CLY
NTO
SOC
Choretrum chrysanthum
Climate
Climate + Soil
Soil
AUC = 0.96
TSS = 0.8
AUC = 0.97
TSS = 0.79
AUC = 0.95
TSS = 0.66
Relative importance
bio01
bio05
bio12
bio14
bio15
CEC
CLY
NTO
PTO
SOC

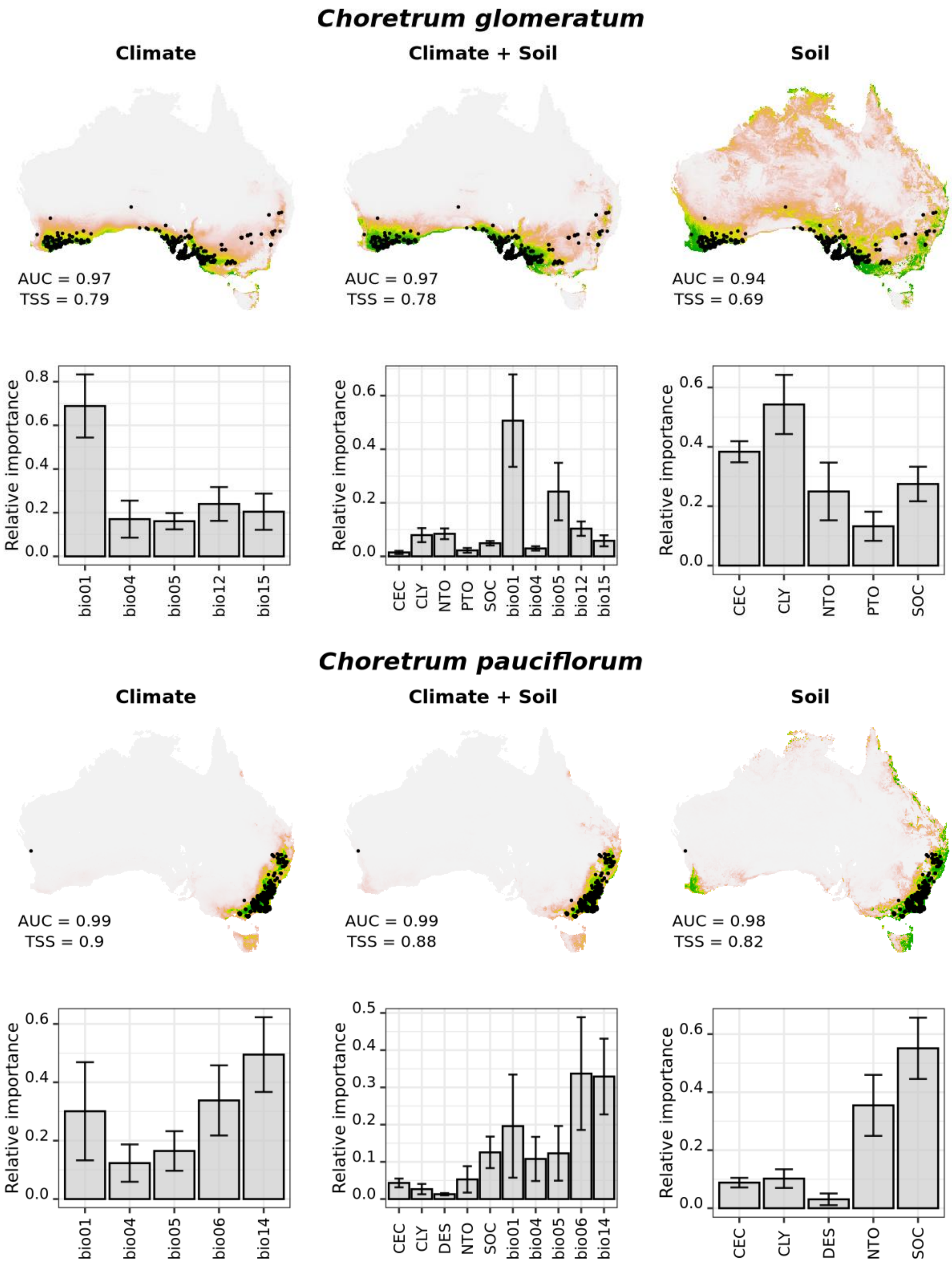
Choretrum glomeratum
Climate
Climate + Soil
Soil
AUC = 0.97
TSS = 0.79
AUC = 0.97
TSS = 0.78
AUC = 0.94
TSS = 0.69
Relative importance
bio01
bio04
bio05
bio12
bio15
CEC
CLY
NTO
PTO
SOC
Choretrum pauciflorum
Climate
Climate + Soil
Soil
AUC = 0.99
TSS = 0.9
AUC = 0.99
TSS = 0.88
AUC = 0.98
TSS = 0.82
Relative importance
bio01
bio04
bio05
bio06
bio14
CEC
CLY
DES
NTO
SOC

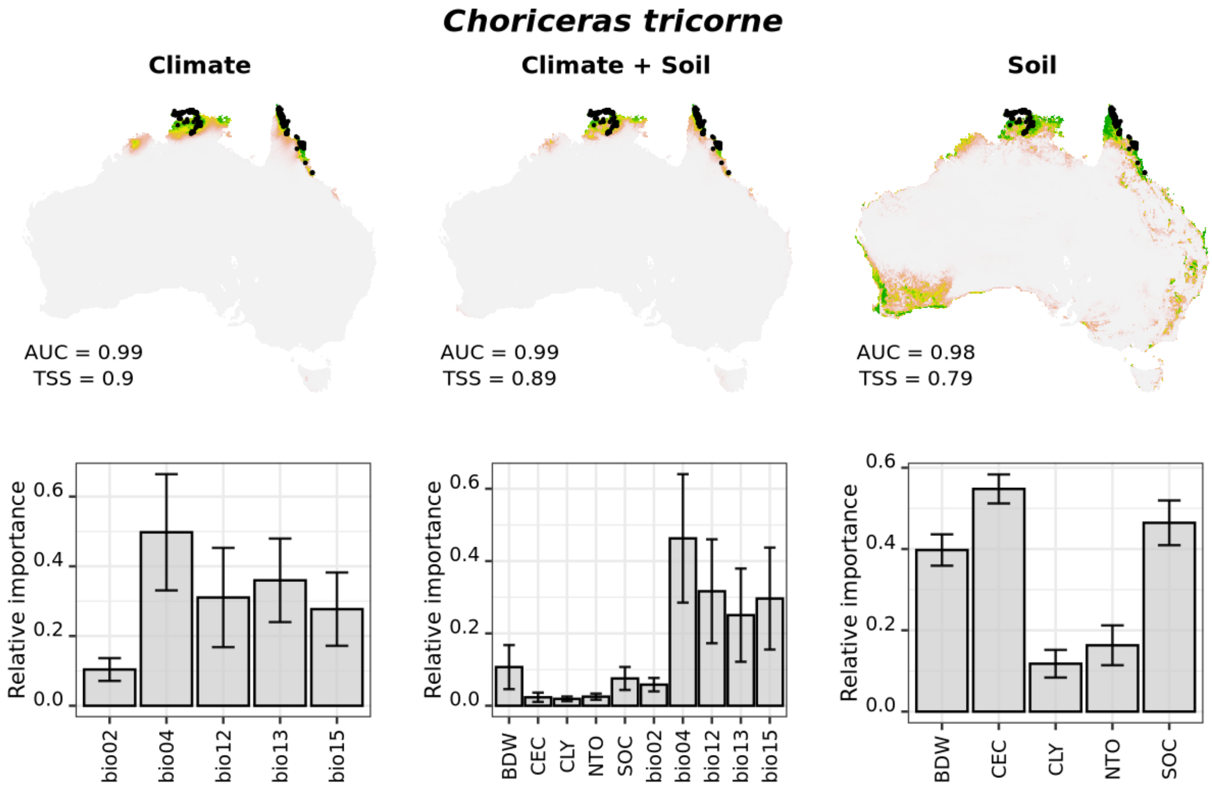
Choriceras tricorne
Climate
Climate + Soil
Soil
AUC = 0.99
TSS = 0.9
AUC = 0.99
TSS = 0.89
AUC = 0.98
TSS = 0.79
Relative importance
0.0
0.2
0.4
0.6
bio02
bio04
bio12
bio13
bio15
BDW
CEC
CLY
NTO
SOC
bio02
bio04
bio12
bio13
bio15
BDW
CEC
CLY
NTO
SOC

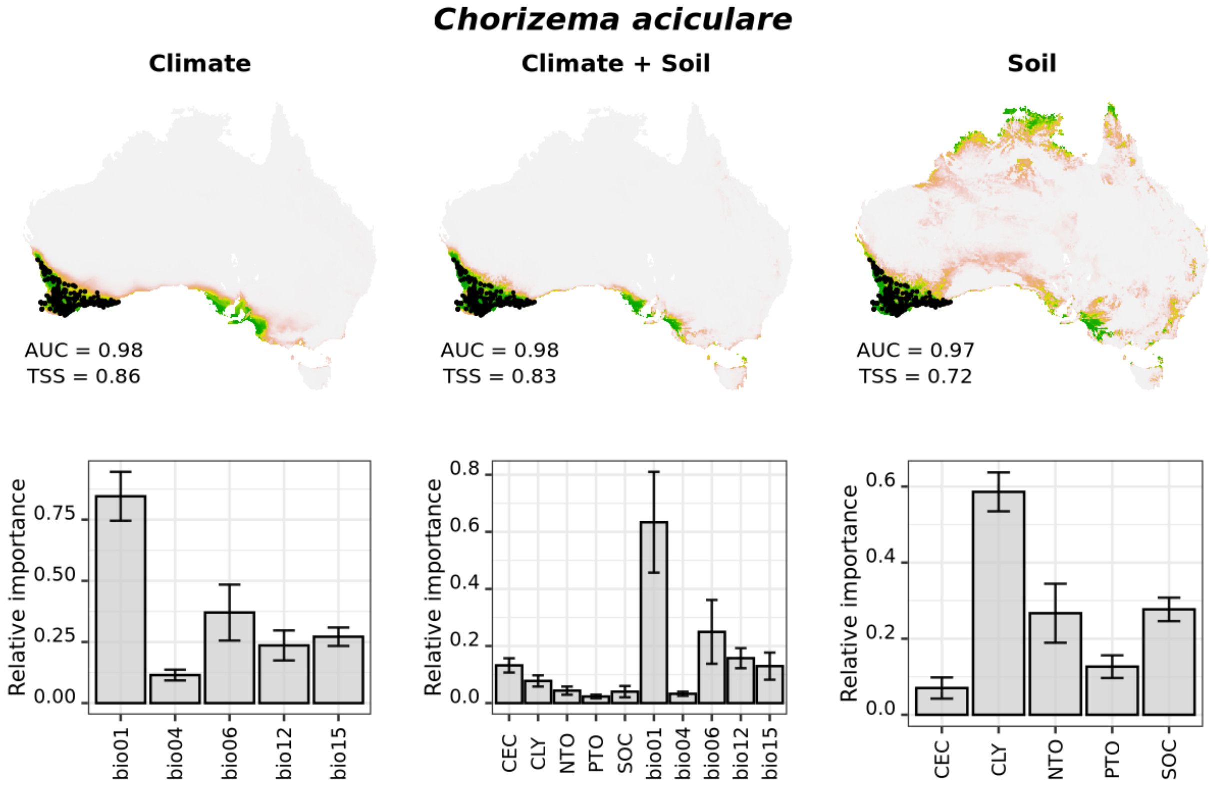
Chorizema aciculare
Climate
Climate + Soil
Soil
AUC = 0.98
TSS = 0.86
AUC = 0.98
TSS = 0.83
AUC = 0.97
TSS = 0.72
Relative importance
0.00
0.25
0.50
0.75
0.0
0.2
0.4
0.6
0.8
bio01
bio04
bio06
bio12
bio15
CEC
CLY
NTO
PTO
SOC
bio01
bio04
bio06
bio12
bio15
CEC
CLY
NTO
PTO
SOC

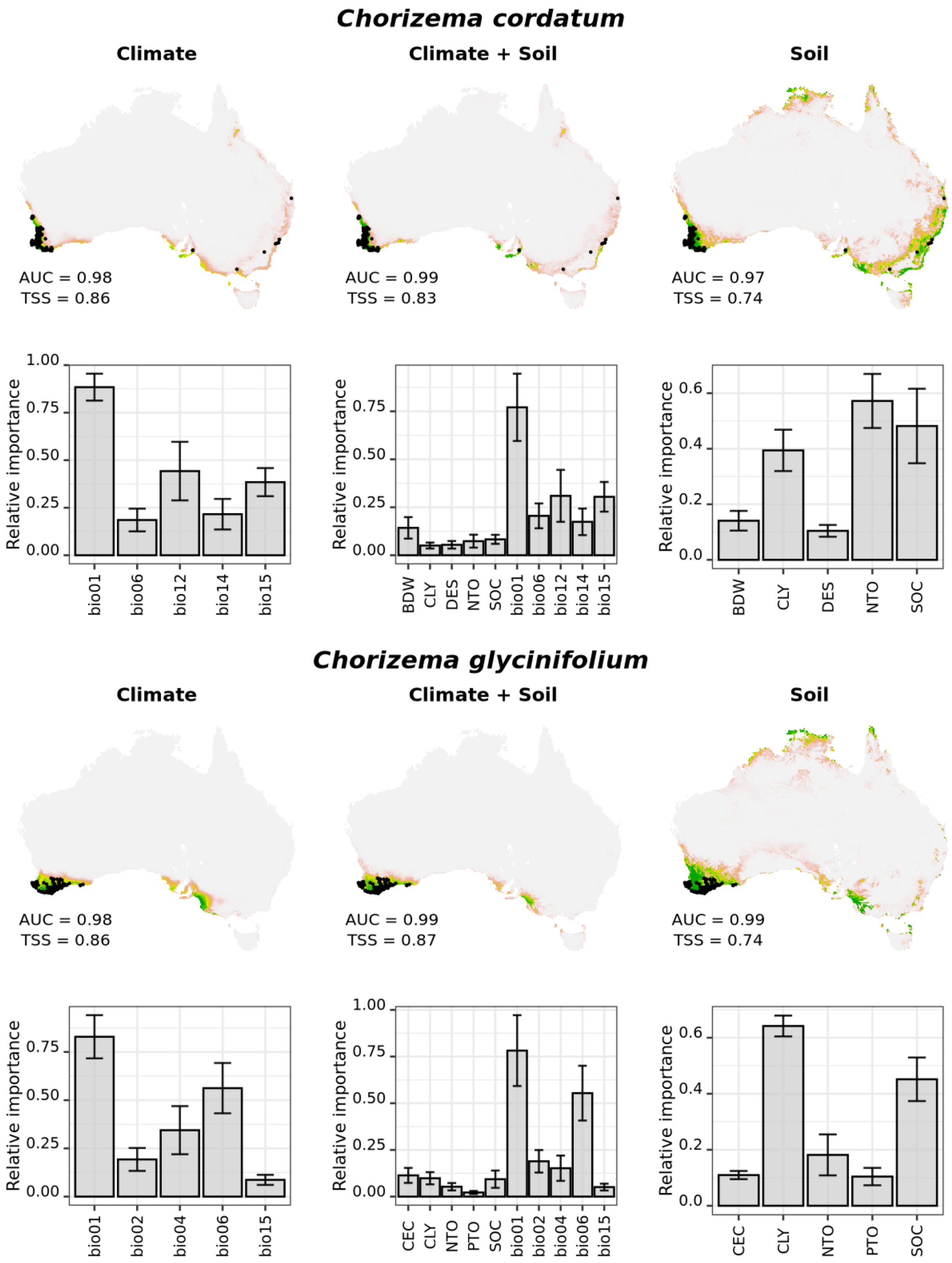

Chorizema cordatum
Climate
Climate + Soil
Soil
AUC = 0.98
TSS = 0.86
AUC = 0.99
TSS = 0.83
AUC = 0.97
TSS = 0.74
Relative importance
1.00
0.75
0.50
0.25
0.00
bio01
bio06
bio12
bio14
bio15
BDW
CLY
DES
NTO
SOC
0.6
0.4
0.2
0.0
Chorizema glycinifolium
Climate
Climate + Soil
Soil
AUC = 0.98
TSS = 0.86
AUC = 0.99
TSS = 0.87
AUC = 0.99
TSS = 0.74
bio01
bio02
bio04
bio06
bio15
CEC
CLY
NTO
PTO
SOC

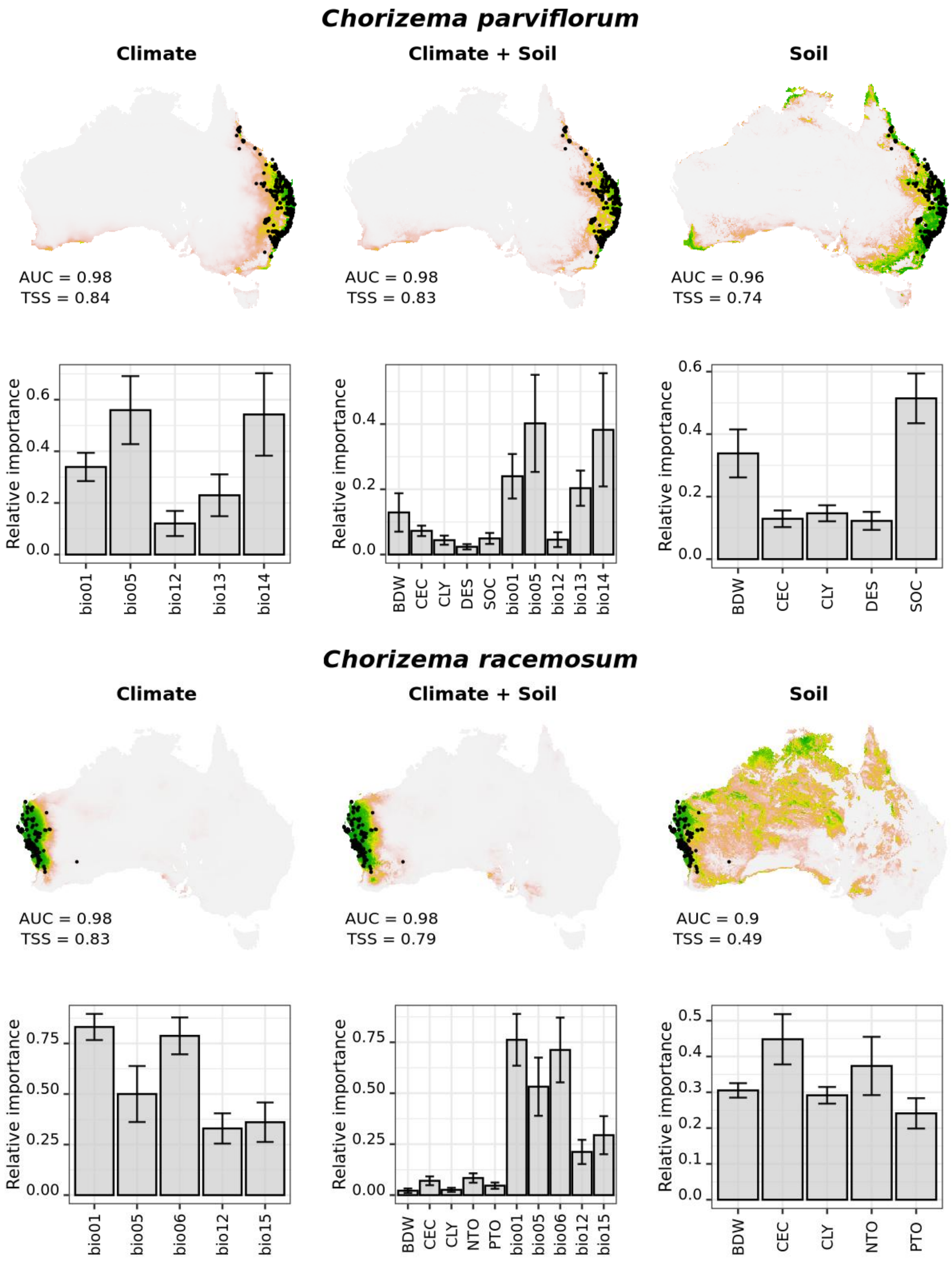
Chorizema parviflorum
Climate
Climate + Soil
Soil
AUC = 0.98
TSS = 0.84
AUC = 0.98
TSS = 0.83
AUC = 0.96
TSS = 0.74
Relative importance
bio01
bio05
bio12
bio13
bio14
BDW
CEC
CLY
DES
SOC
Chorizema racemosum
Climate
Climate + Soil
Soil
AUC = 0.98
TSS = 0.83
AUC = 0.98
TSS = 0.79
AUC = 0.9
TSS = 0.49
Relative importance
bio01
bio05
bio06
bio12
bio15
BDW
CEC
CLY
NTO
PTO

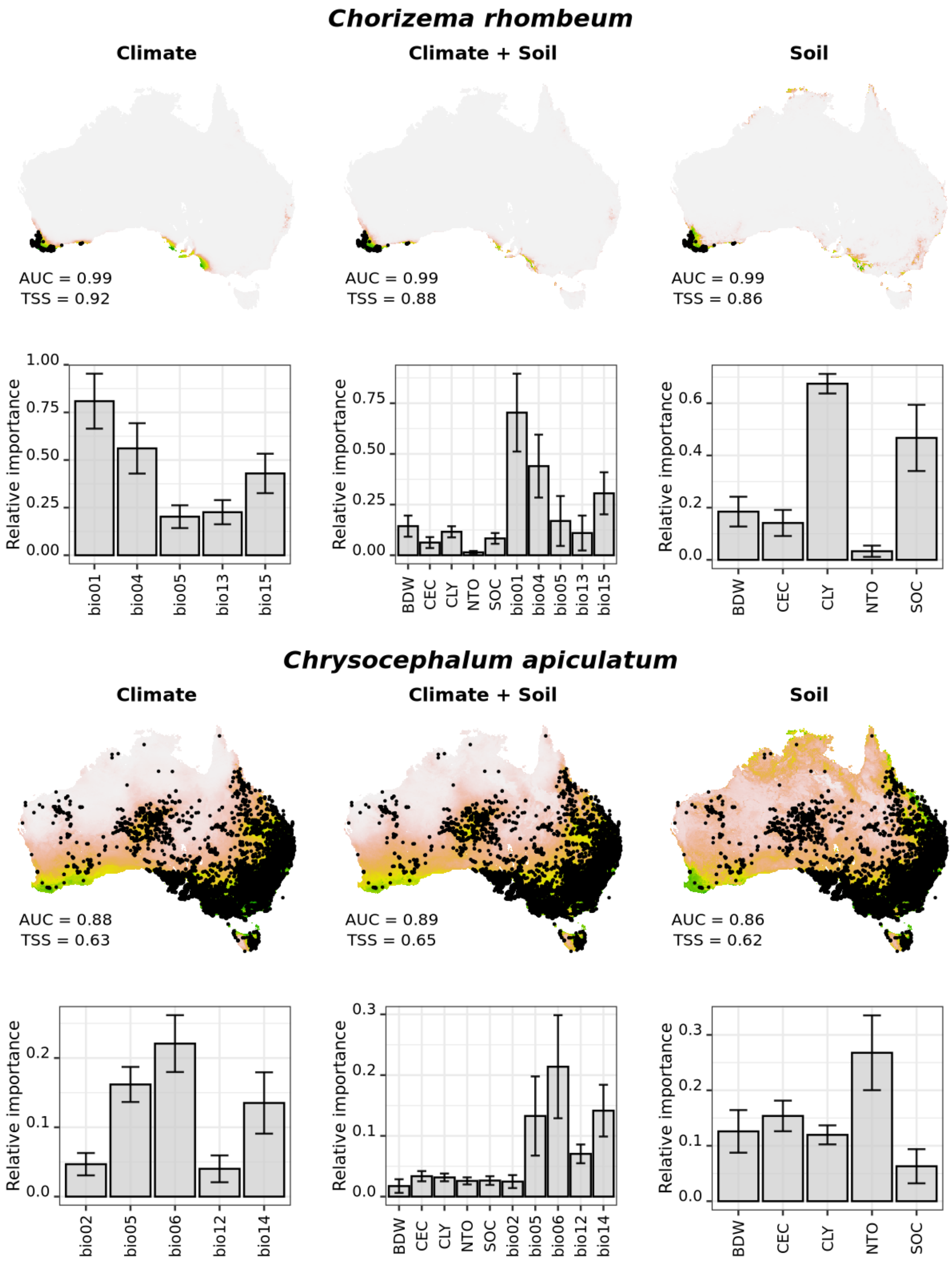

Chorizema rhombeum
Climate
Climate + Soil
Soil
AUC = 0.99
TSS = 0.92
AUC = 0.99
TSS = 0.88
AUC = 0.99
TSS = 0.86
Relative importance
bio01
bio04
bio05
bio13
bio15
BDW
CEC
CLY
NTO
SOC
Chrysocephalum apiculatum
Climate
Climate + Soil
Soil
AUC = 0.88
TSS = 0.63
AUC = 0.89
TSS = 0.65
AUC = 0.86
TSS = 0.62
Relative importance
bio02
bio05
bio06
bio12
bio14
BDW
CEC
CLY
NTO
SOC

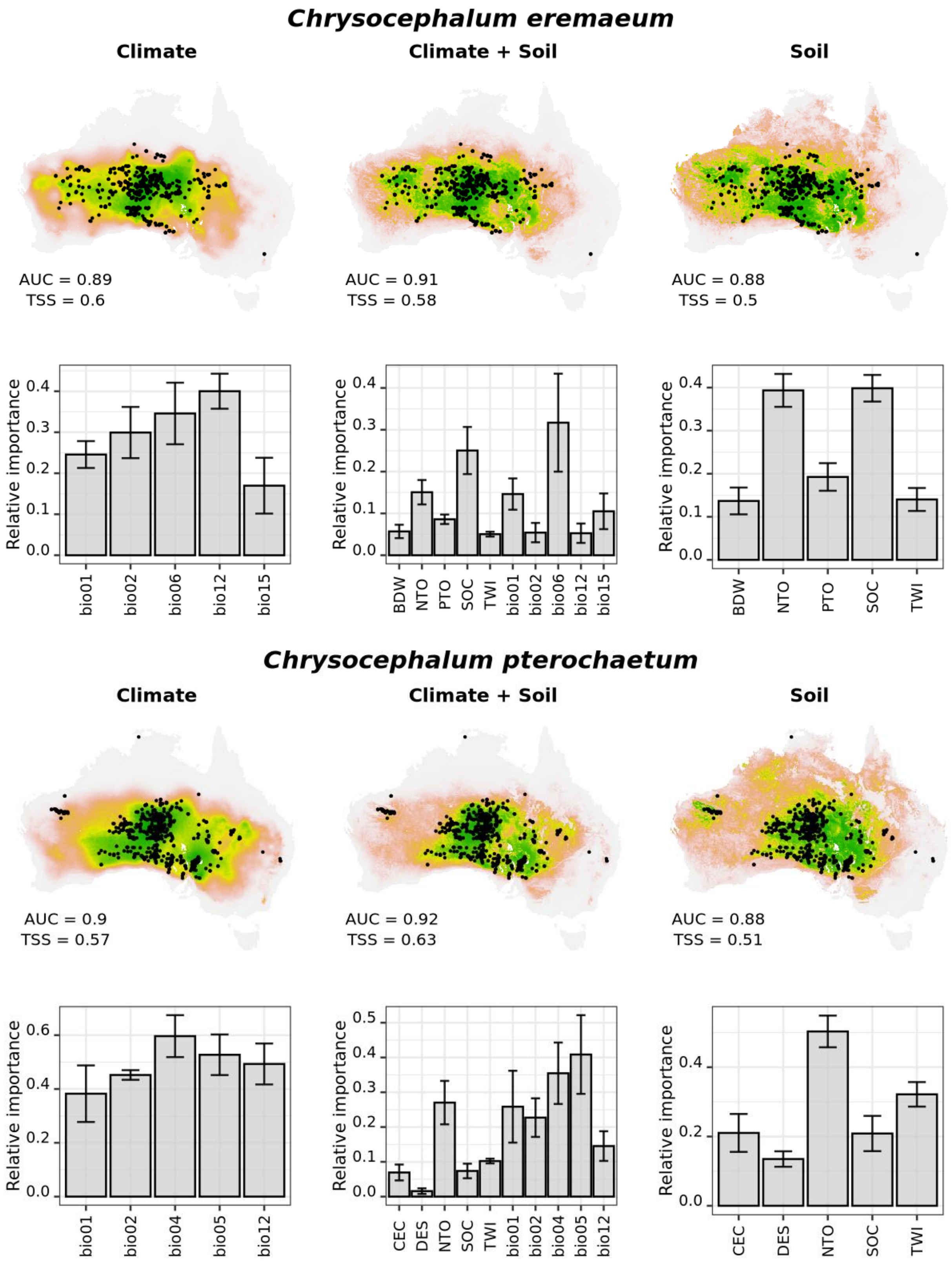
Chrysocephalum eremaeum
Climate
Climate + Soil
Soil
AUC = 0.89
TSS = 0.6
AUC = 0.91
TSS = 0.58
AUC = 0.88
TSS = 0.5
Relative importance
0.0
0.1
0.2
0.3
0.4
bio01
bio02
bio06
bio12
bio15
BDW
NTO
PTO
SOC
TWI
bio01
bio02
bio06
bio12
bio15
BDW
NTO
PTO
SOC
TWI
Chrysocephalum pterochaetum
Climate
Climate + Soil
Soil
AUC = 0.9
TSS = 0.57
AUC = 0.92
TSS = 0.63
AUC = 0.88
TSS = 0.51
Relative importance
0.0
0.2
0.4
0.6
0.0
0.1
0.2
0.3
0.4
0.5
bio01
bio02
bio04
bio05
bio12
CEC
DES
NTO
SOC
TWI
bio01
bio02
bio04
bio05
bio12
CEC
DES
NTO
SOC
TWI

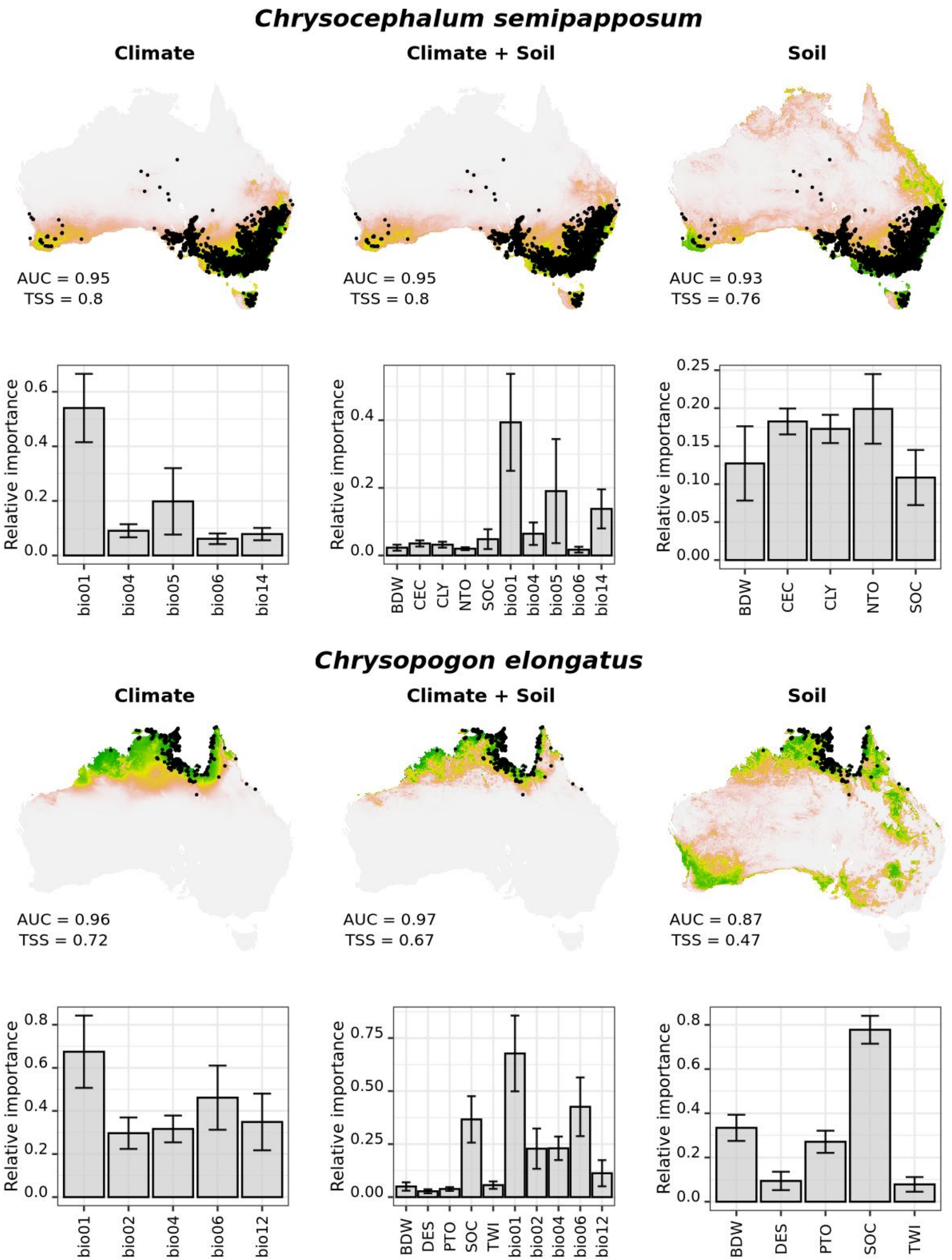
Chrysocephalum semipapposum
Climate
Climate + Soil
Soil
AUC = 0.95
TSS = 0.8
AUC = 0.95
TSS = 0.8
AUC = 0.93
TSS = 0.76
Relative importance
bio01
bio04
bio05
bio06
bio14
BDW
CEC
CLY
NTO
SOC
Chrysopogon elongatus
Climate
Climate + Soil
Soil
AUC = 0.96
TSS = 0.72
AUC = 0.97
TSS = 0.67
AUC = 0.87
TSS = 0.47
Relative importance
bio01
bio02
bio04
bio06
bio12
BDW
DES
PTO
SOC
TWI

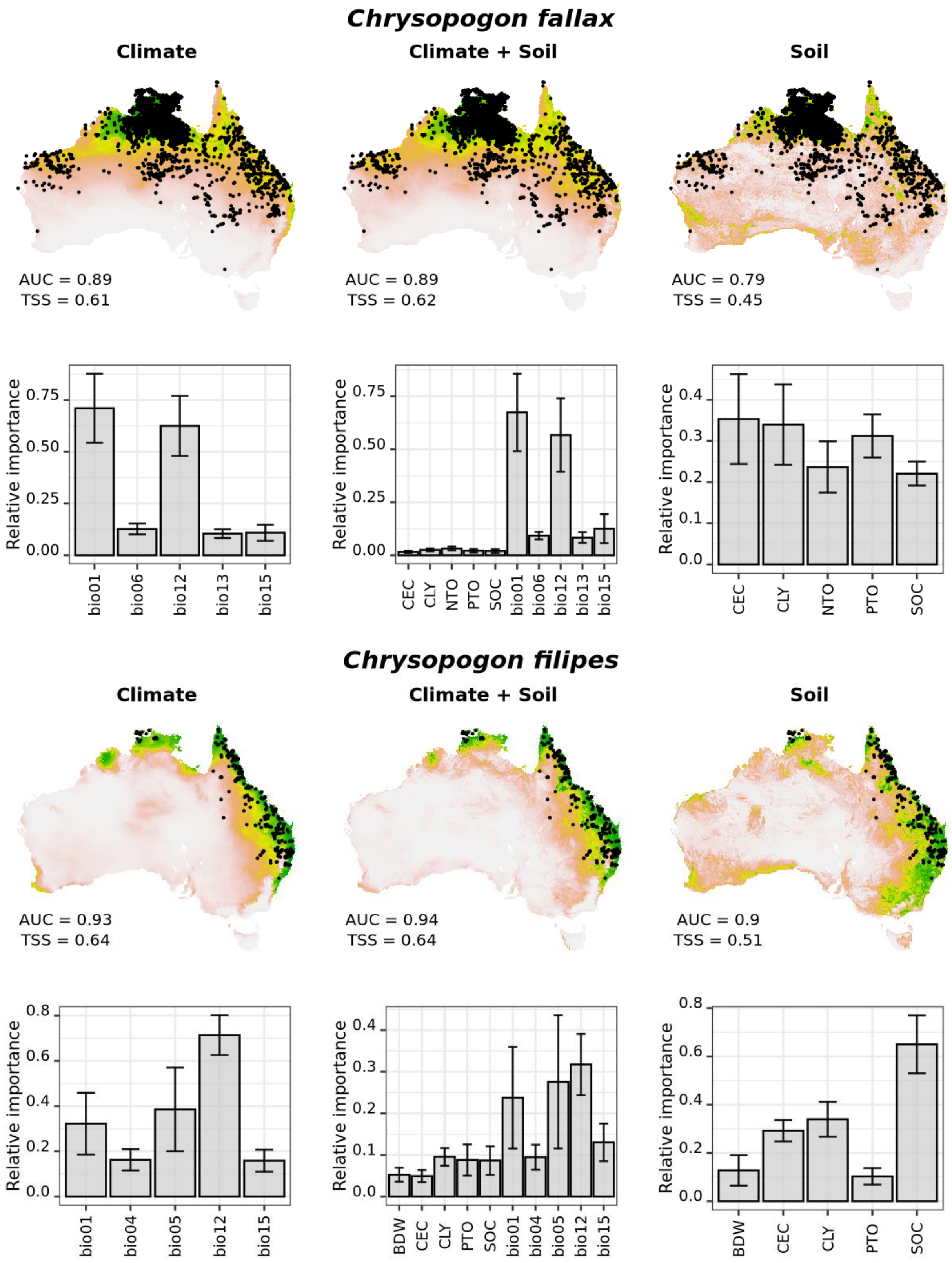
Chrysopogon fallax
Climate
Climate + Soil
Soil
AUC = 0.89
TSS = 0.61
AUC = 0.89
TSS = 0.62
AUC = 0.79
TSS = 0.45
Relative importance
bio01
bio06
bio12
bio13
bio15
CEC
CLY
NTO
PTO
SOC
Chrysopogon filipes
Climate
Climate + Soil
Soil
AUC = 0.93
TSS = 0.64
AUC = 0.94
TSS = 0.64
AUC = 0.9
TSS = 0.51
Relative importance
bio01
bio04
bio05
bio12
bio15
BDW
CEC
CLY
PTO
SOC

Chrysopogon latifolius

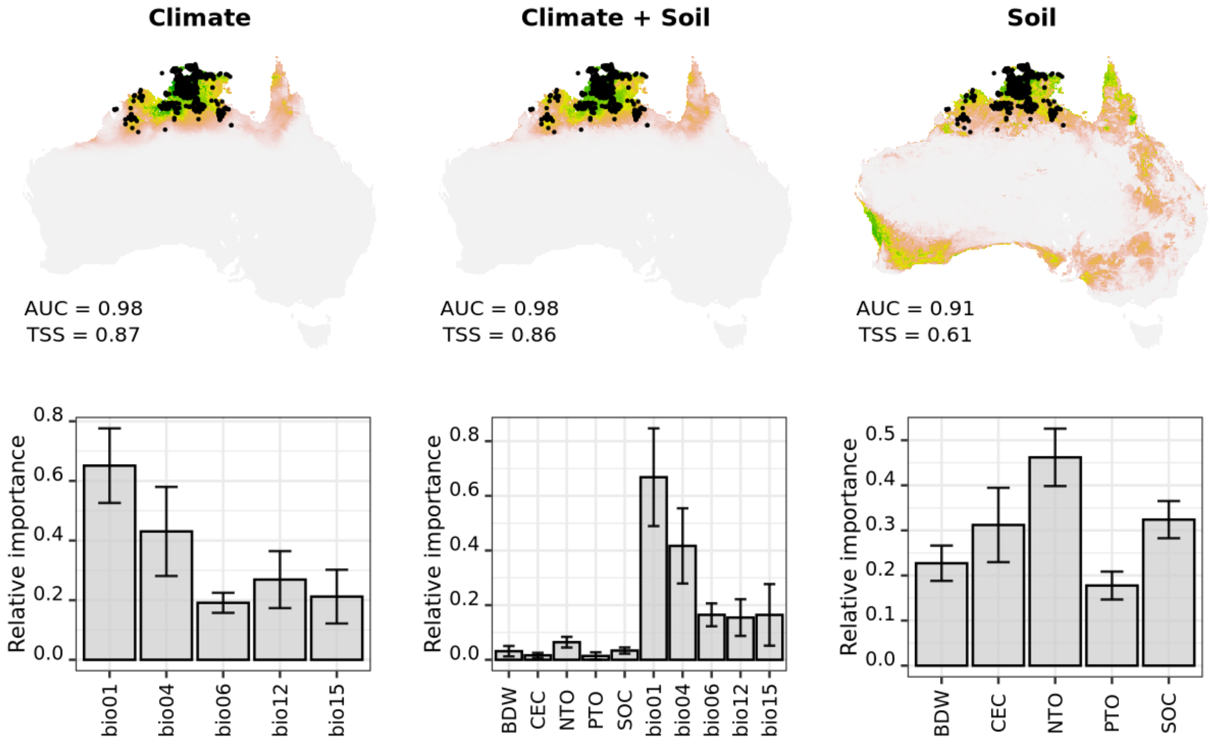

Chrysopogon oliganthus

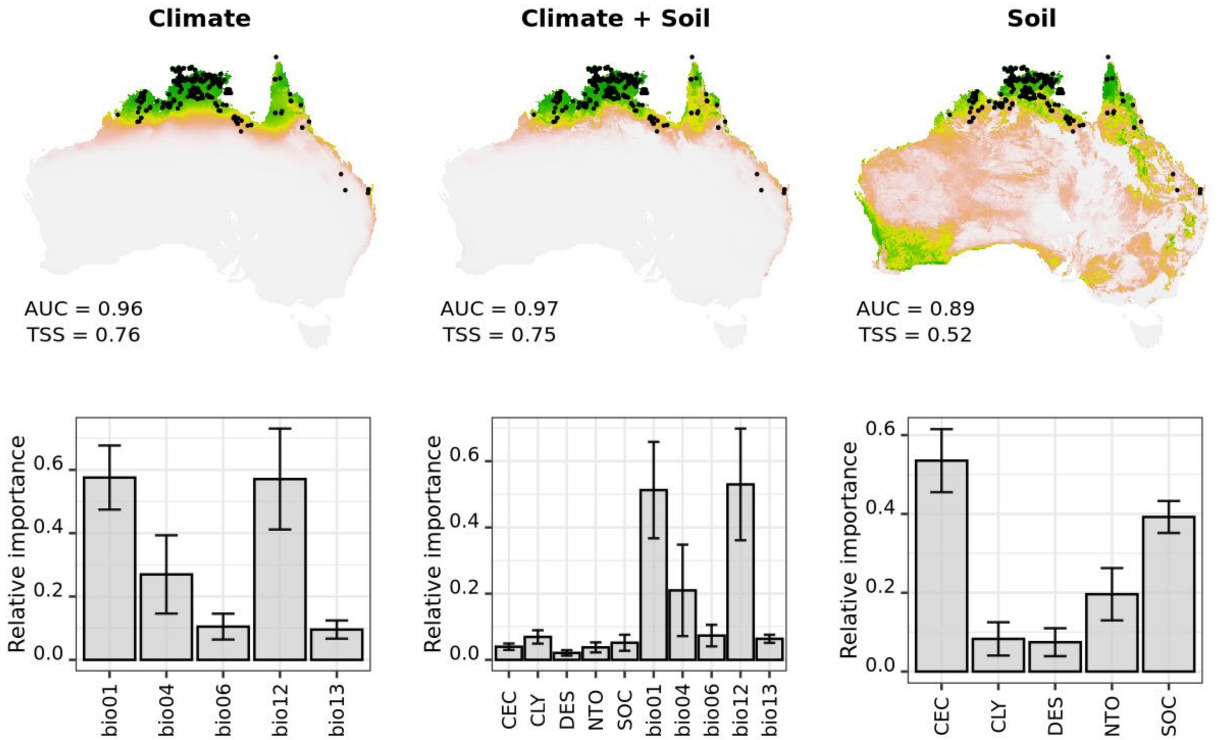

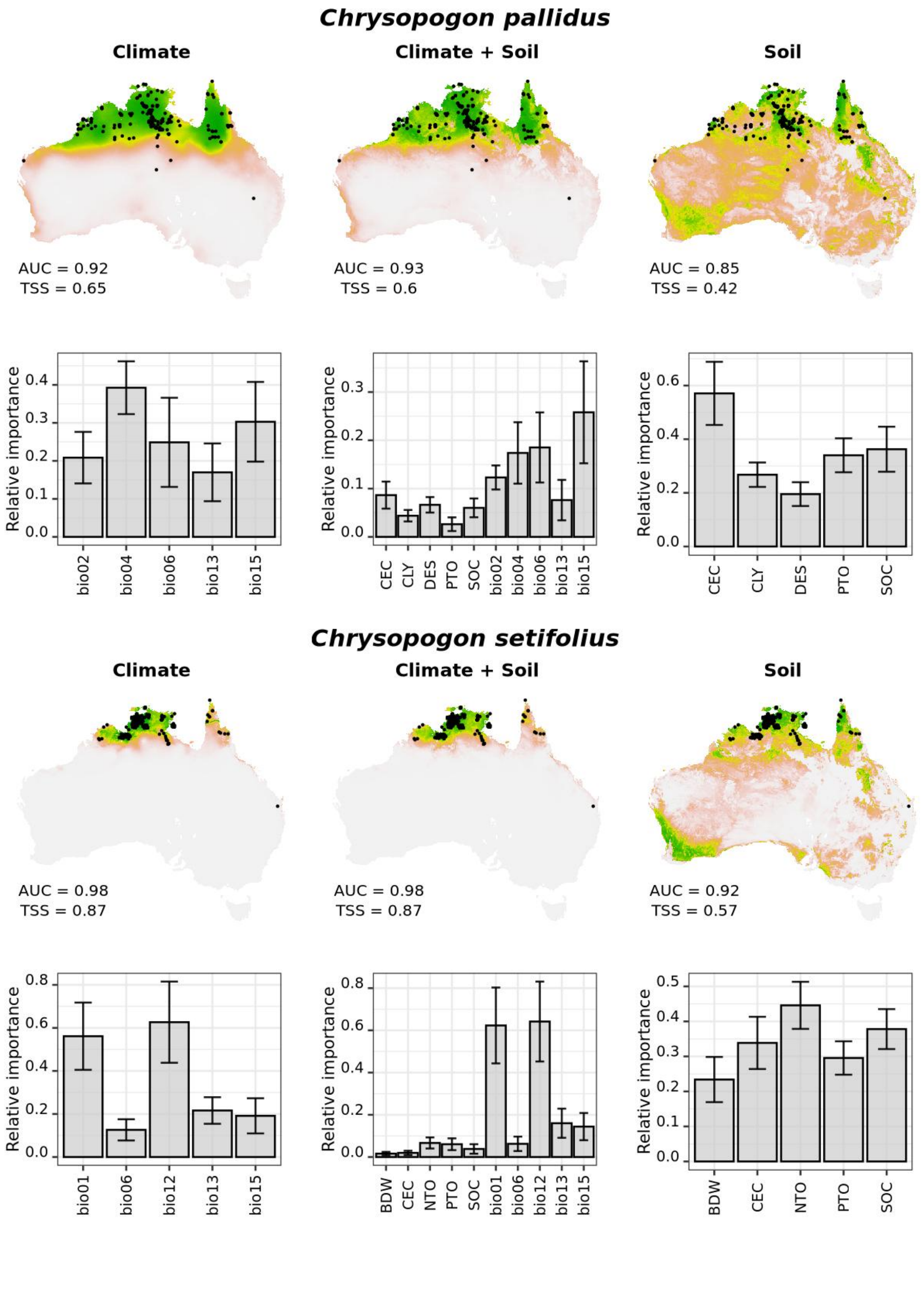
Chrysopogon pallidus
Climate
Climate + Soil
Soil
AUC = 0.92
TSS = 0.65
AUC = 0.93
TSS = 0.6
AUC = 0.85
TSS = 0.42
Relative importance
bio02
bio04
bio06
bio13
bio15
CEC
CLY
DES
PTO
SOC
Chrysopogon setifolius
Climate
Climate + Soil
Soil
AUC = 0.98
TSS = 0.87
AUC = 0.98
TSS = 0.87
AUC = 0.92
TSS = 0.57
Relative importance
bio01
bio06
bio12
bio13
bio15
BDW
CEC
NTO
PTO
SOC

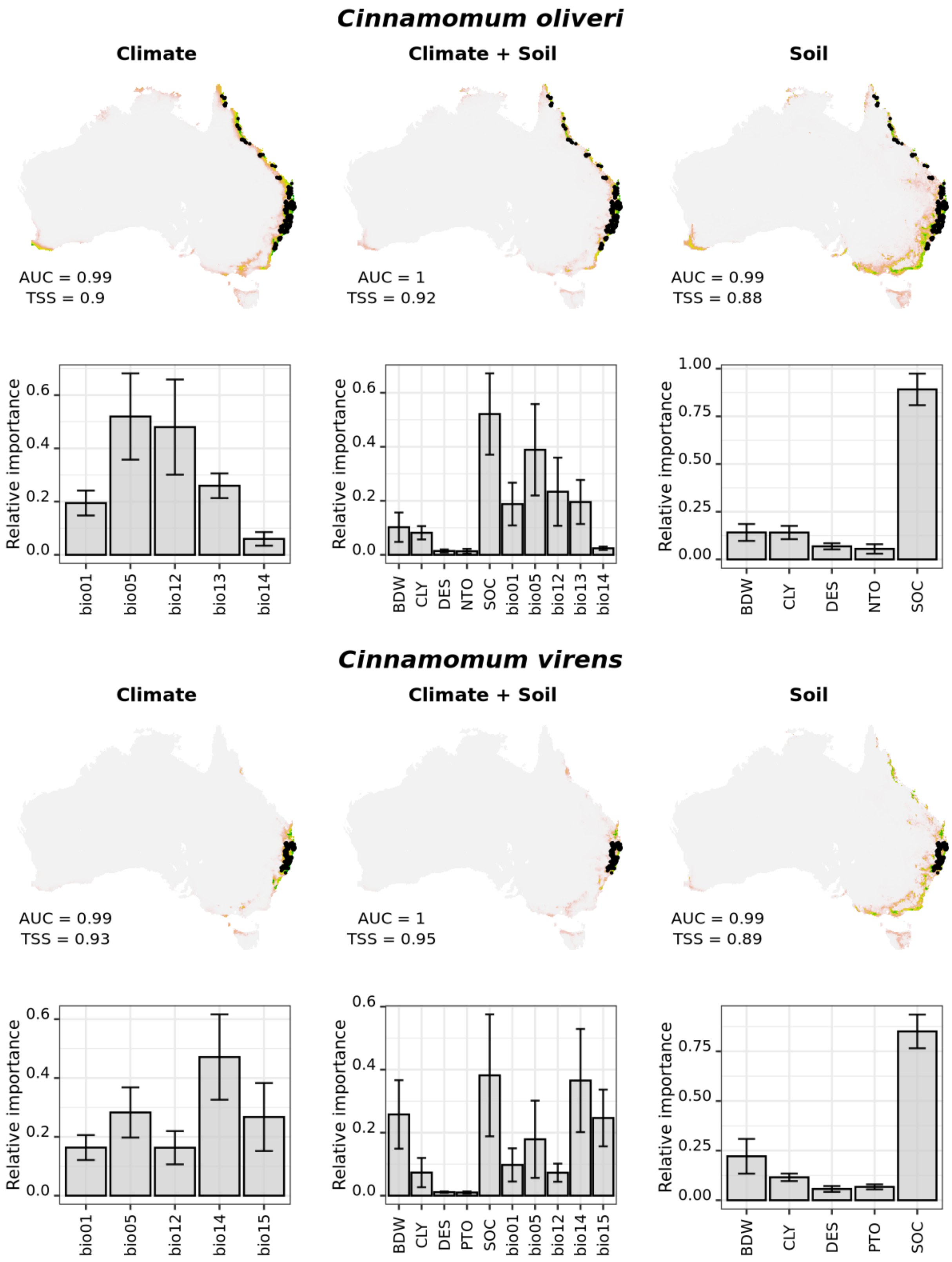

Cinnamomum oliveri
Climate
Climate + Soil
Soil
AUC = 0.99
TSS = 0.9
AUC = 1
TSS = 0.92
AUC = 0.99
TSS = 0.88
Relative importance
bio01
bio05
bio12
bio13
bio14
BDW
CLY
DES
NTO
SOC
Cinnamomum virens
Climate
Climate + Soil
Soil
AUC = 0.99
TSS = 0.93
AUC = 1
TSS = 0.95
AUC = 0.99
TSS = 0.89
Relative importance
bio01
bio05
bio12
bio14
bio15
BDW
CLY
DES
PTO
SOC

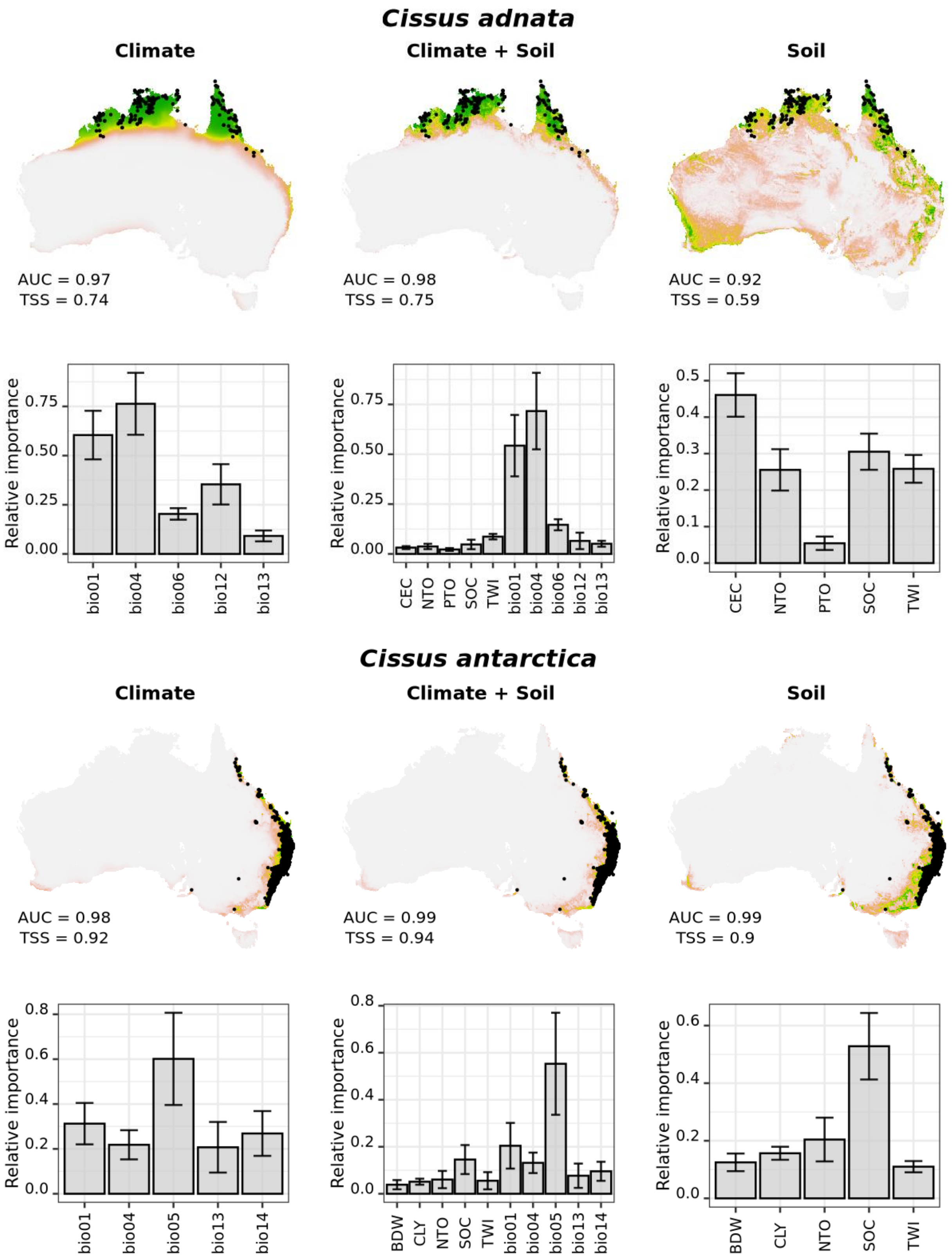

Cissus adnata
Climate
Climate + Soil
Soil
AUC = 0.97
TSS = 0.74
AUC = 0.98
TSS = 0.75
AUC = 0.92
TSS = 0.59
Relative importance
bio01 bio04 bio06 bio12 bio13
CEC NTO PTO SOC TWI bio01 bio04 bio06 bio12 bio13
CEC NTO PTO SOC TWI
Cissus antarctica
Climate
Climate + Soil
Soil
AUC = 0.98
TSS = 0.92
AUC = 0.99
TSS = 0.94
AUC = 0.99
TSS = 0.9
Relative importance
bio01 bio04 bio05 bio13 bio14
BDW CLY NTO SOC TWI bio01 bio04 bio05 bio13 bio14
BDW CLY NTO SOC TWI

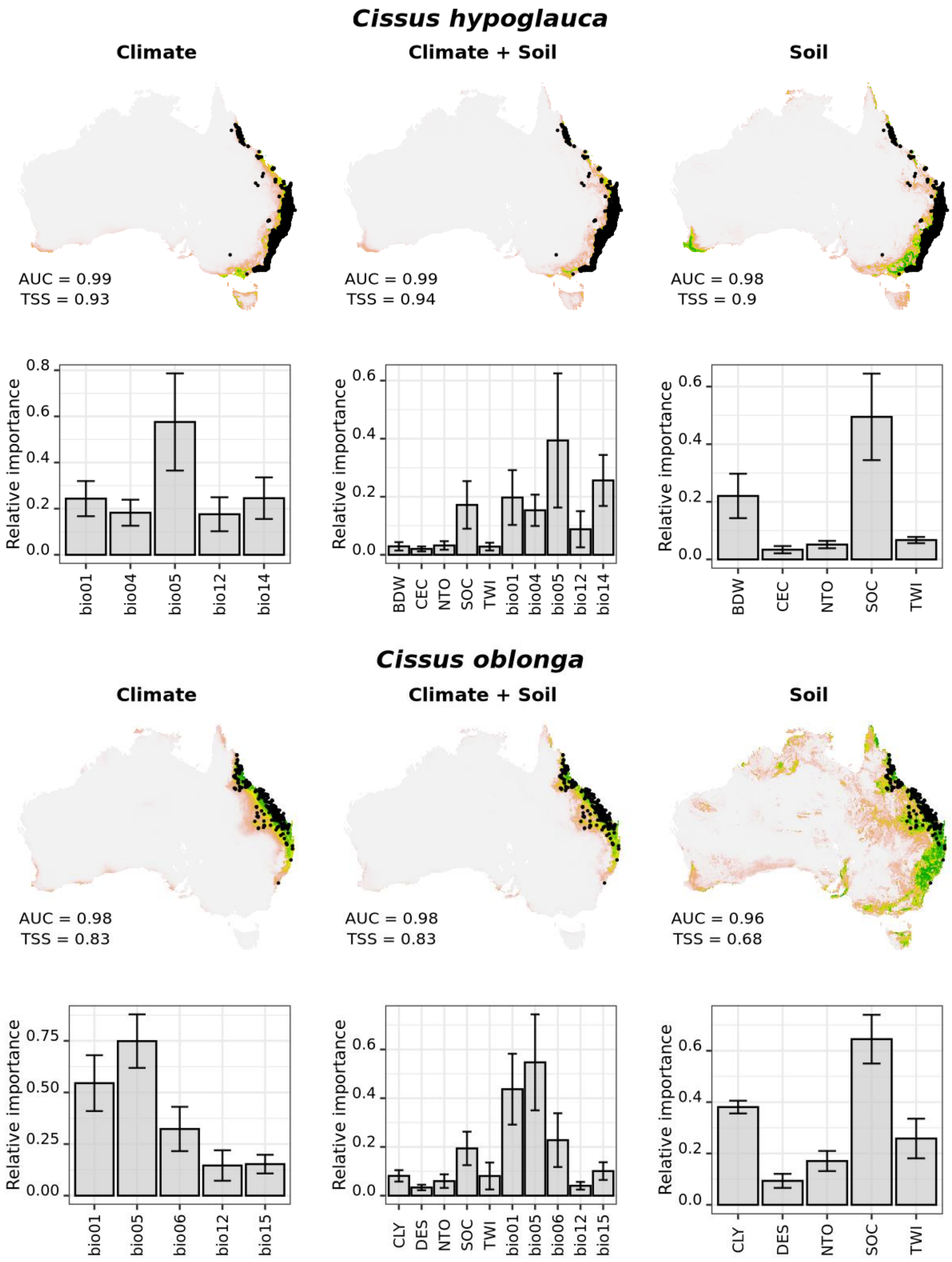
Cissus hypoglauca
Climate
Climate + Soil
Soil
AUC = 0.99
TSS = 0.93
AUC = 0.99
TSS = 0.94
AUC = 0.98
TSS = 0.9
Relative importance
bio01
bio04
bio05
bio12
bio14
BDW
CEC
NTO
SOC
TWI
Cissus oblonga
Climate
Climate + Soil
Soil
AUC = 0.98
TSS = 0.83
AUC = 0.98
TSS = 0.83
AUC = 0.96
TSS = 0.68
Relative importance
bio01
bio05
bio06
bio12
bio15
CLY
DES
NTO
SOC
TWI

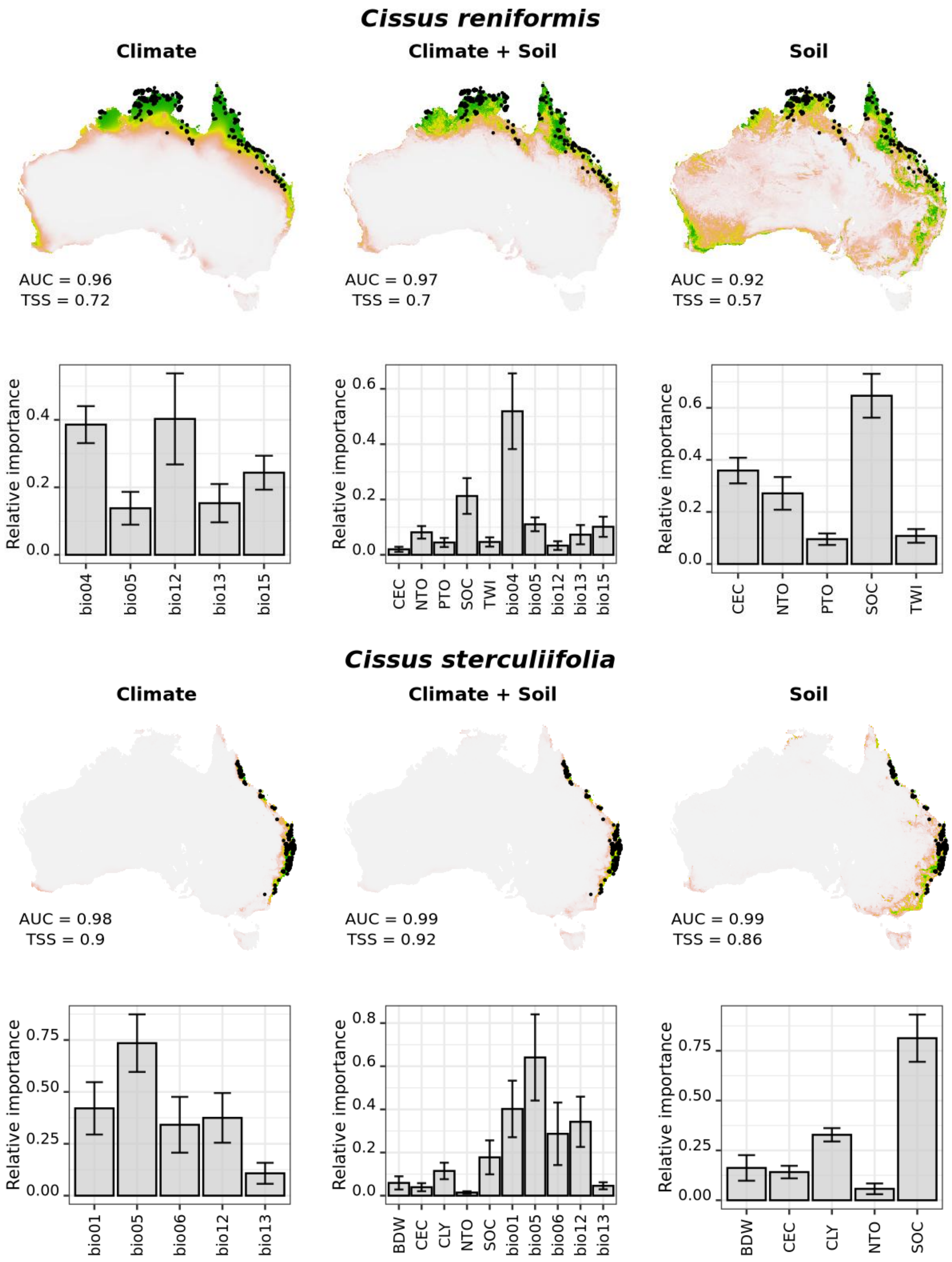

Cissus reniformis
Climate
Climate + Soil
Soil
AUC = 0.96
TSS = 0.72
AUC = 0.97
TSS = 0.7
AUC = 0.92
TSS = 0.57
Relative importance
bio04
bio05
bio12
bio13
bio15
CEC
NTO
PTO
SOC
TWI
Cissus sterculiifolia
Climate
Climate + Soil
Soil
AUC = 0.98
TSS = 0.9
AUC = 0.99
TSS = 0.92
AUC = 0.99
TSS = 0.86
Relative importance
bio01
bio05
bio06
bio12
bio13
BDW
CEC
CLY
NTO
SOC

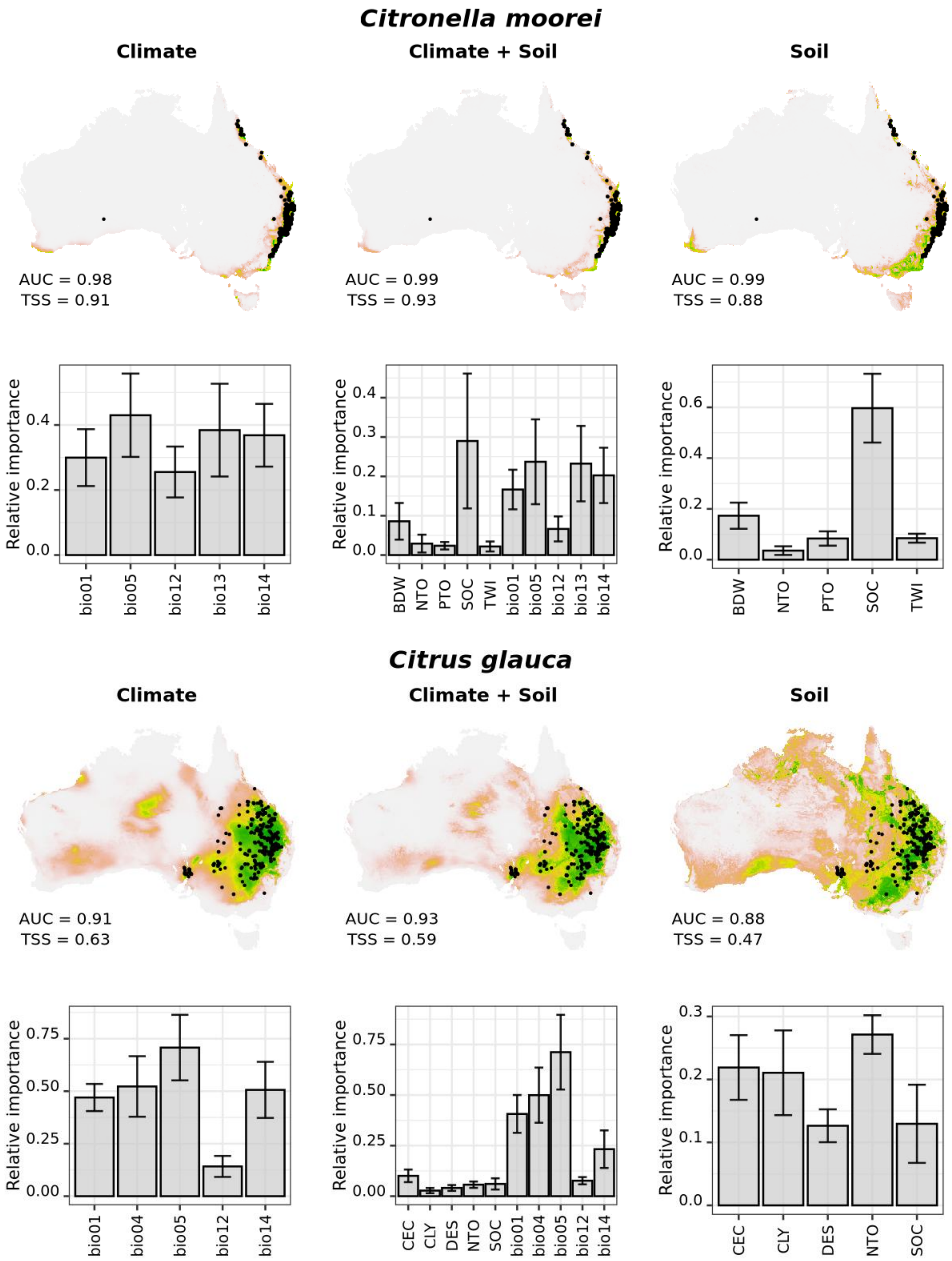
Citronella moorei
Climate
Climate + Soil
Soil
AUC = 0.98
TSS = 0.91
AUC = 0.99
TSS = 0.93
AUC = 0.99
TSS = 0.88
Relative importance
0.0
0.2
0.4
bio01
bio05
bio12
bio13
bio14
0.0
0.1
0.2
0.3
0.4
BDW
NTO
PTO
SOC
TWI
bio01
bio05
bio12
bio13
bio14
0.0
0.2
0.4
0.6
BDW
NTO
PTO
SOC
TWI
Citrus glauca
Climate
Climate + Soil
Soil
AUC = 0.91
TSS = 0.63
AUC = 0.93
TSS = 0.59
AUC = 0.88
TSS = 0.47
Relative importance
0.00
0.25
0.50
0.75
bio01
bio04
bio05
bio12
bio14
CEC
CLY
DES
NTO
SOC
bio01
bio04
bio05
bio12
bio14
0.0
0.1
0.2
0.3
CEC
CLY
DES
NTO
SOC

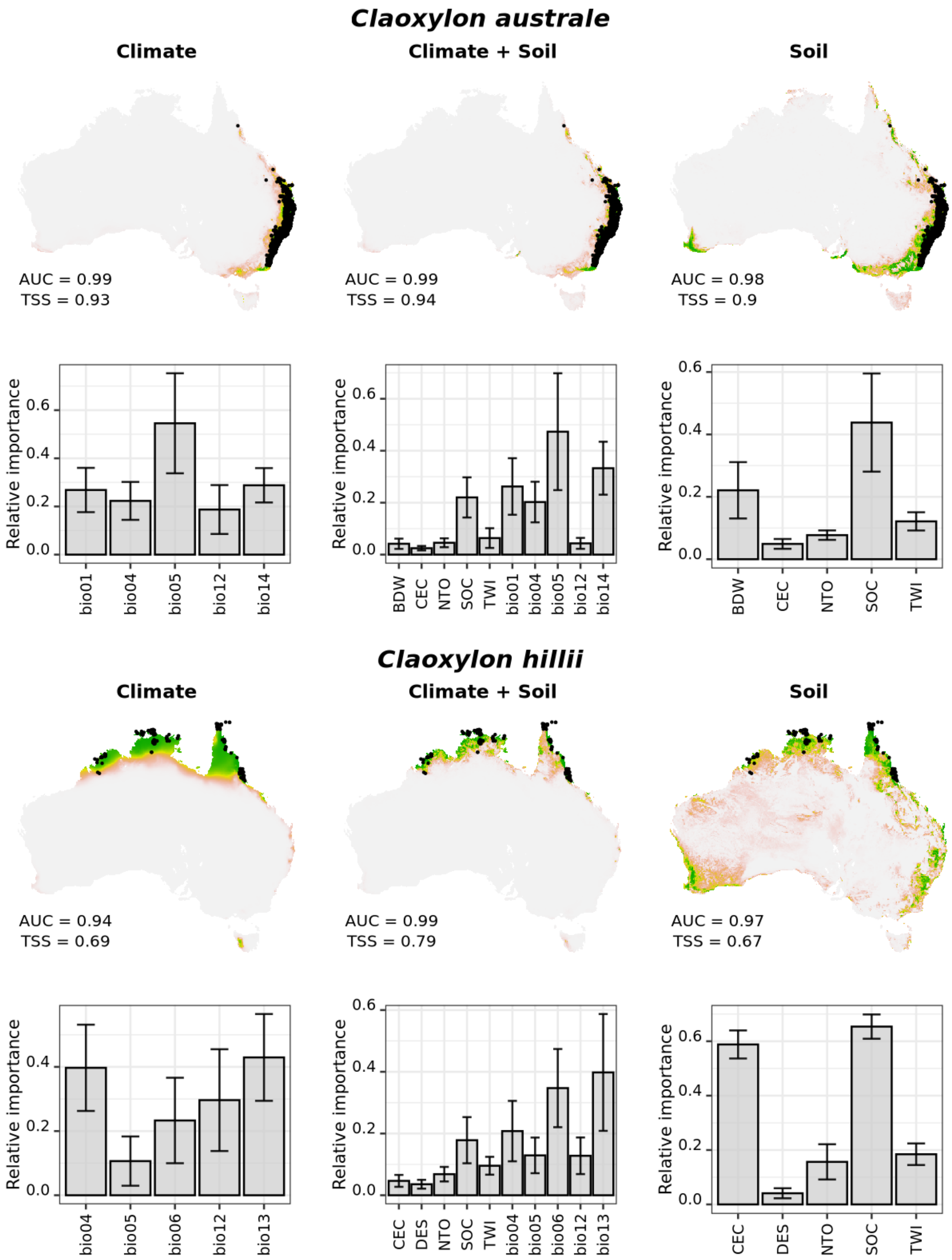

Claoxylon australe
Climate
Climate + Soil
Soil
AUC = 0.99
TSS = 0.93
AUC = 0.99
TSS = 0.94
AUC = 0.98
TSS = 0.9
Relative importance
0.0
0.2
0.4
0.6
bio01
bio04
bio05
bio12
bio14
BDW
CEC
NTO
SOC
TWI
Claoxylon hillii
Climate
Climate + Soil
Soil
AUC = 0.94
TSS = 0.69
AUC = 0.99
TSS = 0.79
AUC = 0.97
TSS = 0.67
Relative importance
bio04
bio05
bio06
bio12
bio13
CEC
DES
NTO
SOC
TWI

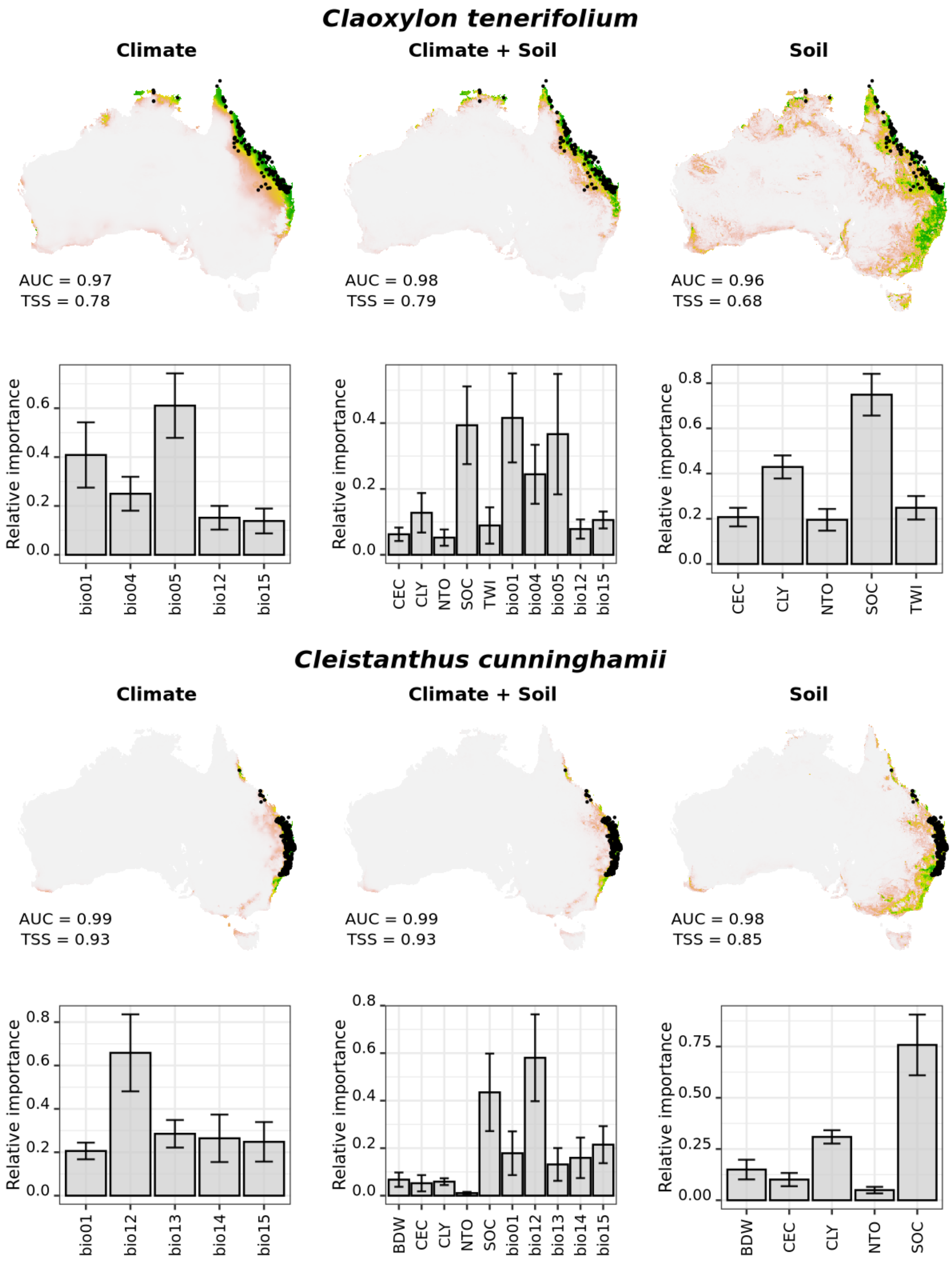
Claoxylon tenerifolium
Climate
Climate + Soil
Soil
AUC = 0.97
TSS = 0.78
AUC = 0.98
TSS = 0.79
AUC = 0.96
TSS = 0.68
Relative importance
bio01
bio04
bio05
bio12
bio15
CEC
CLY
NTO
SOC
TWI
Cleistanthus cunninghamii
AUC = 0.99
TSS = 0.93
AUC = 0.99
TSS = 0.93
AUC = 0.98
TSS = 0.85
bio13
bio14
BDW

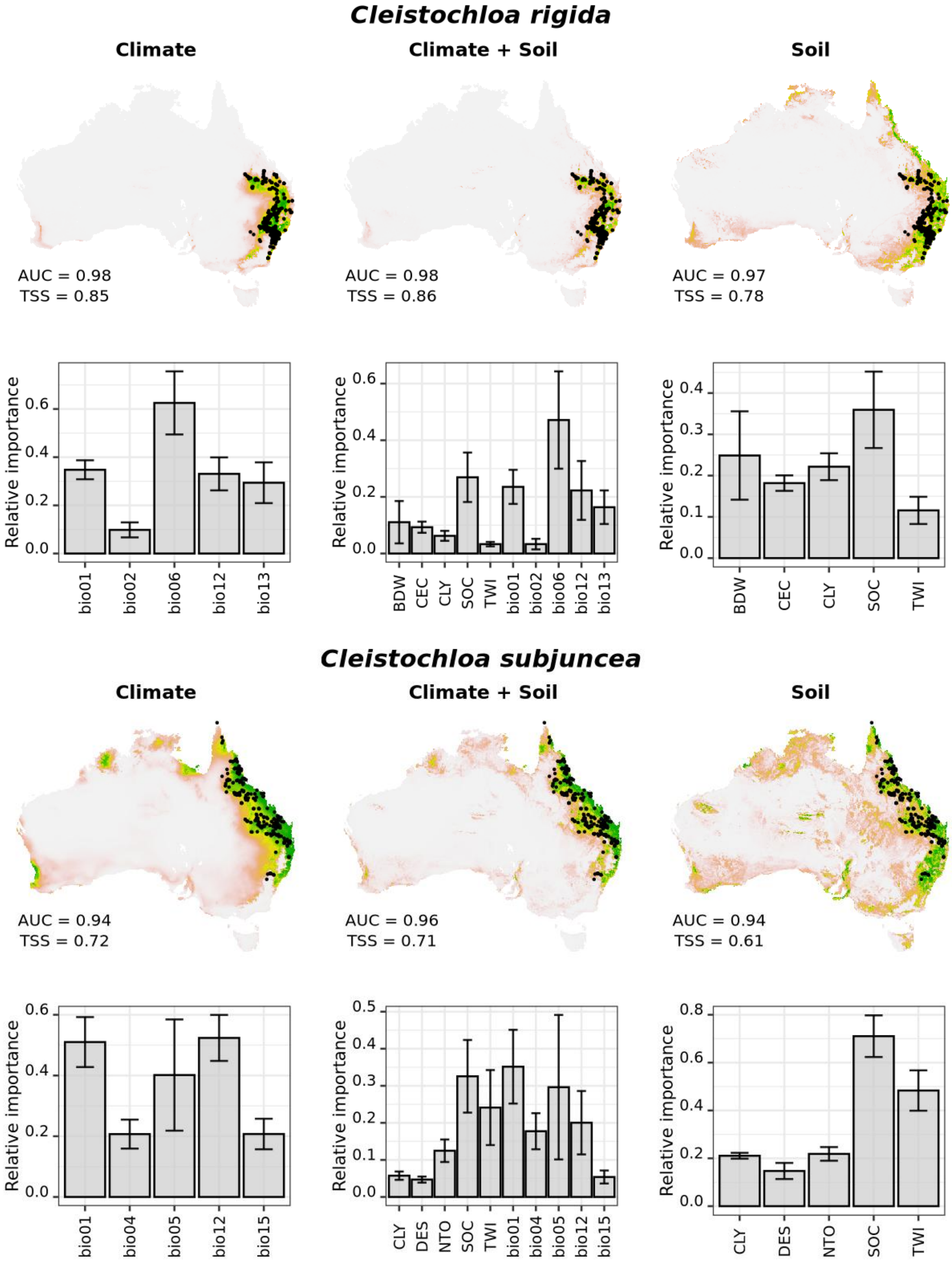

Cleistochloa rigida
Climate
Climate + Soil
Soil
AUC = 0.98
TSS = 0.85
AUC = 0.98
TSS = 0.86
AUC = 0.97
TSS = 0.78
Relative importance
bio01 bio02 bio06 bio12 bio13
BDW CEC CLY SOC TWI bio01 bio02 bio06 bio12 bio13
BDW CEC CLY SOC TWI
Cleistochloa subjuncea
Climate
Climate + Soil
Soil
AUC = 0.94
TSS = 0.72
AUC = 0.96
TSS = 0.71
AUC = 0.94
TSS = 0.61
Relative importance
bio01 bio04 bio05 bio12 bio15
CLY DES NTO SOC TWI bio01 bio04 bio05 bio12 bio15
CLY DES NTO SOC TWI

Clematicissus opaca

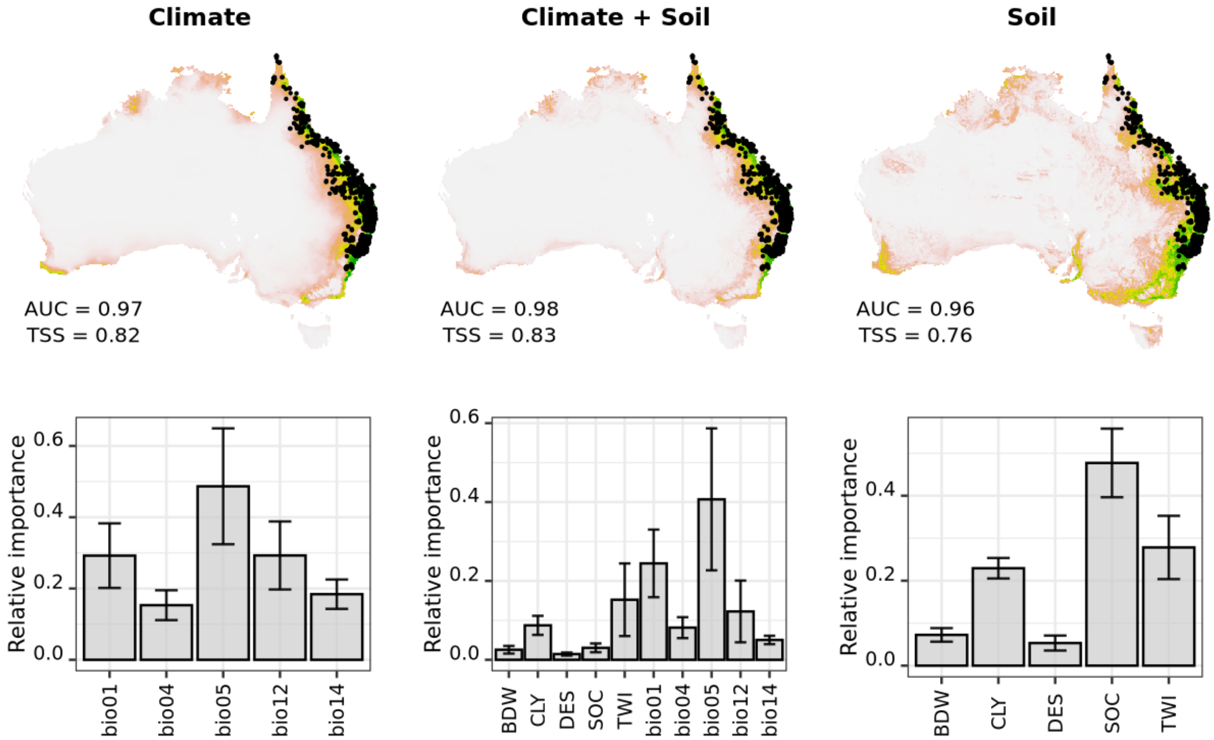

Clematis aristata

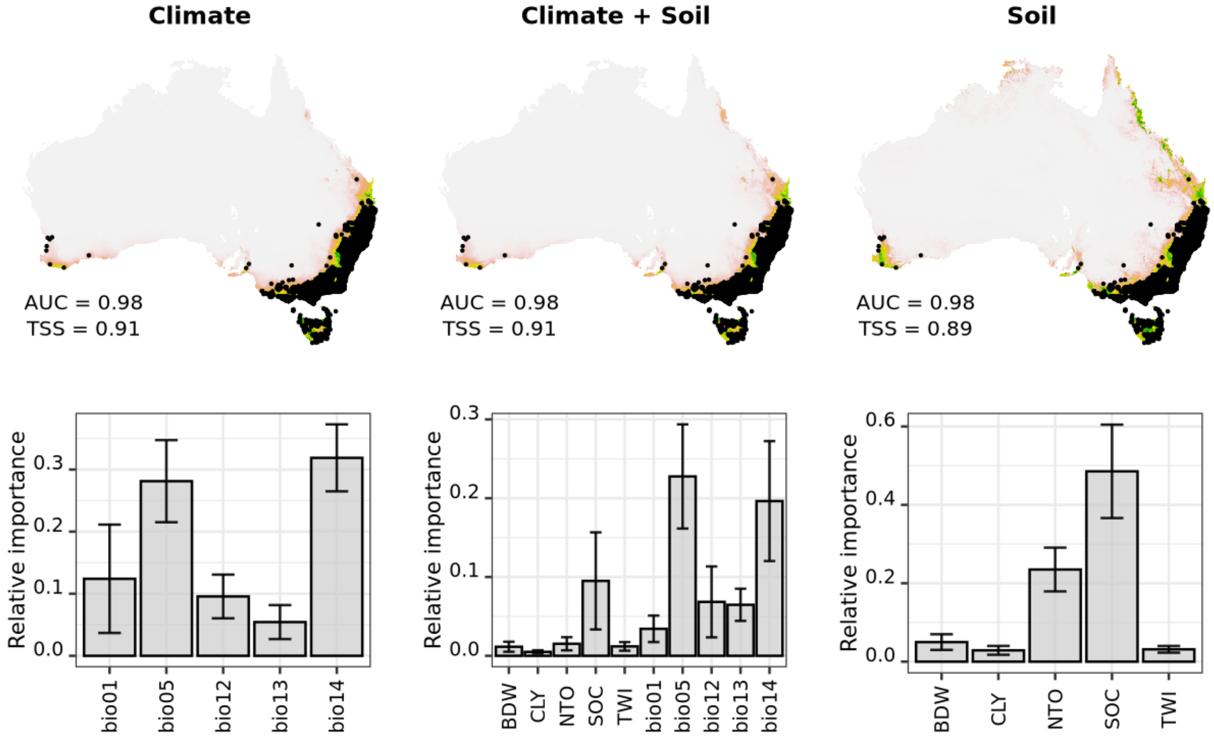

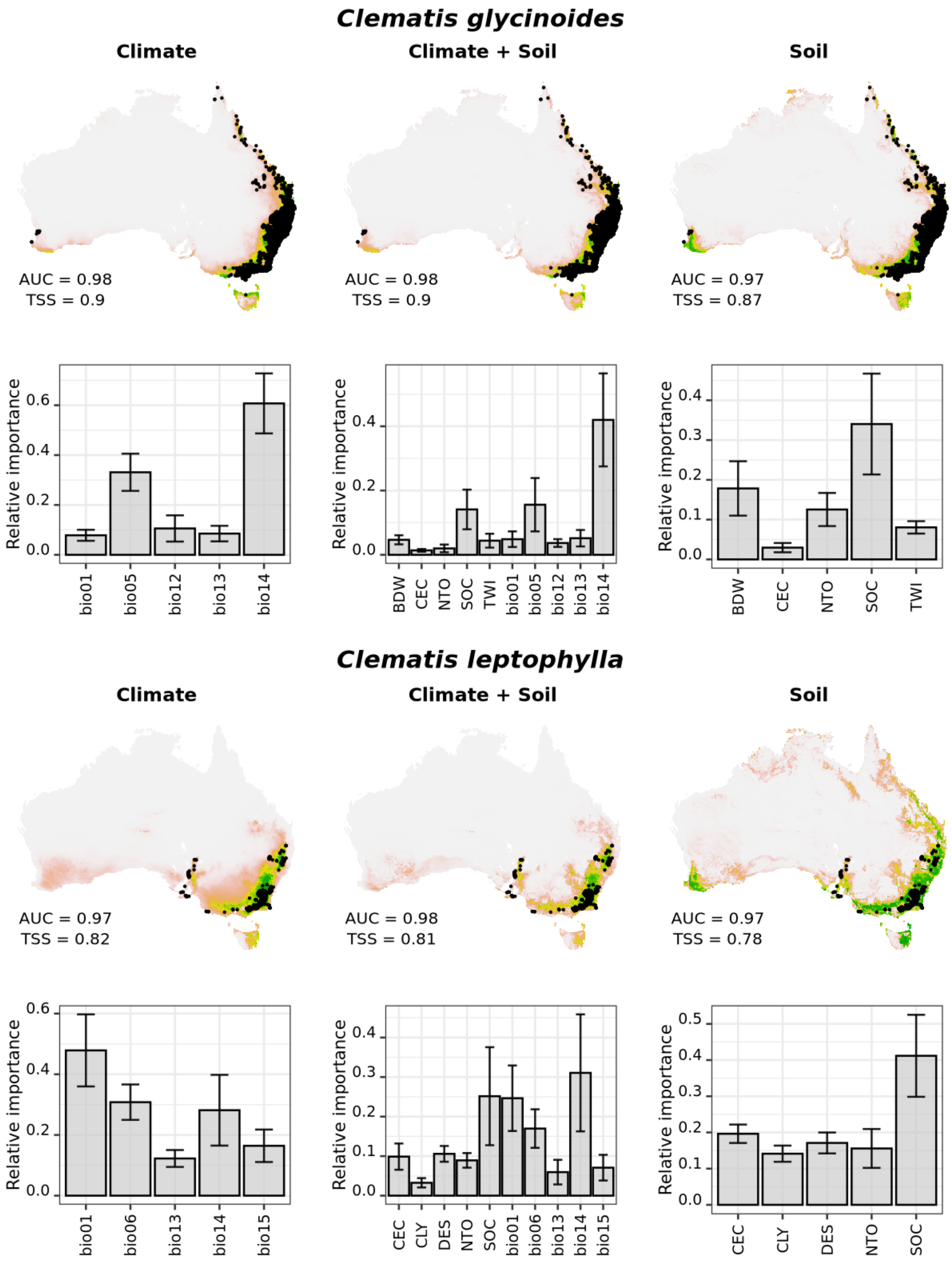

Clematis glycinoides
Climate
Climate + Soil
Soil
AUC = 0.98
TSS = 0.9
AUC = 0.98
TSS = 0.9
AUC = 0.97
TSS = 0.87
Relative importance
bio01
bio05
bio12
bio13
bio14
BDW
CEC
NTO
SOC
TWI
Clematis leptophylla
Climate
Climate + Soil
Soil
AUC = 0.97
TSS = 0.82
AUC = 0.98
TSS = 0.81
AUC = 0.97
TSS = 0.78
Relative importance
bio01
bio06
bio13
bio14
bio15
CEC
CLY
DES
NTO
SOC

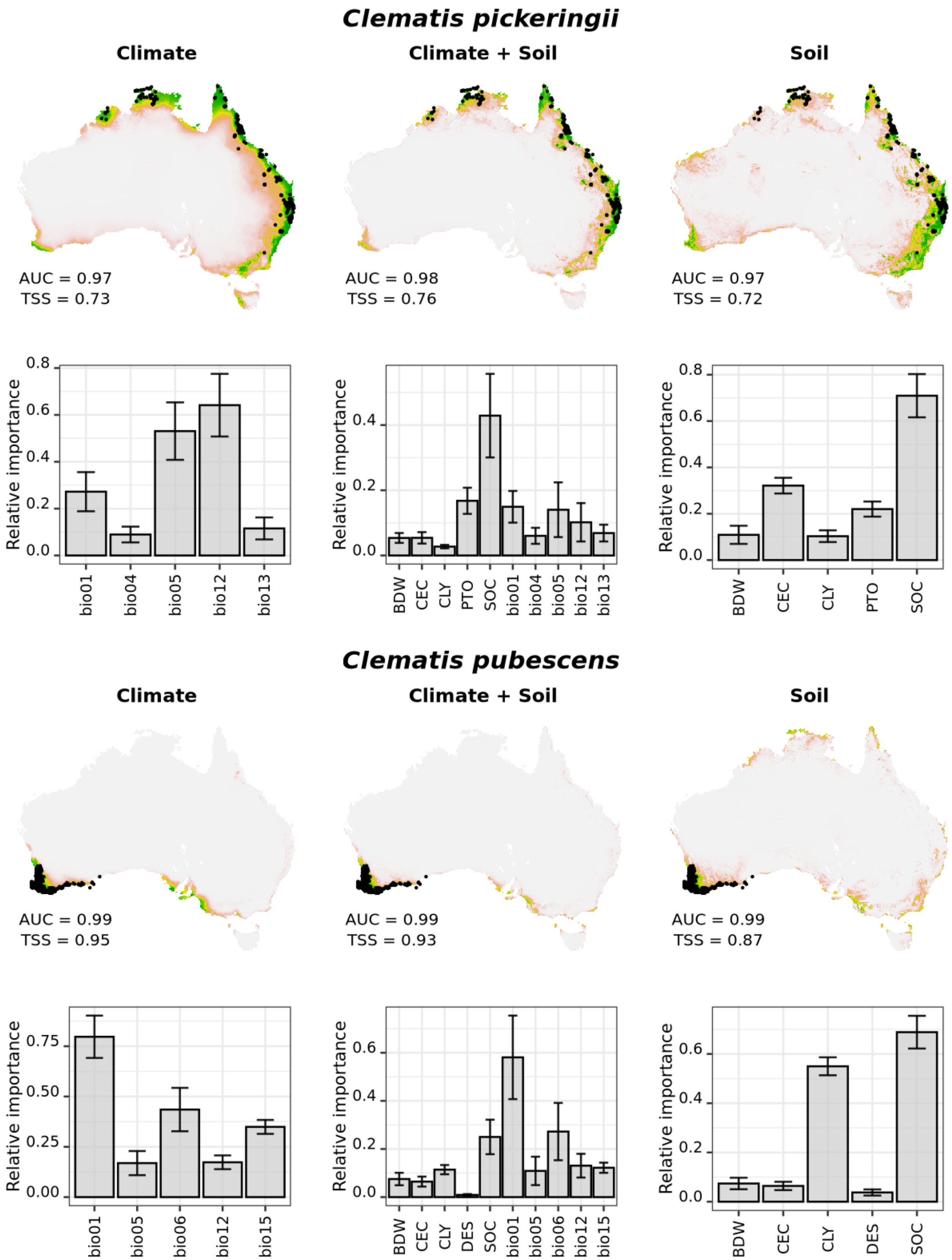

Clematis pickeringii
Climate
Climate + Soil
Soil
AUC = 0.97
TSS = 0.73
AUC = 0.98
TSS = 0.76
AUC = 0.97
TSS = 0.72
Relative importance
0.0
0.2
0.4
0.6
0.8
bio01
bio04
bio05
bio12
bio13
BDW
CEC
CLY
PTO
SOC
bio01
bio04
bio05
bio12
bio13
BDW
CEC
CLY
PTO
SOC
Clematis pubescens
Climate
Climate + Soil
Soil
AUC = 0.99
TSS = 0.95
AUC = 0.99
TSS = 0.93
AUC = 0.99
TSS = 0.87
Relative importance
0.00
0.25
0.50
0.75
bio01
bio05
bio06
bio12
bio15
BDW
CEC
CLY
DES
SOC
bio01
bio05
bio06
bio12
bio15
BDW
CEC
CLY
DES
SOC

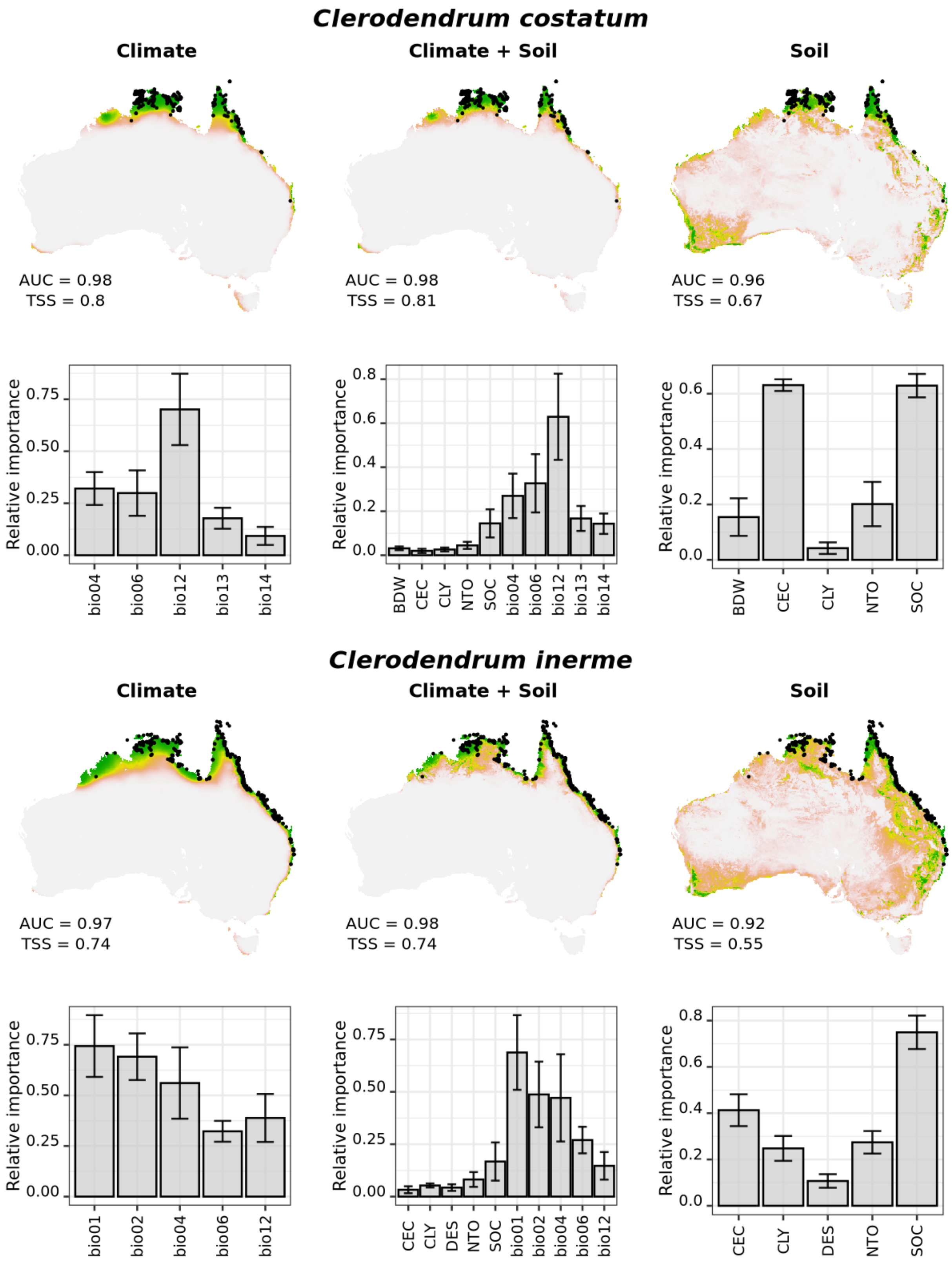
Clerodendrum costatum
Climate
Climate + Soil
Soil
AUC = 0.98
TSS = 0.8
AUC = 0.98
TSS = 0.81
AUC = 0.96
TSS = 0.67
Relative importance
0.00
0.25
0.50
0.75
bio04
bio06
bio12
bio13
bio14
0.0
0.2
0.4
0.6
0.8
BDW
CEC
CLY
NTO
SOC
bio04
bio06
bio12
bio13
bio14
0.0
0.2
0.4
0.6
BDW
CEC
CLY
NTO
SOC
Clerodendrum inerme
Climate
Climate + Soil
Soil
AUC = 0.97
TSS = 0.74
AUC = 0.98
TSS = 0.74
AUC = 0.92
TSS = 0.55
Relative importance
0.00
0.25
0.50
0.75
bio01
bio02
bio04
bio06
bio12
CEC
CLY
DES
NTO
SOC
bio01
bio02
bio04
bio06
bio12
0.0
0.2
0.4
0.6
0.8
CEC
CLY
DES
NTO
SOC

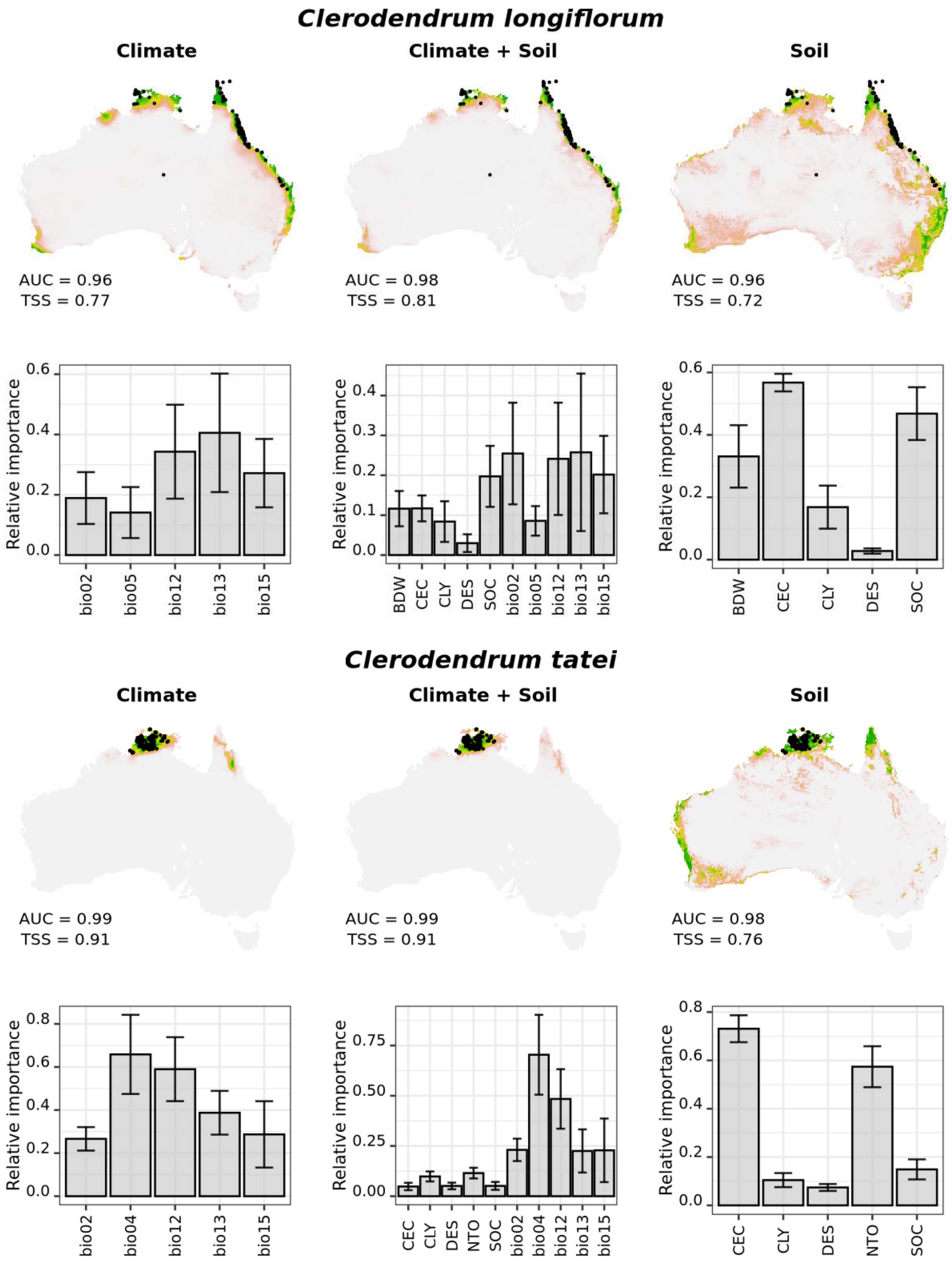
Clerodendrum longiflorum
Climate
Climate + Soil
Soil
AUC = 0.96
TSS = 0.77
AUC = 0.98
TSS = 0.81
AUC = 0.96
TSS = 0.72
Relative importance
0.0
0.2
0.4
0.6
bio02
bio05
bio12
bio13
bio15
0.0
0.1
0.2
0.3
0.4
BDW
CEC
CLY
DES
SOC
bio02
bio05
bio12
bio13
bio15
0.0
0.2
0.4
0.6
BDW
CEC
CLY
DES
SOC
Clerodendrum tatei
Climate
Climate + Soil
Soil
AUC = 0.99
TSS = 0.91
AUC = 0.99
TSS = 0.91
AUC = 0.98
TSS = 0.76
Relative importance
0.0
0.2
0.4
0.6
0.8
bio02
bio04
bio12
bio13
bio15
0.00
0.25
0.50
0.75
CEC
CLY
DES
NTO
SOC
bio02
bio04
bio12
bio13
bio15
0.0
0.2
0.4
0.6
0.8
CEC
CLY
DES
NTO
SOC

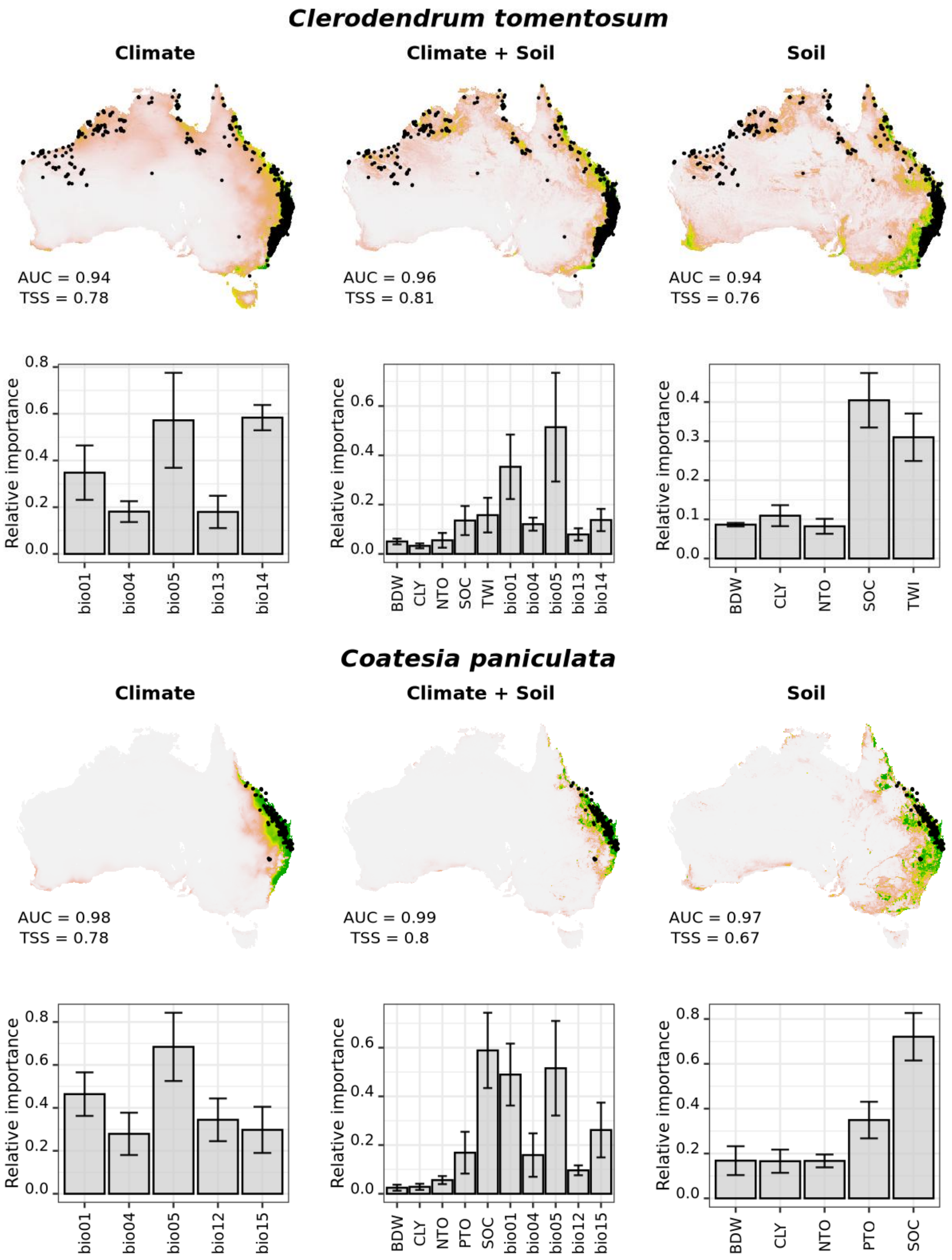
Clerodendrum tomentosum
Climate
Climate + Soil
Soil
AUC = 0.94
TSS = 0.78
AUC = 0.96
TSS = 0.81
AUC = 0.94
TSS = 0.76
Relative importance
bio01
bio04
bio05
bio13
bio14
BDW
CLY
NTO
SOC
TWI
Coatesia paniculata
AUC = 0.98
TSS = 0.78
AUC = 0.99
TSS = 0.8
AUC = 0.97
TSS = 0.67
bio12
bio15
PTO

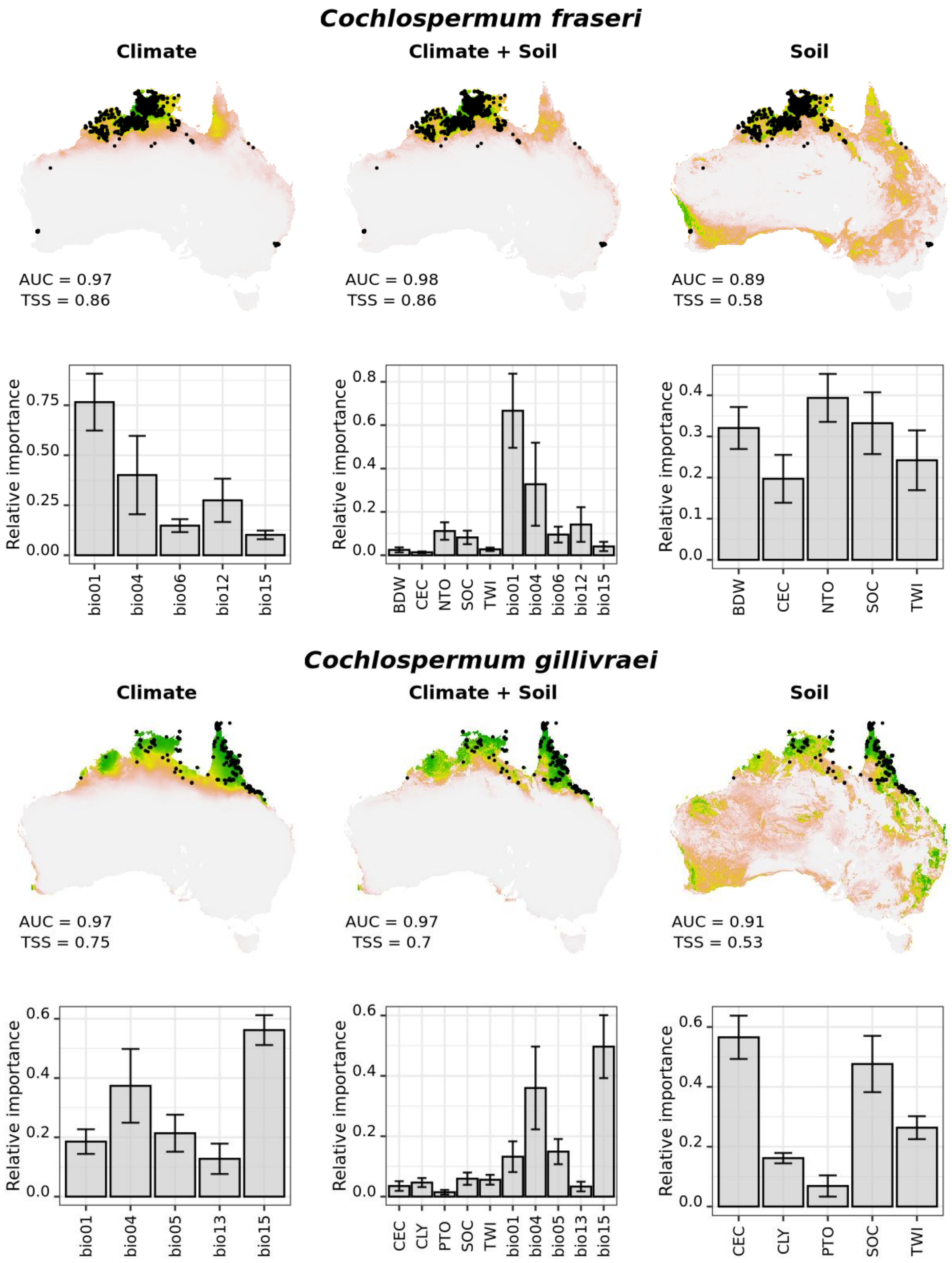
Cochlospermum fraseri
Climate
Climate + Soil
Soil
AUC = 0.97
TSS = 0.86
AUC = 0.98
TSS = 0.86
AUC = 0.89
TSS = 0.58
Relative importance
0.00
0.25
0.50
0.75
bio01
bio04
bio06
bio12
bio15
0.0
0.2
0.4
0.6
0.8
BDW
CEC
NTO
SOC
TWI
bio01
bio04
bio06
bio12
bio15
0.0
0.1
0.2
0.3
0.4
BDW
CEC
NTO
SOC
TWI
Cochlospermum gillivraei
Climate
Climate + Soil
Soil
AUC = 0.97
TSS = 0.75
AUC = 0.97
TSS = 0.7
AUC = 0.91
TSS = 0.53
Relative importance
0.0
0.2
0.4
0.6
bio01
bio04
bio05
bio13
bio15
CEC
CLY
PTO
SOC
TWI
bio01
bio04
bio05
bio13
bio15
CEC
CLY
PTO
SOC
TWI

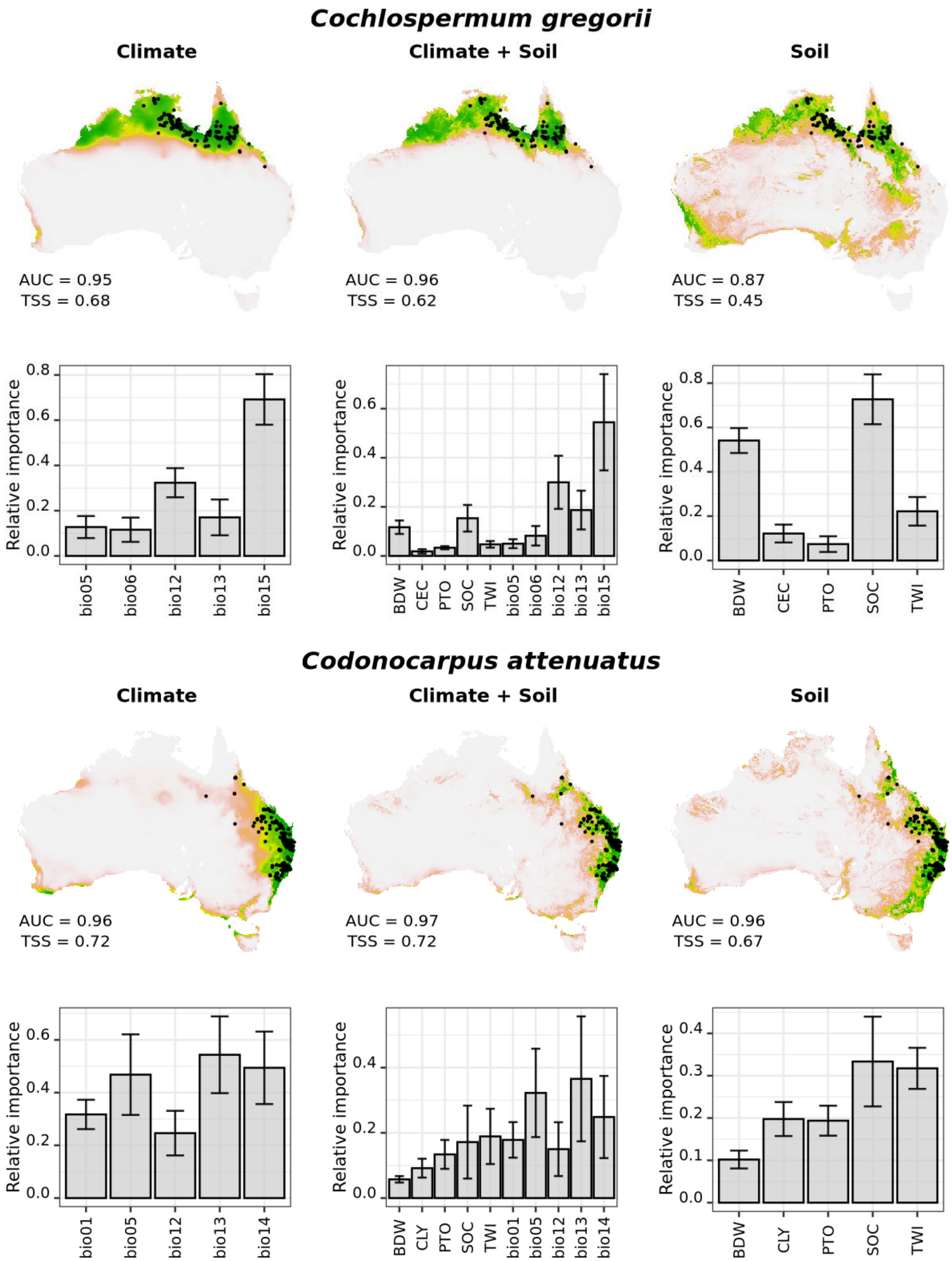
Cochlospermum gregorii
Climate
Climate + Soil
Soil
AUC = 0.95
TSS = 0.68
AUC = 0.96
TSS = 0.62
AUC = 0.87
TSS = 0.45
Relative importance
bio05
bio06
bio12
bio13
bio15
BDW
CEC
PTO
SOC
TWI
Codonocarpus attenuatus
Climate
Climate + Soil
Soil
AUC = 0.96
TSS = 0.72
AUC = 0.97
TSS = 0.72
AUC = 0.96
TSS = 0.67
Relative importance
bio01
bio05
bio12
bio13
bio14
BDW
CLY
PTO
SOC
TWI

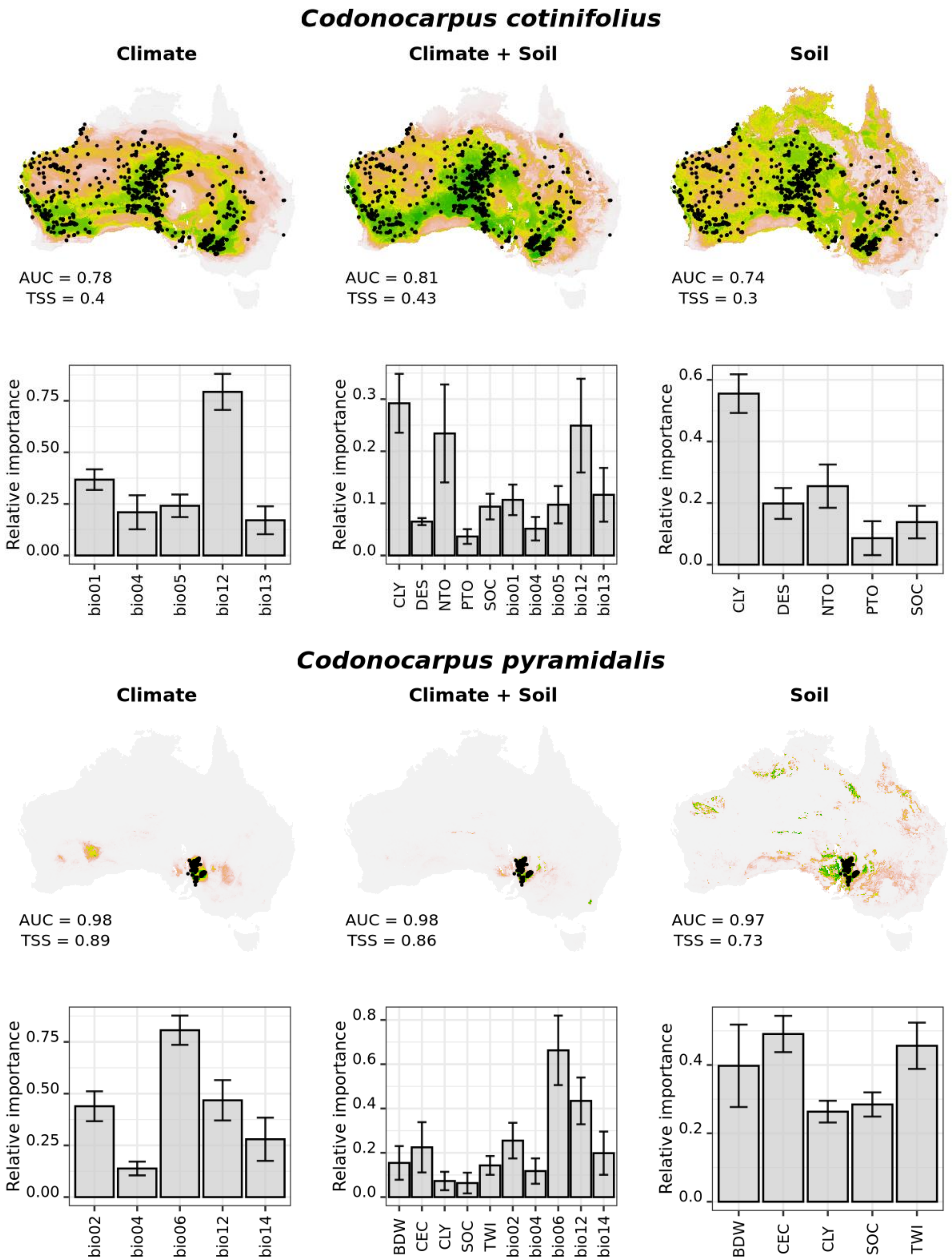
Codonocarpus cotinifolius
Climate
Climate + Soil
Soil
AUC = 0.78
TSS = 0.4
AUC = 0.81
TSS = 0.43
AUC = 0.74
TSS = 0.3
Relative importance
0.00
0.25
0.50
0.75
bio01
bio04
bio05
bio12
bio13
Relative importance
0.0
0.1
0.2
0.3
CLY
DES
NTO
PTO
SOC
bio01
bio04
bio05
bio12
bio13
Relative importance
0.0
0.2
0.4
0.6
CLY
DES
NTO
PTO
SOC
Codonocarpus pyramidalis
Climate
Climate + Soil
Soil
AUC = 0.98
TSS = 0.89
AUC = 0.98
TSS = 0.86
AUC = 0.97
TSS = 0.73
Relative importance
0.00
0.25
0.50
0.75
bio02
bio04
bio06
bio12
bio14
Relative importance
0.0
0.2
0.4
0.6
0.8
BDW
CEC
CLY
SOC
TWI
bio02
bio04
bio06
bio12
bio14
Relative importance
0.0
0.2
0.4
BDW
CEC
CLY
SOC
TWI

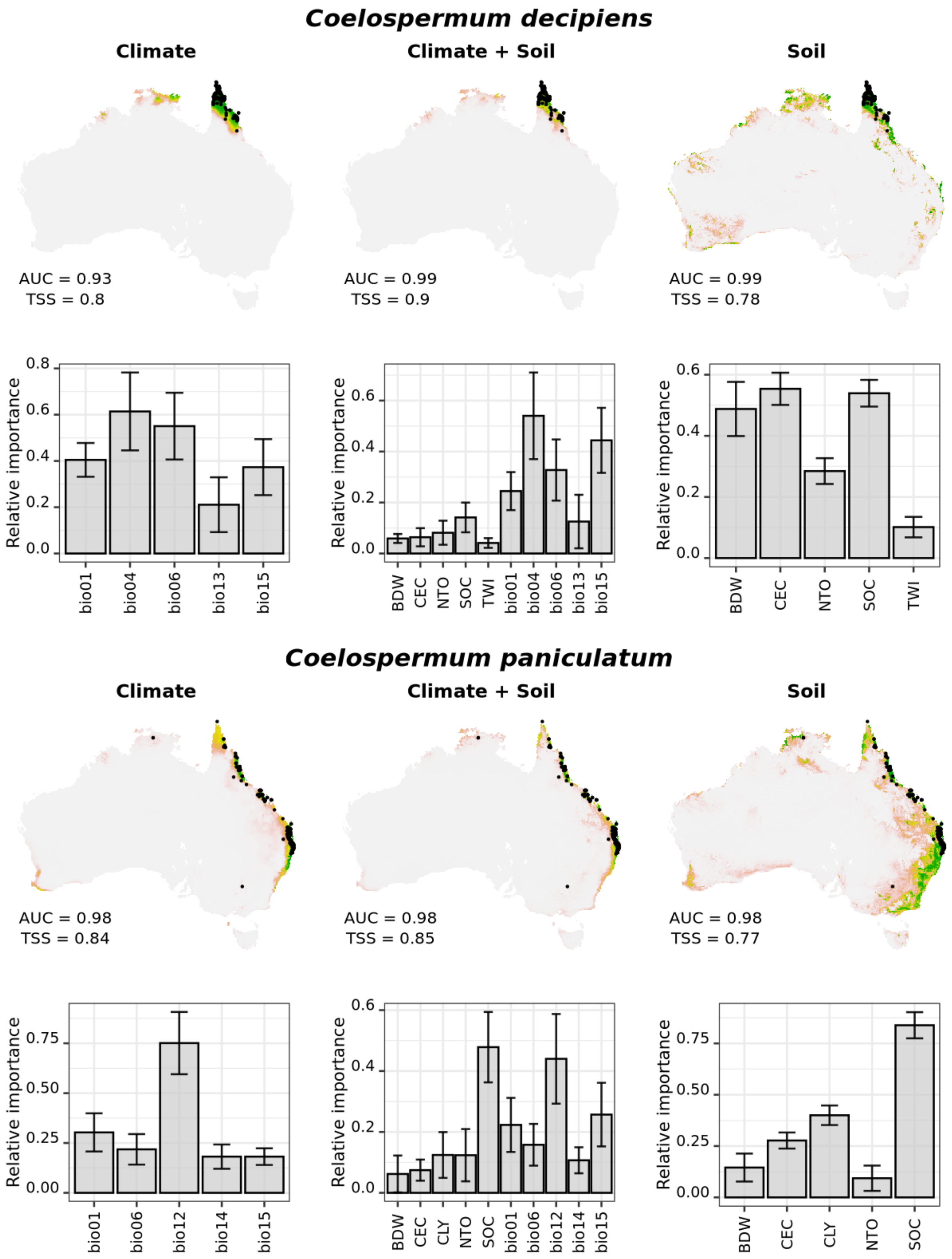
Coelospermum decipiens
Climate
Climate + Soil
Soil
AUC = 0.93
TSS = 0.8
AUC = 0.99
TSS = 0.9
AUC = 0.99
TSS = 0.78
Relative importance
bio01
bio04
bio06
bio13
bio15
BDW
CEC
NTO
SOC
TWI
Coelospermum paniculatum
Climate
Climate + Soil
Soil
AUC = 0.98
TSS = 0.84
AUC = 0.98
TSS = 0.85
AUC = 0.98
TSS = 0.77
Relative importance
bio01
bio06
bio12
bio14
bio15
BDW
CEC
CLY
NTO
SOC

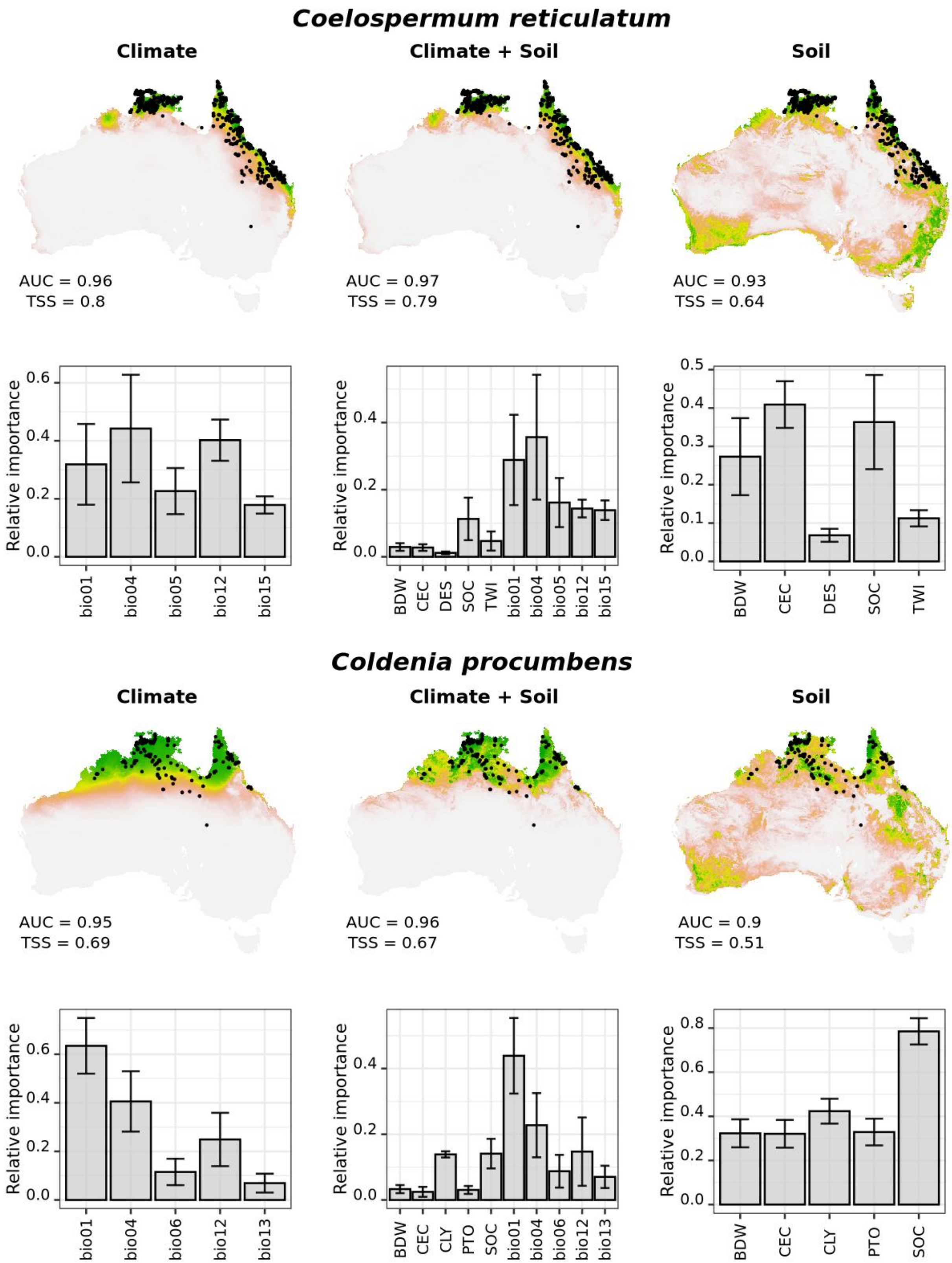
Coelospermum reticulatum
Climate
Climate + Soil
Soil
AUC = 0.96
TSS = 0.8
AUC = 0.97
TSS = 0.79
AUC = 0.93
TSS = 0.64
Relative importance
0.0
0.2
0.4
0.6
bio01
bio04
bio05
bio12
bio15
0.0
0.2
0.4
BDW
CEC
DES
SOC
TWI
bio01
bio04
bio05
bio12
bio15
0.0
0.1
0.2
0.3
0.4
0.5
BDW
CEC
DES
SOC
TWI
Coldenia procumbens
Climate
Climate + Soil
Soil
AUC = 0.95
TSS = 0.69
AUC = 0.96
TSS = 0.67
AUC = 0.9
TSS = 0.51
Relative importance
0.0
0.2
0.4
0.6
bio01
bio04
bio06
bio12
bio13
0.0
0.2
0.4
BDW
CEC
CLY
PTO
SOC
bio01
bio04
bio06
bio12
bio13
0.0
0.2
0.4
0.6
0.8
BDW
CEC
CLY
PTO
SOC

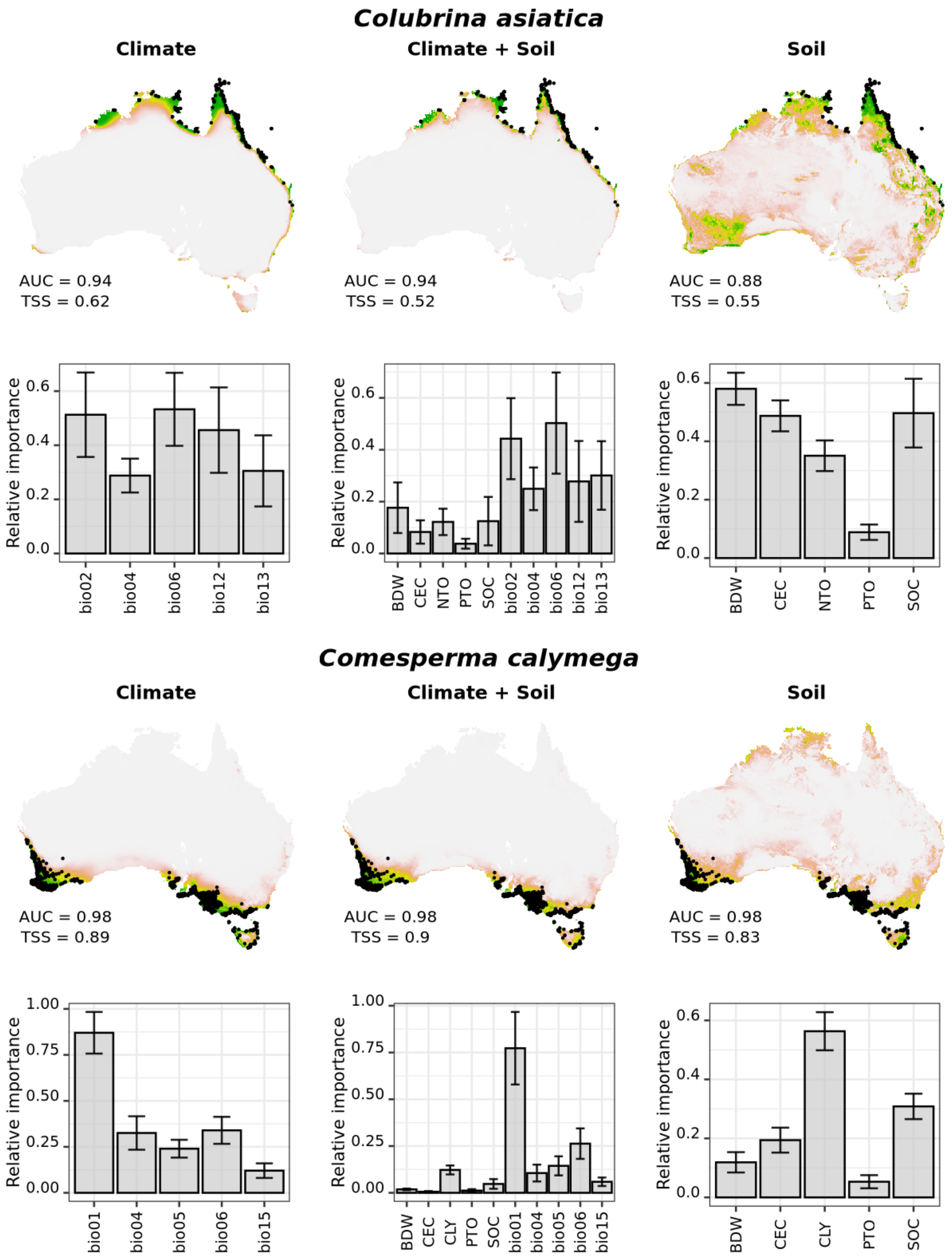
Colubrina asiatica
Climate
Climate + Soil
Soil
AUC = 0.94
TSS = 0.62
AUC = 0.94
TSS = 0.52
AUC = 0.88
TSS = 0.55
Relative importance
0.0
0.2
0.4
0.6
bio02
bio04
bio06
bio12
bio13
BDW
CEC
NTO
PTO
SOC
Comesperma calymega
Climate
Climate + Soil
Soil
AUC = 0.98
TSS = 0.89
AUC = 0.98
TSS = 0.9
AUC = 0.98
TSS = 0.83
0.00
0.25
0.50
0.75
1.00
bio01
bio05
bio15
CLY

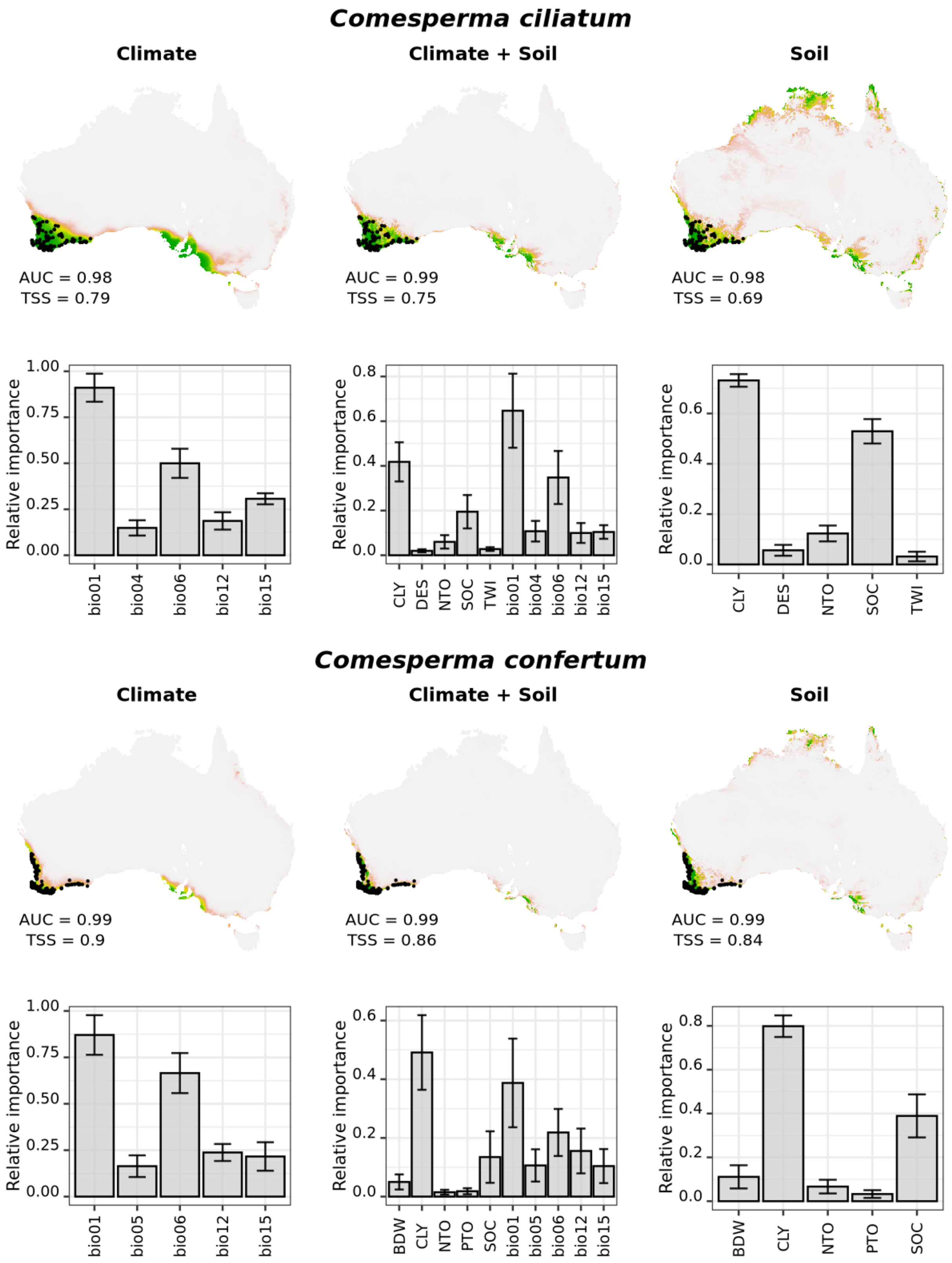
Comesperma ciliatum
Climate
Climate + Soil
Soil
AUC = 0.98
TSS = 0.79
AUC = 0.99
TSS = 0.75
AUC = 0.98
TSS = 0.69
Relative importance
bio01
bio04
bio06
bio12
bio15
CLY
DES
NTO
SOC
TWI
Comesperma confertum
Climate
Climate + Soil
Soil
AUC = 0.99
TSS = 0.9
AUC = 0.99
TSS = 0.86
AUC = 0.99
TSS = 0.84
Relative importance
bio01
bio05
bio06
bio12
bio15
BDW
CLY
NTO
PTO
SOC

Comesperma defoliatum

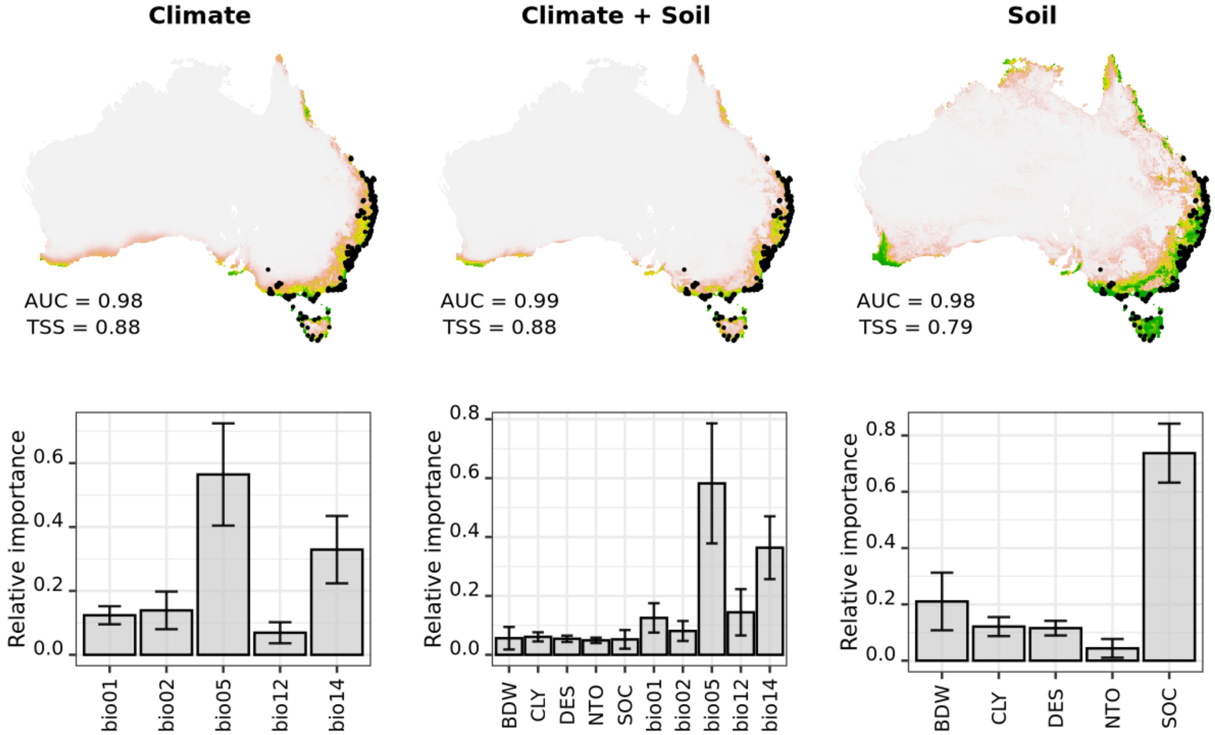

Comesperma ericinum

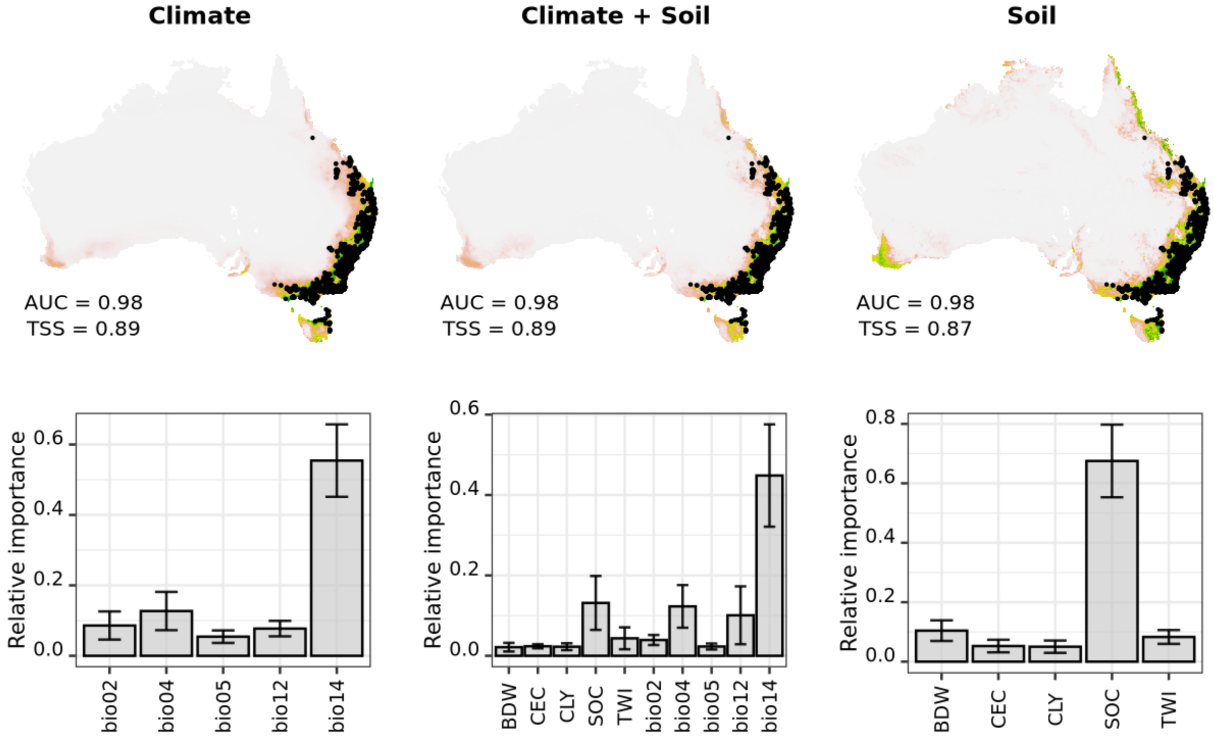

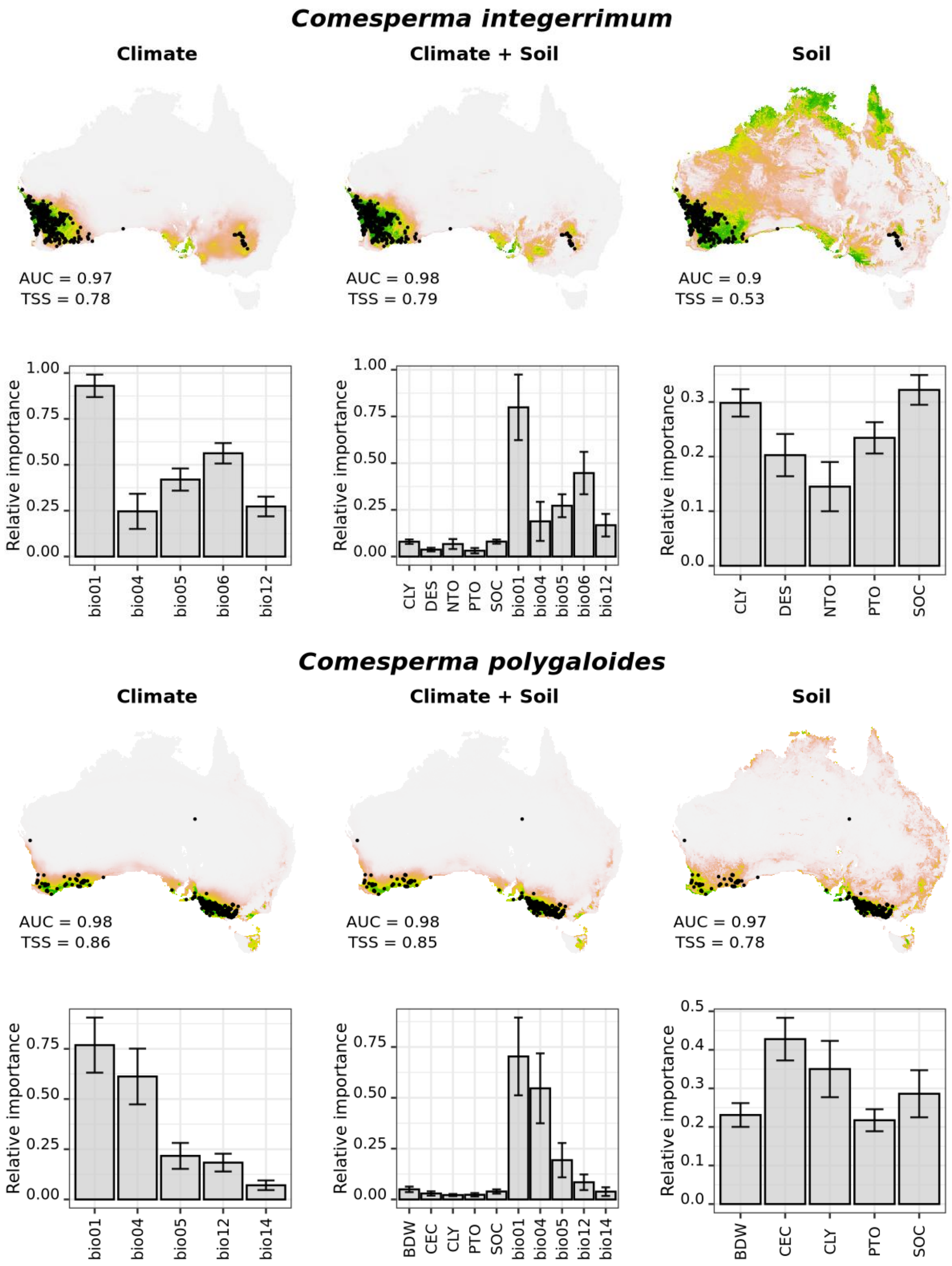
Comesperma integerrimum
Climate
Climate + Soil
Soil
AUC = 0.97
TSS = 0.78
AUC = 0.98
TSS = 0.79
AUC = 0.9
TSS = 0.53
Relative importance
1.00
0.75
0.50
0.25
0.00
bio01
bio04
bio05
bio06
bio12
CLY
DES
NTO
PTO
SOC
0.3
0.2
0.1
0.0
Comesperma polygaloides
Climate
Climate + Soil
Soil
AUC = 0.98
TSS = 0.86
AUC = 0.98
TSS = 0.85
AUC = 0.97
TSS = 0.78
Relative importance
0.75
0.50
0.25
0.00
bio01
bio04
bio05
bio12
bio14
BDW
CEC
CLY
PTO
SOC
0.5
0.4
0.3
0.2
0.1
0.0

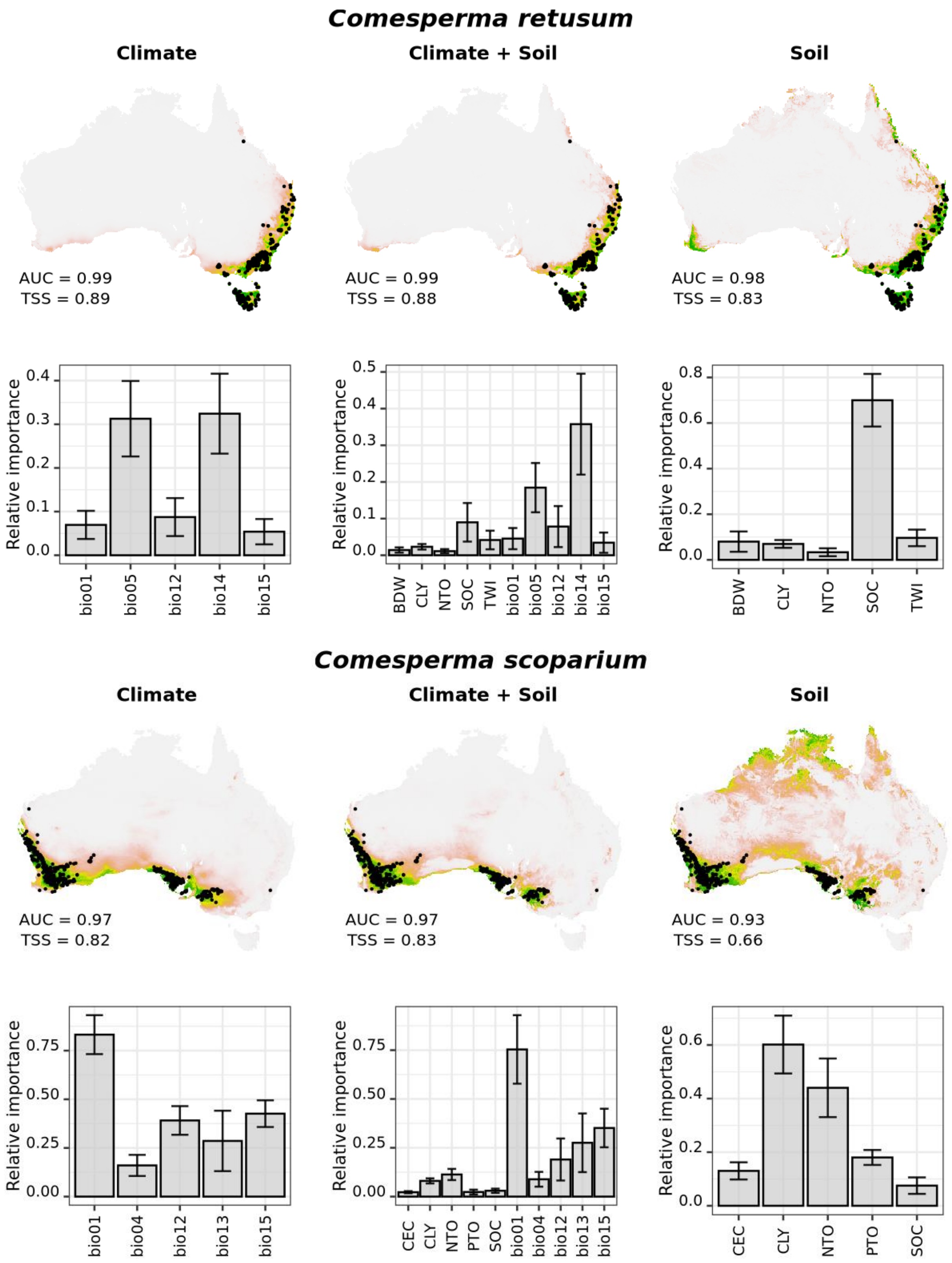

Comesperma retusum
Climate
Climate + Soil
Soil
AUC = 0.99
TSS = 0.89
AUC = 0.99
TSS = 0.88
AUC = 0.98
TSS = 0.83
Relative importance
bio01
bio05
bio12
bio14
bio15
BDW
CLY
NTO
SOC
TWI
Comesperma scoparium
Climate
Climate + Soil
Soil
AUC = 0.97
TSS = 0.82
AUC = 0.97
TSS = 0.83
AUC = 0.93
TSS = 0.66
Relative importance
bio01
bio04
bio12
bio13
bio15
CEC
CLY
NTO
PTO
SOC

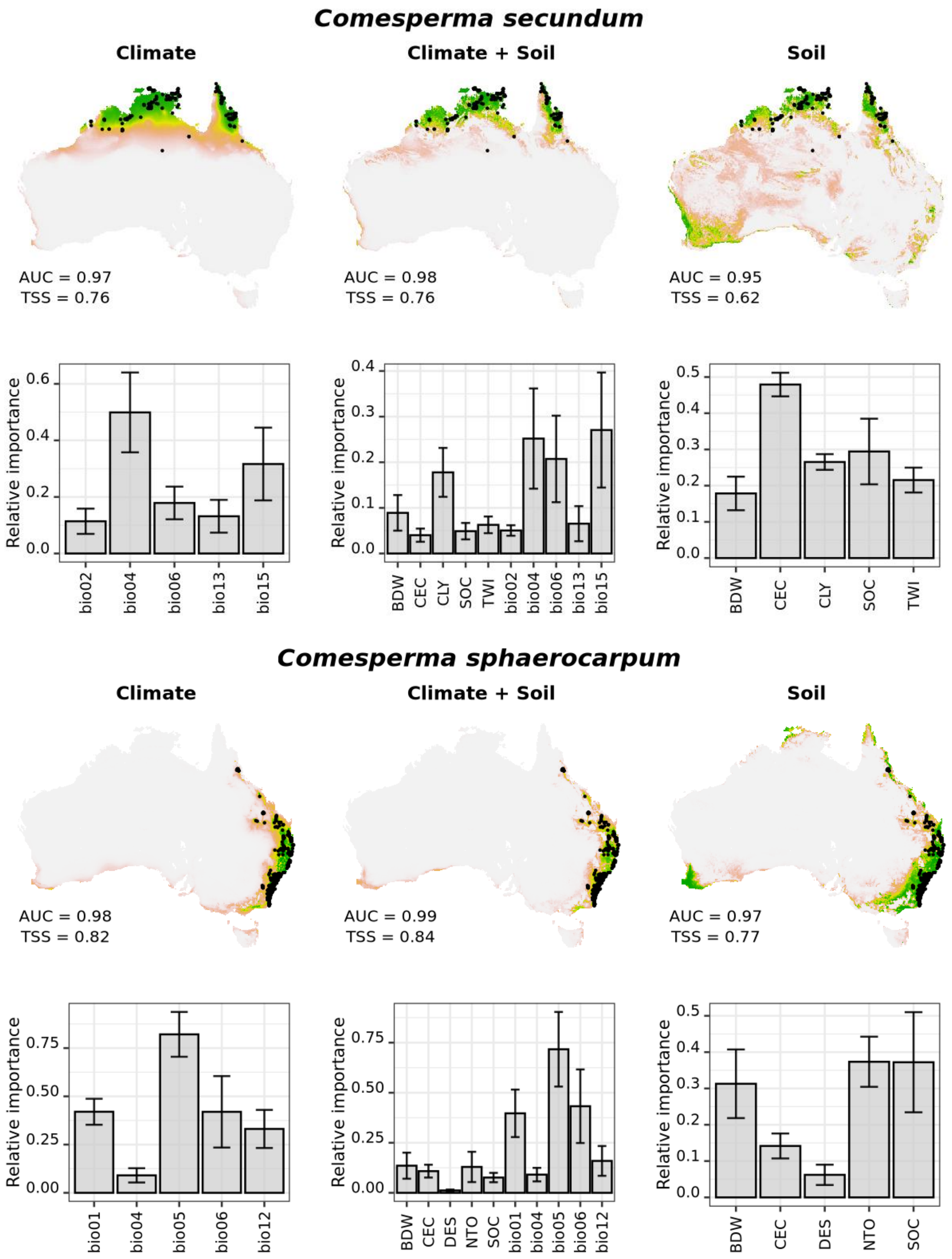

Comesperma secundum
Climate
Climate + Soil
Soil
AUC = 0.97
TSS = 0.76
AUC = 0.98
TSS = 0.76
AUC = 0.95
TSS = 0.62
Relative importance
bio02
bio04
bio06
bio13
bio15
BDW
CEC
CLY
SOC
TWI
bio02
bio04
bio06
bio13
bio15
BDW
CEC
CLY
SOC
TWI
Comesperma sphaerocarpum
Climate
Climate + Soil
Soil
AUC = 0.98
TSS = 0.82
AUC = 0.99
TSS = 0.84
AUC = 0.97
TSS = 0.77
Relative importance
bio01
bio04
bio05
bio06
bio12
BDW
CEC
DES
NTO
SOC
bio01
bio04
bio05
bio06
bio12
BDW
CEC
DES
NTO
SOC

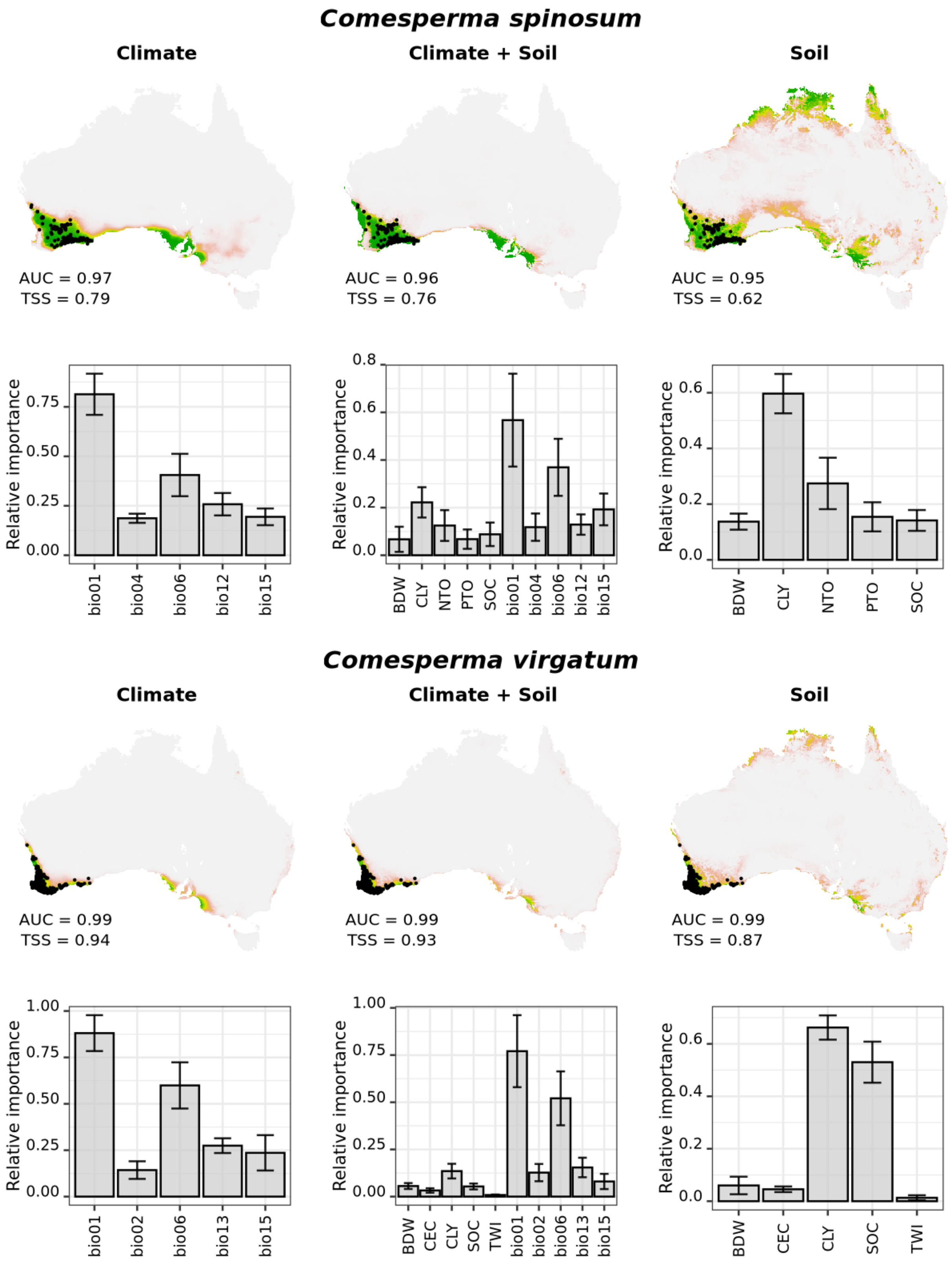
Comesperma spinosum
Climate
Climate + Soil
Soil
AUC = 0.97
TSS = 0.79
AUC = 0.96
TSS = 0.76
AUC = 0.95
TSS = 0.62
Relative importance
0.00
0.25
0.50
0.75
bio01
bio04
bio06
bio12
bio15
0.0
0.2
0.4
0.6
0.8
BDW
CLY
NTO
PTO
SOC
bio01
bio04
bio06
bio12
bio15
0.0
0.2
0.4
0.6
BDW
CLY
NTO
PTO
SOC
Comesperma virgatum
Climate
Climate + Soil
Soil
AUC = 0.99
TSS = 0.94
AUC = 0.99
TSS = 0.93
AUC = 0.99
TSS = 0.87
Relative importance
0.00
0.25
0.50
0.75
1.00
bio01
bio02
bio06
bio13
bio15
BDW
CEC
CLY
SOC
TWI
bio01
bio02
bio06
bio13
bio15
0.0
0.2
0.4
0.6
BDW
CEC
CLY
SOC
TWI

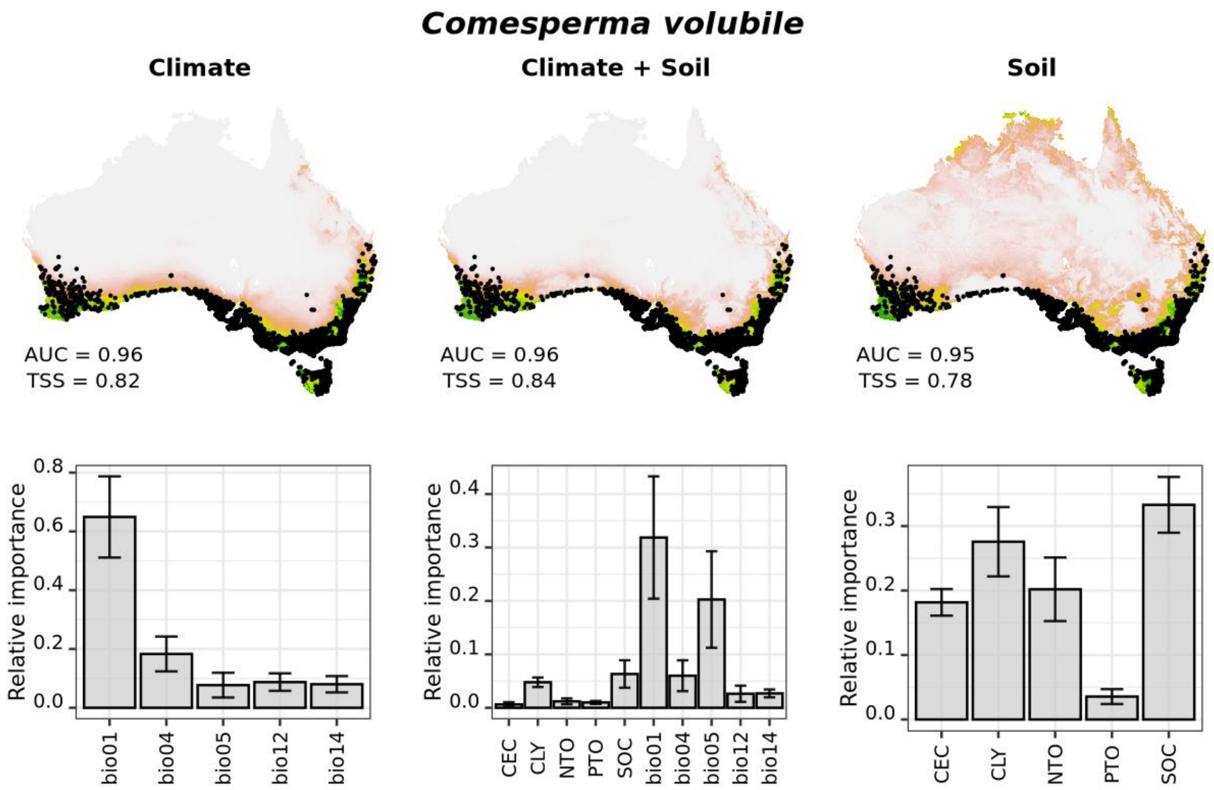
Comesperma volubile
Climate
Climate + Soil
Soil
AUC = 0.96
TSS = 0.82
AUC = 0.96
TSS = 0.84
AUC = 0.95
TSS = 0.78
Relative importance
bio01
bio04
bio05
bio12
bio14
CEC
CLY
NTO
PTO
SOC

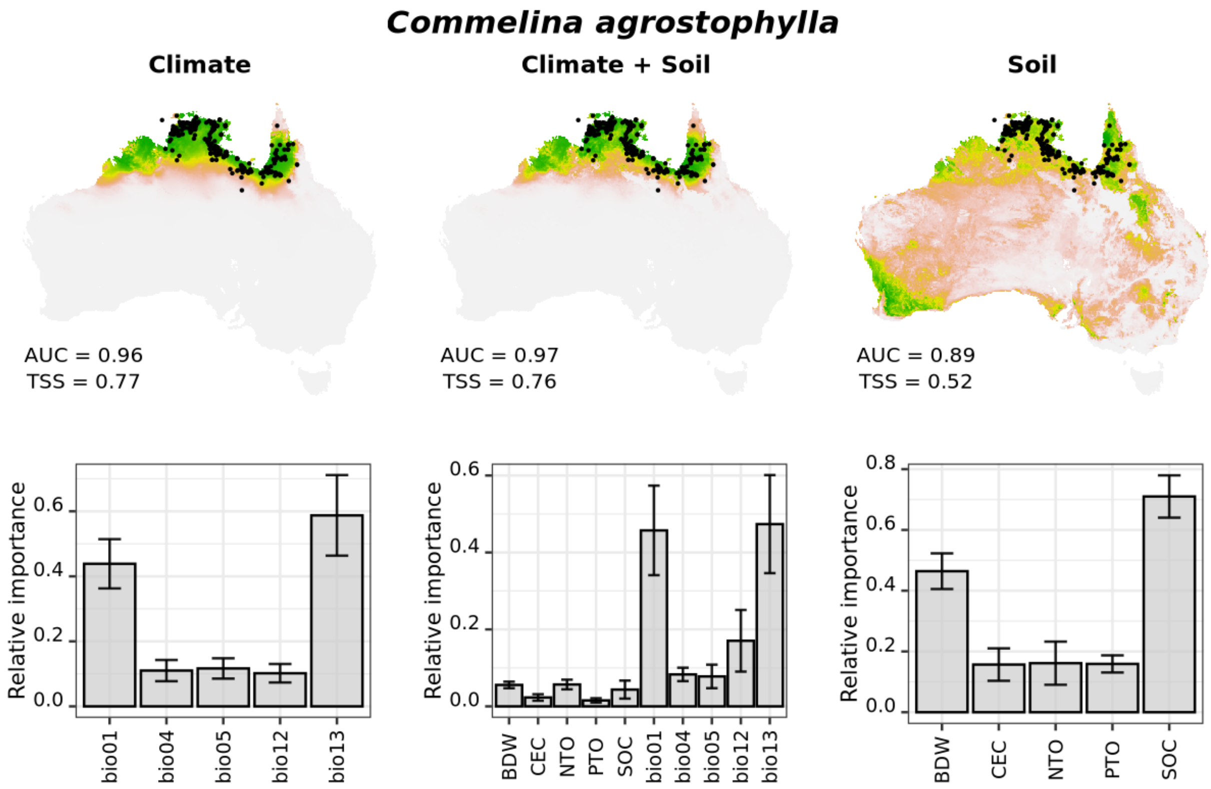
Commelina agrostophylla
Climate
Climate + Soil
Soil
AUC = 0.96
TSS = 0.77
AUC = 0.97
TSS = 0.76
AUC = 0.89
TSS = 0.52
Relative importance
bio01
bio04
bio05
bio12
bio13
BDW
CEC
NTO
PTO
SOC

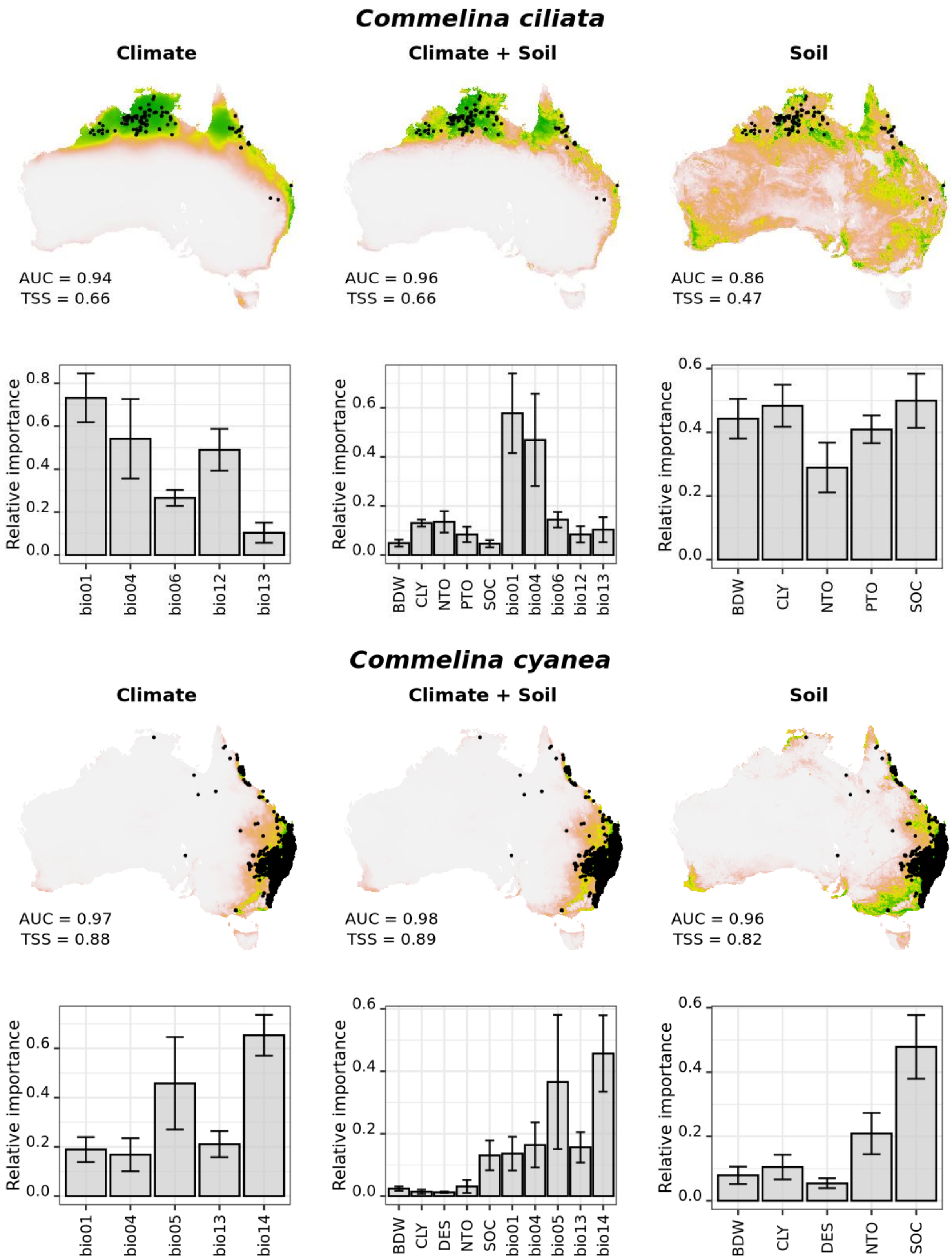
Commelina ciliata
Climate
Climate + Soil
Soil
AUC = 0.94
TSS = 0.66
AUC = 0.96
TSS = 0.66
AUC = 0.86
TSS = 0.47
Relative importance
bio01 bio04 bio06 bio12 bio13
BDW CLY NTO PTO SOC bio01 bio04 bio06 bio12 bio13
BDW CLY NTO PTO SOC
Commelina cyanea
Climate
Climate + Soil
Soil
AUC = 0.97
TSS = 0.88
AUC = 0.98
TSS = 0.89
AUC = 0.96
TSS = 0.82
Relative importance
bio01 bio04 bio05 bio13 bio14
BDW CLY DES NTO SOC bio01 bio04 bio05 bio13 bio14
BDW CLY DES NTO SOC

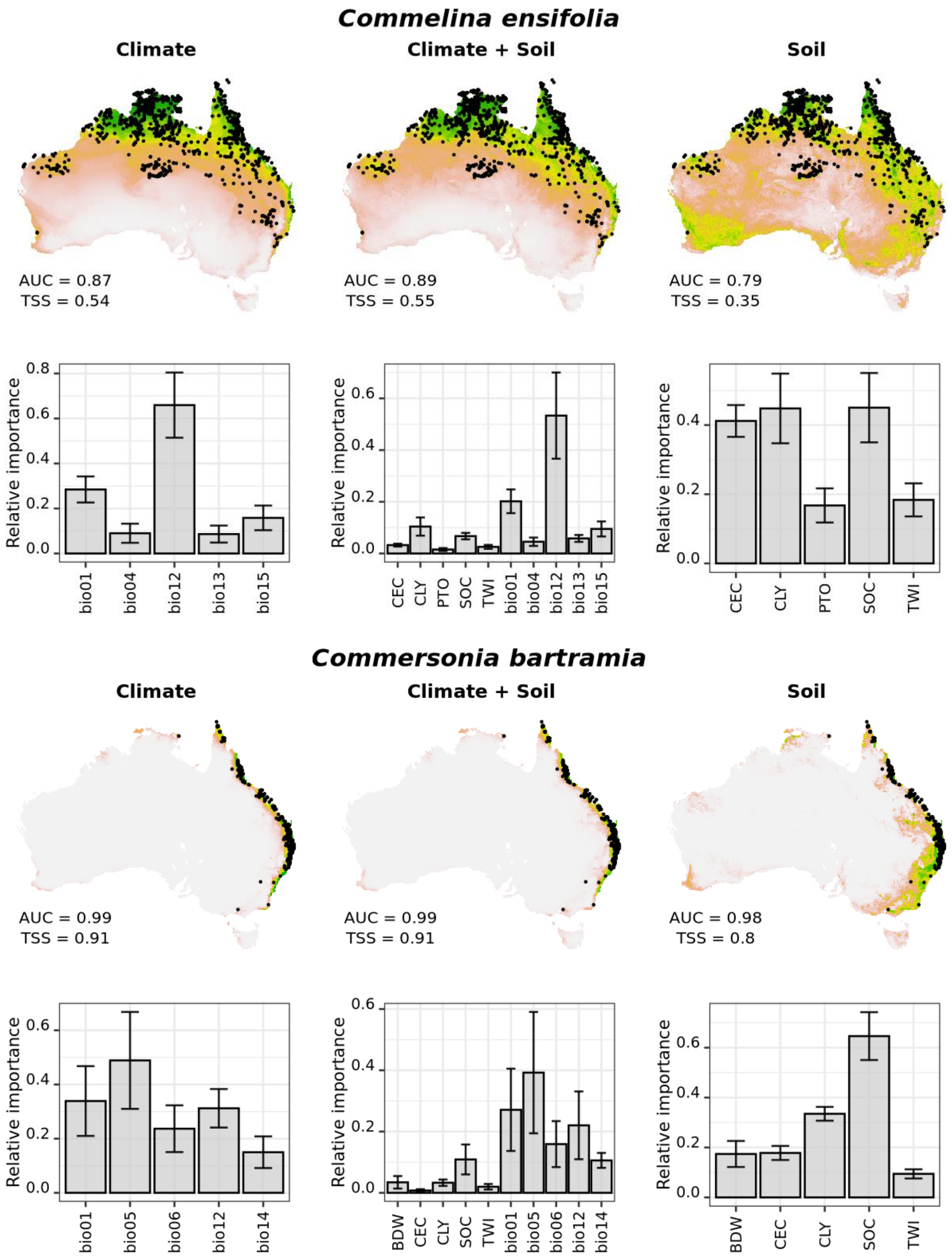
Commelina ensifolia
Climate
Climate + Soil
Soil
AUC = 0.87
TSS = 0.54
AUC = 0.89
TSS = 0.55
AUC = 0.79
TSS = 0.35
Relative importance
bio01 bio04 bio12 bio13 bio15
CEC CLY PTO SOC TWI bio01 bio04 bio12 bio13 bio15
CEC CLY PTO SOC TWI
Commersonia bartramia
Climate
Climate + Soil
Soil
AUC = 0.99
TSS = 0.91
AUC = 0.99
TSS = 0.91
AUC = 0.98
TSS = 0.8
Relative importance
bio01 bio05 bio06 bio12 bio14
BDW CEC CLY SOC TWI bio01 bio05 bio06 bio12 bio14
BDW CEC CLY SOC TWI

Commersonia dasyphylla

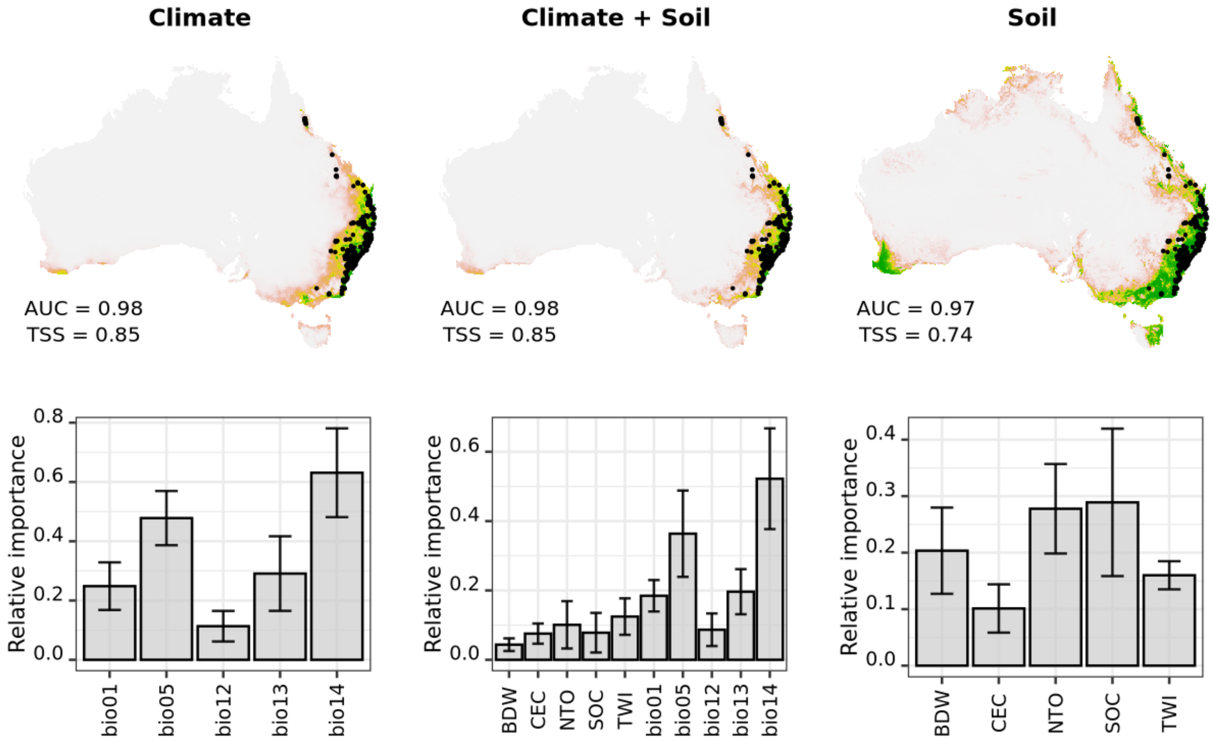

Commicarpus australis

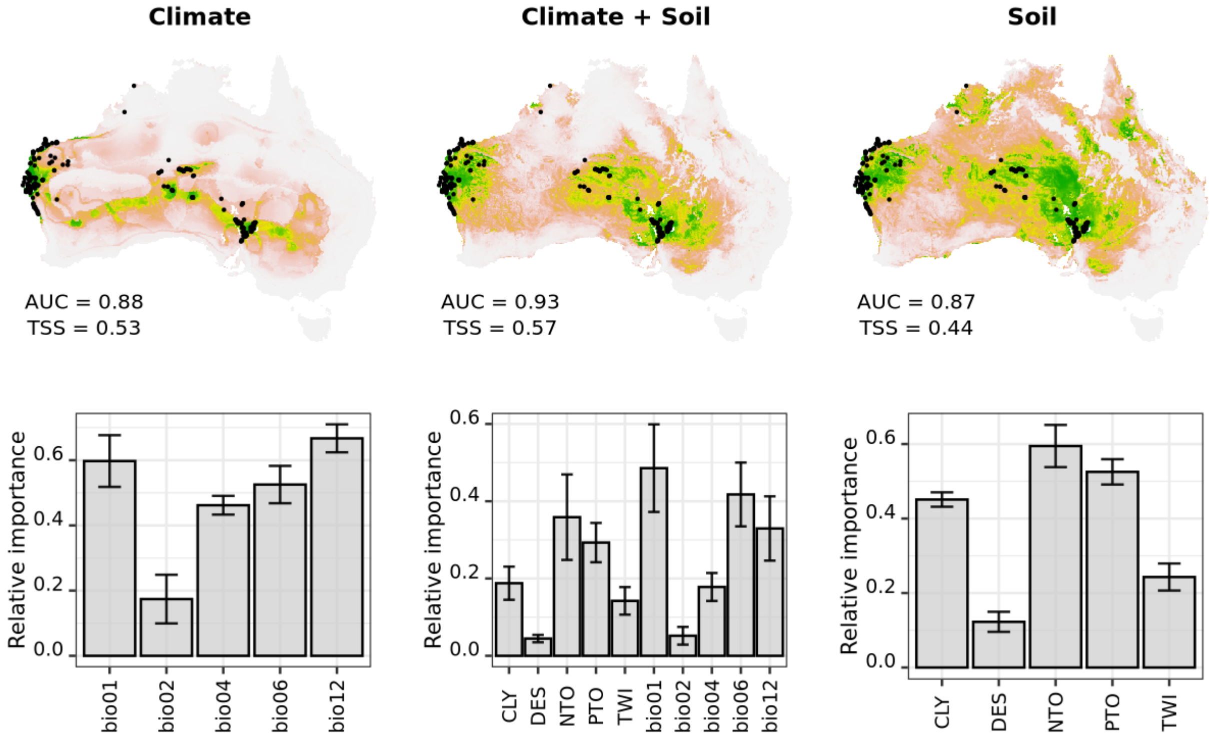

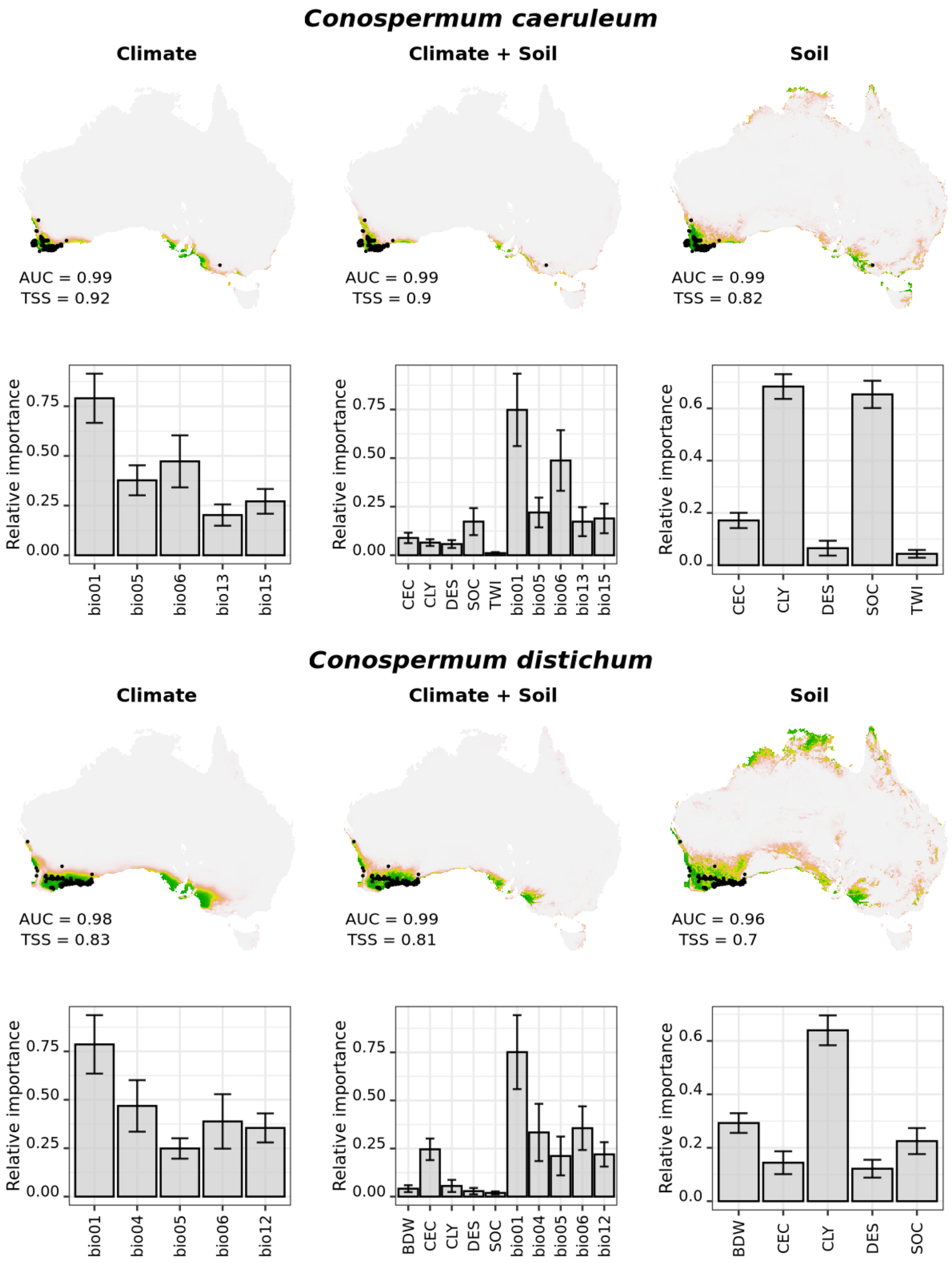
Conospermum caeruleum
Climate
Climate + Soil
Soil
AUC = 0.99
TSS = 0.92
AUC = 0.99
TSS = 0.9
AUC = 0.99
TSS = 0.82
Relative importance
0.00
0.25
0.50
0.75
bio01
bio05
bio06
bio13
bio15
CEC
CLY
DES
SOC
TWI
bio01
bio05
bio06
bio13
bio15
0.0
0.2
0.4
0.6
CEC
CLY
DES
SOC
TWI
Conospermum distichum
Climate
Climate + Soil
Soil
AUC = 0.98
TSS = 0.83
AUC = 0.99
TSS = 0.81
AUC = 0.96
TSS = 0.7
Relative importance
bio01
bio04
bio05
bio06
bio12
BDW
CEC
CLY
DES
SOC
bio01
bio04
bio05
bio06
bio12
BDW
CEC
CLY
DES
SOC

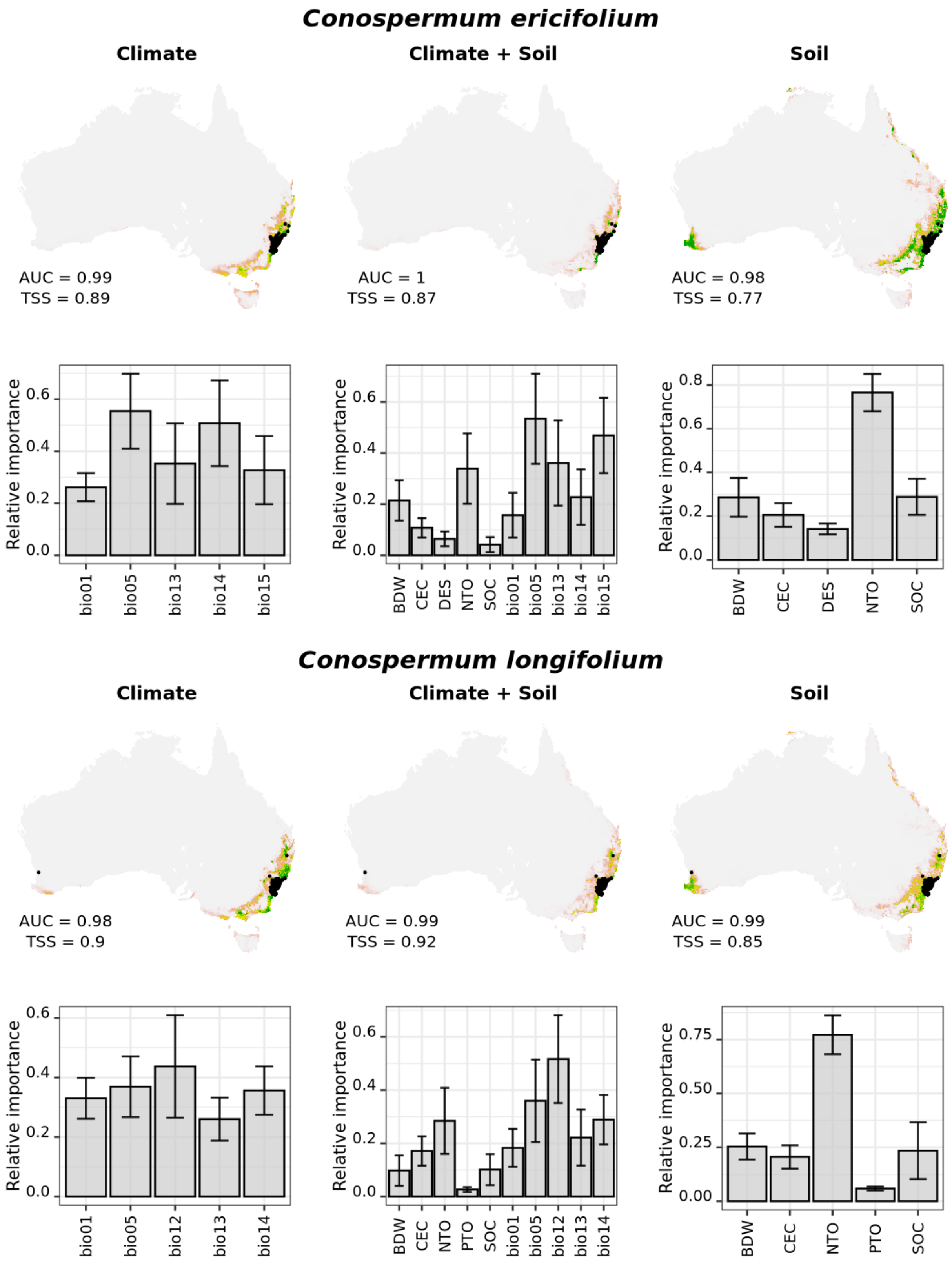
Conospermum ericifolium
Climate
Climate + Soil
Soil
AUC = 0.99
TSS = 0.89
AUC = 1
TSS = 0.87
AUC = 0.98
TSS = 0.77
Relative importance
bio01
bio05
bio13
bio14
bio15
BDW
CEC
DES
NTO
SOC
Conospermum longifolium
AUC = 0.98
TSS = 0.9
AUC = 0.99
TSS = 0.92
AUC = 0.99
TSS = 0.85
bio12
PTO

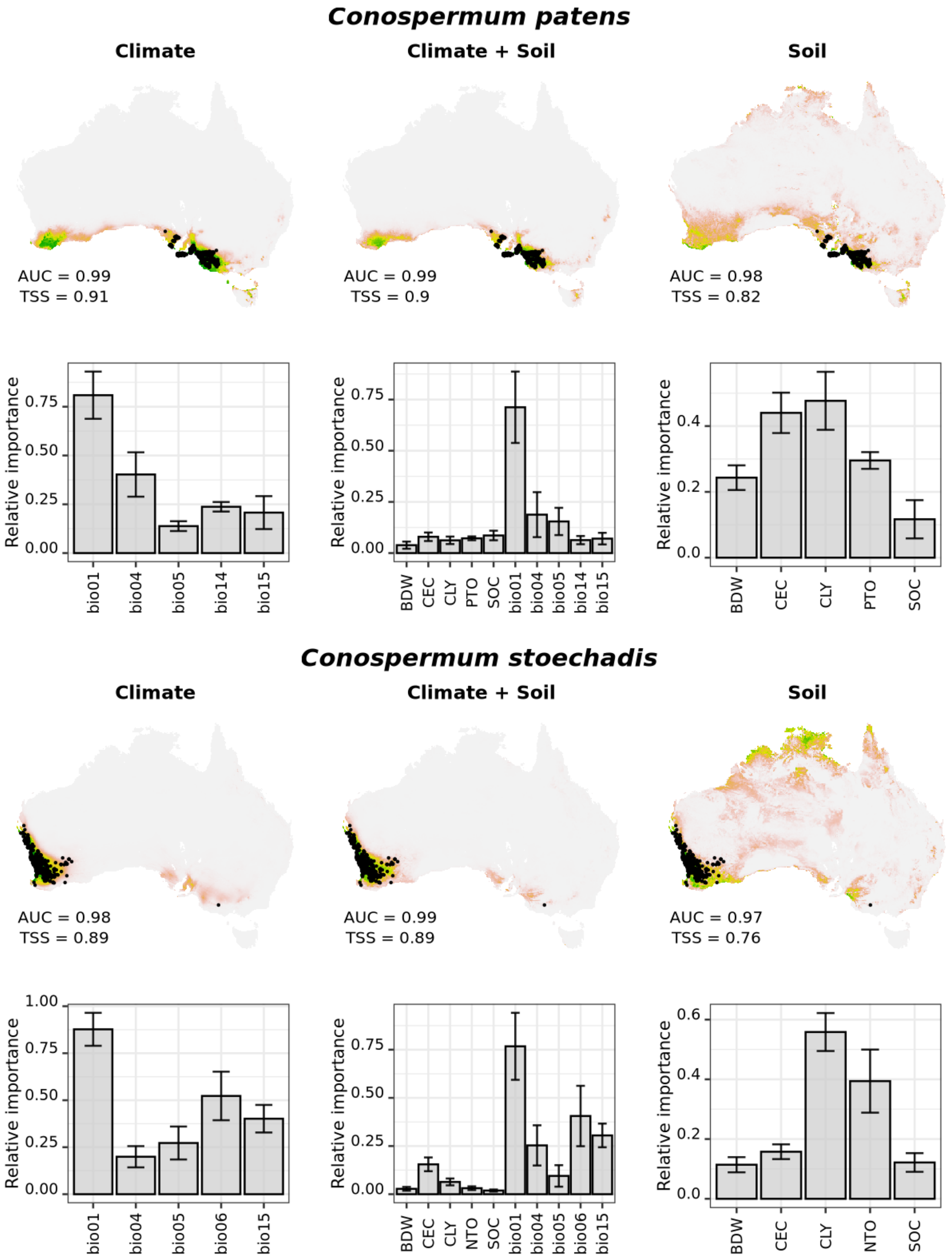
Conospermum patens
Climate
Climate + Soil
Soil
AUC = 0.99
TSS = 0.91
AUC = 0.99
TSS = 0.9
AUC = 0.98
TSS = 0.82
Relative importance
0.00
0.25
0.50
0.75
bio01
bio04
bio05
bio14
bio15
BDW
CEC
CLY
PTO
SOC
0.0
0.2
0.4
Conospermum stoechadis
Climate
Climate + Soil
Soil
AUC = 0.98
TSS = 0.89
AUC = 0.99
TSS = 0.89
AUC = 0.97
TSS = 0.76
1.00
bio06
NTO
0.6

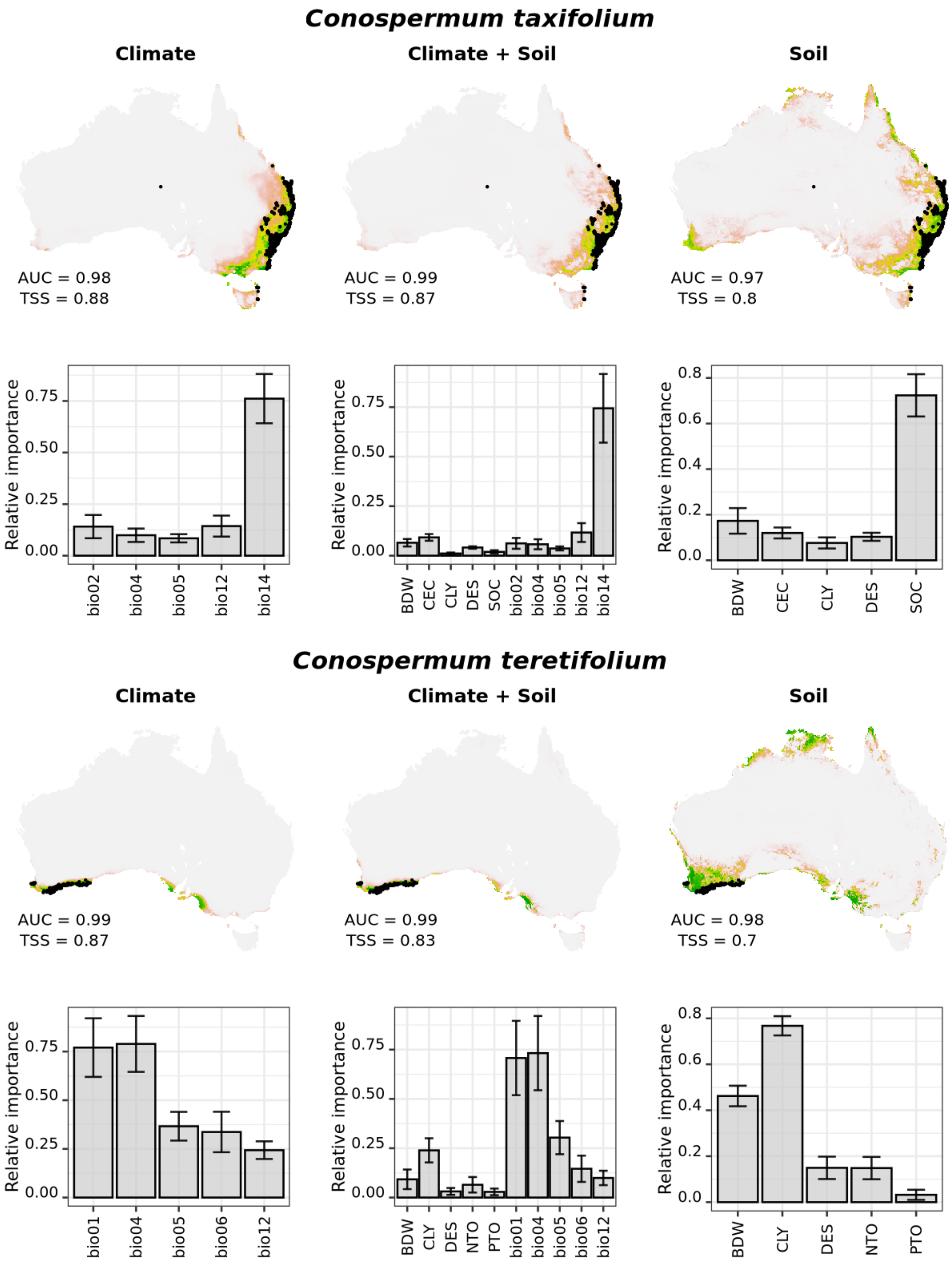
Conospermum taxifolium
Climate
Climate + Soil
Soil
AUC = 0.98
TSS = 0.88
AUC = 0.99
TSS = 0.87
AUC = 0.97
TSS = 0.8
Relative importance
0.00
0.25
0.50
0.75
bio02
bio04
bio05
bio12
bio14
BDW
CEC
CLY
DES
SOC
0.0
0.2
0.4
0.6
0.8
Conospermum teretifolium
Climate
Climate + Soil
Soil
AUC = 0.99
TSS = 0.87
AUC = 0.99
TSS = 0.83
AUC = 0.98
TSS = 0.7
bio01
bio06
NTO
PTO

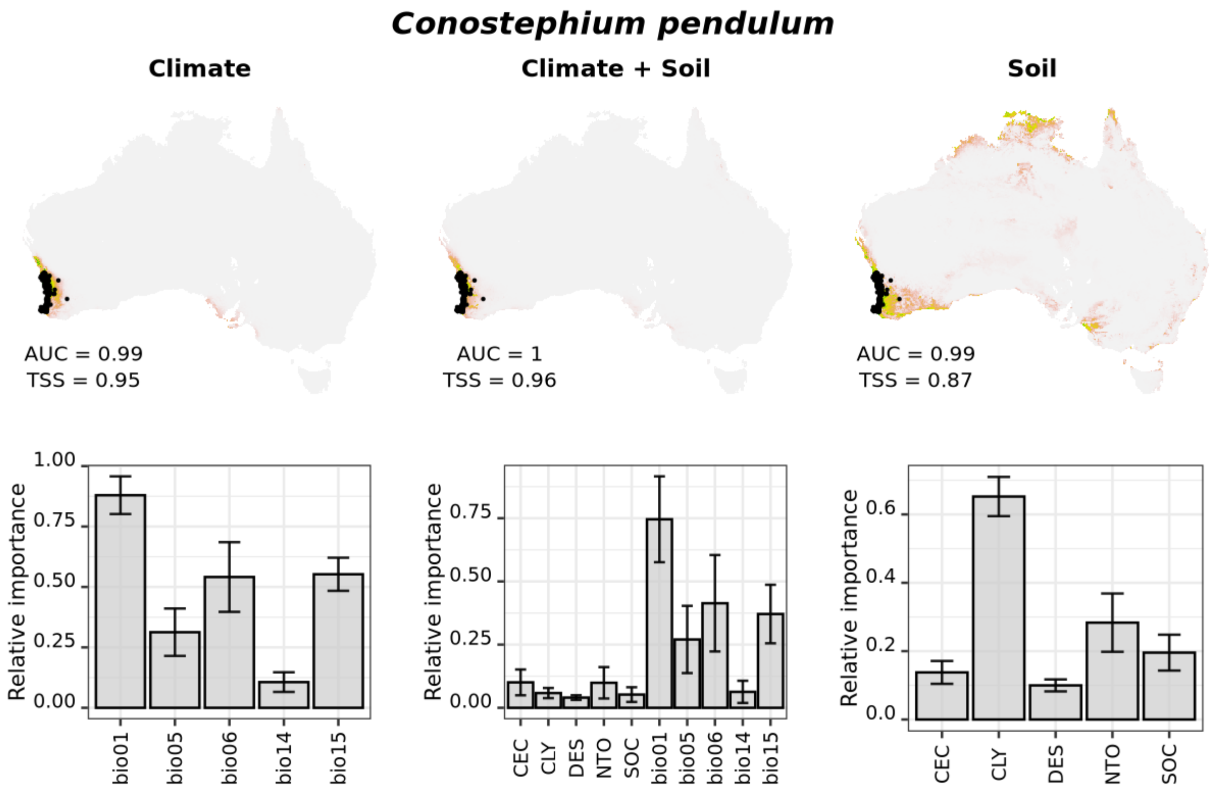
Conostephium pendulum
Climate
Climate + Soil
Soil
AUC = 0.99
TSS = 0.95
AUC = 1
TSS = 0.96
AUC = 0.99
TSS = 0.87
Relative importance
1.00
0.75
0.50
0.25
0.00
bio01
bio05
bio06
bio14
bio15
CEC
CLY
DES
NTO
SOC
0.6
0.4
0.2
0.0

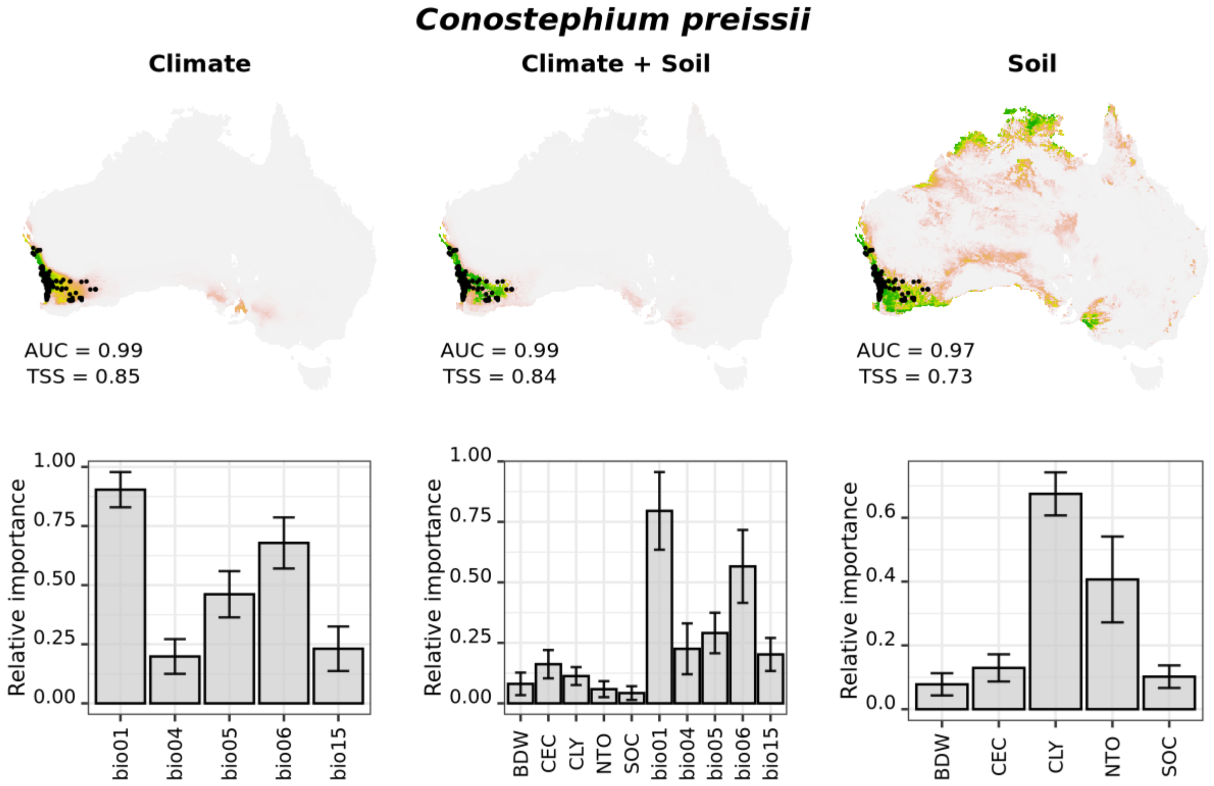
Conostephium preissii
Climate
Climate + Soil
Soil
AUC = 0.99
TSS = 0.85
AUC = 0.99
TSS = 0.84
AUC = 0.97
TSS = 0.73
Relative importance
1.00
0.75
0.50
0.25
0.00
bio01
bio04
bio05
bio06
bio15
BDW
CEC
CLY
NTO
SOC
0.6
0.4
0.2
0.0

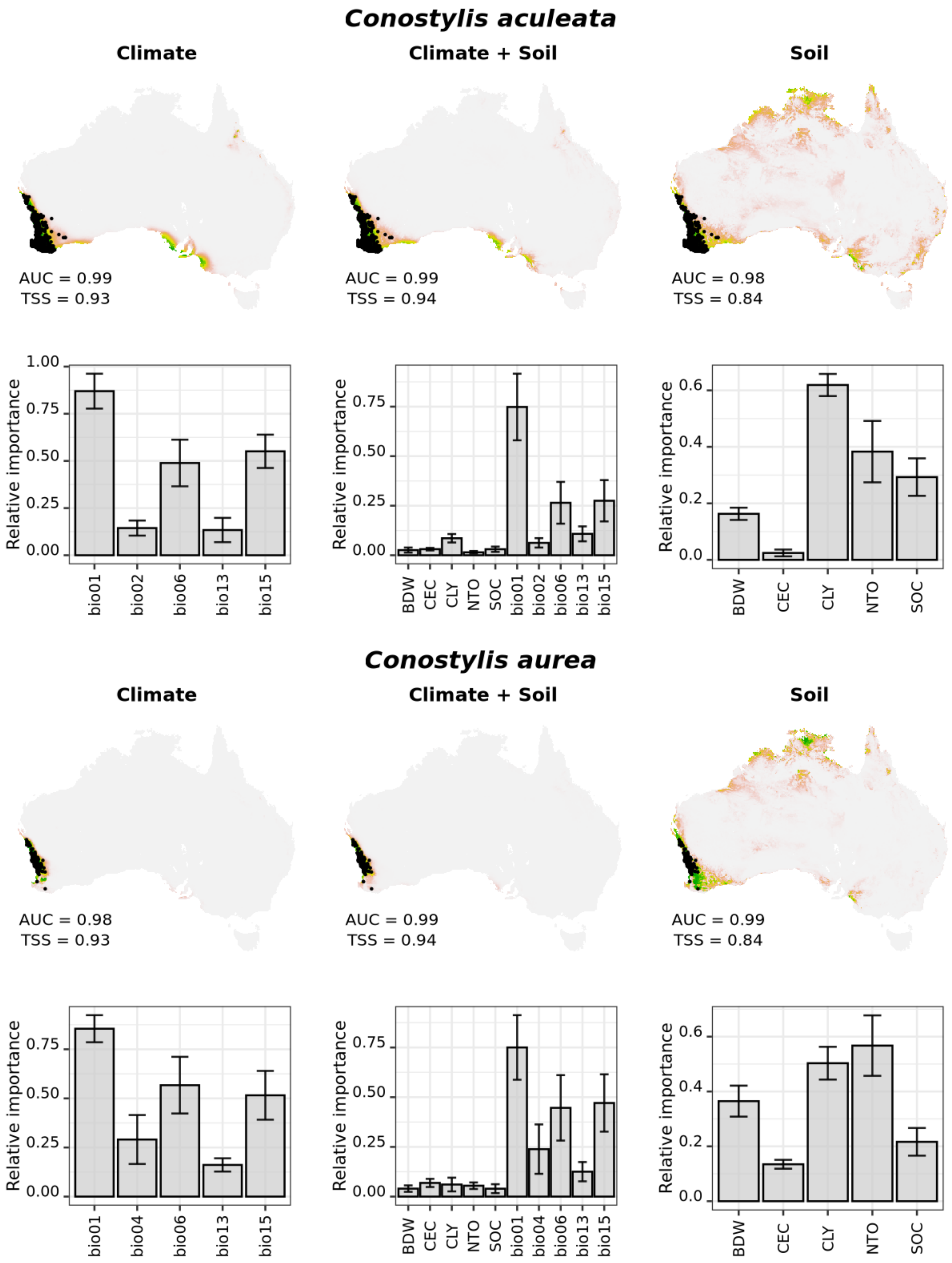
Conostylis aculeata
Climate
Climate + Soil
Soil
AUC = 0.99
TSS = 0.93
AUC = 0.99
TSS = 0.94
AUC = 0.98
TSS = 0.84
Relative importance
bio01
bio02
bio06
bio13
bio15
BDW
CEC
CLY
NTO
SOC
Conostylis aurea
Climate
Climate + Soil
Soil
AUC = 0.98
TSS = 0.93
AUC = 0.99
TSS = 0.94
AUC = 0.99
TSS = 0.84
Relative importance
bio01
bio04
bio06
bio13
bio15
BDW
CEC
CLY
NTO
SOC

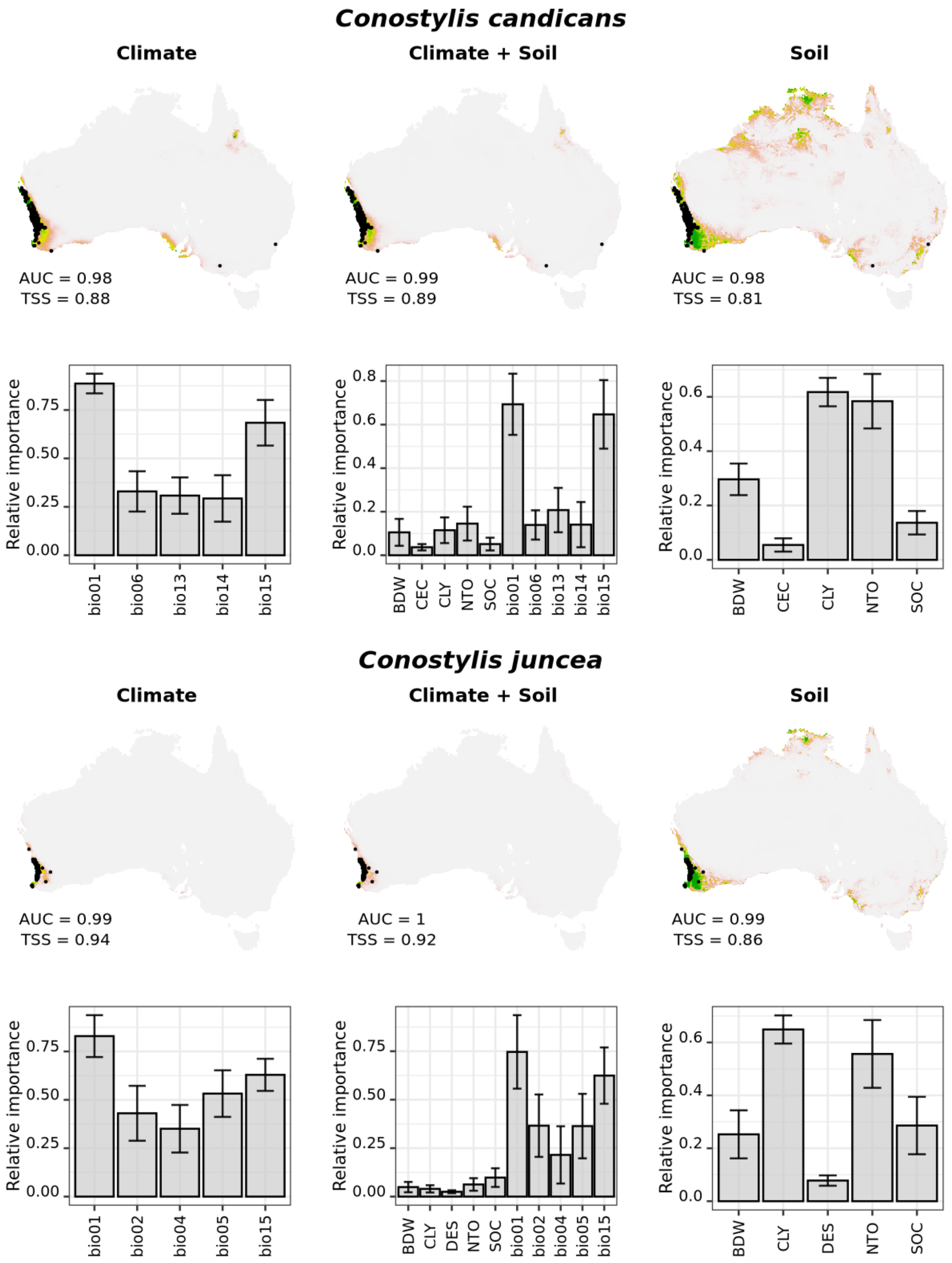
Conostylis candicans
Climate
Climate + Soil
Soil
AUC = 0.98
TSS = 0.88
AUC = 0.99
TSS = 0.89
AUC = 0.98
TSS = 0.81
Relative importance
bio01
bio06
bio13
bio14
bio15
BDW
CEC
CLY
NTO
SOC
Conostylis juncea
Climate
Climate + Soil
Soil
AUC = 0.99
TSS = 0.94
AUC = 1
TSS = 0.92
AUC = 0.99
TSS = 0.86
Relative importance
bio01
bio02
bio04
bio05
bio15
BDW
CLY
DES
NTO
SOC

Conostylis prolifera

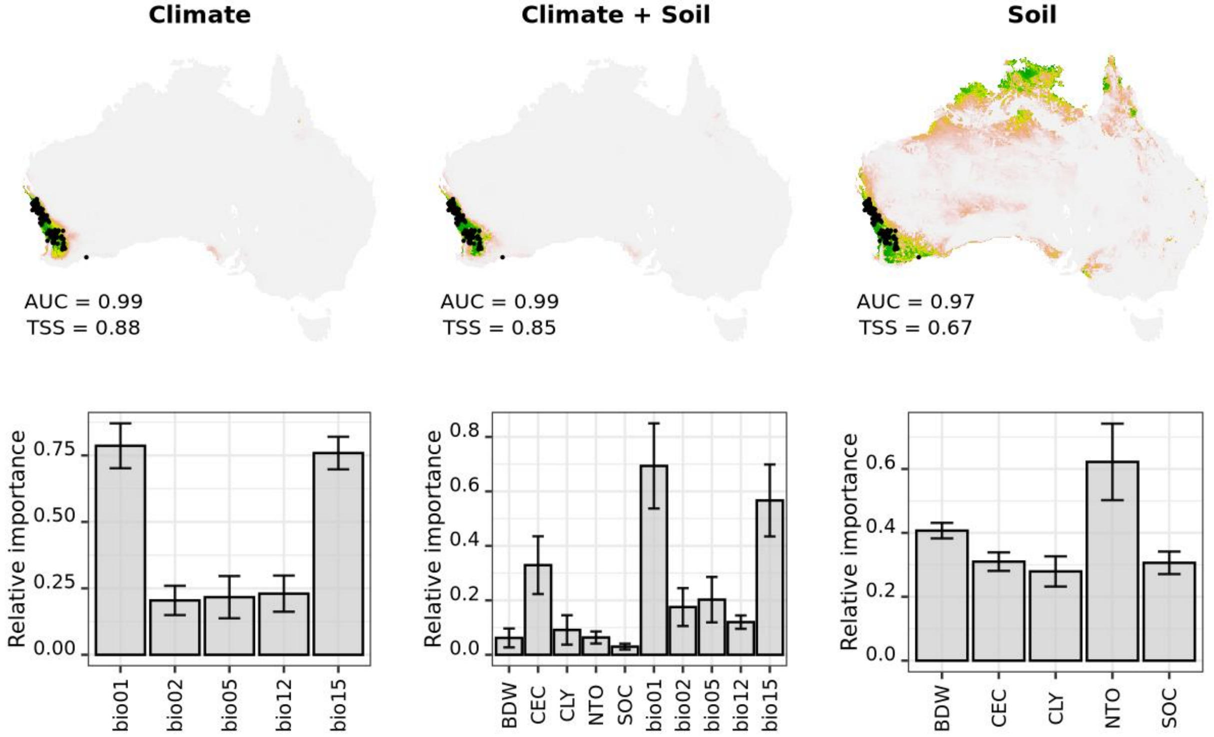

Conostylis pusilla

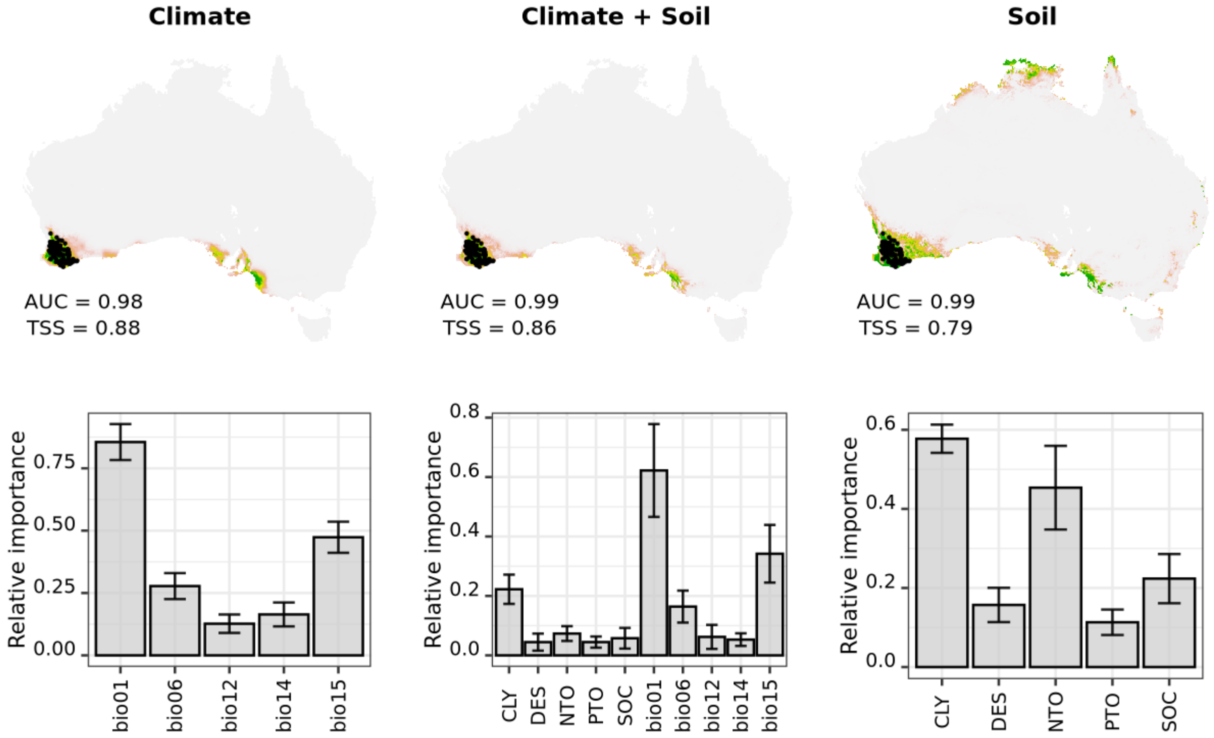

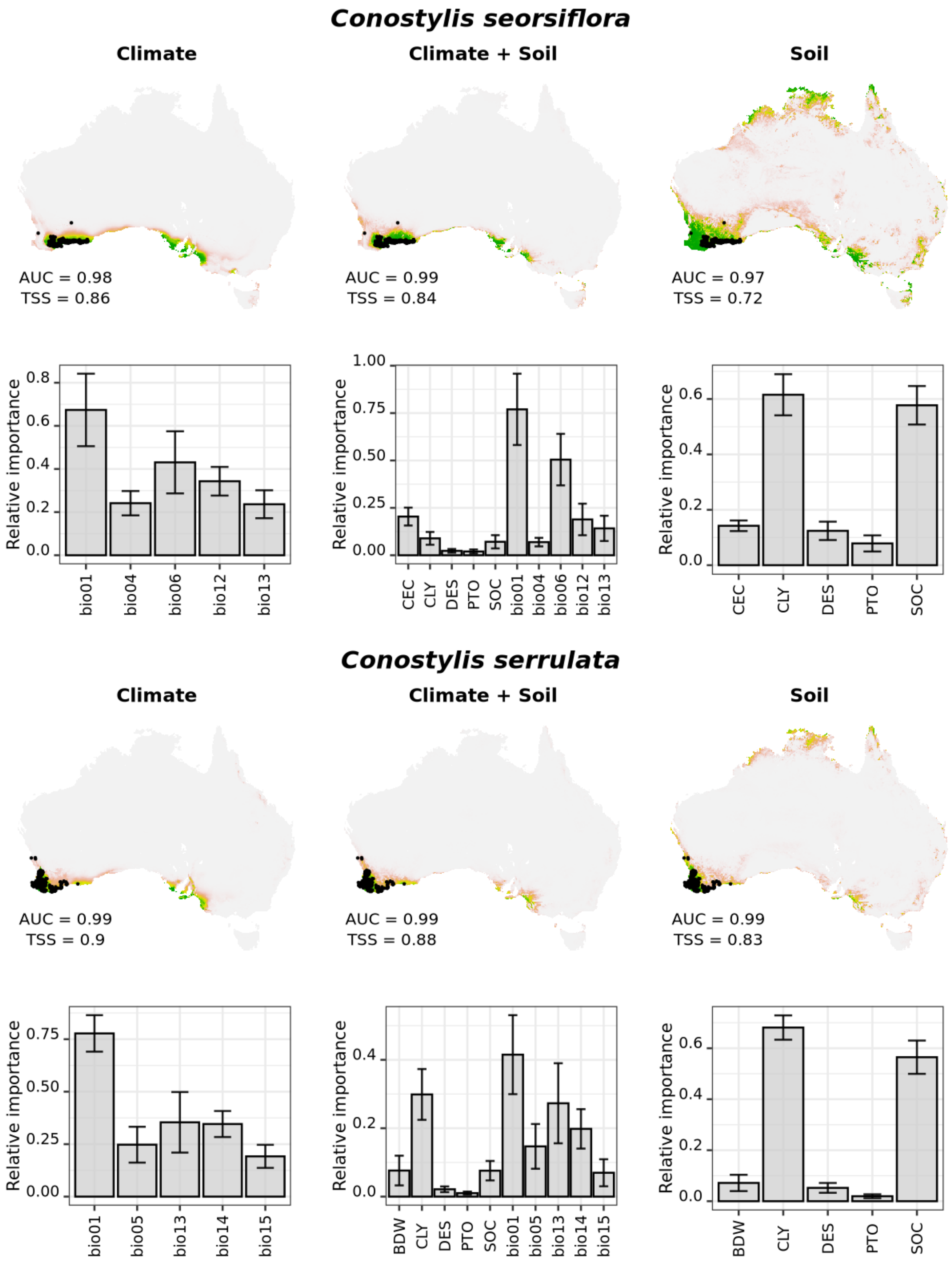
Conostylis seorsiflora
Climate
Climate + Soil
Soil
AUC = 0.98
TSS = 0.86
AUC = 0.99
TSS = 0.84
AUC = 0.97
TSS = 0.72
Relative importance
bio01
bio04
bio06
bio12
bio13
CEC
CLY
DES
PTO
SOC
Conostylis serrulata
Climate
Climate + Soil
Soil
AUC = 0.99
TSS = 0.9
AUC = 0.99
TSS = 0.88
AUC = 0.99
TSS = 0.83
Relative importance
bio01
bio05
bio13
bio14
bio15
BDW
CLY
DES
PTO
SOC

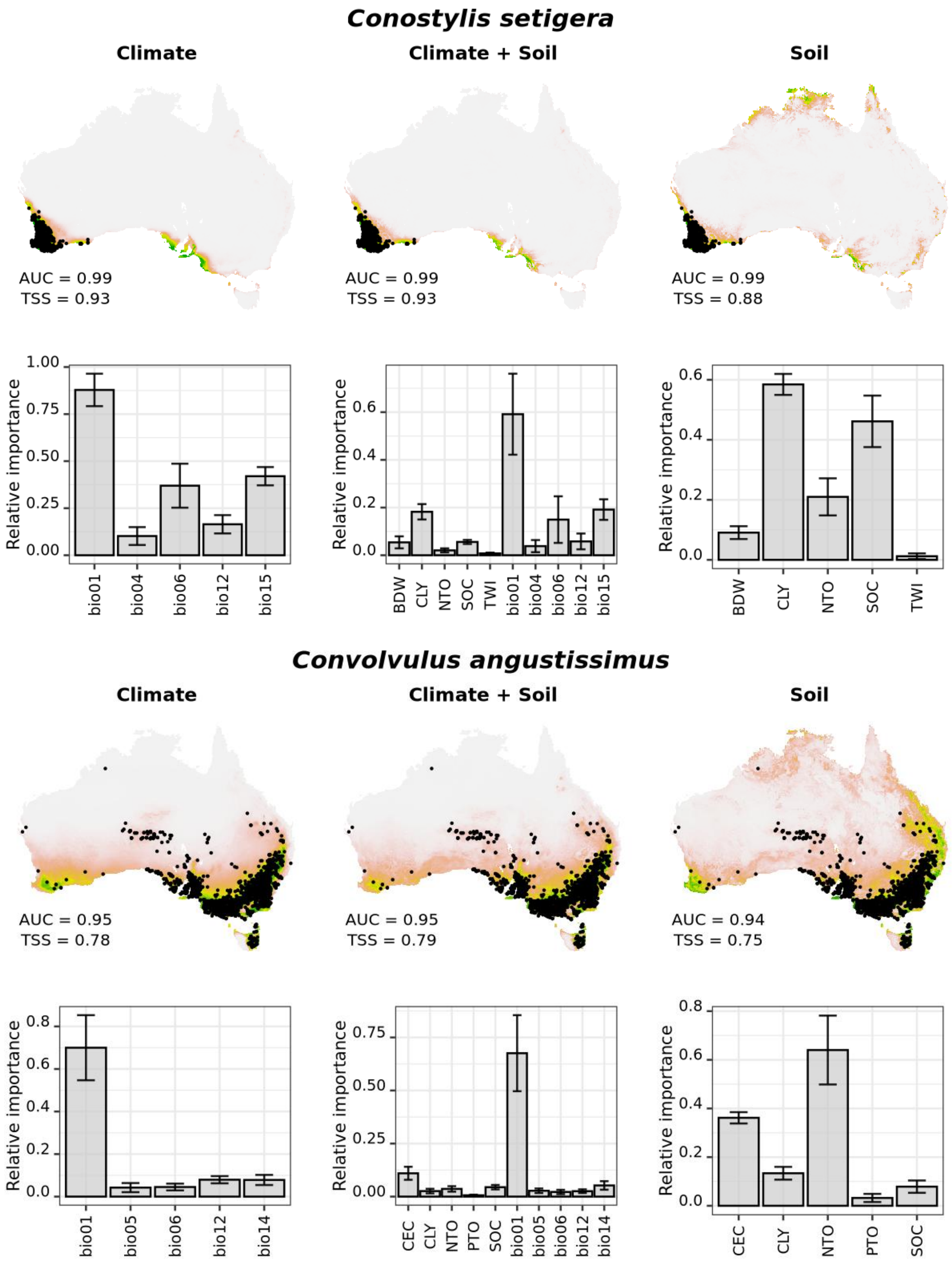
Conostylis setigera
Climate
Climate + Soil
Soil
AUC = 0.99
TSS = 0.93
AUC = 0.99
TSS = 0.93
AUC = 0.99
TSS = 0.88
Relative importance
1.00
0.75
0.50
0.25
0.00
bio01
bio04
bio06
bio12
bio15
Relative importance
0.6
0.4
0.2
0.0
BDW
CLY
NTO
SOC
TWI
bio01
bio04
bio06
bio12
bio15
Relative importance
0.6
0.4
0.2
0.0
BDW
CLY
NTO
SOC
TWI
Convolvulus angustissimus
Climate
Climate + Soil
Soil
AUC = 0.95
TSS = 0.78
AUC = 0.95
TSS = 0.79
AUC = 0.94
TSS = 0.75
Relative importance
0.8
0.6
0.4
0.2
0.0
bio01
bio05
bio06
bio12
bio14
Relative importance
0.75
0.50
0.25
0.00
CEC
CLY
NTO
PTO
SOC
bio01
bio05
bio06
bio12
bio14
Relative importance
0.8
0.6
0.4
0.2
0.0
CEC
CLY
NTO
PTO
SOC

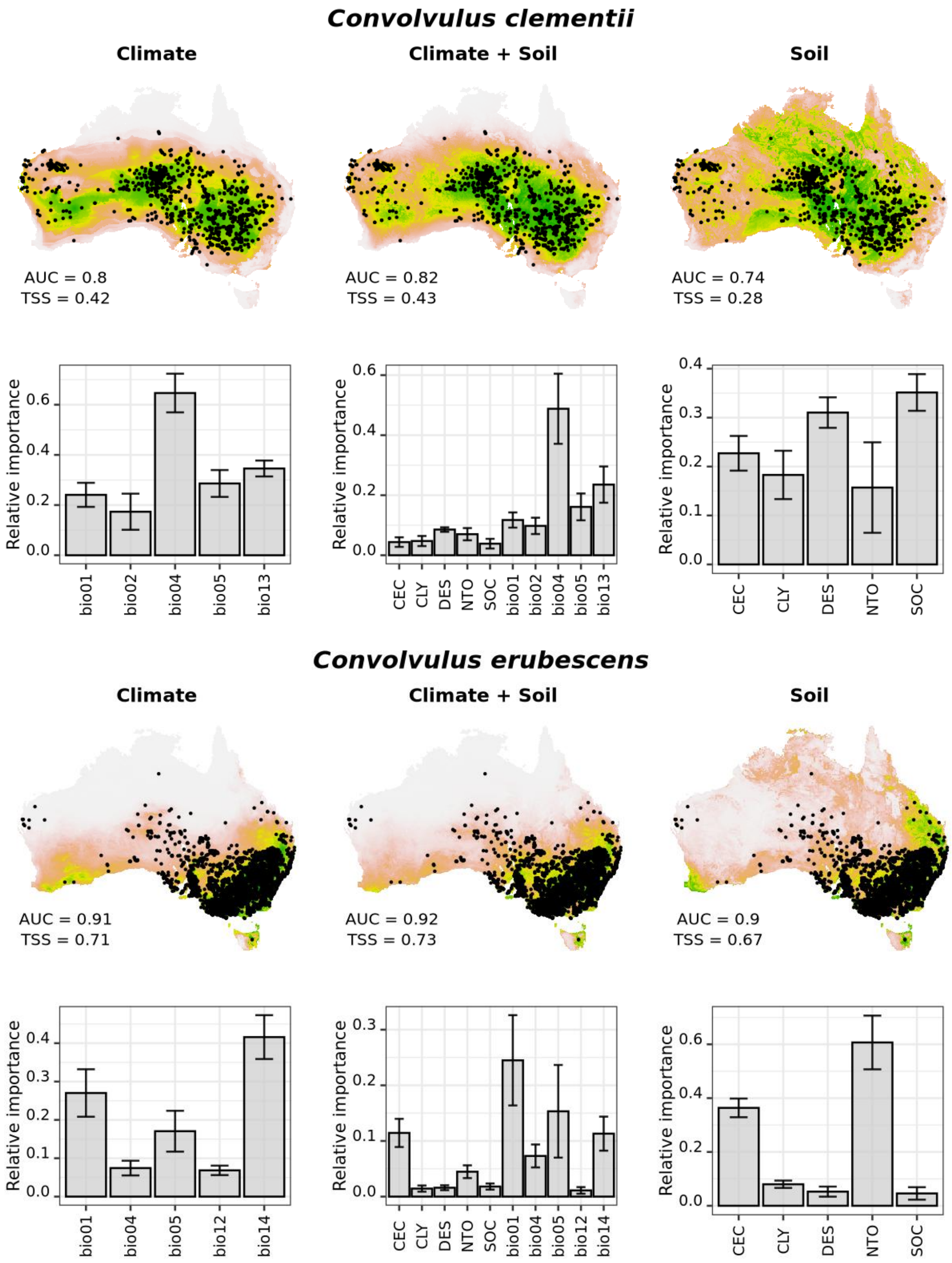
Convolvulus clementii
Climate
Climate + Soil
Soil
AUC = 0.8
TSS = 0.42
AUC = 0.82
TSS = 0.43
AUC = 0.74
TSS = 0.28
Relative importance
bio01
bio02
bio04
bio05
bio13
CEC
CLY
DES
NTO
SOC
Convolvulus erubescens
Climate
Climate + Soil
Soil
AUC = 0.91
TSS = 0.71
AUC = 0.92
TSS = 0.73
AUC = 0.9
TSS = 0.67
Relative importance
bio01
bio04
bio05
bio12
bio14
CEC
CLY
DES
NTO
SOC

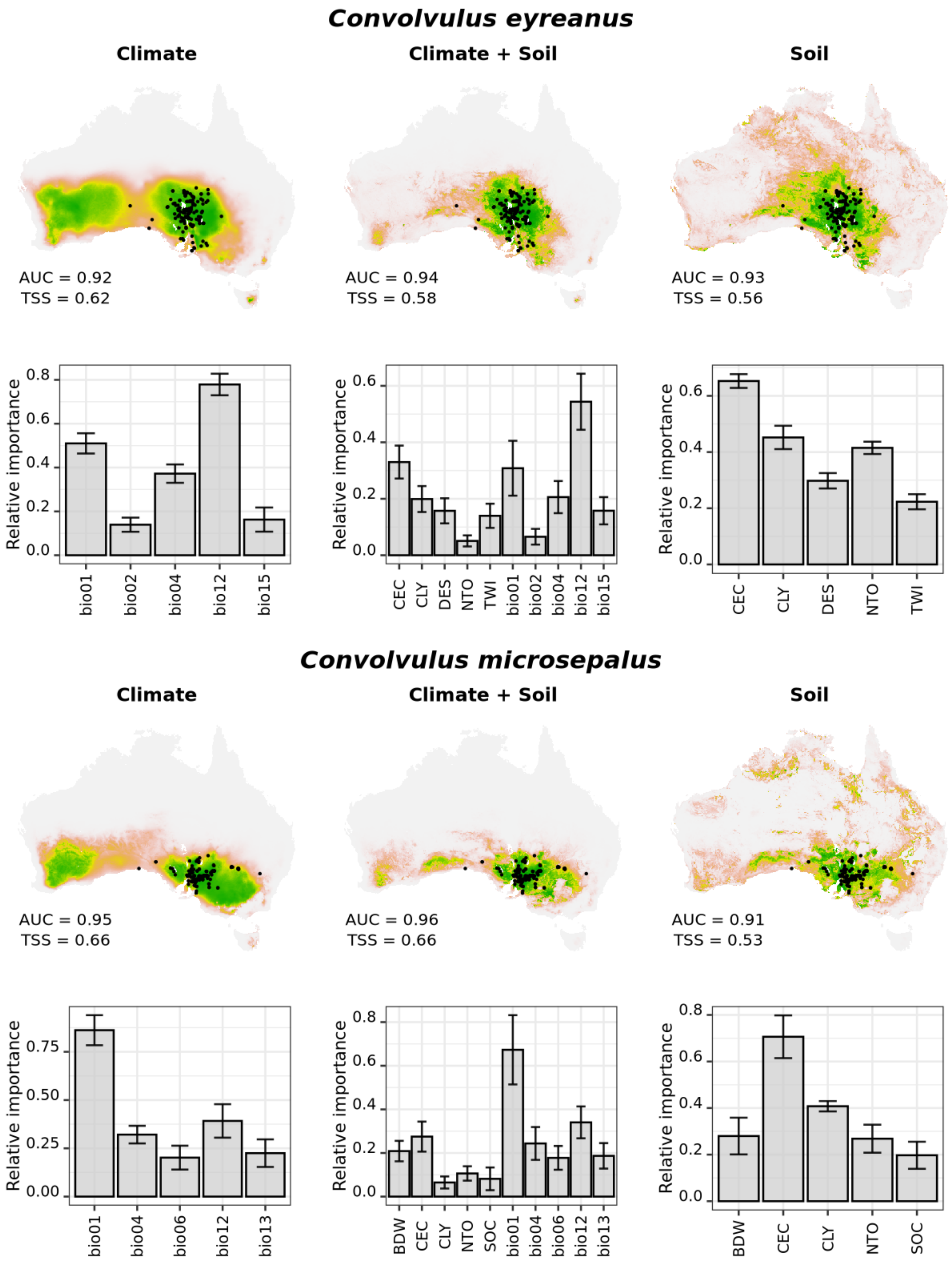

Convolvulus eyreanus
Climate
Climate + Soil
Soil
AUC = 0.92
TSS = 0.62
AUC = 0.94
TSS = 0.58
AUC = 0.93
TSS = 0.56
Relative importance
0.0
0.2
0.4
0.6
0.8
bio01
bio02
bio04
bio12
bio15
CEC
CLY
DES
NTO
TWI
Convolvulus microsepalus
AUC = 0.95
TSS = 0.66
AUC = 0.96
TSS = 0.66
AUC = 0.91
TSS = 0.53
0.00
0.25
0.50
0.75
bio06
bio13
BDW
SOC

Coopernookia barbata

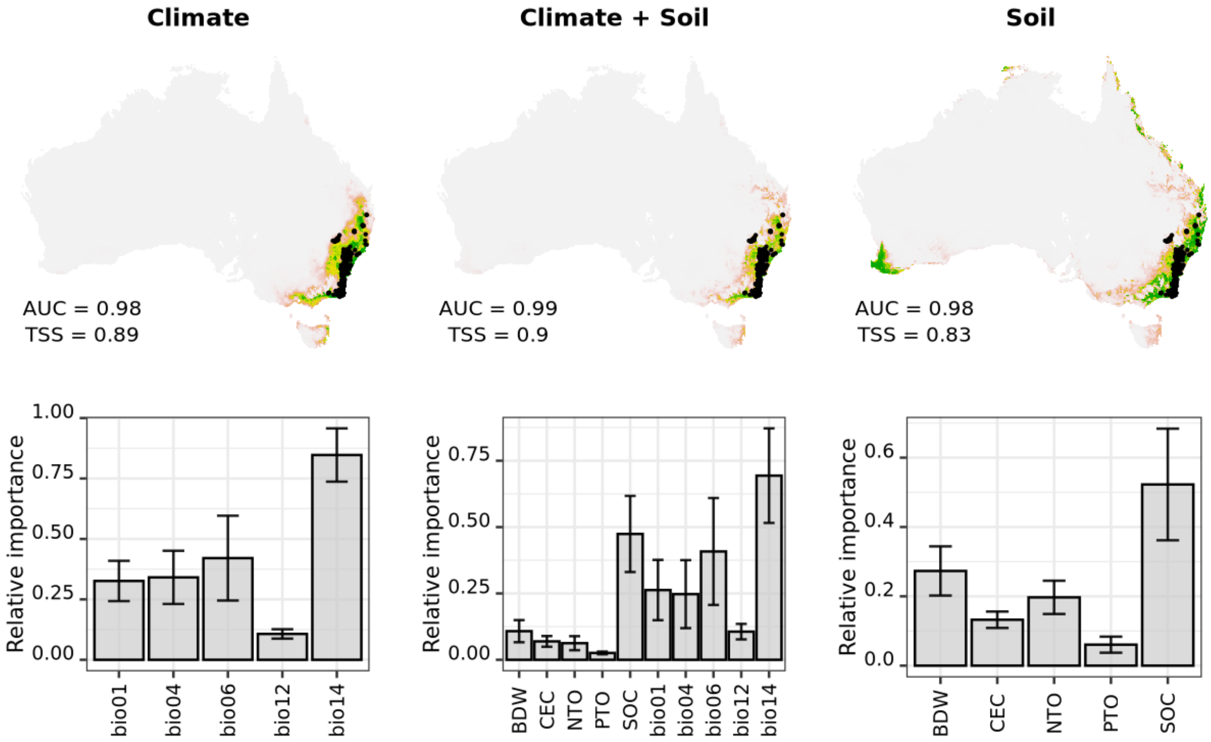

Coopernookia strophiolata

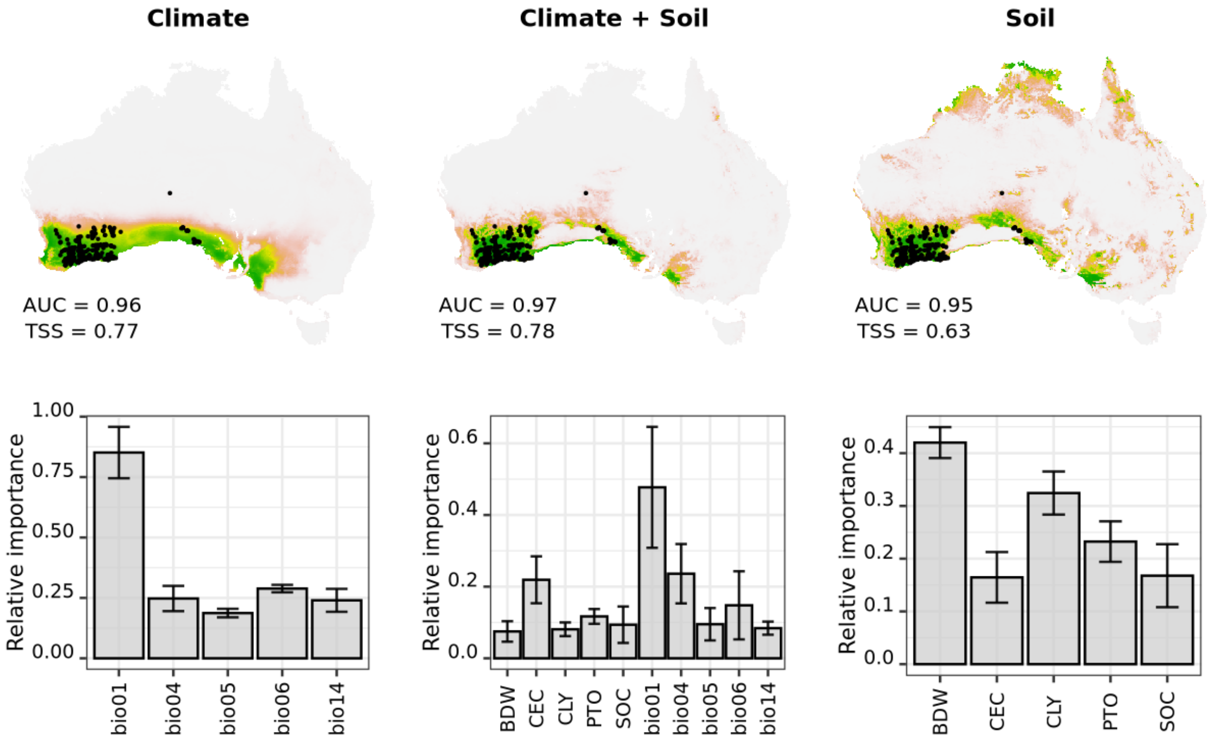

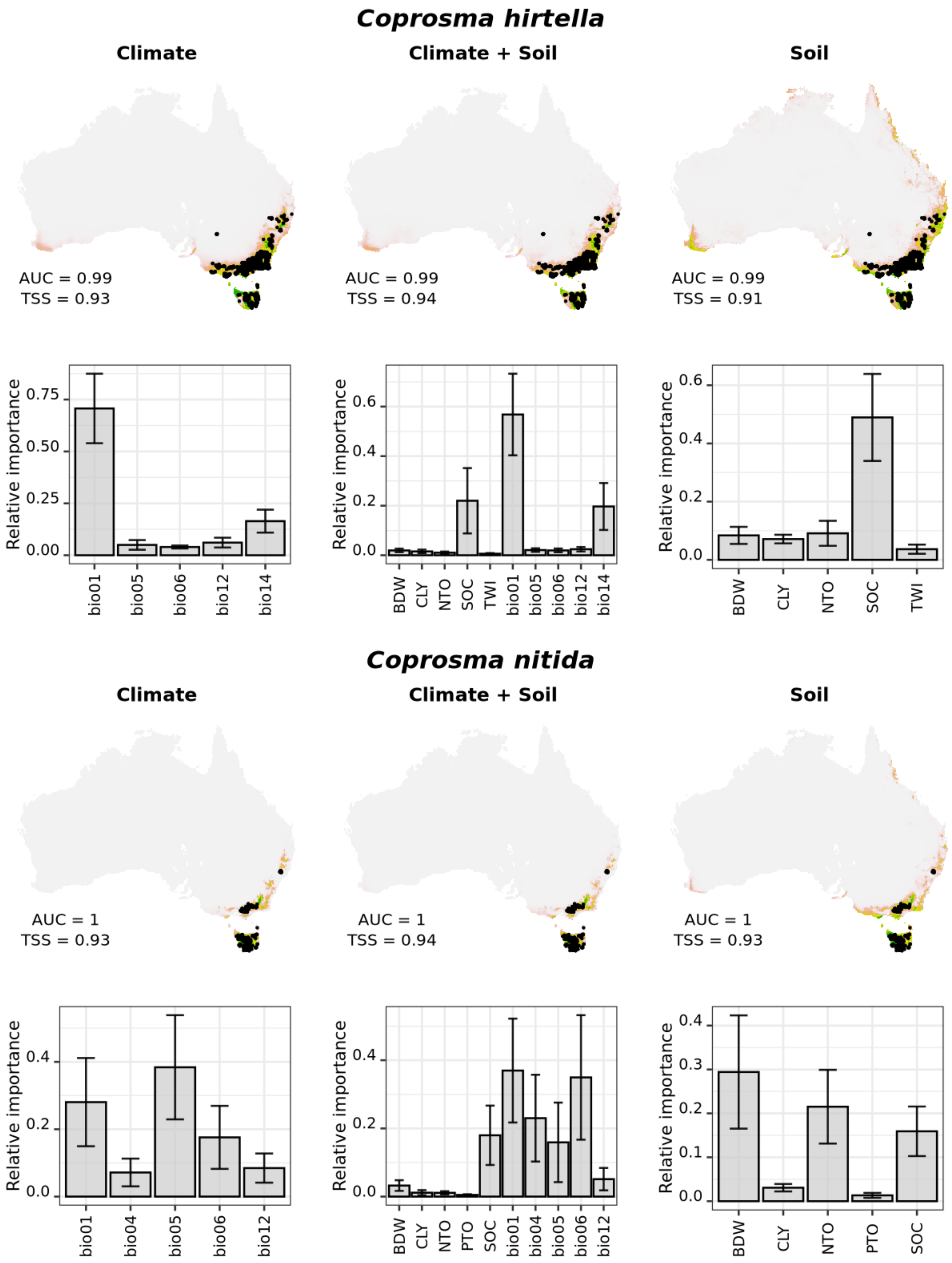

Coprosma hirtella
Climate
Climate + Soil
Soil
AUC = 0.99
TSS = 0.93
AUC = 0.99
TSS = 0.94
AUC = 0.99
TSS = 0.91
Relative importance
bio01
bio05
bio06
bio12
bio14
BDW
CLY
NTO
SOC
TWI
Coprosma nitida
AUC = 1
TSS = 0.93
AUC = 1
TSS = 0.94
AUC = 1
TSS = 0.93
bio04
PTO

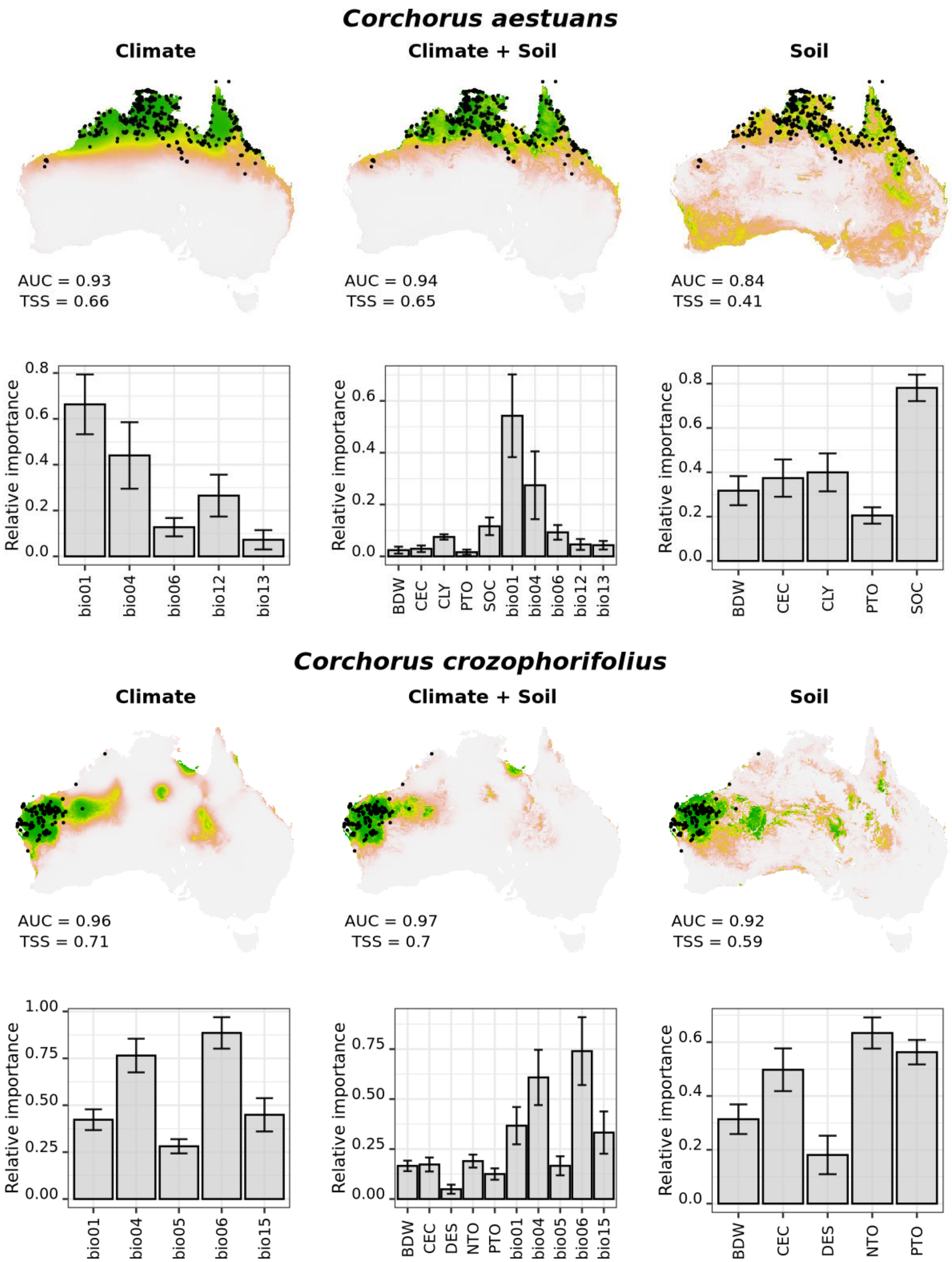

Corchorus aestuans
Climate
Climate + Soil
Soil
AUC = 0.93
TSS = 0.66
AUC = 0.94
TSS = 0.65
AUC = 0.84
TSS = 0.41
Relative importance
bio01
bio04
bio06
bio12
bio13
BDW
CEC
CLY
PTO
SOC
Corchorus crozophorifolius
Climate
Climate + Soil
Soil
AUC = 0.96
TSS = 0.71
AUC = 0.97
TSS = 0.7
AUC = 0.92
TSS = 0.59
Relative importance
bio01
bio04
bio05
bio06
bio15
BDW
CEC
DES
NTO
PTO

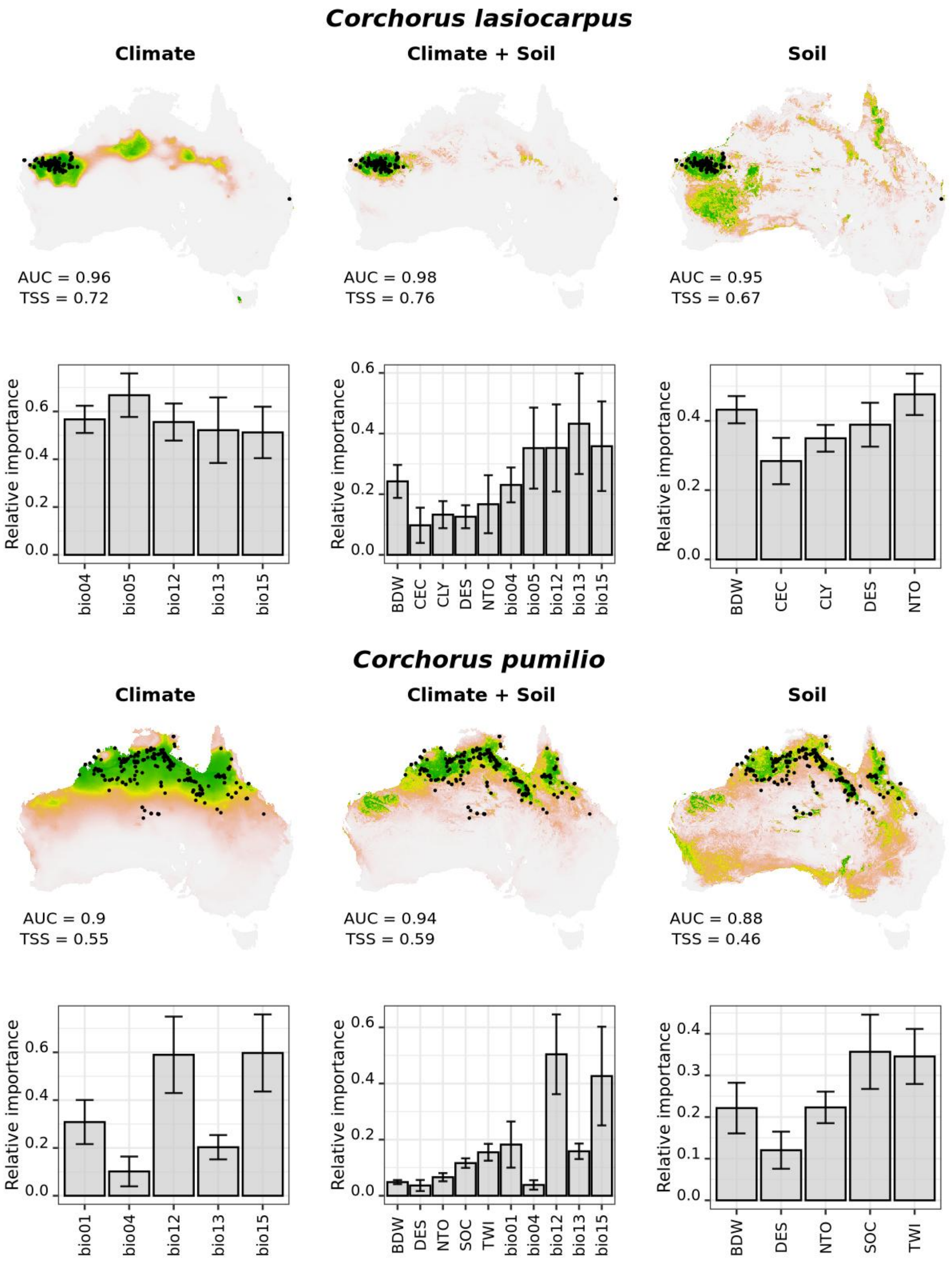
Corchorus lasiocarpus
Climate
Climate + Soil
Soil
AUC = 0.96
TSS = 0.72
AUC = 0.98
TSS = 0.76
AUC = 0.95
TSS = 0.67
Relative importance
bio04 bio05 bio12 bio13 bio15
BDW CEC CLY DES NTO bio04 bio05 bio12 bio13 bio15
BDW CEC CLY DES NTO
Corchorus pumilio
Climate
Climate + Soil
Soil
AUC = 0.9
TSS = 0.55
AUC = 0.94
TSS = 0.59
AUC = 0.88
TSS = 0.46
Relative importance
bio01 bio04 bio12 bio13 bio15
BDW DES NTO SOC TWI bio01 bio04 bio12 bio13 bio15
BDW DES NTO SOC TWI

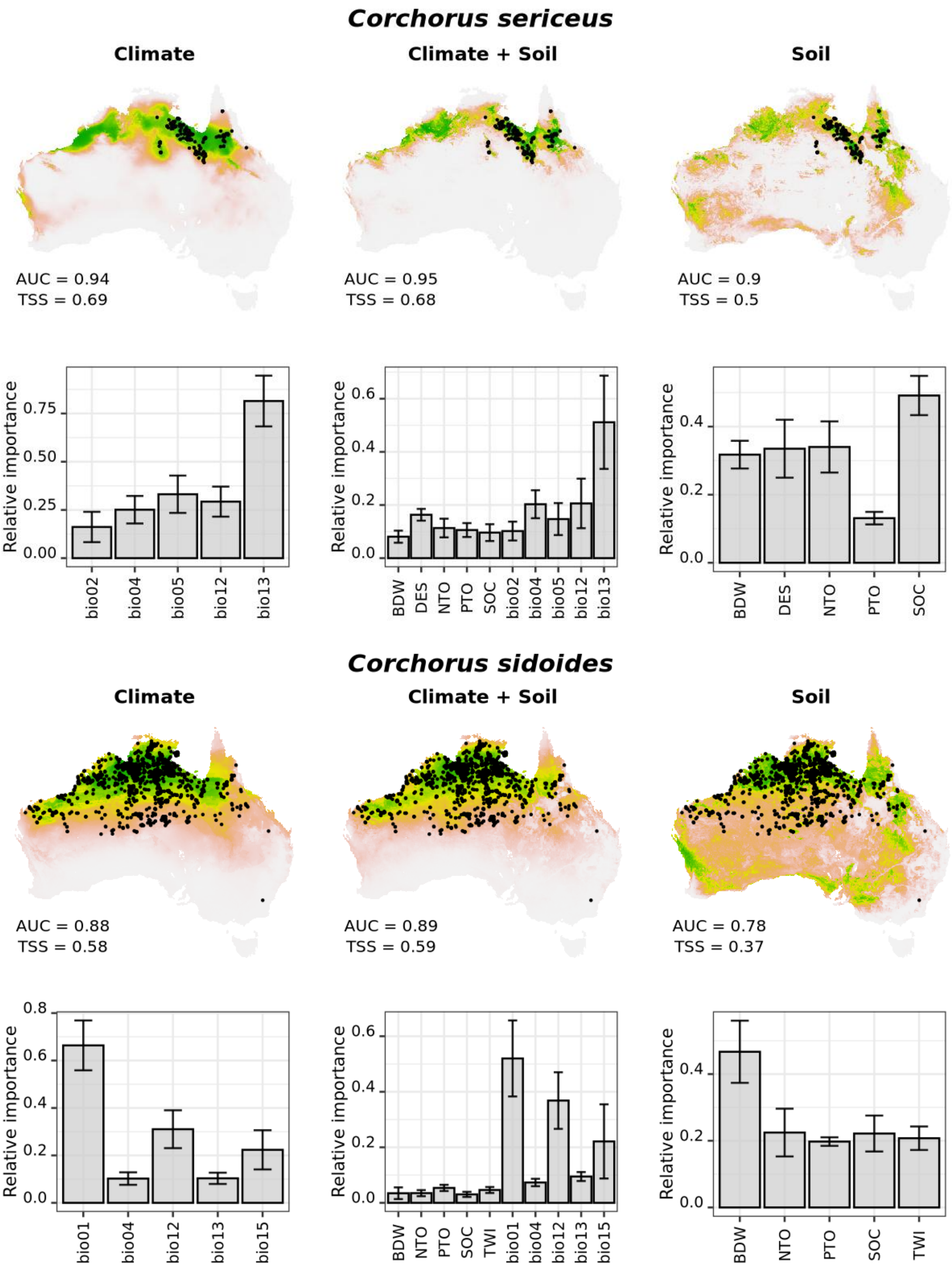
Corchorus sericeus
Climate
Climate + Soil
Soil
AUC = 0.94
TSS = 0.69
AUC = 0.95
TSS = 0.68
AUC = 0.9
TSS = 0.5
Relative importance
0.00
0.25
0.50
0.75
bio02
bio04
bio05
bio12
bio13
0.0
0.2
0.4
0.6
BDW
DES
NTO
PTO
SOC
bio02
bio04
bio05
bio12
bio13
0.0
0.2
0.4
BDW
DES
NTO
PTO
SOC
Corchorus sidoides
Climate
Climate + Soil
Soil
AUC = 0.88
TSS = 0.58
AUC = 0.89
TSS = 0.59
AUC = 0.78
TSS = 0.37
Relative importance
0.0
0.2
0.4
0.6
0.8
bio01
bio04
bio12
bio13
bio15
0.0
0.2
0.4
0.6
BDW
NTO
PTO
SOC
TWI
bio01
bio04
bio12
bio13
bio15
0.0
0.2
0.4
BDW
NTO
PTO
SOC
TWI

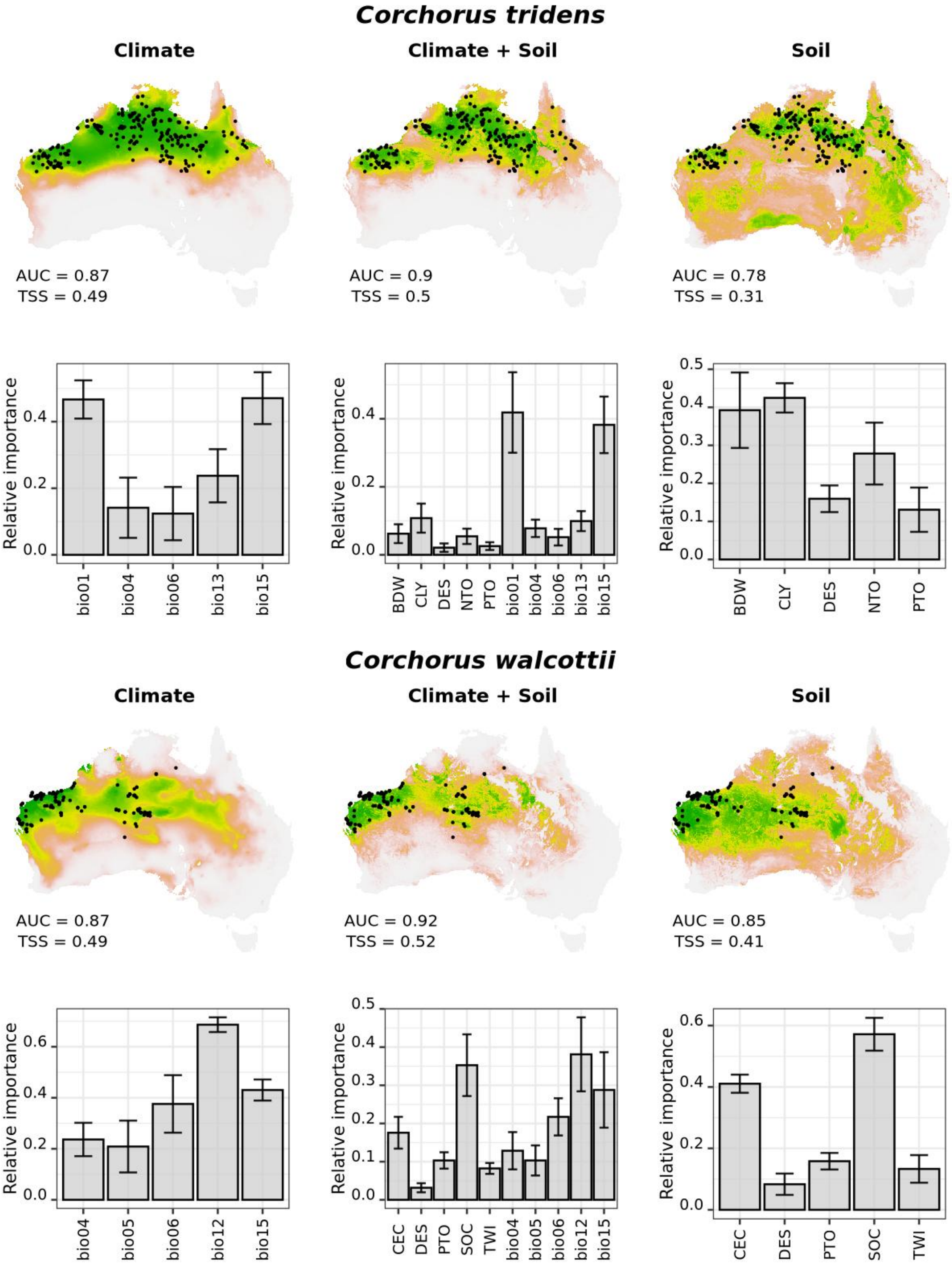
Corchorus tridens
Climate
Climate + Soil
Soil
AUC = 0.87
TSS = 0.49
AUC = 0.9
TSS = 0.5
AUC = 0.78
TSS = 0.31
Relative importance
bio01 bio04 bio06 bio13 bio15
BDW CLY DES NTO PTO bio01 bio04 bio06 bio13 bio15
BDW CLY DES NTO PTO
Corchorus walcottii
Climate
Climate + Soil
Soil
AUC = 0.87
TSS = 0.49
AUC = 0.92
TSS = 0.52
AUC = 0.85
TSS = 0.41
Relative importance
bio04 bio05 bio06 bio12 bio15
CEC DES PTO SOC TWI bio04 bio05 bio06 bio12 bio15
CEC DES PTO SOC TWI

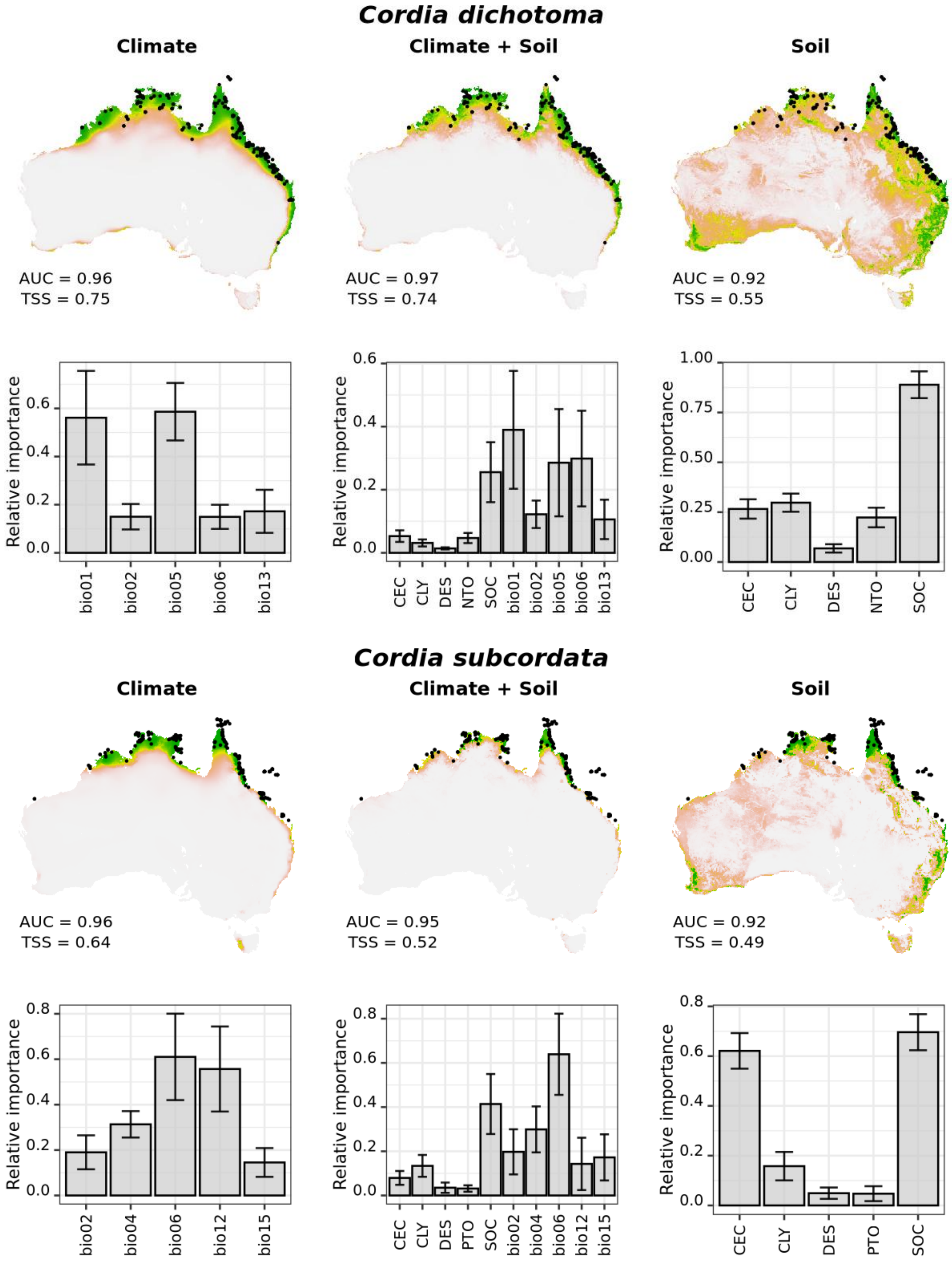

Cordia dichotoma
Climate
Climate + Soil
Soil
AUC = 0.96
TSS = 0.75
AUC = 0.97
TSS = 0.74
AUC = 0.92
TSS = 0.55
Relative importance
bio01
bio02
bio05
bio06
bio13
CEC
CLY
DES
NTO
SOC
Cordia subcordata
Climate
Climate + Soil
Soil
AUC = 0.96
TSS = 0.64
AUC = 0.95
TSS = 0.52
AUC = 0.92
TSS = 0.49
bio02
bio04
bio06
bio12
bio15
PTO

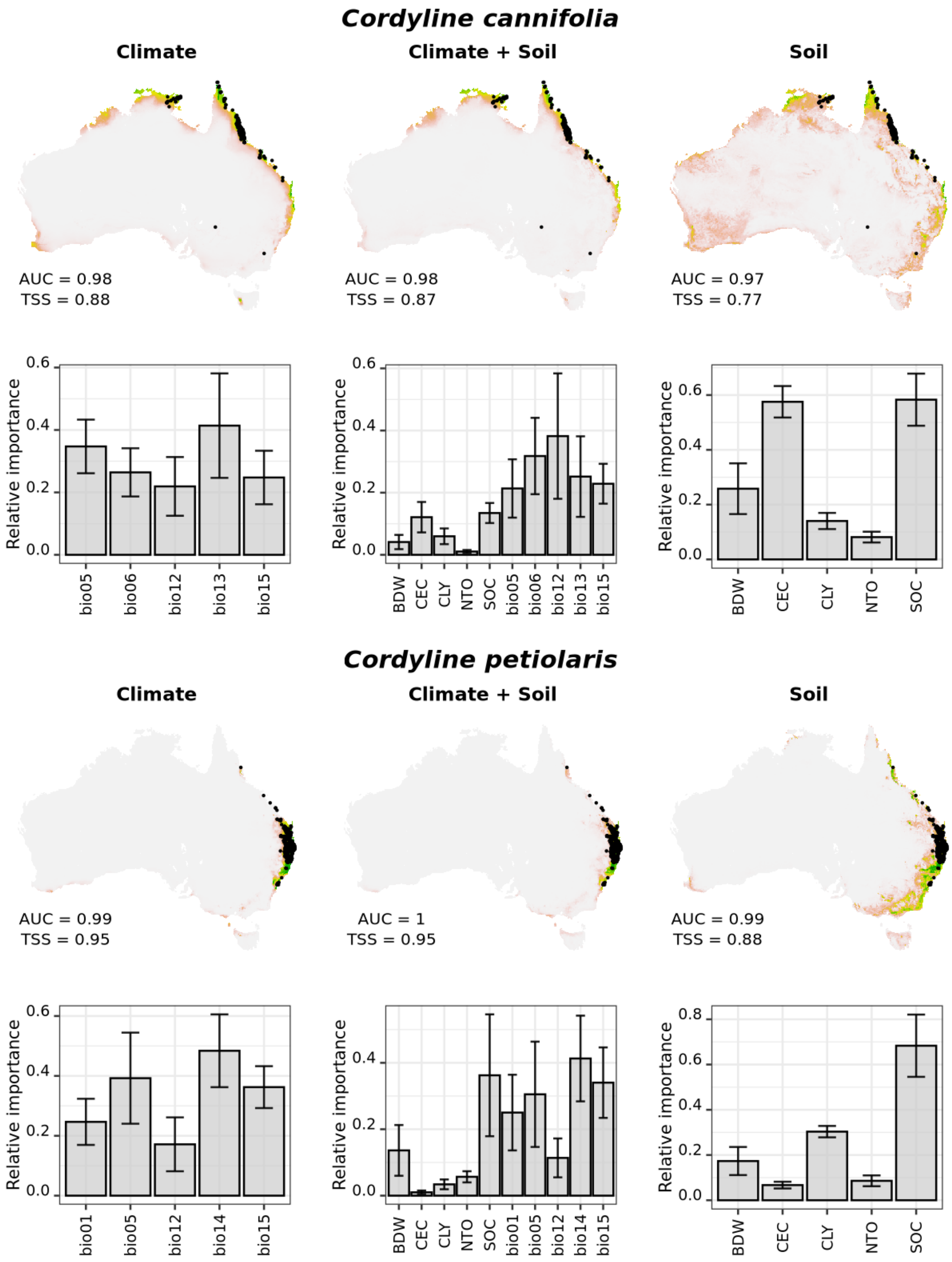

Cordyline cannifolia
Climate
Climate + Soil
Soil
AUC = 0.98
TSS = 0.88
AUC = 0.98
TSS = 0.87
AUC = 0.97
TSS = 0.77
Relative importance
bio05
bio06
bio12
bio13
bio15
BDW
CEC
CLY
NTO
SOC
Cordyline petiolaris
Climate
Climate + Soil
Soil
AUC = 0.99
TSS = 0.95
AUC = 1
TSS = 0.95
AUC = 0.99
TSS = 0.88
bio01
bio14

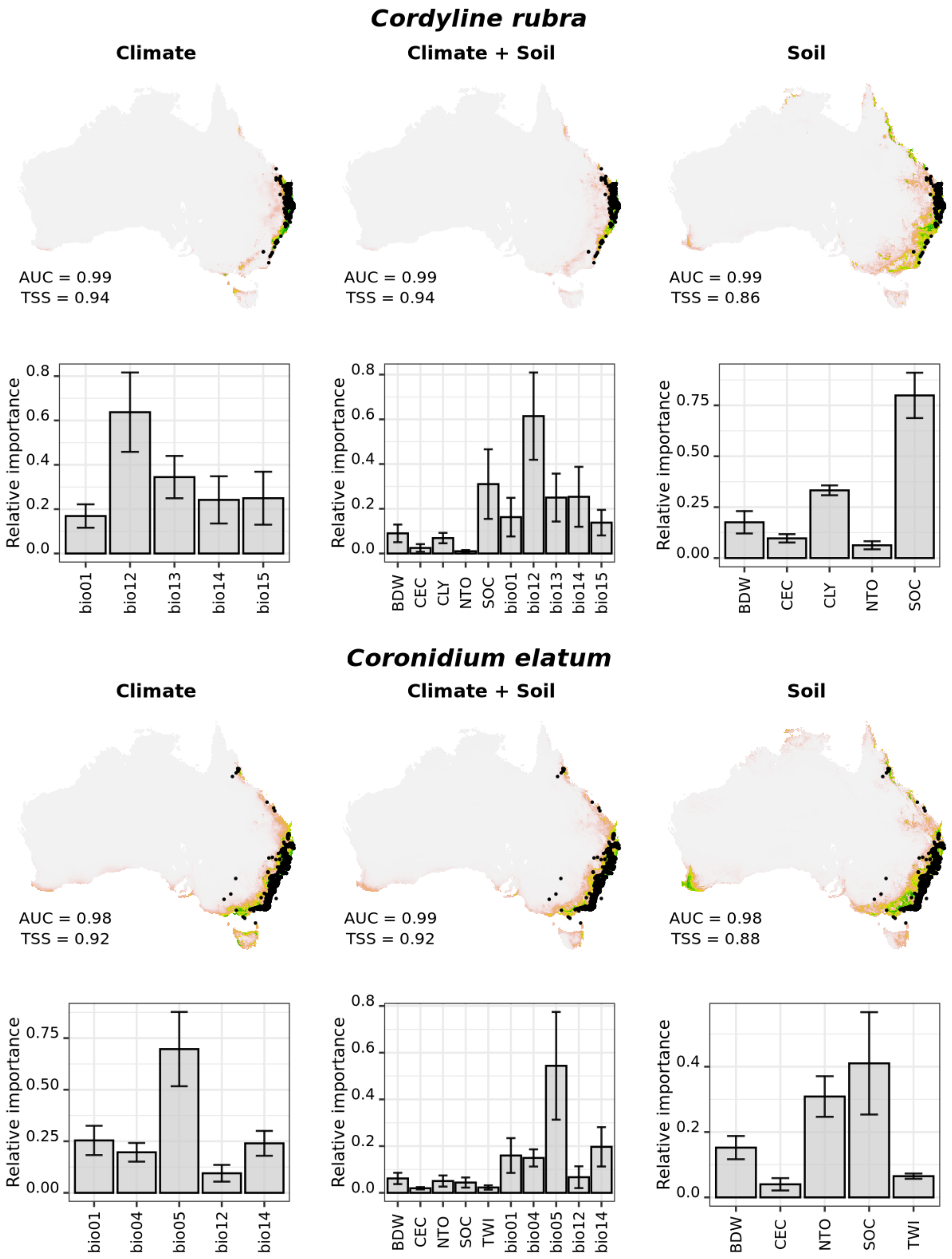
Cordyline rubra
Climate
Climate + Soil
Soil
AUC = 0.99
TSS = 0.94
AUC = 0.99
TSS = 0.94
AUC = 0.99
TSS = 0.86
Relative importance
bio01
bio12
bio13
bio14
bio15
BDW
CEC
CLY
NTO
SOC
Coronidium elatum
Climate
Climate + Soil
Soil
AUC = 0.98
TSS = 0.92
AUC = 0.99
TSS = 0.92
AUC = 0.98
TSS = 0.88
Relative importance
bio01
bio04
bio05
bio12
bio14
BDW
CEC
NTO
SOC
TWI

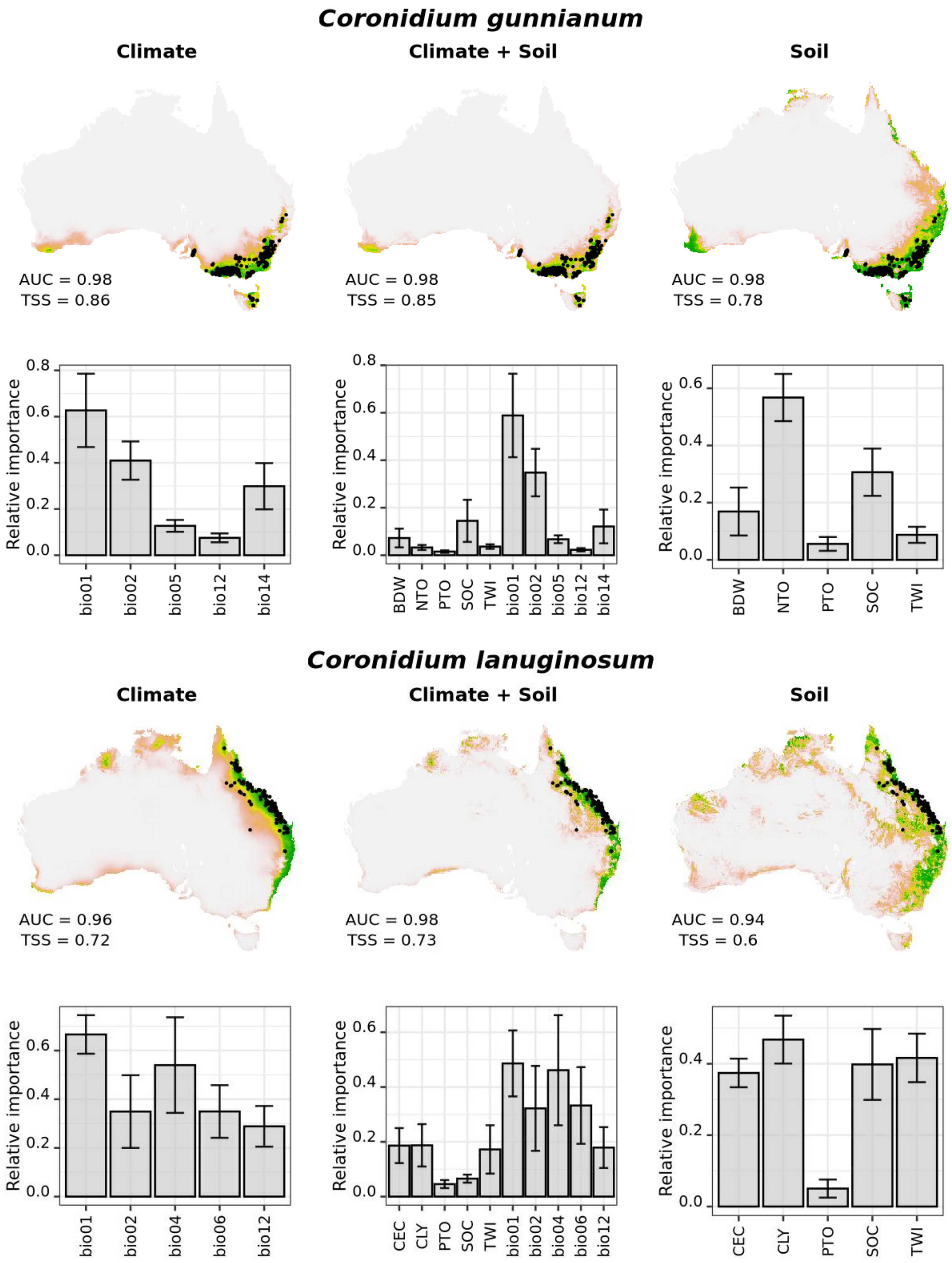

Coronidium gunnianum
Climate
Climate + Soil
Soil
AUC = 0.98
TSS = 0.86
AUC = 0.98
TSS = 0.85
AUC = 0.98
TSS = 0.78
Relative importance
0.0
0.2
0.4
0.6
0.8
bio01
bio02
bio05
bio12
bio14
BDW
NTO
PTO
SOC
TWI
bio01
bio02
bio05
bio12
bio14
BDW
NTO
PTO
SOC
TWI
Coronidium lanuginosum
Climate
Climate + Soil
Soil
AUC = 0.96
TSS = 0.72
AUC = 0.98
TSS = 0.73
AUC = 0.94
TSS = 0.6
Relative importance
bio01
bio02
bio04
bio06
bio12
CEC
CLY
PTO
SOC
TWI
bio01
bio02
bio04
bio06
bio12
CEC
CLY
PTO
SOC
TWI

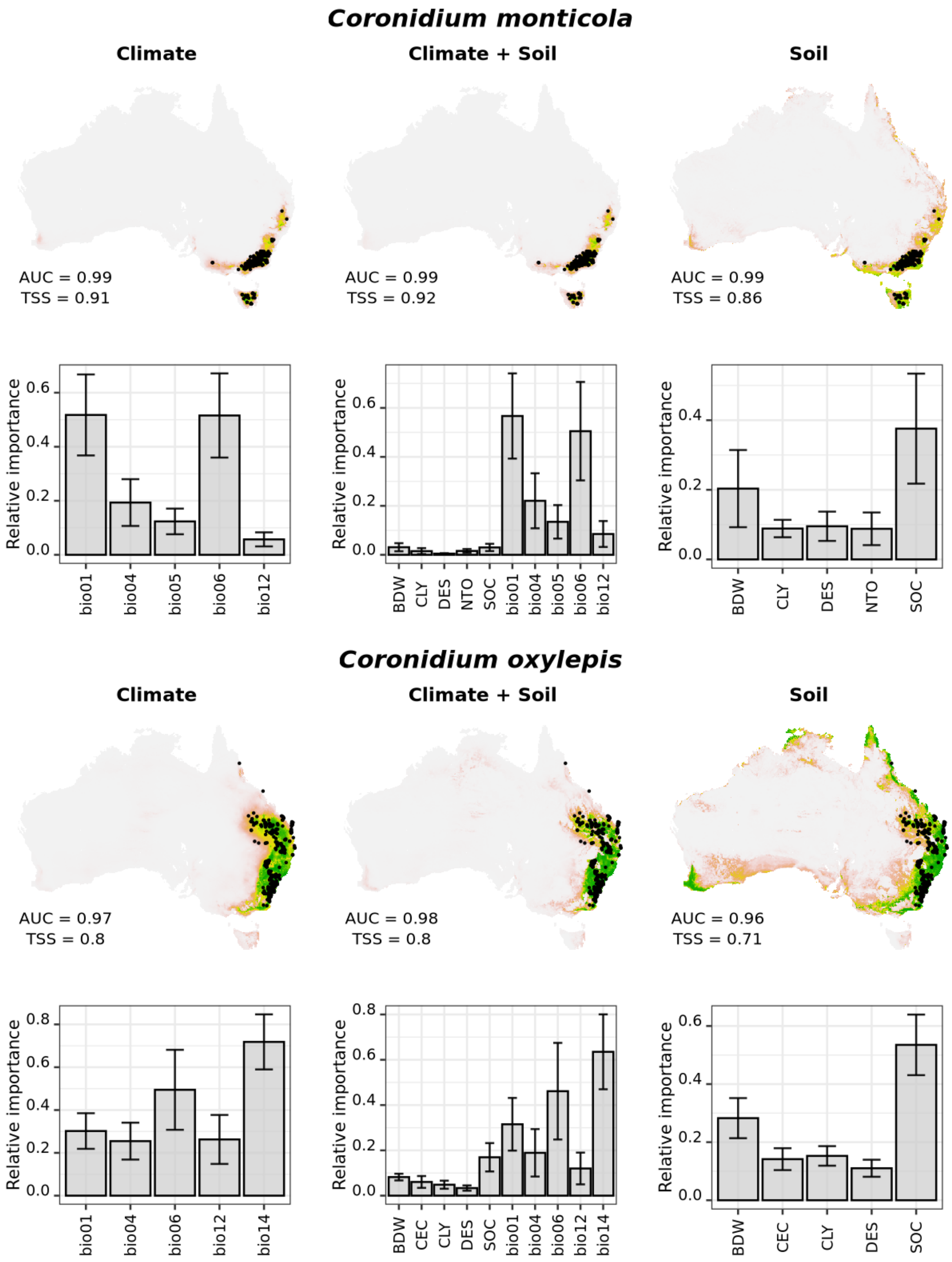
Coronidium monticola
Climate
Climate + Soil
Soil
AUC = 0.99
TSS = 0.91
AUC = 0.99
TSS = 0.92
AUC = 0.99
TSS = 0.86
Relative importance
bio01
bio04
bio05
bio06
bio12
BDW
CLY
DES
NTO
SOC
Coronidium oxylepis
Climate
Climate + Soil
Soil
AUC = 0.97
TSS = 0.8
AUC = 0.98
TSS = 0.8
AUC = 0.96
TSS = 0.71
Relative importance
bio01
bio04
bio06
bio12
bio14
BDW
CEC
CLY
DES
SOC

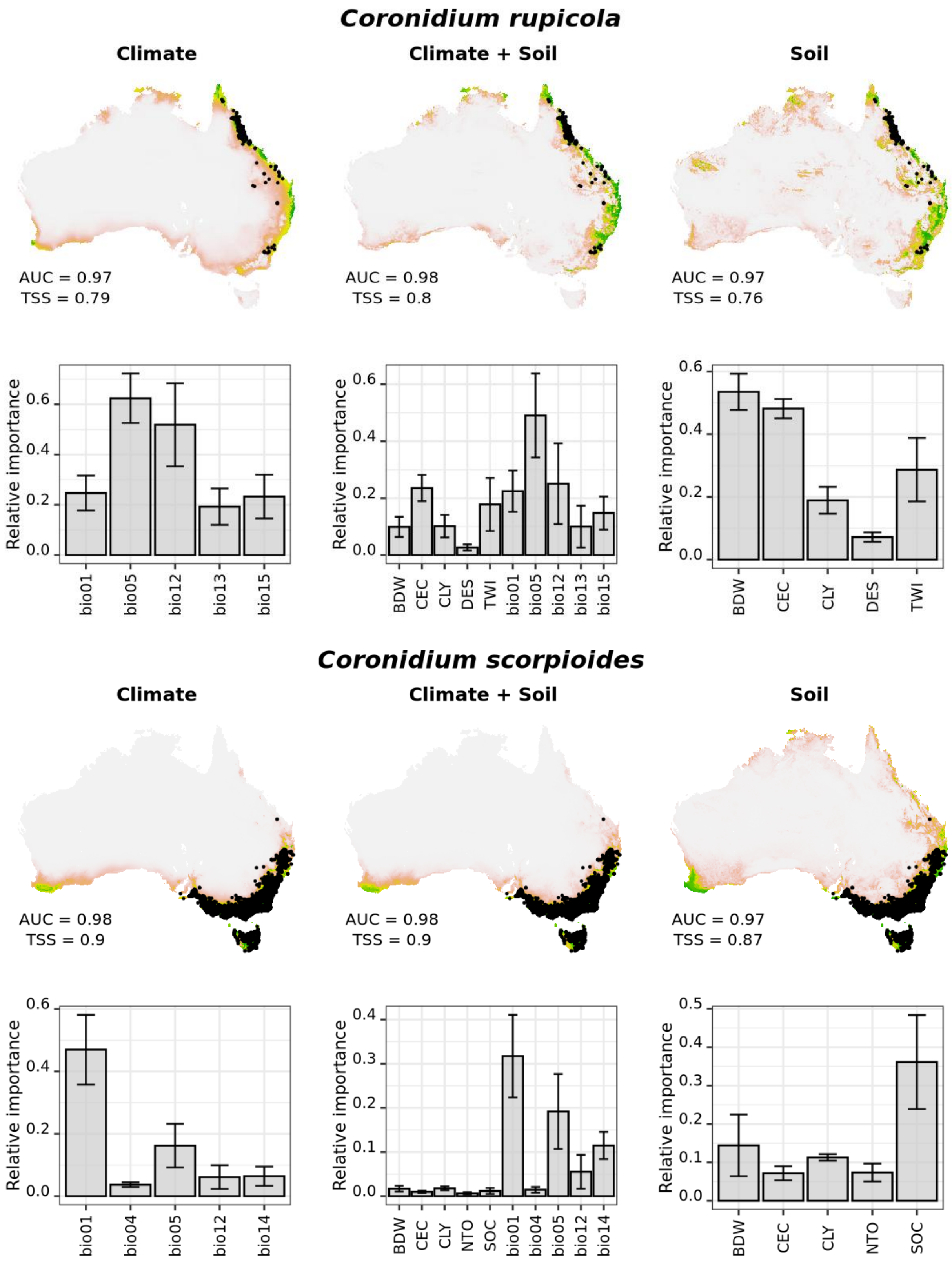

Coronidium rupicola
Climate
Climate + Soil
Soil
AUC = 0.97
TSS = 0.79
AUC = 0.98
TSS = 0.8
AUC = 0.97
TSS = 0.76
Relative importance
bio01
bio05
bio12
bio13
bio15
BDW
CEC
CLY
DES
TWI
Coronidium scorpioides
Climate
Climate + Soil
Soil
AUC = 0.98
TSS = 0.9
AUC = 0.98
TSS = 0.9
AUC = 0.97
TSS = 0.87
Relative importance
bio01
bio04
bio05
bio12
bio14
BDW
CEC
CLY
NTO
SOC

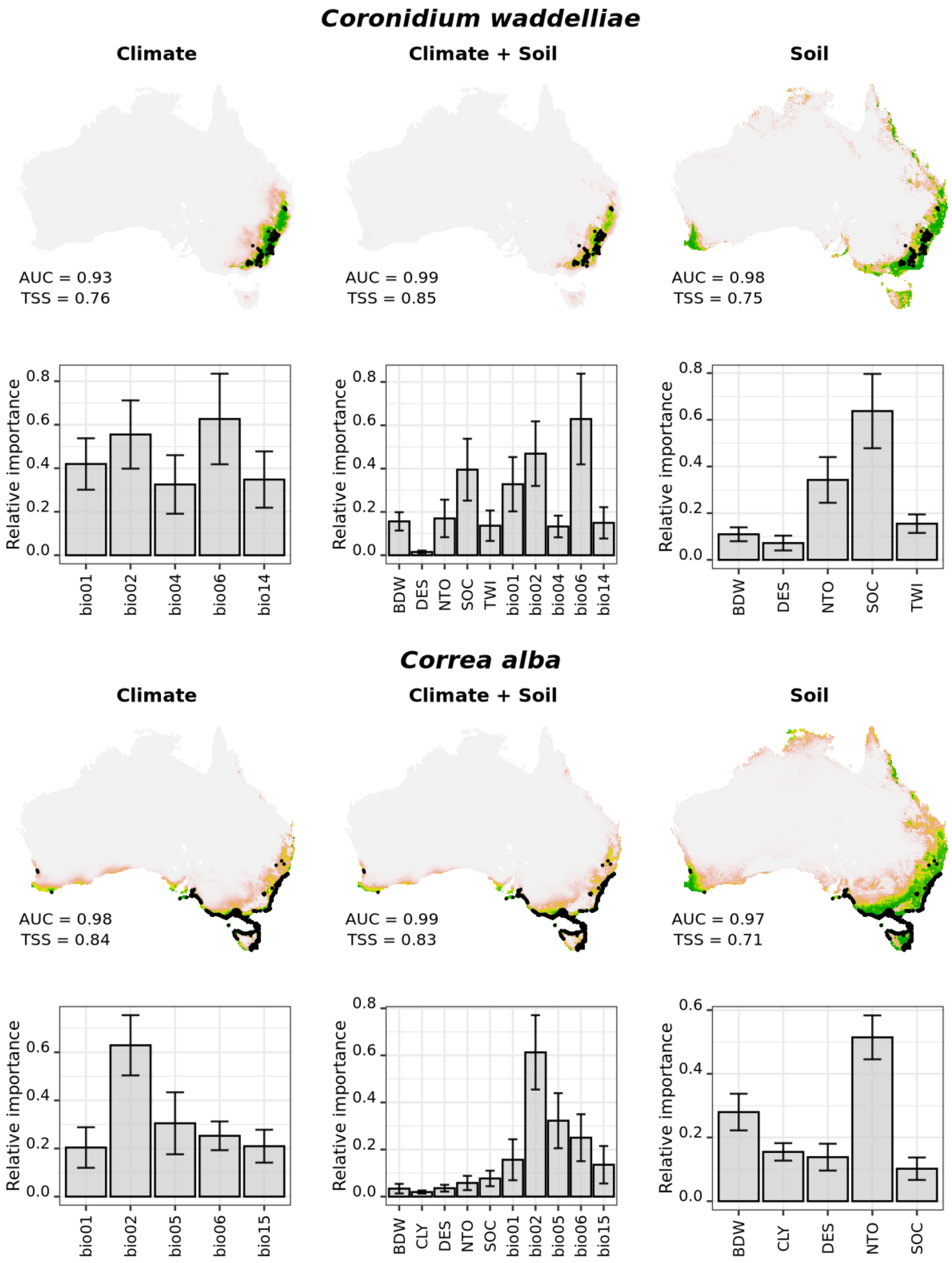

Coronidium waddelliae
Climate
Climate + Soil
Soil
AUC = 0.93
TSS = 0.76
AUC = 0.99
TSS = 0.85
AUC = 0.98
TSS = 0.75
Relative importance
0.0
0.2
0.4
0.6
0.8
bio01
bio02
bio04
bio06
bio14
BDW
DES
NTO
SOC
TWI
Correa alba
Climate
Climate + Soil
Soil
AUC = 0.98
TSS = 0.84
AUC = 0.99
TSS = 0.83
AUC = 0.97
TSS = 0.71
Relative importance
bio01
bio02
bio05
bio06
bio15
BDW
CLY
DES
NTO
SOC

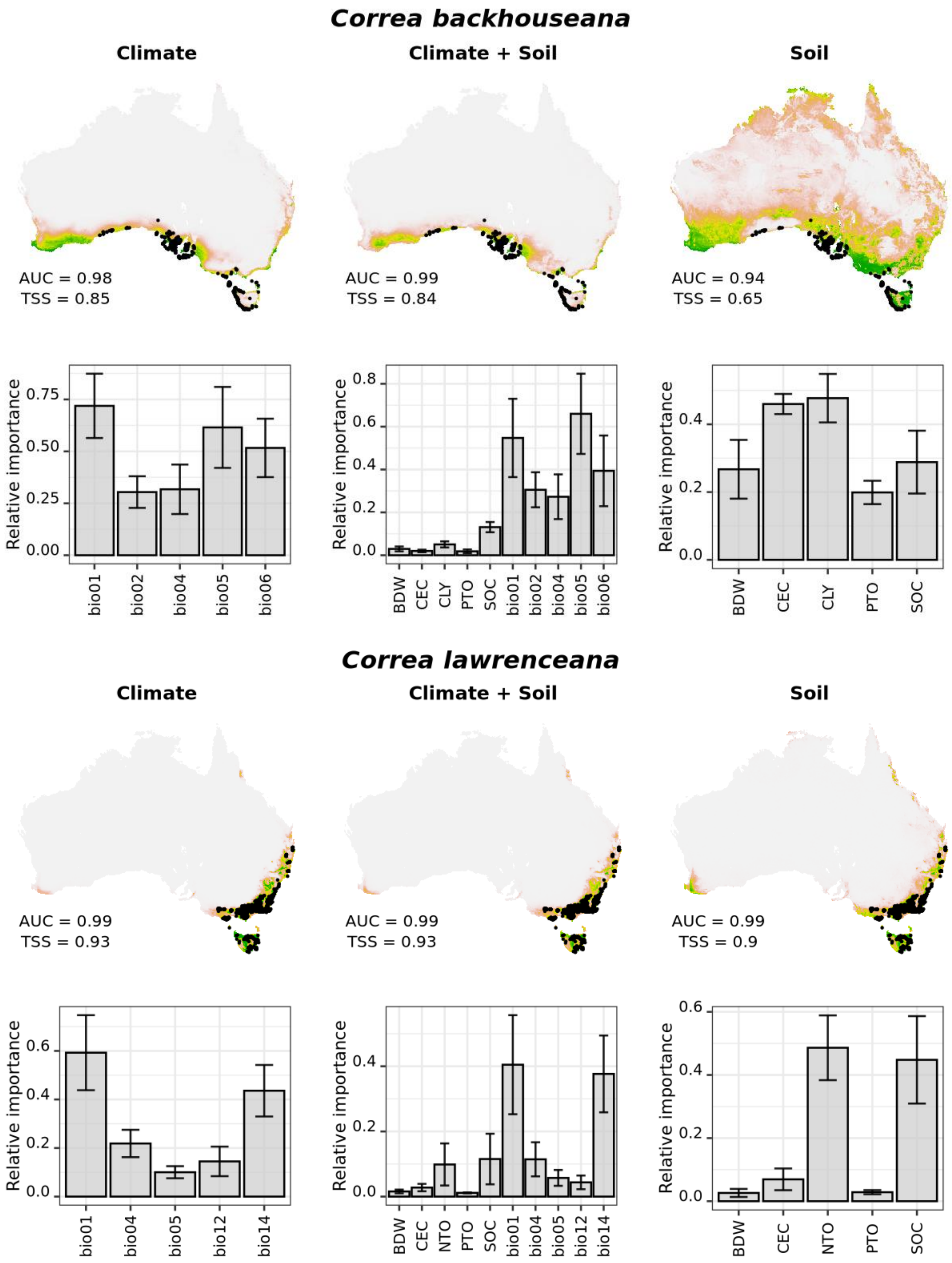
Correrea backhouseana
Climate
Climate + Soil
Soil
AUC = 0.98
TSS = 0.85
AUC = 0.99
TSS = 0.84
AUC = 0.94
TSS = 0.65
Relative importance
bio01 bio02 bio04 bio05 bio06
BDW CEC CLY PTO SOC bio01 bio02 bio04 bio05 bio06
BDW CEC CLY PTO SOC
Correa lawrenceana
Climate
Climate + Soil
Soil
AUC = 0.99
TSS = 0.93
AUC = 0.99
TSS = 0.93
AUC = 0.99
TSS = 0.9
Relative importance
bio01 bio04 bio05 bio12 bio14
BDW CEC NTO PTO SOC bio01 bio04 bio05 bio12 bio14
BDW CEC NTO PTO SOC

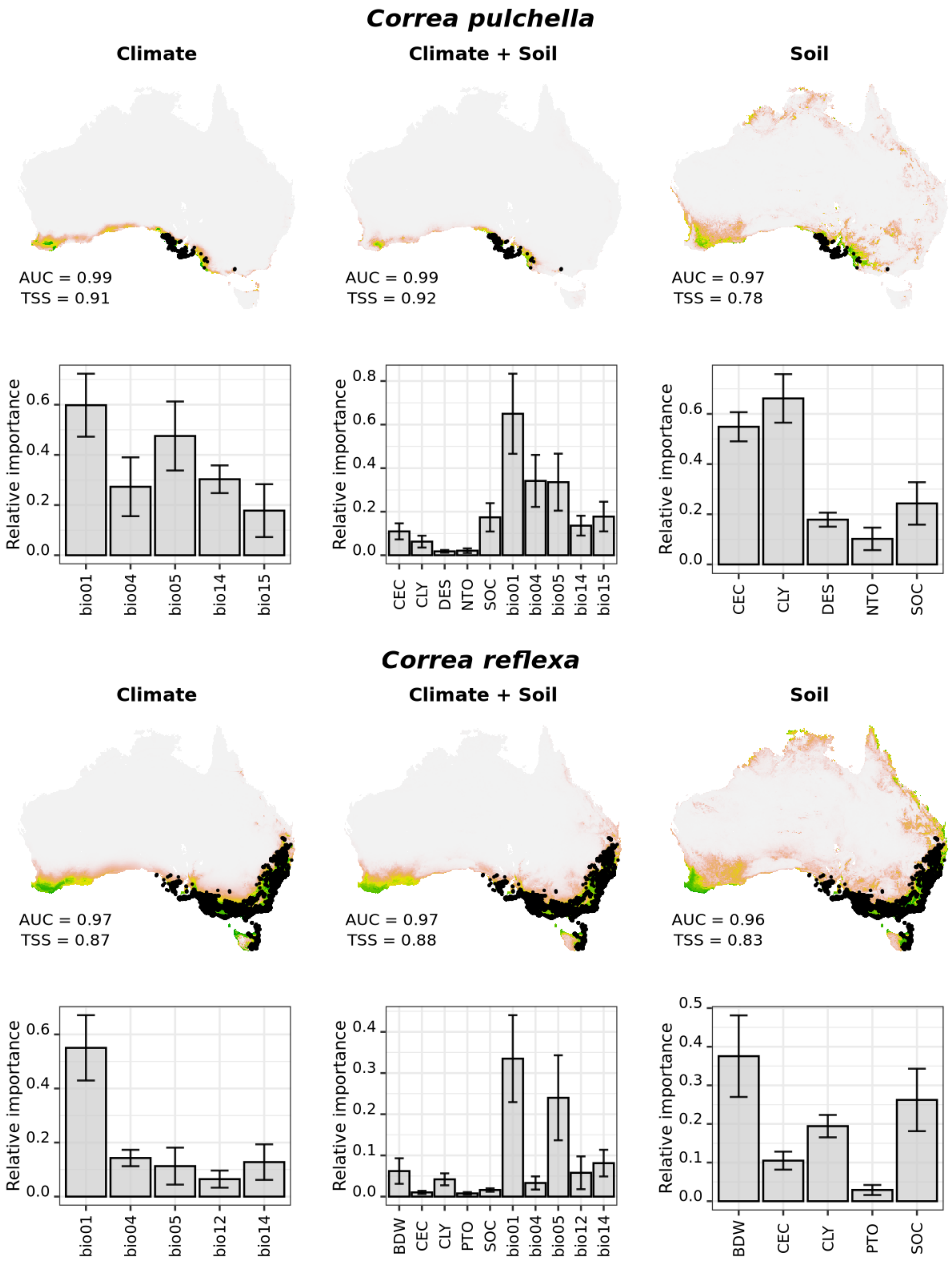
Correa pulchella
Climate
Climate + Soil
Soil
AUC = 0.99
TSS = 0.91
AUC = 0.99
TSS = 0.92
AUC = 0.97
TSS = 0.78
Relative importance
bio01
bio04
bio05
bio14
bio15
CEC
CLY
DES
NTO
SOC
Correa reflexa
Climate
Climate + Soil
Soil
AUC = 0.97
TSS = 0.87
AUC = 0.97
TSS = 0.88
AUC = 0.96
TSS = 0.83
Relative importance
bio01
bio04
bio05
bio12
bio14
BDW
CEC
CLY
PTO
SOC

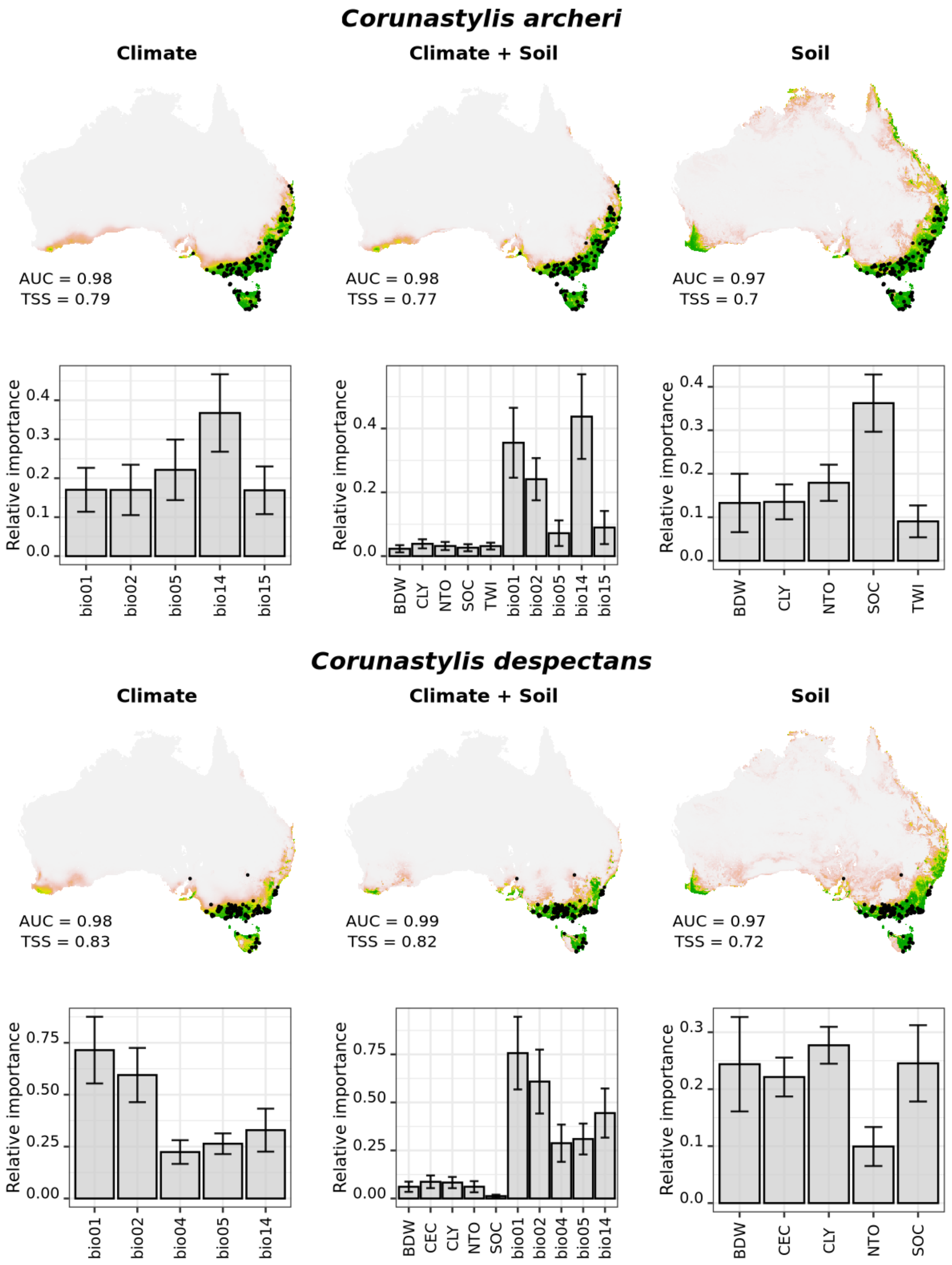

Corunastylis archeri
Climate
Climate + Soil
Soil
AUC = 0.98
TSS = 0.79
AUC = 0.98
TSS = 0.77
AUC = 0.97
TSS = 0.7
Relative importance
0.0
0.1
0.2
0.3
0.4
bio01
bio02
bio05
bio14
bio15
BDW
CLY
NTO
SOC
TWI
Corunastylis despectans
AUC = 0.98
TSS = 0.83
AUC = 0.99
TSS = 0.82
AUC = 0.97
TSS = 0.72
0.00
0.25
0.50
0.75
bio04
CEC

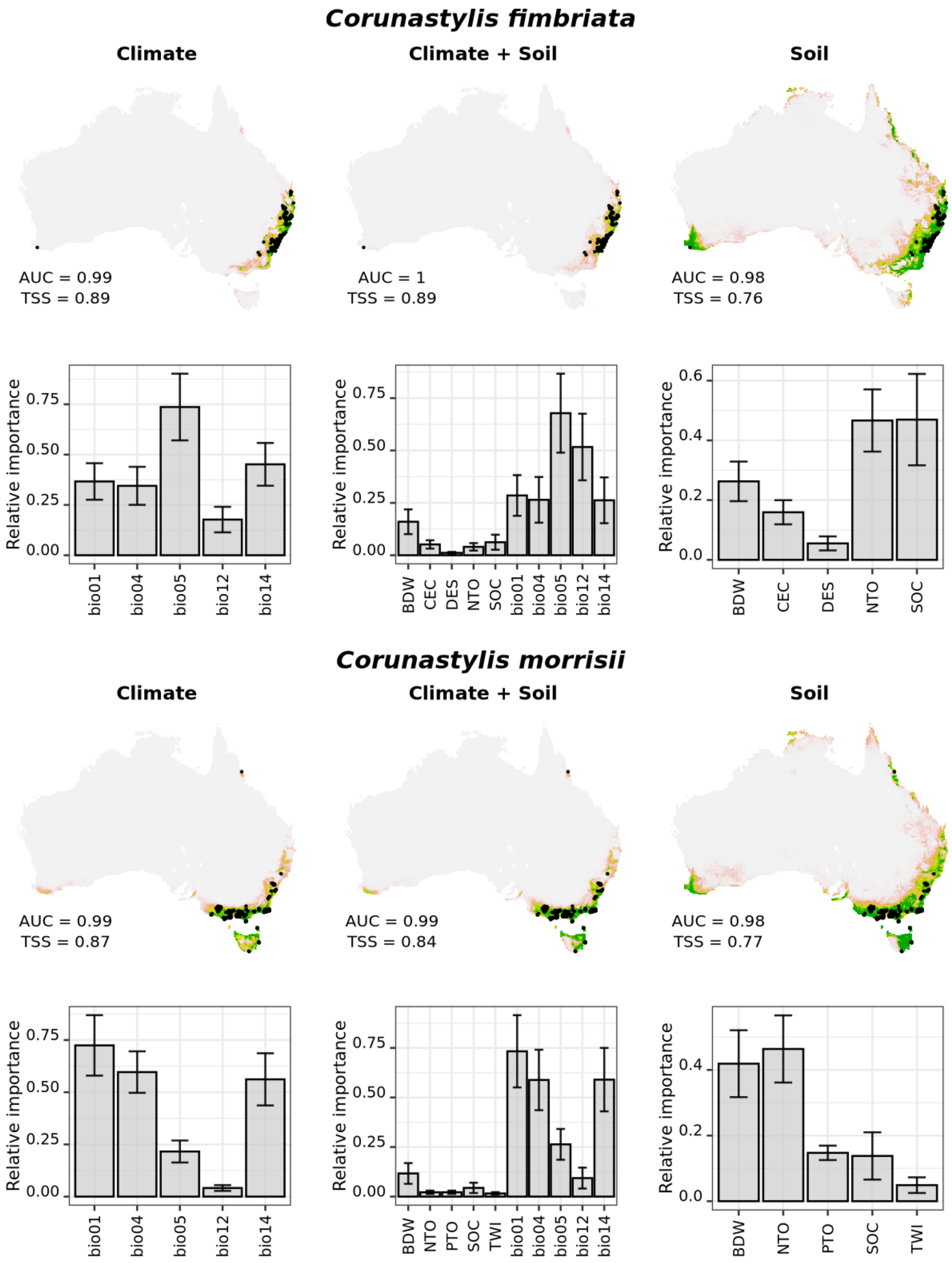
Corunastylis fimbriata
Climate
Climate + Soil
Soil
AUC = 0.99
TSS = 0.89
AUC = 1
TSS = 0.89
AUC = 0.98
TSS = 0.76
Relative importance
0.00
0.25
0.50
0.75
bio01
bio04
bio05
bio12
bio14
BDW
CEC
DES
NTO
SOC
0.0
0.2
0.4
0.6
Corunastylis morrisii
Climate
Climate + Soil
Soil
AUC = 0.99
TSS = 0.87
AUC = 0.99
TSS = 0.84
AUC = 0.98
TSS = 0.77
PTO
TWI

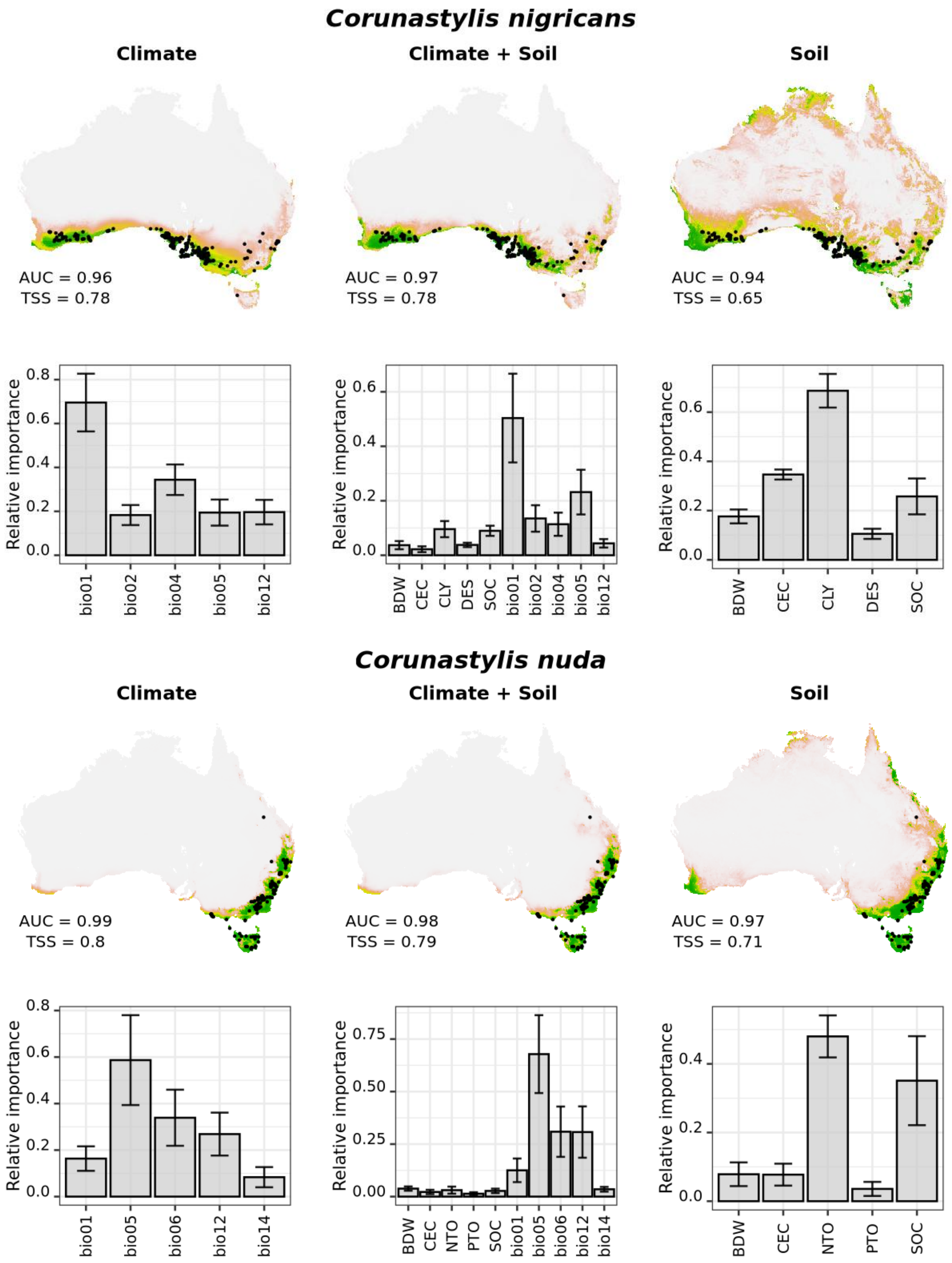

Corunastylis nigricans
Climate
Climate + Soil
Soil
AUC = 0.96
TSS = 0.78
AUC = 0.97
TSS = 0.78
AUC = 0.94
TSS = 0.65
Relative importance
bio01
bio02
bio04
bio05
bio12
BDW
CEC
CLY
DES
SOC
Corunastylis nuda
Climate
Climate + Soil
Soil
AUC = 0.99
TSS = 0.8
AUC = 0.98
TSS = 0.79
AUC = 0.97
TSS = 0.71
Relative importance
bio01
bio05
bio06
bio12
bio14
BDW
CEC
NTO
PTO
SOC

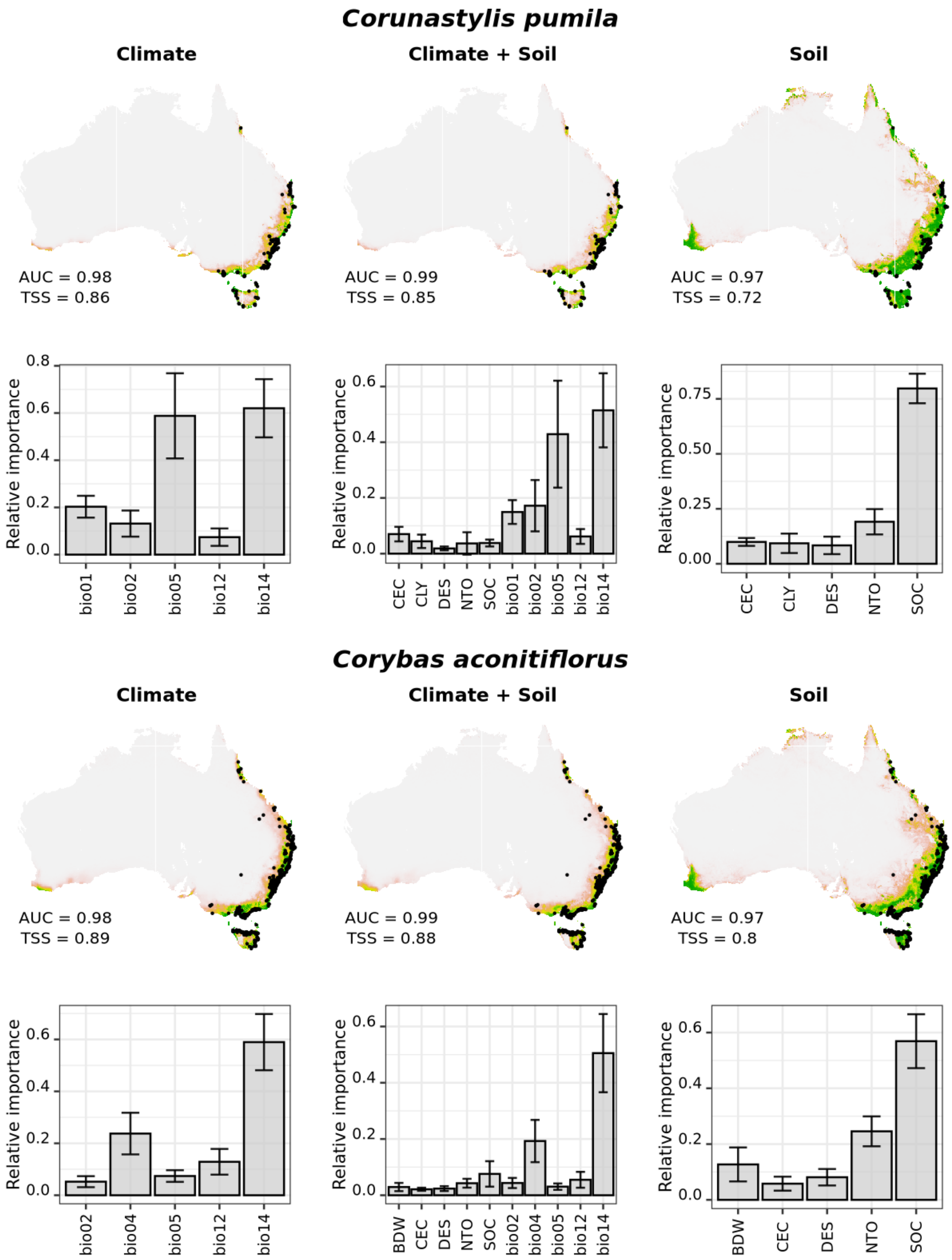
Corunastylis pumila
Climate
Climate + Soil
Soil
AUC = 0.98
TSS = 0.86
AUC = 0.99
TSS = 0.85
AUC = 0.97
TSS = 0.72
Relative importance
bio01
bio02
bio05
bio12
bio14
CEC
CLY
DES
NTO
SOC
Corybas aconitiflorus
Climate
Climate + Soil
Soil
AUC = 0.98
TSS = 0.89
AUC = 0.99
TSS = 0.88
AUC = 0.97
TSS = 0.8
Relative importance
bio02
bio04
bio05
bio12
bio14
BDW
CEC
DES
NTO
SOC

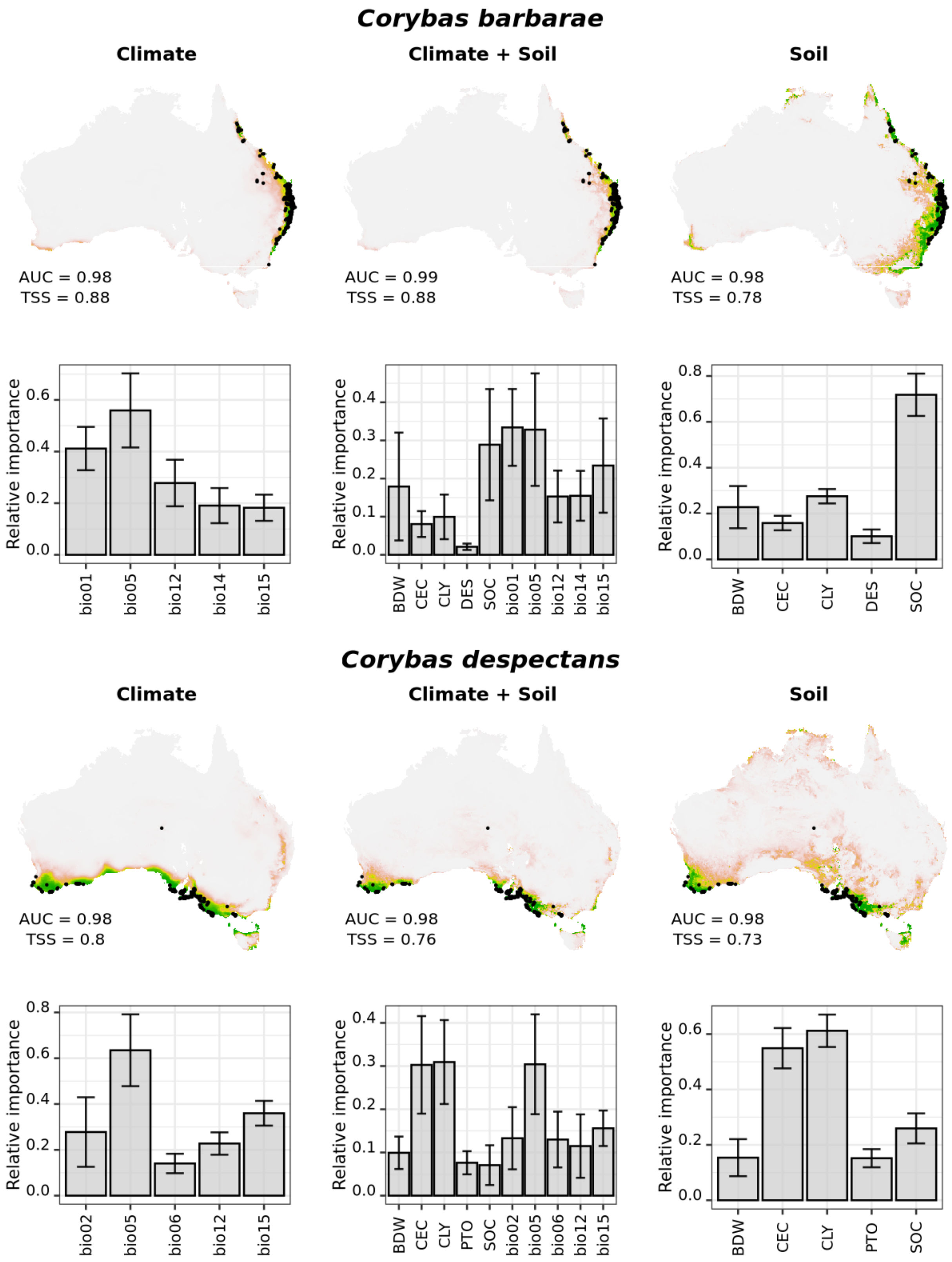
Corybas barbarae
Climate
Climate + Soil
Soil
AUC = 0.98
TSS = 0.88
AUC = 0.99
TSS = 0.88
AUC = 0.98
TSS = 0.78
Relative importance
bio01
bio05
bio12
bio14
bio15
BDW
CEC
CLY
DES
SOC
Corybas despectans
AUC = 0.98
TSS = 0.8
AUC = 0.98
TSS = 0.76
AUC = 0.98
TSS = 0.73
bio02
bio06
PTO

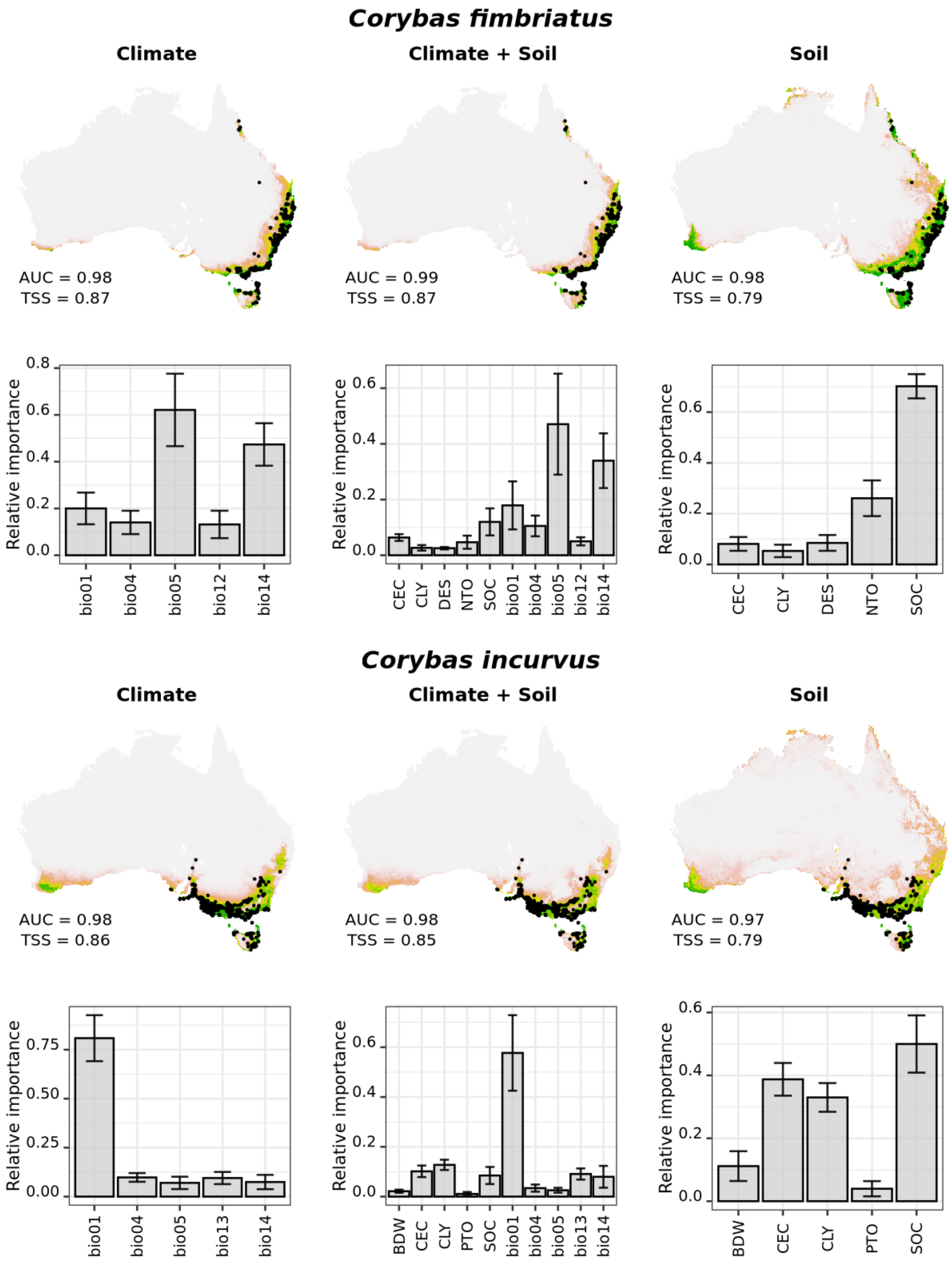
Corybas fimbriatus
Climate
Climate + Soil
Soil
AUC = 0.98
TSS = 0.87
AUC = 0.99
TSS = 0.87
AUC = 0.98
TSS = 0.79
Relative importance
0.0
0.2
0.4
0.6
0.8
bio01
bio04
bio05
bio12
bio14
CEC
CLY
DES
NTO
SOC
Corybas incurvus
Climate
Climate + Soil
Soil
AUC = 0.98
TSS = 0.86
AUC = 0.98
TSS = 0.85
AUC = 0.97
TSS = 0.79
Relative importance
0.00
0.25
0.50
0.75
bio01
bio04
bio05
bio13
bio14
BDW
CEC
CLY
PTO
SOC

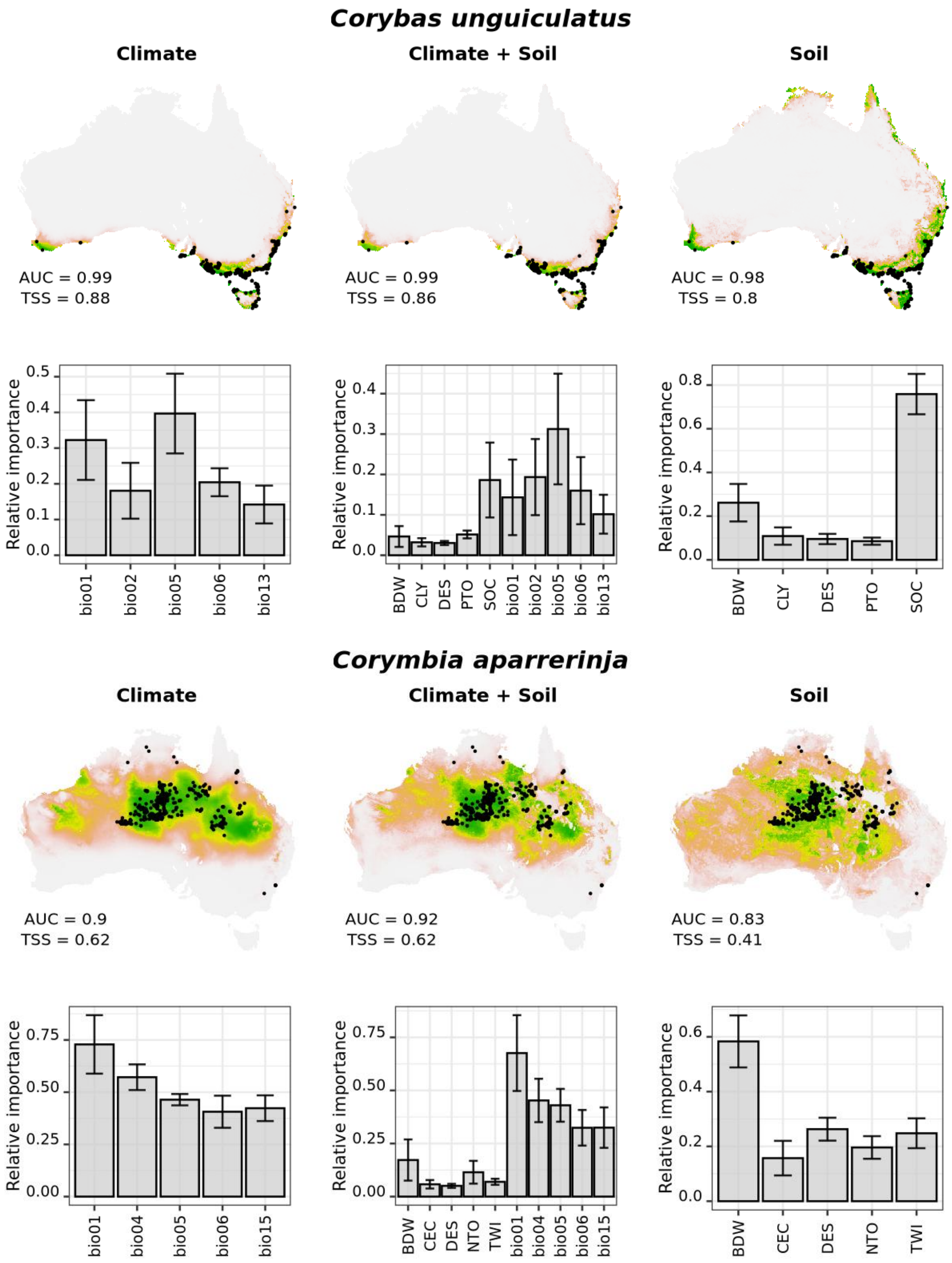

Corybas unguiculatus
Climate
Climate + Soil
Soil
AUC = 0.99
TSS = 0.88
AUC = 0.99
TSS = 0.86
AUC = 0.98
TSS = 0.8
Relative importance
0.0
0.1
0.2
0.3
0.4
0.5
bio01
bio02
bio05
bio06
bio13
Relative importance
0.0
0.1
0.2
0.3
0.4
BDW
CLY
DES
PTO
SOC
bio01
bio02
bio05
bio06
bio13
Relative importance
0.0
0.2
0.4
0.6
0.8
BDW
CLY
DES
PTO
SOC
Corymbia aparrerinja
Climate
Climate + Soil
Soil
AUC = 0.9
TSS = 0.62
AUC = 0.92
TSS = 0.62
AUC = 0.83
TSS = 0.41
Relative importance
0.00
0.25
0.50
0.75
bio01
bio04
bio05
bio06
bio15
Relative importance
0.00
0.25
0.50
0.75
BDW
CEC
DES
NTO
TWI
bio01
bio04
bio05
bio06
bio15
Relative importance
0.0
0.2
0.4
0.6
BDW
CEC
DES
NTO
TWI

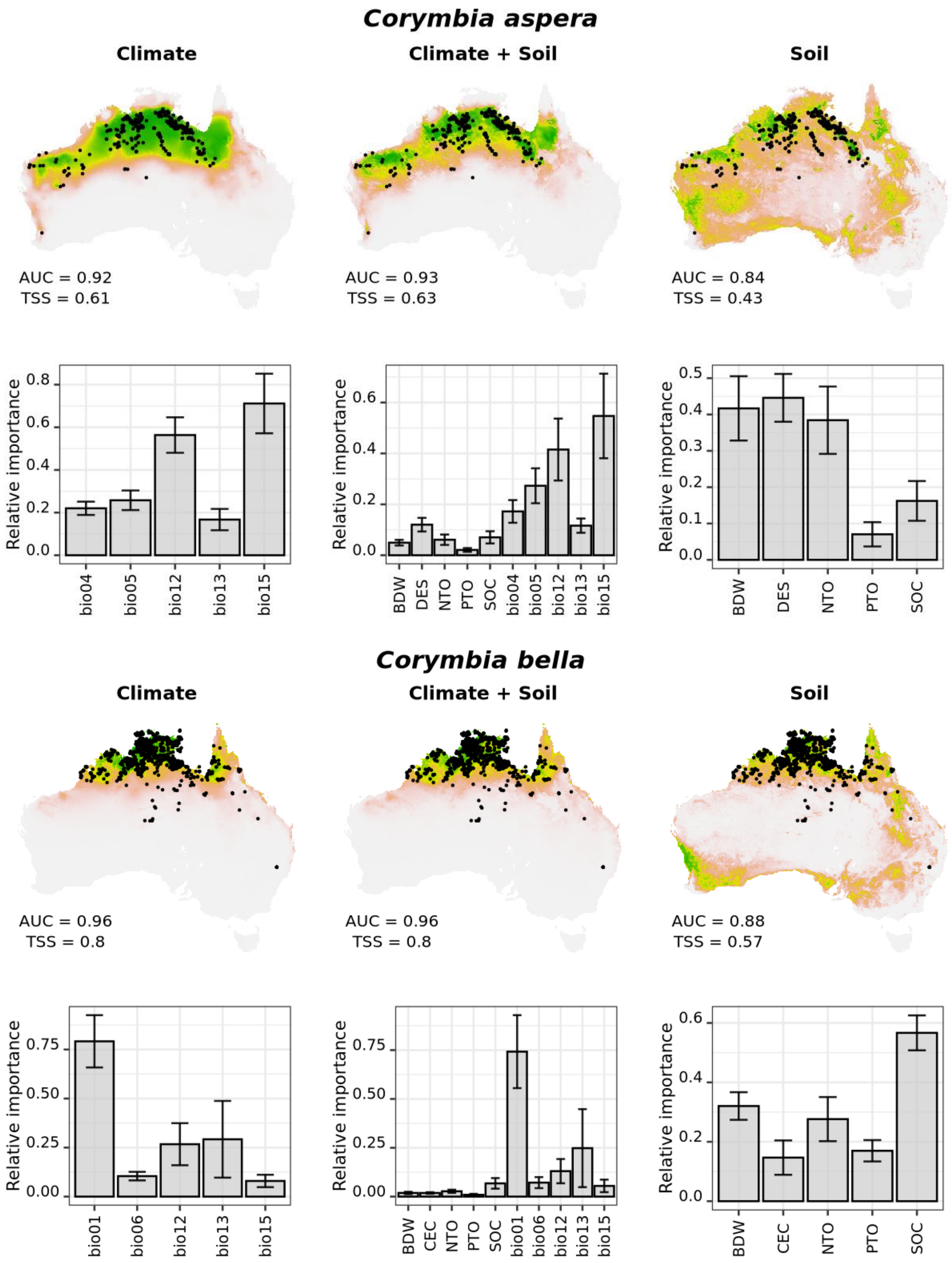
Corymbia aspera
Climate
Climate + Soil
Soil
AUC = 0.92
TSS = 0.61
AUC = 0.93
TSS = 0.63
AUC = 0.84
TSS = 0.43
Relative importance
bio04 bio05 bio12 bio13 bio15
BDW DES NTO PTO SOC bio04 bio05 bio12 bio13 bio15
BDW DES NTO PTO SOC
Corymbia bella
Climate
Climate + Soil
Soil
AUC = 0.96
TSS = 0.8
AUC = 0.96
TSS = 0.8
AUC = 0.88
TSS = 0.57
Relative importance
bio01 bio06 bio12 bio13 bio15
BDW CEC NTO PTO SOC bio01 bio06 bio12 bio13 bio15
BDW CEC NTO PTO SOC

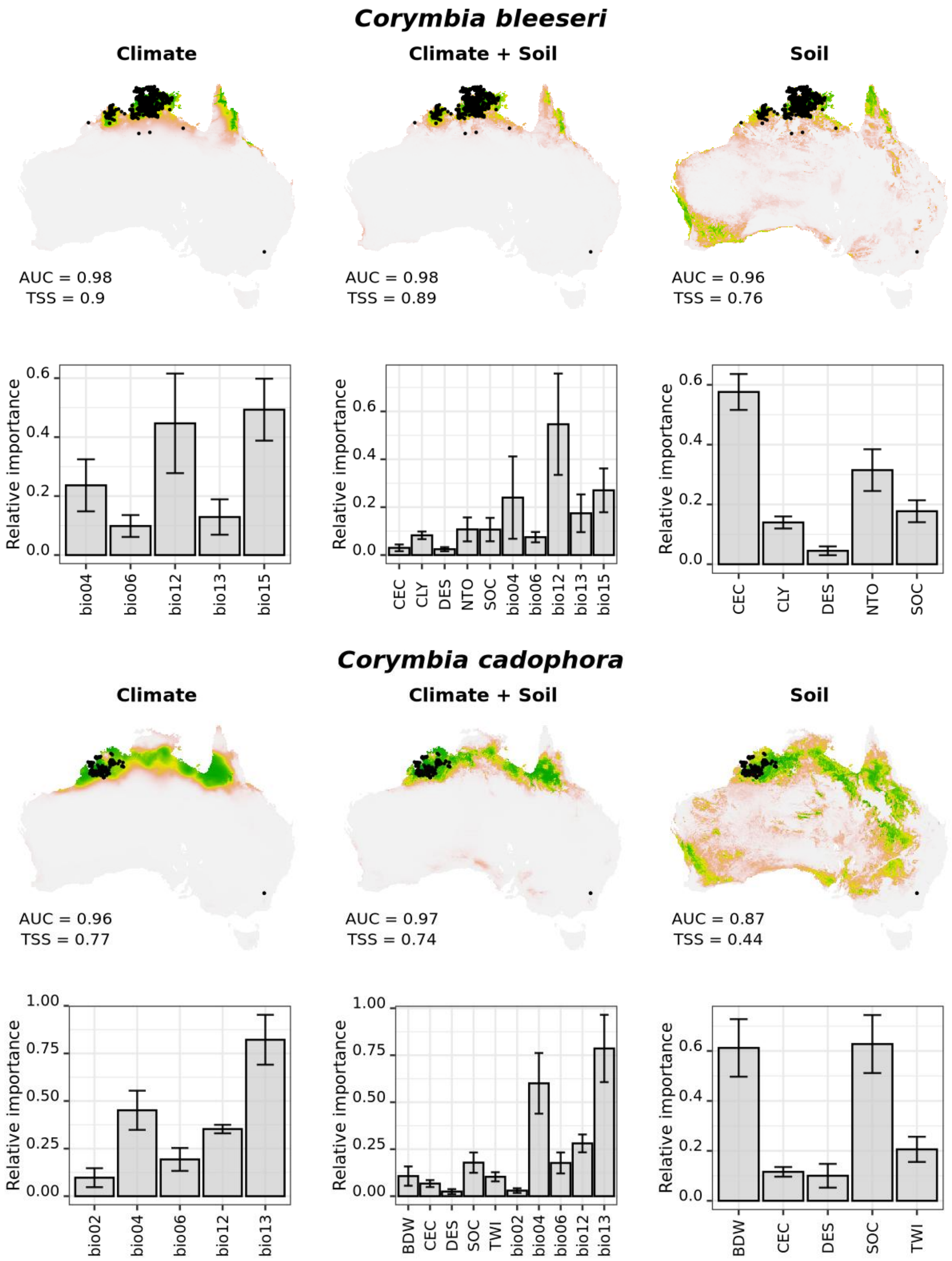
Corymbia bleeseri
Climate
Climate + Soil
Soil
AUC = 0.98
TSS = 0.9
AUC = 0.98
TSS = 0.89
AUC = 0.96
TSS = 0.76
Relative importance
0.0
0.2
0.4
0.6
bio04
bio06
bio12
bio13
bio15
CEC
CLY
DES
NTO
SOC
Corymbia cadophora
AUC = 0.96
TSS = 0.77
AUC = 0.97
TSS = 0.74
AUC = 0.87
TSS = 0.44
0.00
0.25
0.50
0.75
1.00
bio02
BDW
TWI

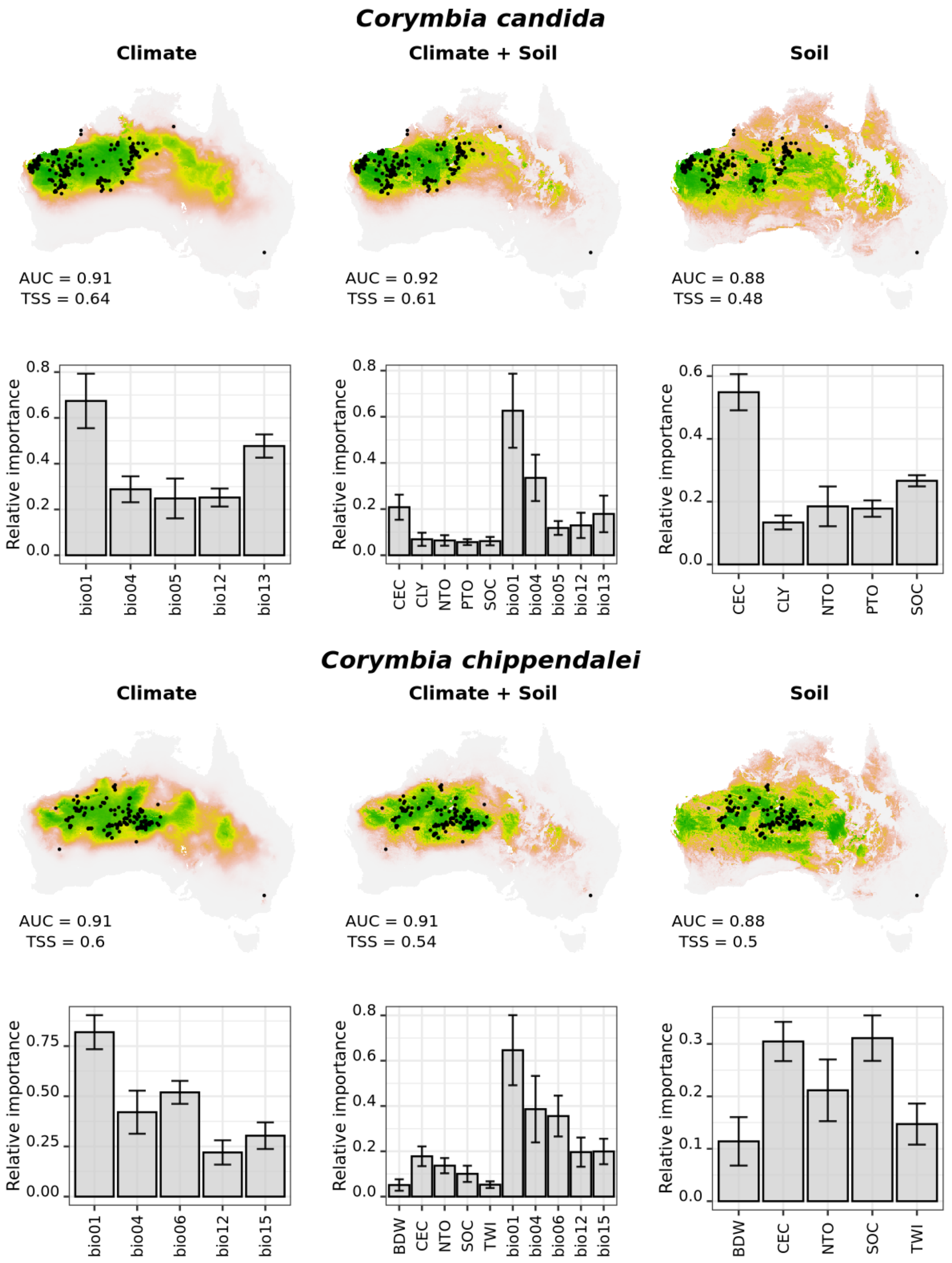
Corymbia candida
Climate
Climate + Soil
Soil
AUC = 0.91
TSS = 0.64
AUC = 0.92
TSS = 0.61
AUC = 0.88
TSS = 0.48
Relative importance
bio01
bio04
bio05
bio12
bio13
CEC
CLY
NTO
PTO
SOC
Corymbia chippendalei
Climate
Climate + Soil
Soil
AUC = 0.91
TSS = 0.6
AUC = 0.91
TSS = 0.54
AUC = 0.88
TSS = 0.5
Relative importance
bio01
bio04
bio06
bio12
bio15
BDW
CEC
NTO
SOC
TWI

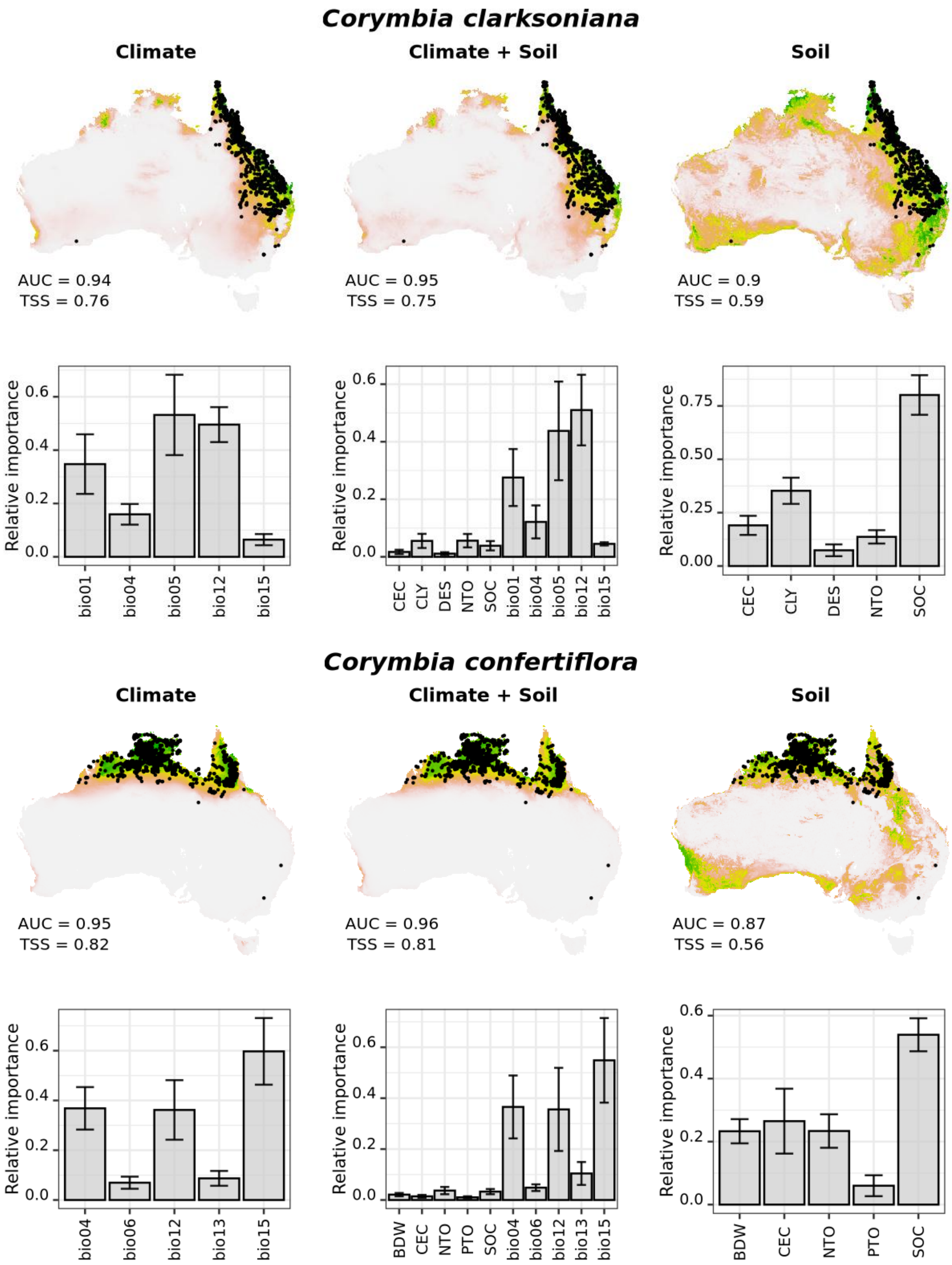
Corymbia clarksoniana
Climate
Climate + Soil
Soil
AUC = 0.94
TSS = 0.76
AUC = 0.95
TSS = 0.75
AUC = 0.9
TSS = 0.59
Relative importance
bio01
bio04
bio05
bio12
bio15
CEC
CLY
DES
NTO
SOC
Corymbia confertiflora
Climate
Climate + Soil
Soil
AUC = 0.95
TSS = 0.82
AUC = 0.96
TSS = 0.81
AUC = 0.87
TSS = 0.56
Relative importance
bio04
bio06
bio12
bio13
bio15
BDW
CEC
NTO
PTO
SOC

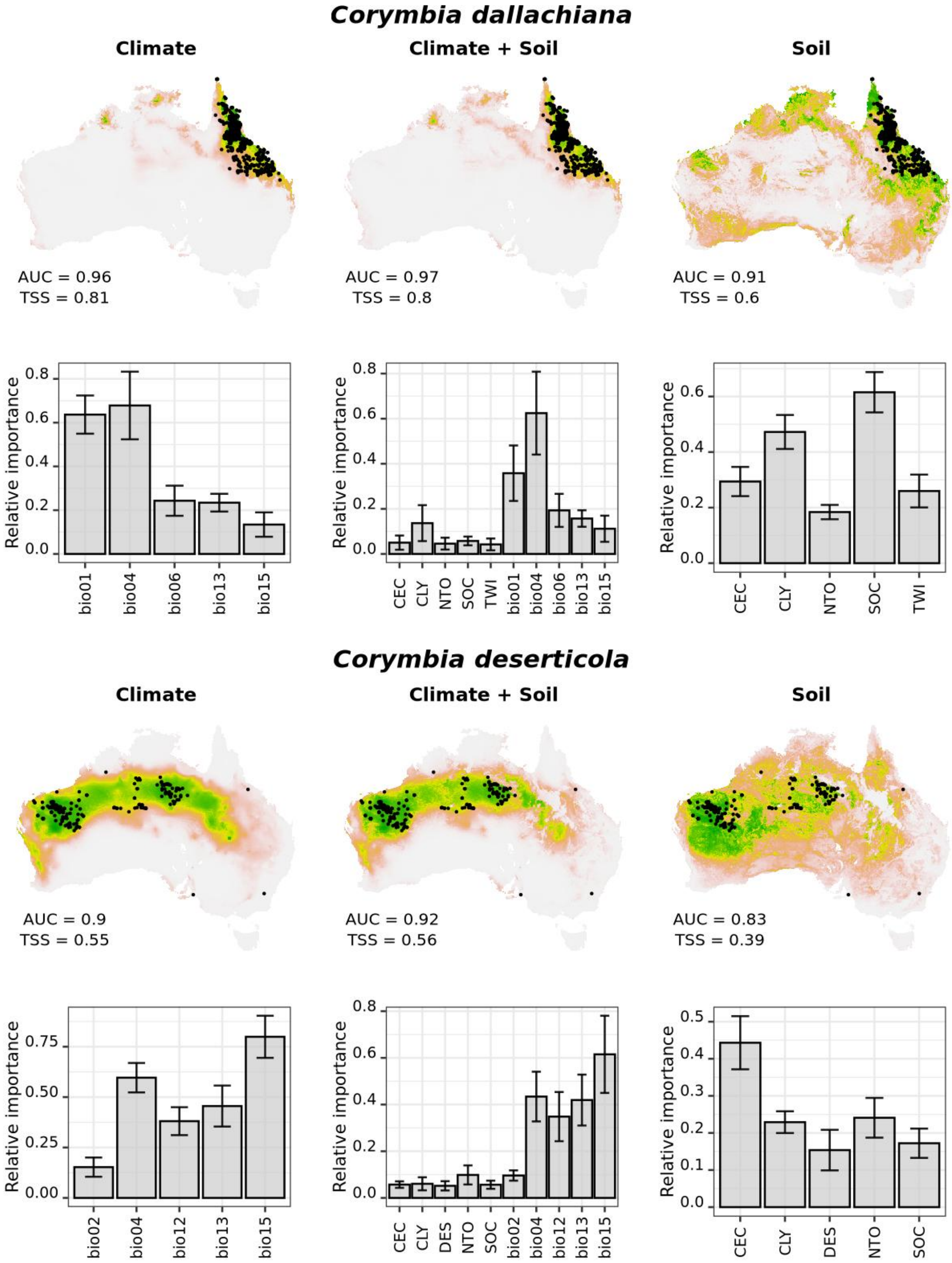

Corymbia dallachiana
Climate
Climate + Soil
Soil
AUC = 0.96
TSS = 0.81
AUC = 0.97
TSS = 0.8
AUC = 0.91
TSS = 0.6
Relative importance
bio01
bio04
bio06
bio13
bio15
CEC
CLY
NTO
SOC
TWI
Corymbia deserticola
Climate
Climate + Soil
Soil
AUC = 0.9
TSS = 0.55
AUC = 0.92
TSS = 0.56
AUC = 0.83
TSS = 0.39
Relative importance
bio02
bio04
bio12
bio13
bio15
CEC
CLY
DES
NTO
SOC

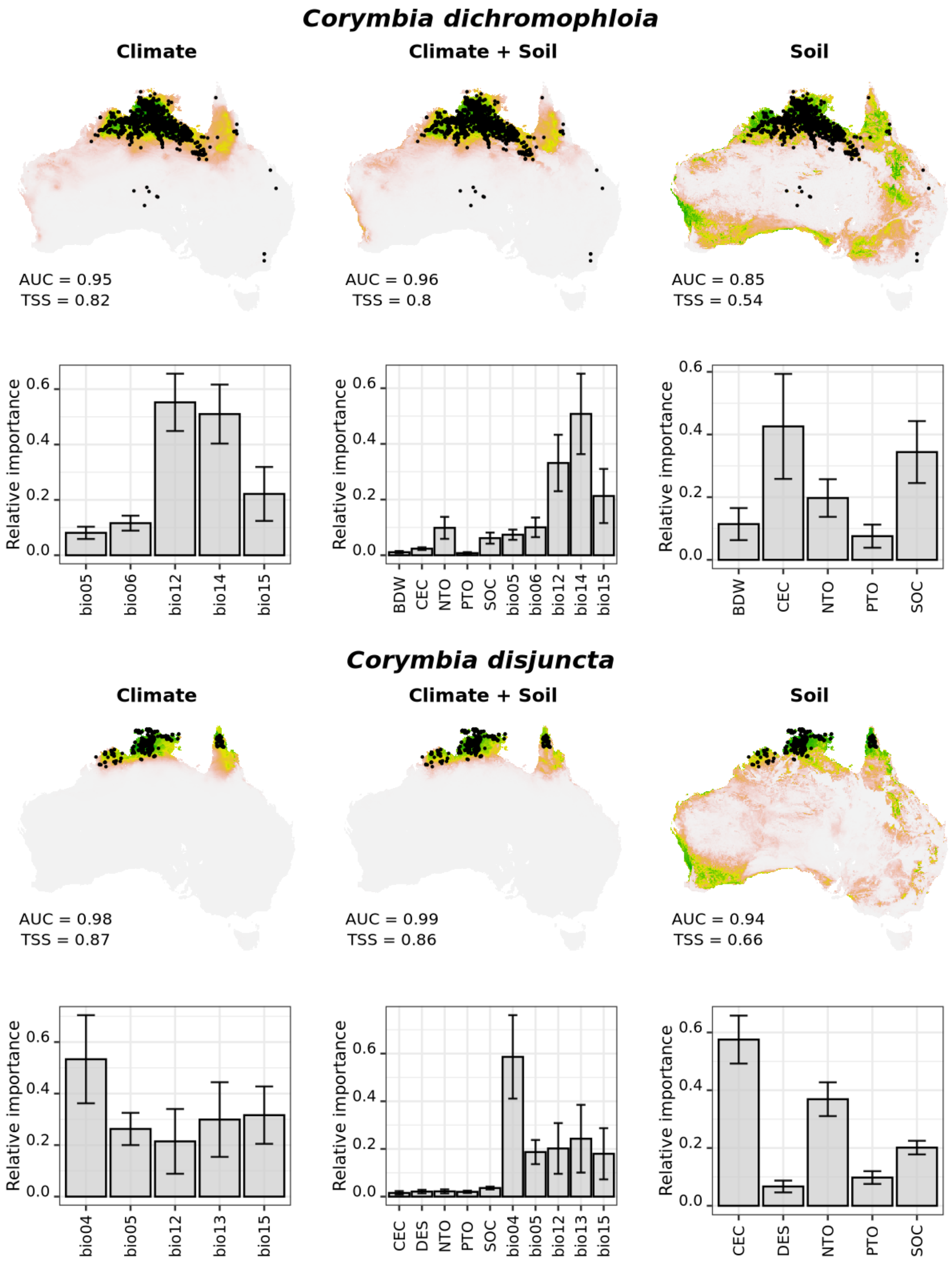

Corymbia dichromophloia
Climate
Climate + Soil
Soil
AUC = 0.95
TSS = 0.82
AUC = 0.96
TSS = 0.8
AUC = 0.85
TSS = 0.54
Relative importance
bio05
bio06
bio12
bio14
bio15
BDW
CEC
NTO
PTO
SOC
Corymbia disjuncta
AUC = 0.98
TSS = 0.87
AUC = 0.99
TSS = 0.86
AUC = 0.94
TSS = 0.66
bio04
bio13
DES

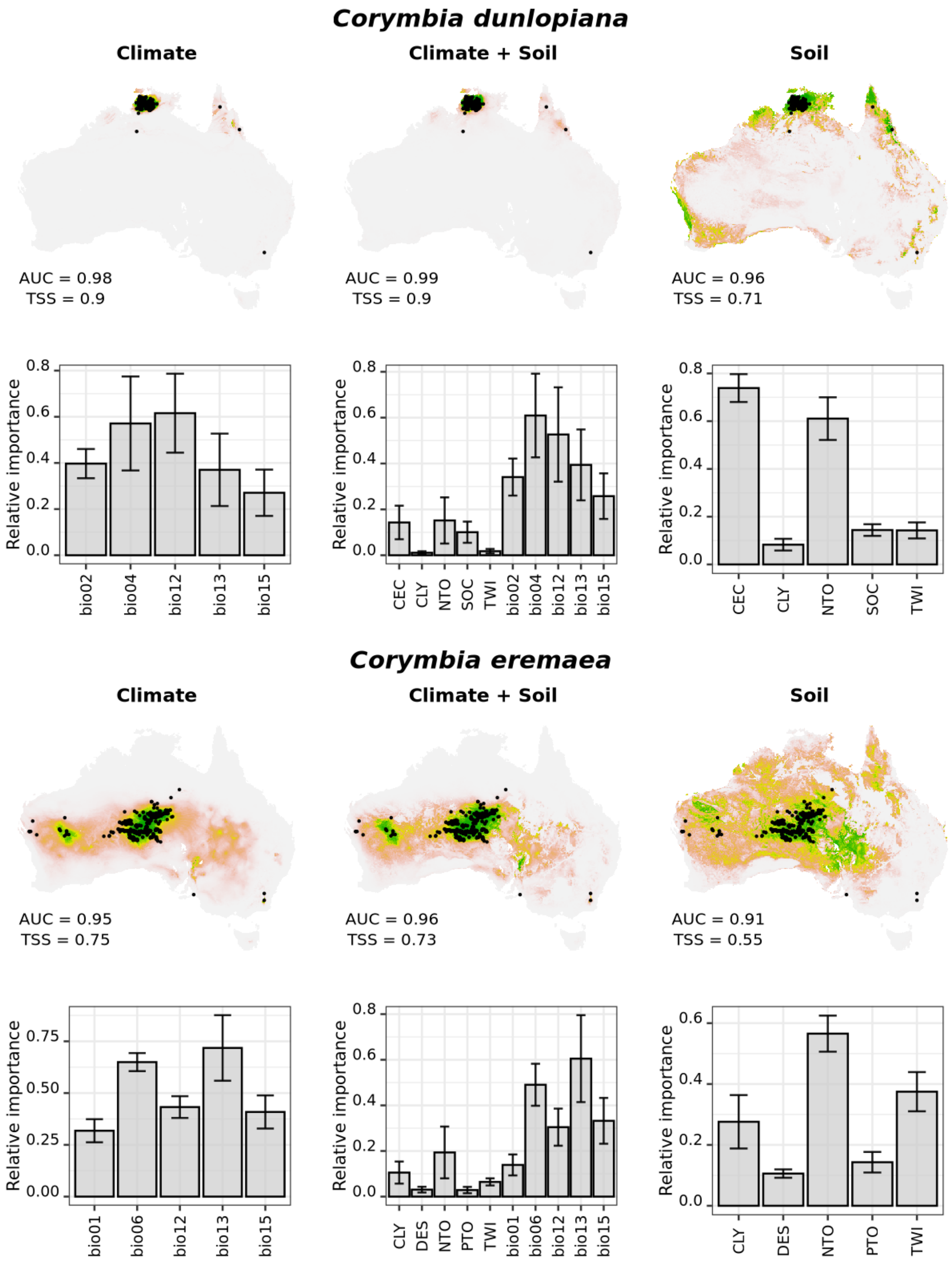
Corymbia dunlopiana
Climate
Climate + Soil
Soil
AUC = 0.98
TSS = 0.9
AUC = 0.99
TSS = 0.9
AUC = 0.96
TSS = 0.71
Relative importance
0.0
0.2
0.4
0.6
0.8
bio02
bio04
bio12
bio13
bio15
CEC
CLY
NTO
SOC
TWI
bio02
bio04
bio12
bio13
bio15
CEC
CLY
NTO
SOC
TWI
Corymbia eremaea
Climate
Climate + Soil
Soil
AUC = 0.95
TSS = 0.75
AUC = 0.96
TSS = 0.73
AUC = 0.91
TSS = 0.55
Relative importance
0.00
0.25
0.50
0.75
bio01
bio06
bio12
bio13
bio15
0.0
0.2
0.4
0.6
0.8
CLY
DES
NTO
PTO
TWI
bio01
bio06
bio12
bio13
bio15
0.0
0.2
0.4
0.6
CLY
DES
NTO
PTO
TWI

Corymbia erythrophloia

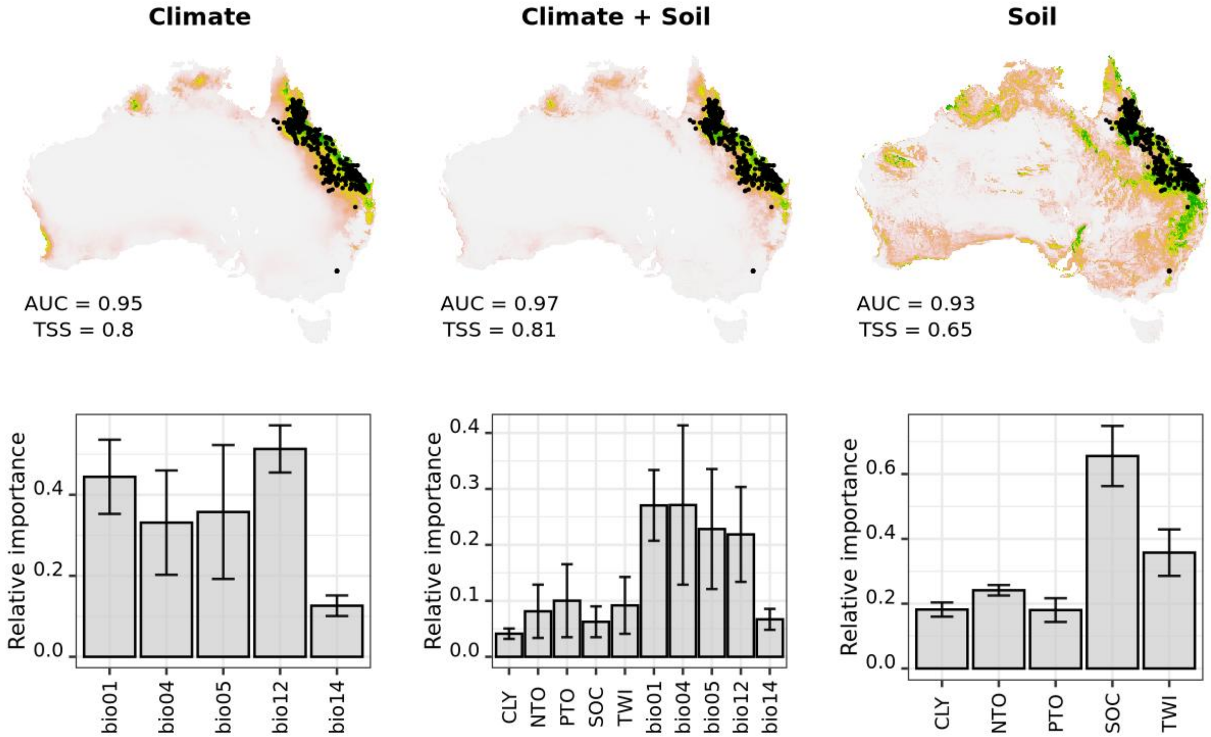

Corymbia eximia

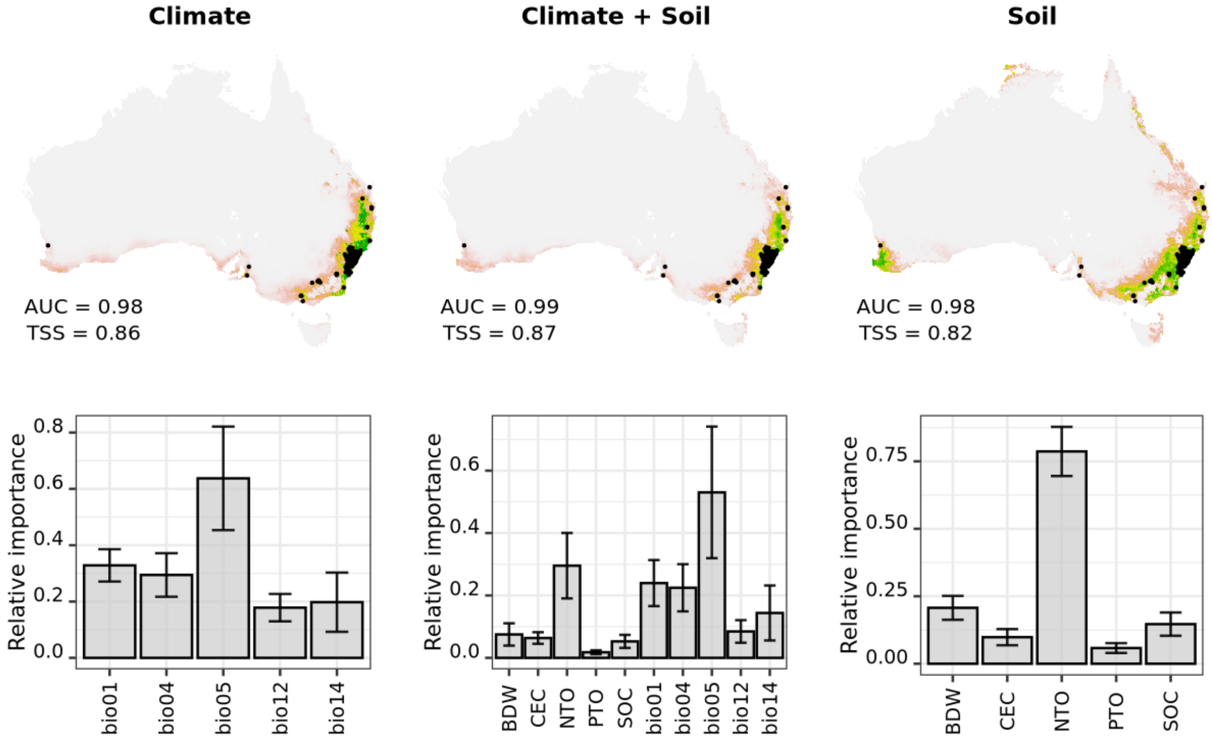

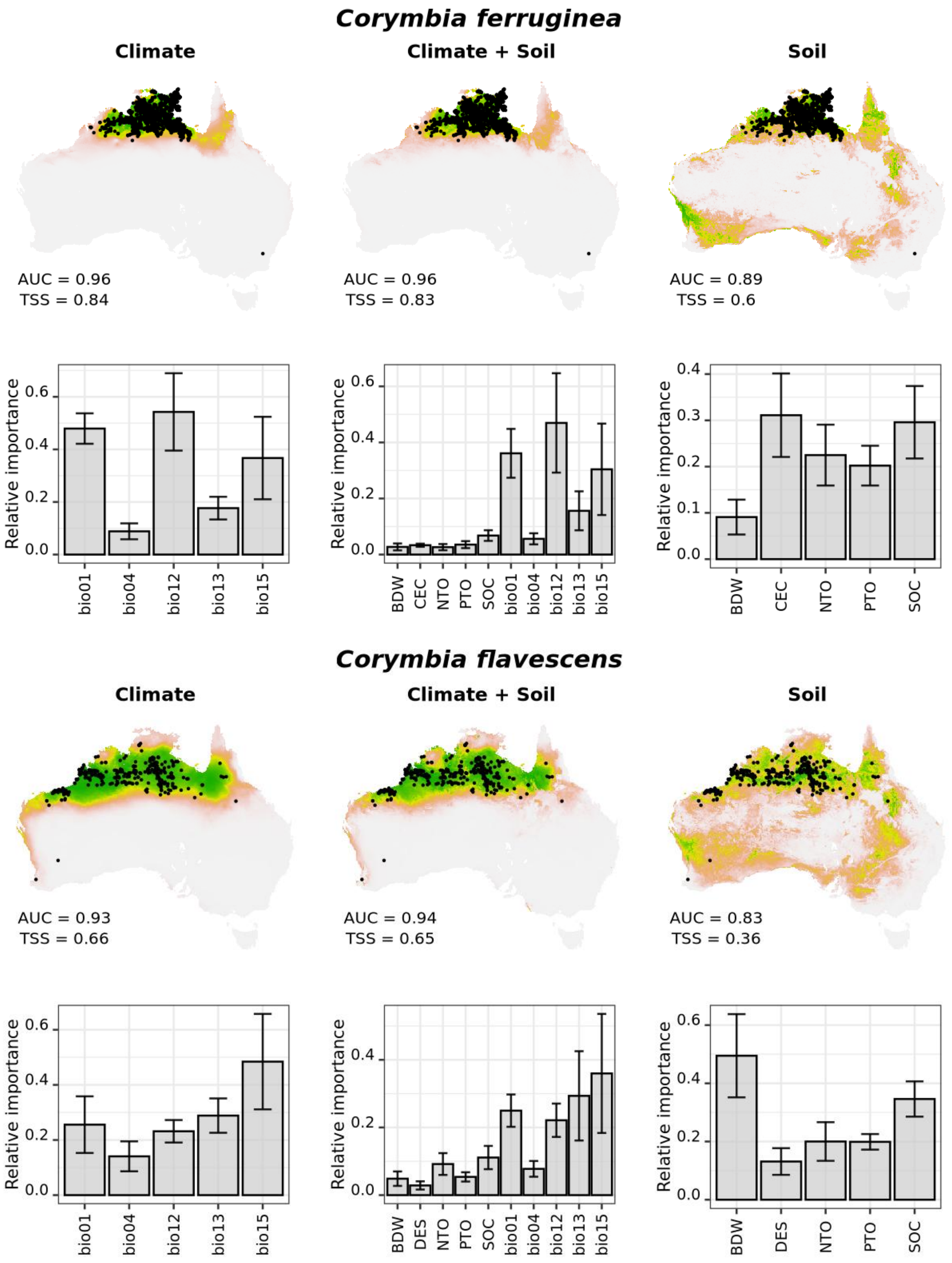
Corymbia ferruginea
Climate
Climate + Soil
Soil
AUC = 0.96
TSS = 0.84
AUC = 0.96
TSS = 0.83
AUC = 0.89
TSS = 0.6
Relative importance
bio01
bio04
bio12
bio13
bio15
BDW
CEC
NTO
PTO
SOC
Corymbia flavescens
Climate
Climate + Soil
Soil
AUC = 0.93
TSS = 0.66
AUC = 0.94
TSS = 0.65
AUC = 0.83
TSS = 0.36
DES

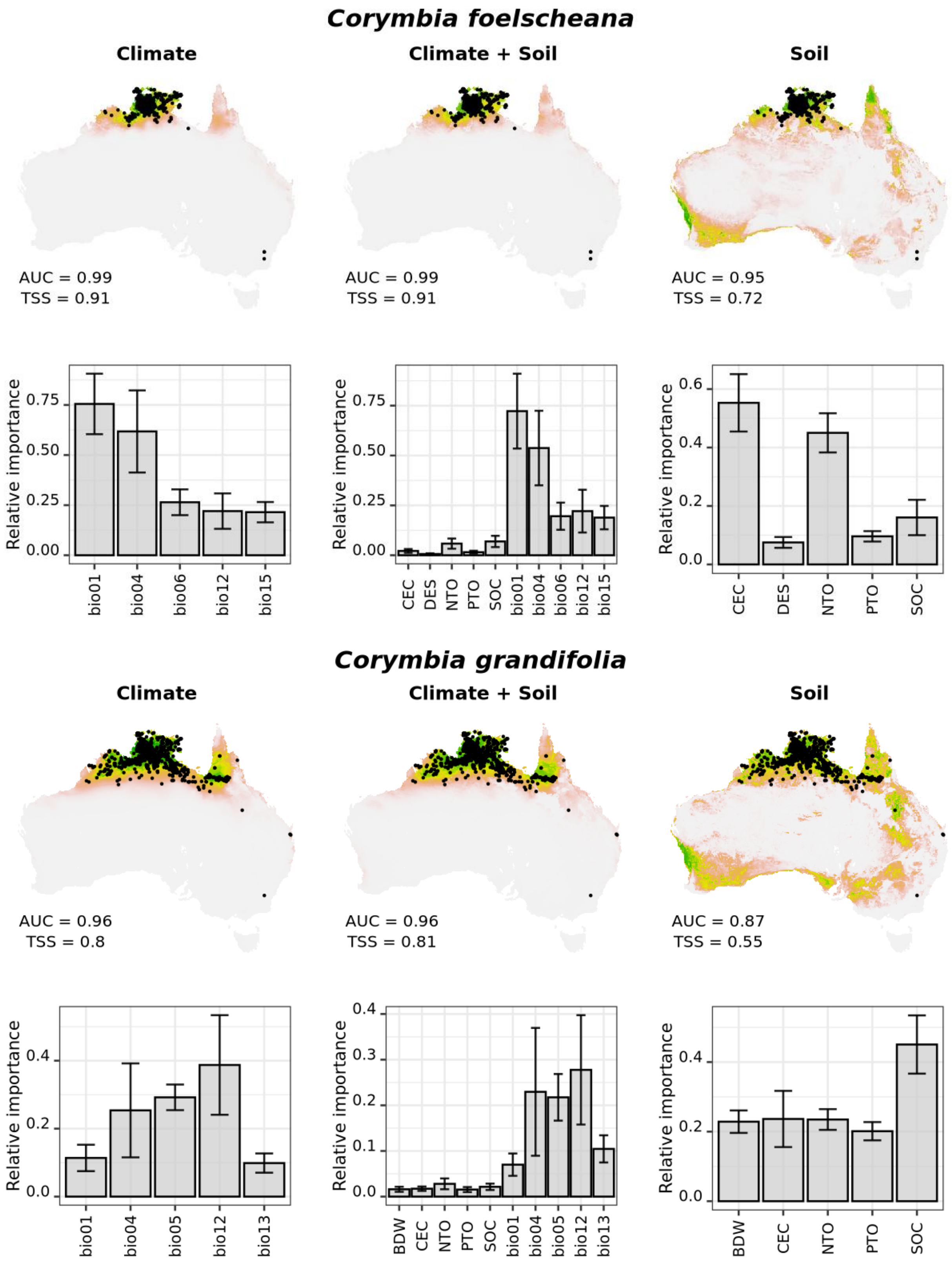

Corymbia foelscheana
Climate
Climate + Soil
Soil
AUC = 0.99
TSS = 0.91
AUC = 0.99
TSS = 0.91
AUC = 0.95
TSS = 0.72
Relative importance
bio01
bio04
bio06
bio12
bio15
CEC
DES
NTO
PTO
SOC
Corymbia grandifolia
Climate
Climate + Soil
Soil
AUC = 0.96
TSS = 0.8
AUC = 0.96
TSS = 0.81
AUC = 0.87
TSS = 0.55
Relative importance
bio01
bio04
bio05
bio12
bio13
BDW
CEC
NTO
PTO
SOC

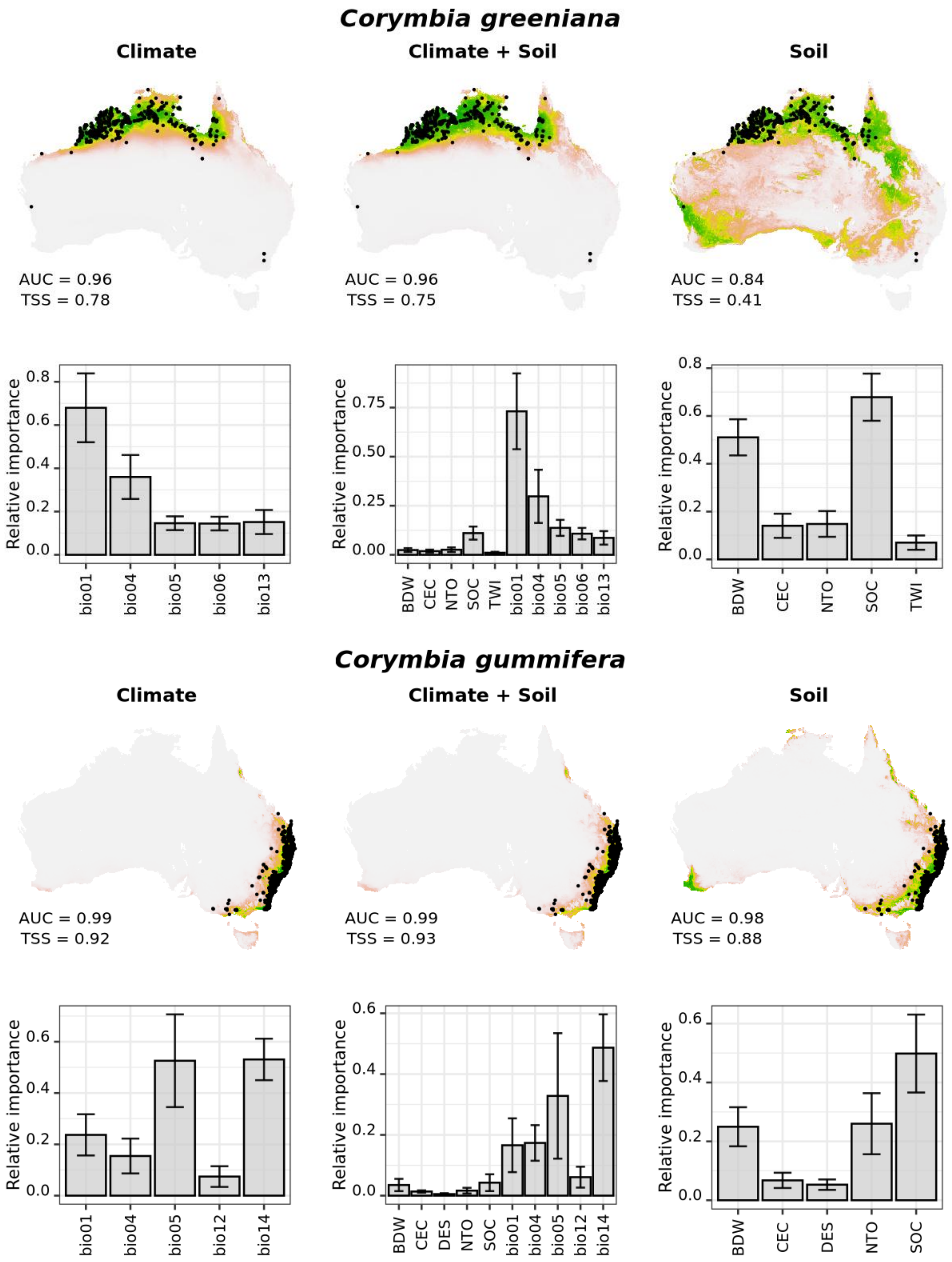
Corymbia greeniana
Climate
Climate + Soil
Soil
AUC = 0.96
TSS = 0.78
AUC = 0.96
TSS = 0.75
AUC = 0.84
TSS = 0.41
Relative importance
0.0
0.2
0.4
0.6
0.8
bio01
bio04
bio05
bio06
bio13
0.00
0.25
0.50
0.75
BDW
CEC
NTO
SOC
TWI
Corymbia gummifera
AUC = 0.99
TSS = 0.92
AUC = 0.99
TSS = 0.93
AUC = 0.98
TSS = 0.88
bio12
bio14
DES

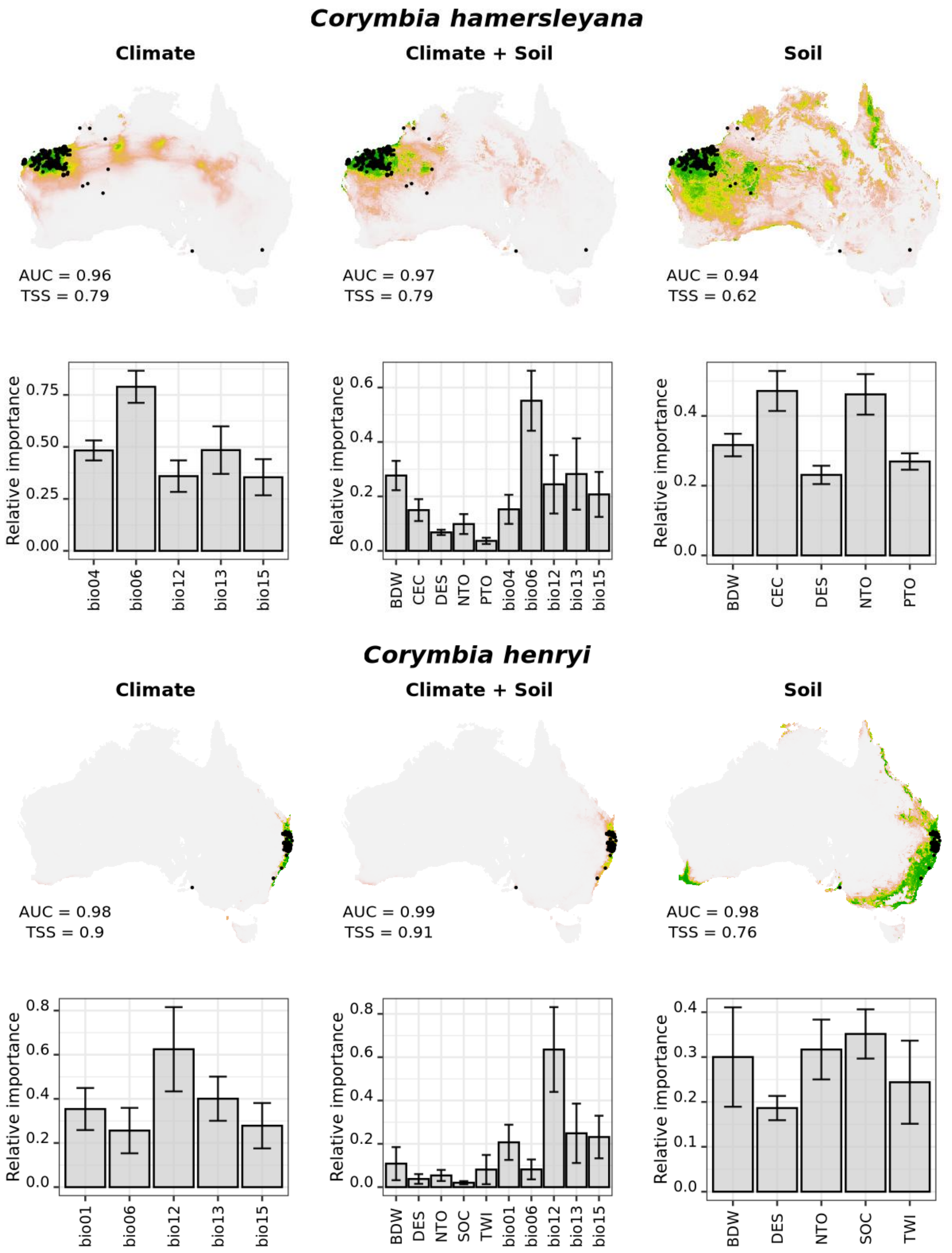

Corymbia hamersleyana
Climate
Climate + Soil
Soil
AUC = 0.96
TSS = 0.79
AUC = 0.97
TSS = 0.79
AUC = 0.94
TSS = 0.62
Relative importance
0.00
0.25
0.50
0.75
bio04
bio06
bio12
bio13
bio15
0.0
0.2
0.4
0.6
BDW
CEC
DES
NTO
PTO
bio04
bio06
bio12
bio13
bio15
0.0
0.2
0.4
BDW
CEC
DES
NTO
PTO
Corymbia henryi
Climate
Climate + Soil
Soil
AUC = 0.98
TSS = 0.9
AUC = 0.99
TSS = 0.91
AUC = 0.98
TSS = 0.76
Relative importance
0.0
0.2
0.4
0.6
0.8
bio01
bio06
bio12
bio13
bio15
BDW
DES
NTO
SOC
TWI
bio01
bio06
bio12
bio13
bio15
0.0
0.1
0.2
0.3
0.4
BDW
DES
NTO
SOC
TWI

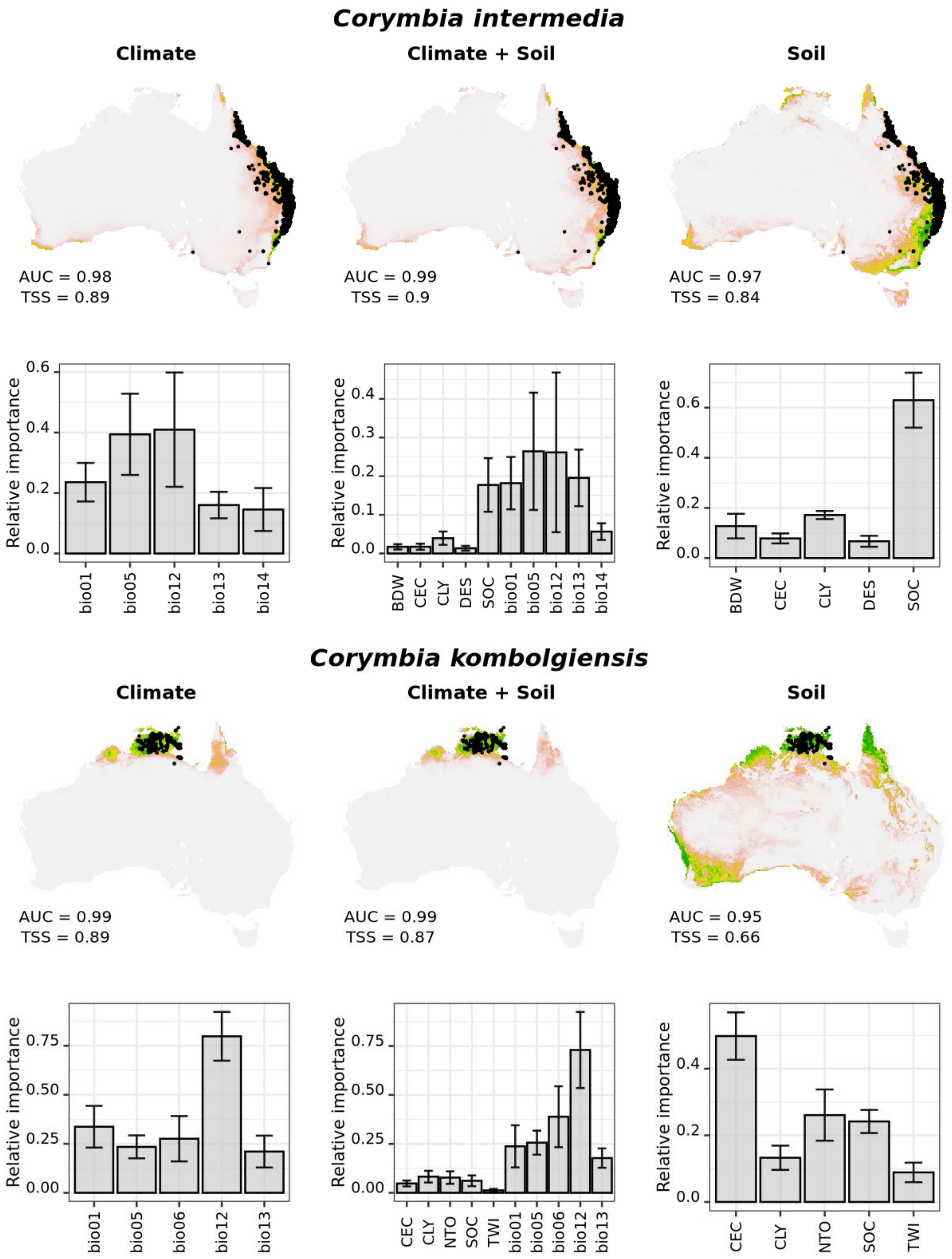
Corymbia intermedia
Climate
Climate + Soil
Soil
AUC = 0.98
TSS = 0.89
AUC = 0.99
TSS = 0.9
AUC = 0.97
TSS = 0.84
Relative importance
0.0
0.2
0.4
0.6
bio01
bio05
bio12
bio13
bio14
0.0
0.1
0.2
0.3
0.4
BDW
CEC
CLY
DES
SOC
bio01
bio05
bio12
bio13
bio14
0.0
0.2
0.4
0.6
BDW
CEC
CLY
DES
SOC
Corymbia kombolgiensis
Climate
Climate + Soil
Soil
AUC = 0.99
TSS = 0.89
AUC = 0.99
TSS = 0.87
AUC = 0.95
TSS = 0.66
Relative importance
0.00
0.25
0.50
0.75
bio01
bio05
bio06
bio12
bio13
CEC
CLY
NTO
SOC
TWI
bio01
bio05
bio06
bio12
bio13
0.0
0.2
0.4
CEC
CLY
NTO
SOC
TWI

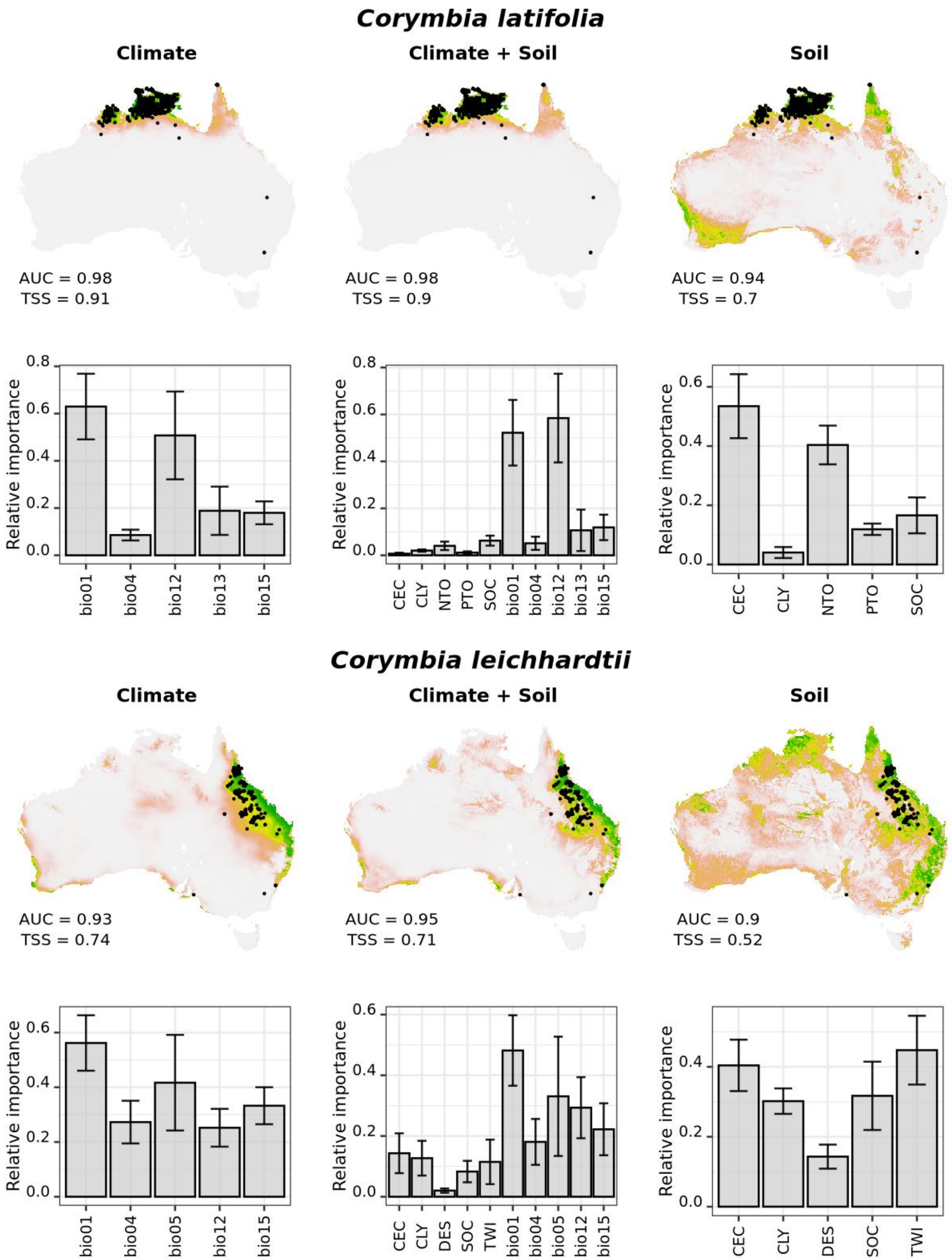
Corymbia latifolia
Climate
Climate + Soil
Soil
AUC = 0.98
TSS = 0.91
AUC = 0.98
TSS = 0.9
AUC = 0.94
TSS = 0.7
Relative importance
0.0 0.2 0.4 0.6 0.8
bio01 bio04 bio12 bio13 bio15
CEC CLY NTO PTO SOC bio01 bio04 bio12 bio13 bio15
0.0 0.2 0.4 0.6
CEC CLY NTO PTO SOC
Corymbia leichhardtii
Climate
Climate + Soil
Soil
AUC = 0.93
TSS = 0.74
AUC = 0.95
TSS = 0.71
AUC = 0.9
TSS = 0.52
bio01 bio04 bio05 bio12 bio15
CEC CLY DES SOC TWI bio01 bio04 bio05 bio12 bio15
CEC CLY DES SOC TWI

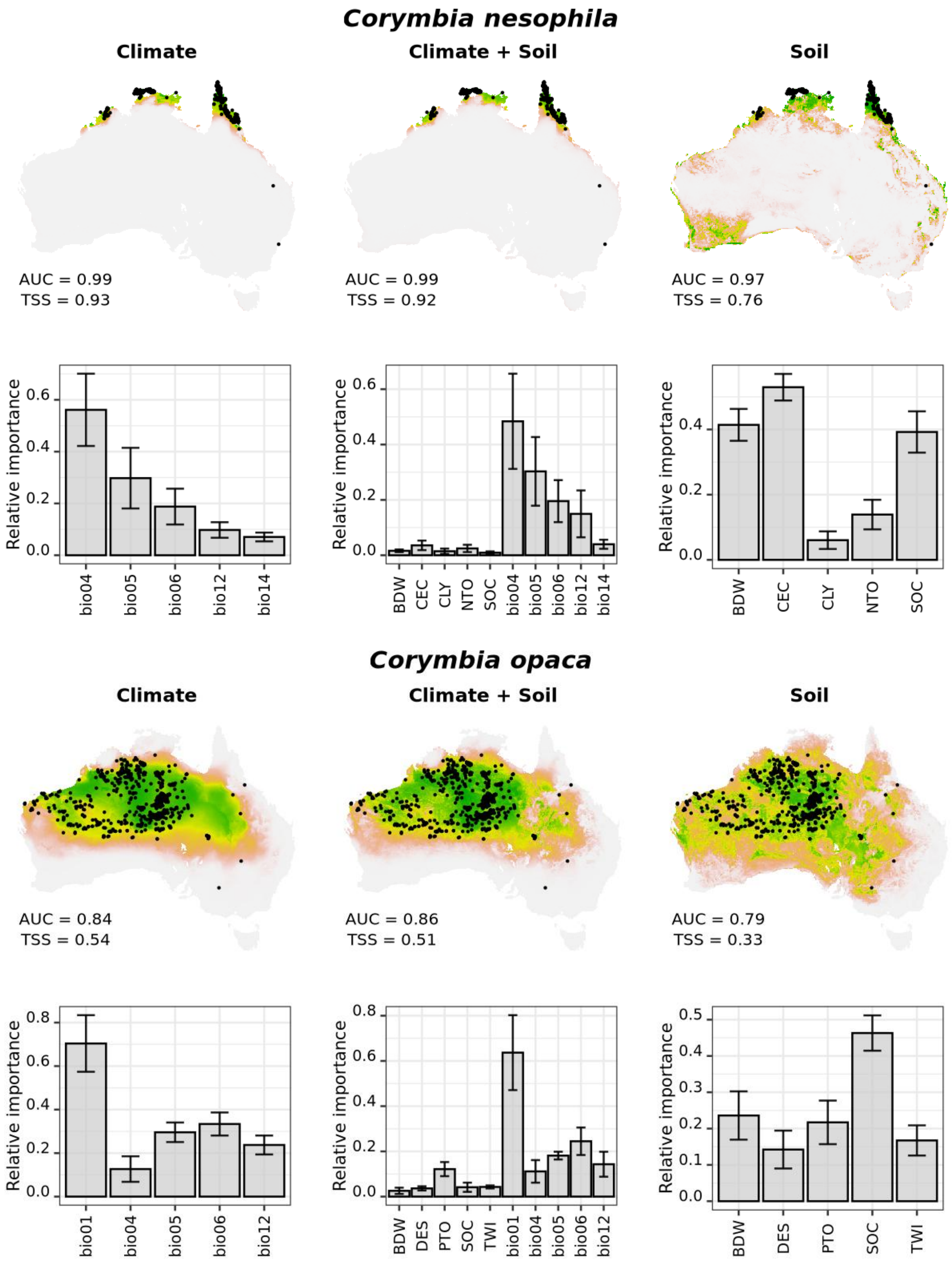

Corymbia nesophila
Climate
Climate + Soil
Soil
AUC = 0.99
TSS = 0.93
AUC = 0.99
TSS = 0.92
AUC = 0.97
TSS = 0.76
Relative importance
bio04
bio05
bio06
bio12
bio14
BDW
CEC
CLY
NTO
SOC
Corymbia opaca
Climate
Climate + Soil
Soil
AUC = 0.84
TSS = 0.54
AUC = 0.86
TSS = 0.51
AUC = 0.79
TSS = 0.33
Relative importance
bio01
bio04
bio05
bio06
bio12
BDW
DES
PTO
SOC
TWI

Corymbia pocillum

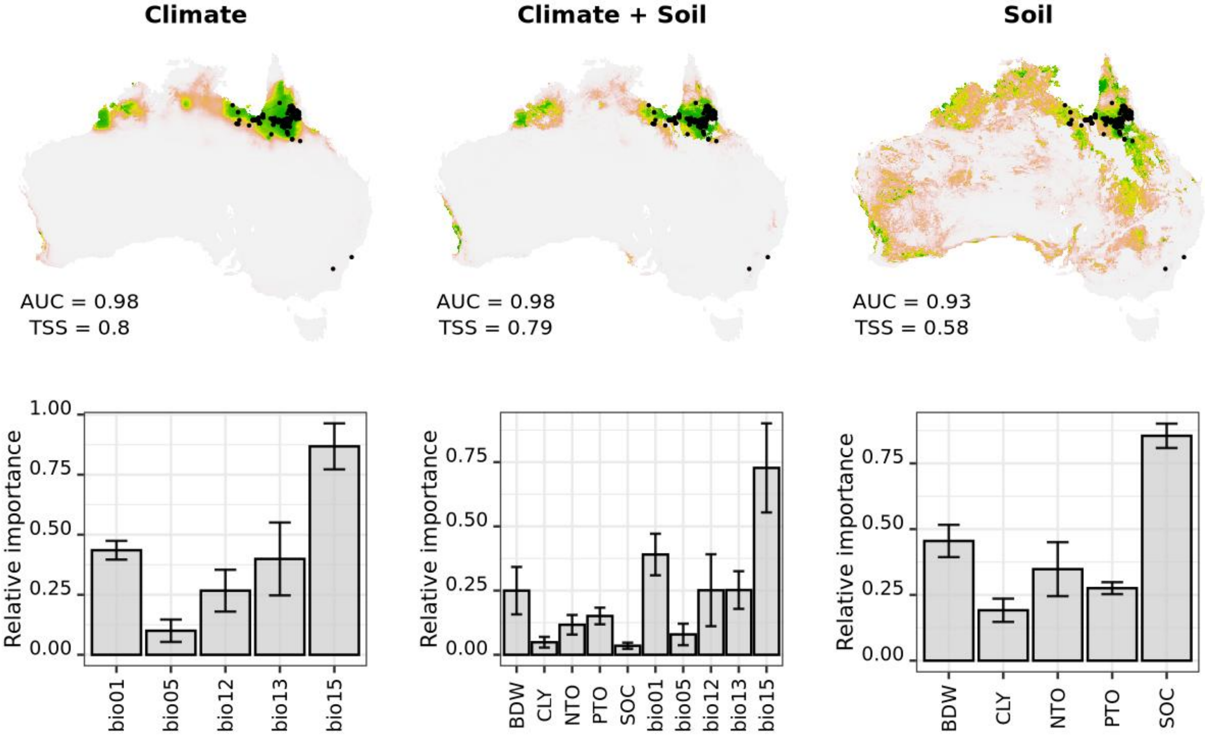

Corymbia polycarpa

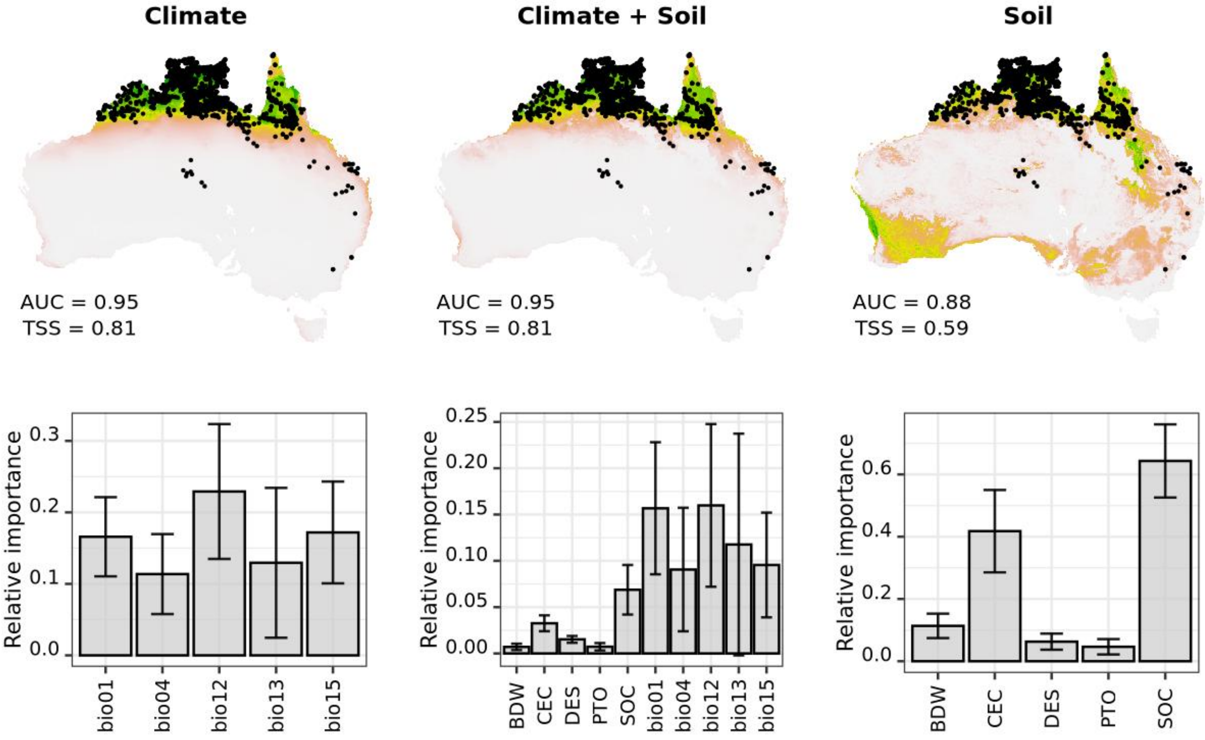

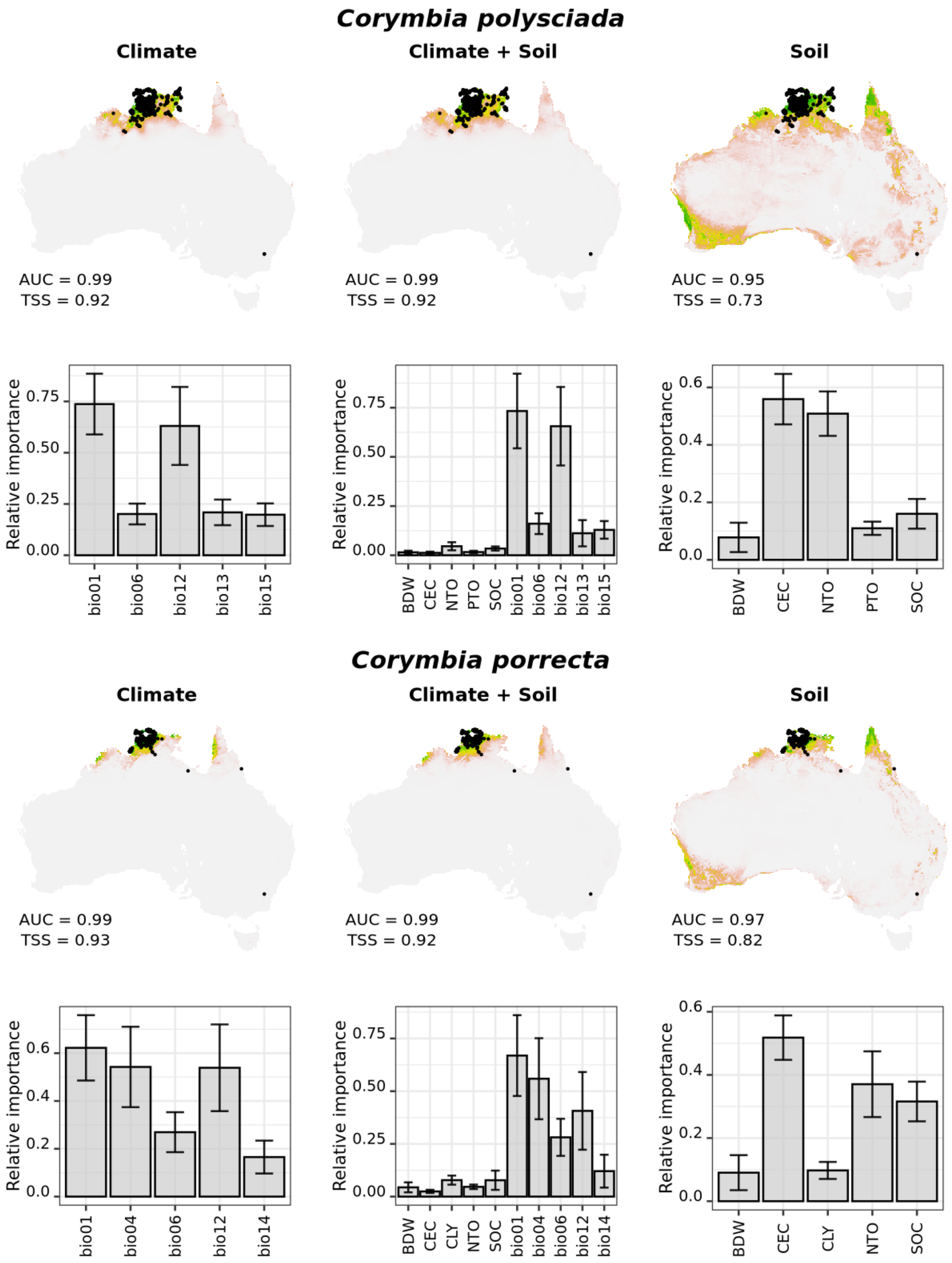

Corymbia polysciada
Climate
Climate + Soil
Soil
AUC = 0.99
TSS = 0.92
AUC = 0.99
TSS = 0.92
AUC = 0.95
TSS = 0.73
Relative importance
bio01
bio06
bio12
bio13
bio15
BDW
CEC
NTO
PTO
SOC
Corymbia porrecta
Climate
Climate + Soil
Soil
AUC = 0.99
TSS = 0.93
AUC = 0.99
TSS = 0.92
AUC = 0.97
TSS = 0.82
Relative importance
bio01
bio04
bio06
bio12
bio14
BDW
CEC
CLY
NTO
SOC

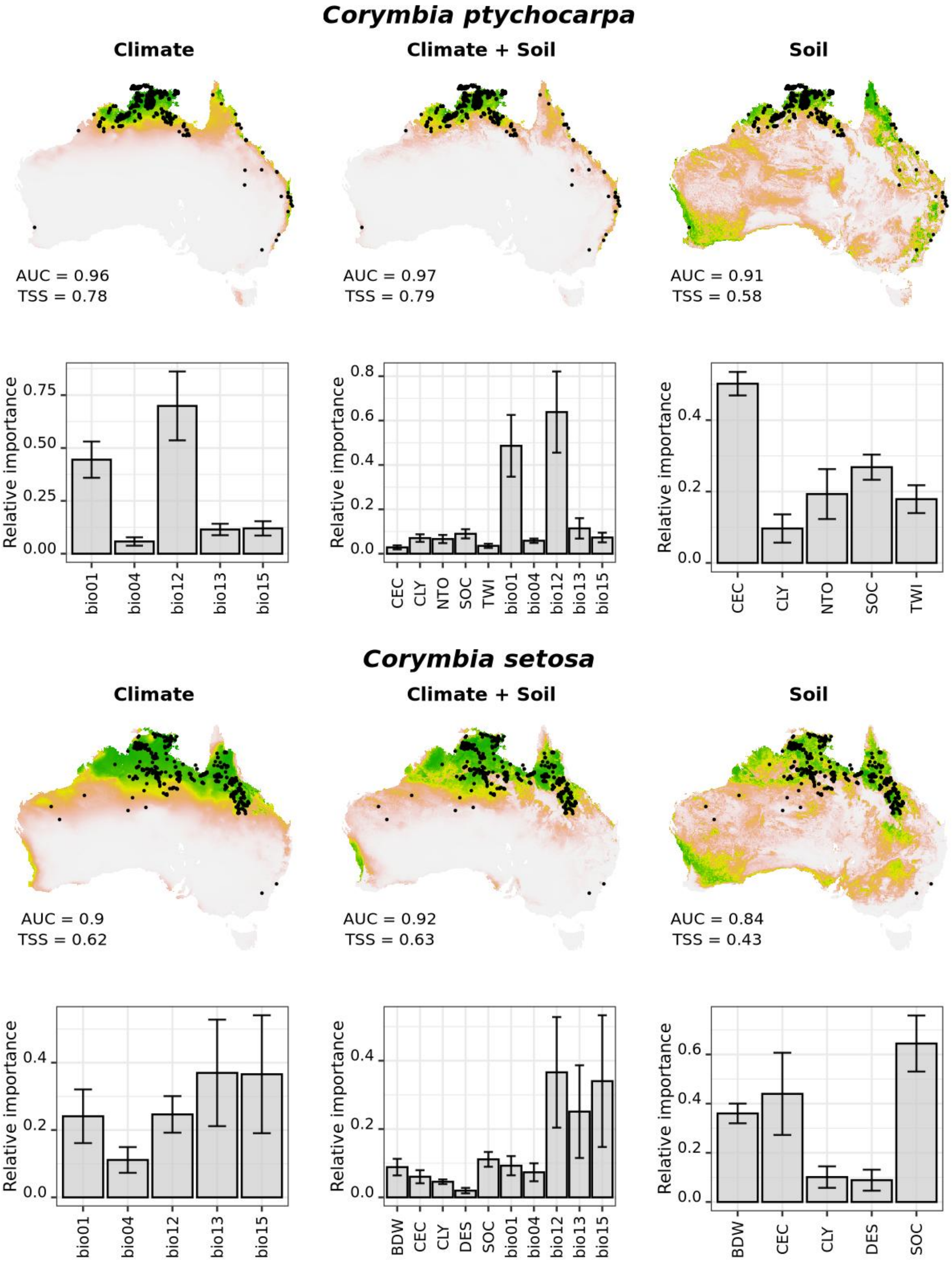
Corymbia ptychocarpa
Climate
Climate + Soil
Soil
AUC = 0.96
TSS = 0.78
AUC = 0.97
TSS = 0.79
AUC = 0.91
TSS = 0.58
Relative importance
bio01 bio04 bio12 bio13 bio15
CEC CLY NTO SOC TWI bio01 bio04 bio12 bio13 bio15
CEC CLY NTO SOC TWI
Corymbia setosa
Climate
Climate + Soil
Soil
AUC = 0.9
TSS = 0.62
AUC = 0.92
TSS = 0.63
AUC = 0.84
TSS = 0.43
Relative importance
bio01 bio04 bio12 bio13 bio15
BDW CEC CLY DES SOC bio01 bio04 bio12 bio13 bio15
BDW CEC CLY DES SOC

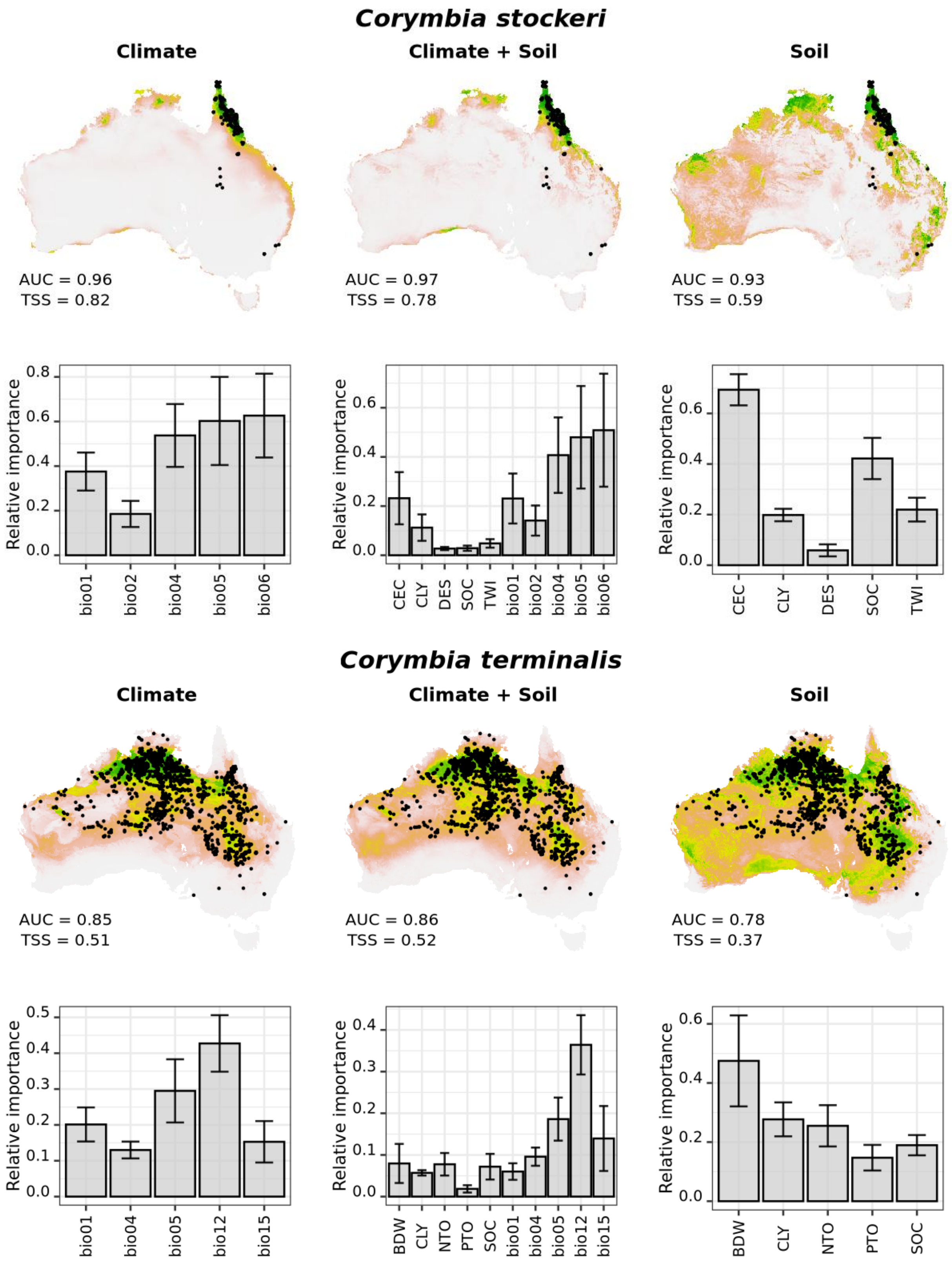
Corymbia stockeri
Climate
Climate + Soil
Soil
AUC = 0.96
TSS = 0.82
AUC = 0.97
TSS = 0.78
AUC = 0.93
TSS = 0.59
Relative importance
bio01 bio02 bio04 bio05 bio06
CEC CLY DES SOC TWI bio01 bio02 bio04 bio05 bio06
CEC CLY DES SOC TWI
Corymbia terminalis
Climate
Climate + Soil
Soil
AUC = 0.85
TSS = 0.51
AUC = 0.86
TSS = 0.52
AUC = 0.78
TSS = 0.37
Relative importance
bio01 bio04 bio05 bio12 bio15
BDW CLY NTO PTO SOC bio01 bio04 bio05 bio12 bio15
BDW CLY NTO PTO SOC

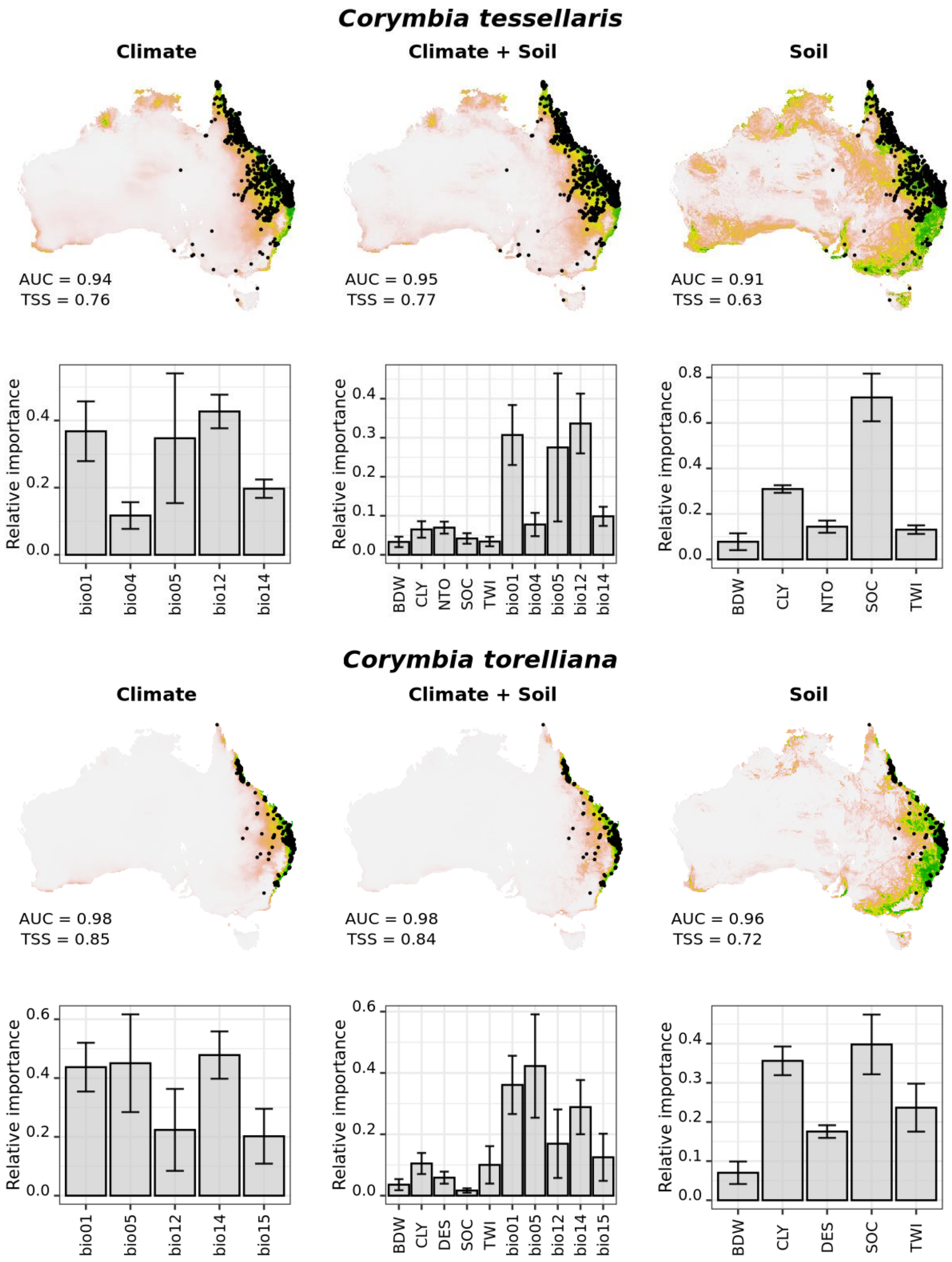

Corymbia tessellaris
Climate
Climate + Soil
Soil
AUC = 0.94
TSS = 0.76
AUC = 0.95
TSS = 0.77
AUC = 0.91
TSS = 0.63
Relative importance
bio01
bio04
bio05
bio12
bio14
BDW
CLY
NTO
SOC
TWI
Corymbia torelliana
Climate
Climate + Soil
Soil
AUC = 0.98
TSS = 0.85
AUC = 0.98
TSS = 0.84
AUC = 0.96
TSS = 0.72
Relative importance
bio01
bio05
bio12
bio14
bio15
BDW
CLY
DES
SOC
TWI

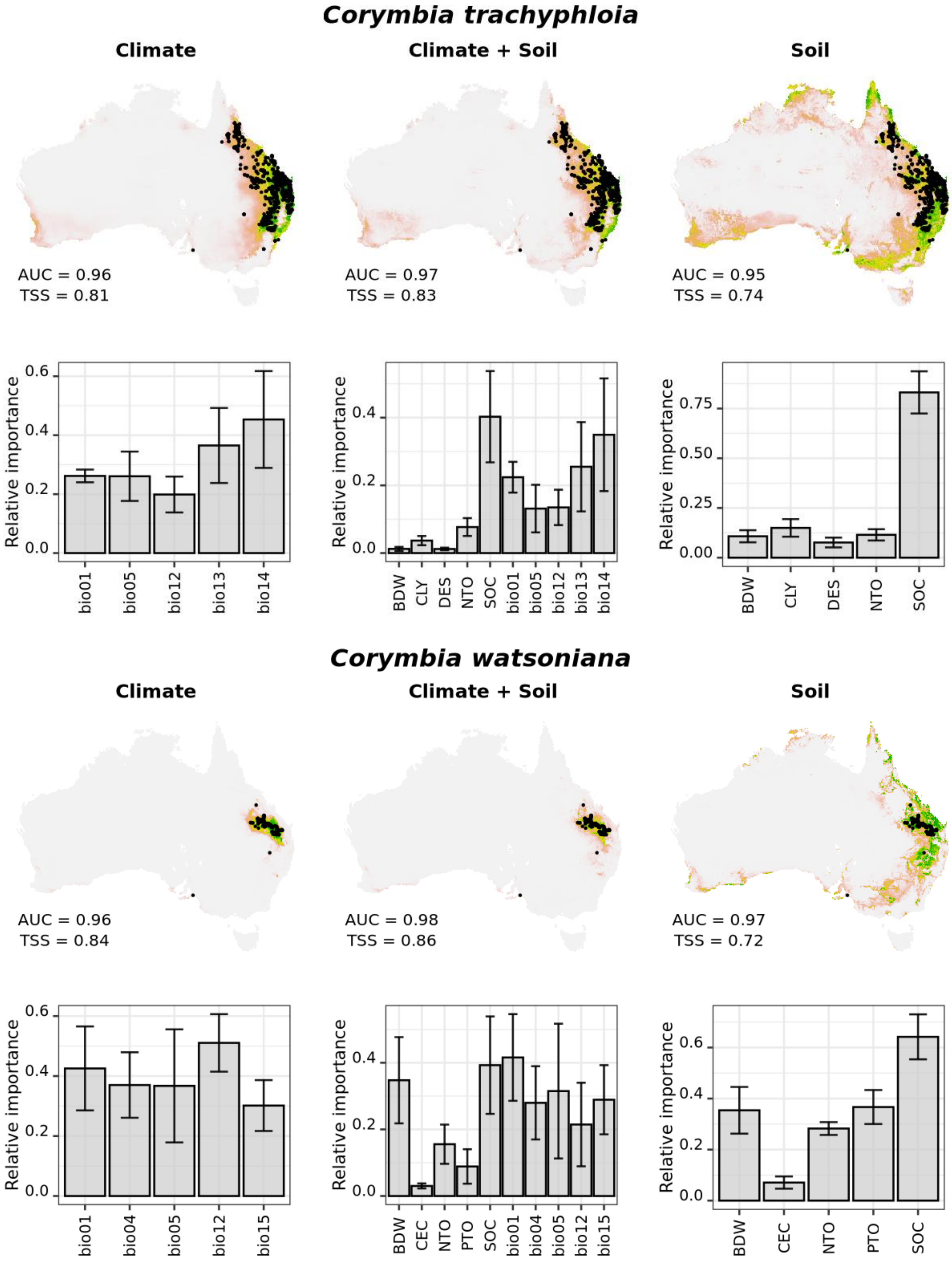
Corymbia trachyphloia
Climate
Climate + Soil
Soil
AUC = 0.96
TSS = 0.81
AUC = 0.97
TSS = 0.83
AUC = 0.95
TSS = 0.74
Relative importance
bio01
bio05
bio12
bio13
bio14
BDW
CLY
DES
NTO
SOC
Corymbia watsoniana
Climate
Climate + Soil
Soil
AUC = 0.96
TSS = 0.84
AUC = 0.98
TSS = 0.86
AUC = 0.97
TSS = 0.72
Relative importance
bio01
bio04
bio05
bio12
bio15
BDW
CEC
NTO
PTO
SOC

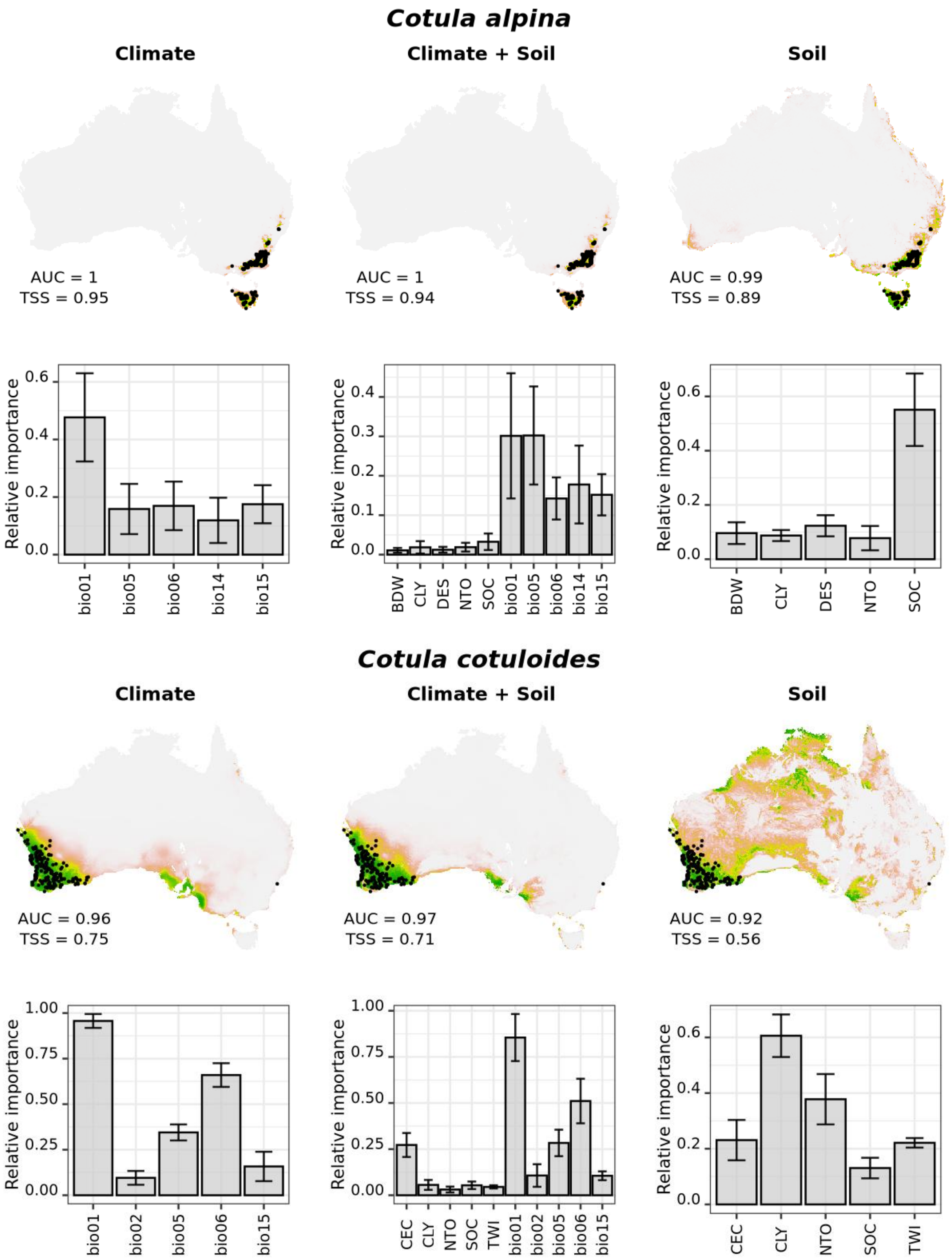
Cotula alpina
Climate
Climate + Soil
Soil
AUC = 1
TSS = 0.95
AUC = 1
TSS = 0.94
AUC = 0.99
TSS = 0.89
Relative importance
bio01 bio05 bio06 bio14 bio15
BDW CLY DES NTO SOC bio01 bio05 bio06 bio14 bio15
BDW CLY DES NTO SOC
Cotula cotuloides
Climate
Climate + Soil
Soil
AUC = 0.96
TSS = 0.75
AUC = 0.97
TSS = 0.71
AUC = 0.92
TSS = 0.56
Relative importance
bio01 bio02 bio05 bio06 bio15
CEC CLY NTO SOC TWI bio01 bio02 bio05 bio06 bio15
CEC CLY NTO SOC TWI

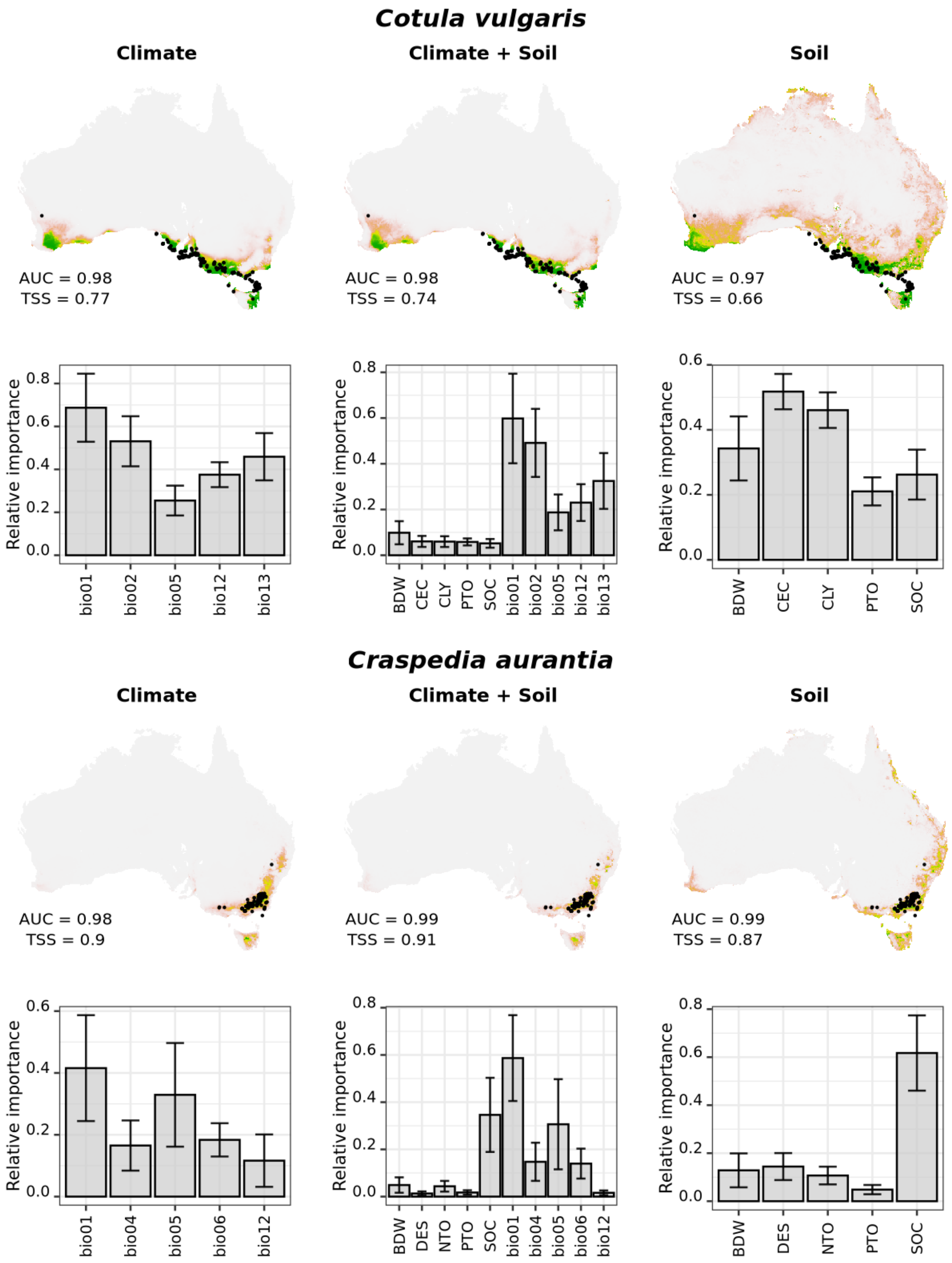

Cotula vulgaris
Climate
Climate + Soil
Soil
AUC = 0.98
TSS = 0.77
AUC = 0.98
TSS = 0.74
AUC = 0.97
TSS = 0.66
Relative importance
bio01
bio02
bio05
bio12
bio13
BDW
CEC
CLY
PTO
SOC
Craspedia aurantia
AUC = 0.98
TSS = 0.9
AUC = 0.99
TSS = 0.91
AUC = 0.99
TSS = 0.87
bio04
bio06
DES
NTO

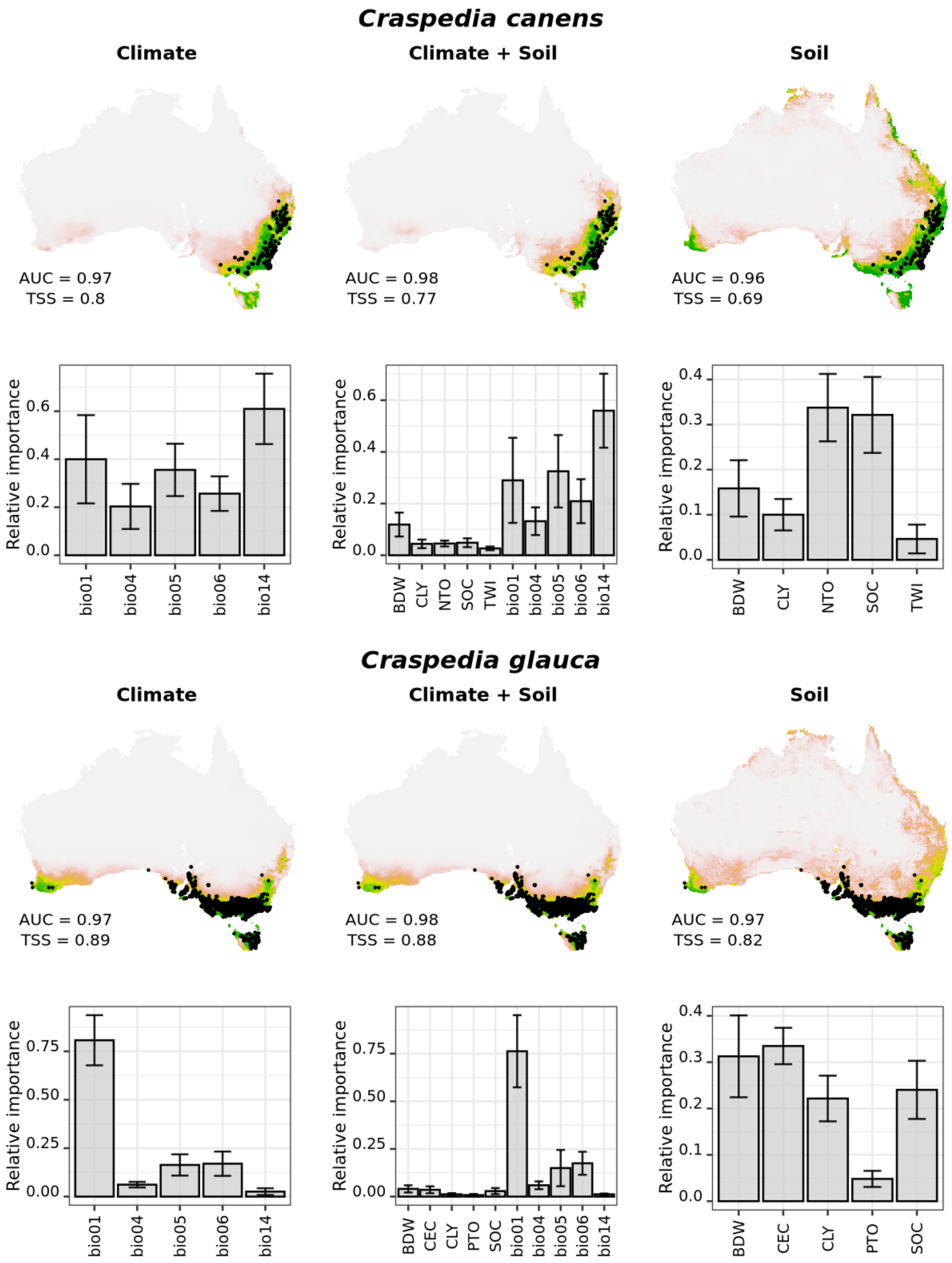
Craspedia canens
Climate
Climate + Soil
Soil
AUC = 0.97
TSS = 0.8
AUC = 0.98
TSS = 0.77
AUC = 0.96
TSS = 0.69
Relative importance
0.0
0.2
0.4
0.6
bio01
bio04
bio05
bio06
bio14
BDW
CLY
NTO
SOC
TWI
0.1
0.3
Craspedia glauca
Climate
Climate + Soil
Soil
AUC = 0.97
TSS = 0.89
AUC = 0.98
TSS = 0.88
AUC = 0.97
TSS = 0.82
Relative importance
0.00
0.25
0.50
0.75
bio01
bio04
bio05
bio06
bio14
BDW
CEC
CLY
PTO
SOC

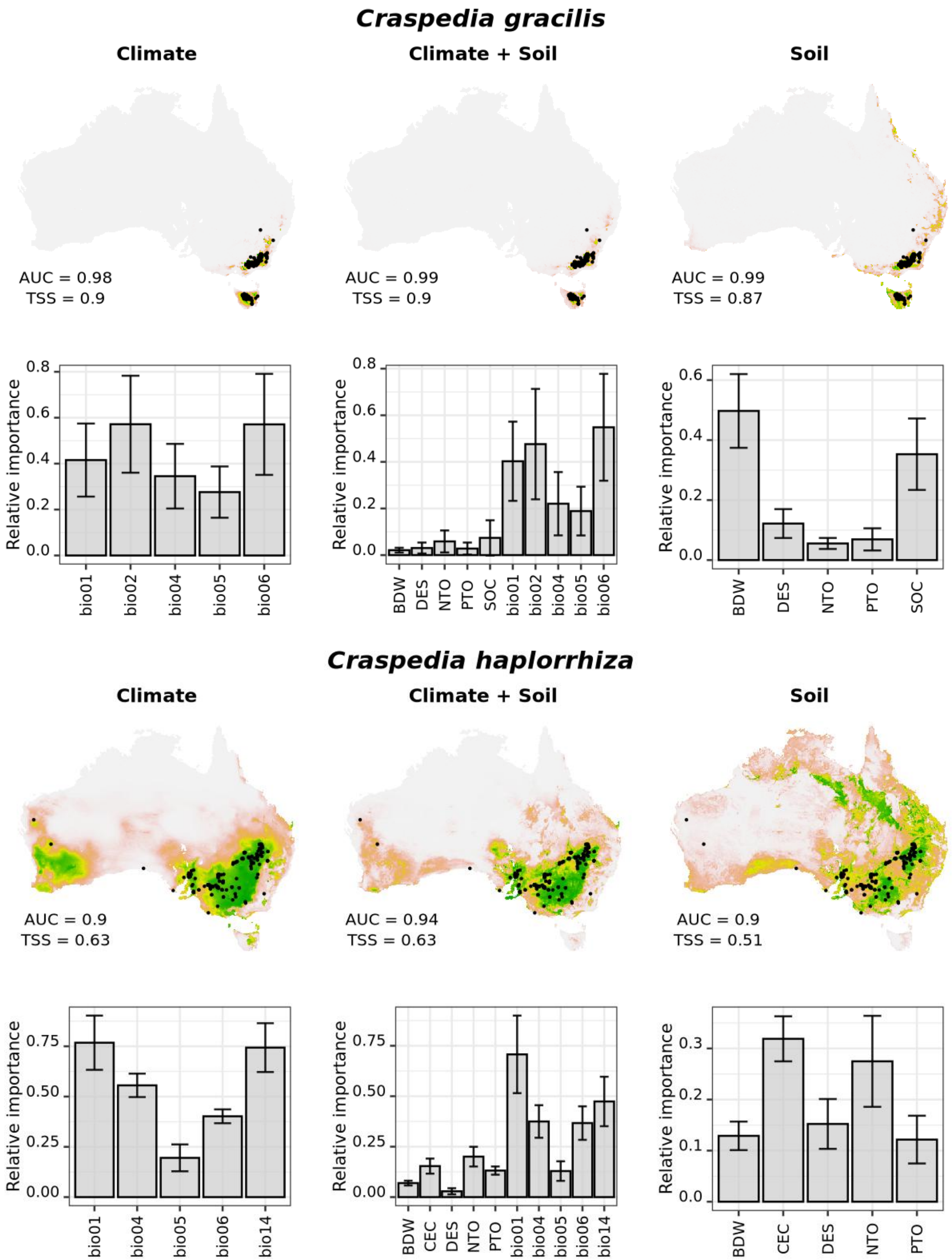

Craspedia gracilis
Climate
Climate + Soil
Soil
AUC = 0.98
TSS = 0.9
AUC = 0.99
TSS = 0.9
AUC = 0.99
TSS = 0.87
Relative importance
bio01
bio02
bio04
bio05
bio06
BDW
DES
NTO
PTO
SOC
Craspedia haplorrhiza
Climate
Climate + Soil
Soil
AUC = 0.9
TSS = 0.63
AUC = 0.94
TSS = 0.63
AUC = 0.9
TSS = 0.51
Relative importance
bio01
bio04
bio05
bio06
bio14
BDW
CEC
DES
NTO
PTO

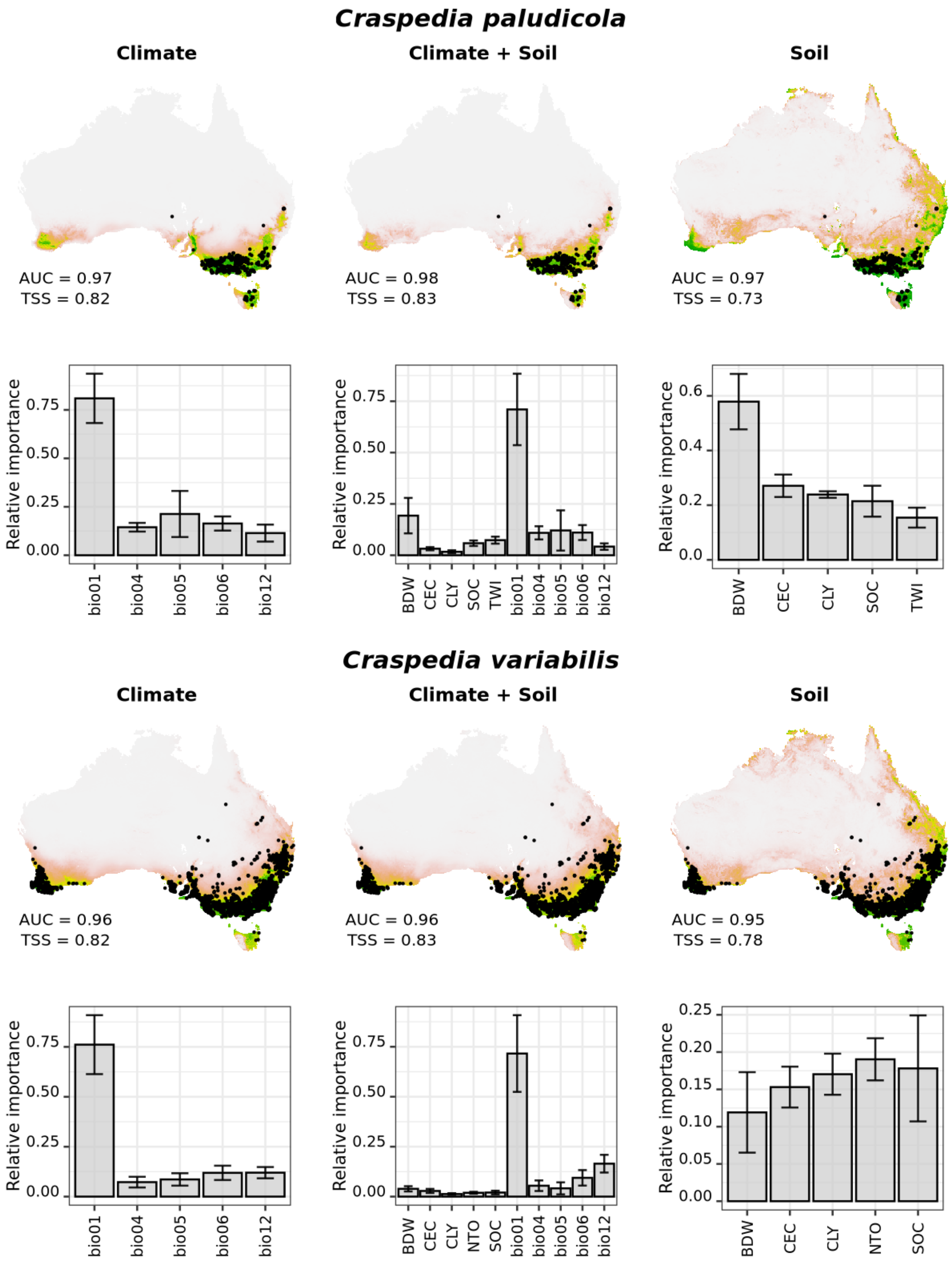

Craspedia paludicola
Climate
Climate + Soil
Soil
AUC = 0.97
TSS = 0.82
AUC = 0.98
TSS = 0.83
AUC = 0.97
TSS = 0.73
Relative importance
0.00
0.25
0.50
0.75
bio01
bio04
bio05
bio06
bio12
BDW
CEC
CLY
SOC
TWI
0.0
0.2
0.4
0.6
Craspedia variabilis
Climate
Climate + Soil
Soil
AUC = 0.96
TSS = 0.82
AUC = 0.96
TSS = 0.83
AUC = 0.95
TSS = 0.78
NTO
0.05
0.10
0.15
0.20
0.25

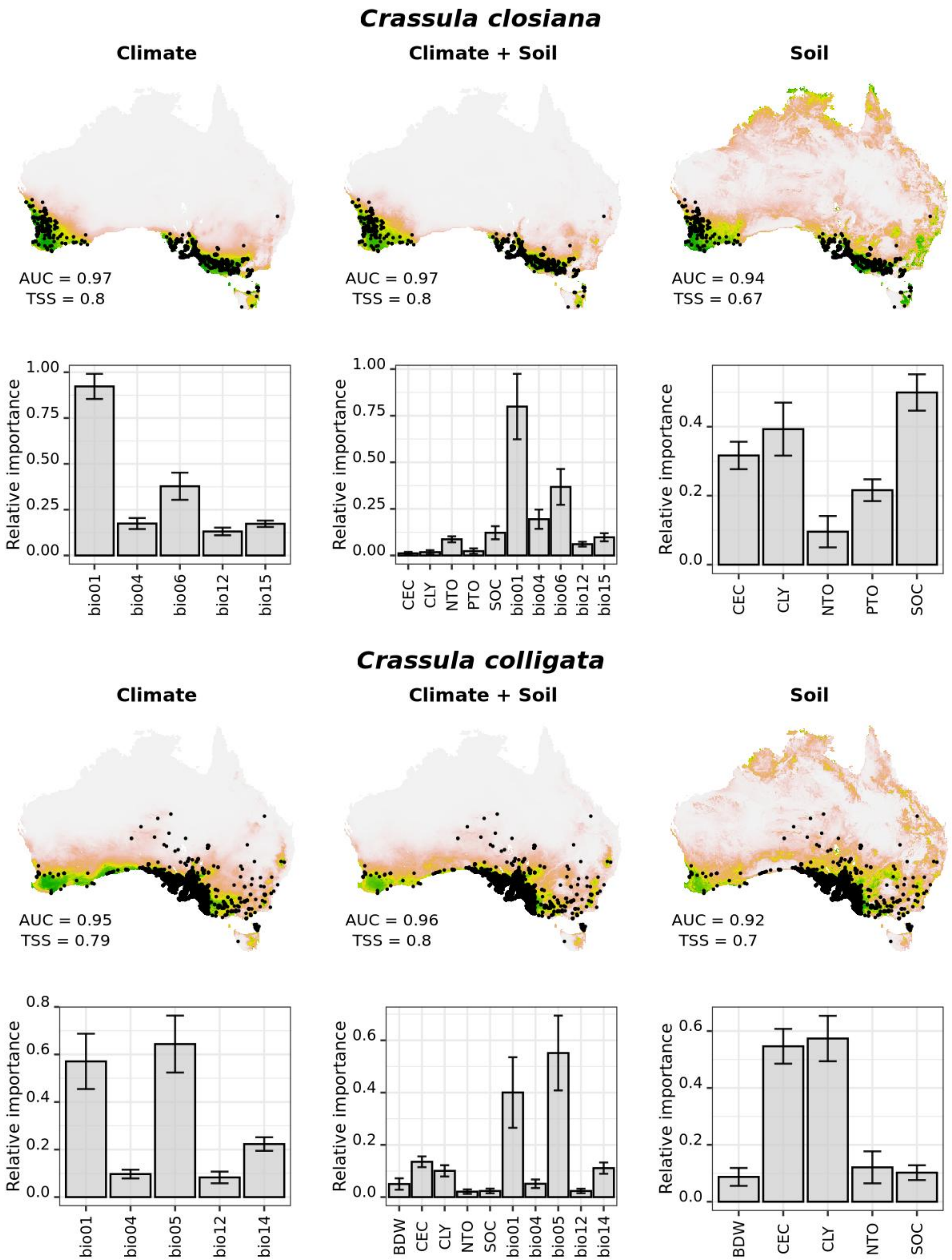
Crassula closiana
Climate
Climate + Soil
Soil
AUC = 0.97
TSS = 0.8
AUC = 0.97
TSS = 0.8
AUC = 0.94
TSS = 0.67
Relative importance
1.00
0.75
0.50
0.25
0.00
bio01
bio04
bio06
bio12
bio15
CEC
CLY
NTO
PTO
SOC
0.4
0.2
0.0
Crassula colligata
Climate
Climate + Soil
Soil
AUC = 0.95
TSS = 0.79
AUC = 0.96
TSS = 0.8
AUC = 0.92
TSS = 0.7
Relative importance
0.8
0.6
0.4
0.2
0.0
bio01
bio04
bio05
bio12
bio14
BDW
CEC
CLY
NTO
SOC

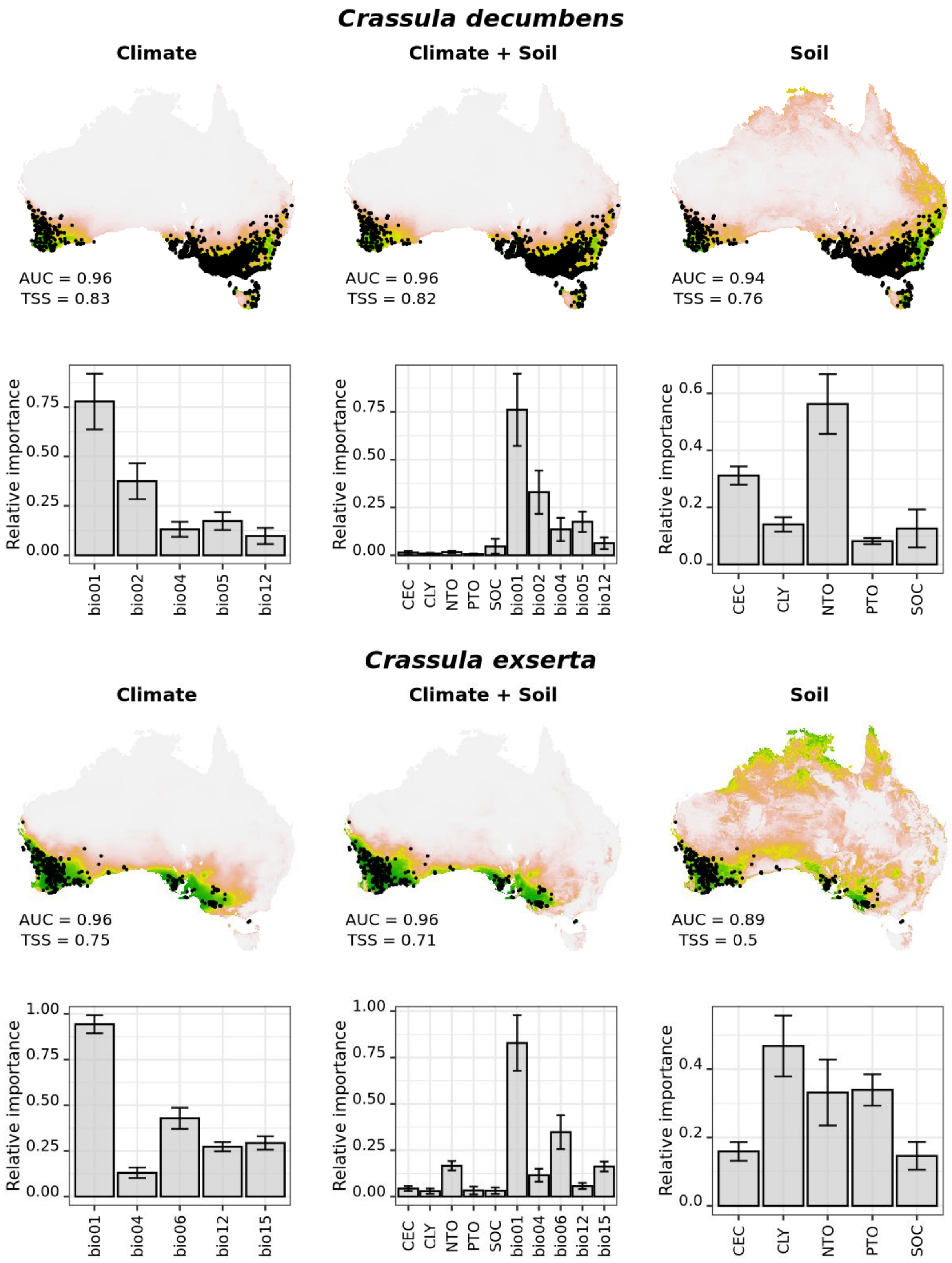
Crassula decumbens
Climate
Climate + Soil
Soil
AUC = 0.96
TSS = 0.83
AUC = 0.96
TSS = 0.82
AUC = 0.94
TSS = 0.76
Relative importance
0.00
0.25
0.50
0.75
bio01
bio02
bio04
bio05
bio12
CEC
CLY
NTO
PTO
SOC
0.0
0.2
0.4
0.6
Crassula exserta
AUC = 0.96
TSS = 0.75
AUC = 0.96
TSS = 0.71
AUC = 0.89
TSS = 0.5
1.00
bio06
bio15

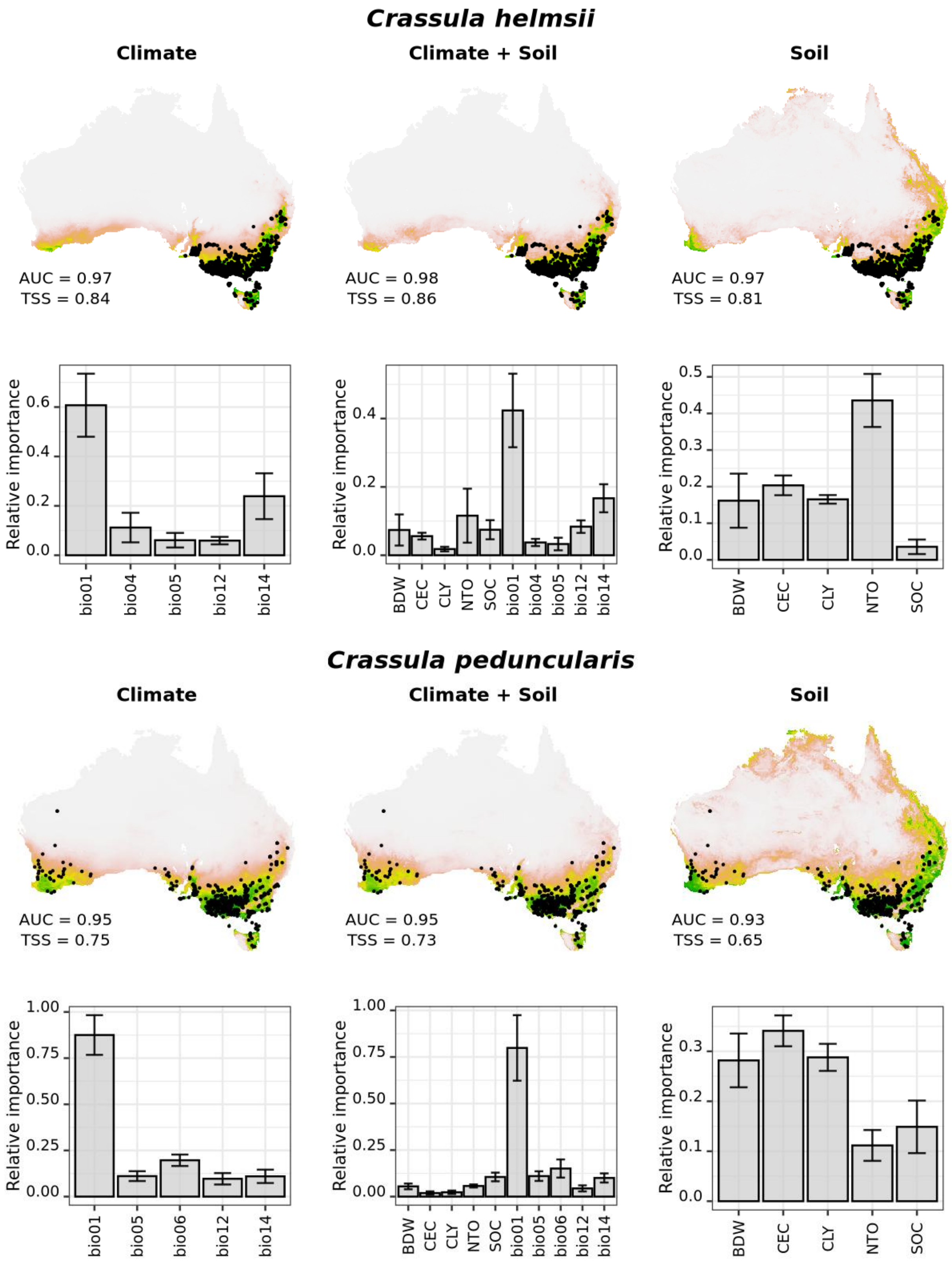

Crassula helmsii
Climate
Climate + Soil
Soil
AUC = 0.97
TSS = 0.84
AUC = 0.98
TSS = 0.86
AUC = 0.97
TSS = 0.81
Relative importance
bio01
bio04
bio05
bio12
bio14
BDW
CEC
CLY
NTO
SOC
Crassula peduncularis
Climate
Climate + Soil
Soil
AUC = 0.95
TSS = 0.75
AUC = 0.95
TSS = 0.73
AUC = 0.93
TSS = 0.65
Relative importance
bio01
bio05
bio06
bio12
bio14
BDW
CEC
CLY
NTO
SOC

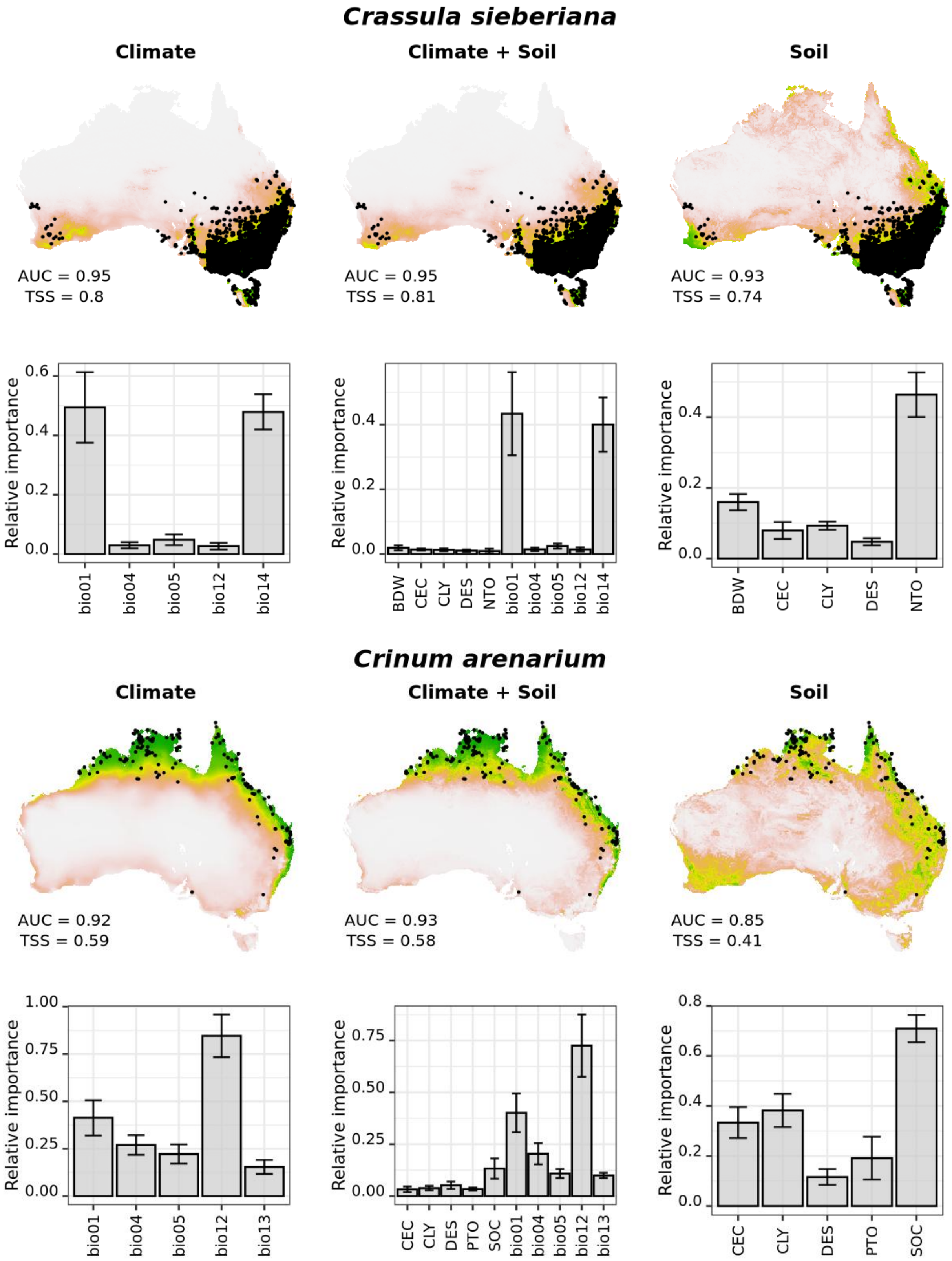
Crassula sieberiana
Climate
Climate + Soil
Soil
AUC = 0.95
TSS = 0.8
AUC = 0.95
TSS = 0.81
AUC = 0.93
TSS = 0.74
Relative importance
0.0
0.2
0.4
0.6
bio01
bio04
bio05
bio12
bio14
BDW
CEC
CLY
DES
NTO
Crinum arenarium
Climate
Climate + Soil
Soil
AUC = 0.92
TSS = 0.59
AUC = 0.93
TSS = 0.58
AUC = 0.85
TSS = 0.41
0.00
0.25
0.50
0.75
1.00
bio13
PTO
SOC
0.8

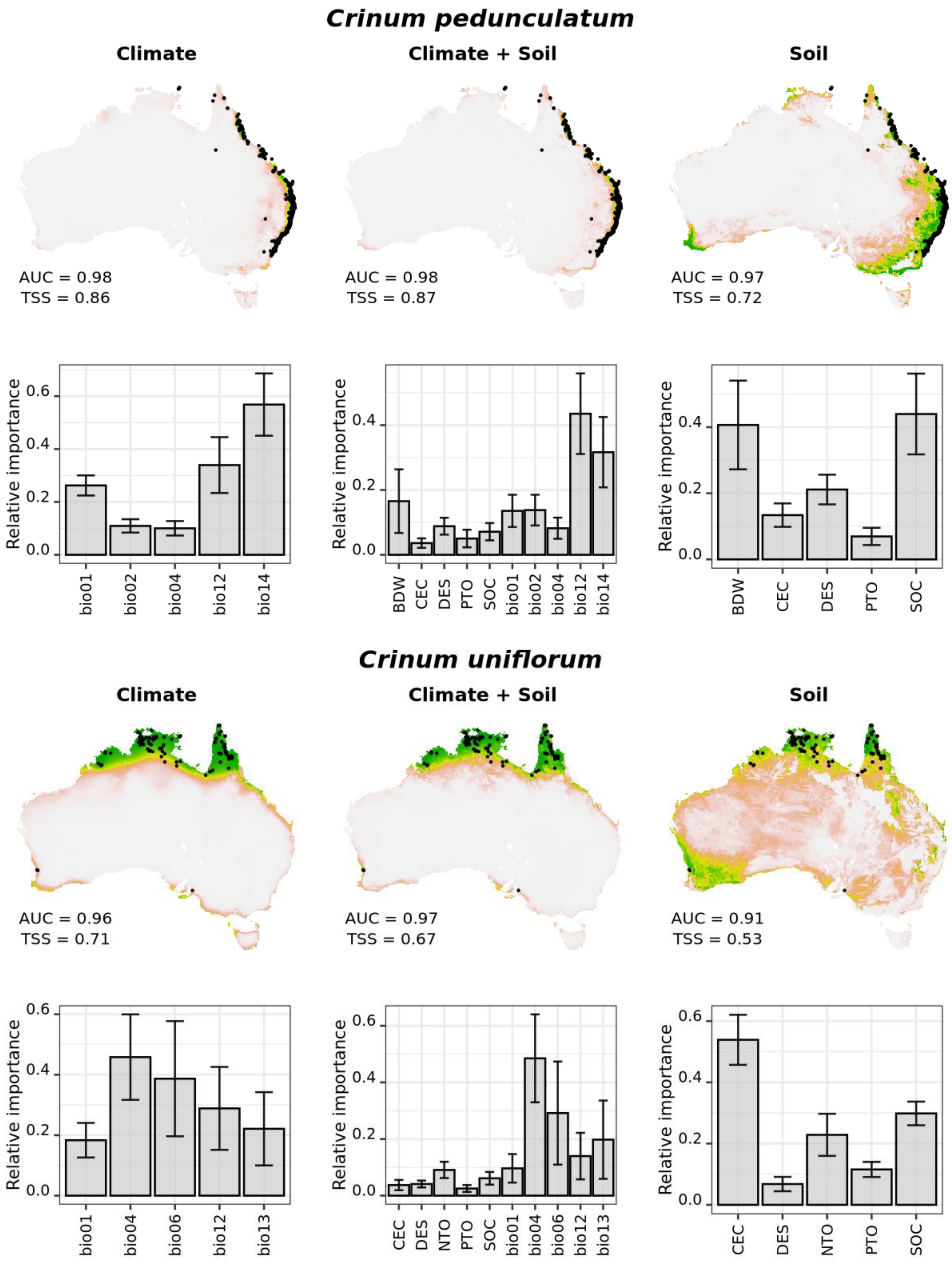

Crinum pedunculatum
Climate
Climate + Soil
Soil
AUC = 0.98
TSS = 0.86
AUC = 0.98
TSS = 0.87
AUC = 0.97
TSS = 0.72
Relative importance
bio01
bio02
bio04
bio12
bio14
BDW
CEC
DES
PTO
SOC
Crinum uniflorum
AUC = 0.96
TSS = 0.71
AUC = 0.97
TSS = 0.67
AUC = 0.91
TSS = 0.53
bio06
bio13
NTO

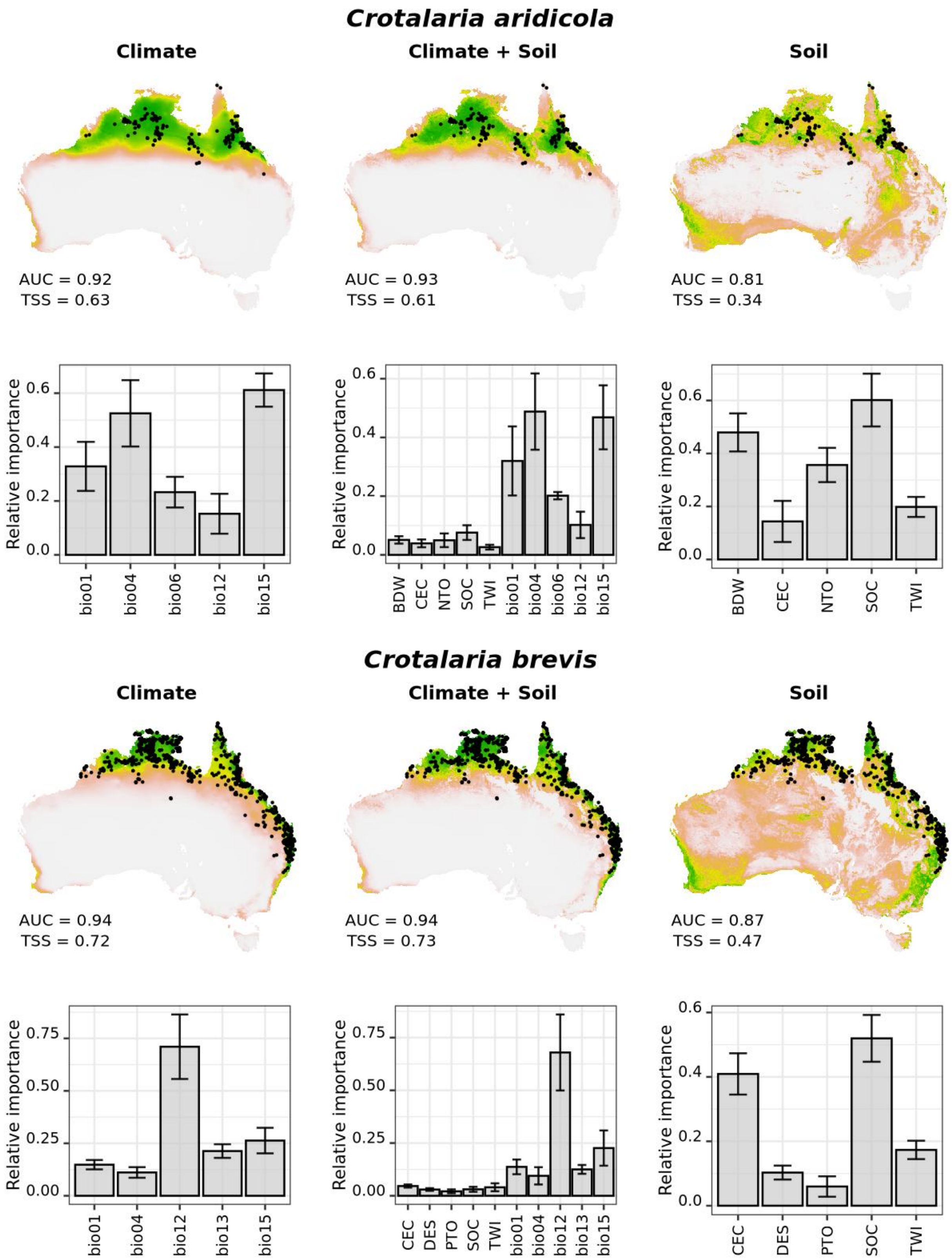
Crotalaria aridicola
Climate
Climate + Soil
Soil
AUC = 0.92
TSS = 0.63
AUC = 0.93
TSS = 0.61
AUC = 0.81
TSS = 0.34
Relative importance
bio01
bio04
bio06
bio12
bio15
BDW
CEC
NTO
SOC
TWI
Crotalaria brevis
Climate
Climate + Soil
Soil
AUC = 0.94
TSS = 0.72
AUC = 0.94
TSS = 0.73
AUC = 0.87
TSS = 0.47
Relative importance
bio01
bio04
bio12
bio13
bio15
CEC
DES
PTO
SOC
TWI

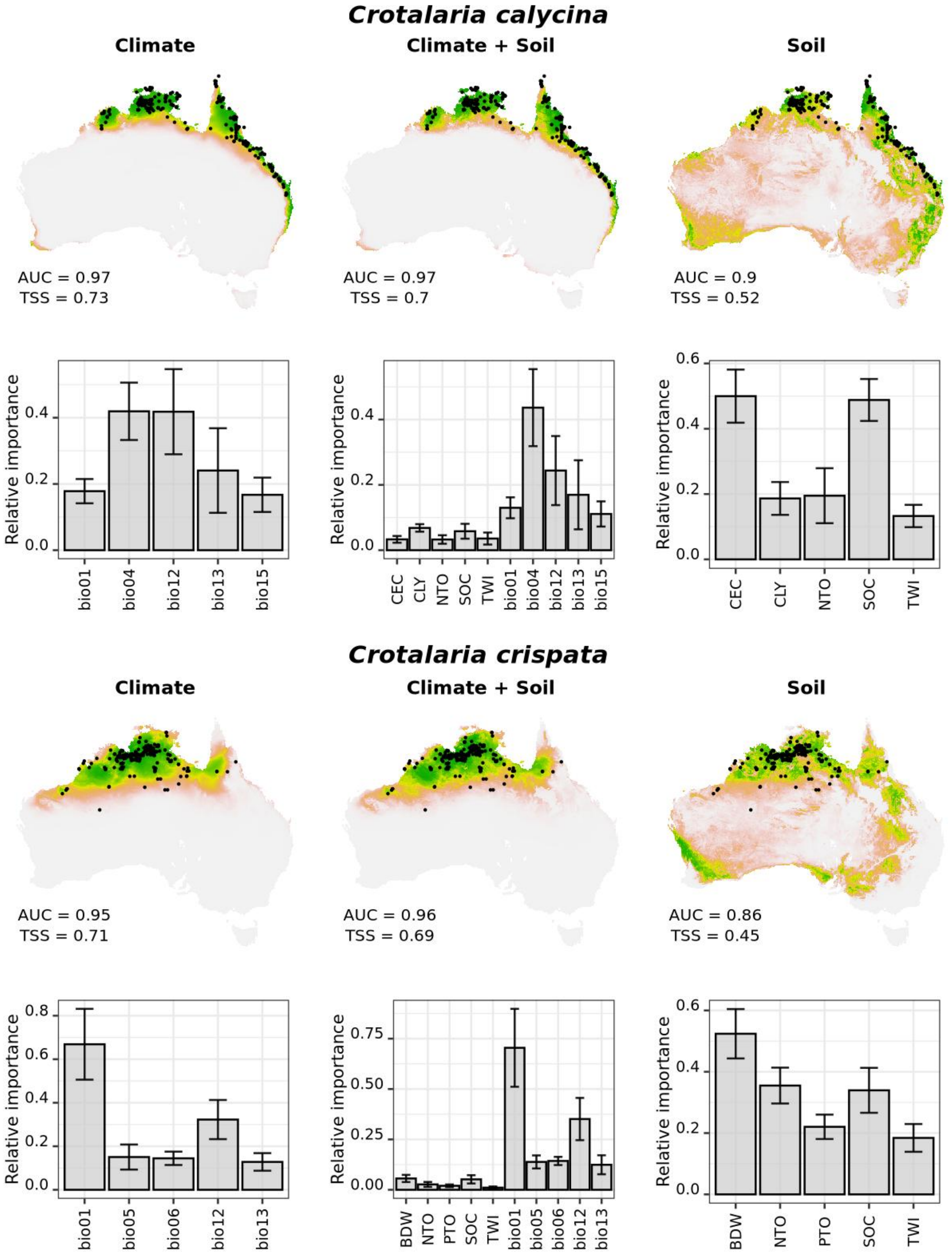
Crotalaria calycina
Climate
Climate + Soil
Soil
AUC = 0.97
TSS = 0.73
AUC = 0.97
TSS = 0.7
AUC = 0.9
TSS = 0.52
Relative importance
bio01
bio04
bio12
bio13
bio15
CEC
CLY
NTO
SOC
TWI
Crotalaria crispata
Climate
Climate + Soil
Soil
AUC = 0.95
TSS = 0.71
AUC = 0.96
TSS = 0.69
AUC = 0.86
TSS = 0.45
Relative importance
bio01
bio05
bio06
bio12
bio13
BDW
NTO
PTO
SOC
TWI

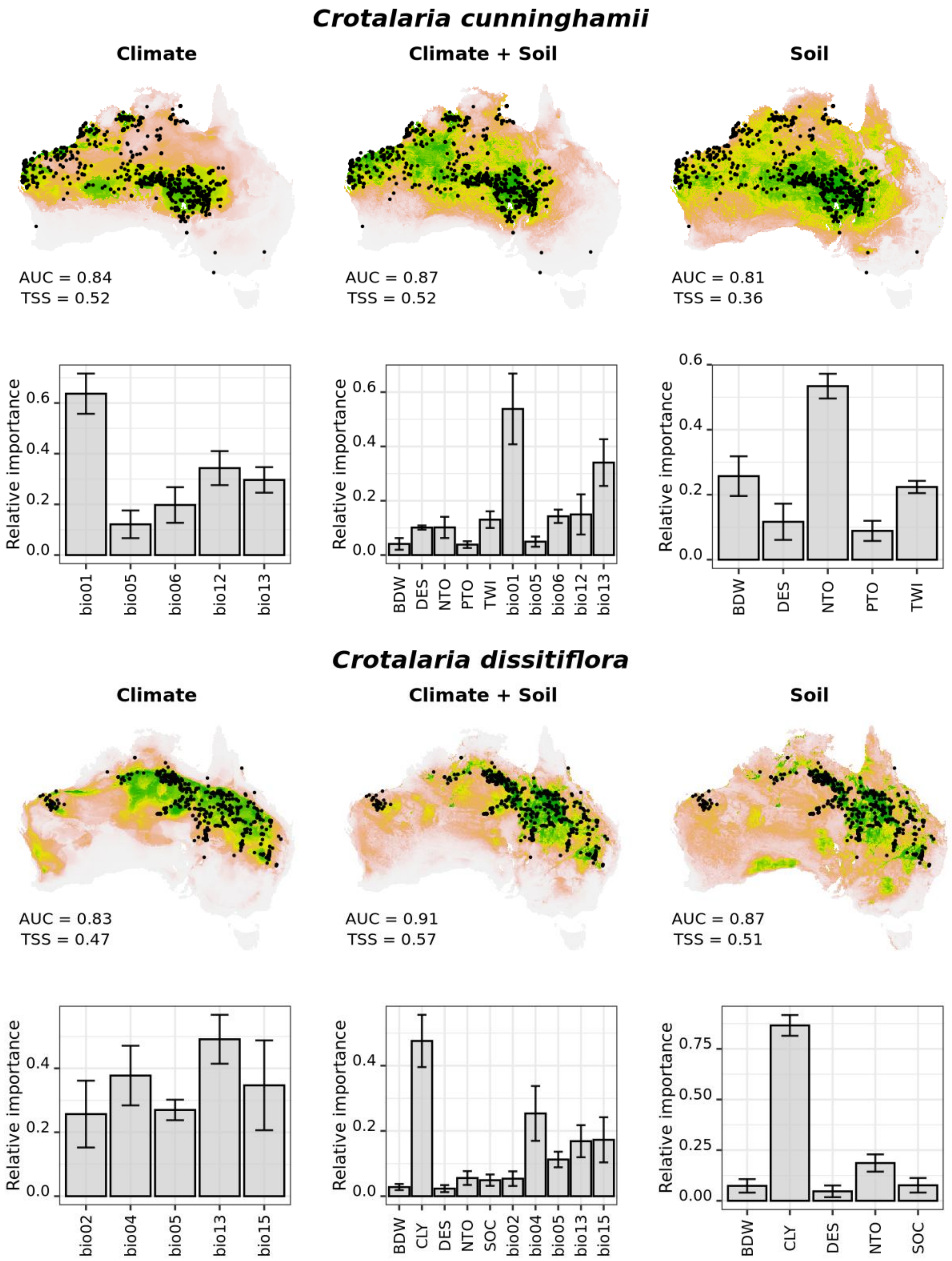
Crotalaria cunninghamii
Climate
Climate + Soil
Soil
AUC = 0.84
TSS = 0.52
AUC = 0.87
TSS = 0.52
AUC = 0.81
TSS = 0.36
Relative importance
bio01
bio05
bio06
bio12
bio13
BDW
DES
NTO
PTO
TWI
Crotalaria dissitiflora
AUC = 0.83
TSS = 0.47
AUC = 0.91
TSS = 0.57
AUC = 0.87
TSS = 0.51
bio02
bio04
bio15
CLY
SOC

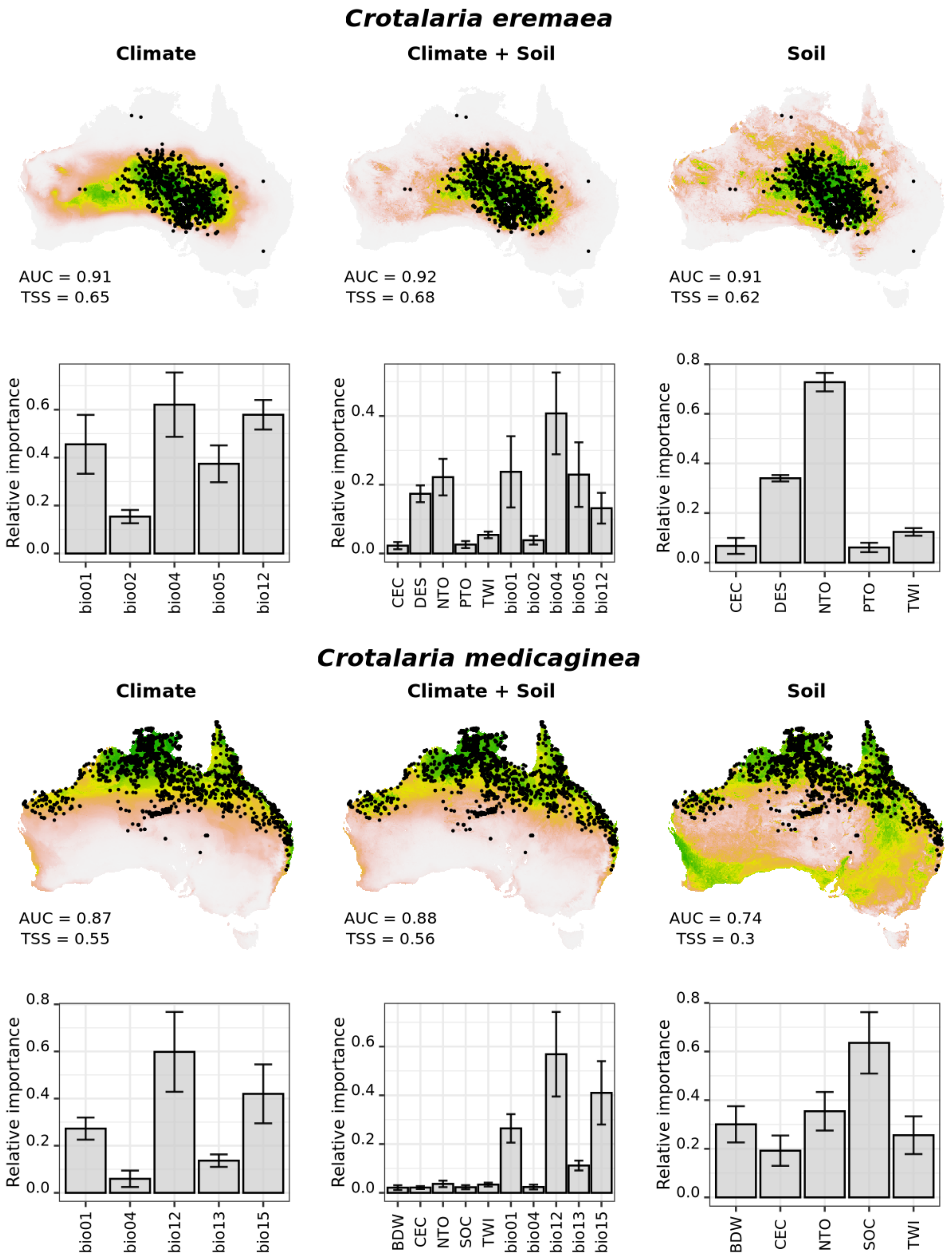

Crotalaria eremaea
Climate
Climate + Soil
Soil
AUC = 0.91
TSS = 0.65
AUC = 0.92
TSS = 0.68
AUC = 0.91
TSS = 0.62
Relative importance
bio01 bio02 bio04 bio05 bio12
CEC DES NTO PTO TWI bio01 bio02 bio04 bio05 bio12
CEC DES NTO PTO TWI
Crotalaria medicaginea
Climate
Climate + Soil
Soil
AUC = 0.87
TSS = 0.55
AUC = 0.88
TSS = 0.56
AUC = 0.74
TSS = 0.3
Relative importance
bio01 bio04 bio12 bio13 bio15
BDW CEC NTO SOC TWI bio01 bio04 bio12 bio13 bio15
BDW CEC NTO SOC TWI

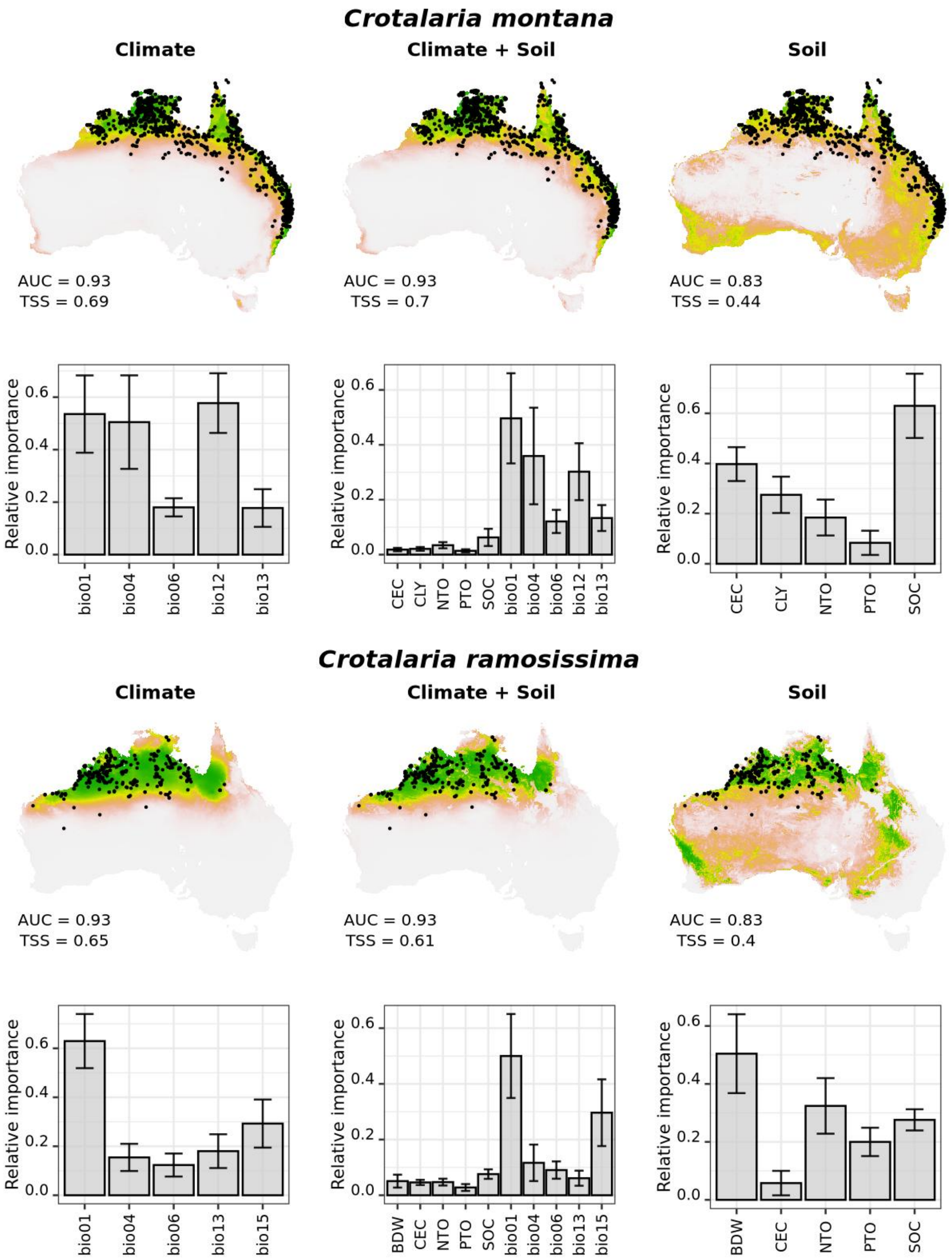

Crotalaria montana
Climate
Climate + Soil
Soil
AUC = 0.93
TSS = 0.69
AUC = 0.93
TSS = 0.7
AUC = 0.83
TSS = 0.44
Relative importance
bio01
bio04
bio06
bio12
bio13
CEC
CLY
NTO
PTO
SOC
Crotalaria ramosissima
Climate
Climate + Soil
Soil
AUC = 0.93
TSS = 0.65
AUC = 0.93
TSS = 0.61
AUC = 0.83
TSS = 0.4
Relative importance
bio01
bio04
bio06
bio13
bio15
BDW
CEC
NTO
PTO
SOC

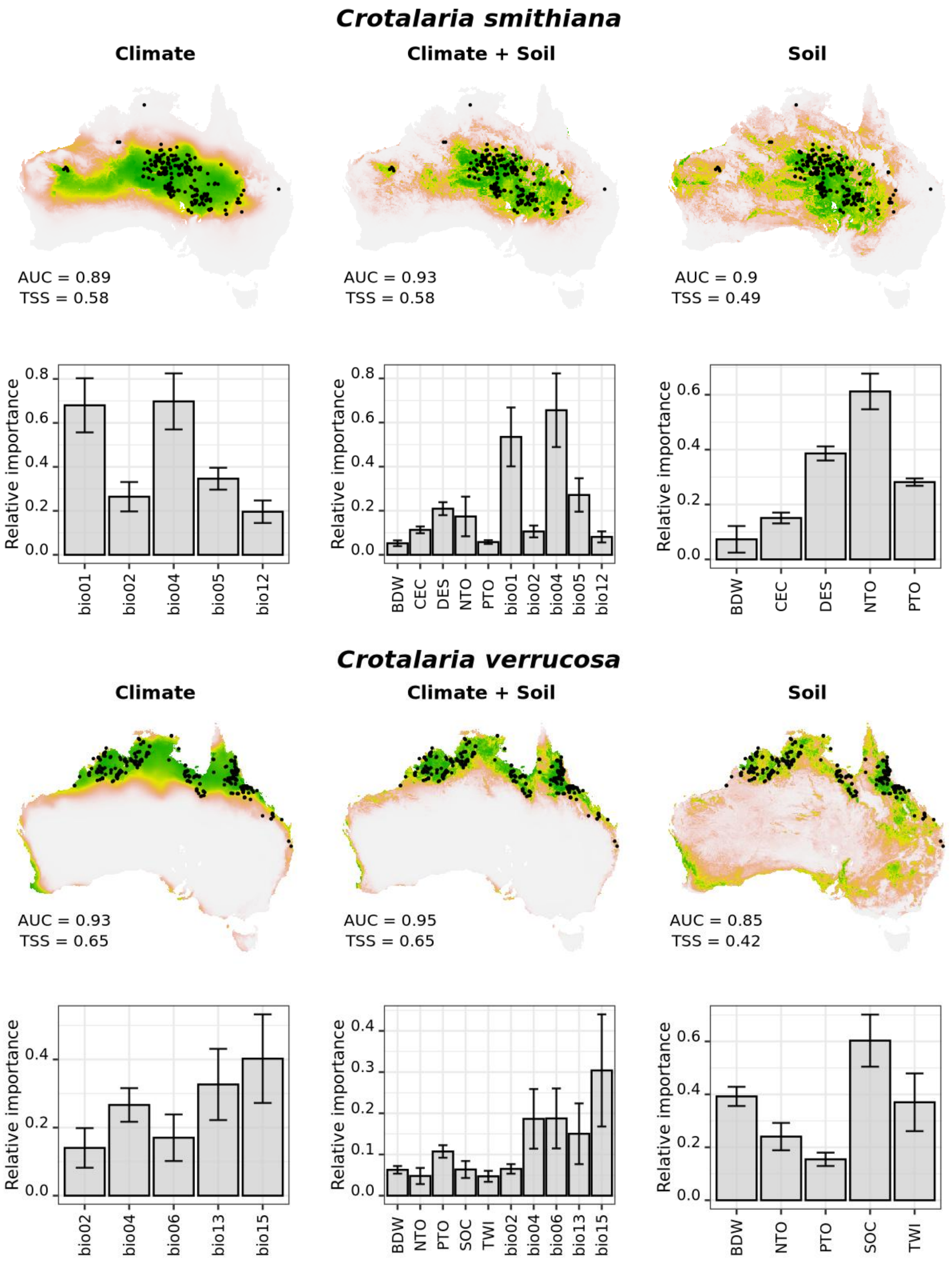
Crotalaria smithiana
Climate
Climate + Soil
Soil
AUC = 0.89
TSS = 0.58
AUC = 0.93
TSS = 0.58
AUC = 0.9
TSS = 0.49
Relative importance
bio01
bio02
bio04
bio05
bio12
BDW
CEC
DES
NTO
PTO
Crotalaria verrucosa
Climate
Climate + Soil
Soil
AUC = 0.93
TSS = 0.65
AUC = 0.95
TSS = 0.65
AUC = 0.85
TSS = 0.42
bio02
bio04
bio06
bio13
bio15
SOC
TWI

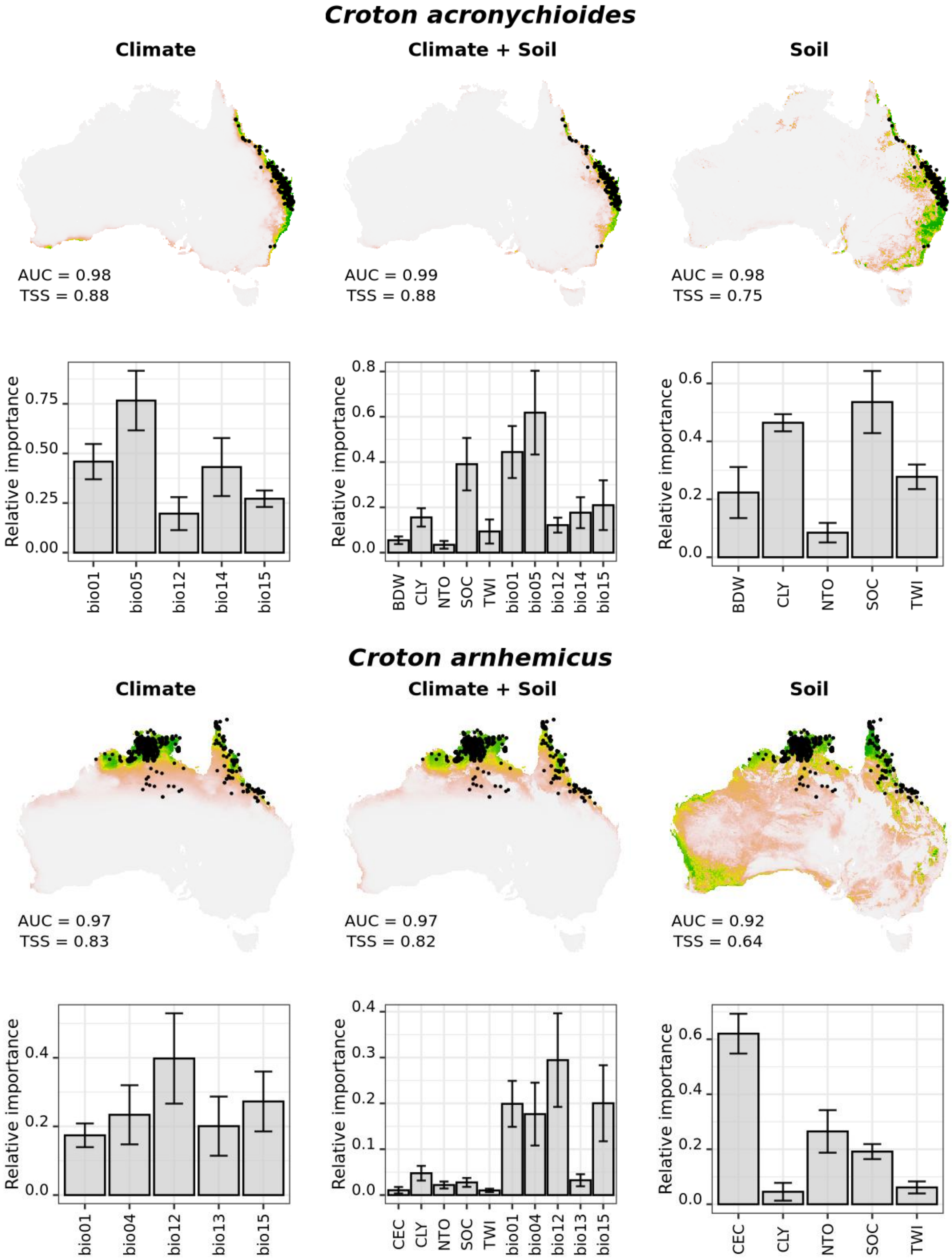
Croton acronychioides
Climate
Climate + Soil
Soil
AUC = 0.98
TSS = 0.88
AUC = 0.99
TSS = 0.88
AUC = 0.98
TSS = 0.75
Relative importance
bio01
bio05
bio12
bio14
bio15
BDW
CLY
NTO
SOC
TWI
Croton arnhemicus
Climate
Climate + Soil
Soil
AUC = 0.97
TSS = 0.83
AUC = 0.97
TSS = 0.82
AUC = 0.92
TSS = 0.64
Relative importance
bio01
bio04
bio12
bio13
bio15
CEC
CLY
NTO
SOC
TWI

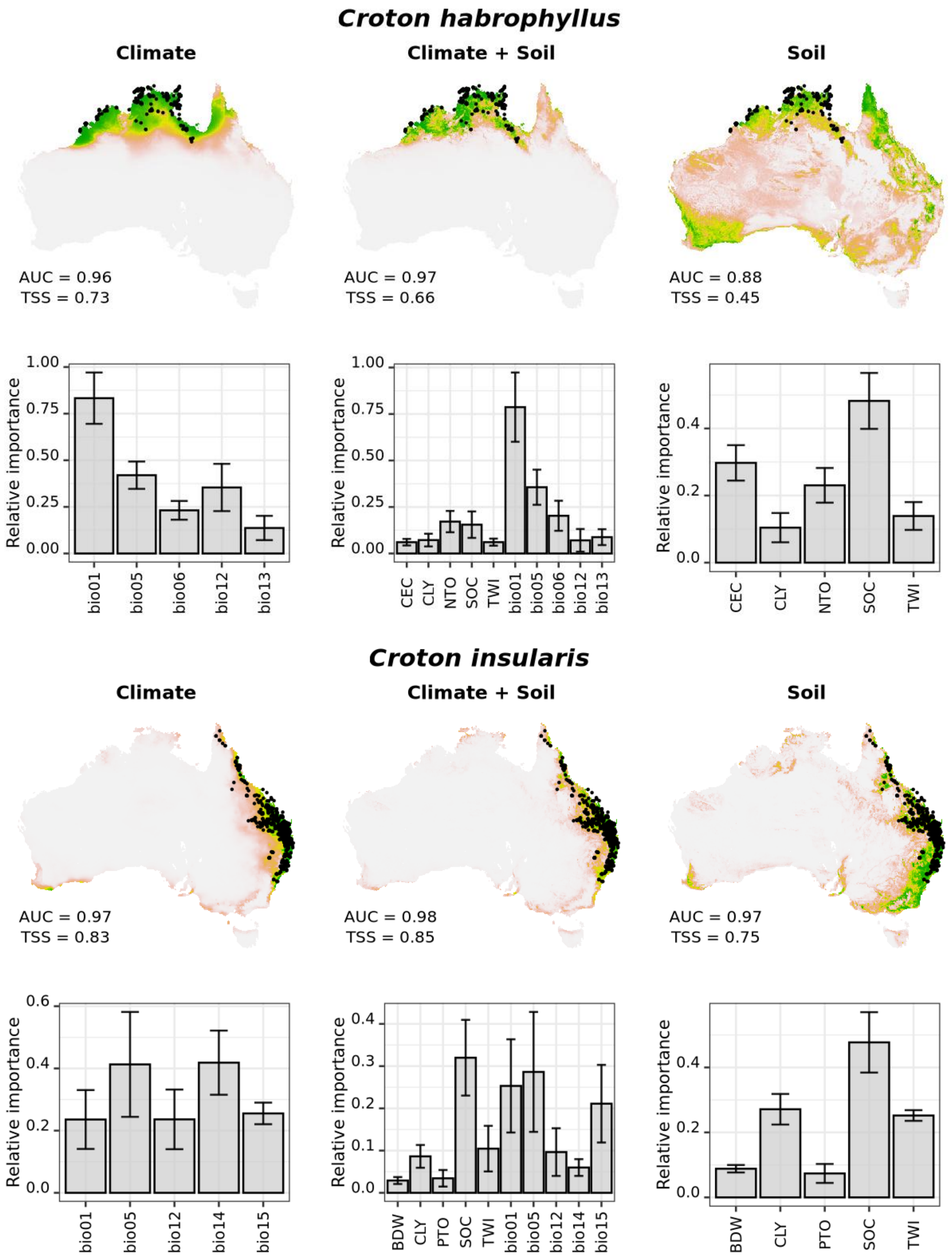

Croton habrophyllus
Climate
Climate + Soil
Soil
AUC = 0.96
TSS = 0.73
AUC = 0.97
TSS = 0.66
AUC = 0.88
TSS = 0.45
Relative importance
bio01
bio05
bio06
bio12
bio13
CEC
CLY
NTO
SOC
TWI
Croton insularis
AUC = 0.97
TSS = 0.83
AUC = 0.98
TSS = 0.85
AUC = 0.97
TSS = 0.75
bio14
bio15
BDW
PTO

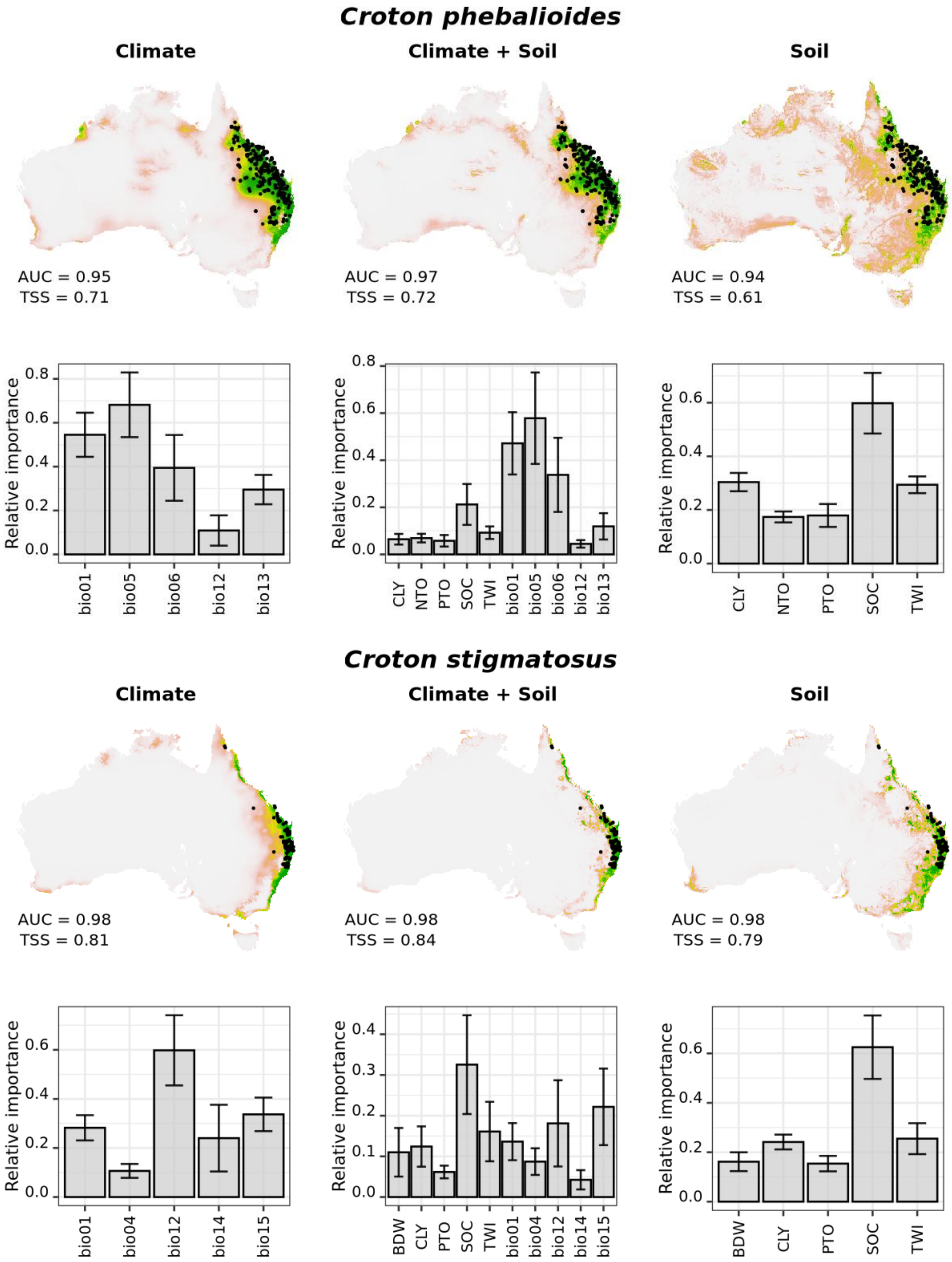
Croton phebalioides
Climate
Climate + Soil
Soil
AUC = 0.95
TSS = 0.71
AUC = 0.97
TSS = 0.72
AUC = 0.94
TSS = 0.61
Relative importance
bio01 bio05 bio06 bio12 bio13
CLY NTO PTO SOC TWI bio01 bio05 bio06 bio12 bio13
CLY NTO PTO SOC TWI
Croton stigmatosus
Climate
Climate + Soil
Soil
AUC = 0.98
TSS = 0.81
AUC = 0.98
TSS = 0.84
AUC = 0.98
TSS = 0.79
Relative importance
bio01 bio04 bio12 bio14 bio15
BDW CLY PTO SOC TWI bio01 bio04 bio12 bio14 bio15
BDW CLY PTO SOC TWI

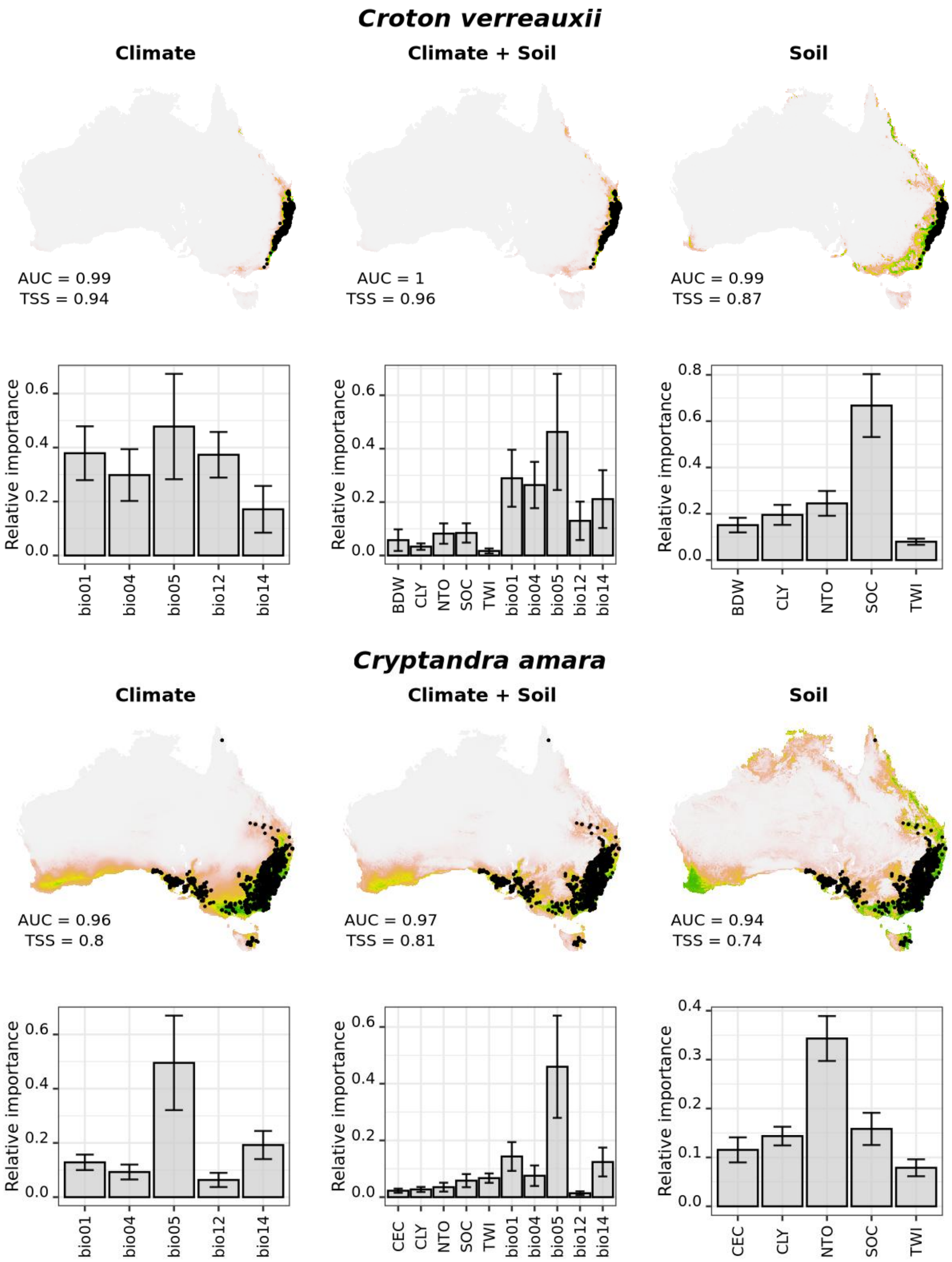

Croton verreauxii
Climate
Climate + Soil
Soil
AUC = 0.99
TSS = 0.94
AUC = 1
TSS = 0.96
AUC = 0.99
TSS = 0.87
Relative importance
bio01
bio04
bio05
bio12
bio14
BDW
CLY
NTO
SOC
TWI
Cryptandra amara
Climate
Climate + Soil
Soil
AUC = 0.96
TSS = 0.8
AUC = 0.97
TSS = 0.81
AUC = 0.94
TSS = 0.74
Relative importance
bio01
bio04
bio05
bio12
bio14
CEC
CLY
NTO
SOC
TWI

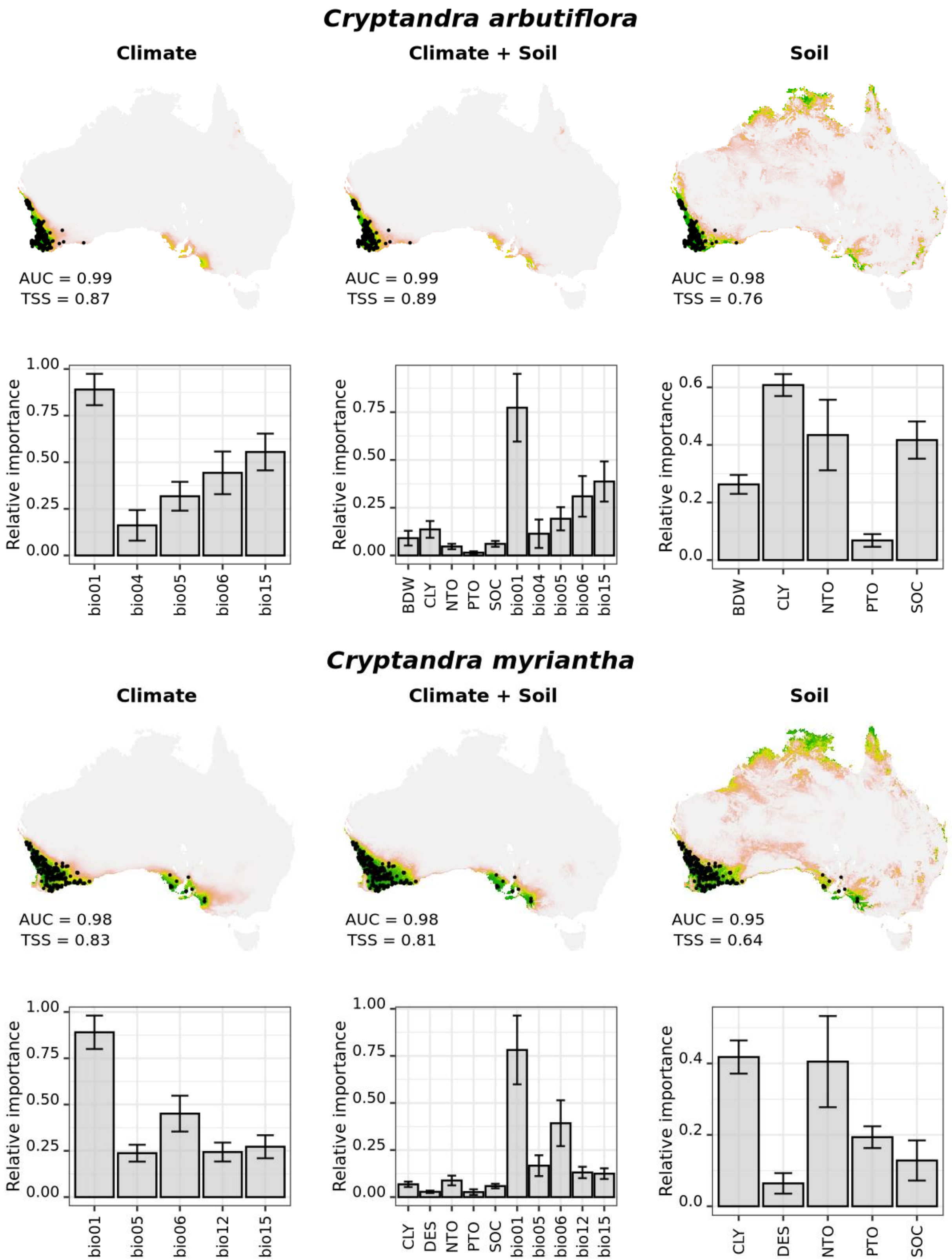
Cryptandra arbutiflora
Climate
Climate + Soil
Soil
AUC = 0.99
TSS = 0.87
AUC = 0.99
TSS = 0.89
AUC = 0.98
TSS = 0.76
Relative importance
1.00
0.75
0.50
0.25
0.00
bio01
bio04
bio05
bio06
bio15
BDW
CLY
NTO
PTO
SOC
0.6
0.4
0.2
0.0
Cryptandra myriantha
Climate
Climate + Soil
Soil
AUC = 0.98
TSS = 0.83
AUC = 0.98
TSS = 0.81
AUC = 0.95
TSS = 0.64
bio12
DES

Cryptandra nutans

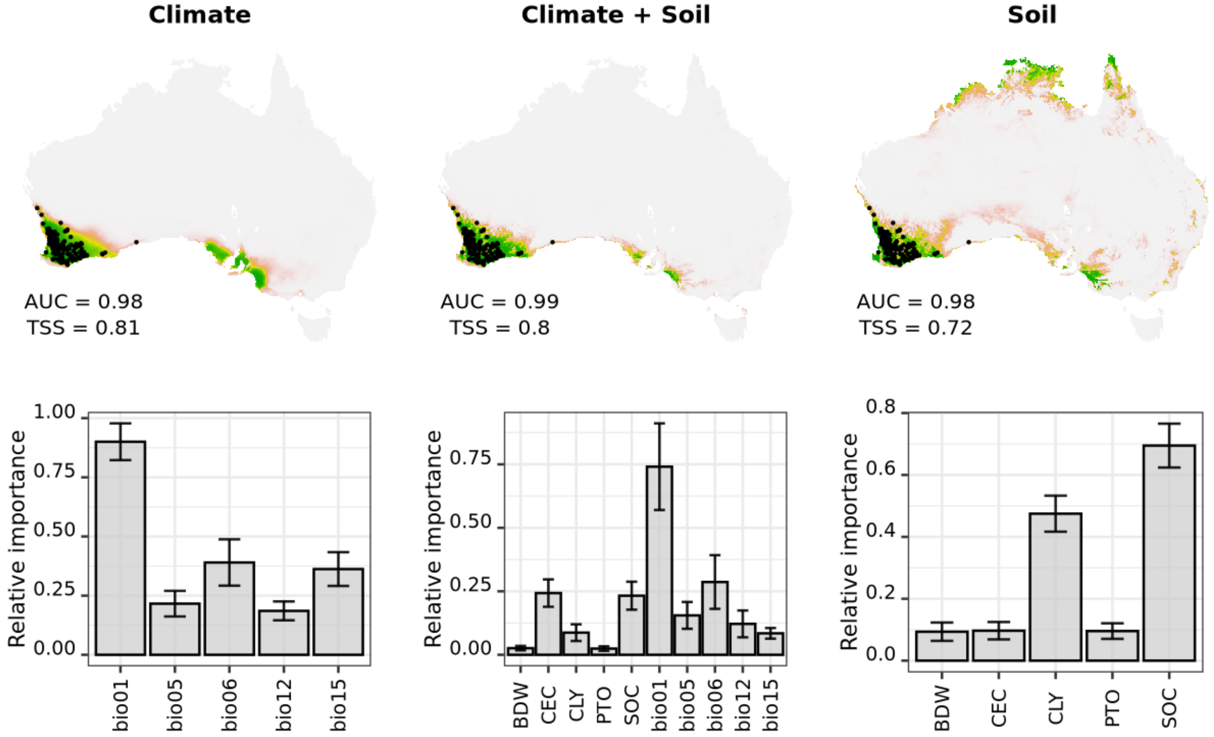

Cryptandra pungens

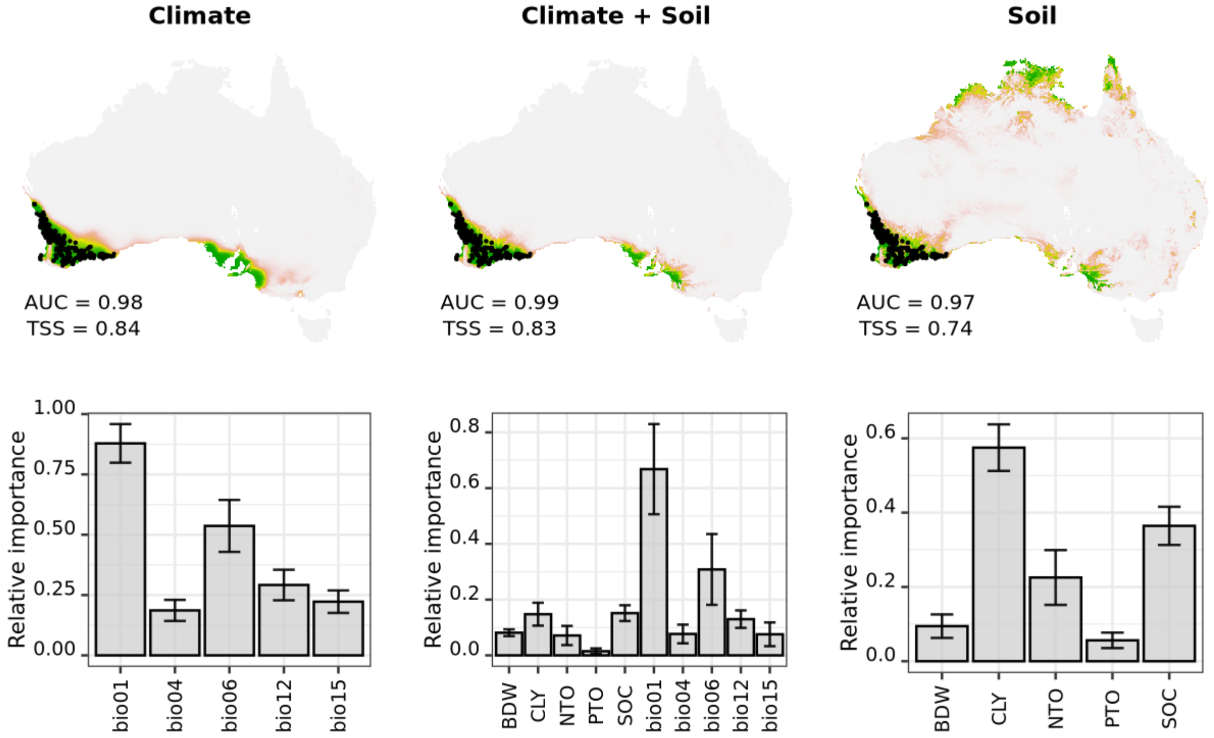

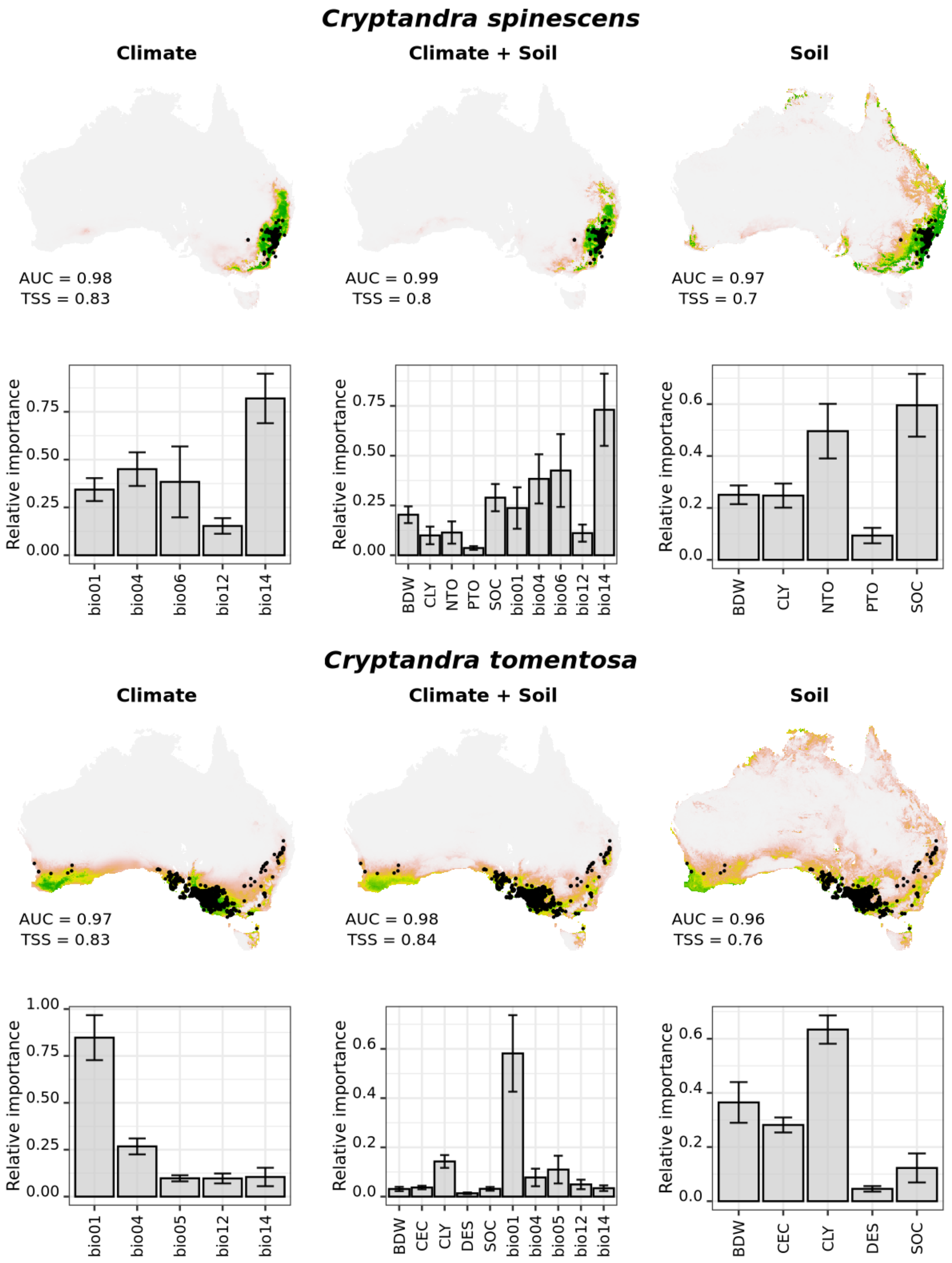

Cryptandra spinescens
Climate
Climate + Soil
Soil
AUC = 0.98
TSS = 0.83
AUC = 0.99
TSS = 0.8
AUC = 0.97
TSS = 0.7
Relative importance
0.00
0.25
0.50
0.75
bio01
bio04
bio06
bio12
bio14
BDW
CLY
NTO
PTO
SOC
0.0
0.2
0.4
0.6
Cryptandra tomentosa
Climate
Climate + Soil
Soil
AUC = 0.97
TSS = 0.83
AUC = 0.98
TSS = 0.84
AUC = 0.96
TSS = 0.76
1.00
bio05
CEC
DES

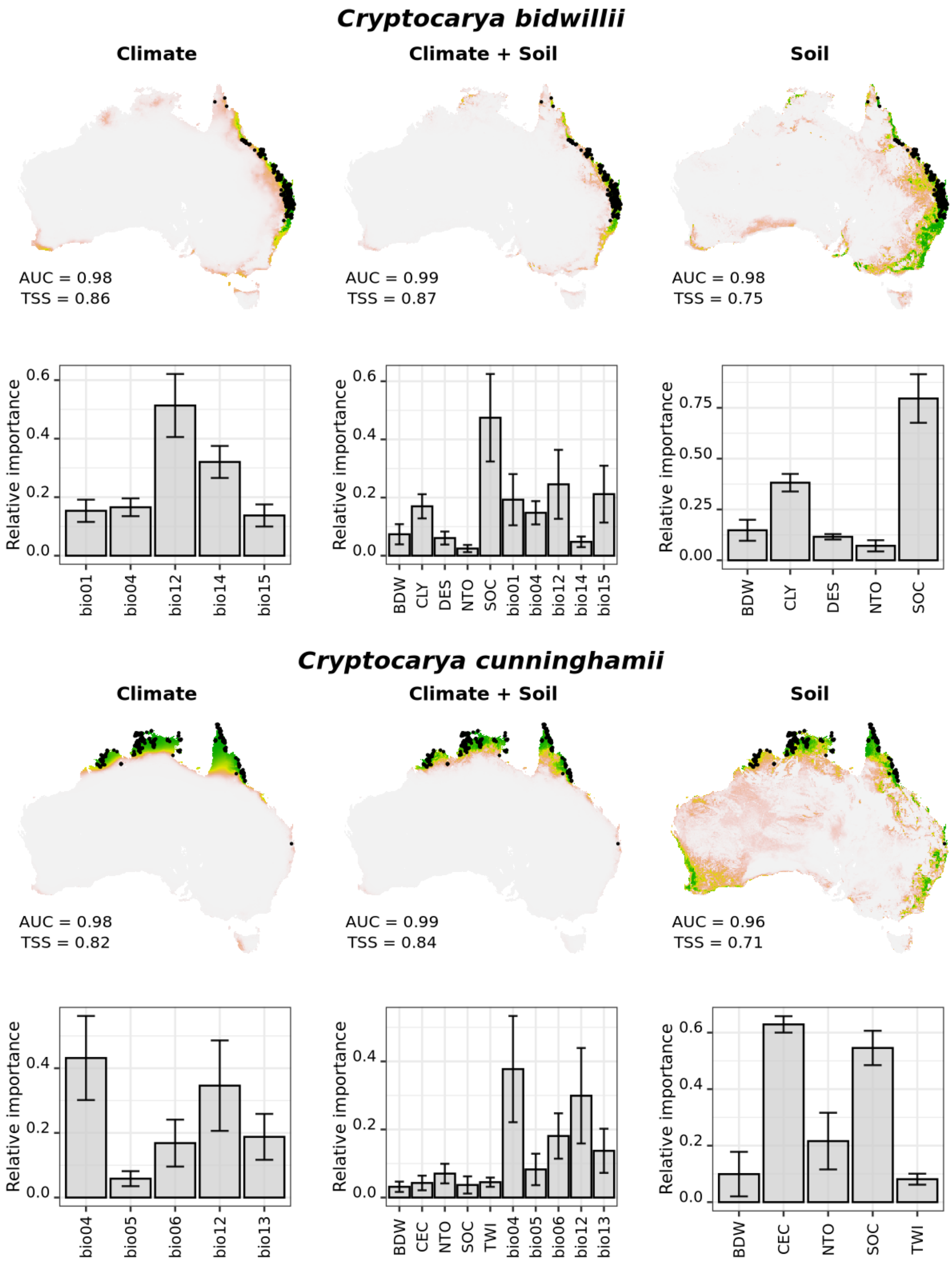
Cryptocarya bidwillii
Climate
Climate + Soil
Soil
AUC = 0.98
TSS = 0.86
AUC = 0.99
TSS = 0.87
AUC = 0.98
TSS = 0.75
Relative importance
0.0
0.2
0.4
0.6
bio01
bio04
bio12
bio14
bio15
BDW
CLY
DES
NTO
SOC
bio01
bio04
bio12
bio14
bio15
0.00
0.25
0.50
0.75
BDW
CLY
DES
NTO
SOC
Cryptocarya cunninghamii
Climate
Climate + Soil
Soil
AUC = 0.98
TSS = 0.82
AUC = 0.99
TSS = 0.84
AUC = 0.96
TSS = 0.71
Relative importance
0.0
0.2
0.4
bio04
bio05
bio06
bio12
bio13
BDW
CEC
NTO
SOC
TWI
bio04
bio05
bio06
bio12
bio13
0.6
BDW
CEC
NTO
SOC
TWI

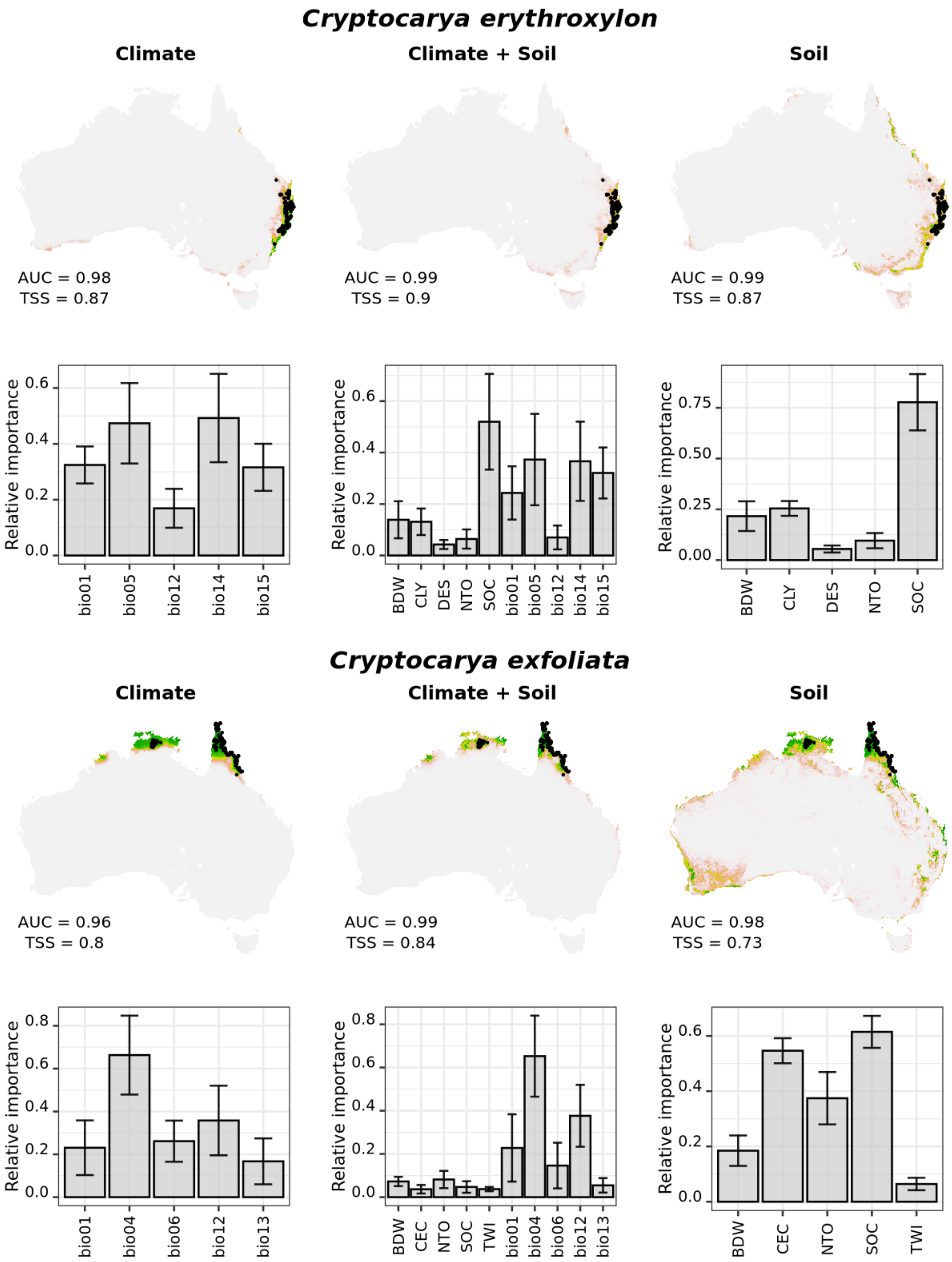

Cryptocarya erythroxylon
Climate
Climate + Soil
Soil
AUC = 0.98
TSS = 0.87
AUC = 0.99
TSS = 0.9
AUC = 0.99
TSS = 0.87
Relative importance
0.0
0.2
0.4
0.6
bio01
bio05
bio12
bio14
bio15
BDW
CLY
DES
NTO
SOC
0.00
0.25
0.50
0.75
Cryptocarya exfoliata
Climate
Climate + Soil
Soil
AUC = 0.96
TSS = 0.8
AUC = 0.99
TSS = 0.84
AUC = 0.98
TSS = 0.73
0.8
bio04
bio06
bio13
CEC
TWI

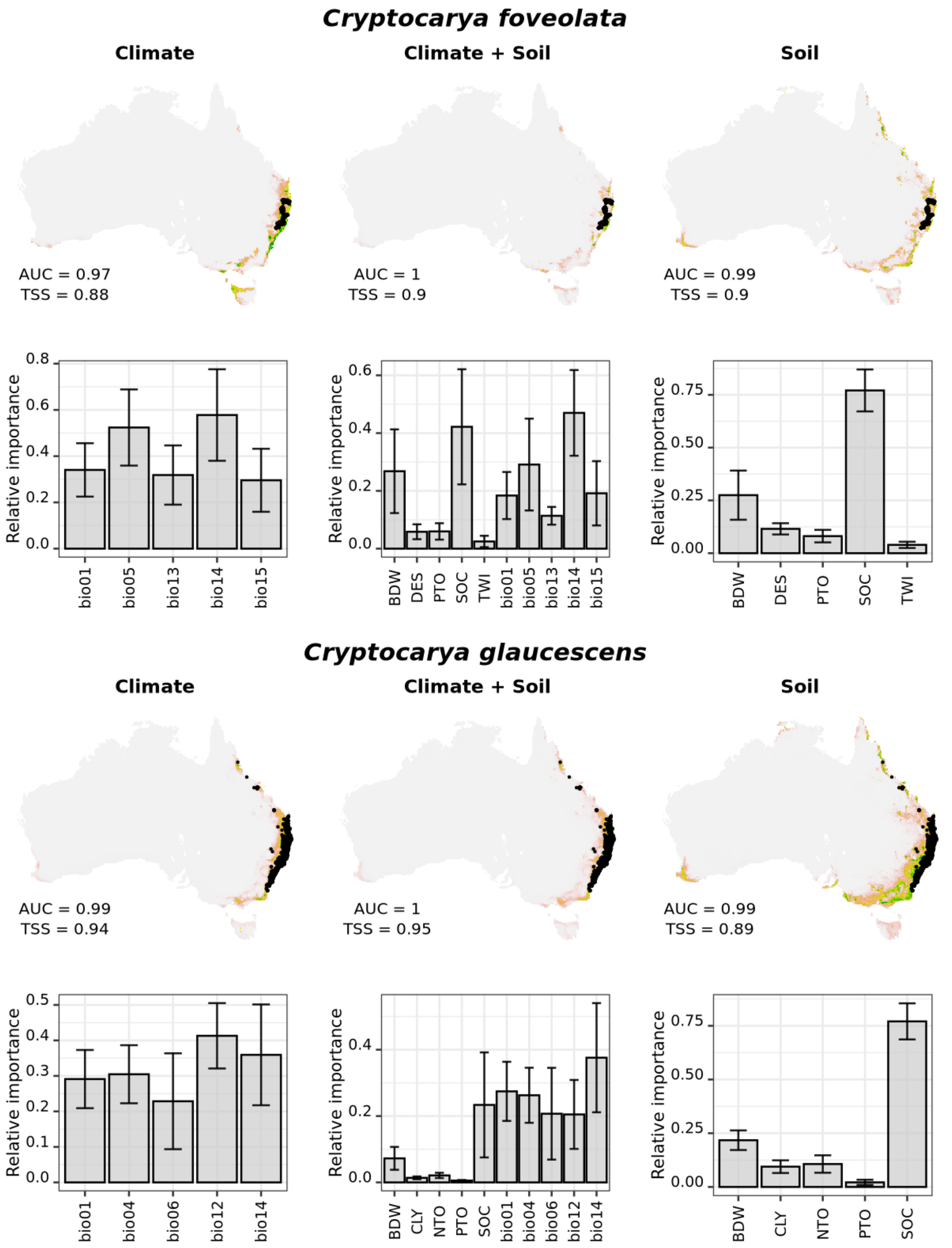

Cryptocarya foveolata
Climate
Climate + Soil
Soil
AUC = 0.97
TSS = 0.88
AUC = 1
TSS = 0.9
AUC = 0.99
TSS = 0.9
Relative importance
bio01 bio05 bio13 bio14 bio15
BDW DES PTO SOC TWI bio01 bio05 bio13 bio14 bio15
BDW DES PTO SOC TWI
Cryptocarya glaucescens
Climate
Climate + Soil
Soil
AUC = 0.99
TSS = 0.94
AUC = 1
TSS = 0.95
AUC = 0.99
TSS = 0.89
Relative importance
bio01 bio04 bio06 bio12 bio14
BDW CLY NTO PTO SOC bio01 bio04 bio06 bio12 bio14
BDW CLY NTO PTO SOC

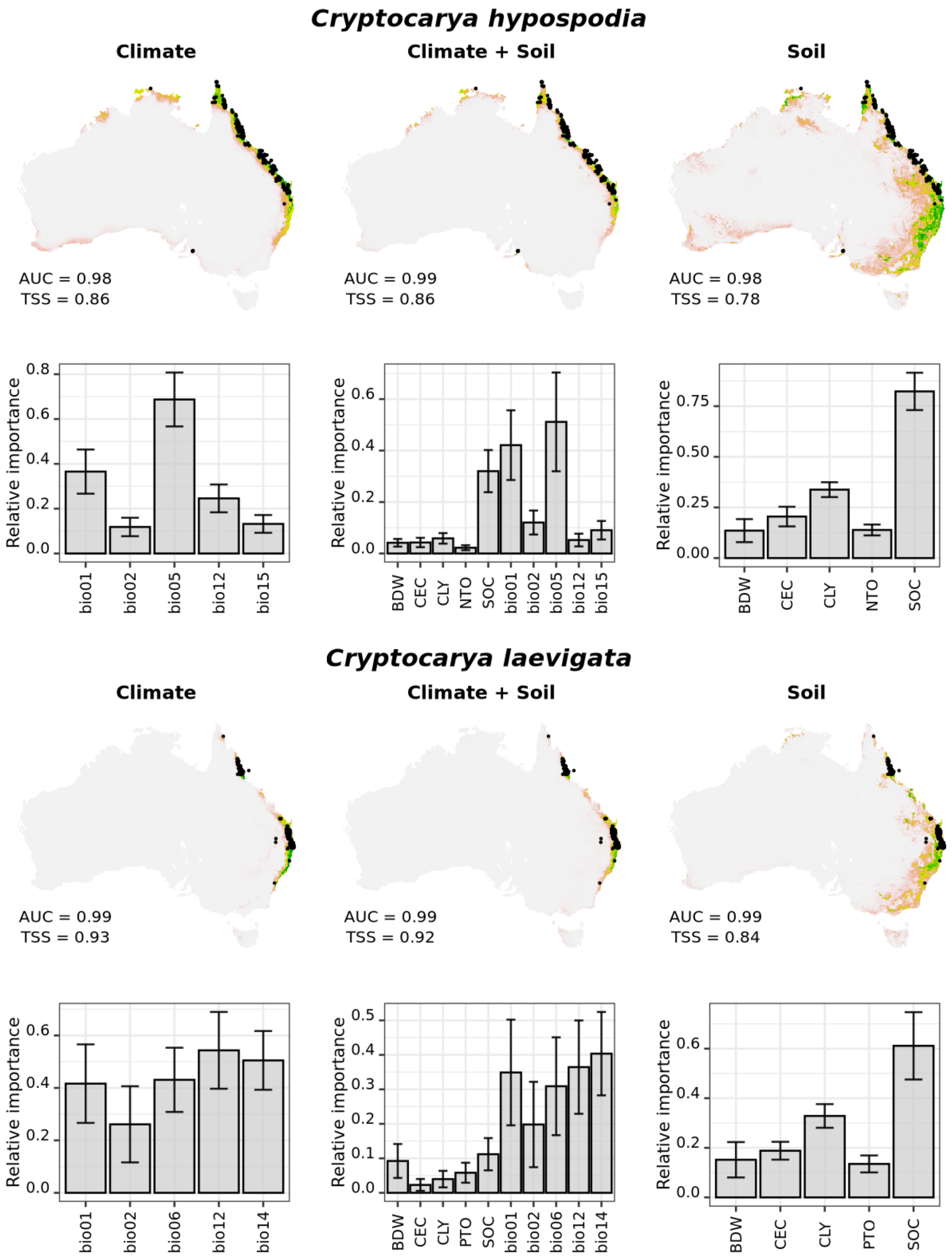

Cryptocarya hypospodia
Climate
Climate + Soil
Soil
AUC = 0.98
TSS = 0.86
AUC = 0.99
TSS = 0.86
AUC = 0.98
TSS = 0.78
Relative importance
bio01
bio02
bio05
bio12
bio15
BDW
CEC
CLY
NTO
SOC
Cryptocarya laevigata
Climate
Climate + Soil
Soil
AUC = 0.99
TSS = 0.93
AUC = 0.99
TSS = 0.92
AUC = 0.99
TSS = 0.84
Relative importance
bio01
bio02
bio06
bio12
bio14
BDW
CEC
CLY
PTO
SOC

Cryptocarya macdonaldii

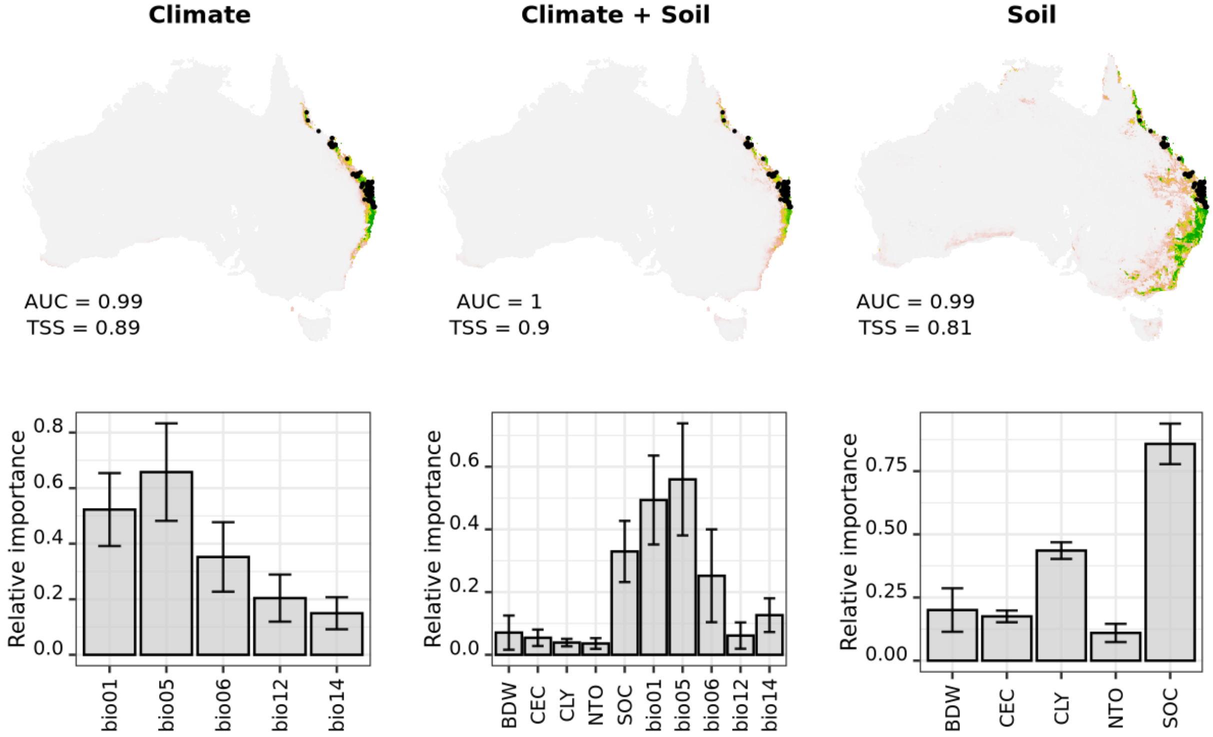

Cryptocarya meisneriana

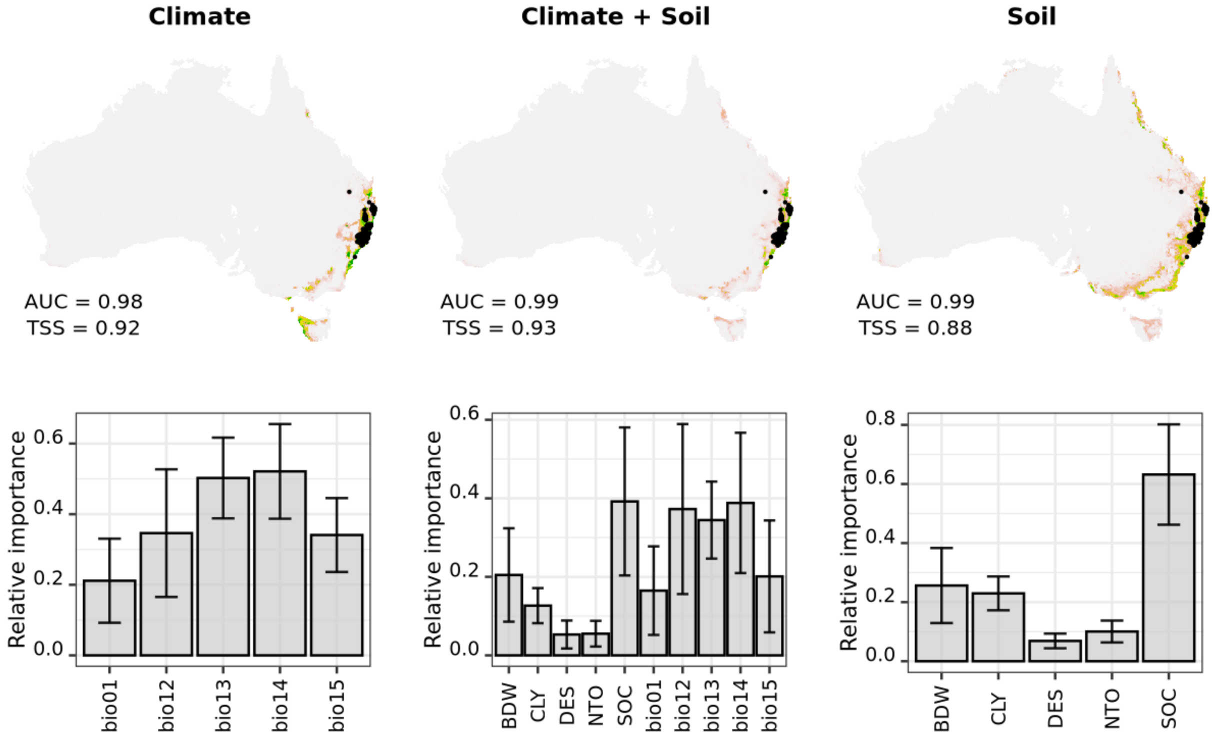

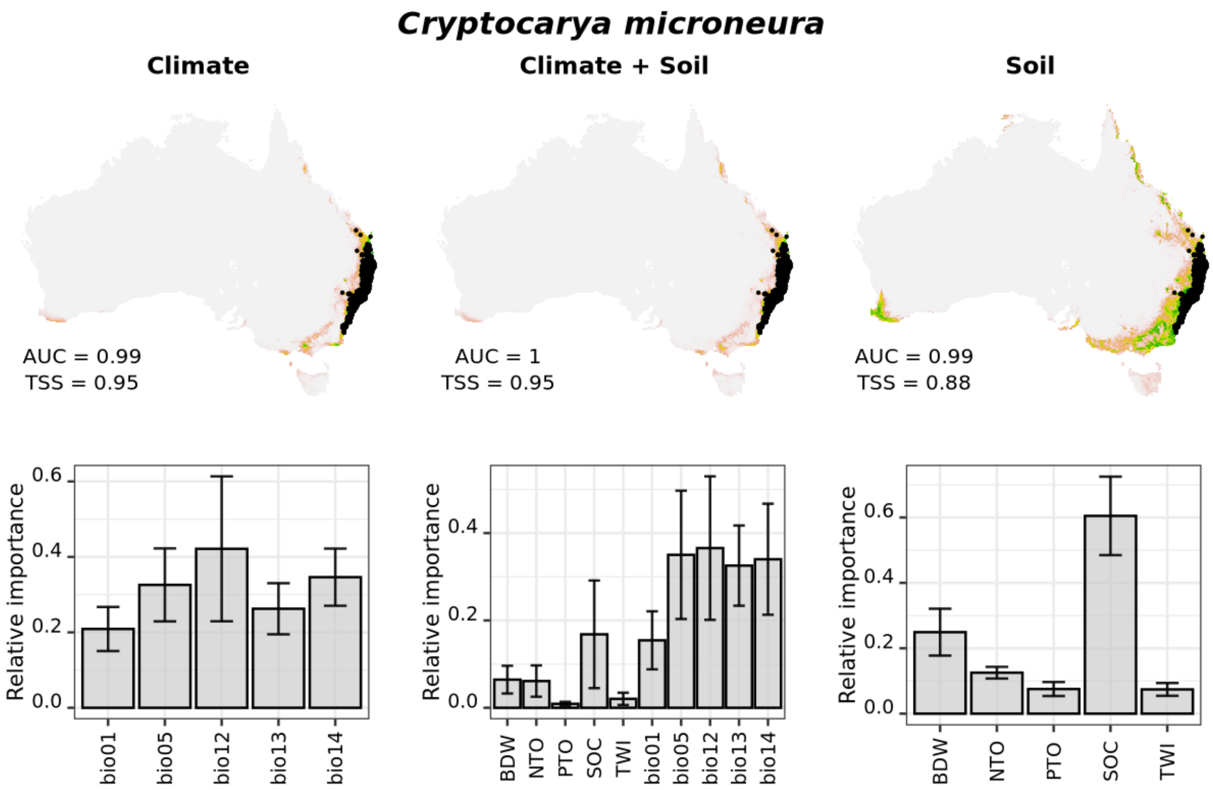
Cryptocarya microneura
Climate
Climate + Soil
Soil
AUC = 0.99
TSS = 0.95
AUC = 1
TSS = 0.95
AUC = 0.99
TSS = 0.88
Relative importance
0.0
0.2
0.4
0.6
bio01
bio05
bio12
bio13
bio14
BDW
NTO
PTO
SOC
TWI

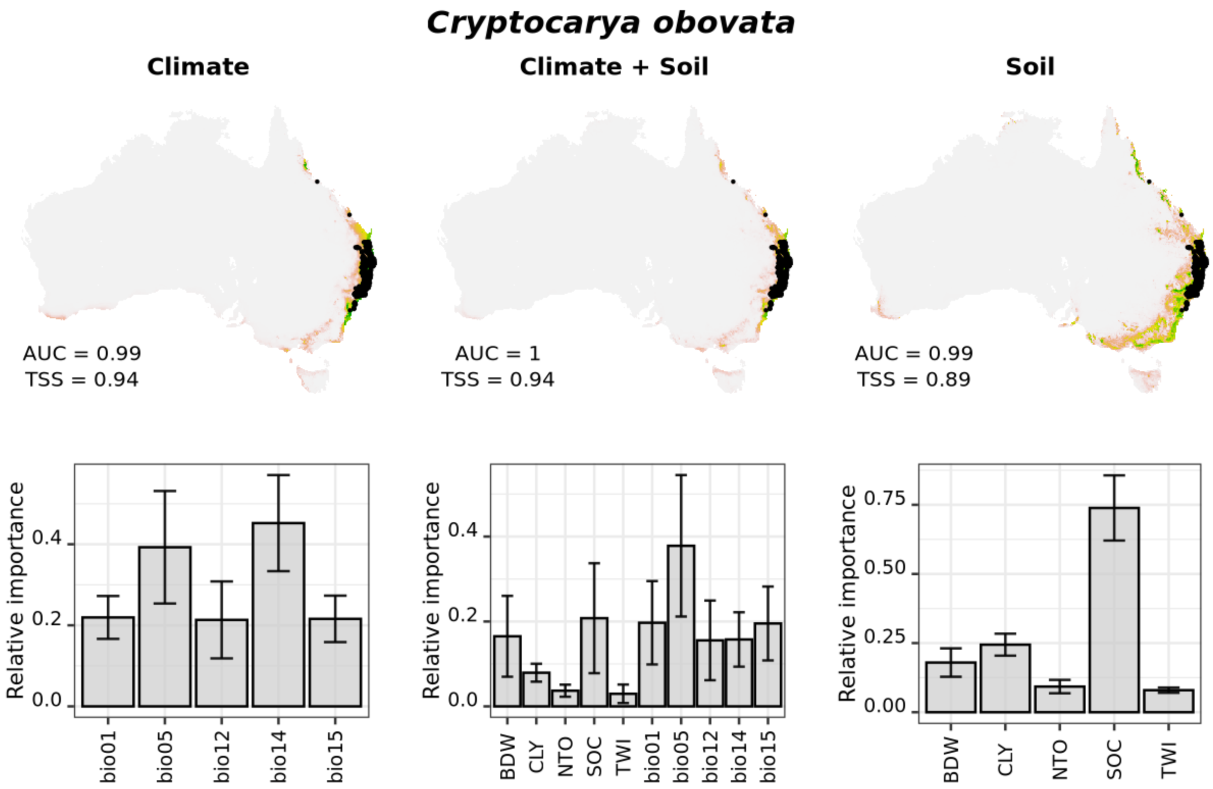
Cryptocarya obovata
Climate
Climate + Soil
Soil
AUC = 0.99
TSS = 0.94
AUC = 1
TSS = 0.94
AUC = 0.99
TSS = 0.89
Relative importance
0.0
0.2
0.4
0.00
0.25
0.50
0.75
bio01
bio05
bio12
bio14
bio15
BDW
CLY
NTO
SOC
TWI

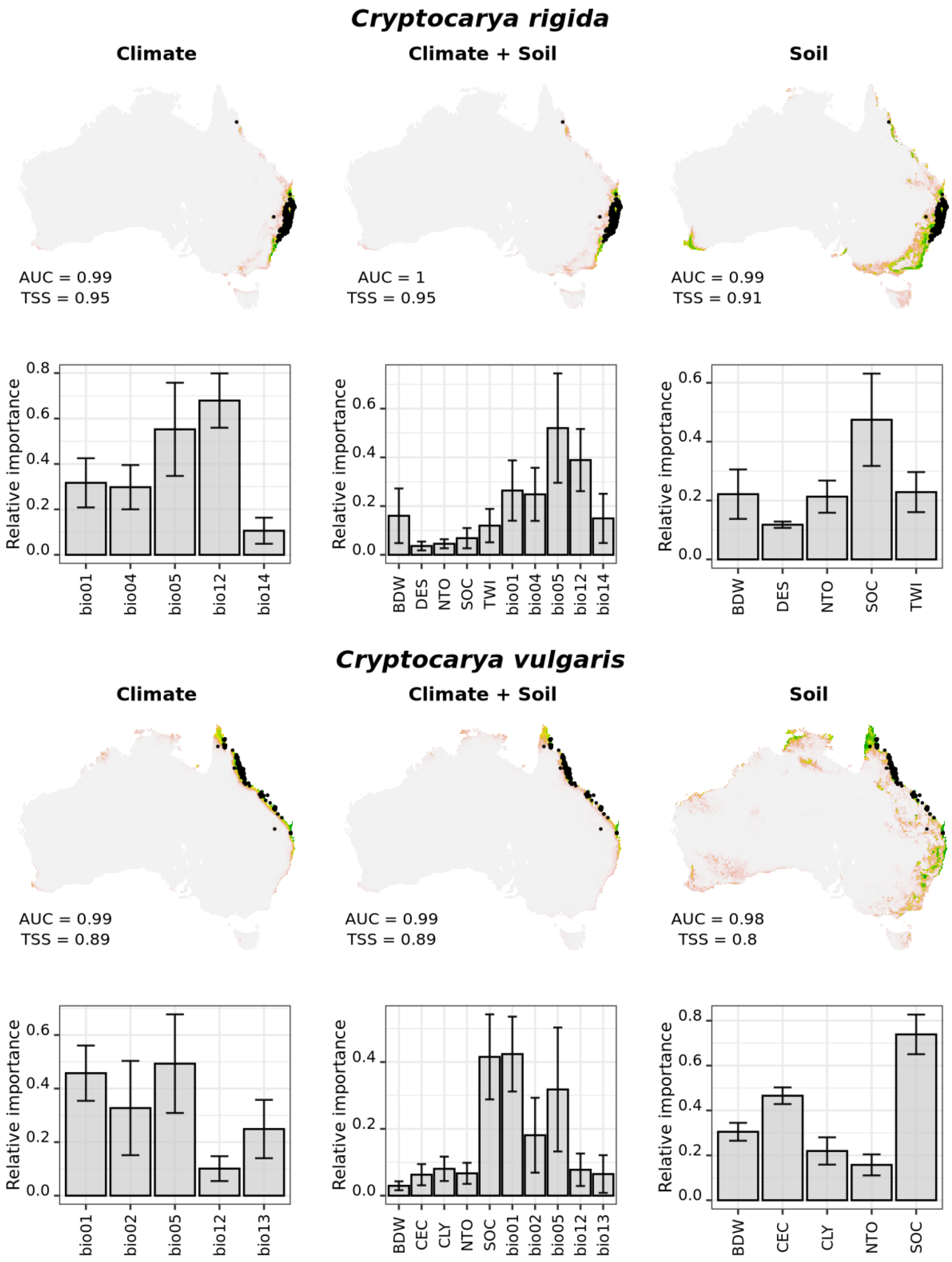

Cryptocarya rigida
Climate
Climate + Soil
Soil
AUC = 0.99
TSS = 0.95
AUC = 1
TSS = 0.95
AUC = 0.99
TSS = 0.91
Relative importance
bio01
bio04
bio05
bio12
bio14
BDW
DES
NTO
SOC
TWI
Cryptocarya vulgaris
Climate
Climate + Soil
Soil
AUC = 0.99
TSS = 0.89
AUC = 0.99
TSS = 0.89
AUC = 0.98
TSS = 0.8
Relative importance
bio01
bio02
bio05
bio12
bio13
BDW
CEC
CLY
NTO
SOC

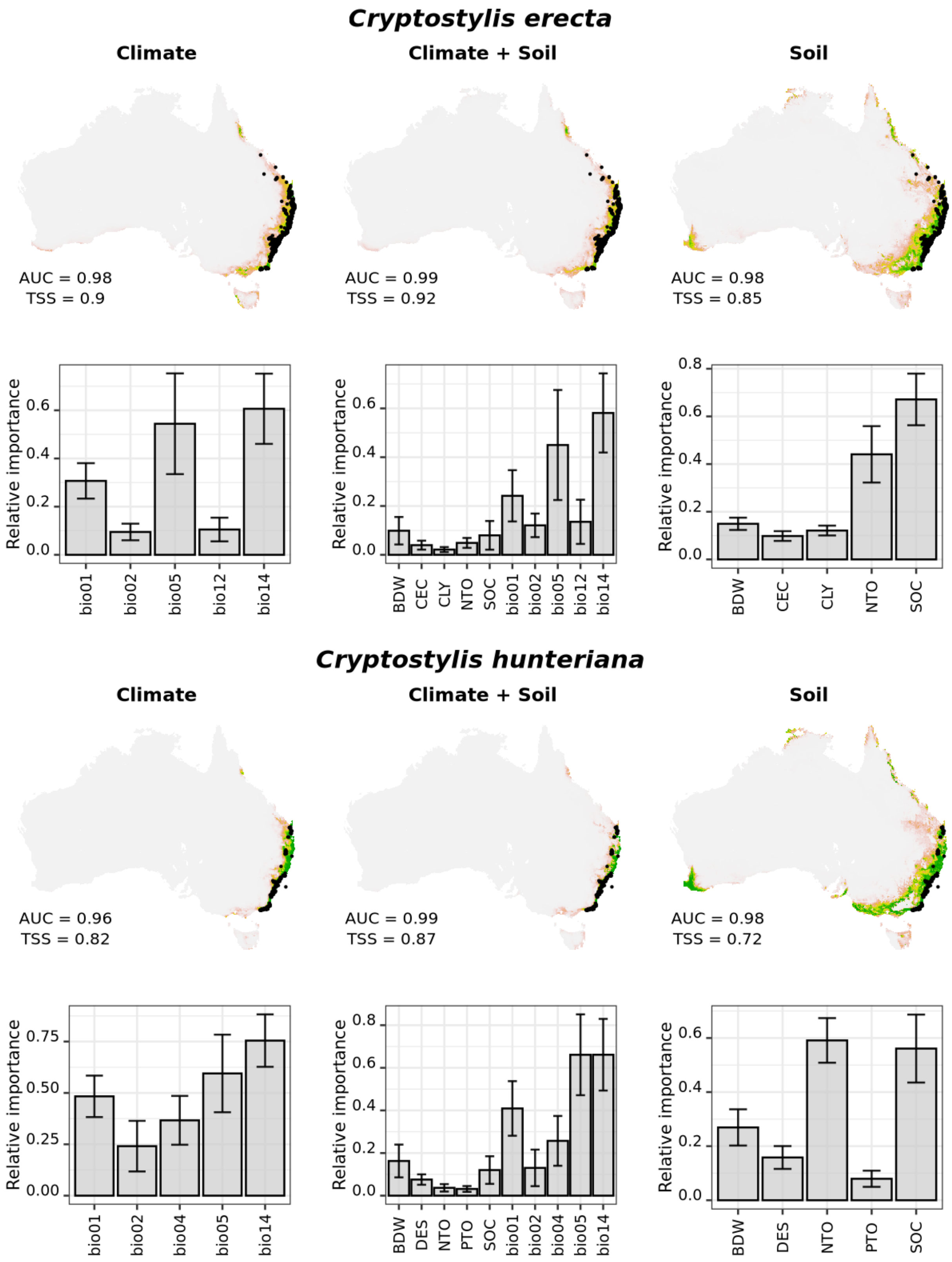
Cryptostylis erecta
Climate
Climate + Soil
Soil
AUC = 0.98
TSS = 0.9
AUC = 0.99
TSS = 0.92
AUC = 0.98
TSS = 0.85
Relative importance
bio01
bio02
bio05
bio12
bio14
BDW
CEC
CLY
NTO
SOC
Cryptostylis hunteriana
Climate
Climate + Soil
Soil
AUC = 0.96
TSS = 0.82
AUC = 0.99
TSS = 0.87
AUC = 0.98
TSS = 0.72
Relative importance
bio01
bio02
bio04
bio05
bio14
BDW
DES
NTO
PTO
SOC

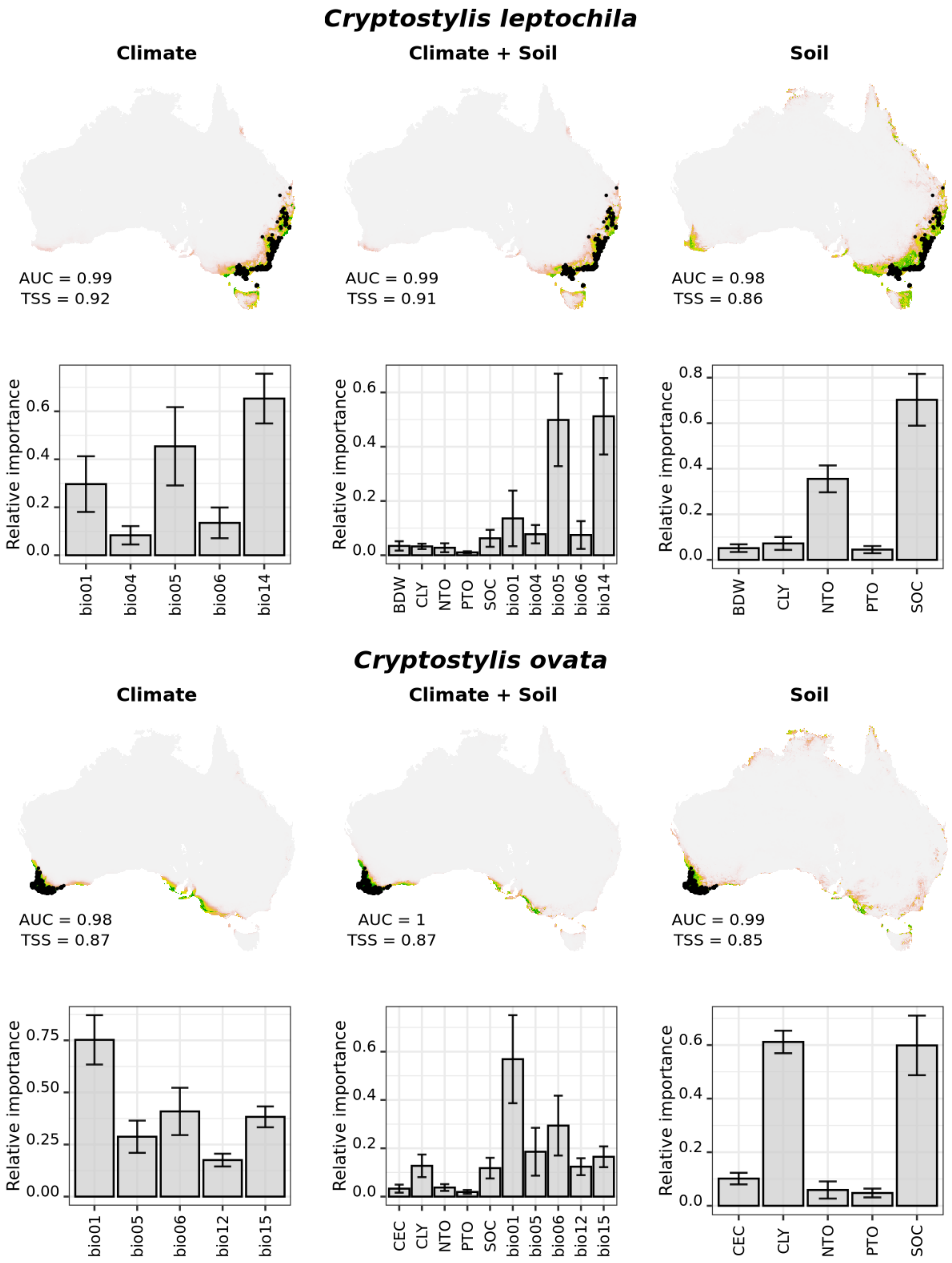
Cryptostylis leptochila
Climate
Climate + Soil
Soil
AUC = 0.99
TSS = 0.92
AUC = 0.99
TSS = 0.91
AUC = 0.98
TSS = 0.86
Relative importance
bio01
bio04
bio05
bio06
bio14
BDW
CLY
NTO
PTO
SOC
Cryptostylis ovata
Climate
Climate + Soil
Soil
AUC = 0.98
TSS = 0.87
AUC = 1
TSS = 0.87
AUC = 0.99
TSS = 0.85
Relative importance
bio01
bio05
bio06
bio12
bio15
CEC
CLY
NTO
PTO
SOC

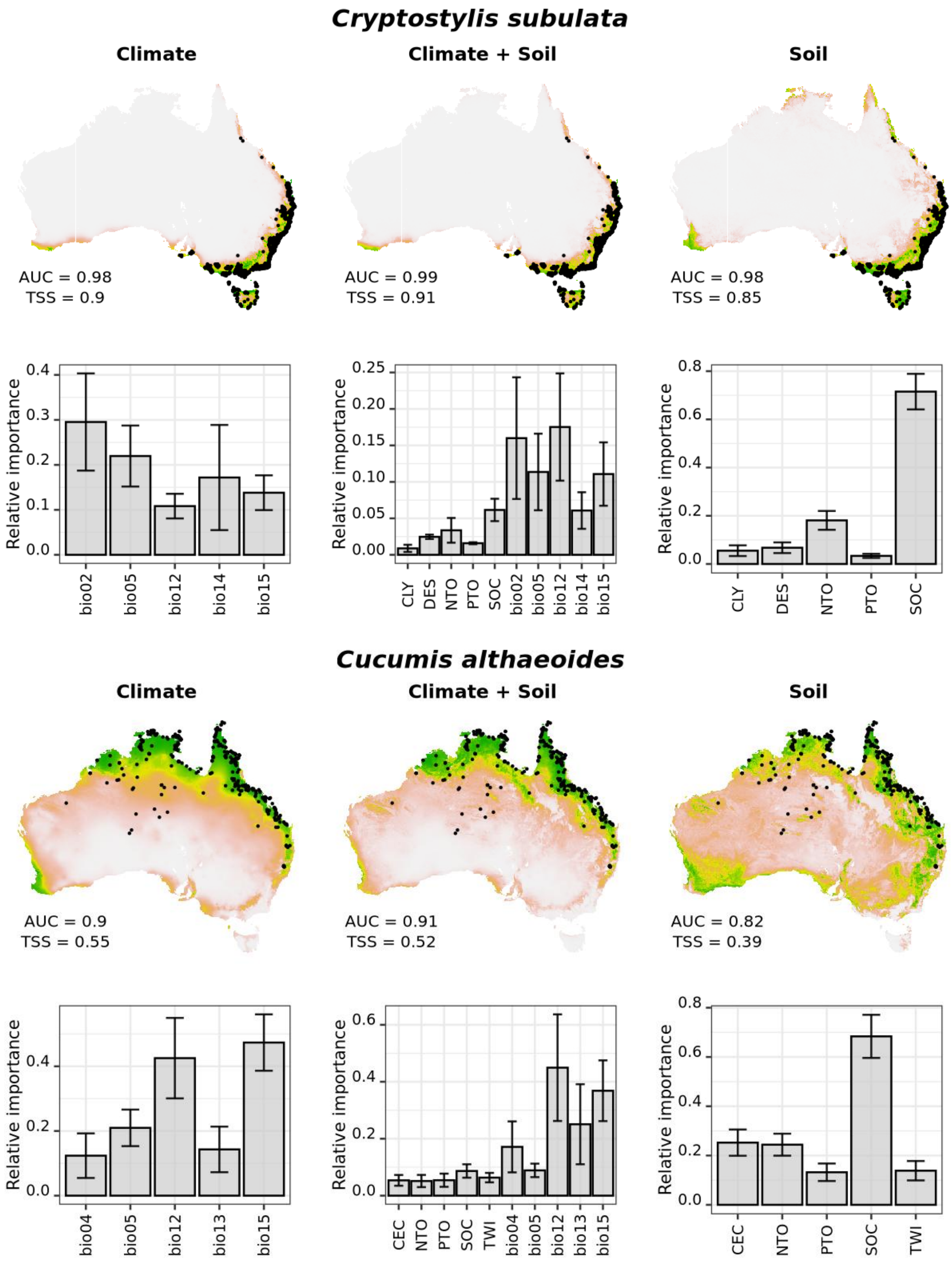

Cryptostylis subulata
Climate
Climate + Soil
Soil
AUC = 0.98
TSS = 0.9
AUC = 0.99
TSS = 0.91
AUC = 0.98
TSS = 0.85
Relative importance
bio02
bio05
bio12
bio14
bio15
CLY
DES
NTO
PTO
SOC
Cucumis althaeoides
Climate
Climate + Soil
Soil
AUC = 0.9
TSS = 0.55
AUC = 0.91
TSS = 0.52
AUC = 0.82
TSS = 0.39
Relative importance
bio04
bio05
bio12
bio13
bio15
CEC
NTO
PTO
SOC
TWI

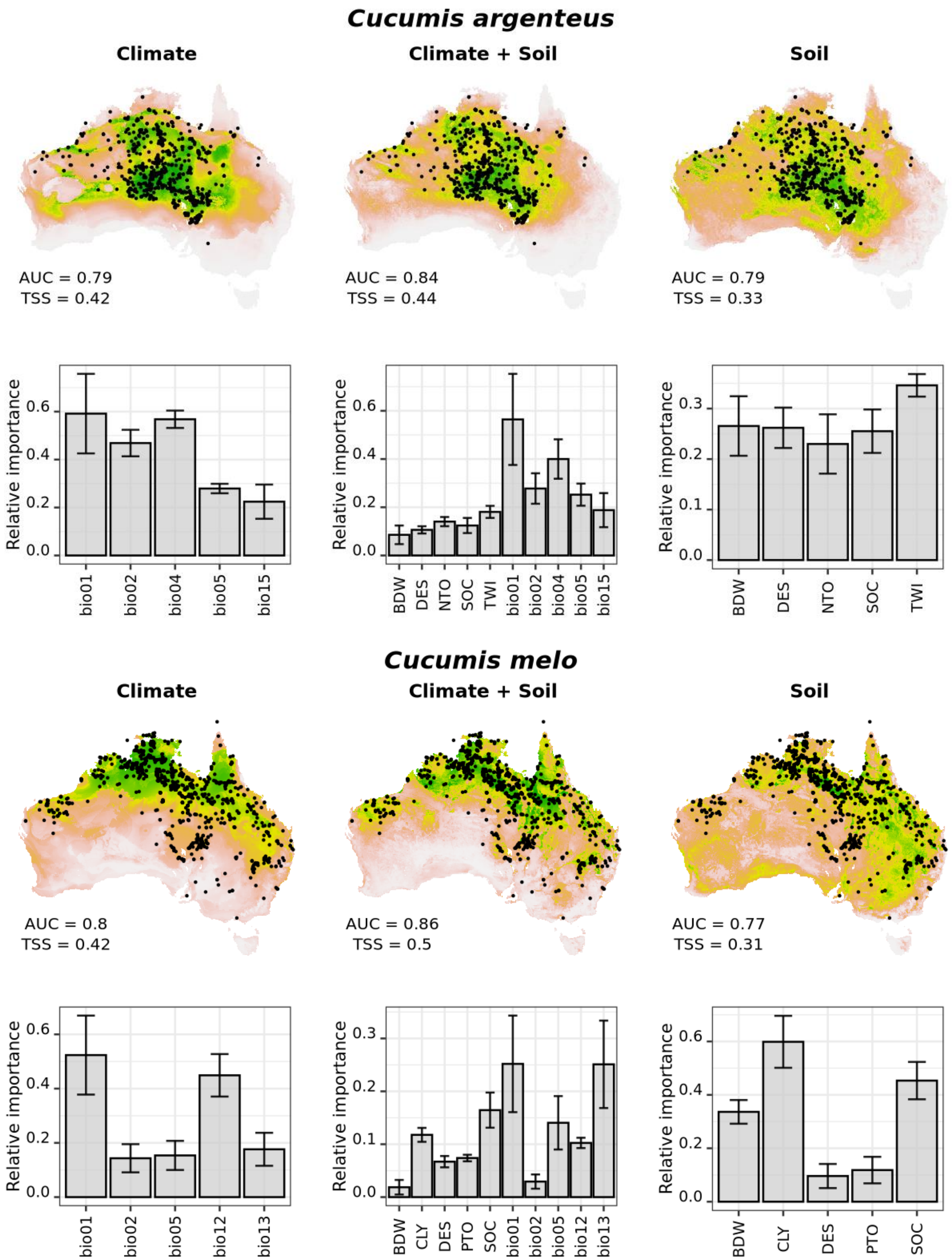

Cucumis argenteus
Climate
Climate + Soil
Soil
AUC = 0.79
TSS = 0.42
AUC = 0.84
TSS = 0.44
AUC = 0.79
TSS = 0.33
Relative importance
0.0 0.2 0.4 0.6
bio01 bio02 bio04 bio05 bio15
BDW DES NTO SOC TWI bio01 bio02 bio04 bio05 bio15
0.0 0.1 0.2 0.3
BDW DES NTO SOC TWI
Cucumis melo
Climate
Climate + Soil
Soil
AUC = 0.8
TSS = 0.42
AUC = 0.86
TSS = 0.5
AUC = 0.77
TSS = 0.31
bio01 bio02 bio05 bio12 bio13
BDW CLY DES PTO SOC bio01 bio02 bio05 bio12 bio13
BDW CLY DES PTO SOC

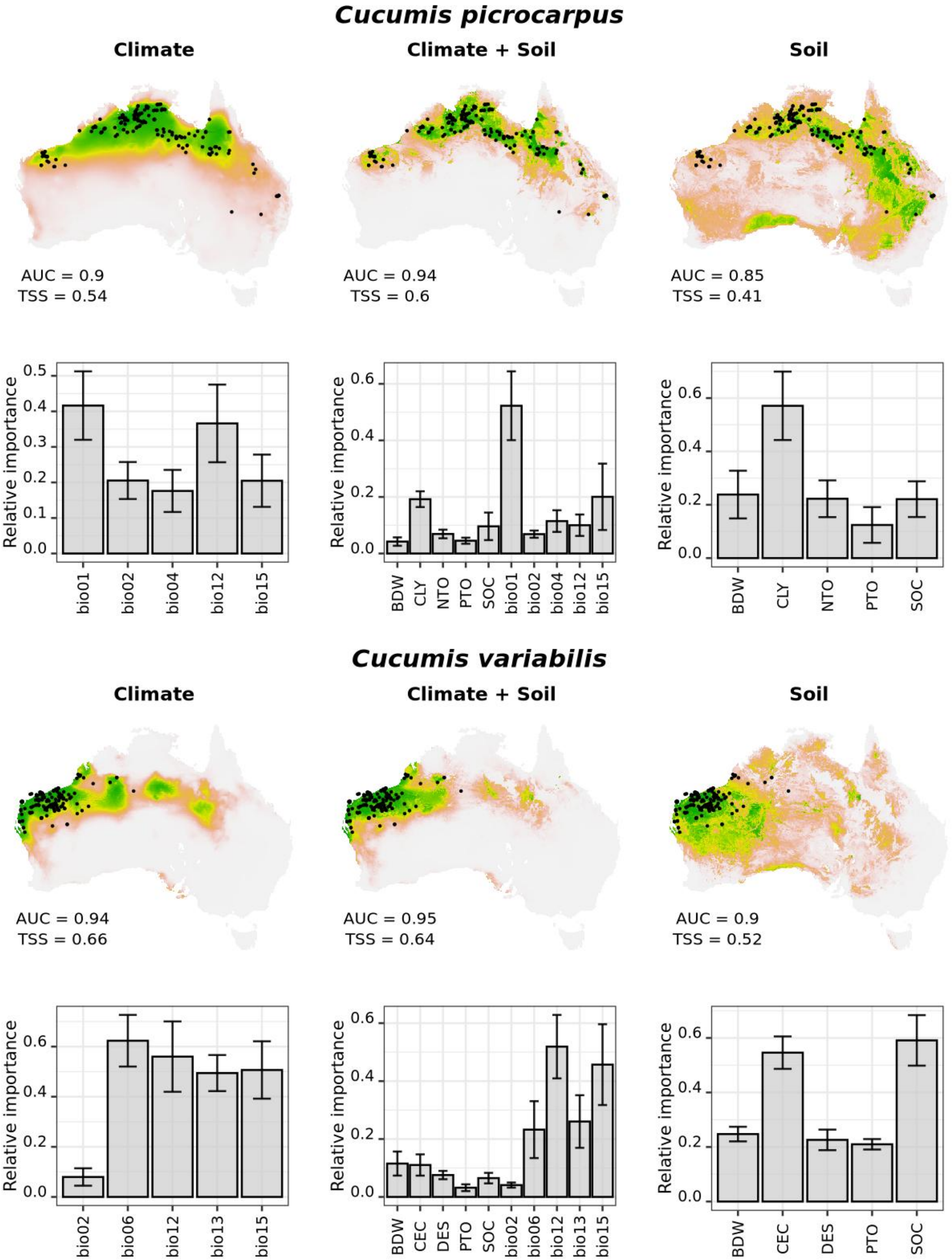

Cucumis picrocarpus
Climate
Climate + Soil
Soil
AUC = 0.9
TSS = 0.54
AUC = 0.94
TSS = 0.6
AUC = 0.85
TSS = 0.41
Relative importance
bio01 bio02 bio04 bio12 bio15
BDW CLY NTO PTO SOC bio01 bio02 bio04 bio12 bio15
BDW CLY NTO PTO SOC
Cucumis variabilis
Climate
Climate + Soil
Soil
AUC = 0.94
TSS = 0.66
AUC = 0.95
TSS = 0.64
AUC = 0.9
TSS = 0.52
Relative importance
bio02 bio06 bio12 bio13 bio15
BDW CEC DES PTO SOC bio02 bio06 bio12 bio13 bio15
BDW CEC DES PTO SOC

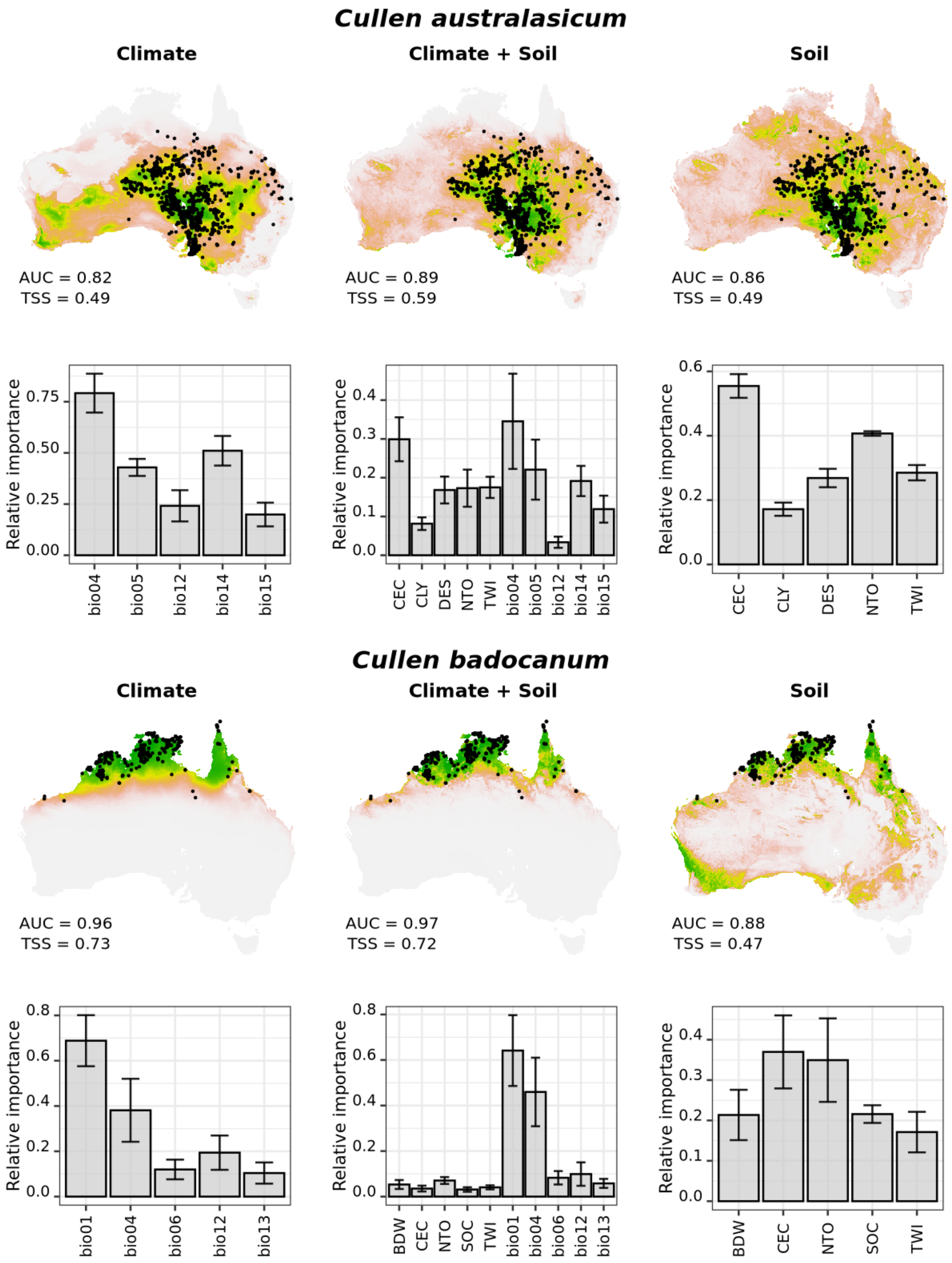
Cullen australasicum
Climate
Climate + Soil
Soil
AUC = 0.82
TSS = 0.49
AUC = 0.89
TSS = 0.59
AUC = 0.86
TSS = 0.49
Relative importance
0.00
0.25
0.50
0.75
bio04
bio05
bio12
bio14
bio15
0.0
0.1
0.2
0.3
0.4
CEC
CLY
DES
NTO
TWI
bio04
bio05
bio12
bio14
bio15
0.0
0.2
0.4
0.6
CEC
CLY
DES
NTO
TWI
Cullen badocanum
Climate
Climate + Soil
Soil
AUC = 0.96
TSS = 0.73
AUC = 0.97
TSS = 0.72
AUC = 0.88
TSS = 0.47
Relative importance
0.0
0.2
0.4
0.6
0.8
bio01
bio04
bio06
bio12
bio13
BDW
CEC
NTO
SOC
TWI
bio01
bio04
bio06
bio12
bio13
0.0
0.1
0.2
0.3
0.4
BDW
CEC
NTO
SOC
TWI

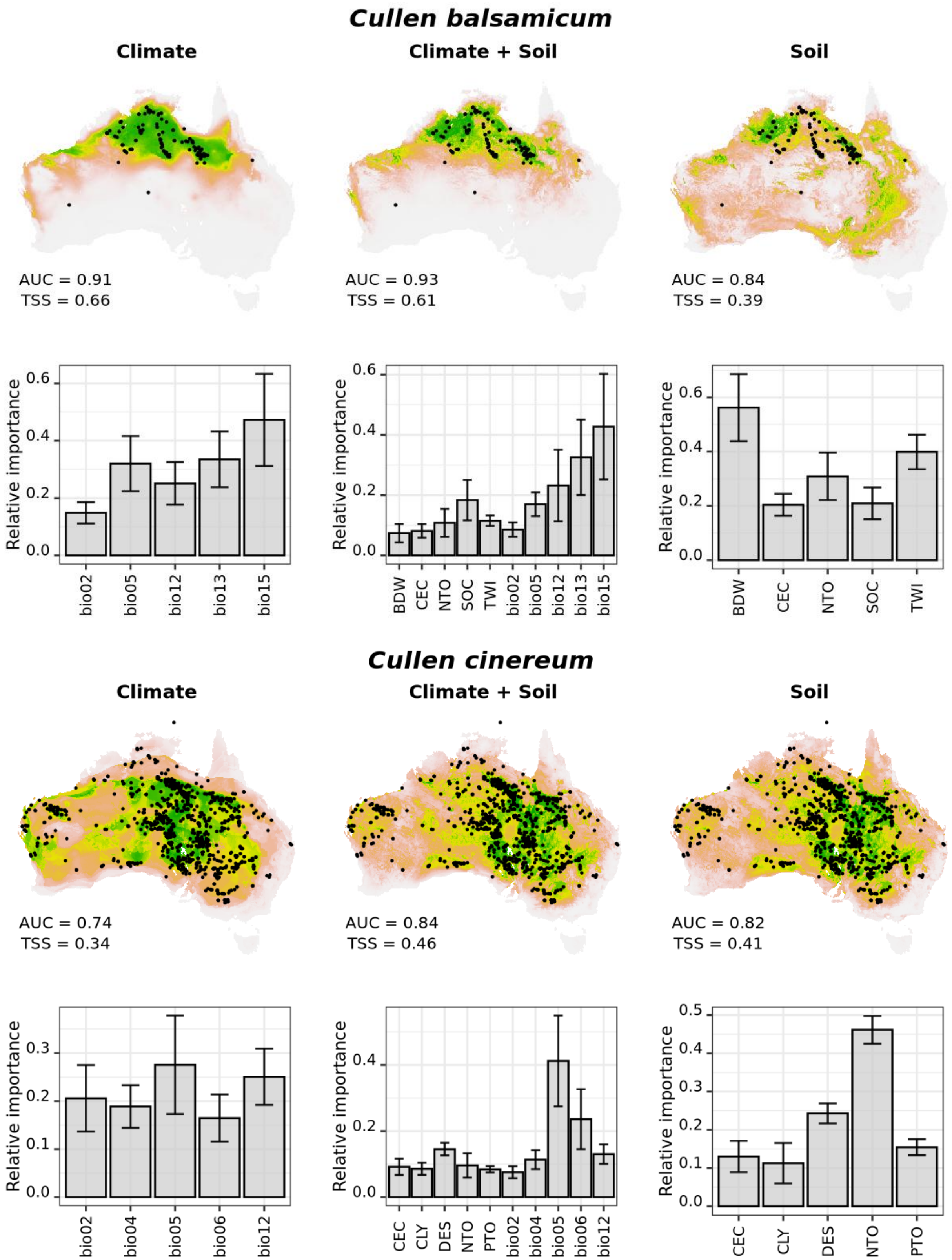
Cullen balsamicum
Climate
Climate + Soil
Soil
AUC = 0.91
TSS = 0.66
AUC = 0.93
TSS = 0.61
AUC = 0.84
TSS = 0.39
Relative importance
bio02
bio05
bio12
bio13
bio15
BDW
CEC
NTO
SOC
TWI
Cullen cinereum
Climate
Climate + Soil
Soil
AUC = 0.74
TSS = 0.34
AUC = 0.84
TSS = 0.46
AUC = 0.82
TSS = 0.41
Relative importance
bio02
bio04
bio05
bio06
bio12
CEC
CLY
DES
NTO
PTO

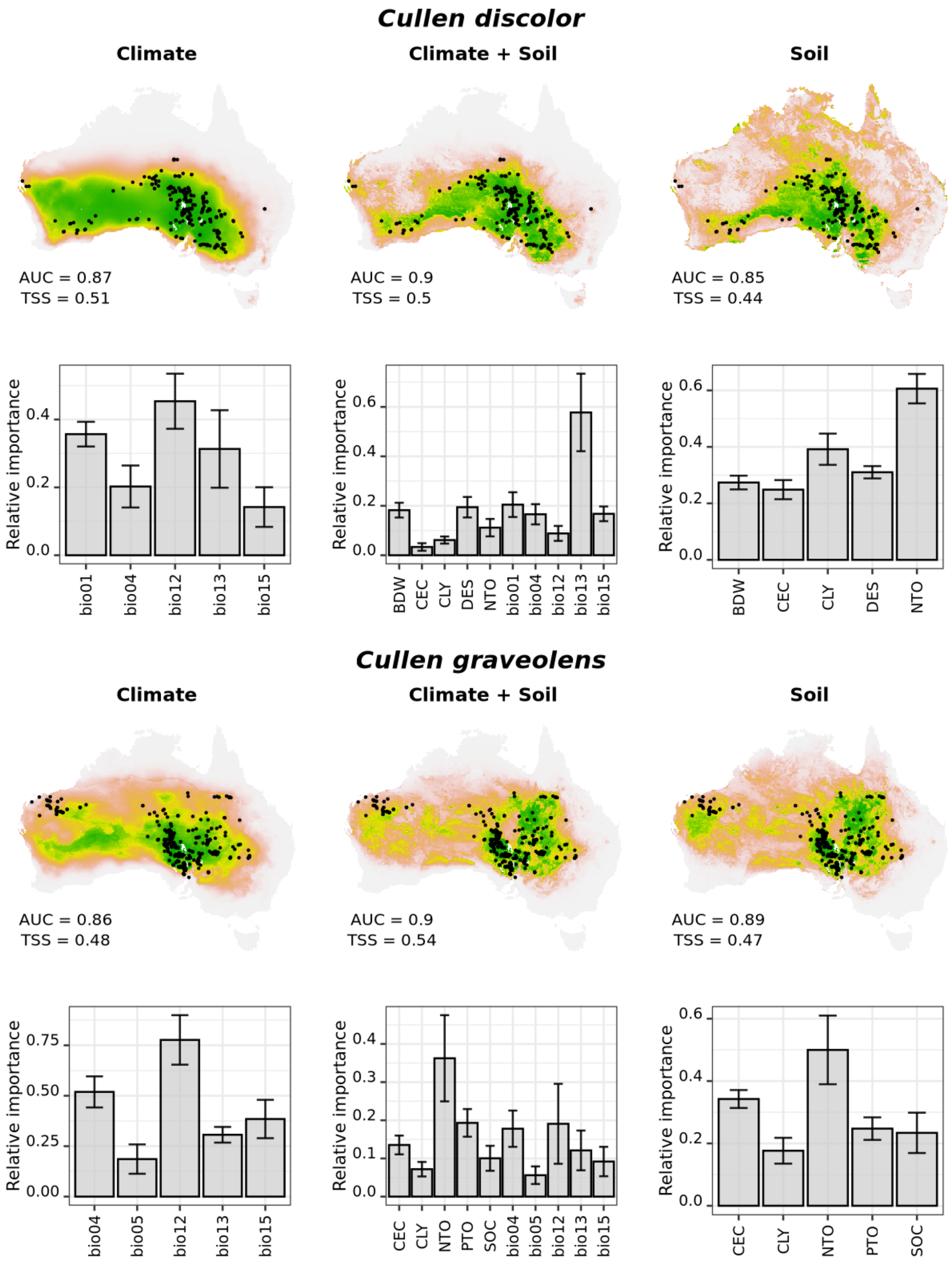
Cullen discolor
Climate
Climate + Soil
Soil
AUC = 0.87
TSS = 0.51
AUC = 0.9
TSS = 0.5
AUC = 0.85
TSS = 0.44
Relative importance
bio01
bio04
bio12
bio13
bio15
BDW
CEC
CLY
DES
NTO
Cullen graveolens
Climate
Climate + Soil
Soil
AUC = 0.86
TSS = 0.48
AUC = 0.9
TSS = 0.54
AUC = 0.89
TSS = 0.47
Relative importance
bio04
bio05
bio12
bio13
bio15
CEC
CLY
NTO
PTO
SOC

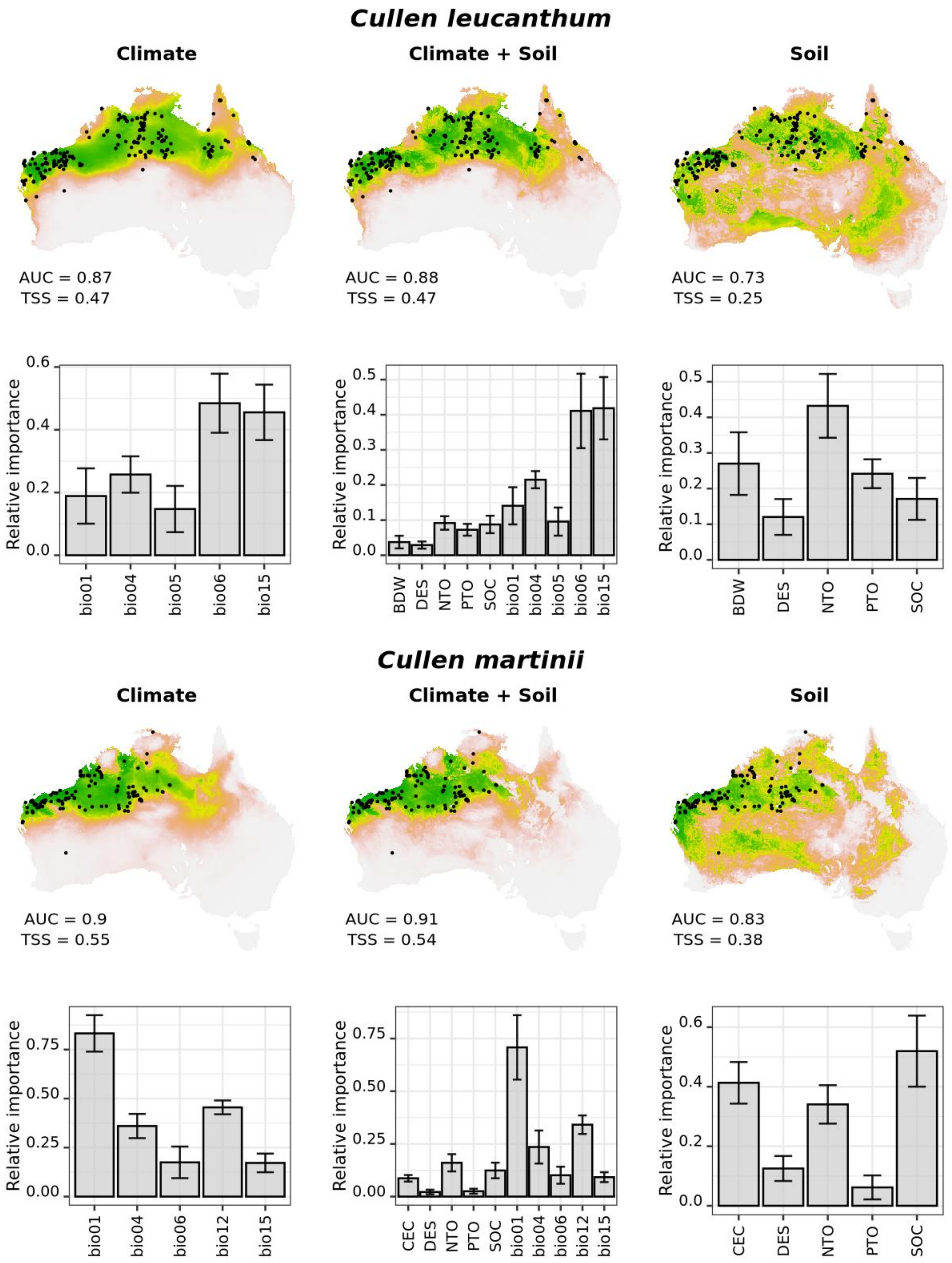
Cullen leucanthum
Climate
Climate + Soil
Soil
AUC = 0.87
TSS = 0.47
AUC = 0.88
TSS = 0.47
AUC = 0.73
TSS = 0.25
Relative importance
0.0 0.2 0.4 0.6
bio01 bio04 bio05 bio06 bio15
0.0 0.1 0.2 0.3 0.4 0.5
BDW DES NTO PTO SOC bio01 bio04 bio05 bio06 bio15
0.0 0.1 0.2 0.3 0.4 0.5
BDW DES NTO PTO SOC
Cullen martinii
Climate
Climate + Soil
Soil
AUC = 0.9
TSS = 0.55
AUC = 0.91
TSS = 0.54
AUC = 0.83
TSS = 0.38
Relative importance
0.00 0.25 0.50 0.75
bio01 bio04 bio06 bio12 bio15
0.00 0.25 0.50 0.75
CEC DES NTO PTO SOC bio01 bio04 bio06 bio12 bio15
0.0 0.2 0.4 0.6
CEC DES NTO PTO SOC

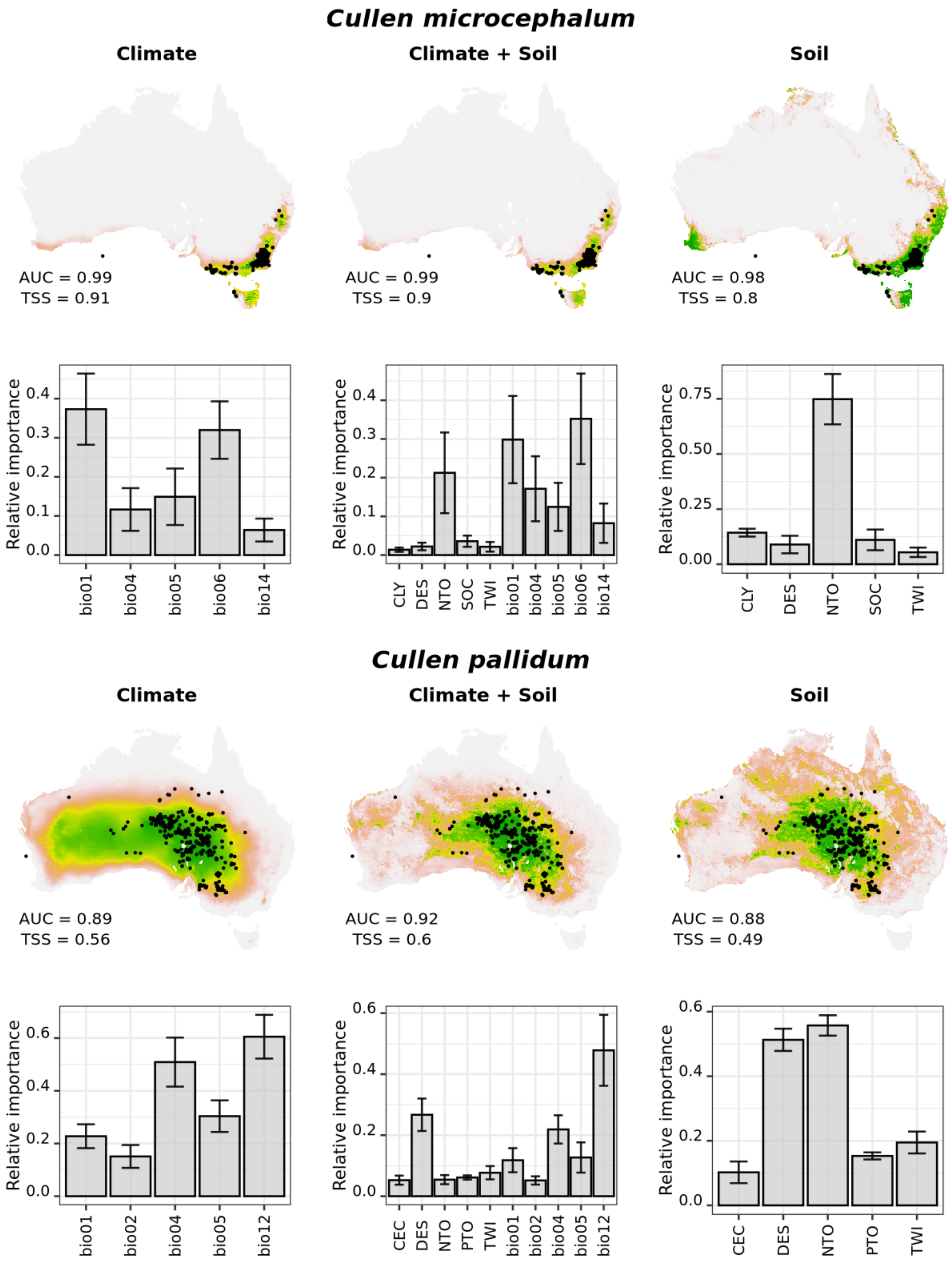
Cullen microcephalum
Climate
Climate + Soil
Soil
AUC = 0.99
TSS = 0.91
AUC = 0.99
TSS = 0.9
AUC = 0.98
TSS = 0.8
Relative importance
bio01
bio04
bio05
bio06
bio14
CLY
DES
NTO
SOC
TWI
Cullen pallidum
Climate
Climate + Soil
Soil
AUC = 0.89
TSS = 0.56
AUC = 0.92
TSS = 0.6
AUC = 0.88
TSS = 0.49
Relative importance
bio01
bio02
bio04
bio05
bio12
CEC
DES
NTO
PTO
TWI

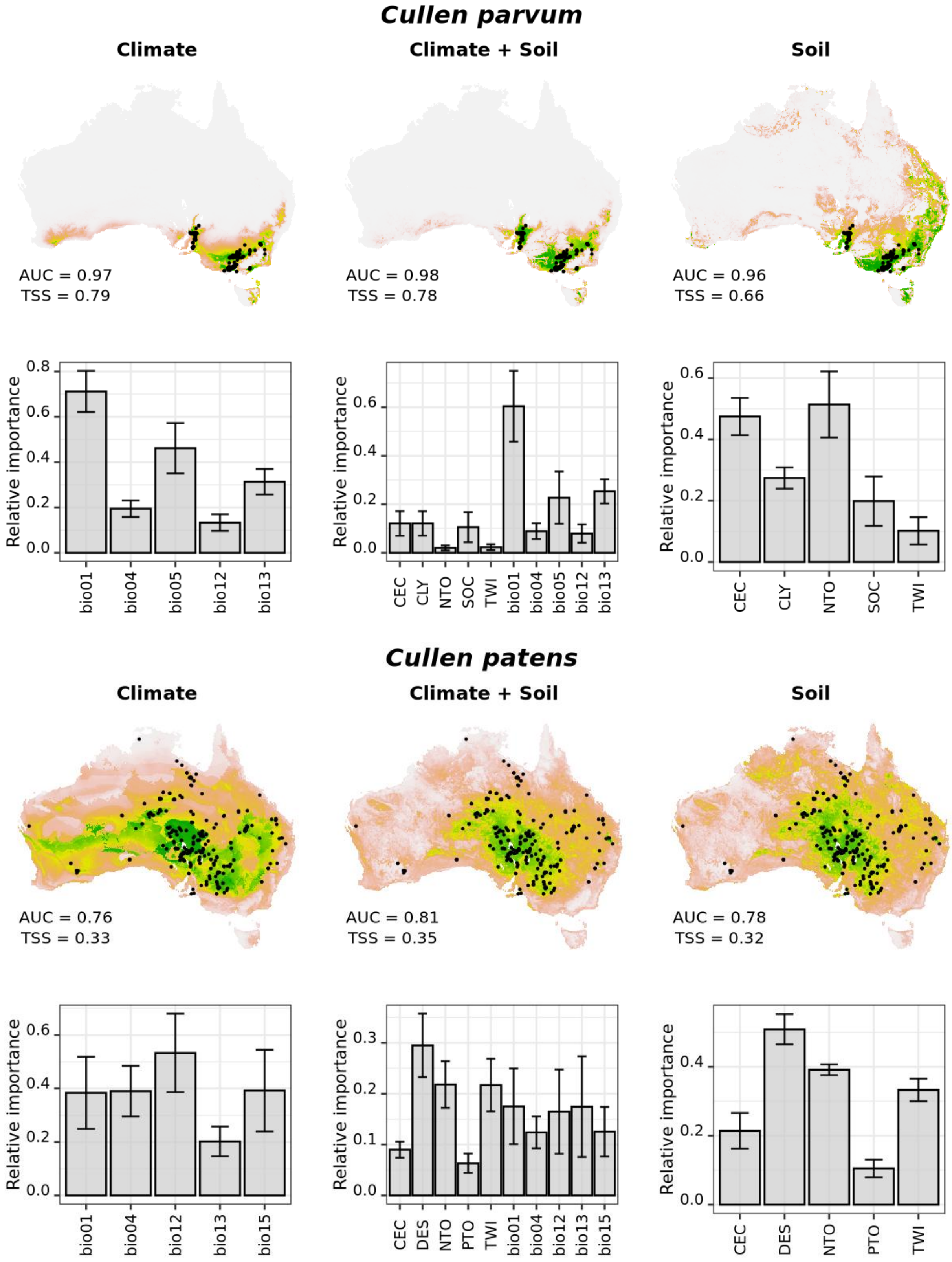
Cullen parvum
Climate
Climate + Soil
Soil
AUC = 0.97
TSS = 0.79
AUC = 0.98
TSS = 0.78
AUC = 0.96
TSS = 0.66
Relative importance
bio01
bio04
bio05
bio12
bio13
CEC
CLY
NTO
SOC
TWI
Cullen patens
Climate
Climate + Soil
Soil
AUC = 0.76
TSS = 0.33
AUC = 0.81
TSS = 0.35
AUC = 0.78
TSS = 0.32
Relative importance
bio01
bio04
bio12
bio13
bio15
CEC
DES
NTO
PTO
TWI

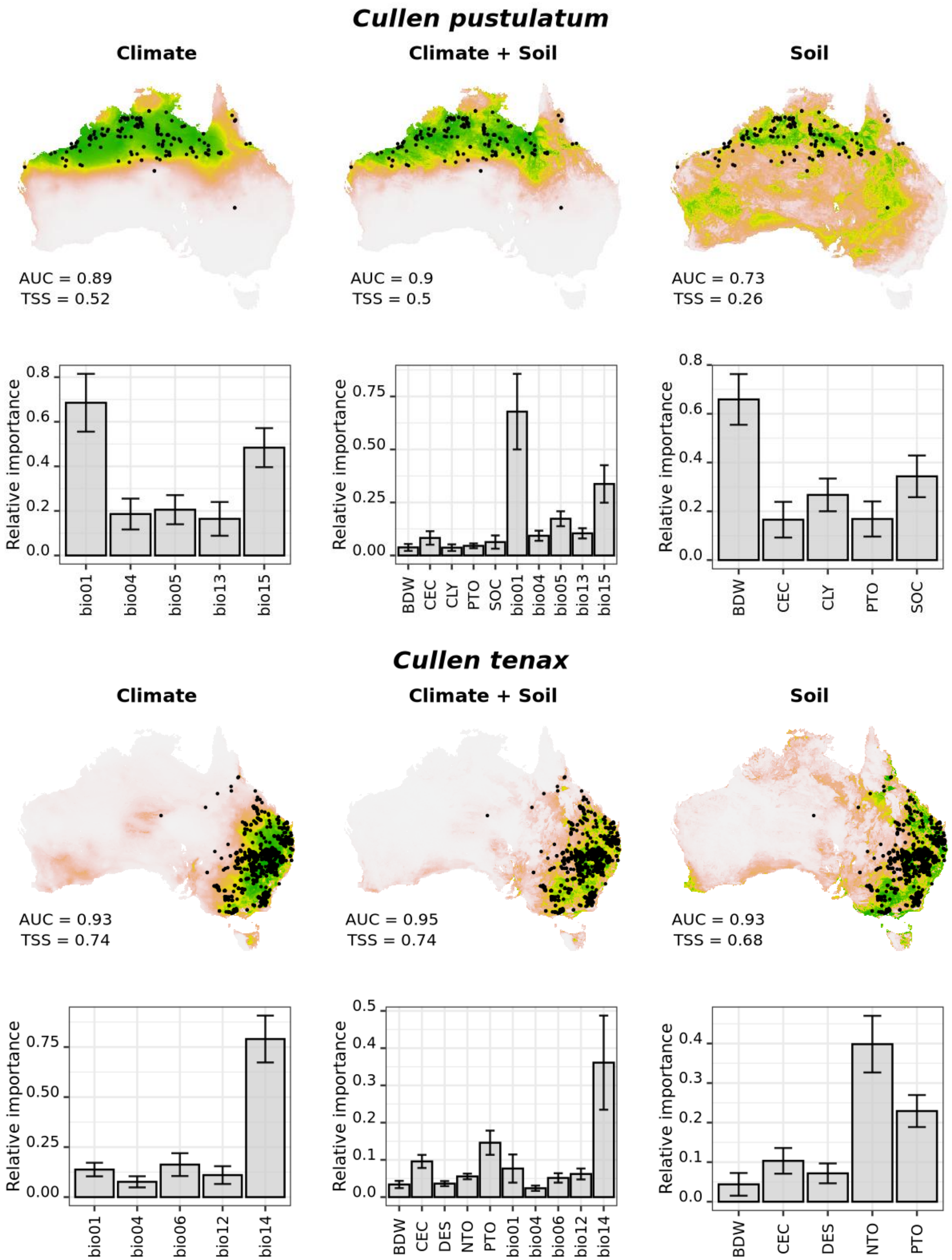
Cullen pustulatum
Climate
Climate + Soil
Soil
AUC = 0.89
TSS = 0.52
AUC = 0.9
TSS = 0.5
AUC = 0.73
TSS = 0.26
Relative importance
bio01
bio04
bio05
bio13
bio15
BDW
CEC
CLY
PTO
SOC
Cullen tenax
Climate
Climate + Soil
Soil
AUC = 0.93
TSS = 0.74
AUC = 0.95
TSS = 0.74
AUC = 0.93
TSS = 0.68
Relative importance
bio01
bio04
bio06
bio12
bio14
BDW
CEC
DES
NTO
PTO

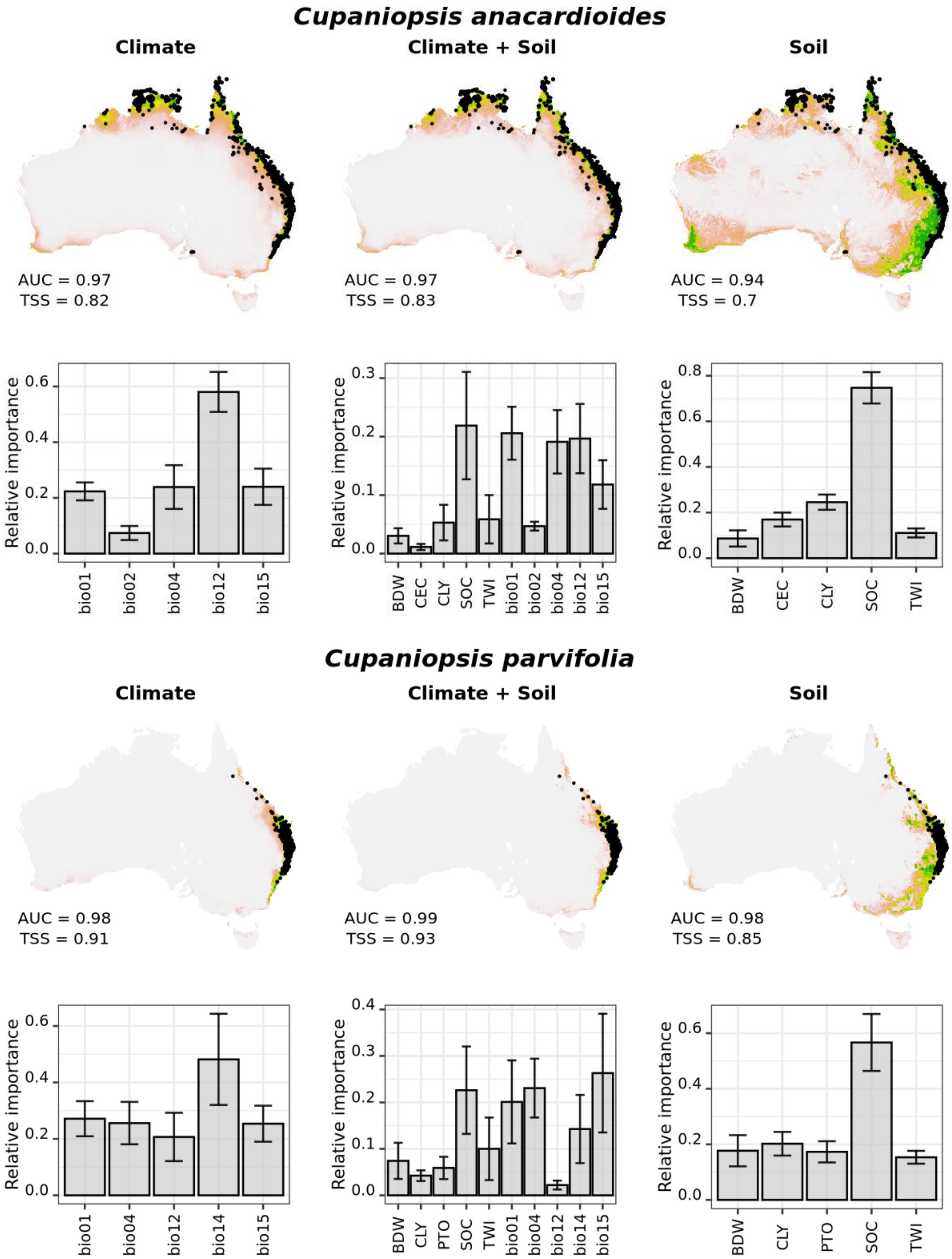

Cupaniopsis anacardioides
Climate
Climate + Soil
Soil
AUC = 0.97
TSS = 0.82
AUC = 0.97
TSS = 0.83
AUC = 0.94
TSS = 0.7
Relative importance
bio01 bio02 bio04 bio12 bio15
BDW CEC CLY SOC TWI bio01 bio02 bio04 bio12 bio15
BDW CEC CLY SOC TWI
Cupaniopsis parvifolia
Climate
Climate + Soil
Soil
AUC = 0.98
TSS = 0.91
AUC = 0.99
TSS = 0.93
AUC = 0.98
TSS = 0.85
Relative importance
bio01 bio04 bio12 bio14 bio15
BDW CLY PTO SOC TWI bio01 bio04 bio12 bio14 bio15
BDW CLY PTO SOC TWI

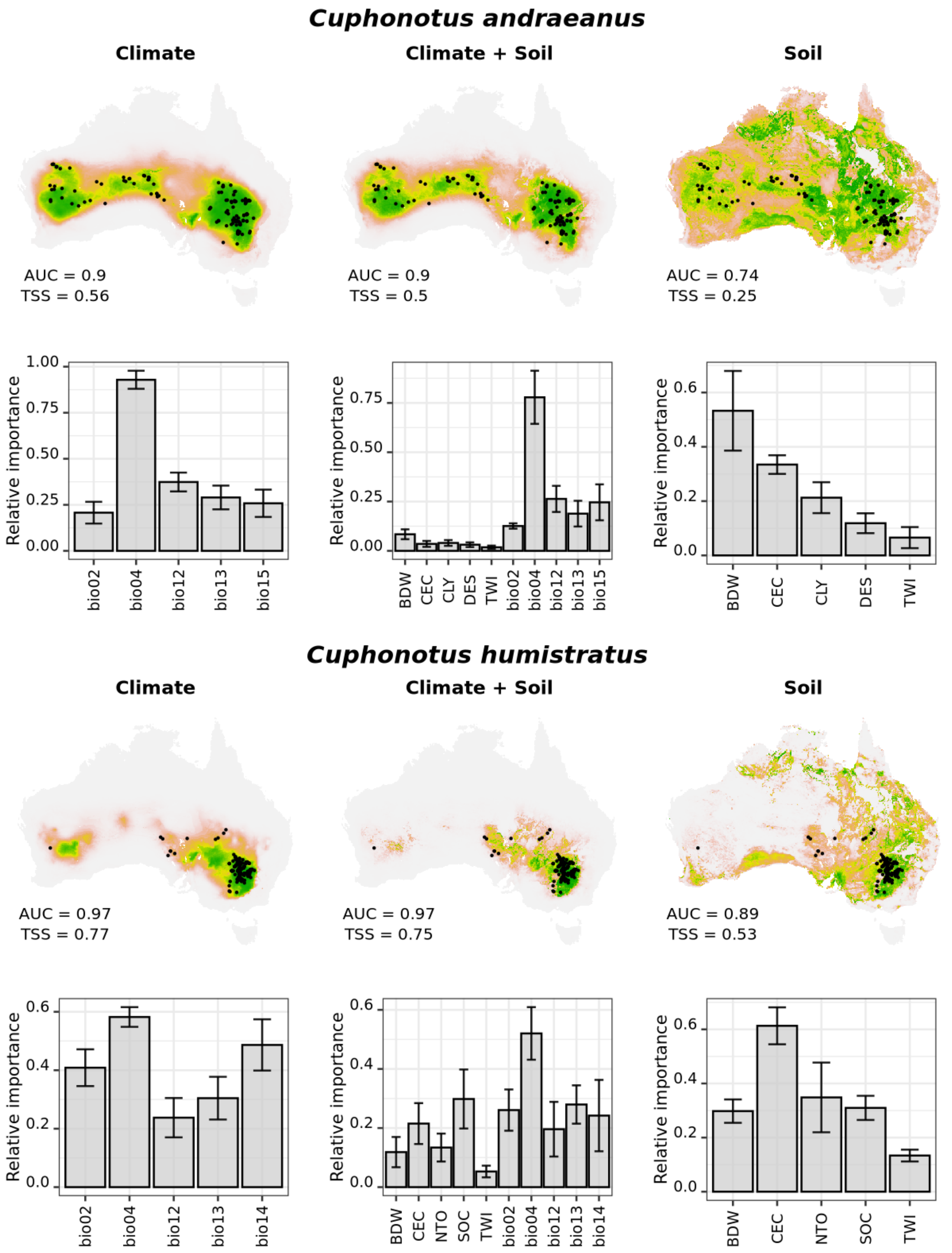
Cuphonotus andraeanus
Climate
Climate + Soil
Soil
AUC = 0.9
TSS = 0.56
AUC = 0.9
TSS = 0.5
AUC = 0.74
TSS = 0.25
Relative importance
bio02
bio04
bio12
bio13
bio15
BDW
CEC
CLY
DES
TWI
Cuphonotus humistratus
Climate
Climate + Soil
Soil
AUC = 0.97
TSS = 0.77
AUC = 0.97
TSS = 0.75
AUC = 0.89
TSS = 0.53
Relative importance
bio02
bio04
bio12
bio13
bio14
BDW
CEC
NTO
SOC
TWI

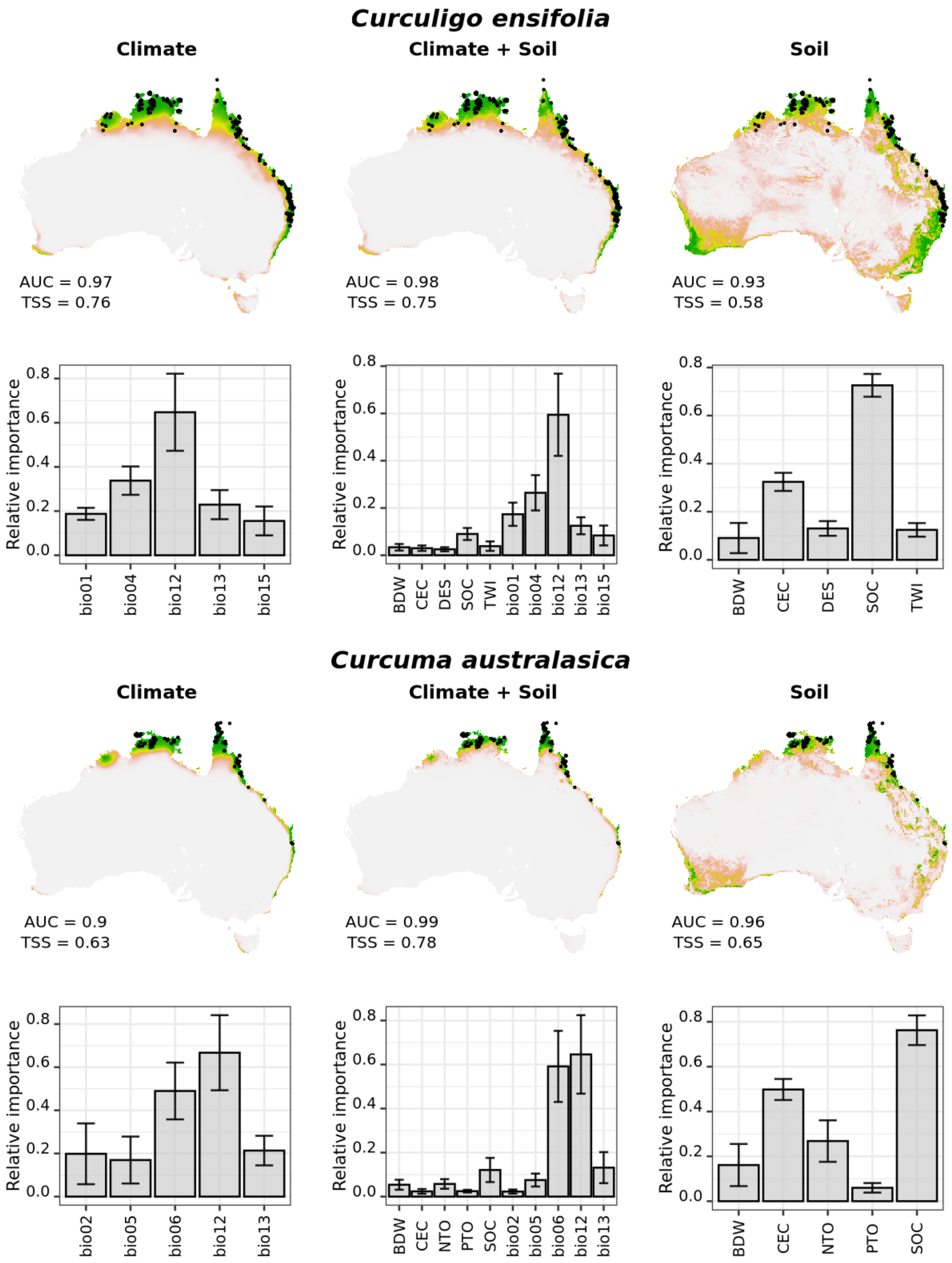

Curculigo ensifolia
Climate
Climate + Soil
Soil
AUC = 0.97
TSS = 0.76
AUC = 0.98
TSS = 0.75
AUC = 0.93
TSS = 0.58
Relative importance
0.0
0.2
0.4
0.6
0.8
bio01
bio04
bio12
bio13
bio15
BDW
CEC
DES
SOC
TWI
Curcuma australasica
AUC = 0.9
TSS = 0.63
AUC = 0.99
TSS = 0.78
AUC = 0.96
TSS = 0.65
bio02
bio05
bio06
NTO
PTO

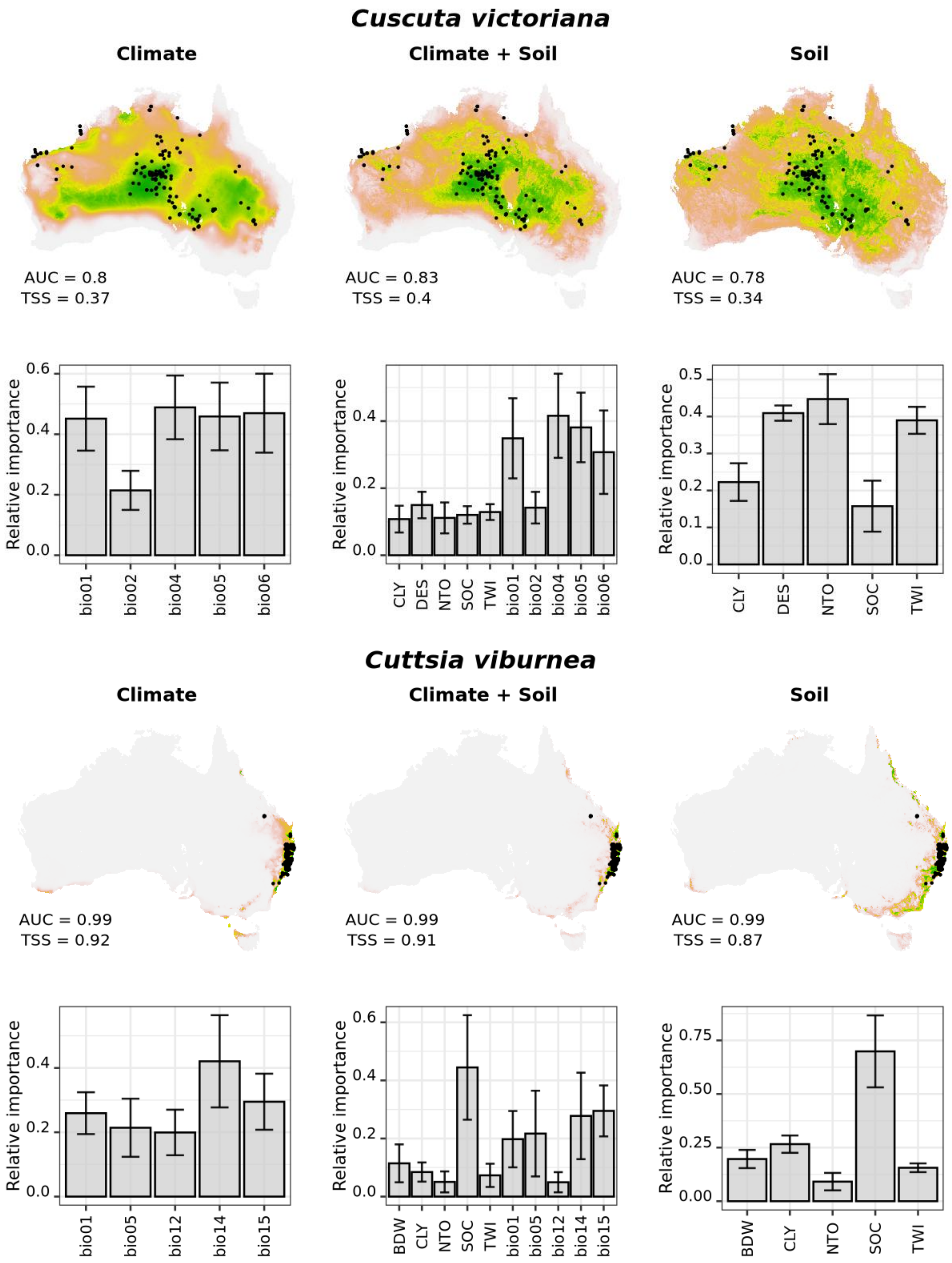
Cuscuta victoriana
Climate
Climate + Soil
Soil
AUC = 0.8
TSS = 0.37
AUC = 0.83
TSS = 0.4
AUC = 0.78
TSS = 0.34
Relative importance
bio01
bio02
bio04
bio05
bio06
CLY
DES
NTO
SOC
TWI
Cuttsia viburnea
Climate
Climate + Soil
Soil
AUC = 0.99
TSS = 0.92
AUC = 0.99
TSS = 0.91
AUC = 0.99
TSS = 0.87
Relative importance
bio01
bio05
bio12
bio14
bio15
BDW
CLY
NTO
SOC
TWI

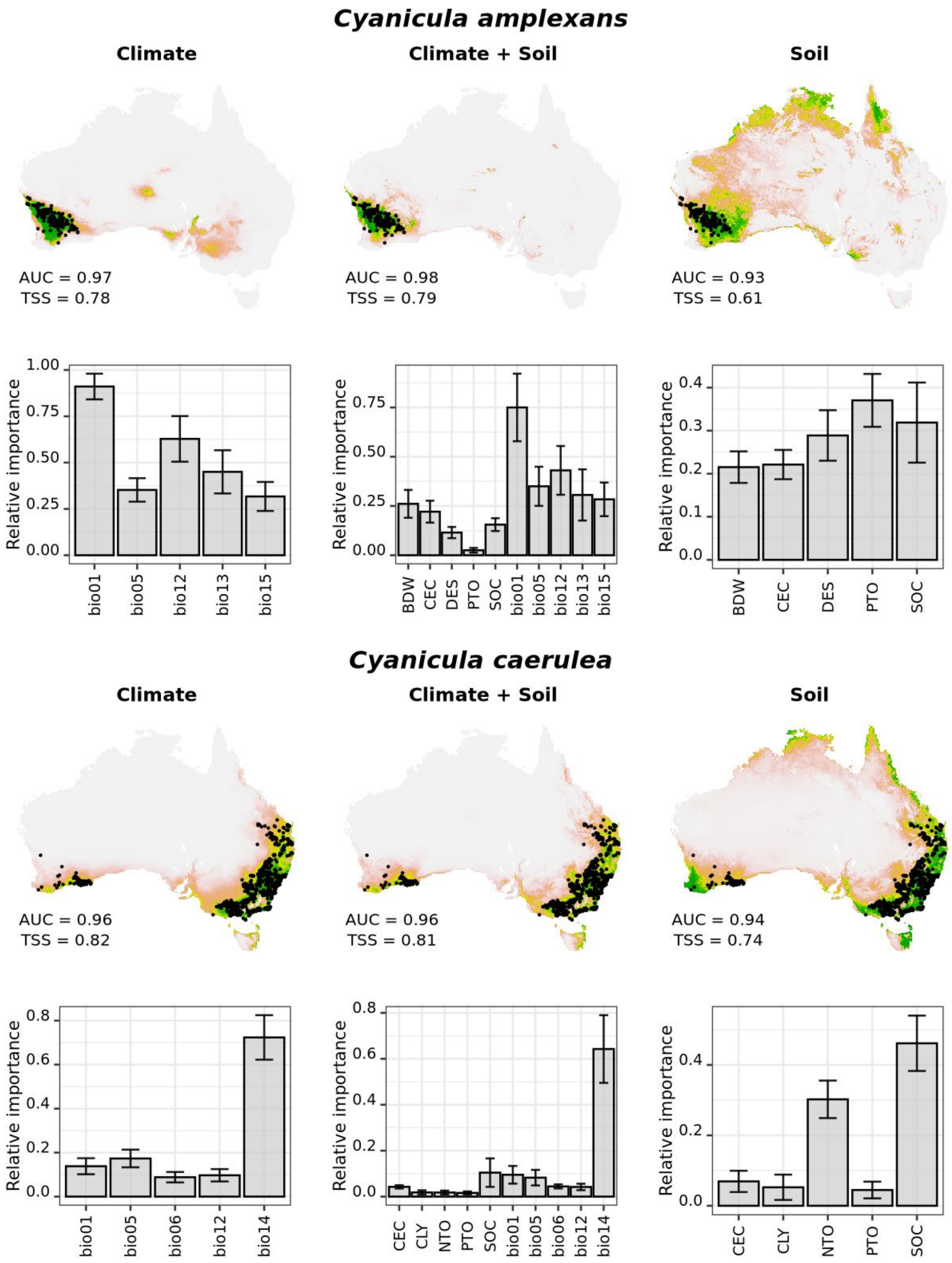

Cyanicula amplexans
Climate
Climate + Soil
Soil
AUC = 0.97
TSS = 0.78
AUC = 0.98
TSS = 0.79
AUC = 0.93
TSS = 0.61
Relative importance
bio01
bio05
bio12
bio13
bio15
BDW
CEC
DES
PTO
SOC
Cyanicula caerulea
Climate
Climate + Soil
Soil
AUC = 0.96
TSS = 0.82
AUC = 0.96
TSS = 0.81
AUC = 0.94
TSS = 0.74
Relative importance
bio01
bio05
bio06
bio12
bio14
CEC
CLY
NTO
PTO
SOC

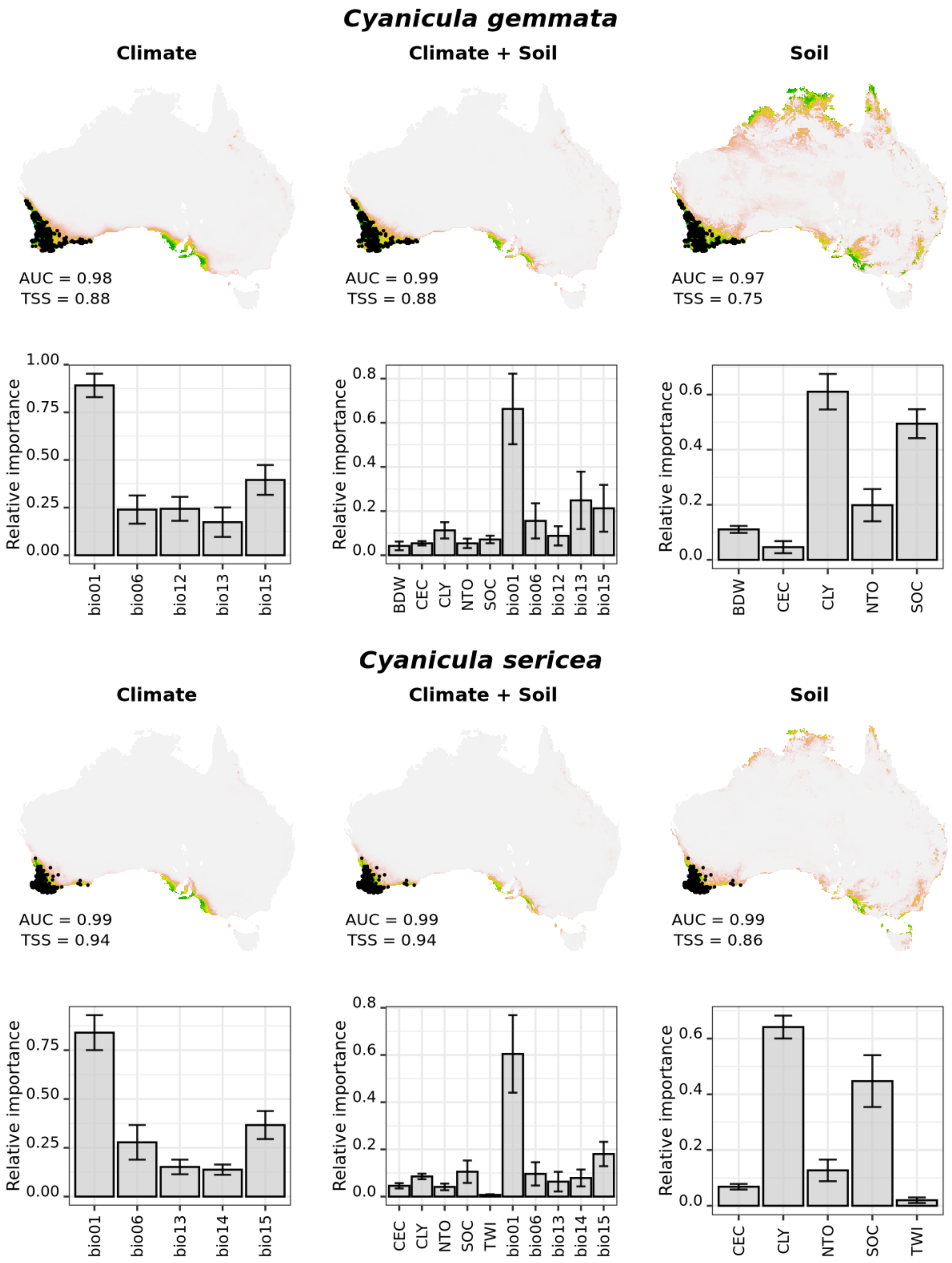
Cyanicula gemmata
Climate
Climate + Soil
Soil
AUC = 0.98
TSS = 0.88
AUC = 0.99
TSS = 0.88
AUC = 0.97
TSS = 0.75
Relative importance
bio01
bio06
bio12
bio13
bio15
BDW
CEC
CLY
NTO
SOC
Cyanicula sericea
Climate
Climate + Soil
Soil
AUC = 0.99
TSS = 0.94
AUC = 0.99
TSS = 0.94
AUC = 0.99
TSS = 0.86
Relative importance
bio01
bio06
bio13
bio14
bio15
CEC
CLY
NTO
SOC
TWI

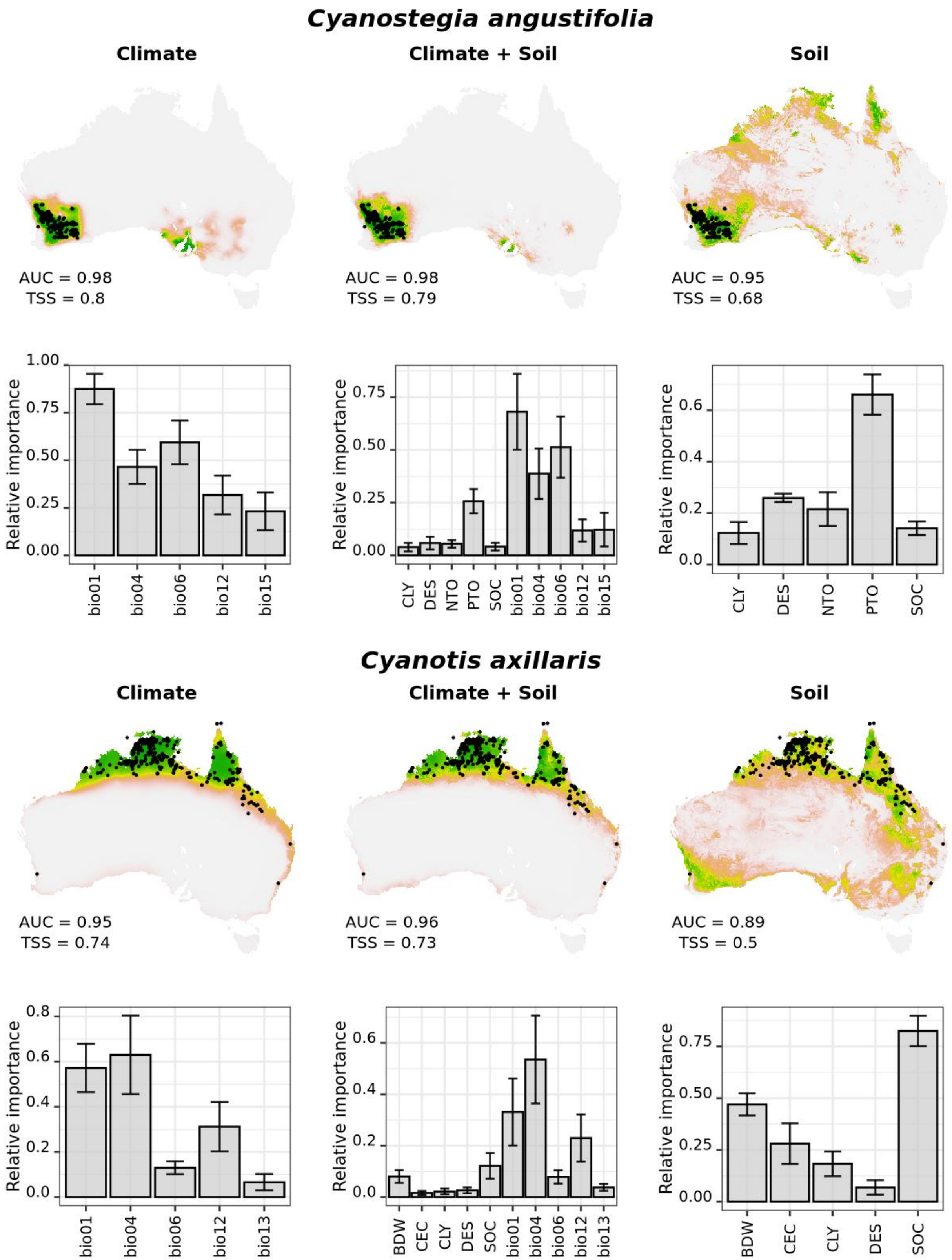

Cyanostegia angustifolia
Climate
Climate + Soil
Soil
AUC = 0.98
TSS = 0.8
AUC = 0.98
TSS = 0.79
AUC = 0.95
TSS = 0.68
Relative importance
bio01
bio04
bio06
bio12
bio15
CLY
DES
NTO
PTO
SOC
Cyanotis axillaris
Climate
Climate + Soil
Soil
AUC = 0.95
TSS = 0.74
AUC = 0.96
TSS = 0.73
AUC = 0.89
TSS = 0.5
Relative importance
bio01
bio04
bio06
bio12
bio13
BDW
CEC
CLY
DES
SOC

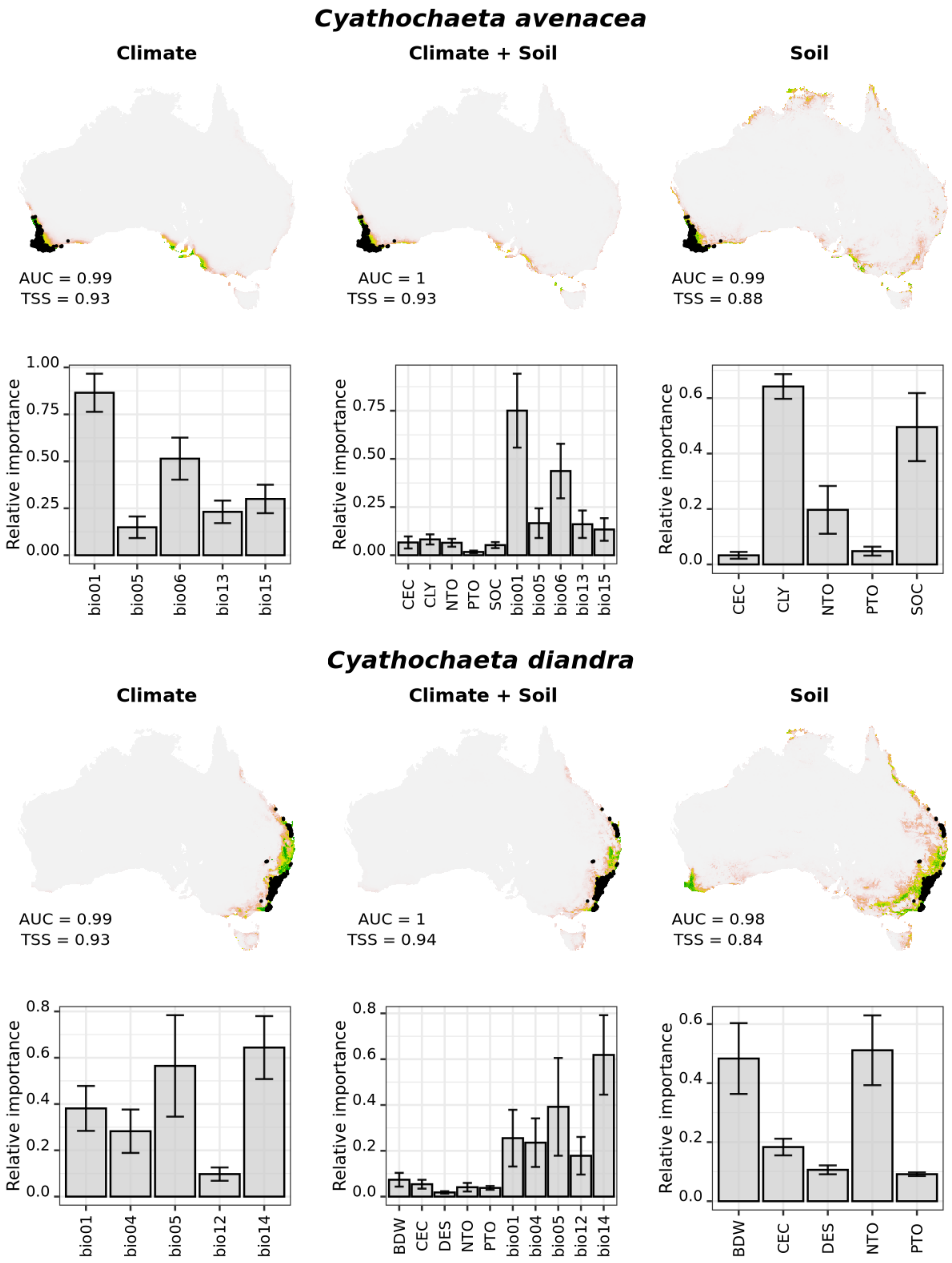
Cyathochaeta avenacea
Climate
Climate + Soil
Soil
AUC = 0.99
TSS = 0.93
AUC = 1
TSS = 0.93
AUC = 0.99
TSS = 0.88
Relative importance
bio01
bio05
bio06
bio13
bio15
CEC
CLY
NTO
PTO
SOC
Cyathochaeta diandra
Climate
Climate + Soil
Soil
AUC = 0.99
TSS = 0.93
AUC = 1
TSS = 0.94
AUC = 0.98
TSS = 0.84
Relative importance
bio01
bio04
bio05
bio12
bio14
BDW
CEC
DES
NTO
PTO

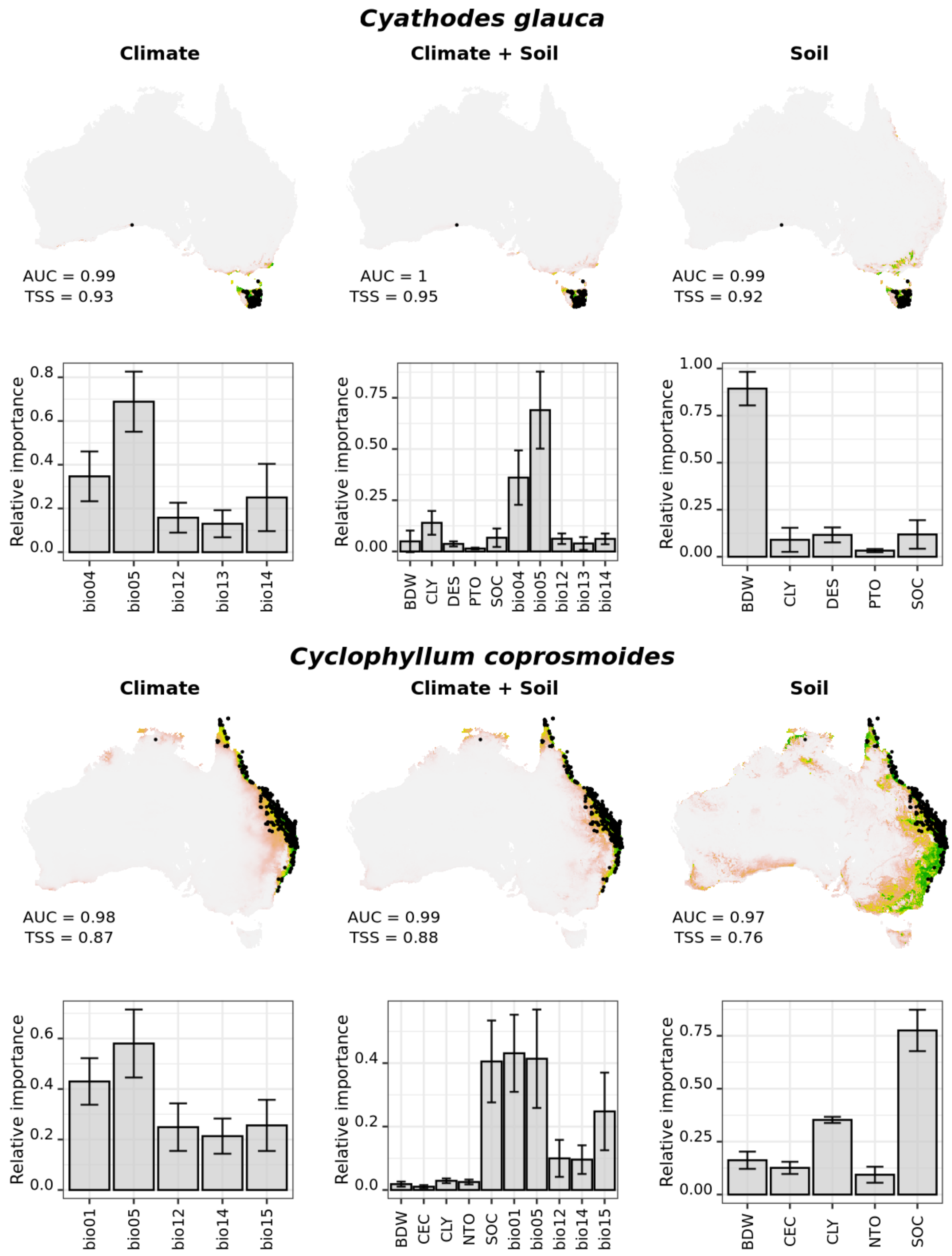

Cyathodes glauca
Climate
Climate + Soil
Soil
AUC = 0.99
TSS = 0.93
AUC = 1
TSS = 0.95
AUC = 0.99
TSS = 0.92
Relative importance
bio04
bio05
bio12
bio13
bio14
BDW
CLY
DES
PTO
SOC
Cyclophyllum coprosmoides
Climate
Climate + Soil
Soil
AUC = 0.98
TSS = 0.87
AUC = 0.99
TSS = 0.88
AUC = 0.97
TSS = 0.76
Relative importance
bio01
bio05
bio12
bio14
bio15
BDW
CEC
CLY
NTO
SOC

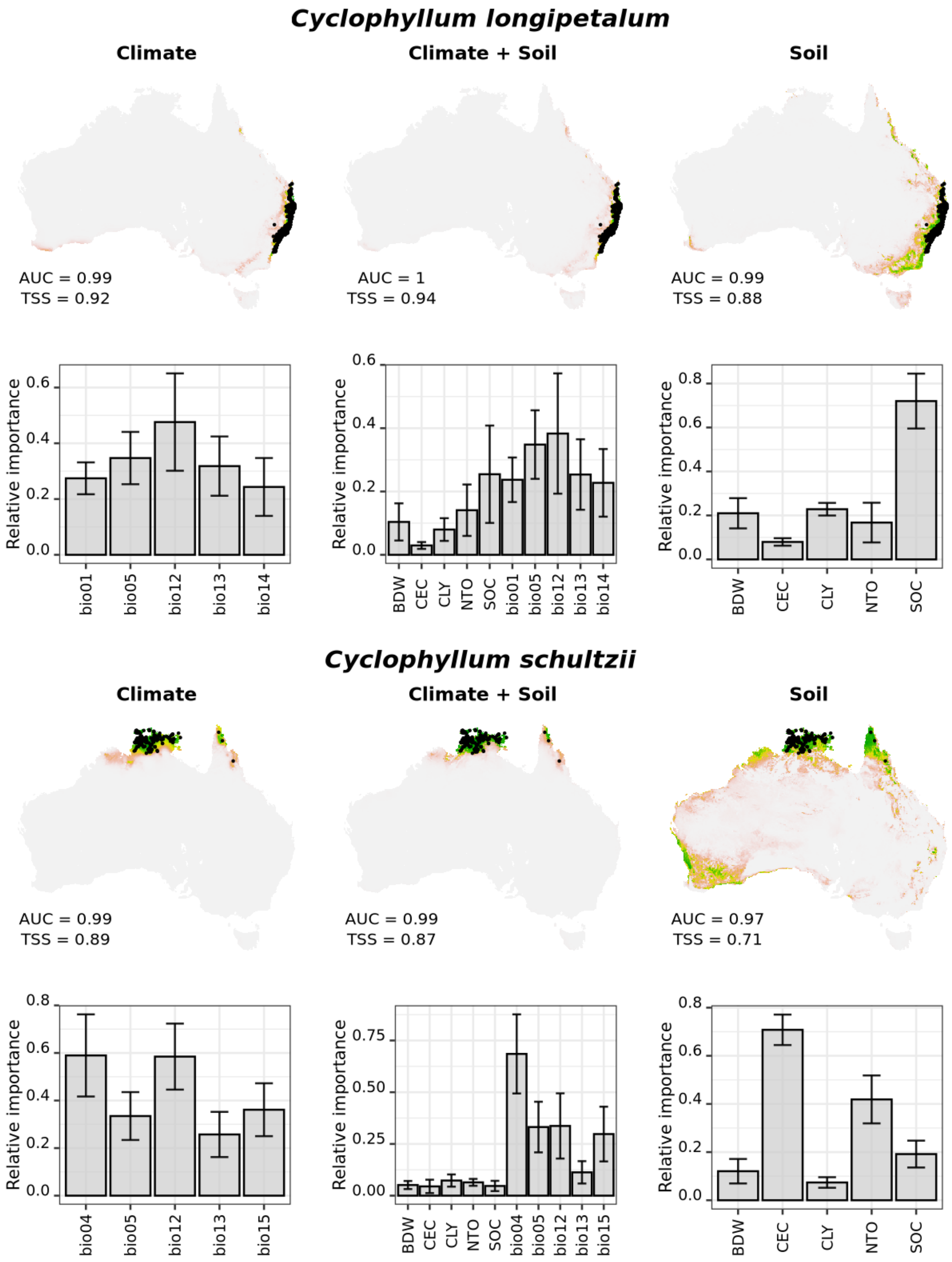
Cyclophyllum longipetalum
Climate
Climate + Soil
Soil
AUC = 0.99
TSS = 0.92
AUC = 1
TSS = 0.94
AUC = 0.99
TSS = 0.88
Relative importance
bio01
bio05
bio12
bio13
bio14
BDW
CEC
CLY
NTO
SOC
Cyclophyllum schultzii
Climate
Climate + Soil
Soil
AUC = 0.99
TSS = 0.89
AUC = 0.99
TSS = 0.87
AUC = 0.97
TSS = 0.71
Relative importance
bio04
bio05
bio12
bio13
bio15
BDW
CEC
CLY
NTO
SOC

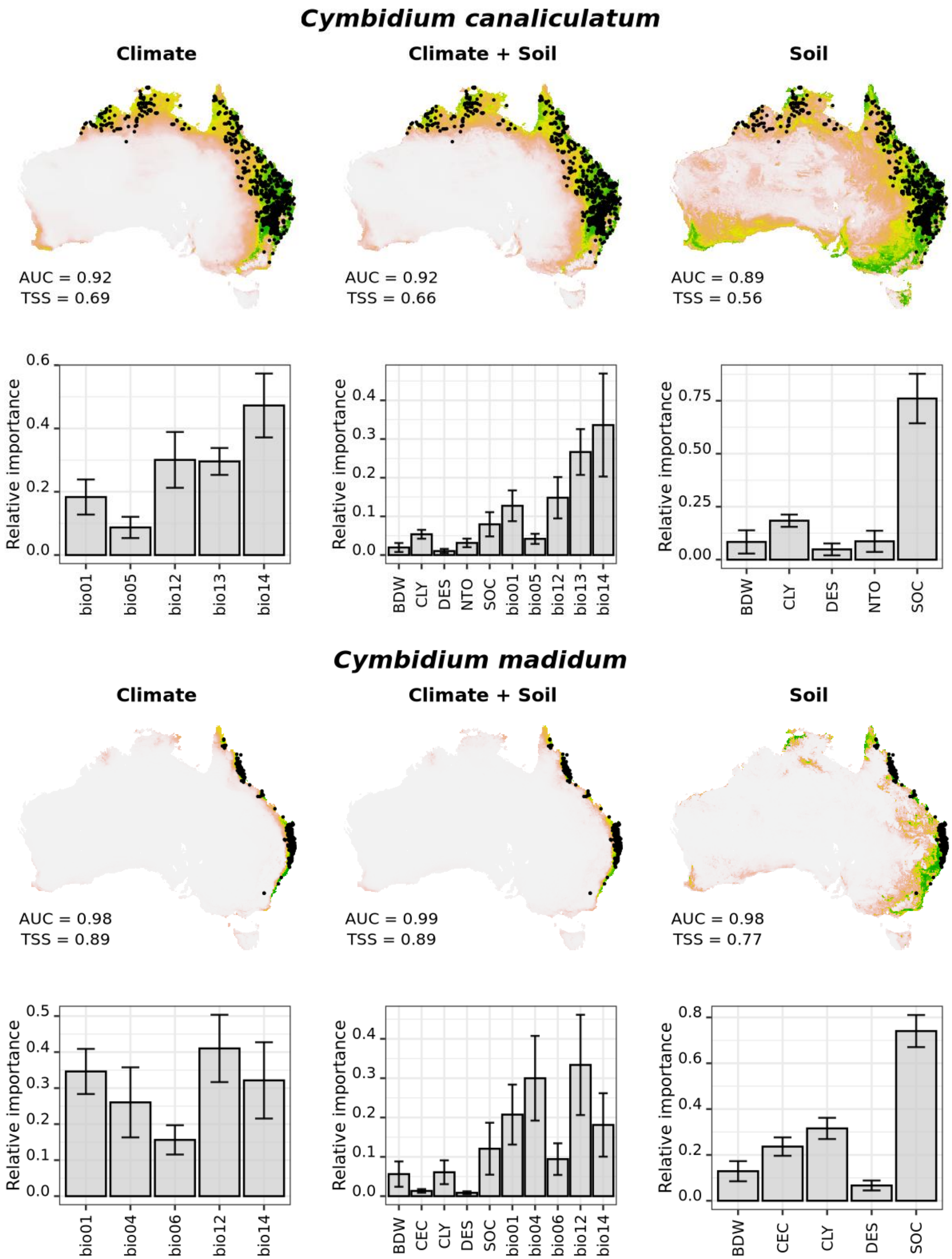
Cymbidium canaliculatum
Climate
Climate + Soil
Soil
AUC = 0.92
TSS = 0.69
AUC = 0.92
TSS = 0.66
AUC = 0.89
TSS = 0.56
Relative importance
bio01
bio05
bio12
bio13
bio14
BDW
CLY
DES
NTO
SOC
Cymbidium madidum
Climate
Climate + Soil
Soil
AUC = 0.98
TSS = 0.89
AUC = 0.99
TSS = 0.89
AUC = 0.98
TSS = 0.77
Relative importance
bio01
bio04
bio06
bio12
bio14
BDW
CEC
CLY
DES
SOC

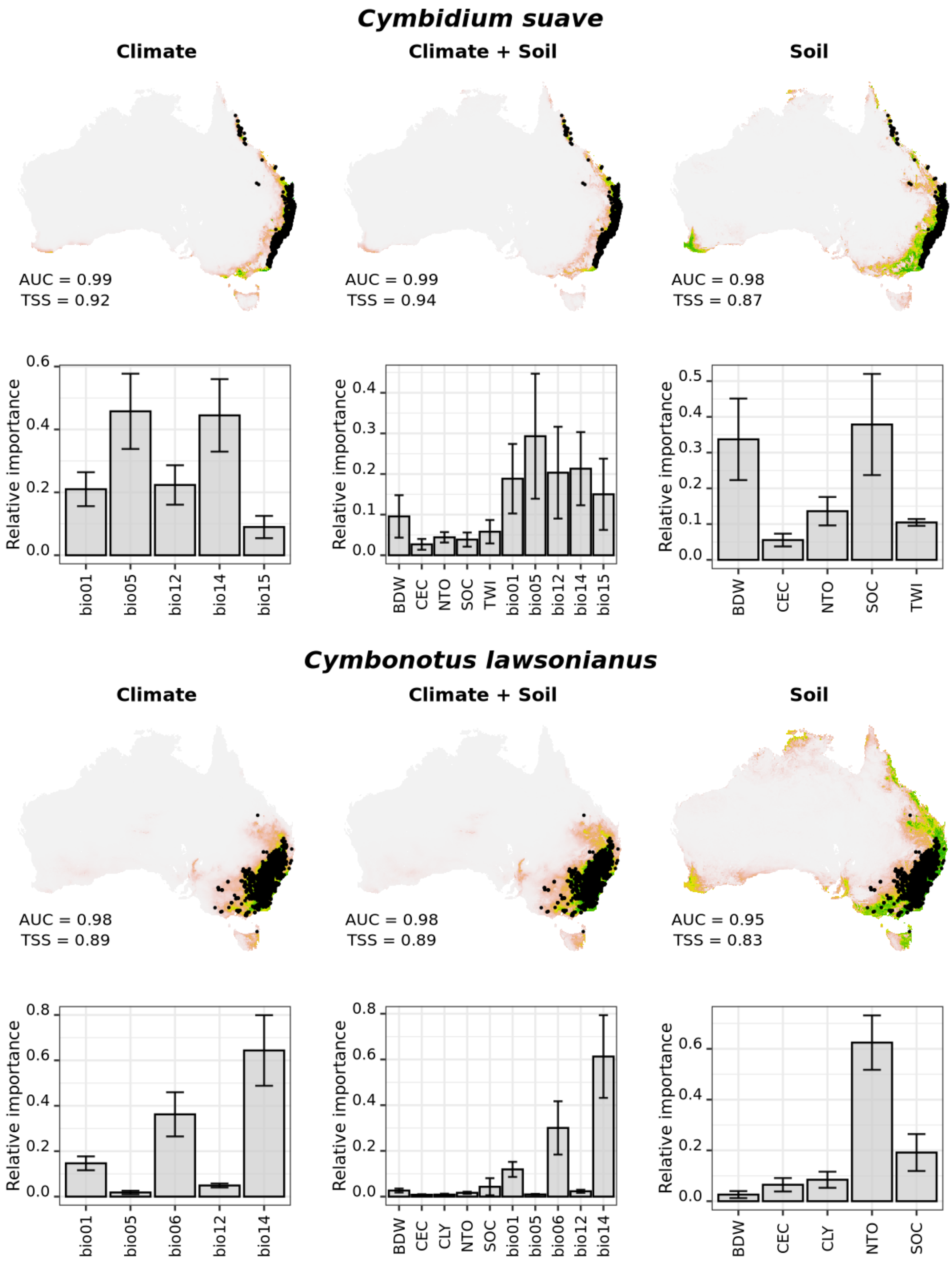

Cymbidium suave
Climate
Climate + Soil
Soil
AUC = 0.99
TSS = 0.92
AUC = 0.99
TSS = 0.94
AUC = 0.98
TSS = 0.87
Relative importance
bio01
bio05
bio12
bio14
bio15
BDW
CEC
NTO
SOC
TWI
Cymbonotus lawsonianus
Climate
Climate + Soil
Soil
AUC = 0.98
TSS = 0.89
AUC = 0.98
TSS = 0.89
AUC = 0.95
TSS = 0.83
Relative importance
bio01
bio05
bio06
bio12
bio14
BDW
CEC
CLY
NTO
SOC

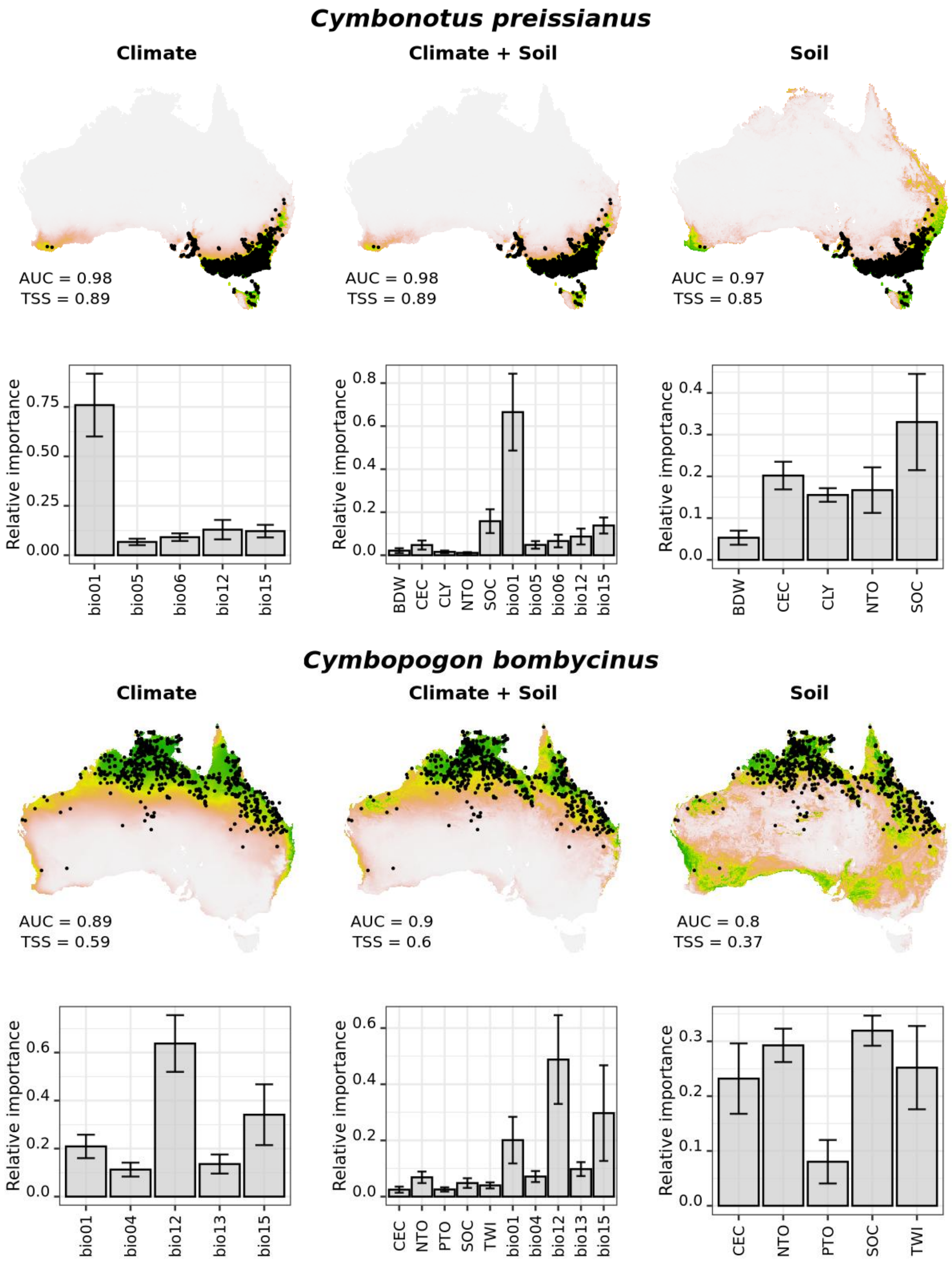
Cymbonotus preissianus
Climate
Climate + Soil
Soil
AUC = 0.98
TSS = 0.89
AUC = 0.98
TSS = 0.89
AUC = 0.97
TSS = 0.85
Relative importance
bio01
bio05
bio06
bio12
bio15
BDW
CEC
CLY
NTO
SOC
Cymbopogon bombycinus
Climate
Climate + Soil
Soil
AUC = 0.89
TSS = 0.59
AUC = 0.9
TSS = 0.6
AUC = 0.8
TSS = 0.37
Relative importance
bio01
bio04
bio12
bio13
bio15
CEC
NTO
PTO
SOC
TWI

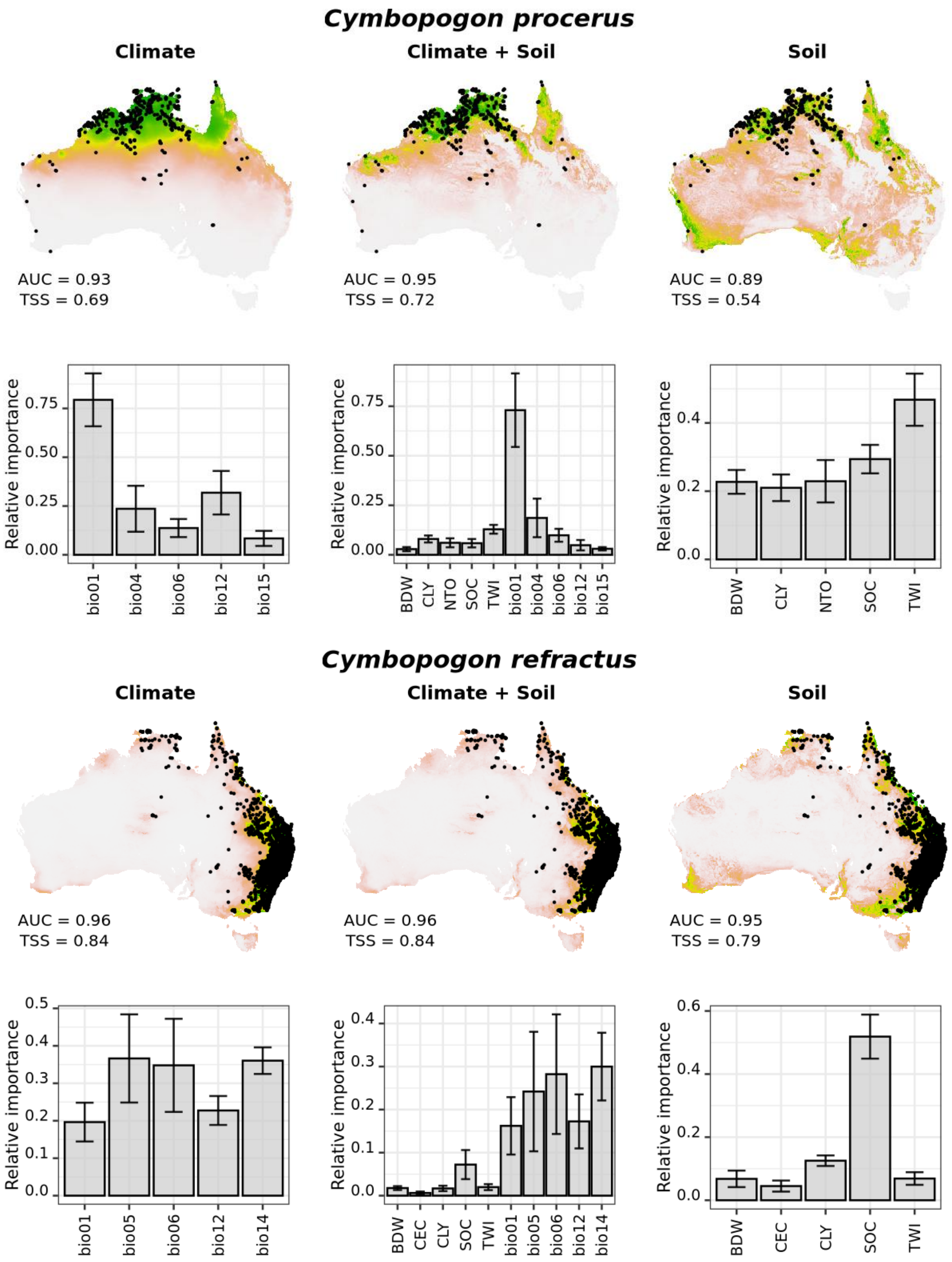
Cymbopogon procerus
Climate
Climate + Soil
Soil
AUC = 0.93
TSS = 0.69
AUC = 0.95
TSS = 0.72
AUC = 0.89
TSS = 0.54
Relative importance
bio01 bio04 bio06 bio12 bio15
BDW CLY NTO SOC TWI bio01 bio04 bio06 bio12 bio15
BDW CLY NTO SOC TWI
Cymbopogon refractus
Climate
Climate + Soil
Soil
AUC = 0.96
TSS = 0.84
AUC = 0.96
TSS = 0.84
AUC = 0.95
TSS = 0.79
Relative importance
bio01 bio05 bio06 bio12 bio14
BDW CEC CLY SOC TWI bio01 bio05 bio06 bio12 bio14
BDW CEC CLY SOC TWI

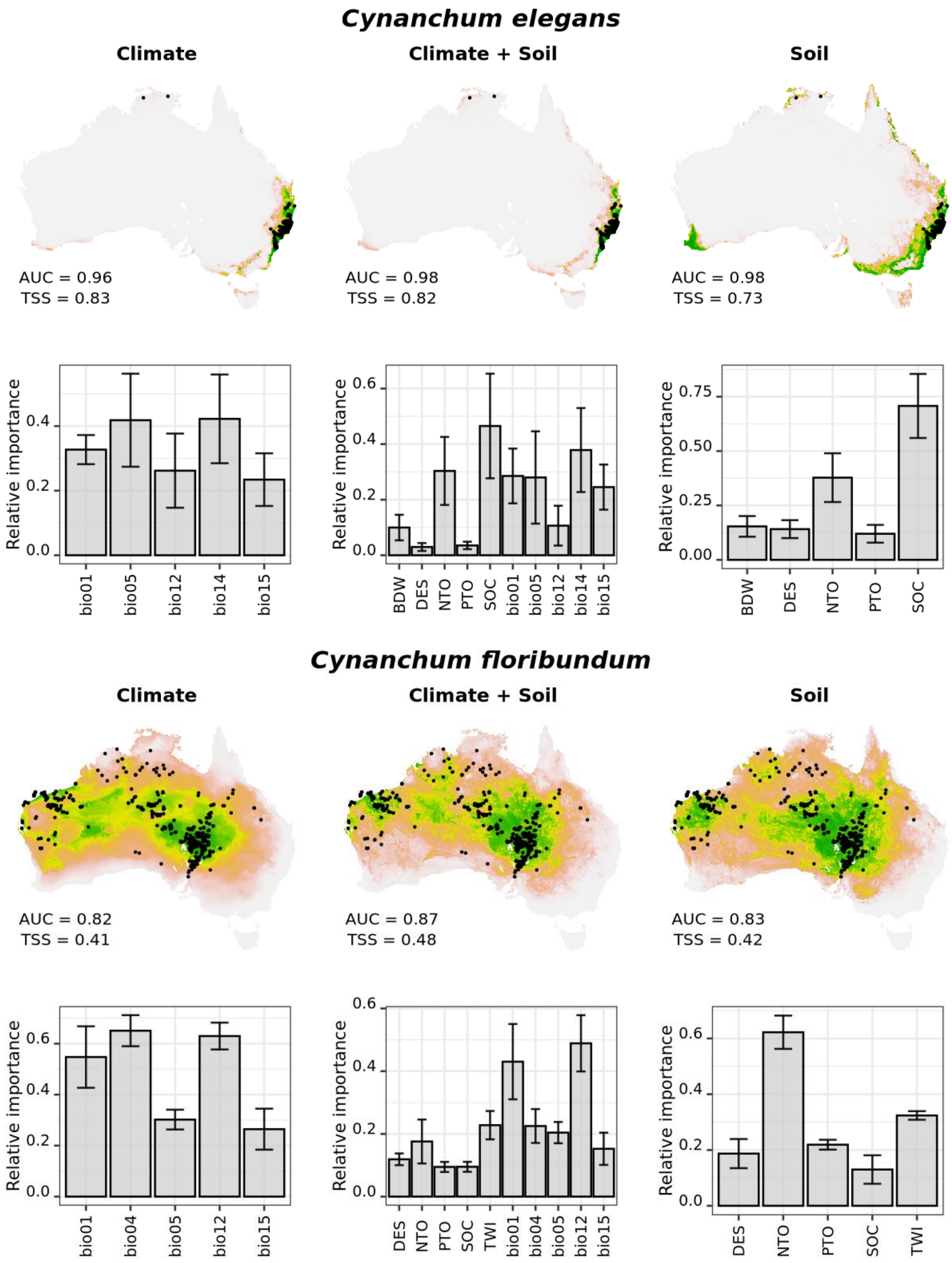
Cynanchum elegans
Climate
Climate + Soil
Soil
AUC = 0.96
TSS = 0.83
AUC = 0.98
TSS = 0.82
AUC = 0.98
TSS = 0.73
Relative importance
bio01
bio05
bio12
bio14
bio15
BDW
DES
NTO
PTO
SOC
Cynanchum floribundum
AUC = 0.82
TSS = 0.41
AUC = 0.87
TSS = 0.48
AUC = 0.83
TSS = 0.42
bio04
TWI

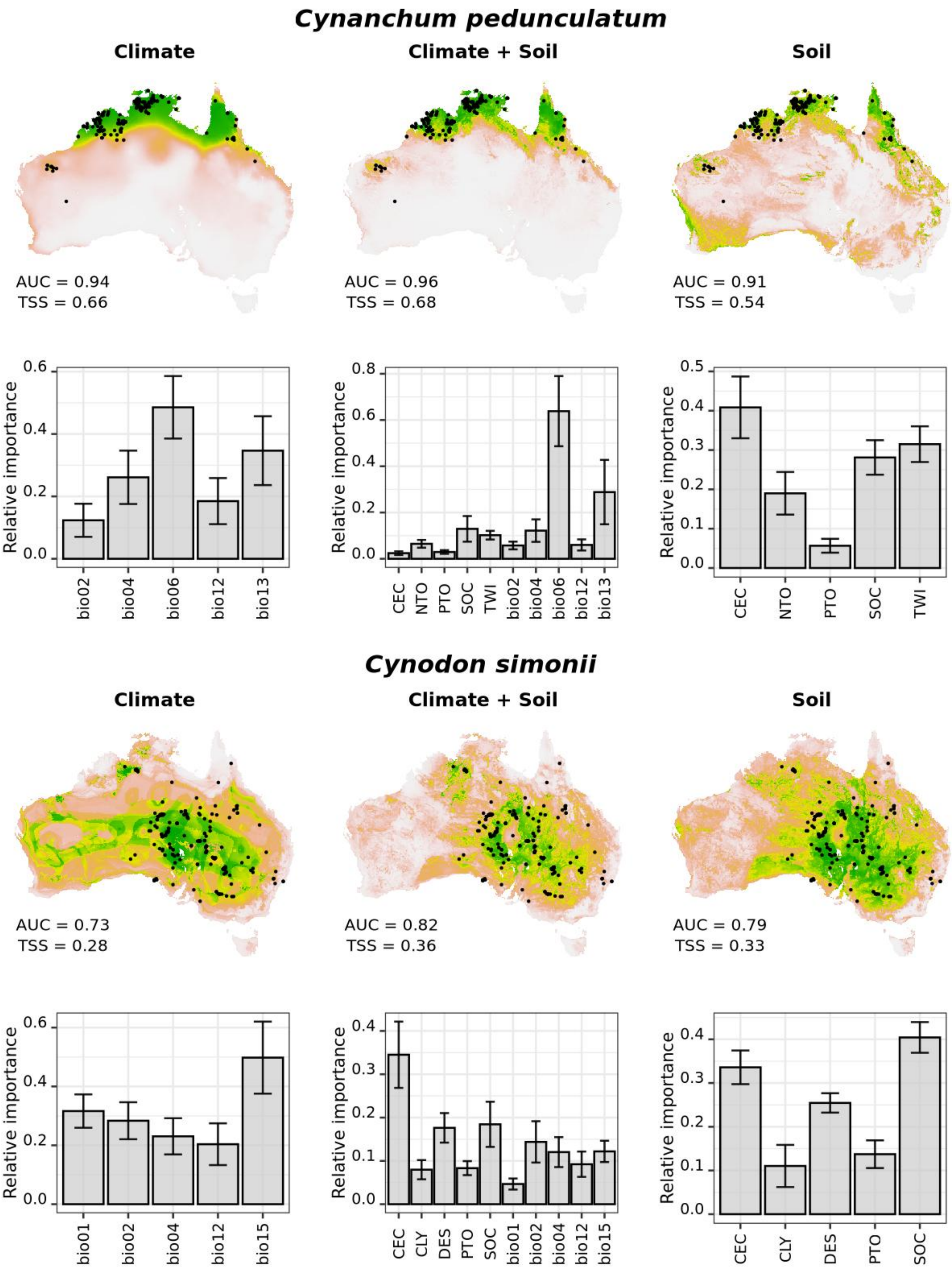
Cynanchum pedunculatum
Climate
Climate + Soil
Soil
AUC = 0.94
TSS = 0.66
AUC = 0.96
TSS = 0.68
AUC = 0.91
TSS = 0.54
Relative importance
bio02
bio04
bio06
bio12
bio13
CEC
NTO
PTO
SOC
TWI
Cynodon simonii
Climate
Climate + Soil
Soil
AUC = 0.73
TSS = 0.28
AUC = 0.82
TSS = 0.36
AUC = 0.79
TSS = 0.33
Relative importance
bio01
bio02
bio04
bio12
bio15
CEC
CLY
DES
PTO
SOC

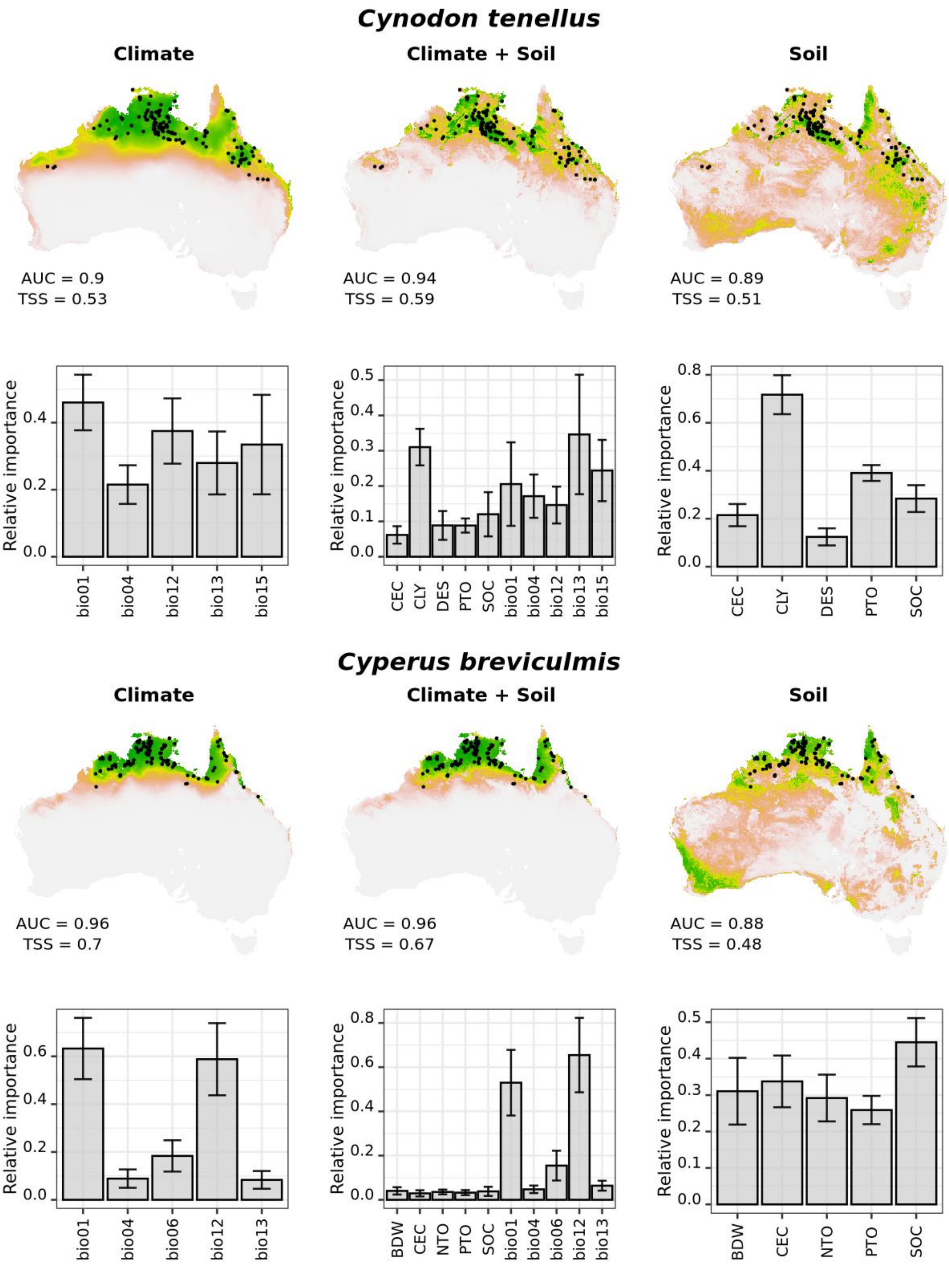
Cynodon tenellus
Climate
Climate + Soil
Soil
AUC = 0.9
TSS = 0.53
AUC = 0.94
TSS = 0.59
AUC = 0.89
TSS = 0.51
Relative importance
bio01
bio04
bio12
bio13
bio15
CEC
CLY
DES
PTO
SOC
Cyperus breviculmis
Climate
Climate + Soil
Soil
AUC = 0.96
TSS = 0.7
AUC = 0.96
TSS = 0.67
AUC = 0.88
TSS = 0.48
Relative importance
bio01
bio04
bio06
bio12
bio13
BDW
CEC
NTO
PTO
SOC

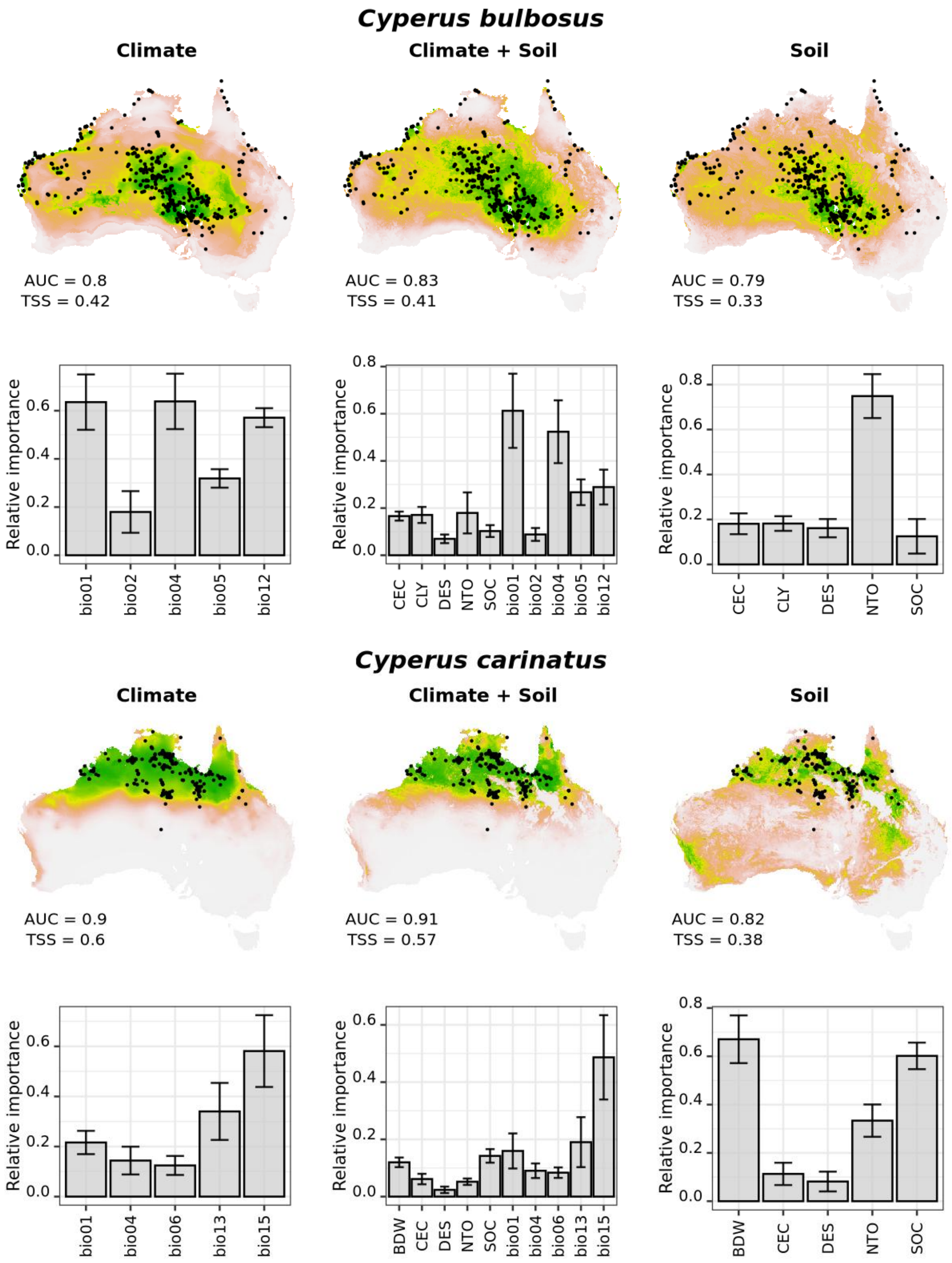
Cyperus bulbosus
Climate
Climate + Soil
Soil
AUC = 0.8
TSS = 0.42
AUC = 0.83
TSS = 0.41
AUC = 0.79
TSS = 0.33
Relative importance
bio01
bio02
bio04
bio05
bio12
CEC
CLY
DES
NTO
SOC
Cyperus carinatus
AUC = 0.9
TSS = 0.6
AUC = 0.91
TSS = 0.57
AUC = 0.82
TSS = 0.38
bio06
bio13
bio15
BDW

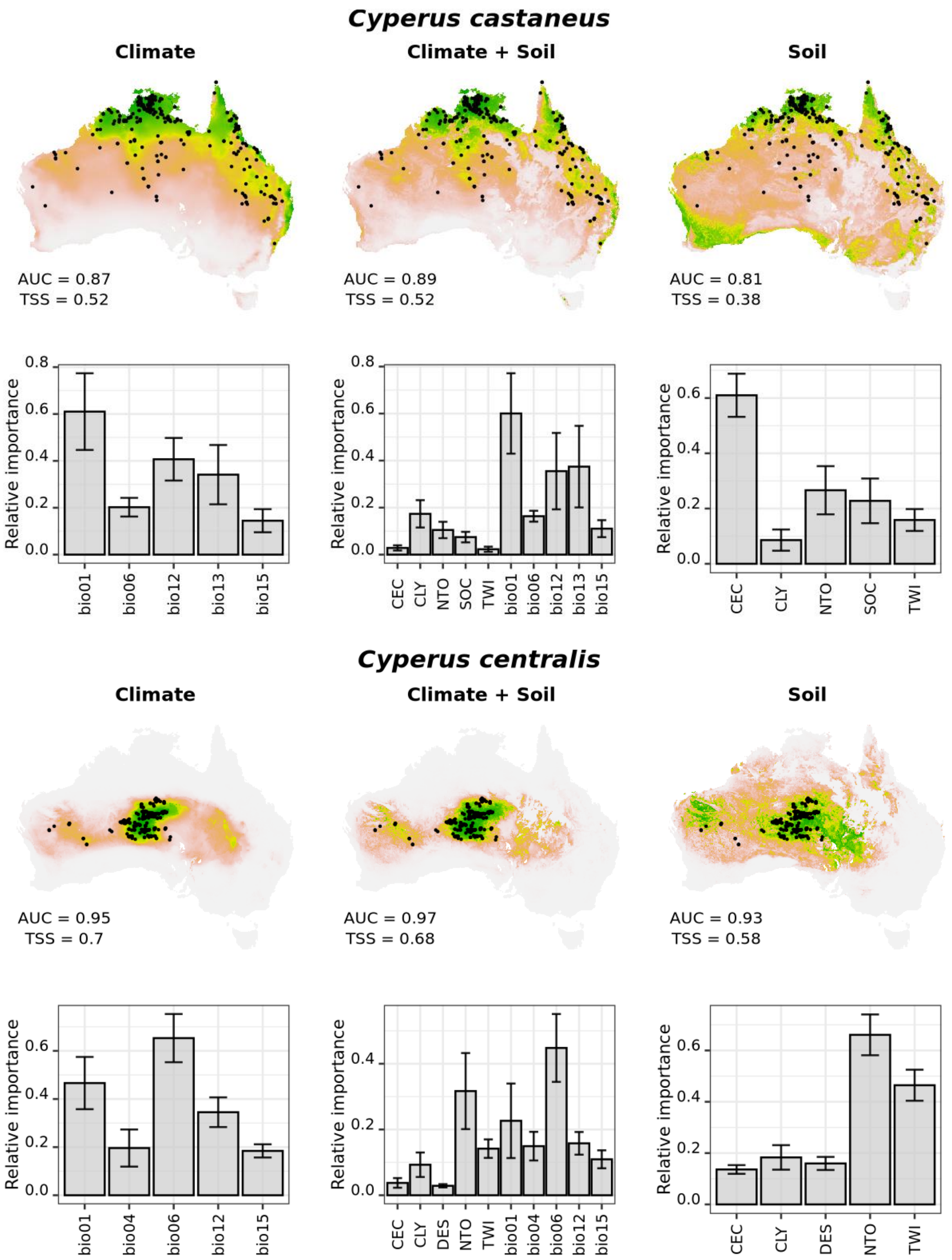
Cyperus castaneus
Climate
Climate + Soil
Soil
AUC = 0.87
TSS = 0.52
AUC = 0.89
TSS = 0.52
AUC = 0.81
TSS = 0.38
Relative importance
bio01 bio06 bio12 bio13 bio15
CEC CLY NTO SOC TWI bio01 bio06 bio12 bio13 bio15
CEC CLY NTO SOC TWI
Cyperus centralis
Climate
Climate + Soil
Soil
AUC = 0.95
TSS = 0.7
AUC = 0.97
TSS = 0.68
AUC = 0.93
TSS = 0.58
Relative importance
bio01 bio04 bio06 bio12 bio15
CEC CLY DES NTO TWI bio01 bio04 bio06 bio12 bio15
CEC CLY DES NTO TWI

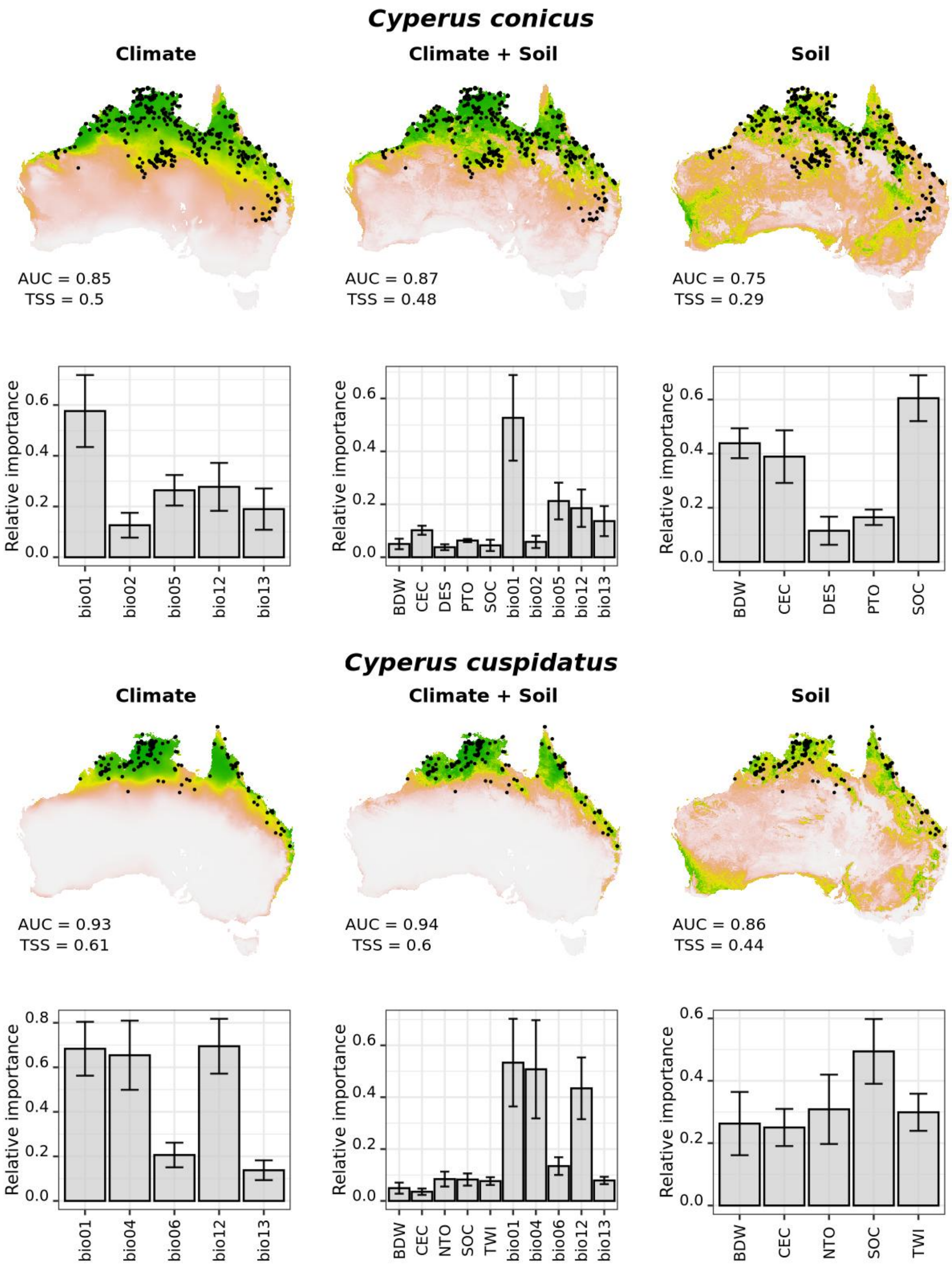

Cyperus conicus
Climate
Climate + Soil
Soil
AUC = 0.85
TSS = 0.5
AUC = 0.87
TSS = 0.48
AUC = 0.75
TSS = 0.29
Relative importance
0.0
0.2
0.4
0.6
bio01
bio02
bio05
bio12
bio13
BDW
CEC
DES
PTO
SOC
Cyperus cuspidatus
Climate
Climate + Soil
Soil
AUC = 0.93
TSS = 0.61
AUC = 0.94
TSS = 0.6
AUC = 0.86
TSS = 0.44
0.8
bio04
bio06
NTO
TWI

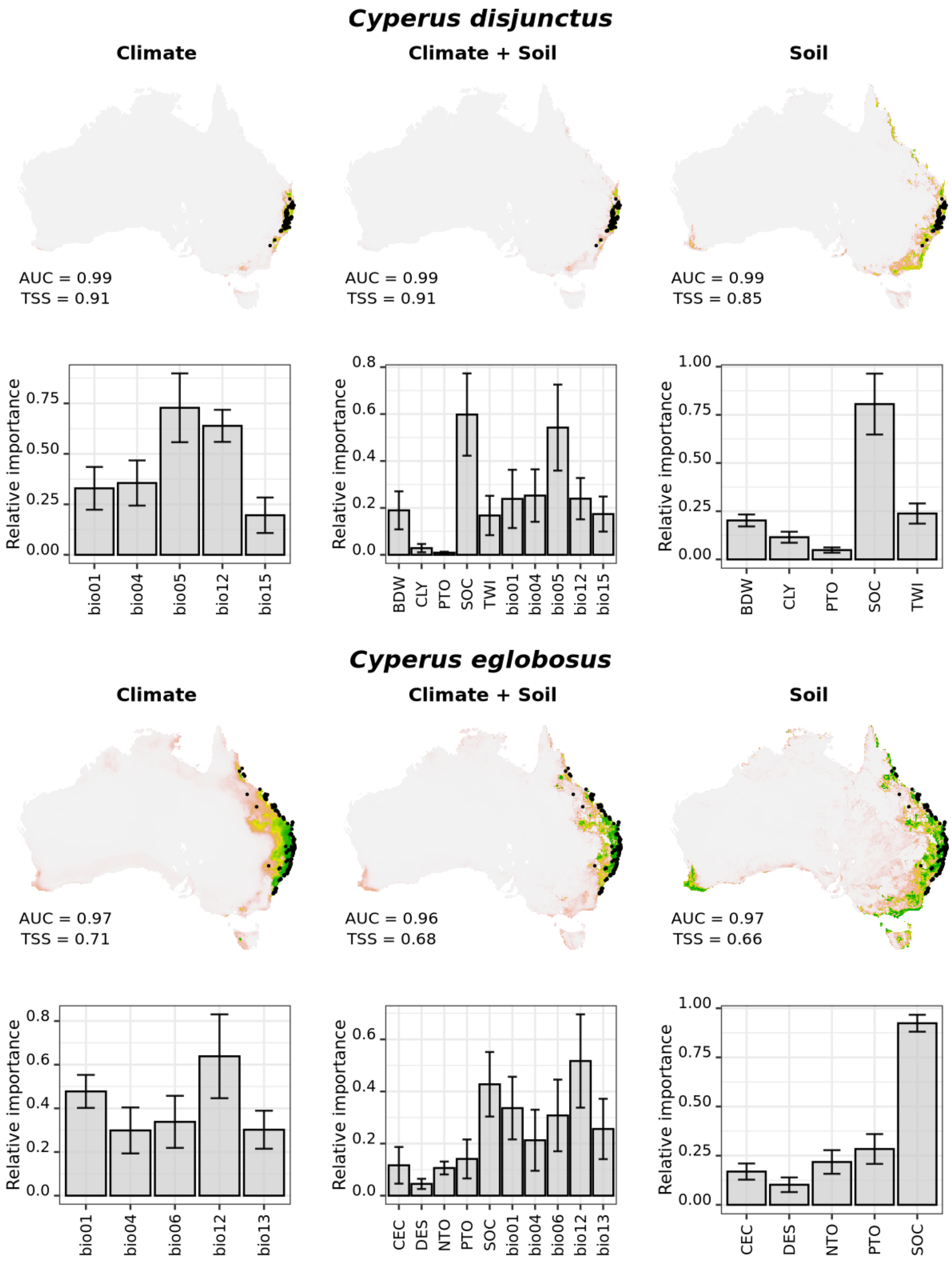
Cyperus disjunctus
Climate
Climate + Soil
Soil
AUC = 0.99
TSS = 0.91
AUC = 0.99
TSS = 0.91
AUC = 0.99
TSS = 0.85
Relative importance
0.00
0.25
0.50
0.75
bio01
bio04
bio05
bio12
bio15
0.0
0.2
0.4
0.6
0.8
BDW
CLY
PTO
SOC
TWI
bio01
bio04
bio05
bio12
bio15
1.00
BDW
CLY
PTO
SOC
TWI
Cyperus eglobosus
Climate
Climate + Soil
Soil
AUC = 0.97
TSS = 0.71
AUC = 0.96
TSS = 0.68
AUC = 0.97
TSS = 0.66
bio01
bio04
bio06
bio12
bio13
CEC
DES
NTO
PTO
SOC
bio01
bio04
bio06
bio12
bio13
CEC
DES
NTO
PTO
SOC

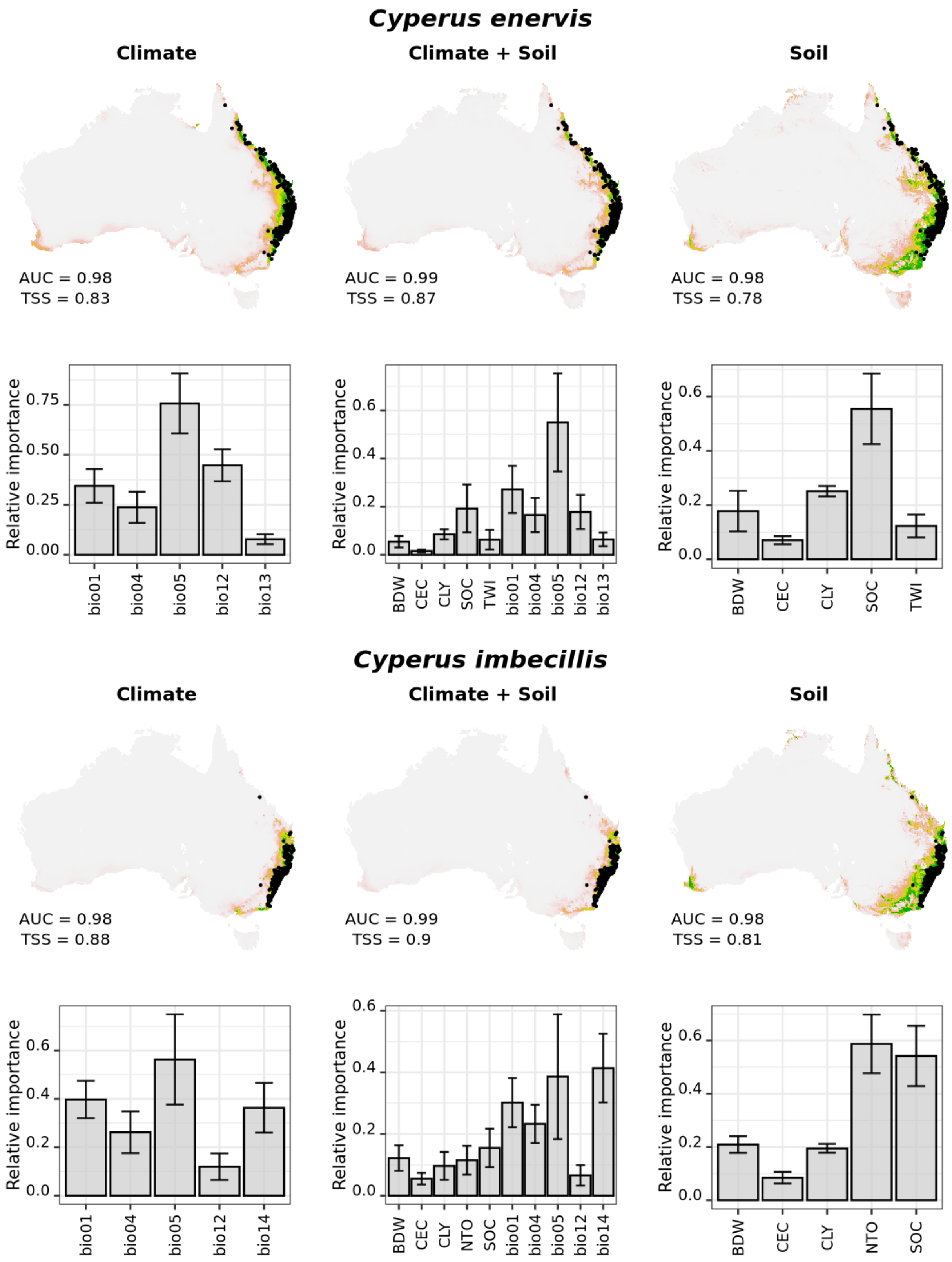
Cyperus enervis
Climate
Climate + Soil
Soil
AUC = 0.98
TSS = 0.83
AUC = 0.99
TSS = 0.87
AUC = 0.98
TSS = 0.78
Relative importance
bio01
bio04
bio05
bio12
bio13
BDW
CEC
CLY
SOC
TWI
Cyperus imbecillis
AUC = 0.98
TSS = 0.88
AUC = 0.99
TSS = 0.9
AUC = 0.98
TSS = 0.81
bio14
NTO

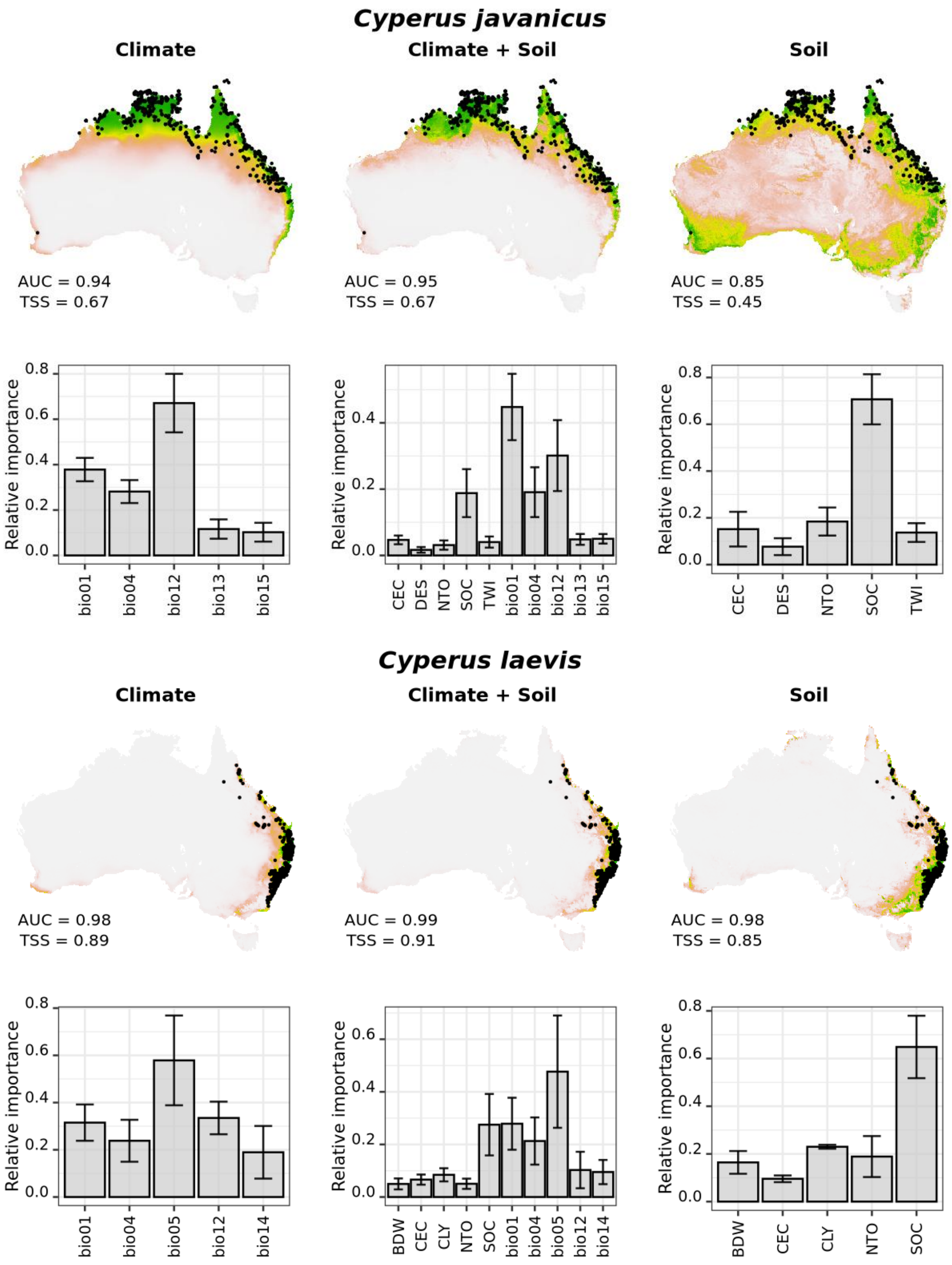
Cyperus javanicus
Climate
Climate + Soil
Soil
AUC = 0.94
TSS = 0.67
AUC = 0.95
TSS = 0.67
AUC = 0.85
TSS = 0.45
Relative importance
bio01
bio04
bio12
bio13
bio15
CEC
DES
NTO
SOC
TWI
Cyperus laevis
Climate
Climate + Soil
Soil
AUC = 0.98
TSS = 0.89
AUC = 0.99
TSS = 0.91
AUC = 0.98
TSS = 0.85
Relative importance
bio01
bio04
bio05
bio12
bio14
BDW
CEC
CLY
NTO
SOC

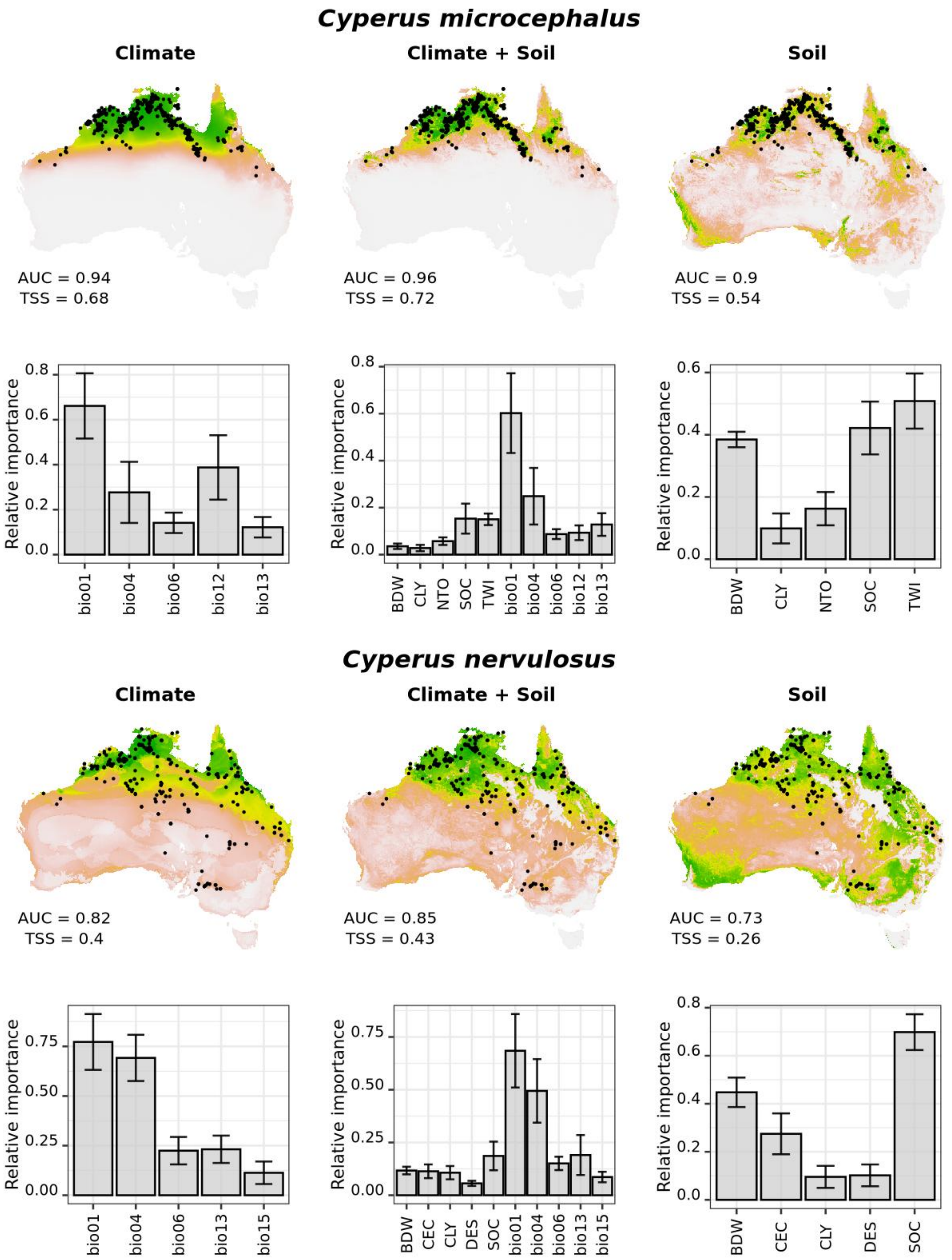

Cyperus microcephalus
Climate
Climate + Soil
Soil
AUC = 0.94
TSS = 0.68
AUC = 0.96
TSS = 0.72
AUC = 0.9
TSS = 0.54
Relative importance
bio01
bio04
bio06
bio12
bio13
BDW
CLY
NTO
SOC
TWI
Cyperus nervulosus
Climate
Climate + Soil
Soil
AUC = 0.82
TSS = 0.4
AUC = 0.85
TSS = 0.43
AUC = 0.73
TSS = 0.26
Relative importance
bio01
bio04
bio06
bio13
bio15
BDW
CEC
CLY
DES
SOC

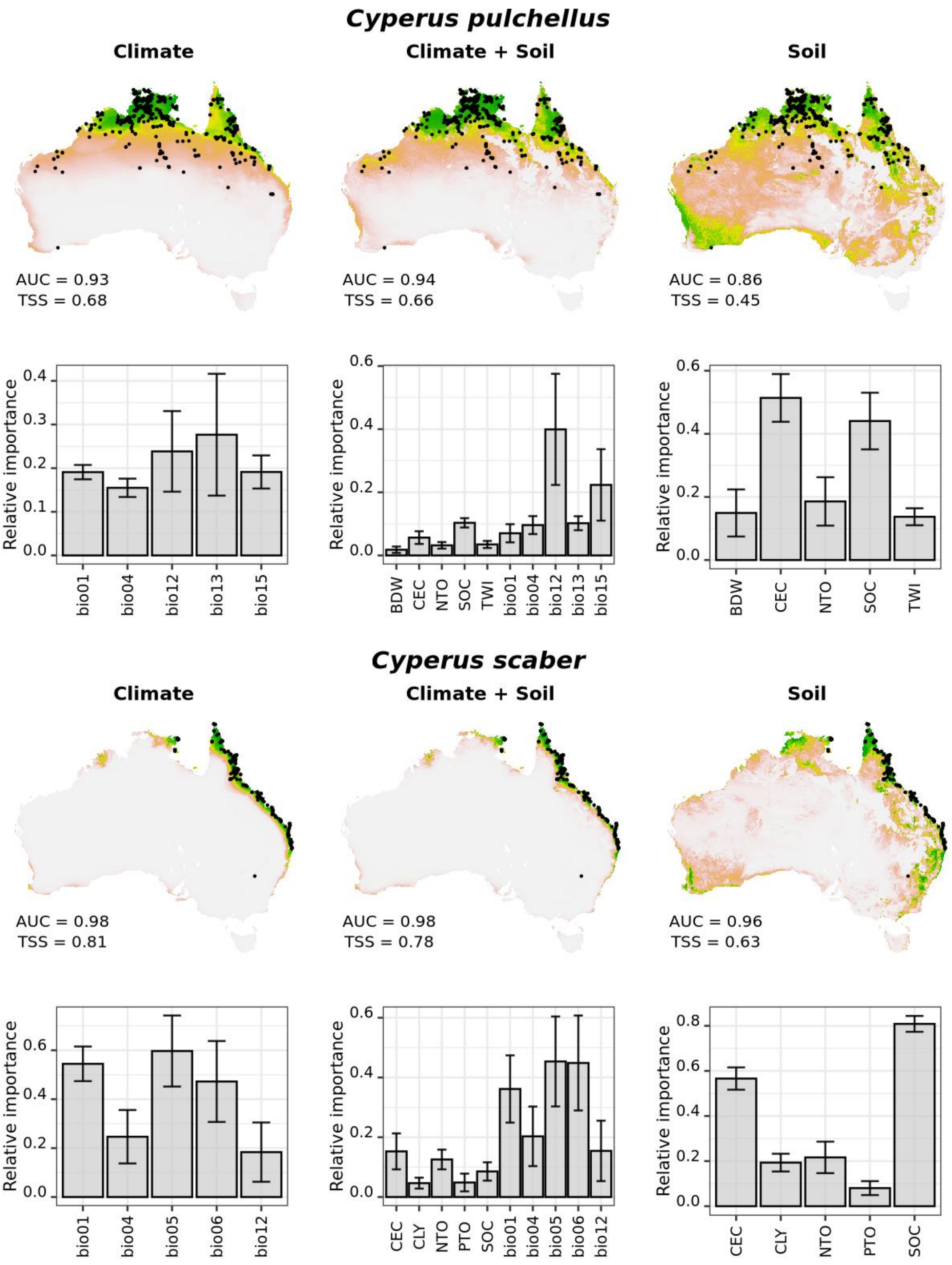
Cyperus pulchellus
Climate
Climate + Soil
Soil
AUC = 0.93
TSS = 0.68
AUC = 0.94
TSS = 0.66
AUC = 0.86
TSS = 0.45
Relative importance
bio01
bio04
bio12
bio13
bio15
BDW
CEC
NTO
SOC
TWI
Cyperus scaber
Climate
Climate + Soil
Soil
AUC = 0.98
TSS = 0.81
AUC = 0.98
TSS = 0.78
AUC = 0.96
TSS = 0.63
Relative importance
bio01
bio04
bio05
bio06
bio12
CEC
CLY
NTO
PTO
SOC

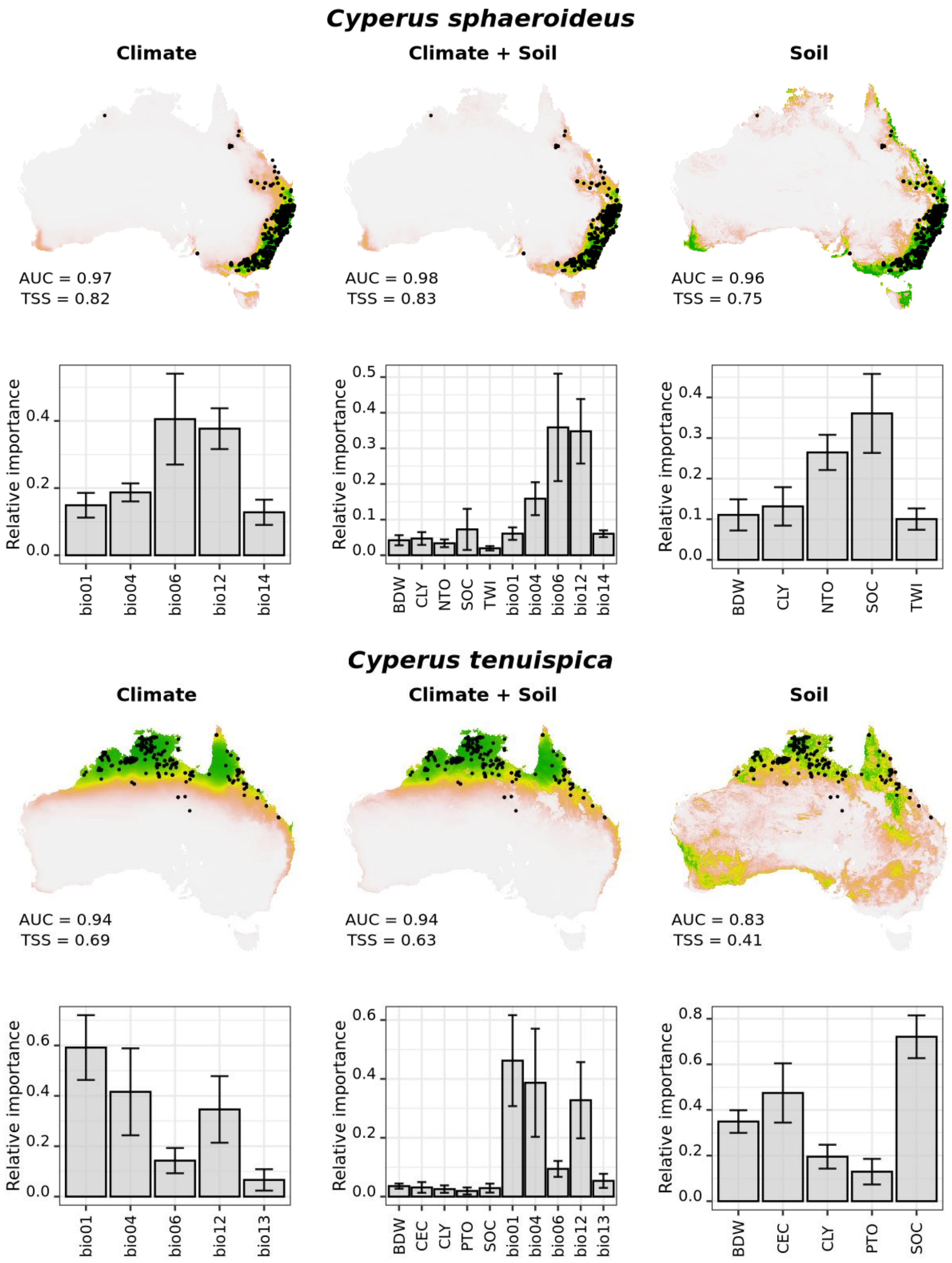

Cyperus sphaeroideus
Climate
Climate + Soil
Soil
AUC = 0.97
TSS = 0.82
AUC = 0.98
TSS = 0.83
AUC = 0.96
TSS = 0.75
Relative importance
bio01 bio04 bio06 bio12 bio14
BDW CLY NTO SOC TWI bio01 bio04 bio06 bio12 bio14
BDW CLY NTO SOC TWI
Cyperus tenuispica
Climate
Climate + Soil
Soil
AUC = 0.94
TSS = 0.69
AUC = 0.94
TSS = 0.63
AUC = 0.83
TSS = 0.41
Relative importance
bio01 bio04 bio06 bio12 bio13
BDW CEC CLY PTO SOC bio01 bio04 bio06 bio12 bio13
BDW CEC CLY PTO SOC

Cyperus tetraphyllus

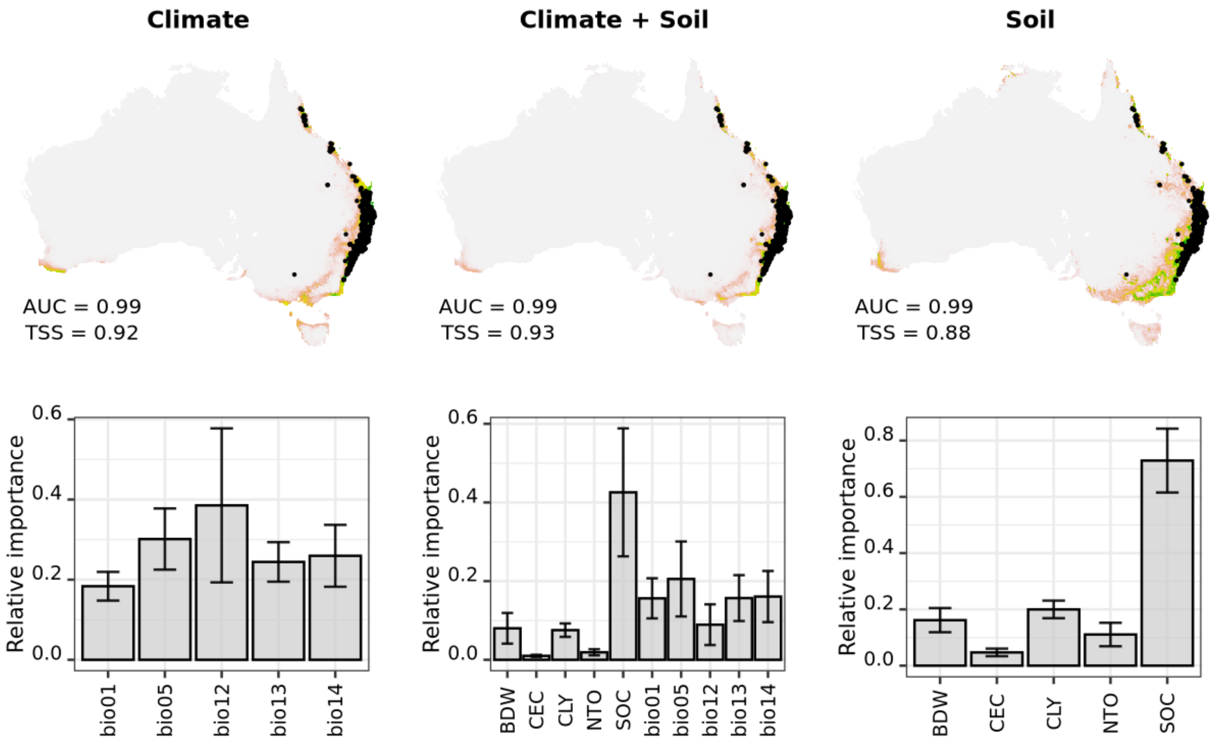

Cyperus trinervis

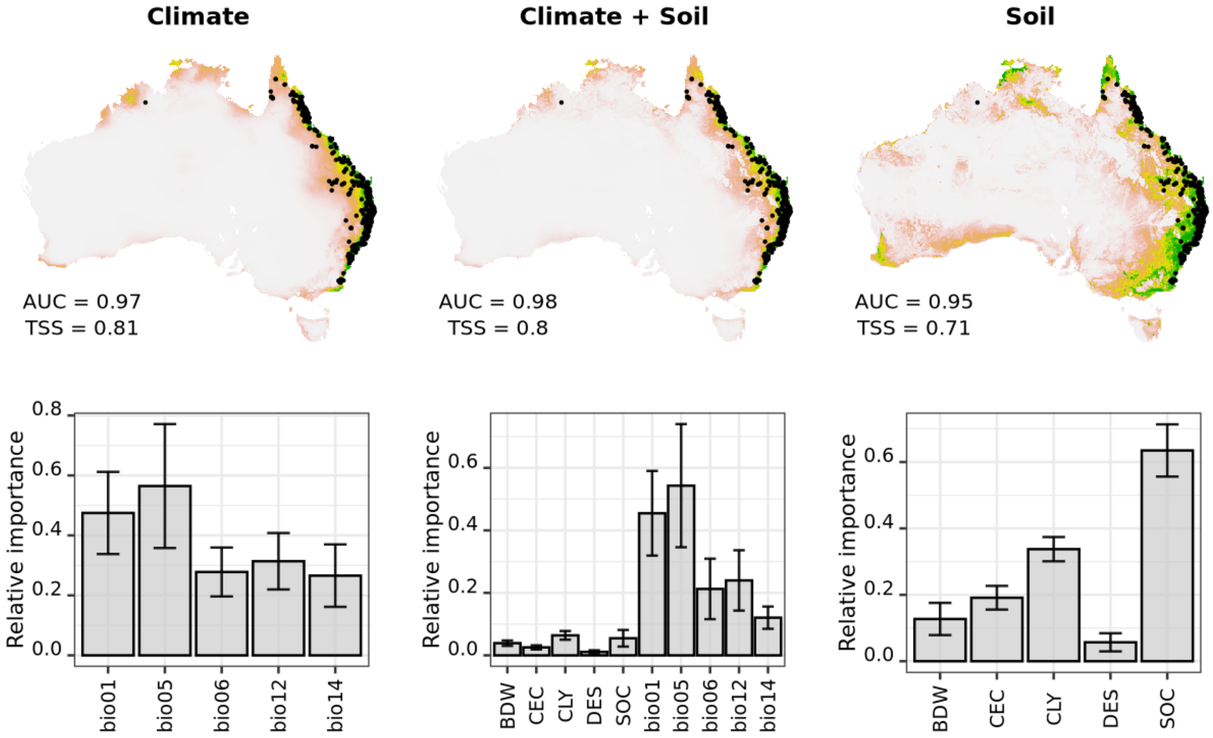

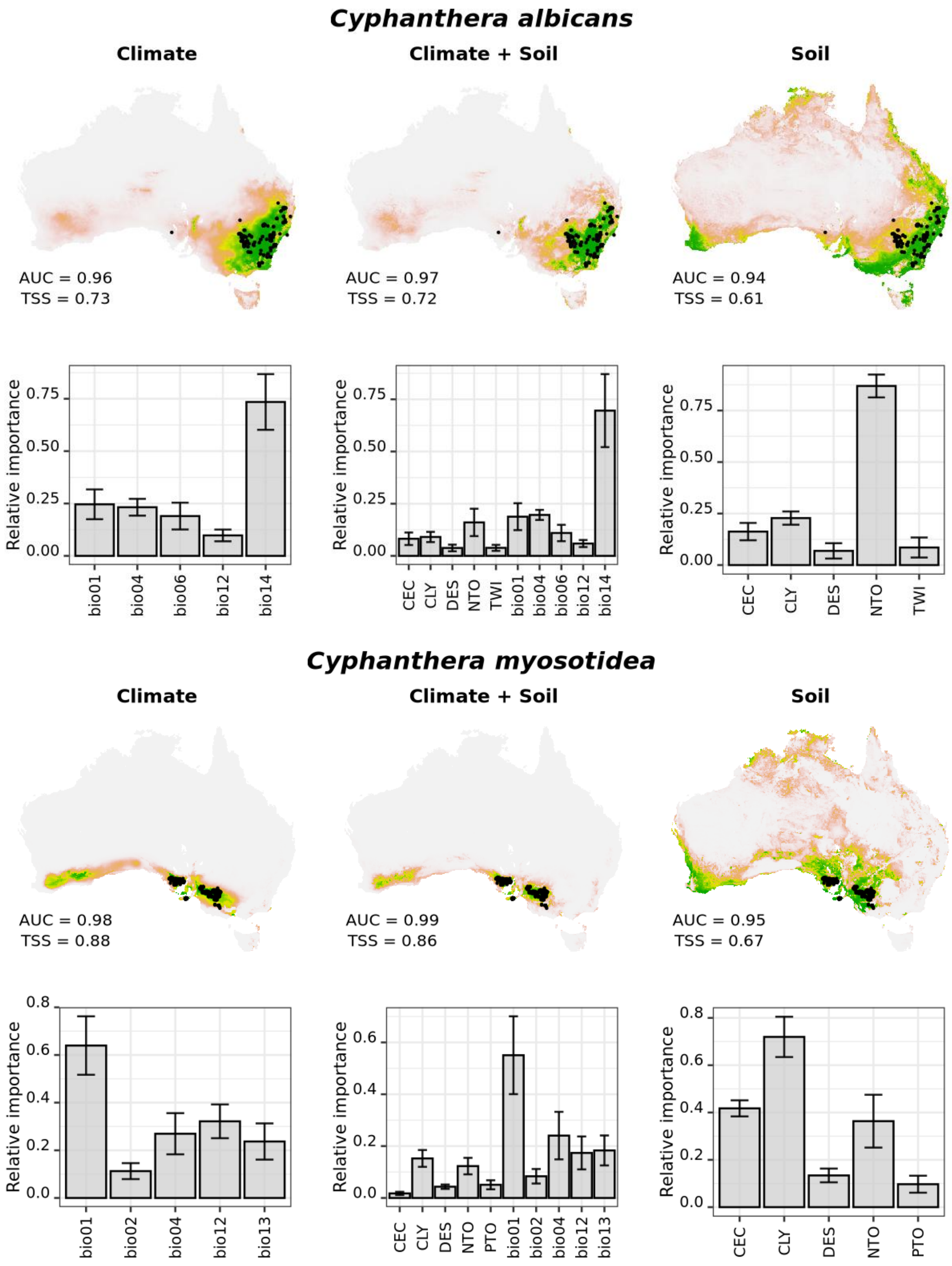

Cyphanthera albicans
Climate
Climate + Soil
Soil
AUC = 0.96
TSS = 0.73
AUC = 0.97
TSS = 0.72
AUC = 0.94
TSS = 0.61
Relative importance
0.00
0.25
0.50
0.75
bio01
bio04
bio06
bio12
bio14
CEC
CLY
DES
NTO
TWI
Cyphanthera myosotidea
Climate
Climate + Soil
Soil
AUC = 0.98
TSS = 0.88
AUC = 0.99
TSS = 0.86
AUC = 0.95
TSS = 0.67
Relative importance
0.0
0.2
0.4
0.6
0.8
bio01
bio02
bio04
bio12
bio13
CEC
CLY
DES
NTO
PTO

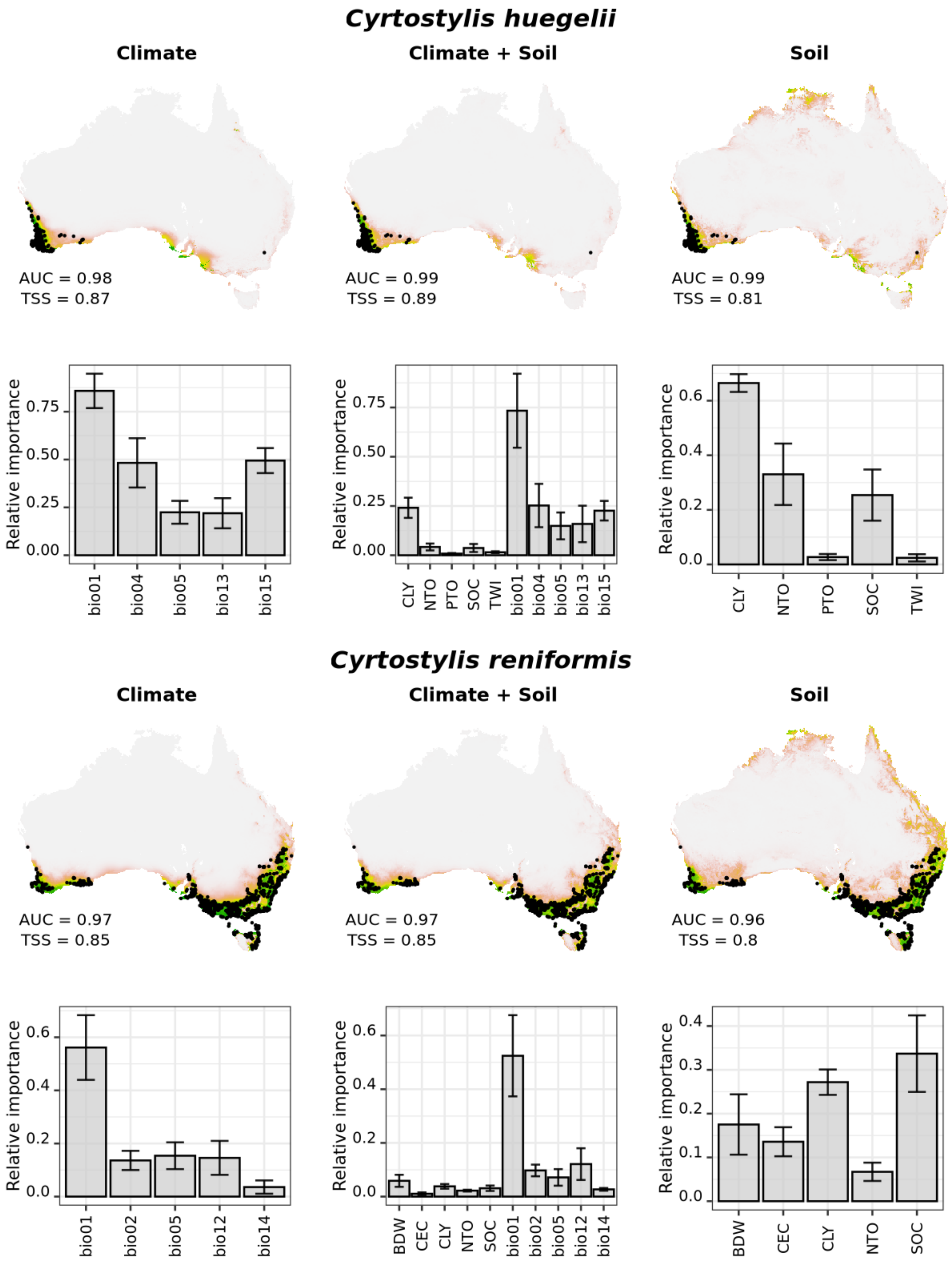

Cyrtostylis huegelii
Climate
Climate + Soil
Soil
AUC = 0.98
TSS = 0.87
AUC = 0.99
TSS = 0.89
AUC = 0.99
TSS = 0.81
Relative importance
bio01
bio04
bio05
bio13
bio15
CLY
NTO
PTO
SOC
TWI
Cyrtostylis reniformis
Climate
Climate + Soil
Soil
AUC = 0.97
TSS = 0.85
AUC = 0.97
TSS = 0.85
AUC = 0.96
TSS = 0.8
bio01
bio02
bio05
bio12
bio14
BDW
CEC
CLY
NTO
SOC

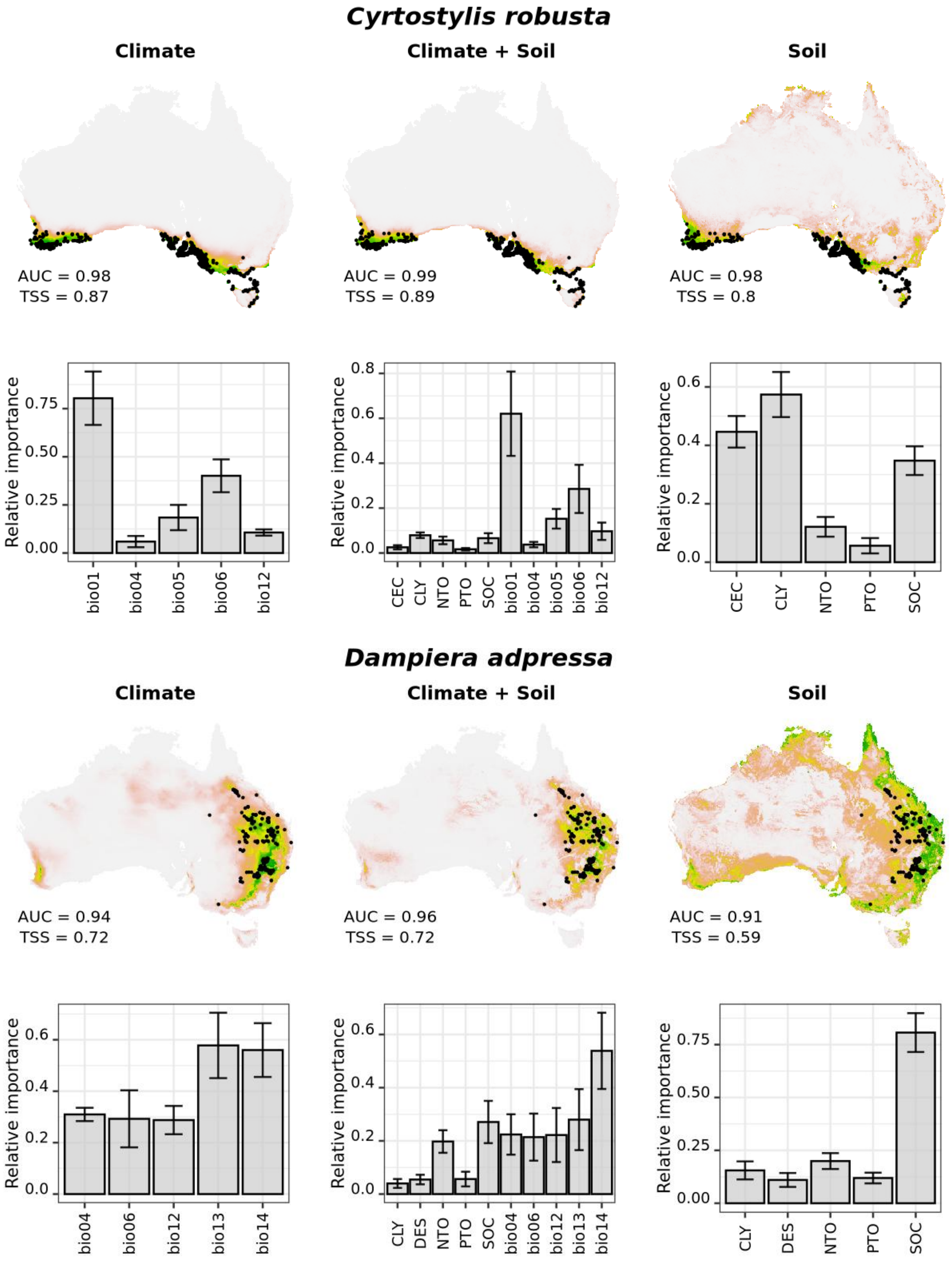
Cyrtostylis robusta
Climate
Climate + Soil
Soil
AUC = 0.98
TSS = 0.87
AUC = 0.99
TSS = 0.89
AUC = 0.98
TSS = 0.8
Relative importance
bio01
bio04
bio05
bio06
bio12
CEC
CLY
NTO
PTO
SOC
Dampiera adpressa
Climate
Climate + Soil
Soil
AUC = 0.94
TSS = 0.72
AUC = 0.96
TSS = 0.72
AUC = 0.91
TSS = 0.59
Relative importance
bio04
bio06
bio12
bio13
bio14
CLY
DES
NTO
PTO
SOC

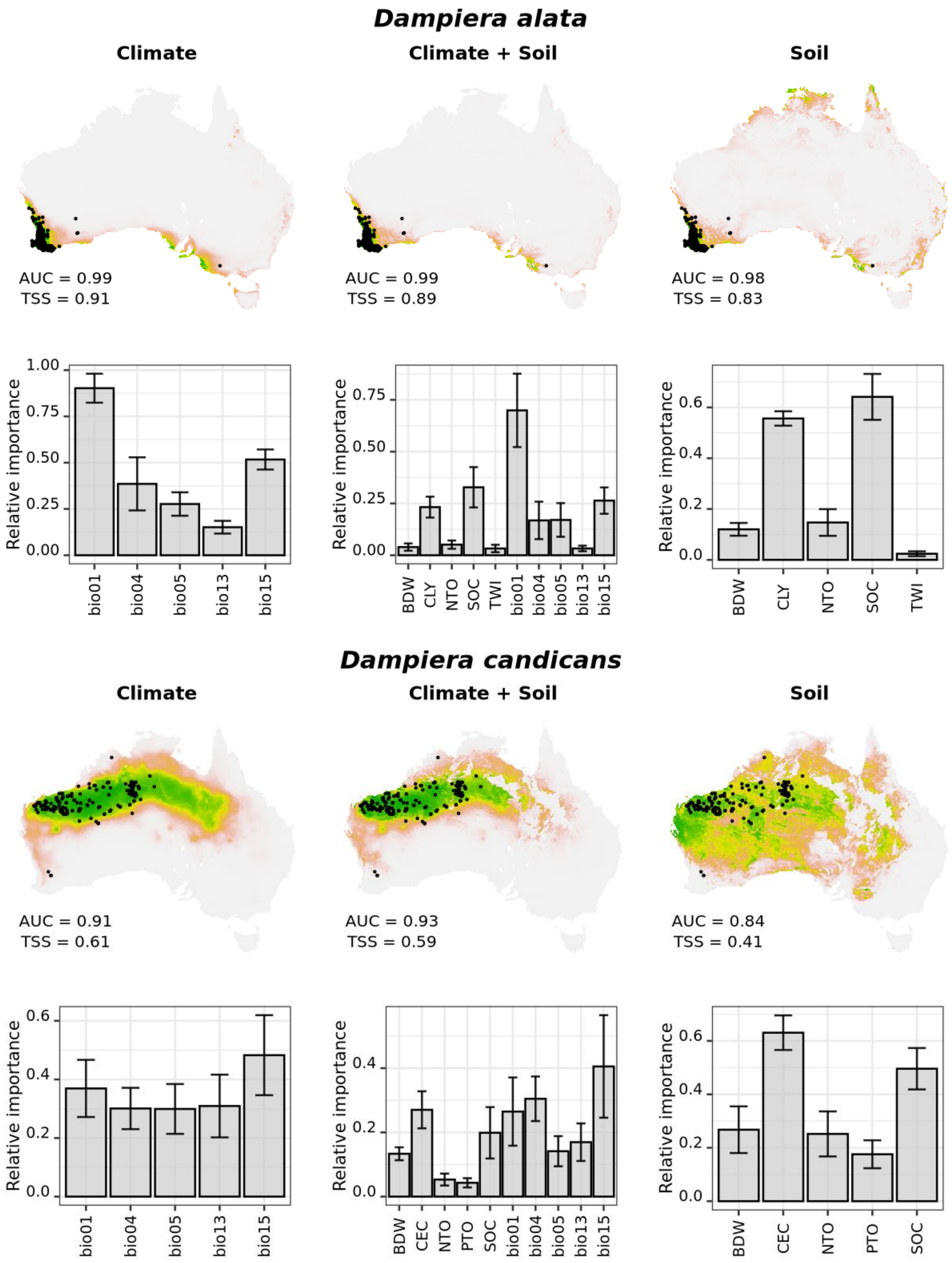

Dampiera alata
Climate
Climate + Soil
Soil
AUC = 0.99
TSS = 0.91
AUC = 0.99
TSS = 0.89
AUC = 0.98
TSS = 0.83
Relative importance
bio01
bio04
bio05
bio13
bio15
BDW
CLY
NTO
SOC
TWI
Dampiera candicans
Climate
Climate + Soil
Soil
AUC = 0.91
TSS = 0.61
AUC = 0.93
TSS = 0.59
AUC = 0.84
TSS = 0.41
CEC
PTO

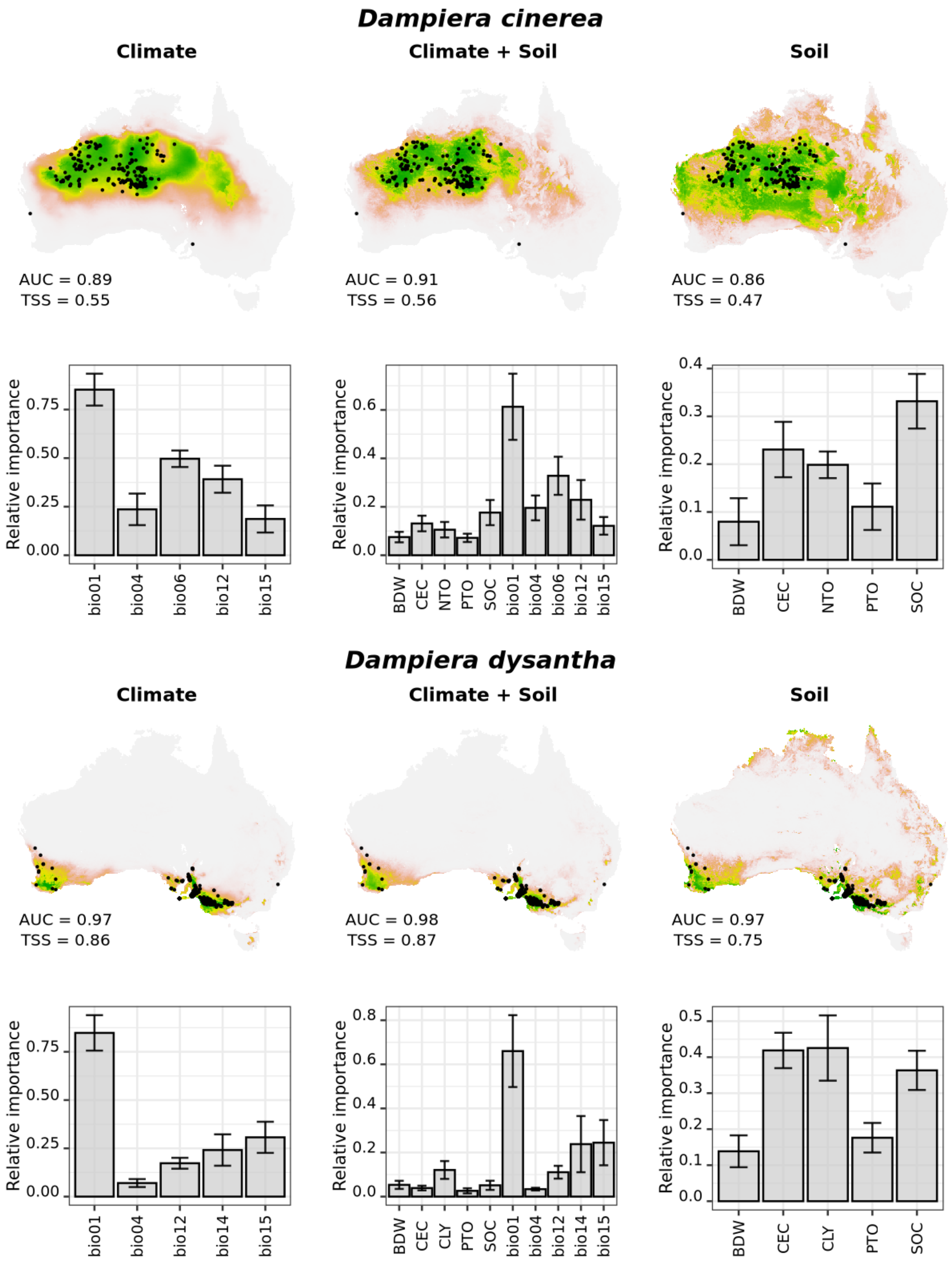
Dampiera cinerea
Climate
Climate + Soil
Soil
AUC = 0.89
TSS = 0.55
AUC = 0.91
TSS = 0.56
AUC = 0.86
TSS = 0.47
Relative importance
bio01
bio04
bio06
bio12
bio15
BDW
CEC
NTO
PTO
SOC
Dampiera dysantha
Climate
Climate + Soil
Soil
AUC = 0.97
TSS = 0.86
AUC = 0.98
TSS = 0.87
AUC = 0.97
TSS = 0.75
Relative importance
bio01
bio04
bio12
bio14
bio15
BDW
CEC
CLY
PTO
SOC

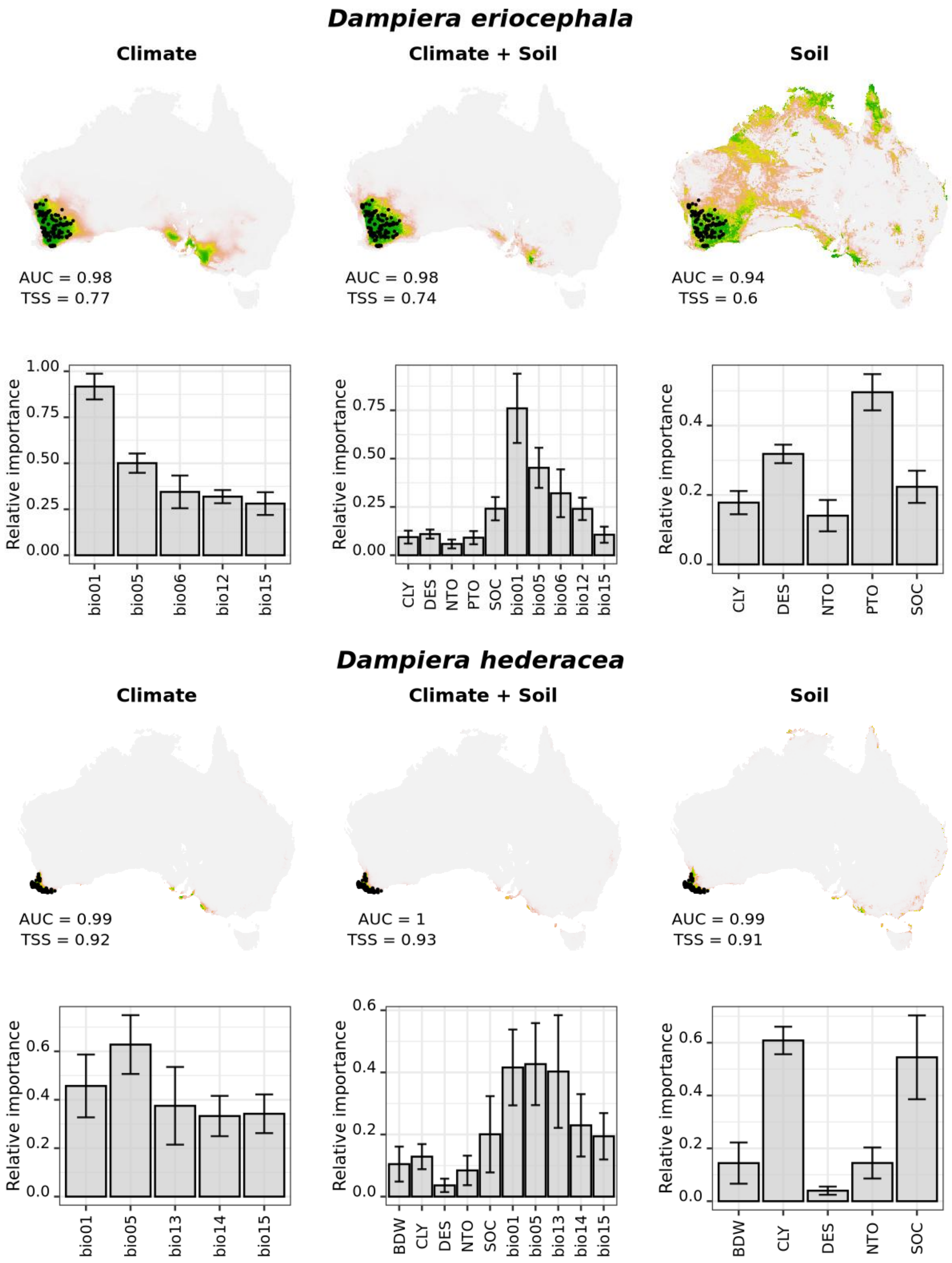
Dampiera eriocephala
Climate
Climate + Soil
Soil
AUC = 0.98
TSS = 0.77
AUC = 0.98
TSS = 0.74
AUC = 0.94
TSS = 0.6
Relative importance
bio01
bio05
bio06
bio12
bio15
CLY
DES
NTO
PTO
SOC
Dampiera hederacea
Climate
Climate + Soil
Soil
AUC = 0.99
TSS = 0.92
AUC = 1
TSS = 0.93
AUC = 0.99
TSS = 0.91
Relative importance
bio01
bio05
bio13
bio14
bio15
BDW
CLY
DES
NTO
SOC

Dampiera incana

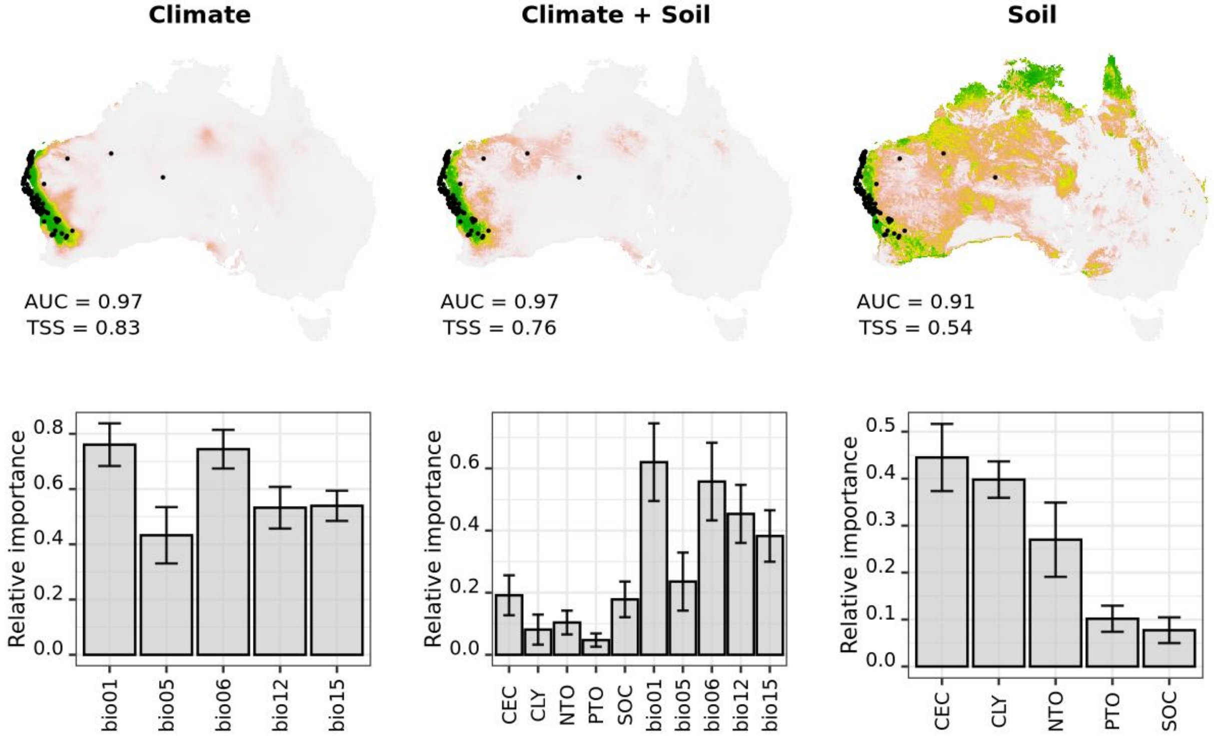

Dampiera juncea

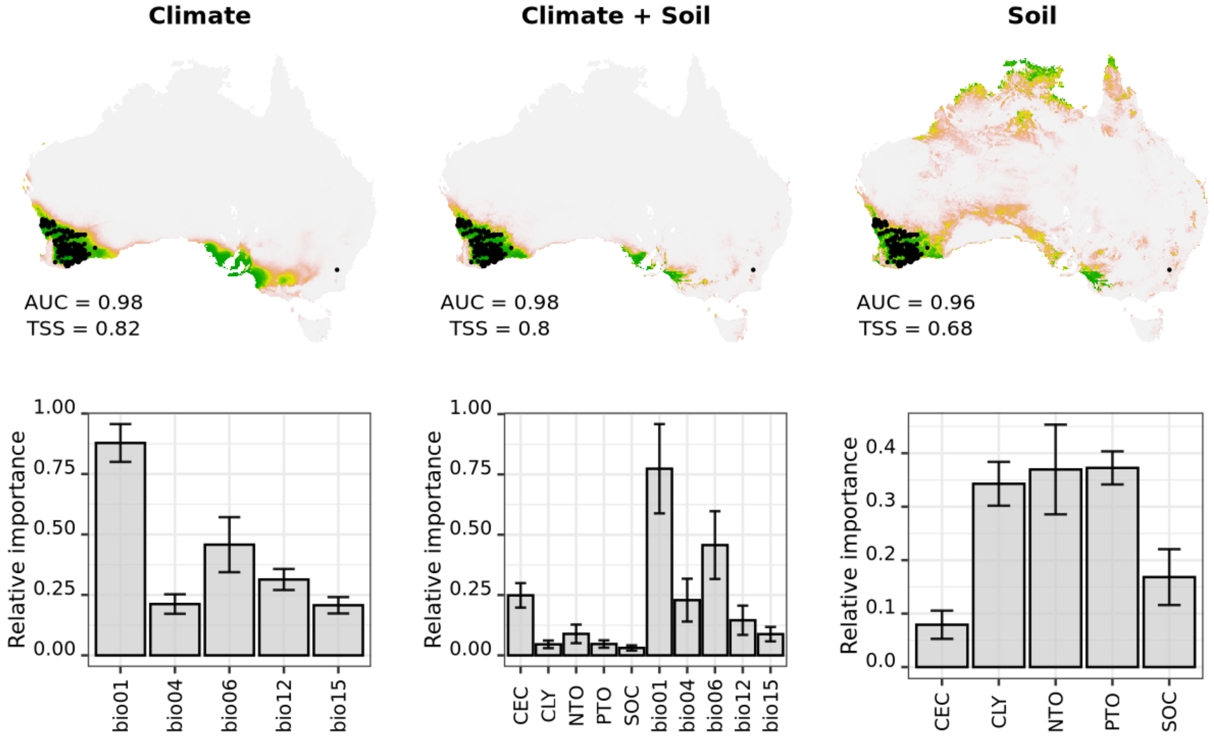

Dampiera lavandulacea

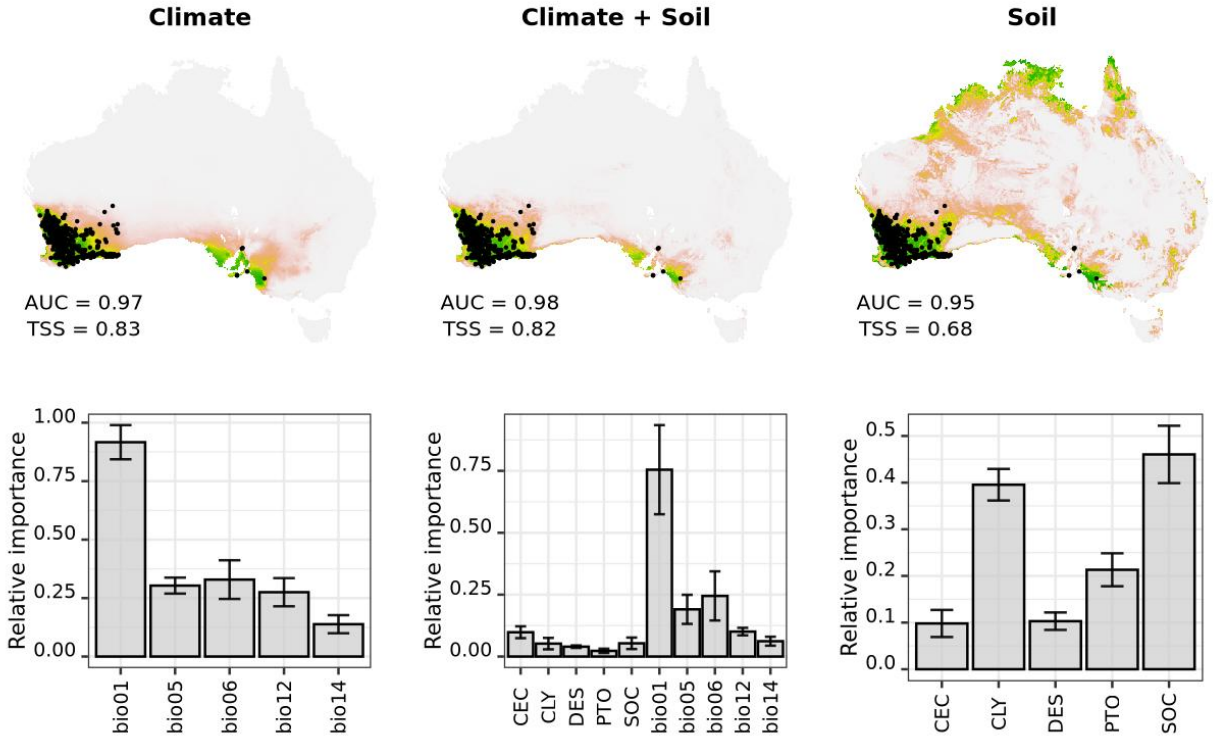

Dampiera lindleyi

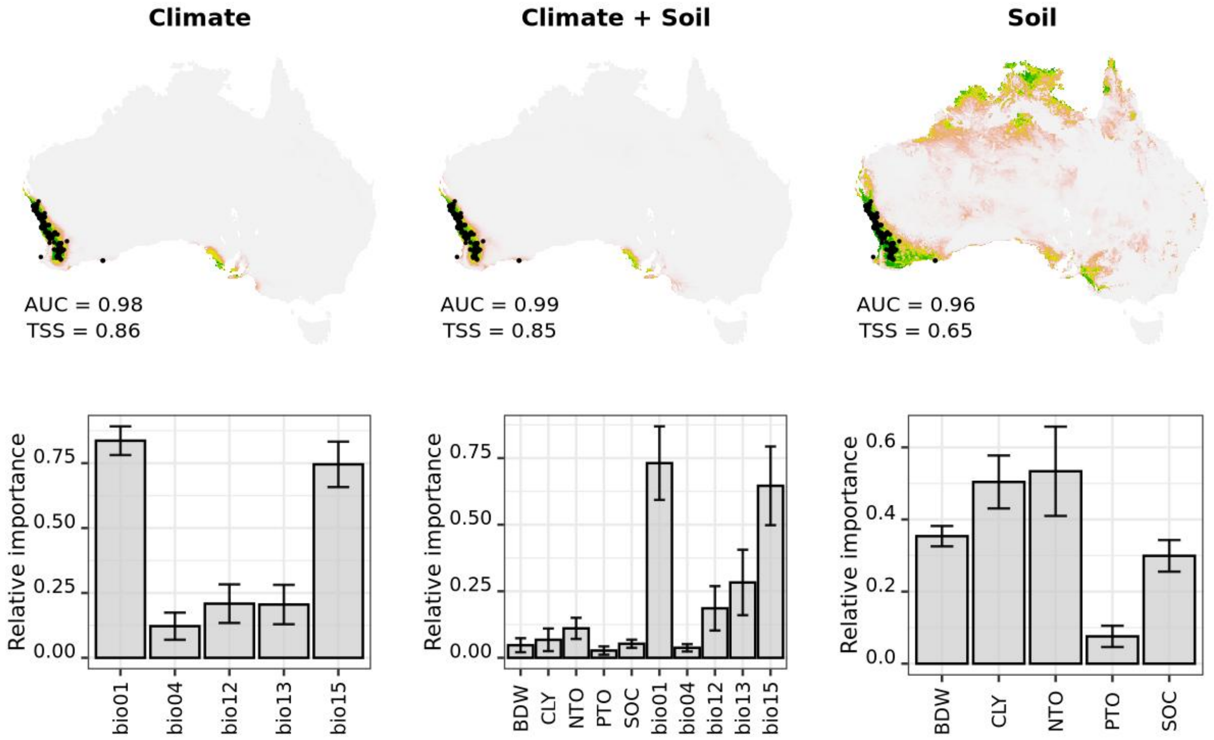

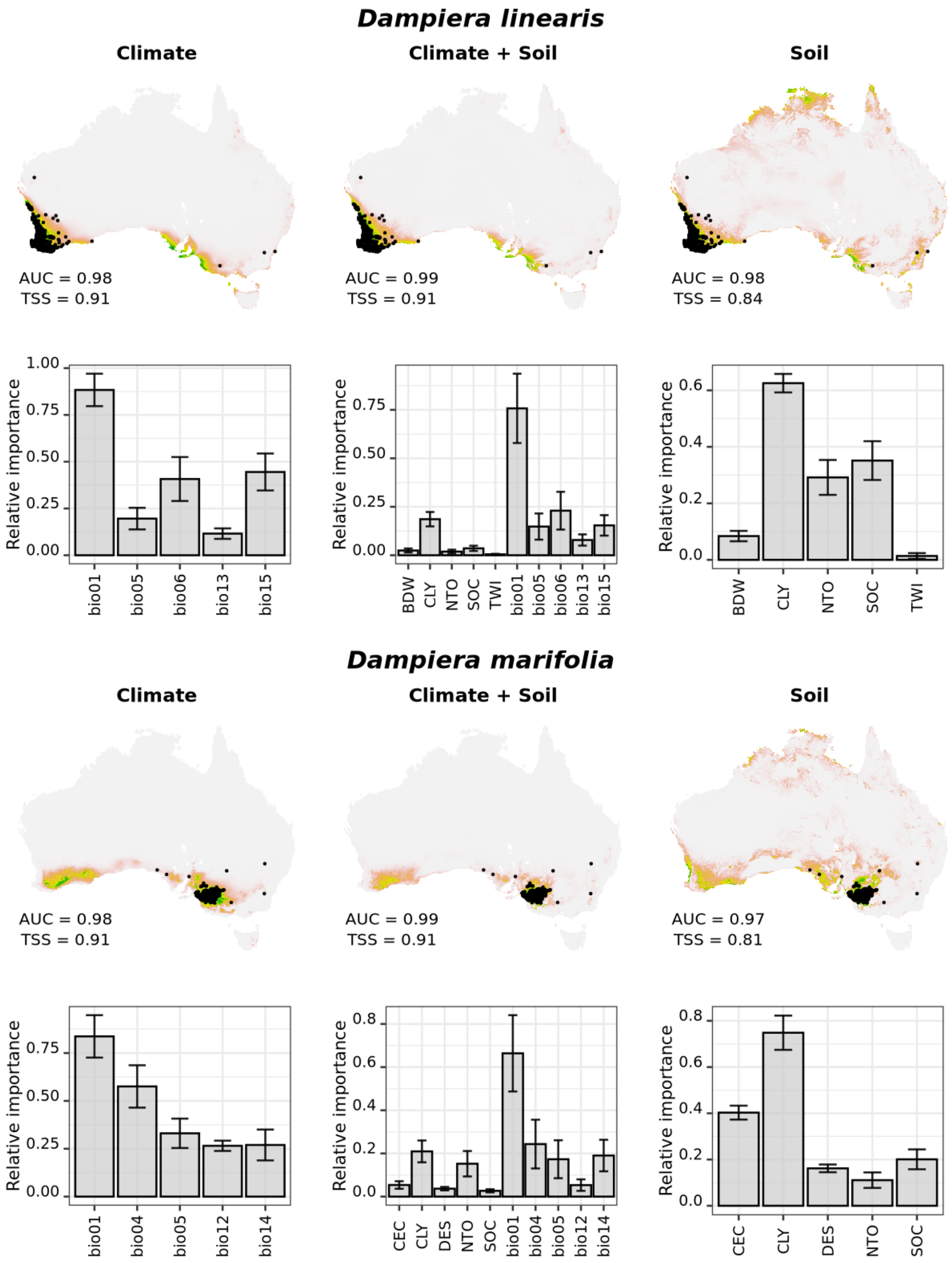

Dampiera linearis
Climate
Climate + Soil
Soil
AUC = 0.98
TSS = 0.91
AUC = 0.99
TSS = 0.91
AUC = 0.98
TSS = 0.84
Relative importance
1.00
0.75
0.50
0.25
0.00
bio01
bio05
bio06
bio13
bio15
Relative importance
0.75
0.50
0.25
0.00
BDW
CLY
NTO
SOC
TWI
bio01
bio05
bio06
bio13
bio15
Relative importance
0.6
0.4
0.2
0.0
BDW
CLY
NTO
SOC
TWI
Dampiera marifolia
Climate
Climate + Soil
Soil
AUC = 0.98
TSS = 0.91
AUC = 0.99
TSS = 0.91
AUC = 0.97
TSS = 0.81
Relative importance
0.75
0.50
0.25
0.00
bio01
bio04
bio05
bio12
bio14
Relative importance
0.8
0.6
0.4
0.2
0.0
CEC
CLY
DES
NTO
SOC
bio01
bio04
bio05
bio12
bio14
Relative importance
0.8
0.6
0.4
0.2
0.0
CEC
CLY
DES
NTO
SOC

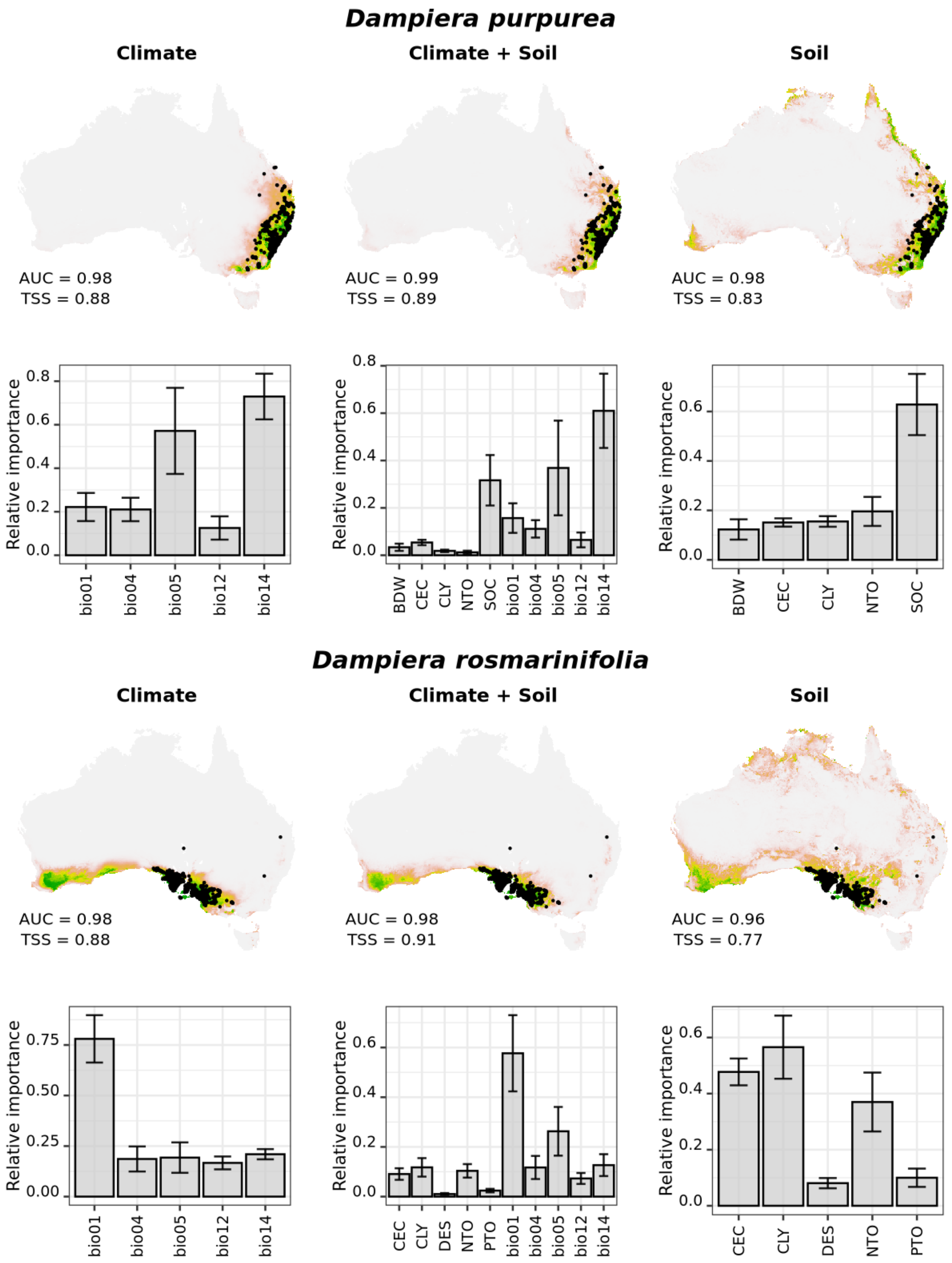

Dampiera purpurea
Climate
Climate + Soil
Soil
AUC = 0.98
TSS = 0.88
AUC = 0.99
TSS = 0.89
AUC = 0.98
TSS = 0.83
Relative importance
bio01
bio04
bio05
bio12
bio14
BDW
CEC
CLY
NTO
SOC
Dampiera rosmarinifolia
Climate
Climate + Soil
Soil
AUC = 0.98
TSS = 0.88
AUC = 0.98
TSS = 0.91
AUC = 0.96
TSS = 0.77
Relative importance
DES
PTO

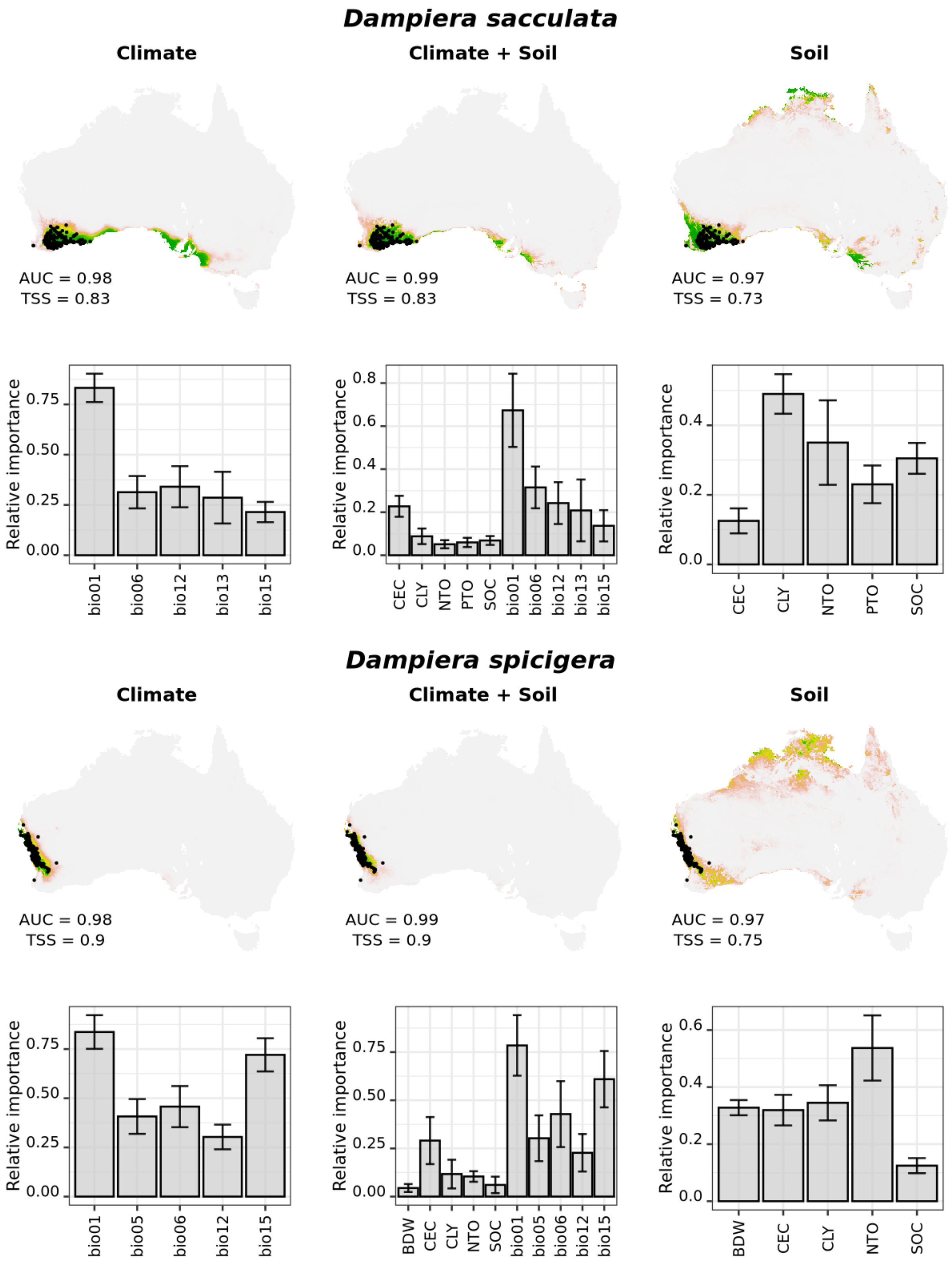
Dampiera sacculata
Climate
Climate + Soil
Soil
AUC = 0.98
TSS = 0.83
AUC = 0.99
TSS = 0.83
AUC = 0.97
TSS = 0.73
Relative importance
bio01
bio06
bio12
bio13
bio15
CEC
CLY
NTO
PTO
SOC
Dampiera spicigera
Climate
Climate + Soil
Soil
AUC = 0.98
TSS = 0.9
AUC = 0.99
TSS = 0.9
AUC = 0.97
TSS = 0.75
Relative importance
bio01
bio05
bio06
bio12
bio15
BDW
CEC
CLY
NTO
SOC

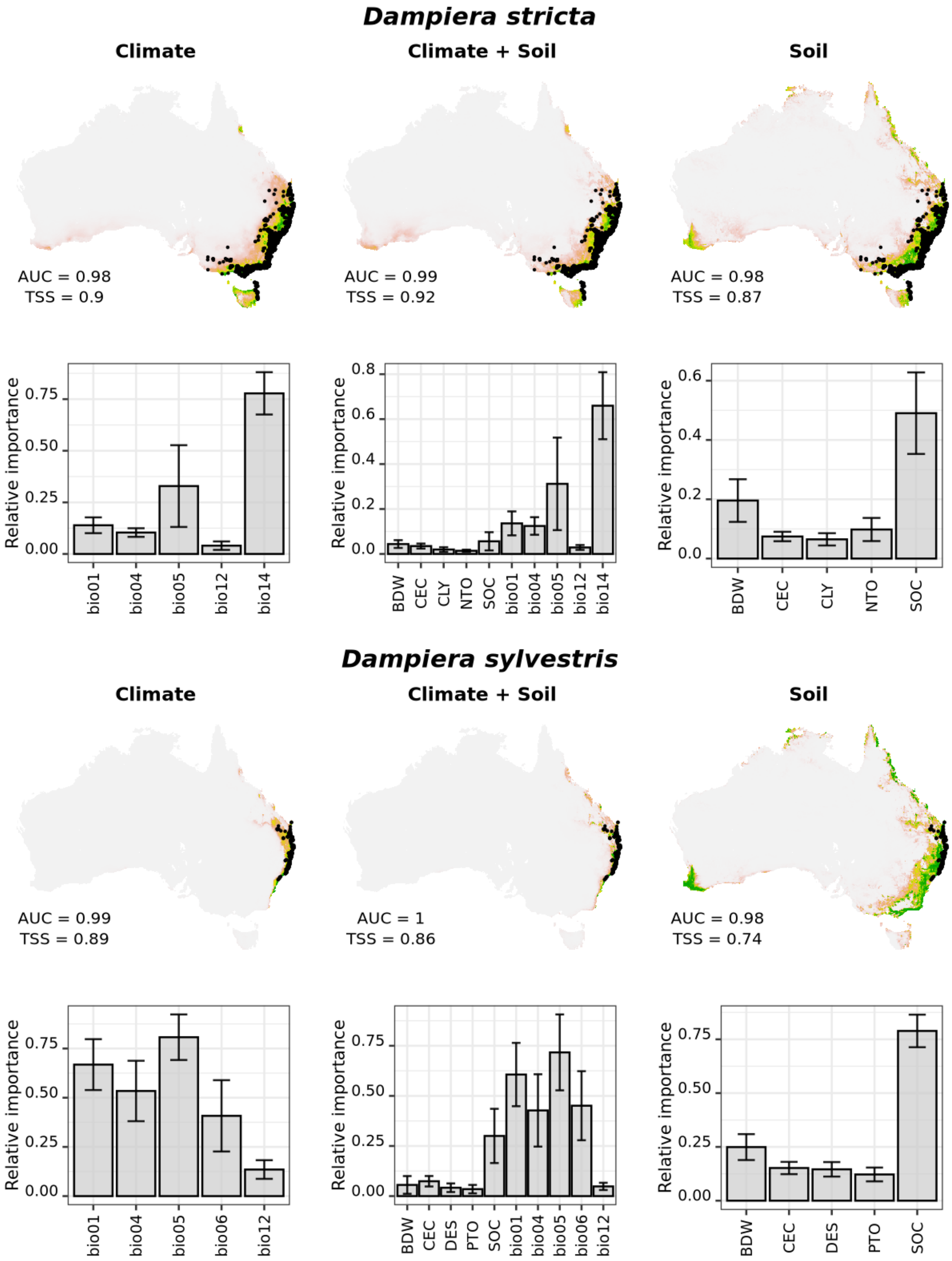

Dampiera stricta
Climate
Climate + Soil
Soil
AUC = 0.98
TSS = 0.9
AUC = 0.99
TSS = 0.92
AUC = 0.98
TSS = 0.87
Relative importance
0.00
0.25
0.50
0.75
bio01
bio04
bio05
bio12
bio14
Relative importance
0.0
0.2
0.4
0.6
0.8
BDW
CEC
CLY
NTO
SOC
bio01
bio04
bio05
bio12
bio14
Relative importance
0.0
0.2
0.4
0.6
BDW
CEC
CLY
NTO
SOC
Dampiera sylvestris
Climate
Climate + Soil
Soil
AUC = 0.99
TSS = 0.89
AUC = 1
TSS = 0.86
AUC = 0.98
TSS = 0.74
Relative importance
0.00
0.25
0.50
0.75
bio01
bio04
bio05
bio06
bio12
Relative importance
0.00
0.25
0.50
0.75
BDW
CEC
DES
PTO
SOC
bio01
bio04
bio05
bio06
bio12
Relative importance
0.00
0.25
0.50
0.75
BDW
CEC
DES
PTO
SOC

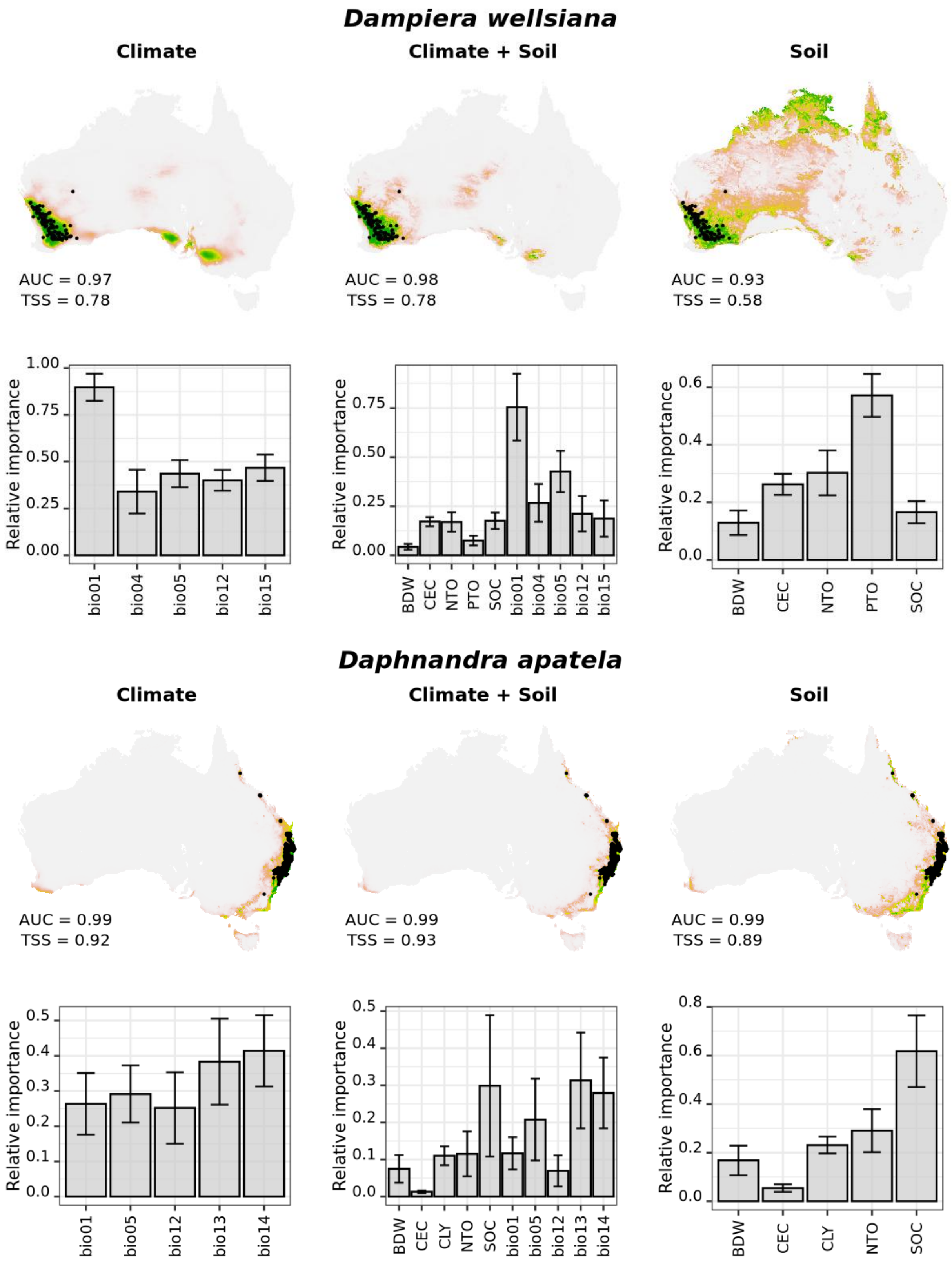

Dampiera wellsiana
Climate
Climate + Soil
Soil
AUC = 0.97
TSS = 0.78
AUC = 0.98
TSS = 0.78
AUC = 0.93
TSS = 0.58
Relative importance
1.00
0.75
0.50
0.25
0.00
bio01
bio04
bio05
bio12
bio15
BDW
CEC
NTO
PTO
SOC
bio01
bio04
bio05
bio12
bio15
0.6
0.4
0.2
0.0
BDW
CEC
NTO
PTO
SOC
Daphnandra apatela
Climate
Climate + Soil
Soil
AUC = 0.99
TSS = 0.92
AUC = 0.99
TSS = 0.93
AUC = 0.99
TSS = 0.89
Relative importance
0.5
0.4
0.3
0.2
0.1
0.0
bio01
bio05
bio12
bio13
bio14
BDW
CEC
CLY
NTO
SOC
bio01
bio05
bio12
bio13
bio14
0.8
0.6
0.4
0.2
0.0
BDW
CEC
CLY
NTO
SOC

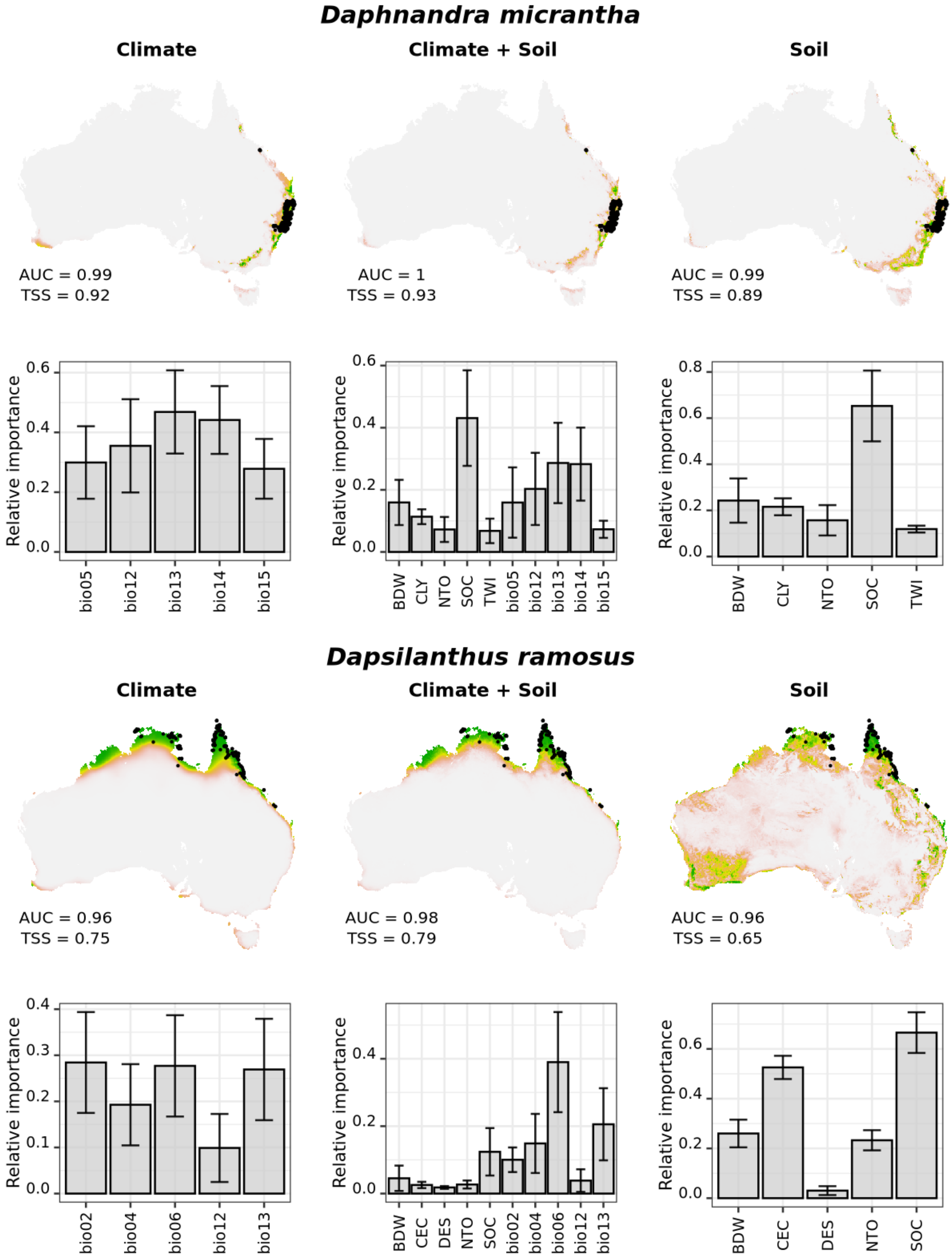
Daphnandra micrantha
Climate
Climate + Soil
Soil
AUC = 0.99
TSS = 0.92
AUC = 1
TSS = 0.93
AUC = 0.99
TSS = 0.89
Relative importance
bio05
bio12
bio13
bio14
bio15
BDW
CLY
NTO
SOC
TWI
Dapsilanthus ramosus
AUC = 0.96
TSS = 0.75
AUC = 0.98
TSS = 0.79
AUC = 0.96
TSS = 0.65
bio02
bio04
bio06
CEC
DES

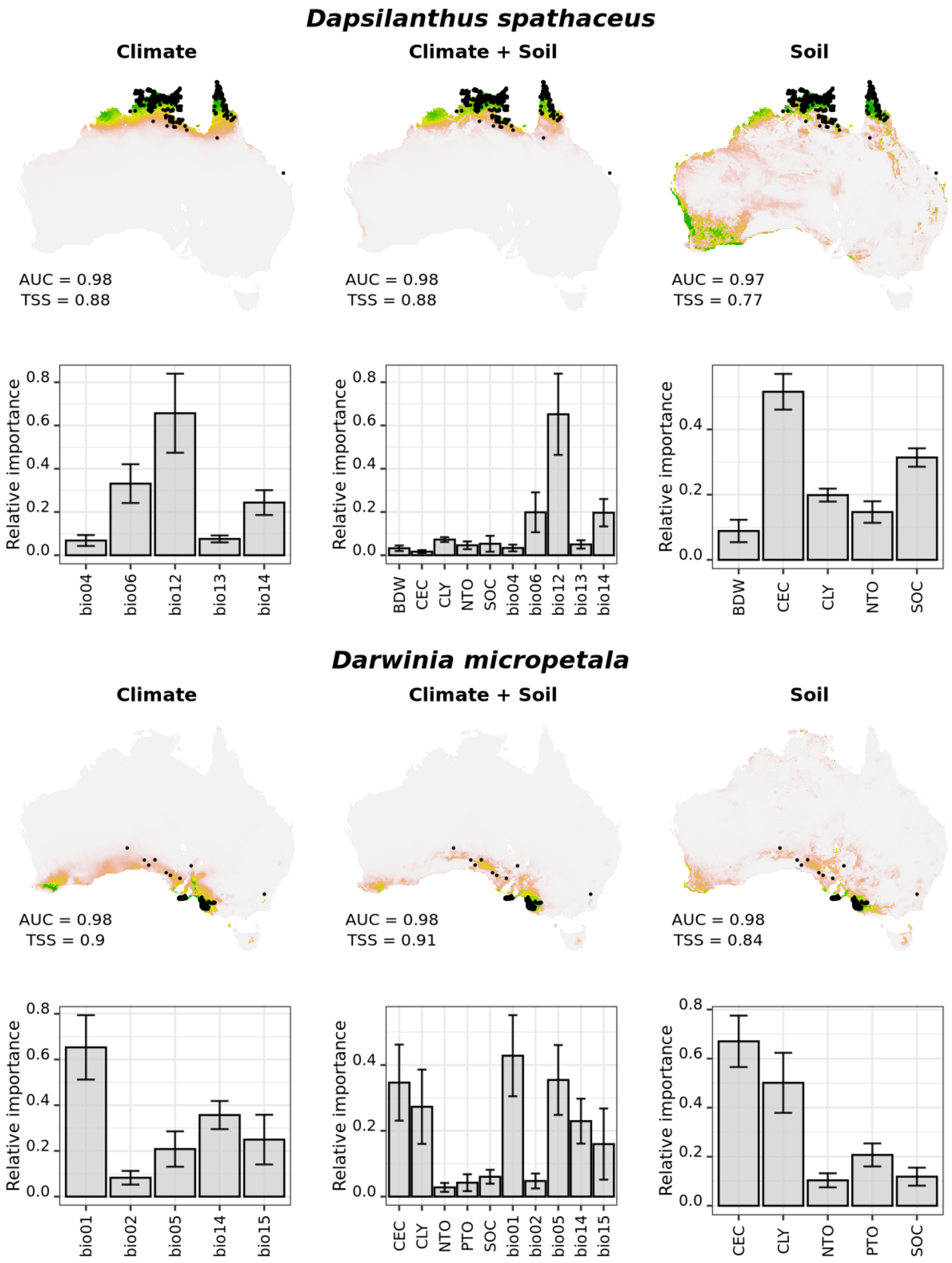

Dapsilanthus spathaceus
Climate
Climate + Soil
Soil
AUC = 0.98
TSS = 0.88
AUC = 0.98
TSS = 0.88
AUC = 0.97
TSS = 0.77
Relative importance
0.0
0.2
0.4
0.6
0.8
bio04
bio06
bio12
bio13
bio14
BDW
CEC
CLY
NTO
SOC
bio04
bio06
bio12
bio13
bio14
BDW
CEC
CLY
NTO
SOC
Darwinia micropetala
Climate
Climate + Soil
Soil
AUC = 0.98
TSS = 0.9
AUC = 0.98
TSS = 0.91
AUC = 0.98
TSS = 0.84
Relative importance
bio01
bio02
bio05
bio14
bio15
CEC
CLY
NTO
PTO
SOC
bio01
bio02
bio05
bio14
bio15
CEC
CLY
NTO
PTO
SOC

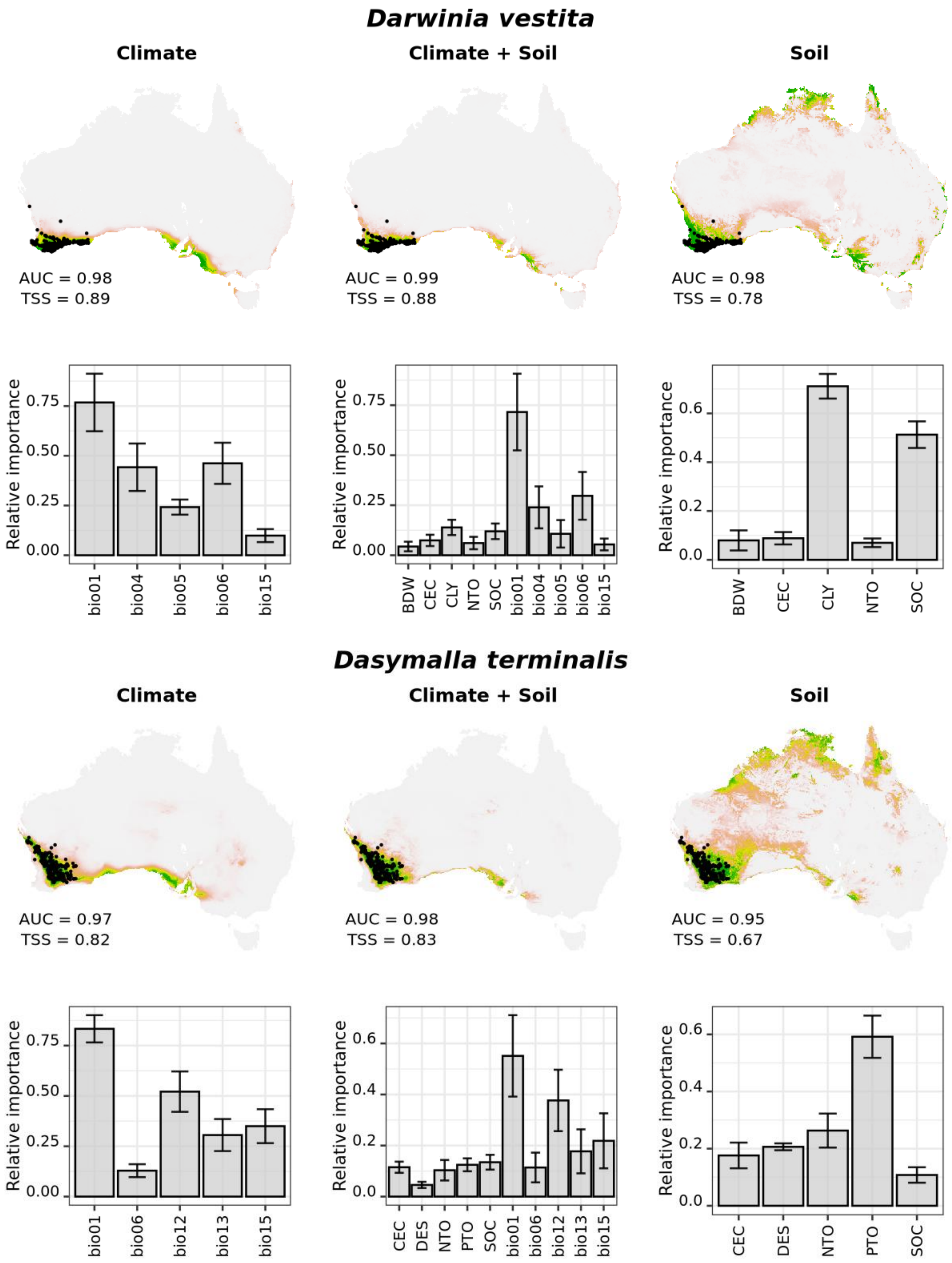

Darwinia vestita
Climate
Climate + Soil
Soil
AUC = 0.98
TSS = 0.89
AUC = 0.99
TSS = 0.88
AUC = 0.98
TSS = 0.78
Relative importance
bio01
bio04
bio05
bio06
bio15
BDW
CEC
CLY
NTO
SOC
Dasymalla terminalis
AUC = 0.97
TSS = 0.82
AUC = 0.98
TSS = 0.83
AUC = 0.95
TSS = 0.67
bio12
bio13
DES
PTO

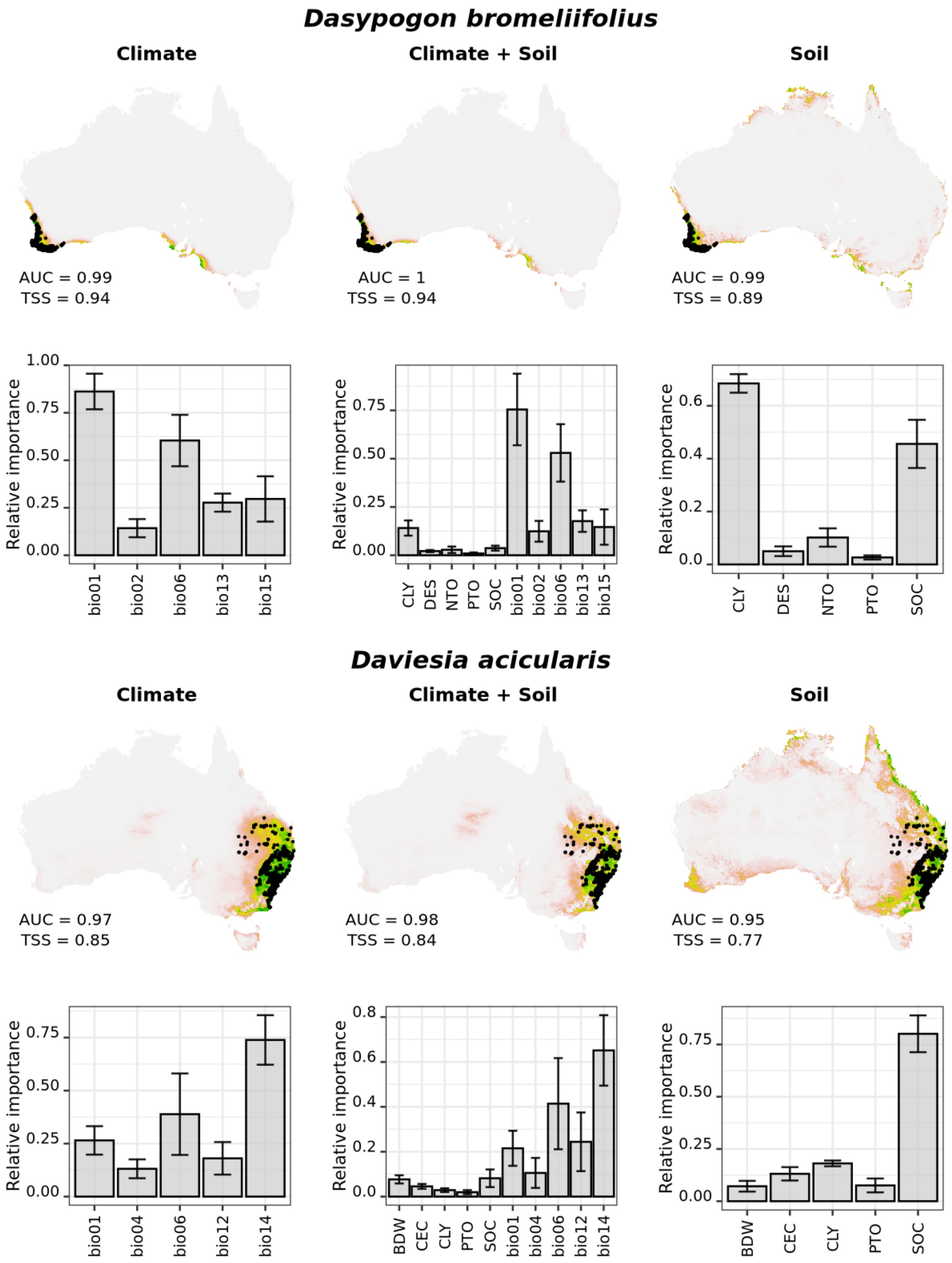
Dasypogon bromeliifolius
Climate
Climate + Soil
Soil
AUC = 0.99
TSS = 0.94
AUC = 1
TSS = 0.94
AUC = 0.99
TSS = 0.89
Relative importance
1.00
0.75
0.50
0.25
0.00
bio01
bio02
bio06
bio13
bio15
0.75
0.50
0.25
0.00
CLY
DES
NTO
PTO
SOC
bio01
bio02
bio06
bio13
bio15
0.6
0.4
0.2
0.0
CLY
DES
NTO
PTO
SOC
Daviesia acicularis
Climate
Climate + Soil
Soil
AUC = 0.97
TSS = 0.85
AUC = 0.98
TSS = 0.84
AUC = 0.95
TSS = 0.77
Relative importance
0.75
0.50
0.25
0.00
bio01
bio04
bio06
bio12
bio14
0.8
0.6
0.4
0.2
0.0
BDW
CEC
CLY
PTO
SOC
bio01
bio04
bio06
bio12
bio14
0.75
0.50
0.25
0.00
BDW
CEC
CLY
PTO
SOC

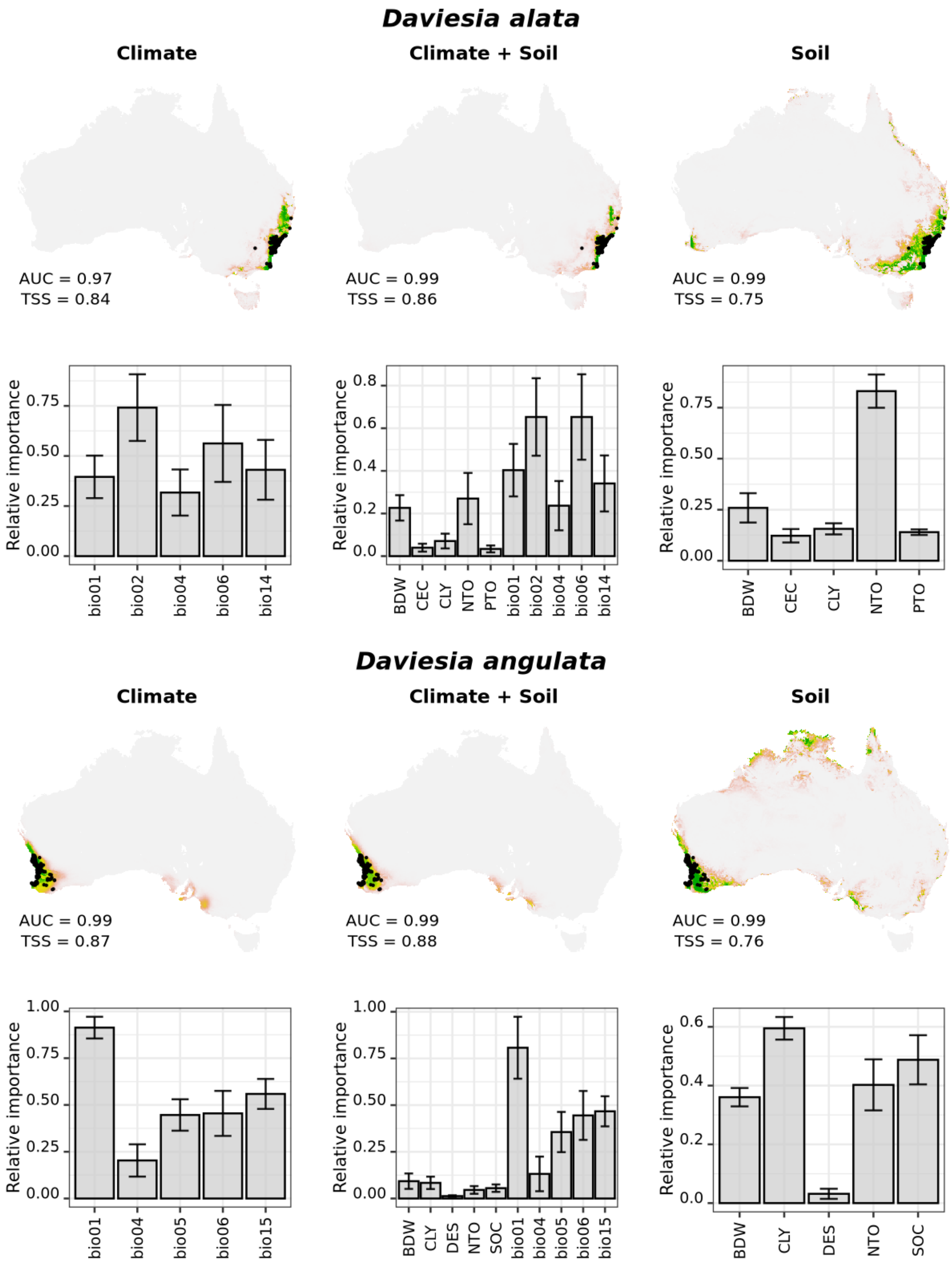
Daviesia alata
Climate
Climate + Soil
Soil
AUC = 0.97
TSS = 0.84
AUC = 0.99
TSS = 0.86
AUC = 0.99
TSS = 0.75
Relative importance
0.00
0.25
0.50
0.75
bio01
bio02
bio04
bio06
bio14
0.0
0.2
0.4
0.6
0.8
BDW
CEC
CLY
NTO
PTO
Daviesia angulata
AUC = 0.99
TSS = 0.87
AUC = 0.99
TSS = 0.88
AUC = 0.99
TSS = 0.76
1.00
bio05
bio15
DES
SOC

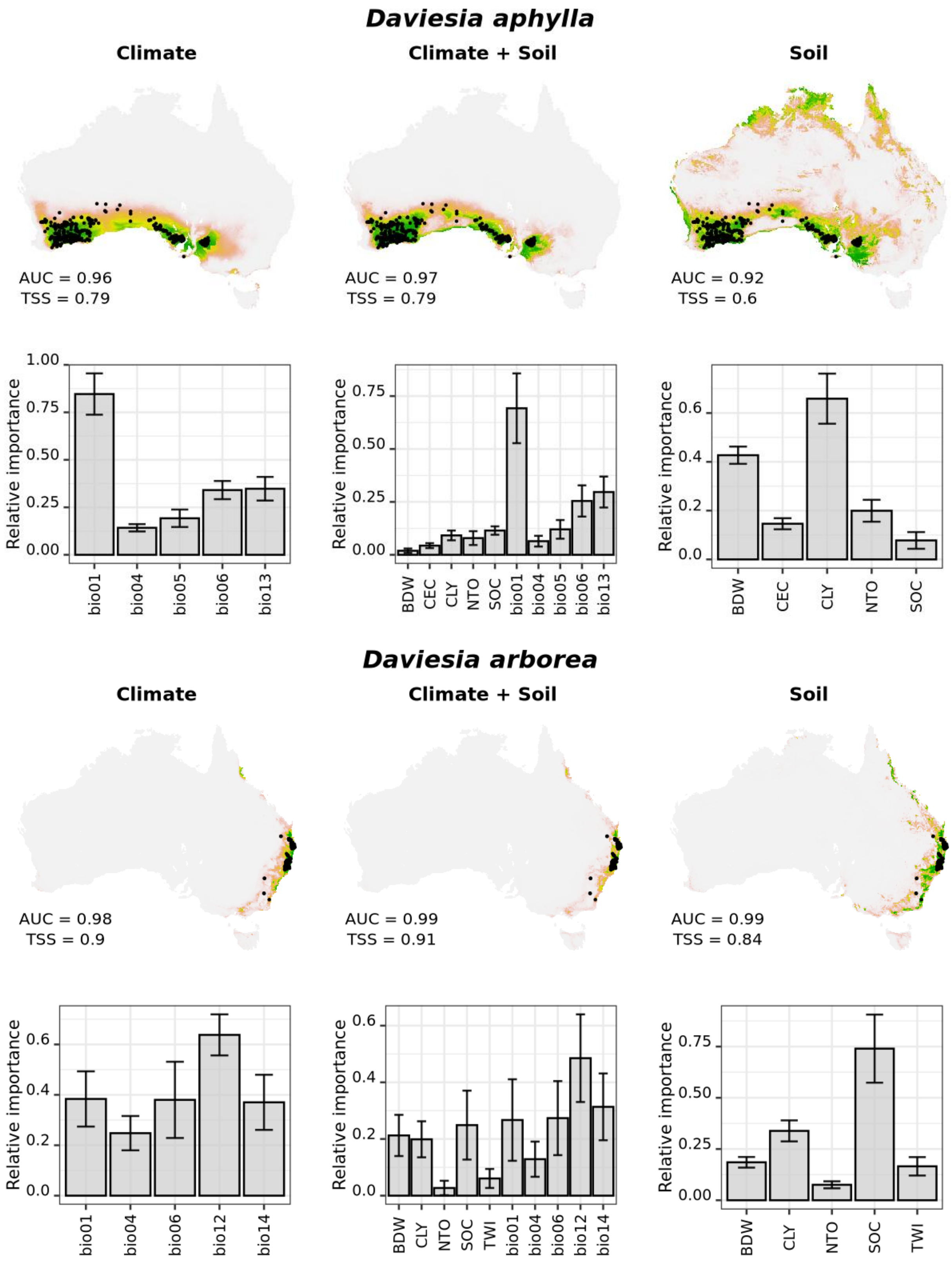
Daviesia aphylla
Climate
Climate + Soil
Soil
AUC = 0.96
TSS = 0.79
AUC = 0.97
TSS = 0.79
AUC = 0.92
TSS = 0.6
Relative importance
bio01
bio04
bio05
bio06
bio13
BDW
CEC
CLY
NTO
SOC
Daviesia arborea
Climate
Climate + Soil
Soil
AUC = 0.98
TSS = 0.9
AUC = 0.99
TSS = 0.91
AUC = 0.99
TSS = 0.84
Relative importance
bio01
bio04
bio06
bio12
bio14
BDW
CLY
NTO
SOC
TWI

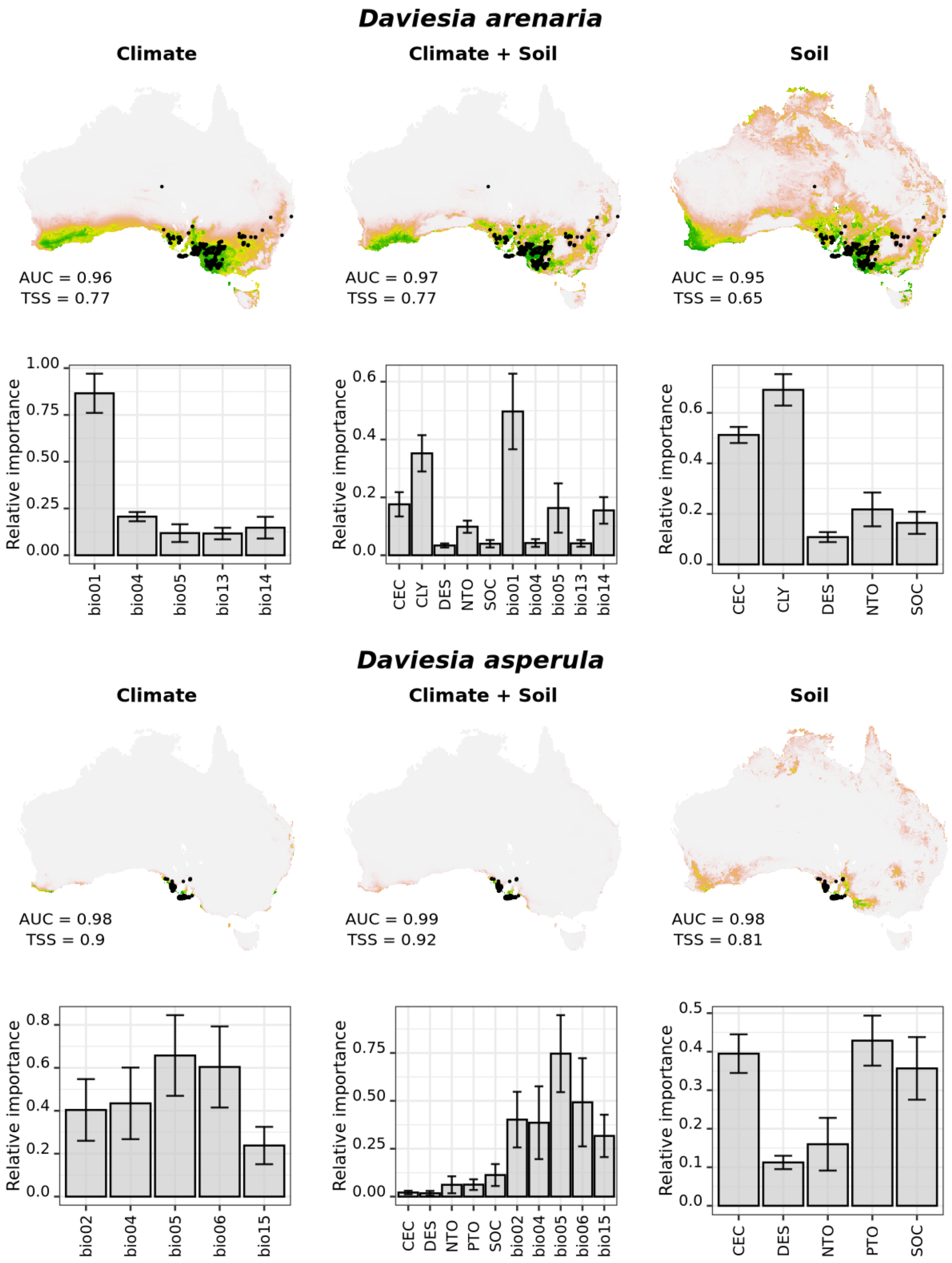
Daviesia arenaria
Climate
Climate + Soil
Soil
AUC = 0.96
TSS = 0.77
AUC = 0.97
TSS = 0.77
AUC = 0.95
TSS = 0.65
Relative importance
1.00
0.75
0.50
0.25
0.00
bio01
bio04
bio05
bio13
bio14
0.6
0.4
0.2
0.0
CEC
CLY
DES
NTO
SOC
bio01
bio04
bio05
bio13
bio14
0.6
0.4
0.2
0.0
CEC
CLY
DES
NTO
SOC
Daviesia asperula
Climate
Climate + Soil
Soil
AUC = 0.98
TSS = 0.9
AUC = 0.99
TSS = 0.92
AUC = 0.98
TSS = 0.81
Relative importance
0.8
0.6
0.4
0.2
0.0
bio02
bio04
bio05
bio06
bio15
0.75
0.50
0.25
0.00
CEC
DES
NTO
PTO
SOC
bio02
bio04
bio05
bio06
bio15
0.5
0.4
0.3
0.2
0.1
0.0
CEC
DES
NTO
PTO
SOC

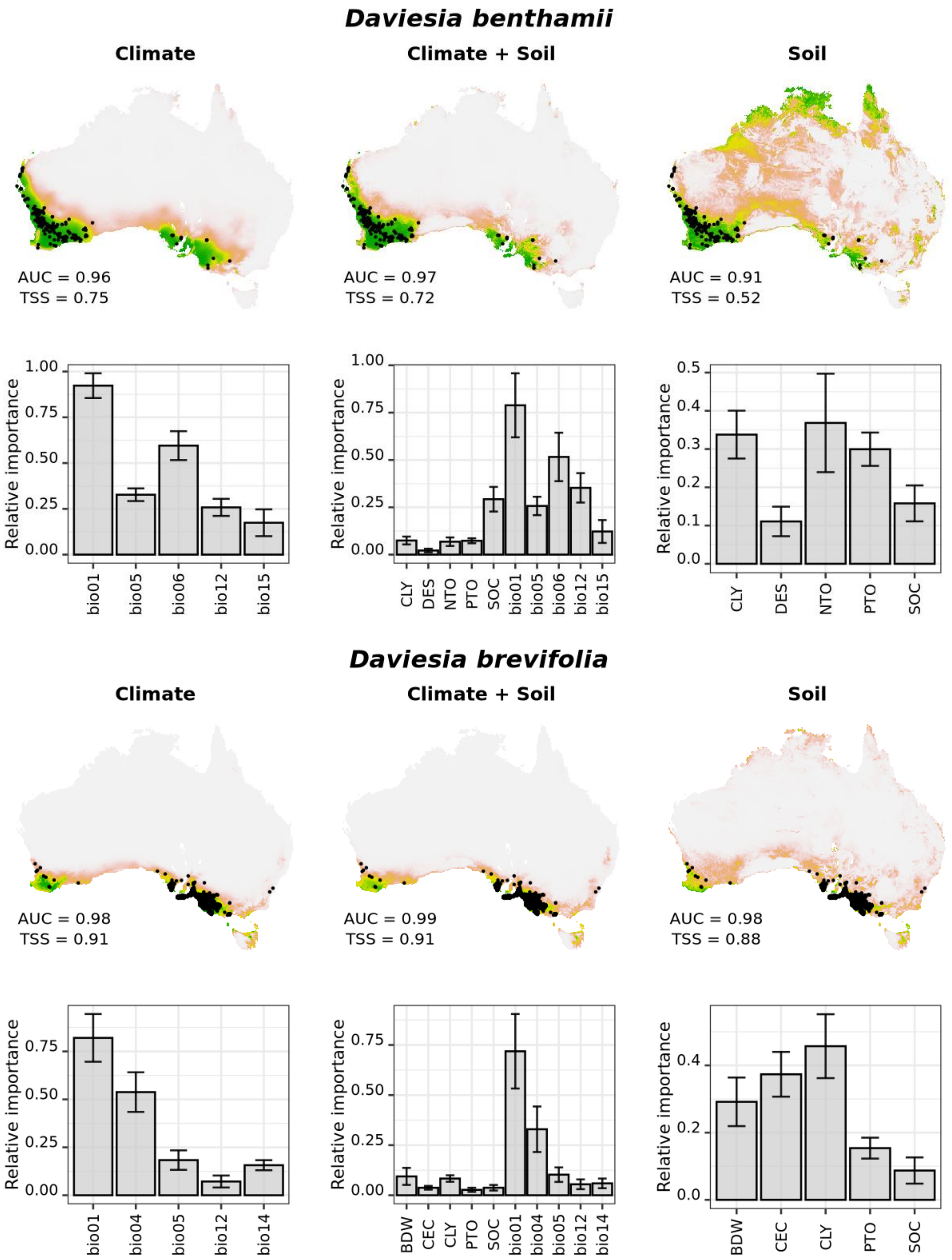
Daviesia benthamii
Climate
Climate + Soil
Soil
AUC = 0.96
TSS = 0.75
AUC = 0.97
TSS = 0.72
AUC = 0.91
TSS = 0.52
Relative importance
bio01
bio05
bio06
bio12
bio15
CLY
DES
NTO
PTO
SOC
Daviesia brevifolia
Climate
Climate + Soil
Soil
AUC = 0.98
TSS = 0.91
AUC = 0.99
TSS = 0.91
AUC = 0.98
TSS = 0.88
Relative importance
bio01
bio04
bio05
bio12
bio14
BDW
CEC
CLY
PTO
SOC

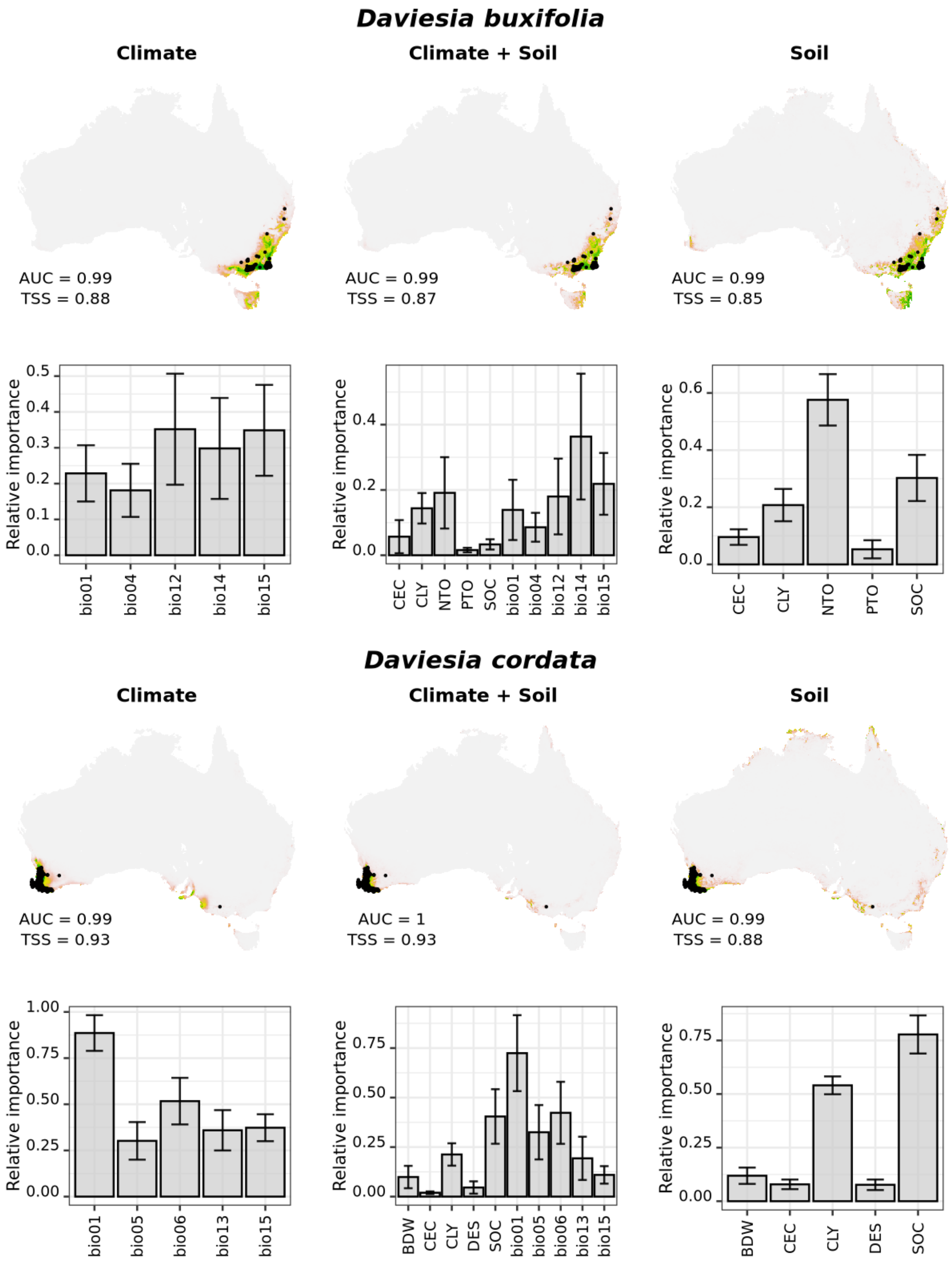
Daviesia buxifolia
Climate
Climate + Soil
Soil
AUC = 0.99
TSS = 0.88
AUC = 0.99
TSS = 0.87
AUC = 0.99
TSS = 0.85
Relative importance
bio01
bio04
bio12
bio14
bio15
CEC
CLY
NTO
PTO
SOC
Daviesia cordata
Climate
Climate + Soil
Soil
AUC = 0.99
TSS = 0.93
AUC = 1
TSS = 0.93
AUC = 0.99
TSS = 0.88
Relative importance
bio01
bio05
bio06
bio13
bio15
BDW
CEC
CLY
DES
SOC

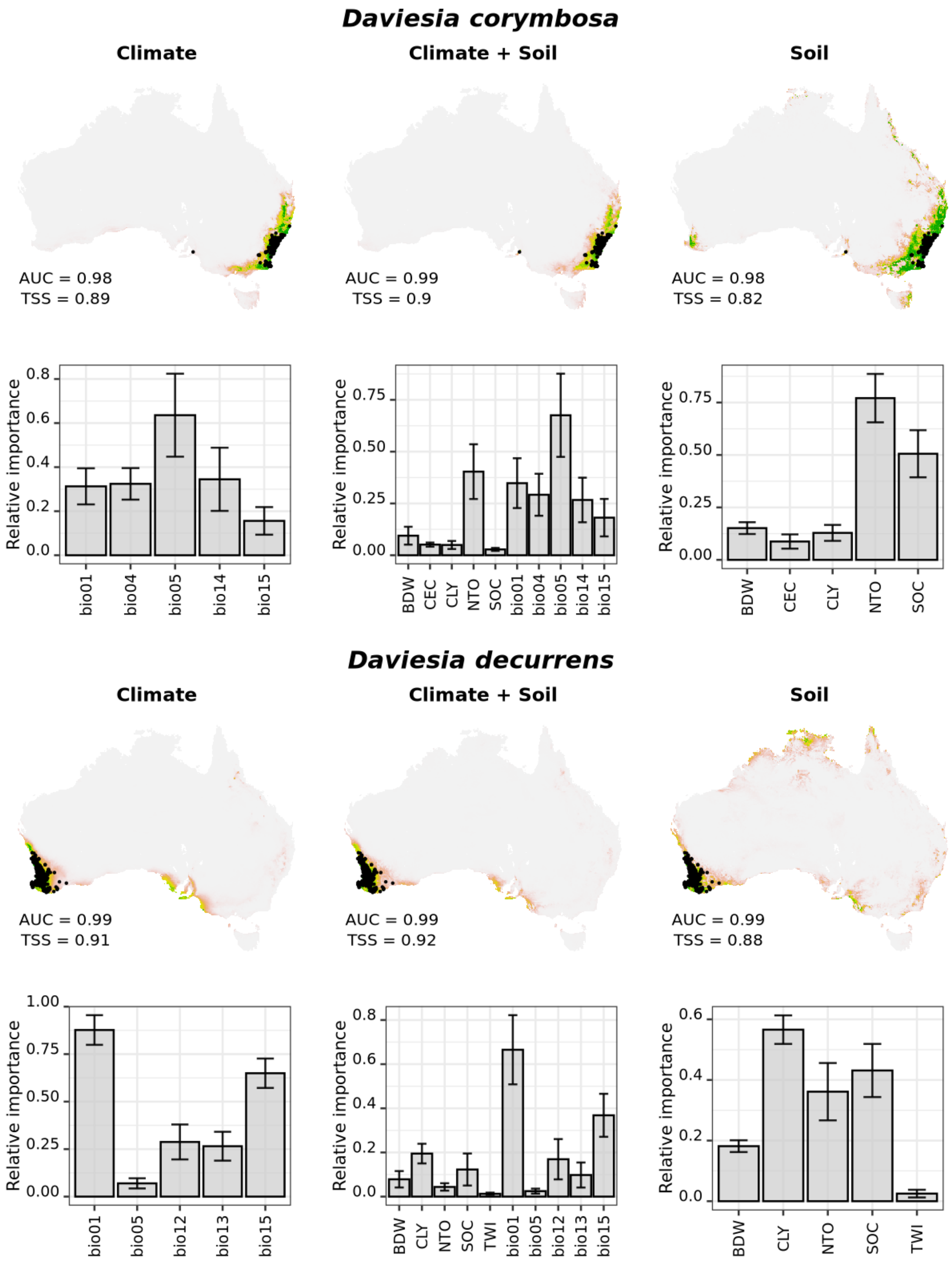

Daviesia corymbosa
Climate
Climate + Soil
Soil
AUC = 0.98
TSS = 0.89
AUC = 0.99
TSS = 0.9
AUC = 0.98
TSS = 0.82
Relative importance
bio01
bio04
bio05
bio14
bio15
BDW
CEC
CLY
NTO
SOC
Daviesia decurrens
Climate
Climate + Soil
Soil
AUC = 0.99
TSS = 0.91
AUC = 0.99
TSS = 0.92
AUC = 0.99
TSS = 0.88
Relative importance
bio01
bio05
bio12
bio13
bio15
BDW
CLY
NTO
SOC
TWI

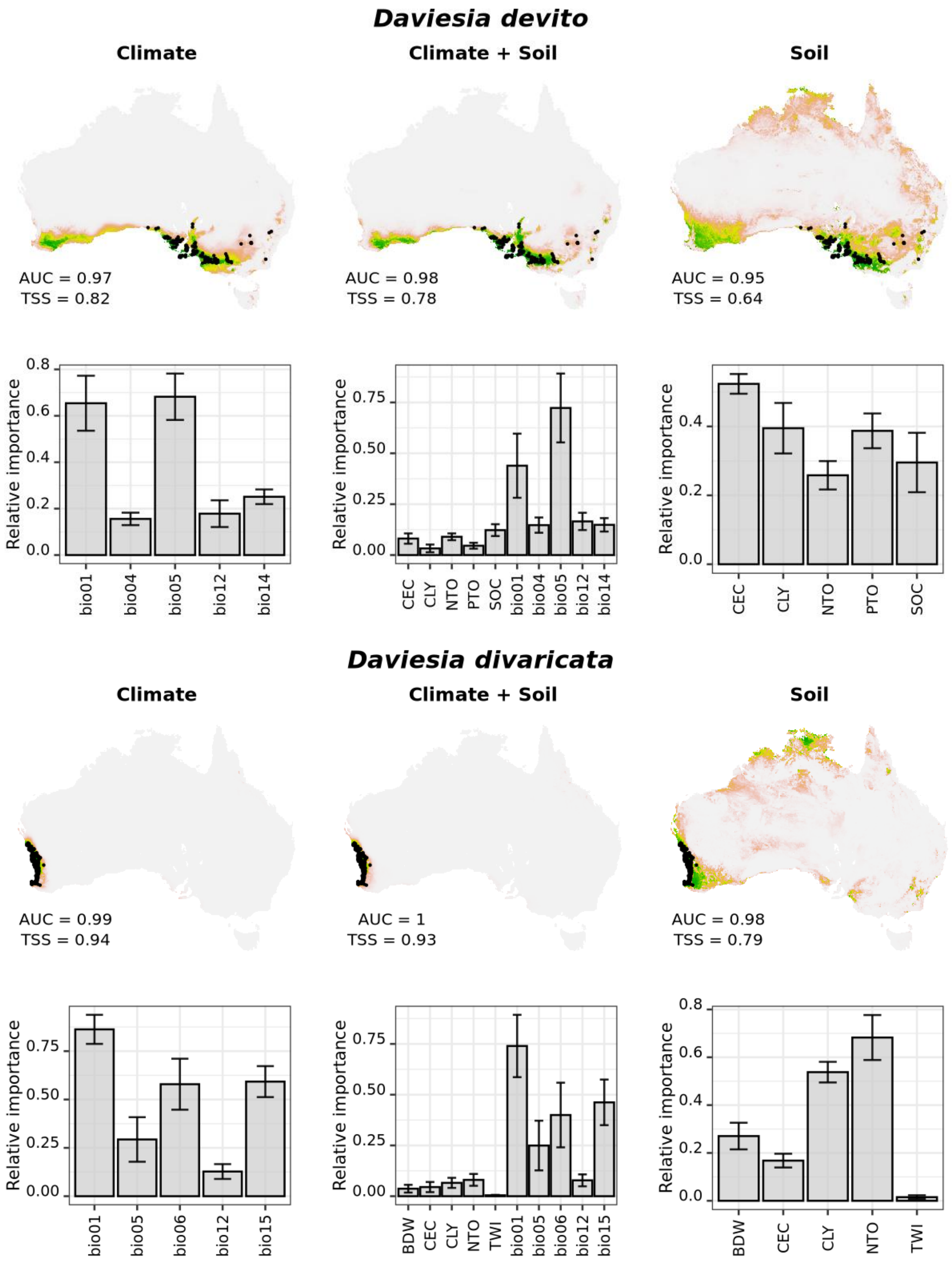
Daviesia devito
Climate
Climate + Soil
Soil
AUC = 0.97
TSS = 0.82
AUC = 0.98
TSS = 0.78
AUC = 0.95
TSS = 0.64
Relative importance
bio01
bio04
bio05
bio12
bio14
CEC
CLY
NTO
PTO
SOC
Daviesia divaricata
AUC = 0.99
TSS = 0.94
AUC = 1
TSS = 0.93
AUC = 0.98
TSS = 0.79
bio06
bio15
BDW
TWI

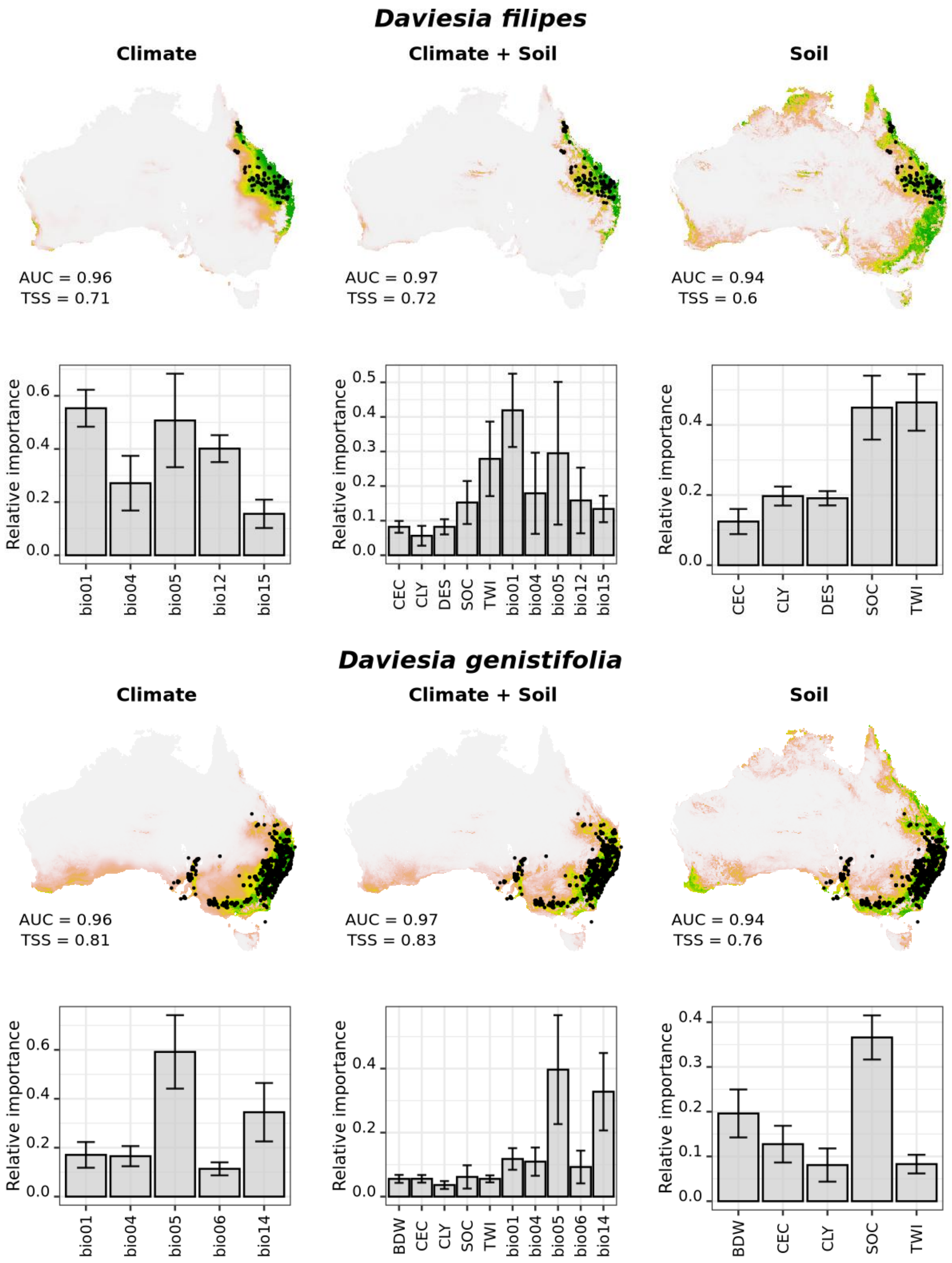

Daviesia filipes
Climate
Climate + Soil
Soil
AUC = 0.96
TSS = 0.71
AUC = 0.97
TSS = 0.72
AUC = 0.94
TSS = 0.6
Relative importance
0.0 0.2 0.4 0.6
bio01 bio04 bio05 bio12 bio15
0.0 0.1 0.2 0.3 0.4 0.5
CEC CLY DES SOC TWI bio01 bio04 bio05 bio12 bio15
0.0 0.2 0.4
CEC CLY DES SOC TWI
Daviesia genistifolia
Climate
Climate + Soil
Soil
AUC = 0.96
TSS = 0.81
AUC = 0.97
TSS = 0.83
AUC = 0.94
TSS = 0.76
Relative importance
0.0 0.2 0.4 0.6
bio01 bio04 bio05 bio06 bio14
0.0 0.2 0.4
BDW CEC CLY SOC TWI bio01 bio04 bio05 bio06 bio14
0.0 0.1 0.2 0.3 0.4
BDW CEC CLY SOC TWI

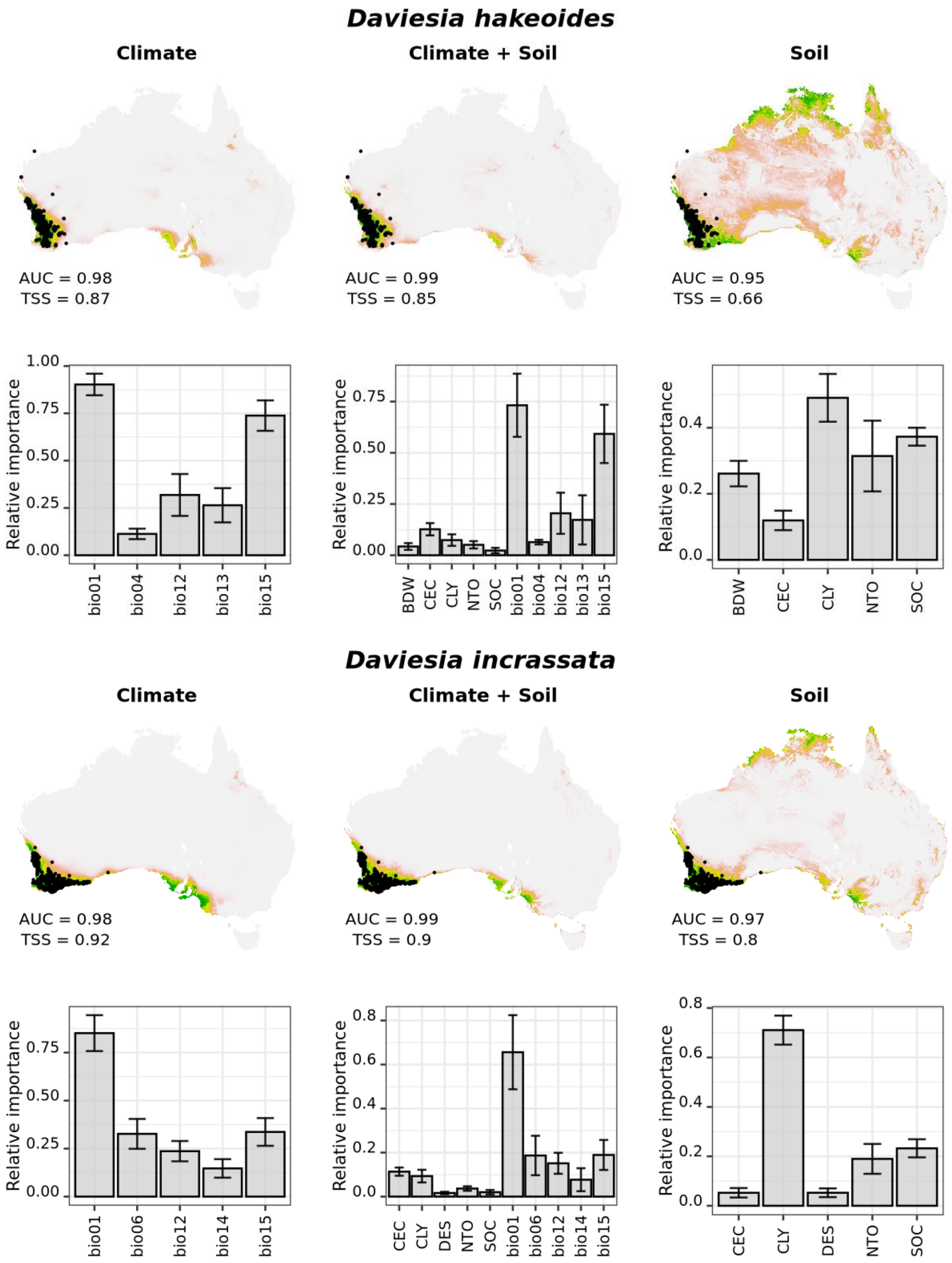

Daviesia hakeoides
Climate
Climate + Soil
Soil
AUC = 0.98
TSS = 0.87
AUC = 0.99
TSS = 0.85
AUC = 0.95
TSS = 0.66
Relative importance
1.00
0.75
0.50
0.25
0.00
bio01
bio04
bio12
bio13
bio15
BDW
CEC
CLY
NTO
SOC
0.4
0.2
0.0
Daviesia incrassata
Climate
Climate + Soil
Soil
AUC = 0.98
TSS = 0.92
AUC = 0.99
TSS = 0.9
AUC = 0.97
TSS = 0.8
Relative importance
bio01
bio06
bio12
bio14
bio15
CEC
CLY
DES
NTO
SOC
0.8
0.6
0.4
0.2
0.0

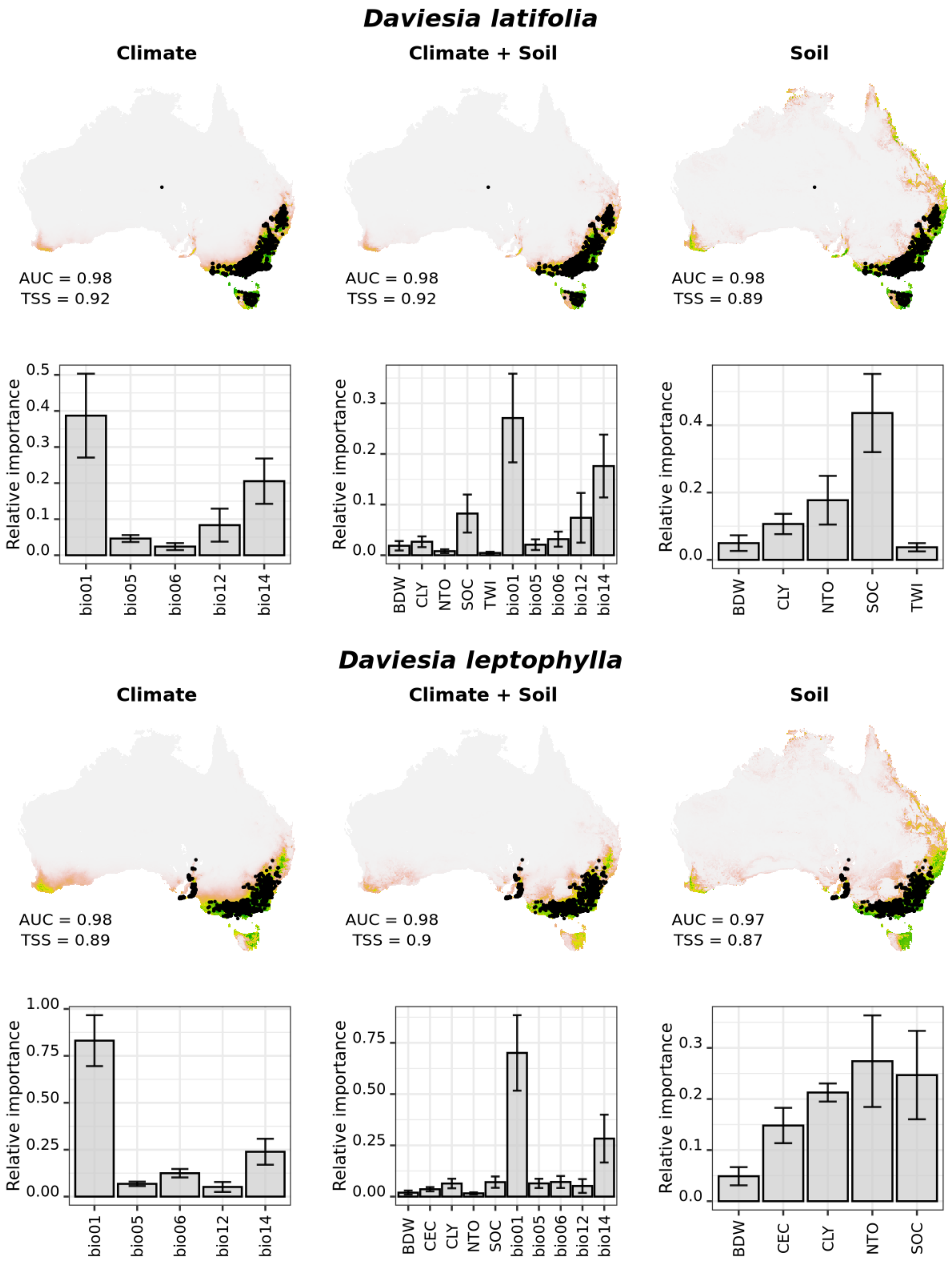
Daviesia latifolia
Climate
Climate + Soil
Soil
AUC = 0.98
TSS = 0.92
AUC = 0.98
TSS = 0.92
AUC = 0.98
TSS = 0.89
Relative importance
0.0
0.1
0.2
0.3
0.4
0.5
bio01
bio05
bio06
bio12
bio14
BDW
CLY
NTO
SOC
TWI
Daviesia leptophylla
Climate
Climate + Soil
Soil
AUC = 0.98
TSS = 0.89
AUC = 0.98
TSS = 0.9
AUC = 0.97
TSS = 0.87
0.00
0.25
0.50
0.75
1.00
CEC

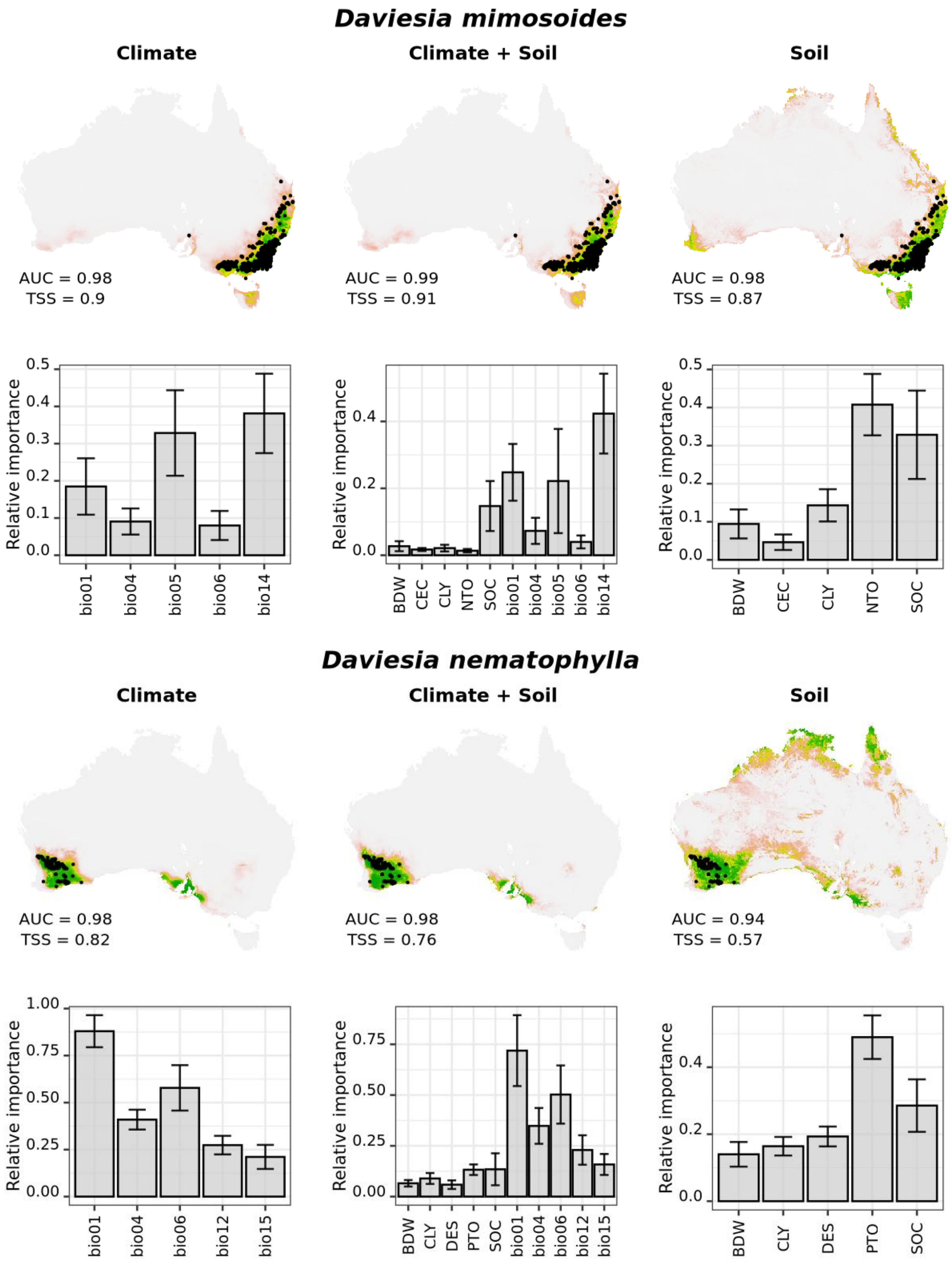
Daviesia mimosoides
Climate
Climate + Soil
Soil
AUC = 0.98
TSS = 0.9
AUC = 0.99
TSS = 0.91
AUC = 0.98
TSS = 0.87
Relative importance
bio01
bio04
bio05
bio06
bio14
BDW
CEC
CLY
NTO
SOC
Daviesia nematophylla
Climate
Climate + Soil
Soil
AUC = 0.98
TSS = 0.82
AUC = 0.98
TSS = 0.76
AUC = 0.94
TSS = 0.57
Relative importance
bio01
bio04
bio06
bio12
bio15
BDW
CLY
DES
PTO
SOC

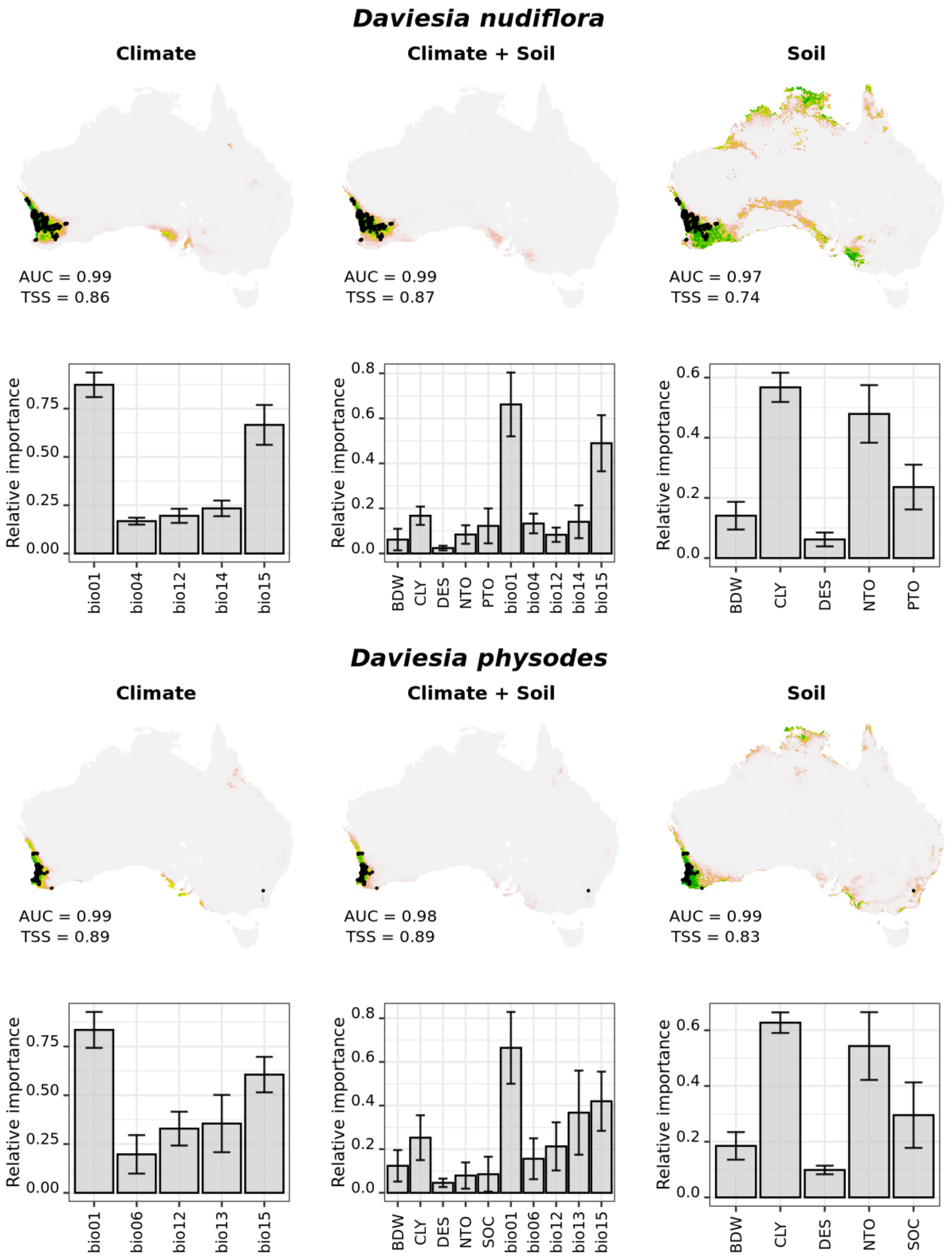
Daviesia nudiflora
Climate
Climate + Soil
Soil
AUC = 0.99
TSS = 0.86
AUC = 0.99
TSS = 0.87
AUC = 0.97
TSS = 0.74
Relative importance
0.00
0.25
0.50
0.75
bio01
bio04
bio12
bio14
bio15
0.0
0.2
0.4
0.6
0.8
BDW
CLY
DES
NTO
PTO
bio01
bio04
bio12
bio14
bio15
0.0
0.2
0.4
0.6
BDW
CLY
DES
NTO
PTO
Daviesia physodes
Climate
Climate + Soil
Soil
AUC = 0.99
TSS = 0.89
AUC = 0.98
TSS = 0.89
AUC = 0.99
TSS = 0.83
Relative importance
0.00
0.25
0.50
0.75
bio01
bio06
bio12
bio13
bio15
0.0
0.2
0.4
0.6
0.8
BDW
CLY
DES
NTO
SOC
bio01
bio06
bio12
bio13
bio15
0.0
0.2
0.4
0.6
BDW
CLY
DES
NTO
SOC

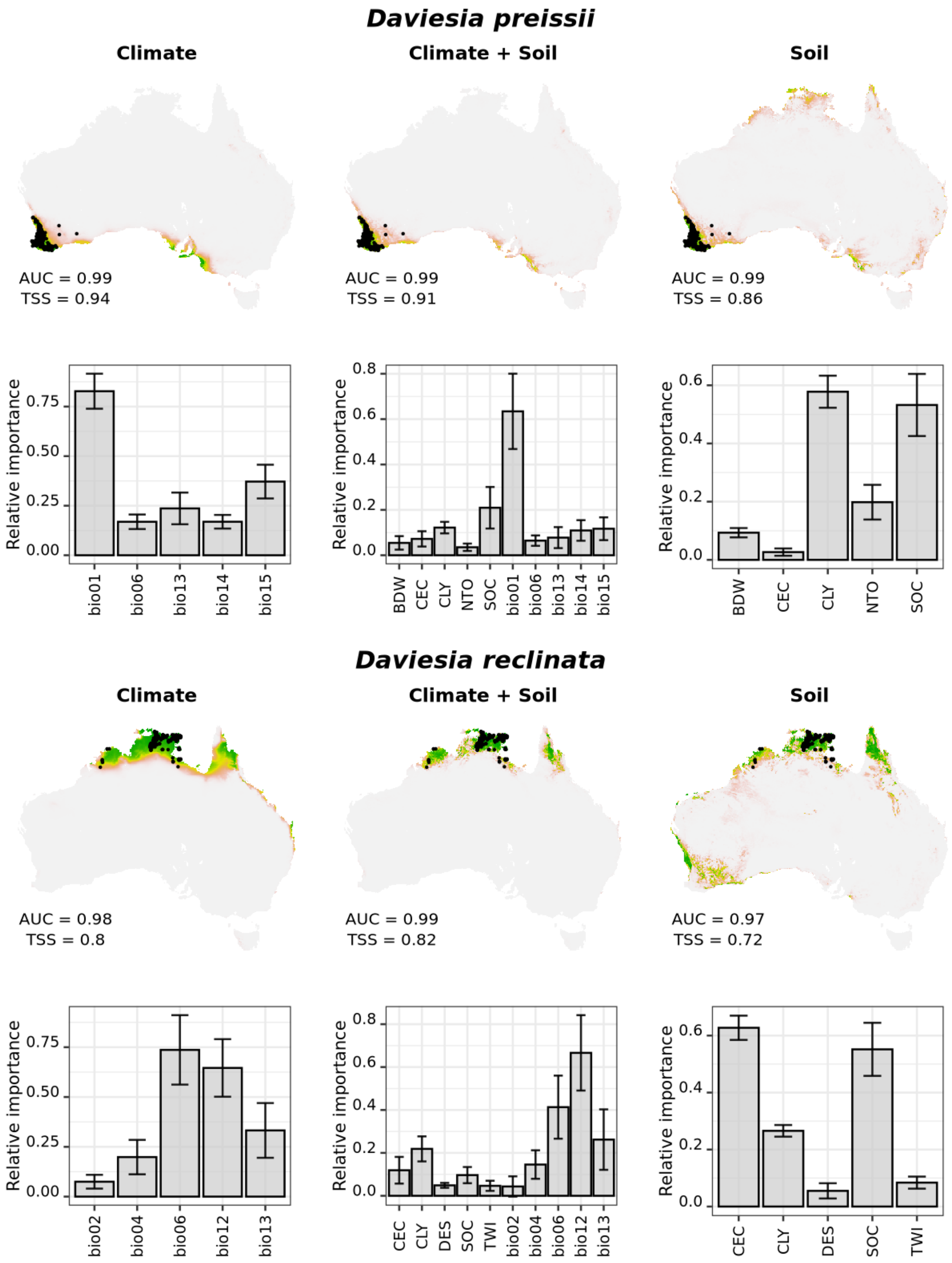
Daviesia preissii
Climate
Climate + Soil
Soil
AUC = 0.99
TSS = 0.94
AUC = 0.99
TSS = 0.91
AUC = 0.99
TSS = 0.86
Relative importance
0.00
0.25
0.50
0.75
bio01
bio06
bio13
bio14
bio15
0.0
0.2
0.4
0.6
0.8
BDW
CEC
CLY
NTO
SOC
bio01
bio06
bio13
bio14
bio15
BDW
CEC
CLY
NTO
SOC
Daviesia reclinata
Climate
Climate + Soil
Soil
AUC = 0.98
TSS = 0.8
AUC = 0.99
TSS = 0.82
AUC = 0.97
TSS = 0.72
bio02
bio04
bio06
bio12
bio13
CEC
CLY
DES
SOC
TWI
bio02
bio04
bio06
bio12
bio13
CEC
CLY
DES
SOC
TWI

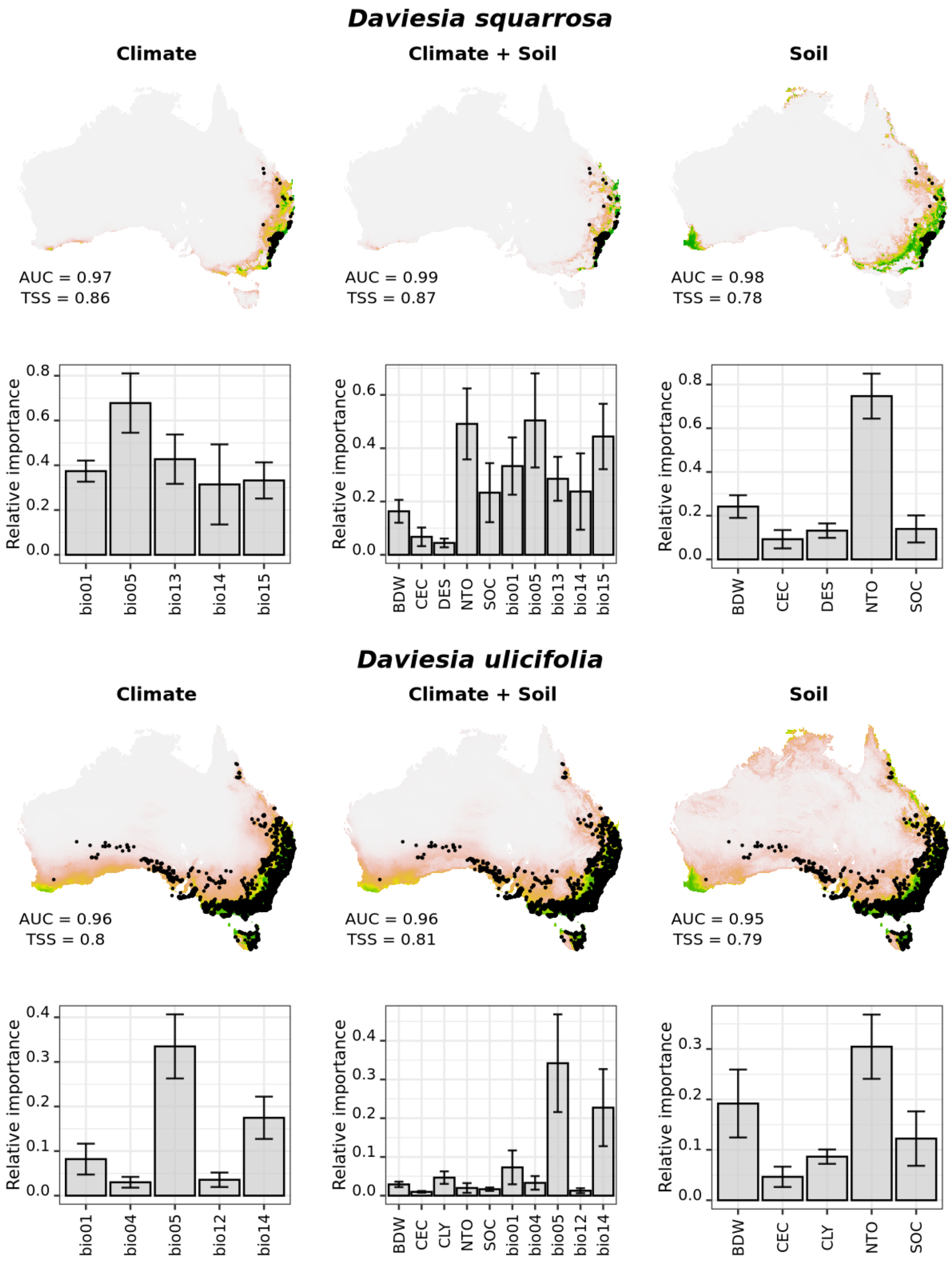
Daviesia squarrosa
Climate
Climate + Soil
Soil
AUC = 0.97
TSS = 0.86
AUC = 0.99
TSS = 0.87
AUC = 0.98
TSS = 0.78
Relative importance
bio01
bio05
bio13
bio14
bio15
BDW
CEC
DES
NTO
SOC
bio01
bio05
bio13
bio14
bio15
BDW
CEC
DES
NTO
SOC
Daviesia ulicifolia
Climate
Climate + Soil
Soil
AUC = 0.96
TSS = 0.8
AUC = 0.96
TSS = 0.81
AUC = 0.95
TSS = 0.79
Relative importance
bio01
bio04
bio05
bio12
bio14
BDW
CEC
CLY
NTO
SOC
bio01
bio04
bio05
bio12
bio14
BDW
CEC
CLY
NTO
SOC

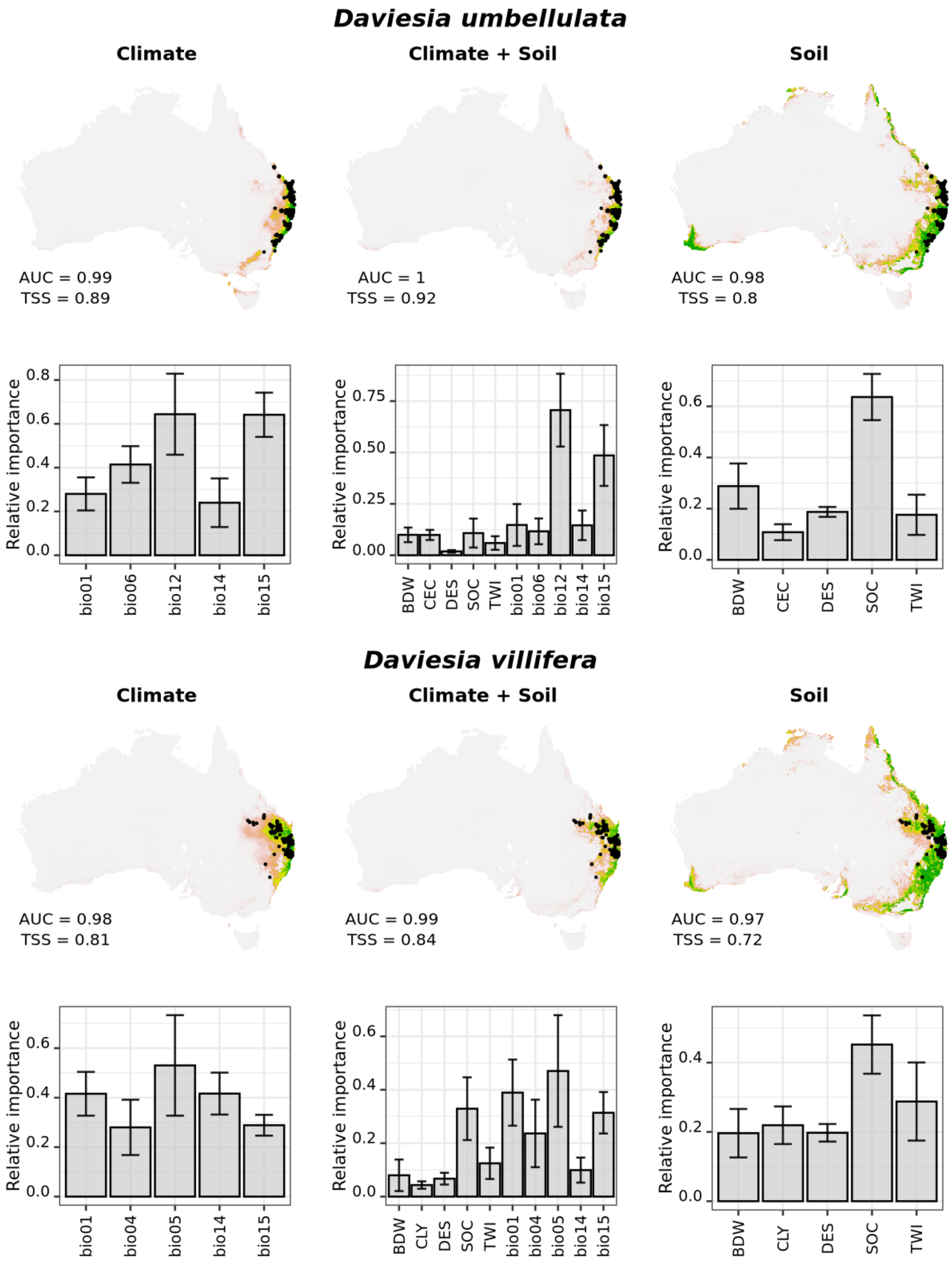
Daviesia umbellulata
Climate
Climate + Soil
Soil
AUC = 0.99
TSS = 0.89
AUC = 1
TSS = 0.92
AUC = 0.98
TSS = 0.8
Relative importance
bio01
bio06
bio12
bio14
bio15
BDW
CEC
DES
SOC
TWI
Daviesia villifera
AUC = 0.98
TSS = 0.81
AUC = 0.99
TSS = 0.84
AUC = 0.97
TSS = 0.72
bio04
bio05
CLY

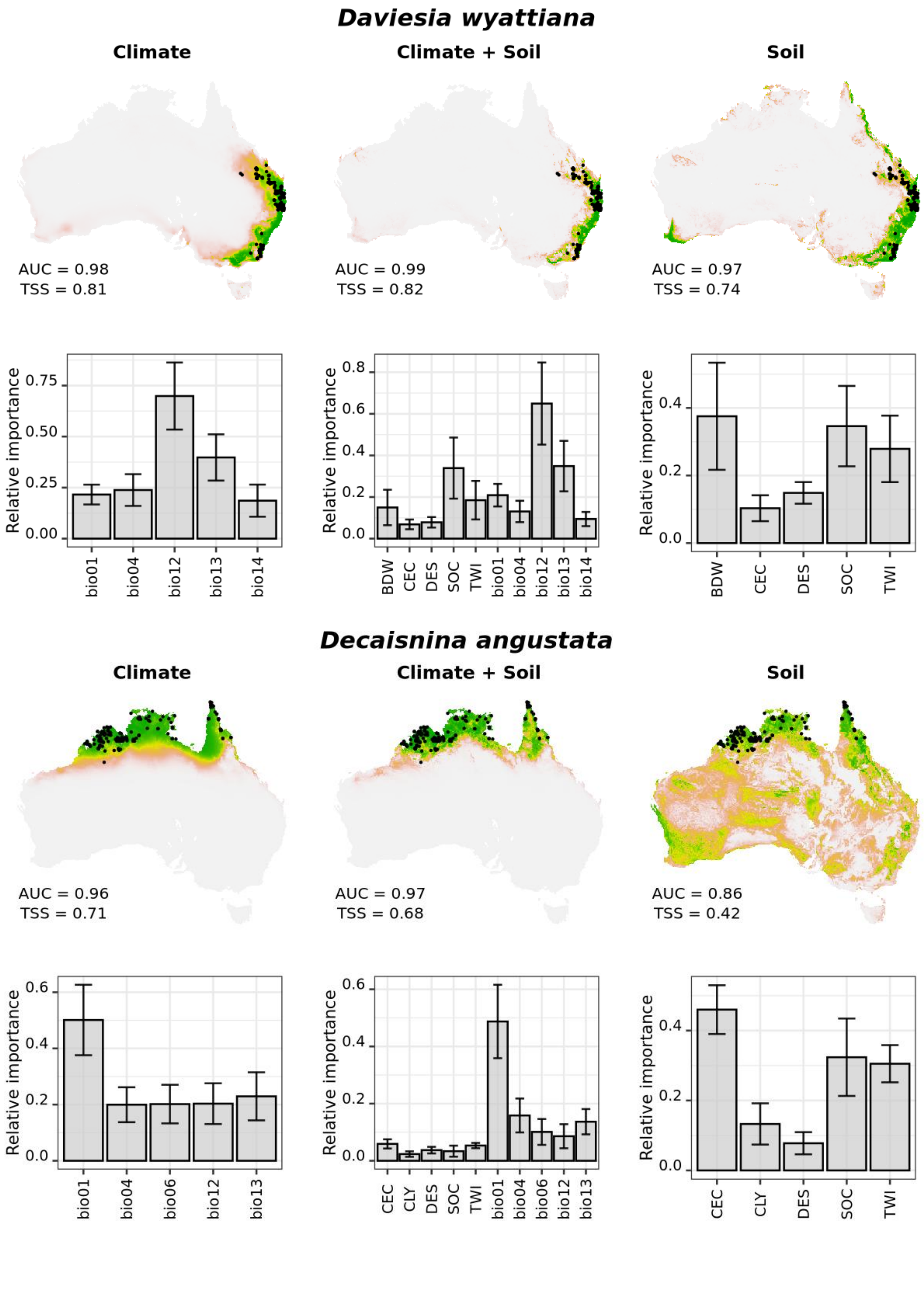
Daviesia wyattiana
Climate
Climate + Soil
Soil
AUC = 0.98
TSS = 0.81
AUC = 0.99
TSS = 0.82
AUC = 0.97
TSS = 0.74
Relative importance
0.00
0.25
0.50
0.75
bio01
bio04
bio12
bio13
bio14
0.0
0.2
0.4
0.6
0.8
BDW
CEC
DES
SOC
TWI
bio01
bio04
bio12
bio13
bio14
0.0
0.2
0.4
BDW
CEC
DES
SOC
TWI
Decaisnina angustata
Climate
Climate + Soil
Soil
AUC = 0.96
TSS = 0.71
AUC = 0.97
TSS = 0.68
AUC = 0.86
TSS = 0.42
Relative importance
0.0
0.2
0.4
0.6
bio01
bio04
bio06
bio12
bio13
CEC
CLY
DES
SOC
TWI
bio01
bio04
bio06
bio12
bio13
CEC
CLY
DES
SOC
TWI

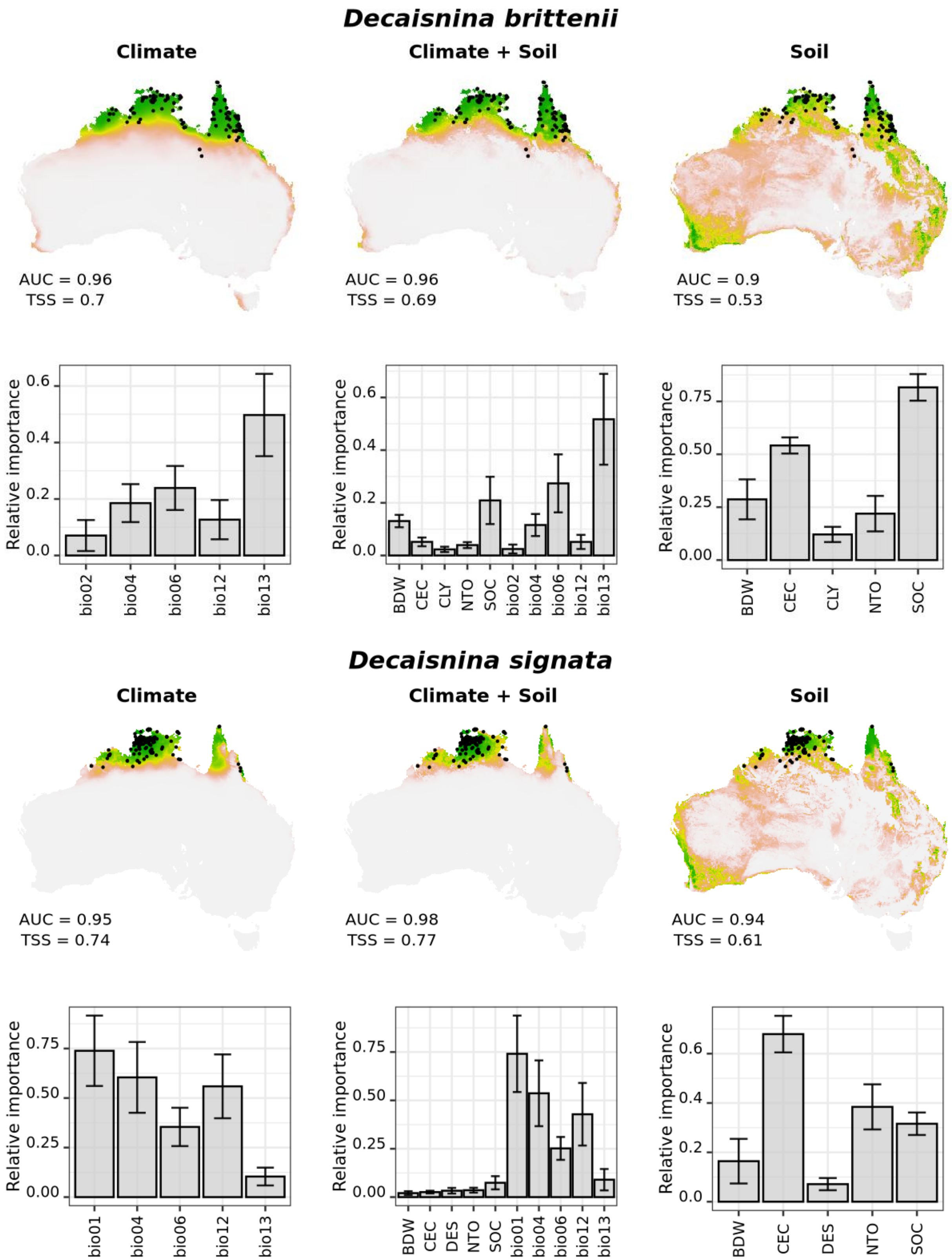
Decaisnina brittenii
Climate
Climate + Soil
Soil
AUC = 0.96
TSS = 0.7
AUC = 0.96
TSS = 0.69
AUC = 0.9
TSS = 0.53
Relative importance
bio02
bio04
bio06
bio12
bio13
BDW
CEC
CLY
NTO
SOC
Decaisnina signata
Climate
Climate + Soil
Soil
AUC = 0.95
TSS = 0.74
AUC = 0.98
TSS = 0.77
AUC = 0.94
TSS = 0.61
Relative importance
bio01
bio04
bio06
bio12
bio13
BDW
CEC
DES
NTO
SOC

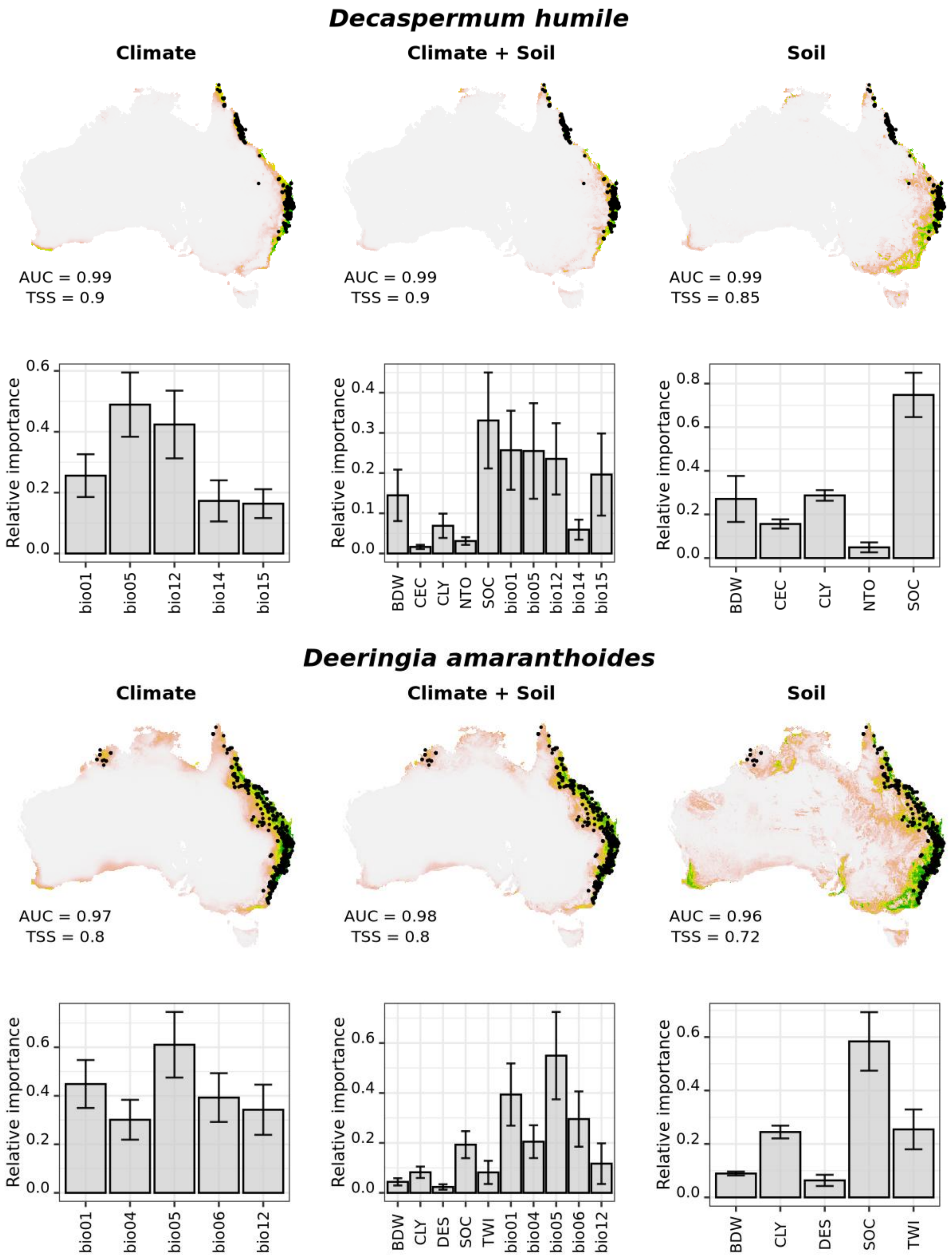
Decaspermum humile
Climate
Climate + Soil
Soil
AUC = 0.99
TSS = 0.9
AUC = 0.99
TSS = 0.9
AUC = 0.99
TSS = 0.85
Relative importance
bio01 bio05 bio12 bio14 bio15
BDW CEC CLY NTO SOC bio01 bio05 bio12 bio14 bio15
BDW CEC CLY NTO SOC
Deeringia amaranthoides
Climate
Climate + Soil
Soil
AUC = 0.97
TSS = 0.8
AUC = 0.98
TSS = 0.8
AUC = 0.96
TSS = 0.72
Relative importance
bio01 bio04 bio05 bio06 bio12
BDW CLY DES SOC TWI bio01 bio04 bio05 bio06 bio12
BDW CLY DES SOC TWI

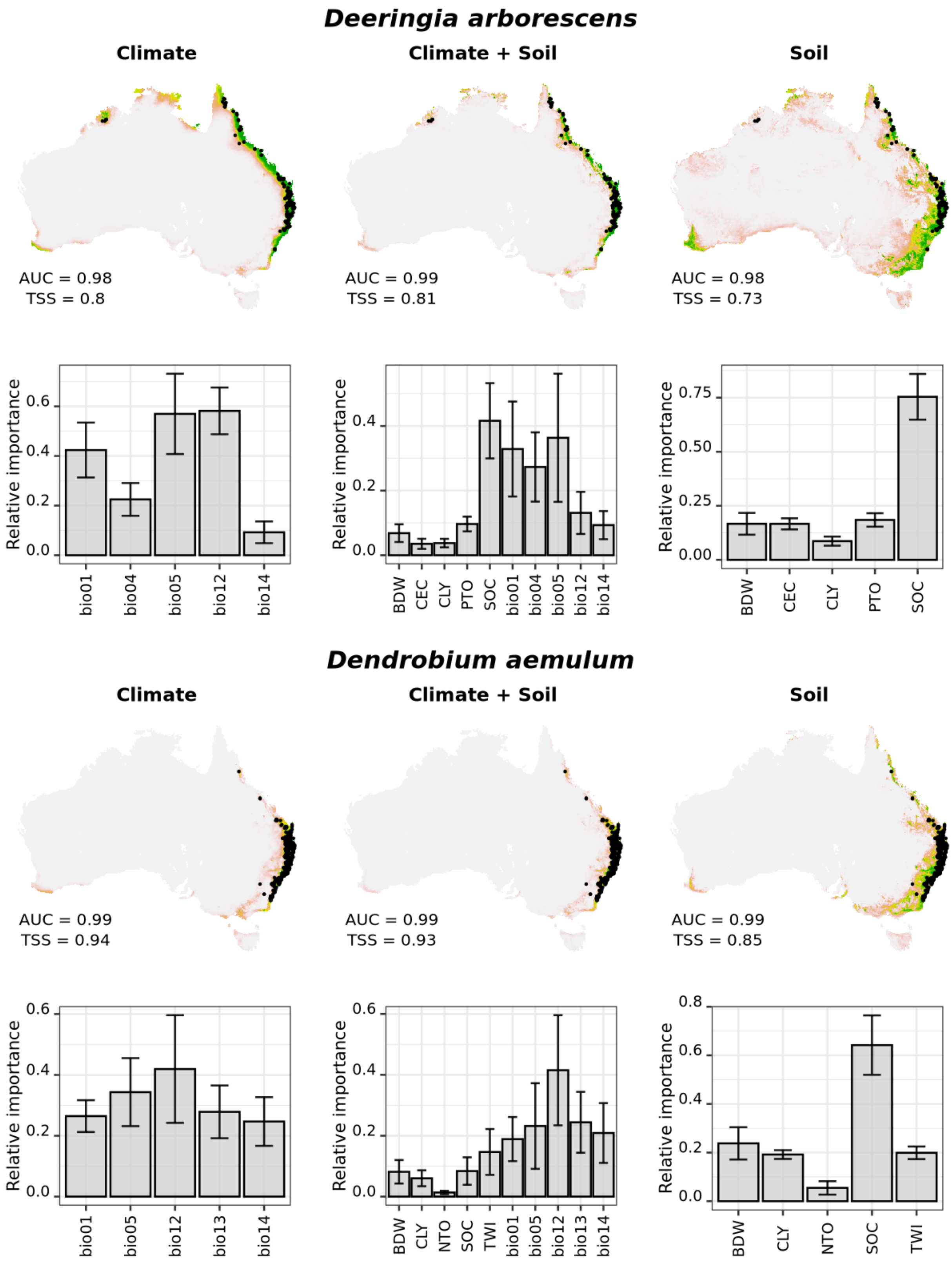

Deeringia arborescens
Climate
Climate + Soil
Soil
AUC = 0.98
TSS = 0.8
AUC = 0.99
TSS = 0.81
AUC = 0.98
TSS = 0.73
Relative importance
bio01
bio04
bio05
bio12
bio14
BDW
CEC
CLY
PTO
SOC
Dendrobium aemulum
Climate
Climate + Soil
Soil
AUC = 0.99
TSS = 0.94
AUC = 0.99
TSS = 0.93
AUC = 0.99
TSS = 0.85
Relative importance
bio01
bio05
bio12
bio13
bio14
BDW
CLY
NTO
SOC
TWI

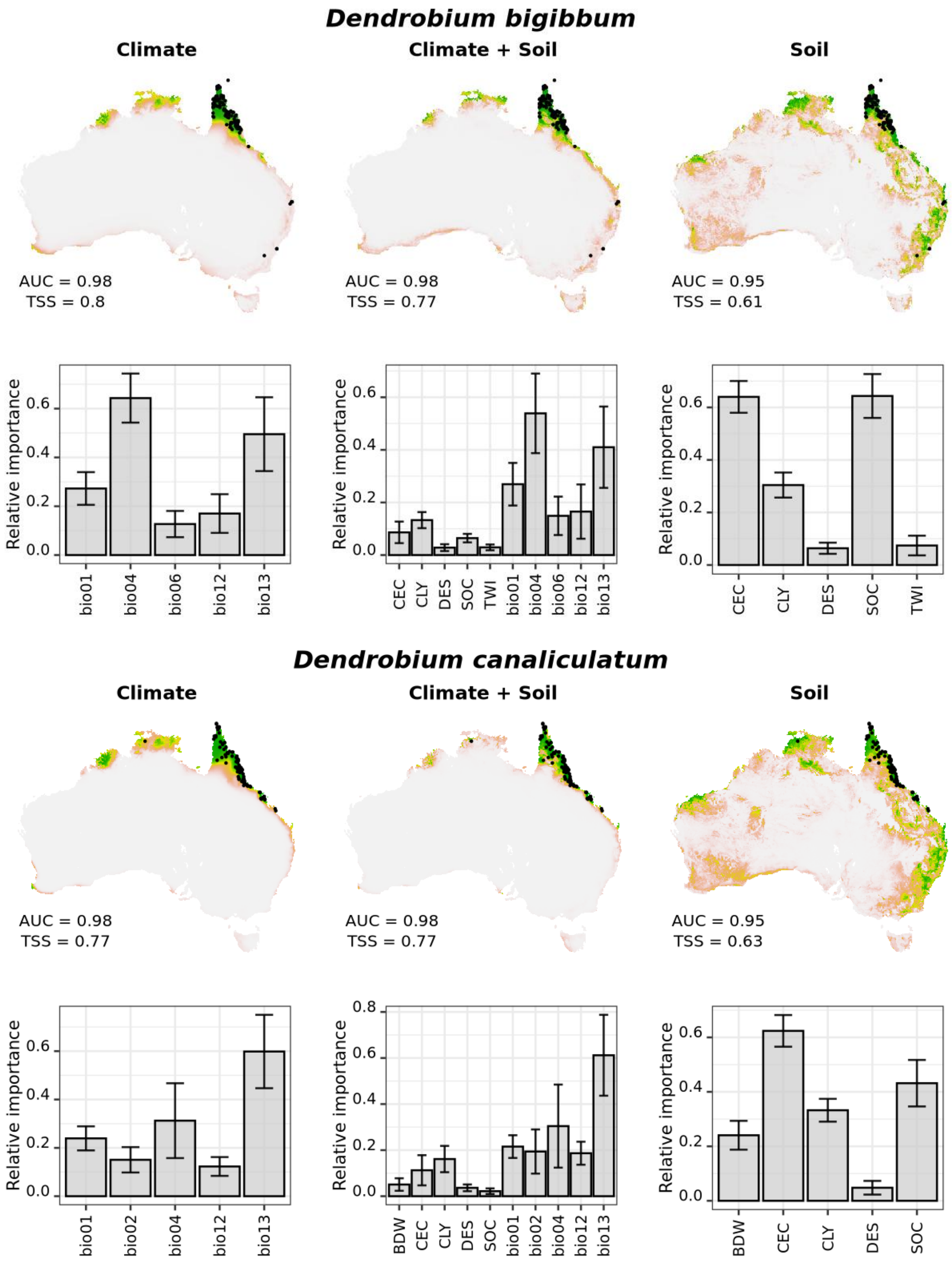

Dendrobium bigibbum
Climate
Climate + Soil
Soil
AUC = 0.98
TSS = 0.8
AUC = 0.98
TSS = 0.77
AUC = 0.95
TSS = 0.61
Relative importance
bio01
bio04
bio06
bio12
bio13
CEC
CLY
DES
SOC
TWI
Dendrobium canaliculatum
Climate
Climate + Soil
Soil
AUC = 0.98
TSS = 0.77
AUC = 0.98
TSS = 0.77
AUC = 0.95
TSS = 0.63
Relative importance
bio01
bio02
bio04
bio12
bio13
BDW
CEC
CLY
DES
SOC

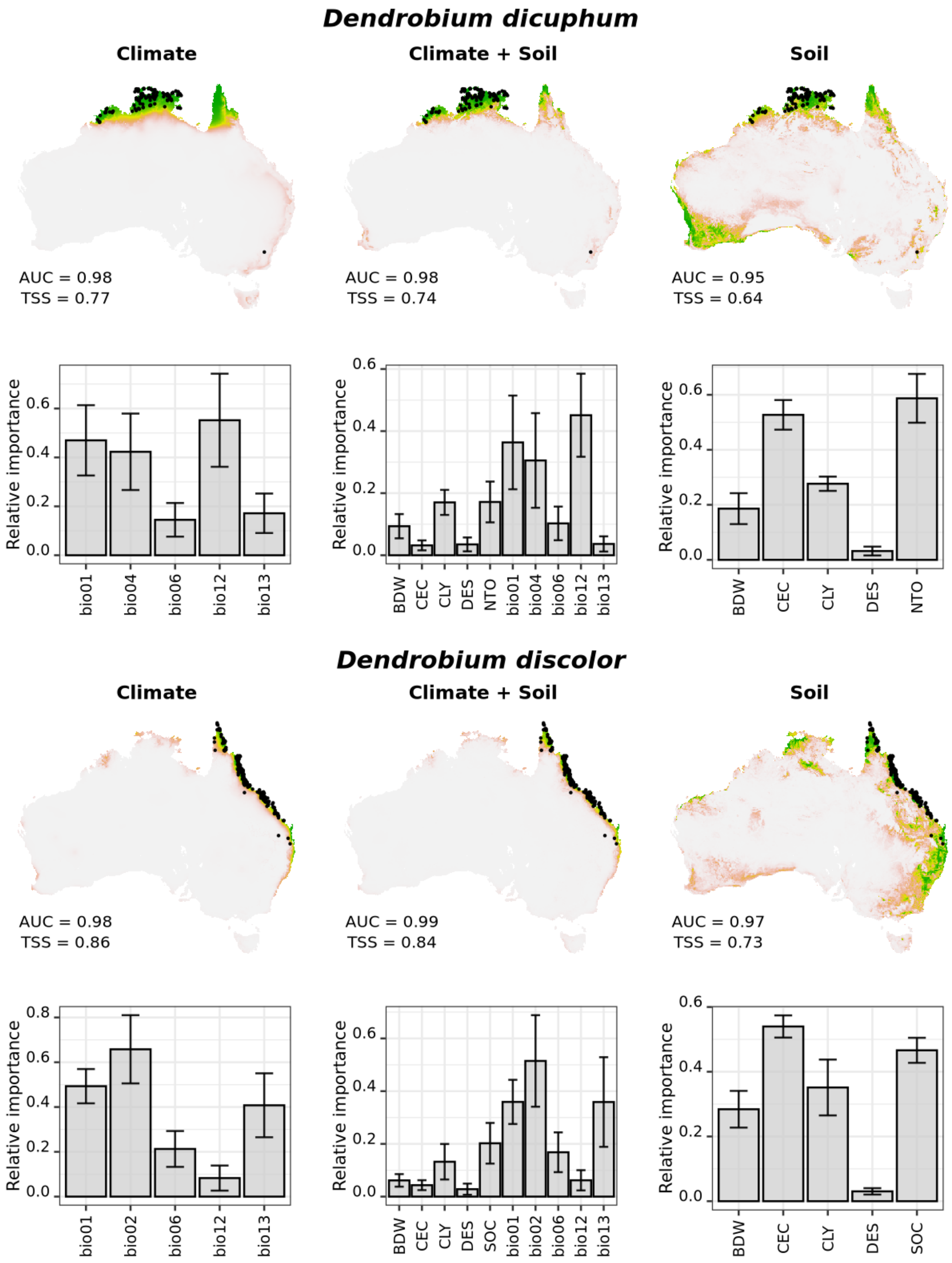

Dendrobium dicuphum
Climate
Climate + Soil
Soil
AUC = 0.98
TSS = 0.77
AUC = 0.98
TSS = 0.74
AUC = 0.95
TSS = 0.64
Relative importance
bio01
bio04
bio06
bio12
bio13
BDW
CEC
CLY
DES
NTO
Dendrobium discolor
Climate
Climate + Soil
Soil
AUC = 0.98
TSS = 0.86
AUC = 0.99
TSS = 0.84
AUC = 0.97
TSS = 0.73
Relative importance
bio01
bio02
bio06
bio12
bio13
BDW
CEC
CLY
DES
SOC

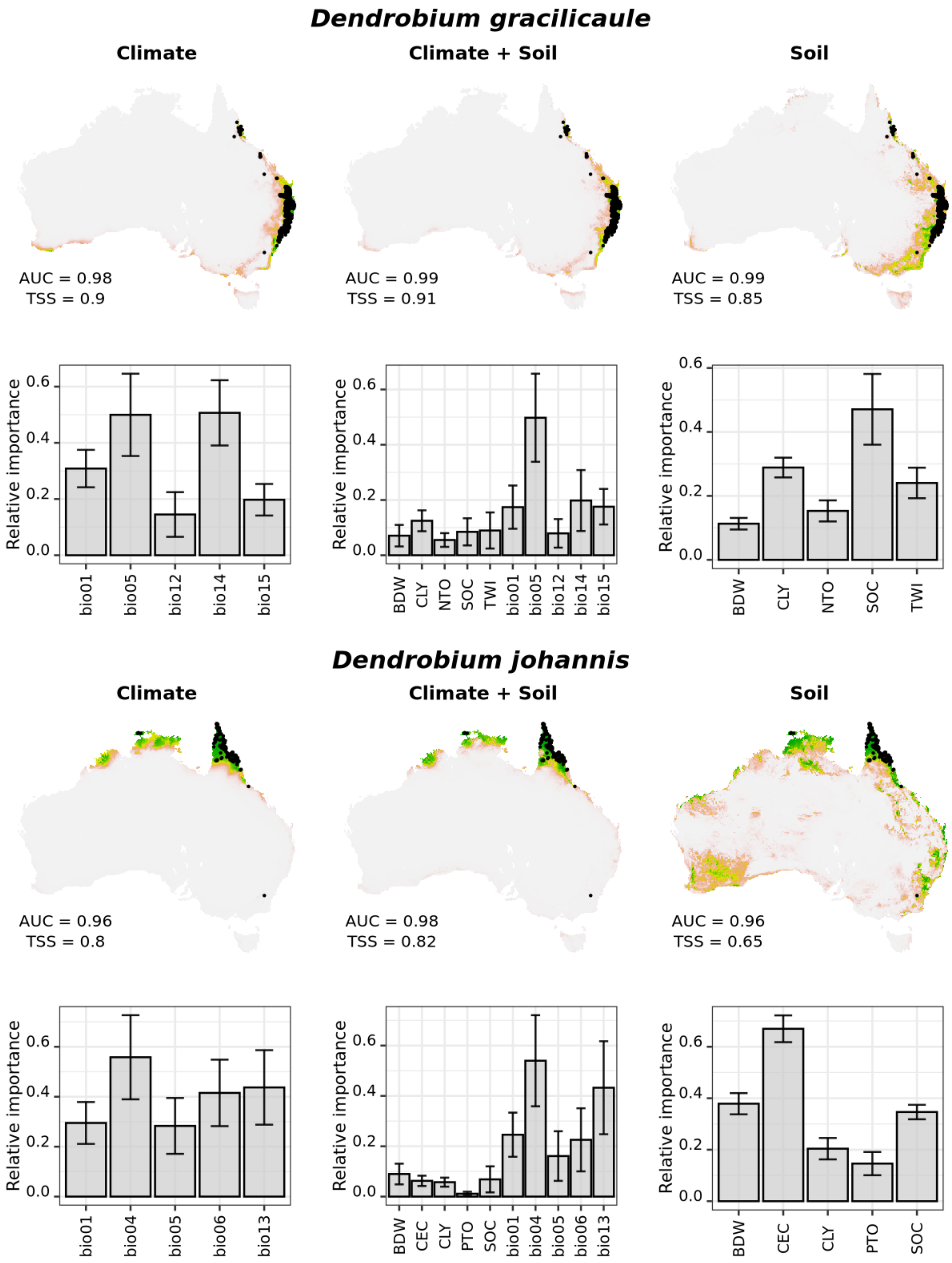
Dendrobium gracilicaule
Climate
Climate + Soil
Soil
AUC = 0.98
TSS = 0.9
AUC = 0.99
TSS = 0.91
AUC = 0.99
TSS = 0.85
Relative importance
0.0
0.2
0.4
0.6
bio01
bio05
bio12
bio14
bio15
BDW
CLY
NTO
SOC
TWI
Dendrobium johannis
Climate
Climate + Soil
Soil
AUC = 0.96
TSS = 0.8
AUC = 0.98
TSS = 0.82
AUC = 0.96
TSS = 0.65
bio04
bio06
bio13
CEC
PTO

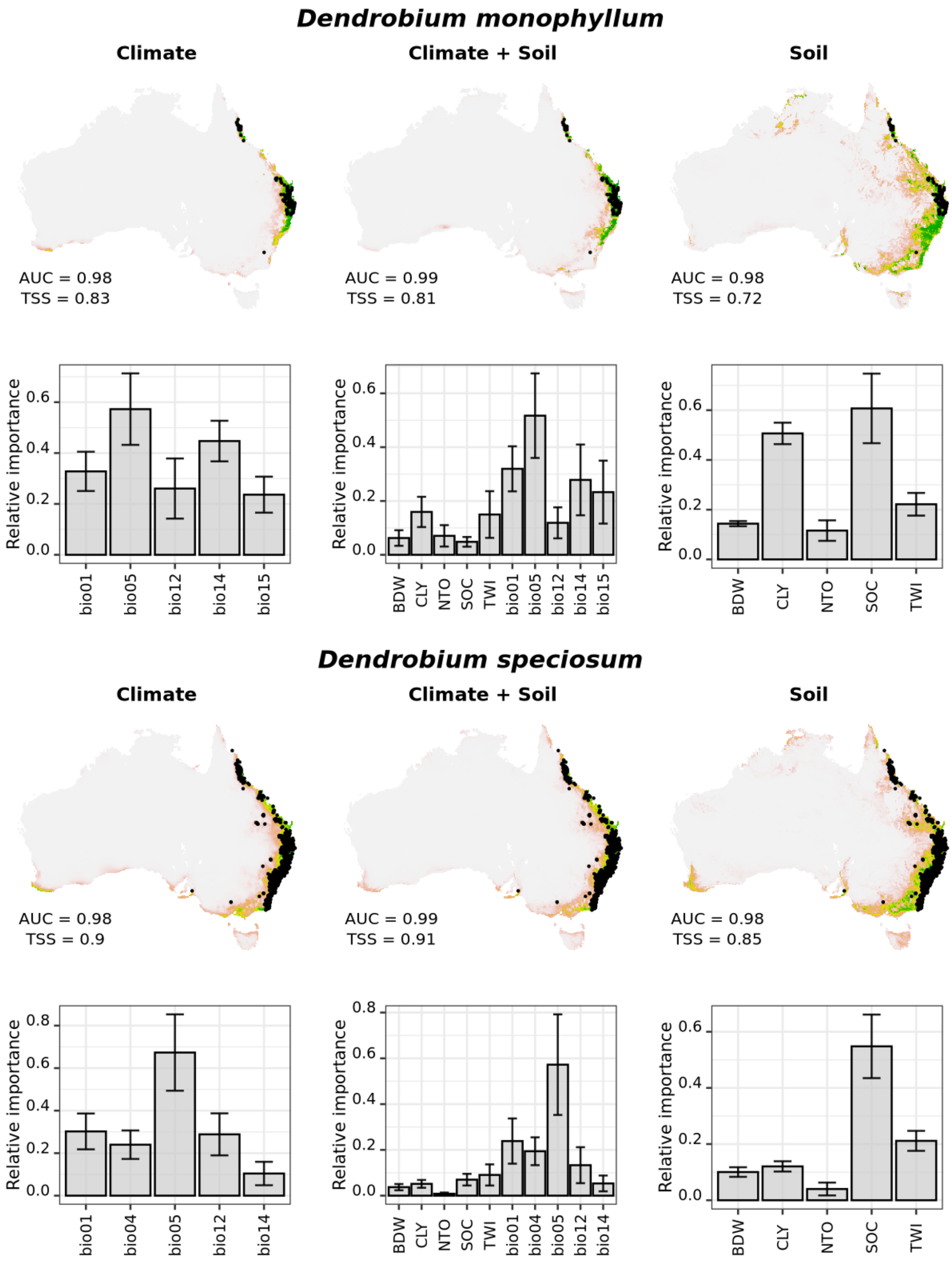

Dendrobium monophyllum
Climate
Climate + Soil
Soil
AUC = 0.98
TSS = 0.83
AUC = 0.99
TSS = 0.81
AUC = 0.98
TSS = 0.72
Relative importance
bio01
bio05
bio12
bio14
bio15
BDW
CLY
NTO
SOC
TWI
Dendrobium speciosum
Climate
Climate + Soil
Soil
AUC = 0.98
TSS = 0.9
AUC = 0.99
TSS = 0.91
AUC = 0.98
TSS = 0.85
Relative importance
bio01
bio04
bio05
bio12
bio14
BDW
CLY
NTO
SOC
TWI

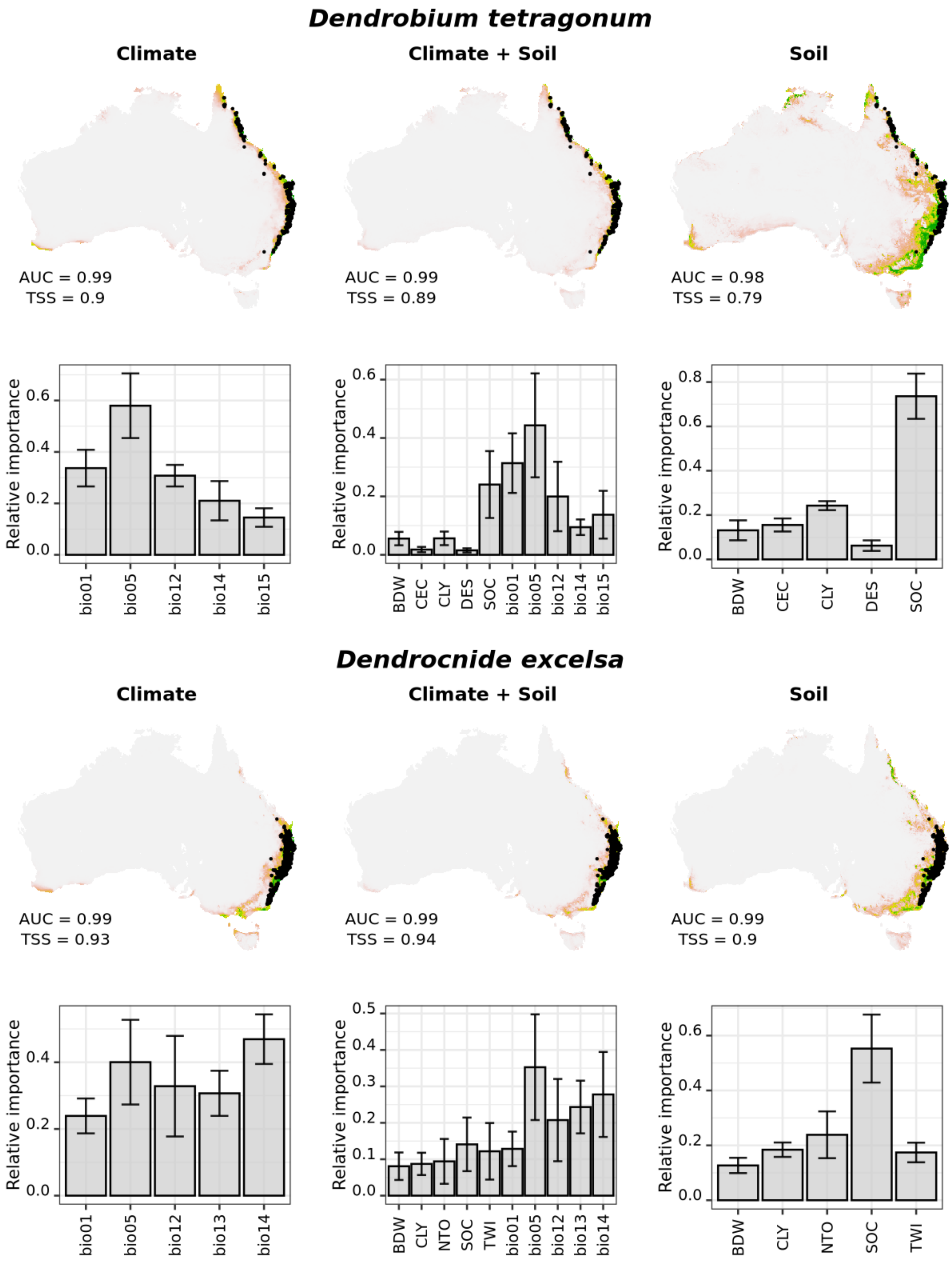
Dendrobium tetragonum
Climate
Climate + Soil
Soil
AUC = 0.99
TSS = 0.9
AUC = 0.99
TSS = 0.89
AUC = 0.98
TSS = 0.79
Relative importance
bio01
bio05
bio12
bio14
bio15
BDW
CEC
CLY
DES
SOC
Dendrocnide excelsa
Climate
Climate + Soil
Soil
AUC = 0.99
TSS = 0.93
AUC = 0.99
TSS = 0.94
AUC = 0.99
TSS = 0.9
Relative importance
bio01
bio05
bio12
bio13
bio14
BDW
CLY
NTO
SOC
TWI

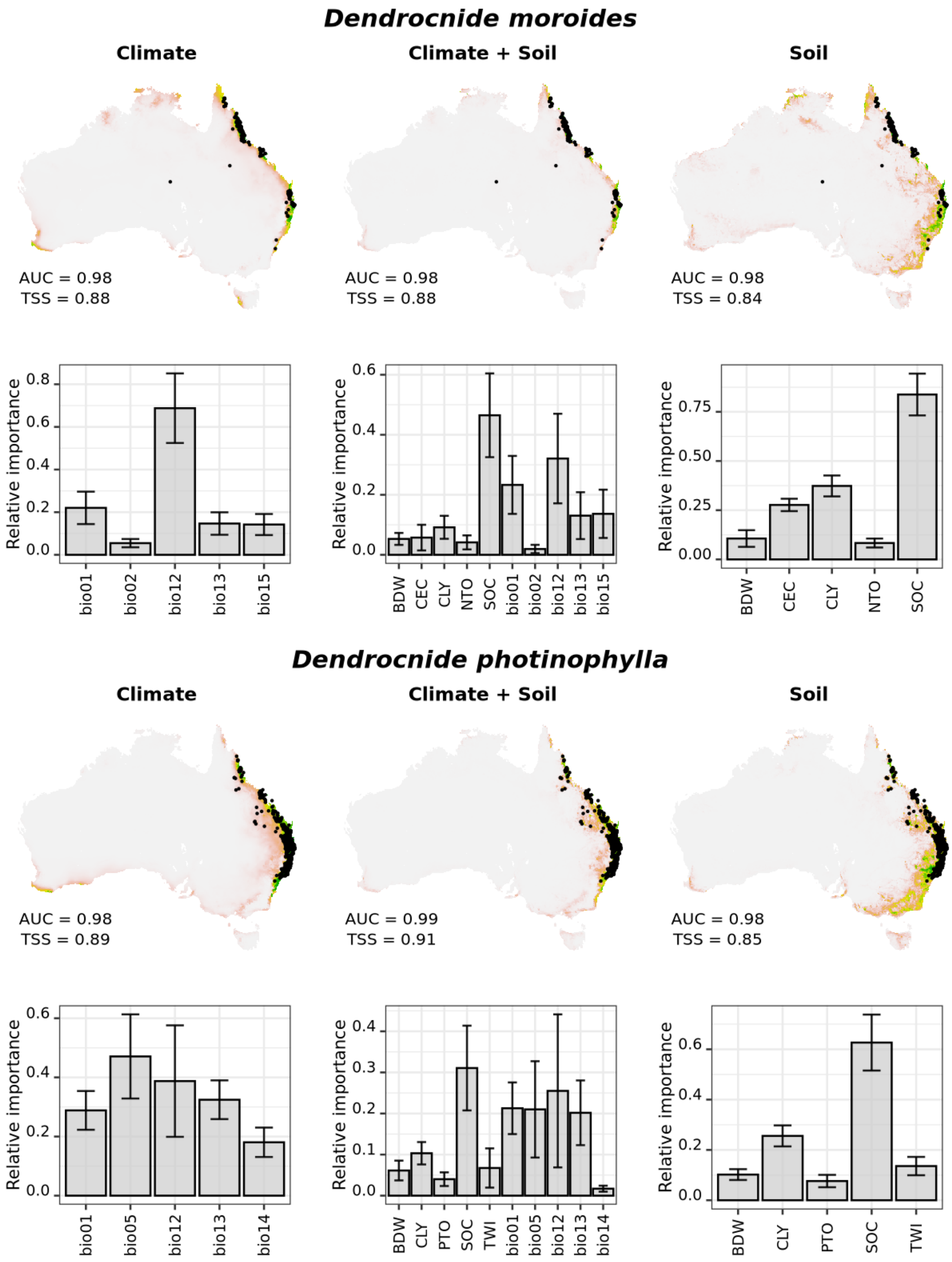
Dendrocnide moroides
Climate
Climate + Soil
Soil
AUC = 0.98
TSS = 0.88
AUC = 0.98
TSS = 0.88
AUC = 0.98
TSS = 0.84
Relative importance
0.0
0.2
0.4
0.6
0.8
bio01
bio02
bio12
bio13
bio15
Relative importance
0.0
0.2
0.4
0.6
BDW
CEC
CLY
NTO
SOC
bio01
bio02
bio12
bio13
bio15
Relative importance
0.00
0.25
0.50
0.75
BDW
CEC
CLY
NTO
SOC
Dendrocnide photinophylla
Climate
Climate + Soil
Soil
AUC = 0.98
TSS = 0.89
AUC = 0.99
TSS = 0.91
AUC = 0.98
TSS = 0.85
Relative importance
0.0
0.2
0.4
0.6
bio01
bio05
bio12
bio13
bio14
Relative importance
0.0
0.1
0.2
0.3
0.4
BDW
CLY
PTO
SOC
TWI
bio01
bio05
bio12
bio13
bio14
Relative importance
0.0
0.2
0.4
0.6
BDW
CLY
PTO
SOC
TWI

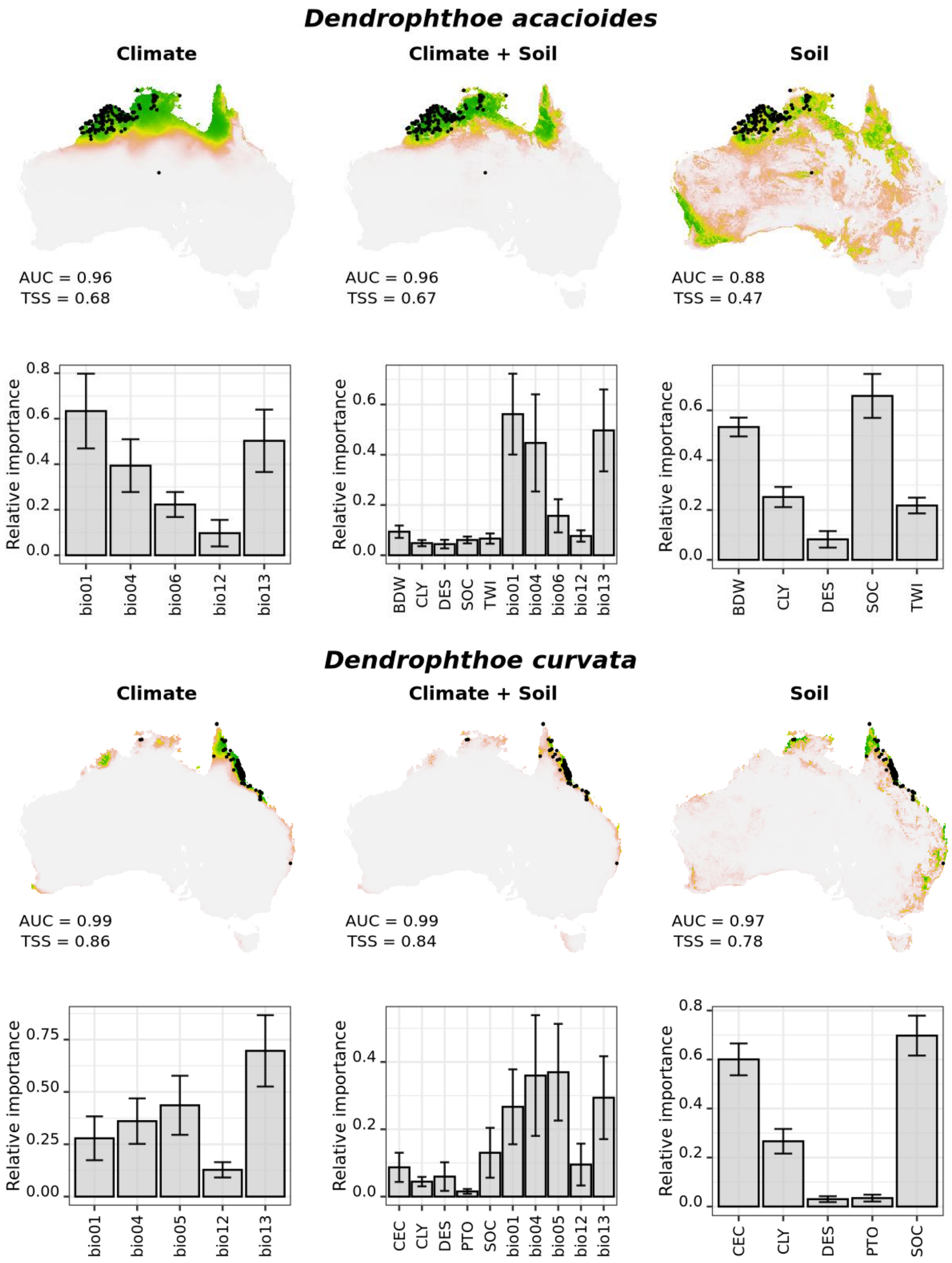
Dendrophthoe acacioides
Climate
Climate + Soil
Soil
AUC = 0.96
TSS = 0.68
AUC = 0.96
TSS = 0.67
AUC = 0.88
TSS = 0.47
Relative importance
bio01
bio04
bio06
bio12
bio13
BDW
CLY
DES
SOC
TWI
Dendrophthoe curvata
Climate
Climate + Soil
Soil
AUC = 0.99
TSS = 0.86
AUC = 0.99
TSS = 0.84
AUC = 0.97
TSS = 0.78
Relative importance
bio01
bio04
bio05
bio12
bio13
CEC
CLY
DES
PTO
SOC

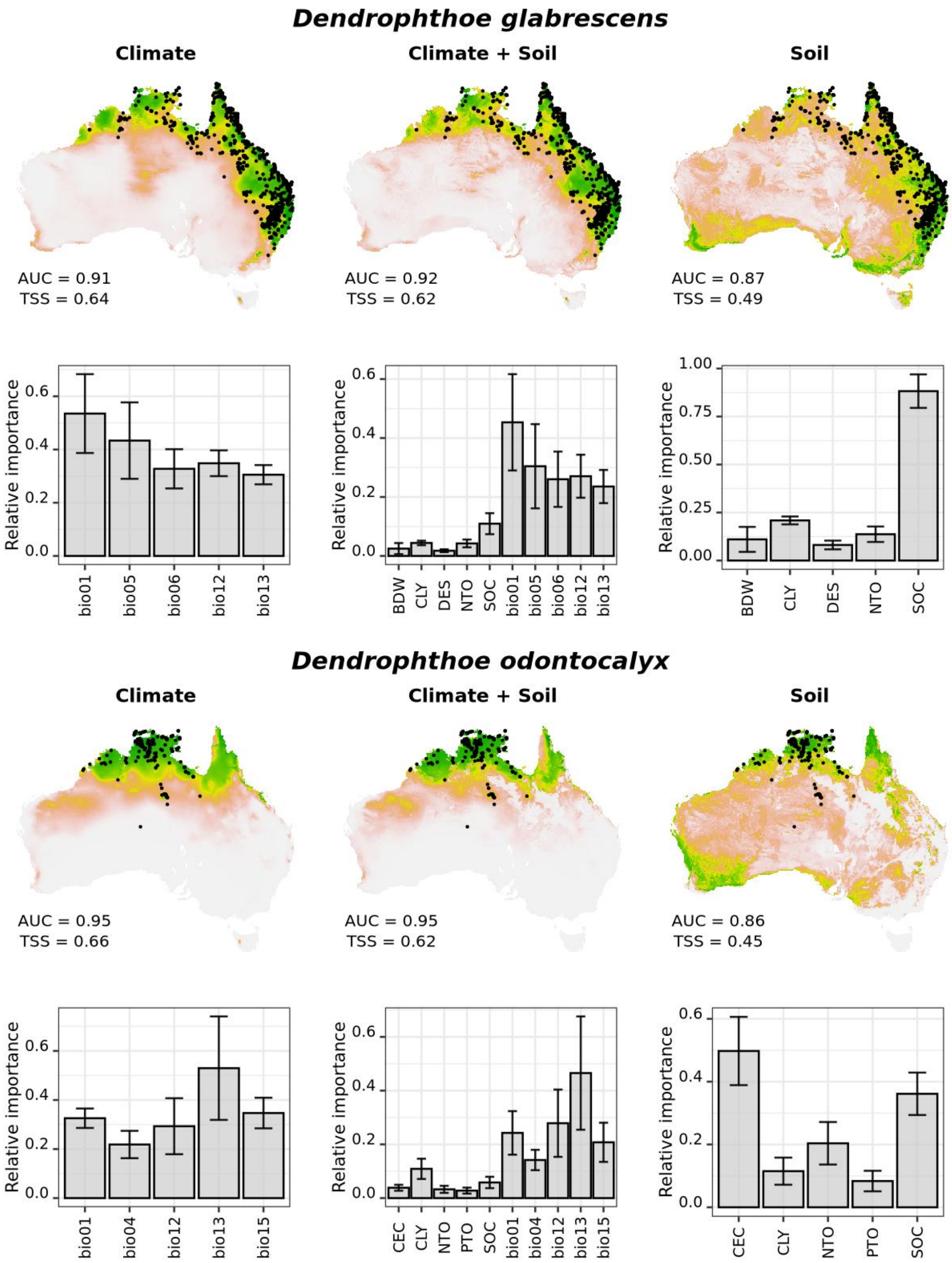
Dendrophthoe glabrescens
Climate
Climate + Soil
Soil
AUC = 0.91
TSS = 0.64
AUC = 0.92
TSS = 0.62
AUC = 0.87
TSS = 0.49
Relative importance
0.0
0.2
0.4
0.6
bio01
bio05
bio06
bio12
bio13
BDW
CLY
DES
NTO
SOC
0.00
0.25
0.50
0.75
1.00
Dendrophthoe odontocalyx
Climate
Climate + Soil
Soil
AUC = 0.95
TSS = 0.66
AUC = 0.95
TSS = 0.62
AUC = 0.86
TSS = 0.45
bio04
bio15
CEC
PTO

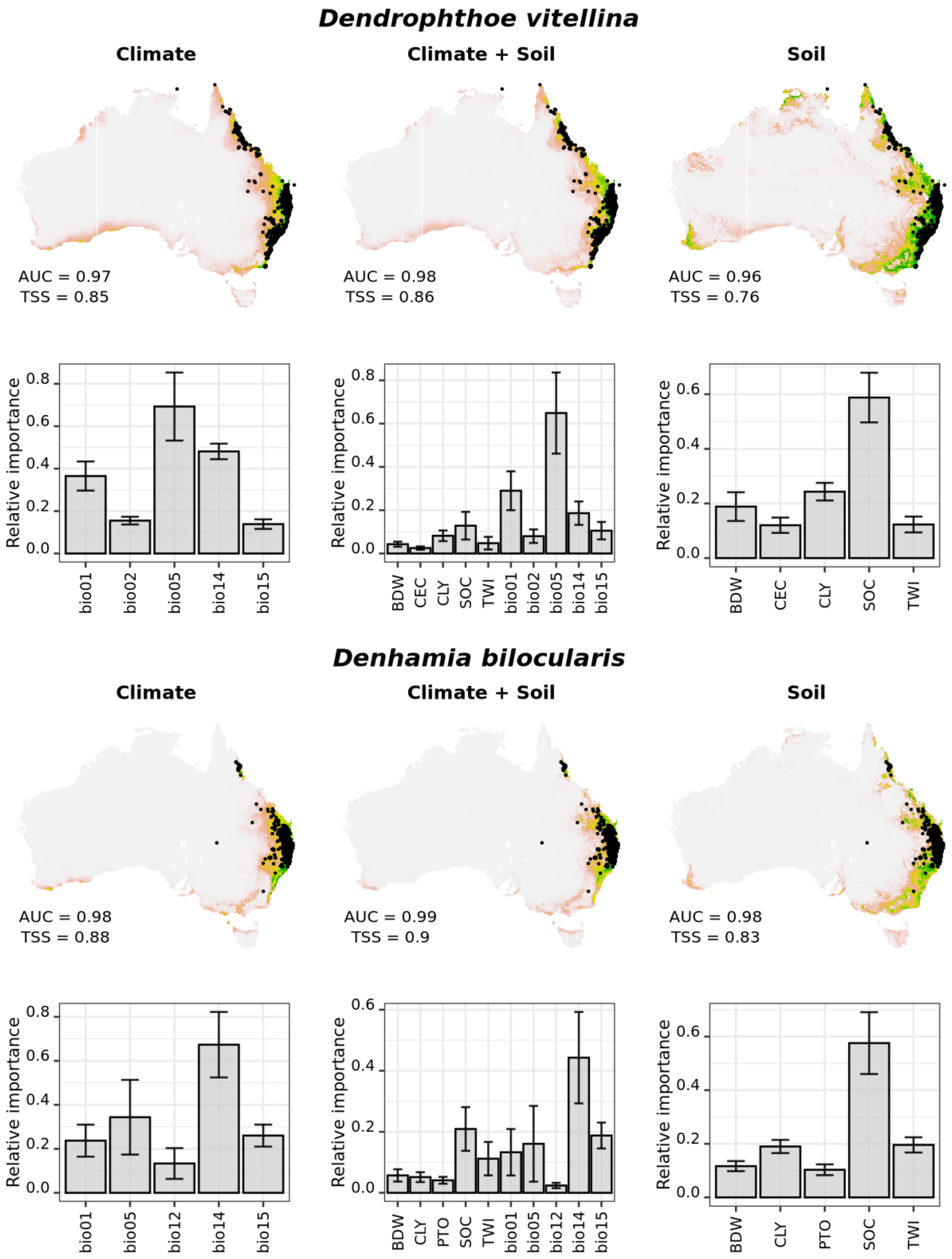
Dendrophthoe vitellina
Climate
Climate + Soil
Soil
AUC = 0.97
TSS = 0.85
AUC = 0.98
TSS = 0.86
AUC = 0.96
TSS = 0.76
Relative importance
bio01
bio02
bio05
bio14
bio15
BDW
CEC
CLY
SOC
TWI
Denhamia bilocularis
Climate
Climate + Soil
Soil
AUC = 0.98
TSS = 0.88
AUC = 0.99
TSS = 0.9
AUC = 0.98
TSS = 0.83
Relative importance
bio01
bio05
bio12
bio14
bio15
BDW
CLY
PTO
SOC
TWI

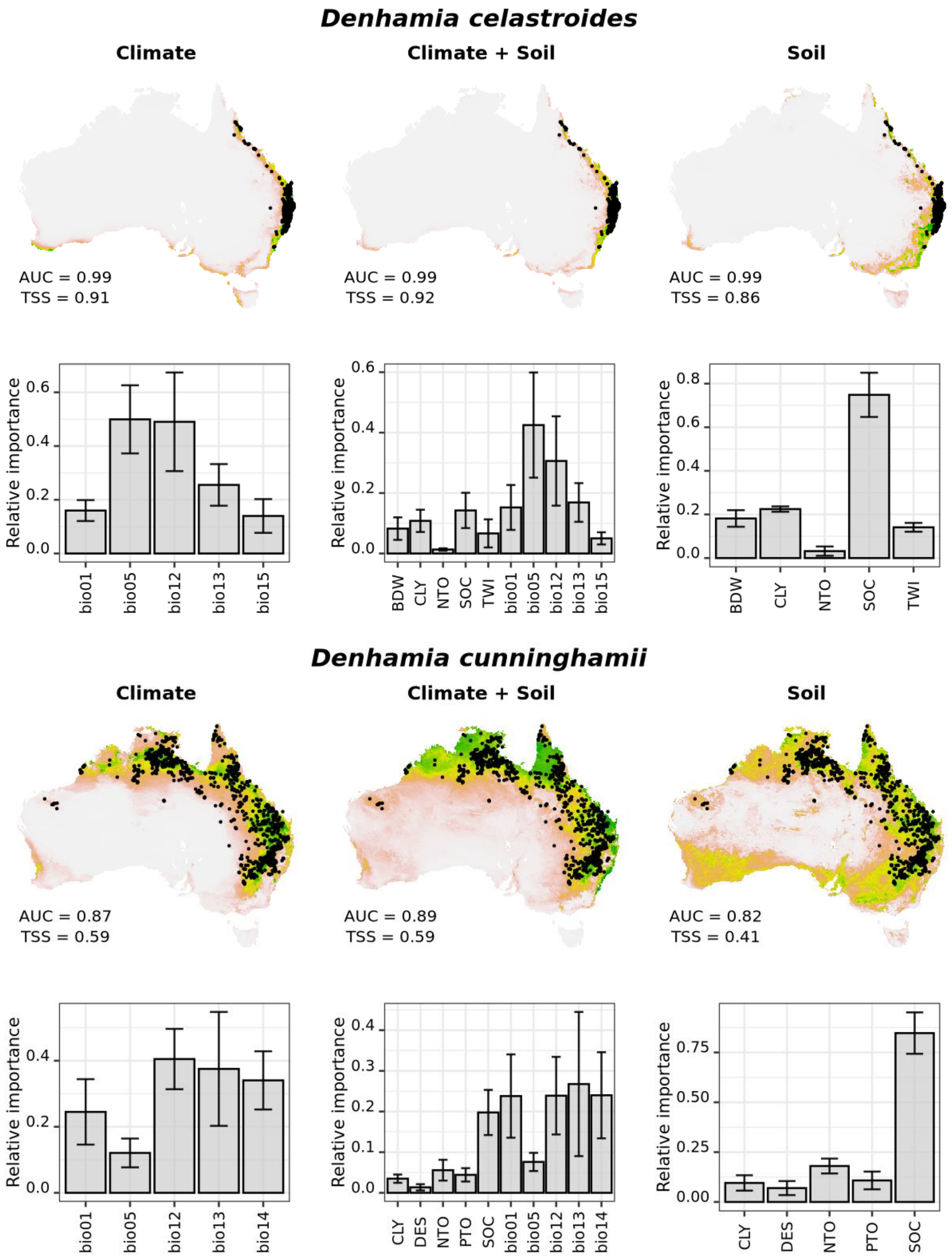
Denhamia celastroides
Climate
Climate + Soil
Soil
AUC = 0.99
TSS = 0.91
AUC = 0.99
TSS = 0.92
AUC = 0.99
TSS = 0.86
Relative importance
bio01
bio05
bio12
bio13
bio15
BDW
CLY
NTO
SOC
TWI
Denhamia cunninghamii
Climate
Climate + Soil
Soil
AUC = 0.87
TSS = 0.59
AUC = 0.89
TSS = 0.59
AUC = 0.82
TSS = 0.41
Relative importance
bio01
bio05
bio12
bio13
bio14
CLY
DES
NTO
PTO
SOC

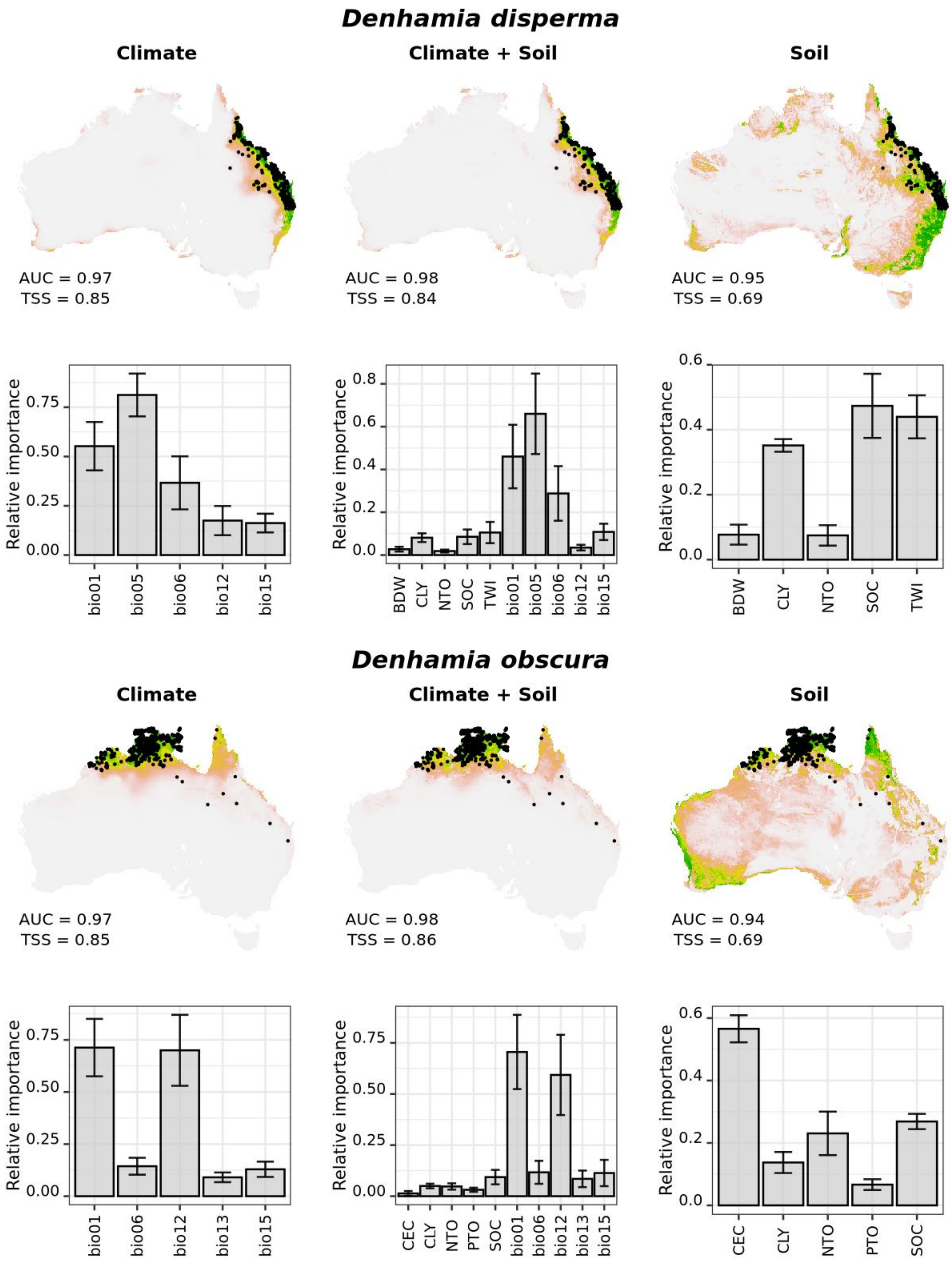
Denhamia disperma
Climate
Climate + Soil
Soil
AUC = 0.97
TSS = 0.85
AUC = 0.98
TSS = 0.84
AUC = 0.95
TSS = 0.69
Relative importance
bio01
bio05
bio06
bio12
bio15
BDW
CLY
NTO
SOC
TWI
Denhamia obscura
Climate
Climate + Soil
Soil
AUC = 0.97
TSS = 0.85
AUC = 0.98
TSS = 0.86
AUC = 0.94
TSS = 0.69
Relative importance
bio01
bio06
bio12
bio13
bio15
CEC
CLY
NTO
PTO
SOC

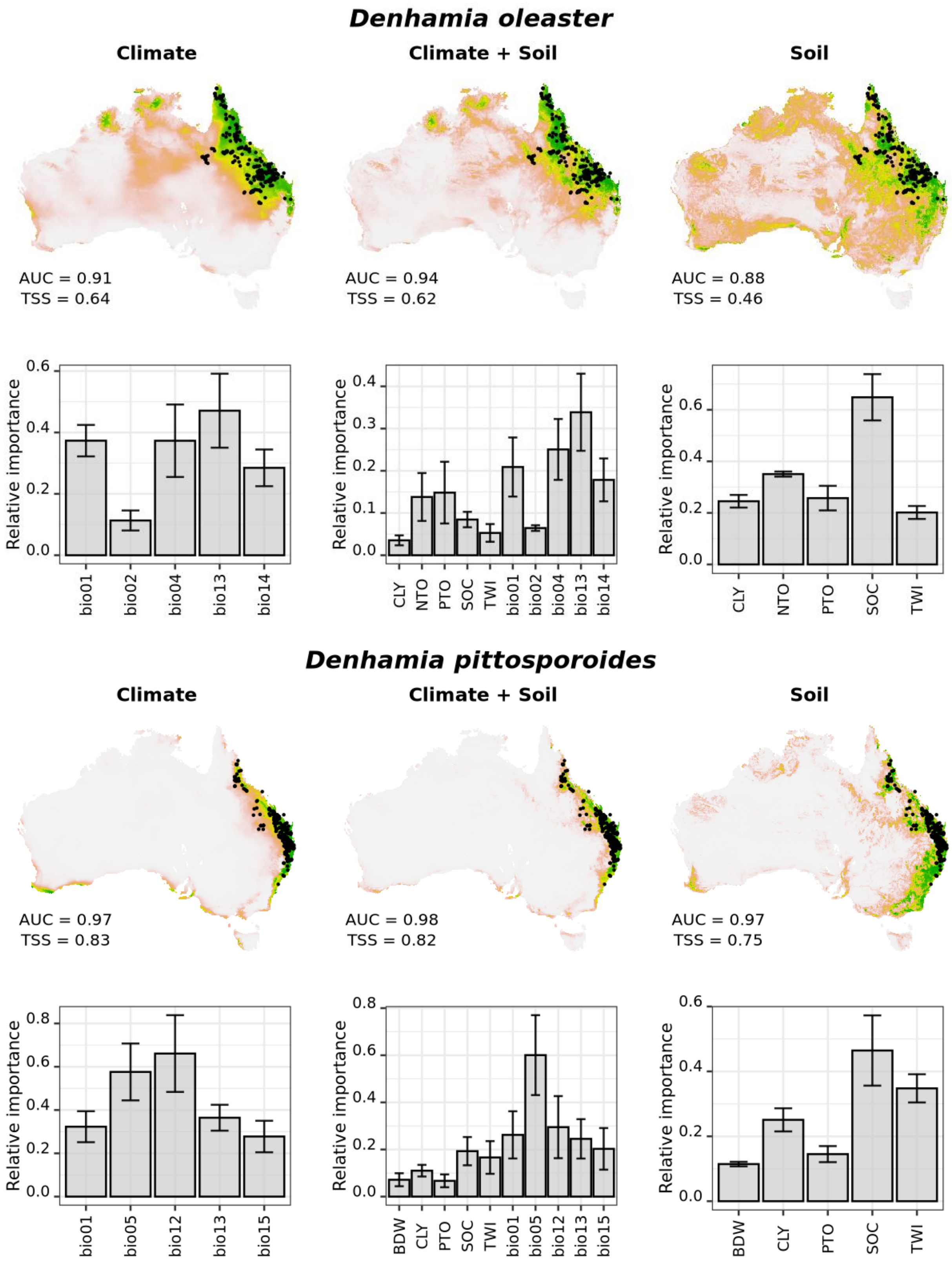

Denhamia oleaster
Climate
Climate + Soil
Soil
AUC = 0.91
TSS = 0.64
AUC = 0.94
TSS = 0.62
AUC = 0.88
TSS = 0.46
Relative importance
0.0
0.2
0.4
0.6
bio01
bio02
bio04
bio13
bio14
0.0
0.1
0.2
0.3
0.4
CLY
NTO
PTO
SOC
TWI
bio01
bio02
bio04
bio13
bio14
0.0
0.2
0.4
0.6
CLY
NTO
PTO
SOC
TWI
Denhamia pittosporoides
Climate
Climate + Soil
Soil
AUC = 0.97
TSS = 0.83
AUC = 0.98
TSS = 0.82
AUC = 0.97
TSS = 0.75
Relative importance
0.0
0.2
0.4
0.6
0.8
bio01
bio05
bio12
bio13
bio15
0.0
0.2
0.4
0.6
0.8
BDW
CLY
PTO
SOC
TWI
bio01
bio05
bio12
bio13
bio15
0.0
0.2
0.4
0.6
BDW
CLY
PTO
SOC
TWI

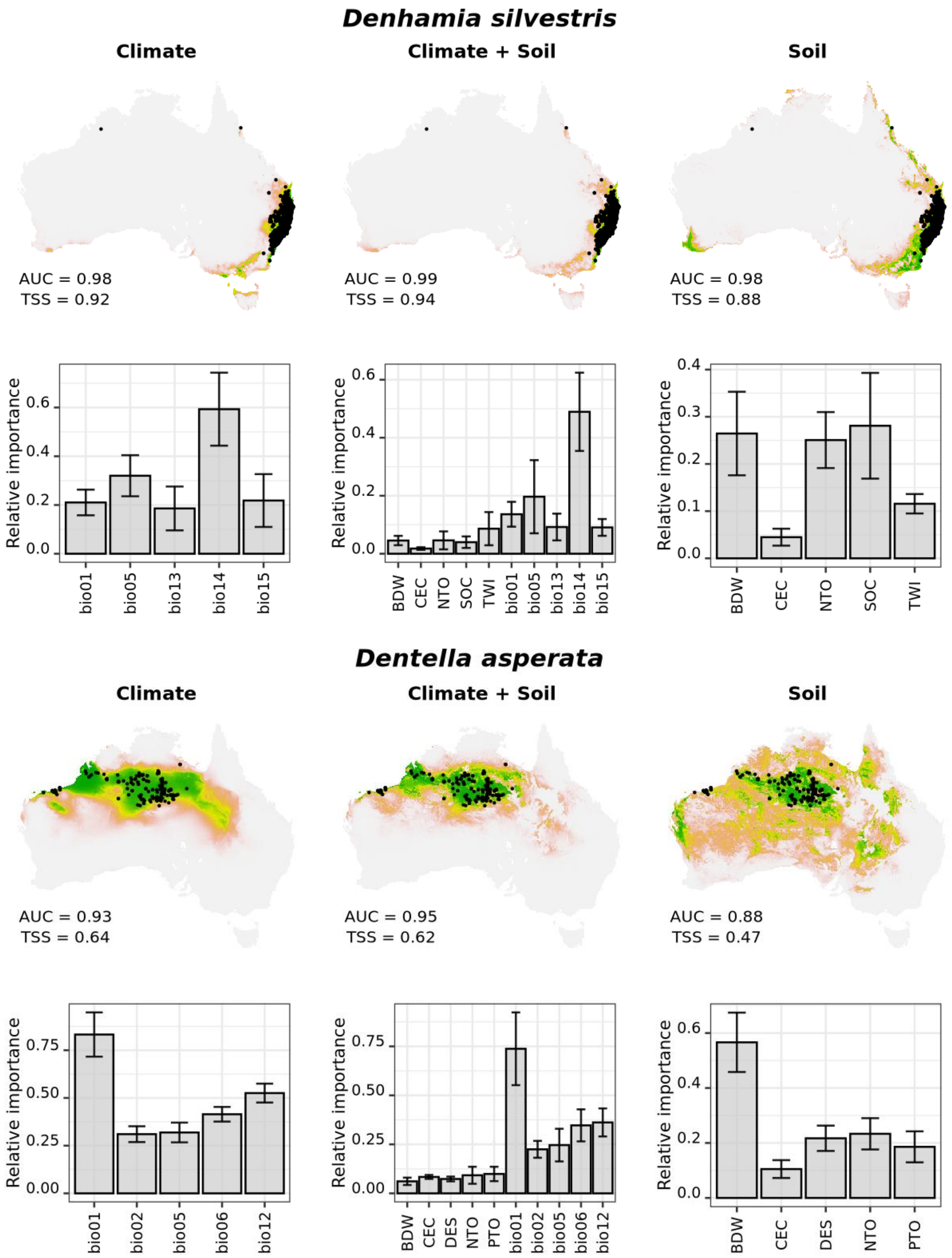

Denhamia silvestris
Climate
Climate + Soil
Soil
AUC = 0.98
TSS = 0.92
AUC = 0.99
TSS = 0.94
AUC = 0.98
TSS = 0.88
Relative importance
0.0
0.2
0.4
0.6
bio01
bio05
bio13
bio14
bio15
BDW
CEC
NTO
SOC
TWI
0.1
0.3
Dentella asperata
AUC = 0.93
TSS = 0.64
AUC = 0.95
TSS = 0.62
AUC = 0.88
TSS = 0.47
0.00
0.25
0.50
0.75
bio02
bio06
bio12
DES
PTO

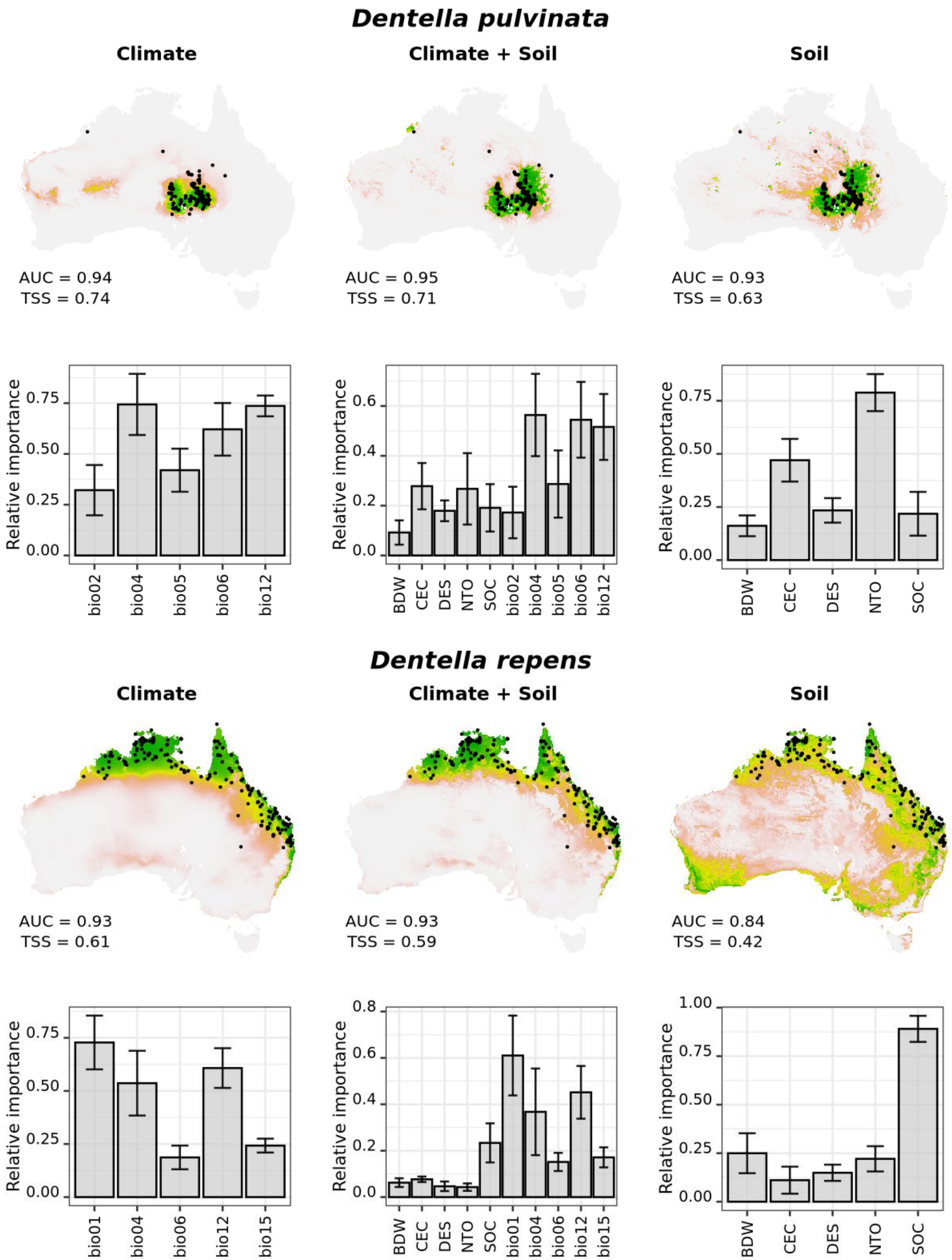
Dentella pulvinata
Climate
Climate + Soil
Soil
AUC = 0.94
TSS = 0.74
AUC = 0.95
TSS = 0.71
AUC = 0.93
TSS = 0.63
Relative importance
0.00
0.25
0.50
0.75
bio02
bio04
bio05
bio06
bio12
0.0
0.2
0.4
0.6
BDW
CEC
DES
NTO
SOC
Dentella repens
AUC = 0.93
TSS = 0.61
AUC = 0.93
TSS = 0.59
AUC = 0.84
TSS = 0.42
bio01
bio15
0.8
1.00

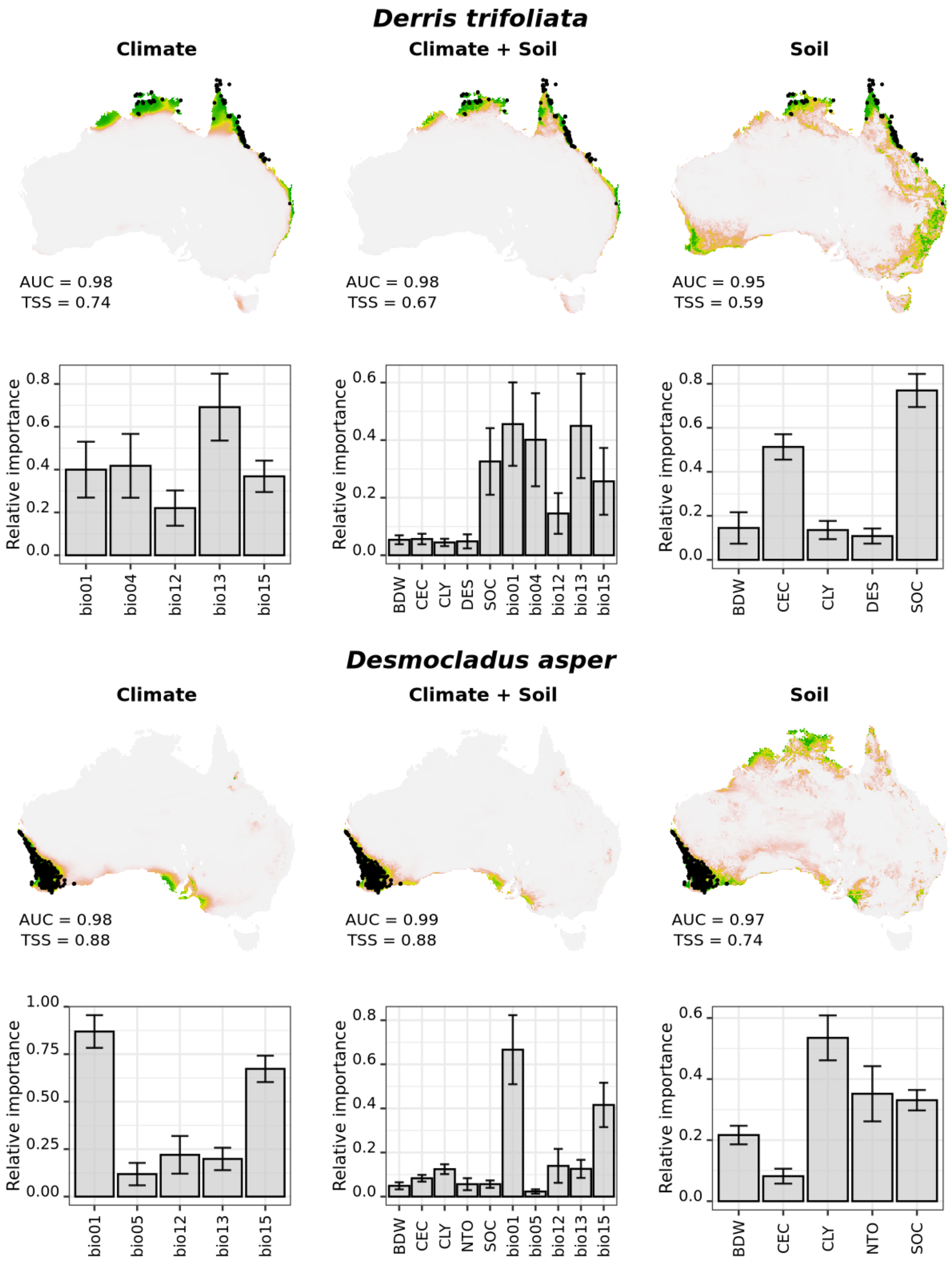

Derris trifoliata
Climate
Climate + Soil
Soil
AUC = 0.98
TSS = 0.74
AUC = 0.98
TSS = 0.67
AUC = 0.95
TSS = 0.59
Relative importance
bio01
bio04
bio12
bio13
bio15
BDW
CEC
CLY
DES
SOC
Desmocladus asper
Climate
Climate + Soil
Soil
AUC = 0.98
TSS = 0.88
AUC = 0.99
TSS = 0.88
AUC = 0.97
TSS = 0.74
Relative importance
bio01
bio05
bio12
bio13
bio15
BDW
CEC
CLY
NTO
SOC

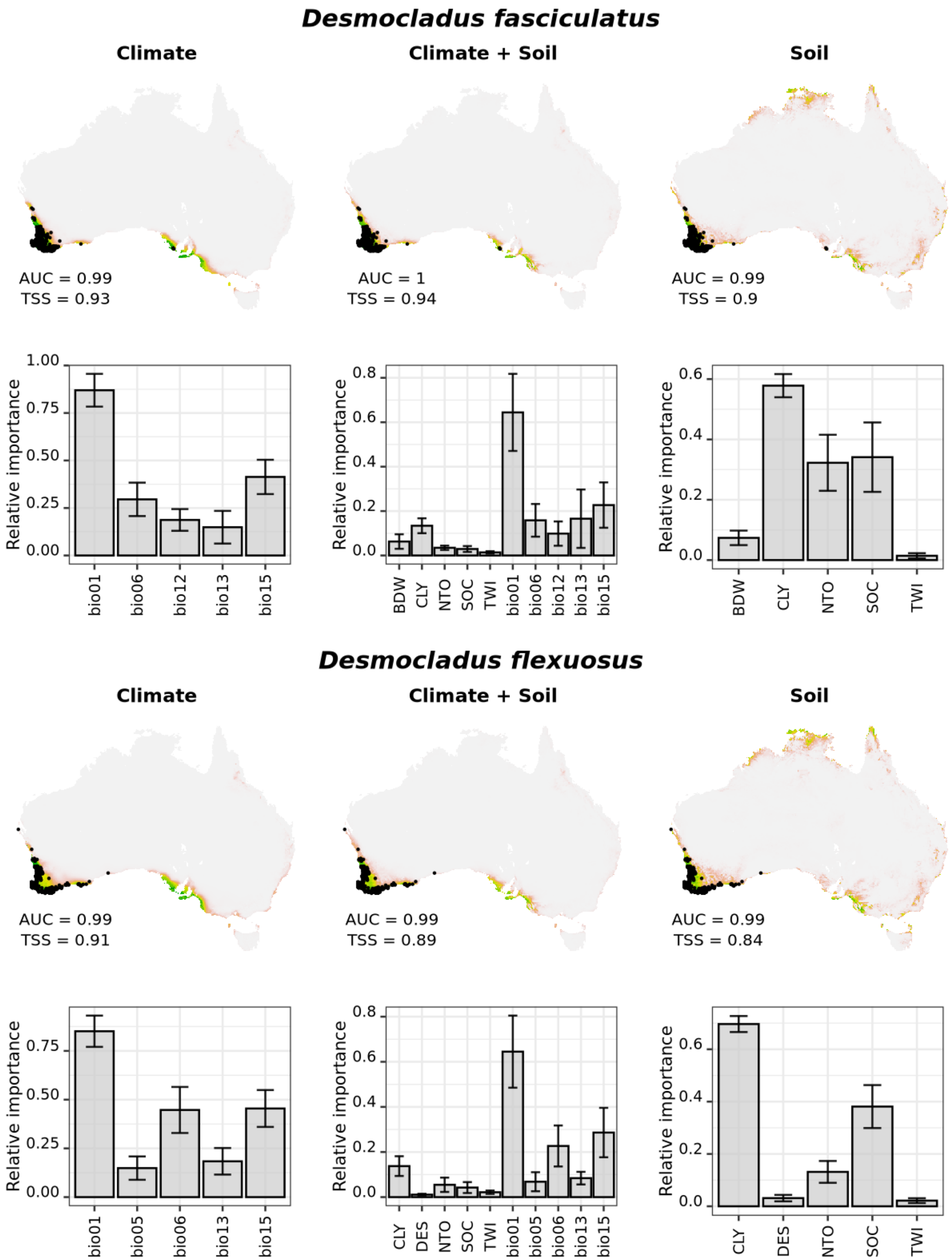
Desmocladus fasciculatus
Climate
Climate + Soil
Soil
AUC = 0.99
TSS = 0.93
AUC = 1
TSS = 0.94
AUC = 0.99
TSS = 0.9
Relative importance
1.00
0.75
0.50
0.25
0.00
bio01
bio06
bio12
bio13
bio15
0.8
0.6
0.4
0.2
0.0
BDW
CLY
NTO
SOC
TWI
bio01
bio06
bio12
bio13
bio15
0.6
0.4
0.2
0.0
BDW
CLY
NTO
SOC
TWI
Desmocladus flexuosus
Climate
Climate + Soil
Soil
AUC = 0.99
TSS = 0.91
AUC = 0.99
TSS = 0.89
AUC = 0.99
TSS = 0.84
Relative importance
0.75
0.50
0.25
0.00
bio01
bio05
bio06
bio13
bio15
0.8
0.6
0.4
0.2
0.0
CLY
DES
NTO
SOC
TWI
bio01
bio05
bio06
bio13
bio15
0.6
0.4
0.2
0.0
CLY
DES
NTO
SOC
TWI

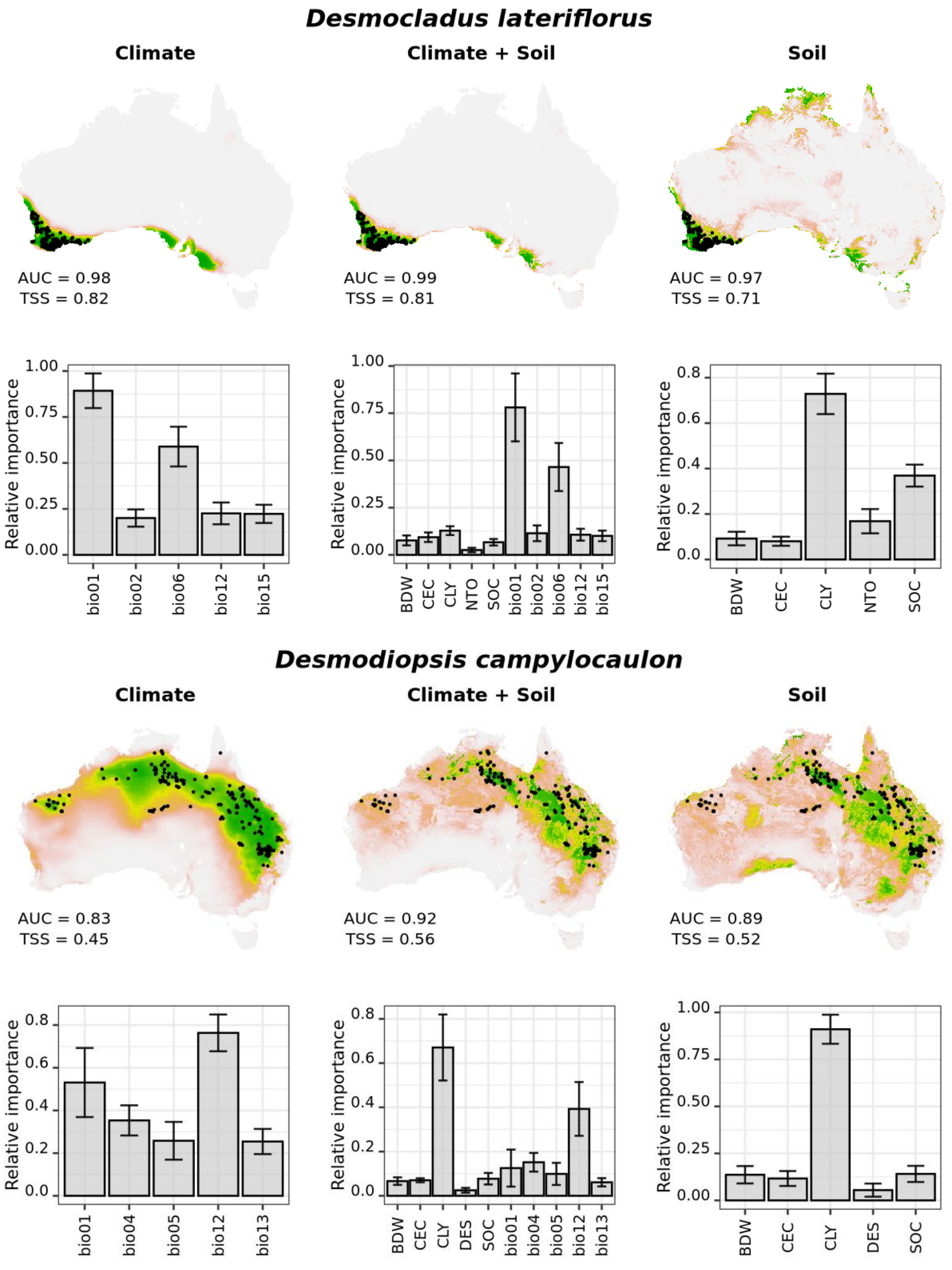
Desmocladus lateriflorus
Climate
Climate + Soil
Soil
AUC = 0.98
TSS = 0.82
AUC = 0.99
TSS = 0.81
AUC = 0.97
TSS = 0.71
Relative importance
bio01
bio02
bio06
bio12
bio15
BDW
CEC
CLY
NTO
SOC
Desmodiopsis campylocaulon
Climate
Climate + Soil
Soil
AUC = 0.83
TSS = 0.45
AUC = 0.92
TSS = 0.56
AUC = 0.89
TSS = 0.52
Relative importance
bio01
bio04
bio05
bio12
bio13
BDW
CEC
CLY
DES
SOC

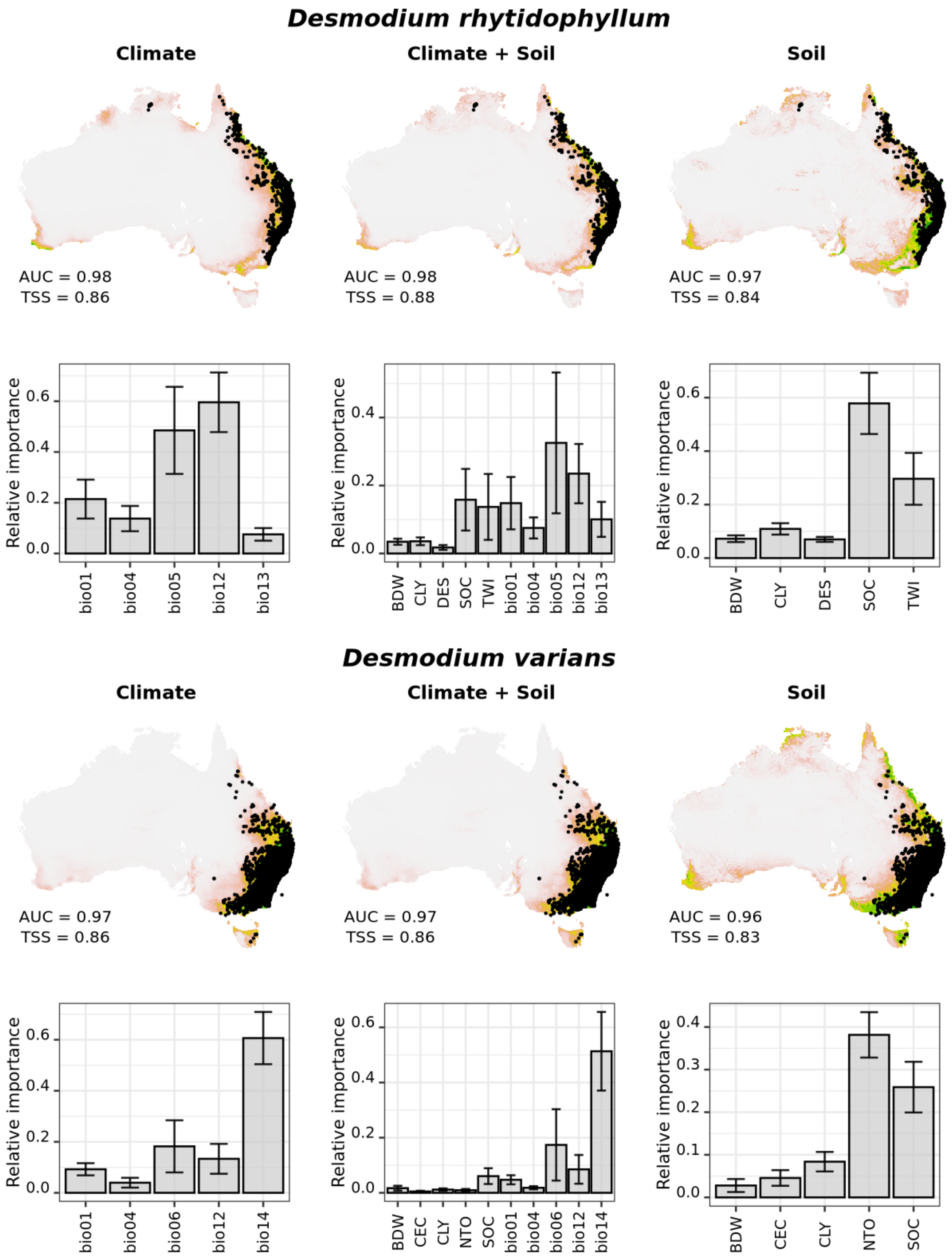

Desmodium rhytidophyllum
Climate
Climate + Soil
Soil
AUC = 0.98
TSS = 0.86
AUC = 0.98
TSS = 0.88
AUC = 0.97
TSS = 0.84
Relative importance
bio01
bio04
bio05
bio12
bio13
BDW
CLY
DES
SOC
TWI
Desmodium varians
AUC = 0.97
TSS = 0.86
AUC = 0.97
TSS = 0.86
AUC = 0.96
TSS = 0.83
bio06
bio14
CEC
NTO

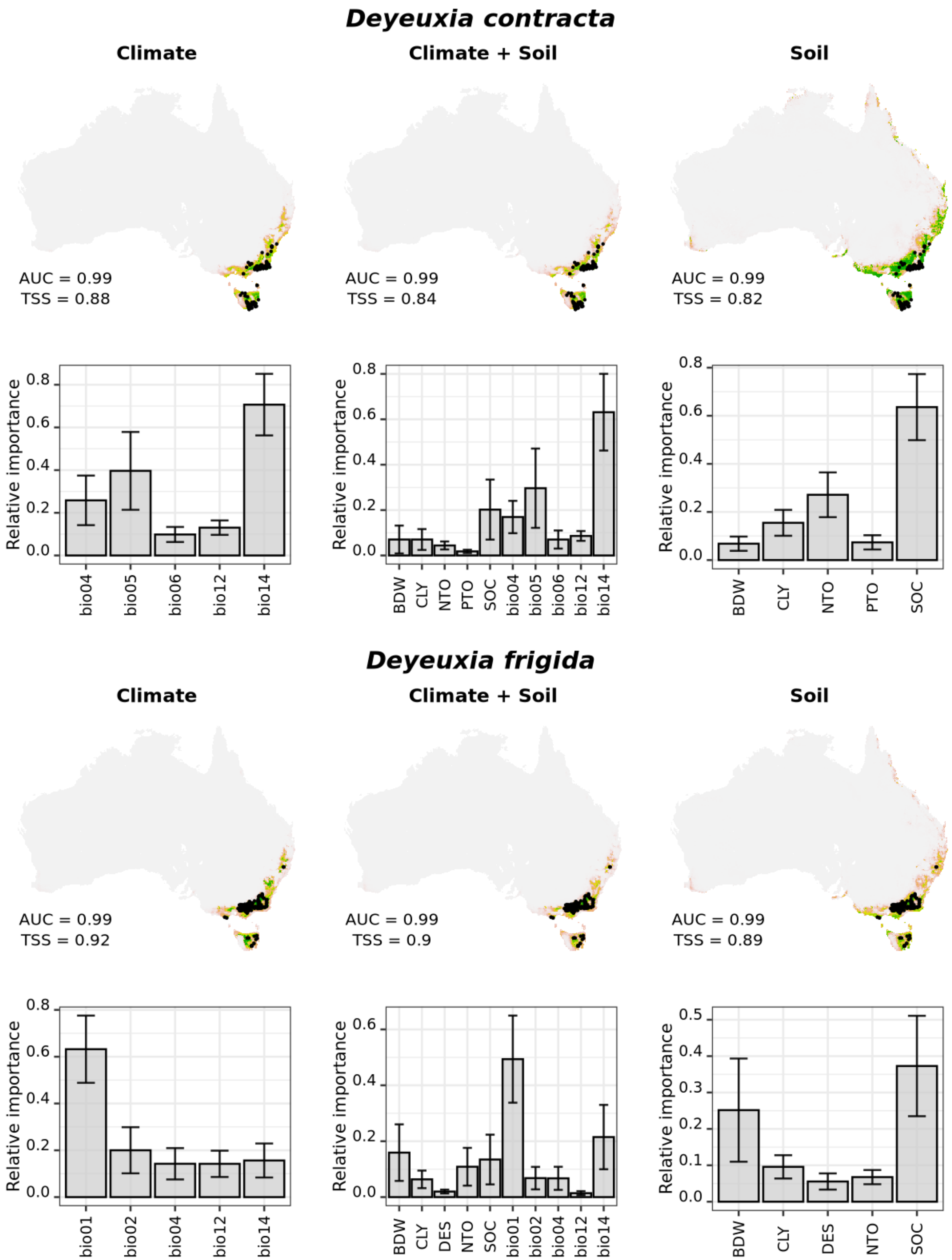
Deyeuxia contracta
Climate
Climate + Soil
Soil
AUC = 0.99
TSS = 0.88
AUC = 0.99
TSS = 0.84
AUC = 0.99
TSS = 0.82
Relative importance
0.0
0.2
0.4
0.6
0.8
bio04
bio05
bio06
bio12
bio14
BDW
CLY
NTO
PTO
SOC
bio04
bio05
bio06
bio12
bio14
BDW
CLY
NTO
PTO
SOC
Deyeuxia frigida
Climate
Climate + Soil
Soil
AUC = 0.99
TSS = 0.92
AUC = 0.99
TSS = 0.9
AUC = 0.99
TSS = 0.89
Relative importance
0.0
0.1
0.2
0.3
0.4
0.5
0.6
0.8
bio01
bio02
bio04
bio12
bio14
BDW
CLY
DES
NTO
SOC
bio01
bio02
bio04
bio12
bio14
BDW
CLY
DES
NTO
SOC

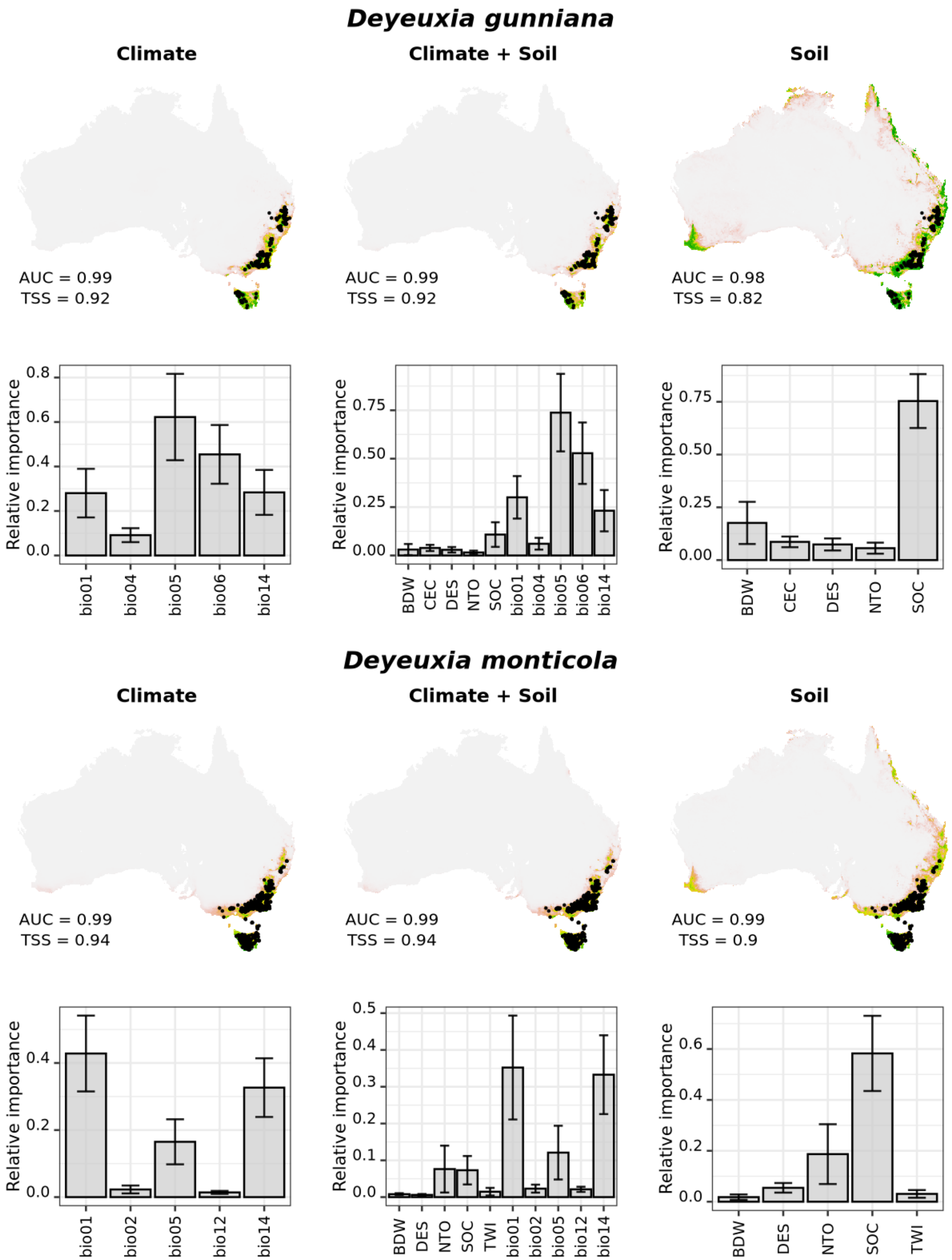
Deyeuxia gunniana
Climate
Climate + Soil
Soil
AUC = 0.99
TSS = 0.92
AUC = 0.99
TSS = 0.92
AUC = 0.98
TSS = 0.82
Relative importance
bio01
bio04
bio05
bio06
bio14
BDW
CEC
DES
NTO
SOC
Deyeuxia monticola
Climate
Climate + Soil
Soil
AUC = 0.99
TSS = 0.94
AUC = 0.99
TSS = 0.94
AUC = 0.99
TSS = 0.9
Relative importance
bio01
bio02
bio05
bio12
bio14
BDW
DES
NTO
SOC
TWI

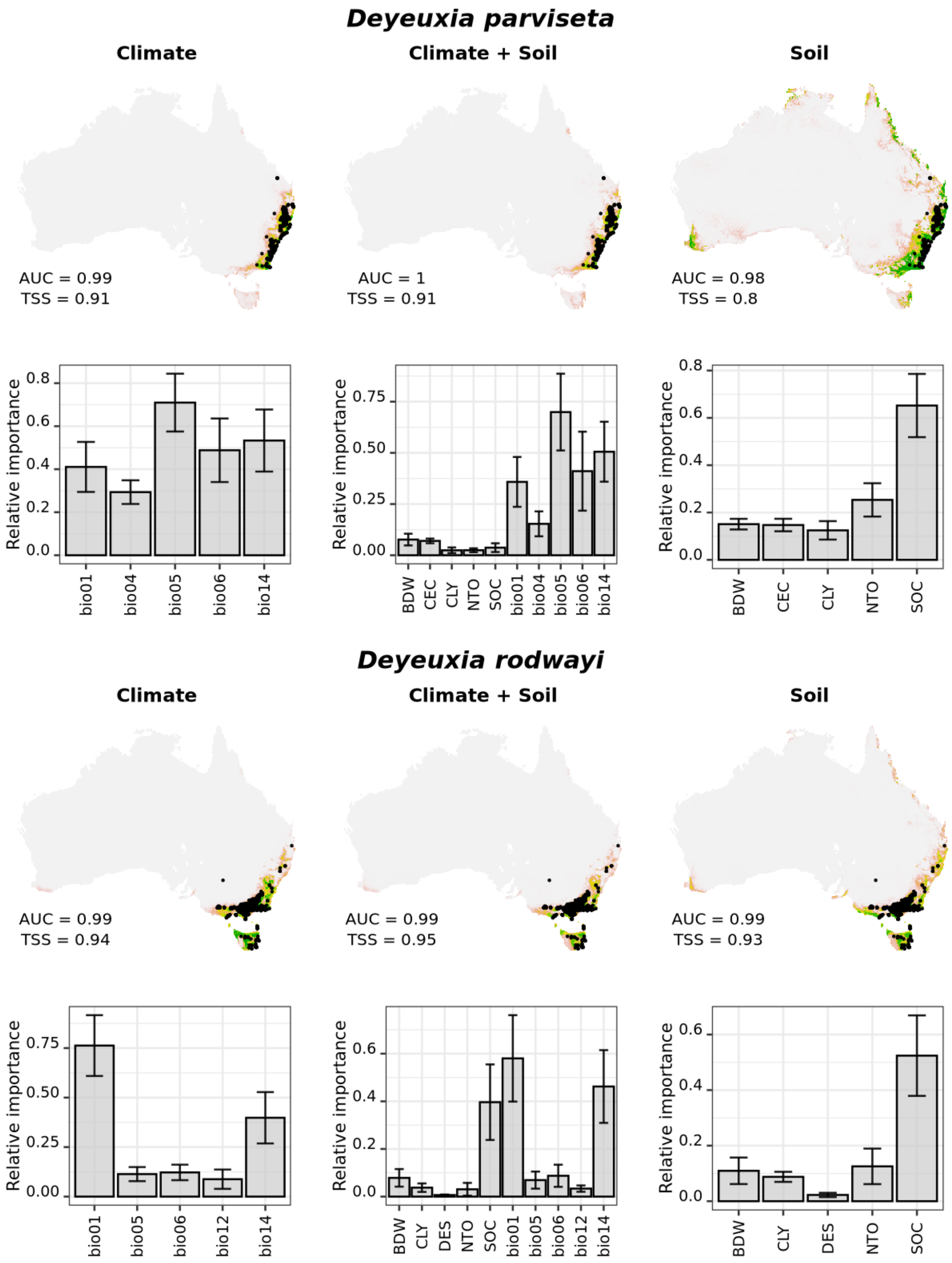
Deyeuxia parviseta
Climate
Climate + Soil
Soil
AUC = 0.99
TSS = 0.91
AUC = 1
TSS = 0.91
AUC = 0.98
TSS = 0.8
Relative importance
0.0
0.2
0.4
0.6
0.8
bio01
bio04
bio05
bio06
bio14
0.00
0.25
0.50
0.75
BDW
CEC
CLY
NTO
SOC
Deyeuxia rodwayi
AUC = 0.99
TSS = 0.94
AUC = 0.99
TSS = 0.95
AUC = 0.99
TSS = 0.93
bio12
DES

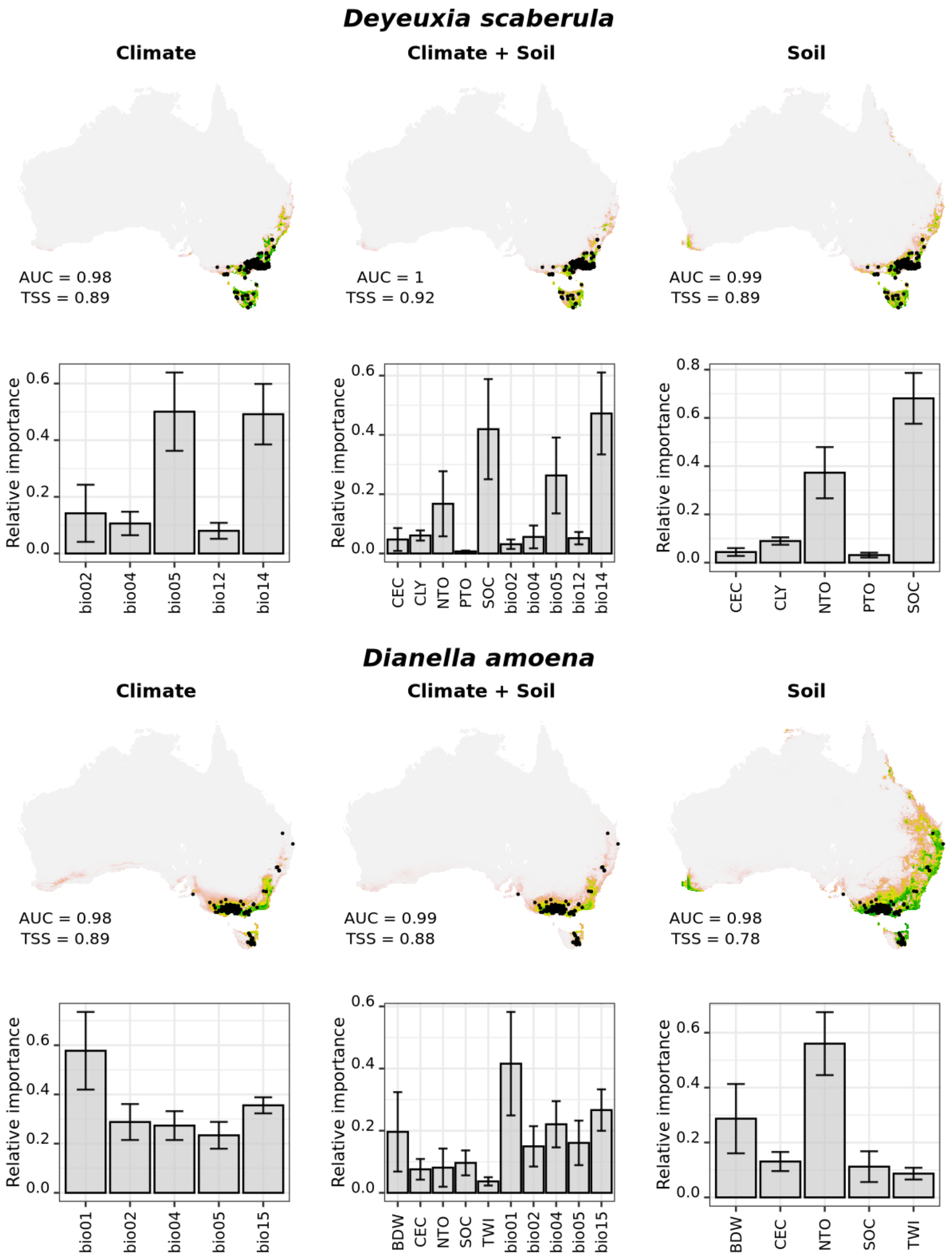
Deyeuxia scaberula
Climate
Climate + Soil
Soil
AUC = 0.98
TSS = 0.89
AUC = 1
TSS = 0.92
AUC = 0.99
TSS = 0.89
Relative importance
bio02 bio04 bio05 bio12 bio14
CEC CLY NTO PTO SOC bio02 bio04 bio05 bio12 bio14
CEC CLY NTO PTO SOC
Dianella amoena
Climate
Climate + Soil
Soil
AUC = 0.98
TSS = 0.89
AUC = 0.99
TSS = 0.88
AUC = 0.98
TSS = 0.78
Relative importance
bio01 bio02 bio04 bio05 bio15
BDW CEC NTO SOC TWI bio01 bio02 bio04 bio05 bio15
BDW CEC NTO SOC TWI

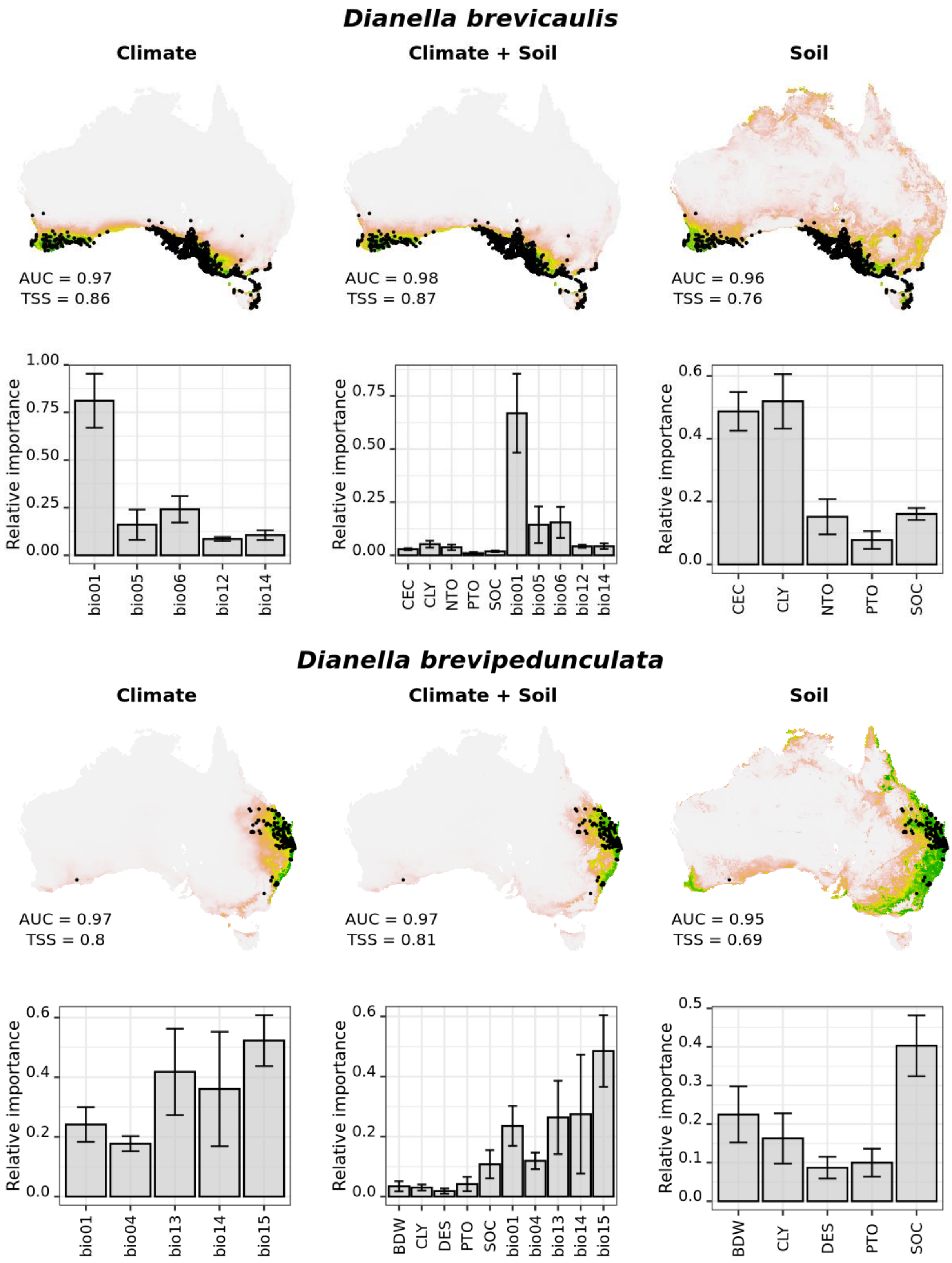
Dianella brevicaulis
Climate
Climate + Soil
Soil
AUC = 0.97
TSS = 0.86
AUC = 0.98
TSS = 0.87
AUC = 0.96
TSS = 0.76
Relative importance
bio01
bio05
bio06
bio12
bio14
CEC
CLY
NTO
PTO
SOC
Dianella brevipedunculata
Climate
Climate + Soil
Soil
AUC = 0.97
TSS = 0.8
AUC = 0.97
TSS = 0.81
AUC = 0.95
TSS = 0.69
Relative importance
bio01
bio04
bio13
bio14
bio15
BDW
CLY
DES
PTO
SOC

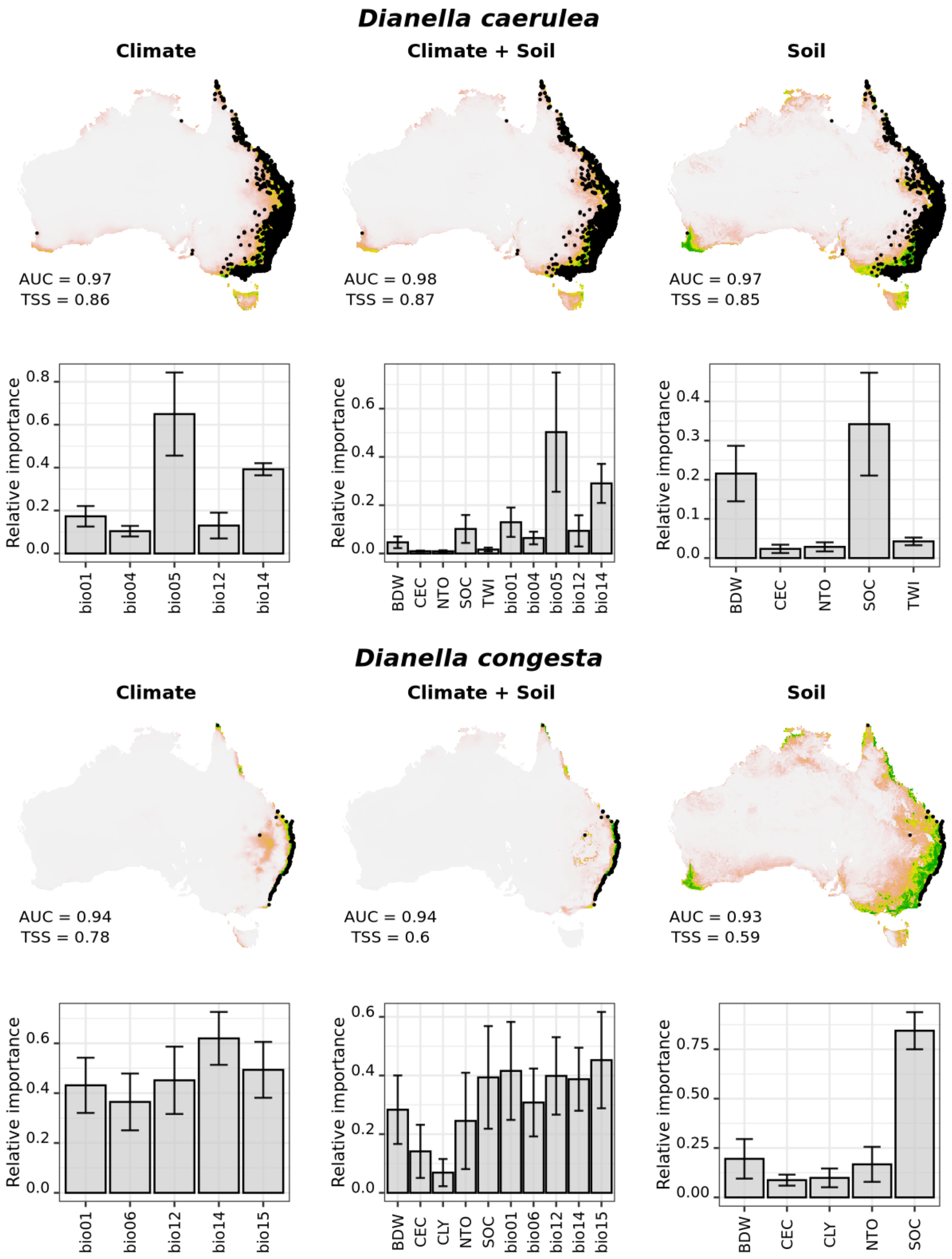
Dianella caerulea
Climate
Climate + Soil
Soil
AUC = 0.97
TSS = 0.86
AUC = 0.98
TSS = 0.87
AUC = 0.97
TSS = 0.85
Relative importance
bio01
bio04
bio05
bio12
bio14
BDW
CEC
NTO
SOC
TWI
Dianella congesta
AUC = 0.94
TSS = 0.78
AUC = 0.94
TSS = 0.6
AUC = 0.93
TSS = 0.59
bio06
bio15
CLY

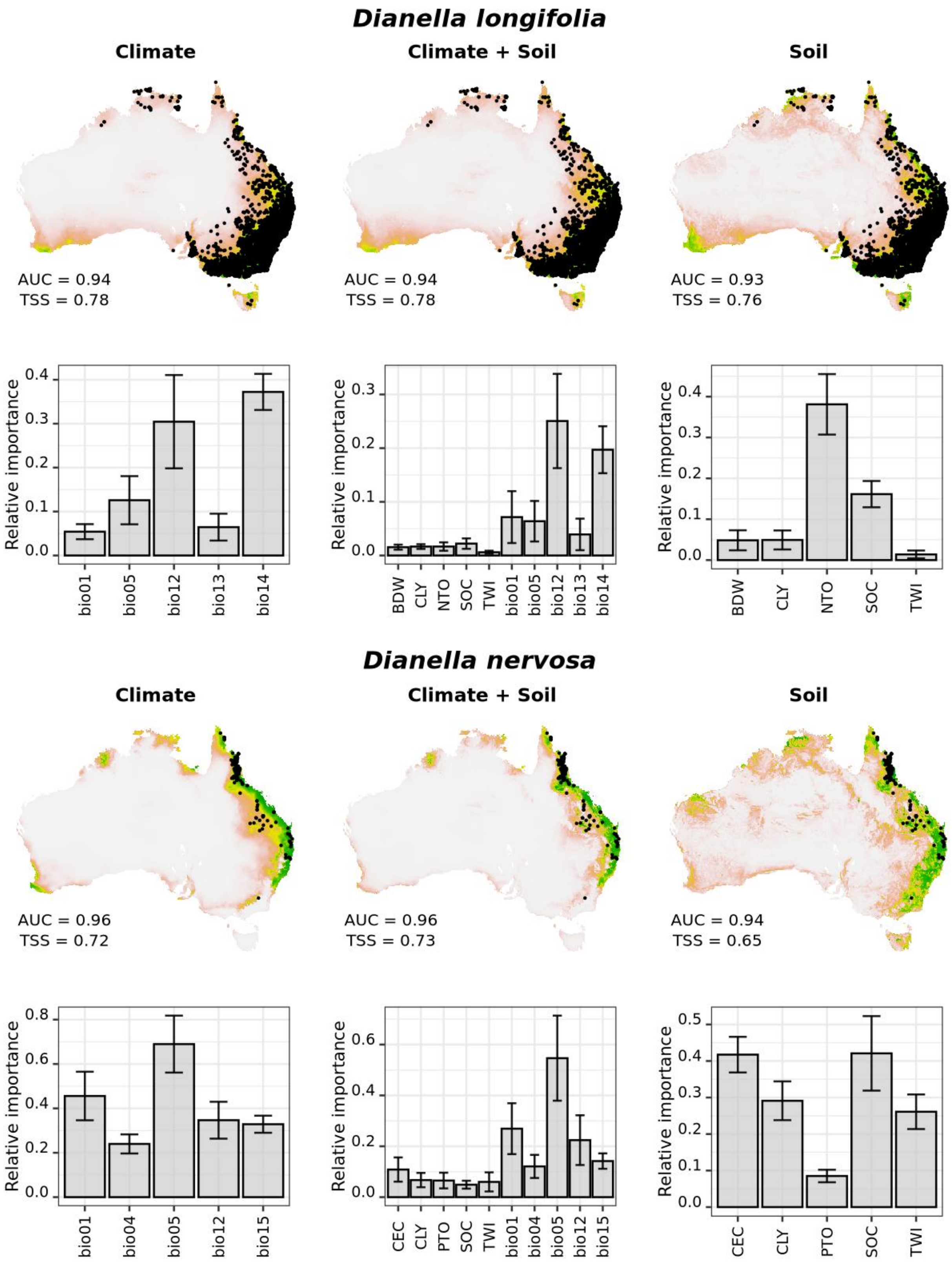
Dianella longifolia
Climate
Climate + Soil
Soil
AUC = 0.94
TSS = 0.78
AUC = 0.94
TSS = 0.78
AUC = 0.93
TSS = 0.76
Relative importance
bio01
bio05
bio12
bio13
bio14
BDW
CLY
NTO
SOC
TWI
Dianella nervosa
Climate
Climate + Soil
Soil
AUC = 0.96
TSS = 0.72
AUC = 0.96
TSS = 0.73
AUC = 0.94
TSS = 0.65
Relative importance
bio01
bio04
bio05
bio12
bio15
CEC
CLY
PTO
SOC
TWI

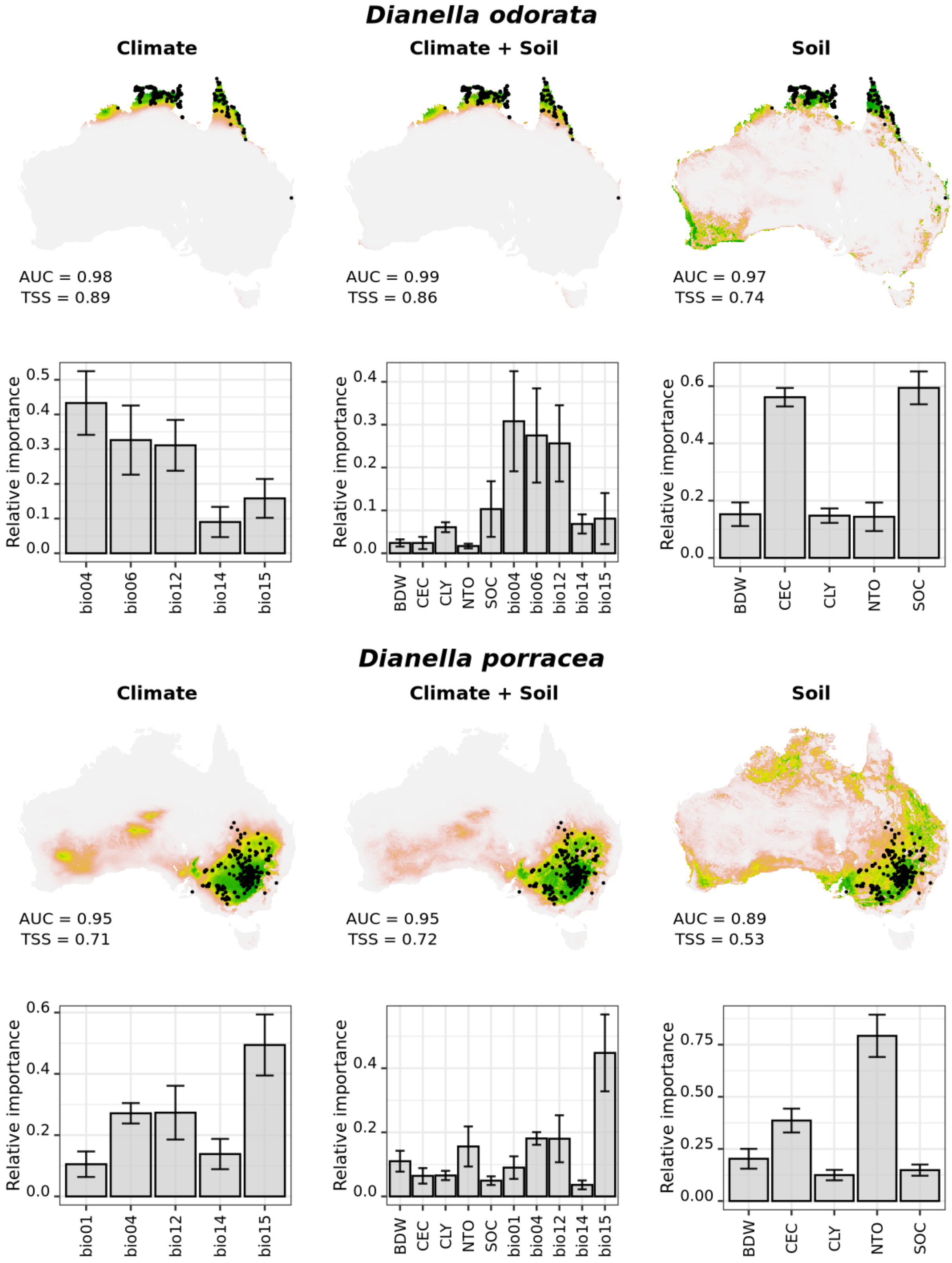
Dianella odorata
Climate
Climate + Soil
Soil
AUC = 0.98
TSS = 0.89
AUC = 0.99
TSS = 0.86
AUC = 0.97
TSS = 0.74
Relative importance
bio04
bio06
bio12
bio14
bio15
BDW
CEC
CLY
NTO
SOC
Dianella porracea
Climate
Climate + Soil
Soil
AUC = 0.95
TSS = 0.71
AUC = 0.95
TSS = 0.72
AUC = 0.89
TSS = 0.53
Relative importance
bio01
bio04
bio12
bio14
bio15
BDW
CEC
CLY
NTO
SOC

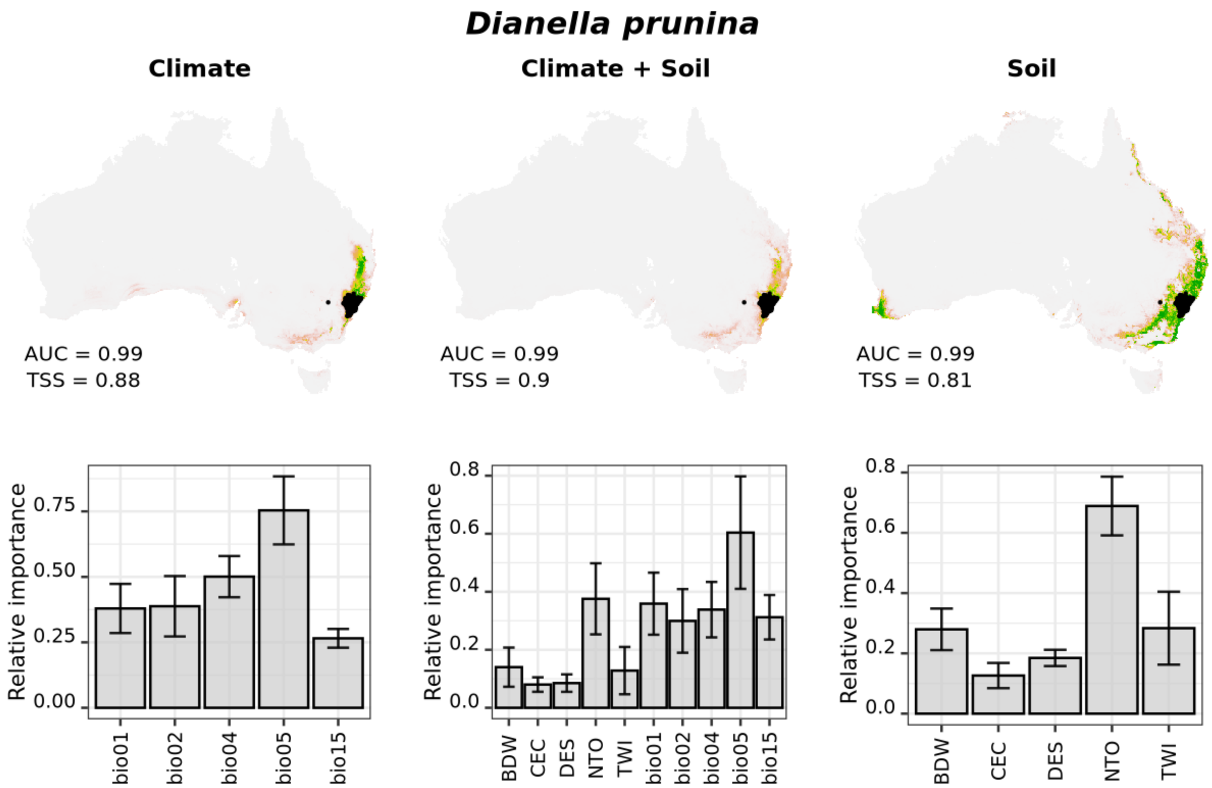
Dianella prunina
Climate
Climate + Soil
Soil
AUC = 0.99
TSS = 0.88
AUC = 0.99
TSS = 0.9
AUC = 0.99
TSS = 0.81
Relative importance
0.00
0.25
0.50
0.75
bio01
bio02
bio04
bio05
bio15
0.0
0.2
0.4
0.6
0.8
BDW
CEC
DES
NTO
TWI
bio01
bio02
bio04
bio05
bio15
BDW
CEC
DES
NTO
TWI

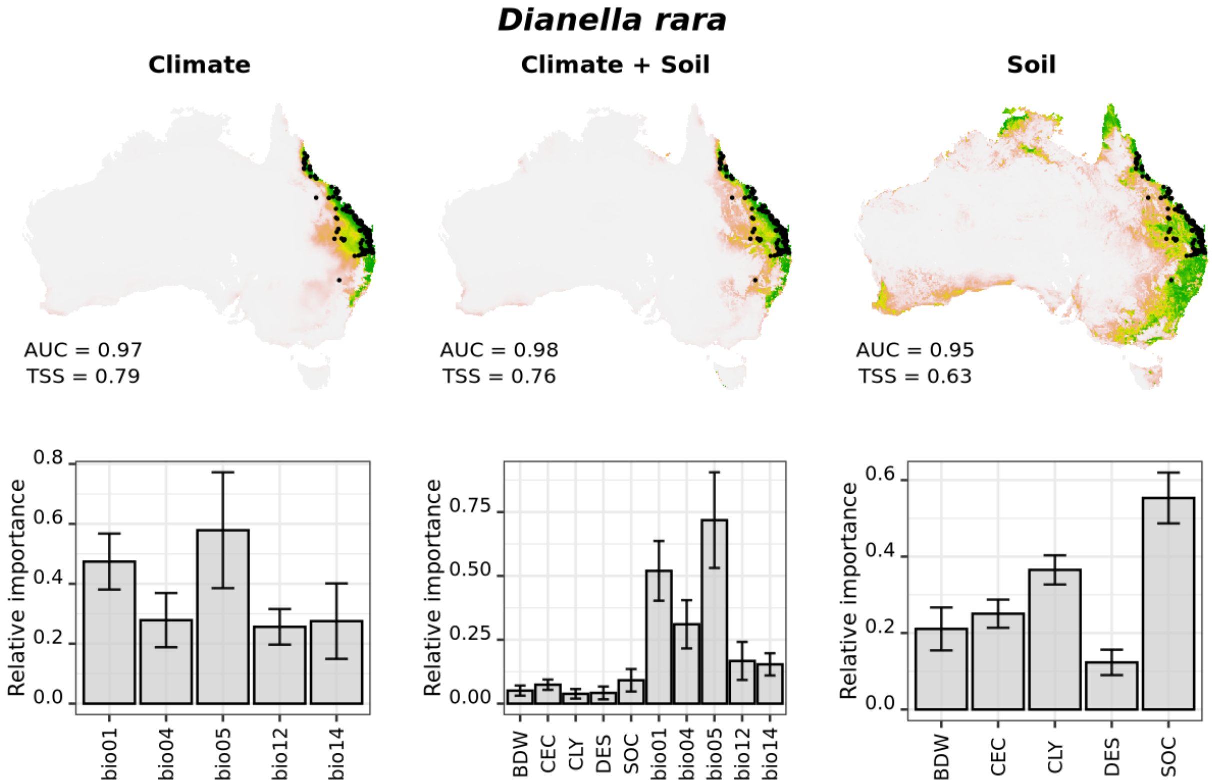
Dianella rara
Climate
Climate + Soil
Soil
AUC = 0.97
TSS = 0.79
AUC = 0.98
TSS = 0.76
AUC = 0.95
TSS = 0.63
Relative importance
0.0
0.2
0.4
0.6
0.8
bio01
bio04
bio05
bio12
bio14
0.00
0.25
0.50
0.75
BDW
CEC
CLY
DES
SOC
bio01
bio04
bio05
bio12
bio14
BDW
CEC
CLY
DES
SOC

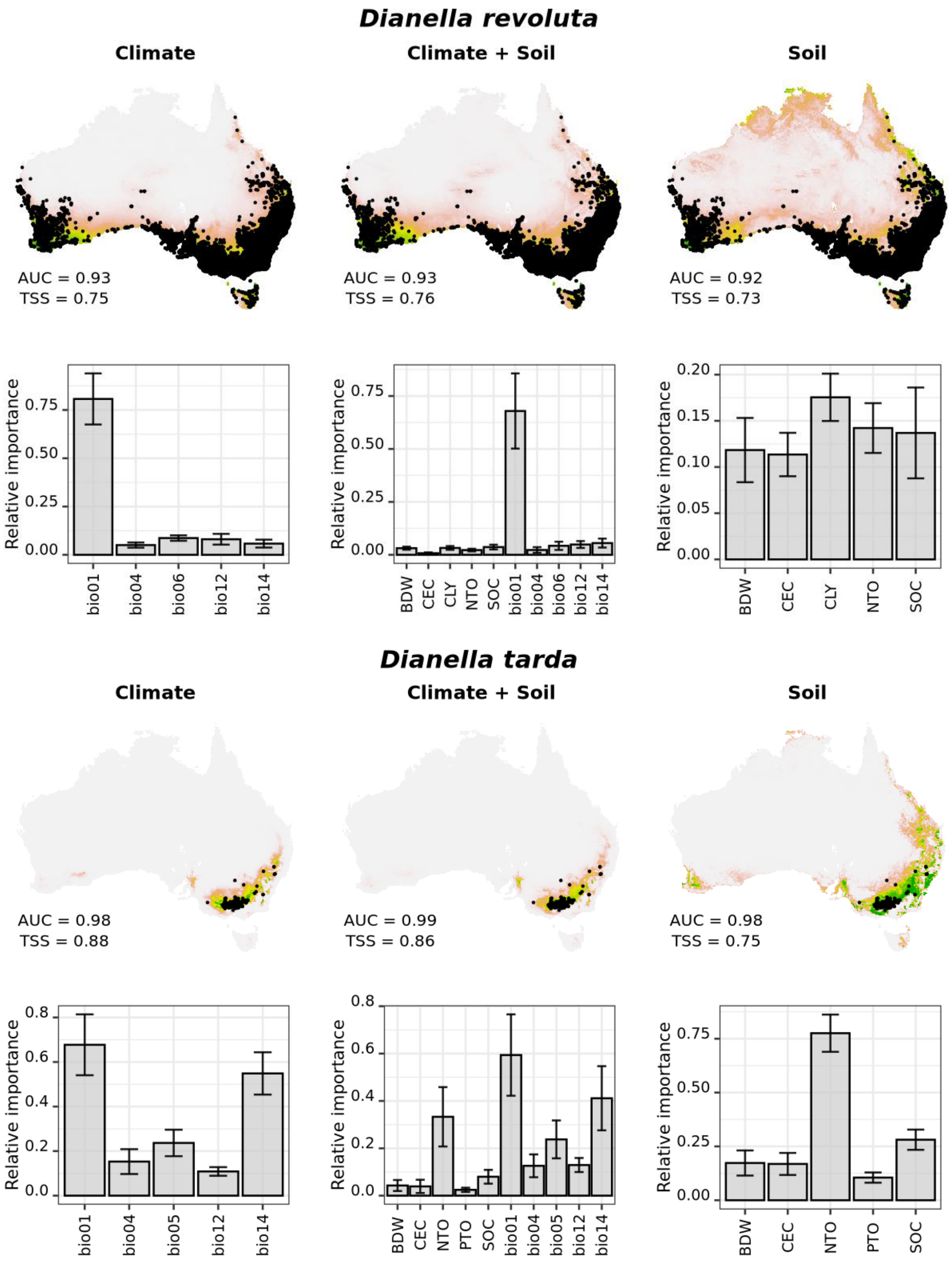
Dianella revoluta
Climate
Climate + Soil
Soil
AUC = 0.93
TSS = 0.75
AUC = 0.93
TSS = 0.76
AUC = 0.92
TSS = 0.73
Relative importance
bio01 bio04 bio06 bio12 bio14
BDW CEC CLY NTO SOC bio01 bio04 bio06 bio12 bio14
BDW CEC CLY NTO SOC
Dianella tarda
Climate
Climate + Soil
Soil
AUC = 0.98
TSS = 0.88
AUC = 0.99
TSS = 0.86
AUC = 0.98
TSS = 0.75
Relative importance
bio01 bio04 bio05 bio12 bio14
BDW CEC NTO PTO SOC bio01 bio04 bio05 bio12 bio14
BDW CEC NTO PTO SOC

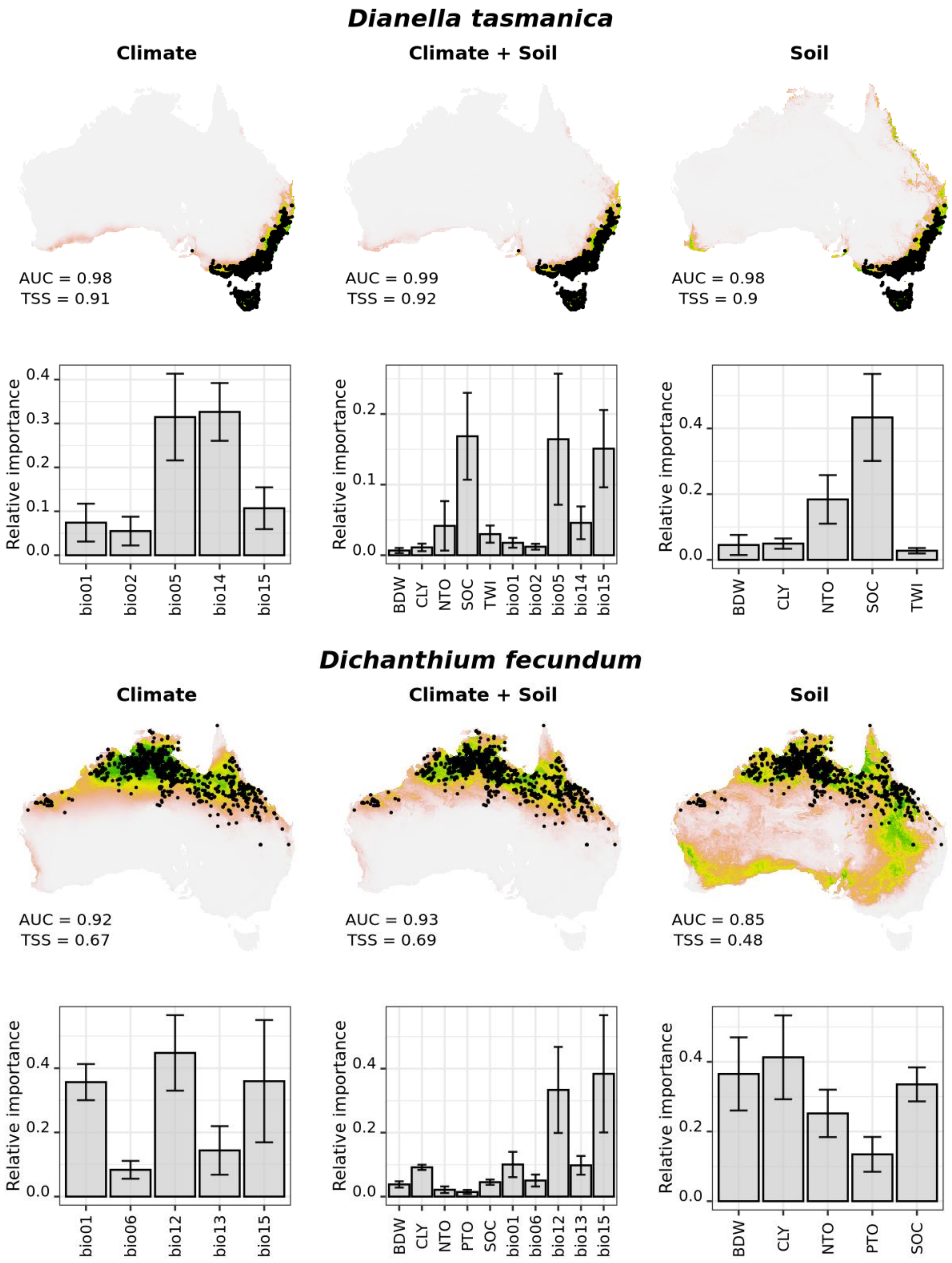

Dianella tasmanica
Climate
Climate + Soil
Soil
AUC = 0.98
TSS = 0.91
AUC = 0.99
TSS = 0.92
AUC = 0.98
TSS = 0.9
Relative importance
bio01
bio02
bio05
bio14
bio15
BDW
CLY
NTO
SOC
TWI
Dichanthium fecundum
AUC = 0.92
TSS = 0.67
AUC = 0.93
TSS = 0.69
AUC = 0.85
TSS = 0.48
bio06
bio12
bio13
PTO

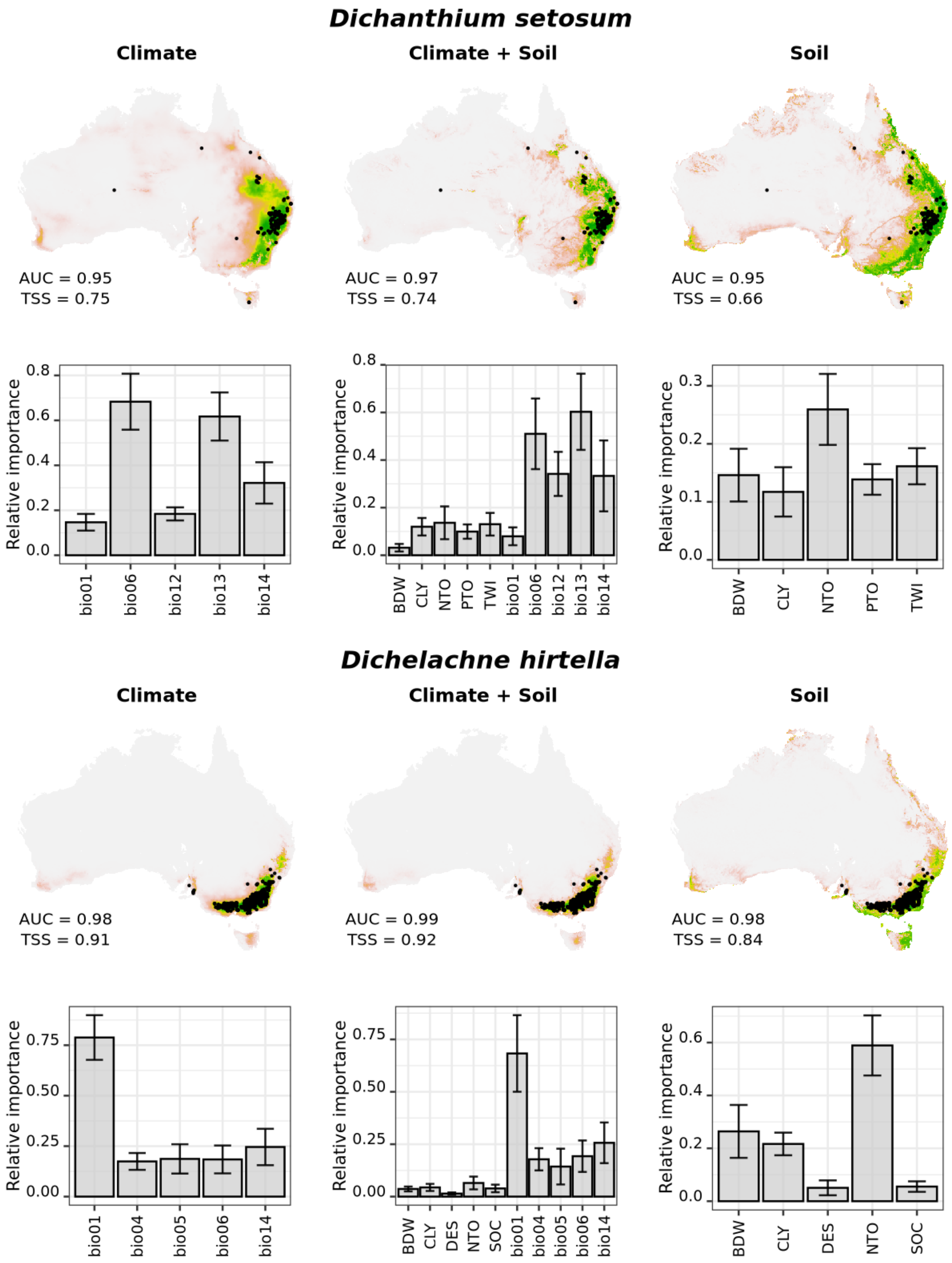
Dichanthium setosum
Climate
Climate + Soil
Soil
AUC = 0.95
TSS = 0.75
AUC = 0.97
TSS = 0.74
AUC = 0.95
TSS = 0.66
Relative importance
bio01 bio06 bio12 bio13 bio14
BDW CLY NTO PTO TWI bio01 bio06 bio12 bio13 bio14
BDW CLY NTO PTO TWI
Dichelachne hirtella
Climate
Climate + Soil
Soil
AUC = 0.98
TSS = 0.91
AUC = 0.99
TSS = 0.92
AUC = 0.98
TSS = 0.84
Relative importance
bio01 bio04 bio05 bio06 bio14
BDW CLY DES NTO SOC bio01 bio04 bio05 bio06 bio14
BDW CLY DES NTO SOC

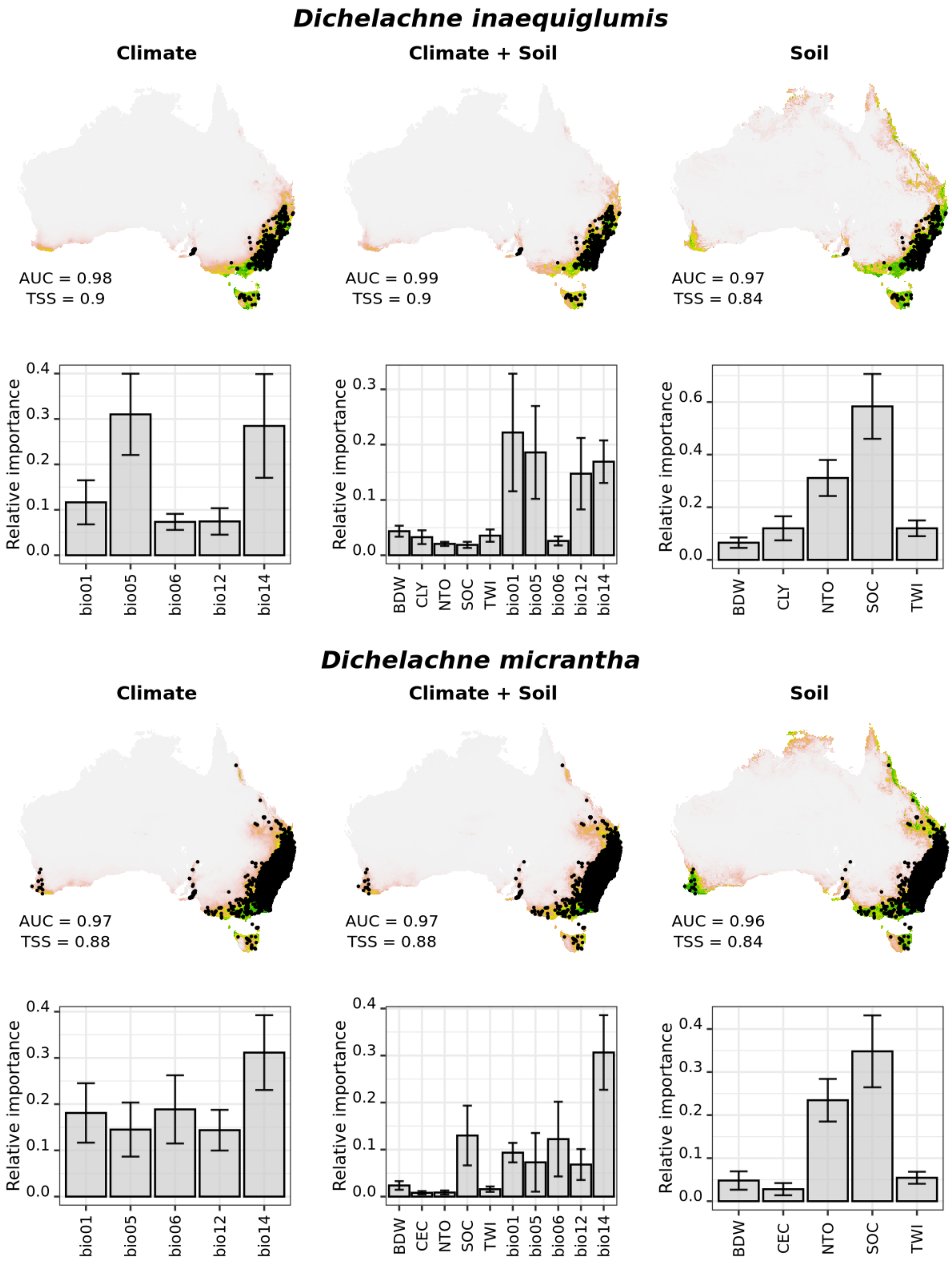
Dichelachne inaequiglumis
Climate
Climate + Soil
Soil
AUC = 0.98
TSS = 0.9
AUC = 0.99
TSS = 0.9
AUC = 0.97
TSS = 0.84
Relative importance
bio01
bio05
bio06
bio12
bio14
BDW
CLY
NTO
SOC
TWI
Dichelachne micrantha
Climate
Climate + Soil
Soil
AUC = 0.97
TSS = 0.88
AUC = 0.97
TSS = 0.88
AUC = 0.96
TSS = 0.84
Relative importance
bio01
bio05
bio06
bio12
bio14
BDW
CEC
NTO
SOC
TWI

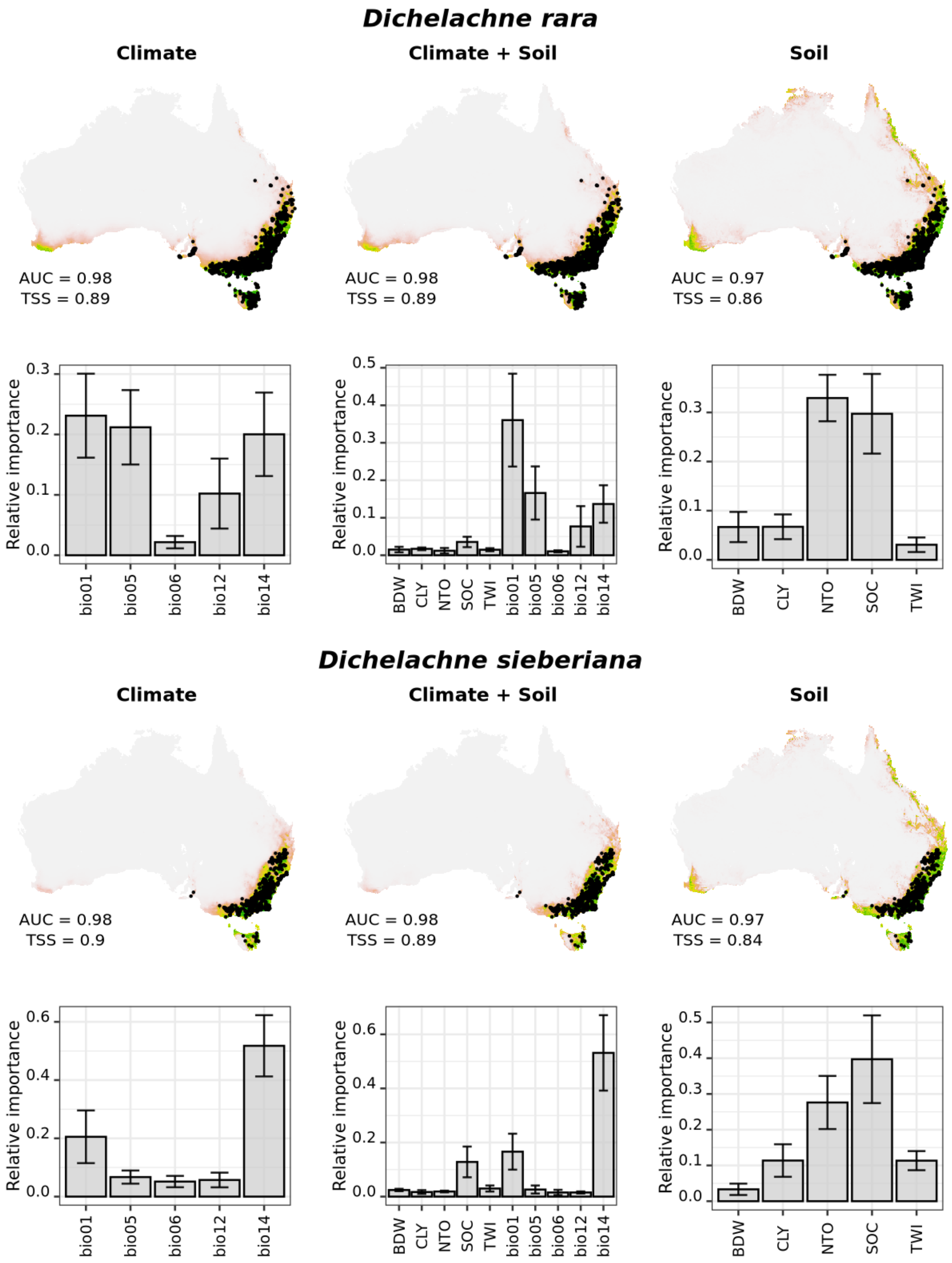

Dichelachne rara
Climate
Climate + Soil
Soil
AUC = 0.98
TSS = 0.89
AUC = 0.98
TSS = 0.89
AUC = 0.97
TSS = 0.86
Relative importance
bio01
bio05
bio06
bio12
bio14
BDW
CLY
NTO
SOC
TWI
Dichelachne sieberiana
Climate
Climate + Soil
Soil
AUC = 0.98
TSS = 0.9
AUC = 0.98
TSS = 0.89
AUC = 0.97
TSS = 0.84

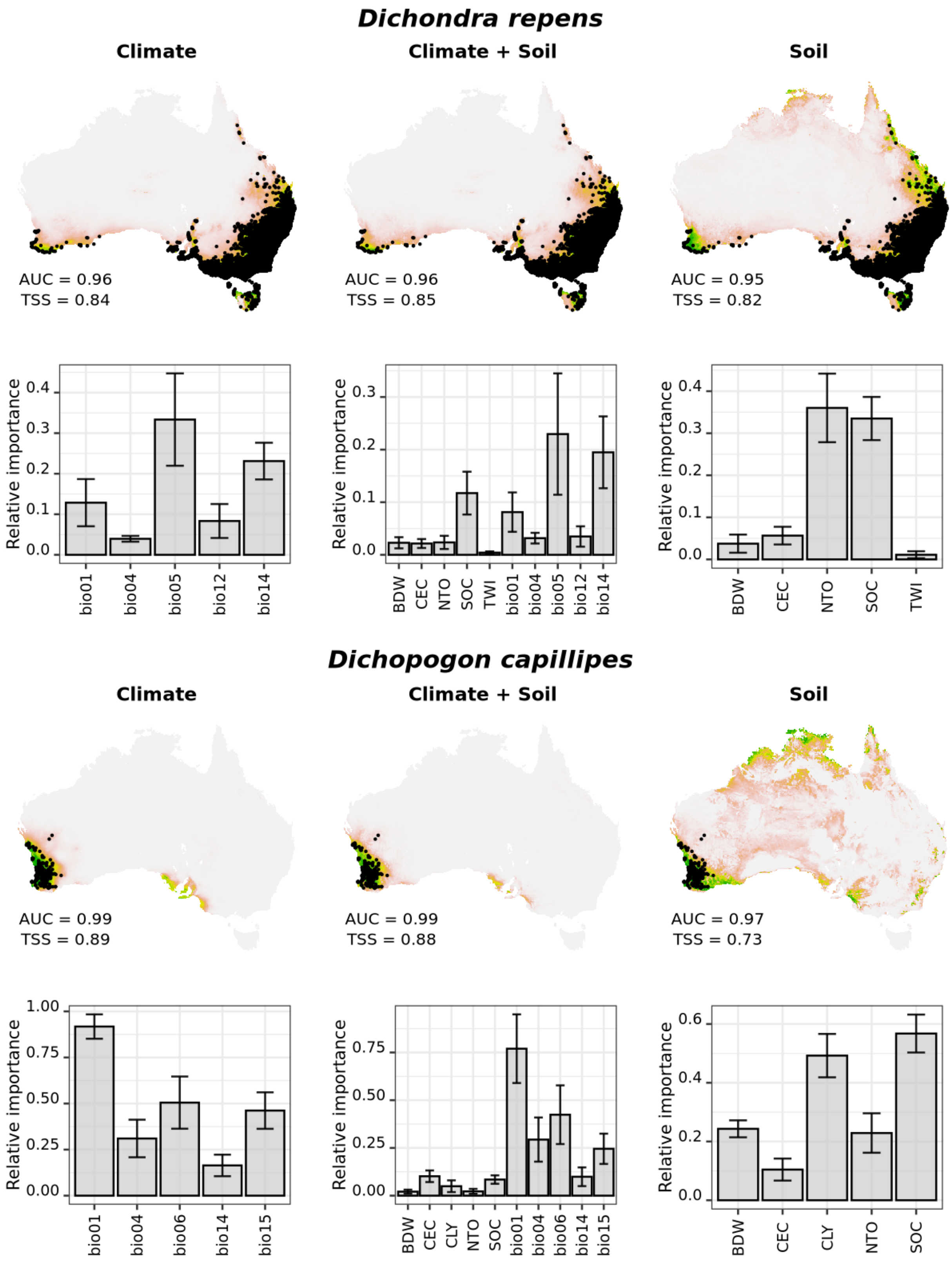
Dichondra repens
Climate
Climate + Soil
Soil
AUC = 0.96
TSS = 0.84
AUC = 0.96
TSS = 0.85
AUC = 0.95
TSS = 0.82
Relative importance
bio01
bio04
bio05
bio12
bio14
BDW
CEC
NTO
SOC
TWI
Dichopogon capillipes
Climate
Climate + Soil
Soil
AUC = 0.99
TSS = 0.89
AUC = 0.99
TSS = 0.88
AUC = 0.97
TSS = 0.73
Relative importance
bio01
bio04
bio06
bio14
bio15
BDW
CEC
CLY
NTO
SOC

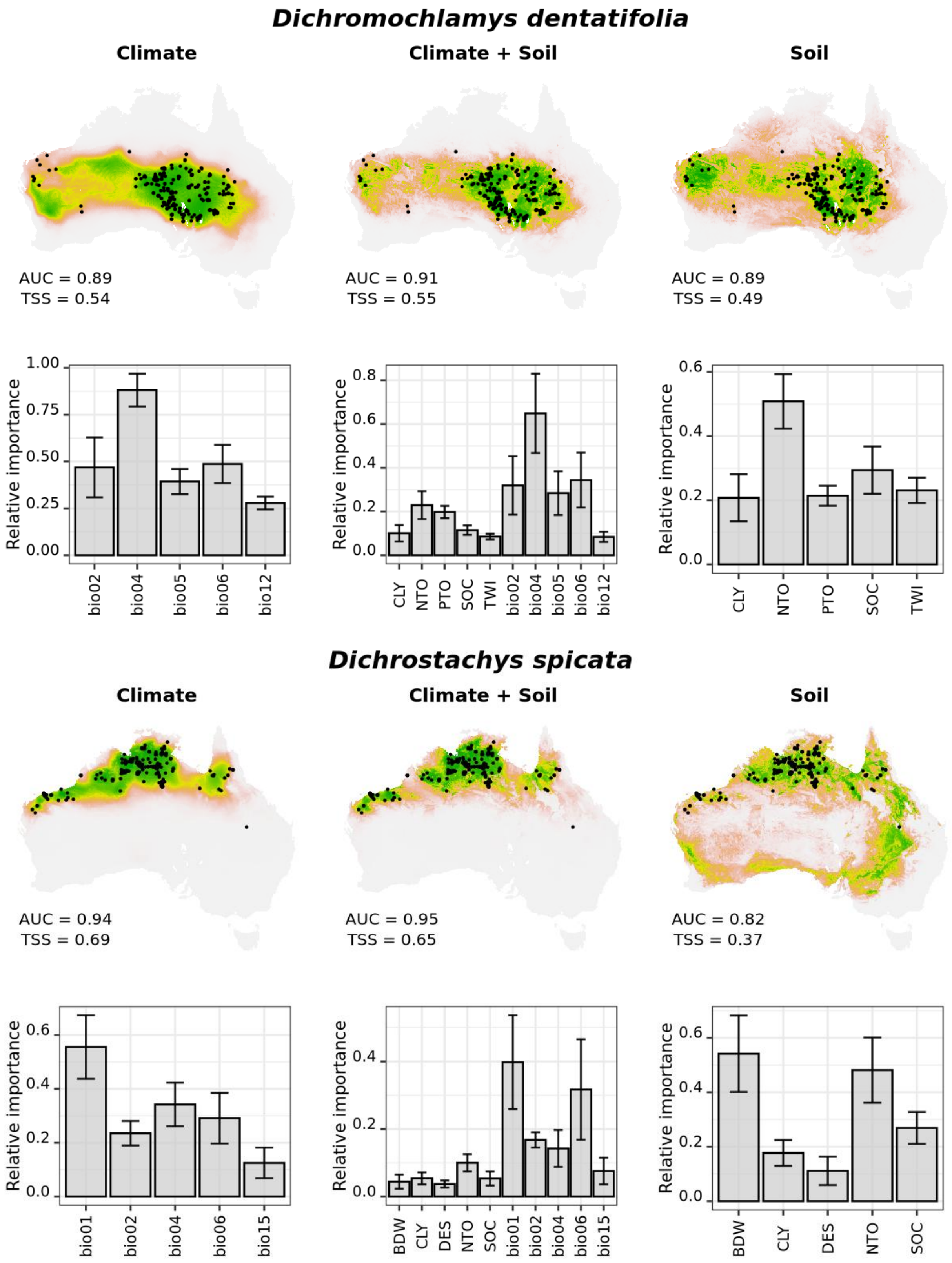
Dichromochlamys dentatifolia
Climate
Climate + Soil
Soil
AUC = 0.89
TSS = 0.54
AUC = 0.91
TSS = 0.55
AUC = 0.89
TSS = 0.49
Relative importance
bio02
bio04
bio05
bio06
bio12
CLY
NTO
PTO
SOC
TWI
Dichrostachys spicata
AUC = 0.94
TSS = 0.69
AUC = 0.95
TSS = 0.65
AUC = 0.82
TSS = 0.37
bio01
bio15
BDW
DES

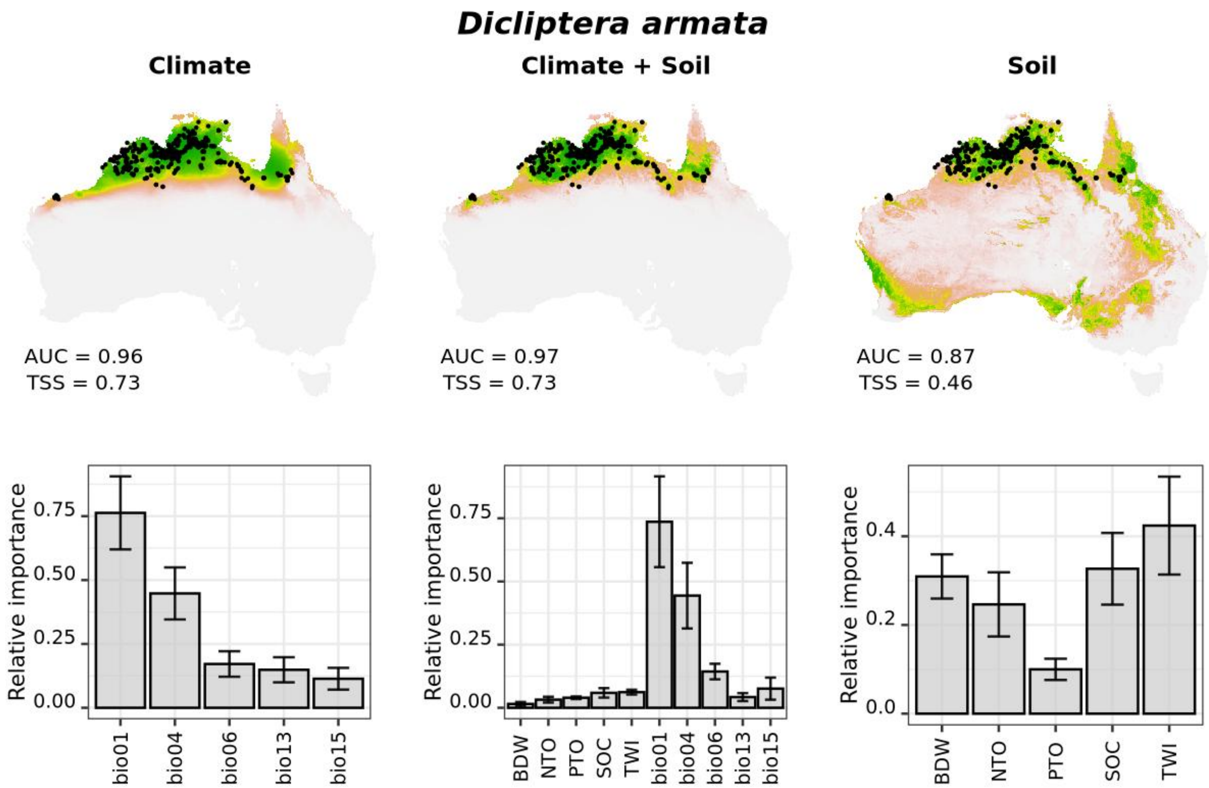

Dicliptera armata
Climate
Climate + Soil
Soil
AUC = 0.96
TSS = 0.73
AUC = 0.97
TSS = 0.73
AUC = 0.87
TSS = 0.46
Relative importance
0.00
0.25
0.50
0.75
bio01
bio04
bio06
bio13
bio15
BDW
NTO
PTO
SOC
TWI
0.0
0.2
0.4

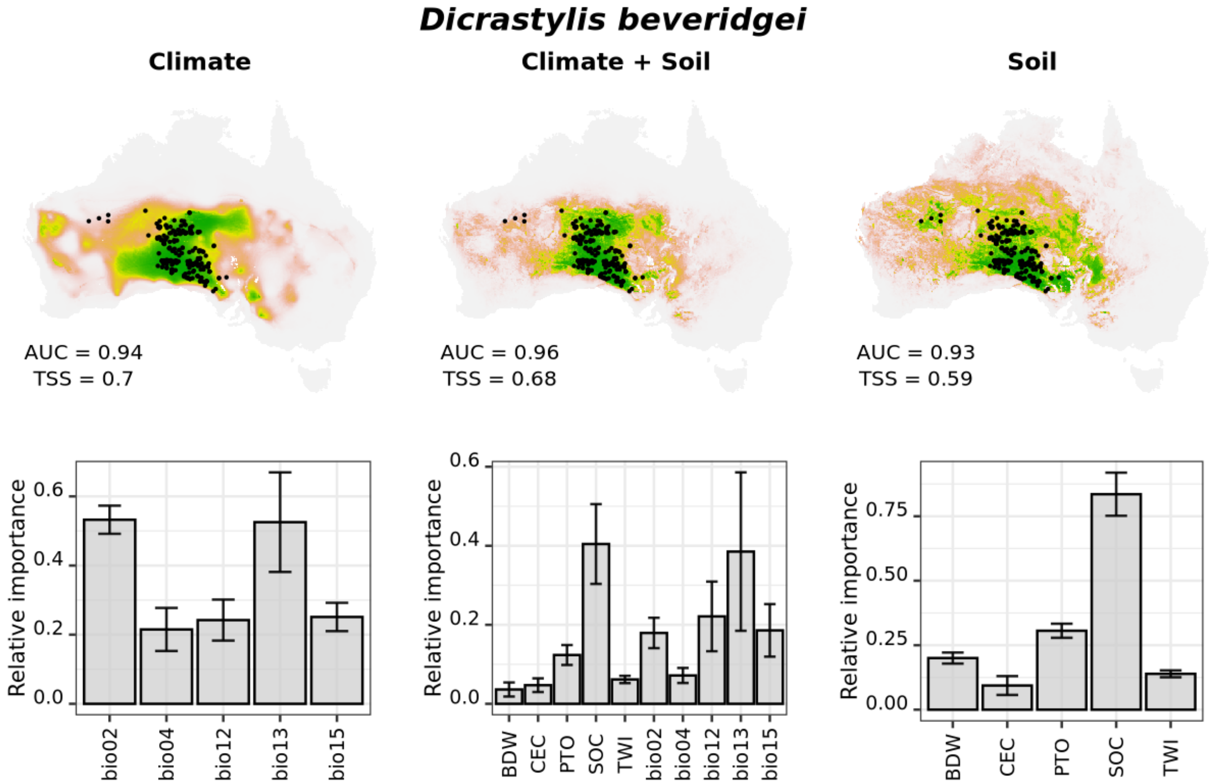

Dicrastylis beveridgei
Climate
Climate + Soil
Soil
AUC = 0.94
TSS = 0.7
AUC = 0.96
TSS = 0.68
AUC = 0.93
TSS = 0.59
Relative importance
0.0
0.2
0.4
0.6
bio02
bio04
bio12
bio13
bio15
BDW
CEC
PTO
SOC
TWI
0.00
0.25
0.50
0.75

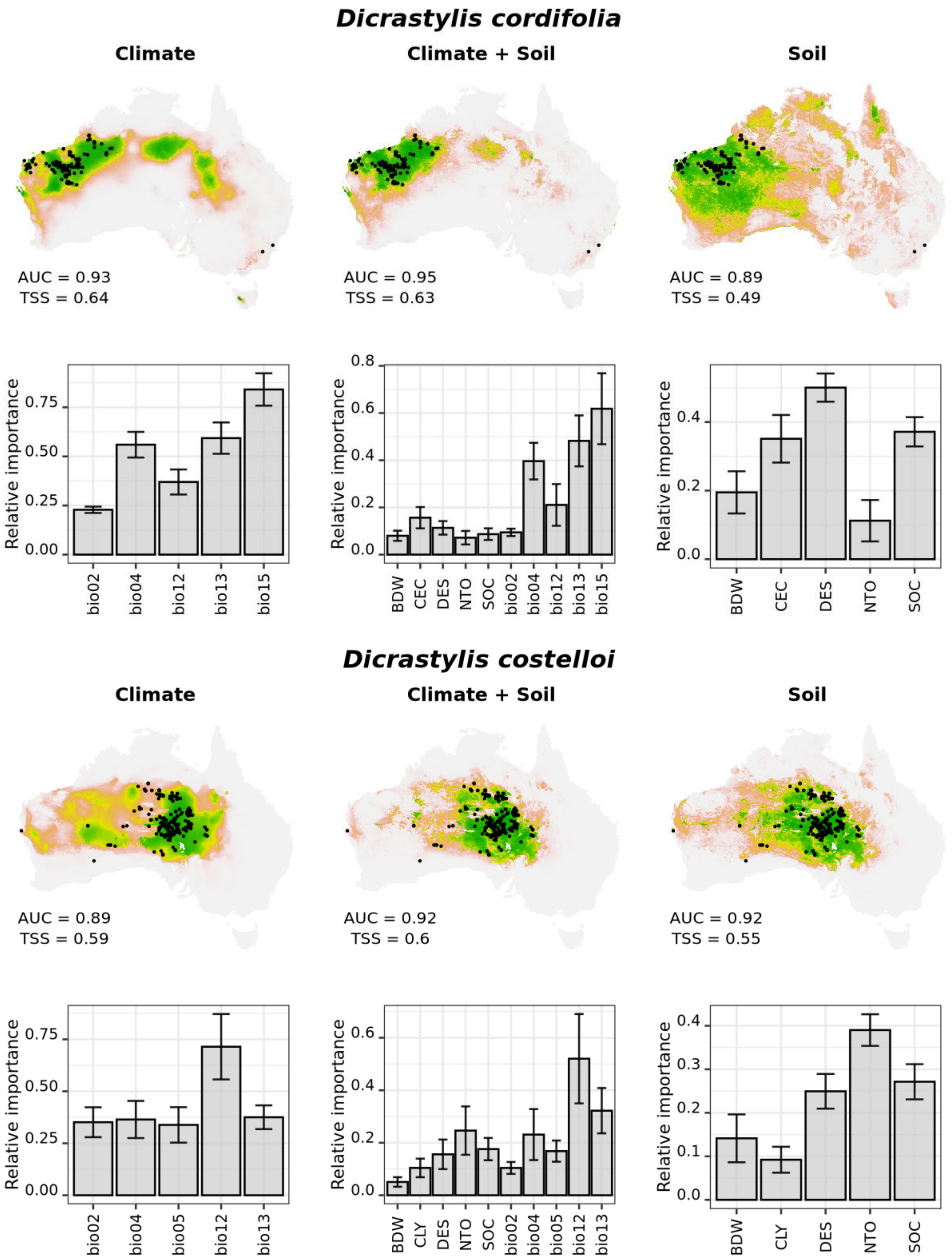
Dicrastylis cordifolia
Climate
Climate + Soil
Soil
AUC = 0.93
TSS = 0.64
AUC = 0.95
TSS = 0.63
AUC = 0.89
TSS = 0.49
Relative importance
0.00
0.25
0.50
0.75
bio02
bio04
bio12
bio13
bio15
0.0
0.2
0.4
0.6
0.8
BDW
CEC
DES
NTO
SOC
bio02
bio04
bio12
bio13
bio15
0.0
0.2
0.4
BDW
CEC
DES
NTO
SOC
Dicrastylis costelloi
Climate
Climate + Soil
Soil
AUC = 0.89
TSS = 0.59
AUC = 0.92
TSS = 0.6
AUC = 0.92
TSS = 0.55
Relative importance
0.00
0.25
0.50
0.75
bio02
bio04
bio05
bio12
bio13
0.0
0.2
0.4
0.6
BDW
CLY
DES
NTO
SOC
bio02
bio04
bio05
bio12
bio13
0.0
0.1
0.2
0.3
0.4
BDW
CLY
DES
NTO
SOC

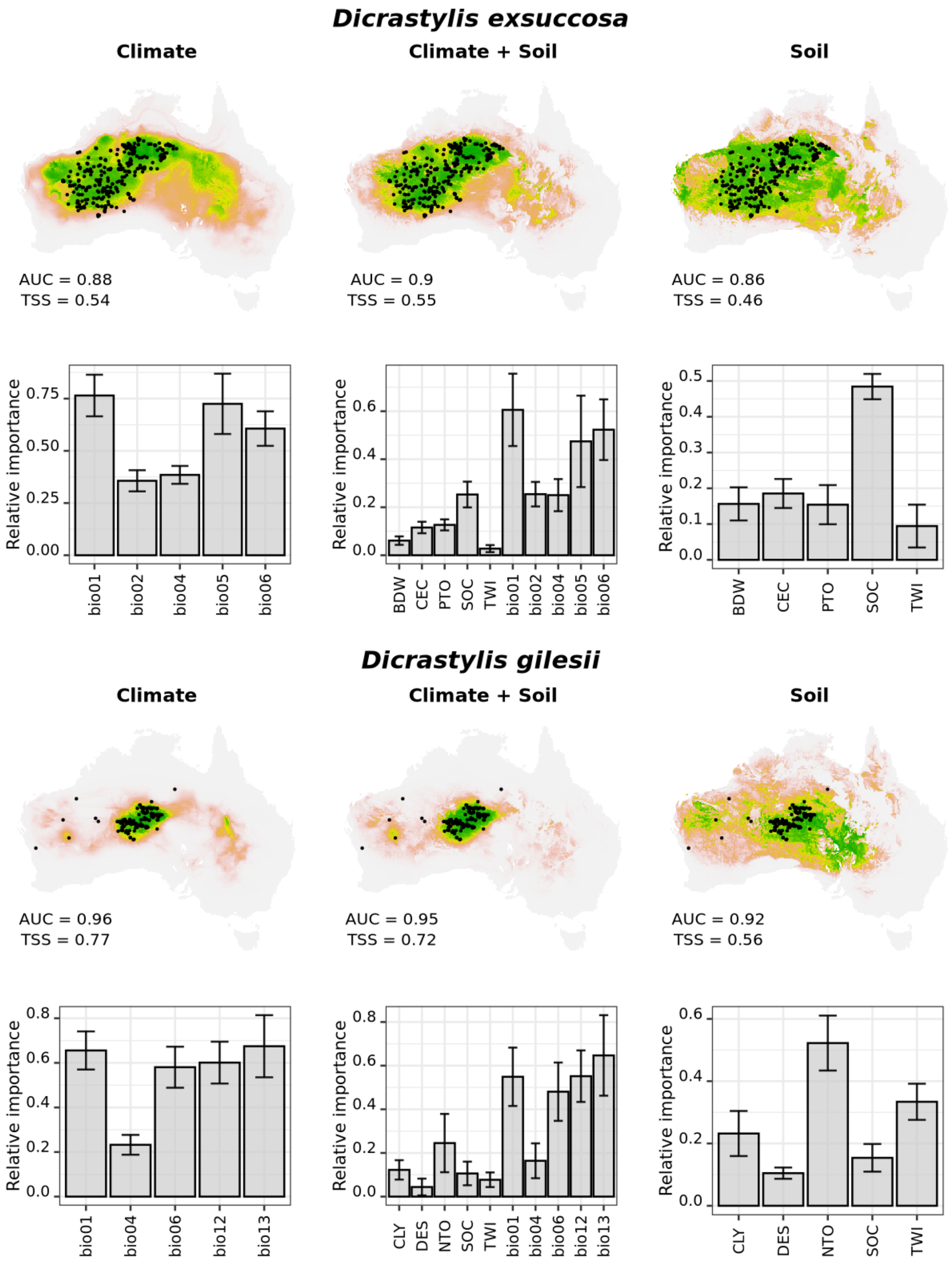
Dicrastylis exsuccosa
Climate
Climate + Soil
Soil
AUC = 0.88
TSS = 0.54
AUC = 0.9
TSS = 0.55
AUC = 0.86
TSS = 0.46
Relative importance
0.00
0.25
0.50
0.75
bio01
bio02
bio04
bio05
bio06
0.0
0.2
0.4
0.6
BDW
CEC
PTO
SOC
TWI
bio01
bio02
bio04
bio05
bio06
0.0
0.1
0.2
0.3
0.4
0.5
BDW
CEC
PTO
SOC
TWI
Dicrastylis gilesii
Climate
Climate + Soil
Soil
AUC = 0.96
TSS = 0.77
AUC = 0.95
TSS = 0.72
AUC = 0.92
TSS = 0.56
Relative importance
0.0
0.2
0.4
0.6
0.8
bio01
bio04
bio06
bio12
bio13
CLY
DES
NTO
SOC
TWI
bio01
bio04
bio06
bio12
bio13
CLY
DES
NTO
SOC
TWI

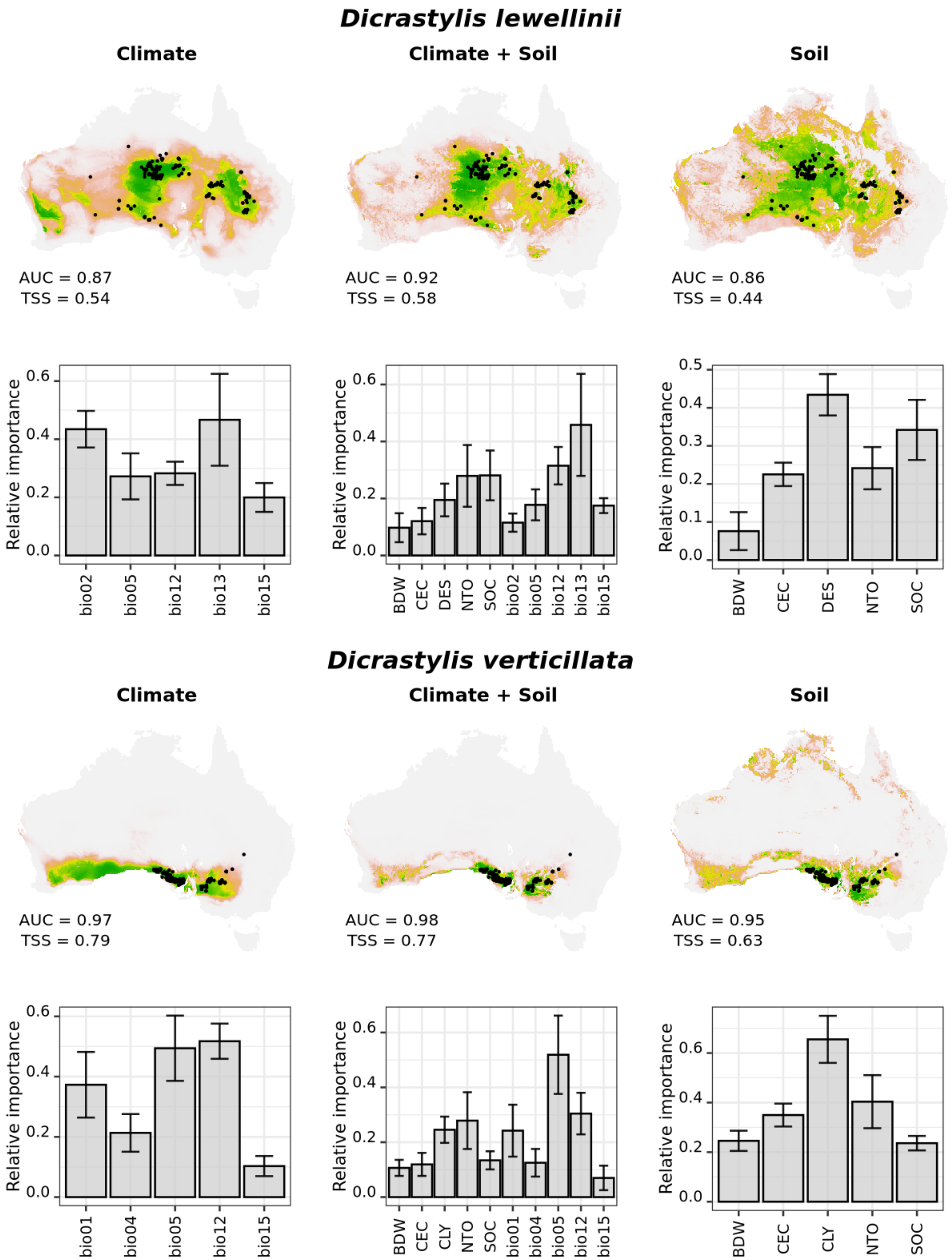
Dicrastylis lewellinii
Climate
Climate + Soil
Soil
AUC = 0.87
TSS = 0.54
AUC = 0.92
TSS = 0.58
AUC = 0.86
TSS = 0.44
Relative importance
bio02 bio05 bio12 bio13 bio15
BDW CEC DES NTO SOC bio02 bio05 bio12 bio13 bio15
BDW CEC DES NTO SOC
Dicrastylis verticillata
Climate
Climate + Soil
Soil
AUC = 0.97
TSS = 0.79
AUC = 0.98
TSS = 0.77
AUC = 0.95
TSS = 0.63
Relative importance
bio01 bio04 bio05 bio12 bio15
BDW CEC CLY NTO SOC bio01 bio04 bio05 bio12 bio15
BDW CEC CLY NTO SOC

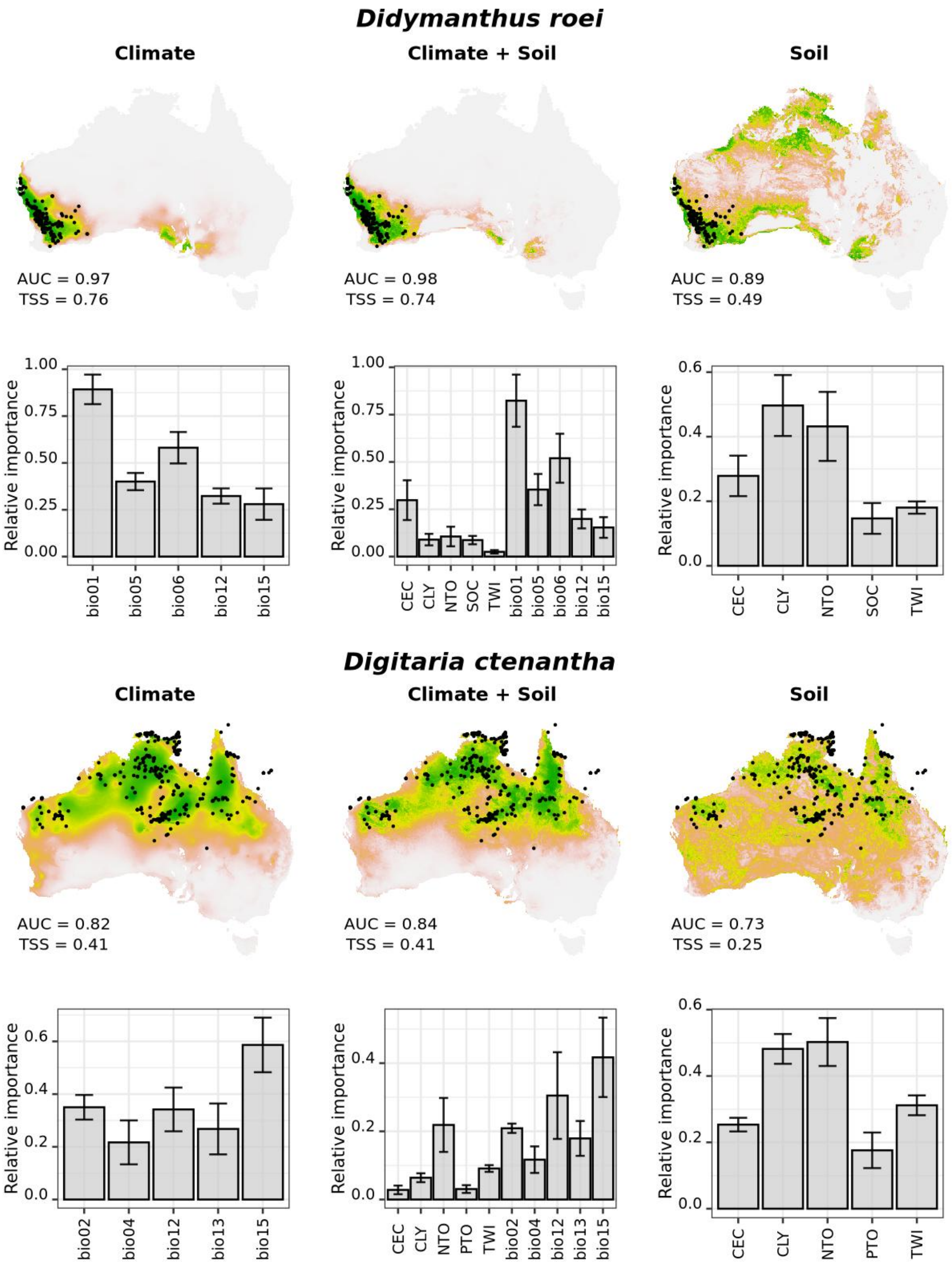
Didymanthus roei
Climate
Climate + Soil
Soil
AUC = 0.97
TSS = 0.76
AUC = 0.98
TSS = 0.74
AUC = 0.89
TSS = 0.49
Relative importance
bio01
bio05
bio06
bio12
bio15
CEC
CLY
NTO
SOC
TWI
Digitaria ctenantha
Climate
Climate + Soil
Soil
AUC = 0.82
TSS = 0.41
AUC = 0.84
TSS = 0.41
AUC = 0.73
TSS = 0.25
Relative importance
bio02
bio04
bio12
bio13
bio15
CEC
CLY
NTO
PTO
TWI

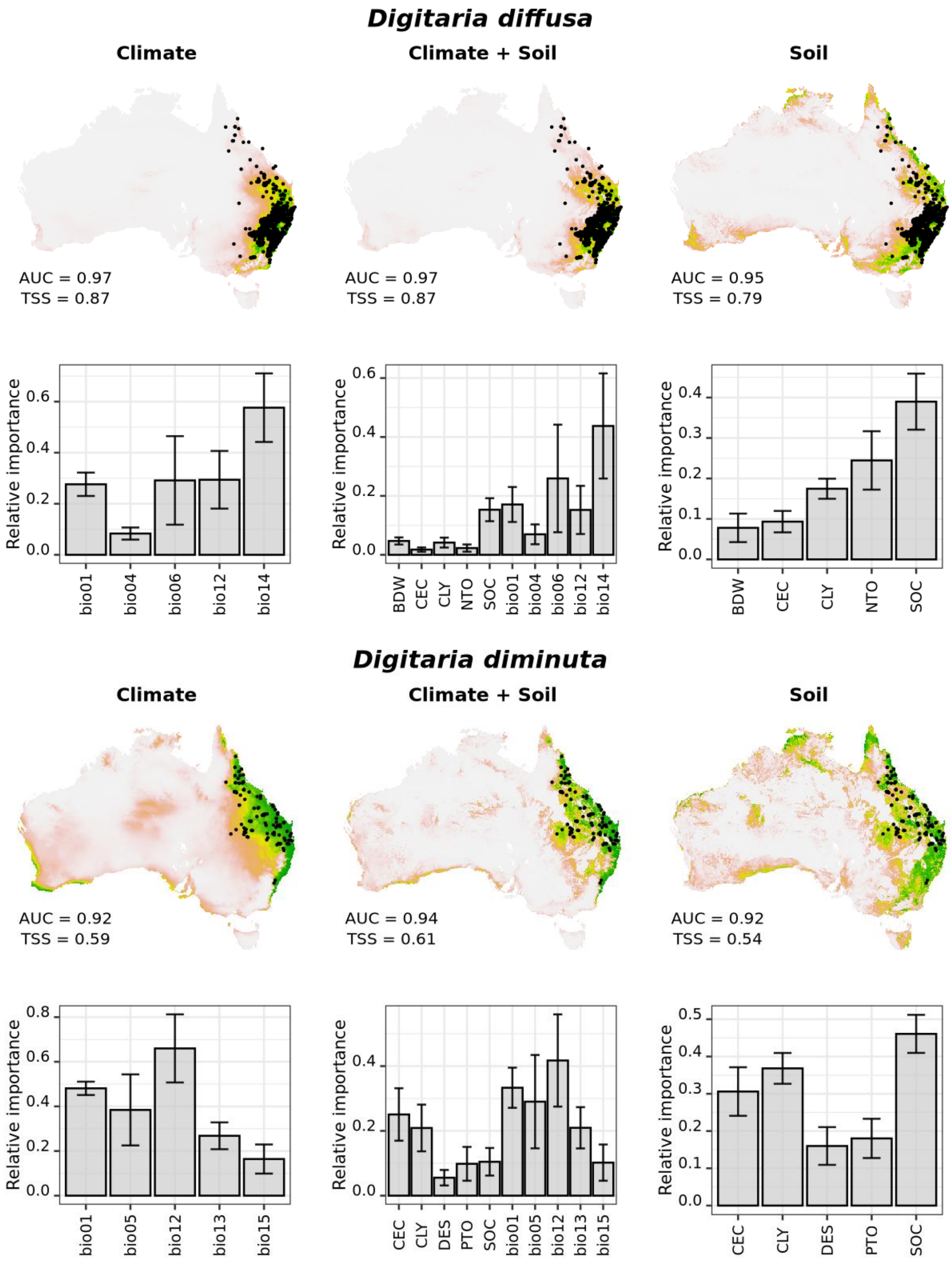

Digitaria diffusa
Climate
Climate + Soil
Soil
AUC = 0.97
TSS = 0.87
AUC = 0.97
TSS = 0.87
AUC = 0.95
TSS = 0.79
Relative importance
bio01
bio04
bio06
bio12
bio14
BDW
CEC
CLY
NTO
SOC
Digitaria diminuta
Climate
Climate + Soil
Soil
AUC = 0.92
TSS = 0.59
AUC = 0.94
TSS = 0.61
AUC = 0.92
TSS = 0.54
Relative importance
bio01
bio05
bio12
bio13
bio15
CEC
CLY
DES
PTO
SOC

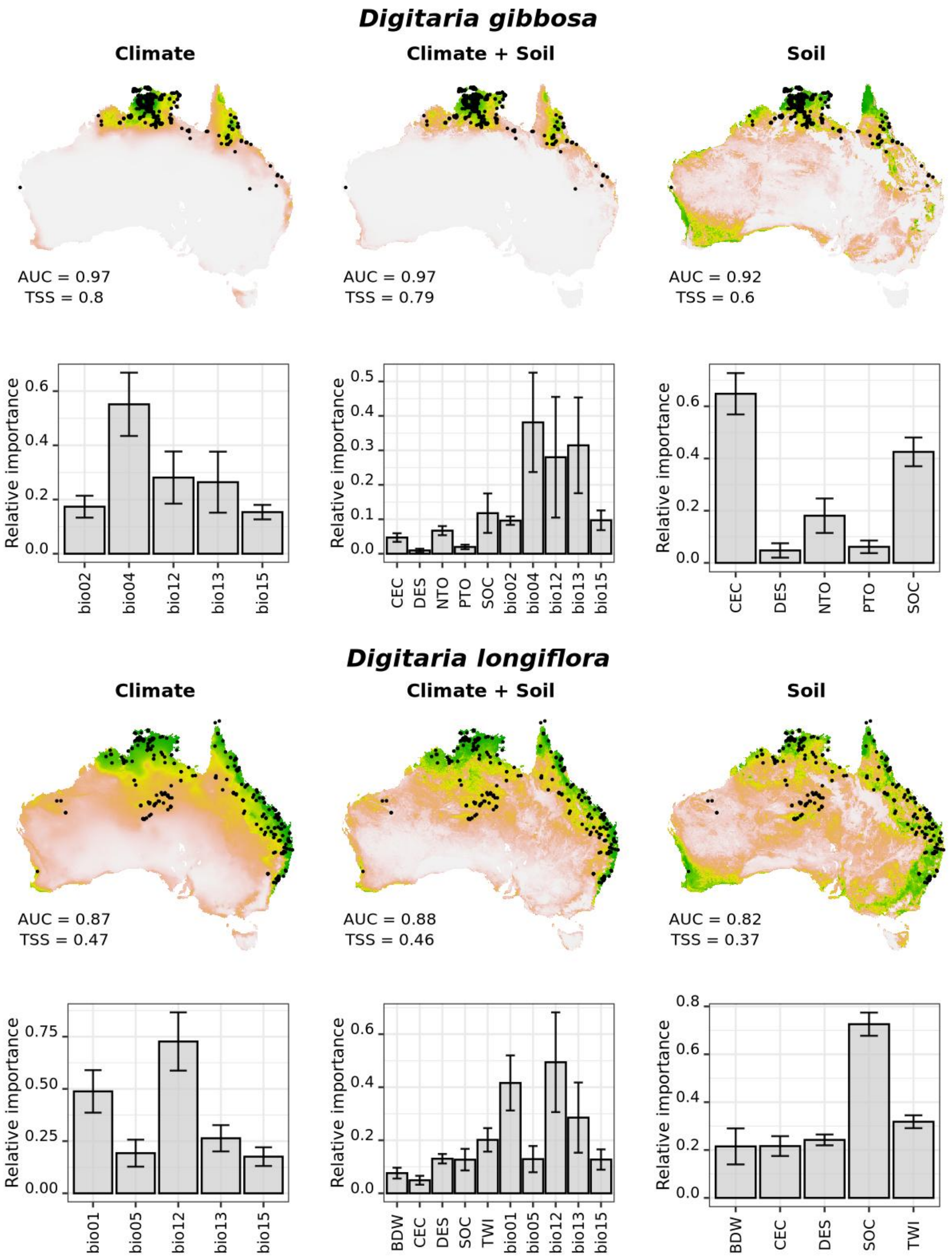

Digitaria gibbosa
Climate
Climate + Soil
Soil
AUC = 0.97
TSS = 0.8
AUC = 0.97
TSS = 0.79
AUC = 0.92
TSS = 0.6
Relative importance
bio02
bio04
bio12
bio13
bio15
CEC
DES
NTO
PTO
SOC
Digitaria longiflora
Climate
Climate + Soil
Soil
AUC = 0.87
TSS = 0.47
AUC = 0.88
TSS = 0.46
AUC = 0.82
TSS = 0.37
Relative importance
bio01
bio05
bio12
bio13
bio15
BDW
CEC
DES
SOC
TWI

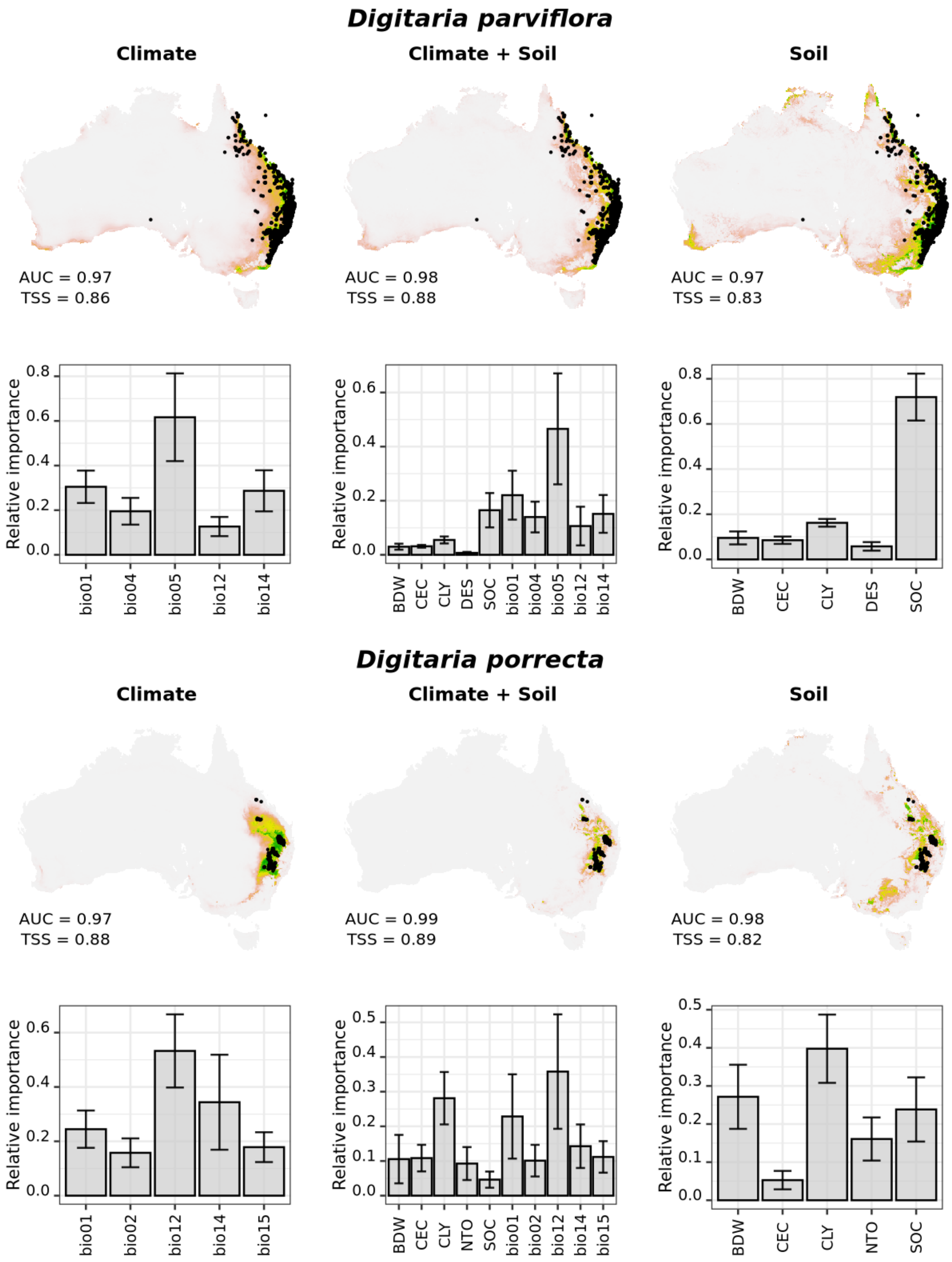

Digitaria parviflora
Climate
Climate + Soil
Soil
AUC = 0.97
TSS = 0.86
AUC = 0.98
TSS = 0.88
AUC = 0.97
TSS = 0.83
Relative importance
0.0
0.2
0.4
0.6
0.8
bio01
bio04
bio05
bio12
bio14
BDW
CEC
CLY
DES
SOC
Digitaria porrecta
Climate
Climate + Soil
Soil
AUC = 0.97
TSS = 0.88
AUC = 0.99
TSS = 0.89
AUC = 0.98
TSS = 0.82
Relative importance
0.0
0.1
0.2
0.3
0.4
0.5
0.6
bio01
bio02
bio12
bio14
bio15
BDW
CEC
CLY
NTO
SOC

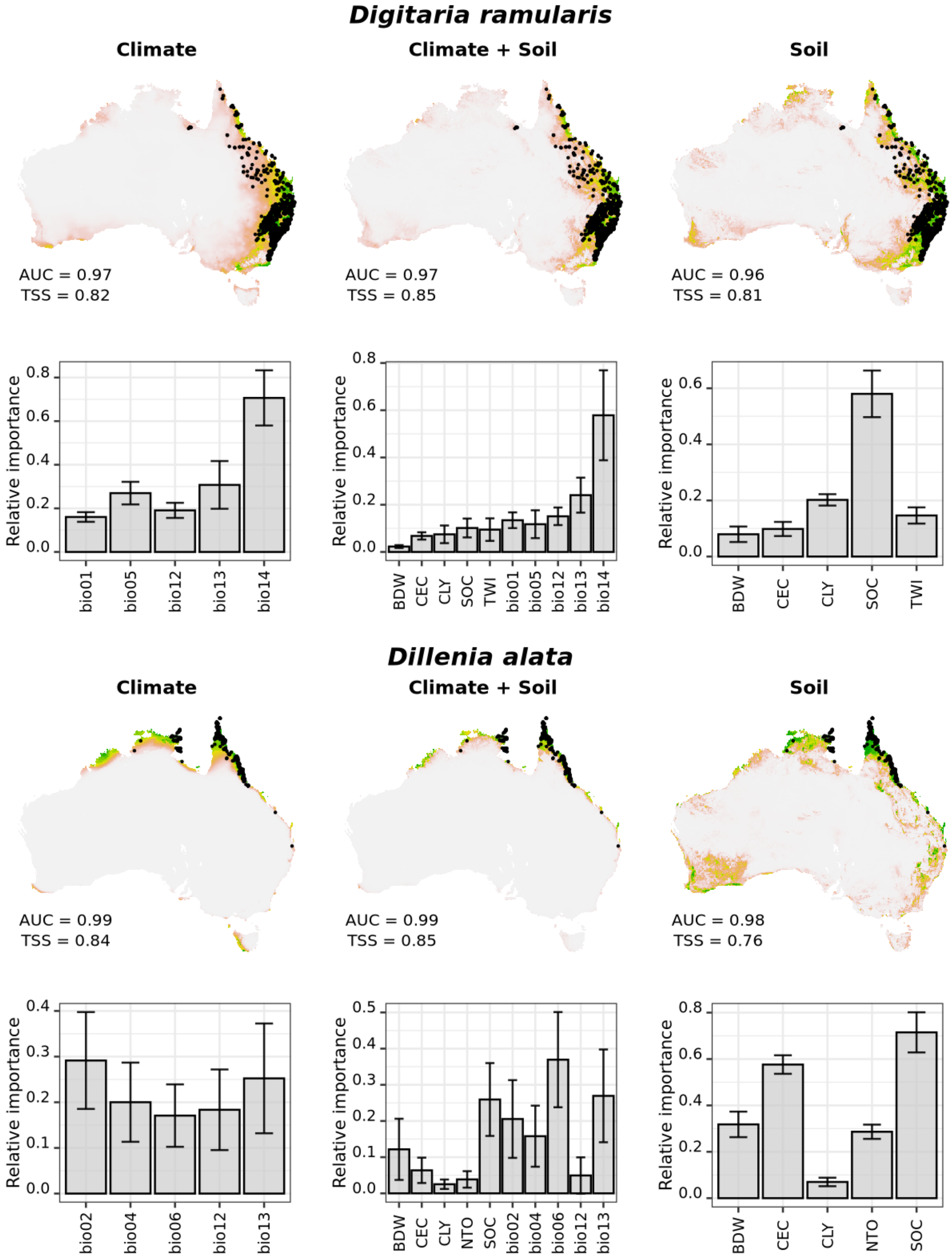

Digitaria ramularis
Climate
Climate + Soil
Soil
AUC = 0.97
TSS = 0.82
AUC = 0.97
TSS = 0.85
AUC = 0.96
TSS = 0.81
Relative importance
bio01
bio05
bio12
bio13
bio14
BDW
CEC
CLY
SOC
TWI
Dillenia alata
AUC = 0.99
TSS = 0.84
AUC = 0.99
TSS = 0.85
AUC = 0.98
TSS = 0.76
bio02
bio04
bio06
NTO

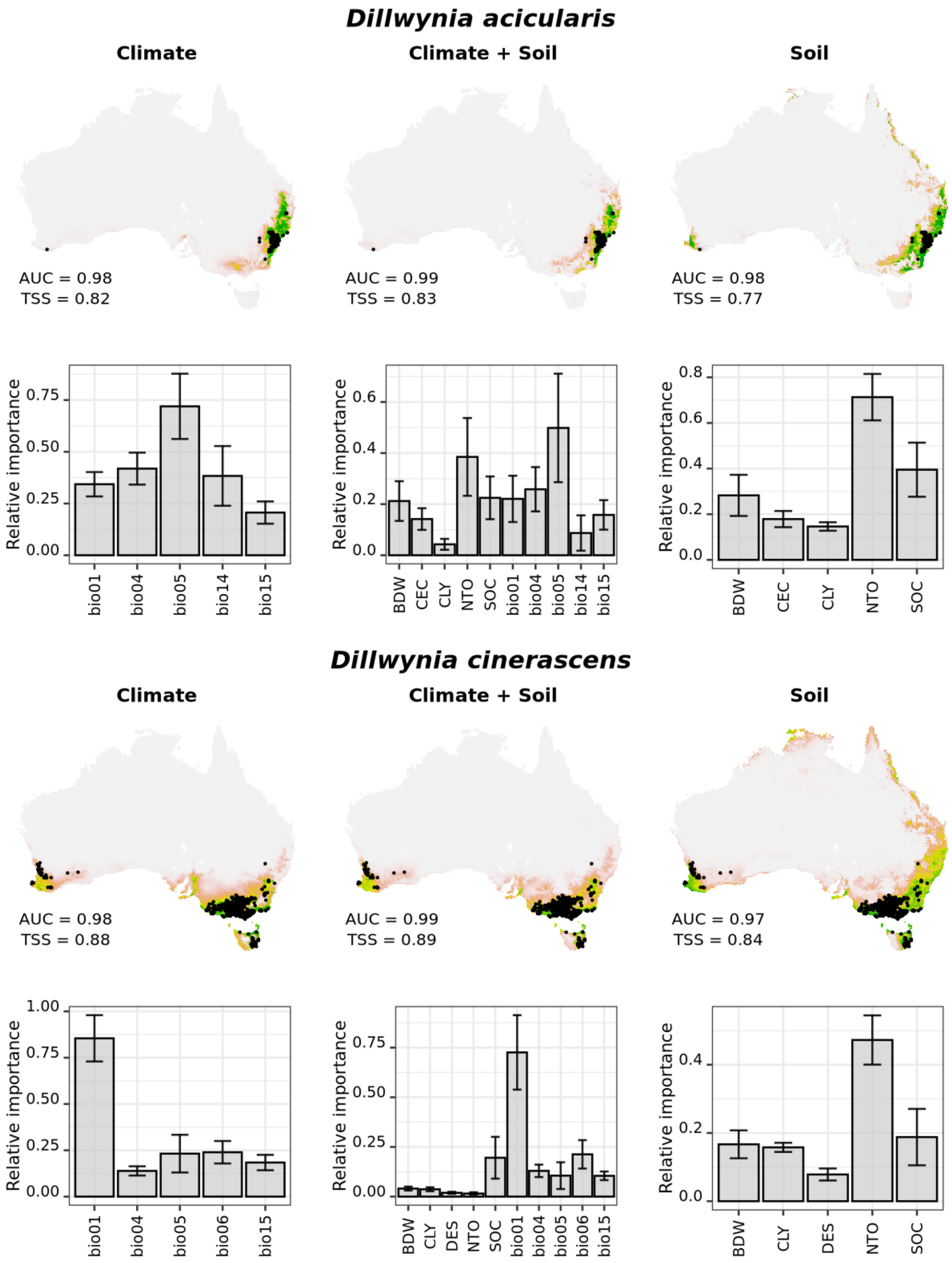

Dillwynia acicularis
Climate
Climate + Soil
Soil
AUC = 0.98
TSS = 0.82
AUC = 0.99
TSS = 0.83
AUC = 0.98
TSS = 0.77
Relative importance
bio01
bio04
bio05
bio14
bio15
BDW
CEC
CLY
NTO
SOC
Dillwynia cinerascens
AUC = 0.98
TSS = 0.88
AUC = 0.99
TSS = 0.89
AUC = 0.97
TSS = 0.84
bio06
DES

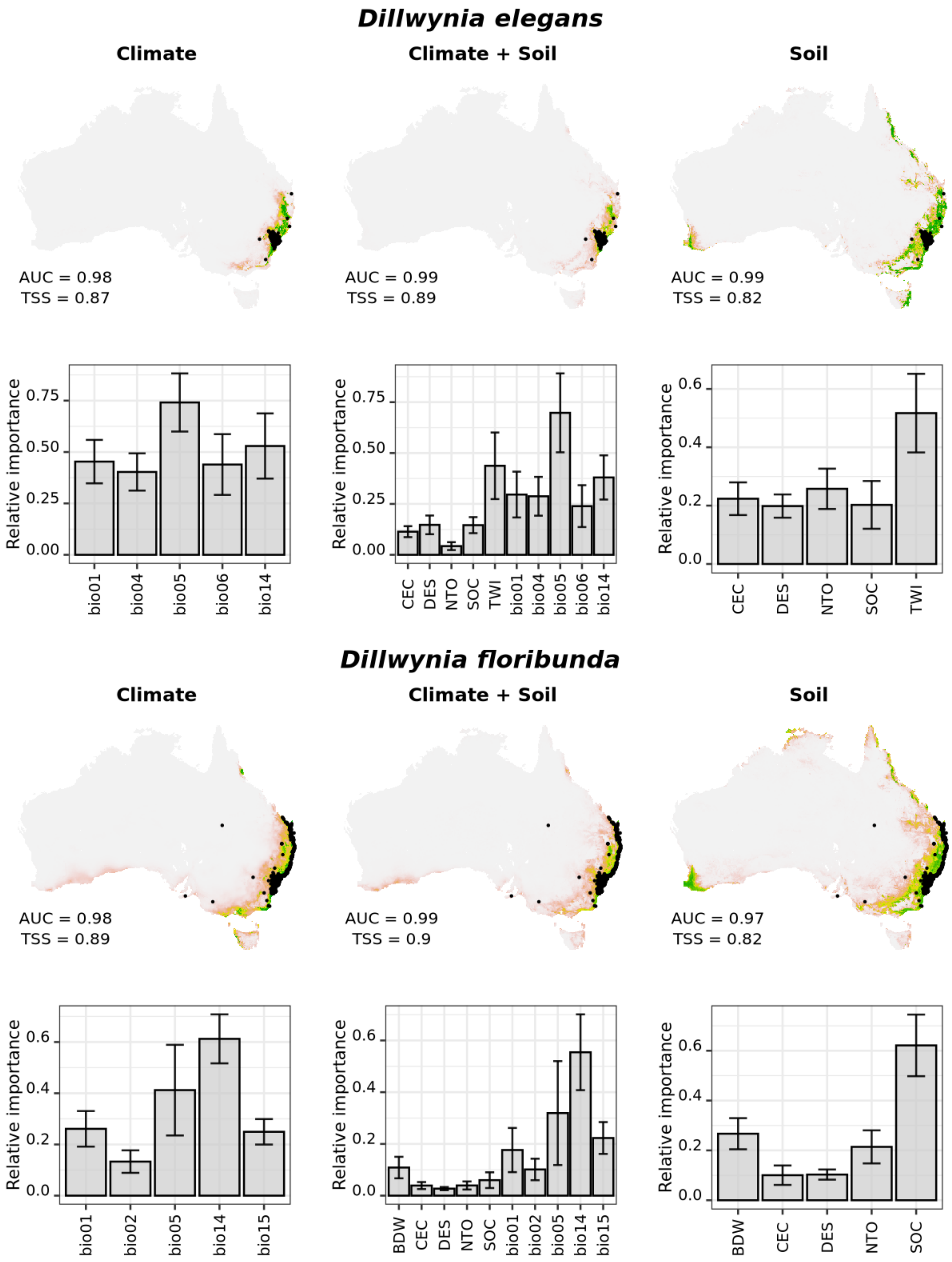

Dillwynia elegans
Climate
Climate + Soil
Soil
AUC = 0.98
TSS = 0.87
AUC = 0.99
TSS = 0.89
AUC = 0.99
TSS = 0.82
Relative importance
0.00
0.25
0.50
0.75
bio01
bio04
bio05
bio06
bio14
CEC
DES
NTO
SOC
TWI
bio01
bio04
bio05
bio06
bio14
0.0
0.2
0.4
0.6
CEC
DES
NTO
SOC
TWI
Dillwynia floribunda
Climate
Climate + Soil
Soil
AUC = 0.98
TSS = 0.89
AUC = 0.99
TSS = 0.9
AUC = 0.97
TSS = 0.82
Relative importance
0.0
0.2
0.4
0.6
bio01
bio02
bio05
bio14
bio15
BDW
CEC
DES
NTO
SOC
bio01
bio02
bio05
bio14
bio15
BDW
CEC
DES
NTO
SOC

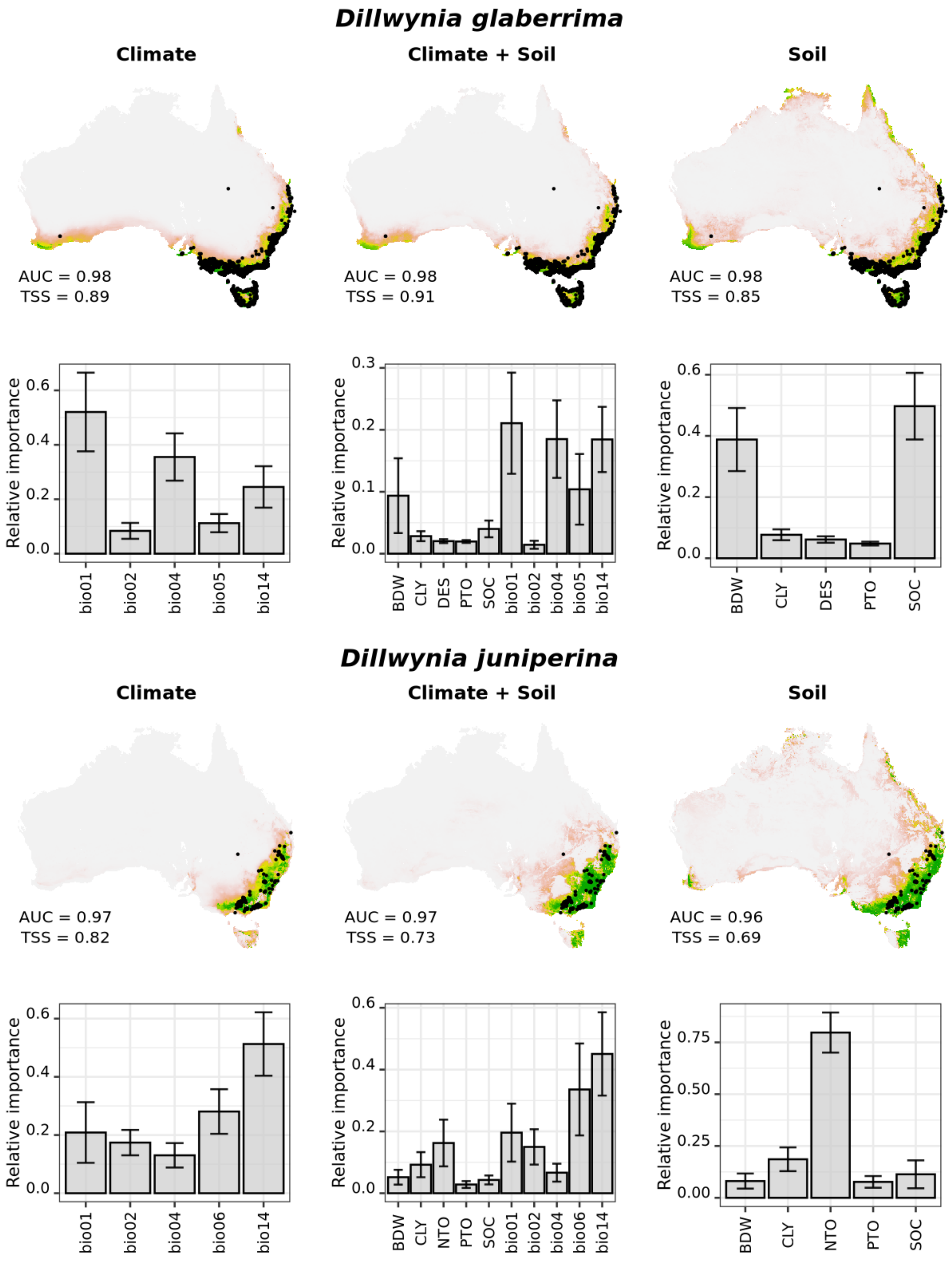

Dillwynia glaberrima
Climate
Climate + Soil
Soil
AUC = 0.98
TSS = 0.89
AUC = 0.98
TSS = 0.91
AUC = 0.98
TSS = 0.85
Relative importance
bio01
bio02
bio04
bio05
bio14
BDW
CLY
DES
PTO
SOC
Dillwynia juniperina
AUC = 0.97
TSS = 0.82
AUC = 0.97
TSS = 0.73
AUC = 0.96
TSS = 0.69
bio06
NTO

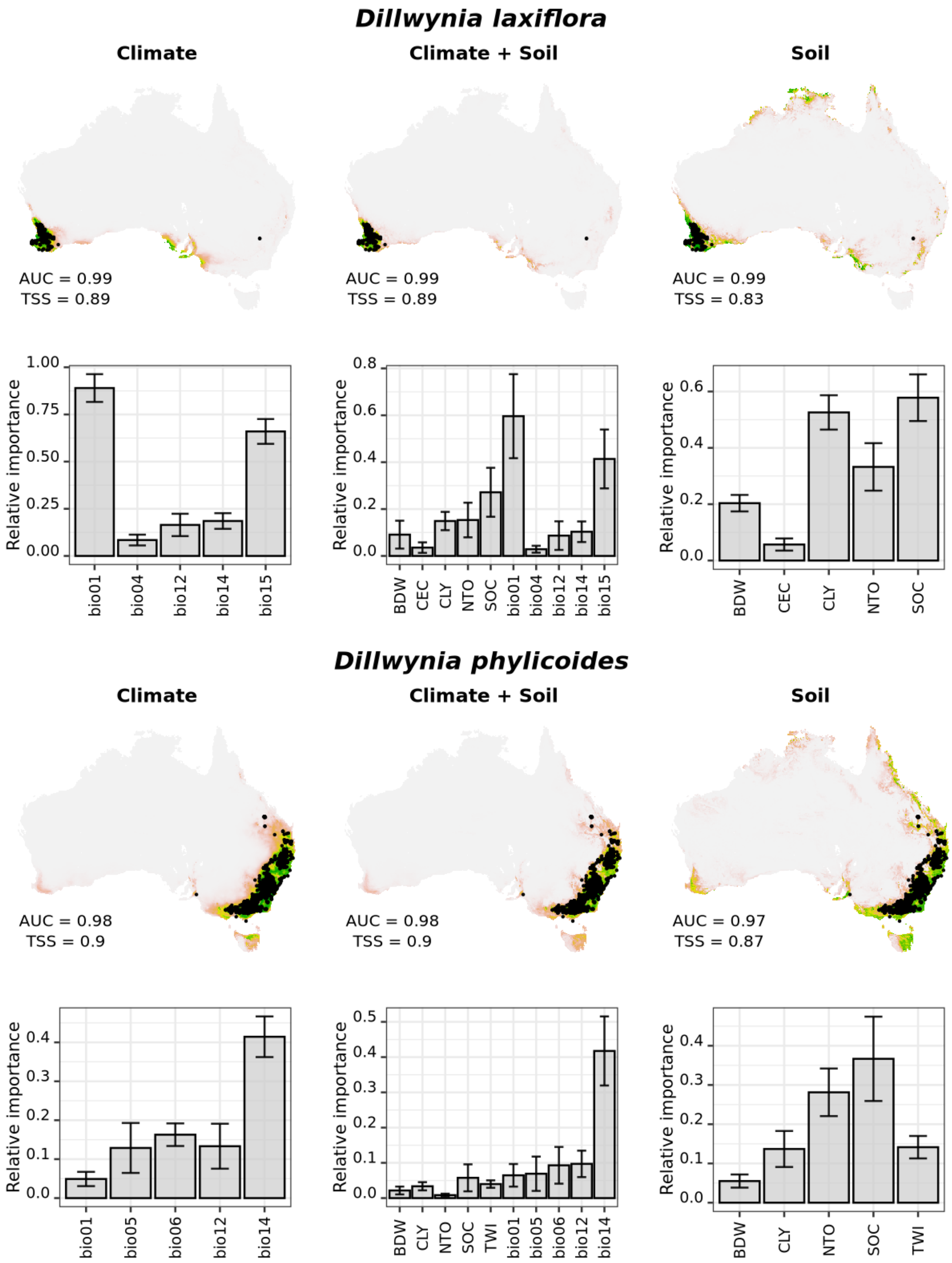
Dillwynia laxiflora
Climate
Climate + Soil
Soil
AUC = 0.99
TSS = 0.89
AUC = 0.99
TSS = 0.89
AUC = 0.99
TSS = 0.83
Relative importance
1.00
0.75
0.50
0.25
0.00
bio01
bio04
bio12
bio14
bio15
0.8
0.6
0.4
0.2
0.0
BDW
CEC
CLY
NTO
SOC
bio01
bio04
bio12
bio14
bio15
0.6
0.4
0.2
0.0
BDW
CEC
CLY
NTO
SOC
Dillwynia phylicoides
Climate
Climate + Soil
Soil
AUC = 0.98
TSS = 0.9
AUC = 0.98
TSS = 0.9
AUC = 0.97
TSS = 0.87
Relative importance
0.4
0.3
0.2
0.1
0.0
bio01
bio05
bio06
bio12
bio14
0.5
0.4
0.3
0.2
0.1
0.0
BDW
CLY
NTO
SOC
TWI
bio01
bio05
bio06
bio12
bio14
0.4
0.3
0.2
0.1
0.0
BDW
CLY
NTO
SOC
TWI

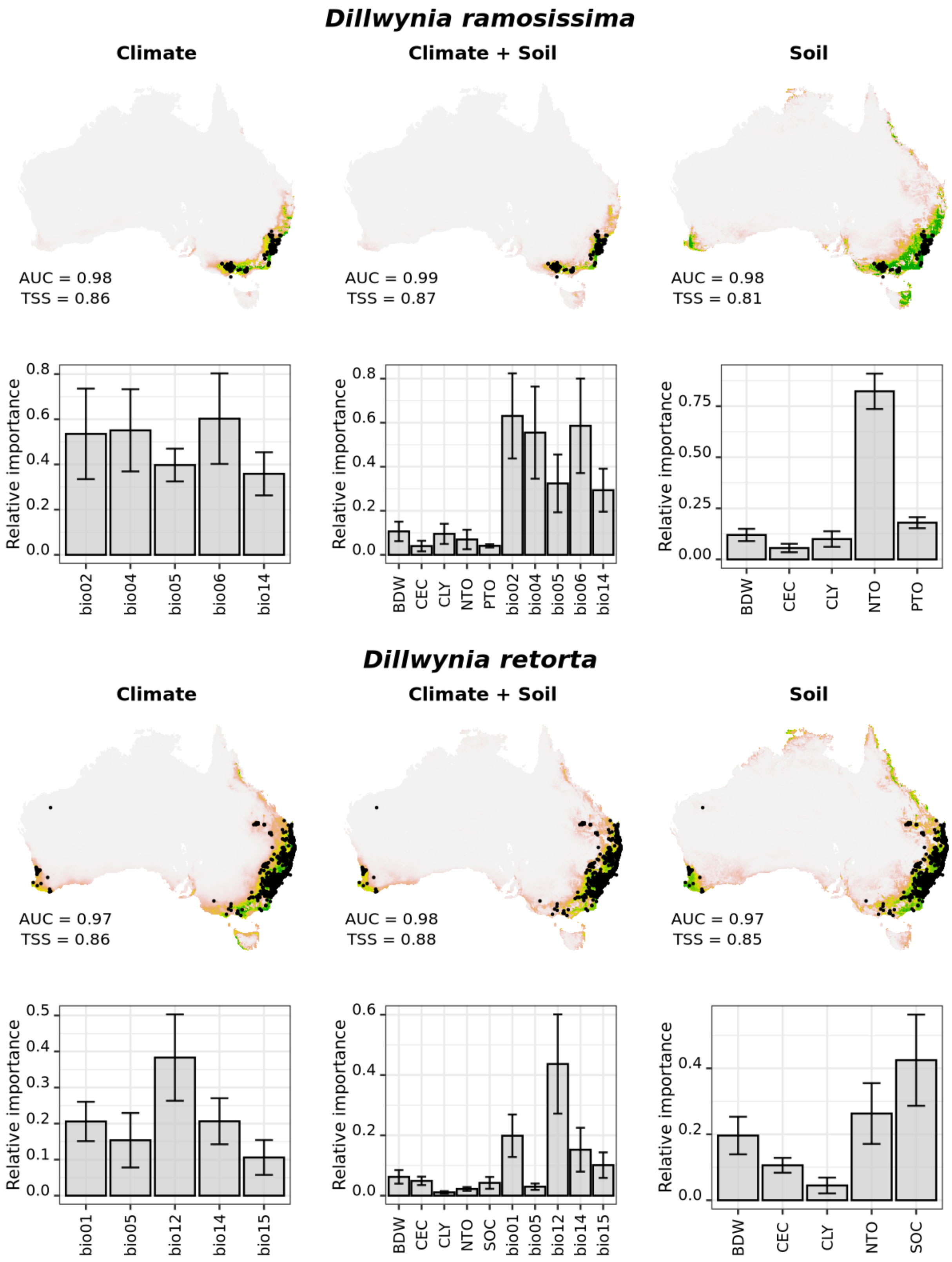
Dillwynia ramosissima
Climate
Climate + Soil
Soil
AUC = 0.98
TSS = 0.86
AUC = 0.99
TSS = 0.87
AUC = 0.98
TSS = 0.81
Relative importance
0.0 0.2 0.4 0.6 0.8
bio02 bio04 bio05 bio06 bio14
BDW CEC CLY NTO PTO bio02 bio04 bio05 bio06 bio14
0.00 0.25 0.50 0.75
BDW CEC CLY NTO PTO
Dillwynia retorta
Climate
Climate + Soil
Soil
AUC = 0.97
TSS = 0.86
AUC = 0.98
TSS = 0.88
AUC = 0.97
TSS = 0.85
0.0 0.1 0.2 0.3 0.4 0.5
bio01 bio05 bio12 bio14 bio15
0.0 0.2 0.4 0.6
BDW CEC CLY NTO SOC bio01 bio05 bio12 bio14 bio15
0.0 0.2 0.4
BDW CEC CLY NTO SOC

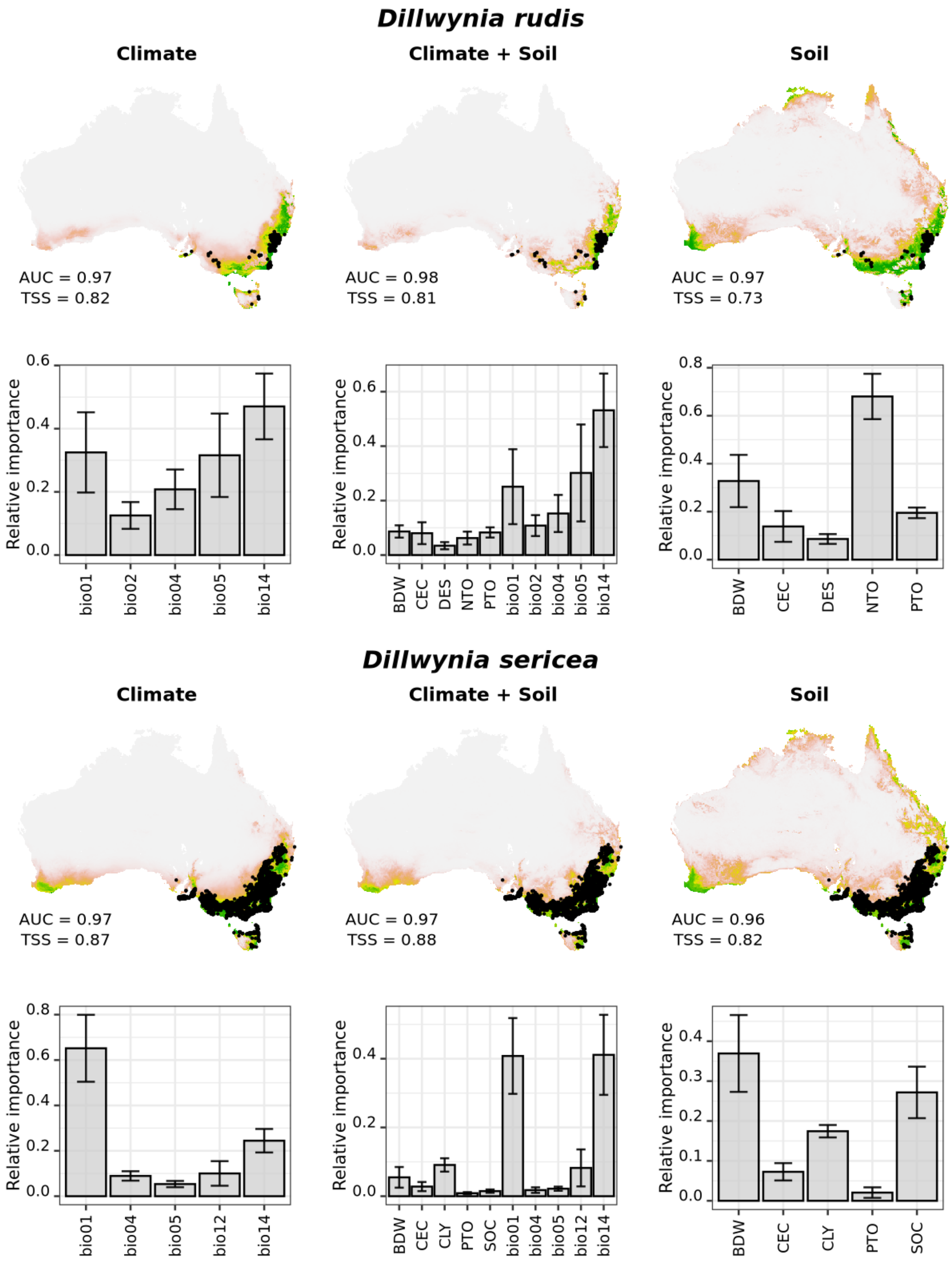

Dillwynia rudis
Climate
Climate + Soil
Soil
AUC = 0.97
TSS = 0.82
AUC = 0.98
TSS = 0.81
AUC = 0.97
TSS = 0.73
Relative importance
bio01
bio02
bio04
bio05
bio14
BDW
CEC
DES
NTO
PTO
Dillwynia sericea
AUC = 0.97
TSS = 0.87
AUC = 0.97
TSS = 0.88
AUC = 0.96
TSS = 0.82
bio12
CLY
SOC

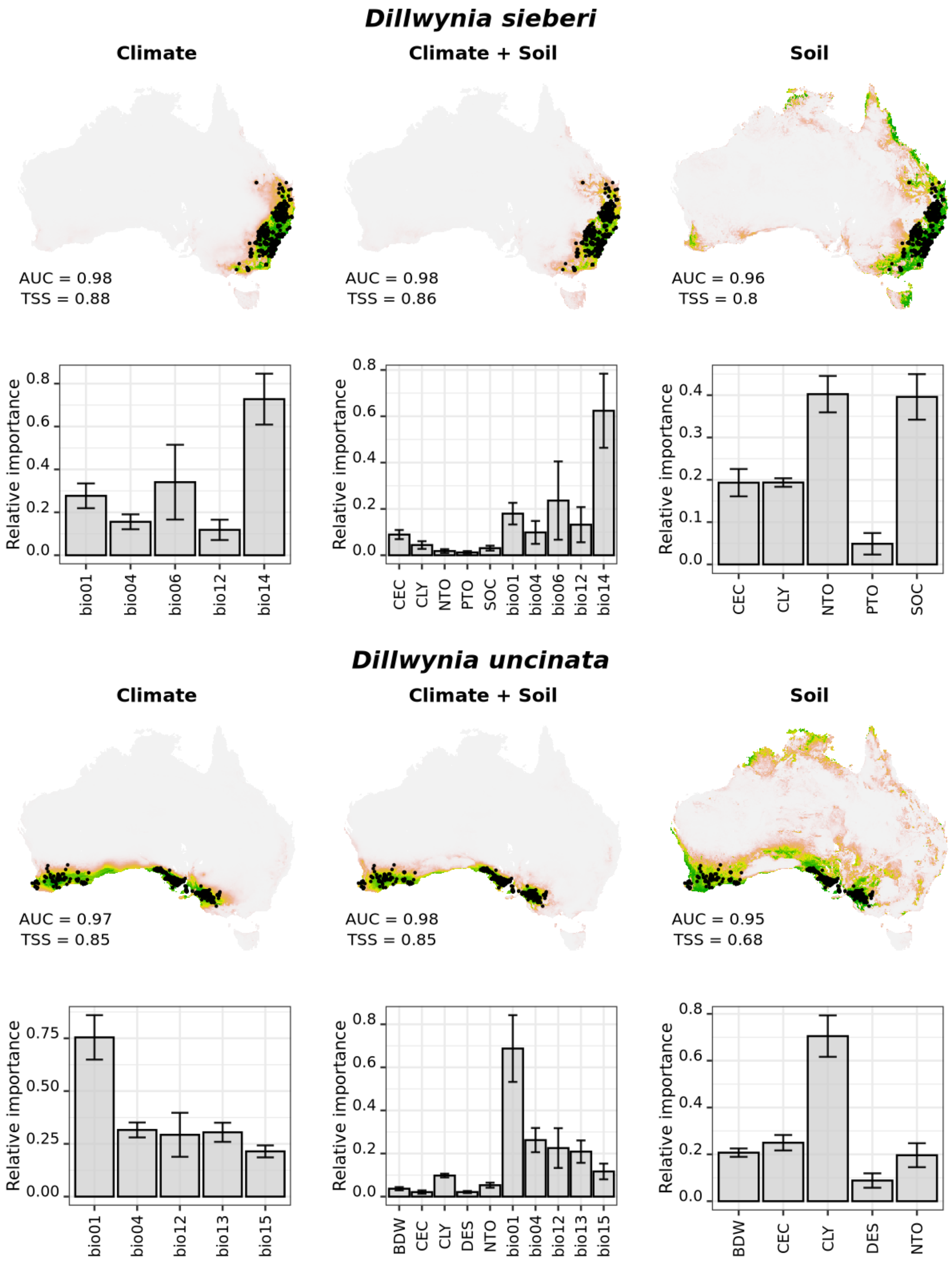
Dillwynia sieberi
Climate
Climate + Soil
Soil
AUC = 0.98
TSS = 0.88
AUC = 0.98
TSS = 0.86
AUC = 0.96
TSS = 0.8
Relative importance
bio01
bio04
bio06
bio12
bio14
CEC
CLY
NTO
PTO
SOC
Dillwynia uncinata
Climate
Climate + Soil
Soil
AUC = 0.97
TSS = 0.85
AUC = 0.98
TSS = 0.85
AUC = 0.95
TSS = 0.68
Relative importance
bio01
bio04
bio12
bio13
bio15
BDW
CEC
CLY
DES
NTO

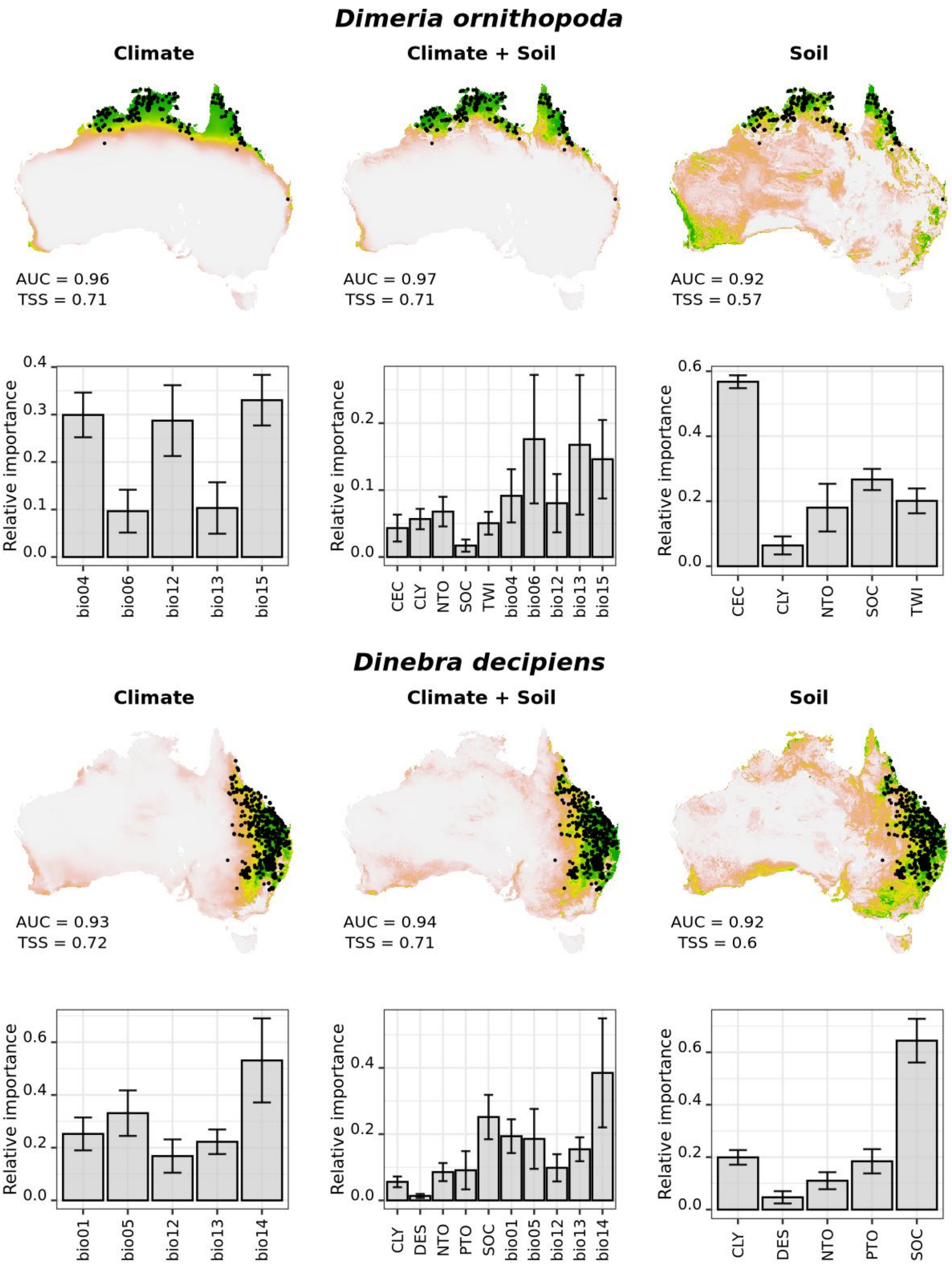
Dimeria ornithopoda
Climate
Climate + Soil
Soil
AUC = 0.96
TSS = 0.71
AUC = 0.97
TSS = 0.71
AUC = 0.92
TSS = 0.57
Relative importance
0.0
0.1
0.2
0.3
0.4
bio04
bio06
bio12
bio13
bio15
Relative importance
0.0
0.1
0.2
CEC
CLY
NTO
SOC
TWI
bio04
bio06
bio12
bio13
bio15
Relative importance
0.0
0.2
0.4
0.6
CEC
CLY
NTO
SOC
TWI
Dinebra decipiens
Climate
Climate + Soil
Soil
AUC = 0.93
TSS = 0.72
AUC = 0.94
TSS = 0.71
AUC = 0.92
TSS = 0.6
Relative importance
0.0
0.2
0.4
0.6
bio01
bio05
bio12
bio13
bio14
Relative importance
0.0
0.2
0.4
CLY
DES
NTO
PTO
SOC
bio01
bio05
bio12
bio13
bio14
Relative importance
0.0
0.2
0.4
0.6
CLY
DES
NTO
PTO
SOC

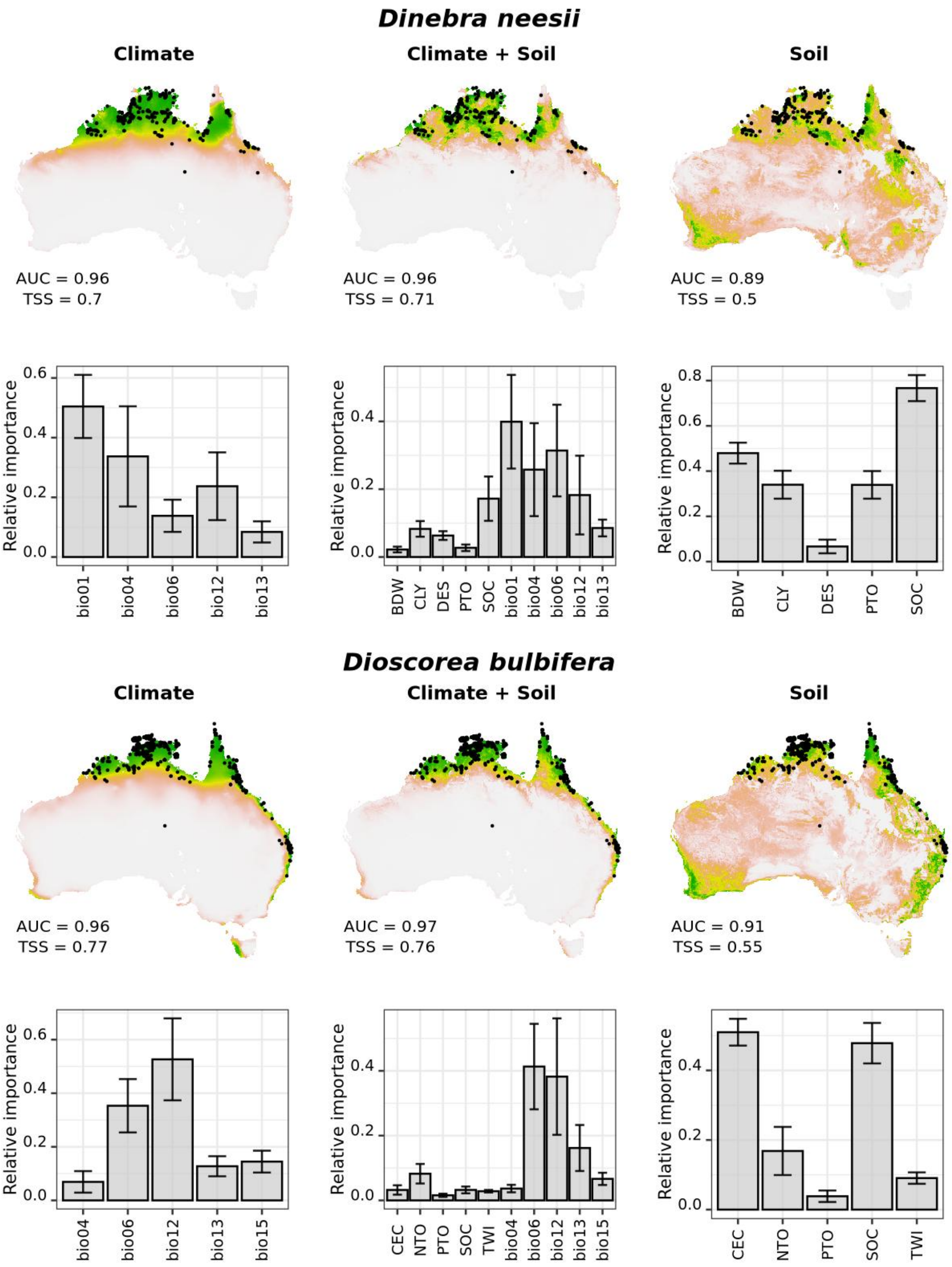
Dinebra neesii
Climate
Climate + Soil
Soil
AUC = 0.96
TSS = 0.7
AUC = 0.96
TSS = 0.71
AUC = 0.89
TSS = 0.5
Relative importance
bio01
bio04
bio06
bio12
bio13
BDW
CLY
DES
PTO
SOC
Dioscorea bulbifera
Climate
Climate + Soil
Soil
AUC = 0.96
TSS = 0.77
AUC = 0.97
TSS = 0.76
AUC = 0.91
TSS = 0.55
Relative importance
bio04
bio06
bio12
bio13
bio15
CEC
NTO
PTO
SOC
TWI

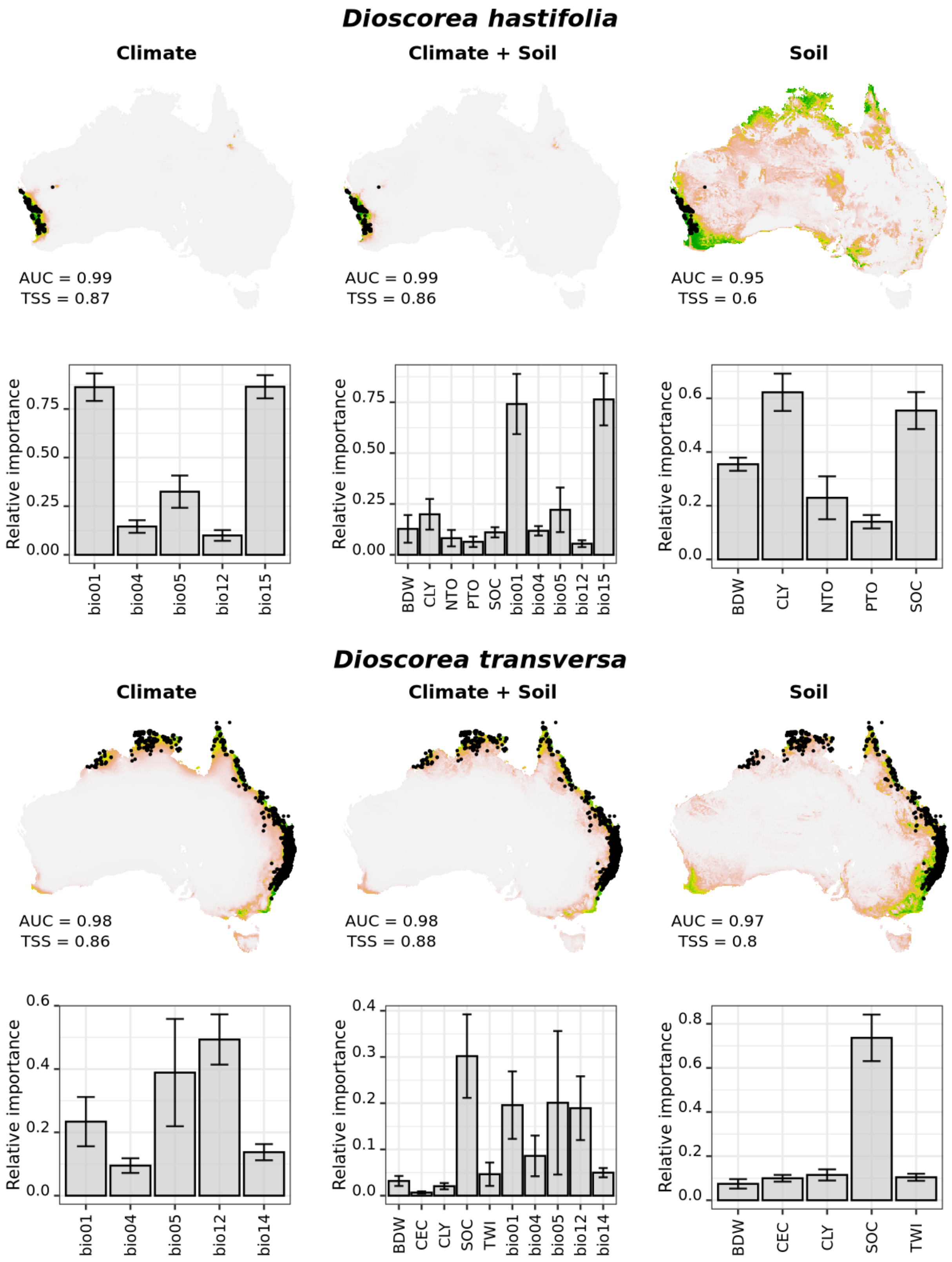
Dioscorea hastifolia
Climate
Climate + Soil
Soil
AUC = 0.99
TSS = 0.87
AUC = 0.99
TSS = 0.86
AUC = 0.95
TSS = 0.6
Relative importance
0.00
0.25
0.50
0.75
bio01
bio04
bio05
bio12
bio15
BDW
CLY
NTO
PTO
SOC
0.0
0.2
0.4
0.6
Dioscorea transversa
Climate
Climate + Soil
Soil
AUC = 0.98
TSS = 0.86
AUC = 0.98
TSS = 0.88
AUC = 0.97
TSS = 0.8
0.1
0.3
0.8
bio14
CEC
TWI

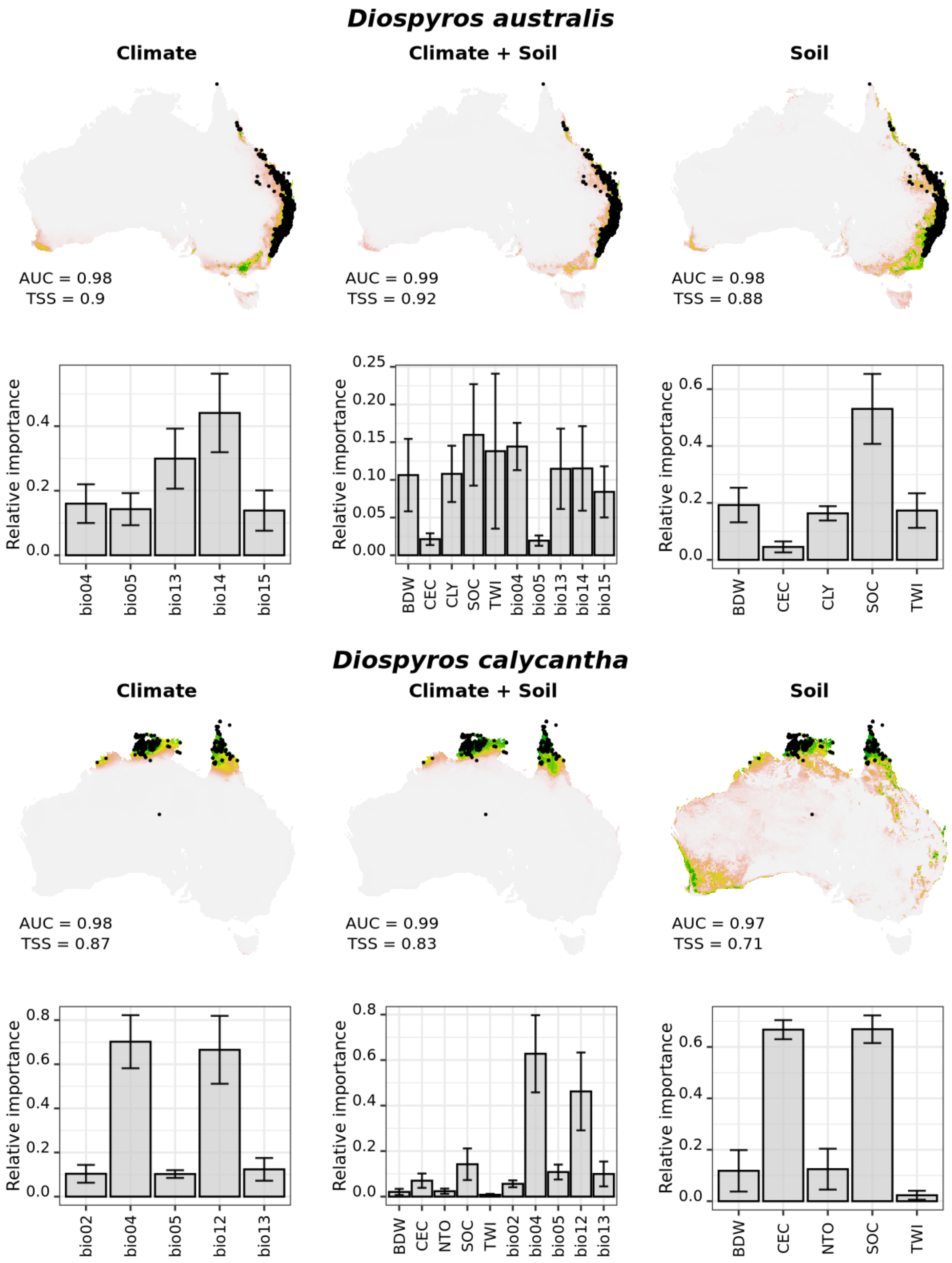
Diospyros australis
Climate
Climate + Soil
Soil
AUC = 0.98
TSS = 0.9
AUC = 0.99
TSS = 0.92
AUC = 0.98
TSS = 0.88
Relative importance
bio04
bio05
bio13
bio14
bio15
BDW
CEC
CLY
SOC
TWI
bio04
bio05
bio13
bio14
bio15
BDW
CEC
CLY
SOC
TWI
Diospyros calycantha
Climate
Climate + Soil
Soil
AUC = 0.98
TSS = 0.87
AUC = 0.99
TSS = 0.83
AUC = 0.97
TSS = 0.71
Relative importance
bio02
bio04
bio05
bio12
bio13
BDW
CEC
NTO
SOC
TWI
bio02
bio04
bio05
bio12
bio13
BDW
CEC
NTO
SOC
TWI

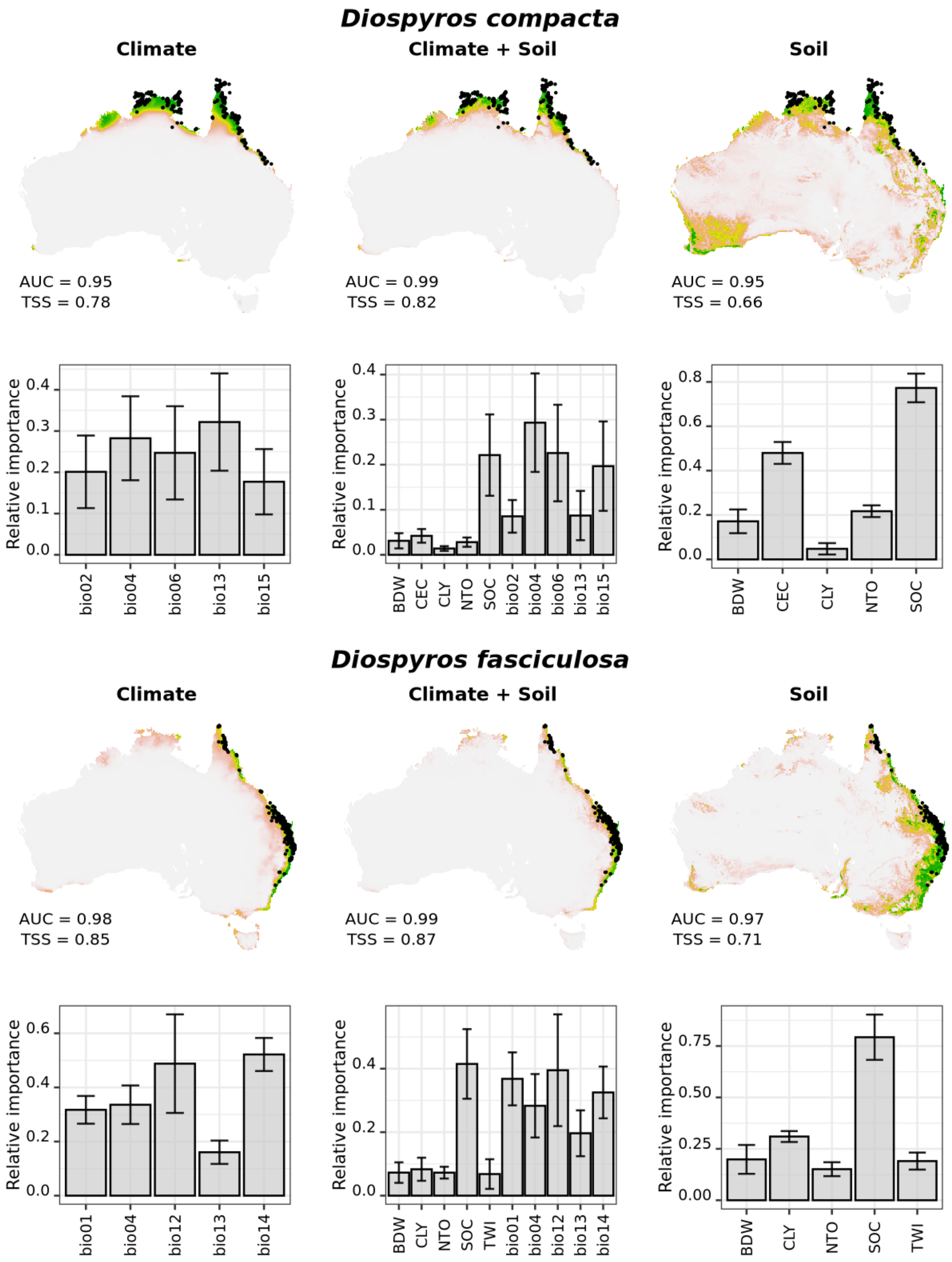

Diospyros compacta
Climate
Climate + Soil
Soil
AUC = 0.95
TSS = 0.78
AUC = 0.99
TSS = 0.82
AUC = 0.95
TSS = 0.66
Relative importance
bio02
bio04
bio06
bio13
bio15
BDW
CEC
CLY
NTO
SOC
Diospyros fasciculosa
Climate
Climate + Soil
Soil
AUC = 0.98
TSS = 0.85
AUC = 0.99
TSS = 0.87
AUC = 0.97
TSS = 0.71
Relative importance
bio01
bio04
bio12
bio13
bio14
BDW
CLY
NTO
SOC
TWI

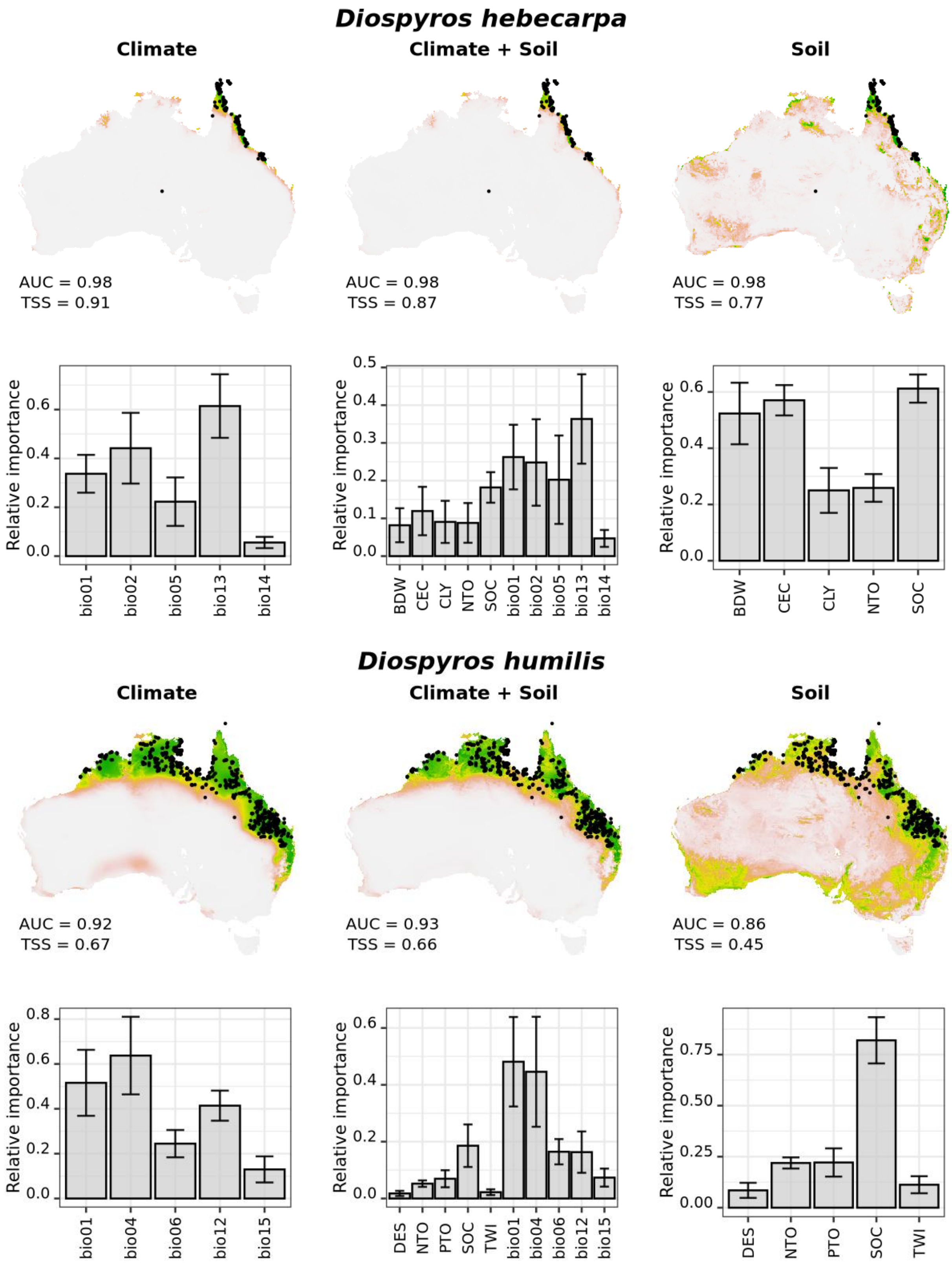
Diospyros hebecarpa
Climate
Climate + Soil
Soil
AUC = 0.98
TSS = 0.91
AUC = 0.98
TSS = 0.87
AUC = 0.98
TSS = 0.77
Relative importance
bio01
bio02
bio05
bio13
bio14
BDW
CEC
CLY
NTO
SOC
bio01
bio02
bio05
bio13
bio14
BDW
CEC
CLY
NTO
SOC
Diospyros humilis
Climate
Climate + Soil
Soil
AUC = 0.92
TSS = 0.67
AUC = 0.93
TSS = 0.66
AUC = 0.86
TSS = 0.45
Relative importance
bio01
bio04
bio06
bio12
bio15
DES
NTO
PTO
SOC
TWI
bio01
bio04
bio06
bio12
bio15
DES
NTO
PTO
SOC
TWI

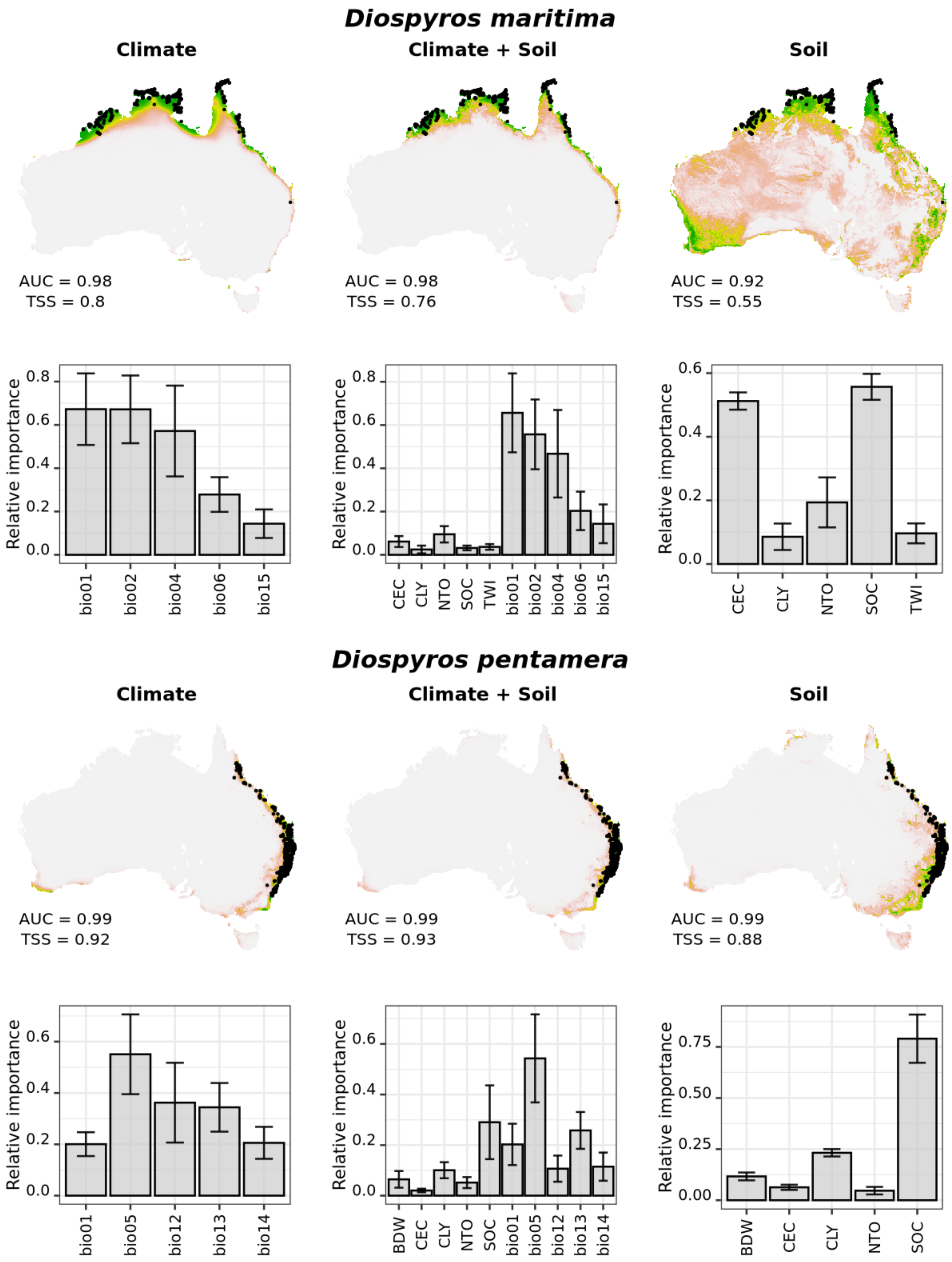

Diospyros maritima
Climate
Climate + Soil
Soil
AUC = 0.98
TSS = 0.8
AUC = 0.98
TSS = 0.76
AUC = 0.92
TSS = 0.55
Relative importance
bio01
bio02
bio04
bio06
bio15
CEC
CLY
NTO
SOC
TWI
Diospyros pentamera
AUC = 0.99
TSS = 0.92
AUC = 0.99
TSS = 0.93
AUC = 0.99
TSS = 0.88
bio05
bio12
bio13
bio14
BDW

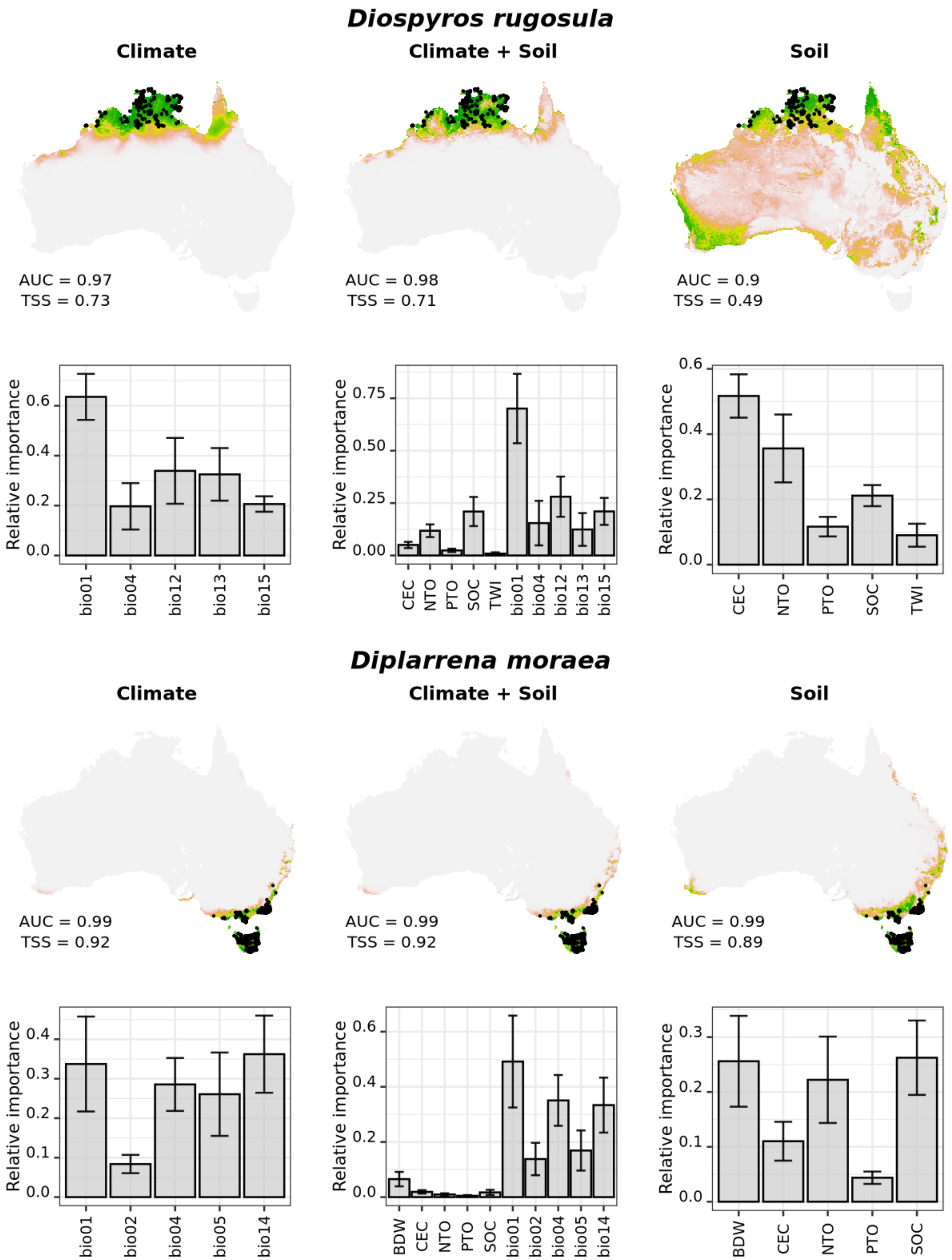
Diospyros rugosula
Climate
Climate + Soil
Soil
AUC = 0.97
TSS = 0.73
AUC = 0.98
TSS = 0.71
AUC = 0.9
TSS = 0.49
Relative importance
bio01
bio04
bio12
bio13
bio15
CEC
NTO
PTO
SOC
TWI
Diplarrena moraea
AUC = 0.99
TSS = 0.92
AUC = 0.99
TSS = 0.92
AUC = 0.99
TSS = 0.89
bio02
bio05
bio14
BDW

Diplatia grandibractea

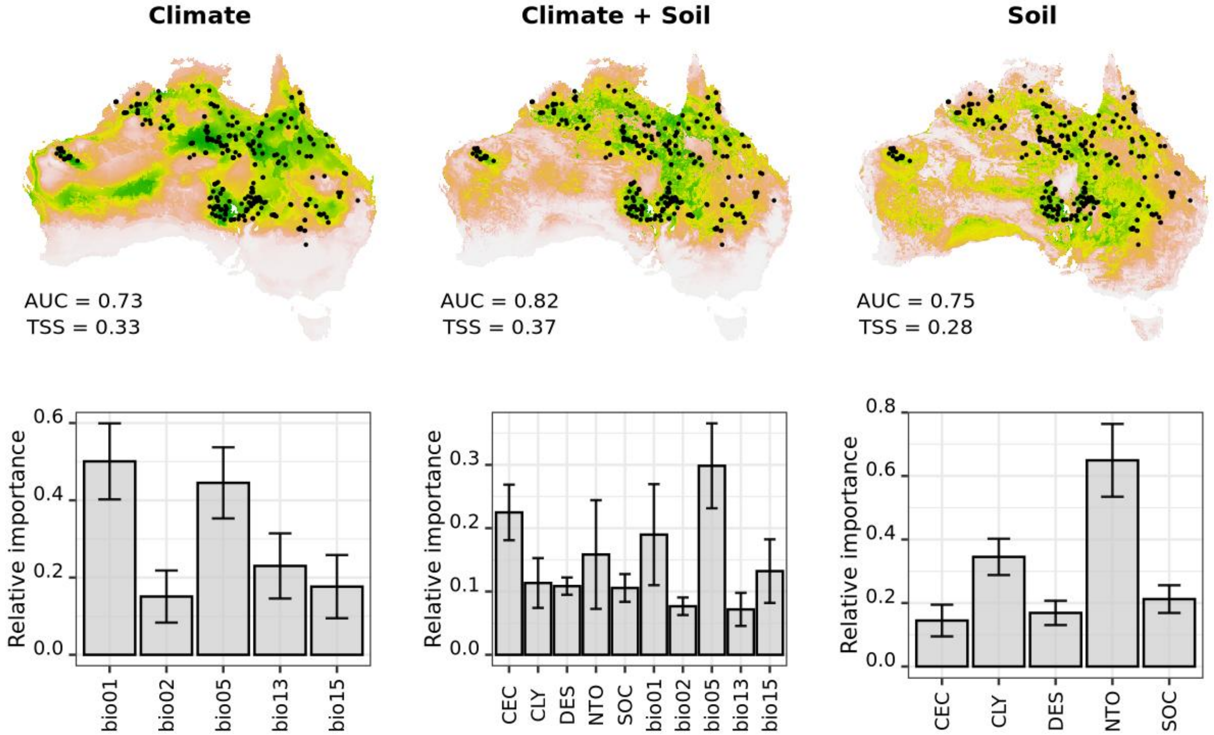

Diploglottis australis

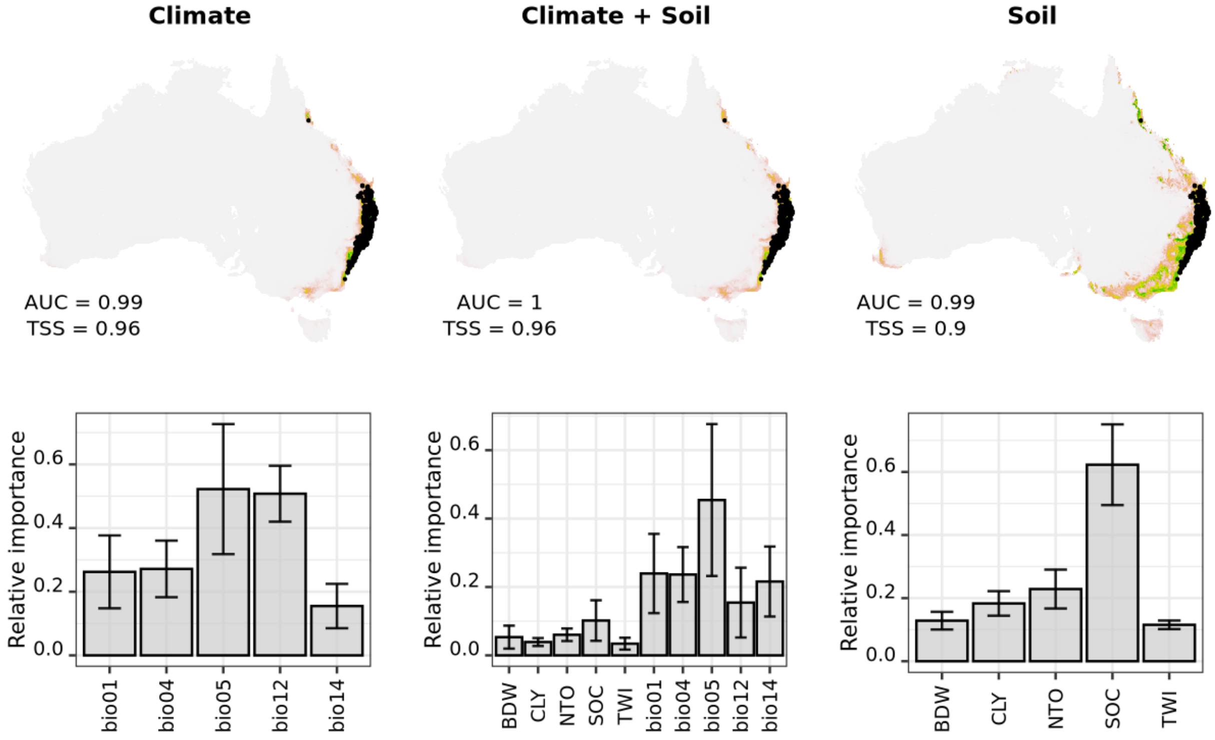

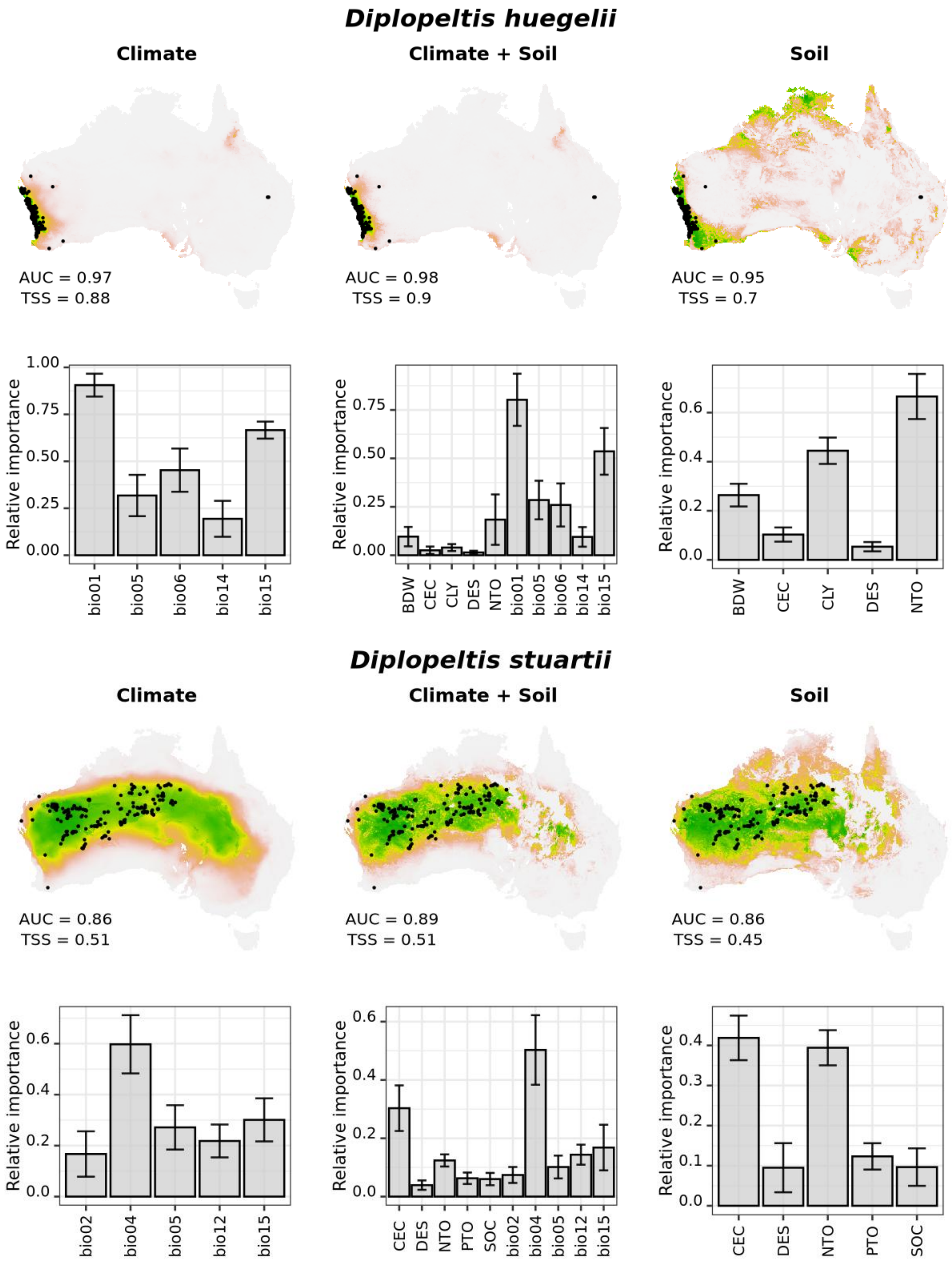

Diplopeltis huegelii
Climate
Climate + Soil
Soil
AUC = 0.97
TSS = 0.88
AUC = 0.98
TSS = 0.9
AUC = 0.95
TSS = 0.7
Relative importance
0.00
0.25
0.50
0.75
1.00
bio01
bio05
bio06
bio14
bio15
BDW
CEC
CLY
DES
NTO
0.0
0.2
0.4
0.6
Diplopeltis stuartii
Climate
Climate + Soil
Soil
AUC = 0.86
TSS = 0.51
AUC = 0.89
TSS = 0.51
AUC = 0.86
TSS = 0.45
Relative importance
bio02
bio04
bio05
bio12
bio15
CEC
DES
NTO
PTO
SOC
0.1
0.3

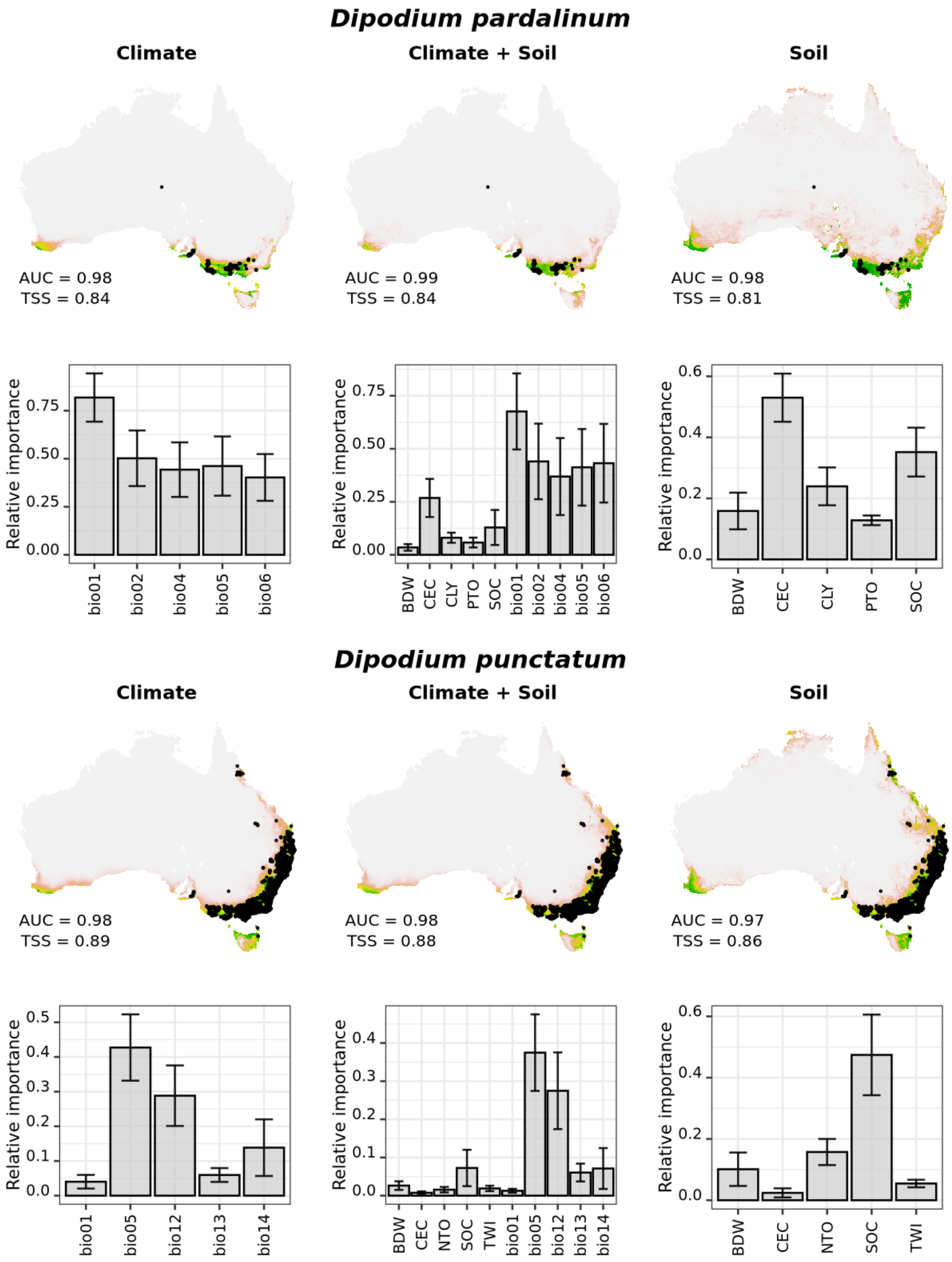

Dipodium pardalinum
Climate
Climate + Soil
Soil
AUC = 0.98
TSS = 0.84
AUC = 0.99
TSS = 0.84
AUC = 0.98
TSS = 0.81
Relative importance
bio01
bio02
bio04
bio05
bio06
BDW
CEC
CLY
PTO
SOC
Dipodium punctatum
Climate
Climate + Soil
Soil
AUC = 0.98
TSS = 0.89
AUC = 0.98
TSS = 0.88
AUC = 0.97
TSS = 0.86
Relative importance
bio01
bio05
bio12
bio13
bio14
BDW
CEC
NTO
SOC
TWI

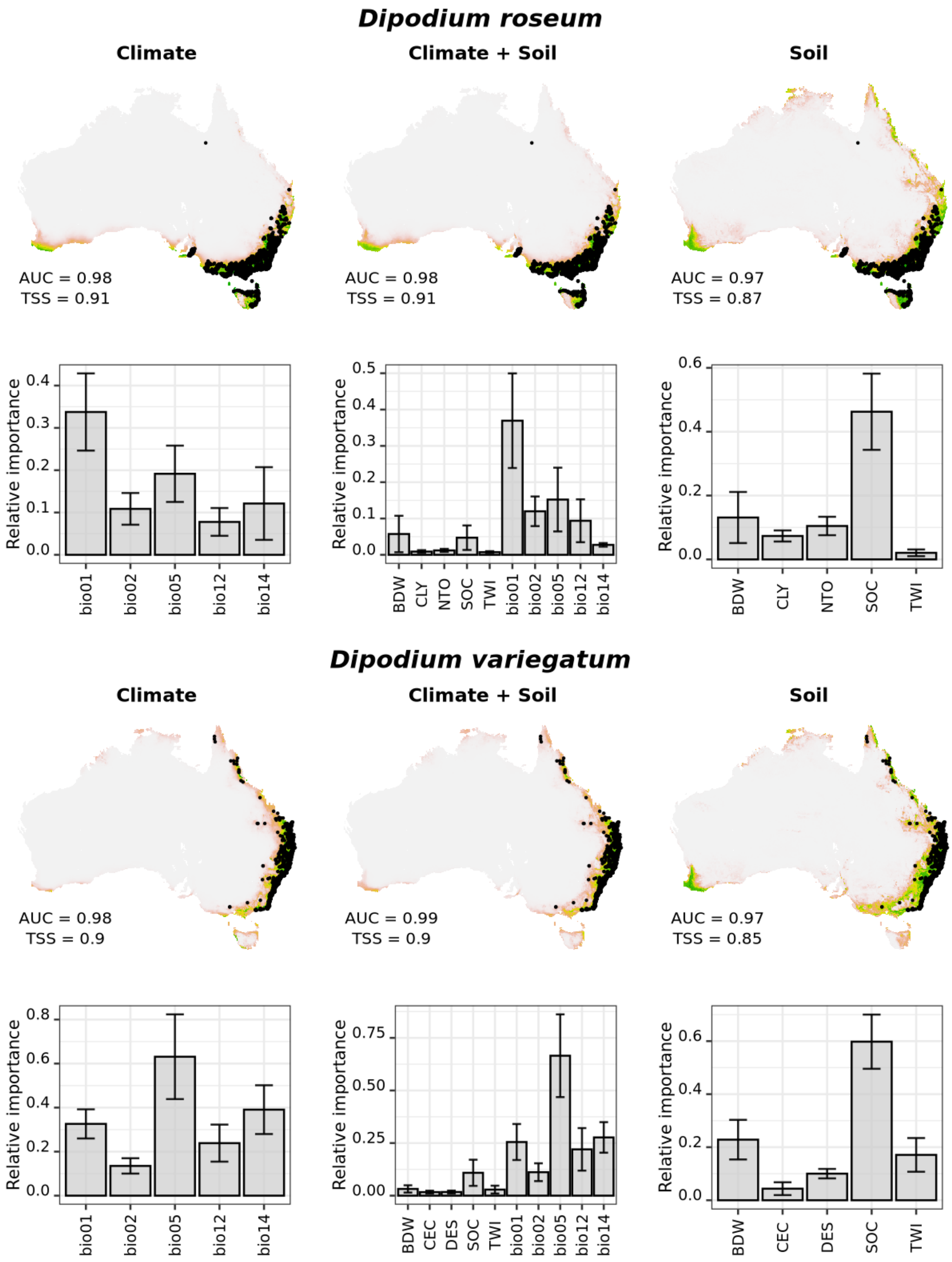
Dipodium roseum
Climate
Climate + Soil
Soil
AUC = 0.98
TSS = 0.91
AUC = 0.98
TSS = 0.91
AUC = 0.97
TSS = 0.87
Relative importance
0.0 0.1 0.2 0.3 0.4
bio01 bio02 bio05 bio12 bio14
0.0 0.1 0.2 0.3 0.4 0.5
BDW CLY NTO SOC TWI bio01 bio02 bio05 bio12 bio14
0.0 0.2 0.4 0.6
BDW CLY NTO SOC TWI
Dipodium variegatum
Climate
Climate + Soil
Soil
AUC = 0.98
TSS = 0.9
AUC = 0.99
TSS = 0.9
AUC = 0.97
TSS = 0.85
0.0 0.2 0.4 0.6 0.8
bio01 bio02 bio05 bio12 bio14
0.00 0.25 0.50 0.75
BDW CEC DES SOC TWI bio01 bio02 bio05 bio12 bio14
0.0 0.2 0.4 0.6
BDW CEC DES SOC TWI

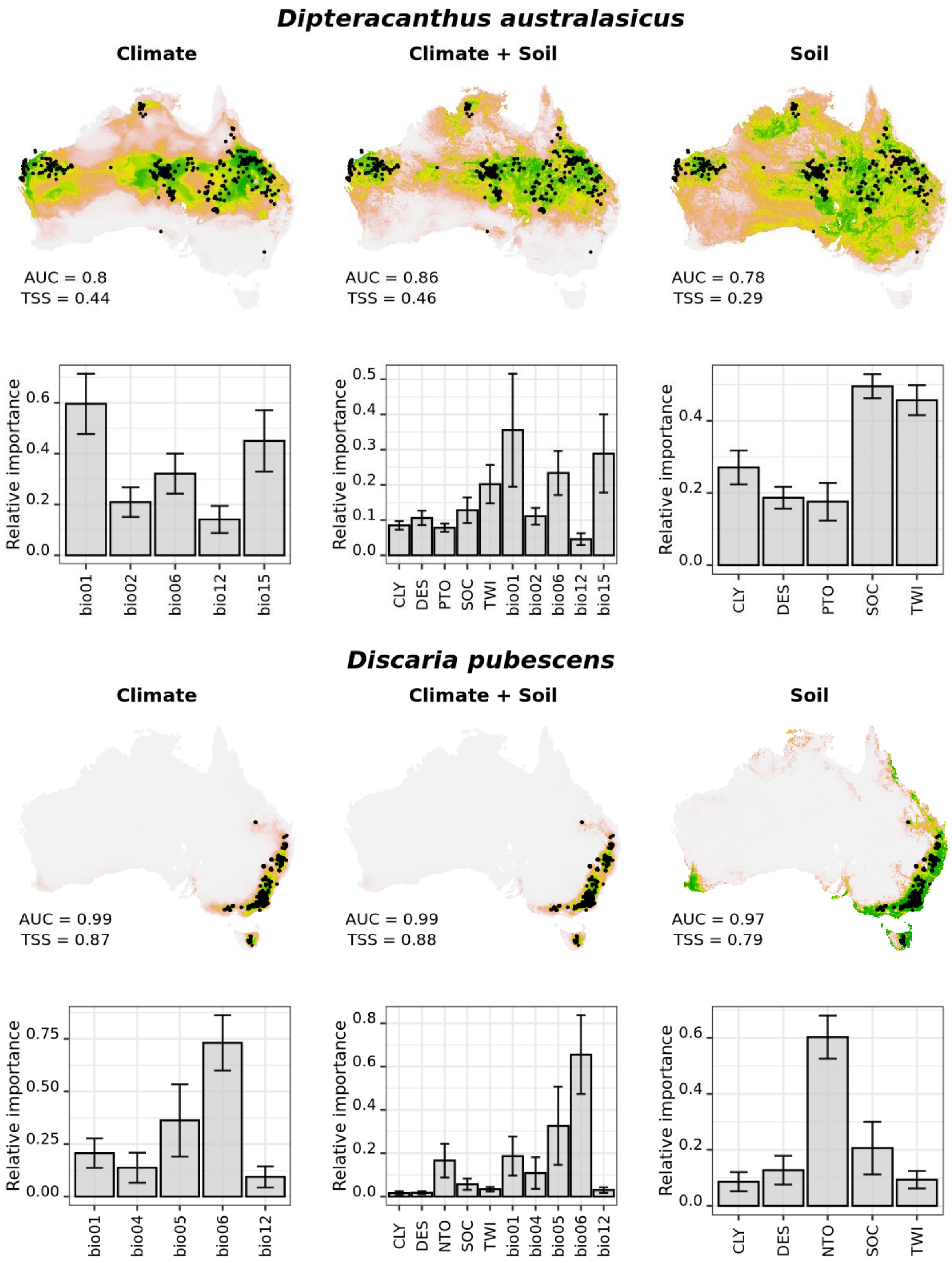

Dipteracanthus australasicus
Climate
Climate + Soil
Soil
AUC = 0.8
TSS = 0.44
AUC = 0.86
TSS = 0.46
AUC = 0.78
TSS = 0.29
Relative importance
bio01 bio02 bio06 bio12 bio15
CLY DES PTO SOC TWI bio01 bio02 bio06 bio12 bio15
CLY DES PTO SOC TWI
Discaria pubescens
Climate
Climate + Soil
Soil
AUC = 0.99
TSS = 0.87
AUC = 0.99
TSS = 0.88
AUC = 0.97
TSS = 0.79
Relative importance
bio01 bio04 bio05 bio06 bio12
CLY DES NTO SOC TWI bio01 bio04 bio05 bio06 bio12
CLY DES NTO SOC TWI

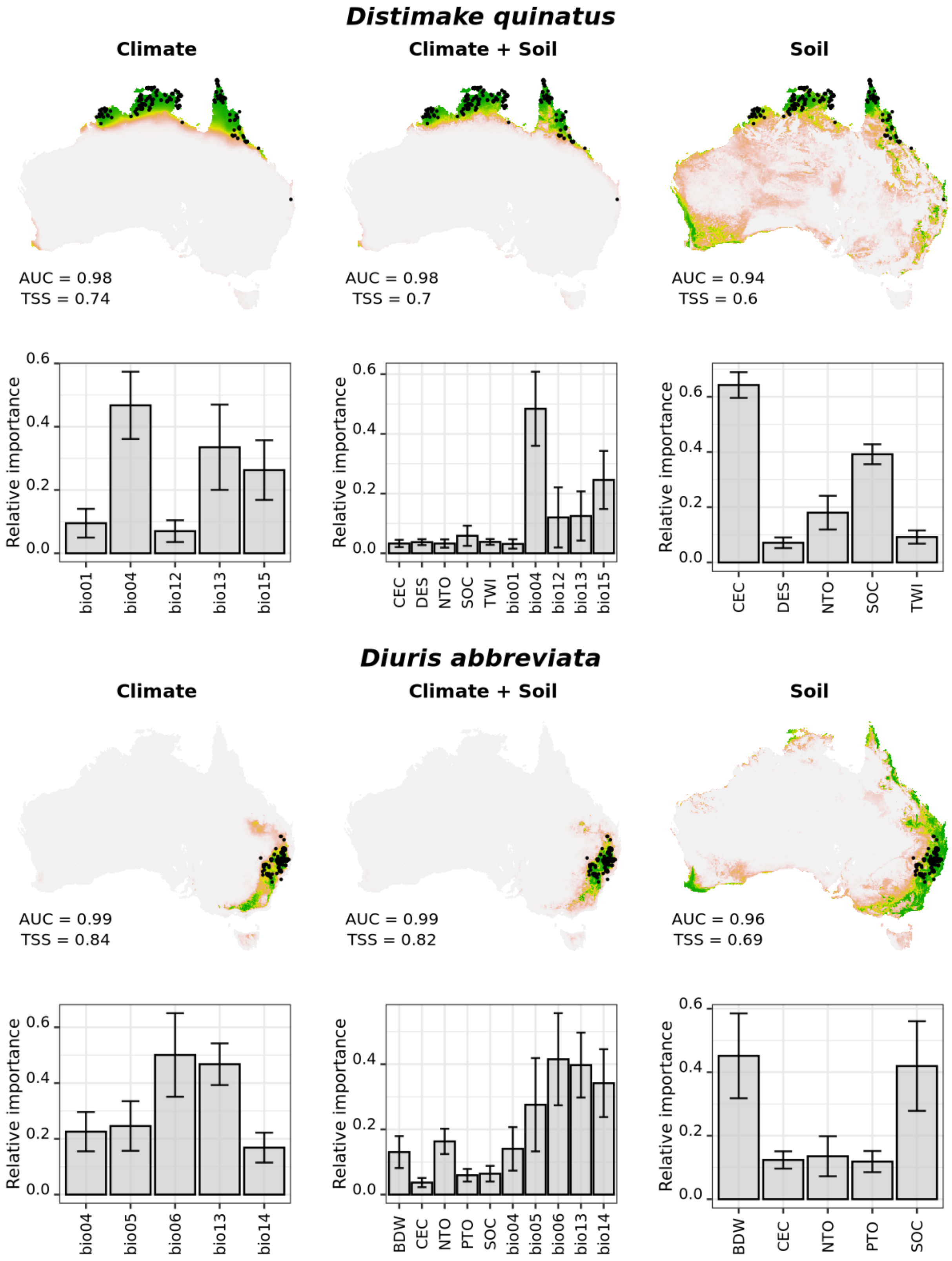

Distimake quinatus
Climate
Climate + Soil
Soil
AUC = 0.98
TSS = 0.74
AUC = 0.98
TSS = 0.7
AUC = 0.94
TSS = 0.6
Relative importance
0.0
0.2
0.4
0.6
bio01
bio04
bio12
bio13
bio15
CEC
DES
NTO
SOC
TWI
bio01
bio04
bio12
bio13
bio15
CEC
DES
NTO
SOC
TWI
Diuris abbreviata
Climate
Climate + Soil
Soil
AUC = 0.99
TSS = 0.84
AUC = 0.99
TSS = 0.82
AUC = 0.96
TSS = 0.69
bio04
bio05
bio06
bio13
bio14
BDW
CEC
NTO
PTO
SOC
bio04
bio05
bio06
bio13
bio14
BDW
CEC
NTO
PTO
SOC

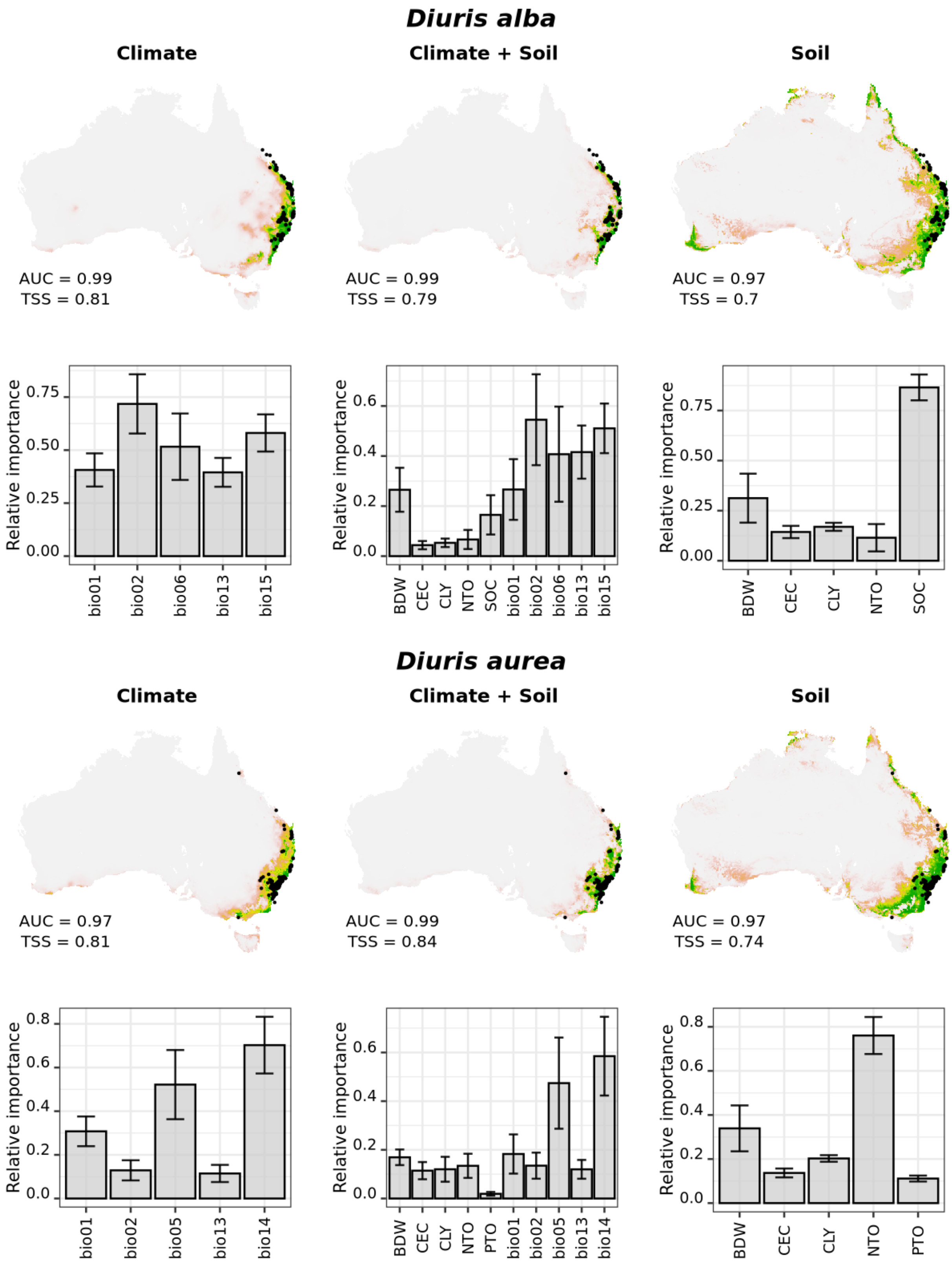

Diuris alba
Climate
Climate + Soil
Soil
AUC = 0.99
TSS = 0.81
AUC = 0.99
TSS = 0.79
AUC = 0.97
TSS = 0.7
Relative importance
bio01
bio02
bio06
bio13
bio15
BDW
CEC
CLY
NTO
SOC
Diuris aurea
Climate
Climate + Soil
Soil
AUC = 0.97
TSS = 0.81
AUC = 0.99
TSS = 0.84
AUC = 0.97
TSS = 0.74
Relative importance
bio01
bio02
bio05
bio13
bio14
BDW
CEC
CLY
NTO
PTO

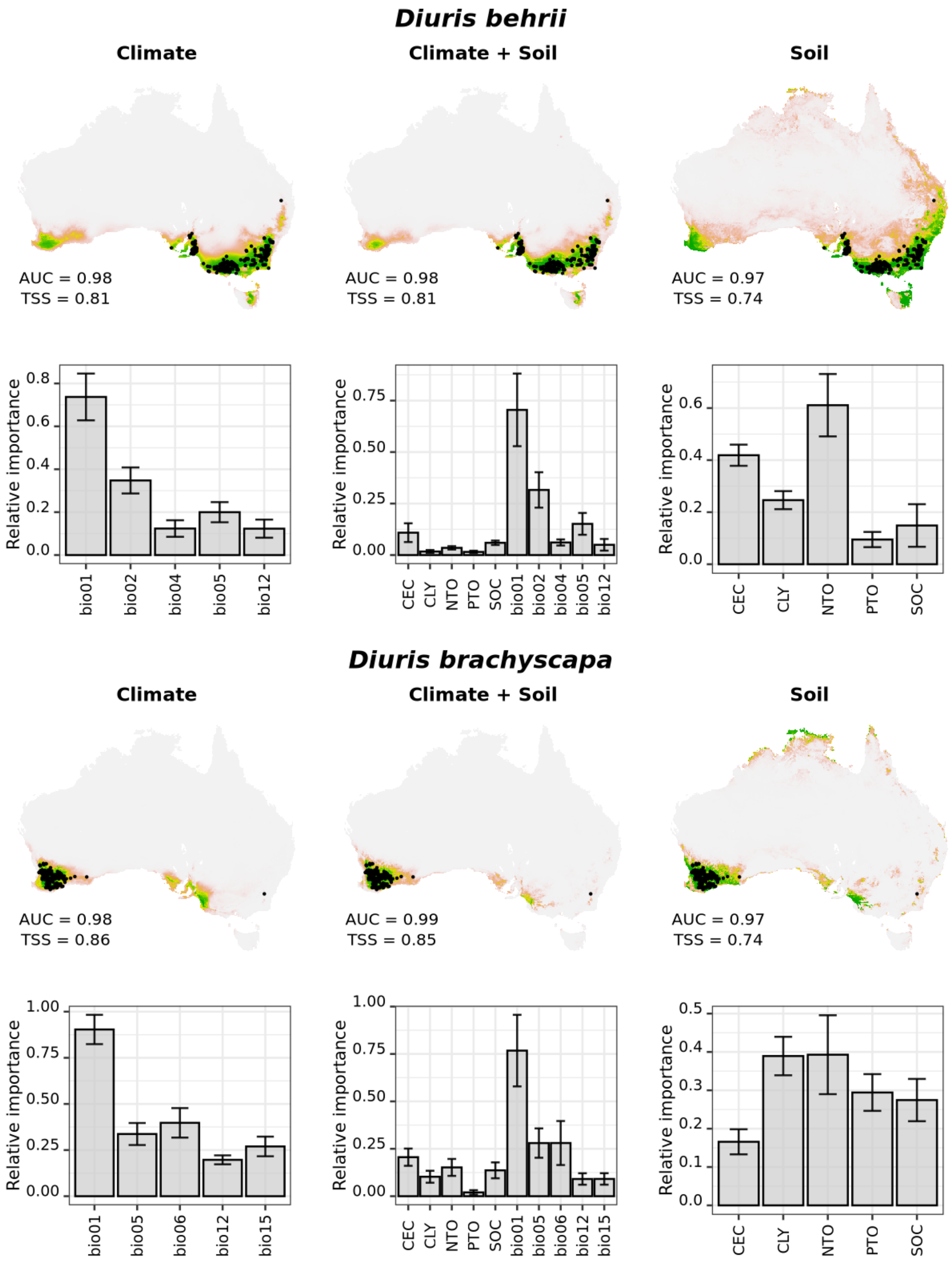
Diuris behrii
Climate
Climate + Soil
Soil
AUC = 0.98
TSS = 0.81
AUC = 0.98
TSS = 0.81
AUC = 0.97
TSS = 0.74
Relative importance
bio01
bio02
bio04
bio05
bio12
CEC
CLY
NTO
PTO
SOC
Diuris brachyscapa
Climate
Climate + Soil
Soil
AUC = 0.98
TSS = 0.86
AUC = 0.99
TSS = 0.85
AUC = 0.97
TSS = 0.74
Relative importance
bio01
bio05
bio06
bio12
bio15
CEC
CLY
NTO
PTO
SOC

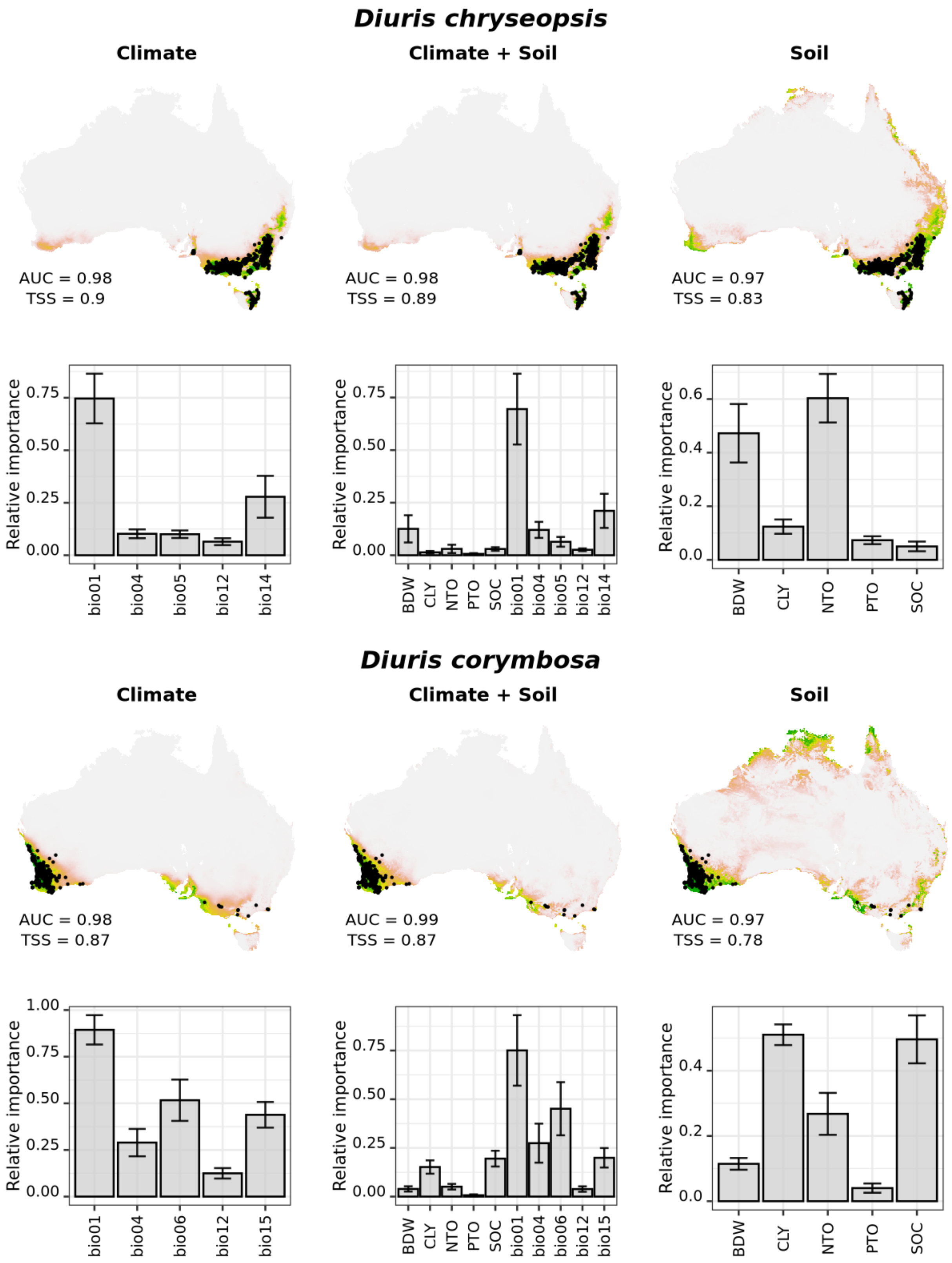

Diuris chryseopsis
Climate
Climate + Soil
Soil
AUC = 0.98
TSS = 0.9
AUC = 0.98
TSS = 0.89
AUC = 0.97
TSS = 0.83
Relative importance
bio01
bio04
bio05
bio12
bio14
BDW
CLY
NTO
PTO
SOC
Diuris corymbosa
Climate
Climate + Soil
Soil
AUC = 0.98
TSS = 0.87
AUC = 0.99
TSS = 0.87
AUC = 0.97
TSS = 0.78
bio06
bio15

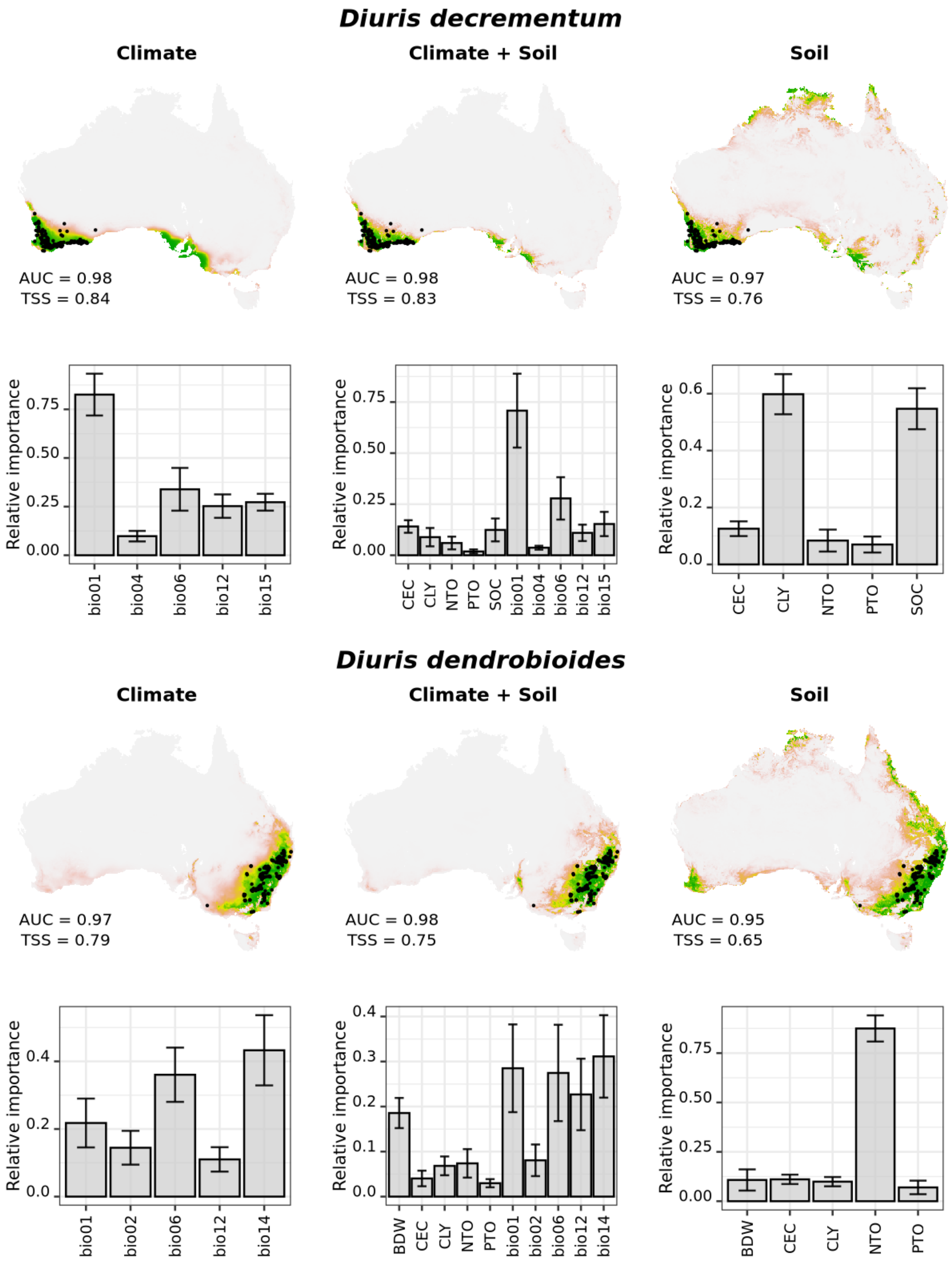

Diuris decrementum
Climate
Climate + Soil
Soil
AUC = 0.98
TSS = 0.84
AUC = 0.98
TSS = 0.83
AUC = 0.97
TSS = 0.76
Relative importance
0.00
0.25
0.50
0.75
bio01
bio04
bio06
bio12
bio15
Relative importance
0.00
0.25
0.50
0.75
CEC
CLY
NTO
PTO
SOC
bio01
bio04
bio06
bio12
bio15
Relative importance
0.0
0.2
0.4
0.6
CEC
CLY
NTO
PTO
SOC
Diuris dendrobioides
Climate
Climate + Soil
Soil
AUC = 0.97
TSS = 0.79
AUC = 0.98
TSS = 0.75
AUC = 0.95
TSS = 0.65
Relative importance
0.0
0.2
0.4
bio01
bio02
bio06
bio12
bio14
Relative importance
0.0
0.1
0.2
0.3
0.4
BDW
CEC
CLY
NTO
PTO
bio01
bio02
bio06
bio12
bio14
Relative importance
0.00
0.25
0.50
0.75
BDW
CEC
CLY
NTO
PTO

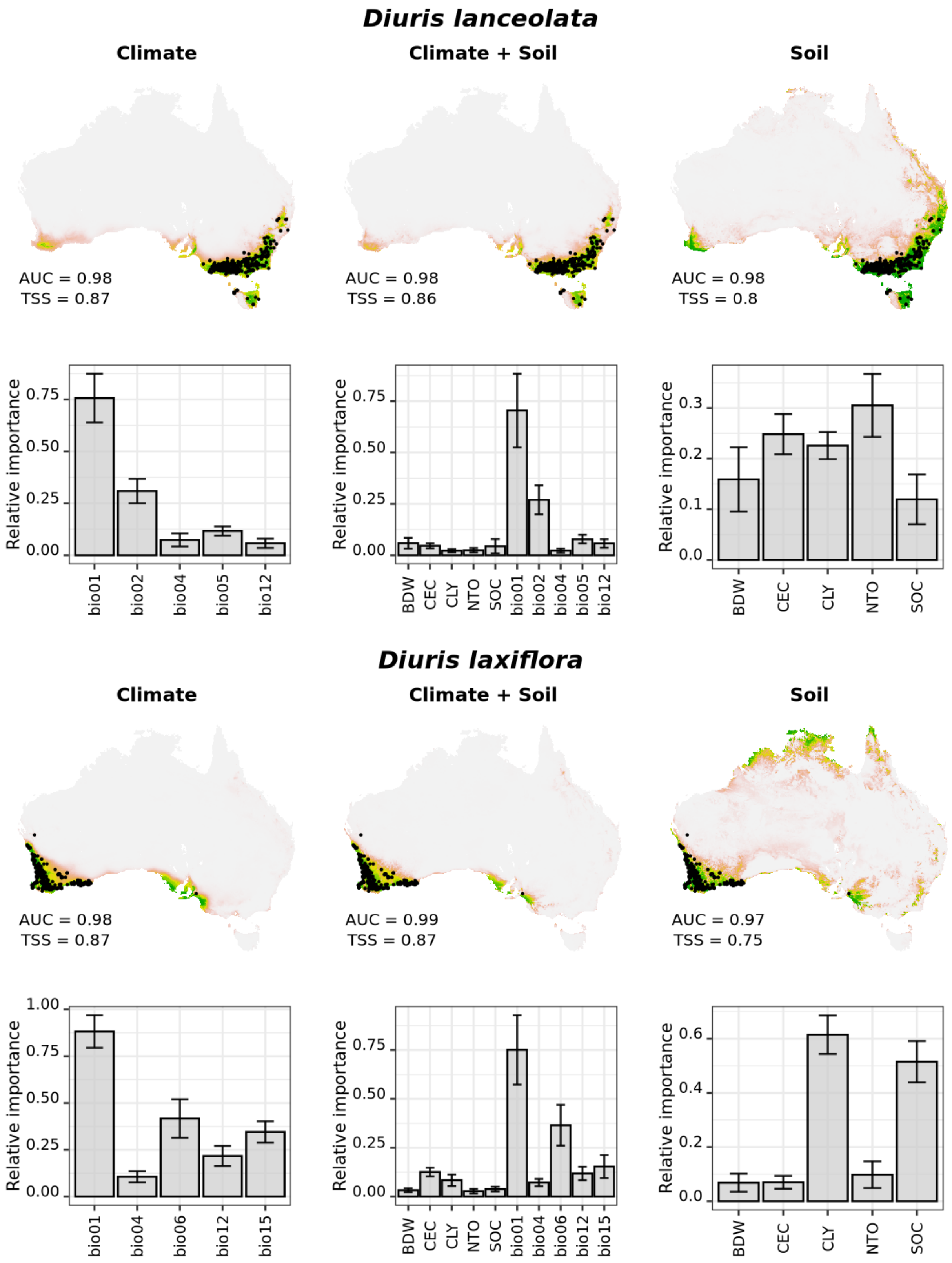

Diuris lanceolata
Climate
Climate + Soil
Soil
AUC = 0.98
TSS = 0.87
AUC = 0.98
TSS = 0.86
AUC = 0.98
TSS = 0.8
Relative importance
bio01
bio02
bio04
bio05
bio12
BDW
CEC
CLY
NTO
SOC
Diuris laxiflora
Climate
Climate + Soil
Soil
AUC = 0.98
TSS = 0.87
AUC = 0.99
TSS = 0.87
AUC = 0.97
TSS = 0.75
Relative importance
bio01
bio04
bio06
bio12
bio15
BDW
CEC
CLY
NTO
SOC

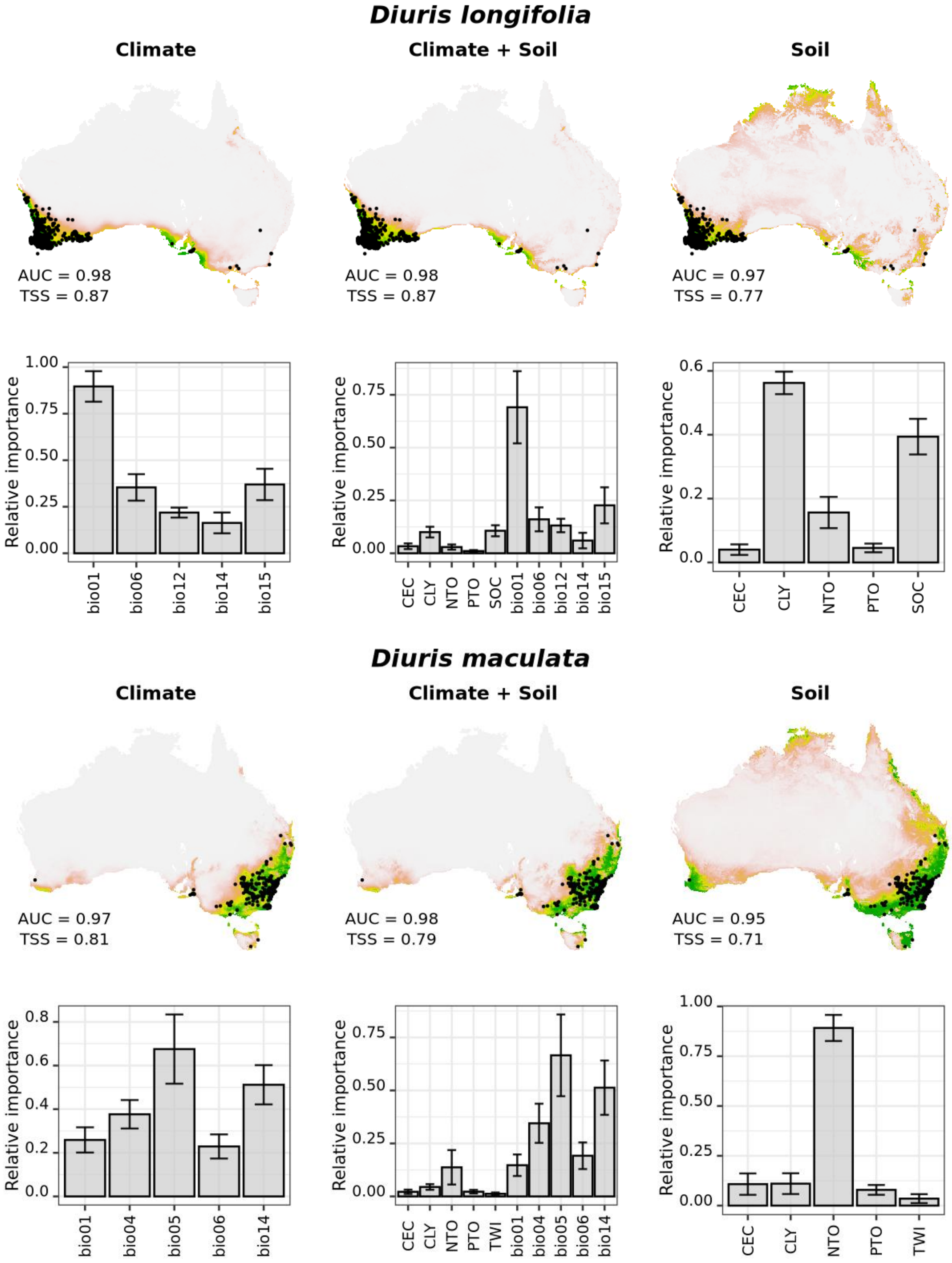
Diuris longifolia
Climate
Climate + Soil
Soil
AUC = 0.98
TSS = 0.87
AUC = 0.98
TSS = 0.87
AUC = 0.97
TSS = 0.77
Relative importance
1.00
0.75
0.50
0.25
0.00
bio01
bio06
bio12
bio14
bio15
Relative importance
0.75
0.50
0.25
0.00
CEC
CLY
NTO
PTO
SOC
bio01
bio06
bio12
bio14
bio15
Relative importance
0.6
0.4
0.2
0.0
CEC
CLY
NTO
PTO
SOC
Diuris maculata
Climate
Climate + Soil
Soil
AUC = 0.97
TSS = 0.81
AUC = 0.98
TSS = 0.79
AUC = 0.95
TSS = 0.71
Relative importance
0.8
0.6
0.4
0.2
0.0
bio01
bio04
bio05
bio06
bio14
Relative importance
0.75
0.50
0.25
0.00
CEC
CLY
NTO
PTO
TWI
bio01
bio04
bio05
bio06
bio14
Relative importance
1.00
0.75
0.50
0.25
0.00
CEC
CLY
NTO
PTO
TWI

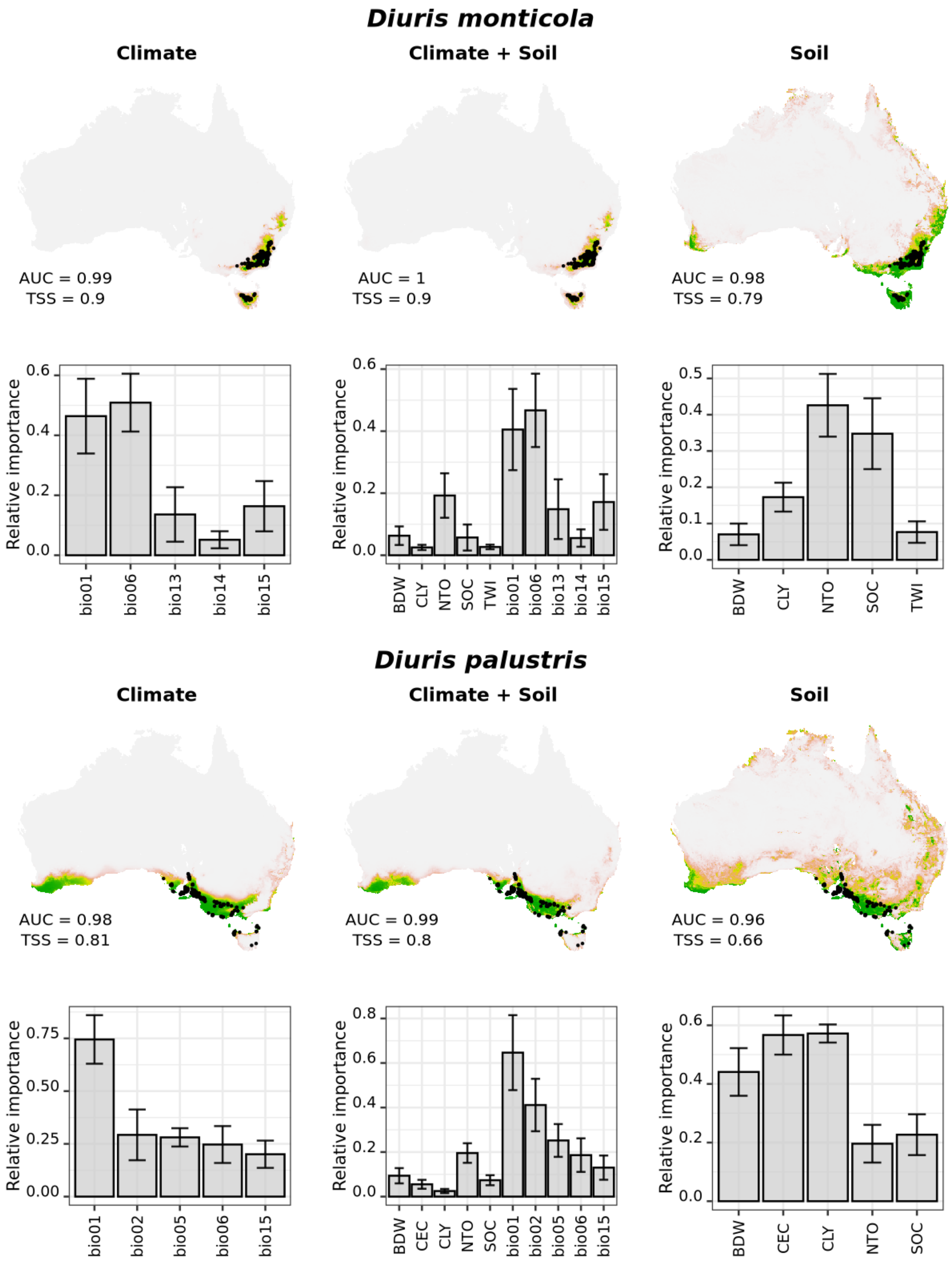

Diuris monticola
Climate
Climate + Soil
Soil
AUC = 0.99
TSS = 0.9
AUC = 1
TSS = 0.9
AUC = 0.98
TSS = 0.79
Relative importance
bio01 bio06 bio13 bio14 bio15
BDW CLY NTO SOC TWI bio01 bio06 bio13 bio14 bio15
BDW CLY NTO SOC TWI
Diuris palustris
Climate
Climate + Soil
Soil
AUC = 0.98
TSS = 0.81
AUC = 0.99
TSS = 0.8
AUC = 0.96
TSS = 0.66
Relative importance
bio01 bio02 bio05 bio06 bio15
BDW CEC CLY NTO SOC bio01 bio02 bio05 bio06 bio15
BDW CEC CLY NTO SOC

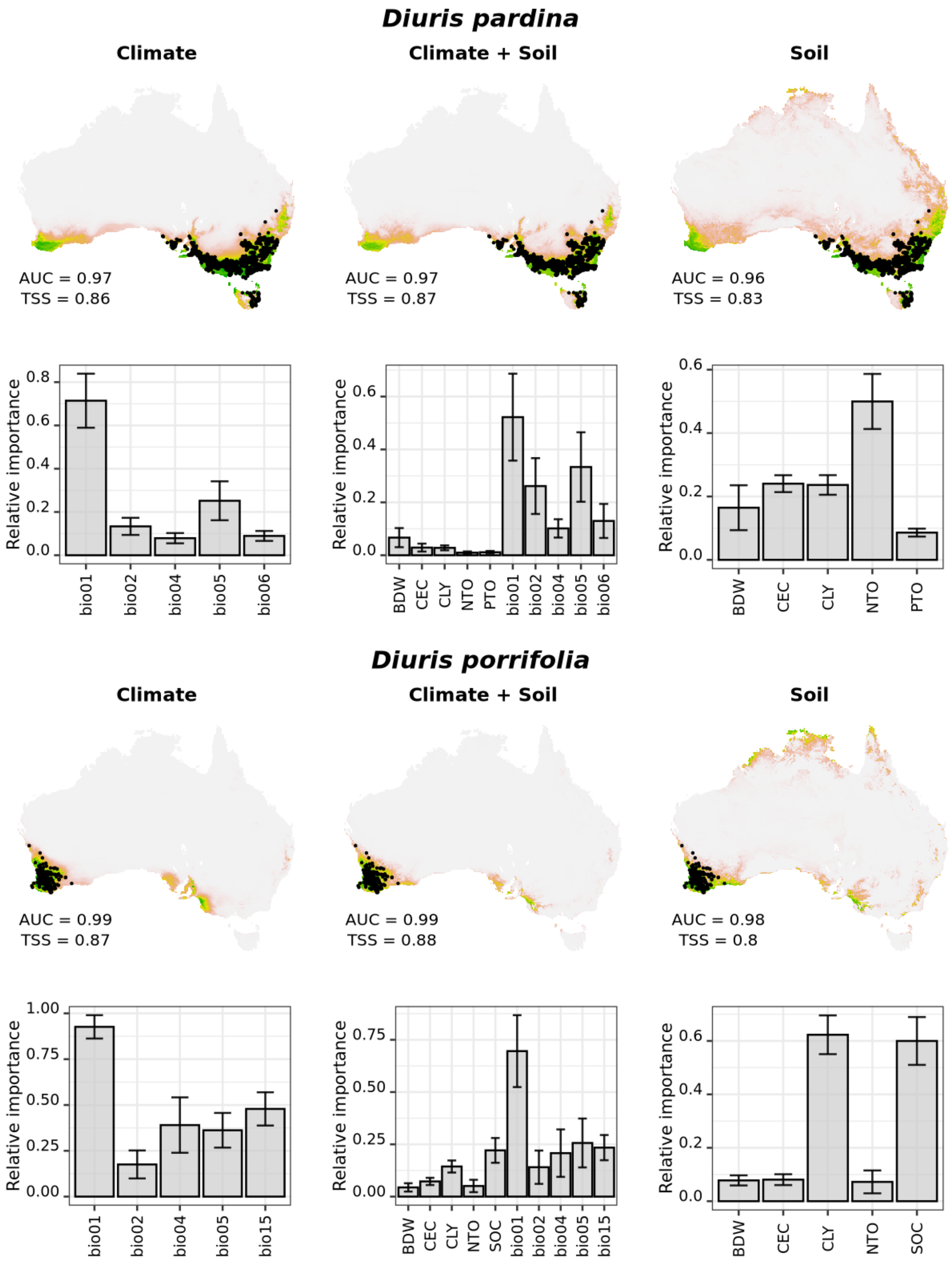
Diuris pardina
Climate
Climate + Soil
Soil
AUC = 0.97
TSS = 0.86
AUC = 0.97
TSS = 0.87
AUC = 0.96
TSS = 0.83
Relative importance
bio01
bio02
bio04
bio05
bio06
BDW
CEC
CLY
NTO
PTO
Diuris porrifolia
Climate
Climate + Soil
Soil
AUC = 0.99
TSS = 0.87
AUC = 0.99
TSS = 0.88
AUC = 0.98
TSS = 0.8
Relative importance
bio01
bio02
bio04
bio05
bio15
BDW
CEC
CLY
NTO
SOC

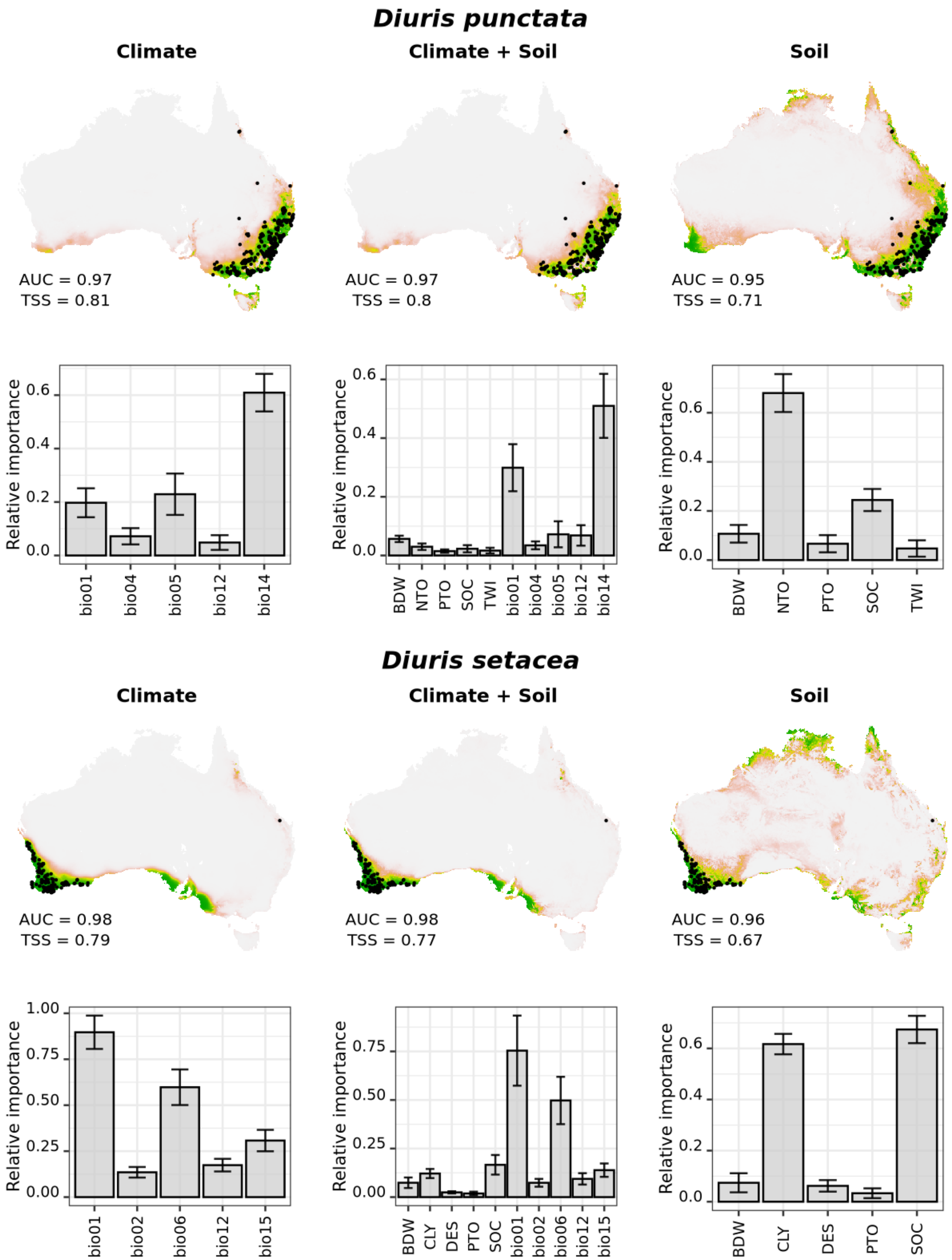

Diuris punctata
Climate
Climate + Soil
Soil
AUC = 0.97
TSS = 0.81
AUC = 0.97
TSS = 0.8
AUC = 0.95
TSS = 0.71
Relative importance
0.0 0.2 0.4 0.6
bio01 bio04 bio05 bio12 bio14
BDW NTO PTO SOC TWI bio01 bio04 bio05 bio12 bio14
BDW NTO PTO SOC TWI
Diuris setacea
Climate
Climate + Soil
Soil
AUC = 0.98
TSS = 0.79
AUC = 0.98
TSS = 0.77
AUC = 0.96
TSS = 0.67
Relative importance
0.00 0.25 0.50 0.75 1.00
bio01 bio02 bio06 bio12 bio15
BDW CLY DES PTO SOC bio01 bio02 bio06 bio12 bio15
BDW CLY DES PTO SOC

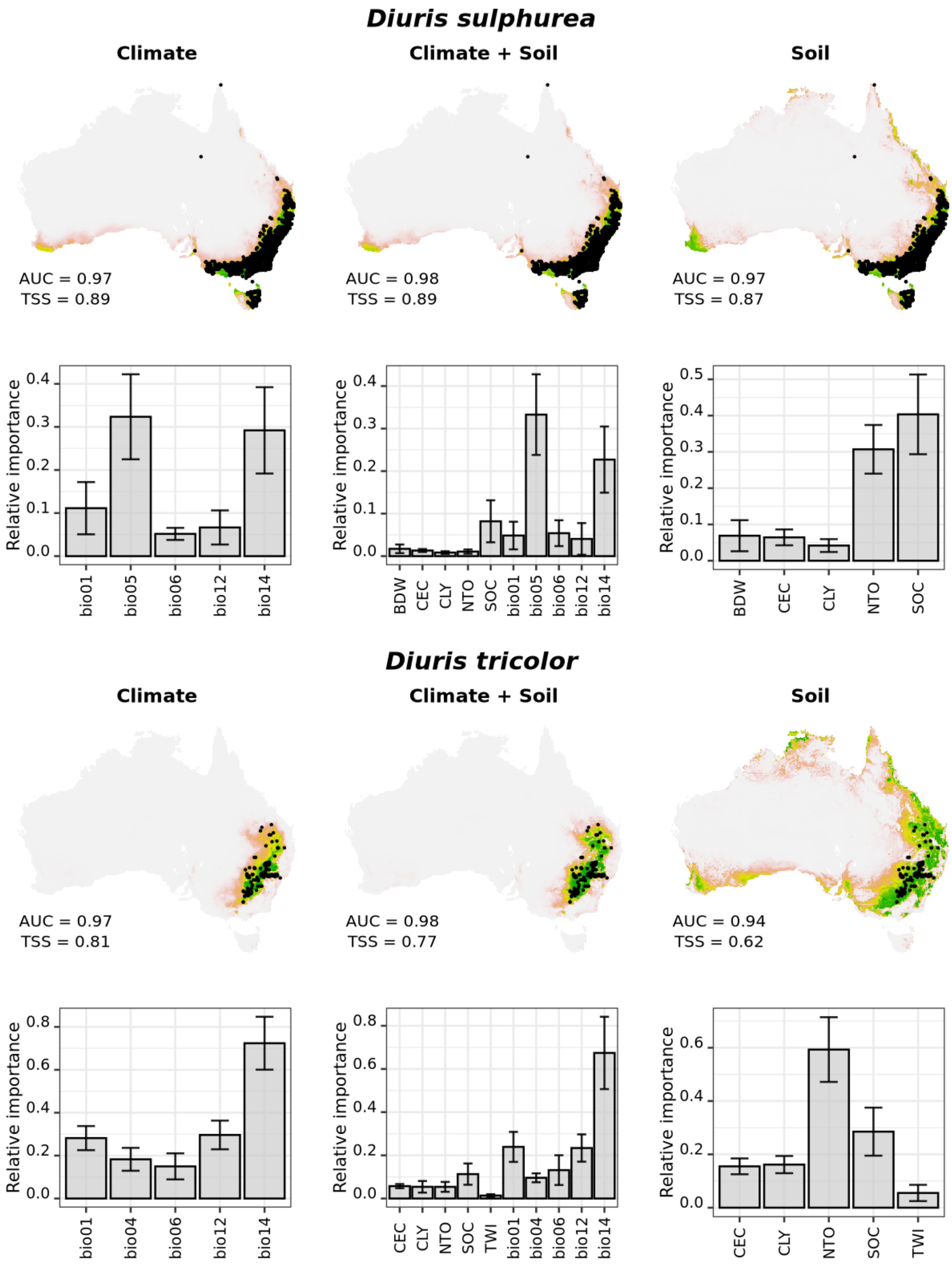

Diuris sulphurea
Climate
Climate + Soil
Soil
AUC = 0.97
TSS = 0.89
AUC = 0.98
TSS = 0.89
AUC = 0.97
TSS = 0.87
Relative importance
0.0
0.1
0.2
0.3
0.4
0.5
bio01
bio05
bio06
bio12
bio14
BDW
CEC
CLY
NTO
SOC
Diuris tricolor
Climate
Climate + Soil
Soil
AUC = 0.97
TSS = 0.81
AUC = 0.98
TSS = 0.77
AUC = 0.94
TSS = 0.62
Relative importance
0.0
0.2
0.4
0.6
0.8
bio01
bio04
bio06
bio12
bio14
CEC
CLY
NTO
SOC
TWI

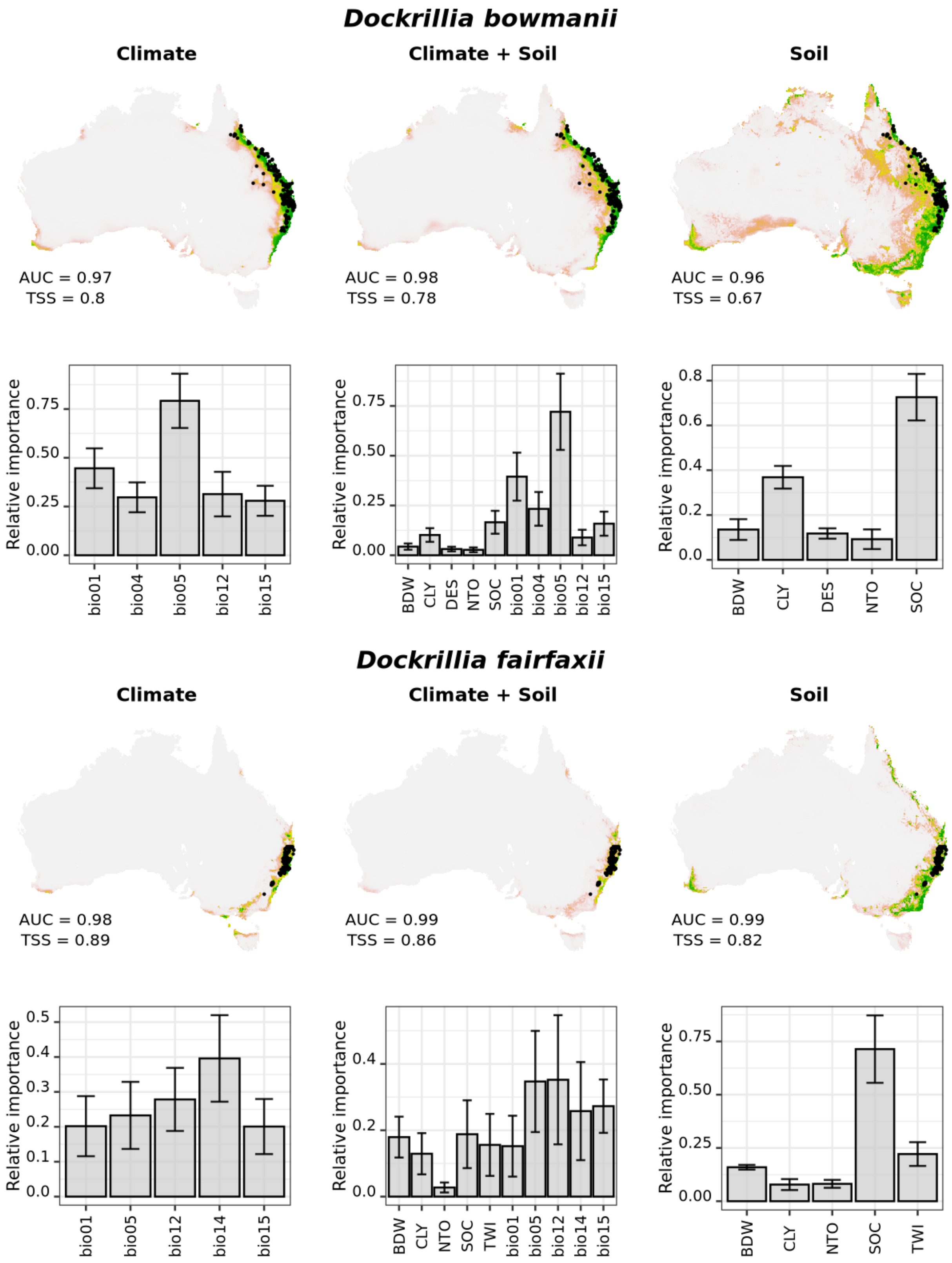

Dockrillia bowmanii
Climate
Climate + Soil
Soil
AUC = 0.97
TSS = 0.8
AUC = 0.98
TSS = 0.78
AUC = 0.96
TSS = 0.67
Relative importance
0.00
0.25
0.50
0.75
bio01
bio04
bio05
bio12
bio15
BDW
CLY
DES
NTO
SOC
0.0
0.2
0.4
0.6
0.8
Dockrillia fairfaxii
Climate
Climate + Soil
Soil
AUC = 0.98
TSS = 0.89
AUC = 0.99
TSS = 0.86
AUC = 0.99
TSS = 0.82
0.1
0.3
0.5
bio14
TWI

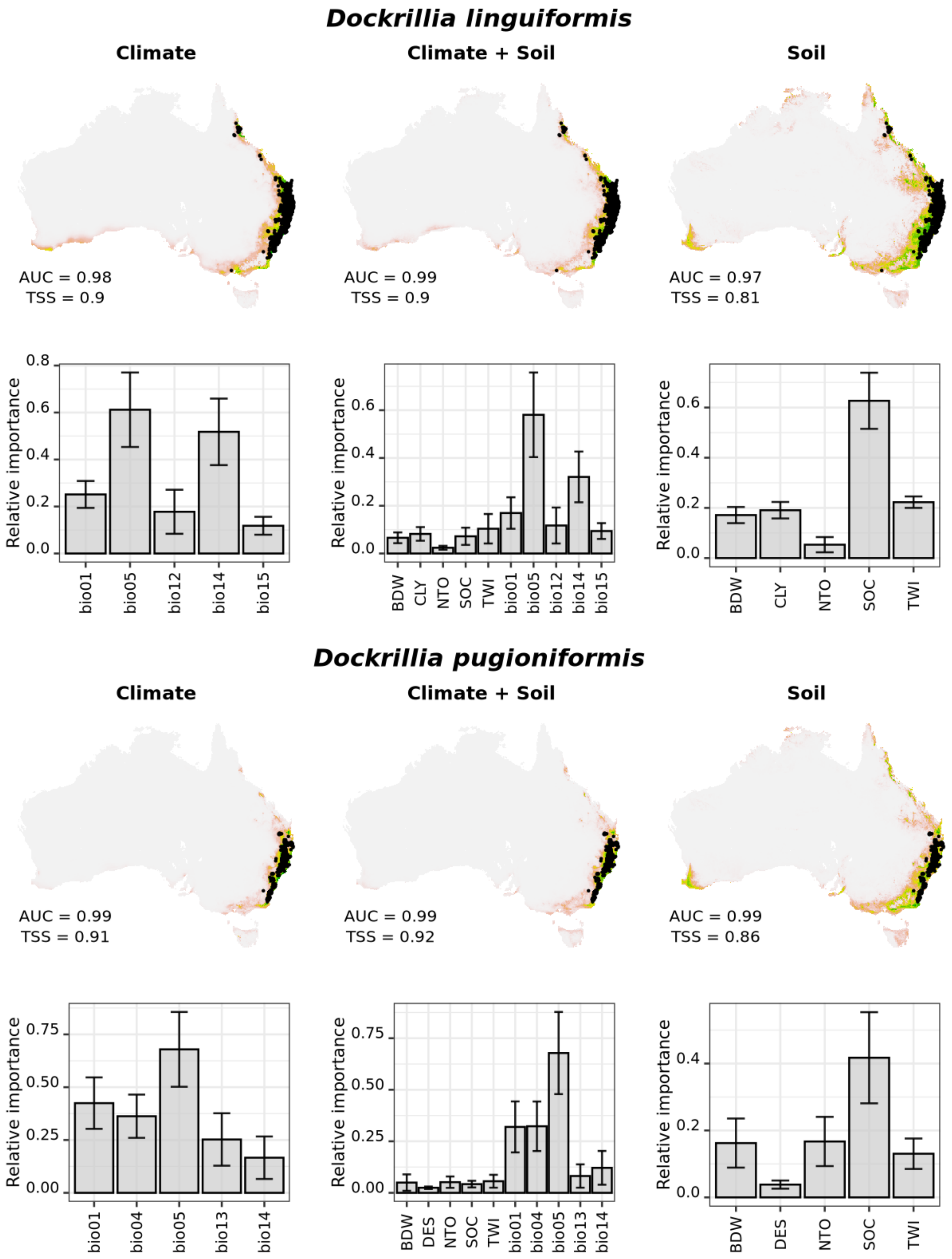

Dockrillia linguiformis
Climate
Climate + Soil
Soil
AUC = 0.98
TSS = 0.9
AUC = 0.99
TSS = 0.9
AUC = 0.97
TSS = 0.81
Relative importance
bio01 bio05 bio12 bio14 bio15
BDW CLY NTO SOC TWI bio01 bio05 bio12 bio14 bio15
BDW CLY NTO SOC TWI
Dockrillia pugioniformis
Climate
Climate + Soil
Soil
AUC = 0.99
TSS = 0.91
AUC = 0.99
TSS = 0.92
AUC = 0.99
TSS = 0.86
Relative importance
bio01 bio04 bio05 bio13 bio14
BDW DES NTO SOC TWI bio01 bio04 bio05 bio13 bio14
BDW DES NTO SOC TWI

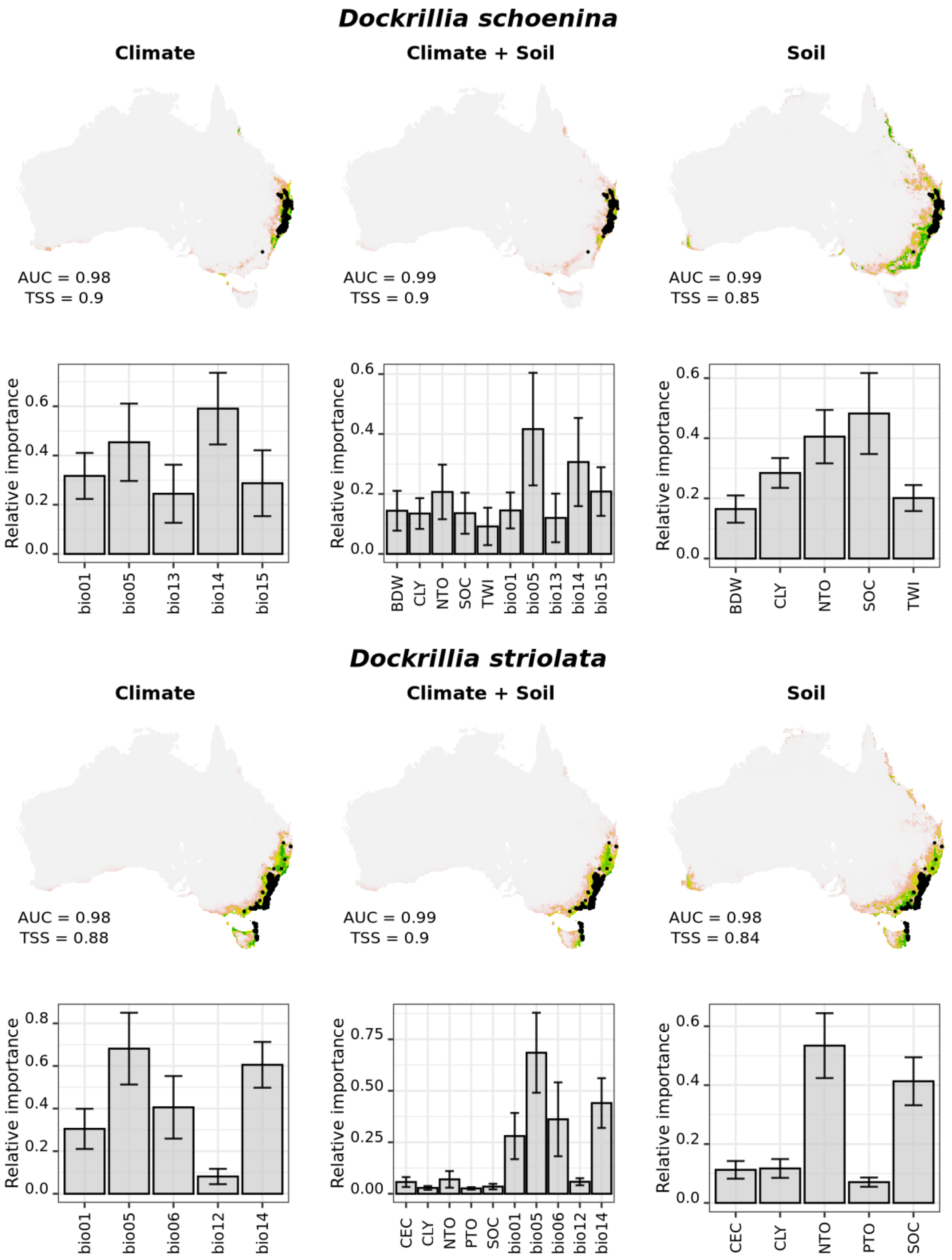

Dockrillia schoenina
Climate
Climate + Soil
Soil
AUC = 0.98
TSS = 0.9
AUC = 0.99
TSS = 0.9
AUC = 0.99
TSS = 0.85
Relative importance
bio01
bio05
bio13
bio14
bio15
BDW
CLY
NTO
SOC
TWI
Dockrillia striolata
Climate
Climate + Soil
Soil
AUC = 0.98
TSS = 0.88
AUC = 0.99
TSS = 0.9
AUC = 0.98
TSS = 0.84
Relative importance
bio01
bio05
bio06
bio12
bio14
CEC
CLY
NTO
PTO
SOC

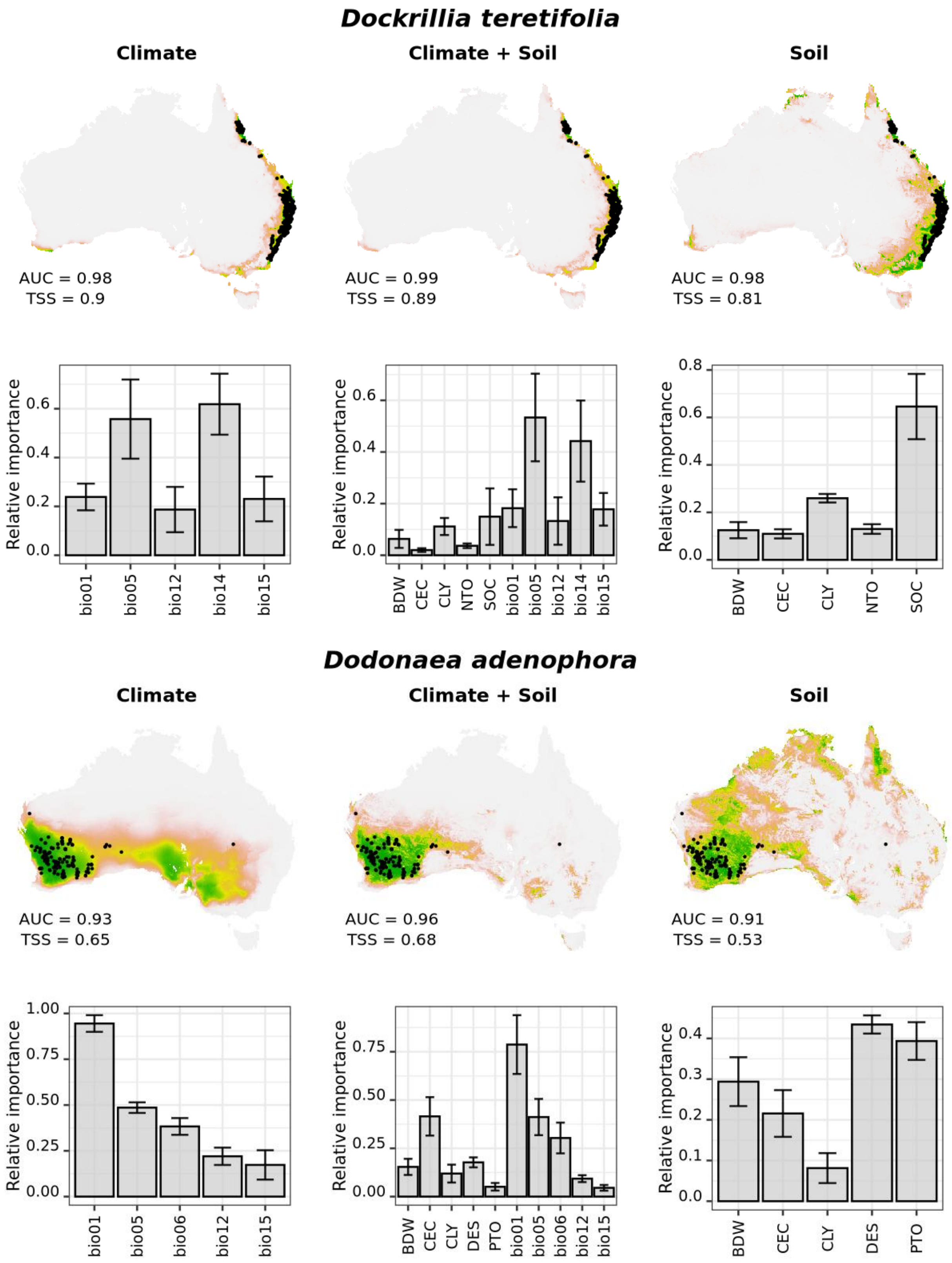

Dockrillia teretifolia
Climate
Climate + Soil
Soil
AUC = 0.98
TSS = 0.9
AUC = 0.99
TSS = 0.89
AUC = 0.98
TSS = 0.81
Relative importance
bio01
bio05
bio12
bio14
bio15
BDW
CEC
CLY
NTO
SOC
Dodonaea adenophora
Climate
Climate + Soil
Soil
AUC = 0.93
TSS = 0.65
AUC = 0.96
TSS = 0.68
AUC = 0.91
TSS = 0.53
Relative importance
bio01
bio05
bio06
bio12
bio15
BDW
CEC
CLY
DES
PTO

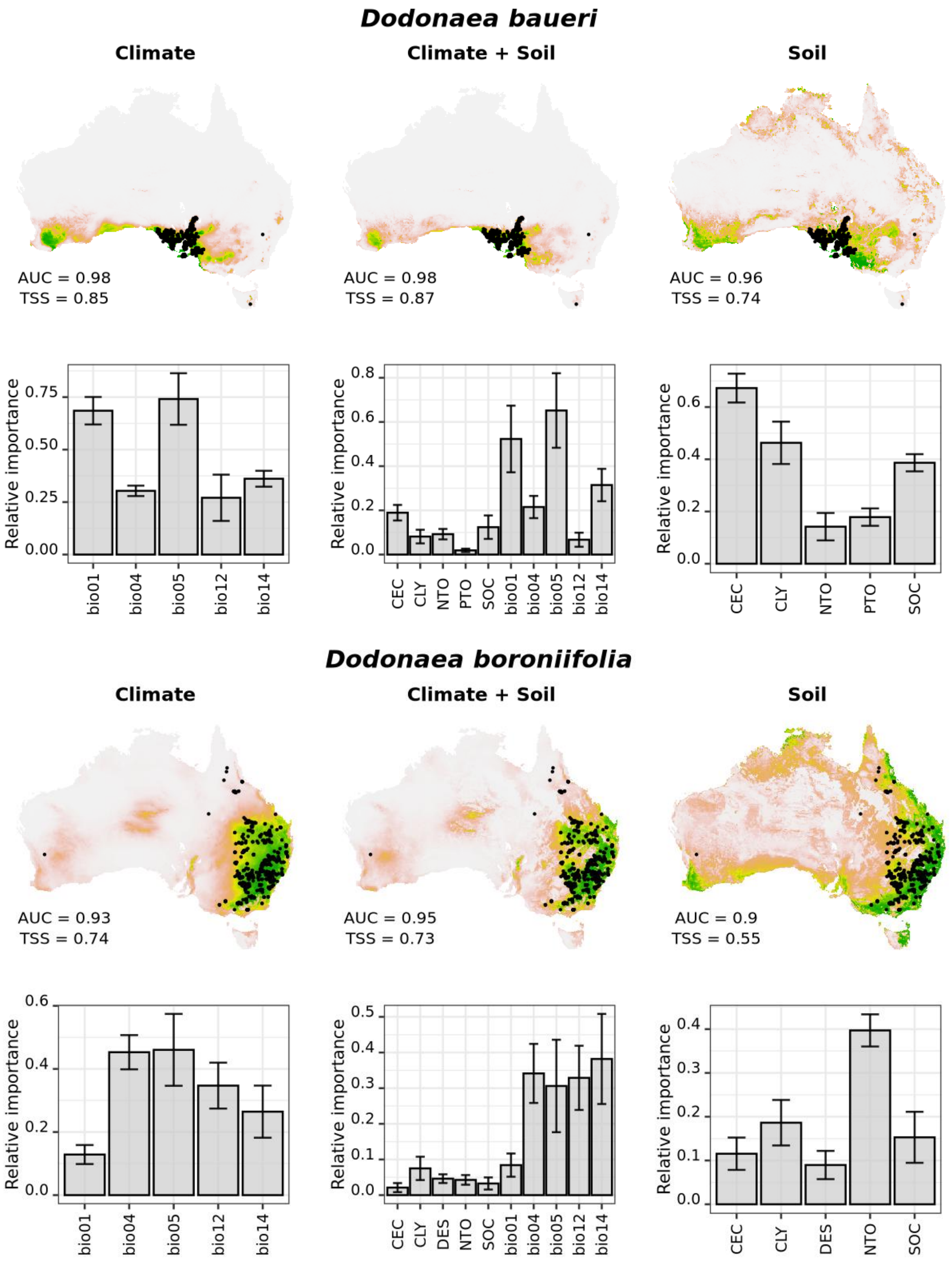
Dodonaea baueri
Climate
Climate + Soil
Soil
AUC = 0.98
TSS = 0.85
AUC = 0.98
TSS = 0.87
AUC = 0.96
TSS = 0.74
Relative importance
bio01 bio04 bio05 bio12 bio14
CEC CLY NTO PTO SOC bio01 bio04 bio05 bio12 bio14
CEC CLY NTO PTO SOC
Dodonaea boroniifolia
Climate
Climate + Soil
Soil
AUC = 0.93
TSS = 0.74
AUC = 0.95
TSS = 0.73
AUC = 0.9
TSS = 0.55
Relative importance
bio01 bio04 bio05 bio12 bio14
CEC CLY DES NTO SOC bio01 bio04 bio05 bio12 bio14
CEC CLY DES NTO SOC

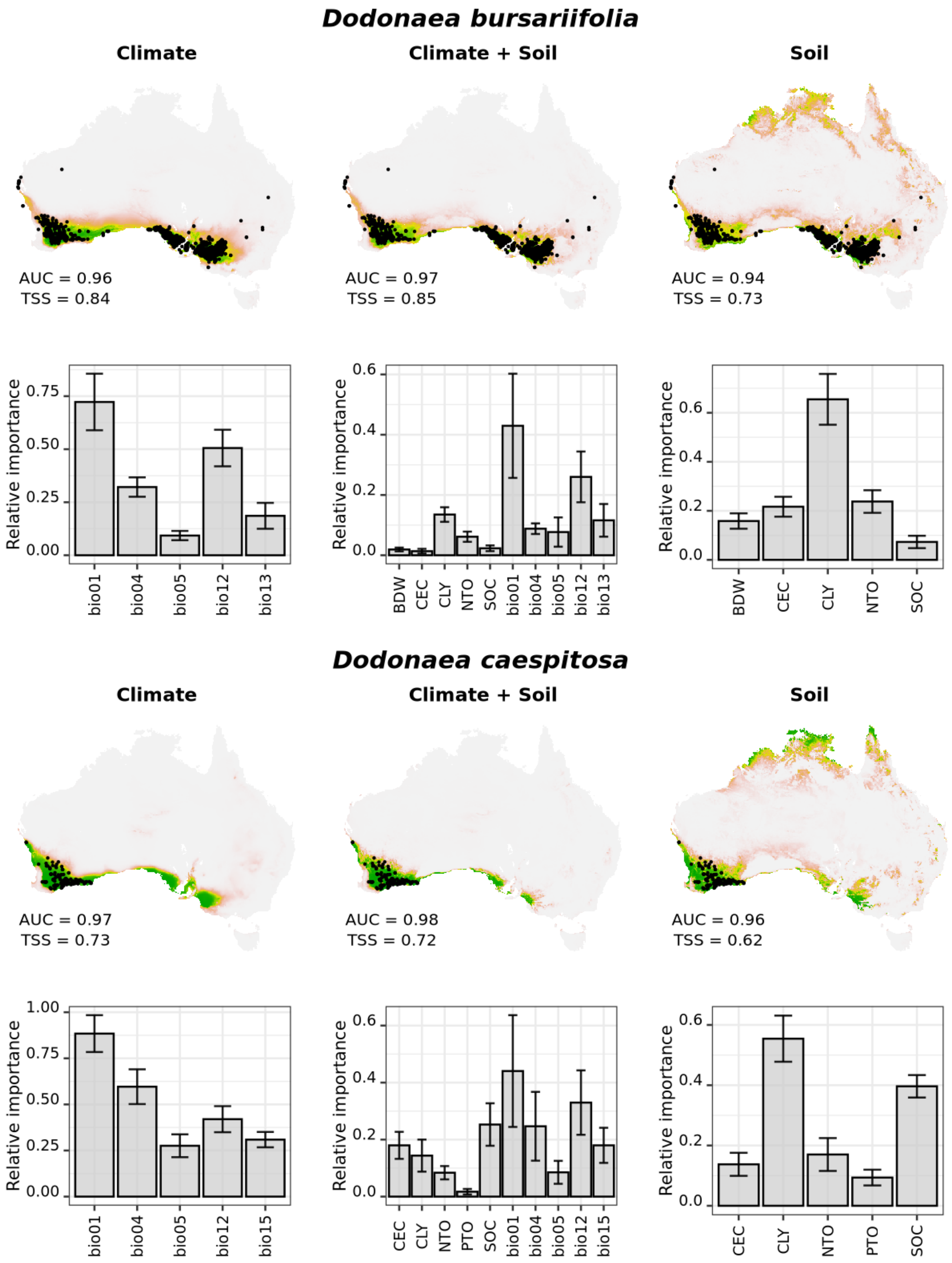
Dodonaea bursariifolia
Climate
Climate + Soil
Soil
AUC = 0.96
TSS = 0.84
AUC = 0.97
TSS = 0.85
AUC = 0.94
TSS = 0.73
Relative importance
0.00
0.25
0.50
0.75
bio01
bio04
bio05
bio12
bio13
0.0
0.2
0.4
0.6
BDW
CEC
CLY
NTO
SOC
bio01
bio04
bio05
bio12
bio13
BDW
CEC
CLY
NTO
SOC
Dodonaea caespitosa
Climate
Climate + Soil
Soil
AUC = 0.97
TSS = 0.73
AUC = 0.98
TSS = 0.72
AUC = 0.96
TSS = 0.62
Relative importance
0.00
0.25
0.50
0.75
1.00
bio01
bio04
bio05
bio12
bio15
CEC
CLY
NTO
PTO
SOC
bio01
bio04
bio05
bio12
bio15
CEC
CLY
NTO
PTO
SOC

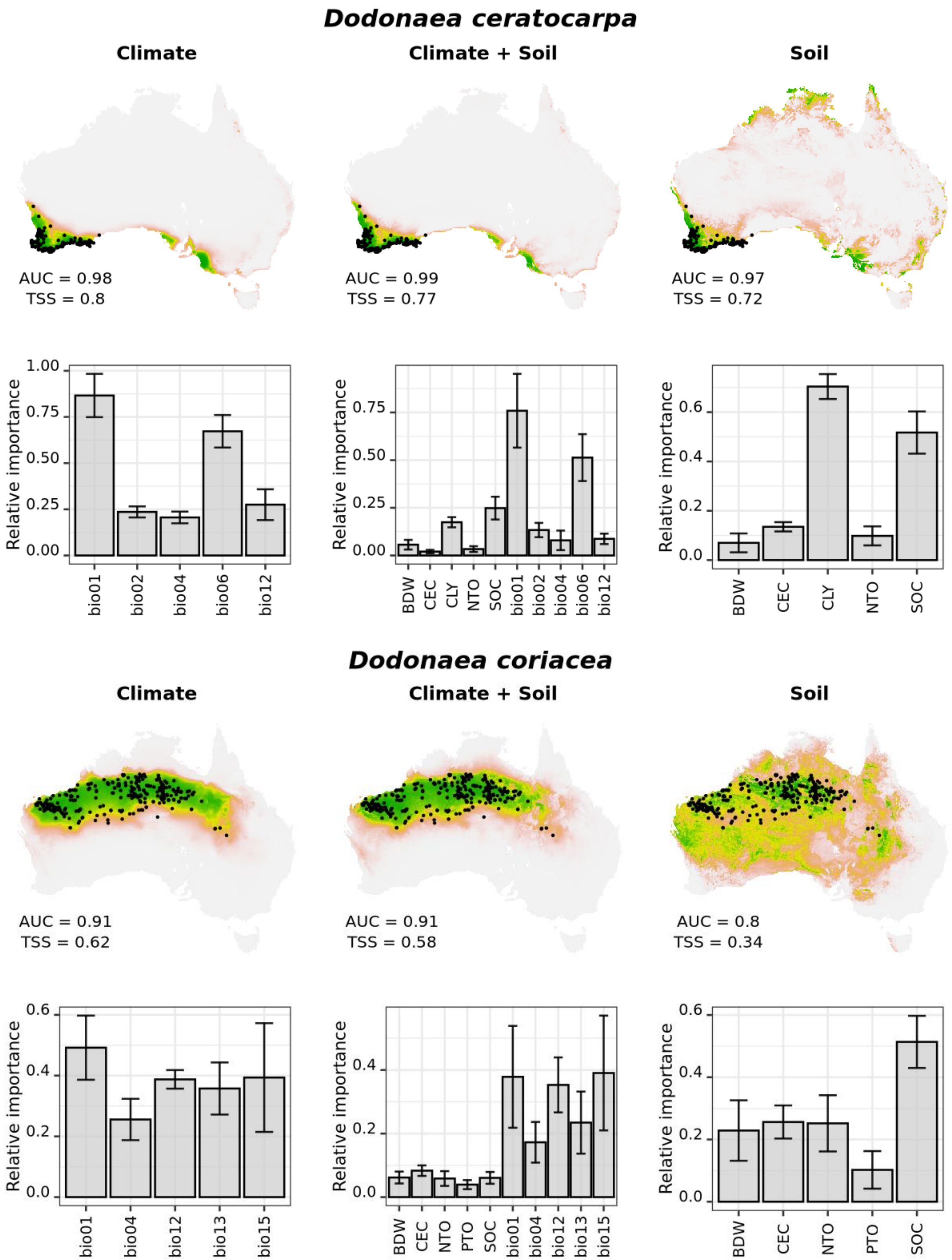

Dodonaea ceratocarpa
Climate
Climate + Soil
Soil
AUC = 0.98
TSS = 0.8
AUC = 0.99
TSS = 0.77
AUC = 0.97
TSS = 0.72
Relative importance
1.00
0.75
0.50
0.25
0.00
bio01
bio02
bio04
bio06
bio12
BDW
CEC
CLY
NTO
SOC
0.6
0.4
0.2
0.0
Dodonaea coriacea
Climate
Climate + Soil
Soil
AUC = 0.91
TSS = 0.62
AUC = 0.91
TSS = 0.58
AUC = 0.8
TSS = 0.34
bio13
bio15
PTO

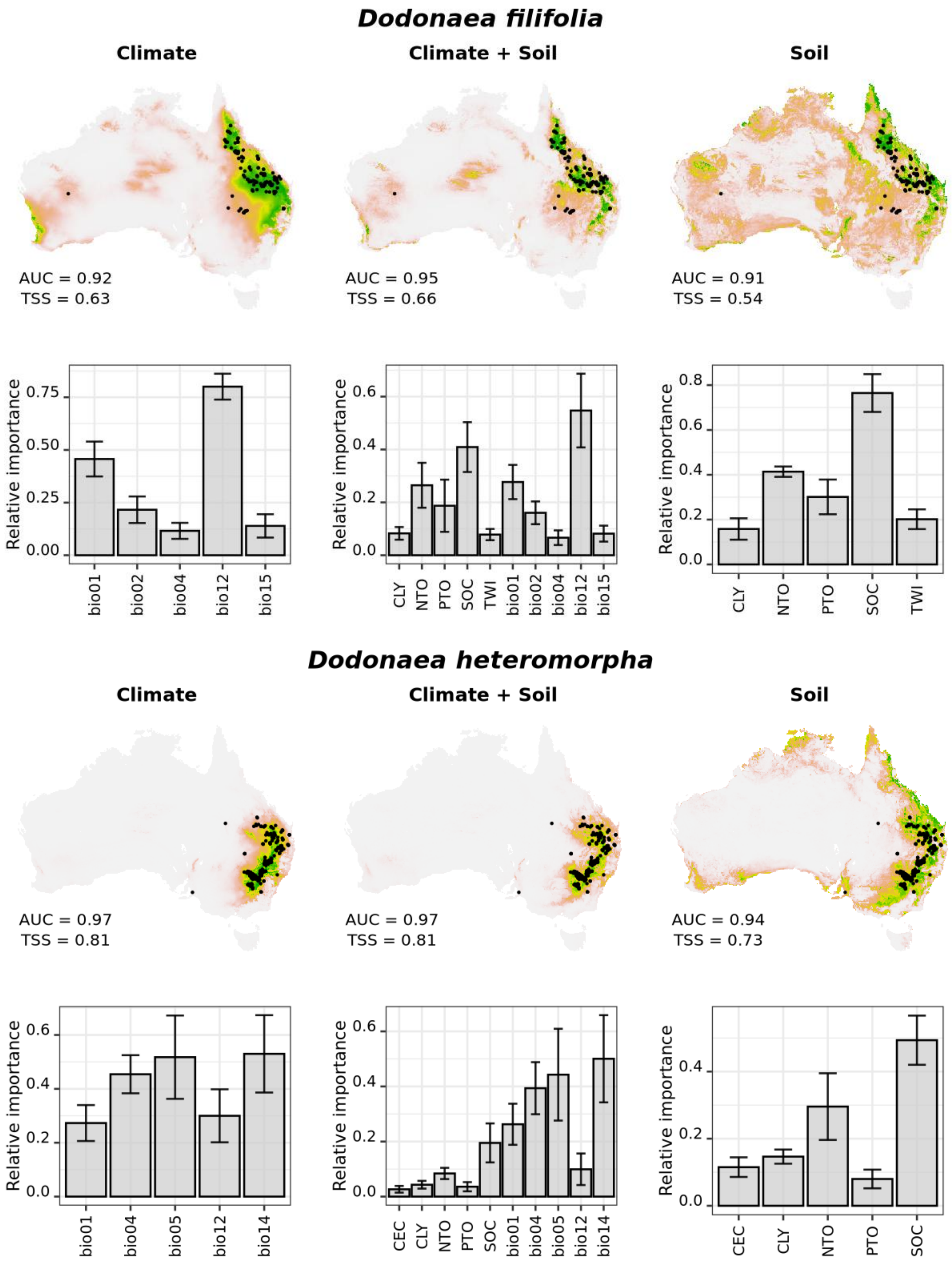
Dodonaea filifolia
Climate
Climate + Soil
Soil
AUC = 0.92
TSS = 0.63
AUC = 0.95
TSS = 0.66
AUC = 0.91
TSS = 0.54
Relative importance
0.00
0.25
0.50
0.75
bio01
bio02
bio04
bio12
bio15
0.0
0.2
0.4
0.6
CLY
NTO
PTO
SOC
TWI
bio01
bio02
bio04
bio12
bio15
0.8
Dodonaea heteromorpha
AUC = 0.97
TSS = 0.81
AUC = 0.97
TSS = 0.81
AUC = 0.94
TSS = 0.73
bio01
bio04
bio05
bio12
bio14
CEC
CLY
NTO
PTO
SOC

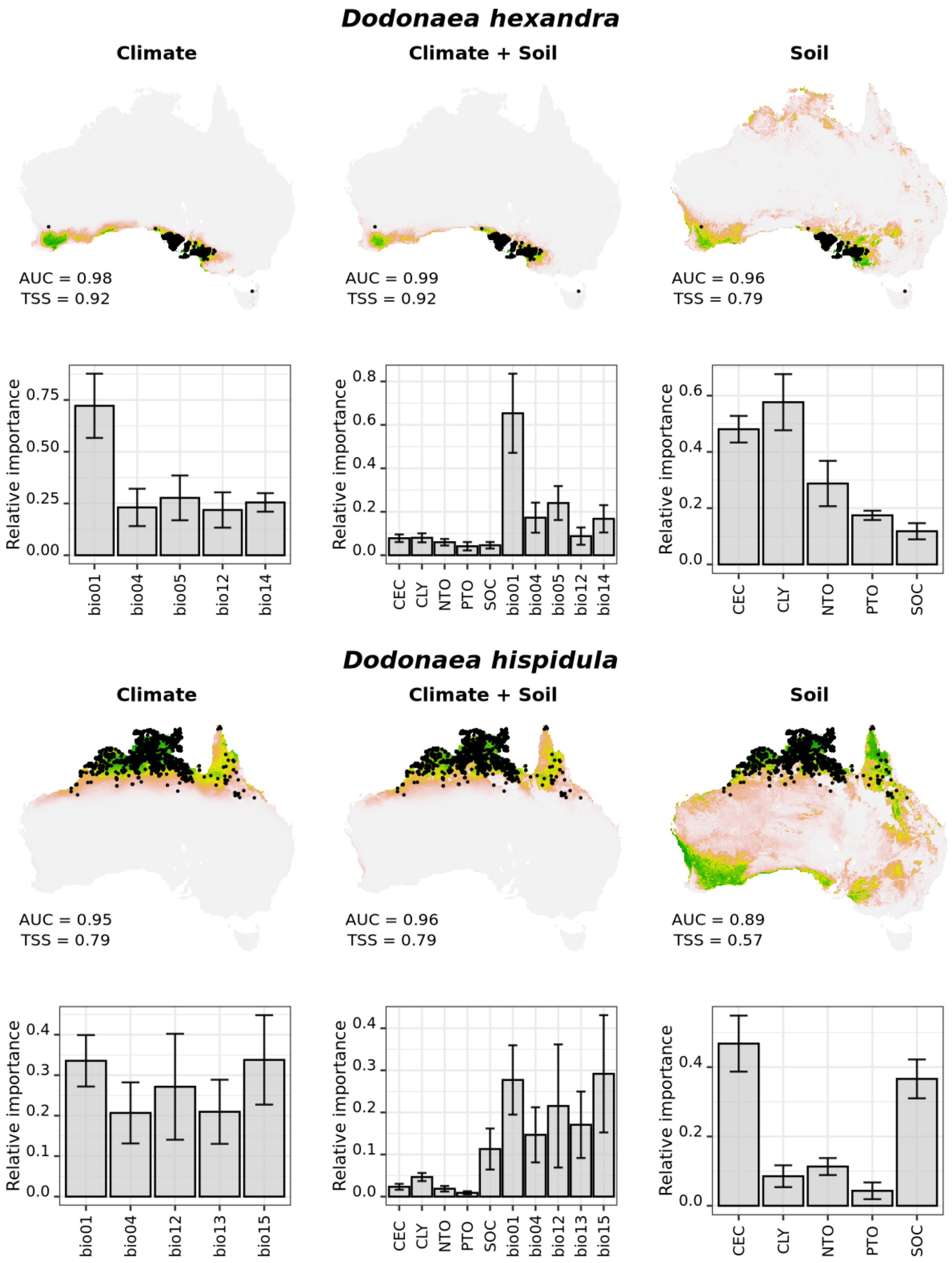

Dodonaea hexandra
Climate
Climate + Soil
Soil
AUC = 0.98
TSS = 0.92
AUC = 0.99
TSS = 0.92
AUC = 0.96
TSS = 0.79
Relative importance
0.00
0.25
0.50
0.75
bio01
bio04
bio05
bio12
bio14
0.0
0.2
0.4
0.6
0.8
CEC
CLY
NTO
PTO
SOC
bio01
bio04
bio05
bio12
bio14
0.0
0.2
0.4
0.6
CEC
CLY
NTO
PTO
SOC
Dodonaea hispidula
Climate
Climate + Soil
Soil
AUC = 0.95
TSS = 0.79
AUC = 0.96
TSS = 0.79
AUC = 0.89
TSS = 0.57
Relative importance
0.0
0.1
0.2
0.3
0.4
bio01
bio04
bio12
bio13
bio15
CEC
CLY
NTO
PTO
SOC
bio01
bio04
bio12
bio13
bio15
0.0
0.2
0.4
CEC
CLY
NTO
PTO
SOC

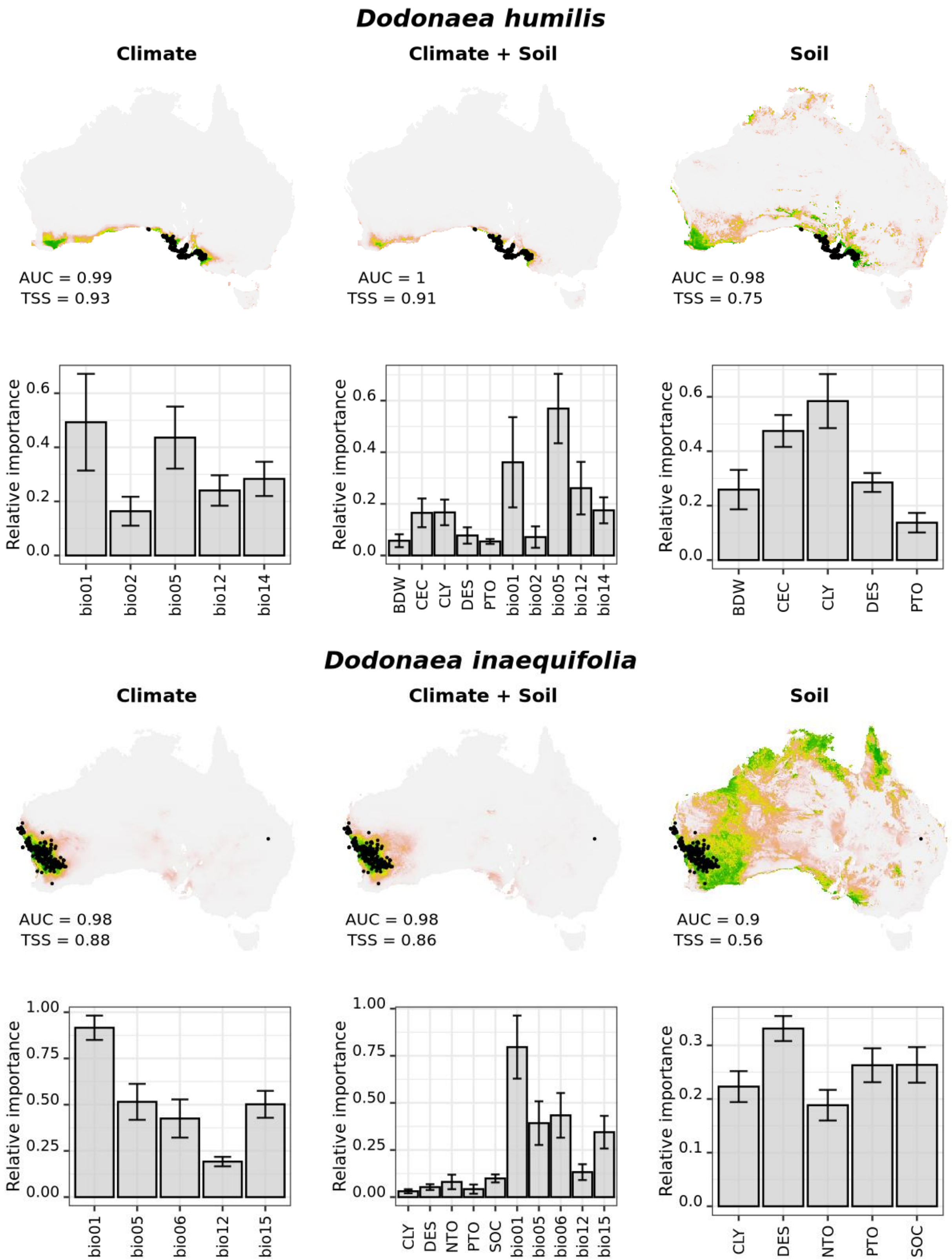

Dodonaea humilis
Climate
Climate + Soil
Soil
AUC = 0.99
TSS = 0.93
AUC = 1
TSS = 0.91
AUC = 0.98
TSS = 0.75
Relative importance
bio01 bio02 bio05 bio12 bio14
BDW CEC CLY DES PTO bio01 bio02 bio05 bio12 bio14
BDW CEC CLY DES PTO
Dodonaea inaequifolia
Climate
Climate + Soil
Soil
AUC = 0.98
TSS = 0.88
AUC = 0.98
TSS = 0.86
AUC = 0.9
TSS = 0.56
Relative importance
bio01 bio05 bio06 bio12 bio15
CLY DES NTO PTO SOC bio01 bio05 bio06 bio12 bio15
CLY DES NTO PTO SOC

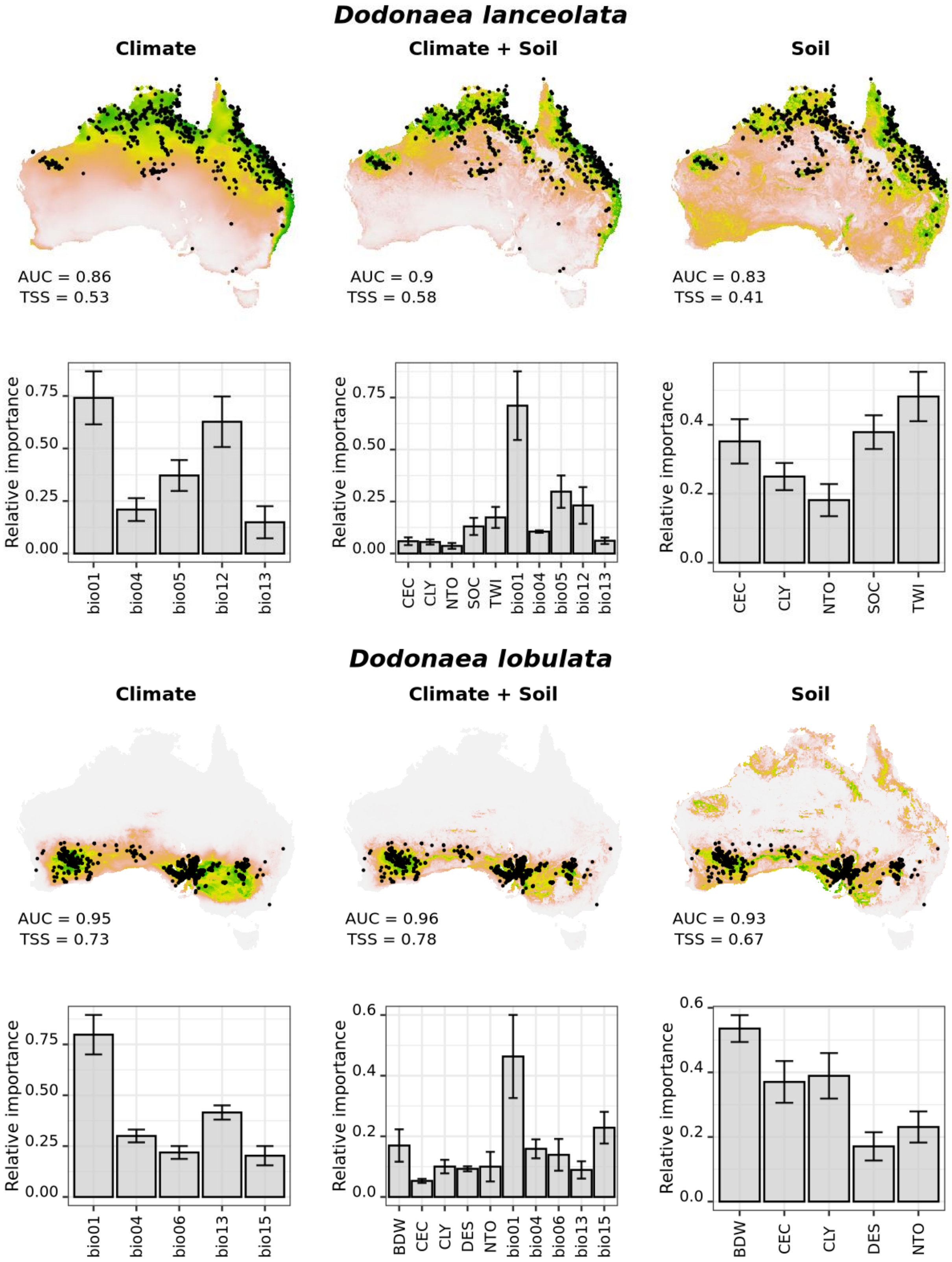
Dodonaea lanceolata
Climate
Climate + Soil
Soil
AUC = 0.86
TSS = 0.53
AUC = 0.9
TSS = 0.58
AUC = 0.83
TSS = 0.41
Relative importance
0.00
0.25
0.50
0.75
bio01
bio04
bio05
bio12
bio13
CEC
CLY
NTO
SOC
TWI
0.0
0.2
0.4
Dodonaea lobulata
AUC = 0.95
TSS = 0.73
AUC = 0.96
TSS = 0.78
AUC = 0.93
TSS = 0.67
bio06
bio15
BDW
DES
0.6

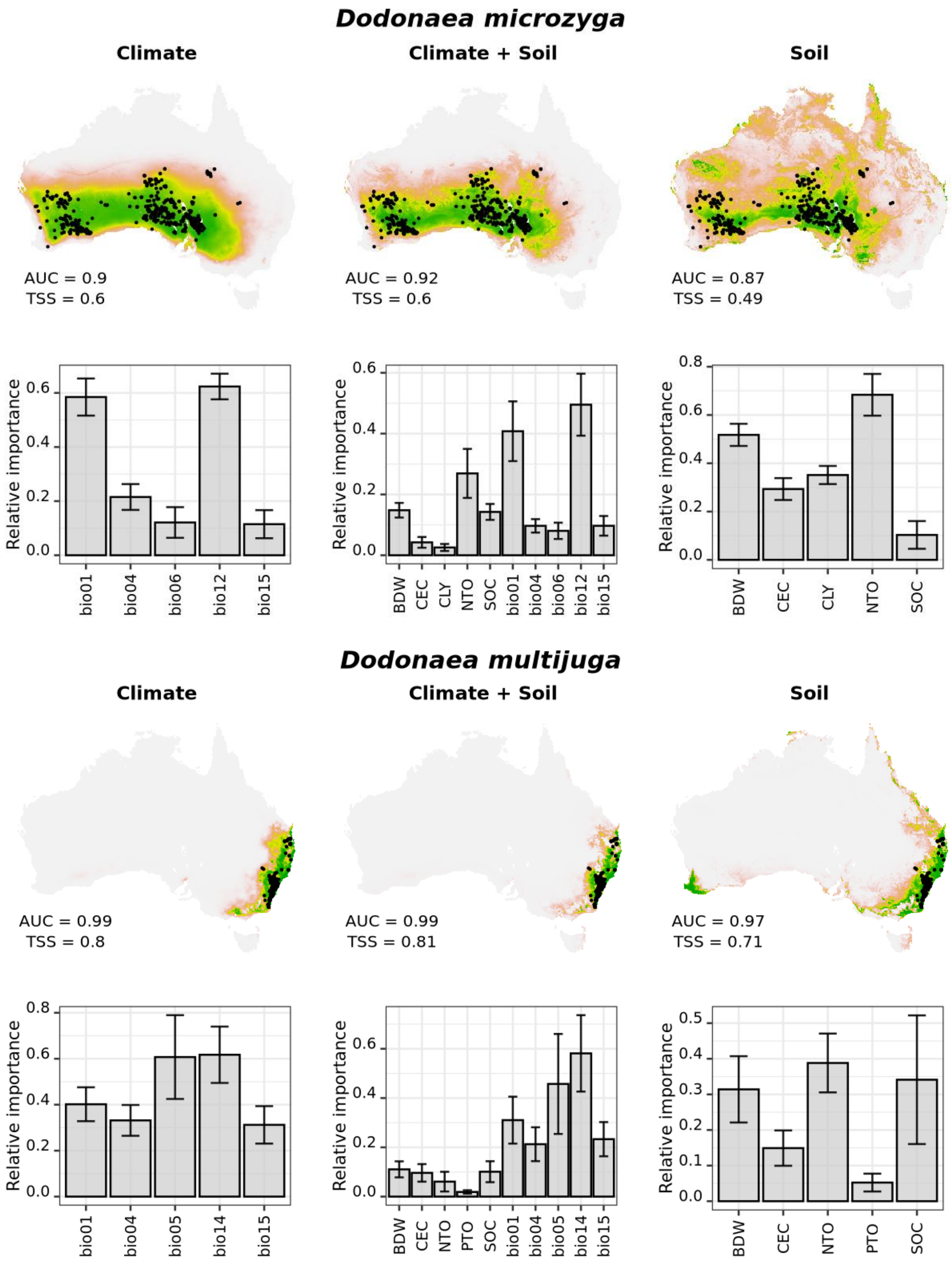
Dodonaea microzyga
Climate
Climate + Soil
Soil
AUC = 0.9
TSS = 0.6
AUC = 0.92
TSS = 0.6
AUC = 0.87
TSS = 0.49
Relative importance
bio01
bio04
bio06
bio12
bio15
BDW
CEC
CLY
NTO
SOC
Dodonaea multijuga
Climate
Climate + Soil
Soil
AUC = 0.99
TSS = 0.8
AUC = 0.99
TSS = 0.81
AUC = 0.97
TSS = 0.71
Relative importance
bio01
bio04
bio05
bio14
bio15
BDW
CEC
NTO
PTO
SOC

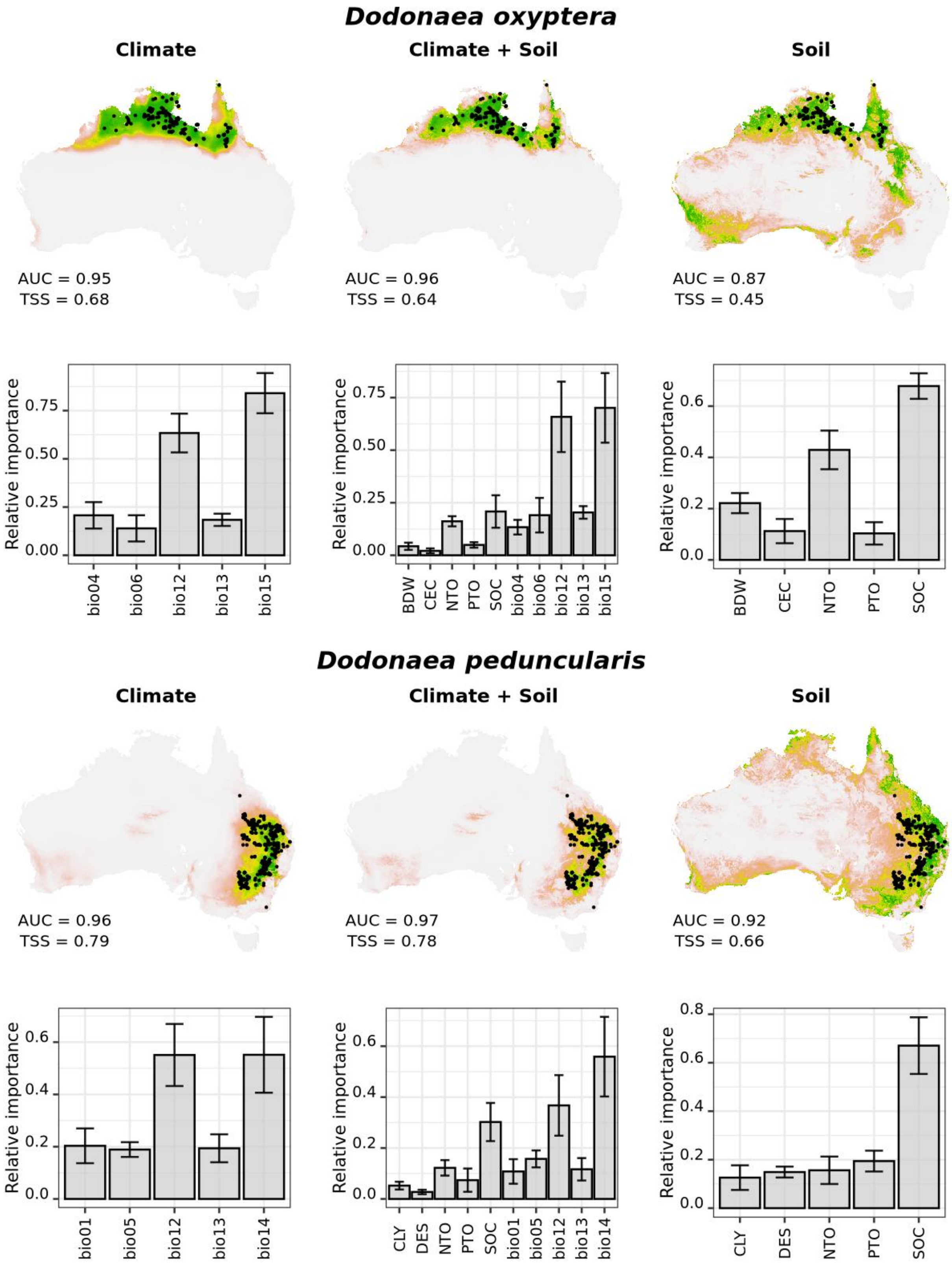

Dodonaea oxyptera
Climate
Climate + Soil
Soil
AUC = 0.95
TSS = 0.68
AUC = 0.96
TSS = 0.64
AUC = 0.87
TSS = 0.45
Relative importance
0.00
0.25
0.50
0.75
bio04
bio06
bio12
bio13
bio15
BDW
CEC
NTO
PTO
SOC
bio04
bio06
bio12
bio13
bio15
0.0
0.2
0.4
0.6
BDW
CEC
NTO
PTO
SOC
Dodonaea peduncularis
Climate
Climate + Soil
Soil
AUC = 0.96
TSS = 0.79
AUC = 0.97
TSS = 0.78
AUC = 0.92
TSS = 0.66
Relative importance
0.0
0.2
0.4
0.6
0.8
bio01
bio05
bio12
bio13
bio14
CLY
DES
NTO
PTO
SOC
bio01
bio05
bio12
bio13
bio14
CLY
DES
NTO
PTO
SOC

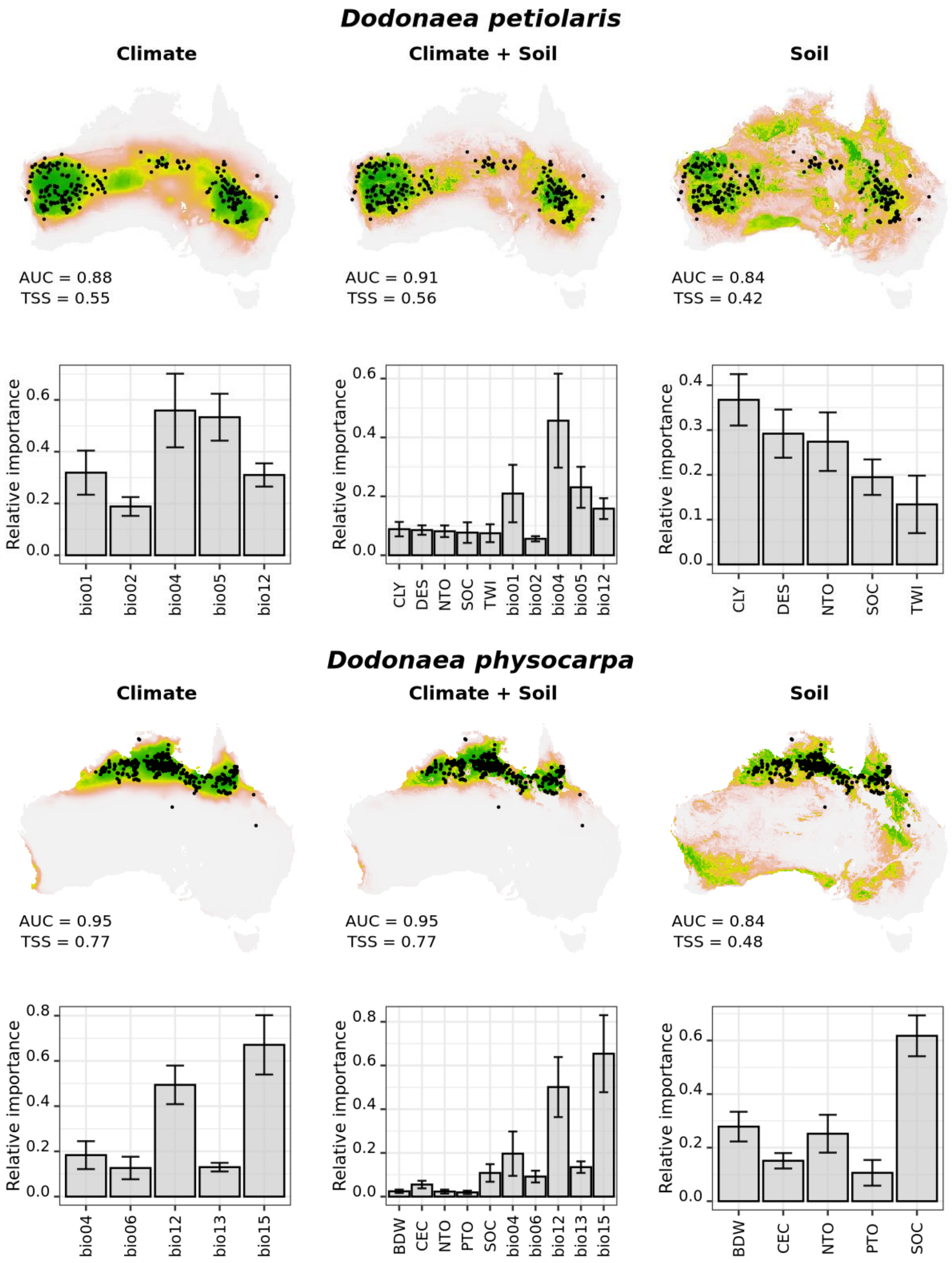
Dodonaea petiolaris
Climate
Climate + Soil
Soil
AUC = 0.88
TSS = 0.55
AUC = 0.91
TSS = 0.56
AUC = 0.84
TSS = 0.42
Relative importance
0.0 0.2 0.4 0.6
bio01 bio02 bio04 bio05 bio12
0.0 0.2 0.4 0.6
CLY DES NTO SOC TWI bio01 bio02 bio04 bio05 bio12
0.0 0.1 0.2 0.3 0.4
CLY DES NTO SOC TWI
Dodonaea physocarpa
Climate
Climate + Soil
Soil
AUC = 0.95
TSS = 0.77
AUC = 0.95
TSS = 0.77
AUC = 0.84
TSS = 0.48
Relative importance
0.0 0.2 0.4 0.6 0.8
bio04 bio06 bio12 bio13 bio15
0.0 0.2 0.4 0.6 0.8
BDW CEC NTO PTO SOC bio04 bio06 bio12 bio13 bio15
0.0 0.2 0.4 0.6
BDW CEC NTO PTO SOC

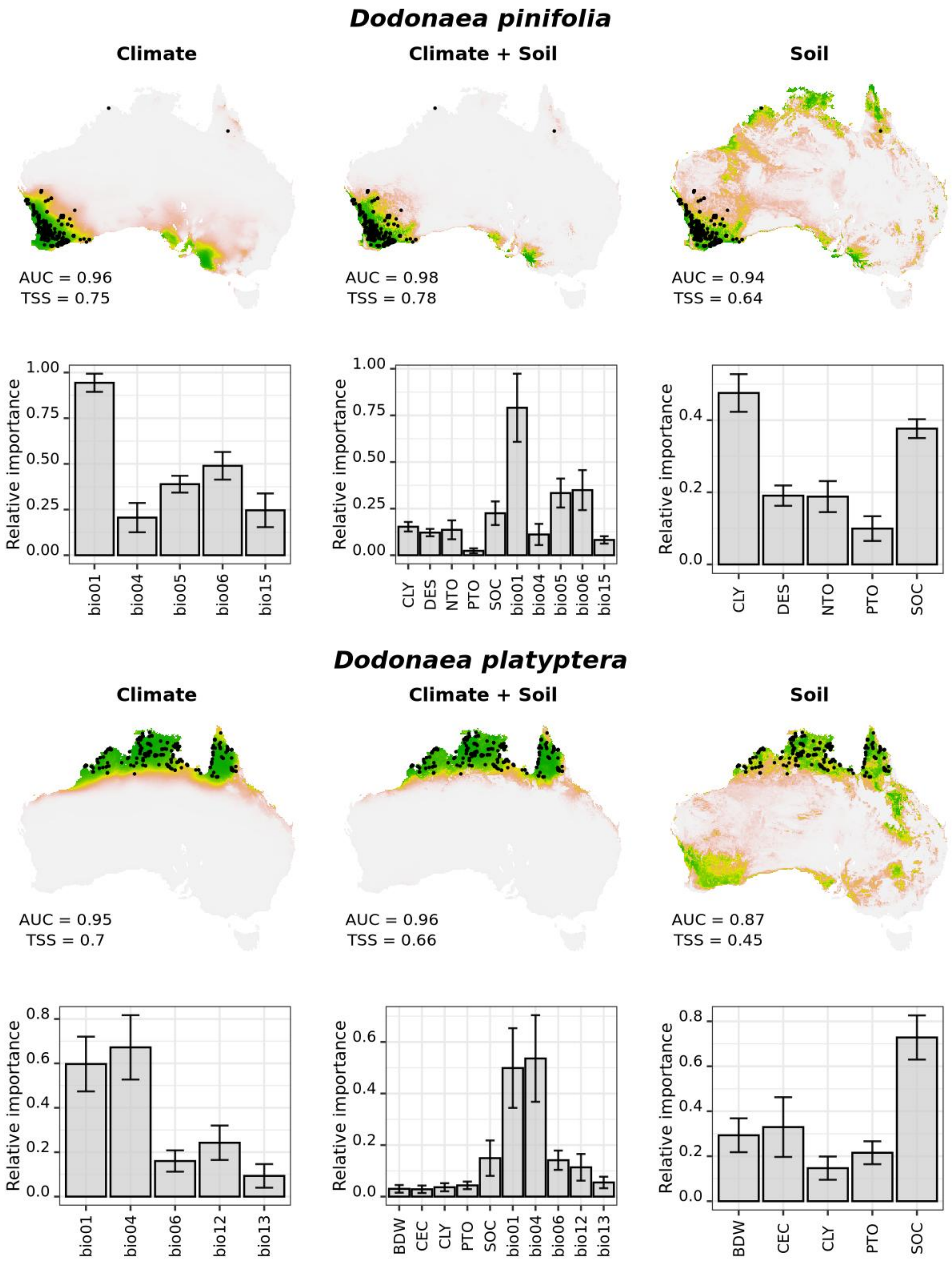
Dodonaea pinifolia
Climate
Climate + Soil
Soil
AUC = 0.96
TSS = 0.75
AUC = 0.98
TSS = 0.78
AUC = 0.94
TSS = 0.64
Relative importance
bio01 bio04 bio05 bio06 bio15
CLY DES NTO PTO SOC bio01 bio04 bio05 bio06 bio15
CLY DES NTO PTO SOC
Dodonaea platyptera
Climate
Climate + Soil
Soil
AUC = 0.95
TSS = 0.7
AUC = 0.96
TSS = 0.66
AUC = 0.87
TSS = 0.45
Relative importance
bio01 bio04 bio06 bio12 bio13
BDW CEC CLY PTO SOC bio01 bio04 bio06 bio12 bio13
BDW CEC CLY PTO SOC

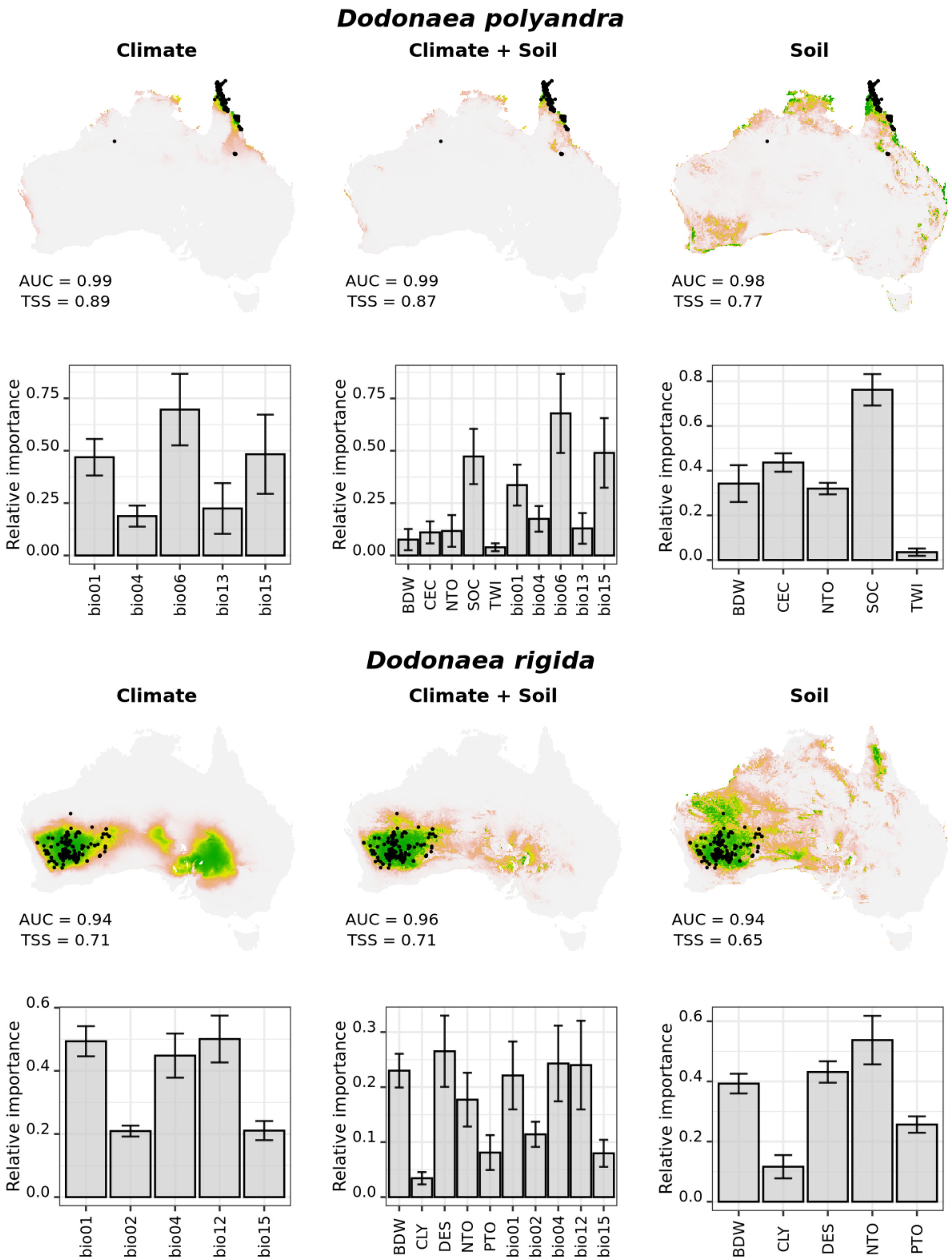

Dodonaea polyandra
Climate
Climate + Soil
Soil
AUC = 0.99
TSS = 0.89
AUC = 0.99
TSS = 0.87
AUC = 0.98
TSS = 0.77
Relative importance
0.00
0.25
0.50
0.75
bio01
bio04
bio06
bio13
bio15
BDW
CEC
NTO
SOC
TWI
0.0
0.2
0.4
0.6
0.8
Dodonaea rigida
Climate
Climate + Soil
Soil
AUC = 0.94
TSS = 0.71
AUC = 0.96
TSS = 0.71
AUC = 0.94
TSS = 0.65
Relative importance
0.0
0.1
0.2
0.3
0.4
0.6
bio01
bio02
bio04
bio12
bio15
BDW
CLY
DES
NTO
PTO

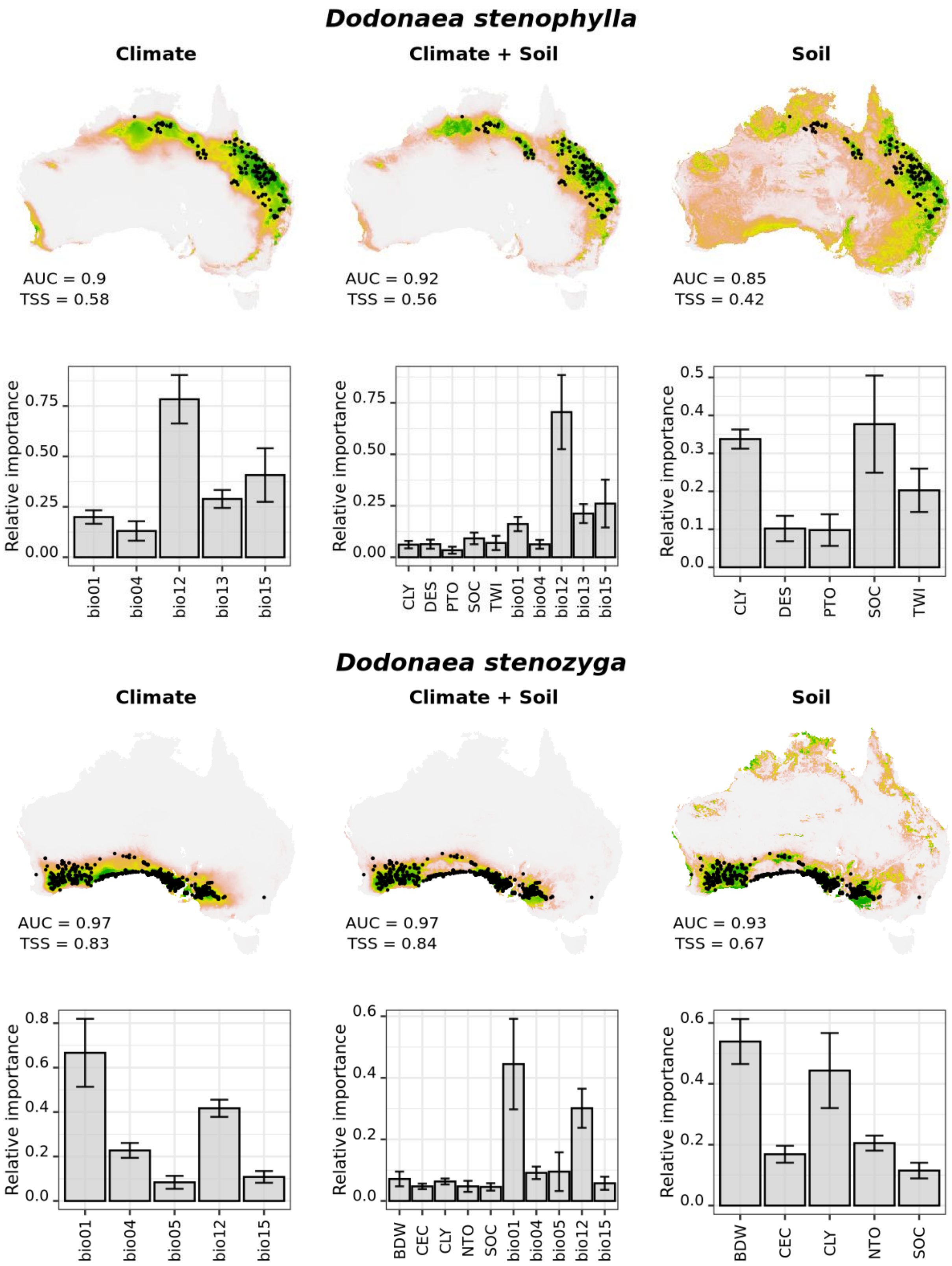
Dodonaea stenophylla
Climate
Climate + Soil
Soil
AUC = 0.9
TSS = 0.58
AUC = 0.92
TSS = 0.56
AUC = 0.85
TSS = 0.42
Relative importance
bio01 bio04 bio12 bio13 bio15
CLY DES PTO SOC TWI bio01 bio04 bio12 bio13 bio15
CLY DES PTO SOC TWI
Dodonaea stenozyga
Climate
Climate + Soil
Soil
AUC = 0.97
TSS = 0.83
AUC = 0.97
TSS = 0.84
AUC = 0.93
TSS = 0.67
Relative importance
bio01 bio04 bio05 bio12 bio15
BDW CEC CLY NTO SOC bio01 bio04 bio05 bio12 bio15
BDW CEC CLY NTO SOC

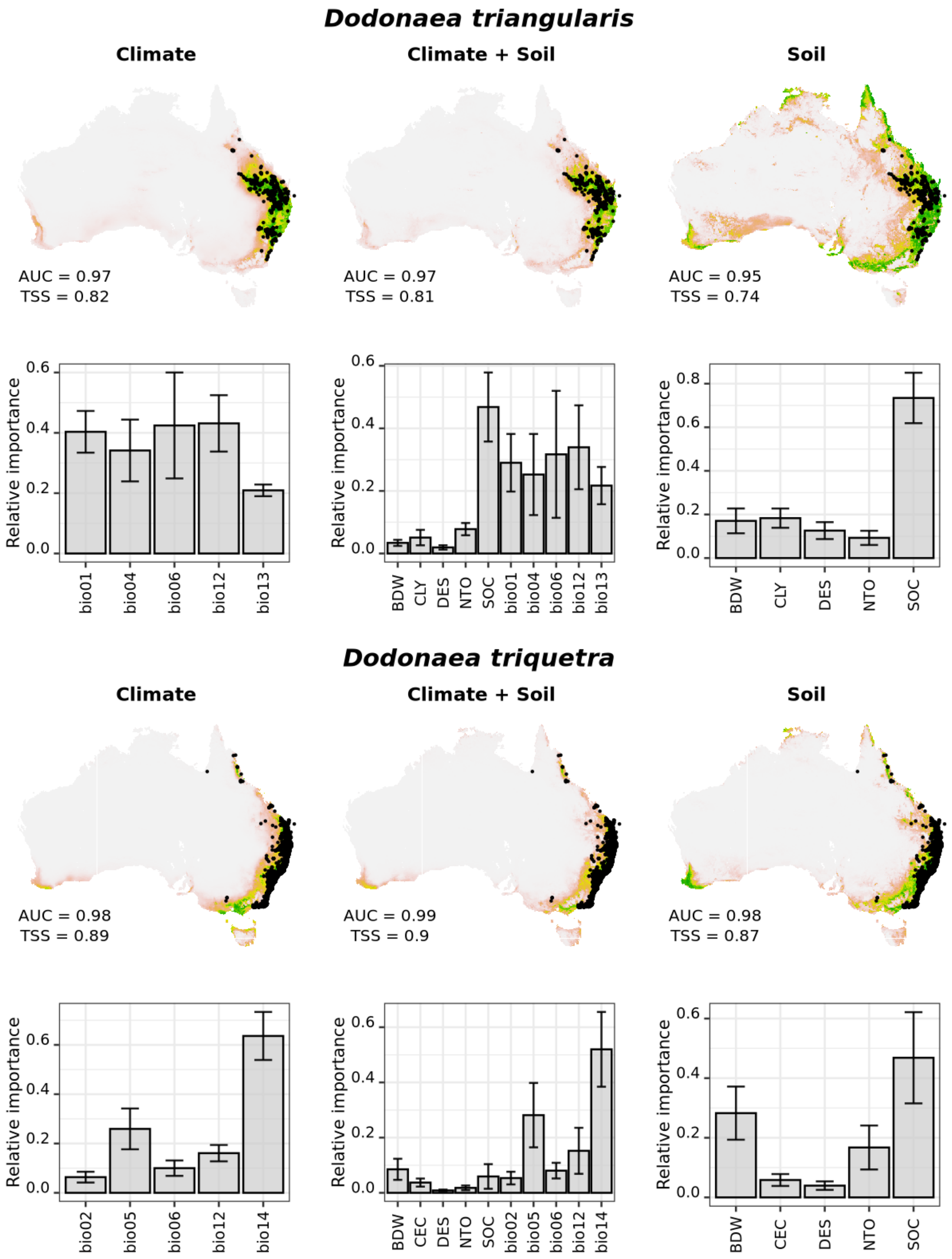

Dodonaea triangularis
Climate
Climate + Soil
Soil
AUC = 0.97
TSS = 0.82
AUC = 0.97
TSS = 0.81
AUC = 0.95
TSS = 0.74
Relative importance
bio01
bio04
bio06
bio12
bio13
BDW
CLY
DES
NTO
SOC
Dodonaea triquetra
Climate
Climate + Soil
Soil
AUC = 0.98
TSS = 0.89
AUC = 0.99
TSS = 0.9
AUC = 0.98
TSS = 0.87
bio02
bio05
bio06
bio12
bio14
CEC

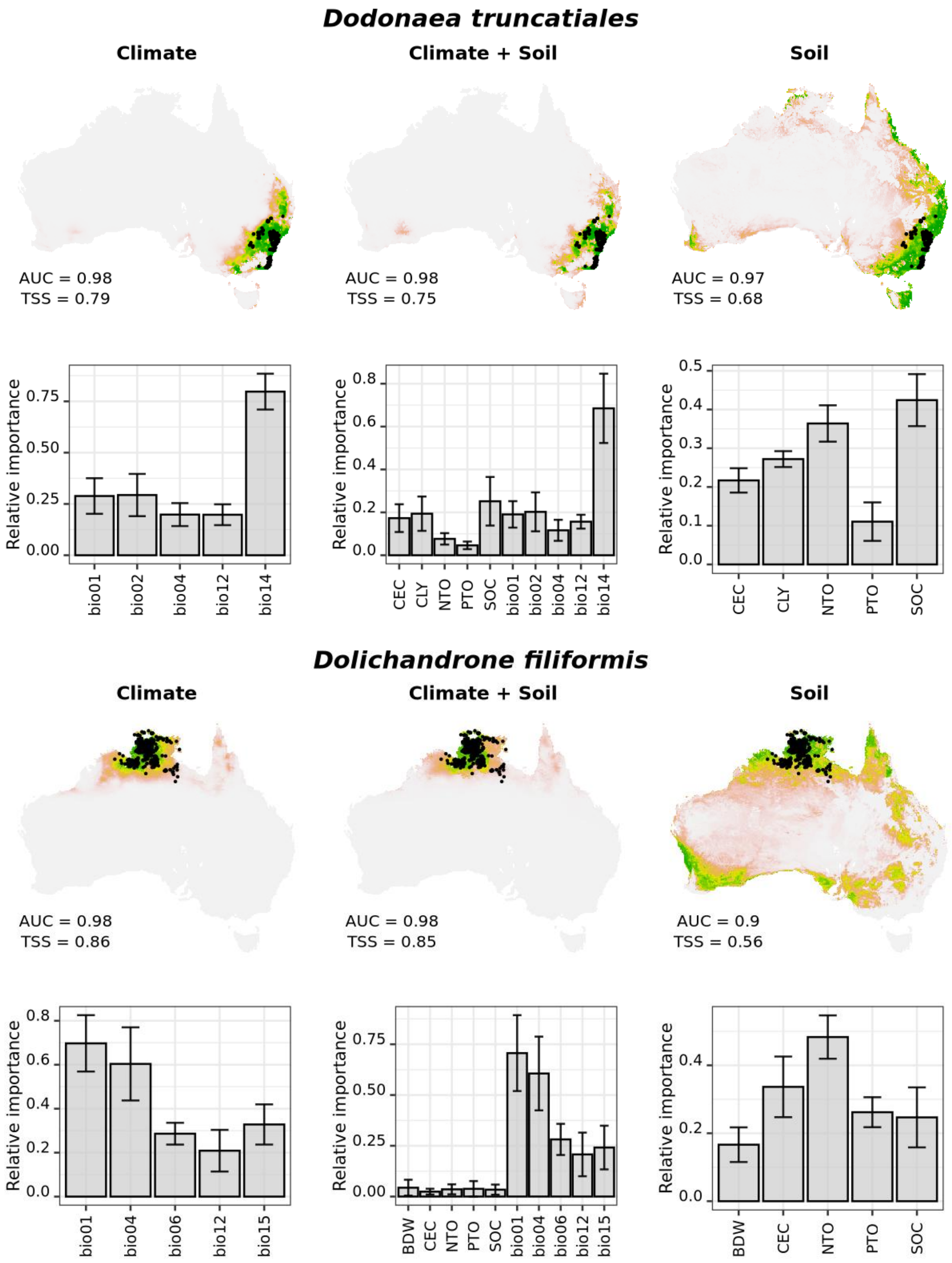
Dodonaea truncatiales
Climate
Climate + Soil
Soil
AUC = 0.98
TSS = 0.79
AUC = 0.98
TSS = 0.75
AUC = 0.97
TSS = 0.68
Relative importance
bio01
bio02
bio04
bio12
bio14
CEC
CLY
NTO
PTO
SOC
Dolichandrone filiformis
Climate
Climate + Soil
Soil
AUC = 0.98
TSS = 0.86
AUC = 0.98
TSS = 0.85
AUC = 0.9
TSS = 0.56
Relative importance
bio01
bio04
bio06
bio12
bio15
BDW
CEC
NTO
PTO
SOC

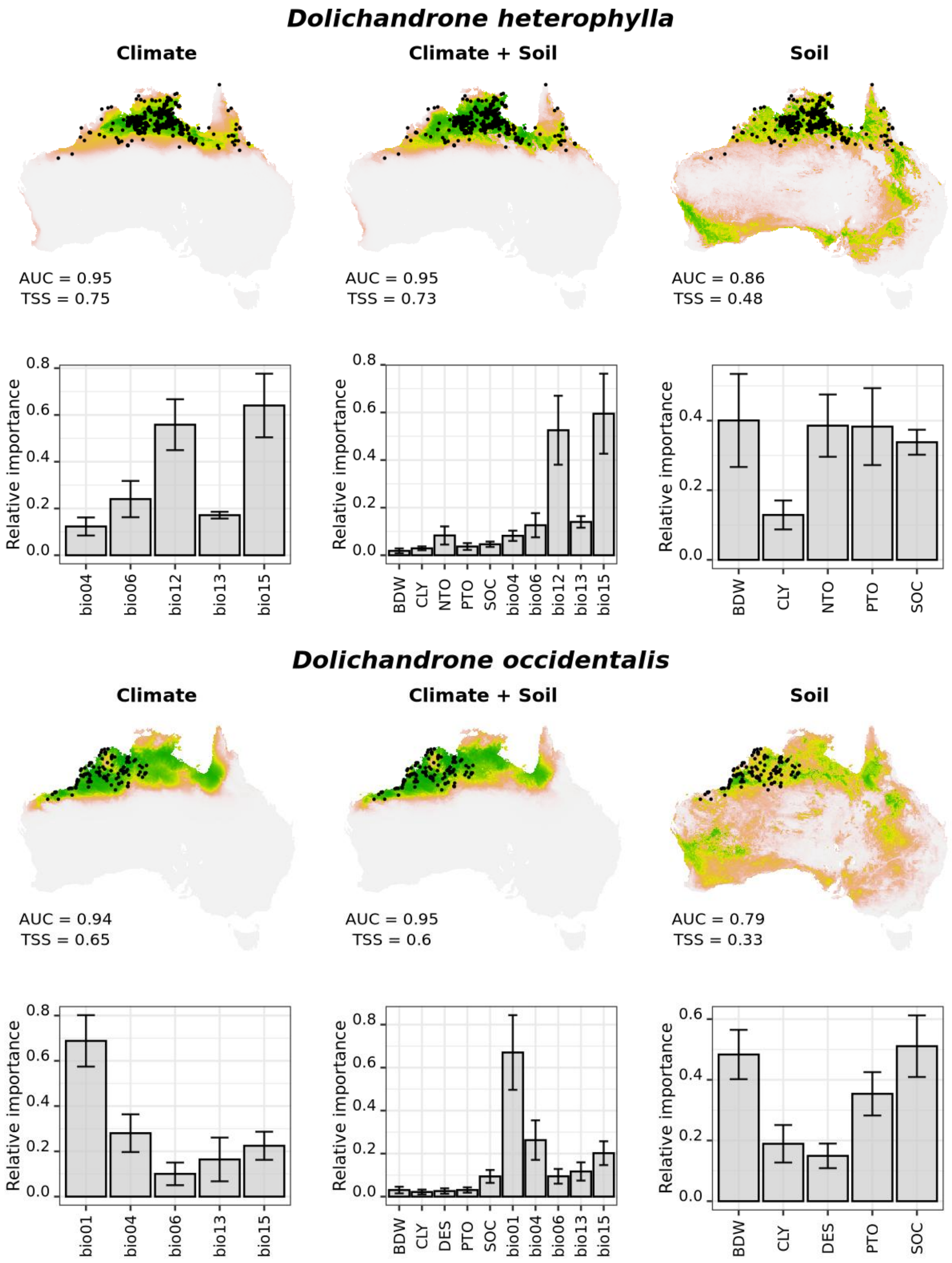

Dolichandrone heterophylla
Climate
Climate + Soil
Soil
AUC = 0.95
TSS = 0.75
AUC = 0.95
TSS = 0.73
AUC = 0.86
TSS = 0.48
Relative importance
bio04
bio06
bio12
bio13
bio15
BDW
CLY
NTO
PTO
SOC
Dolichandrone occidentalis
Climate
Climate + Soil
Soil
AUC = 0.94
TSS = 0.65
AUC = 0.95
TSS = 0.6
AUC = 0.79
TSS = 0.33
Relative importance
bio01
bio04
bio06
bio13
bio15
BDW
CLY
DES
PTO
SOC

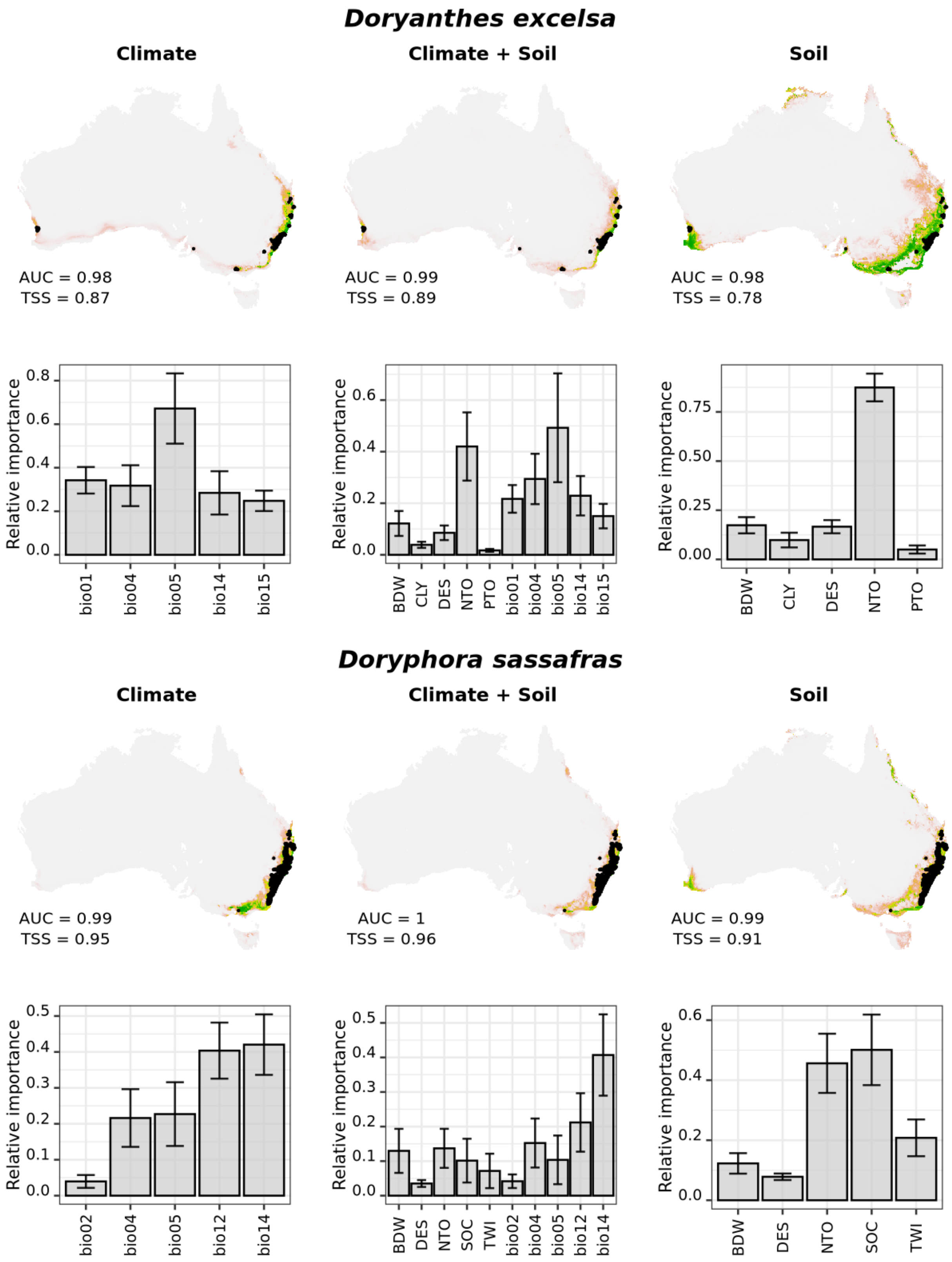

Doryanthes excelsa
Climate
Climate + Soil
Soil
AUC = 0.98
TSS = 0.87
AUC = 0.99
TSS = 0.89
AUC = 0.98
TSS = 0.78
Relative importance
0.0
0.2
0.4
0.6
0.8
bio01
bio04
bio05
bio14
bio15
Relative importance
0.0
0.2
0.4
0.6
BDW
CLY
DES
NTO
PTO
bio01
bio04
bio05
bio14
bio15
Relative importance
0.00
0.25
0.50
0.75
BDW
CLY
DES
NTO
PTO
Doryphora sassafras
Climate
Climate + Soil
Soil
AUC = 0.99
TSS = 0.95
AUC = 1
TSS = 0.96
AUC = 0.99
TSS = 0.91
Relative importance
0.0
0.1
0.2
0.3
0.4
0.5
bio02
bio04
bio05
bio12
bio14
Relative importance
0.0
0.1
0.2
0.3
0.4
0.5
BDW
DES
NTO
SOC
TWI
bio02
bio04
bio05
bio12
bio14
Relative importance
0.0
0.2
0.4
0.6
BDW
DES
NTO
SOC
TWI

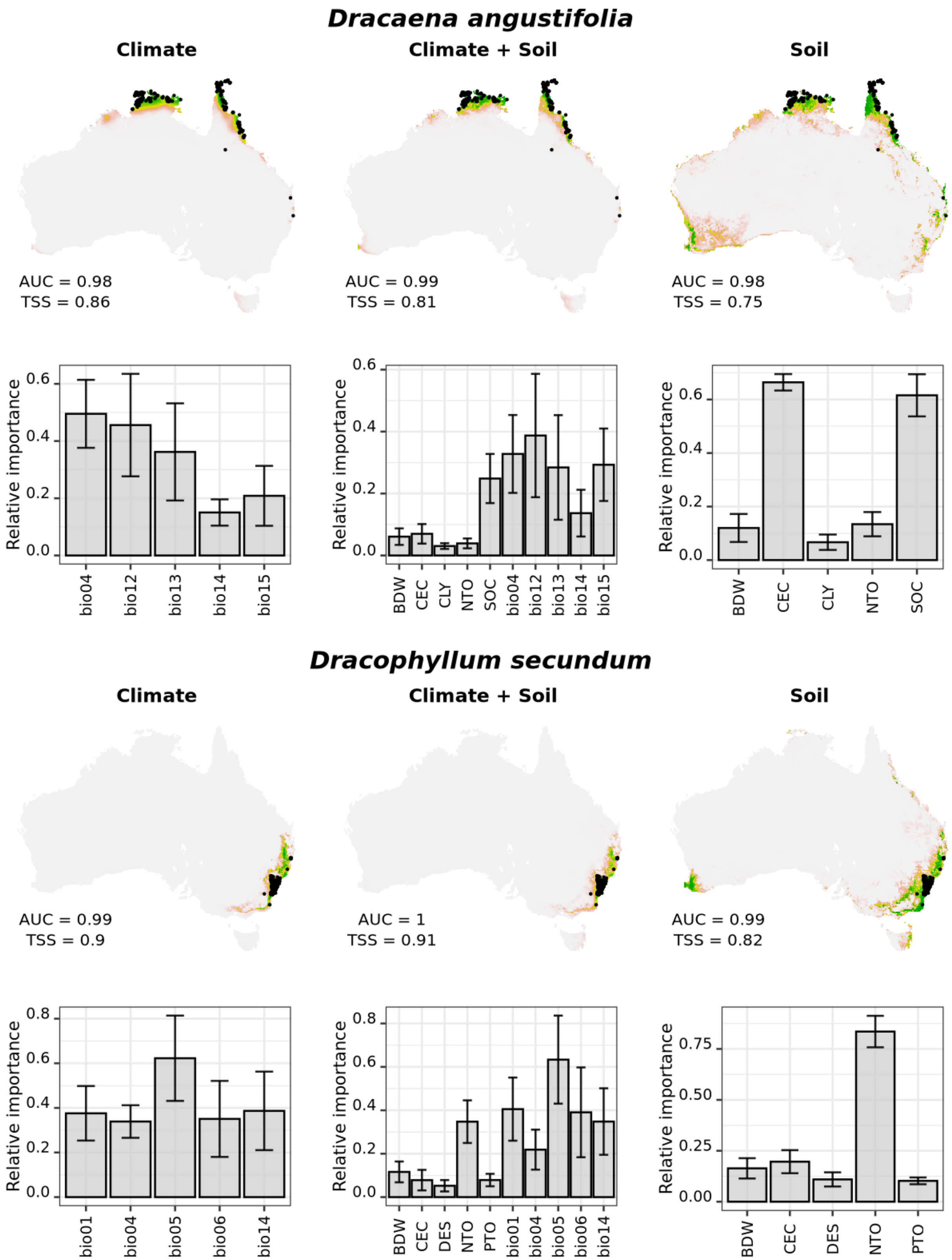
Dracaena angustifolia
Climate
Climate + Soil
Soil
AUC = 0.98
TSS = 0.86
AUC = 0.99
TSS = 0.81
AUC = 0.98
TSS = 0.75
Relative importance
bio04
bio12
bio13
bio14
bio15
BDW
CEC
CLY
NTO
SOC
Dracophyllum secundum
Climate
Climate + Soil
Soil
AUC = 0.99
TSS = 0.9
AUC = 1
TSS = 0.91
AUC = 0.99
TSS = 0.82
Relative importance
bio01
bio04
bio05
bio06
bio14
BDW
CEC
DES
NTO
PTO

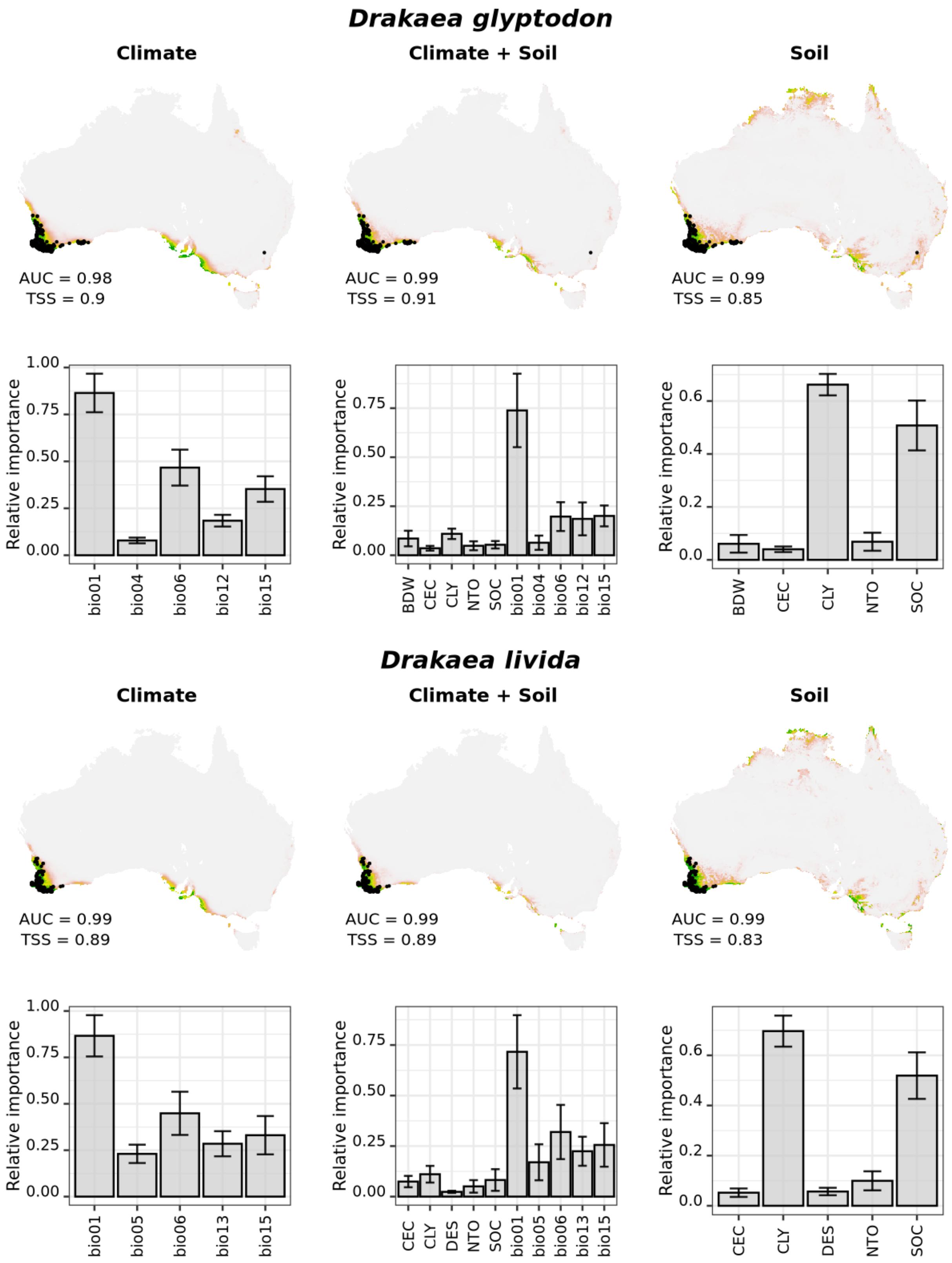

Drakaea glyptodon
Climate
Climate + Soil
Soil
AUC = 0.98
TSS = 0.9
AUC = 0.99
TSS = 0.91
AUC = 0.99
TSS = 0.85
Relative importance
1.00
0.75
0.50
0.25
0.00
bio01
bio04
bio06
bio12
bio15
BDW
CEC
CLY
NTO
SOC
0.6
0.4
0.2
0.0
Drakaea livida
Climate
Climate + Soil
Soil
AUC = 0.99
TSS = 0.89
AUC = 0.99
TSS = 0.89
AUC = 0.99
TSS = 0.83
bio05
bio13
DES

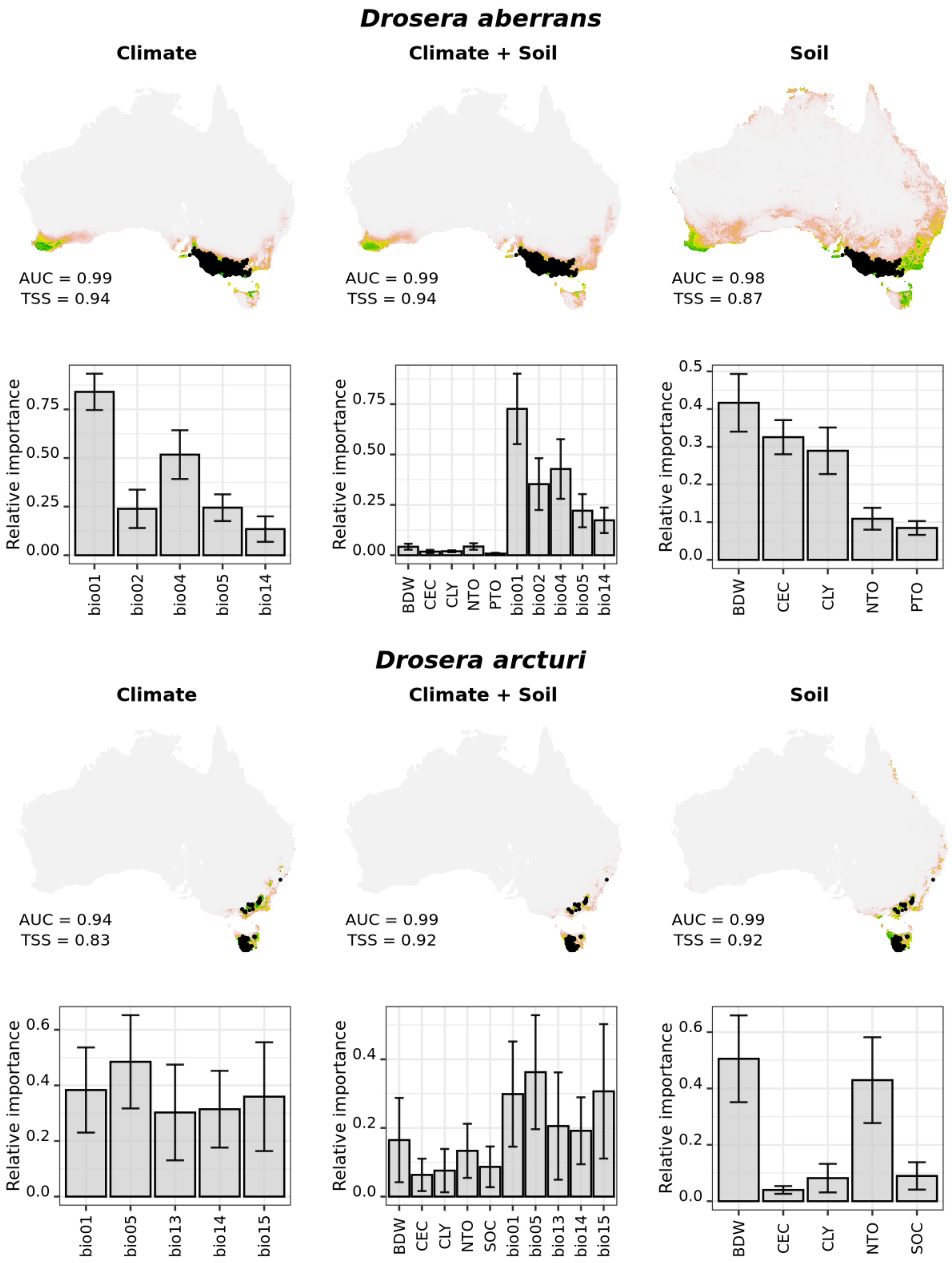

Drosera aberrans
Climate
Climate + Soil
Soil
AUC = 0.99
TSS = 0.94
AUC = 0.99
TSS = 0.94
AUC = 0.98
TSS = 0.87
Relative importance
0.00
0.25
0.50
0.75
bio01
bio02
bio04
bio05
bio14
BDW
CEC
CLY
NTO
PTO
0.0
0.1
0.2
0.3
0.4
0.5
Drosera arcturi
Climate
Climate + Soil
Soil
AUC = 0.94
TSS = 0.83
AUC = 0.99
TSS = 0.92
AUC = 0.99
TSS = 0.92
Relative importance
0.0
0.2
0.4
0.6
bio01
bio05
bio13
bio14
bio15
BDW
CEC
CLY
NTO
SOC

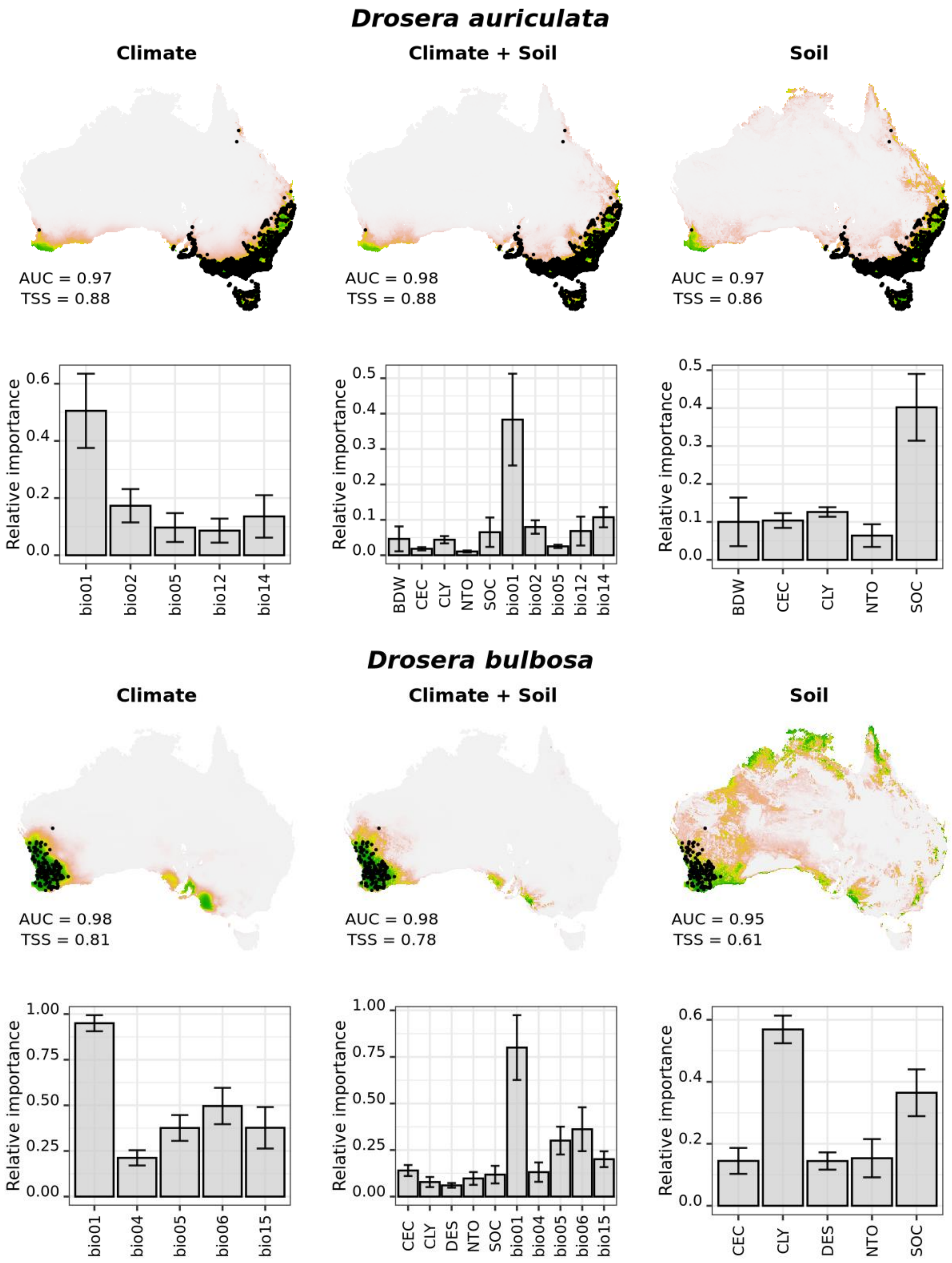
Drosera auriculata
Climate
Climate + Soil
Soil
AUC = 0.97
TSS = 0.88
AUC = 0.98
TSS = 0.88
AUC = 0.97
TSS = 0.86
Relative importance
0.0
0.2
0.4
0.6
bio01
bio02
bio05
bio12
bio14
Relative importance
0.0
0.1
0.2
0.3
0.4
0.5
BDW
CEC
CLY
NTO
SOC
bio01
bio02
bio05
bio12
bio14
Relative importance
0.0
0.1
0.2
0.3
0.4
0.5
BDW
CEC
CLY
NTO
SOC
Drosera bulbosa
Climate
Climate + Soil
Soil
AUC = 0.98
TSS = 0.81
AUC = 0.98
TSS = 0.78
AUC = 0.95
TSS = 0.61
Relative importance
0.00
0.25
0.50
0.75
1.00
bio01
bio04
bio05
bio06
bio15
Relative importance
0.00
0.25
0.50
0.75
1.00
CEC
CLY
DES
NTO
SOC
bio01
bio04
bio05
bio06
bio15
Relative importance
0.0
0.2
0.4
0.6
CEC
CLY
DES
NTO
SOC

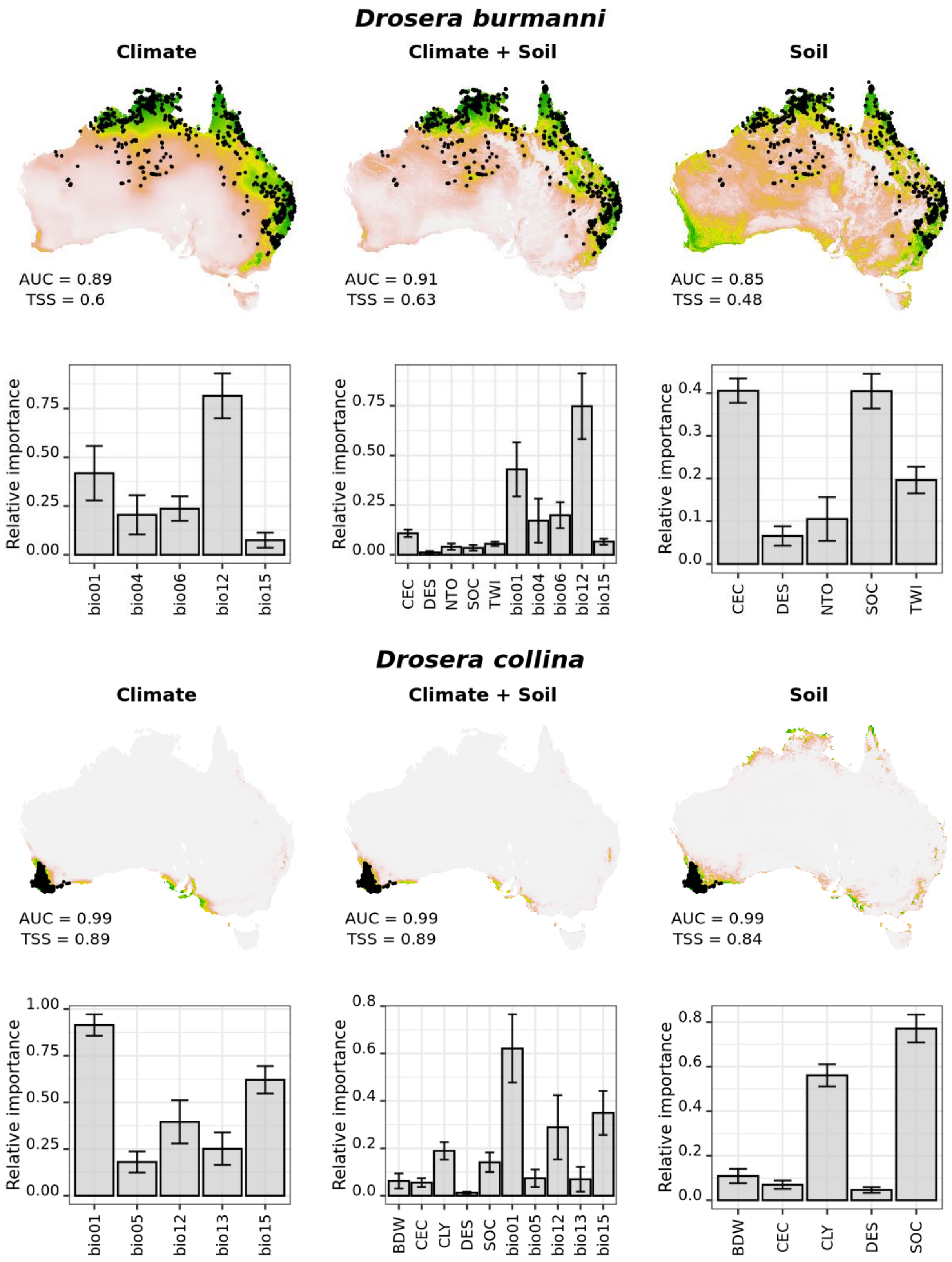
Drosera burmanni
Climate
Climate + Soil
Soil
AUC = 0.89
TSS = 0.6
AUC = 0.91
TSS = 0.63
AUC = 0.85
TSS = 0.48
Relative importance
0.00
0.25
0.50
0.75
bio01
bio04
bio06
bio12
bio15
CEC
DES
NTO
SOC
TWI
0.0
0.1
0.2
0.3
0.4
Drosera collina
Climate
Climate + Soil
Soil
AUC = 0.99
TSS = 0.89
AUC = 0.99
TSS = 0.89
AUC = 0.99
TSS = 0.84
1.00
bio05
bio13
0.6
0.8
BDW
CLY

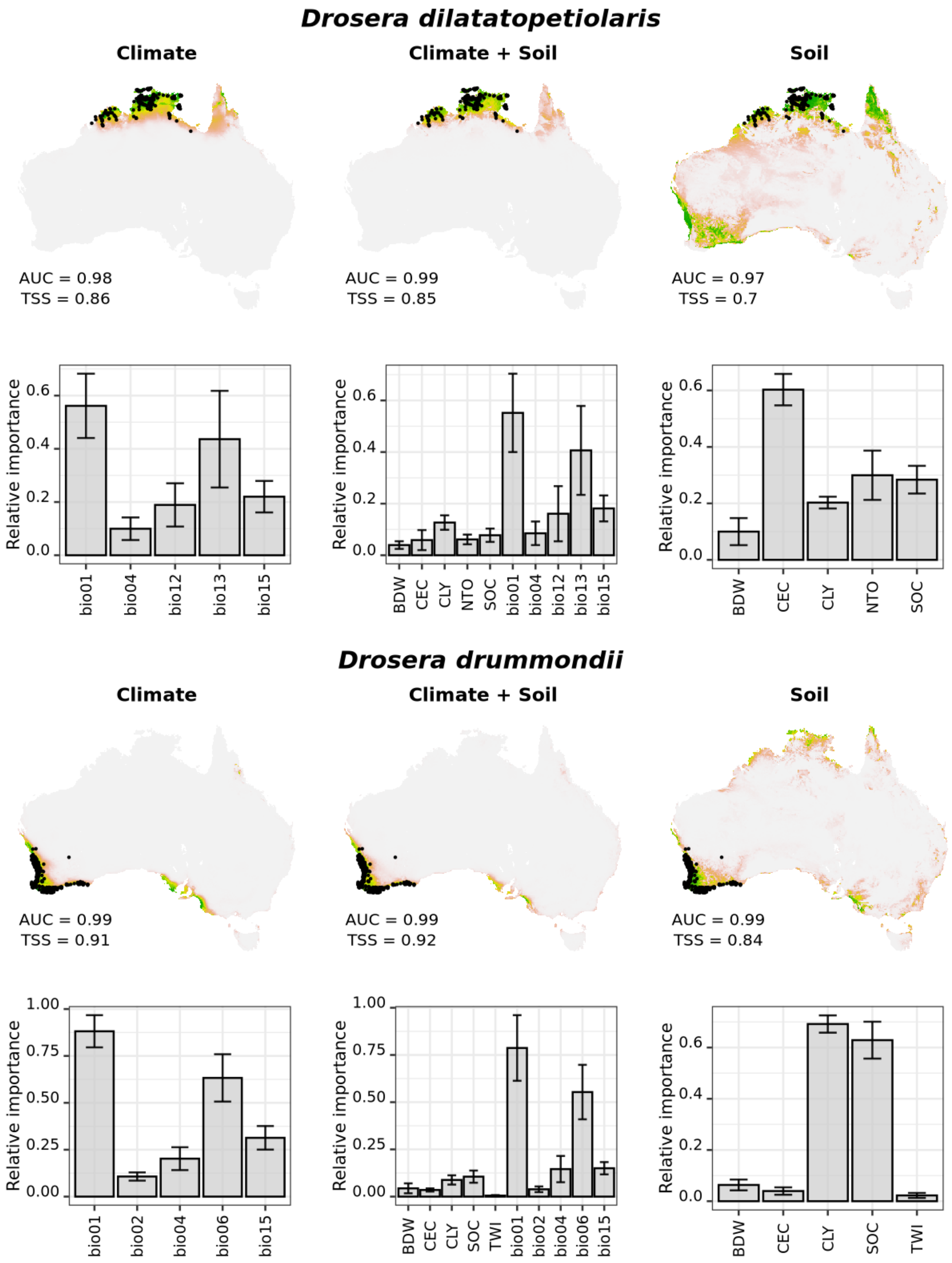
Drosera dilatatopetiolaris
Climate
Climate + Soil
Soil
AUC = 0.98
TSS = 0.86
AUC = 0.99
TSS = 0.85
AUC = 0.97
TSS = 0.7
Relative importance
bio01
bio04
bio12
bio13
bio15
BDW
CEC
CLY
NTO
SOC
Drosera drummondii
Climate
Climate + Soil
Soil
AUC = 0.99
TSS = 0.91
AUC = 0.99
TSS = 0.92
AUC = 0.99
TSS = 0.84
Relative importance
bio01
bio02
bio04
bio06
bio15
BDW
CEC
CLY
SOC
TWI

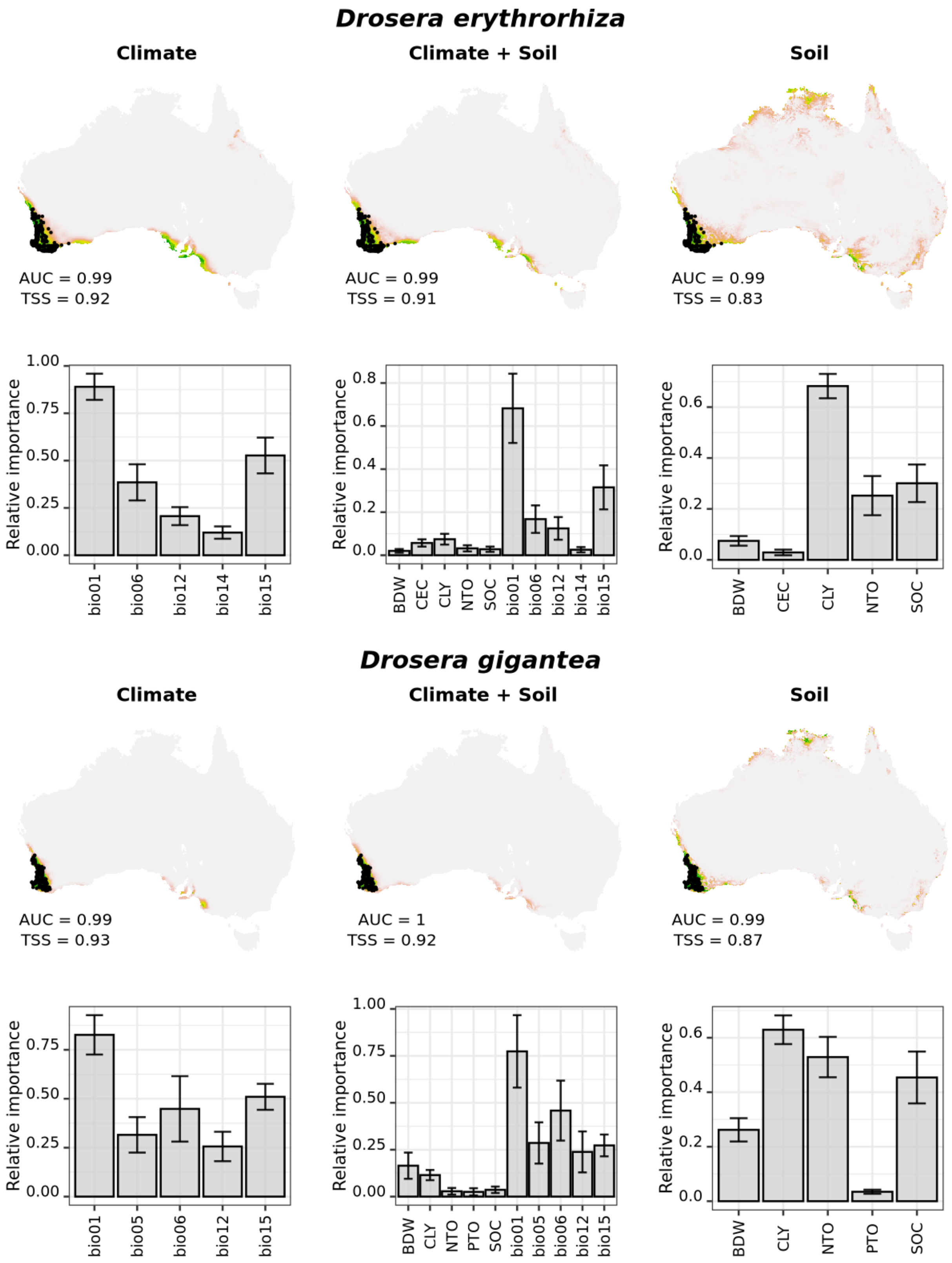
Drosera erythrorhiza
Climate
Climate + Soil
Soil
AUC = 0.99
TSS = 0.92
AUC = 0.99
TSS = 0.91
AUC = 0.99
TSS = 0.83
Relative importance
bio01
bio06
bio12
bio14
bio15
BDW
CEC
CLY
NTO
SOC
Drosera gigantea
Climate
Climate + Soil
Soil
AUC = 0.99
TSS = 0.93
AUC = 1
TSS = 0.92
AUC = 0.99
TSS = 0.87
Relative importance
bio01
bio05
bio06
bio12
bio15
BDW
CLY
NTO
PTO
SOC

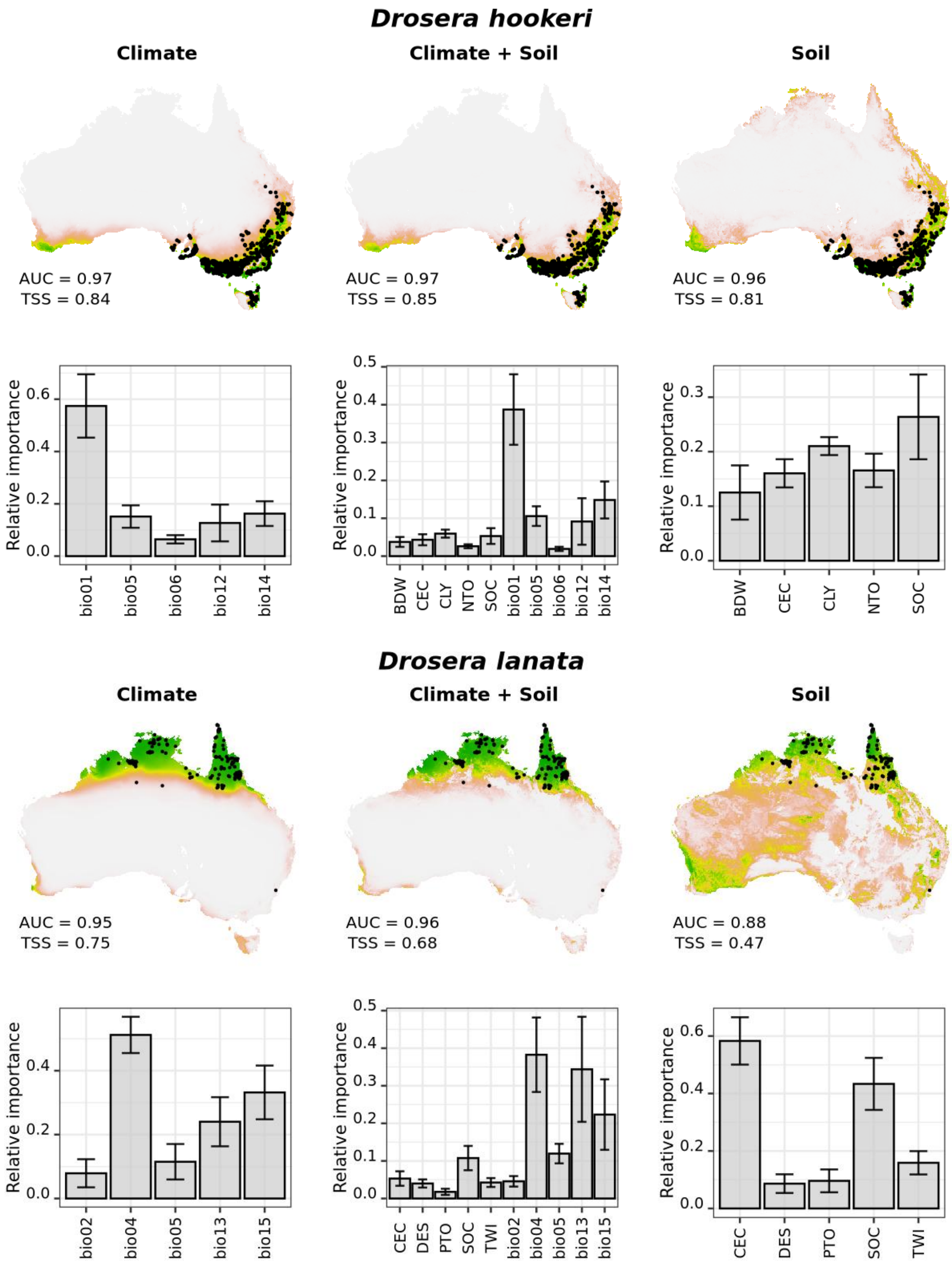

Drosera hookeri
Climate
Climate + Soil
Soil
AUC = 0.97
TSS = 0.84
AUC = 0.97
TSS = 0.85
AUC = 0.96
TSS = 0.81
Relative importance
bio01
bio05
bio06
bio12
bio14
BDW
CEC
CLY
NTO
SOC
Drosera lanata
AUC = 0.95
TSS = 0.75
AUC = 0.96
TSS = 0.68
AUC = 0.88
TSS = 0.47
bio02
bio04
bio13
bio15
DES
PTO
TWI

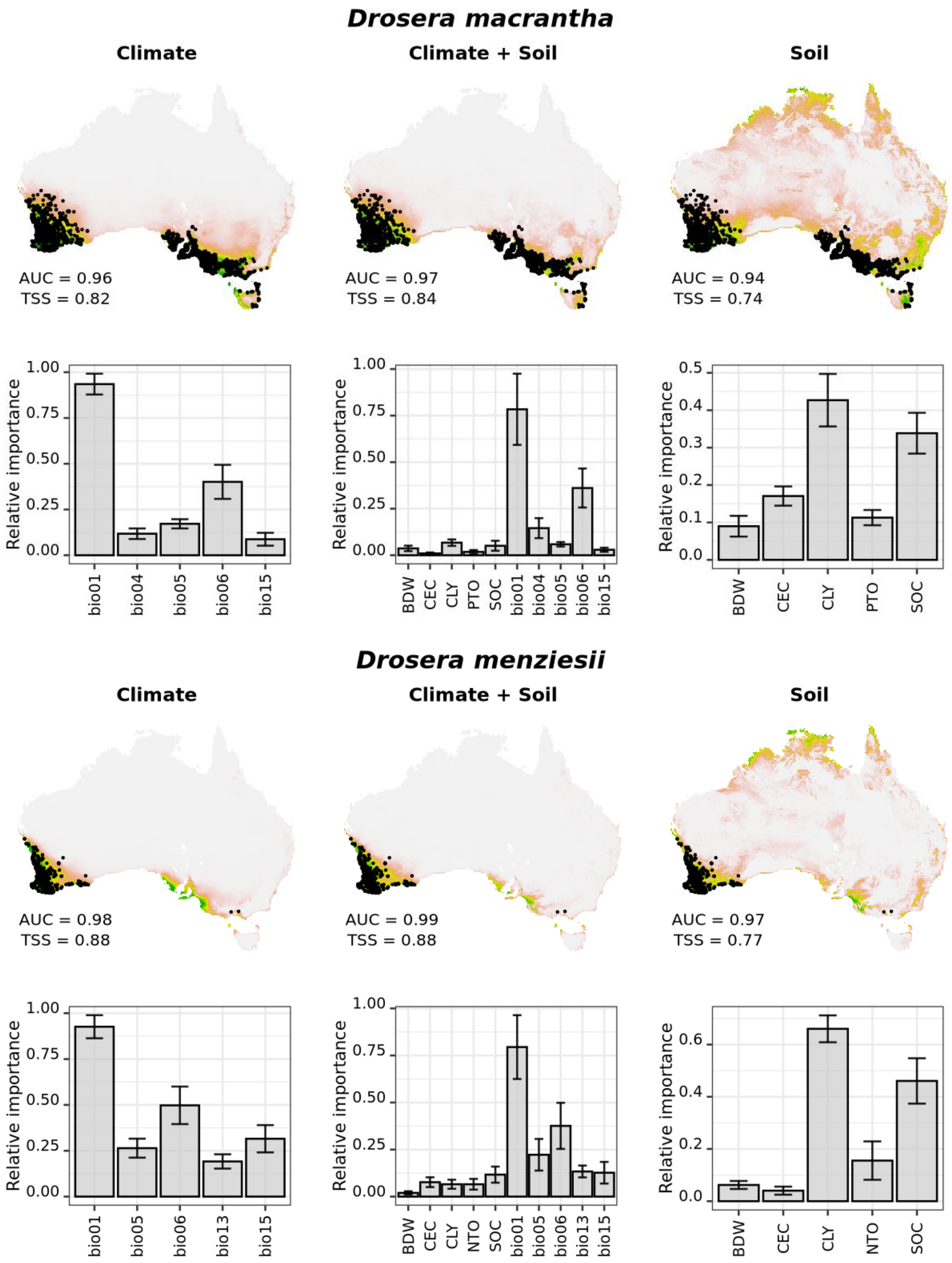

Drosera macrantha
Climate
Climate + Soil
Soil
AUC = 0.96
TSS = 0.82
AUC = 0.97
TSS = 0.84
AUC = 0.94
TSS = 0.74
Relative importance
1.00
0.75
0.50
0.25
0.00
bio01
bio04
bio05
bio06
bio15
BDW
CEC
CLY
PTO
SOC
0.5
0.4
0.3
0.2
0.1
0.0
Drosera menziesii
Climate
Climate + Soil
Soil
AUC = 0.98
TSS = 0.88
AUC = 0.99
TSS = 0.88
AUC = 0.97
TSS = 0.77
Relative importance
bio01
bio05
bio06
bio13
bio15
BDW
CEC
CLY
NTO
SOC
0.6
0.4
0.2
0.0

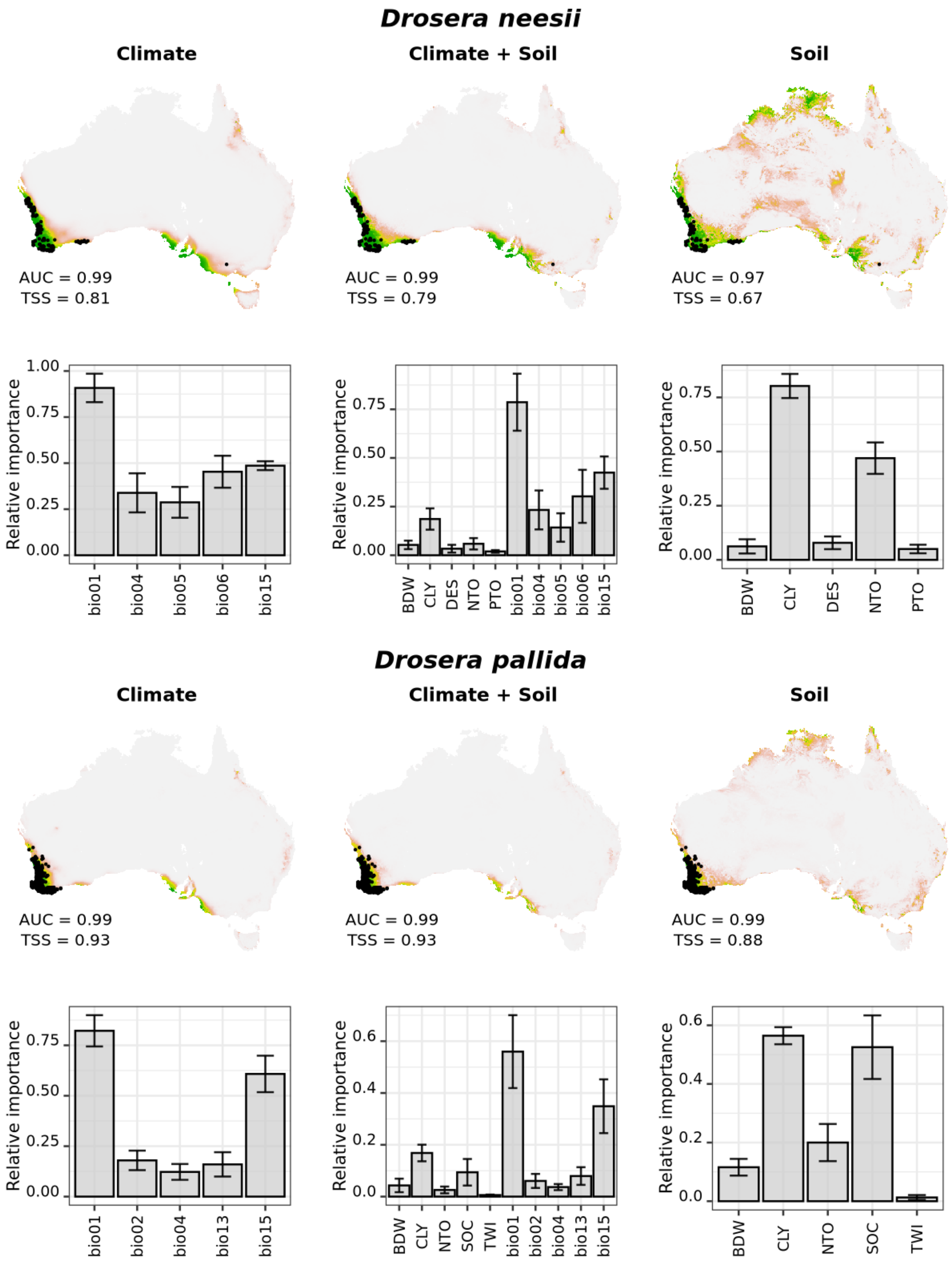
Drosera neesii
Climate
Climate + Soil
Soil
AUC = 0.99
TSS = 0.81
AUC = 0.99
TSS = 0.79
AUC = 0.97
TSS = 0.67
Relative importance
1.00
0.75
0.50
0.25
0.00
bio01
bio04
bio05
bio06
bio15
BDW
CLY
DES
NTO
PTO
Drosera pallida
Climate
Climate + Soil
Soil
AUC = 0.99
TSS = 0.93
AUC = 0.99
TSS = 0.93
AUC = 0.99
TSS = 0.88
0.6
0.4
0.2
0.0
bio01
bio02
bio04
bio13
bio15
BDW
CLY
NTO
SOC
TWI

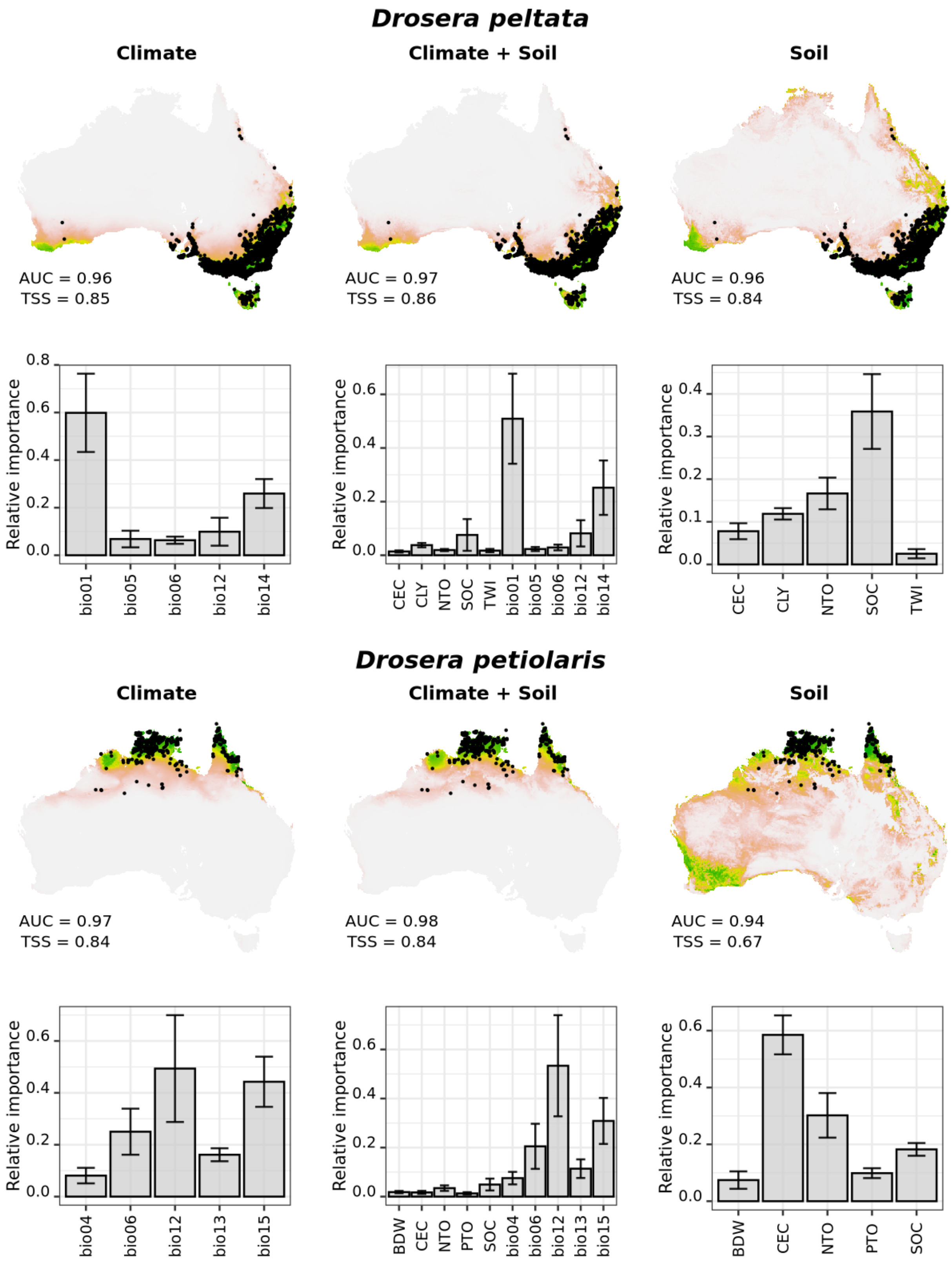
Drosera peltata
Climate
Climate + Soil
Soil
AUC = 0.96
TSS = 0.85
AUC = 0.97
TSS = 0.86
AUC = 0.96
TSS = 0.84
Relative importance
bio01
bio05
bio06
bio12
bio14
CEC
CLY
NTO
SOC
TWI
Drosera petiolaris
Climate
Climate + Soil
Soil
AUC = 0.97
TSS = 0.84
AUC = 0.98
TSS = 0.84
AUC = 0.94
TSS = 0.67
Relative importance
bio04
bio06
bio12
bio13
bio15
BDW
CEC
NTO
PTO
SOC

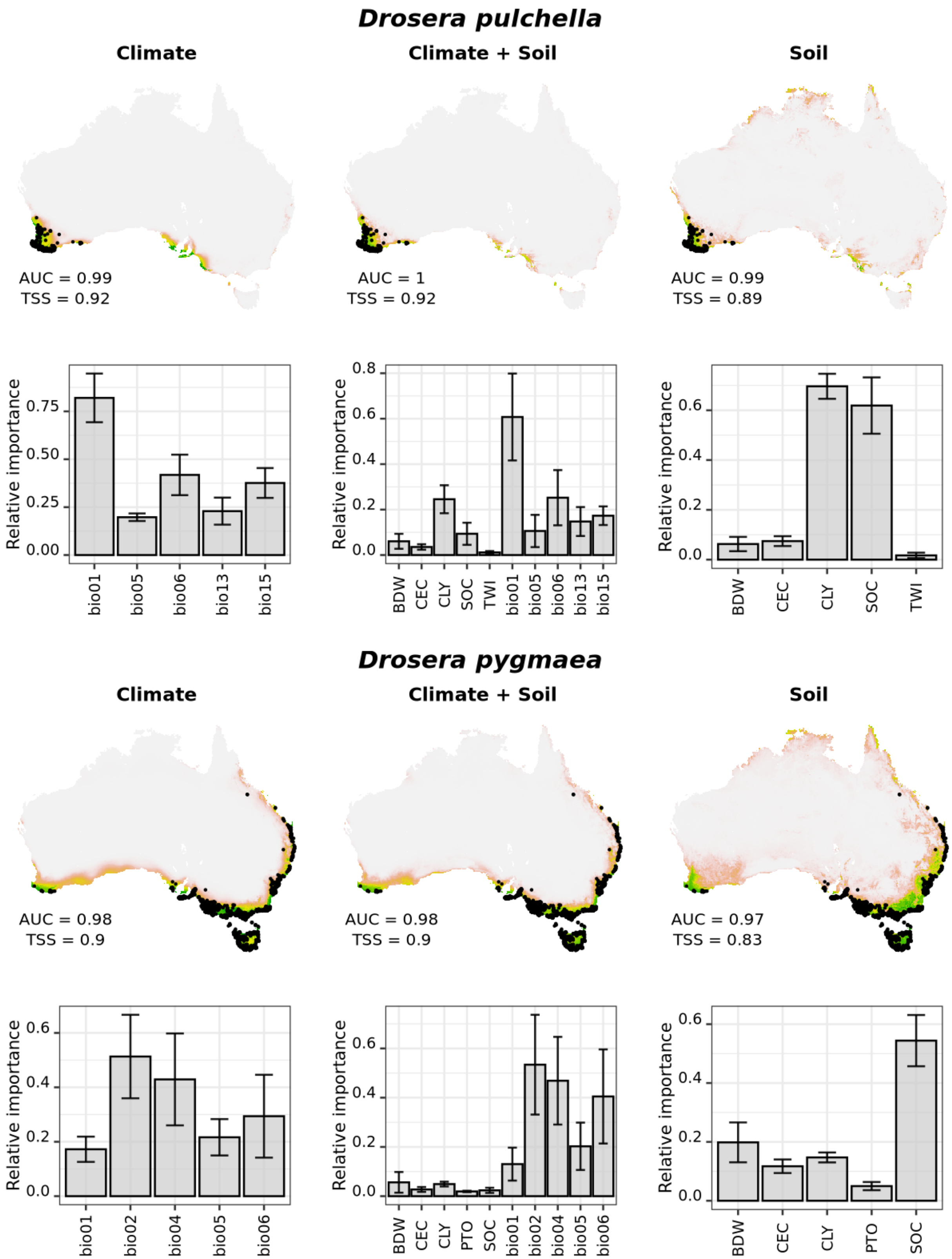
Drosera pulchella
Climate
Climate + Soil
Soil
AUC = 0.99
TSS = 0.92
AUC = 1
TSS = 0.92
AUC = 0.99
TSS = 0.89
Relative importance
0.00
0.25
0.50
0.75
bio01
bio05
bio06
bio13
bio15
0.0
0.2
0.4
0.6
0.8
BDW
CEC
CLY
SOC
TWI
bio01
bio05
bio06
bio13
bio15
0.0
0.2
0.4
0.6
BDW
CEC
CLY
SOC
TWI
Drosera pygmaea
Climate
Climate + Soil
Soil
AUC = 0.98
TSS = 0.9
AUC = 0.98
TSS = 0.9
AUC = 0.97
TSS = 0.83
Relative importance
0.0
0.2
0.4
0.6
bio01
bio02
bio04
bio05
bio06
BDW
CEC
CLY
PTO
SOC
bio01
bio02
bio04
bio05
bio06
BDW
CEC
CLY
PTO
SOC

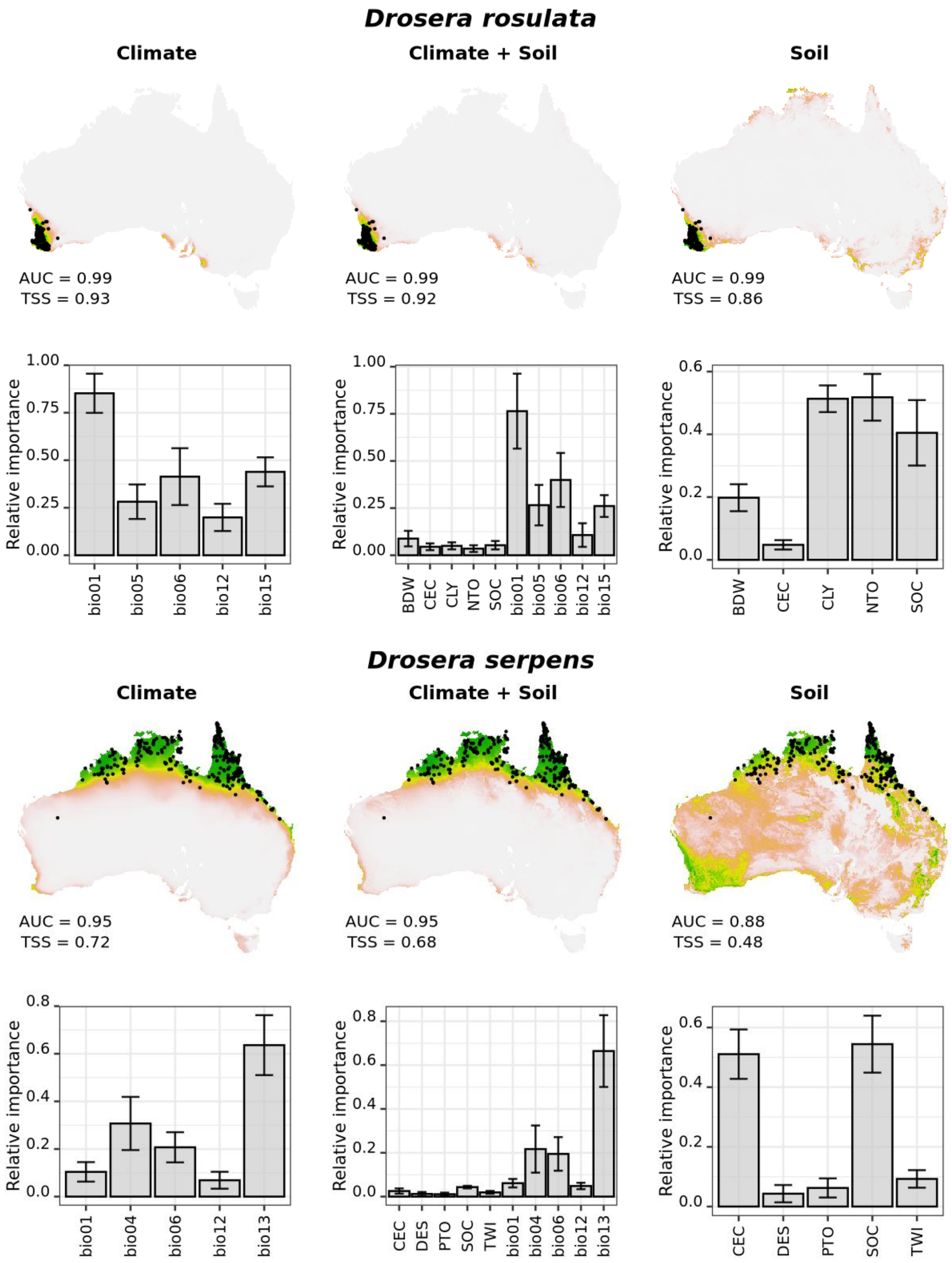
Drosera rosulata
Climate
Climate + Soil
Soil
AUC = 0.99
TSS = 0.93
AUC = 0.99
TSS = 0.92
AUC = 0.99
TSS = 0.86
Relative importance
1.00
0.75
0.50
0.25
0.00
bio01
bio05
bio06
bio12
bio15
BDW
CEC
CLY
NTO
SOC
0.6
0.4
0.2
0.0
Drosera serpens
Climate
Climate + Soil
Soil
AUC = 0.95
TSS = 0.72
AUC = 0.95
TSS = 0.68
AUC = 0.88
TSS = 0.48
0.8
0.6
0.4
0.2
0.0
bio01
bio04
bio06
bio12
bio13
CEC
DES
PTO
SOC
TWI

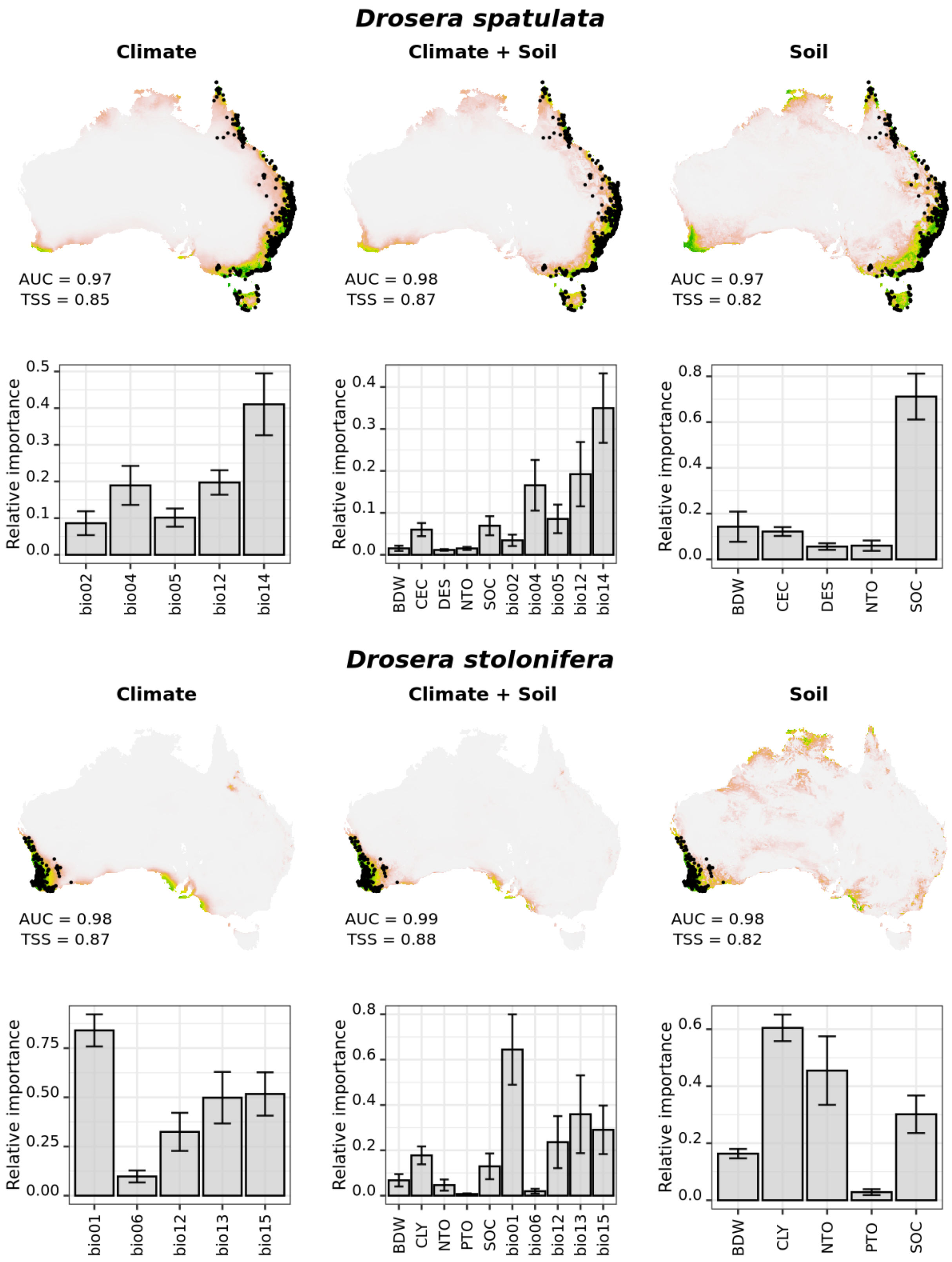

Drosera spatulata
Climate
Climate + Soil
Soil
AUC = 0.97
TSS = 0.85
AUC = 0.98
TSS = 0.87
AUC = 0.97
TSS = 0.82
Relative importance
bio02
bio04
bio05
bio12
bio14
BDW
CEC
DES
NTO
SOC
Drosera stolonifera
Climate
Climate + Soil
Soil
AUC = 0.98
TSS = 0.87
AUC = 0.99
TSS = 0.88
AUC = 0.98
TSS = 0.82
bio01
bio06
bio13
bio15
CLY
PTO

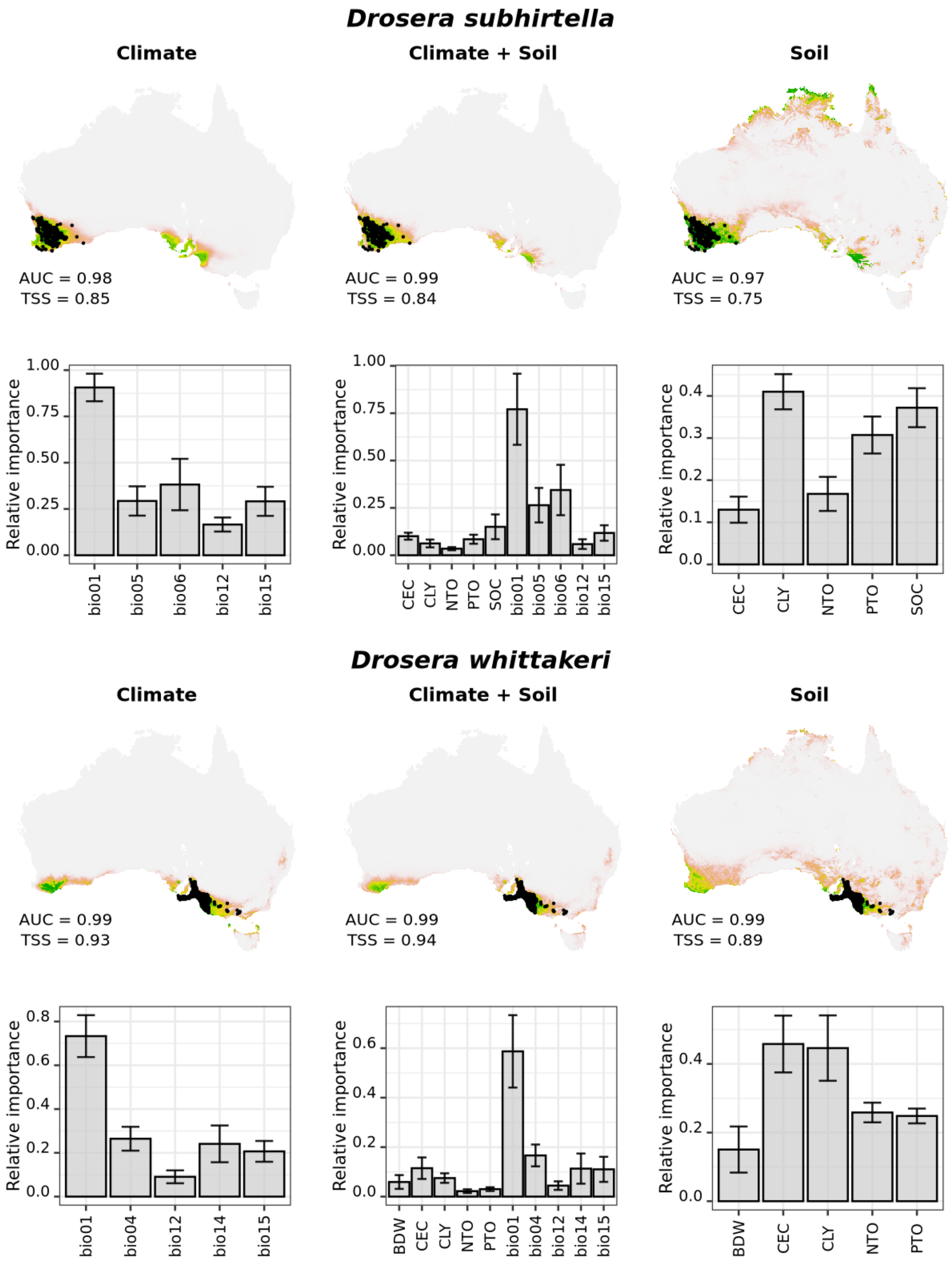
Drosera subhirtella
Climate
Climate + Soil
Soil
AUC = 0.98
TSS = 0.85
AUC = 0.99
TSS = 0.84
AUC = 0.97
TSS = 0.75
Relative importance
1.00
0.75
0.50
0.25
0.00
bio01
bio05
bio06
bio12
bio15
CEC
CLY
NTO
PTO
SOC
0.4
0.3
0.2
0.1
0.0
Drosera whittakeri
Climate
Climate + Soil
Soil
AUC = 0.99
TSS = 0.93
AUC = 0.99
TSS = 0.94
AUC = 0.99
TSS = 0.89
0.8
0.6
0.4
0.2
0.0
bio01
bio04
bio12
bio14
bio15
BDW
CEC
CLY
NTO
PTO

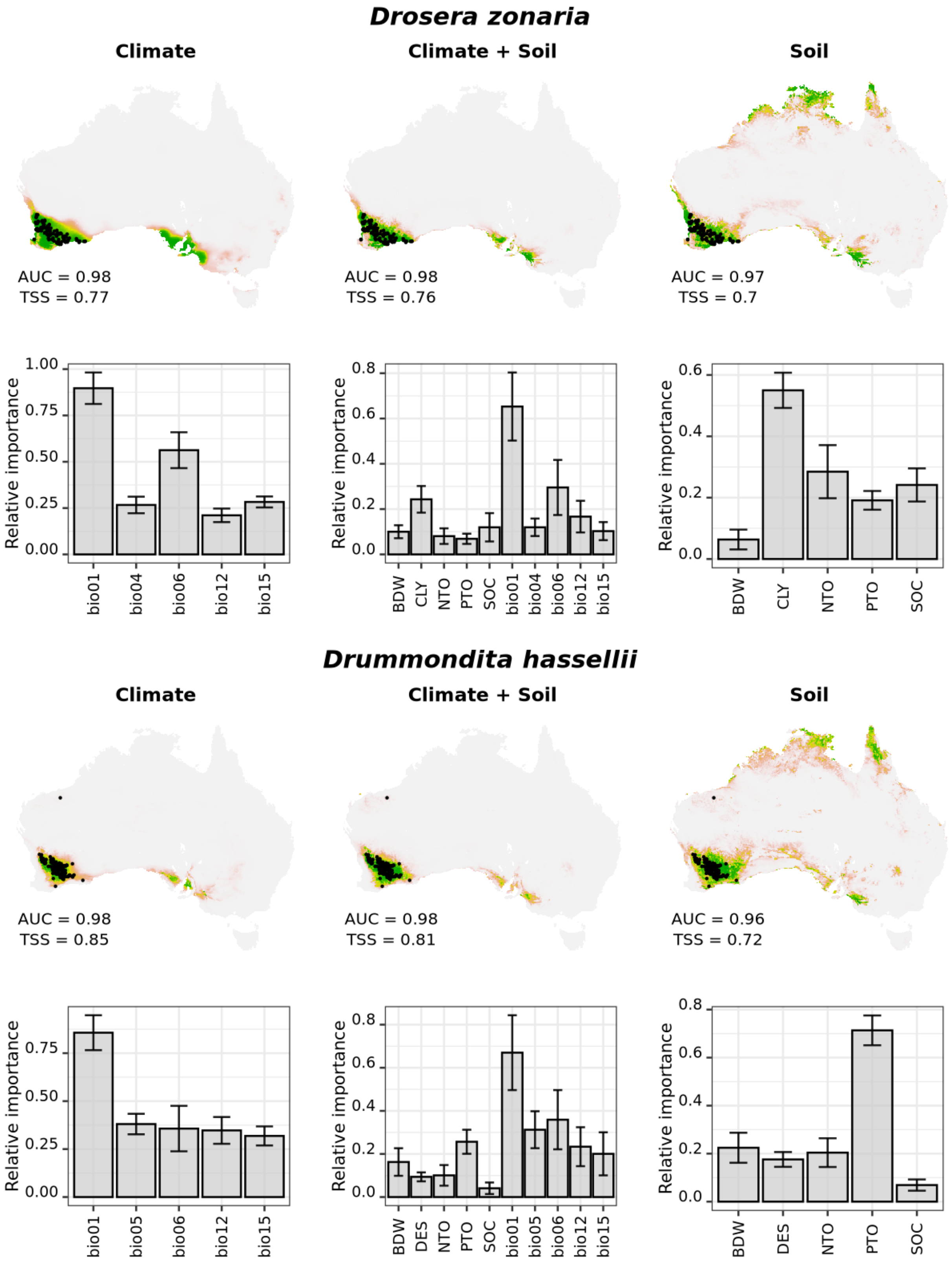
Drosera zonaria
Climate
Climate + Soil
Soil
AUC = 0.98
TSS = 0.77
AUC = 0.98
TSS = 0.76
AUC = 0.97
TSS = 0.7
Relative importance
1.00
0.75
0.50
0.25
0.00
bio01
bio04
bio06
bio12
bio15
0.8
0.6
0.4
0.2
0.0
BDW
CLY
NTO
PTO
SOC
bio01
bio04
bio06
bio12
bio15
0.6
0.4
0.2
0.0
BDW
CLY
NTO
PTO
SOC
Drummondita hassellii
Climate
Climate + Soil
Soil
AUC = 0.98
TSS = 0.85
AUC = 0.98
TSS = 0.81
AUC = 0.96
TSS = 0.72
Relative importance
0.75
0.50
0.25
0.00
bio01
bio05
bio06
bio12
bio15
0.8
0.6
0.4
0.2
0.0
BDW
DES
NTO
PTO
SOC
bio01
bio05
bio06
bio12
bio15
0.8
0.6
0.4
0.2
0.0
BDW
DES
NTO
PTO
SOC

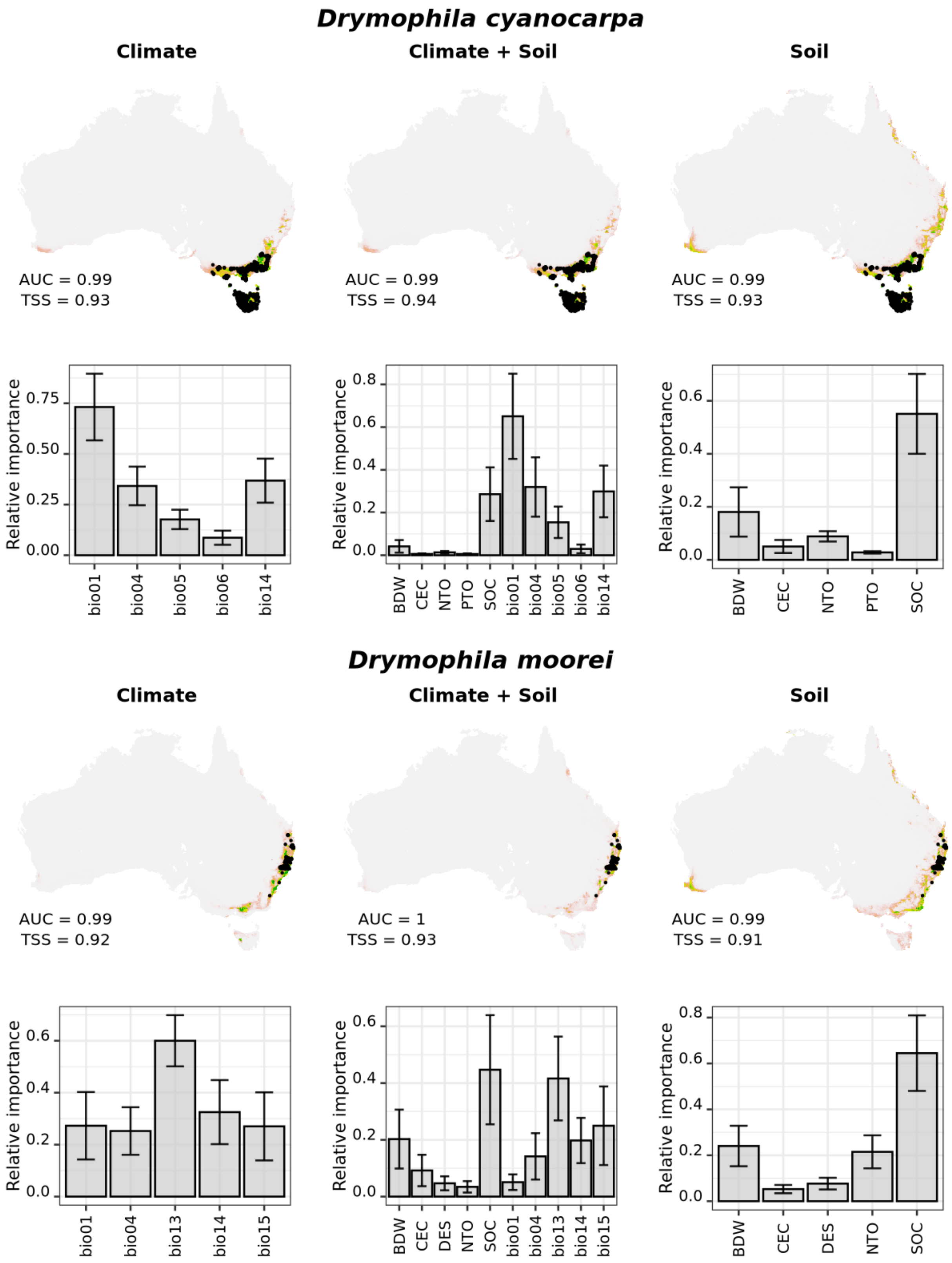
Drymophila cyanocarpa
Climate
Climate + Soil
Soil
AUC = 0.99
TSS = 0.93
AUC = 0.99
TSS = 0.94
AUC = 0.99
TSS = 0.93
Relative importance
0.00
0.25
0.50
0.75
bio01
bio04
bio05
bio06
bio14
0.0
0.2
0.4
0.6
0.8
BDW
CEC
NTO
PTO
SOC
bio01
bio04
bio05
bio06
bio14
0.0
0.2
0.4
0.6
BDW
CEC
NTO
PTO
SOC
Drymophila moorei
Climate
Climate + Soil
Soil
AUC = 0.99
TSS = 0.92
AUC = 1
TSS = 0.93
AUC = 0.99
TSS = 0.91
Relative importance
0.0
0.2
0.4
0.6
bio01
bio04
bio13
bio14
bio15
0.0
0.2
0.4
0.6
BDW
CEC
DES
NTO
SOC
bio01
bio04
bio13
bio14
bio15
0.0
0.2
0.4
0.6
0.8
BDW
CEC
DES
NTO
SOC

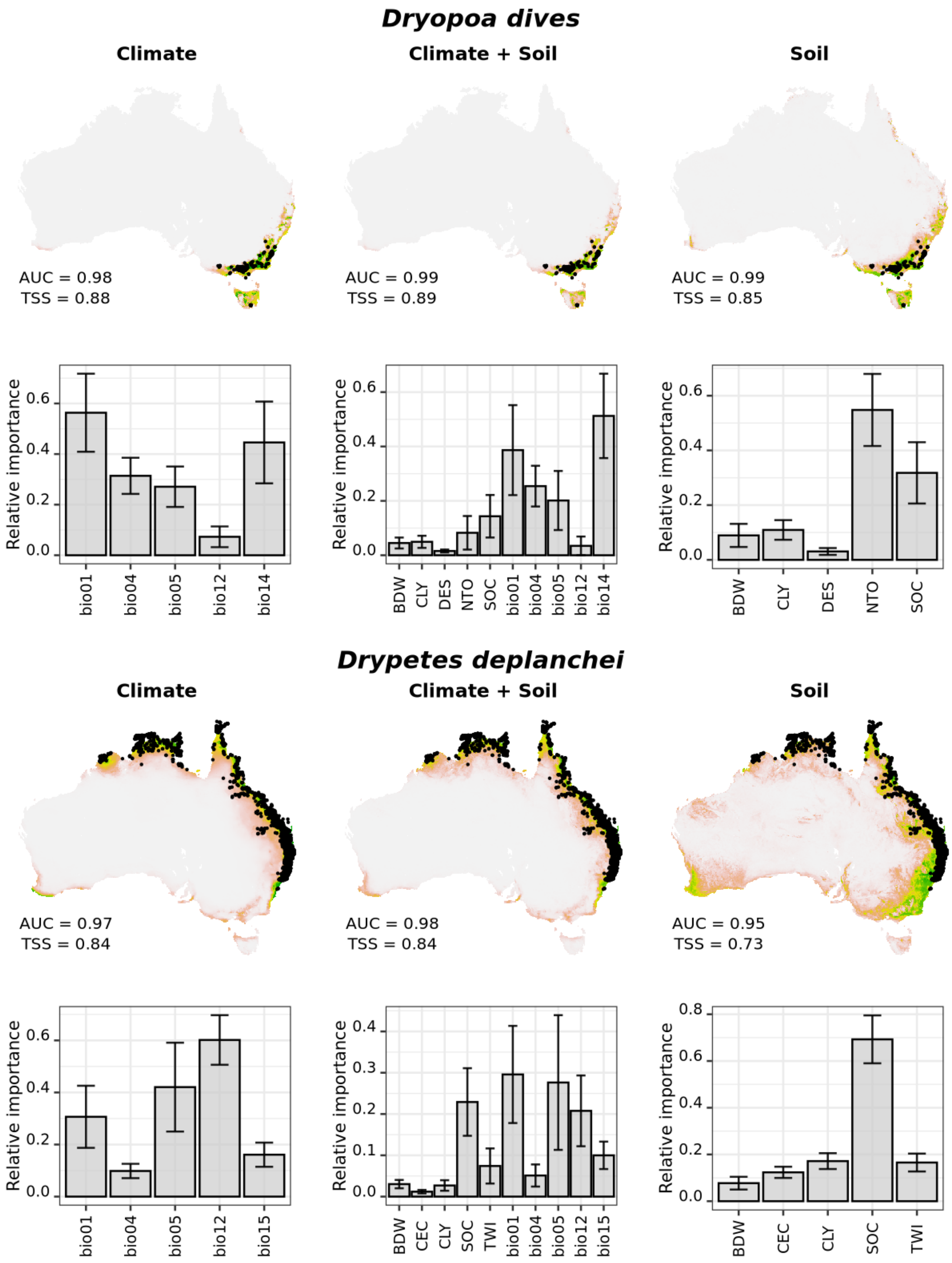
Dryopoa dives
Climate
Climate + Soil
Soil
AUC = 0.98
TSS = 0.88
AUC = 0.99
TSS = 0.89
AUC = 0.99
TSS = 0.85
Relative importance
0.0
0.2
0.4
0.6
bio01
bio04
bio05
bio12
bio14
BDW
CLY
DES
NTO
SOC
Drypetes deplanchei
Climate
Climate + Soil
Soil
AUC = 0.97
TSS = 0.84
AUC = 0.98
TSS = 0.84
AUC = 0.95
TSS = 0.73
0.1
0.3
0.8
bio15
CEC
TWI

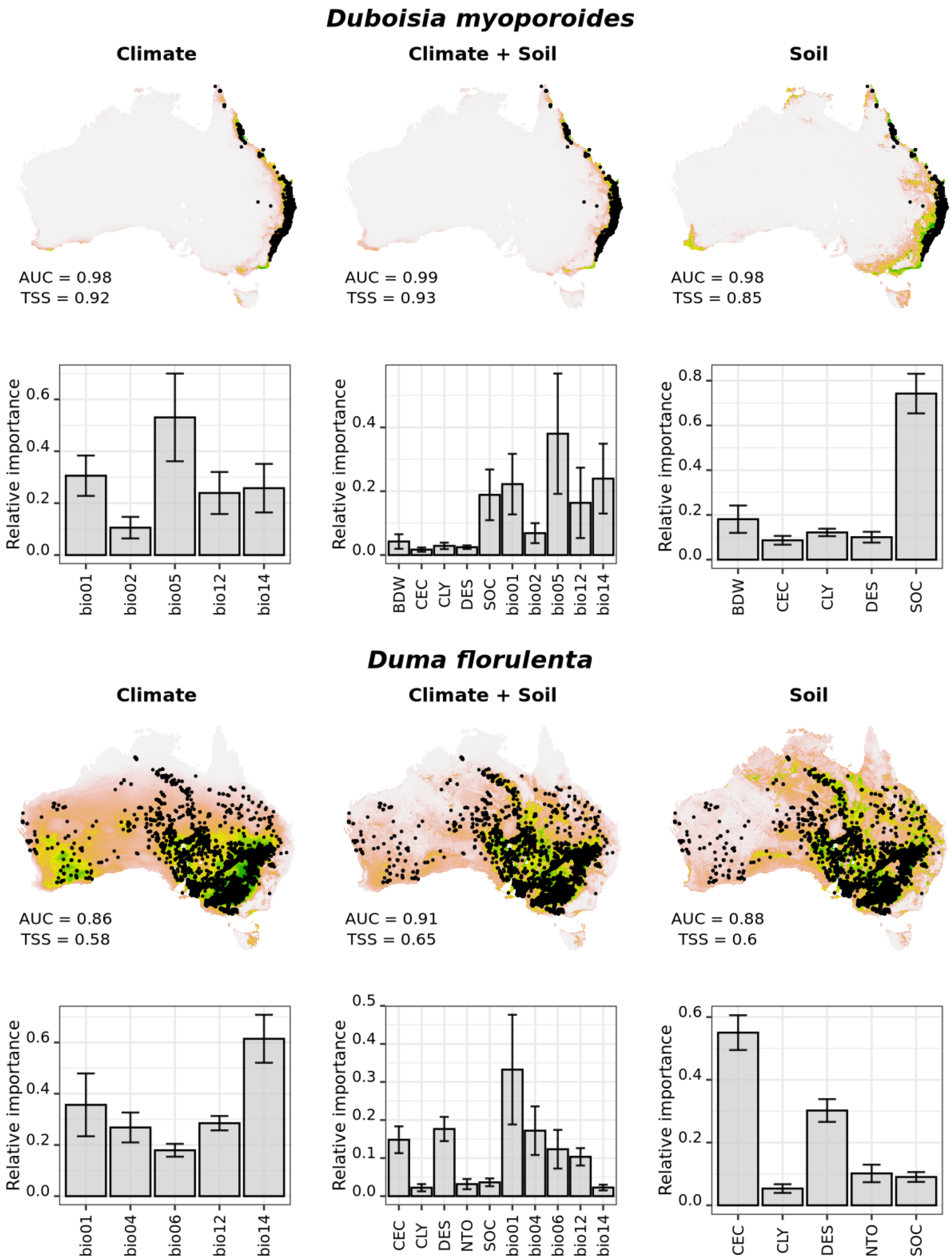

Duboisia myoporoides
Climate
Climate + Soil
Soil
AUC = 0.98
TSS = 0.92
AUC = 0.99
TSS = 0.93
AUC = 0.98
TSS = 0.85
Relative importance
0.0
0.2
0.4
0.6
0.8
bio01
bio02
bio05
bio12
bio14
BDW
CEC
CLY
DES
SOC
Duma florulenta
Climate
Climate + Soil
Soil
AUC = 0.86
TSS = 0.58
AUC = 0.91
TSS = 0.65
AUC = 0.88
TSS = 0.6
Relative importance
0.0
0.1
0.2
0.3
0.4
0.5
0.6
bio01
bio04
bio06
bio12
bio14
CEC
CLY
DES
NTO
SOC

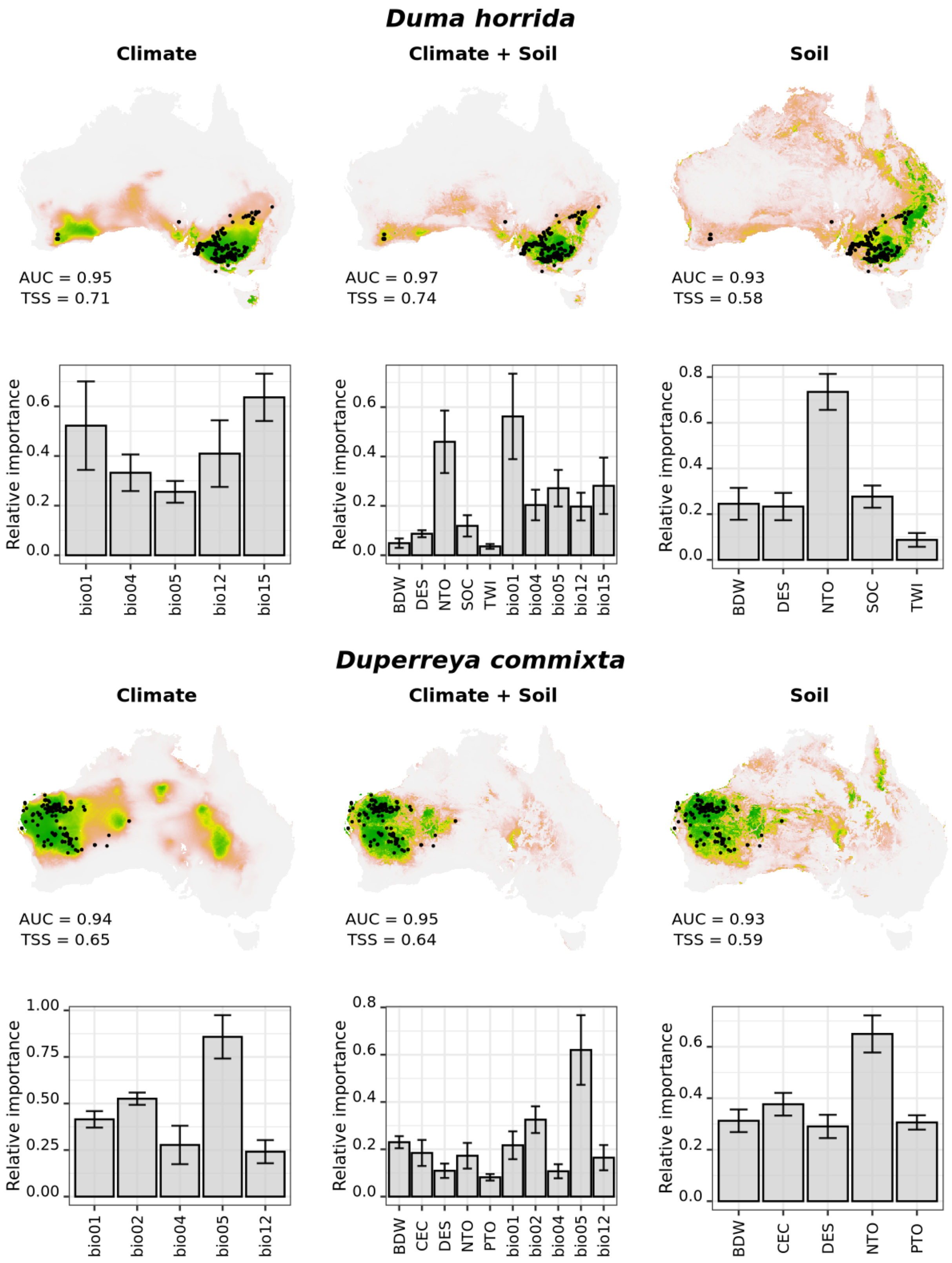

Duma horrida
Climate
Climate + Soil
Soil
AUC = 0.95
TSS = 0.71
AUC = 0.97
TSS = 0.74
AUC = 0.93
TSS = 0.58
Relative importance
bio01
bio04
bio05
bio12
bio15
BDW
DES
NTO
SOC
TWI
Duperreya commixta
AUC = 0.94
TSS = 0.65
AUC = 0.95
TSS = 0.64
AUC = 0.93
TSS = 0.59
bio02
CEC
PTO

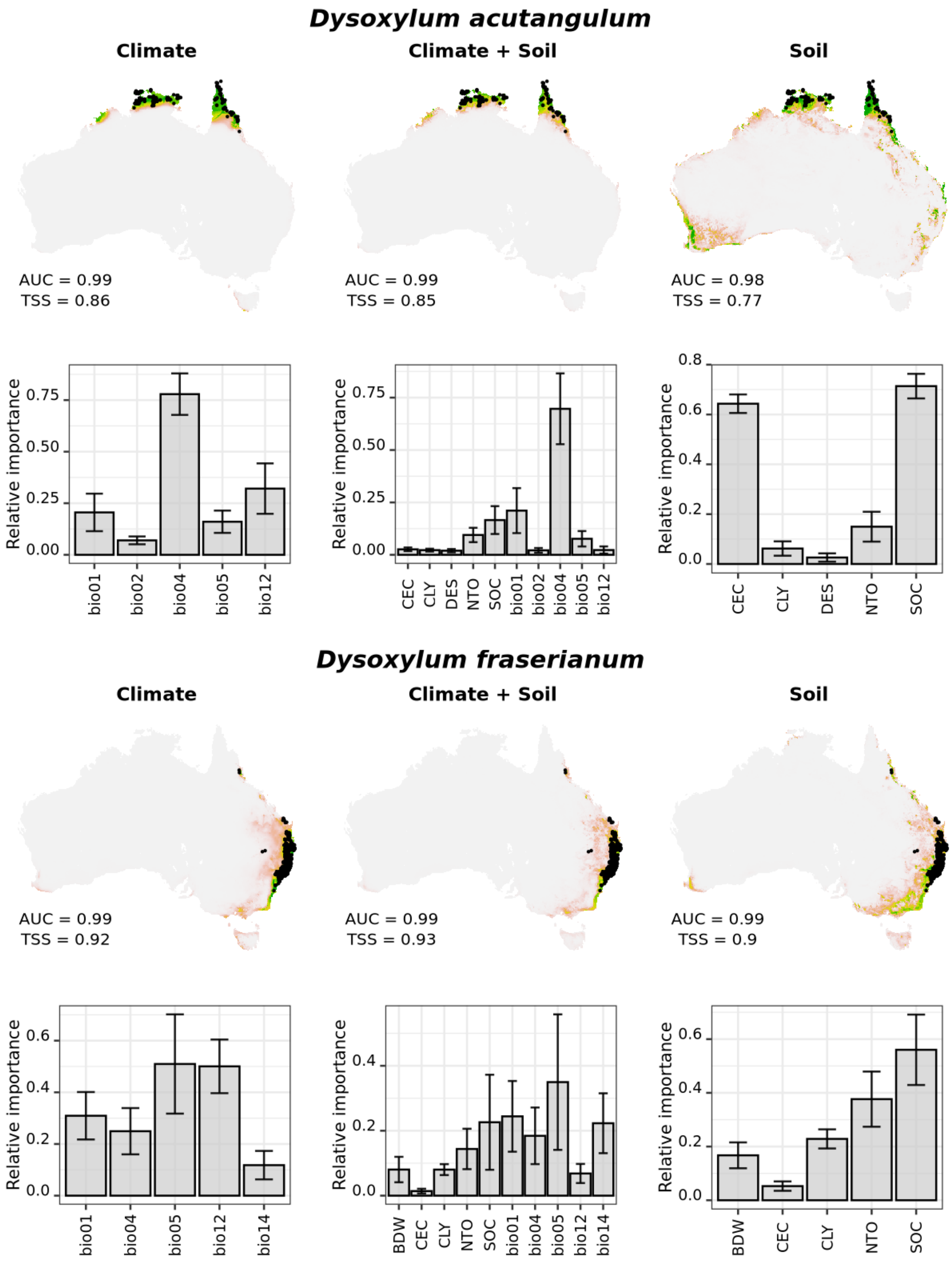

Dysoxylum acutangulum
Climate
Climate + Soil
Soil
AUC = 0.99
TSS = 0.86
AUC = 0.99
TSS = 0.85
AUC = 0.98
TSS = 0.77
Relative importance
0.00
0.25
0.50
0.75
bio01
bio02
bio04
bio05
bio12
CEC
CLY
DES
NTO
SOC
bio01
bio02
bio04
bio05
bio12
0.0
0.2
0.4
0.6
0.8
CEC
CLY
DES
NTO
SOC
Dysoxylum fraserianum
Climate
Climate + Soil
Soil
AUC = 0.99
TSS = 0.92
AUC = 0.99
TSS = 0.93
AUC = 0.99
TSS = 0.9
Relative importance
0.0
0.2
0.4
0.6
bio01
bio04
bio05
bio12
bio14
BDW
CEC
CLY
NTO
SOC
bio01
bio04
bio05
bio12
bio14
BDW
CEC
CLY
NTO
SOC

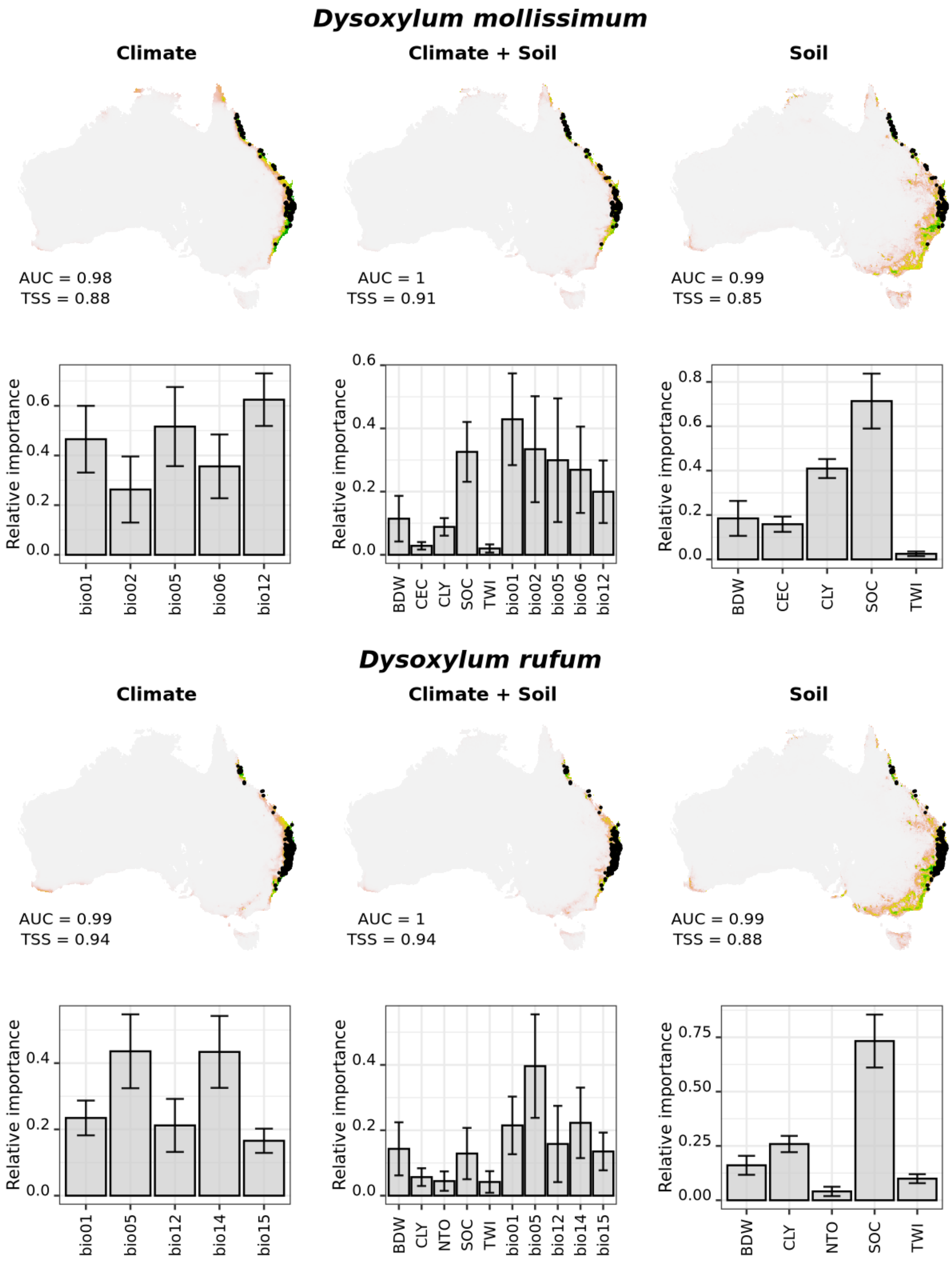

Dysoxylum mollissimum
Climate
Climate + Soil
Soil
AUC = 0.98
TSS = 0.88
AUC = 1
TSS = 0.91
AUC = 0.99
TSS = 0.85
Relative importance
bio01 bio02 bio05 bio06 bio12
BDW CEC CLY SOC TWI bio01 bio02 bio05 bio06 bio12
BDW CEC CLY SOC TWI
Dysoxylum rufum
Climate
Climate + Soil
Soil
AUC = 0.99
TSS = 0.94
AUC = 1
TSS = 0.94
AUC = 0.99
TSS = 0.88
Relative importance
bio01 bio05 bio12 bio14 bio15
BDW CLY NTO SOC TWI bio01 bio05 bio12 bio14 bio15
BDW CLY NTO SOC TWI

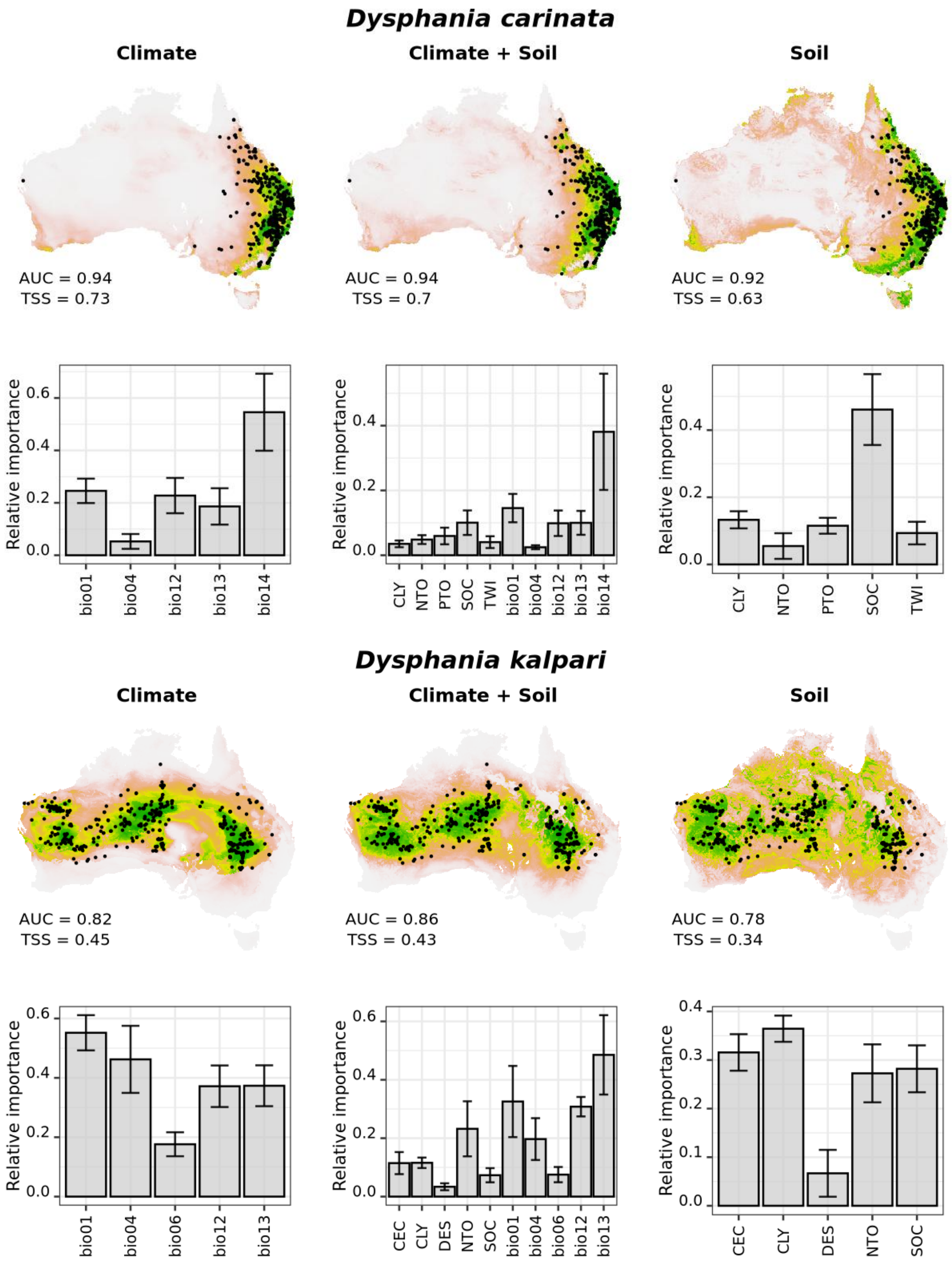
Dysphania carinata
Climate
Climate + Soil
Soil
AUC = 0.94
TSS = 0.73
AUC = 0.94
TSS = 0.7
AUC = 0.92
TSS = 0.63
Relative importance
bio01
bio04
bio12
bio13
bio14
CLY
NTO
PTO
SOC
TWI
Dysphania kalpari
Climate
Climate + Soil
Soil
AUC = 0.82
TSS = 0.45
AUC = 0.86
TSS = 0.43
AUC = 0.78
TSS = 0.34
bio06
CEC
DES

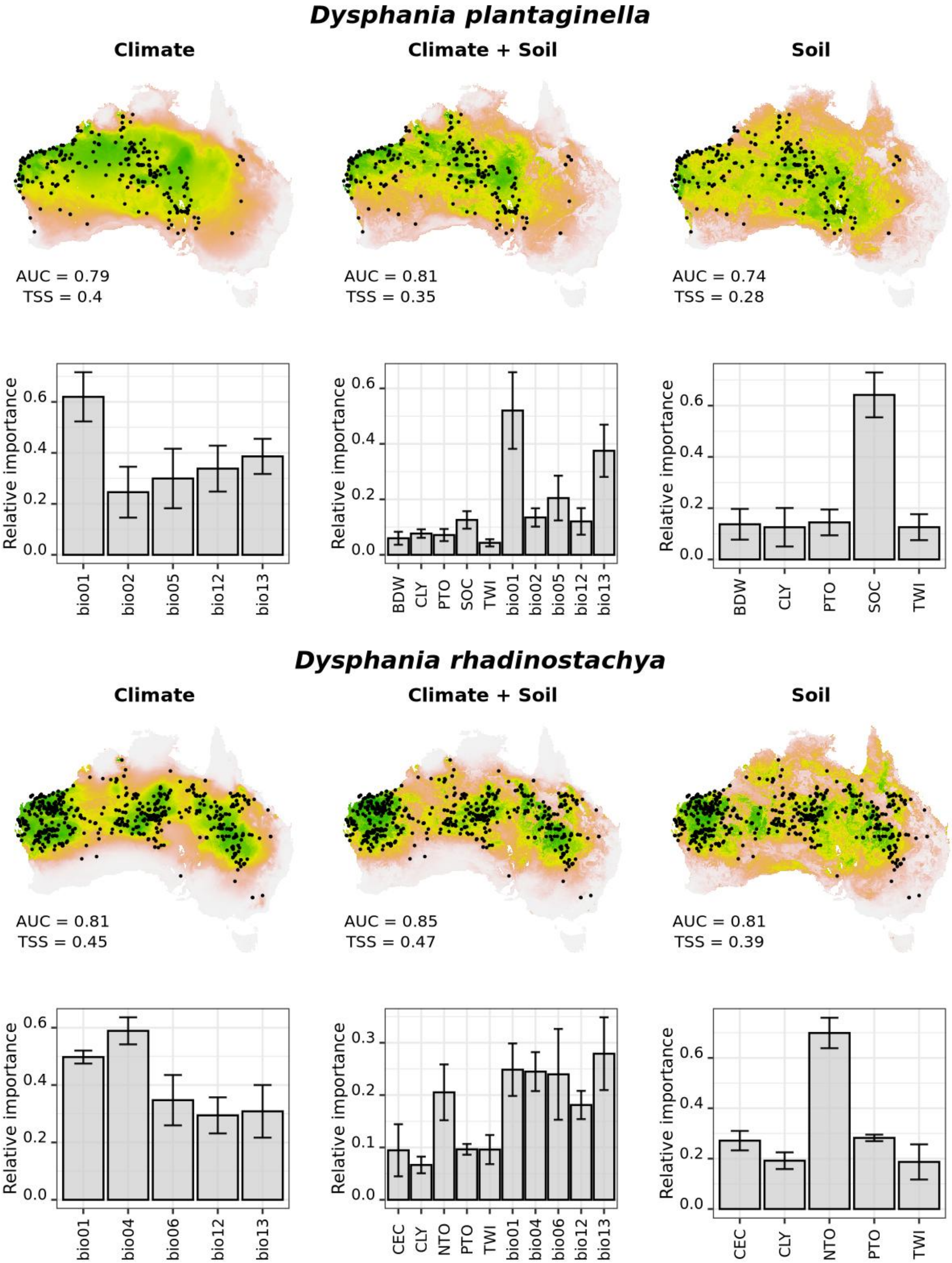
Dysphania plantaginella
Climate
Climate + Soil
Soil
AUC = 0.79
TSS = 0.4
AUC = 0.81
TSS = 0.35
AUC = 0.74
TSS = 0.28
Relative importance
bio01
bio02
bio05
bio12
bio13
BDW
CLY
PTO
SOC
TWI
Dysphania rhadinostachya
Climate
Climate + Soil
Soil
AUC = 0.81
TSS = 0.45
AUC = 0.85
TSS = 0.47
AUC = 0.81
TSS = 0.39
Relative importance
bio01
bio04
bio06
bio12
bio13
CEC
CLY
NTO
PTO
TWI

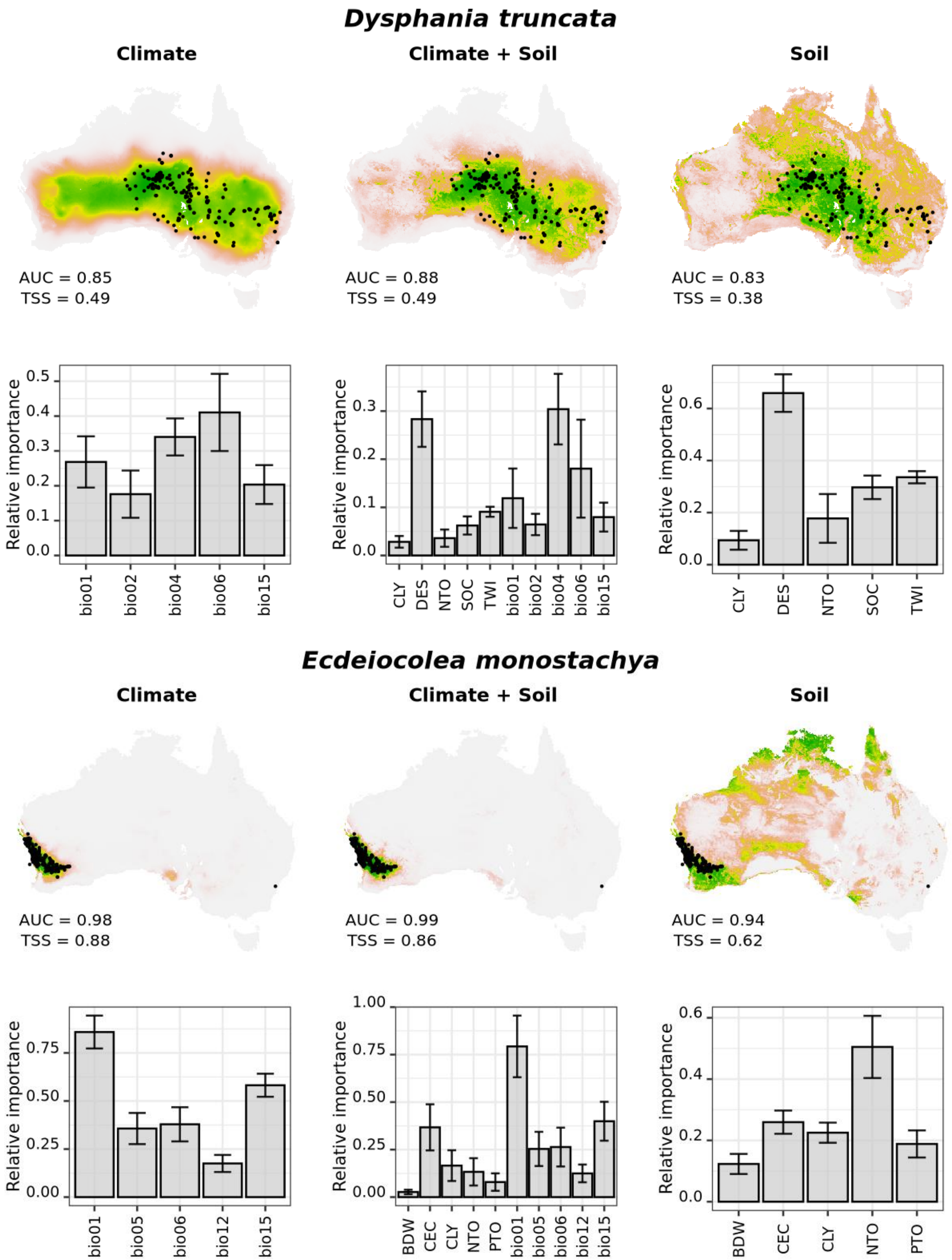
Dysphania truncata
Climate
Climate + Soil
Soil
AUC = 0.85
TSS = 0.49
AUC = 0.88
TSS = 0.49
AUC = 0.83
TSS = 0.38
Relative importance
bio01
bio02
bio04
bio06
bio15
CLY
DES
NTO
SOC
TWI
Ecdeiocolea monostachya
Climate
Climate + Soil
Soil
AUC = 0.98
TSS = 0.88
AUC = 0.99
TSS = 0.86
AUC = 0.94
TSS = 0.62
Relative importance
bio01
bio05
bio06
bio12
bio15
BDW
CEC
CLY
NTO
PTO

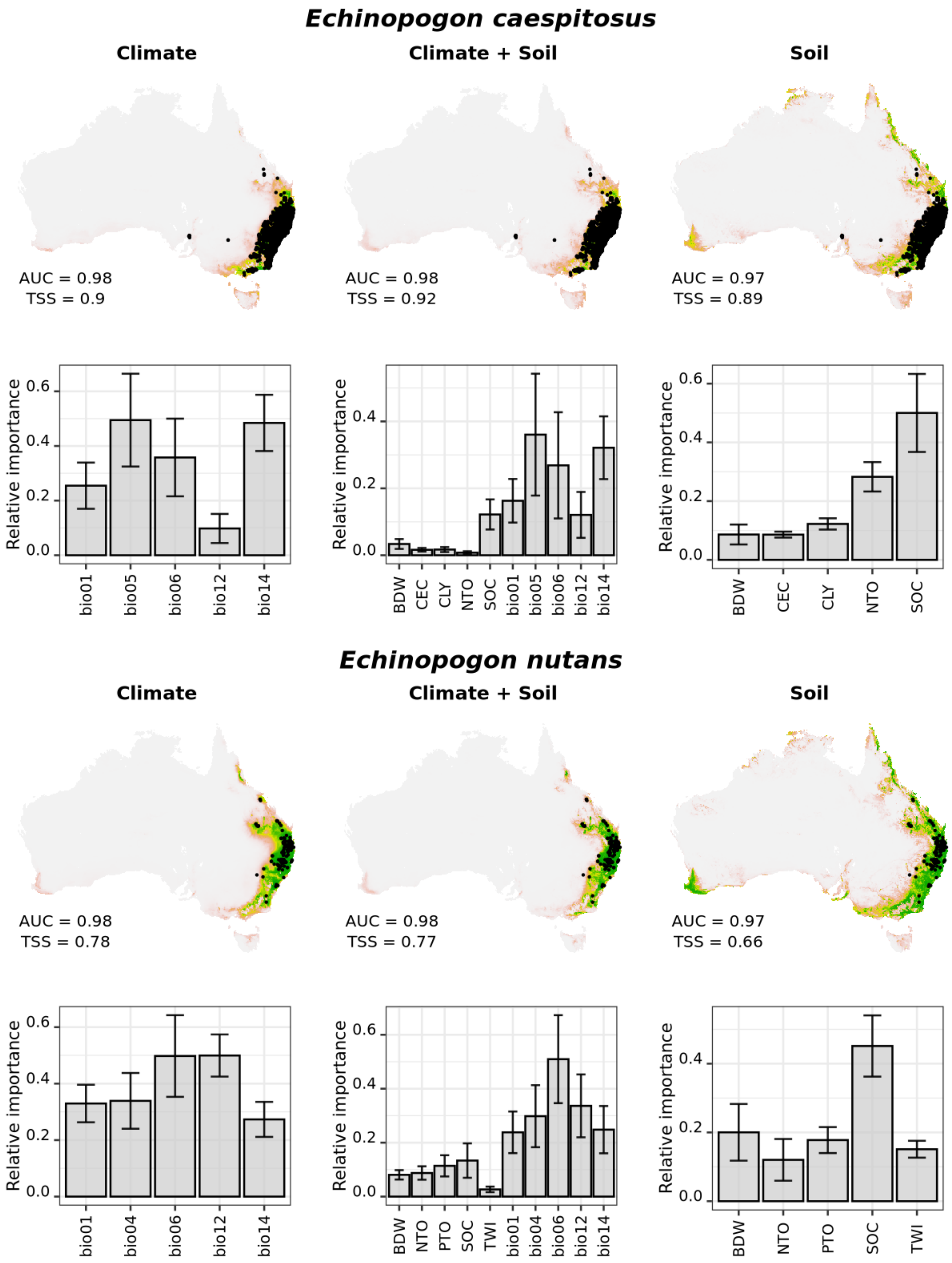
Echinopogon caespitosus
Climate
Climate + Soil
Soil
AUC = 0.98
TSS = 0.9
AUC = 0.98
TSS = 0.92
AUC = 0.97
TSS = 0.89
Relative importance
bio01
bio05
bio06
bio12
bio14
BDW
CEC
CLY
NTO
SOC
Echinopogon nutans
Climate
Climate + Soil
Soil
AUC = 0.98
TSS = 0.78
AUC = 0.98
TSS = 0.77
AUC = 0.97
TSS = 0.66
bio04
PTO
TWI

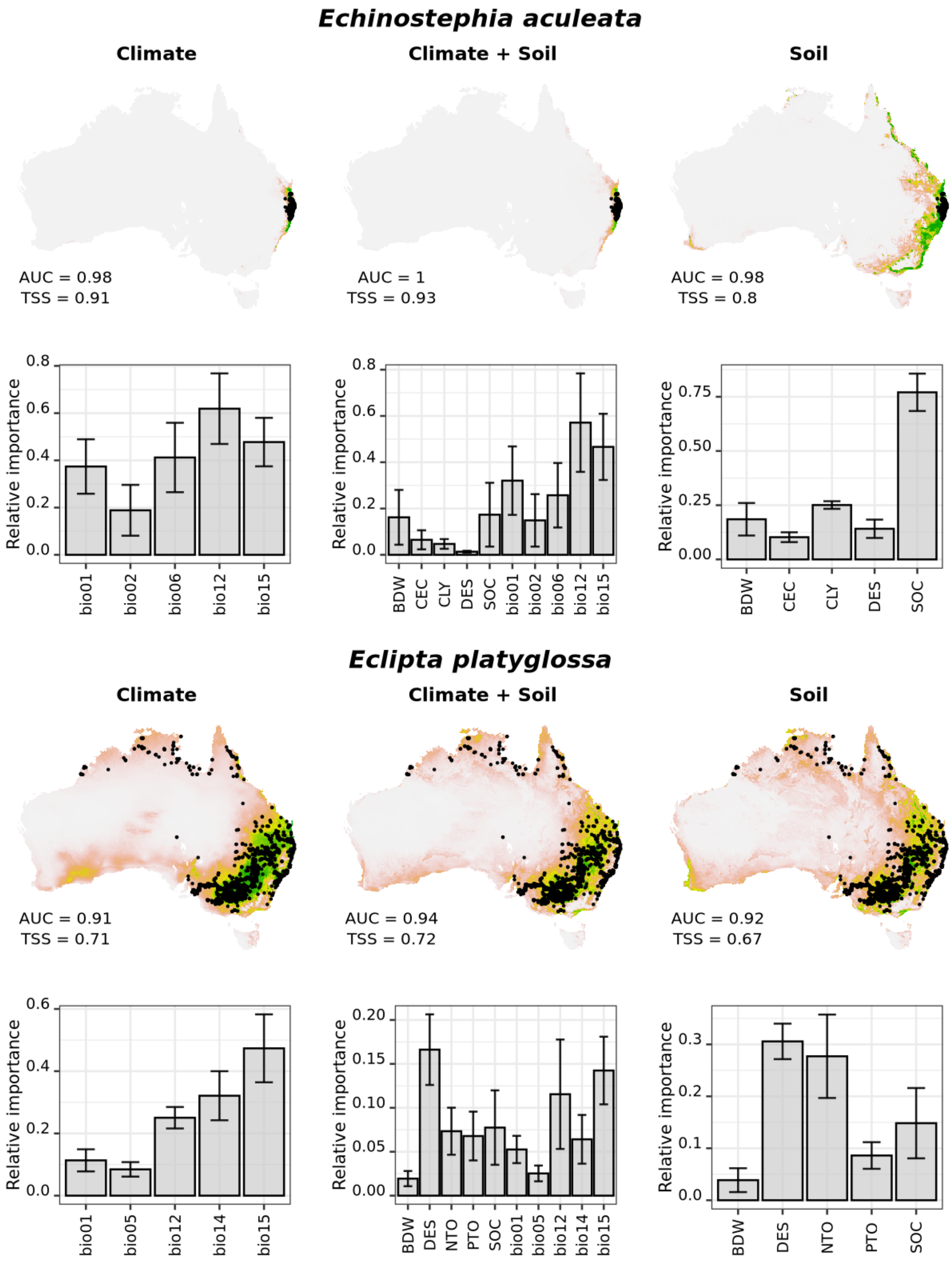
Echinostephia aculeata
Climate
Climate + Soil
Soil
AUC = 0.98
TSS = 0.91
AUC = 1
TSS = 0.93
AUC = 0.98
TSS = 0.8
Relative importance
bio01
bio02
bio06
bio12
bio15
BDW
CEC
CLY
DES
SOC
Eclipta platyglossa
AUC = 0.91
TSS = 0.71
AUC = 0.94
TSS = 0.72
AUC = 0.92
TSS = 0.67
bio05
bio14
NTO
PTO

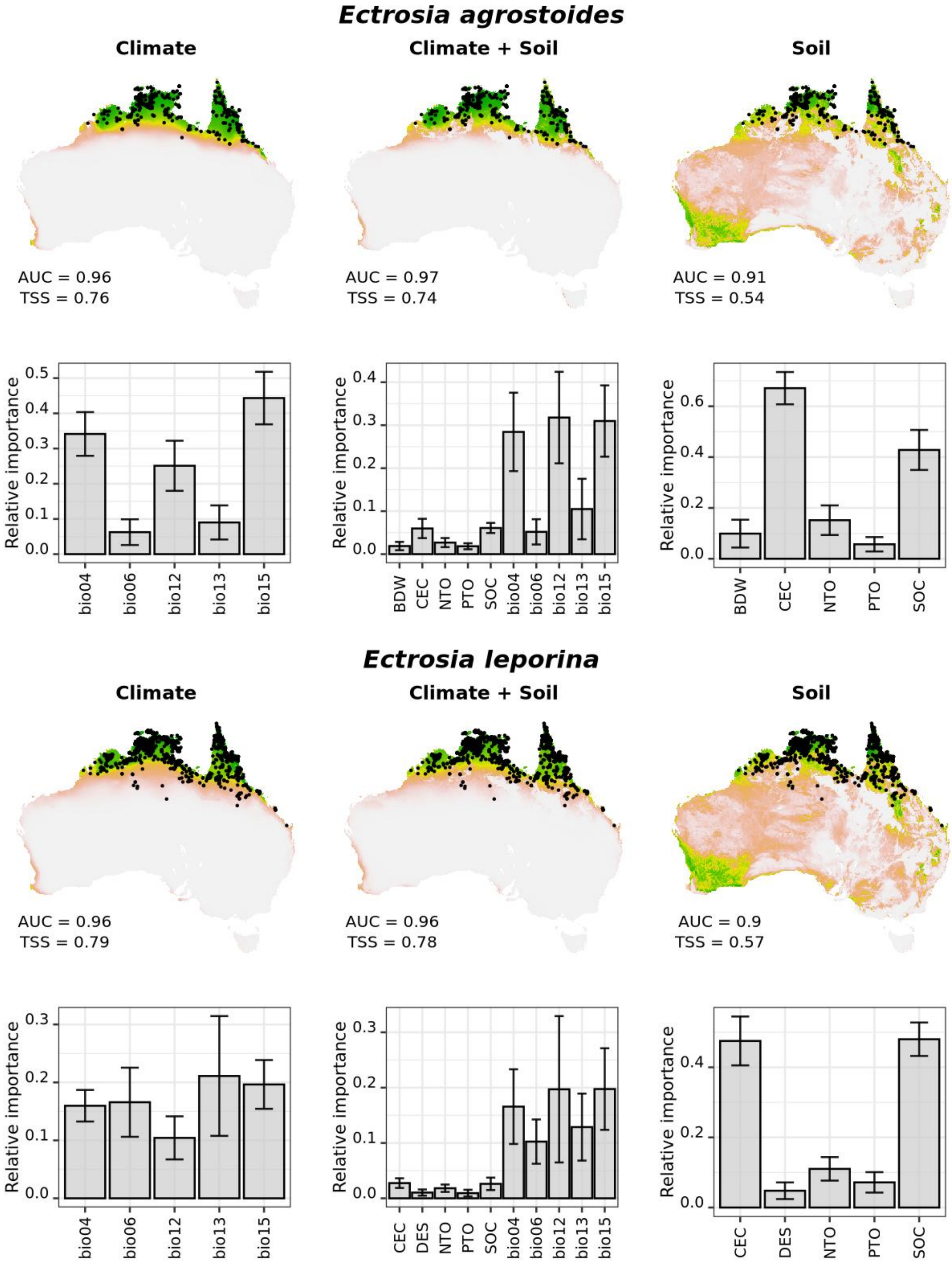
Ectrosia agrostoides
Climate
Climate + Soil
Soil
AUC = 0.96
TSS = 0.76
AUC = 0.97
TSS = 0.74
AUC = 0.91
TSS = 0.54
Relative importance
bio04
bio06
bio12
bio13
bio15
BDW
CEC
NTO
PTO
SOC
Ectrosia leporina
Climate
Climate + Soil
Soil
AUC = 0.96
TSS = 0.79
AUC = 0.96
TSS = 0.78
AUC = 0.9
TSS = 0.57
Relative importance
DES

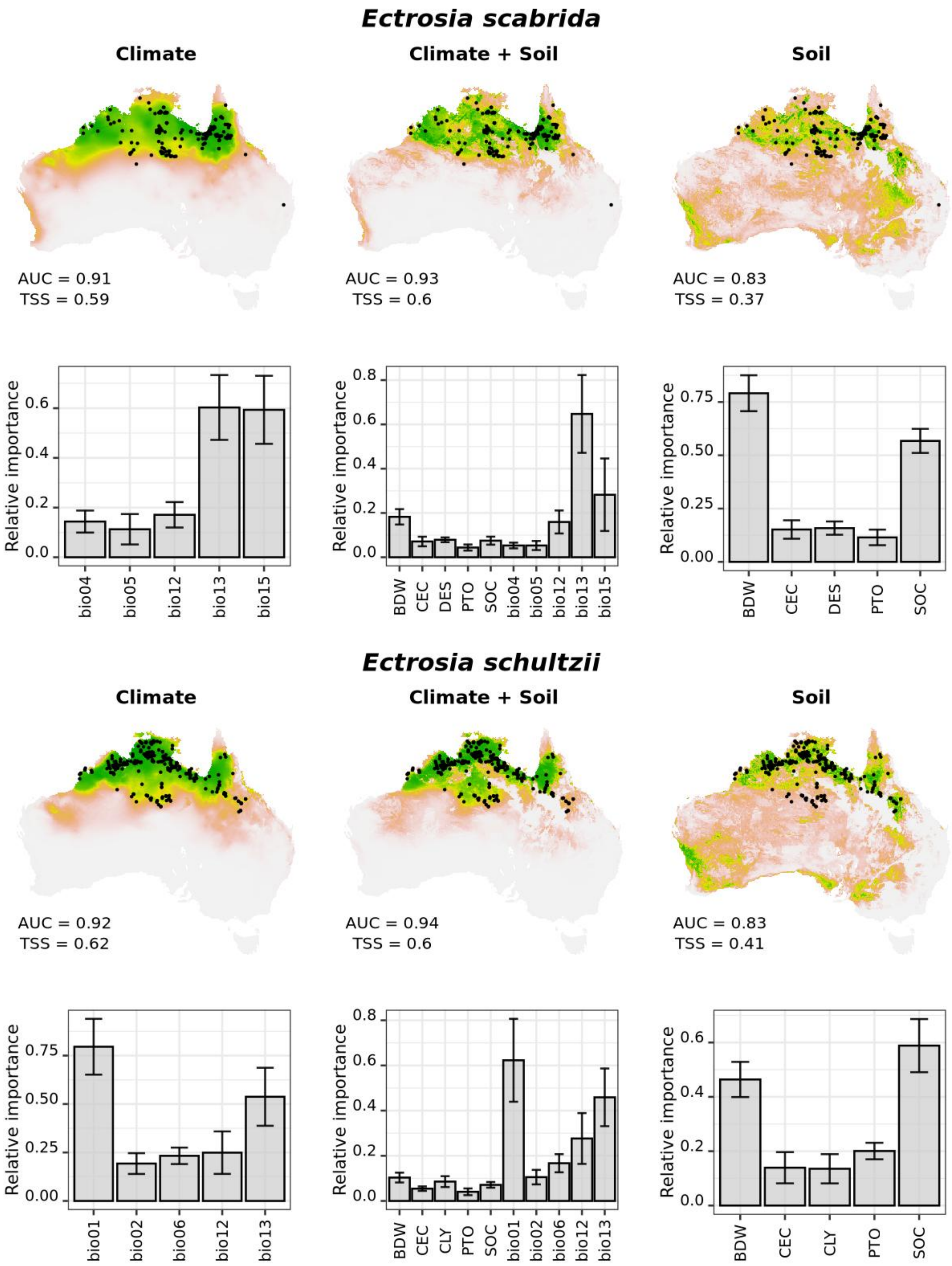
Ectrosia scabrida
Climate
Climate + Soil
Soil
AUC = 0.91
TSS = 0.59
AUC = 0.93
TSS = 0.6
AUC = 0.83
TSS = 0.37
Relative importance
bio04
bio05
bio12
bio13
bio15
BDW
CEC
DES
PTO
SOC
Ectrosia schultzii
Climate
Climate + Soil
Soil
AUC = 0.92
TSS = 0.62
AUC = 0.94
TSS = 0.6
AUC = 0.83
TSS = 0.41
Relative importance
bio01
bio02
bio06
bio12
bio13
BDW
CEC
CLY
PTO
SOC

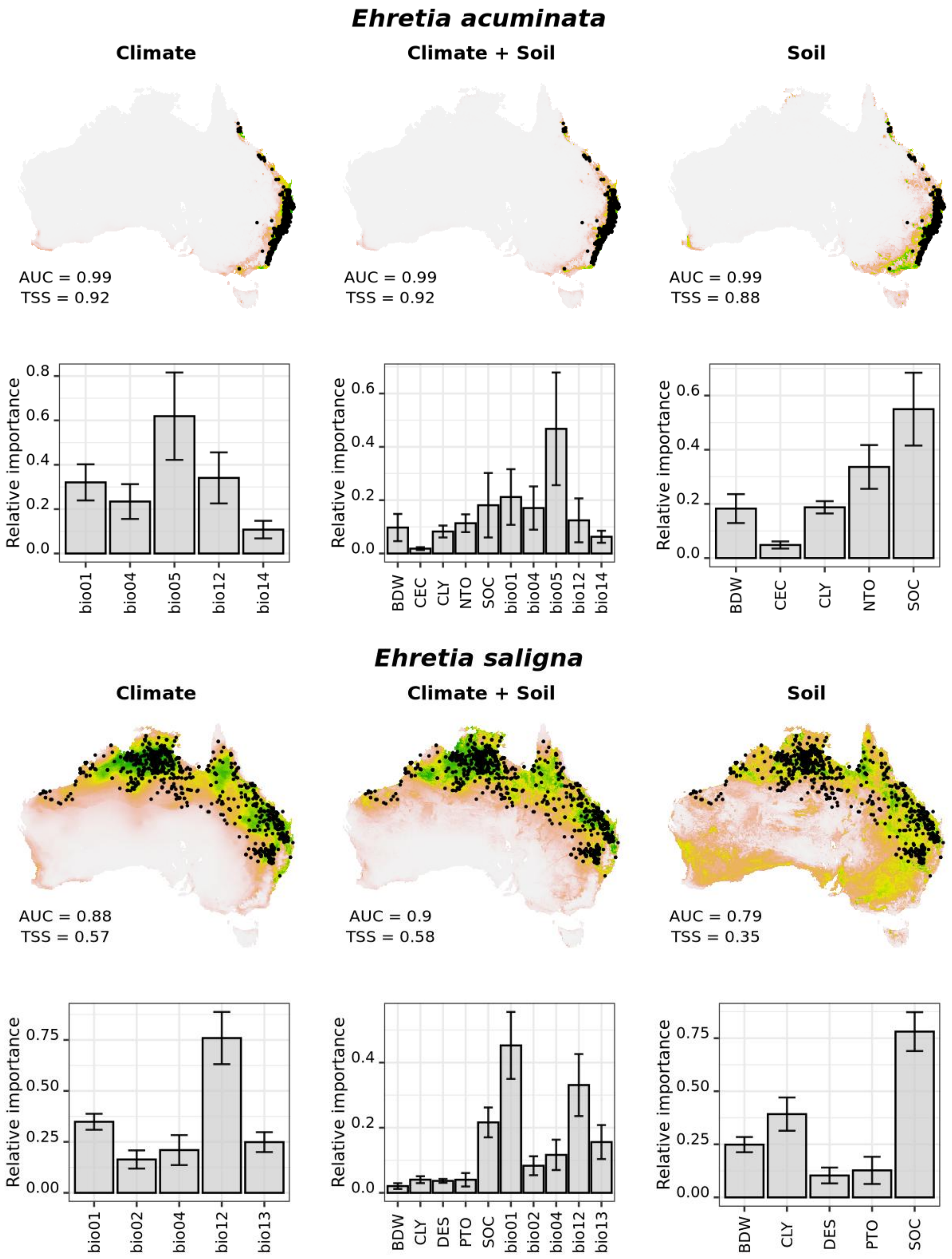
Ehretia acuminata
Climate
Climate + Soil
Soil
AUC = 0.99
TSS = 0.92
AUC = 0.99
TSS = 0.92
AUC = 0.99
TSS = 0.88
Relative importance
0.0 0.2 0.4 0.6 0.8
bio01 bio04 bio05 bio12 bio14
Relative importance
0.0 0.2 0.4 0.6
BDW CEC CLY NTO SOC bio01 bio04 bio05 bio12 bio14
Relative importance
0.0 0.2 0.4 0.6
BDW CEC CLY NTO SOC
Ehretia saligna
Climate
Climate + Soil
Soil
AUC = 0.88
TSS = 0.57
AUC = 0.9
TSS = 0.58
AUC = 0.79
TSS = 0.35
Relative importance
0.00 0.25 0.50 0.75
bio01 bio02 bio04 bio12 bio13
Relative importance
0.0 0.2 0.4
BDW CLY DES PTO SOC bio01 bio02 bio04 bio12 bio13
Relative importance
0.00 0.25 0.50 0.75
BDW CLY DES PTO SOC

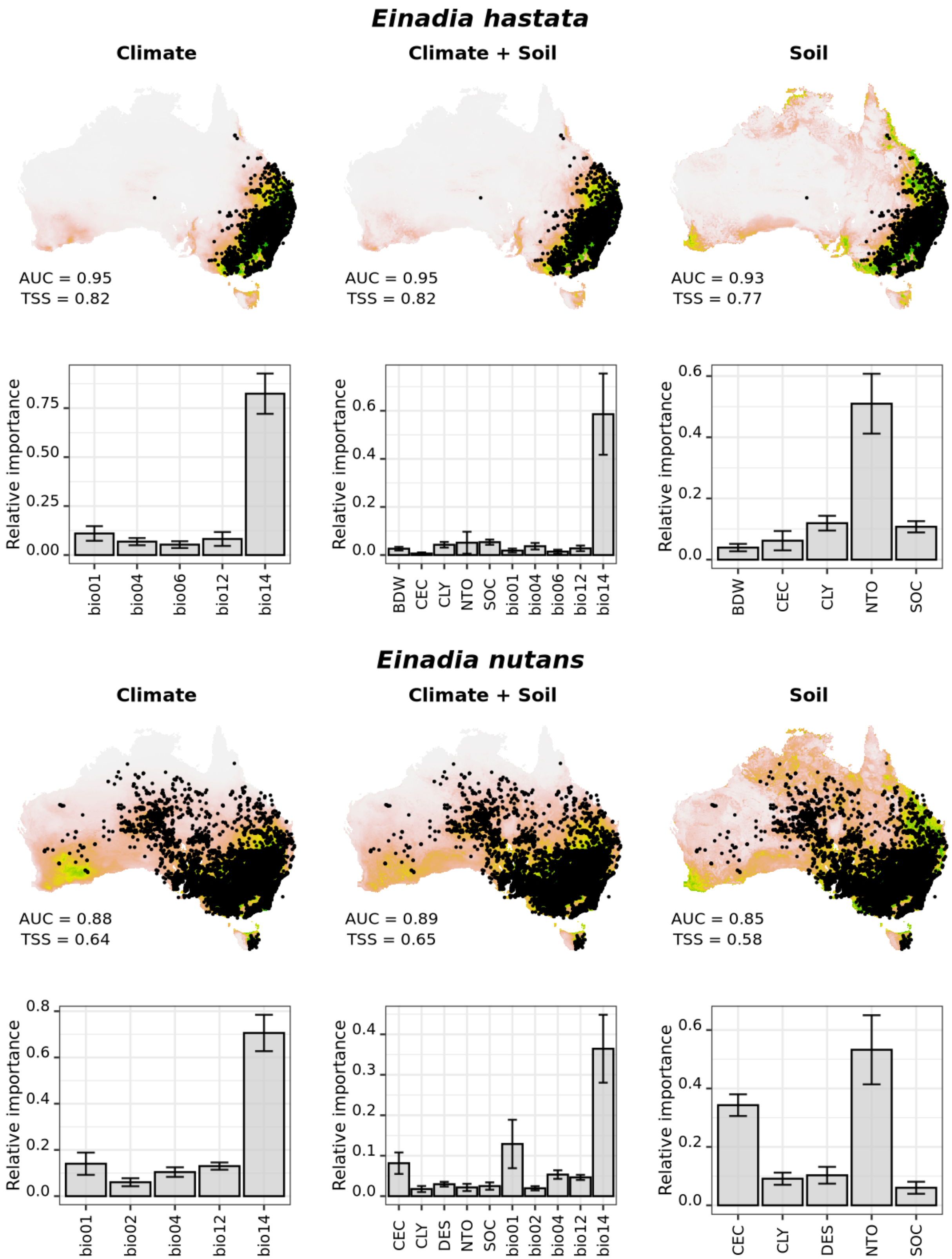
Einadia hastata
Climate
Climate + Soil
Soil
AUC = 0.95
TSS = 0.82
AUC = 0.95
TSS = 0.82
AUC = 0.93
TSS = 0.77
Relative importance
0.00
0.25
0.50
0.75
bio01
bio04
bio06
bio12
bio14
0.0
0.2
0.4
0.6
BDW
CEC
CLY
NTO
SOC
bio01
bio04
bio06
bio12
bio14
0.0
0.2
0.4
0.6
BDW
CEC
CLY
NTO
SOC
Einadia nutans
Climate
Climate + Soil
Soil
AUC = 0.88
TSS = 0.64
AUC = 0.89
TSS = 0.65
AUC = 0.85
TSS = 0.58
0.0
0.2
0.4
0.6
0.8
bio01
bio02
bio04
bio12
bio14
0.0
0.1
0.2
0.3
0.4
CEC
CLY
DES
NTO
SOC
bio01
bio02
bio04
bio12
bio14
0.0
0.2
0.4
0.6
CEC
CLY
DES
NTO
SOC

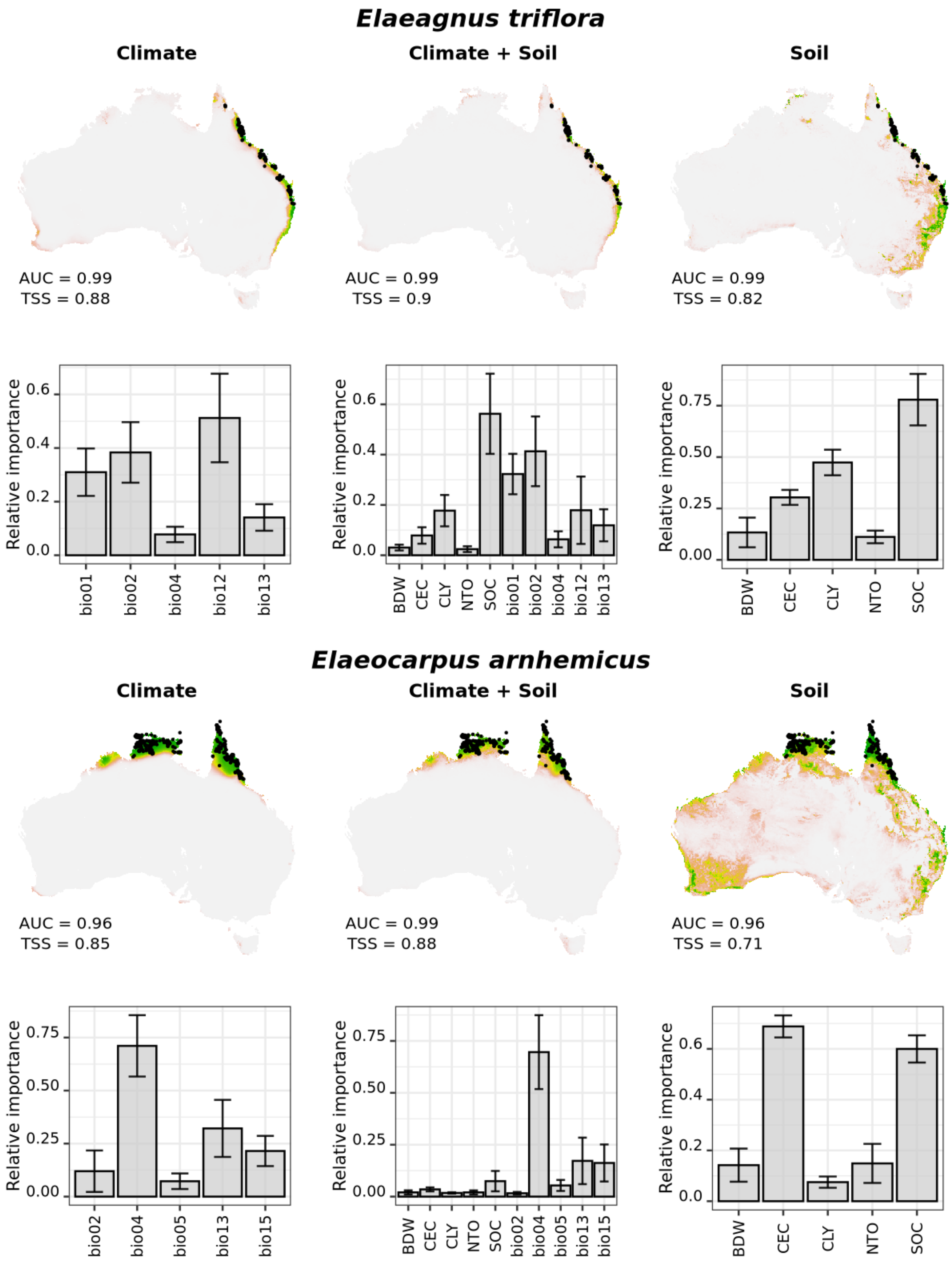

Elaeagnus triflora
Climate
Climate + Soil
Soil
AUC = 0.99
TSS = 0.88
AUC = 0.99
TSS = 0.9
AUC = 0.99
TSS = 0.82
Relative importance
0.0
0.2
0.4
0.6
bio01
bio02
bio04
bio12
bio13
Relative importance
0.0
0.2
0.4
0.6
BDW
CEC
CLY
NTO
SOC
bio01
bio02
bio04
bio12
bio13
Relative importance
0.00
0.25
0.50
0.75
BDW
CEC
CLY
NTO
SOC
Elaeocarpus arnhemicus
Climate
Climate + Soil
Soil
AUC = 0.96
TSS = 0.85
AUC = 0.99
TSS = 0.88
AUC = 0.96
TSS = 0.71
Relative importance
0.00
0.25
0.50
0.75
bio02
bio04
bio05
bio13
bio15
Relative importance
0.00
0.25
0.50
0.75
BDW
CEC
CLY
NTO
SOC
bio02
bio04
bio05
bio13
bio15
Relative importance
0.0
0.2
0.4
0.6
BDW
CEC
CLY
NTO
SOC

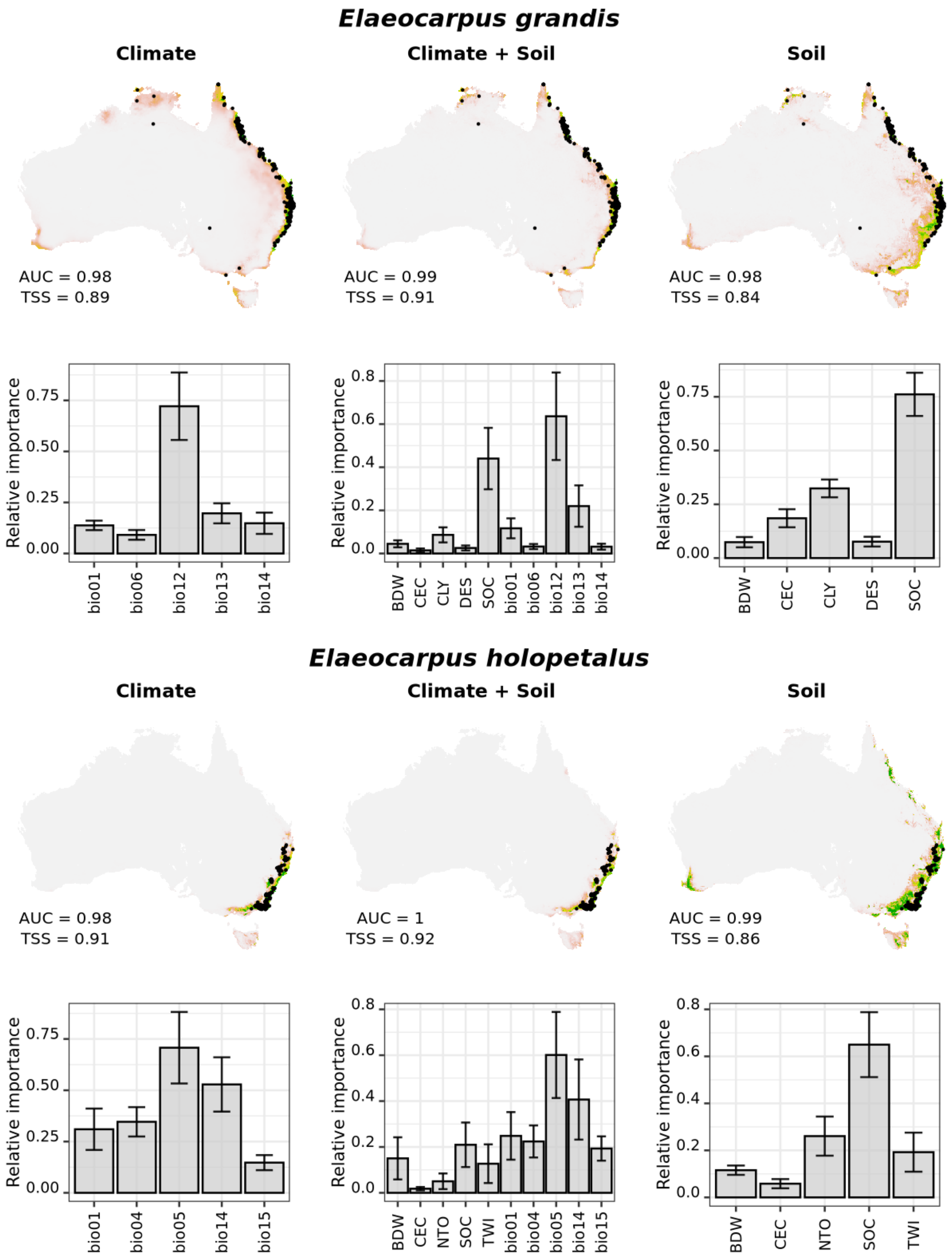
Elaeocarpus grandis
Climate
Climate + Soil
Soil
AUC = 0.98
TSS = 0.89
AUC = 0.99
TSS = 0.91
AUC = 0.98
TSS = 0.84
Relative importance
bio01
bio06
bio12
bio13
bio14
BDW
CEC
CLY
DES
SOC
Elaeocarpus holopetalus
Climate
Climate + Soil
Soil
AUC = 0.98
TSS = 0.91
AUC = 1
TSS = 0.92
AUC = 0.99
TSS = 0.86
Relative importance
bio01
bio04
bio05
bio14
bio15
BDW
CEC
NTO
SOC
TWI

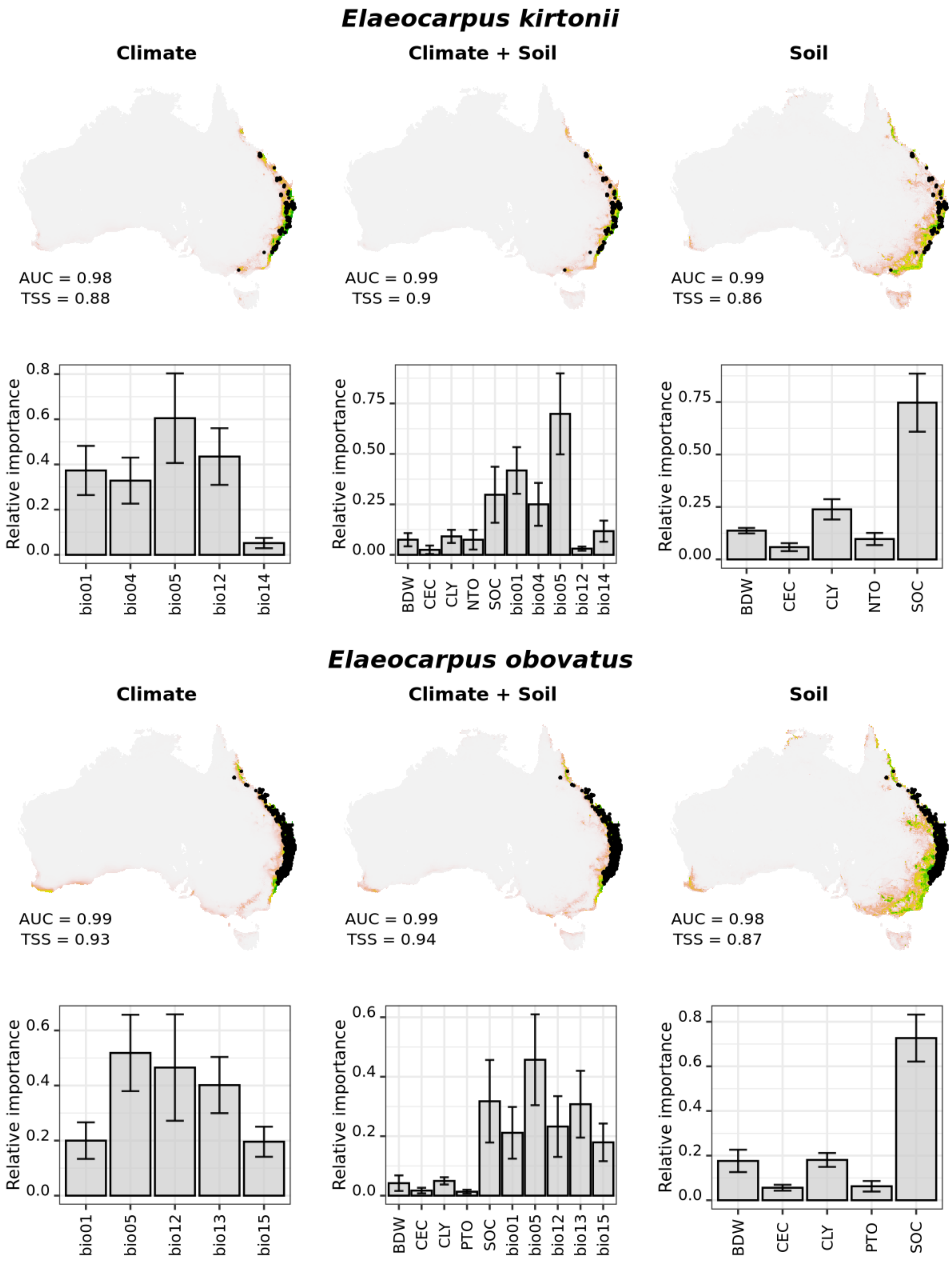
Elaeocarpus kirtonii
Climate
Climate + Soil
Soil
AUC = 0.98
TSS = 0.88
AUC = 0.99
TSS = 0.9
AUC = 0.99
TSS = 0.86
Relative importance
bio01
bio04
bio05
bio12
bio14
BDW
CEC
CLY
NTO
SOC
Elaeocarpus obovatus
AUC = 0.99
TSS = 0.93
AUC = 0.99
TSS = 0.94
AUC = 0.98
TSS = 0.87
bio13
bio15
PTO

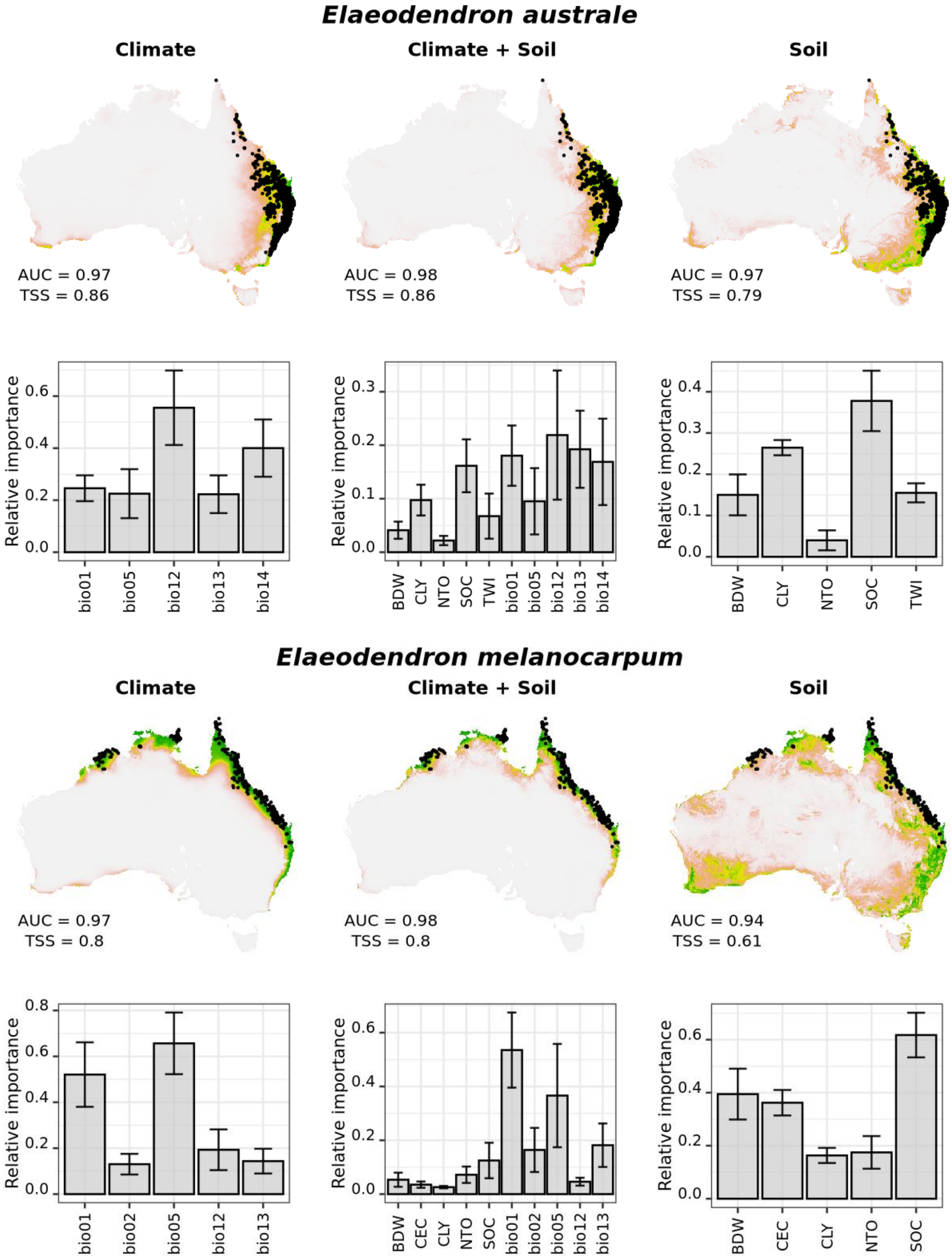
Elaeodendron australe
Climate
Climate + Soil
Soil
AUC = 0.97
TSS = 0.86
AUC = 0.98
TSS = 0.86
AUC = 0.97
TSS = 0.79
Relative importance
bio01 bio05 bio12 bio13 bio14
BDW CLY NTO SOC TWI bio01 bio05 bio12 bio13 bio14
BDW CLY NTO SOC TWI
Elaeodendron melanocarpum
Climate
Climate + Soil
Soil
AUC = 0.97
TSS = 0.8
AUC = 0.98
TSS = 0.8
AUC = 0.94
TSS = 0.61
Relative importance
bio01 bio02 bio05 bio12 bio13
BDW CEC CLY NTO SOC bio01 bio02 bio05 bio12 bio13
BDW CEC CLY NTO SOC

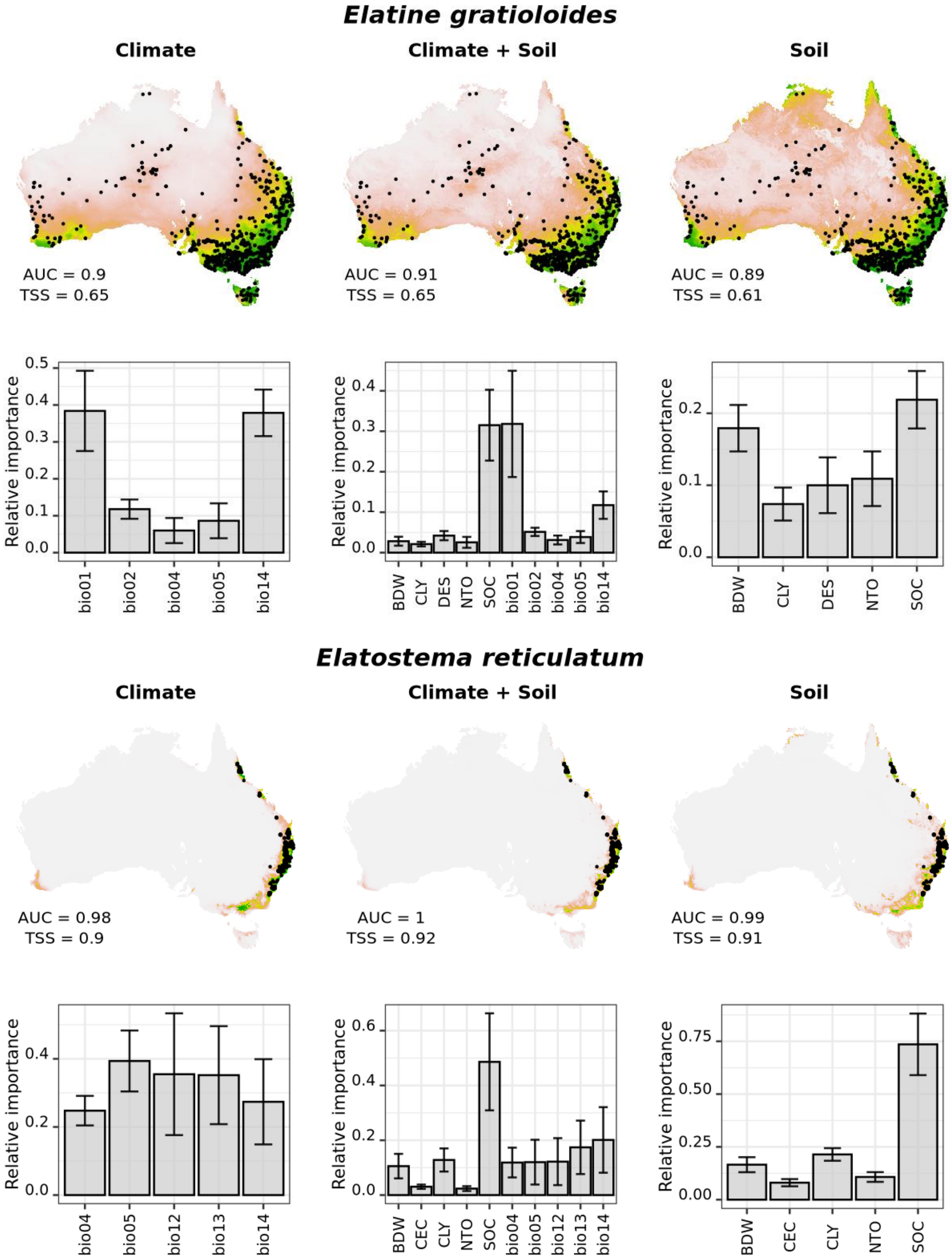

Elatine gratioloides
Climate
Climate + Soil
Soil
AUC = 0.9
TSS = 0.65
AUC = 0.91
TSS = 0.65
AUC = 0.89
TSS = 0.61
Relative importance
bio01
bio02
bio04
bio05
bio14
BDW
CLY
DES
NTO
SOC
Elatostema reticulatum
AUC = 0.98
TSS = 0.9
AUC = 1
TSS = 0.92
AUC = 0.99
TSS = 0.91
bio12
bio13
CEC

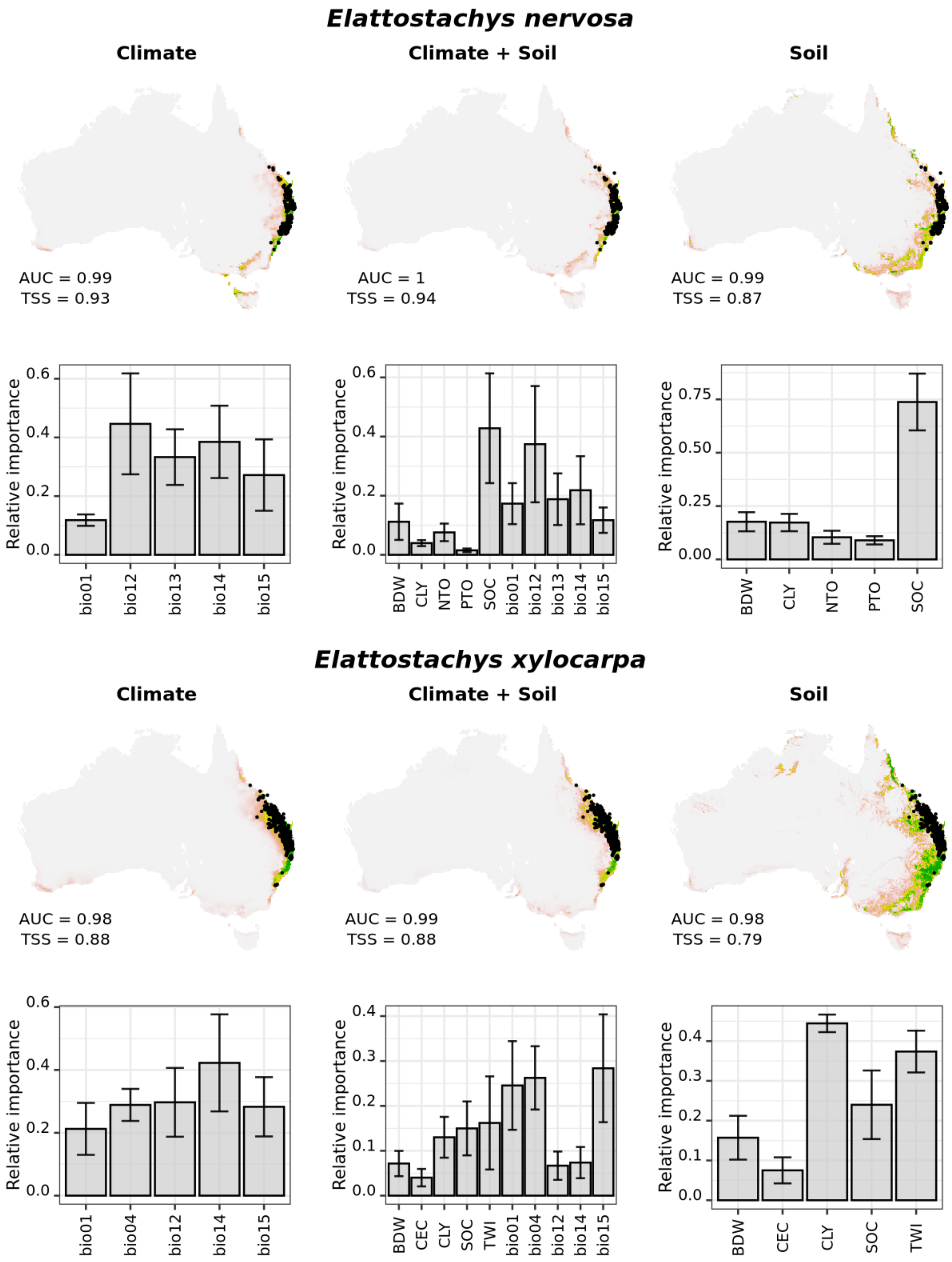
Elattostachys nervosa
Climate
Climate + Soil
Soil
AUC = 0.99
TSS = 0.93
AUC = 1
TSS = 0.94
AUC = 0.99
TSS = 0.87
Relative importance
0.0
0.2
0.4
0.6
bio01
bio12
bio13
bio14
bio15
Relative importance
0.0
0.2
0.4
0.6
BDW
CLY
NTO
PTO
SOC
bio01
bio12
bio13
bio14
bio15
Relative importance
0.00
0.25
0.50
0.75
BDW
CLY
NTO
PTO
SOC
Elattostachys xylocarpa
Climate
Climate + Soil
Soil
AUC = 0.98
TSS = 0.88
AUC = 0.99
TSS = 0.88
AUC = 0.98
TSS = 0.79
Relative importance
0.0
0.2
0.4
0.6
bio01
bio04
bio12
bio14
bio15
Relative importance
0.0
0.1
0.2
0.3
0.4
BDW
CEC
CLY
SOC
TWI
bio01
bio04
bio12
bio14
bio15
Relative importance
0.0
0.1
0.2
0.3
0.4
BDW
CEC
CLY
SOC
TWI

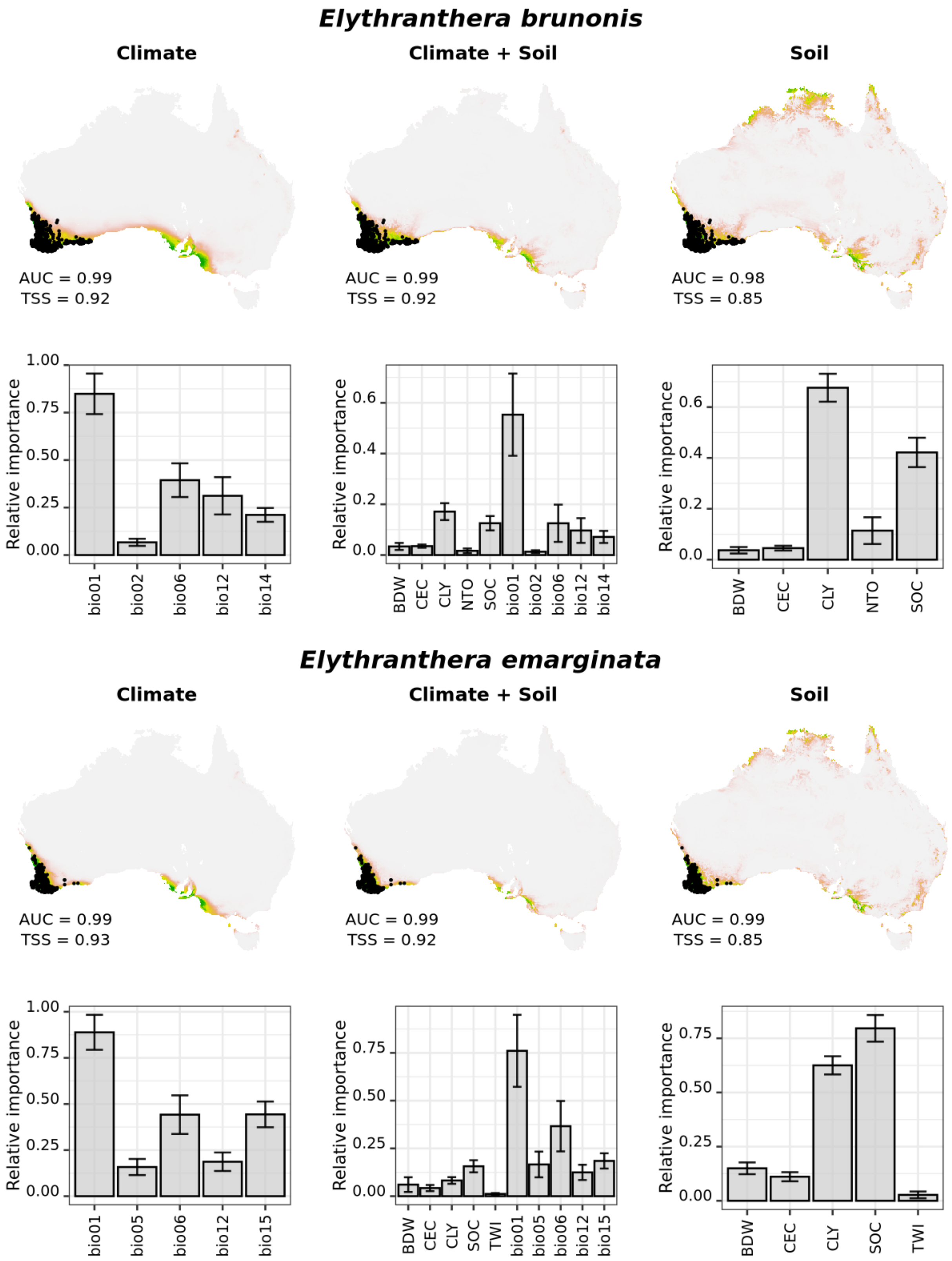
Elythranthera brunonis
Climate
Climate + Soil
Soil
AUC = 0.99
TSS = 0.92
AUC = 0.99
TSS = 0.92
AUC = 0.98
TSS = 0.85
Relative importance
bio01
bio02
bio06
bio12
bio14
BDW
CEC
CLY
NTO
SOC
Elythranthera emarginata
AUC = 0.99
TSS = 0.93
AUC = 0.99
TSS = 0.92
AUC = 0.99
TSS = 0.85
bio05
bio15
TWI

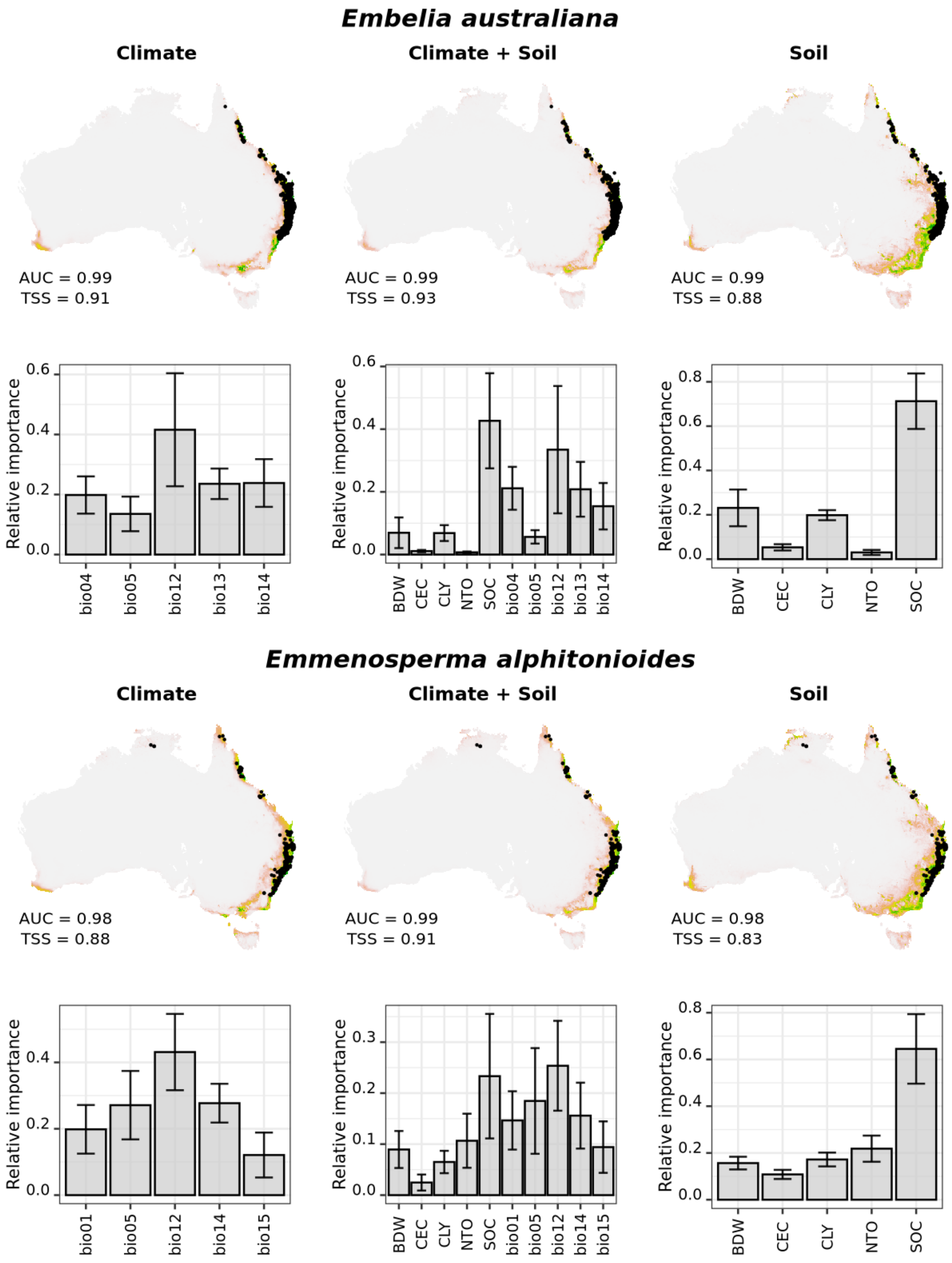
Embelia australiana
Climate
Climate + Soil
Soil
AUC = 0.99
TSS = 0.91
AUC = 0.99
TSS = 0.93
AUC = 0.99
TSS = 0.88
Relative importance
bio04
bio05
bio12
bio13
bio14
BDW
CEC
CLY
NTO
SOC
bio04
bio05
bio12
bio13
bio14
BDW
CEC
CLY
NTO
SOC
Emmenosperma alphitonioides
Climate
Climate + Soil
Soil
AUC = 0.98
TSS = 0.88
AUC = 0.99
TSS = 0.91
AUC = 0.98
TSS = 0.83
Relative importance
bio01
bio05
bio12
bio14
bio15
BDW
CEC
CLY
NTO
SOC
bio01
bio05
bio12
bio14
bio15
BDW
CEC
CLY
NTO
SOC

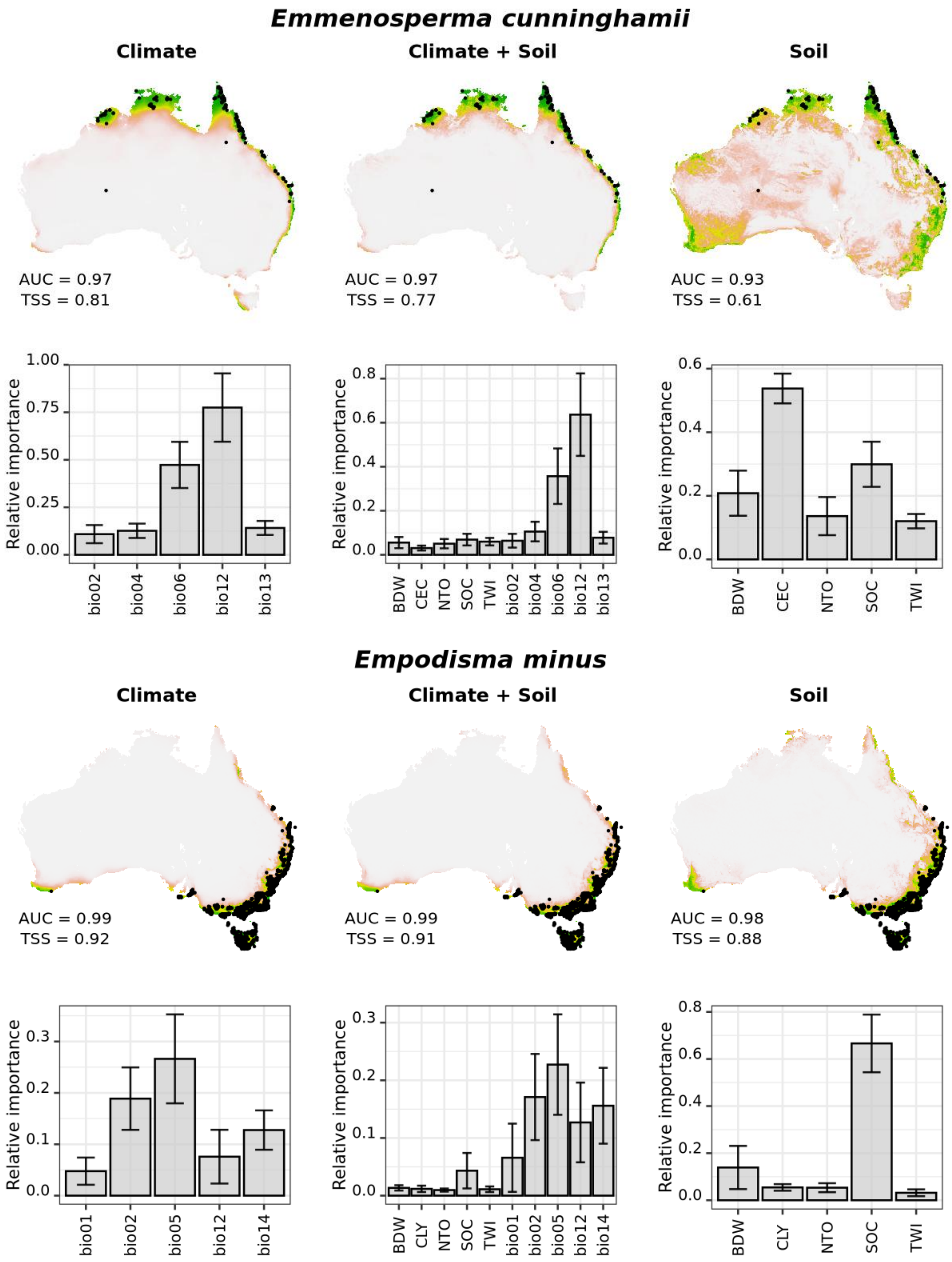

Emmenosperma cunninghamii
Climate
Climate + Soil
Soil
AUC = 0.97
TSS = 0.81
AUC = 0.97
TSS = 0.77
AUC = 0.93
TSS = 0.61
Relative importance
bio02
bio04
bio06
bio12
bio13
BDW
CEC
NTO
SOC
TWI
Empodisma minus
Climate
Climate + Soil
Soil
AUC = 0.99
TSS = 0.92
AUC = 0.99
TSS = 0.91
AUC = 0.98
TSS = 0.88
Relative importance
bio01
bio02
bio05
bio12
bio14
BDW
CLY
NTO
SOC
TWI

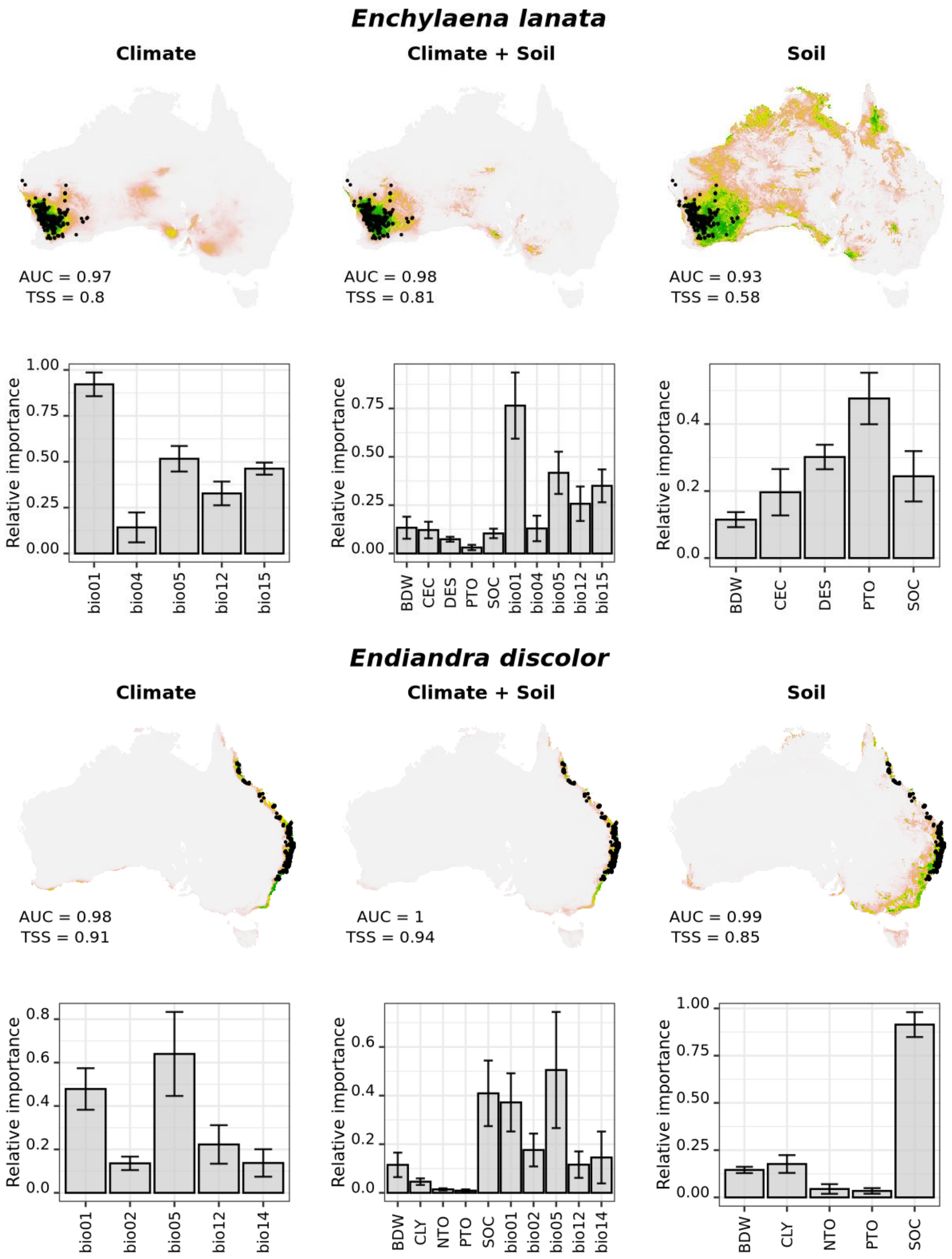
Enchylaena lanata
Climate
Climate + Soil
Soil
AUC = 0.97
TSS = 0.8
AUC = 0.98
TSS = 0.81
AUC = 0.93
TSS = 0.58
Relative importance
bio01
bio04
bio05
bio12
bio15
BDW
CEC
DES
PTO
SOC
Endiandra discolor
Climate
Climate + Soil
Soil
AUC = 0.98
TSS = 0.91
AUC = 1
TSS = 0.94
AUC = 0.99
TSS = 0.85
bio02
bio14
CLY
NTO

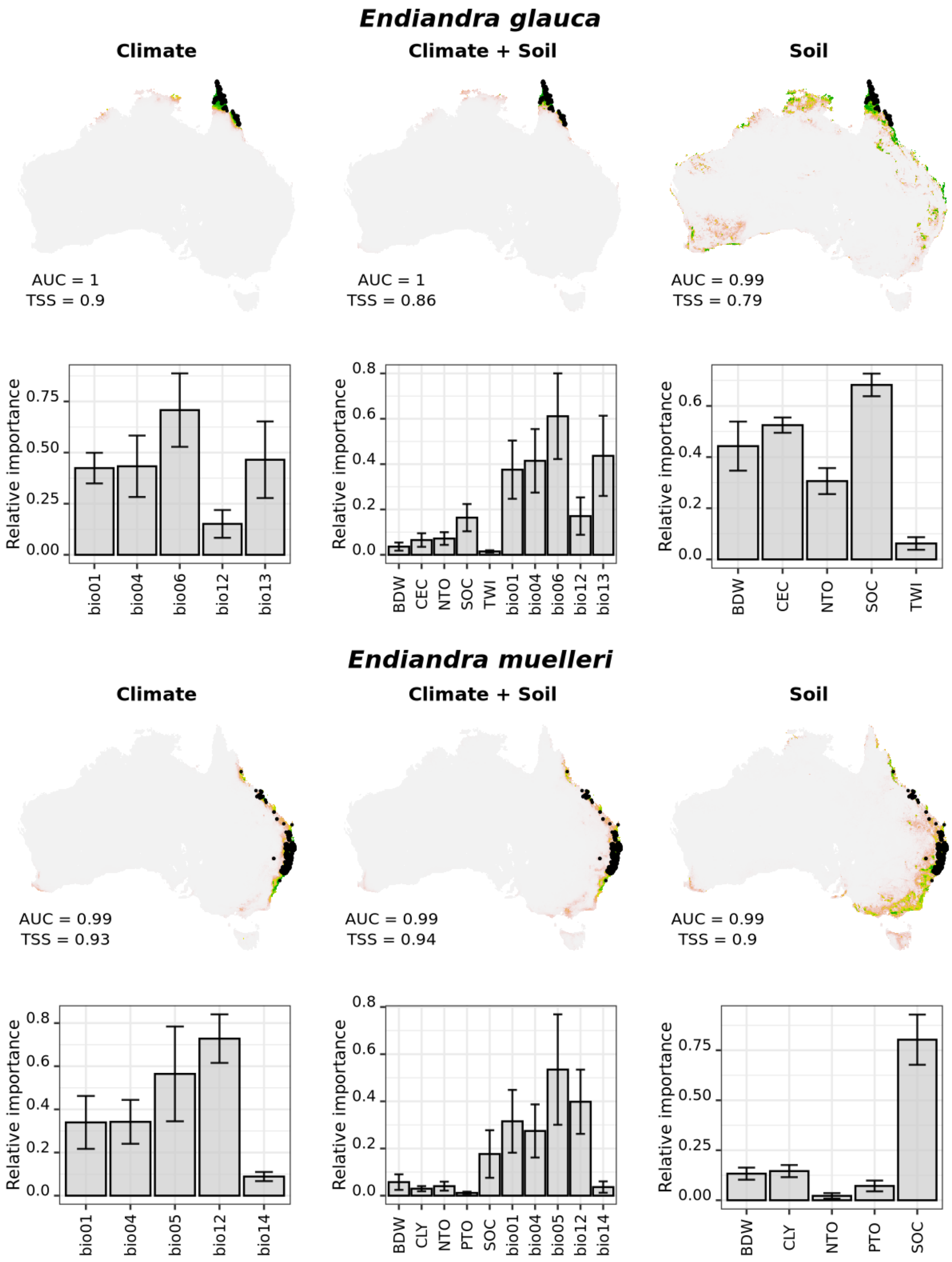
Endiandra glauca
Climate
Climate + Soil
Soil
AUC = 1
TSS = 0.9
AUC = 1
TSS = 0.86
AUC = 0.99
TSS = 0.79
Relative importance
bio01
bio04
bio06
bio12
bio13
BDW
CEC
NTO
SOC
TWI
Endiandra muelleri
AUC = 0.99
TSS = 0.93
AUC = 0.99
TSS = 0.94
AUC = 0.99
TSS = 0.9
bio05
bio14
CLY
PTO

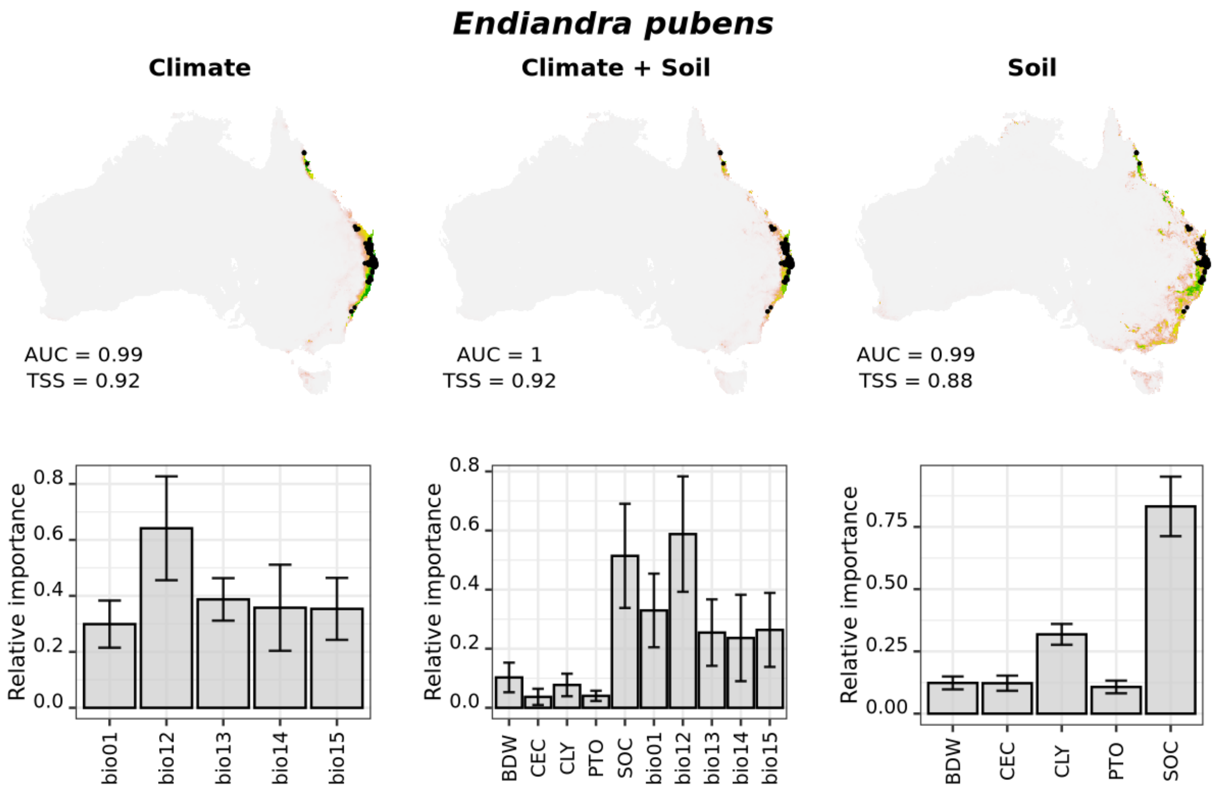
Endiandra pubens
Climate
Climate + Soil
Soil
AUC = 0.99
TSS = 0.92
AUC = 1
TSS = 0.92
AUC = 0.99
TSS = 0.88
Relative importance
bio01
bio12
bio13
bio14
bio15
BDW
CEC
CLY
PTO
SOC
0.0
0.2
0.4
0.6
0.8
0.00
0.25
0.50
0.75

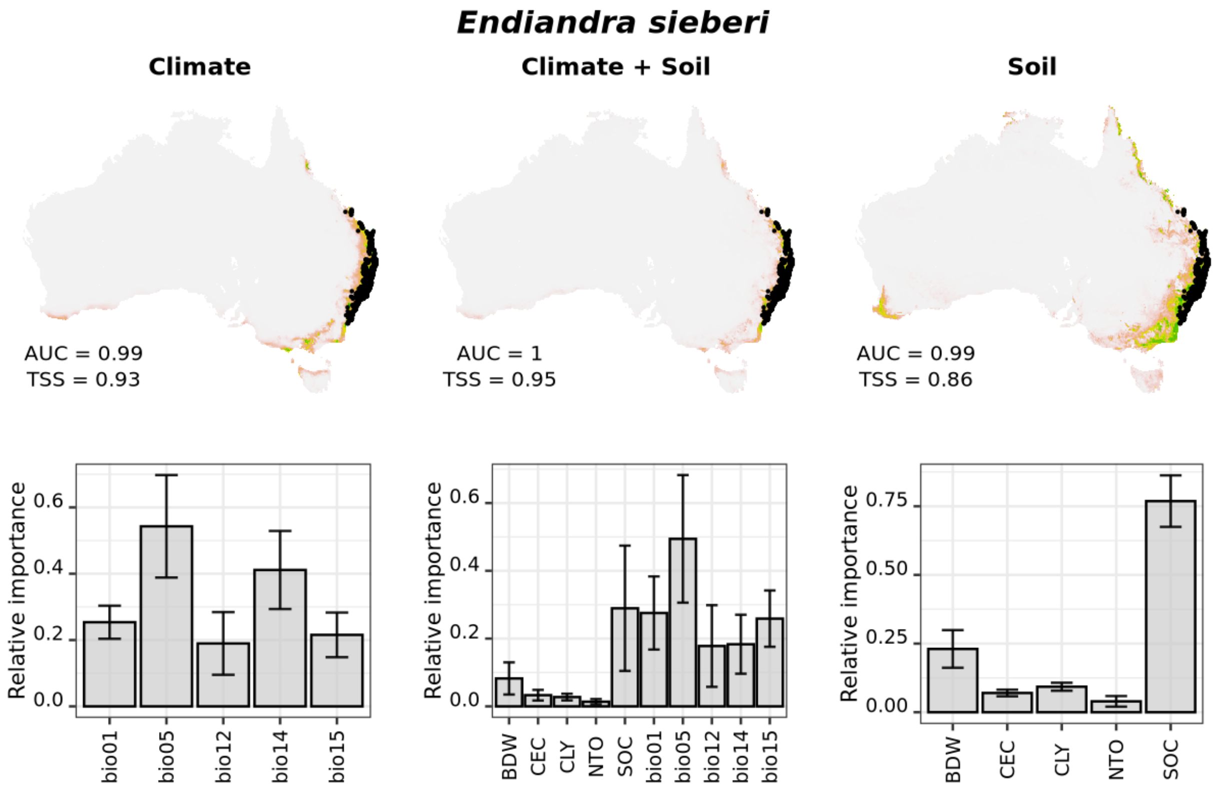
Endiandra sieberi
Climate
Climate + Soil
Soil
AUC = 0.99
TSS = 0.93
AUC = 1
TSS = 0.95
AUC = 0.99
TSS = 0.86
Relative importance
bio01
bio05
bio12
bio14
bio15
BDW
CEC
CLY
NTO
SOC
0.0
0.2
0.4
0.6
0.00
0.25
0.50
0.75

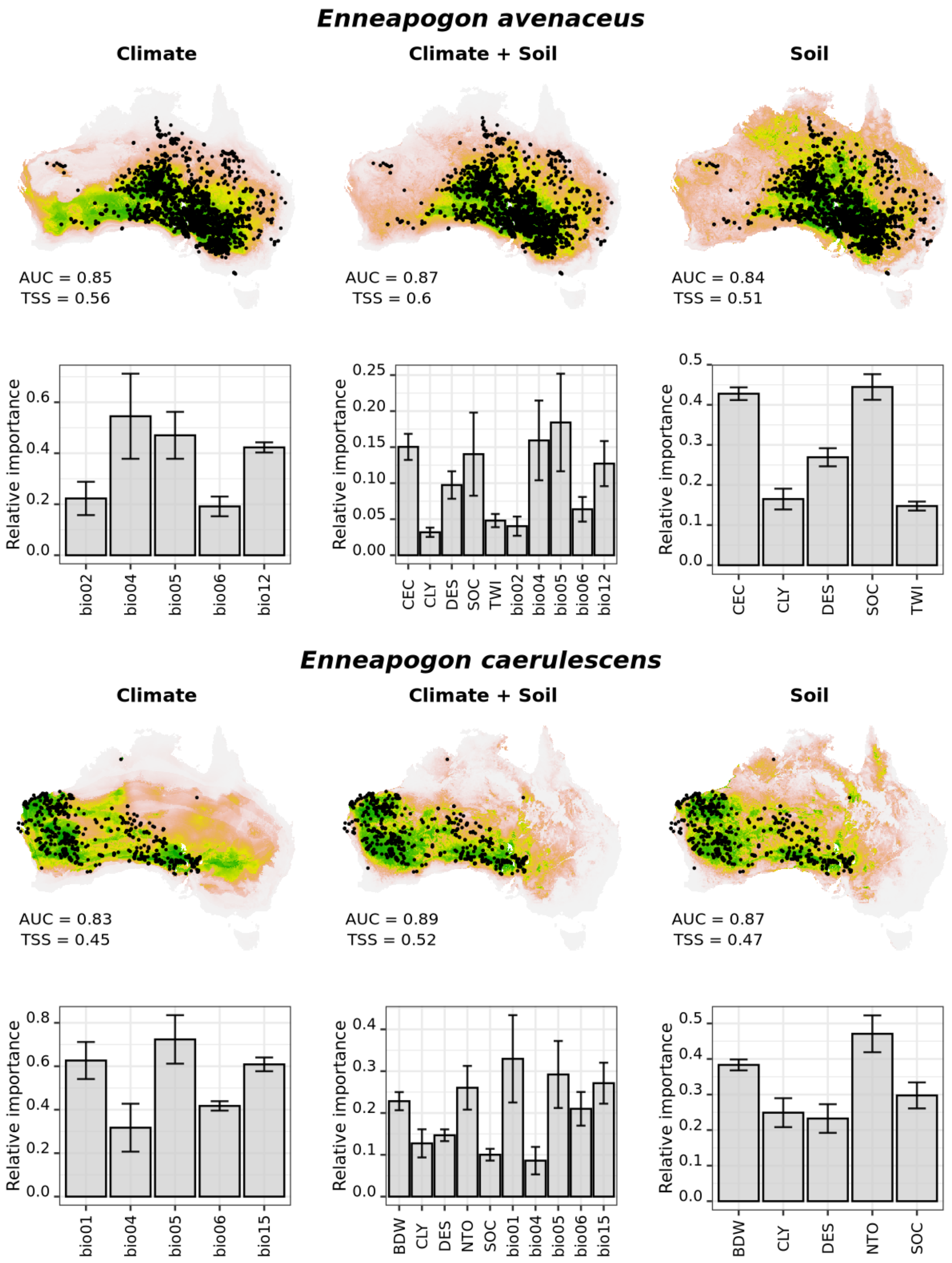
Enneapogon avenaceus
Climate
Climate + Soil
Soil
AUC = 0.85
TSS = 0.56
AUC = 0.87
TSS = 0.6
AUC = 0.84
TSS = 0.51
Relative importance
bio02
bio04
bio05
bio06
bio12
CEC
CLY
DES
SOC
TWI
Enneapogon caerulescens
Climate
Climate + Soil
Soil
AUC = 0.83
TSS = 0.45
AUC = 0.89
TSS = 0.52
AUC = 0.87
TSS = 0.47
Relative importance
bio01
bio04
bio05
bio06
bio15
BDW
CLY
DES
NTO
SOC

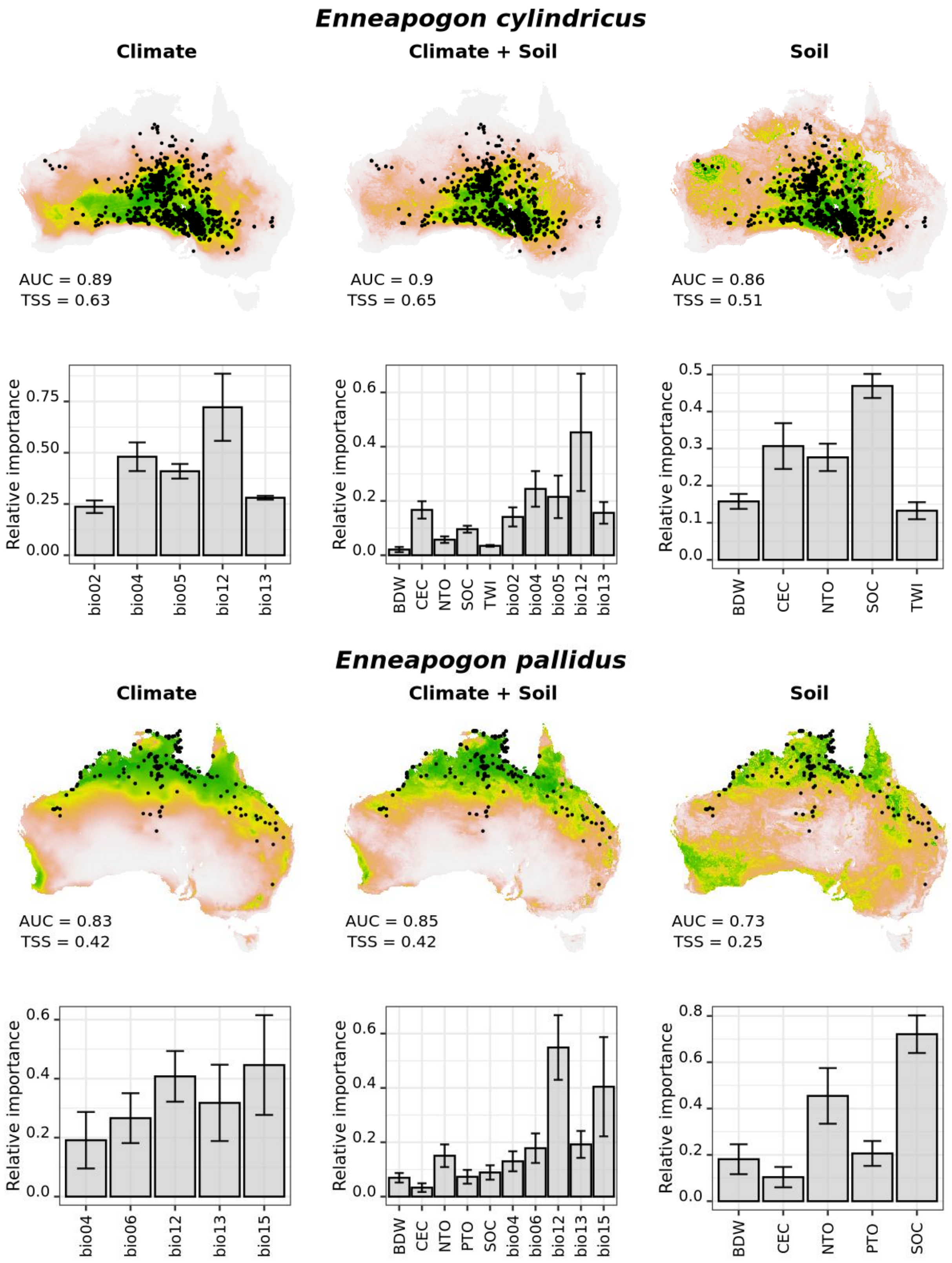
Enneapogon cylindricus
Climate
Climate + Soil
Soil
AUC = 0.89
TSS = 0.63
AUC = 0.9
TSS = 0.65
AUC = 0.86
TSS = 0.51
Relative importance
bio02
bio04
bio05
bio12
bio13
BDW
CEC
NTO
SOC
TWI
Enneapogon pallidus
Climate
Climate + Soil
Soil
AUC = 0.83
TSS = 0.42
AUC = 0.85
TSS = 0.42
AUC = 0.73
TSS = 0.25
bio04
bio06
bio12
bio13
bio15
PTO

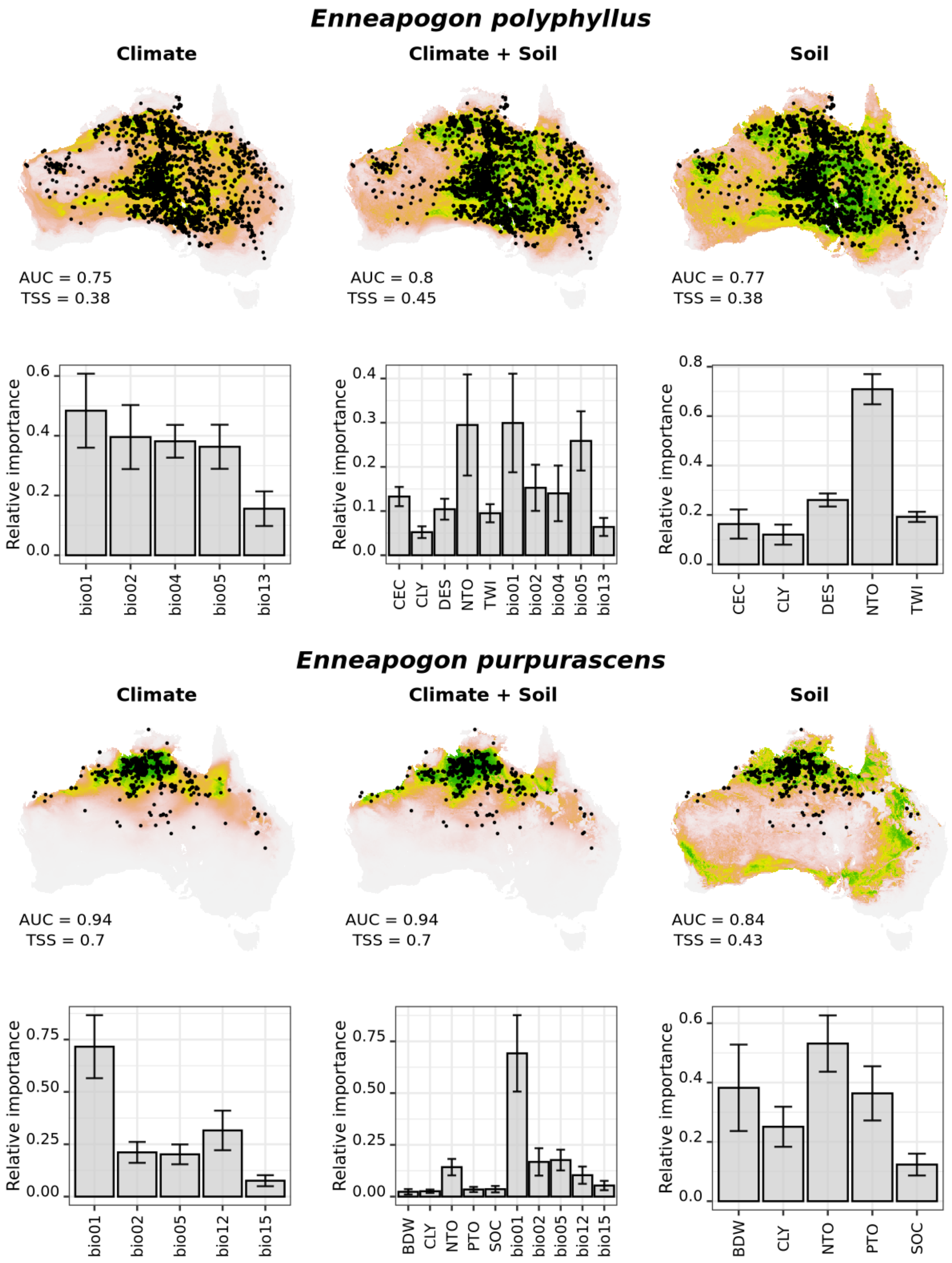
Enneapogon polyphyllus
Climate
Climate + Soil
Soil
AUC = 0.75
TSS = 0.38
AUC = 0.8
TSS = 0.45
AUC = 0.77
TSS = 0.38
Relative importance
bio01
bio02
bio04
bio05
bio13
CEC
CLY
DES
NTO
TWI
bio01
bio02
bio04
bio05
bio13
CEC
CLY
DES
NTO
TWI
Enneapogon purpurascens
Climate
Climate + Soil
Soil
AUC = 0.94
TSS = 0.7
AUC = 0.94
TSS = 0.7
AUC = 0.84
TSS = 0.43
Relative importance
bio01
bio02
bio05
bio12
bio15
BDW
CLY
NTO
PTO
SOC
bio01
bio02
bio05
bio12
bio15
BDW
CLY
NTO
PTO
SOC

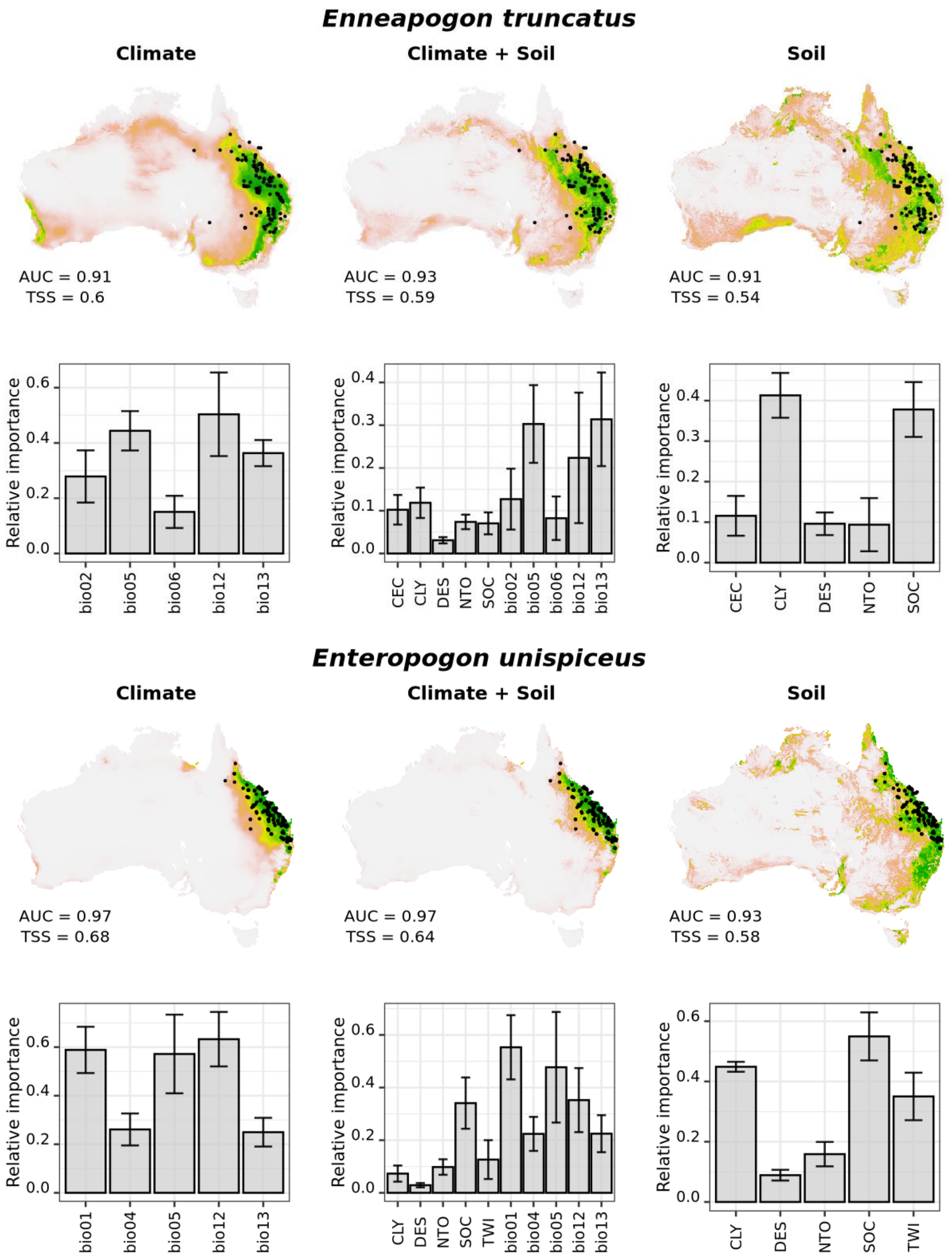

Enneapogon truncatus
Climate
Climate + Soil
Soil
AUC = 0.91
TSS = 0.6
AUC = 0.93
TSS = 0.59
AUC = 0.91
TSS = 0.54
Relative importance
bio02
bio05
bio06
bio12
bio13
CEC
CLY
DES
NTO
SOC
Enteropogon unispiceus
Climate
Climate + Soil
Soil
AUC = 0.97
TSS = 0.68
AUC = 0.97
TSS = 0.64
AUC = 0.93
TSS = 0.58
Relative importance
bio01
bio04
bio05
bio12
bio13
CLY
DES
NTO
SOC
TWI

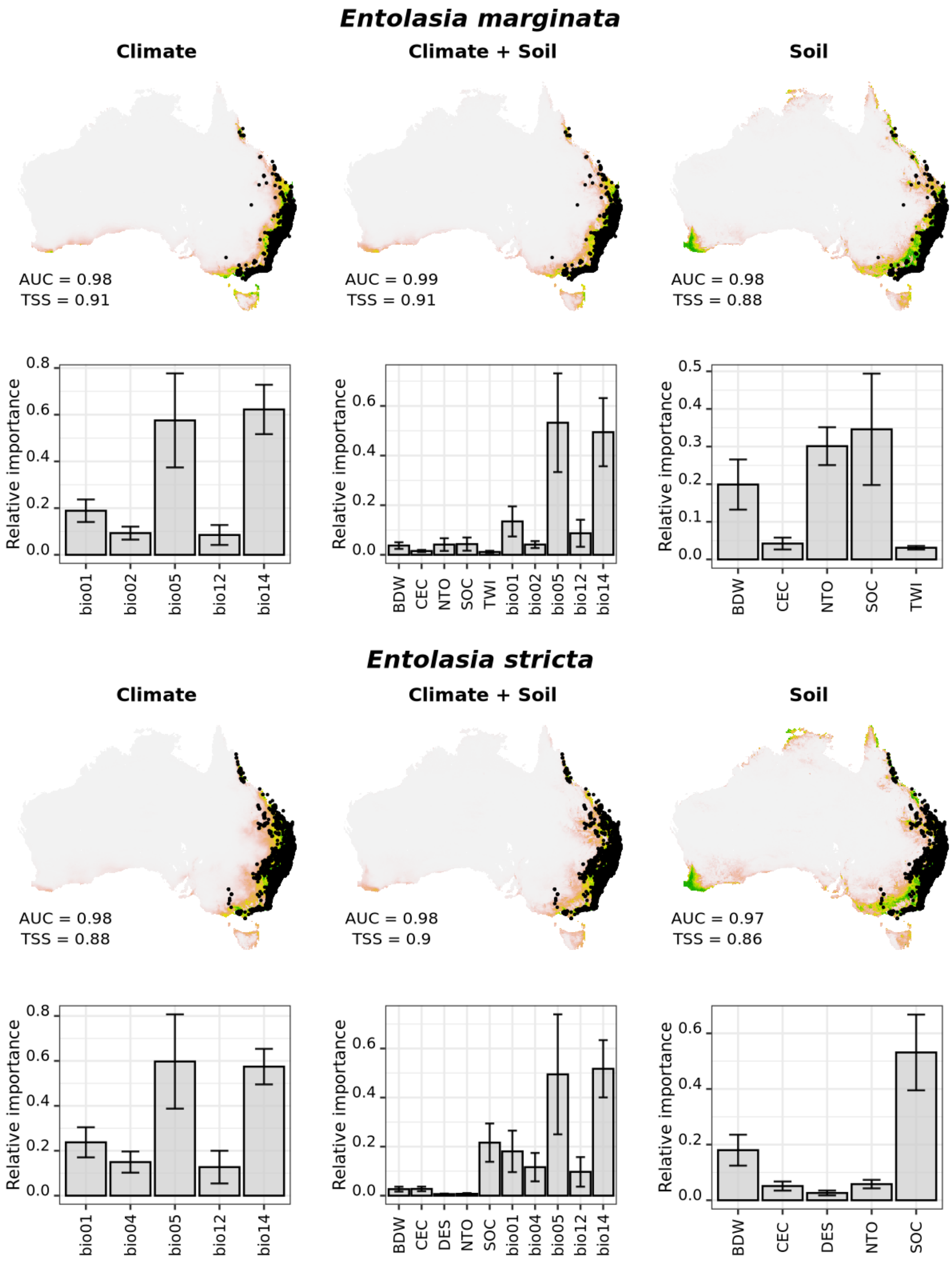

Entolasia marginata
Climate
Climate + Soil
Soil
AUC = 0.98
TSS = 0.91
AUC = 0.99
TSS = 0.91
AUC = 0.98
TSS = 0.88
Relative importance
0.0 0.2 0.4 0.6 0.8
bio01 bio02 bio05 bio12 bio14
0.0 0.2 0.4 0.6
BDW CEC NTO SOC TWI bio01 bio02 bio05 bio12 bio14
0.0 0.1 0.2 0.3 0.4 0.5
BDW CEC NTO SOC TWI
Entolasia stricta
Climate
Climate + Soil
Soil
AUC = 0.98
TSS = 0.88
AUC = 0.98
TSS = 0.9
AUC = 0.97
TSS = 0.86
Relative importance
0.0 0.2 0.4 0.6 0.8
bio01 bio04 bio05 bio12 bio14
0.0 0.2 0.4 0.6
BDW CEC DES NTO SOC bio01 bio04 bio05 bio12 bio14
0.0 0.2 0.4 0.6
BDW CEC DES NTO SOC

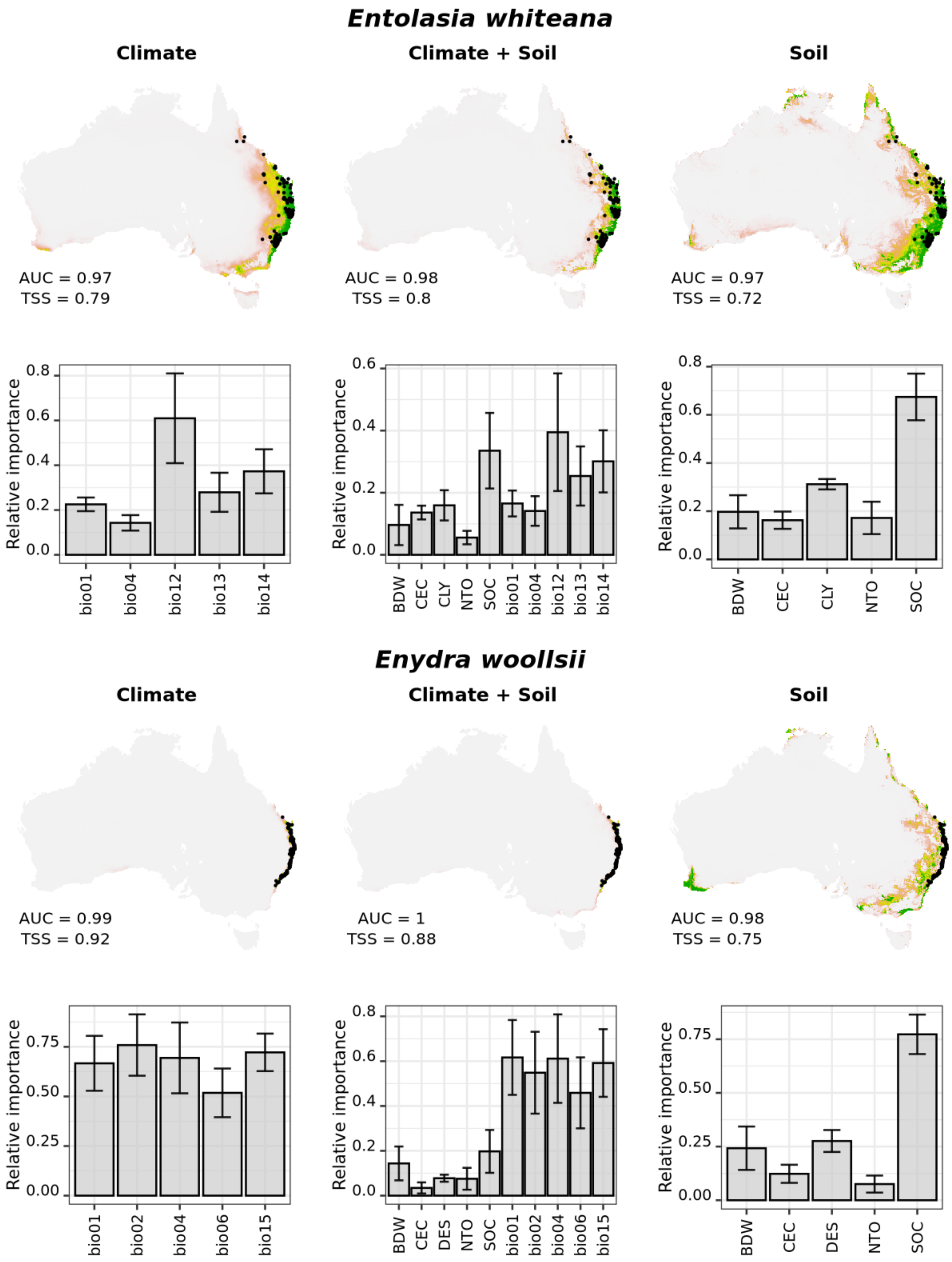

Entolasia whiteana
Climate
Climate + Soil
Soil
AUC = 0.97
TSS = 0.79
AUC = 0.98
TSS = 0.8
AUC = 0.97
TSS = 0.72
Relative importance
bio01 bio04 bio12 bio13 bio14
BDW CEC CLY NTO SOC bio01 bio04 bio12 bio13 bio14
BDW CEC CLY NTO SOC
Enydra woollsii
Climate
Climate + Soil
Soil
AUC = 0.99
TSS = 0.92
AUC = 1
TSS = 0.88
AUC = 0.98
TSS = 0.75
Relative importance
bio01 bio02 bio04 bio06 bio15
BDW CEC DES NTO SOC bio01 bio02 bio04 bio06 bio15
BDW CEC DES NTO SOC

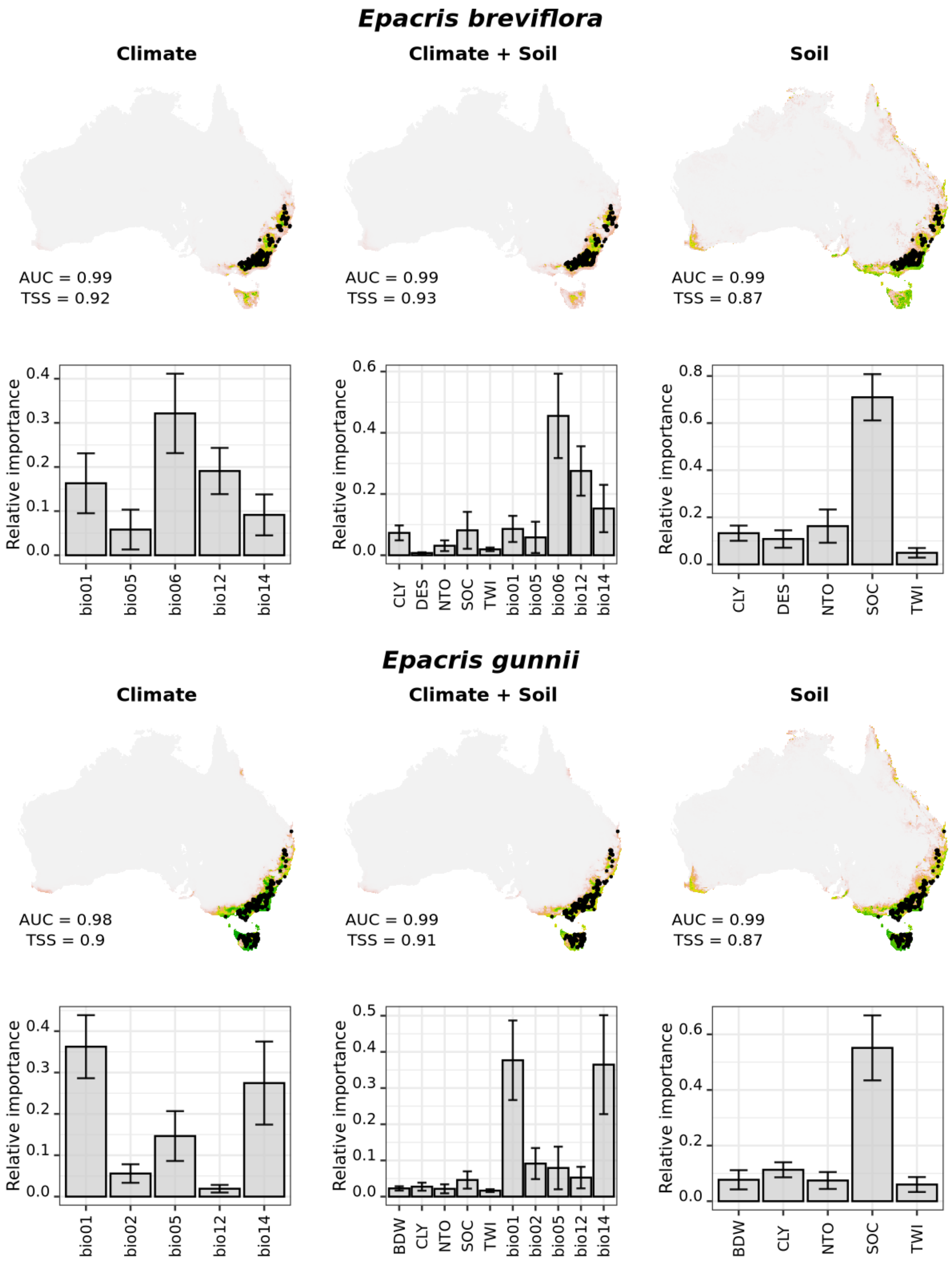
Epacris breviflora
Climate
Climate + Soil
Soil
AUC = 0.99
TSS = 0.92
AUC = 0.99
TSS = 0.93
AUC = 0.99
TSS = 0.87
Relative importance
0.0
0.1
0.2
0.3
0.4
bio01
bio05
bio06
bio12
bio14
0.0
0.2
0.4
0.6
CLY
DES
NTO
SOC
TWI
bio01
bio05
bio06
bio12
bio14
0.0
0.2
0.4
0.6
0.8
CLY
DES
NTO
SOC
TWI
Epacris gunnii
Climate
Climate + Soil
Soil
AUC = 0.98
TSS = 0.9
AUC = 0.99
TSS = 0.91
AUC = 0.99
TSS = 0.87
Relative importance
0.0
0.1
0.2
0.3
0.4
bio01
bio02
bio05
bio12
bio14
0.0
0.1
0.2
0.3
0.4
0.5
BDW
CLY
NTO
SOC
TWI
bio01
bio02
bio05
bio12
bio14
0.0
0.2
0.4
0.6
BDW
CLY
NTO
SOC
TWI

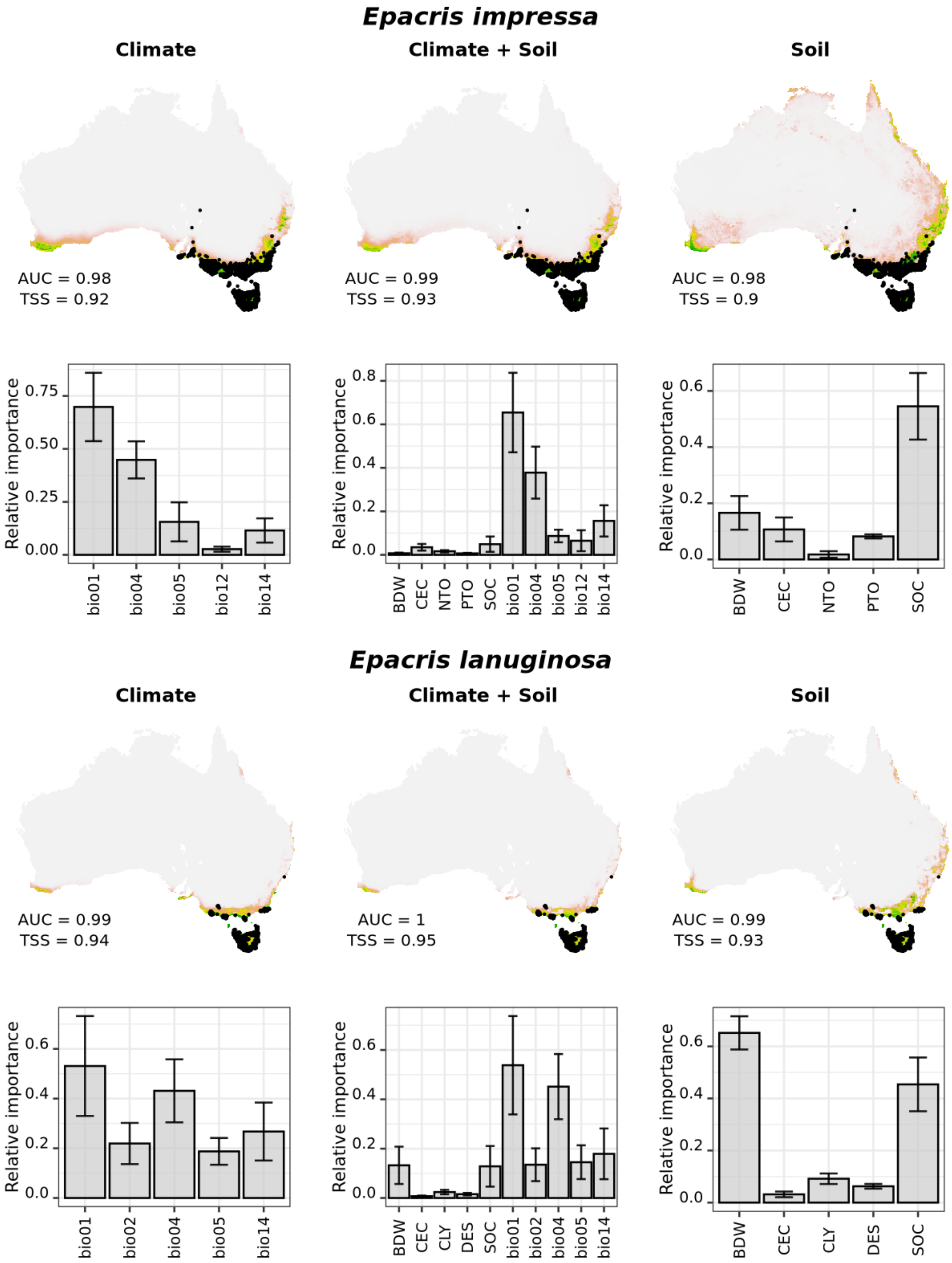
Epacris impressa
Climate
Climate + Soil
Soil
AUC = 0.98
TSS = 0.92
AUC = 0.99
TSS = 0.93
AUC = 0.98
TSS = 0.9
Relative importance
0.00
0.25
0.50
0.75
bio01
bio04
bio05
bio12
bio14
0.0
0.2
0.4
0.6
0.8
BDW
CEC
NTO
PTO
SOC
bio01
bio04
bio05
bio12
bio14
0.0
0.2
0.4
0.6
BDW
CEC
NTO
PTO
SOC
Epacris lanuginosa
Climate
Climate + Soil
Soil
AUC = 0.99
TSS = 0.94
AUC = 1
TSS = 0.95
AUC = 0.99
TSS = 0.93
Relative importance
0.0
0.2
0.4
0.6
bio01
bio02
bio04
bio05
bio14
BDW
CEC
CLY
DES
SOC
bio01
bio02
bio04
bio05
bio14
BDW
CEC
CLY
DES
SOC

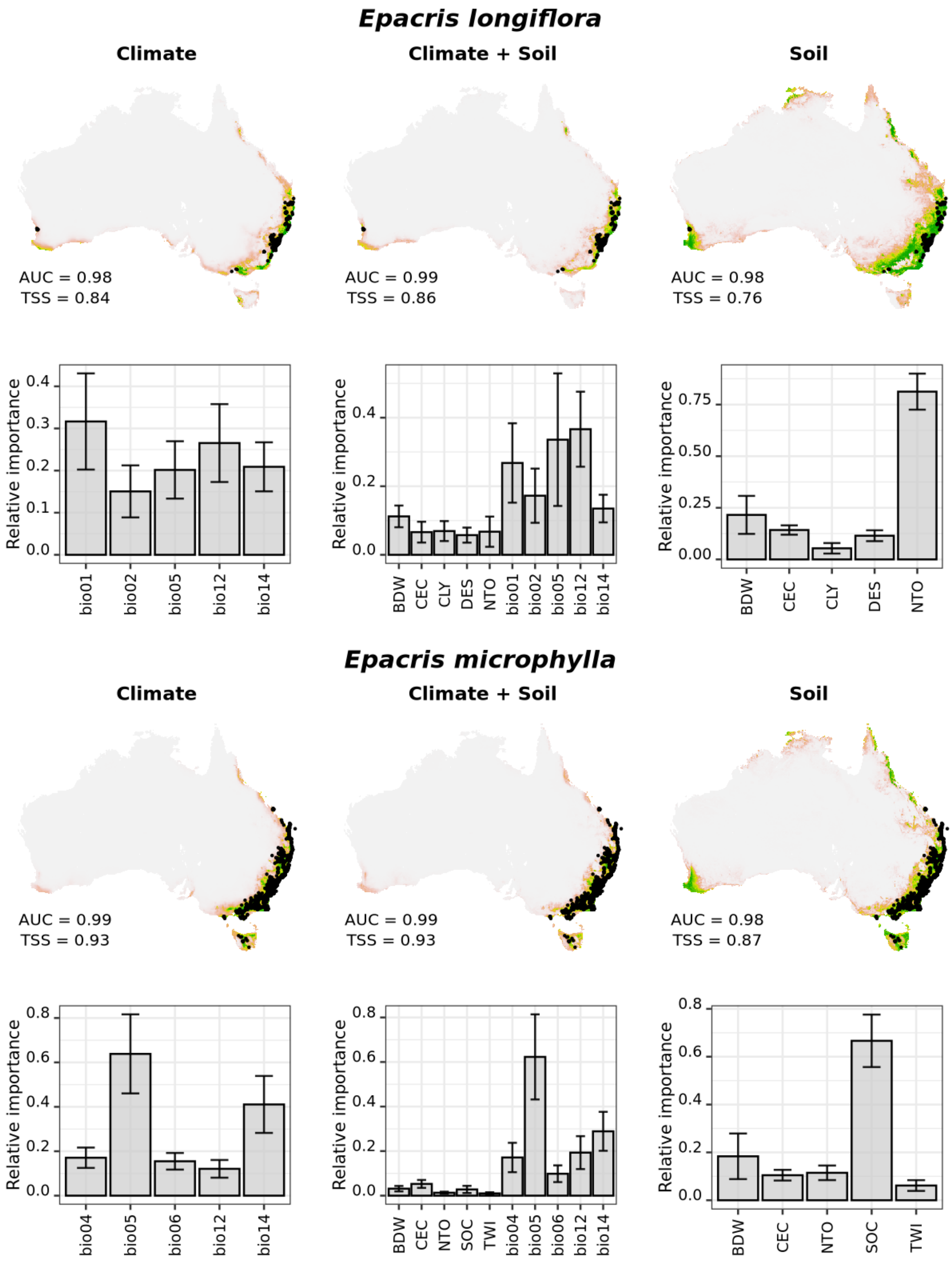

Epacris longiflora
Climate
Climate + Soil
Soil
AUC = 0.98
TSS = 0.84
AUC = 0.99
TSS = 0.86
AUC = 0.98
TSS = 0.76
Relative importance
bio01
bio02
bio05
bio12
bio14
BDW
CEC
CLY
DES
NTO
Epacris microphylla
Climate
Climate + Soil
Soil
AUC = 0.99
TSS = 0.93
AUC = 0.99
TSS = 0.93
AUC = 0.98
TSS = 0.87
Relative importance
bio04
bio05
bio06
bio12
bio14
BDW
CEC
NTO
SOC
TWI

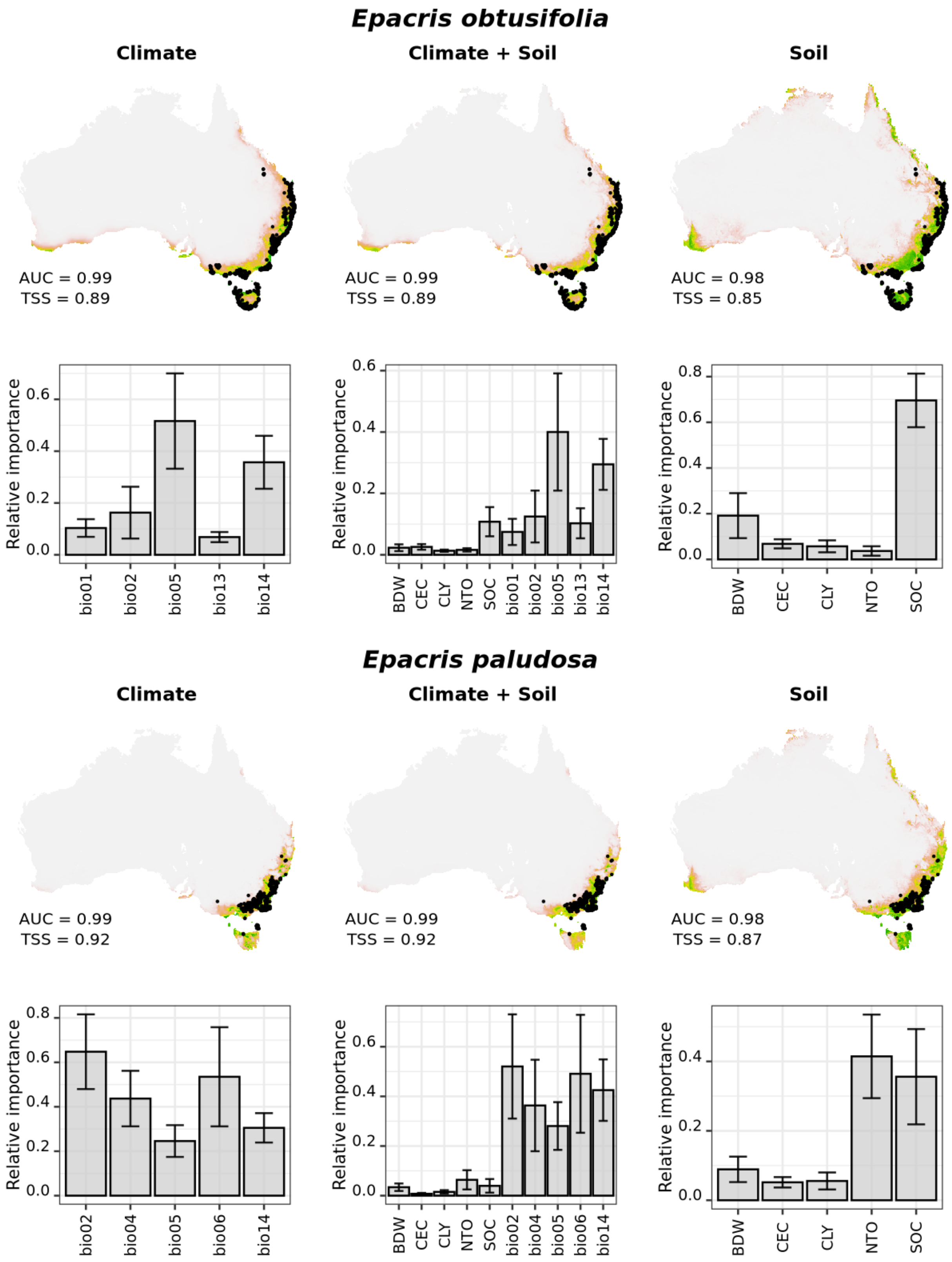
Epacris obtusifolia
Climate
Climate + Soil
Soil
AUC = 0.99
TSS = 0.89
AUC = 0.99
TSS = 0.89
AUC = 0.98
TSS = 0.85
Relative importance
bio01
bio02
bio05
bio13
bio14
BDW
CEC
CLY
NTO
SOC
Epacris paludosa
Climate
Climate + Soil
Soil
AUC = 0.99
TSS = 0.92
AUC = 0.99
TSS = 0.92
AUC = 0.98
TSS = 0.87
Relative importance
bio02
bio04
bio05
bio06
bio14
BDW
CEC
CLY
NTO
SOC

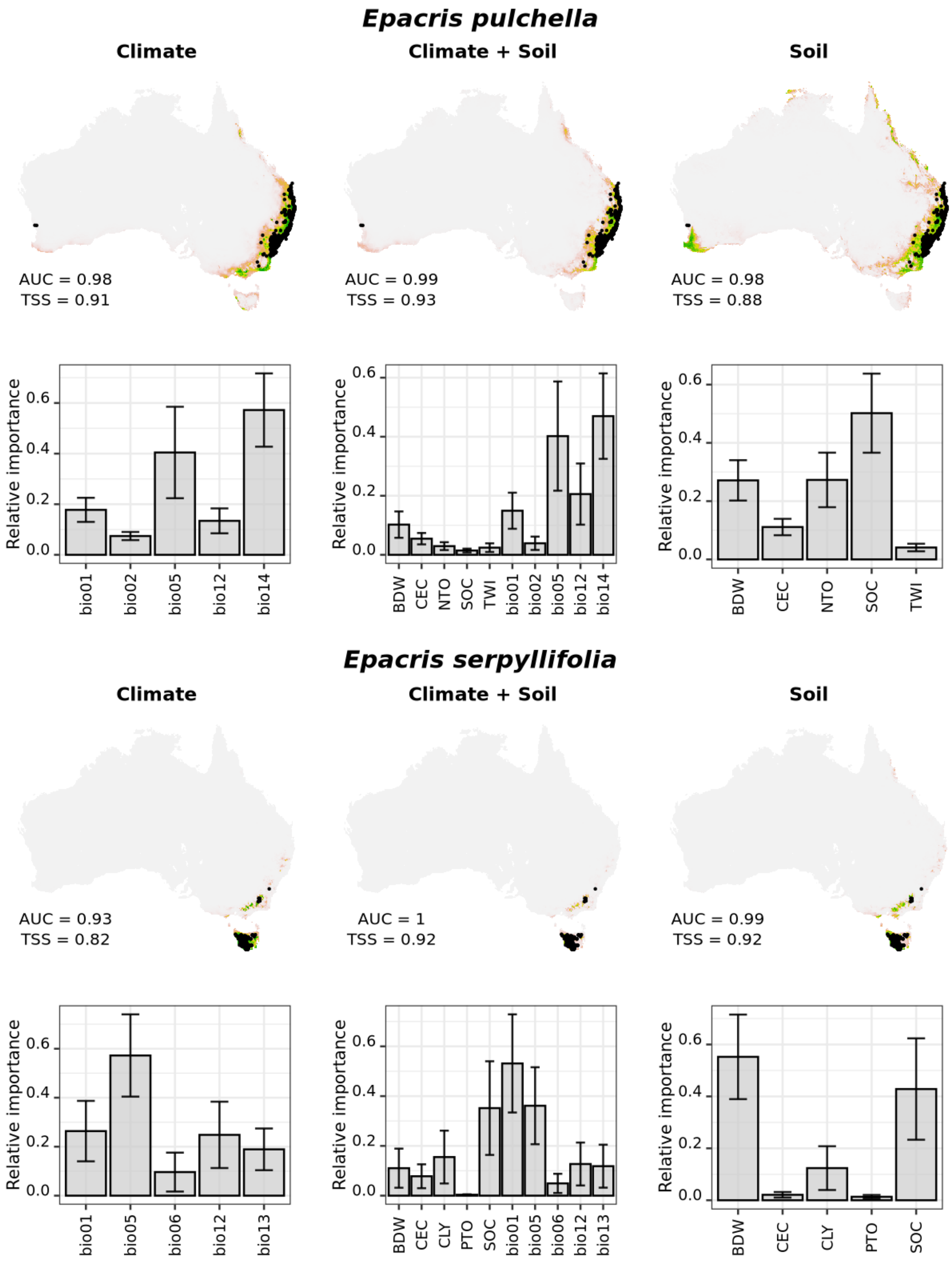

Epacris pulchella
Climate
Climate + Soil
Soil
AUC = 0.98
TSS = 0.91
AUC = 0.99
TSS = 0.93
AUC = 0.98
TSS = 0.88
Relative importance
0.0
0.2
0.4
0.6
bio01
bio02
bio05
bio12
bio14
BDW
CEC
NTO
SOC
TWI
Epacris serpyllifolia
Climate
Climate + Soil
Soil
AUC = 0.93
TSS = 0.82
AUC = 1
TSS = 0.92
AUC = 0.99
TSS = 0.92
bio01
bio05
bio06
bio12
bio13
BDW
CEC
CLY
PTO
SOC

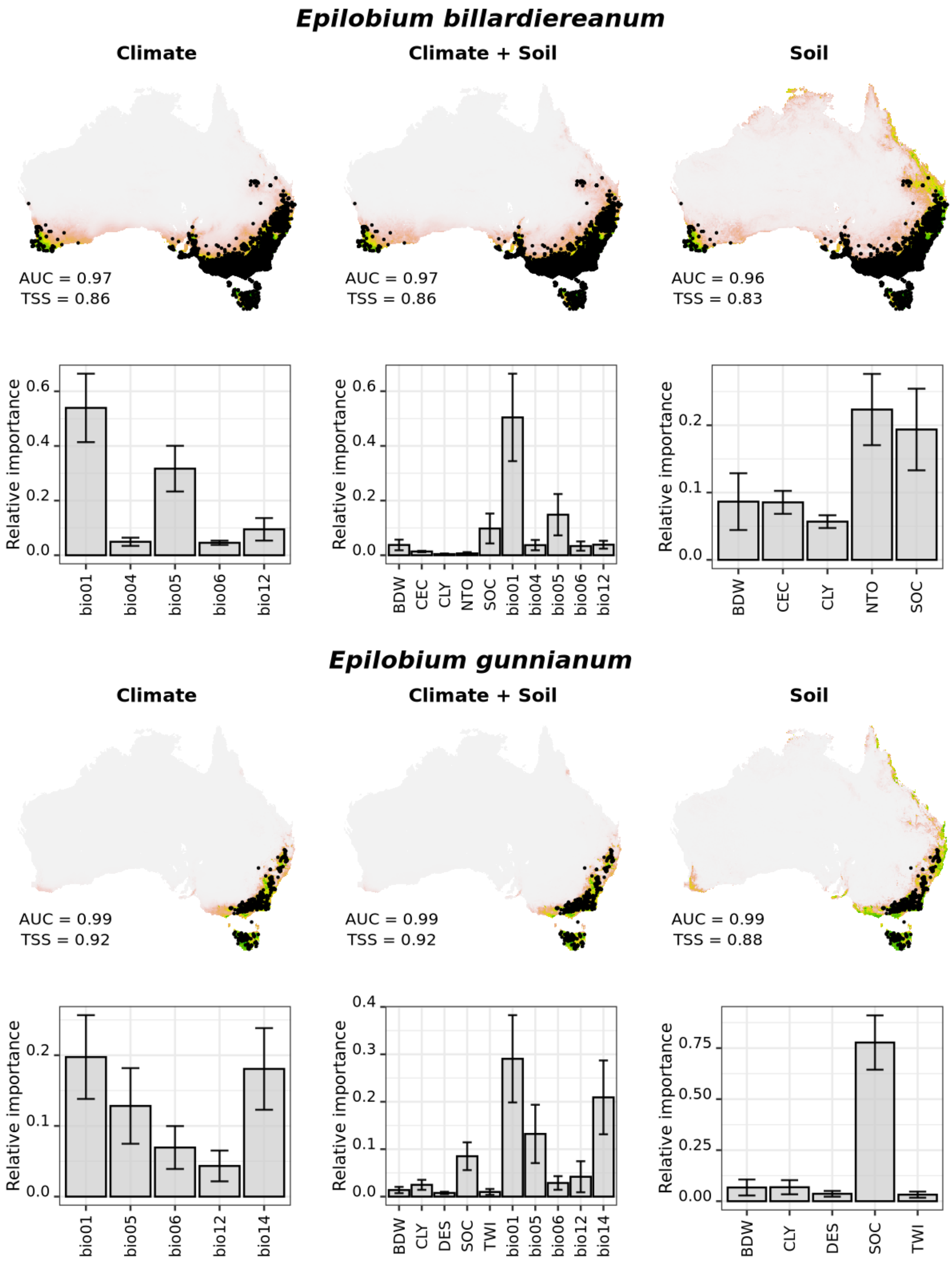

Epilobium billardiereanum
Climate
Climate + Soil
Soil
AUC = 0.97
TSS = 0.86
AUC = 0.97
TSS = 0.86
AUC = 0.96
TSS = 0.83
Relative importance
bio01
bio04
bio05
bio06
bio12
BDW
CEC
CLY
NTO
SOC
Epilobium gunnianum
AUC = 0.99
TSS = 0.92
AUC = 0.99
TSS = 0.92
AUC = 0.99
TSS = 0.88
bio14
DES
TWI

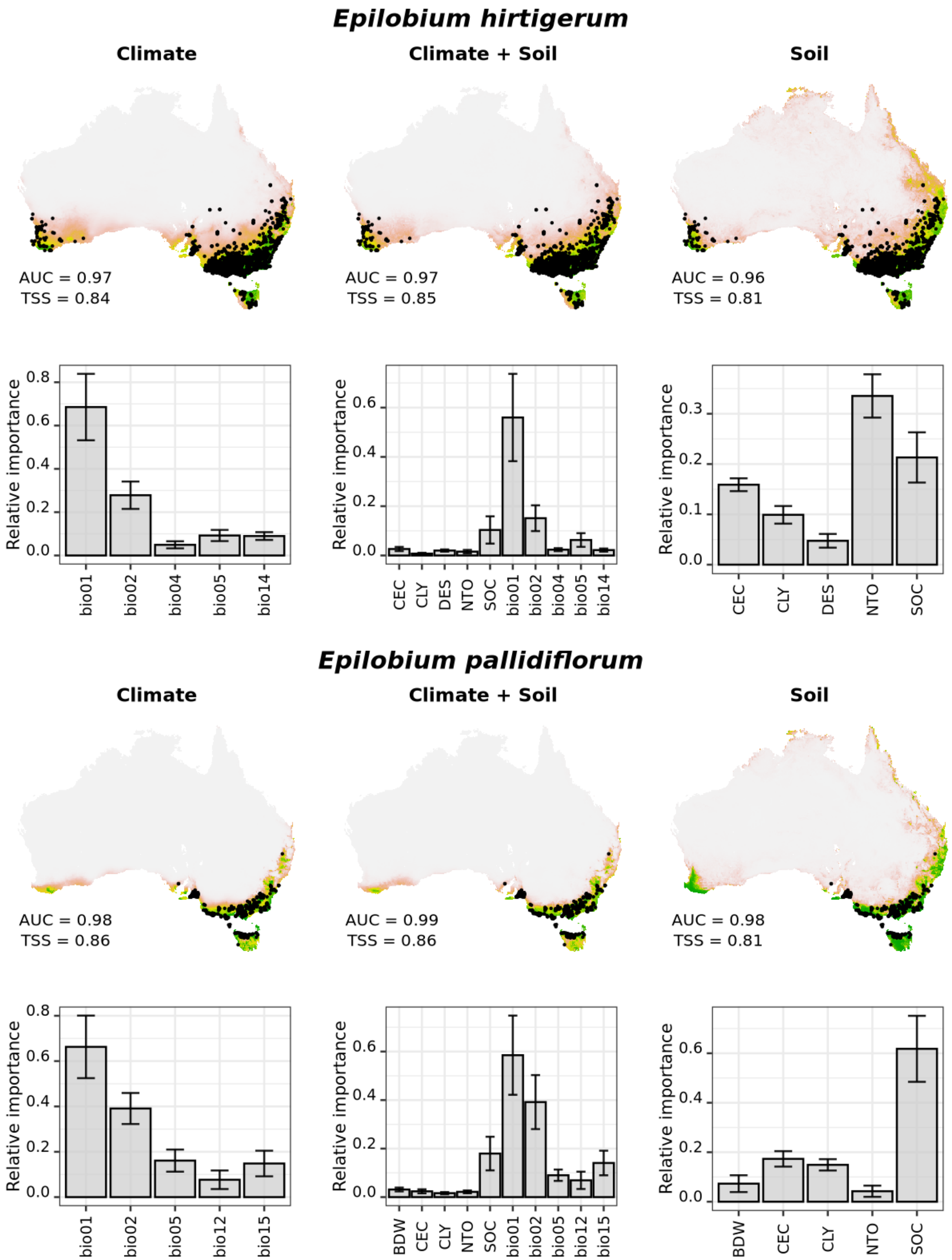
Epilobium hirtigerum
Climate
Climate + Soil
Soil
AUC = 0.97
TSS = 0.84
AUC = 0.97
TSS = 0.85
AUC = 0.96
TSS = 0.81
Relative importance
bio01
bio02
bio04
bio05
bio14
CEC
CLY
DES
NTO
SOC
bio01
bio02
bio04
bio05
bio14
CEC
CLY
DES
NTO
SOC
Epilobium pallidiflorum
Climate
Climate + Soil
Soil
AUC = 0.98
TSS = 0.86
AUC = 0.99
TSS = 0.86
AUC = 0.98
TSS = 0.81
Relative importance
bio01
bio02
bio05
bio12
bio15
BDW
CEC
CLY
NTO
SOC
bio01
bio02
bio05
bio12
bio15
BDW
CEC
CLY
NTO
SOC

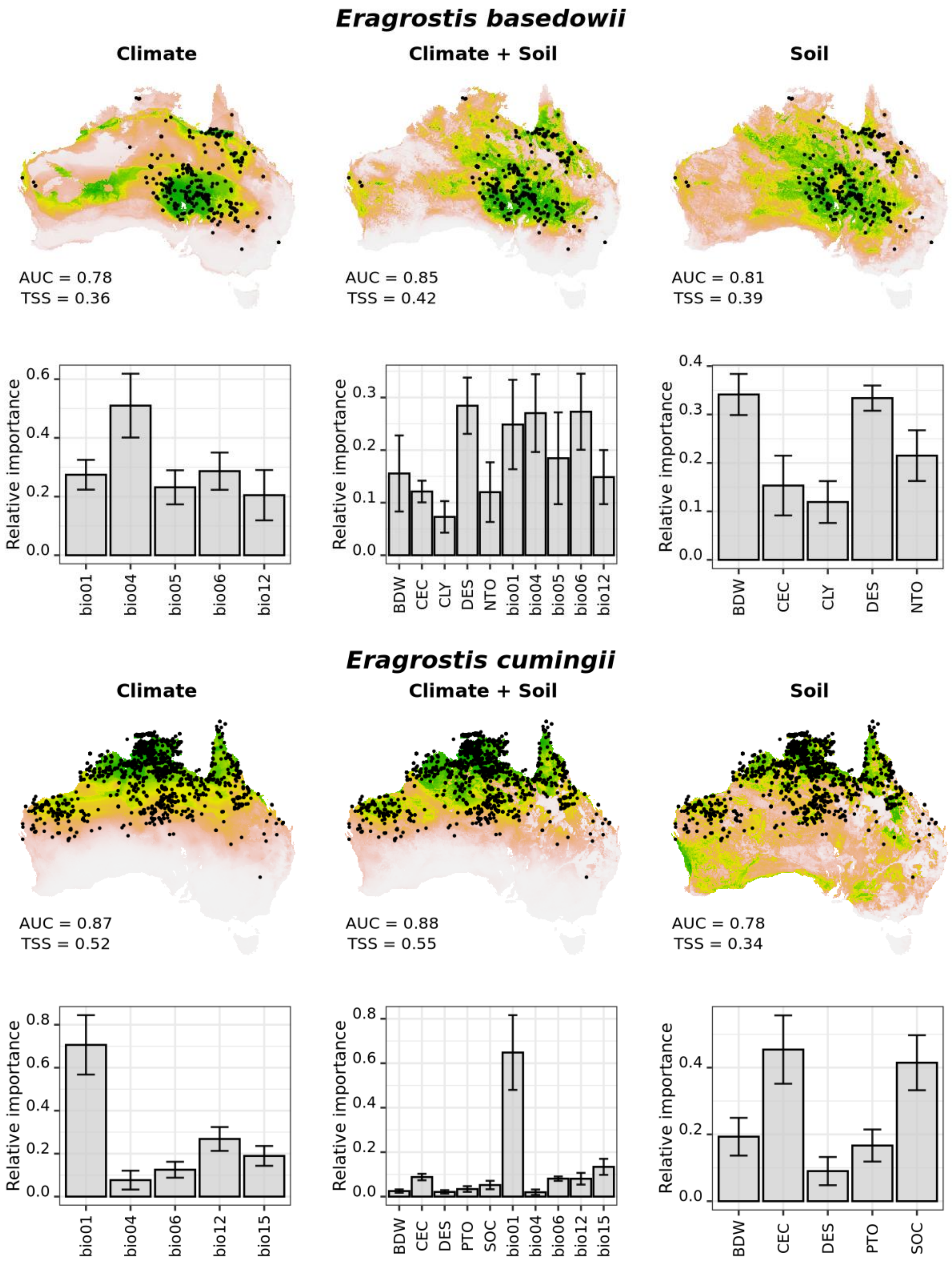
Eragrostis basedowii
Climate
Climate + Soil
Soil
AUC = 0.78
TSS = 0.36
AUC = 0.85
TSS = 0.42
AUC = 0.81
TSS = 0.39
Relative importance
bio01
bio04
bio05
bio06
bio12
BDW
CEC
CLY
DES
NTO
Eragrostis cumingii
Climate
Climate + Soil
Soil
AUC = 0.87
TSS = 0.52
AUC = 0.88
TSS = 0.55
AUC = 0.78
TSS = 0.34
Relative importance
bio01
bio04
bio06
bio12
bio15
BDW
CEC
DES
PTO
SOC

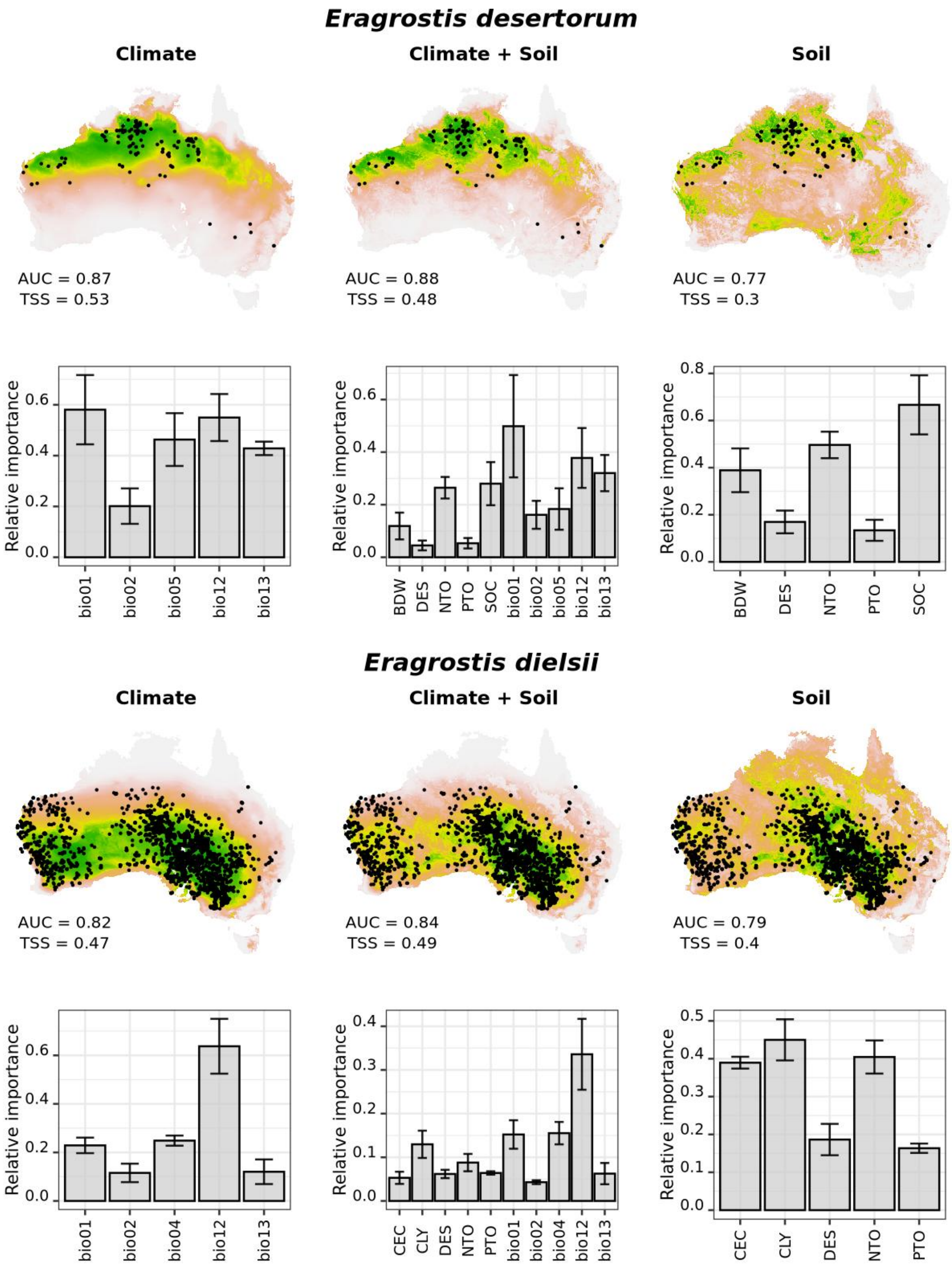

Eragrostis desertorum
Climate
Climate + Soil
Soil
AUC = 0.87
TSS = 0.53
AUC = 0.88
TSS = 0.48
AUC = 0.77
TSS = 0.3
Relative importance
0.0
0.2
0.4
0.6
0.8
bio01
bio02
bio05
bio12
bio13
BDW
DES
NTO
PTO
SOC
Eragrostis dielsii
Climate
Climate + Soil
Soil
AUC = 0.82
TSS = 0.47
AUC = 0.84
TSS = 0.49
AUC = 0.79
TSS = 0.4
Relative importance
0.0
0.1
0.2
0.3
0.4
0.5
0.6
bio01
bio02
bio04
bio12
bio13
CEC
CLY
DES
NTO
PTO

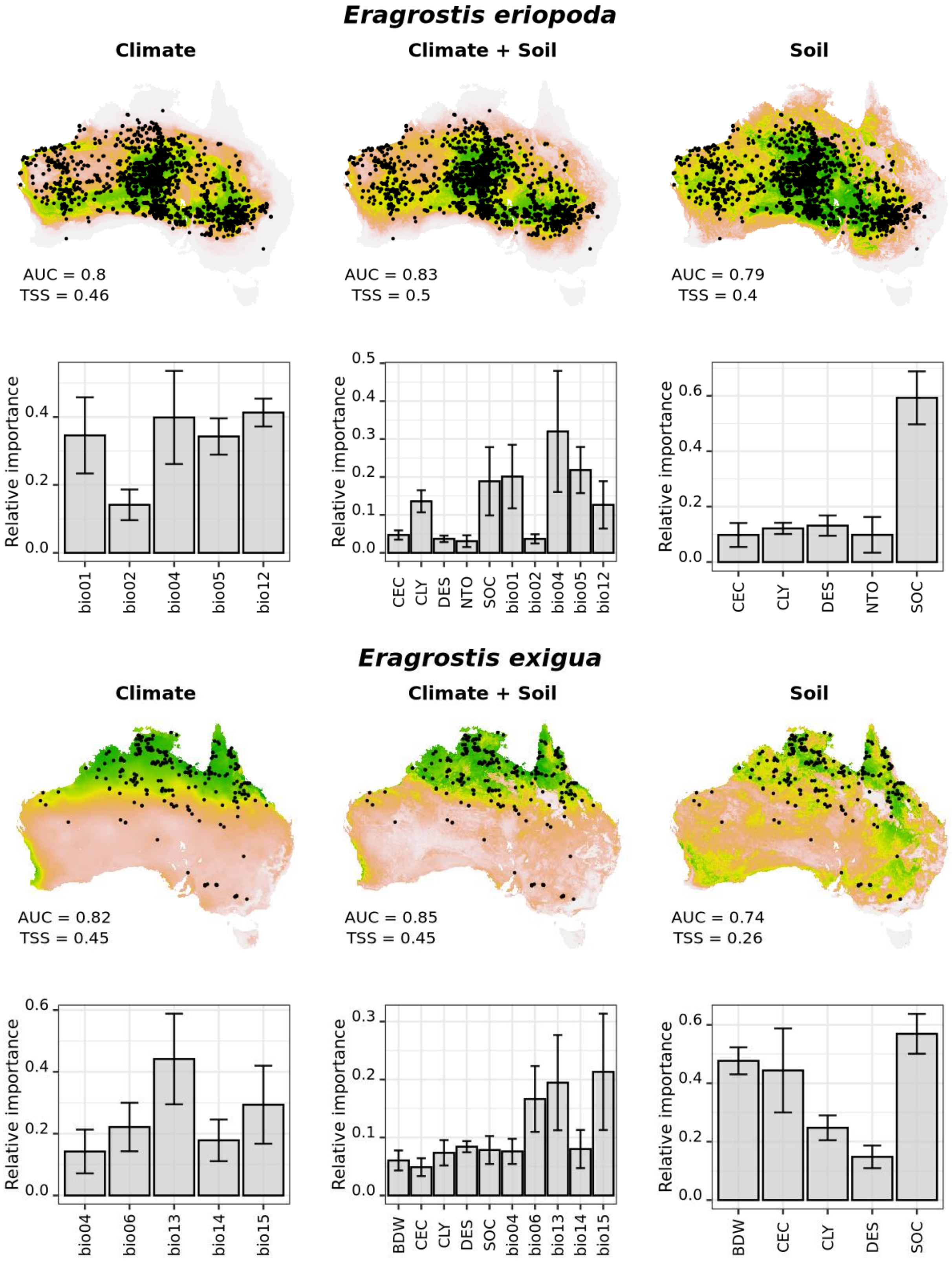

Eragrostis eriopoda
Climate
Climate + Soil
Soil
AUC = 0.8
TSS = 0.46
AUC = 0.83
TSS = 0.5
AUC = 0.79
TSS = 0.4
Relative importance
bio01
bio02
bio04
bio05
bio12
CEC
CLY
DES
NTO
SOC
Eragrostis exigua
Climate
Climate + Soil
Soil
AUC = 0.82
TSS = 0.45
AUC = 0.85
TSS = 0.45
AUC = 0.74
TSS = 0.26
Relative importance
bio04
bio06
bio13
bio14
bio15
BDW
CEC
CLY
DES
SOC

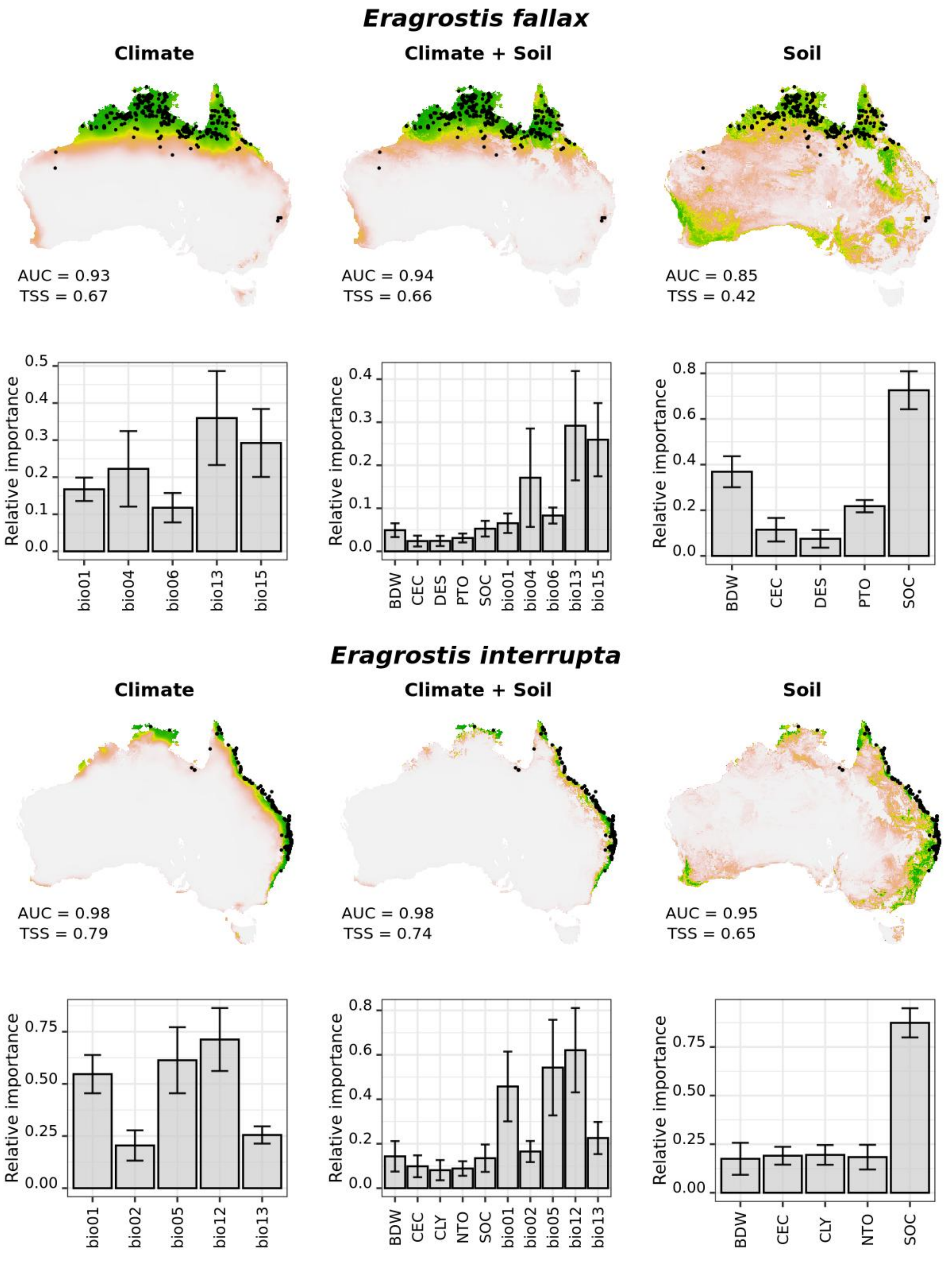
Eragrostis fallax
Climate
Climate + Soil
Soil
AUC = 0.93
TSS = 0.67
AUC = 0.94
TSS = 0.66
AUC = 0.85
TSS = 0.42
Relative importance
bio01 bio04 bio06 bio13 bio15
BDW CEC DES PTO SOC bio01 bio04 bio06 bio13 bio15
BDW CEC DES PTO SOC
Eragrostis interrupta
Climate
Climate + Soil
Soil
AUC = 0.98
TSS = 0.79
AUC = 0.98
TSS = 0.74
AUC = 0.95
TSS = 0.65
Relative importance
bio01 bio02 bio05 bio12 bio13
BDW CEC CLY NTO SOC bio01 bio02 bio05 bio12 bio13
BDW CEC CLY NTO SOC

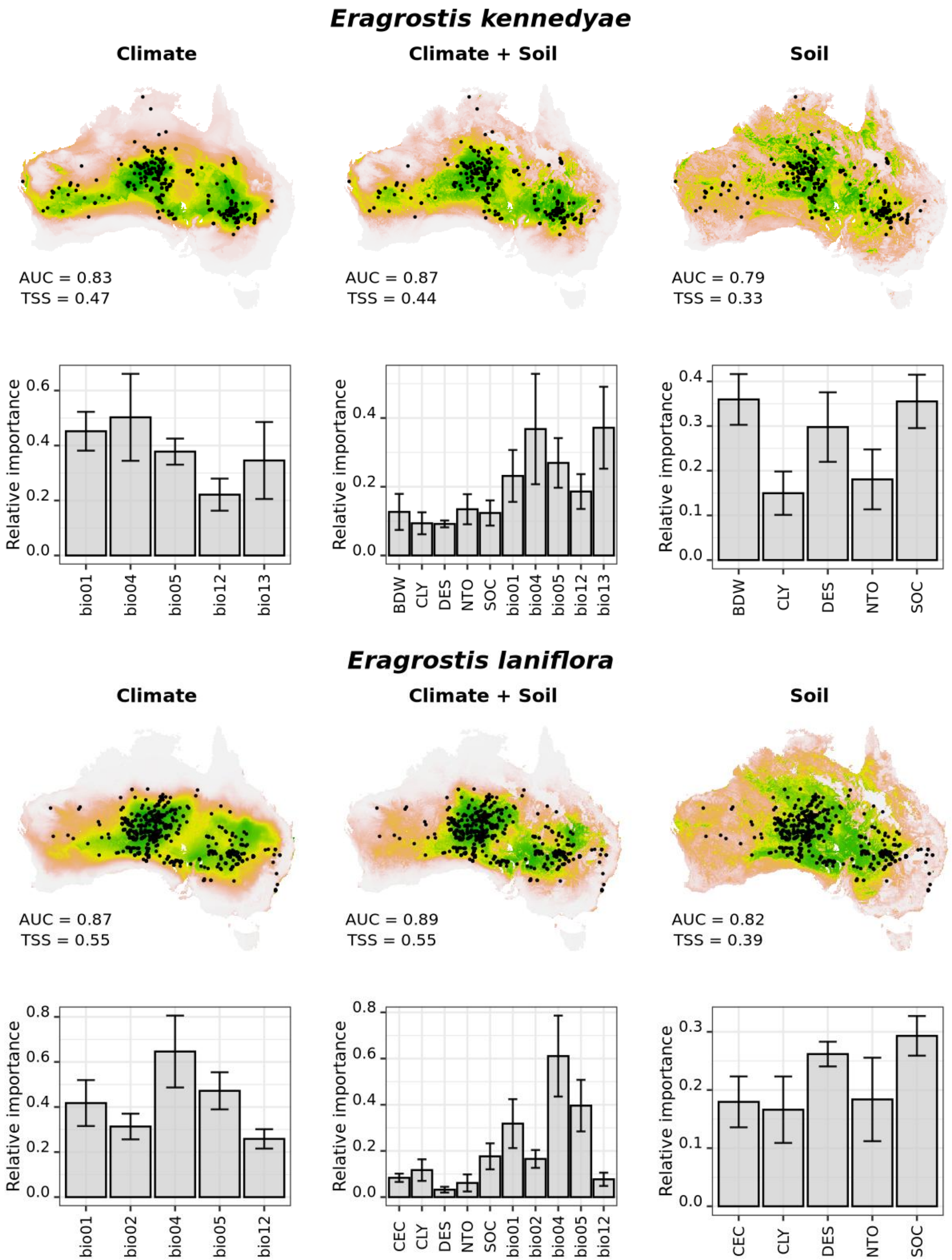

Eragrostis kennedyae
Climate
Climate + Soil
Soil
AUC = 0.83
TSS = 0.47
AUC = 0.87
TSS = 0.44
AUC = 0.79
TSS = 0.33
Relative importance
0.0
0.2
0.4
0.6
bio01
bio04
bio05
bio12
bio13
BDW
CLY
DES
NTO
SOC
0.1
0.3
Eragrostis laniflora
AUC = 0.87
TSS = 0.55
AUC = 0.89
TSS = 0.55
AUC = 0.82
TSS = 0.39
0.8
bio02
CEC

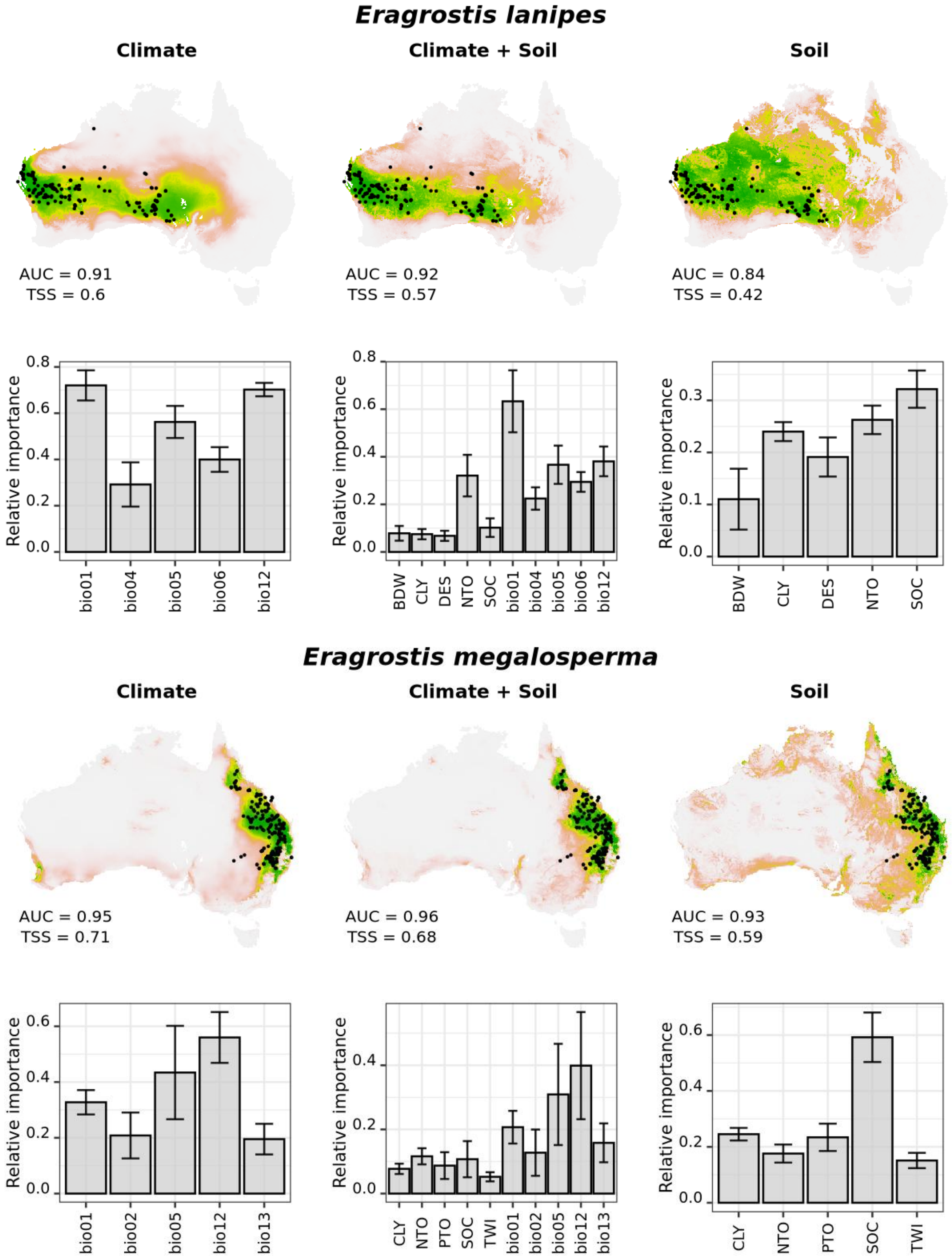
Eragrostis lanipes
Climate
Climate + Soil
Soil
AUC = 0.91
TSS = 0.6
AUC = 0.92
TSS = 0.57
AUC = 0.84
TSS = 0.42
Relative importance
bio01
bio04
bio05
bio06
bio12
BDW
CLY
DES
NTO
SOC
Eragrostis megalosperma
AUC = 0.95
TSS = 0.71
AUC = 0.96
TSS = 0.68
AUC = 0.93
TSS = 0.59
bio02
bio13
PTO
TWI

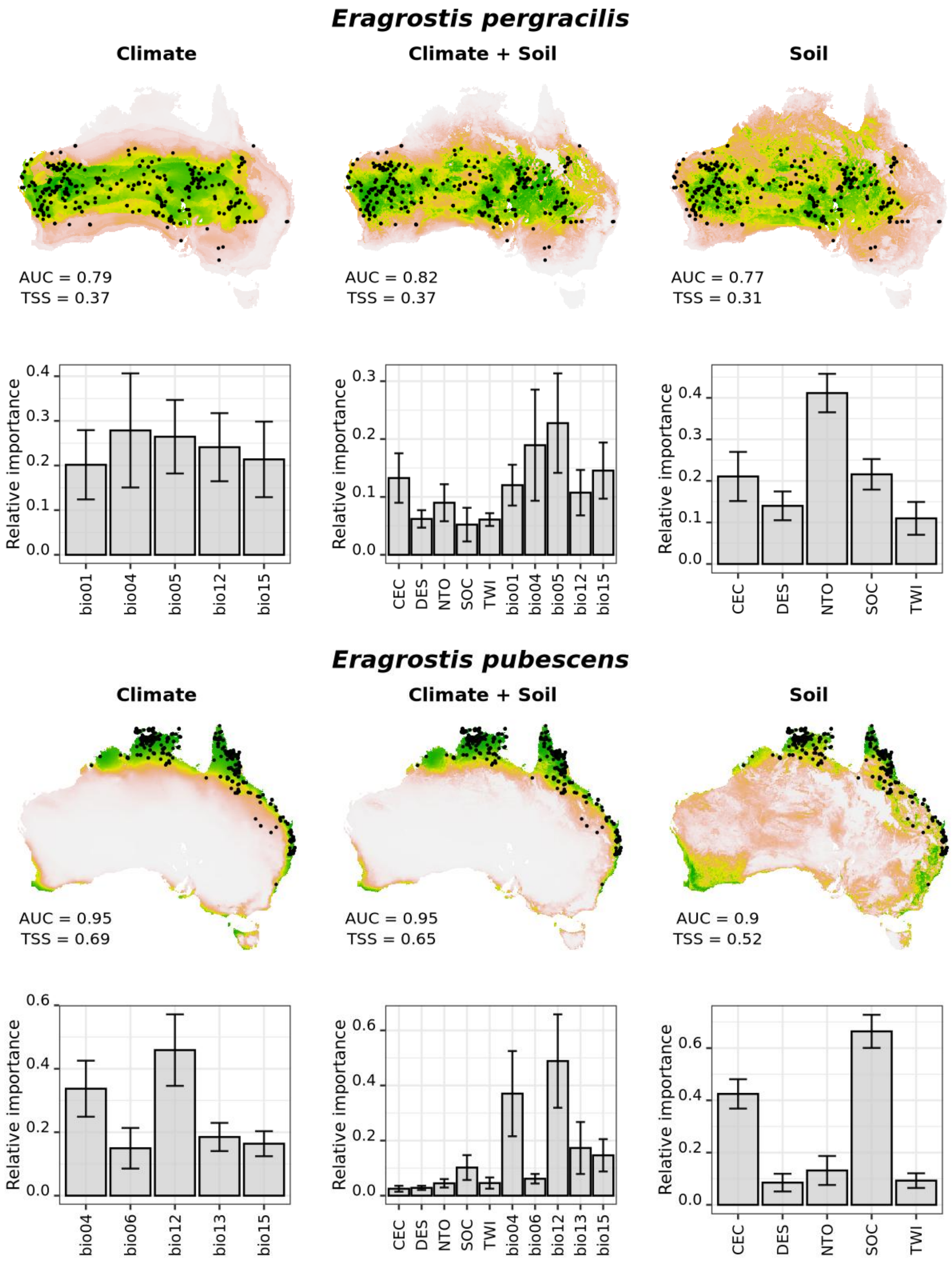
Eragrostis pergracilis
Climate
Climate + Soil
Soil
AUC = 0.79
TSS = 0.37
AUC = 0.82
TSS = 0.37
AUC = 0.77
TSS = 0.31
Relative importance
bio01
bio04
bio05
bio12
bio15
CEC
DES
NTO
SOC
TWI
Eragrostis pubescens
Climate
Climate + Soil
Soil
AUC = 0.95
TSS = 0.69
AUC = 0.95
TSS = 0.65
AUC = 0.9
TSS = 0.52
Relative importance
bio04
bio06
bio12
bio13
bio15
CEC
DES
NTO
SOC
TWI

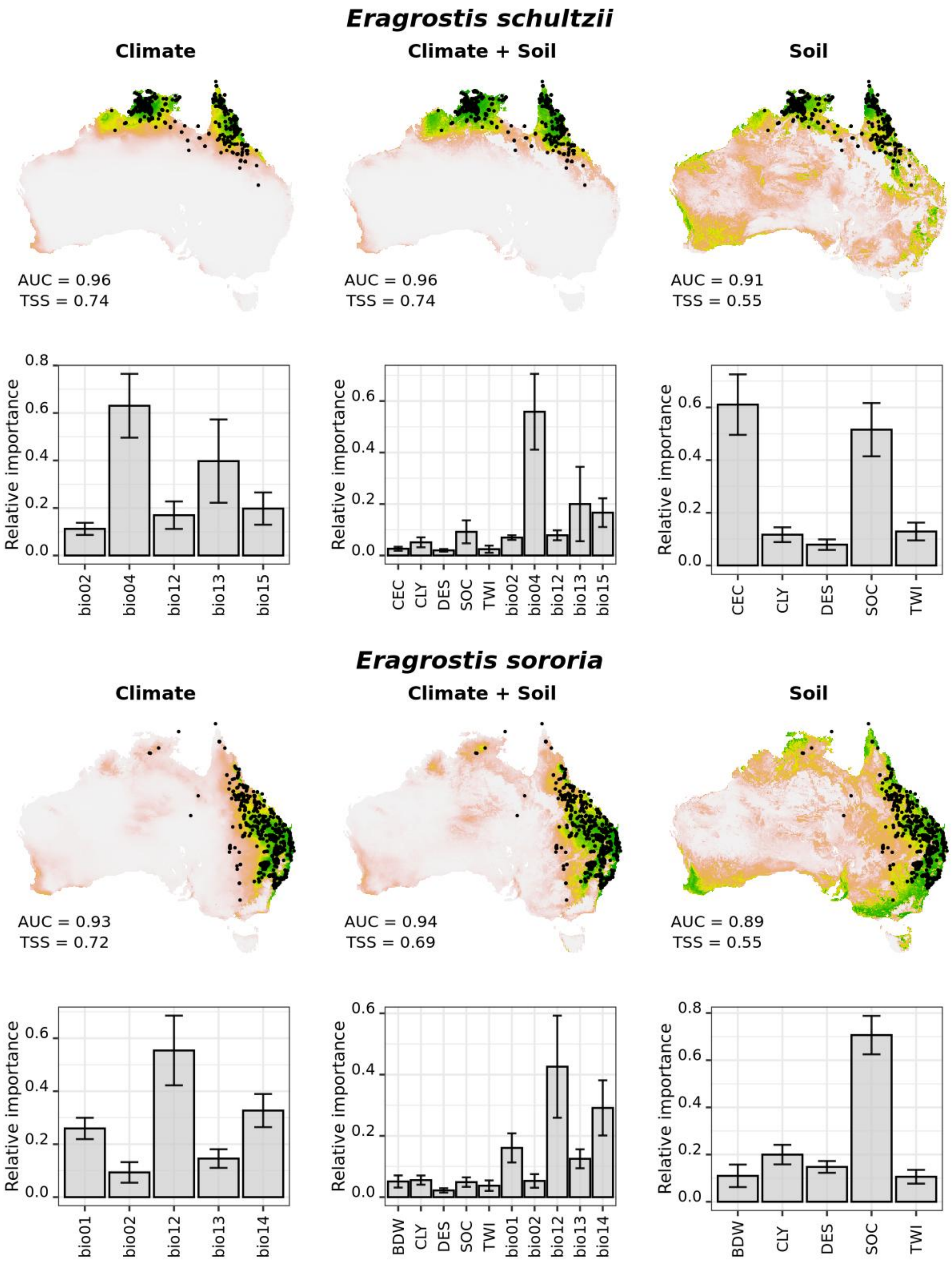
Eragrostis schultzii
Climate
Climate + Soil
Soil
AUC = 0.96
TSS = 0.74
AUC = 0.96
TSS = 0.74
AUC = 0.91
TSS = 0.55
Relative importance
bio02 bio04 bio12 bio13 bio15
CEC CLY DES SOC TWI bio02 bio04 bio12 bio13 bio15
CEC CLY DES SOC TWI
Eragrostis sororia
Climate
Climate + Soil
Soil
AUC = 0.93
TSS = 0.72
AUC = 0.94
TSS = 0.69
AUC = 0.89
TSS = 0.55
Relative importance
bio01 bio02 bio12 bio13 bio14
BDW CLY DES SOC TWI bio01 bio02 bio12 bio13 bio14
BDW CLY DES SOC TWI

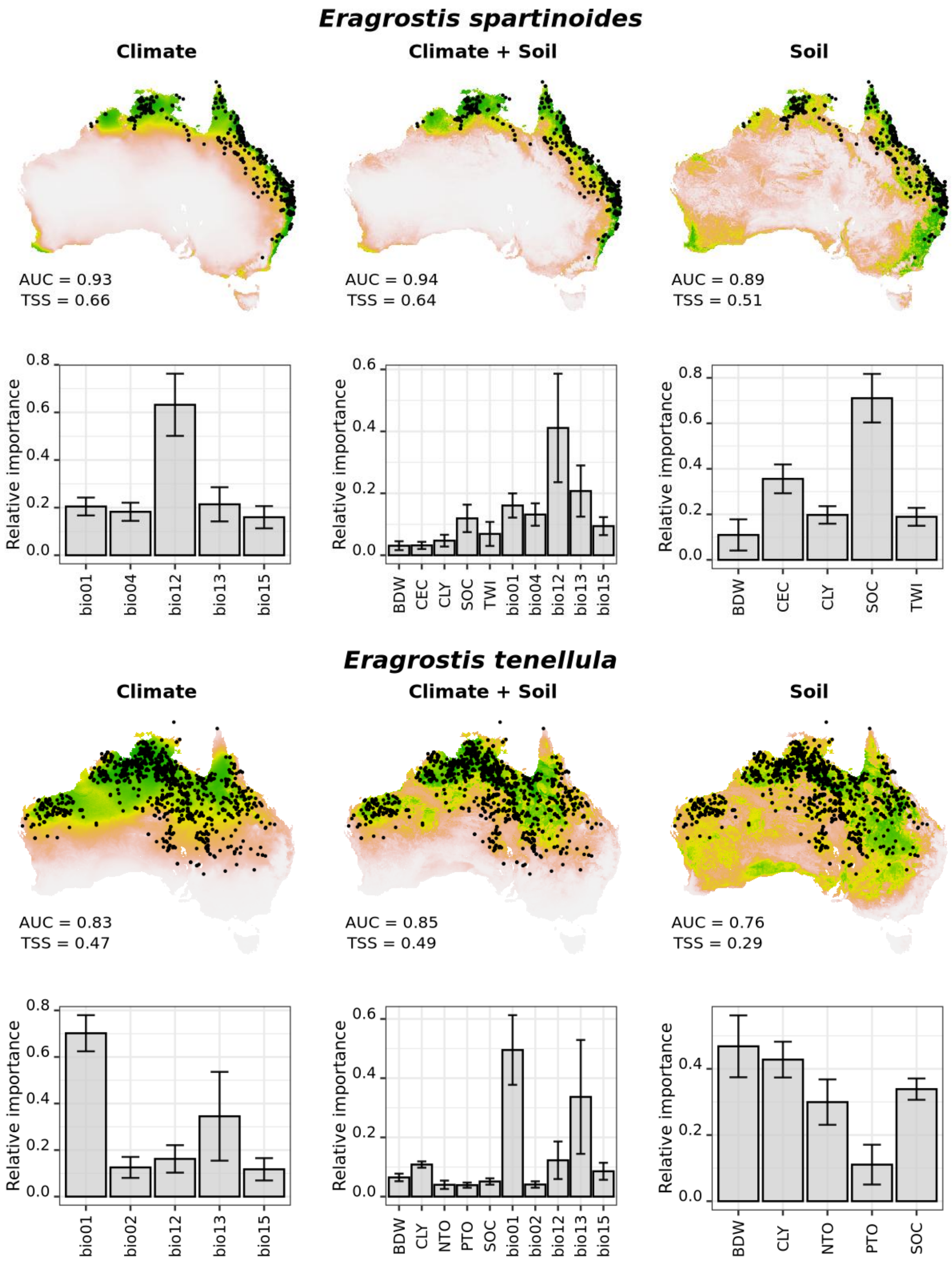

Eragrostis spartinoides
Climate
Climate + Soil
Soil
AUC = 0.93
TSS = 0.66
AUC = 0.94
TSS = 0.64
AUC = 0.89
TSS = 0.51
Relative importance
bio01
bio04
bio12
bio13
bio15
BDW
CEC
CLY
SOC
TWI
Eragrostis tenellula
AUC = 0.83
TSS = 0.47
AUC = 0.85
TSS = 0.49
AUC = 0.76
TSS = 0.29
bio02
NTO
PTO

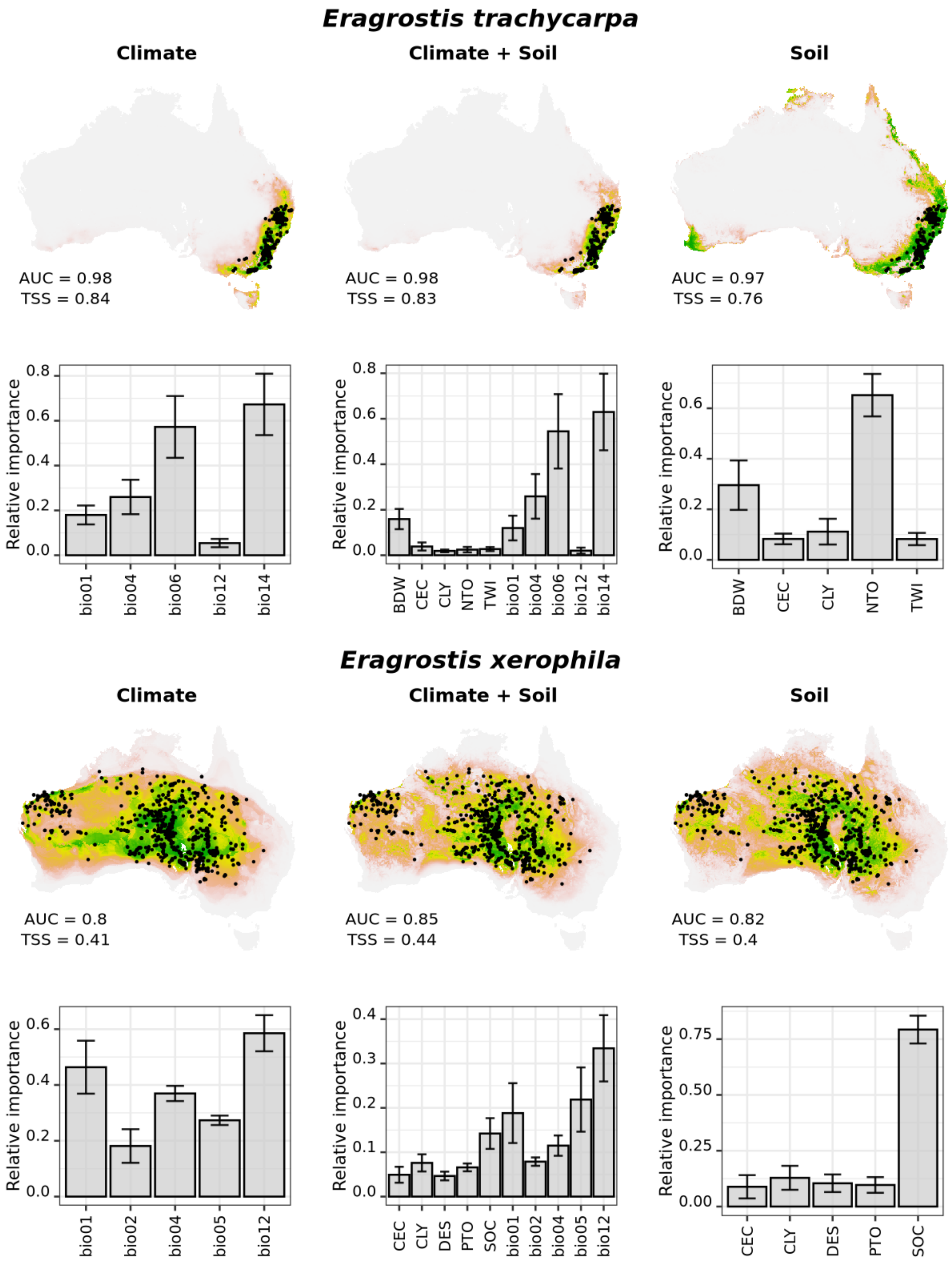

Eragrostis trachycarpa
Climate
Climate + Soil
Soil
AUC = 0.98
TSS = 0.84
AUC = 0.98
TSS = 0.83
AUC = 0.97
TSS = 0.76
Relative importance
bio01
bio04
bio06
bio12
bio14
BDW
CEC
CLY
NTO
TWI
Eragrostis xerophila
Climate
Climate + Soil
Soil
AUC = 0.8
TSS = 0.41
AUC = 0.85
TSS = 0.44
AUC = 0.82
TSS = 0.4
Relative importance
bio01
bio02
bio04
bio05
bio12
CEC
CLY
DES
PTO
SOC

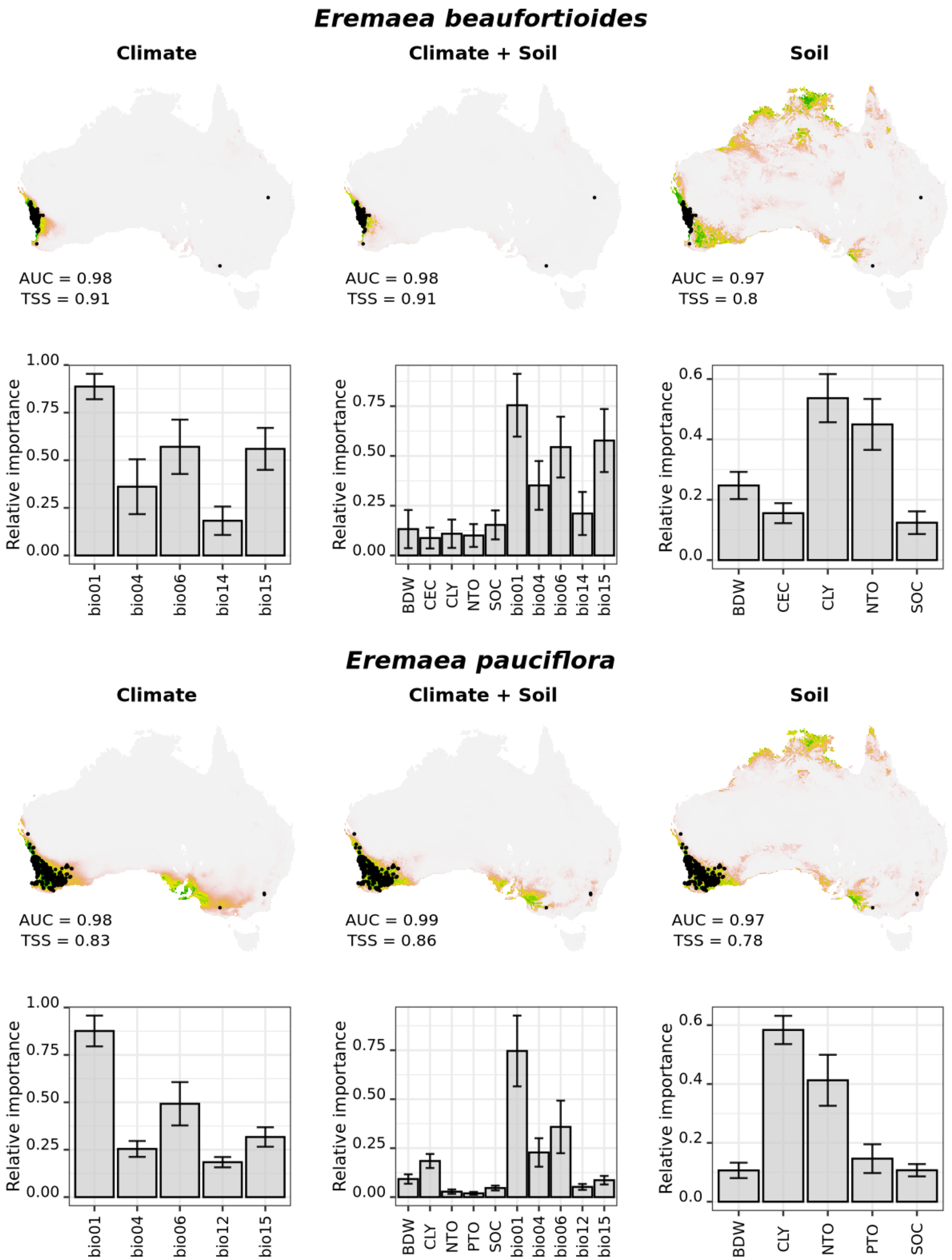
Eremaea beaufortioides
Climate
Climate + Soil
Soil
AUC = 0.98
TSS = 0.91
AUC = 0.98
TSS = 0.91
AUC = 0.97
TSS = 0.8
Relative importance
1.00
0.75
0.50
0.25
0.00
bio01
bio04
bio06
bio14
bio15
BDW
CEC
CLY
NTO
SOC
0.6
0.4
0.2
0.0
Eremaea pauciflora
Climate
Climate + Soil
Soil
AUC = 0.98
TSS = 0.83
AUC = 0.99
TSS = 0.86
AUC = 0.97
TSS = 0.78
bio12
PTO

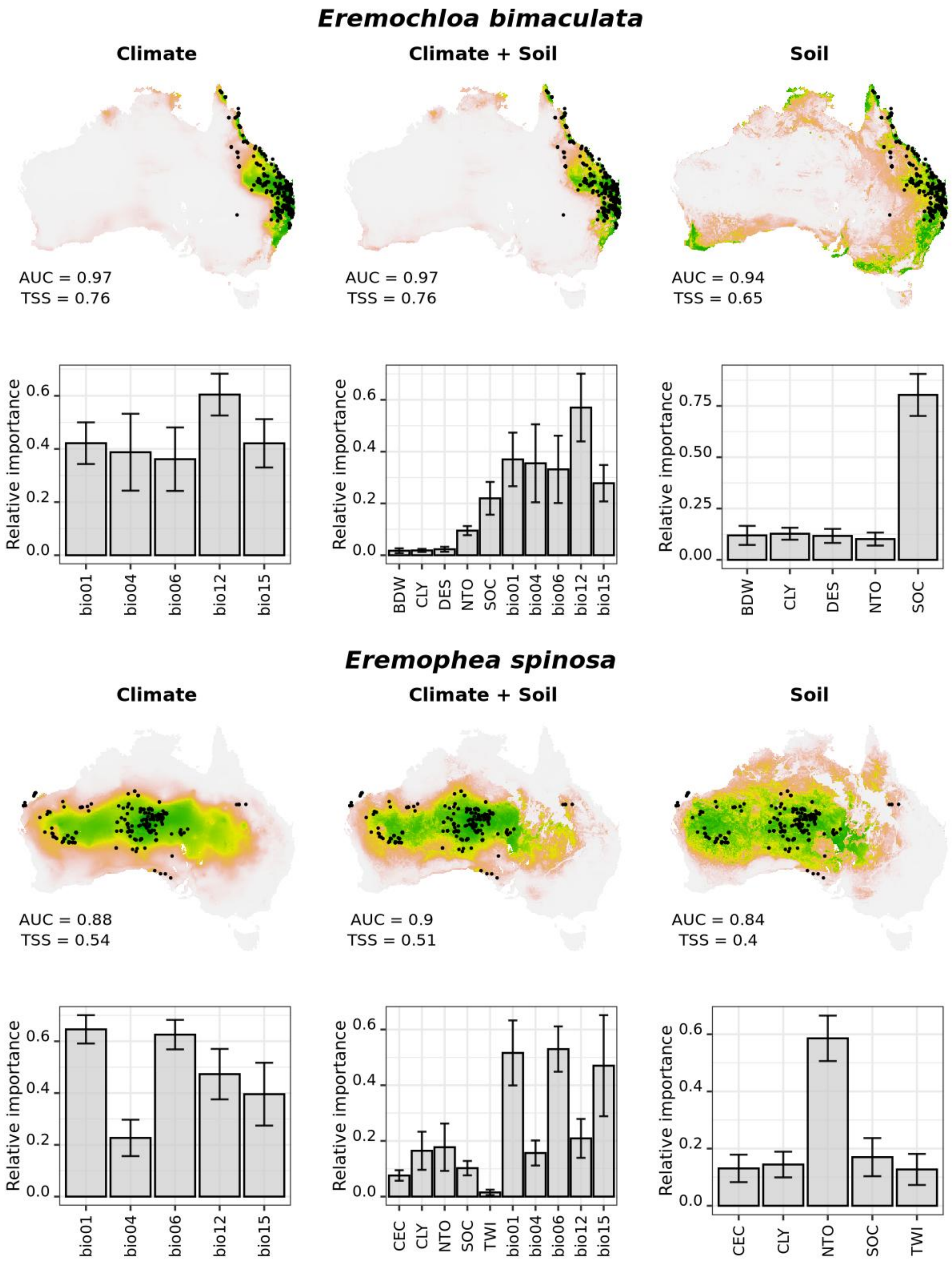
Eremochloa bimaculata
Climate
Climate + Soil
Soil
AUC = 0.97
TSS = 0.76
AUC = 0.97
TSS = 0.76
AUC = 0.94
TSS = 0.65
Relative importance
bio01
bio04
bio06
bio12
bio15
BDW
CLY
DES
NTO
SOC
Eremophea spinosa
Climate
Climate + Soil
Soil
AUC = 0.88
TSS = 0.54
AUC = 0.9
TSS = 0.51
AUC = 0.84
TSS = 0.4
Relative importance
CEC
CLY
NTO
SOC
TWI
bio01
bio04
bio06
bio12
bio15

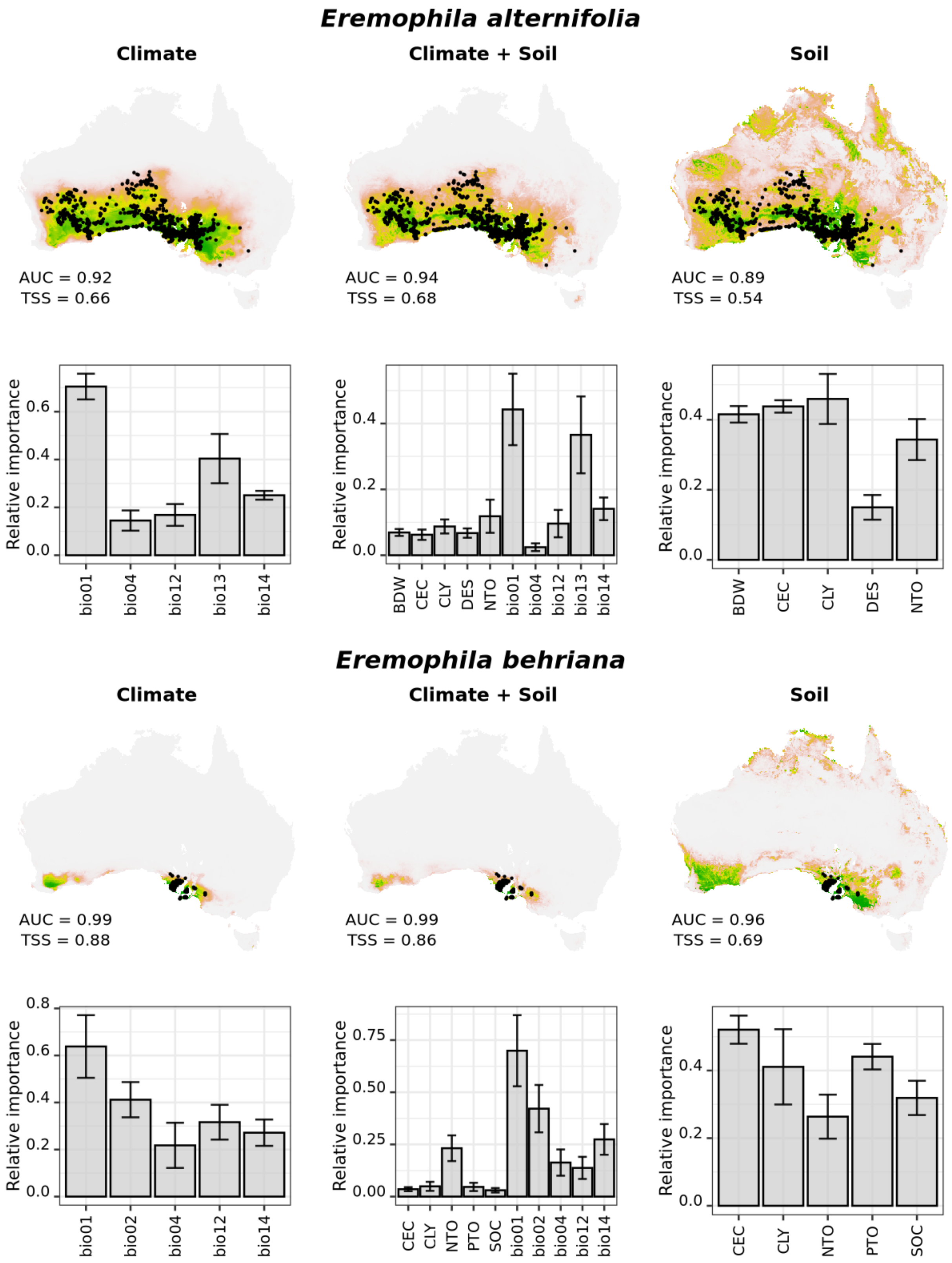
Eremophila alternifolia
Climate
Climate + Soil
Soil
AUC = 0.92
TSS = 0.66
AUC = 0.94
TSS = 0.68
AUC = 0.89
TSS = 0.54
Relative importance
bio01 bio04 bio12 bio13 bio14
BDW CEC CLY DES NTO bio01 bio04 bio12 bio13 bio14
BDW CEC CLY DES NTO
Eremophila behriana
Climate
Climate + Soil
Soil
AUC = 0.99
TSS = 0.88
AUC = 0.99
TSS = 0.86
AUC = 0.96
TSS = 0.69
Relative importance
bio01 bio02 bio04 bio12 bio14
CEC CLY NTO PTO SOC bio01 bio02 bio04 bio12 bio14
CEC CLY NTO PTO SOC

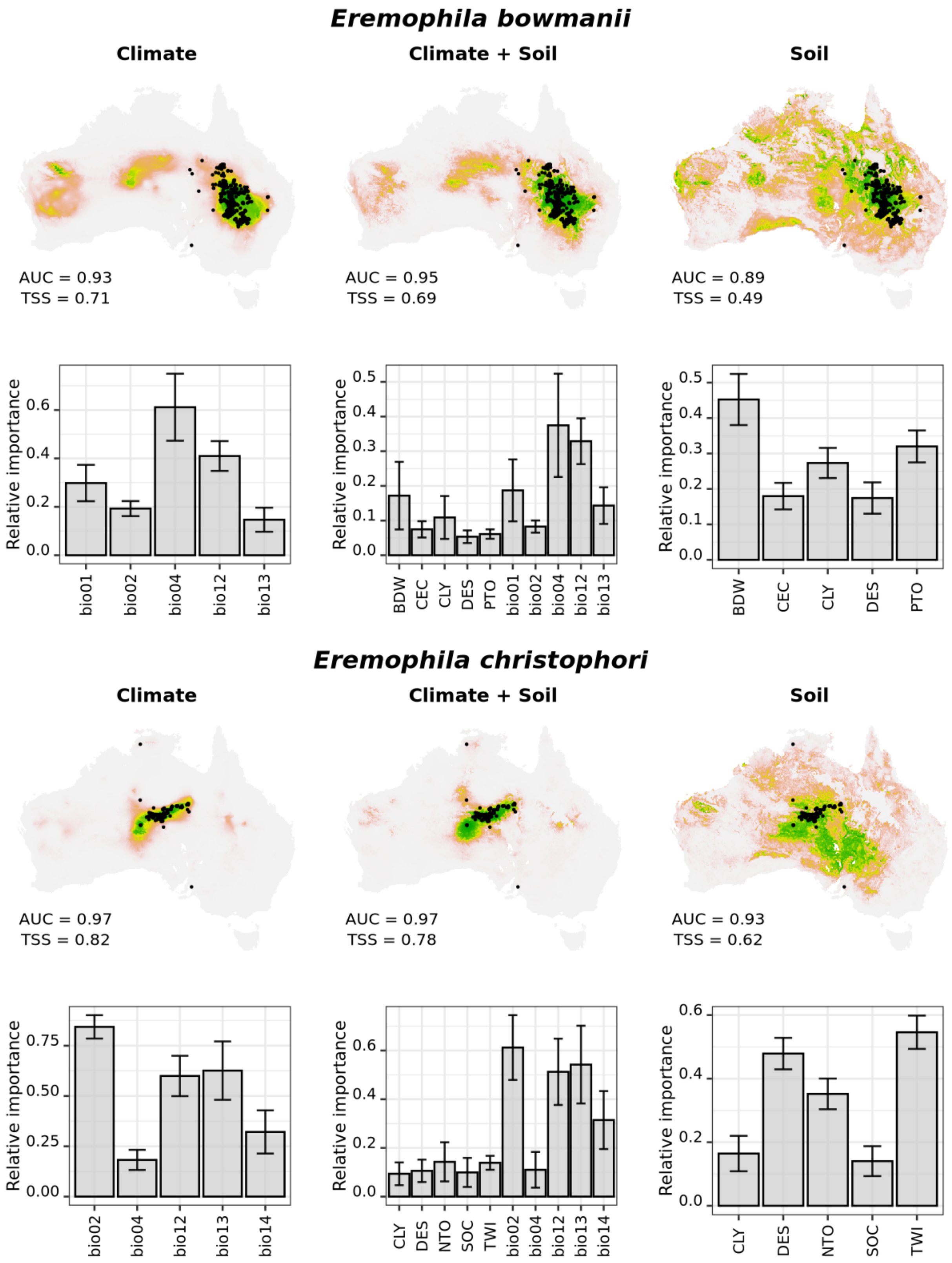

Eremophila bowmanii
Climate
Climate + Soil
Soil
AUC = 0.93
TSS = 0.71
AUC = 0.95
TSS = 0.69
AUC = 0.89
TSS = 0.49
Relative importance
bio01
bio02
bio04
bio12
bio13
BDW
CEC
CLY
DES
PTO
Eremophila christophori
Climate
Climate + Soil
Soil
AUC = 0.97
TSS = 0.82
AUC = 0.97
TSS = 0.78
AUC = 0.93
TSS = 0.62
Relative importance
bio02
bio04
bio12
bio13
bio14
CLY
DES
NTO
SOC
TWI

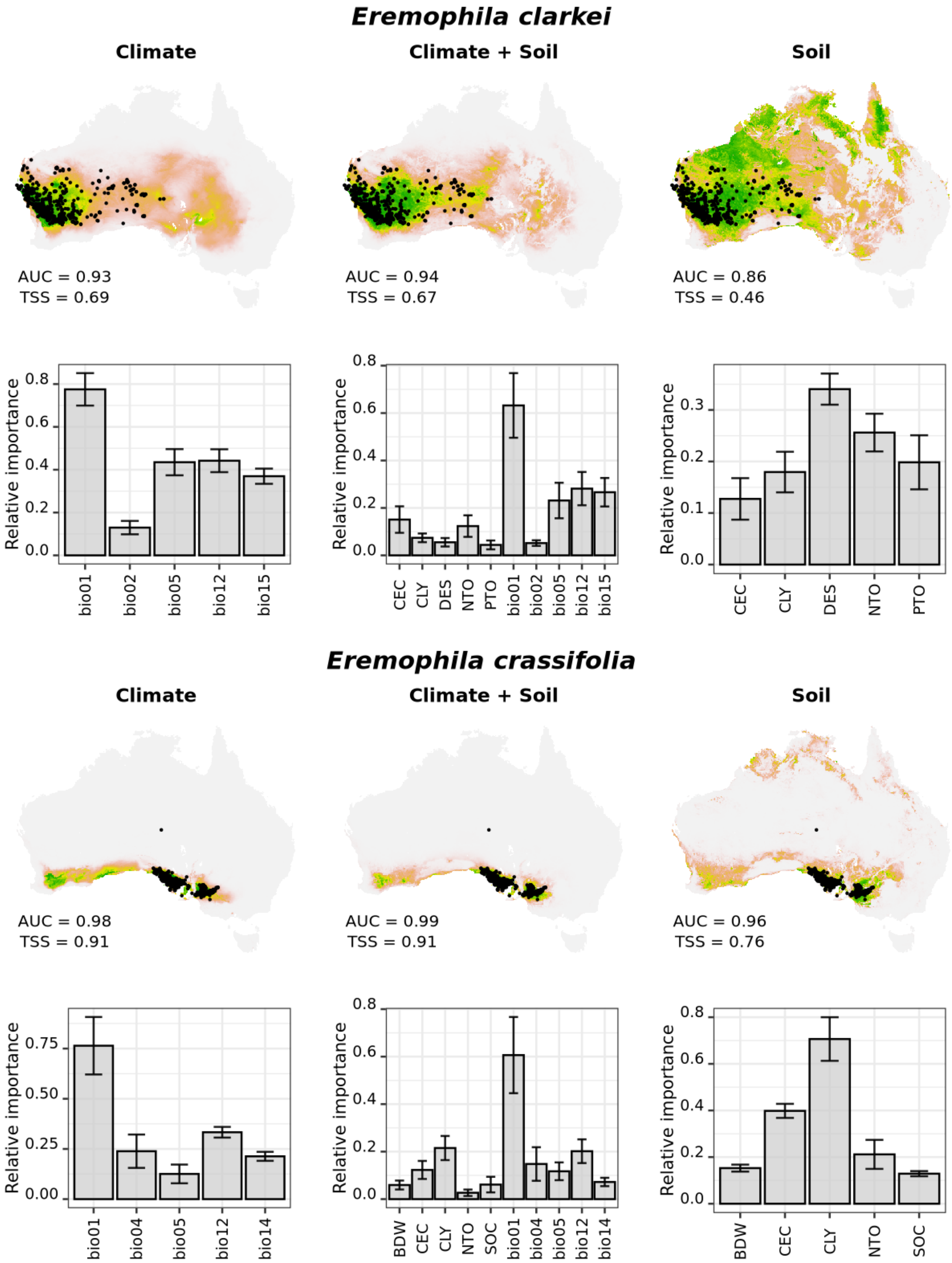

Eremophila clarkei
Climate
Climate + Soil
Soil
AUC = 0.93
TSS = 0.69
AUC = 0.94
TSS = 0.67
AUC = 0.86
TSS = 0.46
Relative importance
0.0
0.2
0.4
0.6
0.8
bio01
bio02
bio05
bio12
bio15
CEC
CLY
DES
NTO
PTO
0.1
0.3
Eremophila crassifolia
AUC = 0.98
TSS = 0.91
AUC = 0.99
TSS = 0.91
AUC = 0.96
TSS = 0.76
0.00
0.25
0.50
0.75
bio04
bio14
BDW
SOC

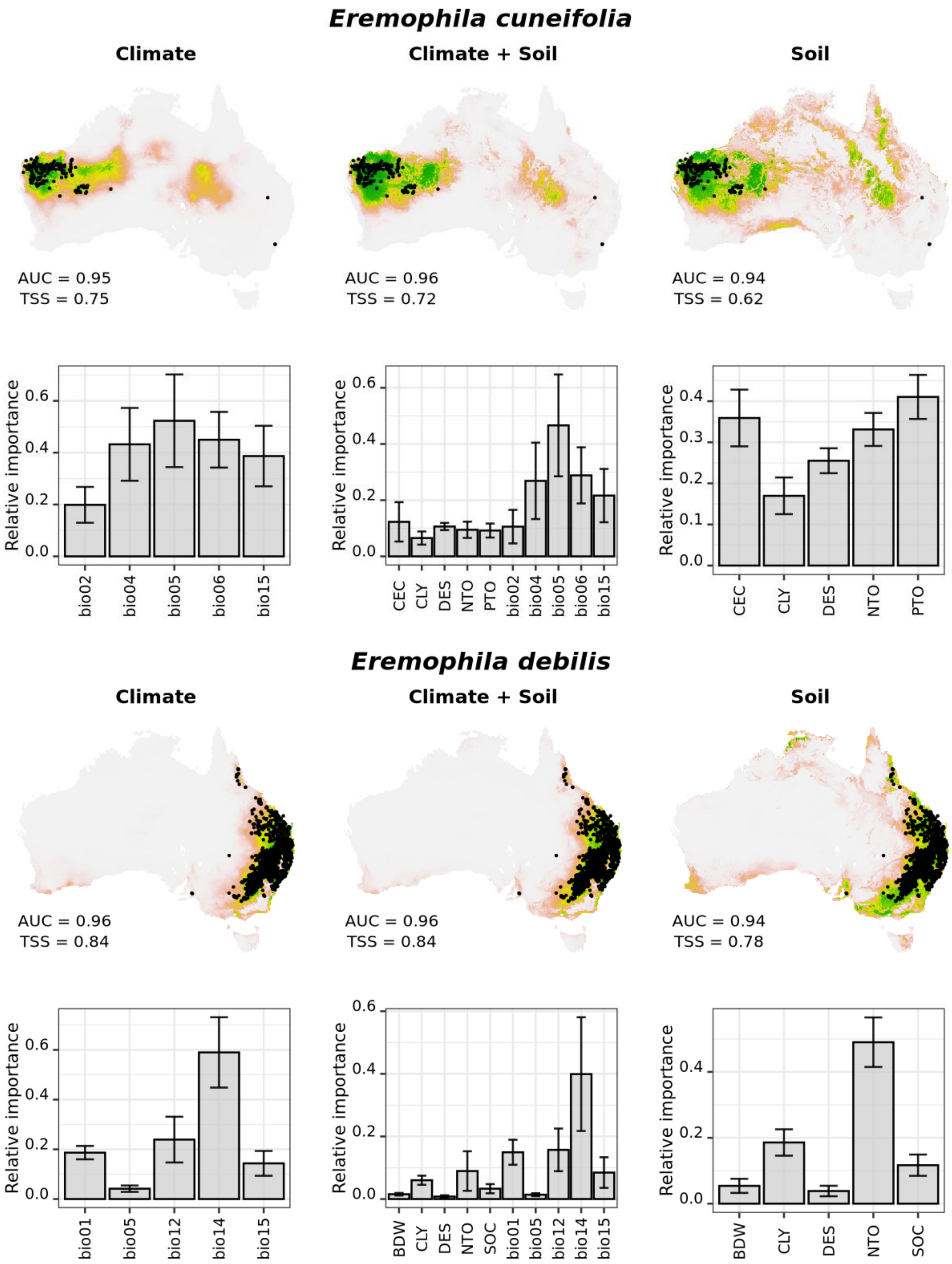

Eremophila cuneifolia
Climate
Climate + Soil
Soil
AUC = 0.95
TSS = 0.75
AUC = 0.96
TSS = 0.72
AUC = 0.94
TSS = 0.62
Relative importance
bio02 bio04 bio05 bio06 bio15
CEC CLY DES NTO PTO bio02 bio04 bio05 bio06 bio15
CEC CLY DES NTO PTO
Eremophila debilis
Climate
Climate + Soil
Soil
AUC = 0.96
TSS = 0.84
AUC = 0.96
TSS = 0.84
AUC = 0.94
TSS = 0.78
Relative importance
bio01 bio05 bio12 bio14 bio15
BDW CLY DES NTO SOC bio01 bio05 bio12 bio14 bio15
BDW CLY DES NTO SOC

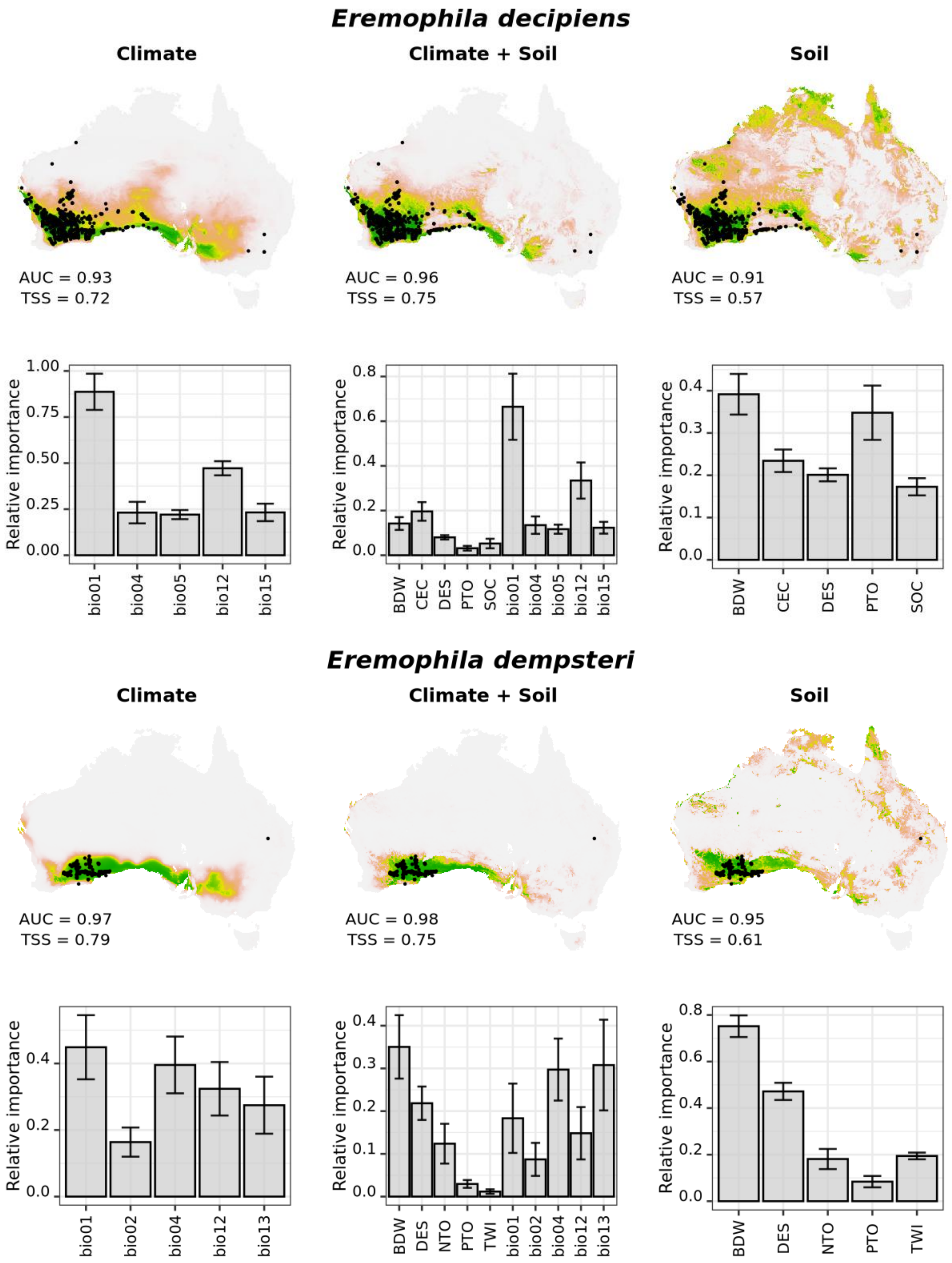

Eremophila decipiens
Climate
Climate + Soil
Soil
AUC = 0.93
TSS = 0.72
AUC = 0.96
TSS = 0.75
AUC = 0.91
TSS = 0.57
Relative importance
1.00
0.75
0.50
0.25
0.00
bio01
bio04
bio05
bio12
bio15
0.8
0.6
0.4
0.2
0.0
BDW
CEC
DES
PTO
SOC
bio01
bio04
bio05
bio12
bio15
0.4
0.3
0.2
0.1
0.0
BDW
CEC
DES
PTO
SOC
Eremophila dempsteri
Climate
Climate + Soil
Soil
AUC = 0.97
TSS = 0.79
AUC = 0.98
TSS = 0.75
AUC = 0.95
TSS = 0.61
Relative importance
0.4
0.2
0.0
bio01
bio02
bio04
bio12
bio13
0.4
0.3
0.2
0.1
0.0
BDW
DES
NTO
PTO
TWI
bio01
bio02
bio04
bio12
bio13
0.8
0.6
0.4
0.2
0.0
BDW
DES
NTO
PTO
TWI

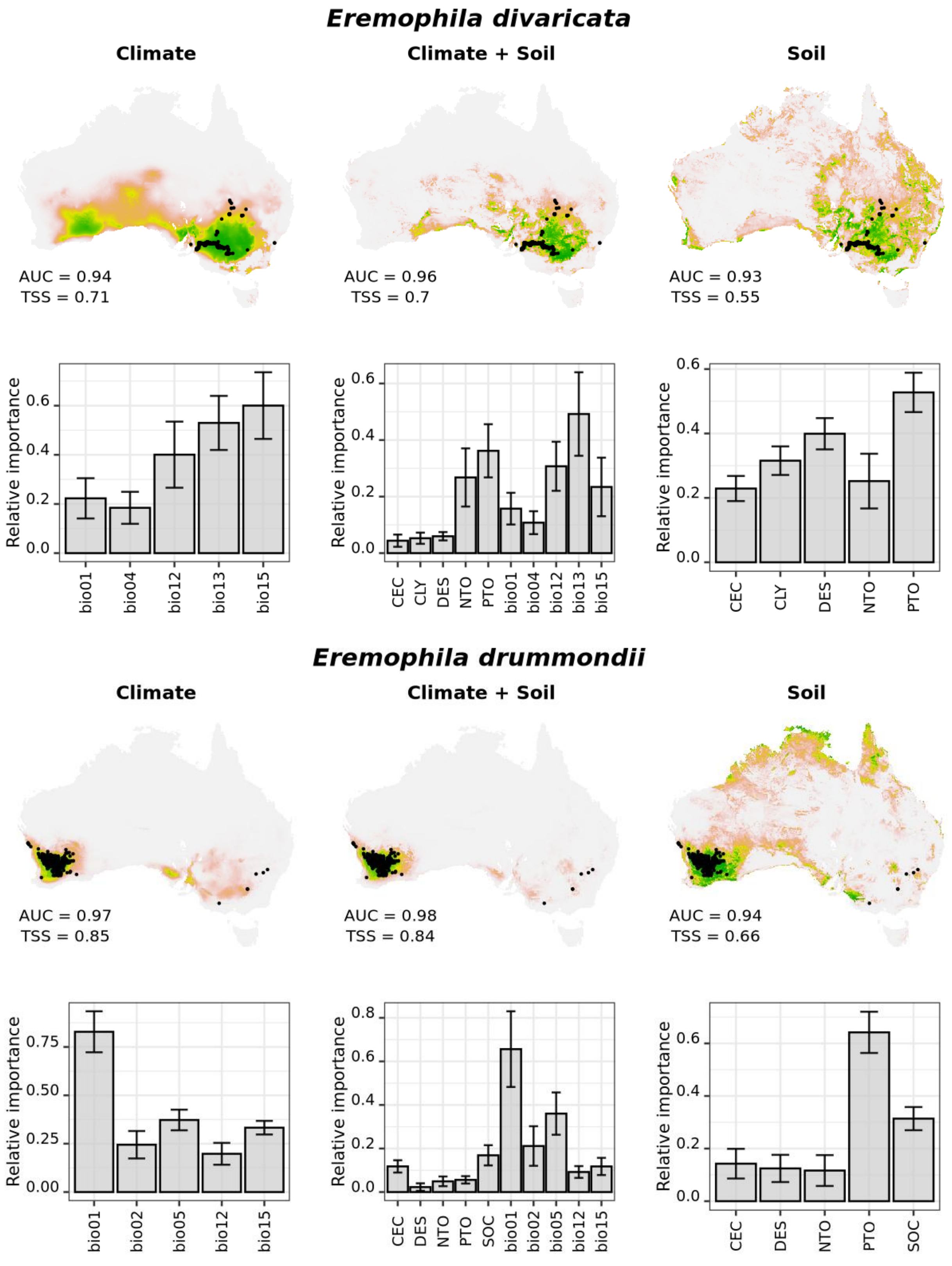

Eremophila divaricata
Climate
Climate + Soil
Soil
AUC = 0.94
TSS = 0.71
AUC = 0.96
TSS = 0.7
AUC = 0.93
TSS = 0.55
Relative importance
bio01 bio04 bio12 bio13 bio15
CEC CLY DES NTO PTO bio01 bio04 bio12 bio13 bio15
CEC CLY DES NTO PTO
Eremophila drummondii
Climate
Climate + Soil
Soil
AUC = 0.97
TSS = 0.85
AUC = 0.98
TSS = 0.84
AUC = 0.94
TSS = 0.66
Relative importance
bio01 bio02 bio05 bio12 bio15
CEC DES NTO PTO SOC bio01 bio02 bio05 bio12 bio15
CEC DES NTO PTO SOC

Eremophila duttonii

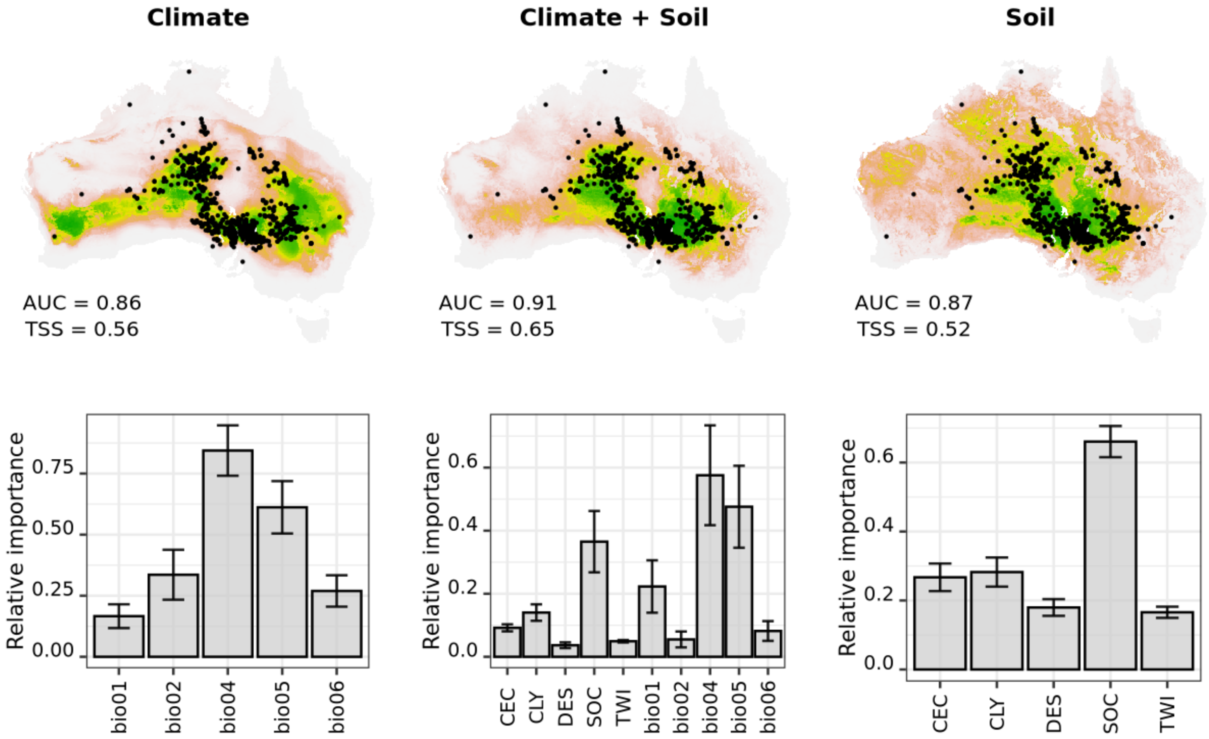

Eremophila eriocalyx

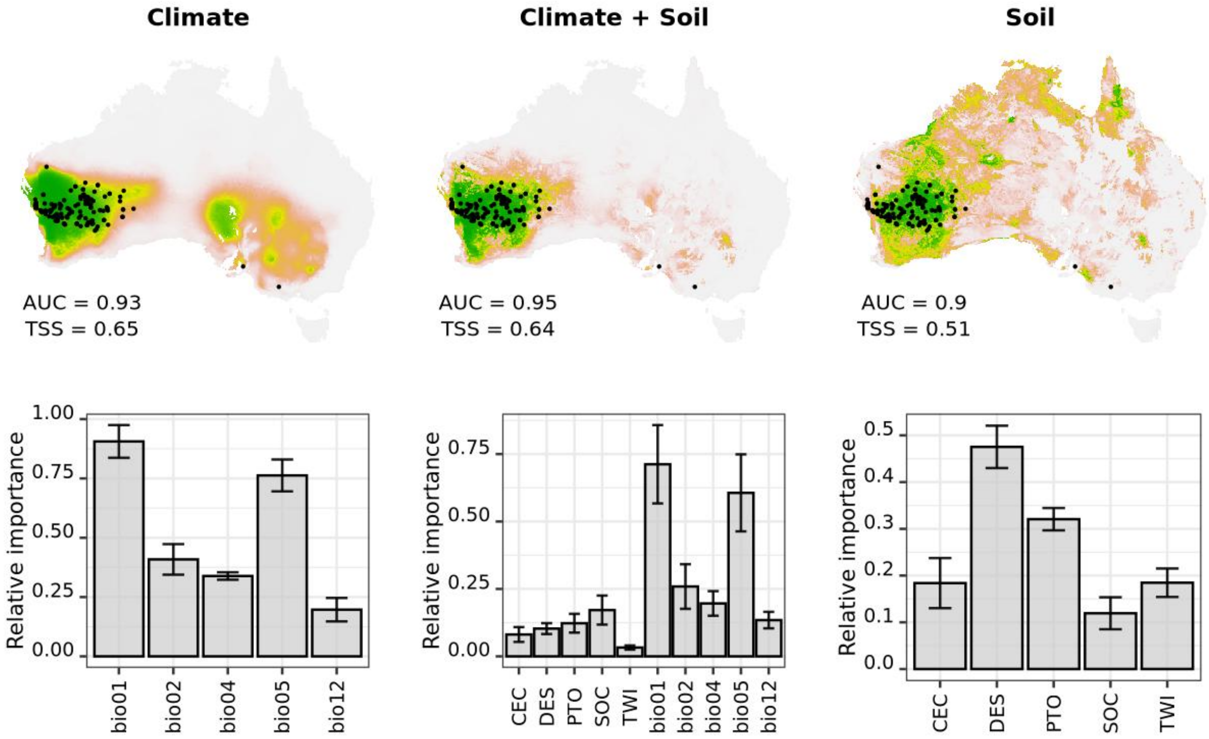

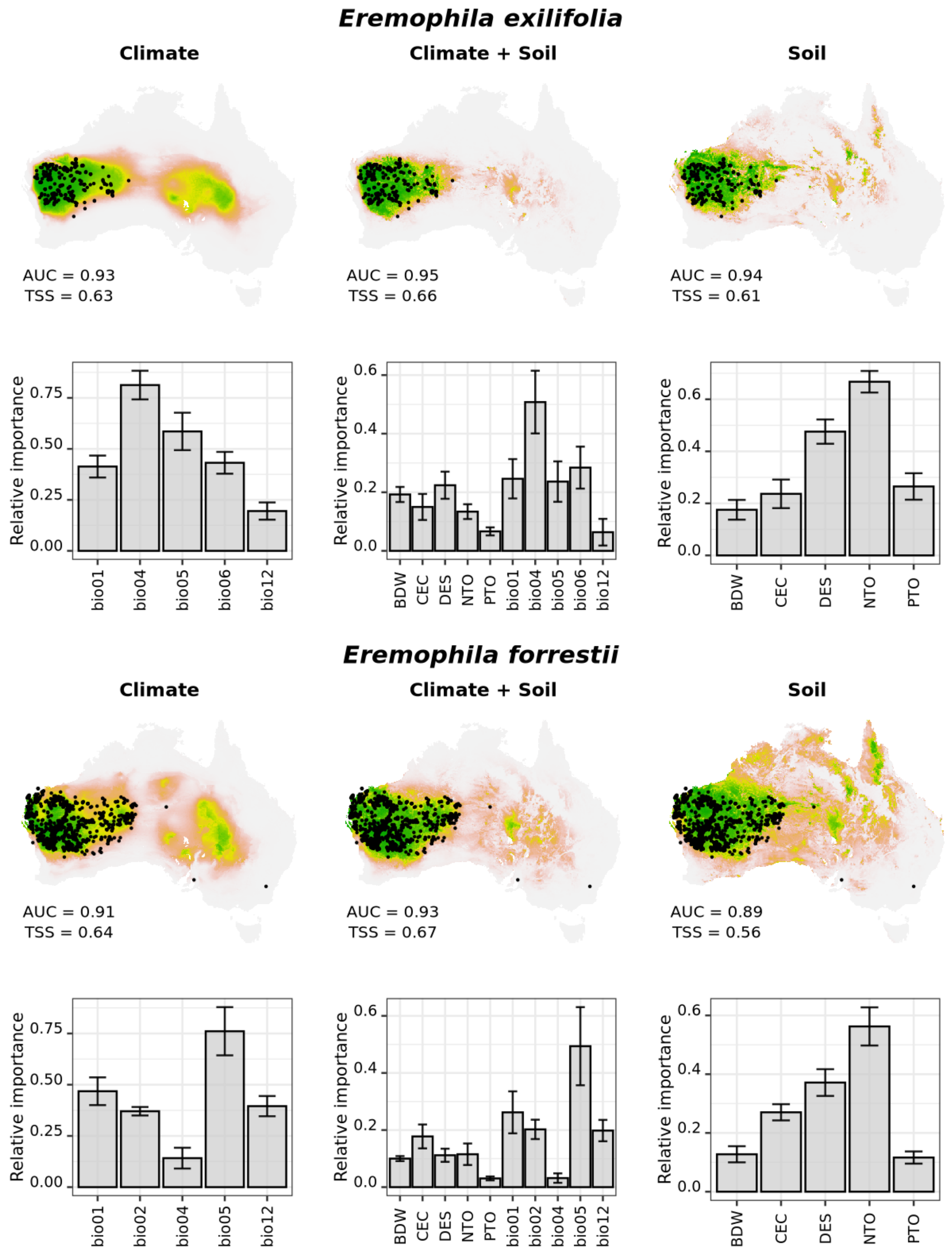

Eremophila exilifolia
Climate
Climate + Soil
Soil
AUC = 0.93
TSS = 0.63
AUC = 0.95
TSS = 0.66
AUC = 0.94
TSS = 0.61
Relative importance
0.00
0.25
0.50
0.75
bio01
bio04
bio05
bio06
bio12
0.0
0.2
0.4
0.6
BDW
CEC
DES
NTO
PTO
bio01
bio04
bio05
bio06
bio12
BDW
CEC
DES
NTO
PTO
Eremophila forrestii
Climate
Climate + Soil
Soil
AUC = 0.91
TSS = 0.64
AUC = 0.93
TSS = 0.67
AUC = 0.89
TSS = 0.56
Relative importance
0.00
0.25
0.50
0.75
bio01
bio02
bio04
bio05
bio12
0.0
0.2
0.4
0.6
BDW
CEC
DES
NTO
PTO
bio01
bio02
bio04
bio05
bio12
BDW
CEC
DES
NTO
PTO

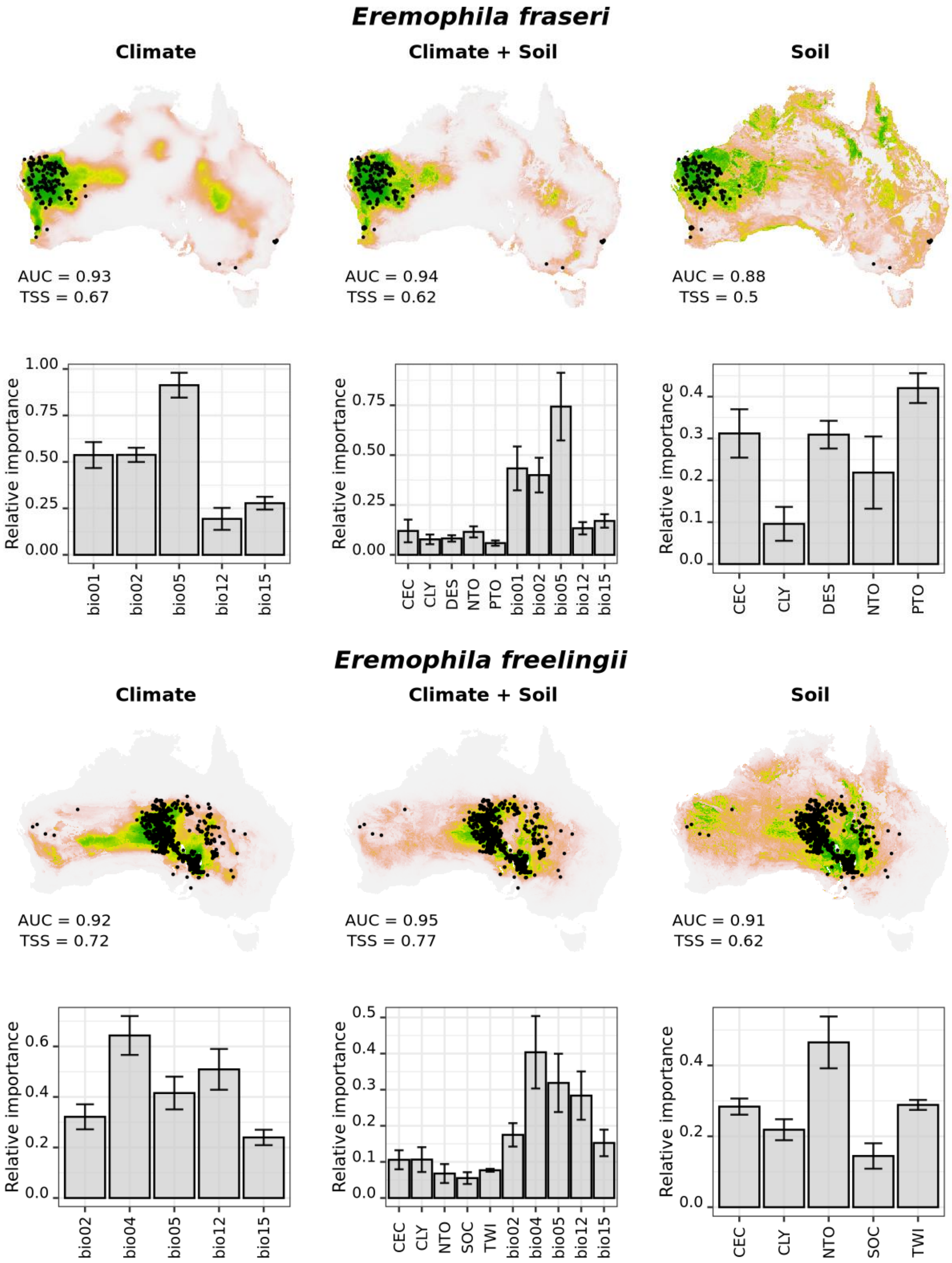
Eremophila fraseri
Climate
Climate + Soil
Soil
AUC = 0.93
TSS = 0.67
AUC = 0.94
TSS = 0.62
AUC = 0.88
TSS = 0.5
Relative importance
bio01 bio02 bio05 bio12 bio15
CEC CLY DES NTO PTO bio01 bio02 bio05 bio12 bio15
CEC CLY DES NTO PTO
Eremophila freelingii
Climate
Climate + Soil
Soil
AUC = 0.92
TSS = 0.72
AUC = 0.95
TSS = 0.77
AUC = 0.91
TSS = 0.62
Relative importance
bio02 bio04 bio05 bio12 bio15
CEC CLY NTO SOC TWI bio02 bio04 bio05 bio12 bio15
CEC CLY NTO SOC TWI

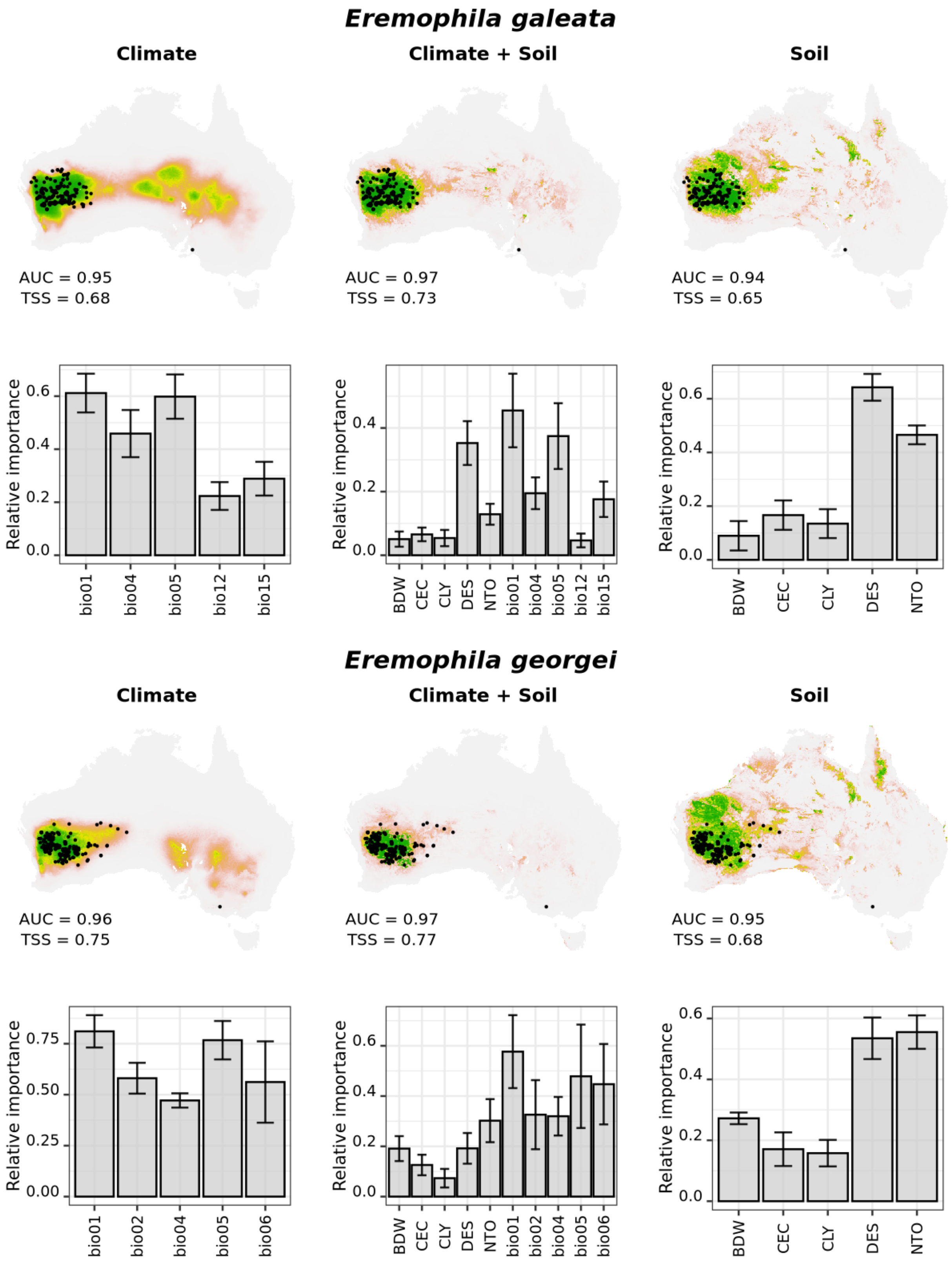
Eremophila galeata
Climate
Climate + Soil
Soil
AUC = 0.95
TSS = 0.68
AUC = 0.97
TSS = 0.73
AUC = 0.94
TSS = 0.65
Relative importance
bio01 bio04 bio05 bio12 bio15
BDW CEC CLY DES NTO bio01 bio04 bio05 bio12 bio15
BDW CEC CLY DES NTO
Eremophila georgei
Climate
Climate + Soil
Soil
AUC = 0.96
TSS = 0.75
AUC = 0.97
TSS = 0.77
AUC = 0.95
TSS = 0.68
Relative importance
bio01 bio02 bio04 bio05 bio06
BDW CEC CLY DES NTO bio01 bio02 bio04 bio05 bio06
BDW CEC CLY DES NTO

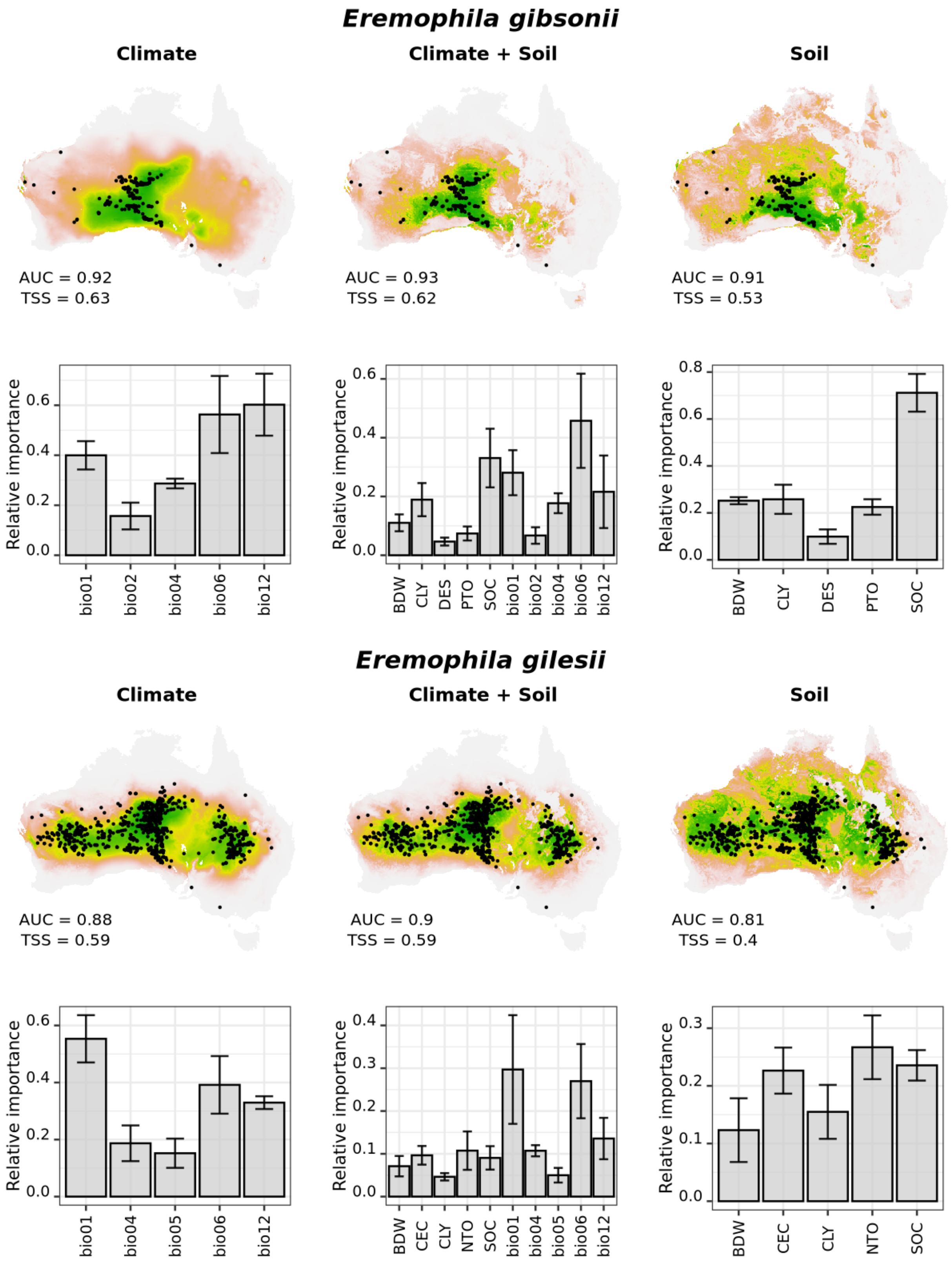
Eremophila gibsonii
Climate
Climate + Soil
Soil
AUC = 0.92
TSS = 0.63
AUC = 0.93
TSS = 0.62
AUC = 0.91
TSS = 0.53
Relative importance
0.0
0.2
0.4
0.6
bio01
bio02
bio04
bio06
bio12
Relative importance
0.0
0.2
0.4
0.6
BDW
CLY
DES
PTO
SOC
bio01
bio02
bio04
bio06
bio12
Relative importance
0.0
0.2
0.4
0.6
0.8
BDW
CLY
DES
PTO
SOC
Eremophila gilesii
Climate
Climate + Soil
Soil
AUC = 0.88
TSS = 0.59
AUC = 0.9
TSS = 0.59
AUC = 0.81
TSS = 0.4
Relative importance
0.0
0.2
0.4
0.6
bio01
bio04
bio05
bio06
bio12
Relative importance
0.0
0.1
0.2
0.3
0.4
BDW
CEC
CLY
NTO
SOC
bio01
bio04
bio05
bio06
bio12
Relative importance
0.0
0.1
0.2
0.3
BDW
CEC
CLY
NTO
SOC

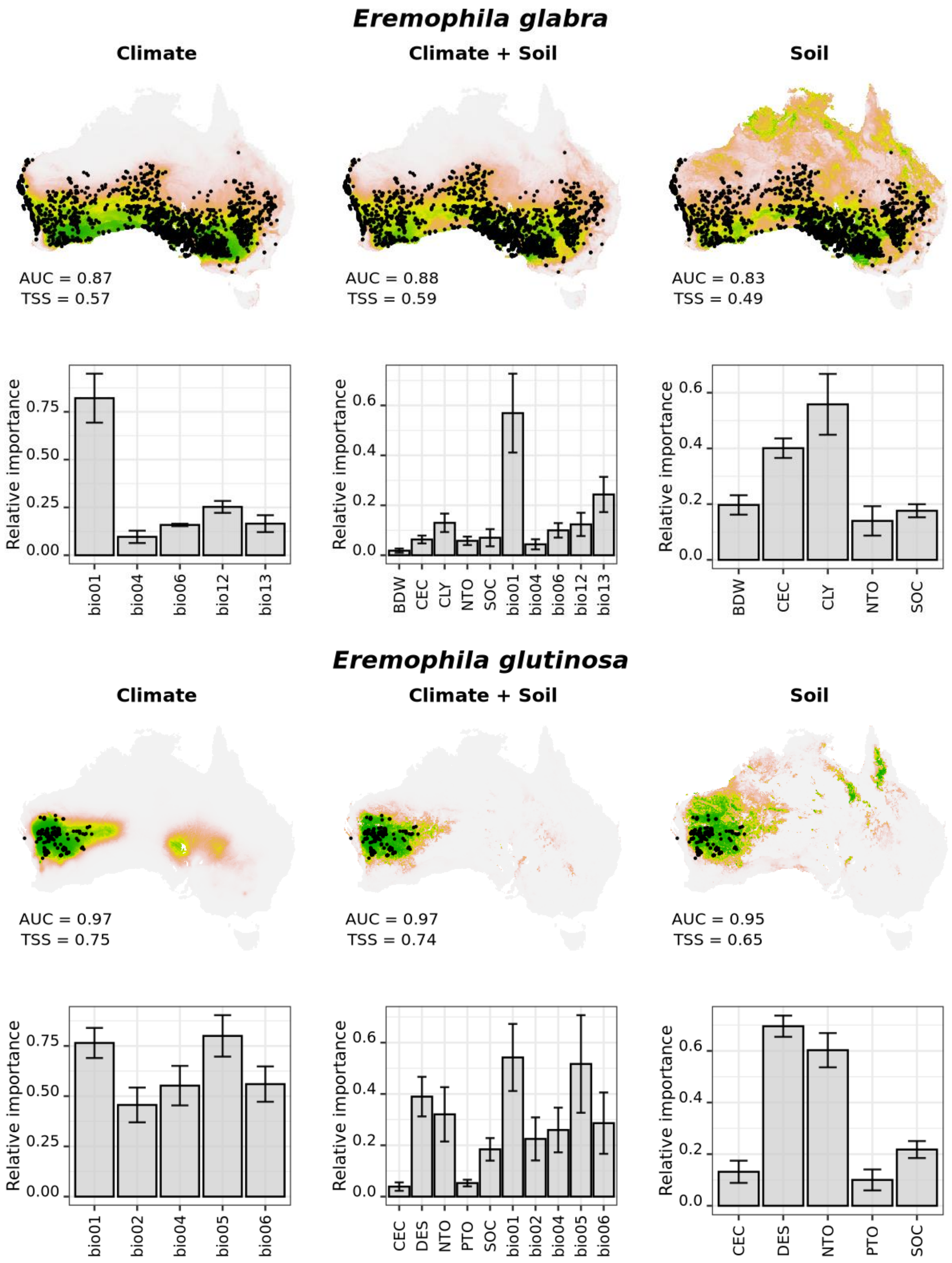

Eremophila glabra
Climate
Climate + Soil
Soil
AUC = 0.87
TSS = 0.57
AUC = 0.88
TSS = 0.59
AUC = 0.83
TSS = 0.49
Relative importance
bio01
bio04
bio06
bio12
bio13
BDW
CEC
CLY
NTO
SOC
Eremophila glutinosa
Climate
Climate + Soil
Soil
AUC = 0.97
TSS = 0.75
AUC = 0.97
TSS = 0.74
AUC = 0.95
TSS = 0.65
bio02
bio05
DES
PTO

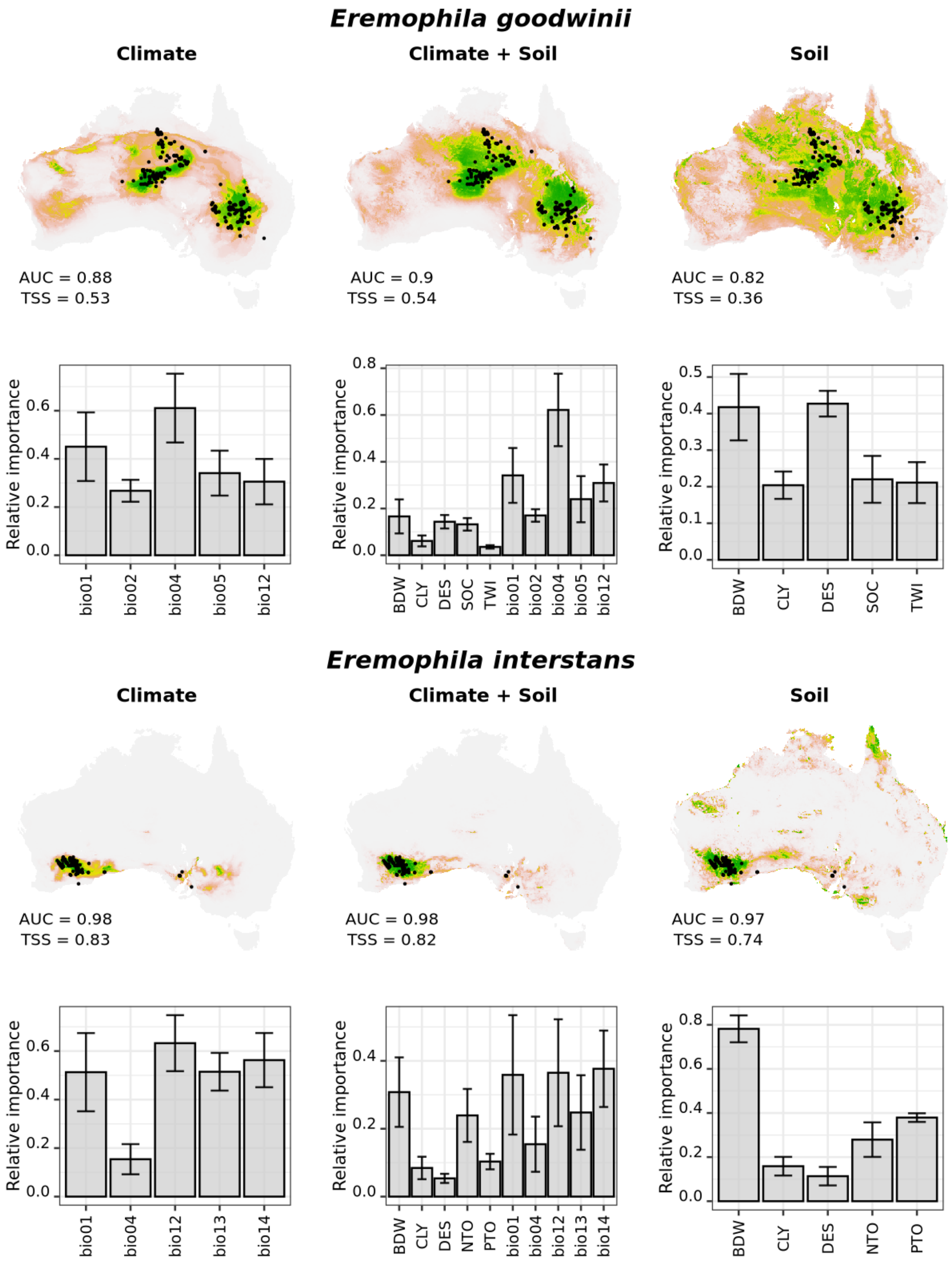
Eremophila goodwinii
Climate
Climate + Soil
Soil
AUC = 0.88
TSS = 0.53
AUC = 0.9
TSS = 0.54
AUC = 0.82
TSS = 0.36
Relative importance
bio01
bio02
bio04
bio05
bio12
BDW
CLY
DES
SOC
TWI
Eremophila interstans
Climate
Climate + Soil
Soil
AUC = 0.98
TSS = 0.83
AUC = 0.98
TSS = 0.82
AUC = 0.97
TSS = 0.74
Relative importance
bio01
bio04
bio12
bio13
bio14
BDW
CLY
DES
NTO
PTO

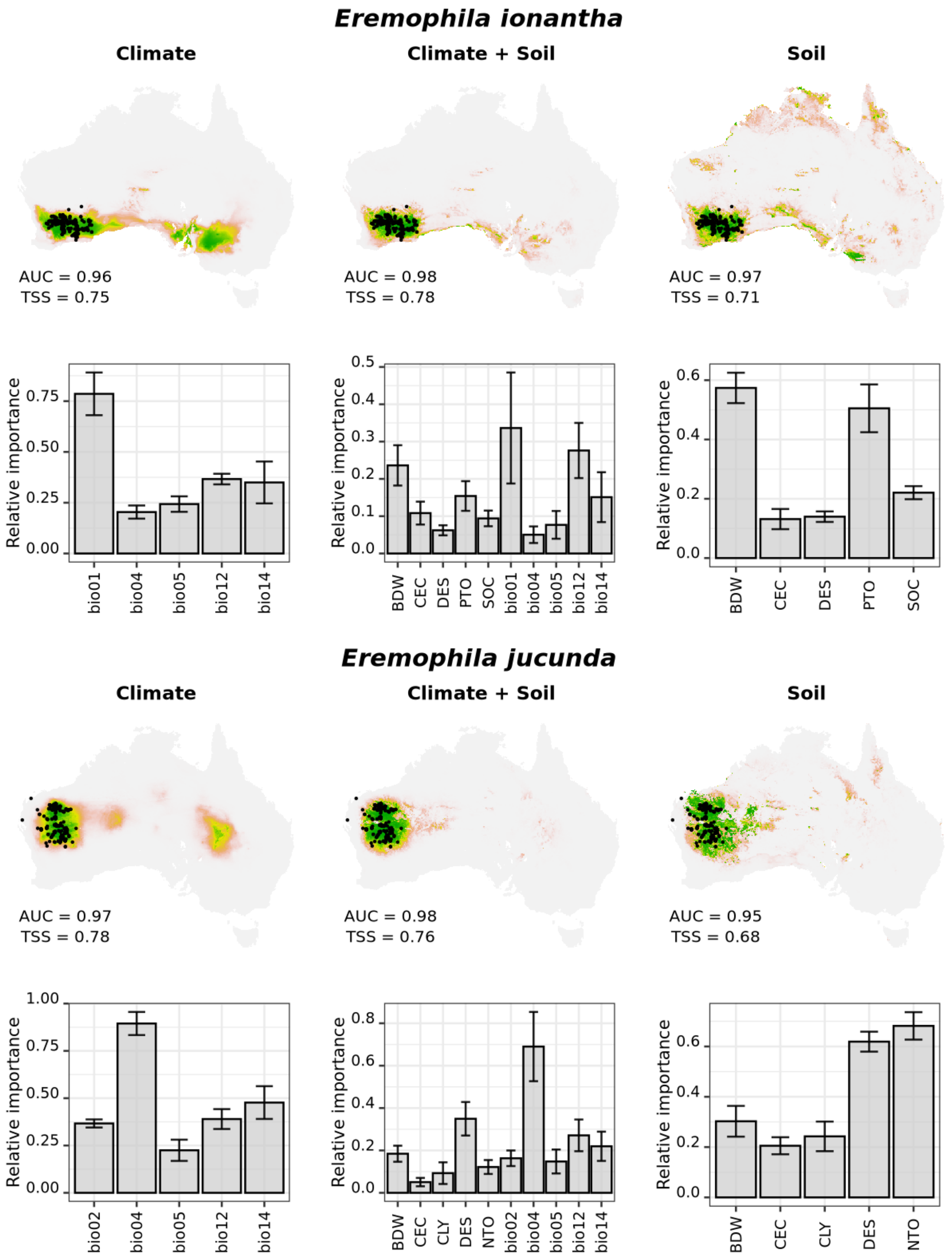

Eremophila ionantha
Climate
Climate + Soil
Soil
AUC = 0.96
TSS = 0.75
AUC = 0.98
TSS = 0.78
AUC = 0.97
TSS = 0.71
Relative importance
0.00
0.25
0.50
0.75
bio01
bio04
bio05
bio12
bio14
0.0
0.1
0.2
0.3
0.4
0.5
BDW
CEC
DES
PTO
SOC
bio01
bio04
bio05
bio12
bio14
0.0
0.2
0.4
0.6
BDW
CEC
DES
PTO
SOC
Eremophila jucunda
Climate
Climate + Soil
Soil
AUC = 0.97
TSS = 0.78
AUC = 0.98
TSS = 0.76
AUC = 0.95
TSS = 0.68
Relative importance
0.00
0.25
0.50
0.75
1.00
bio02
bio04
bio05
bio12
bio14
0.0
0.2
0.4
0.6
0.8
BDW
CEC
CLY
DES
NTO
bio02
bio04
bio05
bio12
bio14
0.0
0.2
0.4
0.6
BDW
CEC
CLY
DES
NTO

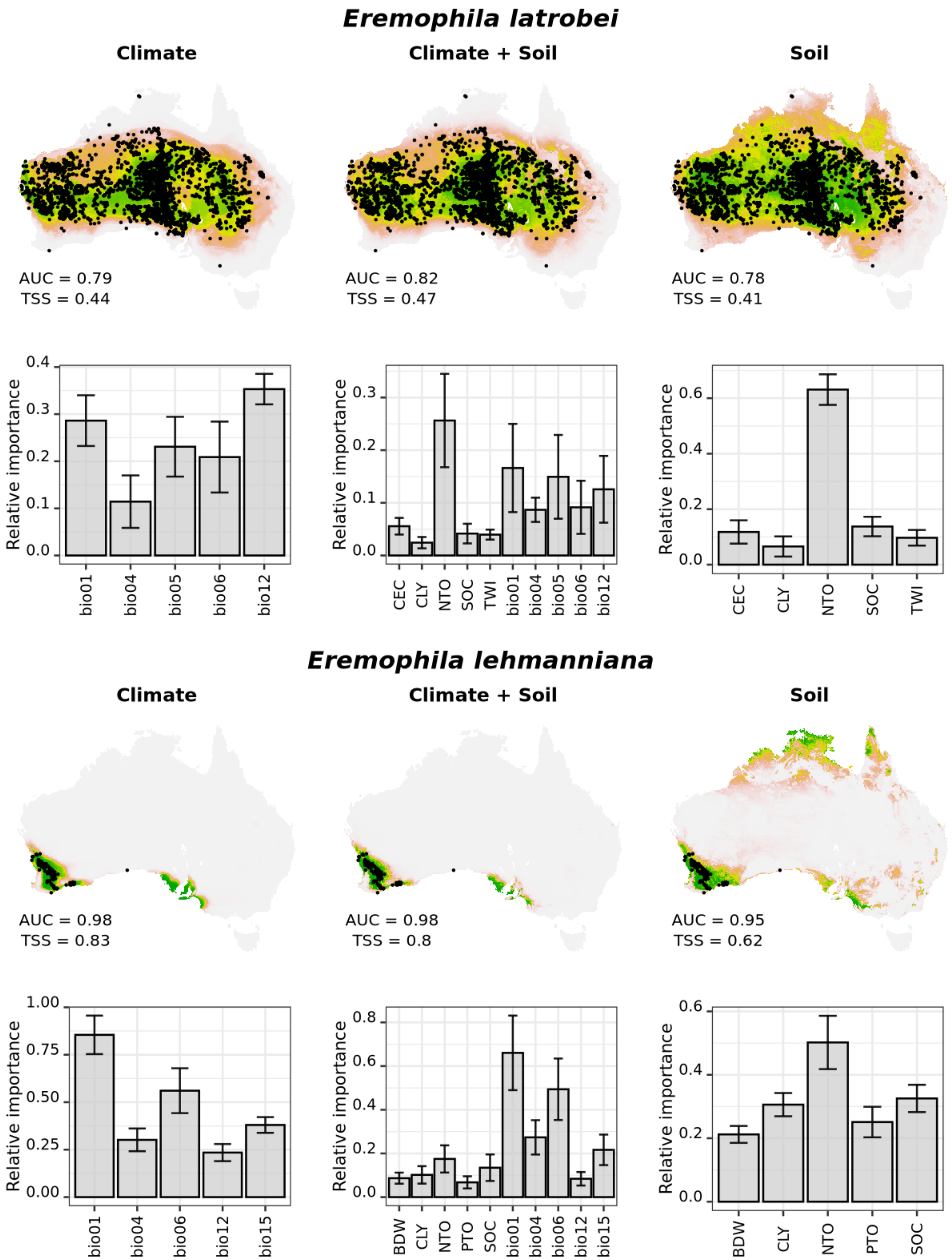
Eremophila latrobei
Climate
Climate + Soil
Soil
AUC = 0.79
TSS = 0.44
AUC = 0.82
TSS = 0.47
AUC = 0.78
TSS = 0.41
Relative importance
0.4
0.3
0.2
0.1
0.0
bio01
bio04
bio05
bio06
bio12
CEC
CLY
NTO
SOC
TWI
0.6
Eremophila lehmanniana
AUC = 0.98
TSS = 0.83
AUC = 0.98
TSS = 0.8
AUC = 0.95
TSS = 0.62
1.00
0.75
0.50
0.25
0.00
bio15
0.8
BDW
PTO

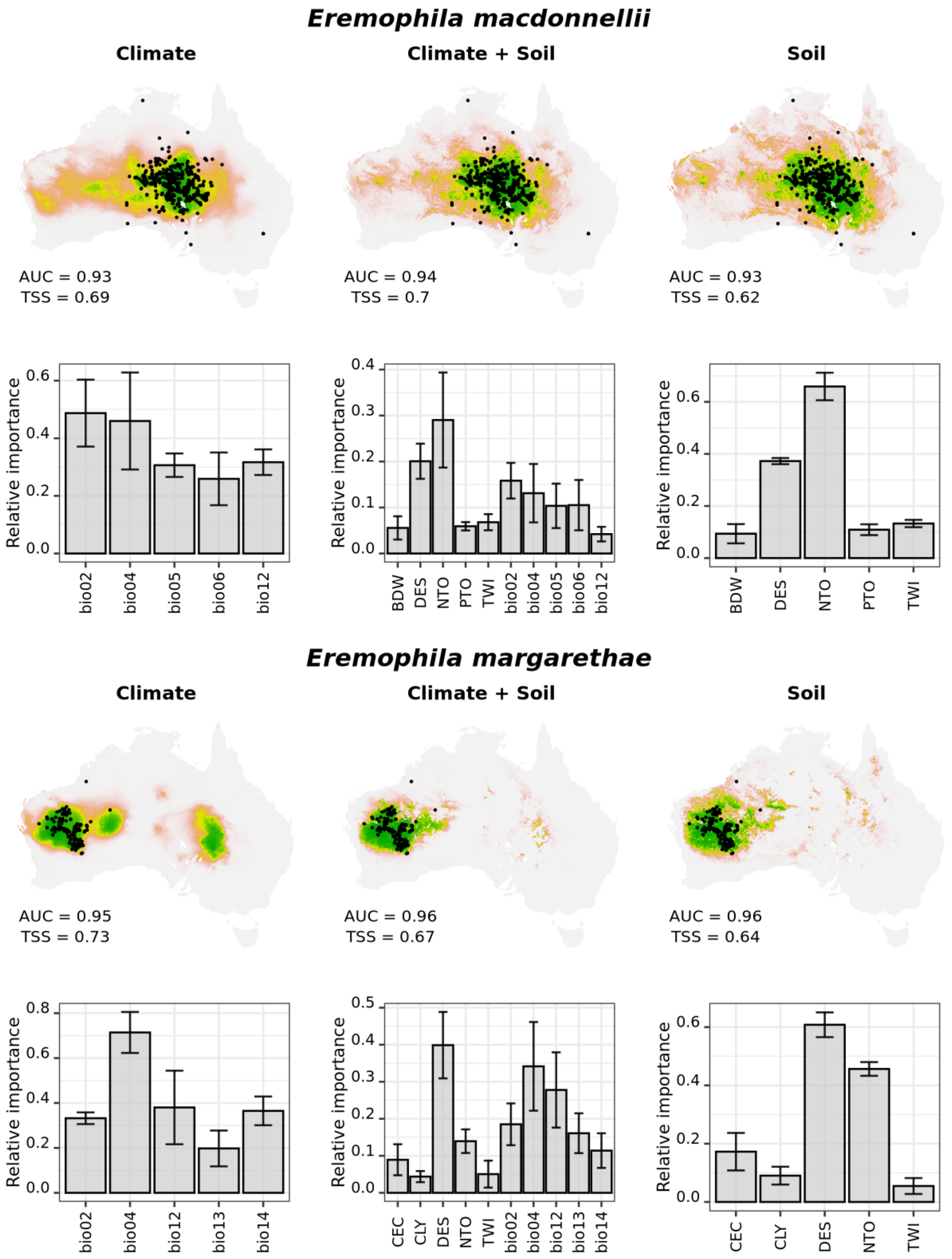
Eremophila macdonnellii
Climate
Climate + Soil
Soil
AUC = 0.93
TSS = 0.69
AUC = 0.94
TSS = 0.7
AUC = 0.93
TSS = 0.62
Relative importance
bio02
bio04
bio05
bio06
bio12
BDW
DES
NTO
PTO
TWI
Eremophila margarethae
Climate
Climate + Soil
Soil
AUC = 0.95
TSS = 0.73
AUC = 0.96
TSS = 0.67
AUC = 0.96
TSS = 0.64
Relative importance
bio02
bio04
bio12
bio13
bio14
CEC
CLY
DES
NTO
TWI

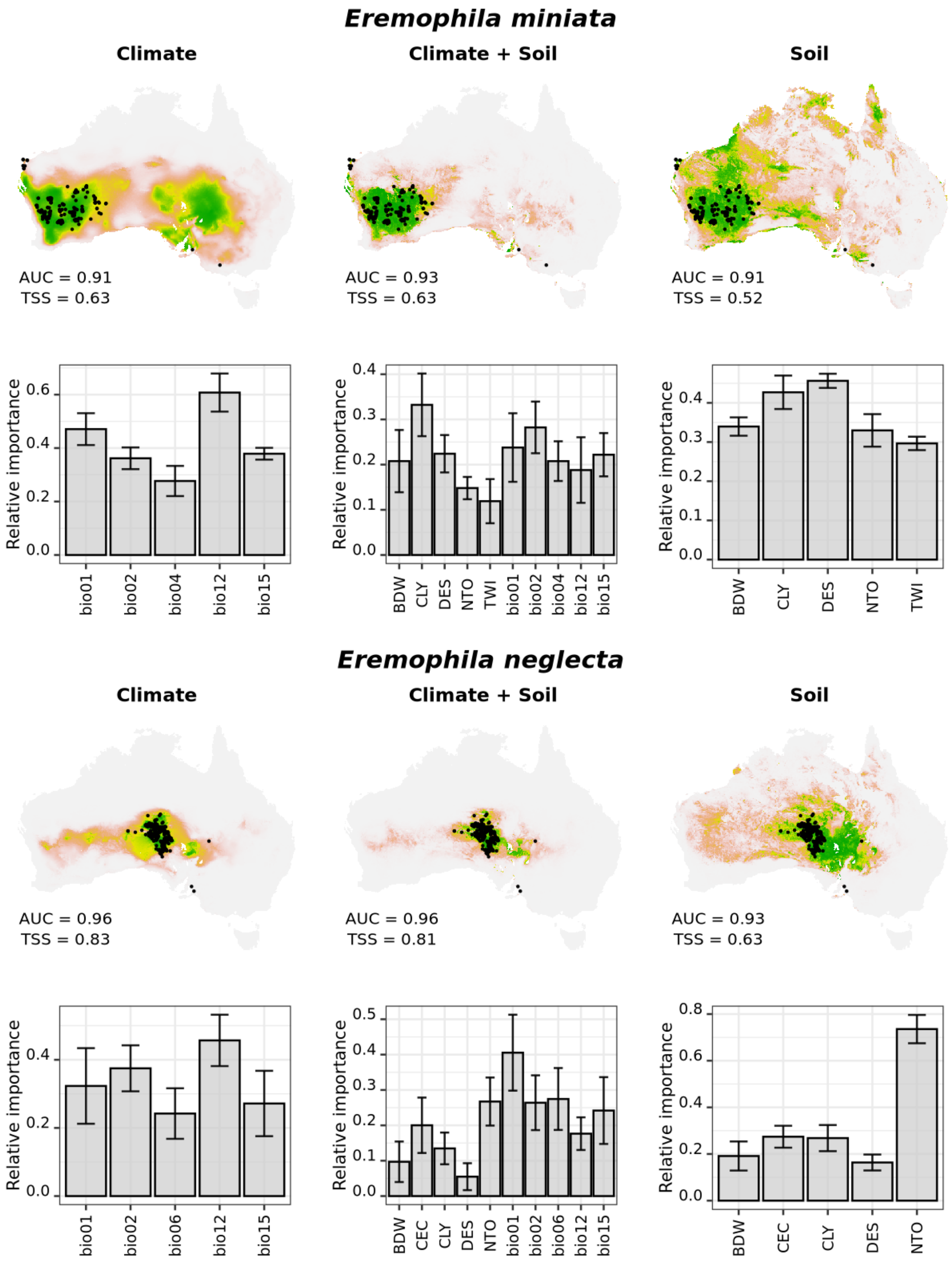
Eremophila miniata
Climate
Climate + Soil
Soil
AUC = 0.91
TSS = 0.63
AUC = 0.93
TSS = 0.63
AUC = 0.91
TSS = 0.52
Relative importance
bio01 bio02 bio04 bio12 bio15
BDW CLY DES NTO TWI bio01 bio02 bio04 bio12 bio15
BDW CLY DES NTO TWI
Eremophila neglecta
Climate
Climate + Soil
Soil
AUC = 0.96
TSS = 0.83
AUC = 0.96
TSS = 0.81
AUC = 0.93
TSS = 0.63
Relative importance
bio01 bio02 bio06 bio12 bio15
BDW CEC CLY DES NTO bio01 bio02 bio06 bio12 bio15
BDW CEC CLY DES NTO

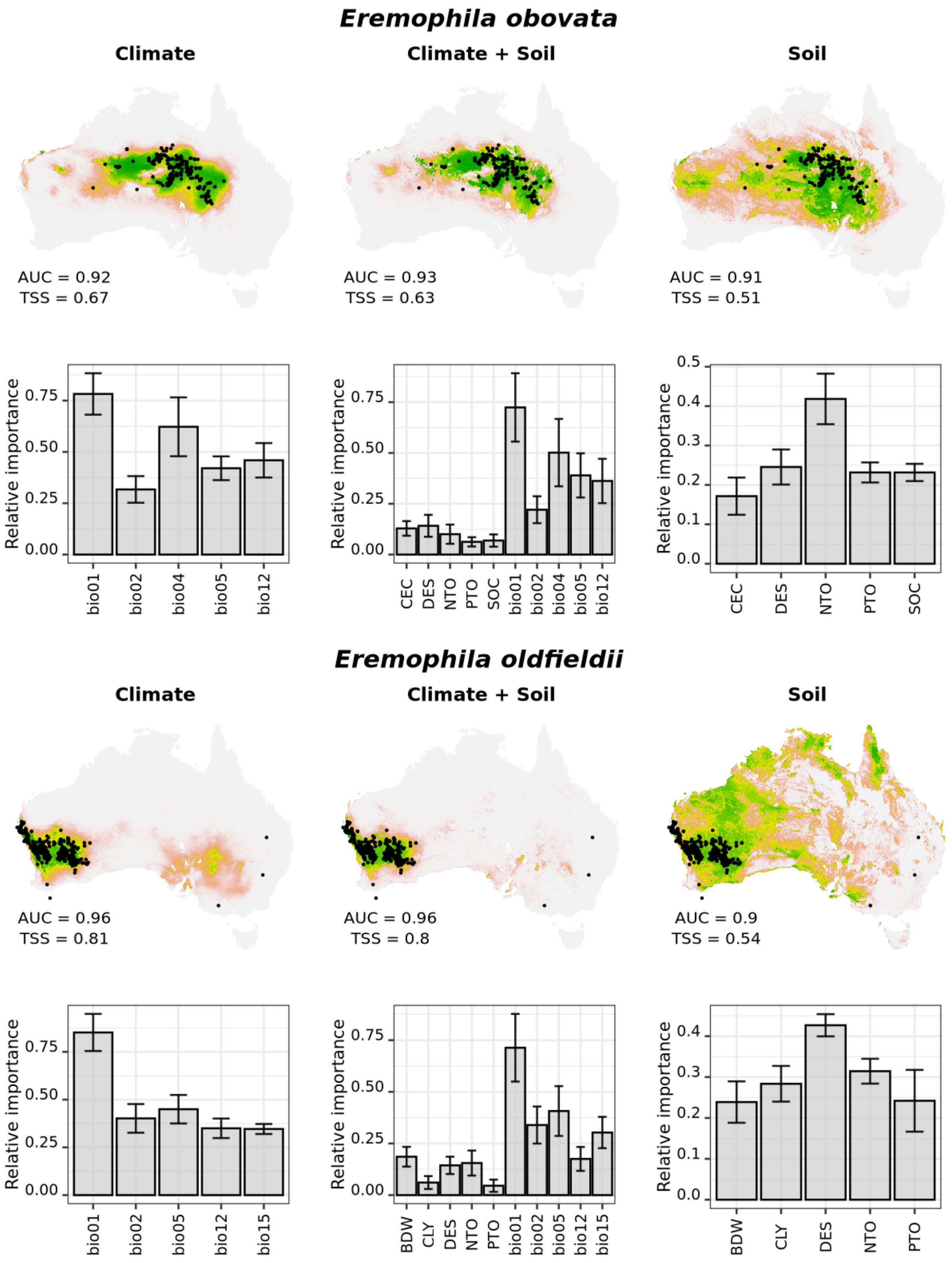

Eremophila obovata
Climate
Climate + Soil
Soil
AUC = 0.92
TSS = 0.67
AUC = 0.93
TSS = 0.63
AUC = 0.91
TSS = 0.51
Relative importance
0.00
0.25
0.50
0.75
bio01
bio02
bio04
bio05
bio12
CEC
DES
NTO
PTO
SOC
0.0
0.1
0.2
0.3
0.4
0.5
Eremophila oldfieldii
Climate
Climate + Soil
Soil
AUC = 0.96
TSS = 0.81
AUC = 0.96
TSS = 0.8
AUC = 0.9
TSS = 0.54
bio15
BDW
CLY

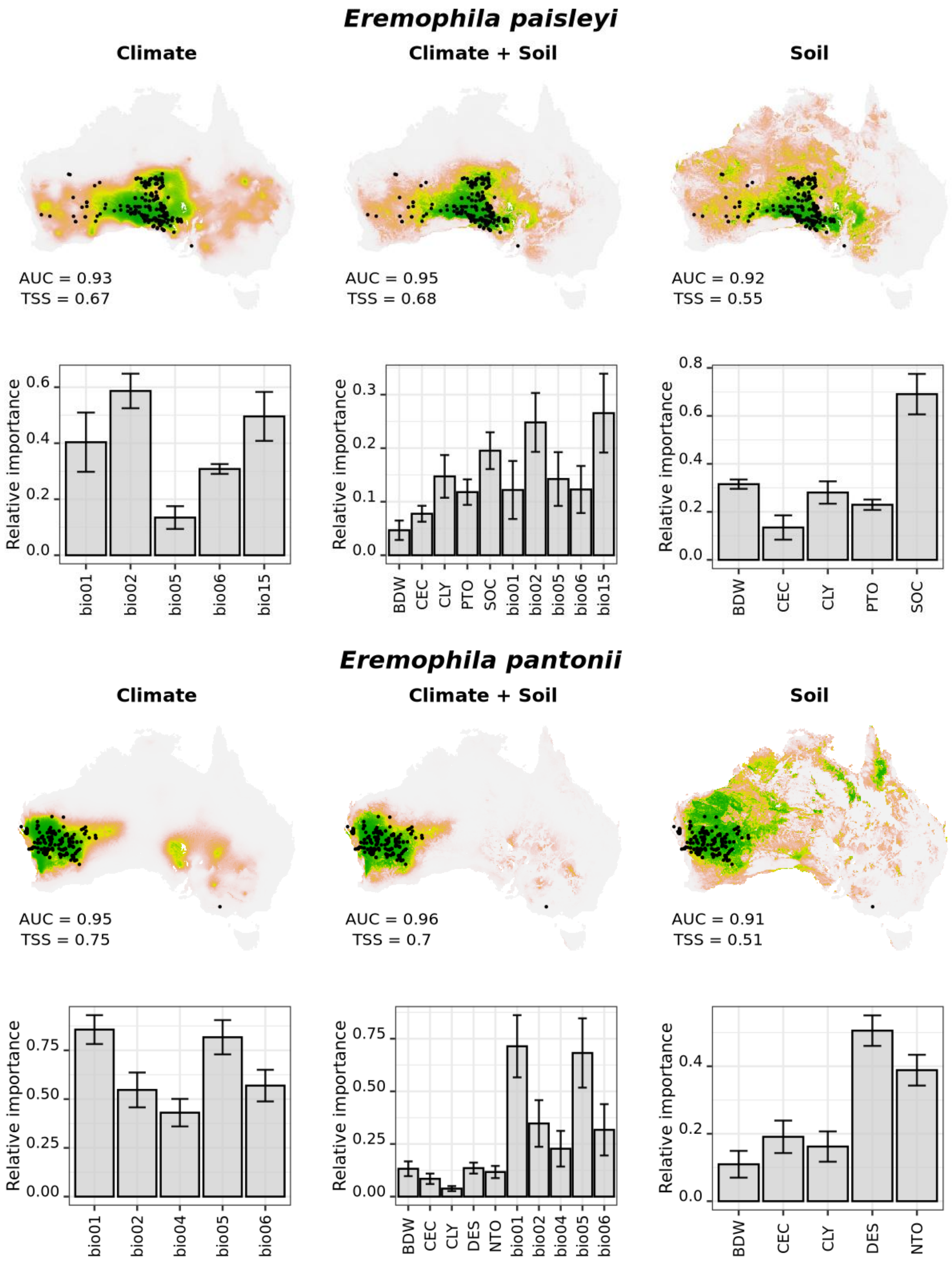
Eremophila paisleyi
Climate
Climate + Soil
Soil
AUC = 0.93
TSS = 0.67
AUC = 0.95
TSS = 0.68
AUC = 0.92
TSS = 0.55
Relative importance
bio01
bio02
bio05
bio06
bio15
BDW
CEC
CLY
PTO
SOC
Eremophila pantonii
Climate
Climate + Soil
Soil
AUC = 0.95
TSS = 0.75
AUC = 0.96
TSS = 0.7
AUC = 0.91
TSS = 0.51
Relative importance
bio01
bio02
bio04
bio05
bio06
BDW
CEC
CLY
DES
NTO

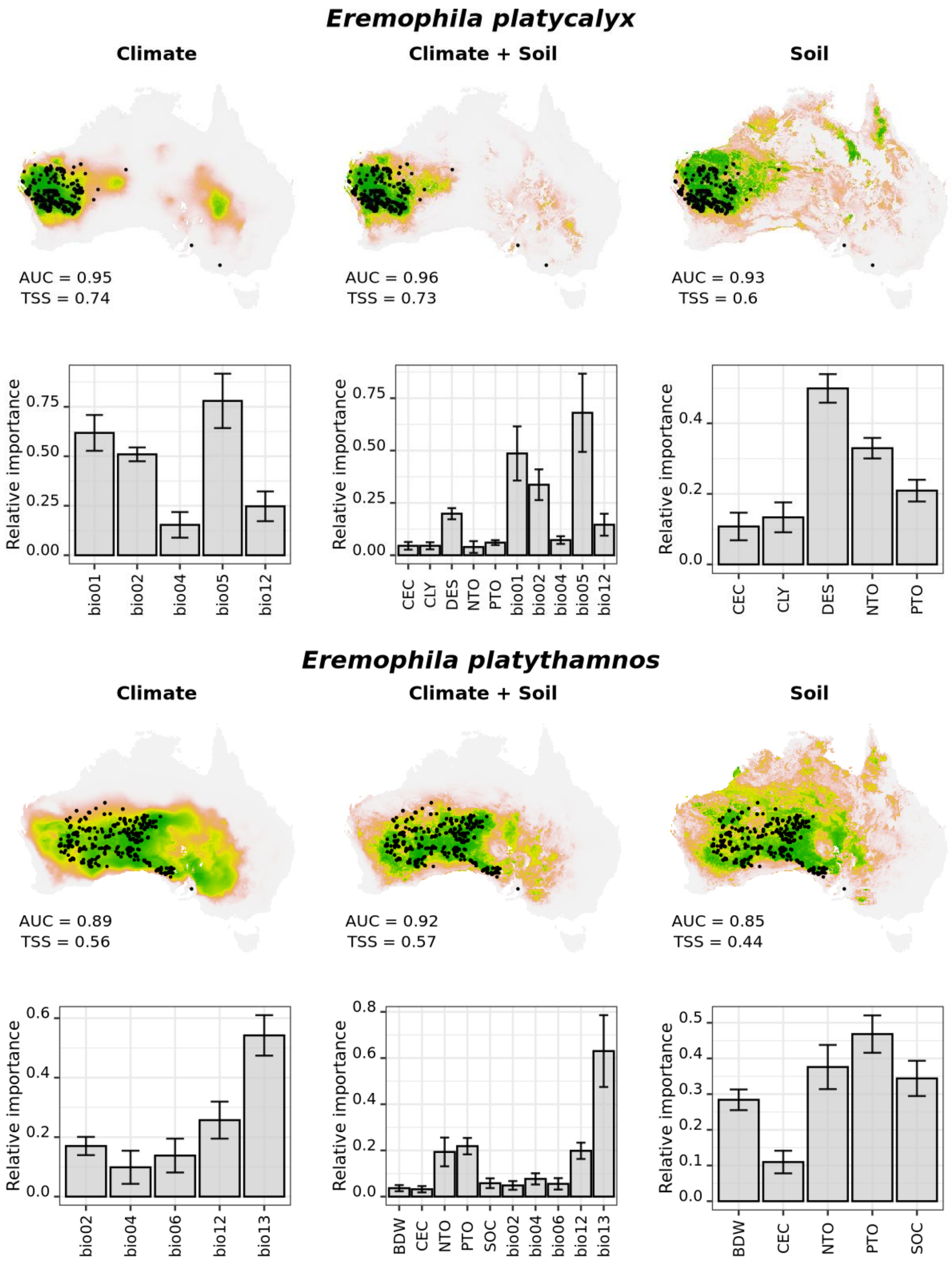

Eremophila platycalyx
Climate
Climate + Soil
Soil
AUC = 0.95
TSS = 0.74
AUC = 0.96
TSS = 0.73
AUC = 0.93
TSS = 0.6
Relative importance
0.00
0.25
0.50
0.75
bio01
bio02
bio04
bio05
bio12
CEC
CLY
DES
NTO
PTO
0.0
0.2
0.4
Eremophila platythamnos
Climate
Climate + Soil
Soil
AUC = 0.89
TSS = 0.56
AUC = 0.92
TSS = 0.57
AUC = 0.85
TSS = 0.44
0.6
0.8
bio06
bio13
BDW
SOC
0.1
0.3
0.5

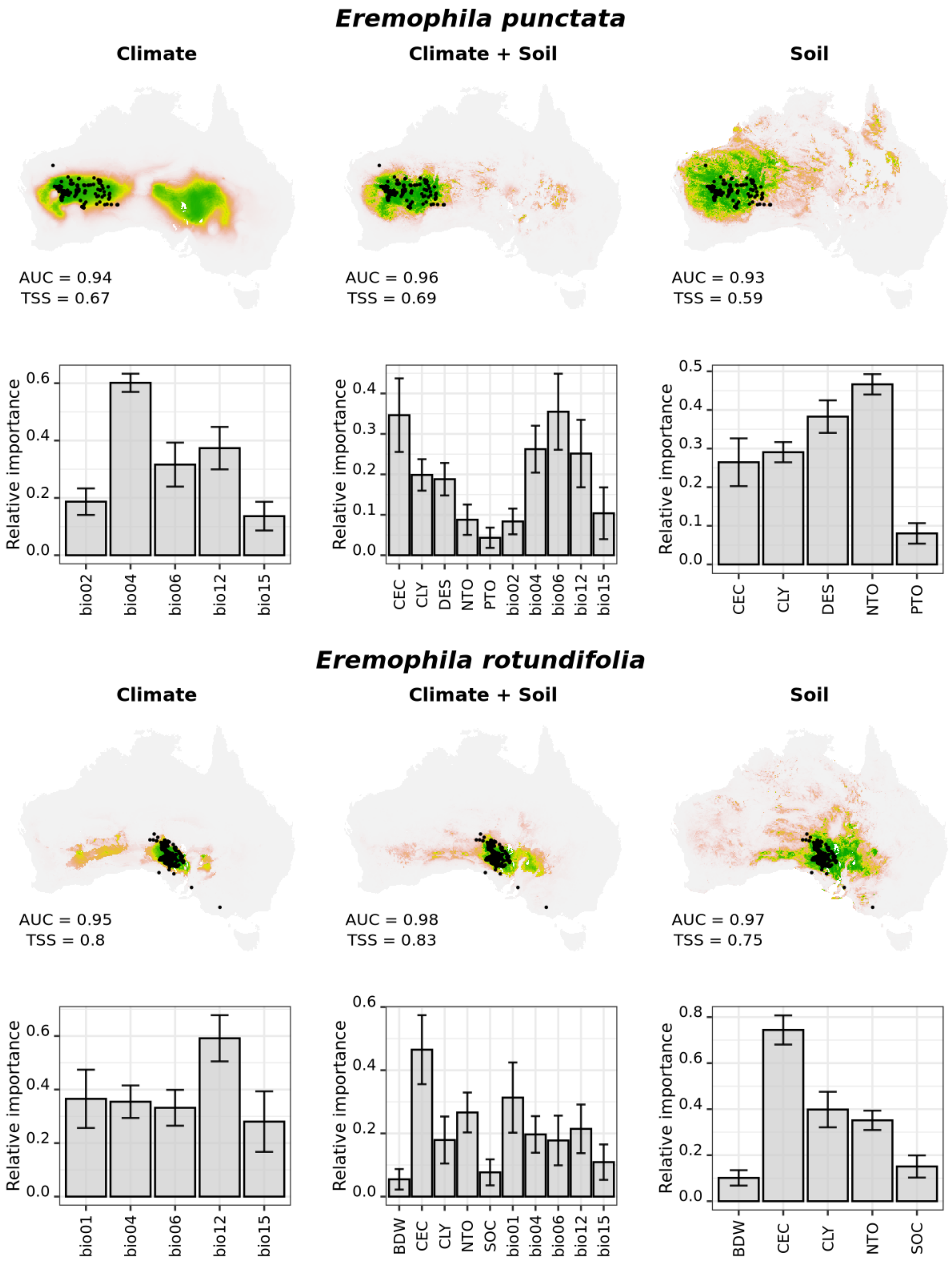

Eremophila punctata
Climate
Climate + Soil
Soil
AUC = 0.94
TSS = 0.67
AUC = 0.96
TSS = 0.69
AUC = 0.93
TSS = 0.59
Relative importance
bio02 bio04 bio06 bio12 bio15
CEC CLY DES NTO PTO bio02 bio04 bio06 bio12 bio15
CEC CLY DES NTO PTO
Eremophila rotundifolia
Climate
Climate + Soil
Soil
AUC = 0.95
TSS = 0.8
AUC = 0.98
TSS = 0.83
AUC = 0.97
TSS = 0.75
Relative importance
bio01 bio04 bio06 bio12 bio15
BDW CEC CLY NTO SOC bio01 bio04 bio06 bio12 bio15
BDW CEC CLY NTO SOC

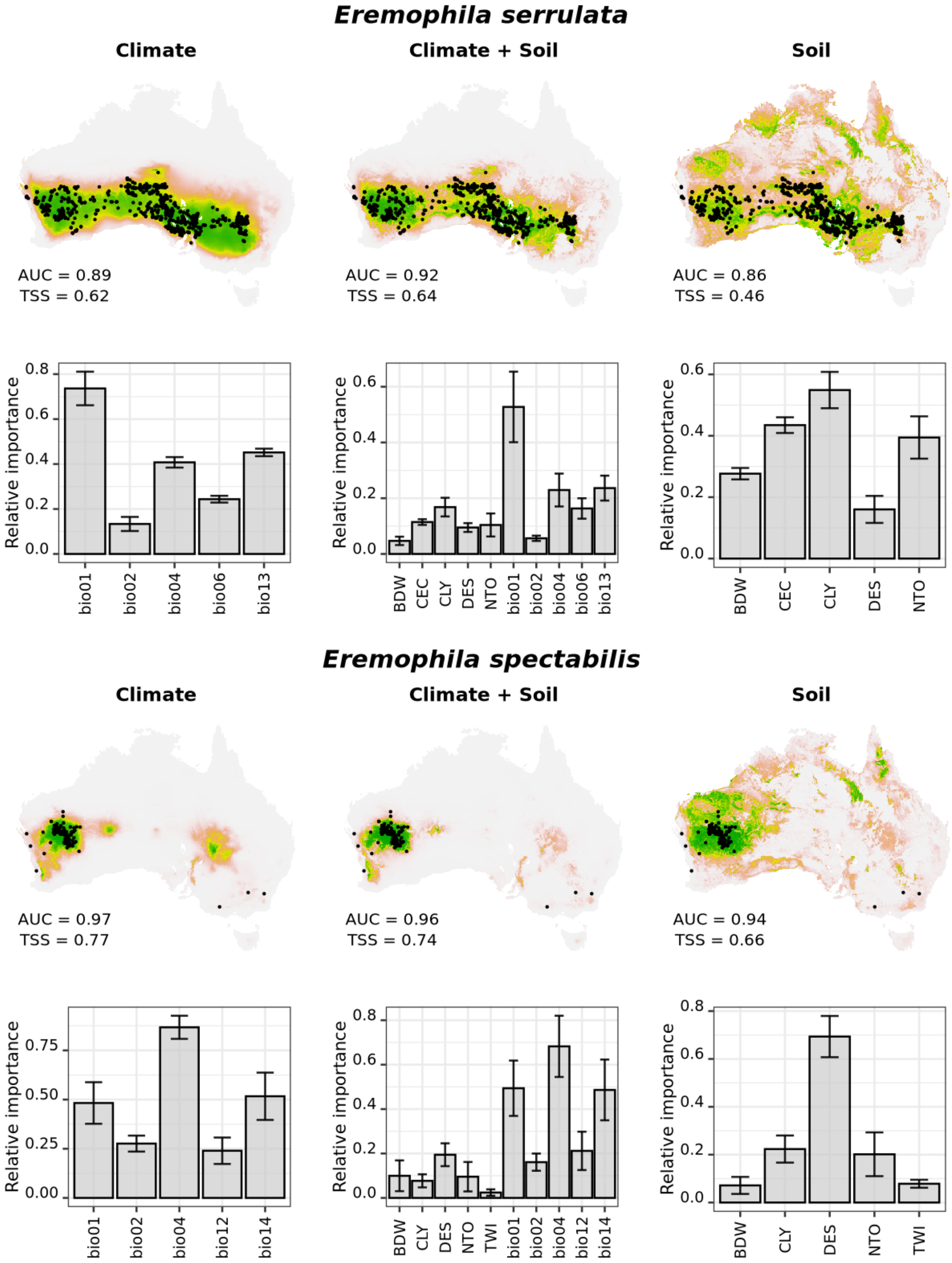
Eremophila serrulata
Climate
Climate + Soil
Soil
AUC = 0.89
TSS = 0.62
AUC = 0.92
TSS = 0.64
AUC = 0.86
TSS = 0.46
Relative importance
bio01
bio02
bio04
bio06
bio13
BDW
CEC
CLY
DES
NTO
Eremophila spectabilis
Climate
Climate + Soil
Soil
AUC = 0.97
TSS = 0.77
AUC = 0.96
TSS = 0.74
AUC = 0.94
TSS = 0.66
Relative importance
bio01
bio02
bio04
bio12
bio14
BDW
CLY
DES
NTO
TWI

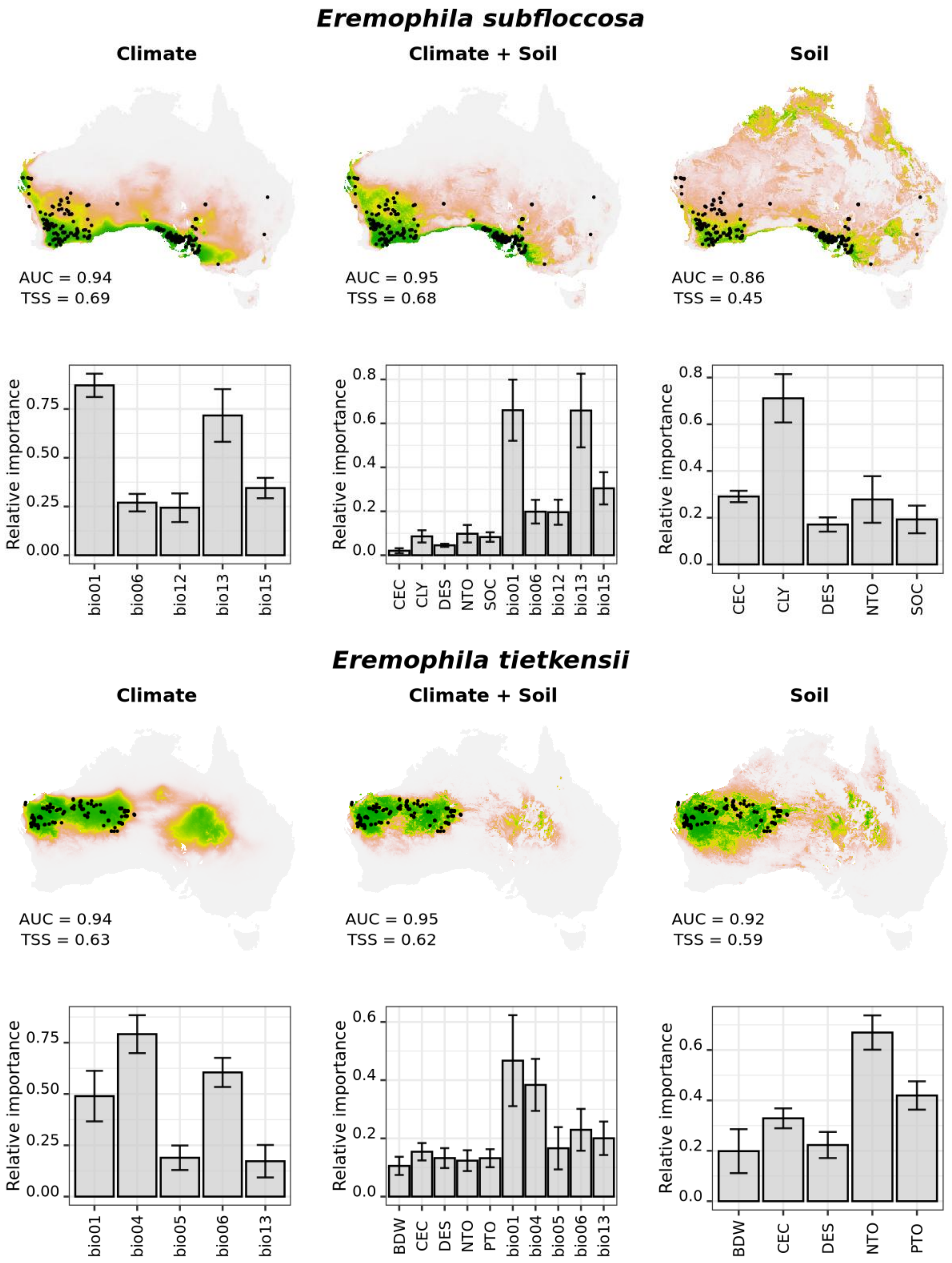
Eremophila subfloccosa
Climate
Climate + Soil
Soil
AUC = 0.94
TSS = 0.69
AUC = 0.95
TSS = 0.68
AUC = 0.86
TSS = 0.45
Relative importance
0.00
0.25
0.50
0.75
bio01
bio06
bio12
bio13
bio15
0.0
0.2
0.4
0.6
0.8
CEC
CLY
DES
NTO
SOC
bio01
bio06
bio12
bio13
bio15
CEC
CLY
DES
NTO
SOC
Eremophila tietkensii
Climate
Climate + Soil
Soil
AUC = 0.94
TSS = 0.63
AUC = 0.95
TSS = 0.62
AUC = 0.92
TSS = 0.59
Relative importance
bio01
bio04
bio05
bio06
bio13
BDW
CEC
DES
NTO
PTO
bio01
bio04
bio05
bio06
bio13
BDW
CEC
DES
NTO
PTO

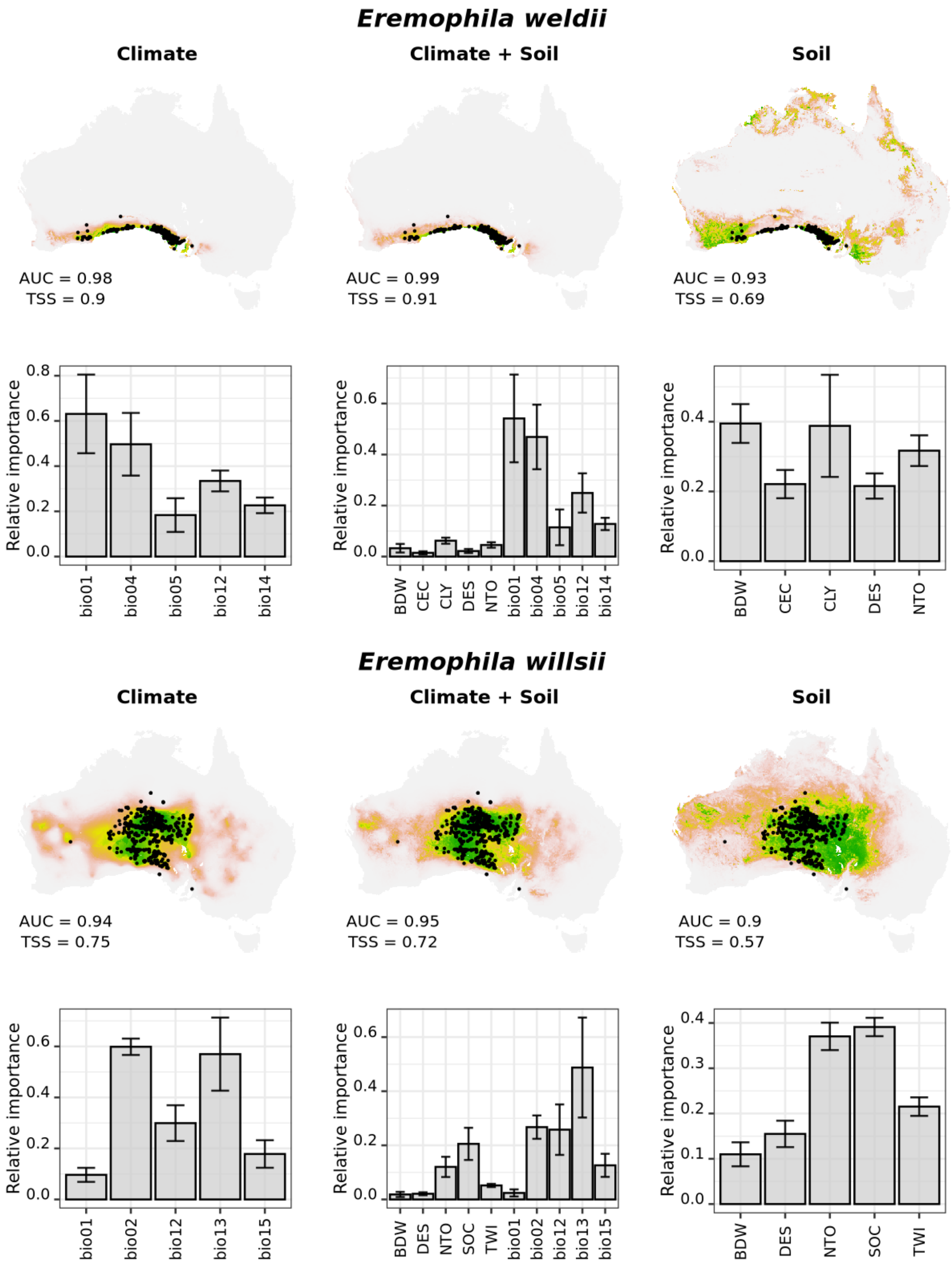
Eremophila weldii
Climate
Climate + Soil
Soil
AUC = 0.98
TSS = 0.9
AUC = 0.99
TSS = 0.91
AUC = 0.93
TSS = 0.69
Relative importance
bio01 bio04 bio05 bio12 bio14
BDW CEC CLY DES NTO bio01 bio04 bio05 bio12 bio14
BDW CEC CLY DES NTO
Eremophila willsii
Climate
Climate + Soil
Soil
AUC = 0.94
TSS = 0.75
AUC = 0.95
TSS = 0.72
AUC = 0.9
TSS = 0.57
Relative importance
bio01 bio02 bio12 bio13 bio15
BDW DES NTO SOC TWI bio01 bio02 bio12 bio13 bio15
BDW DES NTO SOC TWI

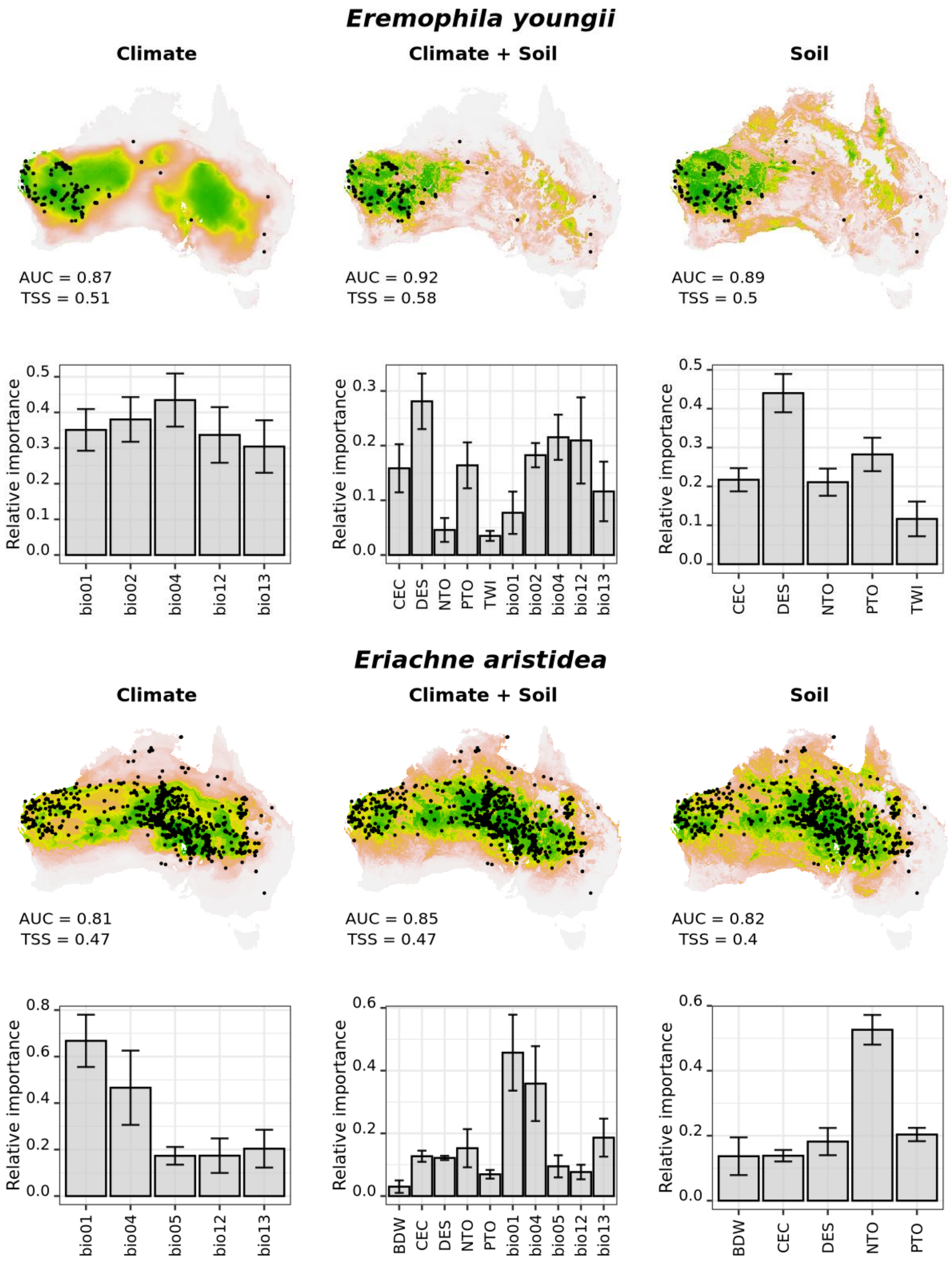
Eremophila youngii
Climate
Climate + Soil
Soil
AUC = 0.87
TSS = 0.51
AUC = 0.92
TSS = 0.58
AUC = 0.89
TSS = 0.5
Relative importance
bio01
bio02
bio04
bio12
bio13
CEC
DES
NTO
PTO
TWI
Eriachne aristidea
AUC = 0.81
TSS = 0.47
AUC = 0.85
TSS = 0.47
AUC = 0.82
TSS = 0.4
bio05
BDW

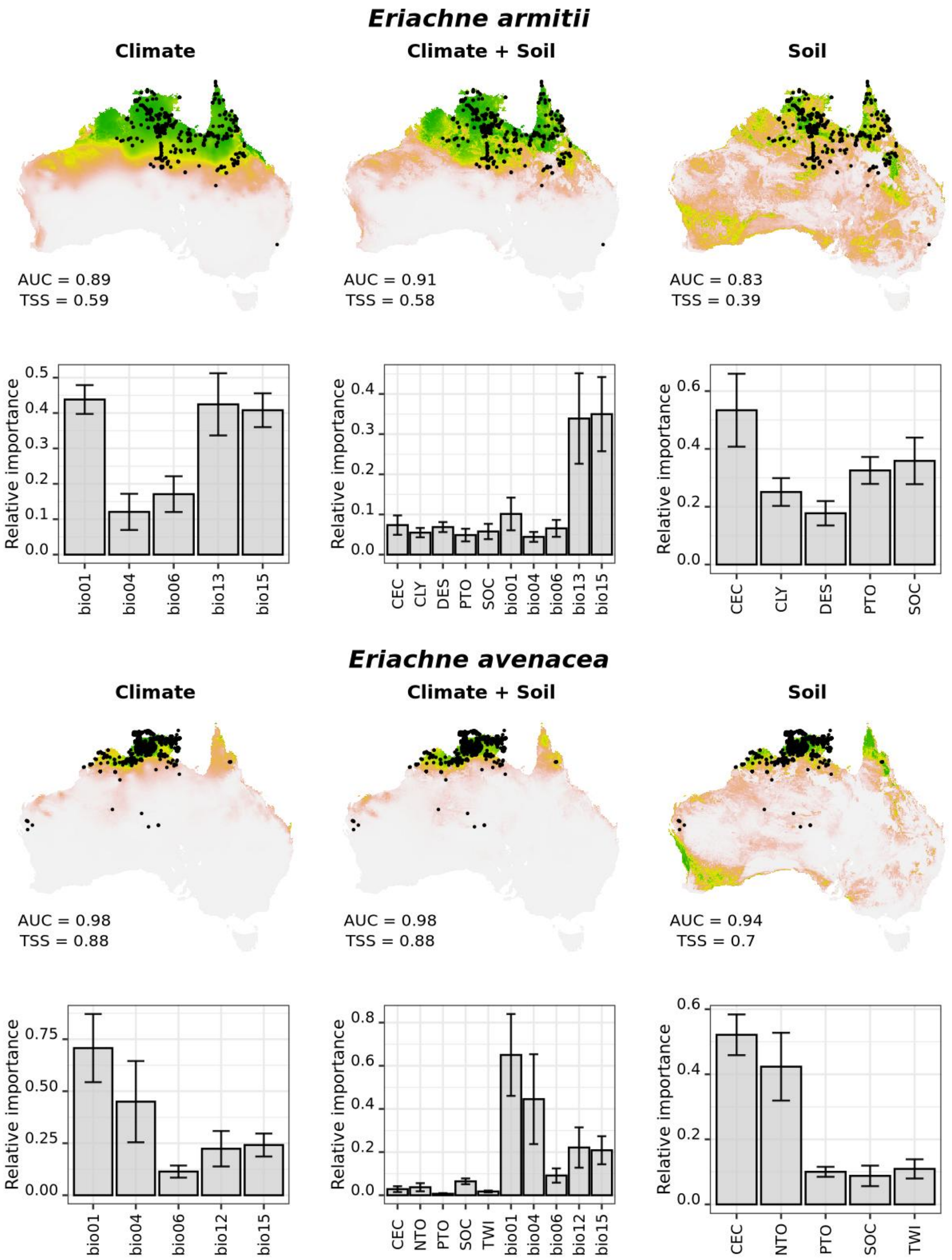
Eriachne armitii
Climate
Climate + Soil
Soil
AUC = 0.89
TSS = 0.59
AUC = 0.91
TSS = 0.58
AUC = 0.83
TSS = 0.39
Relative importance
bio01
bio04
bio06
bio13
bio15
CEC
CLY
DES
PTO
SOC
Eriachne avenacea
Climate
Climate + Soil
Soil
AUC = 0.98
TSS = 0.88
AUC = 0.98
TSS = 0.88
AUC = 0.94
TSS = 0.7
Relative importance
bio01
bio04
bio06
bio12
bio15
CEC
NTO
PTO
SOC
TWI

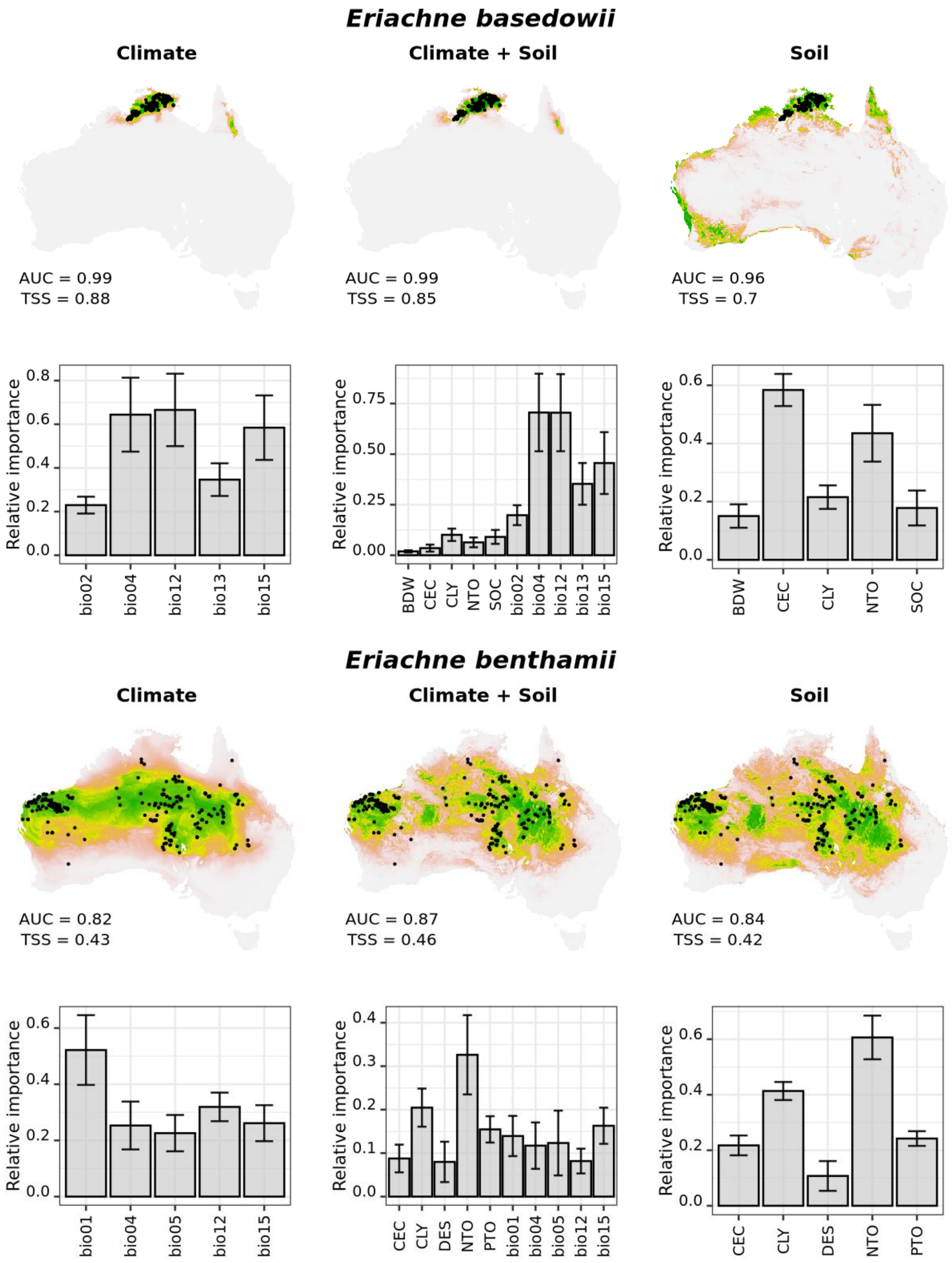
Eriachne basedowii
Climate
Climate + Soil
Soil
AUC = 0.99
TSS = 0.88
AUC = 0.99
TSS = 0.85
AUC = 0.96
TSS = 0.7
Relative importance
bio02
bio04
bio12
bio13
bio15
BDW
CEC
CLY
NTO
SOC
bio02
bio04
bio12
bio13
bio15
BDW
CEC
CLY
NTO
SOC
Eriachne benthamii
Climate
Climate + Soil
Soil
AUC = 0.82
TSS = 0.43
AUC = 0.87
TSS = 0.46
AUC = 0.84
TSS = 0.42
Relative importance
bio01
bio04
bio05
bio12
bio15
CEC
CLY
DES
NTO
PTO
bio01
bio04
bio05
bio12
bio15
CEC
CLY
DES
NTO
PTO

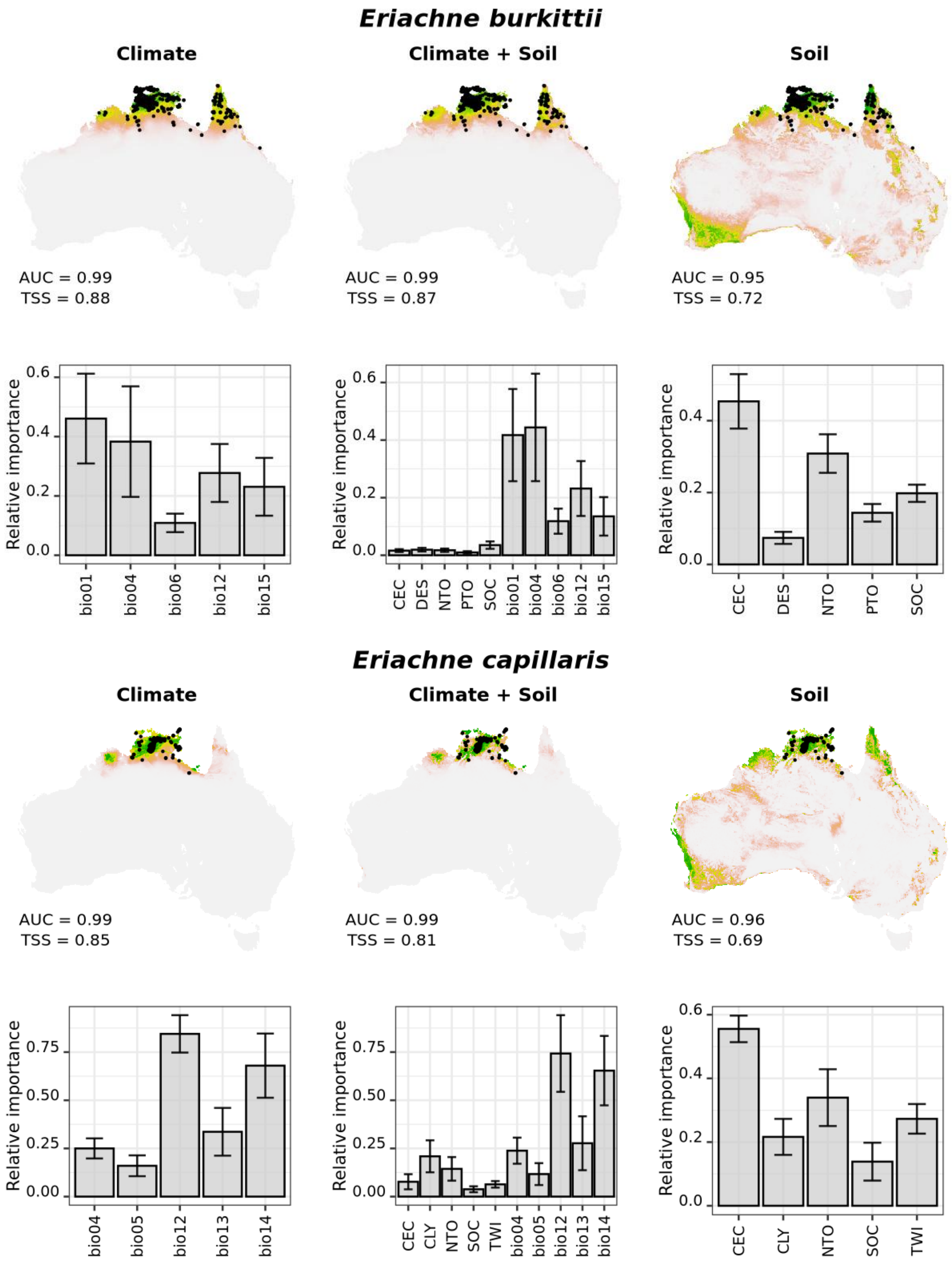
Eriachne burkittii
Climate
Climate + Soil
Soil
AUC = 0.99
TSS = 0.88
AUC = 0.99
TSS = 0.87
AUC = 0.95
TSS = 0.72
Relative importance
0.0
0.2
0.4
0.6
bio01
bio04
bio06
bio12
bio15
CEC
DES
NTO
PTO
SOC
Eriachne capillaris
Climate
Climate + Soil
Soil
AUC = 0.99
TSS = 0.85
AUC = 0.99
TSS = 0.81
AUC = 0.96
TSS = 0.69
Relative importance
0.00
0.25
0.50
0.75
bio04
bio05
bio12
bio13
bio14
CEC
CLY
NTO
SOC
TWI

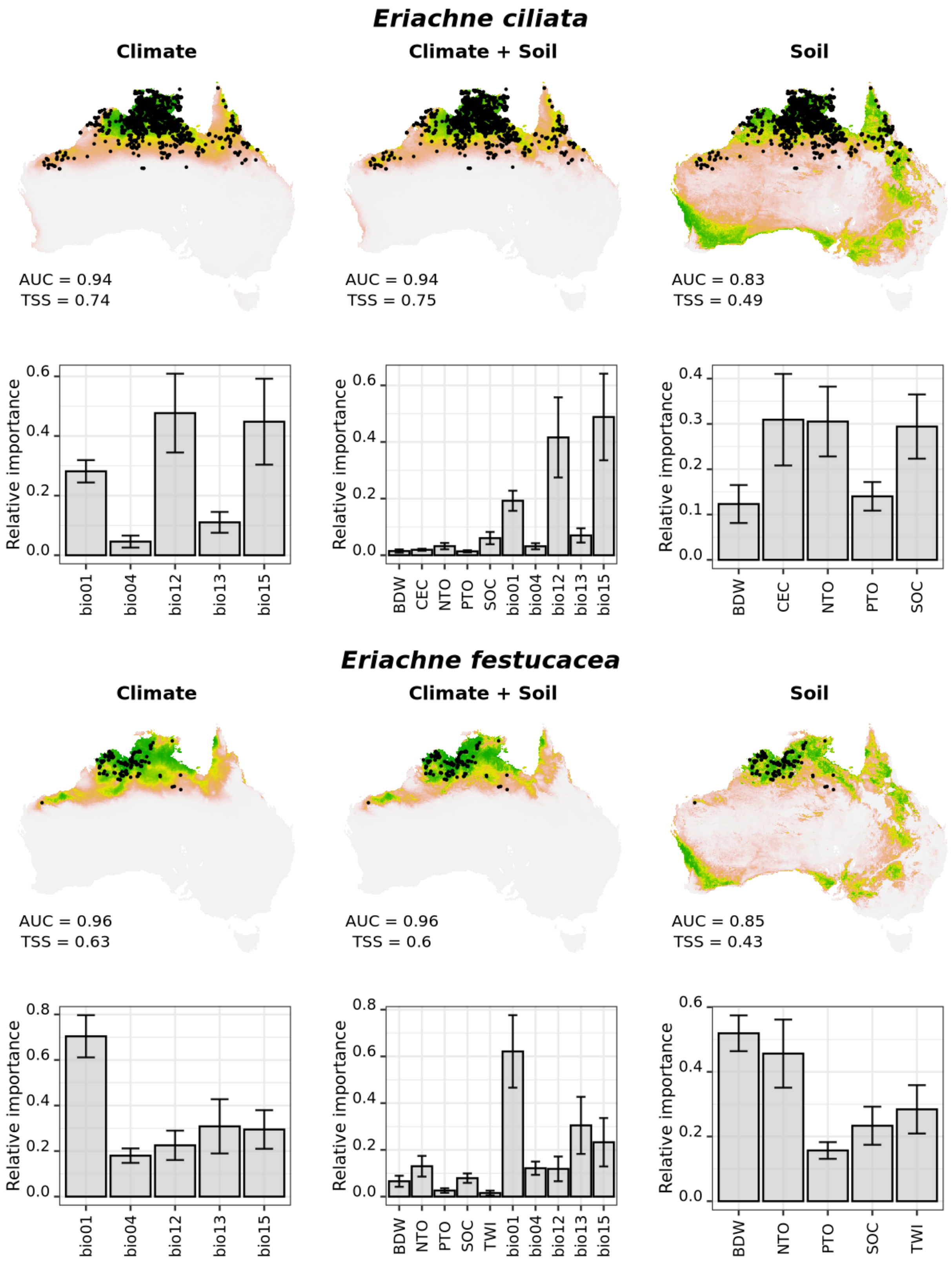
Eriachne ciliata
Climate
Climate + Soil
Soil
AUC = 0.94
TSS = 0.74
AUC = 0.94
TSS = 0.75
AUC = 0.83
TSS = 0.49
Relative importance
bio01 bio04 bio12 bio13 bio15
BDW CEC NTO PTO SOC bio01 bio04 bio12 bio13 bio15
BDW CEC NTO PTO SOC
Eriachne festucacea
Climate
Climate + Soil
Soil
AUC = 0.96
TSS = 0.63
AUC = 0.96
TSS = 0.6
AUC = 0.85
TSS = 0.43
Relative importance
bio01 bio04 bio12 bio13 bio15
BDW NTO PTO SOC TWI bio01 bio04 bio12 bio13 bio15
BDW NTO PTO SOC TWI

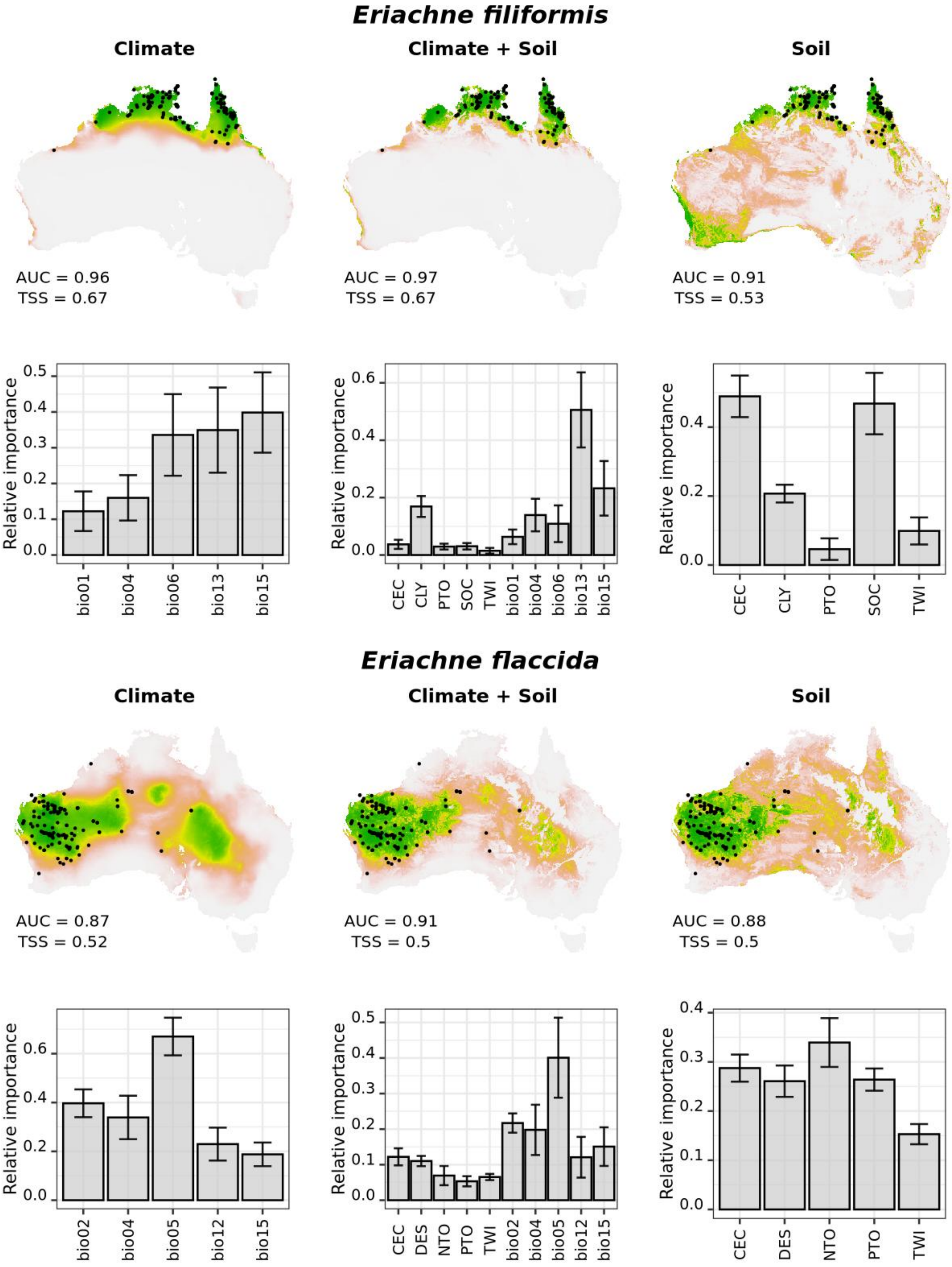

Eriachne filiformis
Climate
Climate + Soil
Soil
AUC = 0.96
TSS = 0.67
AUC = 0.97
TSS = 0.67
AUC = 0.91
TSS = 0.53
Relative importance
bio01 bio04 bio06 bio13 bio15
CEC CLY PTO SOC TWI bio01 bio04 bio06 bio13 bio15
CEC CLY PTO SOC TWI
Eriachne flaccida
Climate
Climate + Soil
Soil
AUC = 0.87
TSS = 0.52
AUC = 0.91
TSS = 0.5
AUC = 0.88
TSS = 0.5
Relative importance
bio02 bio04 bio05 bio12 bio15
CEC DES NTO PTO TWI bio02 bio04 bio05 bio12 bio15
CEC DES NTO PTO TWI

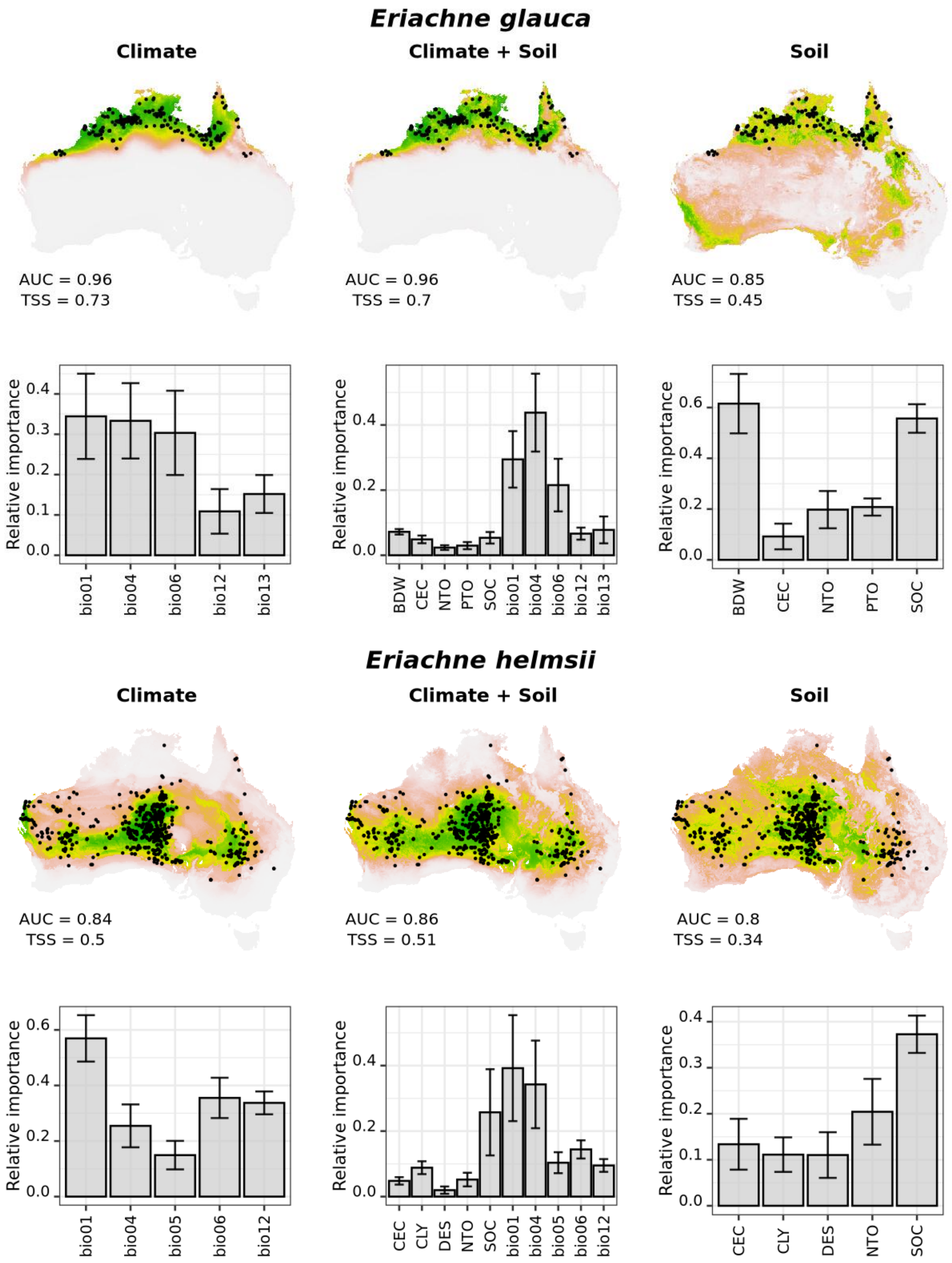
Eriachne glauca
Climate
Climate + Soil
Soil
AUC = 0.96
TSS = 0.73
AUC = 0.96
TSS = 0.7
AUC = 0.85
TSS = 0.45
Relative importance
bio01
bio04
bio06
bio12
bio13
BDW
CEC
NTO
PTO
SOC
Eriachne helmsii
Climate
Climate + Soil
Soil
AUC = 0.84
TSS = 0.5
AUC = 0.86
TSS = 0.51
AUC = 0.8
TSS = 0.34
Relative importance
bio01
bio04
bio05
bio06
bio12
CEC
CLY
DES
NTO
SOC

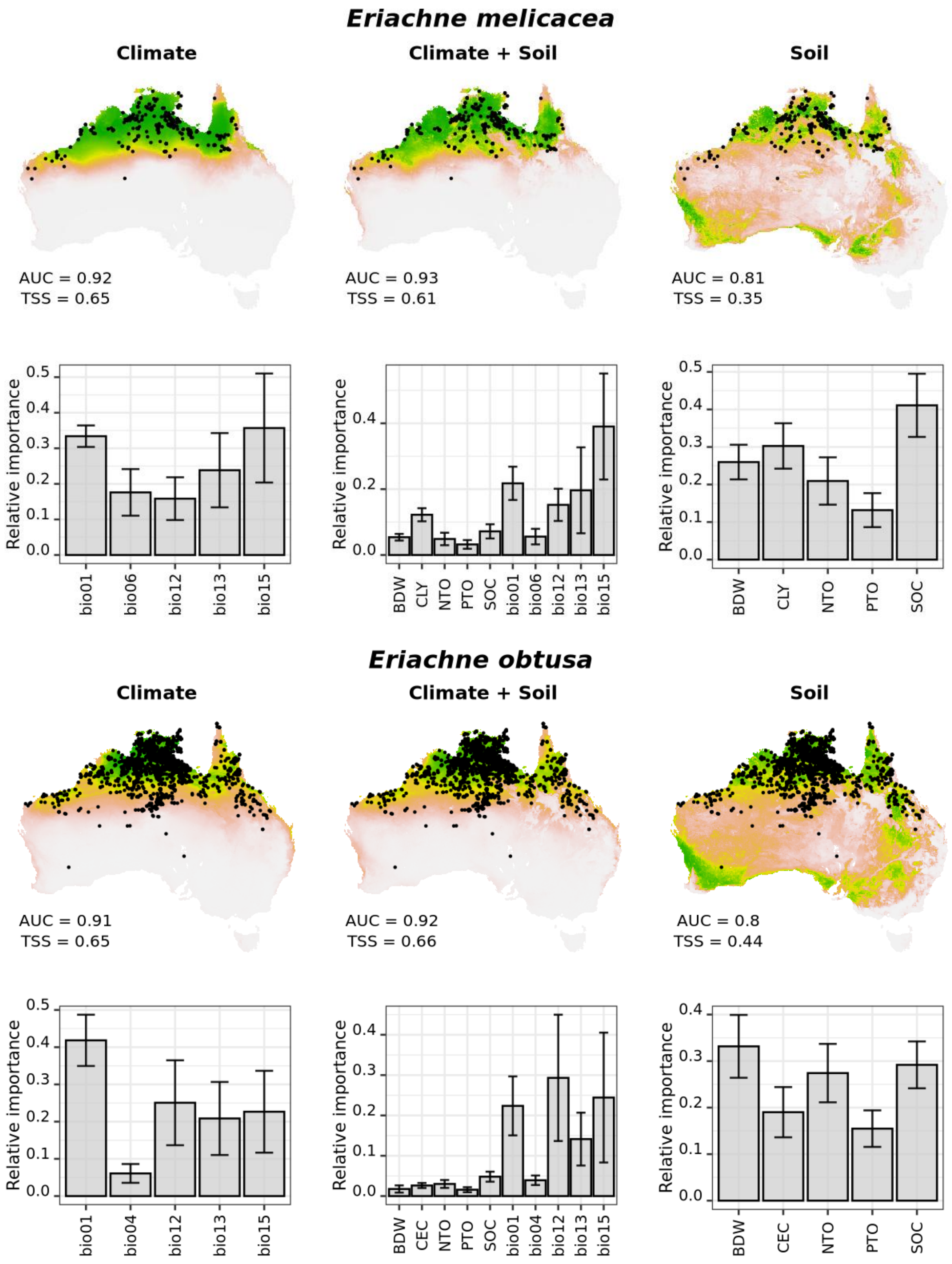

Eriachne melicacea
Climate
Climate + Soil
Soil
AUC = 0.92
TSS = 0.65
AUC = 0.93
TSS = 0.61
AUC = 0.81
TSS = 0.35
Relative importance
0.0
0.1
0.2
0.3
0.4
0.5
bio01
bio06
bio12
bio13
bio15
BDW
CLY
NTO
PTO
SOC
Eriachne obtusa
Climate
Climate + Soil
Soil
AUC = 0.91
TSS = 0.65
AUC = 0.92
TSS = 0.66
AUC = 0.8
TSS = 0.44
Relative importance
bio01
bio04
bio12
bio13
bio15
BDW
CEC
NTO
PTO
SOC

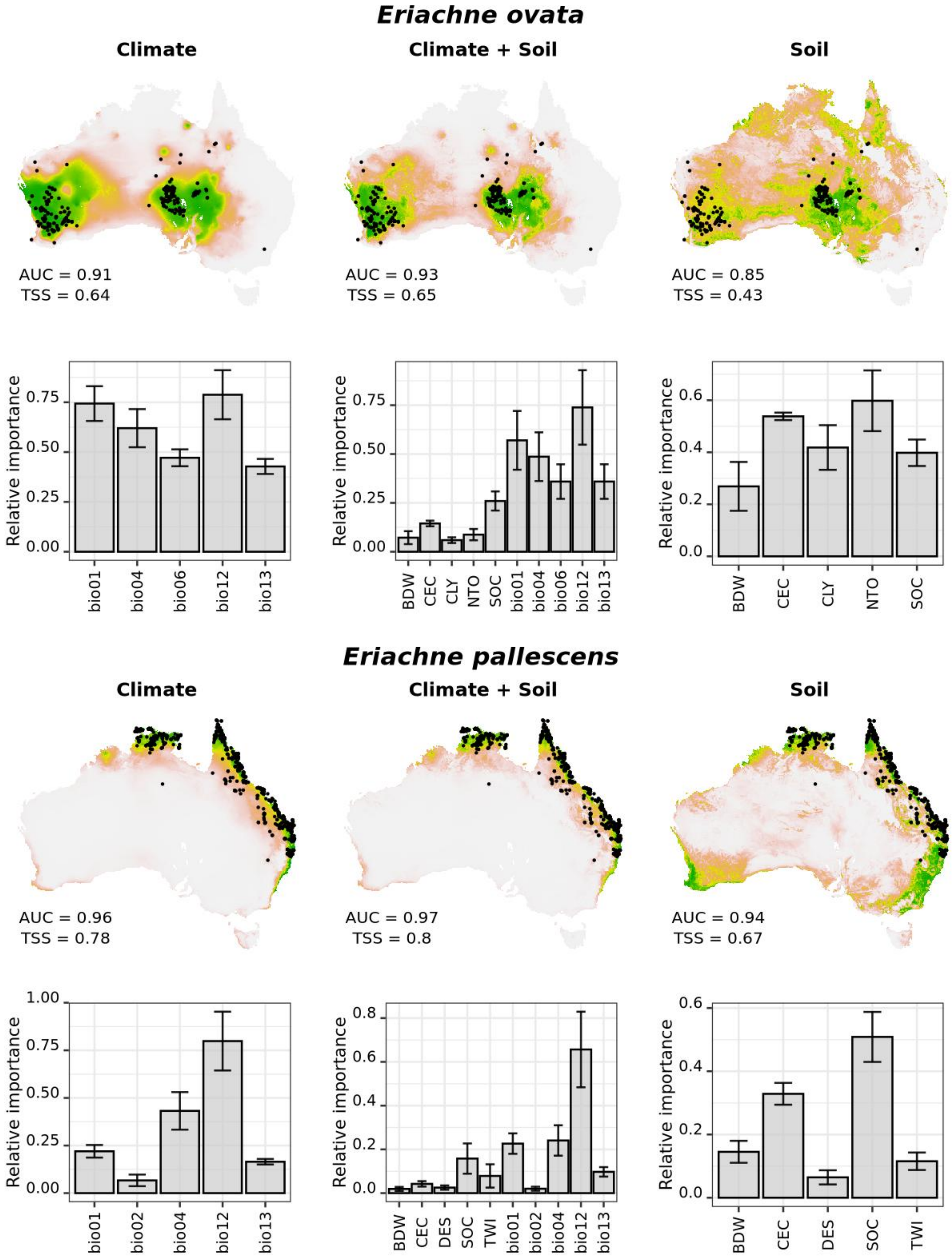
Eriachne ovata
Climate
Climate + Soil
Soil
AUC = 0.91
TSS = 0.64
AUC = 0.93
TSS = 0.65
AUC = 0.85
TSS = 0.43
Relative importance
0.00
0.25
0.50
0.75
bio01
bio04
bio06
bio12
bio13
BDW
CEC
CLY
NTO
SOC
0.0
0.2
0.4
0.6
Eriachne pallescens
Climate
Climate + Soil
Soil
AUC = 0.96
TSS = 0.78
AUC = 0.97
TSS = 0.8
AUC = 0.94
TSS = 0.67
1.00
bio02
DES
TWI
0.8

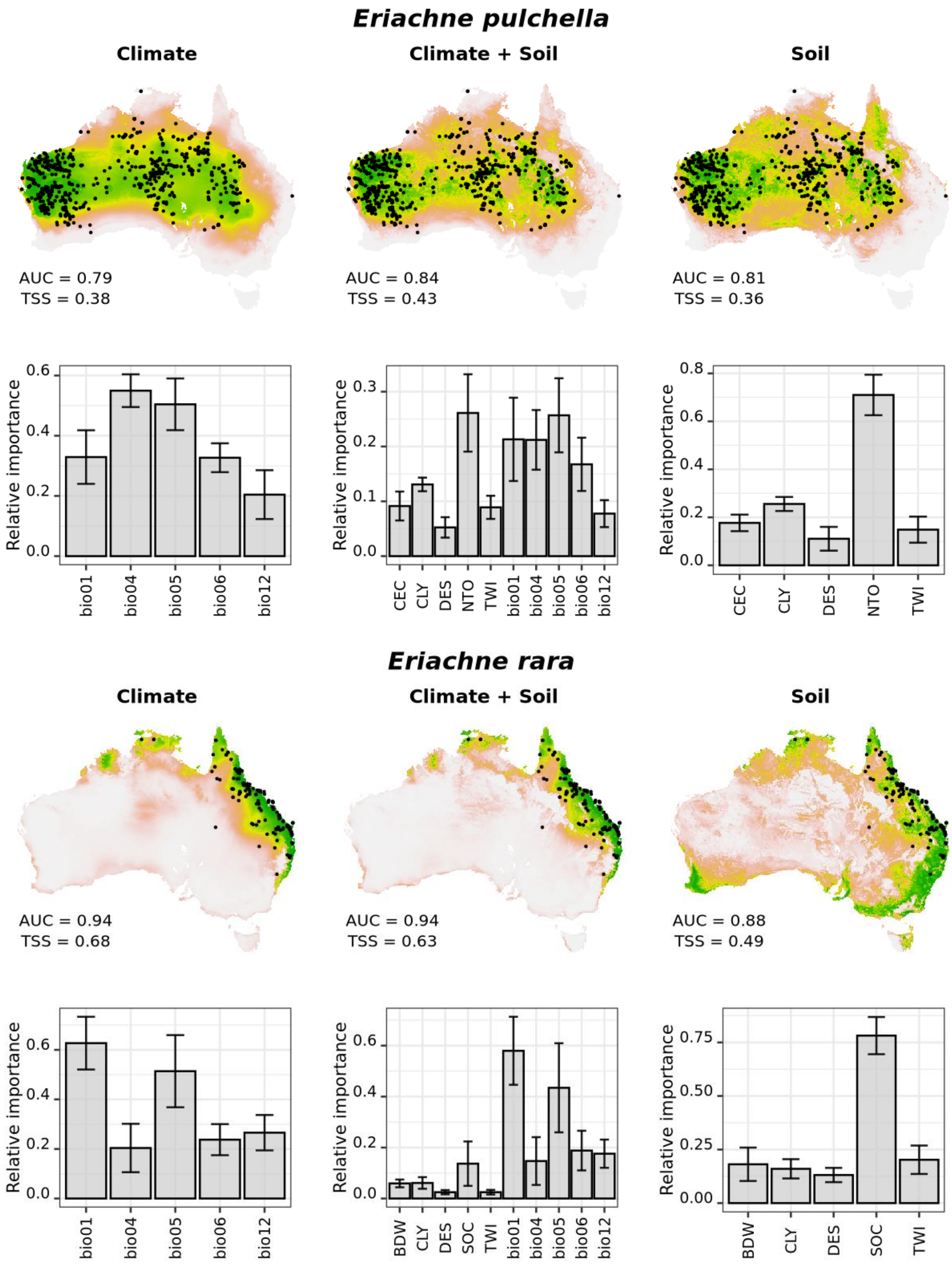
Eriachne pulchella
Climate
Climate + Soil
Soil
AUC = 0.79
TSS = 0.38
AUC = 0.84
TSS = 0.43
AUC = 0.81
TSS = 0.36
Relative importance
bio01
bio04
bio05
bio06
bio12
CEC
CLY
DES
NTO
TWI
Eriachne rara
Climate
Climate + Soil
Soil
AUC = 0.94
TSS = 0.68
AUC = 0.94
TSS = 0.63
AUC = 0.88
TSS = 0.49
Relative importance
BDW
CLY
DES
SOC
TWI

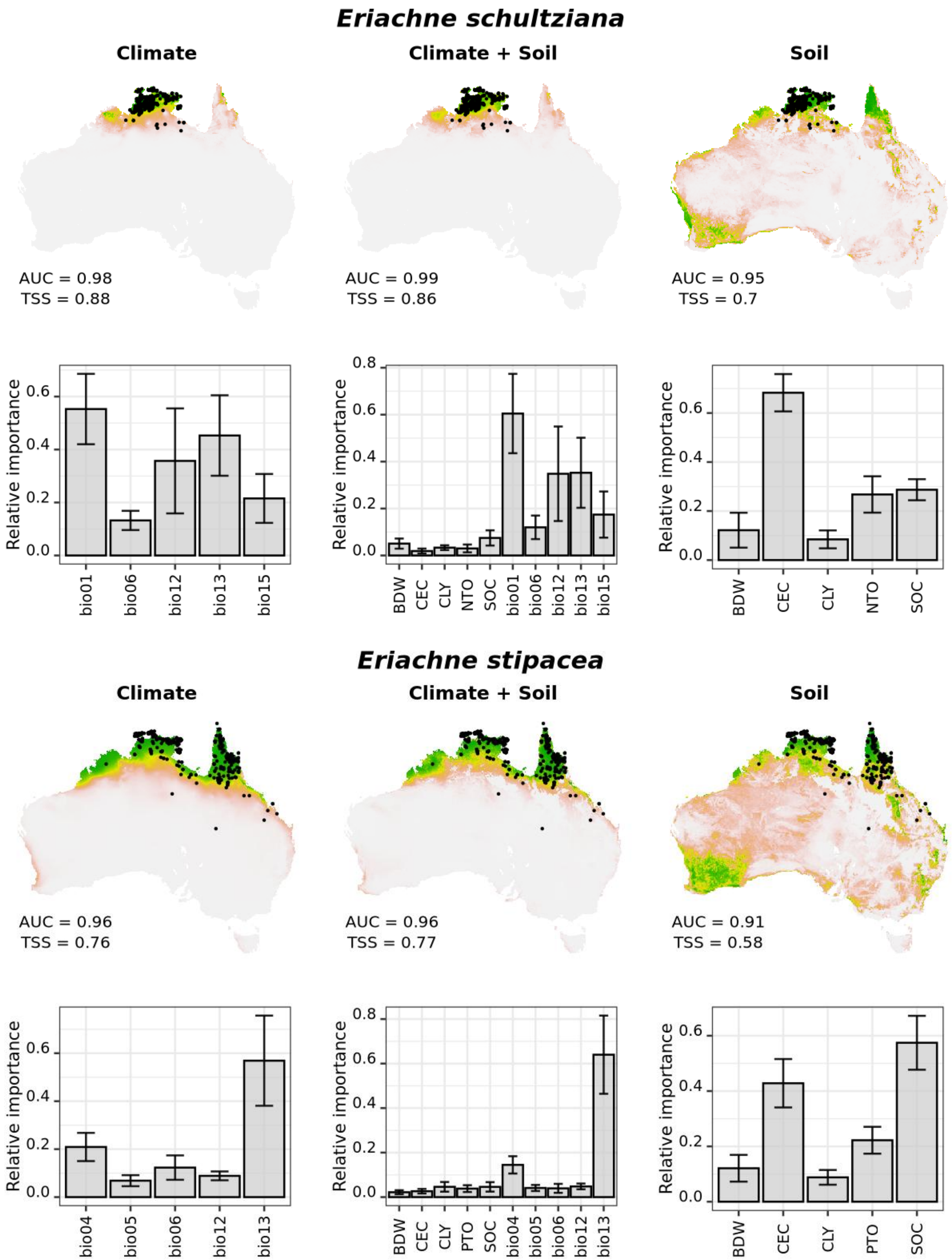

Eriachne schultziana
Climate
Climate + Soil
Soil
AUC = 0.98
TSS = 0.88
AUC = 0.99
TSS = 0.86
AUC = 0.95
TSS = 0.7
Relative importance
0.0
0.2
0.4
0.6
0.8
bio01
bio06
bio12
bio13
bio15
BDW
CEC
CLY
NTO
SOC
Eriachne stipacea
Climate
Climate + Soil
Soil
AUC = 0.96
TSS = 0.76
AUC = 0.96
TSS = 0.77
AUC = 0.91
TSS = 0.58
Relative importance
bio04
bio05
bio06
bio12
bio13
BDW
CEC
CLY
PTO
SOC

Eriachne sulcata

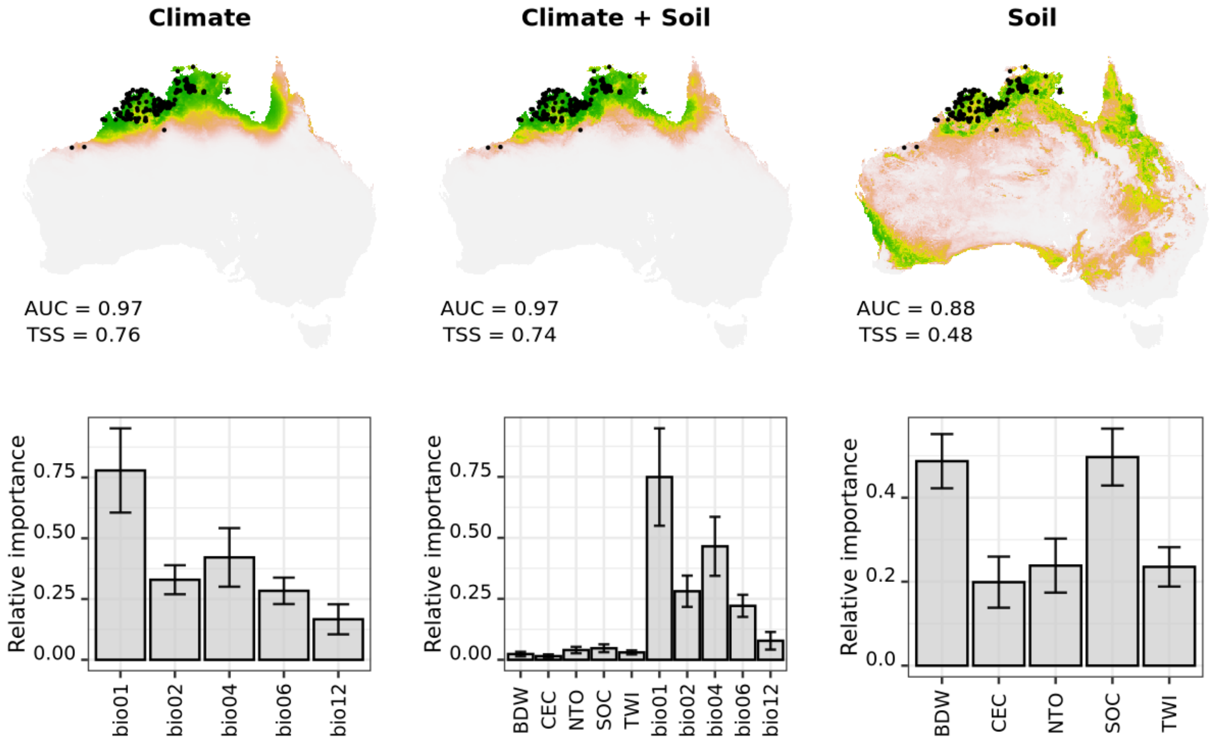

Eriachne triseta

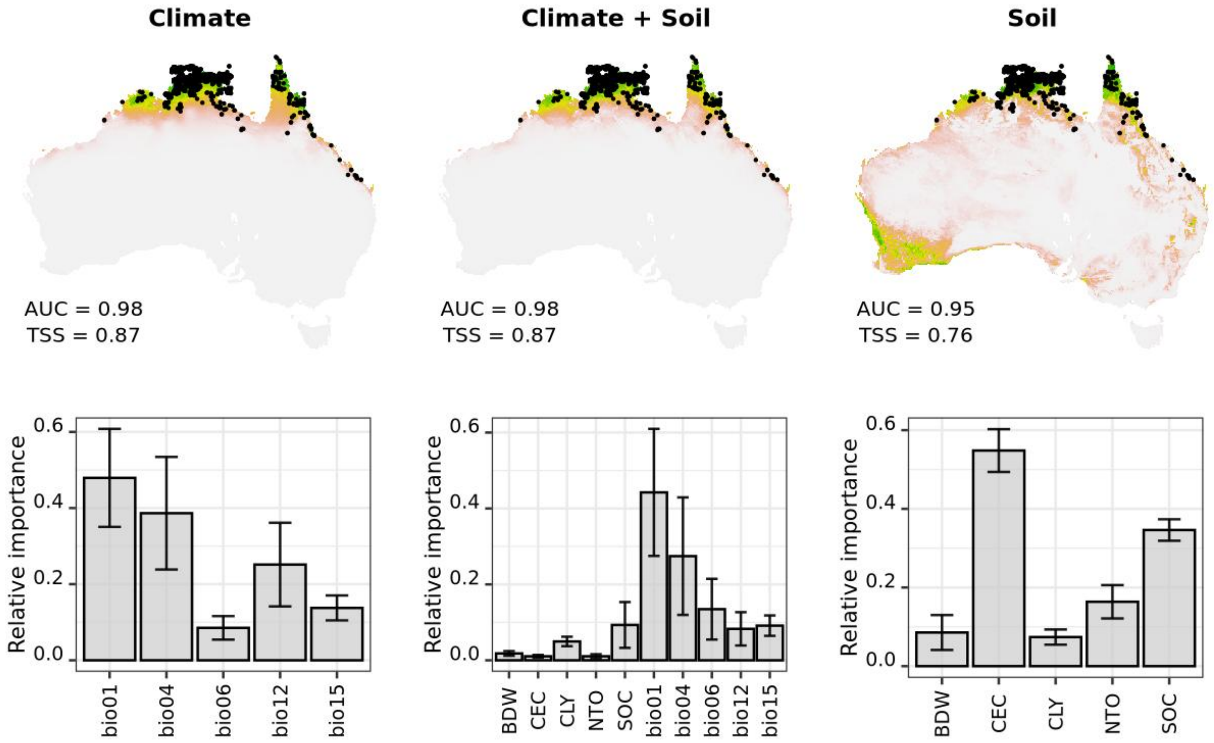

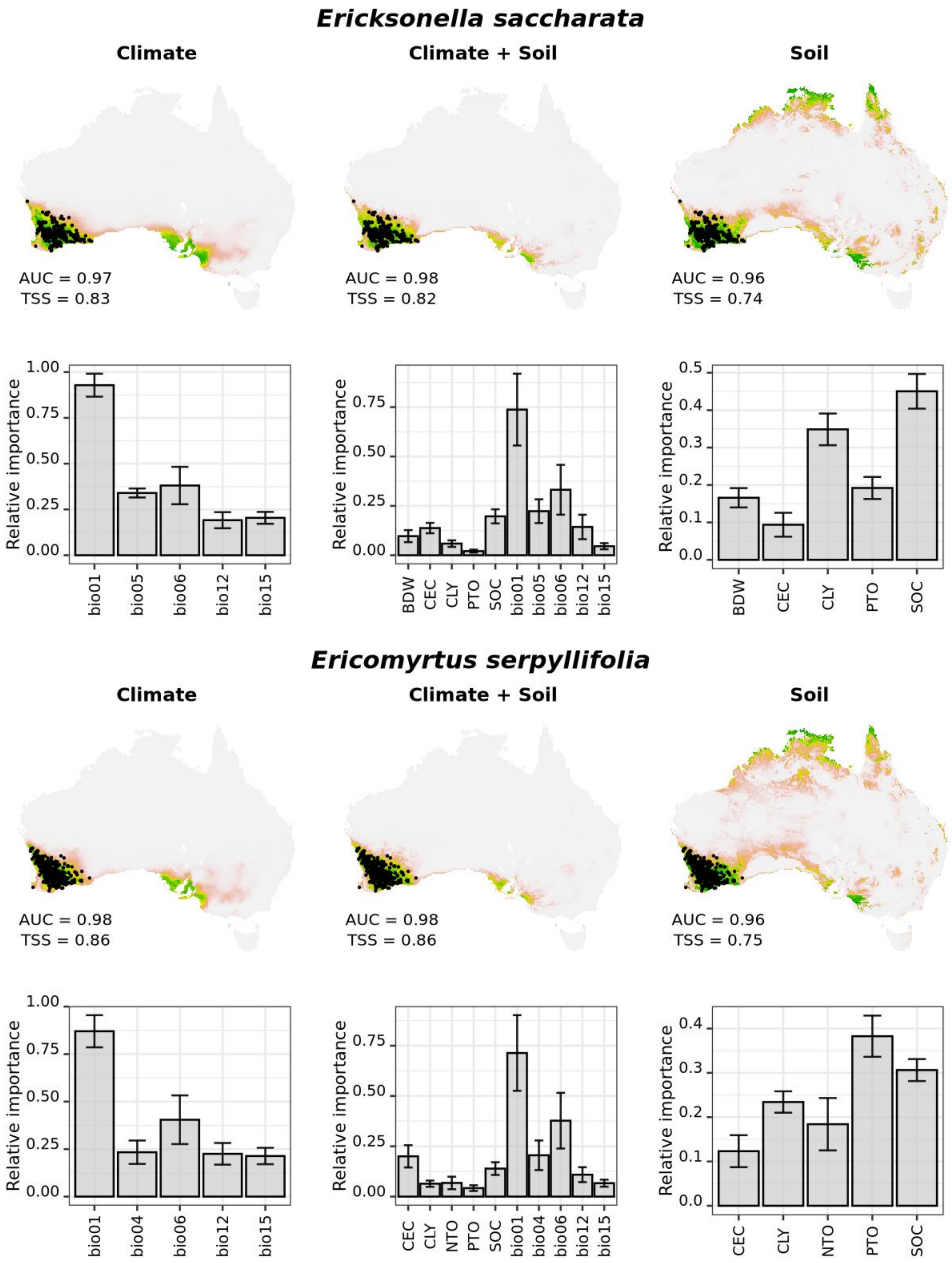
Ericksonella saccharata
Climate
Climate + Soil
Soil
AUC = 0.97
TSS = 0.83
AUC = 0.98
TSS = 0.82
AUC = 0.96
TSS = 0.74
Relative importance
bio01
bio05
bio06
bio12
bio15
BDW
CEC
CLY
PTO
SOC
Ericomyrtus serpyllifolia
Climate
Climate + Soil
Soil
AUC = 0.98
TSS = 0.86
AUC = 0.98
TSS = 0.86
AUC = 0.96
TSS = 0.75
Relative importance
bio01
bio04
bio06
bio12
bio15
CEC
CLY
NTO
PTO
SOC

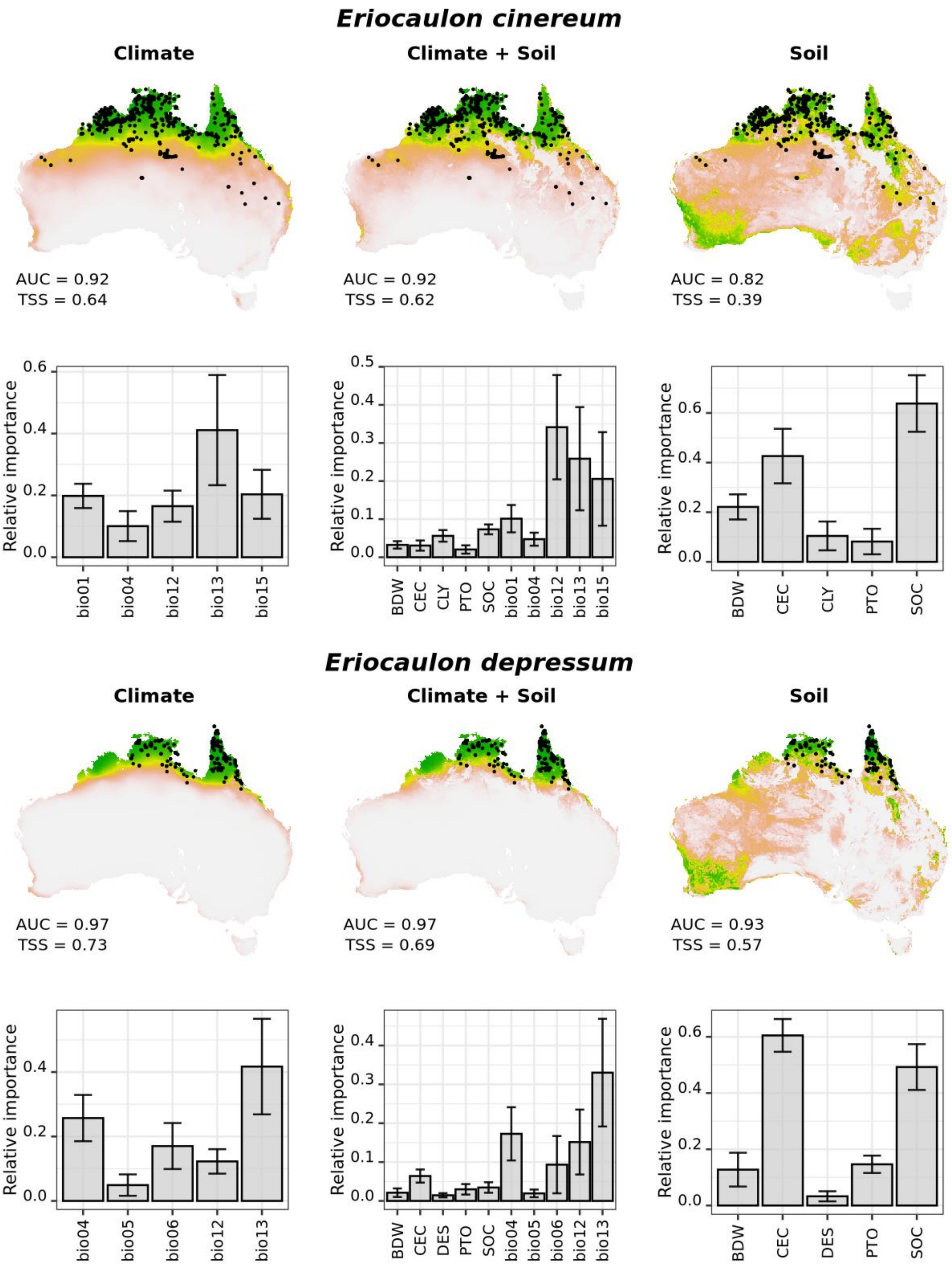

Eriocaulon cinereum
Climate
Climate + Soil
Soil
AUC = 0.92
TSS = 0.64
AUC = 0.92
TSS = 0.62
AUC = 0.82
TSS = 0.39
Relative importance
0.0
0.2
0.4
0.6
bio01
bio04
bio12
bio13
bio15
0.0
0.1
0.2
0.3
0.4
0.5
BDW
CEC
CLY
PTO
SOC
bio01
bio04
bio12
bio13
bio15
0.0
0.2
0.4
0.6
BDW
CEC
CLY
PTO
SOC
Eriocaulon depressum
Climate
Climate + Soil
Soil
AUC = 0.97
TSS = 0.73
AUC = 0.97
TSS = 0.69
AUC = 0.93
TSS = 0.57
Relative importance
0.0
0.2
0.4
bio04
bio05
bio06
bio12
bio13
0.0
0.1
0.2
0.3
0.4
BDW
CEC
DES
PTO
SOC
bio04
bio05
bio06
bio12
bio13
0.0
0.2
0.4
0.6
BDW
CEC
DES
PTO
SOC

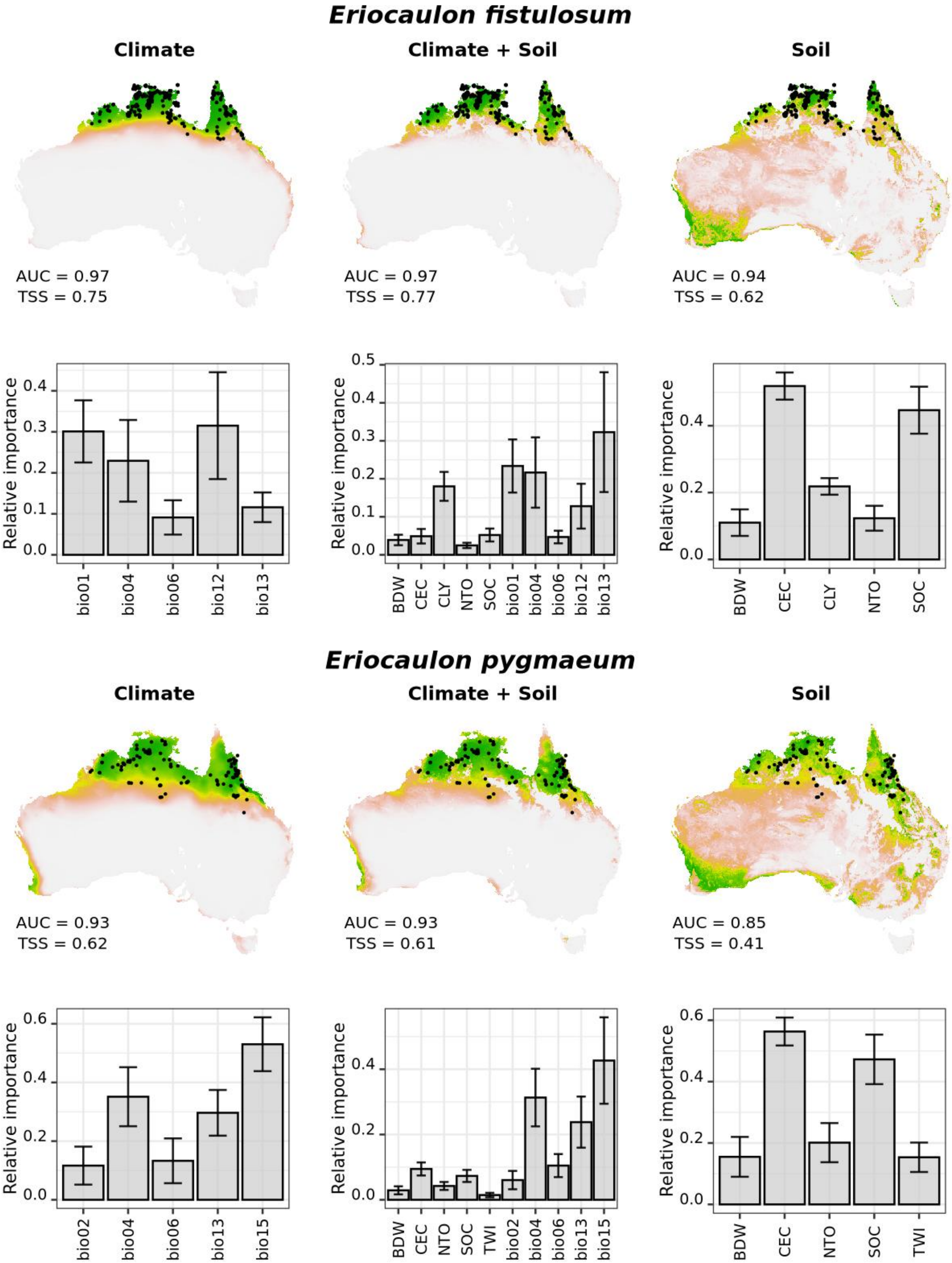
Eriocaulon fistulosum
Climate
Climate + Soil
Soil
AUC = 0.97
TSS = 0.75
AUC = 0.97
TSS = 0.77
AUC = 0.94
TSS = 0.62
Relative importance
0.0
0.1
0.2
0.3
0.4
0.5
bio01
bio04
bio06
bio12
bio13
BDW
CEC
CLY
NTO
SOC
Eriocaulon pygmaeum
Climate
Climate + Soil
Soil
AUC = 0.93
TSS = 0.62
AUC = 0.93
TSS = 0.61
AUC = 0.85
TSS = 0.41
Relative importance
0.0
0.2
0.4
0.6
bio02
bio04
bio06
bio13
bio15
BDW
CEC
NTO
SOC
TWI

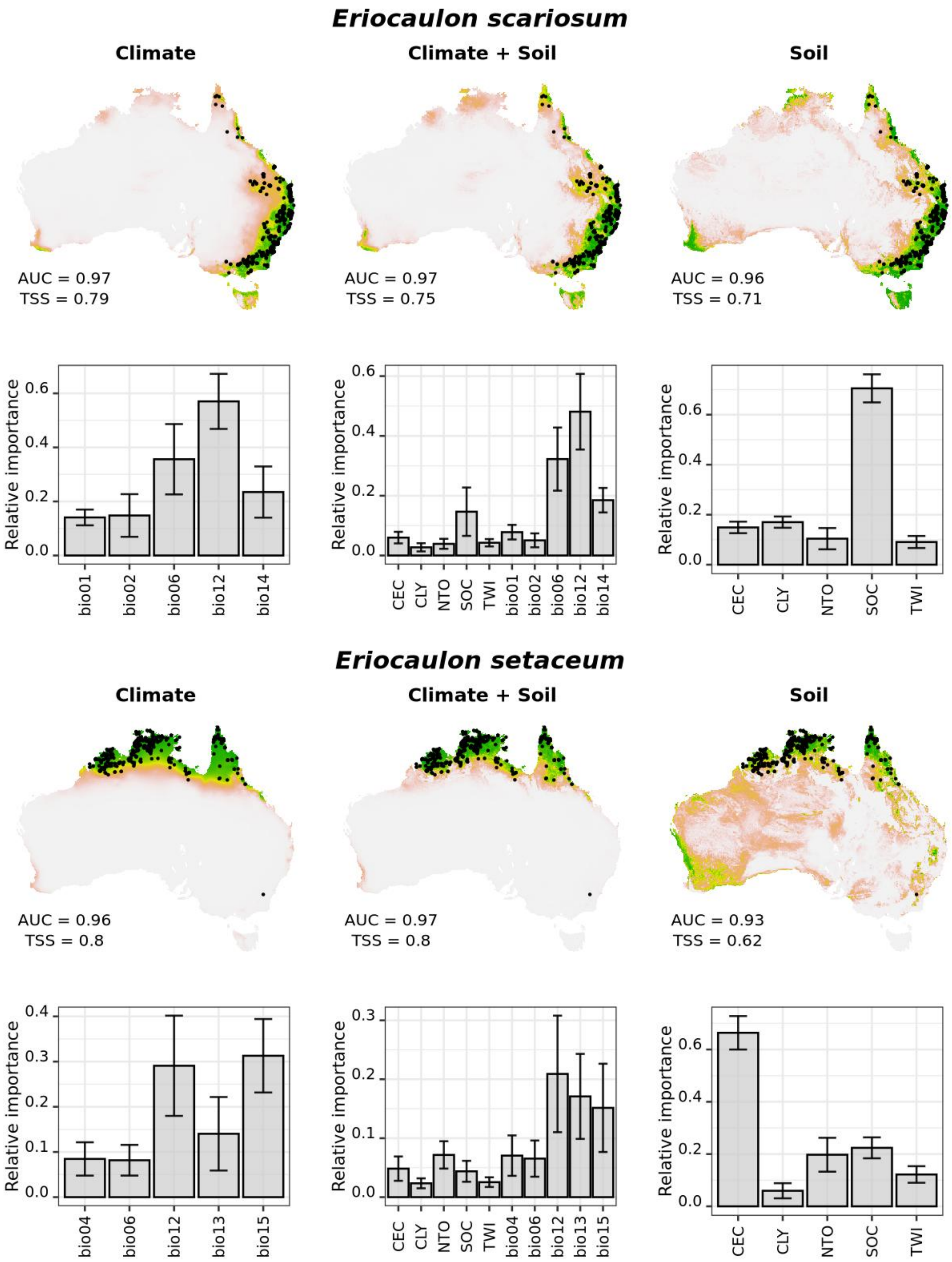

Eriocaulon scariosum
Climate
Climate + Soil
Soil
AUC = 0.97
TSS = 0.79
AUC = 0.97
TSS = 0.75
AUC = 0.96
TSS = 0.71
Relative importance
bio01
bio02
bio06
bio12
bio14
CEC
CLY
NTO
SOC
TWI
Eriocaulon setaceum
Climate
Climate + Soil
Soil
AUC = 0.96
TSS = 0.8
AUC = 0.97
TSS = 0.8
AUC = 0.93
TSS = 0.62
Relative importance
bio04
bio06
bio12
bio13
bio15
CEC
CLY
NTO
SOC
TWI

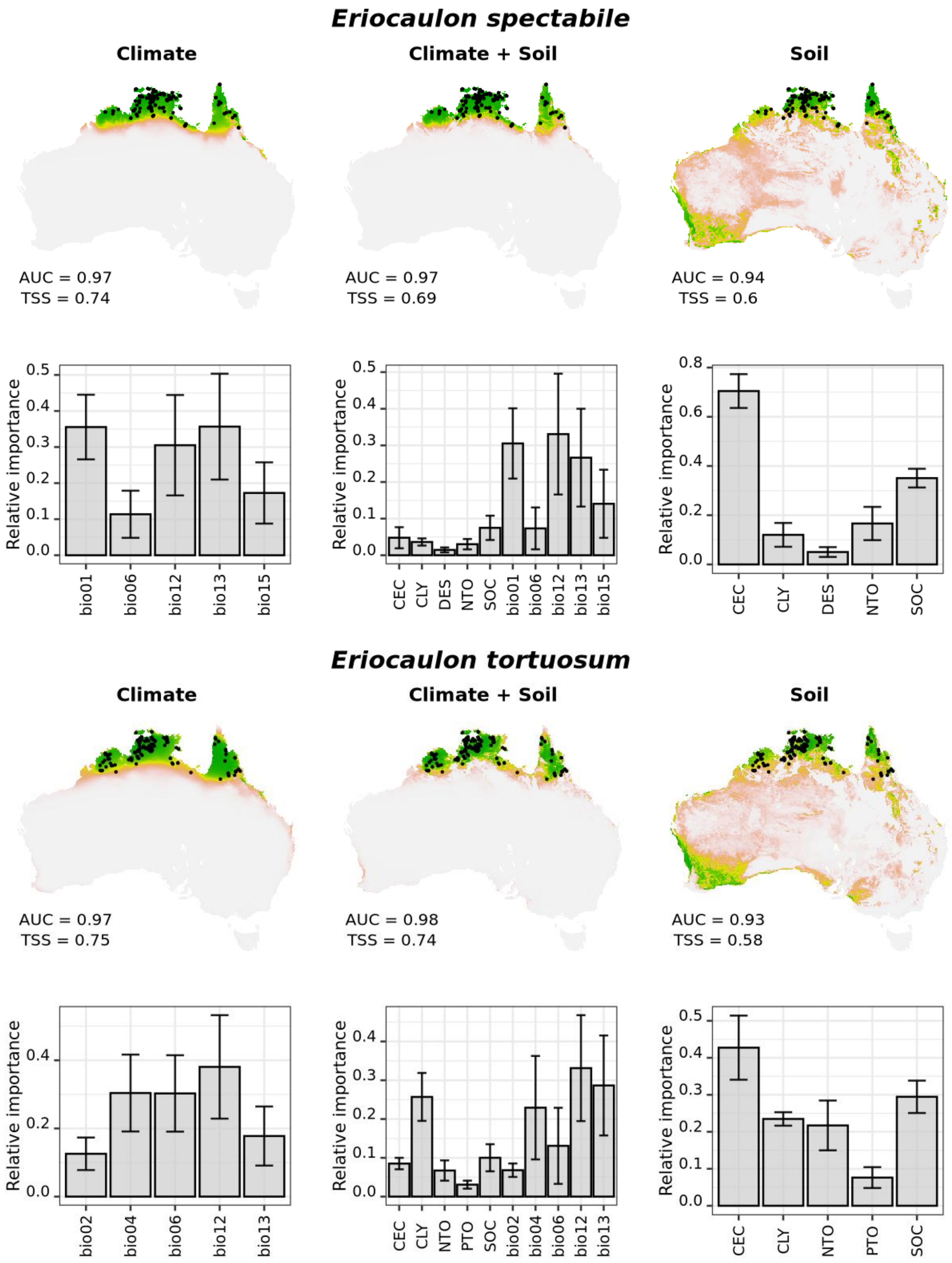
Eriocaulon spectabile
Climate
Climate + Soil
Soil
AUC = 0.97
TSS = 0.74
AUC = 0.97
TSS = 0.69
AUC = 0.94
TSS = 0.6
Relative importance
bio01 bio06 bio12 bio13 bio15
CEC CLY DES NTO SOC bio01 bio06 bio12 bio13 bio15
CEC CLY DES NTO SOC
Eriocaulon tortuosum
Climate
Climate + Soil
Soil
AUC = 0.97
TSS = 0.75
AUC = 0.98
TSS = 0.74
AUC = 0.93
TSS = 0.58
Relative importance
bio02 bio04 bio06 bio12 bio13
CEC CLY NTO PTO SOC bio02 bio04 bio06 bio12 bio13
CEC CLY NTO PTO SOC

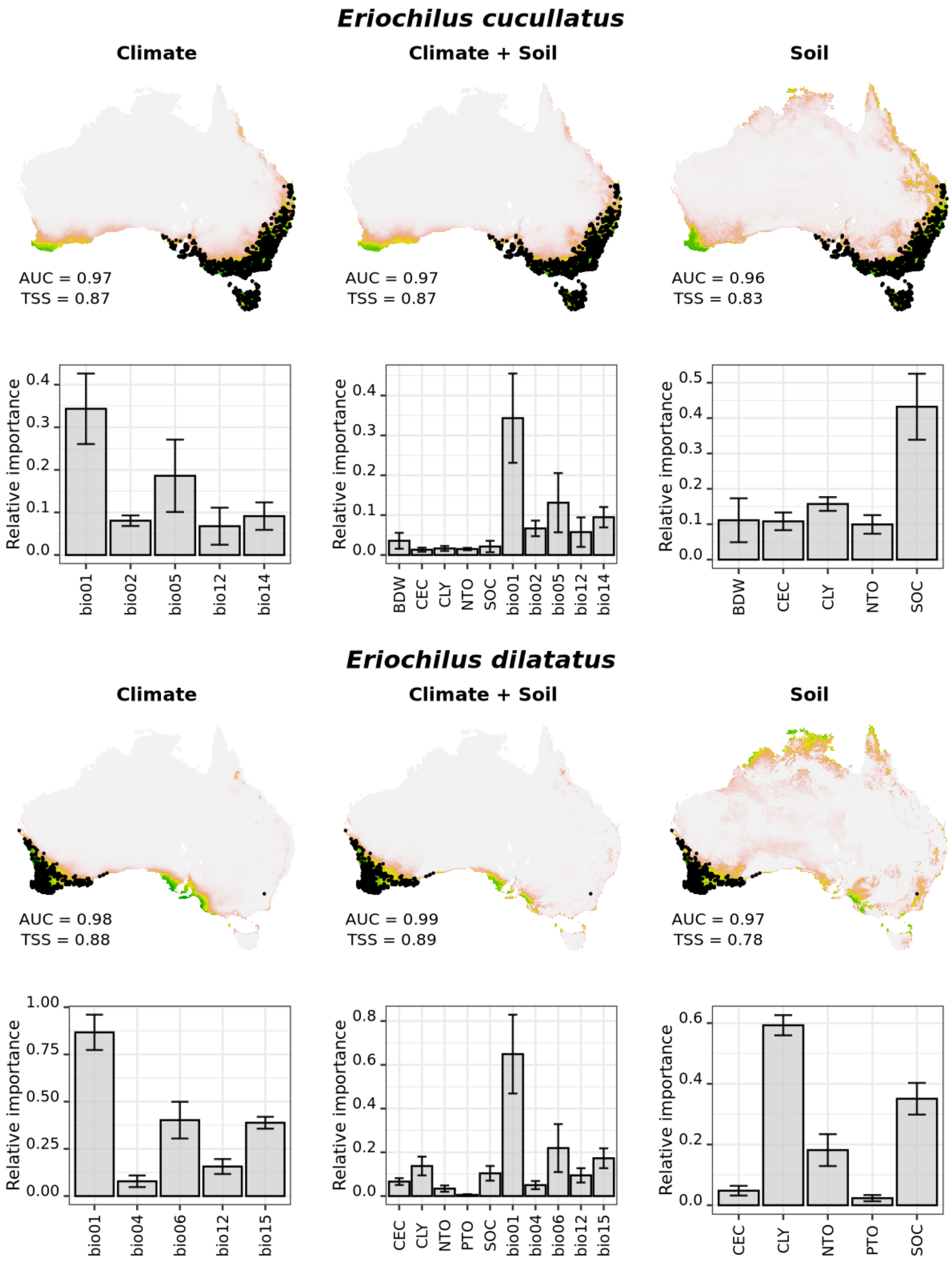

Eriochilus cucullatus
Climate
Climate + Soil
Soil
AUC = 0.97
TSS = 0.87
AUC = 0.97
TSS = 0.87
AUC = 0.96
TSS = 0.83
Relative importance
0.0
0.1
0.2
0.3
0.4
0.5
bio01
bio02
bio05
bio12
bio14
BDW
CEC
CLY
NTO
SOC
Eriochilus dilatatus
Climate
Climate + Soil
Soil
AUC = 0.98
TSS = 0.88
AUC = 0.99
TSS = 0.89
AUC = 0.97
TSS = 0.78
Relative importance
0.00
0.25
0.50
0.75
1.00
0.0
0.2
0.4
0.6
0.8
bio01
bio04
bio06
bio12
bio15
CEC
CLY
NTO
PTO
SOC

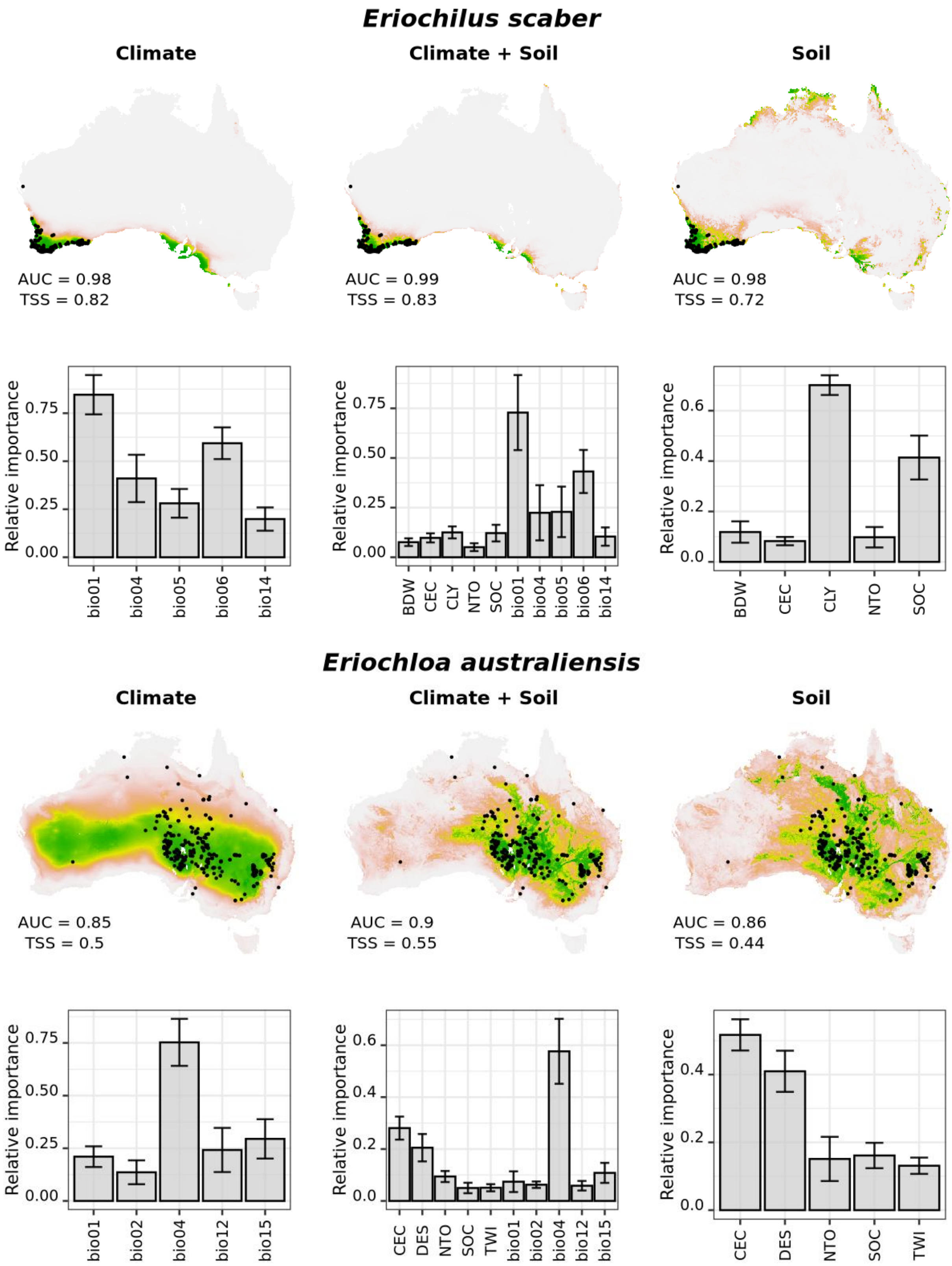

Eriochilus scaber
Climate
Climate + Soil
Soil
AUC = 0.98
TSS = 0.82
AUC = 0.99
TSS = 0.83
AUC = 0.98
TSS = 0.72
Relative importance
0.00
0.25
0.50
0.75
bio01
bio04
bio05
bio06
bio14
BDW
CEC
CLY
NTO
SOC
0.0
0.2
0.4
0.6
Eriochloa australiensis
AUC = 0.85
TSS = 0.5
AUC = 0.9
TSS = 0.55
AUC = 0.86
TSS = 0.44
bio02
bio12
bio15
DES
TWI

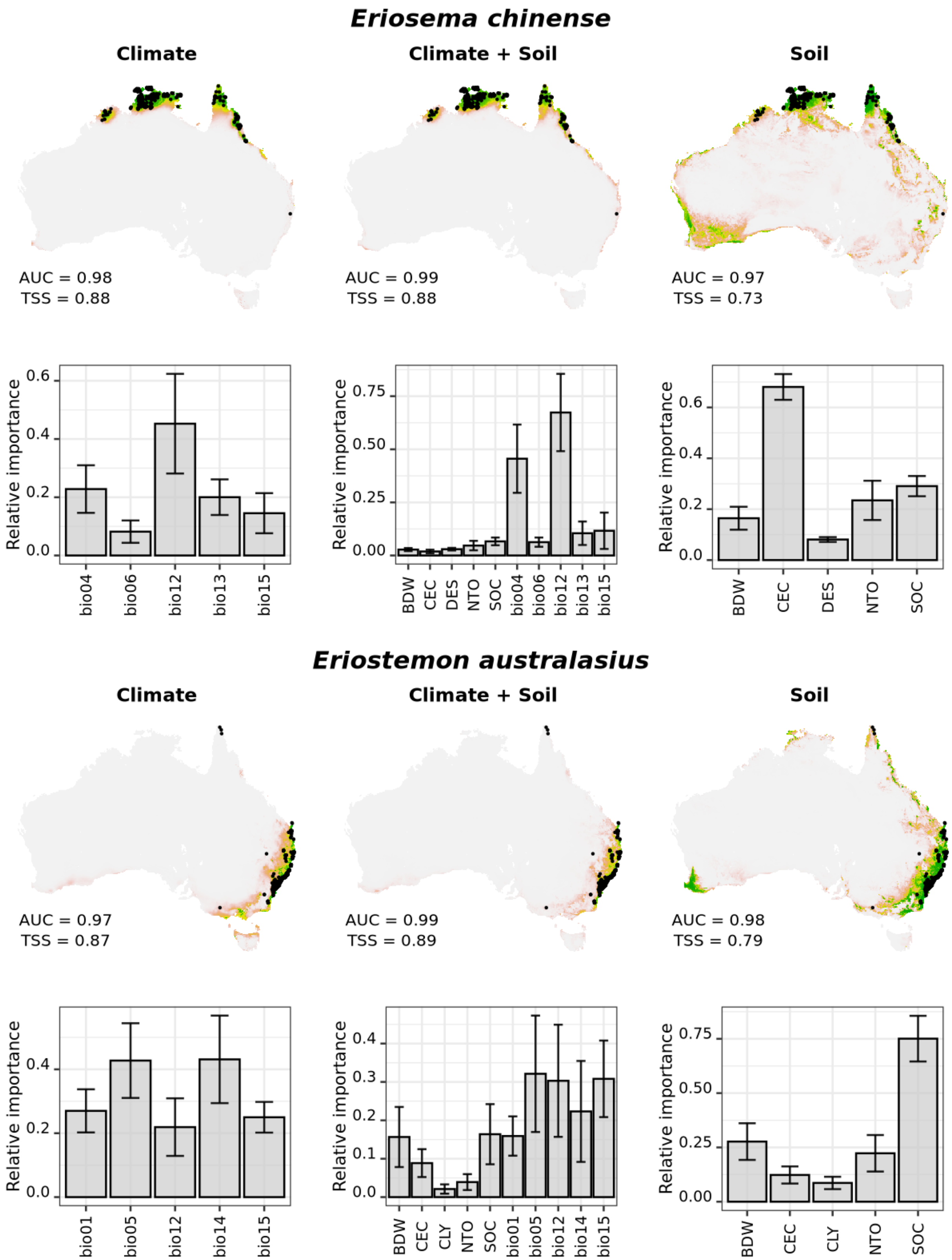

Eriosema chinense
Climate
Climate + Soil
Soil
AUC = 0.98
TSS = 0.88
AUC = 0.99
TSS = 0.88
AUC = 0.97
TSS = 0.73
Relative importance
bio04
bio06
bio12
bio13
bio15
BDW
CEC
DES
NTO
SOC
Eriostemon australasius
Climate
Climate + Soil
Soil
AUC = 0.97
TSS = 0.87
AUC = 0.99
TSS = 0.89
AUC = 0.98
TSS = 0.79
Relative importance
bio01
bio05
bio12
bio14
bio15
BDW
CEC
CLY
NTO
SOC

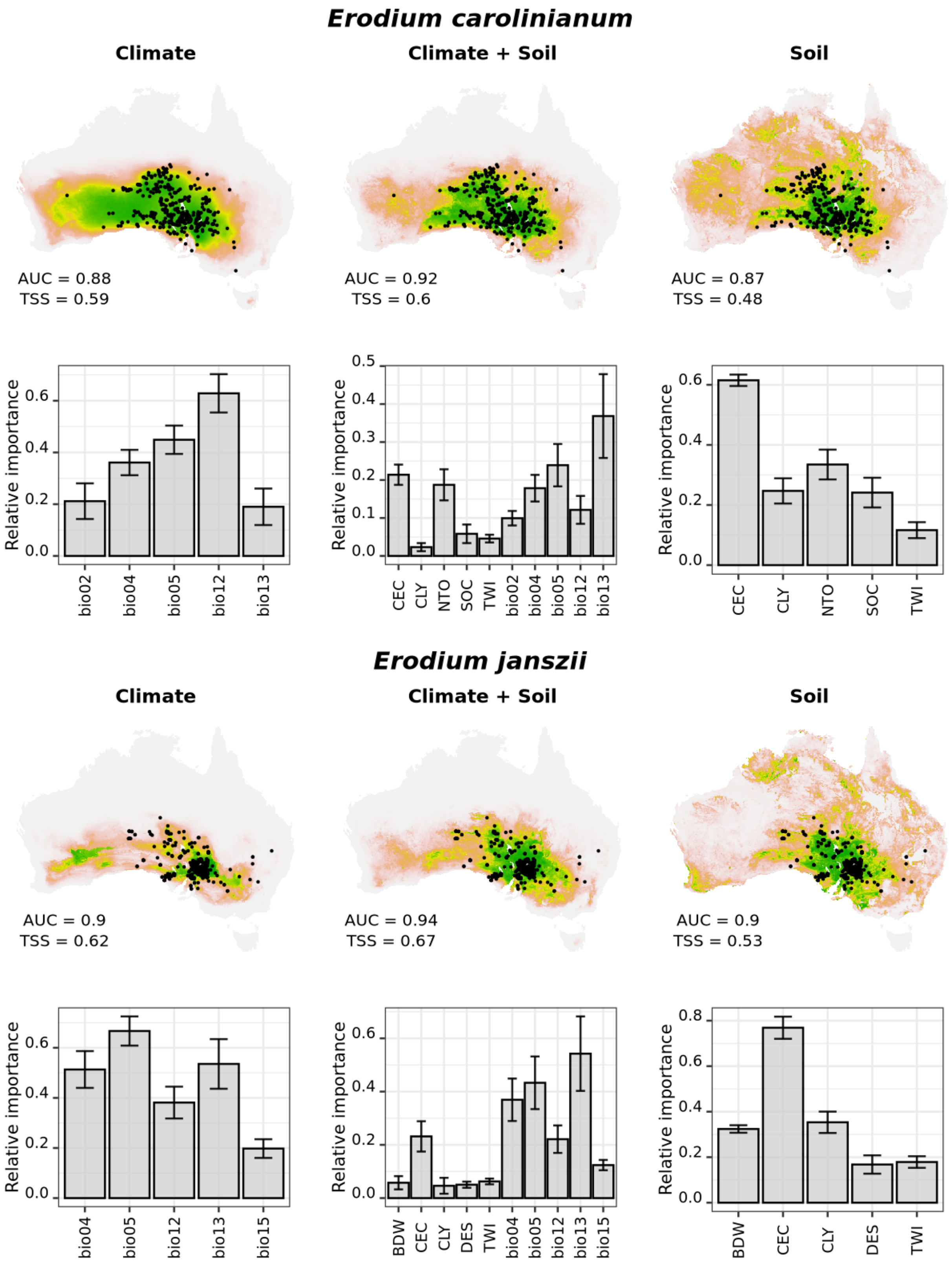

Erodium carolinianum
Climate
Climate + Soil
Soil
AUC = 0.88
TSS = 0.59
AUC = 0.92
TSS = 0.6
AUC = 0.87
TSS = 0.48
Relative importance
bio02
bio04
bio05
bio12
bio13
CEC
CLY
NTO
SOC
TWI
Erodium janszii
Climate
Climate + Soil
Soil
AUC = 0.9
TSS = 0.62
AUC = 0.94
TSS = 0.67
AUC = 0.9
TSS = 0.53
Relative importance
bio04
bio05
bio12
bio13
bio15
BDW
CEC
CLY
DES
TWI

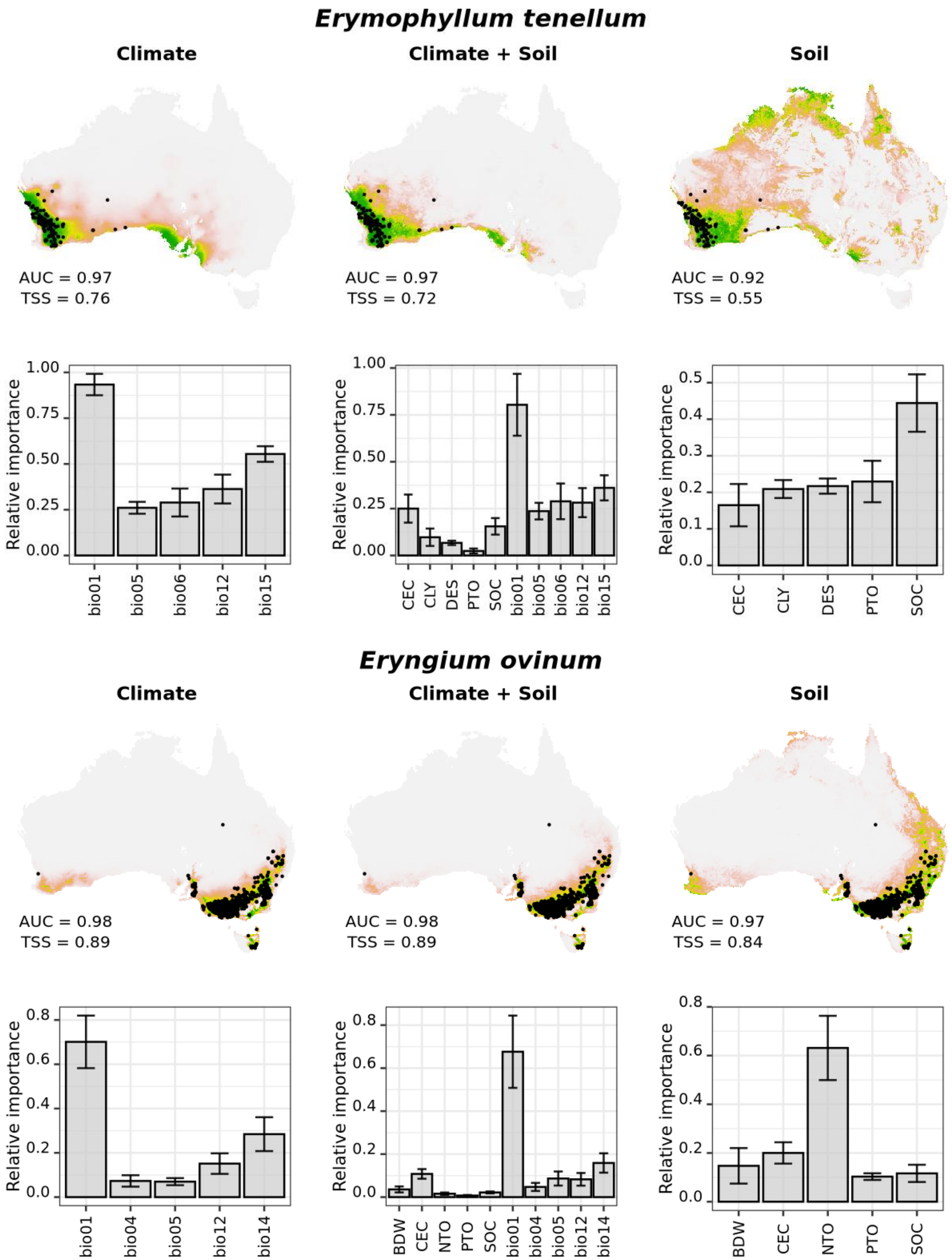

Erymophyllum tenellum
Climate
Climate + Soil
Soil
AUC = 0.97
TSS = 0.76
AUC = 0.97
TSS = 0.72
AUC = 0.92
TSS = 0.55
Relative importance
1.00
0.75
0.50
0.25
0.00
bio01
bio05
bio06
bio12
bio15
CEC
CLY
DES
PTO
SOC
0.5
0.4
0.3
0.2
0.1
0.0
Eryngium ovinum
Climate
Climate + Soil
Soil
AUC = 0.98
TSS = 0.89
AUC = 0.98
TSS = 0.89
AUC = 0.97
TSS = 0.84
0.8
0.6
0.4
0.2
0.0
bio01
bio04
bio05
bio12
bio14
BDW
CEC
NTO
PTO
SOC

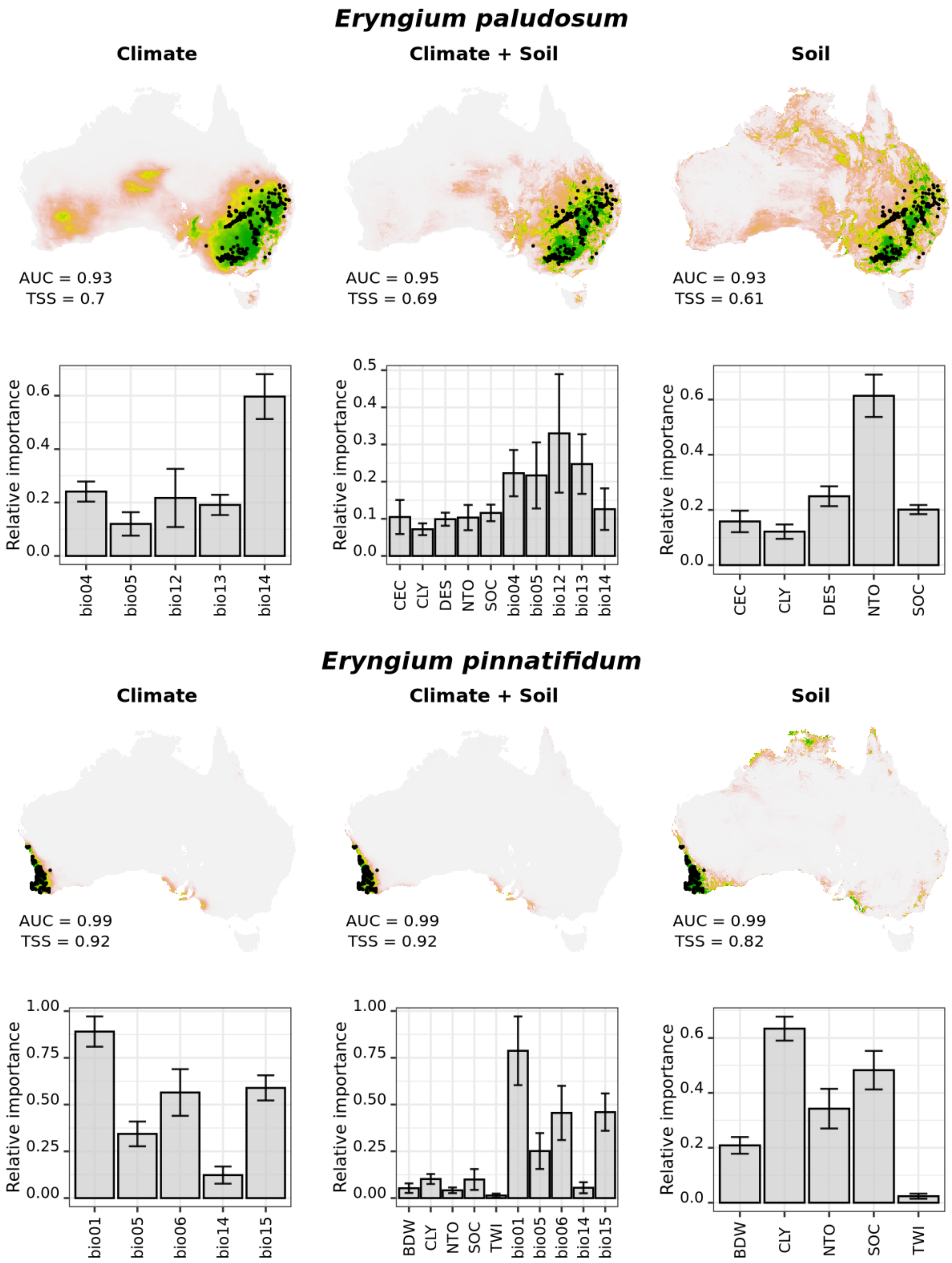
Eryngium paludosum
Climate
Climate + Soil
Soil
AUC = 0.93
TSS = 0.7
AUC = 0.95
TSS = 0.69
AUC = 0.93
TSS = 0.61
Relative importance
bio04
bio05
bio12
bio13
bio14
CEC
CLY
DES
NTO
SOC
Eryngium pinnatifidum
AUC = 0.99
TSS = 0.92
AUC = 0.99
TSS = 0.92
AUC = 0.99
TSS = 0.82
bio01
bio06
bio15
BDW
TWI

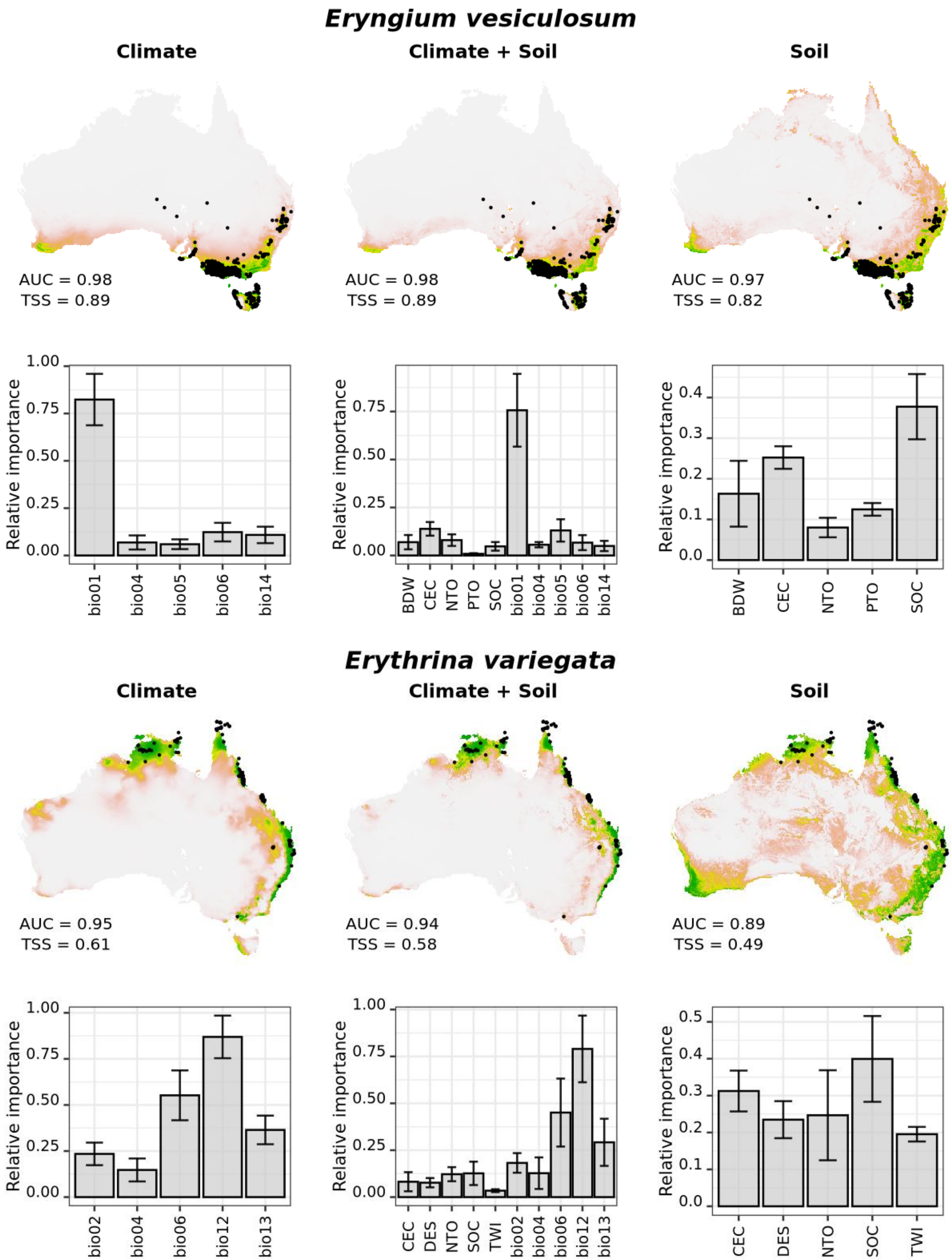

Eryngium vesiculosum
Climate
Climate + Soil
Soil
AUC = 0.98
TSS = 0.89
AUC = 0.98
TSS = 0.89
AUC = 0.97
TSS = 0.82
Relative importance
bio01
bio04
bio05
bio06
bio14
BDW
CEC
NTO
PTO
SOC
Erythrina variegata
AUC = 0.95
TSS = 0.61
AUC = 0.94
TSS = 0.58
AUC = 0.89
TSS = 0.49
bio02
bio12
bio13
DES
TWI

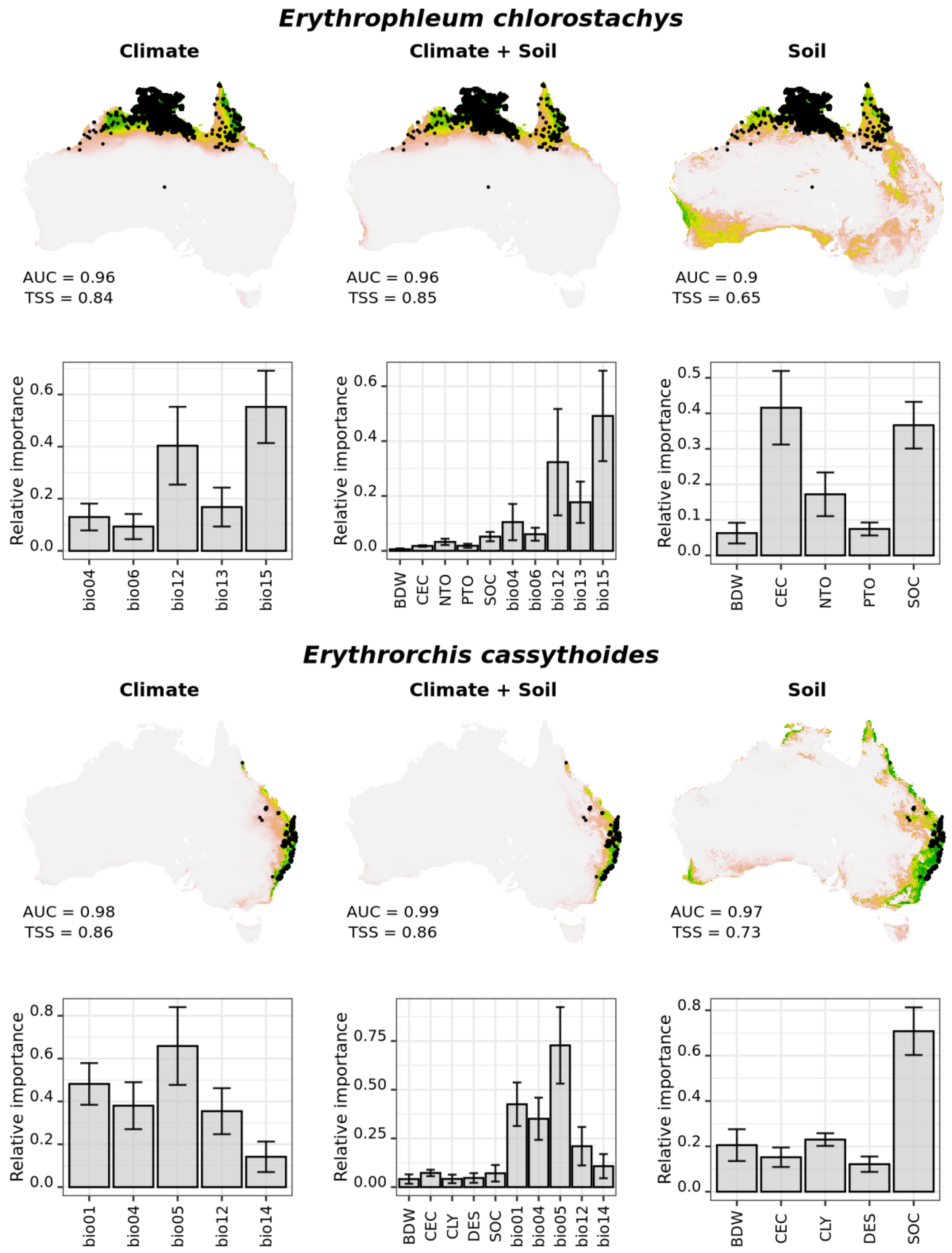
Erythrophleum chlorostachys
Climate
Climate + Soil
Soil
AUC = 0.96
TSS = 0.84
AUC = 0.96
TSS = 0.85
AUC = 0.9
TSS = 0.65
Relative importance
0.0
0.2
0.4
0.6
bio04
bio06
bio12
bio13
bio15
BDW
CEC
NTO
PTO
SOC
0.1
0.3
0.5
Erythrorchis cassythoides
AUC = 0.98
TSS = 0.86
AUC = 0.99
TSS = 0.86
AUC = 0.97
TSS = 0.73
0.8
0.00
0.25
0.50
0.75
bio01
bio05
bio14
CLY
DES

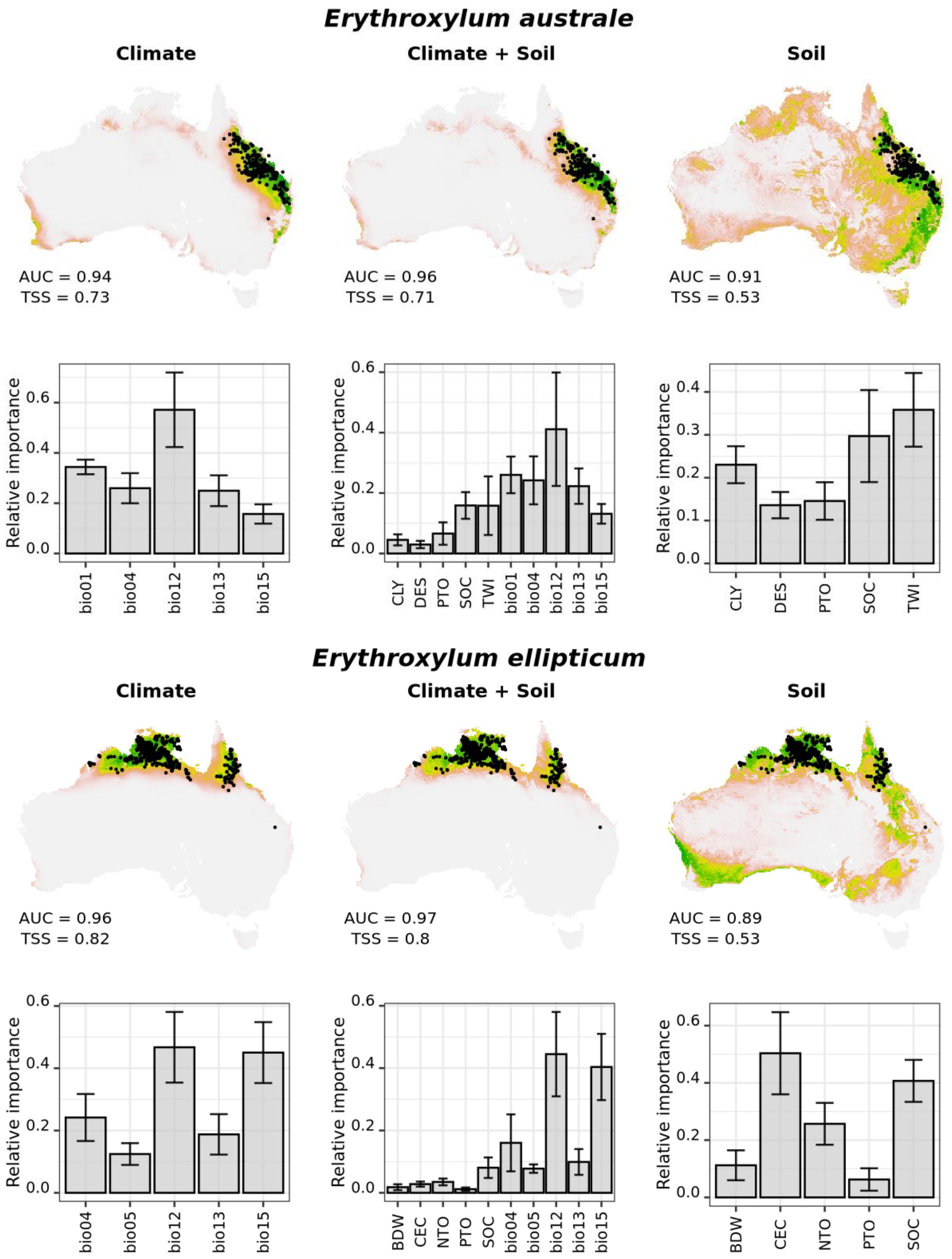
Erythroxylum australe
Climate
Climate + Soil
Soil
AUC = 0.94
TSS = 0.73
AUC = 0.96
TSS = 0.71
AUC = 0.91
TSS = 0.53
Relative importance
bio01 bio04 bio12 bio13 bio15
CLY DES PTO SOC TWI bio01 bio04 bio12 bio13 bio15
CLY DES PTO SOC TWI
Erythroxylum ellipticum
Climate
Climate + Soil
Soil
AUC = 0.96
TSS = 0.82
AUC = 0.97
TSS = 0.8
AUC = 0.89
TSS = 0.53
Relative importance
bio04 bio05 bio12 bio13 bio15
BDW CEC NTO PTO SOC bio04 bio05 bio12 bio13 bio15
BDW CEC NTO PTO SOC

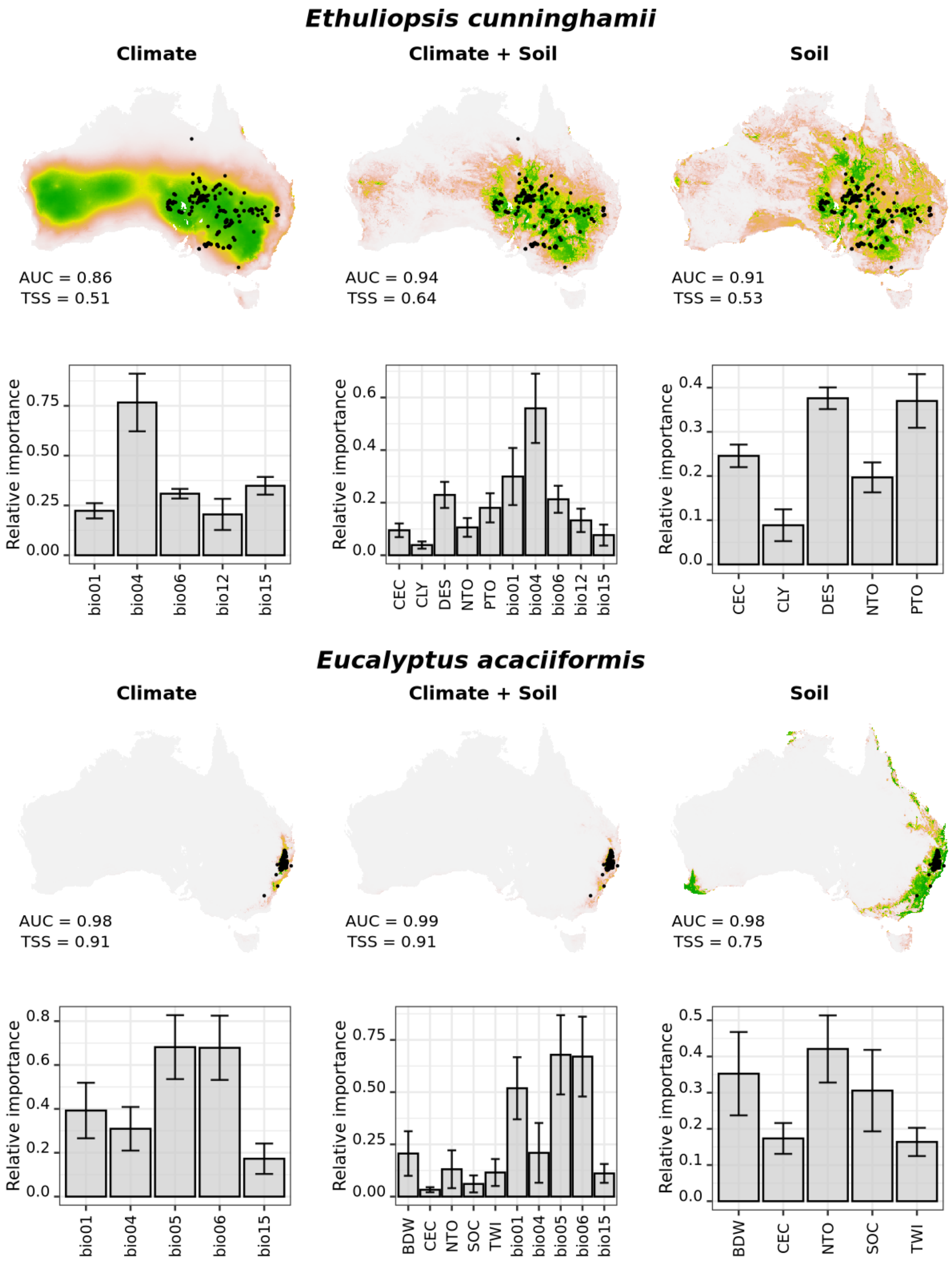
Ethuliopsis cunninghamii
Climate
Climate + Soil
Soil
AUC = 0.86
TSS = 0.51
AUC = 0.94
TSS = 0.64
AUC = 0.91
TSS = 0.53
Relative importance
0.00 0.25 0.50 0.75
bio01 bio04 bio06 bio12 bio15
0.0 0.2 0.4 0.6
CEC CLY DES NTO PTO bio01 bio04 bio06 bio12 bio15
0.0 0.1 0.2 0.3 0.4
CEC CLY DES NTO PTO
Eucalyptus acaciiformis
Climate
Climate + Soil
Soil
AUC = 0.98
TSS = 0.91
AUC = 0.99
TSS = 0.91
AUC = 0.98
TSS = 0.75
Relative importance
0.0 0.2 0.4 0.6 0.8
bio01 bio04 bio05 bio06 bio15
0.00 0.25 0.50 0.75
BDW CEC NTO SOC TWI bio01 bio04 bio05 bio06 bio15
0.0 0.1 0.2 0.3 0.4 0.5
BDW CEC NTO SOC TWI

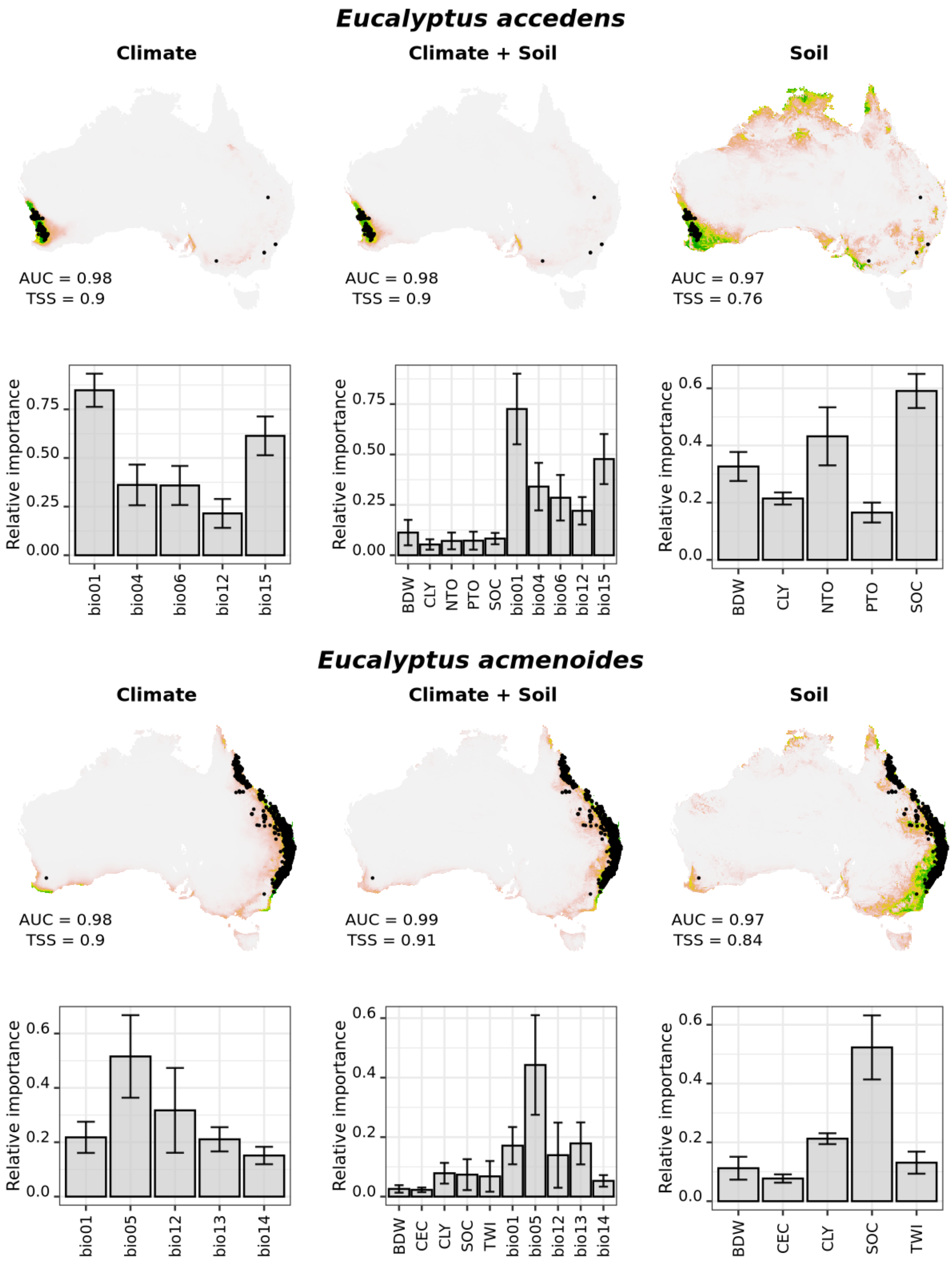
Eucalyptus accedens
Climate
Climate + Soil
Soil
AUC = 0.98
TSS = 0.9
AUC = 0.98
TSS = 0.9
AUC = 0.97
TSS = 0.76
Relative importance
0.00
0.25
0.50
0.75
bio01
bio04
bio06
bio12
bio15
BDW
CLY
NTO
PTO
SOC
0.0
0.2
0.4
0.6
Eucalyptus acmenoides
AUC = 0.98
TSS = 0.9
AUC = 0.99
TSS = 0.91
AUC = 0.97
TSS = 0.84
bio05
bio13
bio14
CEC
TWI

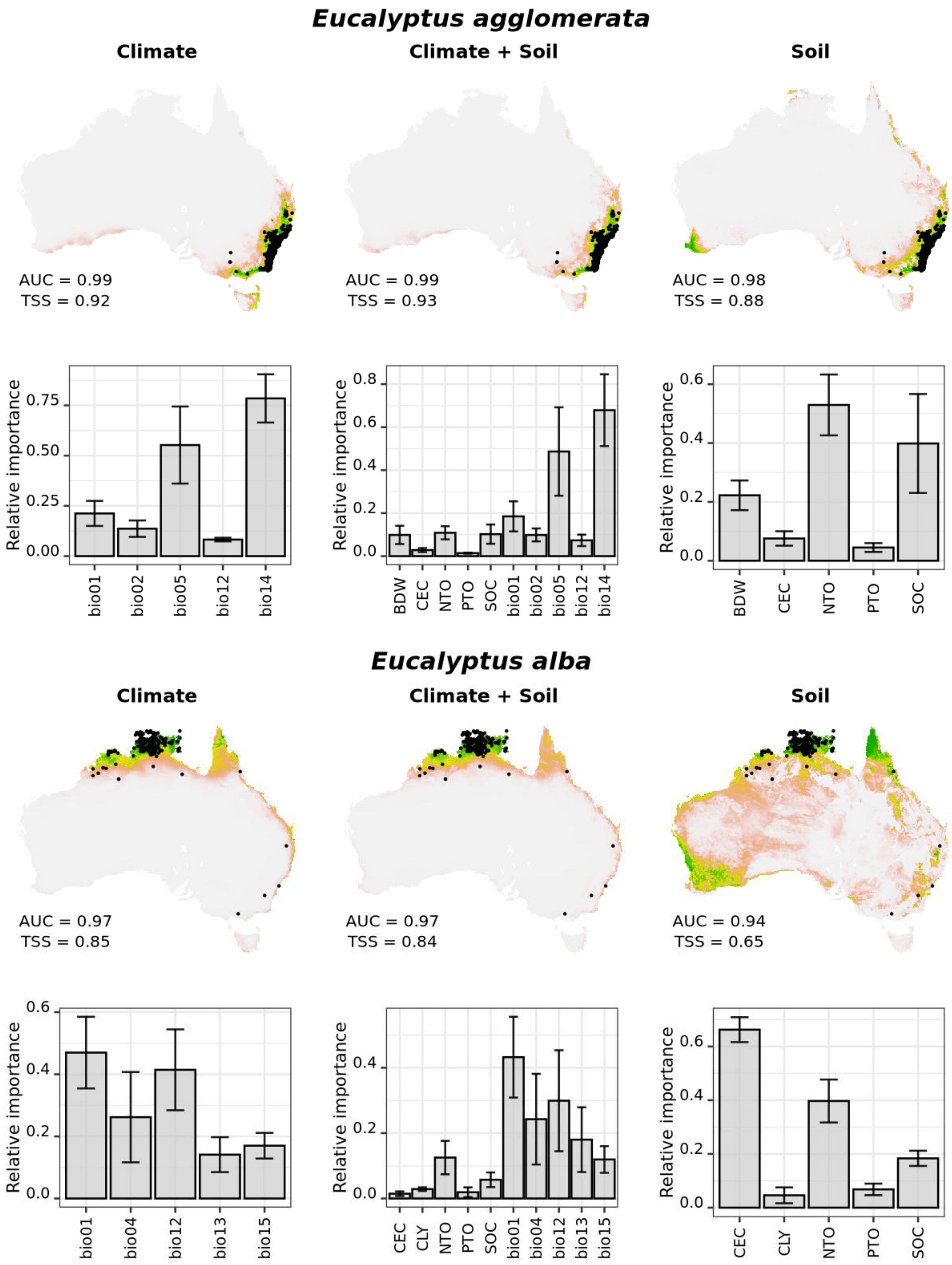

Eucalyptus agglomerata
Climate
Climate + Soil
Soil
AUC = 0.99
TSS = 0.92
AUC = 0.99
TSS = 0.93
AUC = 0.98
TSS = 0.88
Relative importance
0.00
0.25
0.50
0.75
bio01
bio02
bio05
bio12
bio14
0.0
0.2
0.4
0.6
0.8
BDW
CEC
NTO
PTO
SOC
bio01
bio02
bio05
bio12
bio14
0.0
0.2
0.4
0.6
BDW
CEC
NTO
PTO
SOC
Eucalyptus alba
Climate
Climate + Soil
Soil
AUC = 0.97
TSS = 0.85
AUC = 0.97
TSS = 0.84
AUC = 0.94
TSS = 0.65
Relative importance
0.0
0.2
0.4
0.6
bio01
bio04
bio12
bio13
bio15
0.0
0.2
0.4
CEC
CLY
NTO
PTO
SOC
bio01
bio04
bio12
bio13
bio15
0.0
0.2
0.4
0.6
CEC
CLY
NTO
PTO
SOC

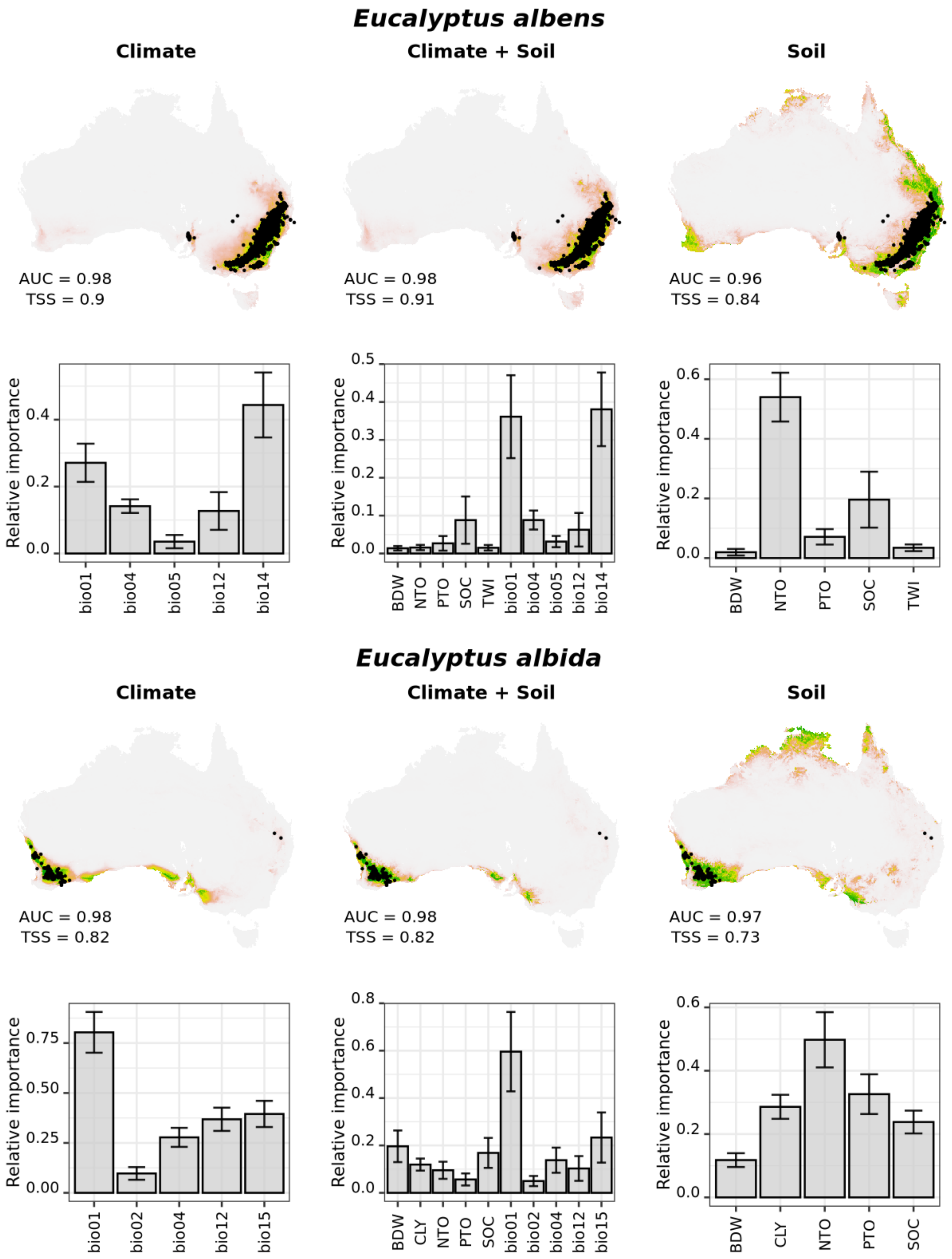

Eucalyptus albens
Climate
Climate + Soil
Soil
AUC = 0.98
TSS = 0.9
AUC = 0.98
TSS = 0.91
AUC = 0.96
TSS = 0.84
Relative importance
bio01 bio04 bio05 bio12 bio14
BDW NTO PTO SOC TWI bio01 bio04 bio05 bio12 bio14
BDW NTO PTO SOC TWI
Eucalyptus albida
Climate
Climate + Soil
Soil
AUC = 0.98
TSS = 0.82
AUC = 0.98
TSS = 0.82
AUC = 0.97
TSS = 0.73
Relative importance
bio01 bio02 bio04 bio12 bio15
BDW CLY NTO PTO SOC bio01 bio02 bio04 bio12 bio15
BDW CLY NTO PTO SOC

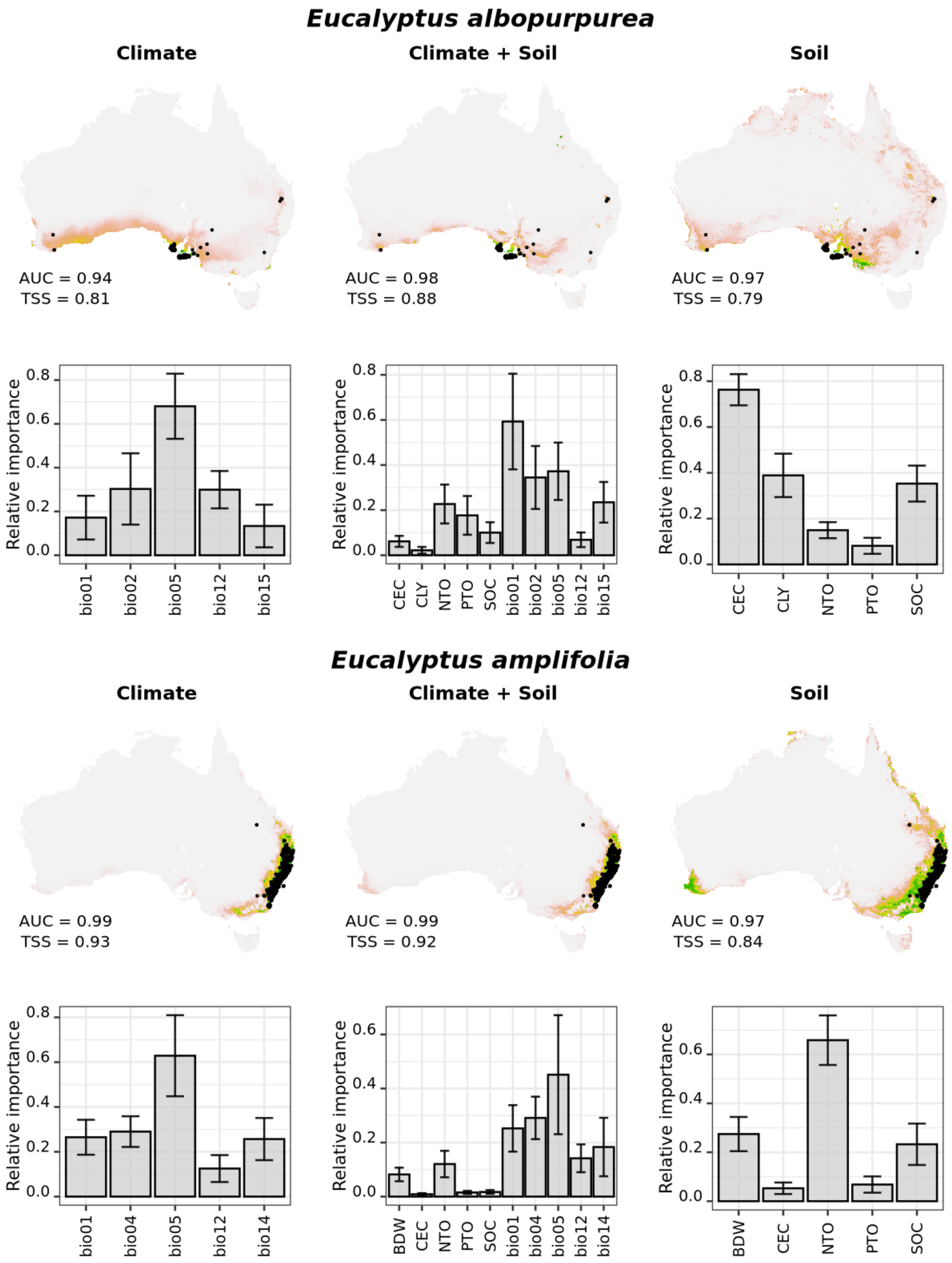
Eucalyptus albopurpurea
Climate
Climate + Soil
Soil
AUC = 0.94
TSS = 0.81
AUC = 0.98
TSS = 0.88
AUC = 0.97
TSS = 0.79
Relative importance
0.0
0.2
0.4
0.6
0.8
bio01
bio02
bio05
bio12
bio15
CEC
CLY
NTO
PTO
SOC
Eucalyptus amplifolia
Climate
Climate + Soil
Soil
AUC = 0.99
TSS = 0.93
AUC = 0.99
TSS = 0.92
AUC = 0.97
TSS = 0.84
bio04
bio14
BDW

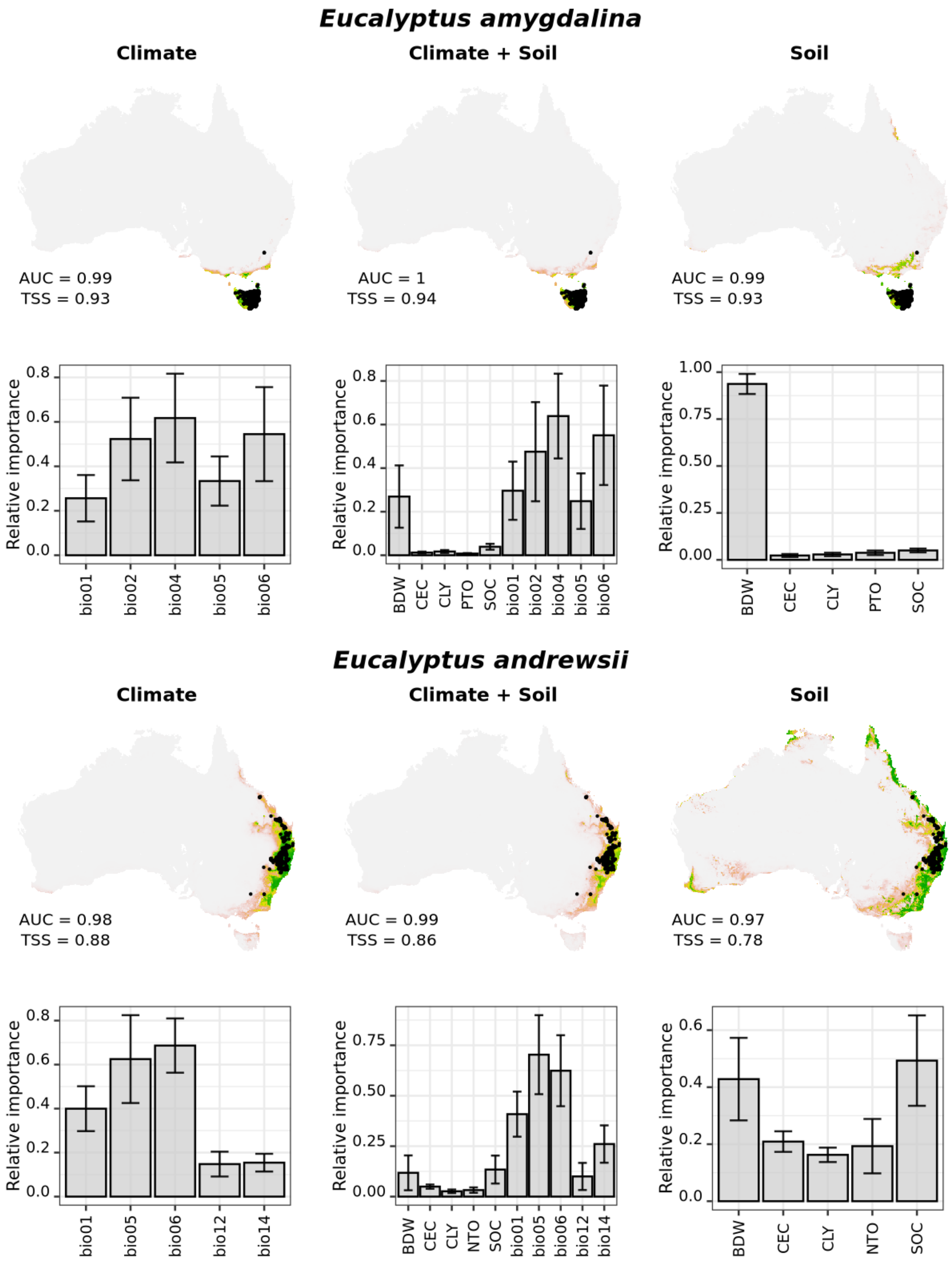
Eucalyptus amygdalina
Climate
Climate + Soil
Soil
AUC = 0.99
TSS = 0.93
AUC = 1
TSS = 0.94
AUC = 0.99
TSS = 0.93
Relative importance
bio01
bio02
bio04
bio05
bio06
BDW
CEC
CLY
PTO
SOC
Eucalyptus andrewsii
Climate
Climate + Soil
Soil
AUC = 0.98
TSS = 0.88
AUC = 0.99
TSS = 0.86
AUC = 0.97
TSS = 0.78
Relative importance
bio01
bio05
bio06
bio12
bio14
BDW
CEC
CLY
NTO
SOC

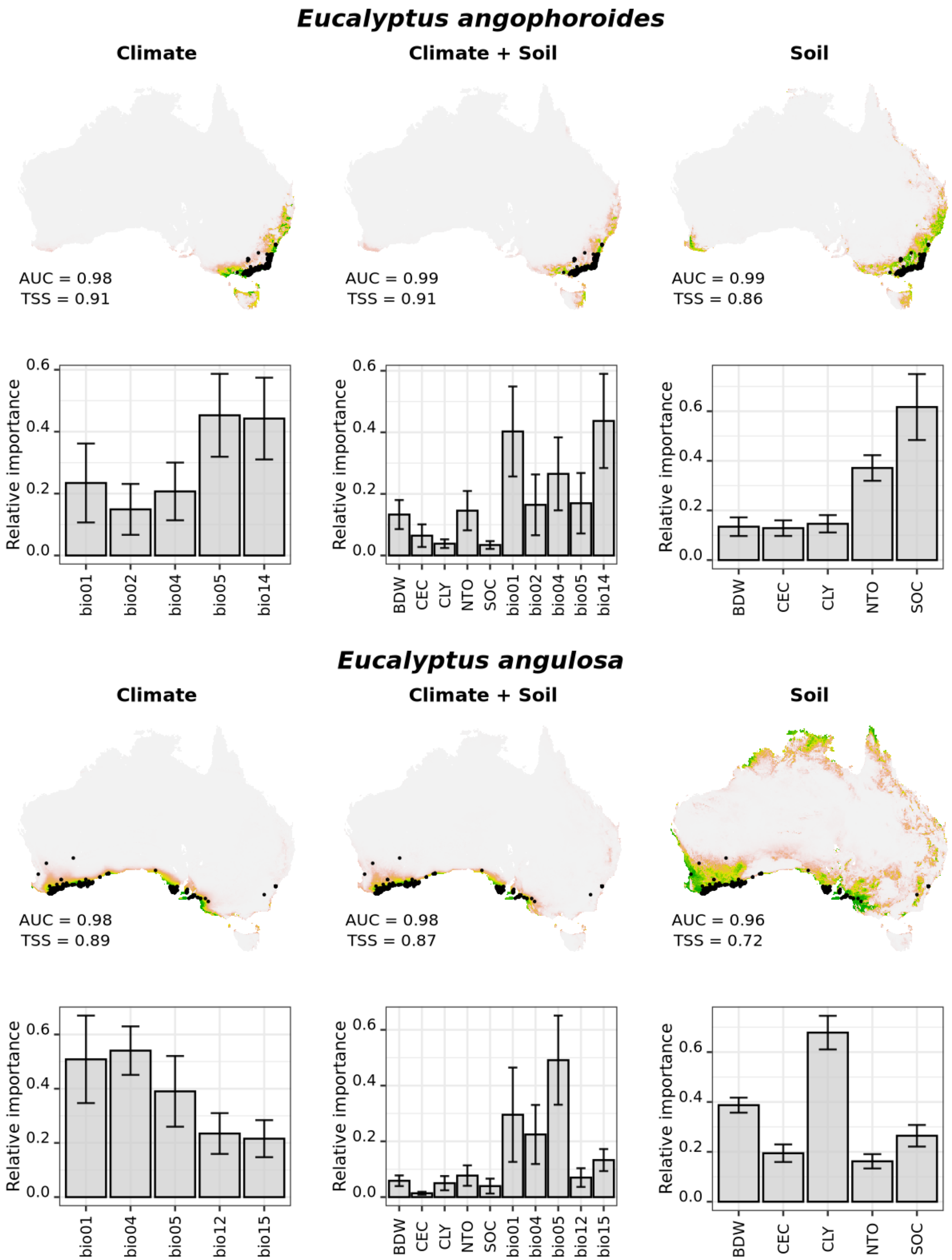
Eucalyptus angophoroides
Climate
Climate + Soil
Soil
AUC = 0.98
TSS = 0.91
AUC = 0.99
TSS = 0.91
AUC = 0.99
TSS = 0.86
Relative importance
0.0
0.2
0.4
0.6
bio01
bio02
bio04
bio05
bio14
BDW
CEC
CLY
NTO
SOC
bio01
bio02
bio04
bio05
bio14
BDW
CEC
CLY
NTO
SOC
Eucalyptus angulosa
Climate
Climate + Soil
Soil
AUC = 0.98
TSS = 0.89
AUC = 0.98
TSS = 0.87
AUC = 0.96
TSS = 0.72
Relative importance
0.0
0.2
0.4
0.6
bio01
bio04
bio05
bio12
bio15
BDW
CEC
CLY
NTO
SOC
bio01
bio04
bio05
bio12
bio15
BDW
CEC
CLY
NTO
SOC

Eucalyptus apodophylla

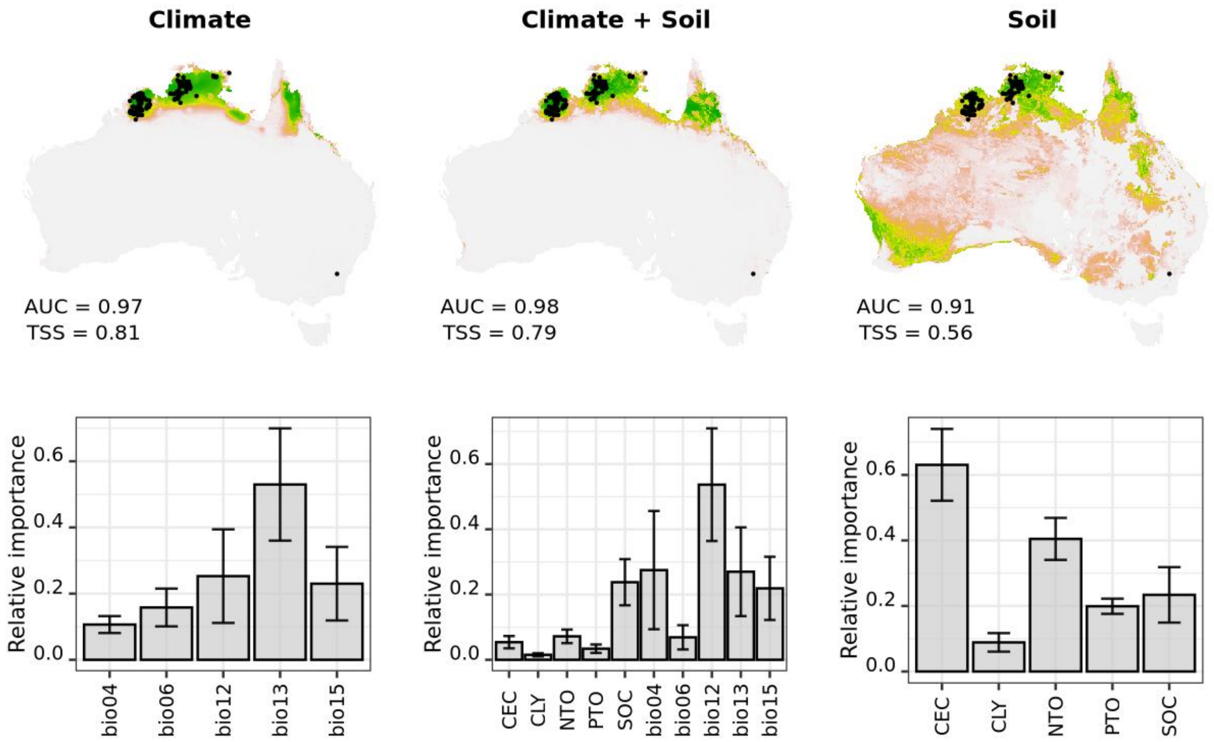

Eucalyptus arachnaea

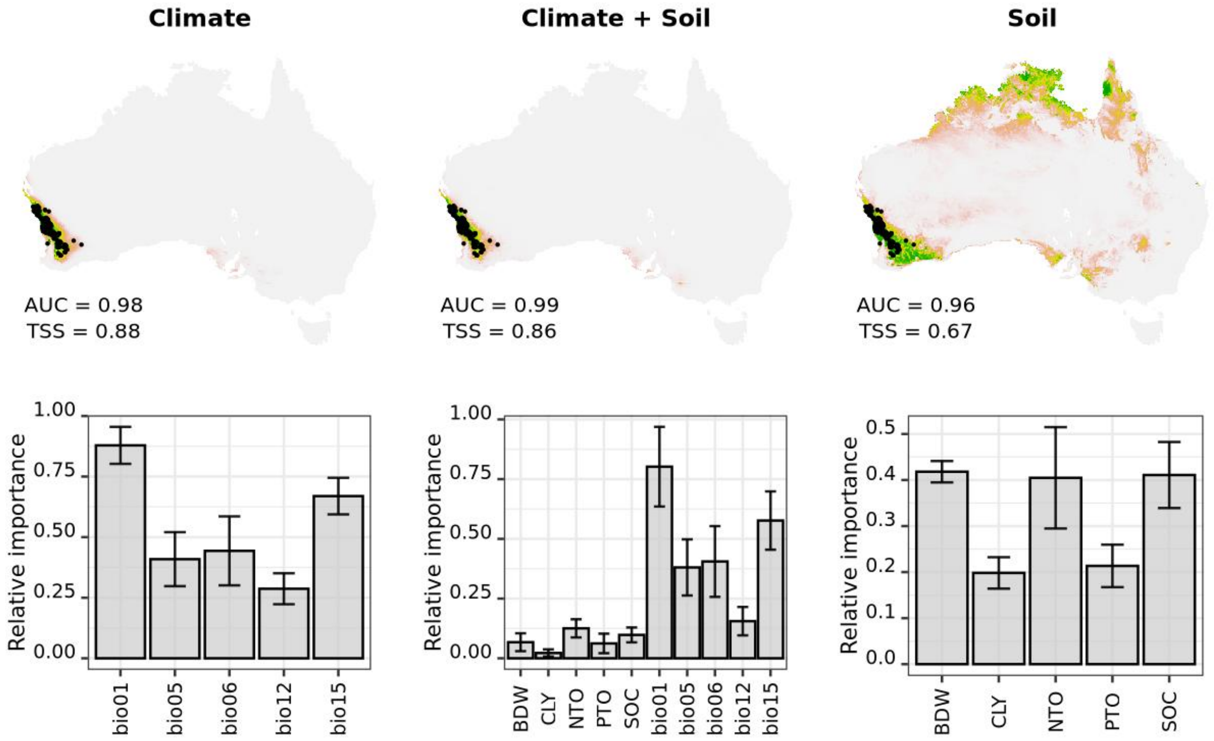

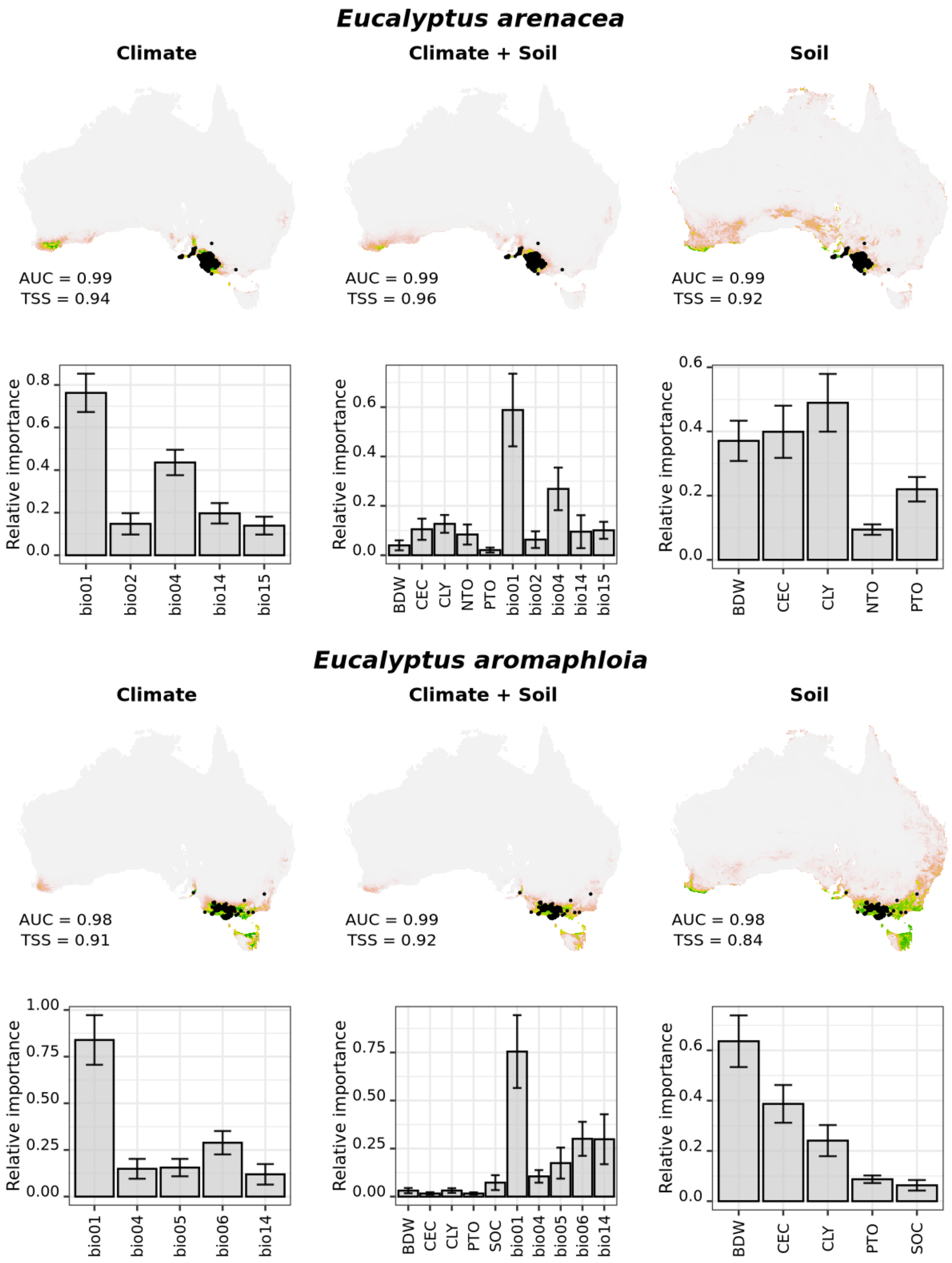

Eucalyptus arenacea
Climate
Climate + Soil
Soil
AUC = 0.99
TSS = 0.94
AUC = 0.99
TSS = 0.96
AUC = 0.99
TSS = 0.92
Relative importance
bio01 bio02 bio04 bio14 bio15
BDW CEC CLY NTO PTO bio01 bio02 bio04 bio14 bio15
BDW CEC CLY NTO PTO
Eucalyptus aromaphloia
Climate
Climate + Soil
Soil
AUC = 0.98
TSS = 0.91
AUC = 0.99
TSS = 0.92
AUC = 0.98
TSS = 0.84
Relative importance
bio01 bio04 bio05 bio06 bio14
BDW CEC CLY PTO SOC bio01 bio04 bio05 bio06 bio14
BDW CEC CLY PTO SOC

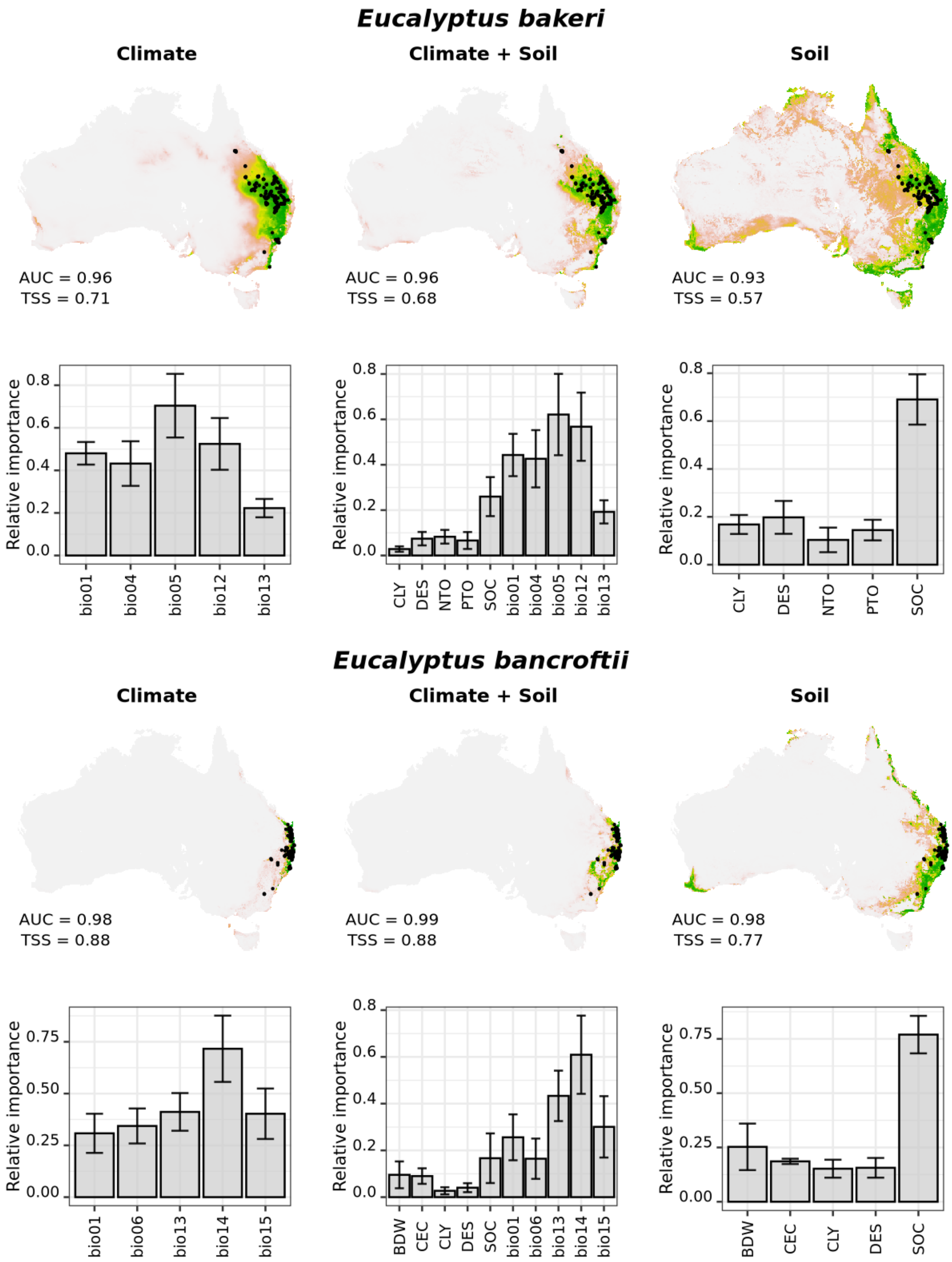

Eucalyptus bakeri
Climate
Climate + Soil
Soil
AUC = 0.96
TSS = 0.71
AUC = 0.96
TSS = 0.68
AUC = 0.93
TSS = 0.57
Relative importance
0.0
0.2
0.4
0.6
0.8
bio01
bio04
bio05
bio12
bio13
CLY
DES
NTO
PTO
SOC
bio01
bio04
bio05
bio12
bio13
CLY
DES
NTO
PTO
SOC
Eucalyptus bancroftii
Climate
Climate + Soil
Soil
AUC = 0.98
TSS = 0.88
AUC = 0.99
TSS = 0.88
AUC = 0.98
TSS = 0.77
Relative importance
0.00
0.25
0.50
0.75
0.0
0.2
0.4
0.6
0.8
bio01
bio06
bio13
bio14
bio15
BDW
CEC
CLY
DES
SOC
bio01
bio06
bio13
bio14
bio15
BDW
CEC
CLY
DES
SOC

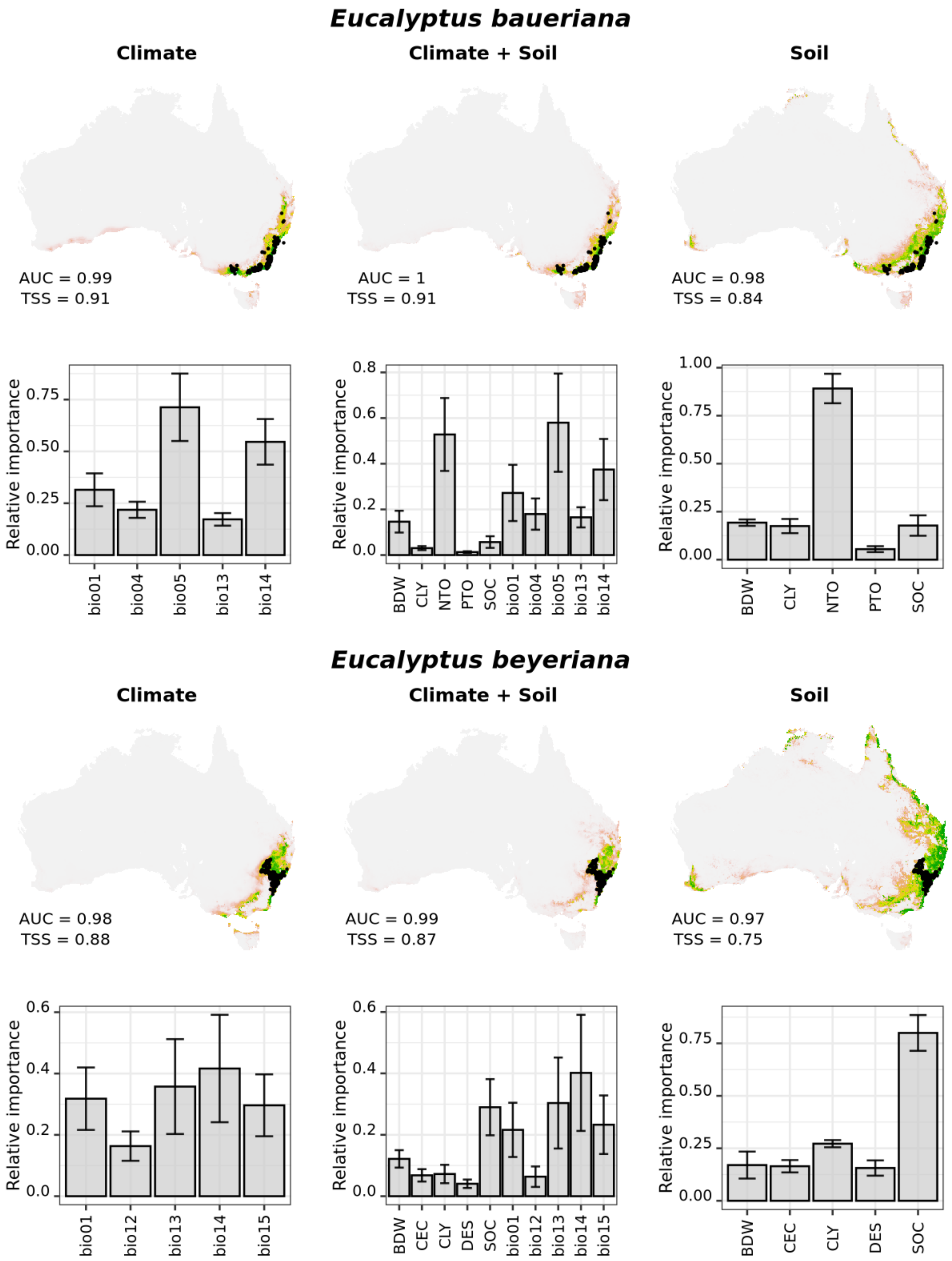
Eucalyptus baueriana
Climate
Climate + Soil
Soil
AUC = 0.99
TSS = 0.91
AUC = 1
TSS = 0.91
AUC = 0.98
TSS = 0.84
Relative importance
0.00
0.25
0.50
0.75
bio01
bio04
bio05
bio13
bio14
0.0
0.2
0.4
0.6
0.8
BDW
CLY
NTO
PTO
SOC
bio01
bio04
bio05
bio13
bio14
1.00
BDW
CLY
NTO
PTO
SOC
Eucalyptus beyeriana
Climate
Climate + Soil
Soil
AUC = 0.98
TSS = 0.88
AUC = 0.99
TSS = 0.87
AUC = 0.97
TSS = 0.75
Relative importance
0.0
0.2
0.4
0.6
bio01
bio12
bio13
bio14
bio15
BDW
CEC
CLY
DES
SOC
bio01
bio12
bio13
bio14
bio15
0.00
0.25
0.50
0.75
BDW
CEC
CLY
DES
SOC

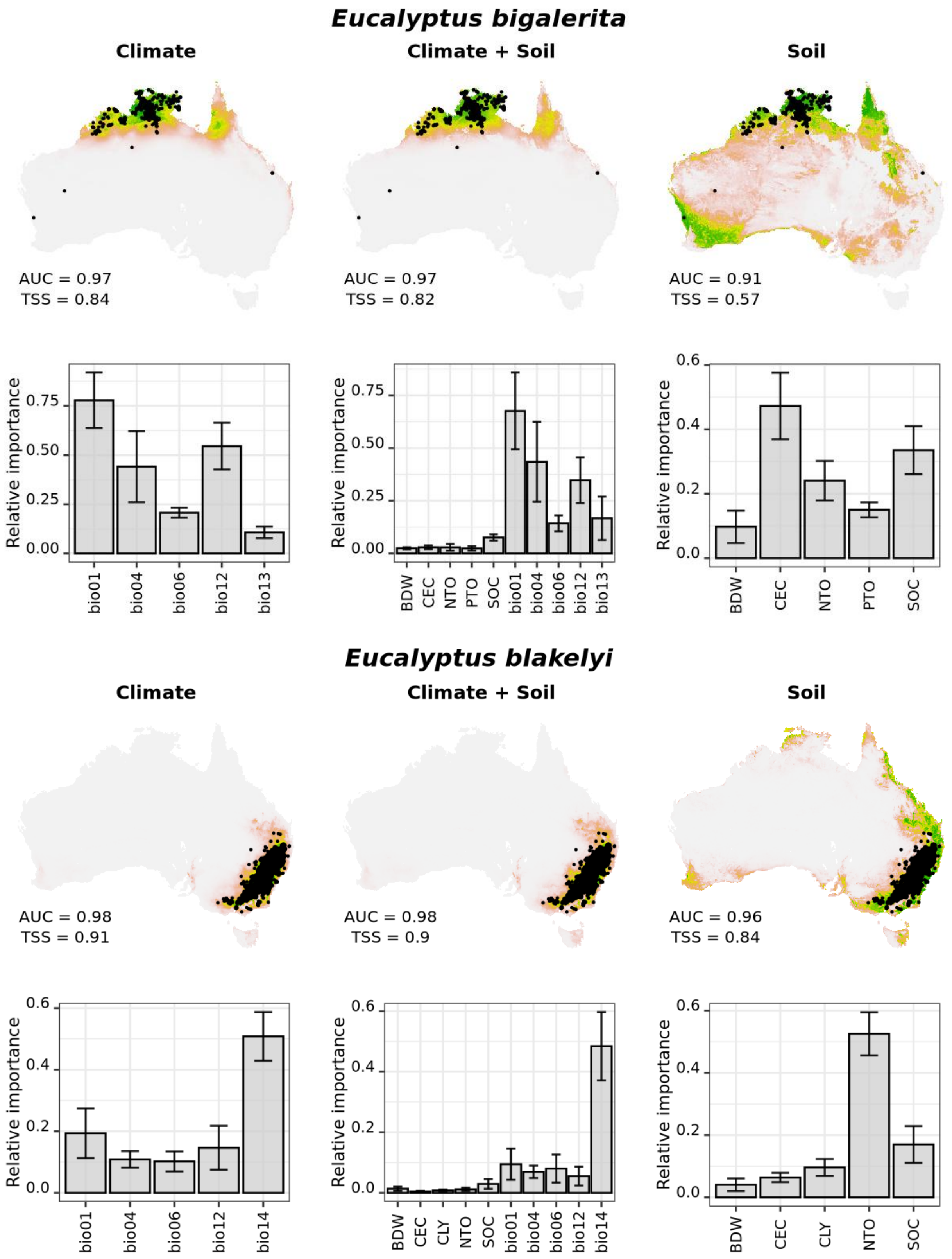

Eucalyptus bigalerita
Climate
Climate + Soil
Soil
AUC = 0.97
TSS = 0.84
AUC = 0.97
TSS = 0.82
AUC = 0.91
TSS = 0.57
Relative importance
0.00
0.25
0.50
0.75
bio01
bio04
bio06
bio12
bio13
BDW
CEC
NTO
PTO
SOC
0.0
0.2
0.4
0.6
Eucalyptus blakelyi
Climate
Climate + Soil
Soil
AUC = 0.98
TSS = 0.91
AUC = 0.98
TSS = 0.9
AUC = 0.96
TSS = 0.84
bio14
CLY

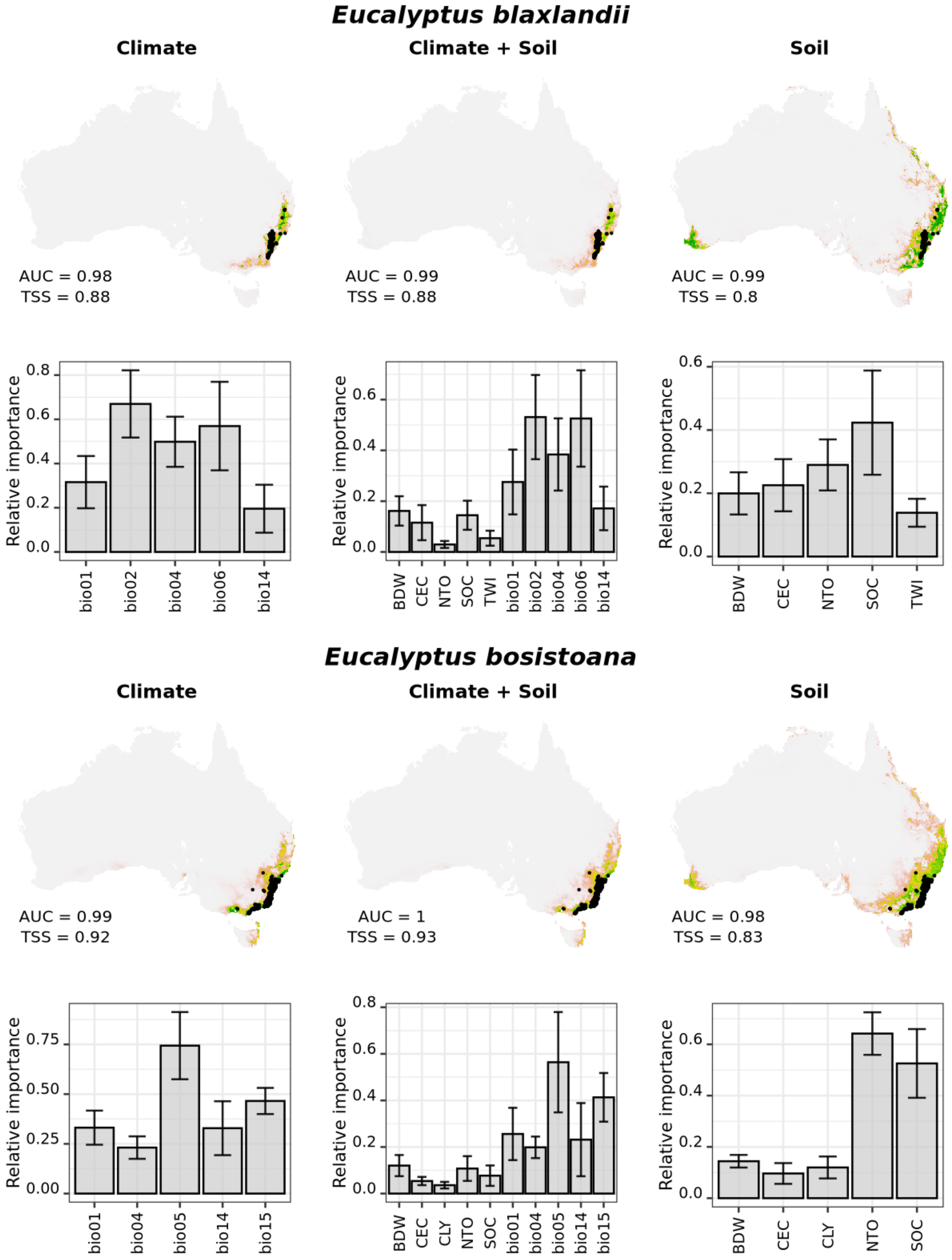

Eucalyptus blaxlandii
Climate
Climate + Soil
Soil
AUC = 0.98
TSS = 0.88
AUC = 0.99
TSS = 0.88
AUC = 0.99
TSS = 0.8
Relative importance
bio01
bio02
bio04
bio06
bio14
BDW
CEC
NTO
SOC
TWI
Eucalyptus bosistoana
Climate
Climate + Soil
Soil
AUC = 0.99
TSS = 0.92
AUC = 1
TSS = 0.93
AUC = 0.98
TSS = 0.83
Relative importance
bio01
bio04
bio05
bio14
bio15
BDW
CEC
CLY
NTO
SOC

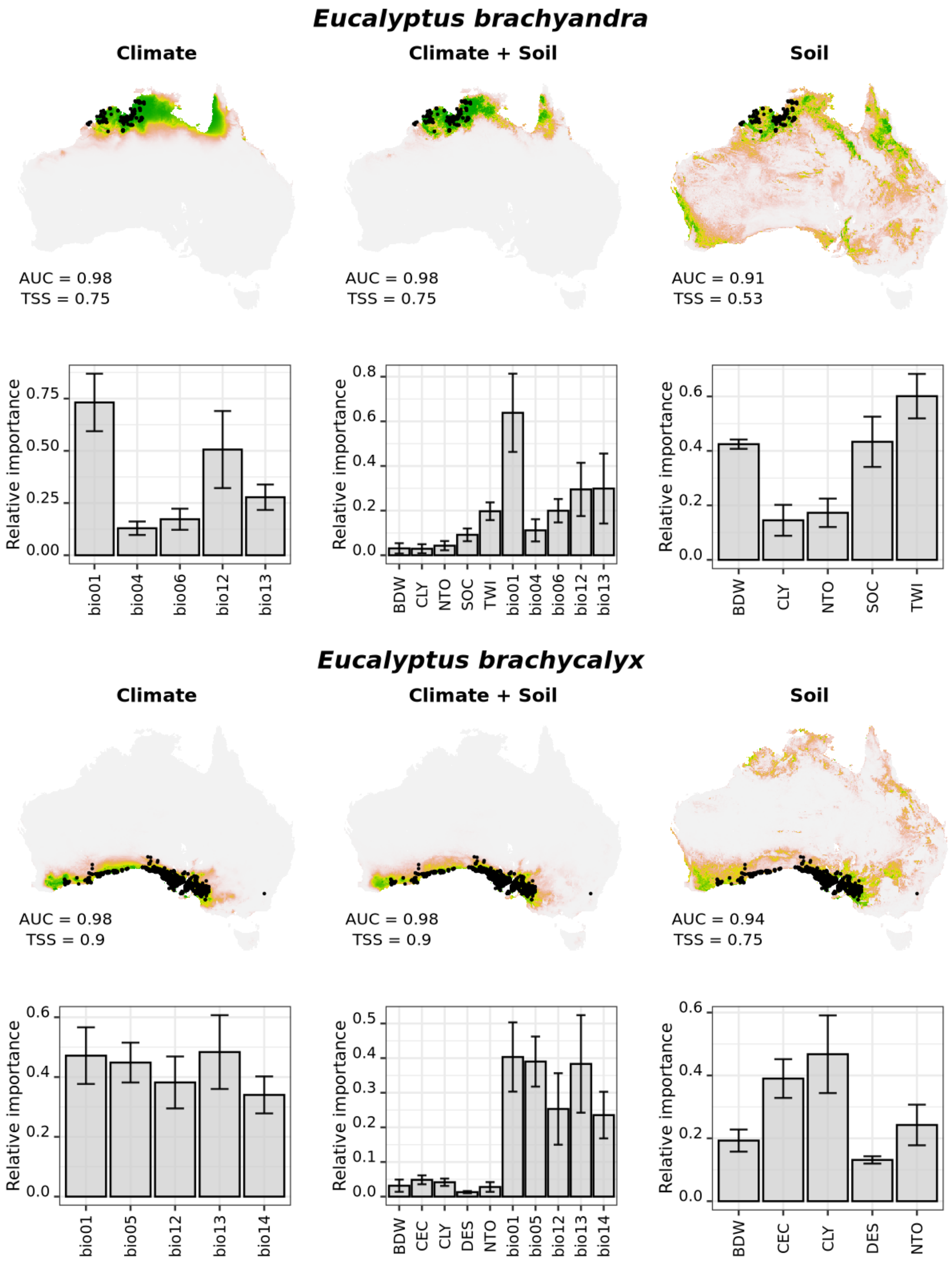

Eucalyptus brachyandra
Climate
Climate + Soil
Soil
AUC = 0.98
TSS = 0.75
AUC = 0.98
TSS = 0.75
AUC = 0.91
TSS = 0.53
Relative importance
0.00
0.25
0.50
0.75
bio01
bio04
bio06
bio12
bio13
Relative importance
0.0
0.2
0.4
0.6
0.8
BDW
CLY
NTO
SOC
TWI
bio01
bio04
bio06
bio12
bio13
Relative importance
0.0
0.2
0.4
0.6
BDW
CLY
NTO
SOC
TWI
Eucalyptus brachycalyx
Climate
Climate + Soil
Soil
AUC = 0.98
TSS = 0.9
AUC = 0.98
TSS = 0.9
AUC = 0.94
TSS = 0.75
Relative importance
0.0
0.2
0.4
0.6
bio01
bio05
bio12
bio13
bio14
Relative importance
0.0
0.1
0.2
0.3
0.4
0.5
BDW
CEC
CLY
DES
NTO
bio01
bio05
bio12
bio13
bio14
Relative importance
0.0
0.2
0.4
0.6
BDW
CEC
CLY
DES
NTO

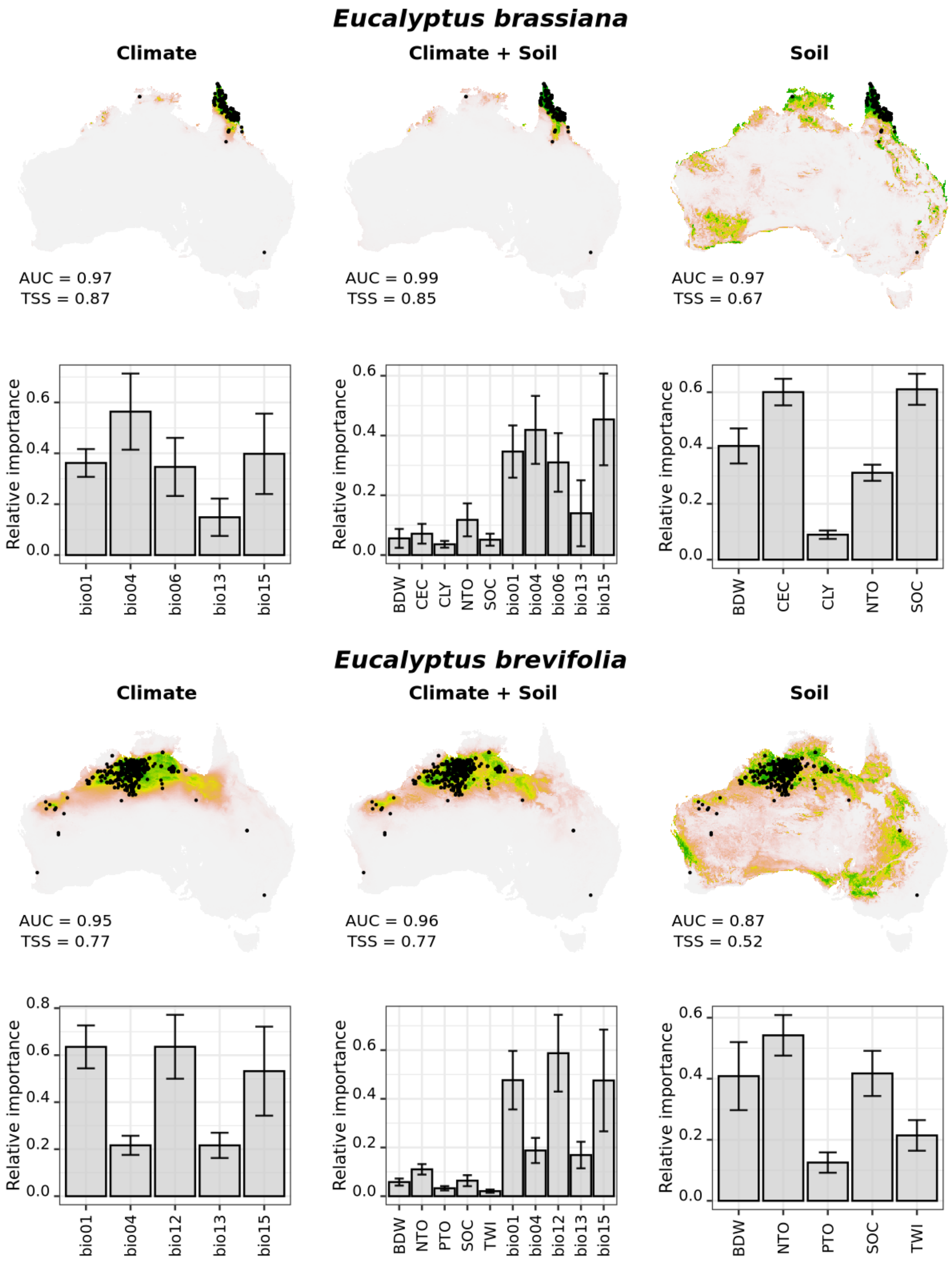

Eucalyptus brassiana
Climate
Climate + Soil
Soil
AUC = 0.97
TSS = 0.87
AUC = 0.99
TSS = 0.85
AUC = 0.97
TSS = 0.67
Relative importance
bio01
bio04
bio06
bio13
bio15
BDW
CEC
CLY
NTO
SOC
Eucalyptus brevifolia
Climate
Climate + Soil
Soil
AUC = 0.95
TSS = 0.77
AUC = 0.96
TSS = 0.77
AUC = 0.87
TSS = 0.52
Relative importance
bio01
bio04
bio12
bio13
bio15
BDW
NTO
PTO
SOC
TWI

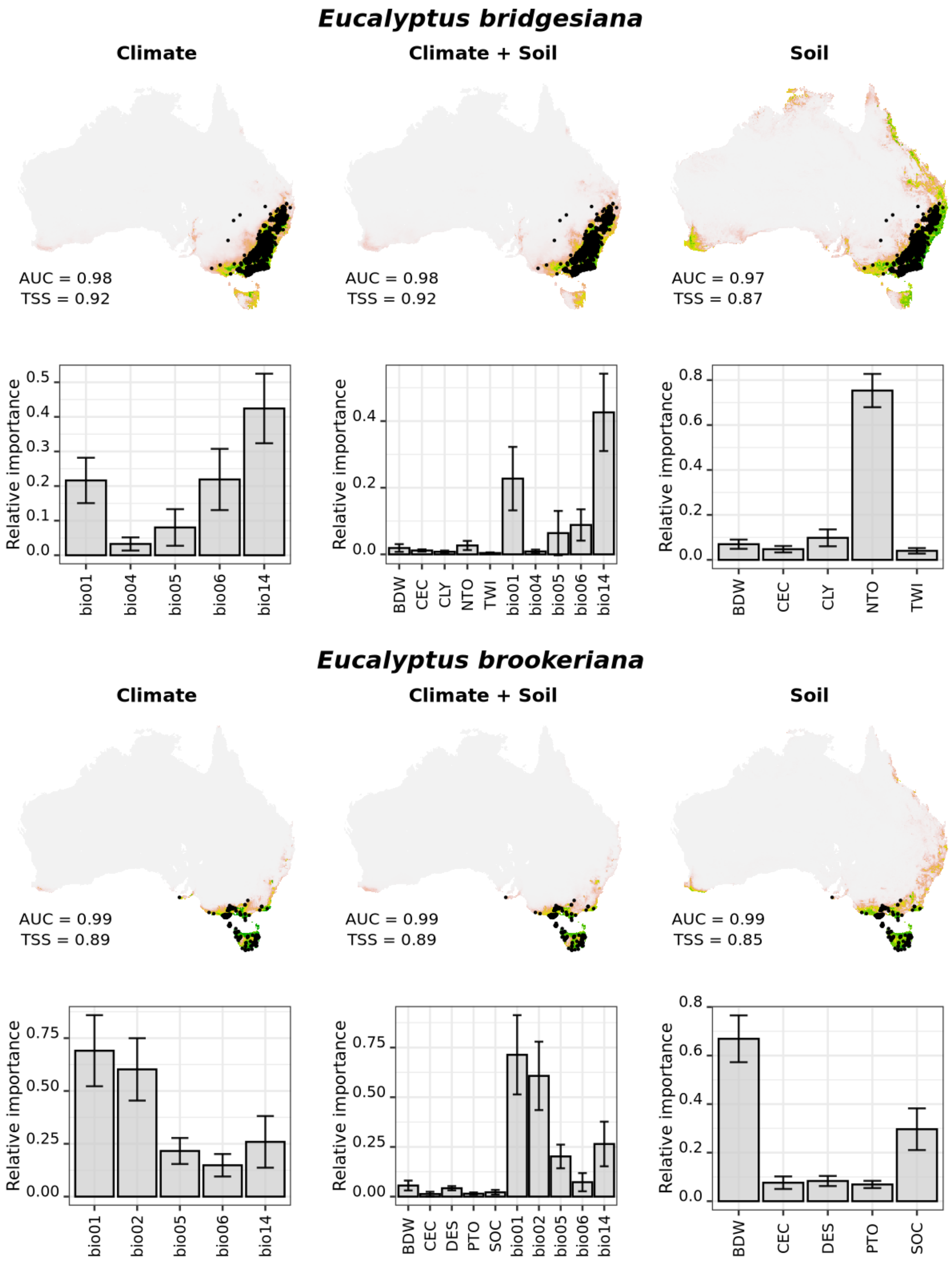
Eucalyptus bridgesiana
Climate
Climate + Soil
Soil
AUC = 0.98
TSS = 0.92
AUC = 0.98
TSS = 0.92
AUC = 0.97
TSS = 0.87
Relative importance
bio01
bio04
bio05
bio06
bio14
BDW
CEC
CLY
NTO
TWI
Eucalyptus brookeriana
Climate
Climate + Soil
Soil
AUC = 0.99
TSS = 0.89
AUC = 0.99
TSS = 0.89
AUC = 0.99
TSS = 0.85
bio01
bio02
bio05
bio06
bio14
BDW
CEC
DES
PTO
SOC

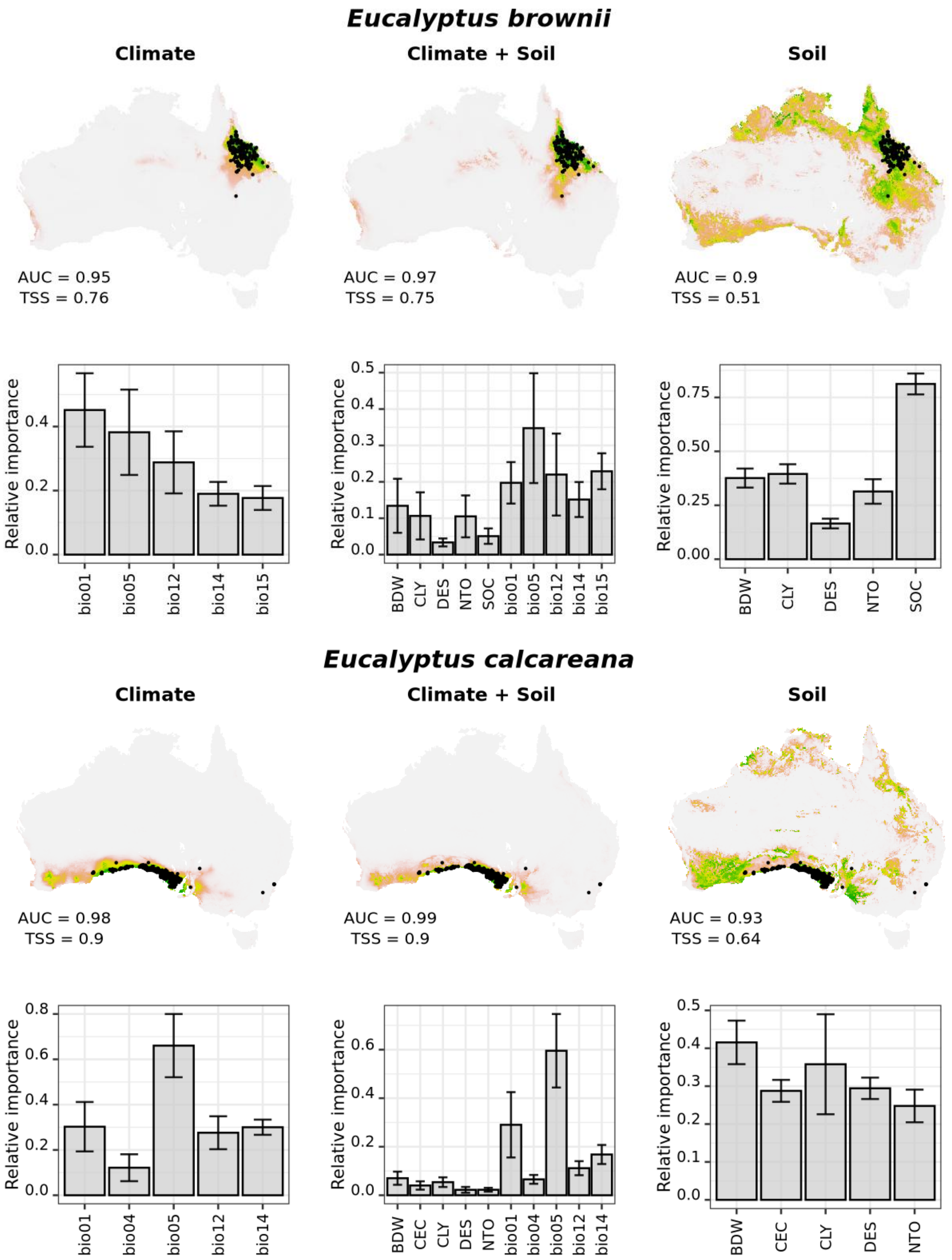

Eucalyptus brownii
Climate
Climate + Soil
Soil
AUC = 0.95
TSS = 0.76
AUC = 0.97
TSS = 0.75
AUC = 0.9
TSS = 0.51
Relative importance
bio01
bio05
bio12
bio14
bio15
BDW
CLY
DES
NTO
SOC
Eucalyptus calcareana
Climate
Climate + Soil
Soil
AUC = 0.98
TSS = 0.9
AUC = 0.99
TSS = 0.9
AUC = 0.93
TSS = 0.64
Relative importance
bio01
bio04
bio05
bio12
bio14
BDW
CEC
CLY
DES
NTO

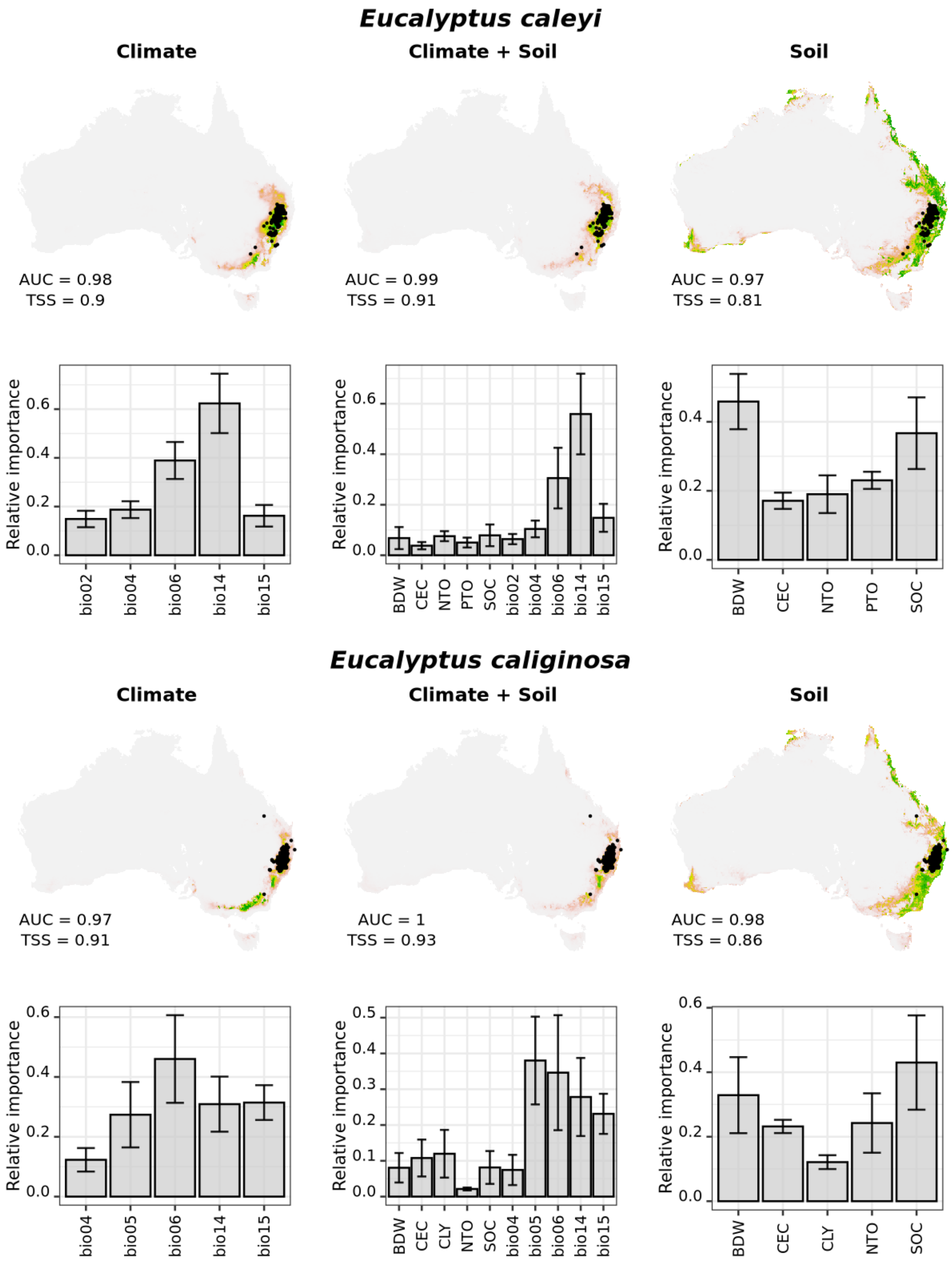
Eucalyptus caleyi
Climate
Climate + Soil
Soil
AUC = 0.98
TSS = 0.9
AUC = 0.99
TSS = 0.91
AUC = 0.97
TSS = 0.81
Relative importance
bio02 bio04 bio06 bio14 bio15
BDW CEC NTO PTO SOC bio02 bio04 bio06 bio14 bio15
BDW CEC NTO PTO SOC
Eucalyptus caliginosa
Climate
Climate + Soil
Soil
AUC = 0.97
TSS = 0.91
AUC = 1
TSS = 0.93
AUC = 0.98
TSS = 0.86
Relative importance
bio04 bio05 bio06 bio14 bio15
BDW CEC CLY NTO SOC bio04 bio05 bio06 bio14 bio15
BDW CEC CLY NTO SOC

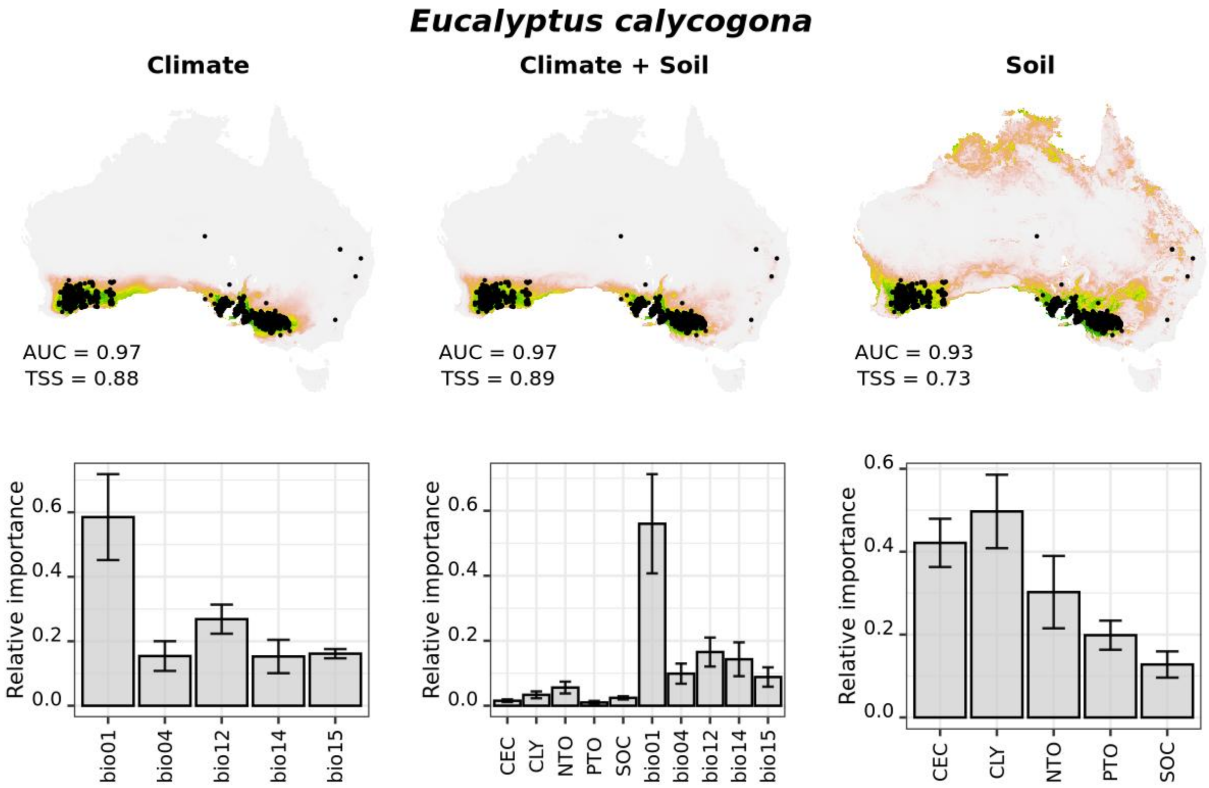
Eucalyptus calycogona
Climate
Climate + Soil
Soil
AUC = 0.97
TSS = 0.88
AUC = 0.97
TSS = 0.89
AUC = 0.93
TSS = 0.73
Relative importance
bio01
bio04
bio12
bio14
bio15
CEC
CLY
NTO
PTO
SOC
0.0
0.2
0.4
0.6

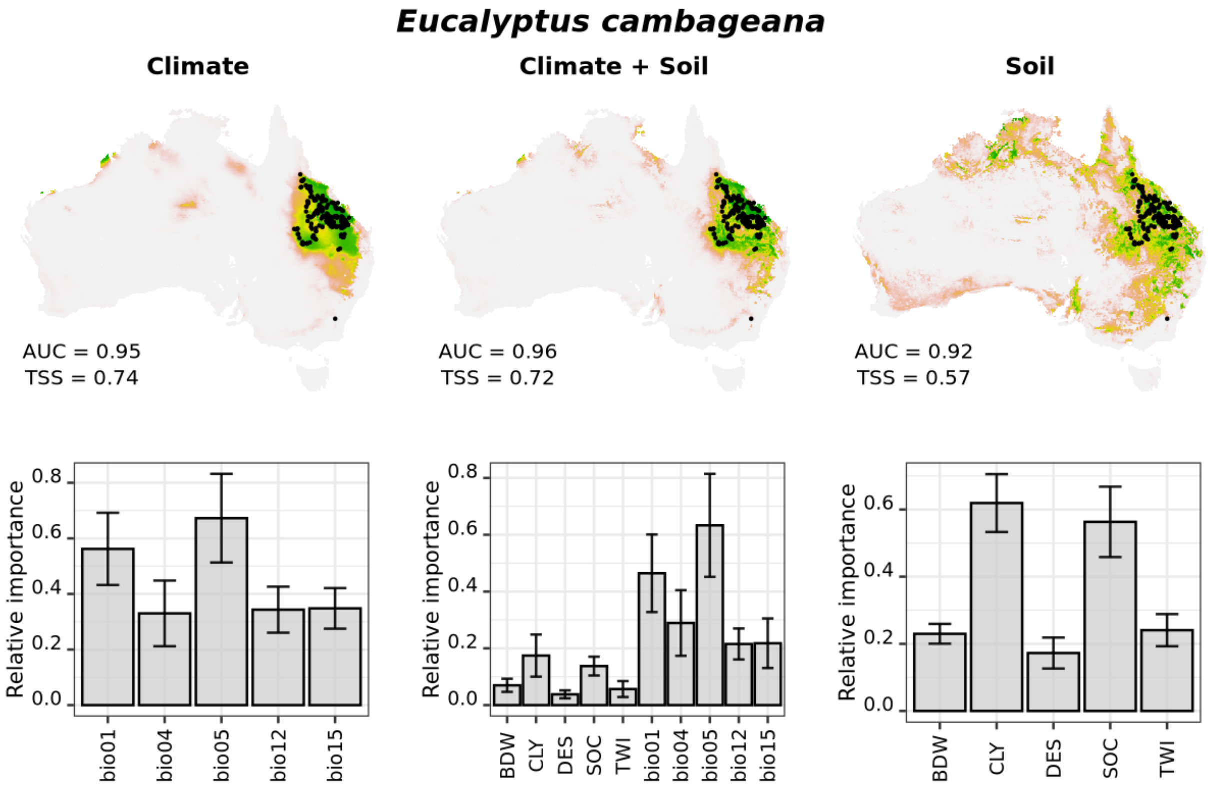
Eucalyptus cambageana
Climate
Climate + Soil
Soil
AUC = 0.95
TSS = 0.74
AUC = 0.96
TSS = 0.72
AUC = 0.92
TSS = 0.57
Relative importance
bio01
bio04
bio05
bio12
bio15
BDW
CLY
DES
SOC
TWI
0.0
0.2
0.4
0.6
0.8

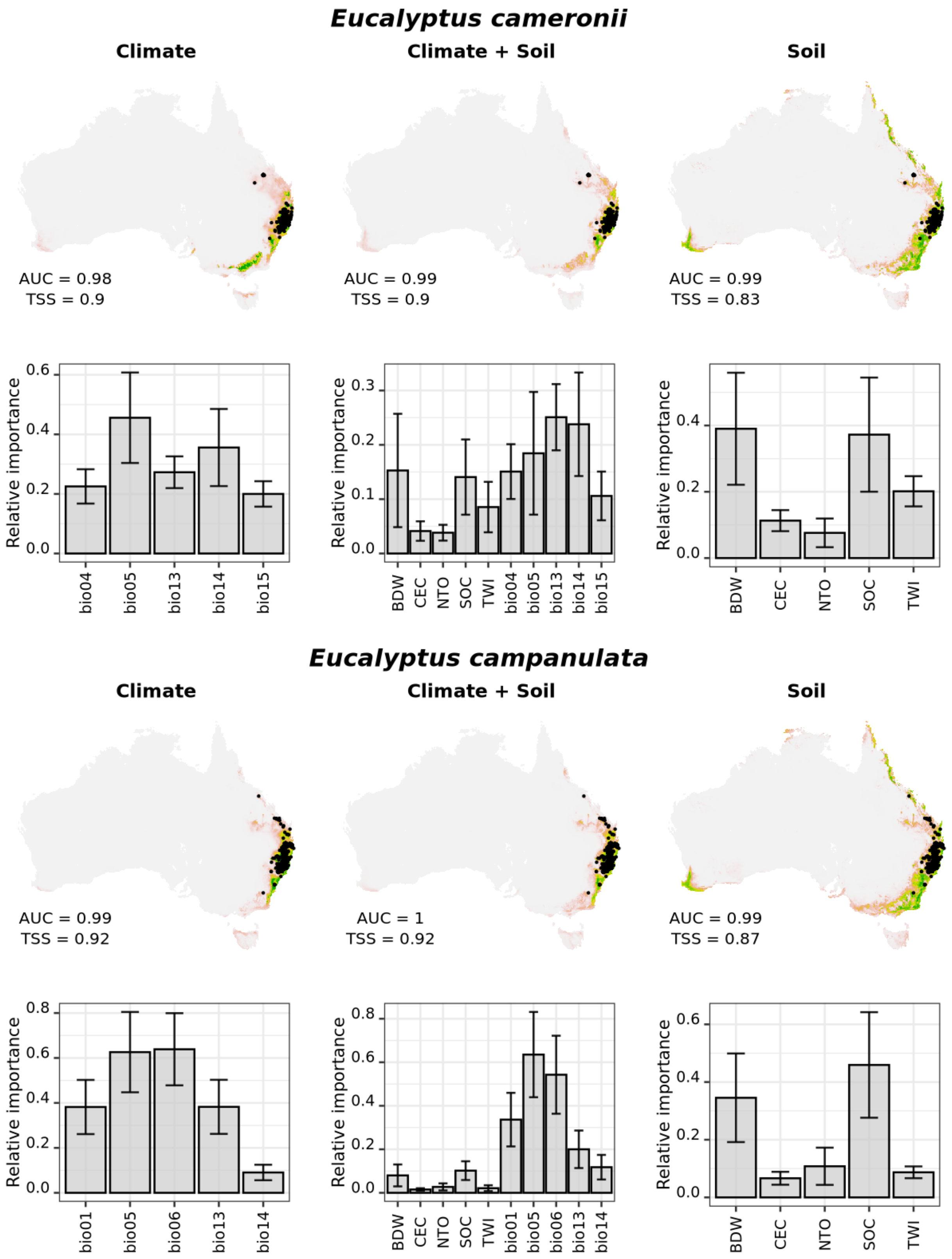

Eucalyptus cameronii
Climate
Climate + Soil
Soil
AUC = 0.98
TSS = 0.9
AUC = 0.99
TSS = 0.9
AUC = 0.99
TSS = 0.83
Relative importance
bio04
bio05
bio13
bio14
bio15
BDW
CEC
NTO
SOC
TWI
Eucalyptus campanulata
Climate
Climate + Soil
Soil
AUC = 0.99
TSS = 0.92
AUC = 1
TSS = 0.92
AUC = 0.99
TSS = 0.87
Relative importance
bio01
bio05
bio06
bio13
bio14
BDW
CEC
NTO
SOC
TWI

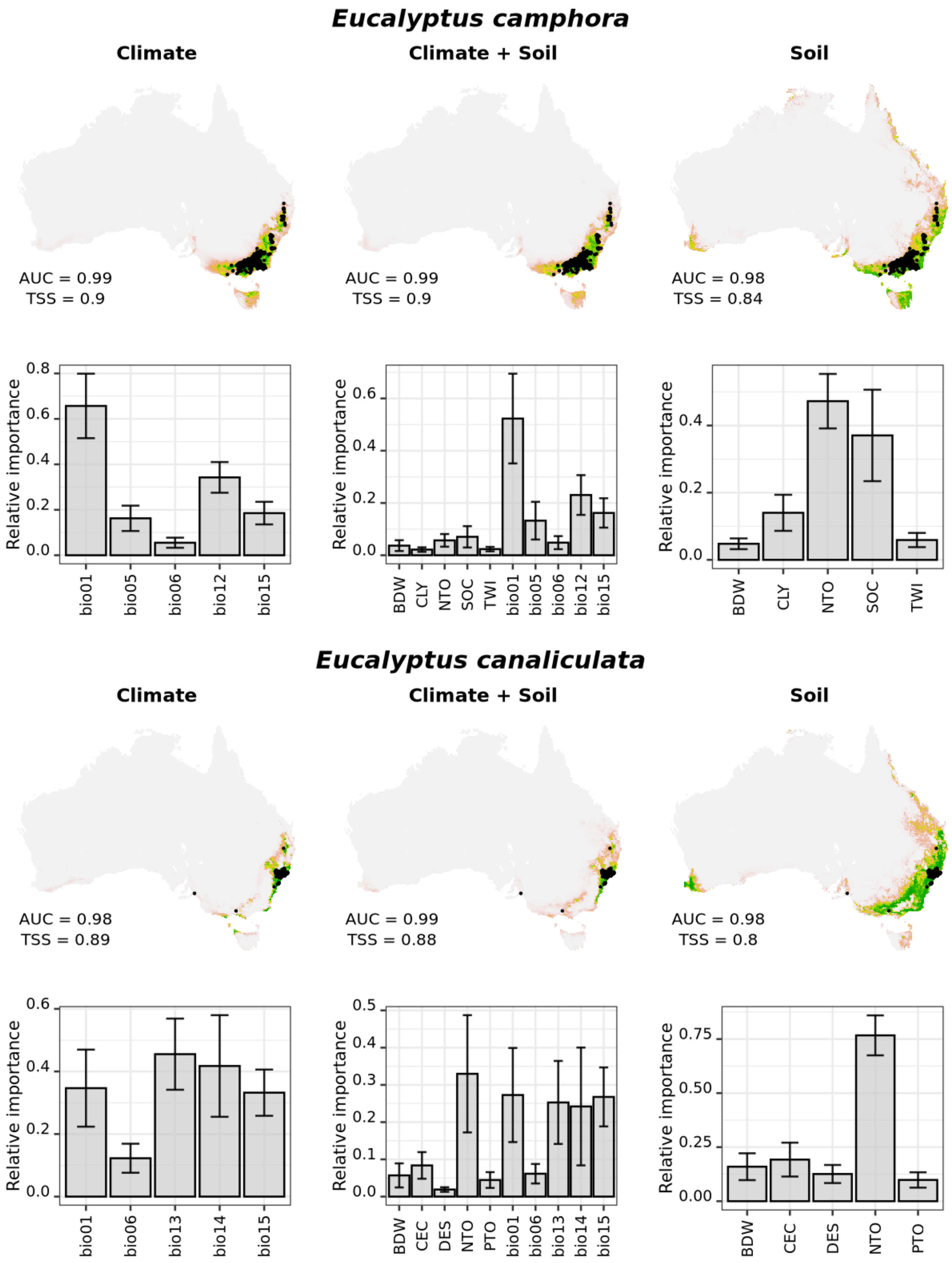
Eucalyptus camphora
Climate
Climate + Soil
Soil
AUC = 0.99
TSS = 0.9
AUC = 0.99
TSS = 0.9
AUC = 0.98
TSS = 0.84
Relative importance
0.0
0.2
0.4
0.6
0.8
bio01
bio05
bio06
bio12
bio15
Relative importance
0.0
0.2
0.4
0.6
BDW
CLY
NTO
SOC
TWI
bio01
bio05
bio06
bio12
bio15
Relative importance
0.0
0.2
0.4
BDW
CLY
NTO
SOC
TWI
Eucalyptus canaliculata
Climate
Climate + Soil
Soil
AUC = 0.98
TSS = 0.89
AUC = 0.99
TSS = 0.88
AUC = 0.98
TSS = 0.8
Relative importance
0.0
0.2
0.4
0.6
bio01
bio06
bio13
bio14
bio15
Relative importance
0.0
0.1
0.2
0.3
0.4
0.5
BDW
CEC
DES
NTO
PTO
bio01
bio06
bio13
bio14
bio15
Relative importance
0.00
0.25
0.50
0.75
BDW
CEC
DES
NTO
PTO

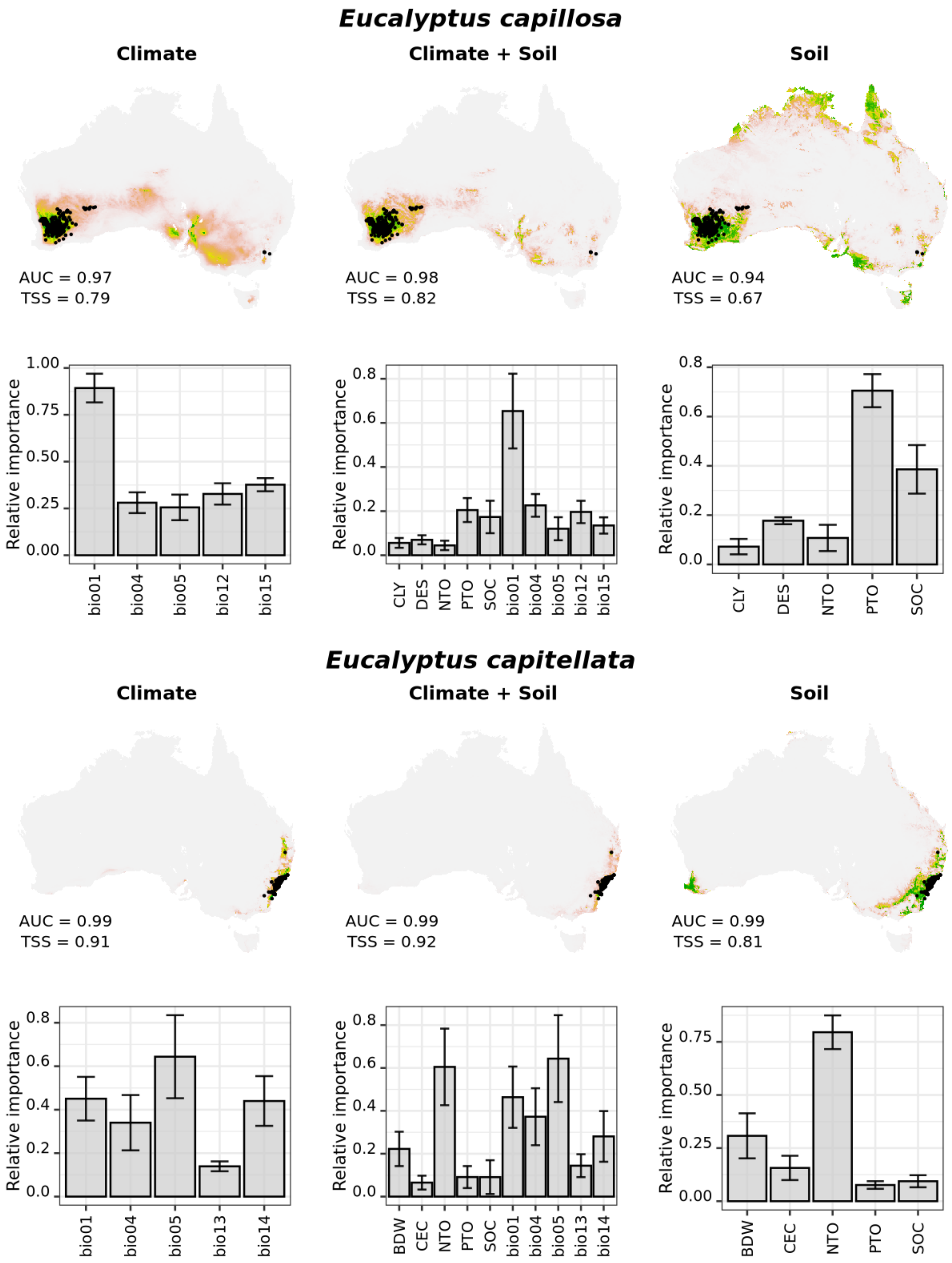
Eucalyptus capillosa
Climate
Climate + Soil
Soil
AUC = 0.97
TSS = 0.79
AUC = 0.98
TSS = 0.82
AUC = 0.94
TSS = 0.67
Relative importance
1.00
0.75
0.50
0.25
0.00
bio01
bio04
bio05
bio12
bio15
0.8
0.6
0.4
0.2
0.0
CLY
DES
NTO
PTO
SOC
bio01
bio04
bio05
bio12
bio15
CLY
DES
NTO
PTO
SOC
Eucalyptus capitellata
Climate
Climate + Soil
Soil
AUC = 0.99
TSS = 0.91
AUC = 0.99
TSS = 0.92
AUC = 0.99
TSS = 0.81
Relative importance
bio01
bio04
bio05
bio13
bio14
BDW
CEC
NTO
PTO
SOC
bio01
bio04
bio05
bio13
bio14
0.75
0.50
0.25
0.00
BDW
CEC
NTO
PTO
SOC

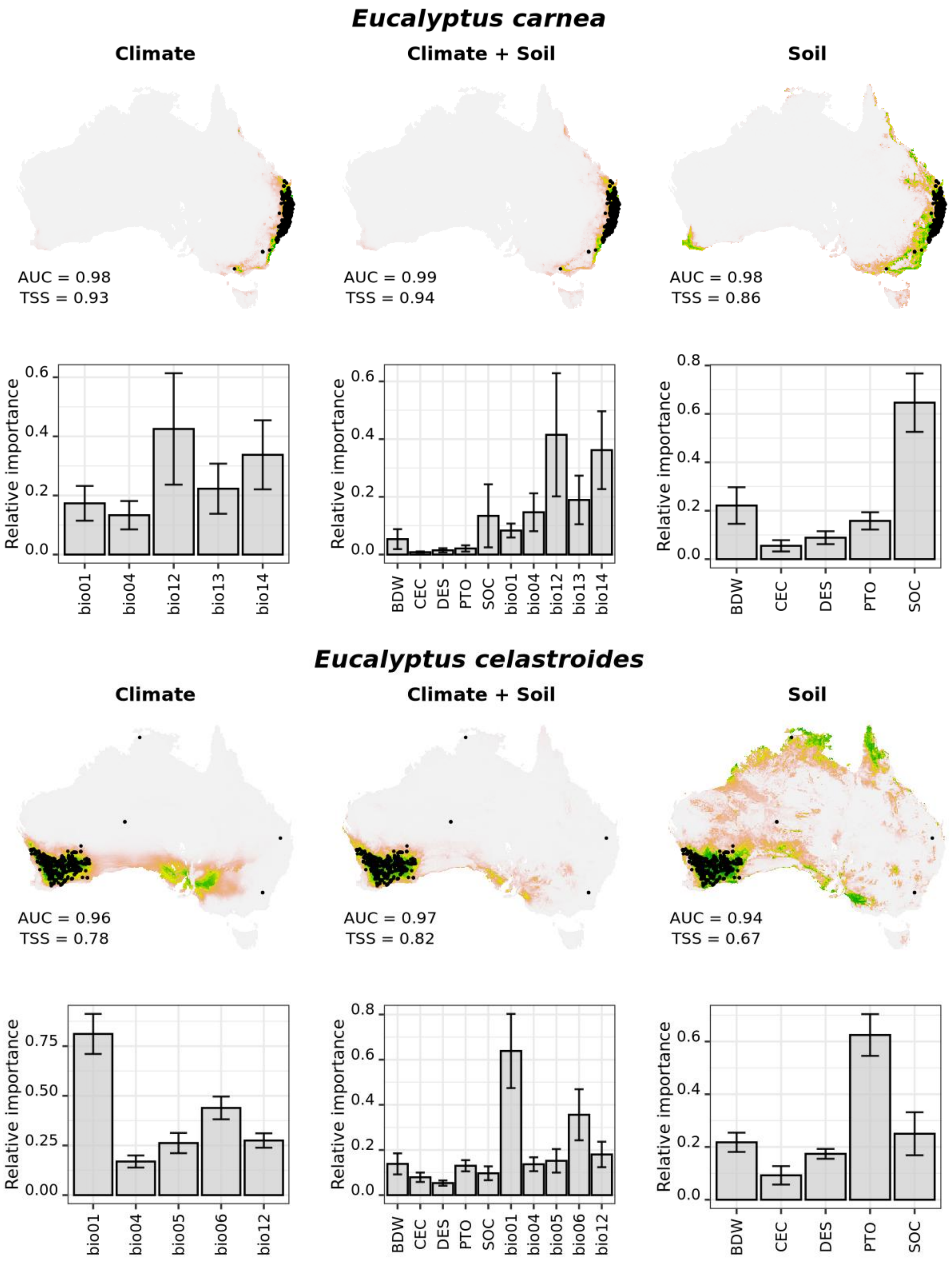
Eucalyptus carnea
Climate
Climate + Soil
Soil
AUC = 0.98
TSS = 0.93
AUC = 0.99
TSS = 0.94
AUC = 0.98
TSS = 0.86
Relative importance
bio01
bio04
bio12
bio13
bio14
BDW
CEC
DES
PTO
SOC
Eucalyptus celastroides
Climate
Climate + Soil
Soil
AUC = 0.96
TSS = 0.78
AUC = 0.97
TSS = 0.82
AUC = 0.94
TSS = 0.67
Relative importance
bio01
bio04
bio05
bio06
bio12
BDW
CEC
DES
PTO
SOC

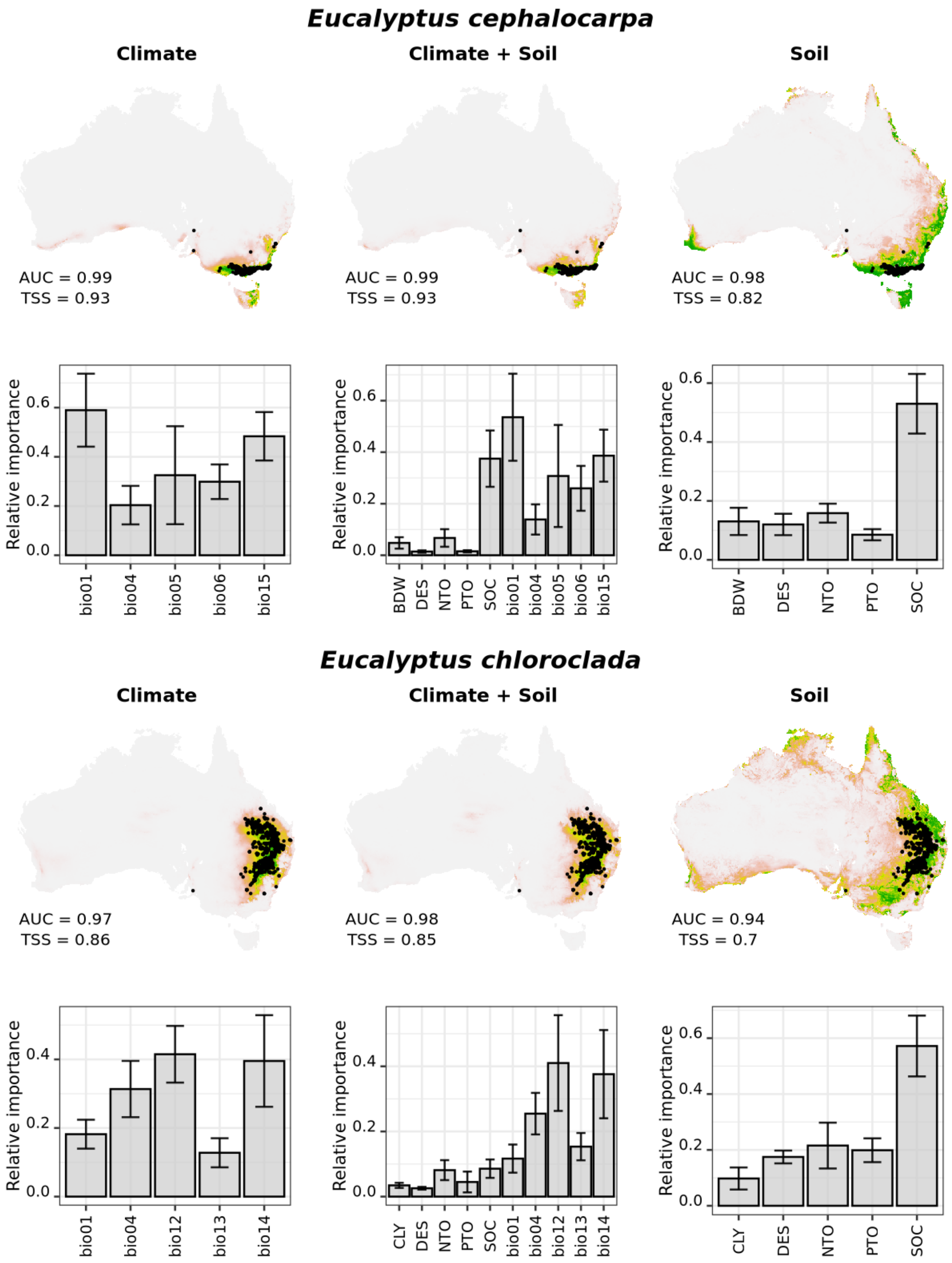

Eucalyptus cephalocarpa
Climate
Climate + Soil
Soil
AUC = 0.99
TSS = 0.93
AUC = 0.99
TSS = 0.93
AUC = 0.98
TSS = 0.82
Relative importance
0.0
0.2
0.4
0.6
bio01
bio04
bio05
bio06
bio15
BDW
DES
NTO
PTO
SOC
Eucalyptus chloroclada
Climate
Climate + Soil
Soil
AUC = 0.97
TSS = 0.86
AUC = 0.98
TSS = 0.85
AUC = 0.94
TSS = 0.7
Relative importance
bio01
bio04
bio12
bio13
bio14
CLY
DES
NTO
PTO
SOC

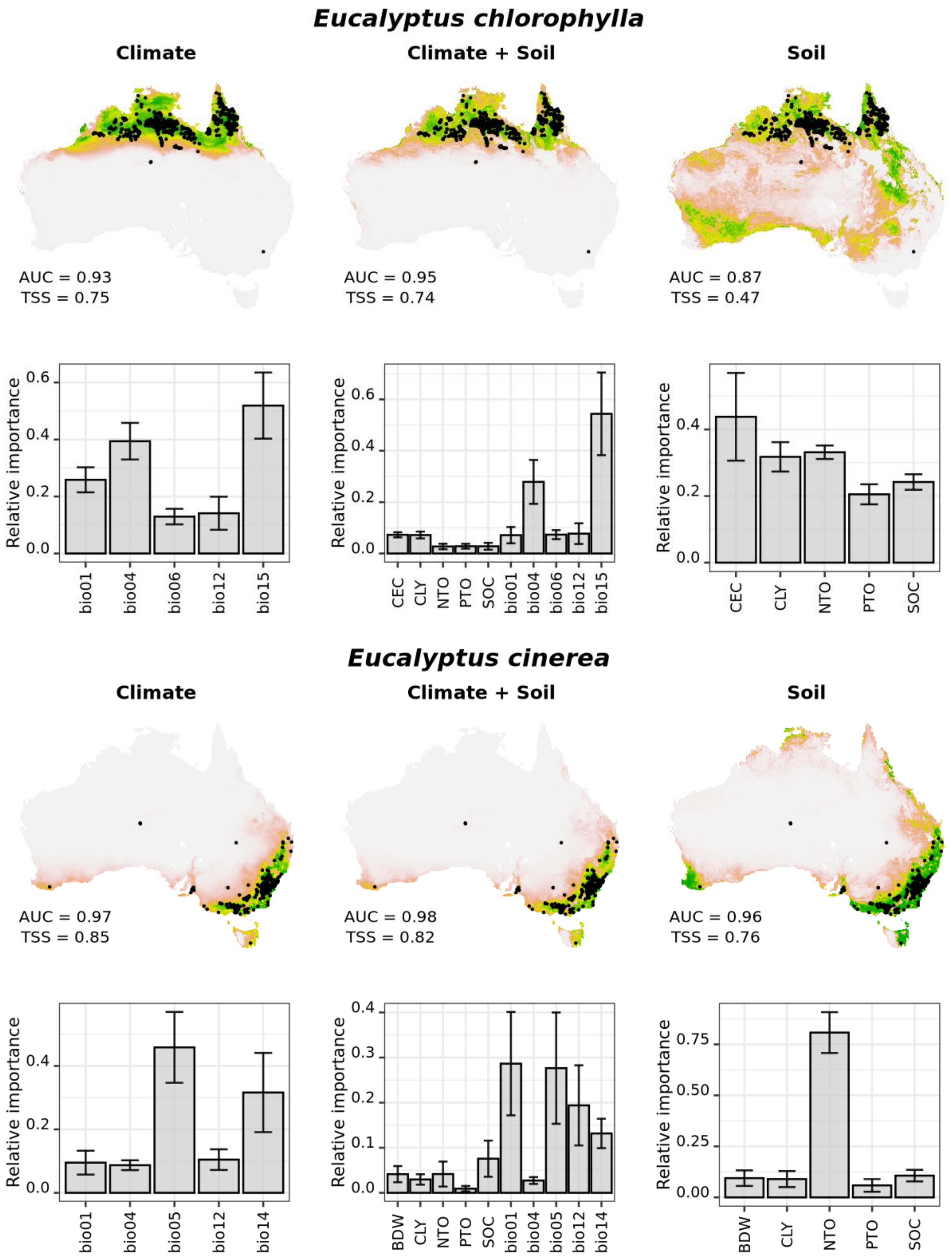

Eucalyptus chlorophylla
Climate
Climate + Soil
Soil
AUC = 0.93
TSS = 0.75
AUC = 0.95
TSS = 0.74
AUC = 0.87
TSS = 0.47
Relative importance
bio01
bio04
bio06
bio12
bio15
CEC
CLY
NTO
PTO
SOC
Eucalyptus cinerea
Climate
Climate + Soil
Soil
AUC = 0.97
TSS = 0.85
AUC = 0.98
TSS = 0.82
AUC = 0.96
TSS = 0.76
Relative importance
bio01
bio04
bio05
bio12
bio14
BDW
CLY
NTO
PTO
SOC

Eucalyptus clelandiorum

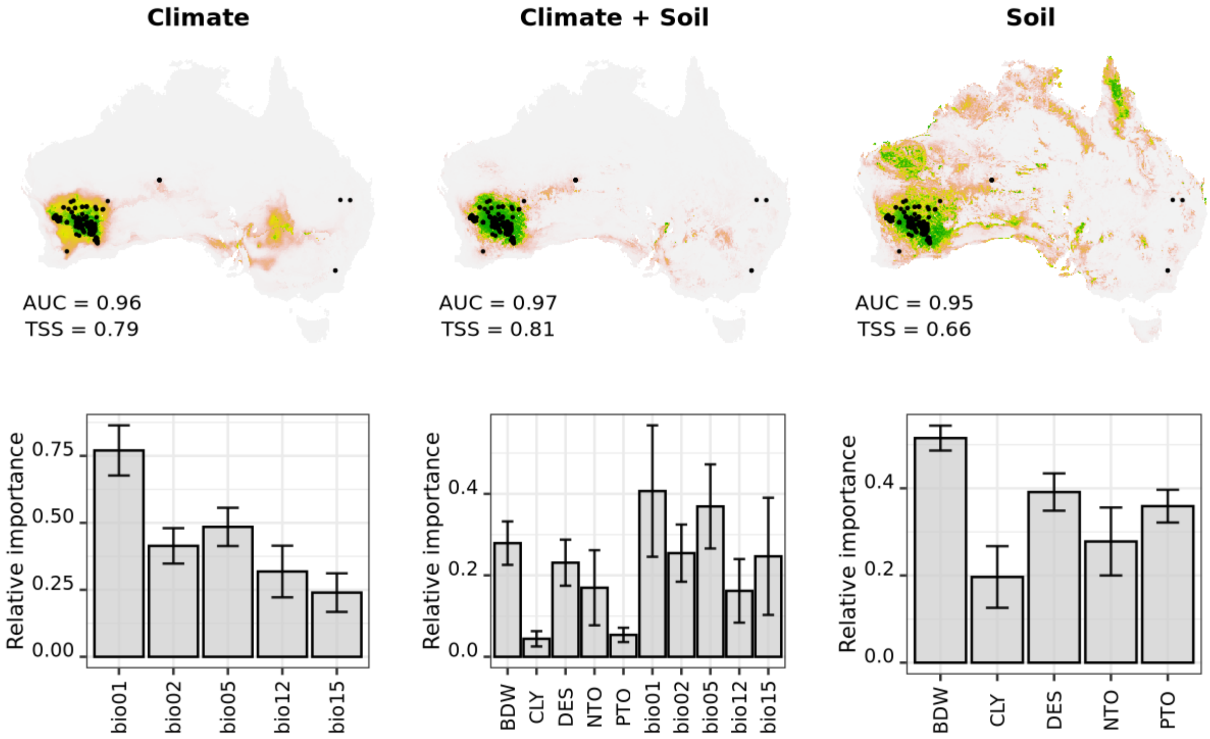

Eucalyptus cloeziana

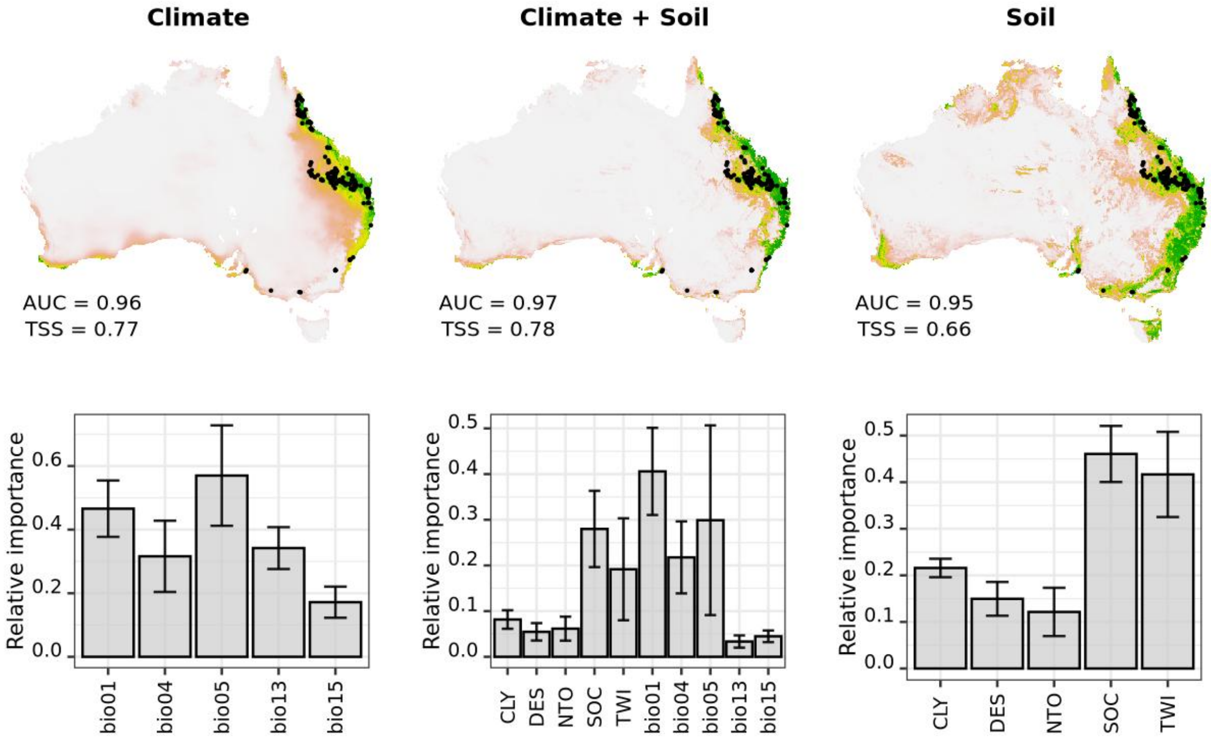

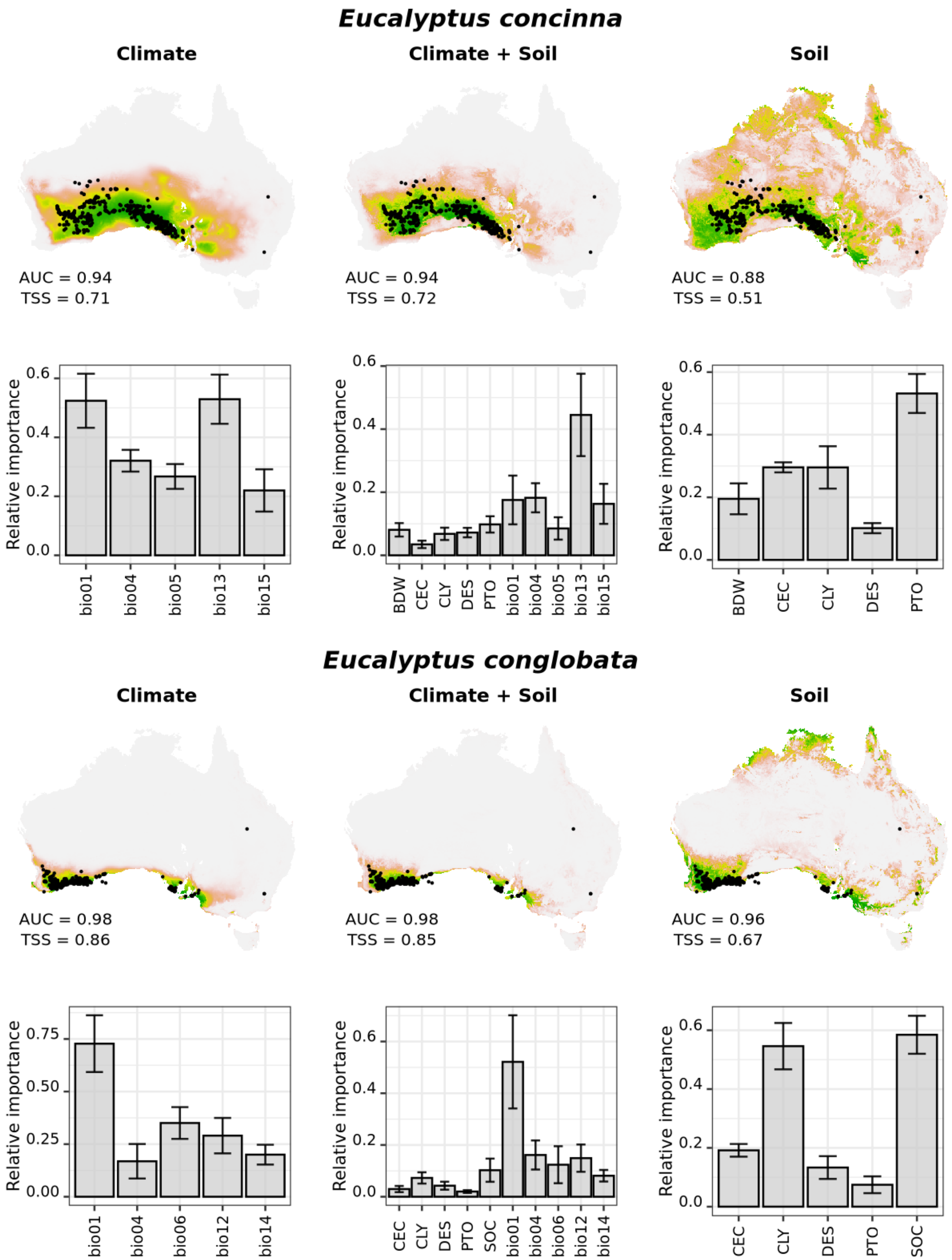
Eucalyptus concinna
Climate
Climate + Soil
Soil
AUC = 0.94
TSS = 0.71
AUC = 0.94
TSS = 0.72
AUC = 0.88
TSS = 0.51
Relative importance
0.0
0.2
0.4
0.6
bio01
bio04
bio05
bio13
bio15
BDW
CEC
CLY
DES
PTO
Eucalyptus conglobata
AUC = 0.98
TSS = 0.86
AUC = 0.98
TSS = 0.85
AUC = 0.96
TSS = 0.67
0.00
0.25
0.50
0.75
bio06
bio12
bio14
SOC

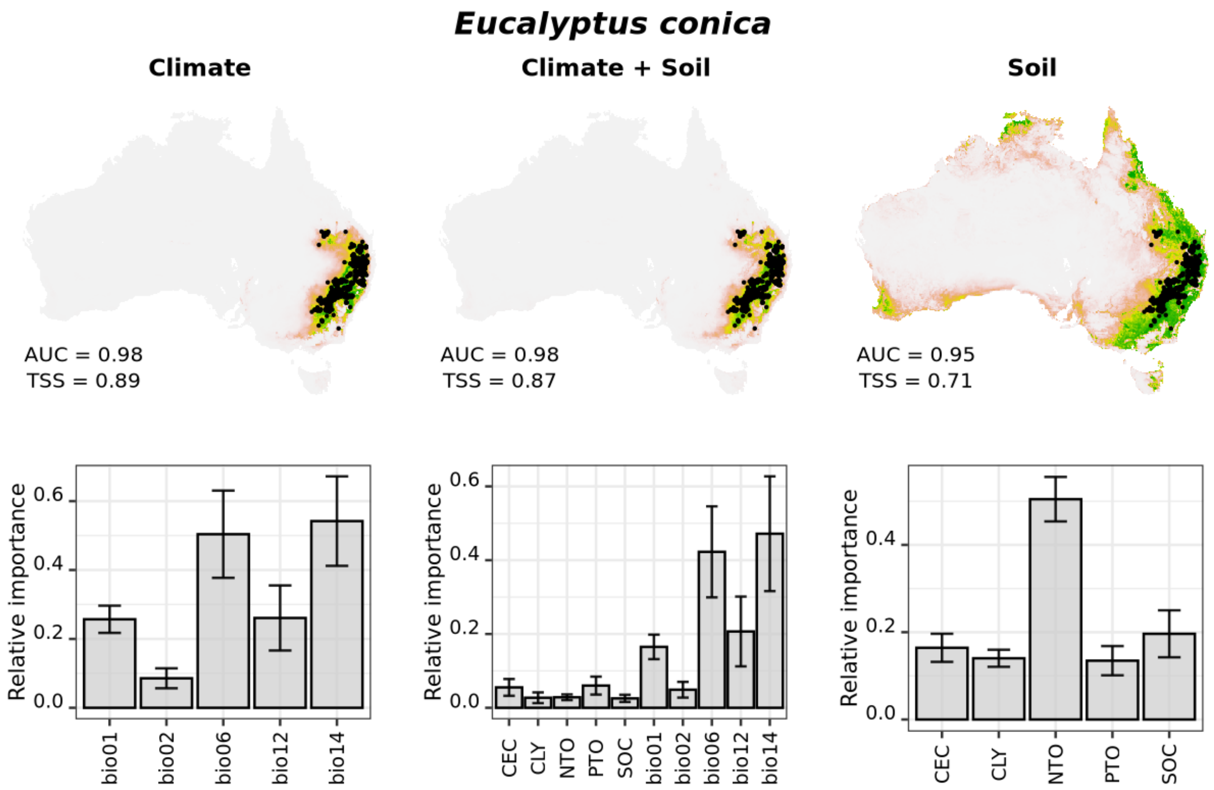
Eucalyptus conica
Climate
Climate + Soil
Soil
AUC = 0.98
TSS = 0.89
AUC = 0.98
TSS = 0.87
AUC = 0.95
TSS = 0.71
Relative importance
bio01
bio02
bio06
bio12
bio14
CEC
CLY
NTO
PTO
SOC
bio01
bio02
bio06
bio12
bio14
CEC
CLY
NTO
PTO
SOC

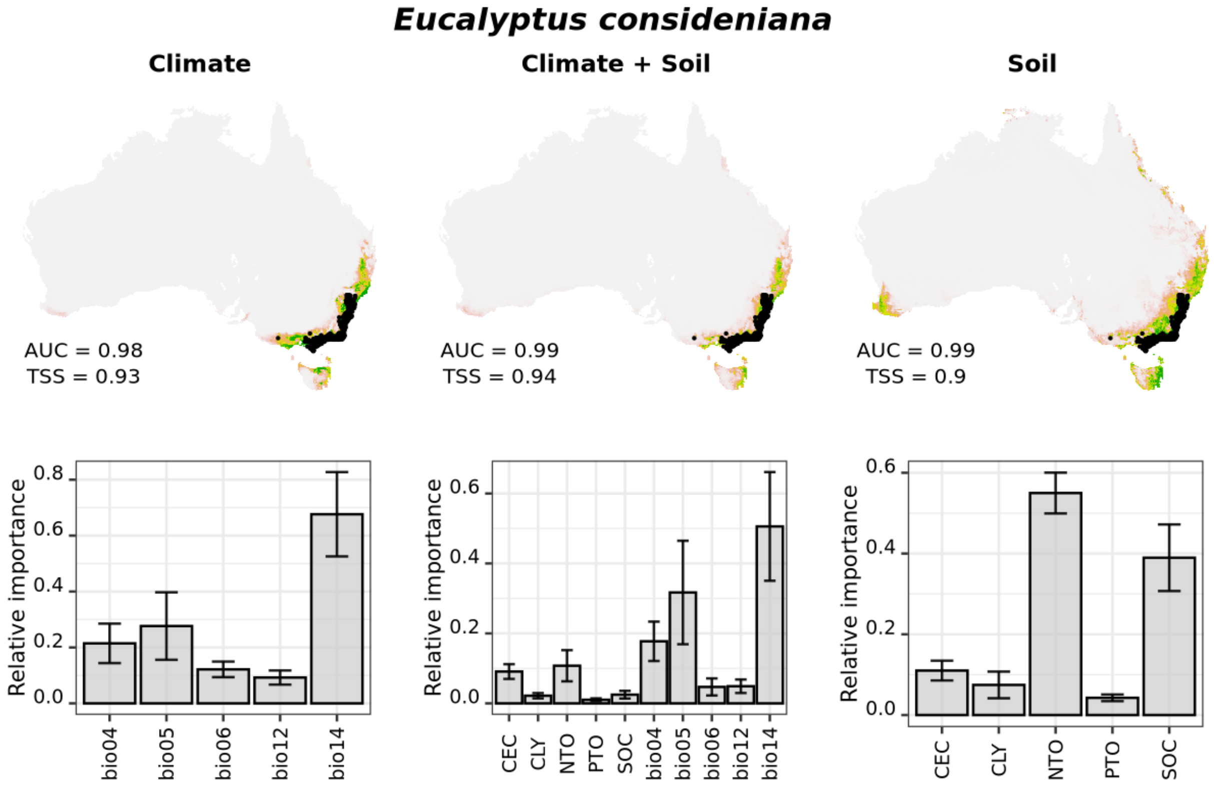
Eucalyptus considениana
Climate
Climate + Soil
Soil
AUC = 0.98
TSS = 0.93
AUC = 0.99
TSS = 0.94
AUC = 0.99
TSS = 0.9
Relative importance
bio04
bio05
bio06
bio12
bio14
CEC
CLY
NTO
PTO
SOC
bio04
bio05
bio06
bio12
bio14
CEC
CLY
NTO
PTO
SOC

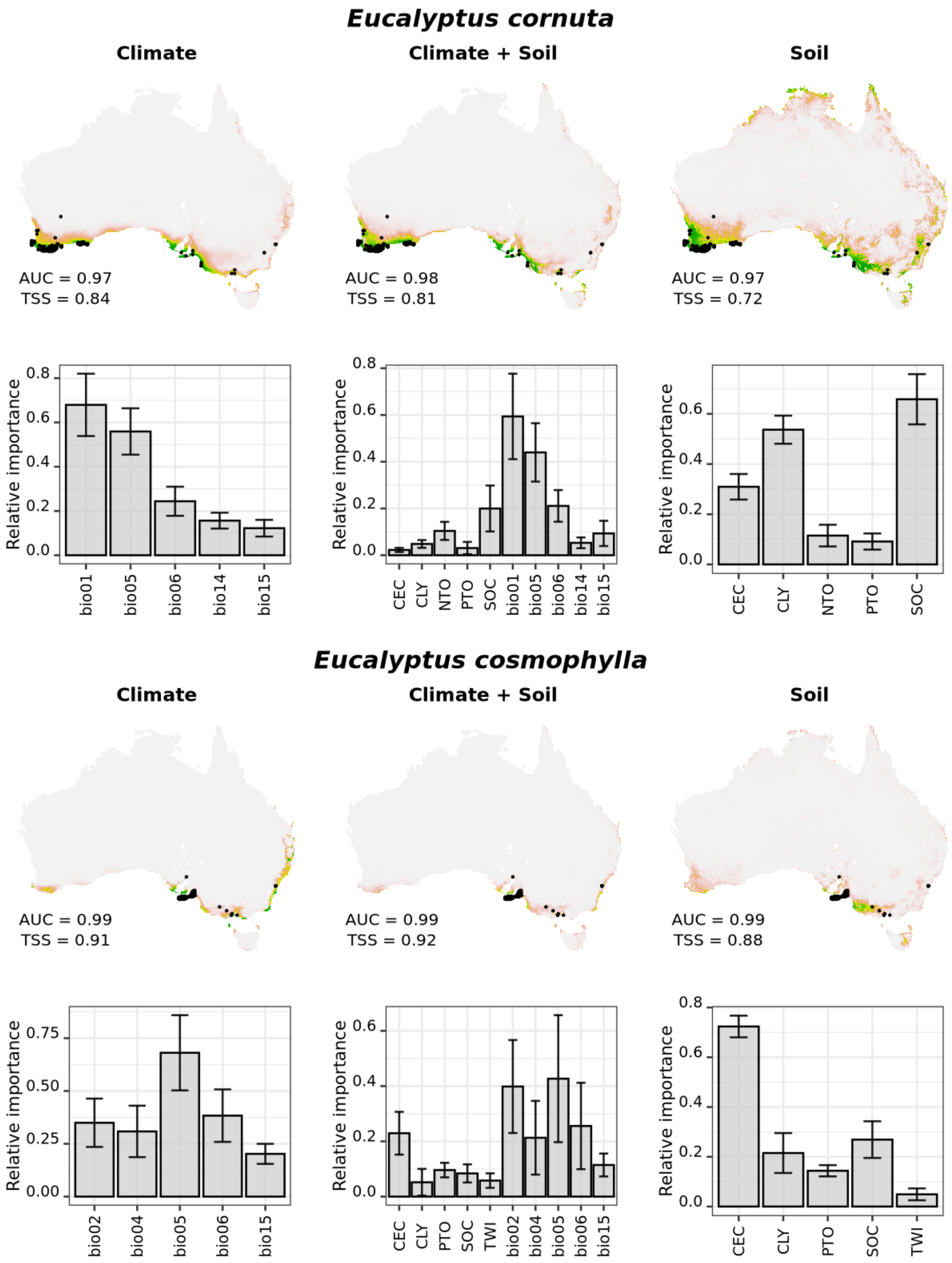

Eucalyptus cornuta
Climate
Climate + Soil
Soil
AUC = 0.97
TSS = 0.84
AUC = 0.98
TSS = 0.81
AUC = 0.97
TSS = 0.72
Relative importance
bio01
bio05
bio06
bio14
bio15
CEC
CLY
NTO
PTO
SOC
Eucalyptus cosmophylla
Climate
Climate + Soil
Soil
AUC = 0.99
TSS = 0.91
AUC = 0.99
TSS = 0.92
AUC = 0.99
TSS = 0.88
Relative importance
bio02
bio04
bio05
bio06
bio15
CEC
CLY
PTO
SOC
TWI

Eucalyptus croajingolensis

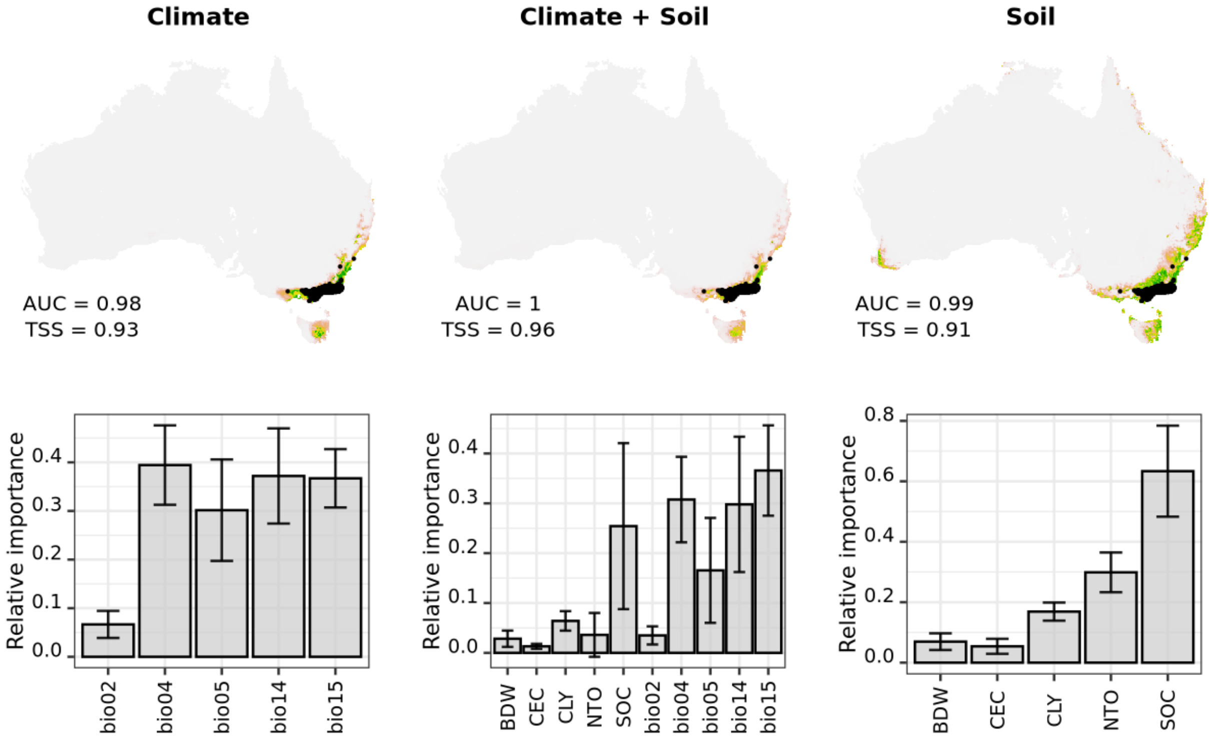

Eucalyptus cullenii

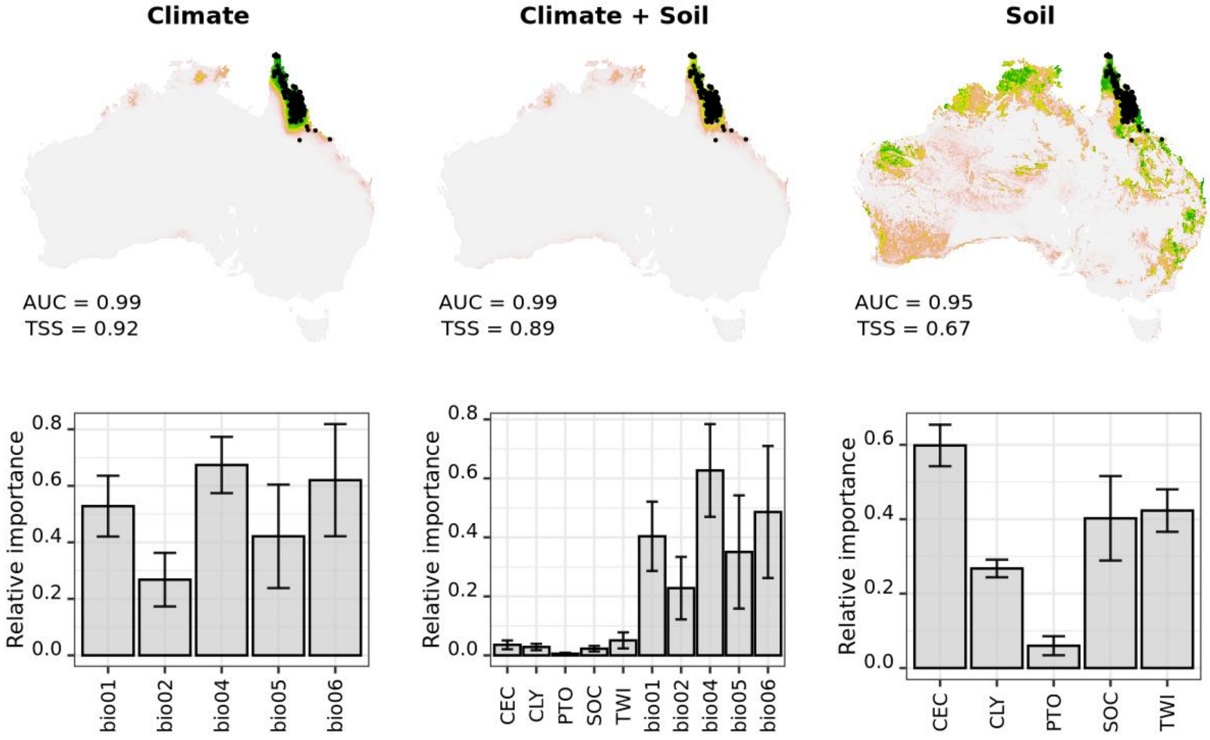

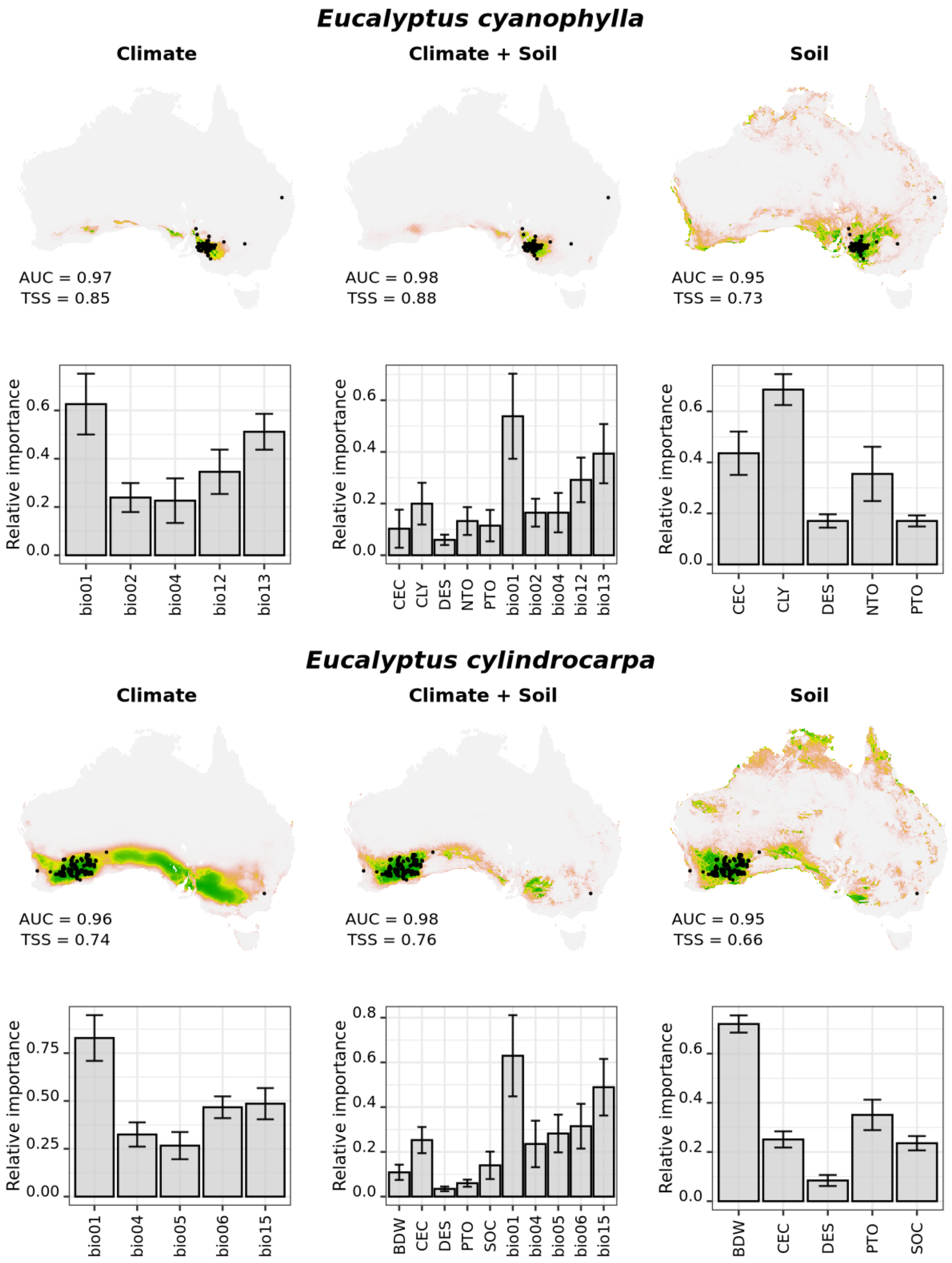

Eucalyptus cyanophylla
Climate
Climate + Soil
Soil
AUC = 0.97
TSS = 0.85
AUC = 0.98
TSS = 0.88
AUC = 0.95
TSS = 0.73
Relative importance
0.0
0.2
0.4
0.6
bio01
bio02
bio04
bio12
bio13
CEC
CLY
DES
NTO
PTO
bio01
bio02
bio04
bio12
bio13
CEC
CLY
DES
NTO
PTO
Eucalyptus cylindrocarpa
Climate
Climate + Soil
Soil
AUC = 0.96
TSS = 0.74
AUC = 0.98
TSS = 0.76
AUC = 0.95
TSS = 0.66
Relative importance
0.00
0.25
0.50
0.75
0.0
0.2
0.4
0.6
0.8
bio01
bio04
bio05
bio06
bio15
BDW
CEC
DES
PTO
SOC
bio01
bio04
bio05
bio06
bio15
BDW
CEC
DES
PTO
SOC

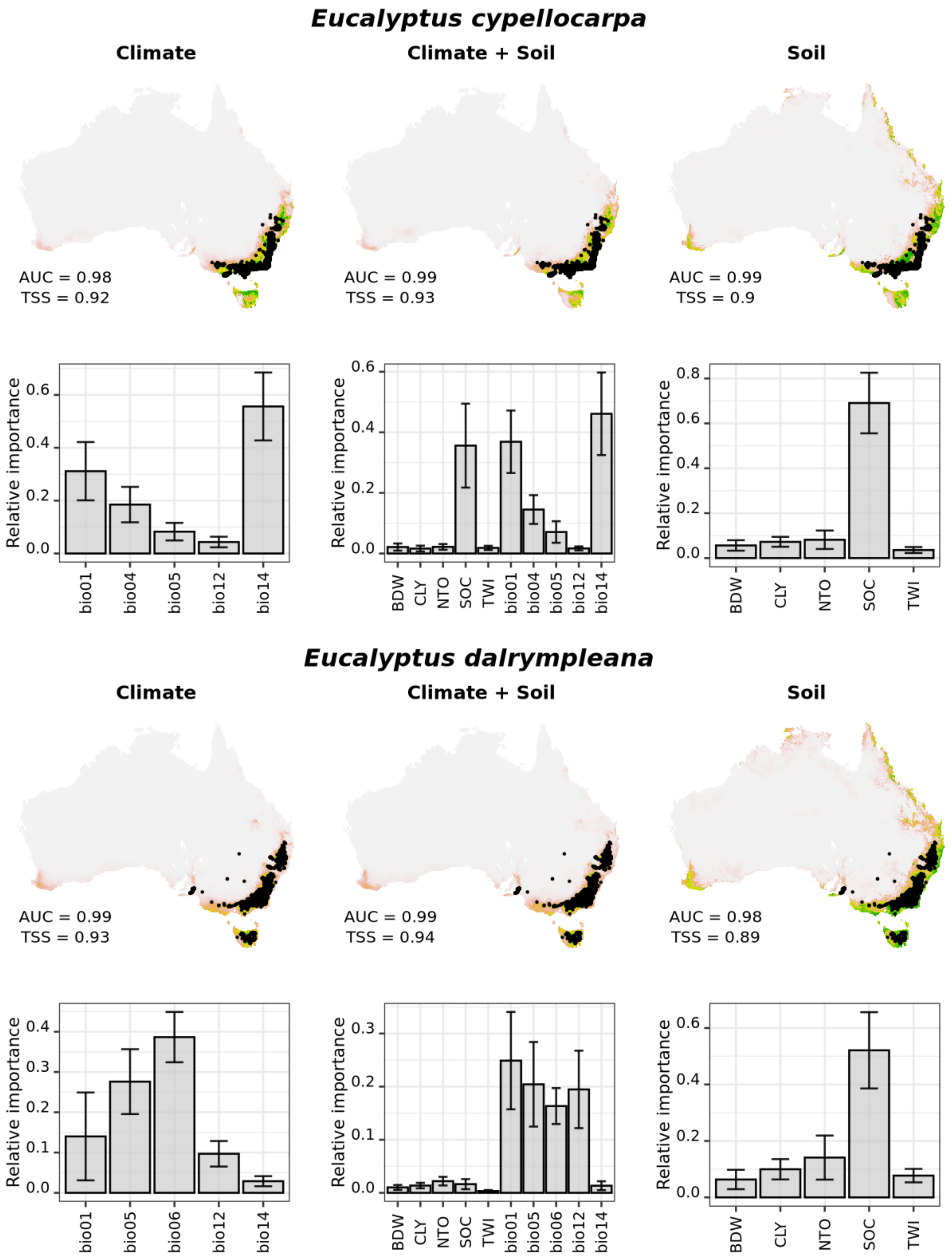
Eucalyptus cypellocarpa
Climate
Climate + Soil
Soil
AUC = 0.98
TSS = 0.92
AUC = 0.99
TSS = 0.93
AUC = 0.99
TSS = 0.9
Relative importance
0.0
0.2
0.4
0.6
0.8
bio01
bio04
bio05
bio12
bio14
BDW
CLY
NTO
SOC
TWI
Eucalyptus dalrympleana
Climate
Climate + Soil
Soil
AUC = 0.99
TSS = 0.93
AUC = 0.99
TSS = 0.94
AUC = 0.98
TSS = 0.89
Relative importance
0.0
0.1
0.2
0.3
0.4
0.6
bio01
bio05
bio06
bio12
bio14
BDW
CLY
NTO
SOC
TWI

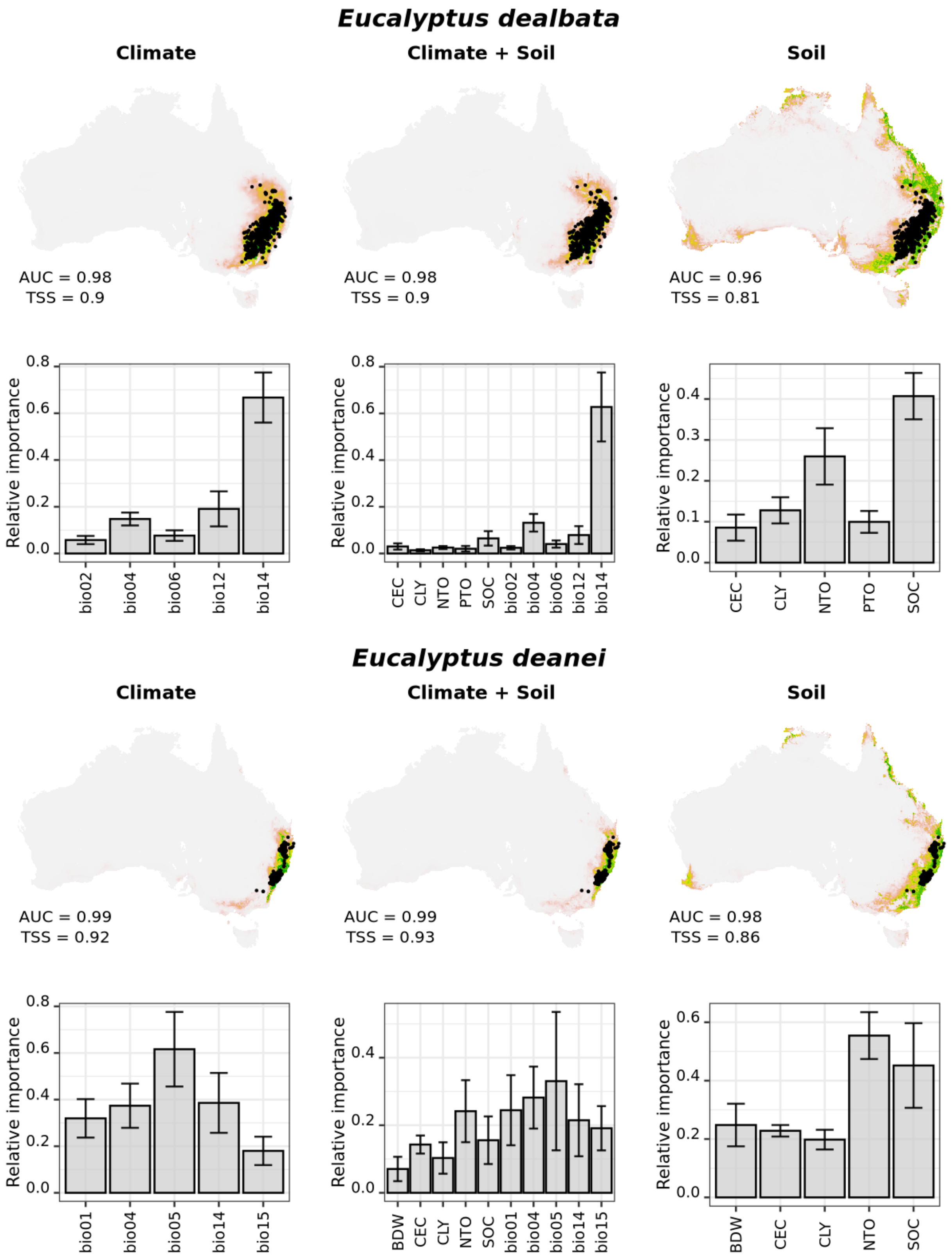
Eucalyptus dealbata
Climate
Climate + Soil
Soil
AUC = 0.98
TSS = 0.9
AUC = 0.98
TSS = 0.9
AUC = 0.96
TSS = 0.81
Relative importance
bio02
bio04
bio06
bio12
bio14
CEC
CLY
NTO
PTO
SOC
bio02
bio04
bio06
bio12
bio14
CEC
CLY
NTO
PTO
SOC
Eucalyptus deanei
Climate
Climate + Soil
Soil
AUC = 0.99
TSS = 0.92
AUC = 0.99
TSS = 0.93
AUC = 0.98
TSS = 0.86
Relative importance
bio01
bio04
bio05
bio14
bio15
BDW
CEC
CLY
NTO
SOC
bio01
bio04
bio05
bio14
bio15
BDW
CEC
CLY
NTO
SOC

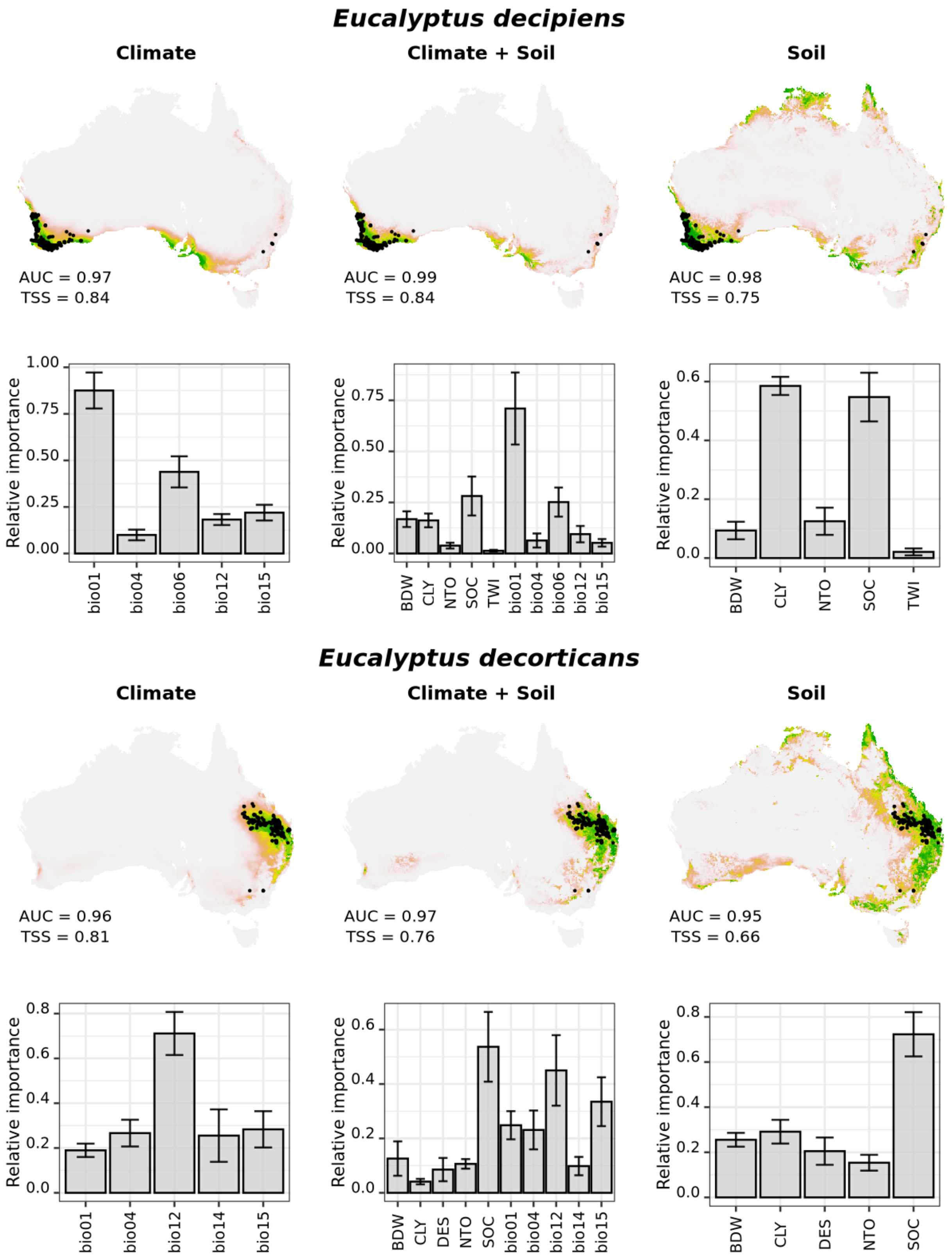
Eucalyptus decipiens
Climate
Climate + Soil
Soil
AUC = 0.97
TSS = 0.84
AUC = 0.99
TSS = 0.84
AUC = 0.98
TSS = 0.75
Relative importance
1.00
0.75
0.50
0.25
0.00
bio01
bio04
bio06
bio12
bio15
BDW
CLY
NTO
SOC
TWI
0.6
0.4
0.2
0.0
Eucalyptus decorticans
AUC = 0.96
TSS = 0.81
AUC = 0.97
TSS = 0.76
AUC = 0.95
TSS = 0.66
0.8
bio14
DES

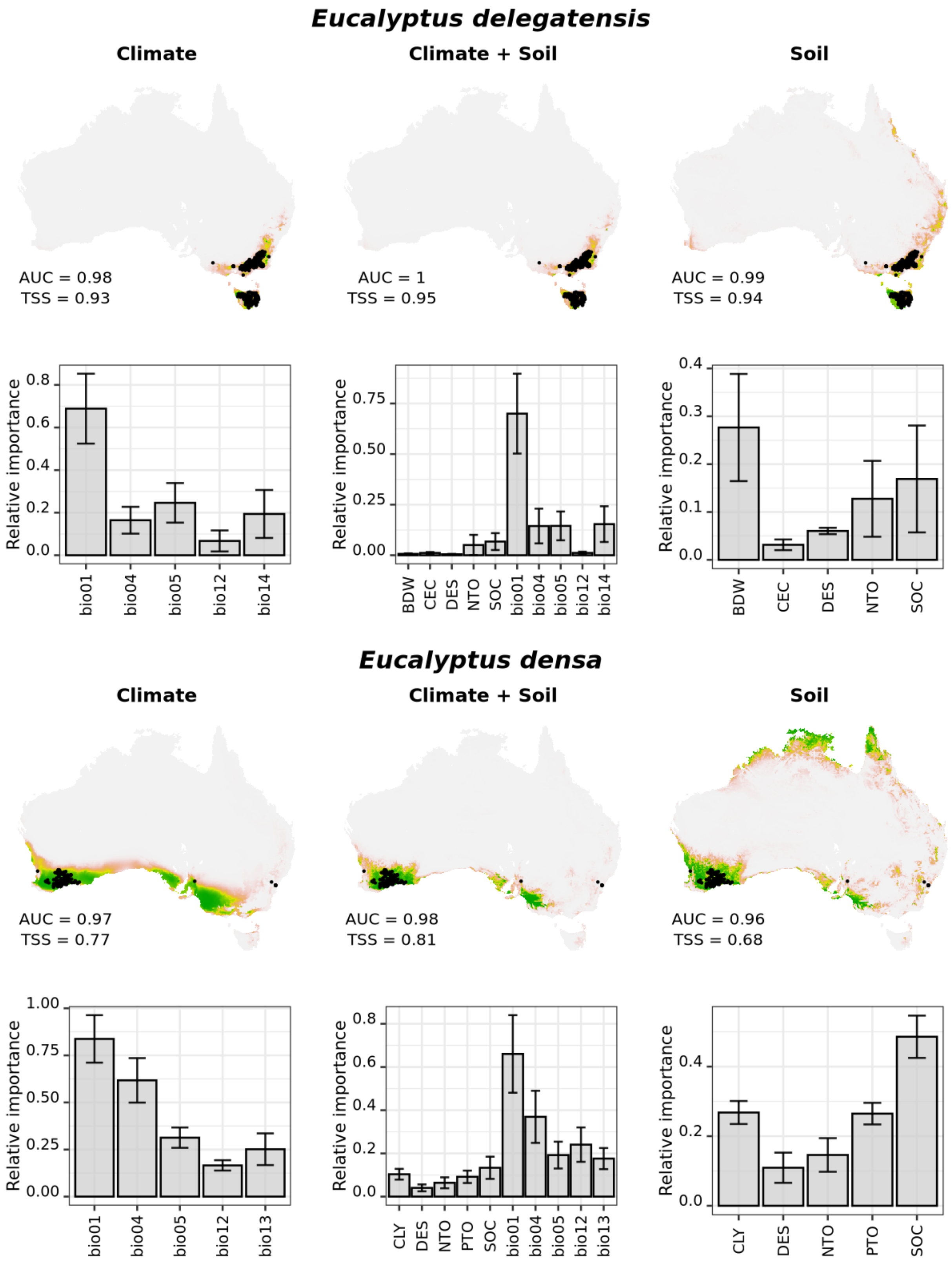

Eucalyptus delegatensis
Climate
Climate + Soil
Soil
AUC = 0.98
TSS = 0.93
AUC = 1
TSS = 0.95
AUC = 0.99
TSS = 0.94
Relative importance
0.0 0.2 0.4 0.6 0.8
bio01 bio04 bio05 bio12 bio14
0.00 0.25 0.50 0.75
BDW CEC DES NTO SOC bio01 bio04 bio05 bio12 bio14
0.0 0.1 0.2 0.3 0.4
BDW CEC DES NTO SOC
Eucalyptus densa
Climate
Climate + Soil
Soil
AUC = 0.97
TSS = 0.77
AUC = 0.98
TSS = 0.81
AUC = 0.96
TSS = 0.68
Relative importance
0.00 0.25 0.50 0.75 1.00
bio01 bio04 bio05 bio12 bio13
0.0 0.2 0.4 0.6 0.8
CLY DES NTO PTO SOC bio01 bio04 bio05 bio12 bio13
0.0 0.2 0.4
CLY DES NTO PTO SOC

Eucalyptus dissimulata

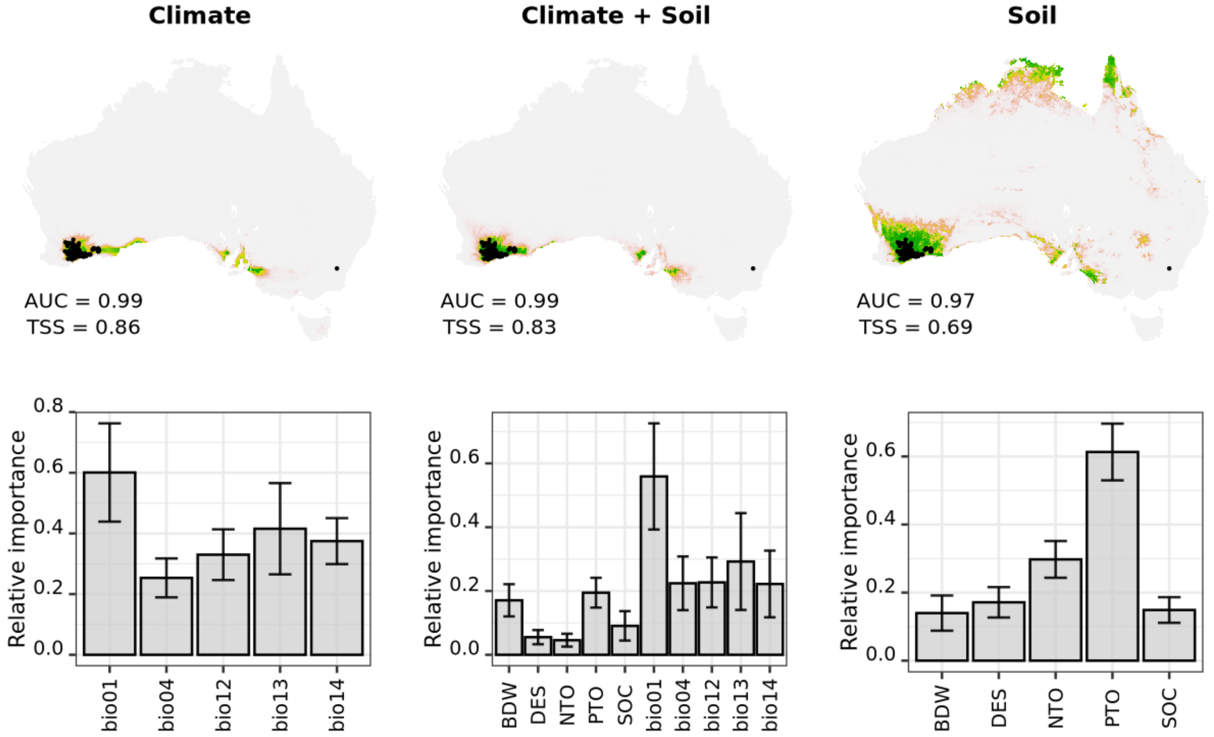

Eucalyptus diversifolia

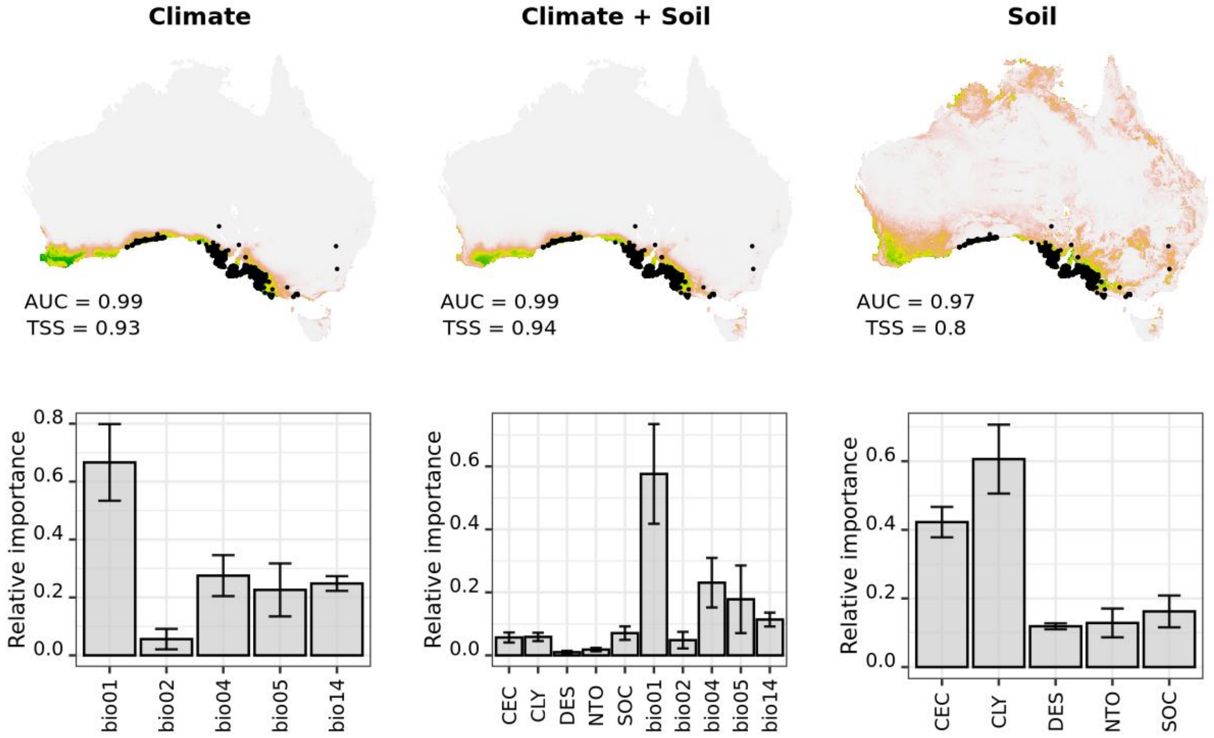

Eucalyptus dives

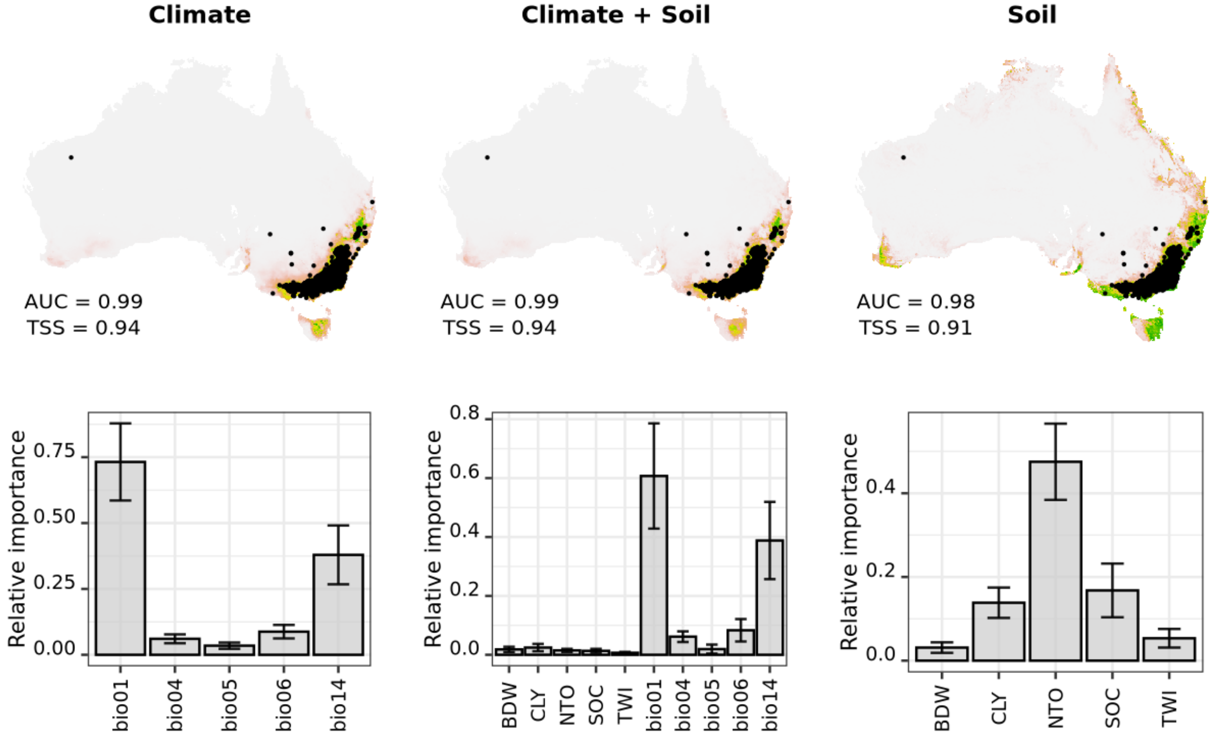

Eucalyptus drummondii

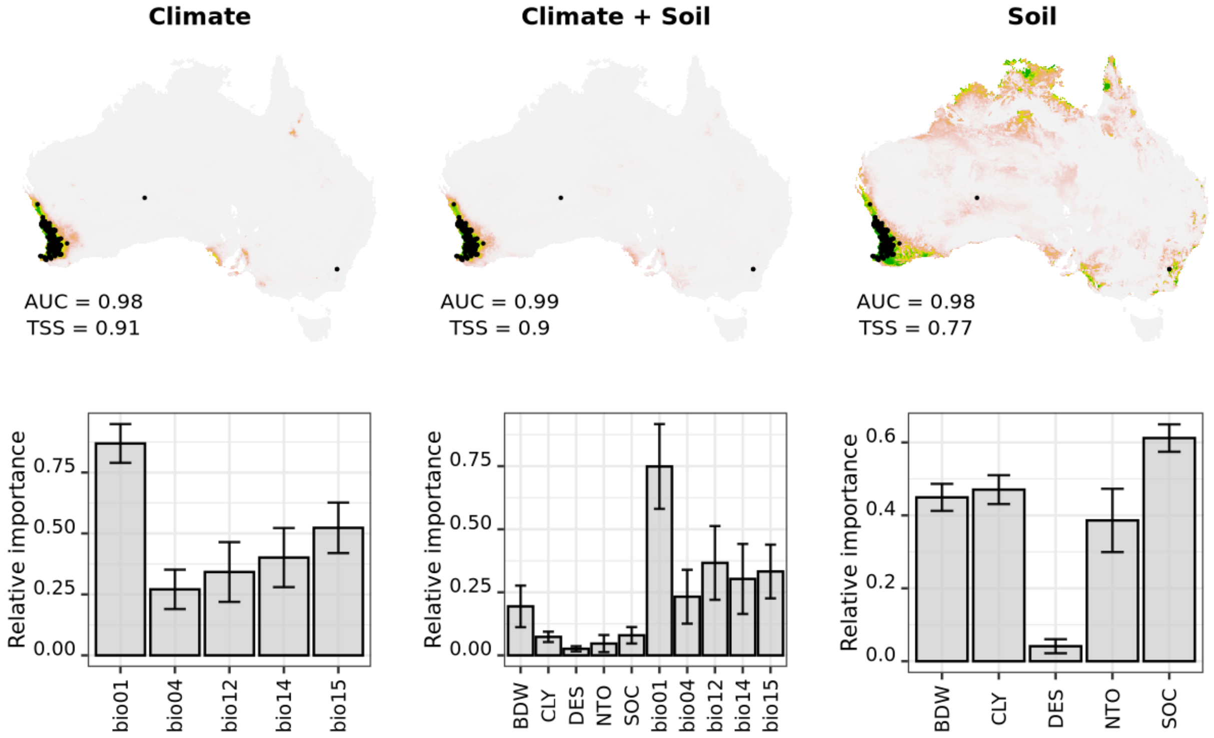

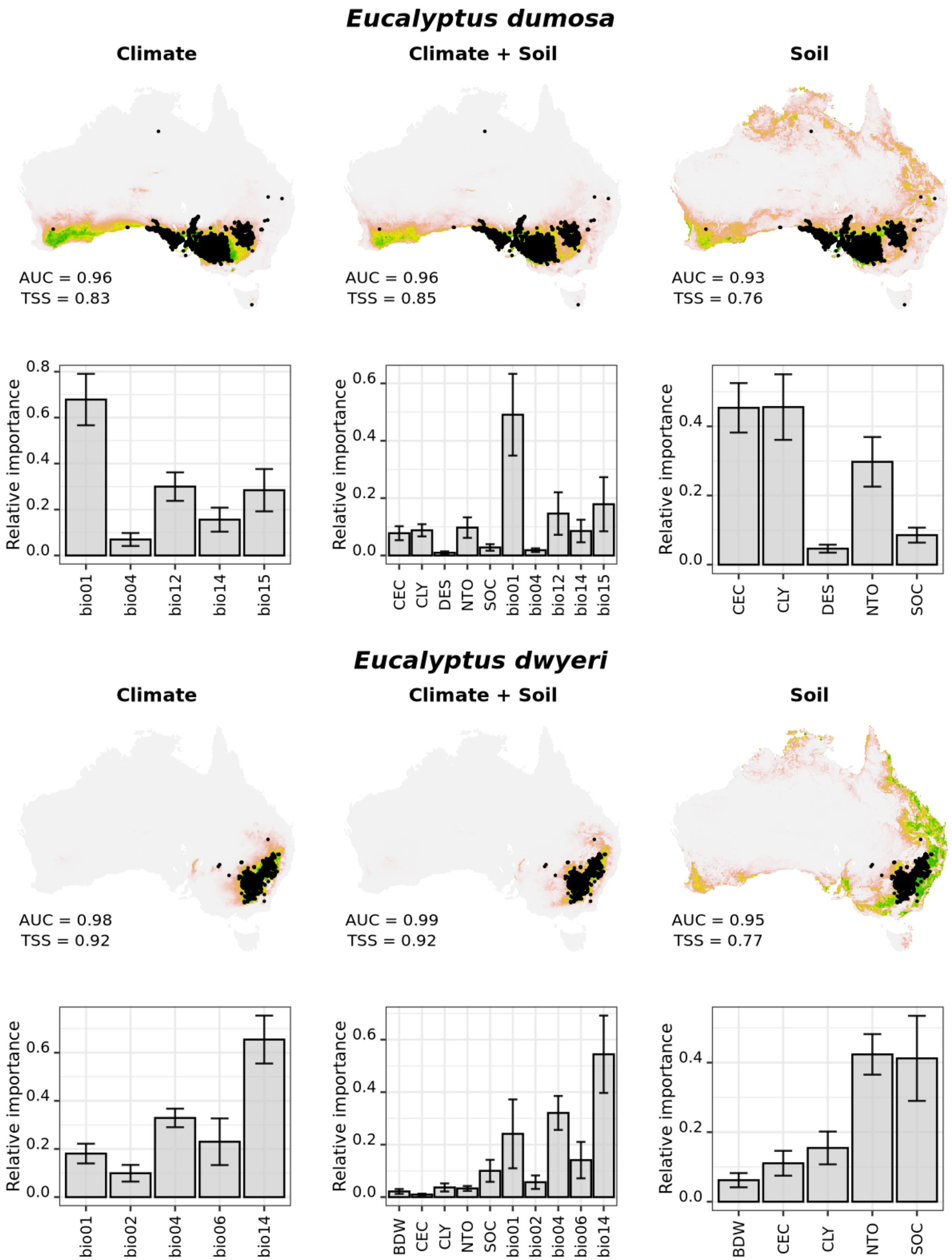
Eucalyptus dumosa
Climate
Climate + Soil
Soil
AUC = 0.96
TSS = 0.83
AUC = 0.96
TSS = 0.85
AUC = 0.93
TSS = 0.76
Relative importance
0.0
0.2
0.4
0.6
0.8
bio01
bio04
bio12
bio14
bio15
CEC
CLY
DES
NTO
SOC
Eucalyptus dwyeri
Climate
Climate + Soil
Soil
AUC = 0.98
TSS = 0.92
AUC = 0.99
TSS = 0.92
AUC = 0.95
TSS = 0.77
Relative importance
bio01
bio02
bio04
bio06
bio14
BDW
CEC
CLY
NTO
SOC

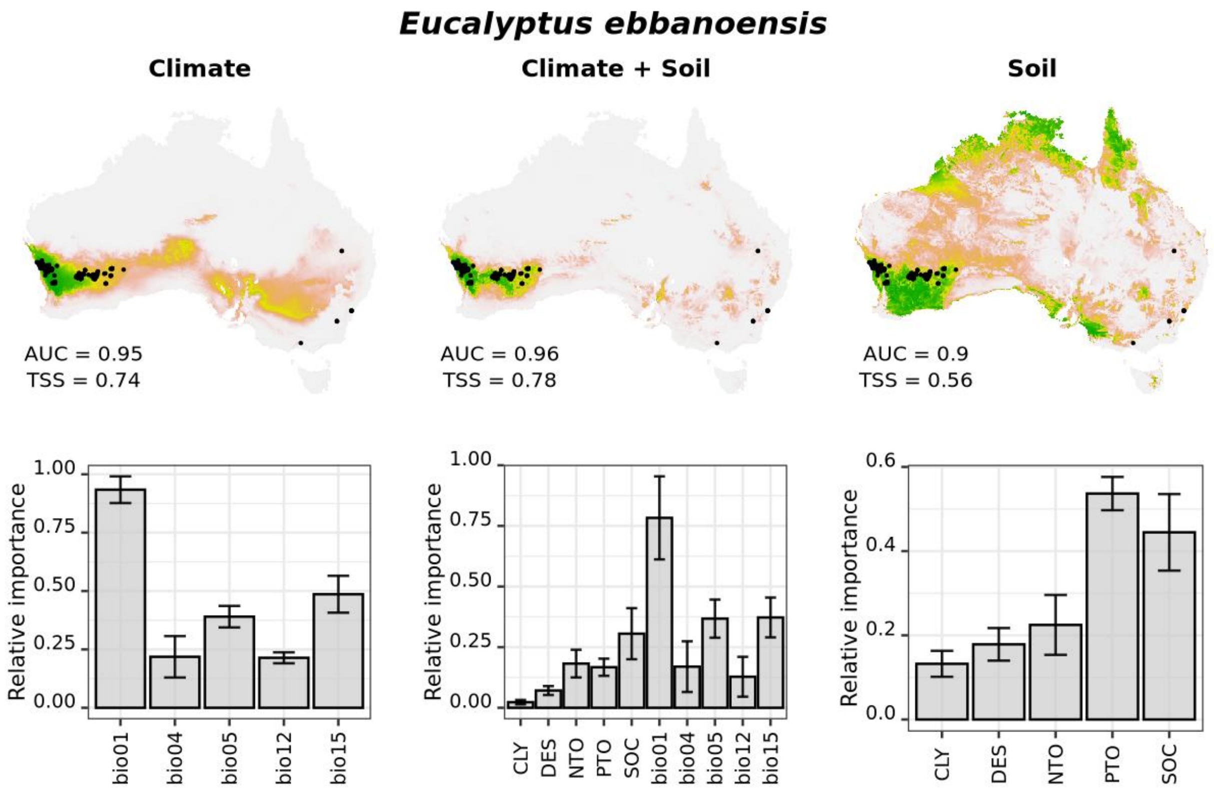
Eucalyptus ebbanoensis
Climate
Climate + Soil
Soil
AUC = 0.95
TSS = 0.74
AUC = 0.96
TSS = 0.78
AUC = 0.9
TSS = 0.56
Relative importance
1.00
0.75
0.50
0.25
0.00
bio01
bio04
bio05
bio12
bio15
CLY
DES
NTO
PTO
SOC
0.6
0.4
0.2
0.0

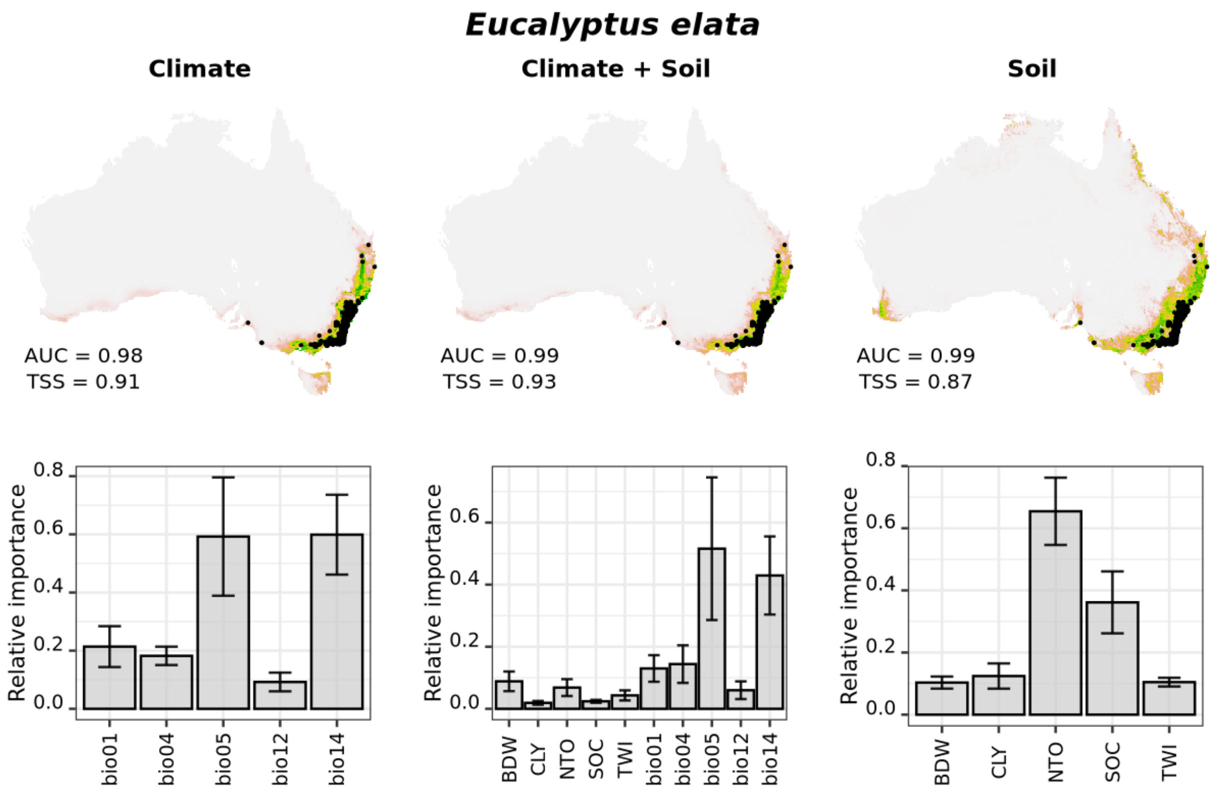
Eucalyptus elata
Climate
Climate + Soil
Soil
AUC = 0.98
TSS = 0.91
AUC = 0.99
TSS = 0.93
AUC = 0.99
TSS = 0.87
Relative importance
0.8
0.6
0.4
0.2
0.0
bio01
bio04
bio05
bio12
bio14
BDW
CLY
NTO
SOC
TWI

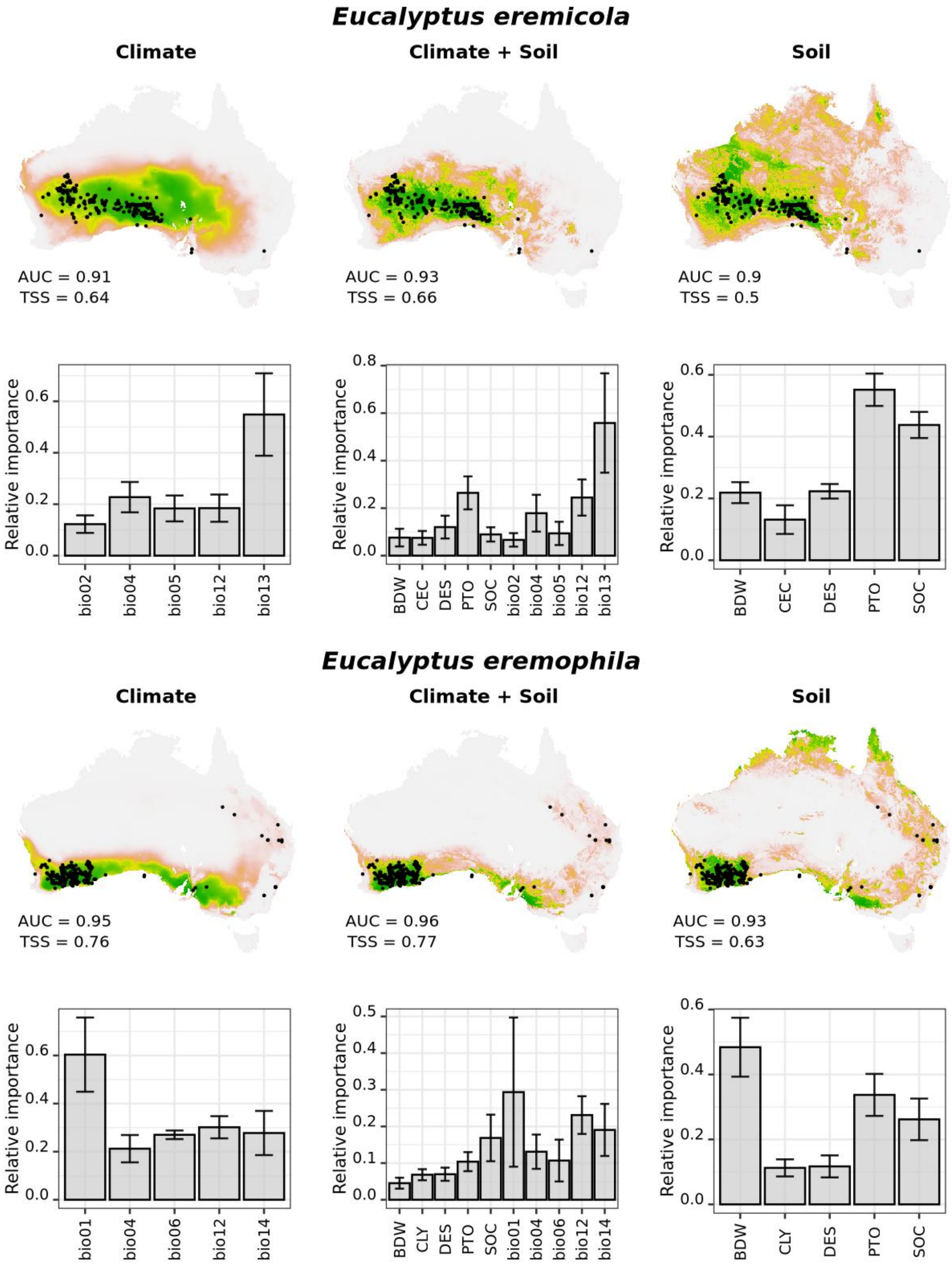
Eucalyptus eremicola
Climate
Climate + Soil
Soil
AUC = 0.91
TSS = 0.64
AUC = 0.93
TSS = 0.66
AUC = 0.9
TSS = 0.5
Relative importance
0.0
0.2
0.4
0.6
0.8
bio02
bio04
bio05
bio12
bio13
BDW
CEC
DES
PTO
SOC
Eucalyptus eremophila
AUC = 0.95
TSS = 0.76
AUC = 0.96
TSS = 0.77
AUC = 0.93
TSS = 0.63
0.1
0.3
0.5
bio01
bio06
bio14
CLY

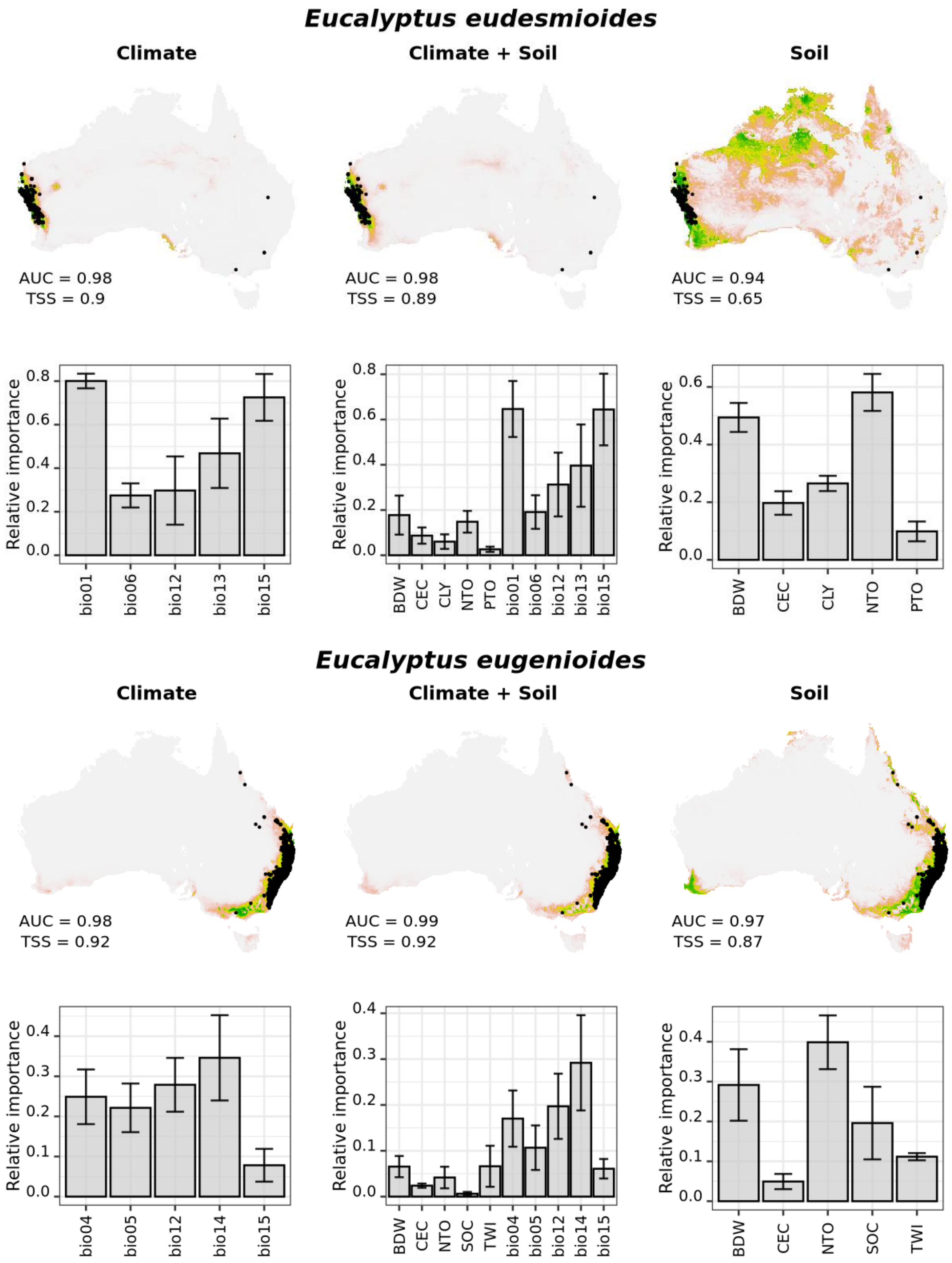

Eucalyptus eudesmioides
Climate
Climate + Soil
Soil
AUC = 0.98
TSS = 0.9
AUC = 0.98
TSS = 0.89
AUC = 0.94
TSS = 0.65
Relative importance
bio01
bio06
bio12
bio13
bio15
BDW
CEC
CLY
NTO
PTO
Eucalyptus eugenioides
Climate
Climate + Soil
Soil
AUC = 0.98
TSS = 0.92
AUC = 0.99
TSS = 0.92
AUC = 0.97
TSS = 0.87
Relative importance
bio04
bio05
bio12
bio14
bio15
BDW
CEC
NTO
SOC
TWI

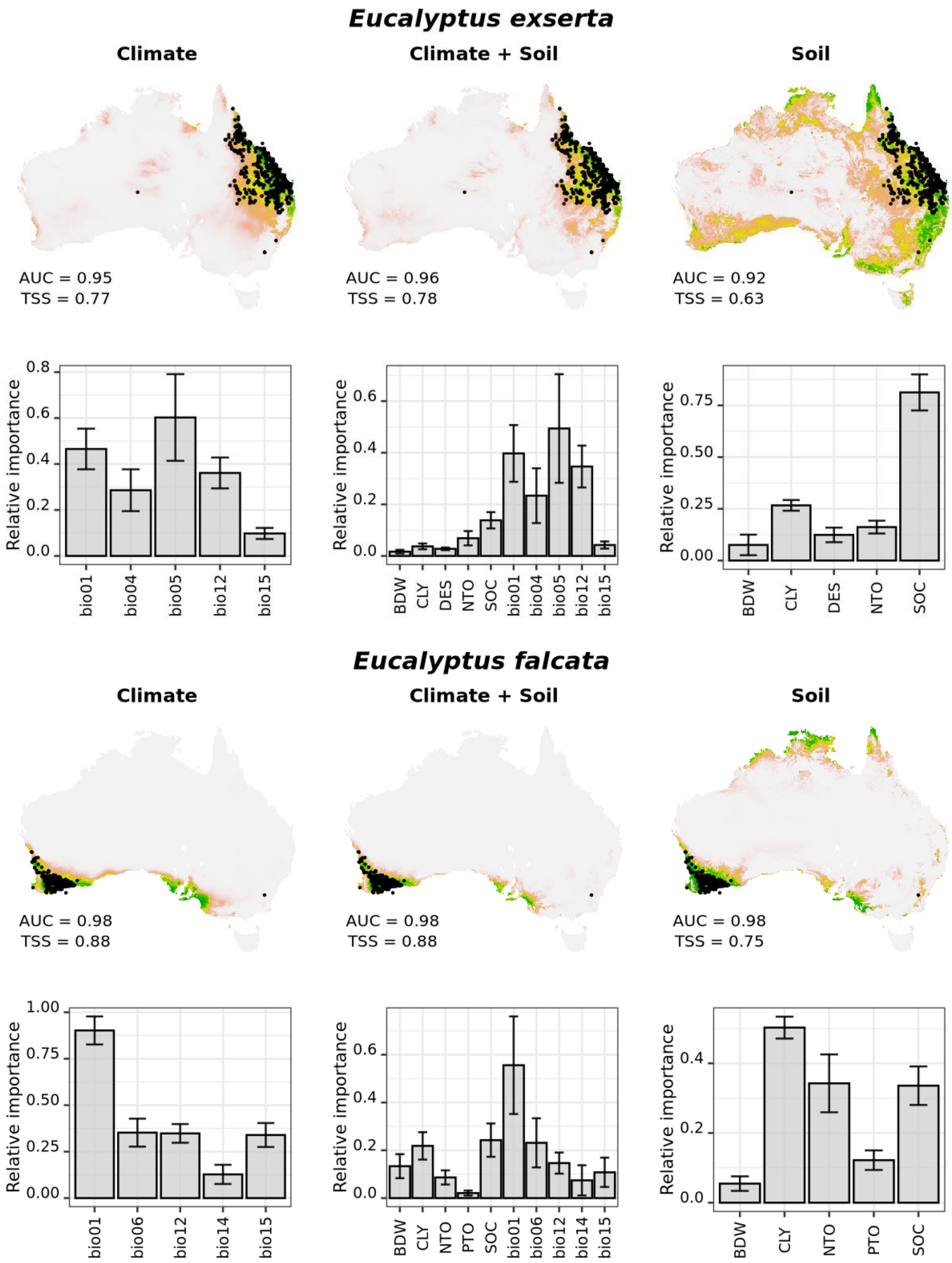

Eucalyptus exserta
Climate
Climate + Soil
Soil
AUC = 0.95
TSS = 0.77
AUC = 0.96
TSS = 0.78
AUC = 0.92
TSS = 0.63
Relative importance
bio01
bio04
bio05
bio12
bio15
BDW
CLY
DES
NTO
SOC
Eucalyptus falcata
Climate
Climate + Soil
Soil
AUC = 0.98
TSS = 0.88
AUC = 0.98
TSS = 0.88
AUC = 0.98
TSS = 0.75
Relative importance
bio01
bio06
bio12
bio14
bio15
BDW
CLY
NTO
PTO
SOC

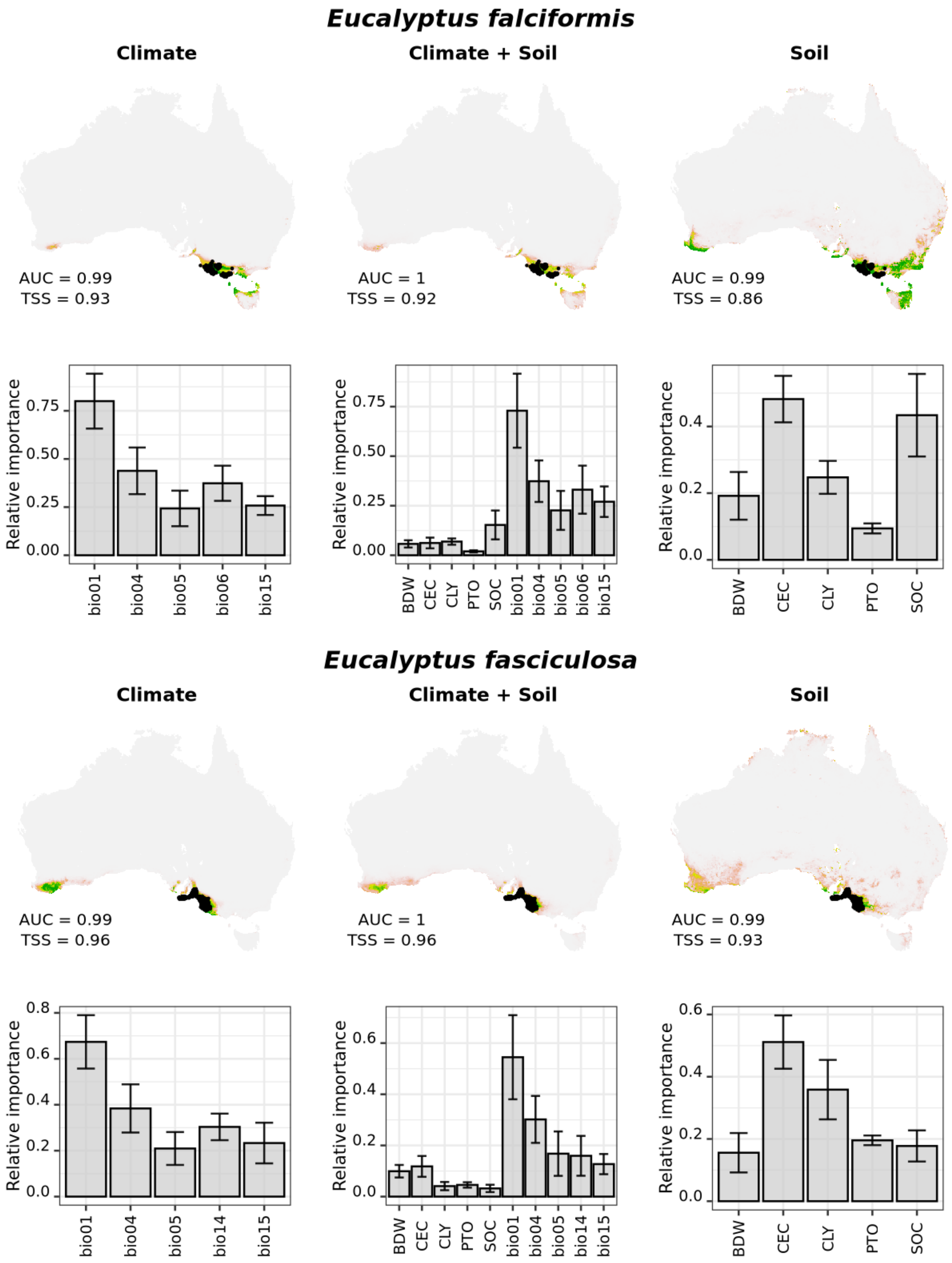
Eucalyptus falciformis
Climate
Climate + Soil
Soil
AUC = 0.99
TSS = 0.93
AUC = 1
TSS = 0.92
AUC = 0.99
TSS = 0.86
Relative importance
0.00
0.25
0.50
0.75
bio01
bio04
bio05
bio06
bio15
BDW
CEC
CLY
PTO
SOC
0.0
0.2
0.4
Eucalyptus fasciculosa
Climate
Climate + Soil
Soil
AUC = 0.99
TSS = 0.96
AUC = 1
TSS = 0.96
AUC = 0.99
TSS = 0.93
0.0
0.2
0.4
0.6
0.8
bio14

Eucalyptus fastigata

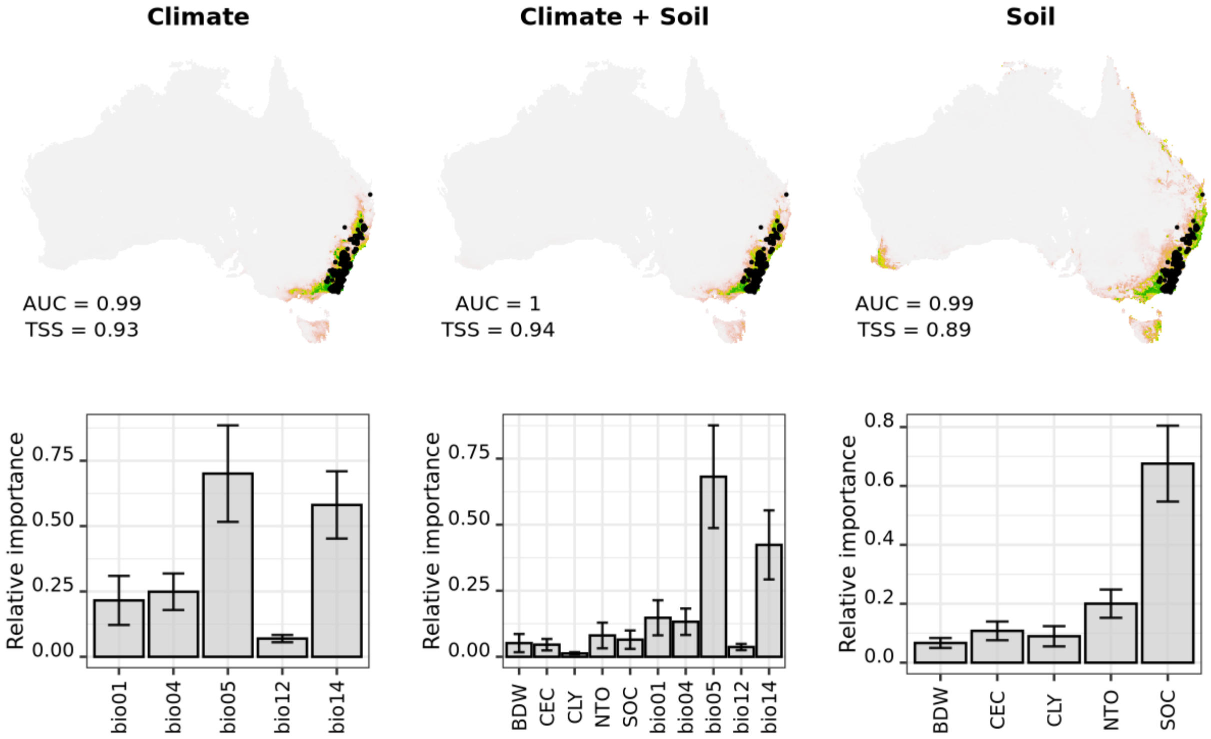

Eucalyptus flocktoniae

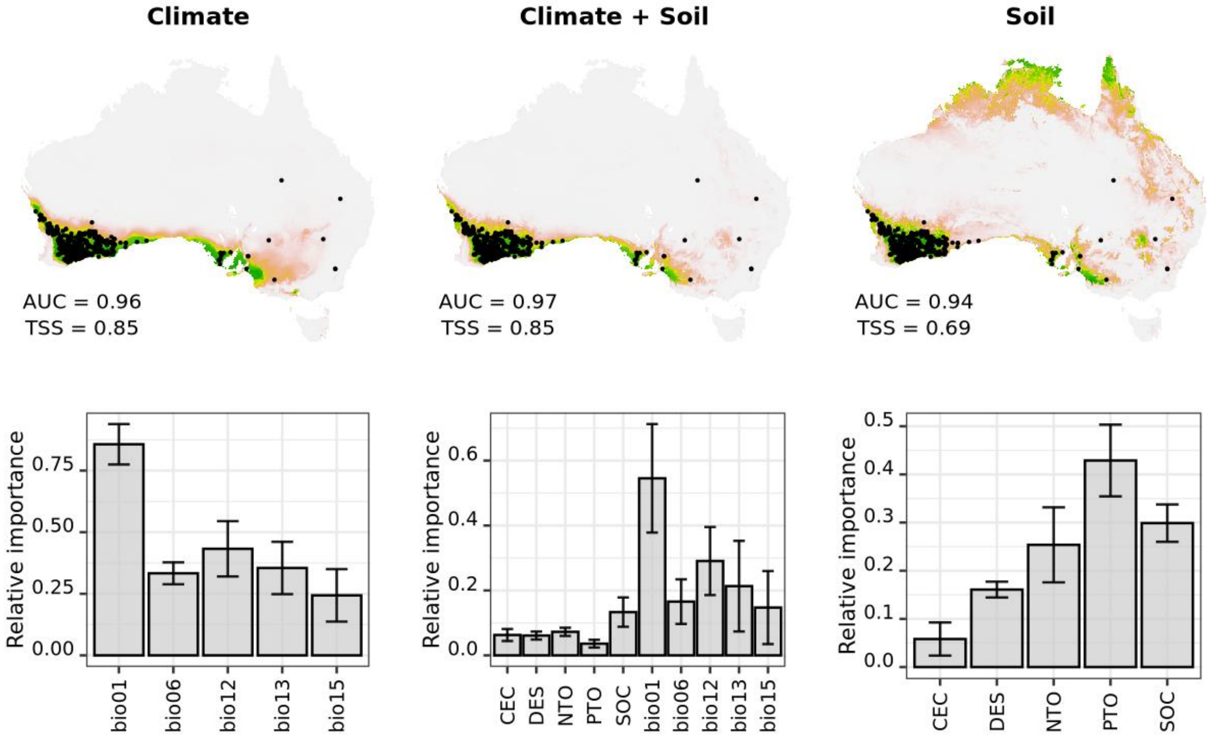

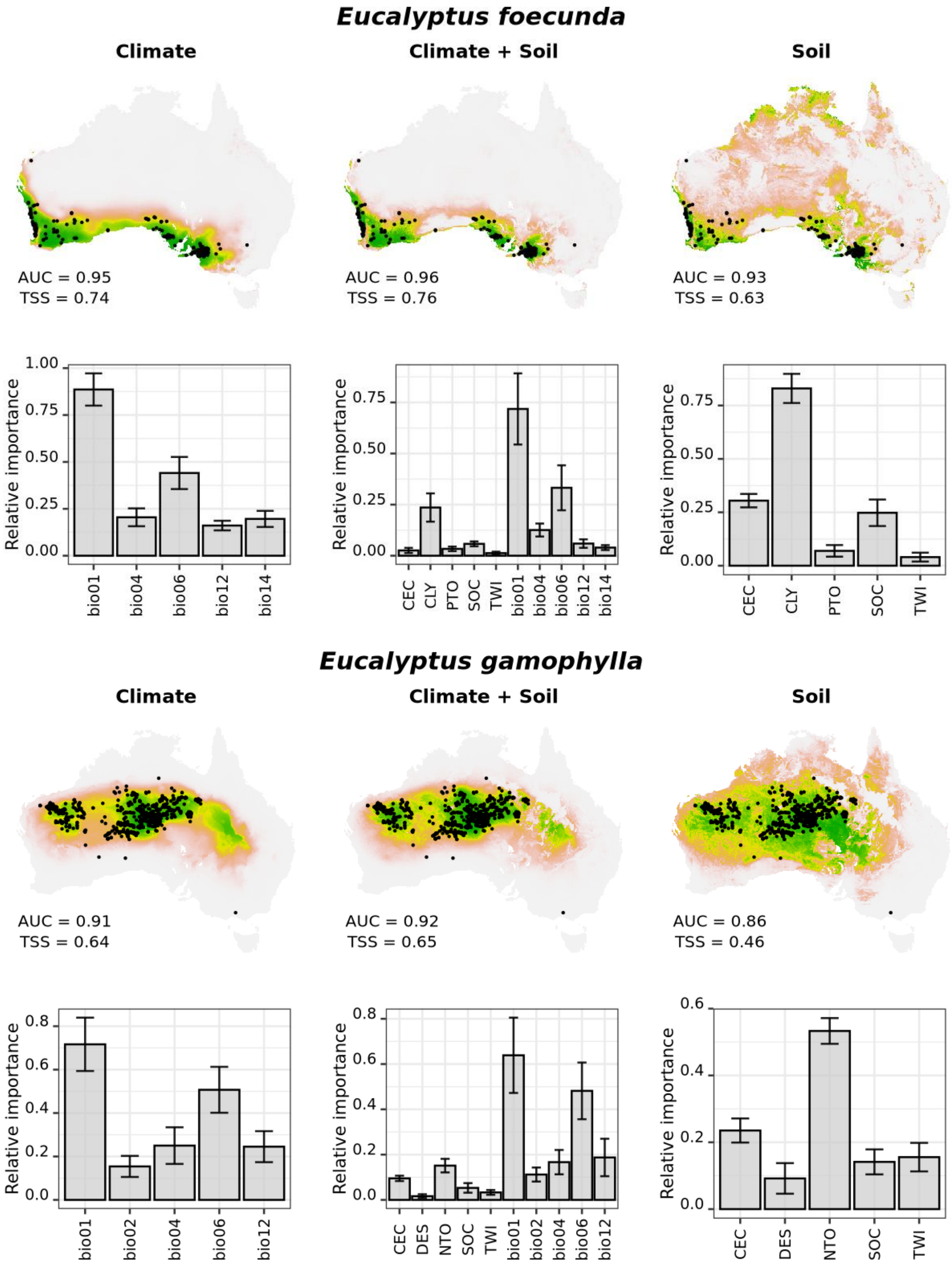

Eucalyptus foecunda
Climate
Climate + Soil
Soil
AUC = 0.95
TSS = 0.74
AUC = 0.96
TSS = 0.76
AUC = 0.93
TSS = 0.63
Relative importance
1.00
0.75
0.50
0.25
0.00
bio01
bio04
bio06
bio12
bio14
CEC
CLY
PTO
SOC
TWI
Eucalyptus gamophylla
Climate
Climate + Soil
Soil
AUC = 0.91
TSS = 0.64
AUC = 0.92
TSS = 0.65
AUC = 0.86
TSS = 0.46
Relative importance
0.8
0.6
0.4
0.2
0.0
bio01
bio02
bio04
bio06
bio12
CEC
DES
NTO
SOC
TWI

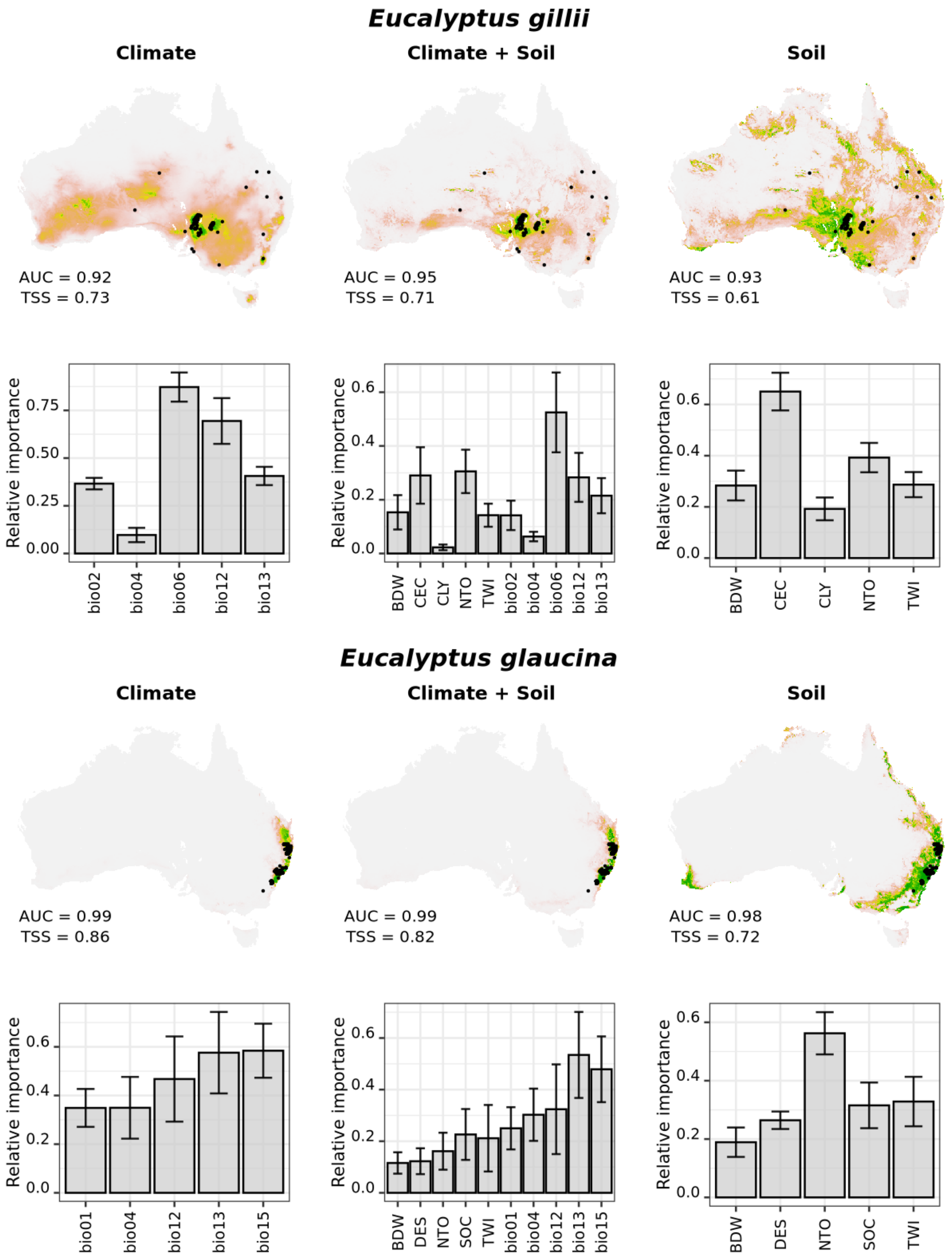

Eucalyptus gillii
Climate
Climate + Soil
Soil
AUC = 0.92
TSS = 0.73
AUC = 0.95
TSS = 0.71
AUC = 0.93
TSS = 0.61
Relative importance
0.00
0.25
0.50
0.75
bio02
bio04
bio06
bio12
bio13
0.0
0.2
0.4
0.6
BDW
CEC
CLY
NTO
TWI
bio02
bio04
bio06
bio12
bio13
BDW
CEC
CLY
NTO
TWI
Eucalyptus glaucina
Climate
Climate + Soil
Soil
AUC = 0.99
TSS = 0.86
AUC = 0.99
TSS = 0.82
AUC = 0.98
TSS = 0.72
Relative importance
bio01
bio04
bio12
bio13
bio15
BDW
DES
NTO
SOC
TWI
bio01
bio04
bio12
bio13
bio15
BDW
DES
NTO
SOC
TWI

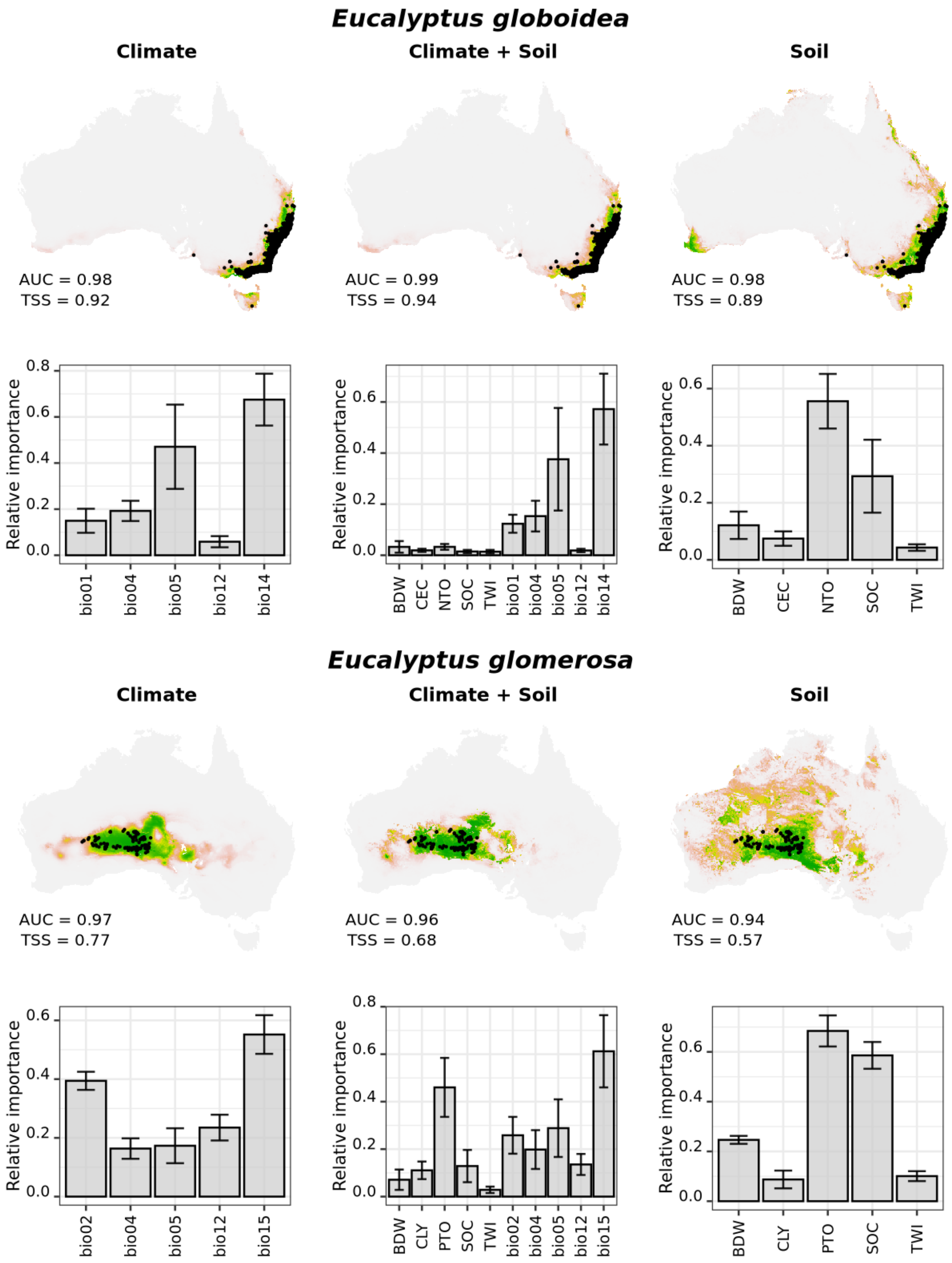
Eucalyptus globoidea
Climate
Climate + Soil
Soil
AUC = 0.98
TSS = 0.92
AUC = 0.99
TSS = 0.94
AUC = 0.98
TSS = 0.89
Relative importance
0.0
0.2
0.4
0.6
0.8
bio01
bio04
bio05
bio12
bio14
BDW
CEC
NTO
SOC
TWI
Eucalyptus glomerosa
AUC = 0.97
TSS = 0.77
AUC = 0.96
TSS = 0.68
AUC = 0.94
TSS = 0.57
bio02
bio15
CLY
PTO

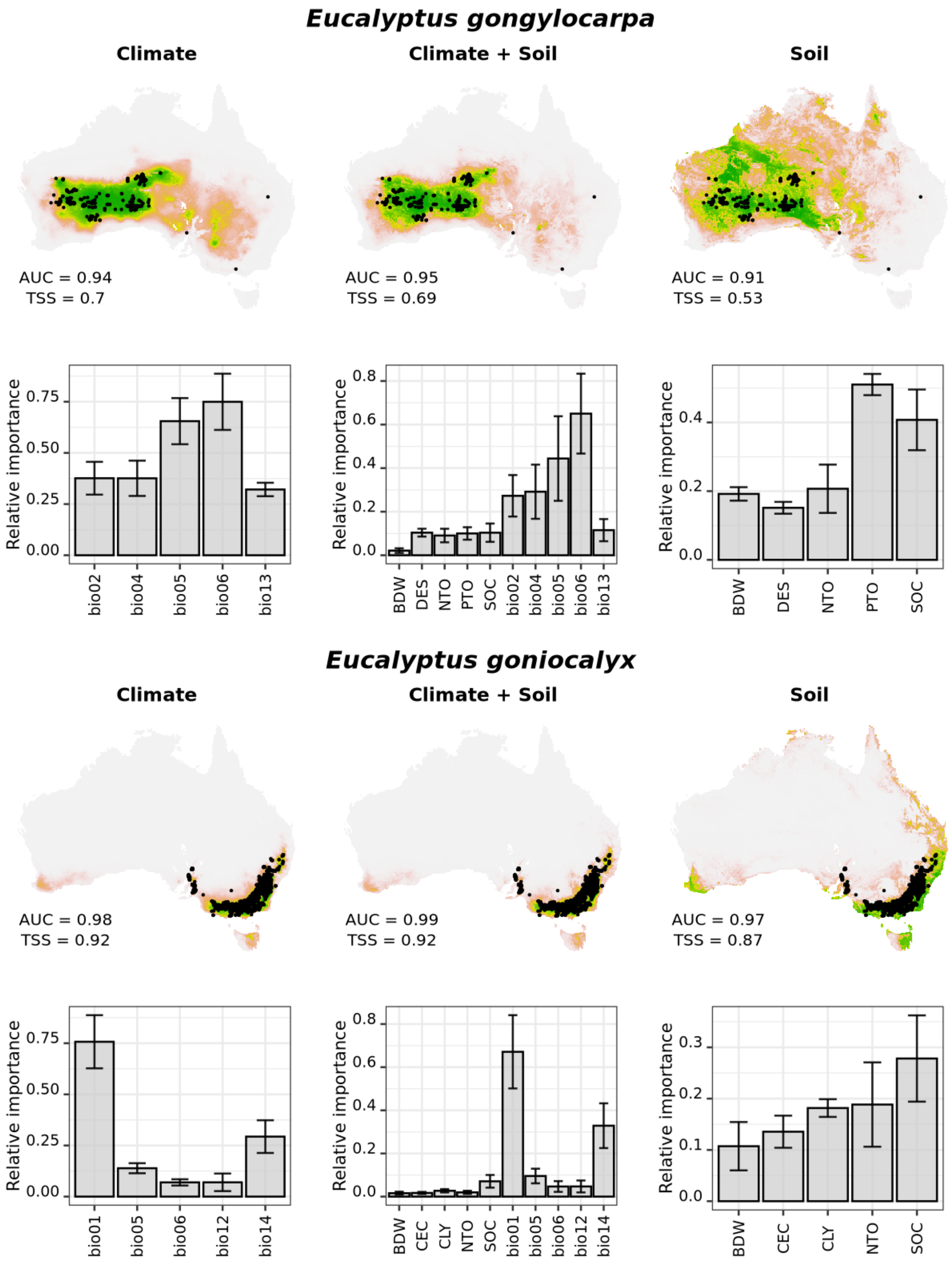
Eucalyptus gongylocarpa
Climate
Climate + Soil
Soil
AUC = 0.94
TSS = 0.7
AUC = 0.95
TSS = 0.69
AUC = 0.91
TSS = 0.53
Relative importance
bio02
bio04
bio05
bio06
bio13
BDW
DES
NTO
PTO
SOC
bio02
bio04
bio05
bio06
bio13
BDW
DES
NTO
PTO
SOC
Eucalyptus goniocalyx
Climate
Climate + Soil
Soil
AUC = 0.98
TSS = 0.92
AUC = 0.99
TSS = 0.92
AUC = 0.97
TSS = 0.87
Relative importance
bio01
bio05
bio06
bio12
bio14
BDW
CEC
CLY
NTO
SOC
bio01
bio05
bio06
bio12
bio14
BDW
CEC
CLY
NTO
SOC

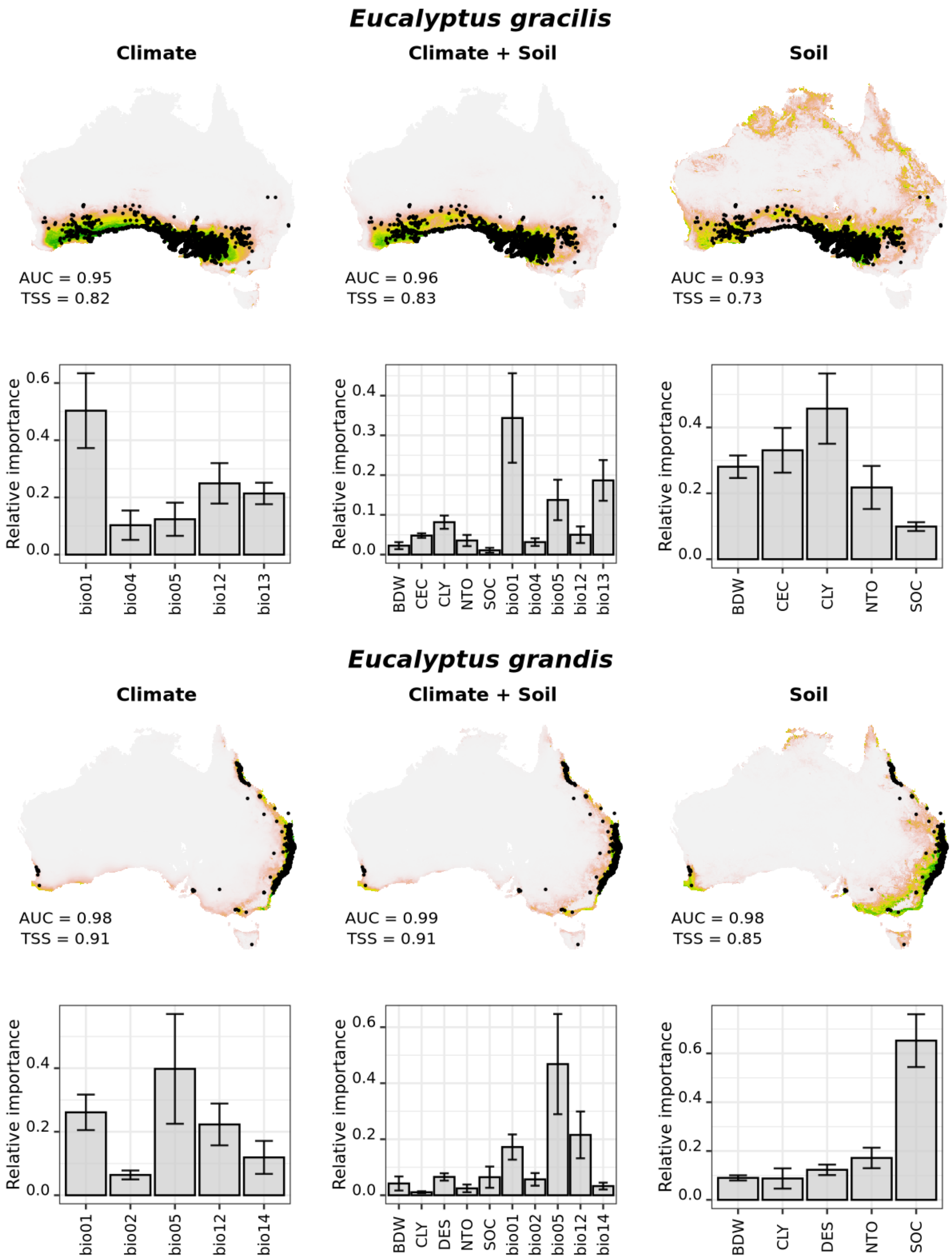
Eucalyptus gracilis
Climate
Climate + Soil
Soil
AUC = 0.95
TSS = 0.82
AUC = 0.96
TSS = 0.83
AUC = 0.93
TSS = 0.73
Relative importance
0.0 0.2 0.4 0.6
bio01 bio04 bio05 bio12 bio13
0.0 0.1 0.2 0.3 0.4
BDW CEC CLY NTO SOC bio01 bio04 bio05 bio12 bio13
0.0 0.2 0.4
BDW CEC CLY NTO SOC
Eucalyptus grandis
Climate
Climate + Soil
Soil
AUC = 0.98
TSS = 0.91
AUC = 0.99
TSS = 0.91
AUC = 0.98
TSS = 0.85
Relative importance
0.0 0.2 0.4
bio01 bio02 bio05 bio12 bio14
0.0 0.2 0.4 0.6
BDW CLY DES NTO SOC bio01 bio02 bio05 bio12 bio14
0.0 0.2 0.4 0.6
BDW CLY DES NTO SOC

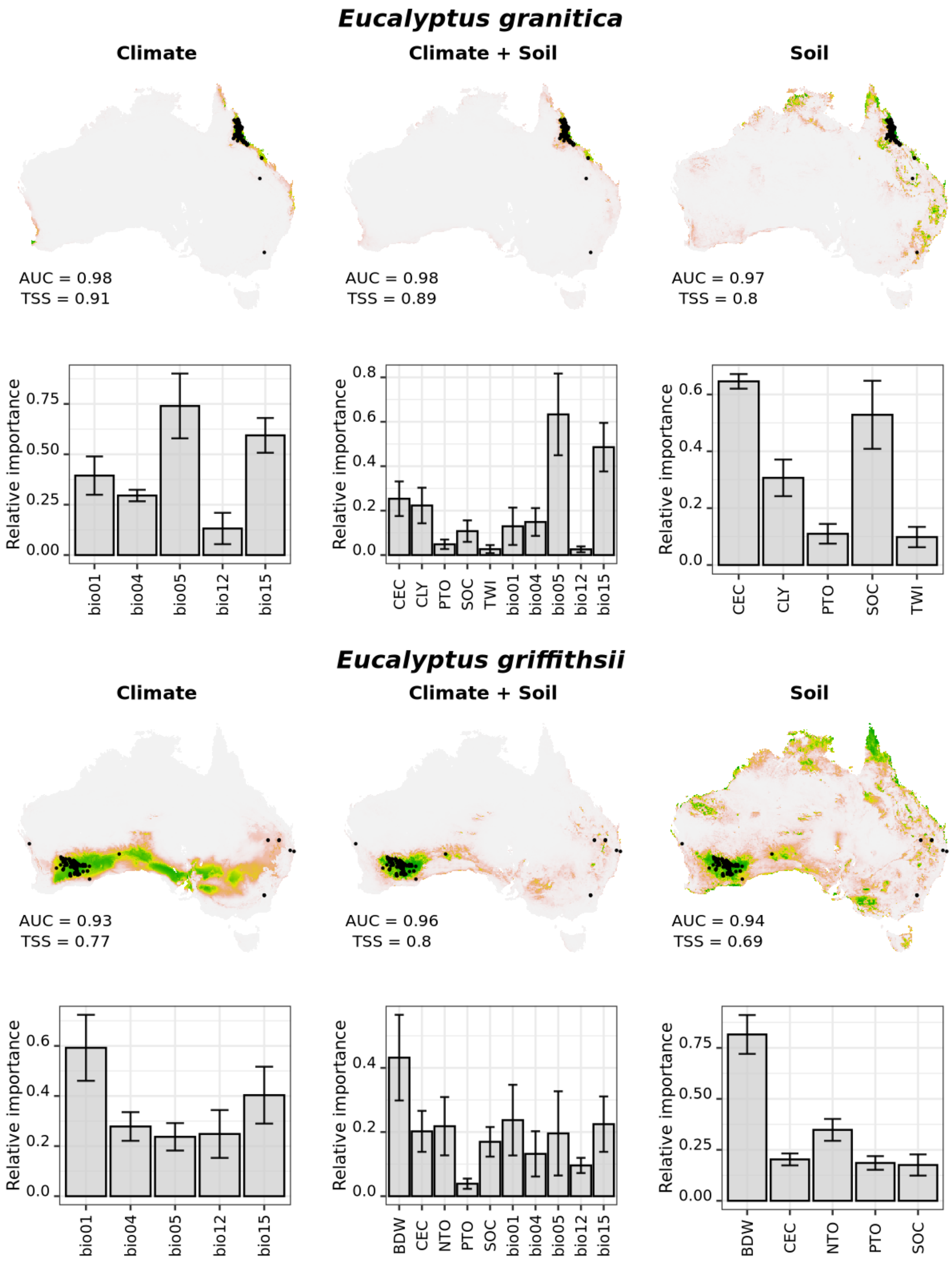

Eucalyptus granitica
Climate
Climate + Soil
Soil
AUC = 0.98
TSS = 0.91
AUC = 0.98
TSS = 0.89
AUC = 0.97
TSS = 0.8
Relative importance
0.00
0.25
0.50
0.75
bio01
bio04
bio05
bio12
bio15
0.0
0.2
0.4
0.6
0.8
CEC
CLY
PTO
SOC
TWI
bio01
bio04
bio05
bio12
bio15
0.0
0.2
0.4
0.6
CEC
CLY
PTO
SOC
TWI
Eucalyptus griffithsii
Climate
Climate + Soil
Soil
AUC = 0.93
TSS = 0.77
AUC = 0.96
TSS = 0.8
AUC = 0.94
TSS = 0.69
Relative importance
0.0
0.2
0.4
0.6
bio01
bio04
bio05
bio12
bio15
0.0
0.2
0.4
BDW
CEC
NTO
PTO
SOC
bio01
bio04
bio05
bio12
bio15
0.00
0.25
0.50
0.75
BDW
CEC
NTO
PTO
SOC

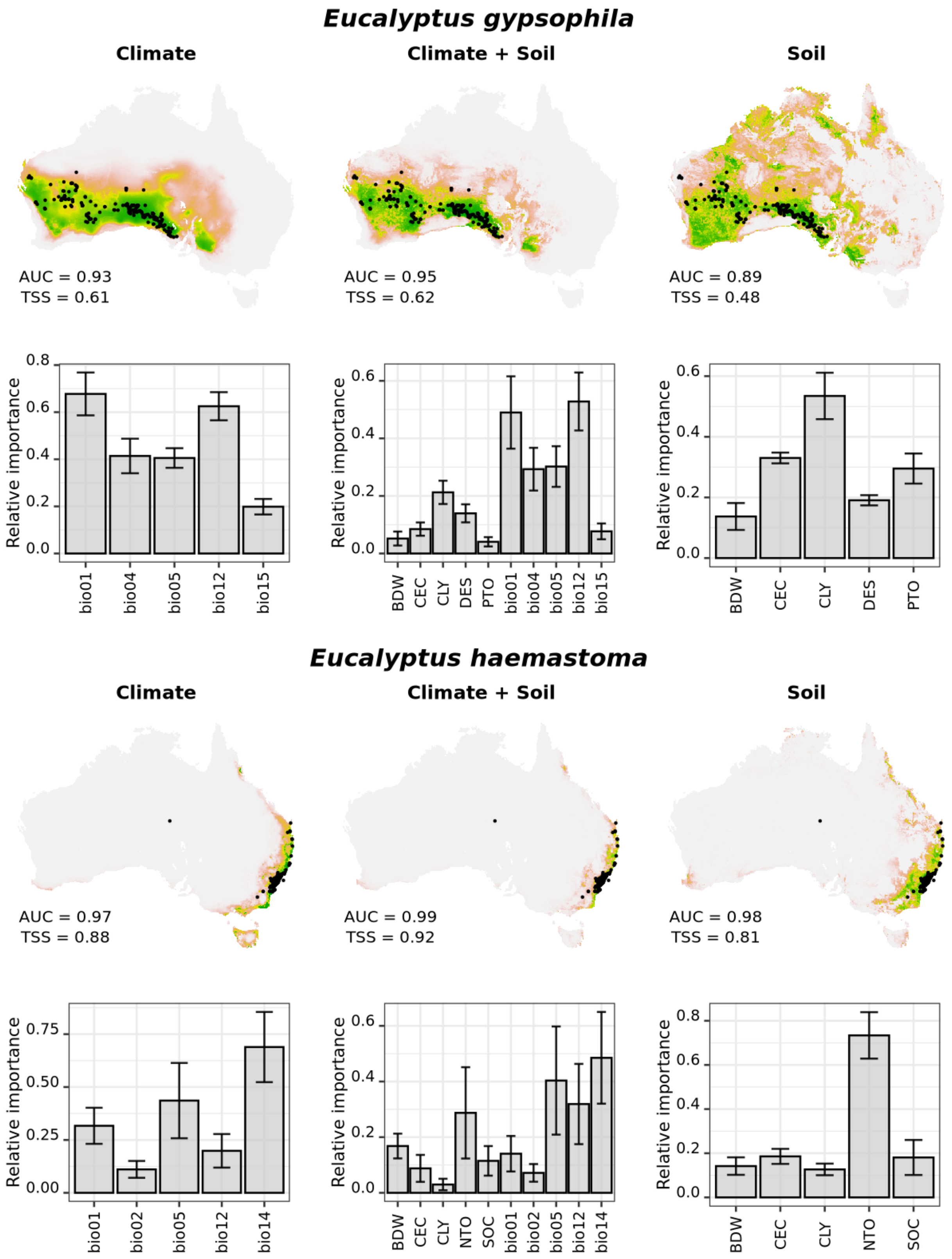

Eucalyptus gypsophila
Climate
Climate + Soil
Soil
AUC = 0.93
TSS = 0.61
AUC = 0.95
TSS = 0.62
AUC = 0.89
TSS = 0.48
Relative importance
0.0
0.2
0.4
0.6
0.8
bio01
bio04
bio05
bio12
bio15
BDW
CEC
CLY
DES
PTO
Eucalyptus haemastoma
AUC = 0.97
TSS = 0.88
AUC = 0.99
TSS = 0.92
AUC = 0.98
TSS = 0.81
0.00
0.25
0.50
0.75
bio01
bio02
bio05
bio12
bio14
BDW
CEC
CLY
NTO
SOC

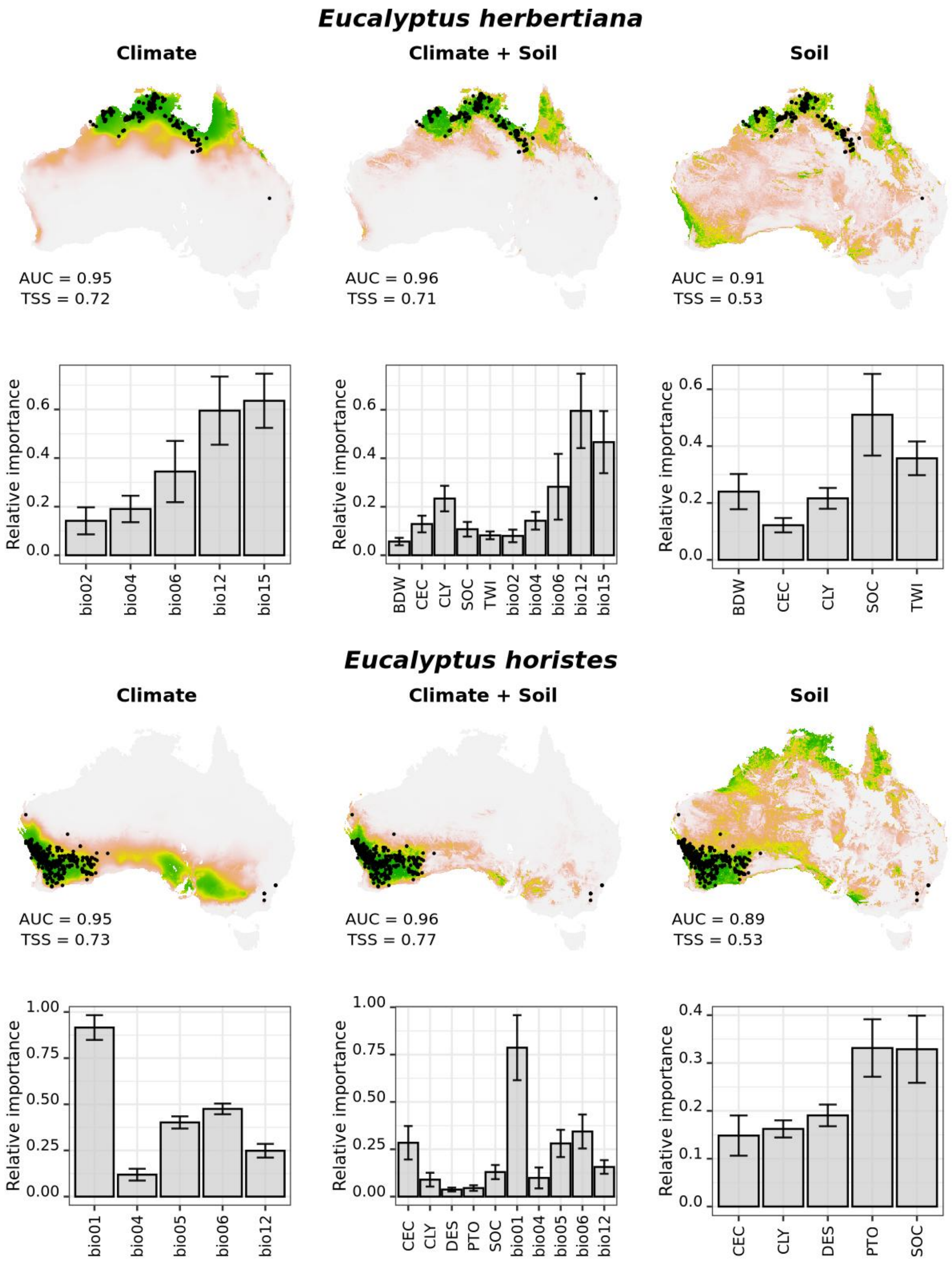
Eucalyptus herbertiana
Climate
Climate + Soil
Soil
AUC = 0.95
TSS = 0.72
AUC = 0.96
TSS = 0.71
AUC = 0.91
TSS = 0.53
Relative importance
bio02
bio04
bio06
bio12
bio15
BDW
CEC
CLY
SOC
TWI
Eucalyptus horistes
Climate
Climate + Soil
Soil
AUC = 0.95
TSS = 0.73
AUC = 0.96
TSS = 0.77
AUC = 0.89
TSS = 0.53
Relative importance
bio01
bio04
bio05
bio06
bio12
CEC
CLY
DES
PTO
SOC

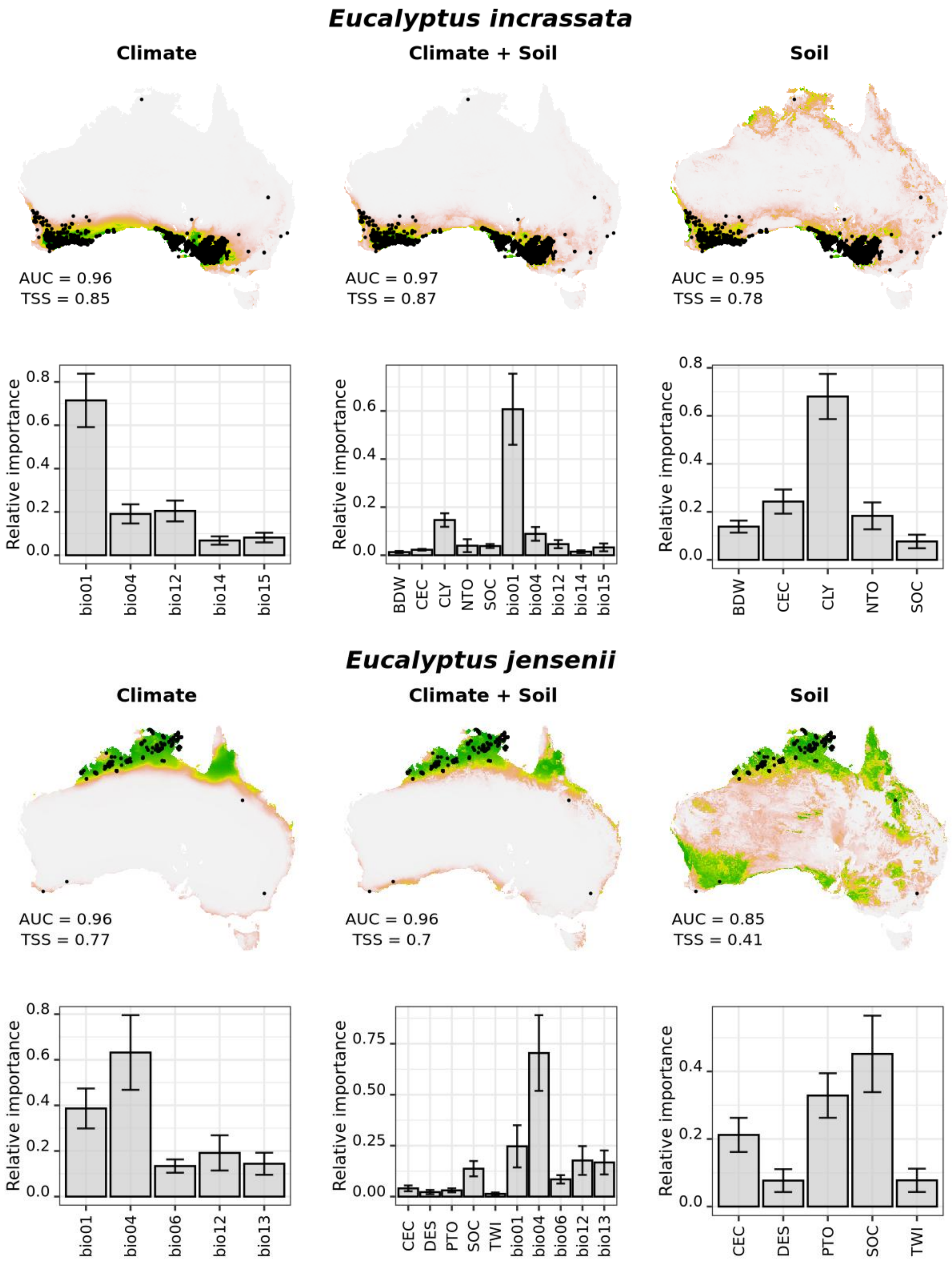

Eucalyptus incrassata
Climate
Climate + Soil
Soil
AUC = 0.96
TSS = 0.85
AUC = 0.97
TSS = 0.87
AUC = 0.95
TSS = 0.78
Relative importance
bio01
bio04
bio12
bio14
bio15
BDW
CEC
CLY
NTO
SOC
Eucalyptus jensenii
AUC = 0.96
TSS = 0.77
AUC = 0.96
TSS = 0.7
AUC = 0.85
TSS = 0.41
bio06
bio13
DES
PTO
TWI

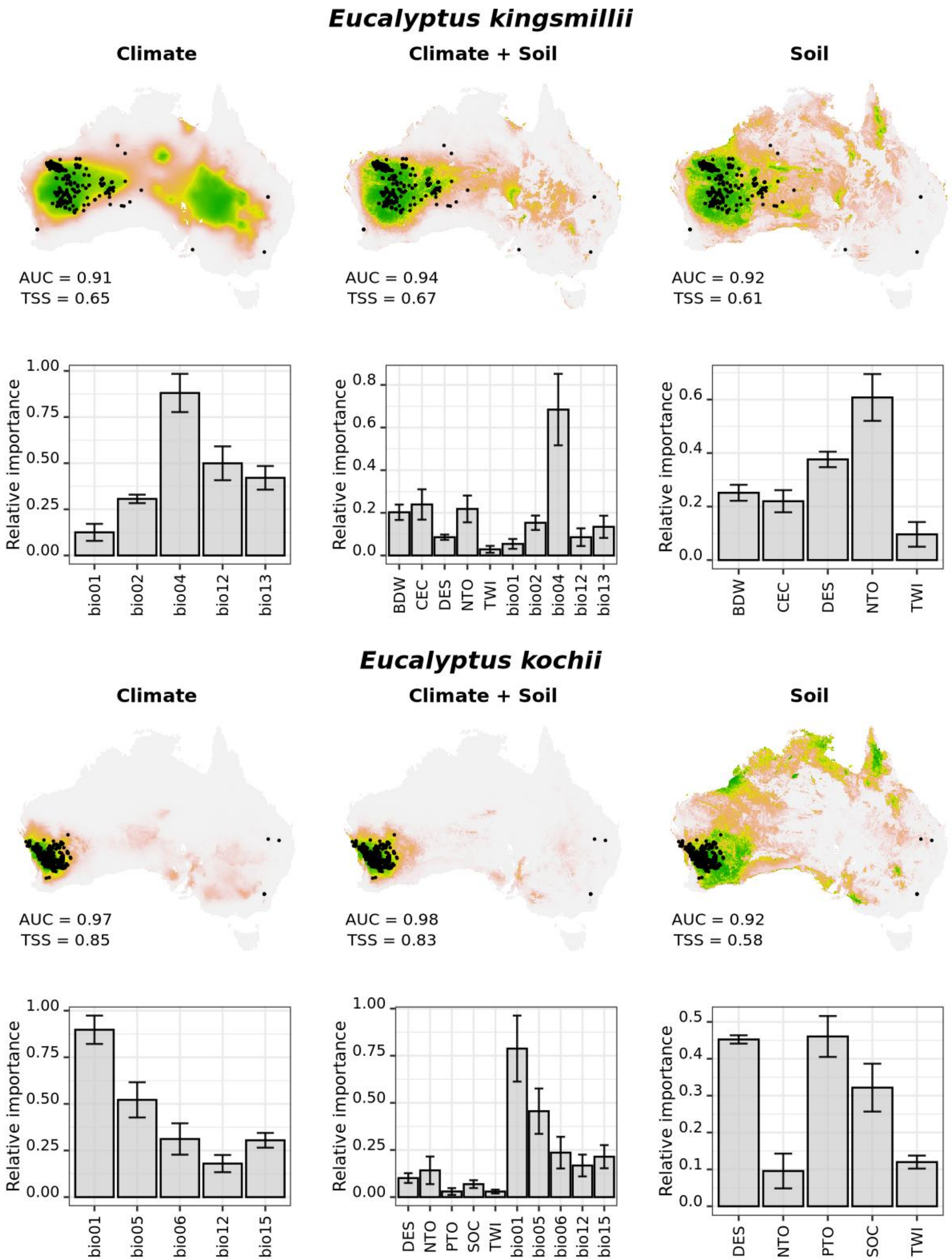
Eucalyptus kingsmillii
Climate
Climate + Soil
Soil
AUC = 0.91
TSS = 0.65
AUC = 0.94
TSS = 0.67
AUC = 0.92
TSS = 0.61
Relative importance
1.00
0.75
0.50
0.25
0.00
bio01
bio02
bio04
bio12
bio13
0.8
0.6
0.4
0.2
0.0
BDW
CEC
DES
NTO
TWI
bio01
bio02
bio04
bio12
bio13
0.6
0.4
0.2
0.0
BDW
CEC
DES
NTO
TWI
Eucalyptus kochii
Climate
Climate + Soil
Soil
AUC = 0.97
TSS = 0.85
AUC = 0.98
TSS = 0.83
AUC = 0.92
TSS = 0.58
Relative importance
1.00
0.75
0.50
0.25
0.00
bio01
bio05
bio06
bio12
bio15
DES
NTO
PTO
SOC
TWI
bio01
bio05
bio06
bio12
bio15
0.5
0.4
0.3
0.2
0.1
0.0
DES
NTO
PTO
SOC
TWI

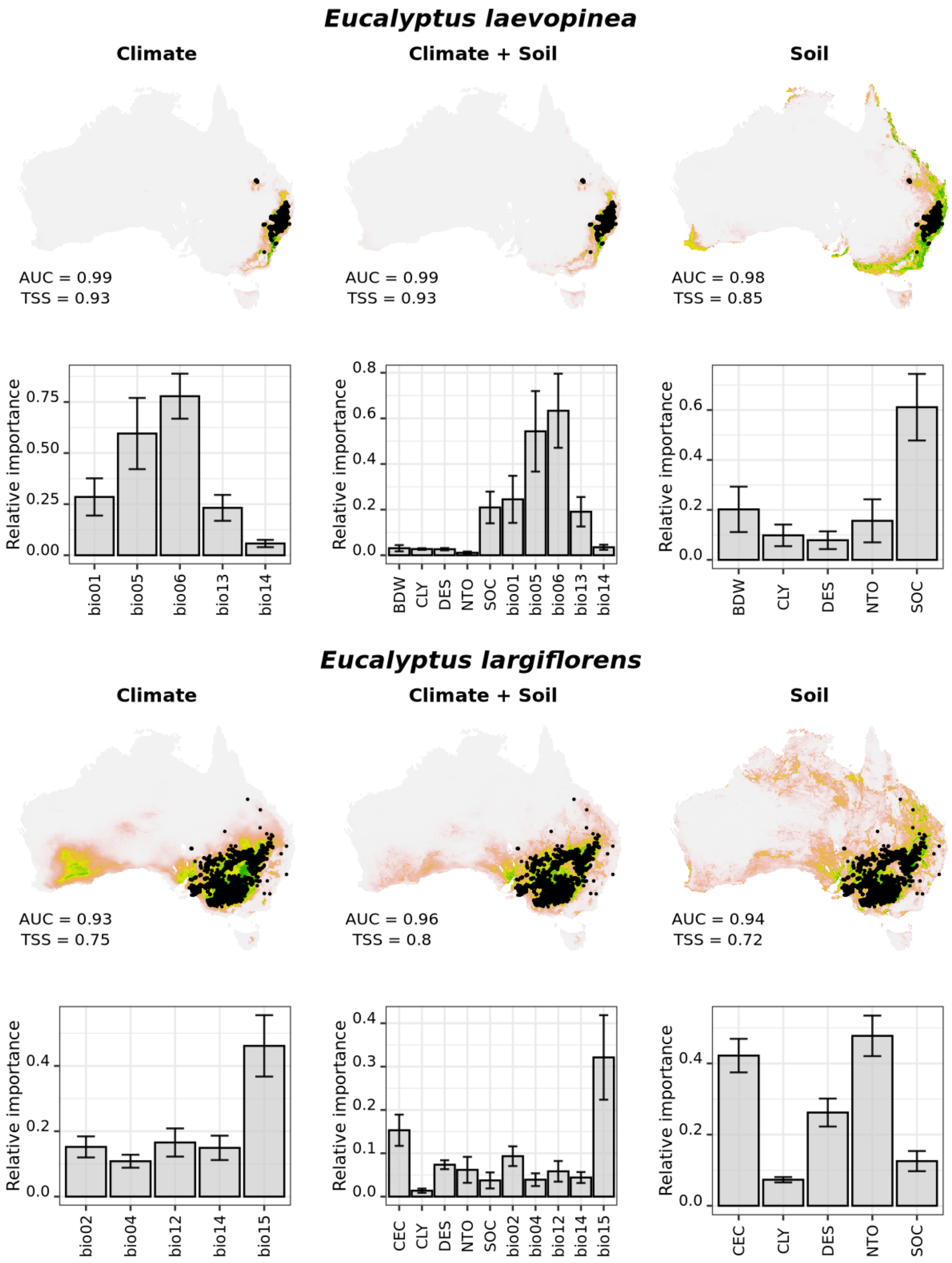
Eucalyptus laevopinea
Climate
Climate + Soil
Soil
AUC = 0.99
TSS = 0.93
AUC = 0.99
TSS = 0.93
AUC = 0.98
TSS = 0.85
Relative importance
0.00
0.25
0.50
0.75
bio01
bio05
bio06
bio13
bio14
0.0
0.2
0.4
0.6
0.8
BDW
CLY
DES
NTO
SOC
bio01
bio05
bio06
bio13
bio14
0.0
0.2
0.4
0.6
BDW
CLY
DES
NTO
SOC
Eucalyptus largiflorens
Climate
Climate + Soil
Soil
AUC = 0.93
TSS = 0.75
AUC = 0.96
TSS = 0.8
AUC = 0.94
TSS = 0.72
Relative importance
0.0
0.2
0.4
bio02
bio04
bio12
bio14
bio15
0.0
0.1
0.2
0.3
0.4
CEC
CLY
DES
NTO
SOC
bio02
bio04
bio12
bio14
bio15
0.0
0.2
0.4
CEC
CLY
DES
NTO
SOC

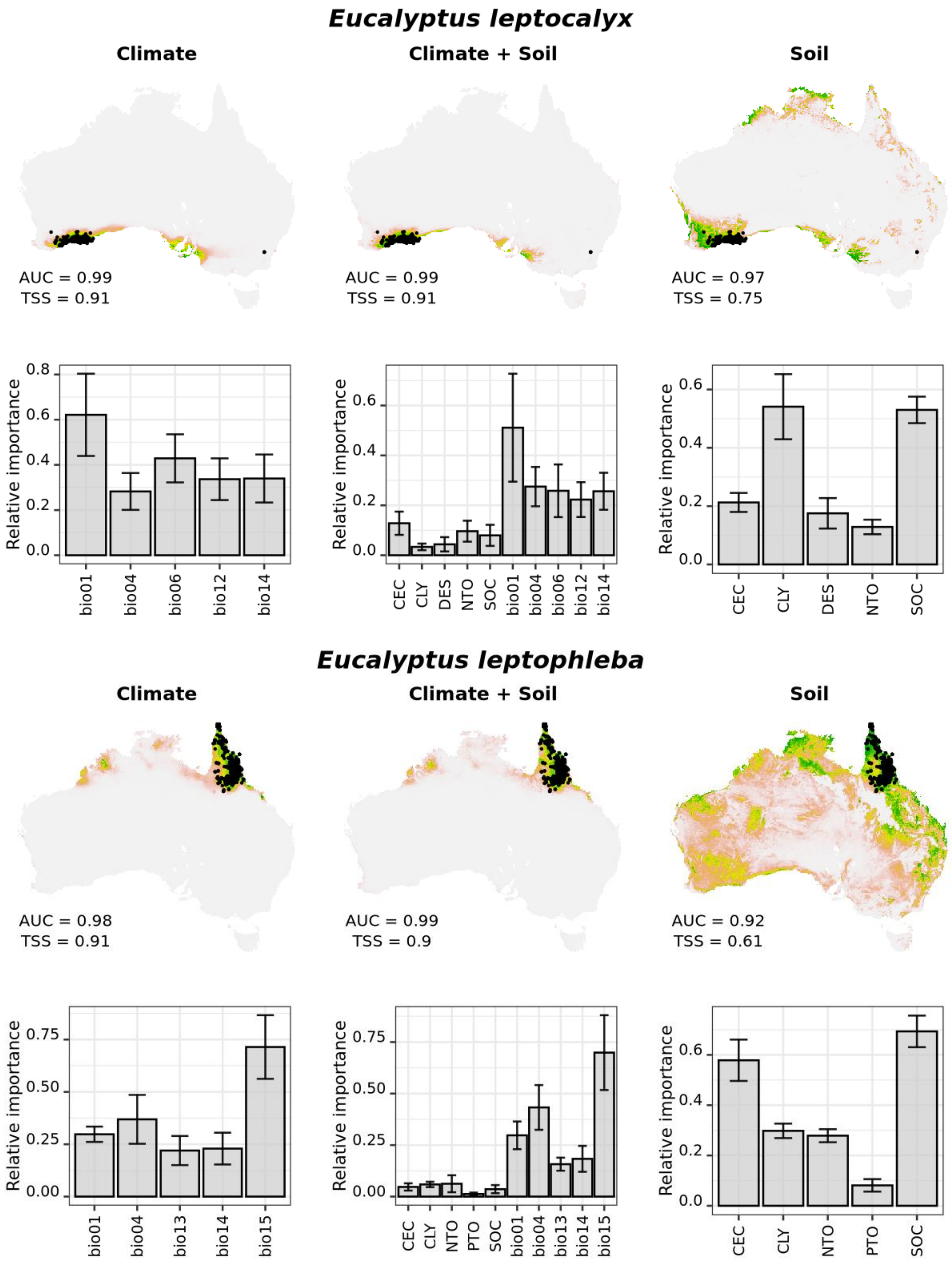
Eucalyptus leptocalyx
Climate
Climate + Soil
Soil
AUC = 0.99
TSS = 0.91
AUC = 0.99
TSS = 0.91
AUC = 0.97
TSS = 0.75
Relative importance
bio01
bio04
bio06
bio12
bio14
CEC
CLY
DES
NTO
SOC
Eucalyptus leptophleba
Climate
Climate + Soil
Soil
AUC = 0.98
TSS = 0.91
AUC = 0.99
TSS = 0.9
AUC = 0.92
TSS = 0.61
Relative importance
bio01
bio04
bio13
bio14
bio15
CEC
CLY
NTO
PTO
SOC

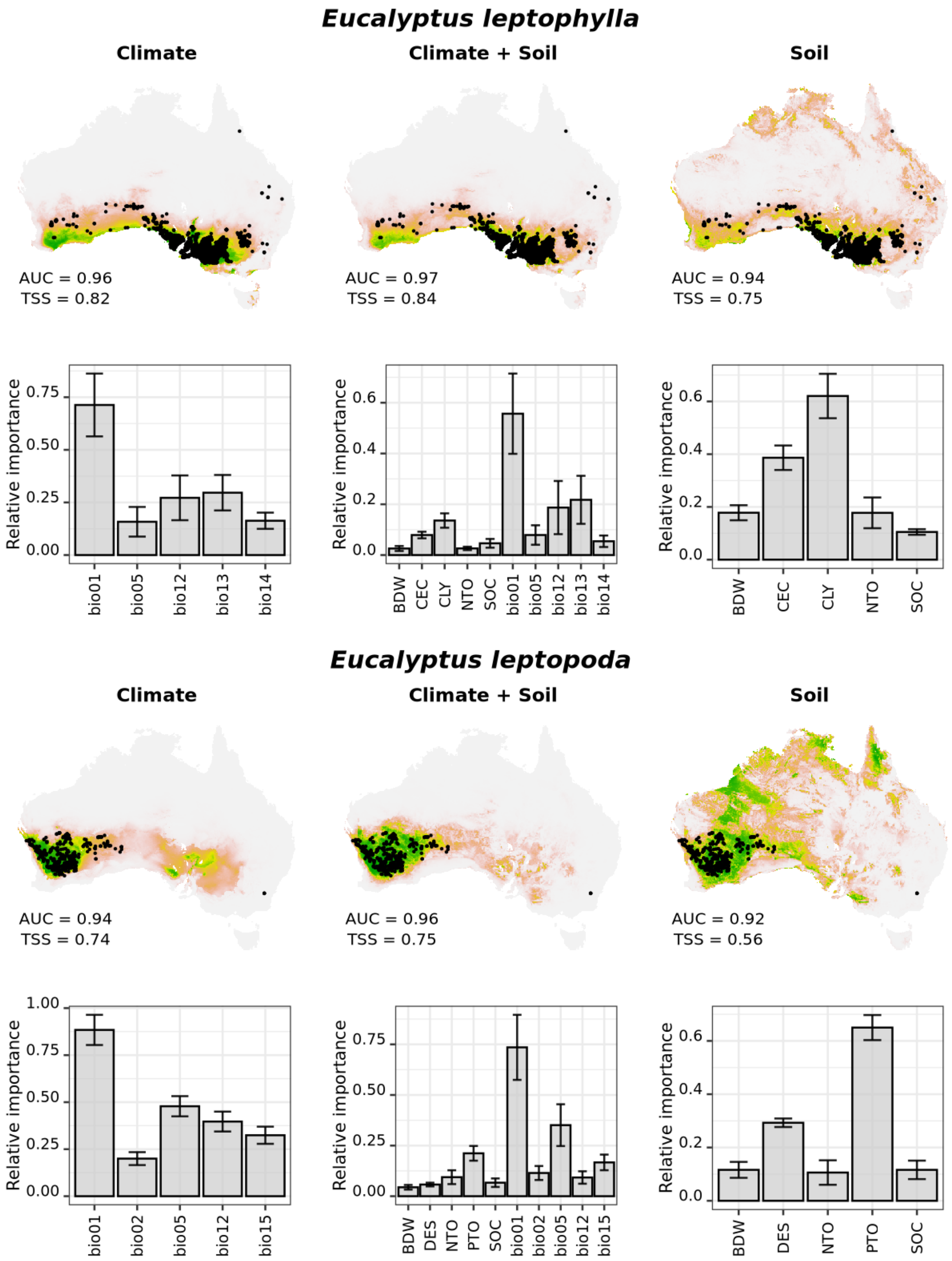

Eucalyptus leptophylla
Climate
Climate + Soil
Soil
AUC = 0.96
TSS = 0.82
AUC = 0.97
TSS = 0.84
AUC = 0.94
TSS = 0.75
Relative importance
bio01 bio05 bio12 bio13 bio14
BDW CEC CLY NTO SOC bio01 bio05 bio12 bio13 bio14
BDW CEC CLY NTO SOC
Eucalyptus leptopoda
Climate
Climate + Soil
Soil
AUC = 0.94
TSS = 0.74
AUC = 0.96
TSS = 0.75
AUC = 0.92
TSS = 0.56
Relative importance
bio01 bio02 bio05 bio12 bio15
BDW DES NTO PTO SOC bio01 bio02 bio05 bio12 bio15
BDW DES NTO PTO SOC

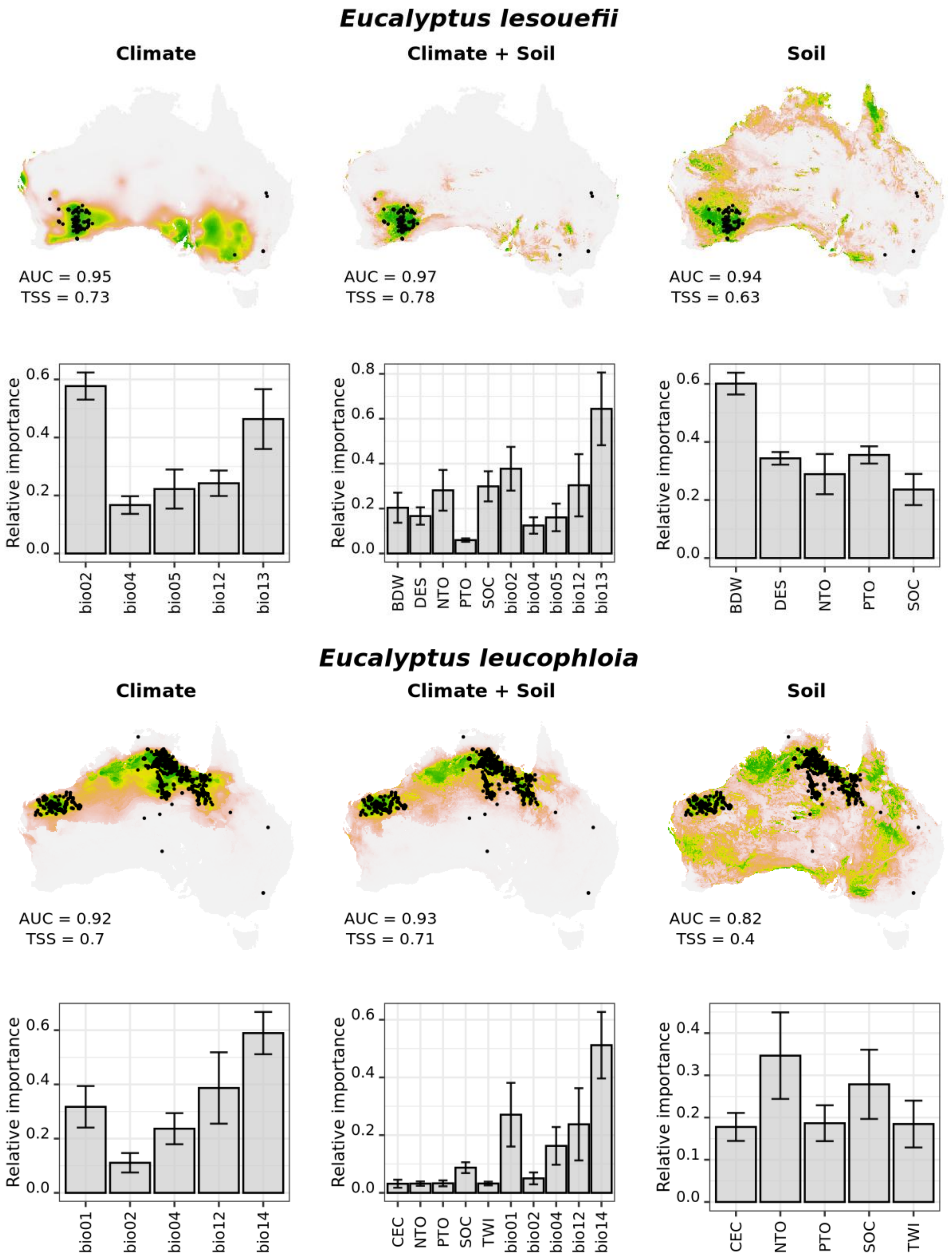
Eucalyptus lesouefii
Climate
Climate + Soil
Soil
AUC = 0.95
TSS = 0.73
AUC = 0.97
TSS = 0.78
AUC = 0.94
TSS = 0.63
Relative importance
0.0
0.2
0.4
0.6
0.8
bio02
bio04
bio05
bio12
bio13
BDW
DES
NTO
PTO
SOC
Eucalyptus leucophloia
Climate
Climate + Soil
Soil
AUC = 0.92
TSS = 0.7
AUC = 0.93
TSS = 0.71
AUC = 0.82
TSS = 0.4
Relative importance
0.0
0.1
0.2
0.3
0.4
0.6
bio01
bio02
bio04
bio12
bio14
CEC
NTO
PTO
SOC
TWI

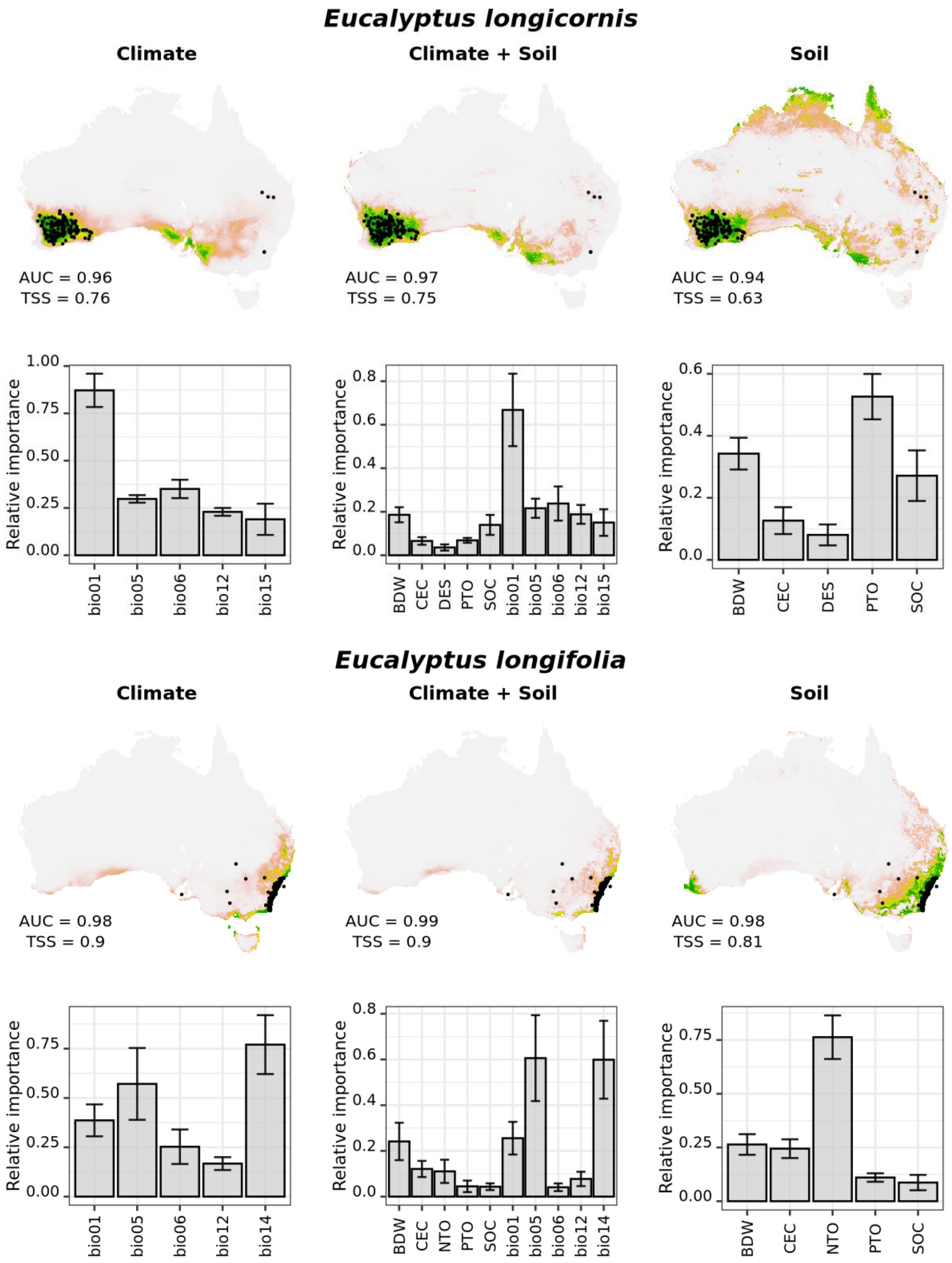

Eucalyptus longicornis
Climate
Climate + Soil
Soil
AUC = 0.96
TSS = 0.76
AUC = 0.97
TSS = 0.75
AUC = 0.94
TSS = 0.63
Relative importance
bio01
bio05
bio06
bio12
bio15
BDW
CEC
DES
PTO
SOC
Eucalyptus longifolia
Climate
Climate + Soil
Soil
AUC = 0.98
TSS = 0.9
AUC = 0.99
TSS = 0.9
AUC = 0.98
TSS = 0.81
Relative importance
bio01
bio05
bio06
bio12
bio14
BDW
CEC
NTO
PTO
SOC

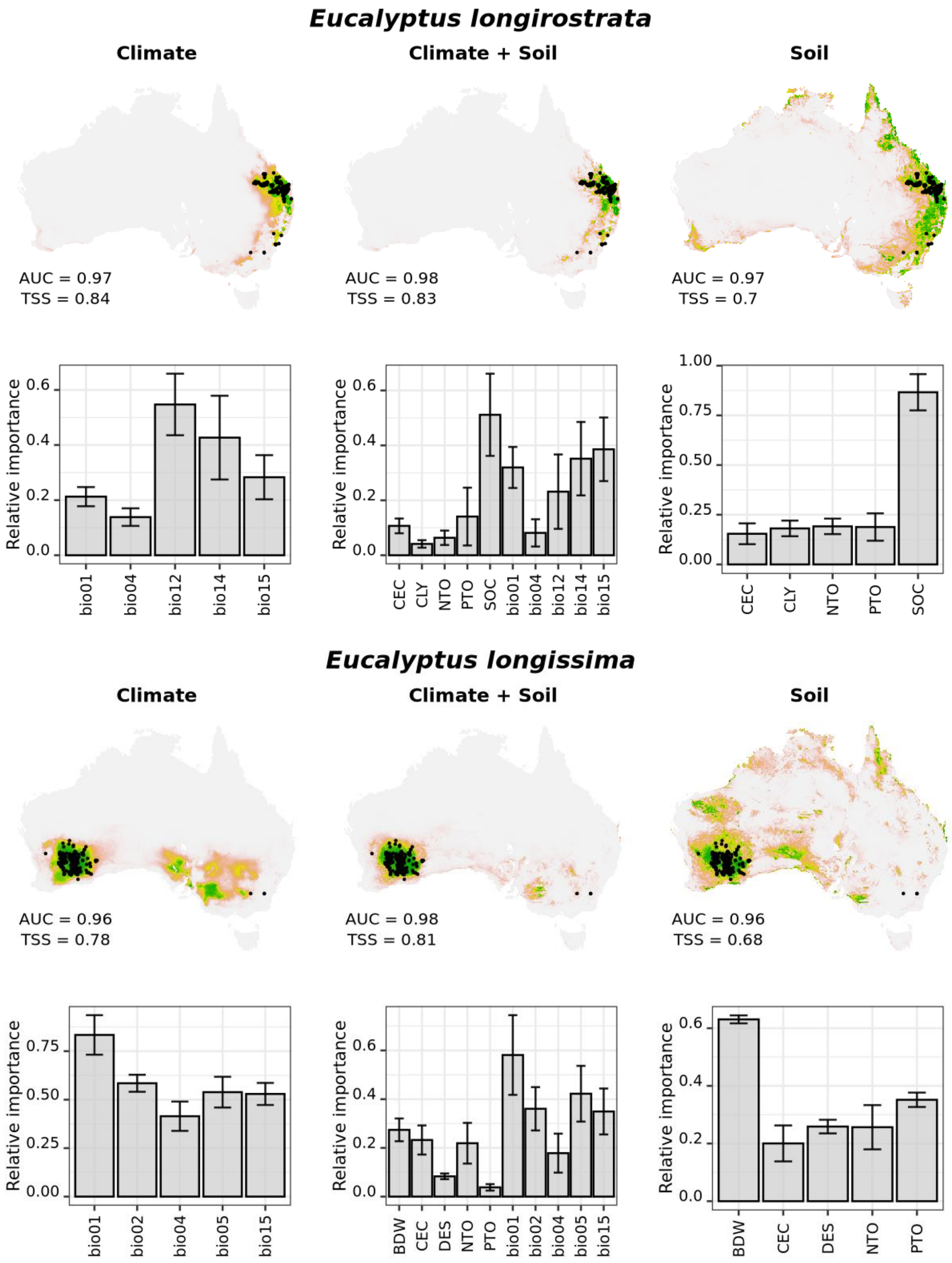
Eucalyptus longirostrata
Climate
Climate + Soil
Soil
AUC = 0.97
TSS = 0.84
AUC = 0.98
TSS = 0.83
AUC = 0.97
TSS = 0.7
Relative importance
bio01 bio04 bio12 bio14 bio15
CEC CLY NTO PTO SOC bio01 bio04 bio12 bio14 bio15
CEC CLY NTO PTO SOC
Eucalyptus longissima
Climate
Climate + Soil
Soil
AUC = 0.96
TSS = 0.78
AUC = 0.98
TSS = 0.81
AUC = 0.96
TSS = 0.68
Relative importance
bio01 bio02 bio04 bio05 bio15
BDW CEC DES NTO PTO bio01 bio02 bio04 bio05 bio15
BDW CEC DES NTO PTO

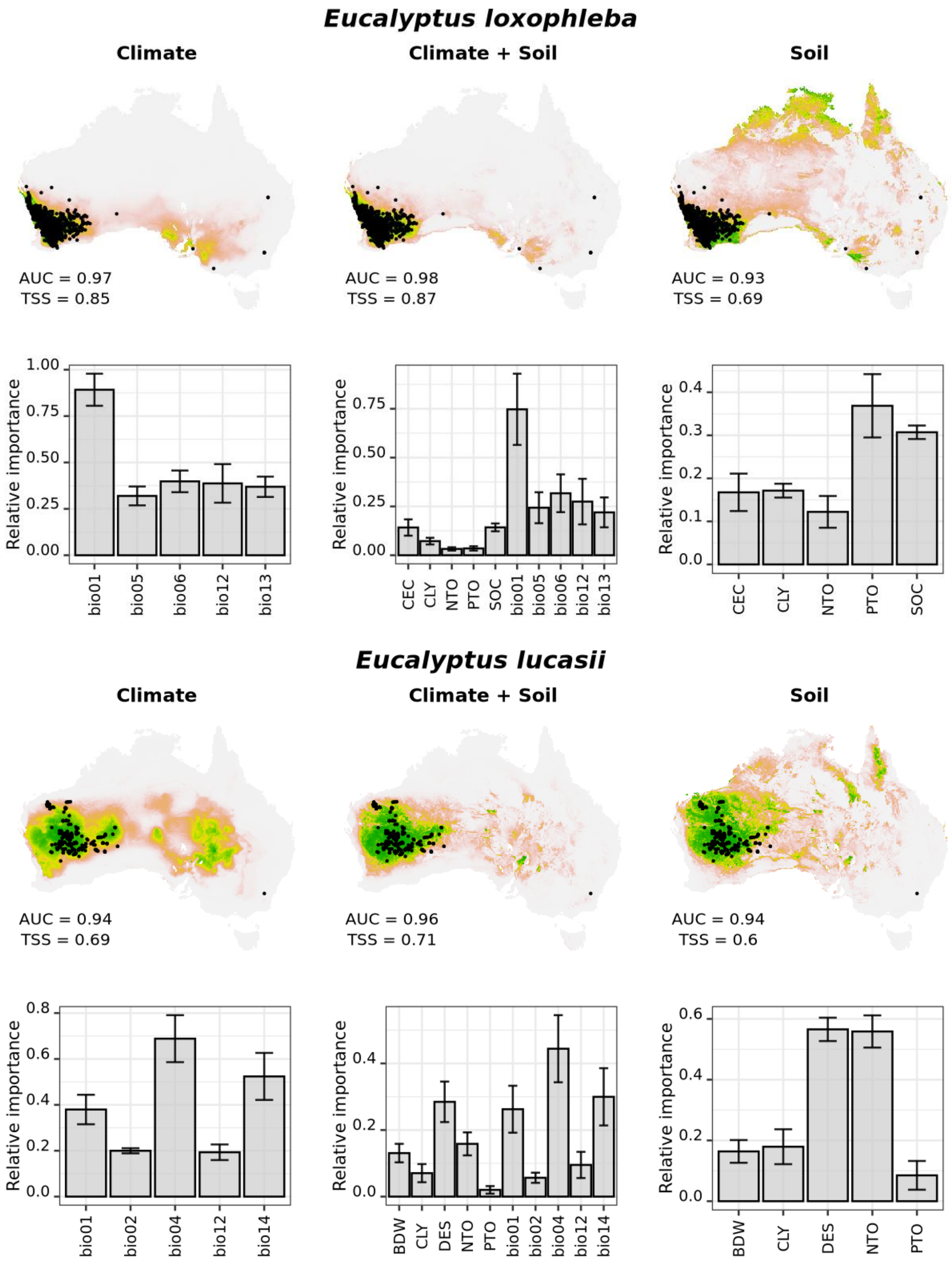
Eucalyptus loxophleba
Climate
Climate + Soil
Soil
AUC = 0.97
TSS = 0.85
AUC = 0.98
TSS = 0.87
AUC = 0.93
TSS = 0.69
Relative importance
bio01
bio05
bio06
bio12
bio13
CEC
CLY
NTO
PTO
SOC
Eucalyptus lucasii
Climate
Climate + Soil
Soil
AUC = 0.94
TSS = 0.69
AUC = 0.96
TSS = 0.71
AUC = 0.94
TSS = 0.6
Relative importance
bio01
bio02
bio04
bio12
bio14
BDW
CLY
DES
NTO
PTO

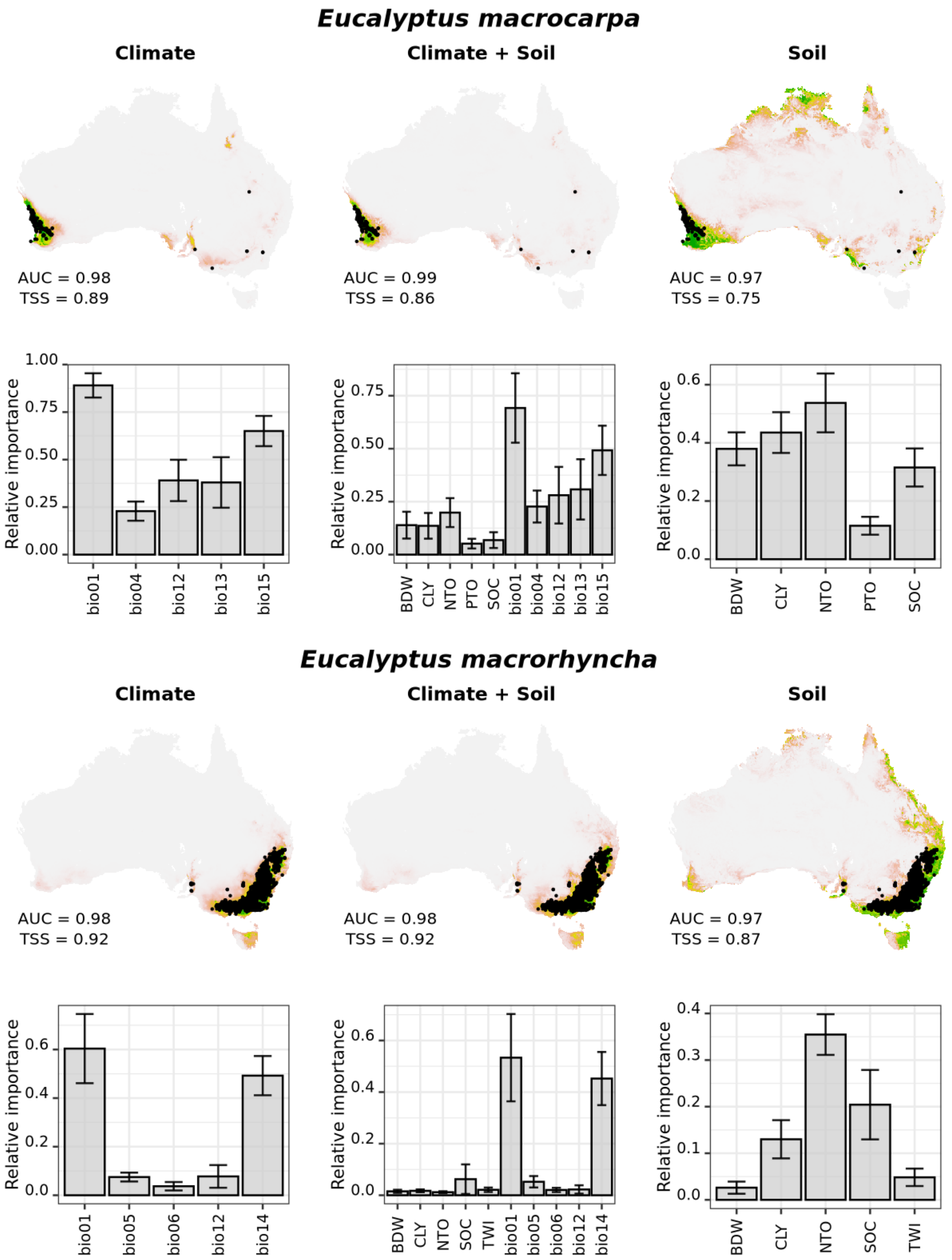
Eucalyptus macrocarpa
Climate
Climate + Soil
Soil
AUC = 0.98
TSS = 0.89
AUC = 0.99
TSS = 0.86
AUC = 0.97
TSS = 0.75
Relative importance
0.00
0.25
0.50
0.75
1.00
bio01
bio04
bio12
bio13
bio15
BDW
CLY
NTO
PTO
SOC
0.0
0.2
0.4
0.6
Eucalyptus macrorhyncha
AUC = 0.98
TSS = 0.92
AUC = 0.98
TSS = 0.92
AUC = 0.97
TSS = 0.87
0.1
0.3
bio05
bio06
bio14
TWI

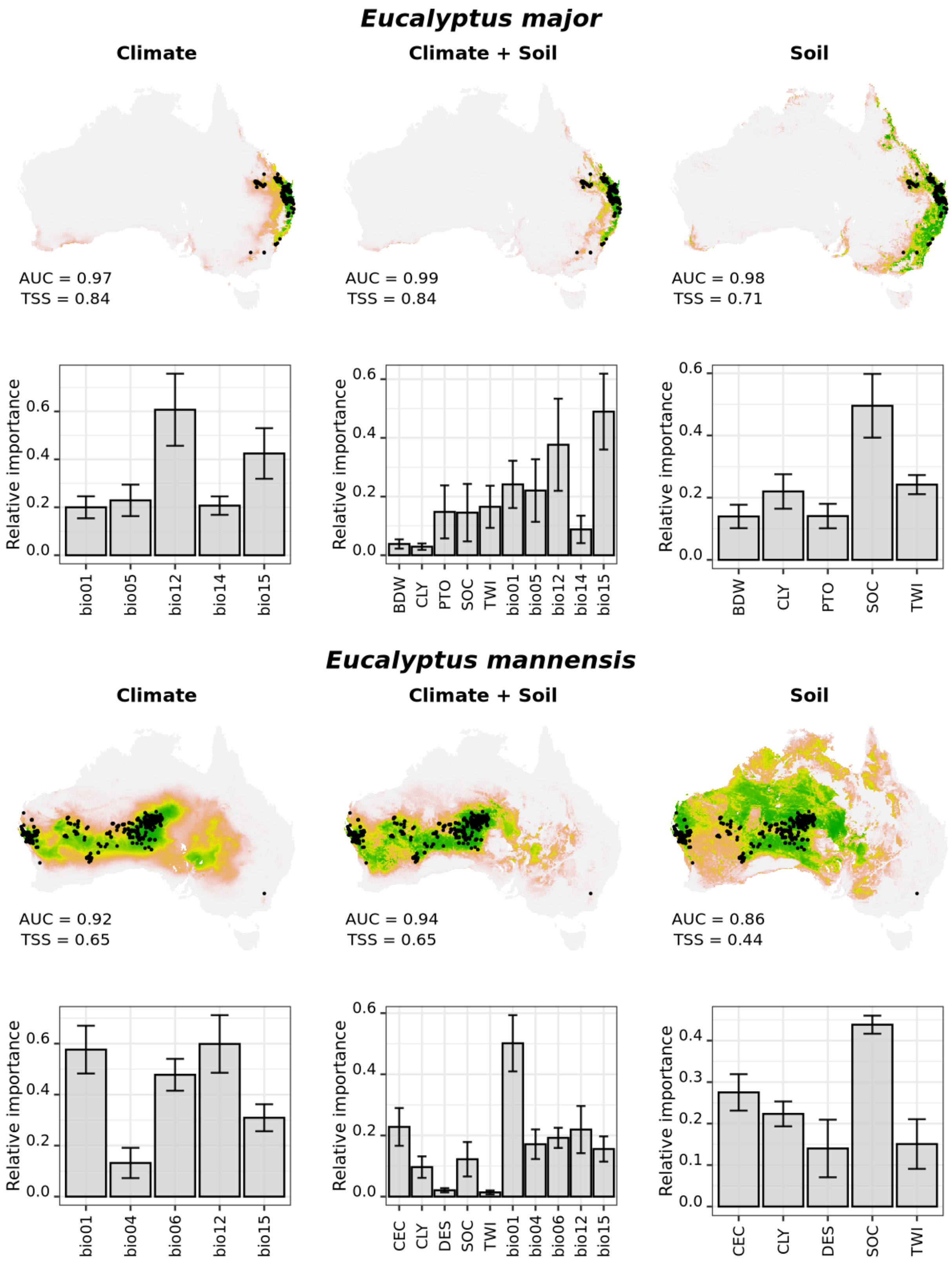

Eucalyptus major
Climate
Climate + Soil
Soil
AUC = 0.97
TSS = 0.84
AUC = 0.99
TSS = 0.84
AUC = 0.98
TSS = 0.71
Relative importance
0.0
0.2
0.4
0.6
bio01
bio05
bio12
bio14
bio15
BDW
CLY
PTO
SOC
TWI
Eucalyptus mannensis
Climate
Climate + Soil
Soil
AUC = 0.92
TSS = 0.65
AUC = 0.94
TSS = 0.65
AUC = 0.86
TSS = 0.44
Relative importance
0.0
0.1
0.2
0.3
0.4
0.6
bio01
bio04
bio06
bio12
bio15
CEC
CLY
DES
SOC
TWI

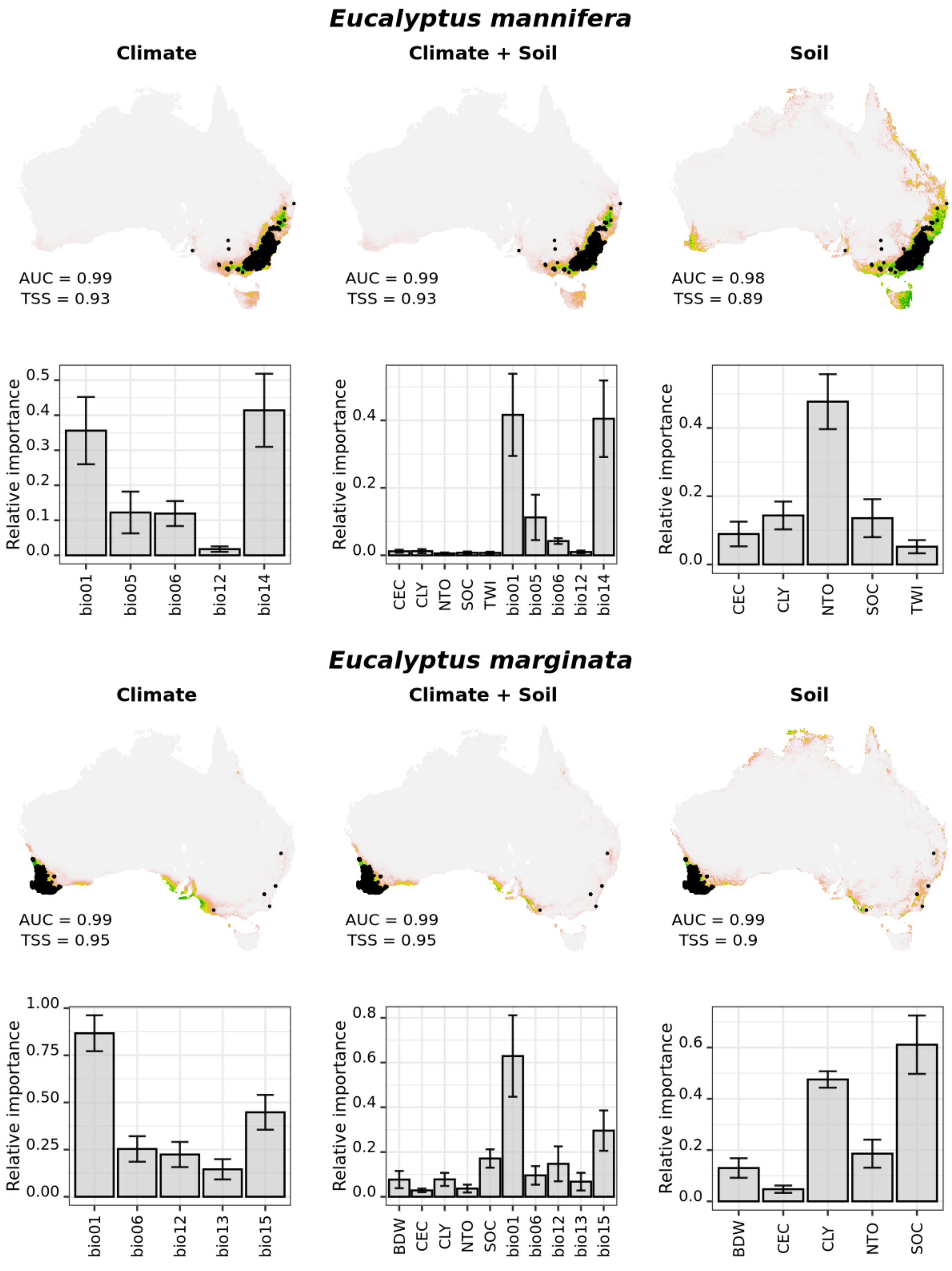

Eucalyptus mannifera
Climate
Climate + Soil
Soil
AUC = 0.99
TSS = 0.93
AUC = 0.99
TSS = 0.93
AUC = 0.98
TSS = 0.89
Relative importance
bio01
bio05
bio06
bio12
bio14
CEC
CLY
NTO
SOC
TWI
Eucalyptus marginata
Climate
Climate + Soil
Soil
AUC = 0.99
TSS = 0.95
AUC = 0.99
TSS = 0.95
AUC = 0.99
TSS = 0.9
Relative importance
bio01
bio06
bio12
bio13
bio15
BDW
CEC
CLY
NTO
SOC

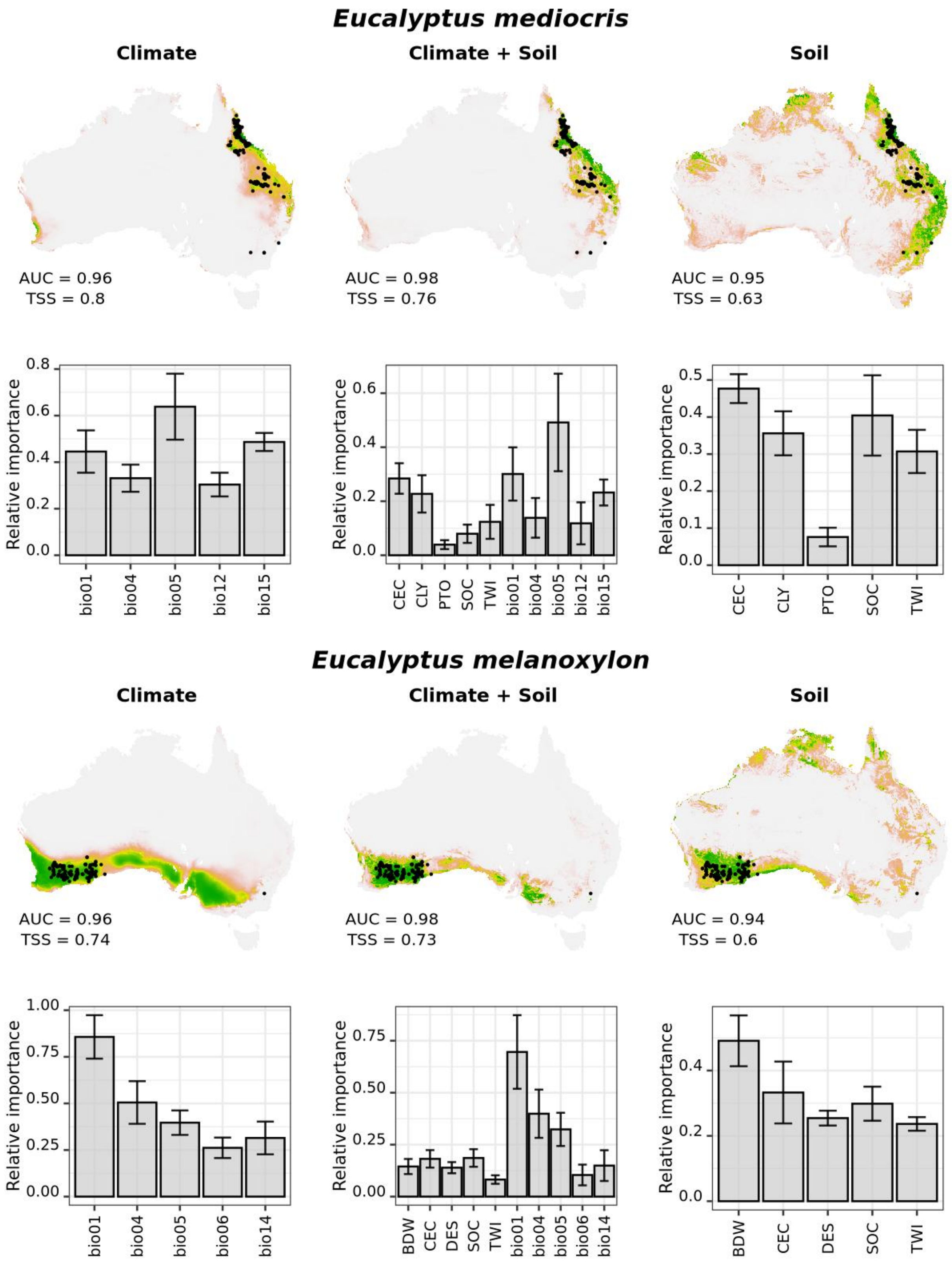

Eucalyptus mediocris
Climate
Climate + Soil
Soil
AUC = 0.96
TSS = 0.8
AUC = 0.98
TSS = 0.76
AUC = 0.95
TSS = 0.63
Relative importance
bio01
bio04
bio05
bio12
bio15
CEC
CLY
PTO
SOC
TWI
Eucalyptus melanoxylon
Climate
Climate + Soil
Soil
AUC = 0.96
TSS = 0.74
AUC = 0.98
TSS = 0.73
AUC = 0.94
TSS = 0.6
Relative importance
bio01
bio04
bio05
bio06
bio14
BDW
CEC
DES
SOC
TWI

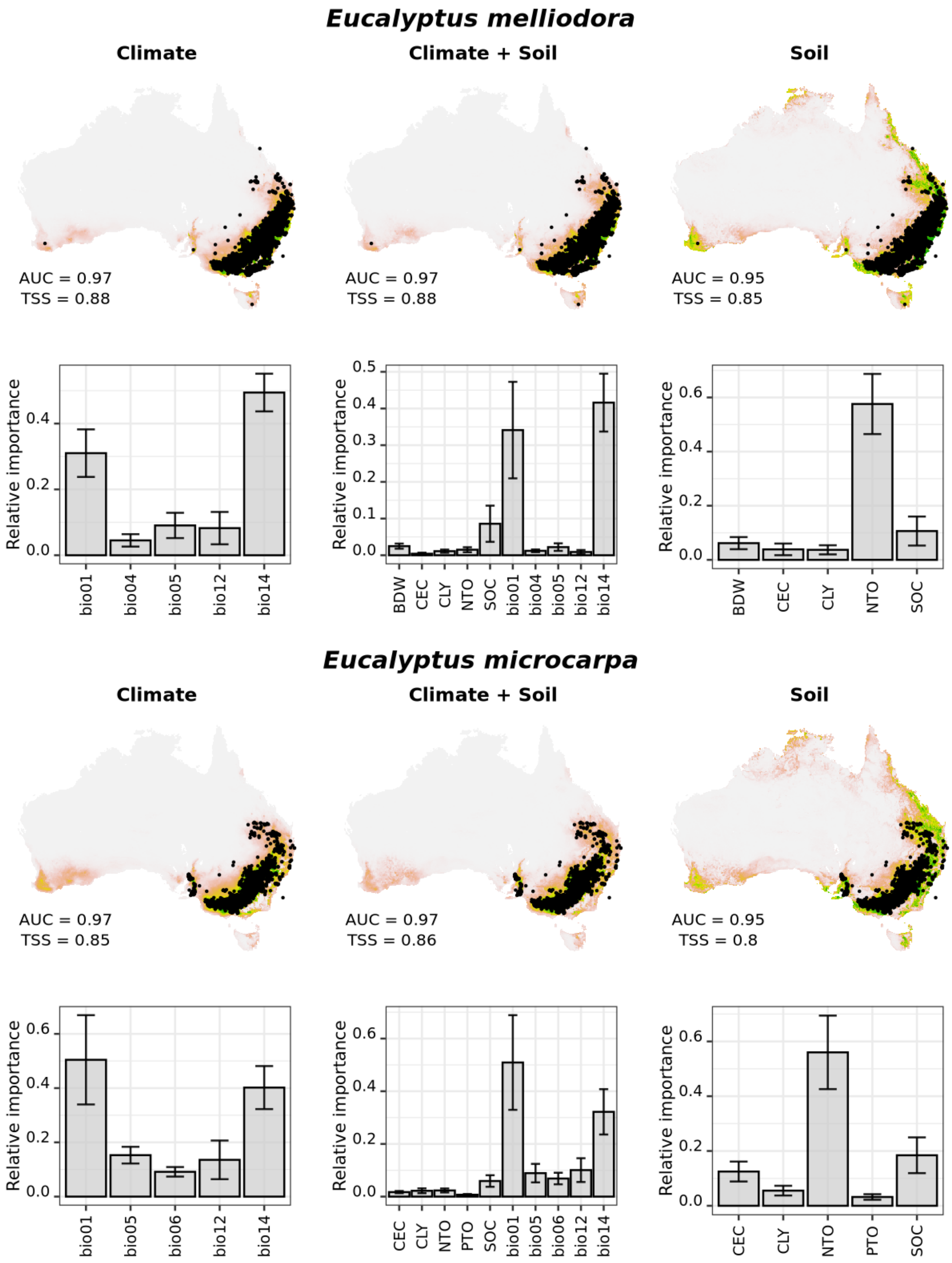

Eucalyptus melliodora
Climate
Climate + Soil
Soil
AUC = 0.97
TSS = 0.88
AUC = 0.97
TSS = 0.88
AUC = 0.95
TSS = 0.85
Relative importance
bio01
bio04
bio05
bio12
bio14
BDW
CEC
CLY
NTO
SOC
Eucalyptus microcarpa
AUC = 0.97
TSS = 0.85
AUC = 0.97
TSS = 0.86
AUC = 0.95
TSS = 0.8
bio06
PTO

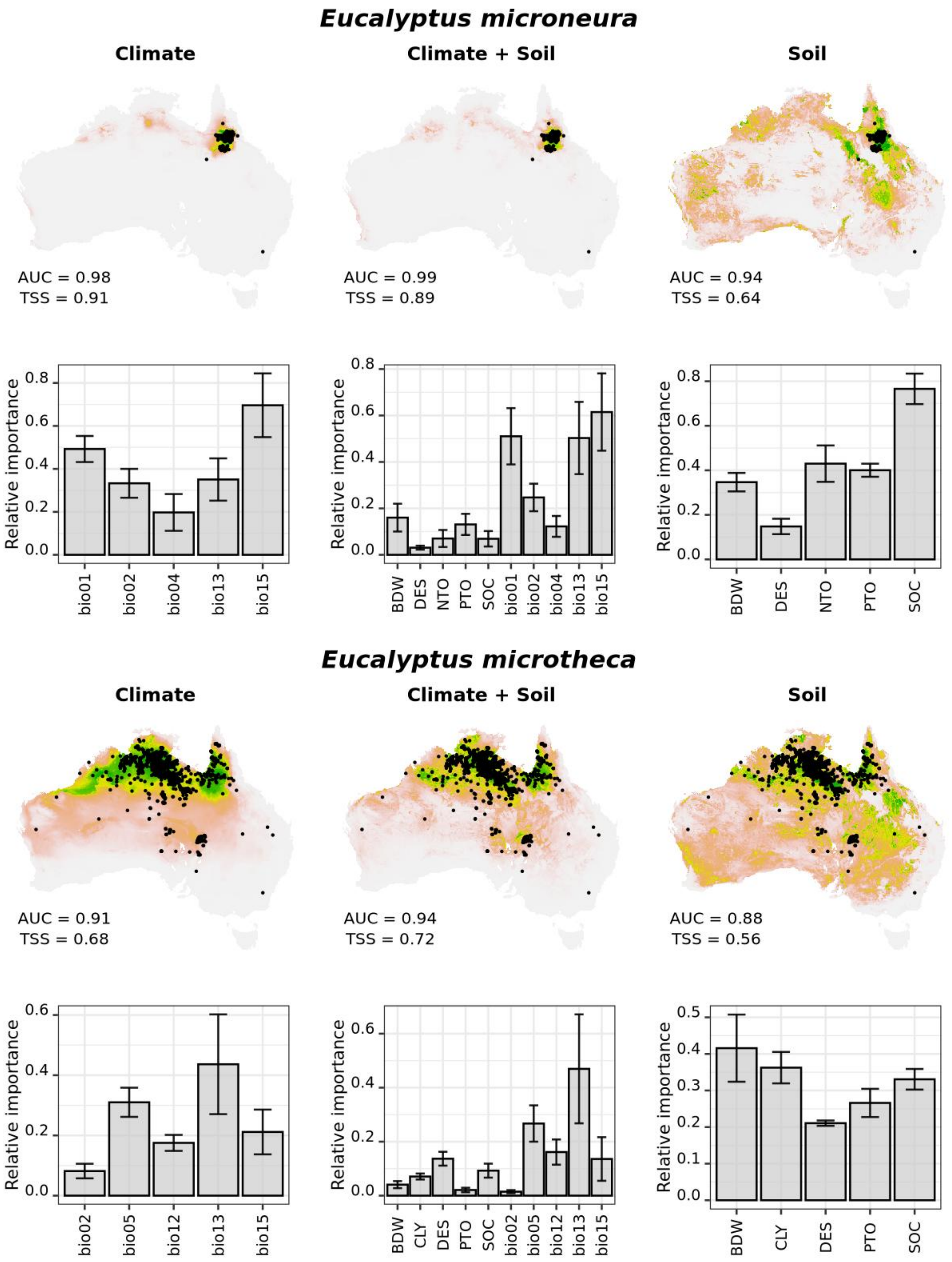
Eucalyptus microneura
Climate
Climate + Soil
Soil
AUC = 0.98
TSS = 0.91
AUC = 0.99
TSS = 0.89
AUC = 0.94
TSS = 0.64
Relative importance
0.0
0.2
0.4
0.6
0.8
bio01
bio02
bio04
bio13
bio15
BDW
DES
NTO
PTO
SOC
Eucalyptus microtheca
AUC = 0.91
TSS = 0.68
AUC = 0.94
TSS = 0.72
AUC = 0.88
TSS = 0.56
bio05
bio12
CLY
0.1
0.3
0.5

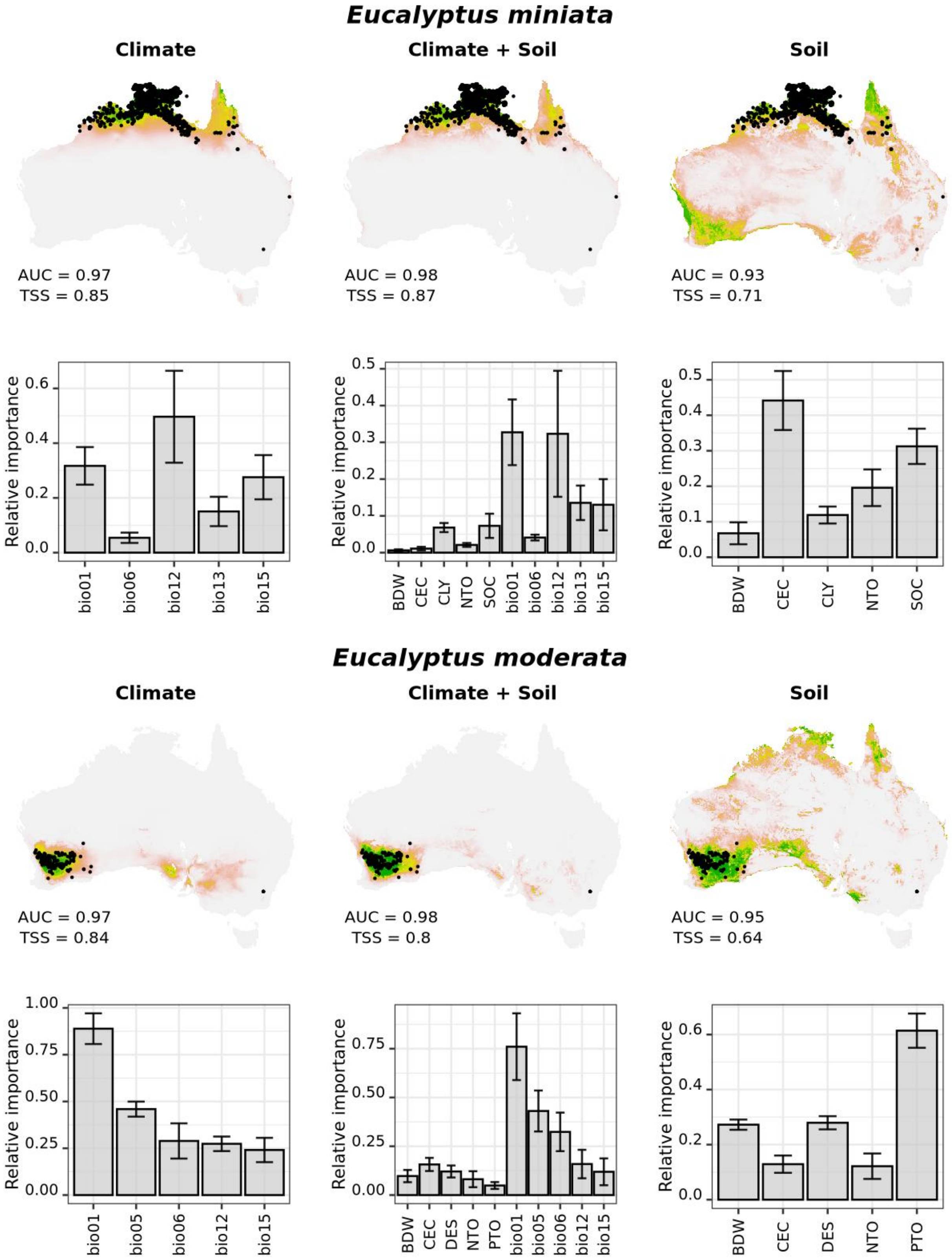
Eucalyptus miniata
Climate
Climate + Soil
Soil
AUC = 0.97
TSS = 0.85
AUC = 0.98
TSS = 0.87
AUC = 0.93
TSS = 0.71
Relative importance
bio01
bio06
bio12
bio13
bio15
BDW
CEC
CLY
NTO
SOC
Eucalyptus moderata
Climate
Climate + Soil
Soil
AUC = 0.97
TSS = 0.84
AUC = 0.98
TSS = 0.8
AUC = 0.95
TSS = 0.64
Relative importance
bio01
bio05
bio06
bio12
bio15
BDW
CEC
DES
NTO
PTO

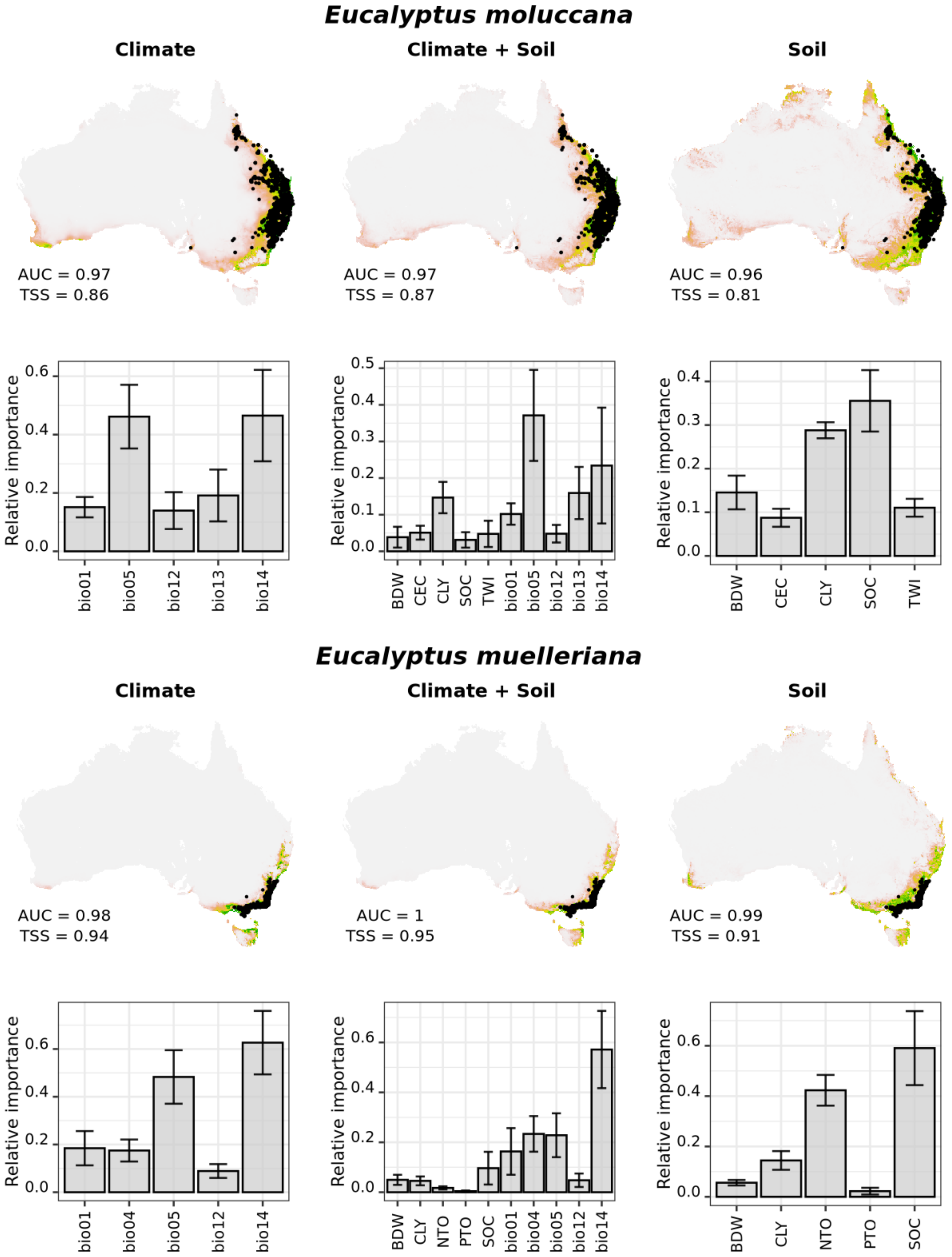
Eucalyptus moluccana
Climate
Climate + Soil
Soil
AUC = 0.97
TSS = 0.86
AUC = 0.97
TSS = 0.87
AUC = 0.96
TSS = 0.81
Relative importance
0.0
0.2
0.4
0.6
bio01
bio05
bio12
bio13
bio14
0.0
0.1
0.2
0.3
0.4
0.5
BDW
CEC
CLY
SOC
TWI
bio01
bio05
bio12
bio13
bio14
0.0
0.1
0.2
0.3
0.4
BDW
CEC
CLY
SOC
TWI
Eucalyptus muelleriana
Climate
Climate + Soil
Soil
AUC = 0.98
TSS = 0.94
AUC = 1
TSS = 0.95
AUC = 0.99
TSS = 0.91
Relative importance
0.0
0.2
0.4
0.6
bio01
bio04
bio05
bio12
bio14
BDW
CLY
NTO
PTO
SOC
bio01
bio04
bio05
bio12
bio14
BDW
CLY
NTO
PTO
SOC

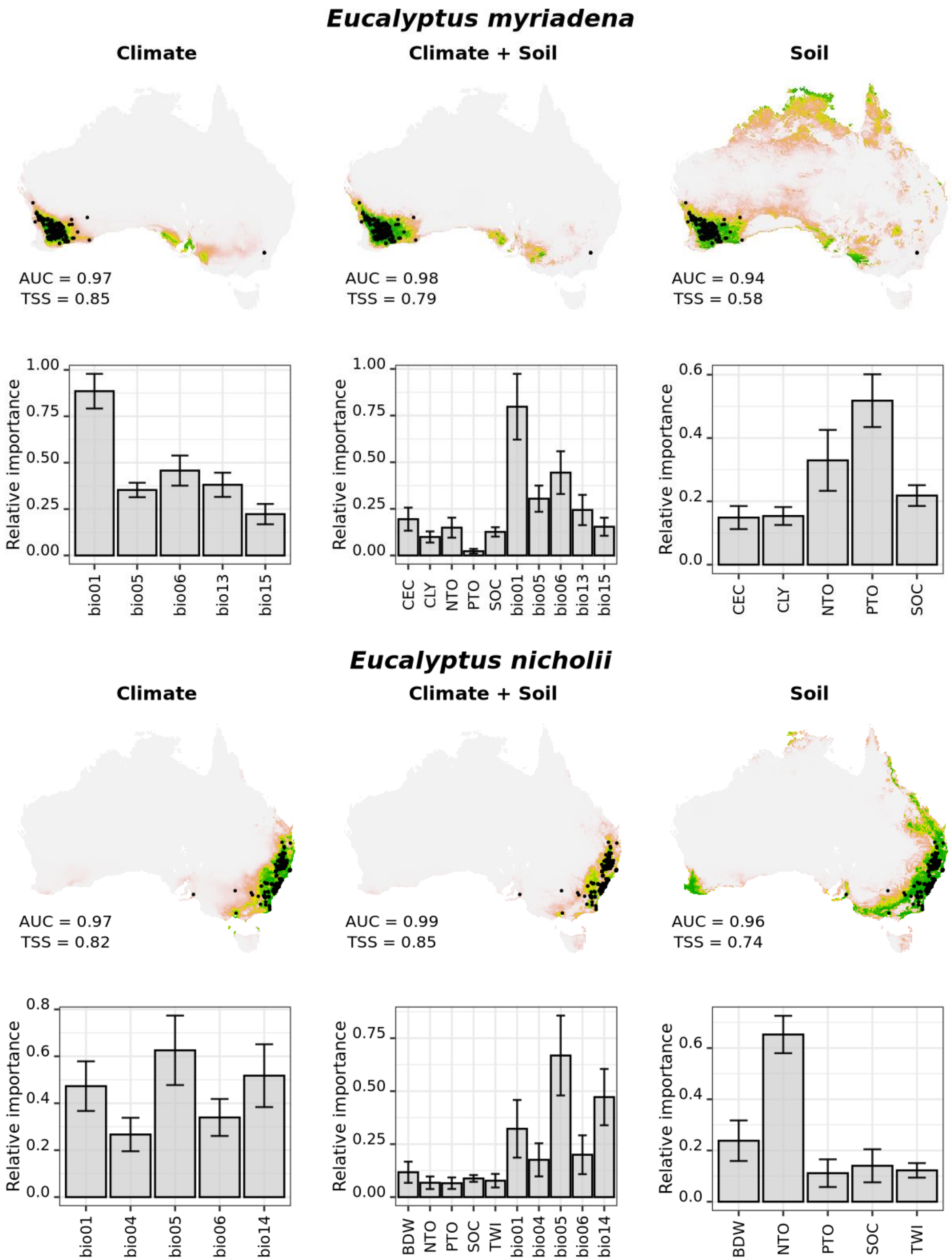
Eucalyptus myriadena
Climate
Climate + Soil
Soil
AUC = 0.97
TSS = 0.85
AUC = 0.98
TSS = 0.79
AUC = 0.94
TSS = 0.58
Relative importance
bio01
bio05
bio06
bio13
bio15
CEC
CLY
NTO
PTO
SOC
Eucalyptus nicholii
AUC = 0.97
TSS = 0.82
AUC = 0.99
TSS = 0.85
AUC = 0.96
TSS = 0.74
bio04
bio14
BDW
TWI

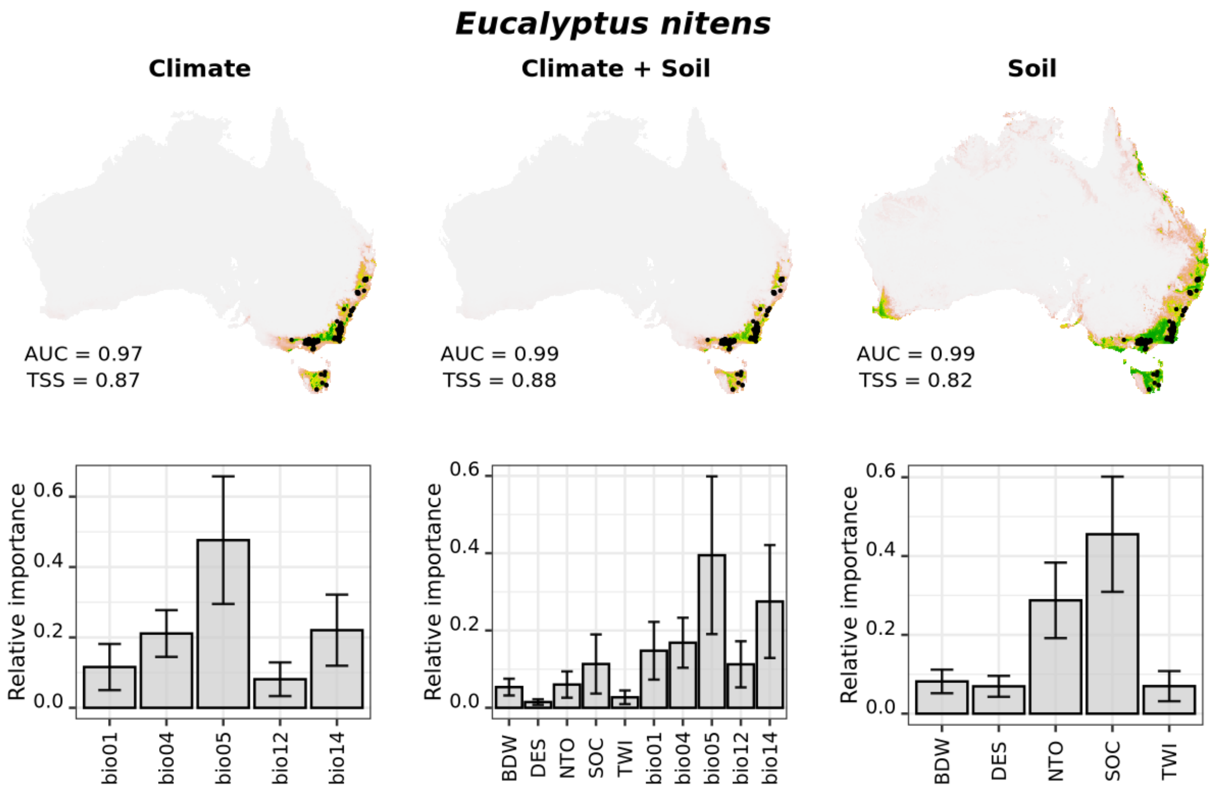
Eucalyptus nitens
Climate
Climate + Soil
Soil
AUC = 0.97
TSS = 0.87
AUC = 0.99
TSS = 0.88
AUC = 0.99
TSS = 0.82
Relative importance
0.0
0.2
0.4
0.6
bio01
bio04
bio05
bio12
bio14
BDW
DES
NTO
SOC
TWI

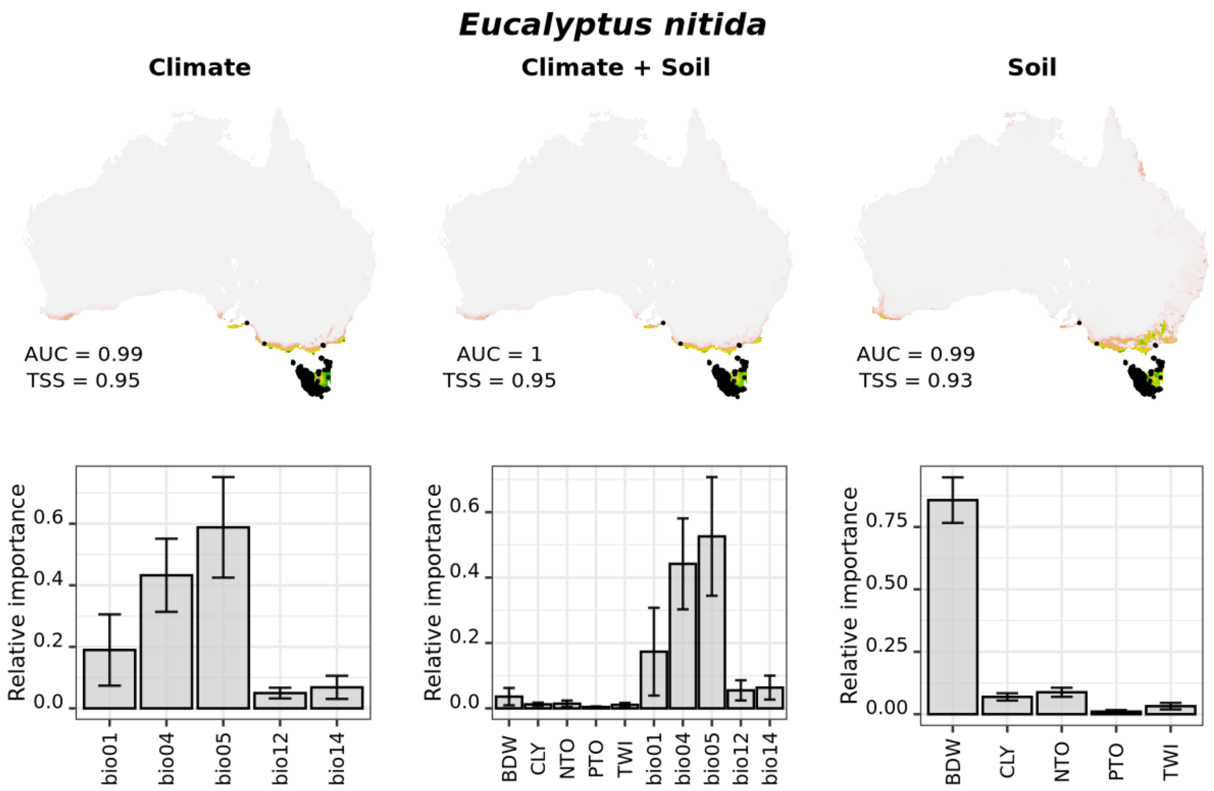
Eucalyptus nitida
Climate
Climate + Soil
Soil
AUC = 0.99
TSS = 0.95
AUC = 1
TSS = 0.95
AUC = 0.99
TSS = 0.93
Relative importance
0.0
0.2
0.4
0.6
0.00
0.25
0.50
0.75
bio01
bio04
bio05
bio12
bio14
BDW
CLY
NTO
PTO
TWI

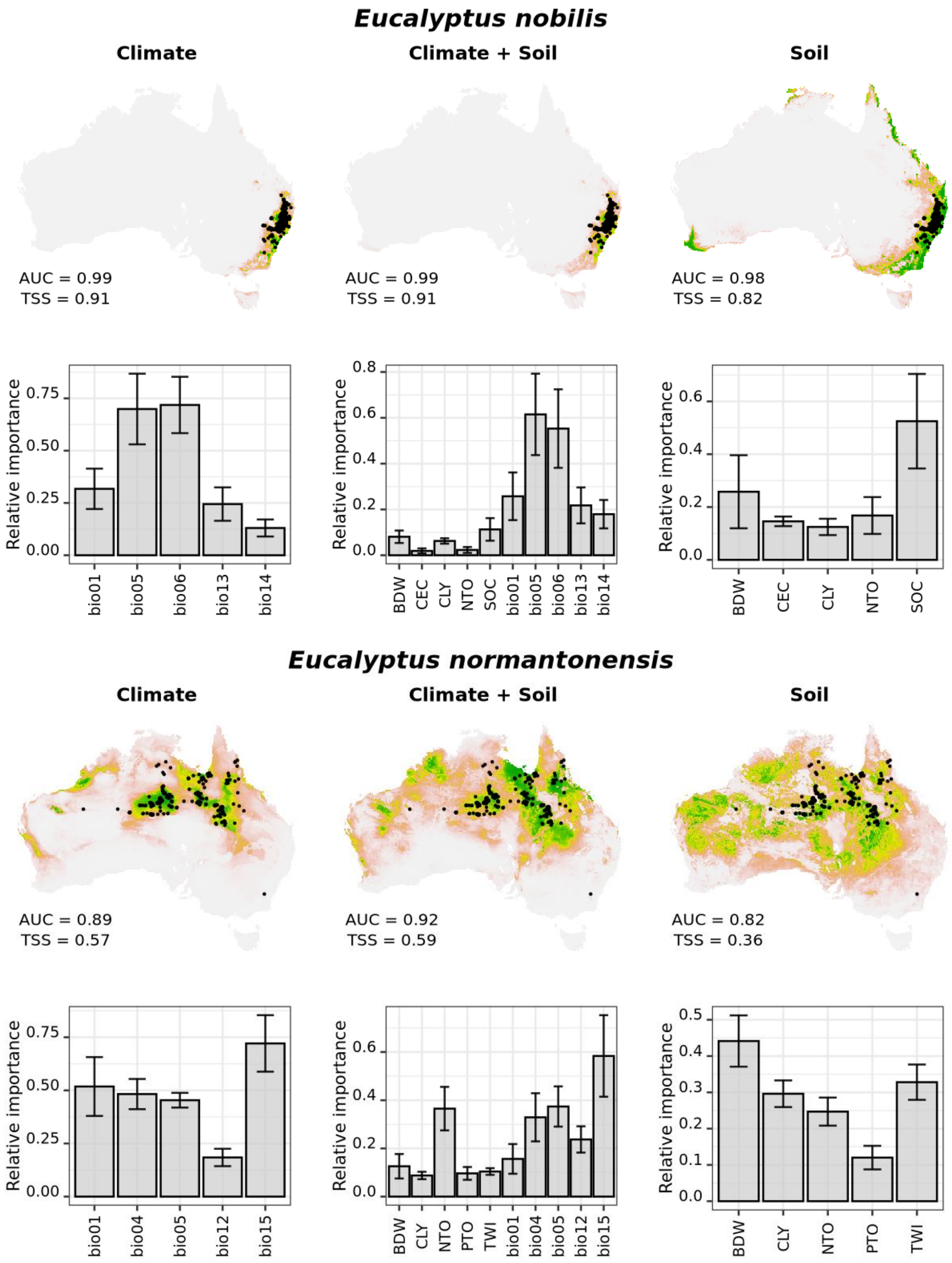
Eucalyptus nobilis
Climate
Climate + Soil
Soil
AUC = 0.99
TSS = 0.91
AUC = 0.99
TSS = 0.91
AUC = 0.98
TSS = 0.82
Relative importance
bio01
bio05
bio06
bio13
bio14
BDW
CEC
CLY
NTO
SOC
Eucalyptus normantonensis
AUC = 0.89
TSS = 0.57
AUC = 0.92
TSS = 0.59
AUC = 0.82
TSS = 0.36
bio04
bio12
bio15
PTO
TWI

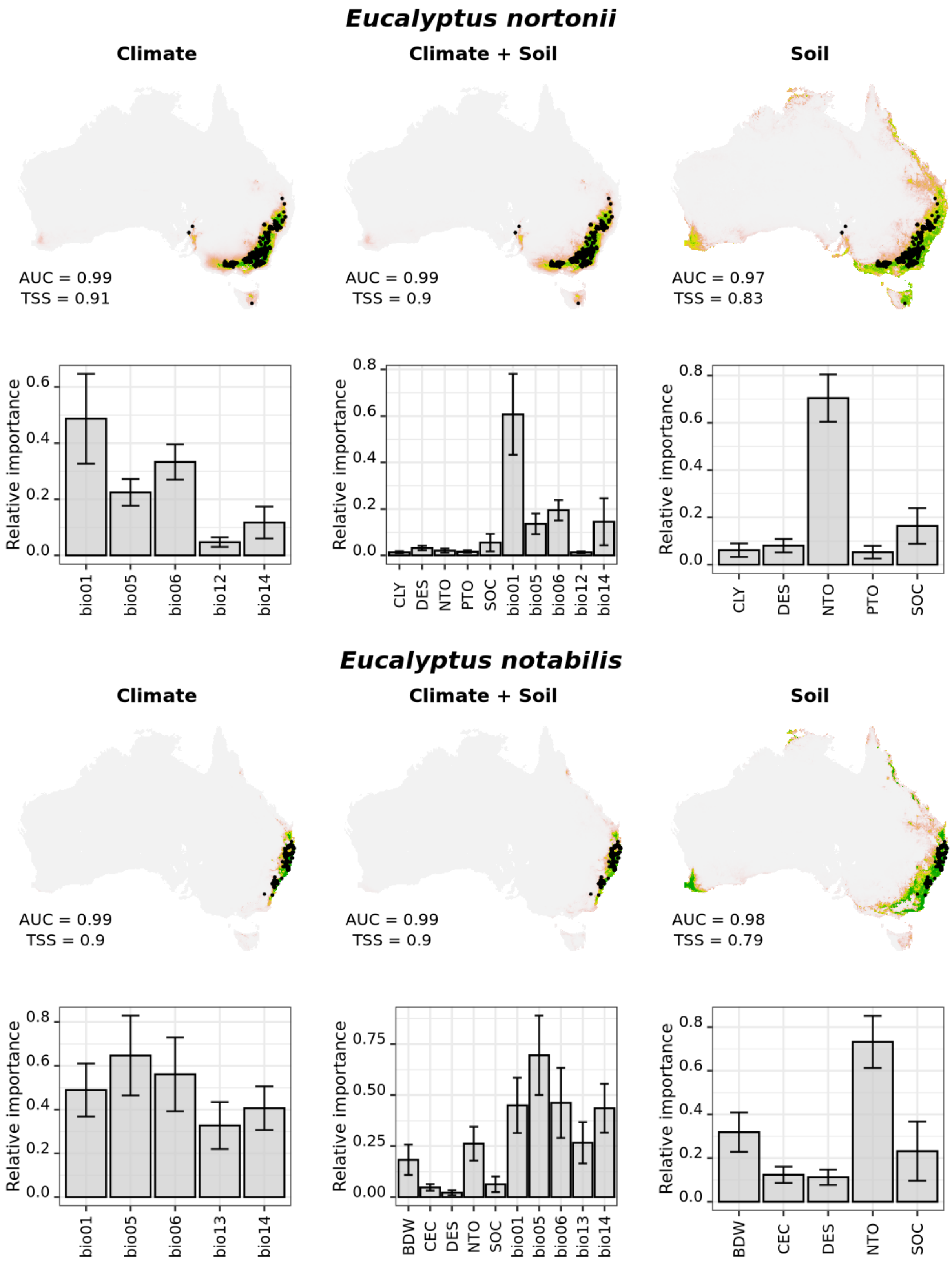
Eucalyptus nortonii
Climate
Climate + Soil
Soil
AUC = 0.99
TSS = 0.91
AUC = 0.99
TSS = 0.9
AUC = 0.97
TSS = 0.83
Relative importance
bio01
bio05
bio06
bio12
bio14
CLY
DES
NTO
PTO
SOC
Eucalyptus notabilis
Climate
Climate + Soil
Soil
AUC = 0.99
TSS = 0.9
AUC = 0.99
TSS = 0.9
AUC = 0.98
TSS = 0.79
Relative importance
bio01
bio05
bio06
bio13
bio14
BDW
CEC
DES
NTO
SOC

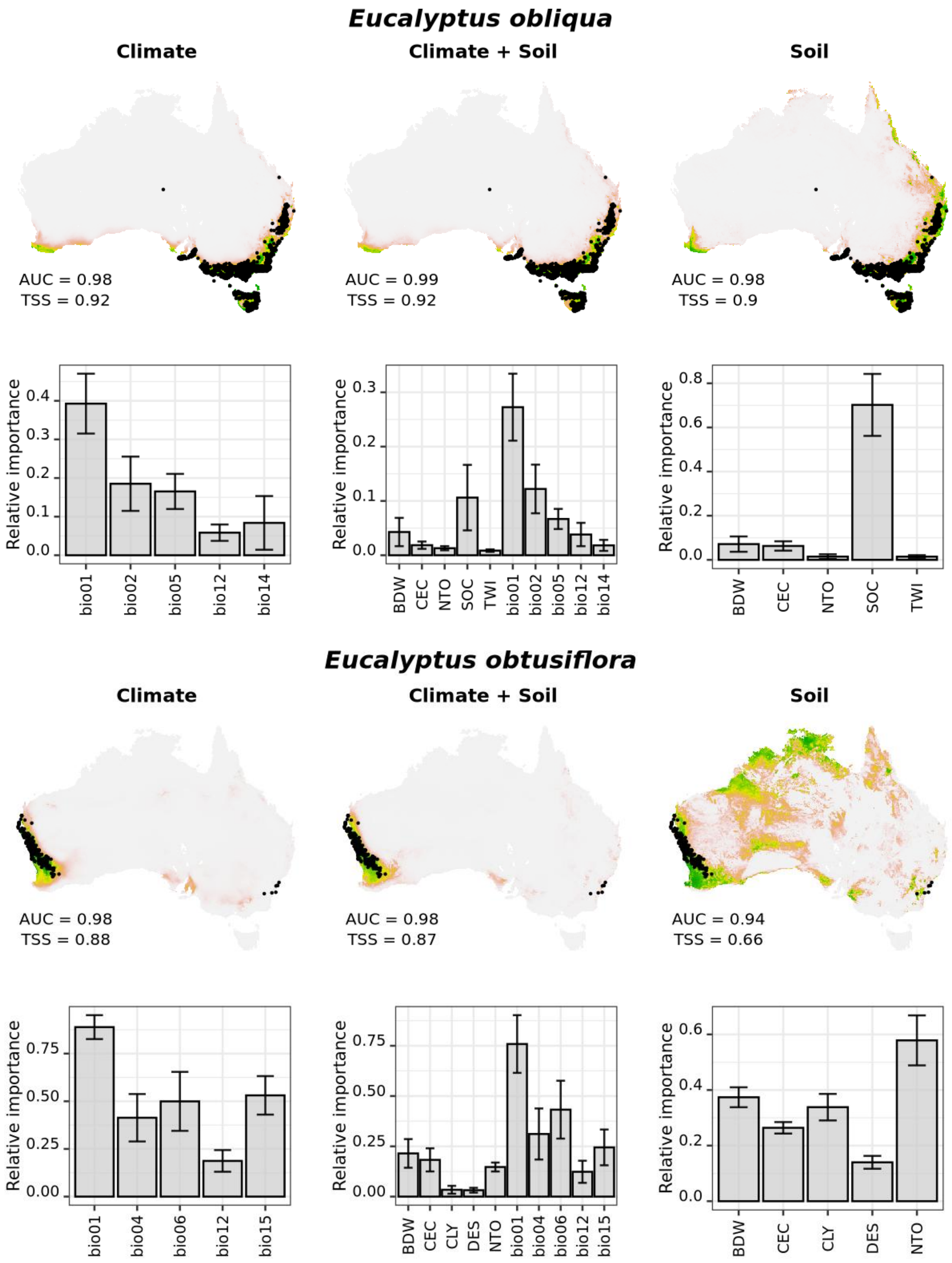

Eucalyptus obliqua
Climate
Climate + Soil
Soil
AUC = 0.98
TSS = 0.92
AUC = 0.99
TSS = 0.92
AUC = 0.98
TSS = 0.9
Relative importance
0.0 0.1 0.2 0.3 0.4
bio01 bio02 bio05 bio12 bio14
Relative importance
0.0 0.1 0.2 0.3
BDW CEC NTO SOC TWI bio01 bio02 bio05 bio12 bio14
Relative importance
0.0 0.2 0.4 0.6 0.8
BDW CEC NTO SOC TWI
Eucalyptus obtusiflora
Climate
Climate + Soil
Soil
AUC = 0.98
TSS = 0.88
AUC = 0.98
TSS = 0.87
AUC = 0.94
TSS = 0.66
Relative importance
0.00 0.25 0.50 0.75
bio01 bio04 bio06 bio12 bio15
Relative importance
0.00 0.25 0.50 0.75
BDW CEC CLY DES NTO bio01 bio04 bio06 bio12 bio15
Relative importance
0.0 0.2 0.4 0.6
BDW CEC CLY DES NTO

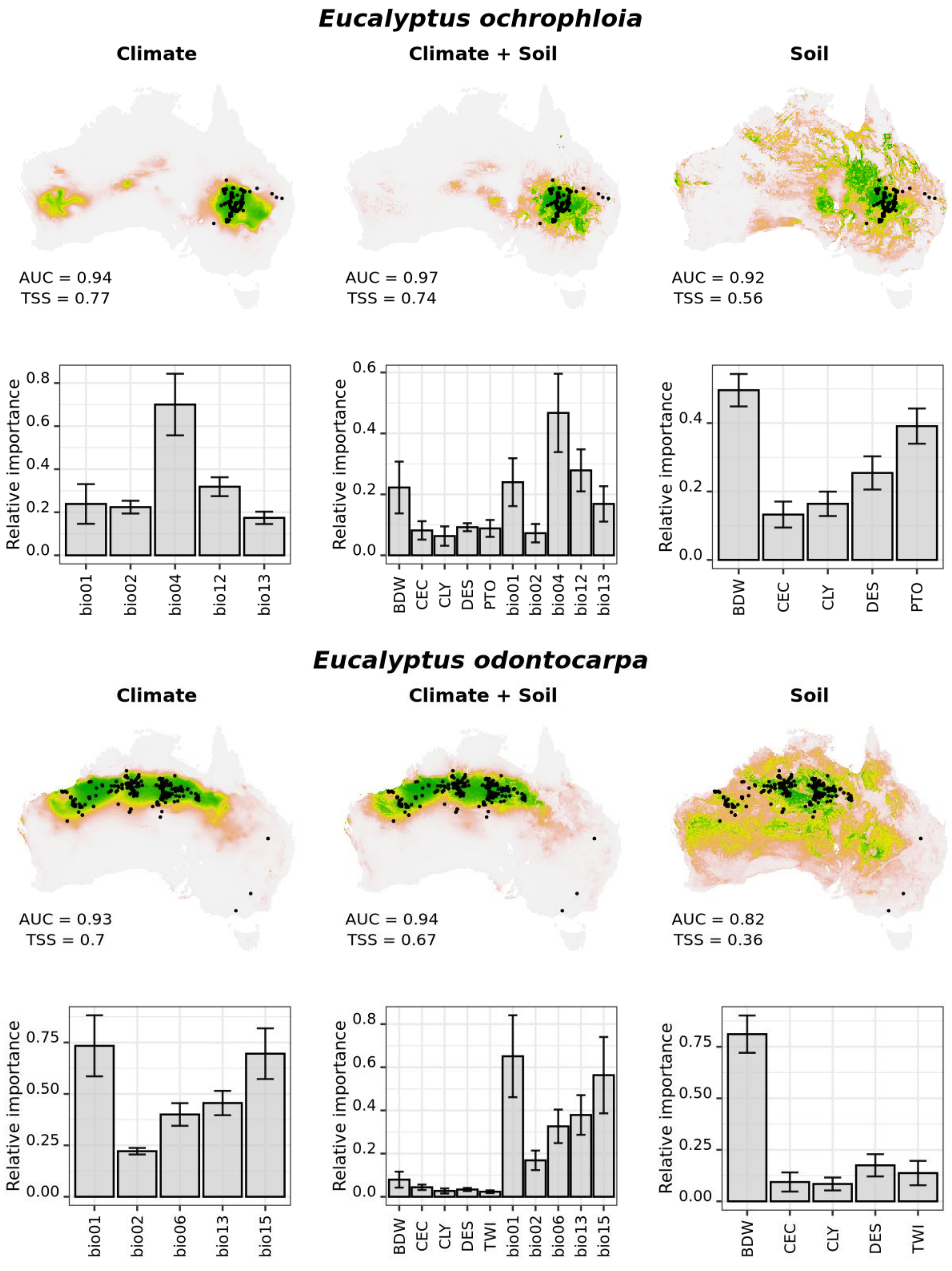
Eucalyptus ochrophloia
Climate
Climate + Soil
Soil
AUC = 0.94
TSS = 0.77
AUC = 0.97
TSS = 0.74
AUC = 0.92
TSS = 0.56
Relative importance
0.0
0.2
0.4
0.6
0.8
bio01
bio02
bio04
bio12
bio13
BDW
CEC
CLY
DES
PTO
Eucalyptus odontocarpa
AUC = 0.93
TSS = 0.7
AUC = 0.94
TSS = 0.67
AUC = 0.82
TSS = 0.36
0.00
0.25
0.50
0.75
bio06
bio15
TWI

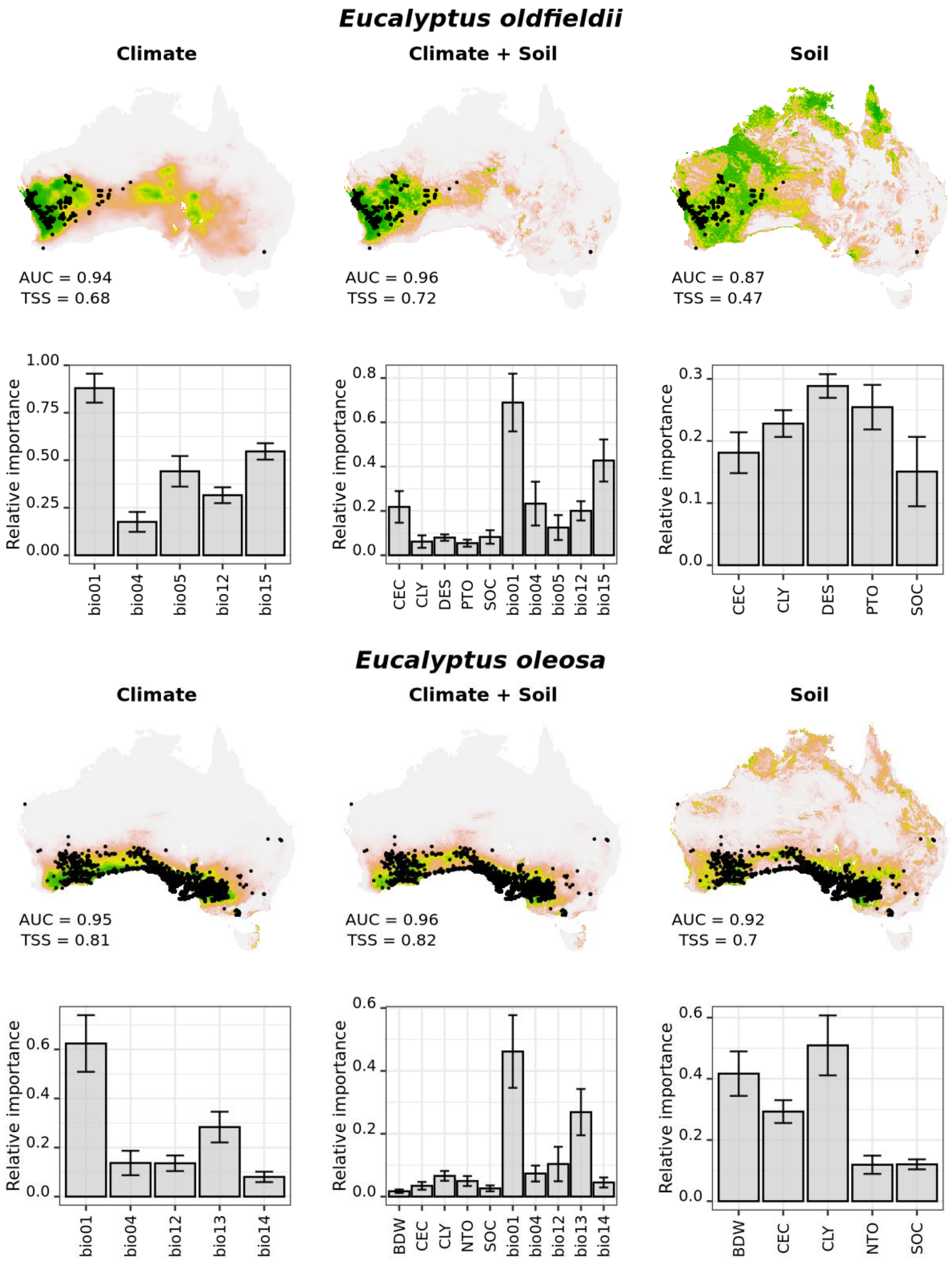

Eucalyptus oldfieldii
Climate
Climate + Soil
Soil
AUC = 0.94
TSS = 0.68
AUC = 0.96
TSS = 0.72
AUC = 0.87
TSS = 0.47
Relative importance
1.00
0.75
0.50
0.25
0.00
bio01
bio04
bio05
bio12
bio15
0.8
0.6
0.4
0.2
0.0
CEC
CLY
DES
PTO
SOC
bio01
bio04
bio05
bio12
bio15
0.3
0.2
0.1
0.0
CEC
CLY
DES
PTO
SOC
Eucalyptus oleosa
Climate
Climate + Soil
Soil
AUC = 0.95
TSS = 0.81
AUC = 0.96
TSS = 0.82
AUC = 0.92
TSS = 0.7
Relative importance
0.6
0.4
0.2
0.0
bio01
bio04
bio12
bio13
bio14
BDW
CEC
CLY
NTO
SOC
bio01
bio04
bio12
bio13
bio14
BDW
CEC
CLY
NTO
SOC

Eucalyptus oligantha

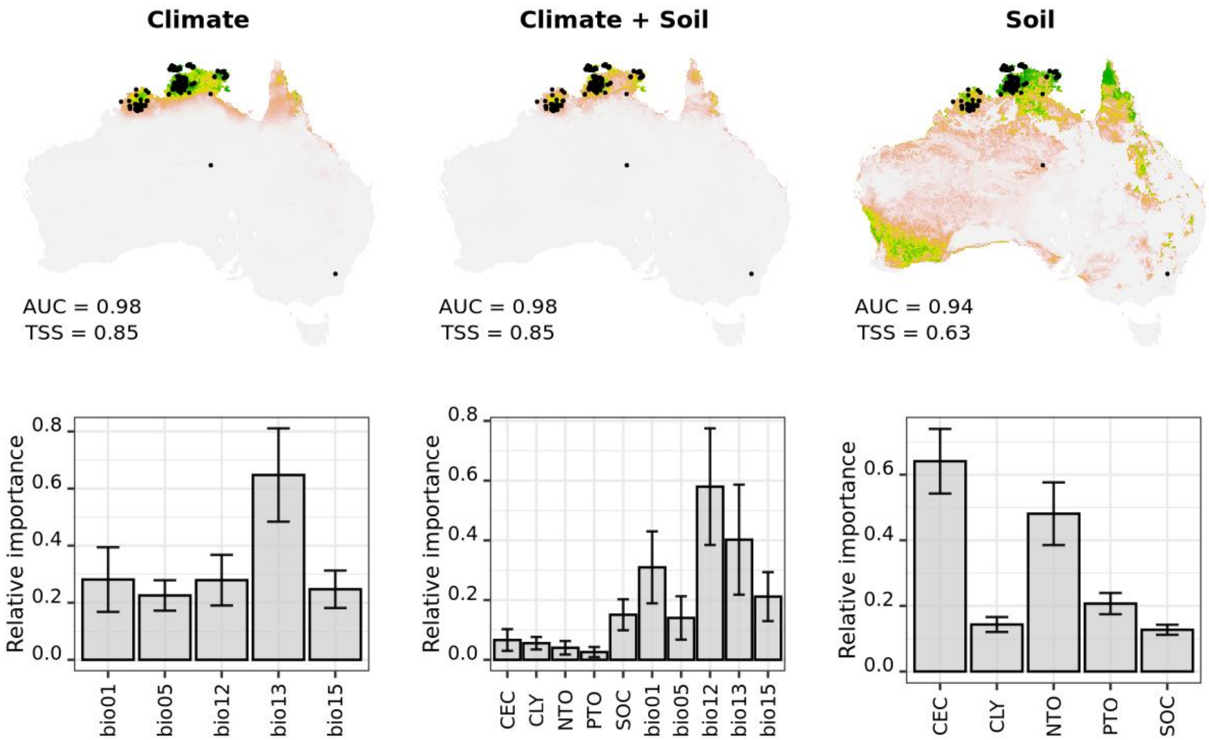

Eucalyptus olivina

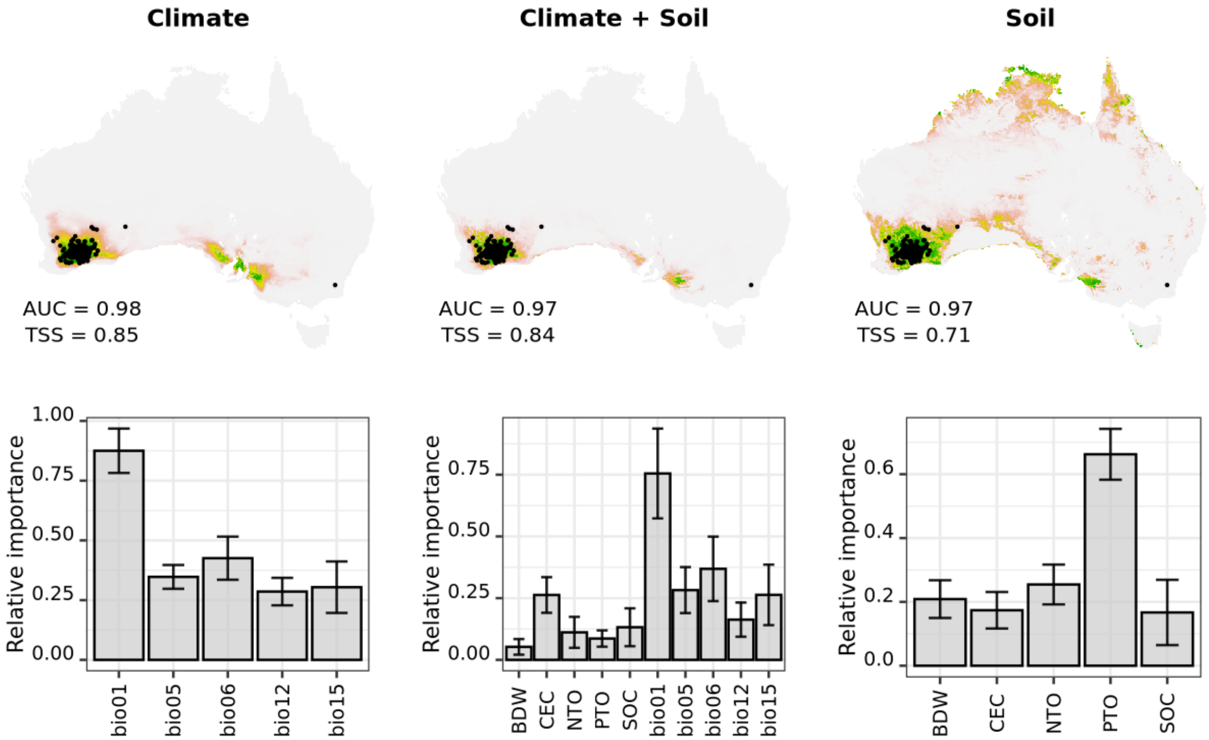

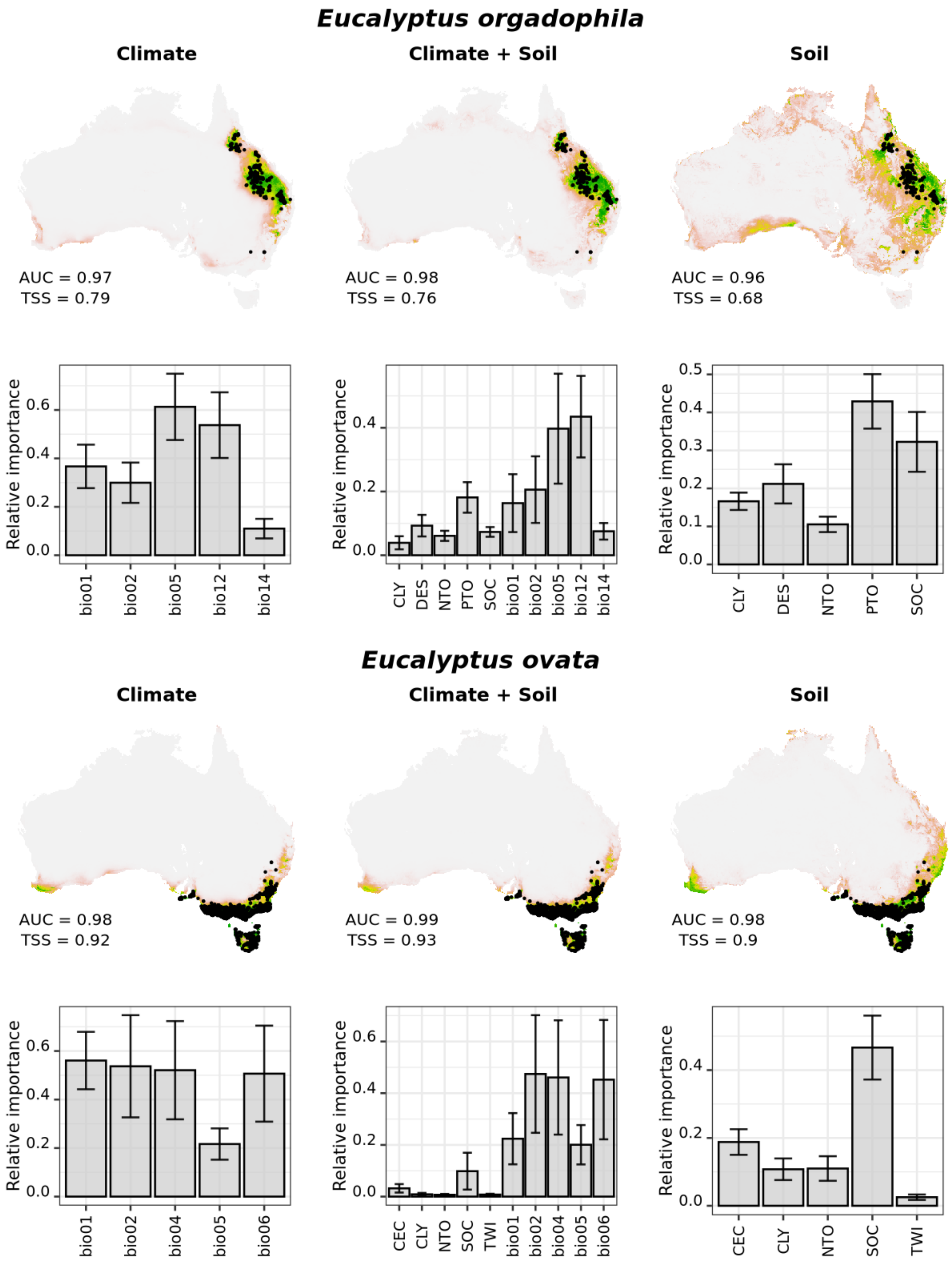

Eucalyptus orgadophila
Climate
Climate + Soil
Soil
AUC = 0.97
TSS = 0.79
AUC = 0.98
TSS = 0.76
AUC = 0.96
TSS = 0.68
Relative importance
bio01
bio02
bio05
bio12
bio14
CLY
DES
NTO
PTO
SOC
bio01
bio02
bio05
bio12
bio14
CLY
DES
NTO
PTO
SOC
Eucalyptus ovata
Climate
Climate + Soil
Soil
AUC = 0.98
TSS = 0.92
AUC = 0.99
TSS = 0.93
AUC = 0.98
TSS = 0.9
Relative importance
bio01
bio02
bio04
bio05
bio06
CEC
CLY
NTO
SOC
TWI
bio01
bio02
bio04
bio05
bio06
CEC
CLY
NTO
SOC
TWI

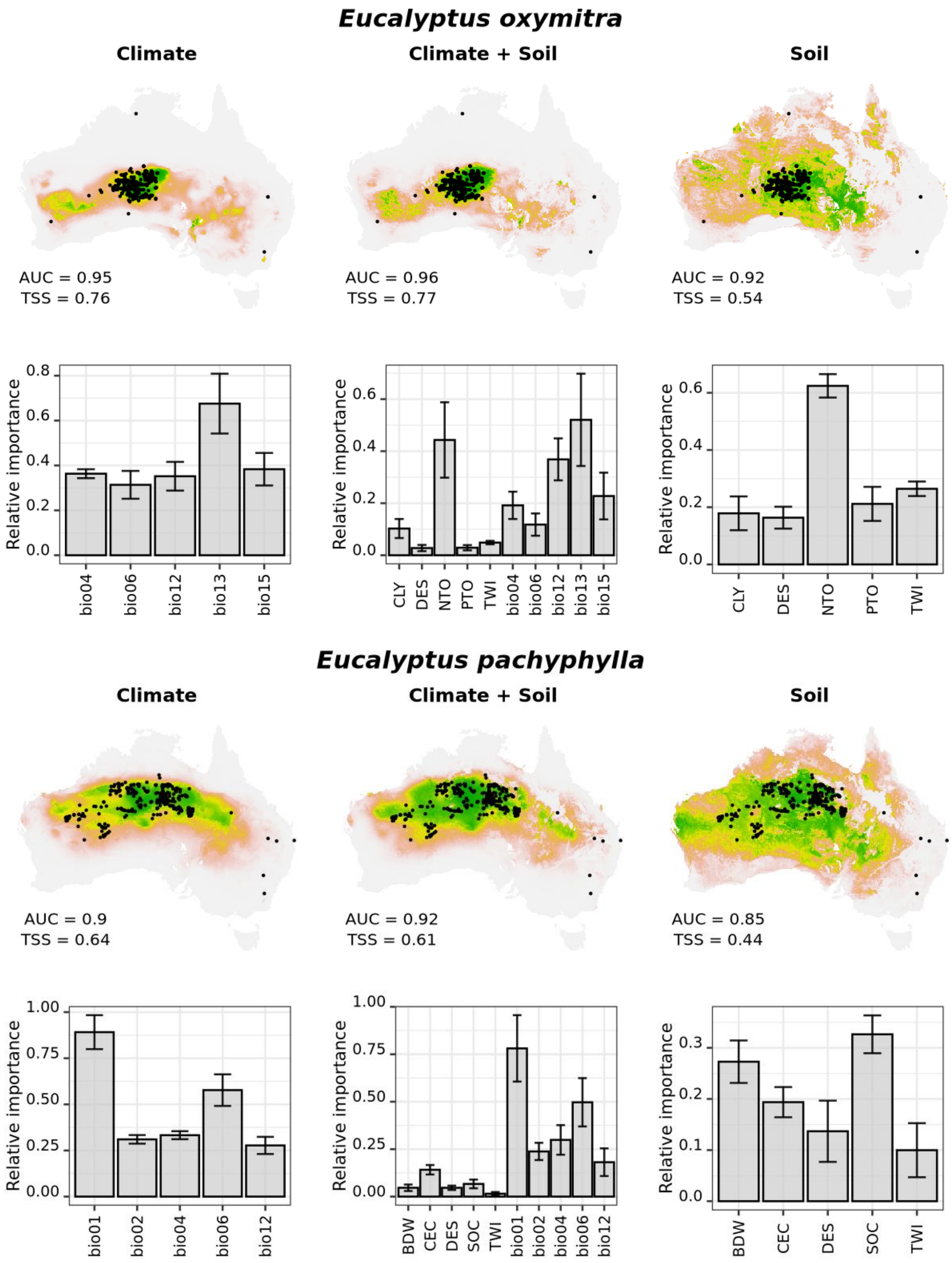

Eucalyptus oxymitra
Climate
Climate + Soil
Soil
AUC = 0.95
TSS = 0.76
AUC = 0.96
TSS = 0.77
AUC = 0.92
TSS = 0.54
Relative importance
bio04
bio06
bio12
bio13
bio15
CLY
DES
NTO
PTO
TWI
bio04
bio06
bio12
bio13
bio15
CLY
DES
NTO
PTO
TWI
Eucalyptus pachyphylla
Climate
Climate + Soil
Soil
AUC = 0.9
TSS = 0.64
AUC = 0.92
TSS = 0.61
AUC = 0.85
TSS = 0.44
Relative importance
bio01
bio02
bio04
bio06
bio12
BDW
CEC
DES
SOC
TWI
bio01
bio02
bio04
bio06
bio12
BDW
CEC
DES
SOC
TWI

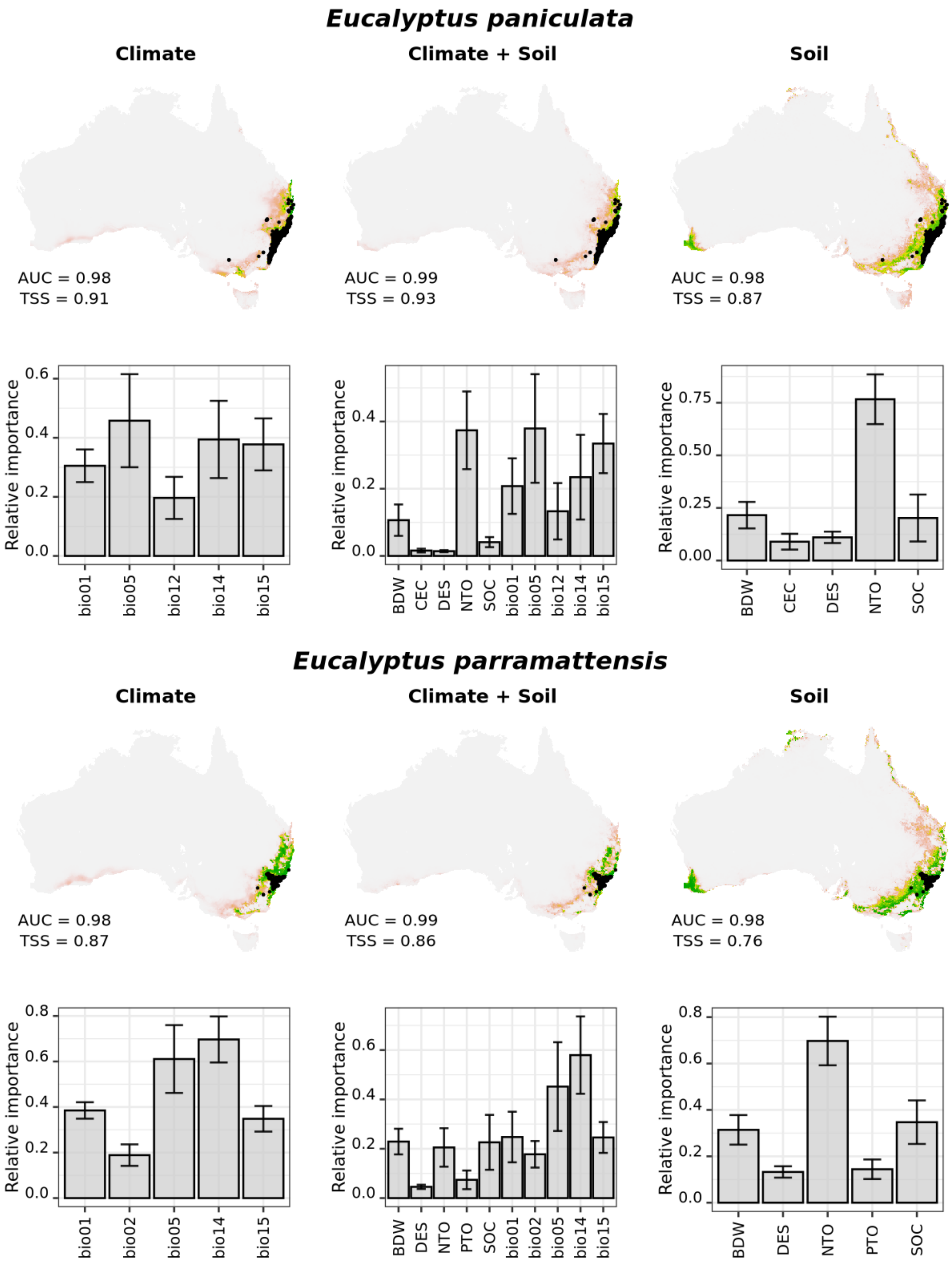

Eucalyptus paniculata
Climate
Climate + Soil
Soil
AUC = 0.98
TSS = 0.91
AUC = 0.99
TSS = 0.93
AUC = 0.98
TSS = 0.87
Relative importance
bio01
bio05
bio12
bio14
bio15
BDW
CEC
DES
NTO
SOC
Eucalyptus parramattensis
Climate
Climate + Soil
Soil
AUC = 0.98
TSS = 0.87
AUC = 0.99
TSS = 0.86
AUC = 0.98
TSS = 0.76
Relative importance
bio01
bio02
bio05
bio14
bio15
BDW
DES
NTO
PTO
SOC

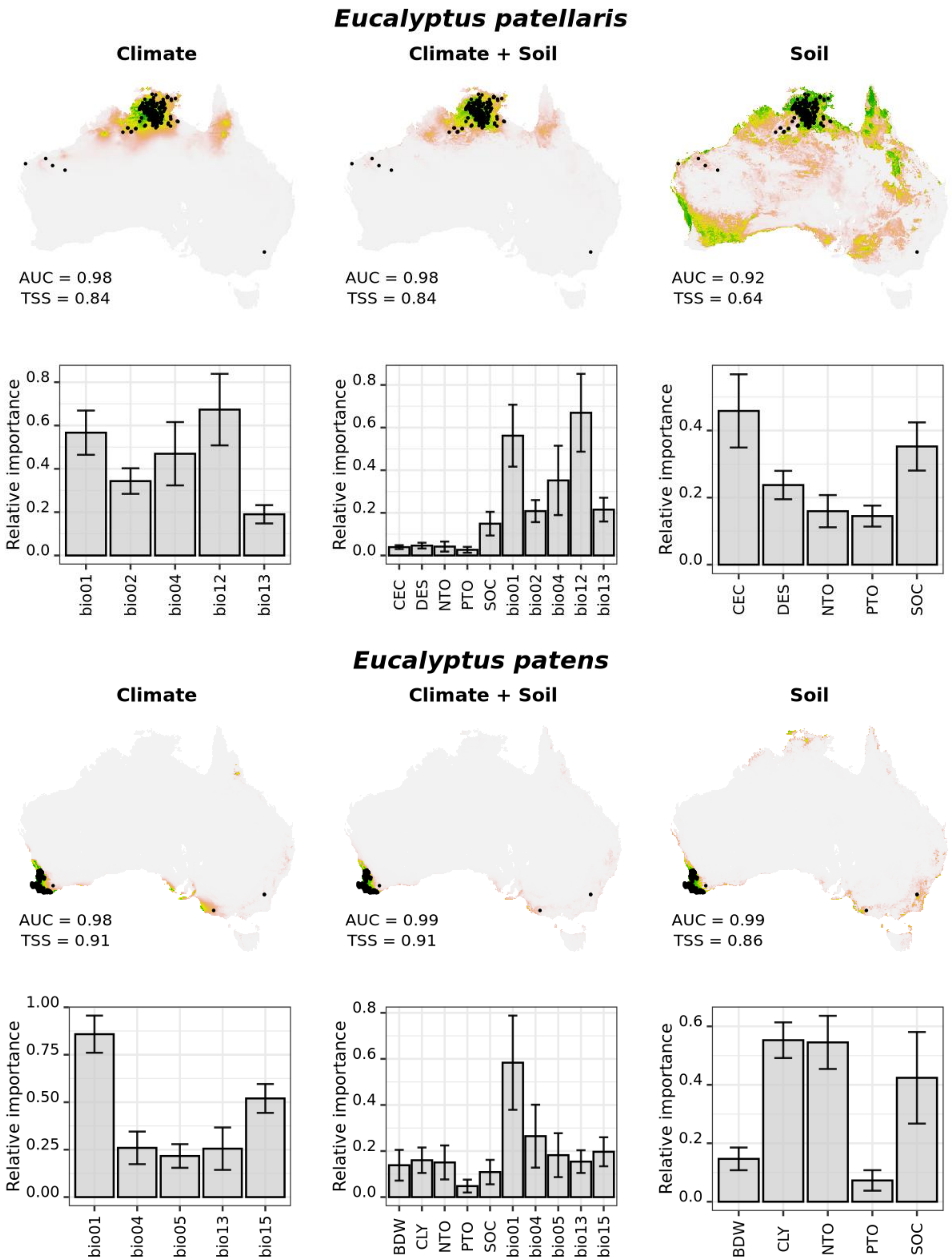

Eucalyptus patellaris
Climate
Climate + Soil
Soil
AUC = 0.98
TSS = 0.84
AUC = 0.98
TSS = 0.84
AUC = 0.92
TSS = 0.64
Relative importance
bio01 bio02 bio04 bio12 bio13
CEC DES NTO PTO SOC bio01 bio02 bio04 bio12 bio13
CEC DES NTO PTO SOC
Eucalyptus patens
Climate
Climate + Soil
Soil
AUC = 0.98
TSS = 0.91
AUC = 0.99
TSS = 0.91
AUC = 0.99
TSS = 0.86
Relative importance
bio01 bio04 bio05 bio13 bio15
BDW CLY NTO PTO SOC bio01 bio04 bio05 bio13 bio15
BDW CLY NTO PTO SOC

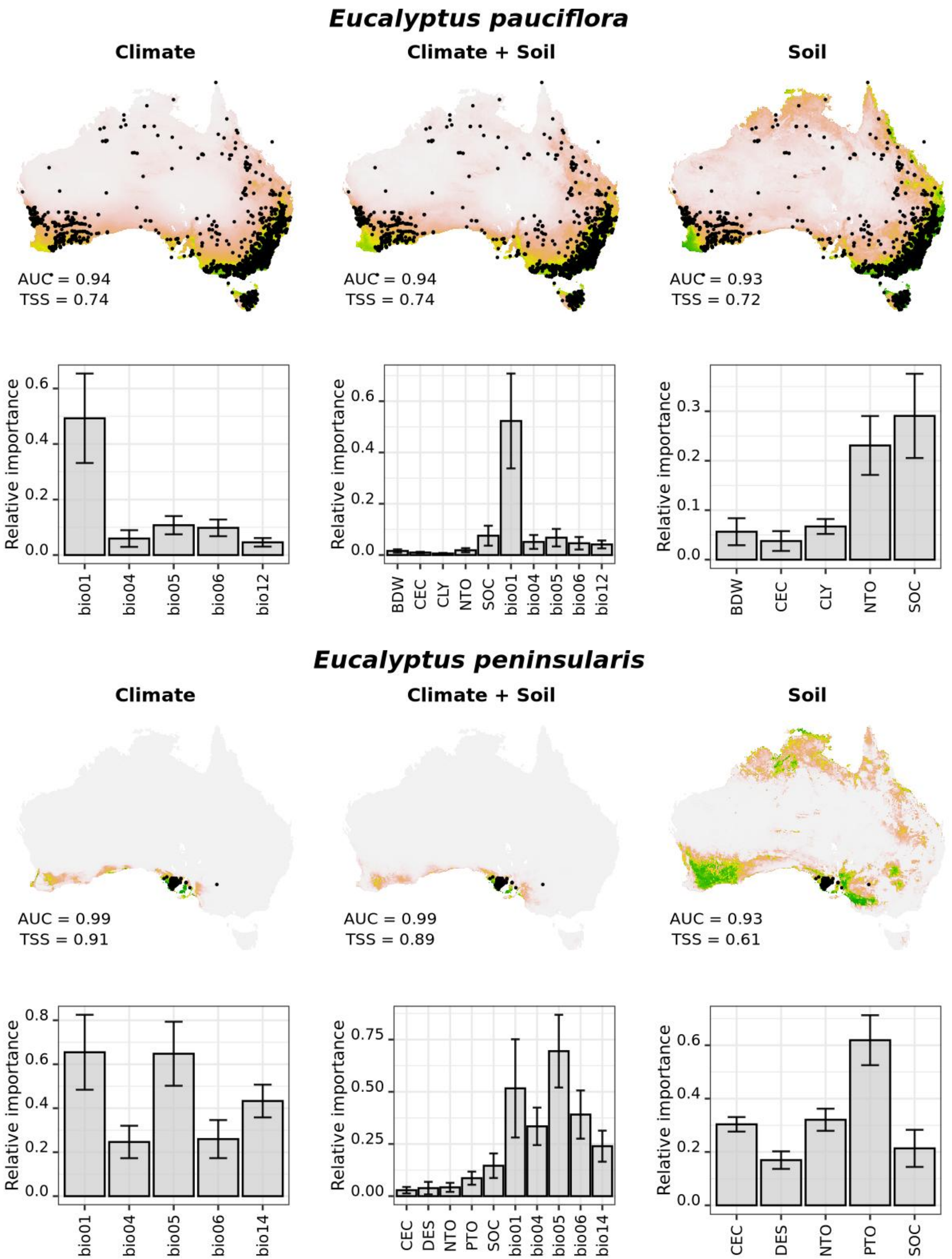
Eucalyptus pauciflora
Climate
Climate + Soil
Soil
AUC = 0.94
TSS = 0.74
AUC = 0.94
TSS = 0.74
AUC = 0.93
TSS = 0.72
Relative importance
bio01 bio04 bio05 bio06 bio12
BDW CEC CLY NTO SOC bio01 bio04 bio05 bio06 bio12
BDW CEC CLY NTO SOC
Eucalyptus peninsularis
Climate
Climate + Soil
Soil
AUC = 0.99
TSS = 0.91
AUC = 0.99
TSS = 0.89
AUC = 0.93
TSS = 0.61
Relative importance
bio01 bio04 bio05 bio06 bio14
CEC DES NTO PTO SOC bio01 bio04 bio05 bio06 bio14
CEC DES NTO PTO SOC

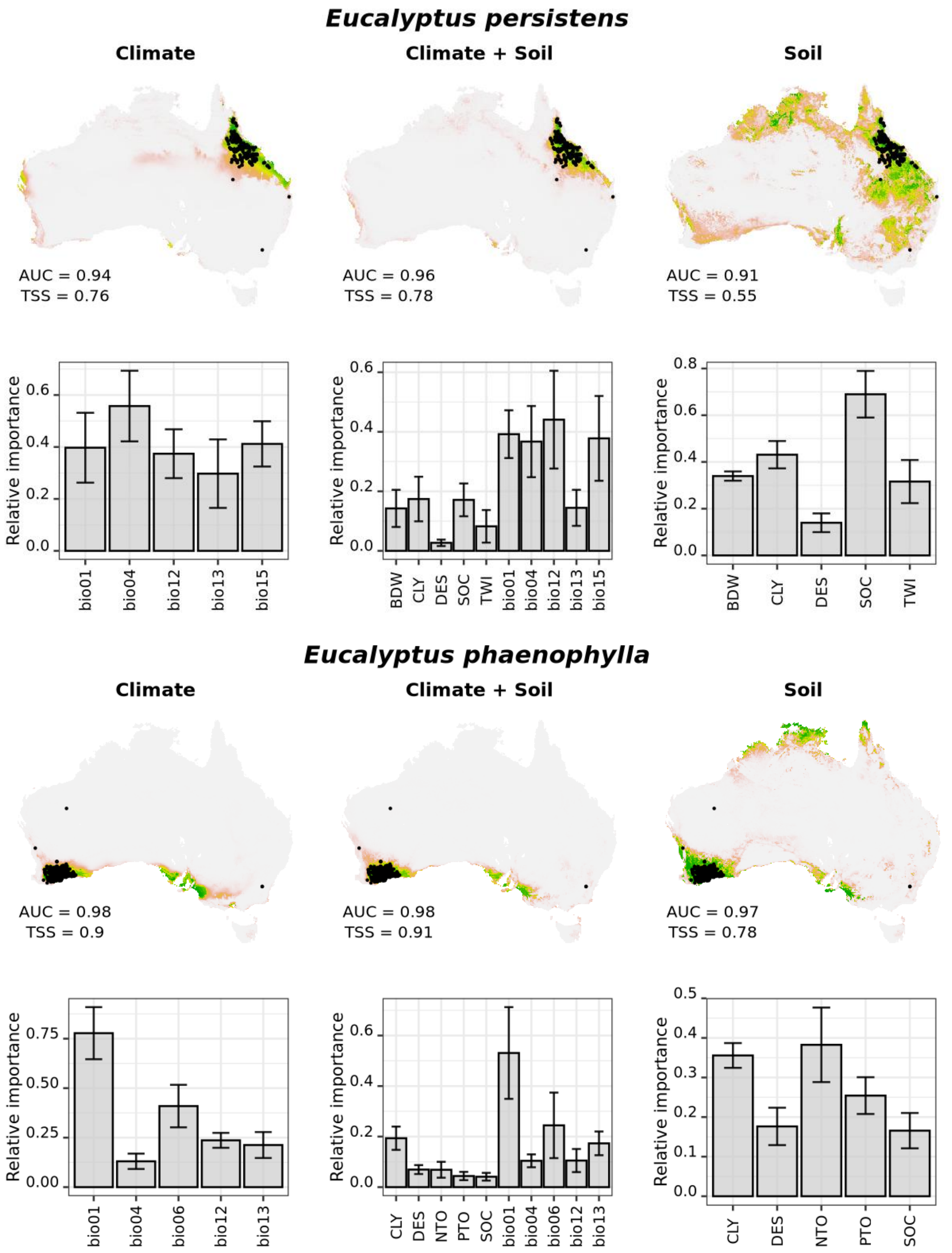

Eucalyptus persistens
Climate
Climate + Soil
Soil
AUC = 0.94
TSS = 0.76
AUC = 0.96
TSS = 0.78
AUC = 0.91
TSS = 0.55
Relative importance
bio01
bio04
bio12
bio13
bio15
BDW
CLY
DES
SOC
TWI
Eucalyptus phaenophylla
Climate
Climate + Soil
Soil
AUC = 0.98
TSS = 0.9
AUC = 0.98
TSS = 0.91
AUC = 0.97
TSS = 0.78
Relative importance
bio01
bio04
bio06
bio12
bio13
CLY
DES
NTO
PTO
SOC

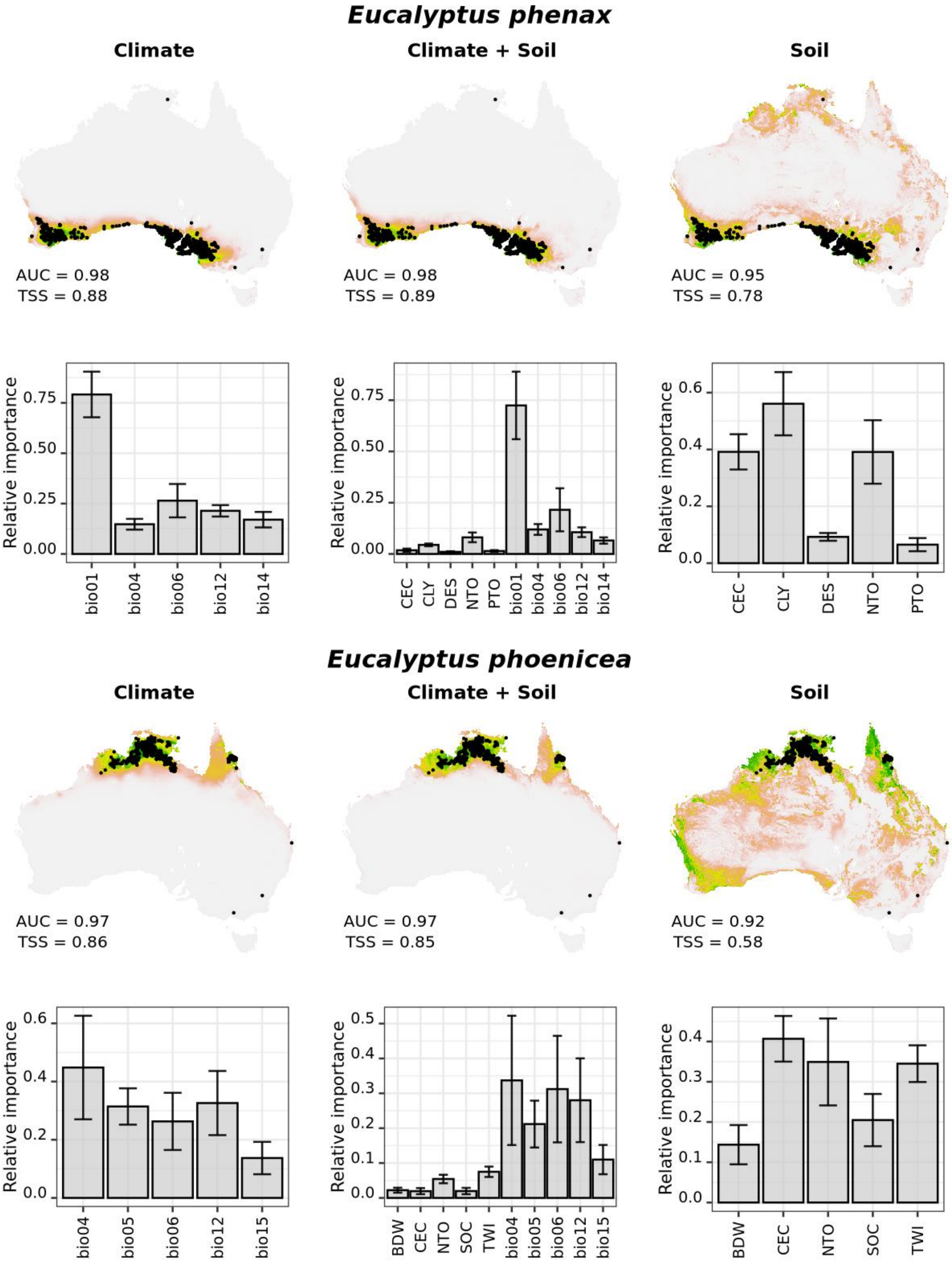
Eucalyptus phenax
Climate
Climate + Soil
Soil
AUC = 0.98
TSS = 0.88
AUC = 0.98
TSS = 0.89
AUC = 0.95
TSS = 0.78
Relative importance
0.00
0.25
0.50
0.75
bio01
bio04
bio06
bio12
bio14
CEC
CLY
DES
NTO
PTO
0.0
0.2
0.4
0.6
Eucalyptus phoenicea
AUC = 0.97
TSS = 0.86
AUC = 0.97
TSS = 0.85
AUC = 0.92
TSS = 0.58
bio05
bio15
BDW
SOC
TWI
0.1
0.3
0.5

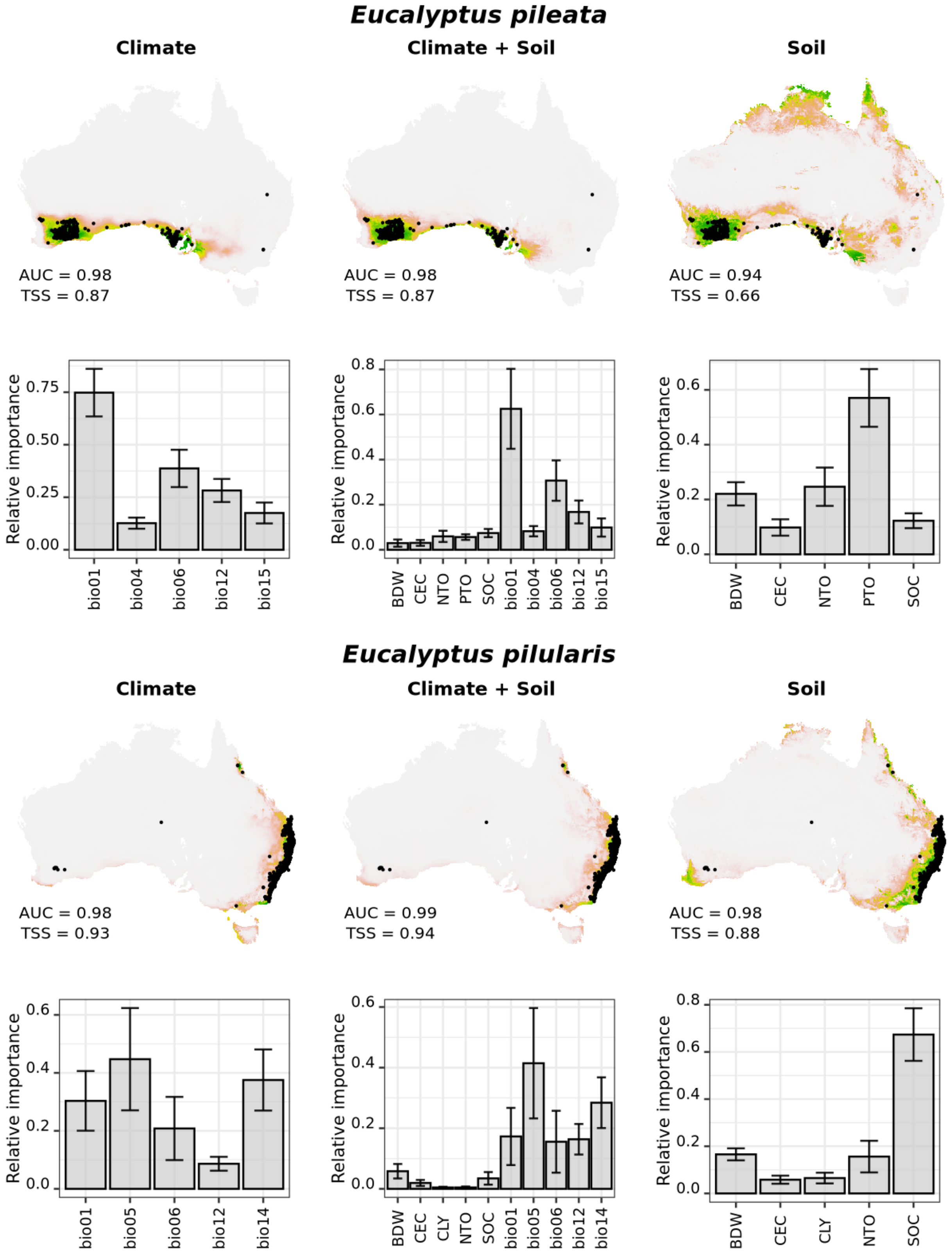

Eucalyptus pileata
Climate
Climate + Soil
Soil
AUC = 0.98
TSS = 0.87
AUC = 0.98
TSS = 0.87
AUC = 0.94
TSS = 0.66
Relative importance
0.00
0.25
0.50
0.75
bio01
bio04
bio06
bio12
bio15
0.0
0.2
0.4
0.6
0.8
BDW
CEC
NTO
PTO
SOC
bio01
bio04
bio06
bio12
bio15
0.0
0.2
0.4
0.6
BDW
CEC
NTO
PTO
SOC
Eucalyptus pilularis
Climate
Climate + Soil
Soil
AUC = 0.98
TSS = 0.93
AUC = 0.99
TSS = 0.94
AUC = 0.98
TSS = 0.88
Relative importance
0.0
0.2
0.4
0.6
bio01
bio05
bio06
bio12
bio14
0.0
0.2
0.4
0.6
BDW
CEC
CLY
NTO
SOC
bio01
bio05
bio06
bio12
bio14
0.0
0.2
0.4
0.6
0.8
BDW
CEC
CLY
NTO
SOC

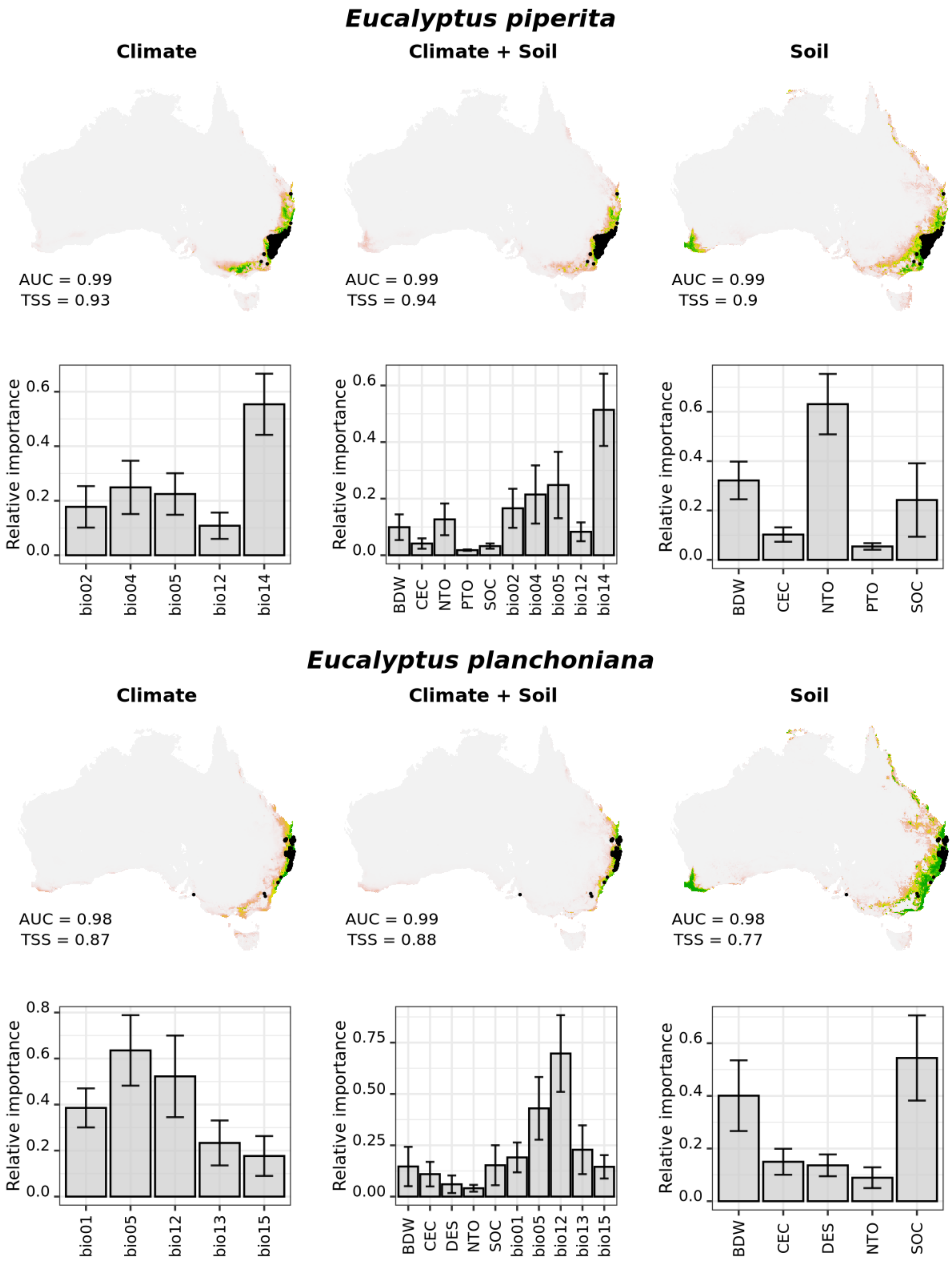

Eucalyptus piperita
Climate
Climate + Soil
Soil
AUC = 0.99
TSS = 0.93
AUC = 0.99
TSS = 0.94
AUC = 0.99
TSS = 0.9
Relative importance
bio02 bio04 bio05 bio12 bio14
BDW CEC NTO PTO SOC bio02 bio04 bio05 bio12 bio14
BDW CEC NTO PTO SOC
Eucalyptus planchoniana
Climate
Climate + Soil
Soil
AUC = 0.98
TSS = 0.87
AUC = 0.99
TSS = 0.88
AUC = 0.98
TSS = 0.77
Relative importance
bio01 bio05 bio12 bio13 bio15
BDW CEC DES NTO SOC bio01 bio05 bio12 bio13 bio15
BDW CEC DES NTO SOC

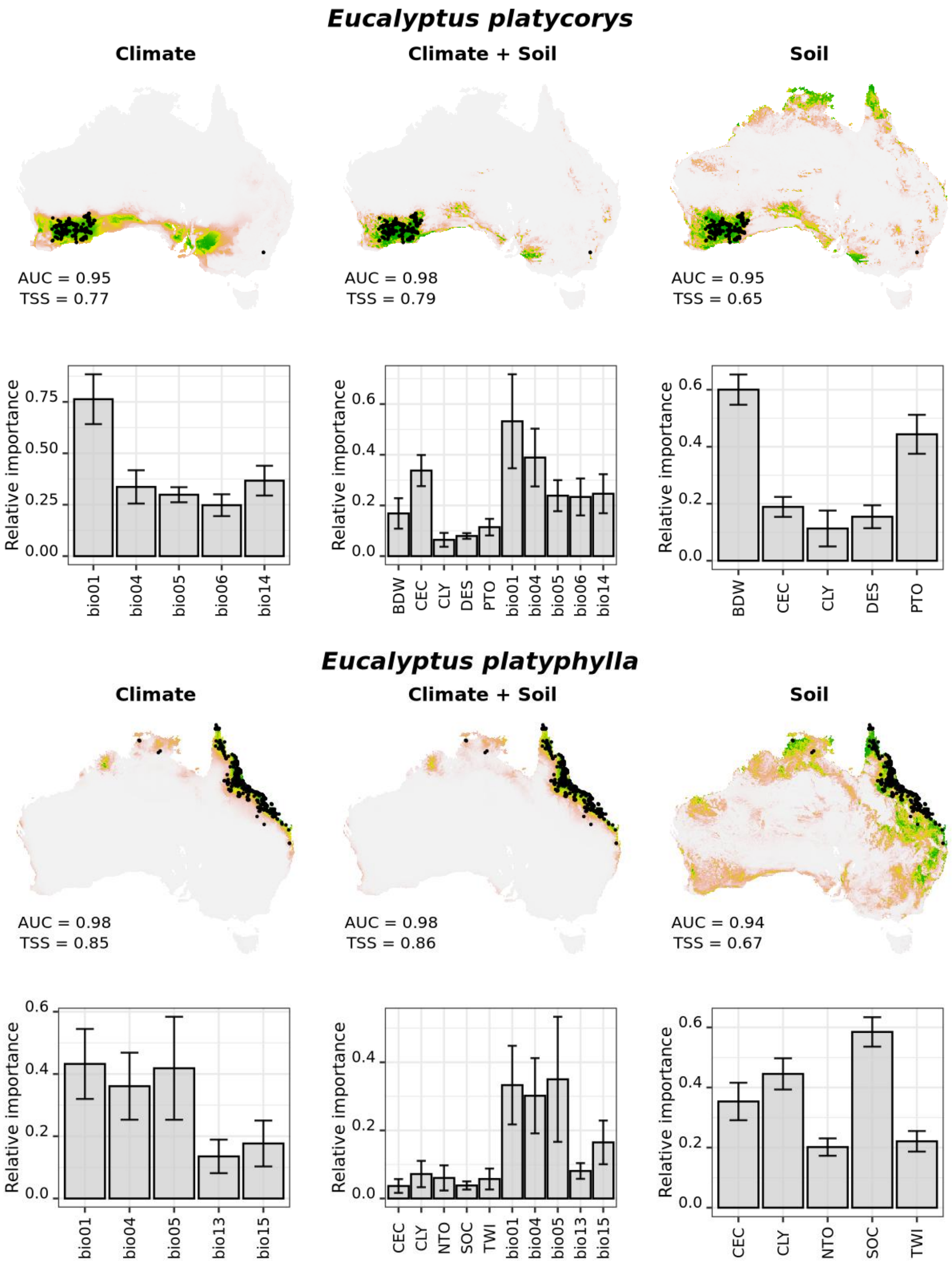

Eucalyptus platycorys
Climate
Climate + Soil
Soil
AUC = 0.95
TSS = 0.77
AUC = 0.98
TSS = 0.79
AUC = 0.95
TSS = 0.65
Relative importance
bio01 bio04 bio05 bio06 bio14
BDW CEC CLY DES PTO bio01 bio04 bio05 bio06 bio14
BDW CEC CLY DES PTO
Eucalyptus platyphylla
Climate
Climate + Soil
Soil
AUC = 0.98
TSS = 0.85
AUC = 0.98
TSS = 0.86
AUC = 0.94
TSS = 0.67
Relative importance
bio01 bio04 bio05 bio13 bio15
CEC CLY NTO SOC TWI bio01 bio04 bio05 bio13 bio15
CEC CLY NTO SOC TWI

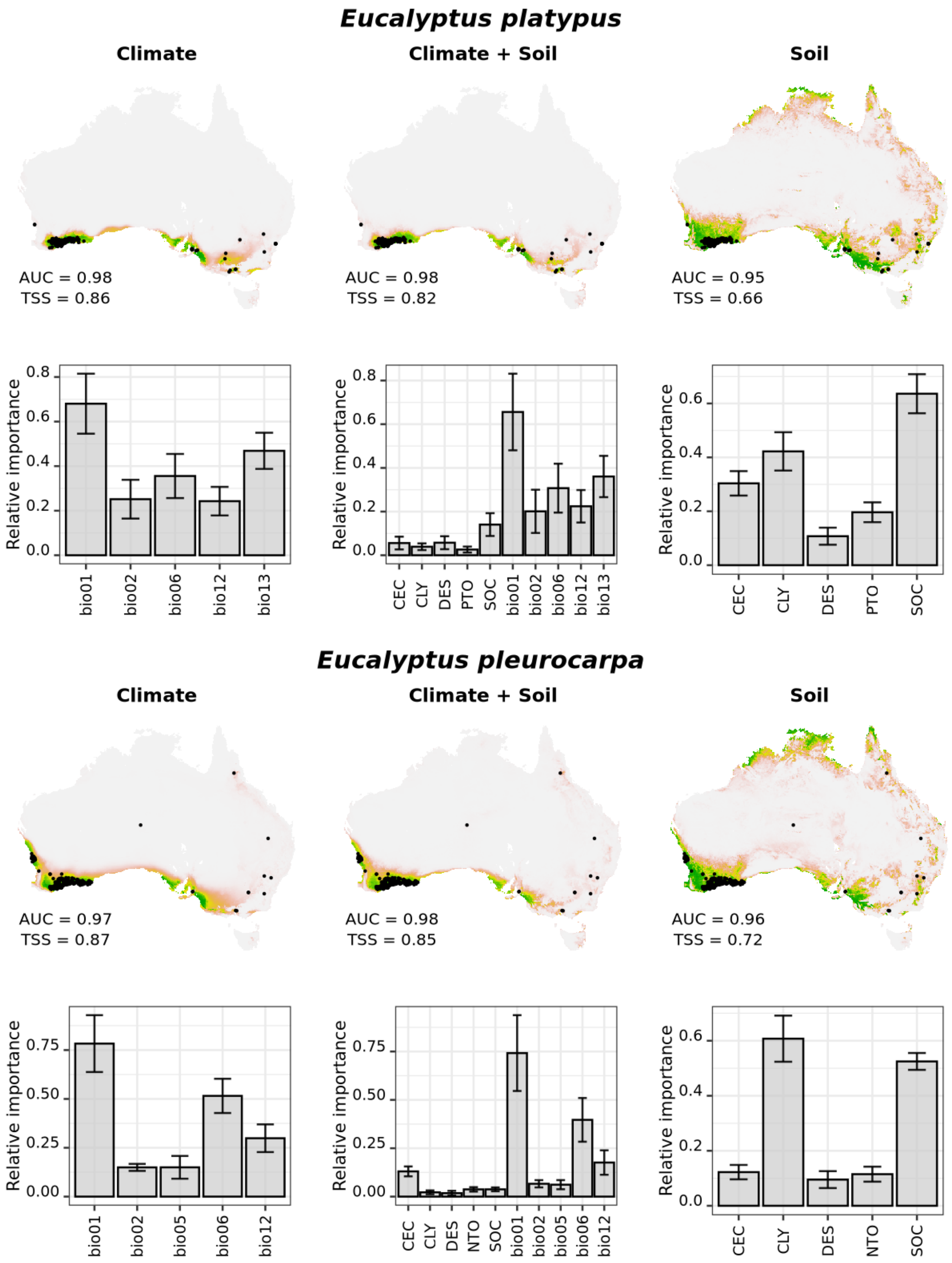

Eucalyptus platypus
Climate
Climate + Soil
Soil
AUC = 0.98
TSS = 0.86
AUC = 0.98
TSS = 0.82
AUC = 0.95
TSS = 0.66
Relative importance
bio01
bio02
bio06
bio12
bio13
CEC
CLY
DES
PTO
SOC
Eucalyptus pleurocarpa
Climate
Climate + Soil
Soil
AUC = 0.97
TSS = 0.87
AUC = 0.98
TSS = 0.85
AUC = 0.96
TSS = 0.72
Relative importance
bio01
bio02
bio05
bio06
bio12
CEC
CLY
DES
NTO
SOC

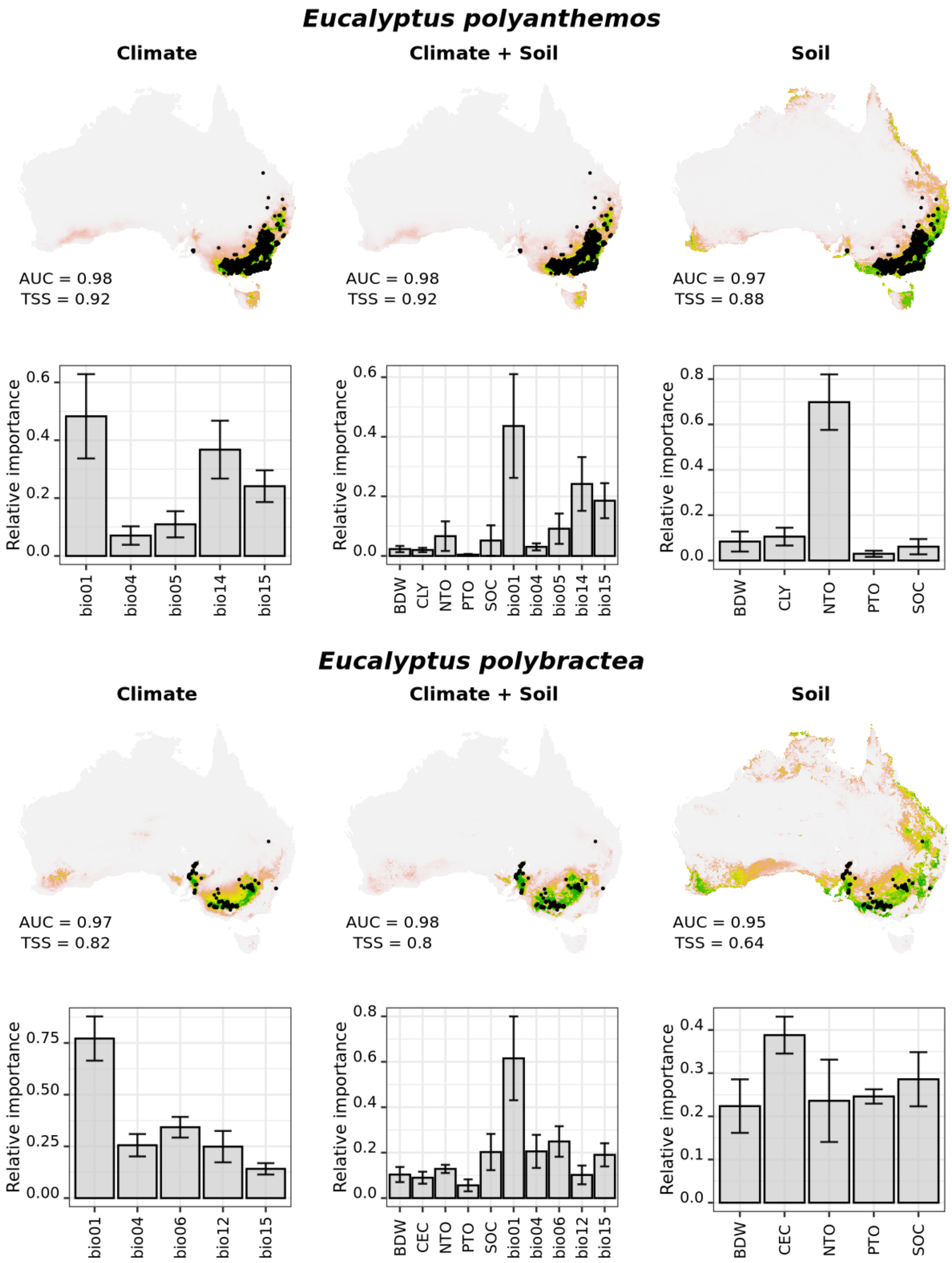
Eucalyptus polyanthemos
Climate
Climate + Soil
Soil
AUC = 0.98
TSS = 0.92
AUC = 0.98
TSS = 0.92
AUC = 0.97
TSS = 0.88
Relative importance
0.0
0.2
0.4
0.6
0.8
bio01
bio04
bio05
bio14
bio15
BDW
CLY
NTO
PTO
SOC
Eucalyptus polybractea
Climate
Climate + Soil
Soil
AUC = 0.97
TSS = 0.82
AUC = 0.98
TSS = 0.8
AUC = 0.95
TSS = 0.64
Relative importance
0.00
0.25
0.50
0.75
0.1
0.3
0.4
bio01
bio04
bio06
bio12
bio15
BDW
CEC
NTO
PTO
SOC

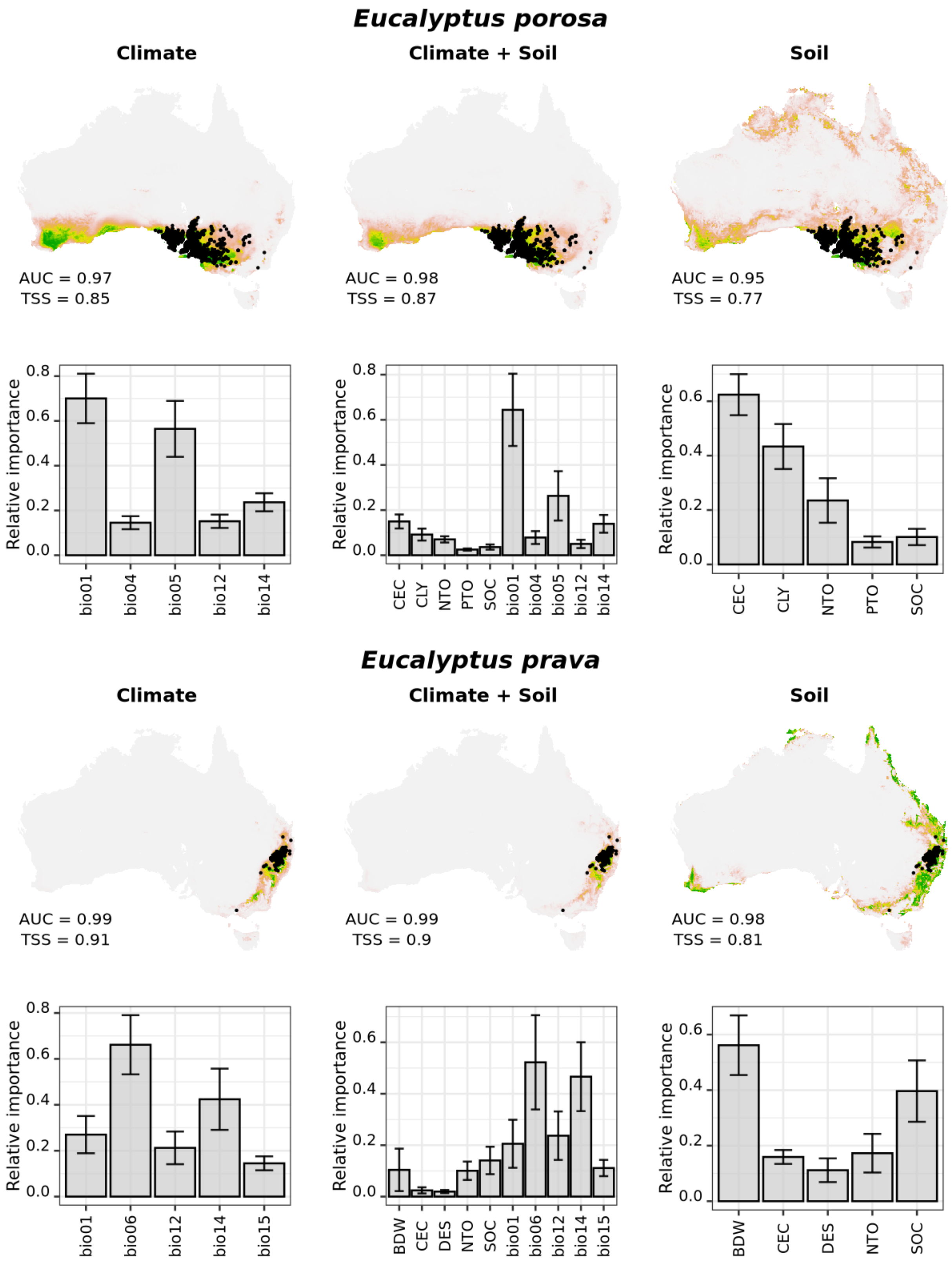
Eucalyptus porosa
Climate
Climate + Soil
Soil
AUC = 0.97
TSS = 0.85
AUC = 0.98
TSS = 0.87
AUC = 0.95
TSS = 0.77
Relative importance
bio01
bio04
bio05
bio12
bio14
CEC
CLY
NTO
PTO
SOC
Eucalyptus prava
AUC = 0.99
TSS = 0.91
AUC = 0.99
TSS = 0.9
AUC = 0.98
TSS = 0.81
bio06
bio15
BDW
DES

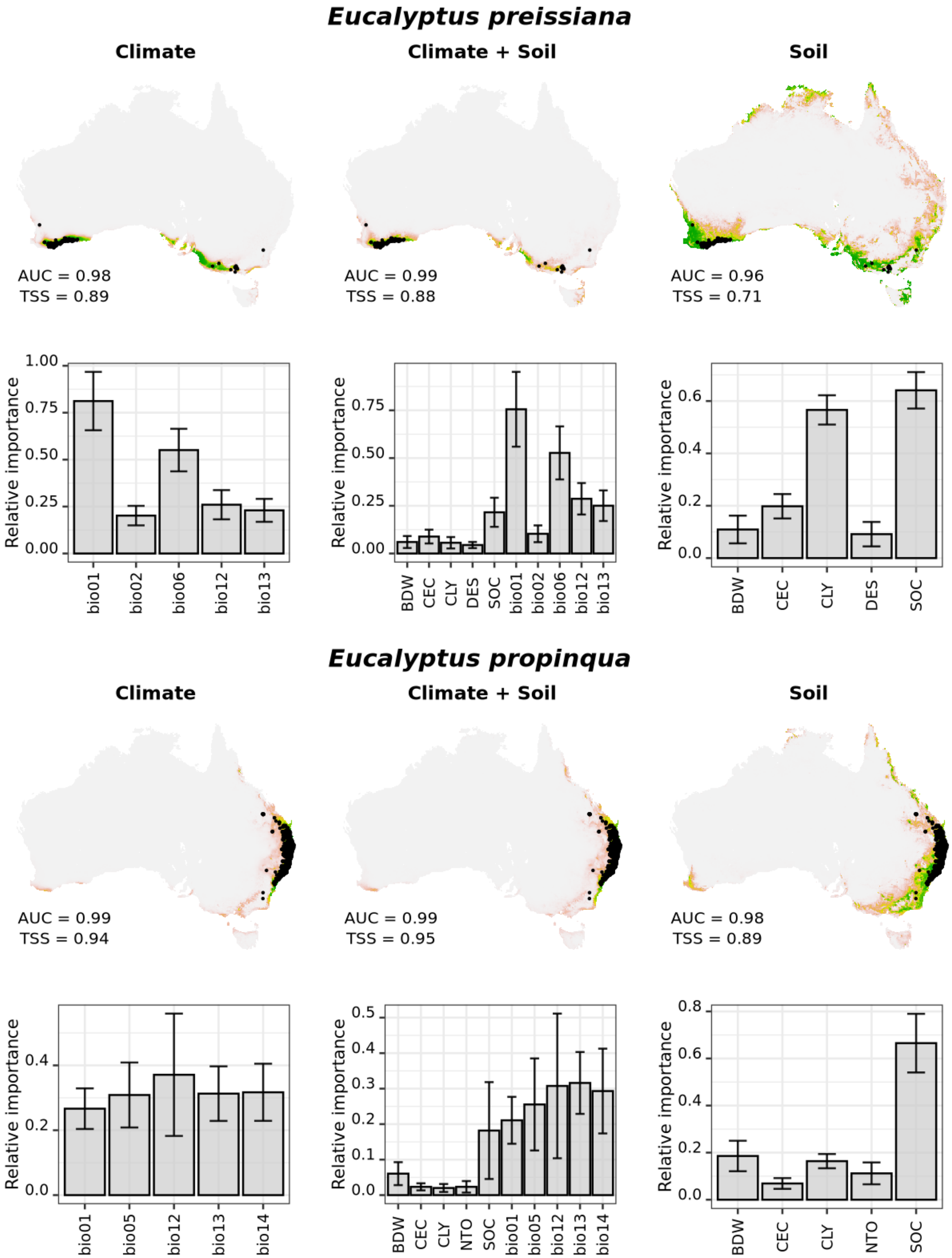
Eucalyptus preissiana
Climate
Climate + Soil
Soil
AUC = 0.98
TSS = 0.89
AUC = 0.99
TSS = 0.88
AUC = 0.96
TSS = 0.71
Relative importance
1.00
0.75
0.50
0.25
0.00
bio01
bio02
bio06
bio12
bio13
Relative importance
0.75
0.50
0.25
0.00
BDW
CEC
CLY
DES
SOC
bio01
bio02
bio06
bio12
bio13
Relative importance
0.6
0.4
0.2
0.0
BDW
CEC
CLY
DES
SOC
Eucalyptus propinqua
Climate
Climate + Soil
Soil
AUC = 0.99
TSS = 0.94
AUC = 0.99
TSS = 0.95
AUC = 0.98
TSS = 0.89
Relative importance
0.4
0.2
0.0
bio01
bio05
bio12
bio13
bio14
Relative importance
0.5
0.4
0.3
0.2
0.1
0.0
BDW
CEC
CLY
NTO
SOC
bio01
bio05
bio12
bio13
bio14
Relative importance
0.8
0.6
0.4
0.2
0.0
BDW
CEC
CLY
NTO
SOC

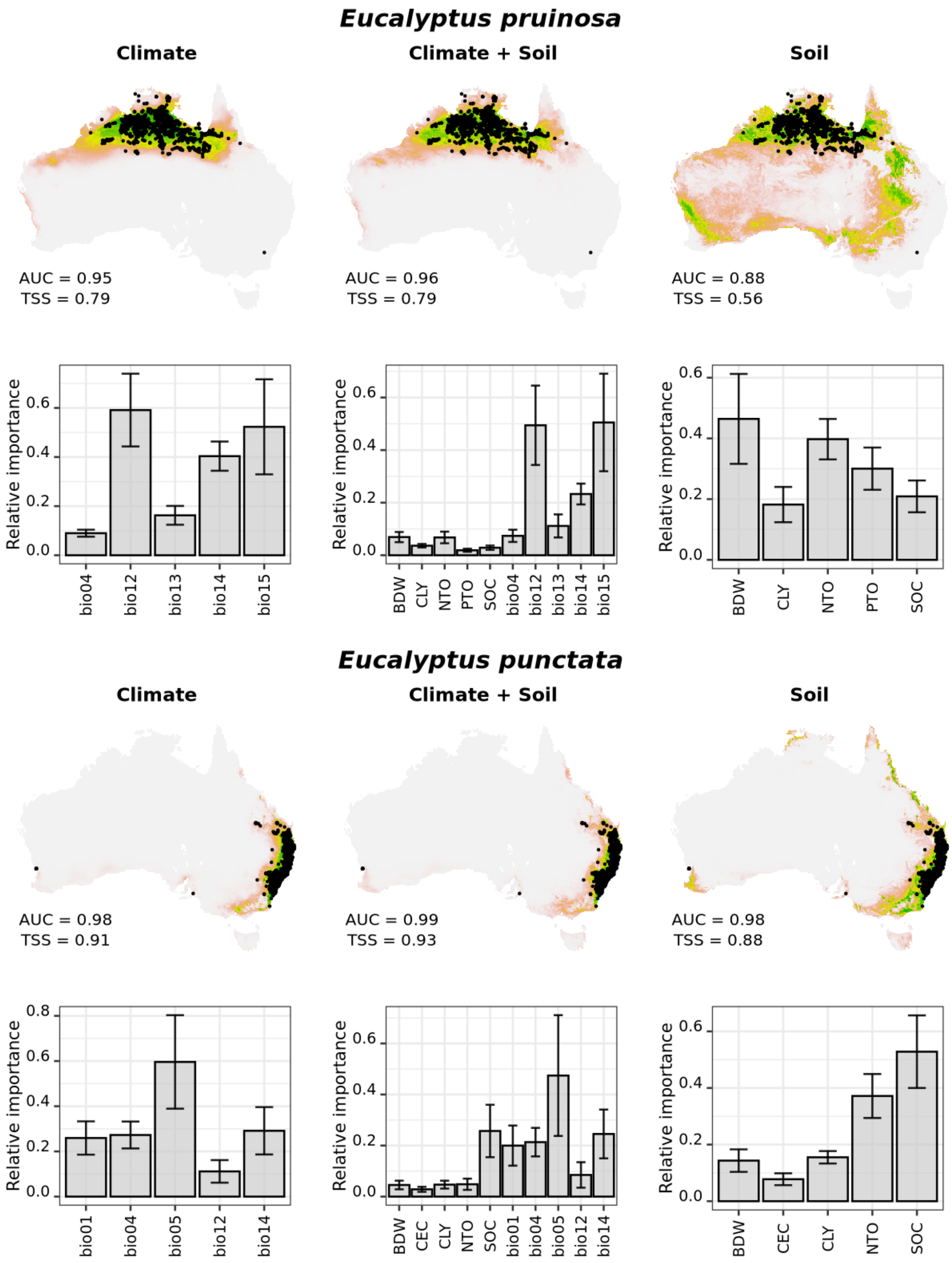
Eucalyptus pruinosa
Climate
Climate + Soil
Soil
AUC = 0.95
TSS = 0.79
AUC = 0.96
TSS = 0.79
AUC = 0.88
TSS = 0.56
Relative importance
0.0
0.2
0.4
0.6
bio04
bio12
bio13
bio14
bio15
BDW
CLY
NTO
PTO
SOC
Eucalyptus punctata
Climate
Climate + Soil
Soil
AUC = 0.98
TSS = 0.91
AUC = 0.99
TSS = 0.93
AUC = 0.98
TSS = 0.88
Relative importance
0.8
bio01
bio05
CEC

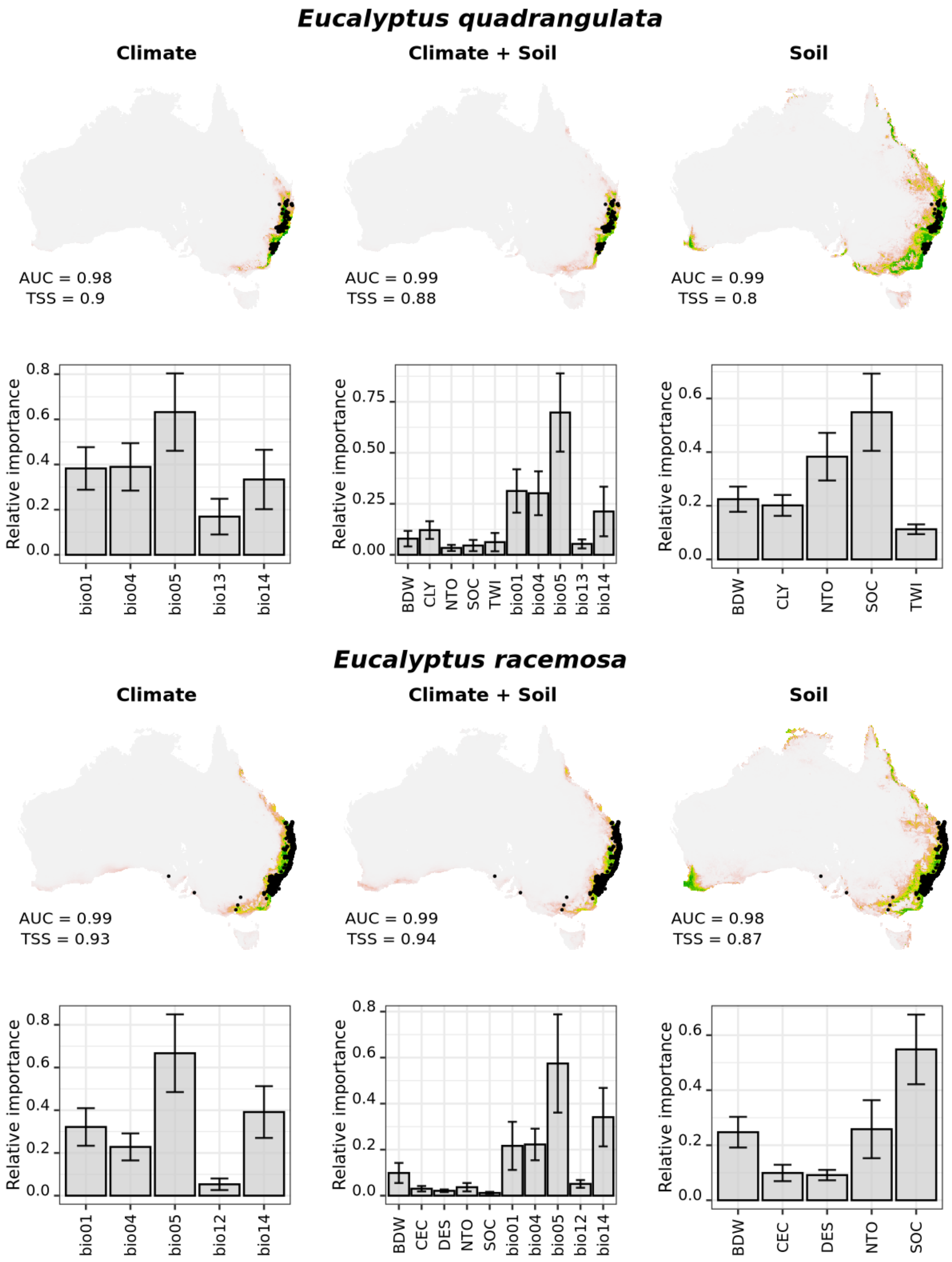
Eucalyptus quadrangulata
Climate
Climate + Soil
Soil
AUC = 0.98
TSS = 0.9
AUC = 0.99
TSS = 0.88
AUC = 0.99
TSS = 0.8
Relative importance
bio01
bio04
bio05
bio13
bio14
BDW
CLY
NTO
SOC
TWI
Eucalyptus racemosa
Climate
Climate + Soil
Soil
AUC = 0.99
TSS = 0.93
AUC = 0.99
TSS = 0.94
AUC = 0.98
TSS = 0.87
Relative importance
bio01
bio04
bio05
bio12
bio14
BDW
CEC
DES
NTO
SOC

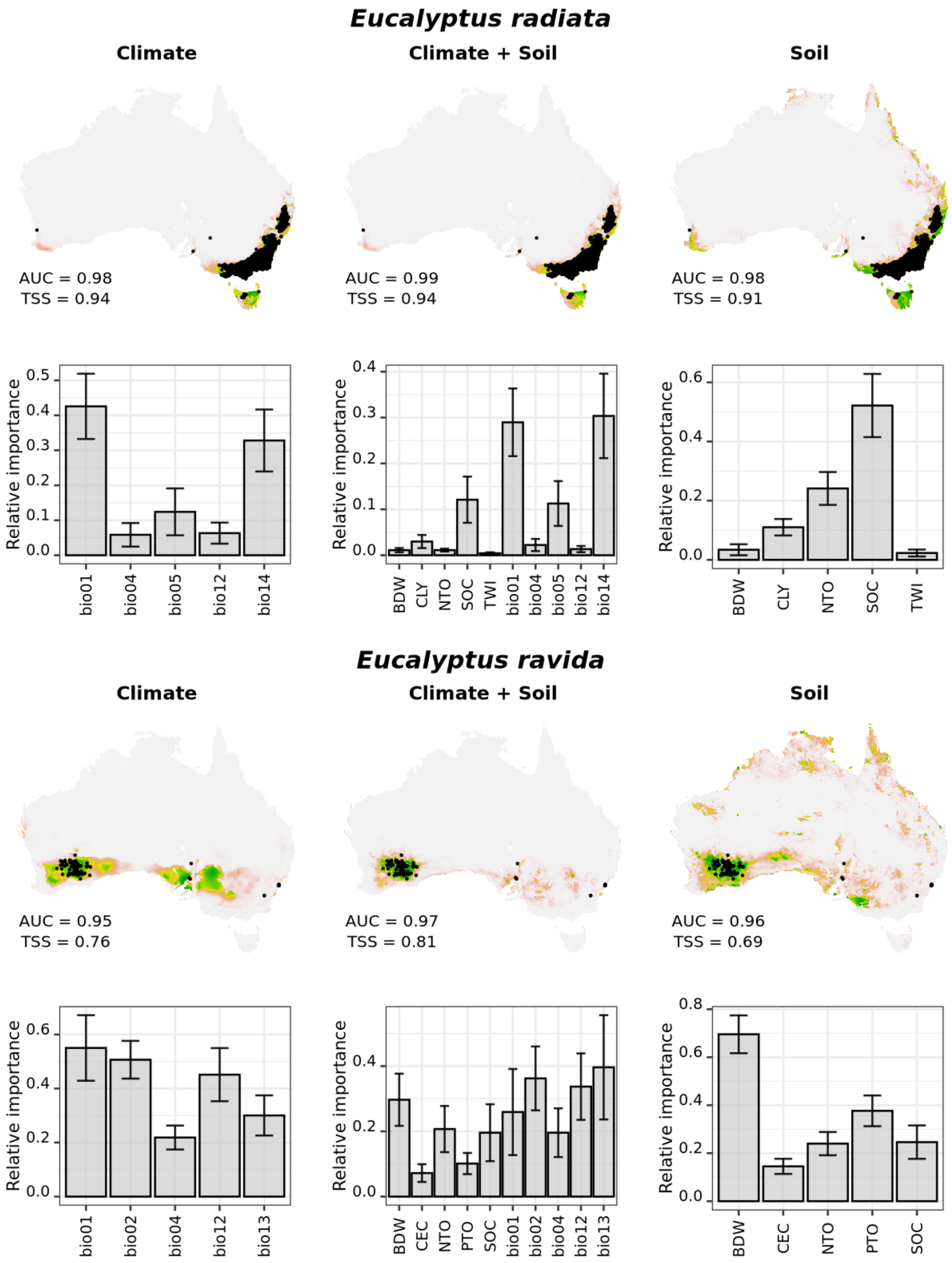
Eucalyptus radiata
Climate
Climate + Soil
Soil
AUC = 0.98
TSS = 0.94
AUC = 0.99
TSS = 0.94
AUC = 0.98
TSS = 0.91
Relative importance
bio01 bio04 bio05 bio12 bio14
BDW CLY NTO SOC TWI bio01 bio04 bio05 bio12 bio14
BDW CLY NTO SOC TWI
Eucalyptus ravida
Climate
Climate + Soil
Soil
AUC = 0.95
TSS = 0.76
AUC = 0.97
TSS = 0.81
AUC = 0.96
TSS = 0.69
Relative importance
bio01 bio02 bio04 bio12 bio13
BDW CEC NTO PTO SOC bio01 bio02 bio04 bio12 bio13
BDW CEC NTO PTO SOC

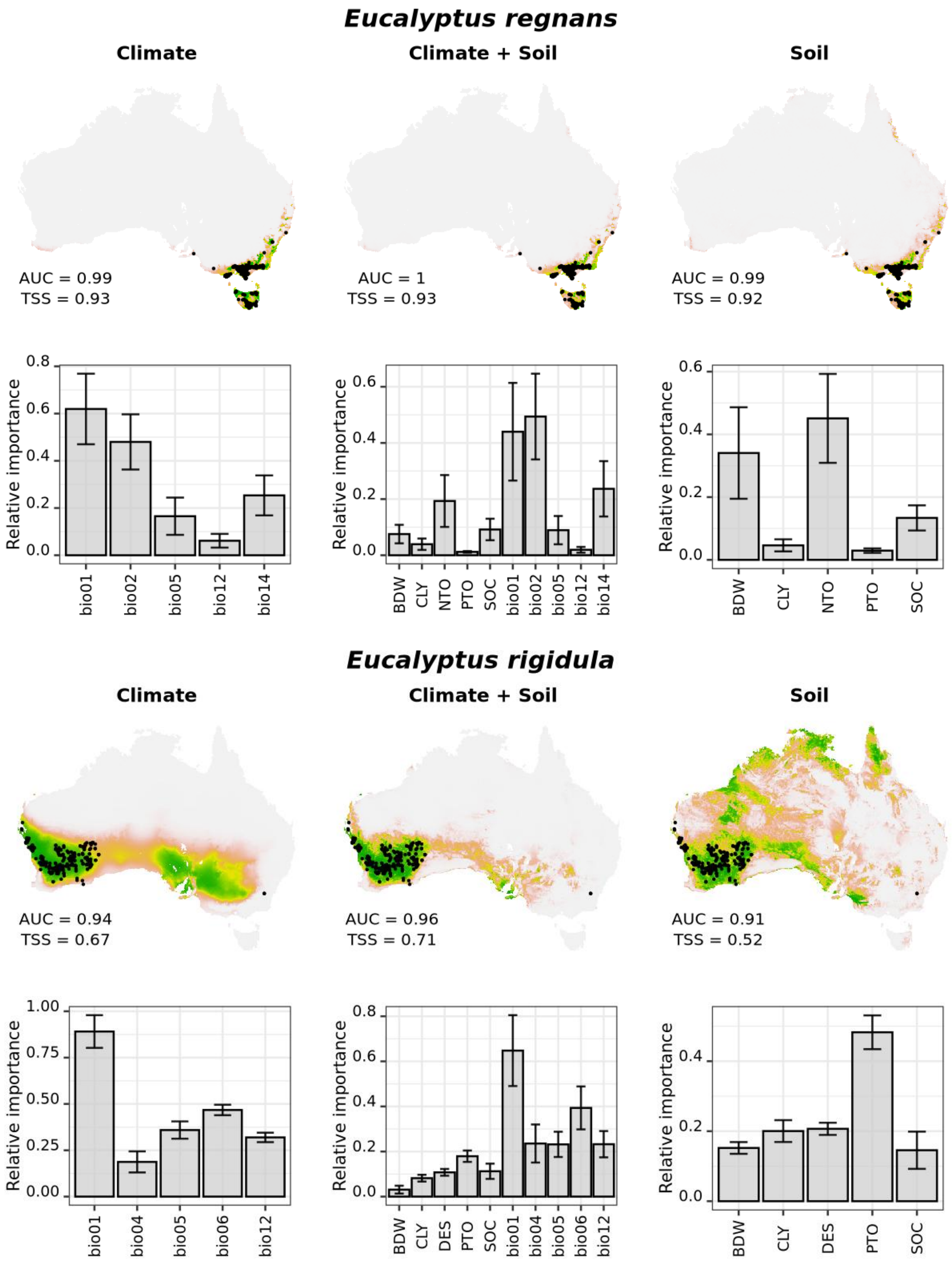
Eucalyptus regnans
Climate
Climate + Soil
Soil
AUC = 0.99
TSS = 0.93
AUC = 1
TSS = 0.93
AUC = 0.99
TSS = 0.92
Relative importance
bio01
bio02
bio05
bio12
bio14
BDW
CLY
NTO
PTO
SOC
Eucalyptus rigidula
Climate
Climate + Soil
Soil
AUC = 0.94
TSS = 0.67
AUC = 0.96
TSS = 0.71
AUC = 0.91
TSS = 0.52
Relative importance
bio01
bio04
bio05
bio06
bio12
BDW
CLY
DES
PTO
SOC

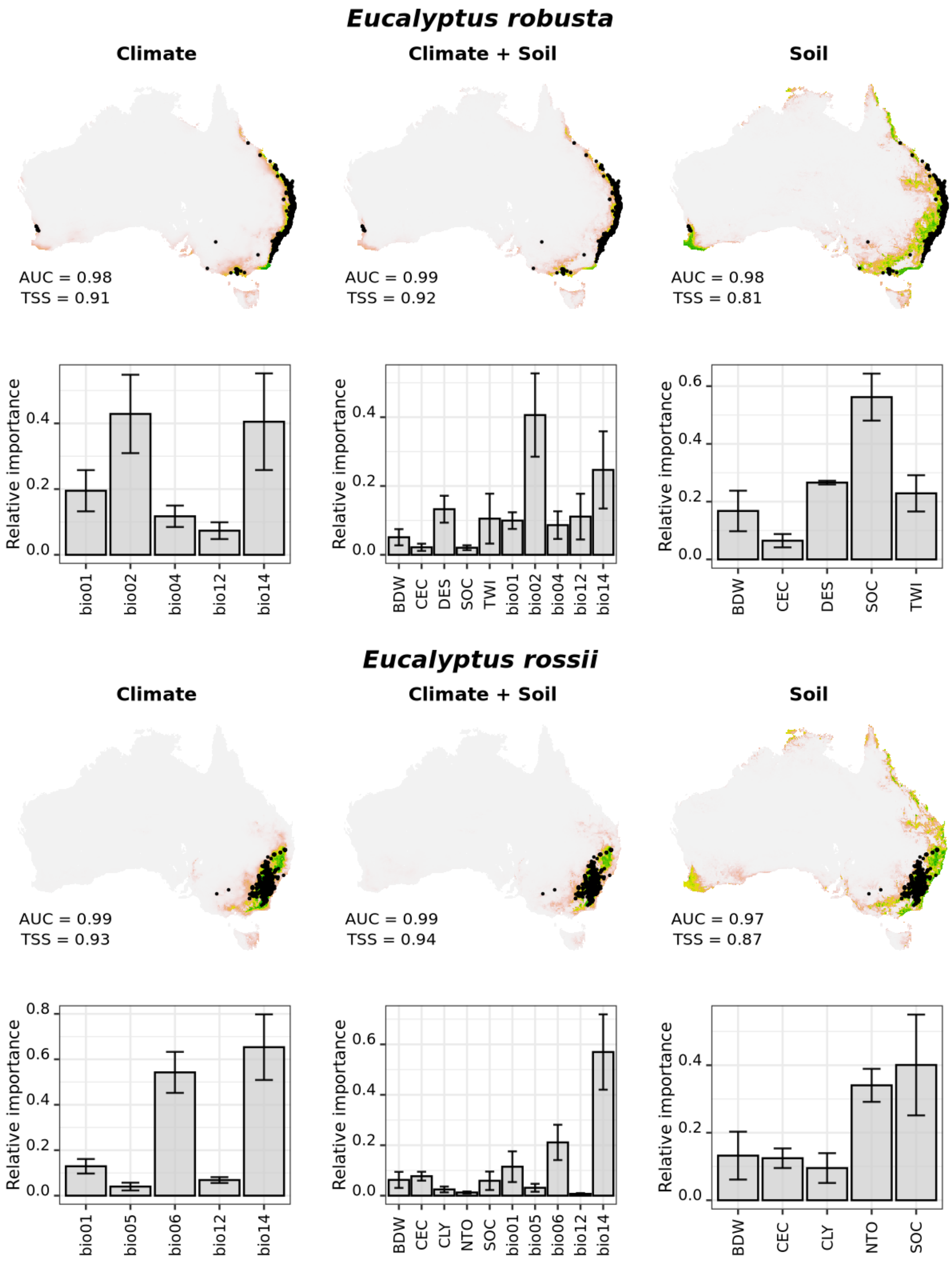
Eucalyptus robusta
Climate
Climate + Soil
Soil
AUC = 0.98
TSS = 0.91
AUC = 0.99
TSS = 0.92
AUC = 0.98
TSS = 0.81
Relative importance
0.0
0.2
0.4
0.6
bio01
bio02
bio04
bio12
bio14
BDW
CEC
DES
SOC
TWI
bio01
bio02
bio04
bio12
bio14
BDW
CEC
DES
SOC
TWI
Eucalyptus rossii
Climate
Climate + Soil
Soil
AUC = 0.99
TSS = 0.93
AUC = 0.99
TSS = 0.94
AUC = 0.97
TSS = 0.87
Relative importance
0.0
0.2
0.4
0.6
0.8
bio01
bio05
bio06
bio12
bio14
BDW
CEC
CLY
NTO
SOC
bio01
bio05
bio06
bio12
bio14
BDW
CEC
CLY
NTO
SOC

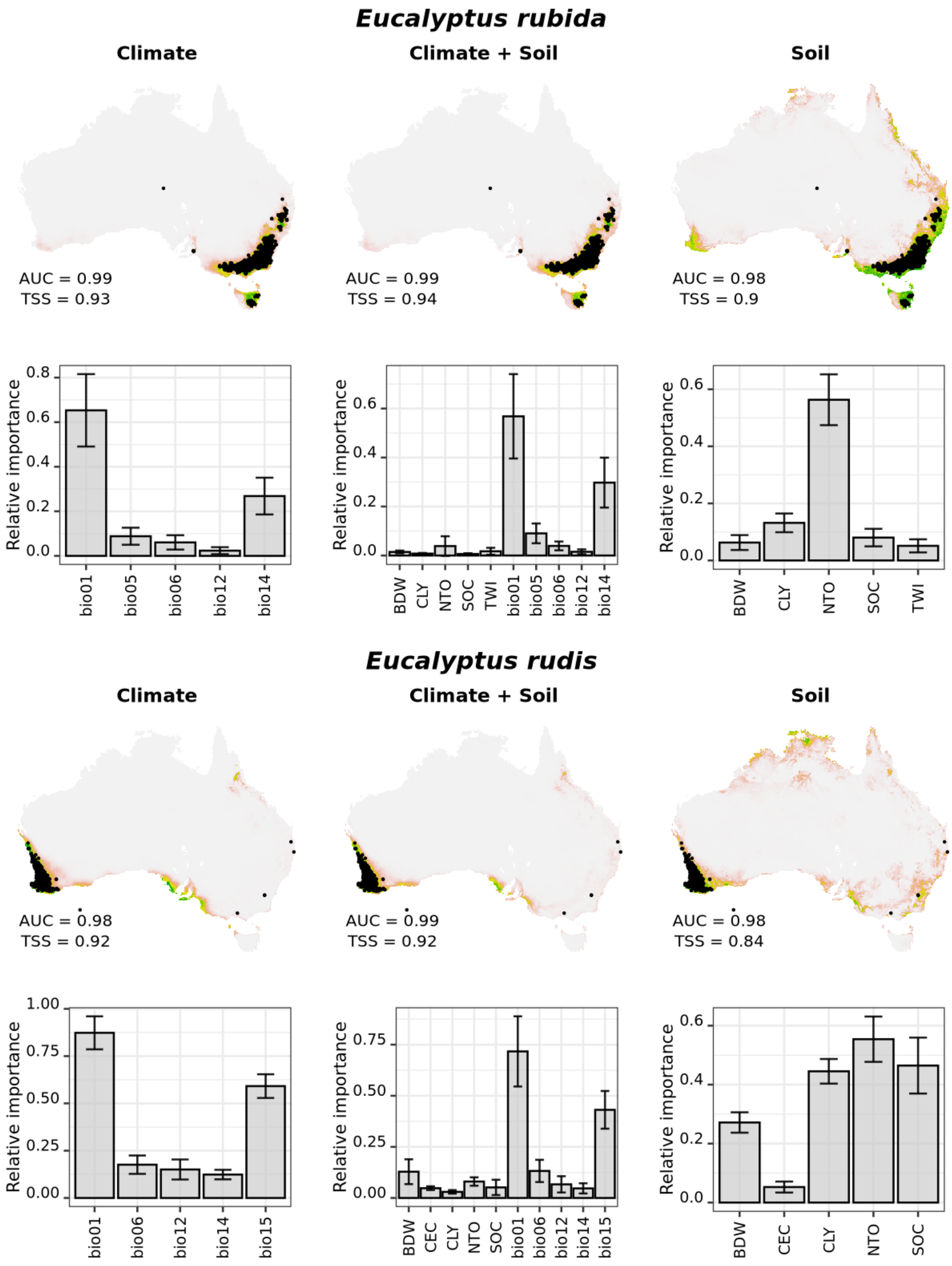
Eucalyptus rubida
Climate
Climate + Soil
Soil
AUC = 0.99
TSS = 0.93
AUC = 0.99
TSS = 0.94
AUC = 0.98
TSS = 0.9
Relative importance
0.0
0.2
0.4
0.6
0.8
bio01
bio05
bio06
bio12
bio14
BDW
CLY
NTO
SOC
TWI
Eucalyptus rudis
Climate
Climate + Soil
Soil
AUC = 0.98
TSS = 0.92
AUC = 0.99
TSS = 0.92
AUC = 0.98
TSS = 0.84
0.00
0.25
0.50
0.75
1.00
bio15
CEC

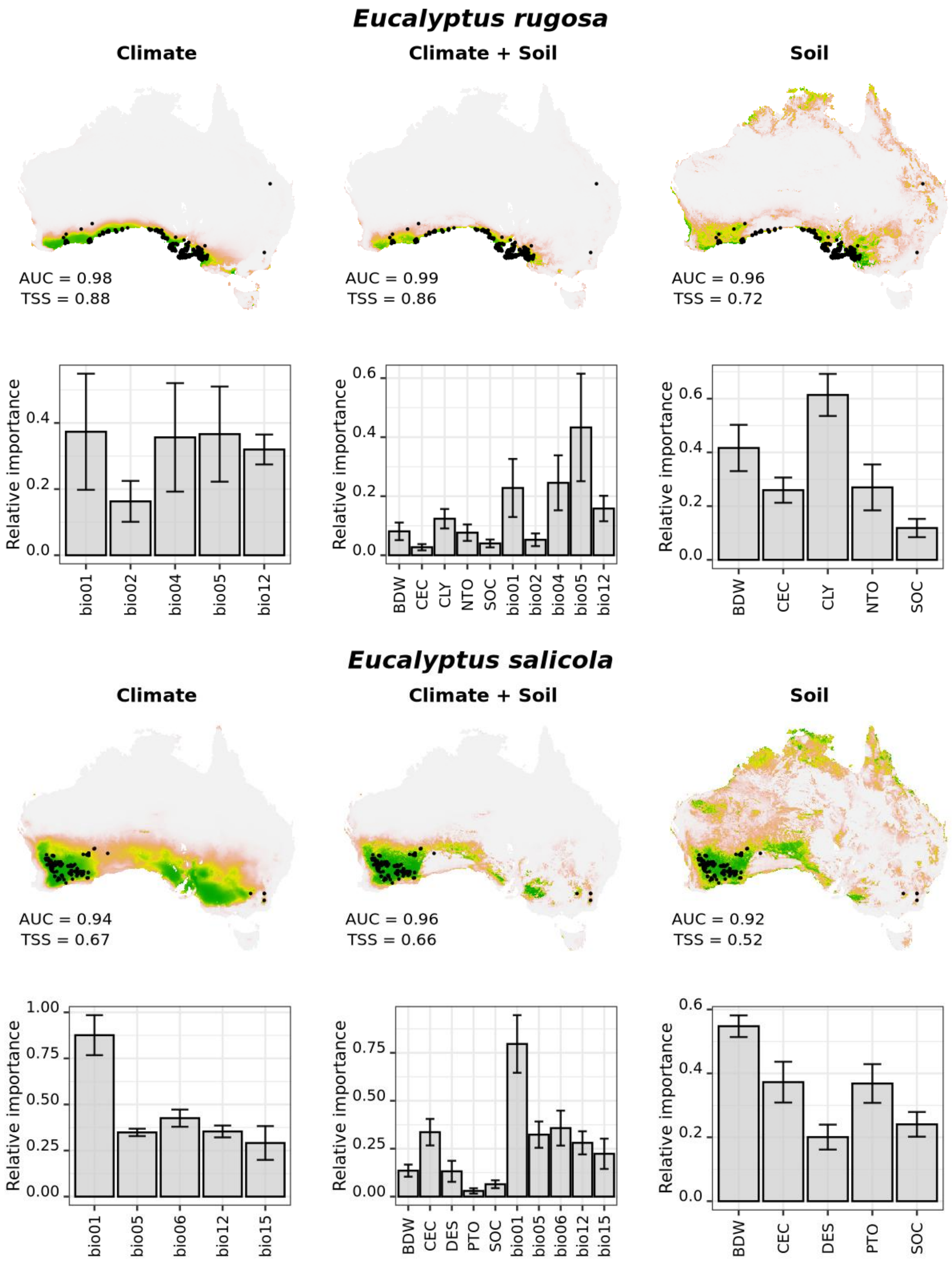
Eucalyptus rugosa
Climate
Climate + Soil
Soil
AUC = 0.98
TSS = 0.88
AUC = 0.99
TSS = 0.86
AUC = 0.96
TSS = 0.72
Relative importance
bio01
bio02
bio04
bio05
bio12
BDW
CEC
CLY
NTO
SOC
Eucalyptus salicola
Climate
Climate + Soil
Soil
AUC = 0.94
TSS = 0.67
AUC = 0.96
TSS = 0.66
AUC = 0.92
TSS = 0.52
Relative importance
bio01
bio05
bio06
bio12
bio15
BDW
CEC
DES
PTO
SOC

Eucalyptus salmonophloia

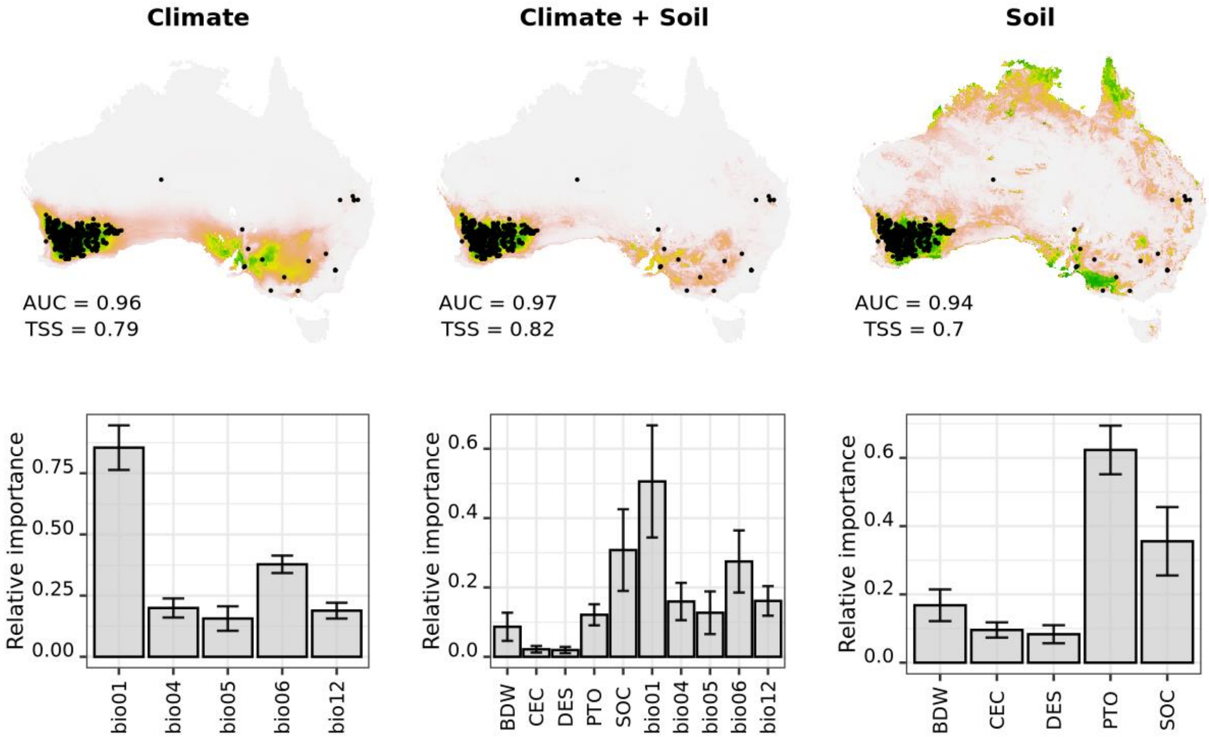

Eucalyptus salubris

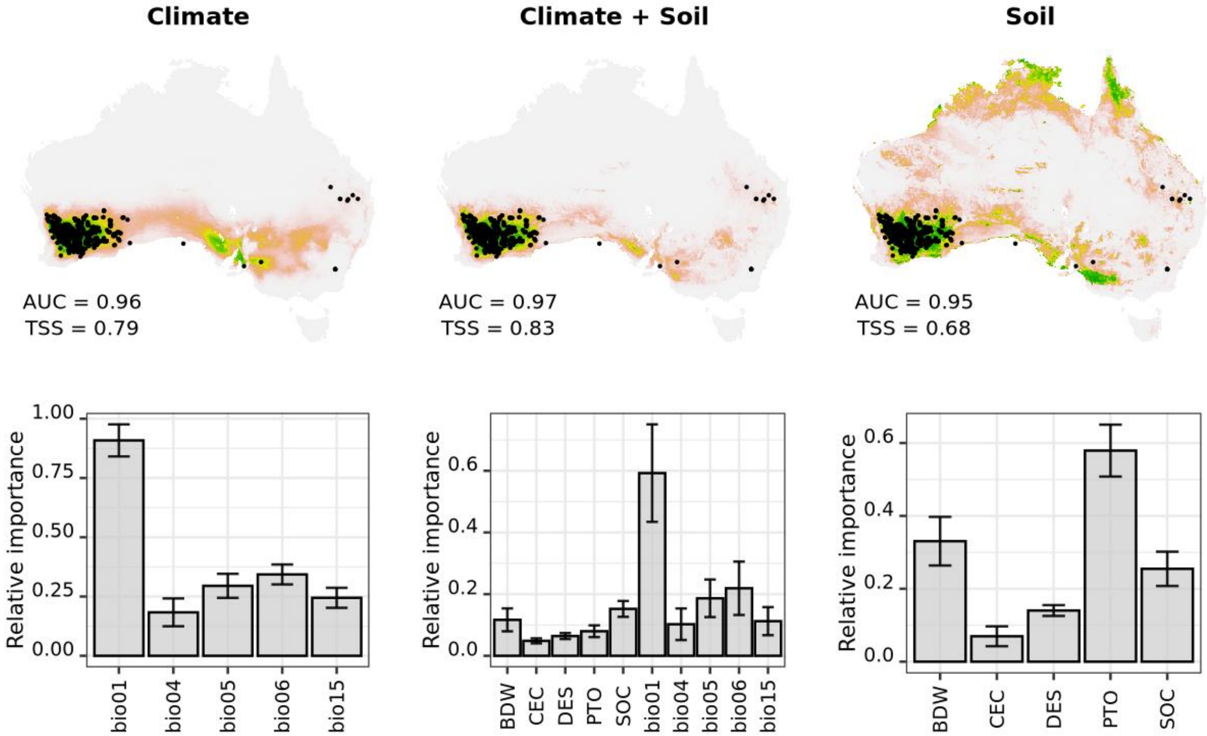

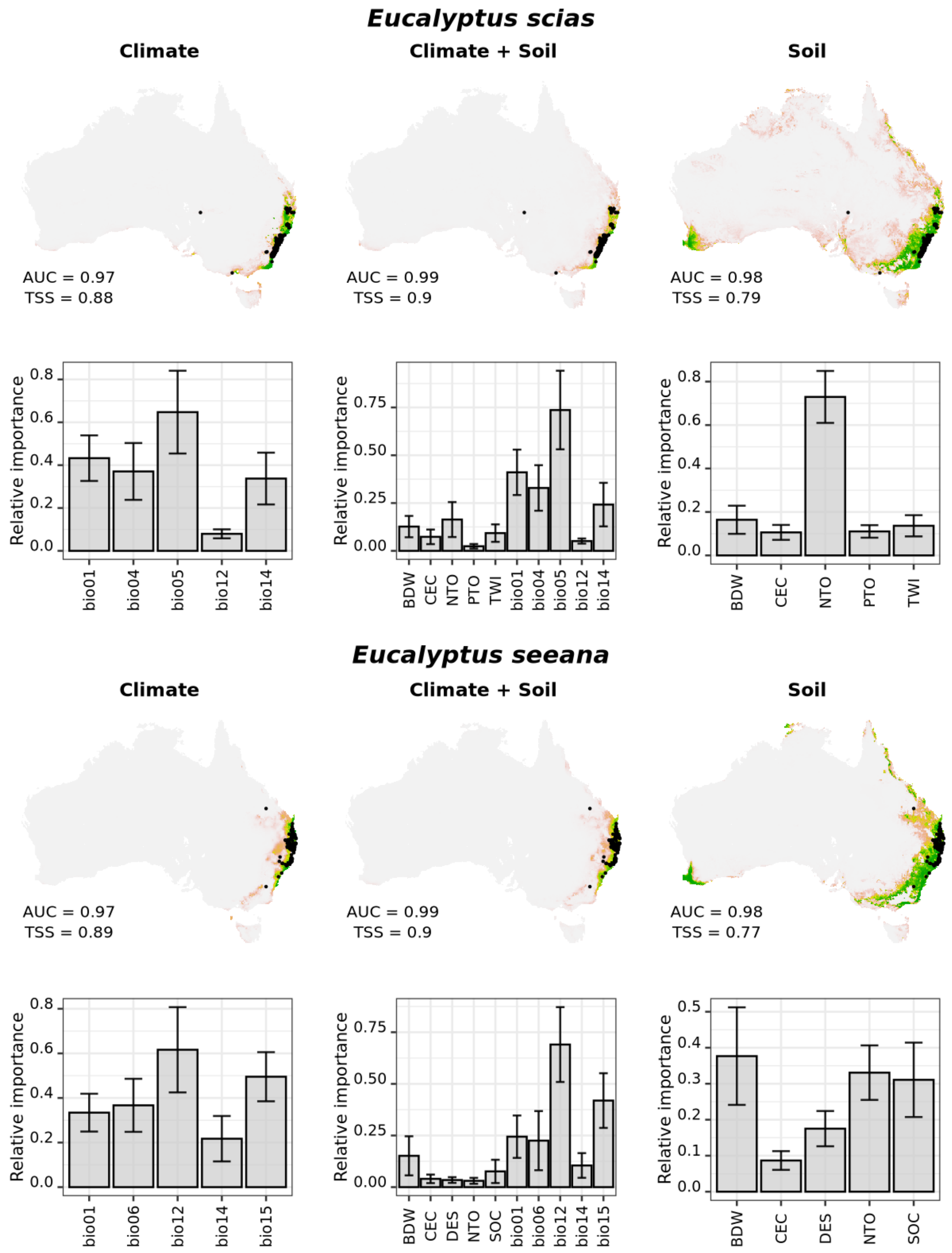

Eucalyptus scias
Climate
Climate + Soil
Soil
AUC = 0.97
TSS = 0.88
AUC = 0.99
TSS = 0.9
AUC = 0.98
TSS = 0.79
Relative importance
bio01
bio04
bio05
bio12
bio14
BDW
CEC
NTO
PTO
TWI
Eucalyptus seeana
Climate
Climate + Soil
Soil
AUC = 0.97
TSS = 0.89
AUC = 0.99
TSS = 0.9
AUC = 0.98
TSS = 0.77
Relative importance
bio01
bio06
bio12
bio14
bio15
BDW
CEC
DES
NTO
SOC

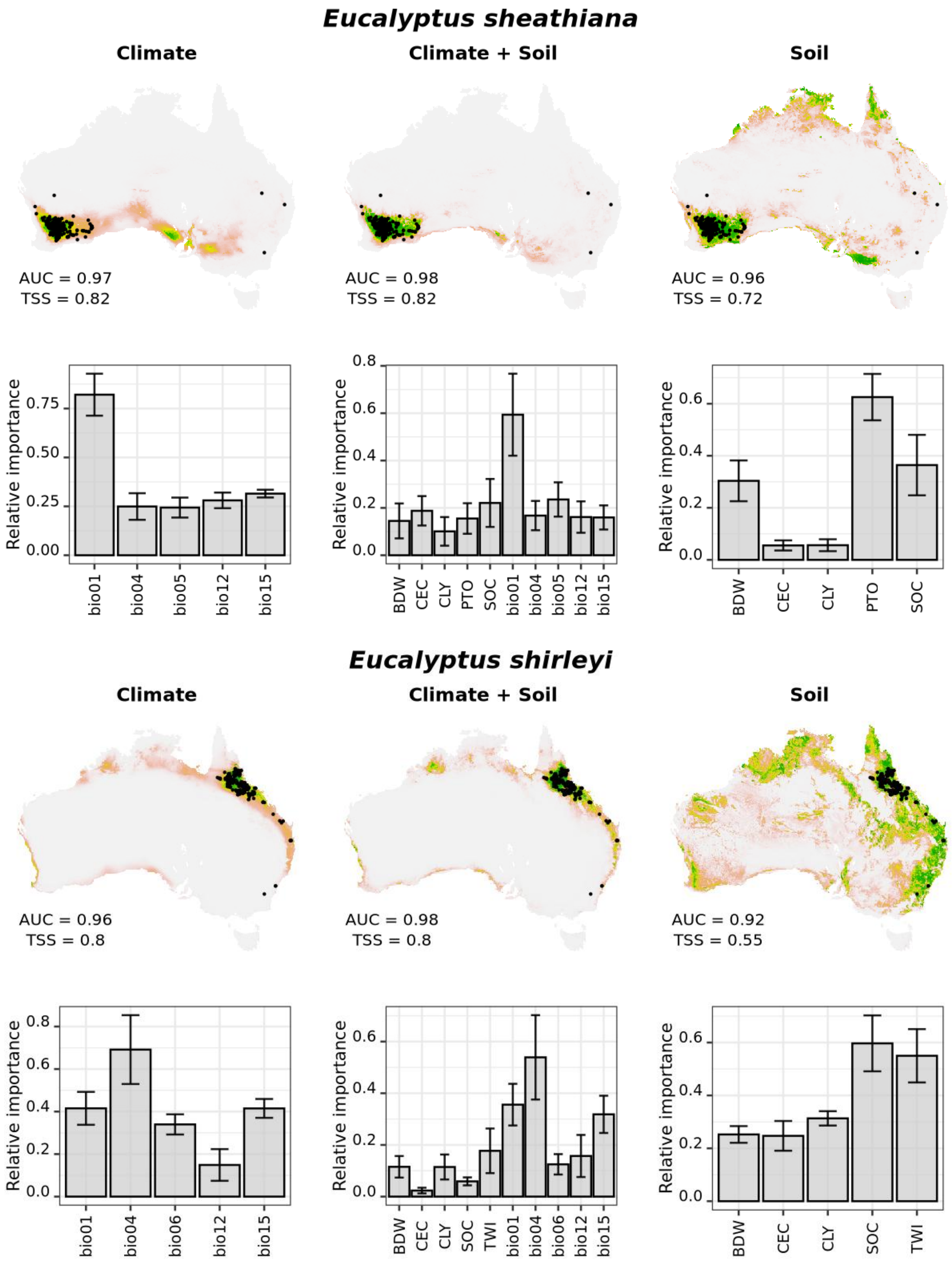
Eucalyptus sheathiana
Climate
Climate + Soil
Soil
AUC = 0.97
TSS = 0.82
AUC = 0.98
TSS = 0.82
AUC = 0.96
TSS = 0.72
Relative importance
0.00
0.25
0.50
0.75
bio01
bio04
bio05
bio12
bio15
0.0
0.2
0.4
0.6
0.8
BDW
CEC
CLY
PTO
SOC
bio01
bio04
bio05
bio12
bio15
0.0
0.2
0.4
0.6
BDW
CEC
CLY
PTO
SOC
Eucalyptus shirleyi
Climate
Climate + Soil
Soil
AUC = 0.96
TSS = 0.8
AUC = 0.98
TSS = 0.8
AUC = 0.92
TSS = 0.55
Relative importance
0.0
0.2
0.4
0.6
0.8
bio01
bio04
bio06
bio12
bio15
0.0
0.2
0.4
0.6
BDW
CEC
CLY
SOC
TWI
bio01
bio04
bio06
bio12
bio15
0.0
0.2
0.4
0.6
BDW
CEC
CLY
SOC
TWI

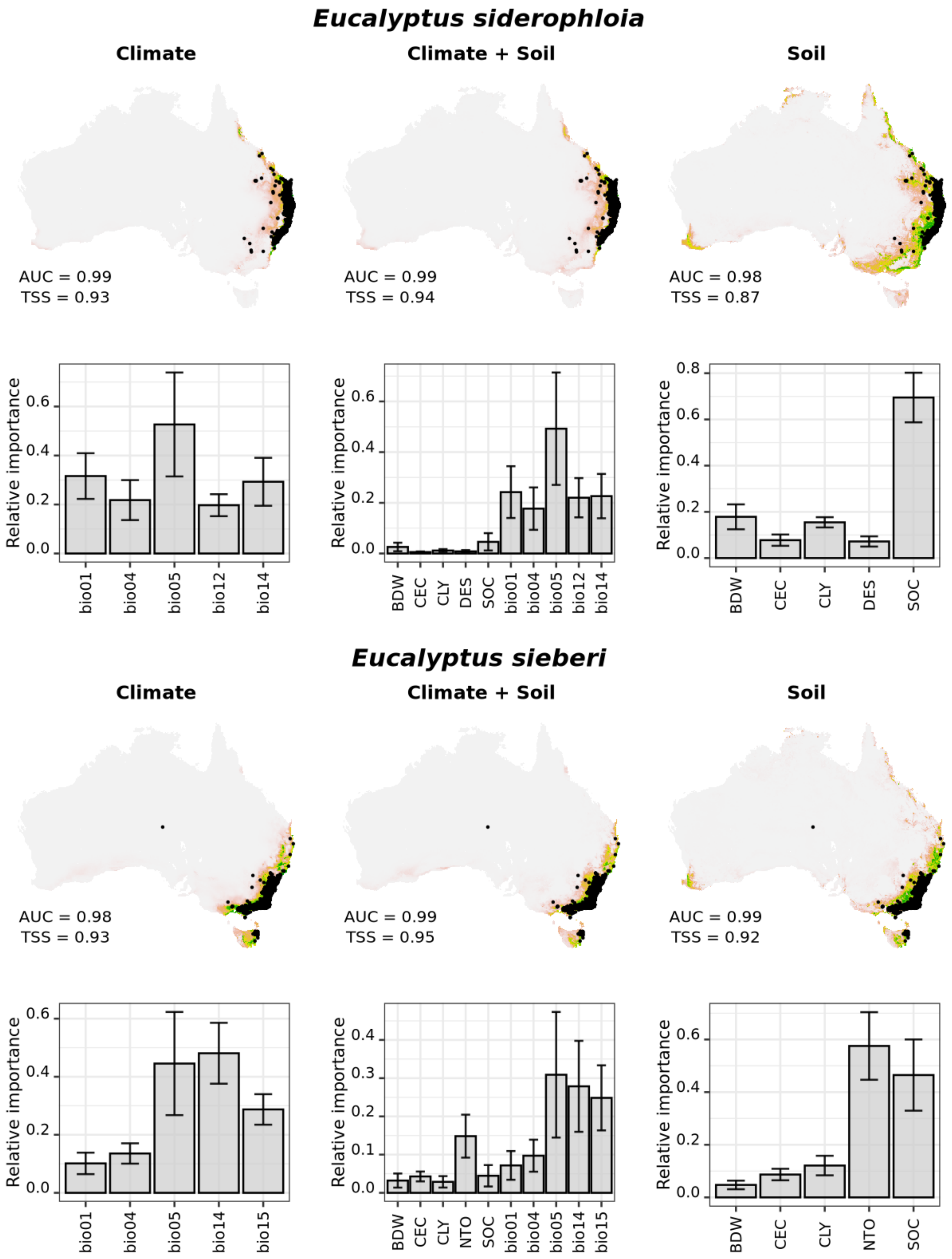
Eucalyptus siderophloia
Climate
Climate + Soil
Soil
AUC = 0.99
TSS = 0.93
AUC = 0.99
TSS = 0.94
AUC = 0.98
TSS = 0.87
Relative importance
bio01
bio04
bio05
bio12
bio14
BDW
CEC
CLY
DES
SOC
Eucalyptus sieberi
Climate
Climate + Soil
Soil
AUC = 0.98
TSS = 0.93
AUC = 0.99
TSS = 0.95
AUC = 0.99
TSS = 0.92
Relative importance
bio01
bio04
bio05
bio14
bio15
BDW
CEC
CLY
NTO
SOC

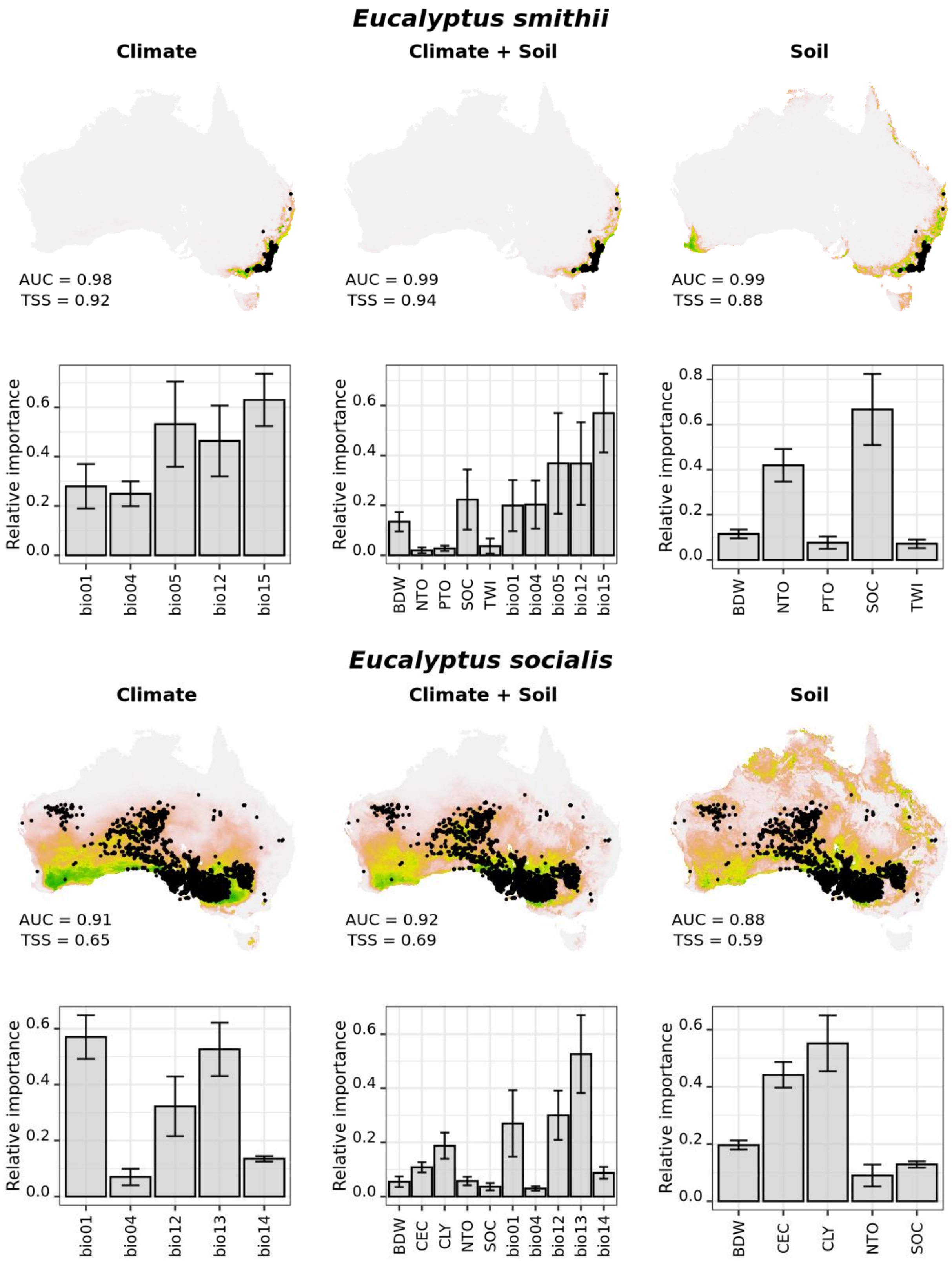

Eucalyptus smithii
Climate
Climate + Soil
Soil
AUC = 0.98
TSS = 0.92
AUC = 0.99
TSS = 0.94
AUC = 0.99
TSS = 0.88
Relative importance
bio01 bio04 bio05 bio12 bio15
BDW NTO PTO SOC TWI bio01 bio04 bio05 bio12 bio15
BDW NTO PTO SOC TWI
Eucalyptus socialis
Climate
Climate + Soil
Soil
AUC = 0.91
TSS = 0.65
AUC = 0.92
TSS = 0.69
AUC = 0.88
TSS = 0.59
Relative importance
bio01 bio04 bio12 bio13 bio14
BDW CEC CLY NTO SOC bio01 bio04 bio12 bio13 bio14
BDW CEC CLY NTO SOC

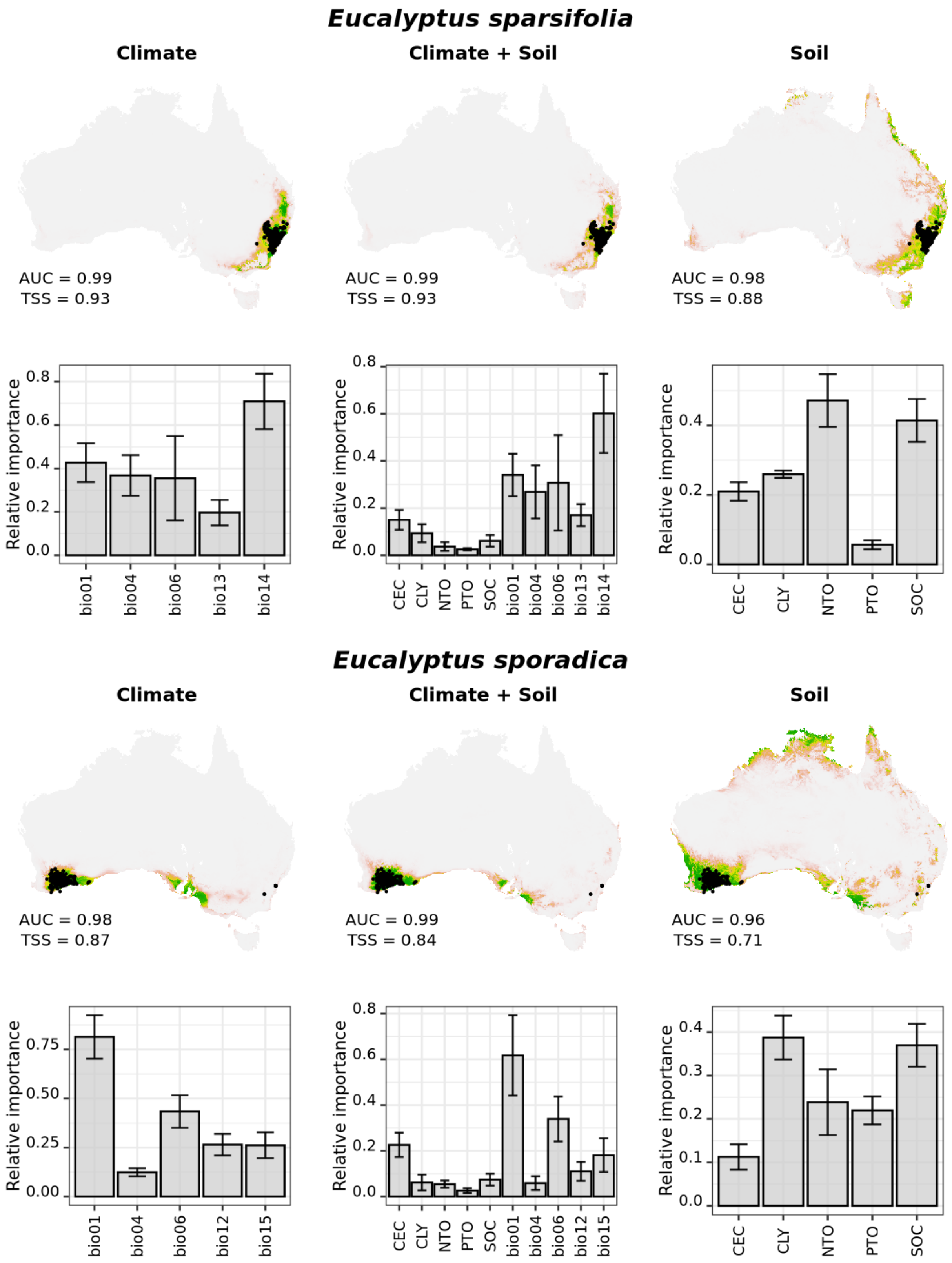
Eucalyptus sparsifolia
Climate
Climate + Soil
Soil
AUC = 0.99
TSS = 0.93
AUC = 0.99
TSS = 0.93
AUC = 0.98
TSS = 0.88
Relative importance
bio01
bio04
bio06
bio13
bio14
CEC
CLY
NTO
PTO
SOC
Eucalyptus sporadica
Climate
Climate + Soil
Soil
AUC = 0.98
TSS = 0.87
AUC = 0.99
TSS = 0.84
AUC = 0.96
TSS = 0.71
Relative importance
bio01
bio04
bio06
bio12
bio15
CEC
CLY
NTO
PTO
SOC

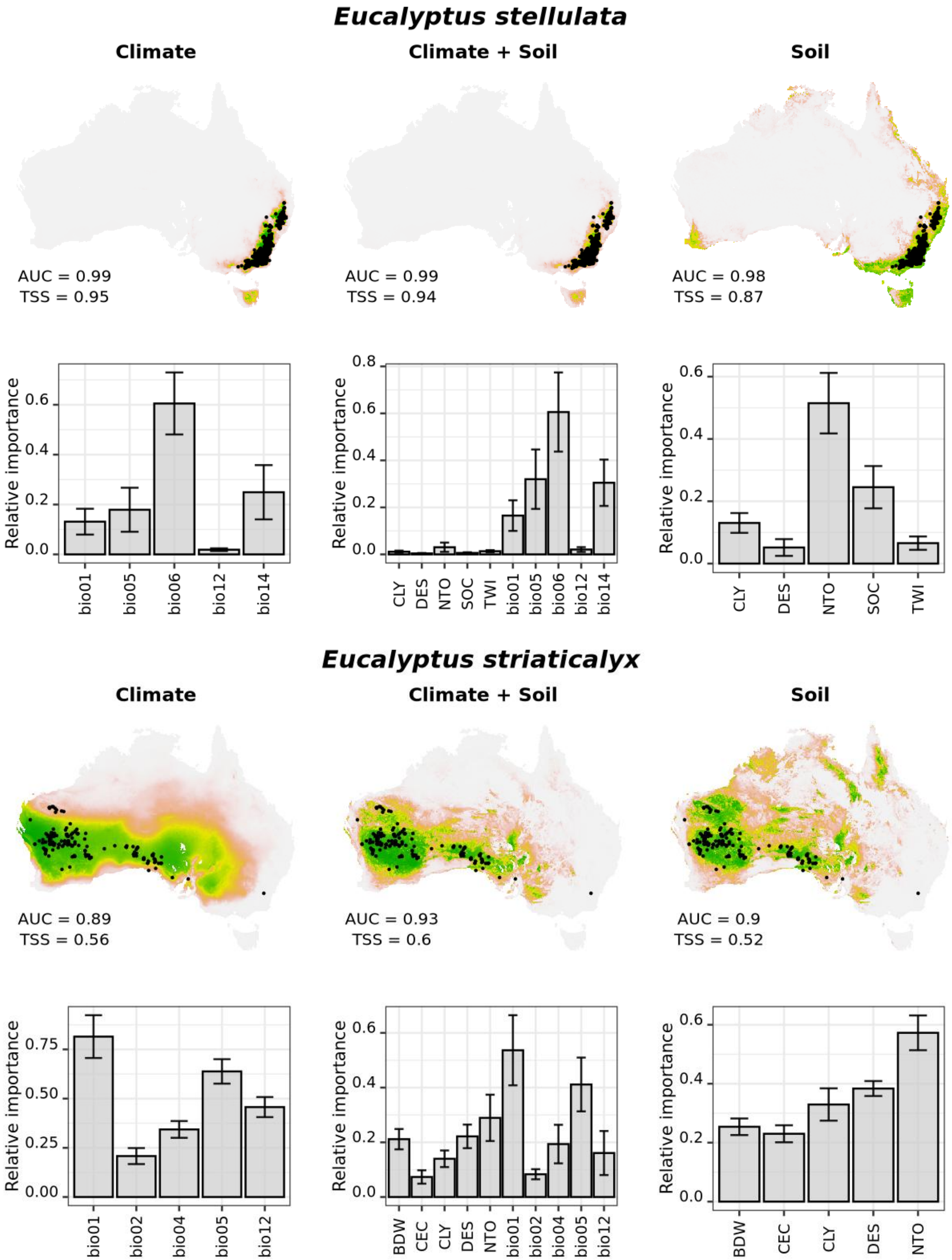
Eucalyptus stellulata
Climate
Climate + Soil
Soil
AUC = 0.99
TSS = 0.95
AUC = 0.99
TSS = 0.94
AUC = 0.98
TSS = 0.87
Relative importance
0.0
0.2
0.4
0.6
0.8
bio01
bio05
bio06
bio12
bio14
CLY
DES
NTO
SOC
TWI
Eucalyptus striaticalyx
AUC = 0.89
TSS = 0.56
AUC = 0.93
TSS = 0.6
AUC = 0.9
TSS = 0.52
0.00
0.25
0.50
0.75
bio02
bio04
BDW
CEC

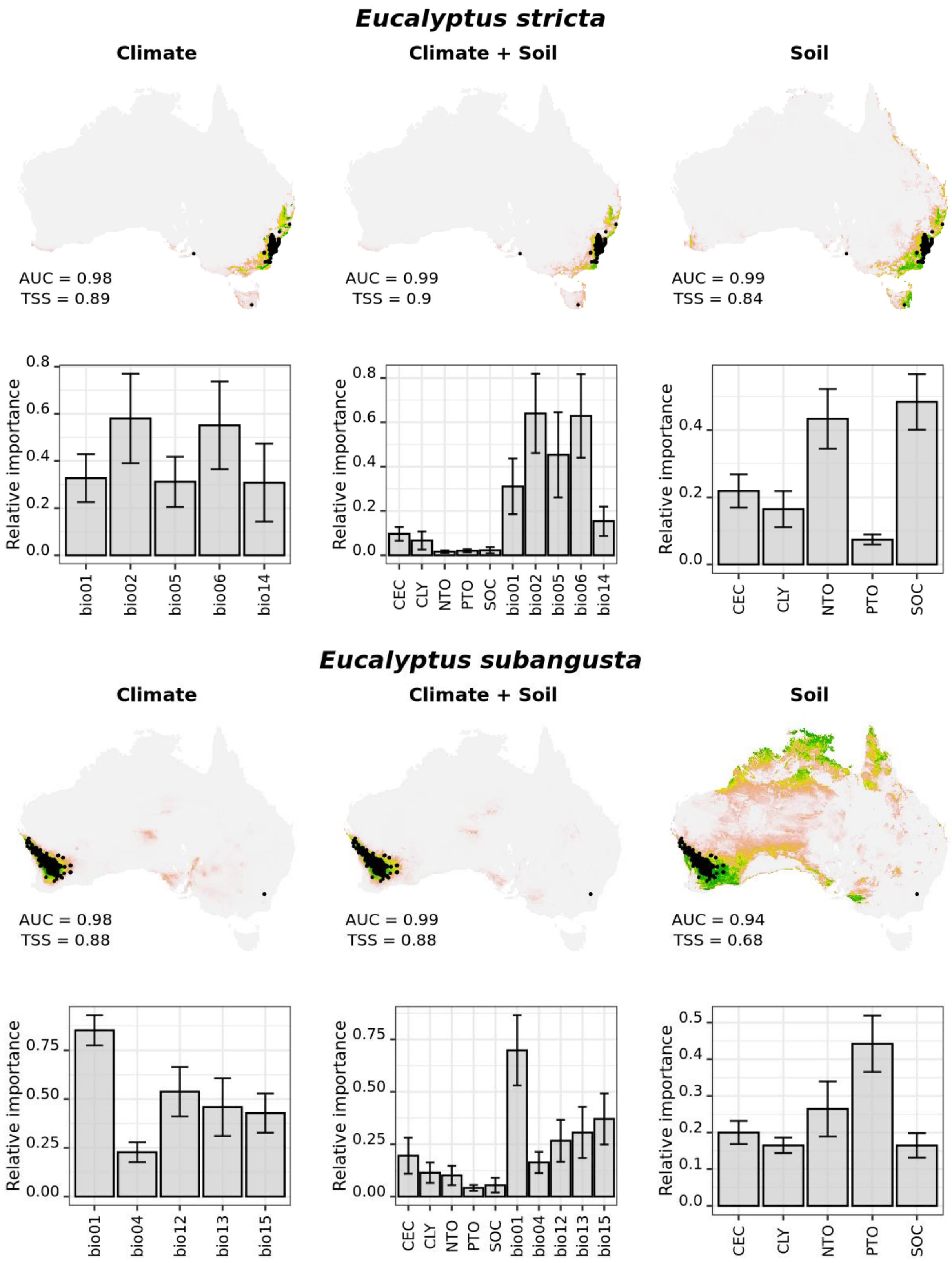
Eucalyptus stricta
Climate
Climate + Soil
Soil
AUC = 0.98
TSS = 0.89
AUC = 0.99
TSS = 0.9
AUC = 0.99
TSS = 0.84
Relative importance
0.0
0.2
0.4
0.6
0.8
bio01
bio02
bio05
bio06
bio14
CEC
CLY
NTO
PTO
SOC
Eucalyptus subangusta
AUC = 0.98
TSS = 0.88
AUC = 0.99
TSS = 0.88
AUC = 0.94
TSS = 0.68
0.00
0.25
0.50
0.75
0.1
0.3
0.5
bio04
bio12
bio13
bio15

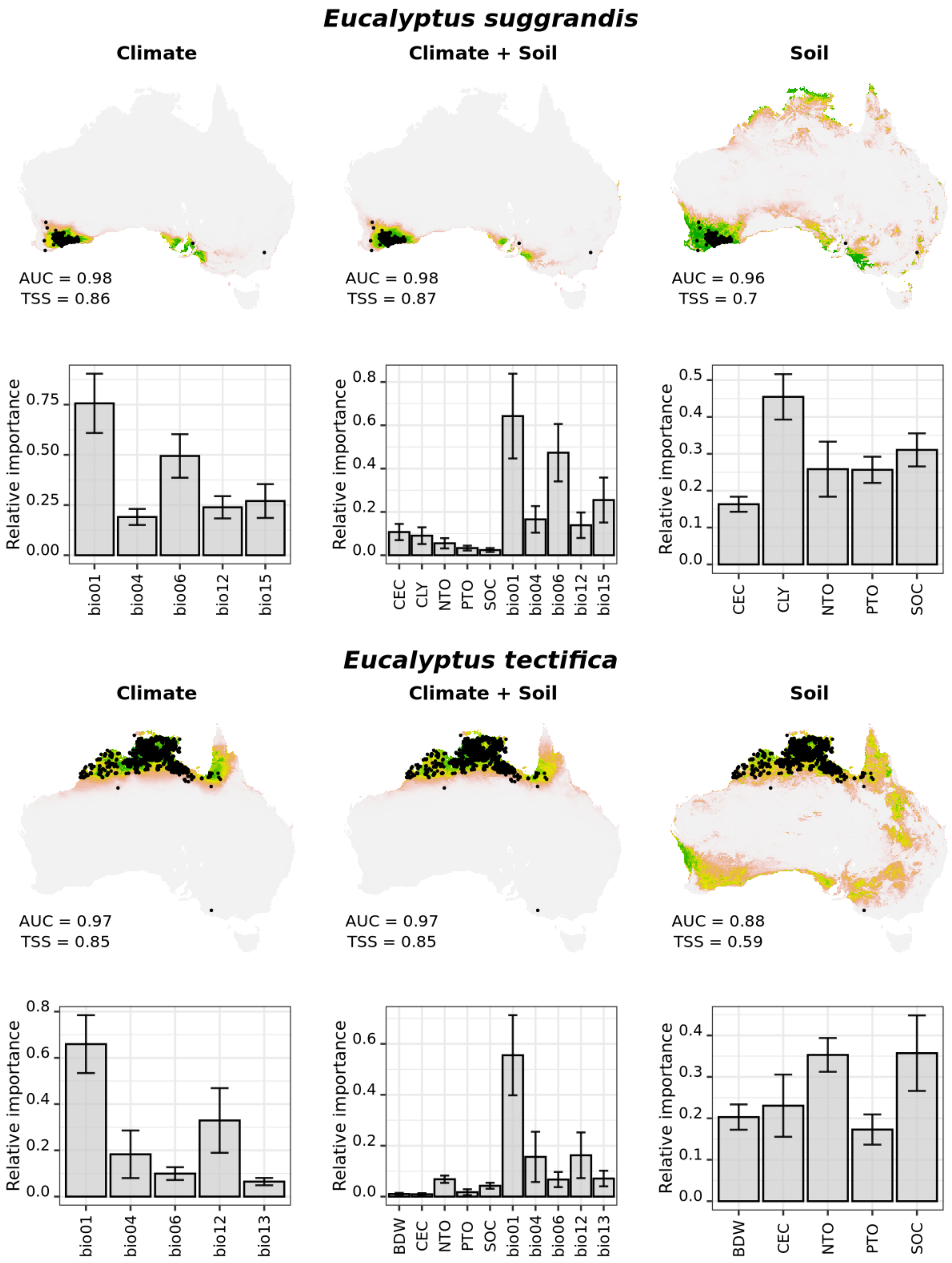
Eucalyptus suggrandis
Climate
Climate + Soil
Soil
AUC = 0.98
TSS = 0.86
AUC = 0.98
TSS = 0.87
AUC = 0.96
TSS = 0.7
Relative importance
bio01
bio04
bio06
bio12
bio15
CEC
CLY
NTO
PTO
SOC
Eucalyptus tectifica
Climate
Climate + Soil
Soil
AUC = 0.97
TSS = 0.85
AUC = 0.97
TSS = 0.85
AUC = 0.88
TSS = 0.59
Relative importance
bio01
bio04
bio06
bio12
bio13
BDW
CEC
NTO
PTO
SOC

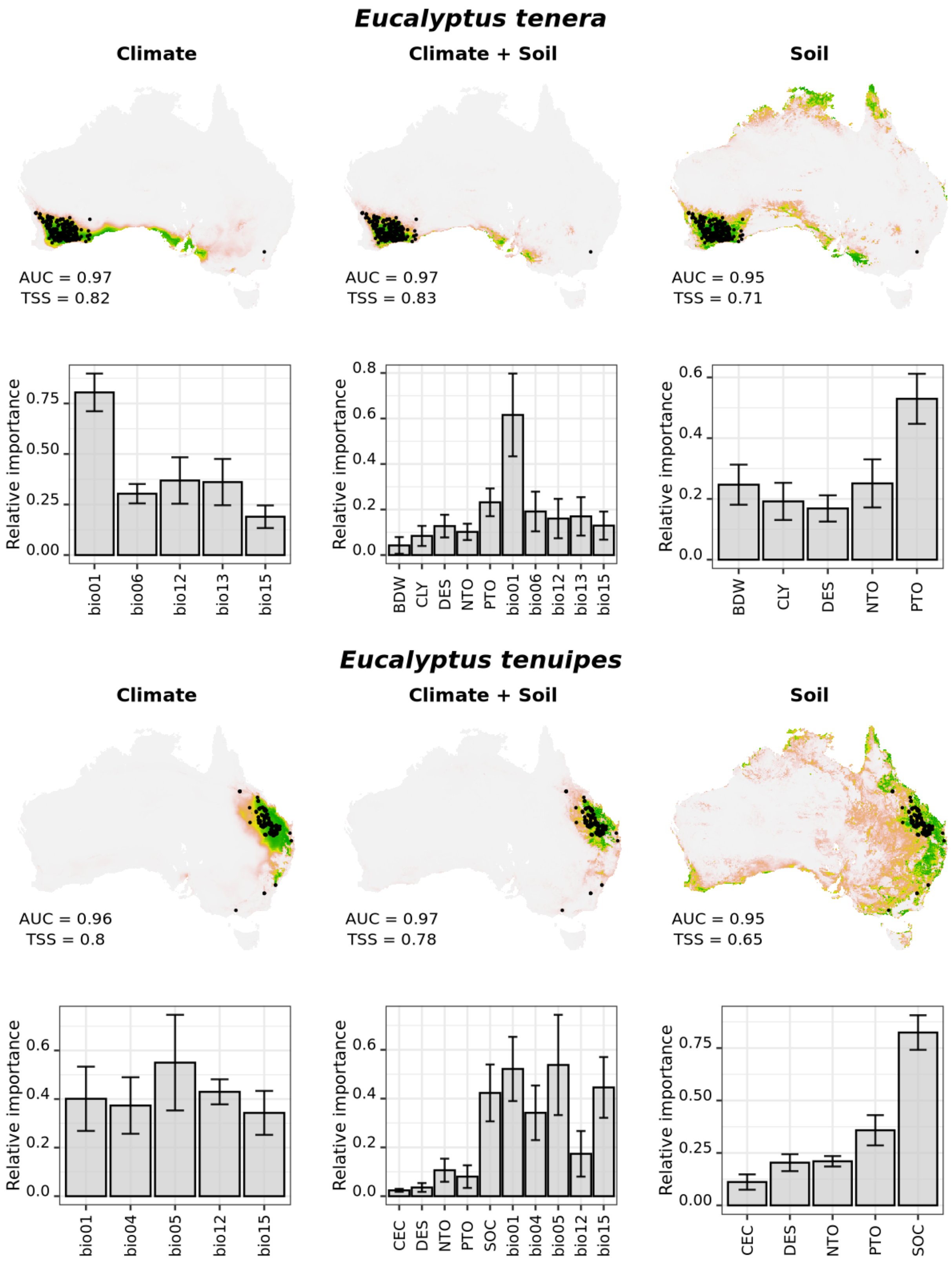
Eucalyptus tenera
Climate
Climate + Soil
Soil
AUC = 0.97
TSS = 0.82
AUC = 0.97
TSS = 0.83
AUC = 0.95
TSS = 0.71
Relative importance
bio01
bio06
bio12
bio13
bio15
BDW
CLY
DES
NTO
PTO
Eucalyptus tenuipes
Climate
Climate + Soil
Soil
AUC = 0.96
TSS = 0.8
AUC = 0.97
TSS = 0.78
AUC = 0.95
TSS = 0.65
Relative importance
bio01
bio04
bio05
bio12
bio15
CEC
DES
NTO
PTO
SOC

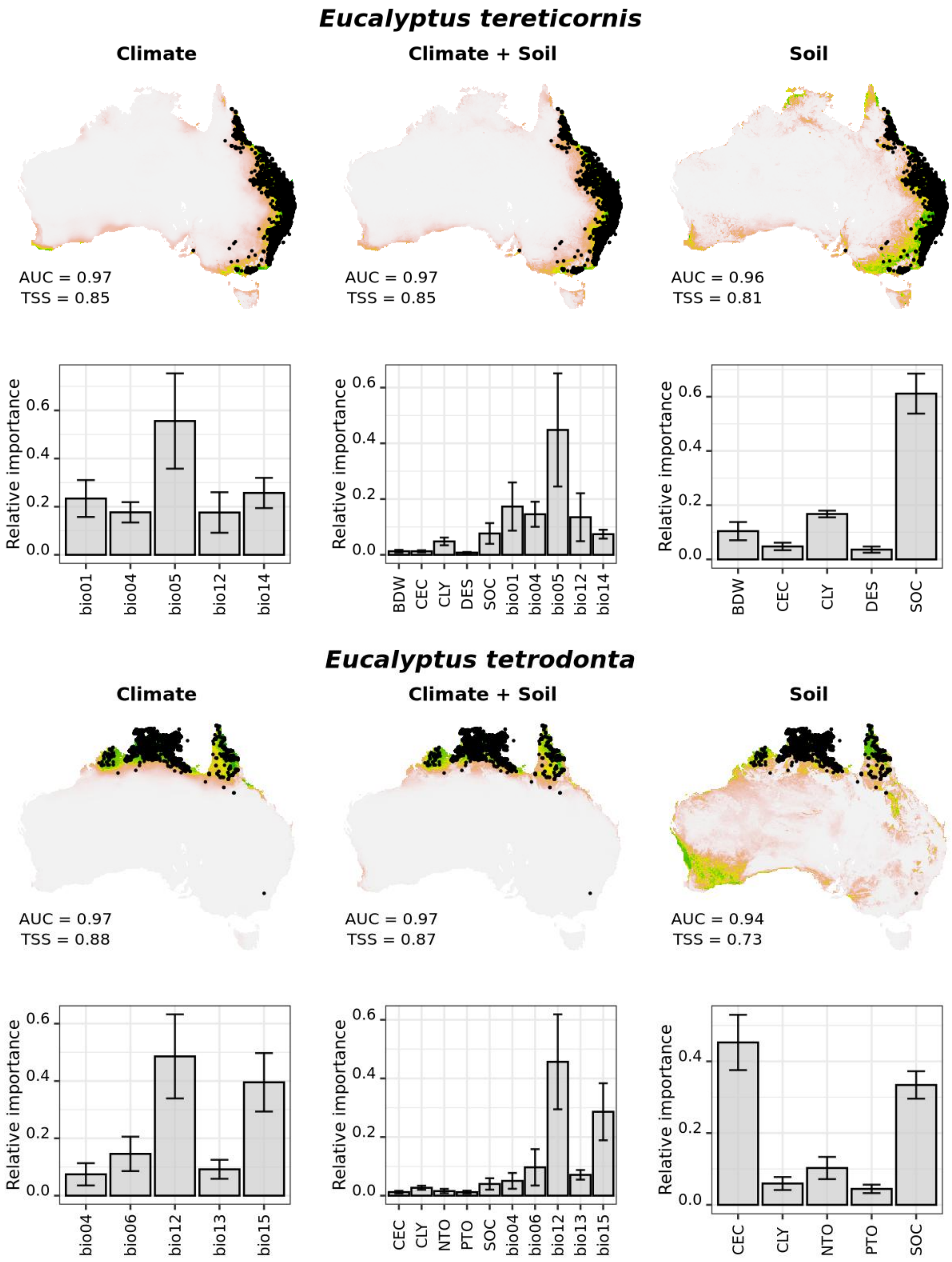

Eucalyptus tereticornis
Climate
Climate + Soil
Soil
AUC = 0.97
TSS = 0.85
AUC = 0.97
TSS = 0.85
AUC = 0.96
TSS = 0.81
Relative importance
bio01 bio04 bio05 bio12 bio14
BDW CEC CLY DES SOC bio01 bio04 bio05 bio12 bio14
BDW CEC CLY DES SOC
Eucalyptus tetrodonta
Climate
Climate + Soil
Soil
AUC = 0.97
TSS = 0.88
AUC = 0.97
TSS = 0.87
AUC = 0.94
TSS = 0.73
Relative importance
bio04 bio06 bio12 bio13 bio15
CEC CLY NTO PTO SOC bio04 bio06 bio12 bio13 bio15
CEC CLY NTO PTO SOC

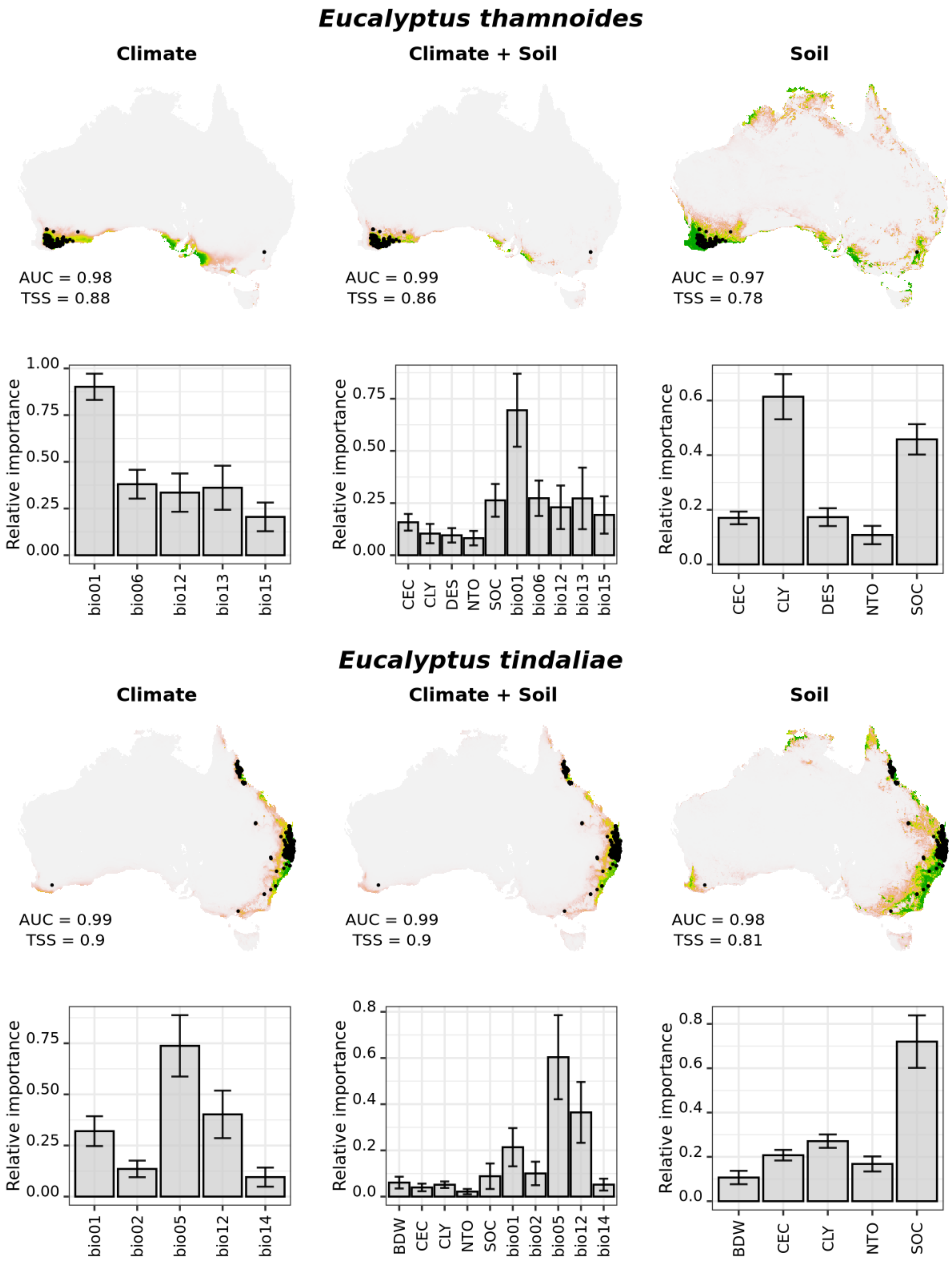

Eucalyptus thamnoides
Climate
Climate + Soil
Soil
AUC = 0.98
TSS = 0.88
AUC = 0.99
TSS = 0.86
AUC = 0.97
TSS = 0.78
Relative importance
bio01
bio06
bio12
bio13
bio15
CEC
CLY
DES
NTO
SOC
Eucalyptus tindaliae
AUC = 0.99
TSS = 0.9
AUC = 0.99
TSS = 0.9
AUC = 0.98
TSS = 0.81
bio02
bio05
bio14
BDW

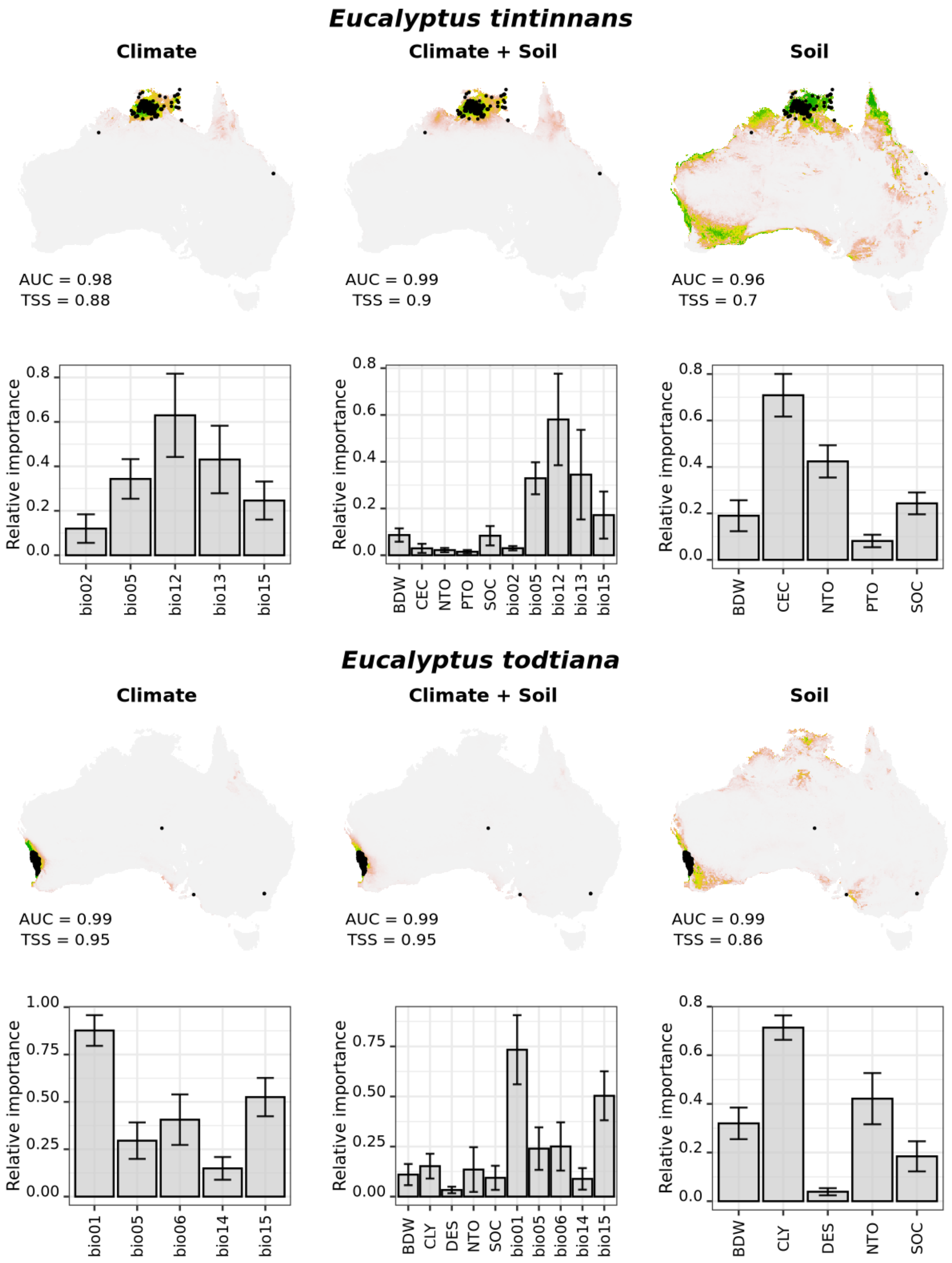
Eucalyptus tintinnans
Climate
Climate + Soil
Soil
AUC = 0.98
TSS = 0.88
AUC = 0.99
TSS = 0.9
AUC = 0.96
TSS = 0.7
Relative importance
bio02
bio05
bio12
bio13
bio15
BDW
CEC
NTO
PTO
SOC
Eucalyptus todtiana
Climate
Climate + Soil
Soil
AUC = 0.99
TSS = 0.95
AUC = 0.99
TSS = 0.95
AUC = 0.99
TSS = 0.86
Relative importance
bio01
bio05
bio06
bio14
bio15
BDW
CLY
DES
NTO
SOC

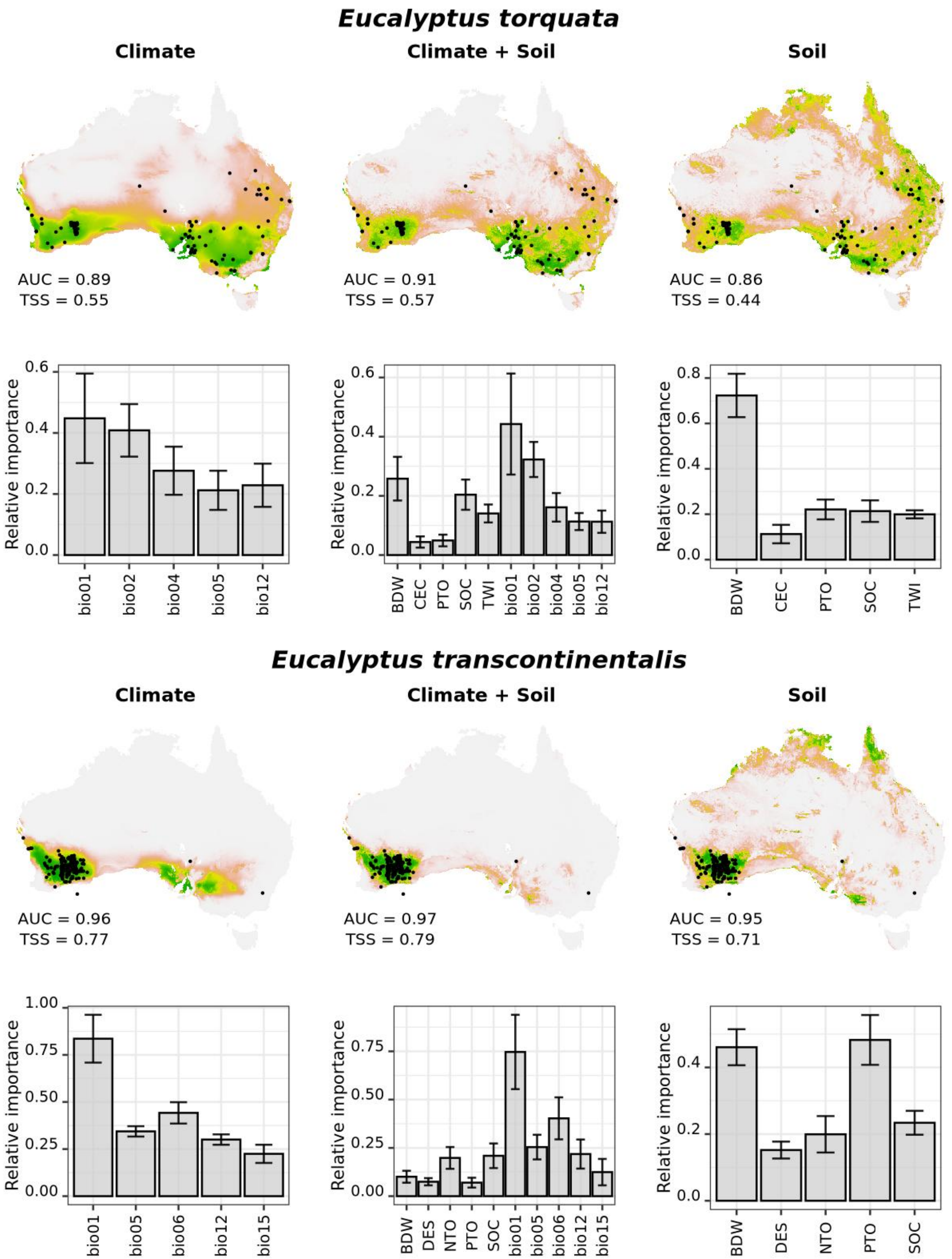
Eucalyptus torquata
Climate
Climate + Soil
Soil
AUC = 0.89
TSS = 0.55
AUC = 0.91
TSS = 0.57
AUC = 0.86
TSS = 0.44
Relative importance
bio01
bio02
bio04
bio05
bio12
BDW
CEC
PTO
SOC
TWI
Eucalyptus transcontinentalis
AUC = 0.96
TSS = 0.77
AUC = 0.97
TSS = 0.79
AUC = 0.95
TSS = 0.71
bio06
bio15
DES
NTO

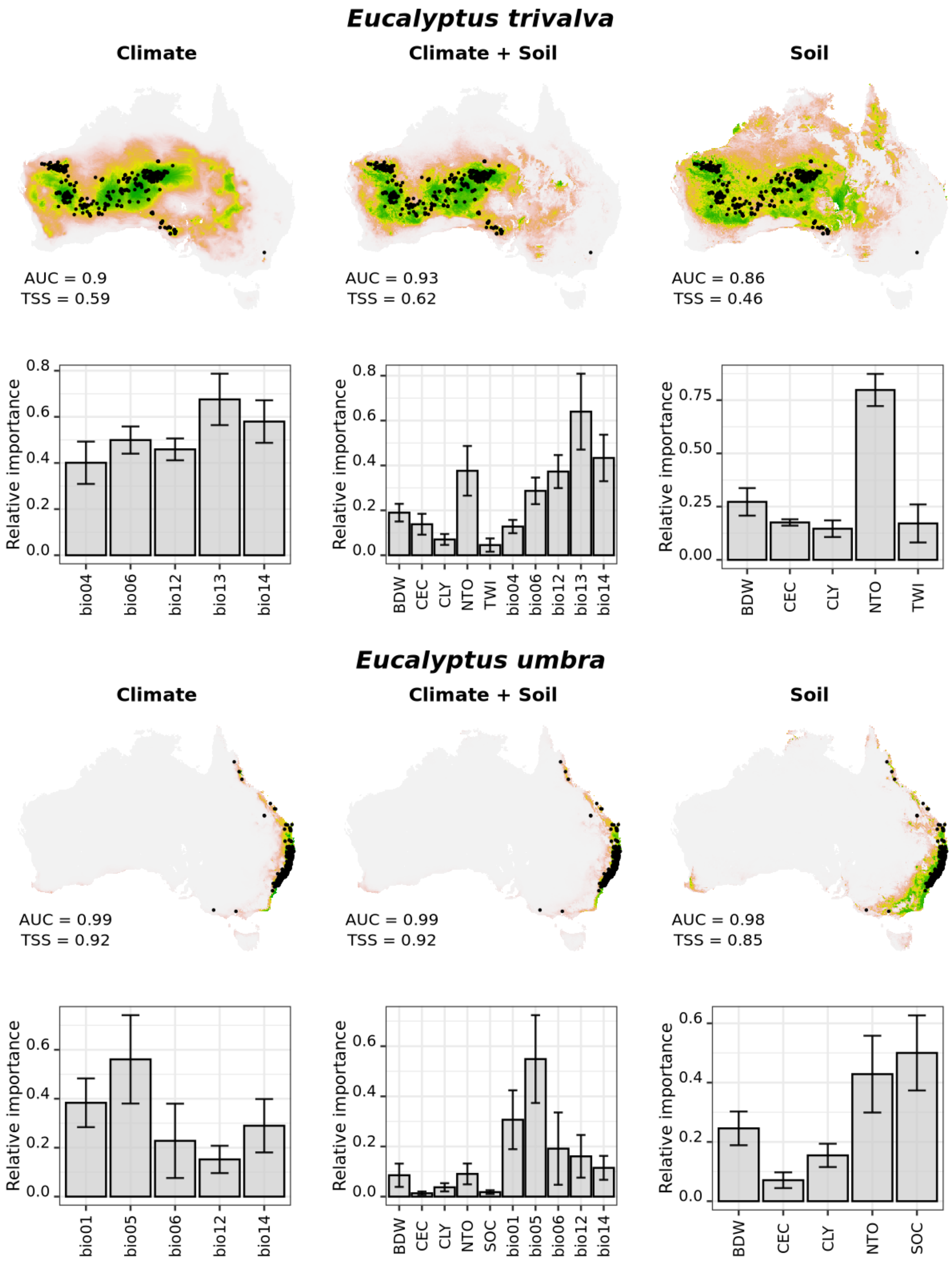
Eucalyptus trivalva
Climate
Climate + Soil
Soil
AUC = 0.9
TSS = 0.59
AUC = 0.93
TSS = 0.62
AUC = 0.86
TSS = 0.46
Relative importance
bio04
bio06
bio12
bio13
bio14
BDW
CEC
CLY
NTO
TWI
Eucalyptus umbra
Climate
Climate + Soil
Soil
AUC = 0.99
TSS = 0.92
AUC = 0.99
TSS = 0.92
AUC = 0.98
TSS = 0.85
Relative importance
bio01
bio05
bio06
bio12
bio14
BDW
CEC
CLY
NTO
SOC

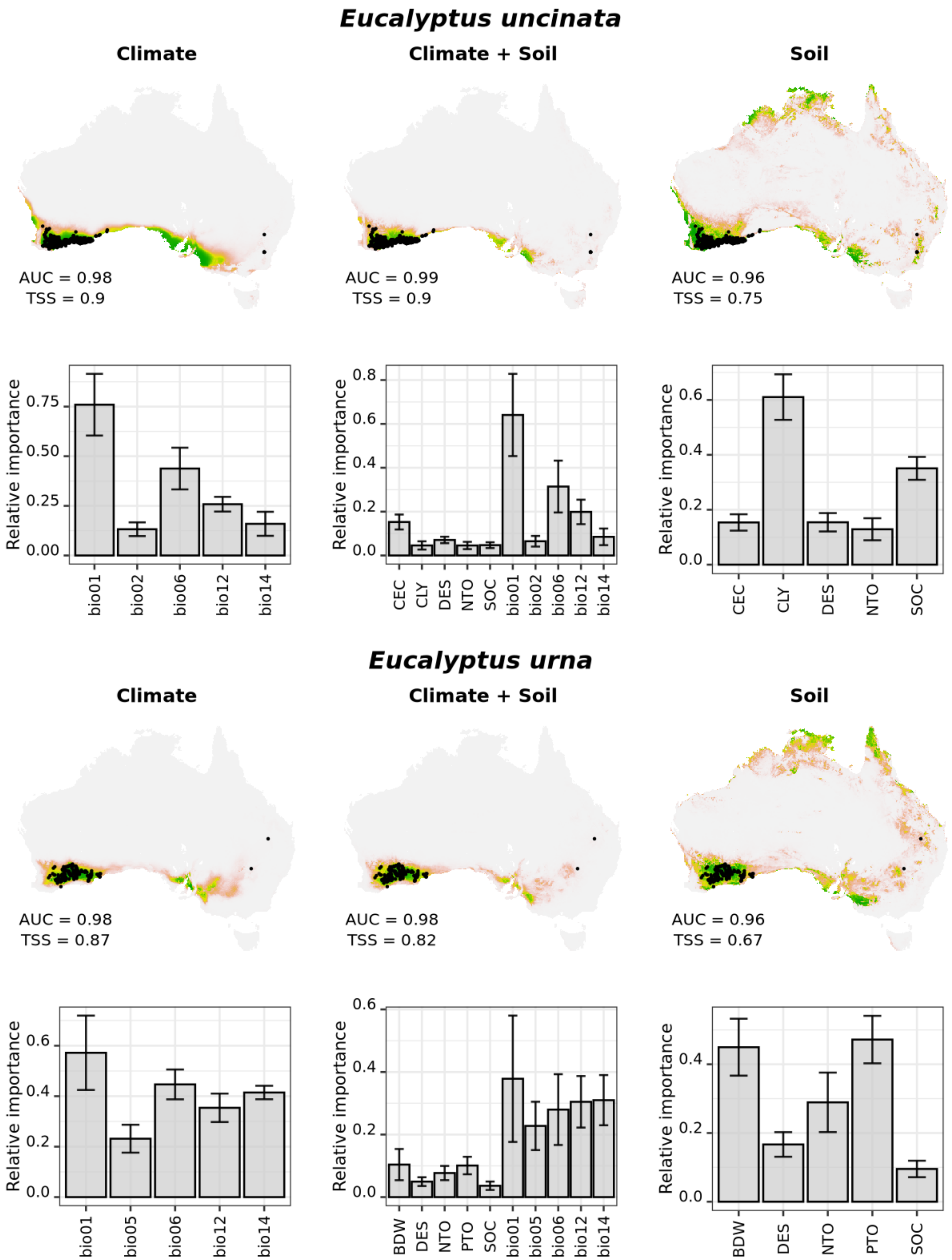

Eucalyptus uncinata
Climate
Climate + Soil
Soil
AUC = 0.98
TSS = 0.9
AUC = 0.99
TSS = 0.9
AUC = 0.96
TSS = 0.75
Relative importance
0.00
0.25
0.50
0.75
bio01
bio02
bio06
bio12
bio14
0.0
0.2
0.4
0.6
0.8
CEC
CLY
DES
NTO
SOC
0.6
Eucalyptus urna
AUC = 0.98
TSS = 0.87
AUC = 0.98
TSS = 0.82
AUC = 0.96
TSS = 0.67
bio05
BDW
PTO

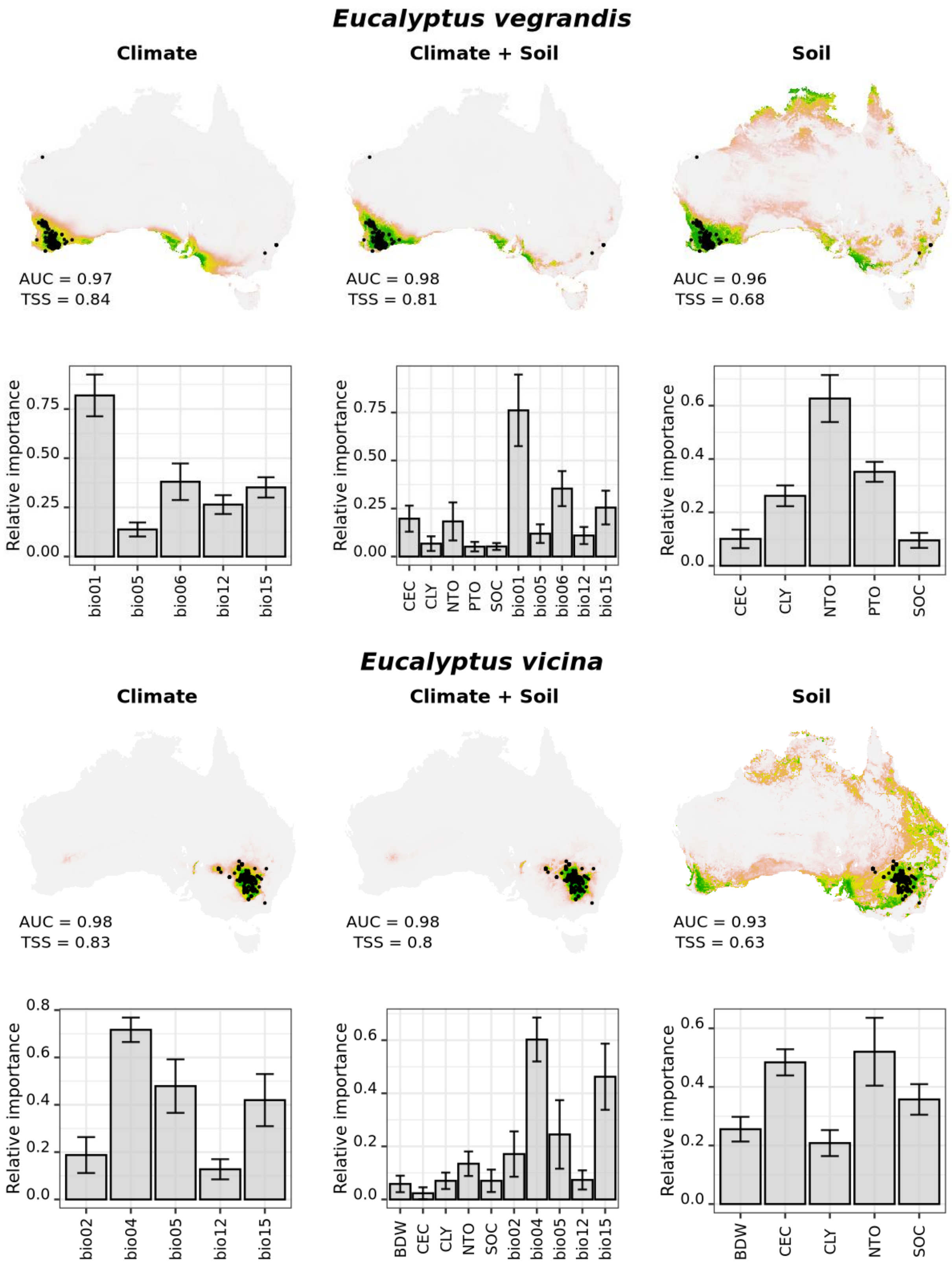
Eucalyptus vegrandis
Climate
Climate + Soil
Soil
AUC = 0.97
TSS = 0.84
AUC = 0.98
TSS = 0.81
AUC = 0.96
TSS = 0.68
Relative importance
0.00
0.25
0.50
0.75
bio01
bio05
bio06
bio12
bio15
CEC
CLY
NTO
PTO
SOC
0.0
0.2
0.4
0.6
Eucalyptus vicina
Climate
Climate + Soil
Soil
AUC = 0.98
TSS = 0.83
AUC = 0.98
TSS = 0.8
AUC = 0.93
TSS = 0.63
0.8
bio02
bio04
BDW

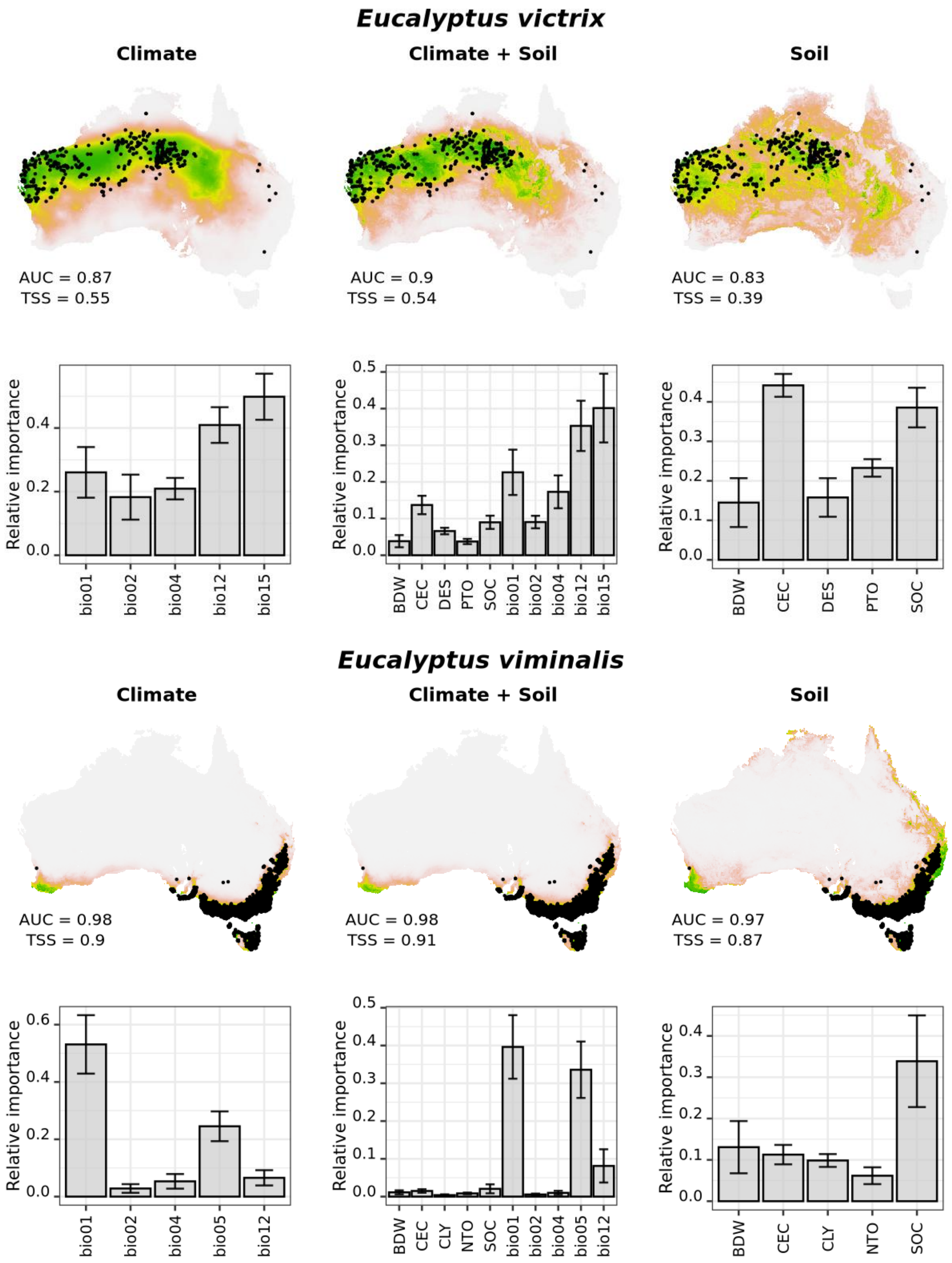

Eucalyptus victrix
Climate
Climate + Soil
Soil
AUC = 0.87
TSS = 0.55
AUC = 0.9
TSS = 0.54
AUC = 0.83
TSS = 0.39
Relative importance
0.0
0.1
0.2
0.3
0.4
0.5
bio01
bio02
bio04
bio12
bio15
BDW
CEC
DES
PTO
SOC
Eucalyptus viminalis
Climate
Climate + Soil
Soil
AUC = 0.98
TSS = 0.9
AUC = 0.98
TSS = 0.91
AUC = 0.97
TSS = 0.87
Relative importance
0.6
bio05
CLY
NTO

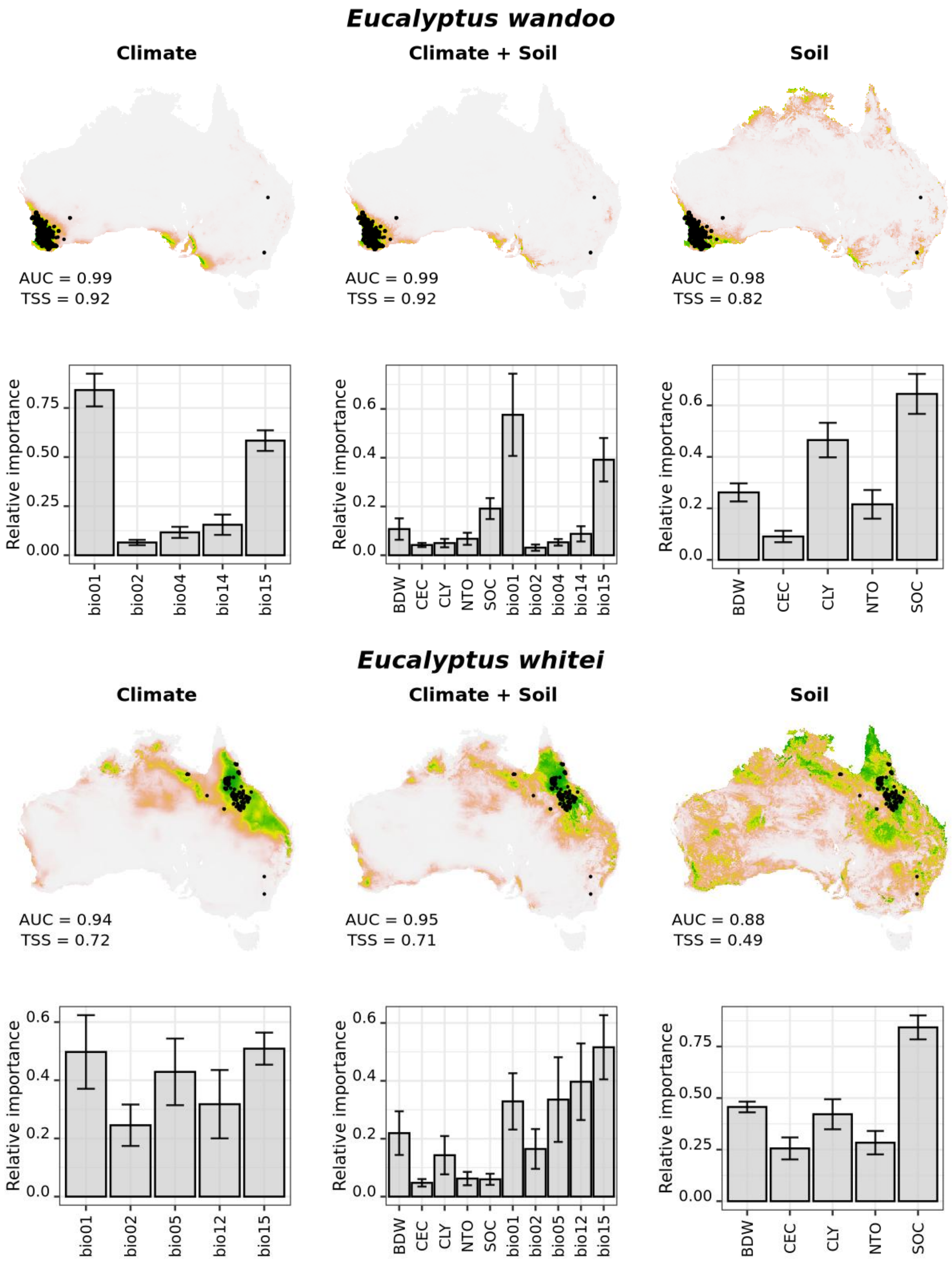

Eucalyptus wandoo
Climate
Climate + Soil
Soil
AUC = 0.99
TSS = 0.92
AUC = 0.99
TSS = 0.92
AUC = 0.98
TSS = 0.82
Relative importance
0.00
0.25
0.50
0.75
bio01
bio02
bio04
bio14
bio15
0.0
0.2
0.4
0.6
BDW
CEC
CLY
NTO
SOC
bio01
bio02
bio04
bio14
bio15
BDW
CEC
CLY
NTO
SOC
Eucalyptus whitei
Climate
Climate + Soil
Soil
AUC = 0.94
TSS = 0.72
AUC = 0.95
TSS = 0.71
AUC = 0.88
TSS = 0.49
Relative importance
bio01
bio02
bio05
bio12
bio15
BDW
CEC
CLY
NTO
SOC
bio01
bio02
bio05
bio12
bio15
BDW
CEC
CLY
NTO
SOC

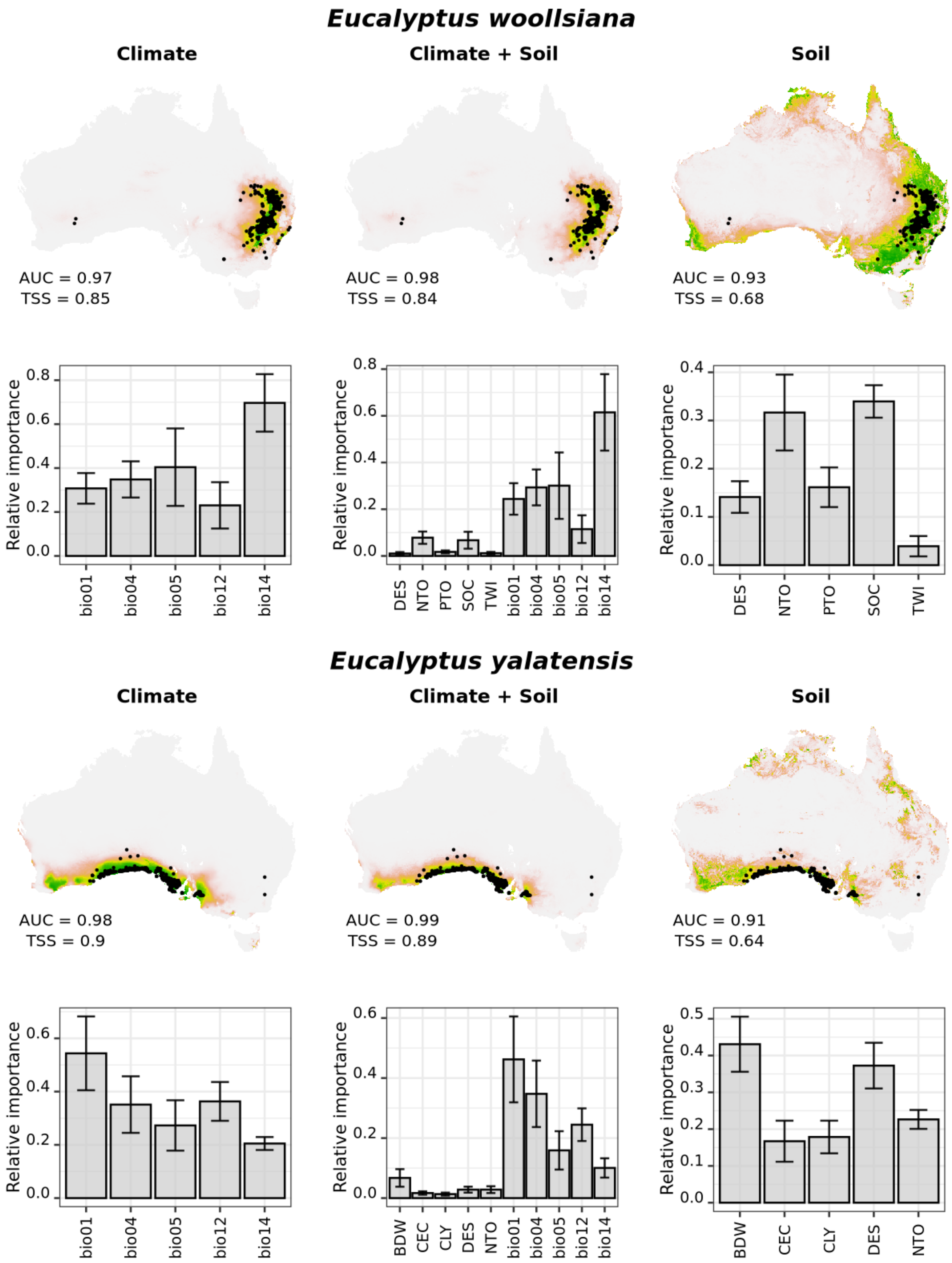
Eucalyptus woollsiana
Climate
Climate + Soil
Soil
AUC = 0.97
TSS = 0.85
AUC = 0.98
TSS = 0.84
AUC = 0.93
TSS = 0.68
Relative importance
0.0 0.2 0.4 0.6 0.8
bio01 bio04 bio05 bio12 bio14
DES NTO PTO SOC TWI bio01 bio04 bio05 bio12 bio14
0.0 0.1 0.2 0.3 0.4
DES NTO PTO SOC TWI
Eucalyptus yalatensis
Climate
Climate + Soil
Soil
AUC = 0.98
TSS = 0.9
AUC = 0.99
TSS = 0.89
AUC = 0.91
TSS = 0.64
Relative importance
0.0 0.2 0.4 0.6
bio01 bio04 bio05 bio12 bio14
BDW CEC CLY DES NTO bio01 bio04 bio05 bio12 bio14
0.0 0.1 0.2 0.3 0.4 0.5
BDW CEC CLY DES NTO

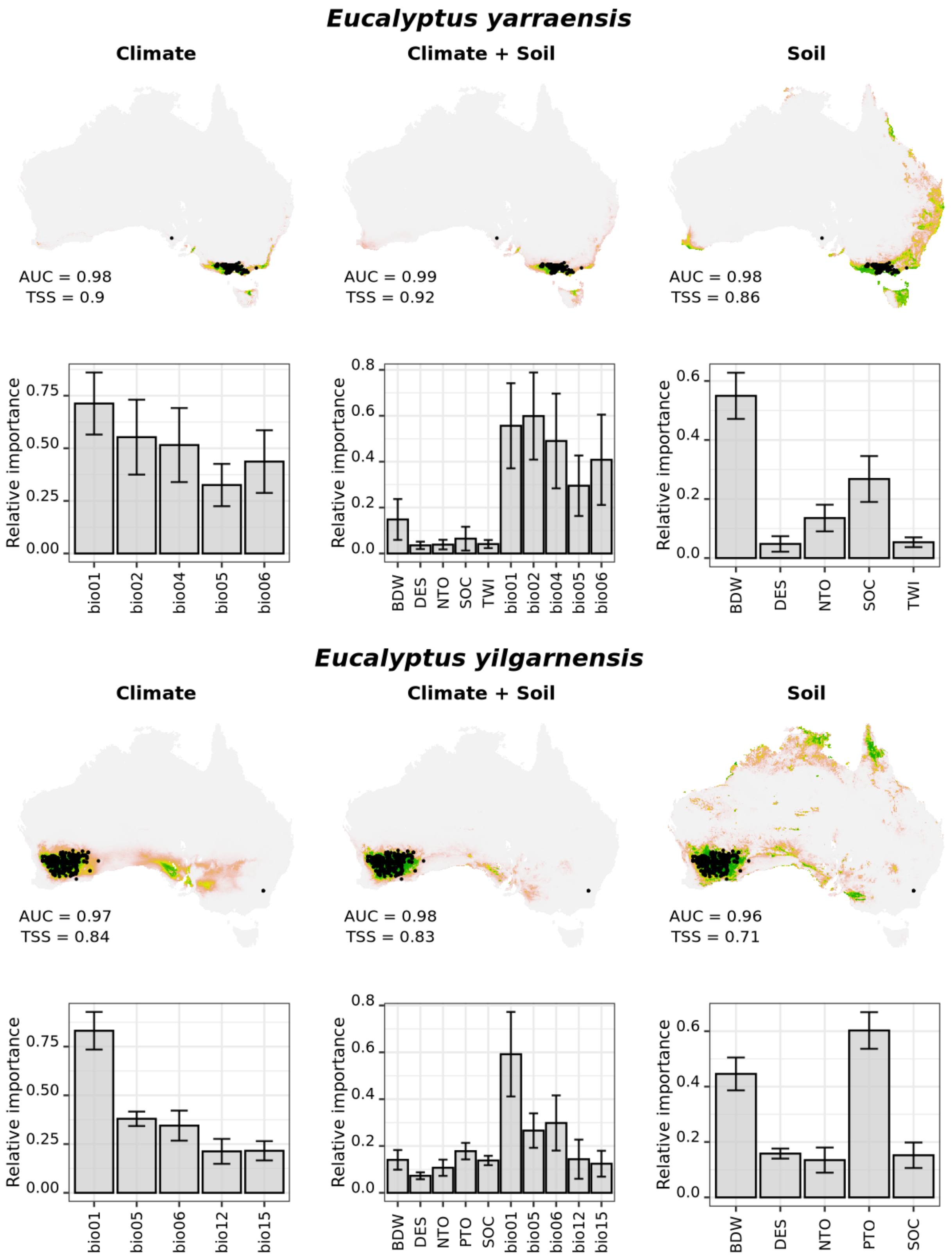

Eucalyptus yarraensis
Climate
Climate + Soil
Soil
AUC = 0.98
TSS = 0.9
AUC = 0.99
TSS = 0.92
AUC = 0.98
TSS = 0.86
Relative importance
0.00
0.25
0.50
0.75
bio01
bio02
bio04
bio05
bio06
0.0
0.2
0.4
0.6
0.8
BDW
DES
NTO
SOC
TWI
bio01
bio02
bio04
bio05
bio06
BDW
DES
NTO
SOC
TWI
Eucalyptus yilgarnensis
Climate
Climate + Soil
Soil
AUC = 0.97
TSS = 0.84
AUC = 0.98
TSS = 0.83
AUC = 0.96
TSS = 0.71
Relative importance
bio01
bio05
bio06
bio12
bio15
BDW
DES
NTO
PTO
SOC
bio01
bio05
bio06
bio12
bio15
BDW
DES
NTO
PTO
SOC

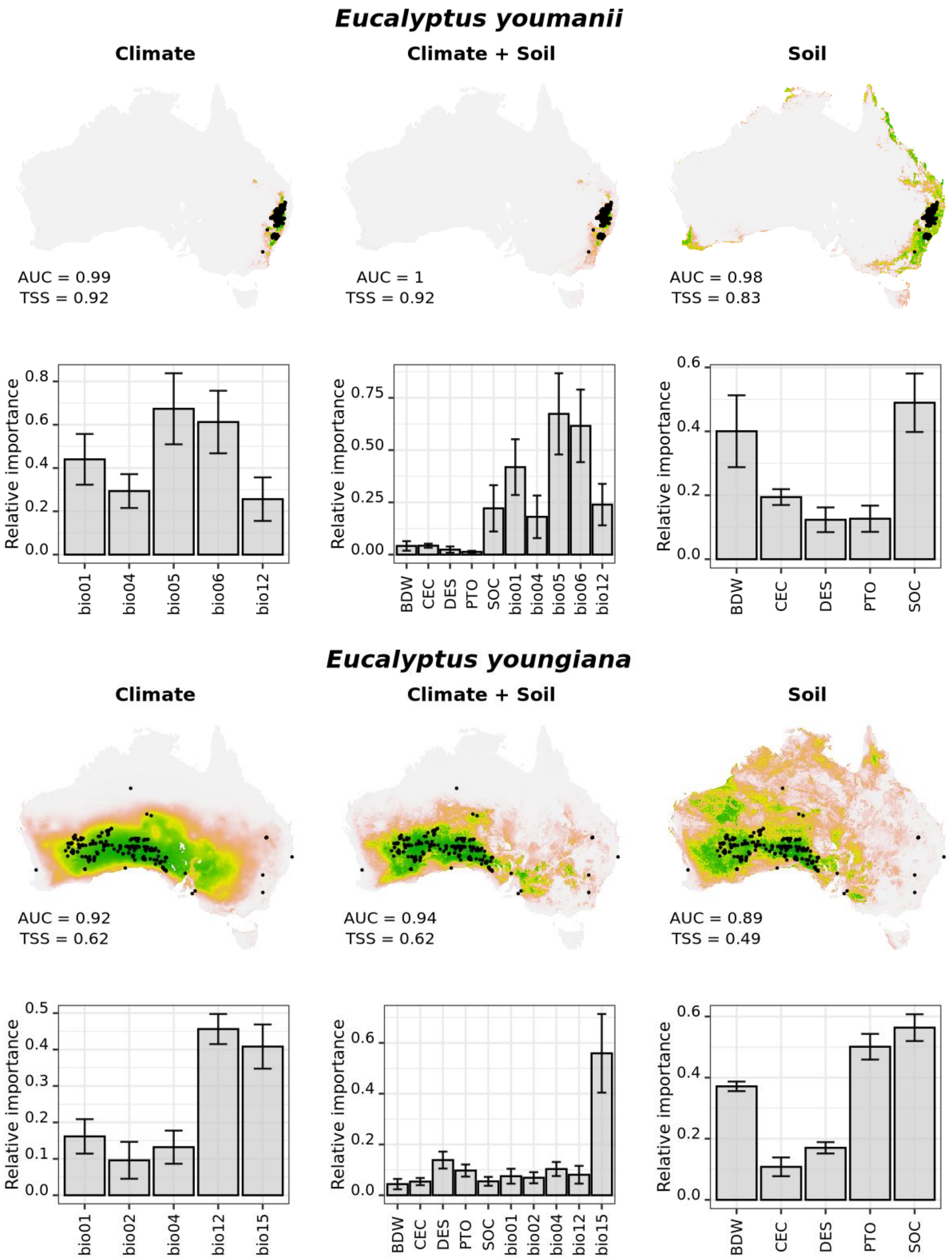
Eucalyptus youmanii
Climate
Climate + Soil
Soil
AUC = 0.99
TSS = 0.92
AUC = 1
TSS = 0.92
AUC = 0.98
TSS = 0.83
Relative importance
0.0
0.2
0.4
0.6
0.8
bio01
bio04
bio05
bio06
bio12
0.00
0.25
0.50
0.75
BDW
CEC
DES
PTO
SOC
bio01
bio04
bio05
bio06
bio12
0.0
0.2
0.4
0.6
BDW
CEC
DES
PTO
SOC
Eucalyptus youngiana
Climate
Climate + Soil
Soil
AUC = 0.92
TSS = 0.62
AUC = 0.94
TSS = 0.62
AUC = 0.89
TSS = 0.49
Relative importance
0.0
0.1
0.2
0.3
0.4
0.5
bio01
bio02
bio04
bio12
bio15
0.0
0.2
0.4
0.6
BDW
CEC
DES
PTO
SOC
bio01
bio02
bio04
bio12
bio15
0.0
0.2
0.4
0.6
BDW
CEC
DES
PTO
SOC

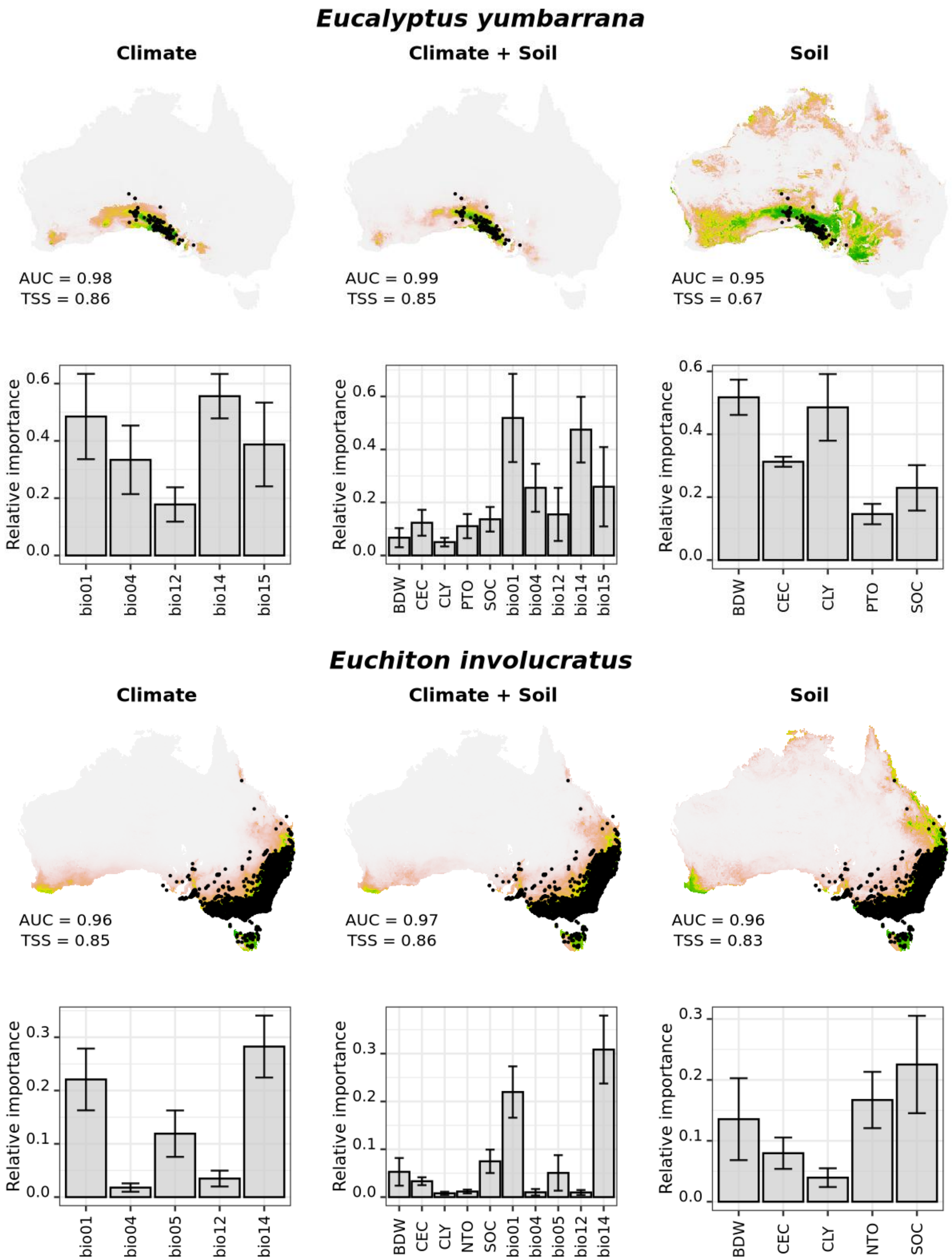

Eucalyptus yumbarrana
Climate
Climate + Soil
Soil
AUC = 0.98
TSS = 0.86
AUC = 0.99
TSS = 0.85
AUC = 0.95
TSS = 0.67
Relative importance
0.0
0.2
0.4
0.6
bio01
bio04
bio12
bio14
bio15
BDW
CEC
CLY
PTO
SOC
Euchiton involucratus
AUC = 0.96
TSS = 0.85
AUC = 0.97
TSS = 0.86
AUC = 0.96
TSS = 0.83
0.1
0.3
bio05
NTO

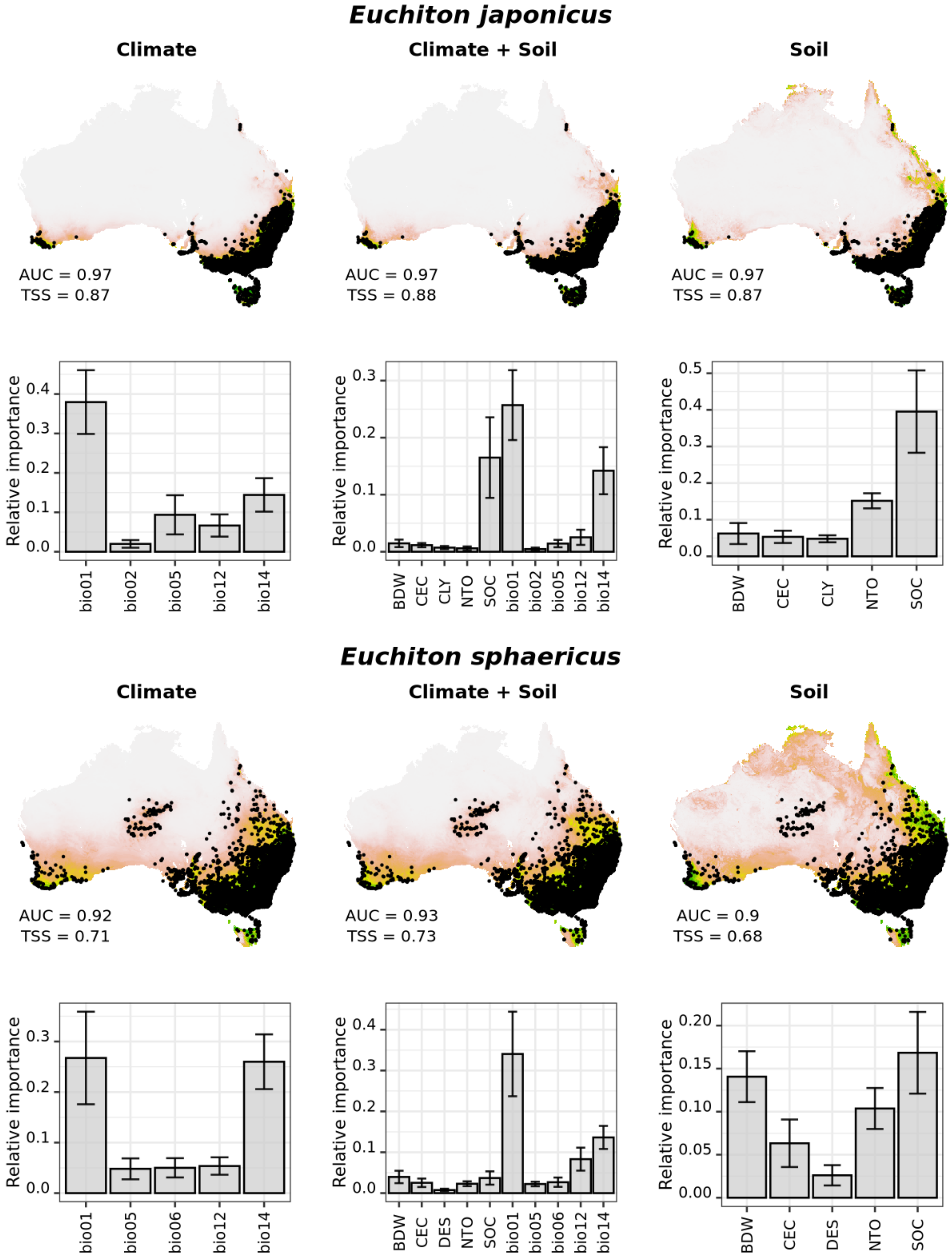
Euchiton japonicus
Climate
Climate + Soil
Soil
AUC = 0.97
TSS = 0.87
AUC = 0.97
TSS = 0.88
AUC = 0.97
TSS = 0.87
Relative importance
bio01
bio02
bio05
bio12
bio14
BDW
CEC
CLY
NTO
SOC
Euchiton sphaericus
AUC = 0.92
TSS = 0.71
AUC = 0.93
TSS = 0.73
AUC = 0.9
TSS = 0.68
bio06
DES

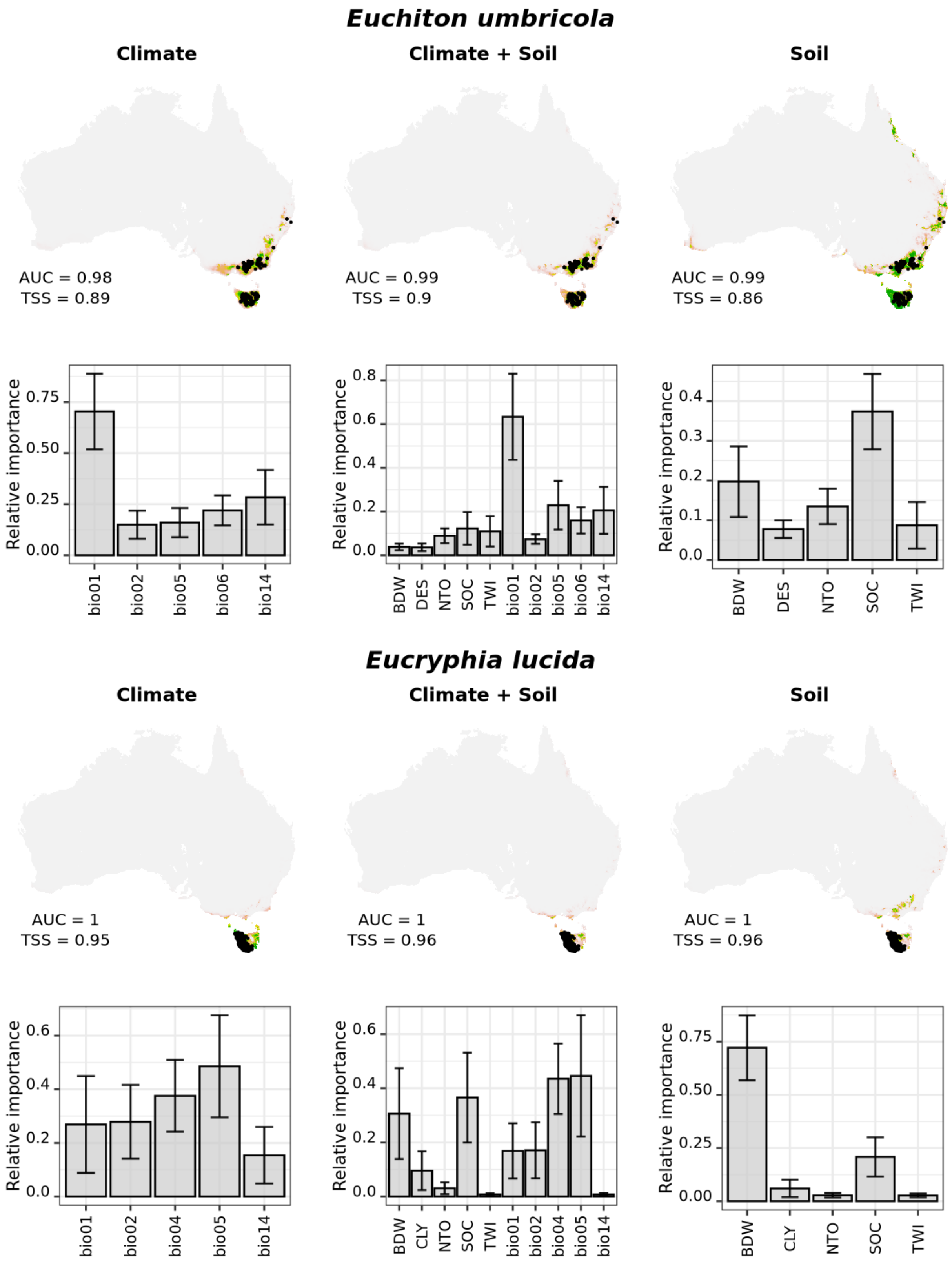
Euchiton umbricola
Climate
Climate + Soil
Soil
AUC = 0.98
TSS = 0.89
AUC = 0.99
TSS = 0.9
AUC = 0.99
TSS = 0.86
Relative importance
0.00 0.25 0.50 0.75
bio01 bio02 bio05 bio06 bio14
0.0 0.2 0.4 0.6 0.8
BDW DES NTO SOC TWI bio01 bio02 bio05 bio06 bio14
0.0 0.1 0.2 0.3 0.4
BDW DES NTO SOC TWI
Eucryphia lucida
Climate
Climate + Soil
Soil
AUC = 1
TSS = 0.95
AUC = 1
TSS = 0.96
AUC = 1
TSS = 0.96
0.0 0.2 0.4 0.6
bio01 bio02 bio04 bio05 bio14
BDW CLY NTO SOC TWI bio01 bio02 bio04 bio05 bio14
0.00 0.25 0.50 0.75
BDW CLY NTO SOC TWI

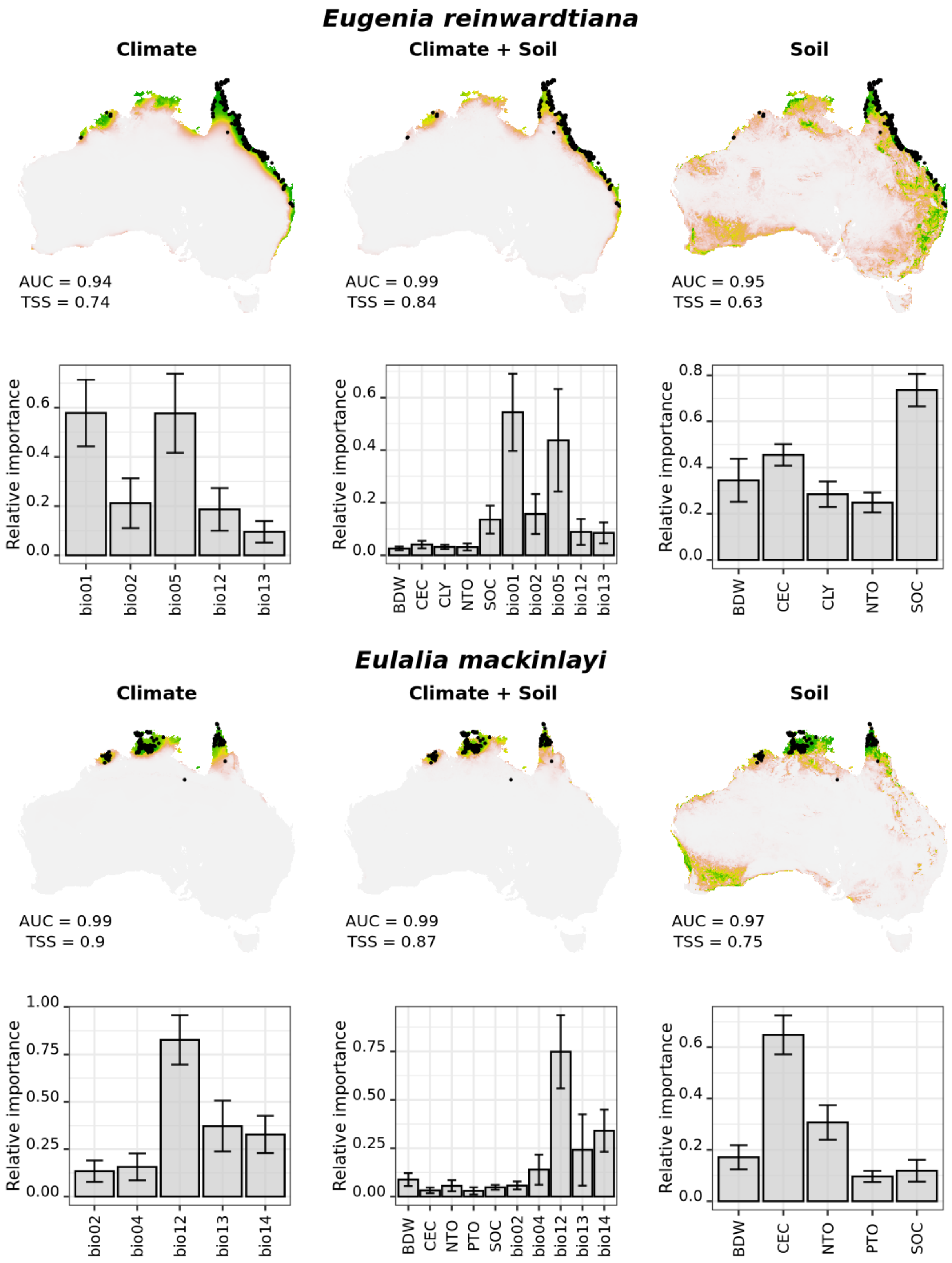
Eugenia reinwardtiana
Climate
Climate + Soil
Soil
AUC = 0.94
TSS = 0.74
AUC = 0.99
TSS = 0.84
AUC = 0.95
TSS = 0.63
Relative importance
0.0
0.2
0.4
0.6
0.8
bio01
bio02
bio05
bio12
bio13
BDW
CEC
CLY
NTO
SOC
Eulalia mackinlayi
Climate
Climate + Soil
Soil
AUC = 0.99
TSS = 0.9
AUC = 0.99
TSS = 0.87
AUC = 0.97
TSS = 0.75
Relative importance
0.00
0.25
0.50
0.75
1.00
bio02
bio04
bio12
bio13
bio14
BDW
CEC
NTO
PTO
SOC

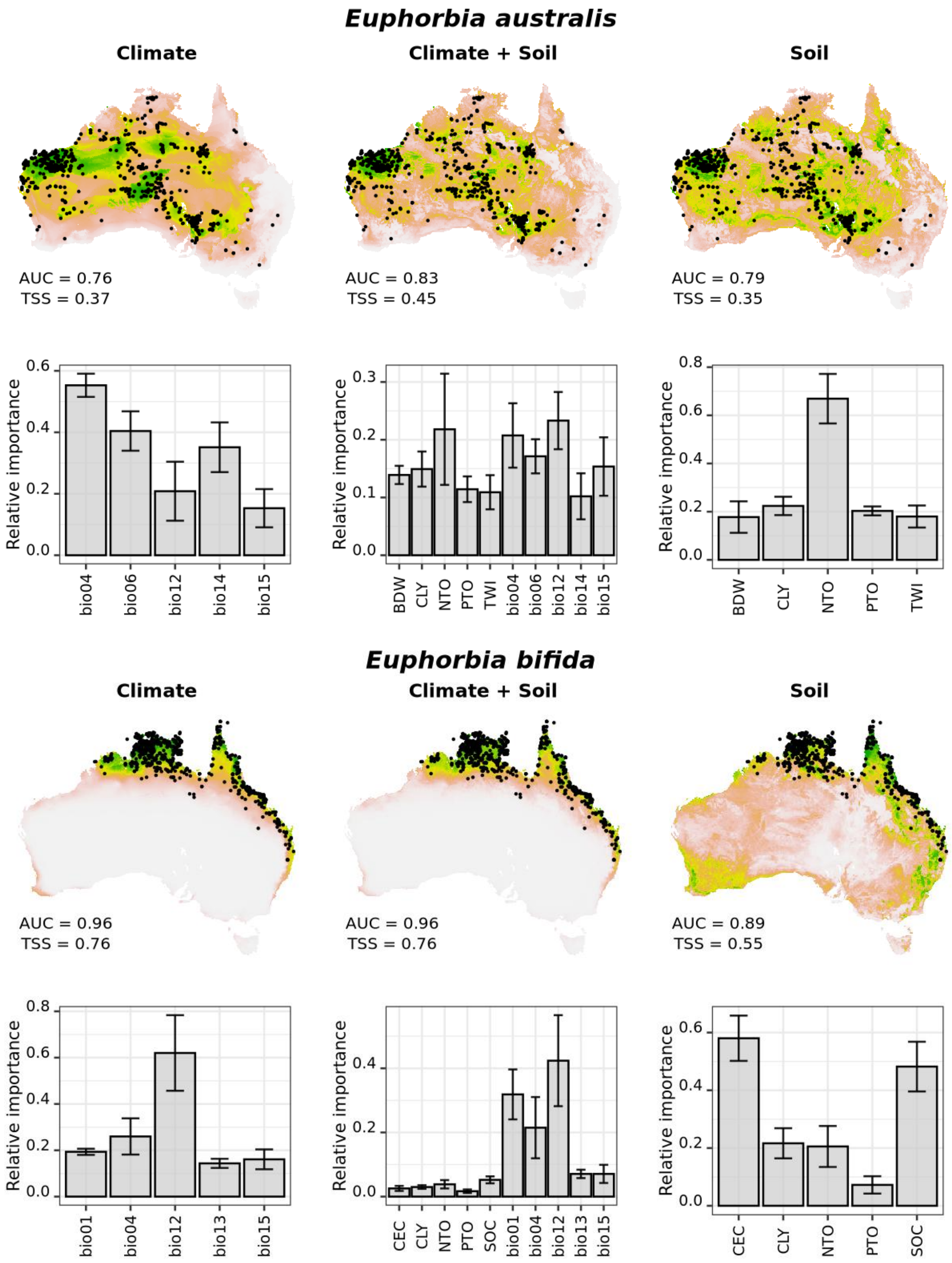
Euphorbia australis
Climate
Climate + Soil
Soil
AUC = 0.76
TSS = 0.37
AUC = 0.83
TSS = 0.45
AUC = 0.79
TSS = 0.35
Relative importance
bio04
bio06
bio12
bio14
bio15
BDW
CLY
NTO
PTO
TWI
Euphorbia bifida
AUC = 0.96
TSS = 0.76
AUC = 0.96
TSS = 0.76
AUC = 0.89
TSS = 0.55
bio01
bio13
CEC
SOC

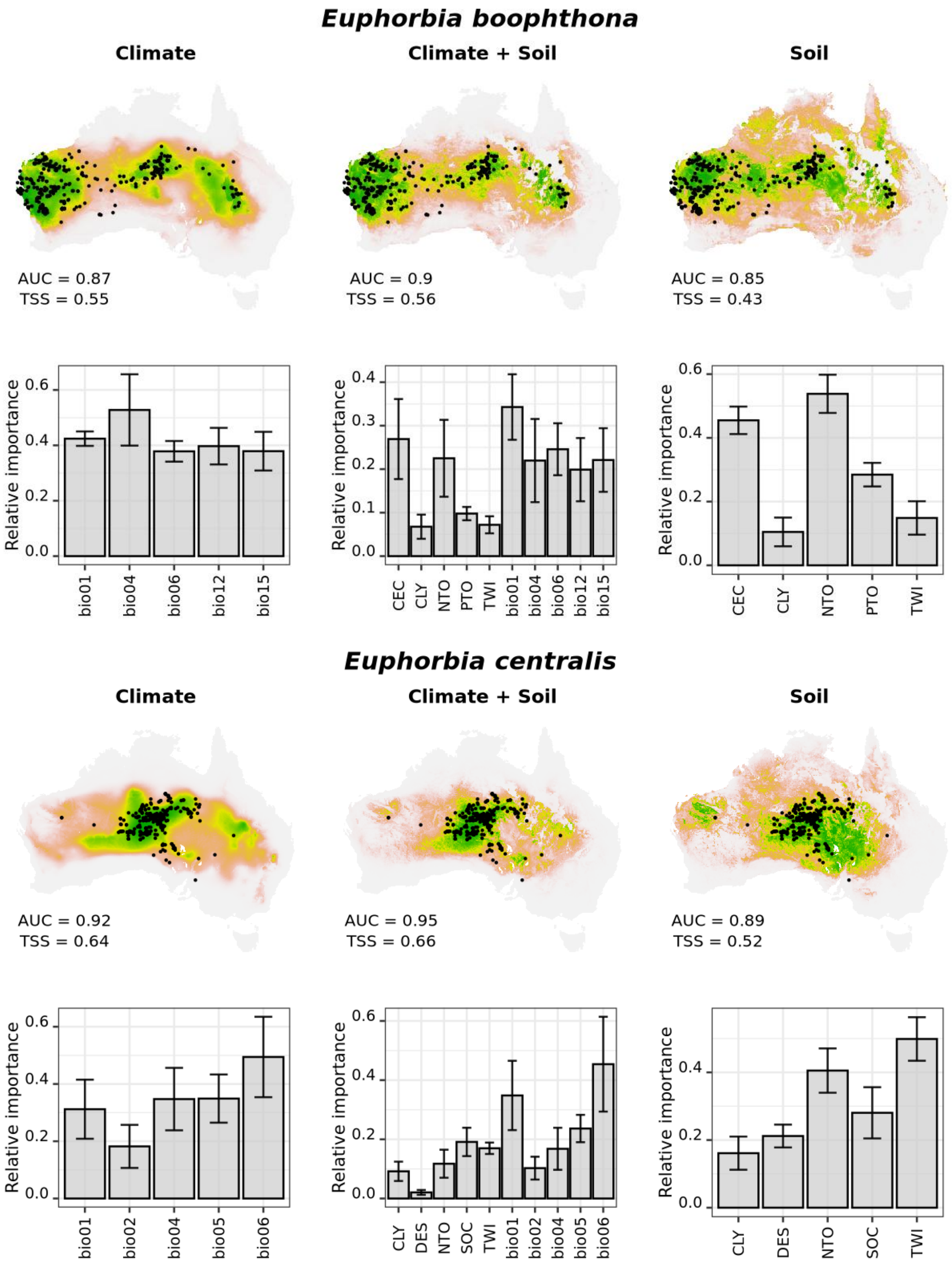
Euphorbia boophthona
Climate
Climate + Soil
Soil
AUC = 0.87
TSS = 0.55
AUC = 0.9
TSS = 0.56
AUC = 0.85
TSS = 0.43
Relative importance
bio01
bio04
bio06
bio12
bio15
CEC
CLY
NTO
PTO
TWI
Euphorbia centralis
Climate
Climate + Soil
Soil
AUC = 0.92
TSS = 0.64
AUC = 0.95
TSS = 0.66
AUC = 0.89
TSS = 0.52
Relative importance
bio01
bio02
bio04
bio05
bio06
CLY
DES
NTO
SOC
TWI

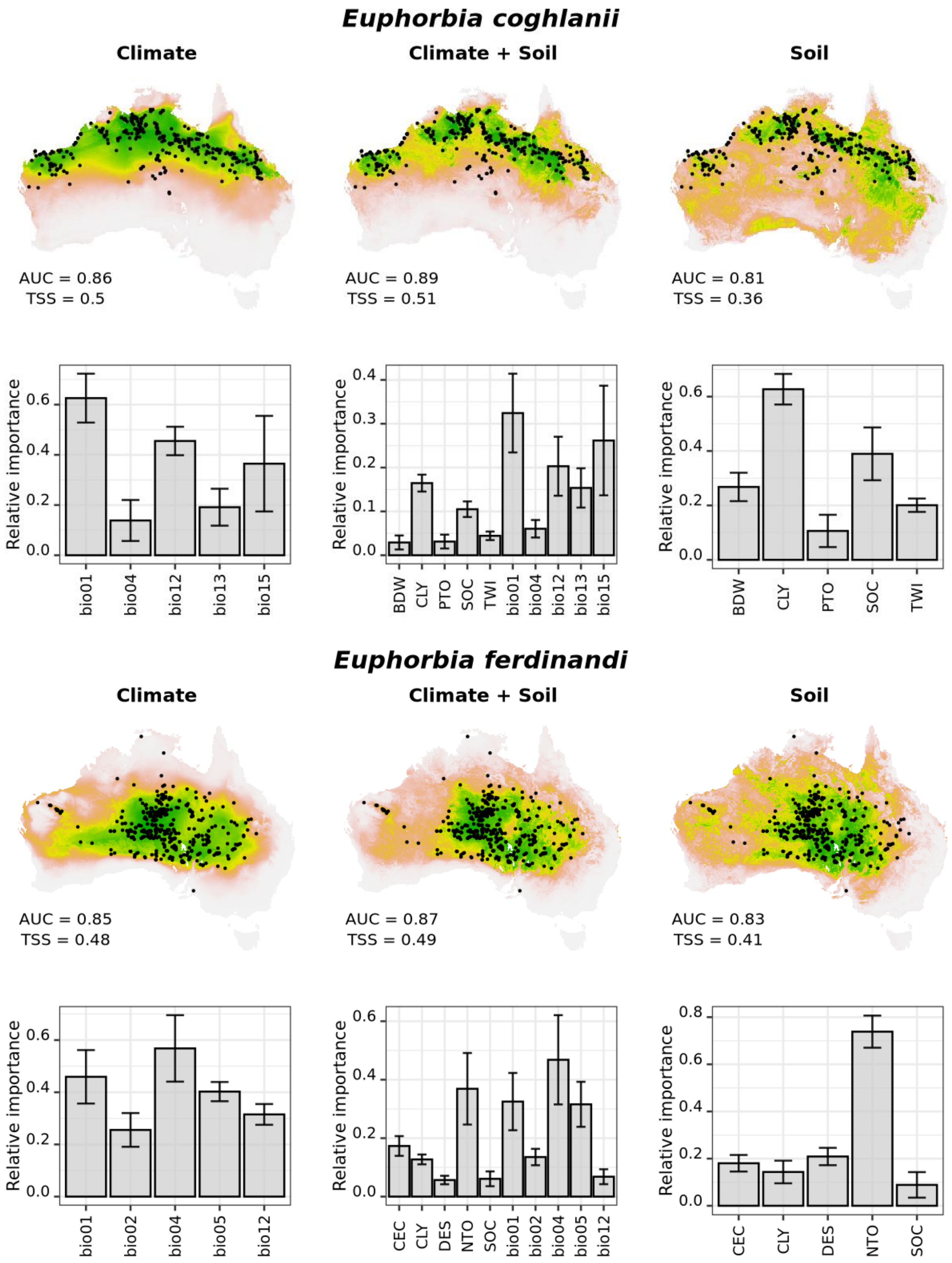
Euphorbia coghlanii
Climate
Climate + Soil
Soil
AUC = 0.86
TSS = 0.5
AUC = 0.89
TSS = 0.51
AUC = 0.81
TSS = 0.36
Relative importance
0.0
0.2
0.4
0.6
bio01
bio04
bio12
bio13
bio15
Relative importance
0.0
0.1
0.2
0.3
0.4
BDW
CLY
PTO
SOC
TWI
bio01
bio04
bio12
bio13
bio15
Relative importance
0.0
0.2
0.4
0.6
BDW
CLY
PTO
SOC
TWI
Euphorbia ferdinandi
Climate
Climate + Soil
Soil
AUC = 0.85
TSS = 0.48
AUC = 0.87
TSS = 0.49
AUC = 0.83
TSS = 0.41
Relative importance
0.0
0.2
0.4
0.6
bio01
bio02
bio04
bio05
bio12
Relative importance
0.0
0.2
0.4
0.6
CEC
CLY
DES
NTO
SOC
bio01
bio02
bio04
bio05
bio12
Relative importance
0.0
0.2
0.4
0.6
0.8
CEC
CLY
DES
NTO
SOC

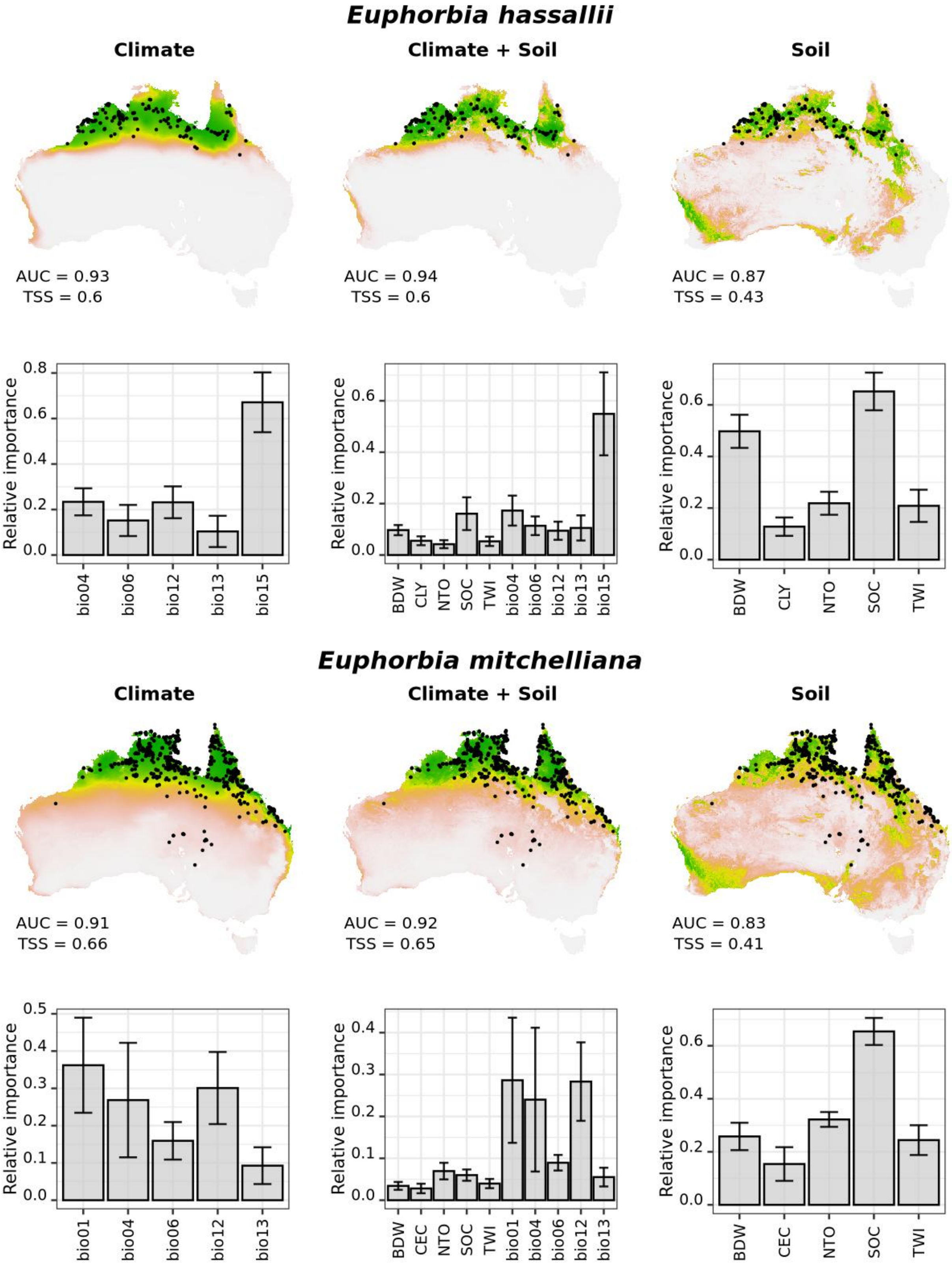
Euphorbia hassallii
Climate
Climate + Soil
Soil
AUC = 0.93
TSS = 0.6
AUC = 0.94
TSS = 0.6
AUC = 0.87
TSS = 0.43
Relative importance
bio04 bio06 bio12 bio13 bio15
BDW CLY NTO SOC TWI bio04 bio06 bio12 bio13 bio15
BDW CLY NTO SOC TWI
Euphorbia mitchelliana
Climate
Climate + Soil
Soil
AUC = 0.91
TSS = 0.66
AUC = 0.92
TSS = 0.65
AUC = 0.83
TSS = 0.41
Relative importance
bio01 bio04 bio06 bio12 bio13
BDW CEC NTO SOC TWI bio01 bio04 bio06 bio12 bio13
BDW CEC NTO SOC TWI

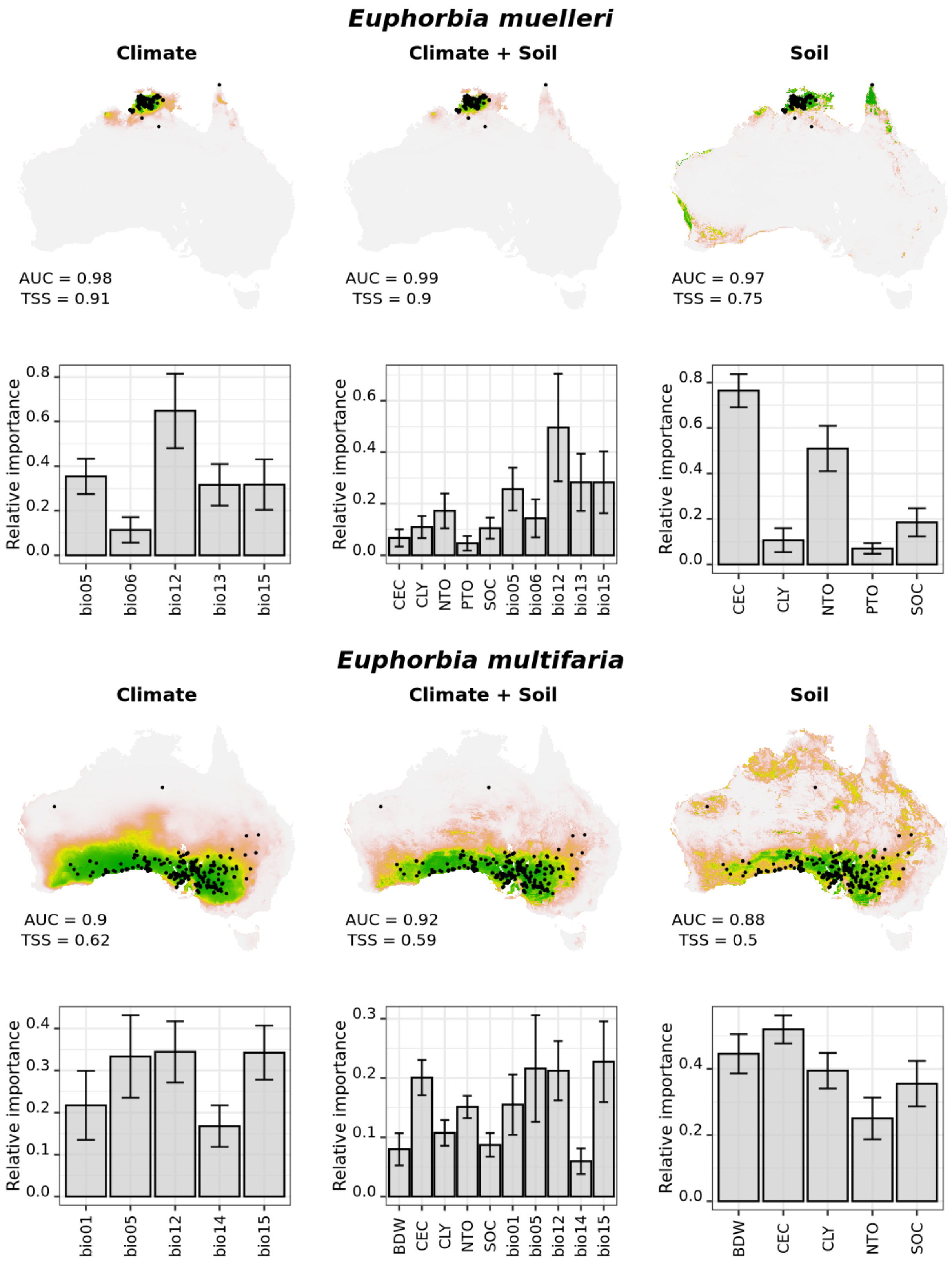
Euphorbia muelleri
Climate
Climate + Soil
Soil
AUC = 0.98
TSS = 0.91
AUC = 0.99
TSS = 0.9
AUC = 0.97
TSS = 0.75
Relative importance
bio05
bio06
bio12
bio13
bio15
CEC
CLY
NTO
PTO
SOC
Euphorbia multifaria
Climate
Climate + Soil
Soil
AUC = 0.9
TSS = 0.62
AUC = 0.92
TSS = 0.59
AUC = 0.88
TSS = 0.5
Relative importance
bio01
bio14
BDW

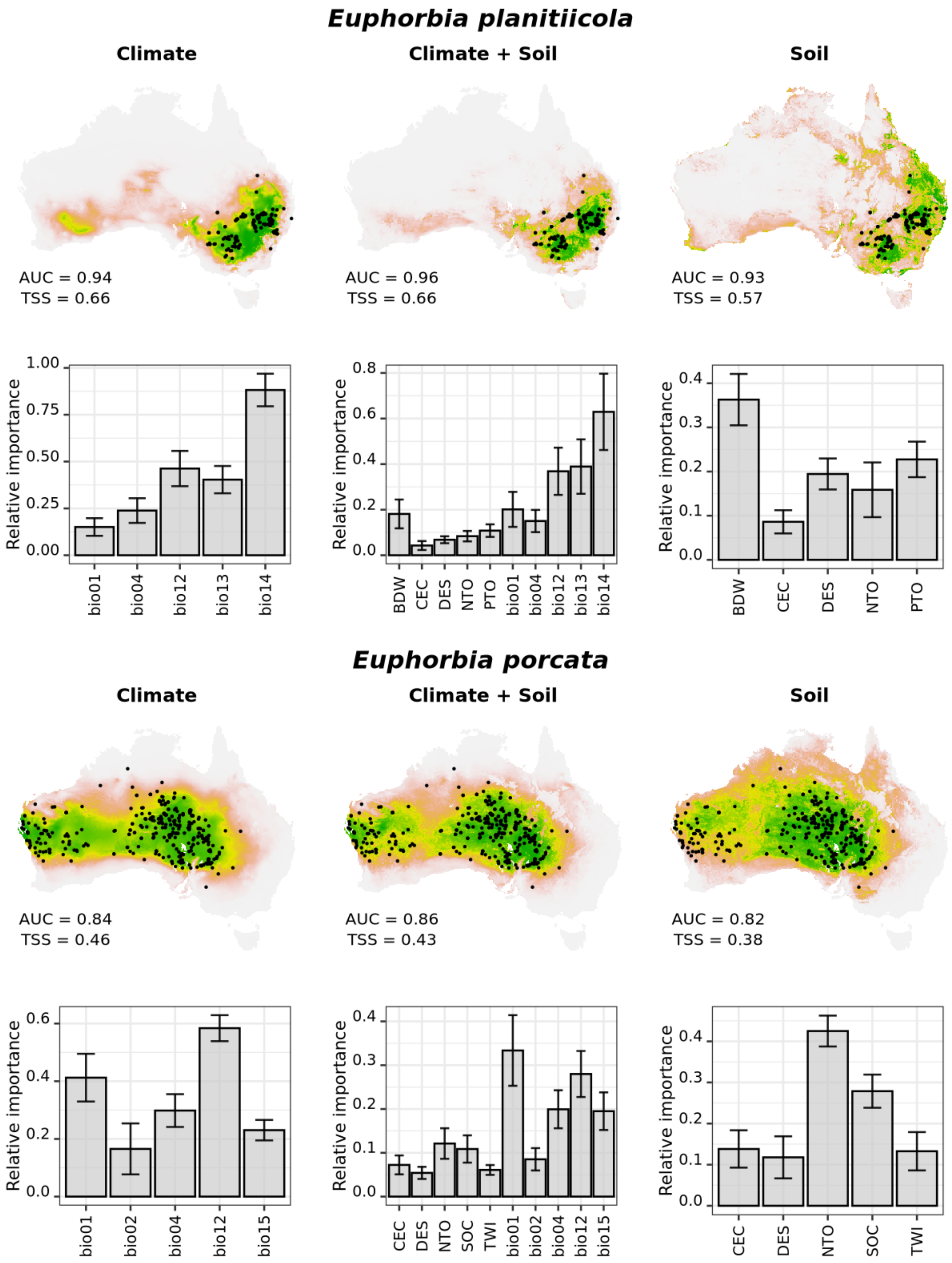
Euphorbia planitiicola
Climate
Climate + Soil
Soil
AUC = 0.94
TSS = 0.66
AUC = 0.96
TSS = 0.66
AUC = 0.93
TSS = 0.57
Relative importance
1.00
0.75
0.50
0.25
0.00
bio01
bio04
bio12
bio13
bio14
0.8
0.6
0.4
0.2
0.0
BDW
CEC
DES
NTO
PTO
bio01
bio04
bio12
bio13
bio14
0.4
0.3
0.2
0.1
0.0
BDW
CEC
DES
NTO
PTO
Euphorbia porcata
Climate
Climate + Soil
Soil
AUC = 0.84
TSS = 0.46
AUC = 0.86
TSS = 0.43
AUC = 0.82
TSS = 0.38
Relative importance
0.6
0.4
0.2
0.0
bio01
bio02
bio04
bio12
bio15
0.4
0.3
0.2
0.1
0.0
CEC
DES
NTO
SOC
TWI
bio01
bio02
bio04
bio12
bio15
0.4
0.3
0.2
0.1
0.0
CEC
DES
NTO
SOC
TWI

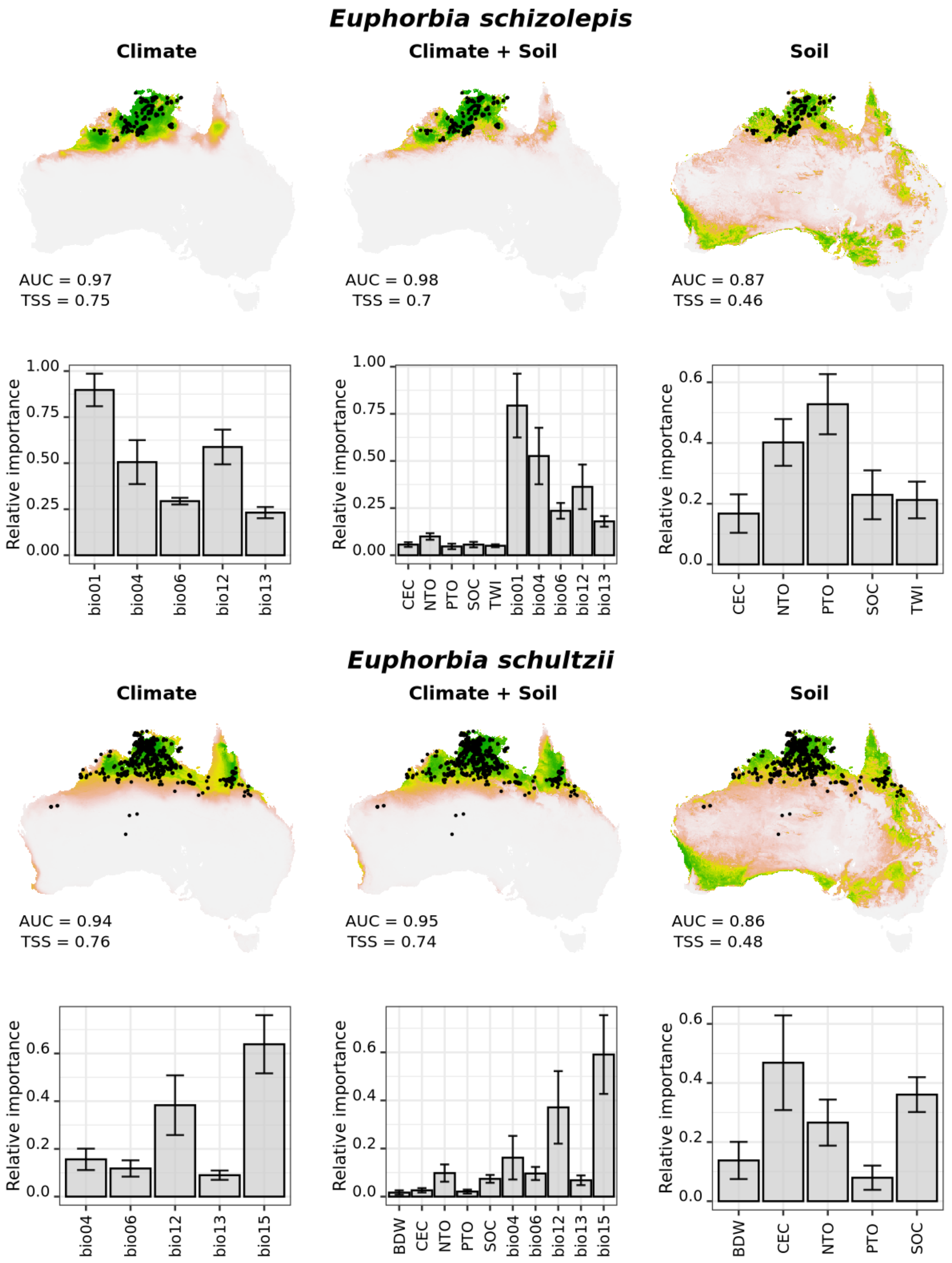
Euphorbia schizolepis
Climate
Climate + Soil
Soil
AUC = 0.97
TSS = 0.75
AUC = 0.98
TSS = 0.7
AUC = 0.87
TSS = 0.46
Relative importance
bio01
bio04
bio06
bio12
bio13
CEC
NTO
PTO
SOC
TWI
Euphorbia schultzii
Climate
Climate + Soil
Soil
AUC = 0.94
TSS = 0.76
AUC = 0.95
TSS = 0.74
AUC = 0.86
TSS = 0.48
Relative importance
bio04
bio06
bio12
bio13
bio15
BDW
CEC
NTO
PTO
SOC

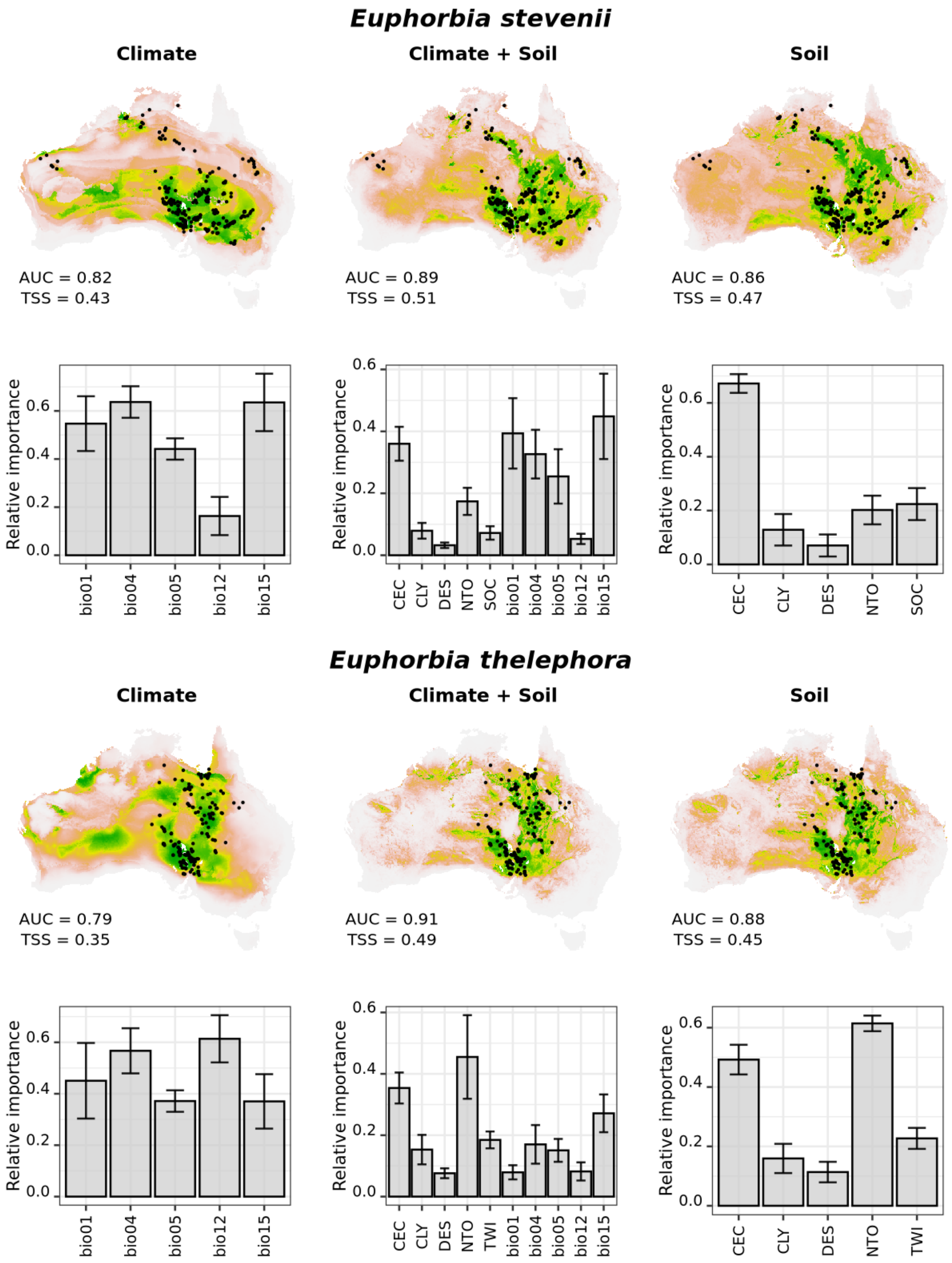

Euphorbia stevenii
Climate
Climate + Soil
Soil
AUC = 0.82
TSS = 0.43
AUC = 0.89
TSS = 0.51
AUC = 0.86
TSS = 0.47
Relative importance
0.0
0.2
0.4
0.6
bio01
bio04
bio05
bio12
bio15
CEC
CLY
DES
NTO
SOC
Euphorbia thelephora
Climate
Climate + Soil
Soil
AUC = 0.79
TSS = 0.35
AUC = 0.91
TSS = 0.49
AUC = 0.88
TSS = 0.45
TWI

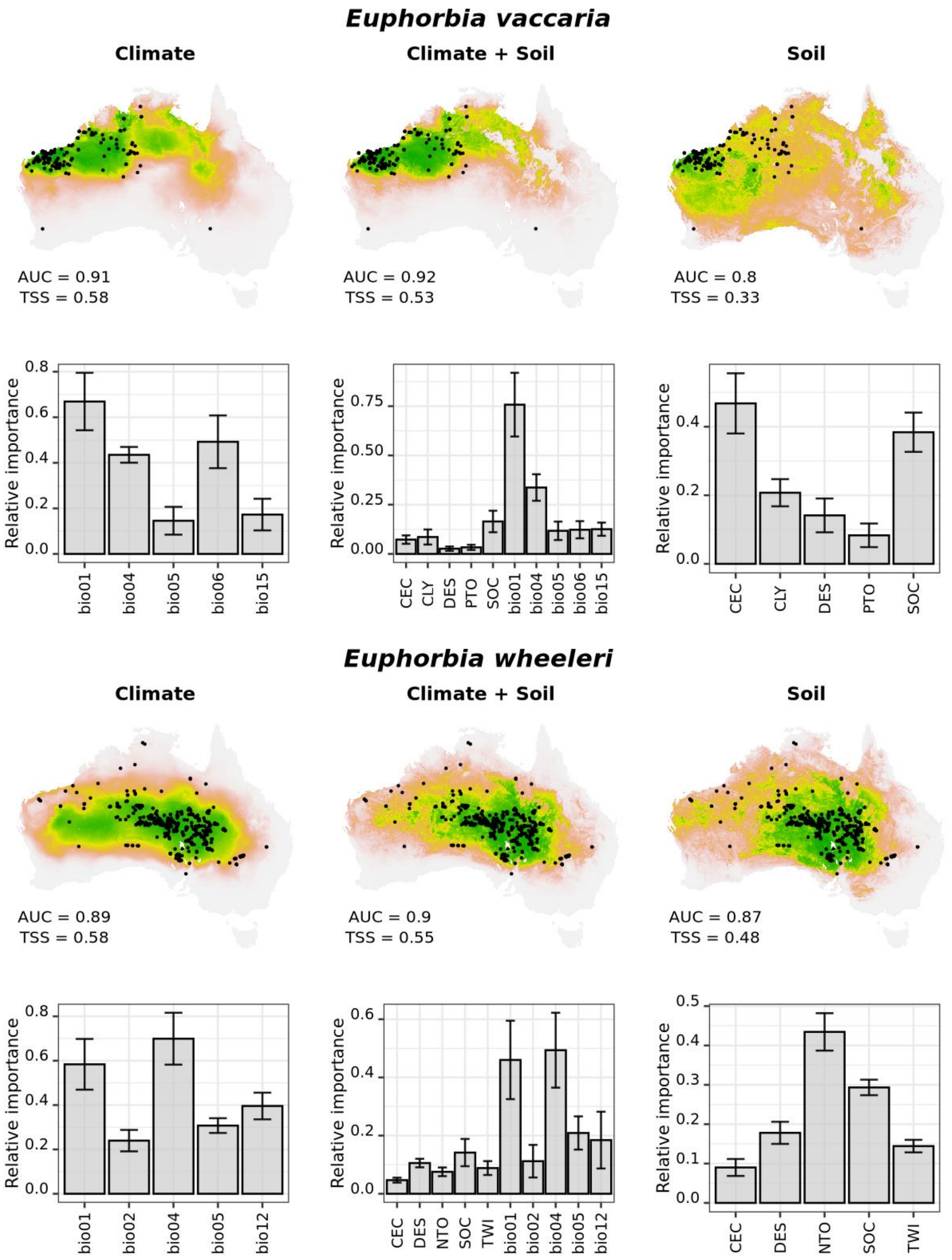
Euphorbia vaccaria
Climate
Climate + Soil
Soil
AUC = 0.91
TSS = 0.58
AUC = 0.92
TSS = 0.53
AUC = 0.8
TSS = 0.33
Relative importance
bio01
bio04
bio05
bio06
bio15
CEC
CLY
DES
PTO
SOC
Euphorbia wheeleri
AUC = 0.89
TSS = 0.58
AUC = 0.9
TSS = 0.55
AUC = 0.87
TSS = 0.48
bio02
bio12
NTO
TWI

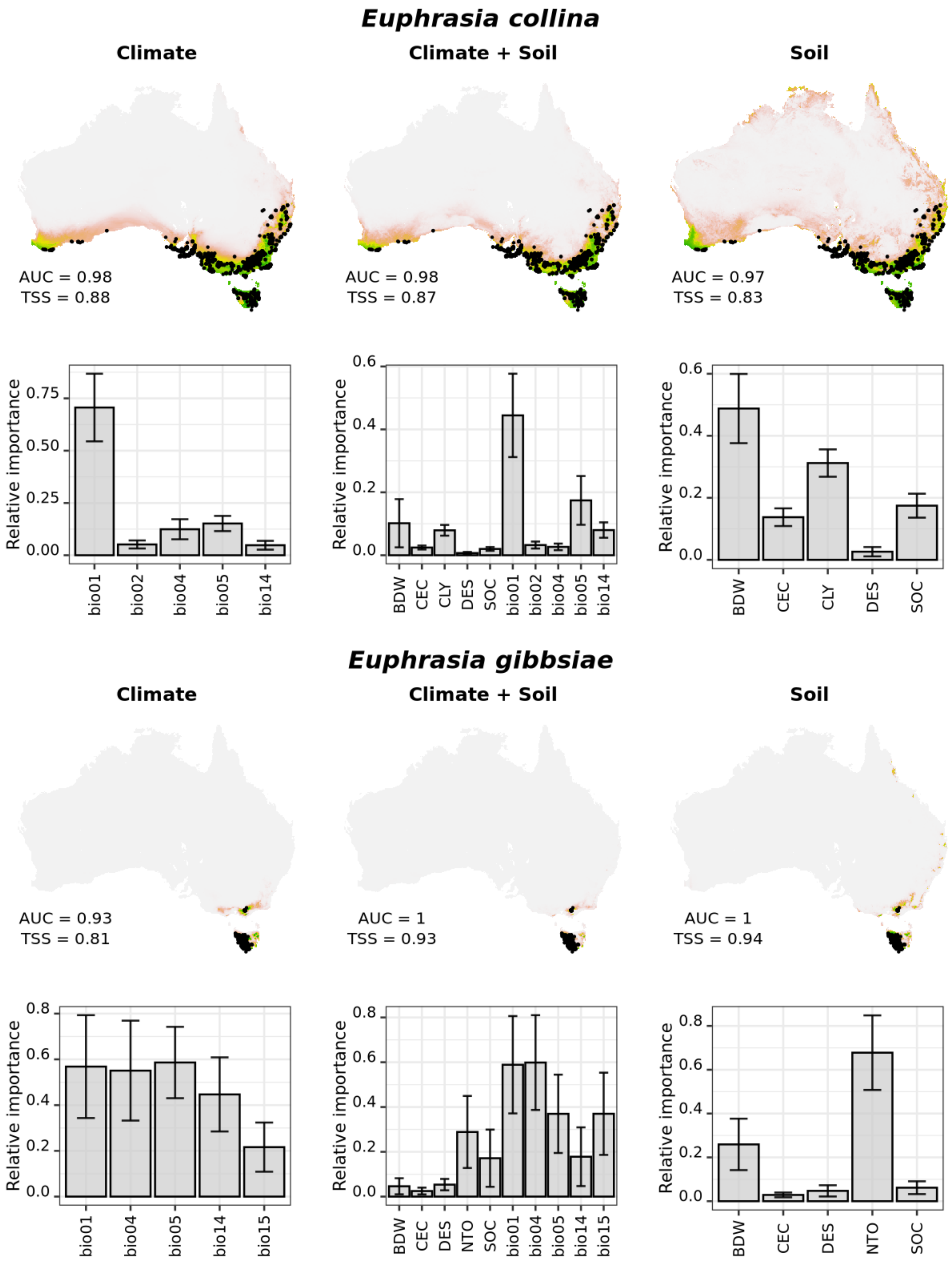
Euphrasia collina
Climate
Climate + Soil
Soil
AUC = 0.98
TSS = 0.88
AUC = 0.98
TSS = 0.87
AUC = 0.97
TSS = 0.83
Relative importance
0.00 0.25 0.50 0.75
bio01 bio02 bio04 bio05 bio14
0.0 0.2 0.4 0.6
BDW CEC CLY DES SOC bio01 bio02 bio04 bio05 bio14
BDW CEC CLY DES SOC
Euphrasia gibbsiae
Climate
Climate + Soil
Soil
AUC = 0.93
TSS = 0.81
AUC = 1
TSS = 0.93
AUC = 1
TSS = 0.94
0.0 0.2 0.4 0.6 0.8
bio01 bio04 bio05 bio14 bio15
BDW CEC DES NTO SOC bio01 bio04 bio05 bio14 bio15
BDW CEC DES NTO SOC

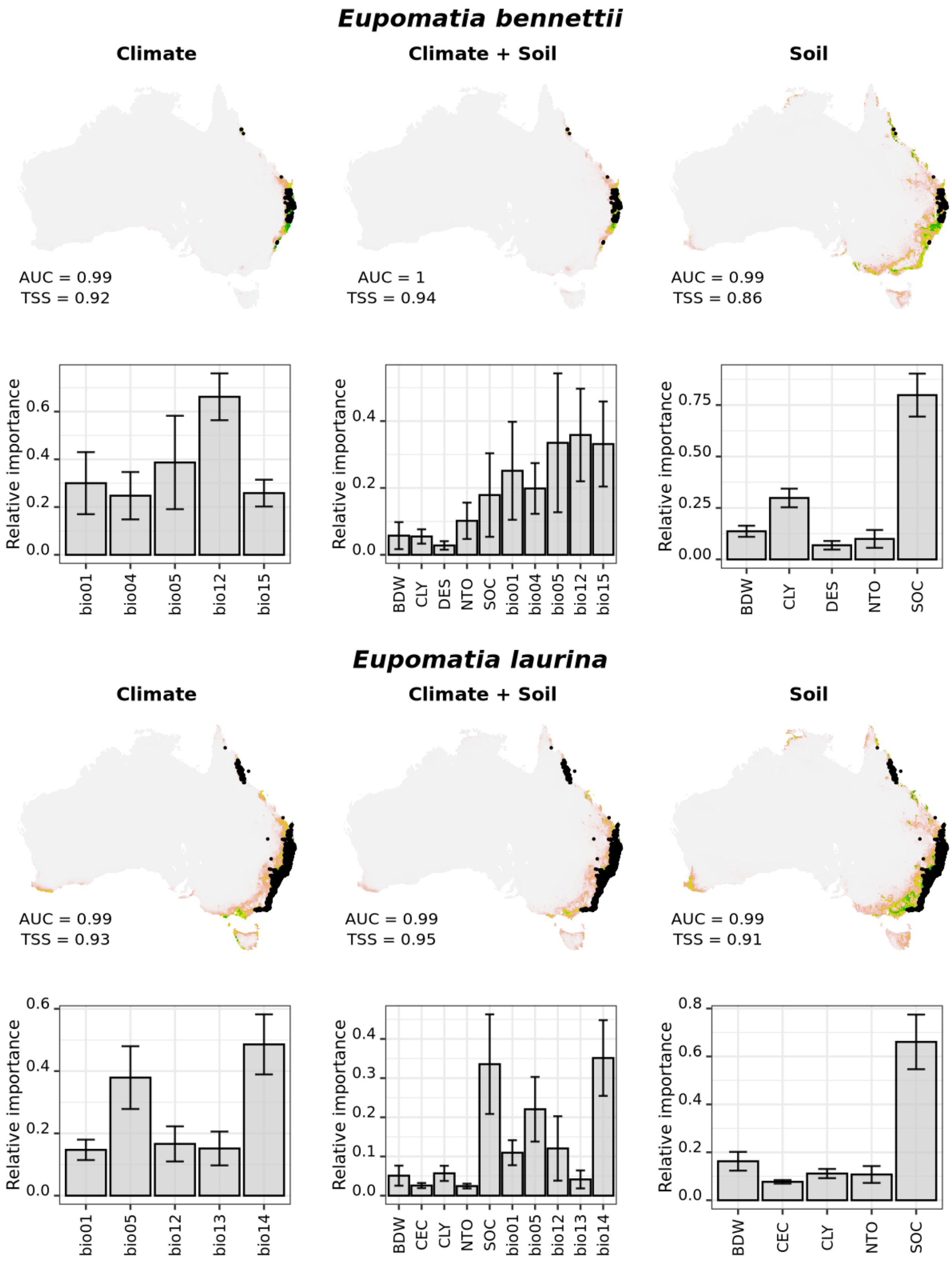
Eupomatia bennettii
Climate
Climate + Soil
Soil
AUC = 0.99
TSS = 0.92
AUC = 1
TSS = 0.94
AUC = 0.99
TSS = 0.86
Relative importance
bio01
bio04
bio05
bio12
bio15
BDW
CLY
DES
NTO
SOC
Eupomatia laurina
Climate
Climate + Soil
Soil
AUC = 0.99
TSS = 0.93
AUC = 0.99
TSS = 0.95
AUC = 0.99
TSS = 0.91
Relative importance
bio01
bio05
bio12
bio13
bio14
BDW
CEC
CLY
NTO
SOC

Euroschinus falcata

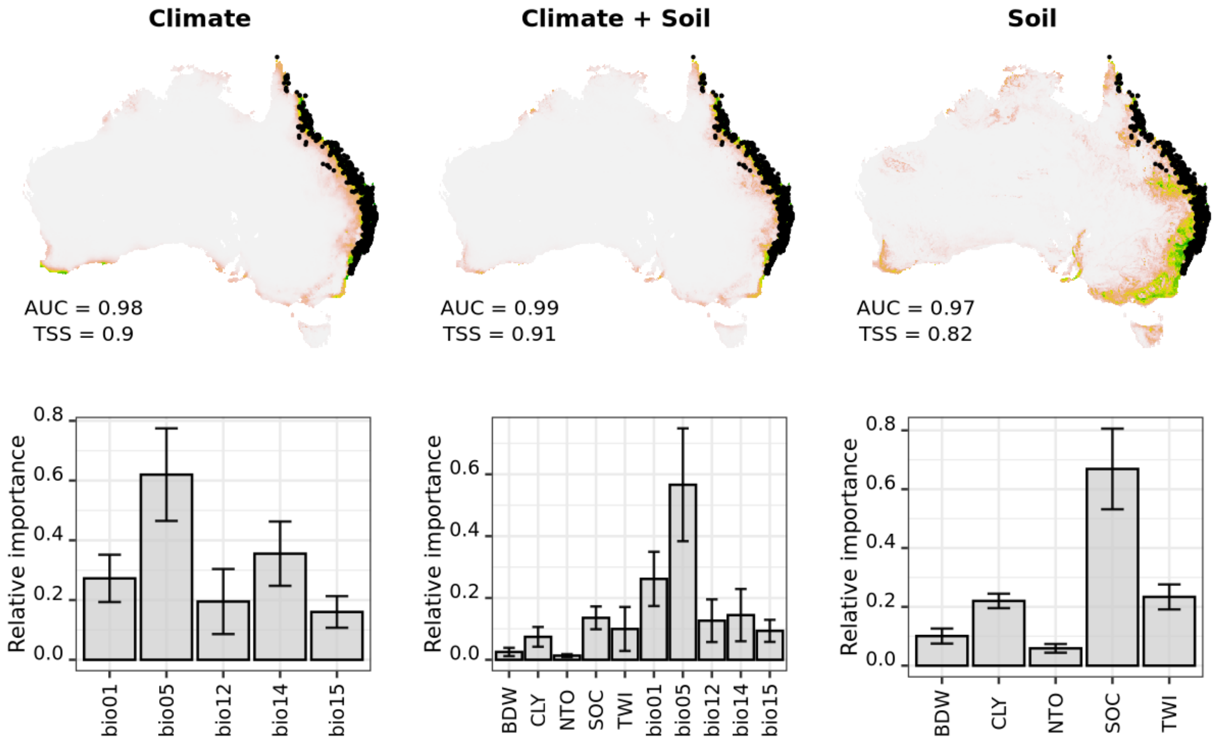

Eurychorda complanata

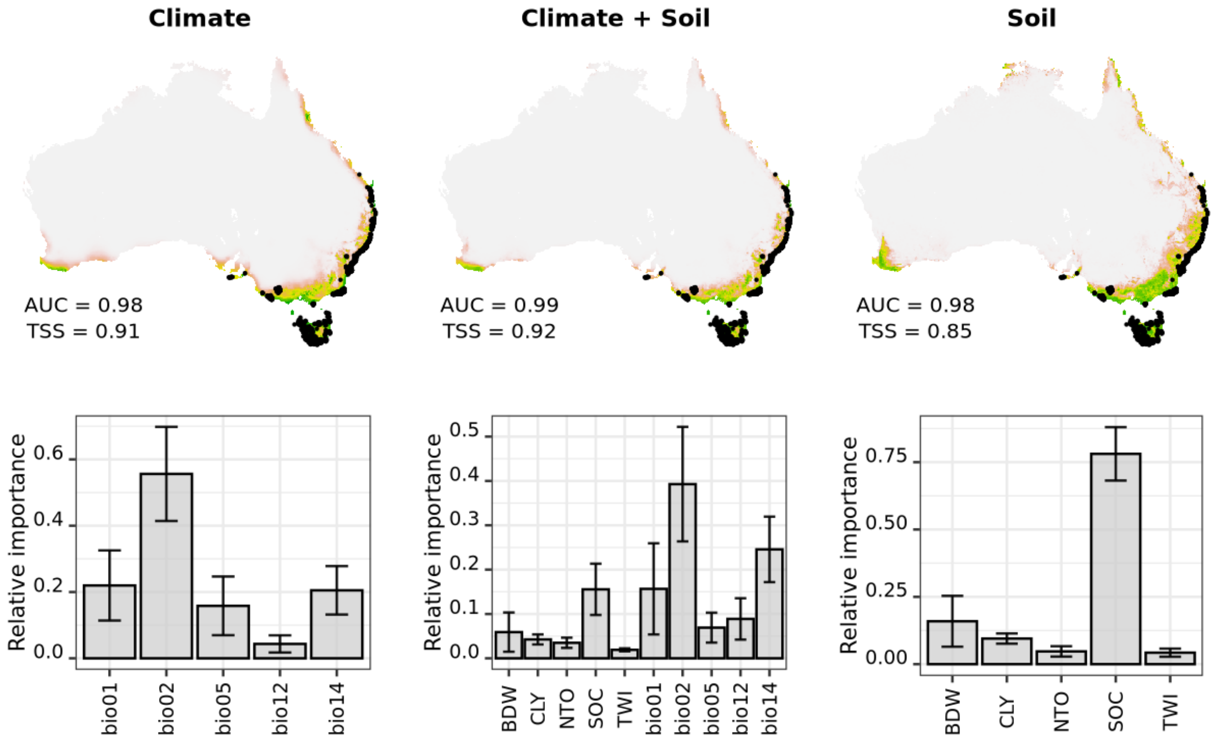

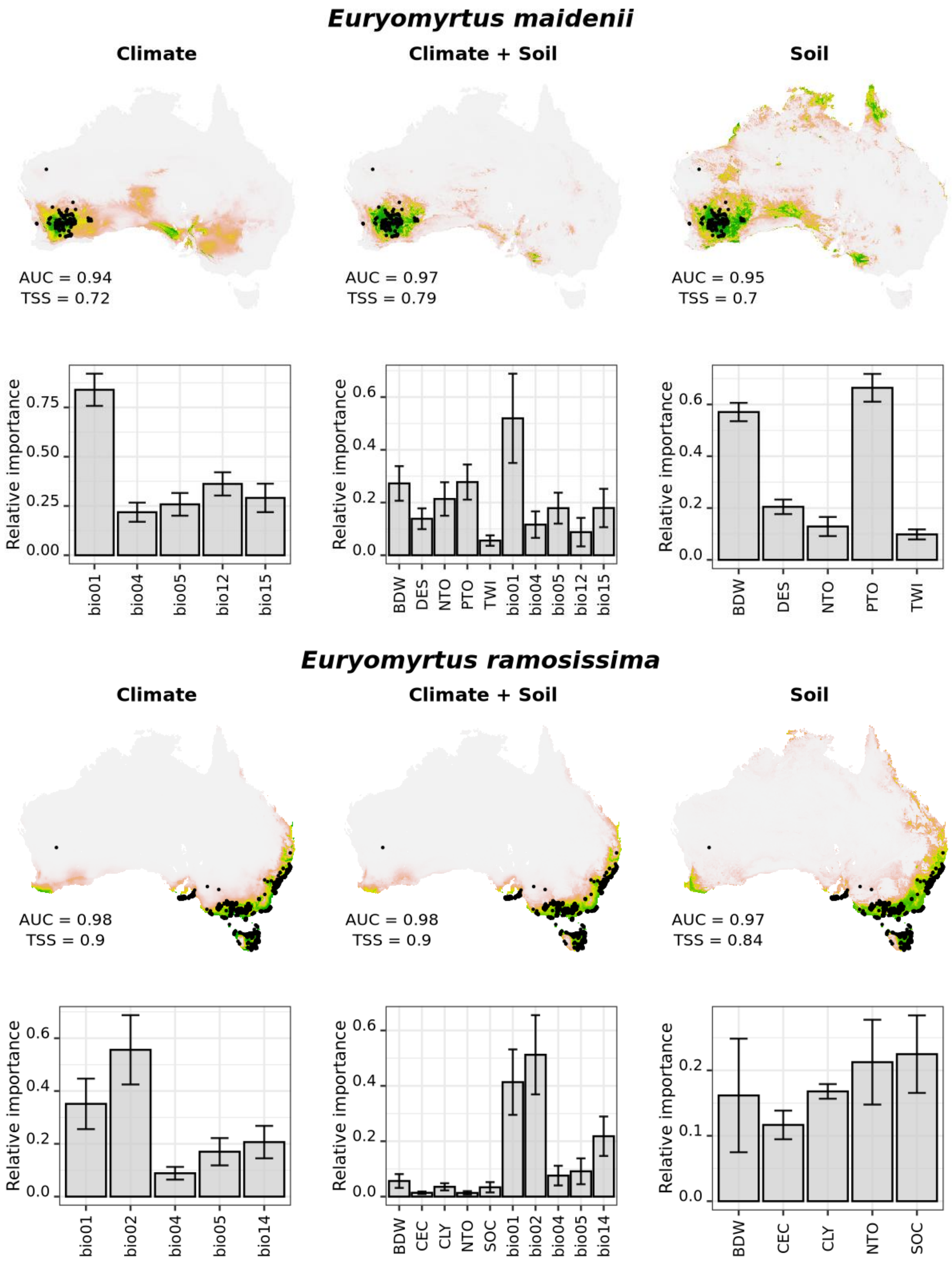

Euryomyrtus maidenii
Climate
Climate + Soil
Soil
AUC = 0.94
TSS = 0.72
AUC = 0.97
TSS = 0.79
AUC = 0.95
TSS = 0.7
Relative importance
0.00
0.25
0.50
0.75
bio01
bio04
bio05
bio12
bio15
0.0
0.2
0.4
0.6
BDW
DES
NTO
PTO
TWI
bio01
bio04
bio05
bio12
bio15
BDW
DES
NTO
PTO
TWI
Euryomyrtus ramosissima
Climate
Climate + Soil
Soil
AUC = 0.98
TSS = 0.9
AUC = 0.98
TSS = 0.9
AUC = 0.97
TSS = 0.84
Relative importance
0.0
0.2
0.4
0.6
bio01
bio02
bio04
bio05
bio14
BDW
CEC
CLY
NTO
SOC
bio01
bio02
bio04
bio05
bio14
0.0
0.1
0.2
BDW
CEC
CLY
NTO
SOC

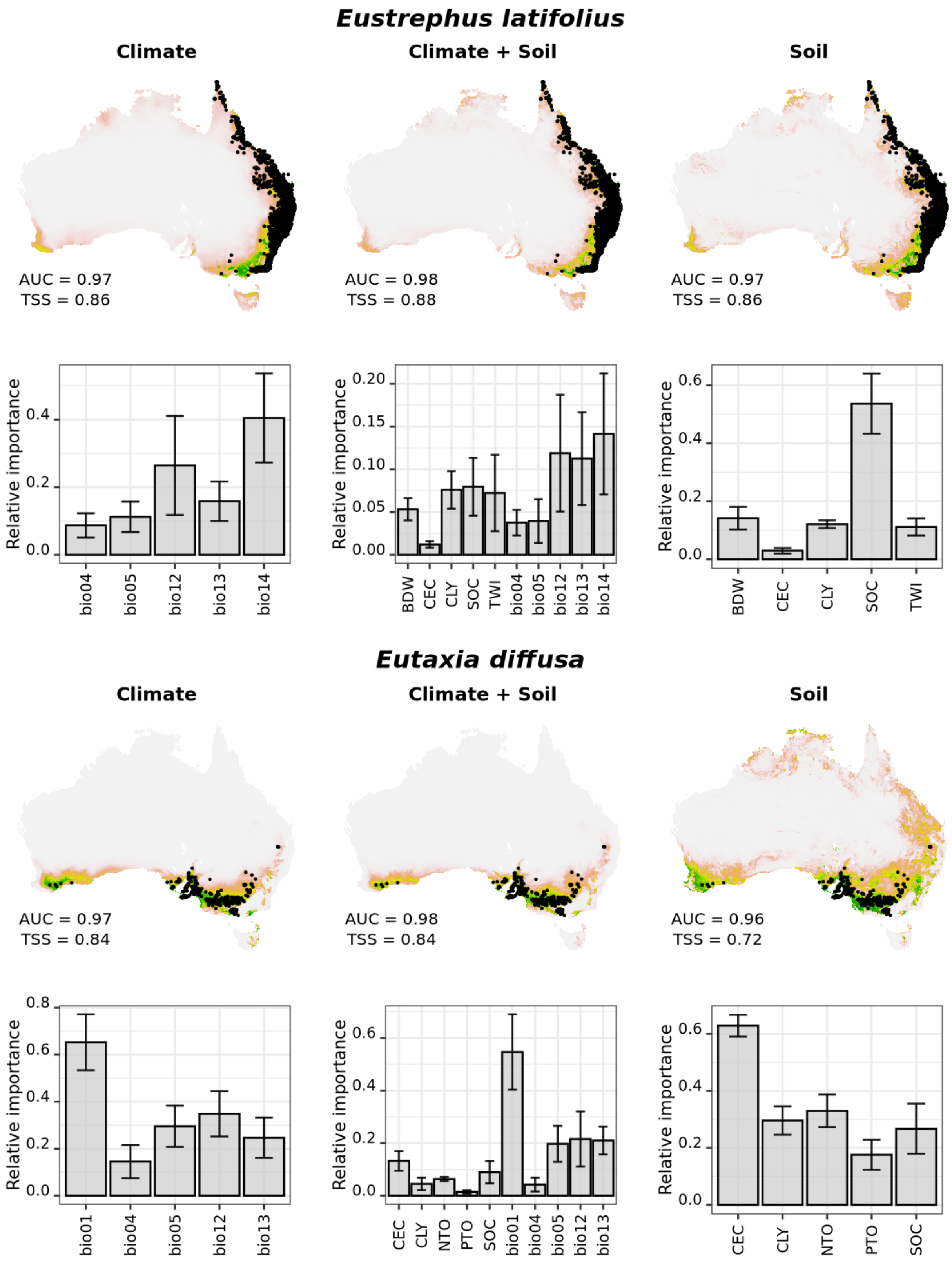
Eustrephus latifolius
Climate
Climate + Soil
Soil
AUC = 0.97
TSS = 0.86
AUC = 0.98
TSS = 0.88
AUC = 0.97
TSS = 0.86
Relative importance
bio04
bio05
bio12
bio13
bio14
BDW
CEC
CLY
SOC
TWI
Eutaxia diffusa
Climate
Climate + Soil
Soil
AUC = 0.97
TSS = 0.84
AUC = 0.98
TSS = 0.84
AUC = 0.96
TSS = 0.72
Relative importance
bio01
bio04
bio05
bio12
bio13
CEC
CLY
NTO
PTO
SOC

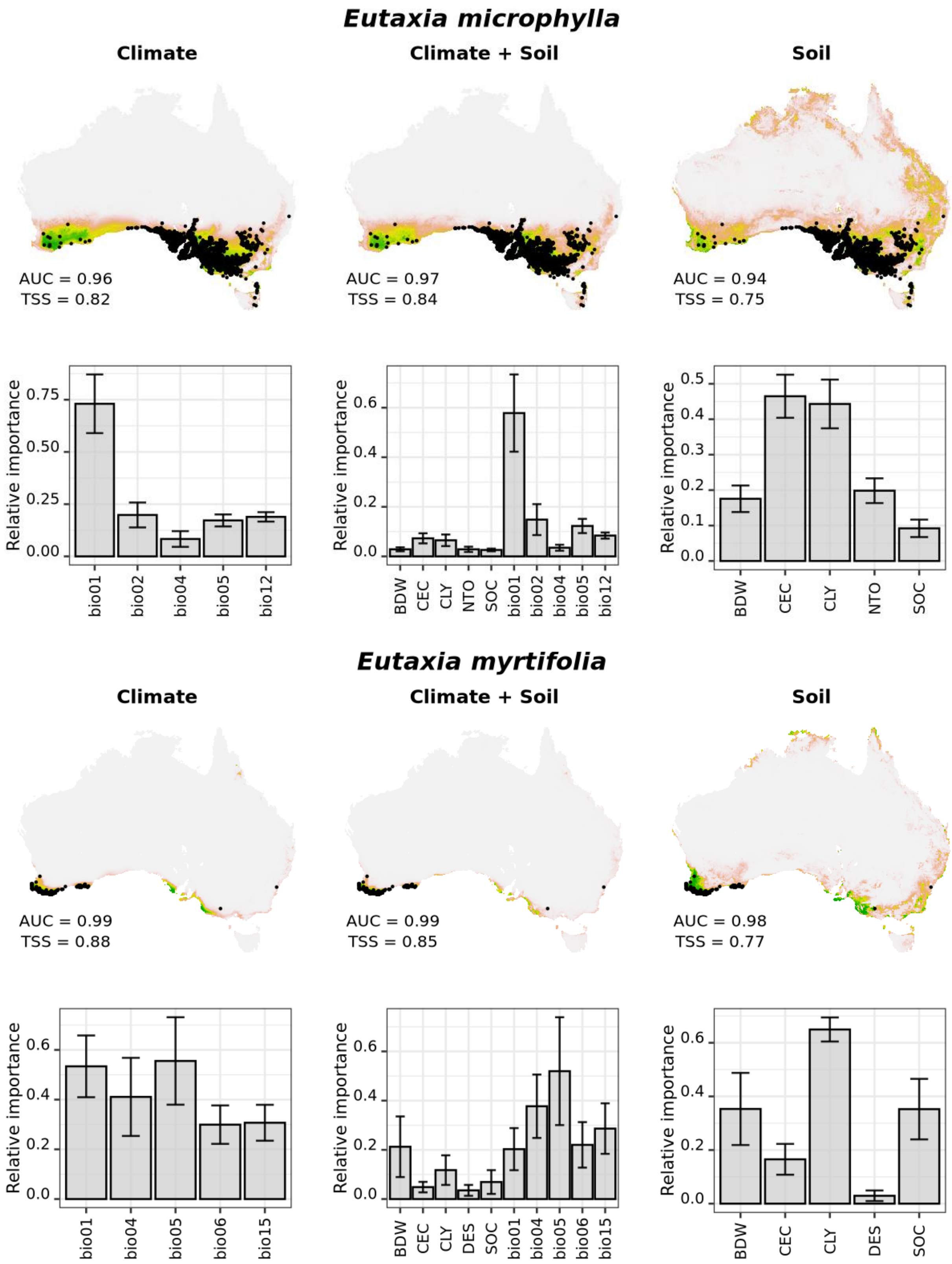
Eutaxia microphylla
Climate
Climate + Soil
Soil
AUC = 0.96
TSS = 0.82
AUC = 0.97
TSS = 0.84
AUC = 0.94
TSS = 0.75
Relative importance
0.00
0.25
0.50
0.75
bio01
bio02
bio04
bio05
bio12
0.0
0.2
0.4
0.6
BDW
CEC
CLY
NTO
SOC
bio01
bio02
bio04
bio05
bio12
0.0
0.1
0.2
0.3
0.4
0.5
BDW
CEC
CLY
NTO
SOC
Eutaxia myrtifolia
Climate
Climate + Soil
Soil
AUC = 0.99
TSS = 0.88
AUC = 0.99
TSS = 0.85
AUC = 0.98
TSS = 0.77
Relative importance
0.0
0.2
0.4
0.6
bio01
bio04
bio05
bio06
bio15
BDW
CEC
CLY
DES
SOC
bio01
bio04
bio05
bio06
bio15
BDW
CEC
CLY
DES
SOC

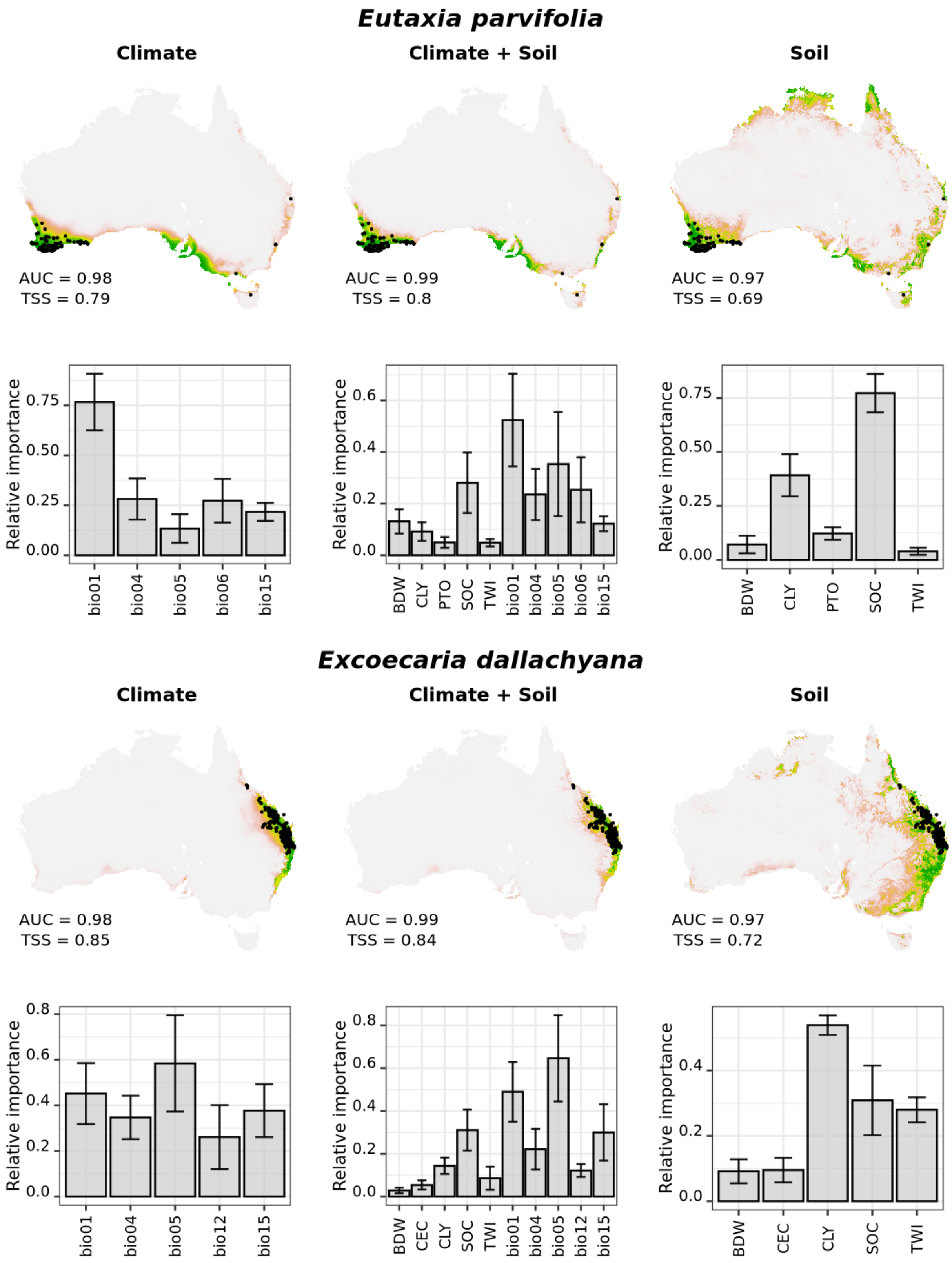

Eutaxia parvifolia
Climate
Climate + Soil
Soil
AUC = 0.98
TSS = 0.79
AUC = 0.99
TSS = 0.8
AUC = 0.97
TSS = 0.69
Relative importance
bio01
bio04
bio05
bio06
bio15
BDW
CLY
PTO
SOC
TWI
Excoecaria dallachyana
AUC = 0.98
TSS = 0.85
AUC = 0.99
TSS = 0.84
AUC = 0.97
TSS = 0.72
bio12
CEC

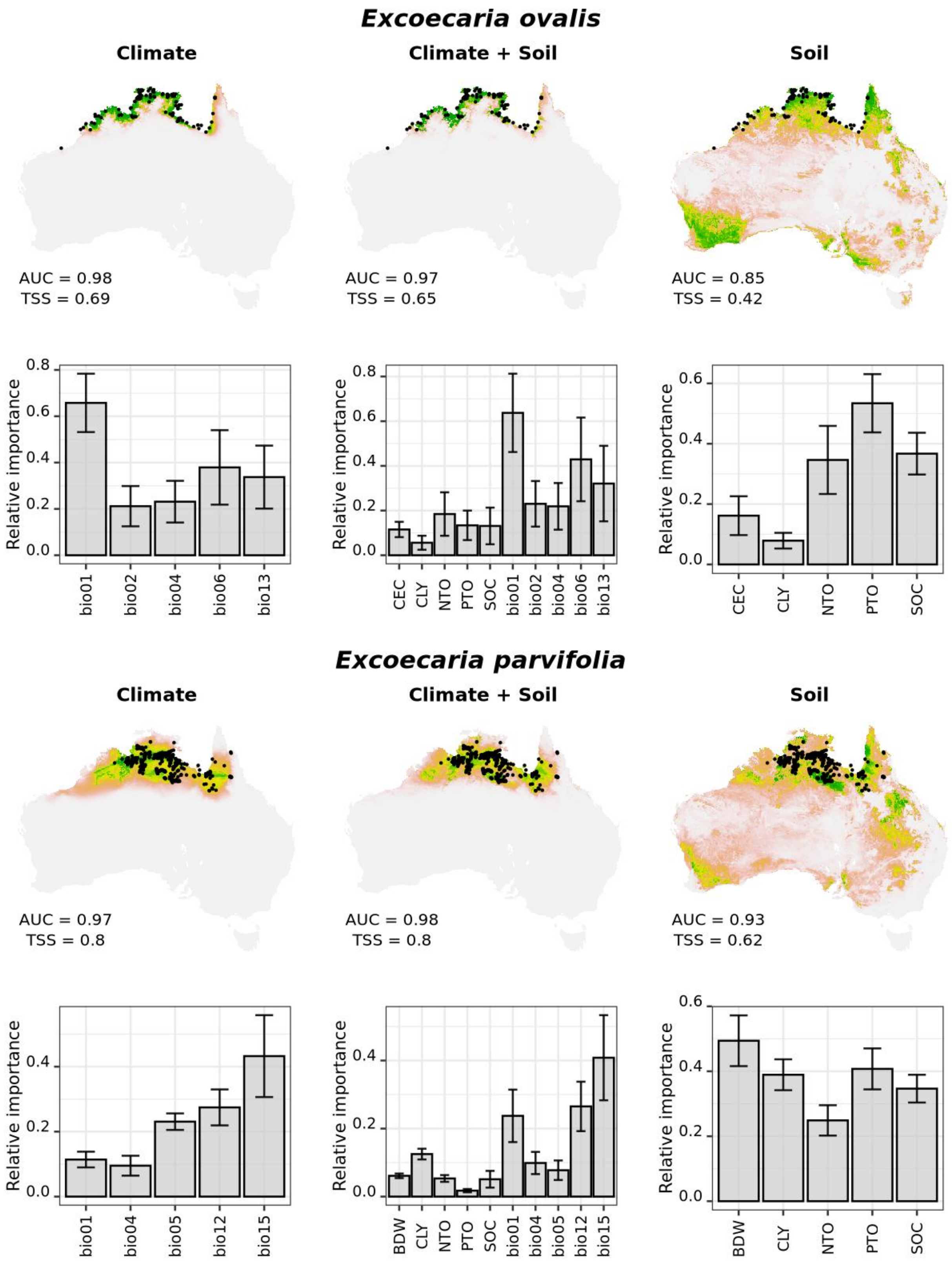
Excoecaria ovalis
Climate
Climate + Soil
Soil
AUC = 0.98
TSS = 0.69
AUC = 0.97
TSS = 0.65
AUC = 0.85
TSS = 0.42
Relative importance
bio01
bio02
bio04
bio06
bio13
CEC
CLY
NTO
PTO
SOC
Excoecaria parvifolia
Climate
Climate + Soil
Soil
AUC = 0.97
TSS = 0.8
AUC = 0.98
TSS = 0.8
AUC = 0.93
TSS = 0.62
Relative importance
bio01
bio04
bio05
bio12
bio15
BDW
CLY
NTO
PTO
SOC

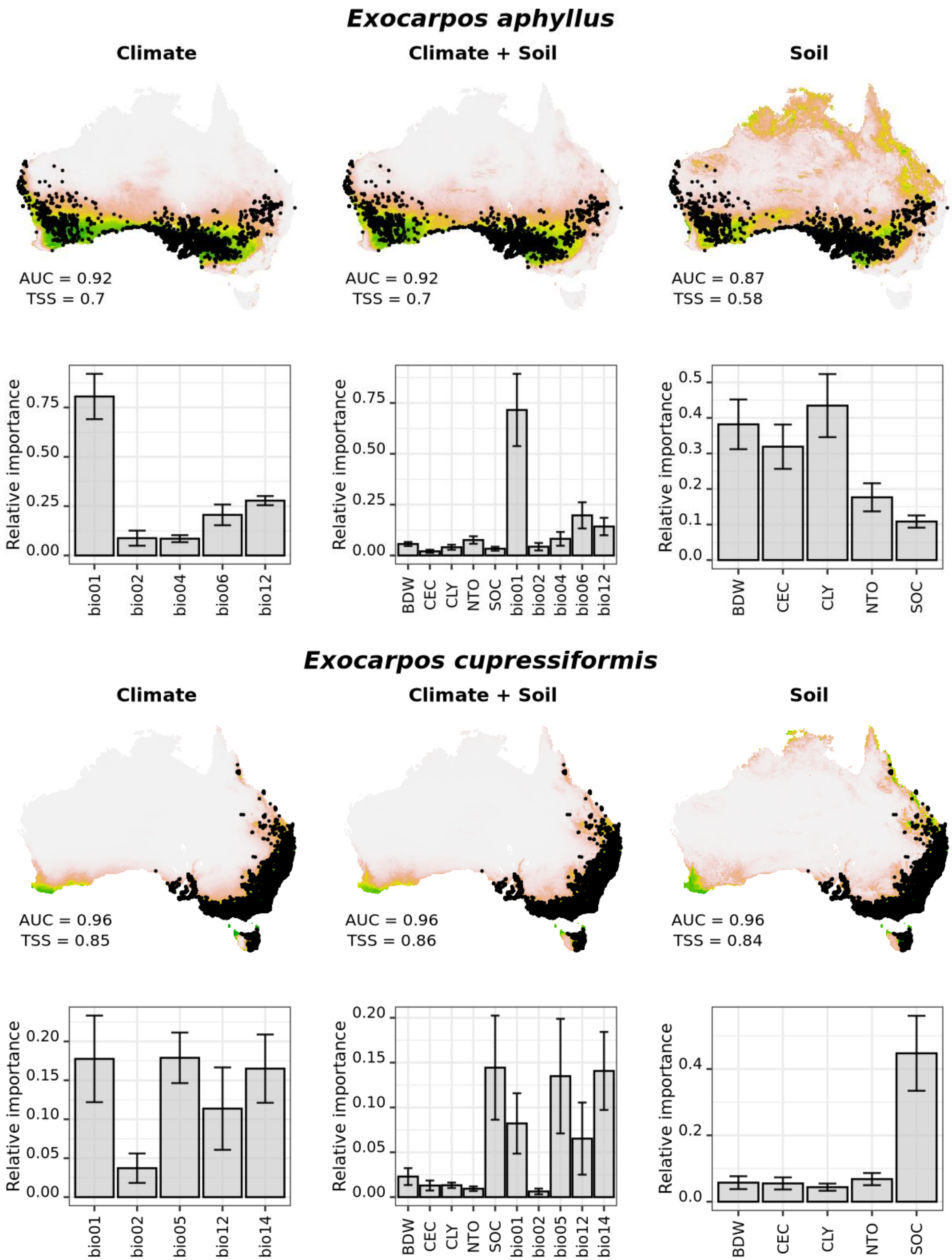

Exocarpos aphyllus
Climate
Climate + Soil
Soil
AUC = 0.92
TSS = 0.7
AUC = 0.92
TSS = 0.7
AUC = 0.87
TSS = 0.58
Relative importance
0.00
0.25
0.50
0.75
bio01
bio02
bio04
bio06
bio12
BDW
CEC
CLY
NTO
SOC
0.0
0.1
0.2
0.3
0.4
0.5
Exocarpos cupressiformis
Climate
Climate + Soil
Soil
AUC = 0.96
TSS = 0.85
AUC = 0.96
TSS = 0.86
AUC = 0.96
TSS = 0.84
Relative importance
0.00
0.05
0.10
0.15
0.20
bio01
bio02
bio05
bio12
bio14
BDW
CEC
CLY
NTO
SOC
0.0
0.2
0.4

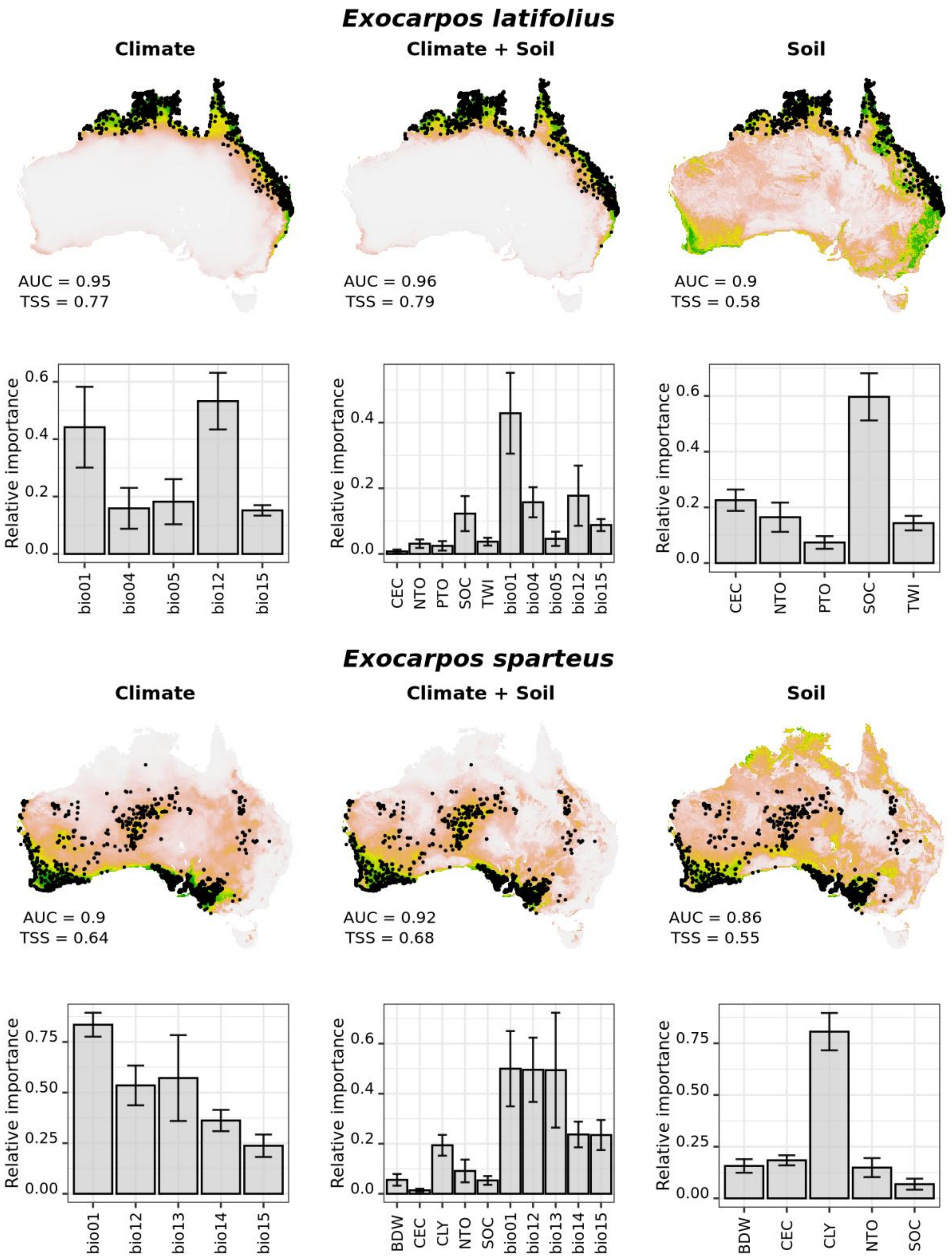

Exocarpos latifolius
Climate
Climate + Soil
Soil
AUC = 0.95
TSS = 0.77
AUC = 0.96
TSS = 0.79
AUC = 0.9
TSS = 0.58
Relative importance
bio01 bio04 bio05 bio12 bio15
CEC NTO PTO SOC TWI bio01 bio04 bio05 bio12 bio15
CEC NTO PTO SOC TWI
Exocarpos sparteus
Climate
Climate + Soil
Soil
AUC = 0.9
TSS = 0.64
AUC = 0.92
TSS = 0.68
AUC = 0.86
TSS = 0.55
Relative importance
bio01 bio12 bio13 bio14 bio15
BDW CEC CLY NTO SOC bio01 bio12 bio13 bio14 bio15
BDW CEC CLY NTO SOC

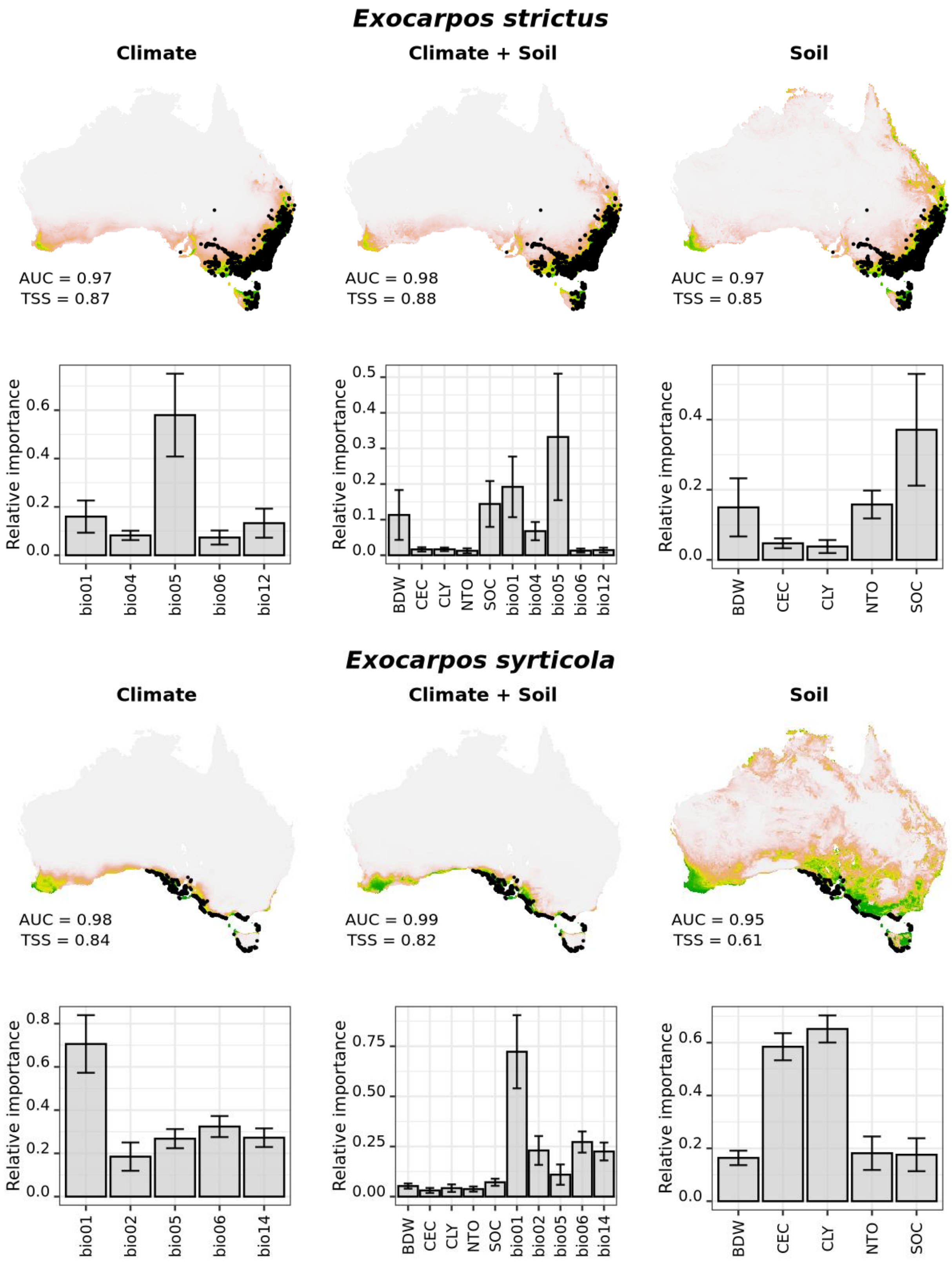

Exocarpos strictus
Climate
Climate + Soil
Soil
AUC = 0.97
TSS = 0.87
AUC = 0.98
TSS = 0.88
AUC = 0.97
TSS = 0.85
Relative importance
bio01
bio04
bio05
bio06
bio12
BDW
CEC
CLY
NTO
SOC
Exocarpos syrticola
Climate
Climate + Soil
Soil
AUC = 0.98
TSS = 0.84
AUC = 0.99
TSS = 0.82
AUC = 0.95
TSS = 0.61
Relative importance
bio01
bio02
bio05
bio06
bio14
BDW
CEC
CLY
NTO
SOC

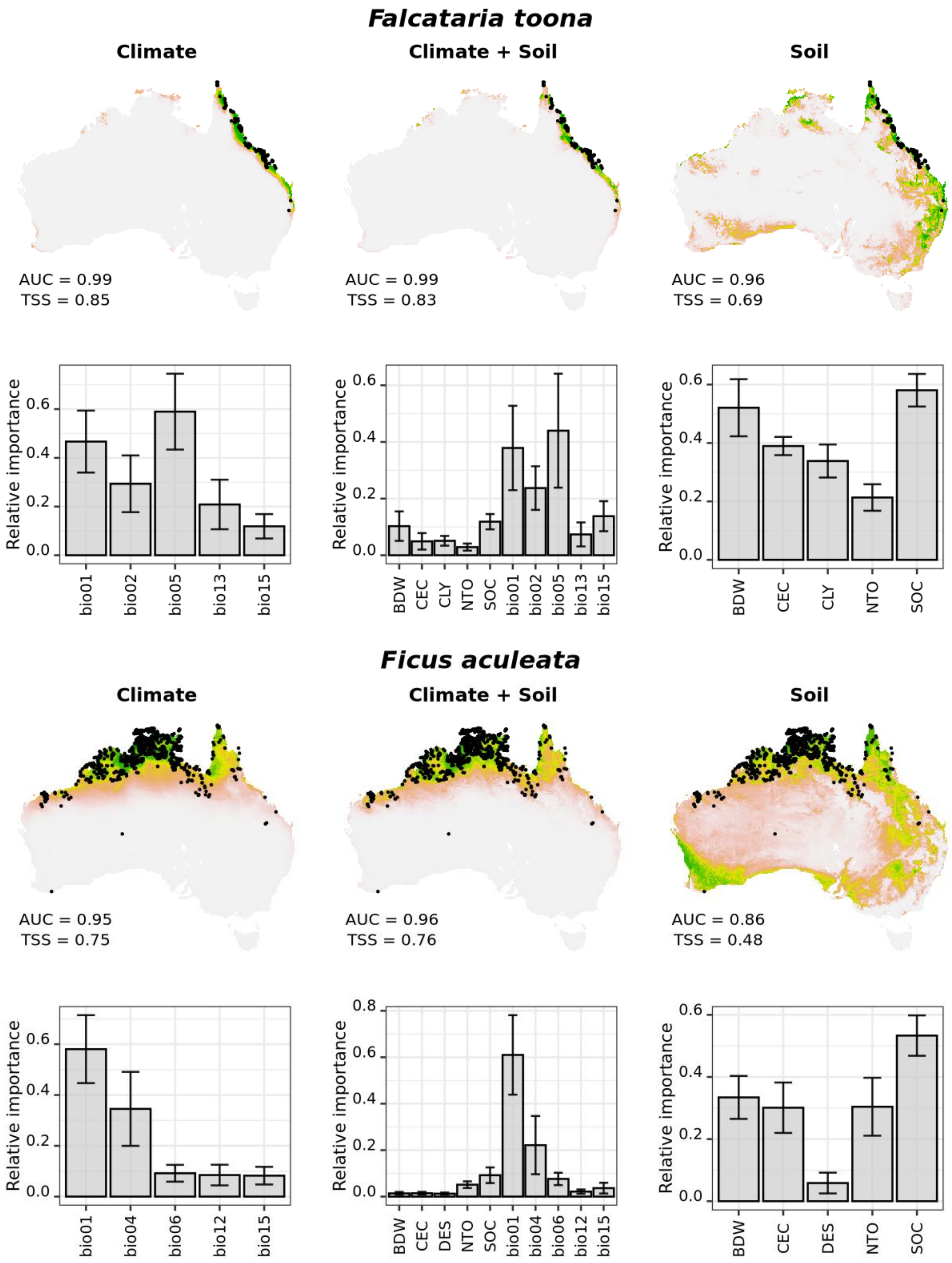

Falcataria toona
Climate
Climate + Soil
Soil
AUC = 0.99
TSS = 0.85
AUC = 0.99
TSS = 0.83
AUC = 0.96
TSS = 0.69
Relative importance
bio01
bio02
bio05
bio13
bio15
BDW
CEC
CLY
NTO
SOC
Ficus aculeata
Climate
Climate + Soil
Soil
AUC = 0.95
TSS = 0.75
AUC = 0.96
TSS = 0.76
AUC = 0.86
TSS = 0.48
Relative importance
bio01
bio04
bio06
bio12
bio15
BDW
CEC
DES
NTO
SOC

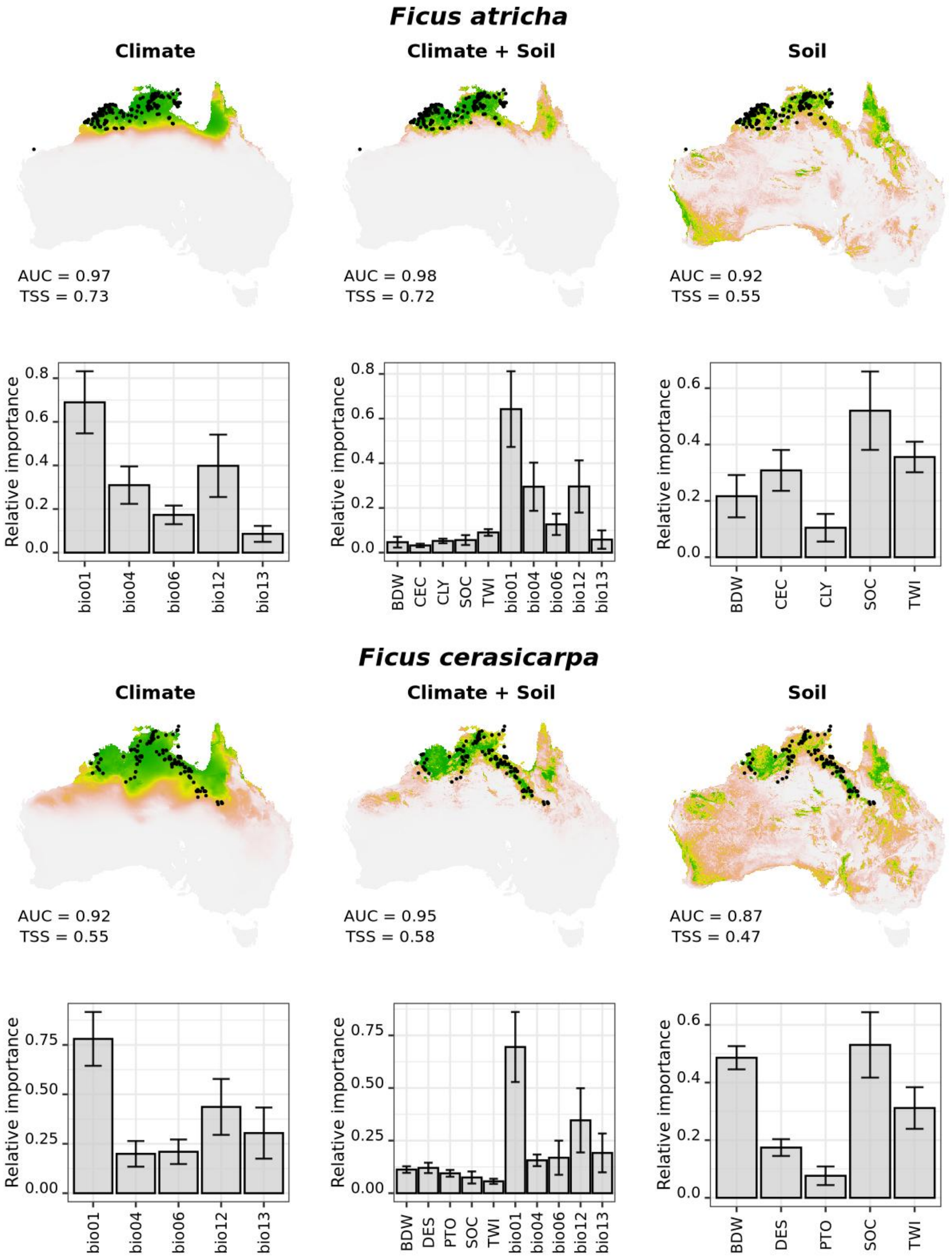

Ficus atricha
Climate
Climate + Soil
Soil
AUC = 0.97
TSS = 0.73
AUC = 0.98
TSS = 0.72
AUC = 0.92
TSS = 0.55
Relative importance
bio01 bio04 bio06 bio12 bio13
BDW CEC CLY SOC TWI bio01 bio04 bio06 bio12 bio13
BDW CEC CLY SOC TWI
Ficus cerasicarpa
Climate
Climate + Soil
Soil
AUC = 0.92
TSS = 0.55
AUC = 0.95
TSS = 0.58
AUC = 0.87
TSS = 0.47
Relative importance
bio01 bio04 bio06 bio12 bio13
BDW DES PTO SOC TWI bio01 bio04 bio06 bio12 bio13
BDW DES PTO SOC TWI

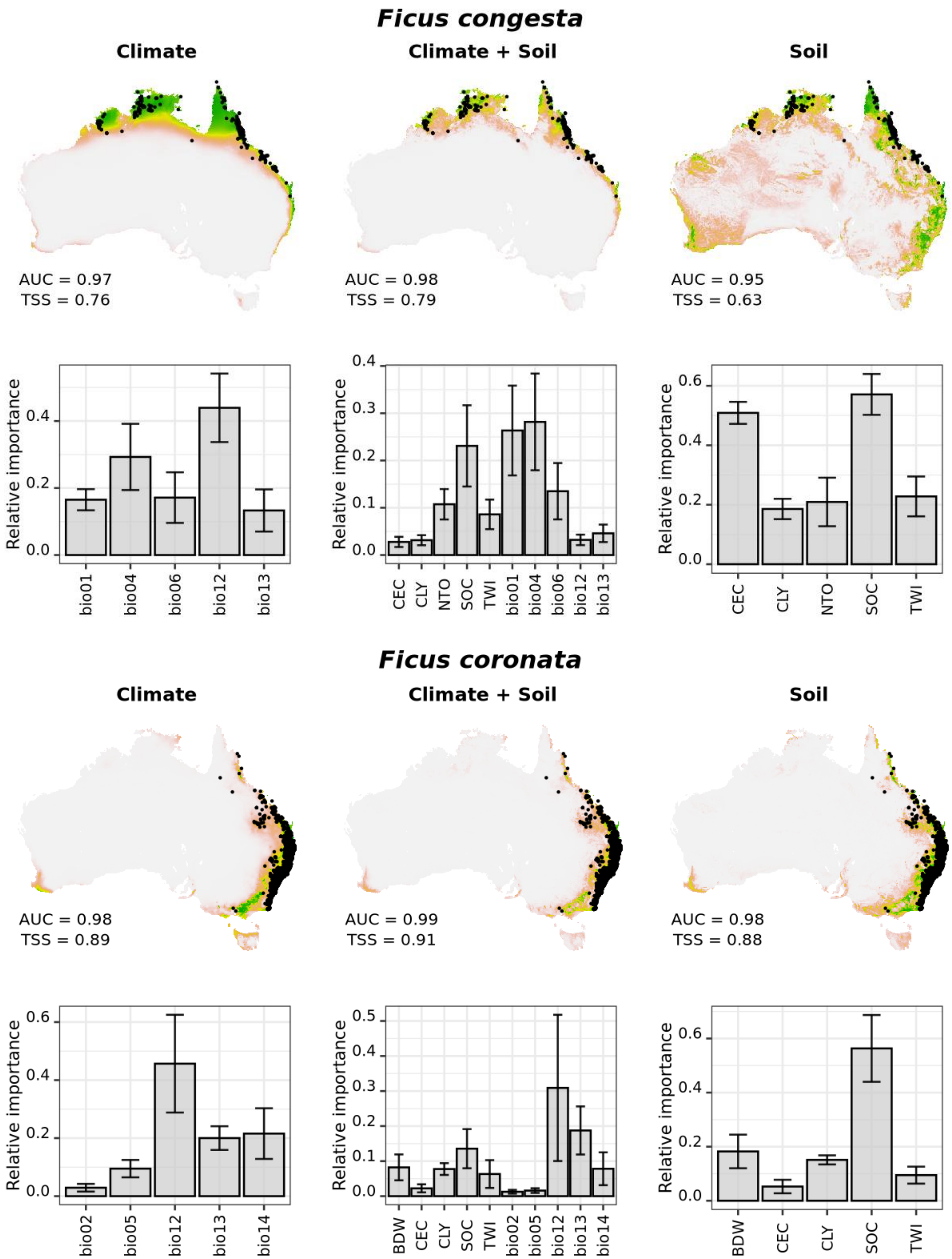

Ficus congesta
Climate
Climate + Soil
Soil
AUC = 0.97
TSS = 0.76
AUC = 0.98
TSS = 0.79
AUC = 0.95
TSS = 0.63
Relative importance
bio01 bio04 bio06 bio12 bio13
CEC CLY NTO SOC TWI bio01 bio04 bio06 bio12 bio13
CEC CLY NTO SOC TWI
Ficus coronata
Climate
Climate + Soil
Soil
AUC = 0.98
TSS = 0.89
AUC = 0.99
TSS = 0.91
AUC = 0.98
TSS = 0.88
Relative importance
bio02 bio05 bio12 bio13 bio14
BDW CEC CLY SOC TWI bio02 bio05 bio12 bio13 bio14
BDW CEC CLY SOC TWI

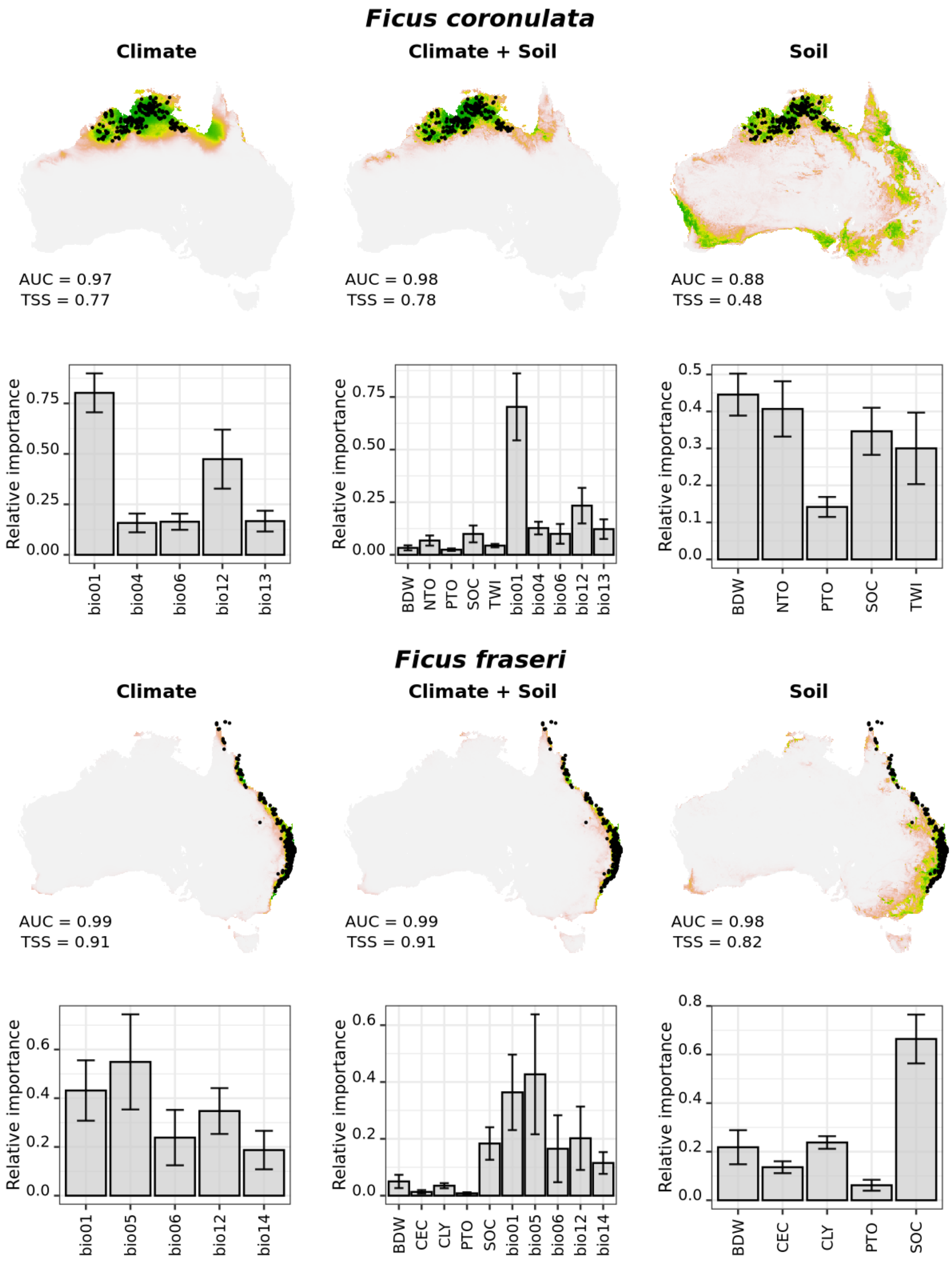
Ficus coronulata
Climate
Climate + Soil
Soil
AUC = 0.97
TSS = 0.77
AUC = 0.98
TSS = 0.78
AUC = 0.88
TSS = 0.48
Relative importance
0.00 0.25 0.50 0.75
bio01 bio04 bio06 bio12 bio13
BDW NTO PTO SOC TWI bio01 bio04 bio06 bio12 bio13
0.0 0.1 0.2 0.3 0.4 0.5
BDW NTO PTO SOC TWI
Ficus fraseri
Climate
Climate + Soil
Soil
AUC = 0.99
TSS = 0.91
AUC = 0.99
TSS = 0.91
AUC = 0.98
TSS = 0.82
Relative importance
0.0 0.2 0.4 0.6
bio01 bio05 bio06 bio12 bio14
BDW CEC CLY PTO SOC bio01 bio05 bio06 bio12 bio14
0.0 0.2 0.4 0.6 0.8
BDW CEC CLY PTO SOC

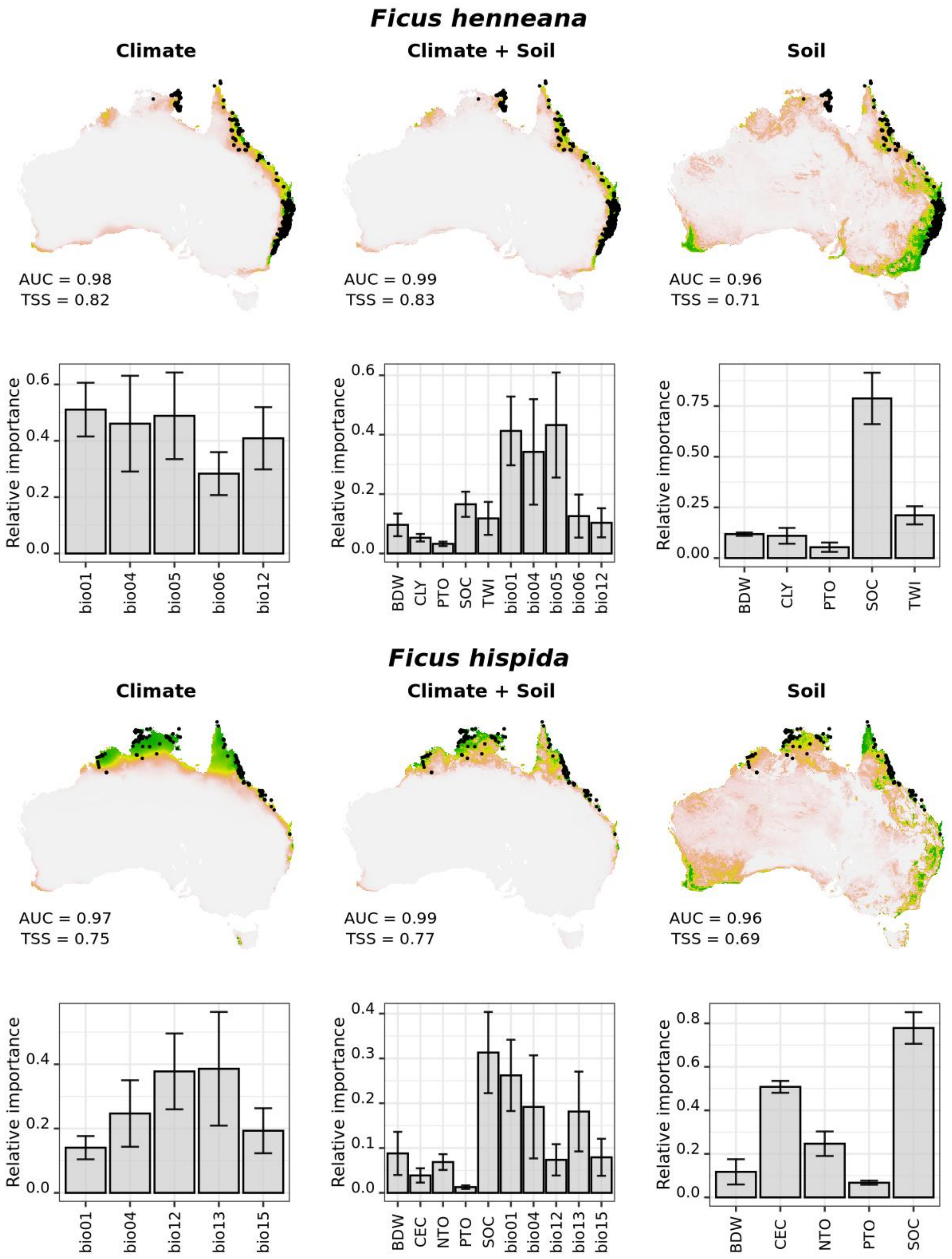
Ficus henneana
Climate
Climate + Soil
Soil
AUC = 0.98
TSS = 0.82
AUC = 0.99
TSS = 0.83
AUC = 0.96
TSS = 0.71
Relative importance
bio01
bio04
bio05
bio06
bio12
BDW
CLY
PTO
SOC
TWI
Ficus hispida
Climate
Climate + Soil
Soil
AUC = 0.97
TSS = 0.75
AUC = 0.99
TSS = 0.77
AUC = 0.96
TSS = 0.69
Relative importance
bio01
bio04
bio12
bio13
bio15
BDW
CEC
NTO
PTO
SOC

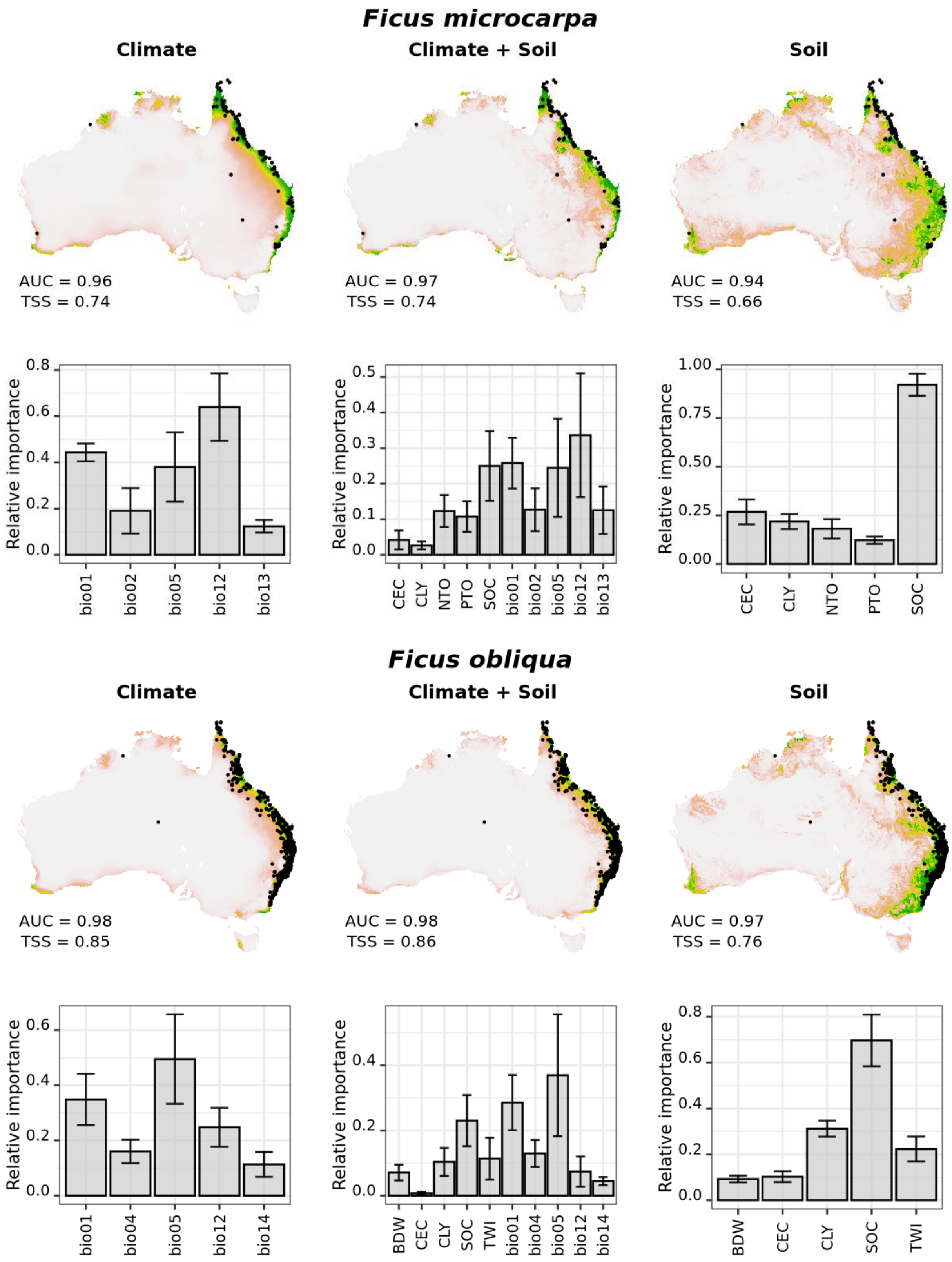

Ficus microcarpa
Climate
Climate + Soil
Soil
AUC = 0.96
TSS = 0.74
AUC = 0.97
TSS = 0.74
AUC = 0.94
TSS = 0.66
Relative importance
bio01
bio02
bio05
bio12
bio13
CEC
CLY
NTO
PTO
SOC
Ficus obliqua
AUC = 0.98
TSS = 0.85
AUC = 0.98
TSS = 0.86
AUC = 0.97
TSS = 0.76
bio04
bio14
BDW
TWI

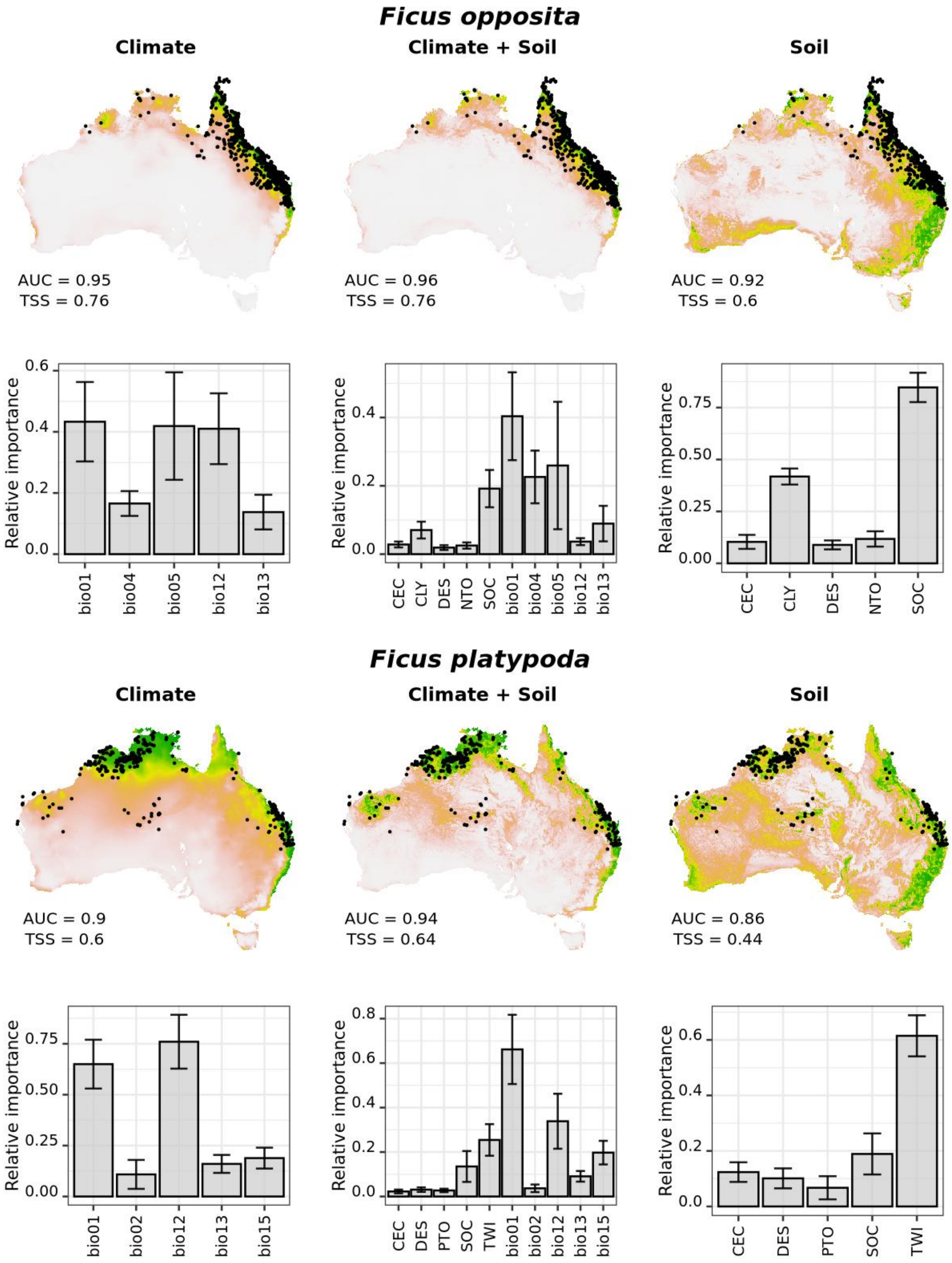
Ficus opposita
Climate
Climate + Soil
Soil
AUC = 0.95
TSS = 0.76
AUC = 0.96
TSS = 0.76
AUC = 0.92
TSS = 0.6
Relative importance
bio01
bio04
bio05
bio12
bio13
CEC
CLY
DES
NTO
SOC
Ficus platypoda
AUC = 0.9
TSS = 0.6
AUC = 0.94
TSS = 0.64
AUC = 0.86
TSS = 0.44
bio02
bio15
PTO
TWI

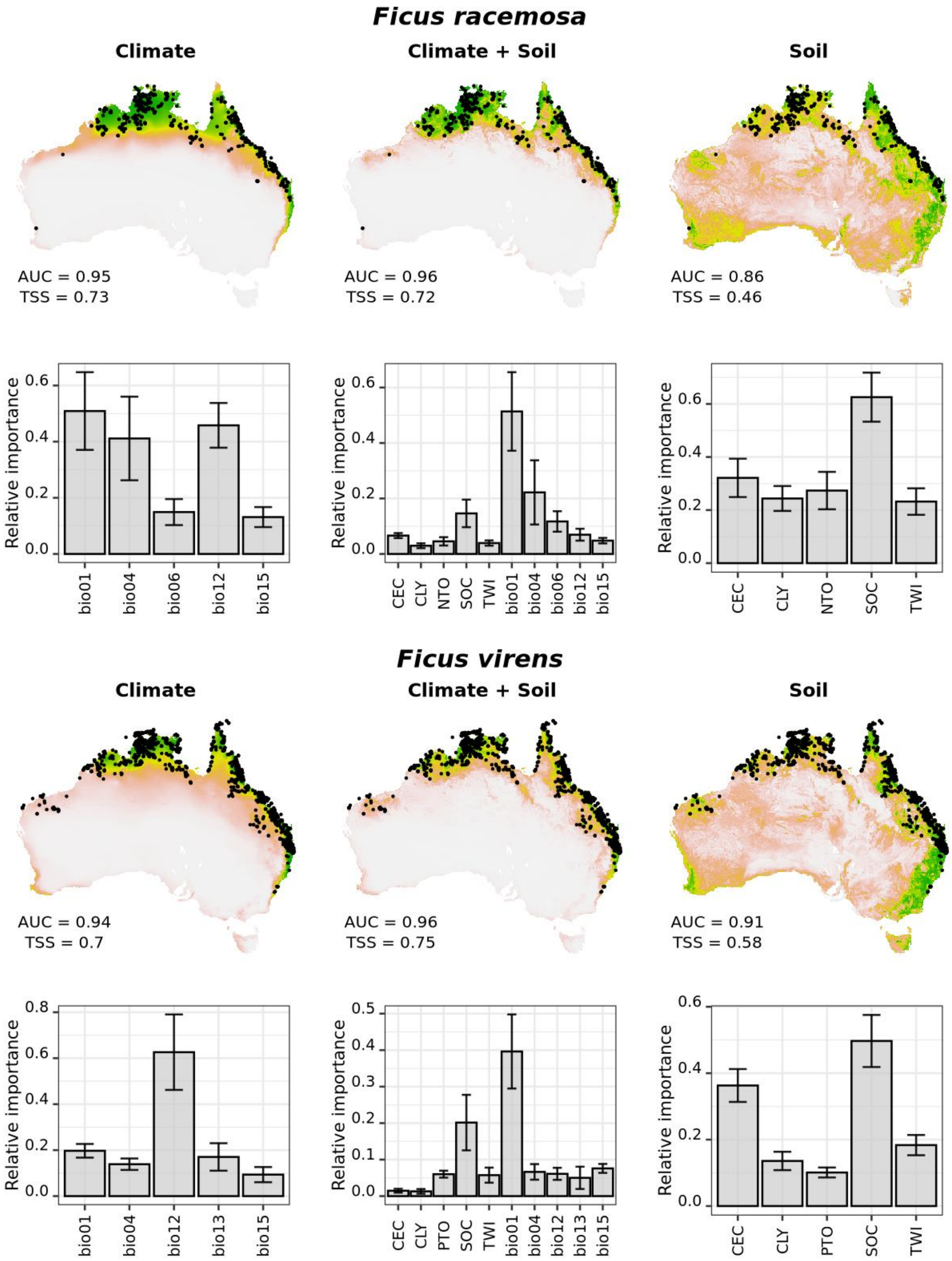

Ficus racemosa
Climate
Climate + Soil
Soil
AUC = 0.95
TSS = 0.73
AUC = 0.96
TSS = 0.72
AUC = 0.86
TSS = 0.46
Relative importance
0.0
0.2
0.4
0.6
bio01
bio04
bio06
bio12
bio15
CEC
CLY
NTO
SOC
TWI
Ficus virens
Climate
Climate + Soil
Soil
AUC = 0.94
TSS = 0.7
AUC = 0.96
TSS = 0.75
AUC = 0.91
TSS = 0.58
0.8
0.1
0.3
0.5
bio13
PTO

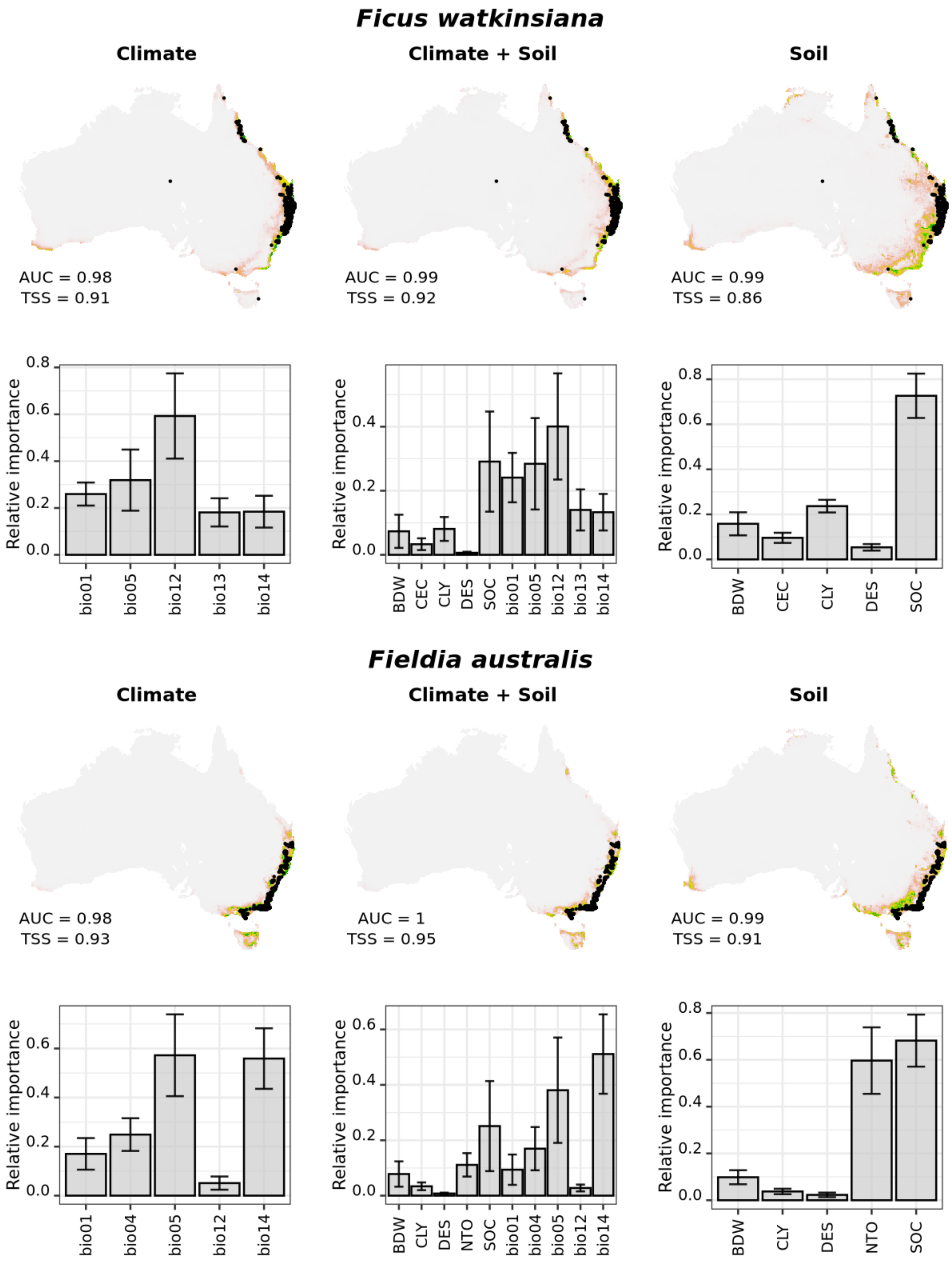

Ficus watkinsiana
Climate
Climate + Soil
Soil
AUC = 0.98
TSS = 0.91
AUC = 0.99
TSS = 0.92
AUC = 0.99
TSS = 0.86
Relative importance
bio01
bio05
bio12
bio13
bio14
BDW
CEC
CLY
DES
SOC
Fieldia australis
Climate
Climate + Soil
Soil
AUC = 0.98
TSS = 0.93
AUC = 1
TSS = 0.95
AUC = 0.99
TSS = 0.91
Relative importance
bio01
bio04
bio05
bio12
bio14
BDW
CLY
DES
NTO
SOC

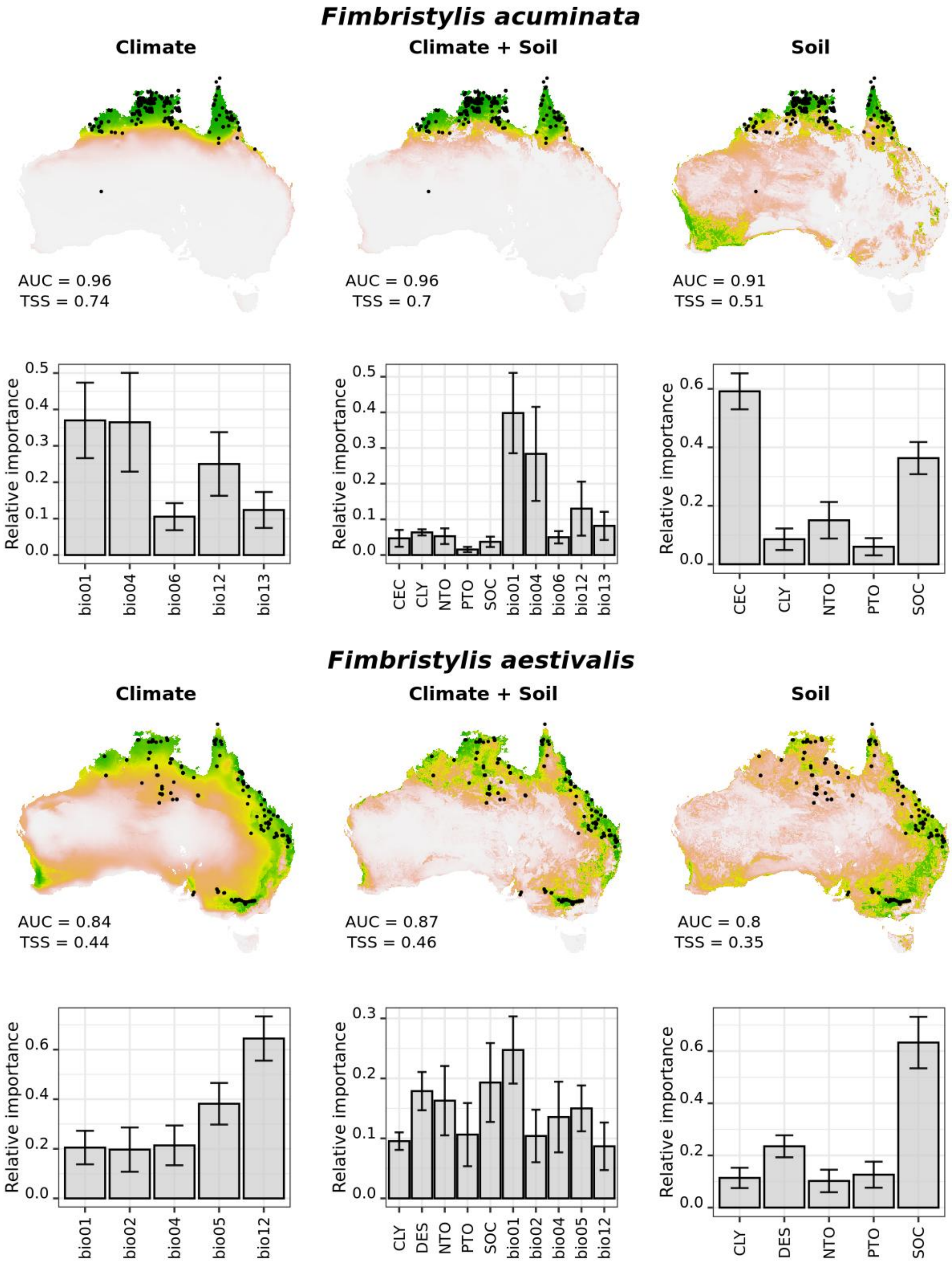

Fimbristylis acuminata
Climate
Climate + Soil
Soil
AUC = 0.96
TSS = 0.74
AUC = 0.96
TSS = 0.7
AUC = 0.91
TSS = 0.51
Relative importance
bio01 bio04 bio06 bio12 bio13
CEC CLY NTO PTO SOC bio01 bio04 bio06 bio12 bio13
CEC CLY NTO PTO SOC
Fimbristylis aestivalis
Climate
Climate + Soil
Soil
AUC = 0.84
TSS = 0.44
AUC = 0.87
TSS = 0.46
AUC = 0.8
TSS = 0.35
Relative importance
bio01 bio02 bio04 bio05 bio12
CLY DES NTO PTO SOC bio01 bio02 bio04 bio05 bio12
CLY DES NTO PTO SOC

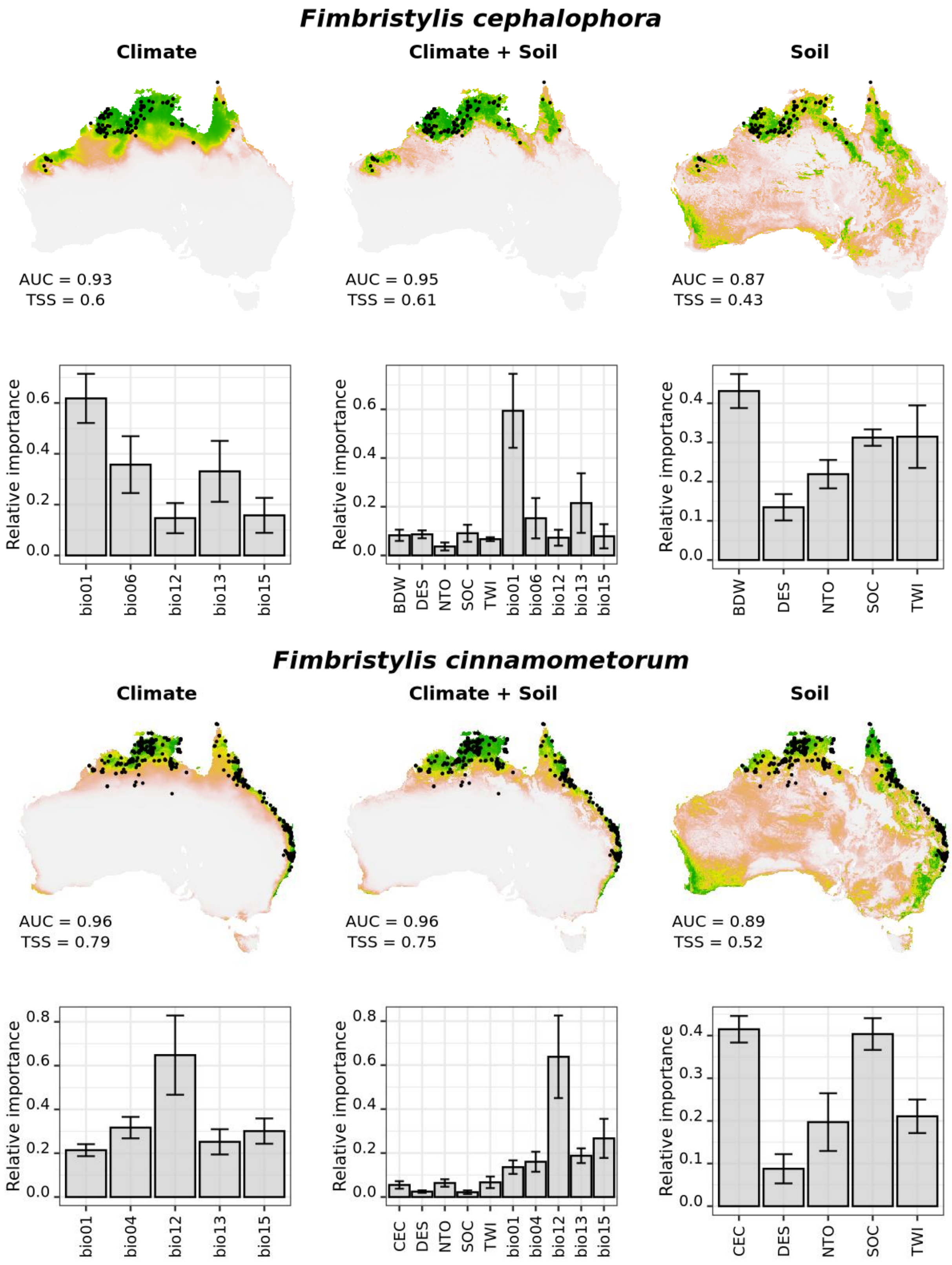

Fimbristylis cephalophora
Climate
Climate + Soil
Soil
AUC = 0.93
TSS = 0.6
AUC = 0.95
TSS = 0.61
AUC = 0.87
TSS = 0.43
Relative importance
bio01
bio06
bio12
bio13
bio15
BDW
DES
NTO
SOC
TWI
Fimbristylis cinnamometorum
Climate
Climate + Soil
Soil
AUC = 0.96
TSS = 0.79
AUC = 0.96
TSS = 0.75
AUC = 0.89
TSS = 0.52
Relative importance
bio01
bio04
bio12
bio13
bio15
CEC
DES
NTO
SOC
TWI

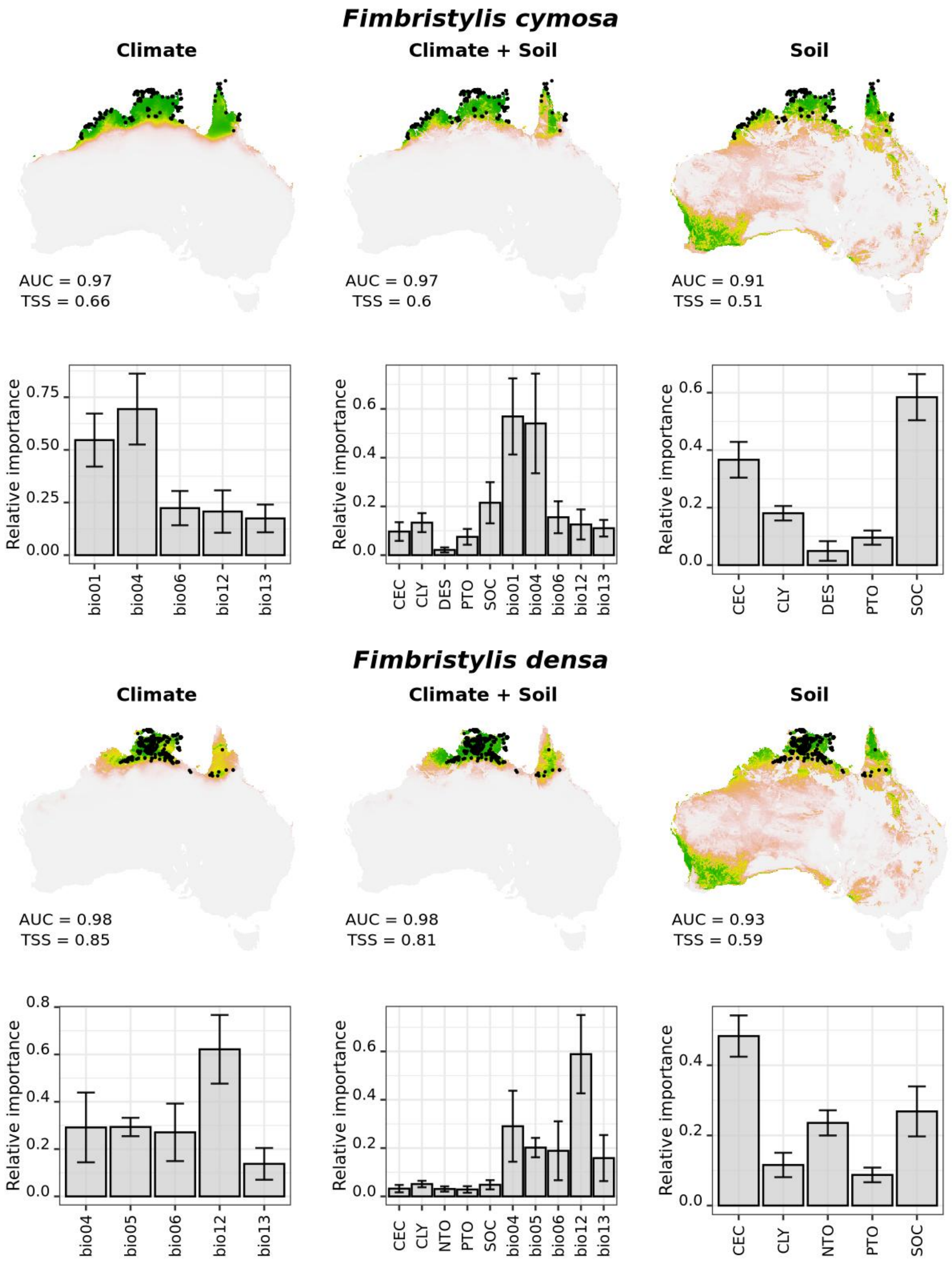
Fimbristylis cymosa
Climate
Climate + Soil
Soil
AUC = 0.97
TSS = 0.66
AUC = 0.97
TSS = 0.6
AUC = 0.91
TSS = 0.51
Relative importance
0.00
0.25
0.50
0.75
bio01
bio04
bio06
bio12
bio13
0.0
0.2
0.4
0.6
CEC
CLY
DES
PTO
SOC
bio01
bio04
bio06
bio12
bio13
CEC
CLY
DES
PTO
SOC
Fimbristylis densa
Climate
Climate + Soil
Soil
AUC = 0.98
TSS = 0.85
AUC = 0.98
TSS = 0.81
AUC = 0.93
TSS = 0.59
Relative importance
0.0
0.2
0.4
0.6
0.8
bio04
bio05
bio06
bio12
bio13
CEC
CLY
NTO
PTO
SOC
bio04
bio05
bio06
bio12
bio13
CEC
CLY
NTO
PTO
SOC

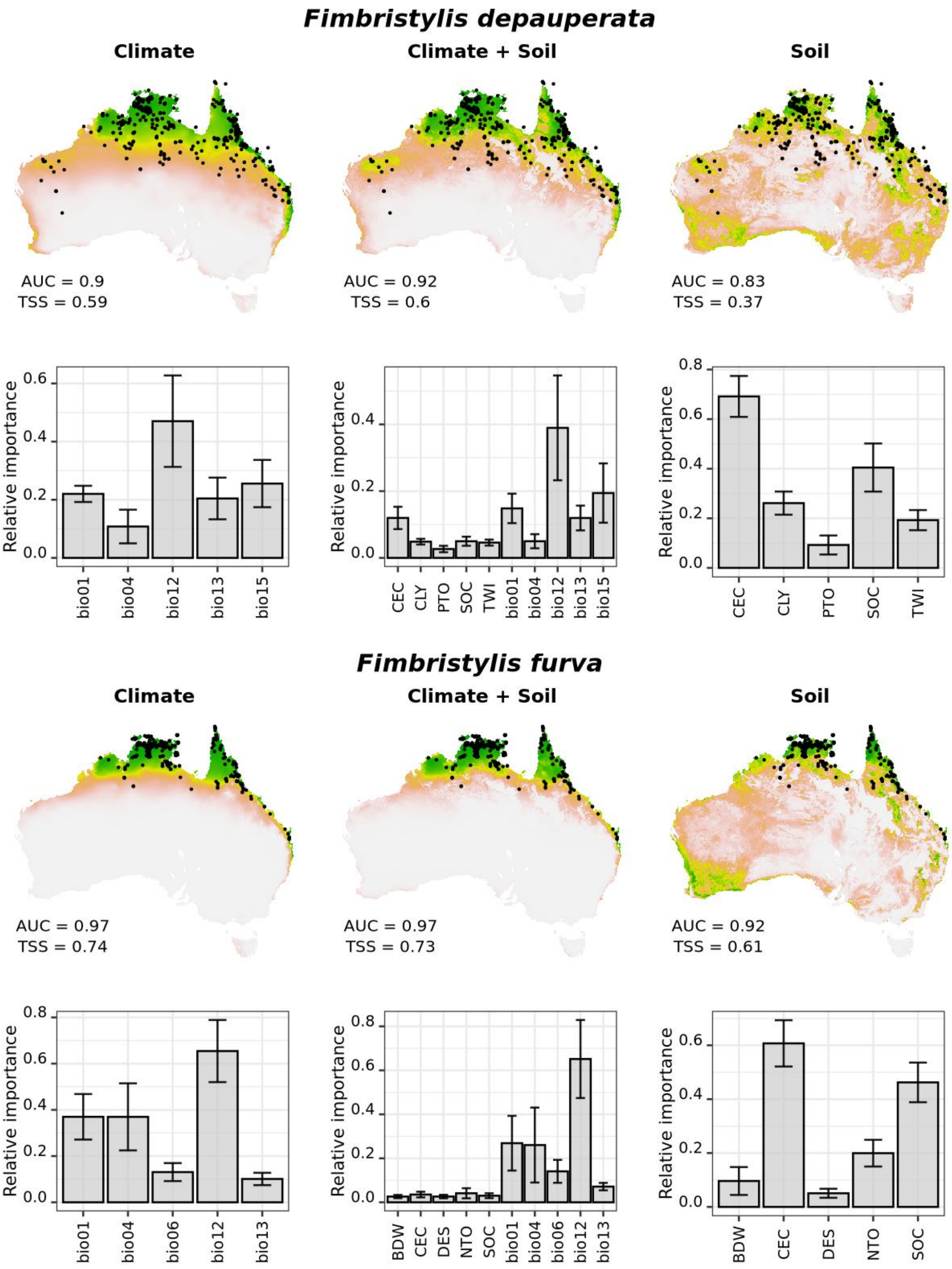

Fimbristylis depauperata
Climate
Climate + Soil
Soil
AUC = 0.9
TSS = 0.59
AUC = 0.92
TSS = 0.6
AUC = 0.83
TSS = 0.37
Relative importance
bio01
bio04
bio12
bio13
bio15
CEC
CLY
PTO
SOC
TWI
Fimbristylis furva
Climate
Climate + Soil
Soil
AUC = 0.97
TSS = 0.74
AUC = 0.97
TSS = 0.73
AUC = 0.92
TSS = 0.61
Relative importance
bio01
bio04
bio06
bio12
bio13
BDW
CEC
DES
NTO
SOC

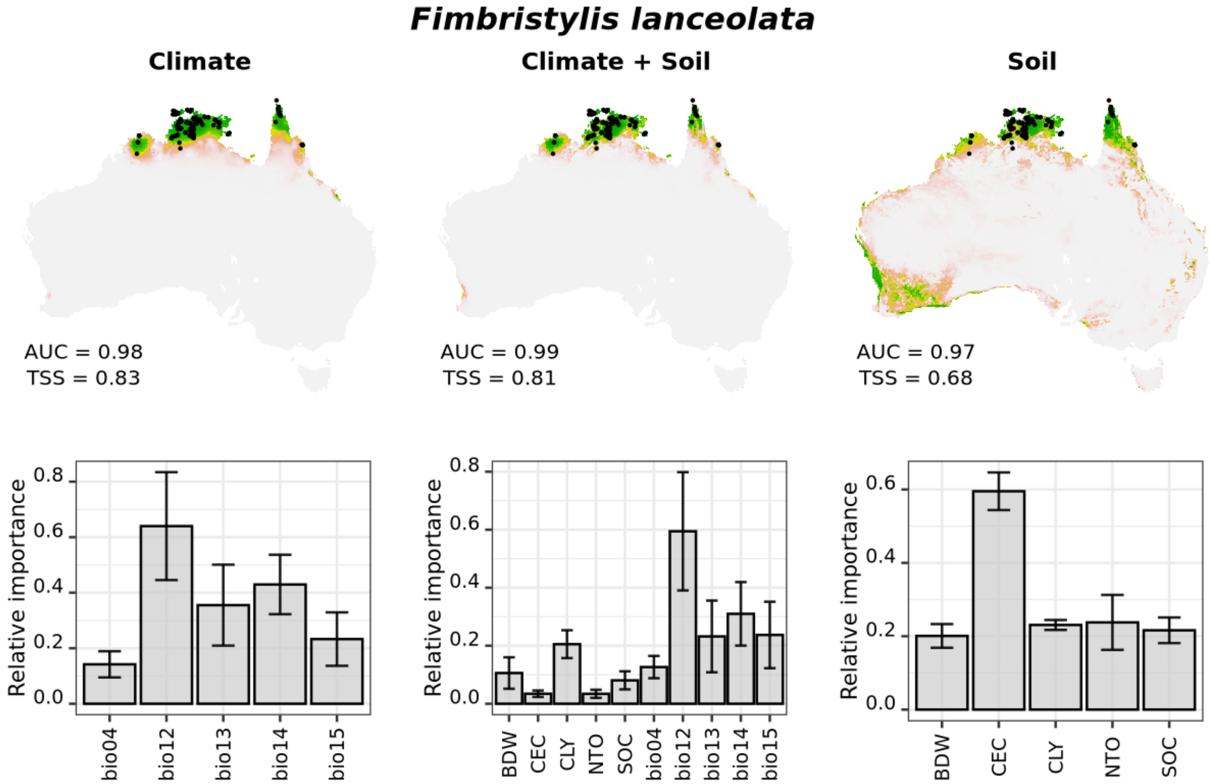
Fimbristylis lanceolata
Climate
Climate + Soil
Soil
AUC = 0.98
TSS = 0.83
AUC = 0.99
TSS = 0.81
AUC = 0.97
TSS = 0.68
Relative importance
0.0
0.2
0.4
0.6
0.8
bio04
bio12
bio13
bio14
bio15
BDW
CEC
CLY
NTO
SOC

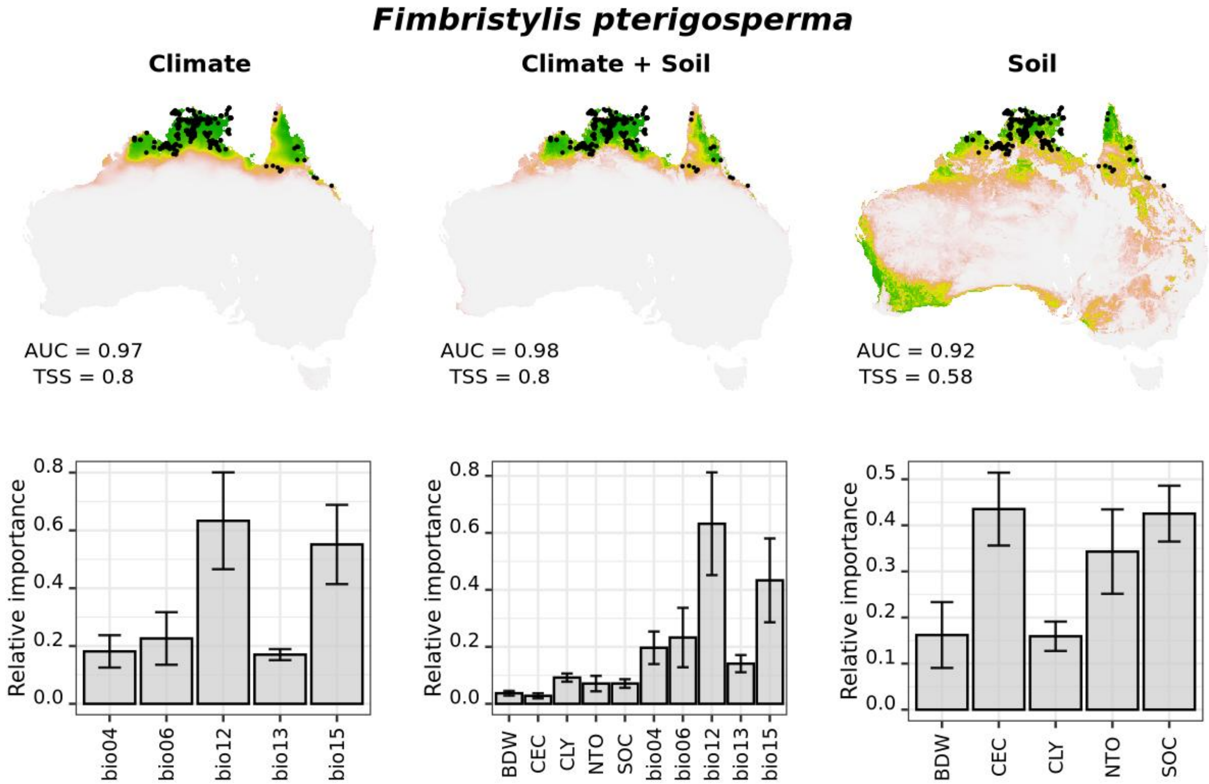
Fimbristylis pterigosperma
Climate
Climate + Soil
Soil
AUC = 0.97
TSS = 0.8
AUC = 0.98
TSS = 0.8
AUC = 0.92
TSS = 0.58
Relative importance
0.0
0.1
0.2
0.3
0.4
0.5
0.6
0.8
bio04
bio06
bio12
bio13
bio15
BDW
CEC
CLY
NTO
SOC

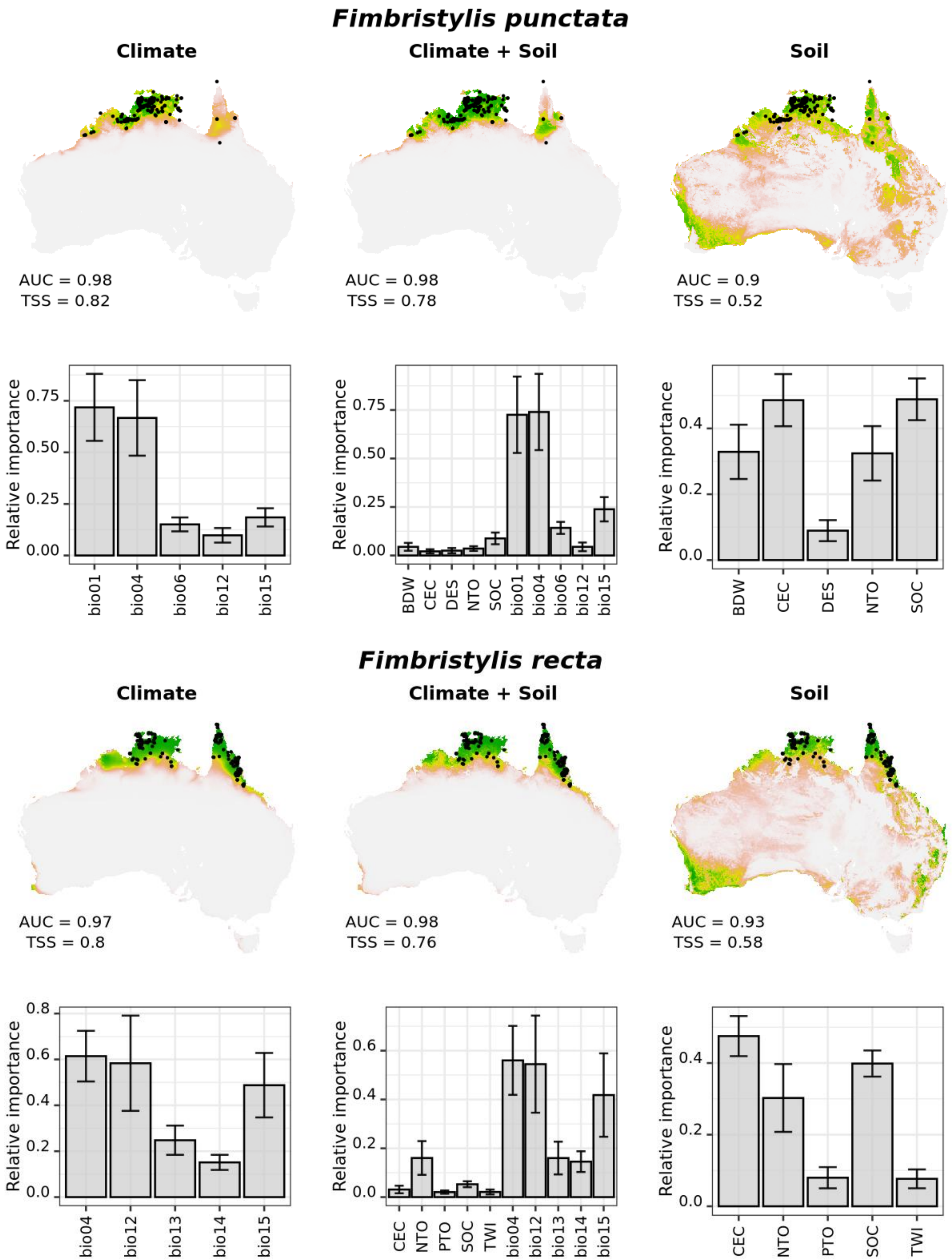

Fimbristylis punctata
Climate
Climate + Soil
Soil
AUC = 0.98
TSS = 0.82
AUC = 0.98
TSS = 0.78
AUC = 0.9
TSS = 0.52
Relative importance
0.00
0.25
0.50
0.75
bio01
bio04
bio06
bio12
bio15
BDW
CEC
DES
NTO
SOC
0.0
0.2
0.4
Fimbristylis recta
Climate
Climate + Soil
Soil
AUC = 0.97
TSS = 0.8
AUC = 0.98
TSS = 0.76
AUC = 0.93
TSS = 0.58
0.0
0.2
0.4
0.6
0.8
bio04
bio12
bio13
bio14
bio15
CEC
NTO
PTO
SOC
TWI

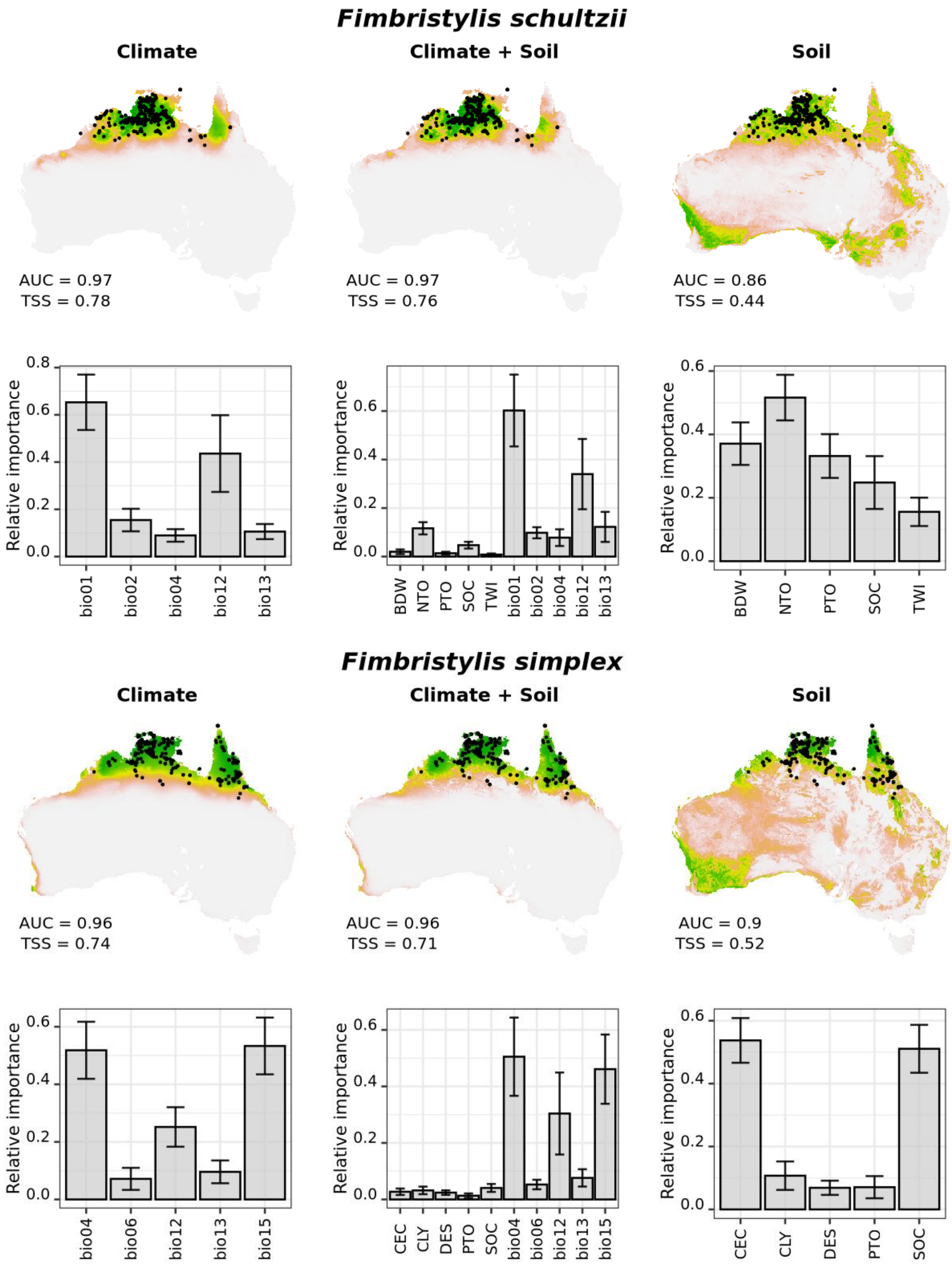
Fimbristylis schultzii
Climate
Climate + Soil
Soil
AUC = 0.97
TSS = 0.78
AUC = 0.97
TSS = 0.76
AUC = 0.86
TSS = 0.44
Relative importance
bio01
bio02
bio04
bio12
bio13
BDW
NTO
PTO
SOC
TWI
Fimbristylis simplex
AUC = 0.96
TSS = 0.74
AUC = 0.96
TSS = 0.71
AUC = 0.9
TSS = 0.52
bio04
bio06
bio12
bio13
bio15
CEC
CLY
DES
PTO
SOC

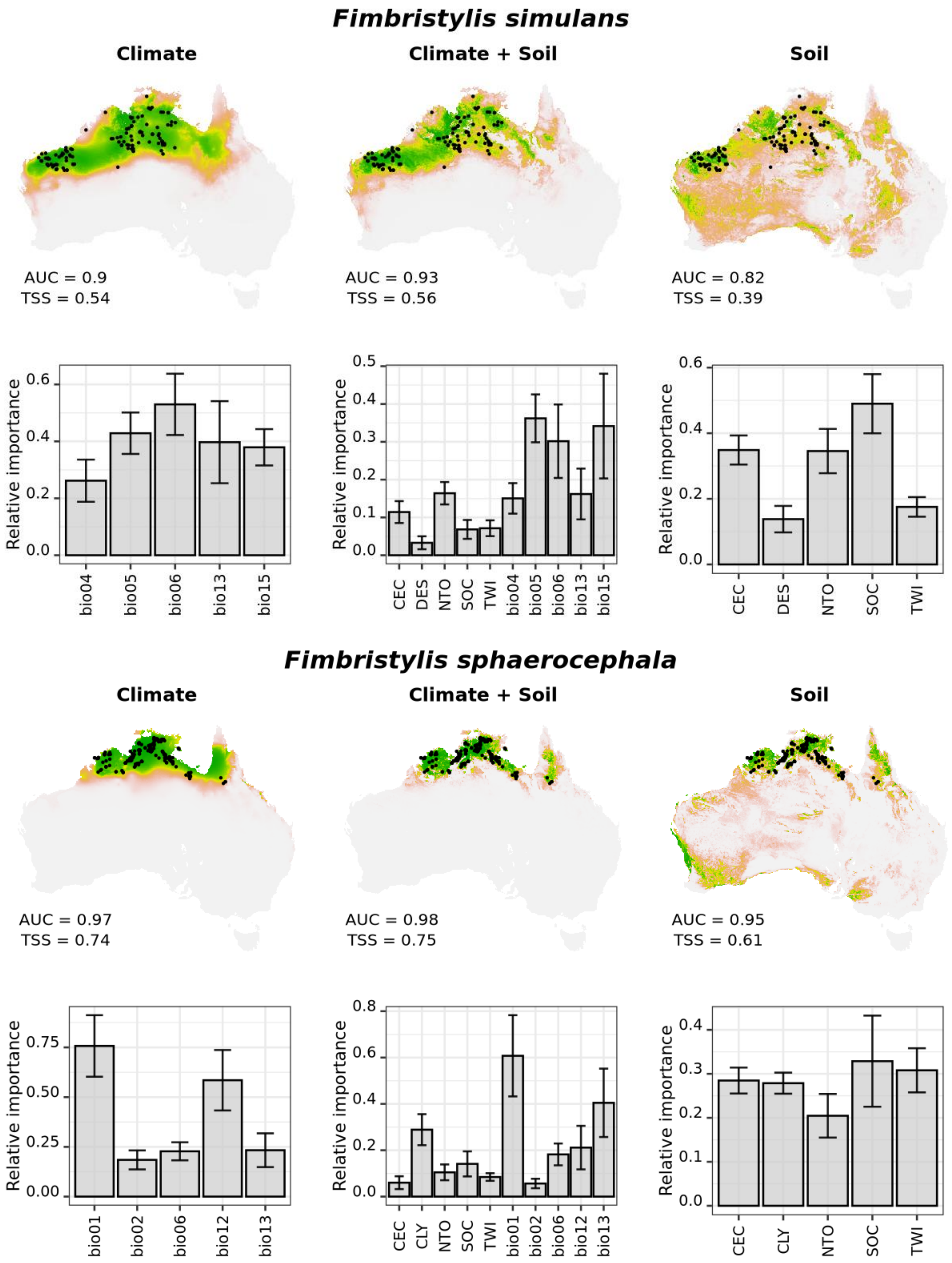

Fimbristylis simulans
Climate
Climate + Soil
Soil
AUC = 0.9
TSS = 0.54
AUC = 0.93
TSS = 0.56
AUC = 0.82
TSS = 0.39
Relative importance
bio04
bio05
bio06
bio13
bio15
CEC
DES
NTO
SOC
TWI
Fimbristylis sphaerocephala
Climate
Climate + Soil
Soil
AUC = 0.97
TSS = 0.74
AUC = 0.98
TSS = 0.75
AUC = 0.95
TSS = 0.61
Relative importance
bio01
bio02
bio06
bio12
bio13
CEC
CLY
NTO
SOC
TWI

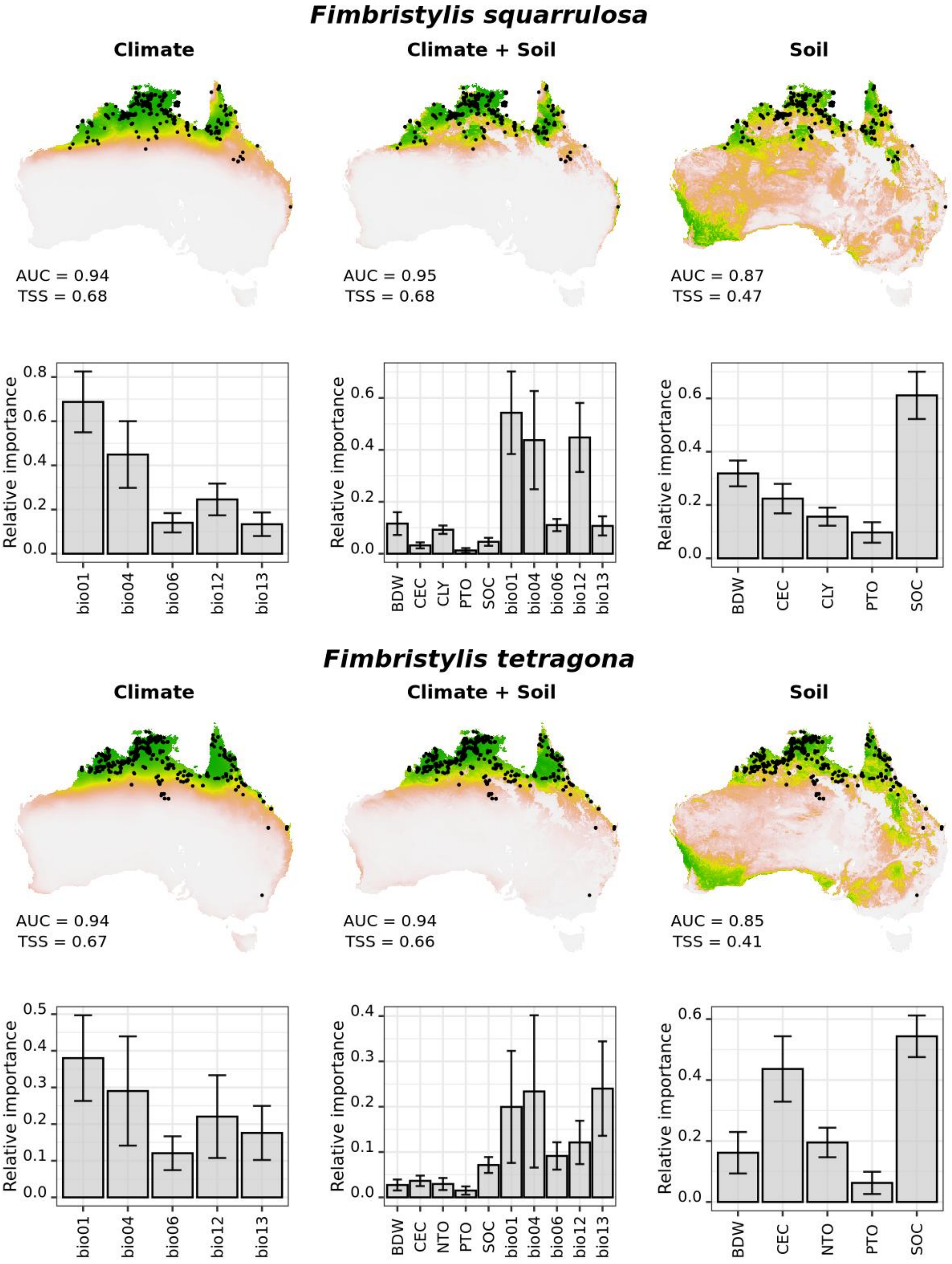

Fimbristylis squarrulosa
Climate
Climate + Soil
Soil
AUC = 0.94
TSS = 0.68
AUC = 0.95
TSS = 0.68
AUC = 0.87
TSS = 0.47
Relative importance
bio01
bio04
bio06
bio12
bio13
BDW
CEC
CLY
PTO
SOC
Fimbristylis tetragona
Climate
Climate + Soil
Soil
AUC = 0.94
TSS = 0.67
AUC = 0.94
TSS = 0.66
AUC = 0.85
TSS = 0.41
Relative importance
NTO

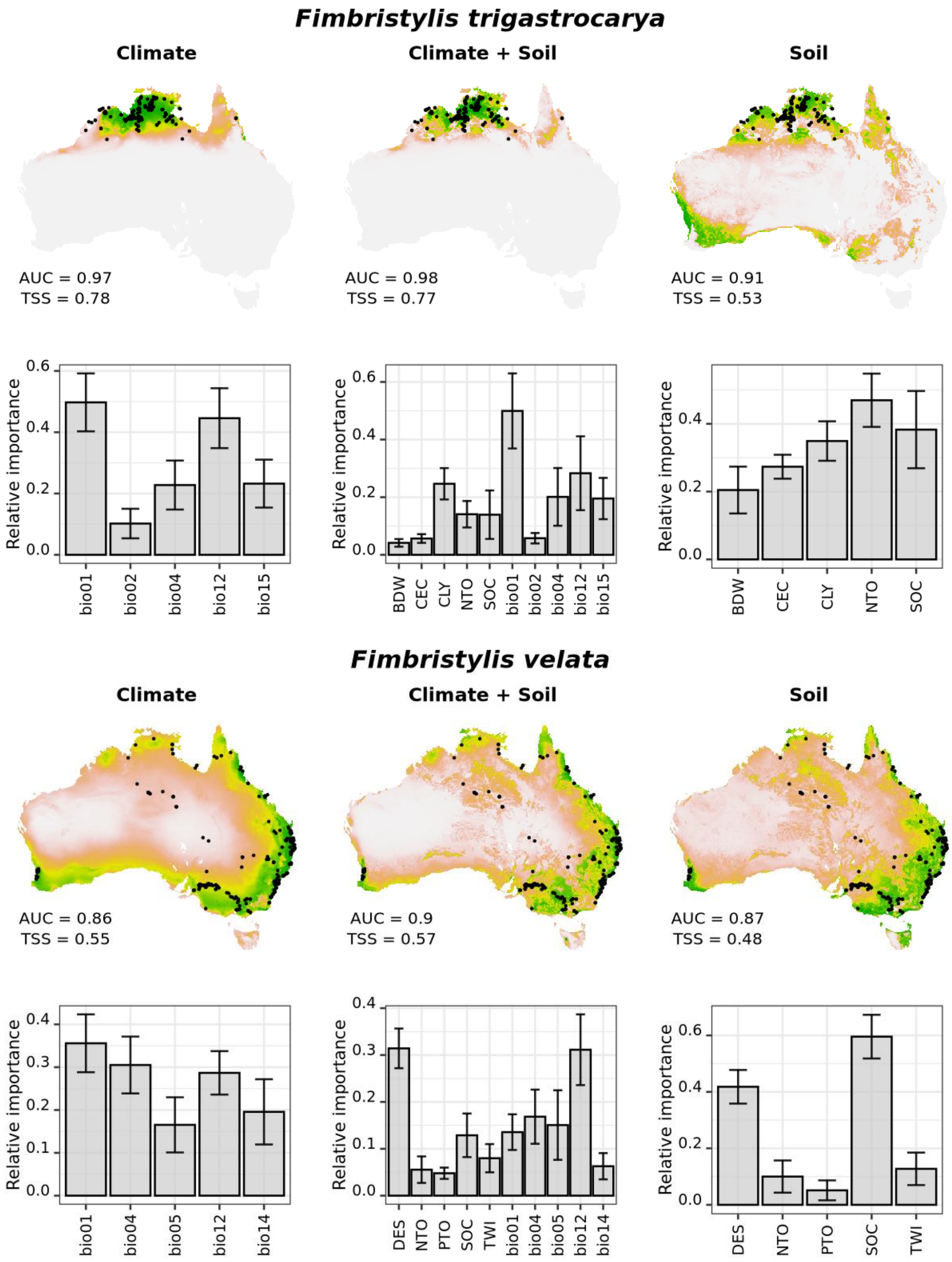

Fimbristylis trigastrocarya
Climate
Climate + Soil
Soil
AUC = 0.97
TSS = 0.78
AUC = 0.98
TSS = 0.77
AUC = 0.91
TSS = 0.53
Relative importance
bio01
bio02
bio04
bio12
bio15
BDW
CEC
CLY
NTO
SOC
Fimbristylis velata
AUC = 0.86
TSS = 0.55
AUC = 0.9
TSS = 0.57
AUC = 0.87
TSS = 0.48
bio05
bio14
DES
PTO
TWI

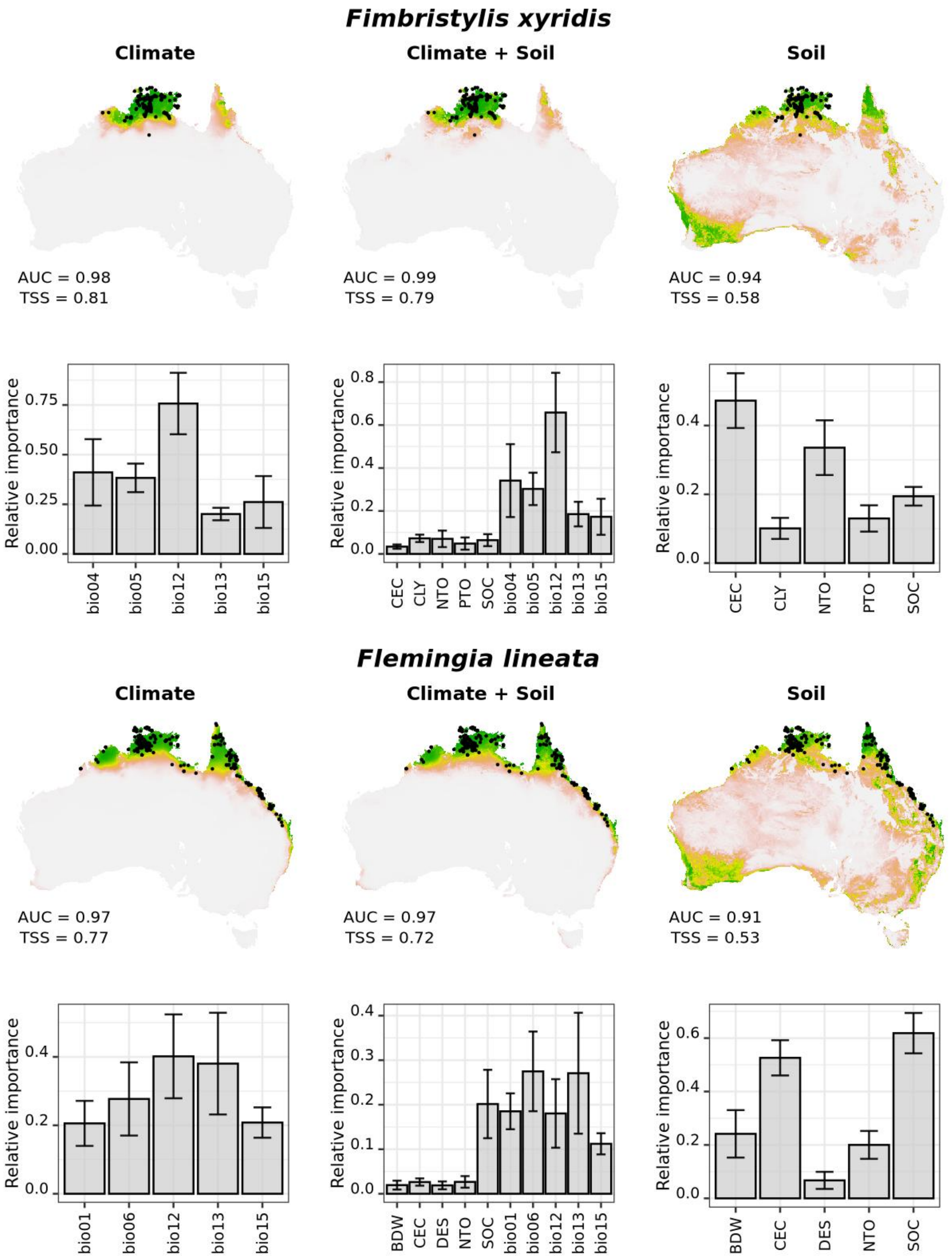
Fimbristylis xyridis
Climate
Climate + Soil
Soil
AUC = 0.98
TSS = 0.81
AUC = 0.99
TSS = 0.79
AUC = 0.94
TSS = 0.58
Relative importance
0.00
0.25
0.50
0.75
bio04
bio05
bio12
bio13
bio15
0.0
0.2
0.4
0.6
0.8
CEC
CLY
NTO
PTO
SOC
Flemingia lineata
AUC = 0.97
TSS = 0.77
AUC = 0.97
TSS = 0.72
AUC = 0.91
TSS = 0.53
0.1
0.3
bio01
bio06
BDW
DES

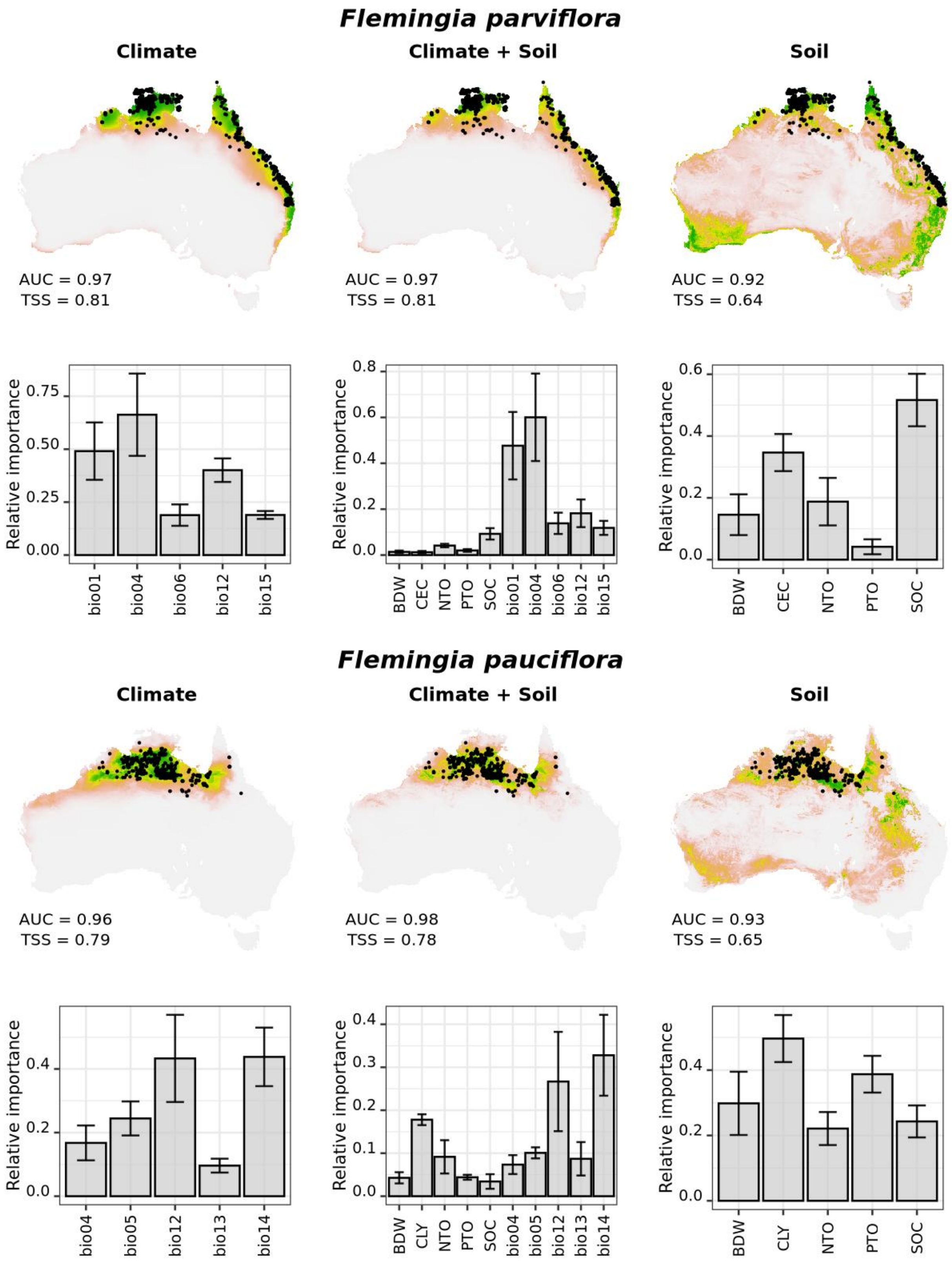
Flemingia parviflora
Climate
Climate + Soil
Soil
AUC = 0.97
TSS = 0.81
AUC = 0.97
TSS = 0.81
AUC = 0.92
TSS = 0.64
Relative importance
bio01 bio04 bio06 bio12 bio15
BDW CEC NTO PTO SOC bio01 bio04 bio06 bio12 bio15
BDW CEC NTO PTO SOC
Flemingia pauciflora
Climate
Climate + Soil
Soil
AUC = 0.96
TSS = 0.79
AUC = 0.98
TSS = 0.78
AUC = 0.93
TSS = 0.65
Relative importance
bio04 bio05 bio12 bio13 bio14
BDW CLY NTO PTO SOC bio04 bio05 bio12 bio13 bio14
BDW CLY NTO PTO SOC

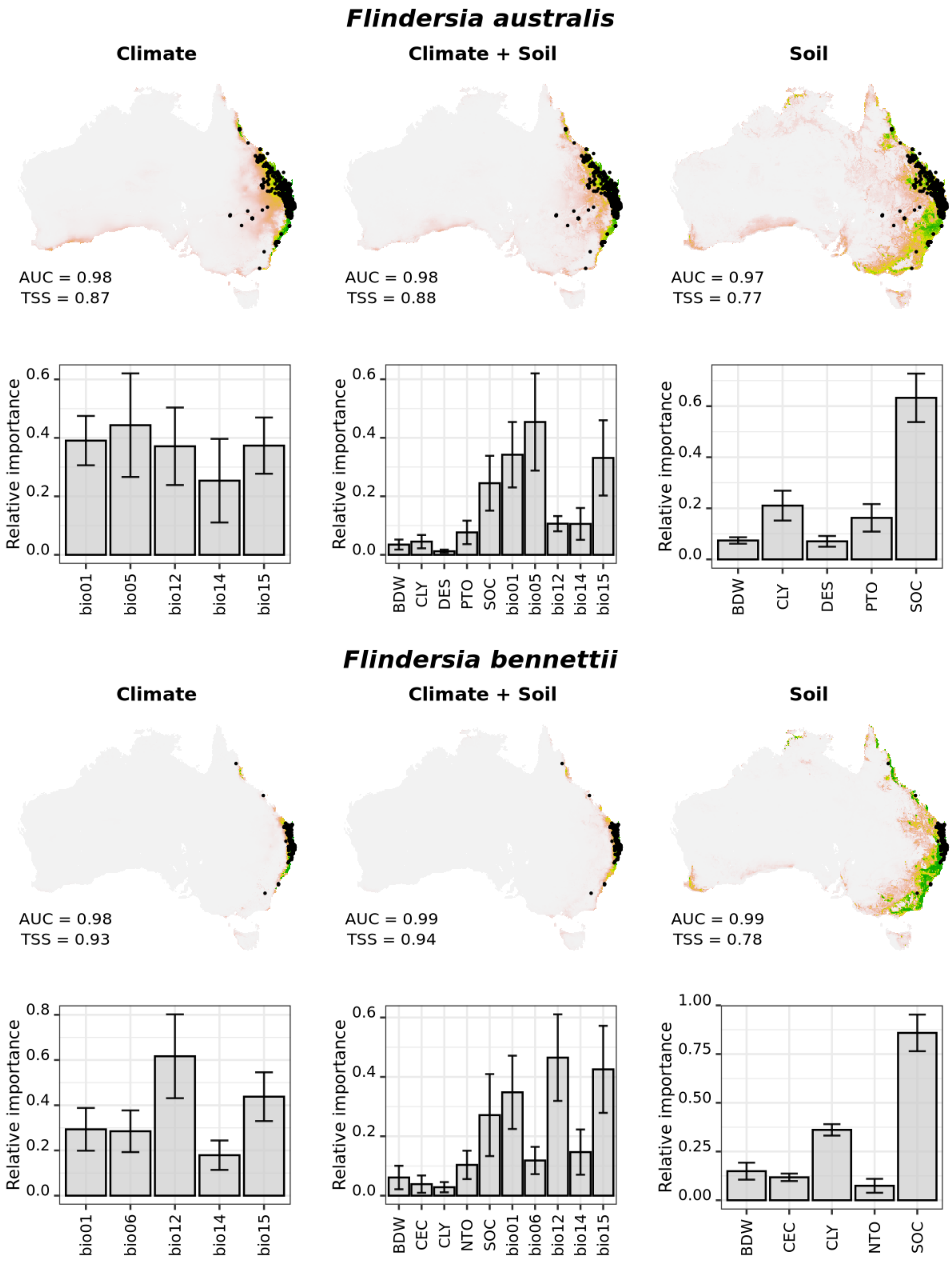
Flindersia australis
Climate
Climate + Soil
Soil
AUC = 0.98
TSS = 0.87
AUC = 0.98
TSS = 0.88
AUC = 0.97
TSS = 0.77
Relative importance
0.0
0.2
0.4
0.6
bio01
bio05
bio12
bio14
bio15
BDW
CLY
DES
PTO
SOC
Flindersia bennettii
Climate
Climate + Soil
Soil
AUC = 0.98
TSS = 0.93
AUC = 0.99
TSS = 0.94
AUC = 0.99
TSS = 0.78
0.8
bio06
CEC
NTO
0.00
0.25
0.50
0.75
1.00

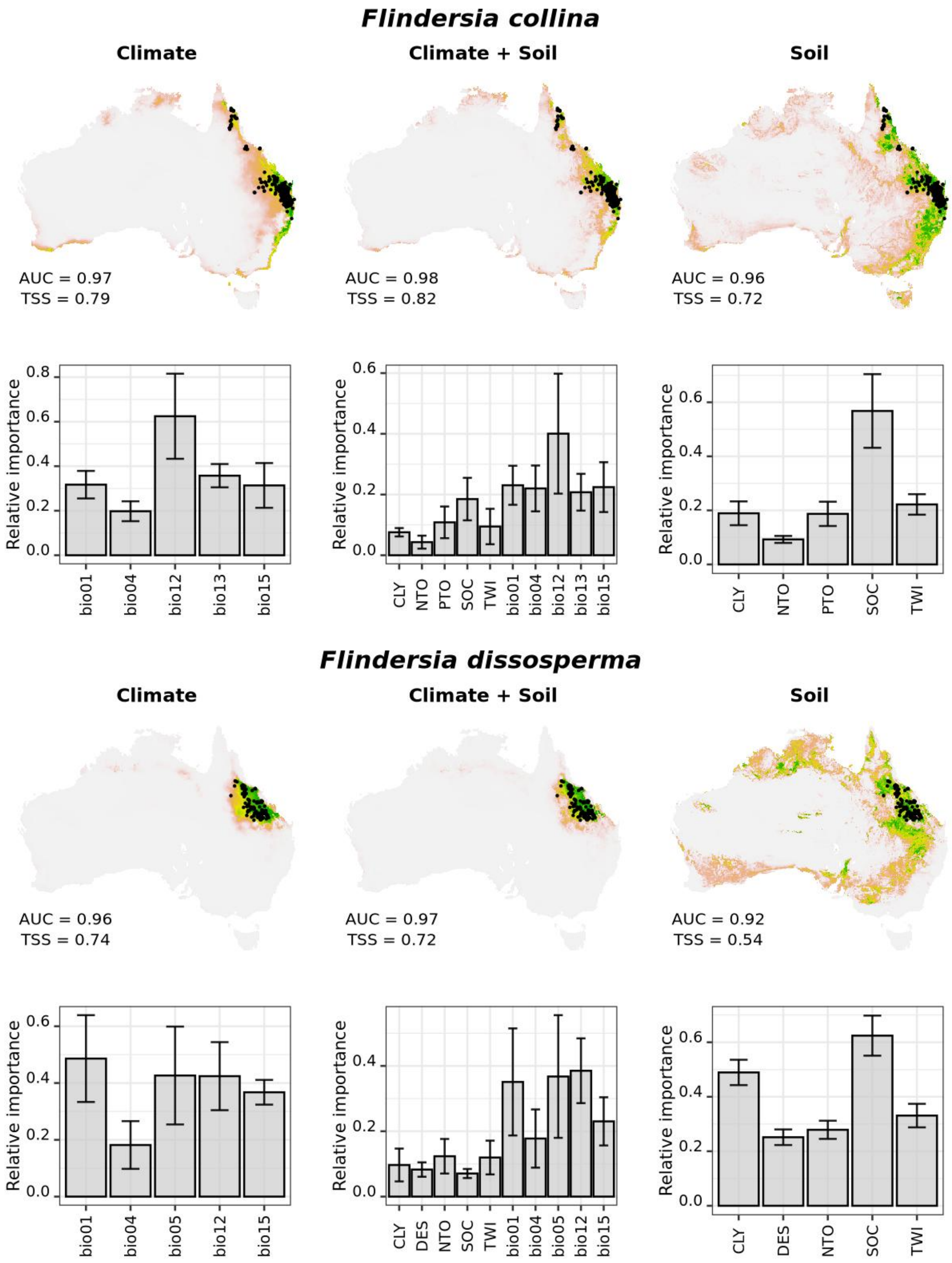
Flindersia collina
Climate
Climate + Soil
Soil
AUC = 0.97
TSS = 0.79
AUC = 0.98
TSS = 0.82
AUC = 0.96
TSS = 0.72
Relative importance
0.0
0.2
0.4
0.6
0.8
bio01
bio04
bio12
bio13
bio15
CLY
NTO
PTO
SOC
TWI
Flindersia dissosperma
Climate
Climate + Soil
Soil
AUC = 0.96
TSS = 0.74
AUC = 0.97
TSS = 0.72
AUC = 0.92
TSS = 0.54
Relative importance
bio01
bio04
bio05
bio12
bio15
CLY
DES
NTO
SOC
TWI

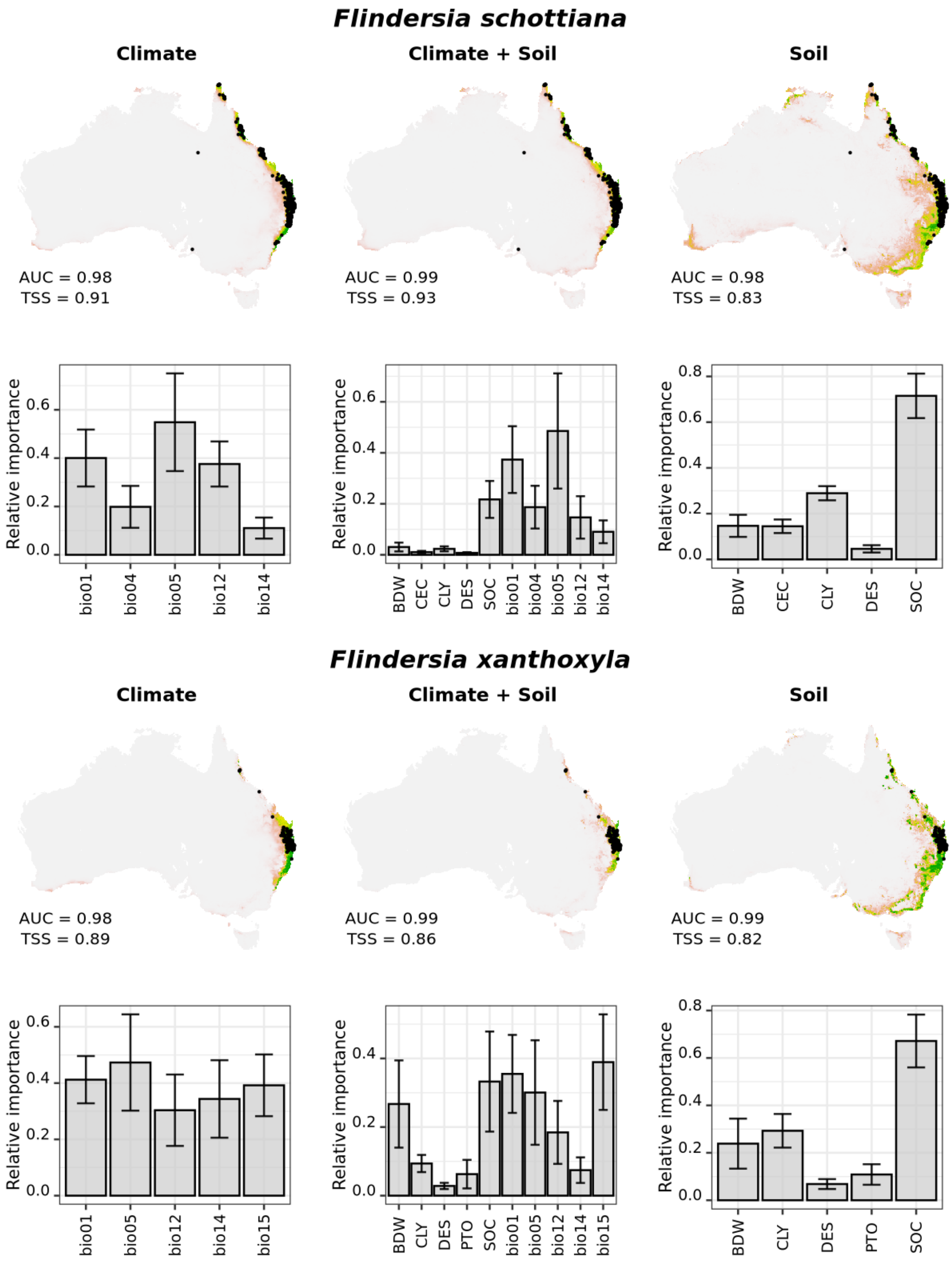
Flindersia schottiana
Climate
Climate + Soil
Soil
AUC = 0.98
TSS = 0.91
AUC = 0.99
TSS = 0.93
AUC = 0.98
TSS = 0.83
Relative importance
0.0
0.2
0.4
0.6
0.8
bio01
bio04
bio05
bio12
bio14
BDW
CEC
CLY
DES
SOC
Flindersia xanthoxyla
Climate
Climate + Soil
Soil
AUC = 0.98
TSS = 0.89
AUC = 0.99
TSS = 0.86
AUC = 0.99
TSS = 0.82
Relative importance
bio01
bio05
bio12
bio14
bio15
BDW
CLY
DES
PTO
SOC

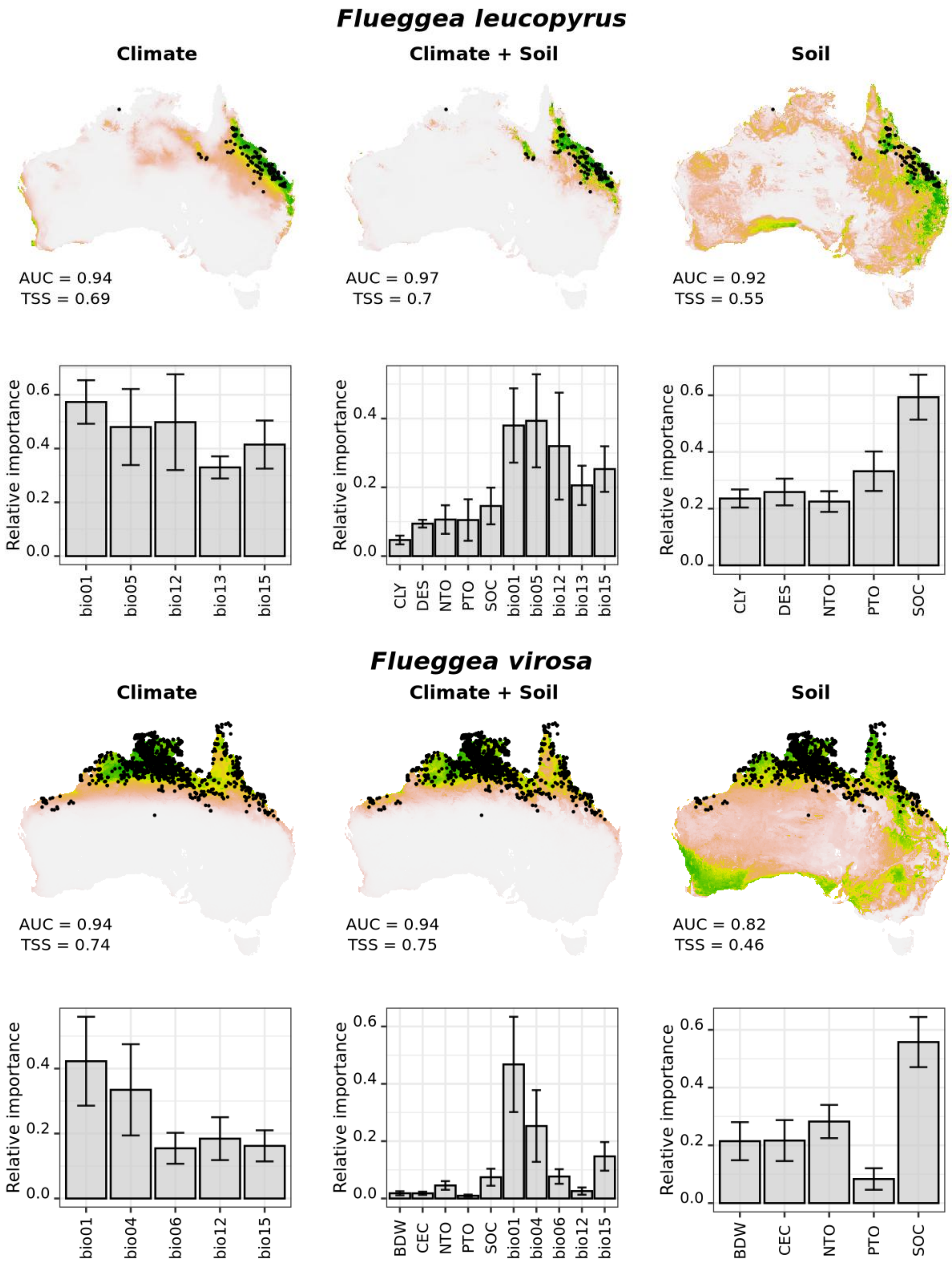
Flueggea leucopyrus
Climate
Climate + Soil
Soil
AUC = 0.94
TSS = 0.69
AUC = 0.97
TSS = 0.7
AUC = 0.92
TSS = 0.55
Relative importance
bio01
bio05
bio12
bio13
bio15
CLY
DES
NTO
PTO
SOC
Flueggea virosa
Climate
Climate + Soil
Soil
AUC = 0.94
TSS = 0.74
AUC = 0.94
TSS = 0.75
AUC = 0.82
TSS = 0.46
Relative importance
bio01
bio04
bio06
bio12
bio15
BDW
CEC
NTO
PTO
SOC

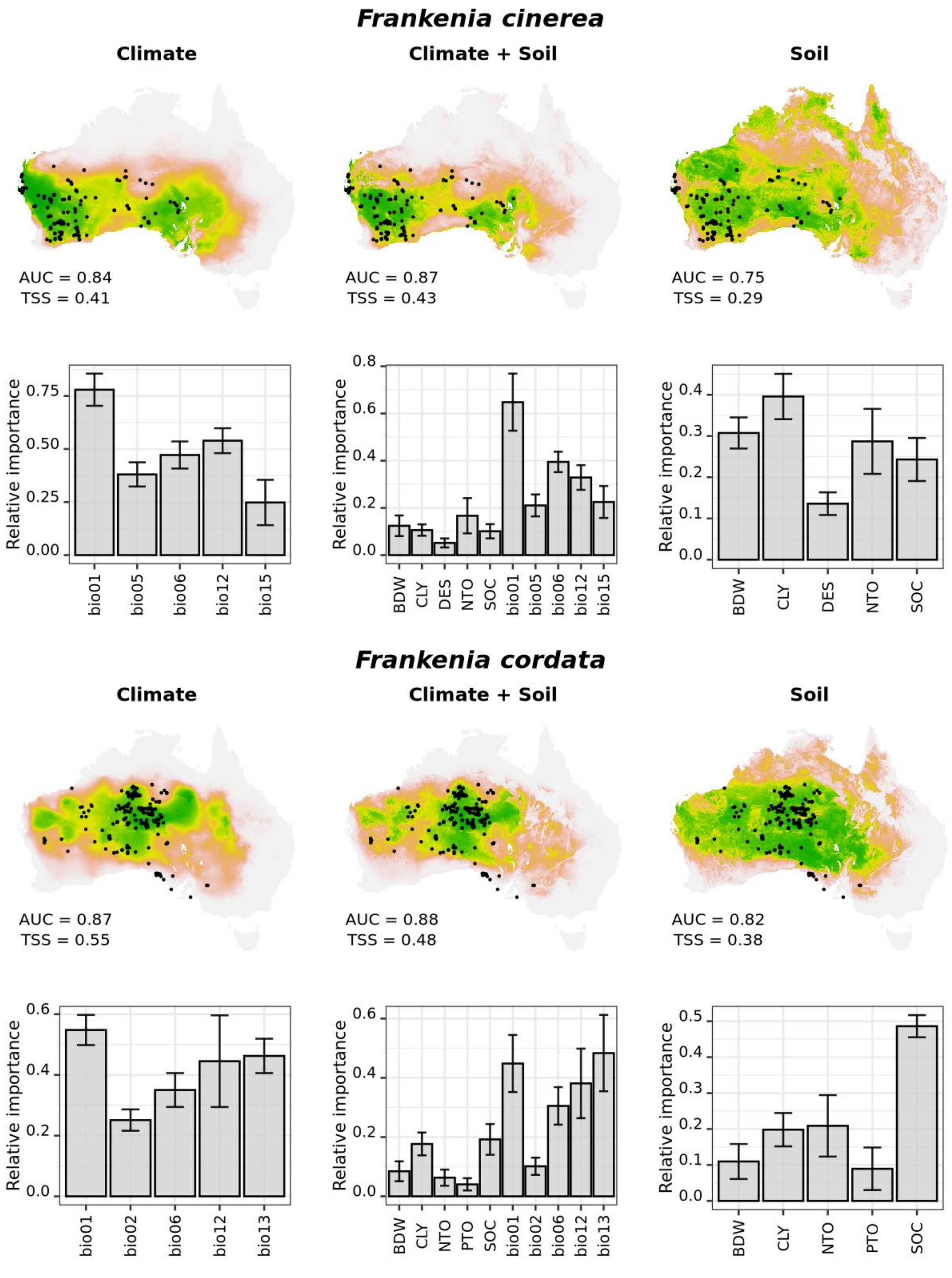

Frankenia cinerea
Climate
Climate + Soil
Soil
AUC = 0.84
TSS = 0.41
AUC = 0.87
TSS = 0.43
AUC = 0.75
TSS = 0.29
Relative importance
bio01
bio05
bio06
bio12
bio15
BDW
CLY
DES
NTO
SOC
Frankenia cordata
Climate
Climate + Soil
Soil
AUC = 0.87
TSS = 0.55
AUC = 0.88
TSS = 0.48
AUC = 0.82
TSS = 0.38
Relative importance
bio01
bio02
bio06
bio12
bio13
BDW
CLY
NTO
PTO
SOC

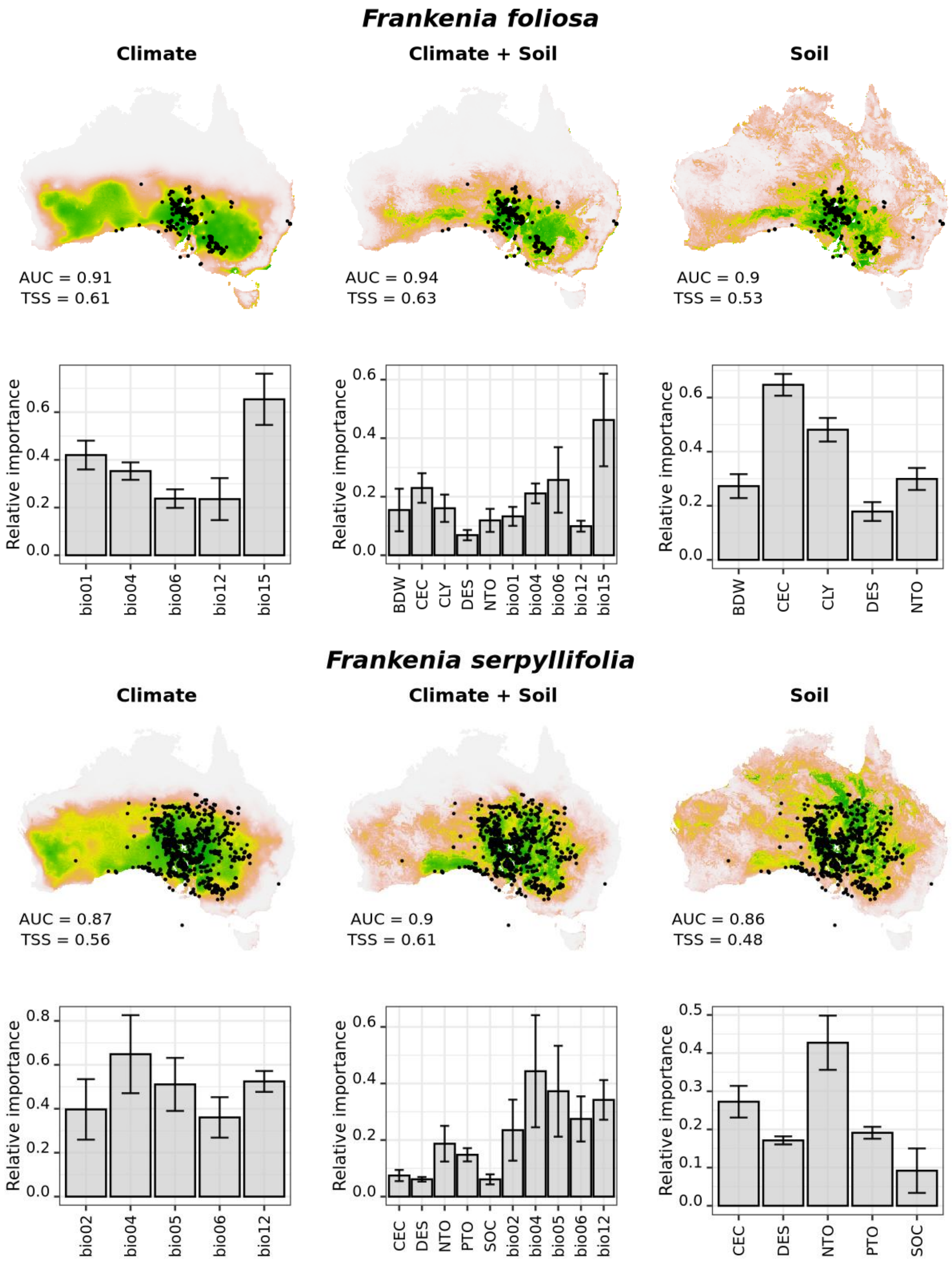

Frankenia foliosa
Climate
Climate + Soil
Soil
AUC = 0.91
TSS = 0.61
AUC = 0.94
TSS = 0.63
AUC = 0.9
TSS = 0.53
Relative importance
bio01
bio04
bio06
bio12
bio15
BDW
CEC
CLY
DES
NTO
Frankenia serpyllifolia
Climate
Climate + Soil
Soil
AUC = 0.87
TSS = 0.56
AUC = 0.9
TSS = 0.61
AUC = 0.86
TSS = 0.48
Relative importance
bio02
bio04
bio05
bio06
bio12
CEC
DES
NTO
PTO
SOC

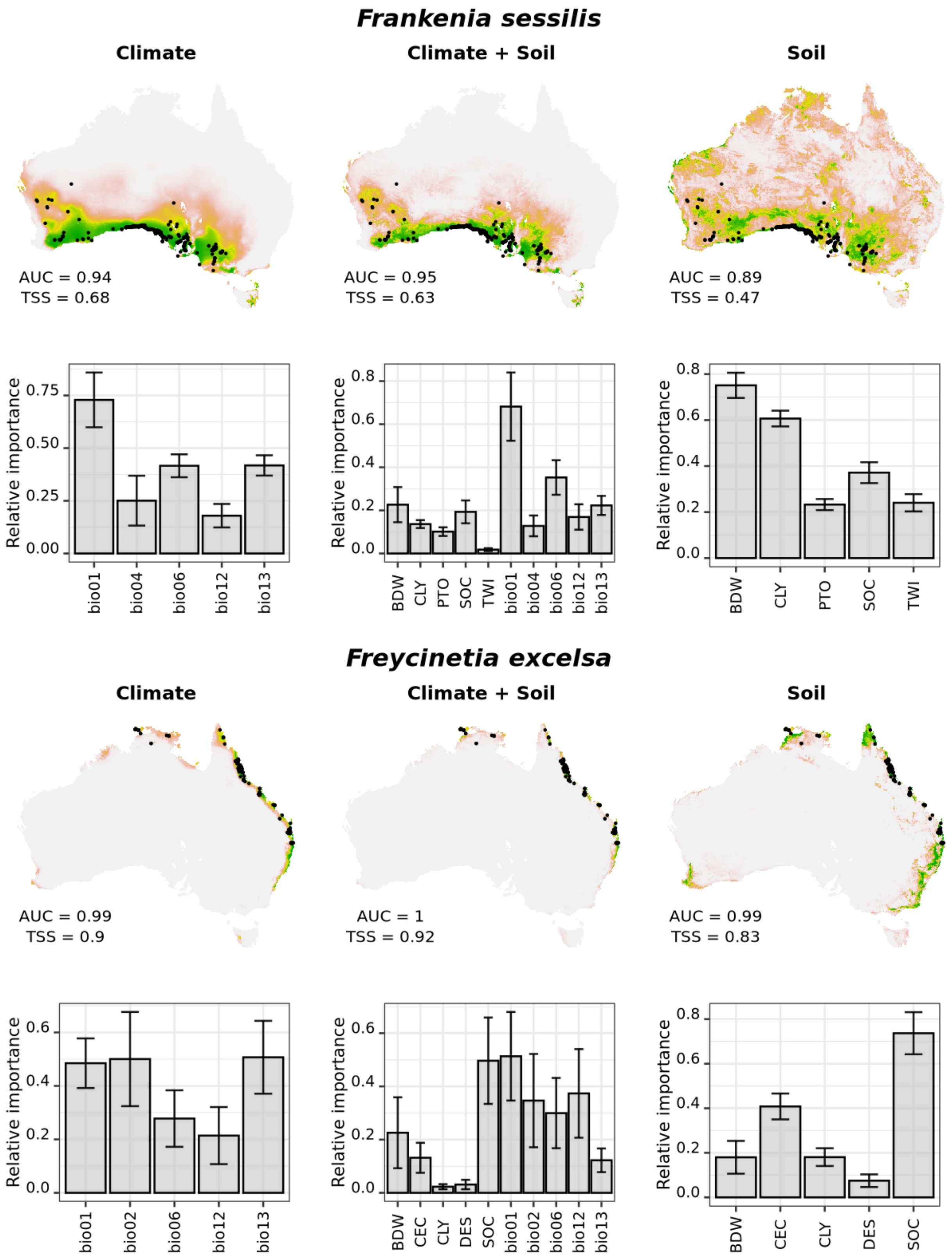

Frankenia sessilis
Climate
Climate + Soil
Soil
AUC = 0.94
TSS = 0.68
AUC = 0.95
TSS = 0.63
AUC = 0.89
TSS = 0.47
Relative importance
bio01
bio04
bio06
bio12
bio13
BDW
CLY
PTO
SOC
TWI
Freycinetia excelsa
AUC = 0.99
TSS = 0.9
AUC = 1
TSS = 0.92
AUC = 0.99
TSS = 0.83
bio02
CEC
DES

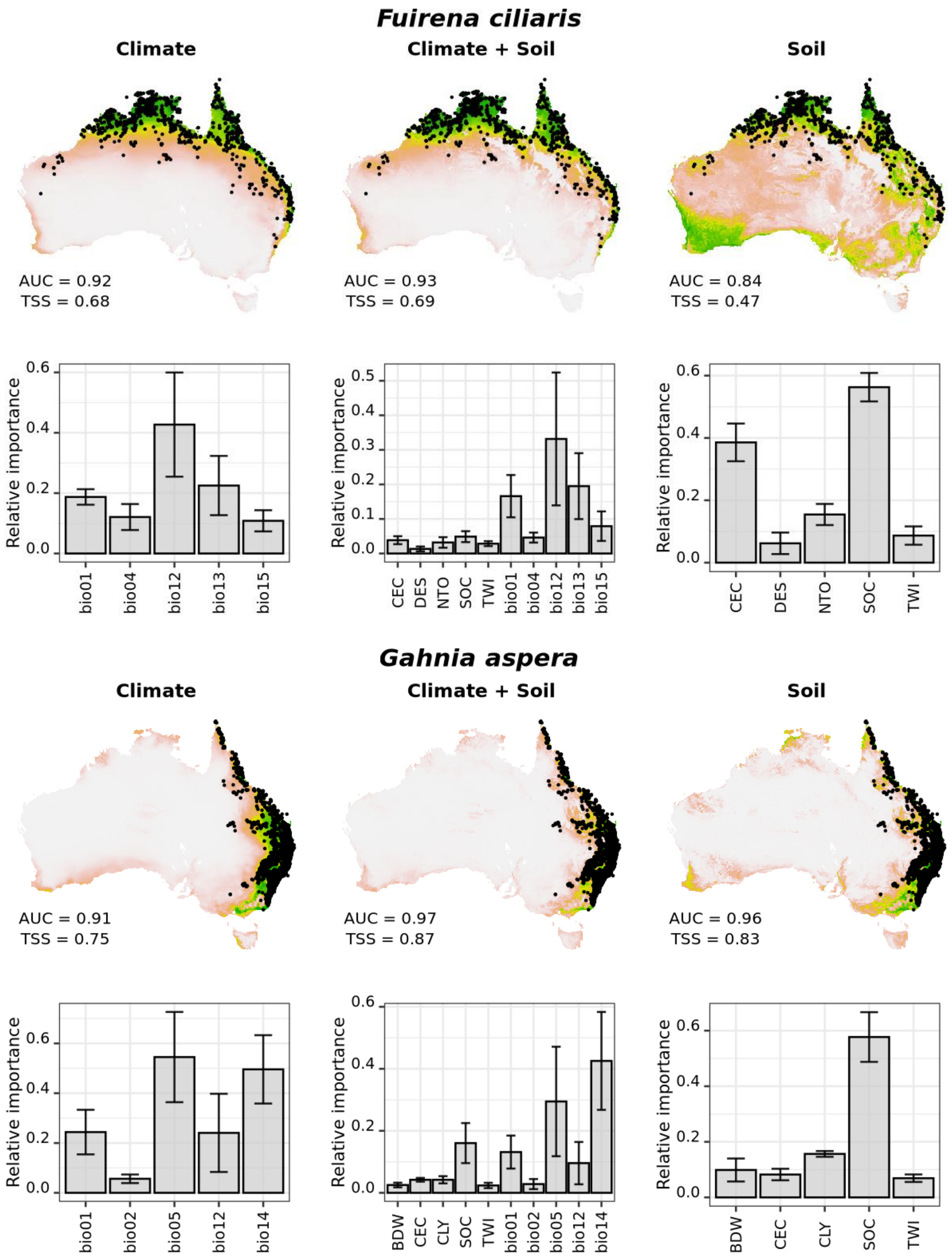

Fuirena ciliaris
Climate
Climate + Soil
Soil
AUC = 0.92
TSS = 0.68
AUC = 0.93
TSS = 0.69
AUC = 0.84
TSS = 0.47
Relative importance
bio01 bio04 bio12 bio13 bio15
CEC DES NTO SOC TWI bio01 bio04 bio12 bio13 bio15
CEC DES NTO SOC TWI
Gahnia aspera
Climate
Climate + Soil
Soil
AUC = 0.91
TSS = 0.75
AUC = 0.97
TSS = 0.87
AUC = 0.96
TSS = 0.83
Relative importance
bio01 bio02 bio05 bio12 bio14
BDW CEC CLY SOC TWI bio01 bio02 bio05 bio12 bio14
BDW CEC CLY SOC TWI

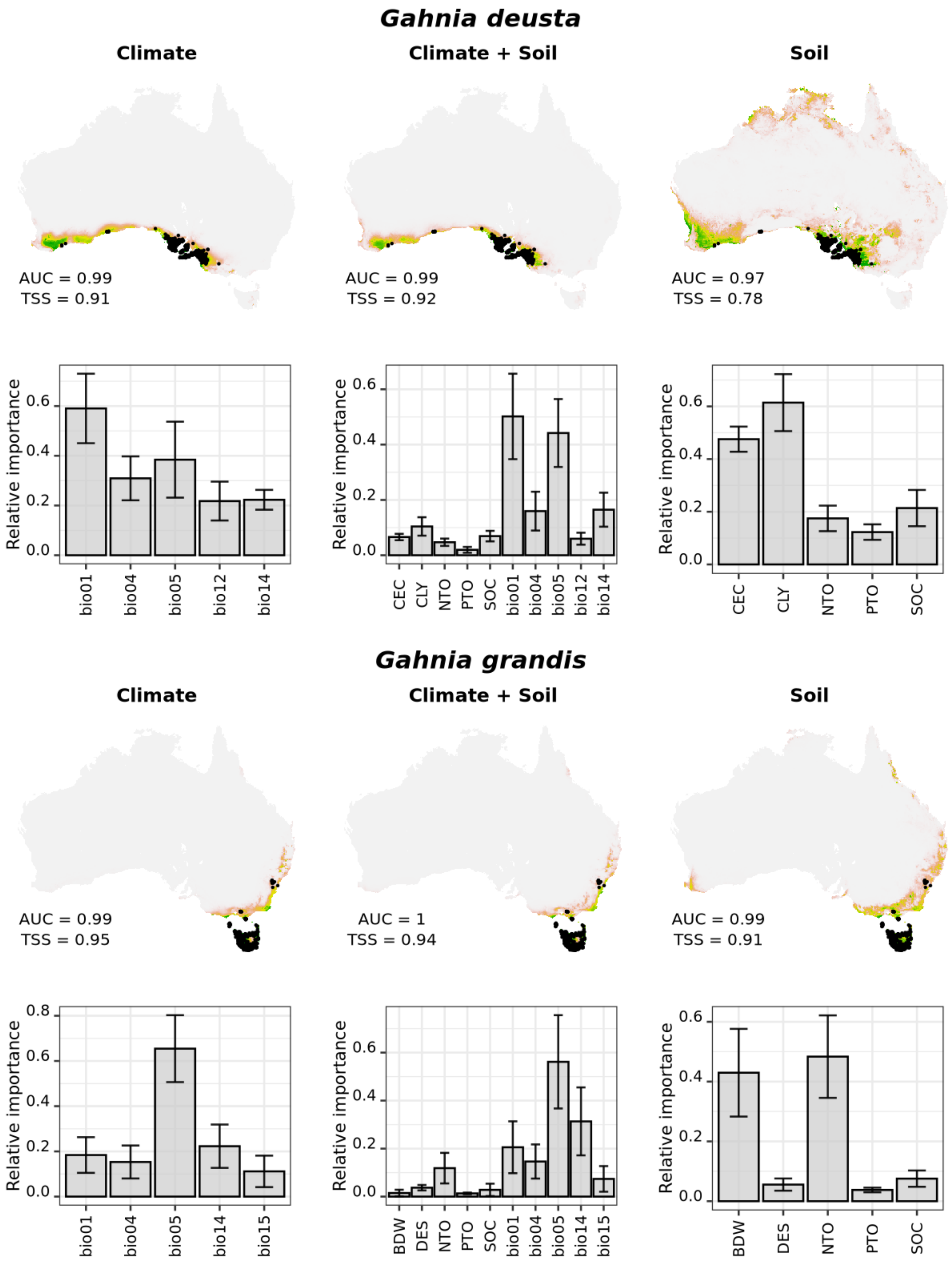

Gahnia deusta
Climate
Climate + Soil
Soil
AUC = 0.99
TSS = 0.91
AUC = 0.99
TSS = 0.92
AUC = 0.97
TSS = 0.78
Relative importance
0.0
0.2
0.4
0.6
bio01
bio04
bio05
bio12
bio14
CEC
CLY
NTO
PTO
SOC
Gahnia grandis
Climate
Climate + Soil
Soil
AUC = 0.99
TSS = 0.95
AUC = 1
TSS = 0.94
AUC = 0.99
TSS = 0.91
0.8
bio15
BDW
DES

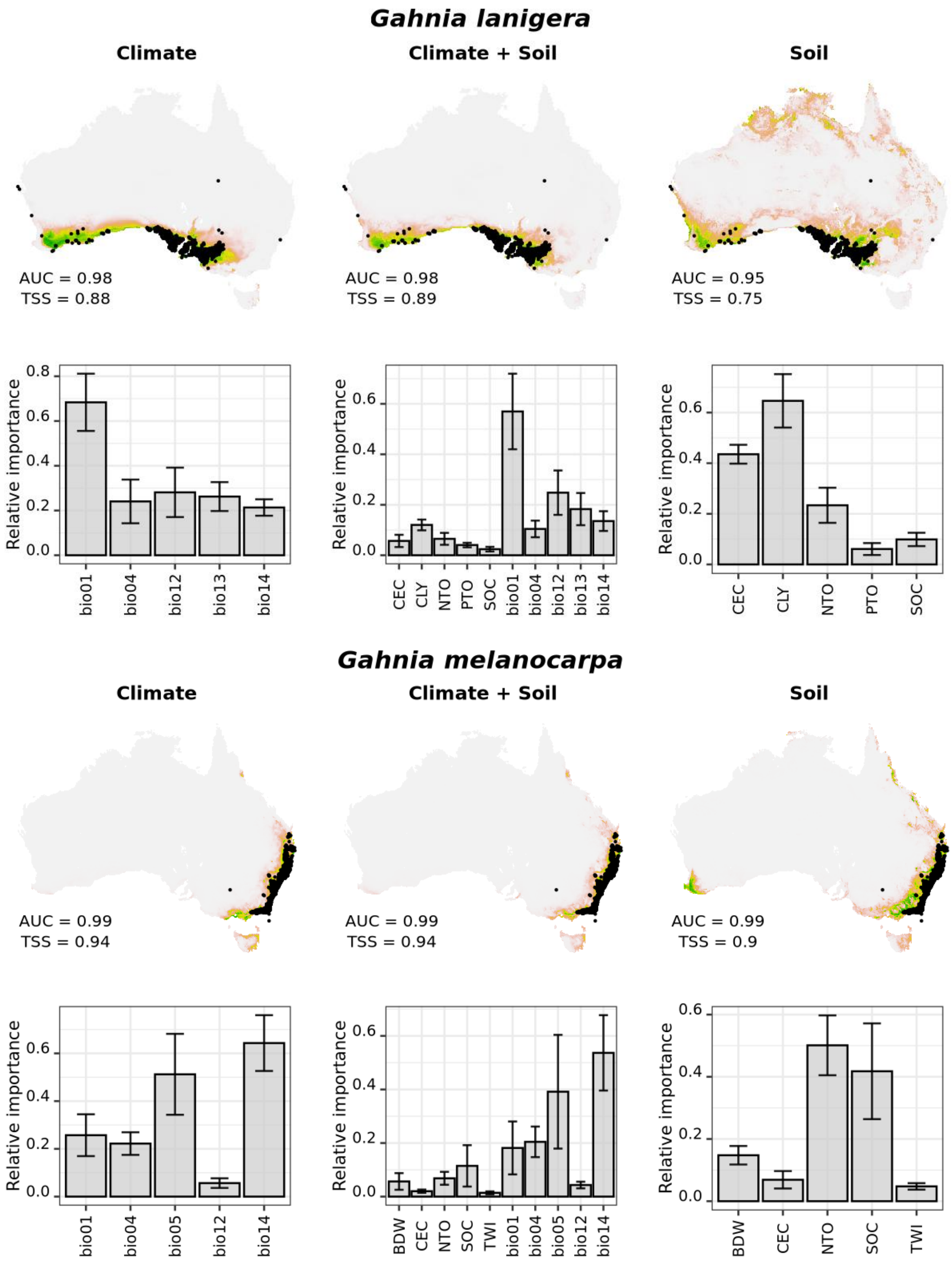
Gahnia lanigera
Climate
Climate + Soil
Soil
AUC = 0.98
TSS = 0.88
AUC = 0.98
TSS = 0.89
AUC = 0.95
TSS = 0.75
Relative importance
bio01
bio04
bio12
bio13
bio14
CEC
CLY
NTO
PTO
SOC
Gahnia melanocarpa
Climate
Climate + Soil
Soil
AUC = 0.99
TSS = 0.94
AUC = 0.99
TSS = 0.94
AUC = 0.99
TSS = 0.9
Relative importance
bio01
bio04
bio05
bio12
bio14
BDW
CEC
NTO
SOC
TWI

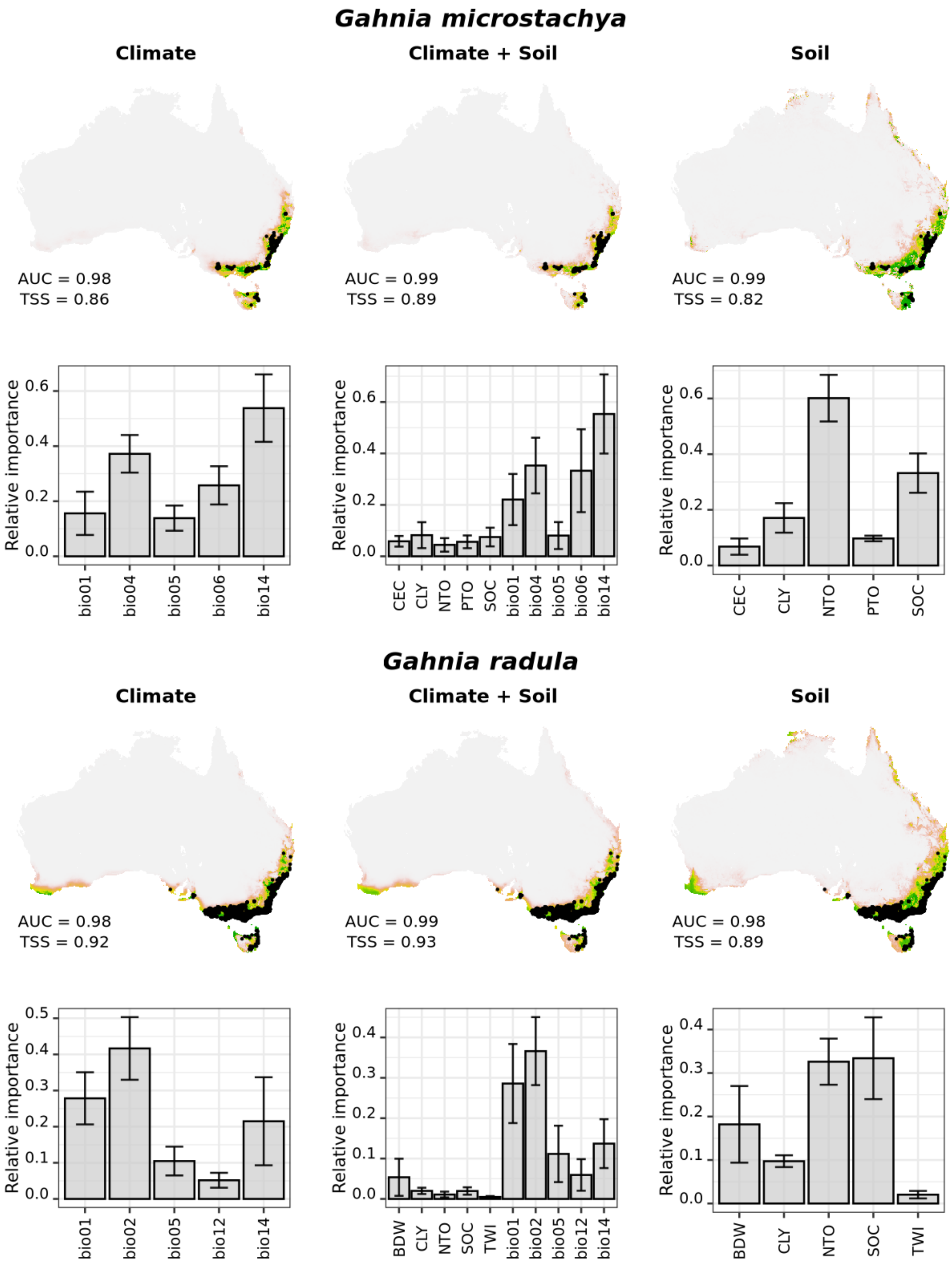
Gahnia microstachya
Climate
Climate + Soil
Soil
AUC = 0.98
TSS = 0.86
AUC = 0.99
TSS = 0.89
AUC = 0.99
TSS = 0.82
Relative importance
0.0
0.2
0.4
0.6
bio01
bio04
bio05
bio06
bio14
CEC
CLY
NTO
PTO
SOC
Gahnia radula
AUC = 0.98
TSS = 0.92
AUC = 0.99
TSS = 0.93
AUC = 0.98
TSS = 0.89
0.1
0.3
0.5
bio02
bio12
BDW
TWI

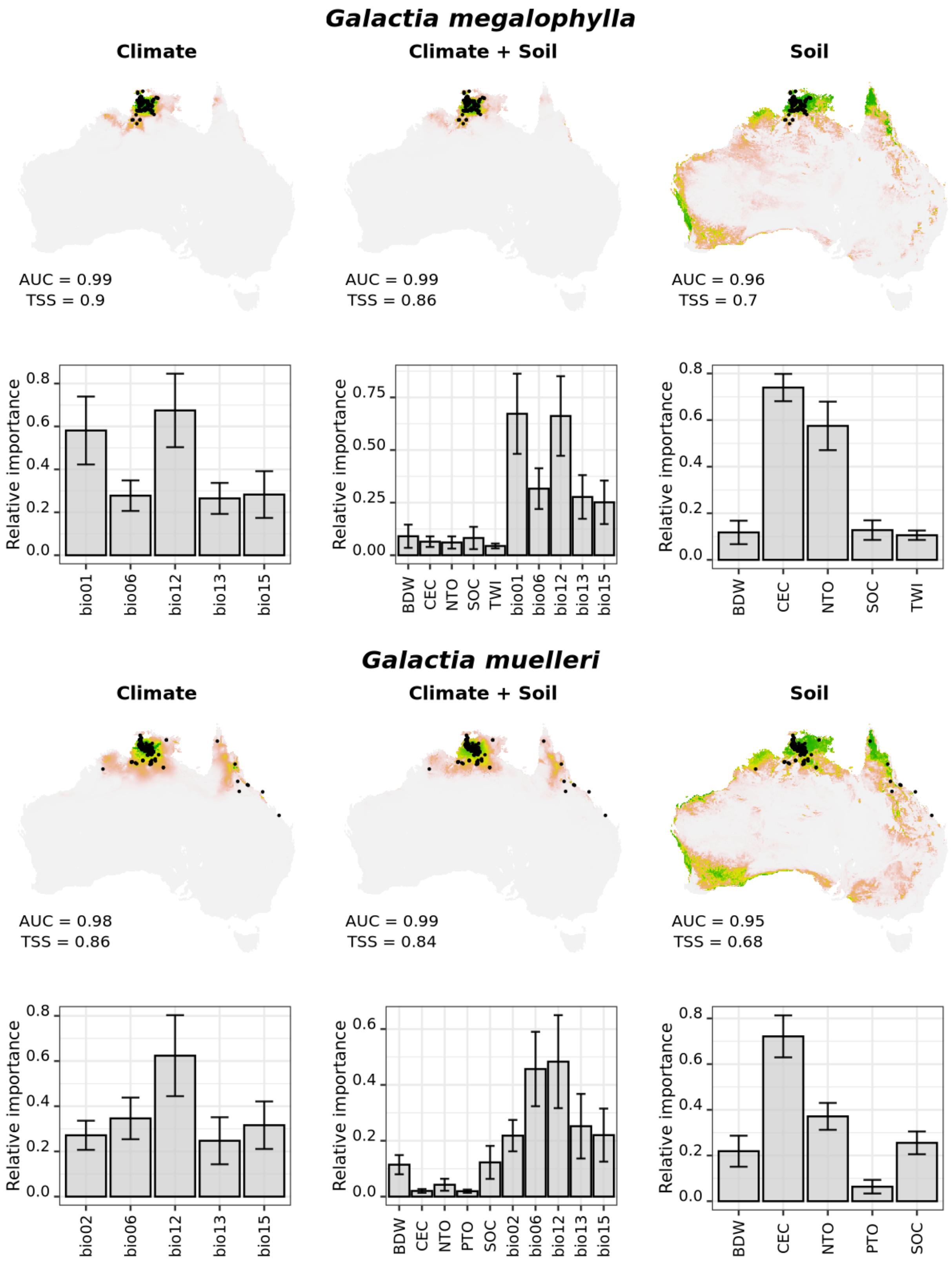
Galactia megalophylla
Climate
Climate + Soil
Soil
AUC = 0.99
TSS = 0.9
AUC = 0.99
TSS = 0.86
AUC = 0.96
TSS = 0.7
Relative importance
bio01 bio06 bio12 bio13 bio15
BDW CEC NTO SOC TWI bio01 bio06 bio12 bio13 bio15
BDW CEC NTO SOC TWI
Galactia muelleri
Climate
Climate + Soil
Soil
AUC = 0.98
TSS = 0.86
AUC = 0.99
TSS = 0.84
AUC = 0.95
TSS = 0.68
Relative importance
bio02 bio06 bio12 bio13 bio15
BDW CEC NTO PTO SOC bio02 bio06 bio12 bio13 bio15
BDW CEC NTO PTO SOC

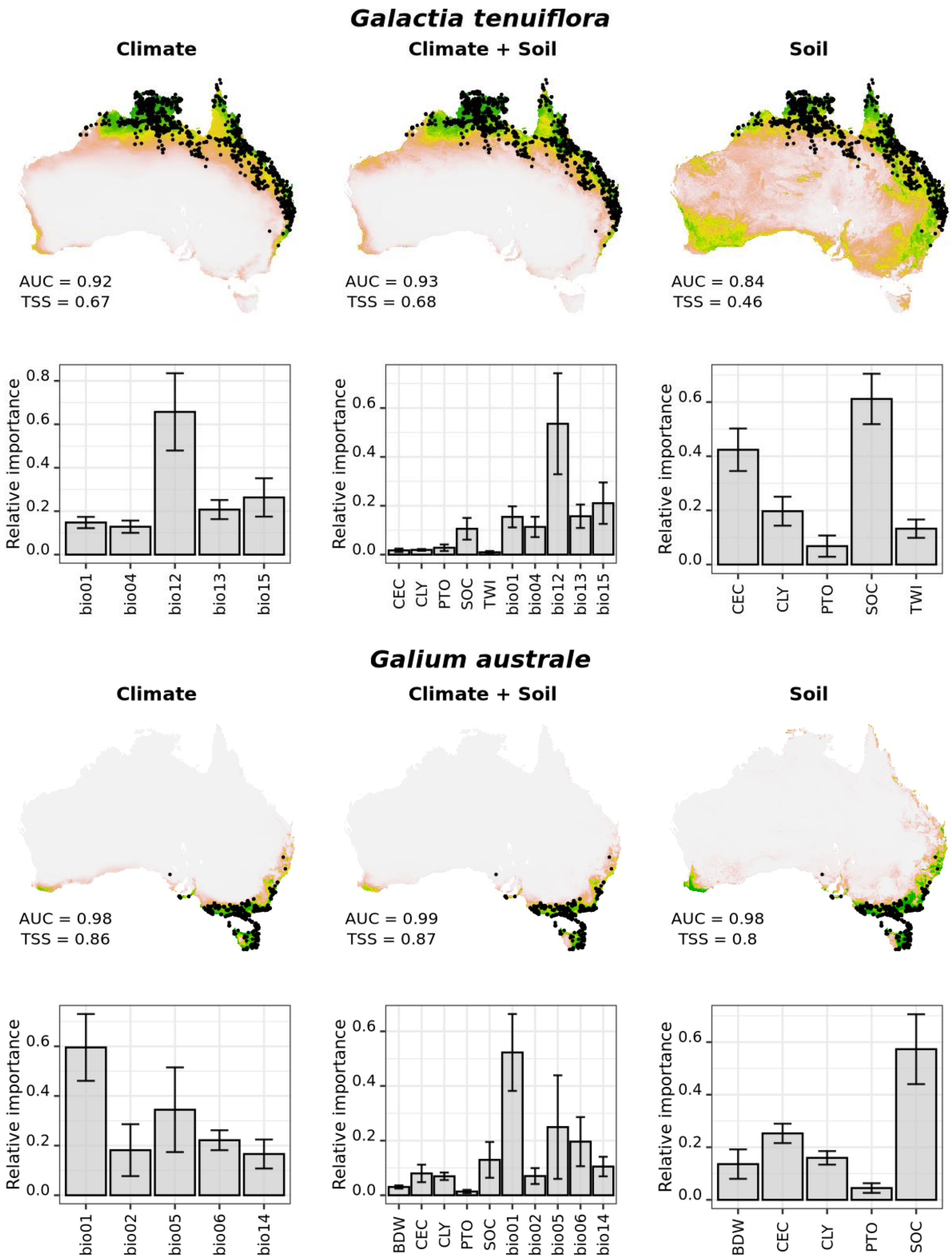
Galactia tenuiflora
Climate
Climate + Soil
Soil
AUC = 0.92
TSS = 0.67
AUC = 0.93
TSS = 0.68
AUC = 0.84
TSS = 0.46
Relative importance
bio01 bio04 bio12 bio13 bio15
CEC CLY PTO SOC TWI bio01 bio04 bio12 bio13 bio15
CEC CLY PTO SOC TWI
Galium australe
Climate
Climate + Soil
Soil
AUC = 0.98
TSS = 0.86
AUC = 0.99
TSS = 0.87
AUC = 0.98
TSS = 0.8
Relative importance
bio01 bio02 bio05 bio06 bio14
BDW CEC CLY PTO SOC bio01 bio02 bio05 bio06 bio14
BDW CEC CLY PTO SOC

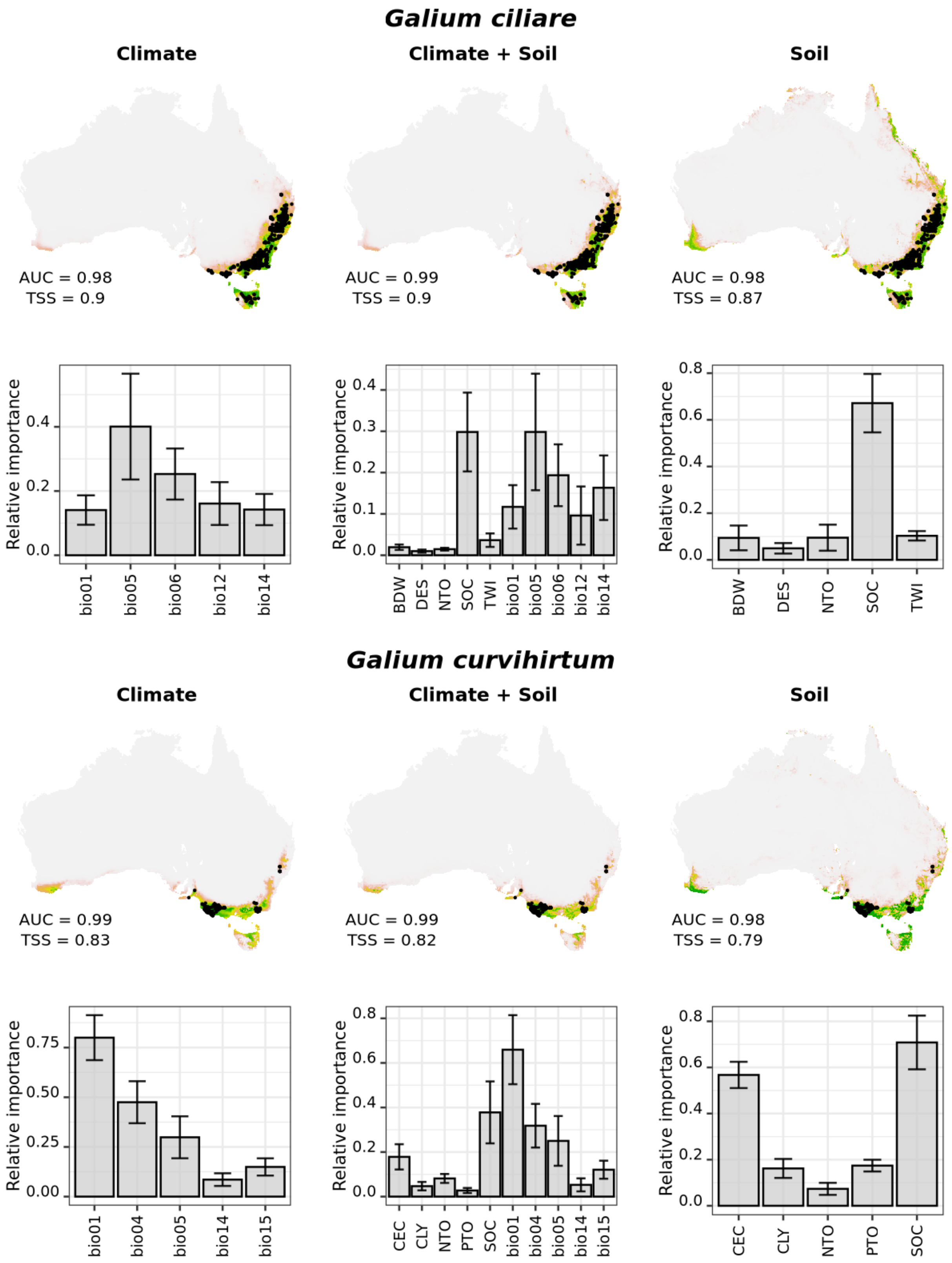

Galium ciliare
Climate
Climate + Soil
Soil
AUC = 0.98
TSS = 0.9
AUC = 0.99
TSS = 0.9
AUC = 0.98
TSS = 0.87
Relative importance
bio01
bio05
bio06
bio12
bio14
BDW
DES
NTO
SOC
TWI
Galium curvihirtum
Climate
Climate + Soil
Soil
AUC = 0.99
TSS = 0.83
AUC = 0.99
TSS = 0.82
AUC = 0.98
TSS = 0.79
Relative importance
bio01
bio04
bio05
bio14
bio15
CEC
CLY
NTO
PTO
SOC

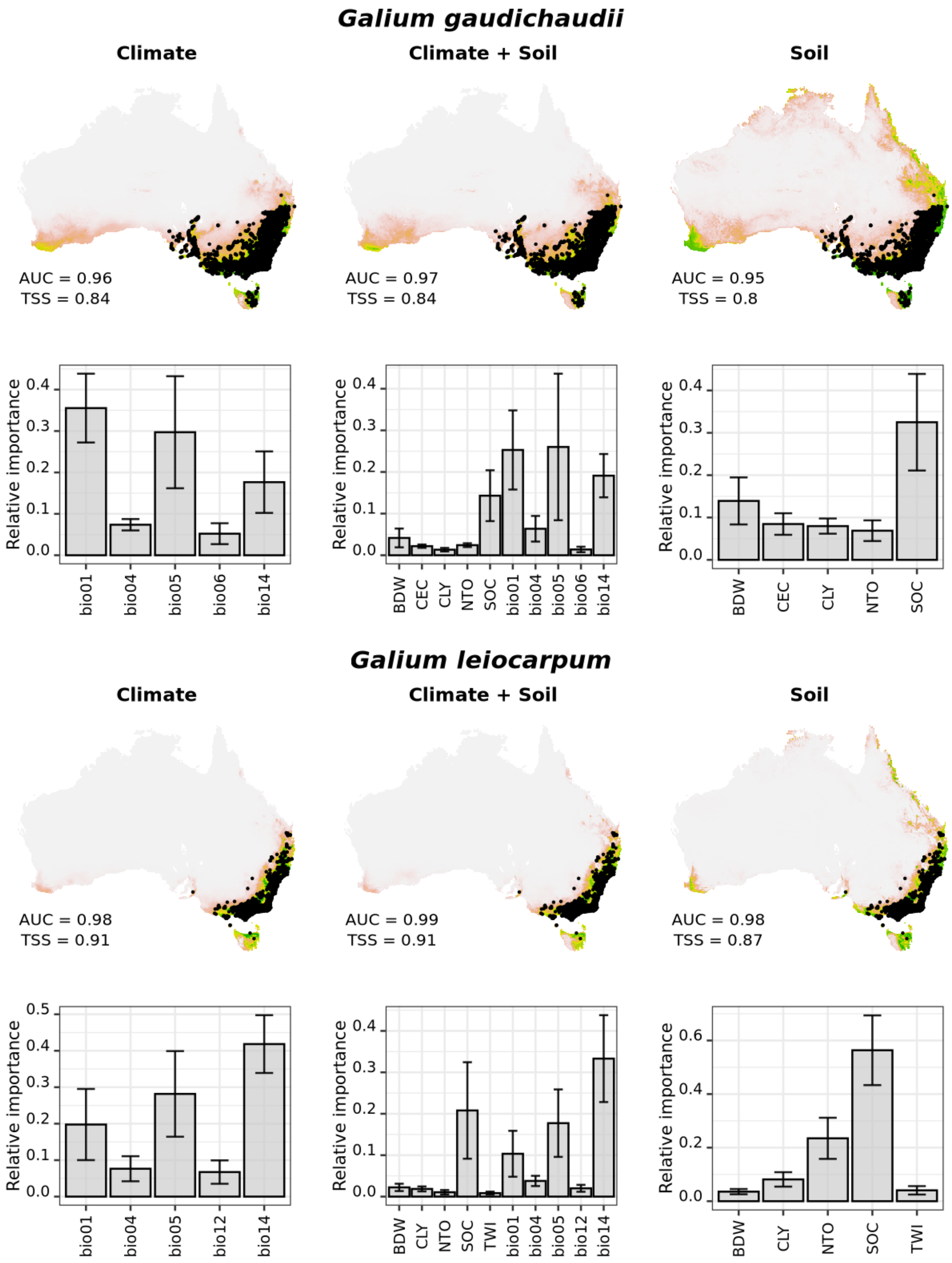
Galium gaudichaudii
Climate
Climate + Soil
Soil
AUC = 0.96
TSS = 0.84
AUC = 0.97
TSS = 0.84
AUC = 0.95
TSS = 0.8
Relative importance
0.0
0.1
0.2
0.3
0.4
bio01
bio04
bio05
bio06
bio14
BDW
CEC
CLY
NTO
SOC
Galium leiocarpum
Climate
Climate + Soil
Soil
AUC = 0.98
TSS = 0.91
AUC = 0.99
TSS = 0.91
AUC = 0.98
TSS = 0.87
0.5
0.6
bio12
TWI

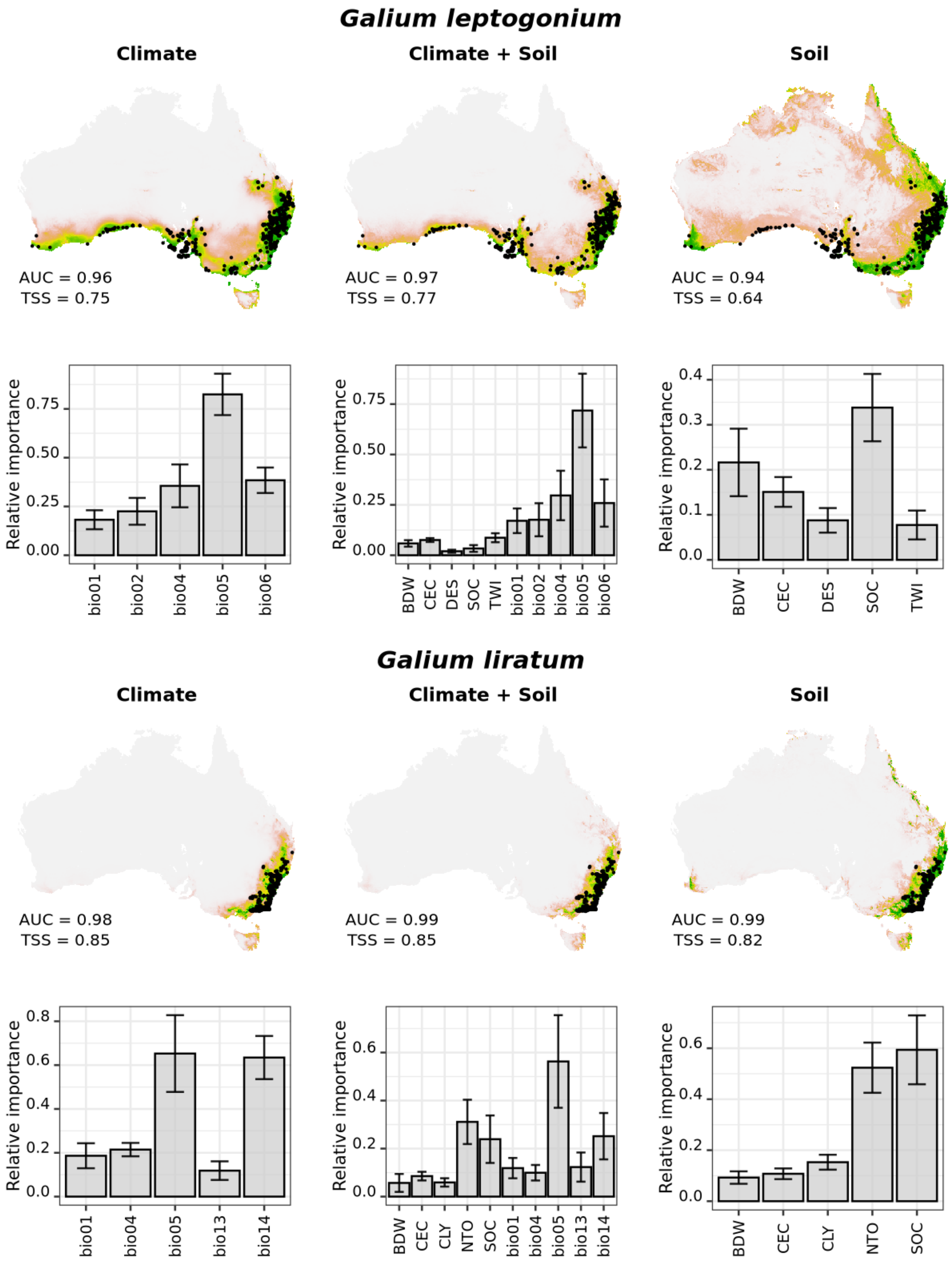
Galium leptogonium
Climate
Climate + Soil
Soil
AUC = 0.96
TSS = 0.75
AUC = 0.97
TSS = 0.77
AUC = 0.94
TSS = 0.64
Relative importance
0.00
0.25
0.50
0.75
bio01
bio02
bio04
bio05
bio06
BDW
CEC
DES
SOC
TWI
0.0
0.1
0.2
0.3
0.4
Galium liratum
Climate
Climate + Soil
Soil
AUC = 0.98
TSS = 0.85
AUC = 0.99
TSS = 0.85
AUC = 0.99
TSS = 0.82
0.0
0.2
0.4
0.6
0.8
bio01
bio04
bio05
bio13
bio14
BDW
CEC
CLY
NTO
SOC

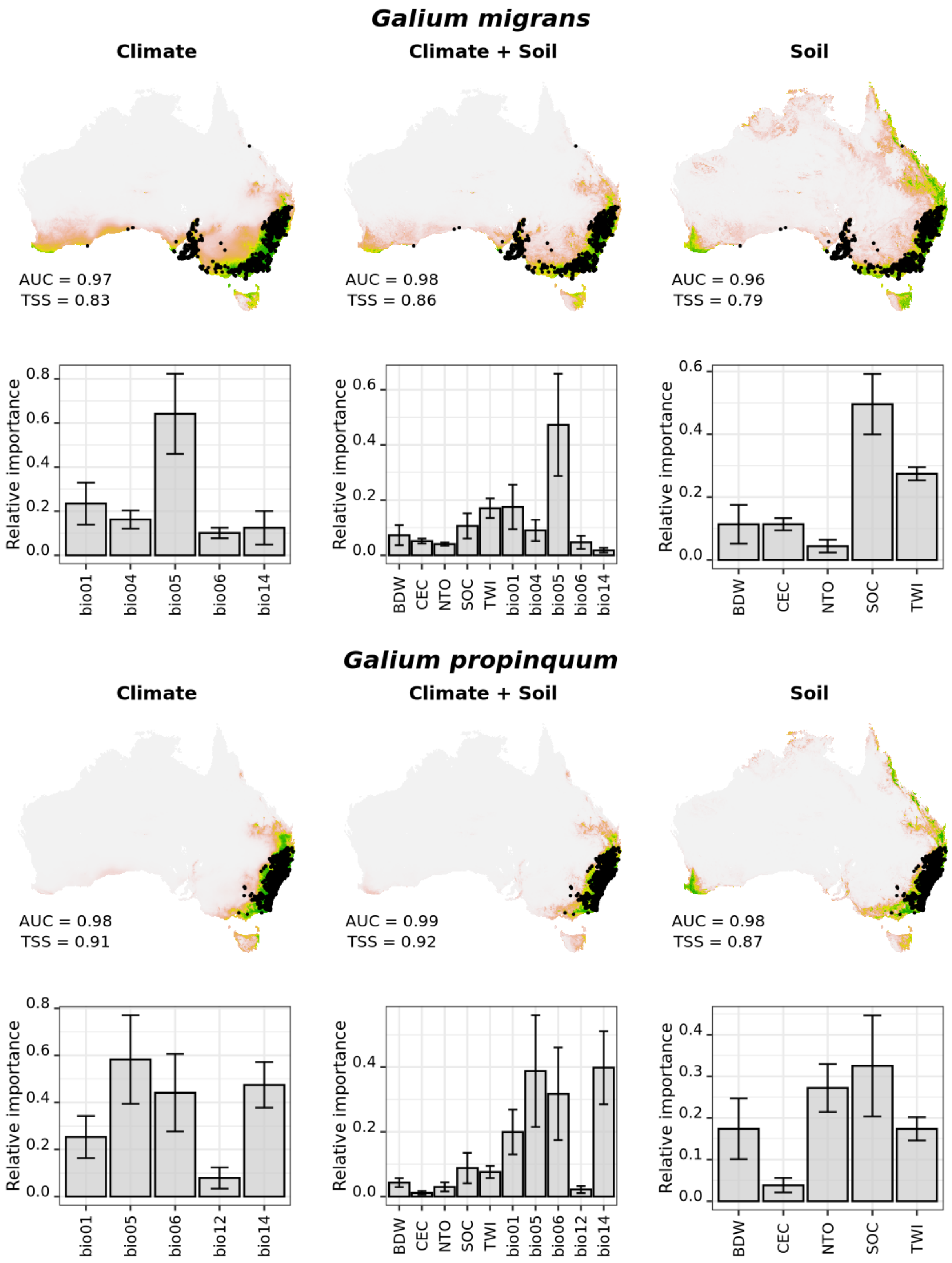

Galium migrans
Climate
Climate + Soil
Soil
AUC = 0.97
TSS = 0.83
AUC = 0.98
TSS = 0.86
AUC = 0.96
TSS = 0.79
Relative importance
bio01
bio04
bio05
bio06
bio14
BDW
CEC
NTO
SOC
TWI
Galium propinquum
Climate
Climate + Soil
Soil
AUC = 0.98
TSS = 0.91
AUC = 0.99
TSS = 0.92
AUC = 0.98
TSS = 0.87
Relative importance
bio01
bio05
bio06
bio12
bio14
BDW
CEC
NTO
SOC
TWI

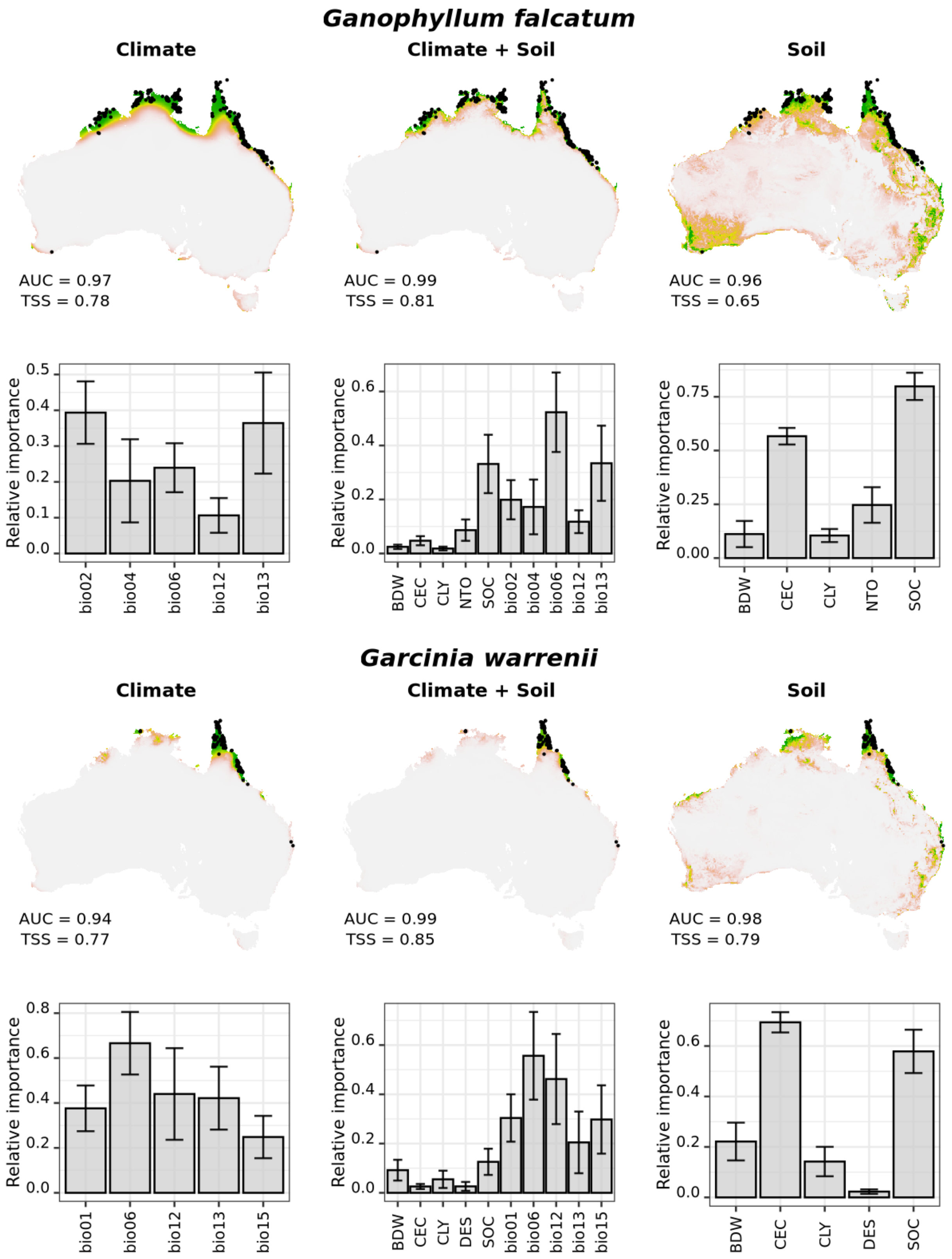
Ganophyllum falcatum
Climate
Climate + Soil
Soil
AUC = 0.97
TSS = 0.78
AUC = 0.99
TSS = 0.81
AUC = 0.96
TSS = 0.65
Relative importance
bio02 bio04 bio06 bio12 bio13
BDW CEC CLY NTO SOC bio02 bio04 bio06 bio12 bio13
BDW CEC CLY NTO SOC
Garcinia warrenii
Climate
Climate + Soil
Soil
AUC = 0.94
TSS = 0.77
AUC = 0.99
TSS = 0.85
AUC = 0.98
TSS = 0.79
Relative importance
bio01 bio06 bio12 bio13 bio15
BDW CEC CLY DES SOC bio01 bio06 bio12 bio13 bio15
BDW CEC CLY DES SOC

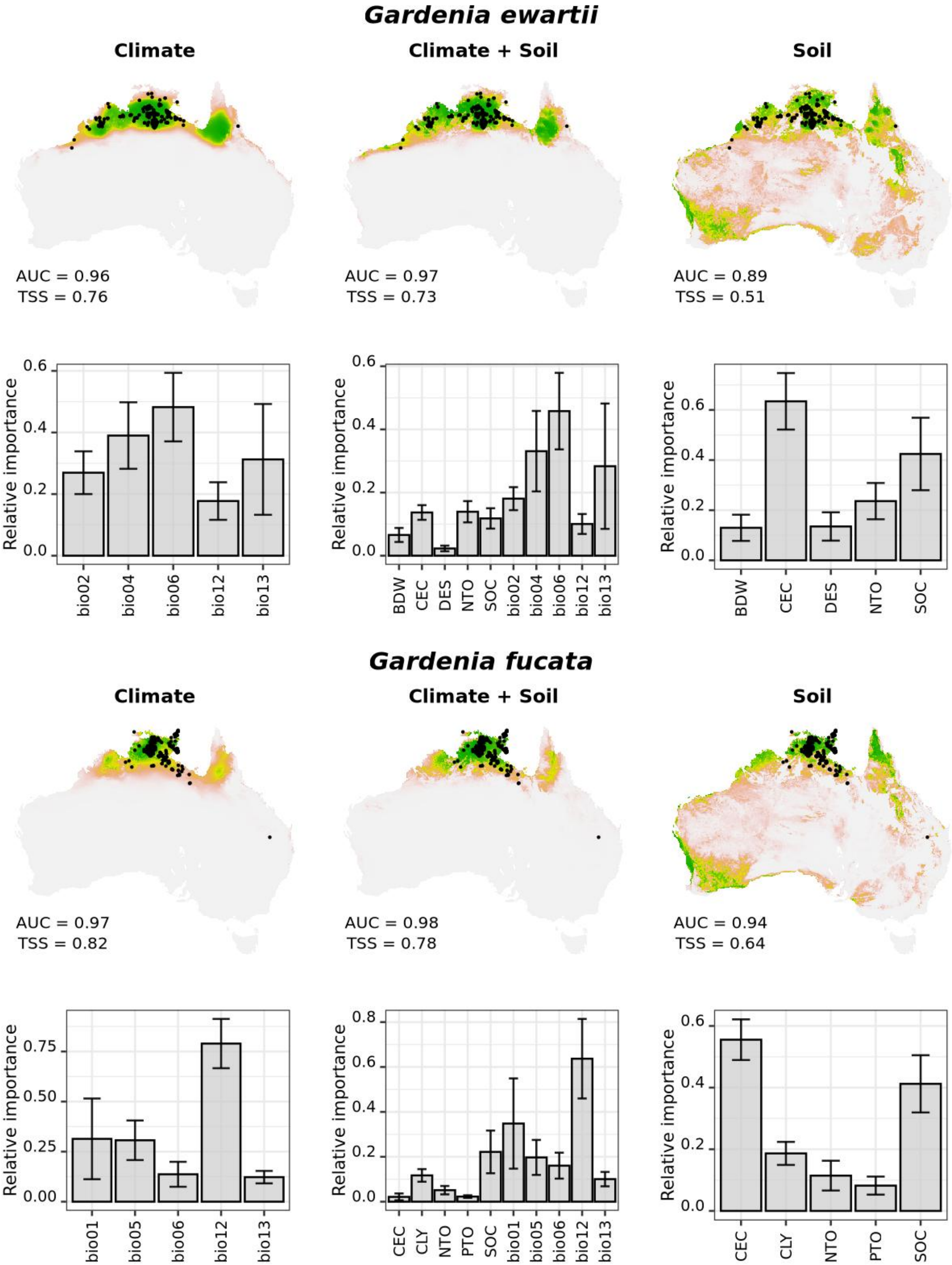
Gardenia ewartii
Climate
Climate + Soil
Soil
AUC = 0.96
TSS = 0.76
AUC = 0.97
TSS = 0.73
AUC = 0.89
TSS = 0.51
Relative importance
0.0
0.2
0.4
0.6
bio02
bio04
bio06
bio12
bio13
BDW
CEC
DES
NTO
SOC
bio02
bio04
bio06
bio12
bio13
BDW
CEC
DES
NTO
SOC
Gardenia fucata
Climate
Climate + Soil
Soil
AUC = 0.97
TSS = 0.82
AUC = 0.98
TSS = 0.78
AUC = 0.94
TSS = 0.64
Relative importance
0.00
0.25
0.50
0.75
0.8
bio01
bio05
bio06
bio12
bio13
CEC
CLY
NTO
PTO
SOC
bio01
bio05
bio06
bio12
bio13
CEC
CLY
NTO
PTO
SOC

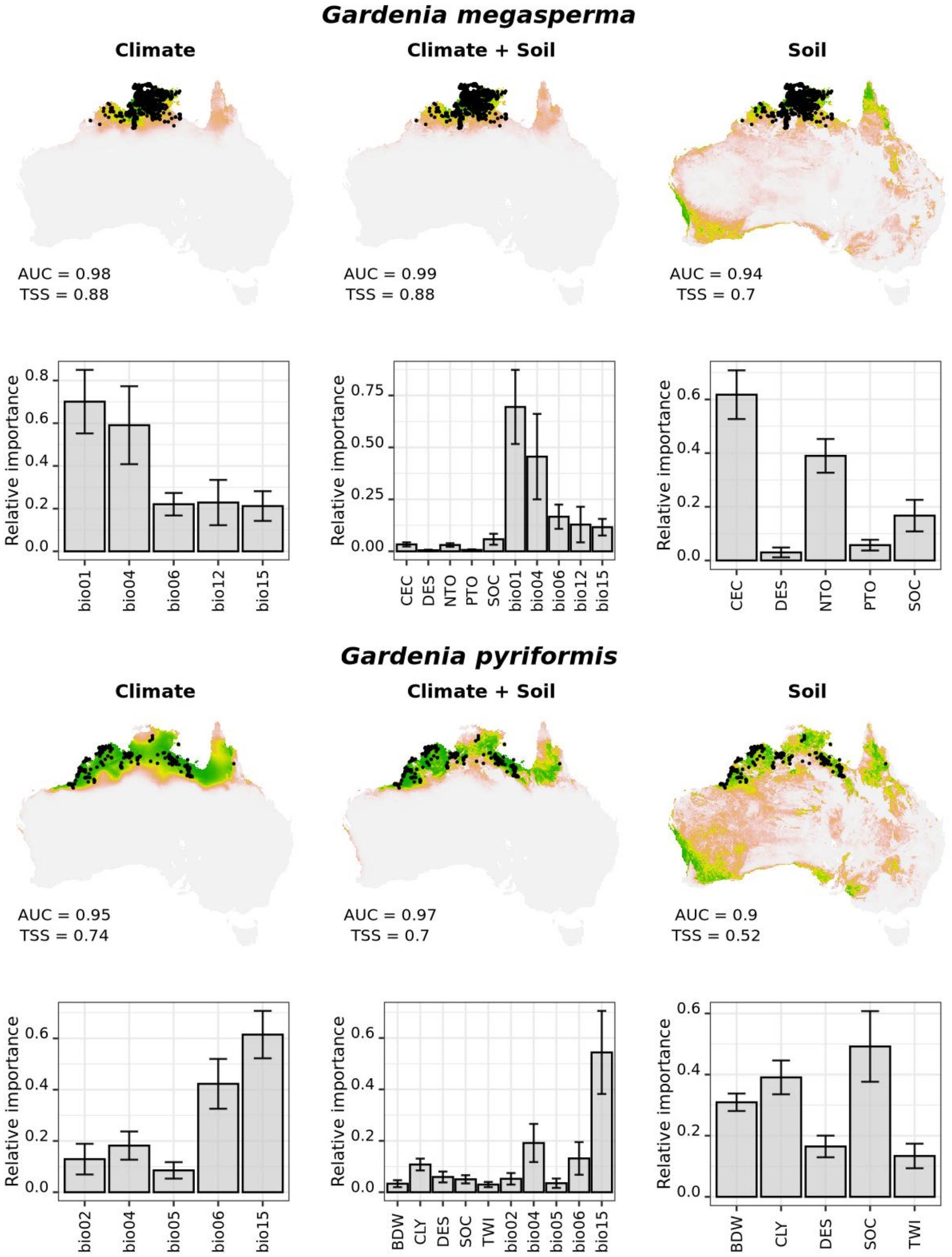
Gardenia megasperma
Climate
Climate + Soil
Soil
AUC = 0.98
TSS = 0.88
AUC = 0.99
TSS = 0.88
AUC = 0.94
TSS = 0.7
Relative importance
bio01
bio04
bio06
bio12
bio15
CEC
DES
NTO
PTO
SOC
Gardenia pyriformis
Climate
Climate + Soil
Soil
AUC = 0.95
TSS = 0.74
AUC = 0.97
TSS = 0.7
AUC = 0.9
TSS = 0.52
Relative importance
bio02
bio04
bio05
bio06
bio15
BDW
CLY
DES
SOC
TWI

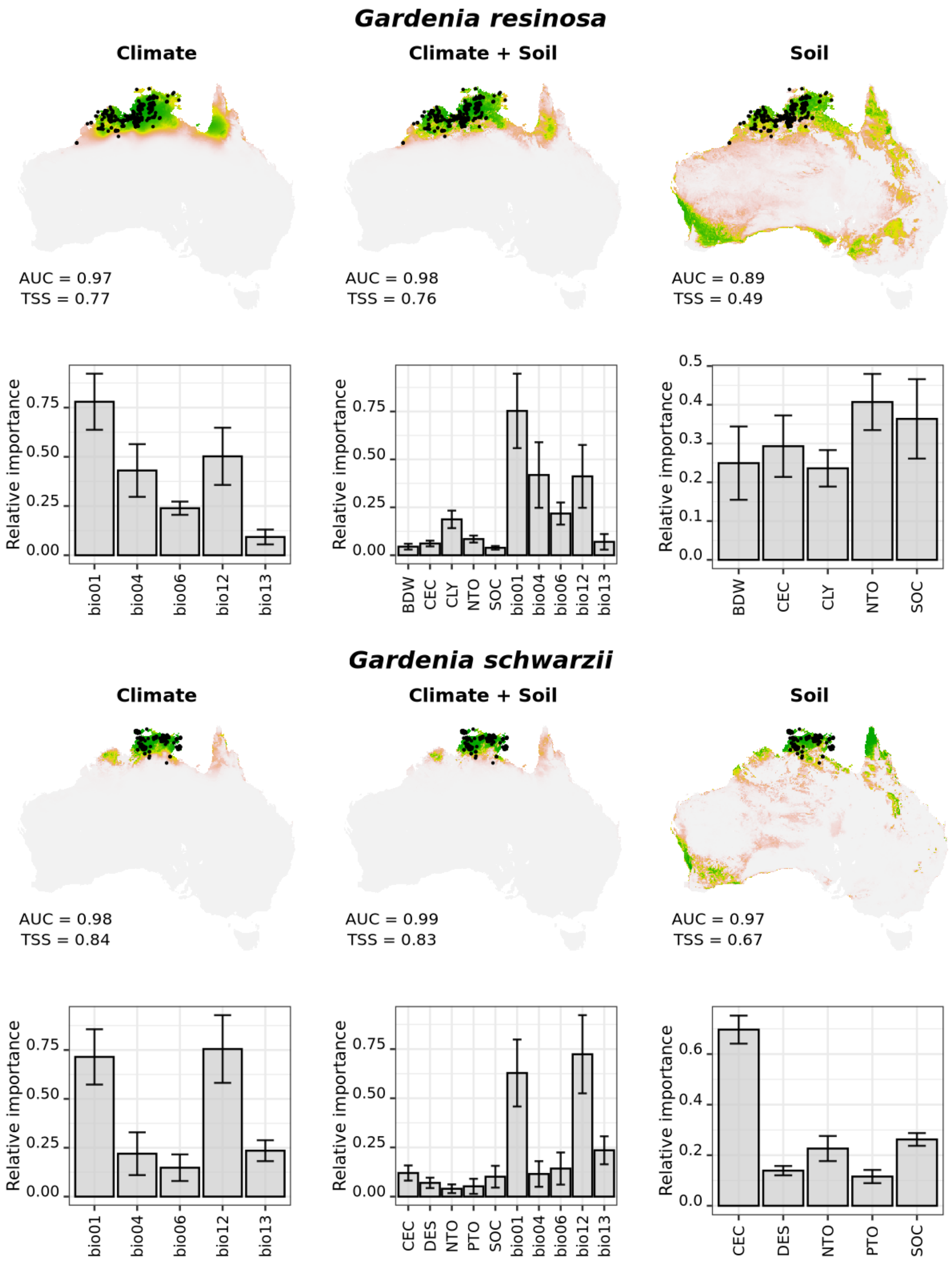

Gardenia resinosa
Climate
Climate + Soil
Soil
AUC = 0.97
TSS = 0.77
AUC = 0.98
TSS = 0.76
AUC = 0.89
TSS = 0.49
Relative importance
bio01
bio04
bio06
bio12
bio13
BDW
CEC
CLY
NTO
SOC
Gardenia schwarzii
AUC = 0.98
TSS = 0.84
AUC = 0.99
TSS = 0.83
AUC = 0.97
TSS = 0.67
DES
PTO

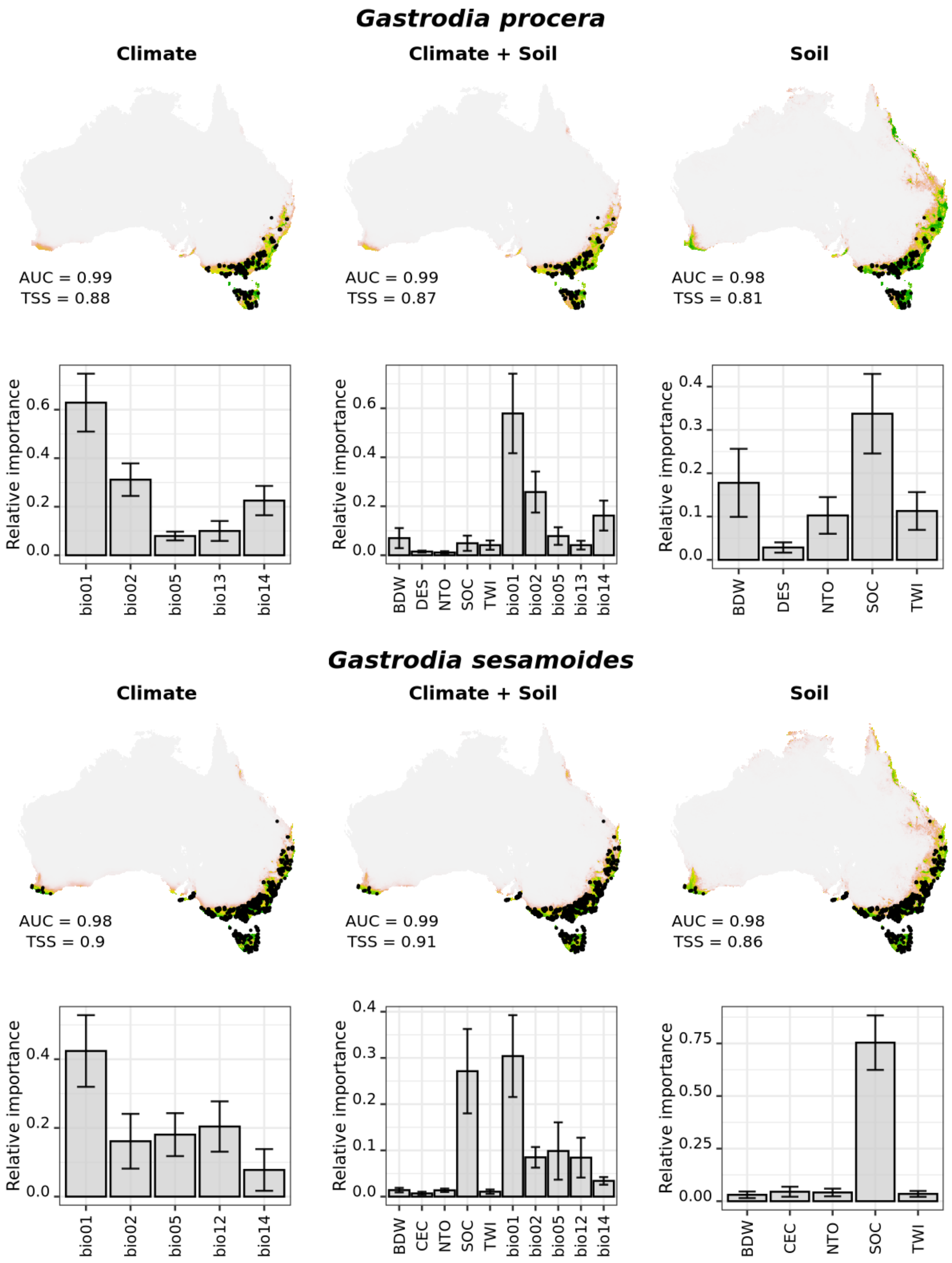
Gastrodia procera
Climate
Climate + Soil
Soil
AUC = 0.99
TSS = 0.88
AUC = 0.99
TSS = 0.87
AUC = 0.98
TSS = 0.81
Relative importance
bio01
bio02
bio05
bio13
bio14
BDW
DES
NTO
SOC
TWI
Gastrodia sesamoides
AUC = 0.98
TSS = 0.9
AUC = 0.99
TSS = 0.91
AUC = 0.98
TSS = 0.86
bio12
CEC

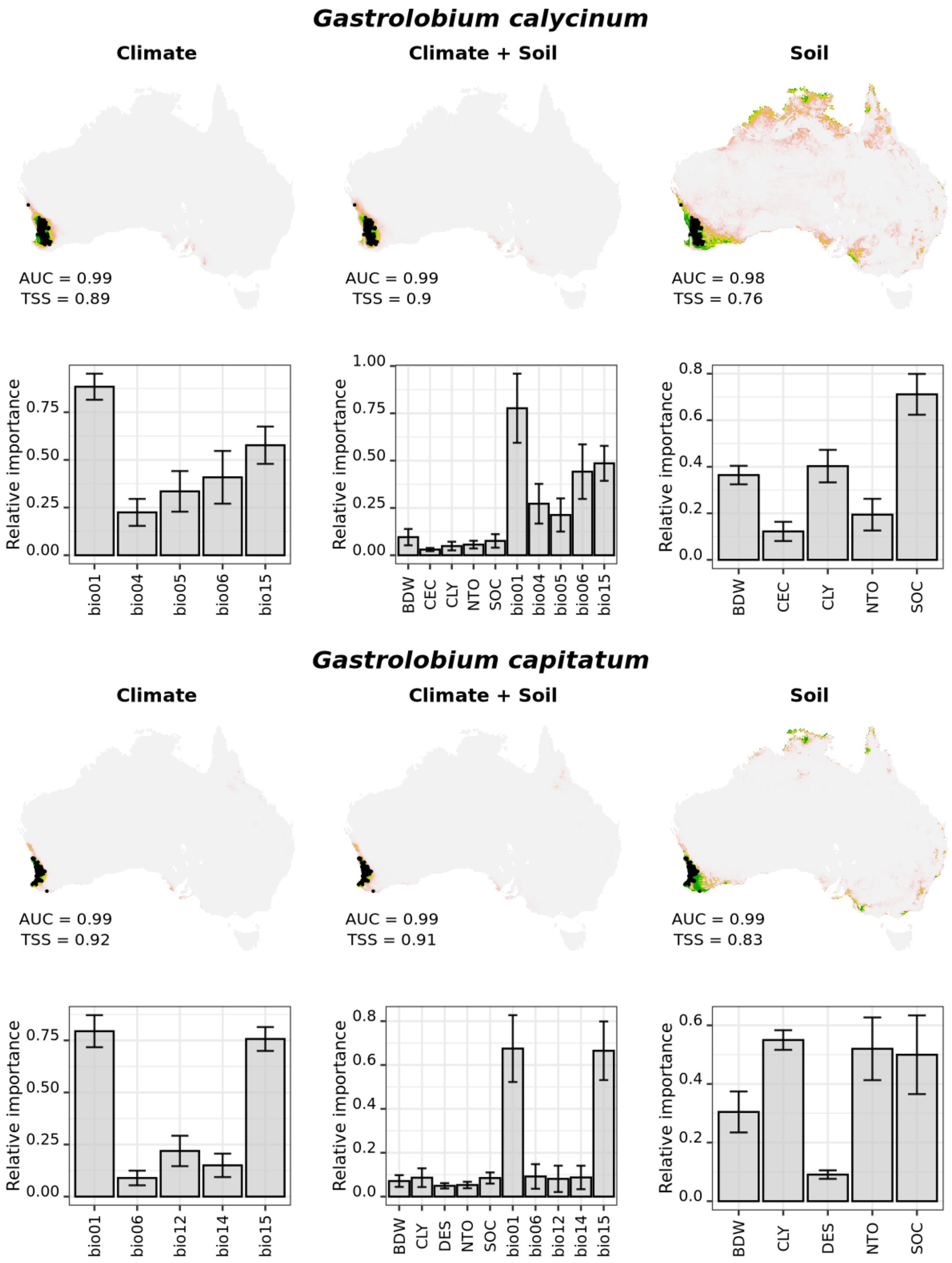

Gastrolobium calycinum
Climate
Climate + Soil
Soil
AUC = 0.99
TSS = 0.89
AUC = 0.99
TSS = 0.9
AUC = 0.98
TSS = 0.76
Relative importance
0.00
0.25
0.50
0.75
1.00
0.0
0.2
0.4
0.6
0.8
bio01
bio04
bio05
bio06
bio15
BDW
CEC
CLY
NTO
SOC
Gastrolobium capitatum
Climate
Climate + Soil
Soil
AUC = 0.99
TSS = 0.92
AUC = 0.99
TSS = 0.91
AUC = 0.99
TSS = 0.83
Relative importance
bio01
bio06
bio12
bio14
bio15
BDW
CLY
DES
NTO
SOC

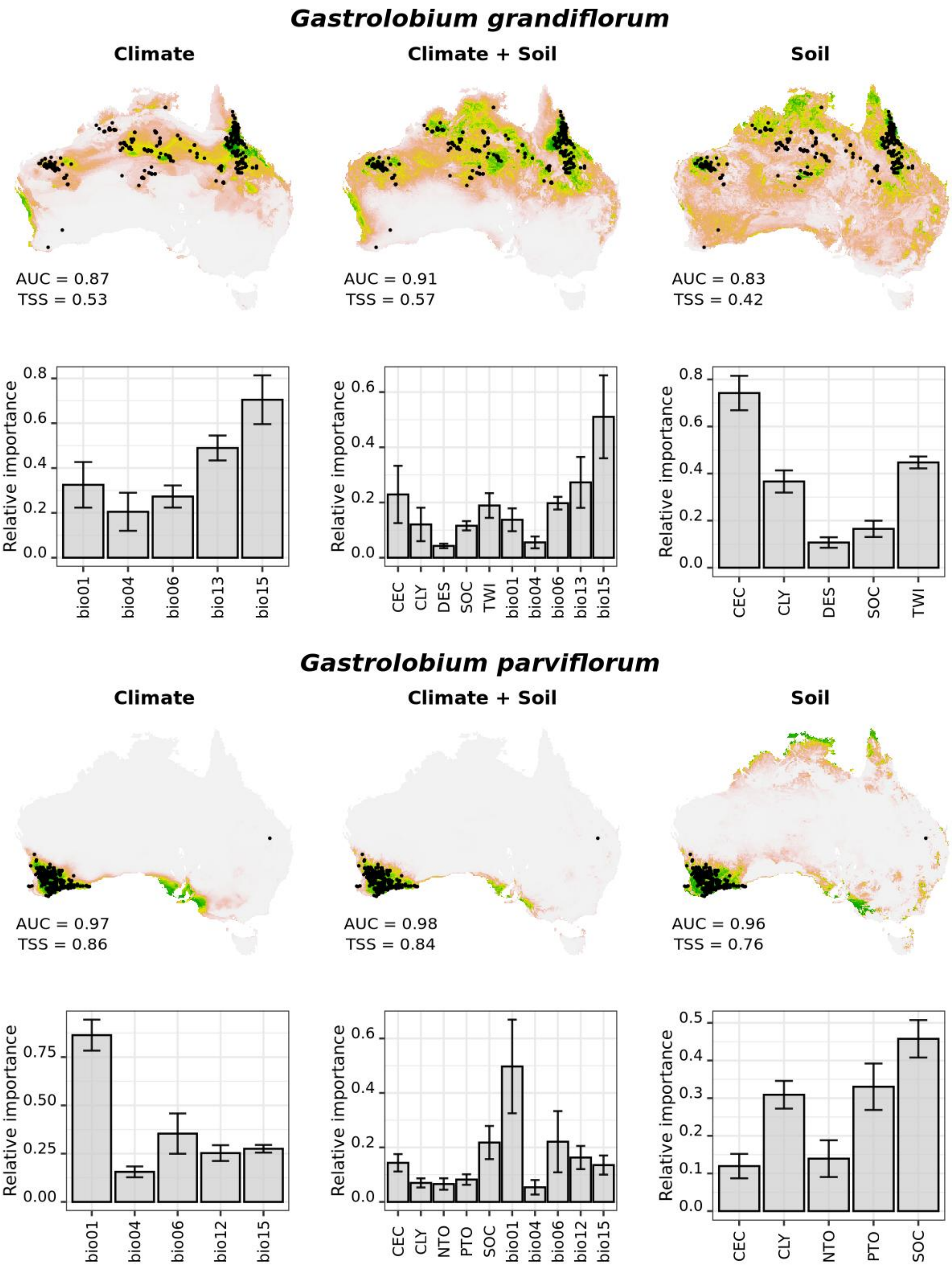
Gastrolobium grandiflorum
Climate
Climate + Soil
Soil
AUC = 0.87
TSS = 0.53
AUC = 0.91
TSS = 0.57
AUC = 0.83
TSS = 0.42
Relative importance
bio01
bio04
bio06
bio13
bio15
CEC
CLY
DES
SOC
TWI
Gastrolobium parviflorum
Climate
Climate + Soil
Soil
AUC = 0.97
TSS = 0.86
AUC = 0.98
TSS = 0.84
AUC = 0.96
TSS = 0.76
Relative importance
bio01
bio04
bio06
bio12
bio15
CEC
CLY
NTO
PTO
SOC

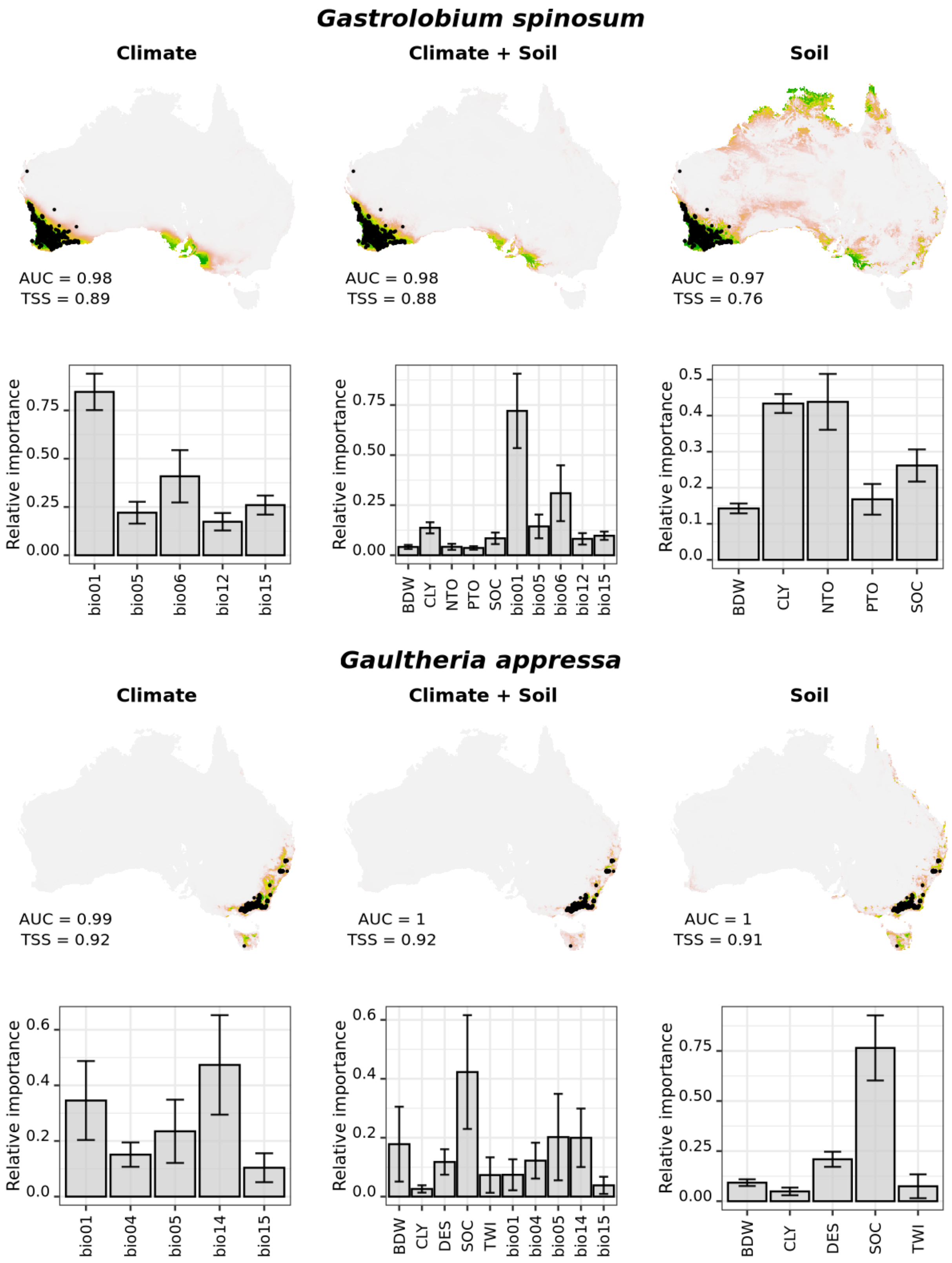
Gastrolobium spinosum
Climate
Climate + Soil
Soil
AUC = 0.98
TSS = 0.89
AUC = 0.98
TSS = 0.88
AUC = 0.97
TSS = 0.76
Relative importance
0.00 0.25 0.50 0.75
bio01 bio05 bio06 bio12 bio15
BDW CLY NTO PTO SOC bio01 bio05 bio06 bio12 bio15
0.0 0.1 0.2 0.3 0.4 0.5
BDW CLY NTO PTO SOC
Gaultheria appressa
Climate
Climate + Soil
Soil
AUC = 0.99
TSS = 0.92
AUC = 1
TSS = 0.92
AUC = 1
TSS = 0.91
Relative importance
0.0 0.2 0.4 0.6
bio01 bio04 bio05 bio14 bio15
BDW CLY DES SOC TWI bio01 bio04 bio05 bio14 bio15
0.00 0.25 0.50 0.75
BDW CLY DES SOC TWI

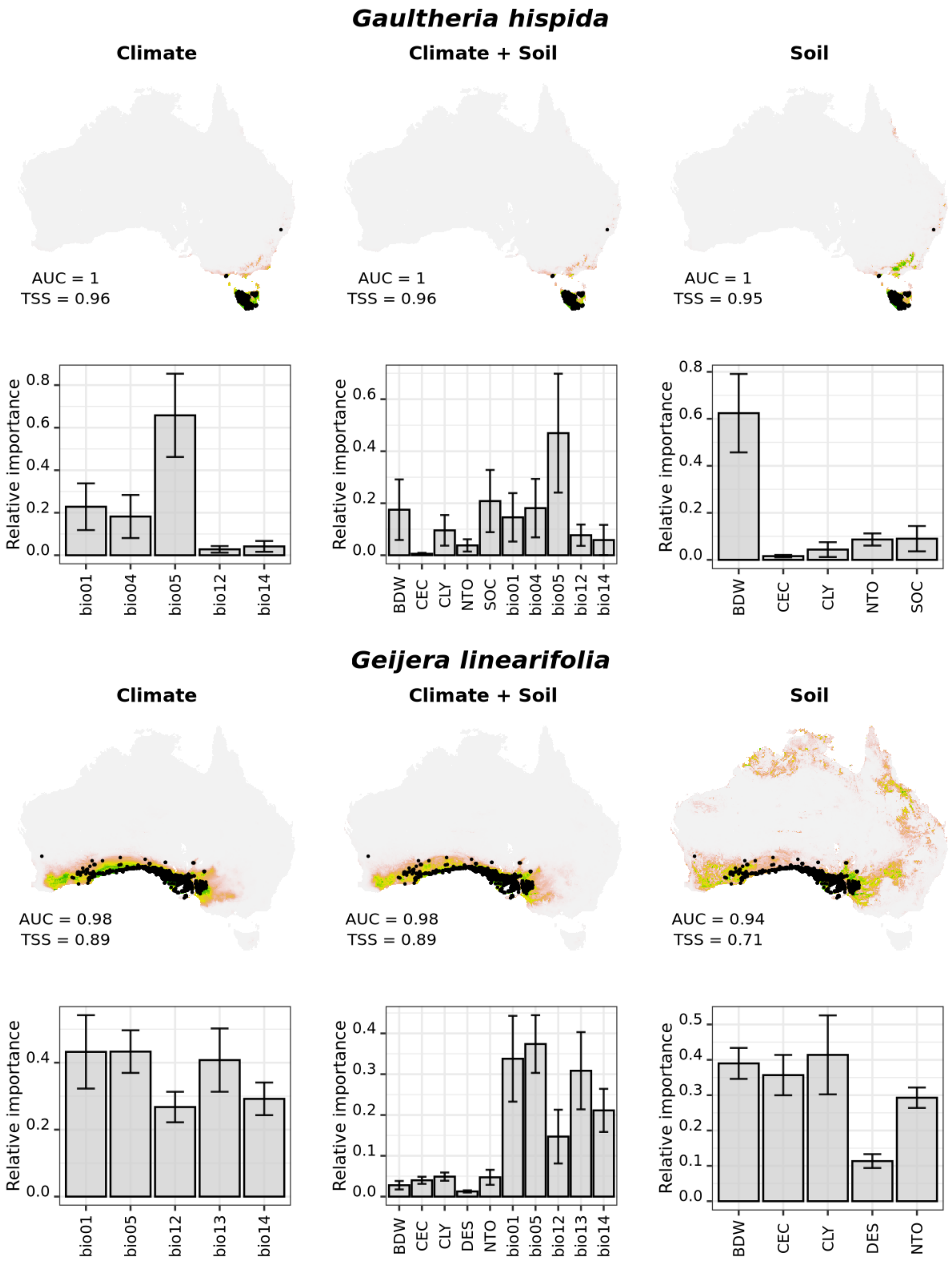

Gaultheria hispida
Climate
Climate + Soil
Soil
AUC = 1
TSS = 0.96
AUC = 1
TSS = 0.96
AUC = 1
TSS = 0.95
Relative importance
bio01
bio04
bio05
bio12
bio14
BDW
CEC
CLY
NTO
SOC
Geijera linearifolia
Climate
Climate + Soil
Soil
AUC = 0.98
TSS = 0.89
AUC = 0.98
TSS = 0.89
AUC = 0.94
TSS = 0.71
Relative importance
bio01
bio05
bio12
bio13
bio14
BDW
CEC
CLY
DES
NTO

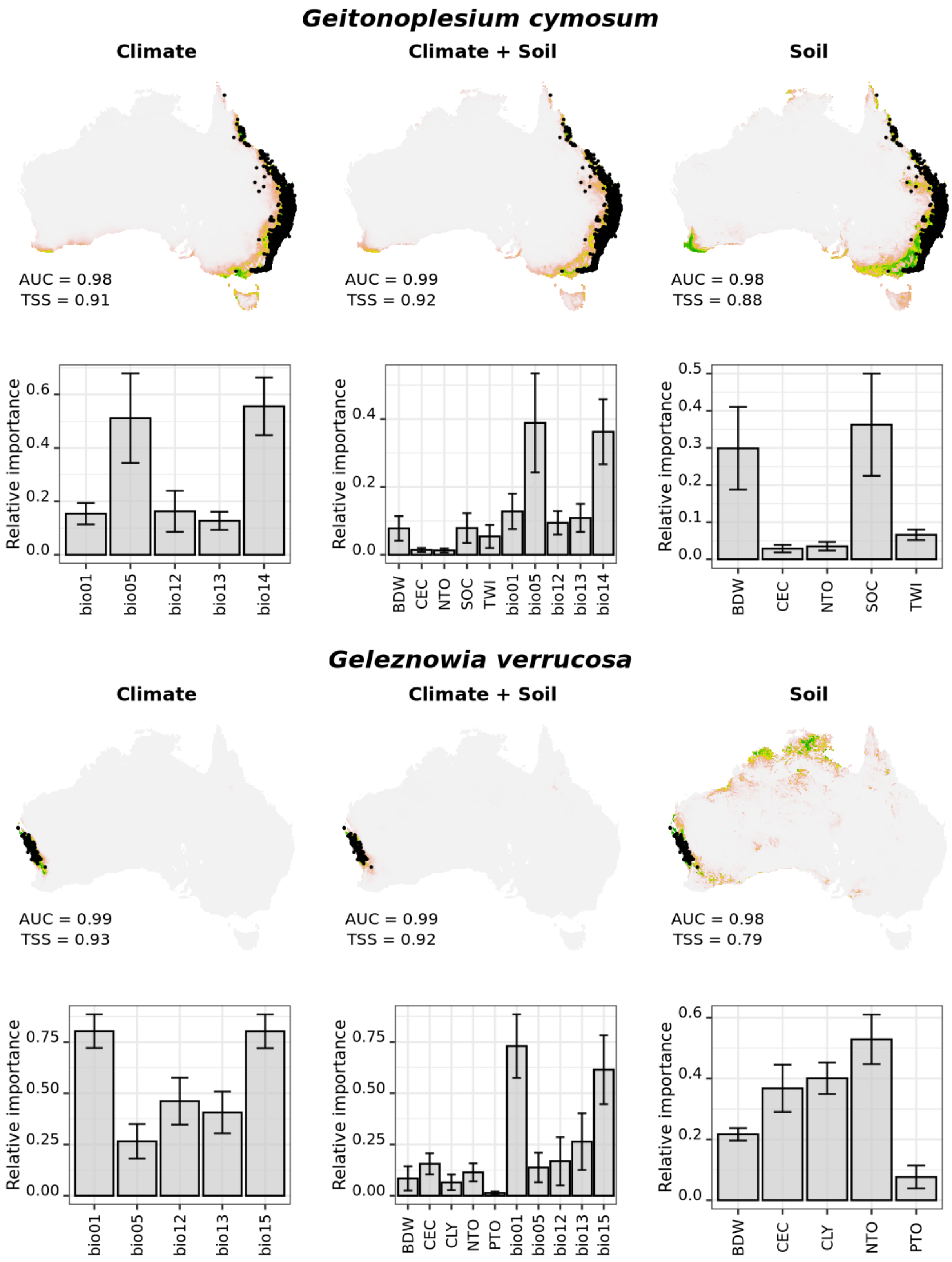
Geitonoplesium cymosum
Climate
Climate + Soil
Soil
AUC = 0.98
TSS = 0.91
AUC = 0.99
TSS = 0.92
AUC = 0.98
TSS = 0.88
Relative importance
bio01
bio05
bio12
bio13
bio14
BDW
CEC
NTO
SOC
TWI
Geleznowia verrucosa
Climate
Climate + Soil
Soil
AUC = 0.99
TSS = 0.93
AUC = 0.99
TSS = 0.92
AUC = 0.98
TSS = 0.79
Relative importance
bio01
bio05
bio12
bio13
bio15
BDW
CEC
CLY
NTO
PTO

Gentianella diemensis

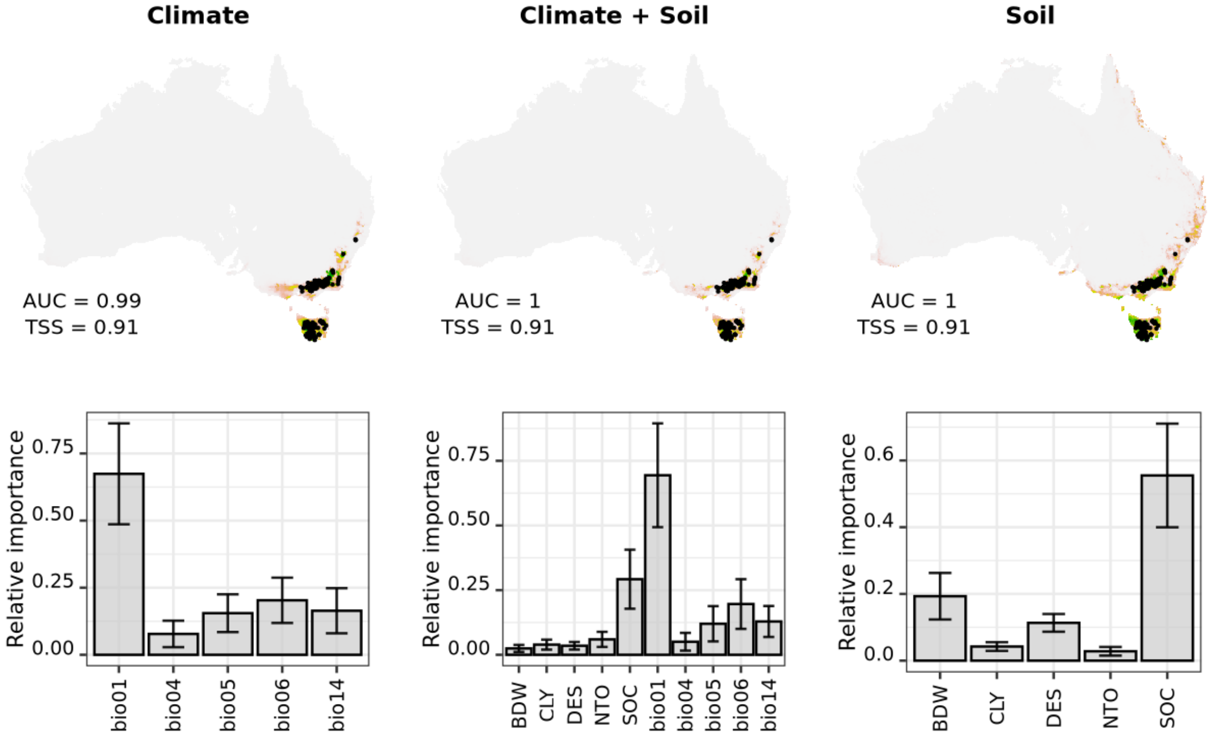

Geodorum densiflorum

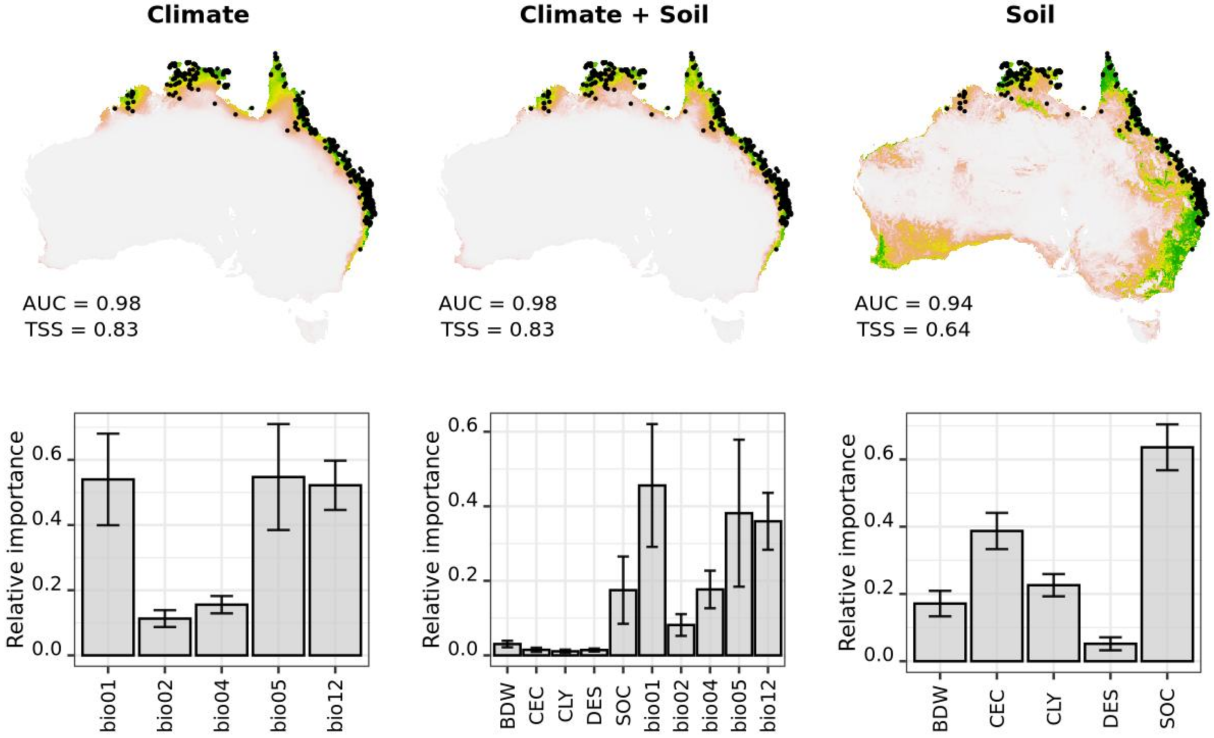

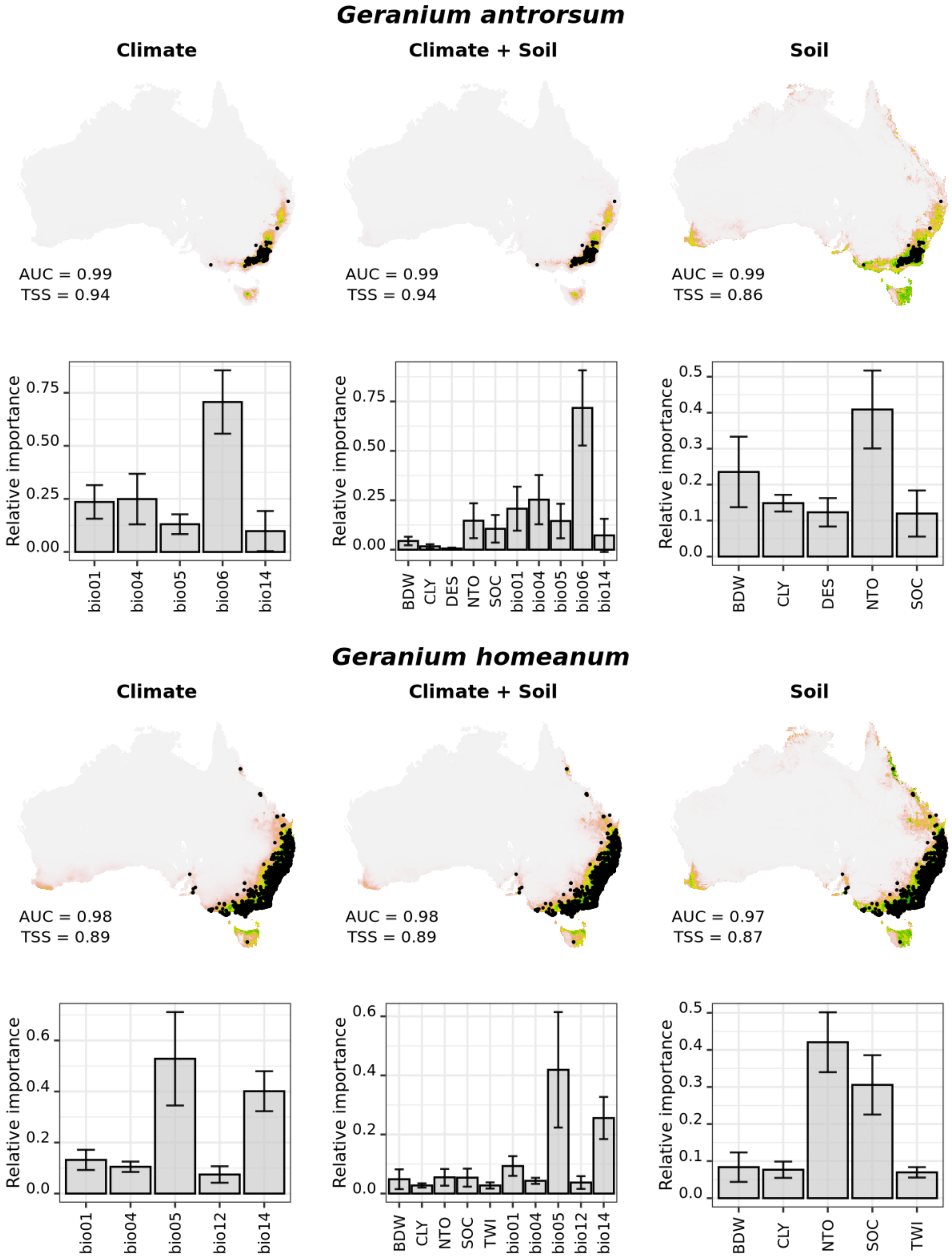
Geranium antrorsum
Climate
Climate + Soil
Soil
AUC = 0.99
TSS = 0.94
AUC = 0.99
TSS = 0.94
AUC = 0.99
TSS = 0.86
Relative importance
0.00
0.25
0.50
0.75
bio01
bio04
bio05
bio06
bio14
BDW
CLY
DES
NTO
SOC
0.0
0.1
0.2
0.3
0.4
0.5
Geranium homeanum
Climate
Climate + Soil
Soil
AUC = 0.98
TSS = 0.89
AUC = 0.98
TSS = 0.89
AUC = 0.97
TSS = 0.87
Relative importance
0.0
0.2
0.4
0.6
bio01
bio04
bio05
bio12
bio14
BDW
CLY
NTO
SOC
TWI

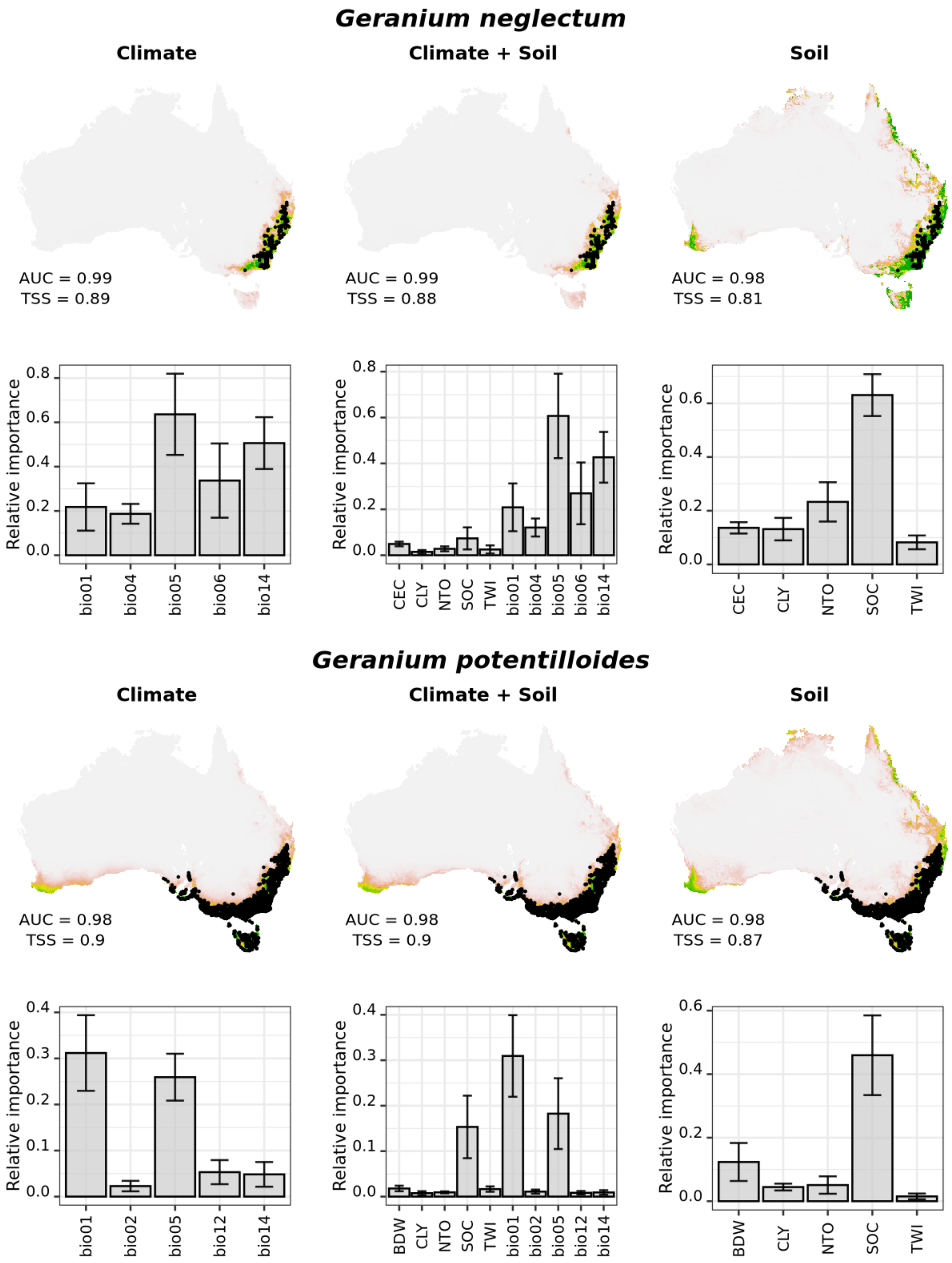

Geranium neglectum
Climate
Climate + Soil
Soil
AUC = 0.99
TSS = 0.89
AUC = 0.99
TSS = 0.88
AUC = 0.98
TSS = 0.81
Relative importance
0.0
0.2
0.4
0.6
0.8
bio01
bio04
bio05
bio06
bio14
CEC
CLY
NTO
SOC
TWI
Geranium potentilloides
AUC = 0.98
TSS = 0.9
AUC = 0.98
TSS = 0.9
AUC = 0.98
TSS = 0.87
0.1
0.3
bio02
bio12
BDW

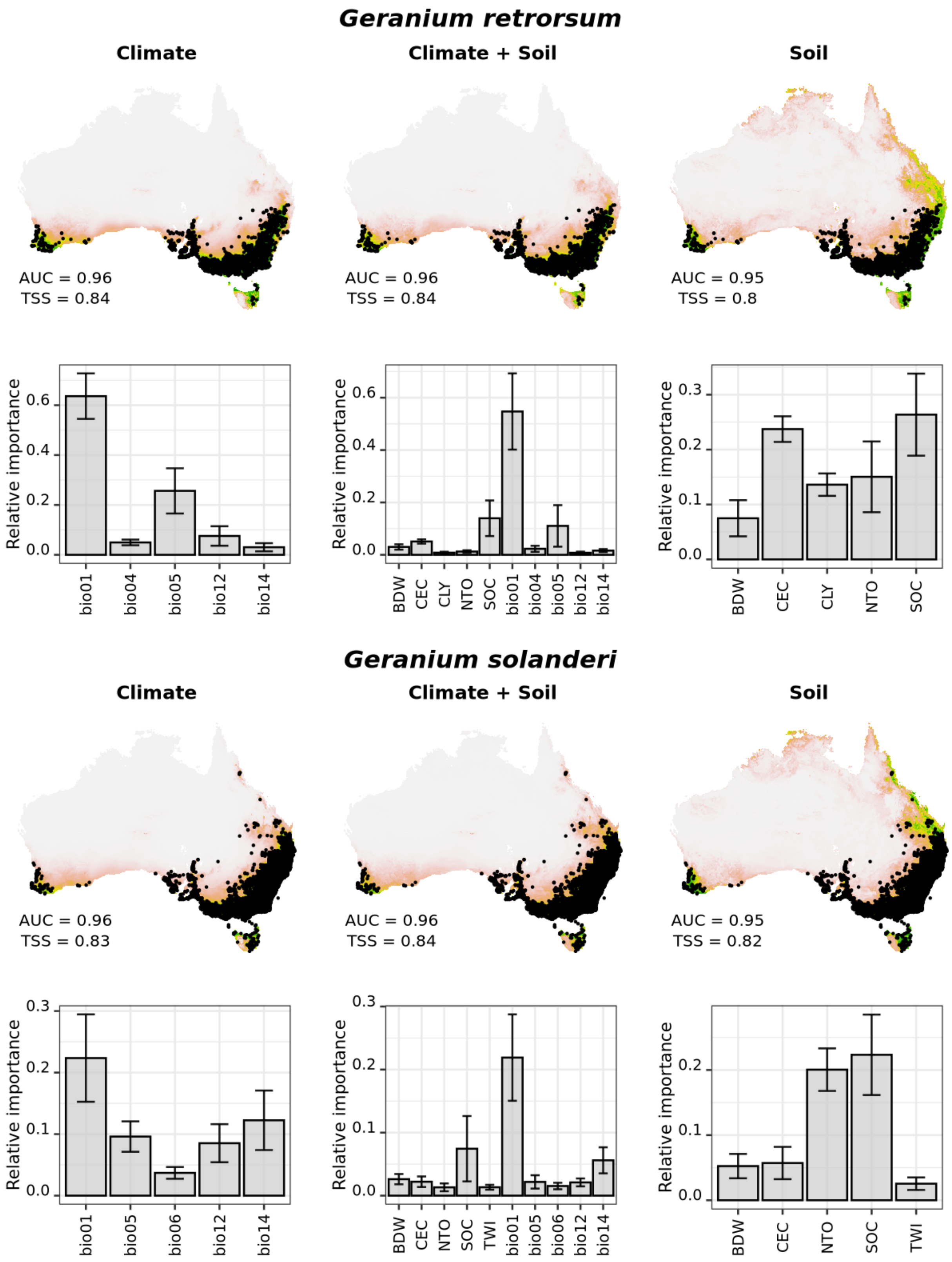

Geranium retrorsum
Climate
Climate + Soil
Soil
AUC = 0.96
TSS = 0.84
AUC = 0.96
TSS = 0.84
AUC = 0.95
TSS = 0.8
Relative importance
0.0
0.2
0.4
0.6
bio01
bio04
bio05
bio12
bio14
Relative importance
0.0
0.2
0.4
0.6
BDW
CEC
CLY
NTO
SOC
bio01
bio04
bio05
bio12
bio14
Relative importance
0.0
0.1
0.2
0.3
BDW
CEC
CLY
NTO
SOC
Geranium solanderi
Climate
Climate + Soil
Soil
AUC = 0.96
TSS = 0.83
AUC = 0.96
TSS = 0.84
AUC = 0.95
TSS = 0.82
Relative importance
0.0
0.1
0.2
0.3
bio01
bio05
bio06
bio12
bio14
Relative importance
0.0
0.1
0.2
0.3
BDW
CEC
NTO
SOC
TWI
bio01
bio05
bio06
bio12
bio14
Relative importance
0.0
0.1
0.2
BDW
CEC
NTO
SOC
TWI

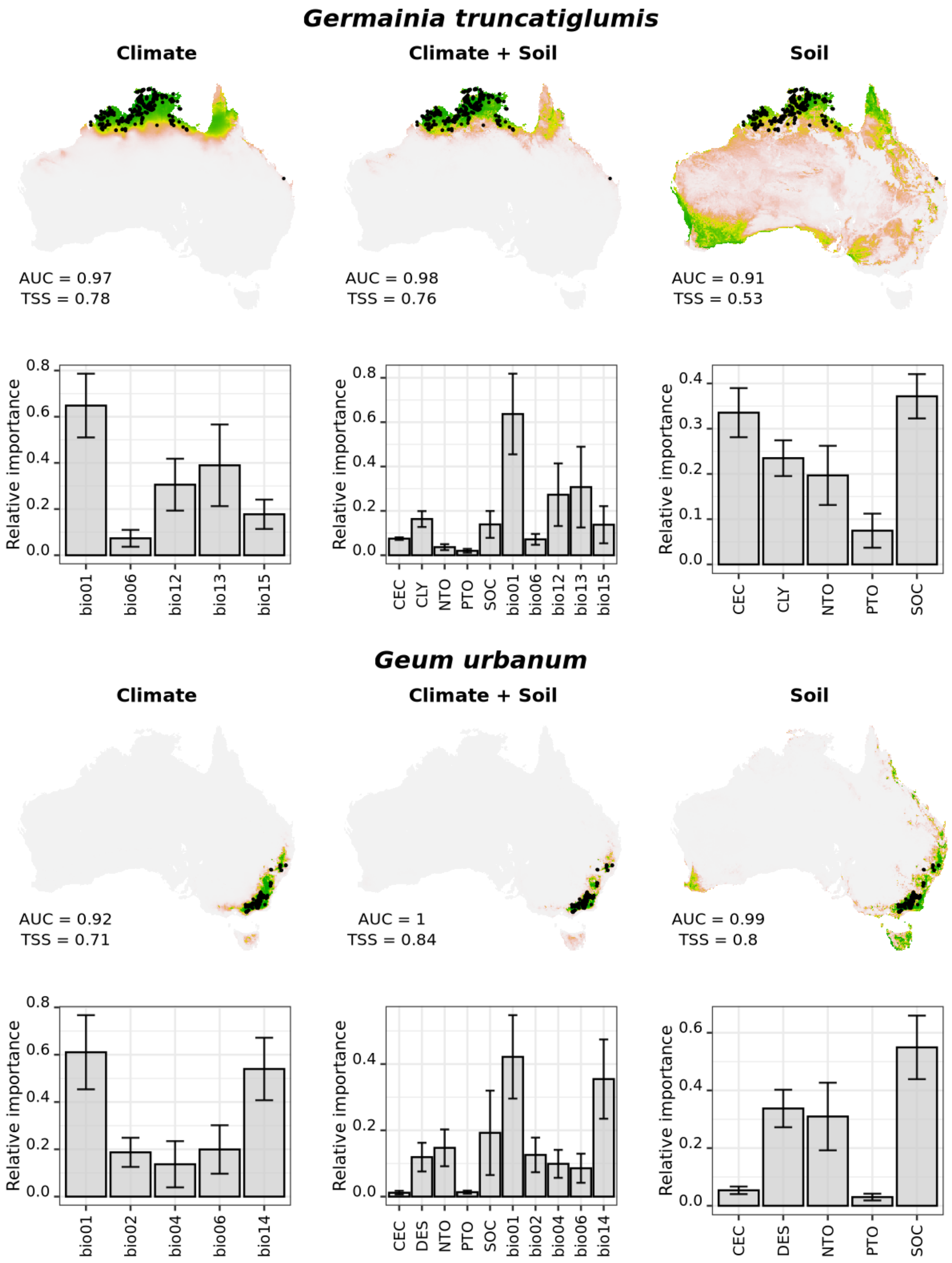
Germainia truncatiglumis
Climate
Climate + Soil
Soil
AUC = 0.97
TSS = 0.78
AUC = 0.98
TSS = 0.76
AUC = 0.91
TSS = 0.53
Relative importance
0.0
0.2
0.4
0.6
0.8
bio01
bio06
bio12
bio13
bio15
Relative importance
0.0
0.2
0.4
0.6
0.8
CEC
CLY
NTO
PTO
SOC
bio01
bio06
bio12
bio13
bio15
Relative importance
0.0
0.1
0.2
0.3
0.4
CEC
CLY
NTO
PTO
SOC
Geum urbanum
Climate
Climate + Soil
Soil
AUC = 0.92
TSS = 0.71
AUC = 1
TSS = 0.84
AUC = 0.99
TSS = 0.8
Relative importance
0.0
0.2
0.4
0.6
0.8
bio01
bio02
bio04
bio06
bio14
Relative importance
0.0
0.2
0.4
CEC
DES
NTO
PTO
SOC
bio01
bio02
bio04
bio06
bio14
Relative importance
0.0
0.2
0.4
0.6
CEC
DES
NTO
PTO
SOC

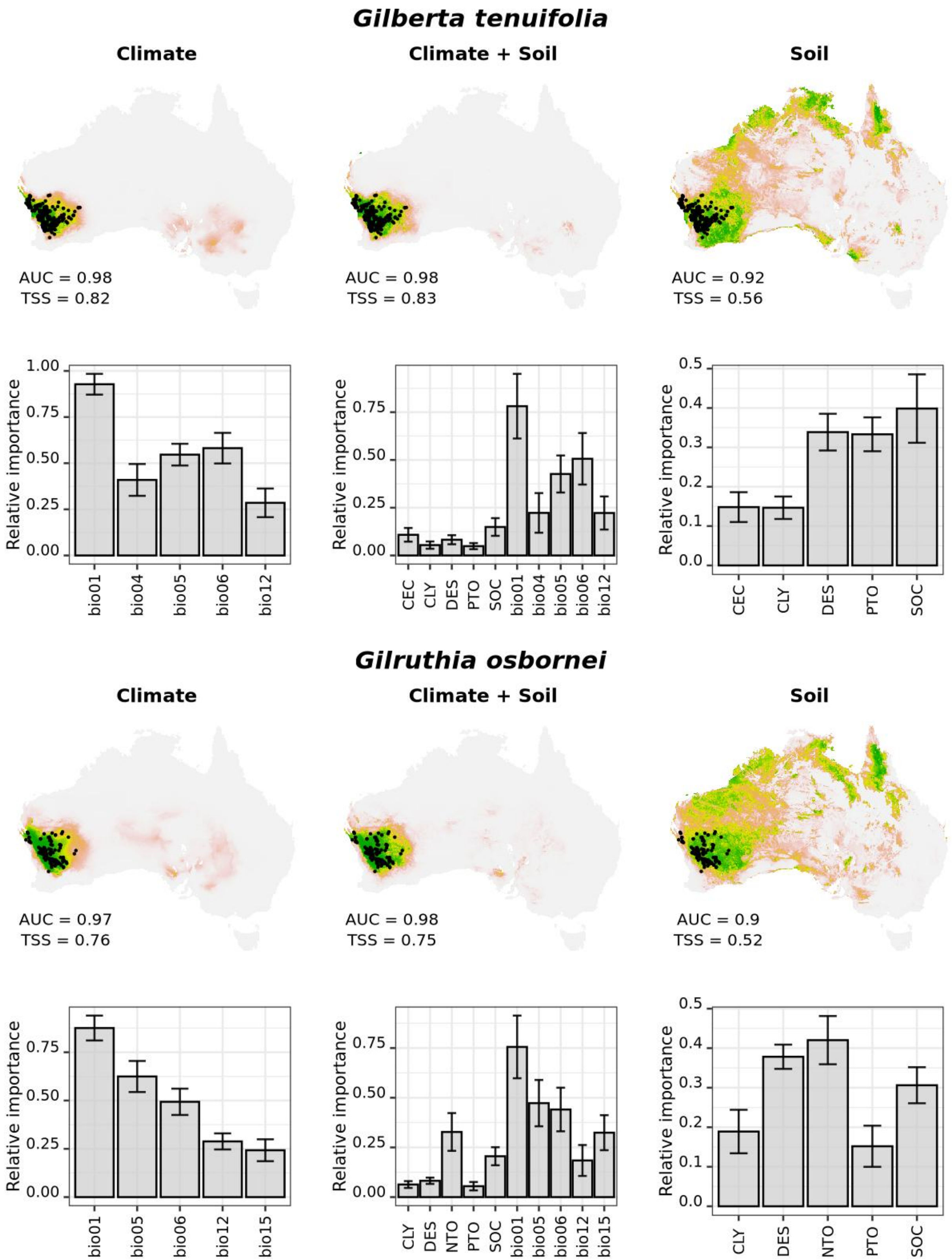
Gilberta tenuifolia
Climate
Climate + Soil
Soil
AUC = 0.98
TSS = 0.82
AUC = 0.98
TSS = 0.83
AUC = 0.92
TSS = 0.56
Relative importance
1.00
0.75
0.50
0.25
0.00
bio01
bio04
bio05
bio06
bio12
Relative importance
0.75
0.50
0.25
0.00
CEC
CLY
DES
PTO
SOC
bio01
bio04
bio05
bio06
bio12
Relative importance
0.5
0.4
0.3
0.2
0.1
0.0
CEC
CLY
DES
PTO
SOC
Gilruthia osbornei
Climate
Climate + Soil
Soil
AUC = 0.97
TSS = 0.76
AUC = 0.98
TSS = 0.75
AUC = 0.9
TSS = 0.52
Relative importance
0.75
0.50
0.25
0.00
bio01
bio05
bio06
bio12
bio15
Relative importance
0.75
0.50
0.25
0.00
CLY
DES
NTO
PTO
SOC
bio01
bio05
bio06
bio12
bio15
Relative importance
0.5
0.4
0.3
0.2
0.1
0.0
CLY
DES
NTO
PTO
SOC

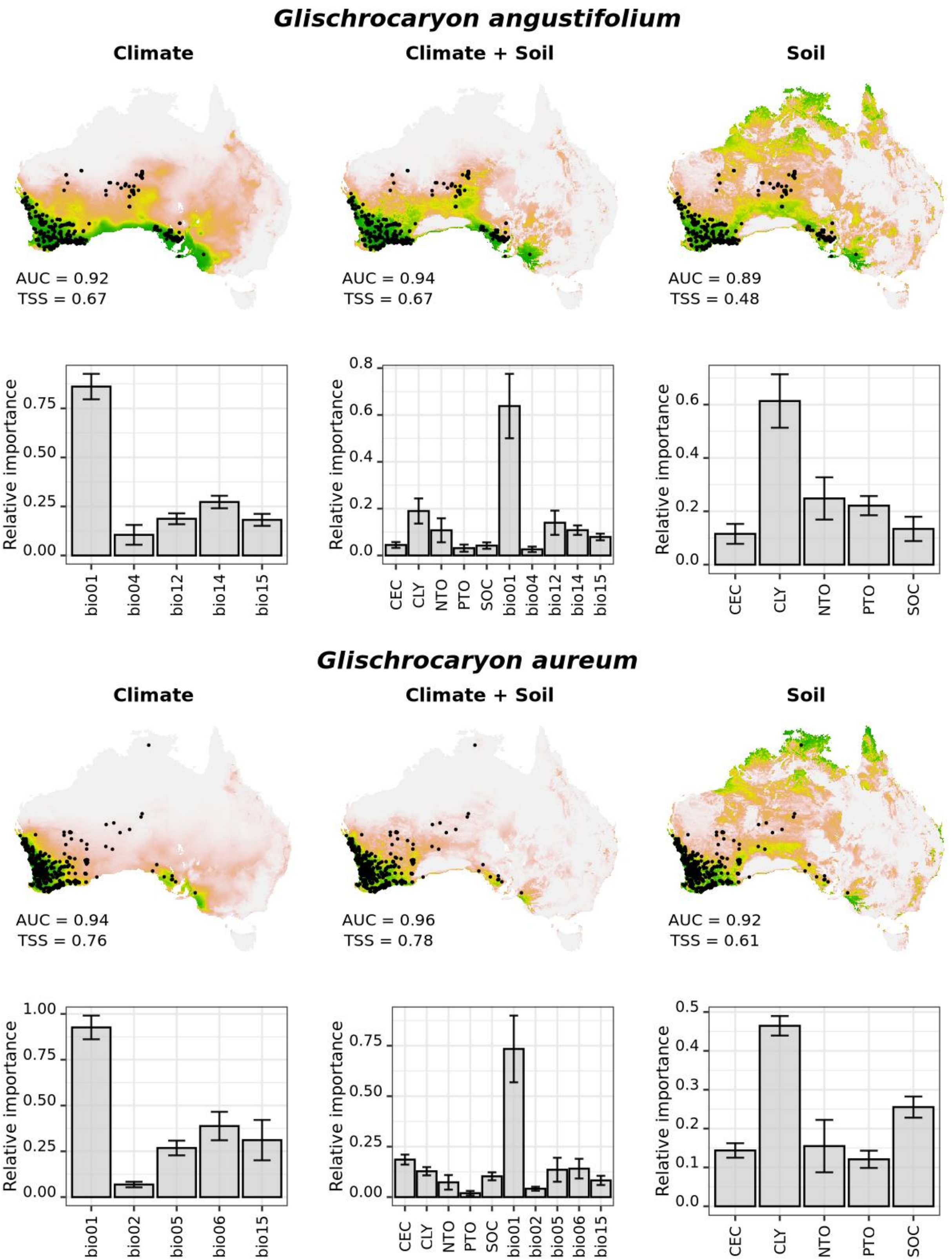

Glischrocaryon angustifolium
Climate
Climate + Soil
Soil
AUC = 0.92
TSS = 0.67
AUC = 0.94
TSS = 0.67
AUC = 0.89
TSS = 0.48
Relative importance
0.00
0.25
0.50
0.75
bio01
bio04
bio12
bio14
bio15
0.0
0.2
0.4
0.6
0.8
CEC
CLY
NTO
PTO
SOC
bio01
bio04
bio12
bio14
bio15
0.0
0.2
0.4
0.6
CEC
CLY
NTO
PTO
SOC
Glischrocaryon aureum
Climate
Climate + Soil
Soil
AUC = 0.94
TSS = 0.76
AUC = 0.96
TSS = 0.78
AUC = 0.92
TSS = 0.61
Relative importance
0.00
0.25
0.50
0.75
1.00
bio01
bio02
bio05
bio06
bio15
0.00
0.25
0.50
0.75
CEC
CLY
NTO
PTO
SOC
bio01
bio02
bio05
bio06
bio15
0.0
0.1
0.2
0.3
0.4
0.5
CEC
CLY
NTO
PTO
SOC

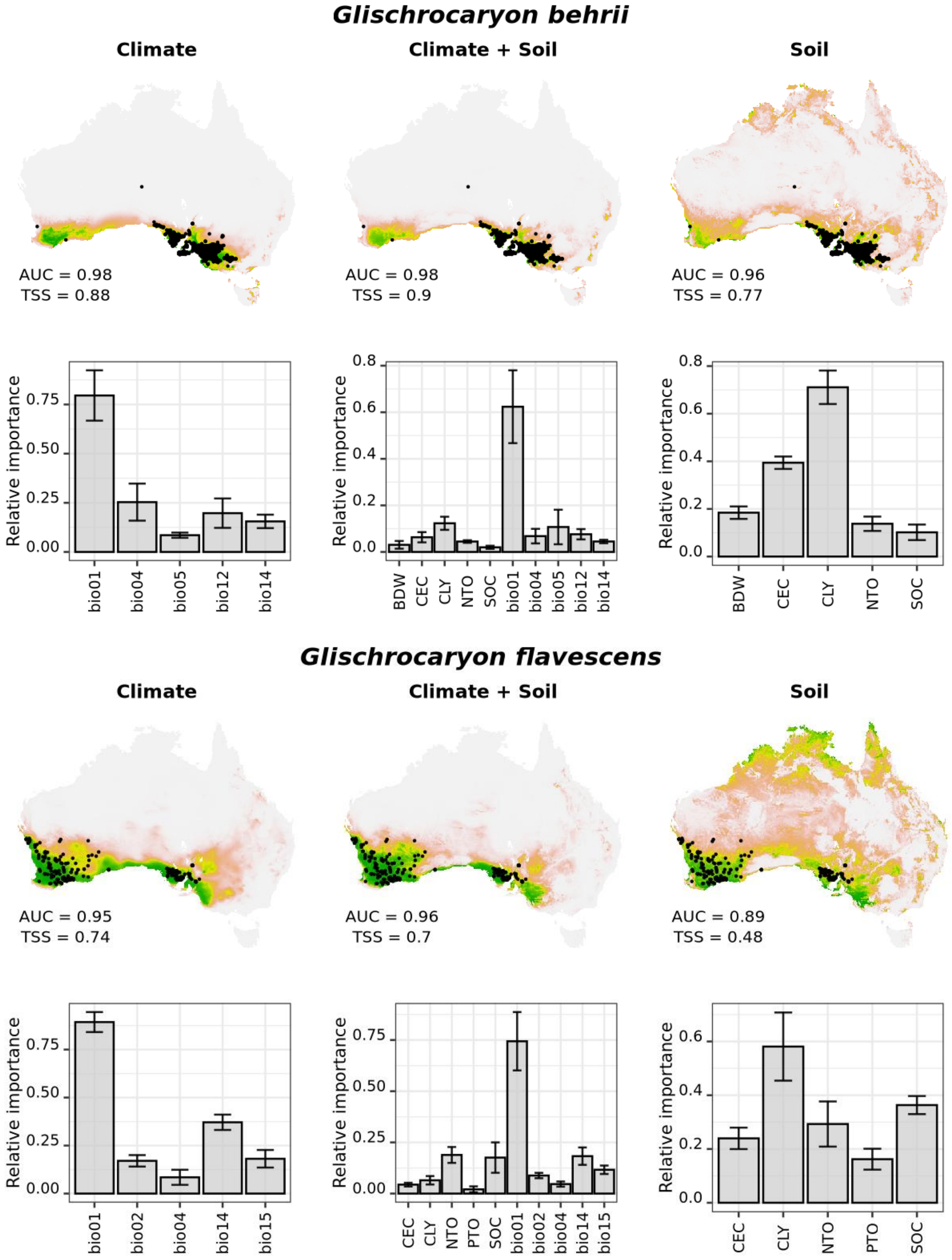
Glischrocaryon behrii
Climate
Climate + Soil
Soil
AUC = 0.98
TSS = 0.88
AUC = 0.98
TSS = 0.9
AUC = 0.96
TSS = 0.77
Relative importance
0.00
0.25
0.50
0.75
bio01
bio04
bio05
bio12
bio14
0.0
0.2
0.4
0.6
0.8
BDW
CEC
CLY
NTO
SOC
bio01
bio04
bio05
bio12
bio14
BDW
CEC
CLY
NTO
SOC
Glischrocaryon flavescens
Climate
Climate + Soil
Soil
AUC = 0.95
TSS = 0.74
AUC = 0.96
TSS = 0.7
AUC = 0.89
TSS = 0.48
Relative importance
bio01
bio02
bio04
bio14
bio15
CEC
CLY
NTO
PTO
SOC
bio01
bio02
bio04
bio14
bio15
CEC
CLY
NTO
PTO
SOC

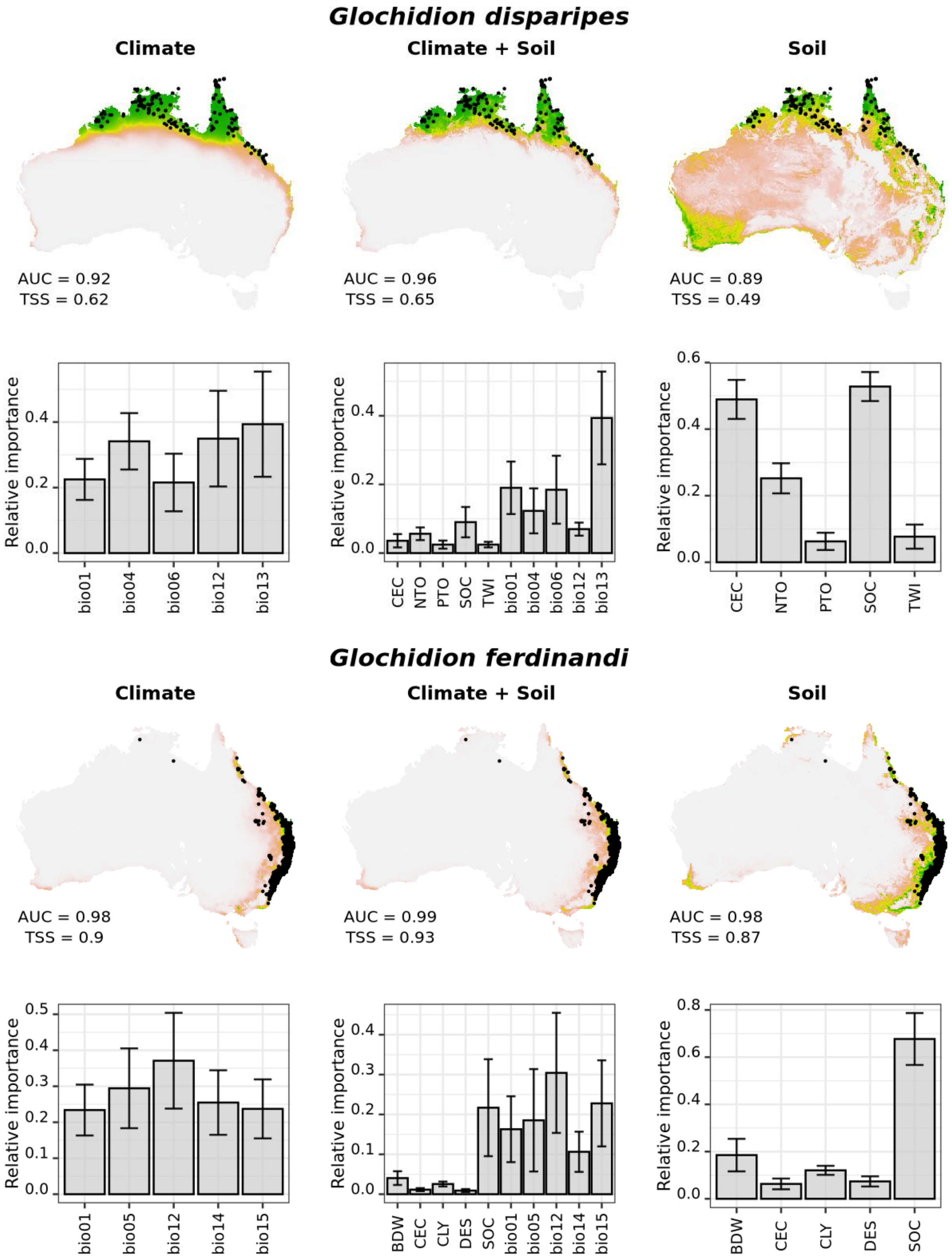
Glochidion disparipes
Climate
Climate + Soil
Soil
AUC = 0.92
TSS = 0.62
AUC = 0.96
TSS = 0.65
AUC = 0.89
TSS = 0.49
Relative importance
0.0
0.2
0.4
0.6
bio01
bio04
bio06
bio12
bio13
CEC
NTO
PTO
SOC
TWI
Glochidion ferdinandi
Climate
Climate + Soil
Soil
AUC = 0.98
TSS = 0.9
AUC = 0.99
TSS = 0.93
AUC = 0.98
TSS = 0.87
Relative importance
0.0
0.1
0.2
0.3
0.4
0.5
0.6
0.8
bio01
bio05
bio12
bio14
bio15
BDW
CEC
CLY
DES
SOC

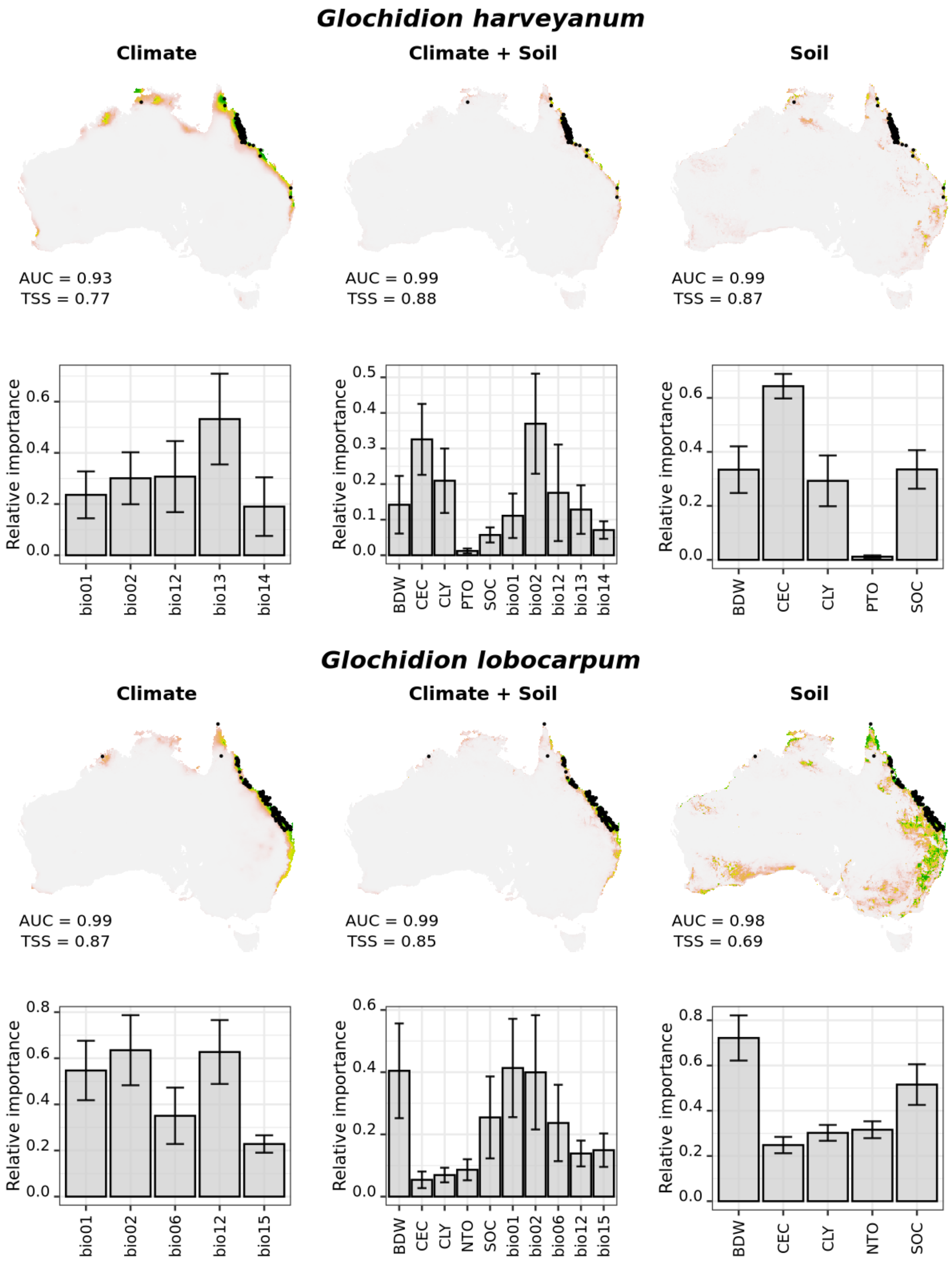
Glochidion harveyanum
Climate
Climate + Soil
Soil
AUC = 0.93
TSS = 0.77
AUC = 0.99
TSS = 0.88
AUC = 0.99
TSS = 0.87
Relative importance
0.0
0.2
0.4
0.6
bio01
bio02
bio12
bio13
bio14
0.0
0.1
0.2
0.3
0.4
0.5
BDW
CEC
CLY
PTO
SOC
bio01
bio02
bio12
bio13
bio14
BDW
CEC
CLY
PTO
SOC
Glochidion lobocarpum
Climate
Climate + Soil
Soil
AUC = 0.99
TSS = 0.87
AUC = 0.99
TSS = 0.85
AUC = 0.98
TSS = 0.69
0.8
bio01
bio02
bio06
bio12
bio15
BDW
CEC
CLY
NTO
SOC
bio01
bio02
bio06
bio12
bio15
BDW
CEC
CLY
NTO
SOC

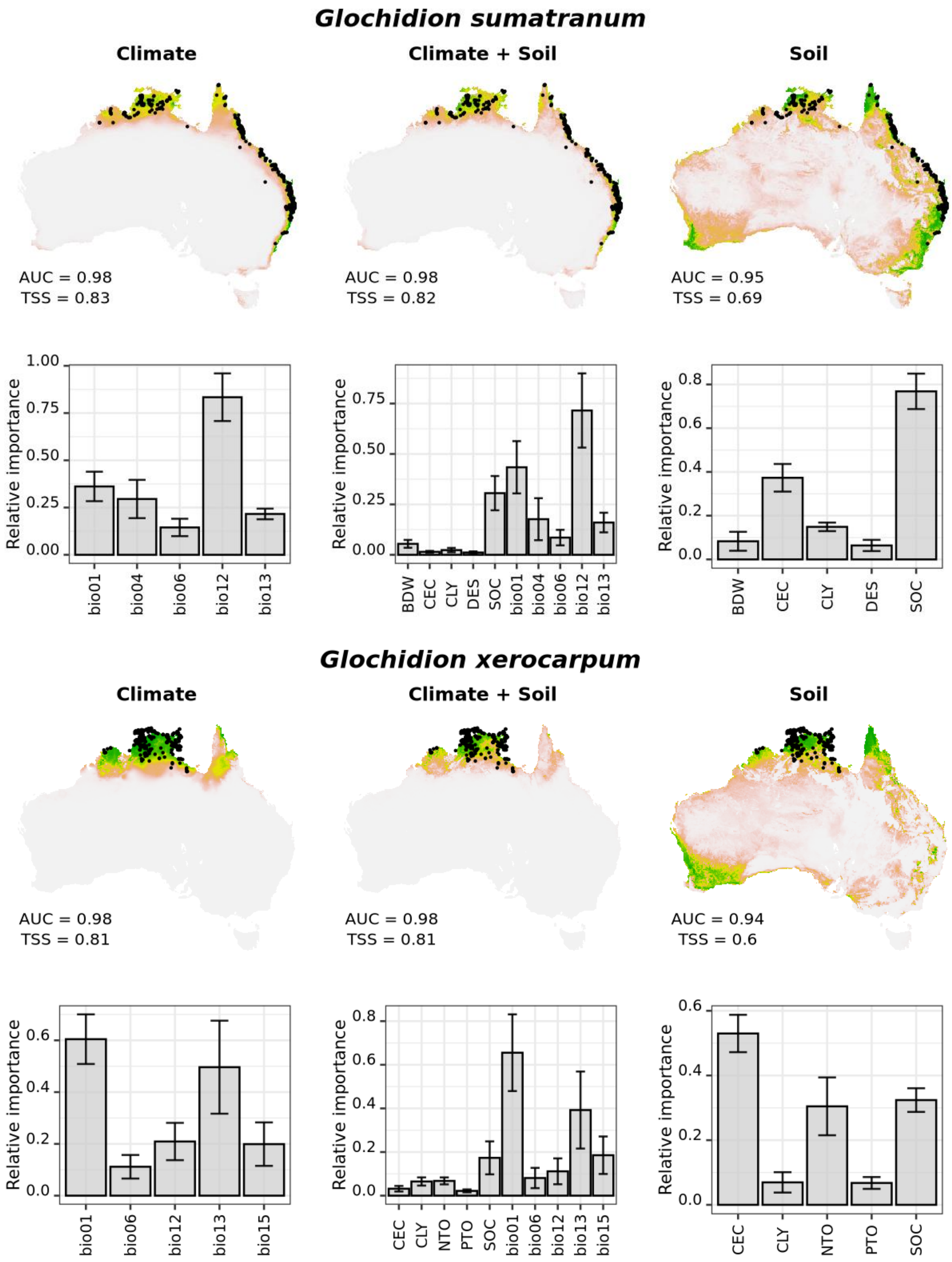
Glochidion sumatranum
Climate
Climate + Soil
Soil
AUC = 0.98
TSS = 0.83
AUC = 0.98
TSS = 0.82
AUC = 0.95
TSS = 0.69
Relative importance
bio01
bio04
bio06
bio12
bio13
BDW
CEC
CLY
DES
SOC
Glochidion xerocarpum
Climate
Climate + Soil
Soil
AUC = 0.98
TSS = 0.81
AUC = 0.98
TSS = 0.81
AUC = 0.94
TSS = 0.6
Relative importance
bio01
bio06
bio12
bio13
bio15
CEC
CLY
NTO
PTO
SOC

Glossocardia bidens

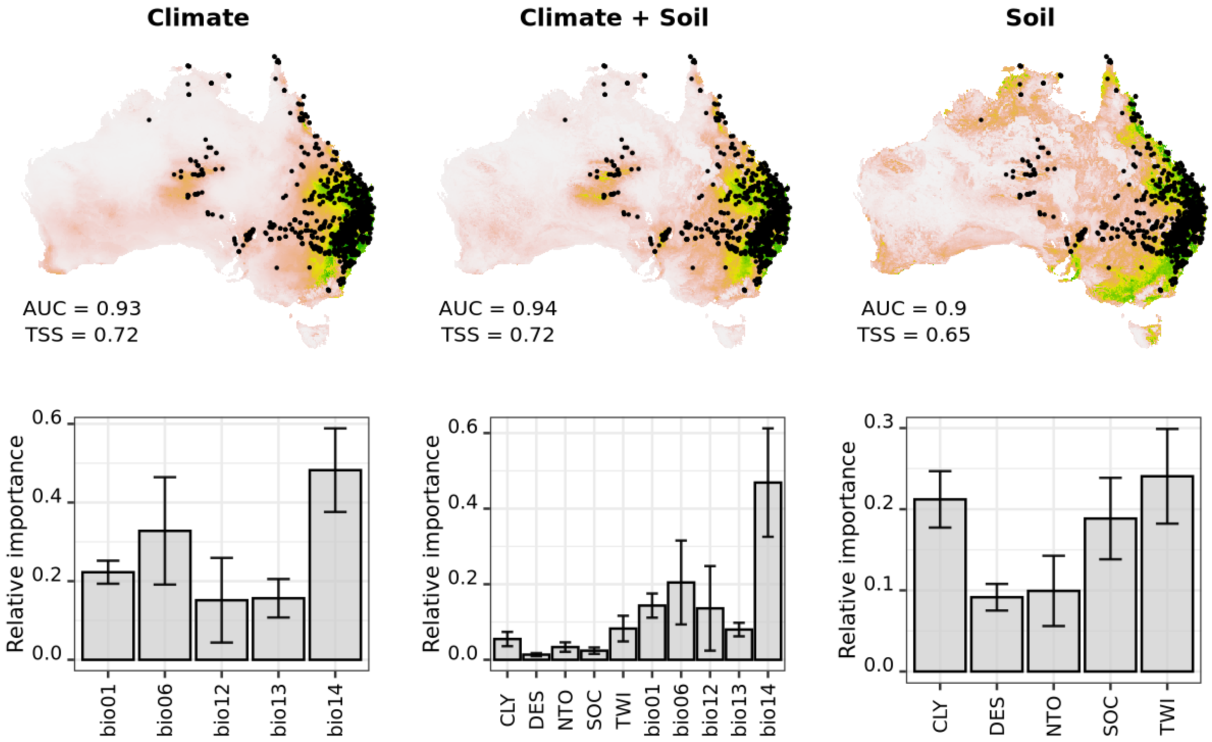

Glossocarya hemiderma

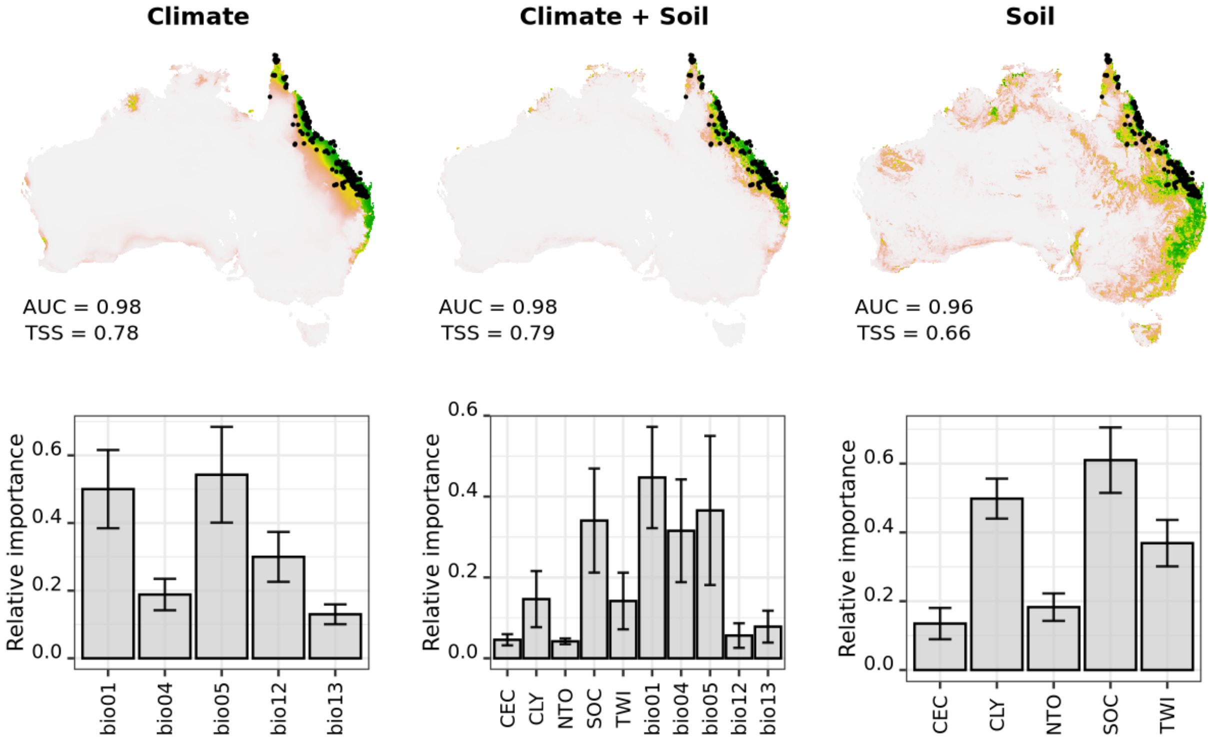

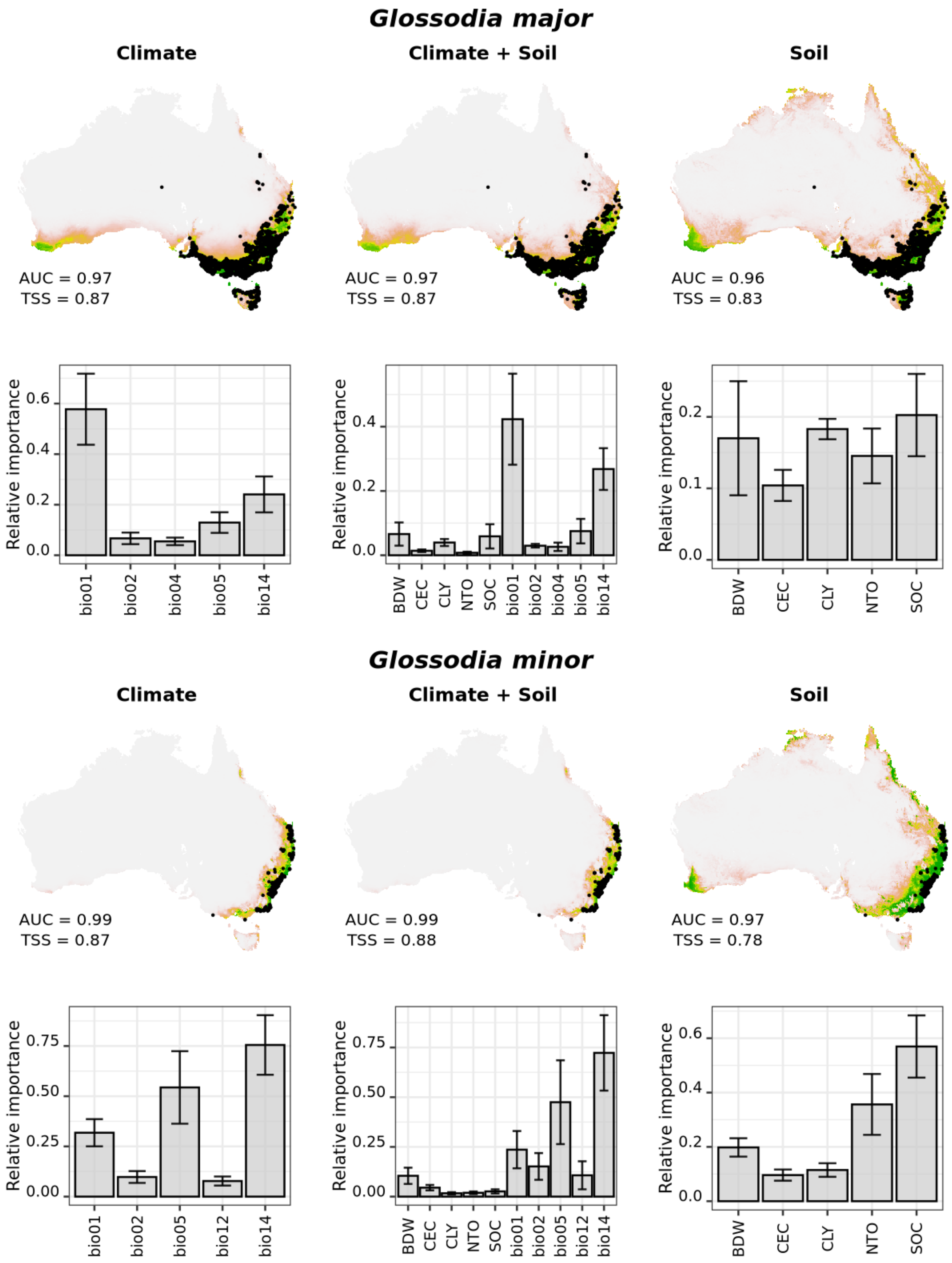
Glossodia major
Climate
Climate + Soil
Soil
AUC = 0.97
TSS = 0.87
AUC = 0.97
TSS = 0.87
AUC = 0.96
TSS = 0.83
Relative importance
bio01
bio02
bio04
bio05
bio14
BDW
CEC
CLY
NTO
SOC
Glossodia minor
AUC = 0.99
TSS = 0.87
AUC = 0.99
TSS = 0.88
AUC = 0.97
TSS = 0.78
bio12

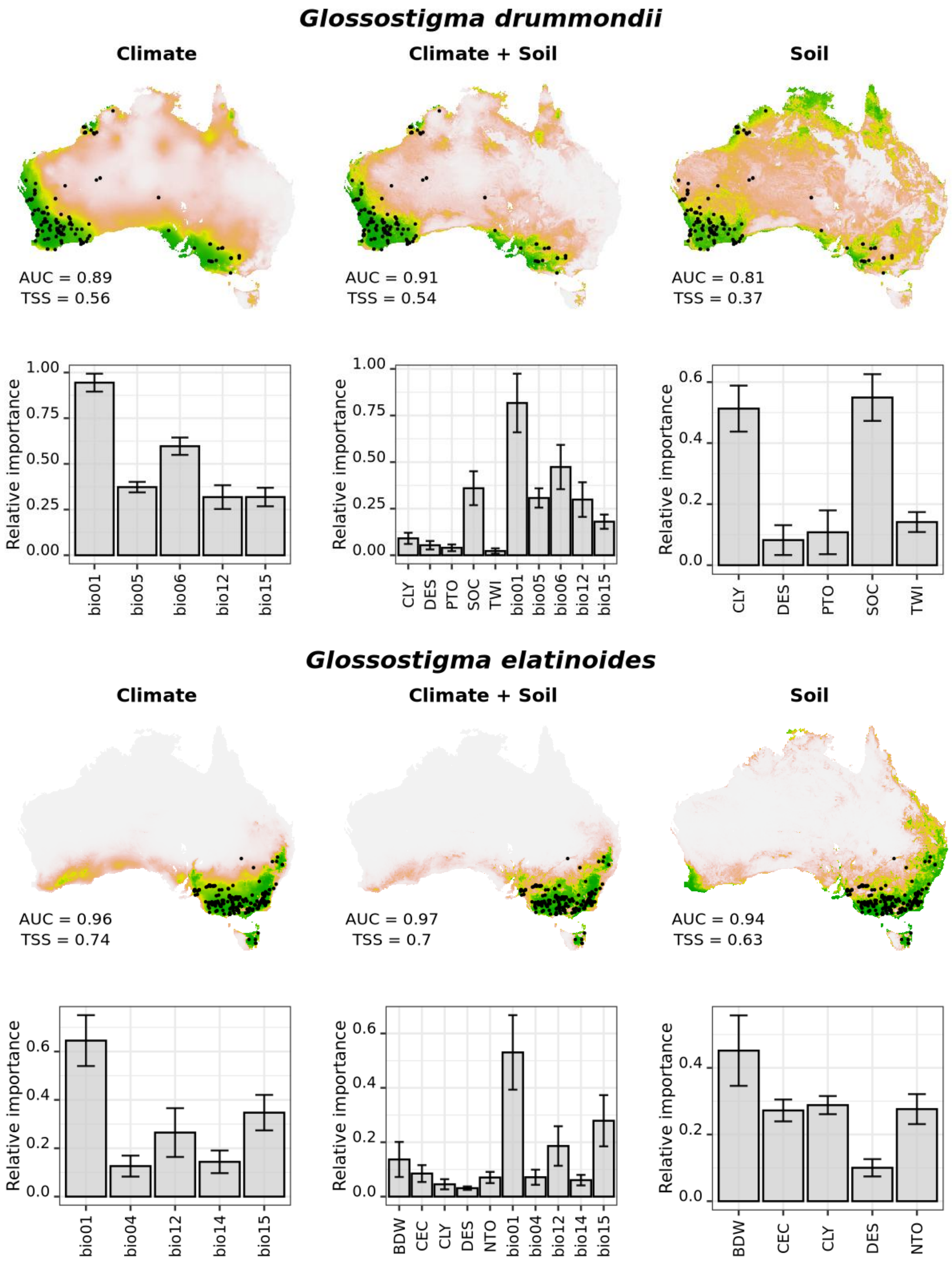

Glossostigma drummondii
Climate
Climate + Soil
Soil
AUC = 0.89
TSS = 0.56
AUC = 0.91
TSS = 0.54
AUC = 0.81
TSS = 0.37
Relative importance
bio01
bio05
bio06
bio12
bio15
CLY
DES
PTO
SOC
TWI
Glossostigma elatinoides
Climate
Climate + Soil
Soil
AUC = 0.96
TSS = 0.74
AUC = 0.97
TSS = 0.7
AUC = 0.94
TSS = 0.63
Relative importance
bio01
bio04
bio12
bio14
bio15
BDW
CEC
CLY
DES
NTO

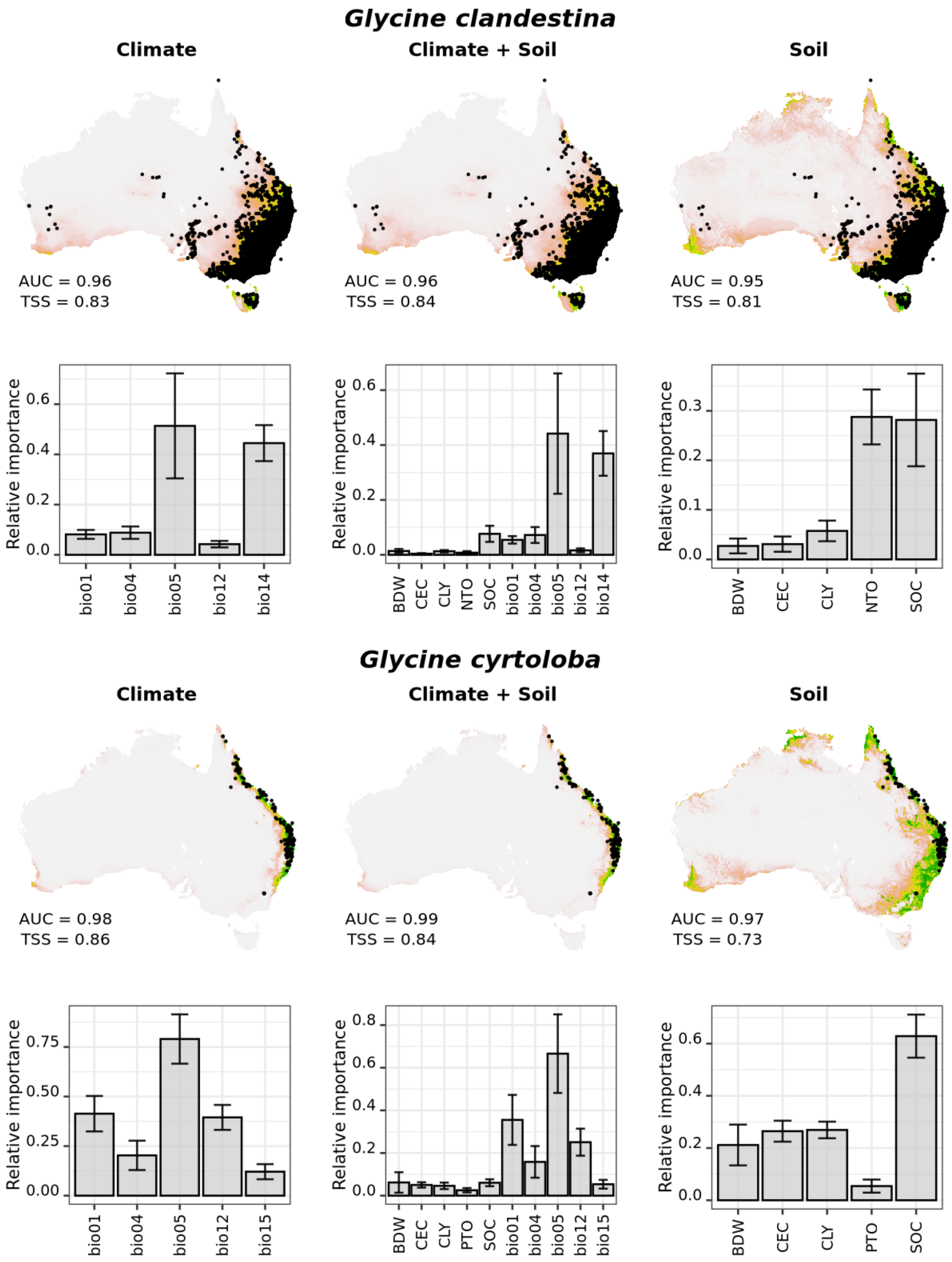

Glycine clandestina
Climate
Climate + Soil
Soil
AUC = 0.96
TSS = 0.83
AUC = 0.96
TSS = 0.84
AUC = 0.95
TSS = 0.81
Relative importance
bio01
bio04
bio05
bio12
bio14
BDW
CEC
CLY
NTO
SOC
Glycine cyrtoloba
Climate
Climate + Soil
Soil
AUC = 0.98
TSS = 0.86
AUC = 0.99
TSS = 0.84
AUC = 0.97
TSS = 0.73
Relative importance
bio01
bio04
bio05
bio12
bio15
BDW
CEC
CLY
PTO
SOC

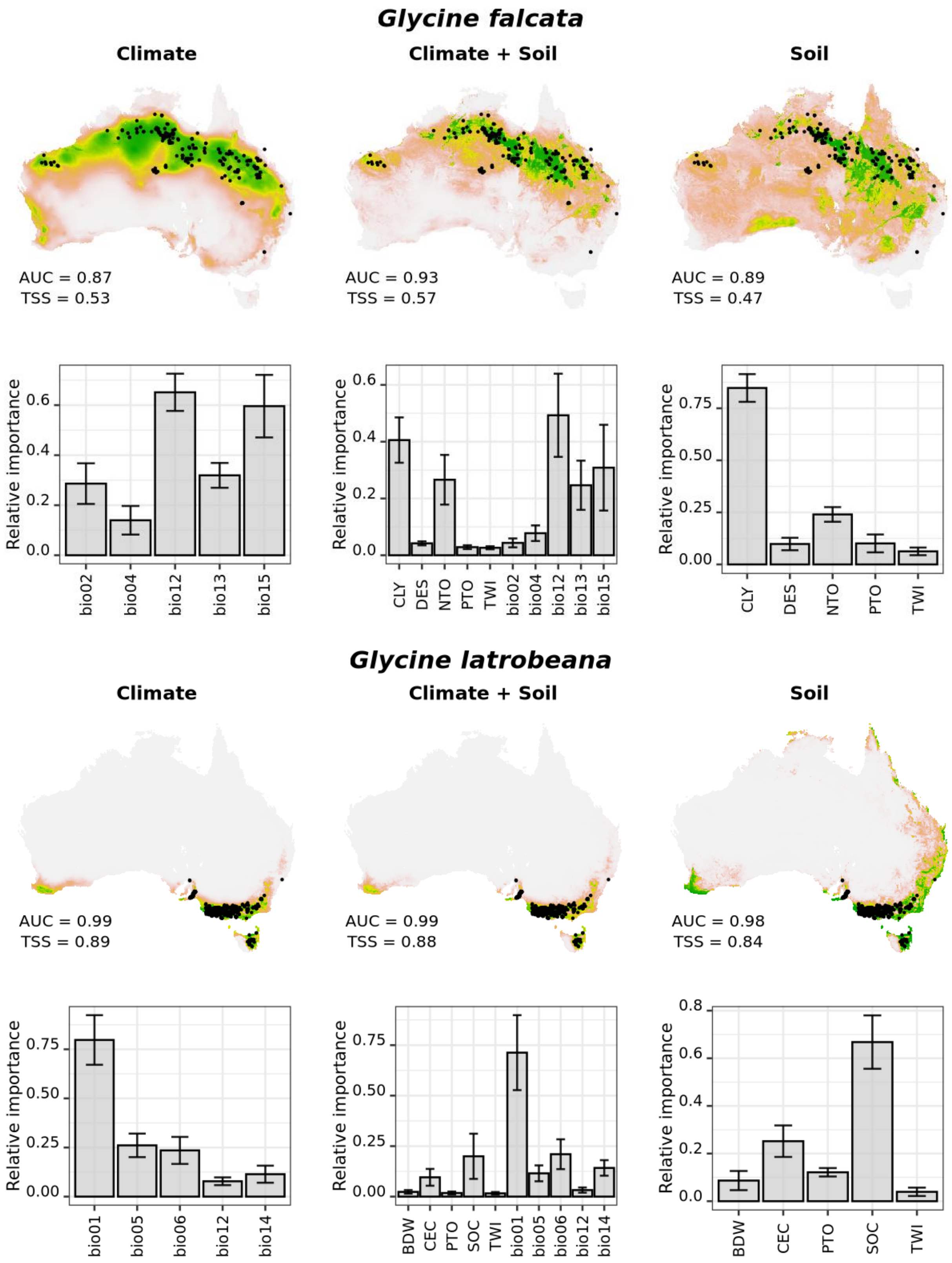

Glycine falcata
Climate
Climate + Soil
Soil
AUC = 0.87
TSS = 0.53
AUC = 0.93
TSS = 0.57
AUC = 0.89
TSS = 0.47
Relative importance
bio02
bio04
bio12
bio13
bio15
CLY
DES
NTO
PTO
TWI
Glycine latrobeana
AUC = 0.99
TSS = 0.89
AUC = 0.99
TSS = 0.88
AUC = 0.98
TSS = 0.84
bio01
bio05
bio06
bio14
BDW
CEC
SOC

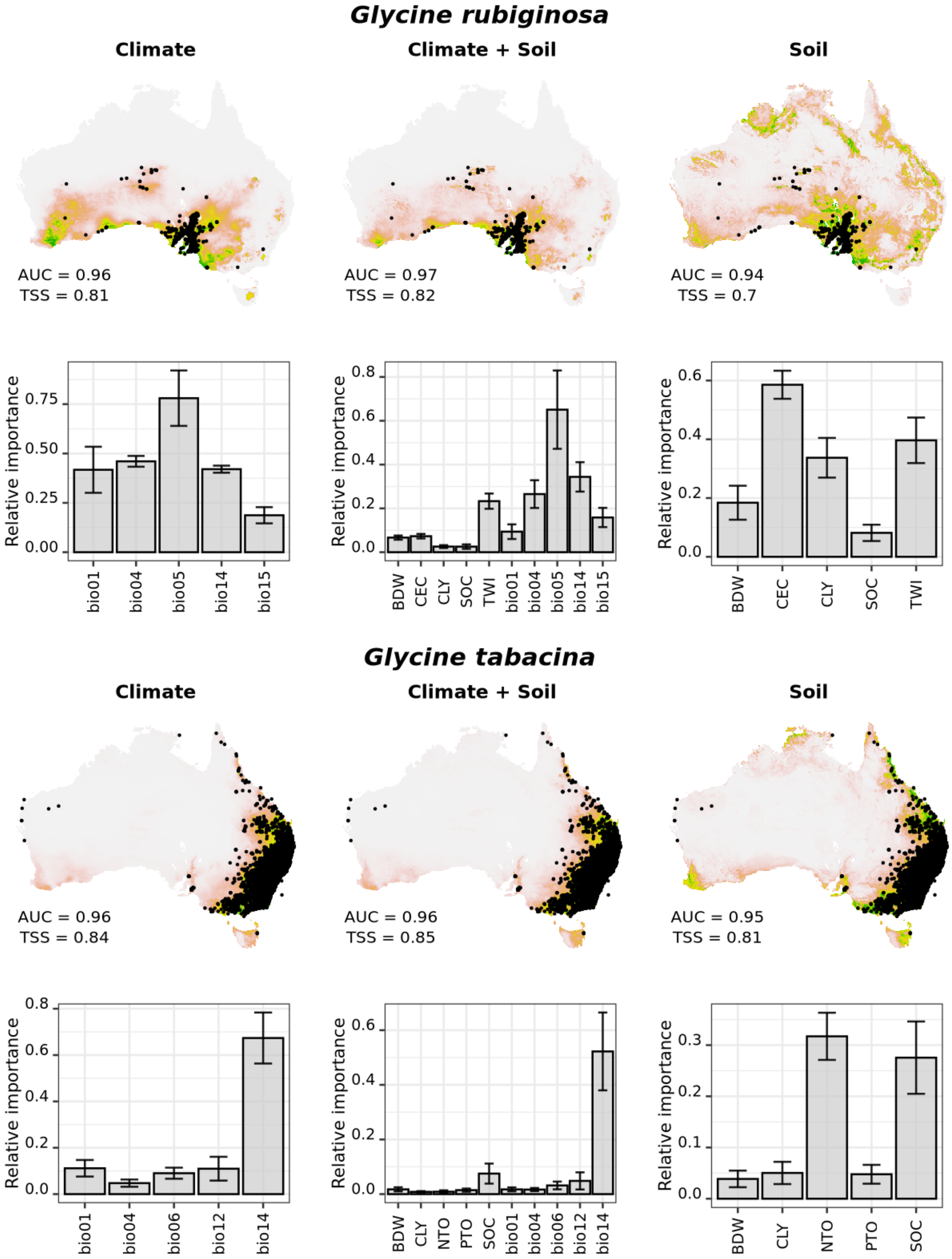
Glycine rubiginosa
Climate
Climate + Soil
Soil
AUC = 0.96
TSS = 0.81
AUC = 0.97
TSS = 0.82
AUC = 0.94
TSS = 0.7
Relative importance
0.00
0.25
0.50
0.75
bio01
bio04
bio05
bio14
bio15
0.0
0.2
0.4
0.6
0.8
BDW
CEC
CLY
SOC
TWI
bio01
bio04
bio05
bio14
bio15
0.0
0.2
0.4
0.6
BDW
CEC
CLY
SOC
TWI
Glycine tabacina
Climate
Climate + Soil
Soil
AUC = 0.96
TSS = 0.84
AUC = 0.96
TSS = 0.85
AUC = 0.95
TSS = 0.81
Relative importance
0.0
0.2
0.4
0.6
0.8
bio01
bio04
bio06
bio12
bio14
0.0
0.2
0.4
0.6
BDW
CLY
NTO
PTO
SOC
bio01
bio04
bio06
bio12
bio14
0.0
0.1
0.2
0.3
BDW
CLY
NTO
PTO
SOC

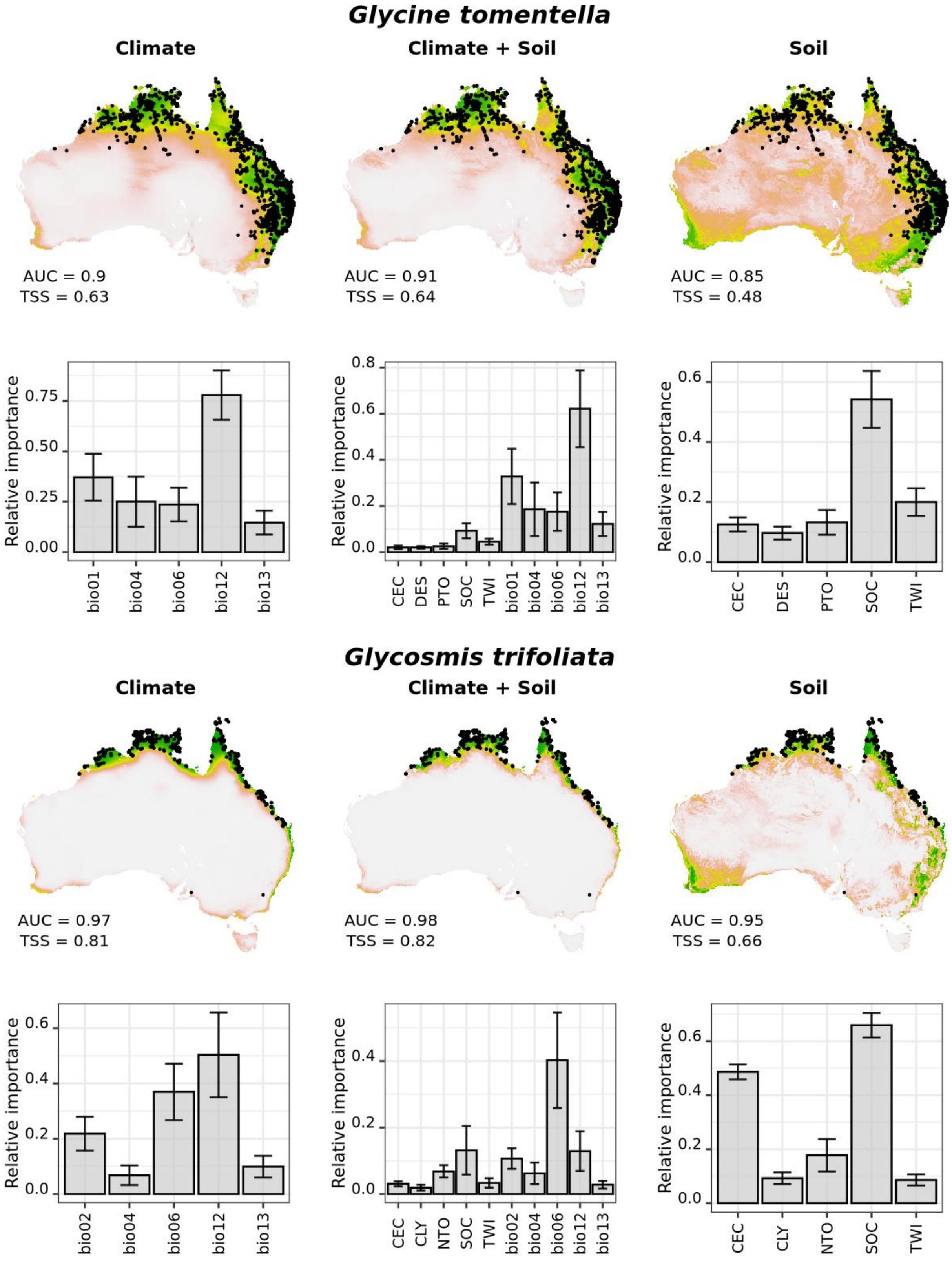
Glycine tomentella
Climate
Climate + Soil
Soil
AUC = 0.9
TSS = 0.63
AUC = 0.91
TSS = 0.64
AUC = 0.85
TSS = 0.48
Relative importance
0.00
0.25
0.50
0.75
bio01
bio04
bio06
bio12
bio13
0.0
0.2
0.4
0.6
0.8
CEC
DES
PTO
SOC
TWI
Glycosmis trifoliata
AUC = 0.97
TSS = 0.81
AUC = 0.98
TSS = 0.82
AUC = 0.95
TSS = 0.66
bio02
CLY
NTO

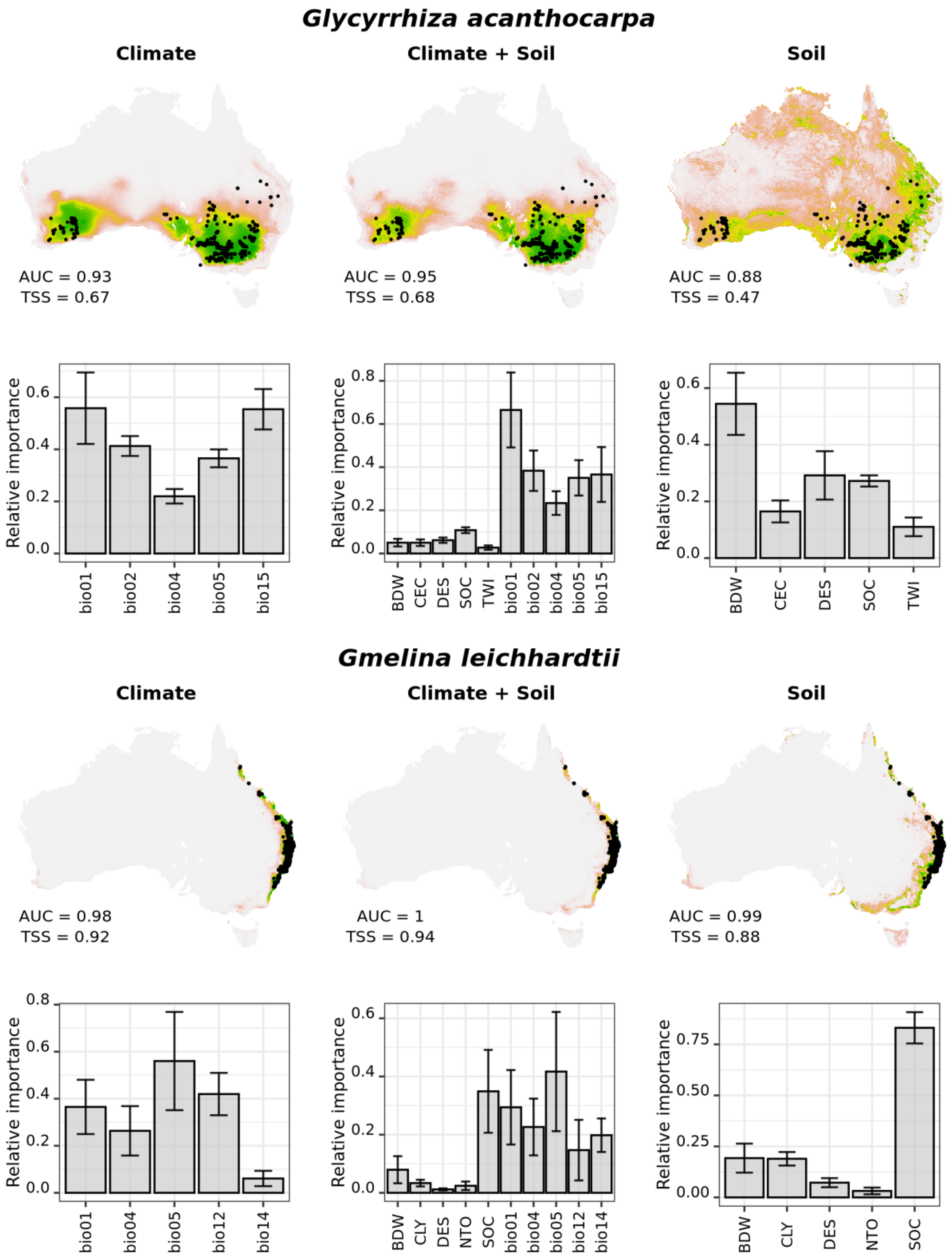
Glycyrrhiza acanthocarpa
Climate
Climate + Soil
Soil
AUC = 0.93
TSS = 0.67
AUC = 0.95
TSS = 0.68
AUC = 0.88
TSS = 0.47
Relative importance
bio01 bio02 bio04 bio05 bio15
BDW CEC DES SOC TWI bio01 bio02 bio04 bio05 bio15
BDW CEC DES SOC TWI
Gmelina leichhardtii
Climate
Climate + Soil
Soil
AUC = 0.98
TSS = 0.92
AUC = 1
TSS = 0.94
AUC = 0.99
TSS = 0.88
Relative importance
bio01 bio04 bio05 bio12 bio14
BDW CLY DES NTO SOC bio01 bio04 bio05 bio12 bio14
BDW CLY DES NTO SOC

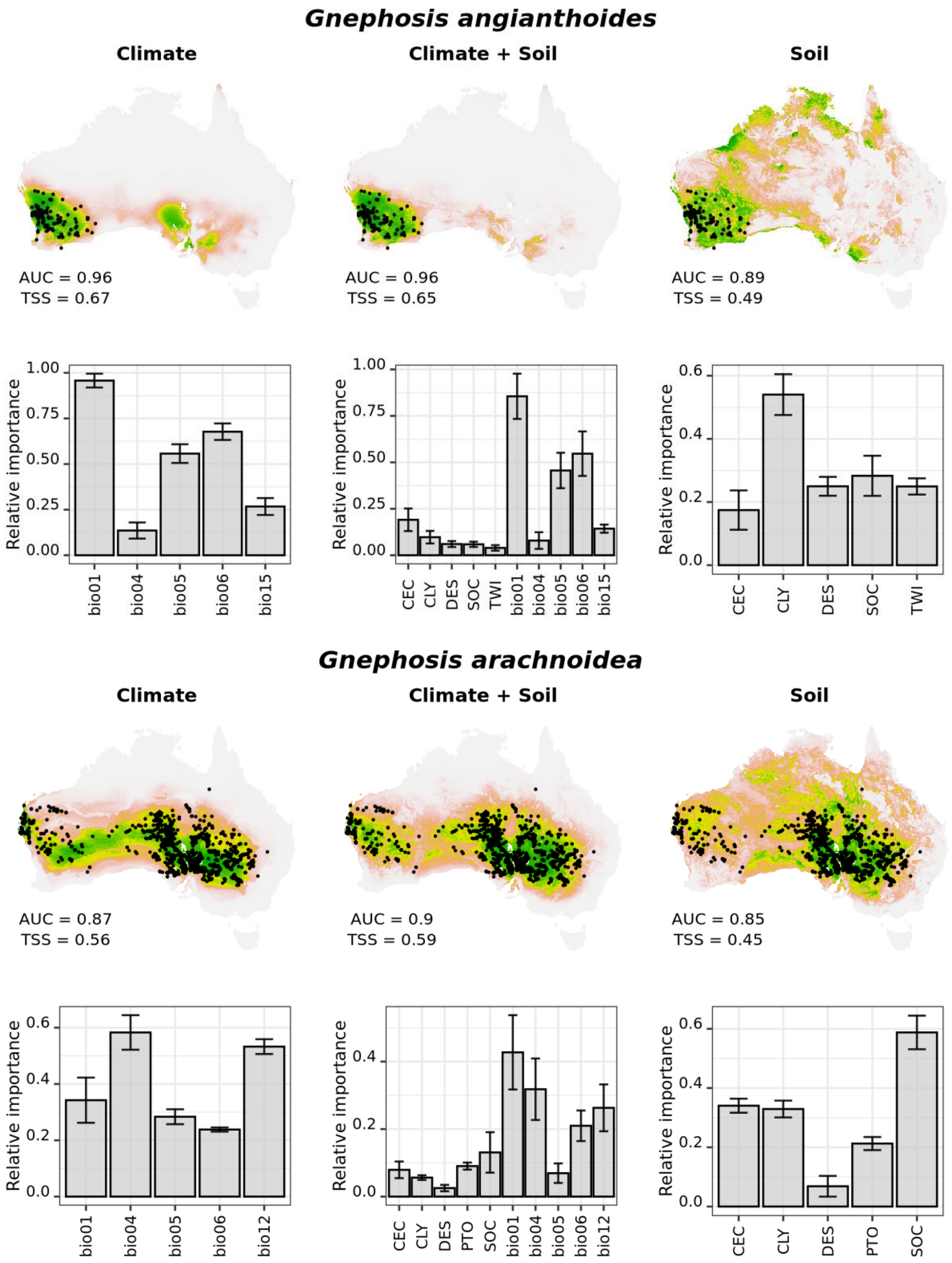
Gnephosis angianthoides
Climate
Climate + Soil
Soil
AUC = 0.96
TSS = 0.67
AUC = 0.96
TSS = 0.65
AUC = 0.89
TSS = 0.49
Relative importance
bio01 bio04 bio05 bio06 bio15
CEC CLY DES SOC TWI bio01 bio04 bio05 bio06 bio15
CEC CLY DES SOC TWI
Gnephosis arachnoidea
Climate
Climate + Soil
Soil
AUC = 0.87
TSS = 0.56
AUC = 0.9
TSS = 0.59
AUC = 0.85
TSS = 0.45
Relative importance
bio01 bio04 bio05 bio06 bio12
CEC CLY DES PTO SOC bio01 bio04 bio05 bio06 bio12
CEC CLY DES PTO SOC

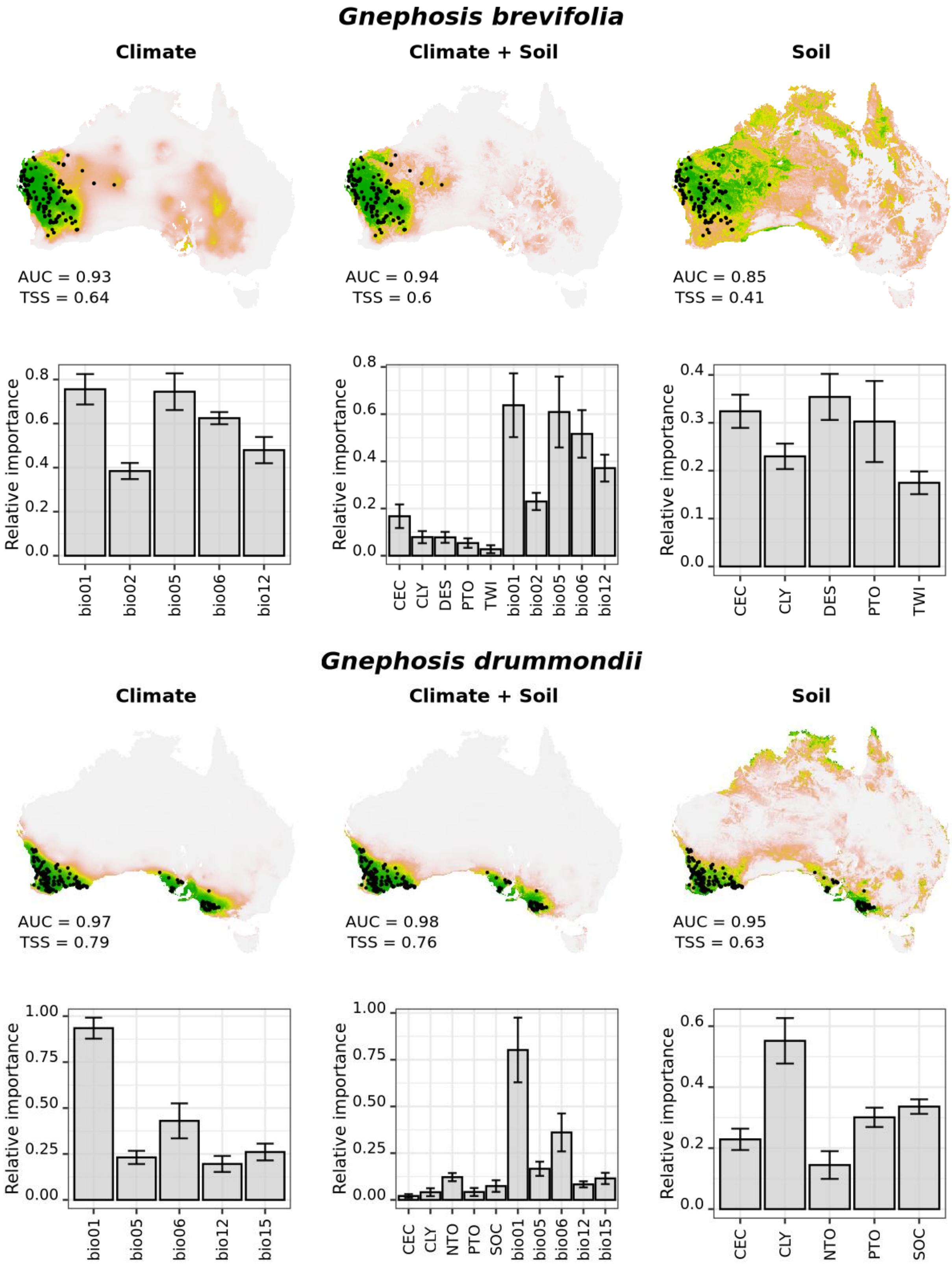
Gnephosis brevifolia
Climate
Climate + Soil
Soil
AUC = 0.93
TSS = 0.64
AUC = 0.94
TSS = 0.6
AUC = 0.85
TSS = 0.41
Relative importance
bio01
bio02
bio05
bio06
bio12
CEC
CLY
DES
PTO
TWI
Gnephosis drummondii
Climate
Climate + Soil
Soil
AUC = 0.97
TSS = 0.79
AUC = 0.98
TSS = 0.76
AUC = 0.95
TSS = 0.63
Relative importance
bio01
bio05
bio06
bio12
bio15
CEC
CLY
NTO
PTO
SOC

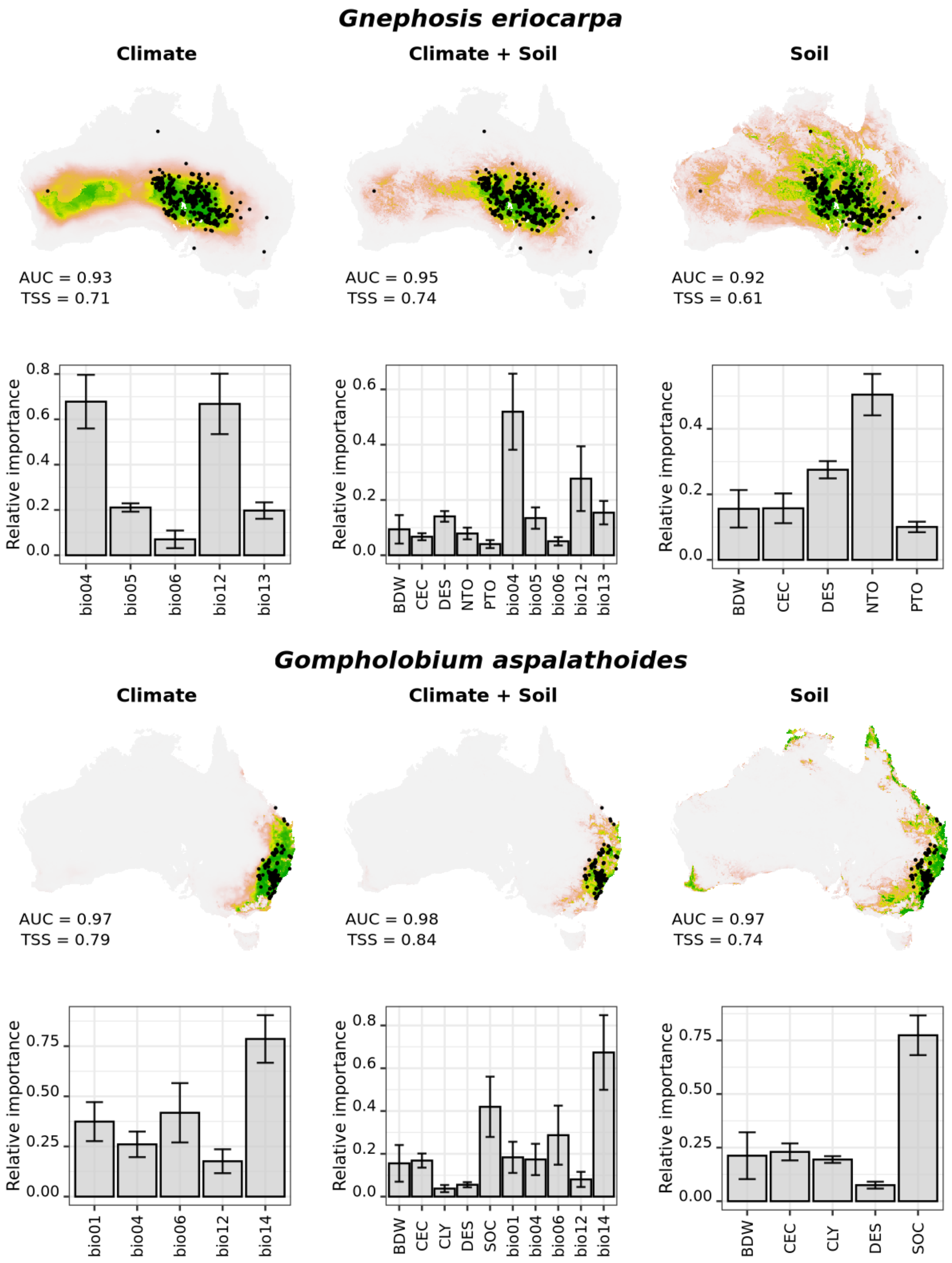
Gnephosis eriocarpa
Climate
Climate + Soil
Soil
AUC = 0.93
TSS = 0.71
AUC = 0.95
TSS = 0.74
AUC = 0.92
TSS = 0.61
Relative importance
bio04
bio05
bio06
bio12
bio13
BDW
CEC
DES
NTO
PTO
Gompholobium aspalathoides
Climate
Climate + Soil
Soil
AUC = 0.97
TSS = 0.79
AUC = 0.98
TSS = 0.84
AUC = 0.97
TSS = 0.74
Relative importance
bio01
bio04
bio06
bio12
bio14
BDW
CEC
CLY
DES
SOC

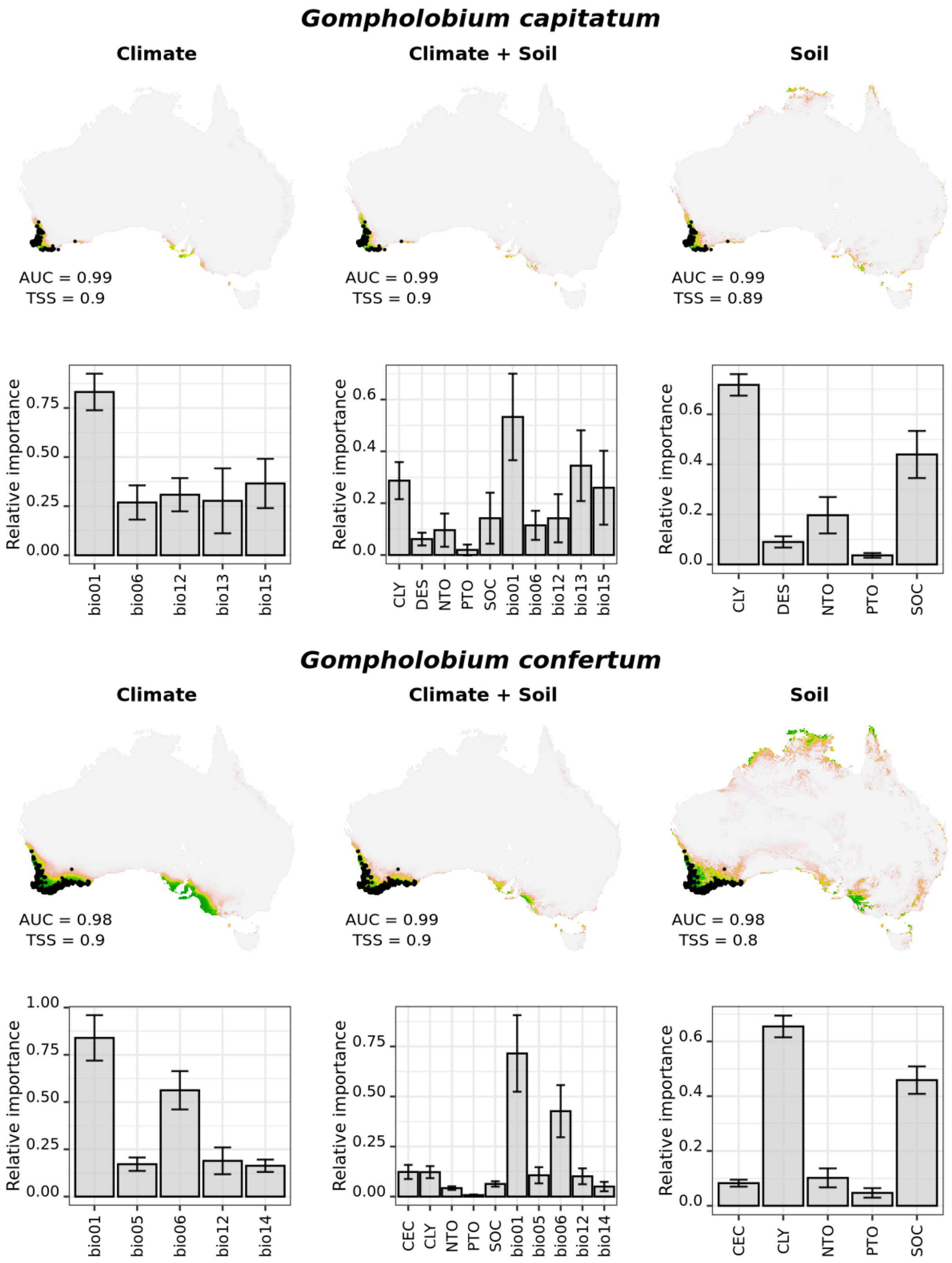
Gompholobium capitatum
Climate
Climate + Soil
Soil
AUC = 0.99
TSS = 0.9
AUC = 0.99
TSS = 0.9
AUC = 0.99
TSS = 0.89
Relative importance
0.00 0.25 0.50 0.75
bio01 bio06 bio12 bio13 bio15
0.0 0.2 0.4 0.6
CLY DES NTO PTO SOC bio01 bio06 bio12 bio13 bio15
0.0 0.2 0.4 0.6
CLY DES NTO PTO SOC
Gompholobium confertum
Climate
Climate + Soil
Soil
AUC = 0.98
TSS = 0.9
AUC = 0.99
TSS = 0.9
AUC = 0.98
TSS = 0.8
0.00 0.25 0.50 0.75 1.00
bio01 bio05 bio06 bio12 bio14
0.00 0.25 0.50 0.75
CEC CLY NTO PTO SOC bio01 bio05 bio06 bio12 bio14
0.0 0.2 0.4 0.6
CEC CLY NTO PTO SOC

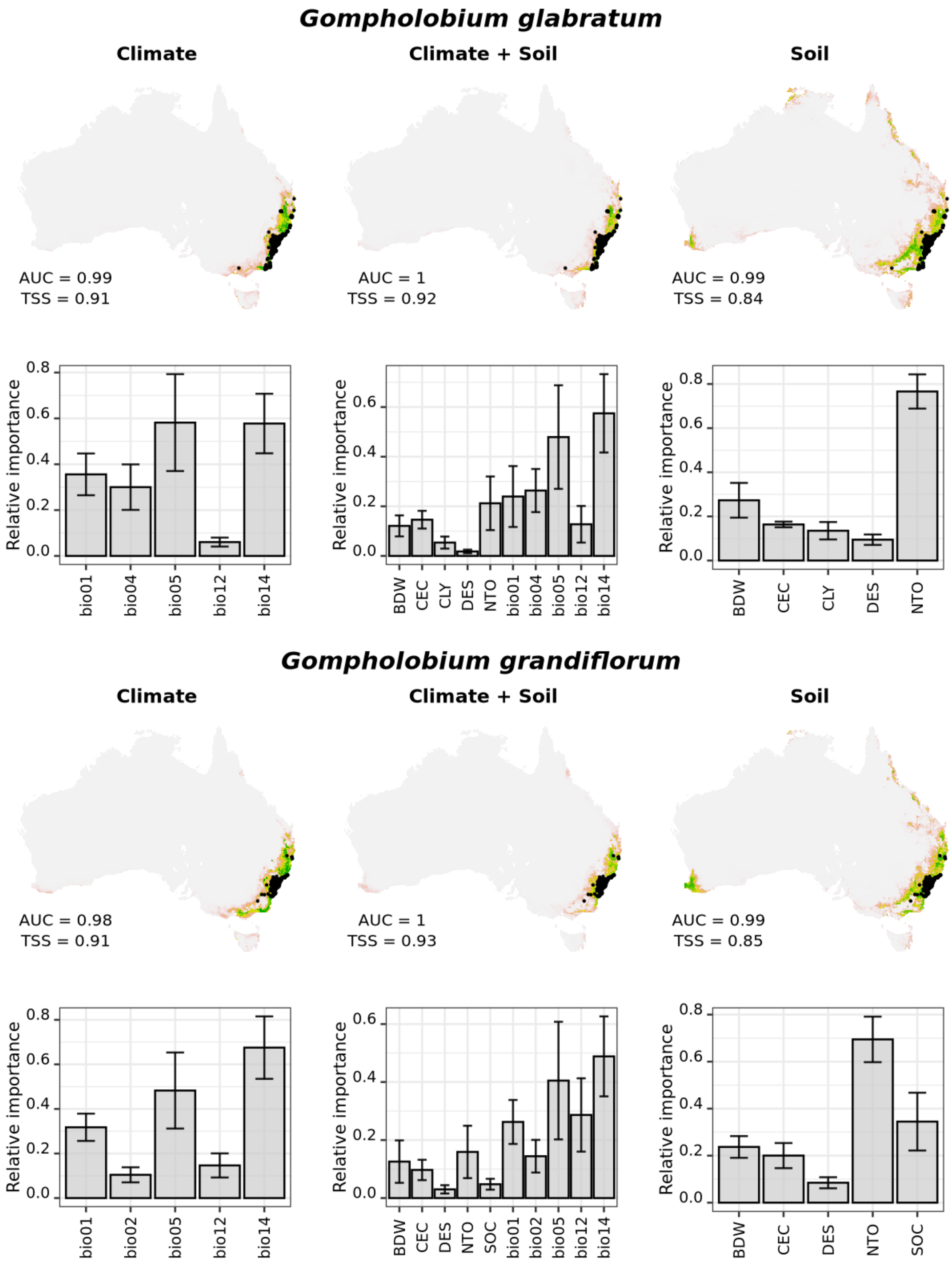

Gompholobium glabratum
Climate
Climate + Soil
Soil
AUC = 0.99
TSS = 0.91
AUC = 1
TSS = 0.92
AUC = 0.99
TSS = 0.84
Relative importance
0.0
0.2
0.4
0.6
0.8
bio01
bio04
bio05
bio12
bio14
BDW
CEC
CLY
DES
NTO
Gompholobium grandiflorum
AUC = 0.98
TSS = 0.91
AUC = 1
TSS = 0.93
AUC = 0.99
TSS = 0.85
bio02
SOC

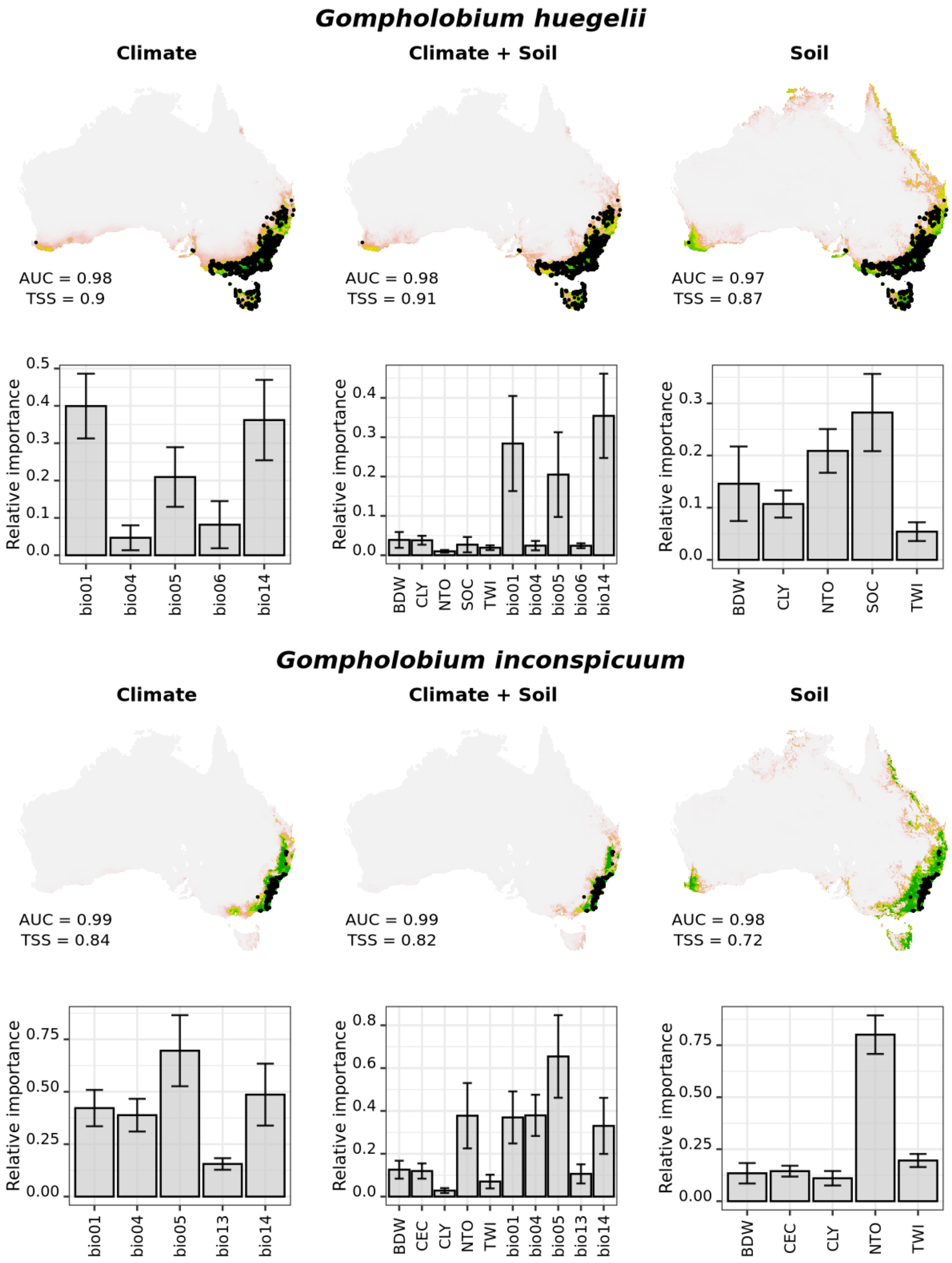
Gompholobium huegelii
Climate
Climate + Soil
Soil
AUC = 0.98
TSS = 0.9
AUC = 0.98
TSS = 0.91
AUC = 0.97
TSS = 0.87
Relative importance
bio01
bio04
bio05
bio06
bio14
BDW
CLY
NTO
SOC
TWI
Gompholobium inconspicuum
Climate
Climate + Soil
Soil
AUC = 0.99
TSS = 0.84
AUC = 0.99
TSS = 0.82
AUC = 0.98
TSS = 0.72
Relative importance
bio01
bio04
bio05
bio13
bio14
BDW
CEC
CLY
NTO
TWI

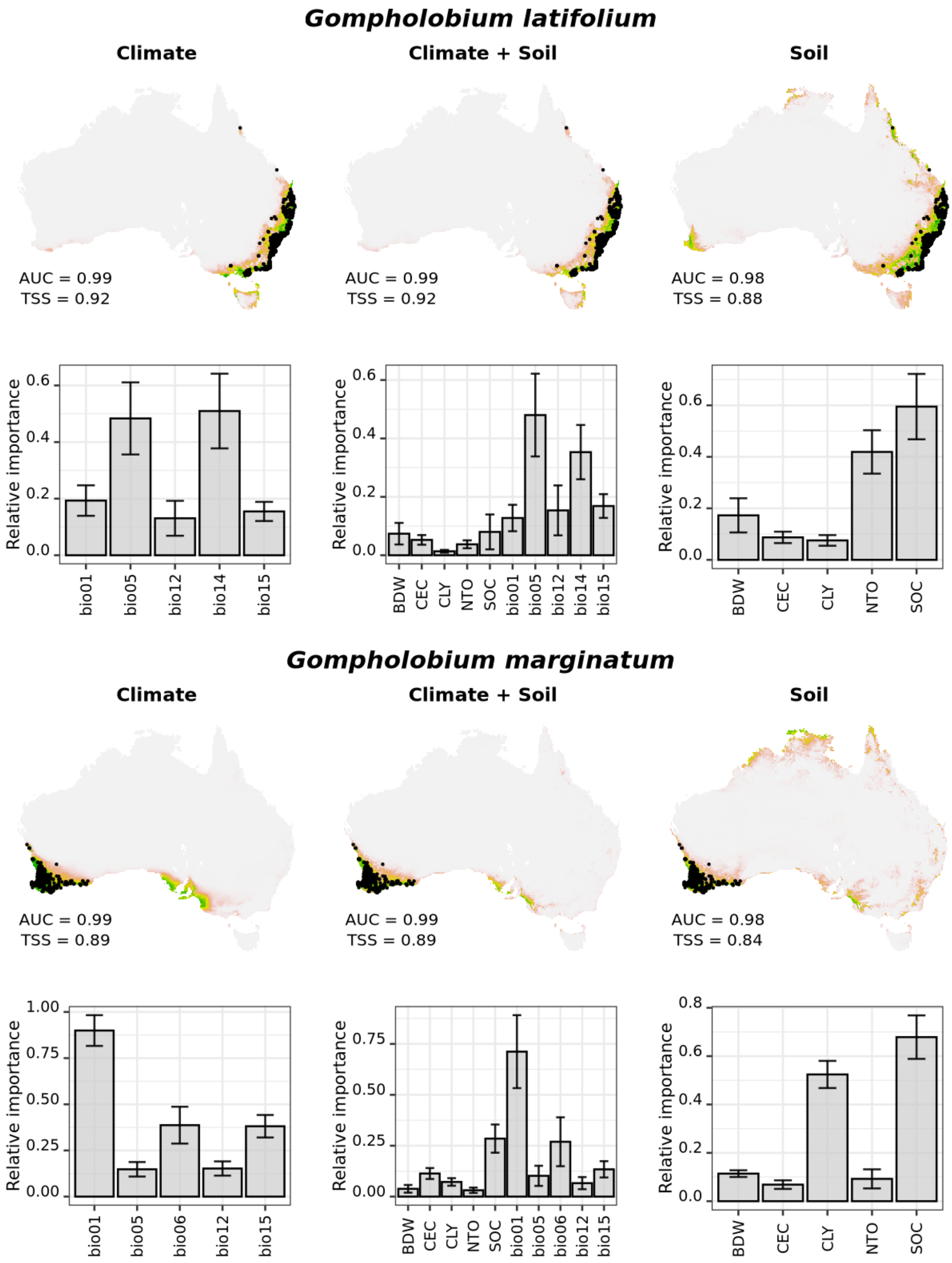

Gompholobium latifolium
Climate
Climate + Soil
Soil
AUC = 0.99
TSS = 0.92
AUC = 0.99
TSS = 0.92
AUC = 0.98
TSS = 0.88
Relative importance
0.0
0.2
0.4
0.6
bio01
bio05
bio12
bio14
bio15
BDW
CEC
CLY
NTO
SOC
Gompholobium marginatum
Climate
Climate + Soil
Soil
AUC = 0.99
TSS = 0.89
AUC = 0.99
TSS = 0.89
AUC = 0.98
TSS = 0.84
Relative importance
0.00
0.25
0.50
0.75
1.00
0.8
bio01
bio05
bio06
bio12
bio15
BDW
CEC
CLY
NTO
SOC

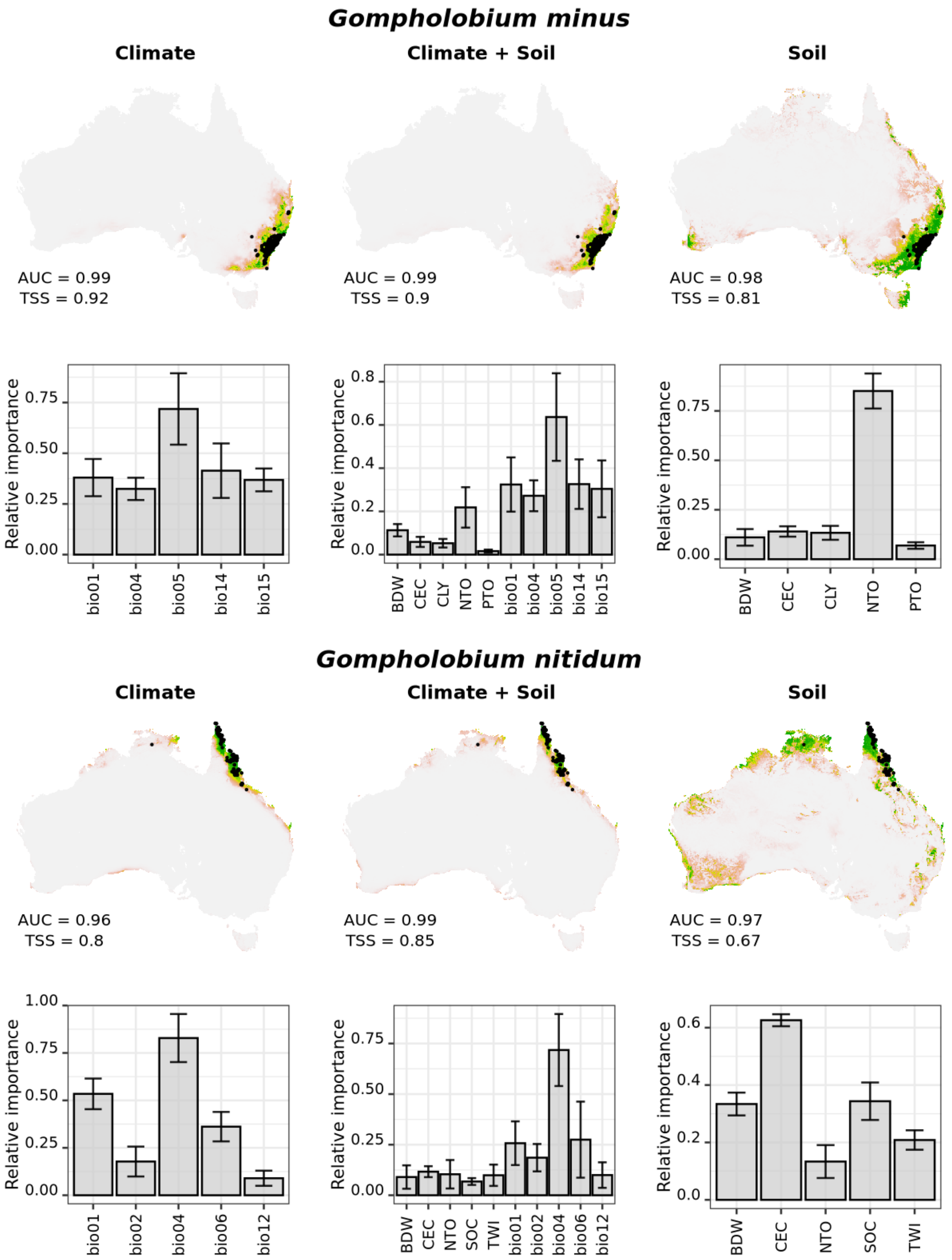

Gompholobium minus
Climate
Climate + Soil
Soil
AUC = 0.99
TSS = 0.92
AUC = 0.99
TSS = 0.9
AUC = 0.98
TSS = 0.81
Relative importance
0.00
0.25
0.50
0.75
bio01
bio04
bio05
bio14
bio15
0.0
0.2
0.4
0.6
0.8
BDW
CEC
CLY
NTO
PTO
Gompholobium nitidum
AUC = 0.96
TSS = 0.8
AUC = 0.99
TSS = 0.85
AUC = 0.97
TSS = 0.67
1.00
bio02
bio06
bio12
SOC
TWI

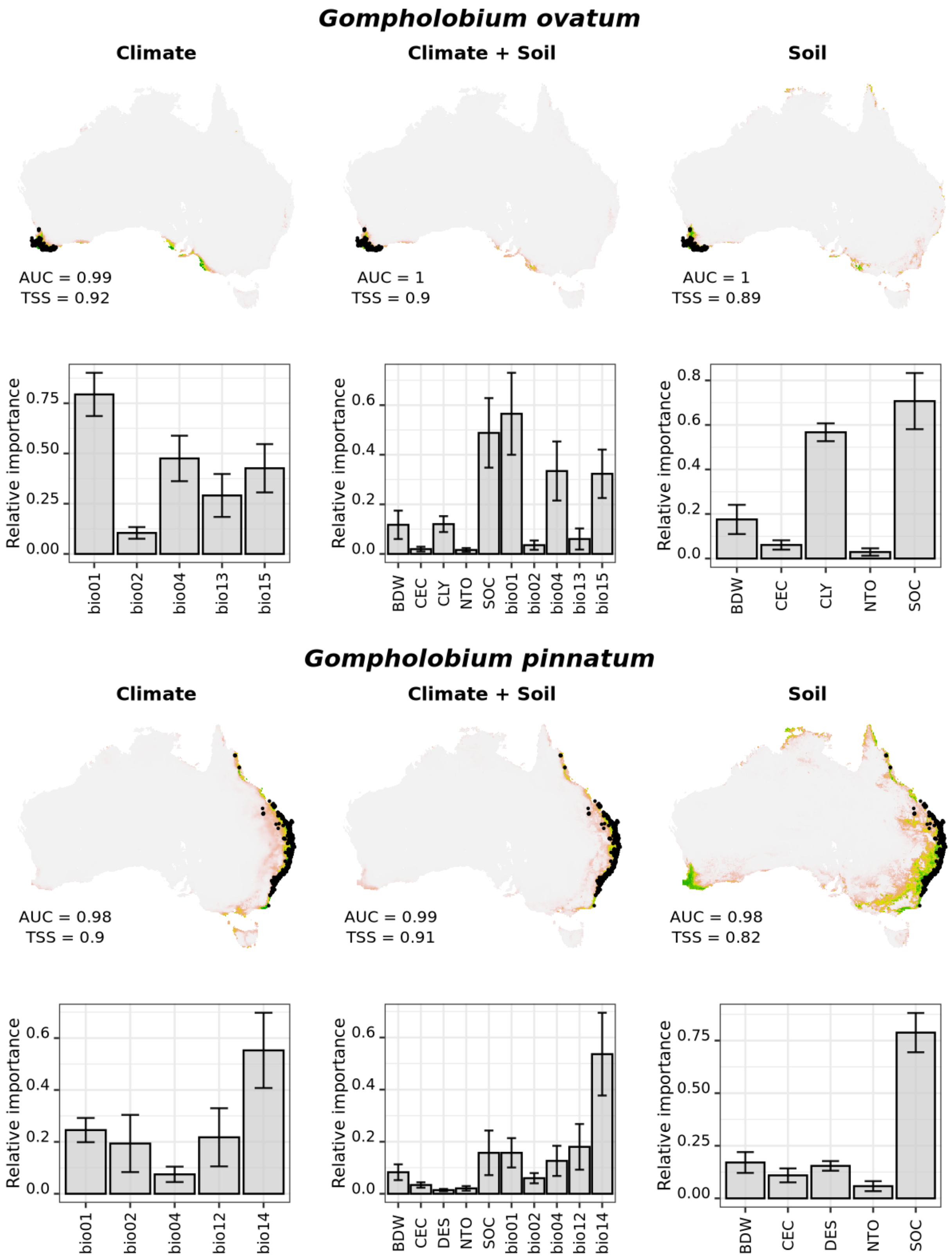

Gompholobium ovatum
Climate
Climate + Soil
Soil
AUC = 0.99
TSS = 0.92
AUC = 1
TSS = 0.9
AUC = 1
TSS = 0.89
Relative importance
bio01
bio02
bio04
bio13
bio15
BDW
CEC
CLY
NTO
SOC
Gompholobium pinnatum
Climate
Climate + Soil
Soil
AUC = 0.98
TSS = 0.9
AUC = 0.99
TSS = 0.91
AUC = 0.98
TSS = 0.82
Relative importance
bio01
bio02
bio04
bio12
bio14
BDW
CEC
DES
NTO
SOC

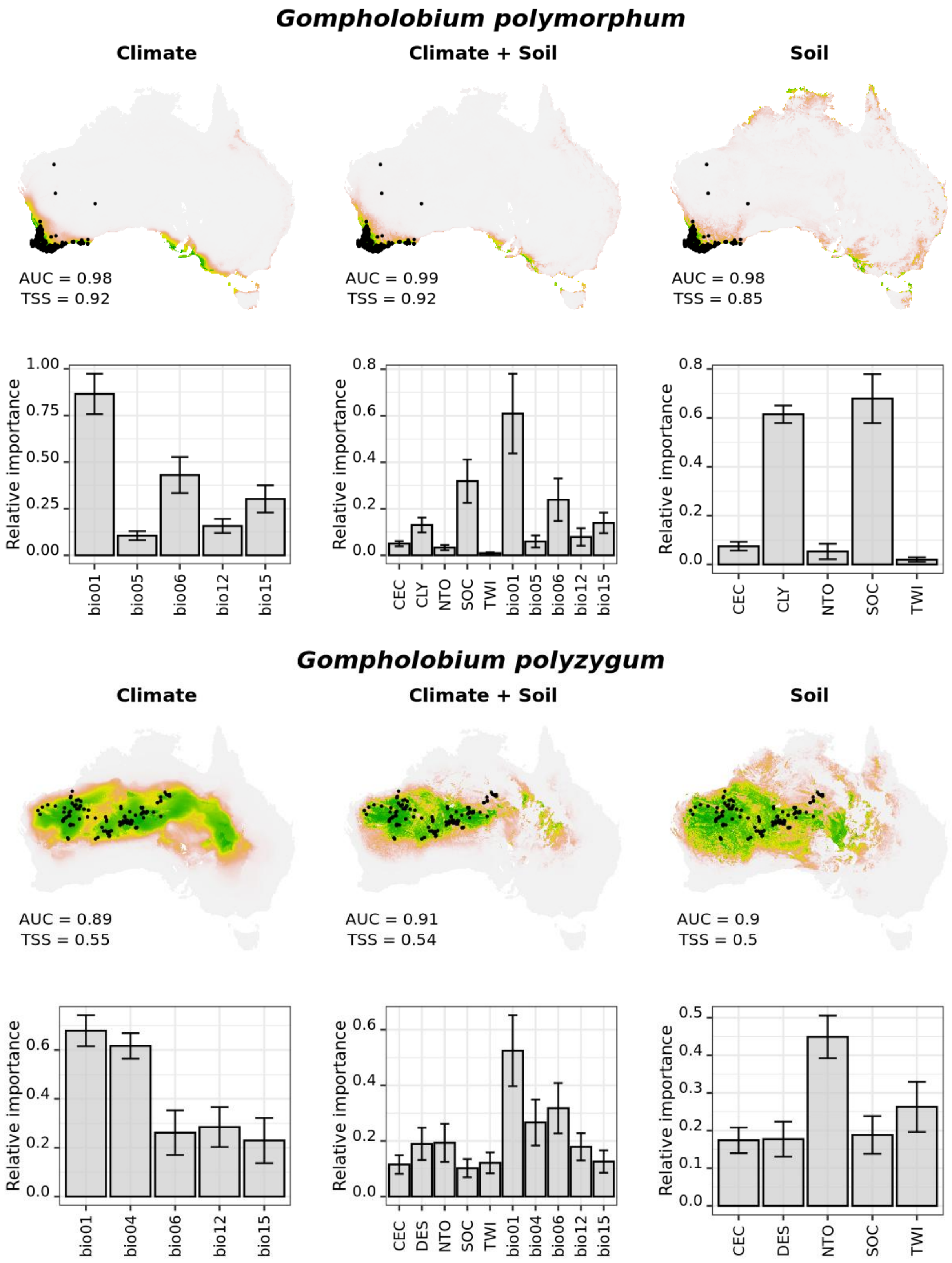
Gompholobium polymorphum
Climate
Climate + Soil
Soil
AUC = 0.98
TSS = 0.92
AUC = 0.99
TSS = 0.92
AUC = 0.98
TSS = 0.85
Relative importance
bio01
bio05
bio06
bio12
bio15
CEC
CLY
NTO
SOC
TWI
Gompholobium polyzygum
Climate
Climate + Soil
Soil
AUC = 0.89
TSS = 0.55
AUC = 0.91
TSS = 0.54
AUC = 0.9
TSS = 0.5
Relative importance
bio01
bio04
bio06
bio12
bio15
CEC
DES
NTO
SOC
TWI

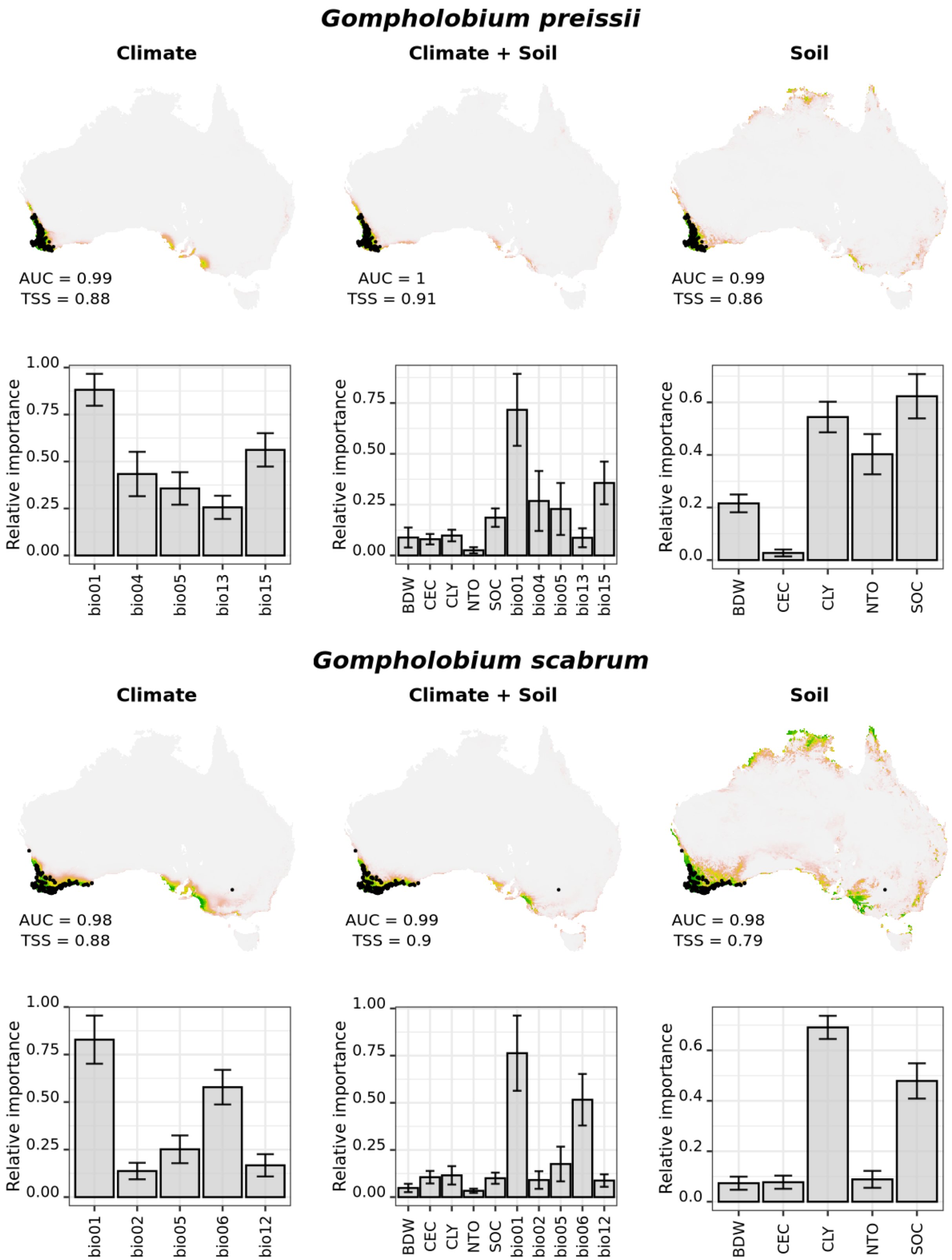
Gompholobium preissii
Climate
Climate + Soil
Soil
AUC = 0.99
TSS = 0.88
AUC = 1
TSS = 0.91
AUC = 0.99
TSS = 0.86
Relative importance
bio01
bio04
bio05
bio13
bio15
BDW
CEC
CLY
NTO
SOC
Gompholobium scabrum
AUC = 0.98
TSS = 0.88
AUC = 0.99
TSS = 0.9
AUC = 0.98
TSS = 0.79
bio02
bio06
bio12

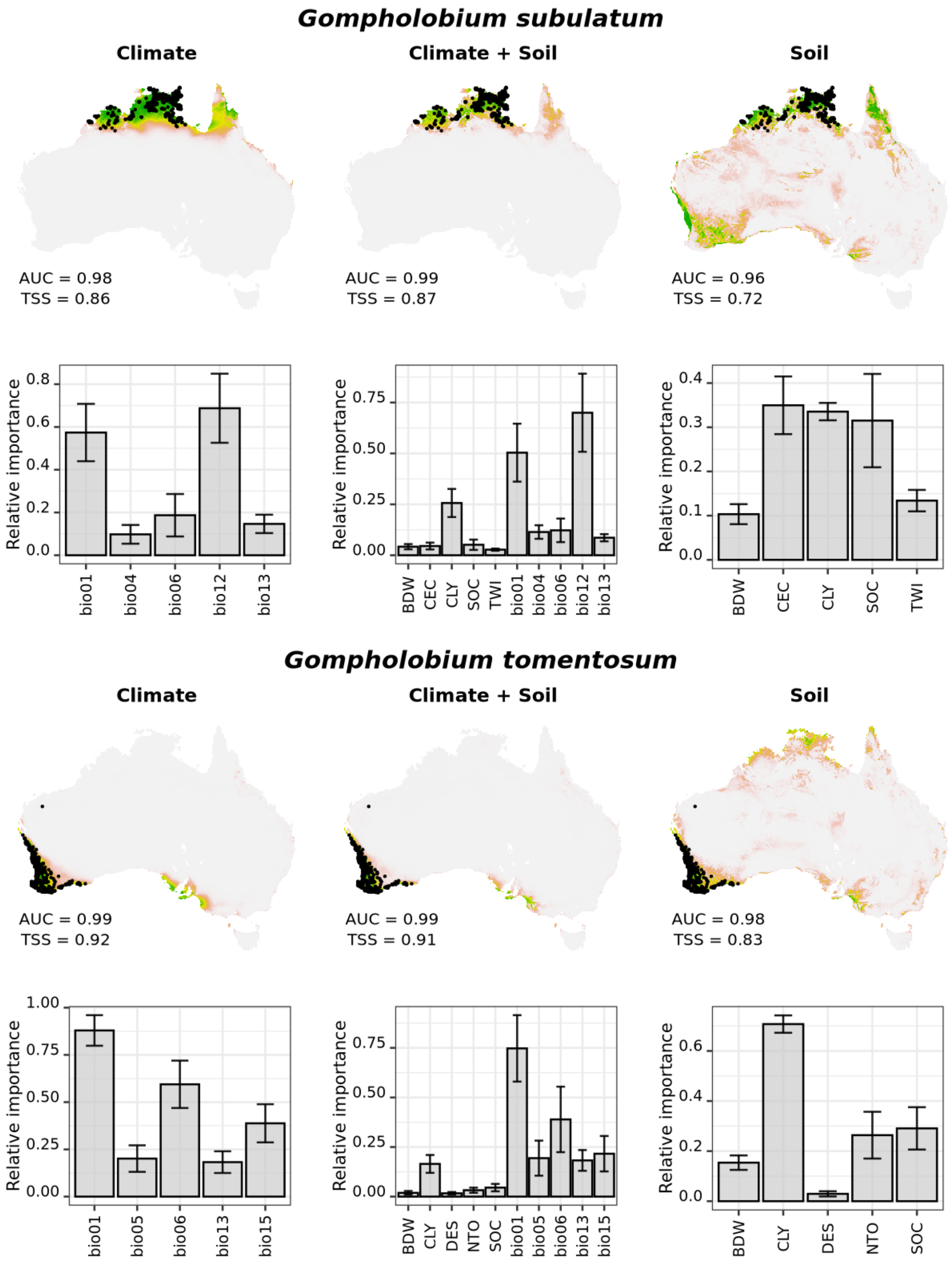

Gompholobium subulatum
Climate
Climate + Soil
Soil
AUC = 0.98
TSS = 0.86
AUC = 0.99
TSS = 0.87
AUC = 0.96
TSS = 0.72
Relative importance
bio01 bio04 bio06 bio12 bio13
BDW CEC CLY SOC TWI bio01 bio04 bio06 bio12 bio13
BDW CEC CLY SOC TWI
Gompholobium tomentosum
Climate
Climate + Soil
Soil
AUC = 0.99
TSS = 0.92
AUC = 0.99
TSS = 0.91
AUC = 0.98
TSS = 0.83
Relative importance
bio01 bio05 bio06 bio13 bio15
BDW CLY DES NTO SOC bio01 bio05 bio06 bio13 bio15
BDW CLY DES NTO SOC

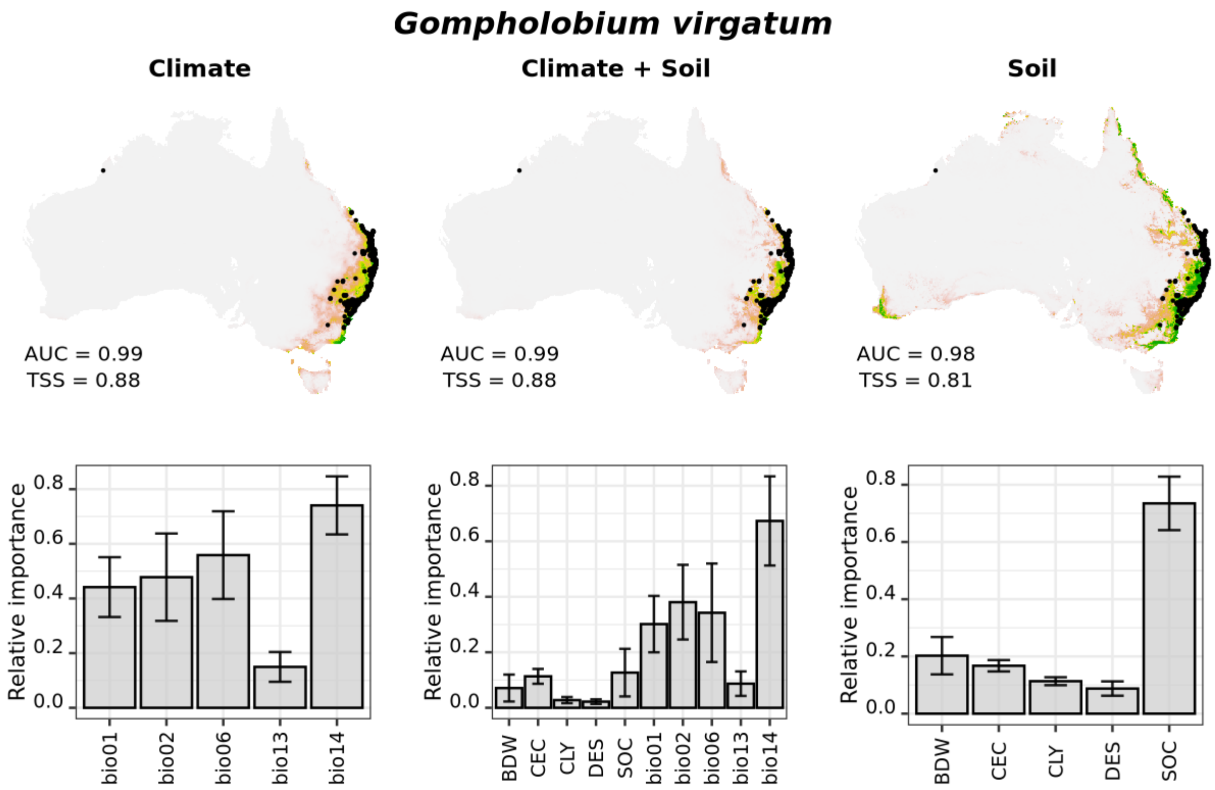
Gompholobium virgatum
Climate
Climate + Soil
Soil
AUC = 0.99
TSS = 0.88
AUC = 0.99
TSS = 0.88
AUC = 0.98
TSS = 0.81
Relative importance
0.0
0.2
0.4
0.6
0.8
bio01
bio02
bio06
bio13
bio14
BDW
CEC
CLY
DES
SOC
bio01
bio02
bio06
bio13
bio14
BDW
CEC
CLY
DES
SOC

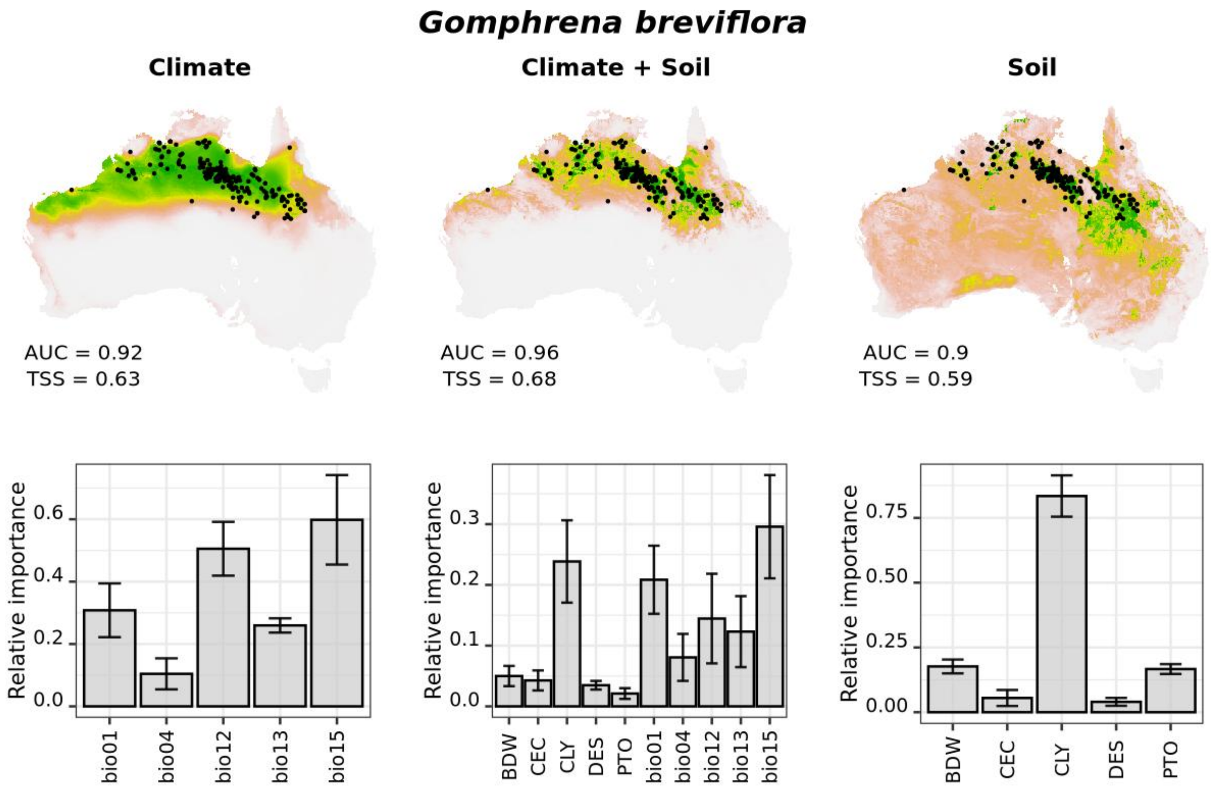
Gomphrena breviflora
Climate
Climate + Soil
Soil
AUC = 0.92
TSS = 0.63
AUC = 0.96
TSS = 0.68
AUC = 0.9
TSS = 0.59
Relative importance
0.0
0.2
0.4
0.6
0.1
0.3
0.00
0.25
0.50
0.75
bio01
bio04
bio12
bio13
bio15
BDW
CEC
CLY
DES
PTO
bio01
bio04
bio12
bio13
bio15
BDW
CEC
CLY
DES
PTO

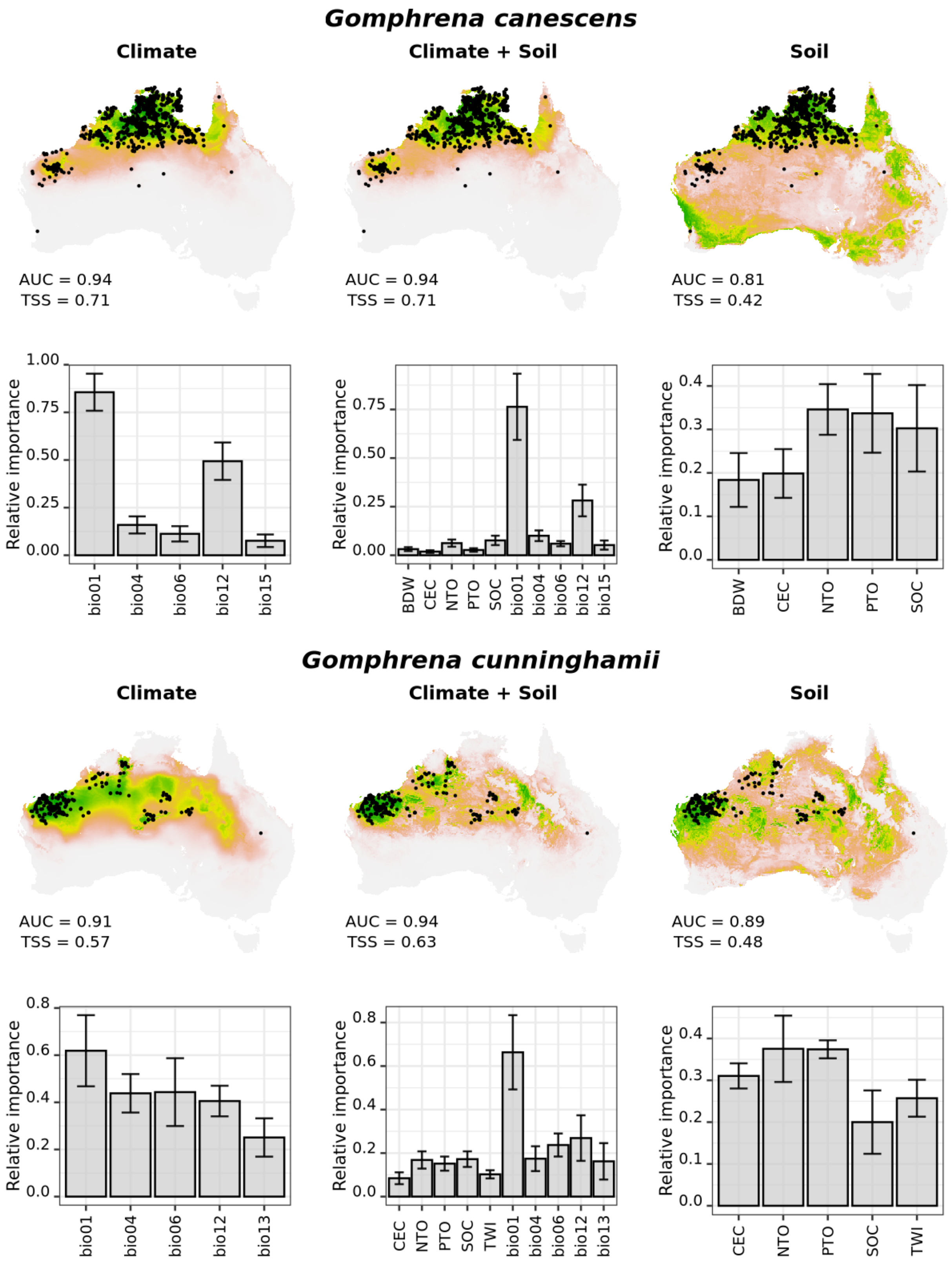

Gomphrena canescens
Climate
Climate + Soil
Soil
AUC = 0.94
TSS = 0.71
AUC = 0.94
TSS = 0.71
AUC = 0.81
TSS = 0.42
Relative importance
1.00
0.75
0.50
0.25
0.00
bio01
bio04
bio06
bio12
bio15
BDW
CEC
NTO
PTO
SOC
0.4
0.3
0.2
0.1
0.0
Gomphrena cunninghamii
Climate
Climate + Soil
Soil
AUC = 0.91
TSS = 0.57
AUC = 0.94
TSS = 0.63
AUC = 0.89
TSS = 0.48
0.8
0.6
0.4
0.2
0.0
bio13
TWI

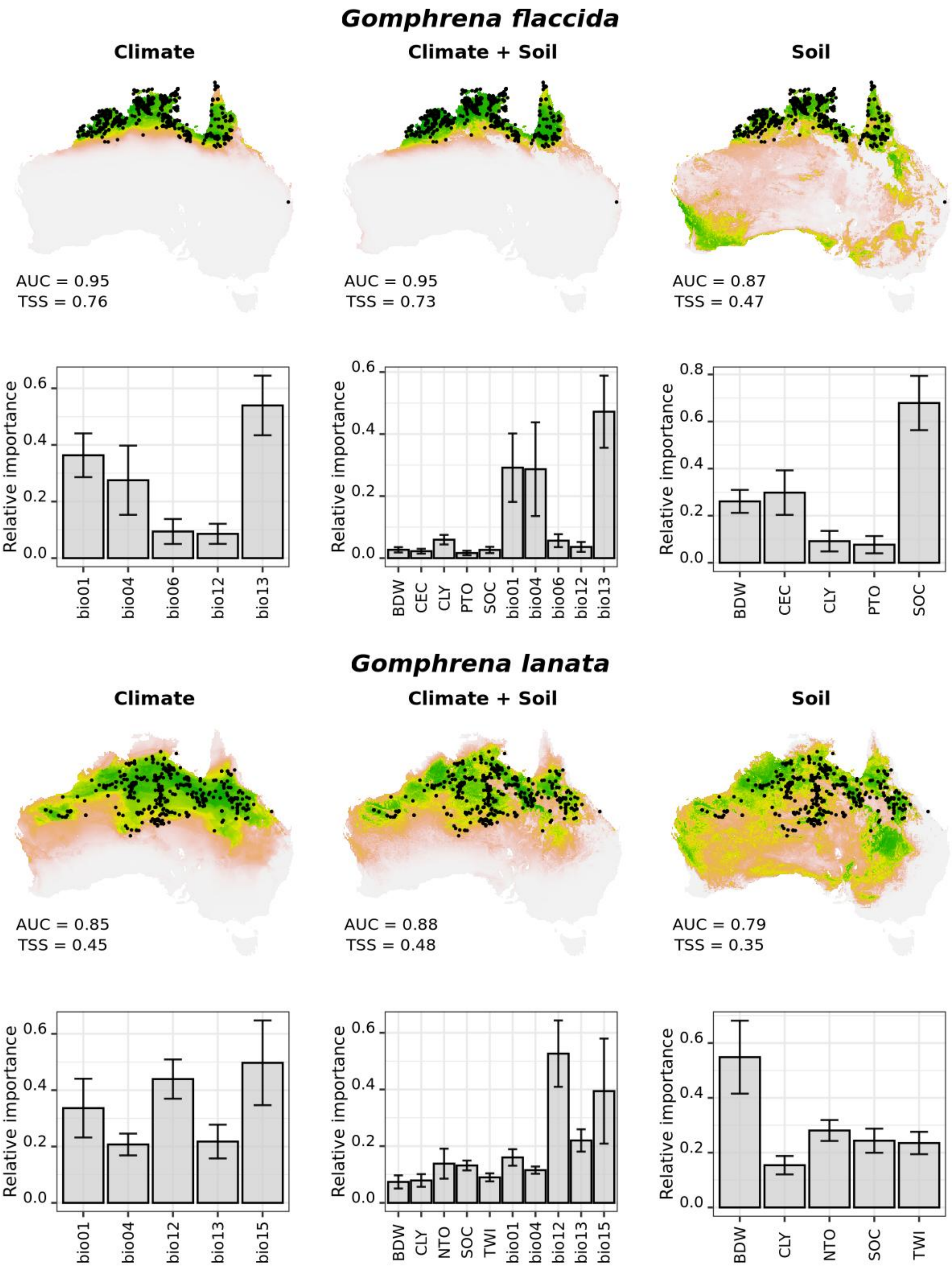

Gomphrena flaccida
Climate
Climate + Soil
Soil
AUC = 0.95
TSS = 0.76
AUC = 0.95
TSS = 0.73
AUC = 0.87
TSS = 0.47
Relative importance
bio01
bio04
bio06
bio12
bio13
BDW
CEC
CLY
PTO
SOC
Gomphrena lanata
Climate
Climate + Soil
Soil
AUC = 0.85
TSS = 0.45
AUC = 0.88
TSS = 0.48
AUC = 0.79
TSS = 0.35
bio15
NTO
TWI

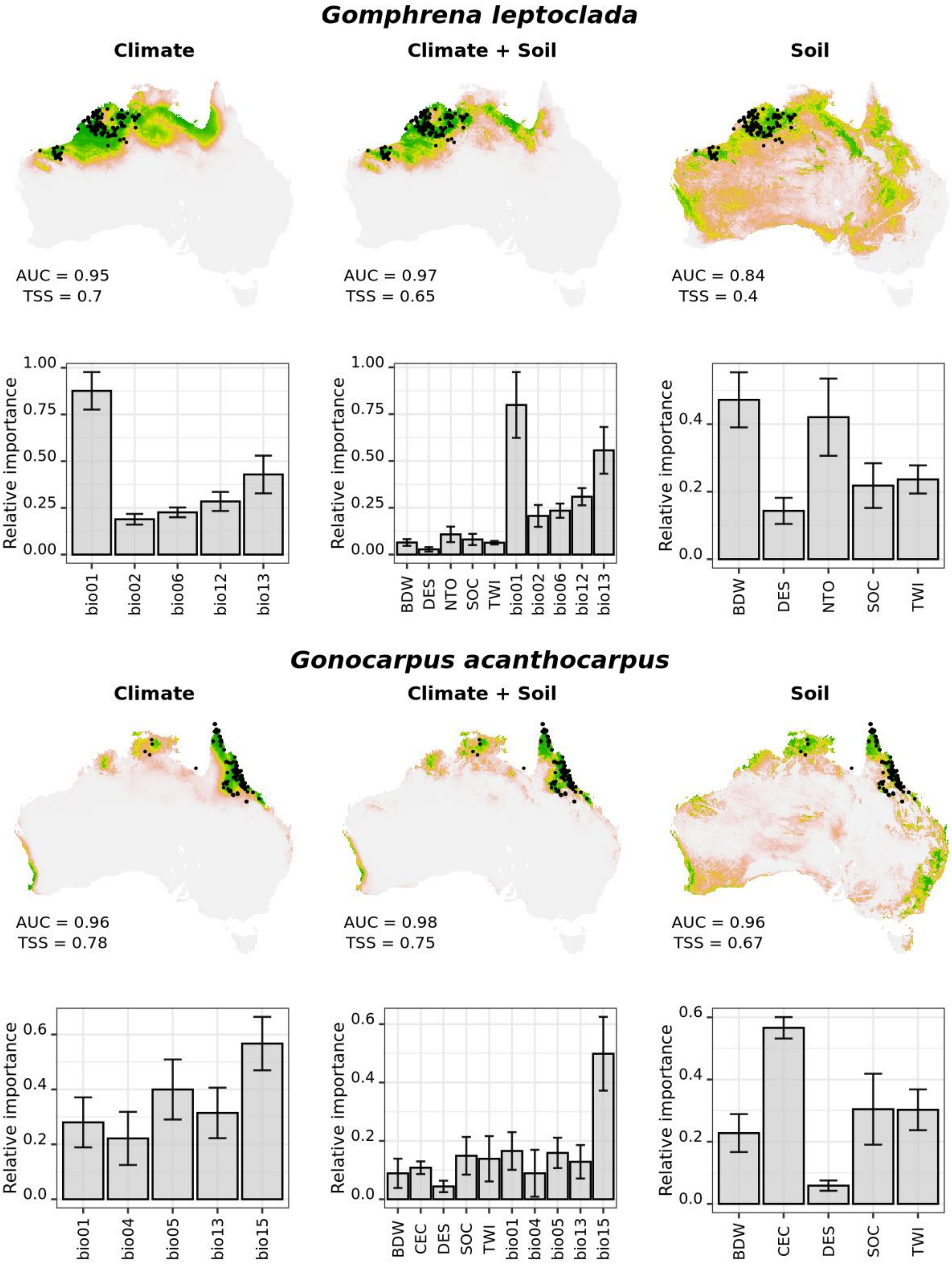

Gomphrena leptoclada
Climate
Climate + Soil
Soil
AUC = 0.95
TSS = 0.7
AUC = 0.97
TSS = 0.65
AUC = 0.84
TSS = 0.4
Relative importance
1.00
0.75
0.50
0.25
0.00
0.4
0.2
0.0
bio01
bio02
bio06
bio12
bio13
BDW
DES
NTO
SOC
TWI
Gonocarpus acanthocarpus
Climate
Climate + Soil
Soil
AUC = 0.96
TSS = 0.78
AUC = 0.98
TSS = 0.75
AUC = 0.96
TSS = 0.67
Relative importance
0.6
0.4
0.2
0.0
bio01
bio04
bio05
bio13
bio15
BDW
CEC
DES
SOC
TWI

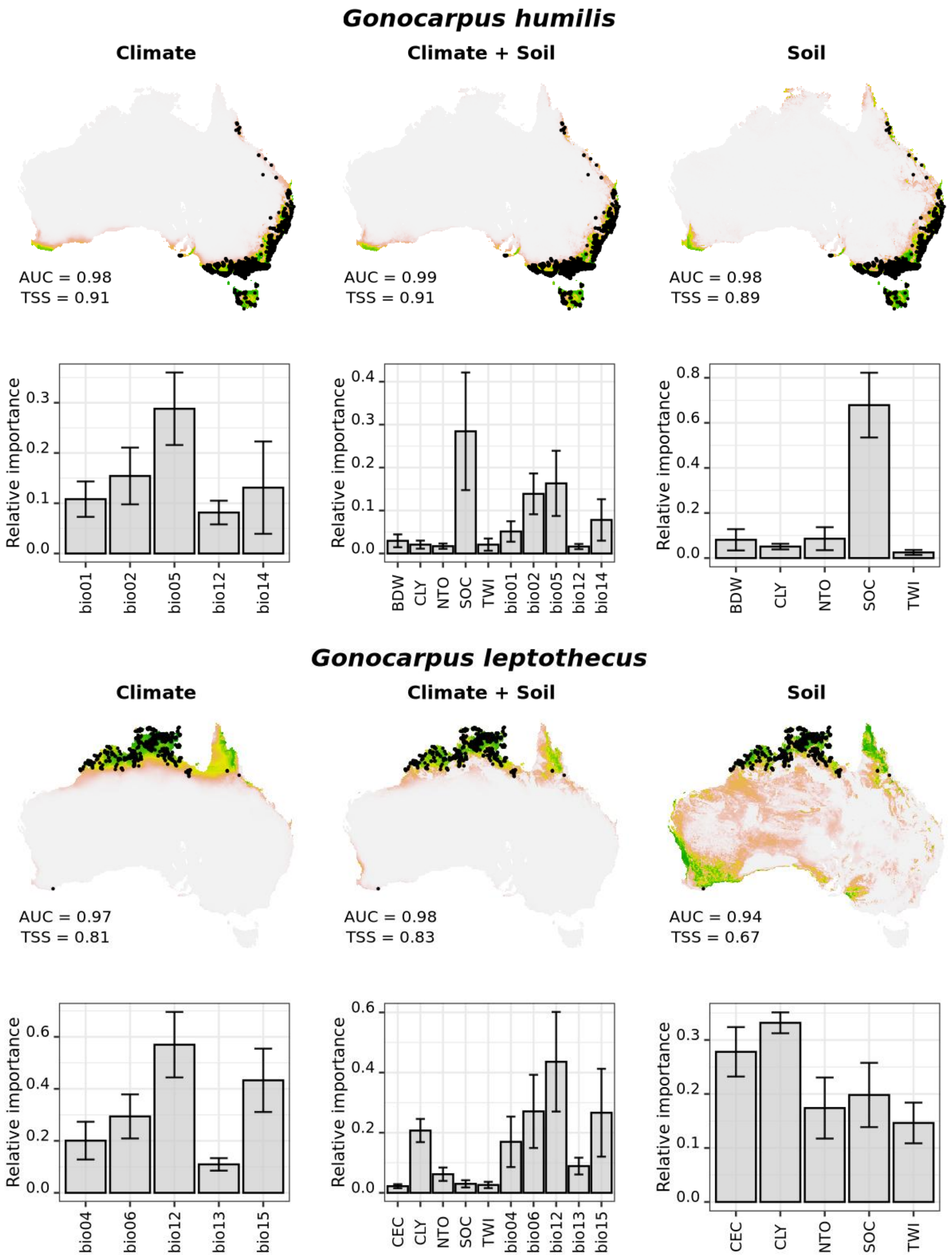

Gonocarpus humilis
Climate
Climate + Soil
Soil
AUC = 0.98
TSS = 0.91
AUC = 0.99
TSS = 0.91
AUC = 0.98
TSS = 0.89
Relative importance
bio01
bio02
bio05
bio12
bio14
BDW
CLY
NTO
SOC
TWI
Gonocarpus leptothecus
Climate
Climate + Soil
Soil
AUC = 0.97
TSS = 0.81
AUC = 0.98
TSS = 0.83
AUC = 0.94
TSS = 0.67
Relative importance
bio04
bio06
bio12
bio13
bio15
CEC
CLY
NTO
SOC
TWI

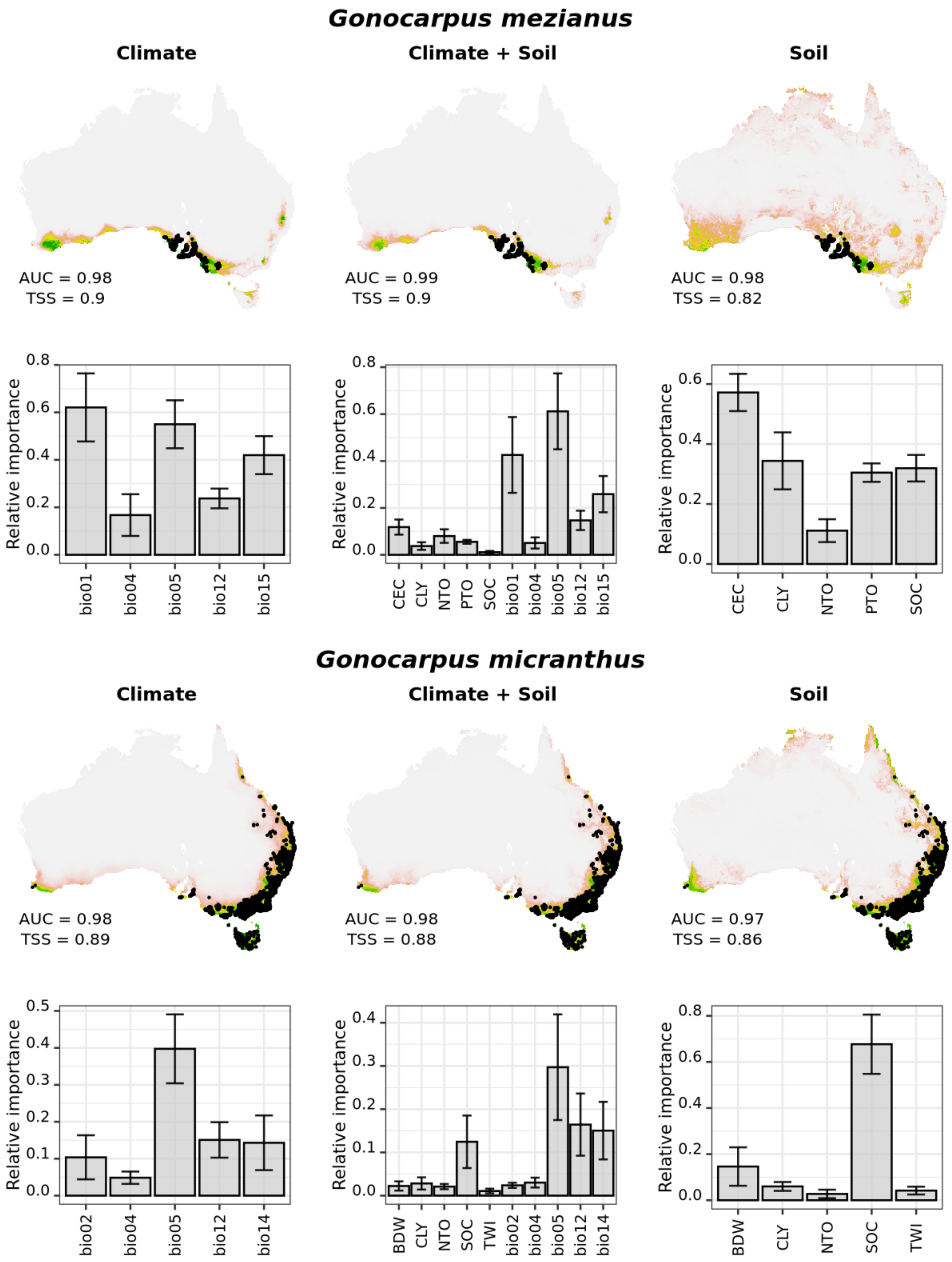
Gonocarpus mezianus
Climate
Climate + Soil
Soil
AUC = 0.98
TSS = 0.9
AUC = 0.99
TSS = 0.9
AUC = 0.98
TSS = 0.82
Relative importance
0.0
0.2
0.4
0.6
0.8
bio01
bio04
bio05
bio12
bio15
CEC
CLY
NTO
PTO
SOC
Gonocarpus micranthus
Climate
Climate + Soil
Soil
AUC = 0.98
TSS = 0.89
AUC = 0.98
TSS = 0.88
AUC = 0.97
TSS = 0.86
Relative importance
0.0
0.1
0.2
0.3
0.4
0.5
bio02
bio04
bio05
bio12
bio14
BDW
CLY
NTO
SOC
TWI

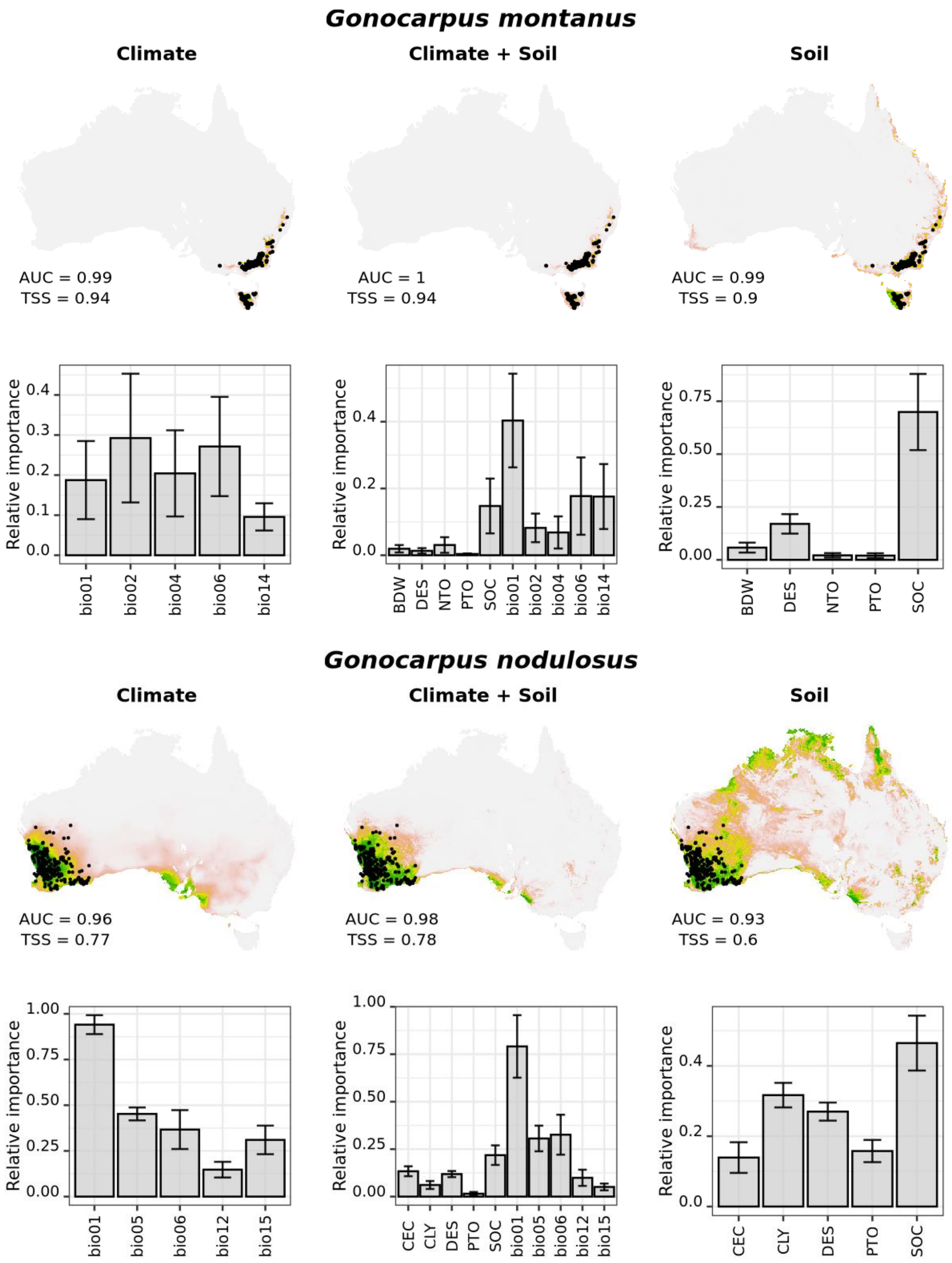
Gonocarpus montanus
Climate
Climate + Soil
Soil
AUC = 0.99
TSS = 0.94
AUC = 1
TSS = 0.94
AUC = 0.99
TSS = 0.9
Relative importance
bio01
bio02
bio04
bio06
bio14
BDW
DES
NTO
PTO
SOC
Gonocarpus nodulosus
AUC = 0.96
TSS = 0.77
AUC = 0.98
TSS = 0.78
AUC = 0.93
TSS = 0.6
bio05
bio12
bio15
CEC
CLY

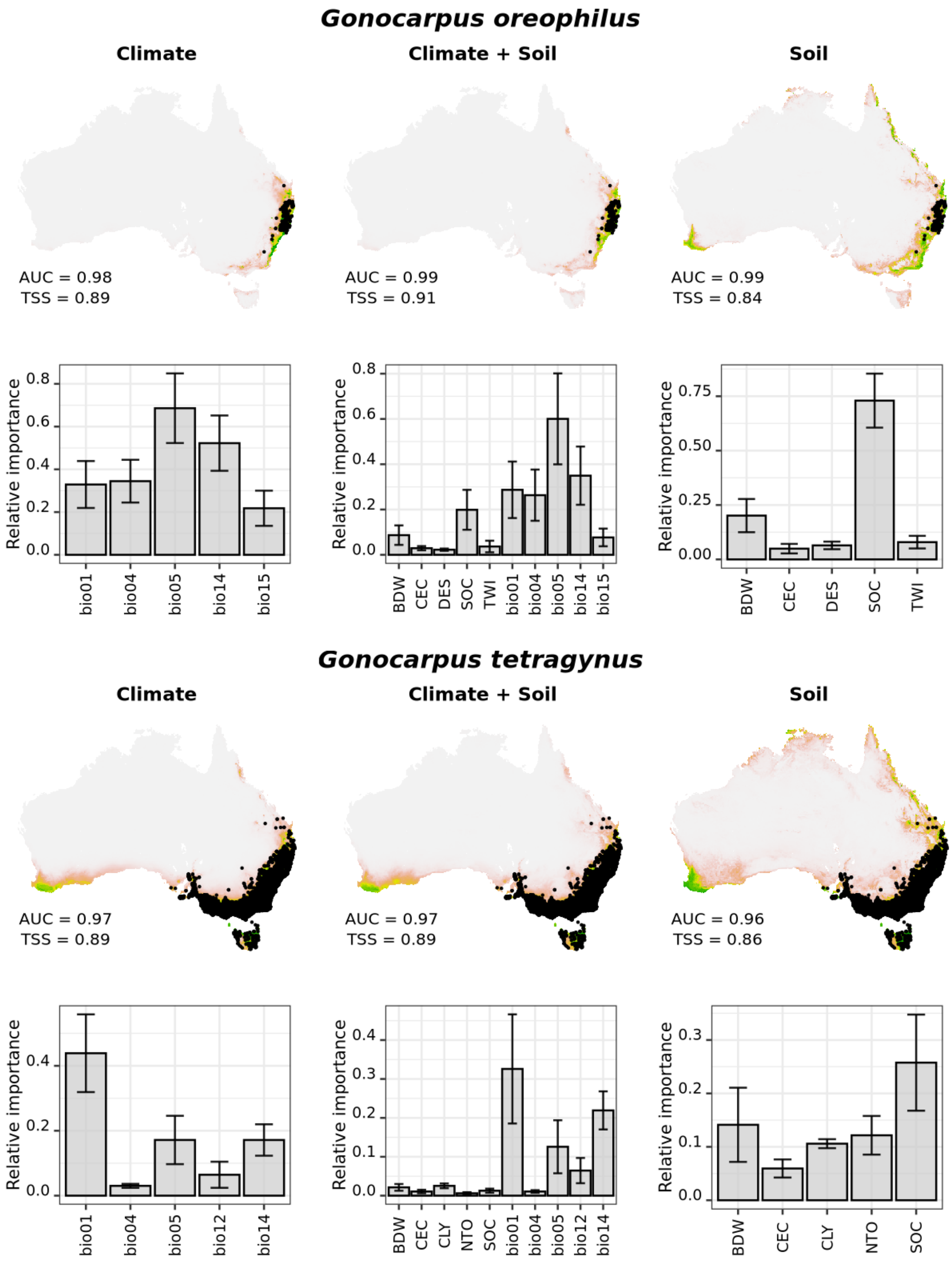
Gonocarpus oreophilus
Climate
Climate + Soil
Soil
AUC = 0.98
TSS = 0.89
AUC = 0.99
TSS = 0.91
AUC = 0.99
TSS = 0.84
Relative importance
bio01
bio04
bio05
bio14
bio15
BDW
CEC
DES
SOC
TWI
Gonocarpus tetragynus
Climate
Climate + Soil
Soil
AUC = 0.97
TSS = 0.89
AUC = 0.97
TSS = 0.89
AUC = 0.96
TSS = 0.86
Relative importance
bio01
bio04
bio05
bio12
bio14
BDW
CEC
CLY
NTO
SOC

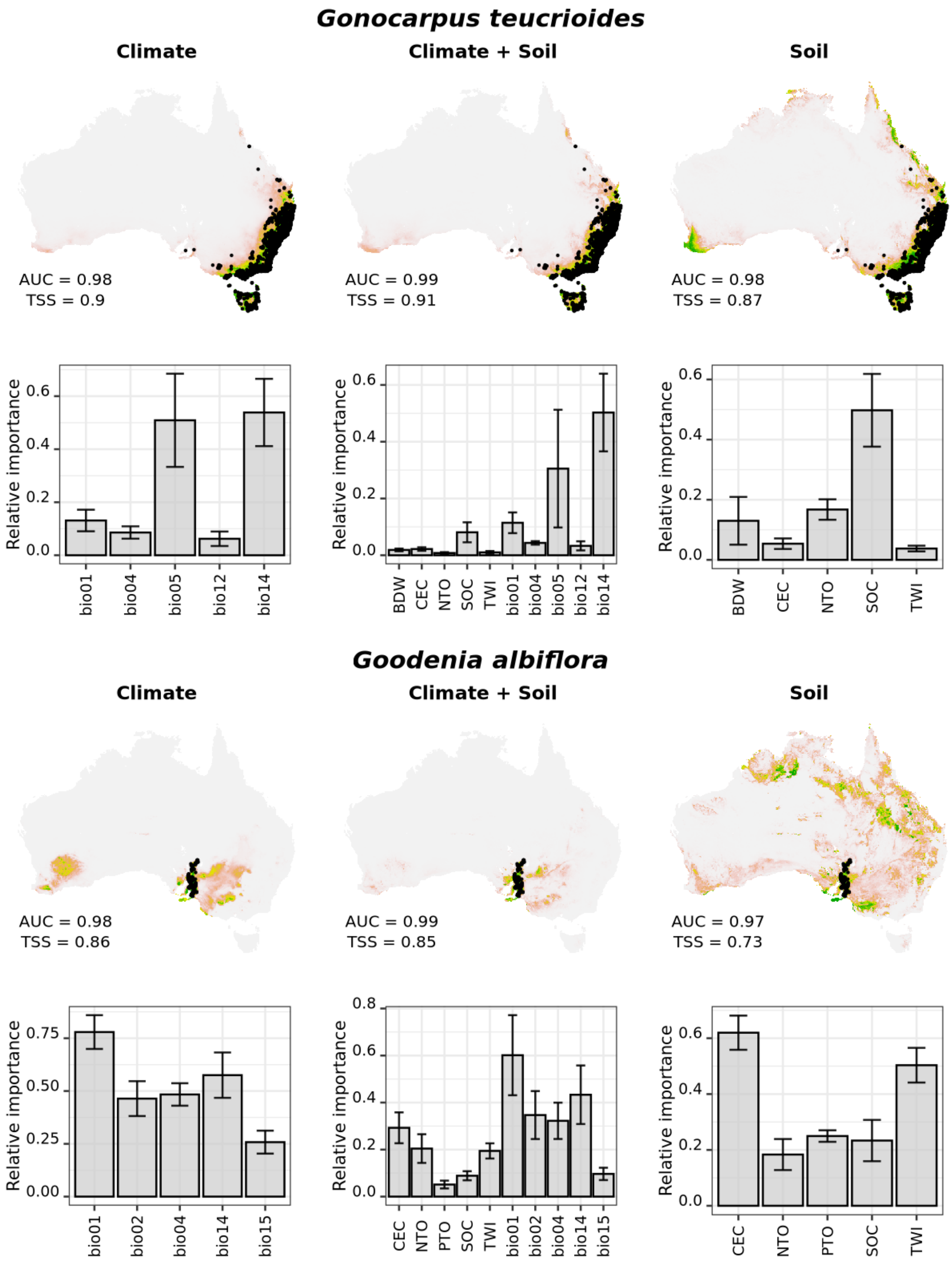
Gonocarpus teucrioides
Climate
Climate + Soil
Soil
AUC = 0.98
TSS = 0.9
AUC = 0.99
TSS = 0.91
AUC = 0.98
TSS = 0.87
Relative importance
0.0
0.2
0.4
0.6
bio01
bio04
bio05
bio12
bio14
BDW
CEC
NTO
SOC
TWI
Goodenia albiflora
Climate
Climate + Soil
Soil
AUC = 0.98
TSS = 0.86
AUC = 0.99
TSS = 0.85
AUC = 0.97
TSS = 0.73
0.00
0.25
0.50
0.75
0.8
bio02
bio15
PTO

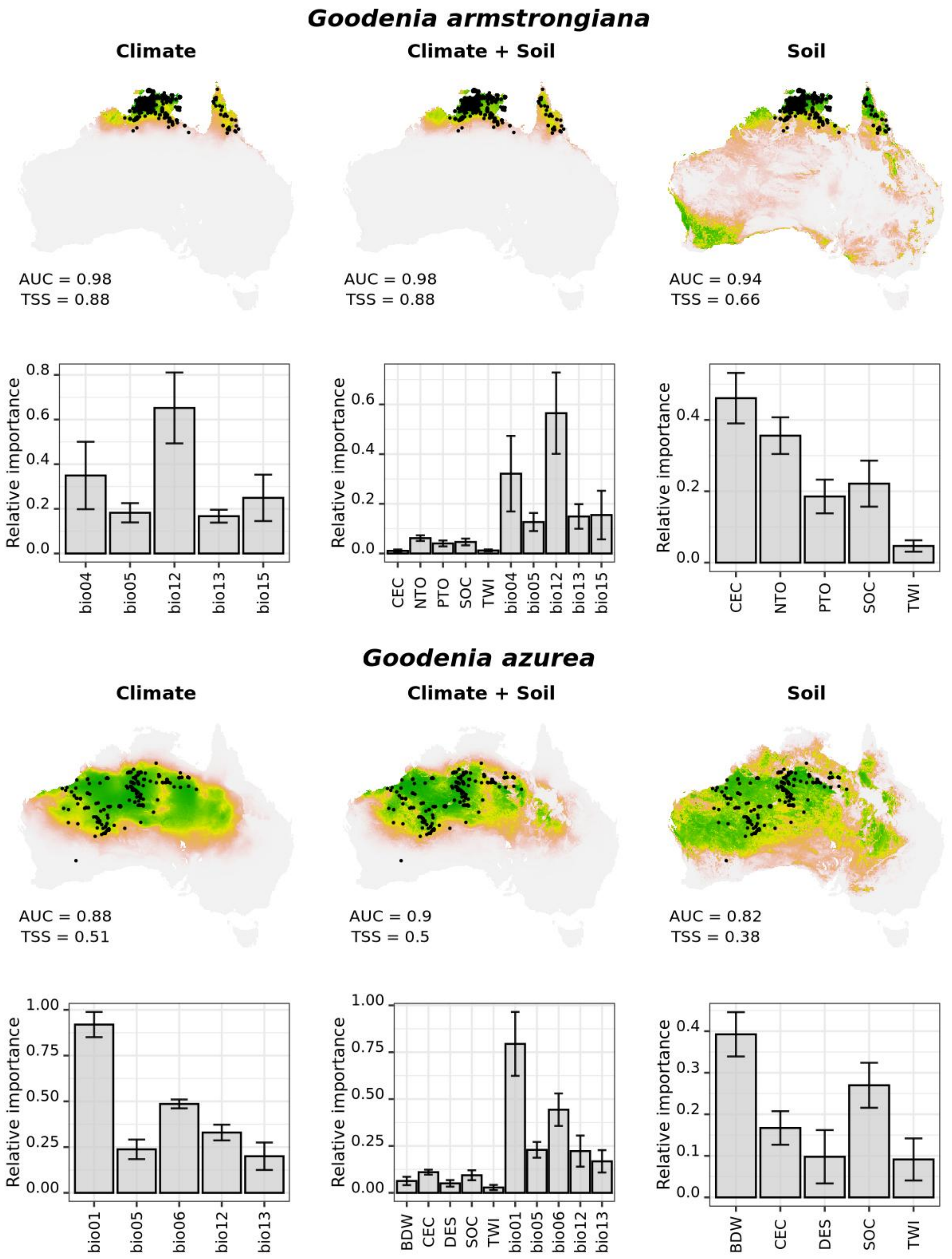

Goodenia armstrongiana
Climate
Climate + Soil
Soil
AUC = 0.98
TSS = 0.88
AUC = 0.98
TSS = 0.88
AUC = 0.94
TSS = 0.66
Relative importance
bio04
bio05
bio12
bio13
bio15
CEC
NTO
PTO
SOC
TWI
Goodenia azurea
Climate
Climate + Soil
Soil
AUC = 0.88
TSS = 0.51
AUC = 0.9
TSS = 0.5
AUC = 0.82
TSS = 0.38
Relative importance
bio01
bio05
bio06
bio12
bio13
BDW
CEC
DES
SOC
TWI

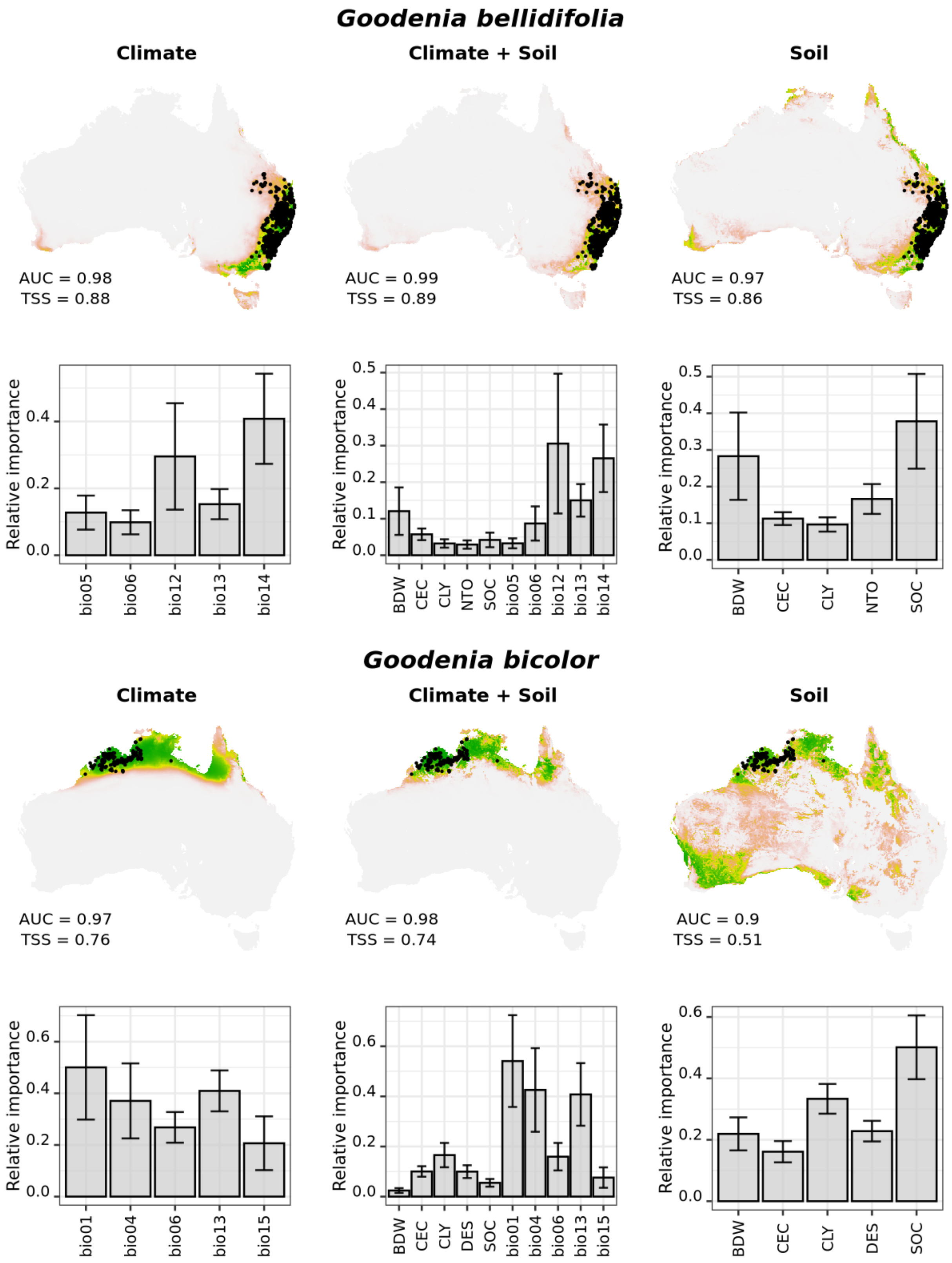
Goodenia bellidifolia
Climate
Climate + Soil
Soil
AUC = 0.98
TSS = 0.88
AUC = 0.99
TSS = 0.89
AUC = 0.97
TSS = 0.86
Relative importance
0.0
0.2
0.4
bio05
bio06
bio12
bio13
bio14
0.0
0.1
0.2
0.3
0.4
0.5
BDW
CEC
CLY
NTO
SOC
bio05
bio06
bio12
bio13
bio14
BDW
CEC
CLY
NTO
SOC
Goodenia bicolor
Climate
Climate + Soil
Soil
AUC = 0.97
TSS = 0.76
AUC = 0.98
TSS = 0.74
AUC = 0.9
TSS = 0.51
Relative importance
0.0
0.2
0.4
0.6
bio01
bio04
bio06
bio13
bio15
BDW
CEC
CLY
DES
SOC
bio01
bio04
bio06
bio13
bio15
BDW
CEC
CLY
DES
SOC

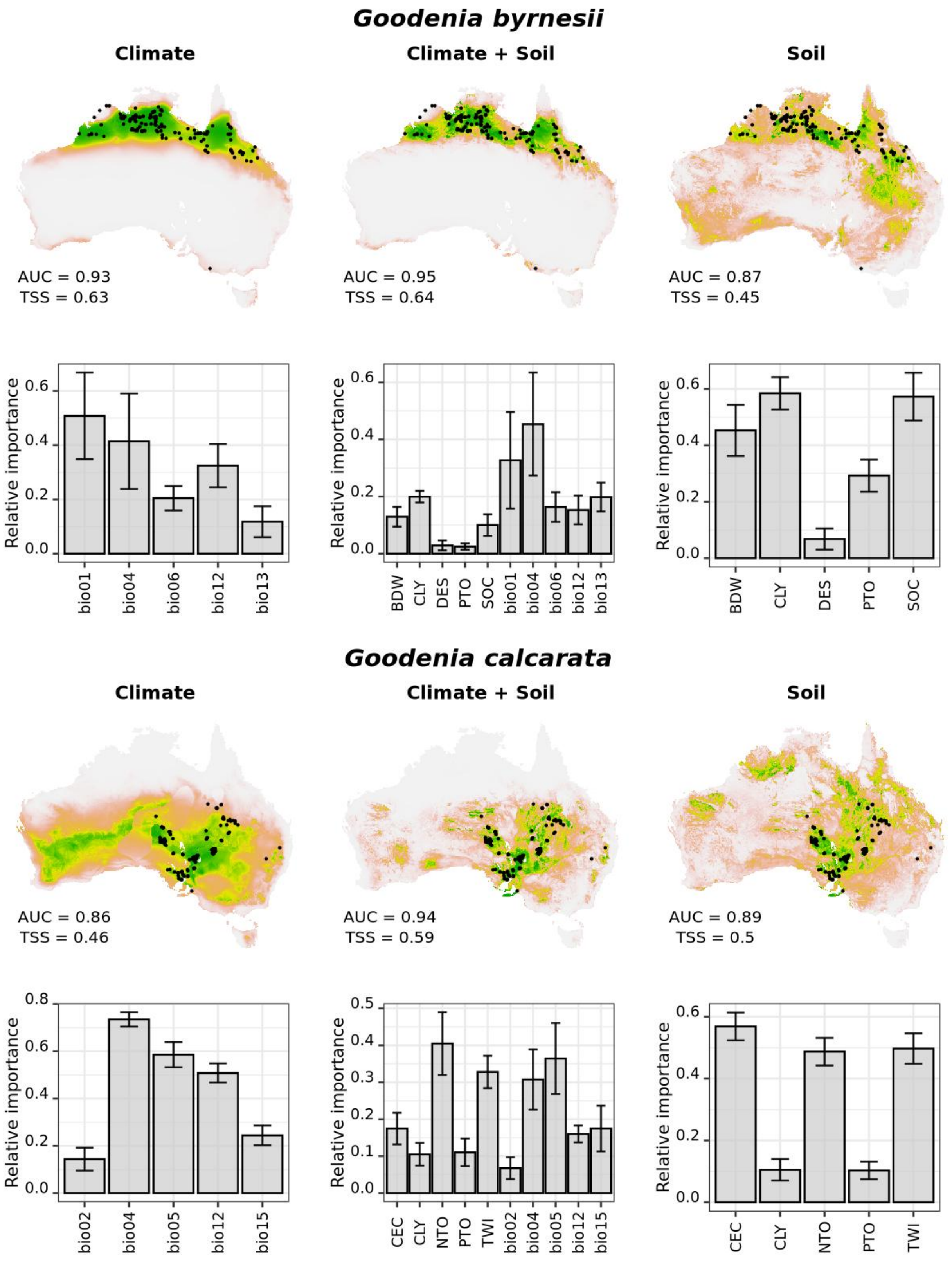
Goodenia byrnesii
Climate
Climate + Soil
Soil
AUC = 0.93
TSS = 0.63
AUC = 0.95
TSS = 0.64
AUC = 0.87
TSS = 0.45
Relative importance
bio01
bio04
bio06
bio12
bio13
BDW
CLY
DES
PTO
SOC
Goodenia calcarata
Climate
Climate + Soil
Soil
AUC = 0.86
TSS = 0.46
AUC = 0.94
TSS = 0.59
AUC = 0.89
TSS = 0.5
Relative importance
bio02
bio04
bio05
bio12
bio15
CEC
CLY
NTO
PTO
TWI

Goodenia centralis

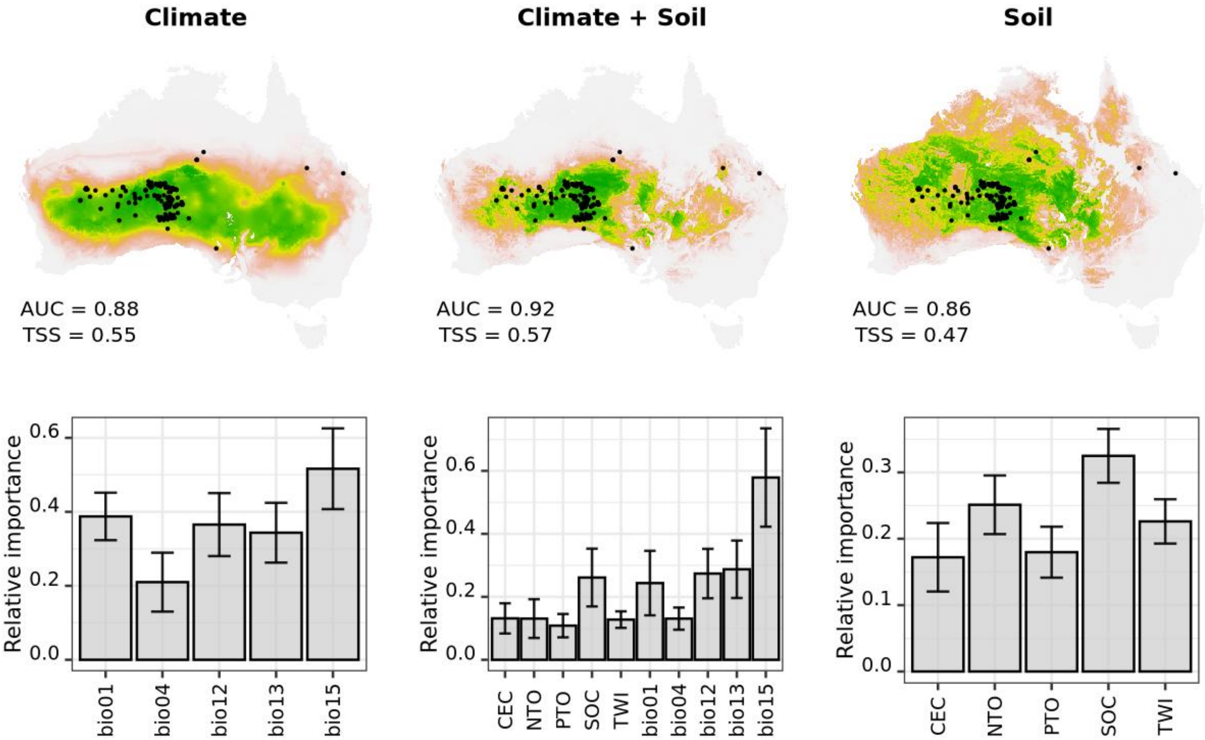

Goodenia concinna

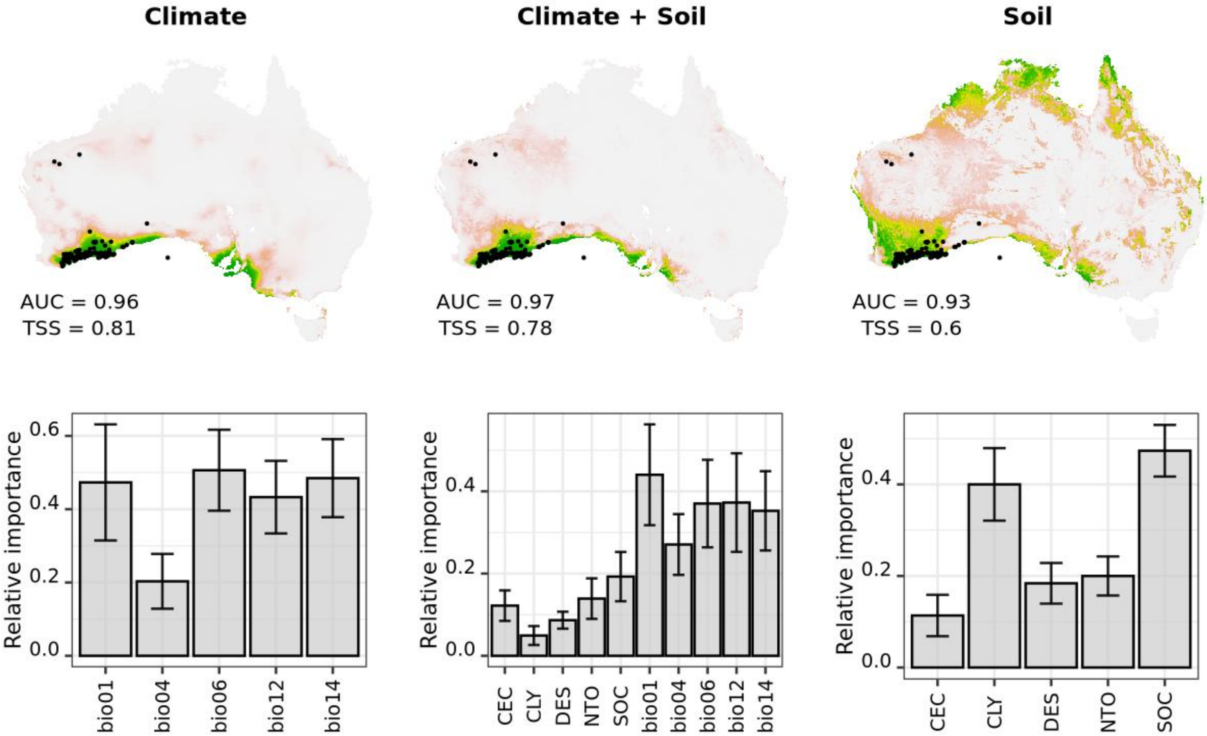

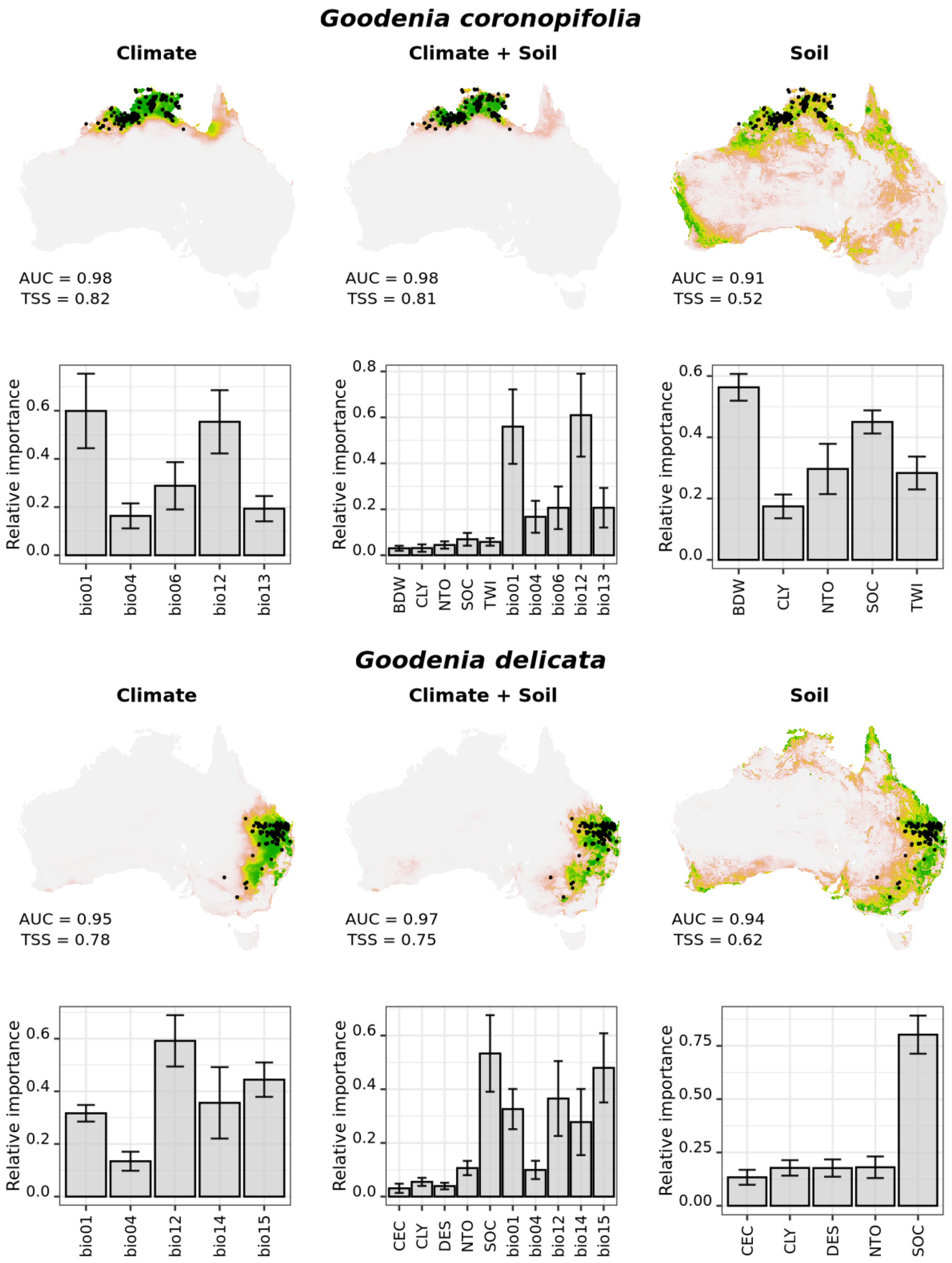
Goodenia coronopifolia
Climate
Climate + Soil
Soil
AUC = 0.98
TSS = 0.82
AUC = 0.98
TSS = 0.81
AUC = 0.91
TSS = 0.52
Relative importance
0.0
0.2
0.4
0.6
0.8
bio01
bio04
bio06
bio12
bio13
BDW
CLY
NTO
SOC
TWI
Goodenia delicata
Climate
Climate + Soil
Soil
AUC = 0.95
TSS = 0.78
AUC = 0.97
TSS = 0.75
AUC = 0.94
TSS = 0.62
Relative importance
0.00
0.25
0.50
0.75
bio01
bio04
bio12
bio14
bio15
CEC
CLY
DES
NTO
SOC

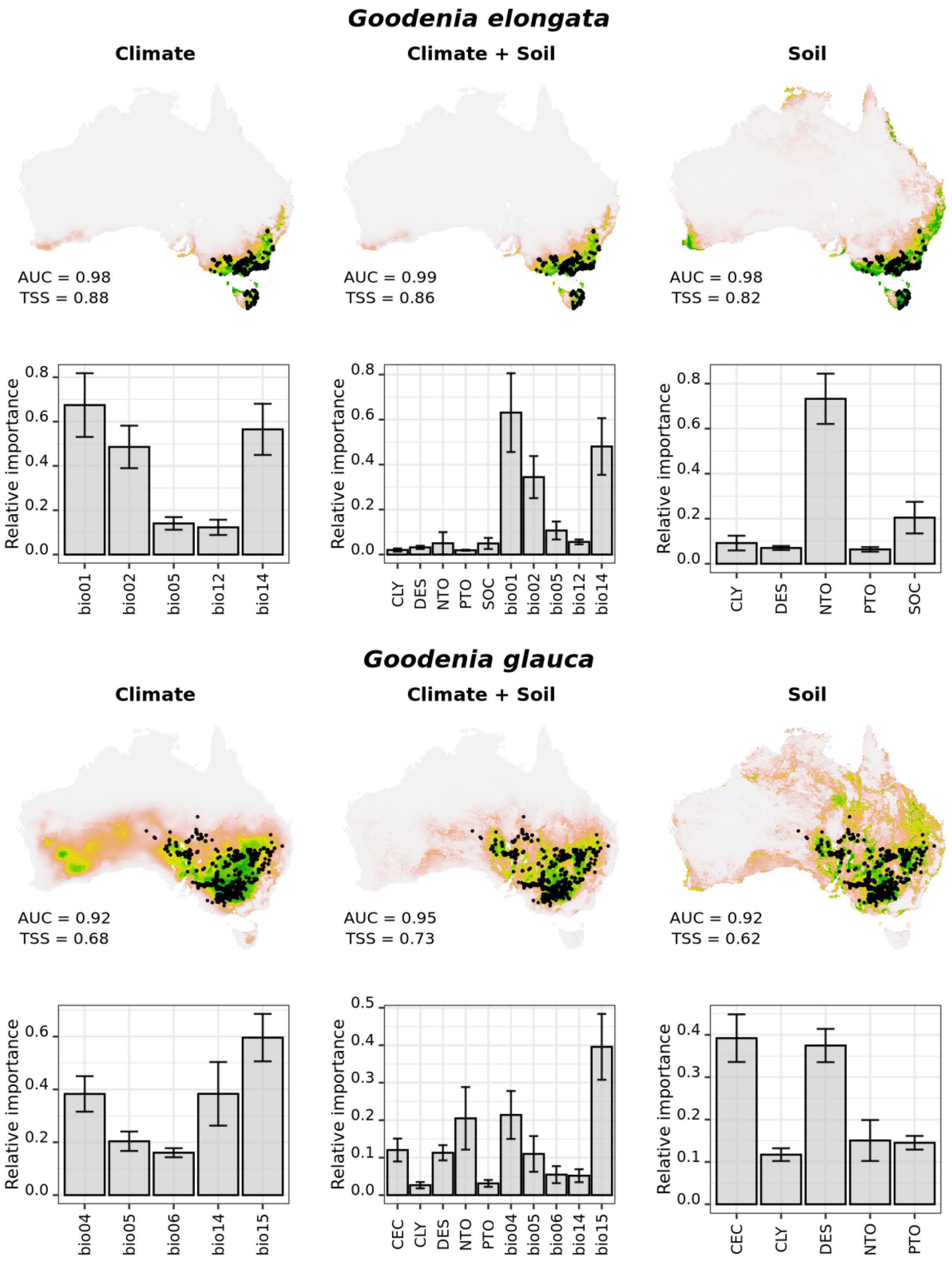
Goodenia elongata
Climate
Climate + Soil
Soil
AUC = 0.98
TSS = 0.88
AUC = 0.99
TSS = 0.86
AUC = 0.98
TSS = 0.82
Relative importance
bio01
bio02
bio05
bio12
bio14
CLY
DES
NTO
PTO
SOC
Goodenia glauca
AUC = 0.92
TSS = 0.68
AUC = 0.95
TSS = 0.73
AUC = 0.92
TSS = 0.62
bio04
bio06
bio15
CEC

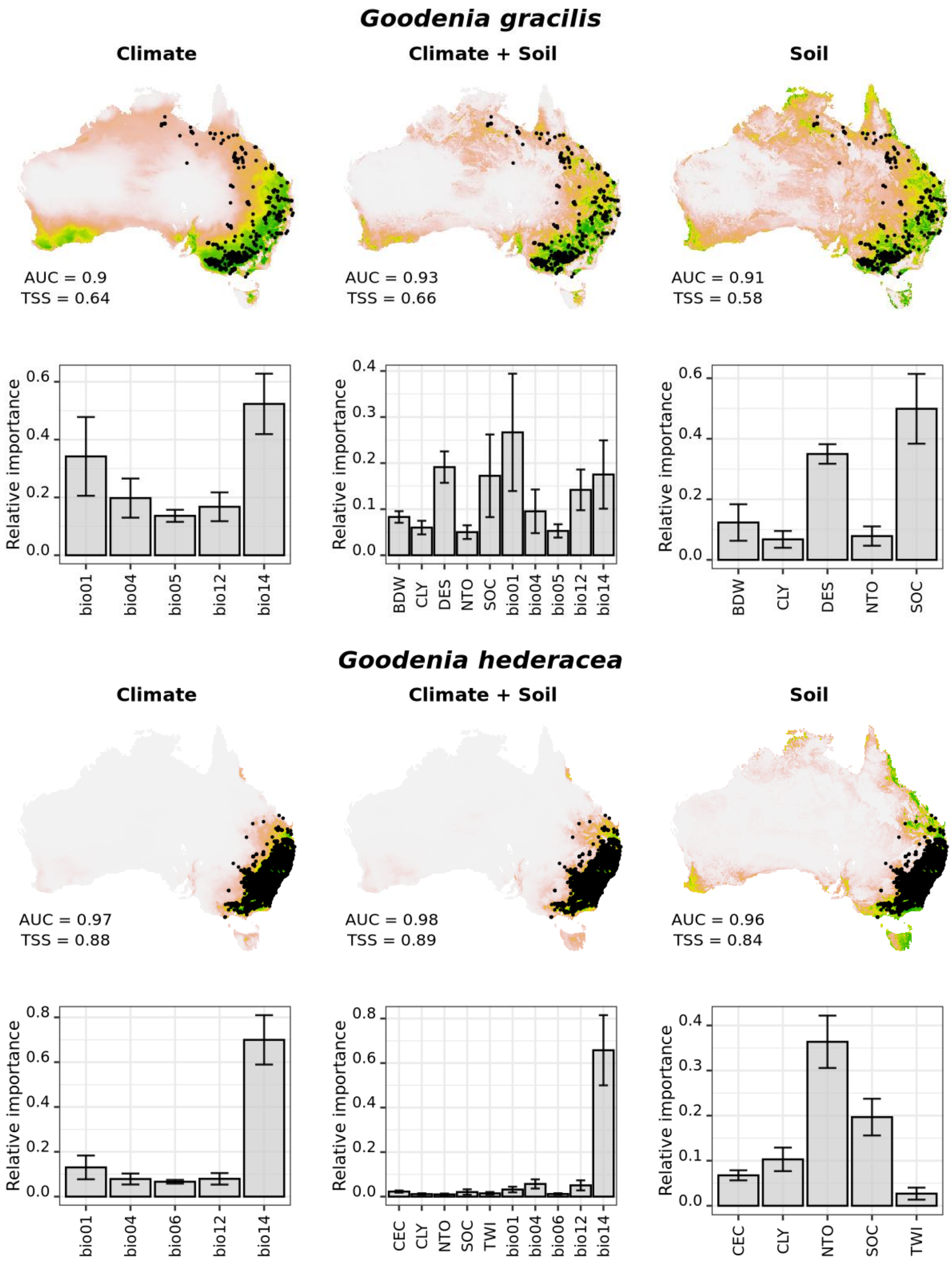
Goodenia gracilis
Climate
Climate + Soil
Soil
AUC = 0.9
TSS = 0.64
AUC = 0.93
TSS = 0.66
AUC = 0.91
TSS = 0.58
Relative importance
0.0
0.2
0.4
0.6
bio01
bio04
bio05
bio12
bio14
Relative importance
0.0
0.1
0.2
0.3
0.4
BDW
CLY
DES
NTO
SOC
bio01
bio04
bio05
bio12
bio14
Relative importance
0.0
0.2
0.4
0.6
BDW
CLY
DES
NTO
SOC
Goodenia hederacea
Climate
Climate + Soil
Soil
AUC = 0.97
TSS = 0.88
AUC = 0.98
TSS = 0.89
AUC = 0.96
TSS = 0.84
Relative importance
0.0
0.2
0.4
0.6
0.8
bio01
bio04
bio06
bio12
bio14
Relative importance
0.0
0.2
0.4
0.6
0.8
CEC
CLY
NTO
SOC
TWI
bio01
bio04
bio06
bio12
bio14
Relative importance
0.0
0.1
0.2
0.3
0.4
CEC
CLY
NTO
SOC
TWI

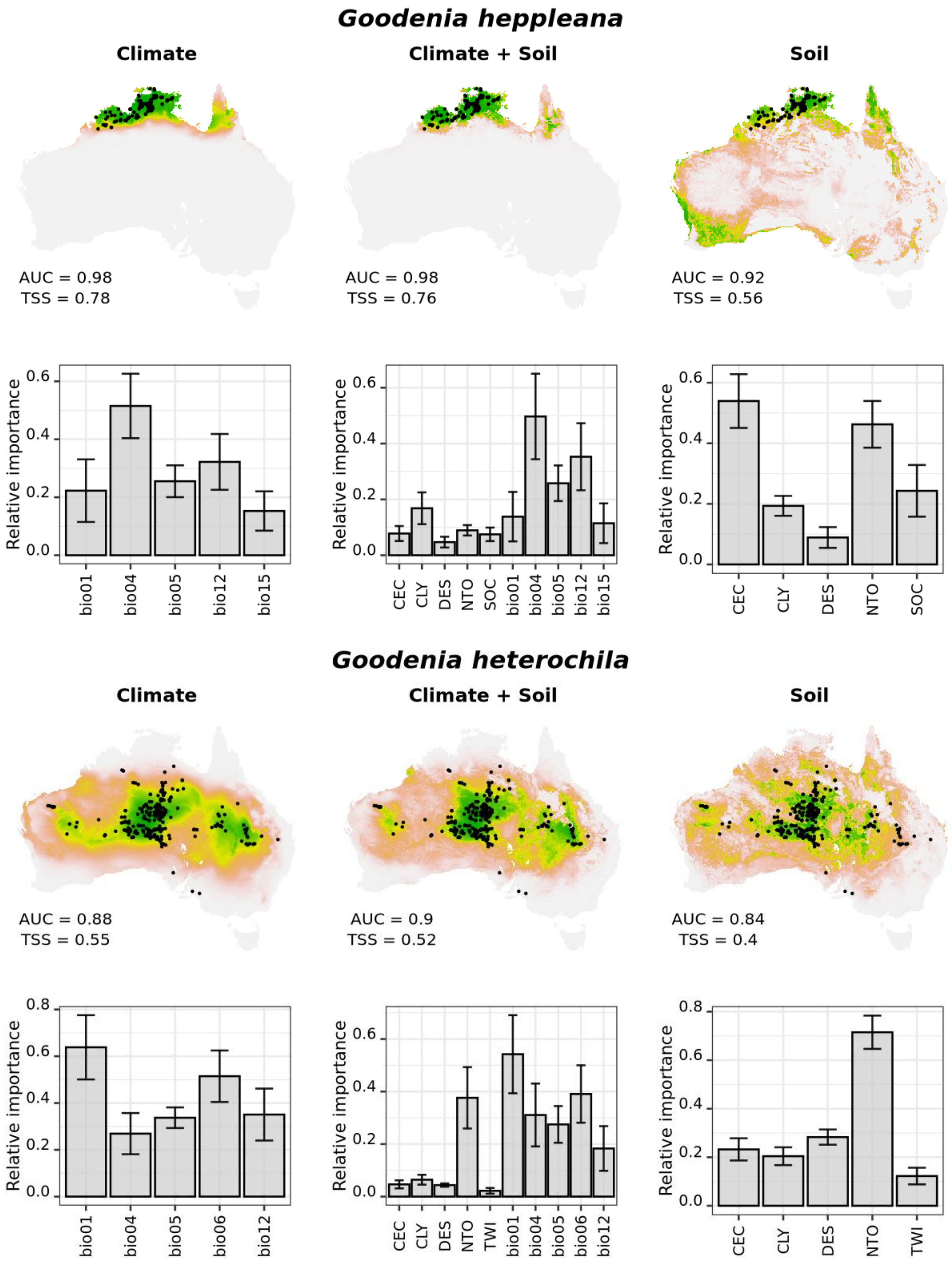
Goodenia heppleana
Climate
Climate + Soil
Soil
AUC = 0.98
TSS = 0.78
AUC = 0.98
TSS = 0.76
AUC = 0.92
TSS = 0.56
Relative importance
bio01 bio04 bio05 bio12 bio15
CEC CLY DES NTO SOC bio01 bio04 bio05 bio12 bio15
CEC CLY DES NTO SOC
Goodenia heterochila
Climate
Climate + Soil
Soil
AUC = 0.88
TSS = 0.55
AUC = 0.9
TSS = 0.52
AUC = 0.84
TSS = 0.4
Relative importance
bio01 bio04 bio05 bio06 bio12
CEC CLY DES NTO TWI bio01 bio04 bio05 bio06 bio12
CEC CLY DES NTO TWI

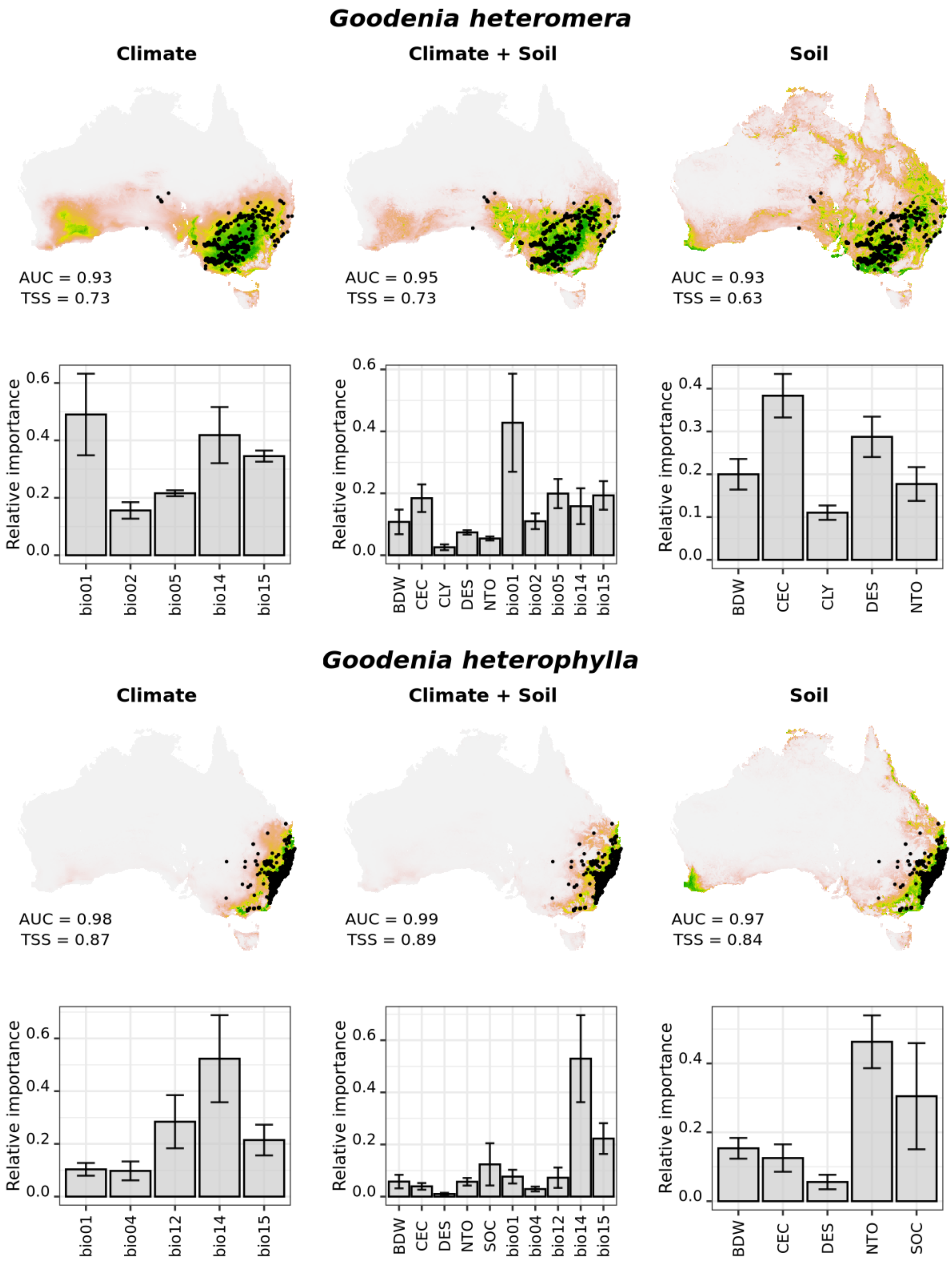
Goodenia heteromera
Climate
Climate + Soil
Soil
AUC = 0.93
TSS = 0.73
AUC = 0.95
TSS = 0.73
AUC = 0.93
TSS = 0.63
Relative importance
bio01
bio02
bio05
bio14
bio15
BDW
CEC
CLY
DES
NTO
bio01
bio02
bio05
bio14
bio15
BDW
CEC
CLY
DES
NTO
Goodenia heterophylla
Climate
Climate + Soil
Soil
AUC = 0.98
TSS = 0.87
AUC = 0.99
TSS = 0.89
AUC = 0.97
TSS = 0.84
Relative importance
bio01
bio04
bio12
bio14
bio15
BDW
CEC
DES
NTO
SOC
bio01
bio04
bio12
bio14
bio15
BDW
CEC
DES
NTO
SOC

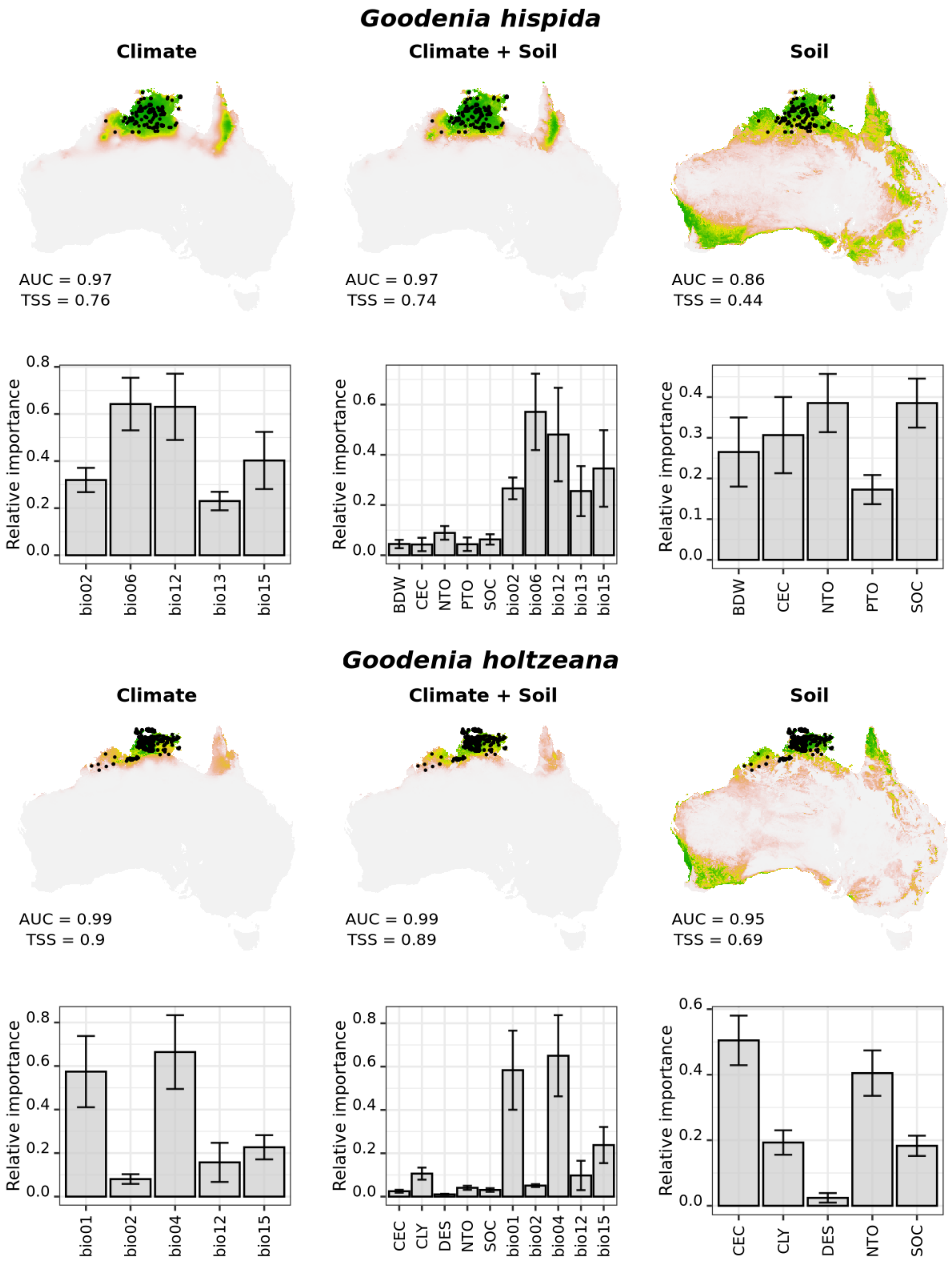
Goodenia hispida
Climate
Climate + Soil
Soil
AUC = 0.97
TSS = 0.76
AUC = 0.97
TSS = 0.74
AUC = 0.86
TSS = 0.44
Relative importance
0.0
0.2
0.4
0.6
0.8
bio02
bio06
bio12
bio13
bio15
BDW
CEC
NTO
PTO
SOC
0.1
0.3
Goodenia holtzeana
Climate
Climate + Soil
Soil
AUC = 0.99
TSS = 0.9
AUC = 0.99
TSS = 0.89
AUC = 0.95
TSS = 0.69
bio01
bio04
CLY
DES

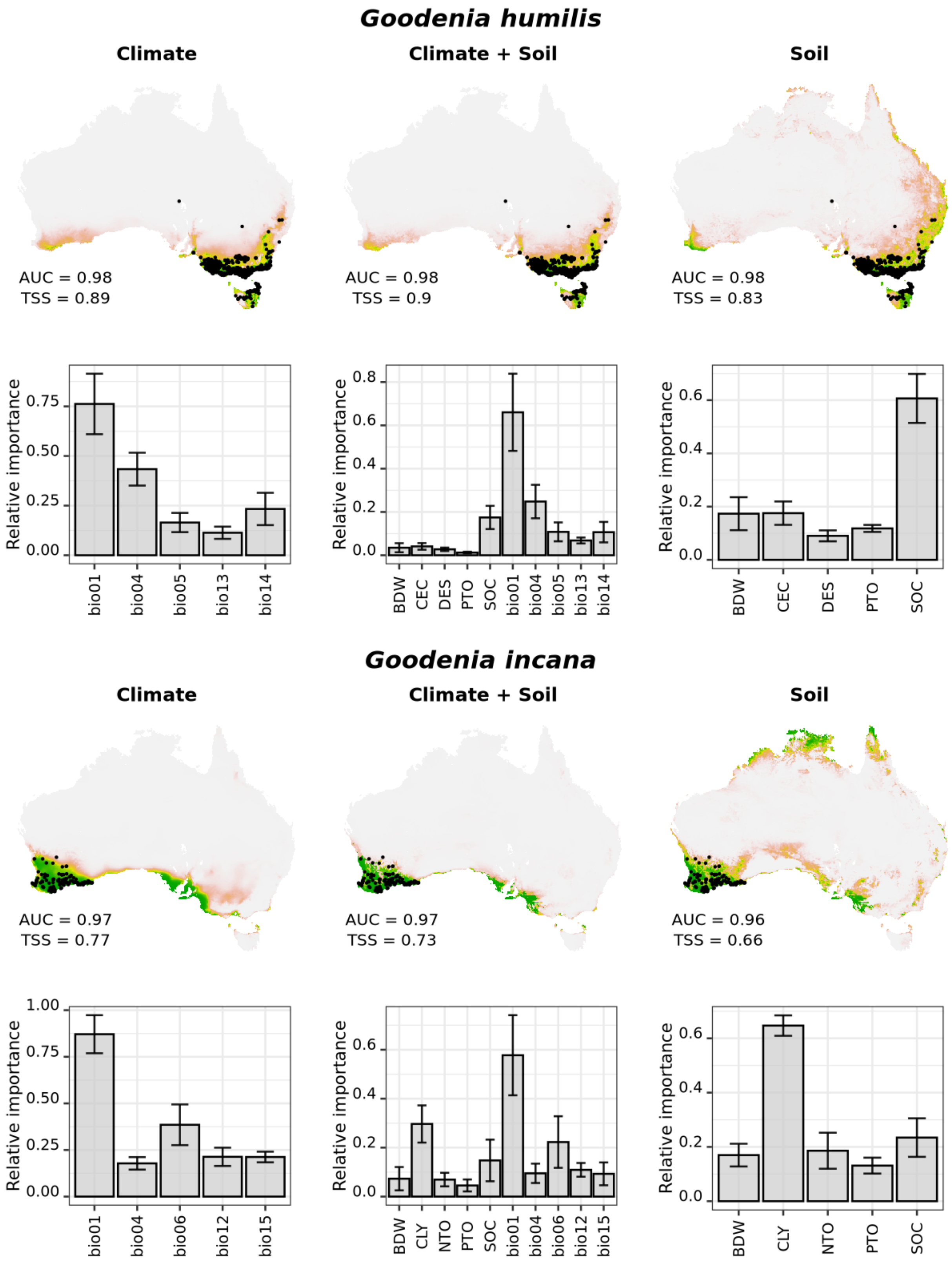

Goodenia humilis
Climate
Climate + Soil
Soil
AUC = 0.98
TSS = 0.89
AUC = 0.98
TSS = 0.9
AUC = 0.98
TSS = 0.83
Relative importance
0.00
0.25
0.50
0.75
bio01
bio04
bio05
bio13
bio14
0.0
0.2
0.4
0.6
0.8
BDW
CEC
DES
PTO
SOC
bio01
bio04
bio05
bio13
bio14
0.0
0.2
0.4
0.6
BDW
CEC
DES
PTO
SOC
Goodenia incana
Climate
Climate + Soil
Soil
AUC = 0.97
TSS = 0.77
AUC = 0.97
TSS = 0.73
AUC = 0.96
TSS = 0.66
Relative importance
0.00
0.25
0.50
0.75
1.00
bio01
bio04
bio06
bio12
bio15
0.0
0.2
0.4
0.6
BDW
CLY
NTO
PTO
SOC
bio01
bio04
bio06
bio12
bio15
0.0
0.2
0.4
0.6
BDW
CLY
NTO
PTO
SOC

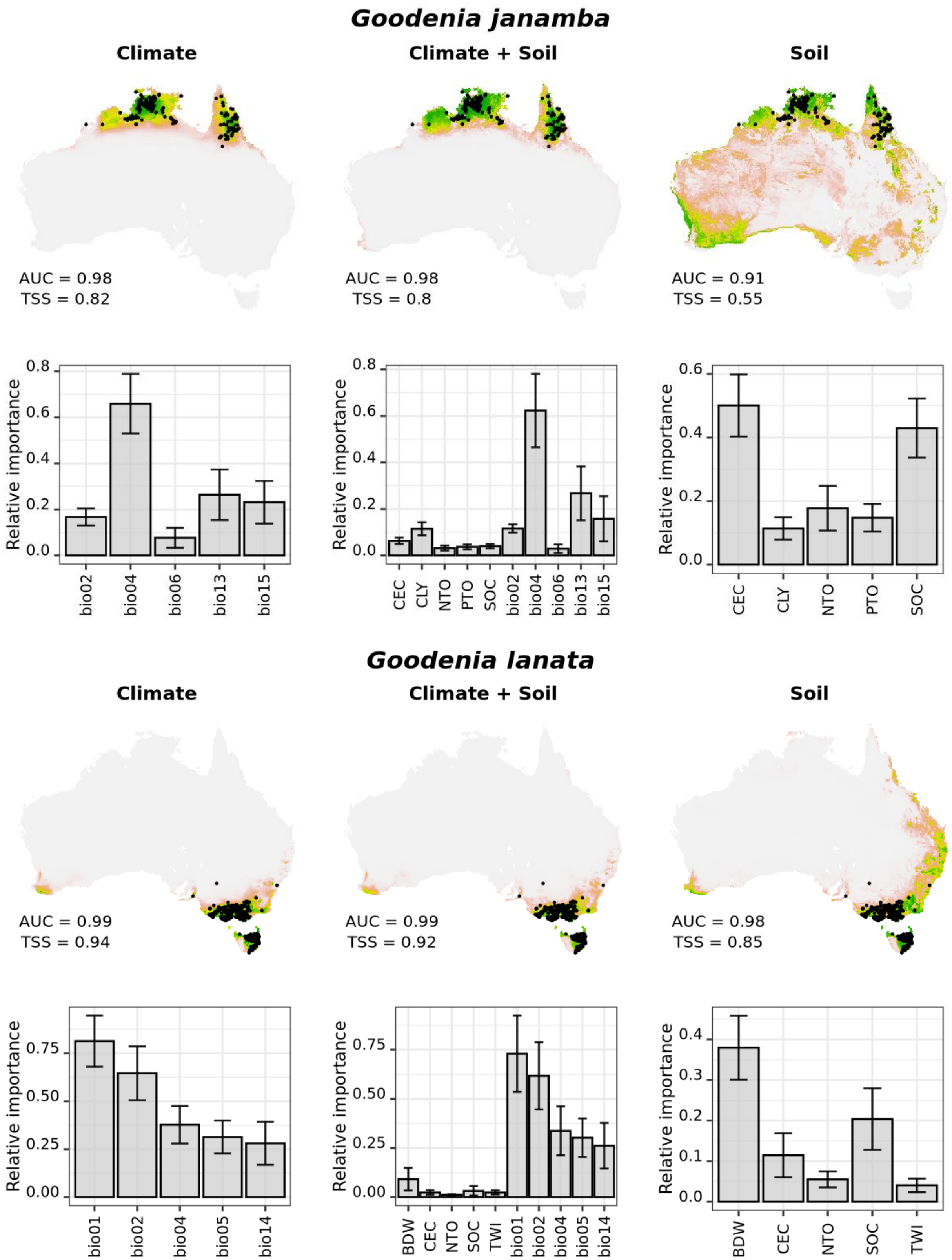

Goodenia janamba
Climate
Climate + Soil
Soil
AUC = 0.98
TSS = 0.82
AUC = 0.98
TSS = 0.8
AUC = 0.91
TSS = 0.55
Relative importance
bio02 bio04 bio06 bio13 bio15
CEC CLY NTO PTO SOC bio02 bio04 bio06 bio13 bio15
CEC CLY NTO PTO SOC
Goodenia lanata
Climate
Climate + Soil
Soil
AUC = 0.99
TSS = 0.94
AUC = 0.99
TSS = 0.92
AUC = 0.98
TSS = 0.85
Relative importance
bio01 bio02 bio04 bio05 bio14
BDW CEC NTO SOC TWI bio01 bio02 bio04 bio05 bio14
BDW CEC NTO SOC TWI

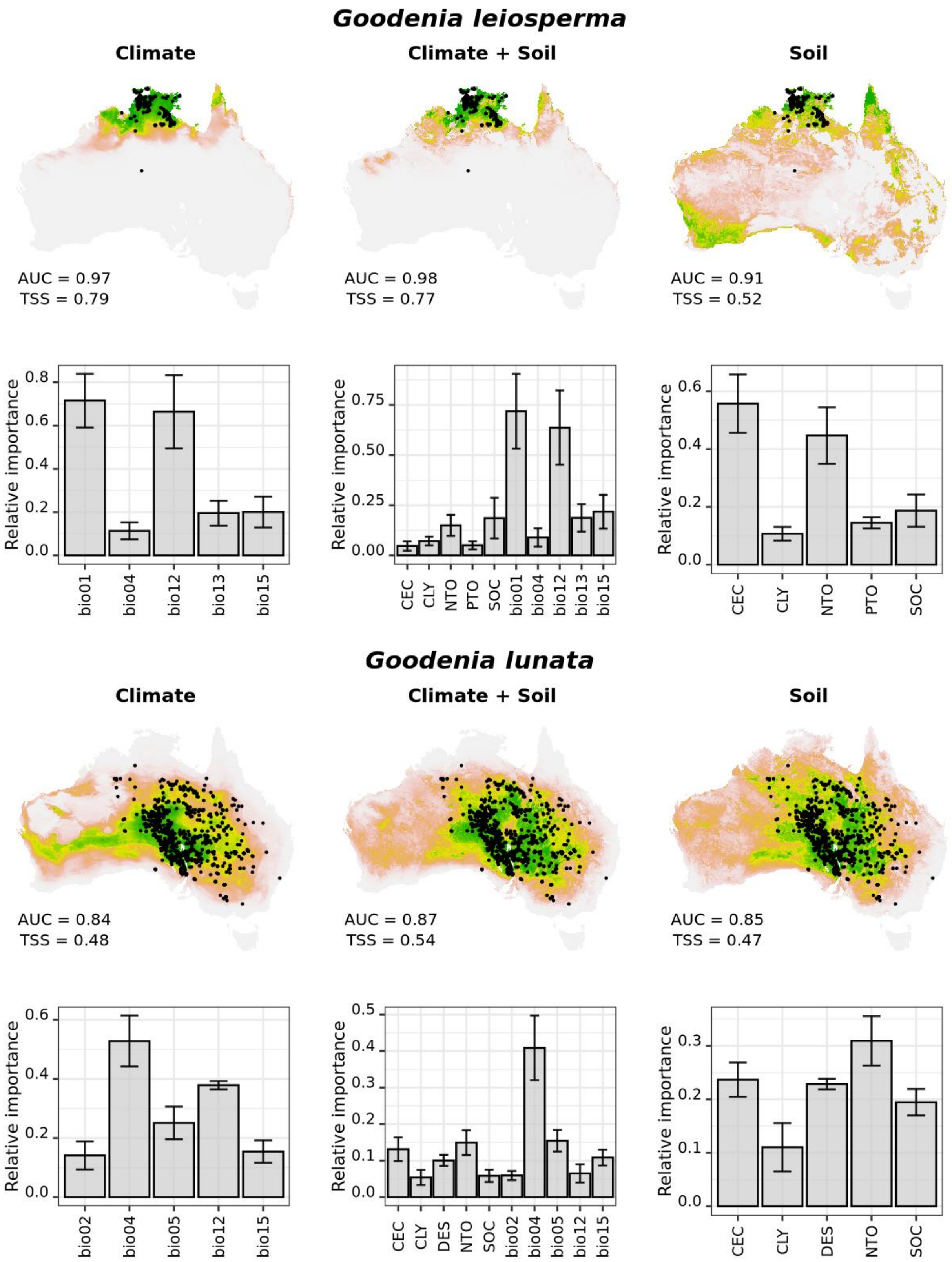
Goodenia leiosperma
Climate
Climate + Soil
Soil
AUC = 0.97
TSS = 0.79
AUC = 0.98
TSS = 0.77
AUC = 0.91
TSS = 0.52
Relative importance
bio01
bio04
bio12
bio13
bio15
CEC
CLY
NTO
PTO
SOC
Goodenia lunata
Climate
Climate + Soil
Soil
AUC = 0.84
TSS = 0.48
AUC = 0.87
TSS = 0.54
AUC = 0.85
TSS = 0.47
Relative importance
bio02
bio04
bio05
bio12
bio15
CEC
CLY
DES
NTO
SOC

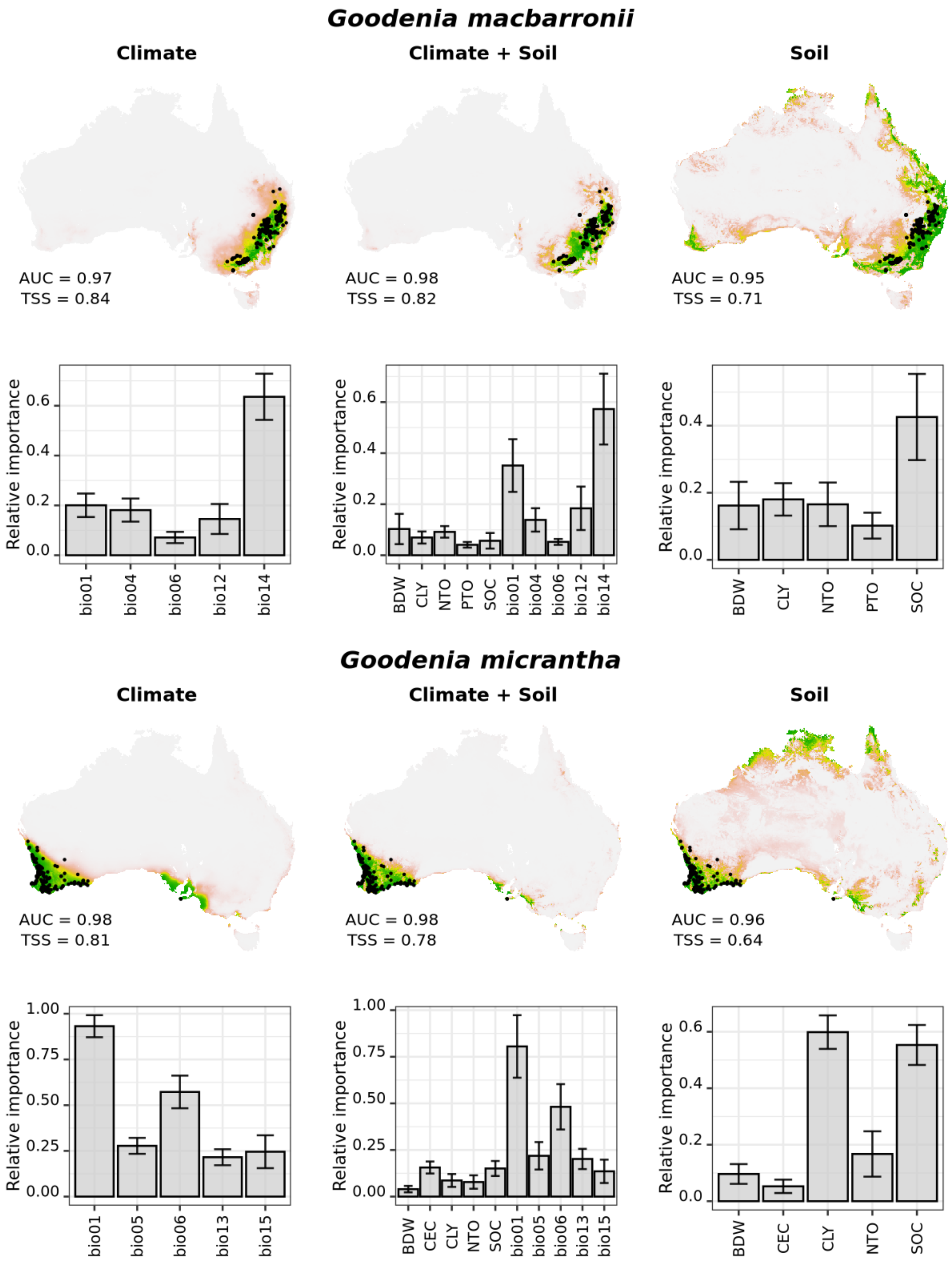

Goodenia macbarronii
Climate
Climate + Soil
Soil
AUC = 0.97
TSS = 0.84
AUC = 0.98
TSS = 0.82
AUC = 0.95
TSS = 0.71
Relative importance
0.0
0.2
0.4
0.6
bio01
bio04
bio06
bio12
bio14
BDW
CLY
NTO
PTO
SOC
Goodenia micrantha
AUC = 0.98
TSS = 0.81
AUC = 0.98
TSS = 0.78
AUC = 0.96
TSS = 0.64
0.00
0.25
0.50
0.75
1.00
bio05
bio13
bio15
CEC

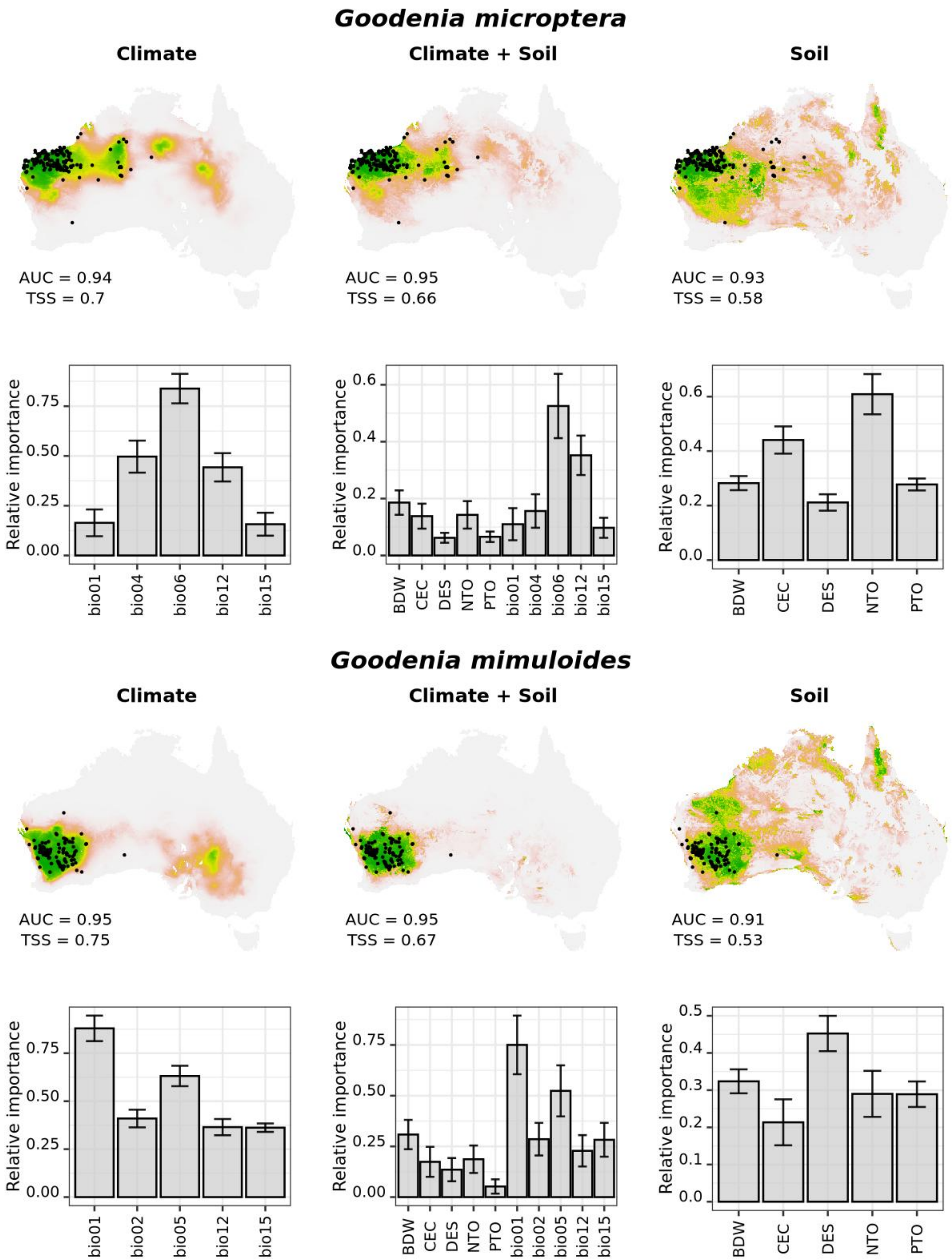

Goodenia microptera
Climate
Climate + Soil
Soil
AUC = 0.94
TSS = 0.7
AUC = 0.95
TSS = 0.66
AUC = 0.93
TSS = 0.58
Relative importance
0.00
0.25
0.50
0.75
bio01
bio04
bio06
bio12
bio15
0.0
0.2
0.4
0.6
BDW
CEC
DES
NTO
PTO
bio01
bio04
bio06
bio12
bio15
BDW
CEC
DES
NTO
PTO
Goodenia mimuloides
Climate
Climate + Soil
Soil
AUC = 0.95
TSS = 0.75
AUC = 0.95
TSS = 0.67
AUC = 0.91
TSS = 0.53
Relative importance
bio01
bio02
bio05
bio12
bio15
BDW
CEC
DES
NTO
PTO
bio01
bio02
bio05
bio12
bio15
0.0
0.1
0.2
0.3
0.4
0.5
BDW
CEC
DES
NTO
PTO

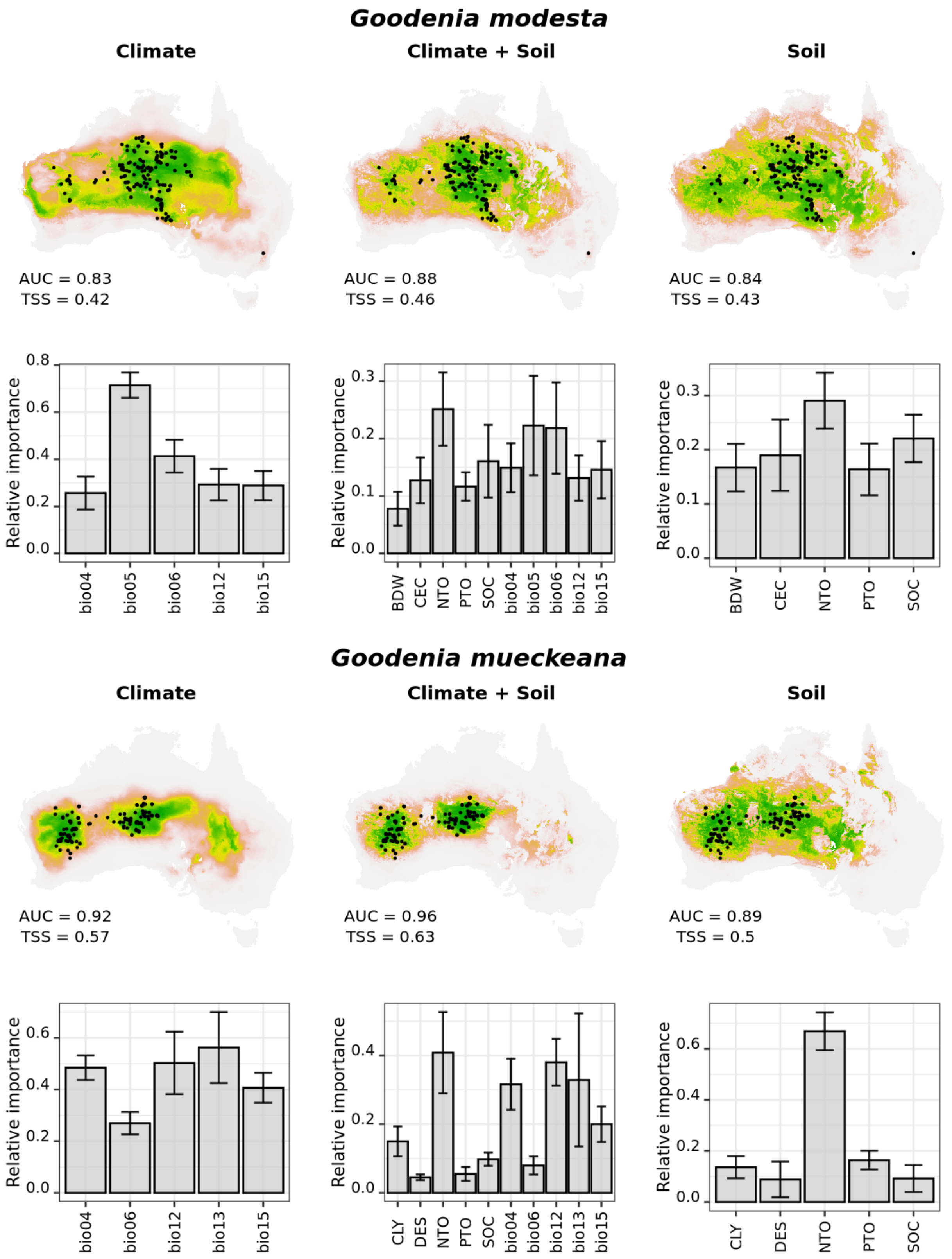

Goodenia modesta
Climate
Climate + Soil
Soil
AUC = 0.83
TSS = 0.42
AUC = 0.88
TSS = 0.46
AUC = 0.84
TSS = 0.43
Relative importance
bio04
bio05
bio06
bio12
bio15
BDW
CEC
NTO
PTO
SOC
bio04
bio05
bio06
bio12
bio15
BDW
CEC
NTO
PTO
SOC
Goodenia mueckeana
Climate
Climate + Soil
Soil
AUC = 0.92
TSS = 0.57
AUC = 0.96
TSS = 0.63
AUC = 0.89
TSS = 0.5
Relative importance
bio04
bio06
bio12
bio13
bio15
CLY
DES
NTO
PTO
SOC
bio04
bio06
bio12
bio13
bio15
CLY
DES
NTO
PTO
SOC

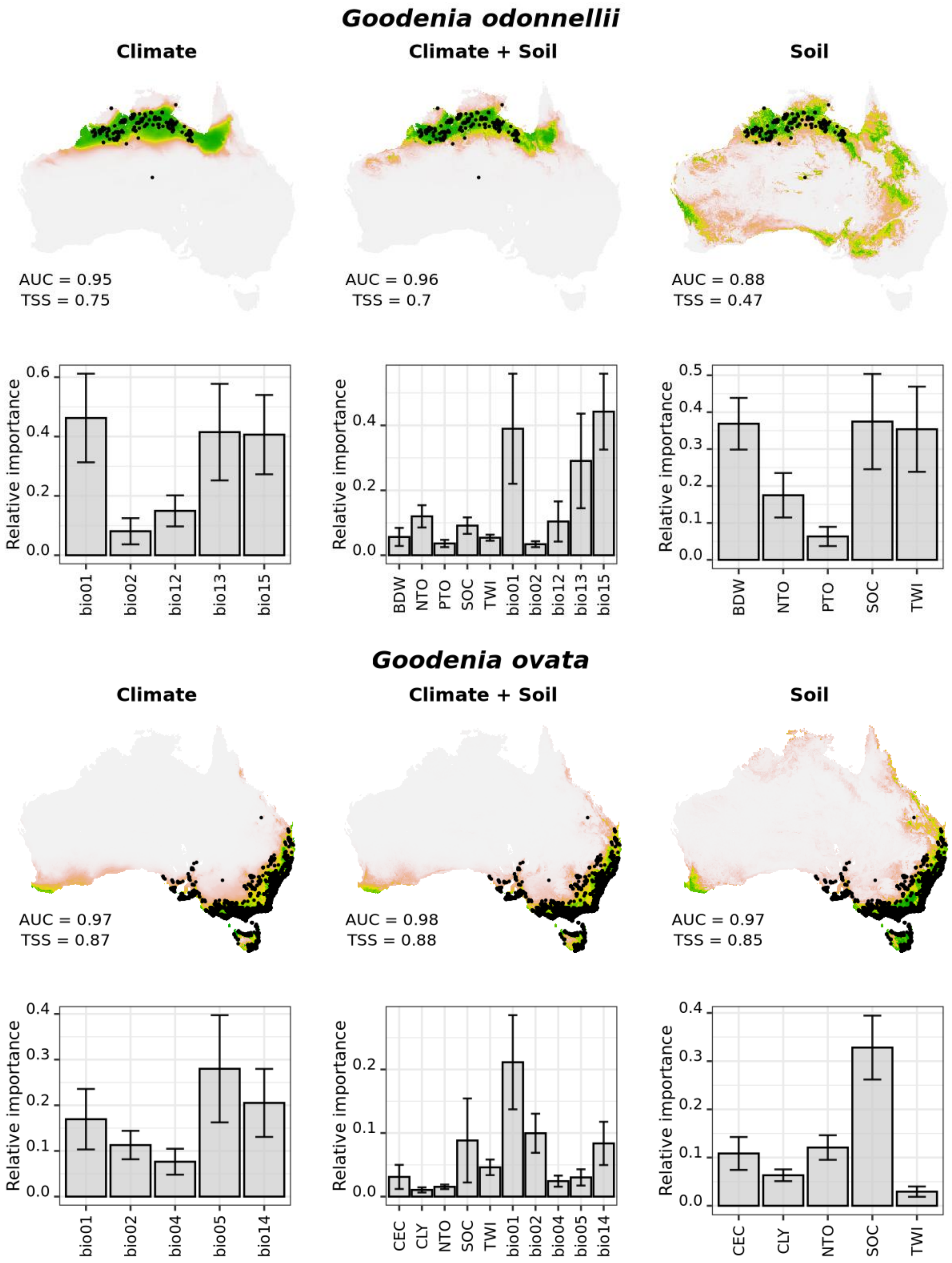

Goodenia odonnellii
Climate
Climate + Soil
Soil
AUC = 0.95
TSS = 0.75
AUC = 0.96
TSS = 0.7
AUC = 0.88
TSS = 0.47
Relative importance
bio01
bio02
bio12
bio13
bio15
BDW
NTO
PTO
SOC
TWI
Goodenia ovata
Climate
Climate + Soil
Soil
AUC = 0.97
TSS = 0.87
AUC = 0.98
TSS = 0.88
AUC = 0.97
TSS = 0.85
Relative importance
bio01
bio02
bio04
bio05
bio14
CEC
CLY
NTO
SOC
TWI

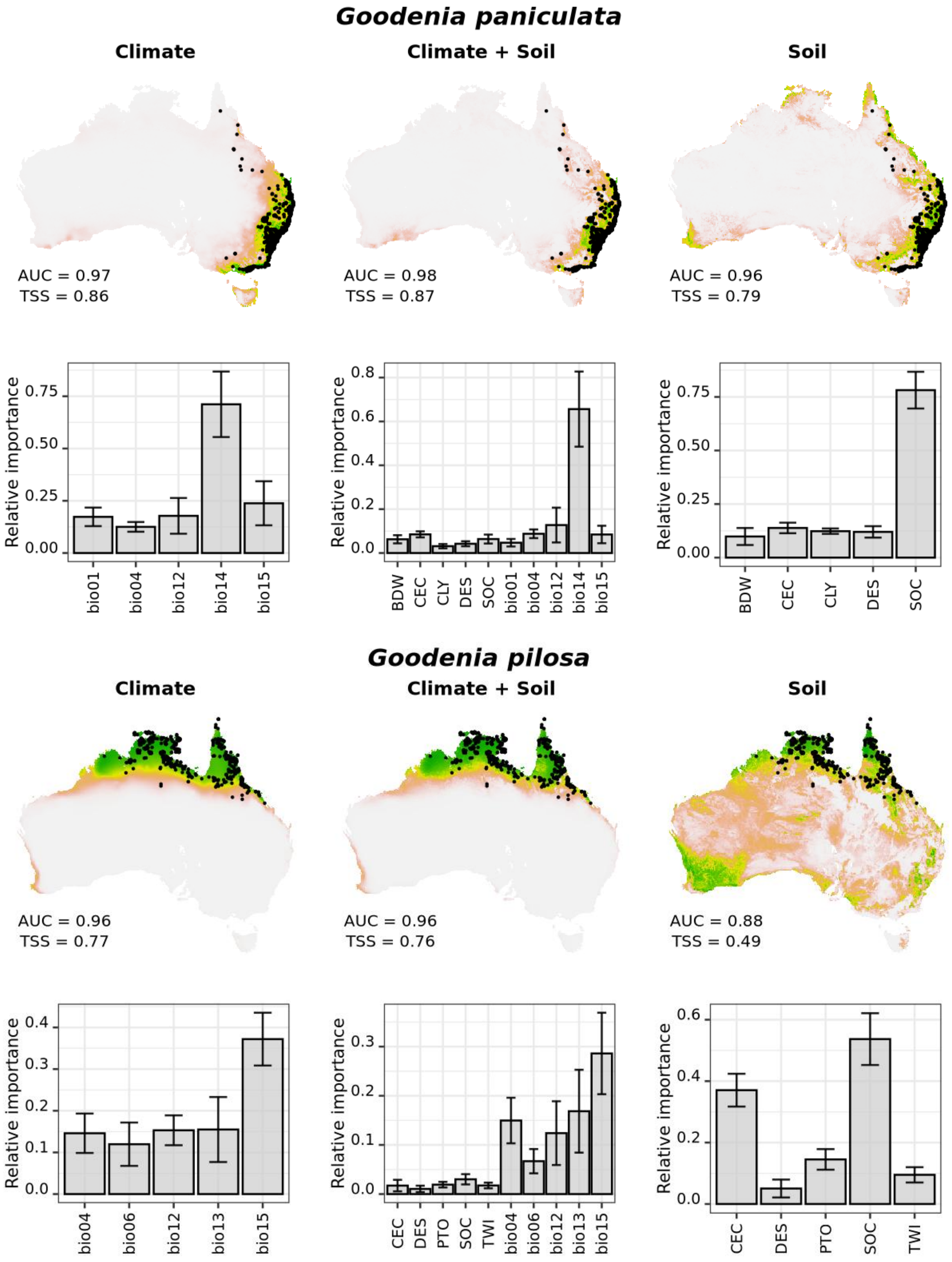
Goodenia paniculata
Climate
Climate + Soil
Soil
AUC = 0.97
TSS = 0.86
AUC = 0.98
TSS = 0.87
AUC = 0.96
TSS = 0.79
Relative importance
0.00
0.25
0.50
0.75
bio01
bio04
bio12
bio14
bio15
0.0
0.2
0.4
0.6
0.8
BDW
CEC
CLY
DES
SOC
bio01
bio04
bio12
bio14
bio15
BDW
CEC
CLY
DES
SOC
Goodenia pilosa
Climate
Climate + Soil
Soil
AUC = 0.96
TSS = 0.77
AUC = 0.96
TSS = 0.76
AUC = 0.88
TSS = 0.49
Relative importance
0.0
0.1
0.2
0.3
0.4
bio04
bio06
bio12
bio13
bio15
CEC
DES
PTO
SOC
TWI
bio04
bio06
bio12
bio13
bio15
0.6
CEC
DES
PTO
SOC
TWI

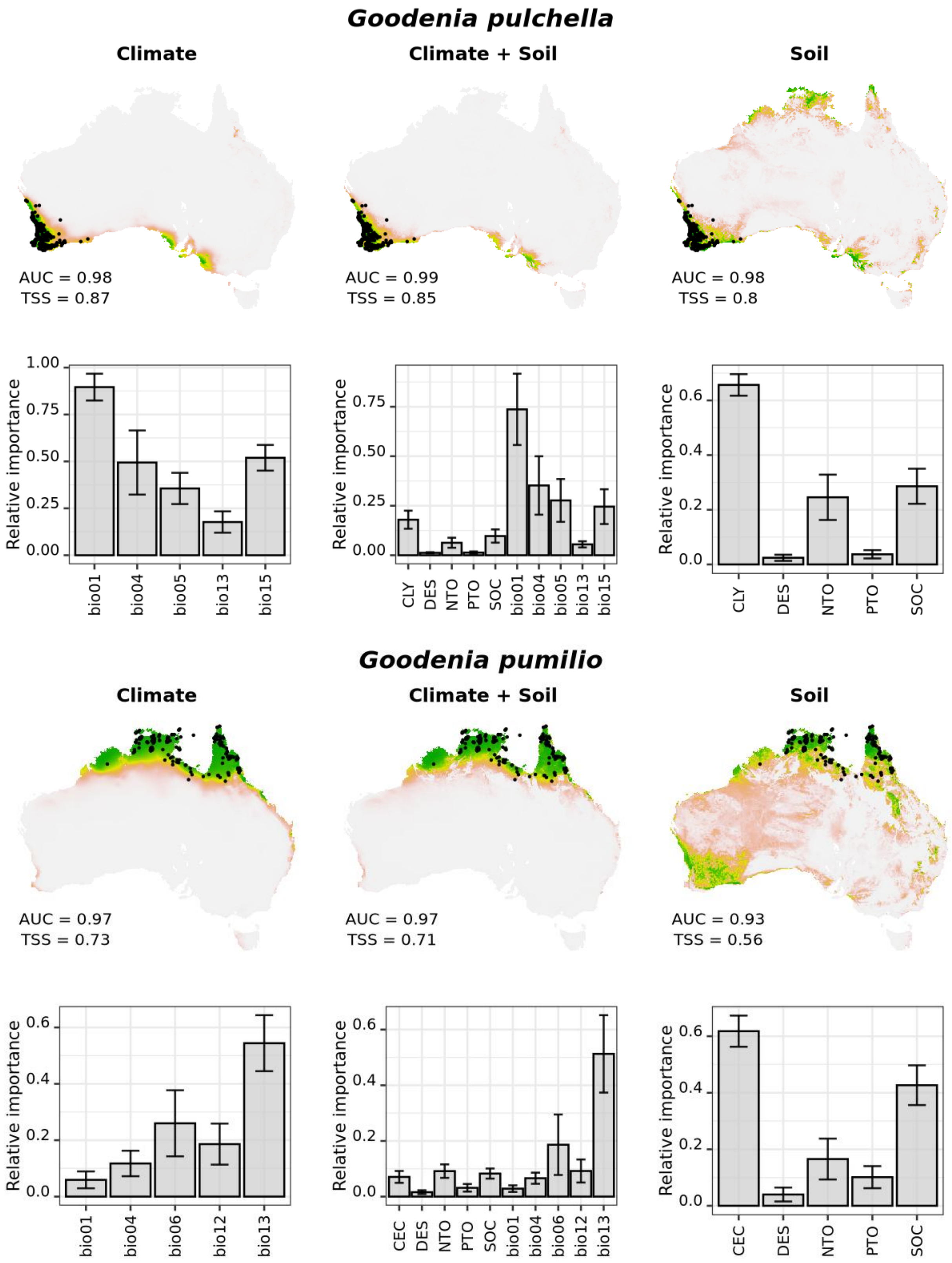
Goodenia pulchella
Climate
Climate + Soil
Soil
AUC = 0.98
TSS = 0.87
AUC = 0.99
TSS = 0.85
AUC = 0.98
TSS = 0.8
Relative importance
bio01 bio04 bio05 bio13 bio15
CLY DES NTO PTO SOC bio01 bio04 bio05 bio13 bio15
CLY DES NTO PTO SOC
Goodenia pumilio
Climate
Climate + Soil
Soil
AUC = 0.97
TSS = 0.73
AUC = 0.97
TSS = 0.71
AUC = 0.93
TSS = 0.56
Relative importance
bio01 bio04 bio06 bio12 bio13
CEC DES NTO PTO SOC bio01 bio04 bio06 bio12 bio13
CEC DES NTO PTO SOC

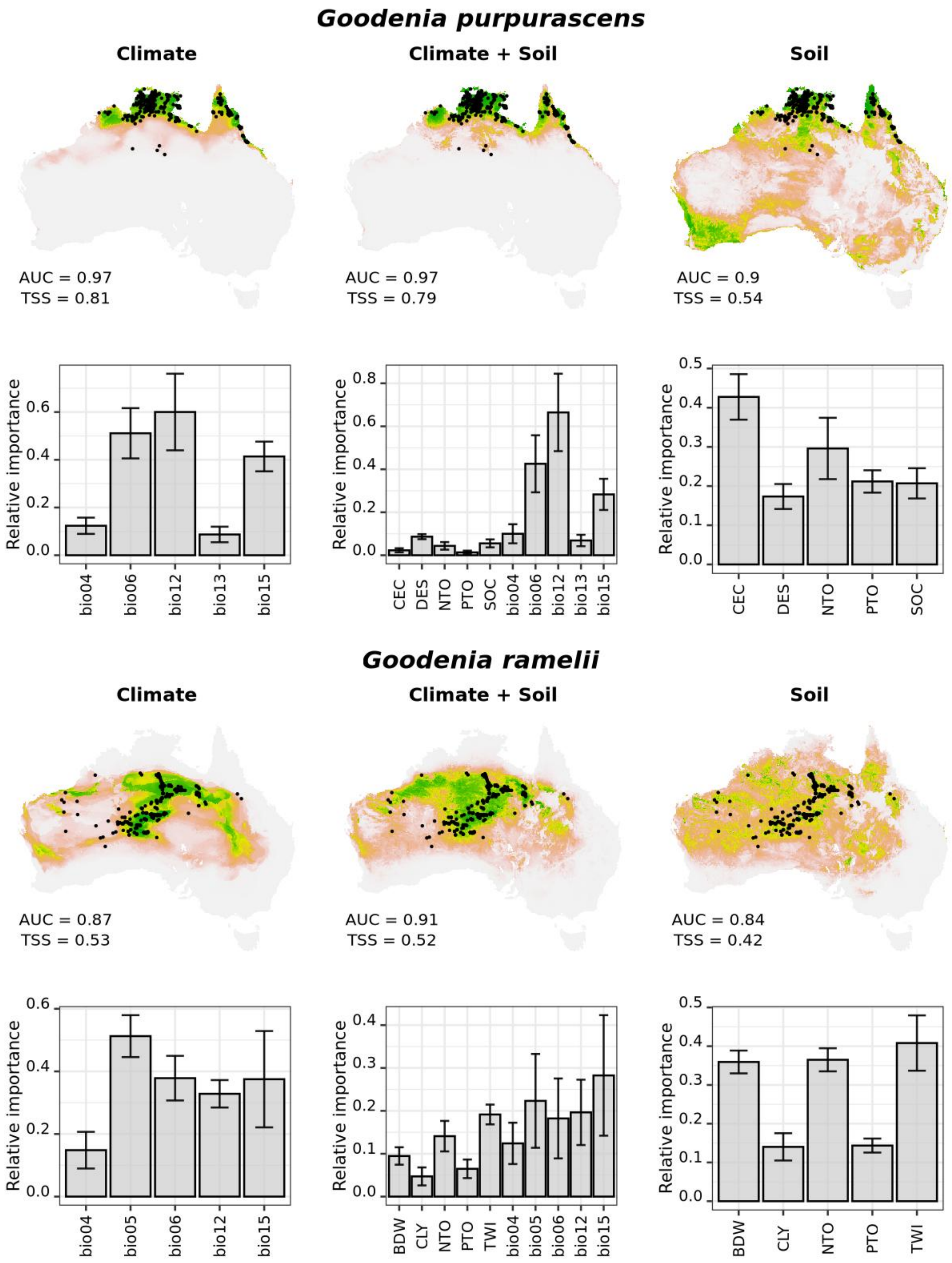
Goodenia purpurascens
Climate
Climate + Soil
Soil
AUC = 0.97
TSS = 0.81
AUC = 0.97
TSS = 0.79
AUC = 0.9
TSS = 0.54
Relative importance
bio04
bio06
bio12
bio13
bio15
CEC
DES
NTO
PTO
SOC
Goodenia ramelii
Climate
Climate + Soil
Soil
AUC = 0.87
TSS = 0.53
AUC = 0.91
TSS = 0.52
AUC = 0.84
TSS = 0.42
bio05
BDW
CLY
TWI

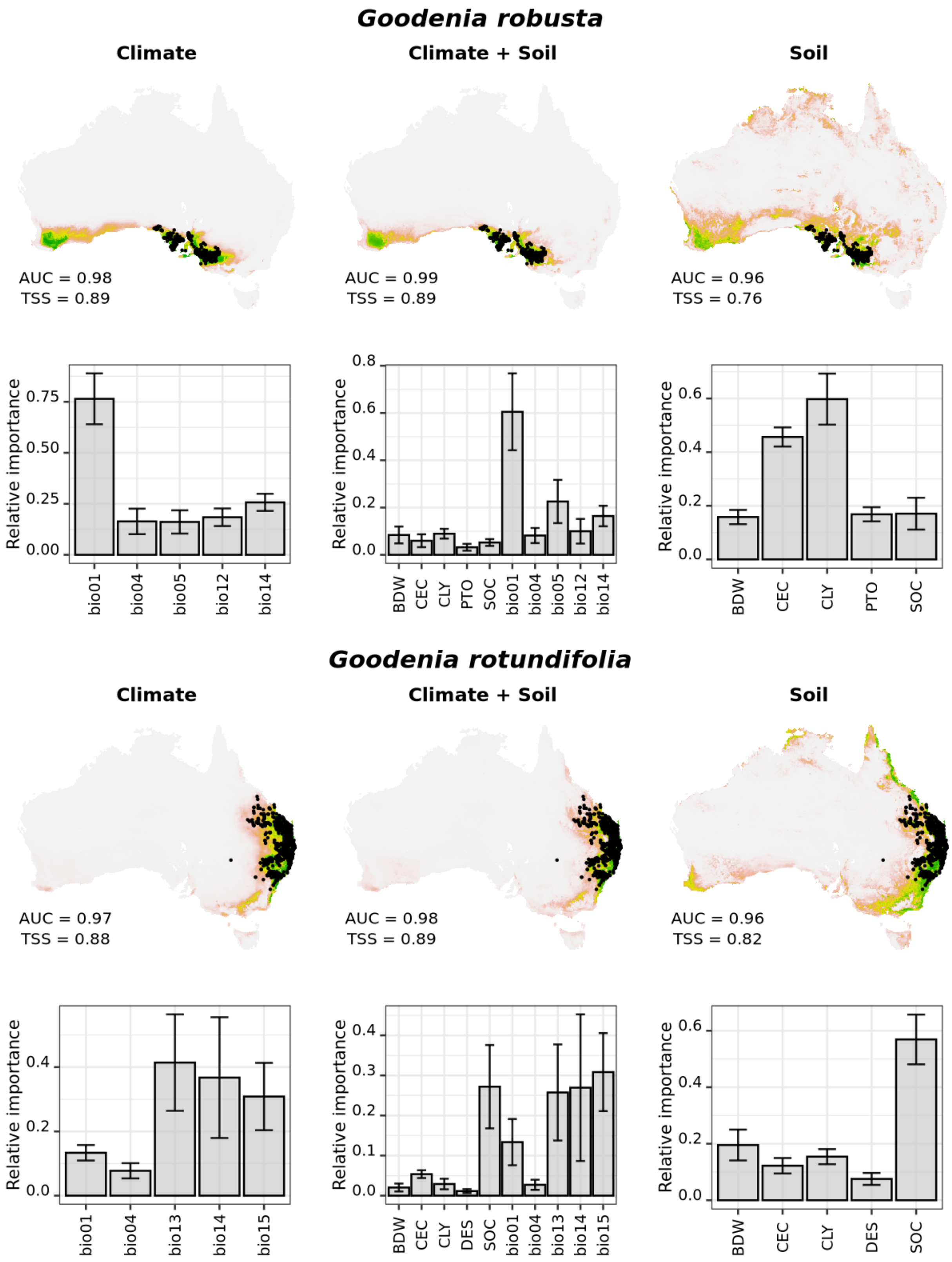
Goodenia robusta
Climate
Climate + Soil
Soil
AUC = 0.98
TSS = 0.89
AUC = 0.99
TSS = 0.89
AUC = 0.96
TSS = 0.76
Relative importance
bio01
bio04
bio05
bio12
bio14
BDW
CEC
CLY
PTO
SOC
Goodenia rotundifolia
Climate
Climate + Soil
Soil
AUC = 0.97
TSS = 0.88
AUC = 0.98
TSS = 0.89
AUC = 0.96
TSS = 0.82
Relative importance
bio01
bio04
bio13
bio14
bio15
BDW
CEC
CLY
DES
SOC

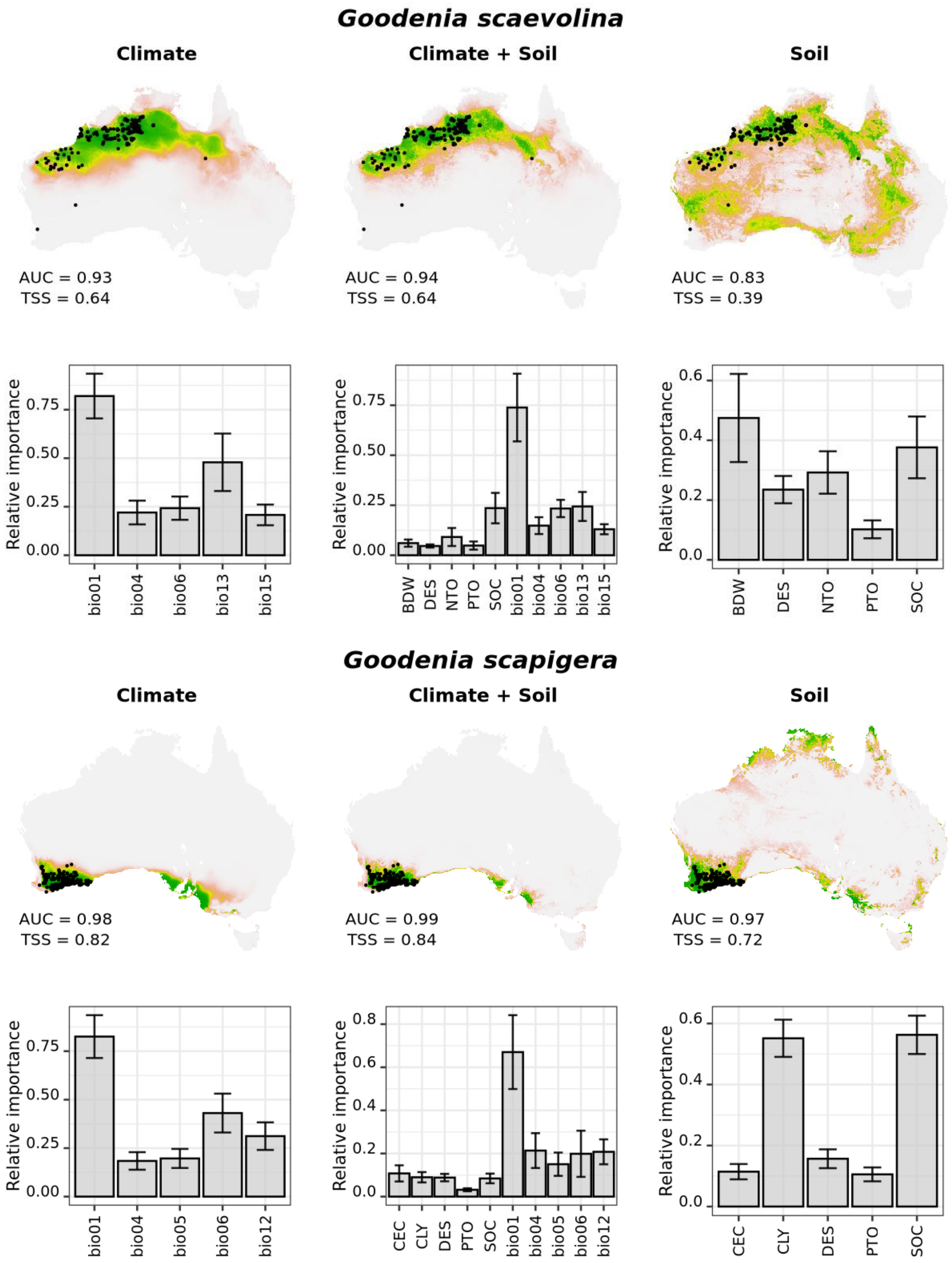
Goodenia scaevolina
Climate
Climate + Soil
Soil
AUC = 0.93
TSS = 0.64
AUC = 0.94
TSS = 0.64
AUC = 0.83
TSS = 0.39
Relative importance
0.00
0.25
0.50
0.75
bio01
bio04
bio06
bio13
bio15
BDW
DES
NTO
PTO
SOC
bio01
bio04
bio06
bio13
bio15
0.0
0.2
0.4
0.6
BDW
DES
NTO
PTO
SOC
Goodenia scapigera
Climate
Climate + Soil
Soil
AUC = 0.98
TSS = 0.82
AUC = 0.99
TSS = 0.84
AUC = 0.97
TSS = 0.72
Relative importance
0.00
0.25
0.50
0.75
bio01
bio04
bio05
bio06
bio12
0.0
0.2
0.4
0.6
0.8
CEC
CLY
DES
PTO
SOC
bio01
bio04
bio05
bio06
bio12
0.0
0.2
0.4
0.6
CEC
CLY
DES
PTO
SOC

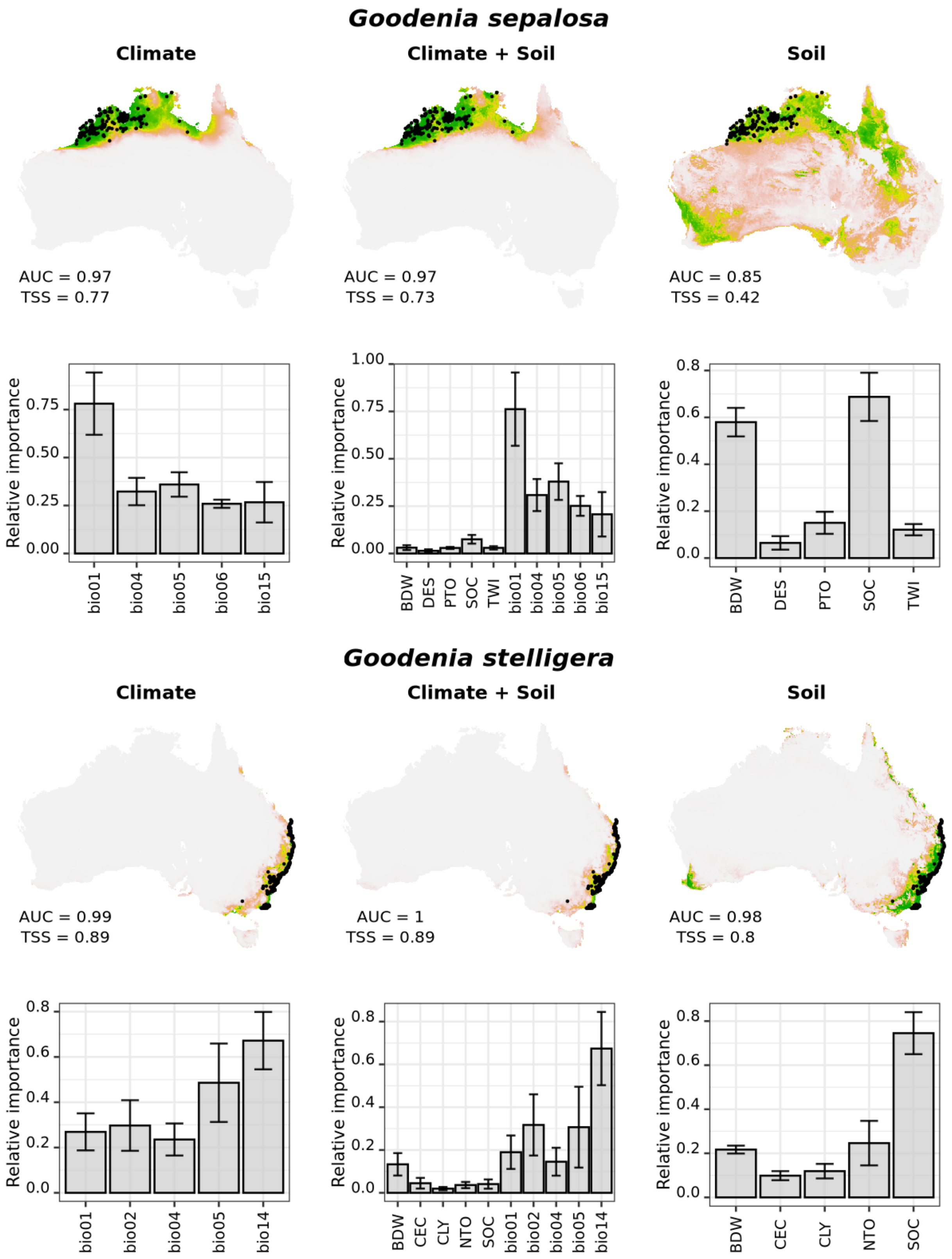

Goodenia sepalosa
Climate
Climate + Soil
Soil
AUC = 0.97
TSS = 0.77
AUC = 0.97
TSS = 0.73
AUC = 0.85
TSS = 0.42
Relative importance
0.00 0.25 0.50 0.75 1.00
0.0 0.2 0.4 0.6 0.8
bio01 bio04 bio05 bio06 bio15
BDW DES PTO SOC TWI bio01 bio04 bio05 bio06 bio15
BDW DES PTO SOC TWI
Goodenia stelligera
Climate
Climate + Soil
Soil
AUC = 0.99
TSS = 0.89
AUC = 1
TSS = 0.89
AUC = 0.98
TSS = 0.8
Relative importance
0.0 0.2 0.4 0.6 0.8
bio01 bio02 bio04 bio05 bio14
BDW CEC CLY NTO SOC bio01 bio02 bio04 bio05 bio14
BDW CEC CLY NTO SOC

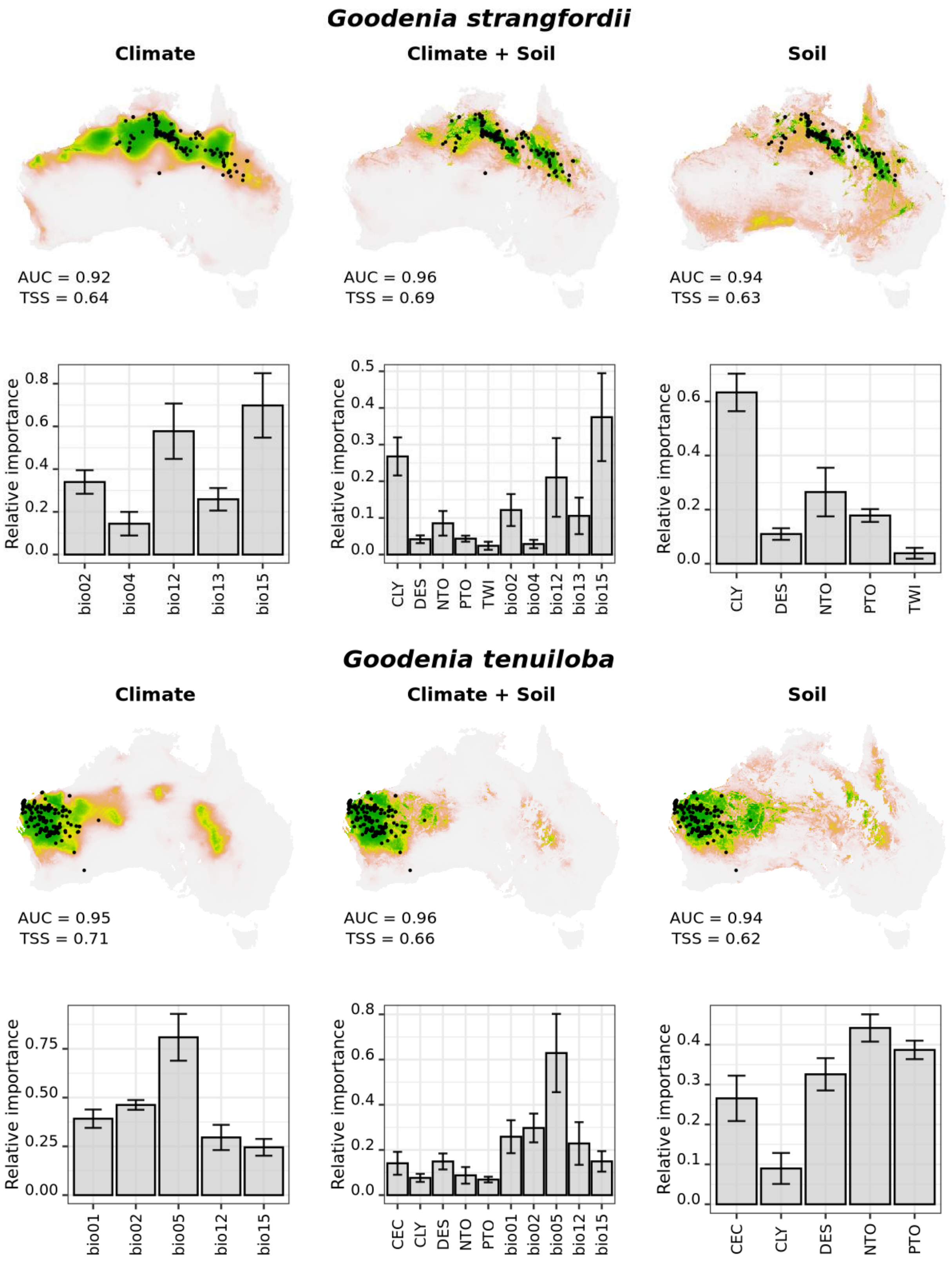
Goodenia strangfordii
Climate
Climate + Soil
Soil
AUC = 0.92
TSS = 0.64
AUC = 0.96
TSS = 0.69
AUC = 0.94
TSS = 0.63
Relative importance
0.0
0.2
0.4
0.6
0.8
bio02
bio04
bio12
bio13
bio15
0.0
0.1
0.2
0.3
0.4
0.5
CLY
DES
NTO
PTO
TWI
bio02
bio04
bio12
bio13
bio15
0.0
0.2
0.4
0.6
CLY
DES
NTO
PTO
TWI
Goodenia tenuiloba
Climate
Climate + Soil
Soil
AUC = 0.95
TSS = 0.71
AUC = 0.96
TSS = 0.66
AUC = 0.94
TSS = 0.62
Relative importance
0.00
0.25
0.50
0.75
bio01
bio02
bio05
bio12
bio15
0.0
0.2
0.4
0.6
0.8
CEC
CLY
DES
NTO
PTO
bio01
bio02
bio05
bio12
bio15
0.0
0.1
0.2
0.3
0.4
CEC
CLY
DES
NTO
PTO

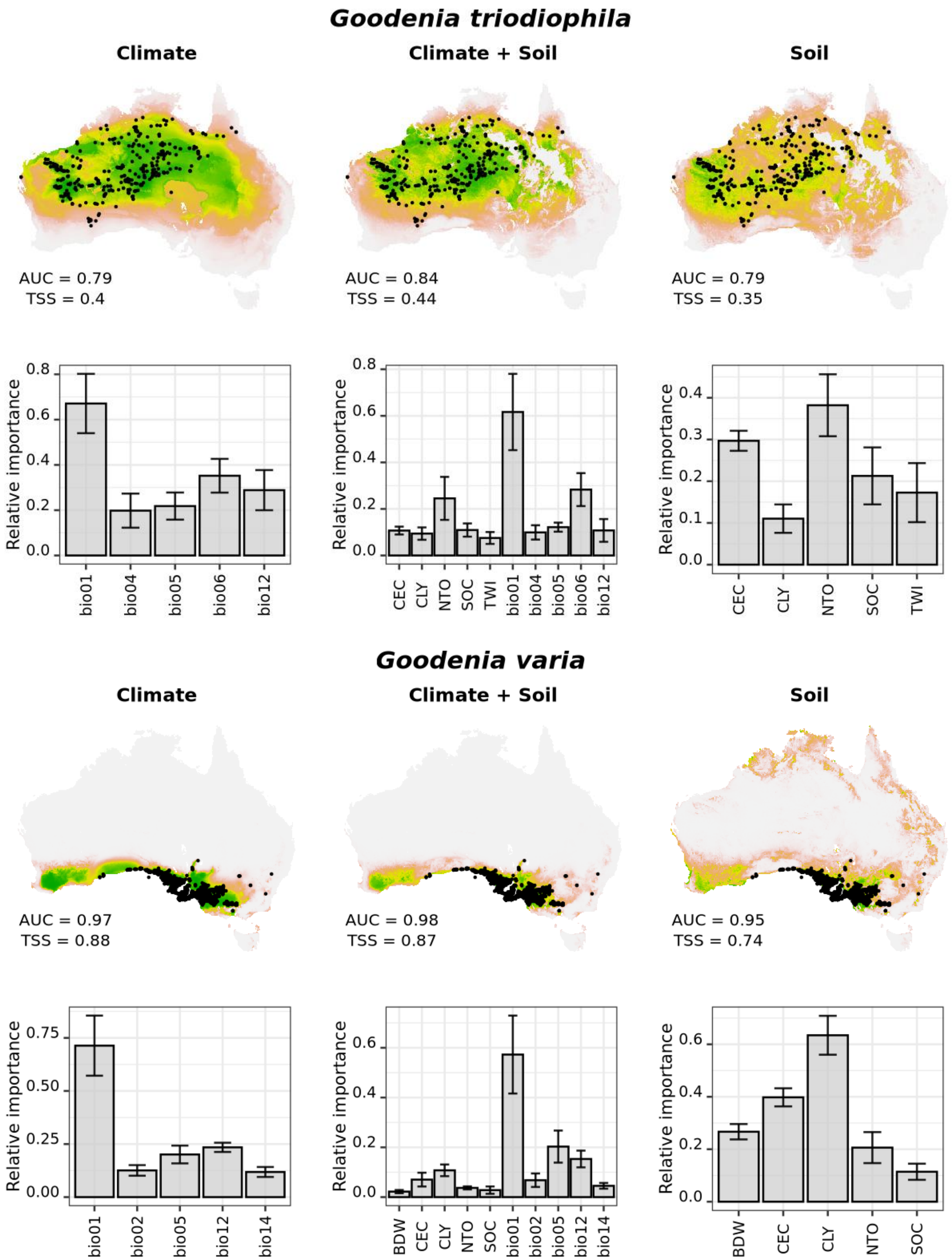
Goodenia triodiophila
Climate
Climate + Soil
Soil
AUC = 0.79
TSS = 0.4
AUC = 0.84
TSS = 0.44
AUC = 0.79
TSS = 0.35
Relative importance
bio01
bio04
bio05
bio06
bio12
CEC
CLY
NTO
SOC
TWI
Goodenia varia
AUC = 0.97
TSS = 0.88
AUC = 0.98
TSS = 0.87
AUC = 0.95
TSS = 0.74
bio02
bio14
BDW

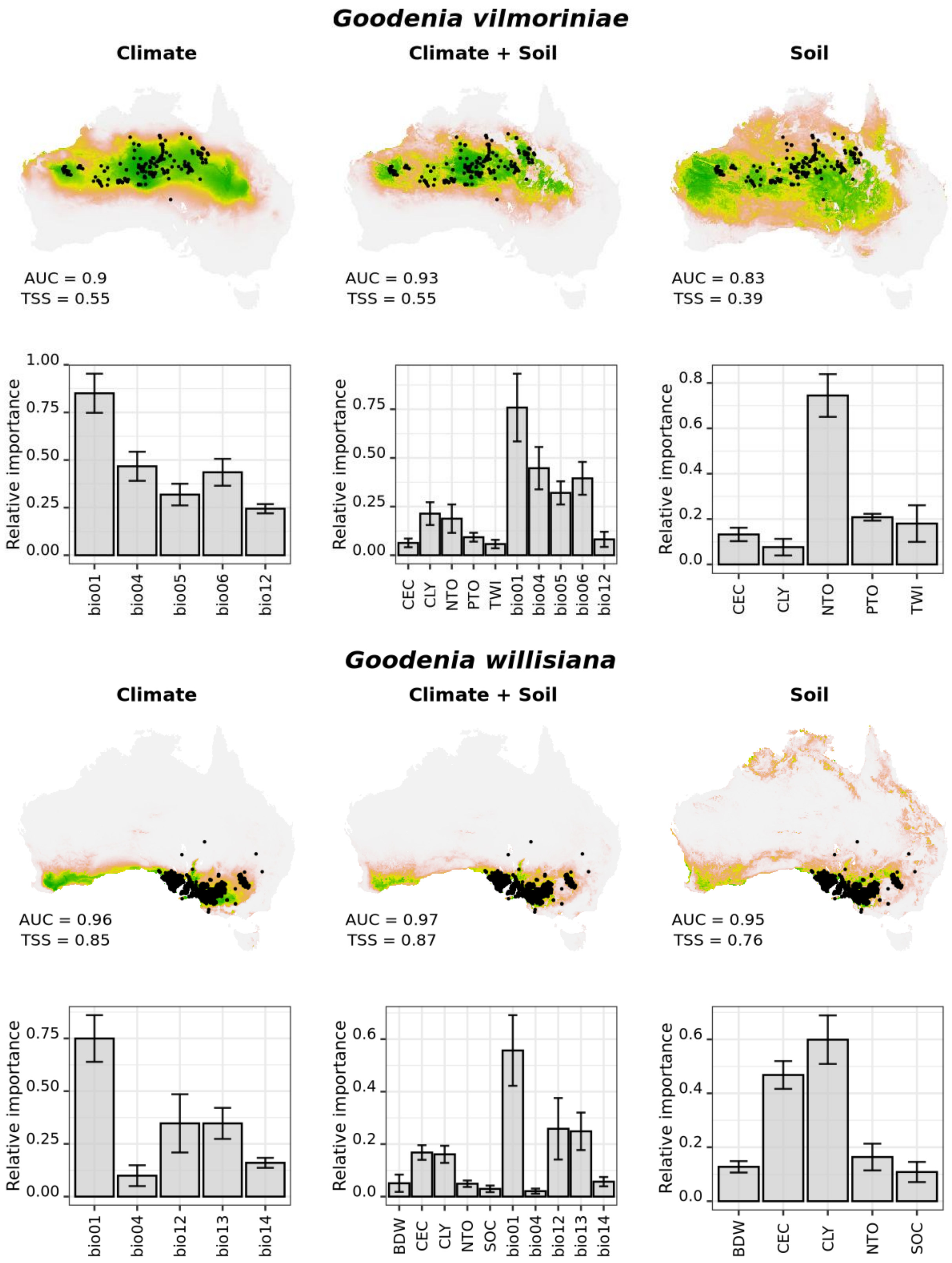
Goodenia vilmoriniae
Climate
Climate + Soil
Soil
AUC = 0.9
TSS = 0.55
AUC = 0.93
TSS = 0.55
AUC = 0.83
TSS = 0.39
Relative importance
1.00
0.75
0.50
0.25
0.00
bio01
bio04
bio05
bio06
bio12
CEC
CLY
NTO
PTO
TWI
0.8
0.6
0.4
0.2
0.0
Goodenia willisiana
Climate
Climate + Soil
Soil
AUC = 0.96
TSS = 0.85
AUC = 0.97
TSS = 0.87
AUC = 0.95
TSS = 0.76
bio13
bio14
BDW
SOC

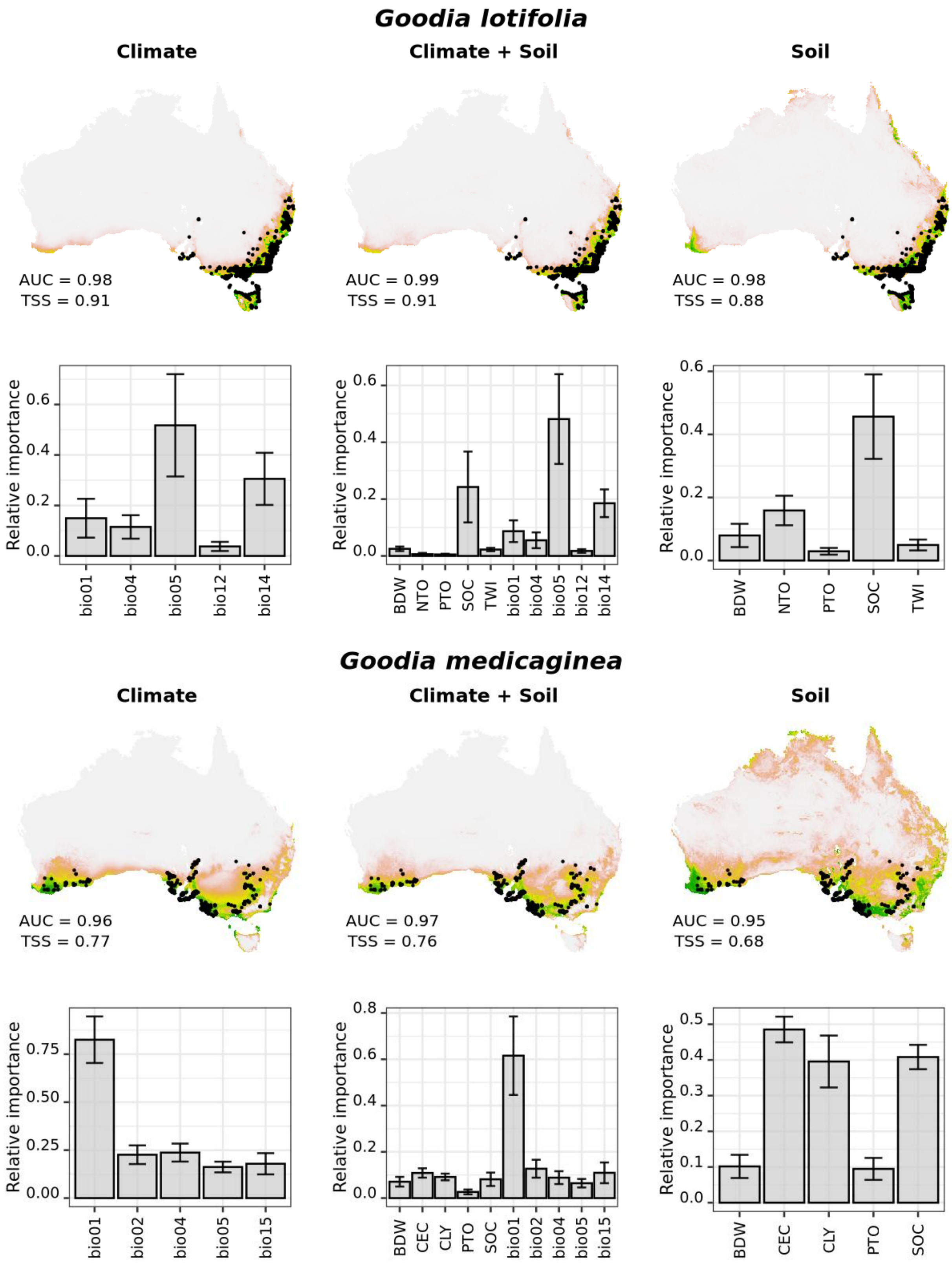
Goodia lotifolia
Climate
Climate + Soil
Soil
AUC = 0.98
TSS = 0.91
AUC = 0.99
TSS = 0.91
AUC = 0.98
TSS = 0.88
Relative importance
0.0
0.2
0.4
0.6
bio01
bio04
bio05
bio12
bio14
BDW
NTO
PTO
SOC
TWI
Goodia medicaginea
Climate
Climate + Soil
Soil
AUC = 0.96
TSS = 0.77
AUC = 0.97
TSS = 0.76
AUC = 0.95
TSS = 0.68
Relative importance
0.00
0.25
0.50
0.75
0.8
0.1
0.3
0.5
bio02
bio15
CEC
CLY

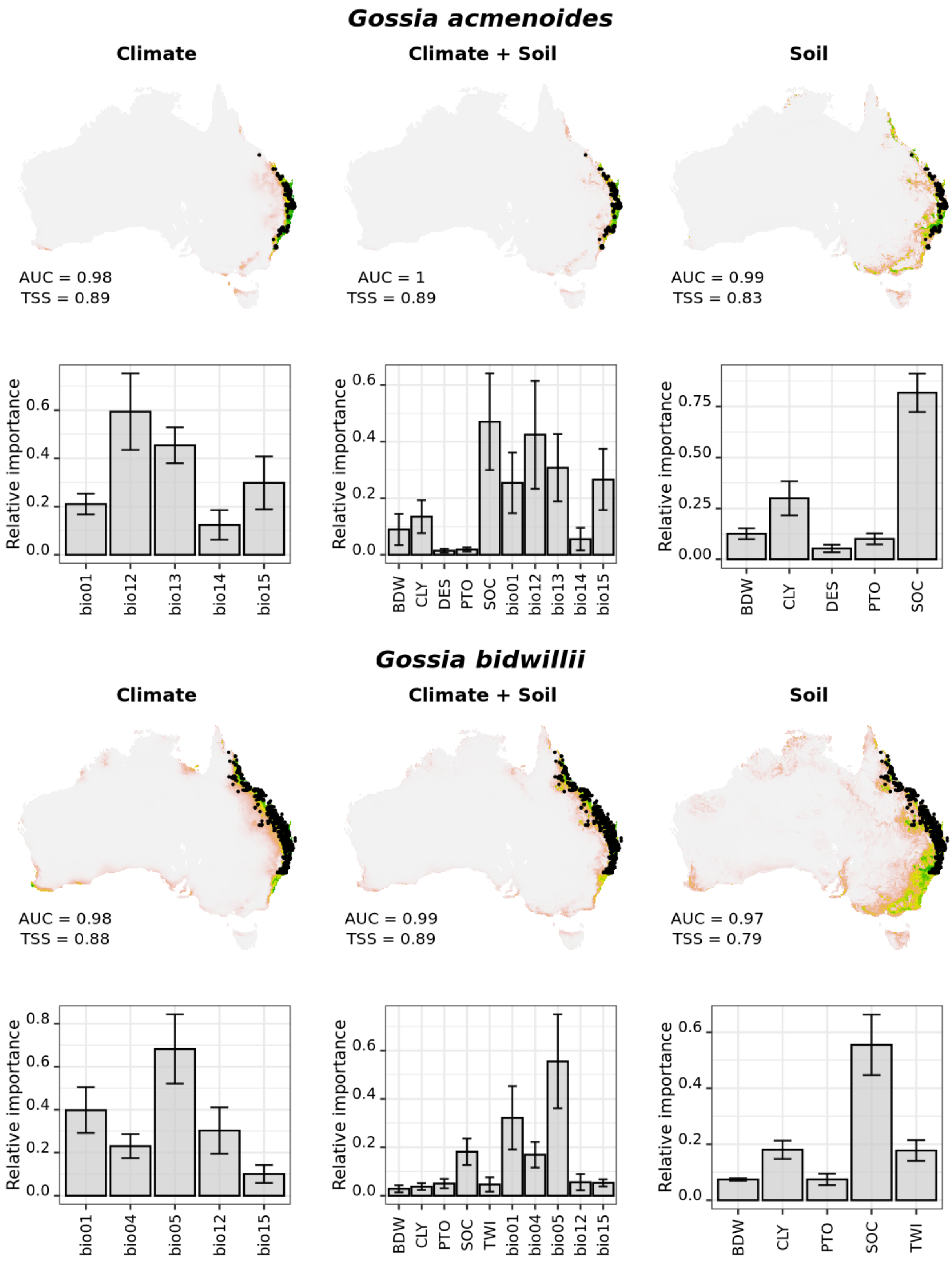
Gossia acmenoides
Climate
Climate + Soil
Soil
AUC = 0.98
TSS = 0.89
AUC = 1
TSS = 0.89
AUC = 0.99
TSS = 0.83
Relative importance
bio01
bio12
bio13
bio14
bio15
BDW
CLY
DES
PTO
SOC
Gossia bidwillii
Climate
Climate + Soil
Soil
AUC = 0.98
TSS = 0.88
AUC = 0.99
TSS = 0.89
AUC = 0.97
TSS = 0.79
Relative importance
bio01
bio04
bio05
bio12
bio15
BDW
CLY
PTO
SOC
TWI

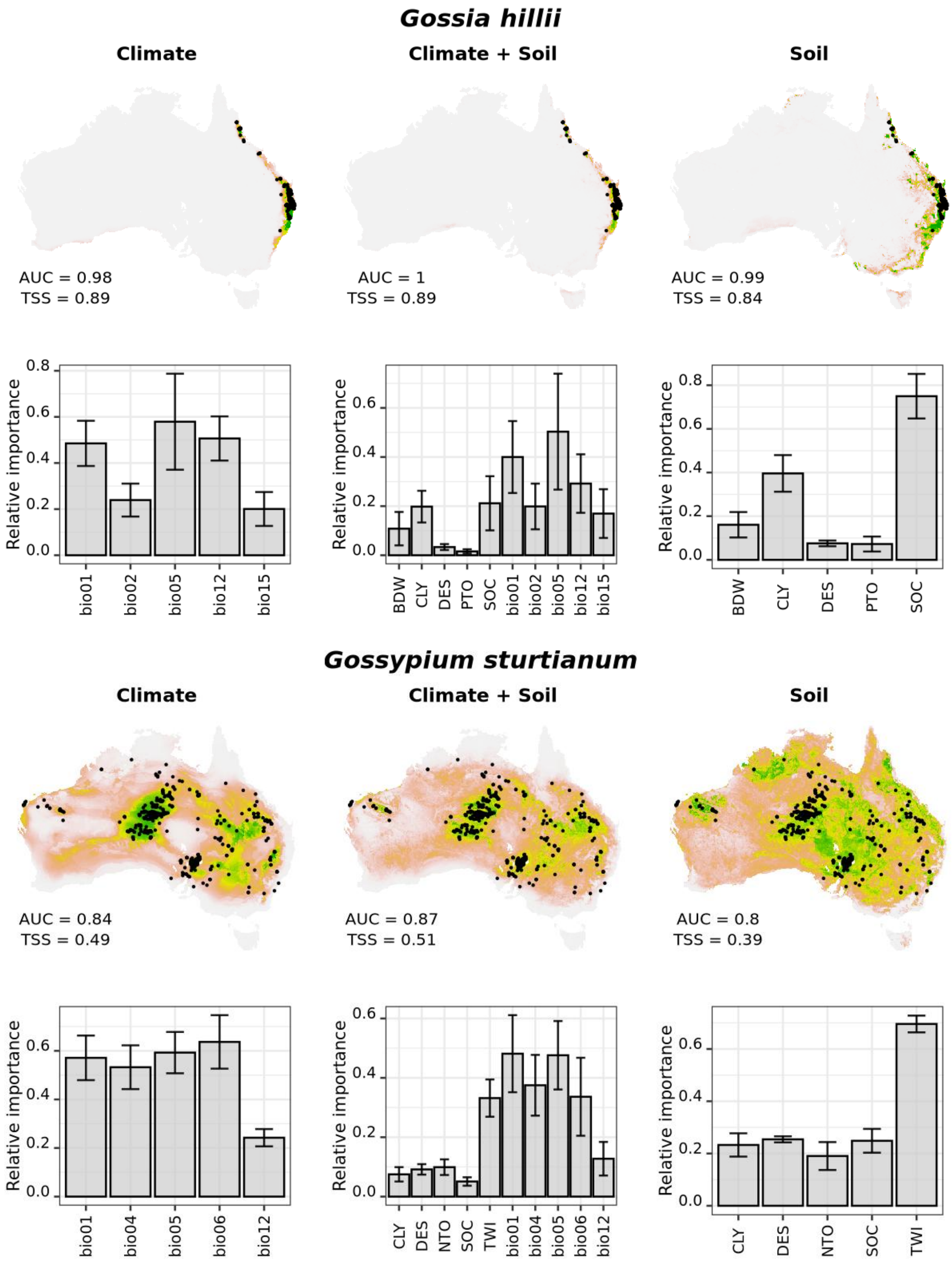
Gossia hillii
Climate
Climate + Soil
Soil
AUC = 0.98
TSS = 0.89
AUC = 1
TSS = 0.89
AUC = 0.99
TSS = 0.84
Relative importance
bio01
bio02
bio05
bio12
bio15
BDW
CLY
DES
PTO
SOC
Gossypium sturtianum
Climate
Climate + Soil
Soil
AUC = 0.84
TSS = 0.49
AUC = 0.87
TSS = 0.51
AUC = 0.8
TSS = 0.39
Relative importance
bio01
bio04
bio05
bio06
bio12
CLY
DES
NTO
SOC
TWI

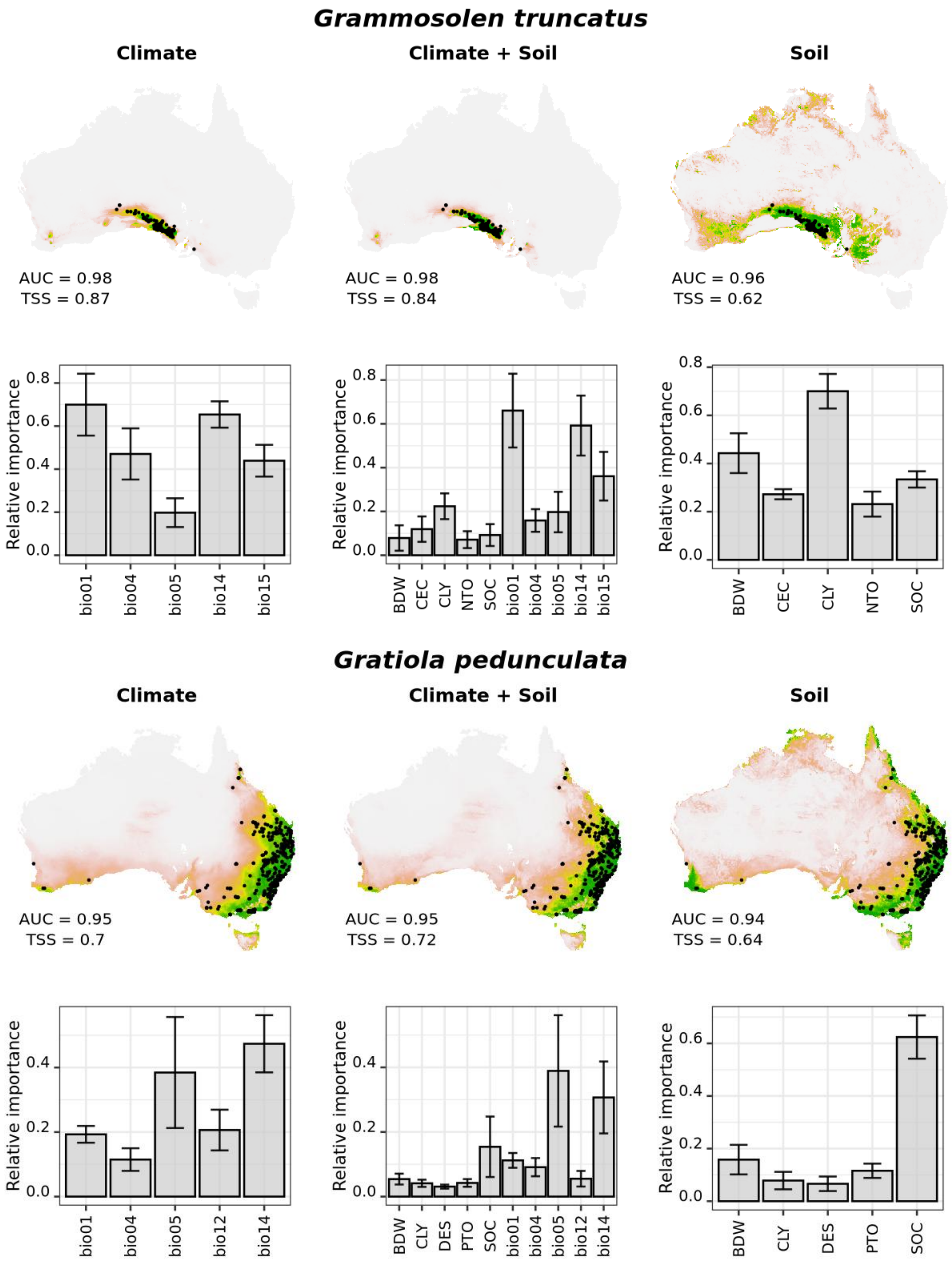
Grammosolen truncatus
Climate
Climate + Soil
Soil
AUC = 0.98
TSS = 0.87
AUC = 0.98
TSS = 0.84
AUC = 0.96
TSS = 0.62
Relative importance
bio01
bio04
bio05
bio14
bio15
BDW
CEC
CLY
NTO
SOC
Gratiola pedunculata
Climate
Climate + Soil
Soil
AUC = 0.95
TSS = 0.7
AUC = 0.95
TSS = 0.72
AUC = 0.94
TSS = 0.64
Relative importance
bio01
bio04
bio05
bio12
bio14
BDW
CLY
DES
PTO
SOC

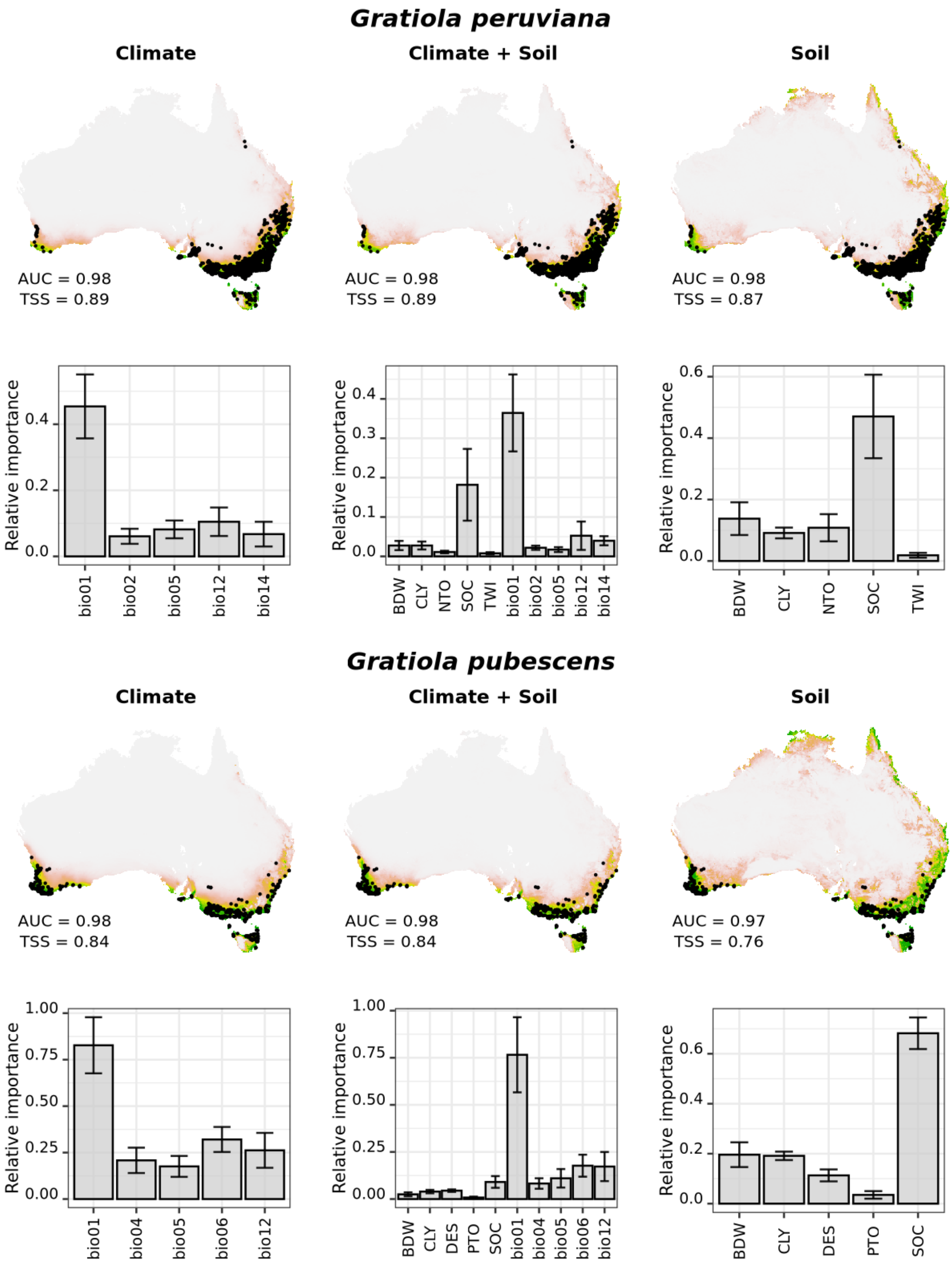
Gratiola peruviana
Climate
Climate + Soil
Soil
AUC = 0.98
TSS = 0.89
AUC = 0.98
TSS = 0.89
AUC = 0.98
TSS = 0.87
Relative importance
0.0
0.2
0.4
bio01
bio02
bio05
bio12
bio14
Relative importance
0.0
0.1
0.2
0.3
0.4
BDW
CLY
NTO
SOC
TWI
bio01
bio02
bio05
bio12
bio14
Relative importance
0.0
0.2
0.4
0.6
BDW
CLY
NTO
SOC
TWI
Gratiola pubescens
Climate
Climate + Soil
Soil
AUC = 0.98
TSS = 0.84
AUC = 0.98
TSS = 0.84
AUC = 0.97
TSS = 0.76
Relative importance
0.00
0.25
0.50
0.75
1.00
bio01
bio04
bio05
bio06
bio12
Relative importance
0.00
0.25
0.50
0.75
1.00
BDW
CLY
DES
PTO
SOC
bio01
bio04
bio05
bio06
bio12
Relative importance
0.0
0.2
0.4
0.6
BDW
CLY
DES
PTO
SOC

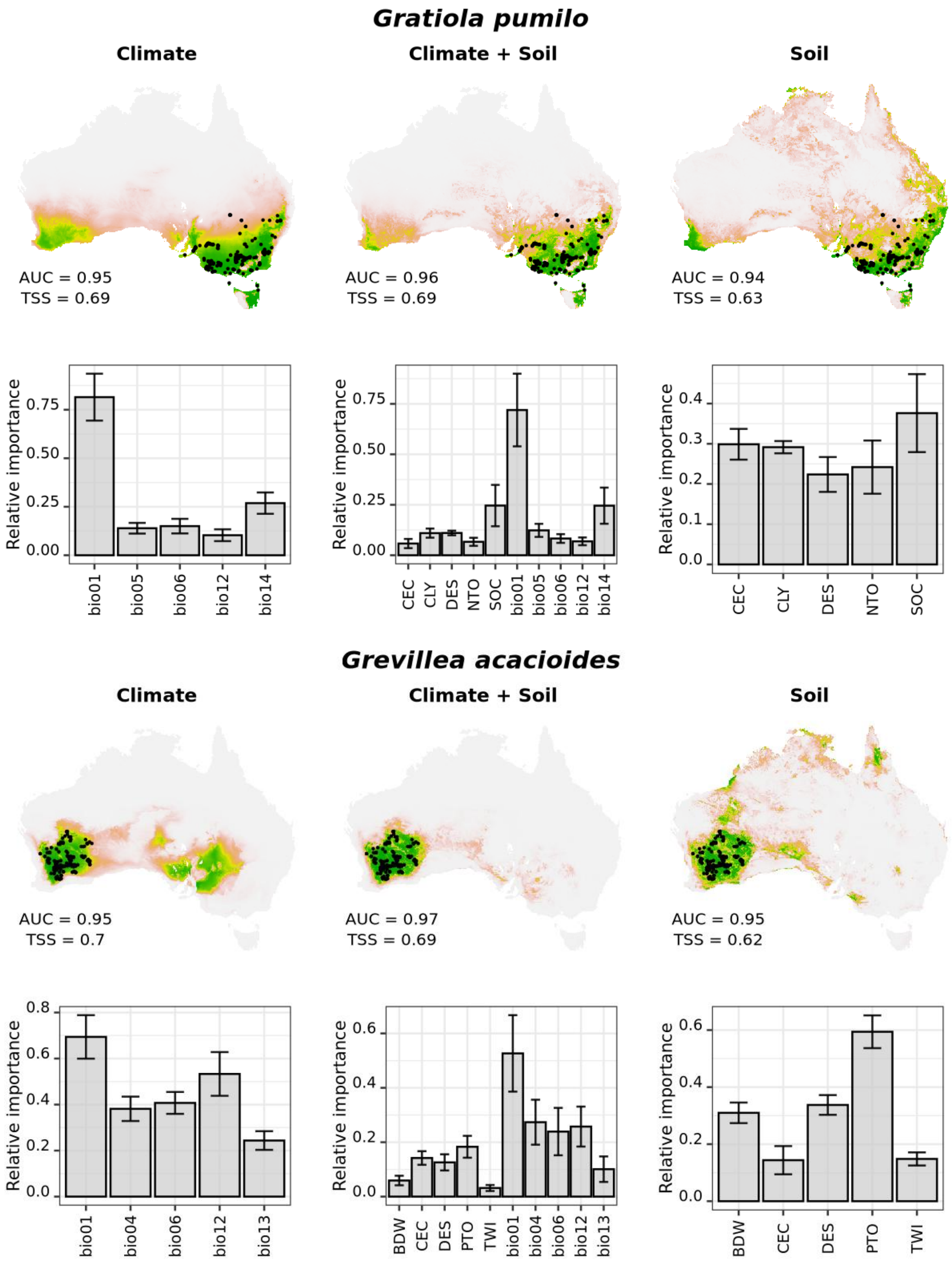

Gratiola pumilo
Climate
Climate + Soil
Soil
AUC = 0.95
TSS = 0.69
AUC = 0.96
TSS = 0.69
AUC = 0.94
TSS = 0.63
Relative importance
0.00
0.25
0.50
0.75
bio01
bio05
bio06
bio12
bio14
CEC
CLY
DES
NTO
SOC
0.0
0.1
0.2
0.3
0.4
Grevillea acacioides
AUC = 0.95
TSS = 0.7
AUC = 0.97
TSS = 0.69
AUC = 0.95
TSS = 0.62
0.2
0.4
0.6
0.8
bio04
bio13
BDW
PTO
TWI

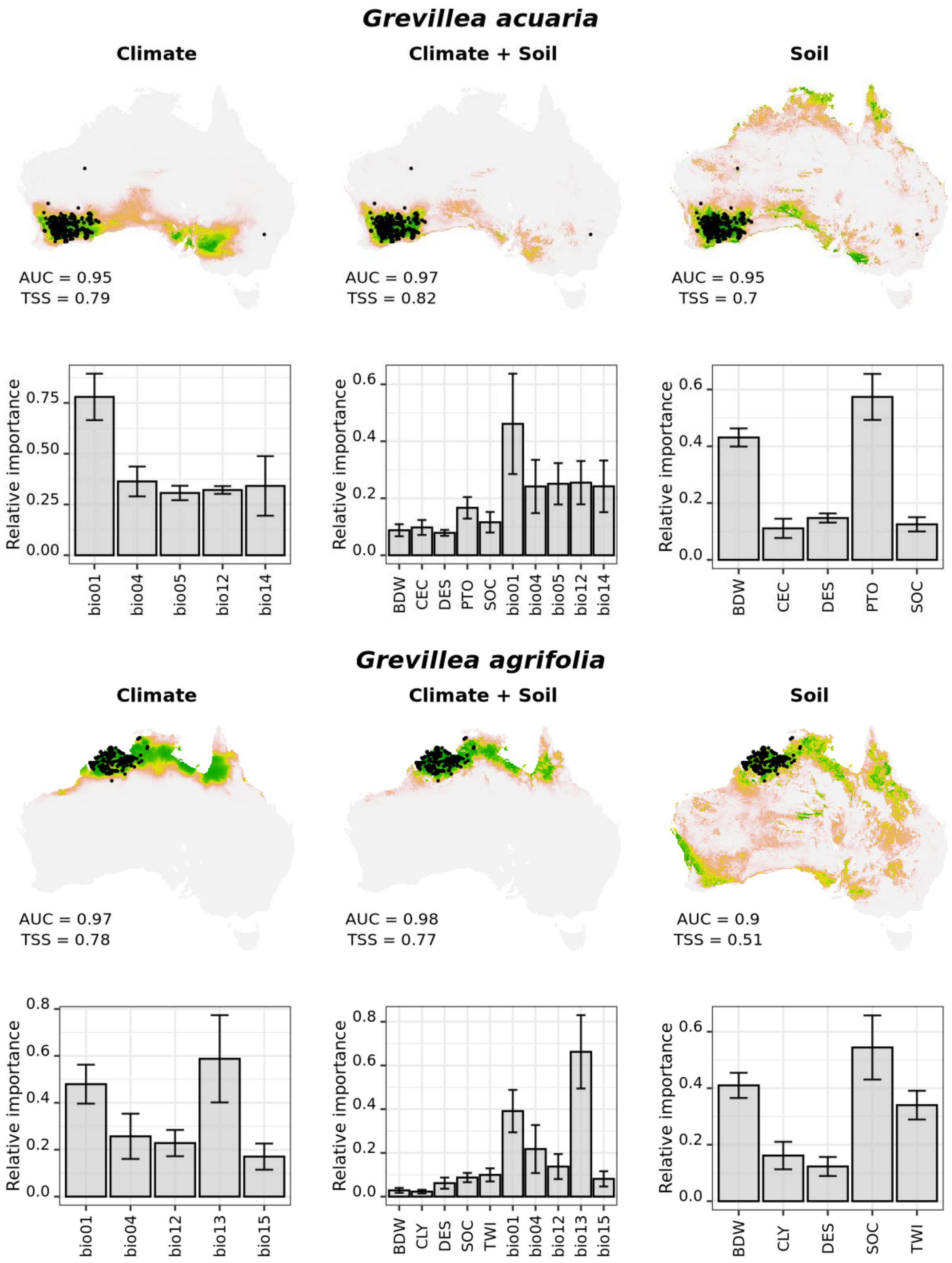

Grevillea acuaria
Climate
Climate + Soil
Soil
AUC = 0.95
TSS = 0.79
AUC = 0.97
TSS = 0.82
AUC = 0.95
TSS = 0.7
Relative importance
bio01
bio04
bio05
bio12
bio14
BDW
CEC
DES
PTO
SOC
Grevillea agrifolia
Climate
Climate + Soil
Soil
AUC = 0.97
TSS = 0.78
AUC = 0.98
TSS = 0.77
AUC = 0.9
TSS = 0.51
Relative importance
bio01
bio04
bio12
bio13
bio15
BDW
CLY
DES
SOC
TWI

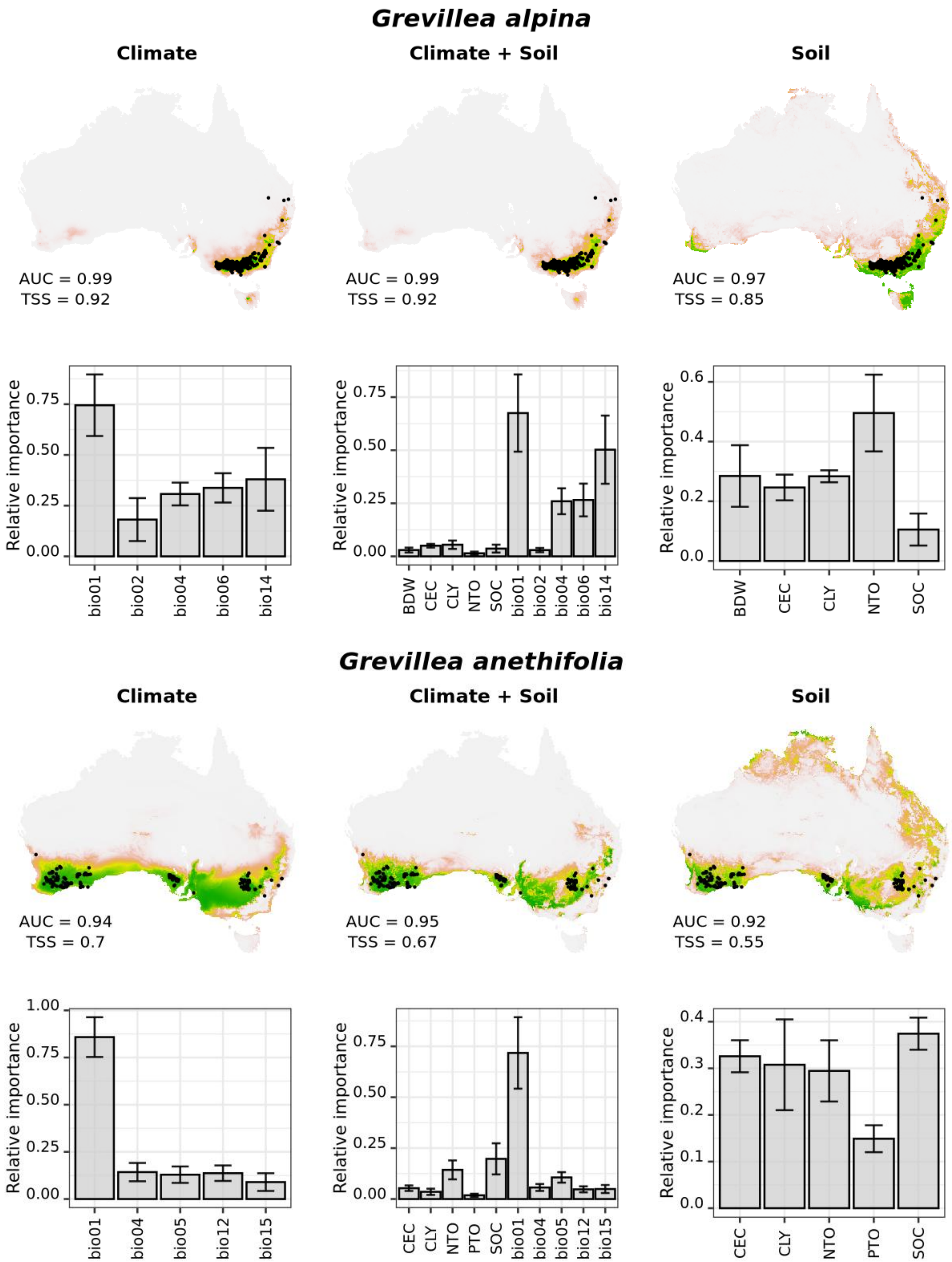
Grevillea alpina
Climate
Climate + Soil
Soil
AUC = 0.99
TSS = 0.92
AUC = 0.99
TSS = 0.92
AUC = 0.97
TSS = 0.85
Relative importance
0.00
0.25
0.50
0.75
bio01
bio02
bio04
bio06
bio14
BDW
CEC
CLY
NTO
SOC
0.0
0.2
0.4
0.6
Grevillea anethifolia
Climate
Climate + Soil
Soil
AUC = 0.94
TSS = 0.7
AUC = 0.95
TSS = 0.67
AUC = 0.92
TSS = 0.55
1.00
bio05
bio12
bio15
PTO
0.1
0.3

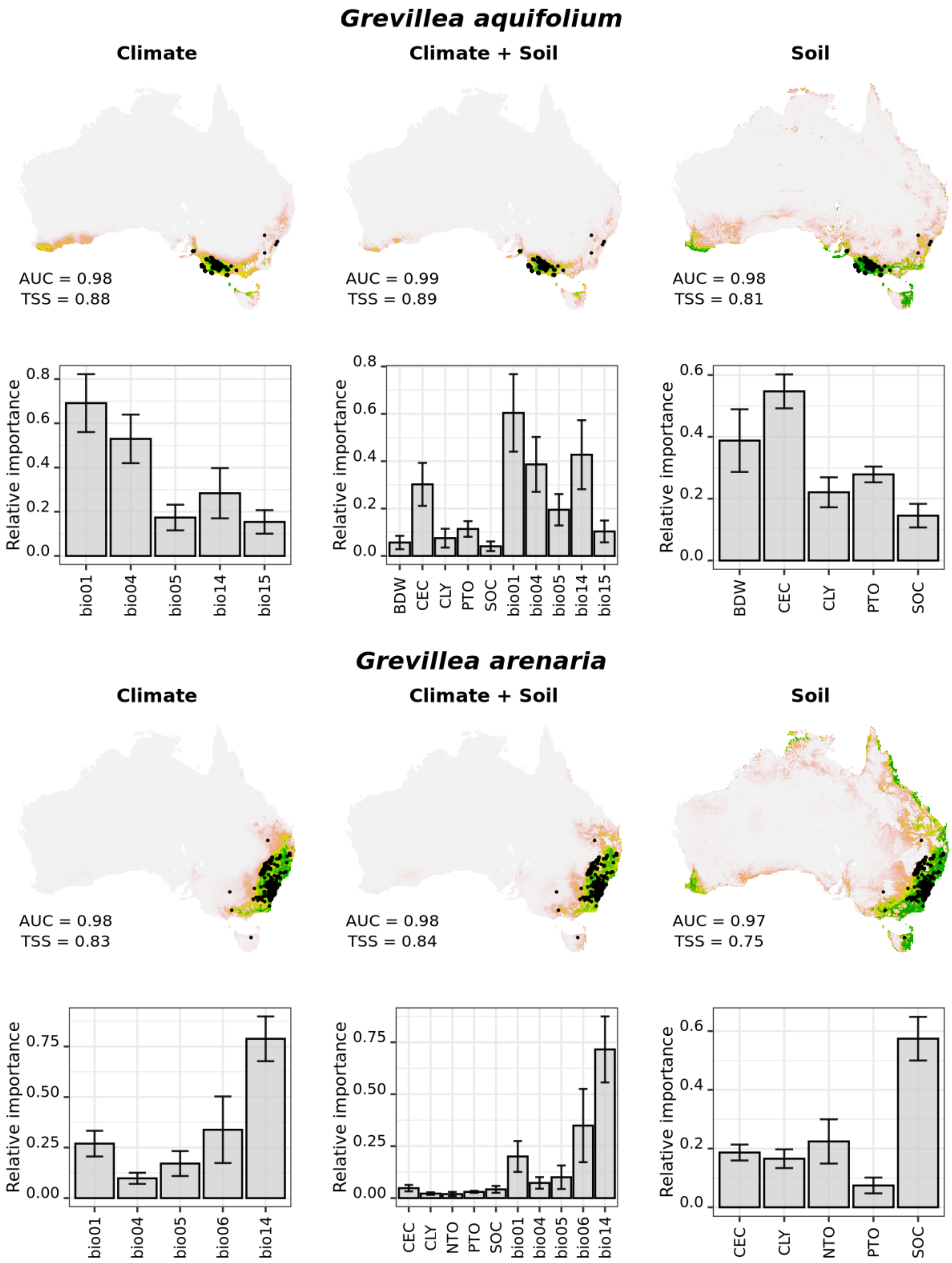
Grevillea aquifolium
Climate
Climate + Soil
Soil
AUC = 0.98
TSS = 0.88
AUC = 0.99
TSS = 0.89
AUC = 0.98
TSS = 0.81
Relative importance
bio01
bio04
bio05
bio14
bio15
BDW
CEC
CLY
PTO
SOC
Grevillea arenaria
AUC = 0.98
TSS = 0.83
AUC = 0.98
TSS = 0.84
AUC = 0.97
TSS = 0.75
bio06
NTO

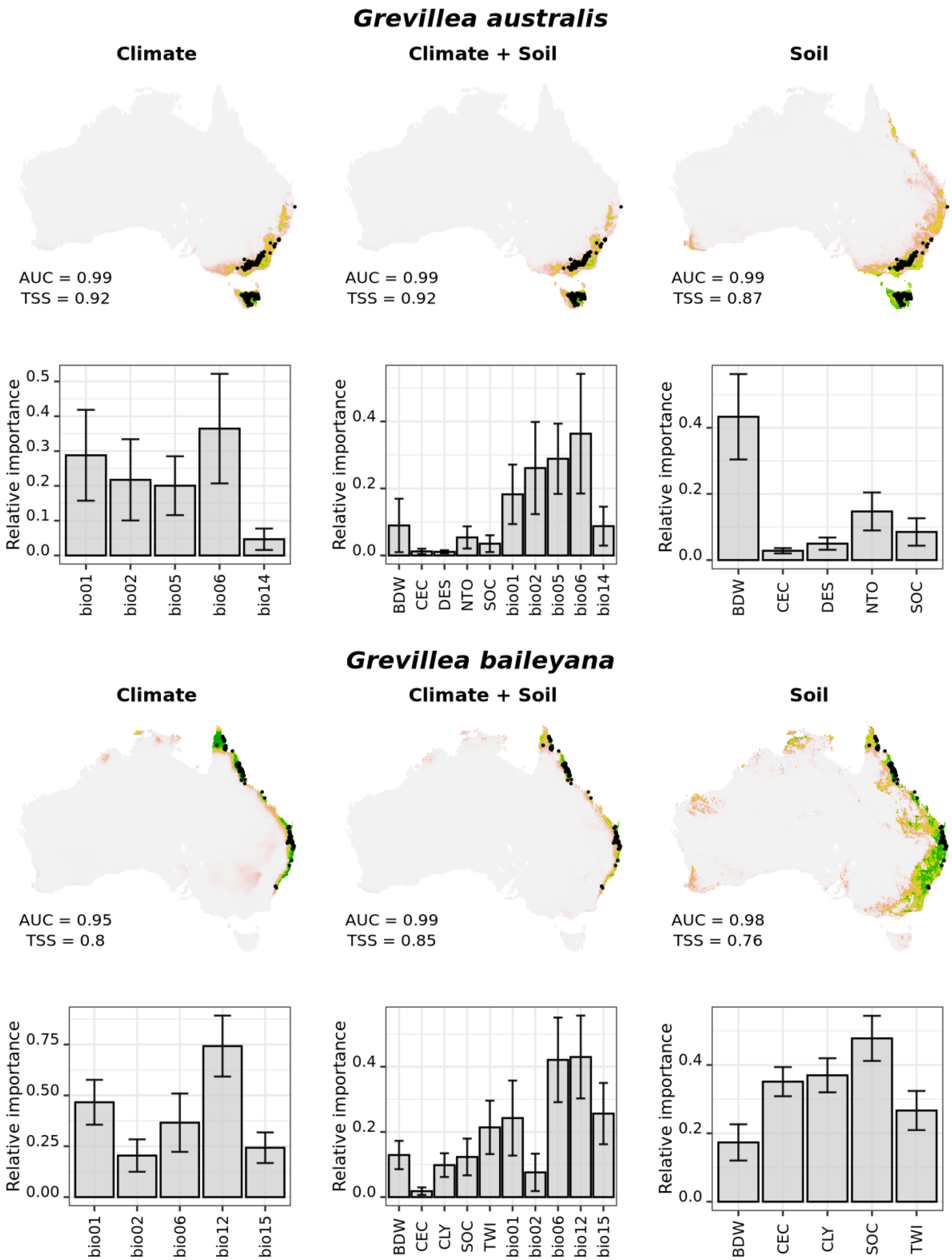
Grevillea australis
Climate
Climate + Soil
Soil
AUC = 0.99
TSS = 0.92
AUC = 0.99
TSS = 0.92
AUC = 0.99
TSS = 0.87
Relative importance
bio01
bio02
bio05
bio06
bio14
BDW
CEC
DES
NTO
SOC
Grevillea baileyana
Climate
Climate + Soil
Soil
AUC = 0.95
TSS = 0.8
AUC = 0.99
TSS = 0.85
AUC = 0.98
TSS = 0.76
Relative importance
bio01
bio02
bio06
bio12
bio15
BDW
CEC
CLY
SOC
TWI

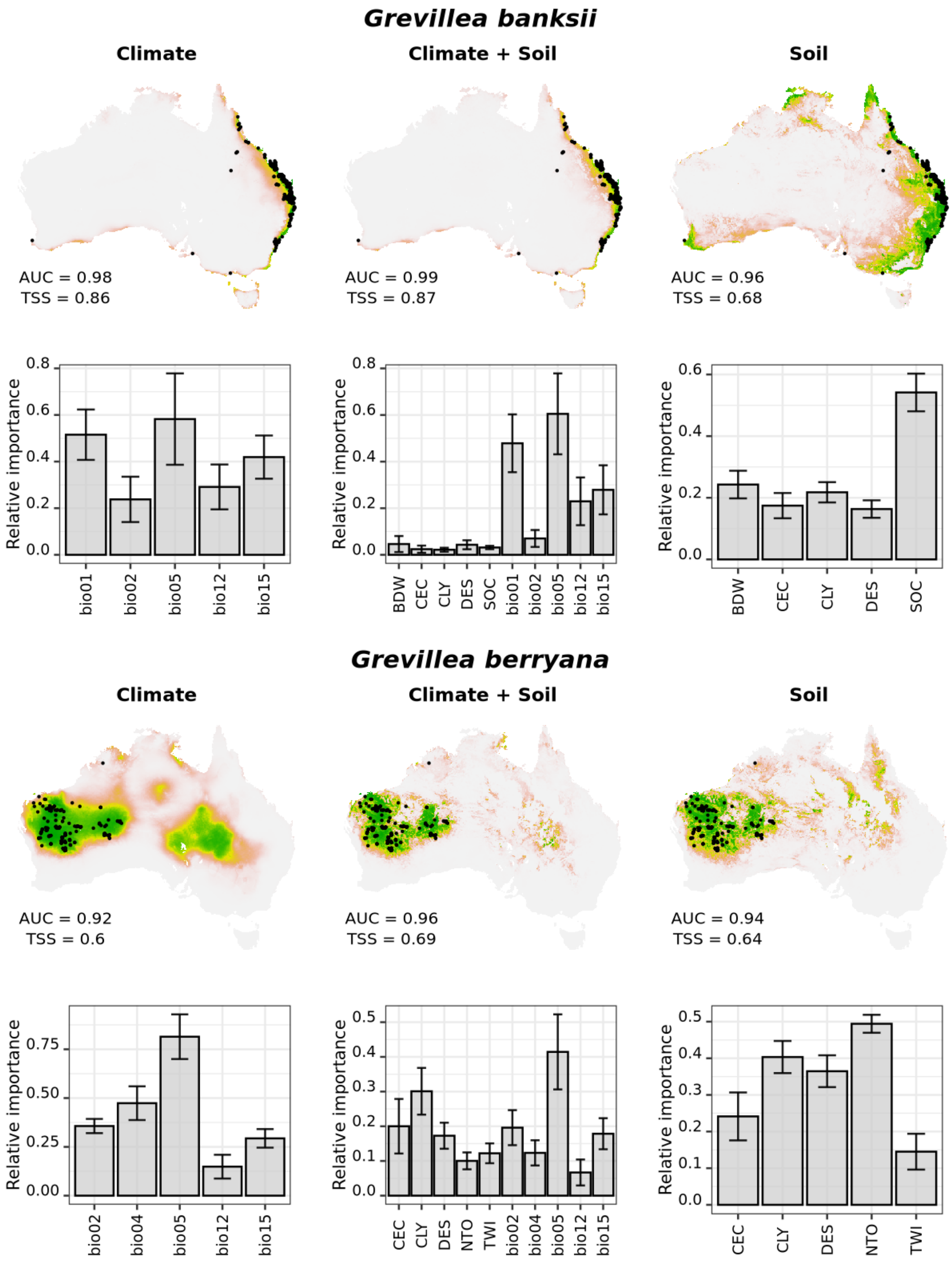
Grevillea banksii
Climate
Climate + Soil
Soil
AUC = 0.98
TSS = 0.86
AUC = 0.99
TSS = 0.87
AUC = 0.96
TSS = 0.68
Relative importance
bio01
bio02
bio05
bio12
bio15
BDW
CEC
CLY
DES
SOC
Grevillea berryana
Climate
Climate + Soil
Soil
AUC = 0.92
TSS = 0.6
AUC = 0.96
TSS = 0.69
AUC = 0.94
TSS = 0.64
Relative importance
bio02
bio04
bio05
bio12
bio15
CEC
CLY
DES
NTO
TWI

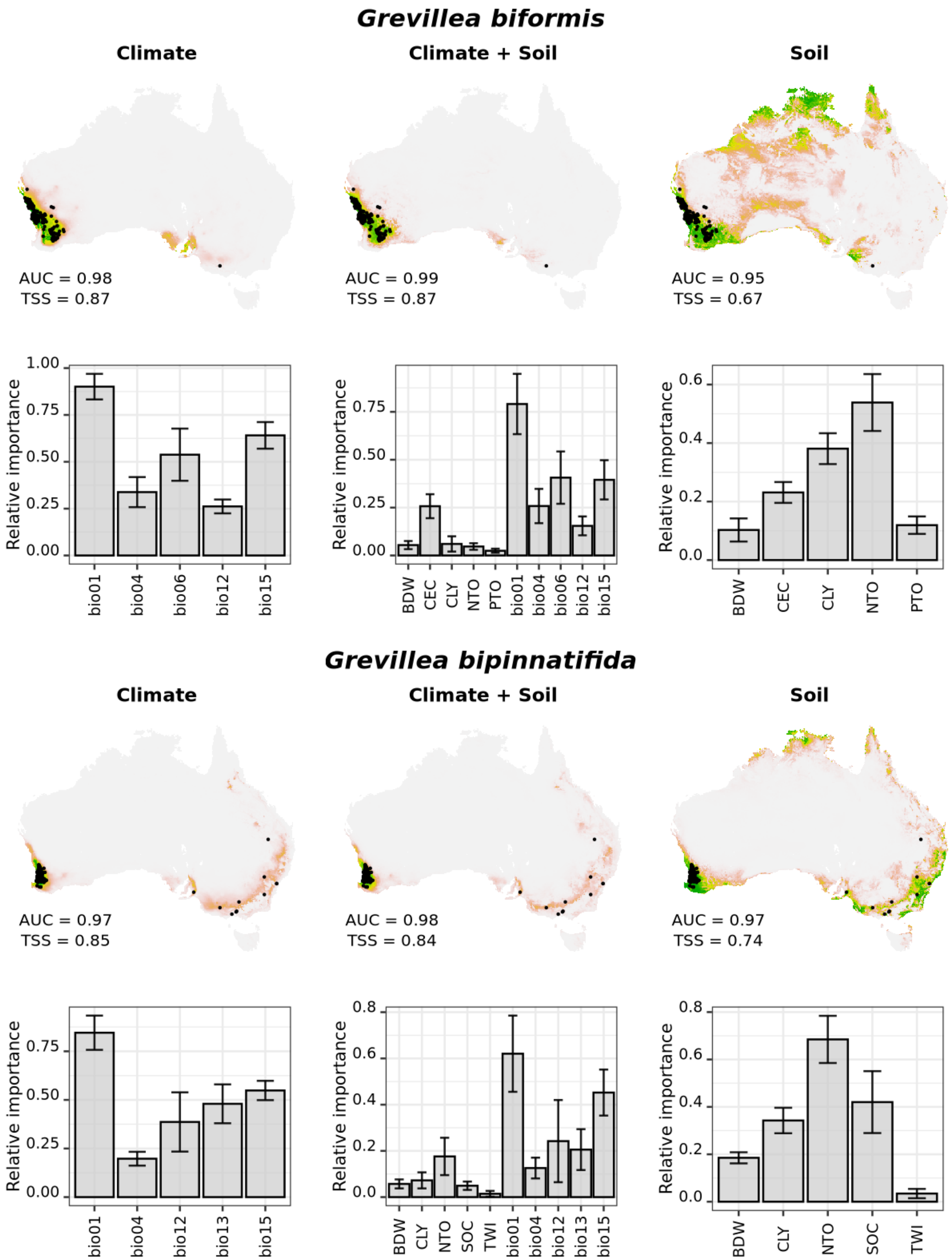

Grevillea biformis
Climate
Climate + Soil
Soil
AUC = 0.98
TSS = 0.87
AUC = 0.99
TSS = 0.87
AUC = 0.95
TSS = 0.67
Relative importance
bio01
bio04
bio06
bio12
bio15
BDW
CEC
CLY
NTO
PTO
Grevillea bipinnatifida
Climate
Climate + Soil
Soil
AUC = 0.97
TSS = 0.85
AUC = 0.98
TSS = 0.84
AUC = 0.97
TSS = 0.74
Relative importance
bio01
bio04
bio12
bio13
bio15
BDW
CLY
NTO
SOC
TWI

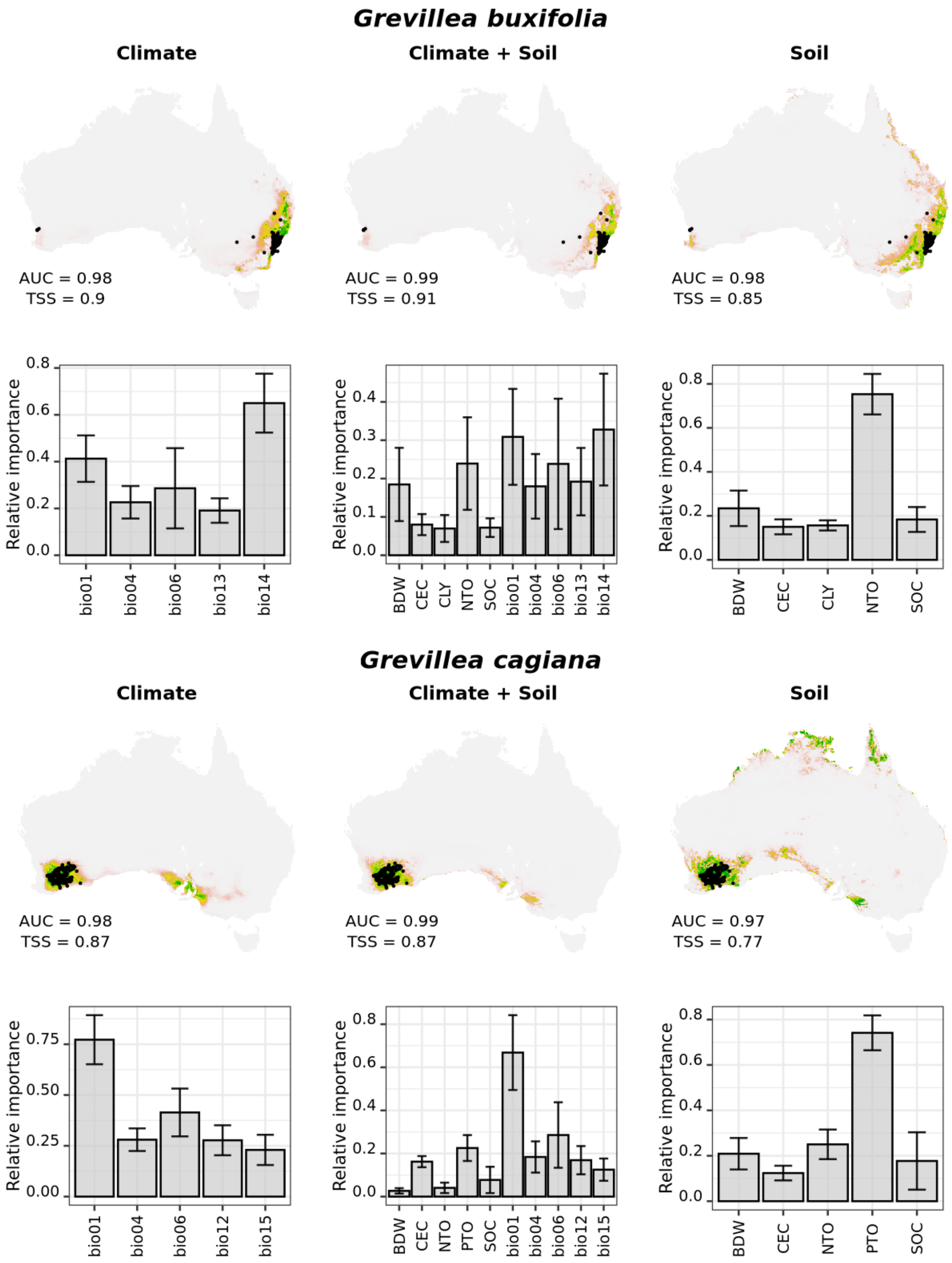
Grevillea buxifolia
Climate
Climate + Soil
Soil
AUC = 0.98
TSS = 0.9
AUC = 0.99
TSS = 0.91
AUC = 0.98
TSS = 0.85
Relative importance
bio01
bio04
bio06
bio13
bio14
BDW
CEC
CLY
NTO
SOC
Grevillea cagiana
AUC = 0.98
TSS = 0.87
AUC = 0.99
TSS = 0.87
AUC = 0.97
TSS = 0.77
bio12
bio15
PTO

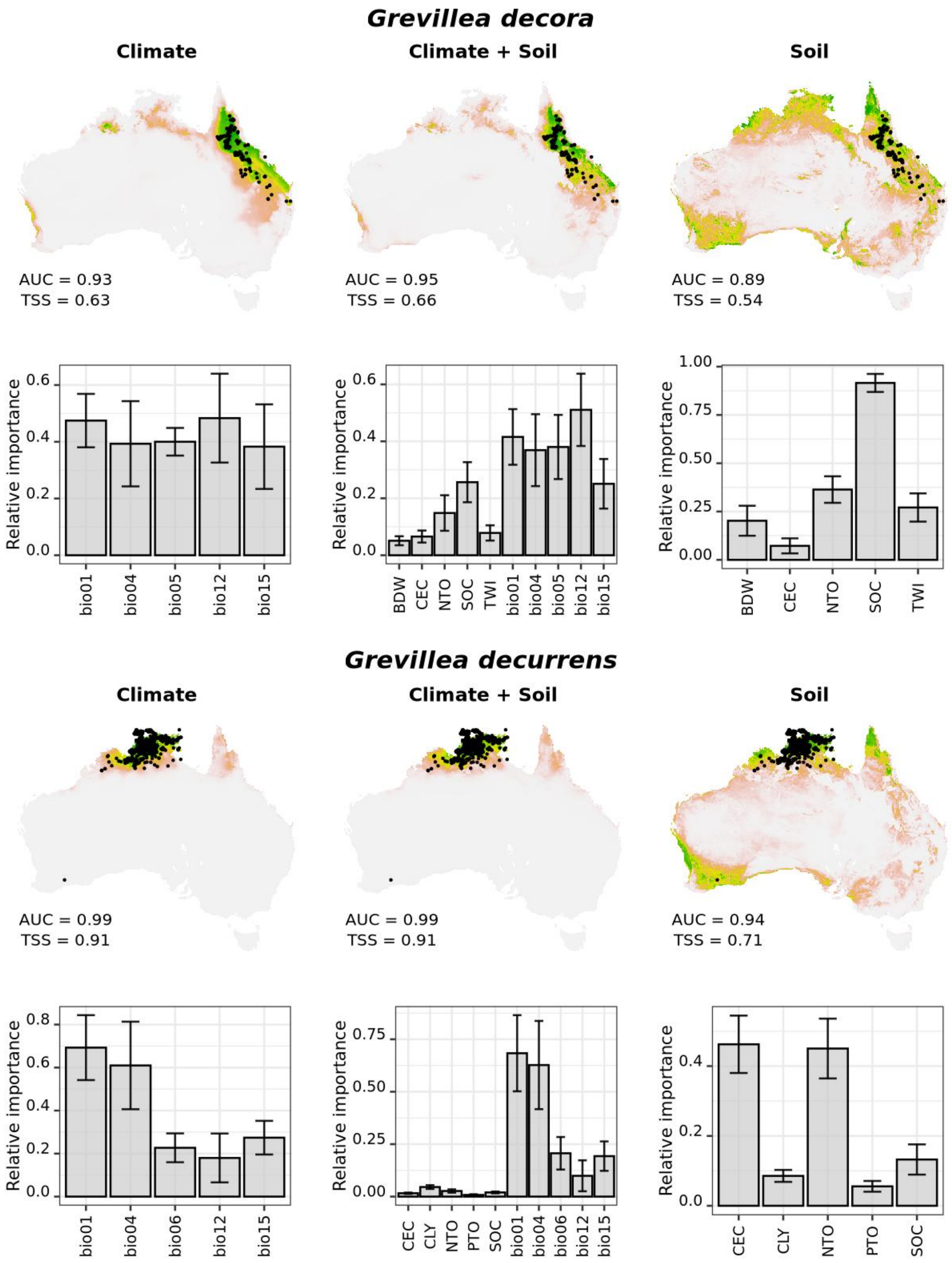
Grevillea decora
Climate
Climate + Soil
Soil
AUC = 0.93
TSS = 0.63
AUC = 0.95
TSS = 0.66
AUC = 0.89
TSS = 0.54
Relative importance
0.0
0.2
0.4
0.6
bio01
bio04
bio05
bio12
bio15
BDW
CEC
NTO
SOC
TWI
0.00
0.25
0.50
0.75
1.00
Grevillea decurrens
Climate
Climate + Soil
Soil
AUC = 0.99
TSS = 0.91
AUC = 0.99
TSS = 0.91
AUC = 0.94
TSS = 0.71
0.8
bio06
CLY
PTO

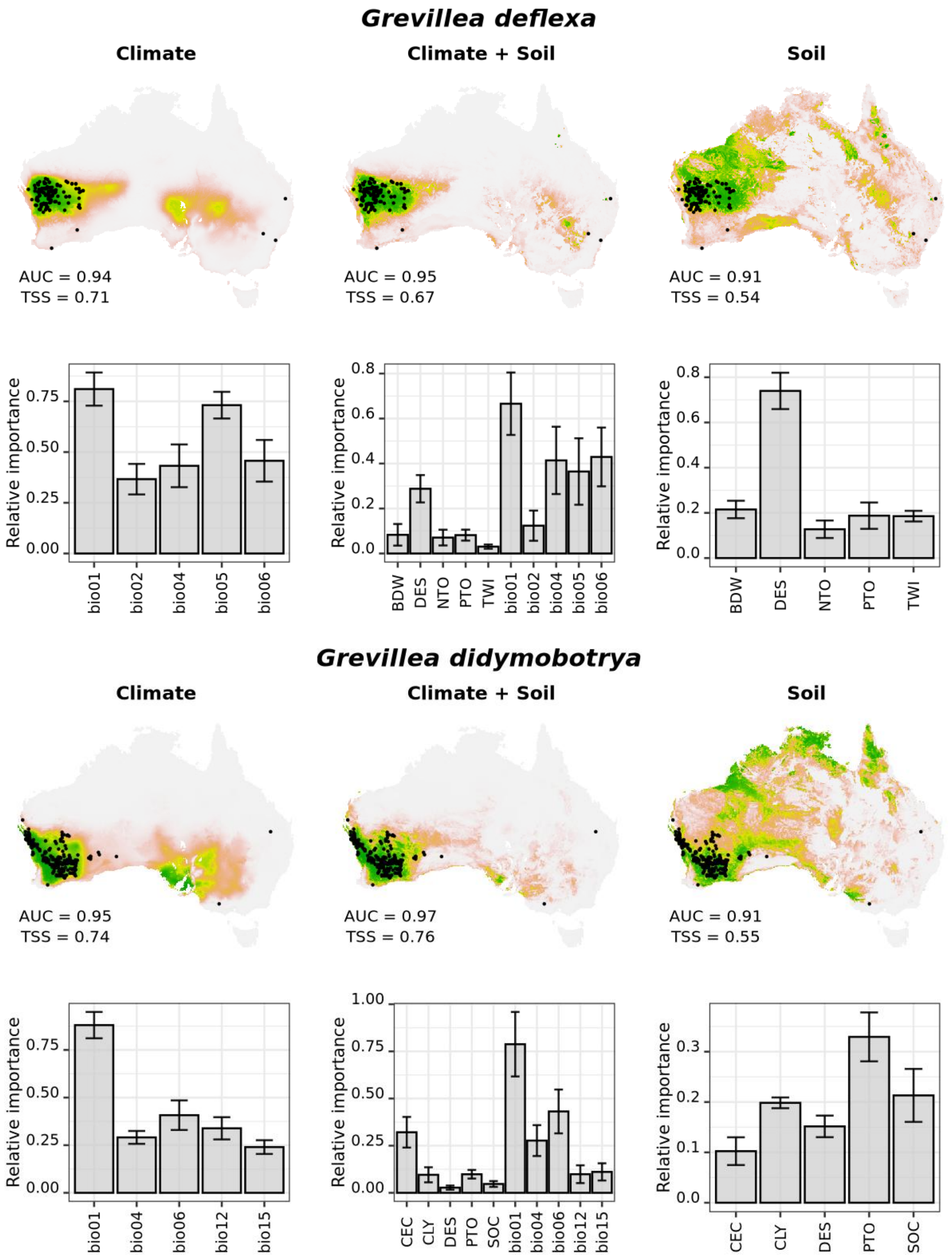
Grevillea deflexa
Climate
Climate + Soil
Soil
AUC = 0.94
TSS = 0.71
AUC = 0.95
TSS = 0.67
AUC = 0.91
TSS = 0.54
Relative importance
bio01 bio02 bio04 bio05 bio06
BDW DES NTO PTO TWI bio01 bio02 bio04 bio05 bio06
BDW DES NTO PTO TWI
Grevillea didymobotrya
Climate
Climate + Soil
Soil
AUC = 0.95
TSS = 0.74
AUC = 0.97
TSS = 0.76
AUC = 0.91
TSS = 0.55
Relative importance
bio01 bio04 bio06 bio12 bio15
CEC CLY DES PTO SOC bio01 bio04 bio06 bio12 bio15
CEC CLY DES PTO SOC

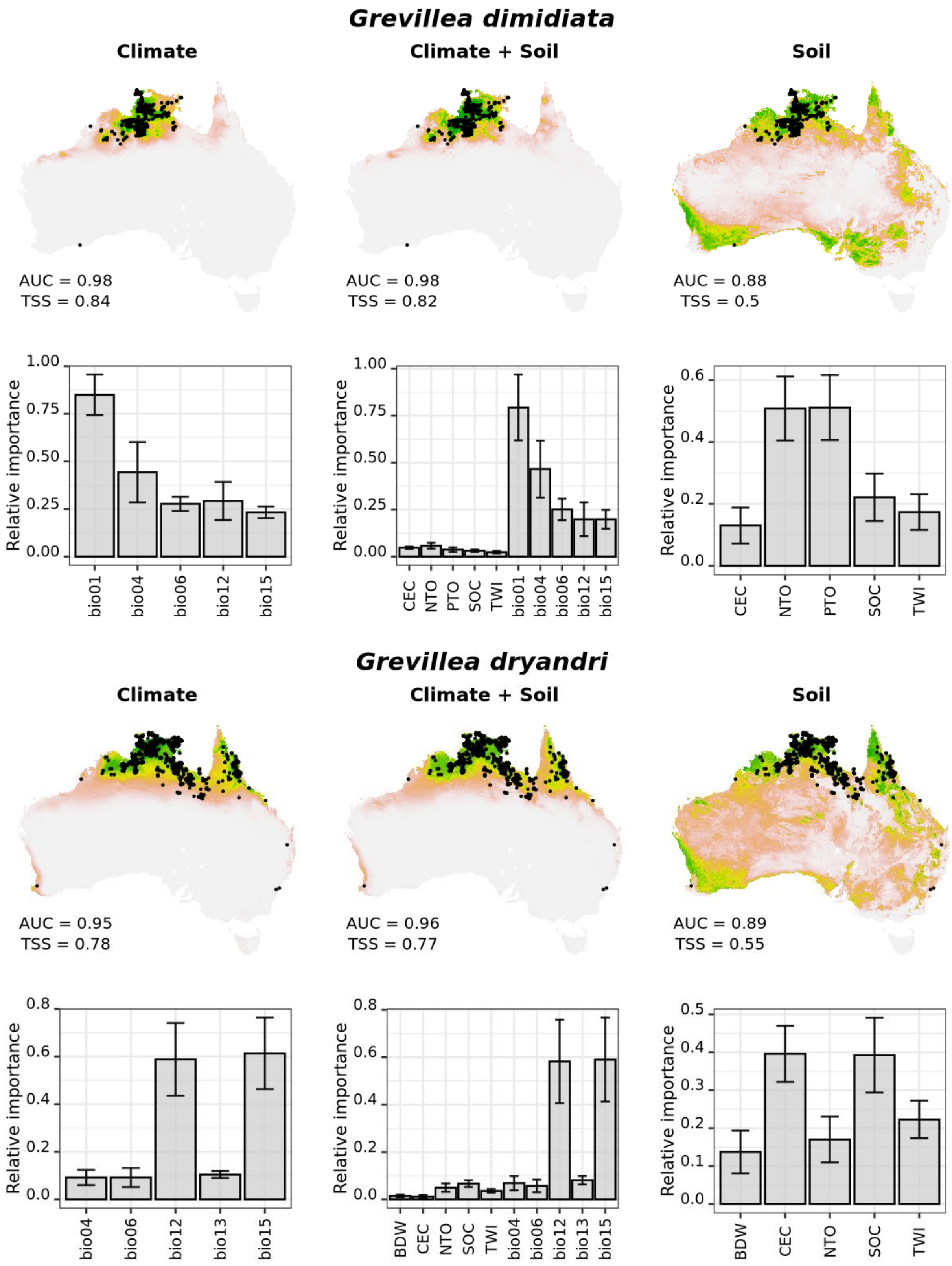

Grevillea dimidiata
Climate
Climate + Soil
Soil
AUC = 0.98
TSS = 0.84
AUC = 0.98
TSS = 0.82
AUC = 0.88
TSS = 0.5
Relative importance
1.00
0.75
0.50
0.25
0.00
bio01
bio04
bio06
bio12
bio15
CEC
NTO
PTO
SOC
TWI
0.6
0.4
0.2
0.0
Grevillea dryandri
Climate
Climate + Soil
Soil
AUC = 0.95
TSS = 0.78
AUC = 0.96
TSS = 0.77
AUC = 0.89
TSS = 0.55
0.8
0.6
0.4
0.2
0.0
bio13
BDW
0.5
0.3
0.1

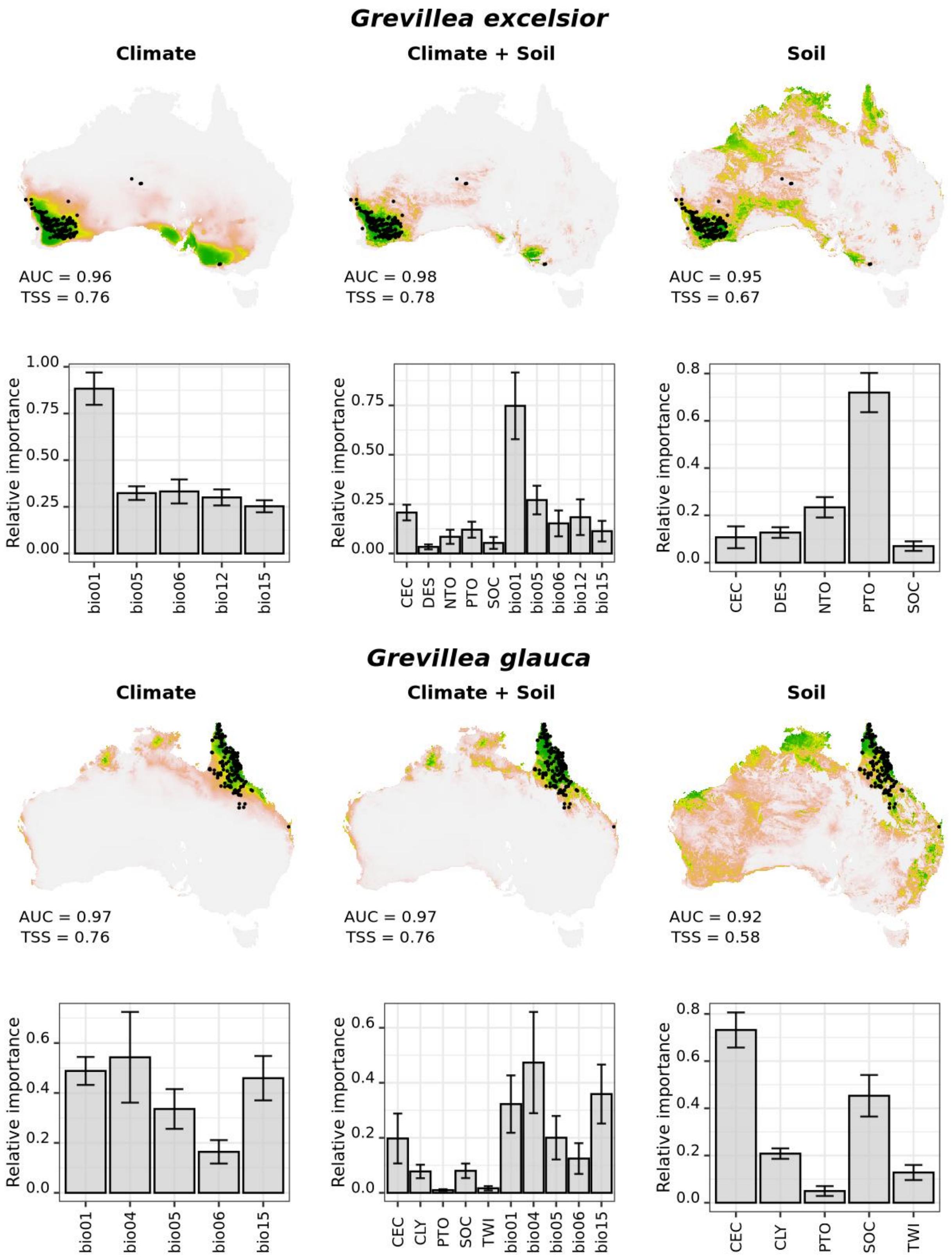

Grevillea excelsior
Climate
Climate + Soil
Soil
AUC = 0.96
TSS = 0.76
AUC = 0.98
TSS = 0.78
AUC = 0.95
TSS = 0.67
Relative importance
bio01
bio05
bio06
bio12
bio15
CEC
DES
NTO
PTO
SOC
Grevillea glauca
Climate
Climate + Soil
Soil
AUC = 0.97
TSS = 0.76
AUC = 0.97
TSS = 0.76
AUC = 0.92
TSS = 0.58
Relative importance
bio01
bio04
bio05
bio06
bio15
CEC
CLY
PTO
SOC
TWI

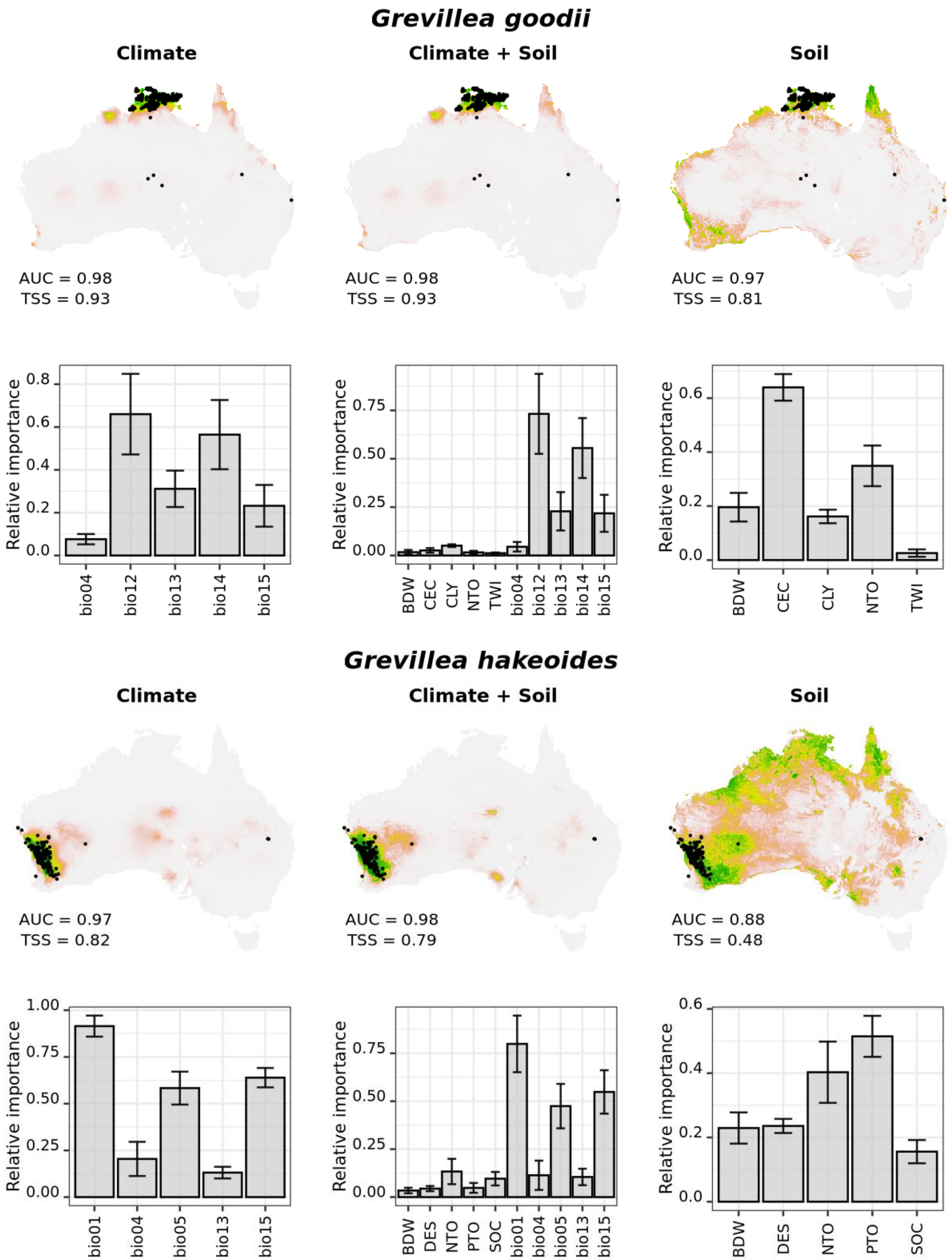
Grevillea goodii
Climate
Climate + Soil
Soil
AUC = 0.98
TSS = 0.93
AUC = 0.98
TSS = 0.93
AUC = 0.97
TSS = 0.81
Relative importance
0.0
0.2
0.4
0.6
0.8
bio04
bio12
bio13
bio14
bio15
Relative importance
0.00
0.25
0.50
0.75
BDW
CEC
CLY
NTO
TWI
bio04
bio12
bio13
bio14
bio15
Relative importance
0.0
0.2
0.4
0.6
BDW
CEC
CLY
NTO
TWI
Grevillea hakeoides
Climate
Climate + Soil
Soil
AUC = 0.97
TSS = 0.82
AUC = 0.98
TSS = 0.79
AUC = 0.88
TSS = 0.48
Relative importance
0.00
0.25
0.50
0.75
1.00
bio01
bio04
bio05
bio13
bio15
Relative importance
0.00
0.25
0.50
0.75
BDW
DES
NTO
PTO
SOC
bio01
bio04
bio05
bio13
bio15
Relative importance
0.0
0.2
0.4
0.6
BDW
DES
NTO
PTO
SOC

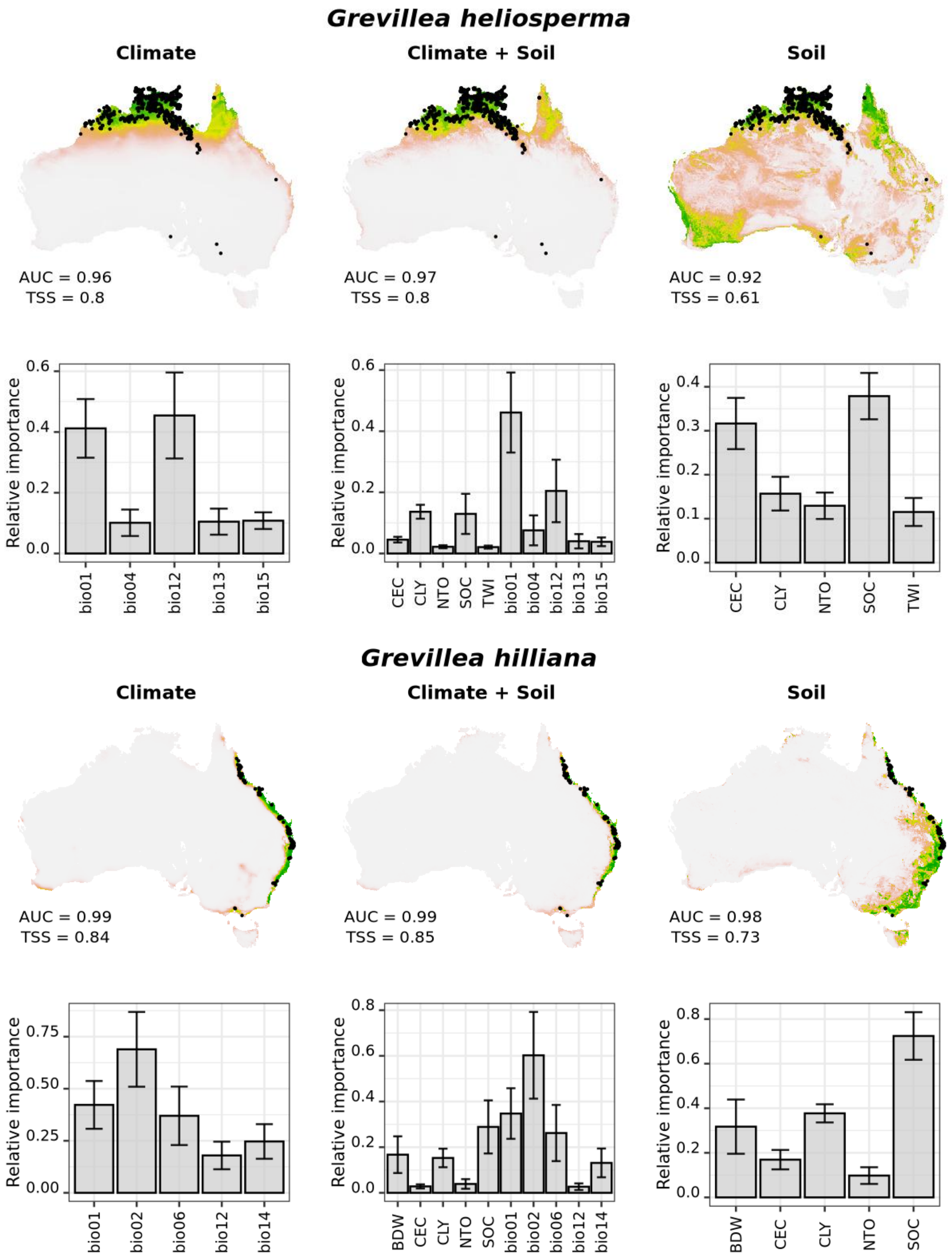
Grevillea heliosperma
Climate
Climate + Soil
Soil
AUC = 0.96
TSS = 0.8
AUC = 0.97
TSS = 0.8
AUC = 0.92
TSS = 0.61
Relative importance
0.0
0.2
0.4
0.6
bio01
bio04
bio12
bio13
bio15
CEC
CLY
NTO
SOC
TWI
bio01
bio04
bio12
bio13
bio15
0.1
0.3
CEC
CLY
NTO
SOC
TWI
Grevillea hilliana
Climate
Climate + Soil
Soil
AUC = 0.99
TSS = 0.84
AUC = 0.99
TSS = 0.85
AUC = 0.98
TSS = 0.73
0.00
0.25
0.50
0.75
bio01
bio02
bio06
bio12
bio14
0.8
BDW
CEC
CLY
NTO
SOC
bio01
bio02
bio06
bio12
bio14
BDW
CEC
CLY
NTO
SOC

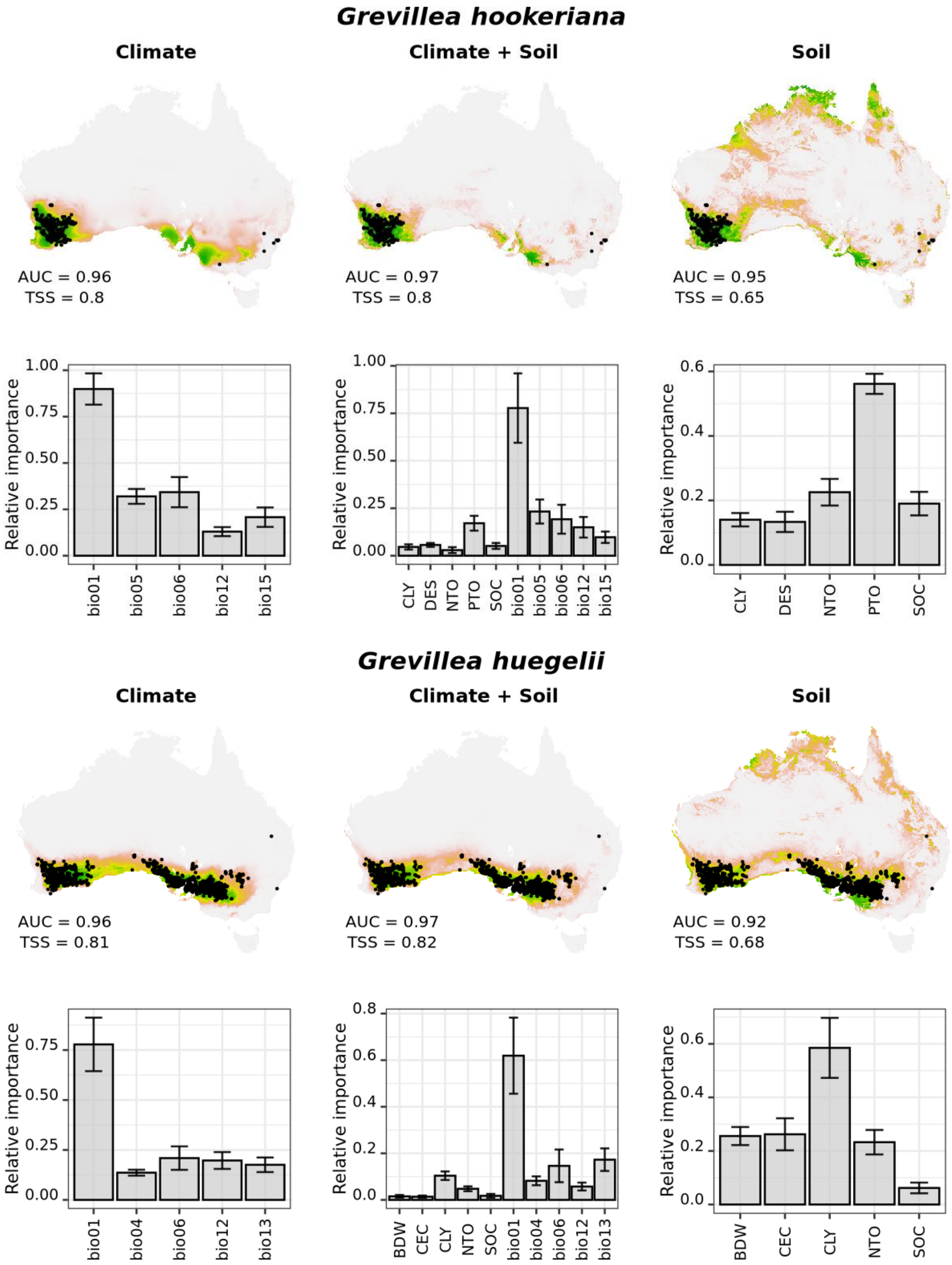
Grevillea hookeriana
Climate
Climate + Soil
Soil
AUC = 0.96
TSS = 0.8
AUC = 0.97
TSS = 0.8
AUC = 0.95
TSS = 0.65
Relative importance
bio01
bio05
bio06
bio12
bio15
CLY
DES
NTO
PTO
SOC
Grevillea huegelii
Climate
Climate + Soil
Soil
AUC = 0.96
TSS = 0.81
AUC = 0.97
TSS = 0.82
AUC = 0.92
TSS = 0.68
Relative importance
bio01
bio04
bio06
bio12
bio13
BDW
CEC
CLY
NTO
SOC

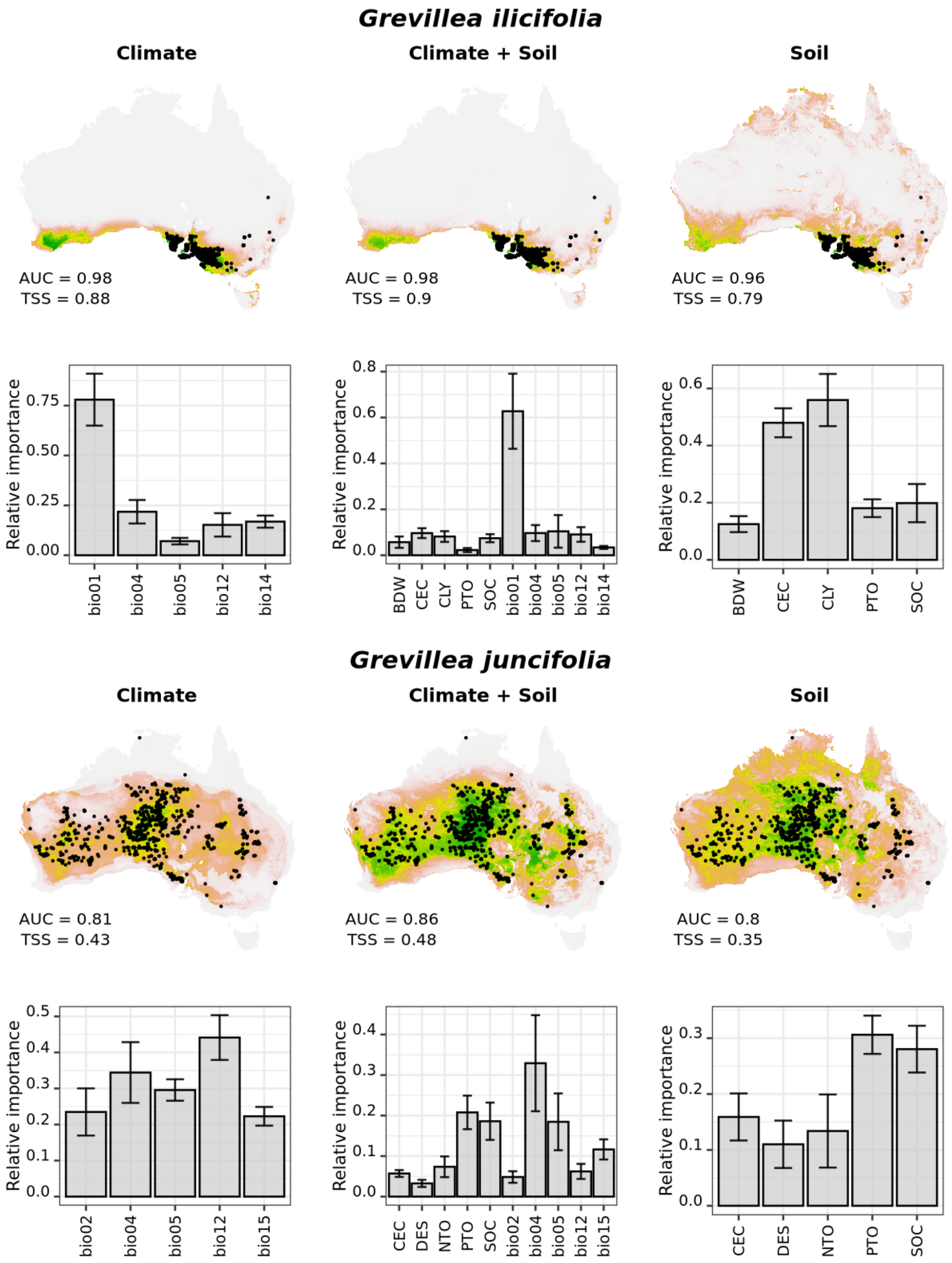
Grevillea ilicifolia
Climate
Climate + Soil
Soil
AUC = 0.98
TSS = 0.88
AUC = 0.98
TSS = 0.9
AUC = 0.96
TSS = 0.79
Relative importance
0.00
0.25
0.50
0.75
bio01
bio04
bio05
bio12
bio14
0.0
0.2
0.4
0.6
0.8
BDW
CEC
CLY
PTO
SOC
bio01
bio04
bio05
bio12
bio14
0.0
0.2
0.4
0.6
BDW
CEC
CLY
PTO
SOC
Grevillea juncifolia
Climate
Climate + Soil
Soil
AUC = 0.81
TSS = 0.43
AUC = 0.86
TSS = 0.48
AUC = 0.8
TSS = 0.35
Relative importance
0.0
0.1
0.2
0.3
0.4
0.5
bio02
bio04
bio05
bio12
bio15
0.0
0.1
0.2
0.3
0.4
CEC
DES
NTO
PTO
SOC
bio02
bio04
bio05
bio12
bio15
0.0
0.1
0.2
0.3
CEC
DES
NTO
PTO
SOC

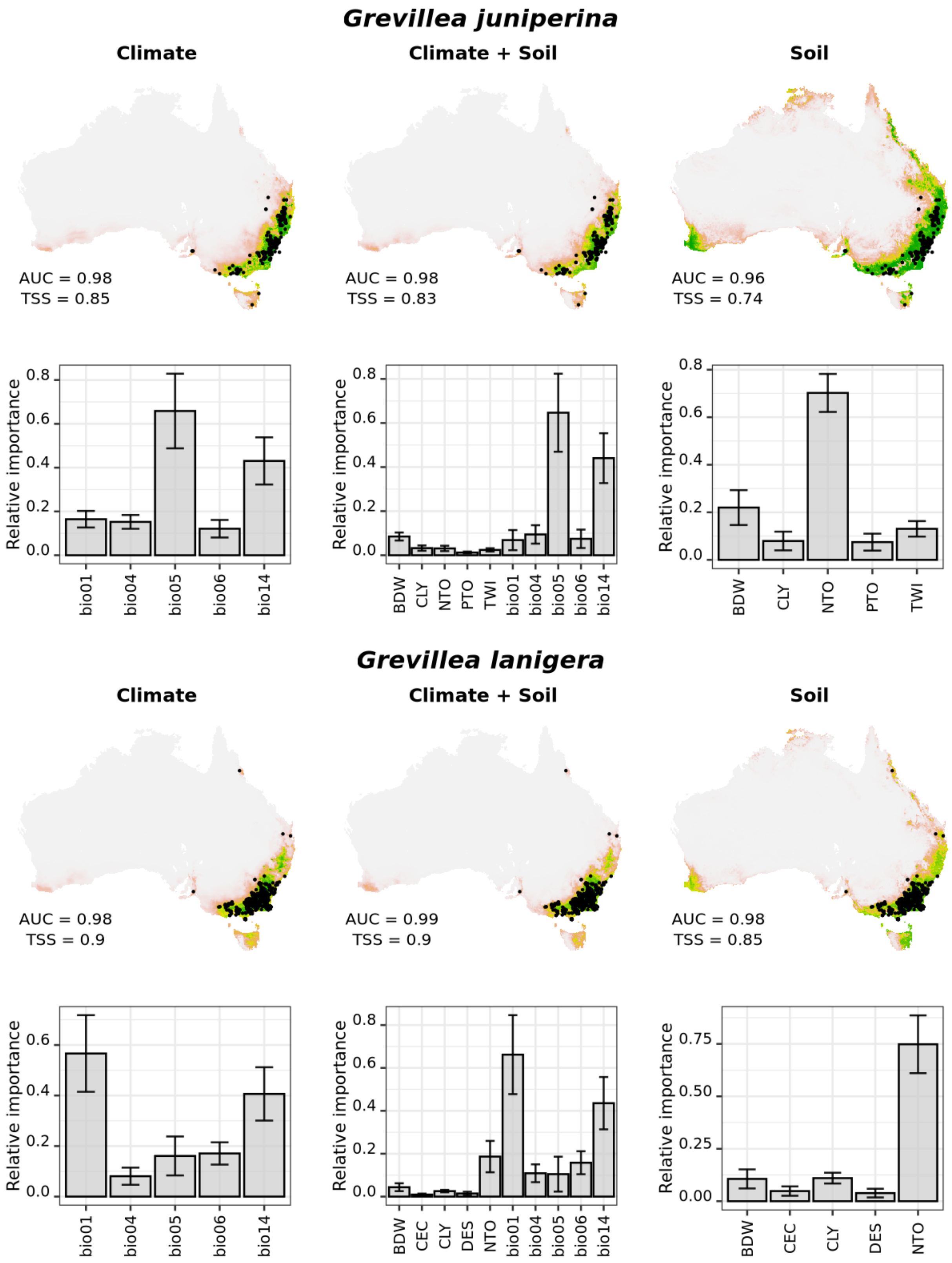

Grevillea juniperina
Climate
Climate + Soil
Soil
AUC = 0.98
TSS = 0.85
AUC = 0.98
TSS = 0.83
AUC = 0.96
TSS = 0.74
Relative importance
bio01
bio04
bio05
bio06
bio14
BDW
CLY
NTO
PTO
TWI
Grevillea lanigera
Climate
Climate + Soil
Soil
AUC = 0.98
TSS = 0.9
AUC = 0.99
TSS = 0.9
AUC = 0.98
TSS = 0.85
Relative importance
bio01
bio04
bio05
bio06
bio14
BDW
CEC
CLY
DES
NTO

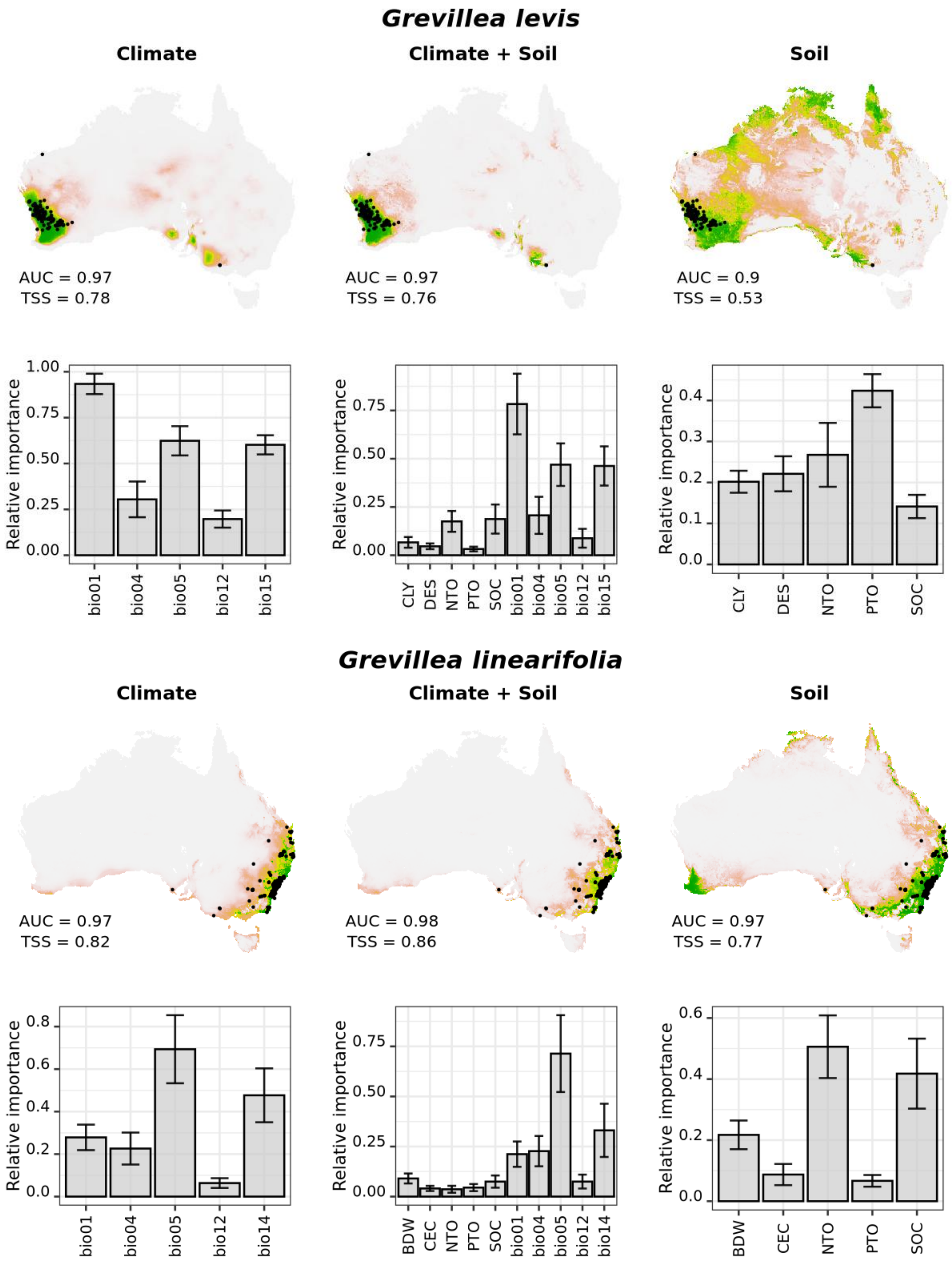

Grevillea levis
Climate
Climate + Soil
Soil
AUC = 0.97
TSS = 0.78
AUC = 0.97
TSS = 0.76
AUC = 0.9
TSS = 0.53
Relative importance
1.00
0.75
0.50
0.25
0.00
bio01
bio04
bio05
bio12
bio15
Relative importance
0.75
0.50
0.25
0.00
CLY
DES
NTO
PTO
SOC
bio01
bio04
bio05
bio12
bio15
Relative importance
0.4
0.3
0.2
0.1
0.0
CLY
DES
NTO
PTO
SOC
Grevillea linearifolia
Climate
Climate + Soil
Soil
AUC = 0.97
TSS = 0.82
AUC = 0.98
TSS = 0.86
AUC = 0.97
TSS = 0.77
Relative importance
0.8
0.6
0.4
0.2
0.0
bio01
bio04
bio05
bio12
bio14
Relative importance
0.75
0.50
0.25
0.00
BDW
CEC
NTO
PTO
SOC
bio01
bio04
bio05
bio12
bio14
Relative importance
0.6
0.4
0.2
0.0
BDW
CEC
NTO
PTO
SOC

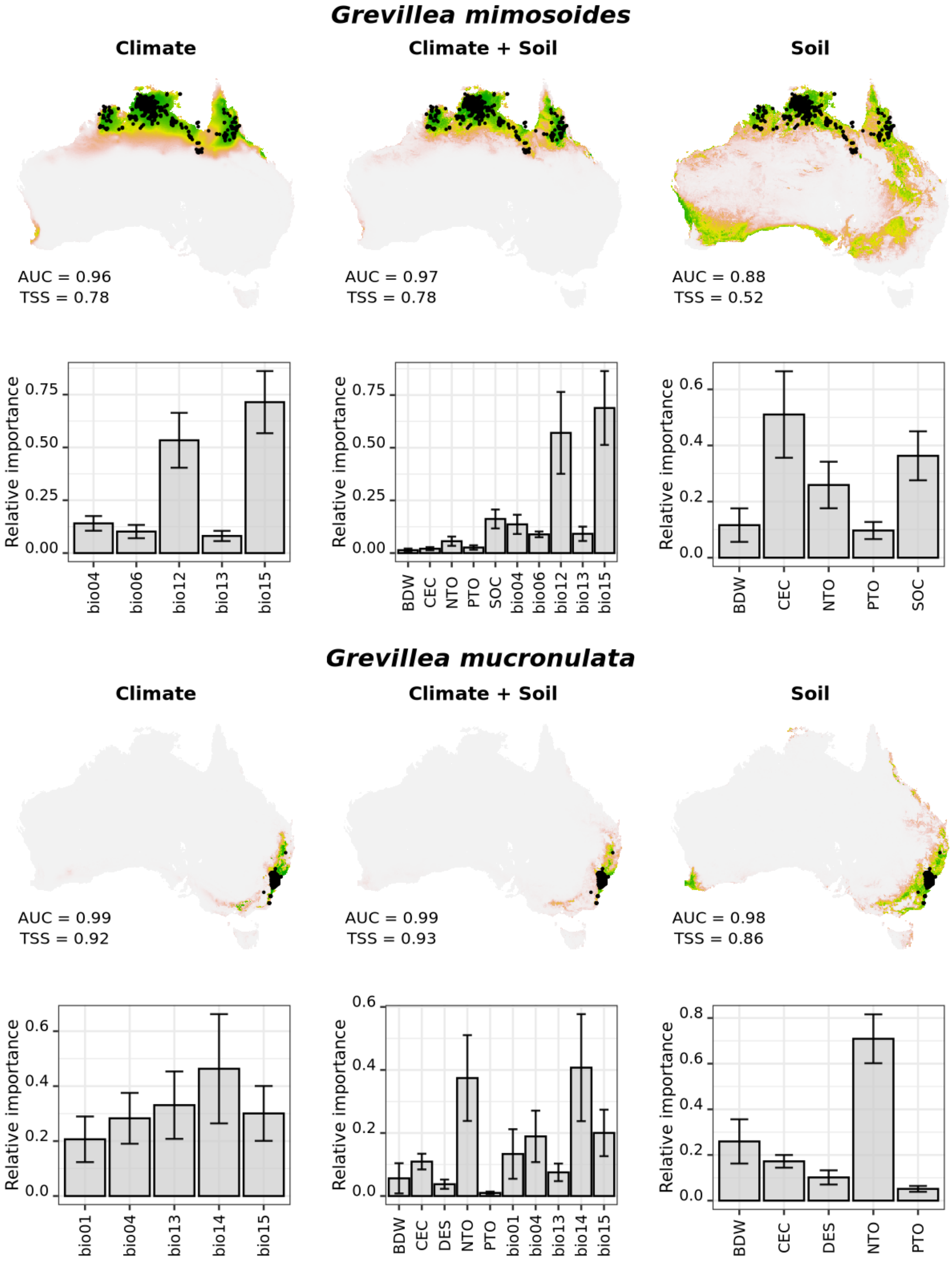
Grevillea mimosoides
Climate
Climate + Soil
Soil
AUC = 0.96
TSS = 0.78
AUC = 0.97
TSS = 0.78
AUC = 0.88
TSS = 0.52
Relative importance
bio04
bio06
bio12
bio13
bio15
BDW
CEC
NTO
PTO
SOC
Grevillea mucronulata
Climate
Climate + Soil
Soil
AUC = 0.99
TSS = 0.92
AUC = 0.99
TSS = 0.93
AUC = 0.98
TSS = 0.86
Relative importance
bio01
bio04
bio13
bio14
bio15
BDW
CEC
DES
NTO
PTO

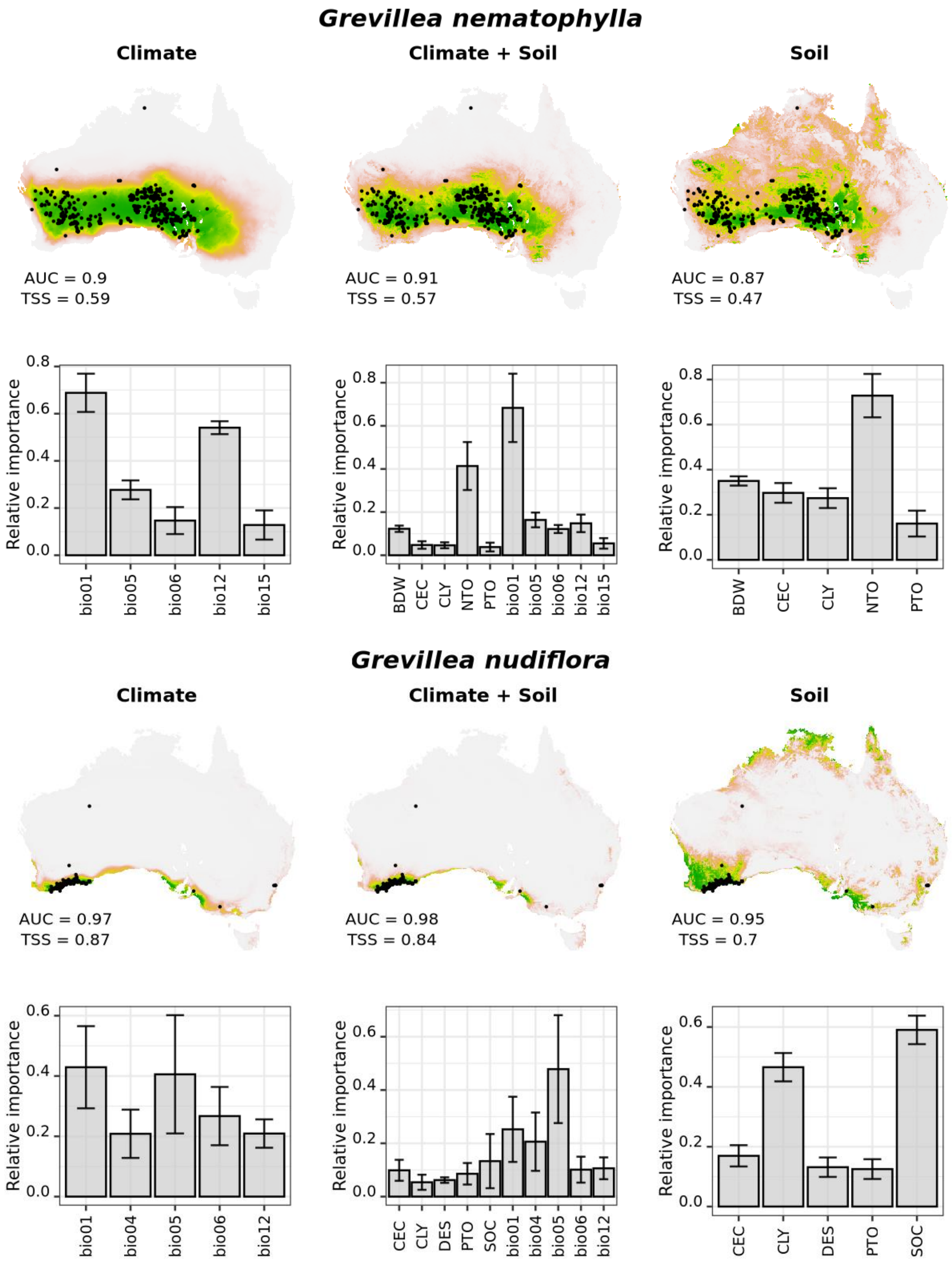

Grevillea nematophylla
Climate
Climate + Soil
Soil
AUC = 0.9
TSS = 0.59
AUC = 0.91
TSS = 0.57
AUC = 0.87
TSS = 0.47
Relative importance
bio01
bio05
bio06
bio12
bio15
BDW
CEC
CLY
NTO
PTO
bio01
bio05
bio06
bio12
bio15
BDW
CEC
CLY
NTO
PTO
Grevillea nudiflora
Climate
Climate + Soil
Soil
AUC = 0.97
TSS = 0.87
AUC = 0.98
TSS = 0.84
AUC = 0.95
TSS = 0.7
Relative importance
bio01
bio04
bio05
bio06
bio12
CEC
CLY
DES
PTO
SOC
bio01
bio04
bio05
bio06
bio12
CEC
CLY
DES
PTO
SOC

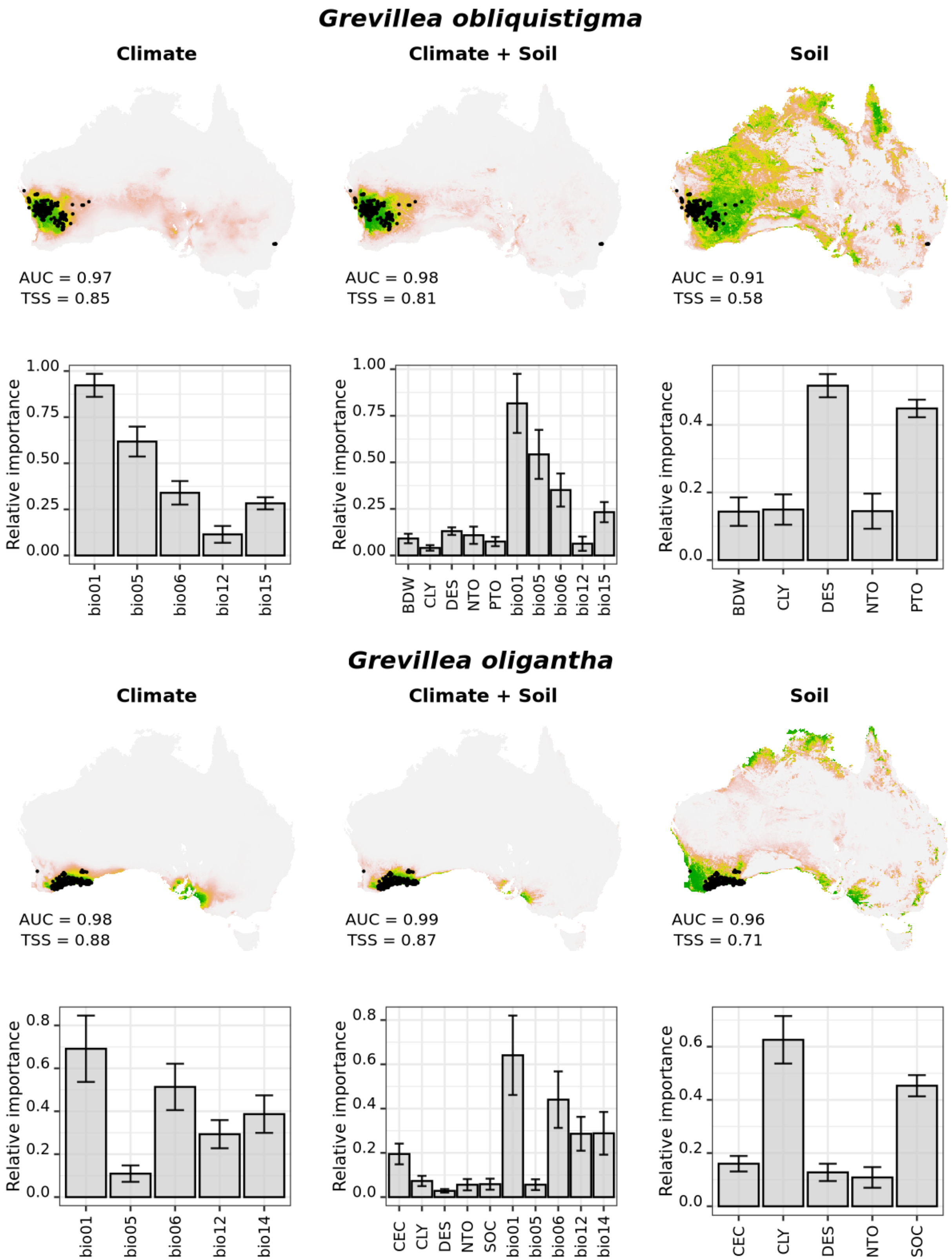

Grevillea obliquistigma
Climate
Climate + Soil
Soil
AUC = 0.97
TSS = 0.85
AUC = 0.98
TSS = 0.81
AUC = 0.91
TSS = 0.58
Relative importance
1.00
0.75
0.50
0.25
0.00
bio01
bio05
bio06
bio12
bio15
BDW
CLY
DES
NTO
PTO
0.4
0.2
0.0
Grevillea oligantha
Climate
Climate + Soil
Soil
AUC = 0.98
TSS = 0.88
AUC = 0.99
TSS = 0.87
AUC = 0.96
TSS = 0.71
Relative importance
0.8
0.6
0.4
0.2
0.0
bio01
bio05
bio06
bio12
bio14
CEC
CLY
DES
NTO
SOC

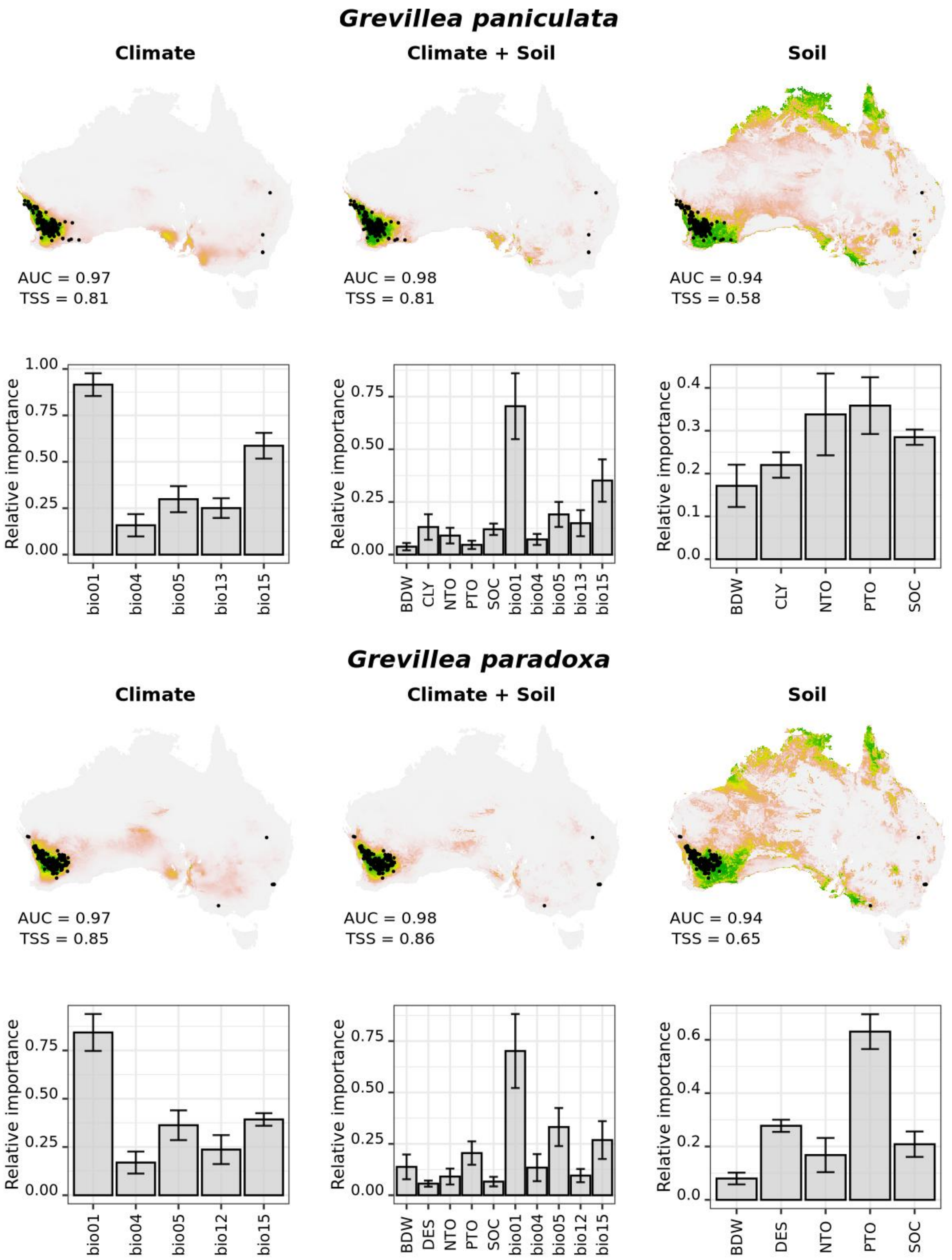
Grevillea paniculata
Climate
Climate + Soil
Soil
AUC = 0.97
TSS = 0.81
AUC = 0.98
TSS = 0.81
AUC = 0.94
TSS = 0.58
Relative importance
bio01
bio04
bio05
bio13
bio15
BDW
CLY
NTO
PTO
SOC
Grevillea paradoxa
Climate
Climate + Soil
Soil
AUC = 0.97
TSS = 0.85
AUC = 0.98
TSS = 0.86
AUC = 0.94
TSS = 0.65
Relative importance
bio01
bio04
bio05
bio12
bio15
BDW
DES
NTO
PTO
SOC

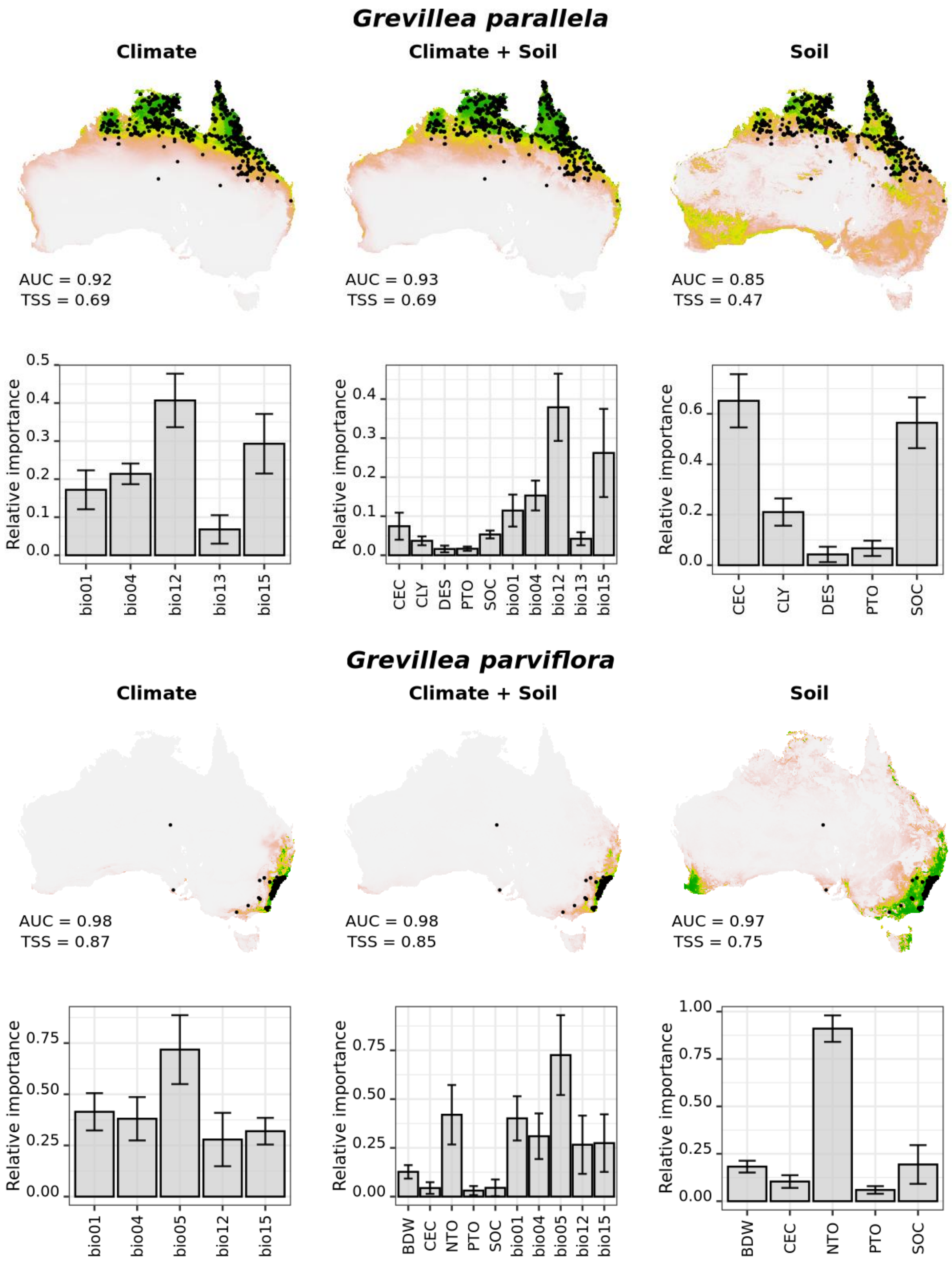

Grevillea parallela
Climate
Climate + Soil
Soil
AUC = 0.92
TSS = 0.69
AUC = 0.93
TSS = 0.69
AUC = 0.85
TSS = 0.47
Relative importance
0.0
0.1
0.2
0.3
0.4
0.5
0.6
bio01
bio04
bio12
bio13
bio15
CEC
CLY
DES
PTO
SOC
Grevillea parviflora
Climate
Climate + Soil
Soil
AUC = 0.98
TSS = 0.87
AUC = 0.98
TSS = 0.85
AUC = 0.97
TSS = 0.75
Relative importance
0.00
0.25
0.50
0.75
1.00
bio01
bio04
bio05
bio12
bio15
BDW
CEC
NTO
PTO
SOC

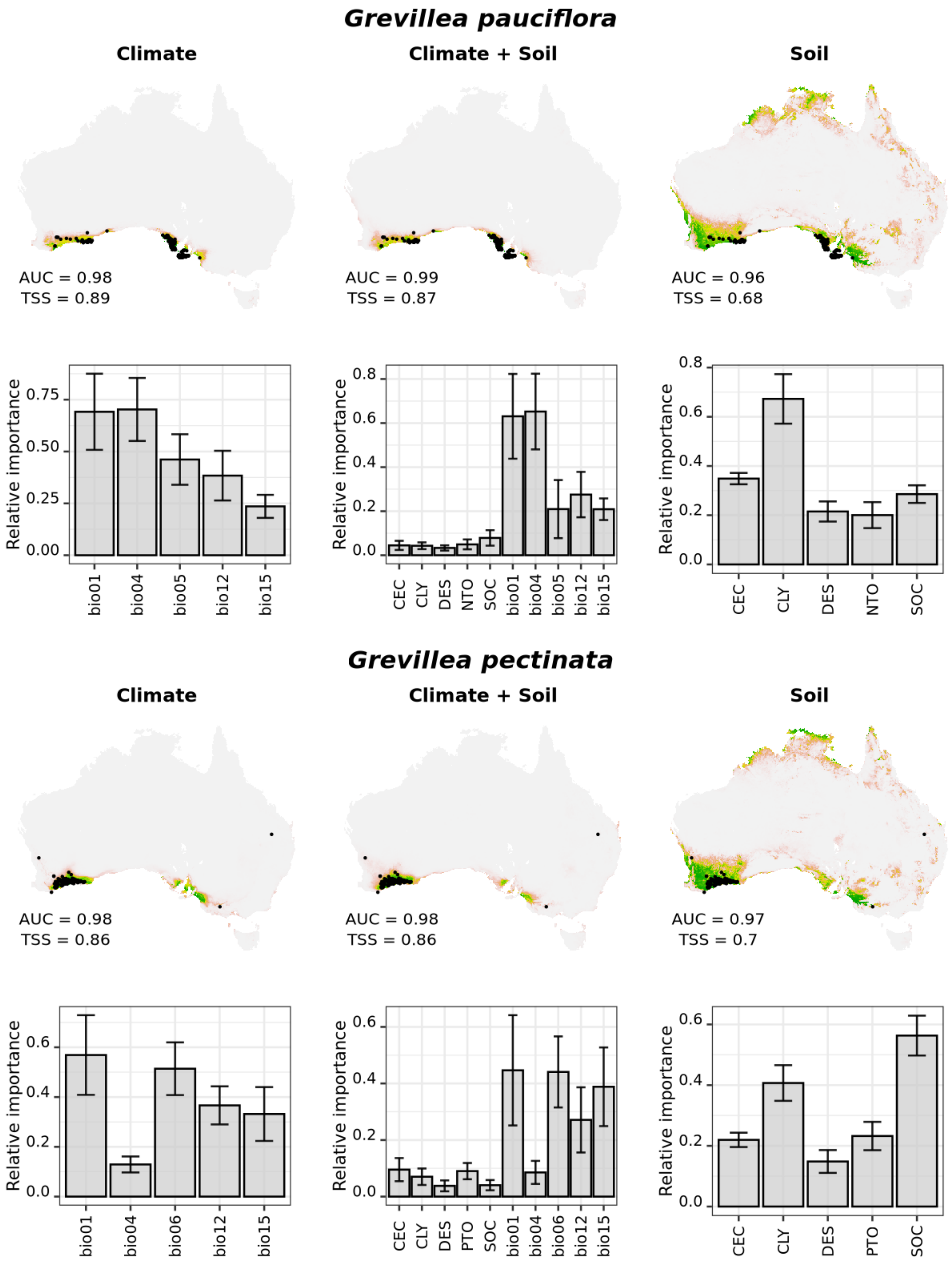
Grevillea pauciflora
Climate
Climate + Soil
Soil
AUC = 0.98
TSS = 0.89
AUC = 0.99
TSS = 0.87
AUC = 0.96
TSS = 0.68
Relative importance
0.00
0.25
0.50
0.75
bio01
bio04
bio05
bio12
bio15
Relative importance
0.0
0.2
0.4
0.6
0.8
CEC
CLY
DES
NTO
SOC
bio01
bio04
bio05
bio12
bio15
Relative importance
0.0
0.2
0.4
0.6
0.8
CEC
CLY
DES
NTO
SOC
Grevillea pectinata
Climate
Climate + Soil
Soil
AUC = 0.98
TSS = 0.86
AUC = 0.98
TSS = 0.86
AUC = 0.97
TSS = 0.7
Relative importance
0.0
0.2
0.4
0.6
bio01
bio04
bio06
bio12
bio15
Relative importance
0.0
0.2
0.4
0.6
CEC
CLY
DES
PTO
SOC
bio01
bio04
bio06
bio12
bio15
Relative importance
0.0
0.2
0.4
0.6
CEC
CLY
DES
PTO
SOC

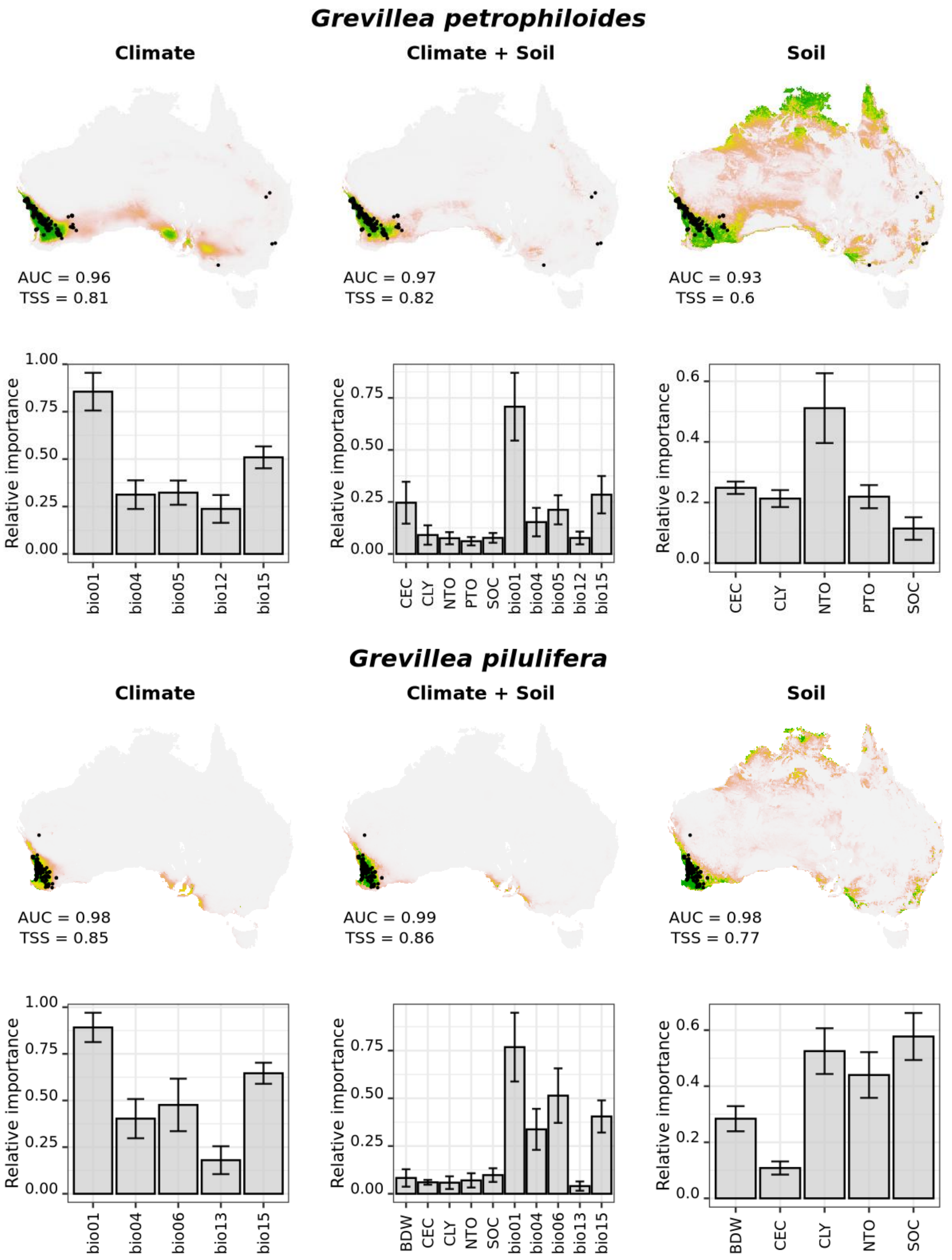
Grevillea petrophiloides
Climate
Climate + Soil
Soil
AUC = 0.96
TSS = 0.81
AUC = 0.97
TSS = 0.82
AUC = 0.93
TSS = 0.6
Relative importance
bio01
bio04
bio05
bio12
bio15
CEC
CLY
NTO
PTO
SOC
Grevillea pilulifera
Climate
Climate + Soil
Soil
AUC = 0.98
TSS = 0.85
AUC = 0.99
TSS = 0.86
AUC = 0.98
TSS = 0.77
Relative importance
bio01
bio04
bio06
bio13
bio15
BDW
CEC
CLY
NTO
SOC

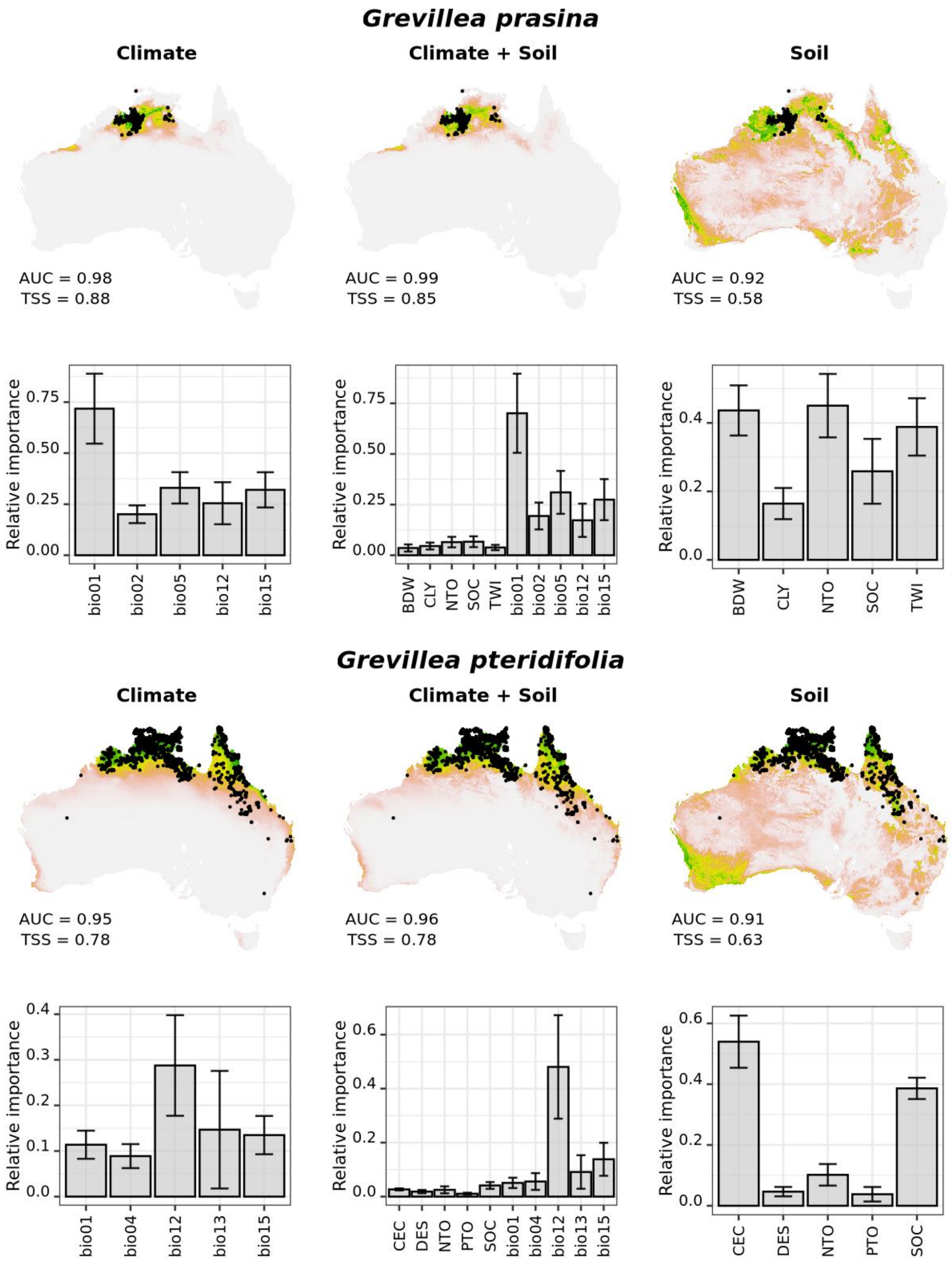

Grevillea prasina
Climate
Climate + Soil
Soil
AUC = 0.98
TSS = 0.88
AUC = 0.99
TSS = 0.85
AUC = 0.92
TSS = 0.58
Relative importance
bio01
bio02
bio05
bio12
bio15
BDW
CLY
NTO
SOC
TWI
Grevillea pteridifolia
Climate
Climate + Soil
Soil
AUC = 0.95
TSS = 0.78
AUC = 0.96
TSS = 0.78
AUC = 0.91
TSS = 0.63
Relative importance
bio01
bio04
bio12
bio13
bio15
CEC
DES
NTO
PTO
SOC

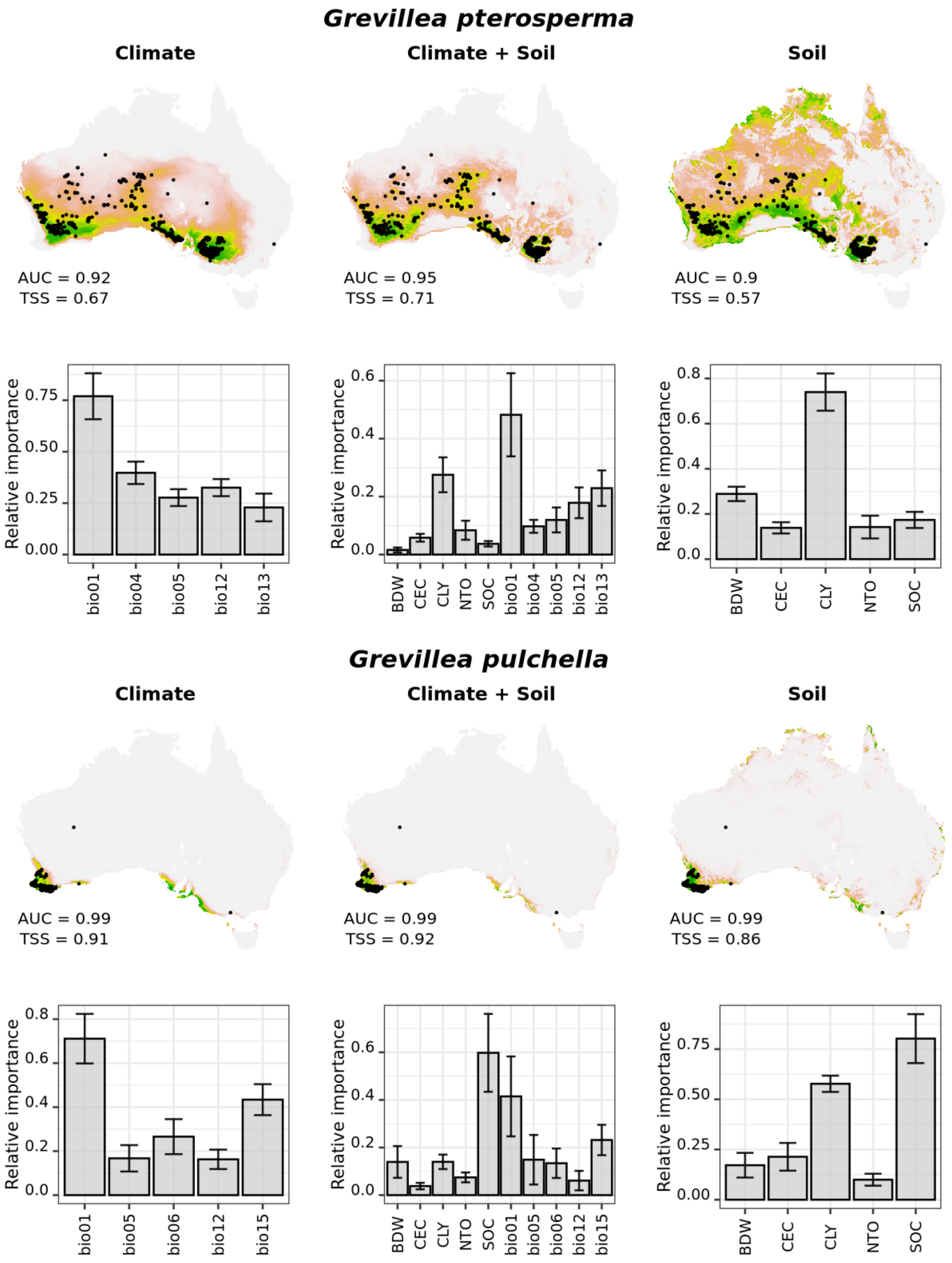
Grevillea pterosperma
Climate
Climate + Soil
Soil
AUC = 0.92
TSS = 0.67
AUC = 0.95
TSS = 0.71
AUC = 0.9
TSS = 0.57
Relative importance
0.00
0.25
0.50
0.75
bio01
bio04
bio05
bio12
bio13
0.0
0.2
0.4
0.6
BDW
CEC
CLY
NTO
SOC
bio01
bio04
bio05
bio12
bio13
0.0
0.2
0.4
0.6
0.8
BDW
CEC
CLY
NTO
SOC
Grevillea pulchella
Climate
Climate + Soil
Soil
AUC = 0.99
TSS = 0.91
AUC = 0.99
TSS = 0.92
AUC = 0.99
TSS = 0.86
Relative importance
0.0
0.2
0.4
0.6
0.8
bio01
bio05
bio06
bio12
bio15
0.0
0.2
0.4
0.6
BDW
CEC
CLY
NTO
SOC
bio01
bio05
bio06
bio12
bio15
0.00
0.25
0.50
0.75
BDW
CEC
CLY
NTO
SOC

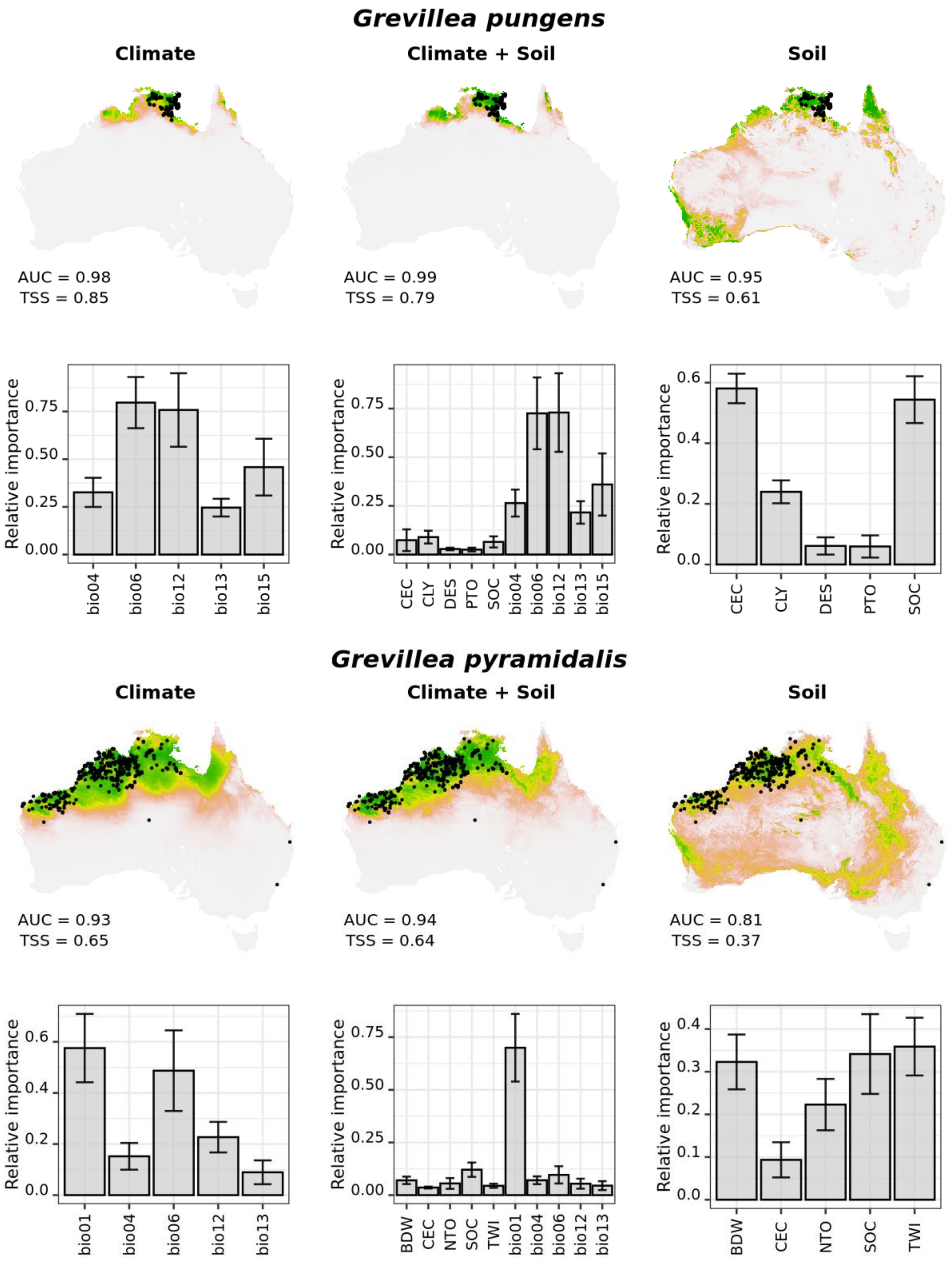
Grevillea pungens
Climate
Climate + Soil
Soil
AUC = 0.98
TSS = 0.85
AUC = 0.99
TSS = 0.79
AUC = 0.95
TSS = 0.61
Relative importance
0.00
0.25
0.50
0.75
bio04
bio06
bio12
bio13
bio15
CEC
CLY
DES
PTO
SOC
0.0
0.2
0.4
0.6
Grevillea pyramidalis
Climate
Climate + Soil
Soil
AUC = 0.93
TSS = 0.65
AUC = 0.94
TSS = 0.64
AUC = 0.81
TSS = 0.37
Relative importance
bio01
bio04
bio06
bio12
bio13
BDW
CEC
NTO
SOC
TWI
0.1
0.3

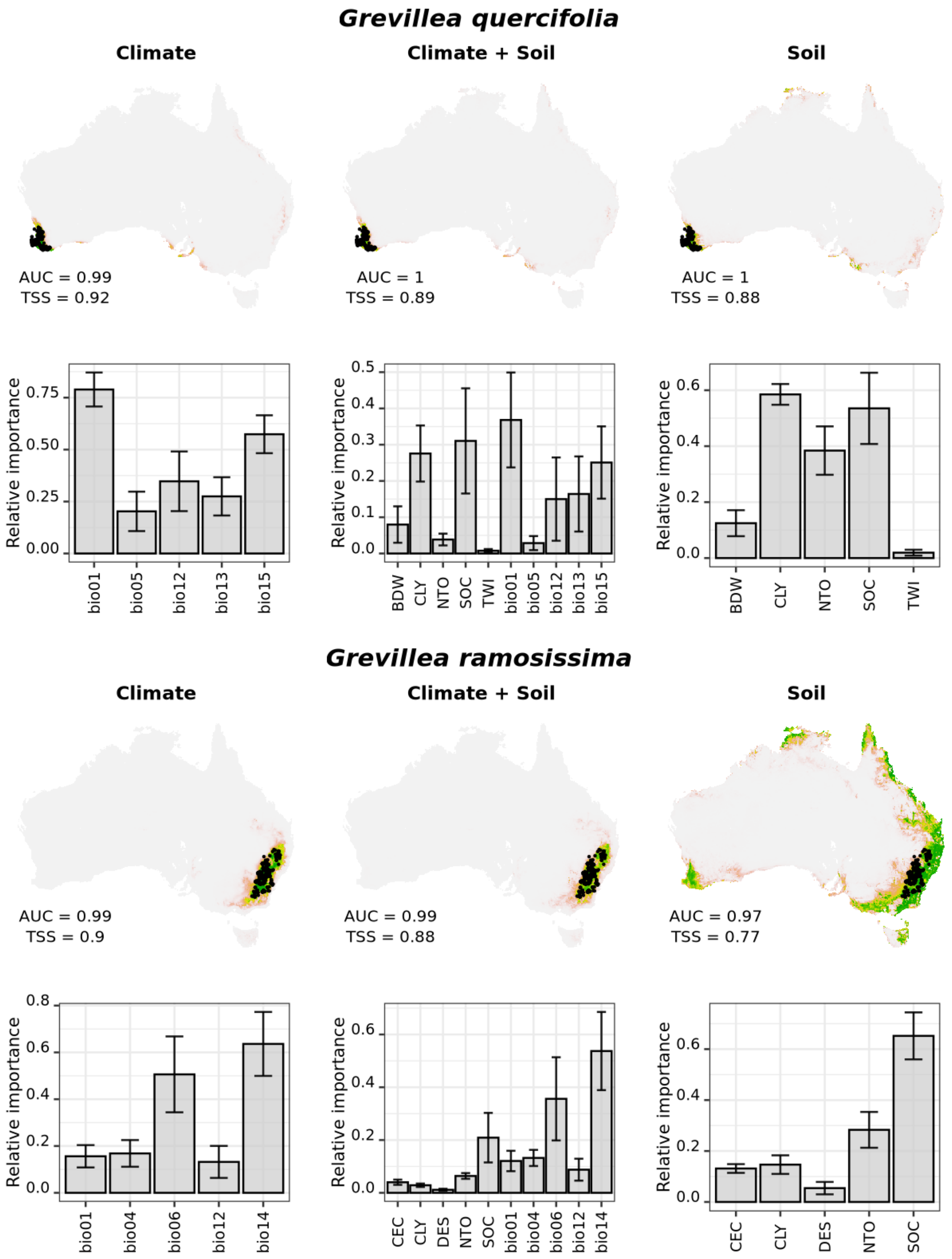
Grevillea quercifolia
Climate
Climate + Soil
Soil
AUC = 0.99
TSS = 0.92
AUC = 1
TSS = 0.89
AUC = 1
TSS = 0.88
Relative importance
0.00
0.25
0.50
0.75
bio01
bio05
bio12
bio13
bio15
0.0
0.1
0.2
0.3
0.4
0.5
BDW
CLY
NTO
SOC
TWI
bio01
bio05
bio12
bio13
bio15
0.0
0.2
0.4
0.6
BDW
CLY
NTO
SOC
TWI
Grevillea ramosissima
Climate
Climate + Soil
Soil
AUC = 0.99
TSS = 0.9
AUC = 0.99
TSS = 0.88
AUC = 0.97
TSS = 0.77
Relative importance
0.0
0.2
0.4
0.6
0.8
bio01
bio04
bio06
bio12
bio14
0.0
0.2
0.4
0.6
CEC
CLY
DES
NTO
SOC
bio01
bio04
bio06
bio12
bio14
0.0
0.2
0.4
0.6
CEC
CLY
DES
NTO
SOC

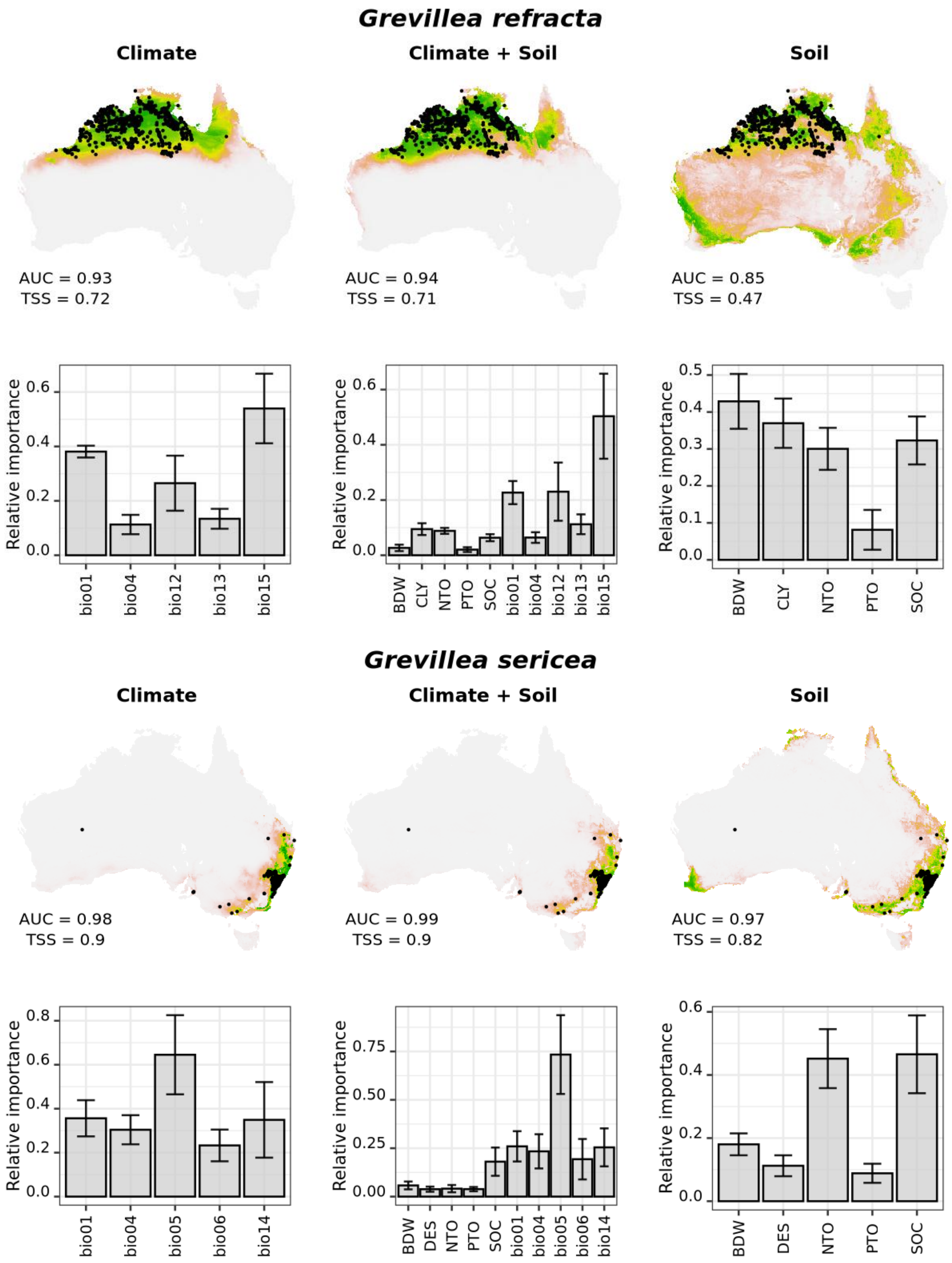
Grevillea refracta
Climate
Climate + Soil
Soil
AUC = 0.93
TSS = 0.72
AUC = 0.94
TSS = 0.71
AUC = 0.85
TSS = 0.47
Relative importance
bio01 bio04 bio12 bio13 bio15
BDW CLY NTO PTO SOC bio01 bio04 bio12 bio13 bio15
BDW CLY NTO PTO SOC
Grevillea sericea
Climate
Climate + Soil
Soil
AUC = 0.98
TSS = 0.9
AUC = 0.99
TSS = 0.9
AUC = 0.97
TSS = 0.82
Relative importance
bio01 bio04 bio05 bio06 bio14
BDW DES NTO PTO SOC bio01 bio04 bio05 bio06 bio14
BDW DES NTO PTO SOC

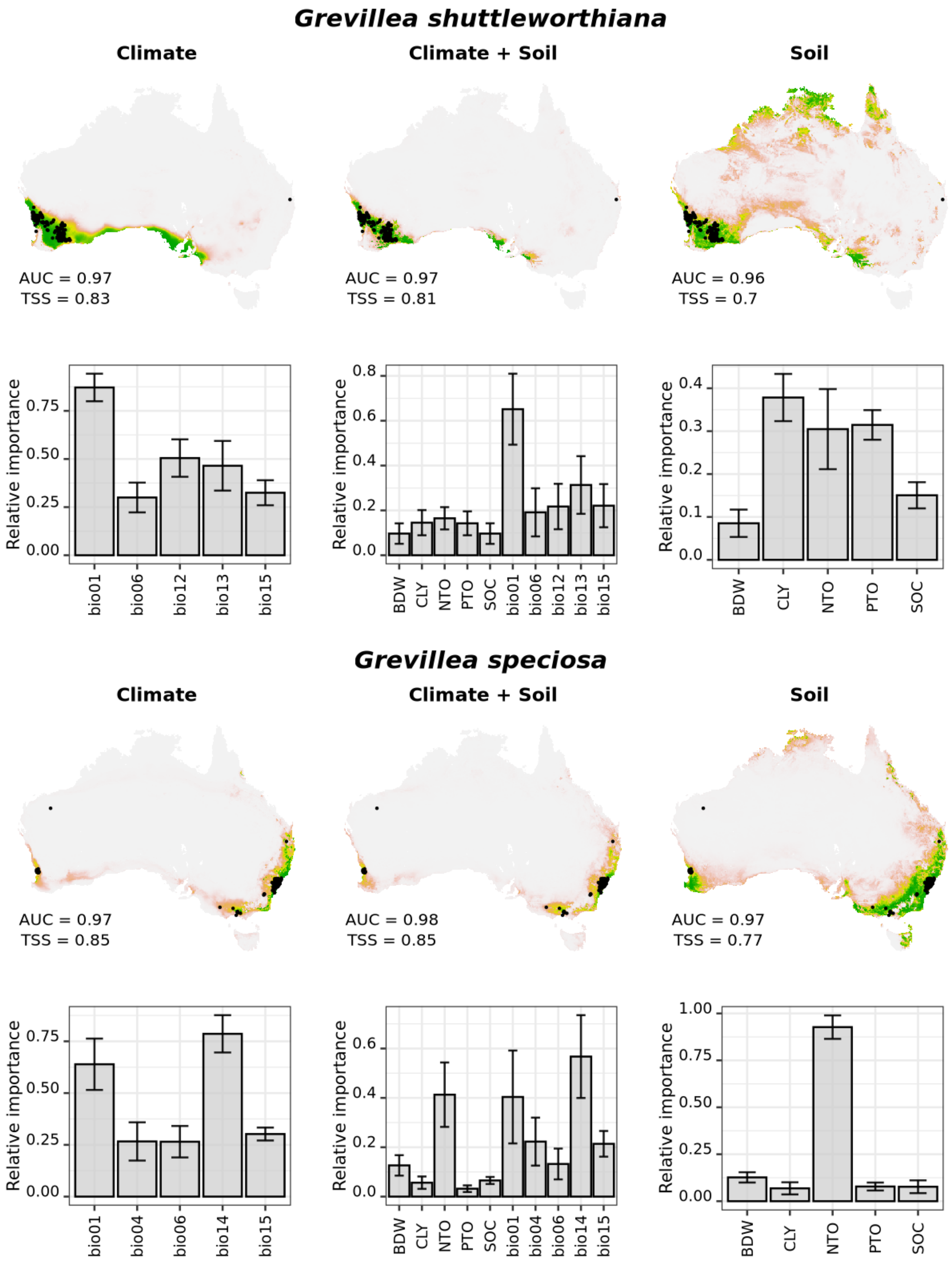

Grevillea shuttleworthiana
Climate
Climate + Soil
Soil
AUC = 0.97
TSS = 0.83
AUC = 0.97
TSS = 0.81
AUC = 0.96
TSS = 0.7
Relative importance
bio01
bio06
bio12
bio13
bio15
BDW
CLY
NTO
PTO
SOC
Grevillea speciosa
Climate
Climate + Soil
Soil
AUC = 0.97
TSS = 0.85
AUC = 0.98
TSS = 0.85
AUC = 0.97
TSS = 0.77
Relative importance
bio01
bio04
bio06
bio14
bio15
BDW
CLY
NTO
PTO
SOC

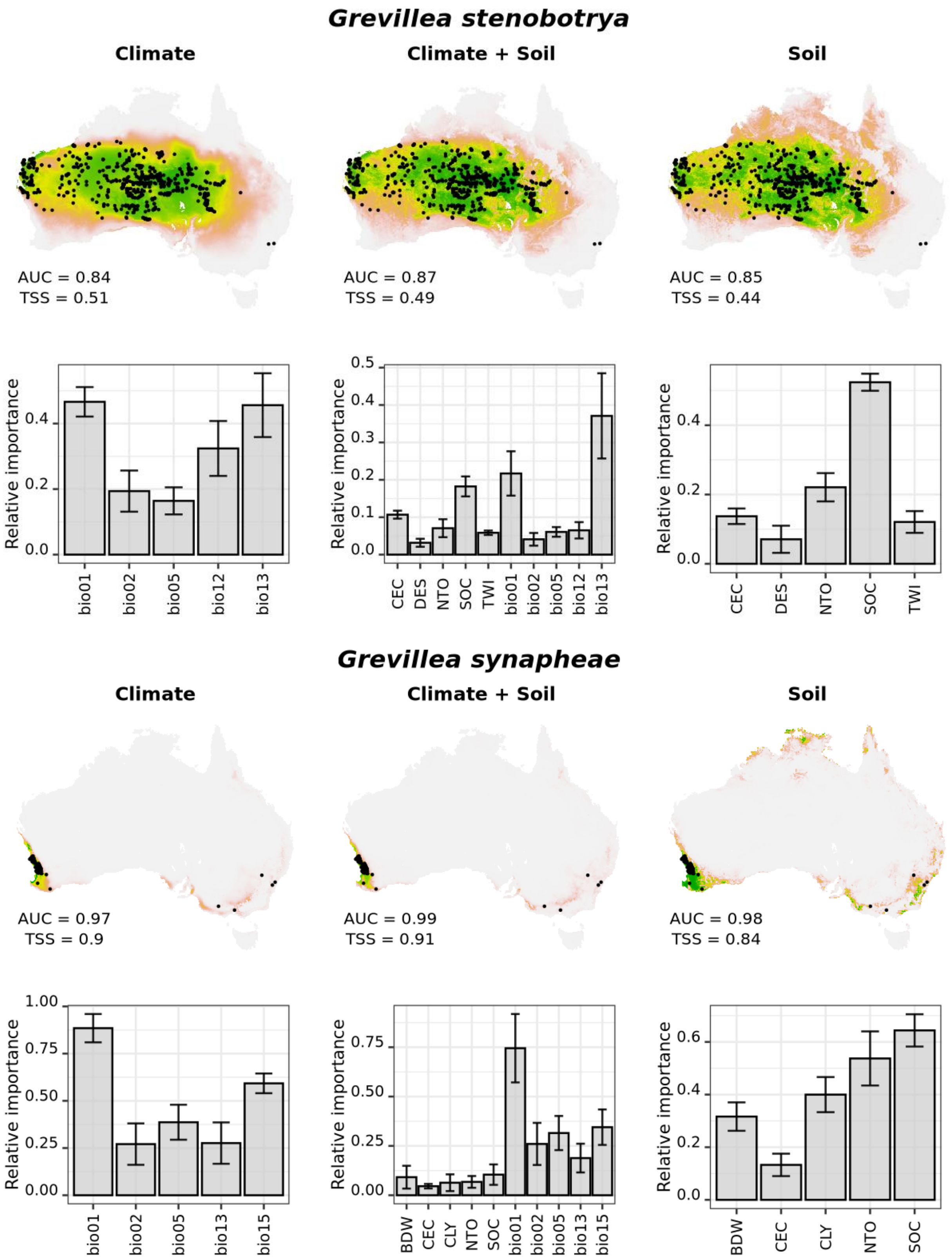

Grevillea stenobotrya
Climate
Climate + Soil
Soil
AUC = 0.84
TSS = 0.51
AUC = 0.87
TSS = 0.49
AUC = 0.85
TSS = 0.44
Relative importance
bio01
bio02
bio05
bio12
bio13
CEC
DES
NTO
SOC
TWI
Grevillea synapheae
Climate
Climate + Soil
Soil
AUC = 0.97
TSS = 0.9
AUC = 0.99
TSS = 0.91
AUC = 0.98
TSS = 0.84
Relative importance
bio01
bio02
bio05
bio13
bio15
BDW
CEC
CLY
NTO
SOC

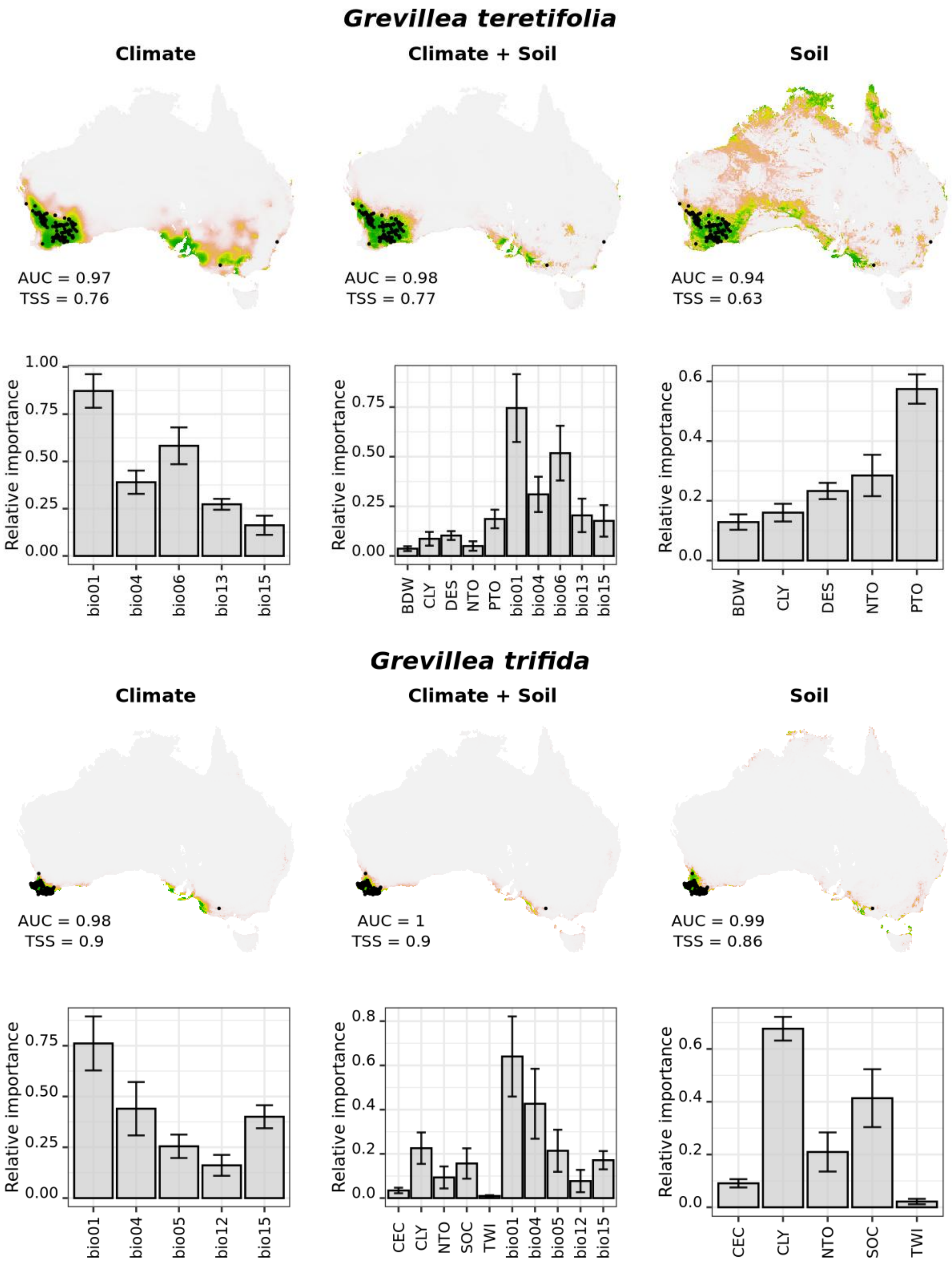
Grevillea teretifolia
Climate
Climate + Soil
Soil
AUC = 0.97
TSS = 0.76
AUC = 0.98
TSS = 0.77
AUC = 0.94
TSS = 0.63
Relative importance
1.00
0.75
0.50
0.25
0.00
bio01
bio04
bio06
bio13
bio15
BDW
CLY
DES
NTO
PTO
0.6
0.4
0.2
0.0
Grevillea trifida
AUC = 0.98
TSS = 0.9
AUC = 1
TSS = 0.9
AUC = 0.99
TSS = 0.86
bio05
bio12
CEC
SOC
TWI
0.8

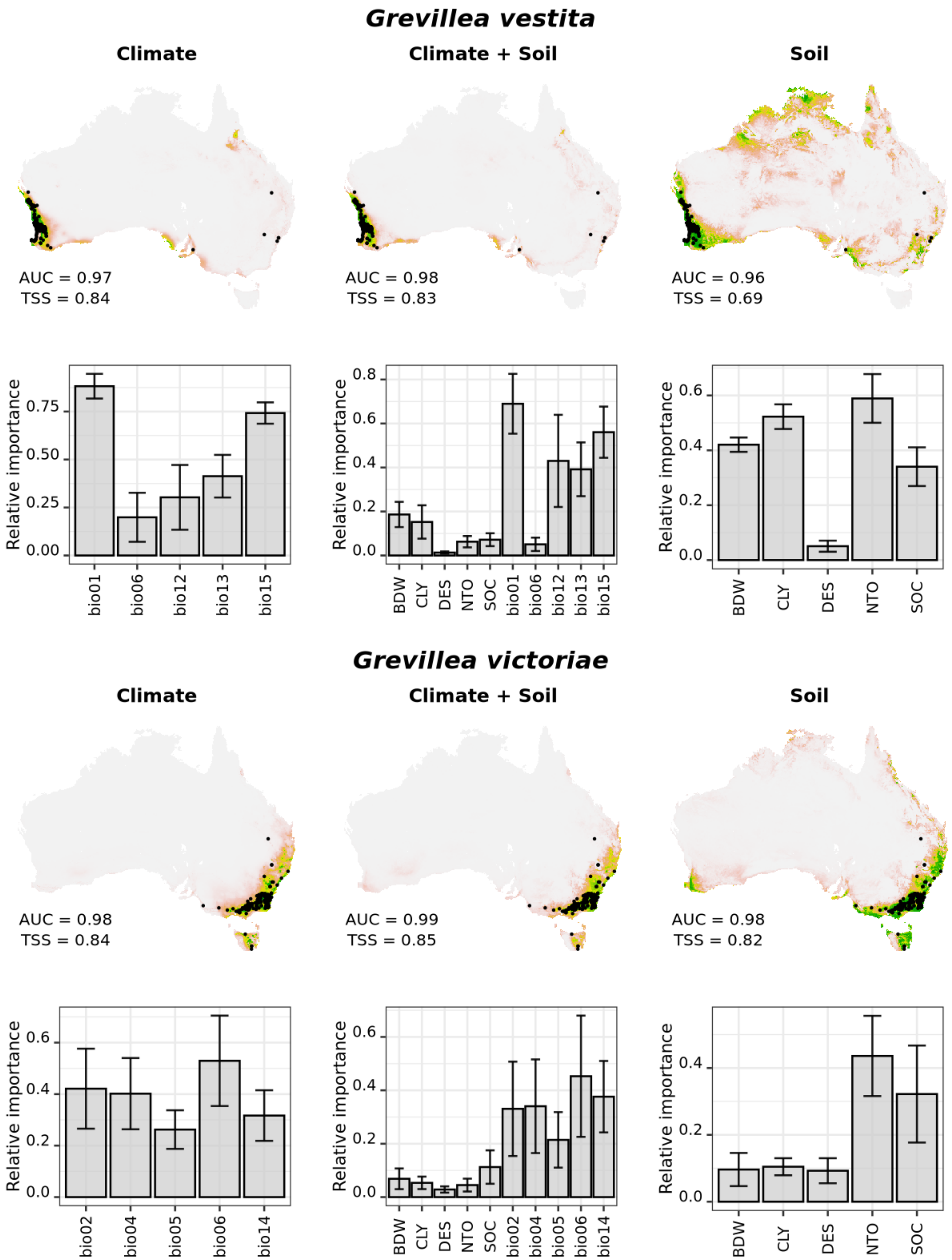

Grevillea vestita
Climate
Climate + Soil
Soil
AUC = 0.97
TSS = 0.84
AUC = 0.98
TSS = 0.83
AUC = 0.96
TSS = 0.69
Relative importance
0.00
0.25
0.50
0.75
bio01
bio06
bio12
bio13
bio15
0.0
0.2
0.4
0.6
0.8
BDW
CLY
DES
NTO
SOC
Grevillea victoriae
Climate
Climate + Soil
Soil
AUC = 0.98
TSS = 0.84
AUC = 0.99
TSS = 0.85
AUC = 0.98
TSS = 0.82
bio02
bio04
bio05
bio06
bio14

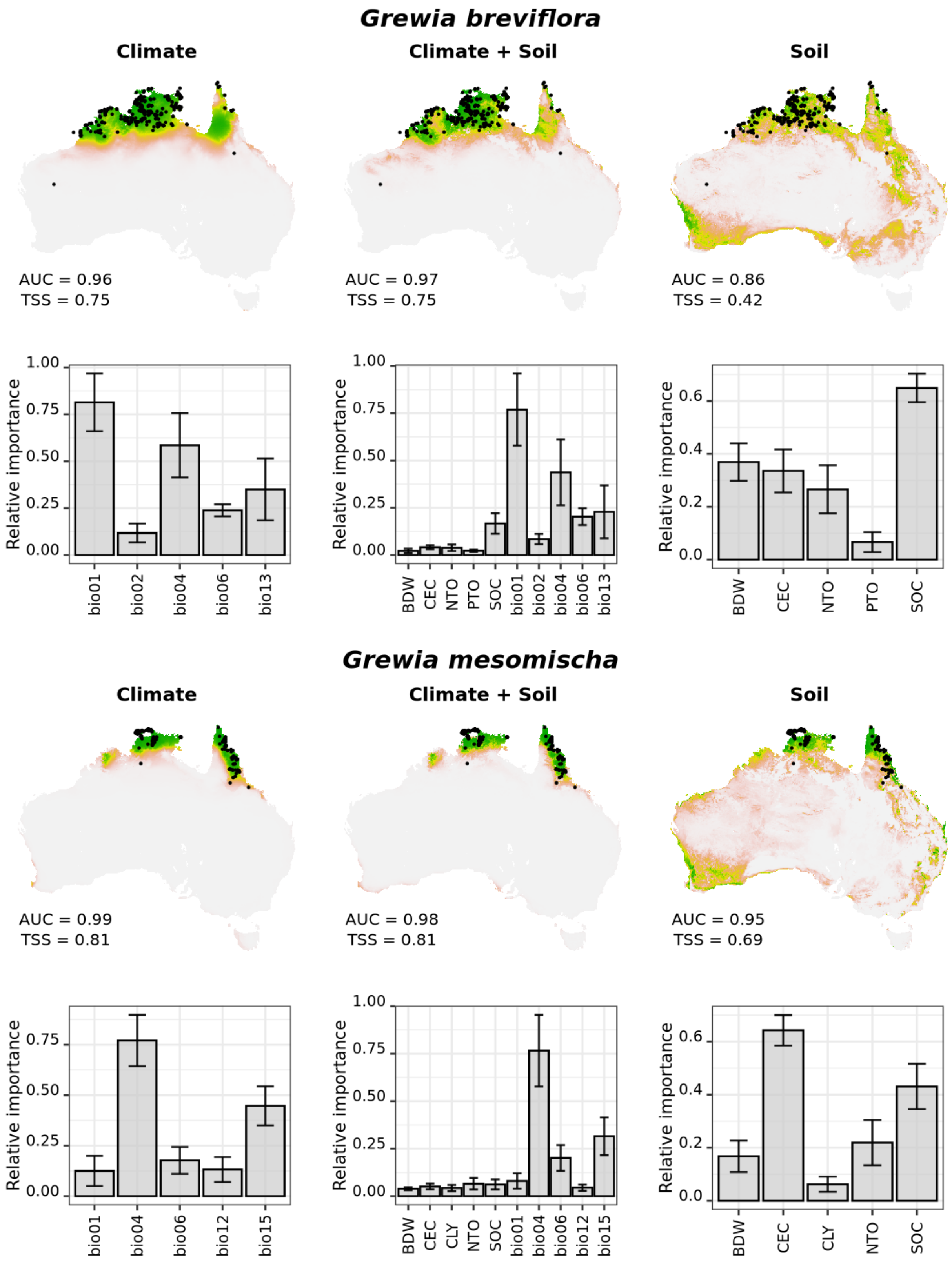
Grewia breviflora
Climate
Climate + Soil
Soil
AUC = 0.96
TSS = 0.75
AUC = 0.97
TSS = 0.75
AUC = 0.86
TSS = 0.42
Relative importance
bio01
bio02
bio04
bio06
bio13
BDW
CEC
NTO
PTO
SOC
bio01
bio02
bio04
bio06
bio13
BDW
CEC
NTO
PTO
SOC
Grewia mesomischa
Climate
Climate + Soil
Soil
AUC = 0.99
TSS = 0.81
AUC = 0.98
TSS = 0.81
AUC = 0.95
TSS = 0.69
Relative importance
bio01
bio04
bio06
bio12
bio15
BDW
CEC
CLY
NTO
SOC
bio01
bio04
bio06
bio12
bio15
BDW
CEC
CLY
NTO
SOC

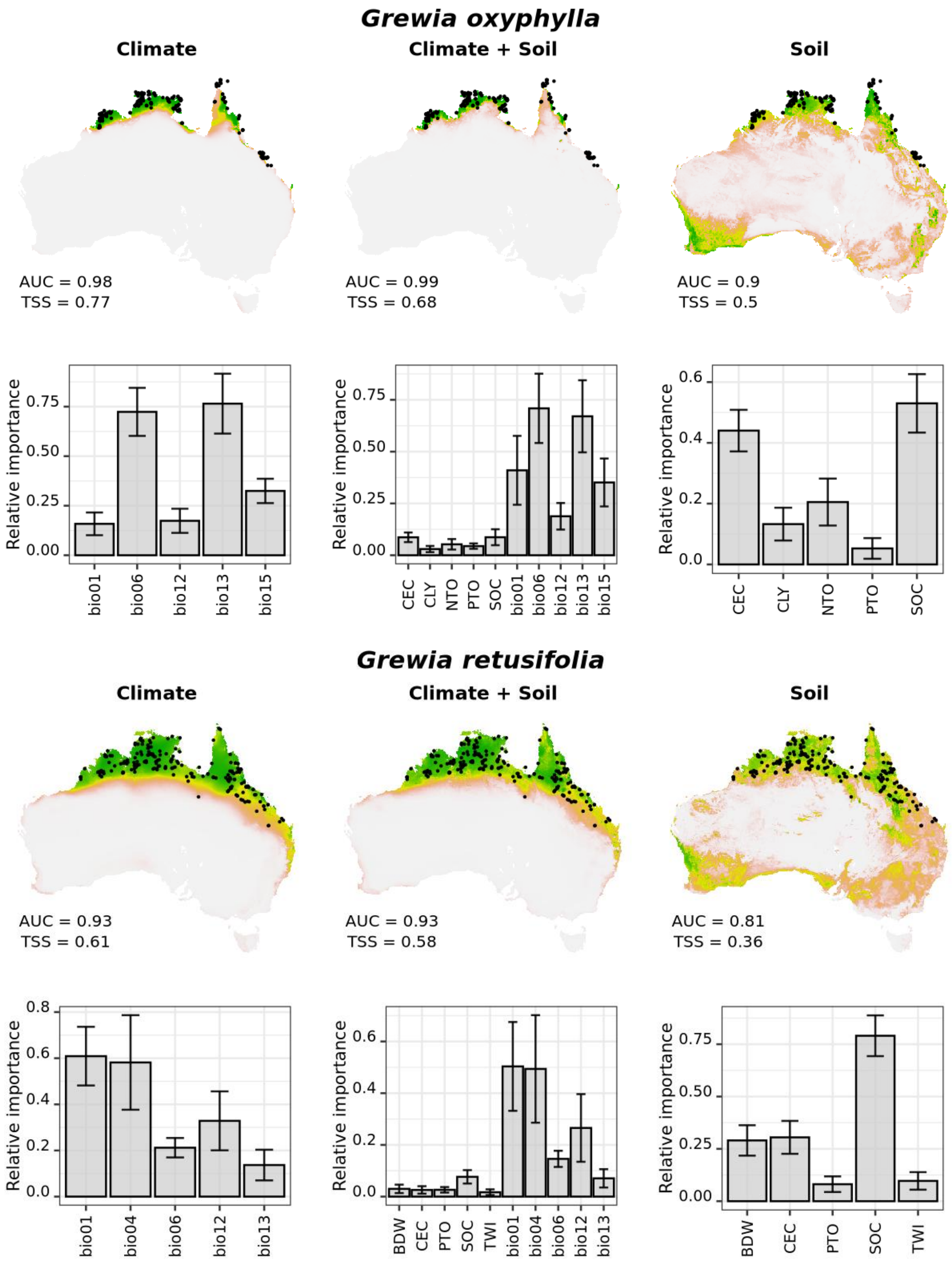
Grewia oxyphylla
Climate
Climate + Soil
Soil
AUC = 0.98
TSS = 0.77
AUC = 0.99
TSS = 0.68
AUC = 0.9
TSS = 0.5
Relative importance
0.00
0.25
0.50
0.75
bio01
bio06
bio12
bio13
bio15
CEC
CLY
NTO
PTO
SOC
0.0
0.2
0.4
0.6
Grewia retusifolia
Climate
Climate + Soil
Soil
AUC = 0.93
TSS = 0.61
AUC = 0.93
TSS = 0.58
AUC = 0.81
TSS = 0.36
0.8
bio04
BDW
TWI

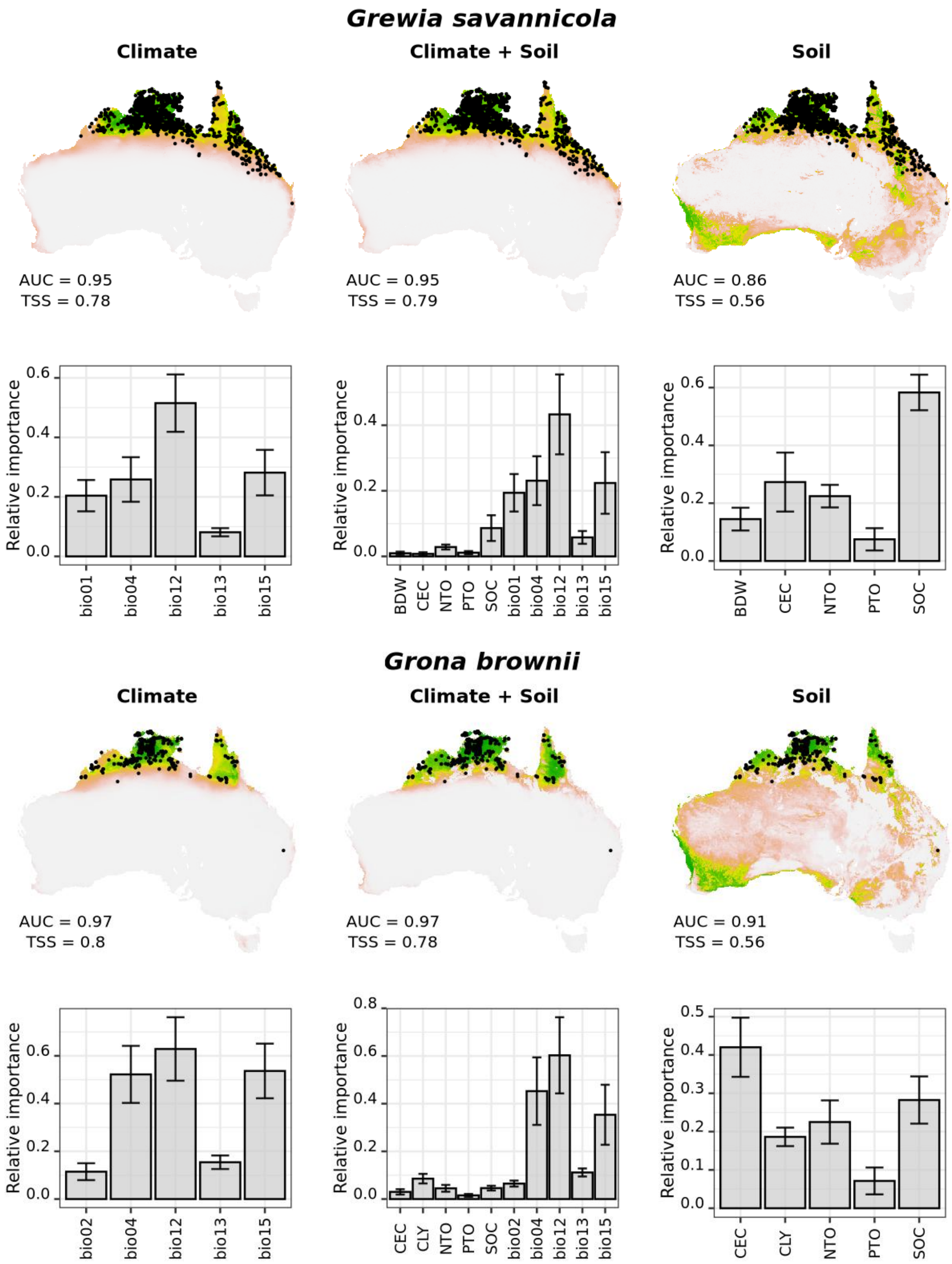
Grewia savannicola
Climate
Climate + Soil
Soil
AUC = 0.95
TSS = 0.78
AUC = 0.95
TSS = 0.79
AUC = 0.86
TSS = 0.56
Relative importance
0.0
0.2
0.4
0.6
bio01
bio04
bio12
bio13
bio15
BDW
CEC
NTO
PTO
SOC
Grona brownii
Climate
Climate + Soil
Soil
AUC = 0.97
TSS = 0.8
AUC = 0.97
TSS = 0.78
AUC = 0.91
TSS = 0.56
Relative importance
0.0
0.1
0.2
0.3
0.4
0.5
0.6
0.8
bio02
bio04
bio12
bio13
bio15
CEC
CLY
NTO
PTO
SOC

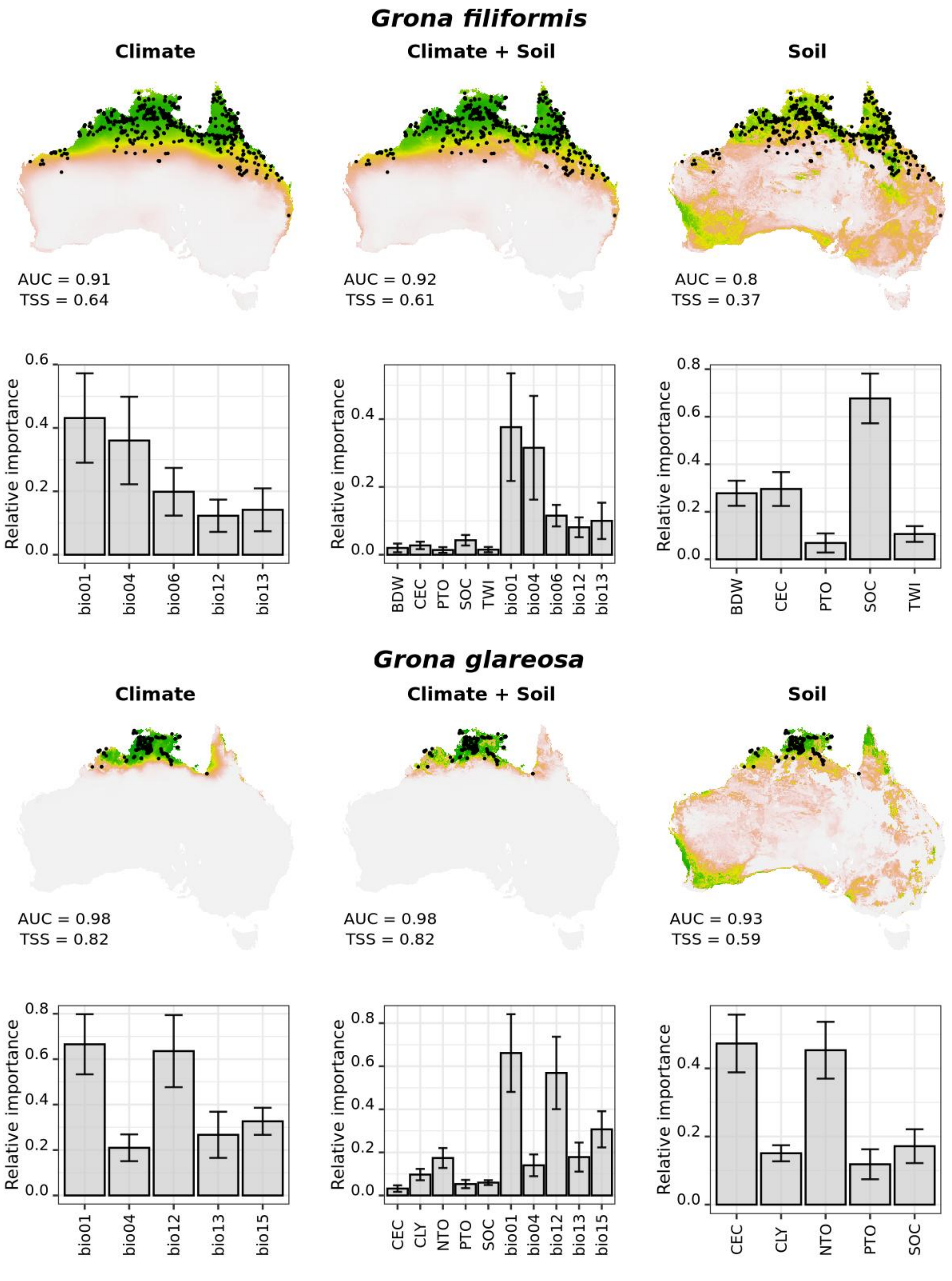
Grona filiformis
Climate
Climate + Soil
Soil
AUC = 0.91
TSS = 0.64
AUC = 0.92
TSS = 0.61
AUC = 0.8
TSS = 0.37
Relative importance
bio01
bio04
bio06
bio12
bio13
BDW
CEC
PTO
SOC
TWI
Grona glareosa
Climate
Climate + Soil
Soil
AUC = 0.98
TSS = 0.82
AUC = 0.98
TSS = 0.82
AUC = 0.93
TSS = 0.59
Relative importance
bio01
bio04
bio12
bio13
bio15
CEC
CLY
NTO
PTO
SOC

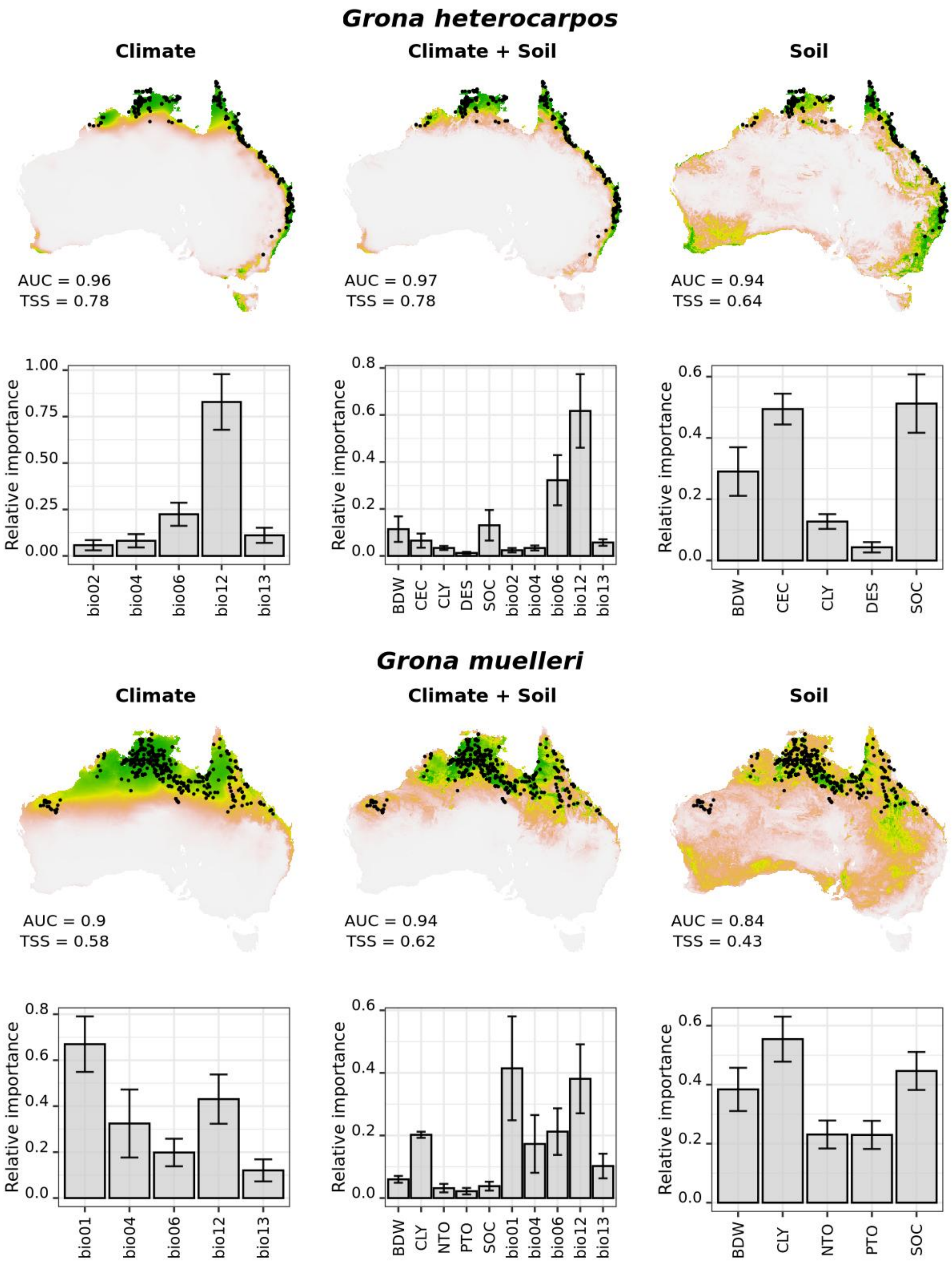

Grona heterocarpos
Climate
Climate + Soil
Soil
AUC = 0.96
TSS = 0.78
AUC = 0.97
TSS = 0.78
AUC = 0.94
TSS = 0.64
Relative importance
1.00
0.75
0.50
0.25
0.00
bio02
bio04
bio06
bio12
bio13
0.8
0.6
0.4
0.2
0.0
BDW
CEC
CLY
DES
SOC
bio02
bio04
bio06
bio12
bio13
0.6
0.4
0.2
0.0
BDW
CEC
CLY
DES
SOC
Grona muelleri
Climate
Climate + Soil
Soil
AUC = 0.9
TSS = 0.58
AUC = 0.94
TSS = 0.62
AUC = 0.84
TSS = 0.43
Relative importance
0.8
0.6
0.4
0.2
0.0
bio01
bio04
bio06
bio12
bio13
0.6
0.4
0.2
0.0
BDW
CLY
NTO
PTO
SOC
bio01
bio04
bio06
bio12
bio13
0.6
0.4
0.2
0.0
BDW
CLY
NTO
PTO
SOC

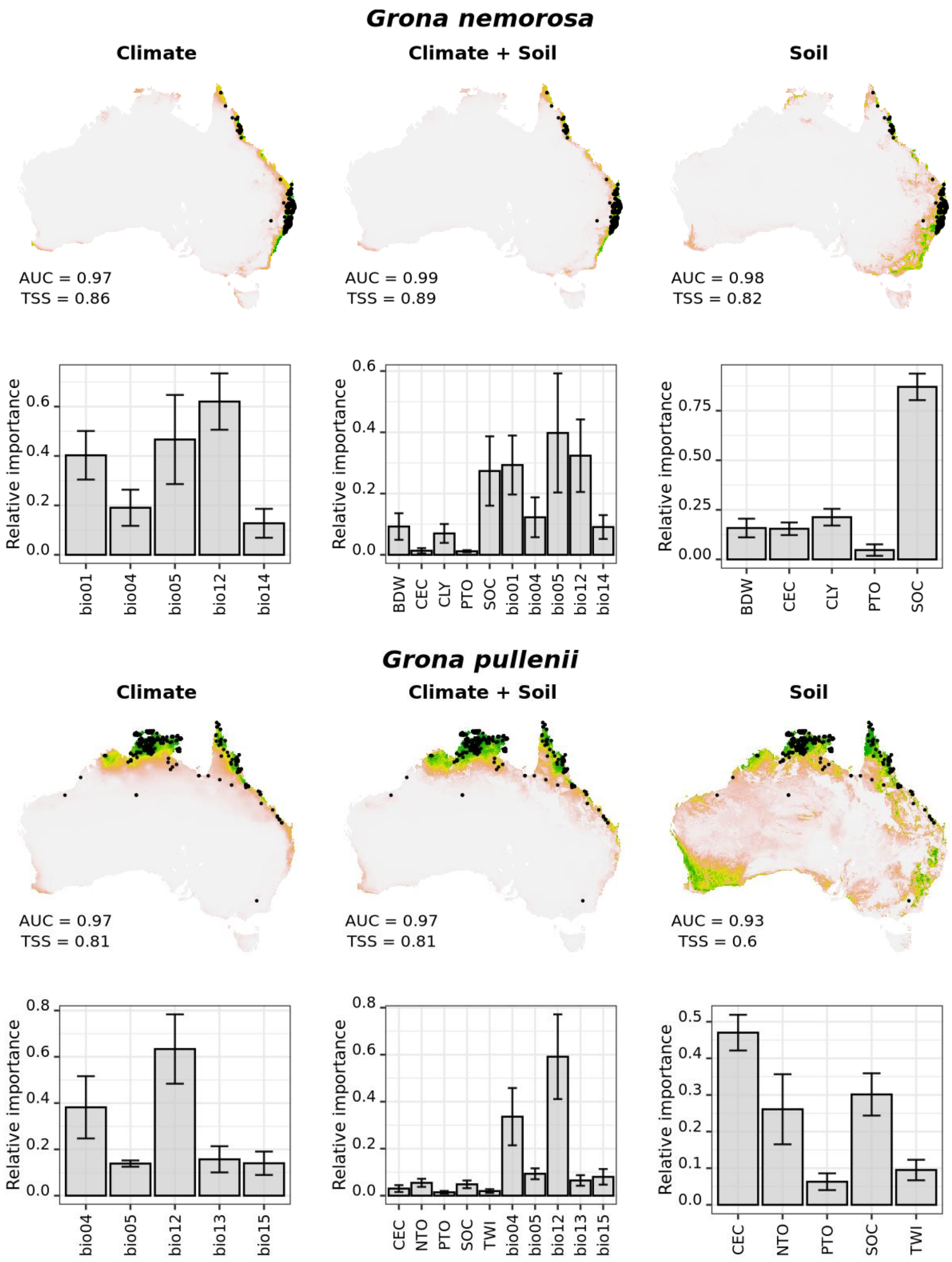
Grona nemorosa
Climate
Climate + Soil
Soil
AUC = 0.97
TSS = 0.86
AUC = 0.99
TSS = 0.89
AUC = 0.98
TSS = 0.82
Relative importance
0.0
0.2
0.4
0.6
bio01
bio04
bio05
bio12
bio14
Relative importance
0.0
0.2
0.4
0.6
BDW
CEC
CLY
PTO
SOC
bio01
bio04
bio05
bio12
bio14
Relative importance
0.00
0.25
0.50
0.75
BDW
CEC
CLY
PTO
SOC
Grona pullenii
Climate
Climate + Soil
Soil
AUC = 0.97
TSS = 0.81
AUC = 0.97
TSS = 0.81
AUC = 0.93
TSS = 0.6
Relative importance
0.0
0.2
0.4
0.6
0.8
bio04
bio05
bio12
bio13
bio15
Relative importance
0.0
0.2
0.4
0.6
0.8
CEC
NTO
PTO
SOC
TWI
bio04
bio05
bio12
bio13
bio15
Relative importance
0.0
0.1
0.2
0.3
0.4
0.5
CEC
NTO
PTO
SOC
TWI

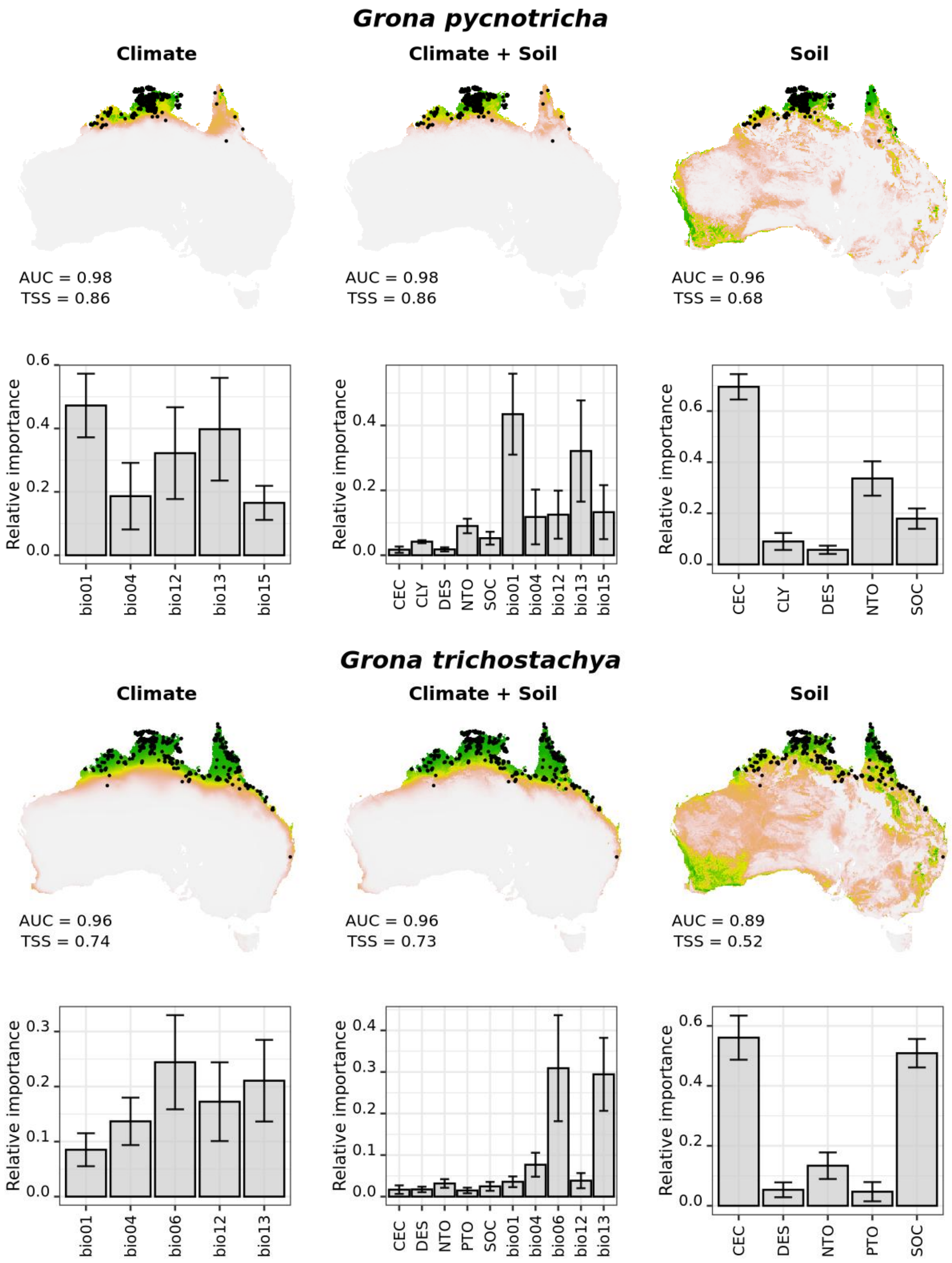
Grona pycnotricha
Climate
Climate + Soil
Soil
AUC = 0.98
TSS = 0.86
AUC = 0.98
TSS = 0.86
AUC = 0.96
TSS = 0.68
Relative importance
0.0
0.2
0.4
0.6
bio01
bio04
bio12
bio13
bio15
CEC
CLY
DES
NTO
SOC
Grona trichostachya
Climate
Climate + Soil
Soil
AUC = 0.96
TSS = 0.74
AUC = 0.96
TSS = 0.73
AUC = 0.89
TSS = 0.52
Relative importance
0.0
0.1
0.2
0.3
0.4
0.6
bio01
bio04
bio06
bio12
bio13
CEC
DES
NTO
PTO
SOC

Guettarda speciosa

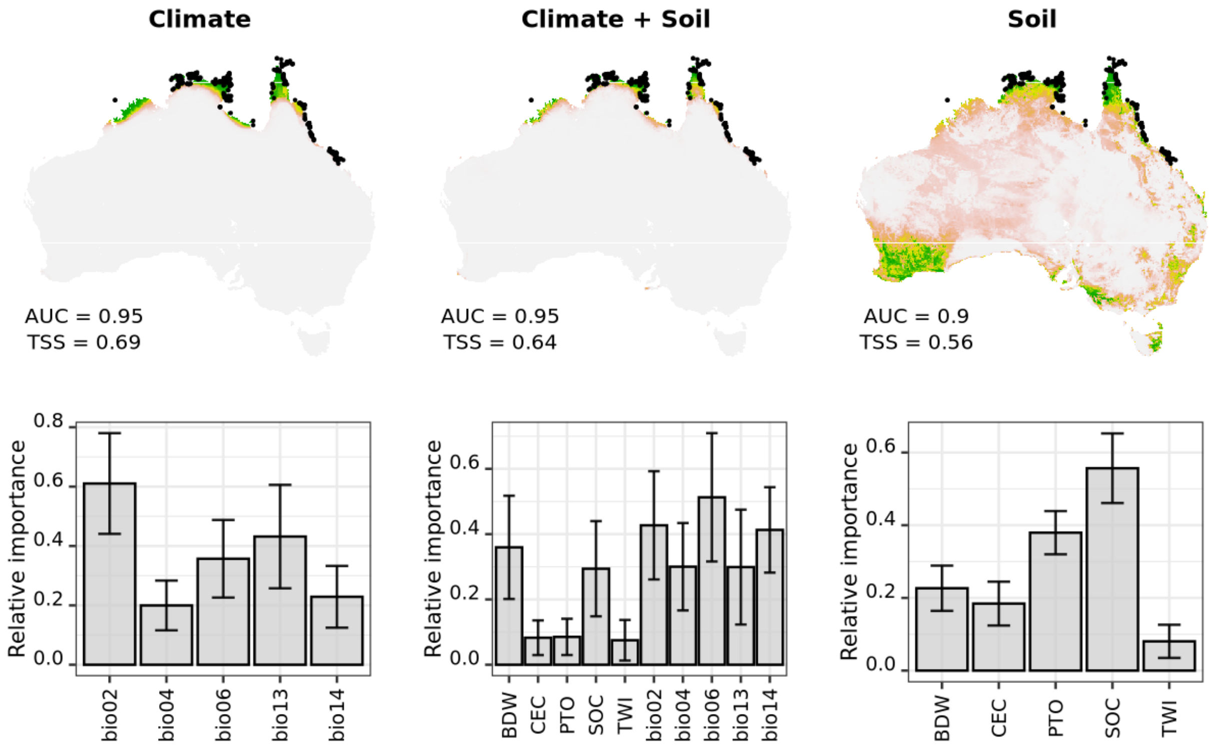

Guichenotia ledifolia

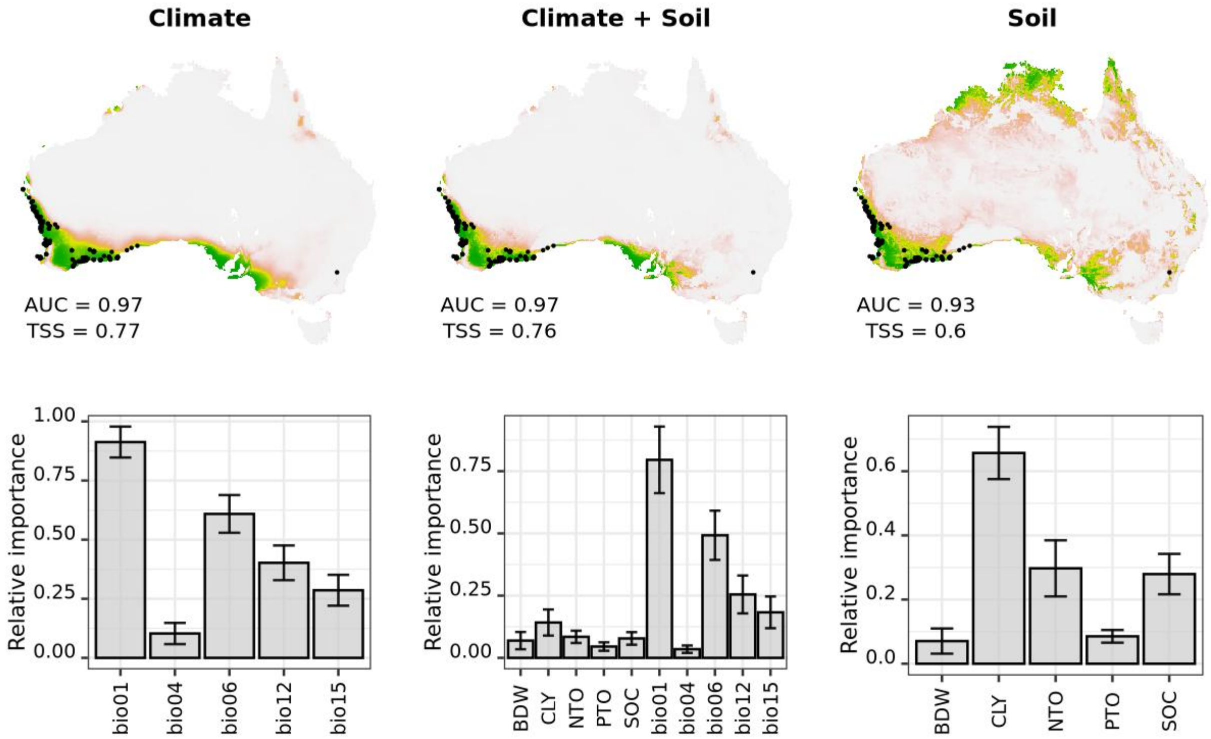

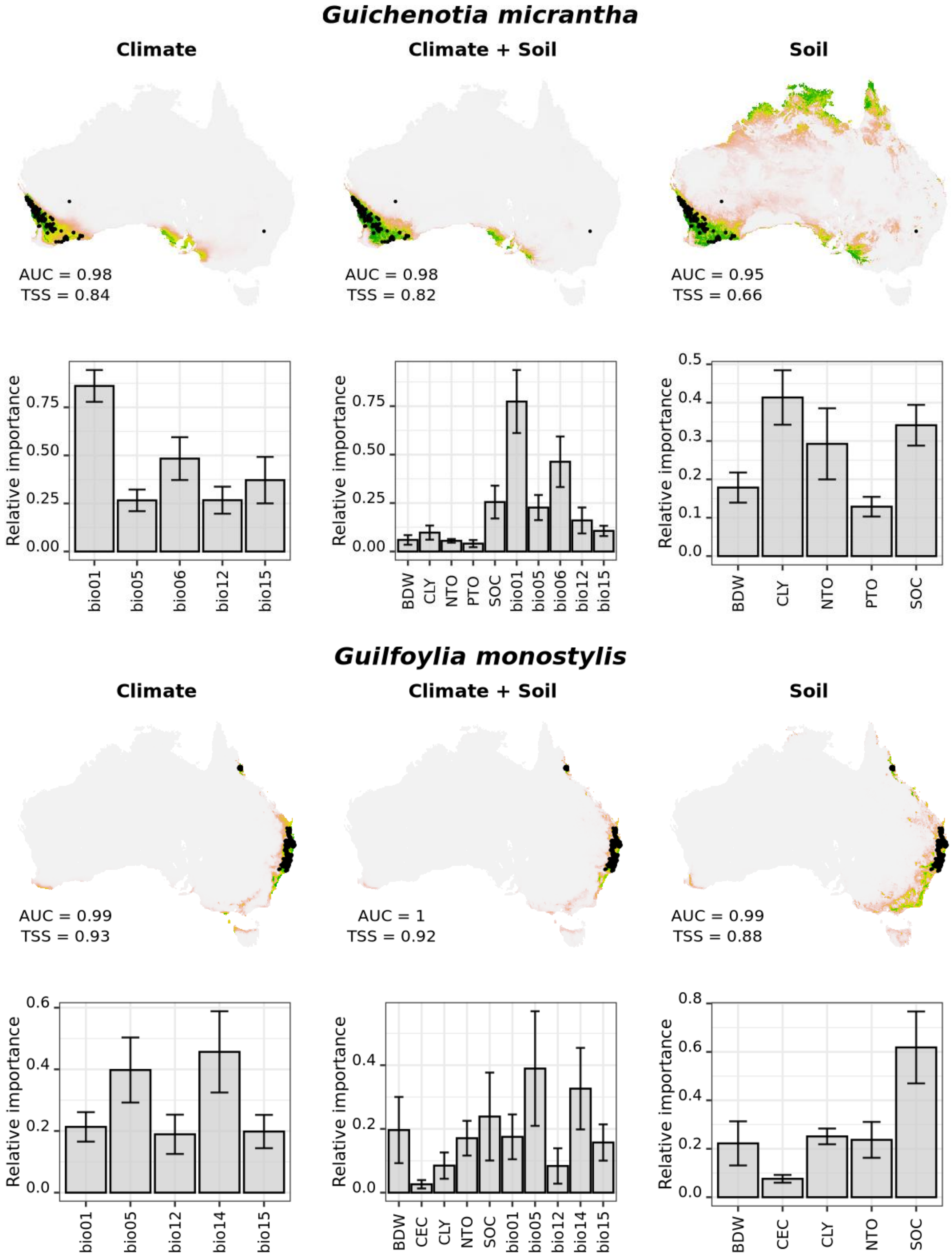
Guichenotia micrantha
Climate
Climate + Soil
Soil
AUC = 0.98
TSS = 0.84
AUC = 0.98
TSS = 0.82
AUC = 0.95
TSS = 0.66
Relative importance
0.00
0.25
0.50
0.75
bio01
bio05
bio06
bio12
bio15
BDW
CLY
NTO
PTO
SOC
0.0
0.1
0.2
0.3
0.4
0.5
Guilfoylia monostylis
Climate
Climate + Soil
Soil
AUC = 0.99
TSS = 0.93
AUC = 1
TSS = 0.92
AUC = 0.99
TSS = 0.88
Relative importance
0.0
0.2
0.4
0.6
0.8
bio01
bio05
bio12
bio14
bio15
BDW
CEC
CLY
NTO
SOC

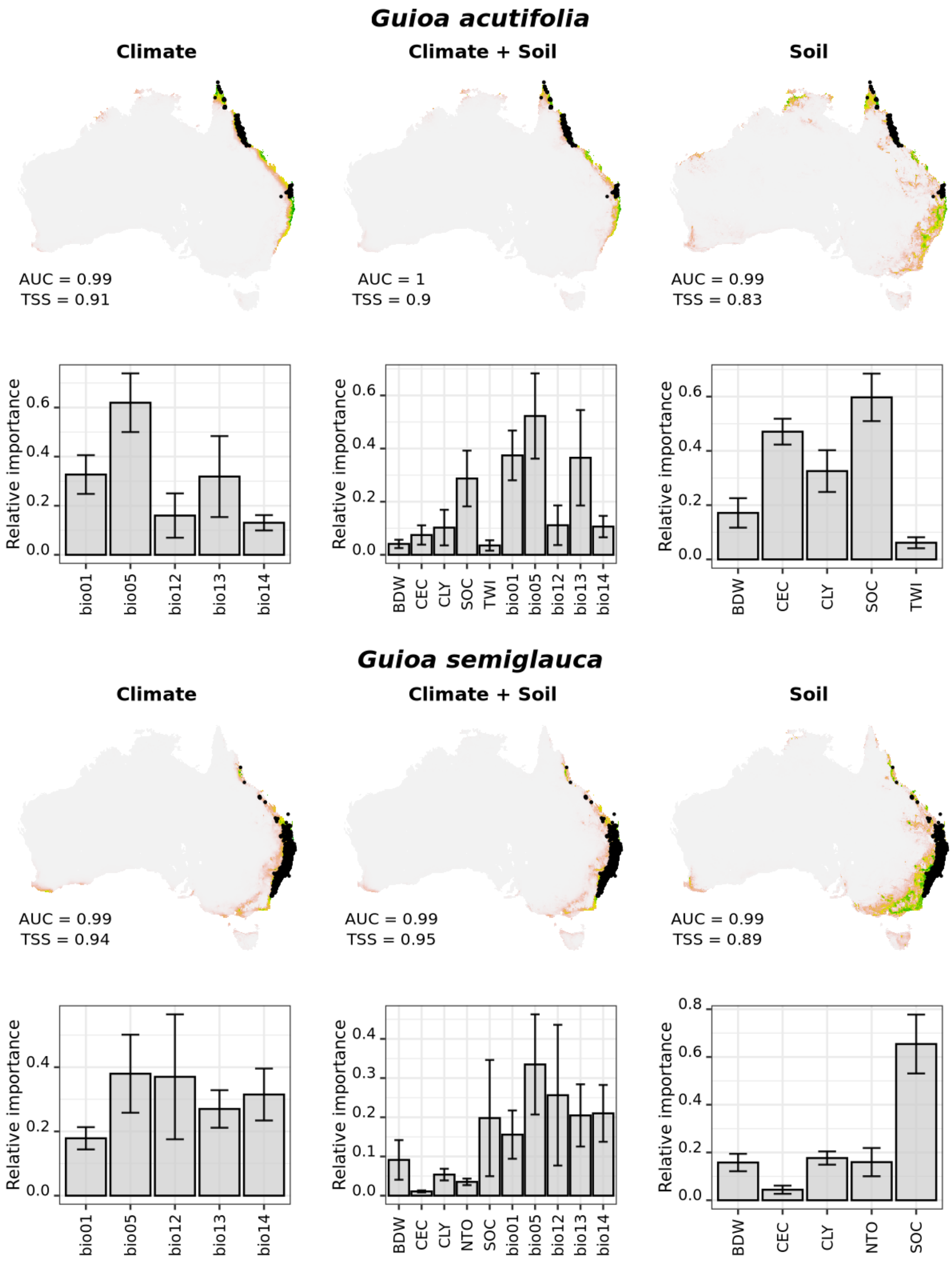
Guioa acutifolia
Climate
Climate + Soil
Soil
AUC = 0.99
TSS = 0.91
AUC = 1
TSS = 0.9
AUC = 0.99
TSS = 0.83
Relative importance
0.0
0.2
0.4
0.6
bio01
bio05
bio12
bio13
bio14
BDW
CEC
CLY
SOC
TWI
Guioa semiglauca
Climate
Climate + Soil
Soil
AUC = 0.99
TSS = 0.94
AUC = 0.99
TSS = 0.95
AUC = 0.99
TSS = 0.89
Relative importance
0.0
0.1
0.2
0.3
0.4
0.6
0.8
bio01
bio05
bio12
bio13
bio14
BDW
CEC
CLY
NTO
SOC

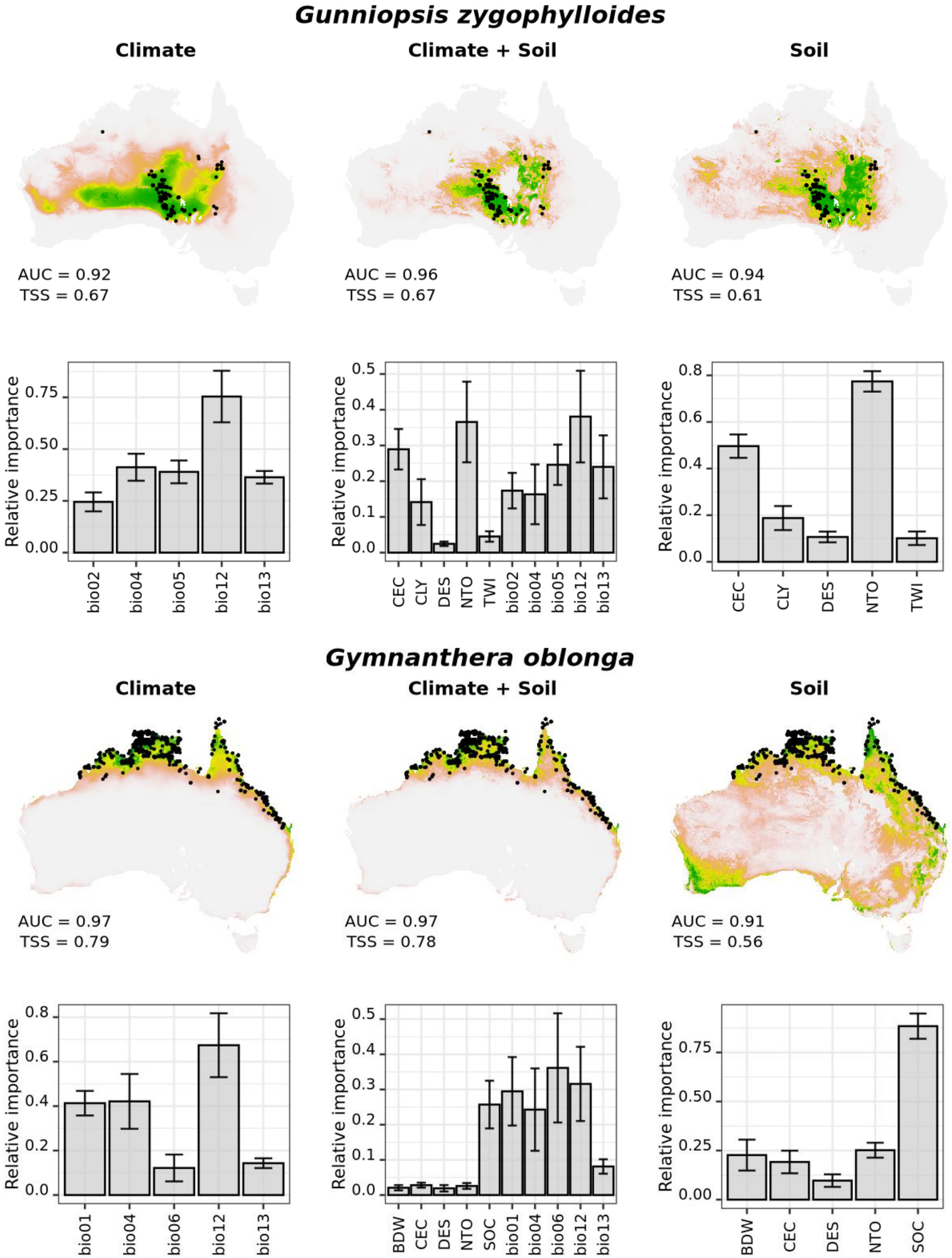

Gunniopsis zygophylloides
Climate
Climate + Soil
Soil
AUC = 0.92
TSS = 0.67
AUC = 0.96
TSS = 0.67
AUC = 0.94
TSS = 0.61
Relative importance
0.00
0.25
0.50
0.75
bio02
bio04
bio05
bio12
bio13
0.0
0.1
0.2
0.3
0.4
0.5
CEC
CLY
DES
NTO
TWI
bio02
bio04
bio05
bio12
bio13
0.0
0.2
0.4
0.6
0.8
CEC
CLY
DES
NTO
TWI
Gymnanthera oblonga
Climate
Climate + Soil
Soil
AUC = 0.97
TSS = 0.79
AUC = 0.97
TSS = 0.78
AUC = 0.91
TSS = 0.56
Relative importance
0.0
0.2
0.4
0.6
0.8
bio01
bio04
bio06
bio12
bio13
0.0
0.1
0.2
0.3
0.4
0.5
BDW
CEC
DES
NTO
SOC
bio01
bio04
bio06
bio12
bio13
0.00
0.25
0.50
0.75
BDW
CEC
DES
NTO
SOC

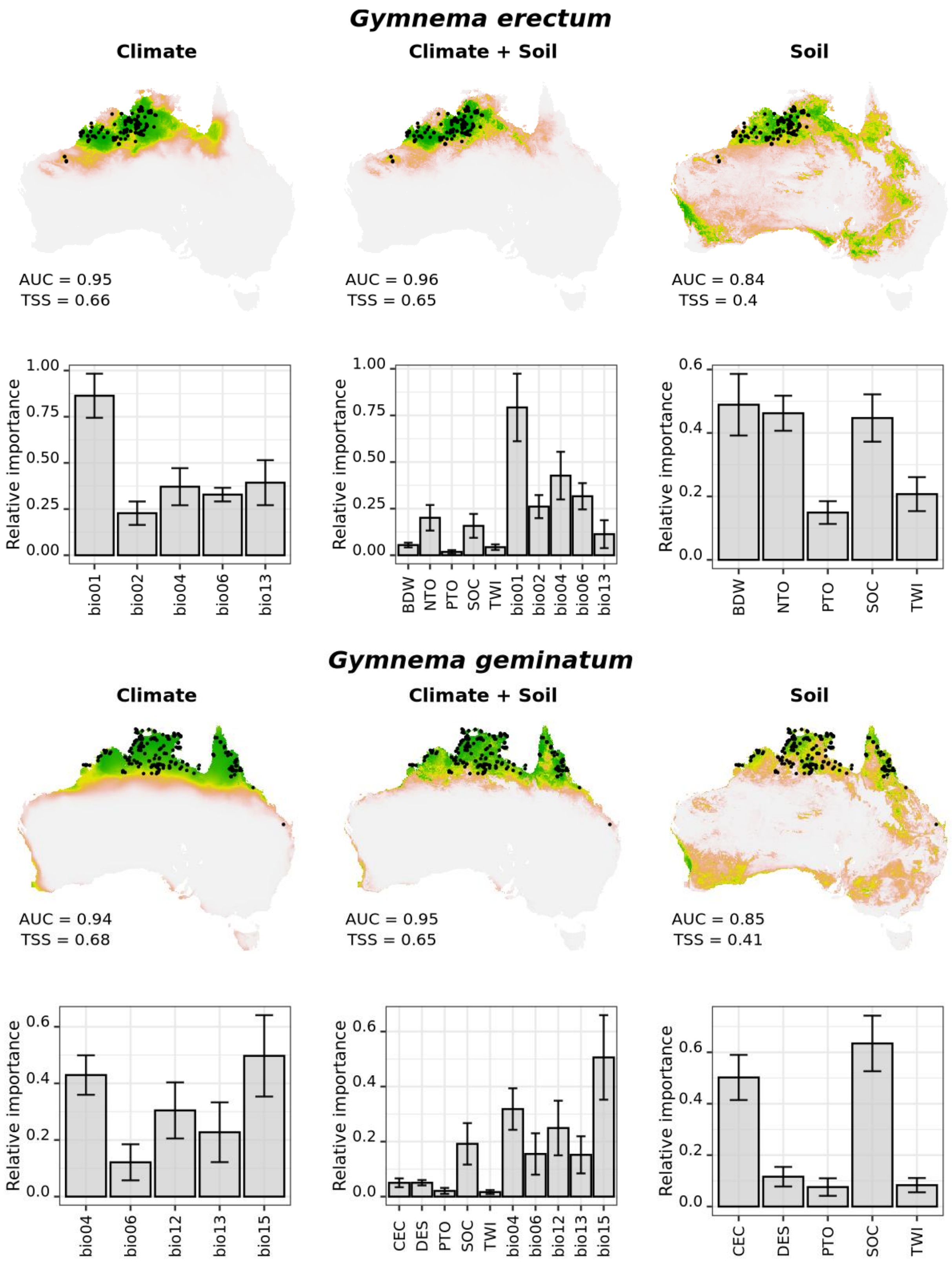

Gymnema erectum
Climate
Climate + Soil
Soil
AUC = 0.95
TSS = 0.66
AUC = 0.96
TSS = 0.65
AUC = 0.84
TSS = 0.4
Relative importance
bio01
bio02
bio04
bio06
bio13
BDW
NTO
PTO
SOC
TWI
Gymnema geminatum
Climate
Climate + Soil
Soil
AUC = 0.94
TSS = 0.68
AUC = 0.95
TSS = 0.65
AUC = 0.85
TSS = 0.41
Relative importance
bio04
bio06
bio12
bio13
bio15
CEC
DES
PTO
SOC
TWI

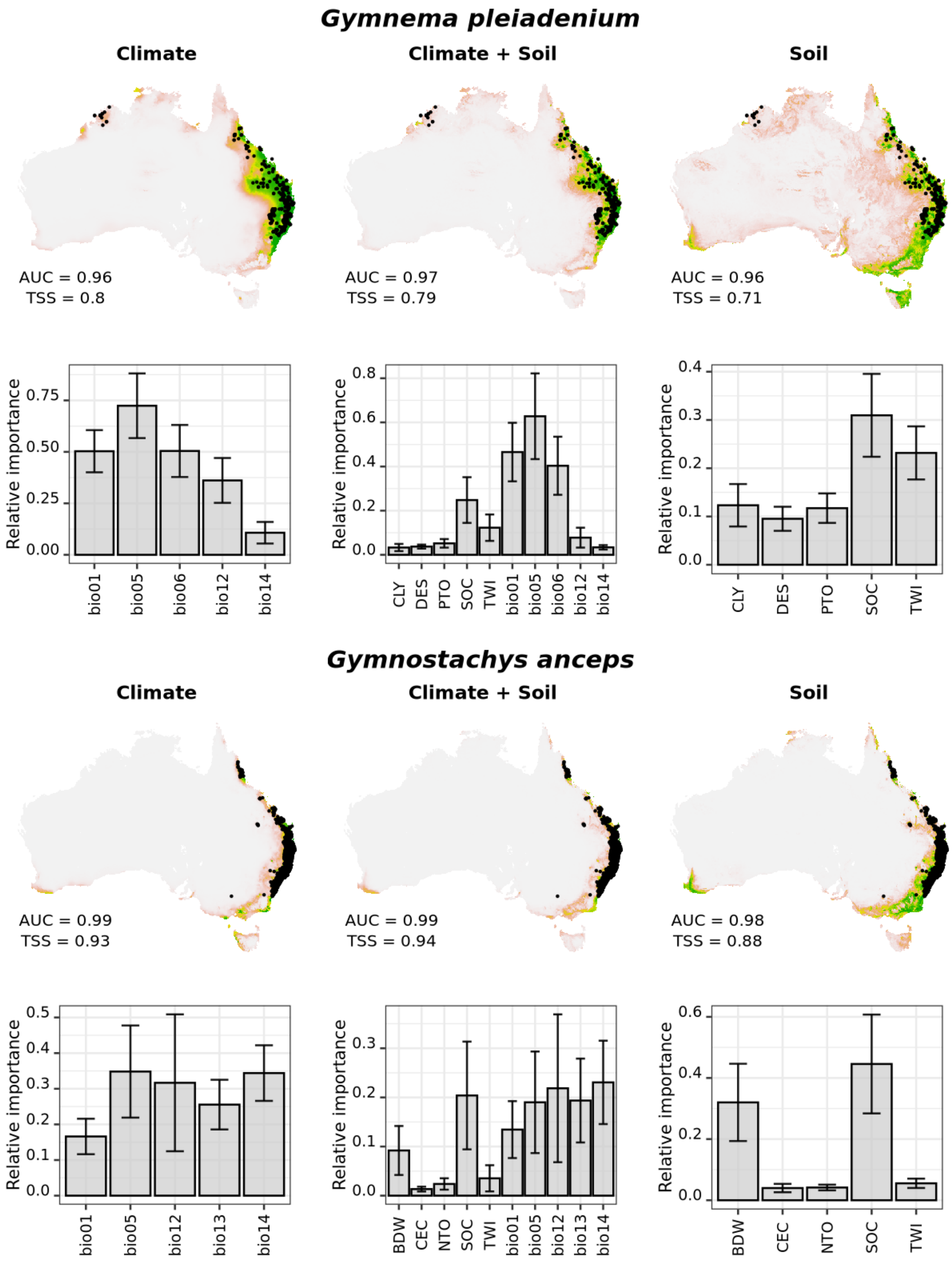
Gymnema pleiadenium
Climate
Climate + Soil
Soil
AUC = 0.96
TSS = 0.8
AUC = 0.97
TSS = 0.79
AUC = 0.96
TSS = 0.71
Relative importance
0.00
0.25
0.50
0.75
bio01
bio05
bio06
bio12
bio14
0.0
0.2
0.4
0.6
0.8
CLY
DES
PTO
SOC
TWI
bio01
bio05
bio06
bio12
bio14
0.0
0.1
0.2
0.3
0.4
CLY
DES
PTO
SOC
TWI
Gymnostachys anceps
Climate
Climate + Soil
Soil
AUC = 0.99
TSS = 0.93
AUC = 0.99
TSS = 0.94
AUC = 0.98
TSS = 0.88
Relative importance
0.0
0.1
0.2
0.3
0.4
0.5
bio01
bio05
bio12
bio13
bio14
0.0
0.1
0.2
0.3
BDW
CEC
NTO
SOC
TWI
bio01
bio05
bio12
bio13
bio14
0.0
0.2
0.4
0.6
BDW
CEC
NTO
SOC
TWI

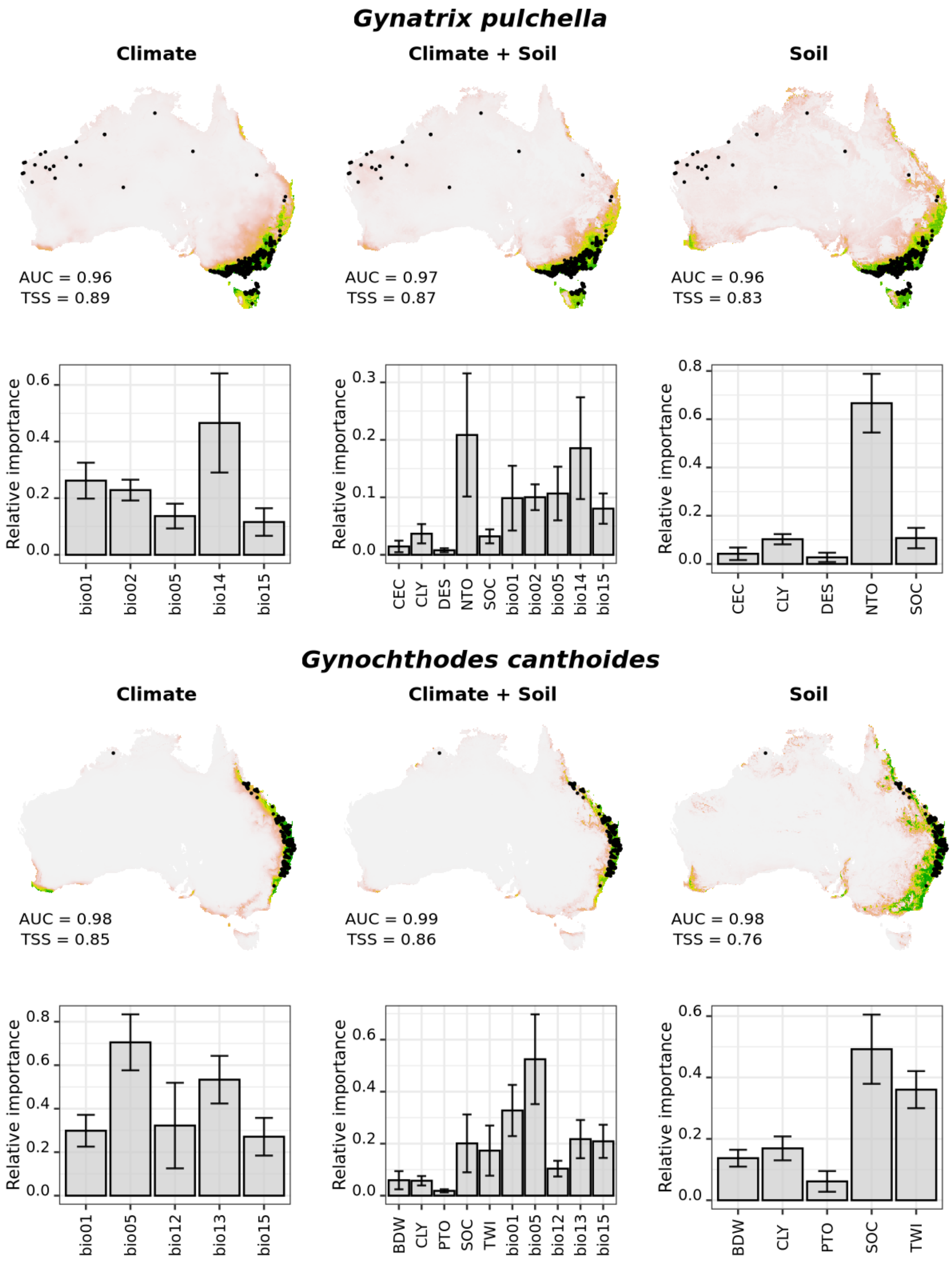
Gynatrix pulchella
Climate
Climate + Soil
Soil
AUC = 0.96
TSS = 0.89
AUC = 0.97
TSS = 0.87
AUC = 0.96
TSS = 0.83
Relative importance
0.0
0.2
0.4
0.6
bio01
bio02
bio05
bio14
bio15
Relative importance
0.0
0.1
0.2
0.3
CEC
CLY
DES
NTO
SOC
bio01
bio02
bio05
bio14
bio15
Relative importance
0.0
0.2
0.4
0.6
0.8
CEC
CLY
DES
NTO
SOC
Gynochthodes canthoides
Climate
Climate + Soil
Soil
AUC = 0.98
TSS = 0.85
AUC = 0.99
TSS = 0.86
AUC = 0.98
TSS = 0.76
Relative importance
0.0
0.2
0.4
0.6
0.8
bio01
bio05
bio12
bio13
bio15
Relative importance
0.0
0.2
0.4
0.6
BDW
CLY
PTO
SOC
TWI
bio01
bio05
bio12
bio13
bio15
Relative importance
0.0
0.2
0.4
0.6
BDW
CLY
PTO
SOC
TWI

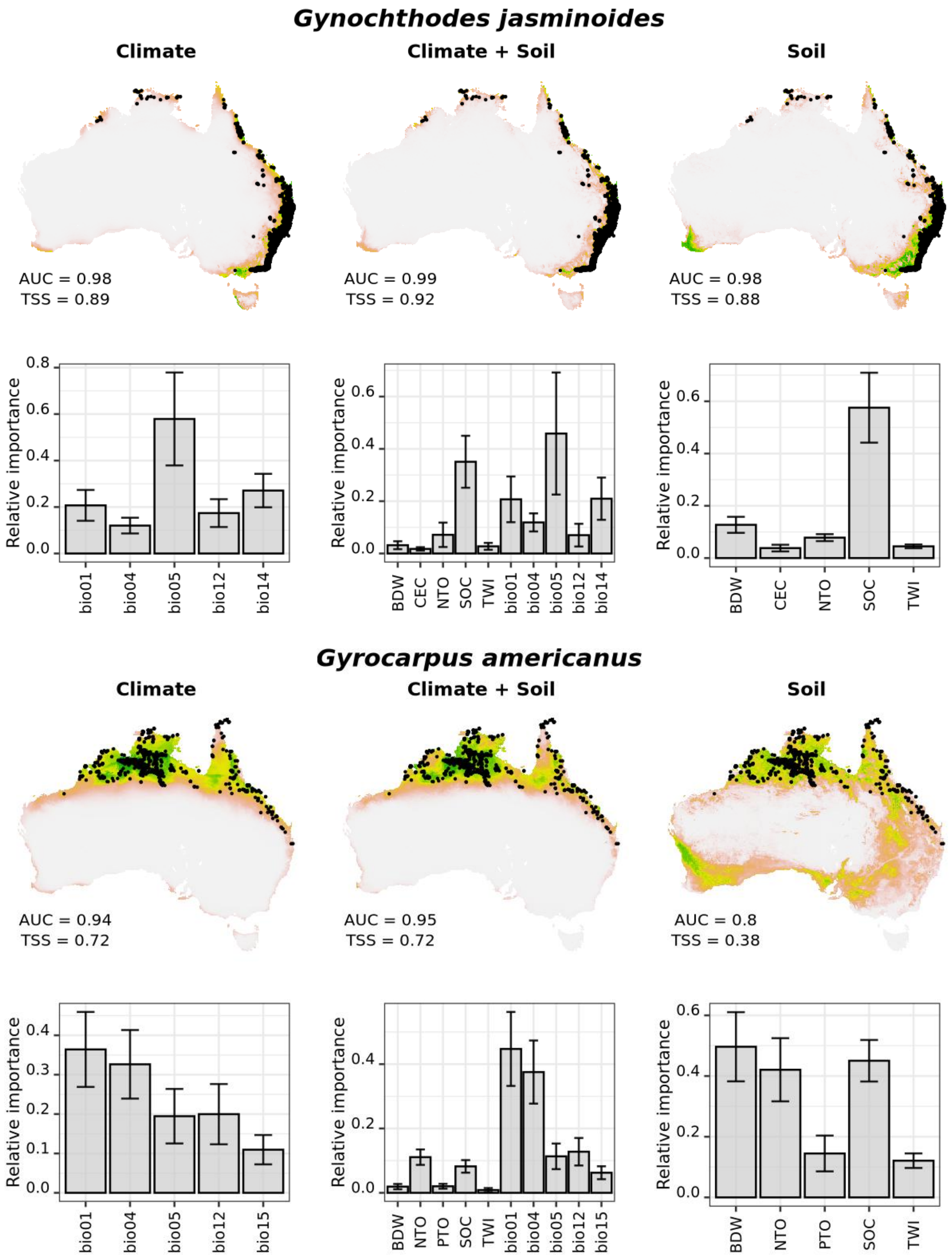
Gynochthodes jasminoides
Climate
Climate + Soil
Soil
AUC = 0.98
TSS = 0.89
AUC = 0.99
TSS = 0.92
AUC = 0.98
TSS = 0.88
Relative importance
bio01
bio04
bio05
bio12
bio14
BDW
CEC
NTO
SOC
TWI
Gyrocarpus americanus
Climate
Climate + Soil
Soil
AUC = 0.94
TSS = 0.72
AUC = 0.95
TSS = 0.72
AUC = 0.8
TSS = 0.38
Relative importance
bio01
bio04
bio05
bio12
bio15
BDW
NTO
PTO
SOC
TWI

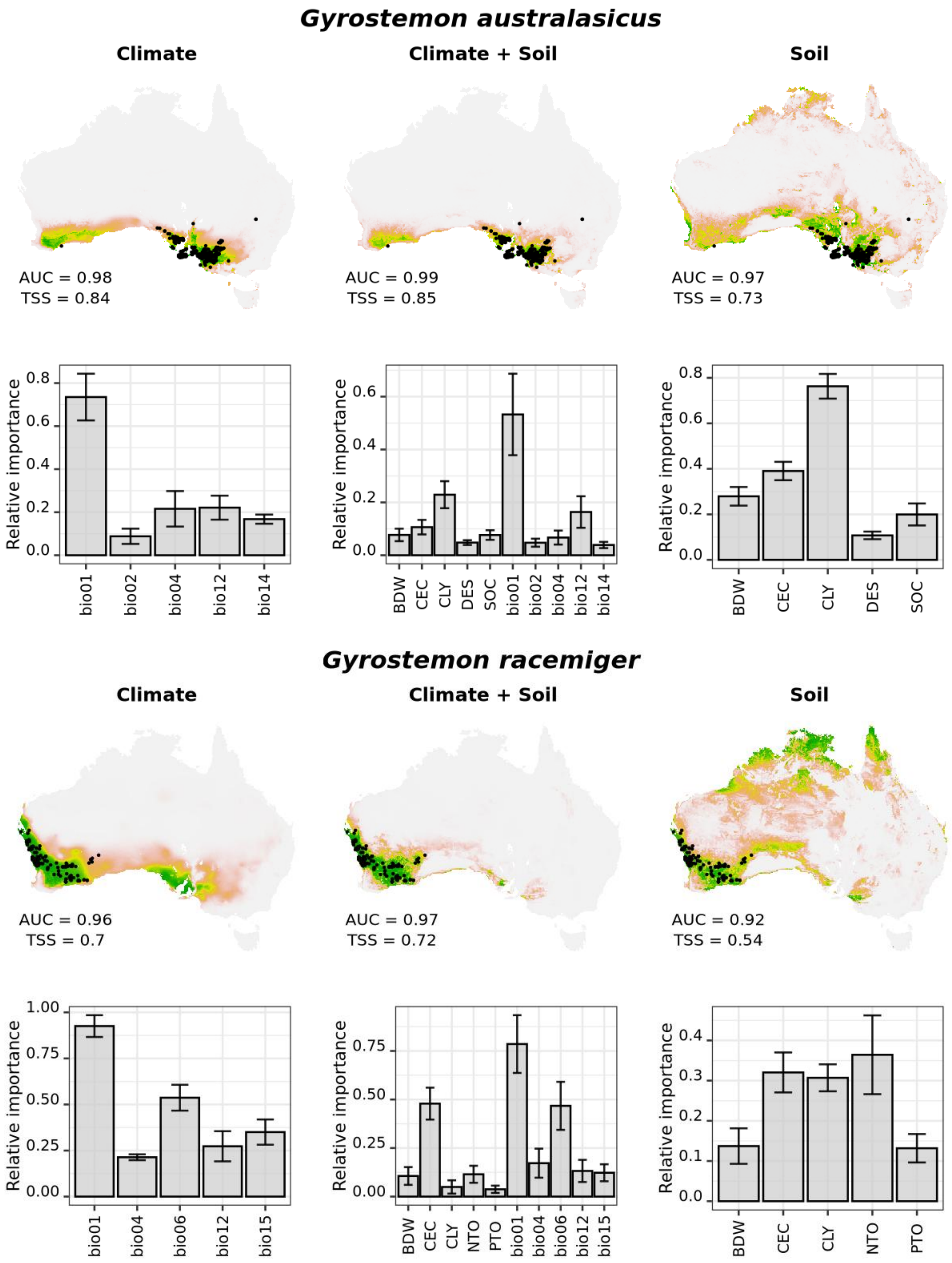

Gyrostemon australasicus
Climate
Climate + Soil
Soil
AUC = 0.98
TSS = 0.84
AUC = 0.99
TSS = 0.85
AUC = 0.97
TSS = 0.73
Relative importance
0.0
0.2
0.4
0.6
0.8
bio01
bio02
bio04
bio12
bio14
BDW
CEC
CLY
DES
SOC
bio01
bio02
bio04
bio12
bio14
BDW
CEC
CLY
DES
SOC
Gyrostemon racemiger
Climate
Climate + Soil
Soil
AUC = 0.96
TSS = 0.7
AUC = 0.97
TSS = 0.72
AUC = 0.92
TSS = 0.54
Relative importance
0.00
0.25
0.50
0.75
1.00
0.0
0.1
0.2
0.3
0.4
bio01
bio04
bio06
bio12
bio15
BDW
CEC
CLY
NTO
PTO
bio01
bio04
bio06
bio12
bio15
BDW
CEC
CLY
NTO
PTO

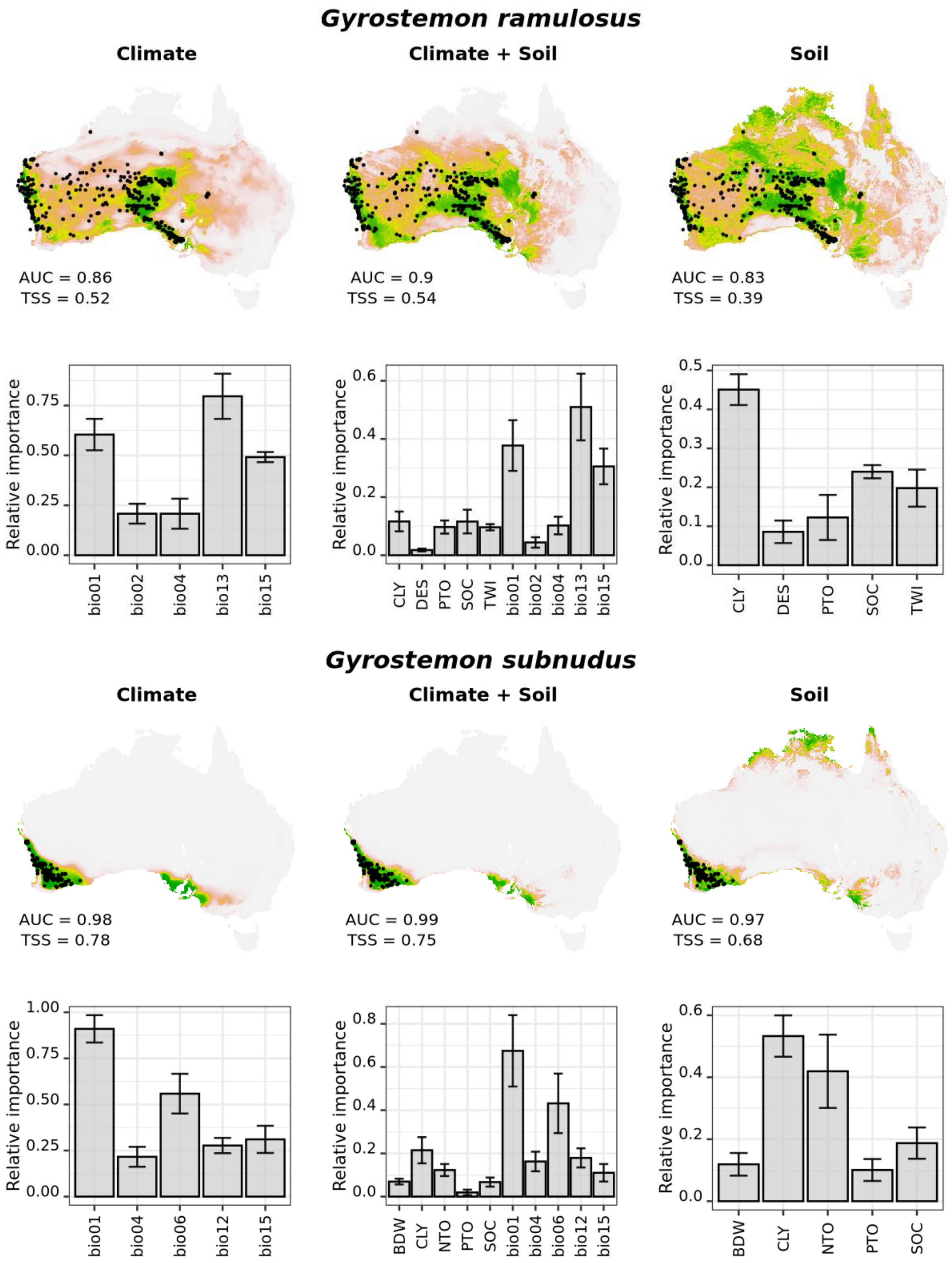
Gyrostemon ramulosus
Climate
Climate + Soil
Soil
AUC = 0.86
TSS = 0.52
AUC = 0.9
TSS = 0.54
AUC = 0.83
TSS = 0.39
Relative importance
bio01
bio02
bio04
bio13
bio15
CLY
DES
PTO
SOC
TWI
Gyrostemon subnudus
Climate
Climate + Soil
Soil
AUC = 0.98
TSS = 0.78
AUC = 0.99
TSS = 0.75
AUC = 0.97
TSS = 0.68
Relative importance
bio01
bio04
bio06
bio12
bio15
BDW
CLY
NTO
PTO
SOC

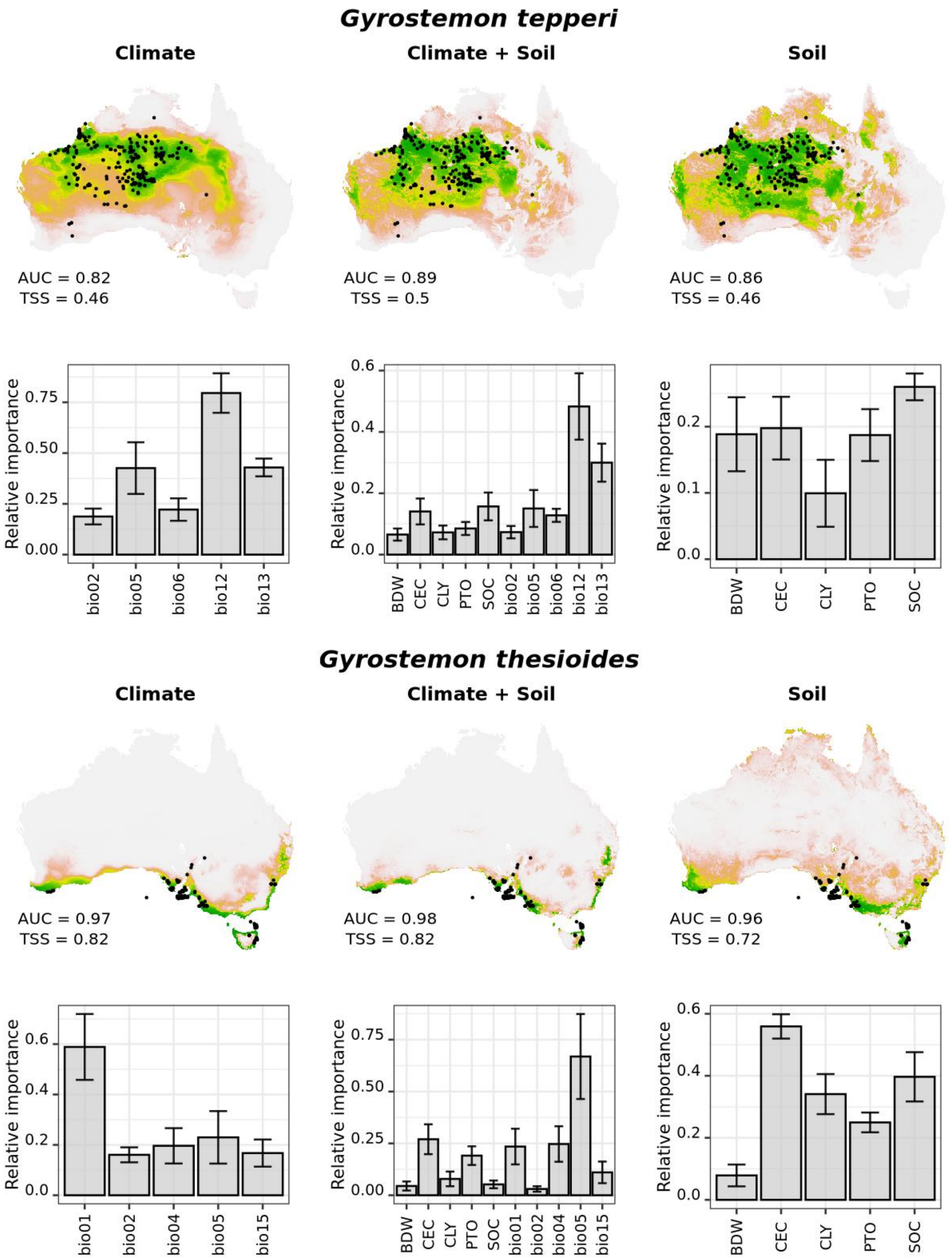

Gyrostemon tepperi
Climate
Climate + Soil
Soil
AUC = 0.82
TSS = 0.46
AUC = 0.89
TSS = 0.5
AUC = 0.86
TSS = 0.46
Relative importance
0.00
0.25
0.50
0.75
bio02
bio05
bio06
bio12
bio13
0.0
0.2
0.4
0.6
BDW
CEC
CLY
PTO
SOC
bio02
bio05
bio06
bio12
bio13
0.0
0.1
0.2
BDW
CEC
CLY
PTO
SOC
Gyrostemon thesioides
Climate
Climate + Soil
Soil
AUC = 0.97
TSS = 0.82
AUC = 0.98
TSS = 0.82
AUC = 0.96
TSS = 0.72
Relative importance
0.0
0.2
0.4
0.6
bio01
bio02
bio04
bio05
bio15
0.00
0.25
0.50
0.75
BDW
CEC
CLY
PTO
SOC
bio01
bio02
bio04
bio05
bio15
0.0
0.2
0.4
0.6
BDW
CEC
CLY
PTO
SOC

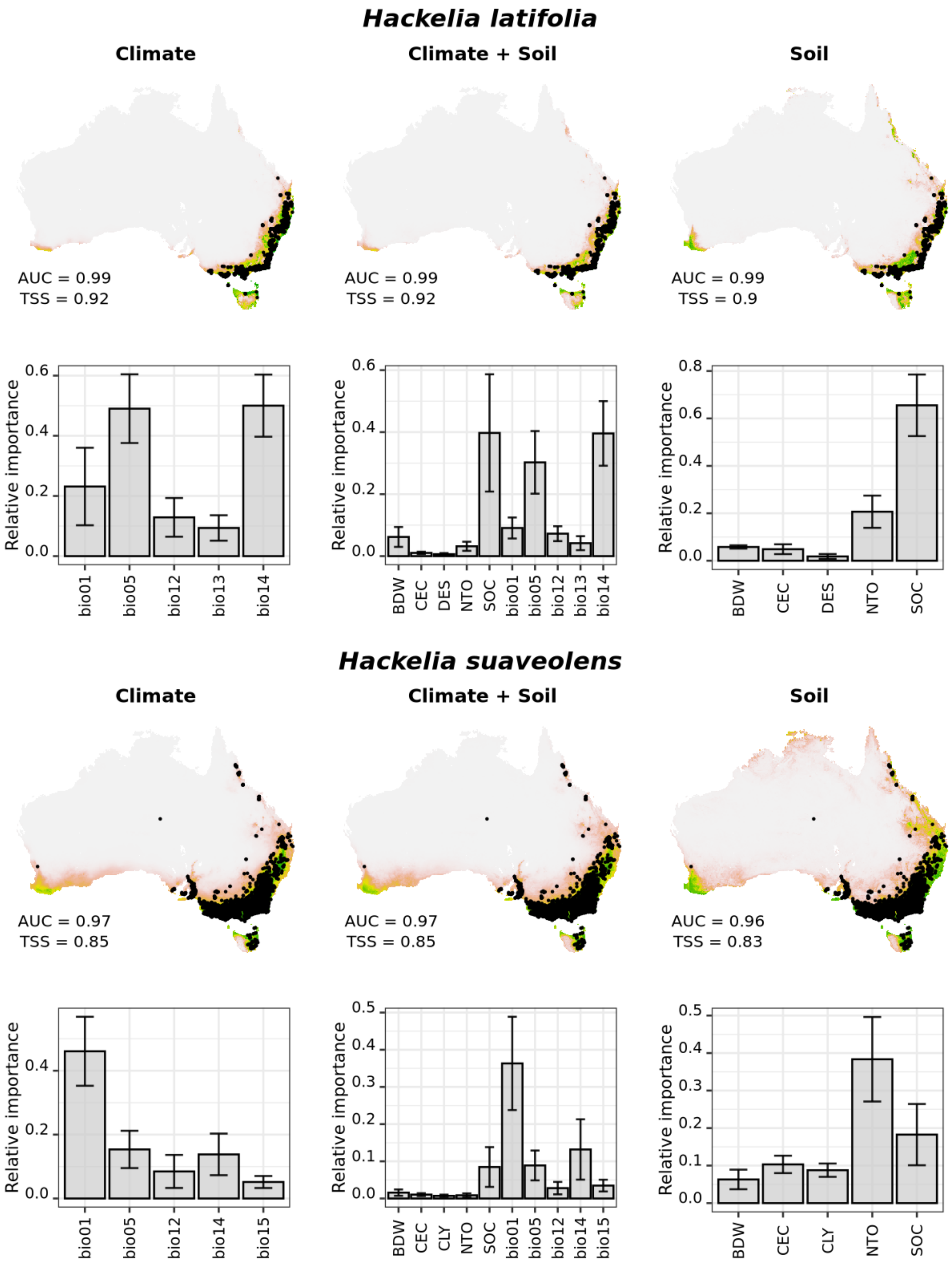
Hackelia latifolia
Climate
Climate + Soil
Soil
AUC = 0.99
TSS = 0.92
AUC = 0.99
TSS = 0.92
AUC = 0.99
TSS = 0.9
Relative importance
bio01
bio05
bio12
bio13
bio14
BDW
CEC
DES
NTO
SOC
Hackelia suaveolens
Climate
Climate + Soil
Soil
AUC = 0.97
TSS = 0.85
AUC = 0.97
TSS = 0.85
AUC = 0.96
TSS = 0.83
Relative importance
bio01
bio05
bio12
bio14
bio15
BDW
CEC
CLY
NTO
SOC

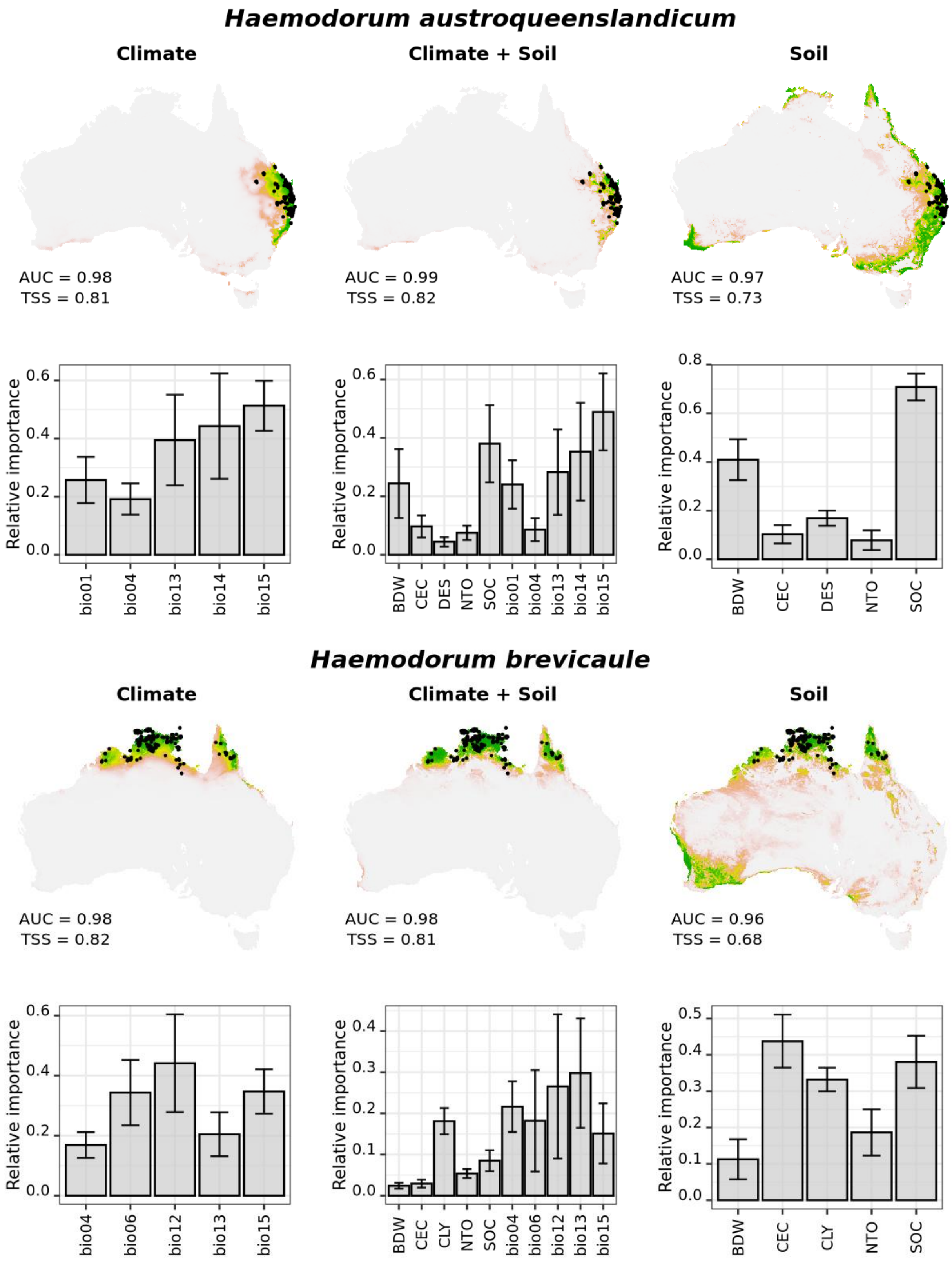
Haemodorum austroqueenslandicum
Climate
Climate + Soil
Soil
AUC = 0.98
TSS = 0.81
AUC = 0.99
TSS = 0.82
AUC = 0.97
TSS = 0.73
Relative importance
0.0
0.2
0.4
0.6
0.8
bio01
bio04
bio13
bio14
bio15
BDW
CEC
DES
NTO
SOC
Haemodorum brevicaule
AUC = 0.98
TSS = 0.82
AUC = 0.98
TSS = 0.81
AUC = 0.96
TSS = 0.68
0.1
0.3
0.5
bio06
bio12
CLY

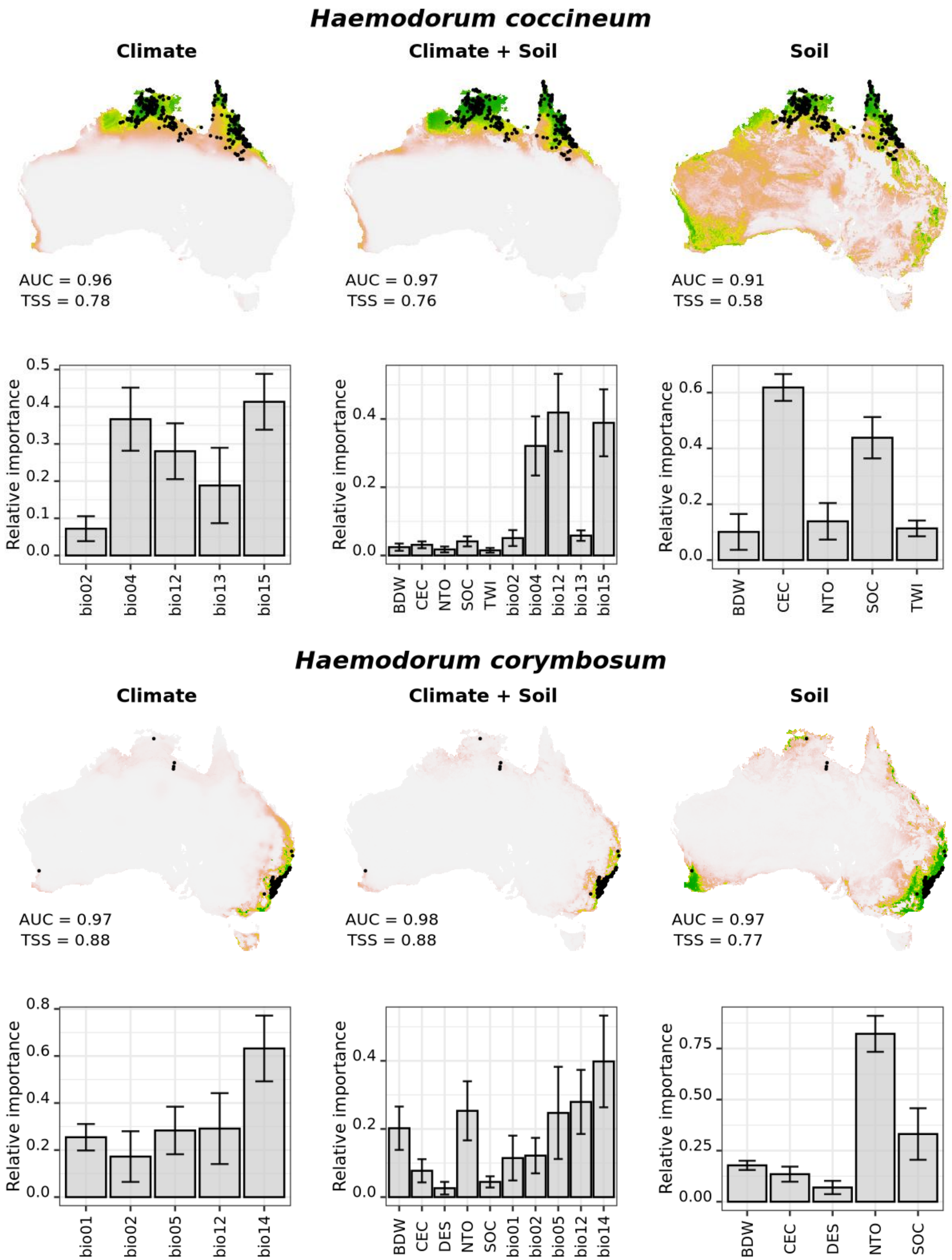
Haemodorum coccineum
Climate
Climate + Soil
Soil
AUC = 0.96
TSS = 0.78
AUC = 0.97
TSS = 0.76
AUC = 0.91
TSS = 0.58
Relative importance
bio02
bio04
bio12
bio13
bio15
BDW
CEC
NTO
SOC
TWI
Haemodorum corymbosum
Climate
Climate + Soil
Soil
AUC = 0.97
TSS = 0.88
AUC = 0.98
TSS = 0.88
AUC = 0.97
TSS = 0.77
Relative importance
bio01
bio02
bio05
bio12
bio14
BDW
CEC
DES
NTO
SOC

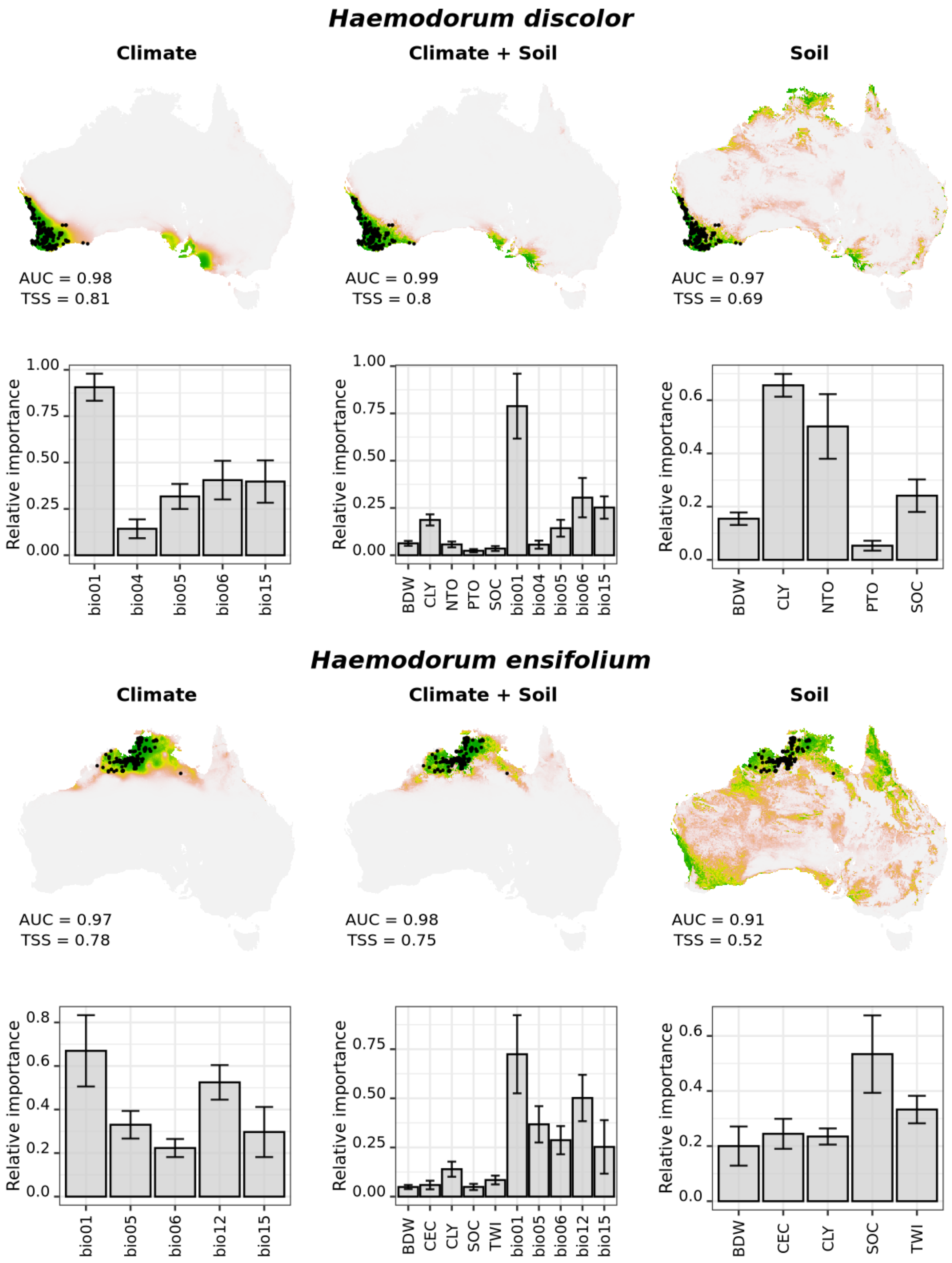

Haemodorum discolor
Climate
Climate + Soil
Soil
AUC = 0.98
TSS = 0.81
AUC = 0.99
TSS = 0.8
AUC = 0.97
TSS = 0.69
Relative importance
1.00
0.75
0.50
0.25
0.00
bio01
bio04
bio05
bio06
bio15
BDW
CLY
NTO
PTO
SOC
0.6
0.4
0.2
0.0
Haemodorum ensifolium
Climate
Climate + Soil
Soil
AUC = 0.97
TSS = 0.78
AUC = 0.98
TSS = 0.75
AUC = 0.91
TSS = 0.52
0.8
bio12
CEC
TWI

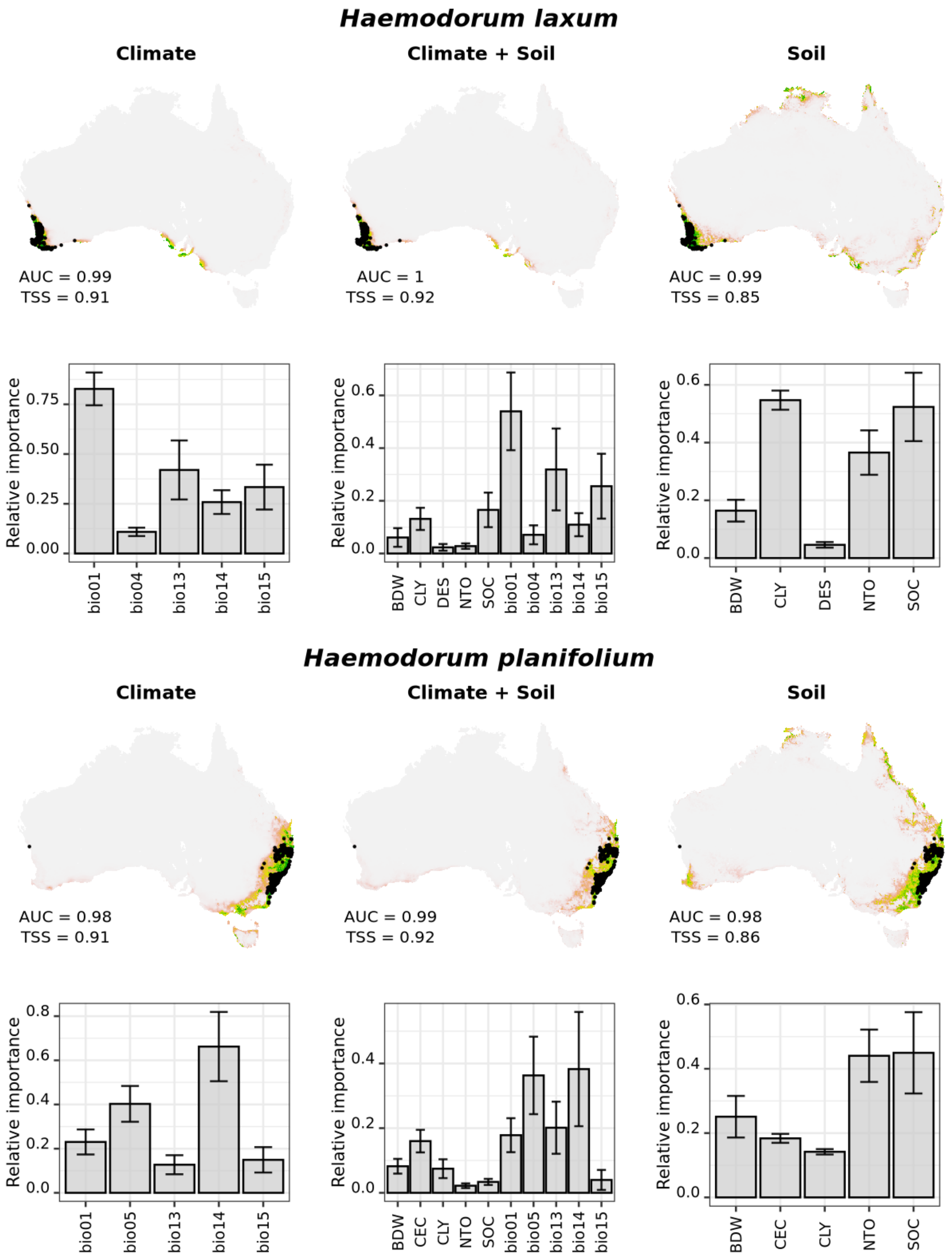
Haemodorum laxum
Climate
Climate + Soil
Soil
AUC = 0.99
TSS = 0.91
AUC = 1
TSS = 0.92
AUC = 0.99
TSS = 0.85
Relative importance
0.00
0.25
0.50
0.75
bio01
bio04
bio13
bio14
bio15
0.0
0.2
0.4
0.6
BDW
CLY
DES
NTO
SOC
bio01
bio04
bio13
bio14
bio15
BDW
CLY
DES
NTO
SOC
Haemodorum planifolium
Climate
Climate + Soil
Soil
AUC = 0.98
TSS = 0.91
AUC = 0.99
TSS = 0.92
AUC = 0.98
TSS = 0.86
0.0
0.2
0.4
0.6
0.8
bio01
bio05
bio13
bio14
bio15
BDW
CEC
CLY
NTO
SOC
bio01
bio05
bio13
bio14
bio15
BDW
CEC
CLY
NTO
SOC

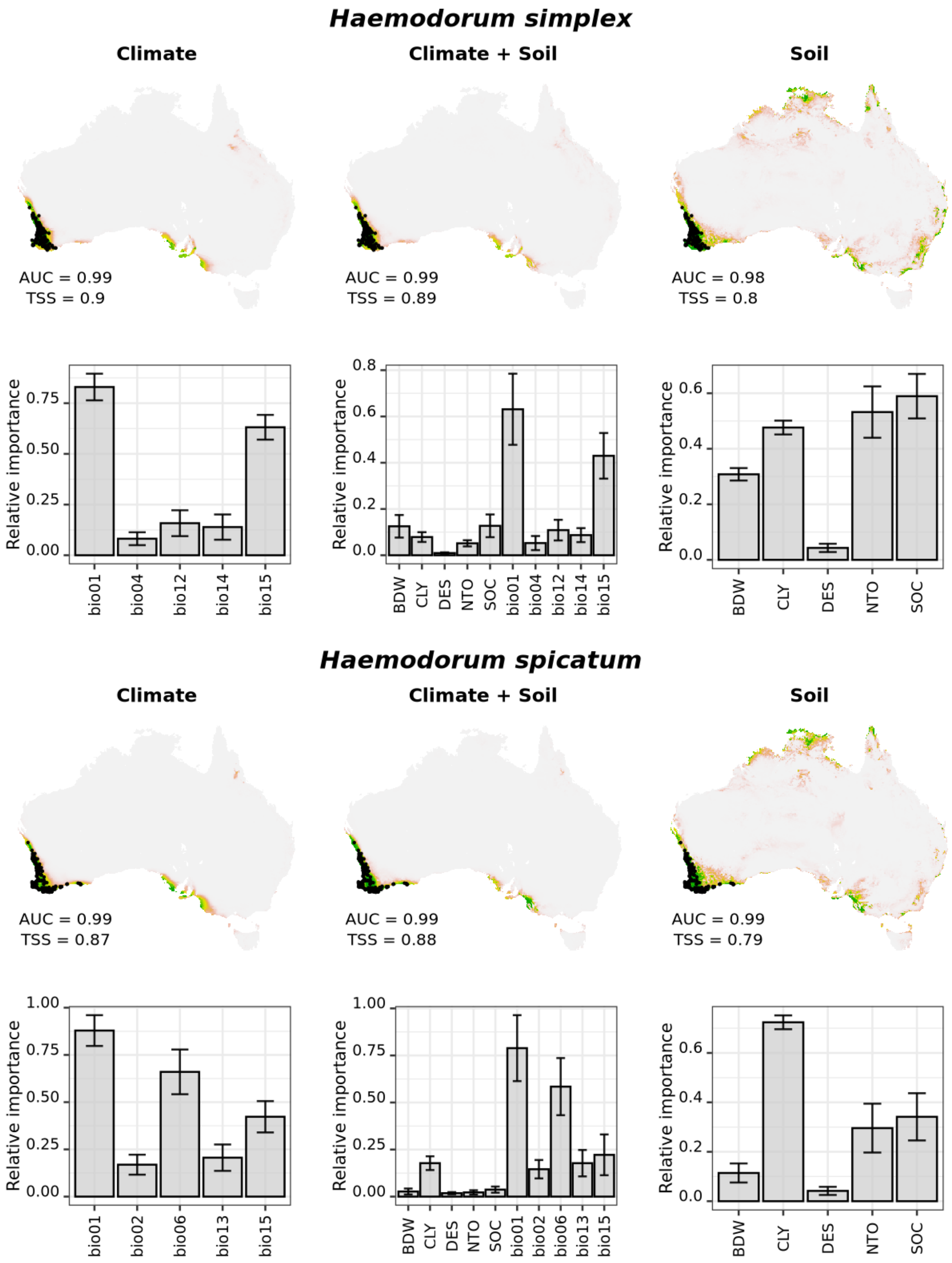
Haemodorum simplex
Climate
Climate + Soil
Soil
AUC = 0.99
TSS = 0.9
AUC = 0.99
TSS = 0.89
AUC = 0.98
TSS = 0.8
Relative importance
bio01
bio04
bio12
bio14
bio15
BDW
CLY
DES
NTO
SOC
Haemodorum spicatum
Climate
Climate + Soil
Soil
AUC = 0.99
TSS = 0.87
AUC = 0.99
TSS = 0.88
AUC = 0.99
TSS = 0.79
Relative importance
bio01
bio02
bio06
bio13
bio15
BDW
CLY
DES
NTO
SOC

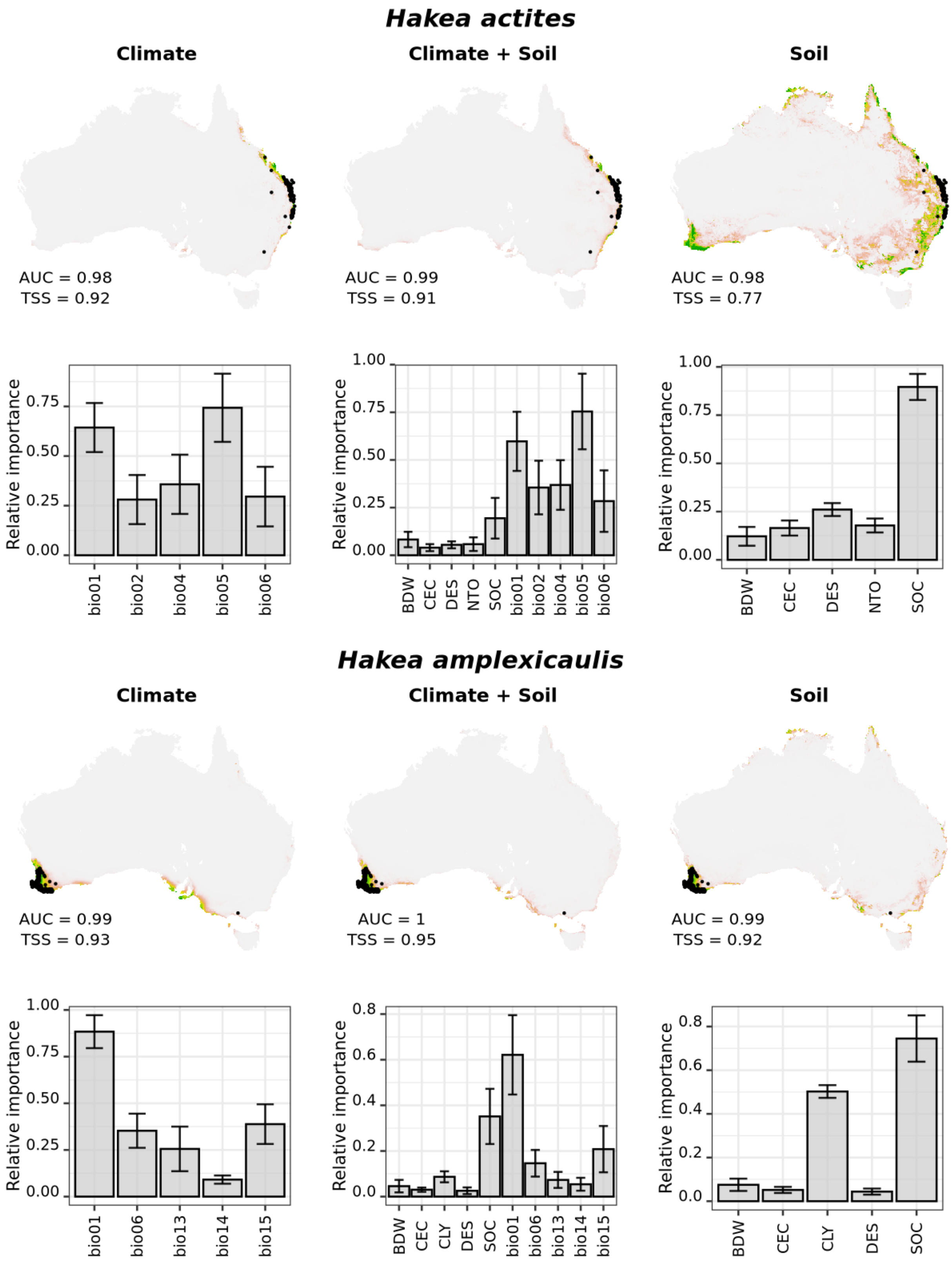
Hakea actites
Climate
Climate + Soil
Soil
AUC = 0.98
TSS = 0.92
AUC = 0.99
TSS = 0.91
AUC = 0.98
TSS = 0.77
Relative importance
bio01
bio02
bio04
bio05
bio06
BDW
CEC
DES
NTO
SOC
Hakea amplexicaulis
Climate
Climate + Soil
Soil
AUC = 0.99
TSS = 0.93
AUC = 1
TSS = 0.95
AUC = 0.99
TSS = 0.92
Relative importance
bio01
bio06
bio13
bio14
bio15
BDW
CEC
CLY
DES
SOC

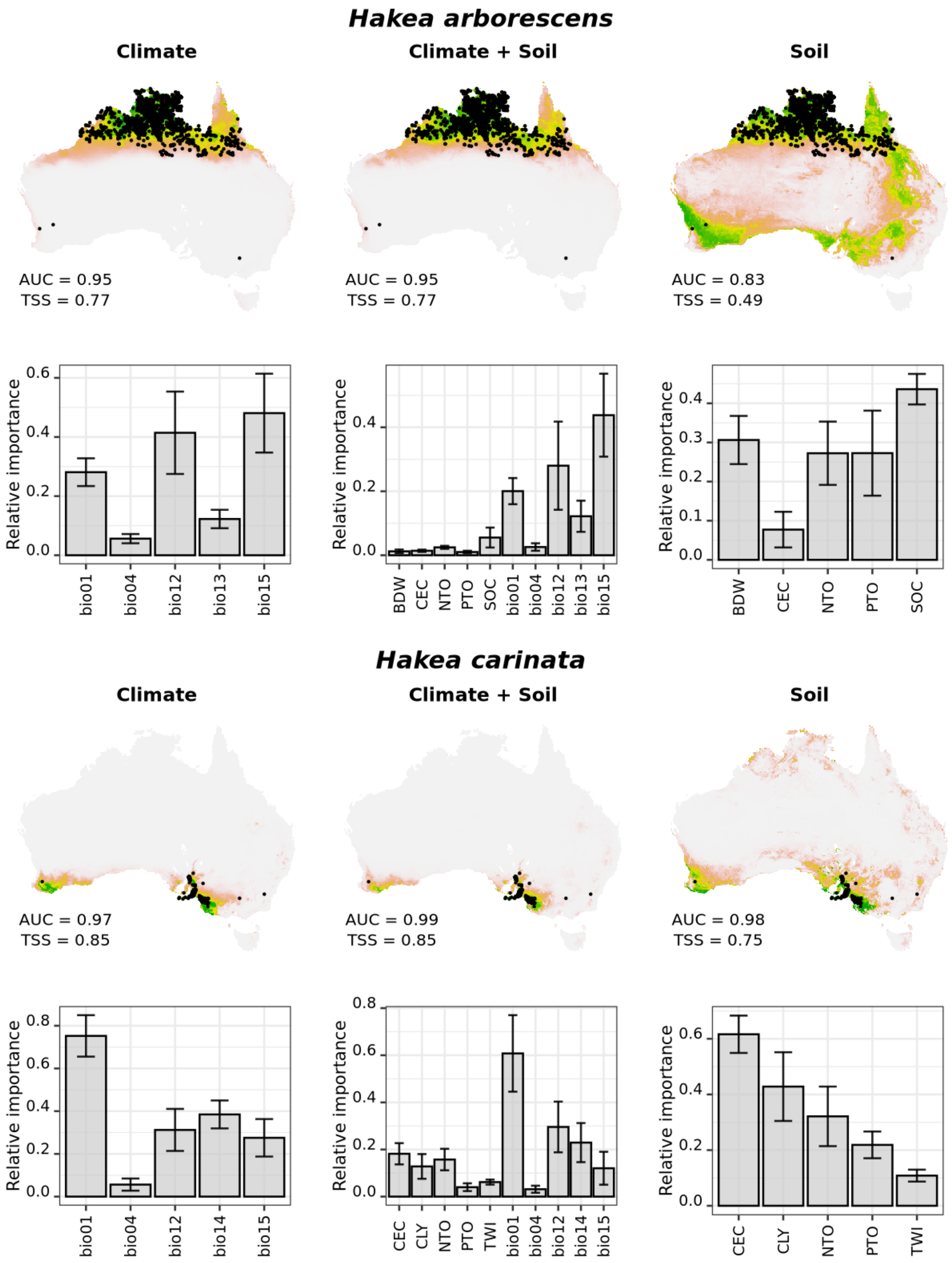

Hakea arborescens
Climate
Climate + Soil
Soil
AUC = 0.95
TSS = 0.77
AUC = 0.95
TSS = 0.77
AUC = 0.83
TSS = 0.49
Relative importance
0.0
0.2
0.4
0.6
0.1
0.3
bio01
bio04
bio12
bio13
bio15
BDW
CEC
NTO
PTO
SOC
Hakea carinata
Climate
Climate + Soil
Soil
AUC = 0.97
TSS = 0.85
AUC = 0.99
TSS = 0.85
AUC = 0.98
TSS = 0.75
Relative importance
0.8
bio14
CLY
TWI

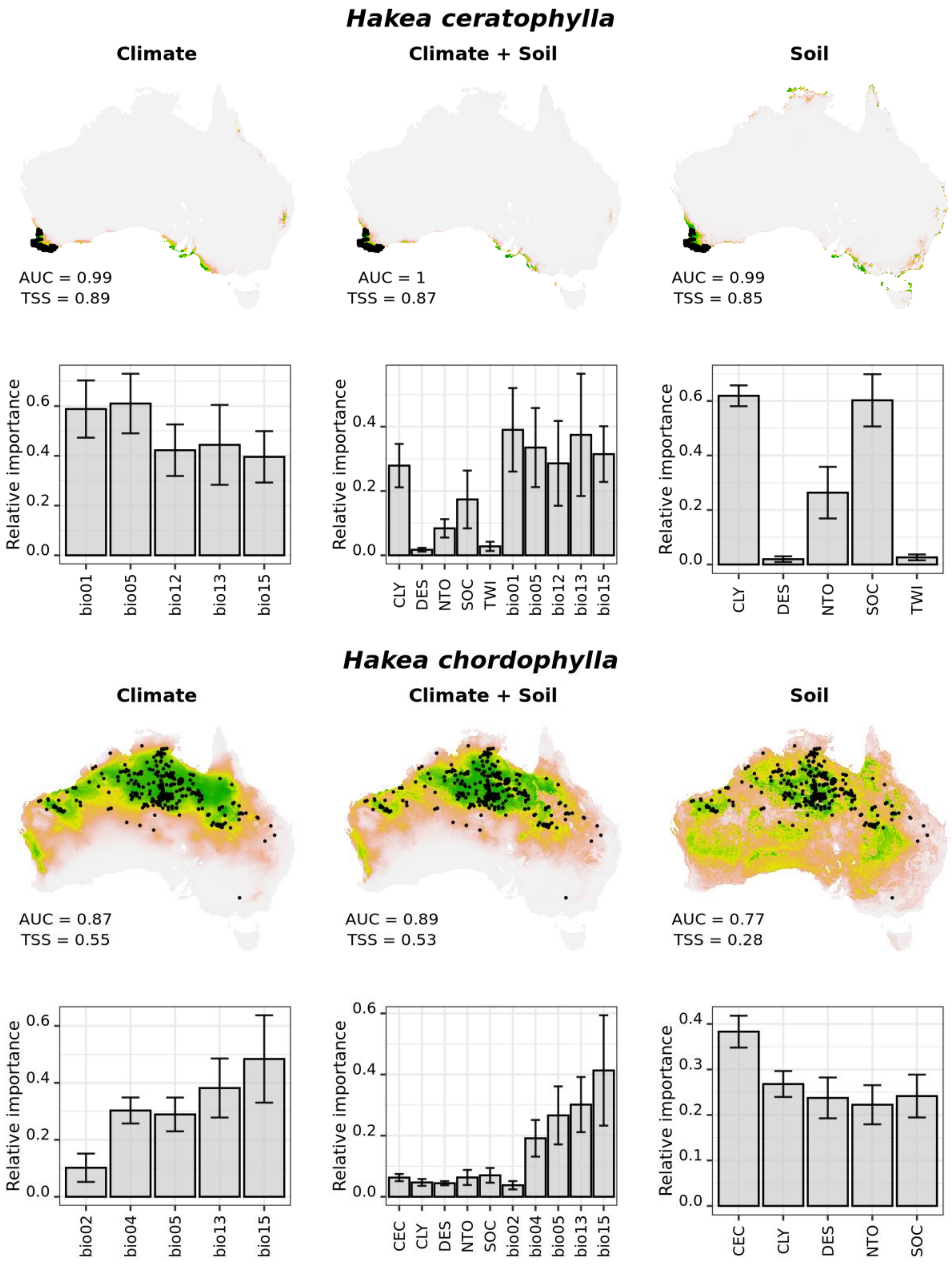

Hakea ceratophylla
Climate
Climate + Soil
Soil
AUC = 0.99
TSS = 0.89
AUC = 1
TSS = 0.87
AUC = 0.99
TSS = 0.85
Relative importance
bio01 bio05 bio12 bio13 bio15
CLY DES NTO SOC TWI bio01 bio05 bio12 bio13 bio15
CLY DES NTO SOC TWI
Hakea chordophylla
Climate
Climate + Soil
Soil
AUC = 0.87
TSS = 0.55
AUC = 0.89
TSS = 0.53
AUC = 0.77
TSS = 0.28
Relative importance
bio02 bio04 bio05 bio13 bio15
CEC CLY DES NTO SOC bio02 bio04 bio05 bio13 bio15
CEC CLY DES NTO SOC

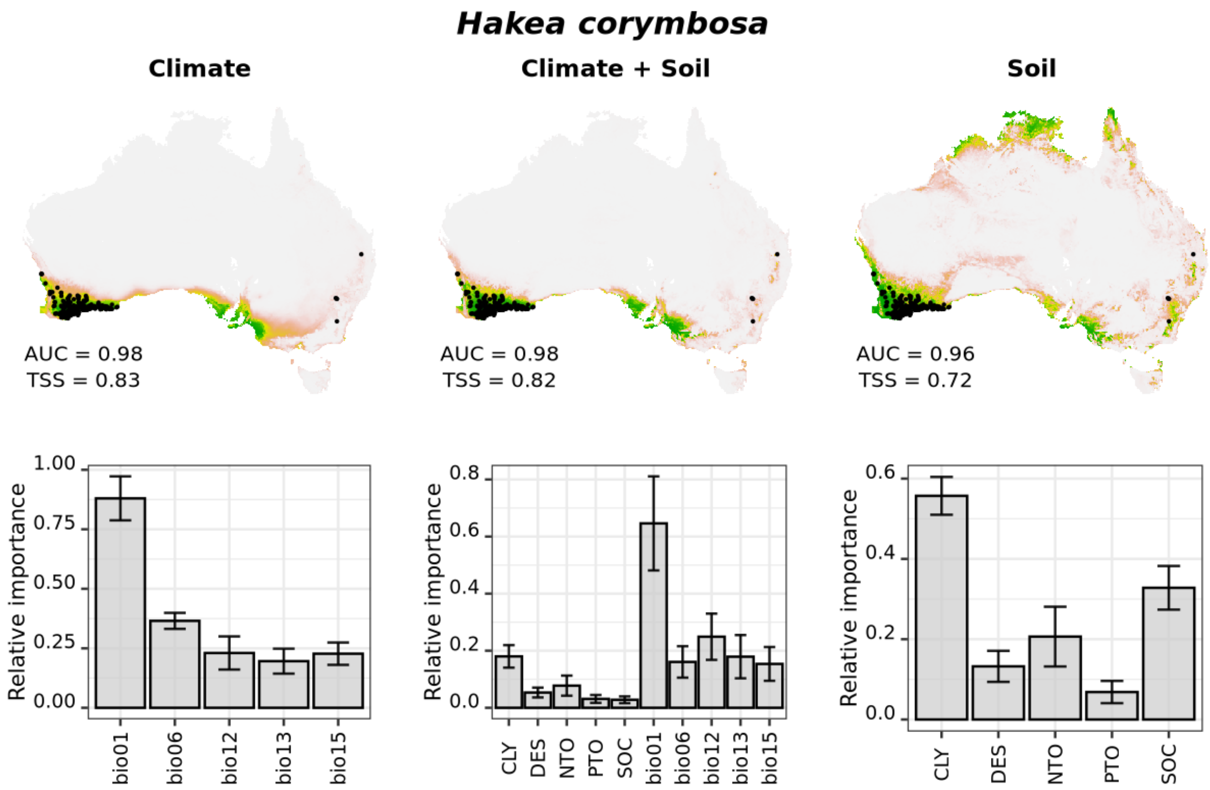
Hakea corymbosa
Climate
Climate + Soil
Soil
AUC = 0.98
TSS = 0.83
AUC = 0.98
TSS = 0.82
AUC = 0.96
TSS = 0.72
Relative importance
1.00
0.75
0.50
0.25
0.00
bio01
bio06
bio12
bio13
bio15
0.8
0.6
0.4
0.2
0.0
CLY
DES
NTO
PTO
SOC
bio01
bio06
bio12
bio13
bio15
0.6
0.4
0.2
0.0
CLY
DES
NTO
PTO
SOC

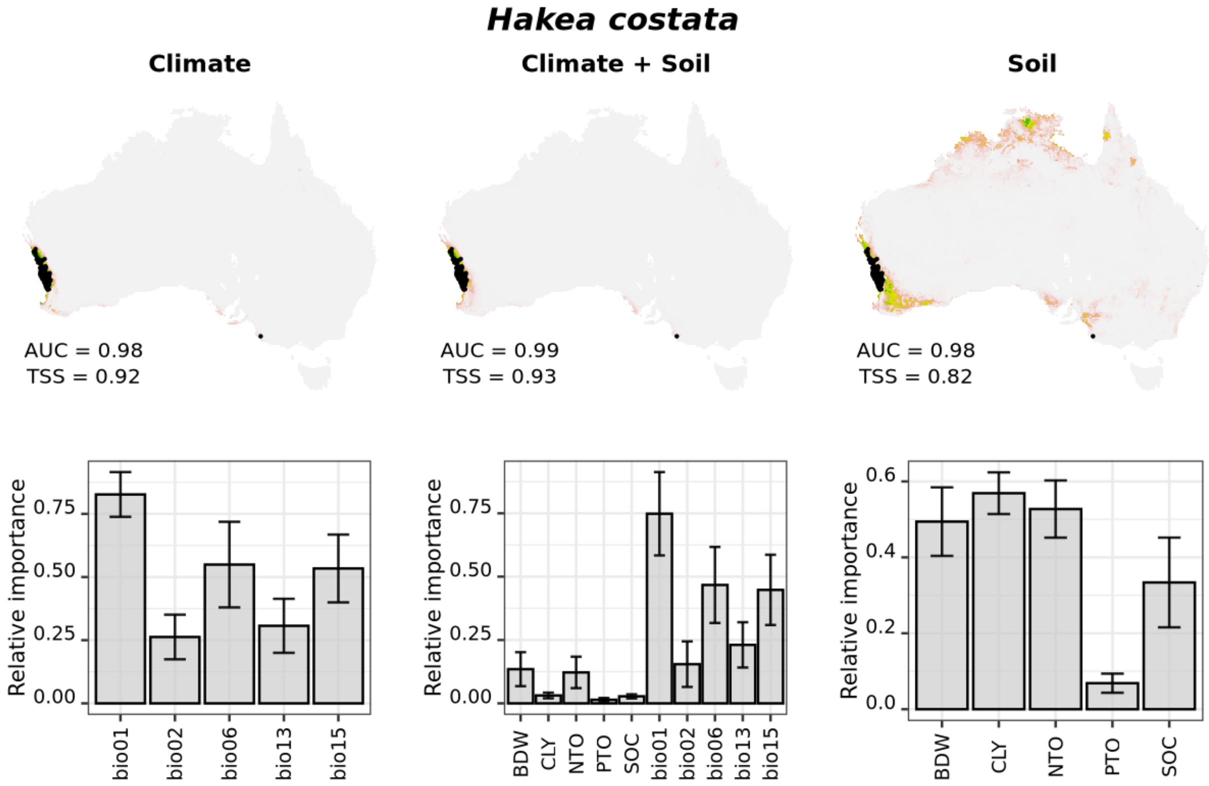
Hakea costata
Climate
Climate + Soil
Soil
AUC = 0.98
TSS = 0.92
AUC = 0.99
TSS = 0.93
AUC = 0.98
TSS = 0.82
Relative importance
0.75
0.50
0.25
0.00
bio01
bio02
bio06
bio13
bio15
0.75
0.50
0.25
0.00
BDW
CLY
NTO
PTO
SOC
bio01
bio02
bio06
bio13
bio15
0.6
0.4
0.2
0.0
BDW
CLY
NTO
PTO
SOC

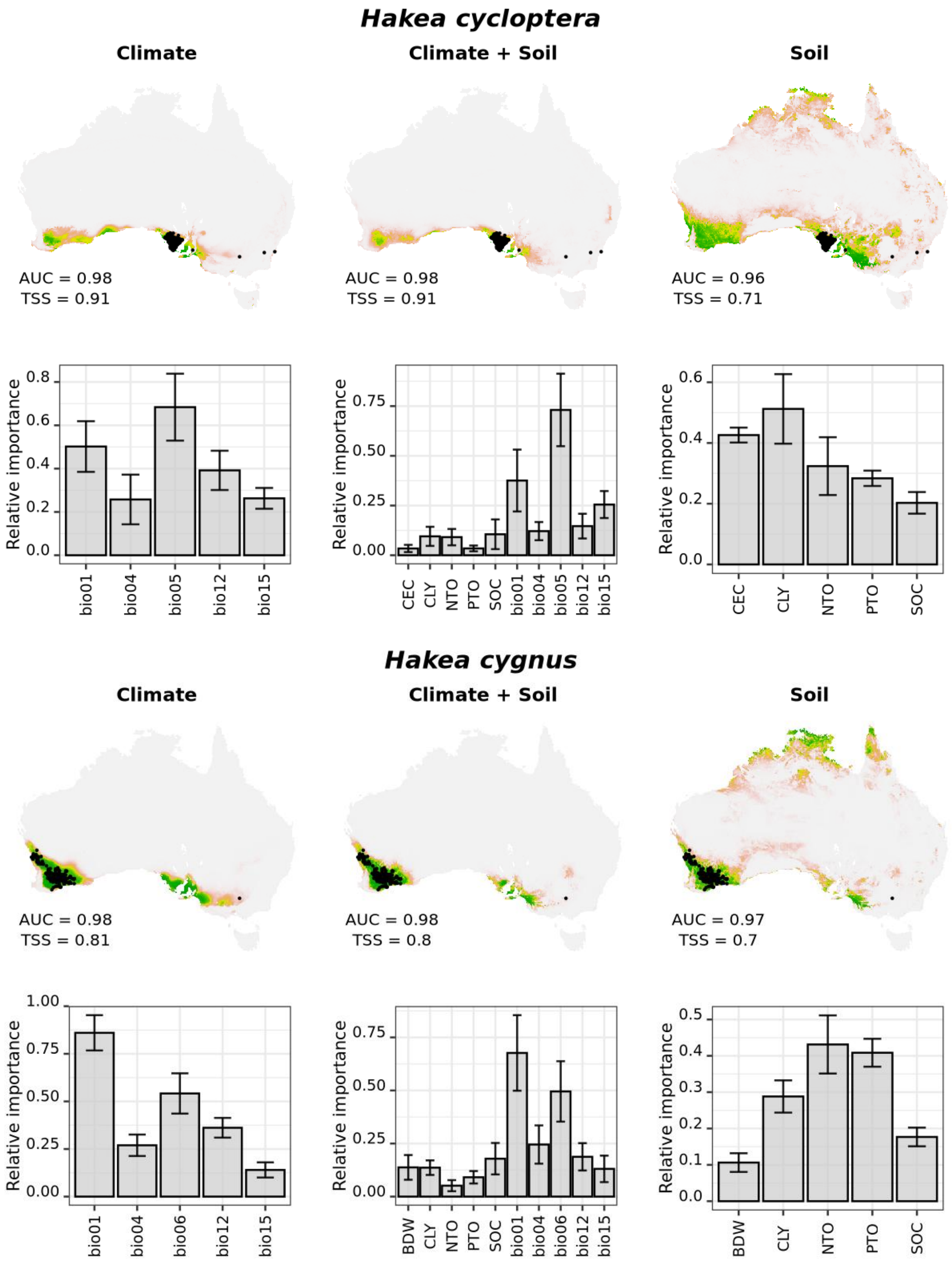
Hakea cycloptera
Climate
Climate + Soil
Soil
AUC = 0.98
TSS = 0.91
AUC = 0.98
TSS = 0.91
AUC = 0.96
TSS = 0.71
Relative importance
bio01
bio04
bio05
bio12
bio15
CEC
CLY
NTO
PTO
SOC
Hakea cygnus
Climate
Climate + Soil
Soil
AUC = 0.98
TSS = 0.81
AUC = 0.98
TSS = 0.8
AUC = 0.97
TSS = 0.7
Relative importance
bio01
bio04
bio06
bio12
bio15
BDW
CLY
NTO
PTO
SOC

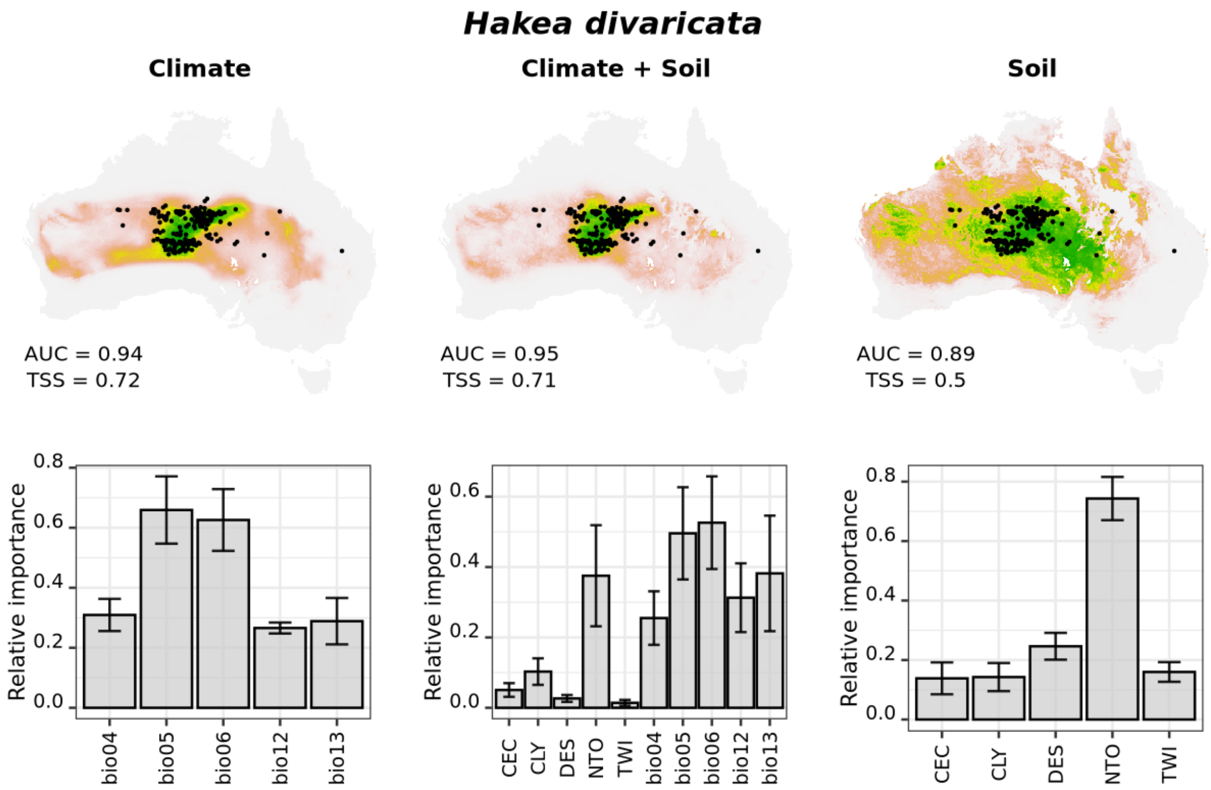

Hakea divaricata
Climate
Climate + Soil
Soil
AUC = 0.94
TSS = 0.72
AUC = 0.95
TSS = 0.71
AUC = 0.89
TSS = 0.5
Relative importance
0.0 0.2 0.4 0.6 0.8
bio04 bio05 bio06 bio12 bio13
0.0 0.2 0.4 0.6
CEC CLY DES NTO TWI bio04 bio05 bio06 bio12 bio13
0.0 0.2 0.4 0.6 0.8
CEC CLY DES NTO TWI

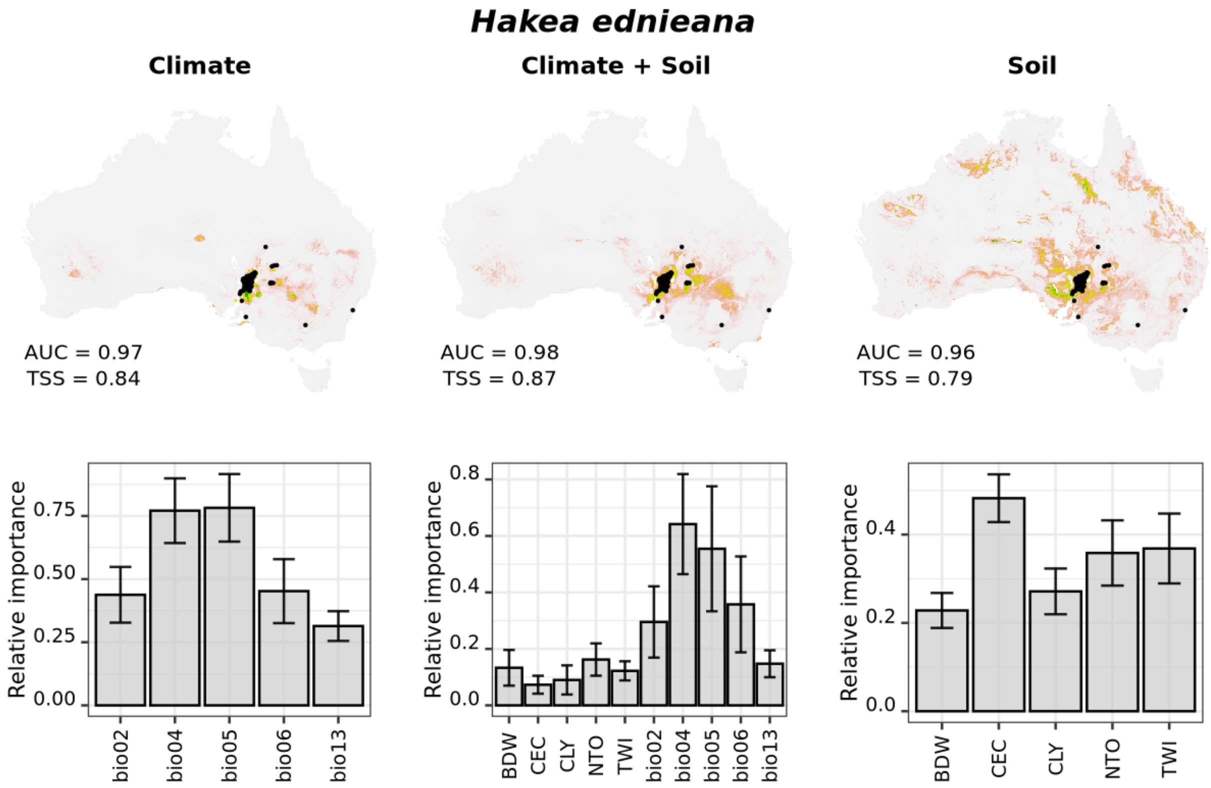

Hakea ednieana
Climate
Climate + Soil
Soil
AUC = 0.97
TSS = 0.84
AUC = 0.98
TSS = 0.87
AUC = 0.96
TSS = 0.79
Relative importance
0.00 0.25 0.50 0.75
bio02 bio04 bio05 bio06 bio13
0.0 0.2 0.4 0.6 0.8
BDW CEC CLY NTO TWI bio02 bio04 bio05 bio06 bio13
0.0 0.2 0.4
BDW CEC CLY NTO TWI

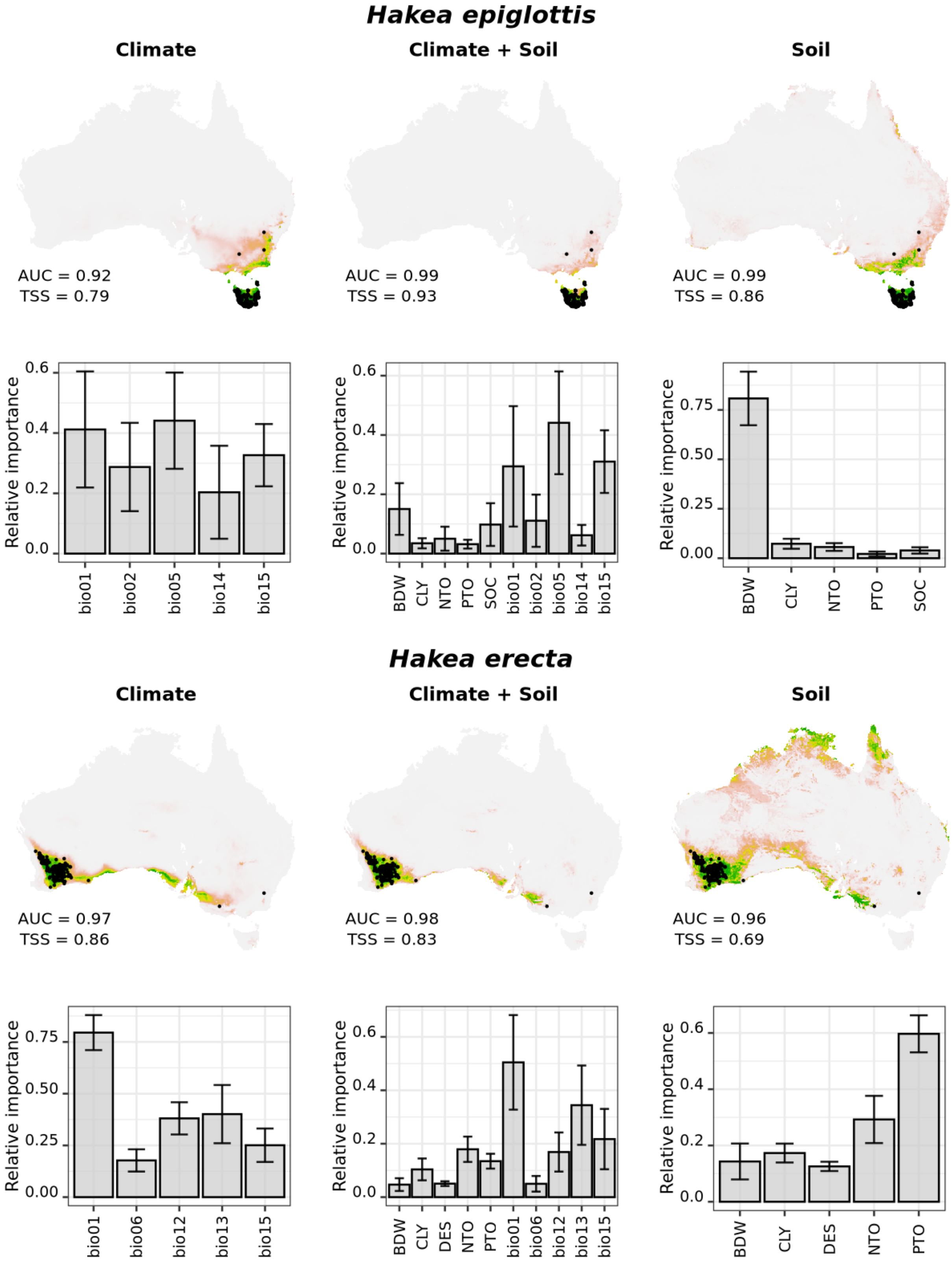
Hakea epiglottis
Climate
Climate + Soil
Soil
AUC = 0.92
TSS = 0.79
AUC = 0.99
TSS = 0.93
AUC = 0.99
TSS = 0.86
Relative importance
bio01 bio02 bio05 bio14 bio15
BDW CLY NTO PTO SOC bio01 bio02 bio05 bio14 bio15
BDW CLY NTO PTO SOC
Hakea erecta
Climate
Climate + Soil
Soil
AUC = 0.97
TSS = 0.86
AUC = 0.98
TSS = 0.83
AUC = 0.96
TSS = 0.69
Relative importance
bio01 bio06 bio12 bio13 bio15
BDW CLY DES NTO PTO bio01 bio06 bio12 bio13 bio15
BDW CLY DES NTO PTO

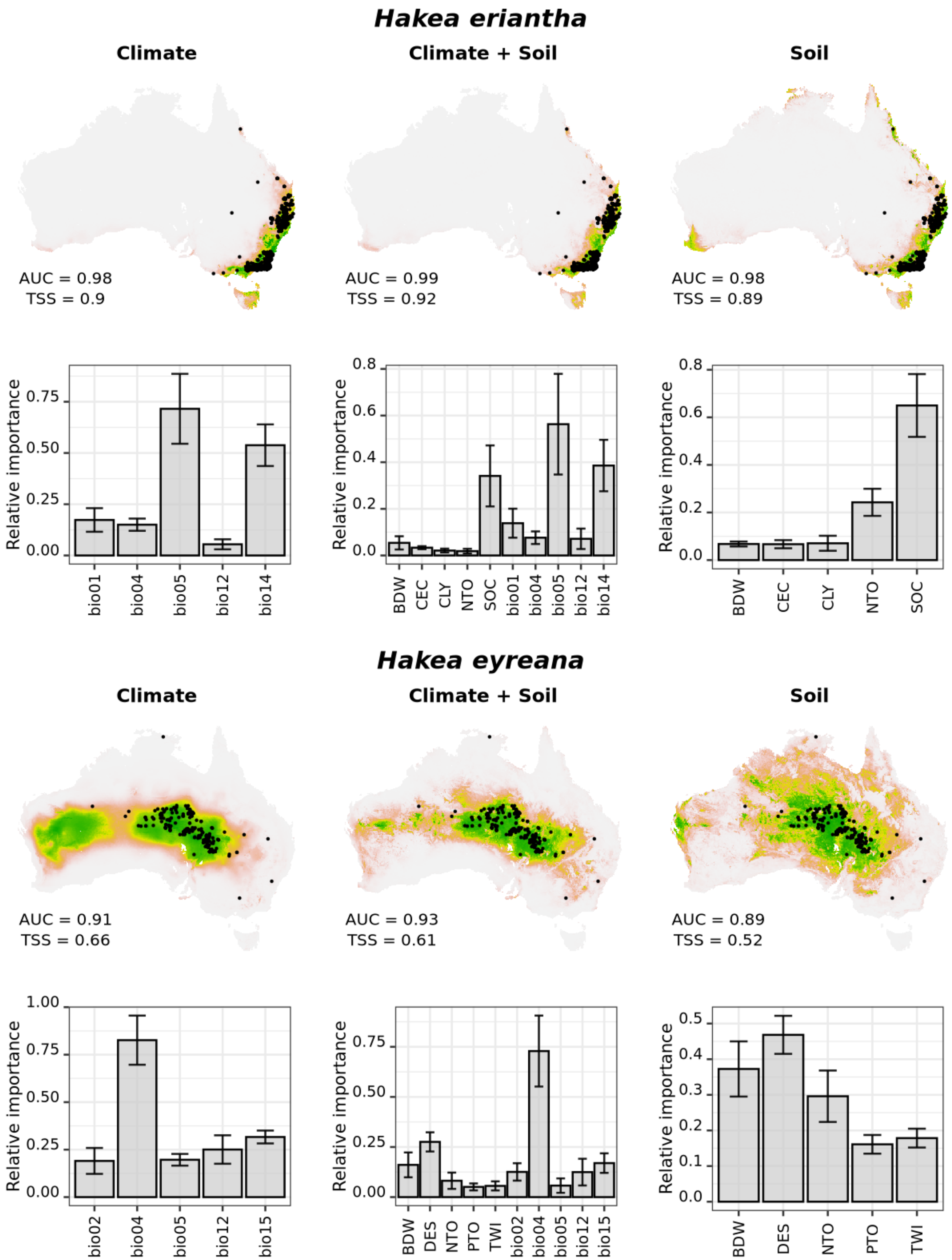
Hakea eriantha
Climate
Climate + Soil
Soil
AUC = 0.98
TSS = 0.9
AUC = 0.99
TSS = 0.92
AUC = 0.98
TSS = 0.89
Relative importance
bio01
bio04
bio05
bio12
bio14
BDW
CEC
CLY
NTO
SOC
Hakea eyreana
Climate
Climate + Soil
Soil
AUC = 0.91
TSS = 0.66
AUC = 0.93
TSS = 0.61
AUC = 0.89
TSS = 0.52
Relative importance
bio02
bio04
bio05
bio12
bio15
BDW
DES
NTO
PTO
TWI

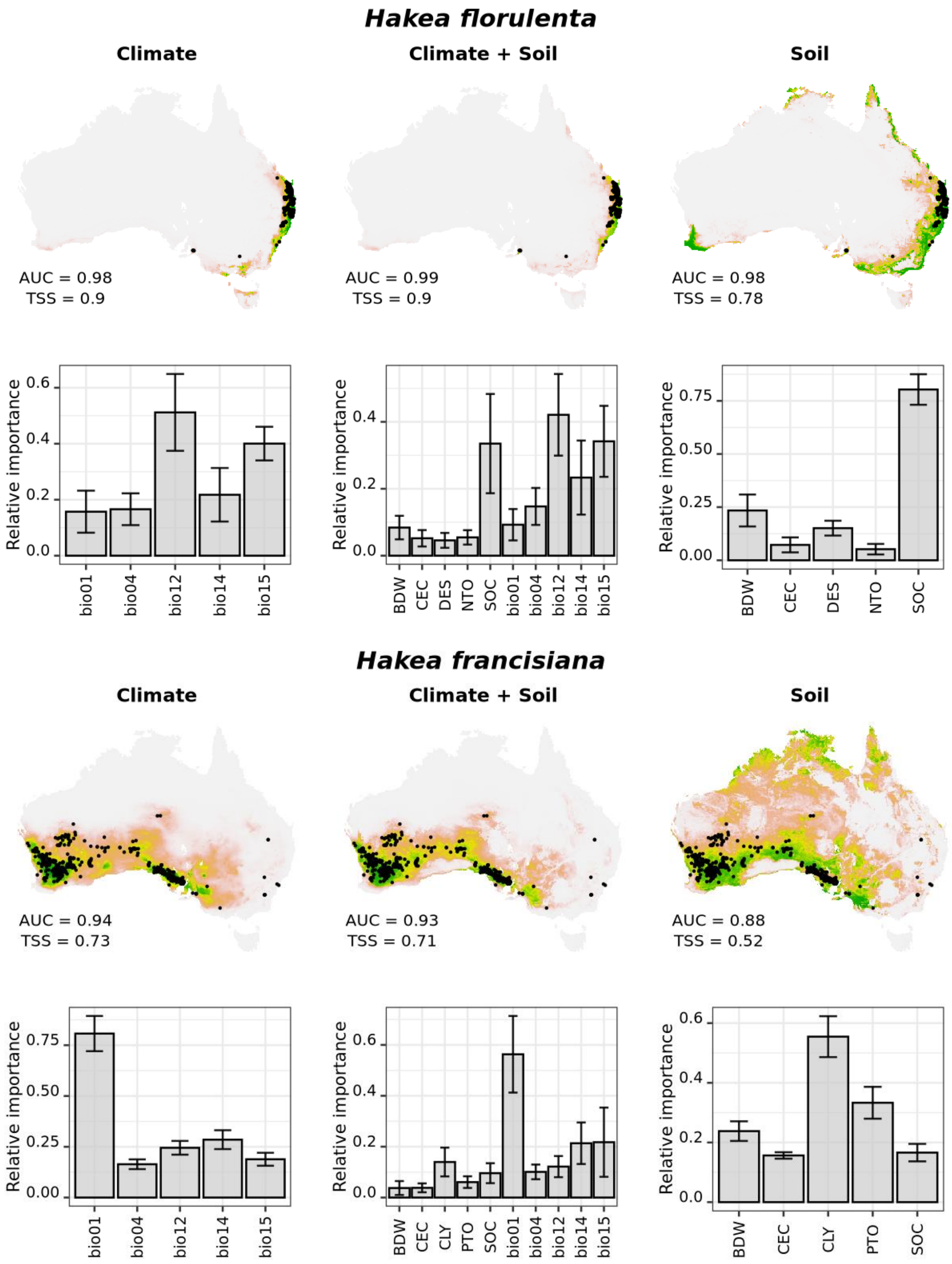
Hakea florulenta
Climate
Climate + Soil
Soil
AUC = 0.98
TSS = 0.9
AUC = 0.99
TSS = 0.9
AUC = 0.98
TSS = 0.78
Relative importance
bio01 bio04 bio12 bio14 bio15
BDW CEC DES NTO SOC bio01 bio04 bio12 bio14 bio15
BDW CEC DES NTO SOC
Hakea francisiana
Climate
Climate + Soil
Soil
AUC = 0.94
TSS = 0.73
AUC = 0.93
TSS = 0.71
AUC = 0.88
TSS = 0.52
Relative importance
bio01 bio04 bio12 bio14 bio15
BDW CEC CLY PTO SOC bio01 bio04 bio12 bio14 bio15
BDW CEC CLY PTO SOC

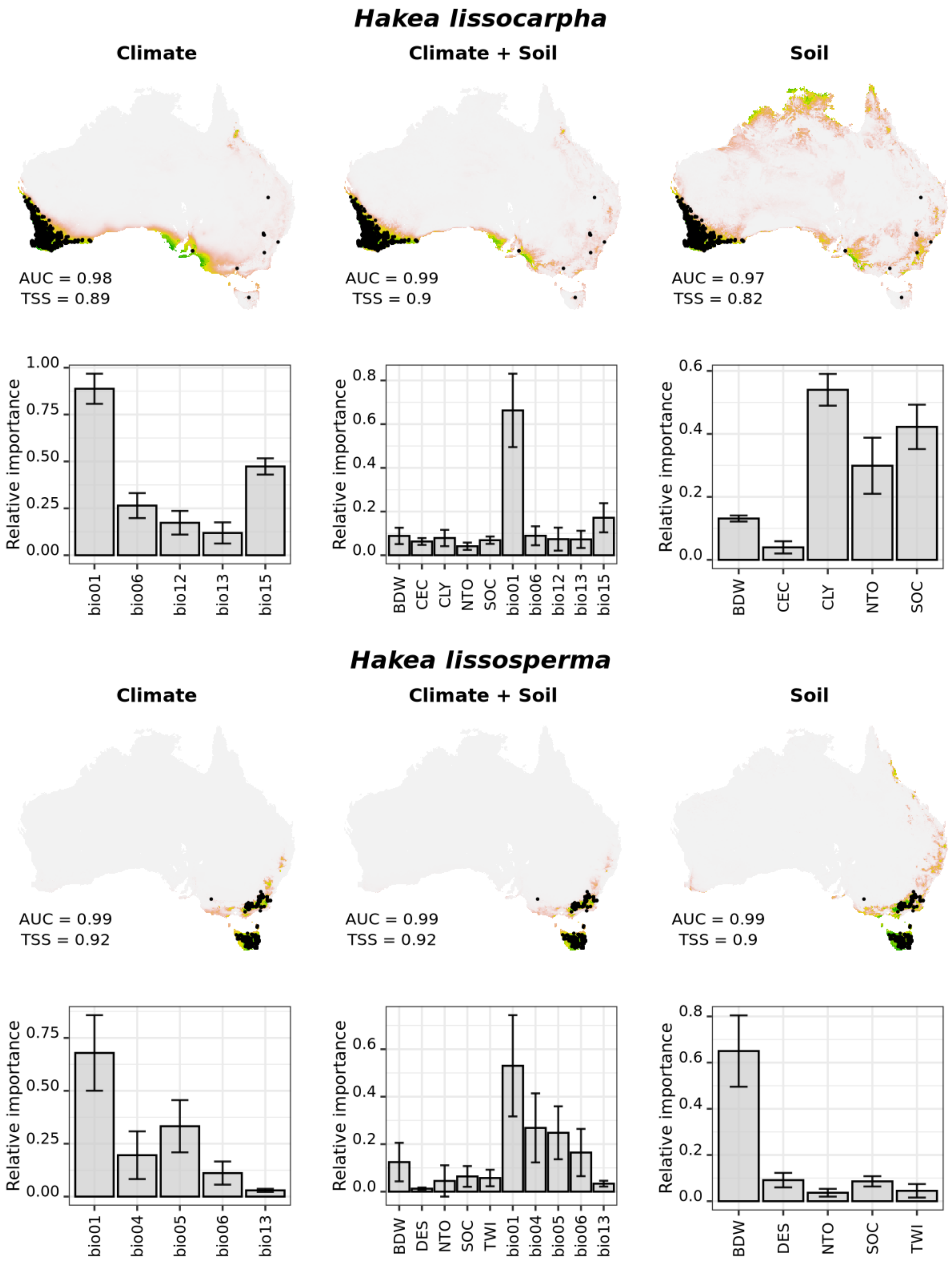
Hakea lissocarpha
Climate
Climate + Soil
Soil
AUC = 0.98
TSS = 0.89
AUC = 0.99
TSS = 0.9
AUC = 0.97
TSS = 0.82
Relative importance
1.00
0.75
0.50
0.25
0.00
bio01
bio06
bio12
bio13
bio15
0.8
0.6
0.4
0.2
0.0
BDW
CEC
CLY
NTO
SOC
bio01
bio06
bio12
bio13
bio15
0.6
0.4
0.2
0.0
BDW
CEC
CLY
NTO
SOC
Hakea lissosperma
Climate
Climate + Soil
Soil
AUC = 0.99
TSS = 0.92
AUC = 0.99
TSS = 0.92
AUC = 0.99
TSS = 0.9
Relative importance
0.75
0.50
0.25
0.00
bio01
bio04
bio05
bio06
bio13
0.6
0.4
0.2
0.0
BDW
DES
NTO
SOC
TWI
bio01
bio04
bio05
bio06
bio13
0.8
0.6
0.4
0.2
0.0
BDW
DES
NTO
SOC
TWI

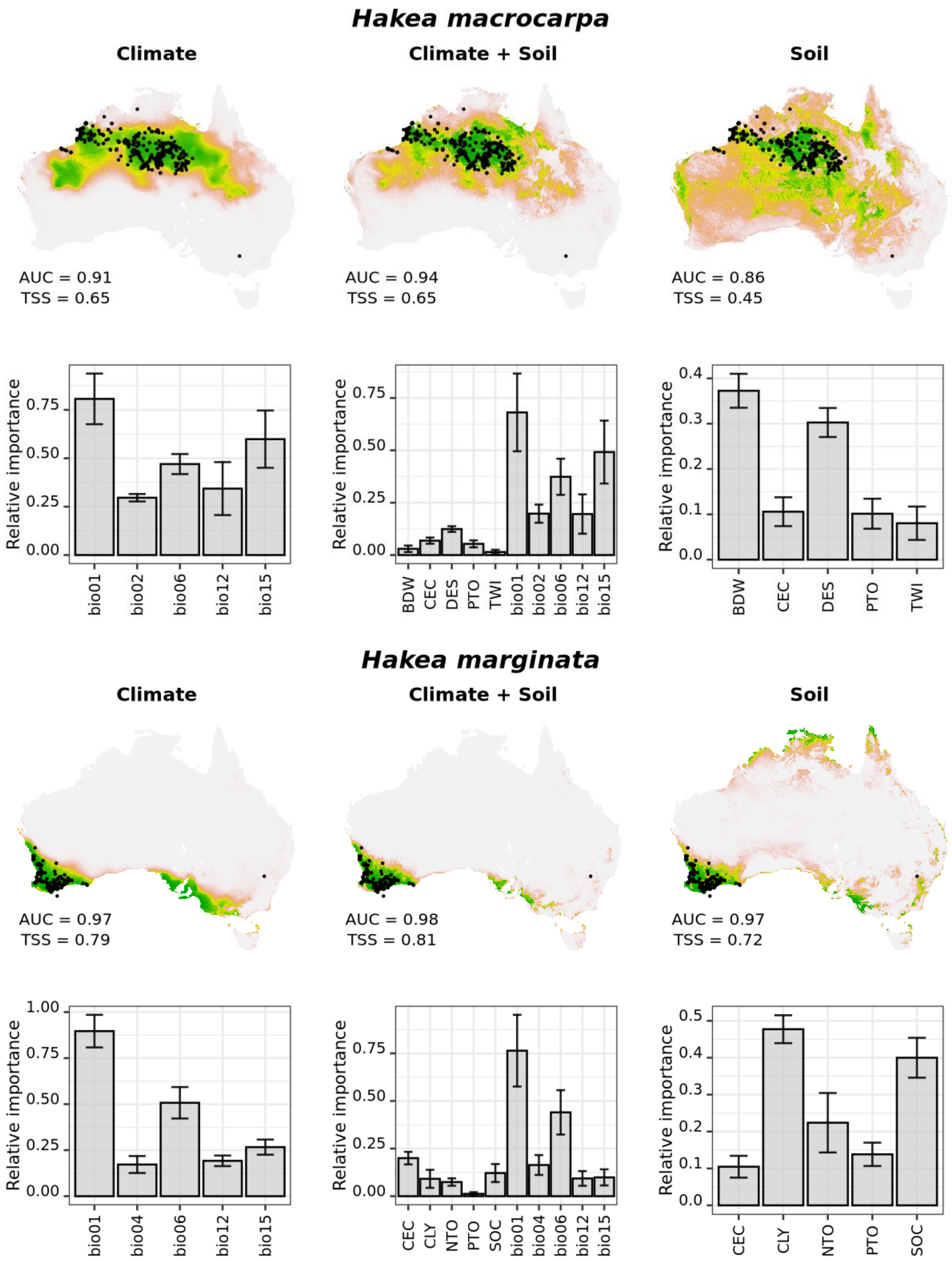
Hakea macrocarpa
Climate
Climate + Soil
Soil
AUC = 0.91
TSS = 0.65
AUC = 0.94
TSS = 0.65
AUC = 0.86
TSS = 0.45
Relative importance
0.00
0.25
0.50
0.75
bio01
bio02
bio06
bio12
bio15
BDW
CEC
DES
PTO
TWI
0.0
0.1
0.2
0.3
0.4
Hakea marginata
Climate
Climate + Soil
Soil
AUC = 0.97
TSS = 0.79
AUC = 0.98
TSS = 0.81
AUC = 0.97
TSS = 0.72
1.00
bio04
CLY
NTO
SOC
0.5

Hakea microcarpa

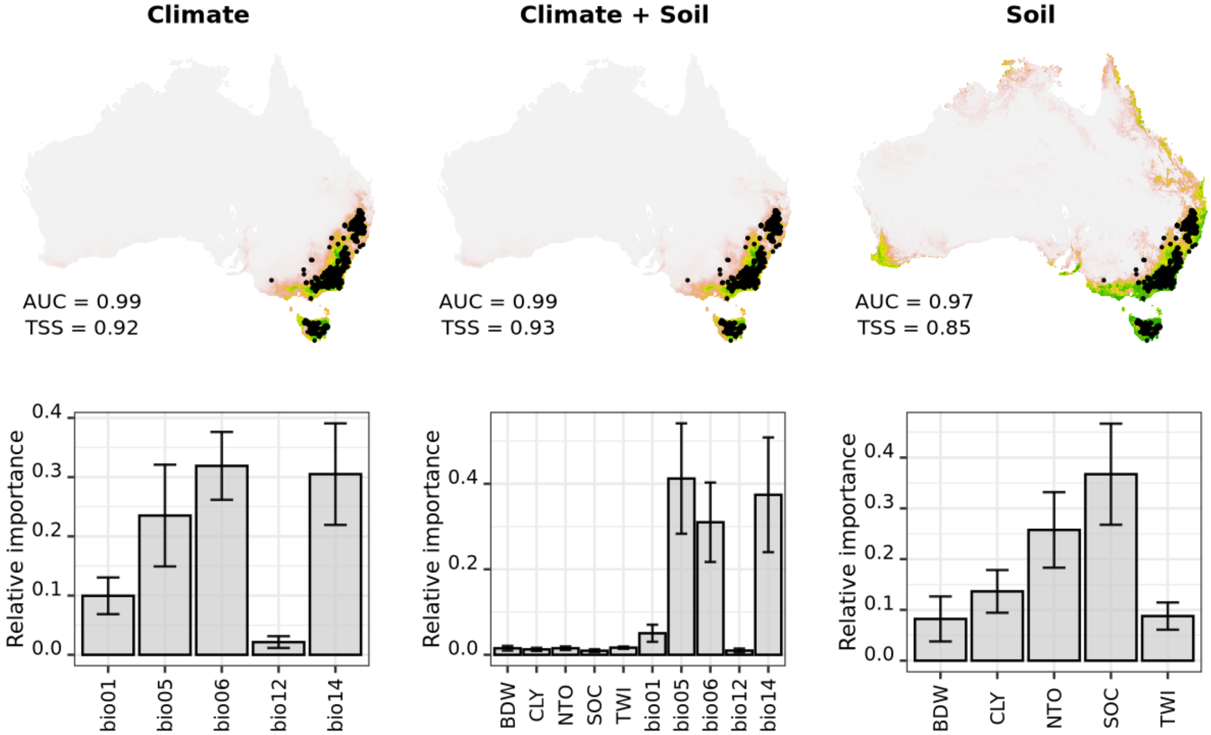

Hakea minyma

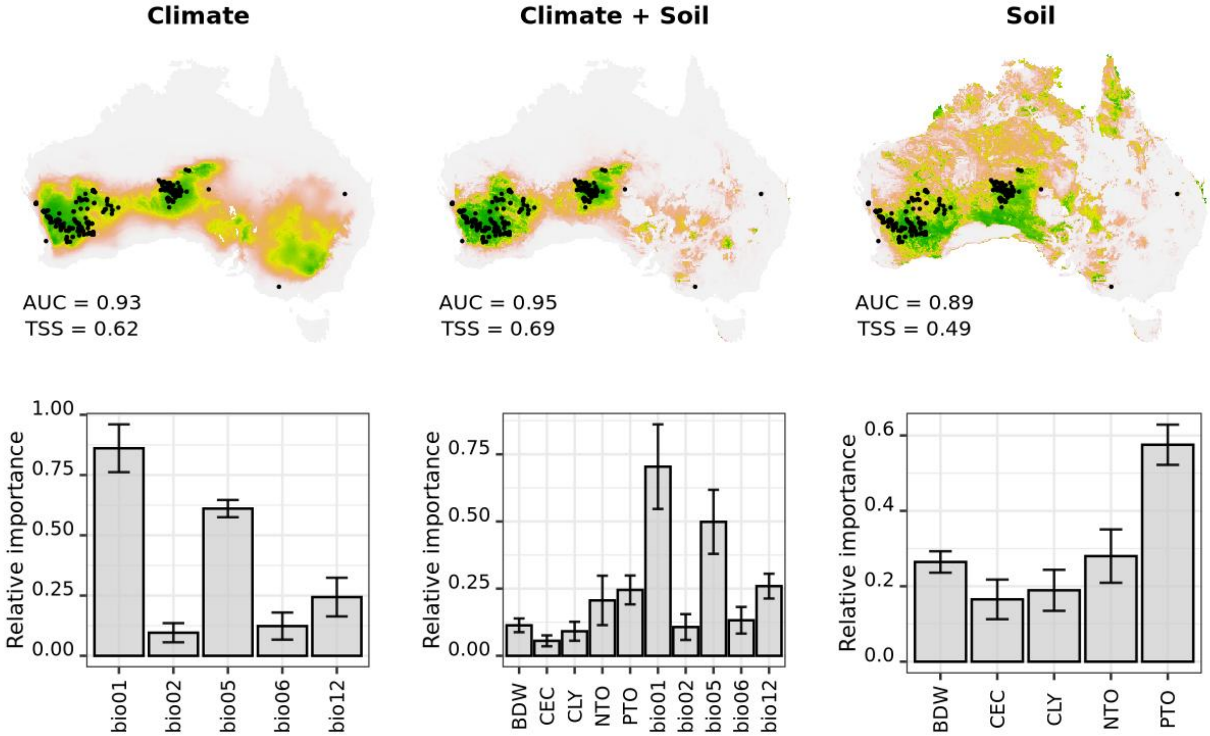

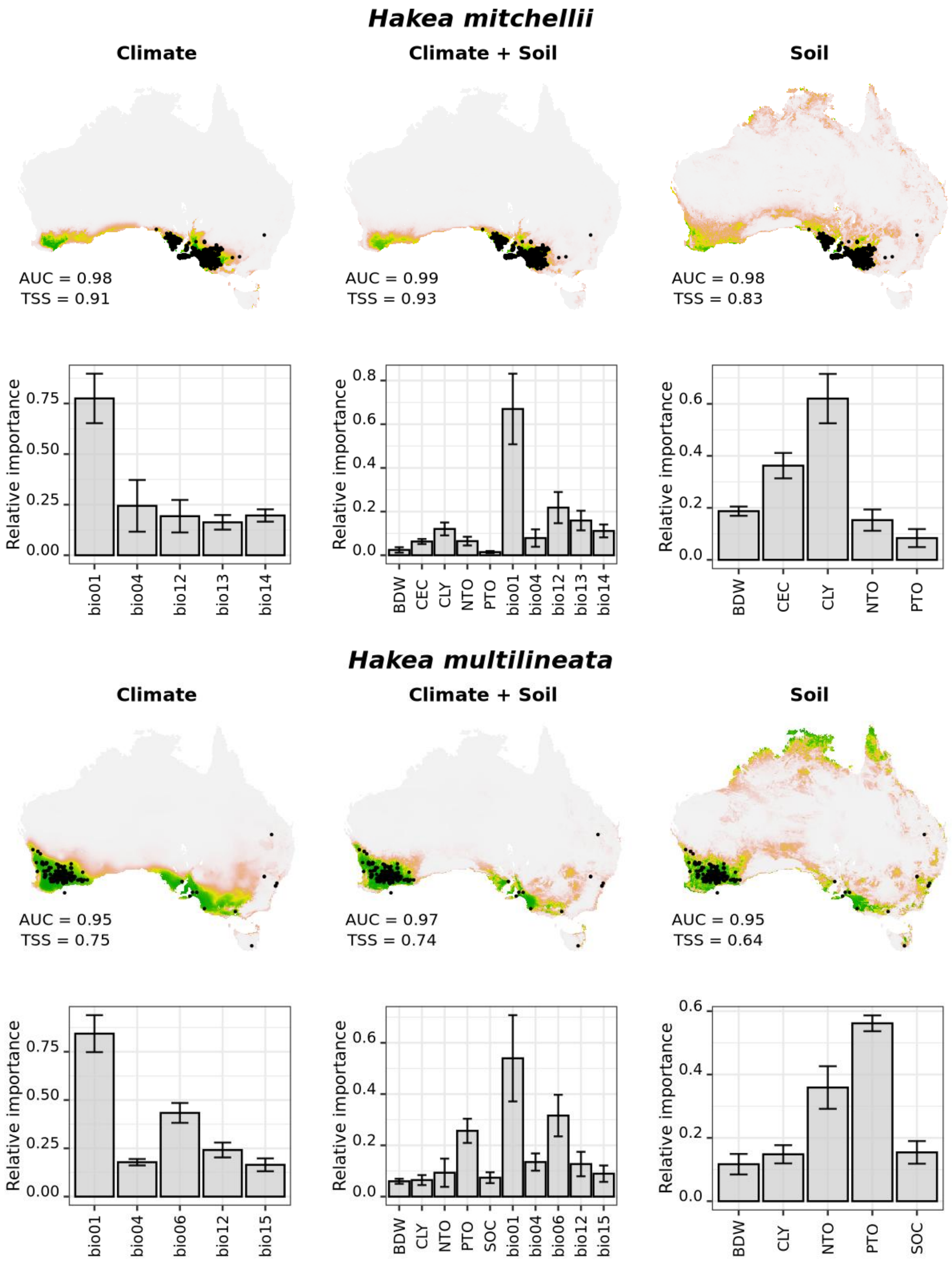

Hakea mitchellii
Climate
Climate + Soil
Soil
AUC = 0.98
TSS = 0.91
AUC = 0.99
TSS = 0.93
AUC = 0.98
TSS = 0.83
Relative importance
0.00
0.25
0.50
0.75
bio01
bio04
bio12
bio13
bio14
Relative importance
0.0
0.2
0.4
0.6
0.8
BDW
CEC
CLY
NTO
PTO
bio01
bio04
bio12
bio13
bio14
Relative importance
0.0
0.2
0.4
0.6
BDW
CEC
CLY
NTO
PTO
Hakea multilineata
Climate
Climate + Soil
Soil
AUC = 0.95
TSS = 0.75
AUC = 0.97
TSS = 0.74
AUC = 0.95
TSS = 0.64
Relative importance
0.00
0.25
0.50
0.75
bio01
bio04
bio06
bio12
bio15
Relative importance
0.0
0.2
0.4
0.6
BDW
CLY
NTO
PTO
SOC
bio01
bio04
bio06
bio12
bio15
Relative importance
0.0
0.2
0.4
0.6
BDW
CLY
NTO
PTO
SOC

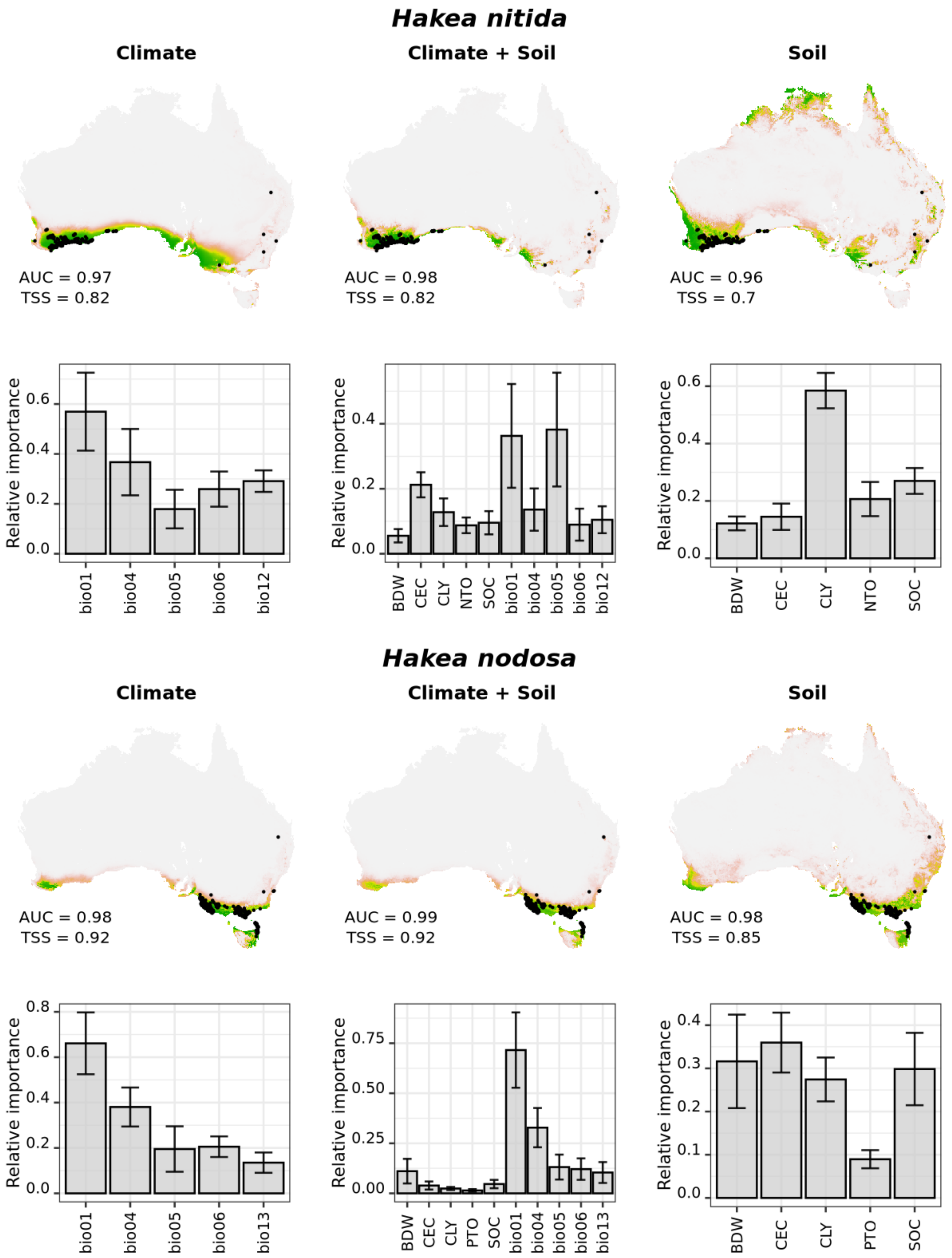
Hakea nitida
Climate
Climate + Soil
Soil
AUC = 0.97
TSS = 0.82
AUC = 0.98
TSS = 0.82
AUC = 0.96
TSS = 0.7
Relative importance
bio01
bio04
bio05
bio06
bio12
BDW
CEC
CLY
NTO
SOC
Hakea nodosa
Climate
Climate + Soil
Soil
AUC = 0.98
TSS = 0.92
AUC = 0.99
TSS = 0.92
AUC = 0.98
TSS = 0.85
Relative importance
bio01
bio04
bio05
bio06
bio13
BDW
CEC
CLY
PTO
SOC

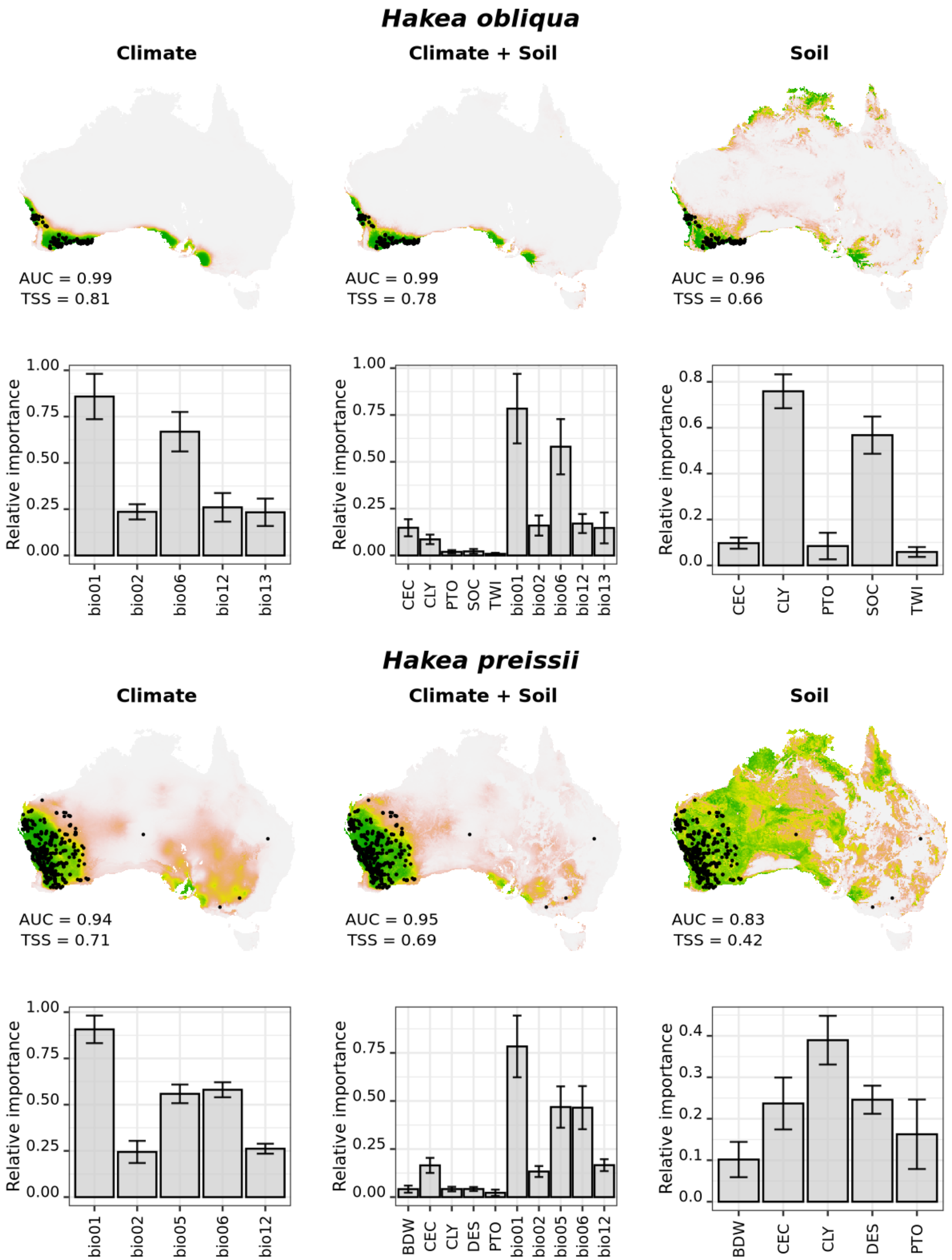
Hakea obliqua
Climate
Climate + Soil
Soil
AUC = 0.99
TSS = 0.81
AUC = 0.99
TSS = 0.78
AUC = 0.96
TSS = 0.66
Relative importance
0.00
0.25
0.50
0.75
1.00
bio01
bio02
bio06
bio12
bio13
CEC
CLY
PTO
SOC
TWI
0.0
0.2
0.4
0.6
0.8
Hakea preissii
Climate
Climate + Soil
Soil
AUC = 0.94
TSS = 0.71
AUC = 0.95
TSS = 0.69
AUC = 0.83
TSS = 0.42
bio05
BDW
DES
0.1
0.3

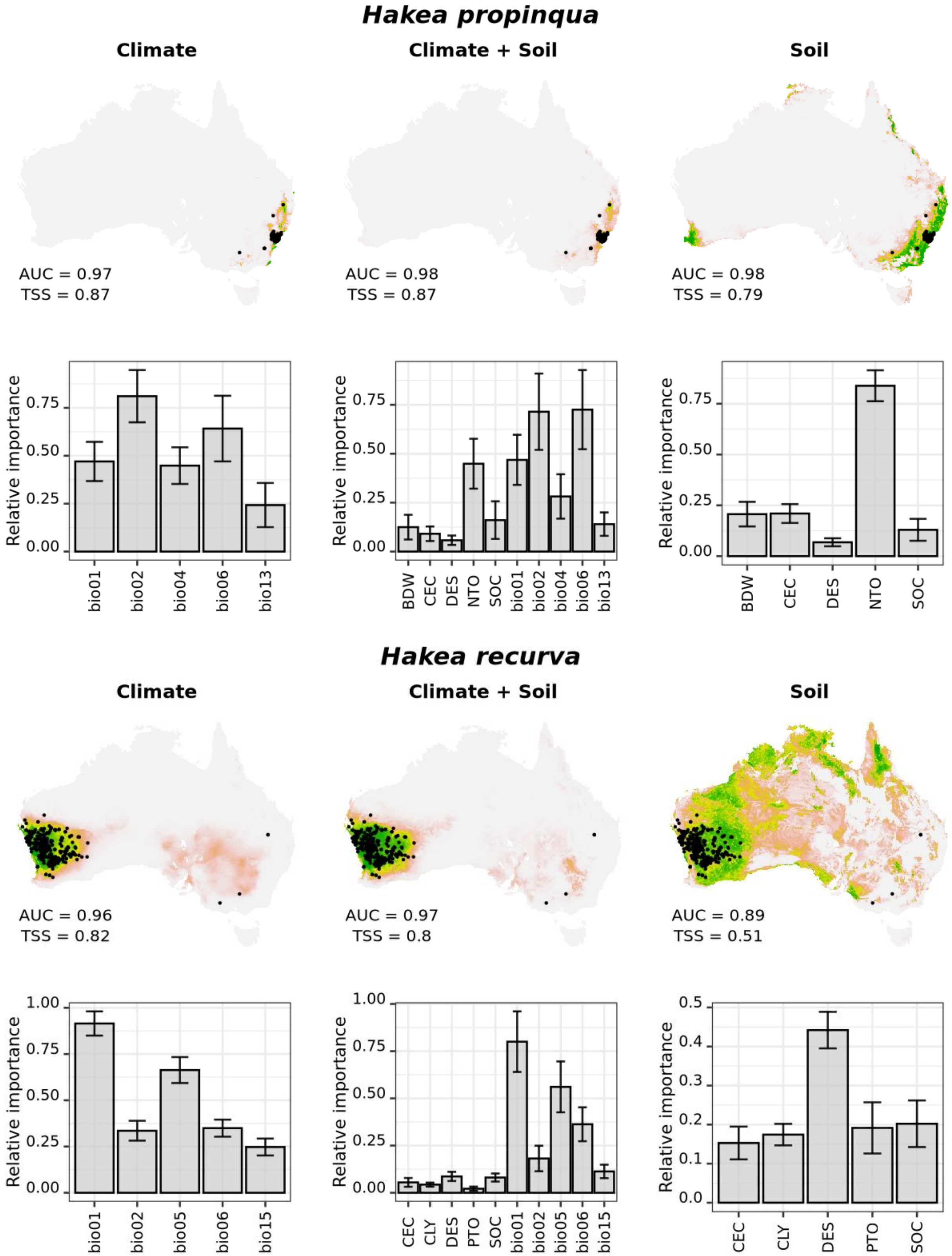

Hakea propinqua
Climate
Climate + Soil
Soil
AUC = 0.97
TSS = 0.87
AUC = 0.98
TSS = 0.87
AUC = 0.98
TSS = 0.79
Relative importance
bio01 bio02 bio04 bio06 bio13
BDW CEC DES NTO SOC bio01 bio02 bio04 bio06 bio13
BDW CEC DES NTO SOC
Hakea recurva
Climate
Climate + Soil
Soil
AUC = 0.96
TSS = 0.82
AUC = 0.97
TSS = 0.8
AUC = 0.89
TSS = 0.51
Relative importance
bio01 bio02 bio05 bio06 bio15
CEC CLY DES PTO SOC bio01 bio02 bio05 bio06 bio15
CEC CLY DES PTO SOC

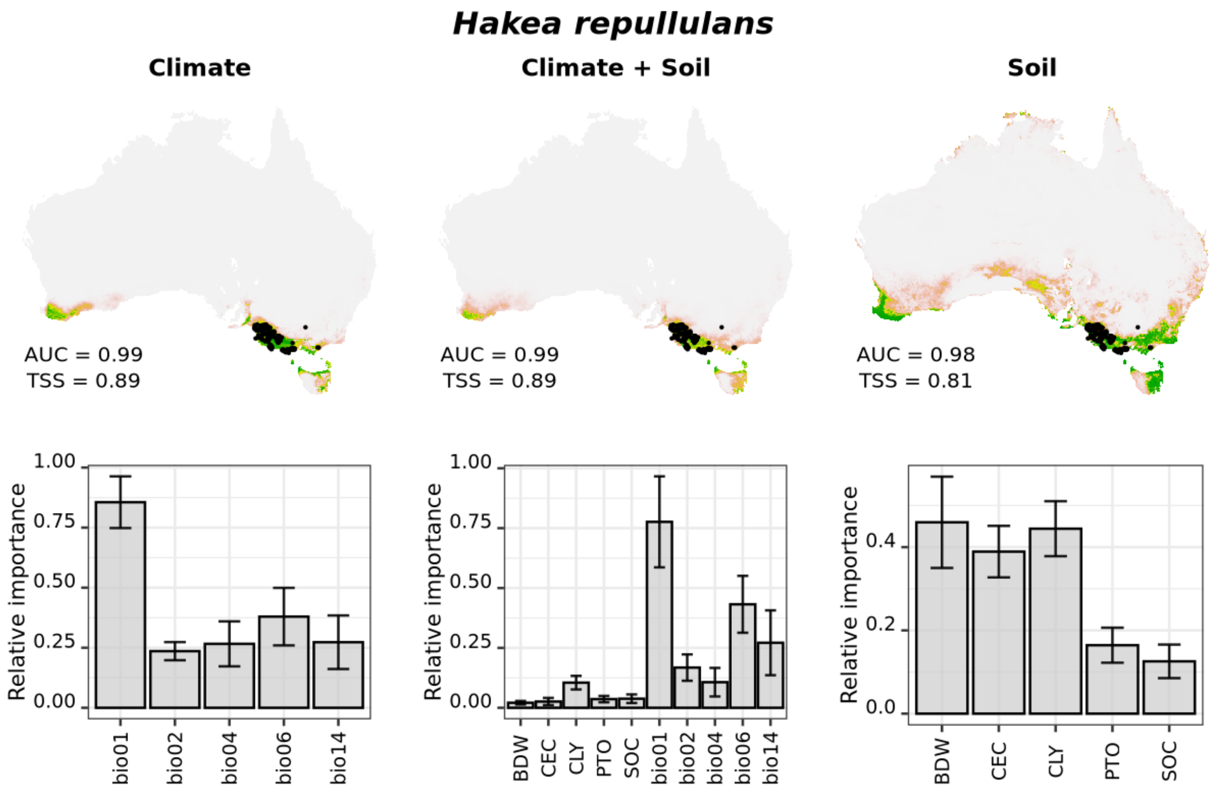
Hakea repullulans
Climate
Climate + Soil
Soil
AUC = 0.99
TSS = 0.89
AUC = 0.99
TSS = 0.89
AUC = 0.98
TSS = 0.81
Relative importance
1.00
0.75
0.50
0.25
0.00
bio01
bio02
bio04
bio06
bio14
BDW
CEC
CLY
PTO
SOC
0.4
0.2
0.0

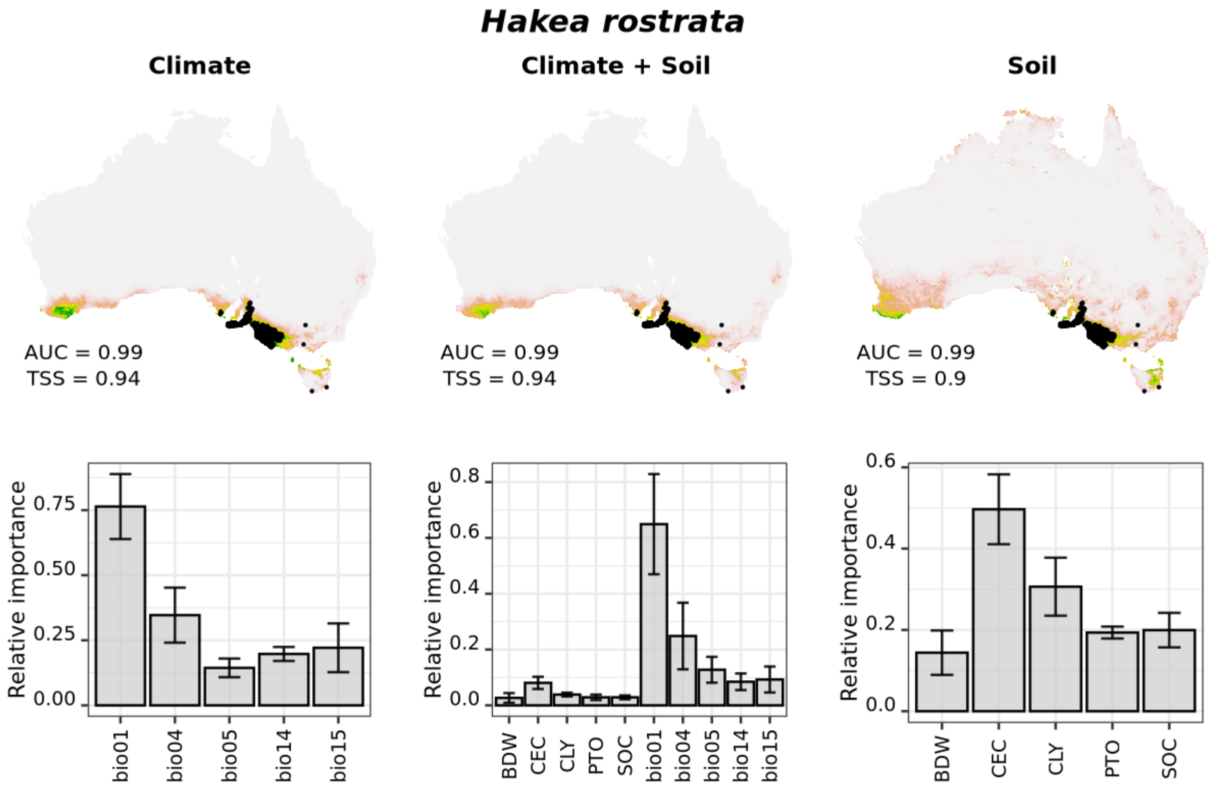
Hakea rostrata
Climate
Climate + Soil
Soil
AUC = 0.99
TSS = 0.94
AUC = 0.99
TSS = 0.94
AUC = 0.99
TSS = 0.9
Relative importance
0.75
0.50
0.25
0.00
bio01
bio04
bio05
bio14
bio15
0.8
0.6
0.4
0.2
0.0
BDW
CEC
CLY
PTO
SOC

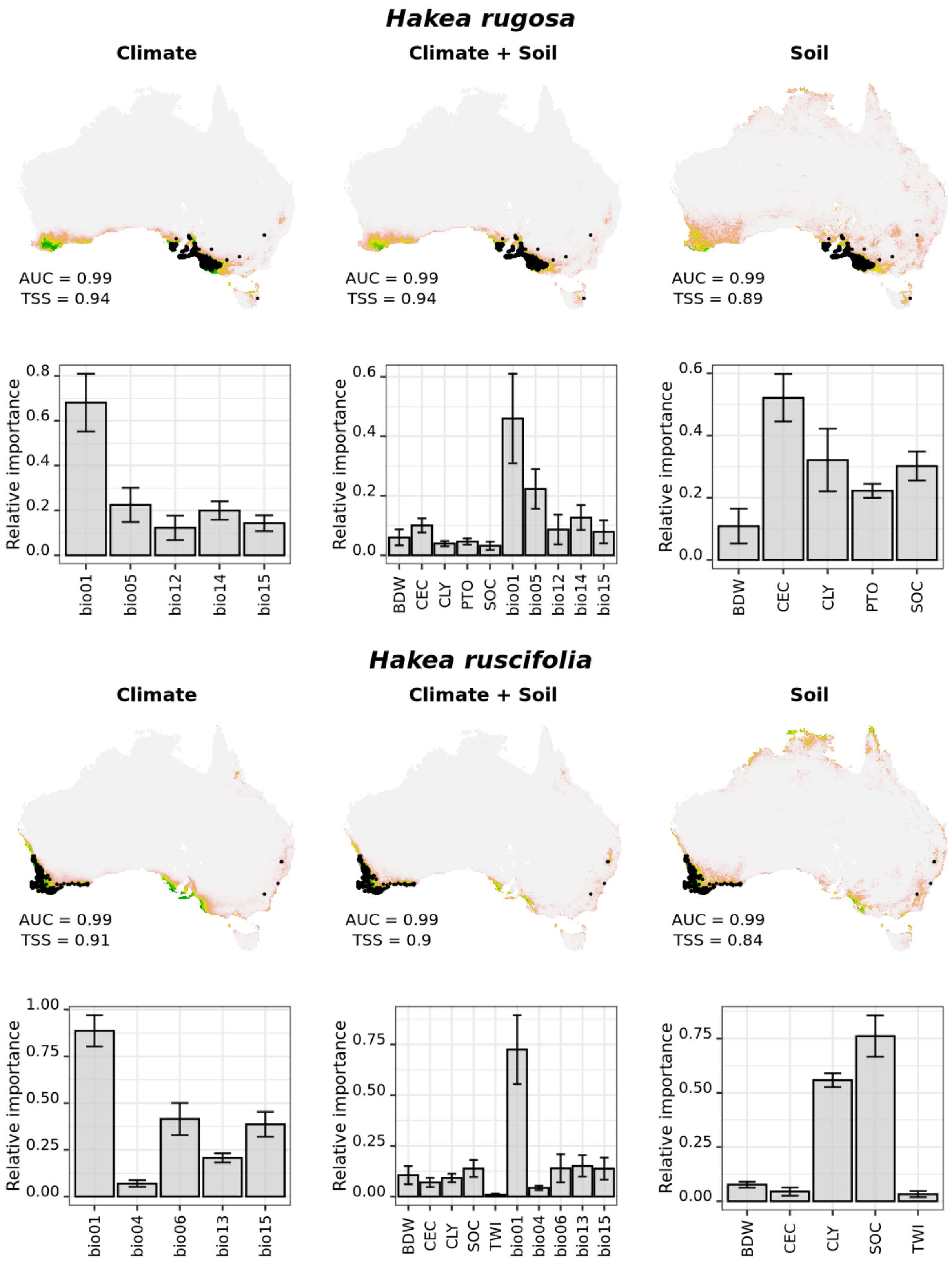
Hakea rugosa
Climate
Climate + Soil
Soil
AUC = 0.99
TSS = 0.94
AUC = 0.99
TSS = 0.94
AUC = 0.99
TSS = 0.89
Relative importance
bio01
bio05
bio12
bio14
bio15
BDW
CEC
CLY
PTO
SOC
Hakea ruscifolia
Climate
Climate + Soil
Soil
AUC = 0.99
TSS = 0.91
AUC = 0.99
TSS = 0.9
AUC = 0.99
TSS = 0.84
bio04
bio06
bio13
TWI

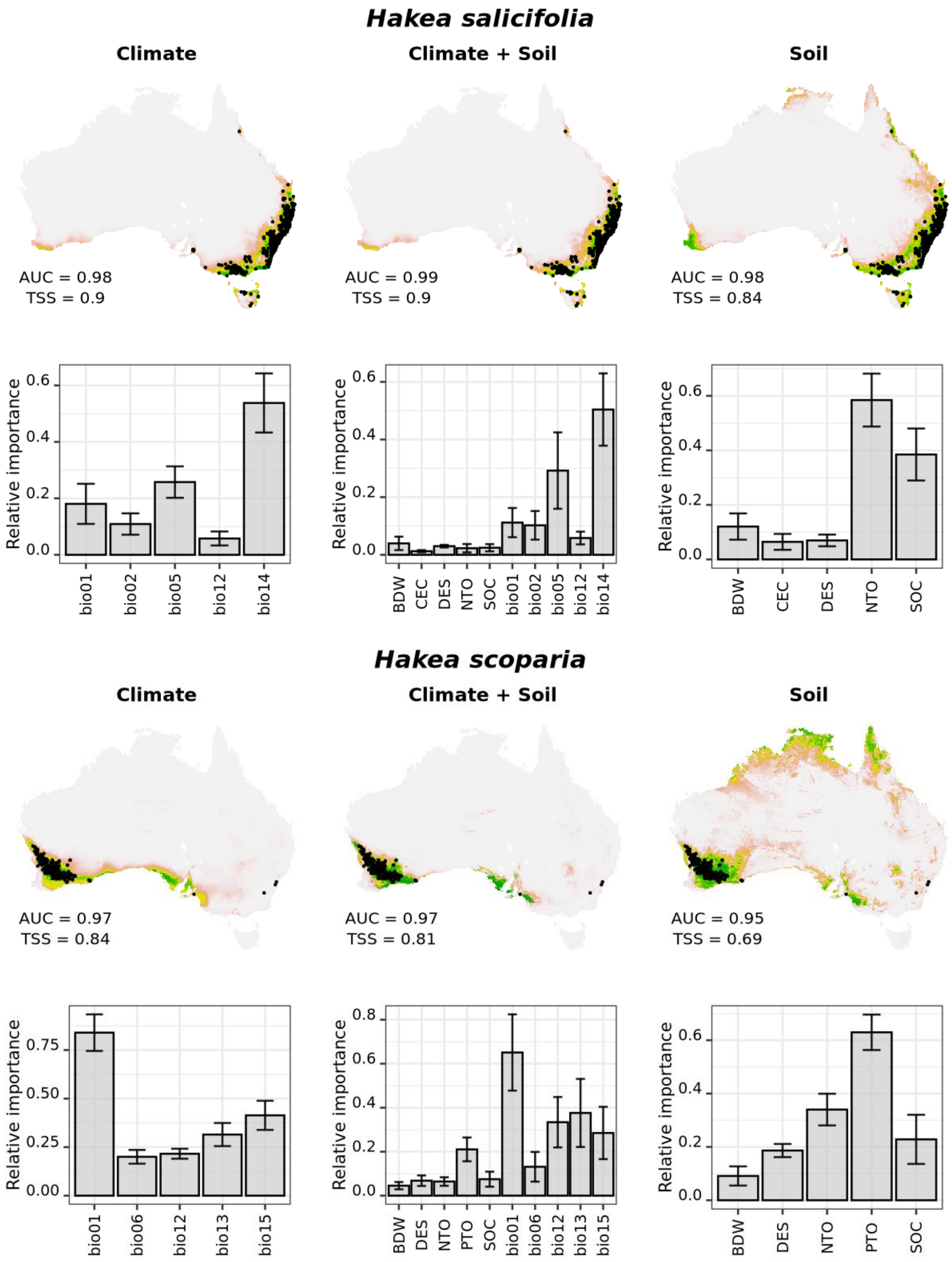
Hakea salicifolia
Climate
Climate + Soil
Soil
AUC = 0.98
TSS = 0.9
AUC = 0.99
TSS = 0.9
AUC = 0.98
TSS = 0.84
Relative importance
bio01 bio02 bio05 bio12 bio14
BDW CEC DES NTO SOC bio01 bio02 bio05 bio12 bio14
BDW CEC DES NTO SOC
Hakea scoparia
Climate
Climate + Soil
Soil
AUC = 0.97
TSS = 0.84
AUC = 0.97
TSS = 0.81
AUC = 0.95
TSS = 0.69
Relative importance
bio01 bio06 bio12 bio13 bio15
BDW DES NTO PTO SOC bio01 bio06 bio12 bio13 bio15
BDW DES NTO PTO SOC

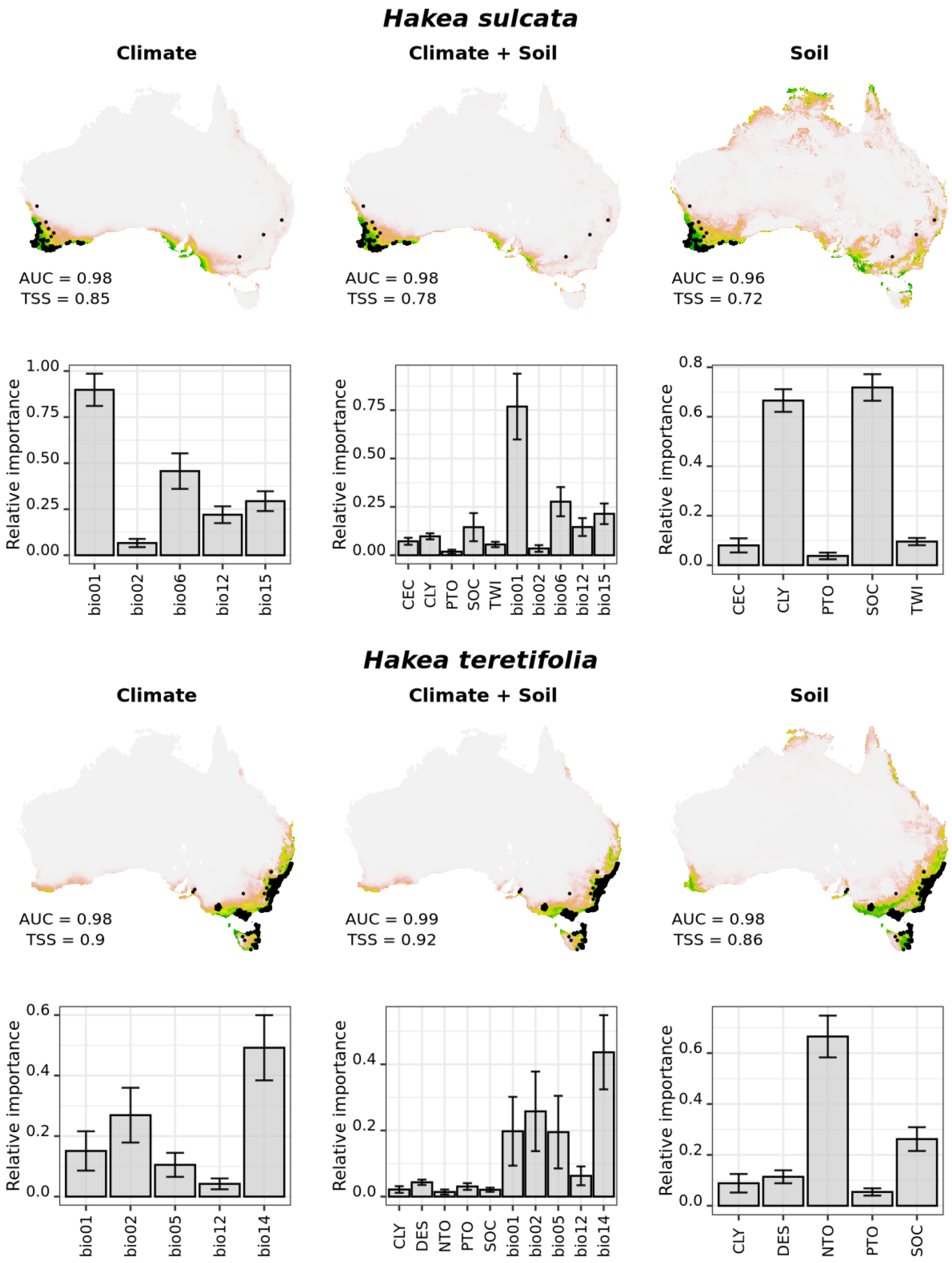
Hakea sulcata
Climate
Climate + Soil
Soil
AUC = 0.98
TSS = 0.85
AUC = 0.98
TSS = 0.78
AUC = 0.96
TSS = 0.72
Relative importance
bio01
bio02
bio06
bio12
bio15
CEC
CLY
PTO
SOC
TWI
Hakea teretifolia
Climate
Climate + Soil
Soil
AUC = 0.98
TSS = 0.9
AUC = 0.99
TSS = 0.92
AUC = 0.98
TSS = 0.86
Relative importance
bio01
bio02
bio05
bio12
bio14
CLY
DES
NTO
PTO
SOC

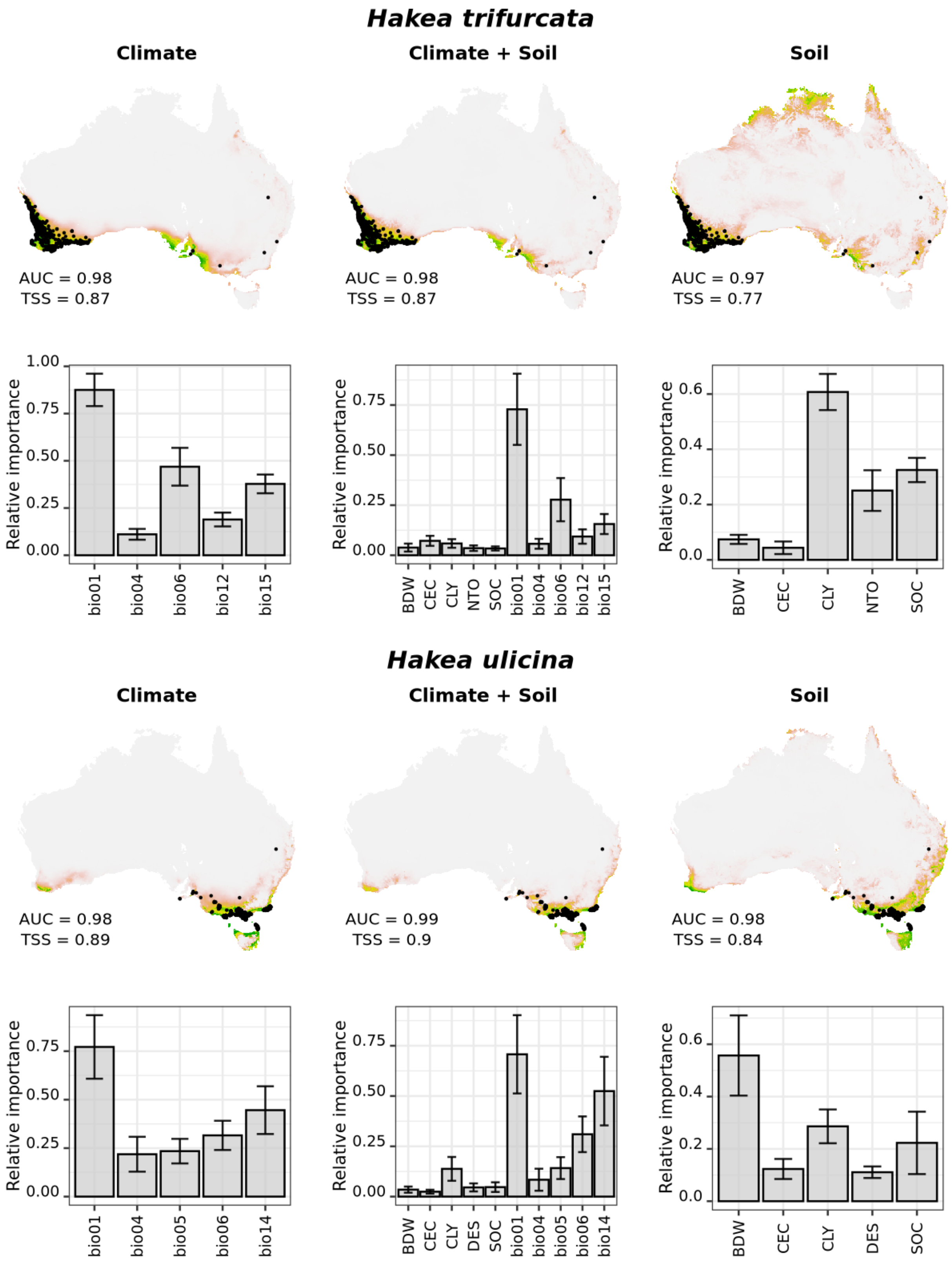

Hakea trifurcata
Climate
Climate + Soil
Soil
AUC = 0.98
TSS = 0.87
AUC = 0.98
TSS = 0.87
AUC = 0.97
TSS = 0.77
Relative importance
bio01
bio04
bio06
bio12
bio15
BDW
CEC
CLY
NTO
SOC
Hakea ulicina
Climate
Climate + Soil
Soil
AUC = 0.98
TSS = 0.89
AUC = 0.99
TSS = 0.9
AUC = 0.98
TSS = 0.84
Relative importance
bio01
bio04
bio05
bio06
bio14
BDW
CEC
CLY
DES
SOC

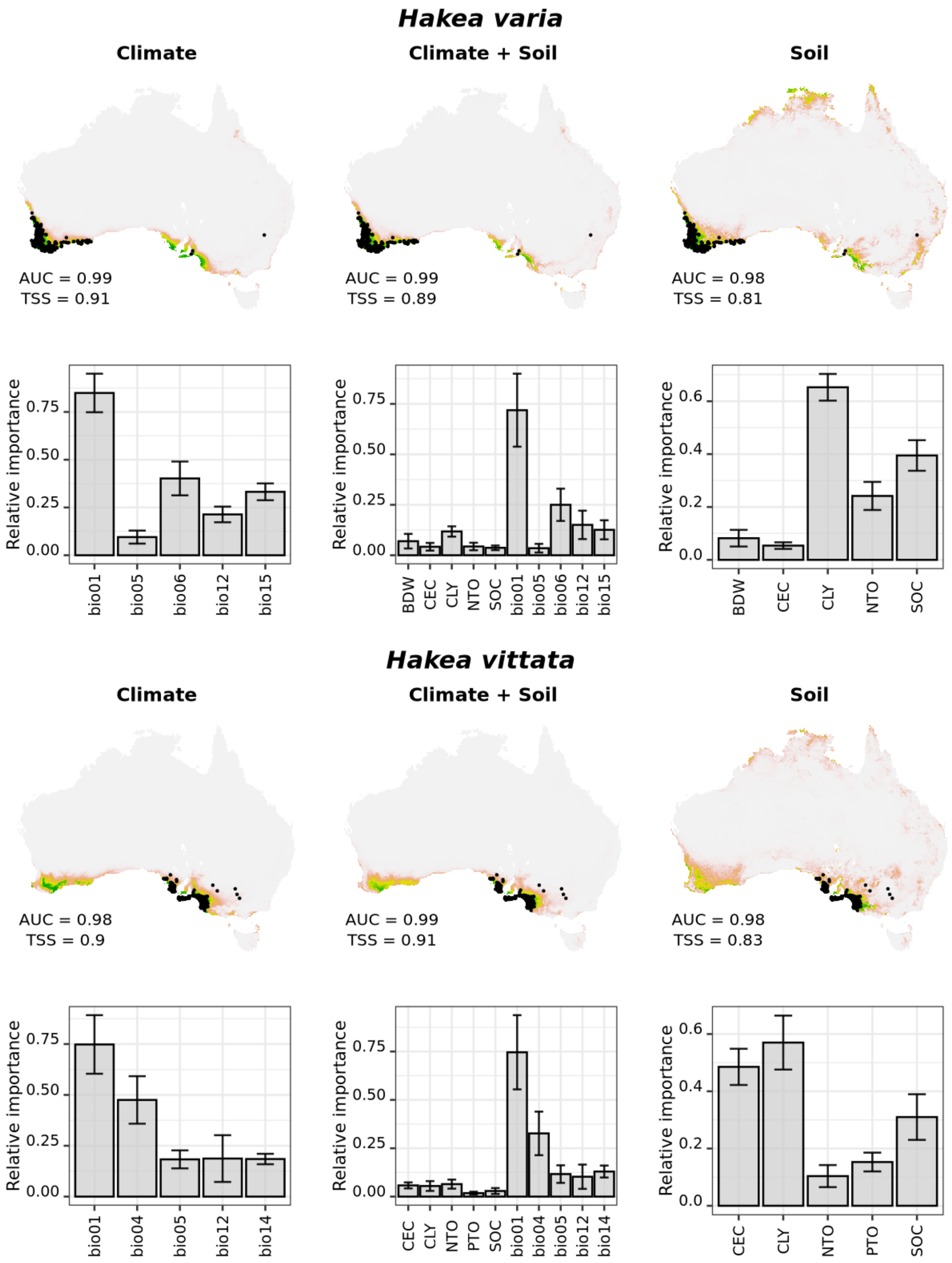

Hakea varia
Climate
Climate + Soil
Soil
AUC = 0.99
TSS = 0.91
AUC = 0.99
TSS = 0.89
AUC = 0.98
TSS = 0.81
Relative importance
0.00
0.25
0.50
0.75
bio01
bio05
bio06
bio12
bio15
BDW
CEC
CLY
NTO
SOC
0.0
0.2
0.4
0.6
Hakea vittata
Climate
Climate + Soil
Soil
AUC = 0.98
TSS = 0.9
AUC = 0.99
TSS = 0.91
AUC = 0.98
TSS = 0.83
bio04
bio14
PTO

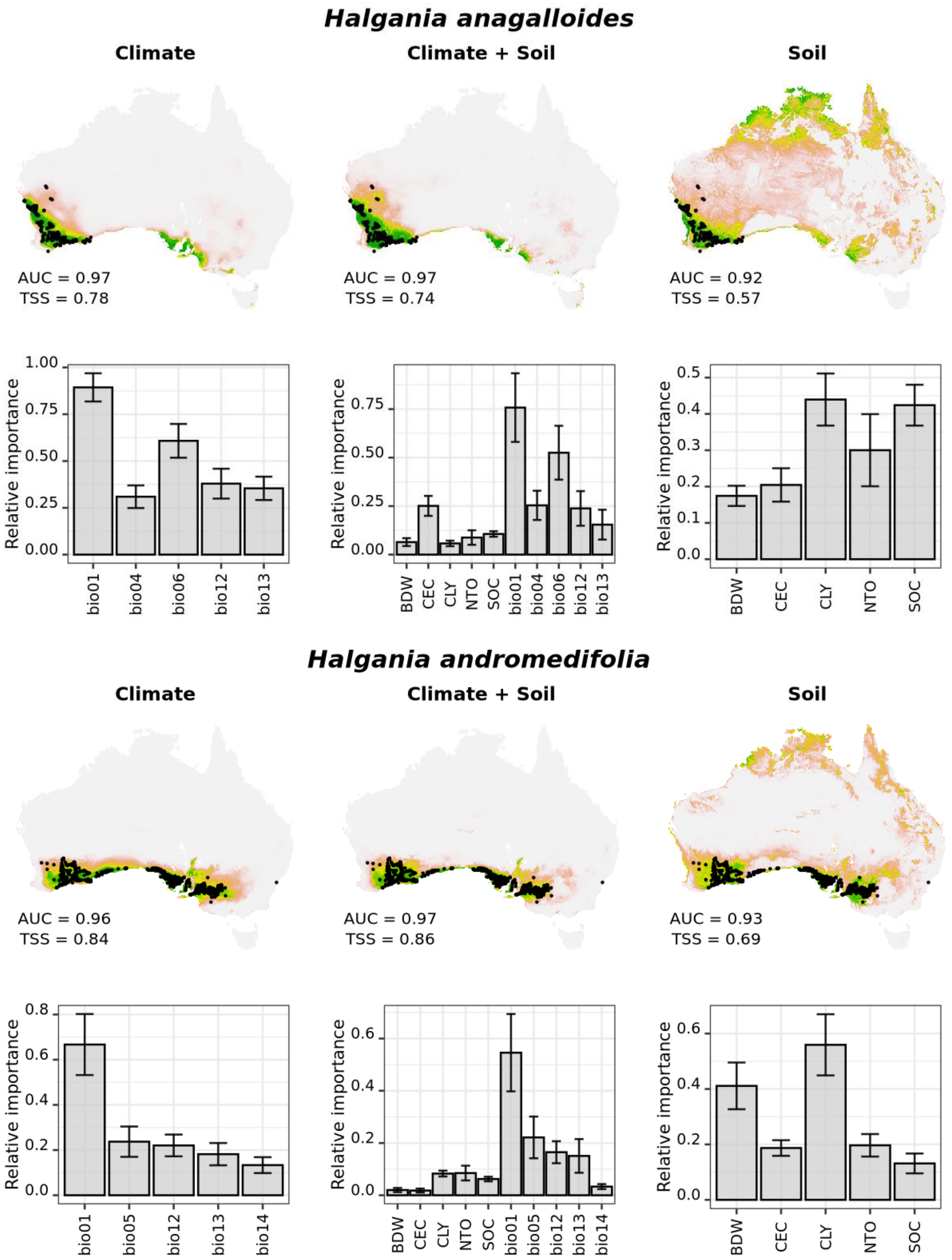

Halgania anagalloides
Climate
Climate + Soil
Soil
AUC = 0.97
TSS = 0.78
AUC = 0.97
TSS = 0.74
AUC = 0.92
TSS = 0.57
Relative importance
1.00
0.75
0.50
0.25
0.00
bio01
bio04
bio06
bio12
bio13
BDW
CEC
CLY
NTO
SOC
0.5
0.4
0.3
0.2
0.1
0.0
Halgania andromedifolia
Climate
Climate + Soil
Soil
AUC = 0.96
TSS = 0.84
AUC = 0.97
TSS = 0.86
AUC = 0.93
TSS = 0.69
Relative importance
0.8
0.6
0.4
0.2
0.0
bio01
bio05
bio12
bio13
bio14
BDW
CEC
CLY
NTO
SOC

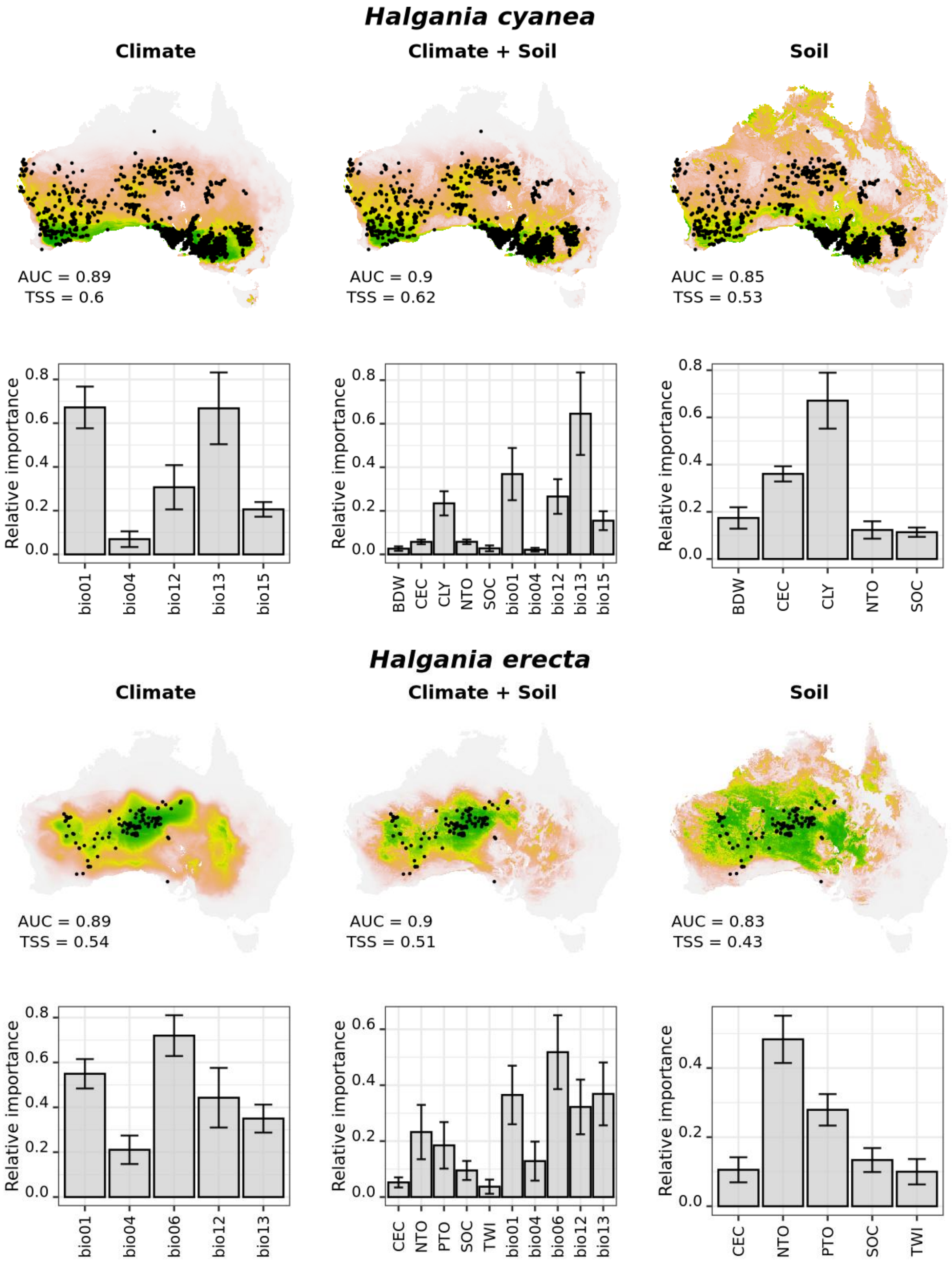

Halgania cyanea
Climate
Climate + Soil
Soil
AUC = 0.89
TSS = 0.6
AUC = 0.9
TSS = 0.62
AUC = 0.85
TSS = 0.53
Relative importance
0.0
0.2
0.4
0.6
0.8
bio01
bio04
bio12
bio13
bio15
BDW
CEC
CLY
NTO
SOC
Halgania erecta
AUC = 0.89
TSS = 0.54
AUC = 0.9
TSS = 0.51
AUC = 0.83
TSS = 0.43
bio06
PTO
TWI

Halgania integerrima

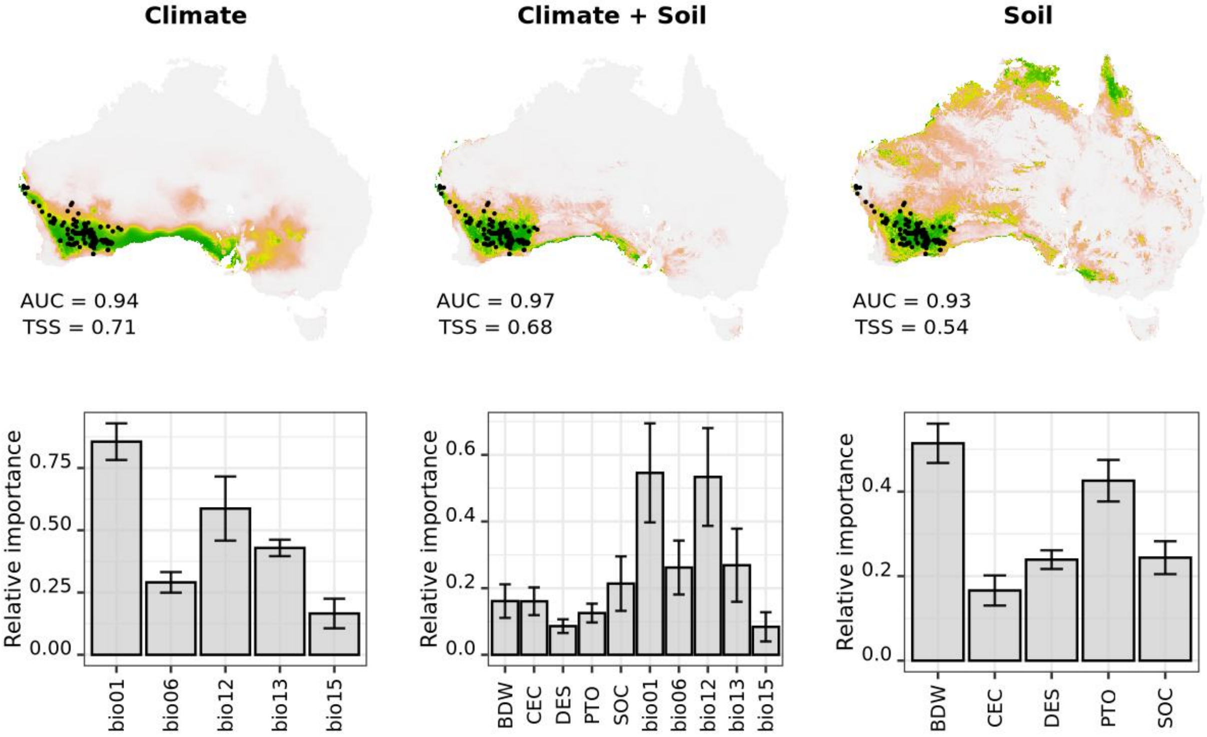

Halgania lavandulacea

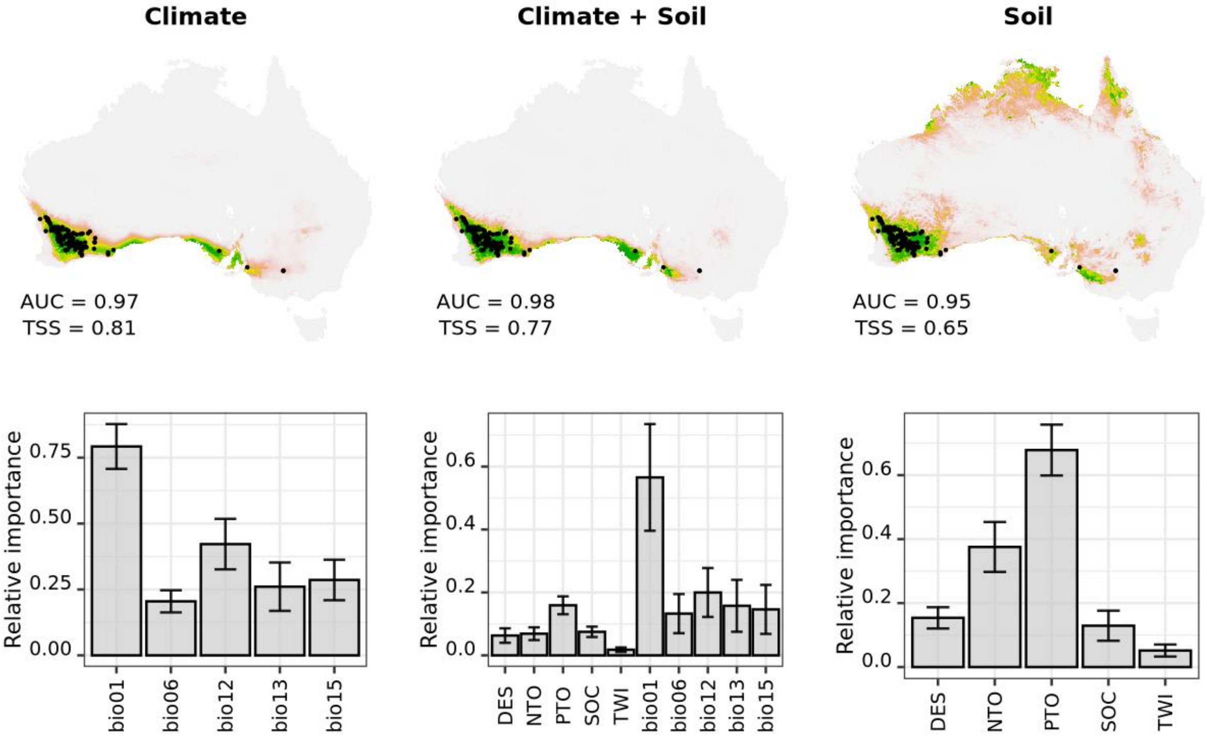

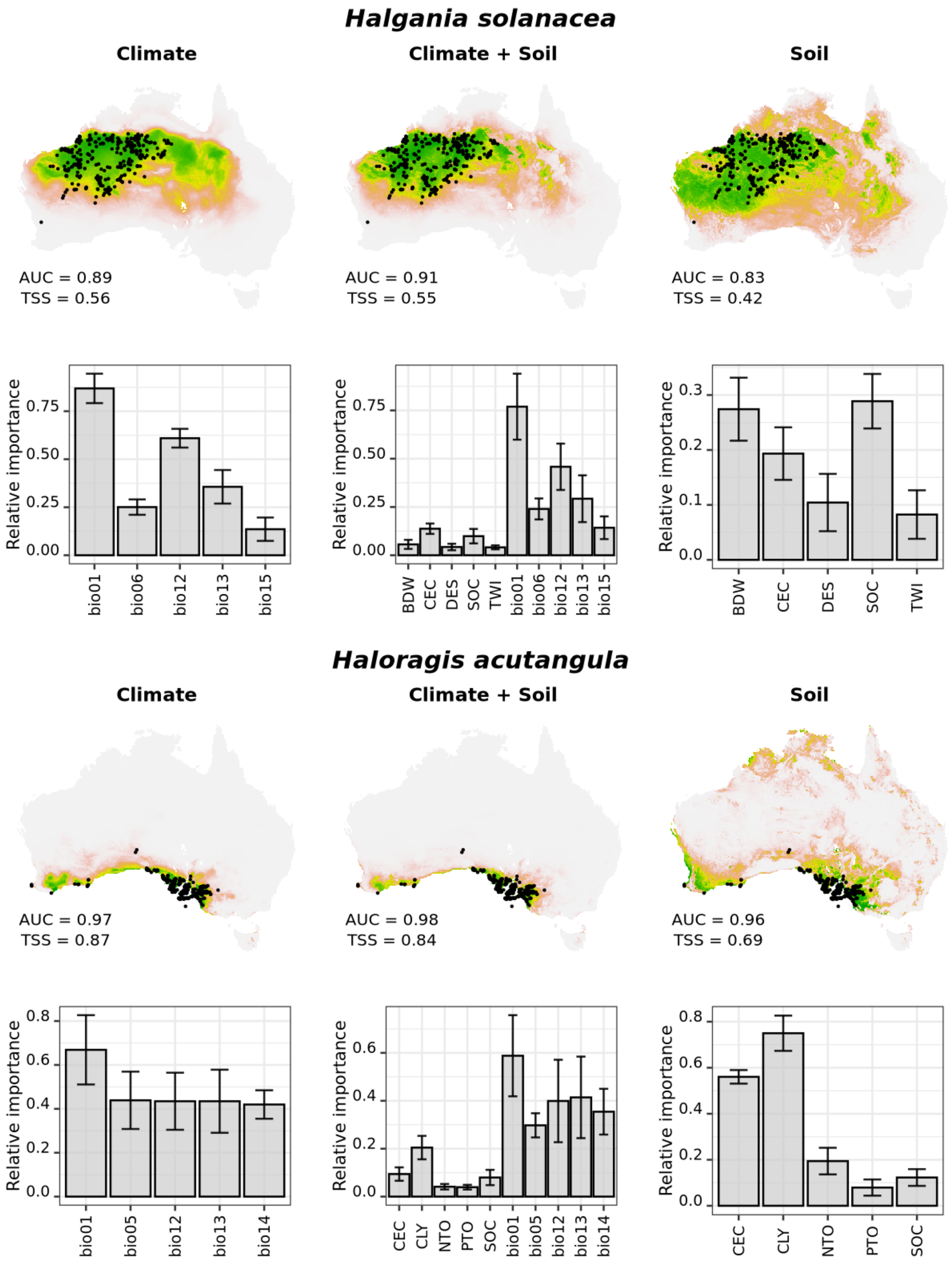
Halgania solanacea
Climate
Climate + Soil
Soil
AUC = 0.89
TSS = 0.56
AUC = 0.91
TSS = 0.55
AUC = 0.83
TSS = 0.42
Relative importance
0.00
0.25
0.50
0.75
bio01
bio06
bio12
bio13
bio15
BDW
CEC
DES
SOC
TWI
0.0
0.1
0.2
0.3
Haloragis acutangula
Climate
Climate + Soil
Soil
AUC = 0.97
TSS = 0.87
AUC = 0.98
TSS = 0.84
AUC = 0.96
TSS = 0.69
Relative importance
0.0
0.2
0.4
0.6
0.8
bio01
bio05
bio12
bio13
bio14
CEC
CLY
NTO
PTO
SOC

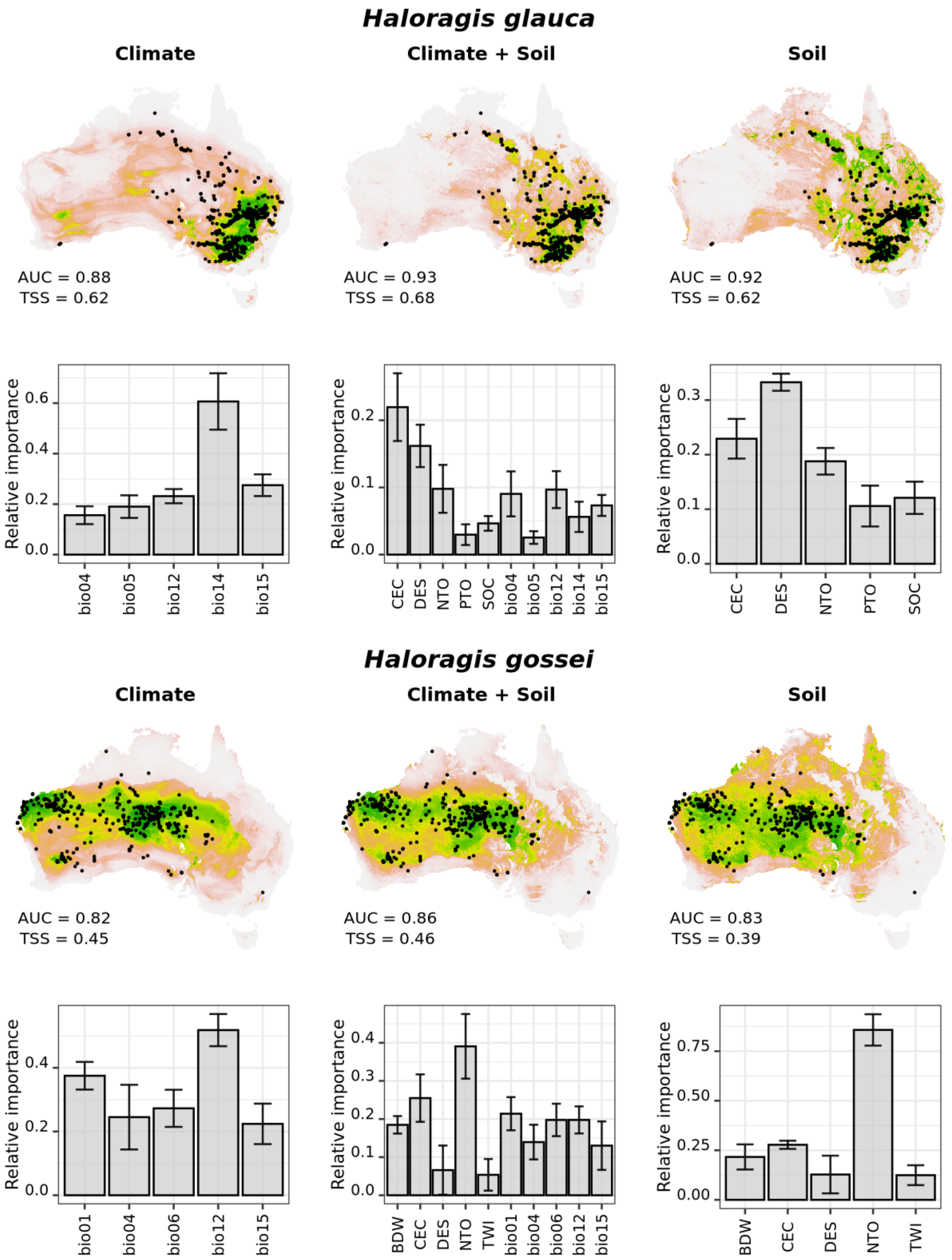
Haloragis glauca
Climate
Climate + Soil
Soil
AUC = 0.88
TSS = 0.62
AUC = 0.93
TSS = 0.68
AUC = 0.92
TSS = 0.62
Relative importance
bio04
bio05
bio12
bio14
bio15
CEC
DES
NTO
PTO
SOC
bio04
bio05
bio12
bio14
bio15
CEC
DES
NTO
PTO
SOC
Haloragis gossei
Climate
Climate + Soil
Soil
AUC = 0.82
TSS = 0.45
AUC = 0.86
TSS = 0.46
AUC = 0.83
TSS = 0.39
Relative importance
bio01
bio04
bio06
bio12
bio15
BDW
CEC
DES
NTO
TWI
bio01
bio04
bio06
bio12
bio15
BDW
CEC
DES
NTO
TWI

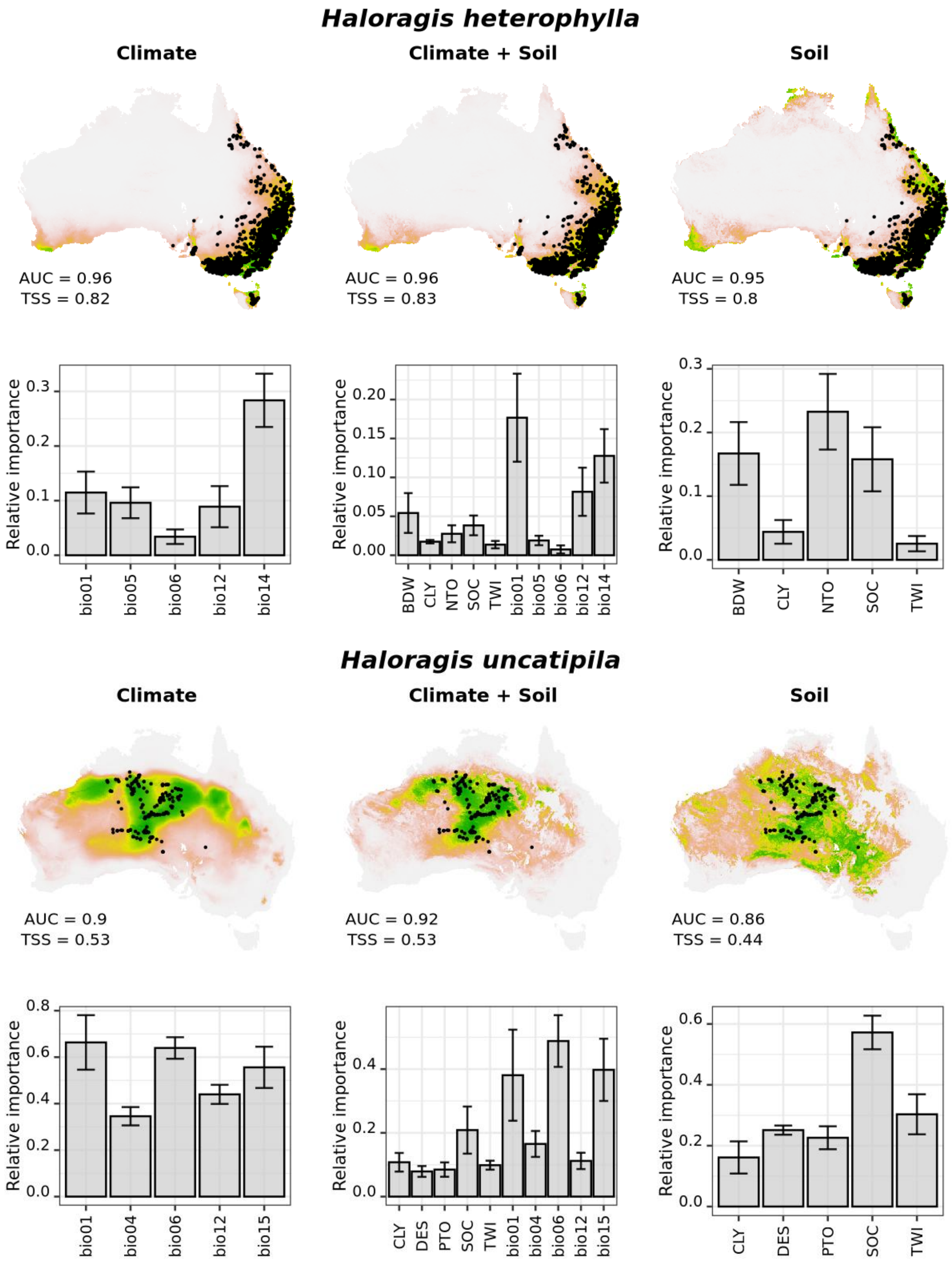
Haloragis heterophylla
Climate
Climate + Soil
Soil
AUC = 0.96
TSS = 0.82
AUC = 0.96
TSS = 0.83
AUC = 0.95
TSS = 0.8
Relative importance
bio01
bio05
bio06
bio12
bio14
BDW
CLY
NTO
SOC
TWI
Haloragis uncatipila
AUC = 0.9
TSS = 0.53
AUC = 0.92
TSS = 0.53
AUC = 0.86
TSS = 0.44
bio04
bio15
DES
PTO

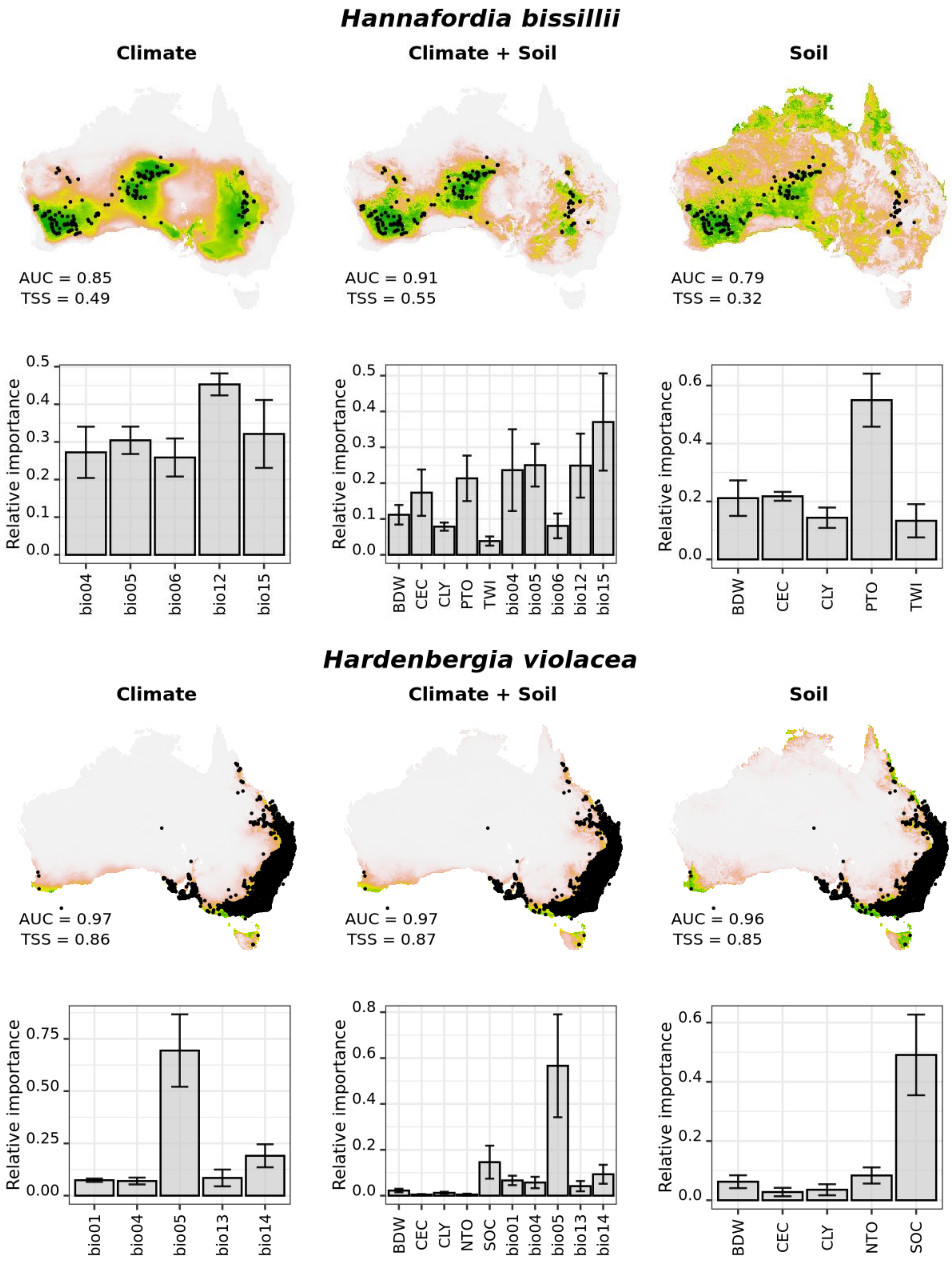
Hannafordia bissillii
Climate
Climate + Soil
Soil
AUC = 0.85
TSS = 0.49
AUC = 0.91
TSS = 0.55
AUC = 0.79
TSS = 0.32
Relative importance
bio04
bio05
bio06
bio12
bio15
BDW
CEC
CLY
PTO
TWI
Hardenbergia violacea
Climate
Climate + Soil
Soil
AUC = 0.97
TSS = 0.86
AUC = 0.97
TSS = 0.87
AUC = 0.96
TSS = 0.85
Relative importance
bio01
bio04
bio05
bio13
bio14
BDW
CEC
CLY
NTO
SOC

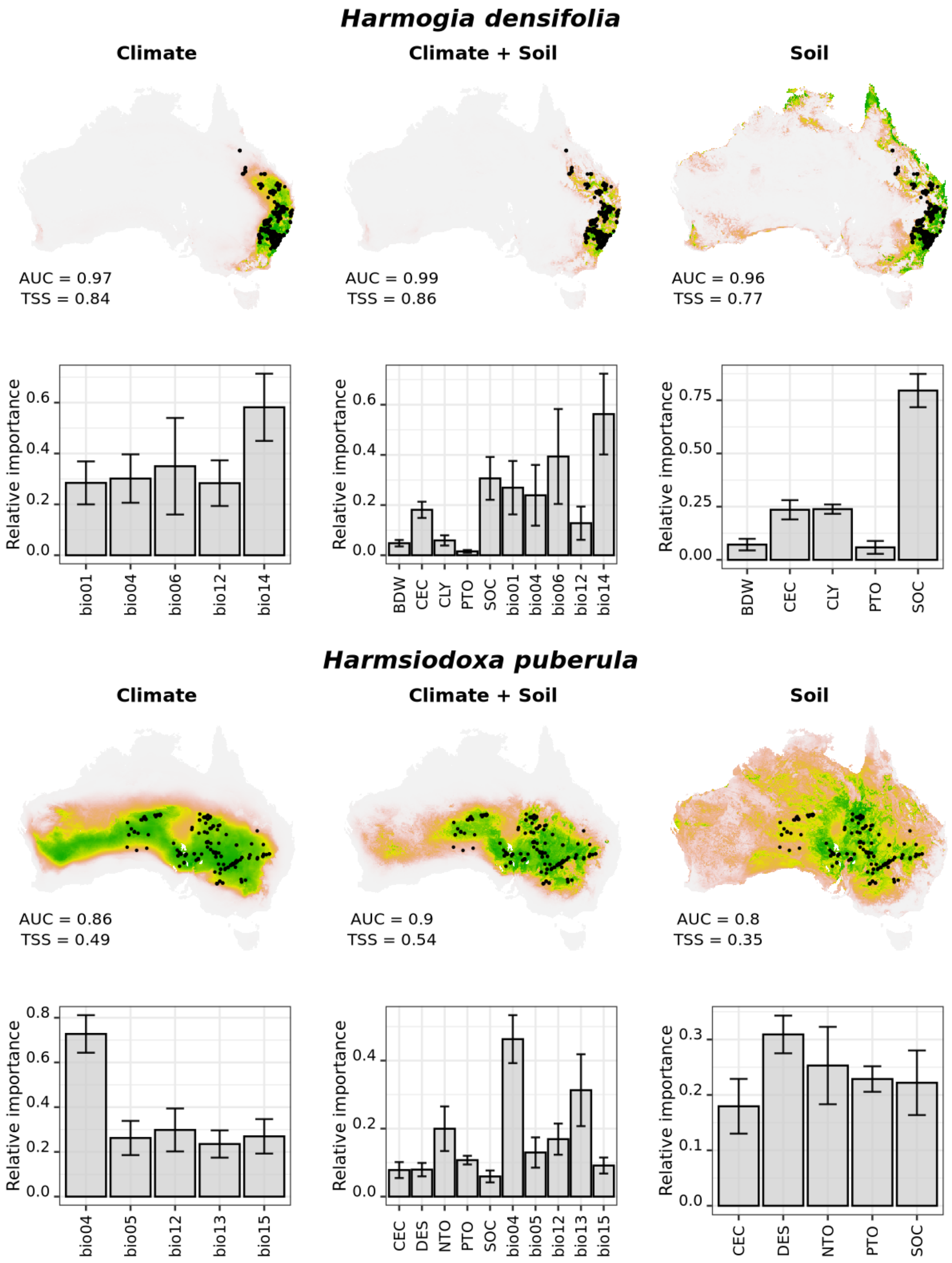

Harmogia densifolia
Climate
Climate + Soil
Soil
AUC = 0.97
TSS = 0.84
AUC = 0.99
TSS = 0.86
AUC = 0.96
TSS = 0.77
Relative importance
0.0
0.2
0.4
0.6
bio01
bio04
bio06
bio12
bio14
BDW
CEC
CLY
PTO
SOC
0.00
0.25
0.50
0.75
Harmsiodoxa puberula
Climate
Climate + Soil
Soil
AUC = 0.86
TSS = 0.49
AUC = 0.9
TSS = 0.54
AUC = 0.8
TSS = 0.35
0.8
bio05
bio13
bio15
DES
NTO
0.1
0.3

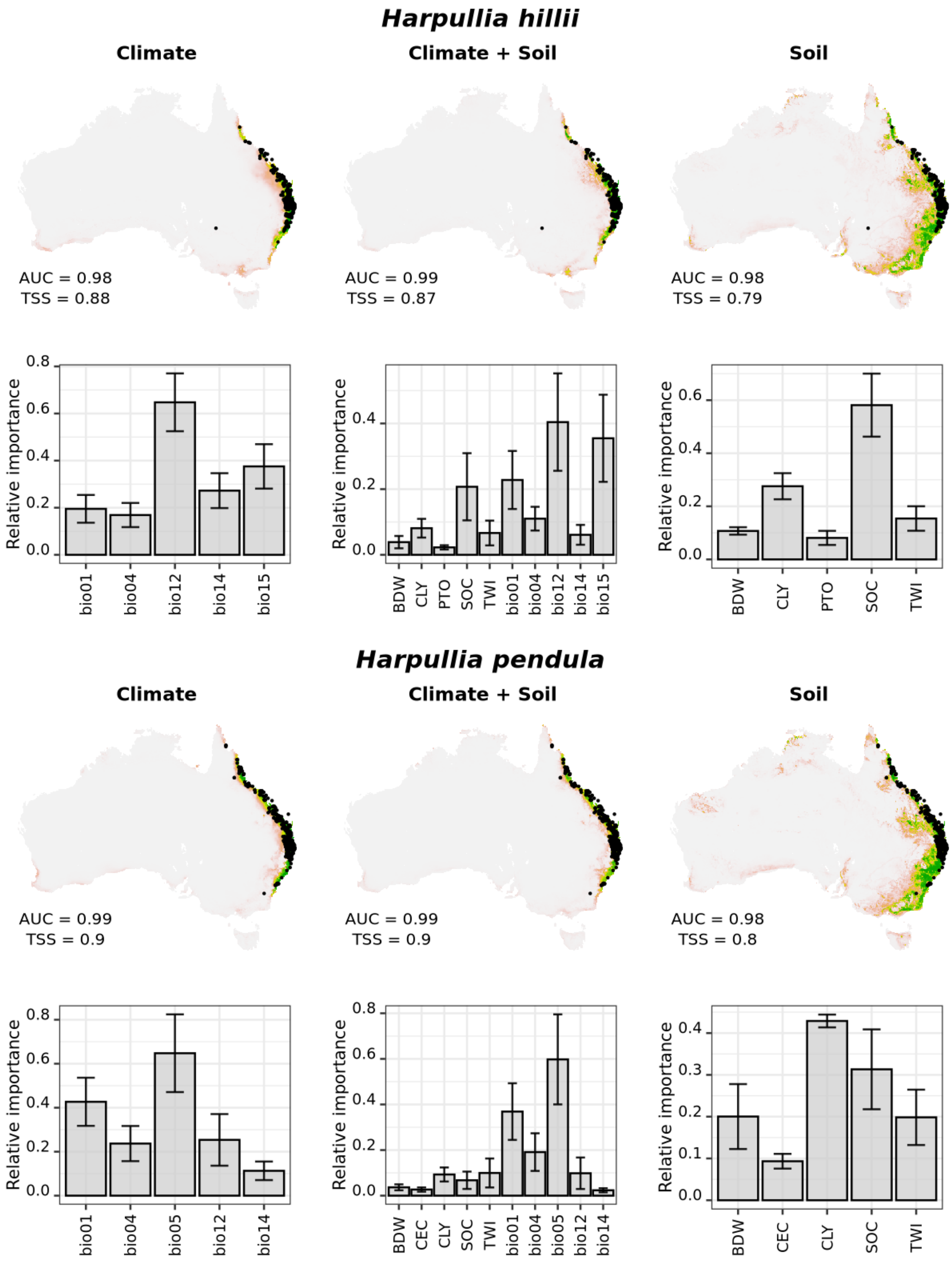
Harpullia hillii
Climate
Climate + Soil
Soil
AUC = 0.98
TSS = 0.88
AUC = 0.99
TSS = 0.87
AUC = 0.98
TSS = 0.79
Relative importance
0.0
0.2
0.4
0.6
0.8
bio01
bio04
bio12
bio14
bio15
BDW
CLY
PTO
SOC
TWI
Harpullia pendula
AUC = 0.99
TSS = 0.9
AUC = 0.99
TSS = 0.9
AUC = 0.98
TSS = 0.8
bio05
CEC
0.1
0.3

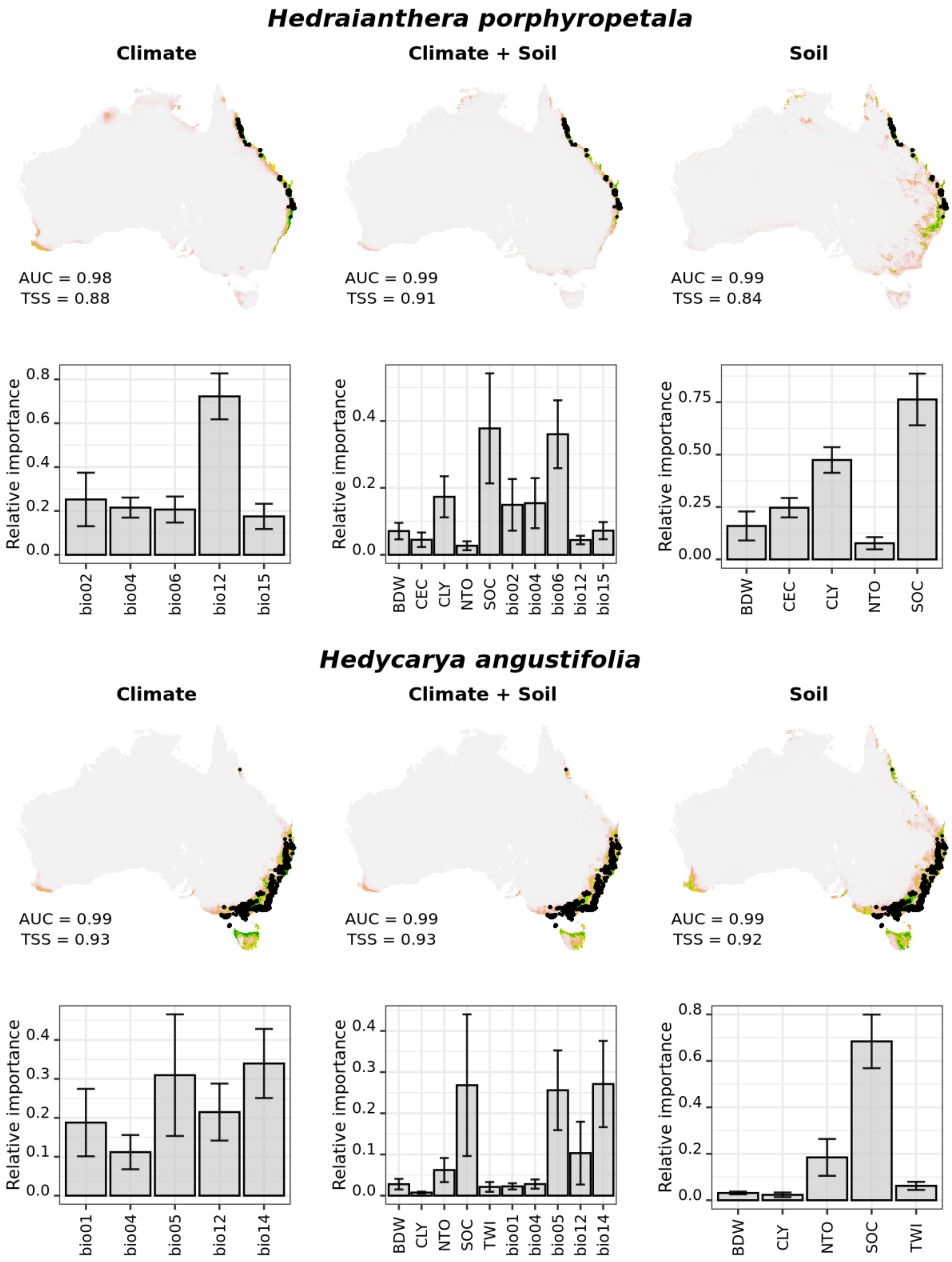

Hedraiantheru porphyropetala
Climate
Climate + Soil
Soil
AUC = 0.98
TSS = 0.88
AUC = 0.99
TSS = 0.91
AUC = 0.99
TSS = 0.84
Relative importance
bio02
bio04
bio06
bio12
bio15
BDW
CEC
CLY
NTO
SOC
bio02
bio04
bio06
bio12
bio15
BDW
CEC
CLY
NTO
SOC
Hedycarya angustifolia
Climate
Climate + Soil
Soil
AUC = 0.99
TSS = 0.93
AUC = 0.99
TSS = 0.93
AUC = 0.99
TSS = 0.92
Relative importance
bio01
bio04
bio05
bio12
bio14
BDW
CLY
NTO
SOC
TWI
bio01
bio04
bio05
bio12
bio14
BDW
CLY
NTO
SOC
TWI

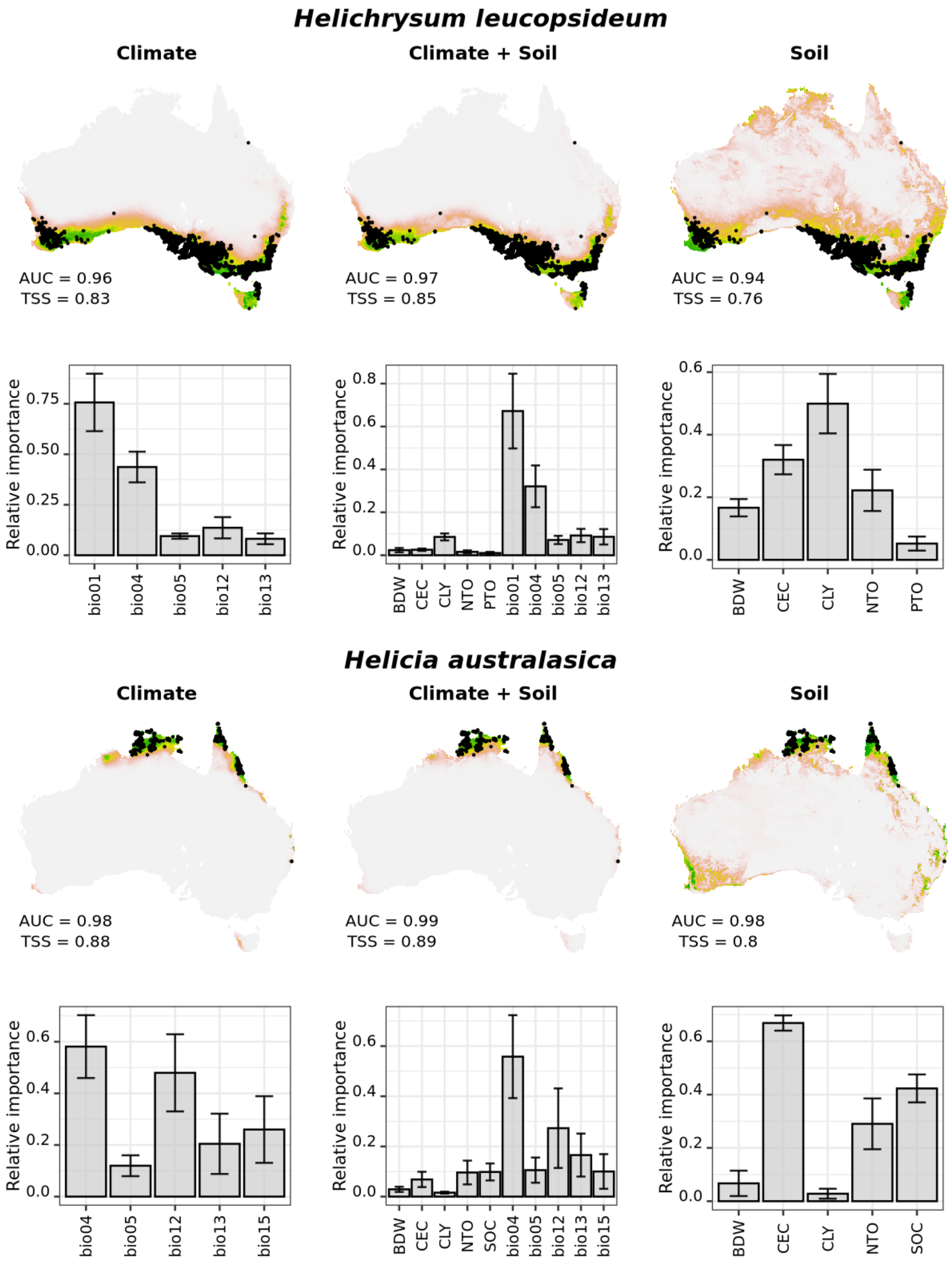
Helichrysum leucopsideum
Climate
Climate + Soil
Soil
AUC = 0.96
TSS = 0.83
AUC = 0.97
TSS = 0.85
AUC = 0.94
TSS = 0.76
Relative importance
0.00
0.25
0.50
0.75
bio01
bio04
bio05
bio12
bio13
0.0
0.2
0.4
0.6
0.8
BDW
CEC
CLY
NTO
PTO
Helicia australasica
AUC = 0.98
TSS = 0.88
AUC = 0.99
TSS = 0.89
AUC = 0.98
TSS = 0.8
bio15
SOC

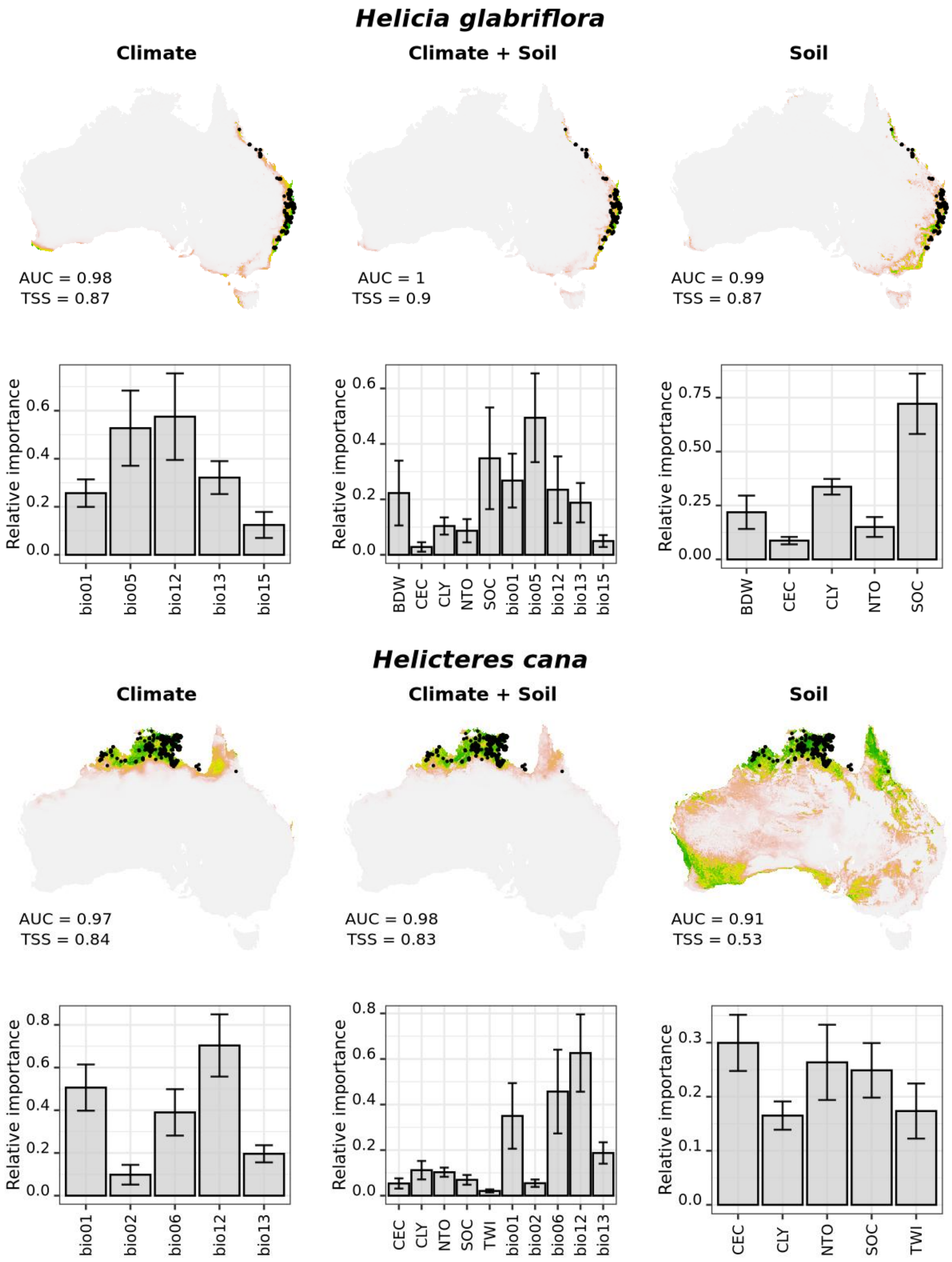

Helicia glabriflora
Climate
Climate + Soil
Soil
AUC = 0.98
TSS = 0.87
AUC = 1
TSS = 0.9
AUC = 0.99
TSS = 0.87
Relative importance
bio01 bio05 bio12 bio13 bio15
BDW CEC CLY NTO SOC bio01 bio05 bio12 bio13 bio15
BDW CEC CLY NTO SOC
Helicteres cana
Climate
Climate + Soil
Soil
AUC = 0.97
TSS = 0.84
AUC = 0.98
TSS = 0.83
AUC = 0.91
TSS = 0.53
Relative importance
bio01 bio02 bio06 bio12 bio13
CEC CLY NTO SOC TWI bio01 bio02 bio06 bio12 bio13
CEC CLY NTO SOC TWI

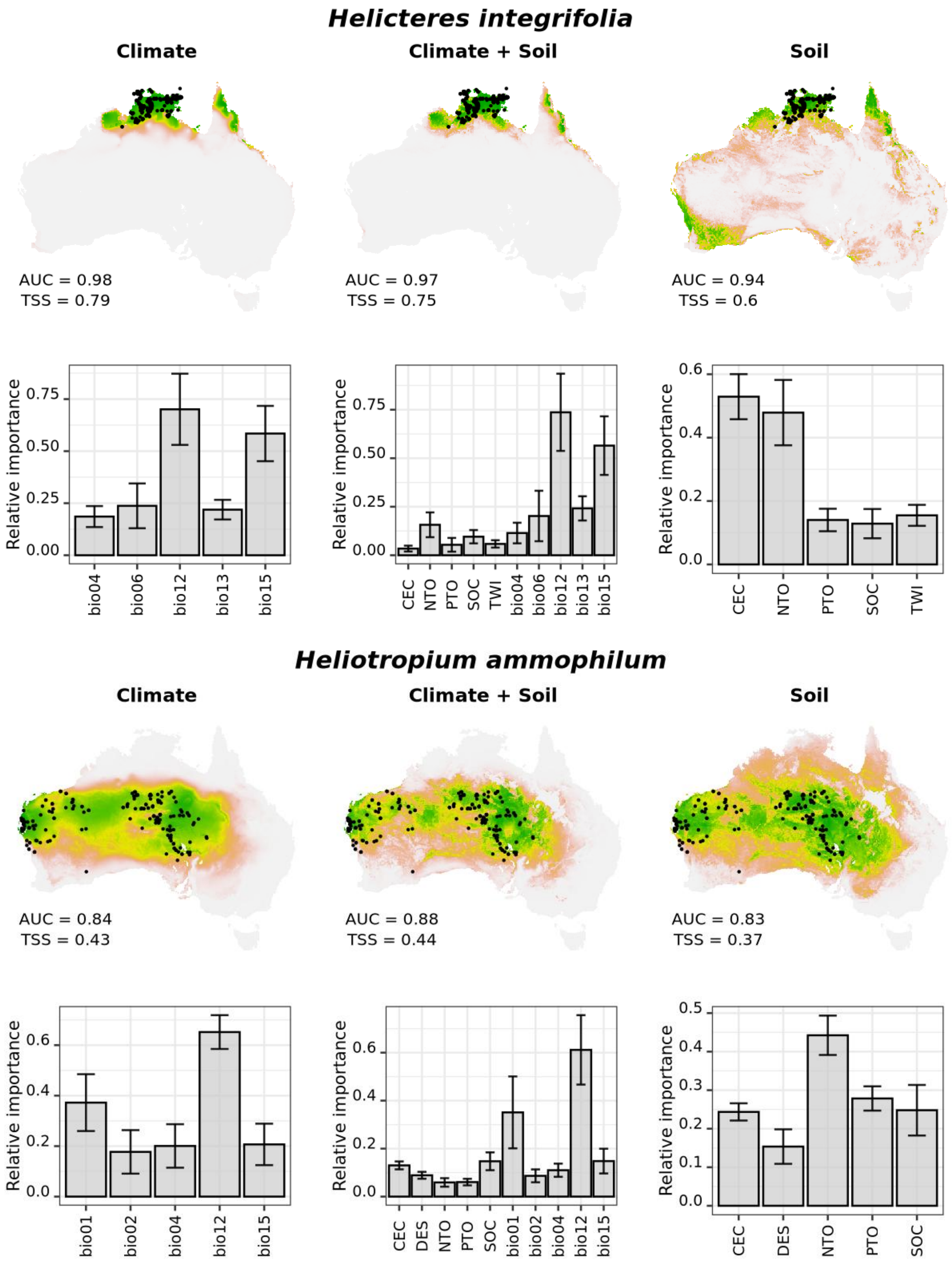
Helicteres integrifolia
Climate
Climate + Soil
Soil
AUC = 0.98
TSS = 0.79
AUC = 0.97
TSS = 0.75
AUC = 0.94
TSS = 0.6
Relative importance
0.00
0.25
0.50
0.75
bio04
bio06
bio12
bio13
bio15
CEC
NTO
PTO
SOC
TWI
0.0
0.2
0.4
0.6
Heliotropium ammophilum
Climate
Climate + Soil
Soil
AUC = 0.84
TSS = 0.43
AUC = 0.88
TSS = 0.44
AUC = 0.83
TSS = 0.37
Relative importance
0.0
0.2
0.4
0.6
bio01
bio02
bio04
bio12
bio15
CEC
DES
NTO
PTO
SOC
0.1
0.3
0.5

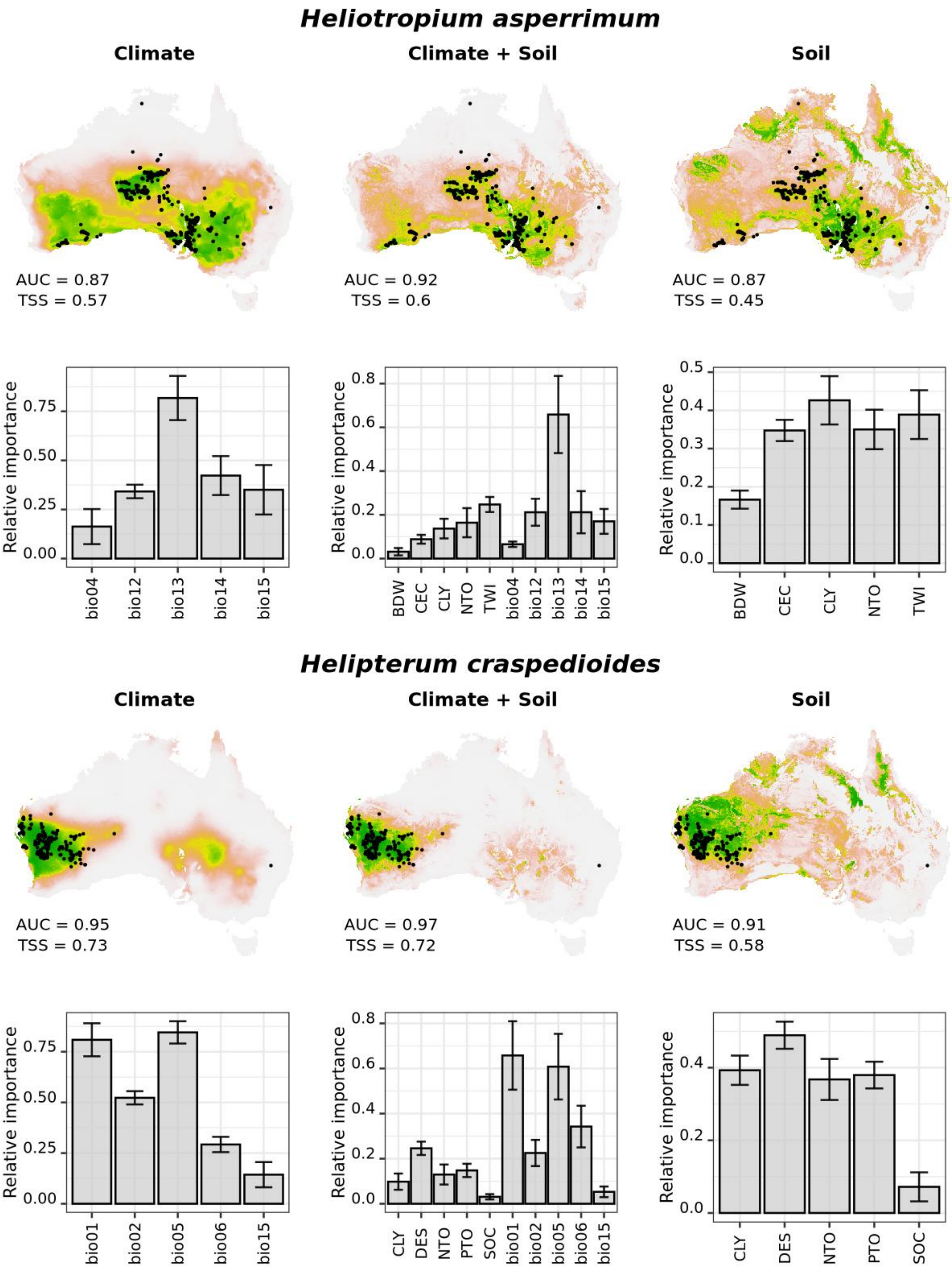
Heliotropium asperrimum
Climate
Climate + Soil
Soil
AUC = 0.87
TSS = 0.57
AUC = 0.92
TSS = 0.6
AUC = 0.87
TSS = 0.45
Relative importance
0.00
0.25
0.50
0.75
bio04
bio12
bio13
bio14
bio15
0.0
0.2
0.4
0.6
0.8
BDW
CEC
CLY
NTO
TWI
bio04
bio12
bio13
bio14
bio15
0.0
0.1
0.2
0.3
0.4
0.5
BDW
CEC
CLY
NTO
TWI
Helipterum craspedioides
Climate
Climate + Soil
Soil
AUC = 0.95
TSS = 0.73
AUC = 0.97
TSS = 0.72
AUC = 0.91
TSS = 0.58
Relative importance
0.00
0.25
0.50
0.75
bio01
bio02
bio05
bio06
bio15
0.0
0.2
0.4
0.6
0.8
CLY
DES
NTO
PTO
SOC
bio01
bio02
bio05
bio06
bio15
0.0
0.2
0.4
CLY
DES
NTO
PTO
SOC

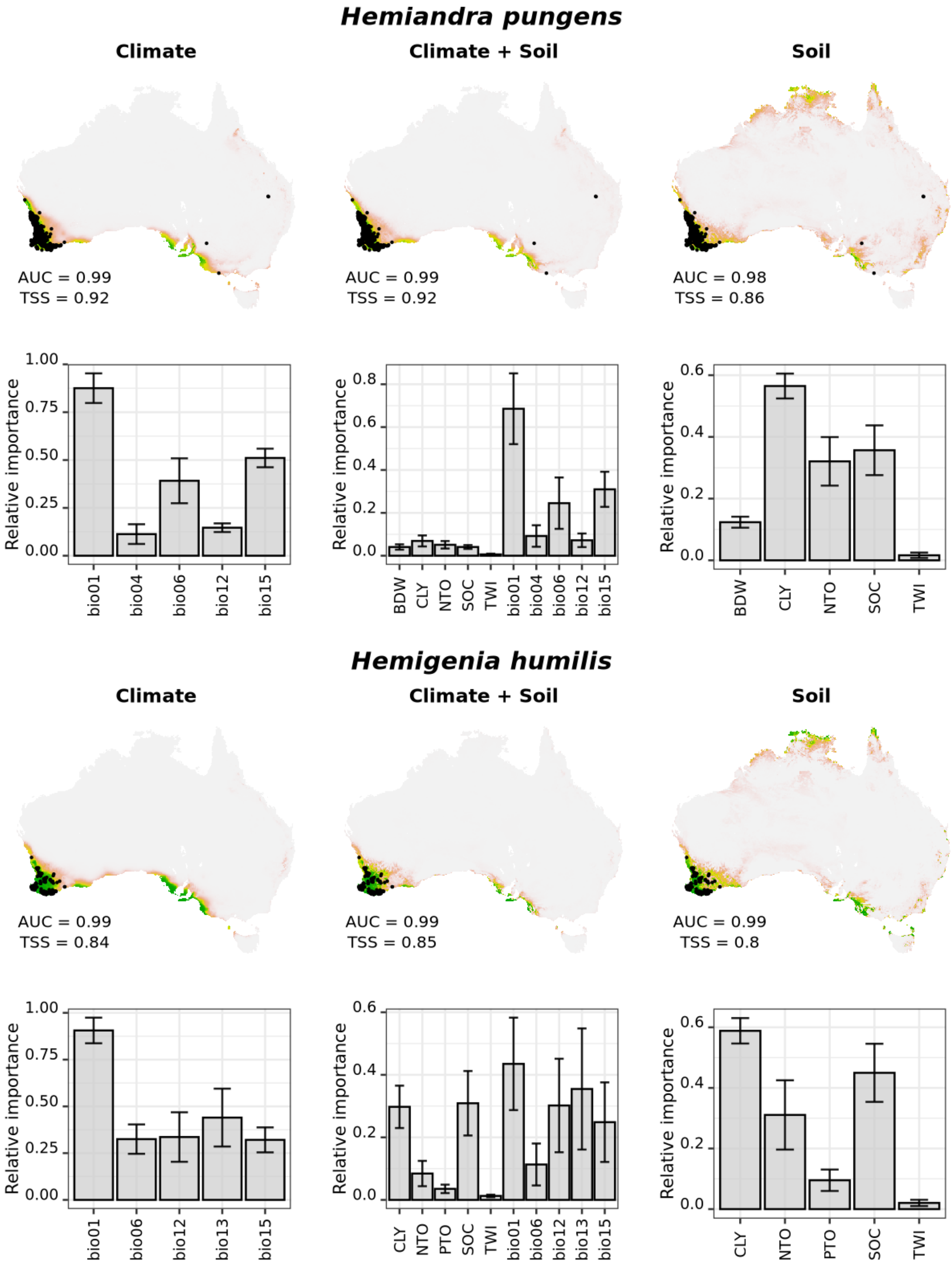
Hemiandra pungens
Climate
Climate + Soil
Soil
AUC = 0.99
TSS = 0.92
AUC = 0.99
TSS = 0.92
AUC = 0.98
TSS = 0.86
Relative importance
1.00
0.75
0.50
0.25
0.00
bio01
bio04
bio06
bio12
bio15
0.8
0.6
0.4
0.2
0.0
BDW
CLY
NTO
SOC
TWI
bio01
bio04
bio06
bio12
bio15
0.6
0.4
0.2
0.0
BDW
CLY
NTO
SOC
TWI
Hemigenia humilis
Climate
Climate + Soil
Soil
AUC = 0.99
TSS = 0.84
AUC = 0.99
TSS = 0.85
AUC = 0.99
TSS = 0.8
Relative importance
1.00
0.75
0.50
0.25
0.00
bio01
bio06
bio12
bio13
bio15
0.6
0.4
0.2
0.0
CLY
NTO
PTO
SOC
TWI
bio01
bio06
bio12
bio13
bio15
0.6
0.4
0.2
0.0
CLY
NTO
PTO
SOC
TWI

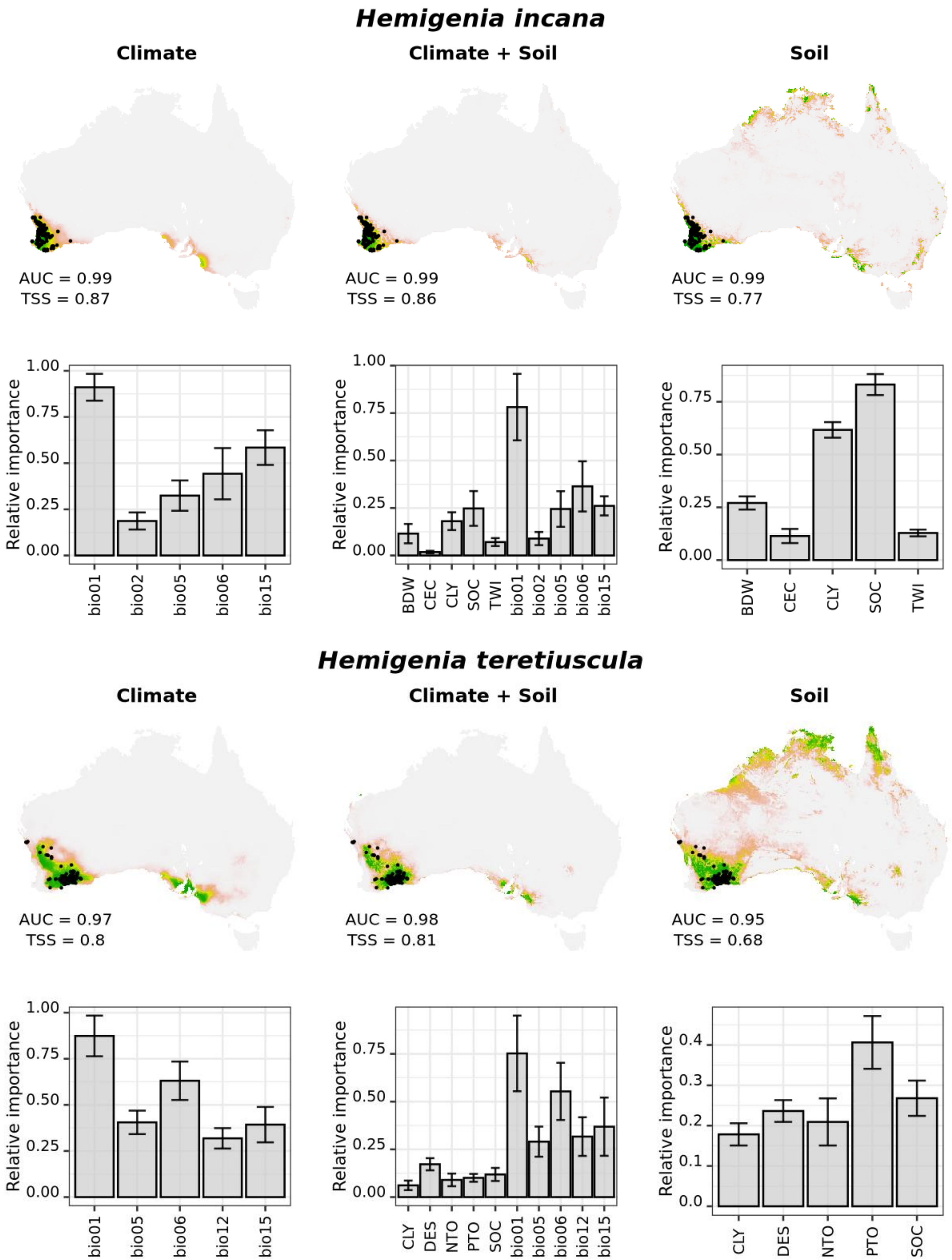

Hemigenia incana
Climate
Climate + Soil
Soil
AUC = 0.99
TSS = 0.87
AUC = 0.99
TSS = 0.86
AUC = 0.99
TSS = 0.77
Relative importance
bio01 bio02 bio05 bio06 bio15
BDW CEC CLY SOC TWI bio01 bio02 bio05 bio06 bio15
BDW CEC CLY SOC TWI
Hemigenia teretiuscula
Climate
Climate + Soil
Soil
AUC = 0.97
TSS = 0.8
AUC = 0.98
TSS = 0.81
AUC = 0.95
TSS = 0.68
Relative importance
bio01 bio05 bio06 bio12 bio15
CLY DES NTO PTO SOC bio01 bio05 bio06 bio12 bio15
CLY DES NTO PTO SOC

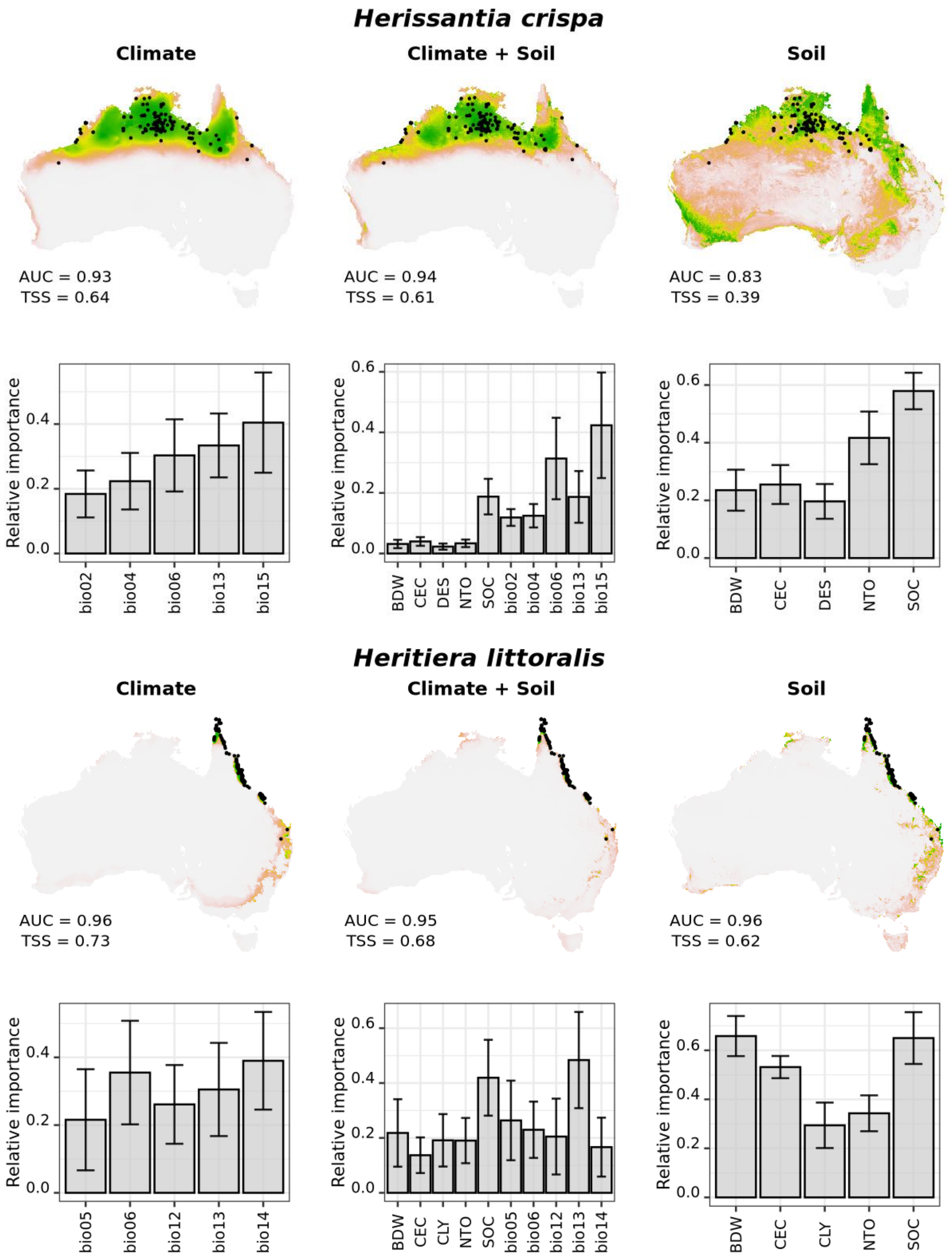

Herissantia crispa
Climate
Climate + Soil
Soil
AUC = 0.93
TSS = 0.64
AUC = 0.94
TSS = 0.61
AUC = 0.83
TSS = 0.39
Relative importance
bio02 bio04 bio06 bio13 bio15
BDW CEC DES NTO SOC bio02 bio04 bio06 bio13 bio15
BDW CEC DES NTO SOC
Heritiera littoralis
Climate
Climate + Soil
Soil
AUC = 0.96
TSS = 0.73
AUC = 0.95
TSS = 0.68
AUC = 0.96
TSS = 0.62
Relative importance
bio05 bio06 bio12 bio13 bio14
BDW CEC CLY NTO SOC bio05 bio06 bio12 bio13 bio14
BDW CEC CLY NTO SOC

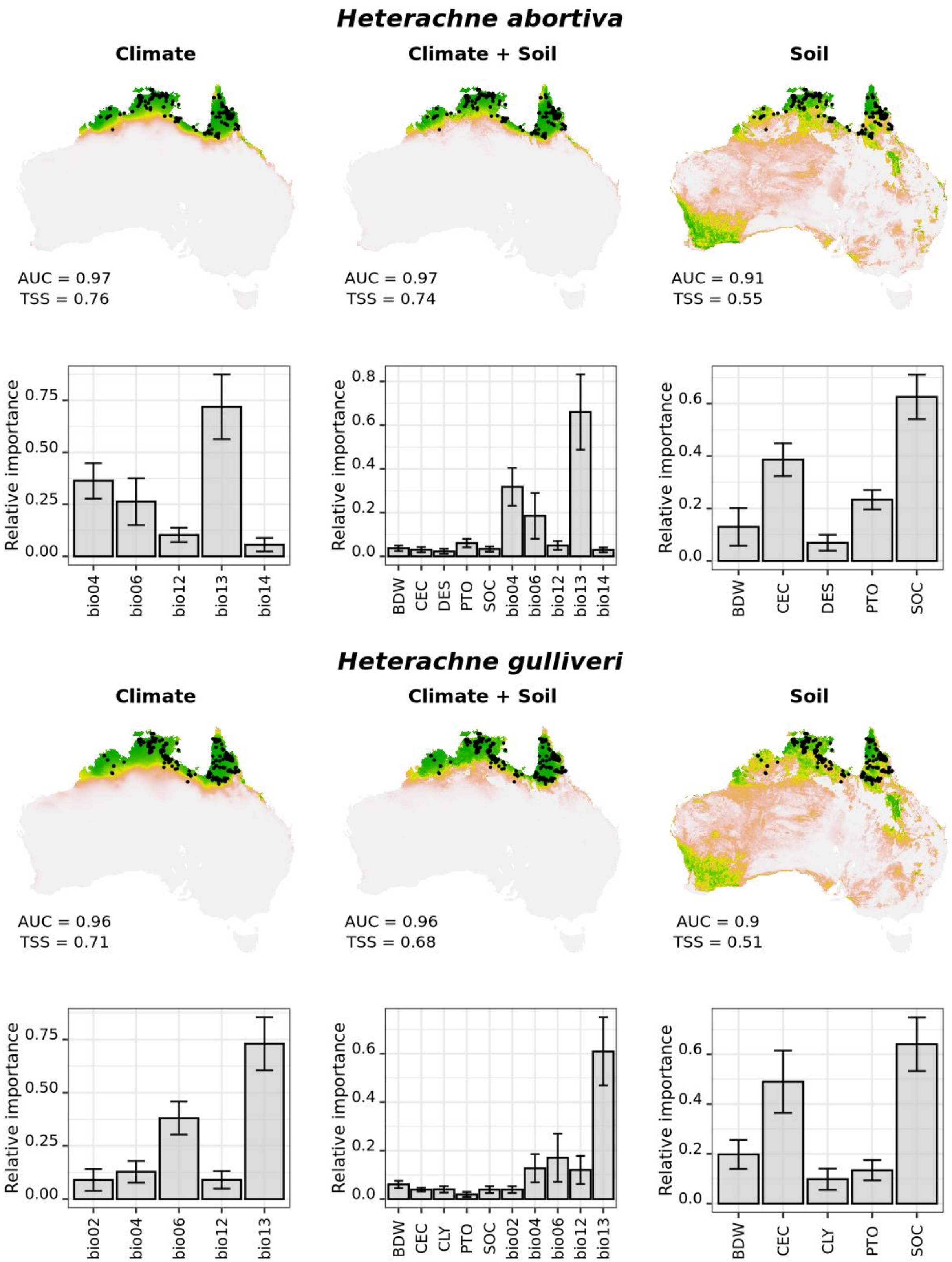
Heterachne abortiva
Climate
Climate + Soil
Soil
AUC = 0.97
TSS = 0.76
AUC = 0.97
TSS = 0.74
AUC = 0.91
TSS = 0.55
Relative importance
bio04
bio06
bio12
bio13
bio14
BDW
CEC
DES
PTO
SOC
Heterachne gulliveri
Climate
Climate + Soil
Soil
AUC = 0.96
TSS = 0.71
AUC = 0.96
TSS = 0.68
AUC = 0.9
TSS = 0.51
Relative importance
bio02
bio04
bio06
bio12
bio13
BDW
CEC
CLY
PTO
SOC

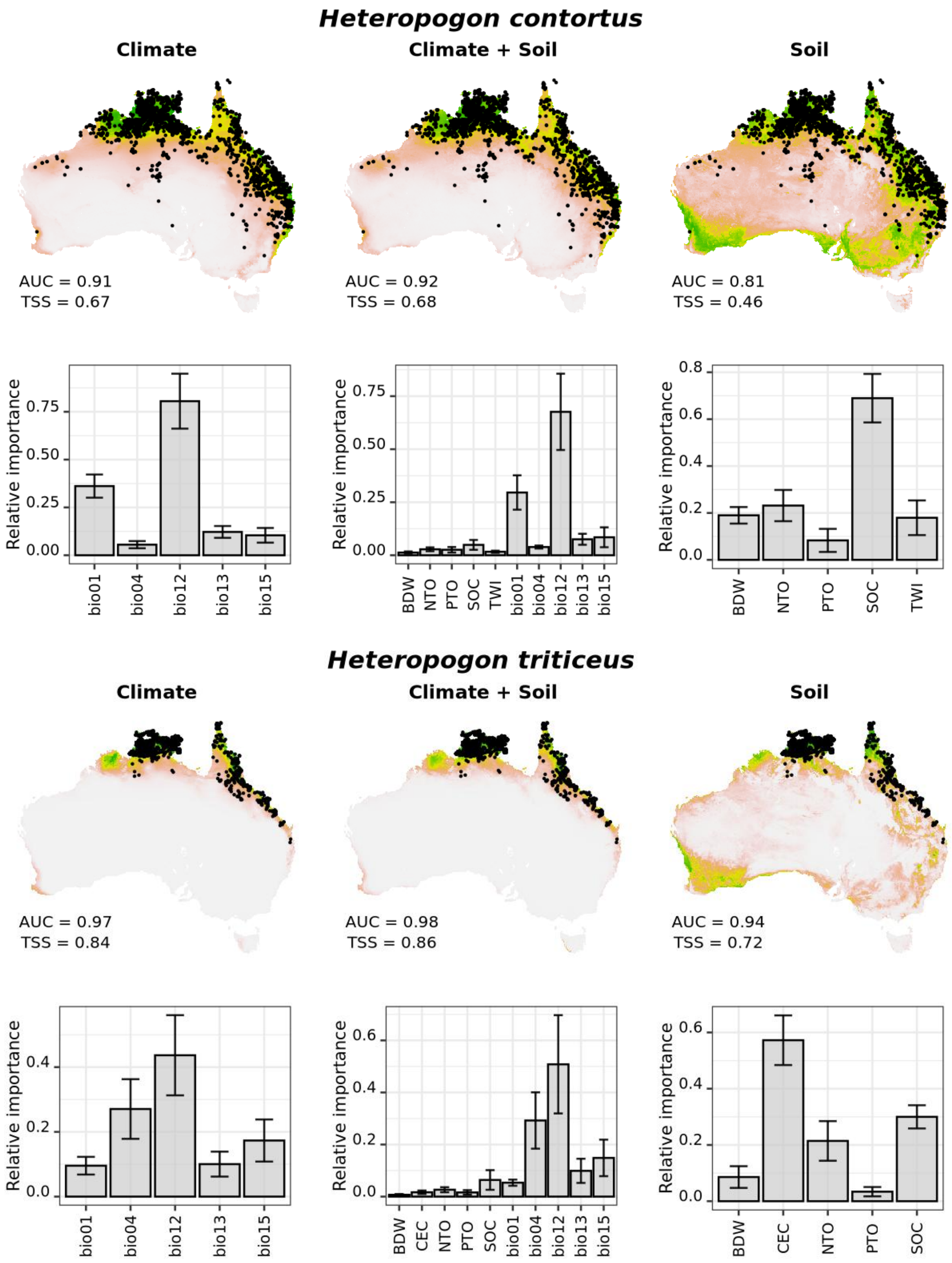
Heteropogon contortus
Climate
Climate + Soil
Soil
AUC = 0.91
TSS = 0.67
AUC = 0.92
TSS = 0.68
AUC = 0.81
TSS = 0.46
Relative importance
0.00
0.25
0.50
0.75
bio01
bio04
bio12
bio13
bio15
BDW
NTO
PTO
SOC
TWI
0.0
0.2
0.4
0.6
0.8
Heteropogon triticeus
Climate
Climate + Soil
Soil
AUC = 0.97
TSS = 0.84
AUC = 0.98
TSS = 0.86
AUC = 0.94
TSS = 0.72
Relative importance
CEC

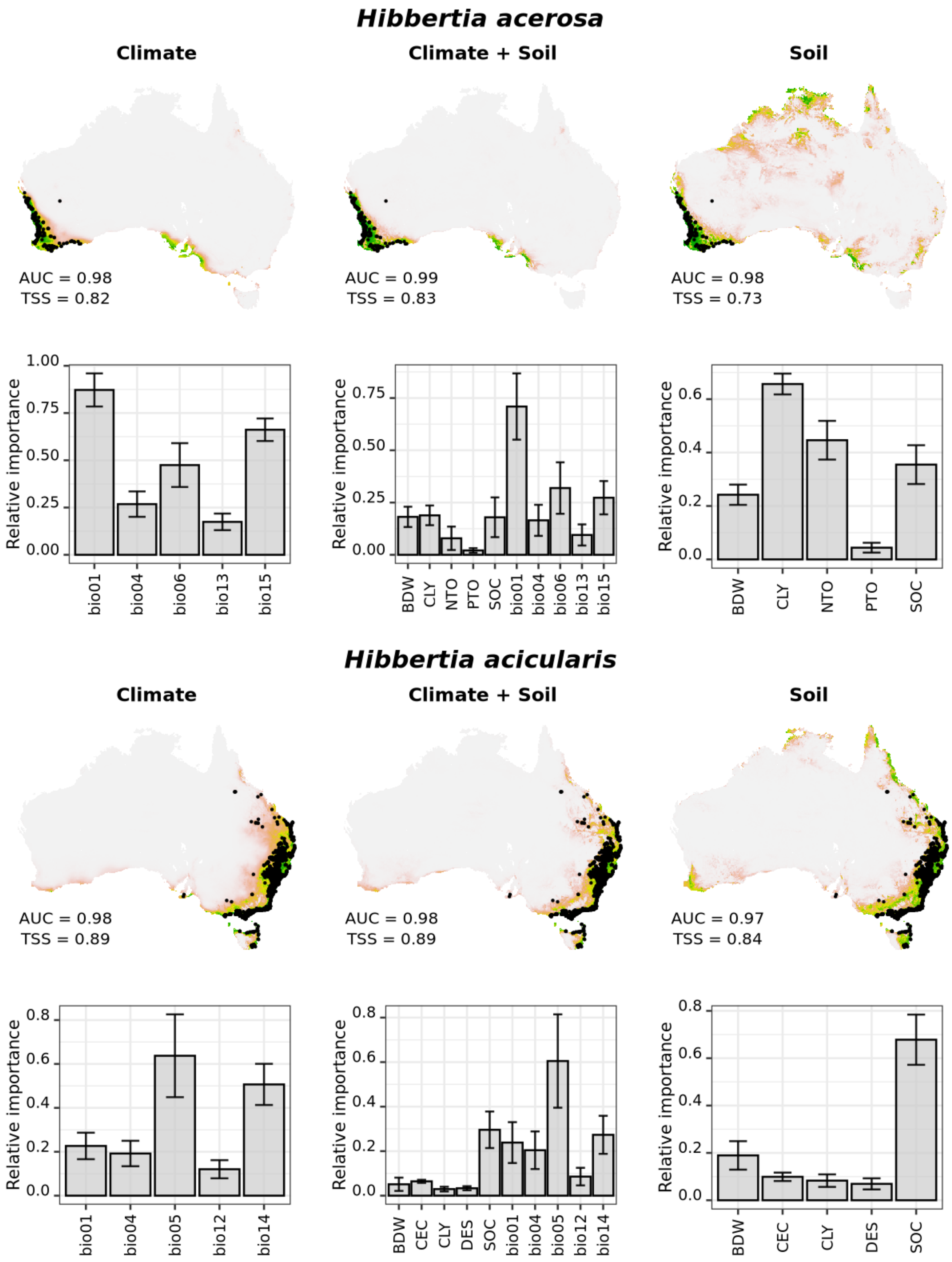
Hibbertia acerosa
Climate
Climate + Soil
Soil
AUC = 0.98
TSS = 0.82
AUC = 0.99
TSS = 0.83
AUC = 0.98
TSS = 0.73
Relative importance
bio01
bio04
bio06
bio13
bio15
BDW
CLY
NTO
PTO
SOC
Hibbertia acicularis
Climate
Climate + Soil
Soil
AUC = 0.98
TSS = 0.89
AUC = 0.98
TSS = 0.89
AUC = 0.97
TSS = 0.84
Relative importance
bio01
bio04
bio05
bio12
bio14
BDW
CEC
CLY
DES
SOC

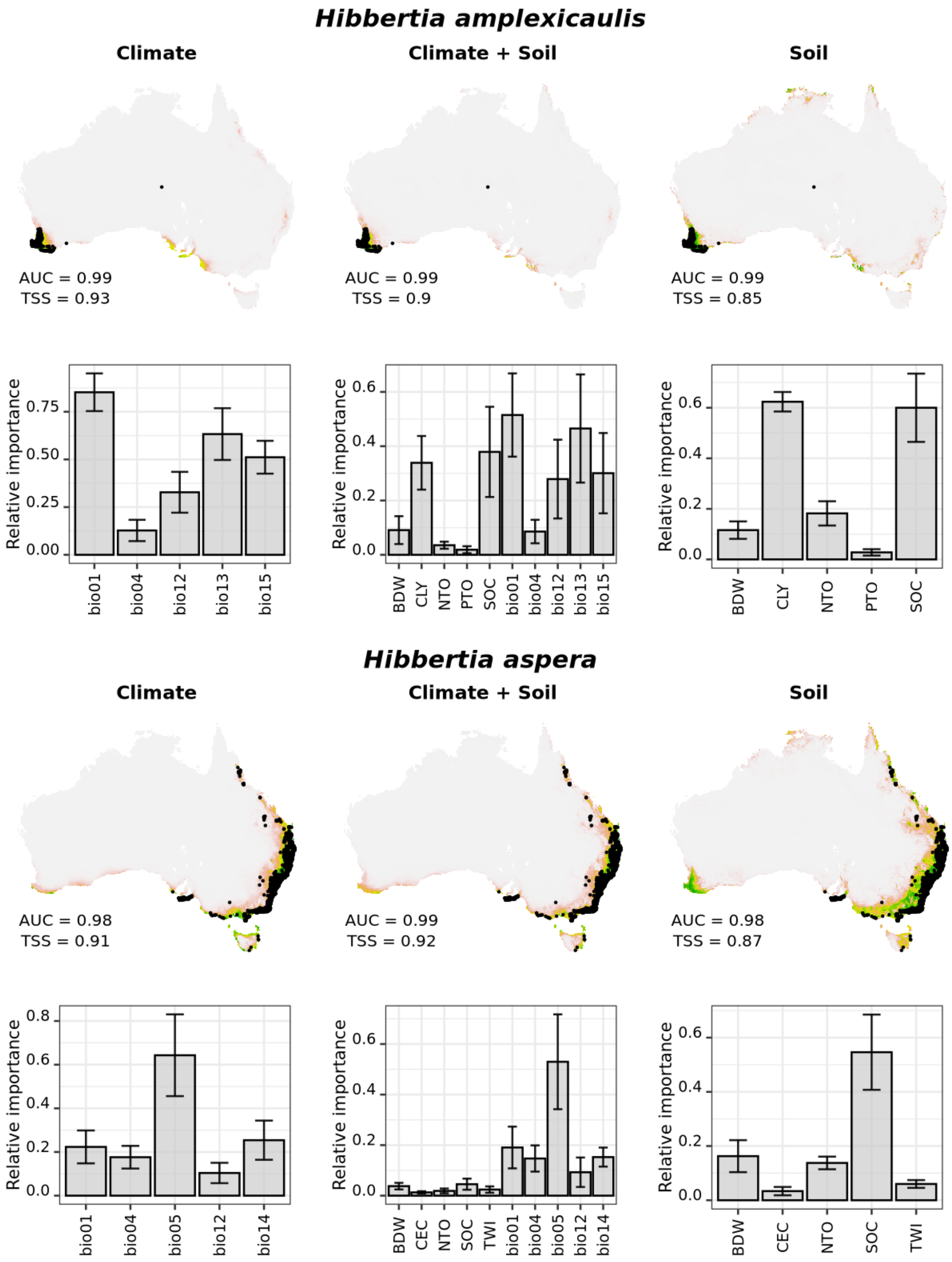
Hibbertia amplexicaulis
Climate
Climate + Soil
Soil
AUC = 0.99
TSS = 0.93
AUC = 0.99
TSS = 0.9
AUC = 0.99
TSS = 0.85
Relative importance
bio01
bio04
bio12
bio13
bio15
BDW
CLY
NTO
PTO
SOC
Hibbertia aspera
Climate
Climate + Soil
Soil
AUC = 0.98
TSS = 0.91
AUC = 0.99
TSS = 0.92
AUC = 0.98
TSS = 0.87
Relative importance
bio01
bio04
bio05
bio12
bio14
BDW
CEC
NTO
SOC
TWI

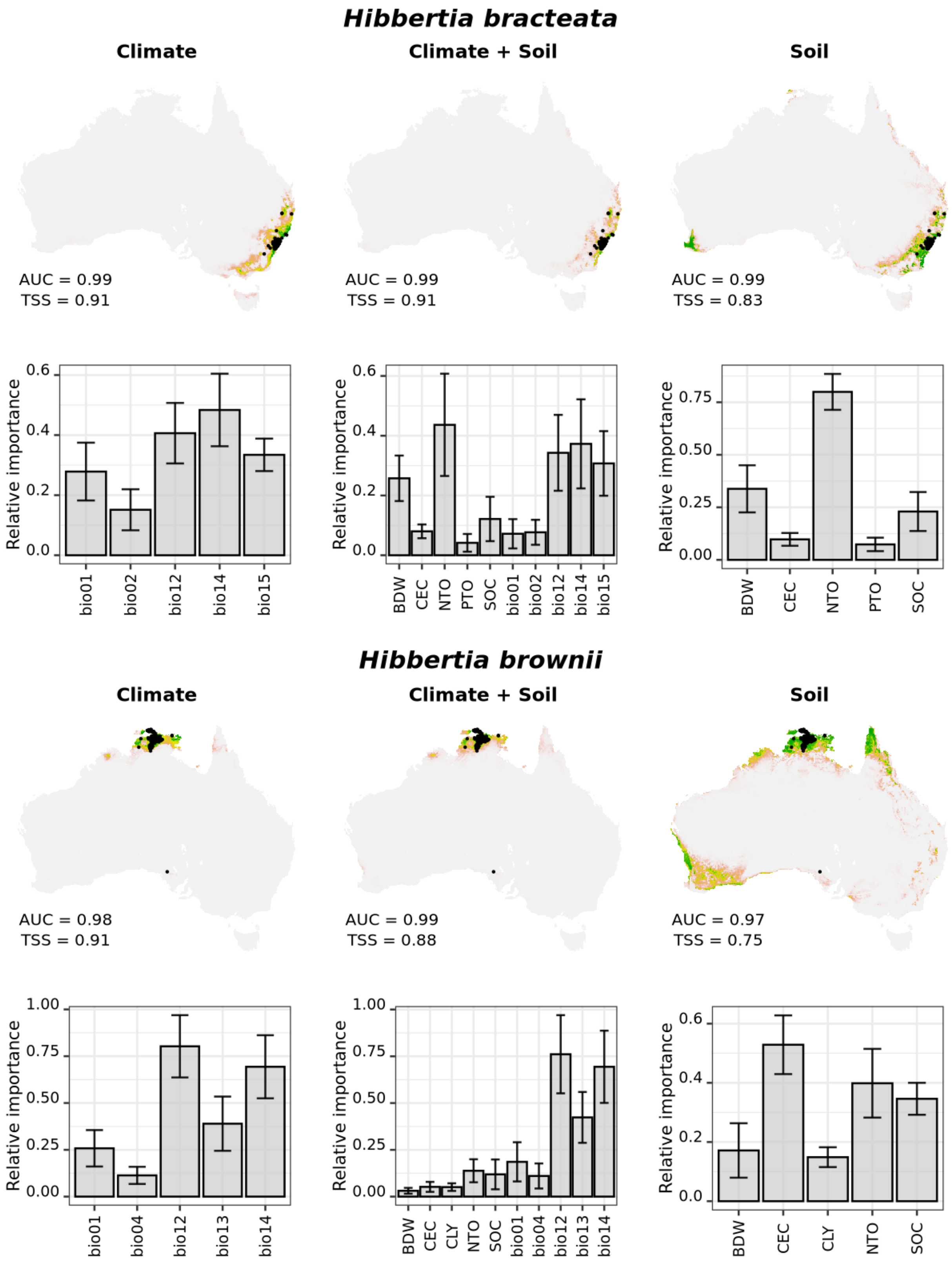
Hibbertia bracteata
Climate
Climate + Soil
Soil
AUC = 0.99
TSS = 0.91
AUC = 0.99
TSS = 0.91
AUC = 0.99
TSS = 0.83
Relative importance
bio01
bio02
bio12
bio14
bio15
BDW
CEC
NTO
PTO
SOC
Hibbertia brownii
Climate
Climate + Soil
Soil
AUC = 0.98
TSS = 0.91
AUC = 0.99
TSS = 0.88
AUC = 0.97
TSS = 0.75
Relative importance
bio01
bio04
bio12
bio13
bio14
BDW
CEC
CLY
NTO
SOC

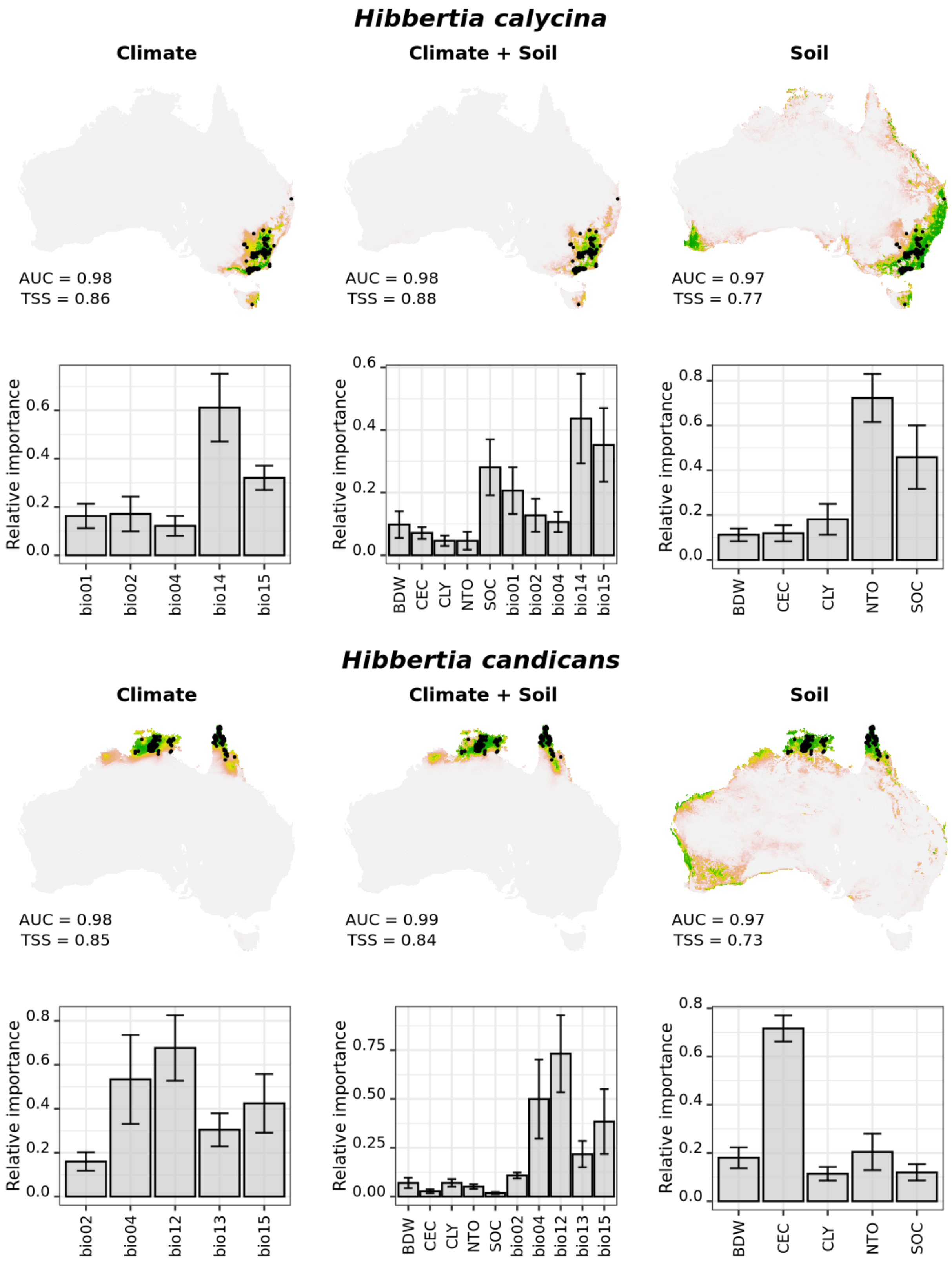
Hibbertia calycina
Climate
Climate + Soil
Soil
AUC = 0.98
TSS = 0.86
AUC = 0.98
TSS = 0.88
AUC = 0.97
TSS = 0.77
Relative importance
bio01
bio02
bio04
bio14
bio15
BDW
CEC
CLY
NTO
SOC
Hibbertia candicans
Climate
Climate + Soil
Soil
AUC = 0.98
TSS = 0.85
AUC = 0.99
TSS = 0.84
AUC = 0.97
TSS = 0.73
bio12
bio13

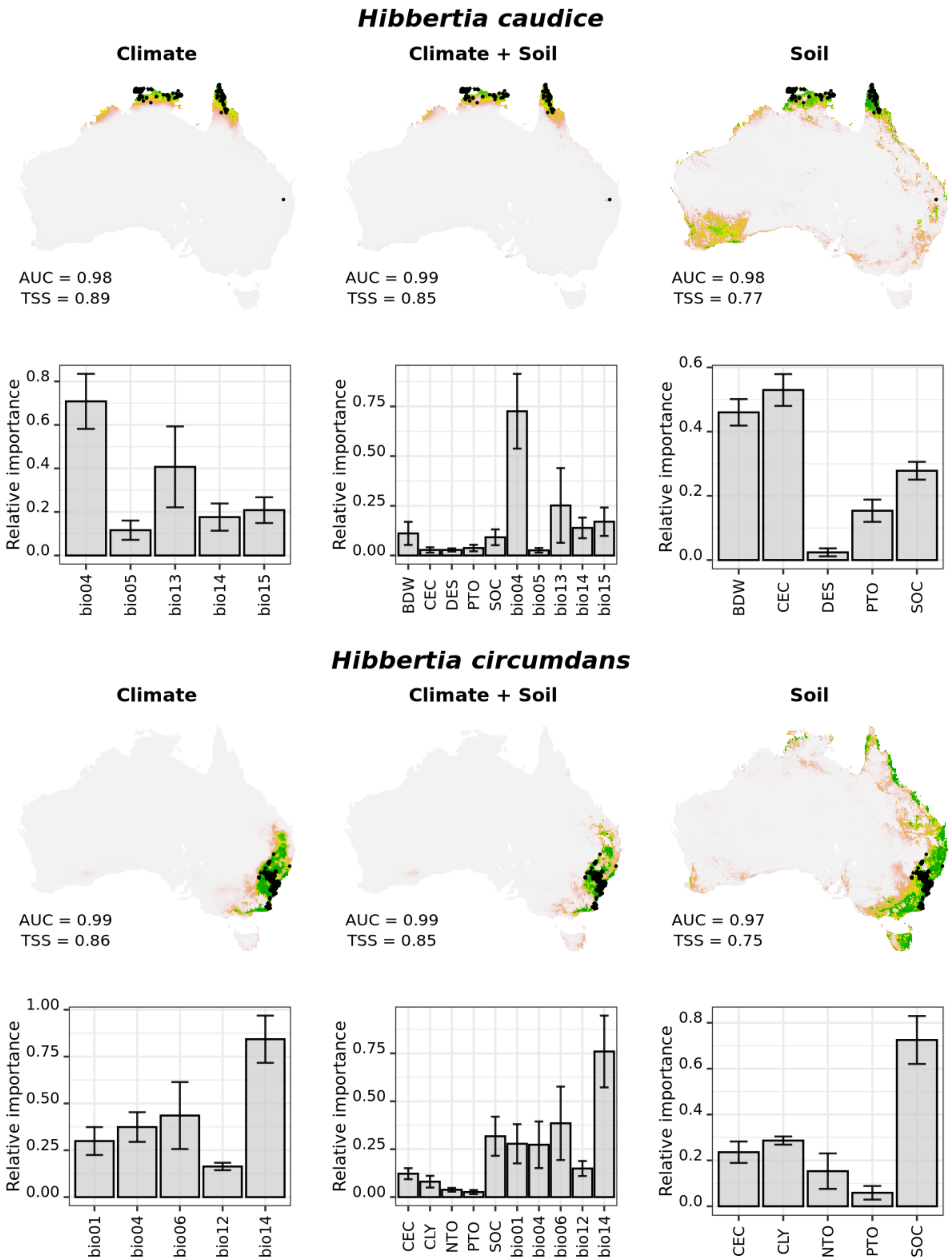
Hibbertia caudice
Climate
Climate + Soil
Soil
AUC = 0.98
TSS = 0.89
AUC = 0.99
TSS = 0.85
AUC = 0.98
TSS = 0.77
Relative importance
0.0
0.2
0.4
0.6
0.8
bio04
bio05
bio13
bio14
bio15
Relative importance
0.00
0.25
0.50
0.75
BDW
CEC
DES
PTO
SOC
bio04
bio05
bio13
bio14
bio15
Relative importance
0.0
0.2
0.4
0.6
BDW
CEC
DES
PTO
SOC
Hibbertia circumdans
Climate
Climate + Soil
Soil
AUC = 0.99
TSS = 0.86
AUC = 0.99
TSS = 0.85
AUC = 0.97
TSS = 0.75
Relative importance
0.00
0.25
0.50
0.75
1.00
bio01
bio04
bio06
bio12
bio14
Relative importance
0.00
0.25
0.50
0.75
CEC
CLY
NTO
PTO
SOC
bio01
bio04
bio06
bio12
bio14
Relative importance
0.0
0.2
0.4
0.6
0.8
CEC
CLY
NTO
PTO
SOC

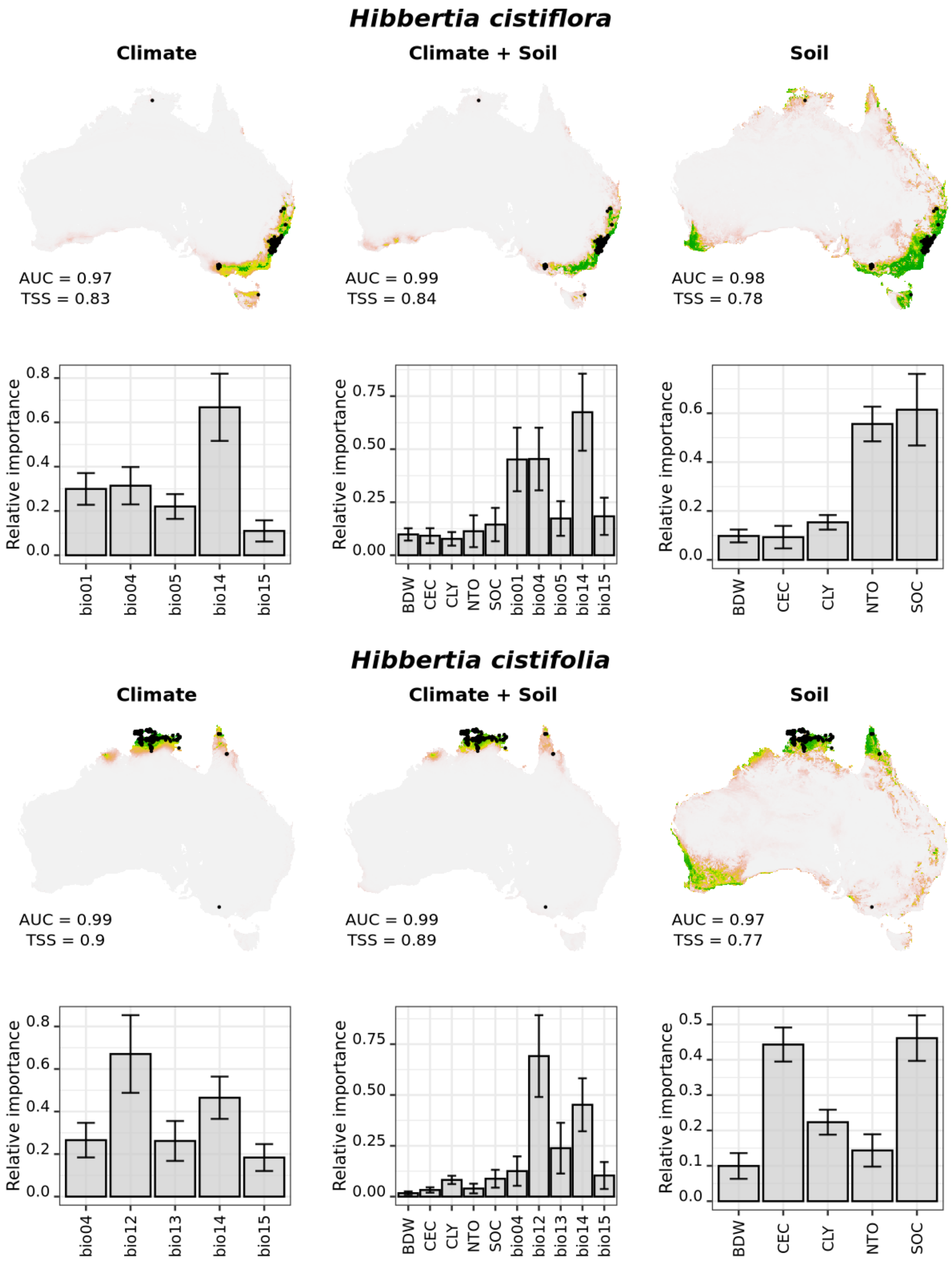
Hibbertia cistiflora
Climate
Climate + Soil
Soil
AUC = 0.97
TSS = 0.83
AUC = 0.99
TSS = 0.84
AUC = 0.98
TSS = 0.78
Relative importance
bio01
bio04
bio05
bio14
bio15
BDW
CEC
CLY
NTO
SOC
Hibbertia cistifolia
AUC = 0.99
TSS = 0.9
AUC = 0.99
TSS = 0.89
AUC = 0.97
TSS = 0.77
bio12
bio13

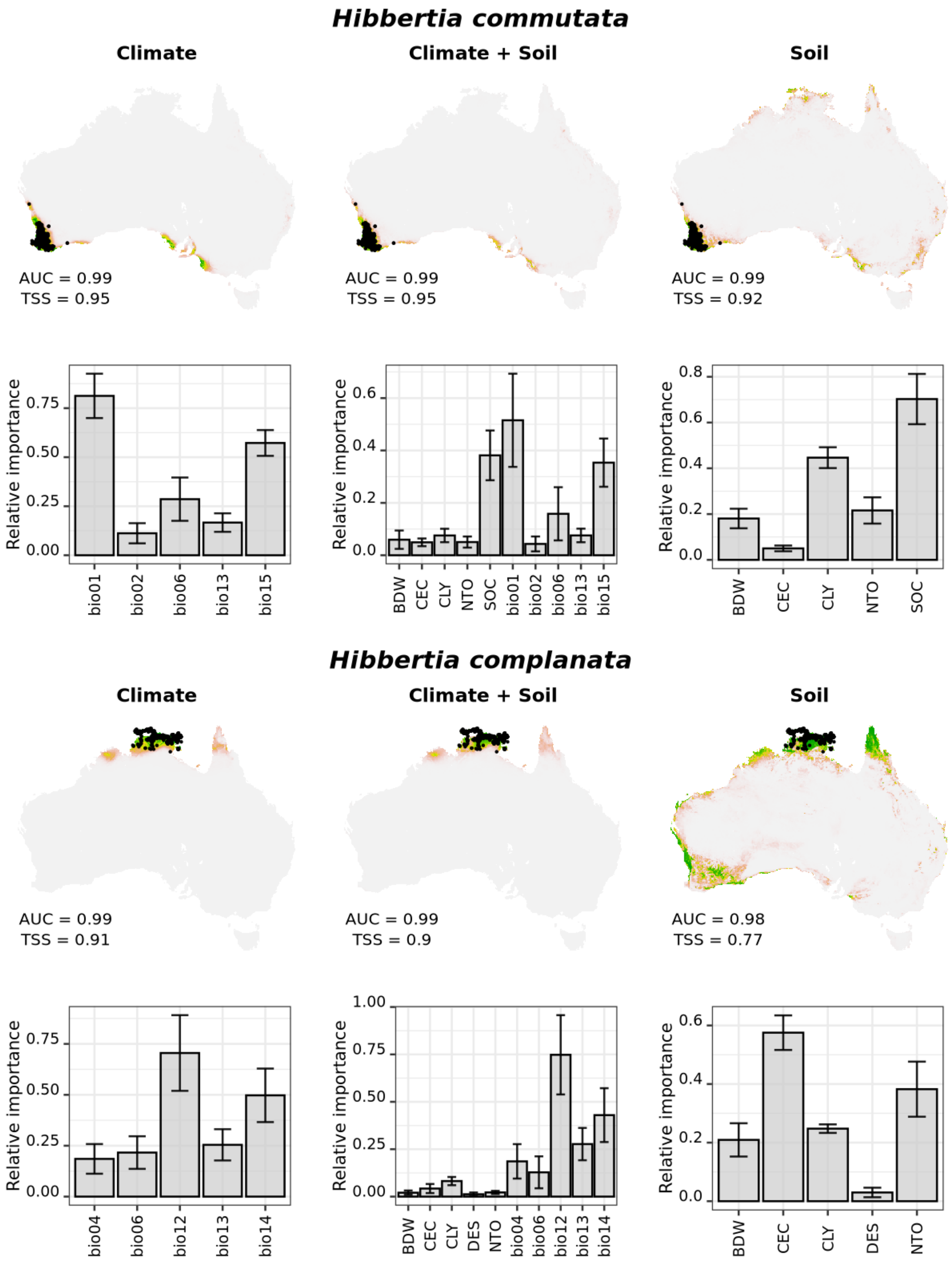
Hibbertia commutata
Climate
Climate + Soil
Soil
AUC = 0.99
TSS = 0.95
AUC = 0.99
TSS = 0.95
AUC = 0.99
TSS = 0.92
Relative importance
bio01
bio02
bio06
bio13
bio15
BDW
CEC
CLY
NTO
SOC
Hibbertia complanata
Climate
Climate + Soil
Soil
AUC = 0.99
TSS = 0.91
AUC = 0.99
TSS = 0.9
AUC = 0.98
TSS = 0.77
Relative importance
bio04
bio06
bio12
bio13
bio14
BDW
CEC
CLY
DES
NTO

Hibbertia cuneiformis

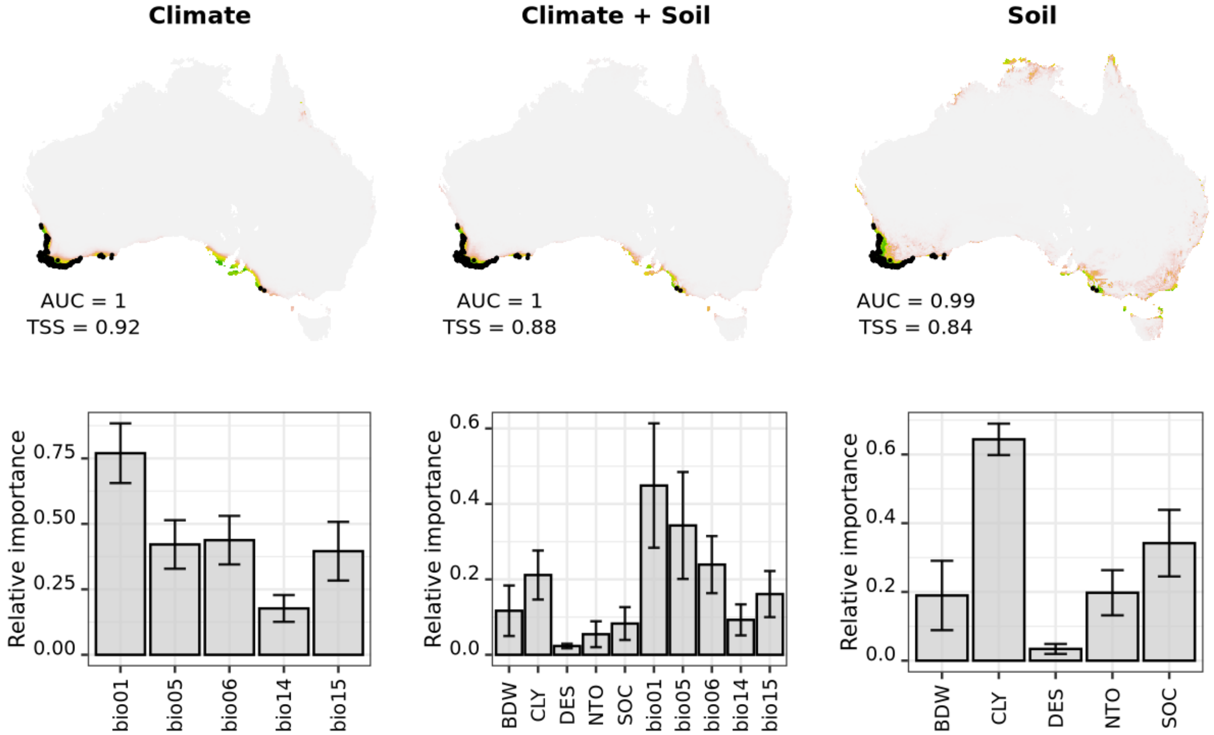

Hibbertia cunninghamii

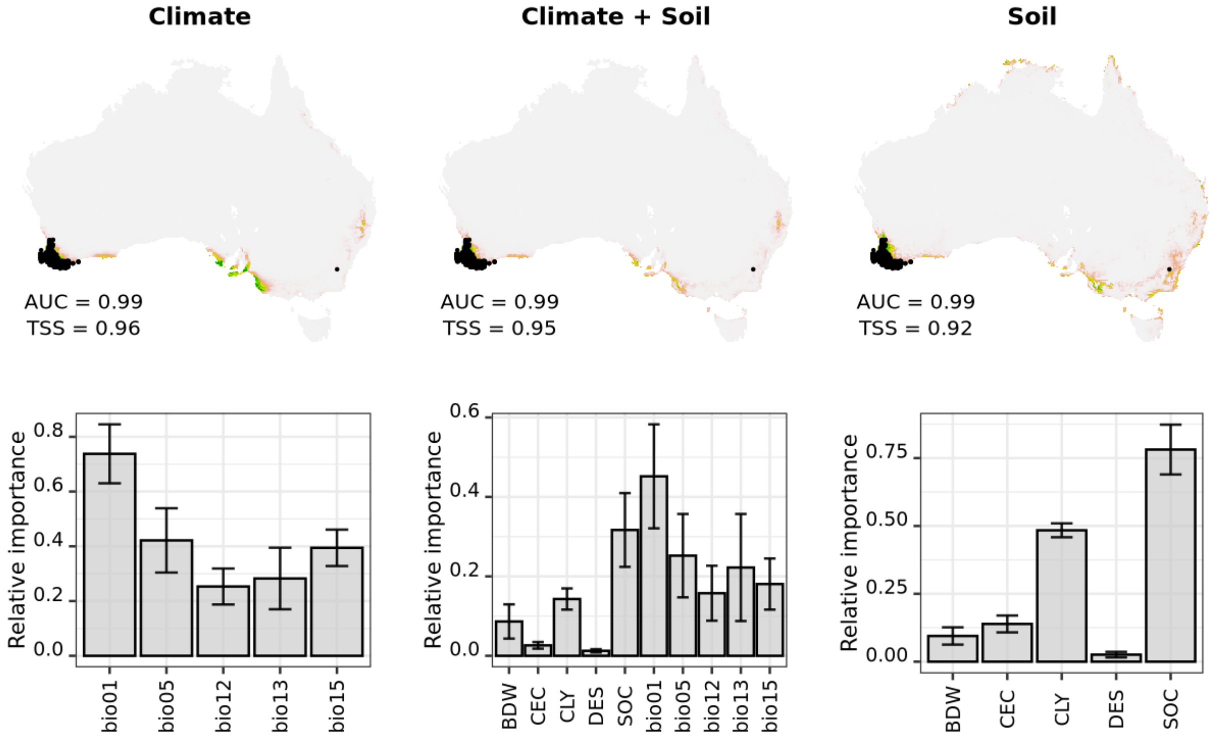

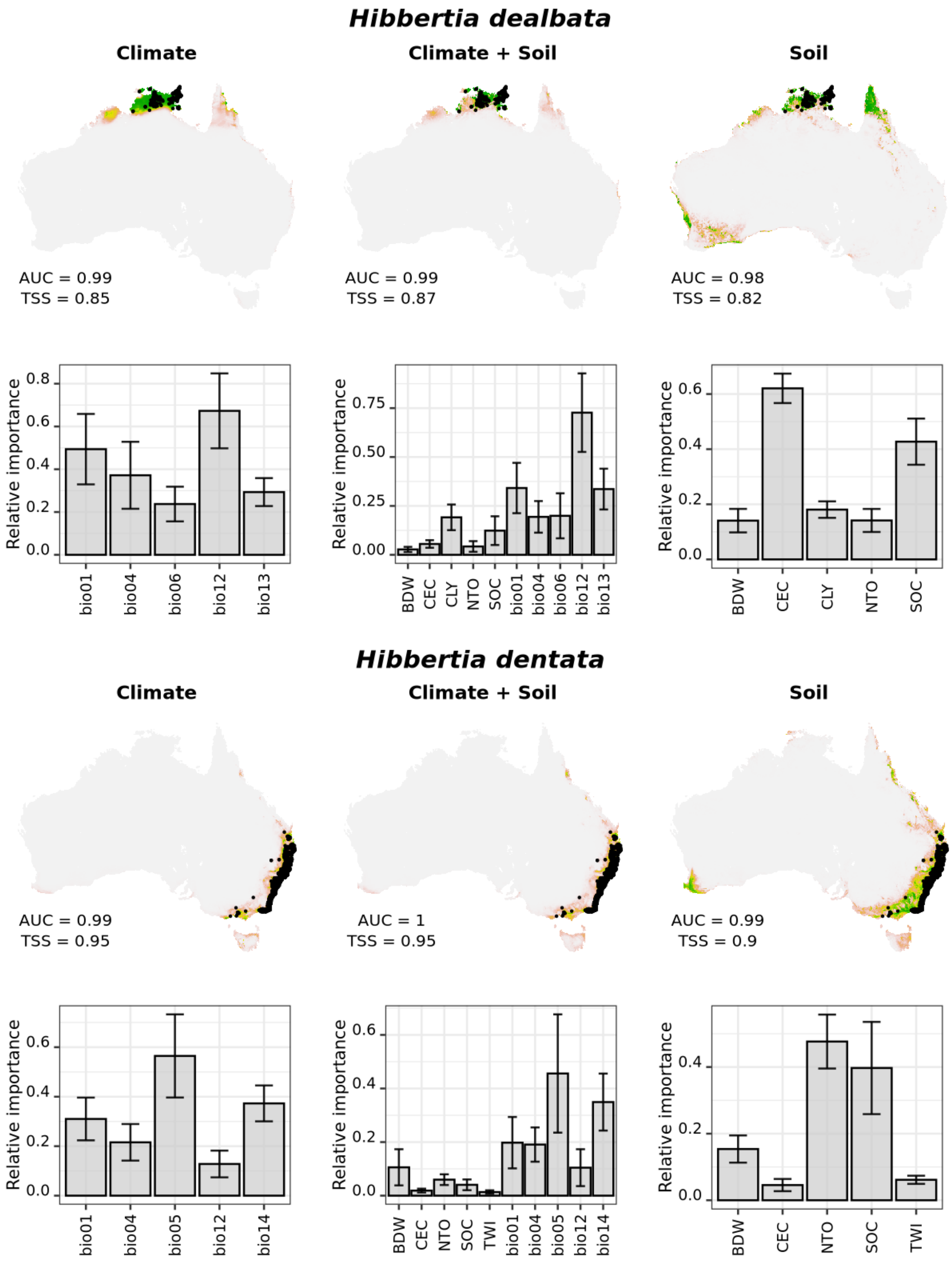

Hibbertia dealbata
Climate
Climate + Soil
Soil
AUC = 0.99
TSS = 0.85
AUC = 0.99
TSS = 0.87
AUC = 0.98
TSS = 0.82
Relative importance
bio01
bio04
bio06
bio12
bio13
BDW
CEC
CLY
NTO
SOC
Hibbertia dentata
Climate
Climate + Soil
Soil
AUC = 0.99
TSS = 0.95
AUC = 1
TSS = 0.95
AUC = 0.99
TSS = 0.9
Relative importance
bio01
bio04
bio05
bio12
bio14
BDW
CEC
NTO
SOC
TWI

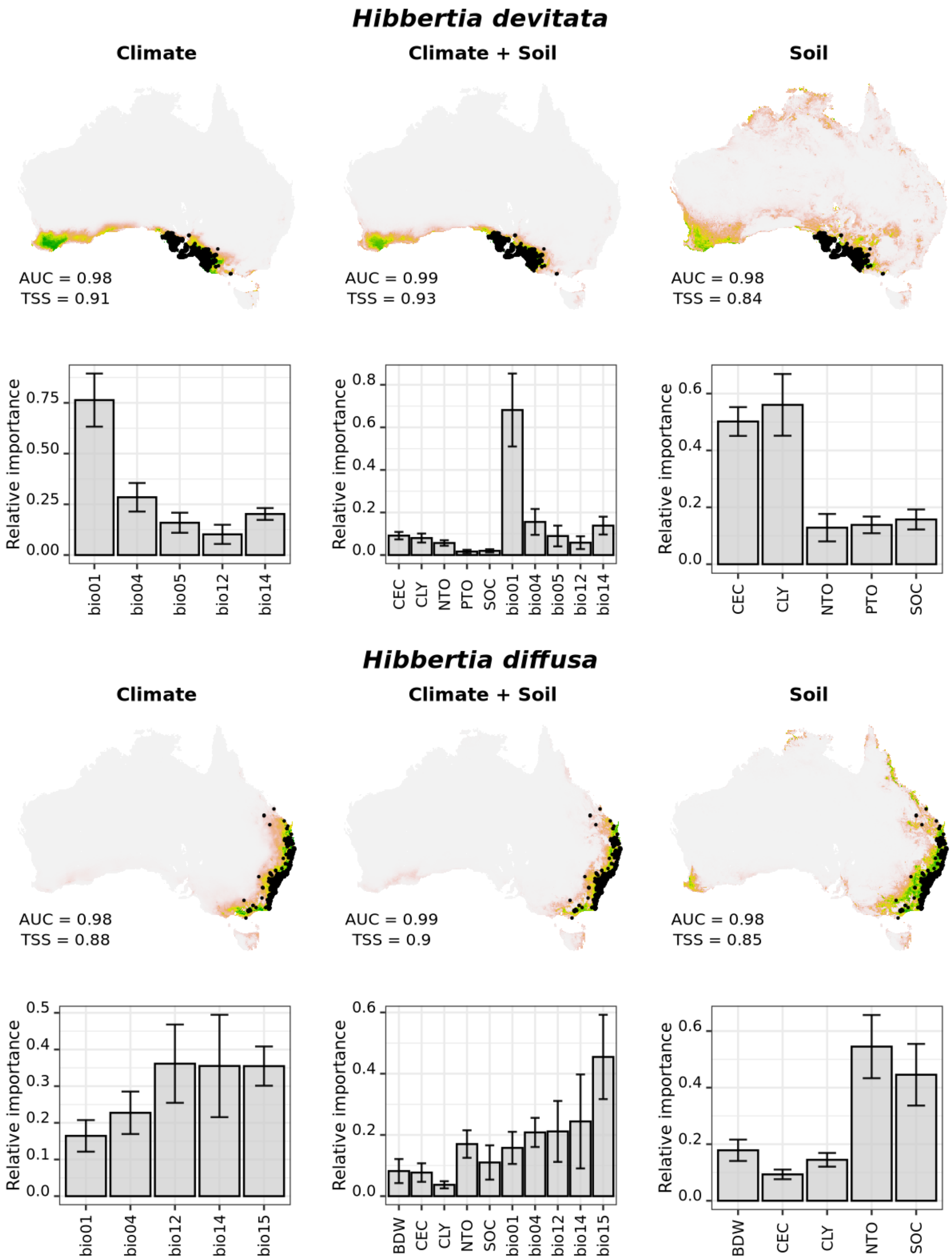

Hibbertia devitata
Climate
Climate + Soil
Soil
AUC = 0.98
TSS = 0.91
AUC = 0.99
TSS = 0.93
AUC = 0.98
TSS = 0.84
Relative importance
0.00
0.25
0.50
0.75
bio01
bio04
bio05
bio12
bio14
0.0
0.2
0.4
0.6
0.8
CEC
CLY
NTO
PTO
SOC
bio01
bio04
bio05
bio12
bio14
0.0
0.2
0.4
0.6
CEC
CLY
NTO
PTO
SOC
Hibbertia diffusa
Climate
Climate + Soil
Soil
AUC = 0.98
TSS = 0.88
AUC = 0.99
TSS = 0.9
AUC = 0.98
TSS = 0.85
Relative importance
0.0
0.1
0.2
0.3
0.4
0.5
bio01
bio04
bio12
bio14
bio15
0.0
0.2
0.4
0.6
BDW
CEC
CLY
NTO
SOC
bio01
bio04
bio12
bio14
bio15
0.0
0.2
0.4
0.6
BDW
CEC
CLY
NTO
SOC

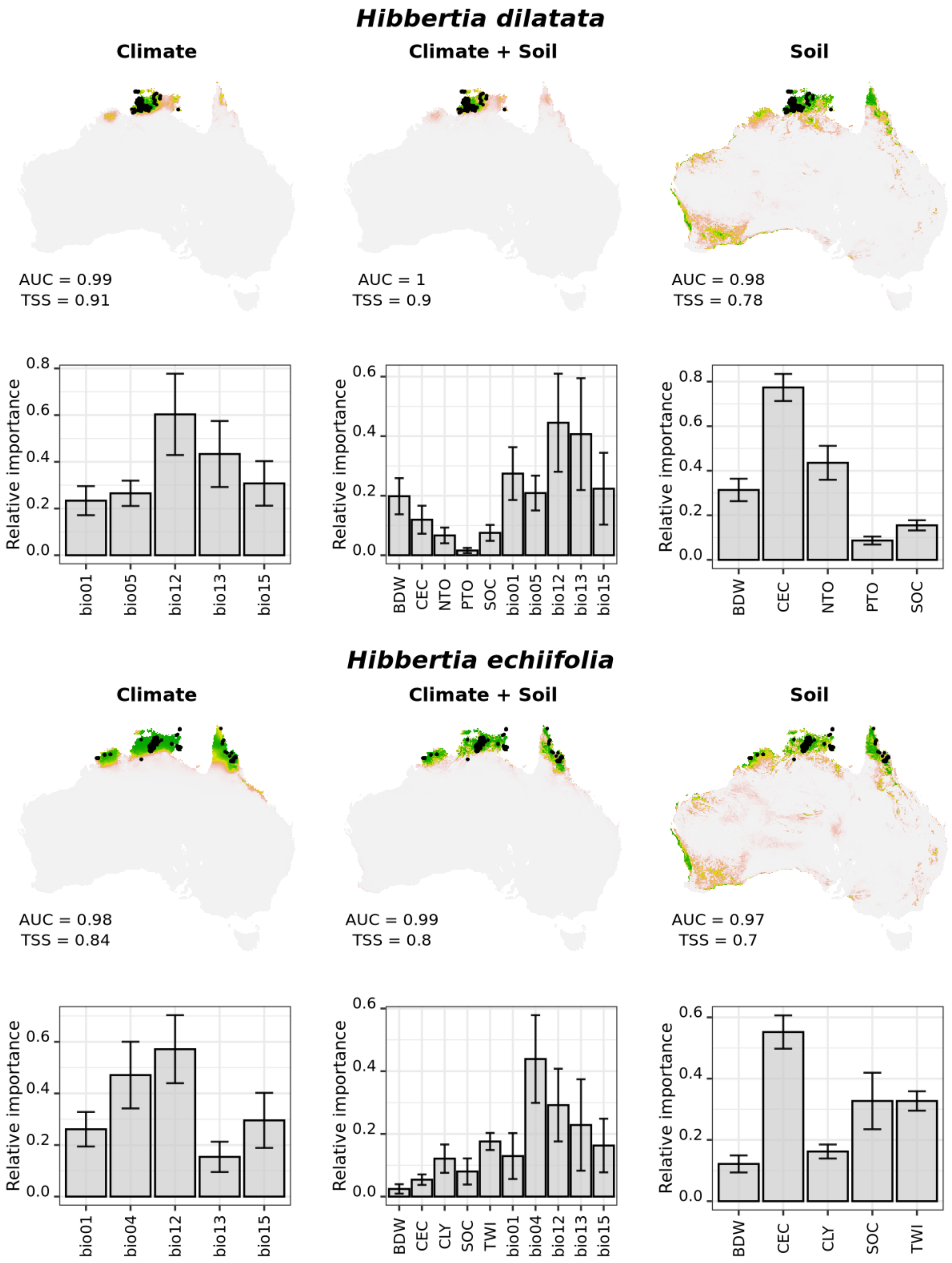
Hibbertia dilatata
Climate
Climate + Soil
Soil
AUC = 0.99
TSS = 0.91
AUC = 1
TSS = 0.9
AUC = 0.98
TSS = 0.78
Relative importance
bio01 bio05 bio12 bio13 bio15
BDW CEC NTO PTO SOC bio01 bio05 bio12 bio13 bio15
BDW CEC NTO PTO SOC
Hibbertia echiifolia
Climate
Climate + Soil
Soil
AUC = 0.98
TSS = 0.84
AUC = 0.99
TSS = 0.8
AUC = 0.97
TSS = 0.7
Relative importance
bio01 bio04 bio12 bio13 bio15
BDW CEC CLY SOC TWI bio01 bio04 bio12 bio13 bio15
BDW CEC CLY SOC TWI

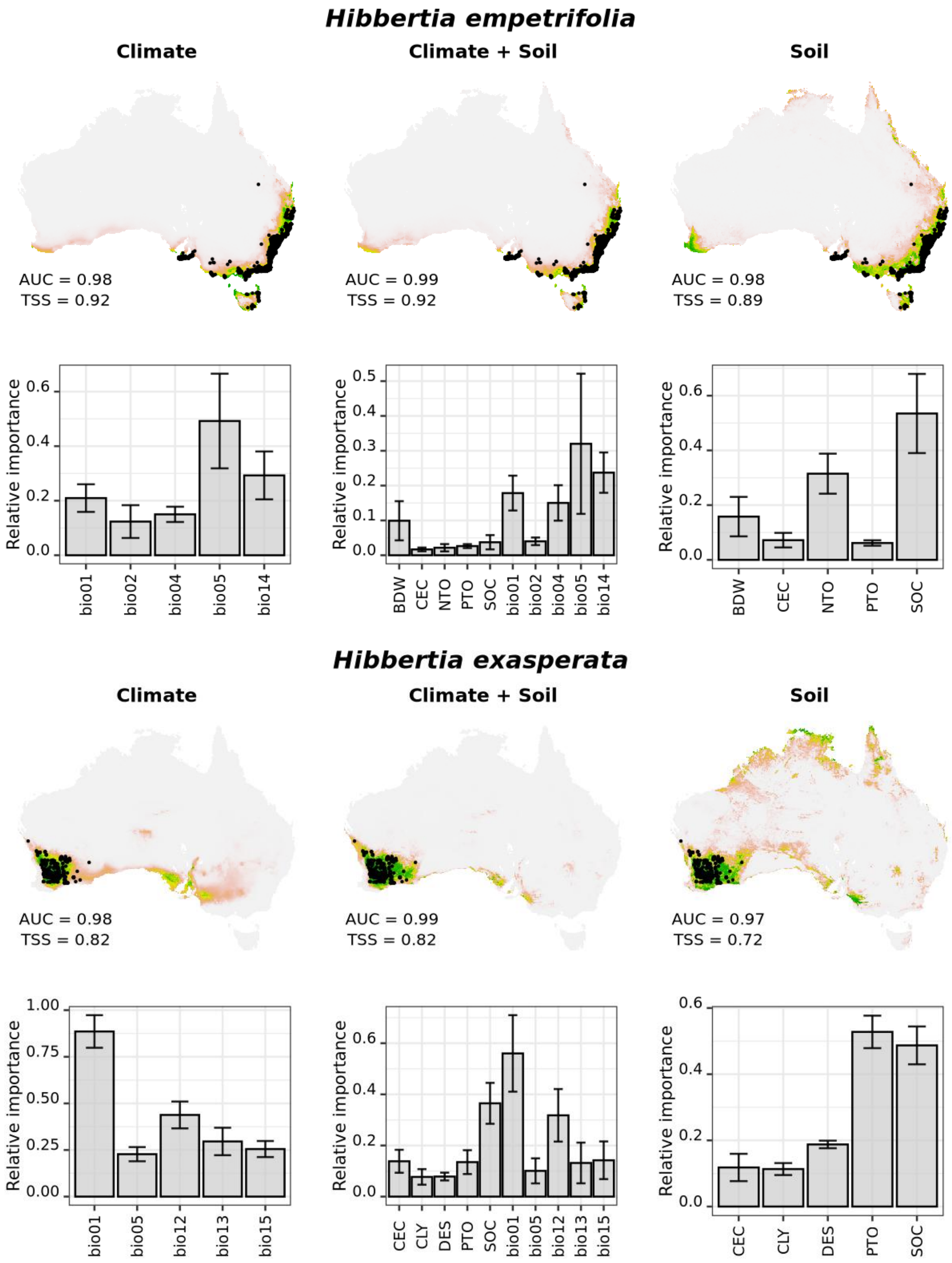

Hibbertia empetrifolia
Climate
Climate + Soil
Soil
AUC = 0.98
TSS = 0.92
AUC = 0.99
TSS = 0.92
AUC = 0.98
TSS = 0.89
Relative importance
bio01
bio02
bio04
bio05
bio14
BDW
CEC
NTO
PTO
SOC
Hibbertia exasperata
Climate
Climate + Soil
Soil
AUC = 0.98
TSS = 0.82
AUC = 0.99
TSS = 0.82
AUC = 0.97
TSS = 0.72
Relative importance
bio01
bio05
bio12
bio13
bio15
CEC
CLY
DES
PTO
SOC

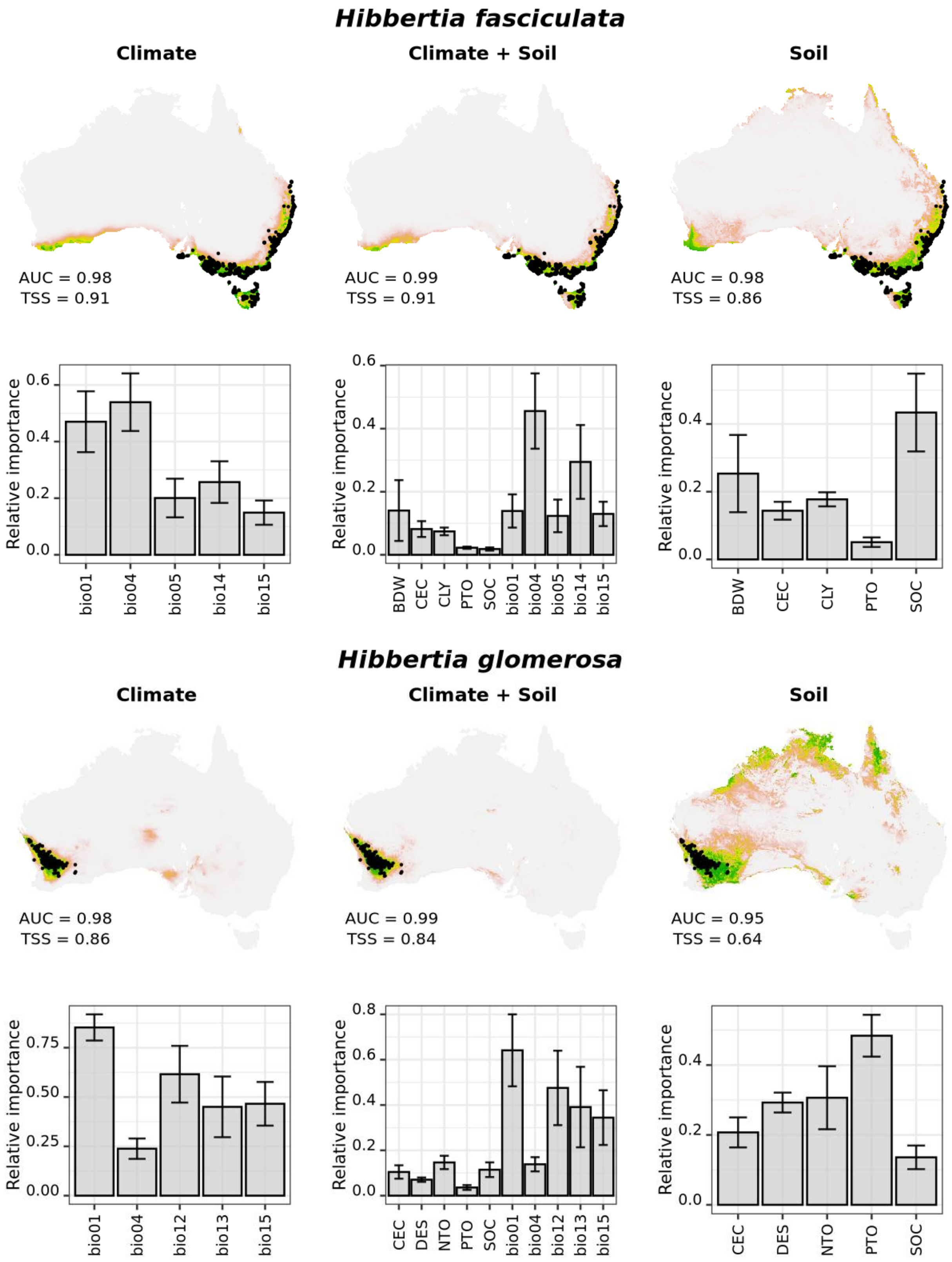

Hibbertia fasciculata
Climate
Climate + Soil
Soil
AUC = 0.98
TSS = 0.91
AUC = 0.99
TSS = 0.91
AUC = 0.98
TSS = 0.86
Relative importance
0.0
0.2
0.4
0.6
bio01
bio04
bio05
bio14
bio15
BDW
CEC
CLY
PTO
SOC
Hibbertia glomerosa
Climate
Climate + Soil
Soil
AUC = 0.98
TSS = 0.86
AUC = 0.99
TSS = 0.84
AUC = 0.95
TSS = 0.64
0.00
0.25
0.50
0.75
0.8
bio12
bio13
DES
NTO

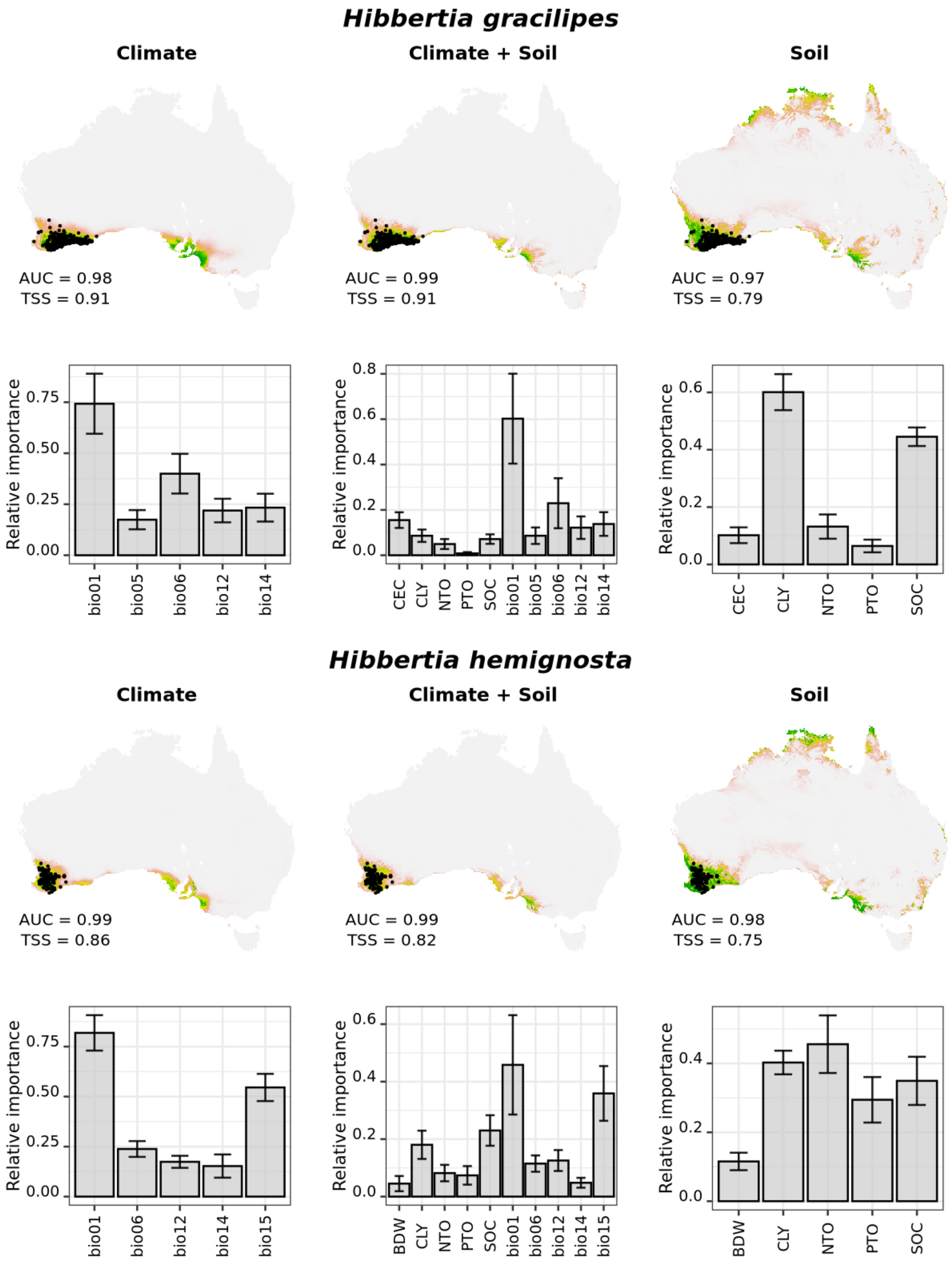

Hibbertia gracilipes
Climate
Climate + Soil
Soil
AUC = 0.98
TSS = 0.91
AUC = 0.99
TSS = 0.91
AUC = 0.97
TSS = 0.79
Relative importance
0.00
0.25
0.50
0.75
bio01
bio05
bio06
bio12
bio14
0.0
0.2
0.4
0.6
0.8
CEC
CLY
NTO
PTO
SOC
bio01
bio05
bio06
bio12
bio14
0.0
0.2
0.4
0.6
CEC
CLY
NTO
PTO
SOC
Hibbertia hemignosta
Climate
Climate + Soil
Soil
AUC = 0.99
TSS = 0.86
AUC = 0.99
TSS = 0.82
AUC = 0.98
TSS = 0.75
Relative importance
0.00
0.25
0.50
0.75
bio01
bio06
bio12
bio14
bio15
0.0
0.2
0.4
0.6
BDW
CLY
NTO
PTO
SOC
bio01
bio06
bio12
bio14
bio15
0.0
0.2
0.4
BDW
CLY
NTO
PTO
SOC

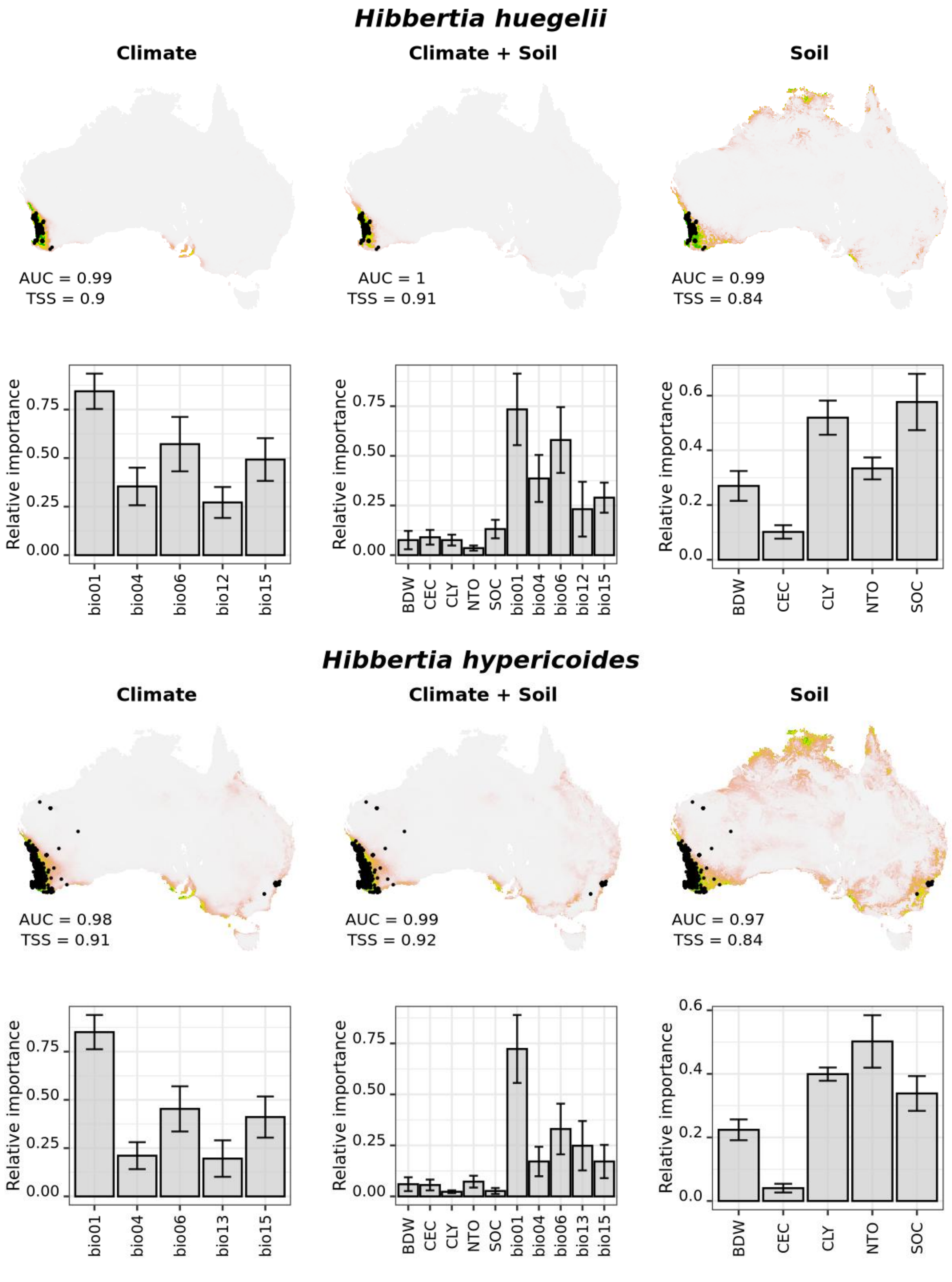

Hibbertia huegelii
Climate
Climate + Soil
Soil
AUC = 0.99
TSS = 0.9
AUC = 1
TSS = 0.91
AUC = 0.99
TSS = 0.84
Relative importance
0.00
0.25
0.50
0.75
bio01
bio04
bio06
bio12
bio15
BDW
CEC
CLY
NTO
SOC
0.0
0.2
0.4
0.6
Hibbertia hypericoides
Climate
Climate + Soil
Soil
AUC = 0.98
TSS = 0.91
AUC = 0.99
TSS = 0.92
AUC = 0.97
TSS = 0.84
Relative importance
bio01
bio04
bio06
bio13
bio15
BDW
CEC
CLY
NTO
SOC

Hibbertia juncea

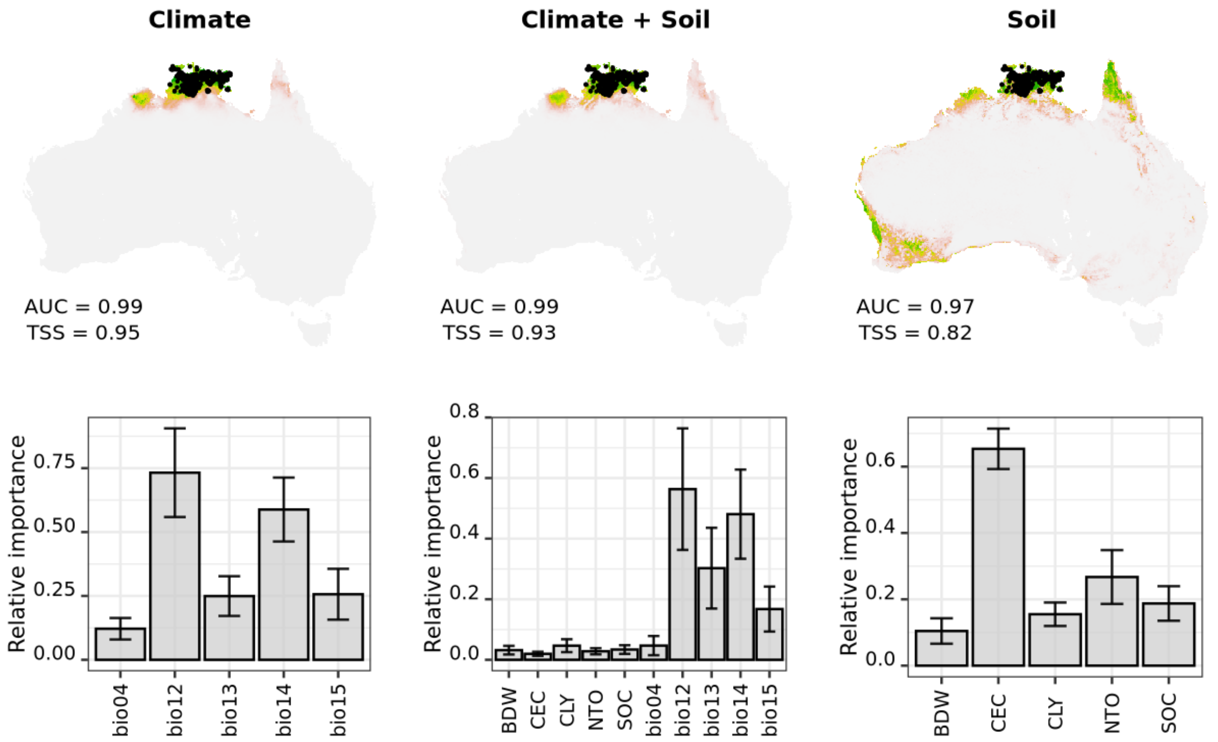

Hibbertia lepidota

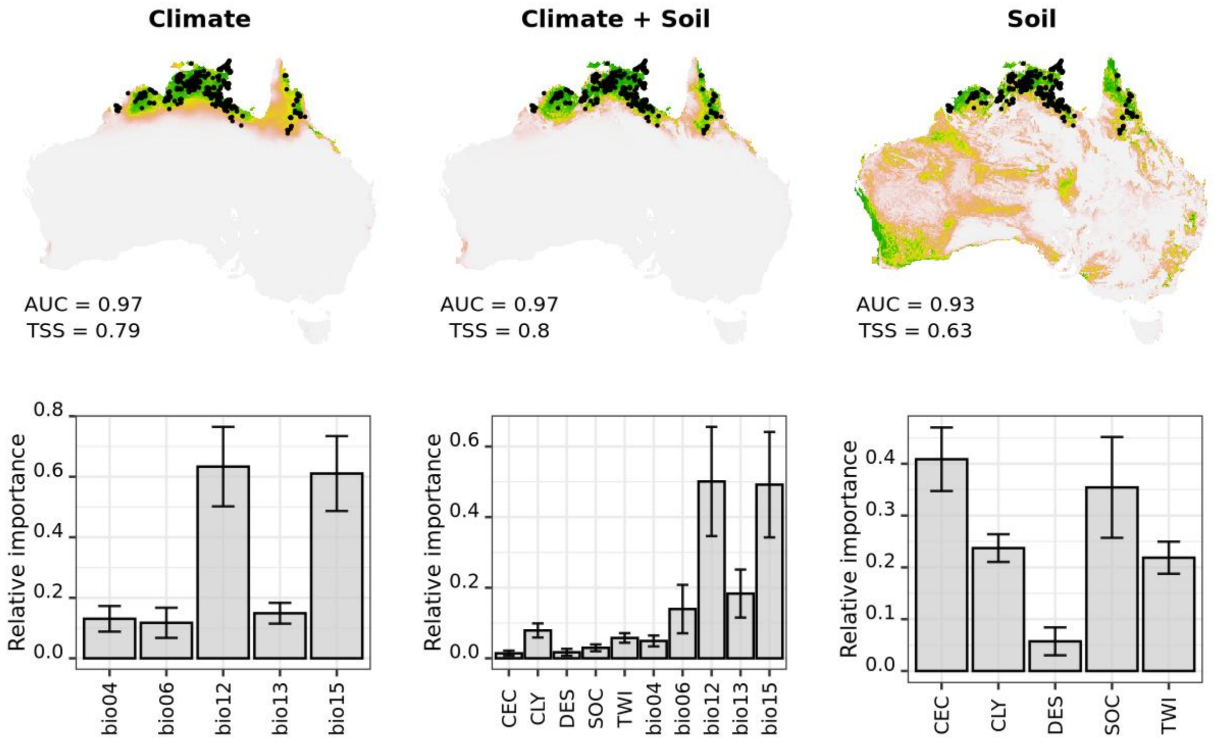

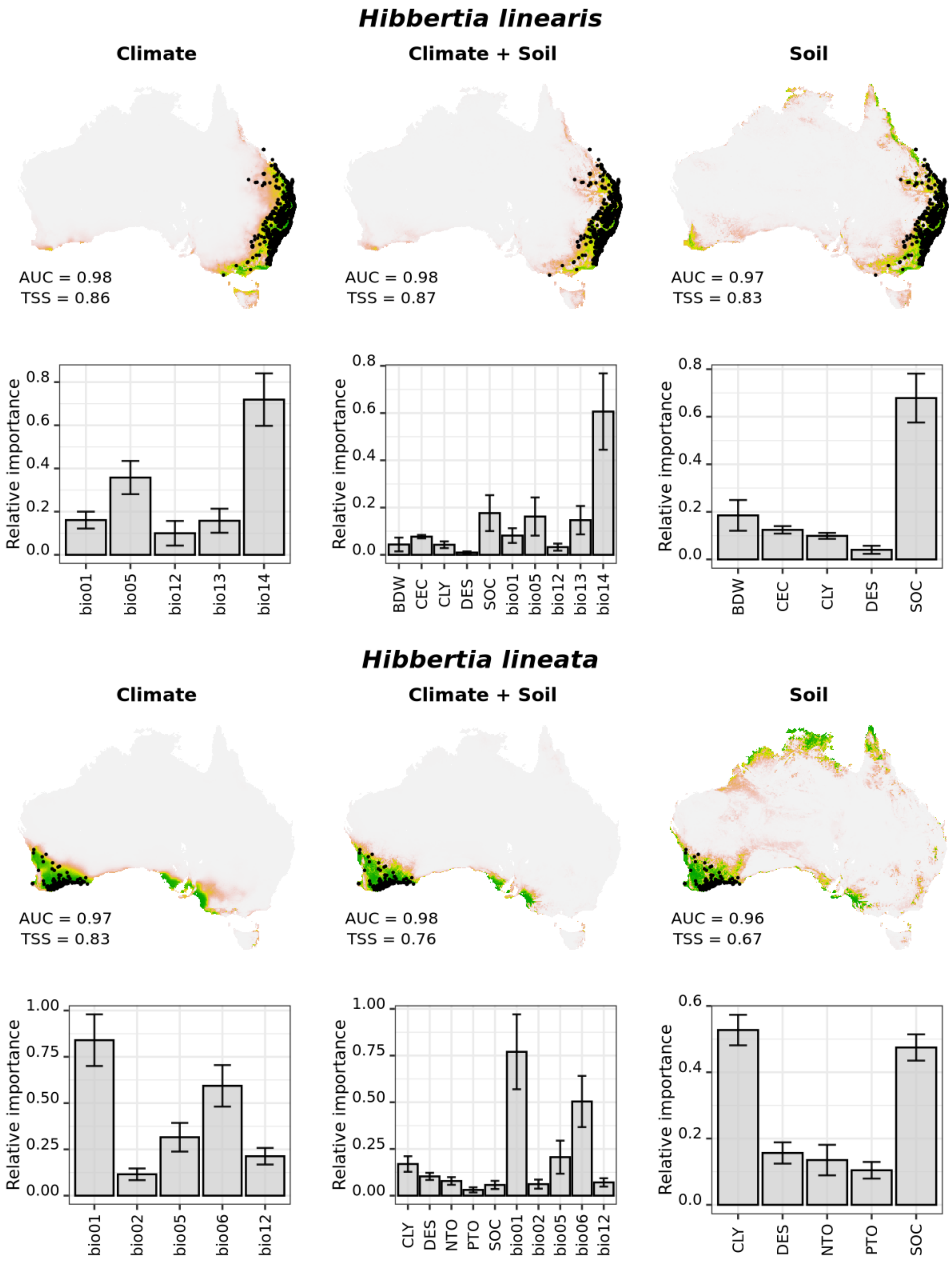
Hibbertia linearis
Climate
Climate + Soil
Soil
AUC = 0.98
TSS = 0.86
AUC = 0.98
TSS = 0.87
AUC = 0.97
TSS = 0.83
Relative importance
bio01
bio05
bio12
bio13
bio14
BDW
CEC
CLY
DES
SOC
Hibbertia lineata
AUC = 0.97
TSS = 0.83
AUC = 0.98
TSS = 0.76
AUC = 0.96
TSS = 0.67
bio02
bio06
NTO
PTO

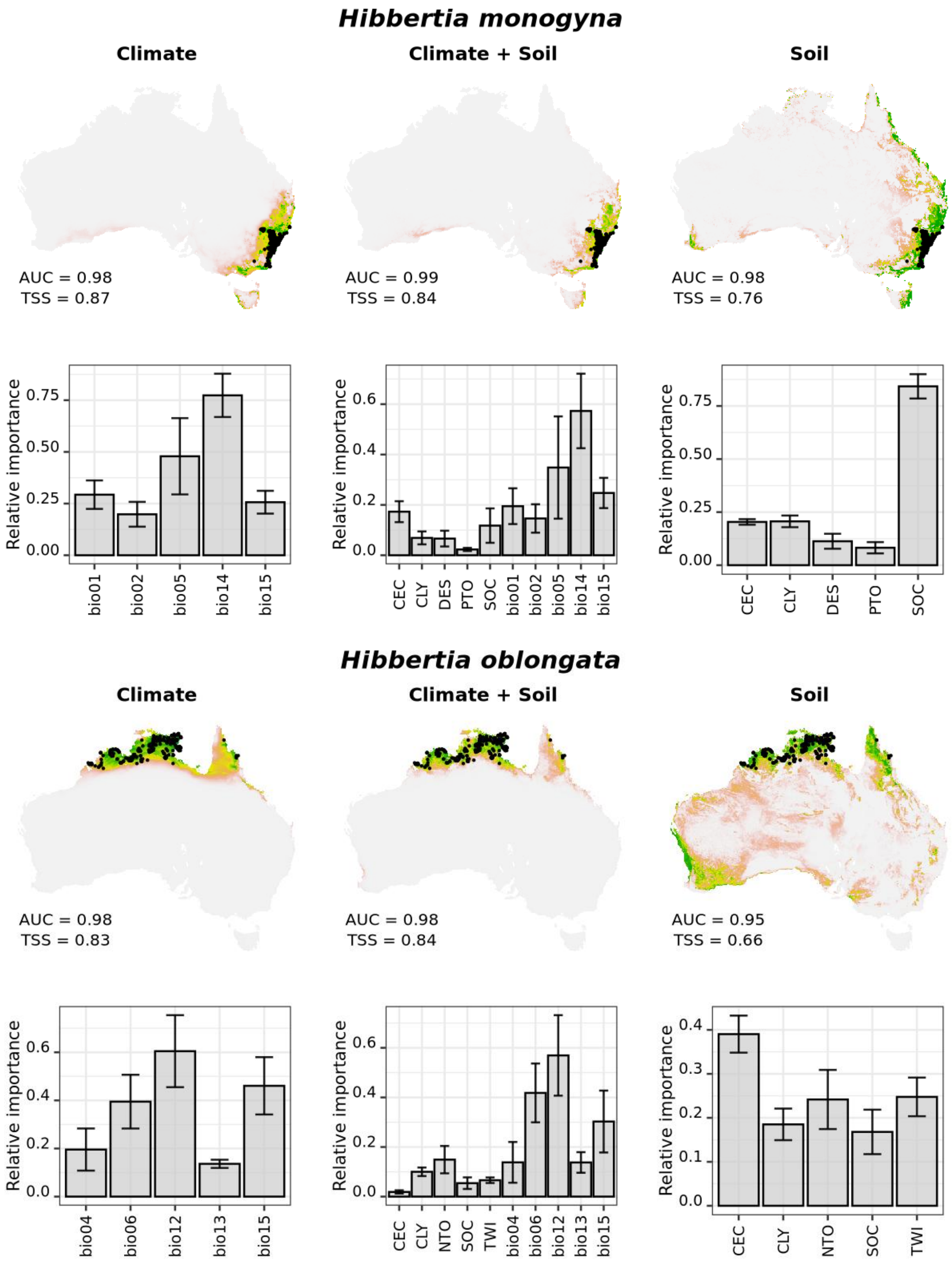

Hibbertia monogyna
Climate
Climate + Soil
Soil
AUC = 0.98
TSS = 0.87
AUC = 0.99
TSS = 0.84
AUC = 0.98
TSS = 0.76
Relative importance
0.00
0.25
0.50
0.75
bio01
bio02
bio05
bio14
bio15
0.0
0.2
0.4
0.6
CEC
CLY
DES
PTO
SOC
bio01
bio02
bio05
bio14
bio15
CEC
CLY
DES
PTO
SOC
Hibbertia oblongata
Climate
Climate + Soil
Soil
AUC = 0.98
TSS = 0.83
AUC = 0.98
TSS = 0.84
AUC = 0.95
TSS = 0.66
bio04
bio06
bio12
bio13
bio15
CEC
CLY
NTO
SOC
TWI
bio04
bio06
bio12
bio13
bio15
0.1
0.2
0.3
0.4
CEC
CLY
NTO
SOC
TWI

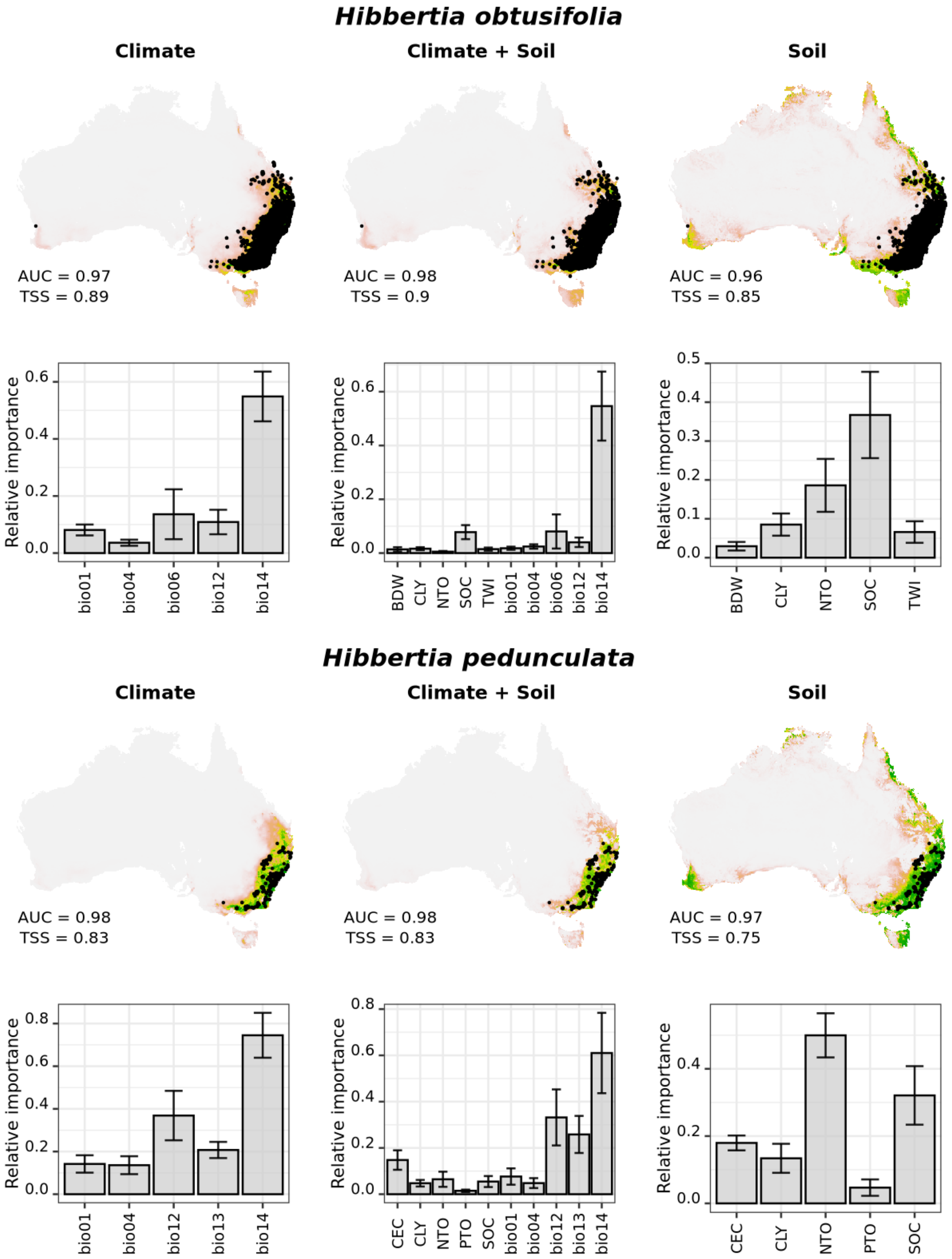

Hibbertia obtusifolia
Climate
Climate + Soil
Soil
AUC = 0.97
TSS = 0.89
AUC = 0.98
TSS = 0.9
AUC = 0.96
TSS = 0.85
Relative importance
bio01 bio04 bio06 bio12 bio14
BDW CLY NTO SOC TWI bio01 bio04 bio06 bio12 bio14
BDW CLY NTO SOC TWI
Hibbertia pedunculata
Climate
Climate + Soil
Soil
AUC = 0.98
TSS = 0.83
AUC = 0.98
TSS = 0.83
AUC = 0.97
TSS = 0.75
Relative importance
bio01 bio04 bio12 bio13 bio14
CEC CLY NTO PTO SOC bio01 bio04 bio12 bio13 bio14
CEC CLY NTO PTO SOC

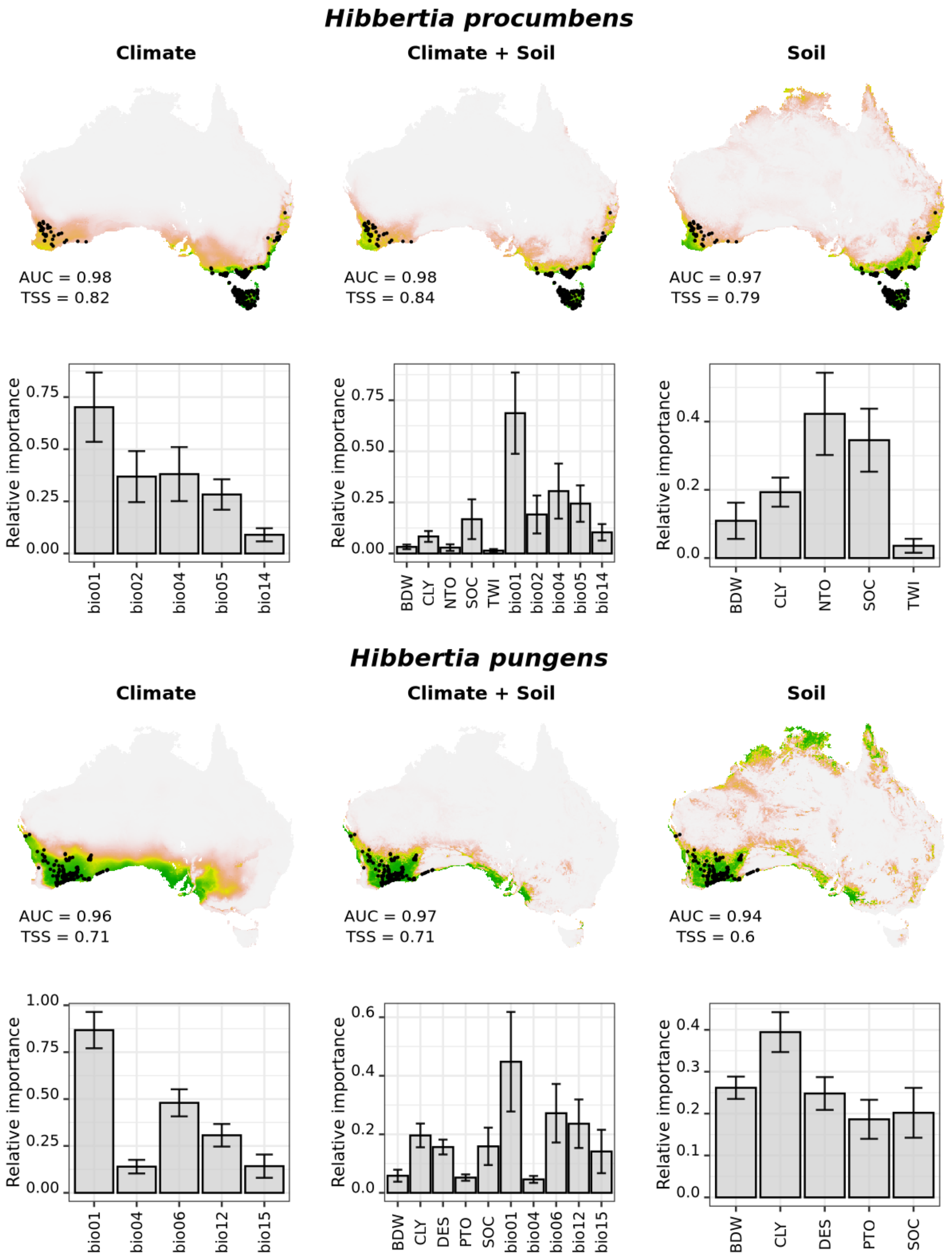
Hibbertia procumbens
Climate
Climate + Soil
Soil
AUC = 0.98
TSS = 0.82
AUC = 0.98
TSS = 0.84
AUC = 0.97
TSS = 0.79
Relative importance
0.00
0.25
0.50
0.75
bio01
bio02
bio04
bio05
bio14
BDW
CLY
NTO
SOC
TWI
0.0
0.2
0.4
Hibbertia pungens
Climate
Climate + Soil
Soil
AUC = 0.96
TSS = 0.71
AUC = 0.97
TSS = 0.71
AUC = 0.94
TSS = 0.6
1.00
bio06
bio12
bio15
DES
PTO
0.1
0.3
0.6

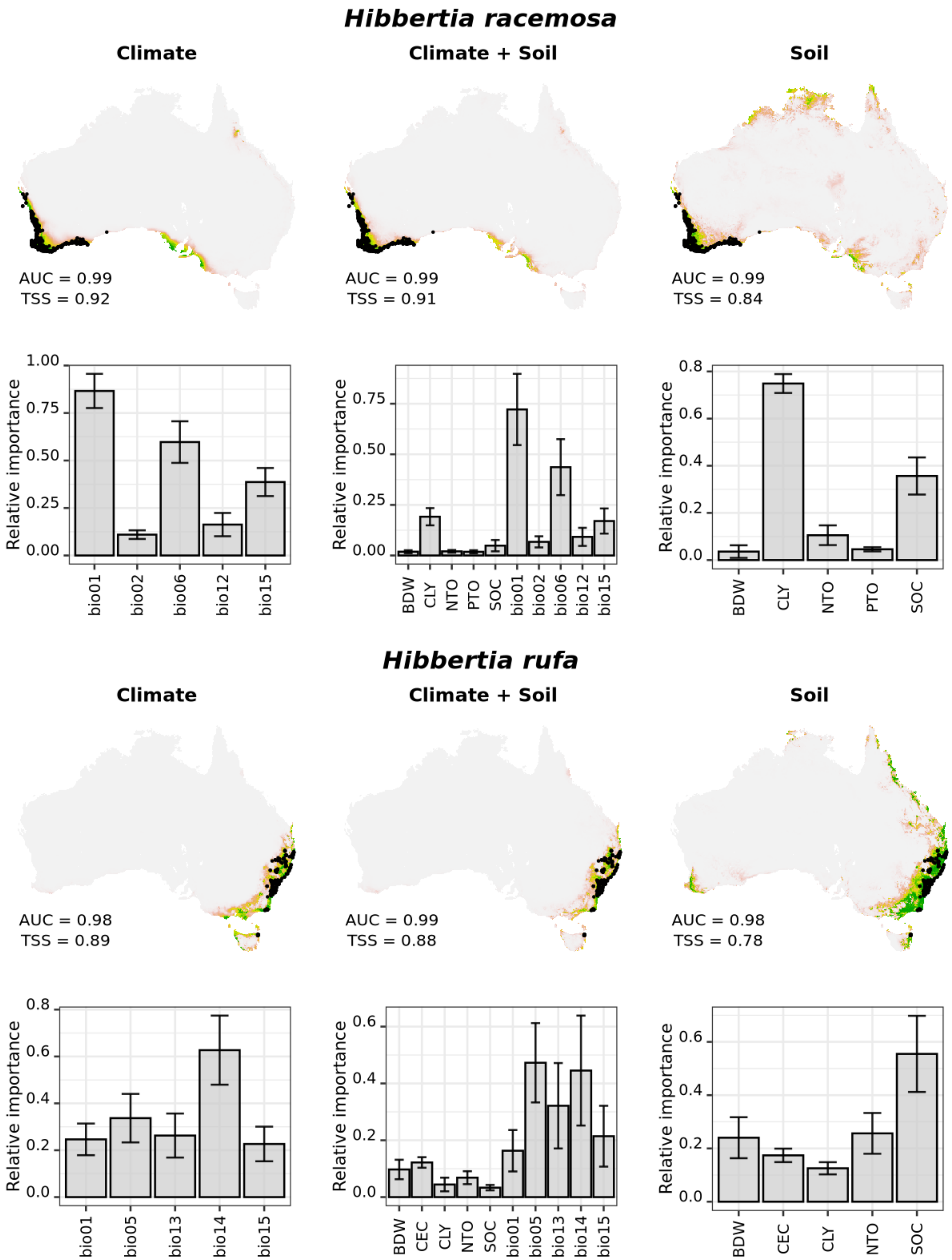
Hibbertia racemosa
Climate
Climate + Soil
Soil
AUC = 0.99
TSS = 0.92
AUC = 0.99
TSS = 0.91
AUC = 0.99
TSS = 0.84
Relative importance
1.00
0.75
0.50
0.25
0.00
bio01
bio02
bio06
bio12
bio15
0.75
0.50
0.25
0.00
BDW
CLY
NTO
PTO
SOC
bio01
bio02
bio06
bio12
bio15
0.8
0.6
0.4
0.2
0.0
BDW
CLY
NTO
PTO
SOC
Hibbertia rufa
Climate
Climate + Soil
Soil
AUC = 0.98
TSS = 0.89
AUC = 0.99
TSS = 0.88
AUC = 0.98
TSS = 0.78
Relative importance
0.8
0.6
0.4
0.2
0.0
bio01
bio05
bio13
bio14
bio15
0.6
0.4
0.2
0.0
BDW
CEC
CLY
NTO
SOC
bio01
bio05
bio13
bio14
bio15
0.6
0.4
0.2
0.0
BDW
CEC
CLY
NTO
SOC

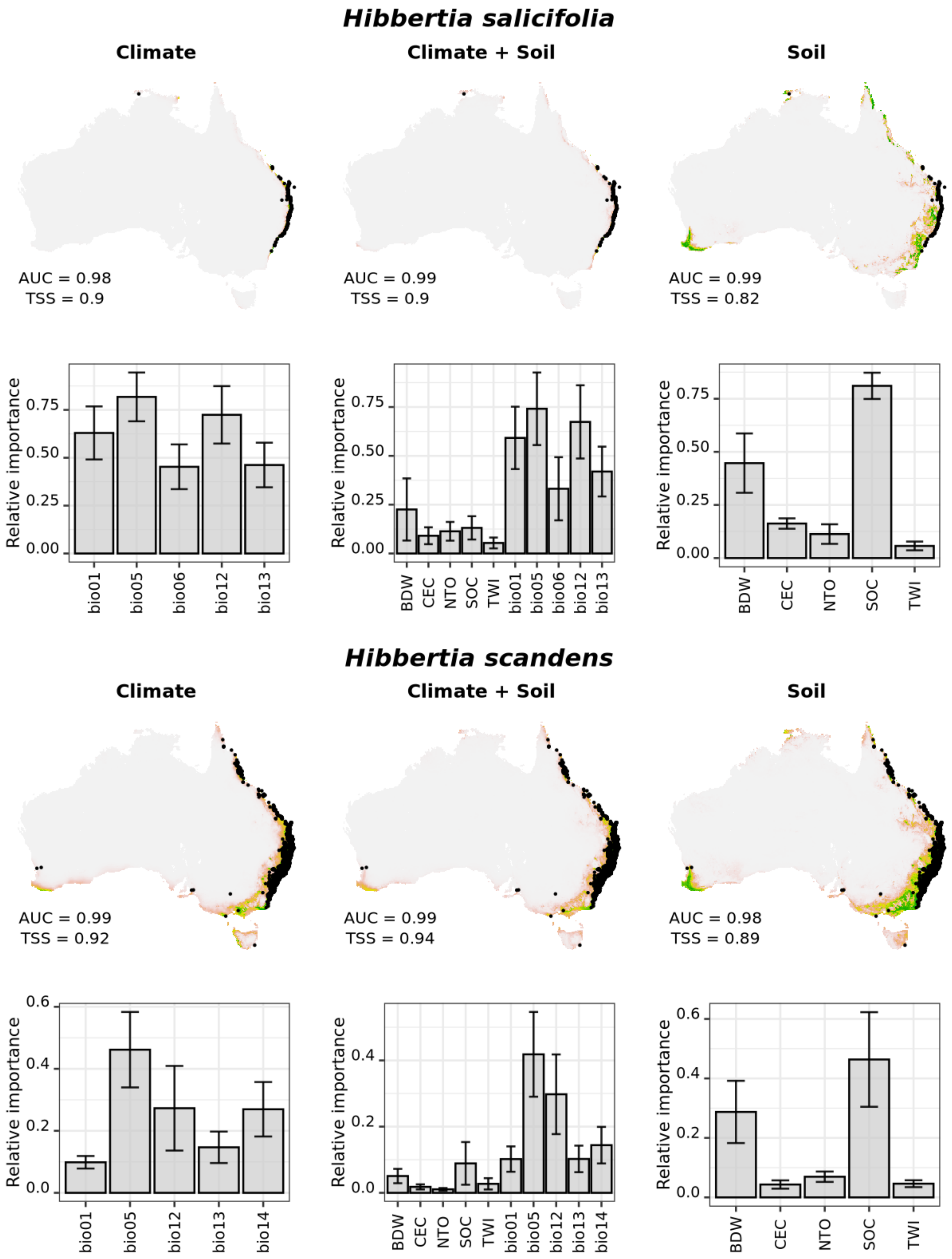
Hibbertia salicifolia
Climate
Climate + Soil
Soil
AUC = 0.98
TSS = 0.9
AUC = 0.99
TSS = 0.9
AUC = 0.99
TSS = 0.82
Relative importance
bio01
bio05
bio06
bio12
bio13
BDW
CEC
NTO
SOC
TWI
Hibbertia scandens
Climate
Climate + Soil
Soil
AUC = 0.99
TSS = 0.92
AUC = 0.99
TSS = 0.94
AUC = 0.98
TSS = 0.89
Relative importance
bio01
bio05
bio12
bio13
bio14
BDW
CEC
NTO
SOC
TWI

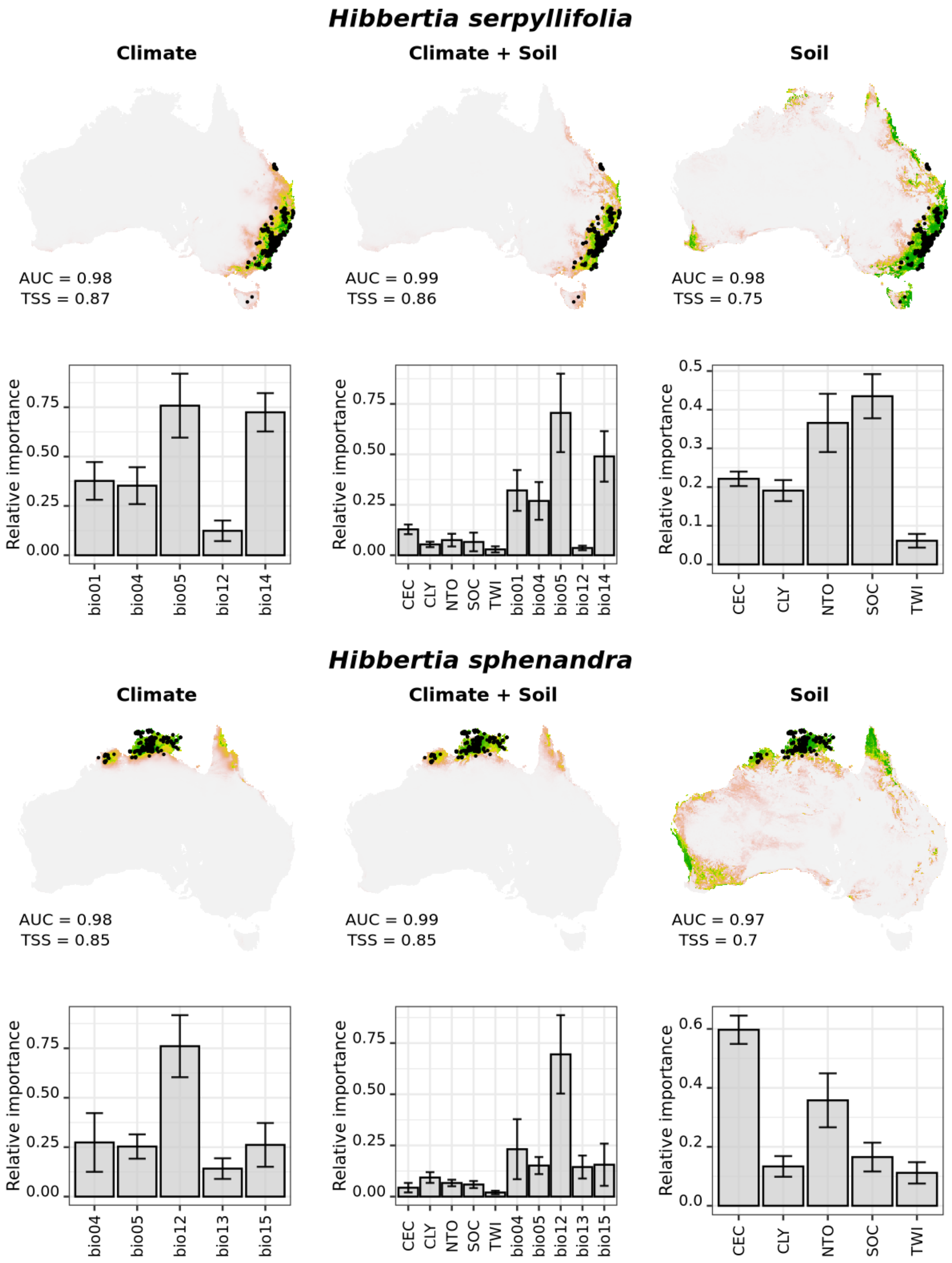
Hibbertia serpyllifolia
Climate
Climate + Soil
Soil
AUC = 0.98
TSS = 0.87
AUC = 0.99
TSS = 0.86
AUC = 0.98
TSS = 0.75
Relative importance
0.00
0.25
0.50
0.75
bio01
bio04
bio05
bio12
bio14
CEC
CLY
NTO
SOC
TWI
0.0
0.1
0.2
0.3
0.4
0.5
Hibbertia sphenandra
AUC = 0.98
TSS = 0.85
AUC = 0.99
TSS = 0.85
AUC = 0.97
TSS = 0.7
bio13
bio15
0.6

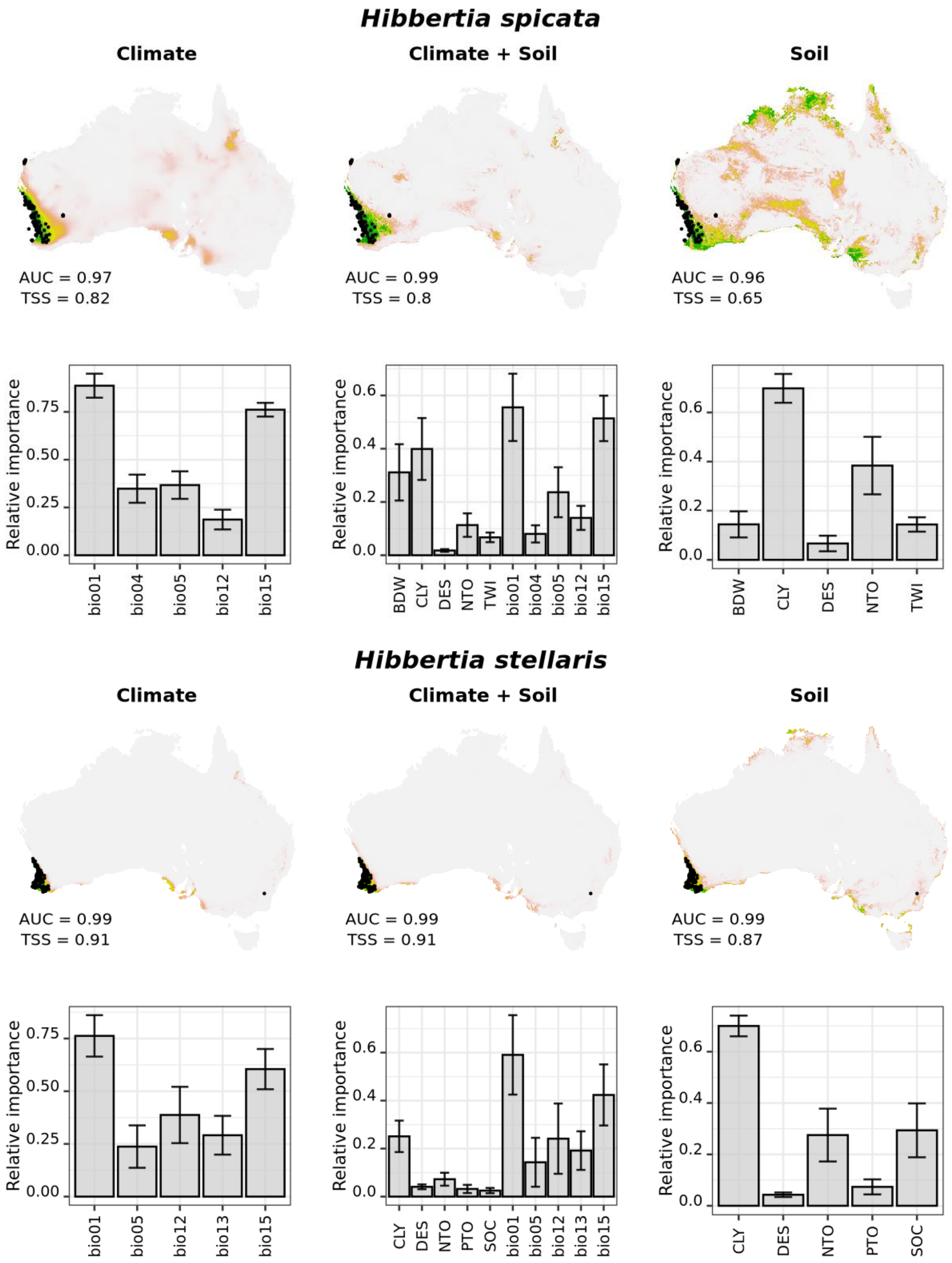
Hibbertia spicata
Climate
Climate + Soil
Soil
AUC = 0.97
TSS = 0.82
AUC = 0.99
TSS = 0.8
AUC = 0.96
TSS = 0.65
Relative importance
bio01
bio04
bio05
bio12
bio15
BDW
CLY
DES
NTO
TWI
Hibbertia stellaris
Climate
Climate + Soil
Soil
AUC = 0.99
TSS = 0.91
AUC = 0.99
TSS = 0.91
AUC = 0.99
TSS = 0.87
Relative importance
bio01
bio05
bio12
bio13
bio15
CLY
DES
NTO
PTO
SOC

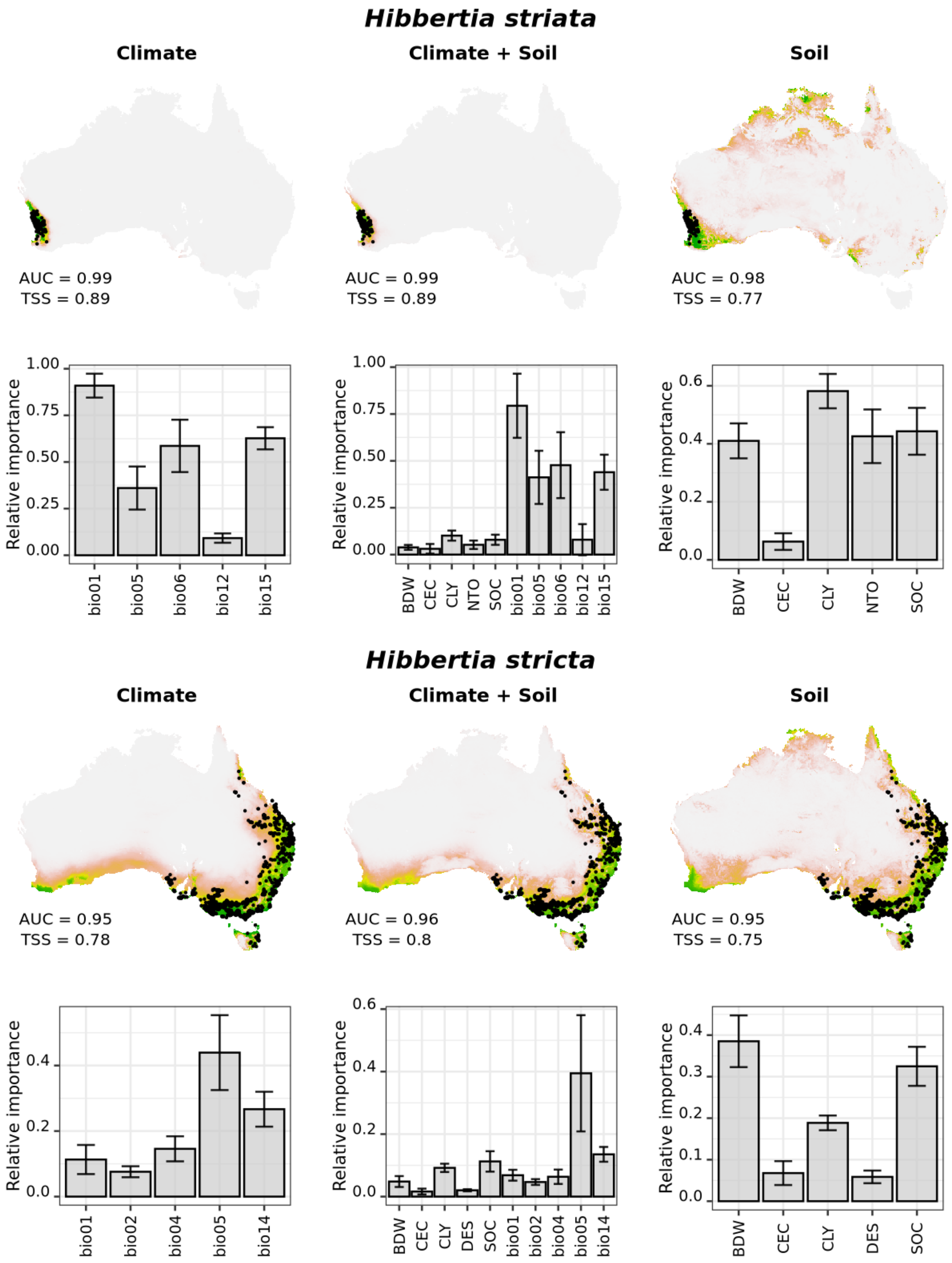

Hibbertia striata
Climate
Climate + Soil
Soil
AUC = 0.99
TSS = 0.89
AUC = 0.99
TSS = 0.89
AUC = 0.98
TSS = 0.77
Relative importance
1.00
0.75
0.50
0.25
0.00
bio01
bio05
bio06
bio12
bio15
BDW
CEC
CLY
NTO
SOC
0.6
0.4
0.2
0.0
Hibbertia stricta
Climate
Climate + Soil
Soil
AUC = 0.95
TSS = 0.78
AUC = 0.96
TSS = 0.8
AUC = 0.95
TSS = 0.75
Relative importance
bio01
bio02
bio04
bio05
bio14
BDW
CEC
CLY
DES
SOC
0.4
0.3
0.2
0.1
0.0

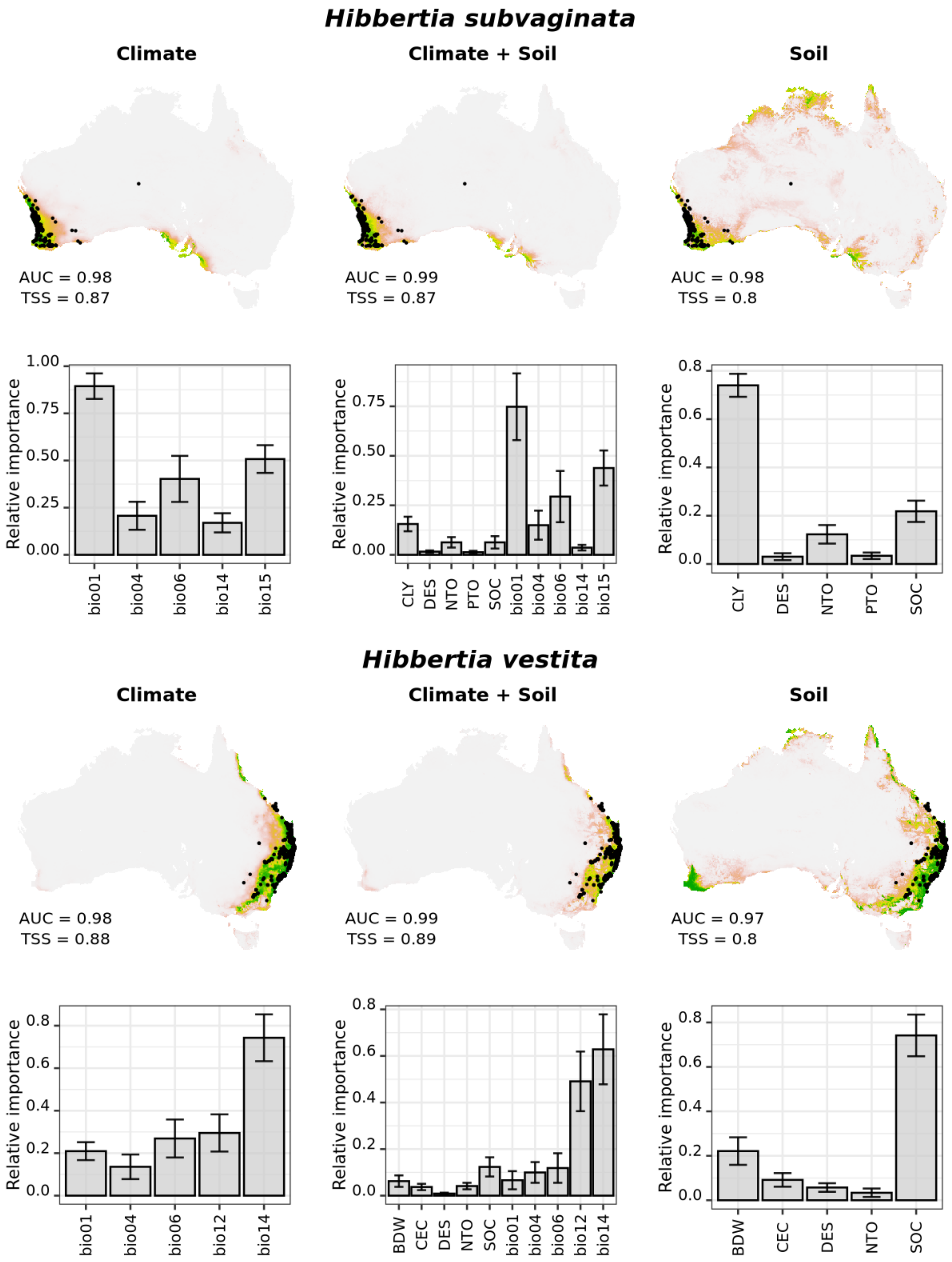
Hibbertia subvaginata
Climate
Climate + Soil
Soil
AUC = 0.98
TSS = 0.87
AUC = 0.99
TSS = 0.87
AUC = 0.98
TSS = 0.8
Relative importance
bio01
bio04
bio06
bio14
bio15
CLY
DES
NTO
PTO
SOC
Hibbertia vestita
Climate
Climate + Soil
Soil
AUC = 0.98
TSS = 0.88
AUC = 0.99
TSS = 0.89
AUC = 0.97
TSS = 0.8
Relative importance
bio01
bio04
bio06
bio12
bio14
BDW
CEC
DES
NTO
SOC

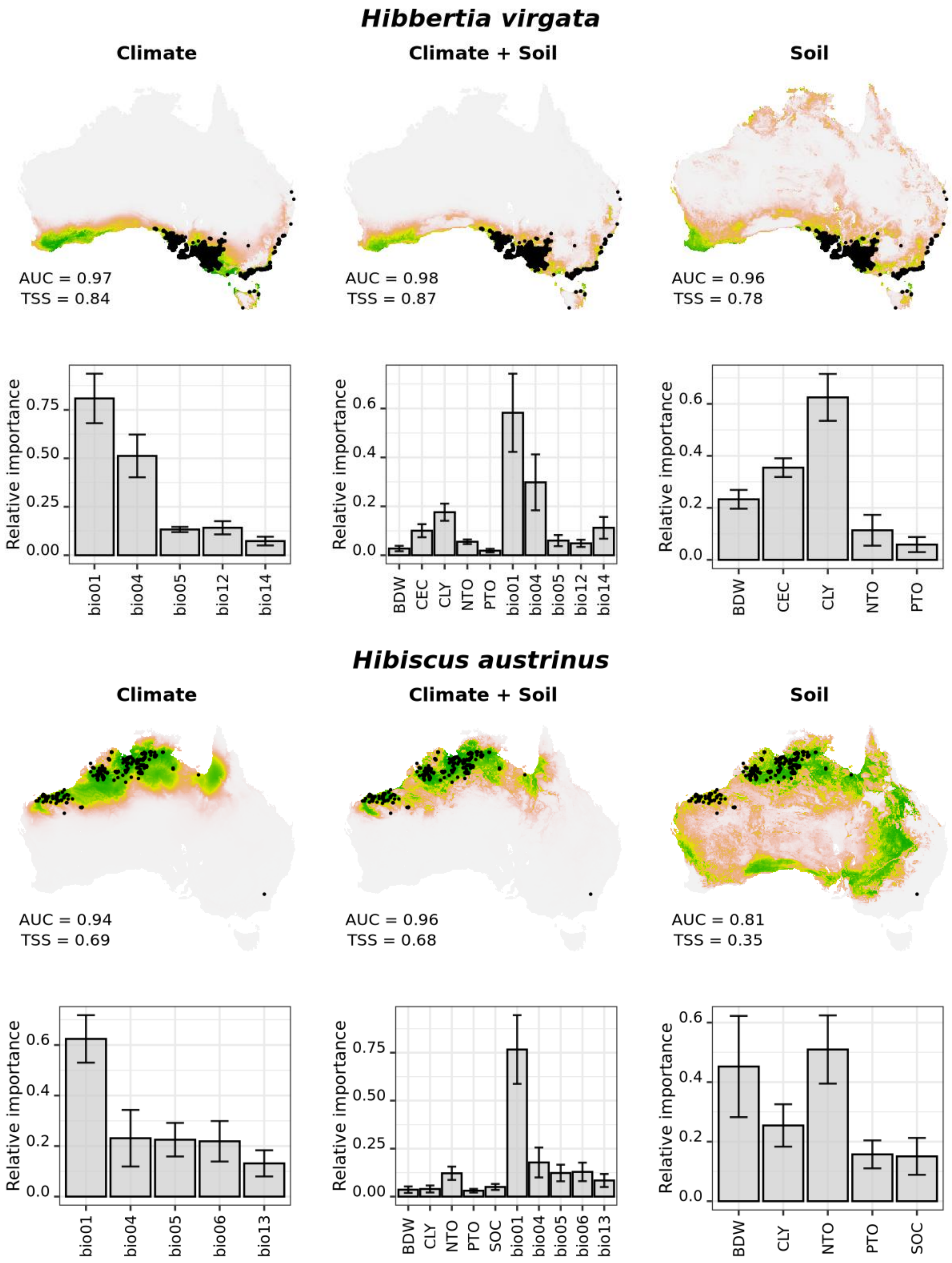

Hibbertia virgata
Climate
Climate + Soil
Soil
AUC = 0.97
TSS = 0.84
AUC = 0.98
TSS = 0.87
AUC = 0.96
TSS = 0.78
Relative importance
0.00
0.25
0.50
0.75
bio01
bio04
bio05
bio12
bio14
0.0
0.2
0.4
0.6
BDW
CEC
CLY
NTO
PTO
bio01
bio04
bio05
bio12
bio14
Hibiscus austrinus
Climate
Climate + Soil
Soil
AUC = 0.94
TSS = 0.69
AUC = 0.96
TSS = 0.68
AUC = 0.81
TSS = 0.35
bio01
bio04
bio05
bio06
bio13
BDW
CLY
NTO
PTO
SOC
bio01
bio04
bio05
bio06
bio13

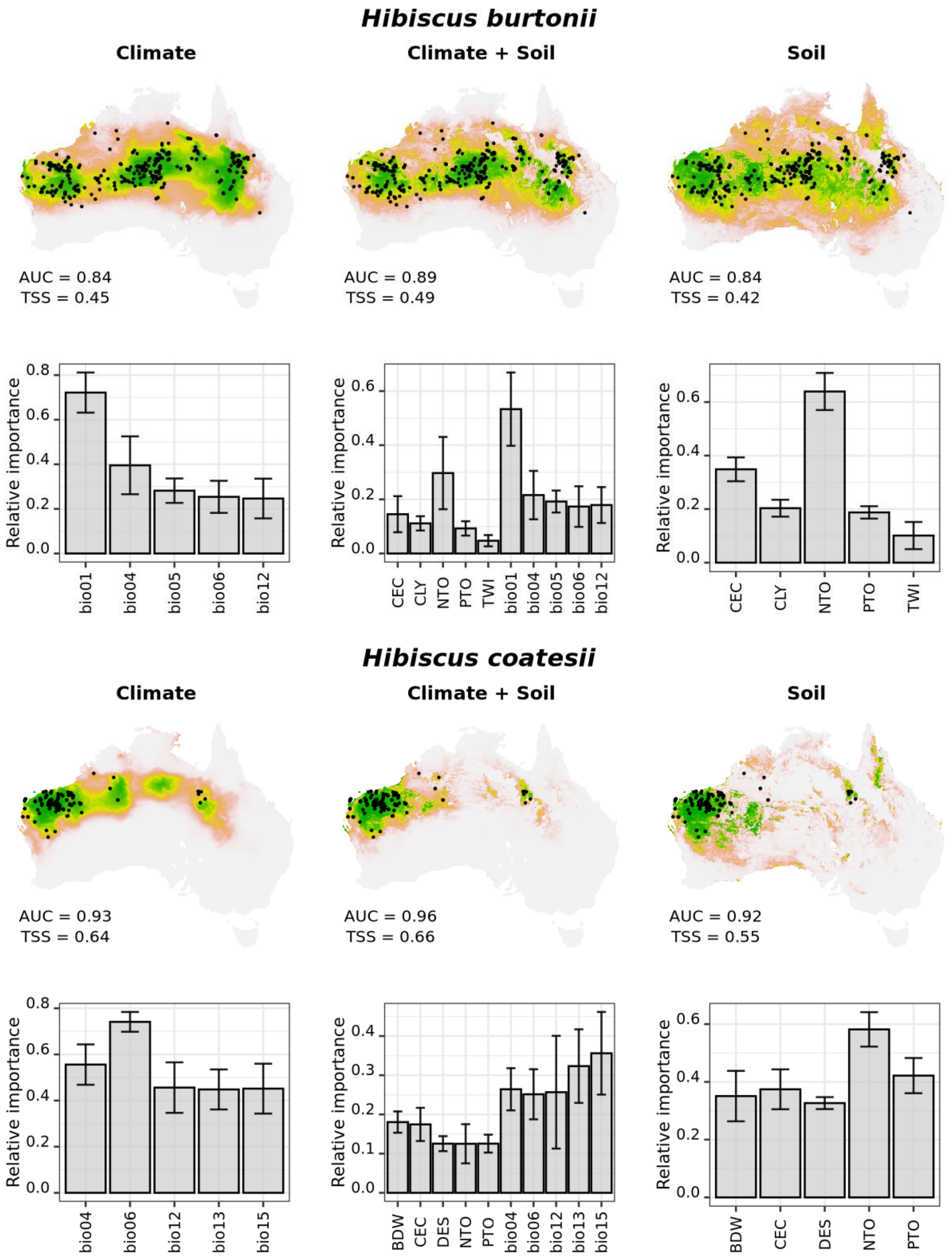

Hibiscus burtonii
Climate
Climate + Soil
Soil
AUC = 0.84
TSS = 0.45
AUC = 0.89
TSS = 0.49
AUC = 0.84
TSS = 0.42
Relative importance
bio01
bio04
bio05
bio06
bio12
CEC
CLY
NTO
PTO
TWI
Hibiscus coatesii
Climate
Climate + Soil
Soil
AUC = 0.93
TSS = 0.64
AUC = 0.96
TSS = 0.66
AUC = 0.92
TSS = 0.55
Relative importance
bio04
bio06
bio12
bio13
bio15
BDW
CEC
DES
NTO
PTO

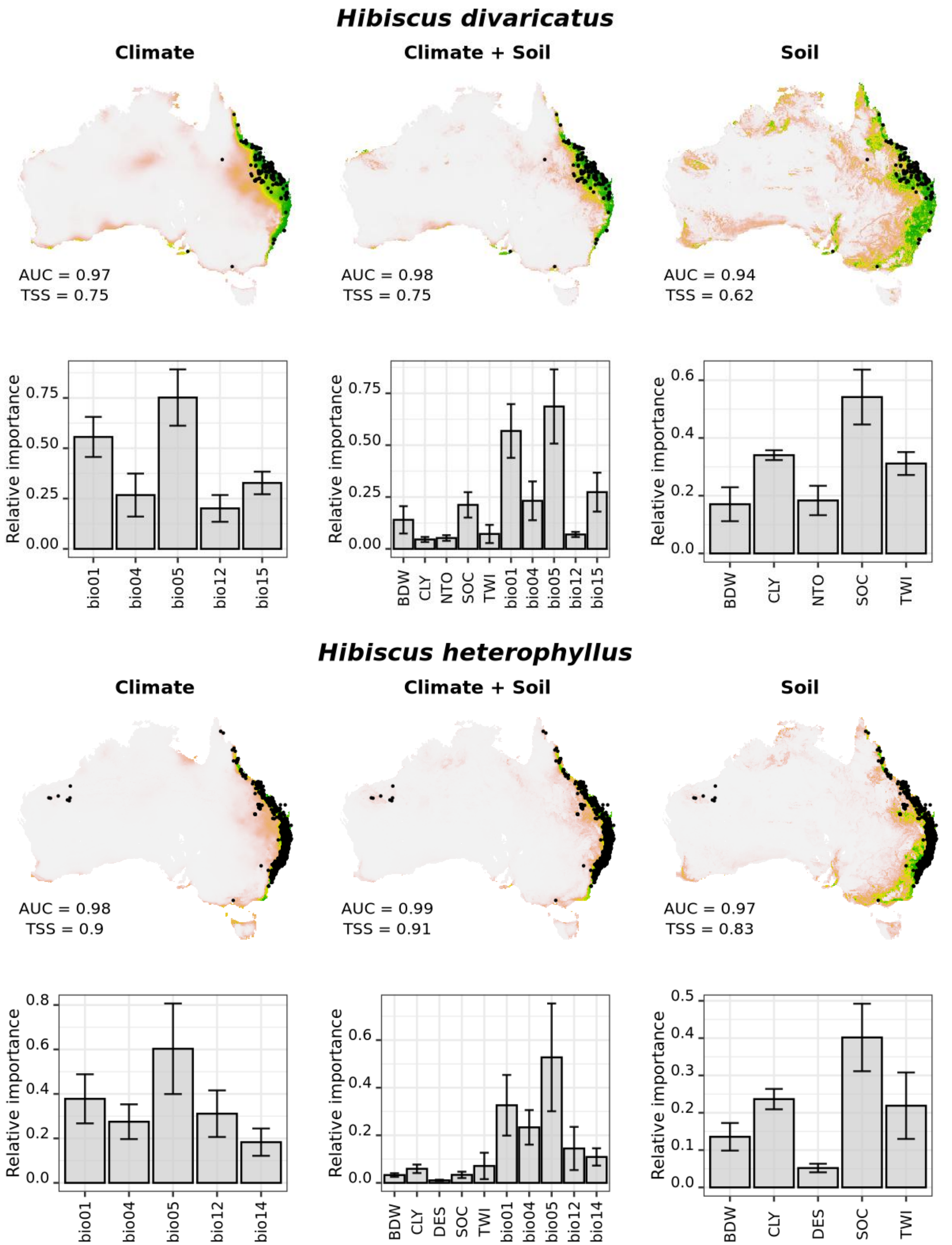
Hibiscus divaricatus
Climate
Climate + Soil
Soil
AUC = 0.97
TSS = 0.75
AUC = 0.98
TSS = 0.75
AUC = 0.94
TSS = 0.62
Relative importance
bio01 bio04 bio05 bio12 bio15
BDW CLY NTO SOC TWI bio01 bio04 bio05 bio12 bio15
BDW CLY NTO SOC TWI
Hibiscus heterophyllus
Climate
Climate + Soil
Soil
AUC = 0.98
TSS = 0.9
AUC = 0.99
TSS = 0.91
AUC = 0.97
TSS = 0.83
Relative importance
bio01 bio04 bio05 bio12 bio14
BDW CLY DES SOC TWI bio01 bio04 bio05 bio12 bio14
BDW CLY DES SOC TWI

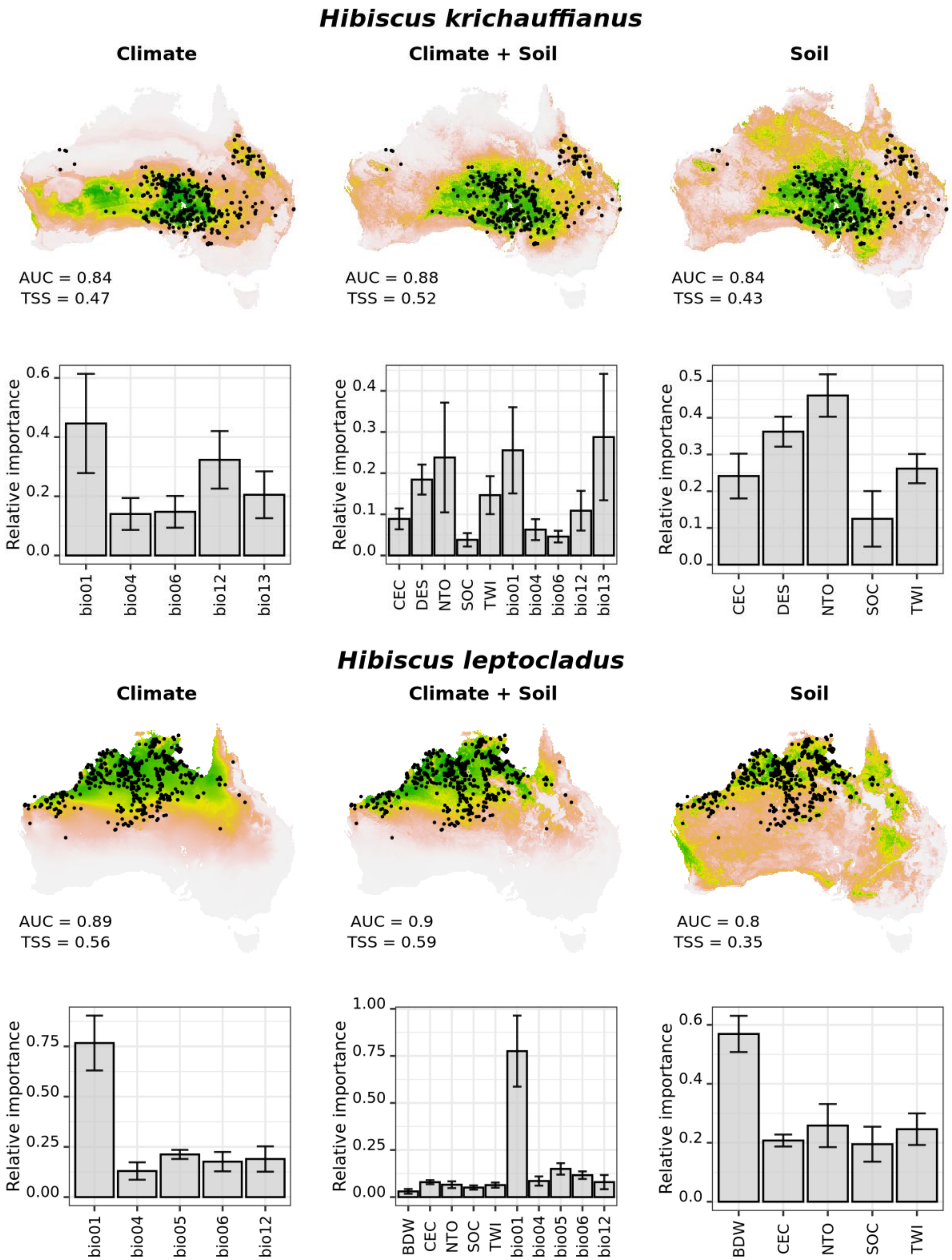
Hibiscus krichauffianus
Climate
Climate + Soil
Soil
AUC = 0.84
TSS = 0.47
AUC = 0.88
TSS = 0.52
AUC = 0.84
TSS = 0.43
Relative importance
bio01
bio04
bio06
bio12
bio13
CEC
DES
NTO
SOC
TWI
Hibiscus leptocladus
Climate
Climate + Soil
Soil
AUC = 0.89
TSS = 0.56
AUC = 0.9
TSS = 0.59
AUC = 0.8
TSS = 0.35
Relative importance
bio01
bio04
bio05
bio06
bio12
BDW
CEC
NTO
SOC
TWI

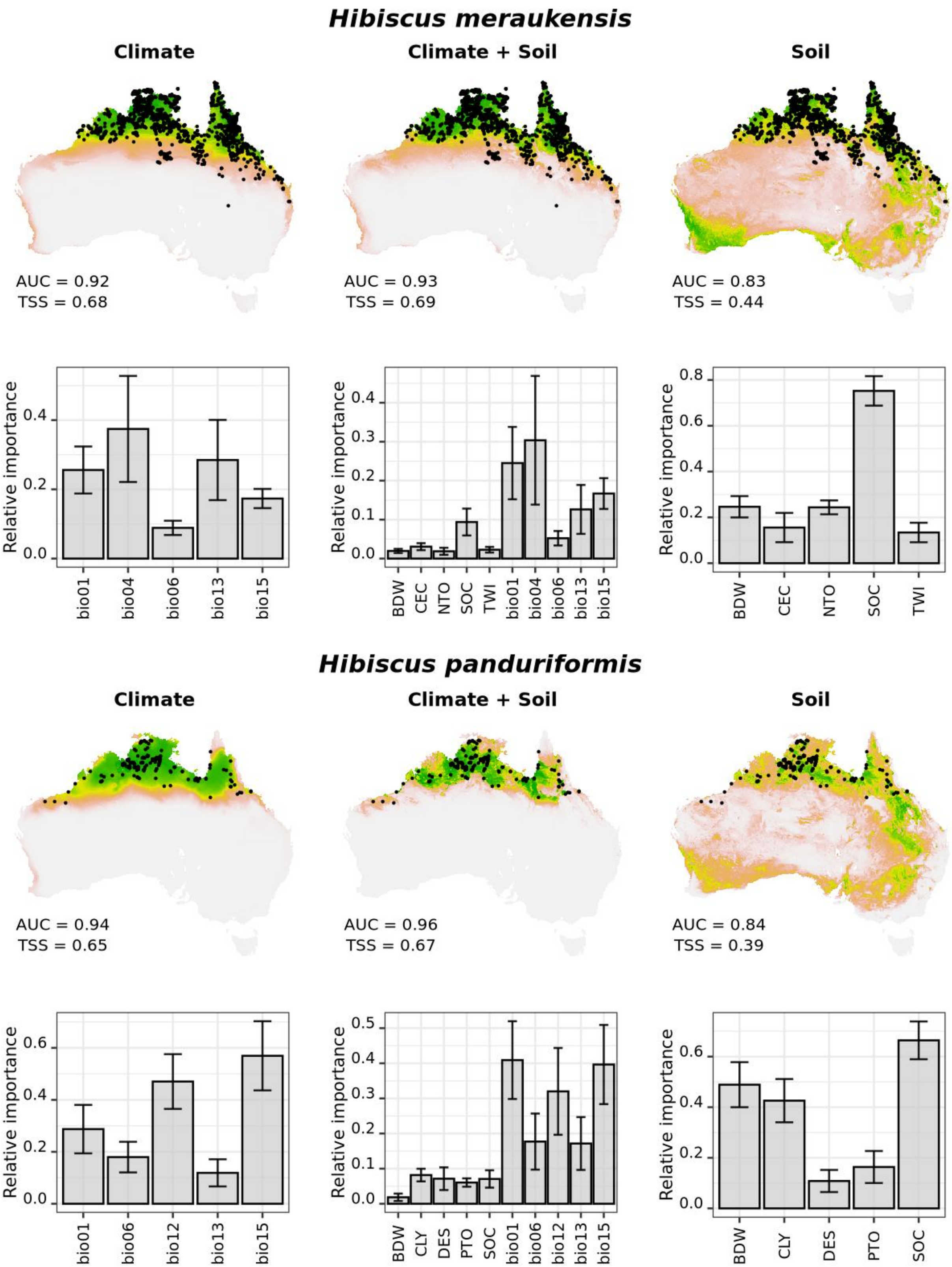
Hibiscus meraukensis
Climate
Climate + Soil
Soil
AUC = 0.92
TSS = 0.68
AUC = 0.93
TSS = 0.69
AUC = 0.83
TSS = 0.44
Relative importance
bio01
bio04
bio06
bio13
bio15
BDW
CEC
NTO
SOC
TWI
Hibiscus panduriformis
Climate
Climate + Soil
Soil
AUC = 0.94
TSS = 0.65
AUC = 0.96
TSS = 0.67
AUC = 0.84
TSS = 0.39
Relative importance
bio01
bio06
bio12
bio13
bio15
BDW
CLY
DES
PTO
SOC

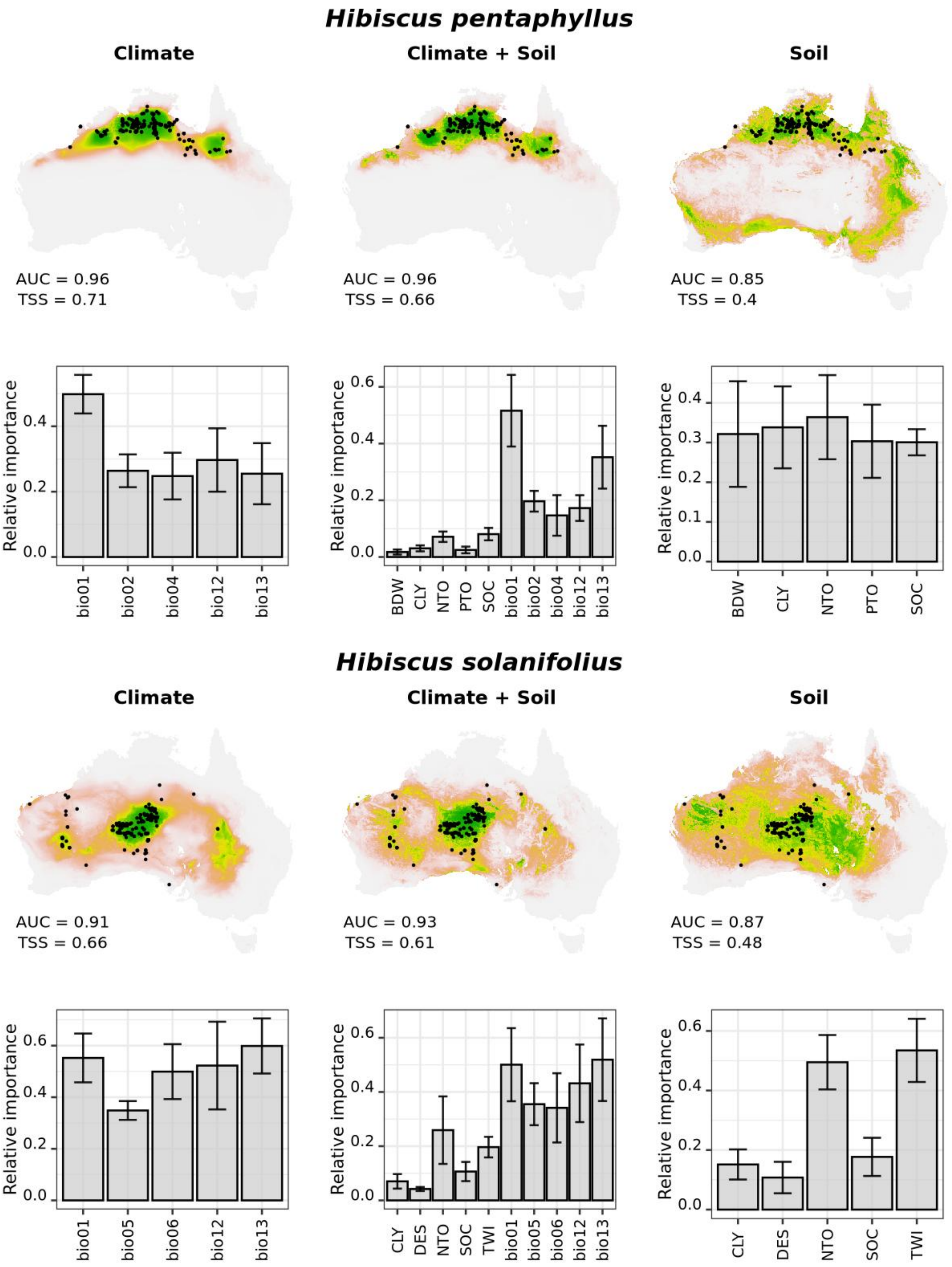

Hibiscus pentaphyllus
Climate
Climate + Soil
Soil
AUC = 0.96
TSS = 0.71
AUC = 0.96
TSS = 0.66
AUC = 0.85
TSS = 0.4
Relative importance
bio01 bio02 bio04 bio12 bio13
BDW CLY NTO PTO SOC bio01 bio02 bio04 bio12 bio13
BDW CLY NTO PTO SOC
Hibiscus solanifolius
Climate
Climate + Soil
Soil
AUC = 0.91
TSS = 0.66
AUC = 0.93
TSS = 0.61
AUC = 0.87
TSS = 0.48
Relative importance
bio01 bio05 bio06 bio12 bio13
CLY DES NTO SOC TWI bio01 bio05 bio06 bio12 bio13
CLY DES NTO SOC TWI

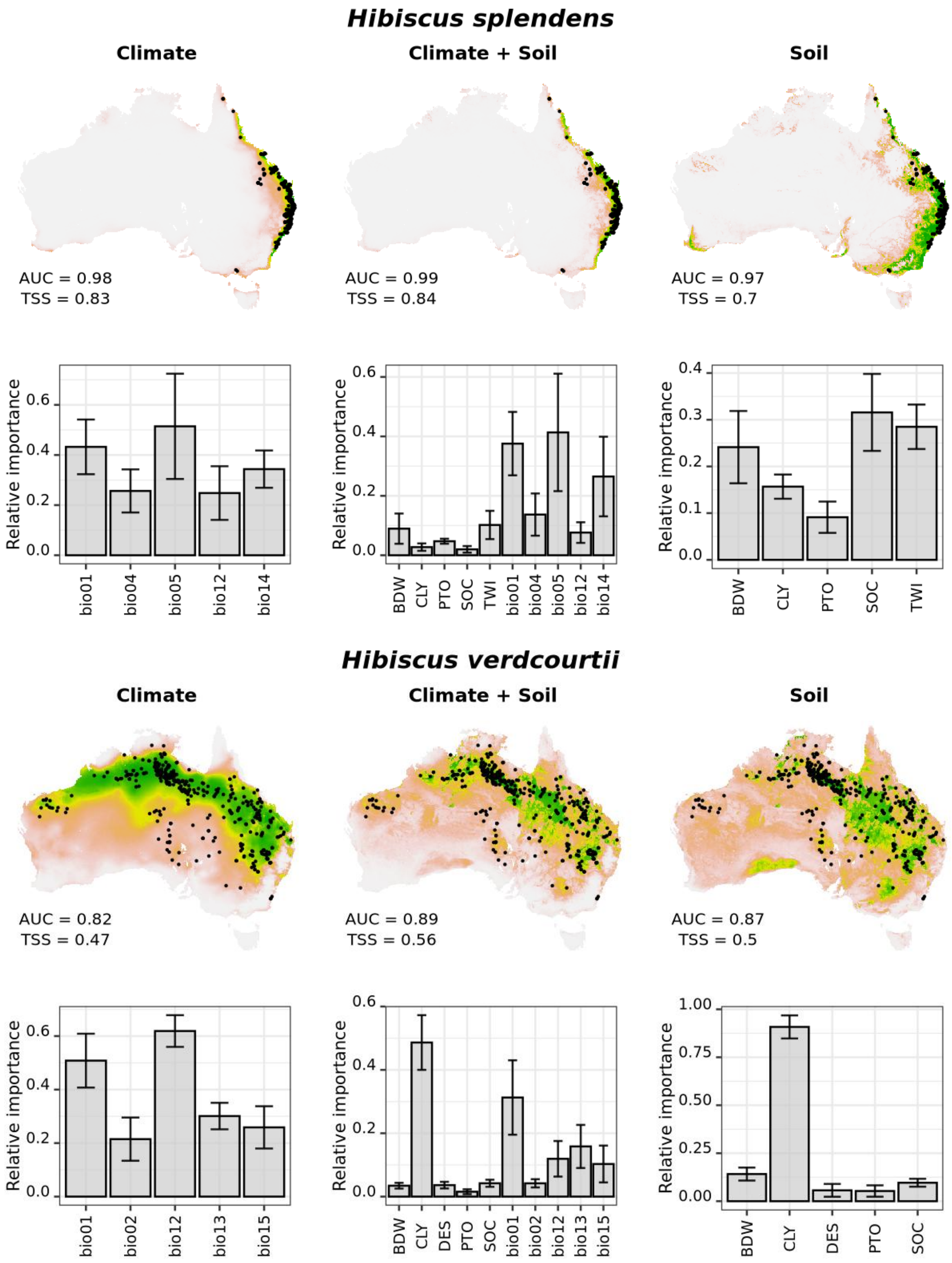

Hibiscus splendens
Climate
Climate + Soil
Soil
AUC = 0.98
TSS = 0.83
AUC = 0.99
TSS = 0.84
AUC = 0.97
TSS = 0.7
Relative importance
bio01
bio04
bio05
bio12
bio14
BDW
CLY
PTO
SOC
TWI
Hibiscus verdcourtii
AUC = 0.82
TSS = 0.47
AUC = 0.89
TSS = 0.56
AUC = 0.87
TSS = 0.5
bio02
bio13
bio15
DES

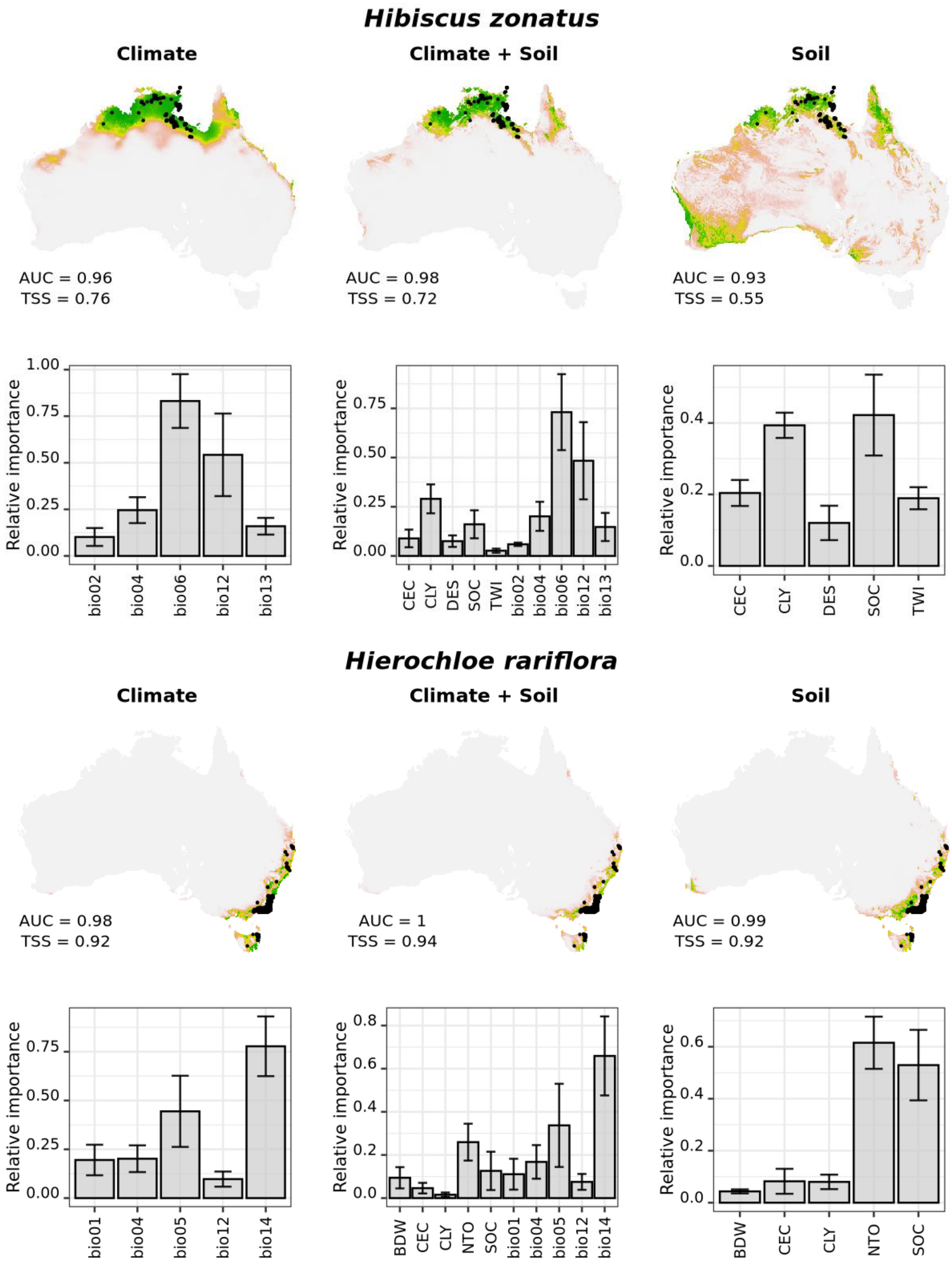

Hibiscus zonatus
Climate
Climate + Soil
Soil
AUC = 0.96
TSS = 0.76
AUC = 0.98
TSS = 0.72
AUC = 0.93
TSS = 0.55
Relative importance
bio02
bio04
bio06
bio12
bio13
CEC
CLY
DES
SOC
TWI
Hierochloe rariflora
Climate
Climate + Soil
Soil
AUC = 0.98
TSS = 0.92
AUC = 1
TSS = 0.94
AUC = 0.99
TSS = 0.92
Relative importance
bio01
bio04
bio05
bio12
bio14
BDW
CEC
CLY
NTO
SOC

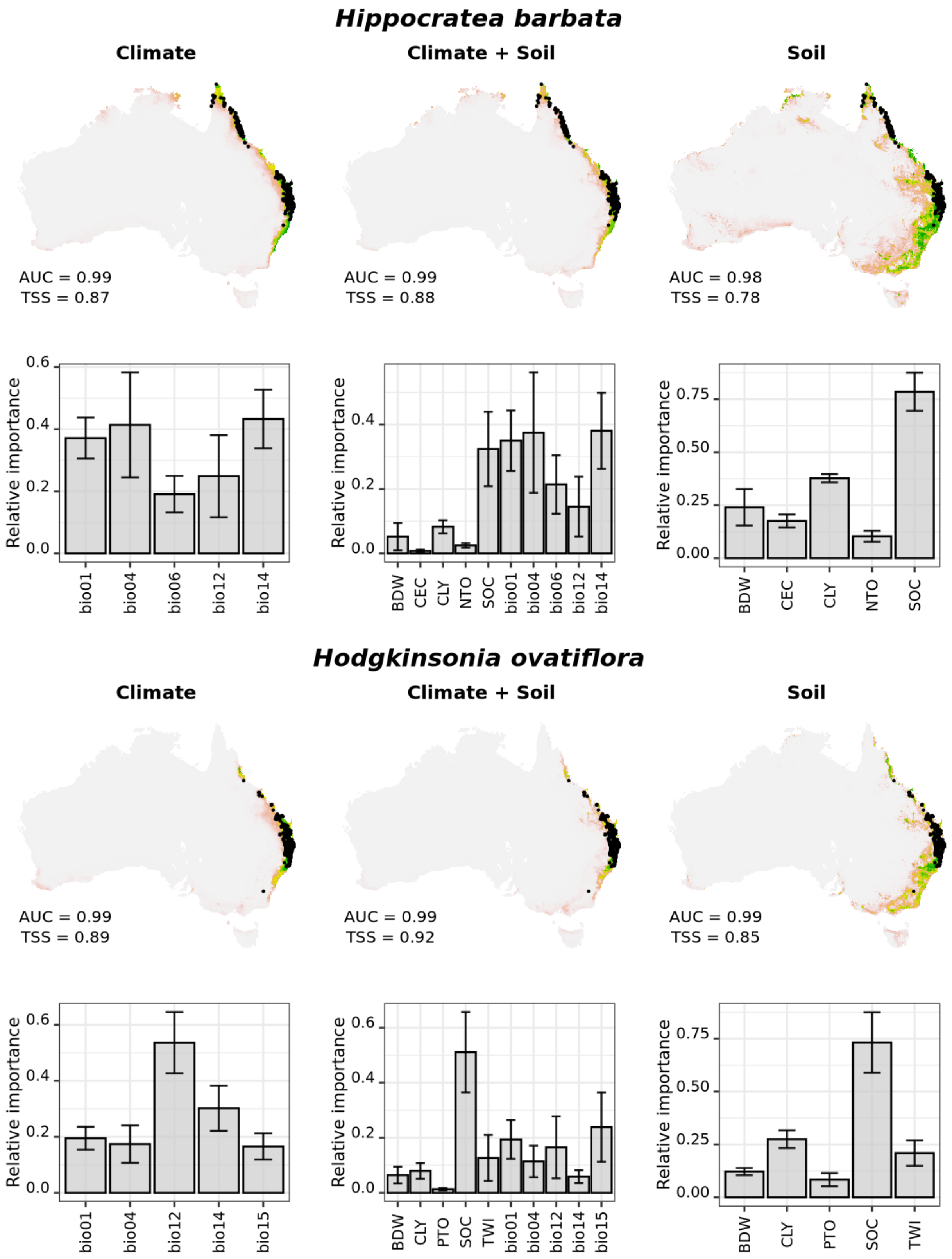
Hippocratea barbata
Climate
Climate + Soil
Soil
AUC = 0.99
TSS = 0.87
AUC = 0.99
TSS = 0.88
AUC = 0.98
TSS = 0.78
Relative importance
0.0
0.2
0.4
0.6
bio01
bio04
bio06
bio12
bio14
BDW
CEC
CLY
NTO
SOC
bio01
bio04
bio06
bio12
bio14
0.00
0.25
0.50
0.75
BDW
CEC
CLY
NTO
SOC
Hodgkinsonia ovatiflora
Climate
Climate + Soil
Soil
AUC = 0.99
TSS = 0.89
AUC = 0.99
TSS = 0.92
AUC = 0.99
TSS = 0.85
Relative importance
0.0
0.2
0.4
0.6
bio01
bio04
bio12
bio14
bio15
BDW
CLY
PTO
SOC
TWI
bio01
bio04
bio12
bio14
bio15
0.00
0.25
0.50
0.75
BDW
CLY
PTO
SOC
TWI

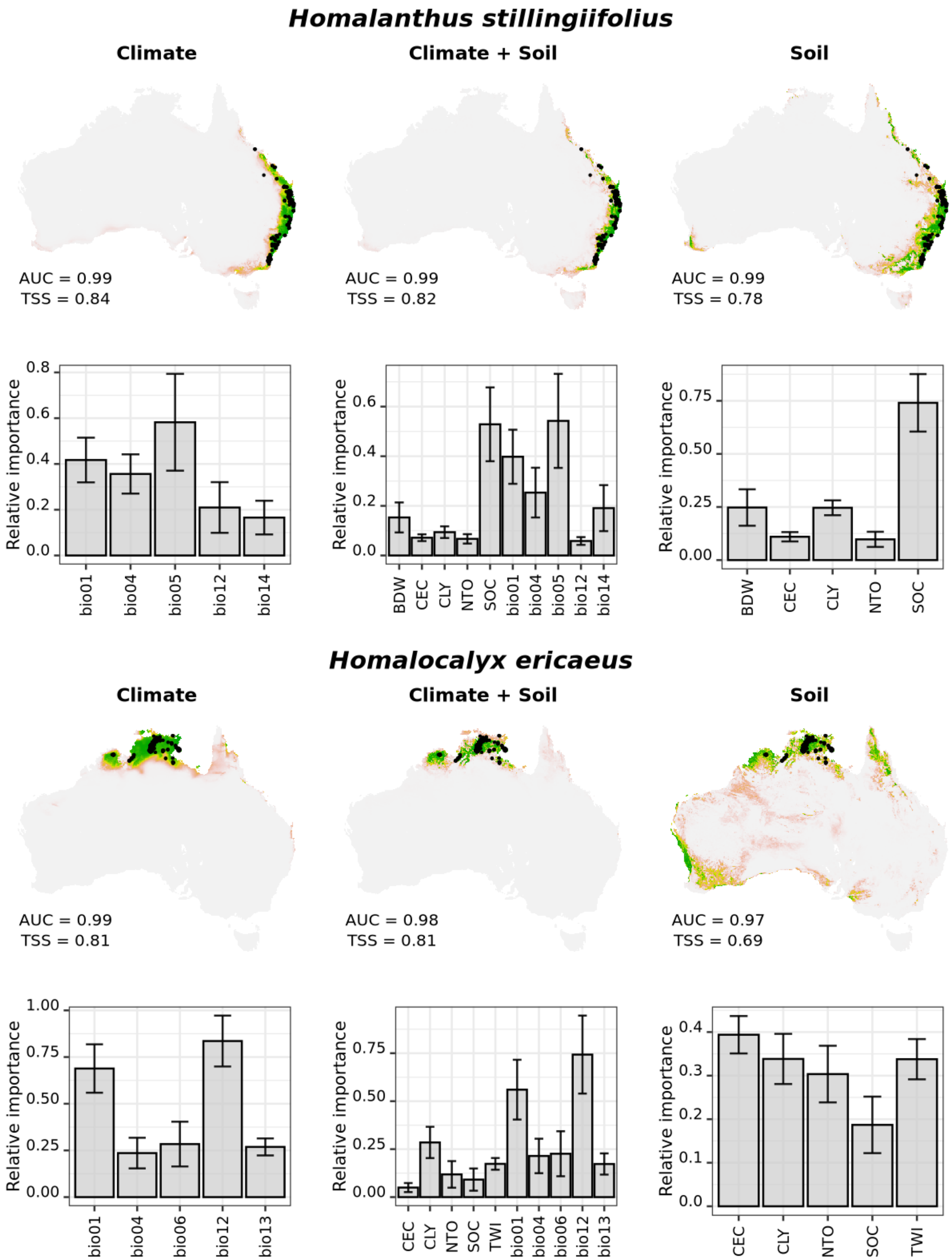
Homalanthus stillingiifolius
Climate
Climate + Soil
Soil
AUC = 0.99
TSS = 0.84
AUC = 0.99
TSS = 0.82
AUC = 0.99
TSS = 0.78
Relative importance
0.0
0.2
0.4
0.6
0.8
bio01
bio04
bio05
bio12
bio14
Relative importance
0.0
0.2
0.4
0.6
BDW
CEC
CLY
NTO
SOC
bio01
bio04
bio05
bio12
bio14
Relative importance
0.00
0.25
0.50
0.75
BDW
CEC
CLY
NTO
SOC
Homalocalyx ericaeus
Climate
Climate + Soil
Soil
AUC = 0.99
TSS = 0.81
AUC = 0.98
TSS = 0.81
AUC = 0.97
TSS = 0.69
Relative importance
0.00
0.25
0.50
0.75
1.00
bio01
bio04
bio06
bio12
bio13
Relative importance
0.00
0.25
0.50
0.75
CEC
CLY
NTO
SOC
TWI
bio01
bio04
bio06
bio12
bio13
Relative importance
0.0
0.1
0.2
0.3
0.4
CEC
CLY
NTO
SOC
TWI

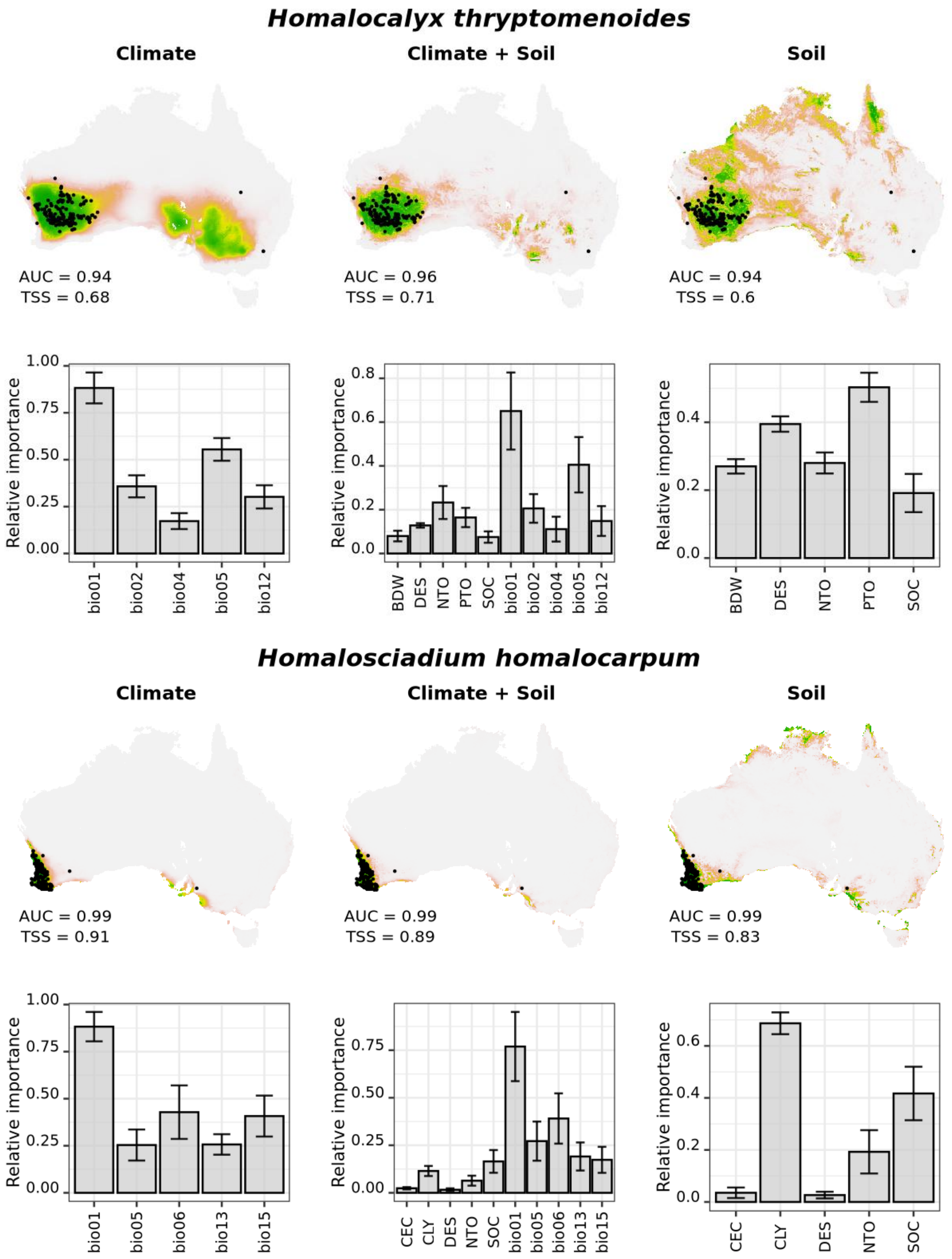
Homalocalyx thryptomenoides
Climate
Climate + Soil
Soil
AUC = 0.94
TSS = 0.68
AUC = 0.96
TSS = 0.71
AUC = 0.94
TSS = 0.6
Relative importance
bio01
bio02
bio04
bio05
bio12
BDW
DES
NTO
PTO
SOC
Homalosciadium homalocarpum
Climate
Climate + Soil
Soil
AUC = 0.99
TSS = 0.91
AUC = 0.99
TSS = 0.89
AUC = 0.99
TSS = 0.83
Relative importance
bio01
bio05
bio06
bio13
bio15
CEC
CLY
DES
NTO
SOC

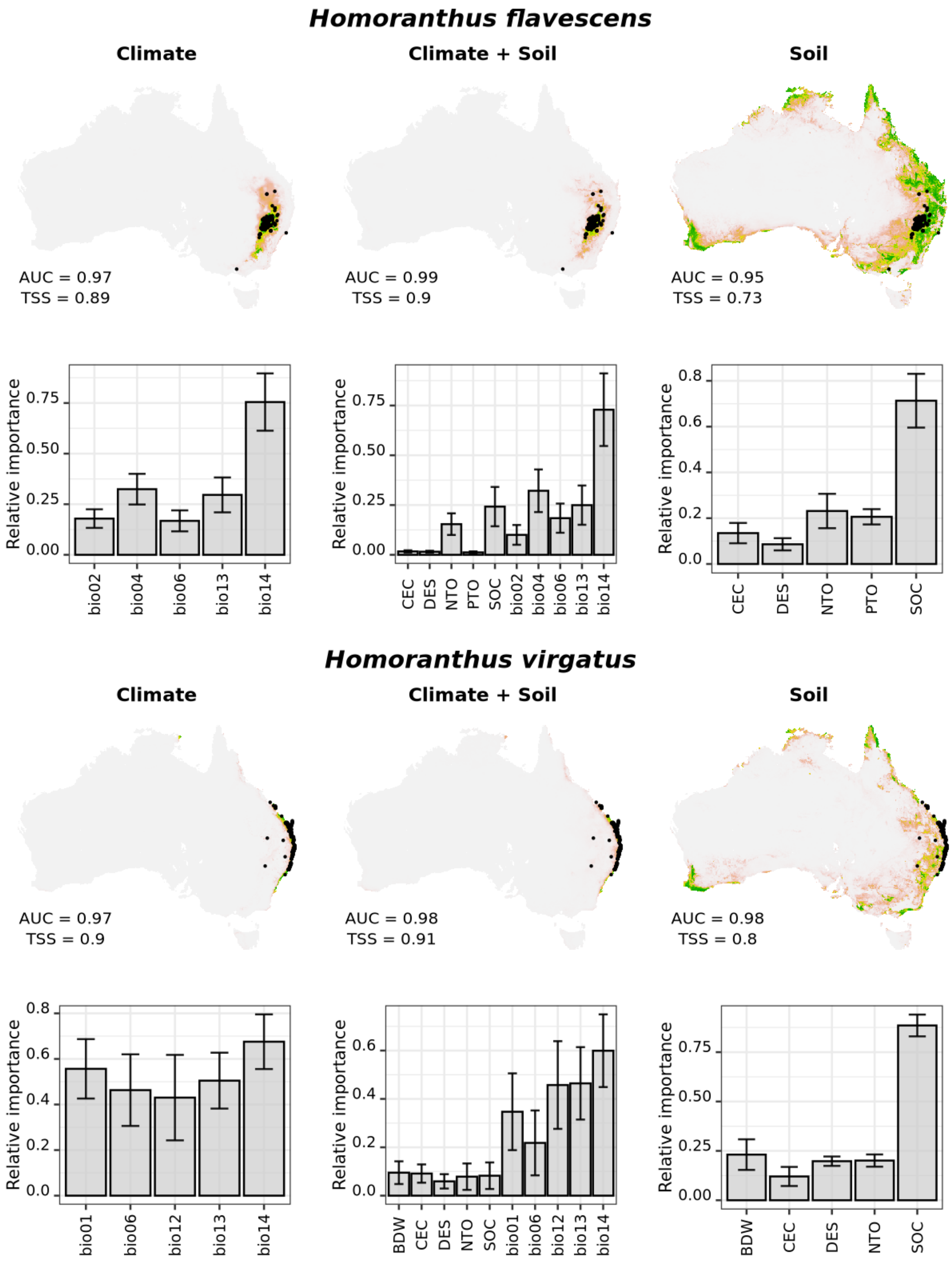
Homoranthus flavescens
Climate
Climate + Soil
Soil
AUC = 0.97
TSS = 0.89
AUC = 0.99
TSS = 0.9
AUC = 0.95
TSS = 0.73
Relative importance
bio02
bio04
bio06
bio13
bio14
CEC
DES
NTO
PTO
SOC
Homoranthus virgatus
Climate
Climate + Soil
Soil
AUC = 0.97
TSS = 0.9
AUC = 0.98
TSS = 0.91
AUC = 0.98
TSS = 0.8
Relative importance
bio01
bio06
bio12
bio13
bio14
BDW
CEC
DES
NTO
SOC

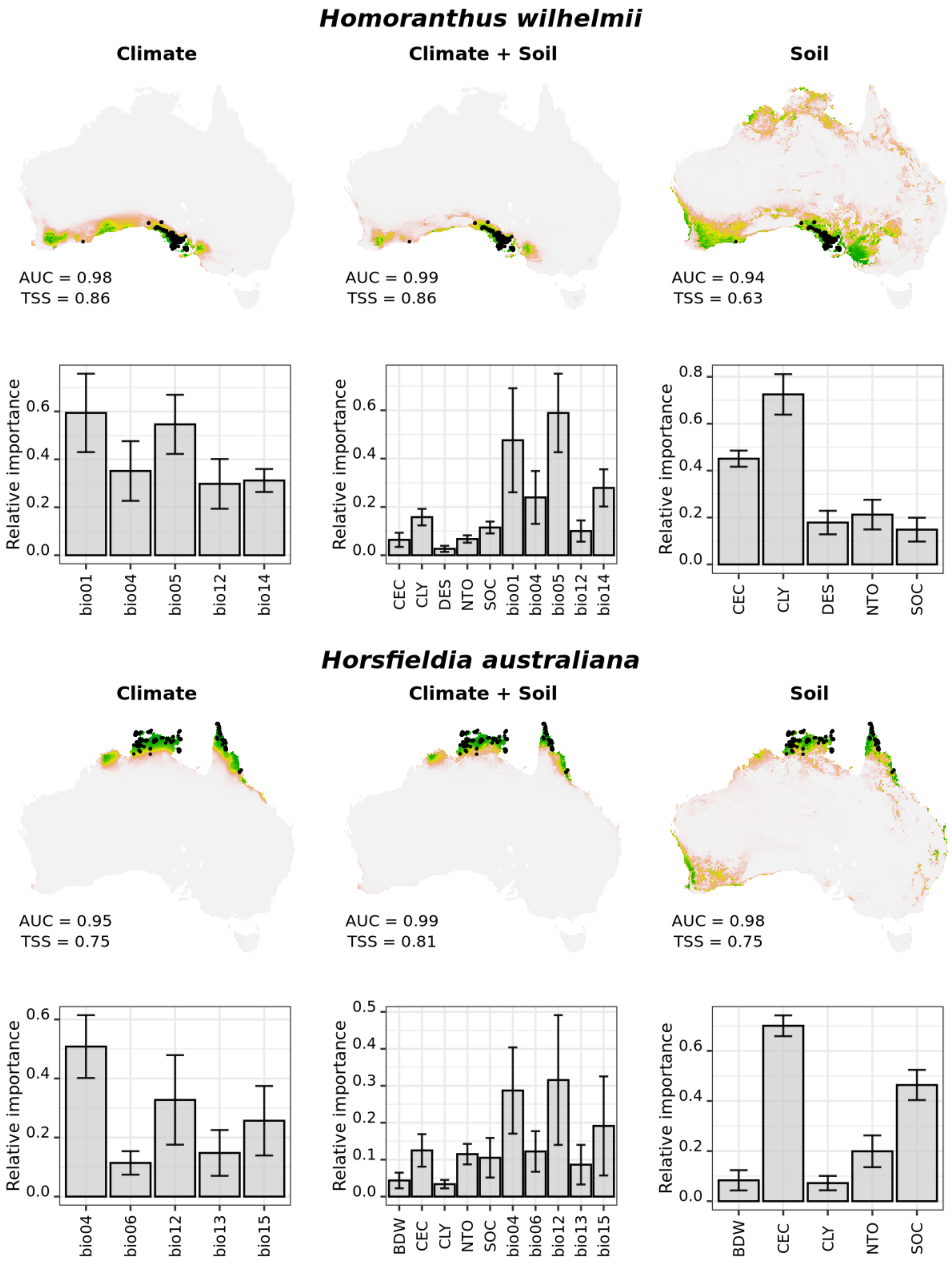

Homoranthus wilhelmii
Climate
Climate + Soil
Soil
AUC = 0.98
TSS = 0.86
AUC = 0.99
TSS = 0.86
AUC = 0.94
TSS = 0.63
Relative importance
bio01
bio04
bio05
bio12
bio14
CEC
CLY
DES
NTO
SOC
Horsfieldia australiana
AUC = 0.95
TSS = 0.75
AUC = 0.99
TSS = 0.81
AUC = 0.98
TSS = 0.75
bio06
bio13
bio15
BDW

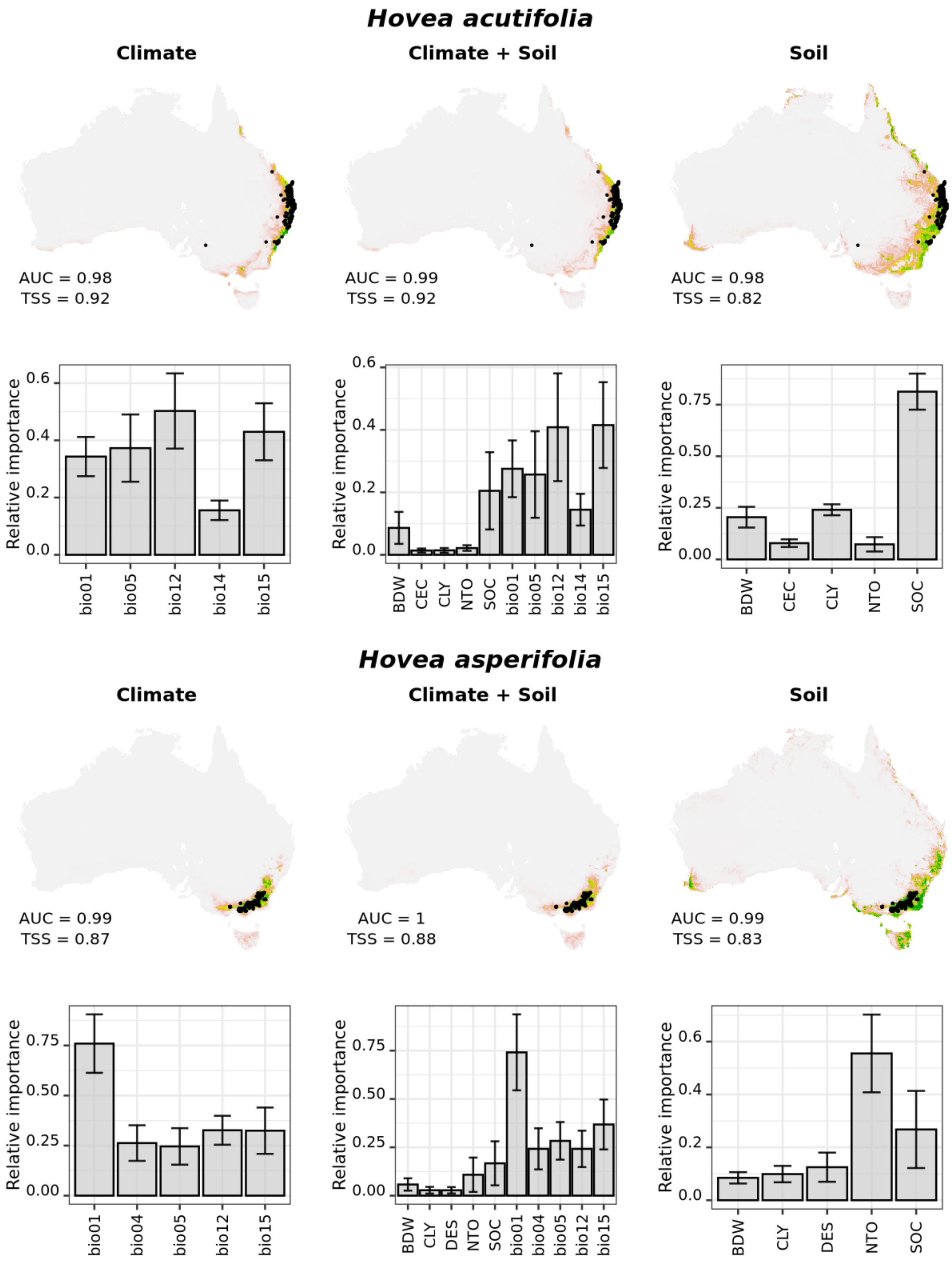
Hovea acutifolia
Climate
Climate + Soil
Soil
AUC = 0.98
TSS = 0.92
AUC = 0.99
TSS = 0.92
AUC = 0.98
TSS = 0.82
Relative importance
bio01
bio05
bio12
bio14
bio15
BDW
CEC
CLY
NTO
SOC
Hovea asperifolia
Climate
Climate + Soil
Soil
AUC = 0.99
TSS = 0.87
AUC = 1
TSS = 0.88
AUC = 0.99
TSS = 0.83
Relative importance
bio01
bio04
bio05
bio12
bio15
BDW
CLY
DES
NTO
SOC

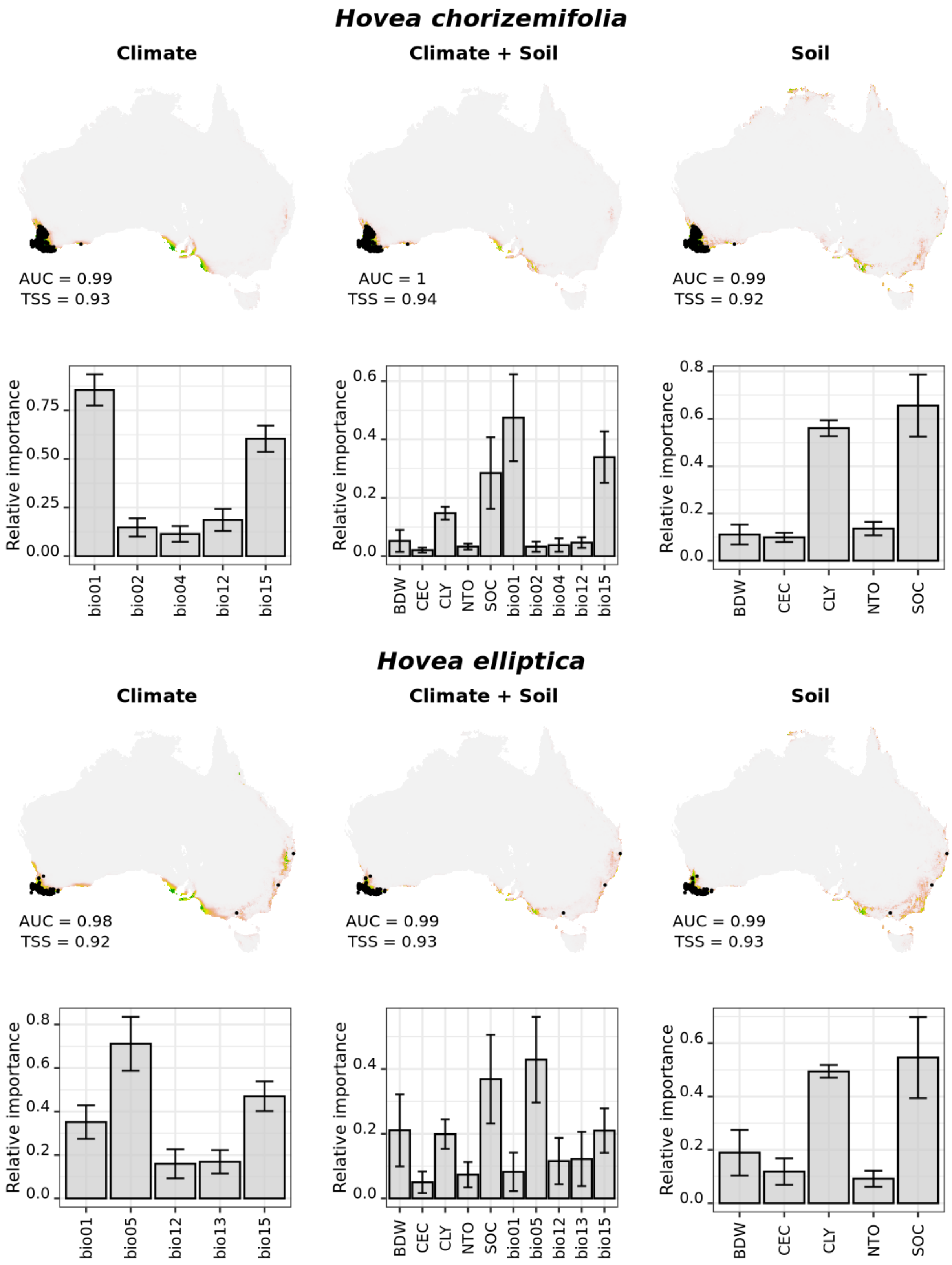
Hovea chorizemifolia
Climate
Climate + Soil
Soil
AUC = 0.99
TSS = 0.93
AUC = 1
TSS = 0.94
AUC = 0.99
TSS = 0.92
Relative importance
0.00
0.25
0.50
0.75
bio01
bio02
bio04
bio12
bio15
0.0
0.2
0.4
0.6
BDW
CEC
CLY
NTO
SOC
bio01
bio02
bio04
bio12
bio15
0.8
Hovea elliptica
Climate
Climate + Soil
Soil
AUC = 0.98
TSS = 0.92
AUC = 0.99
TSS = 0.93
AUC = 0.99
TSS = 0.93
bio01
bio05
bio12
bio13
bio15

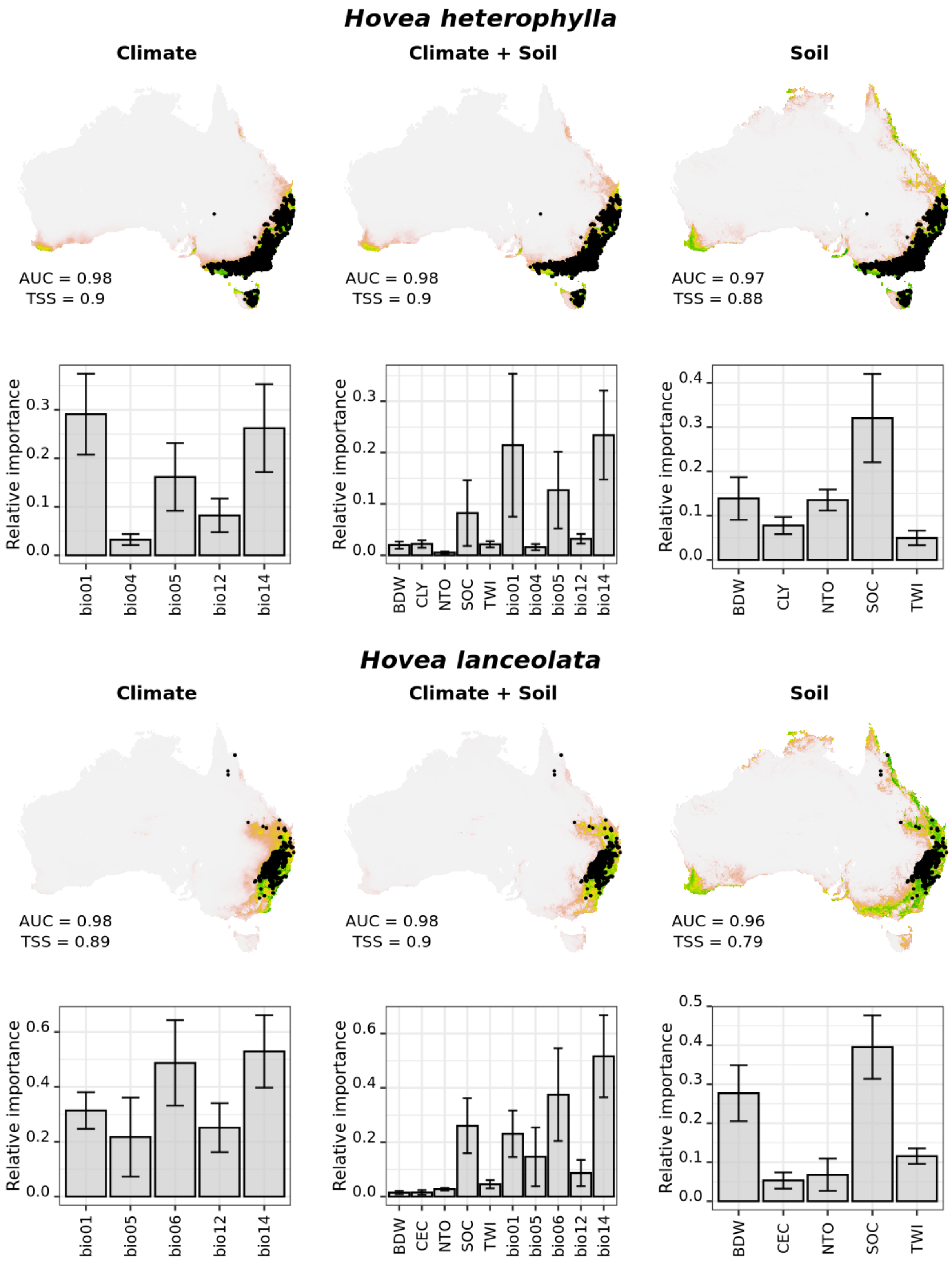
Hovea heterophylla
Climate
Climate + Soil
Soil
AUC = 0.98
TSS = 0.9
AUC = 0.98
TSS = 0.9
AUC = 0.97
TSS = 0.88
Relative importance
bio01
bio04
bio05
bio12
bio14
BDW
CLY
NTO
SOC
TWI
Hovea lanceolata
Climate
Climate + Soil
Soil
AUC = 0.98
TSS = 0.89
AUC = 0.98
TSS = 0.9
AUC = 0.96
TSS = 0.79
Relative importance
bio01
bio05
bio06
bio12
bio14
BDW
CEC
NTO
SOC
TWI

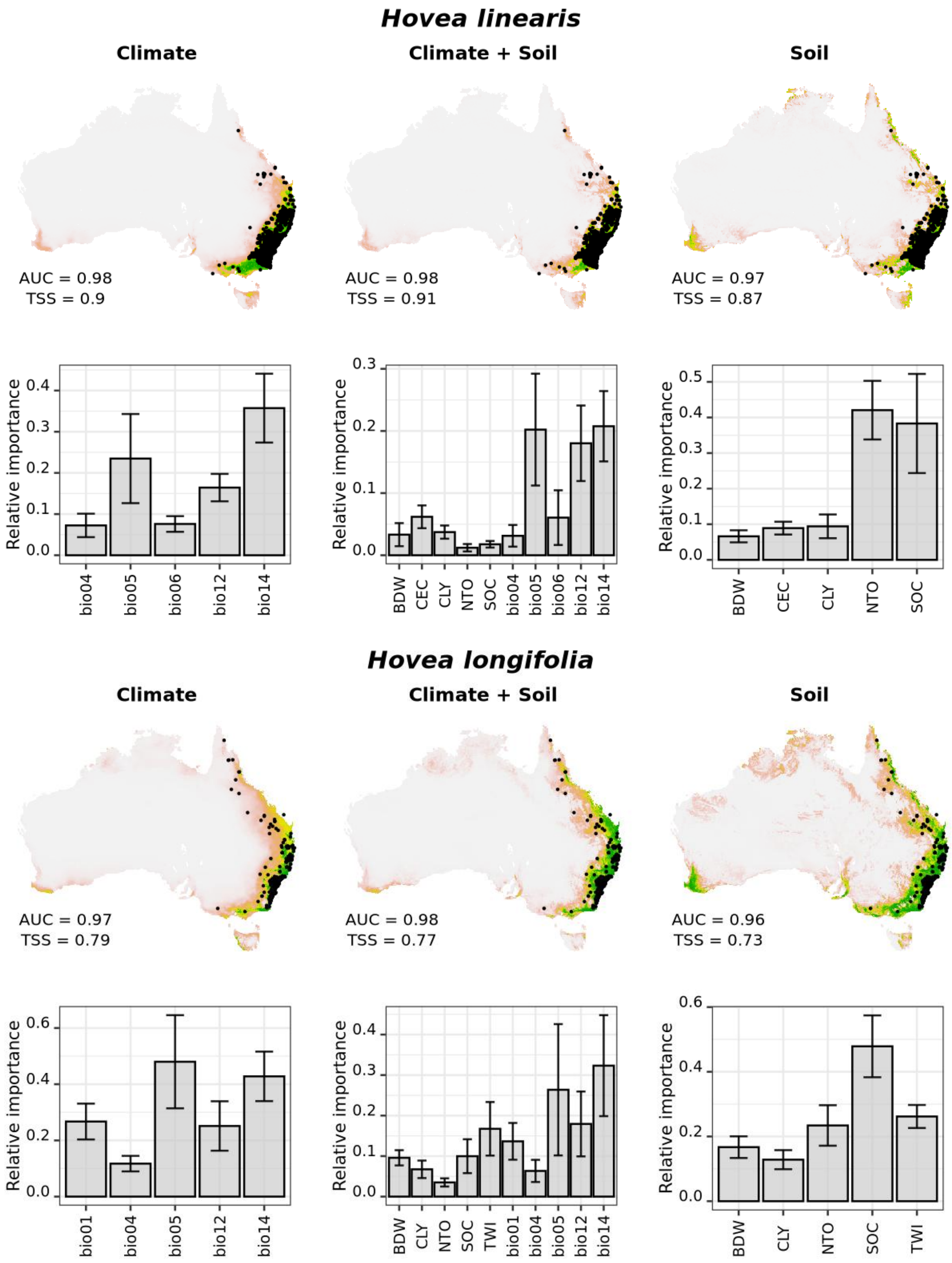

Hovea linearis
Climate
Climate + Soil
Soil
AUC = 0.98
TSS = 0.9
AUC = 0.98
TSS = 0.91
AUC = 0.97
TSS = 0.87
Relative importance
bio04
bio05
bio06
bio12
bio14
BDW
CEC
CLY
NTO
SOC
Hovea longifolia
AUC = 0.97
TSS = 0.79
AUC = 0.98
TSS = 0.77
AUC = 0.96
TSS = 0.73
bio01
TWI

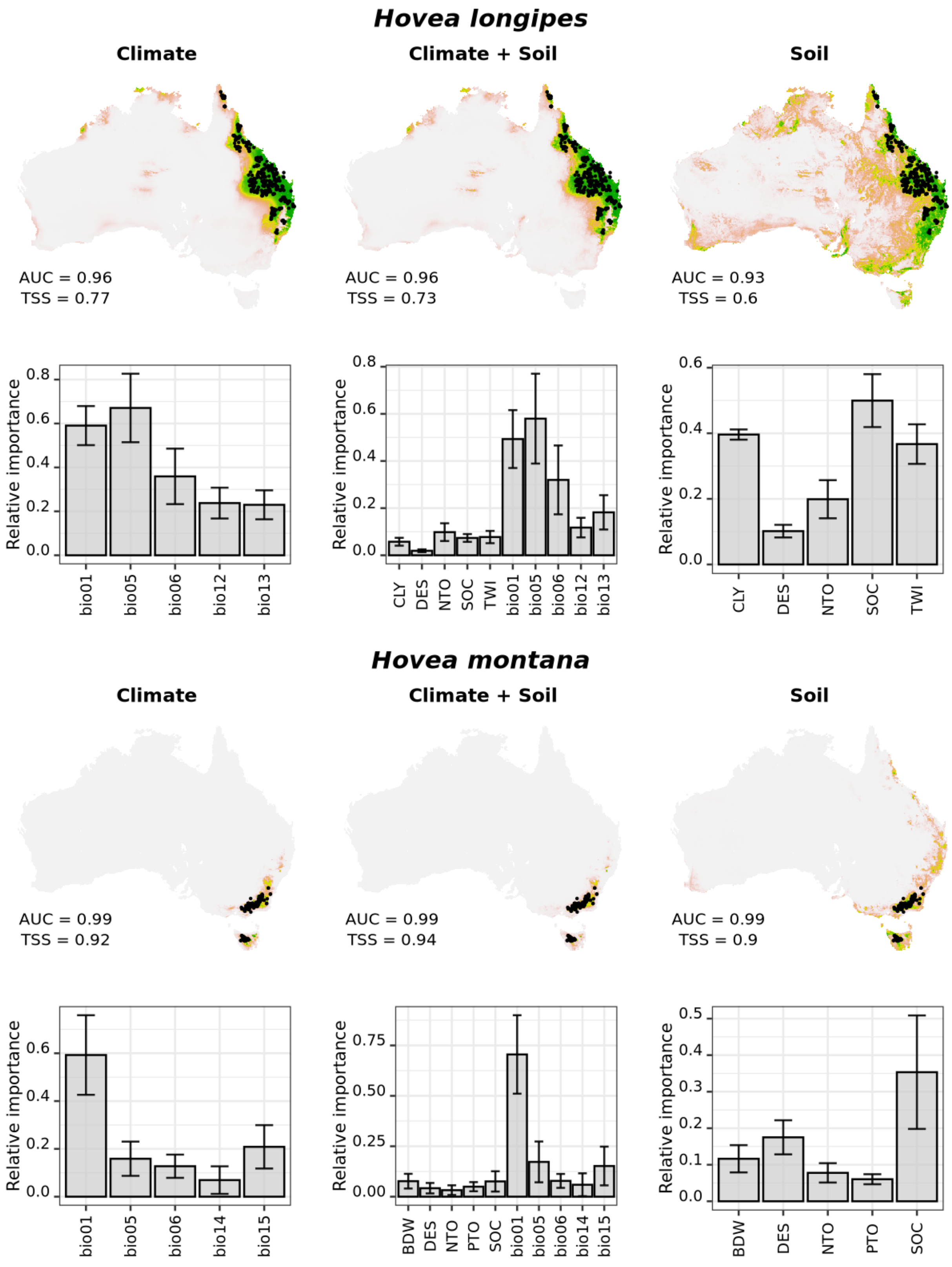

Hovea longipes
Climate
Climate + Soil
Soil
AUC = 0.96
TSS = 0.77
AUC = 0.96
TSS = 0.73
AUC = 0.93
TSS = 0.6
Relative importance
bio01
bio05
bio06
bio12
bio13
CLY
DES
NTO
SOC
TWI
Hovea montana
Climate
Climate + Soil
Soil
AUC = 0.99
TSS = 0.92
AUC = 0.99
TSS = 0.94
AUC = 0.99
TSS = 0.9
Relative importance
bio01
bio05
bio06
bio14
bio15
BDW
DES
NTO
PTO
SOC

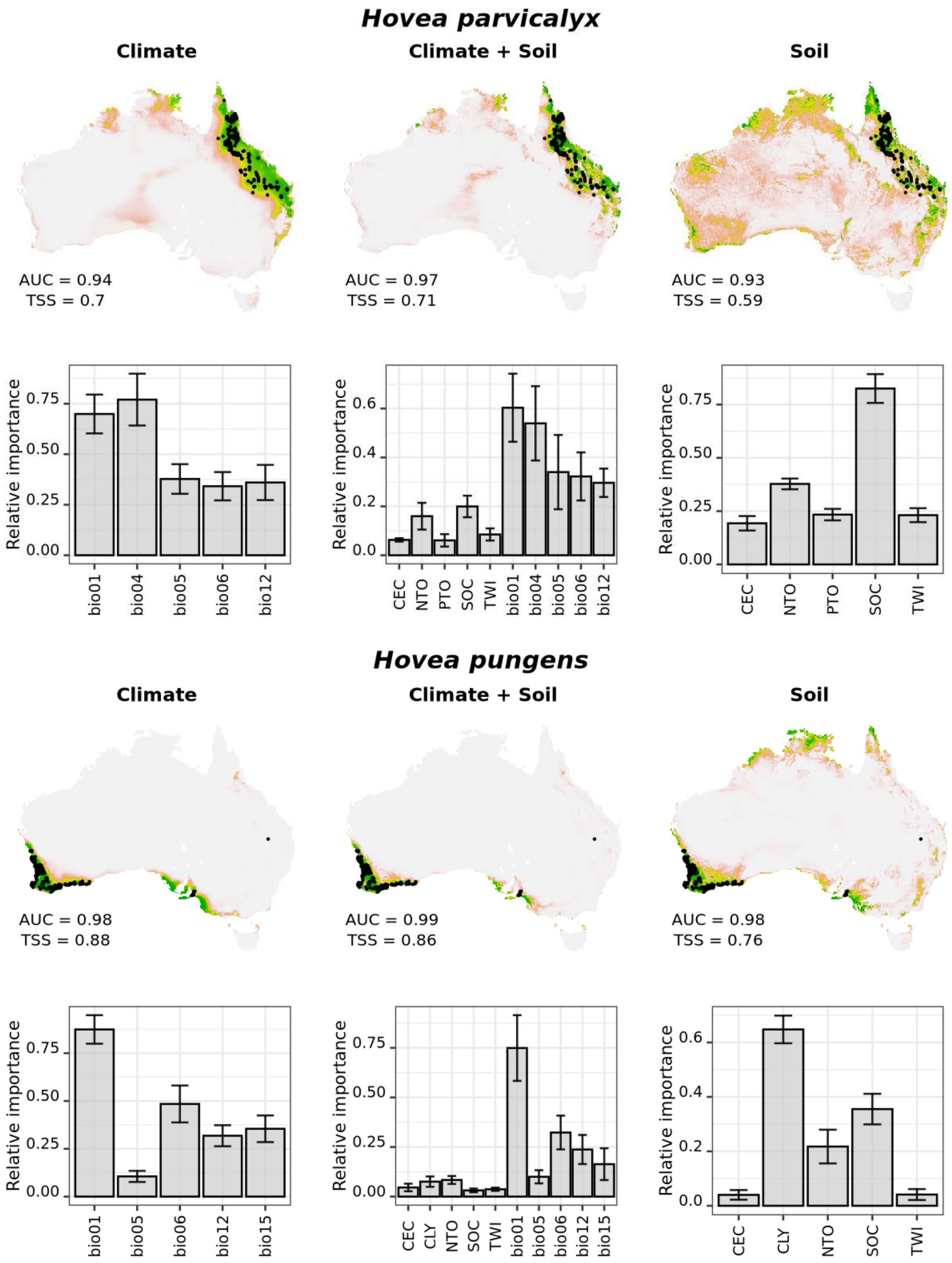
Hovea parvicalyx
Climate
Climate + Soil
Soil
AUC = 0.94
TSS = 0.7
AUC = 0.97
TSS = 0.71
AUC = 0.93
TSS = 0.59
Relative importance
bio01
bio04
bio05
bio06
bio12
CEC
NTO
PTO
SOC
TWI
Hovea pungens
Climate
Climate + Soil
Soil
AUC = 0.98
TSS = 0.88
AUC = 0.99
TSS = 0.86
AUC = 0.98
TSS = 0.76
Relative importance
bio01
bio05
bio06
bio12
bio15
CEC
CLY
NTO
SOC
TWI

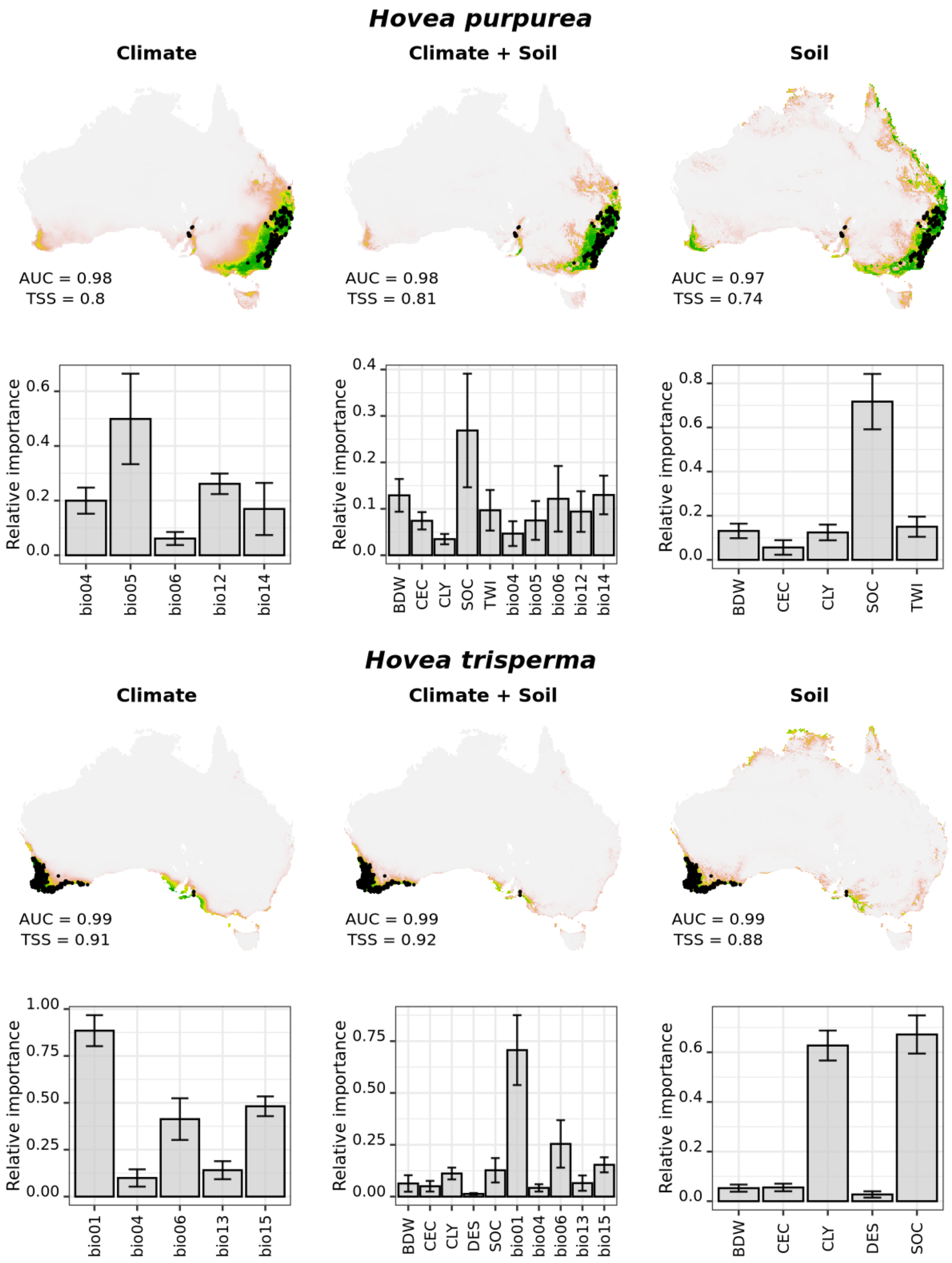
Hovea purpurea
Climate
Climate + Soil
Soil
AUC = 0.98
TSS = 0.8
AUC = 0.98
TSS = 0.81
AUC = 0.97
TSS = 0.74
Relative importance
bio04 bio05 bio06 bio12 bio14
BDW CEC CLY SOC TWI bio04 bio05 bio06 bio12 bio14
BDW CEC CLY SOC TWI
Hovea trisperma
Climate
Climate + Soil
Soil
AUC = 0.99
TSS = 0.91
AUC = 0.99
TSS = 0.92
AUC = 0.99
TSS = 0.88
Relative importance
bio01 bio04 bio06 bio13 bio15
BDW CEC CLY DES SOC bio01 bio04 bio06 bio13 bio15
BDW CEC CLY DES SOC

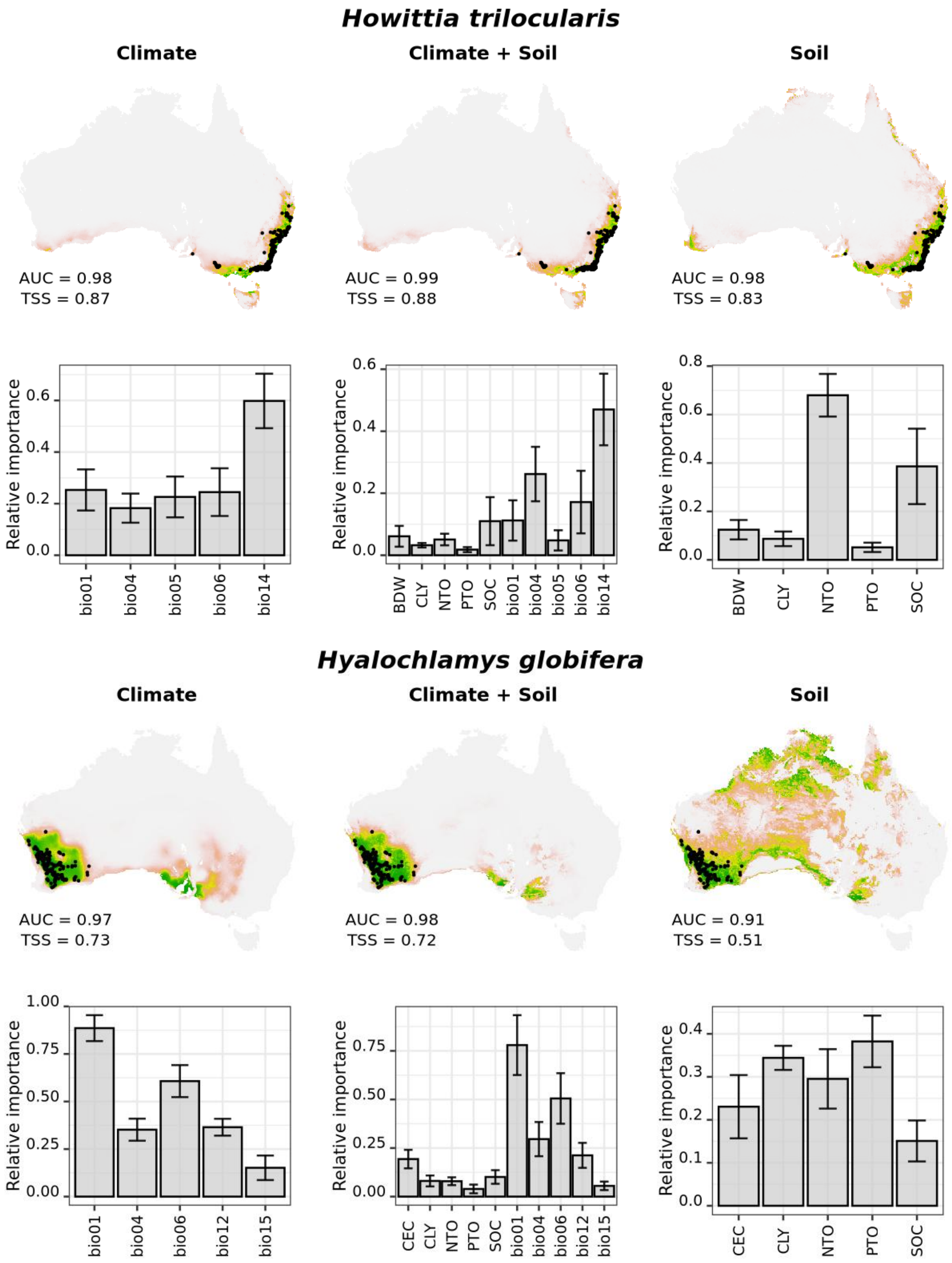
Howittia trilocularis
Climate
Climate + Soil
Soil
AUC = 0.98
TSS = 0.87
AUC = 0.99
TSS = 0.88
AUC = 0.98
TSS = 0.83
Relative importance
0.0
0.2
0.4
0.6
0.8
bio01
bio04
bio05
bio06
bio14
BDW
CLY
NTO
PTO
SOC
Hyalochlamys globifera
Climate
Climate + Soil
Soil
AUC = 0.97
TSS = 0.73
AUC = 0.98
TSS = 0.72
AUC = 0.91
TSS = 0.51
Relative importance
0.00
0.25
0.50
0.75
1.00
0.0
0.1
0.2
0.3
0.4
bio01
bio04
bio06
bio12
bio15
CEC
CLY
NTO
PTO
SOC

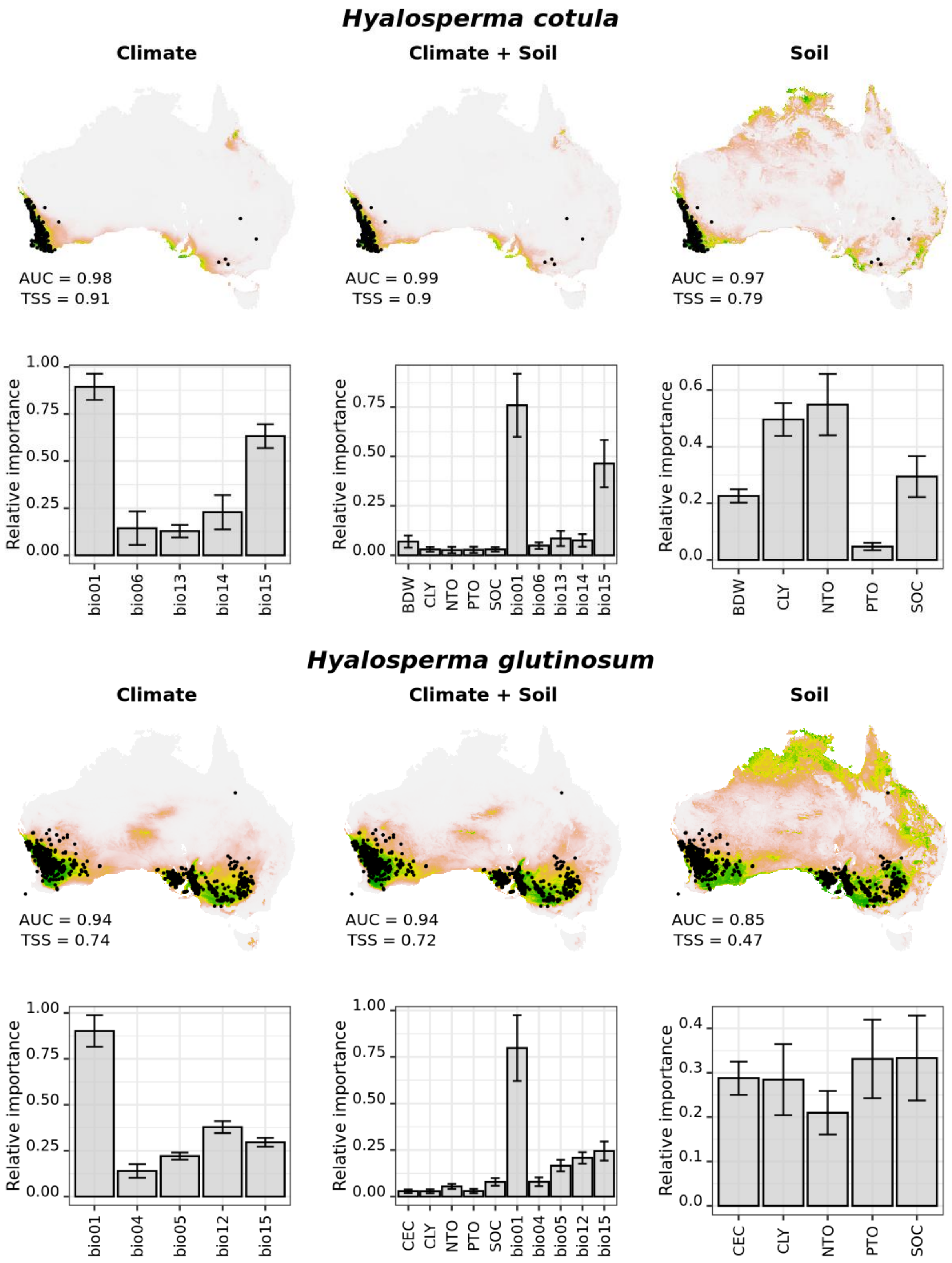

Hyalosperma cotula
Climate
Climate + Soil
Soil
AUC = 0.98
TSS = 0.91
AUC = 0.99
TSS = 0.9
AUC = 0.97
TSS = 0.79
Relative importance
bio01 bio06 bio13 bio14 bio15
BDW CLY NTO PTO SOC bio01 bio06 bio13 bio14 bio15
BDW CLY NTO PTO SOC
Hyalosperma glutinosum
Climate
Climate + Soil
Soil
AUC = 0.94
TSS = 0.74
AUC = 0.94
TSS = 0.72
AUC = 0.85
TSS = 0.47
Relative importance
bio01 bio04 bio05 bio12 bio15
CEC CLY NTO PTO SOC bio01 bio04 bio05 bio12 bio15
CEC CLY NTO PTO SOC

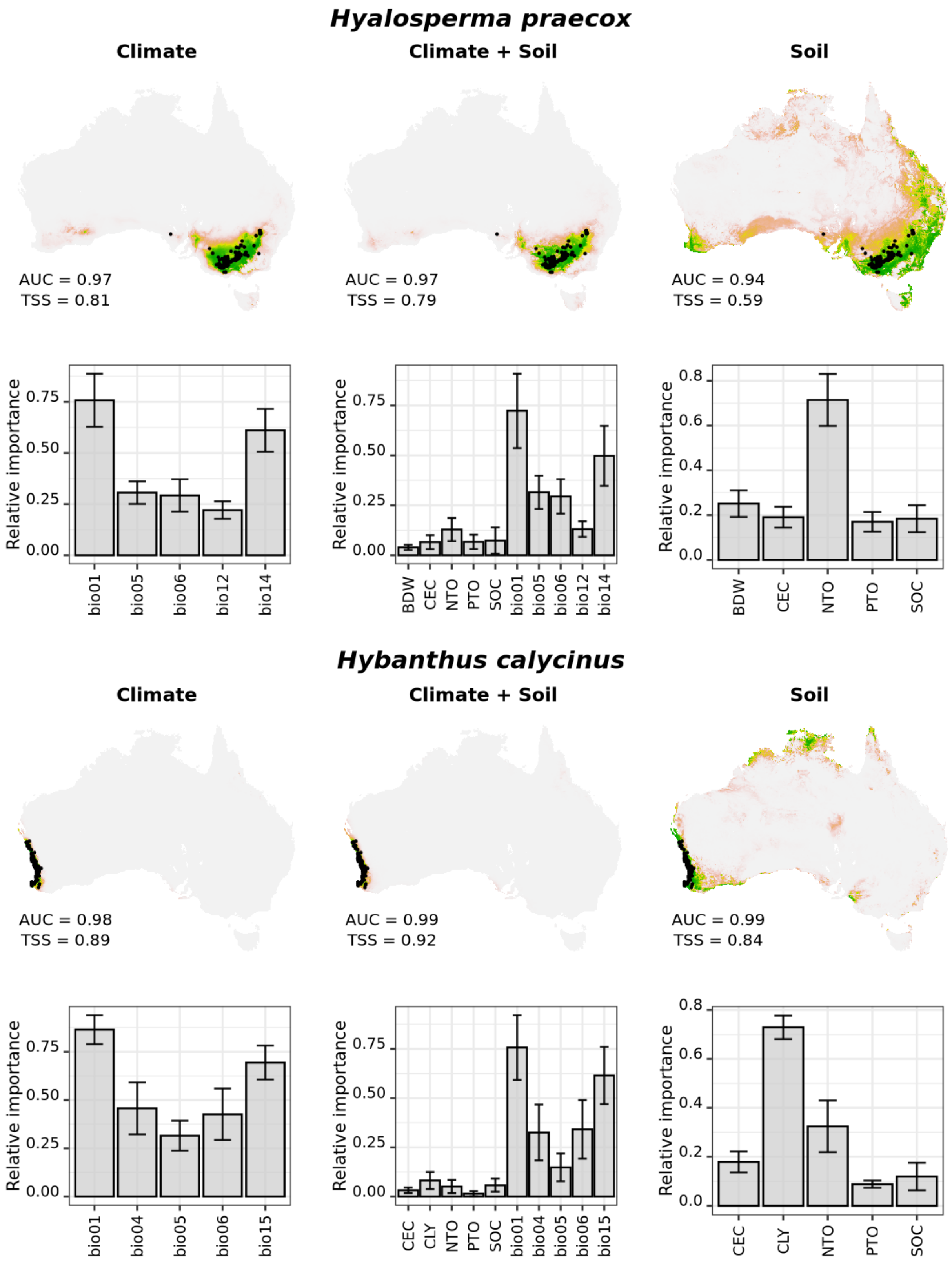
Hyalosperma praecox
Climate
Climate + Soil
Soil
AUC = 0.97
TSS = 0.81
AUC = 0.97
TSS = 0.79
AUC = 0.94
TSS = 0.59
Relative importance
0.00
0.25
0.50
0.75
bio01
bio05
bio06
bio12
bio14
BDW
CEC
NTO
PTO
SOC
0.0
0.2
0.4
0.6
0.8
Hybanthus calycinus
AUC = 0.98
TSS = 0.89
AUC = 0.99
TSS = 0.92
AUC = 0.99
TSS = 0.84
bio04
bio15
CLY

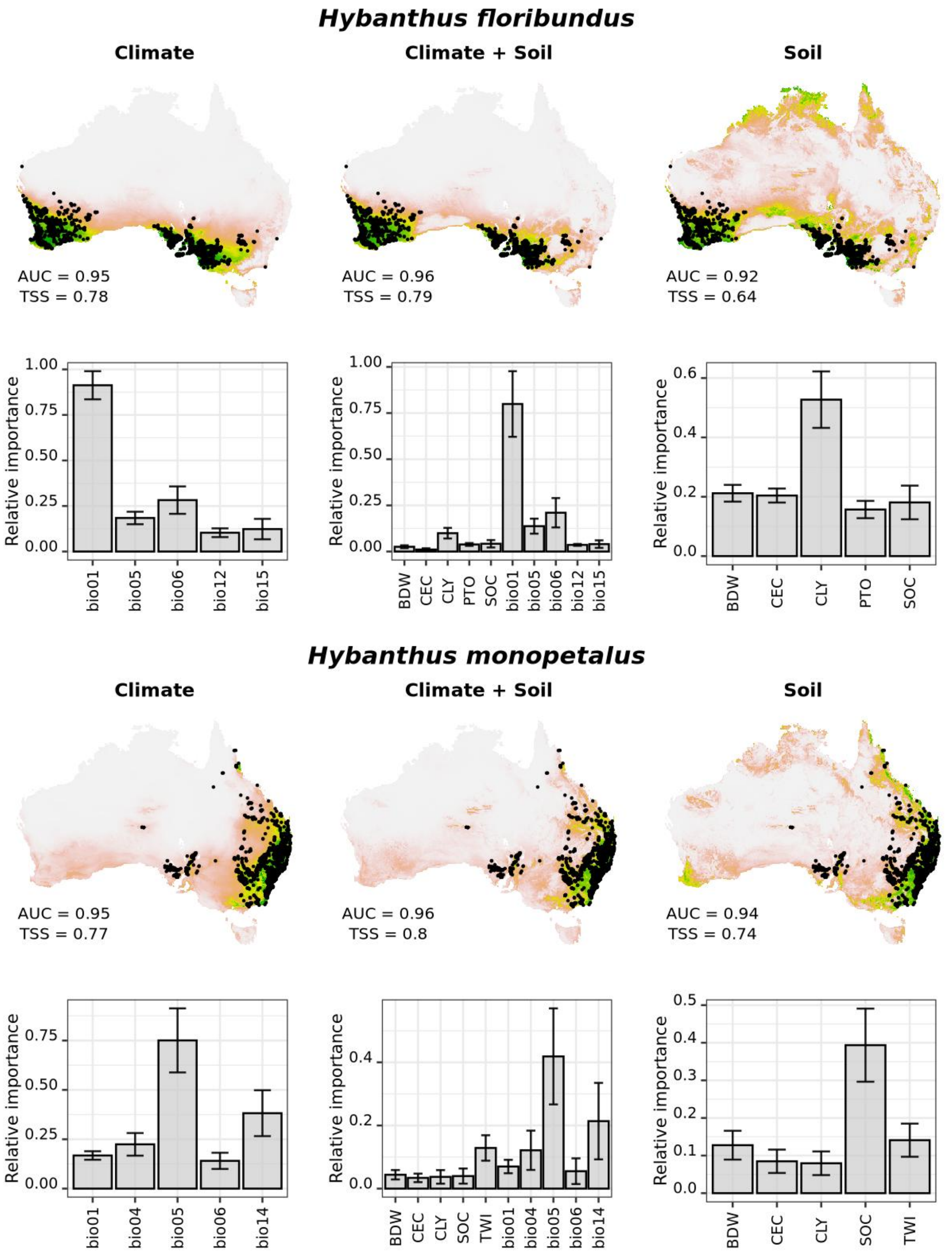
Hybanthus floribundus
Climate
Climate + Soil
Soil
AUC = 0.95
TSS = 0.78
AUC = 0.96
TSS = 0.79
AUC = 0.92
TSS = 0.64
Relative importance
bio01
bio05
bio06
bio12
bio15
BDW
CEC
CLY
PTO
SOC
Hybanthus monopetalus
Climate
Climate + Soil
Soil
AUC = 0.95
TSS = 0.77
AUC = 0.96
TSS = 0.8
AUC = 0.94
TSS = 0.74
Relative importance
bio01
bio04
bio05
bio06
bio14
BDW
CEC
CLY
SOC
TWI

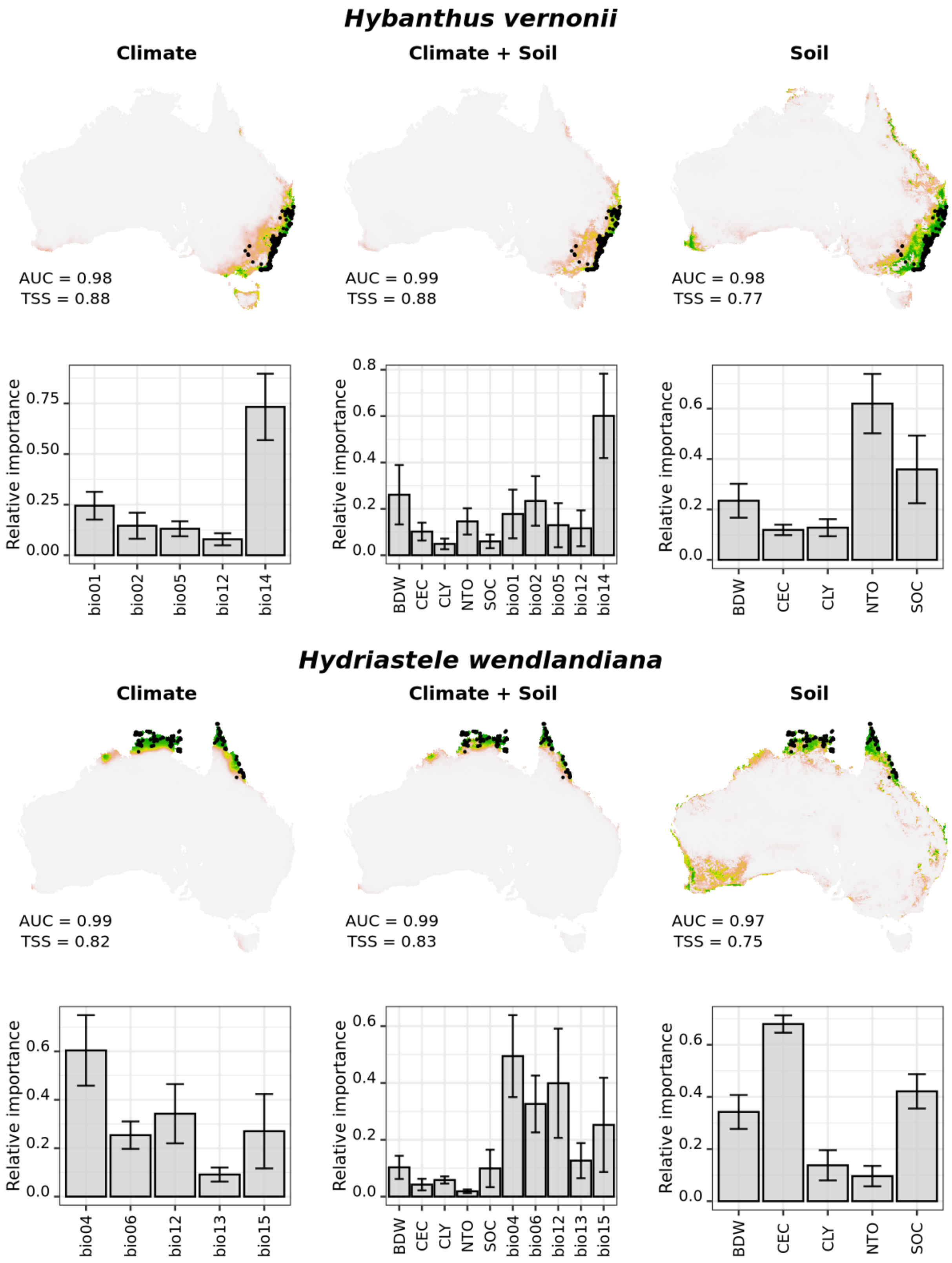

Hybanthus vernonii
Climate
Climate + Soil
Soil
AUC = 0.98
TSS = 0.88
AUC = 0.99
TSS = 0.88
AUC = 0.98
TSS = 0.77
Relative importance
0.00
0.25
0.50
0.75
bio01
bio02
bio05
bio12
bio14
0.0
0.2
0.4
0.6
0.8
BDW
CEC
CLY
NTO
SOC
bio01
bio02
bio05
bio12
bio14
BDW
CEC
CLY
NTO
SOC
Hydriastele wendlandiana
Climate
Climate + Soil
Soil
AUC = 0.99
TSS = 0.82
AUC = 0.99
TSS = 0.83
AUC = 0.97
TSS = 0.75
Relative importance
0.0
0.2
0.4
0.6
bio04
bio06
bio12
bio13
bio15
BDW
CEC
CLY
NTO
SOC
bio04
bio06
bio12
bio13
bio15
BDW
CEC
CLY
NTO
SOC

Hydrocotyle acutiloba

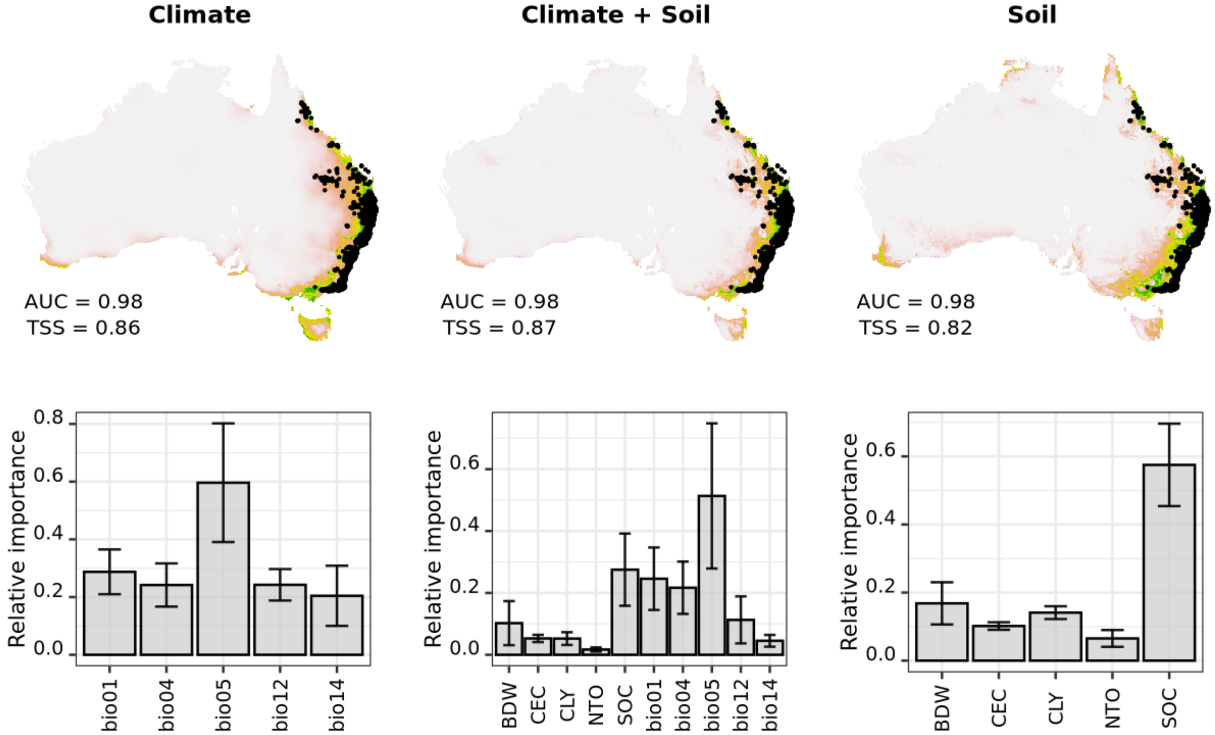

Hydrocotyle alata

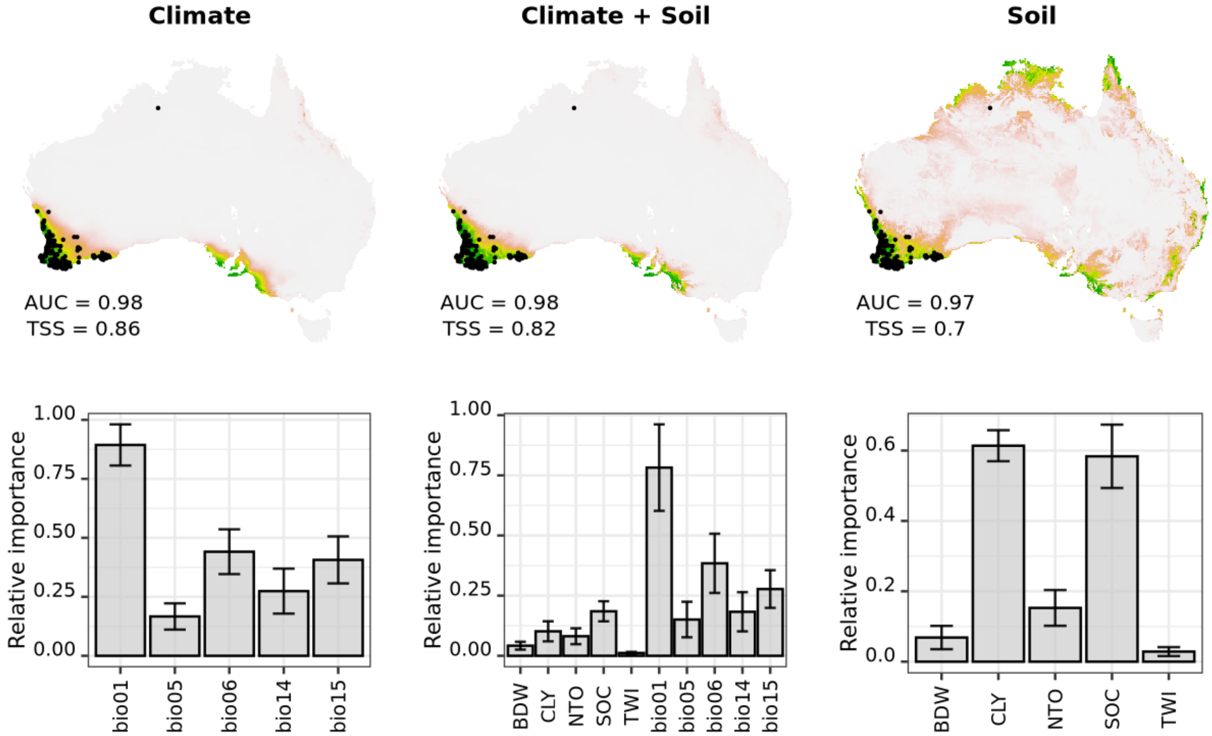

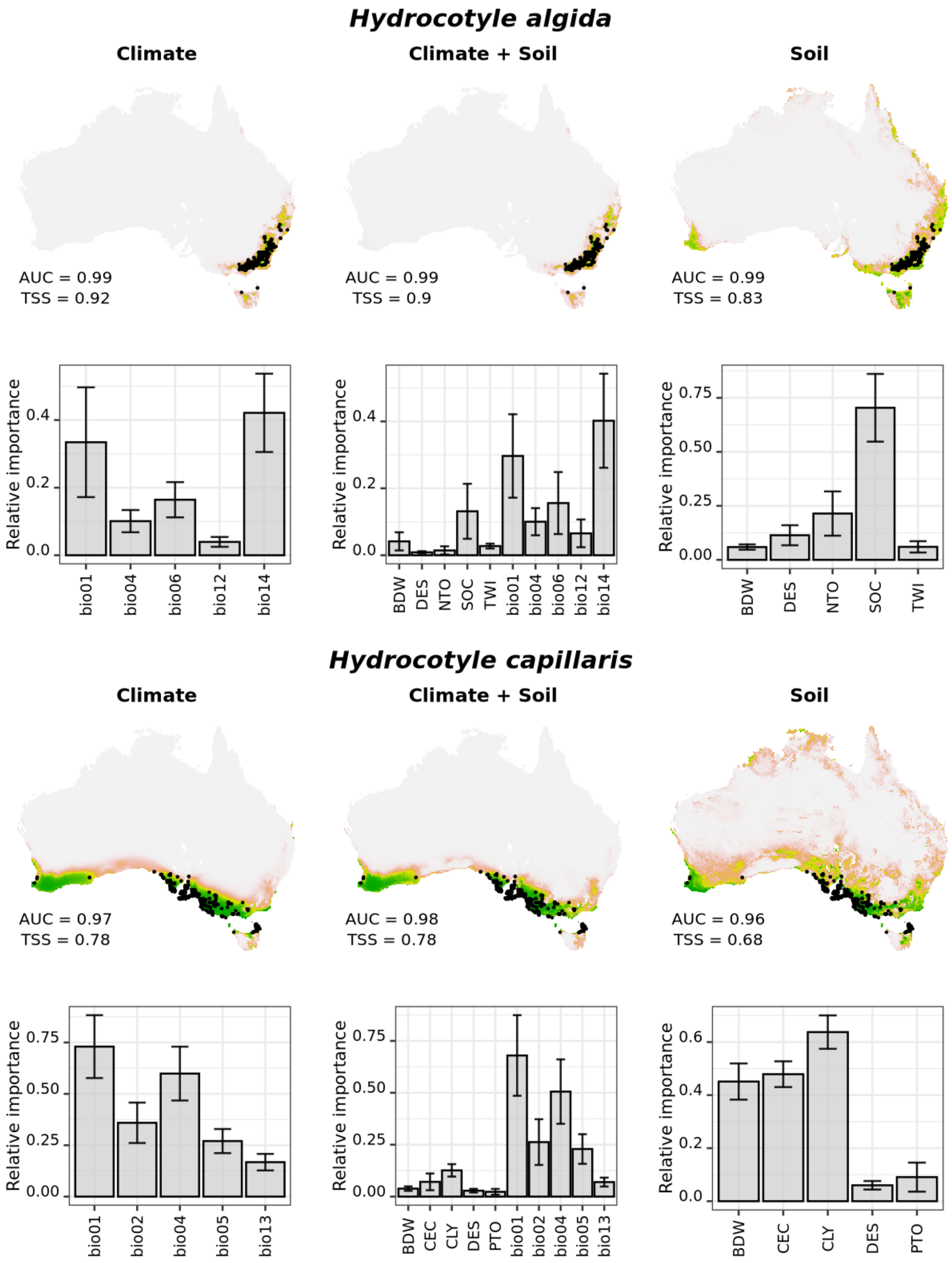

Hydrocotyle algida
Climate
Climate + Soil
Soil
AUC = 0.99
TSS = 0.92
AUC = 0.99
TSS = 0.9
AUC = 0.99
TSS = 0.83
Relative importance
bio01
bio04
bio06
bio12
bio14
BDW
DES
NTO
SOC
TWI
Hydrocotyle capillaris
Climate
Climate + Soil
Soil
AUC = 0.97
TSS = 0.78
AUC = 0.98
TSS = 0.78
AUC = 0.96
TSS = 0.68
Relative importance
bio01
bio02
bio04
bio05
bio13
BDW
CEC
CLY
DES
PTO

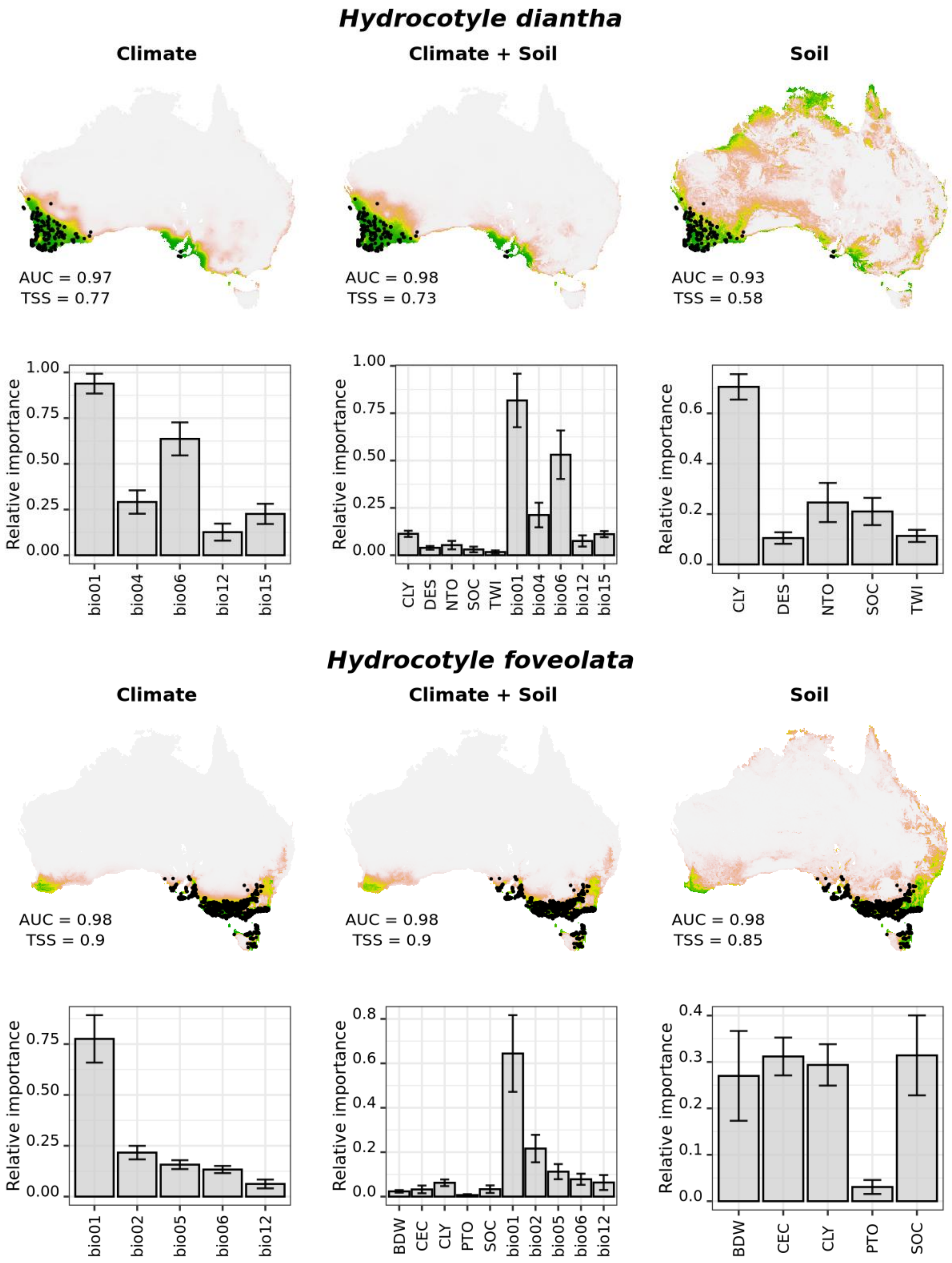
Hydrocotyle diantha
Climate
Climate + Soil
Soil
AUC = 0.97
TSS = 0.77
AUC = 0.98
TSS = 0.73
AUC = 0.93
TSS = 0.58
Relative importance
bio01
bio04
bio06
bio12
bio15
CLY
DES
NTO
SOC
TWI
Hydrocotyle foveolata
Climate
Climate + Soil
Soil
AUC = 0.98
TSS = 0.9
AUC = 0.98
TSS = 0.9
AUC = 0.98
TSS = 0.85
Relative importance
bio01
bio02
bio05
bio06
bio12
BDW
CEC
CLY
PTO
SOC

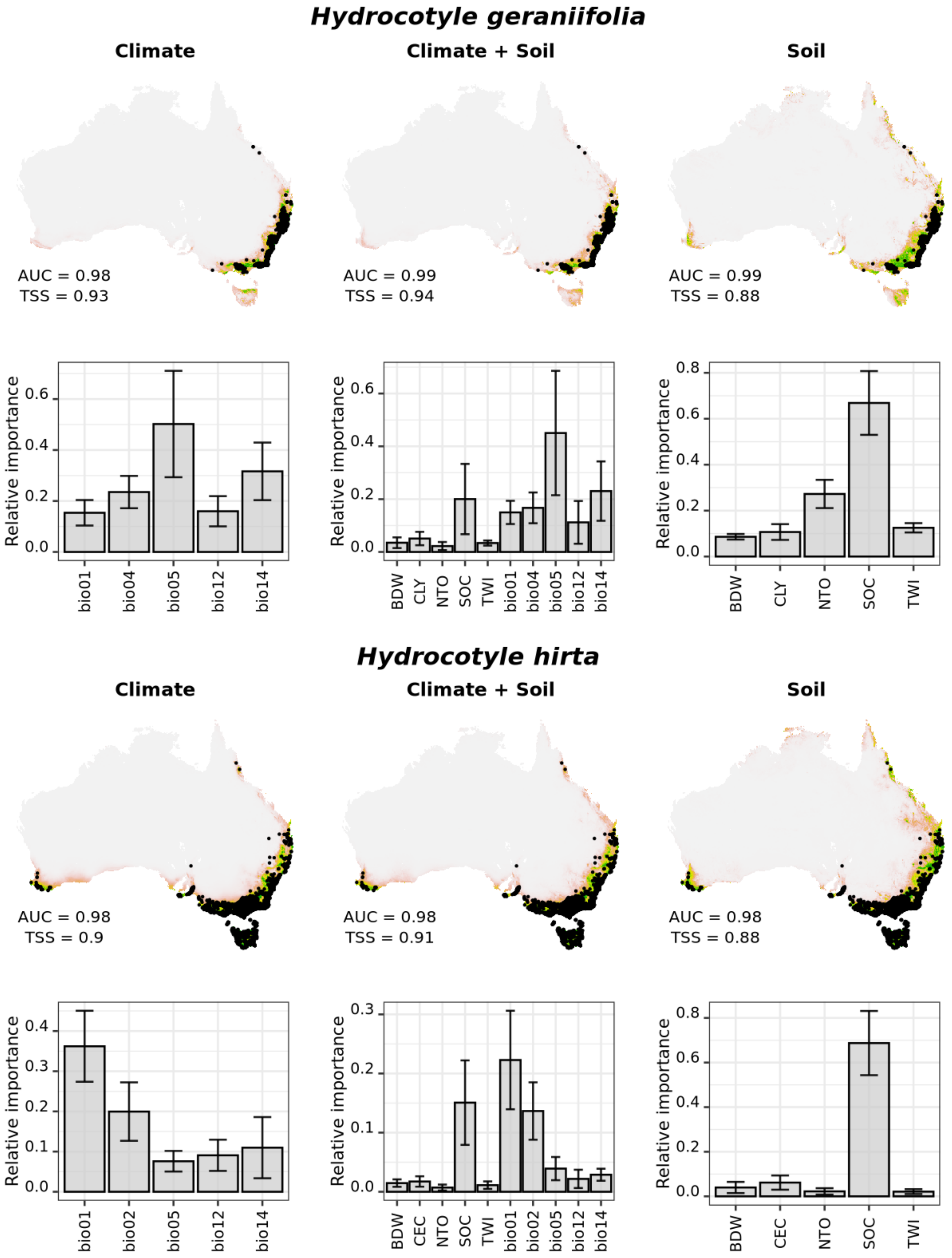

Hydrocotyle geraniifolia
Climate
Climate + Soil
Soil
AUC = 0.98
TSS = 0.93
AUC = 0.99
TSS = 0.94
AUC = 0.99
TSS = 0.88
Relative importance
bio01
bio04
bio05
bio12
bio14
BDW
CLY
NTO
SOC
TWI
Hydrocotyle hirta
Climate
Climate + Soil
Soil
AUC = 0.98
TSS = 0.9
AUC = 0.98
TSS = 0.91
AUC = 0.98
TSS = 0.88
Relative importance
bio01
bio02
bio05
bio12
bio14
BDW
CEC
NTO
SOC
TWI

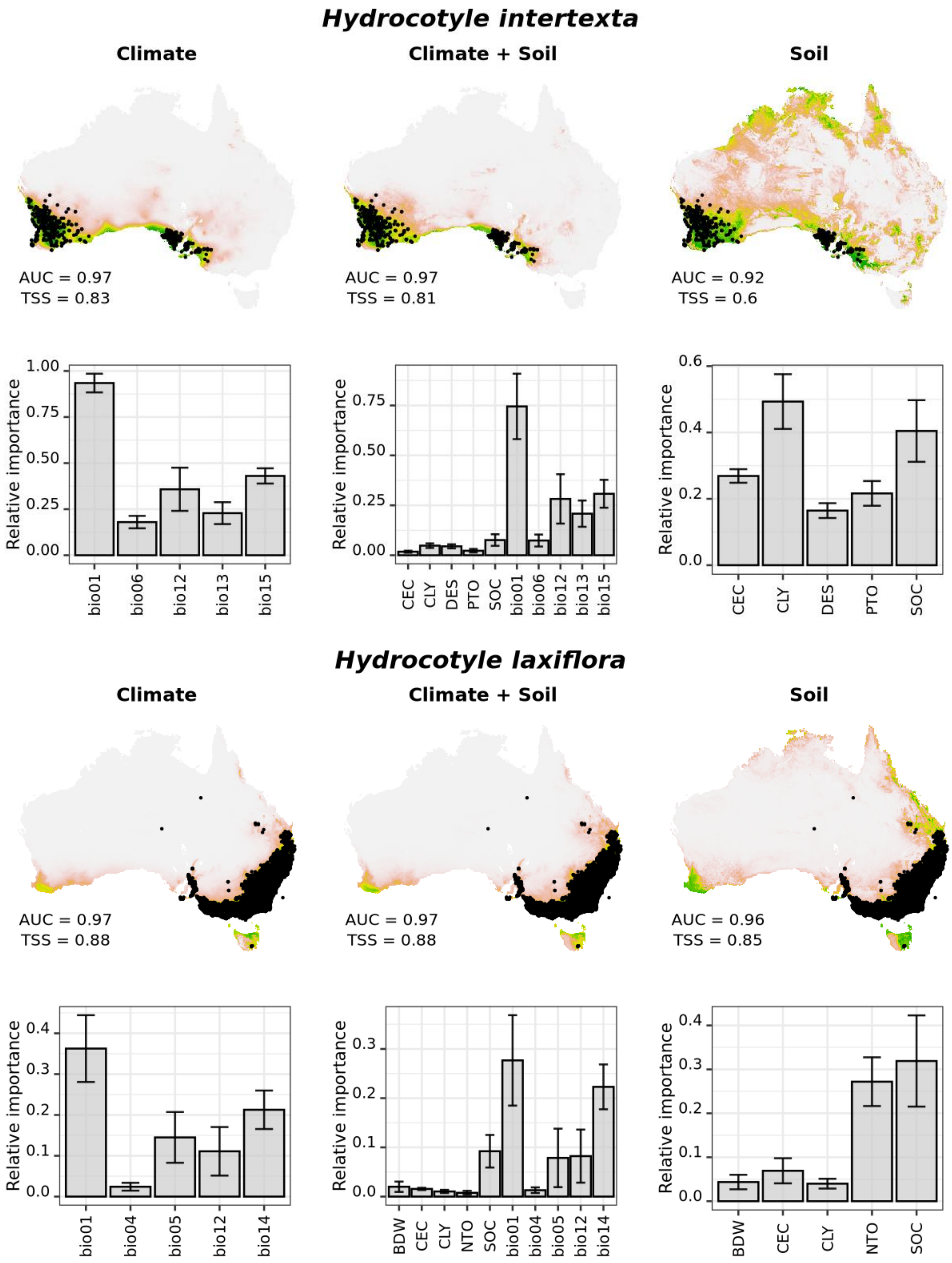
Hydrocotyle intertexta
Climate
Climate + Soil
Soil
AUC = 0.97
TSS = 0.83
AUC = 0.97
TSS = 0.81
AUC = 0.92
TSS = 0.6
Relative importance
bio01 bio06 bio12 bio13 bio15
CEC CLY DES PTO SOC bio01 bio06 bio12 bio13 bio15
CEC CLY DES PTO SOC
Hydrocotyle laxiflora
Climate
Climate + Soil
Soil
AUC = 0.97
TSS = 0.88
AUC = 0.97
TSS = 0.88
AUC = 0.96
TSS = 0.85
Relative importance
bio01 bio04 bio05 bio12 bio14
BDW CEC CLY NTO SOC bio01 bio04 bio05 bio12 bio14
BDW CEC CLY NTO SOC

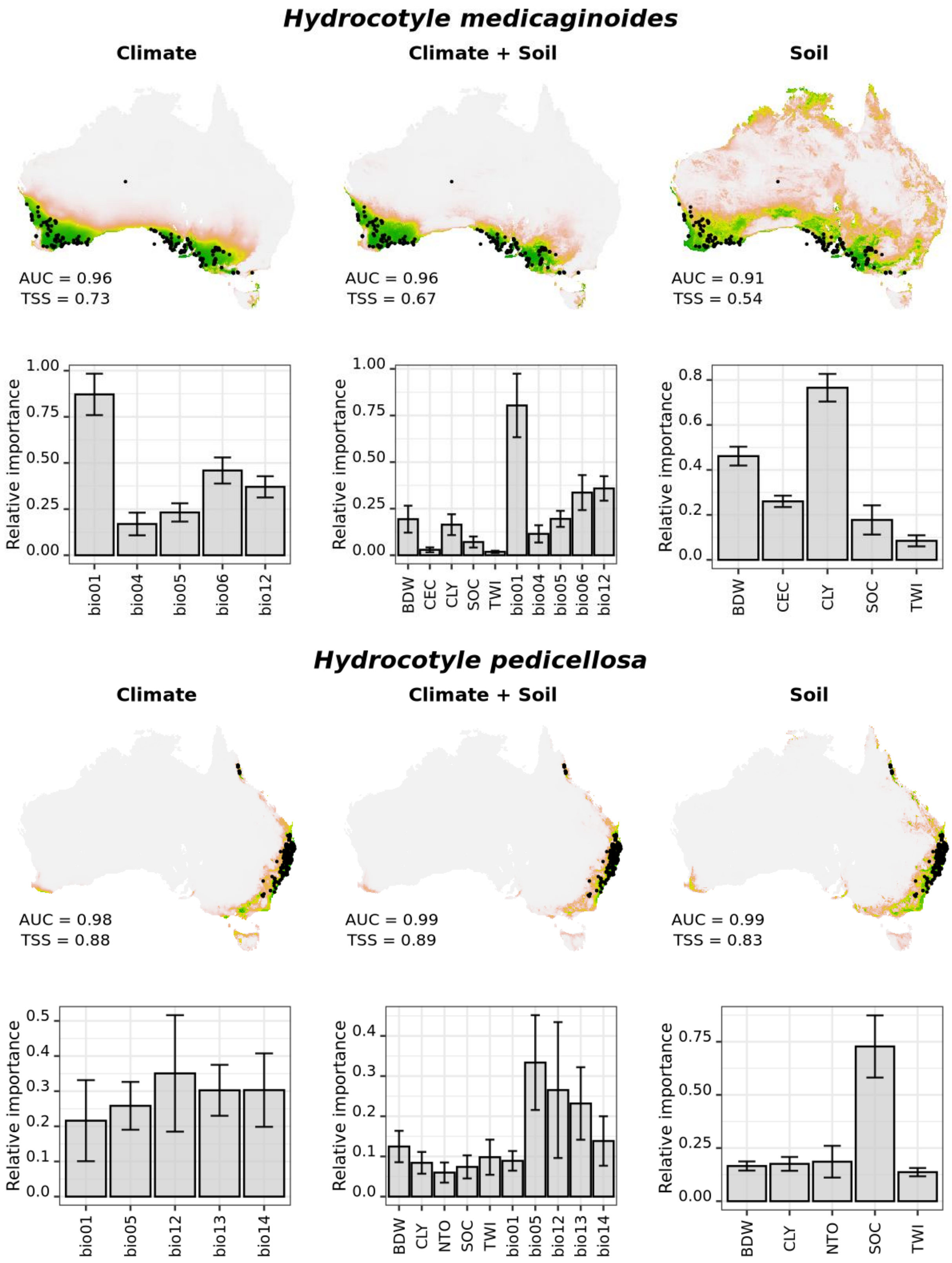
Hydrocotyle medicaginoides
Climate
Climate + Soil
Soil
AUC = 0.96
TSS = 0.73
AUC = 0.96
TSS = 0.67
AUC = 0.91
TSS = 0.54
Relative importance
bio01
bio04
bio05
bio06
bio12
BDW
CEC
CLY
SOC
TWI
Hydrocotyle pedicellosa
Climate
Climate + Soil
Soil
AUC = 0.98
TSS = 0.88
AUC = 0.99
TSS = 0.89
AUC = 0.99
TSS = 0.83
Relative importance
bio01
bio05
bio12
bio13
bio14
BDW
CLY
NTO
SOC
TWI

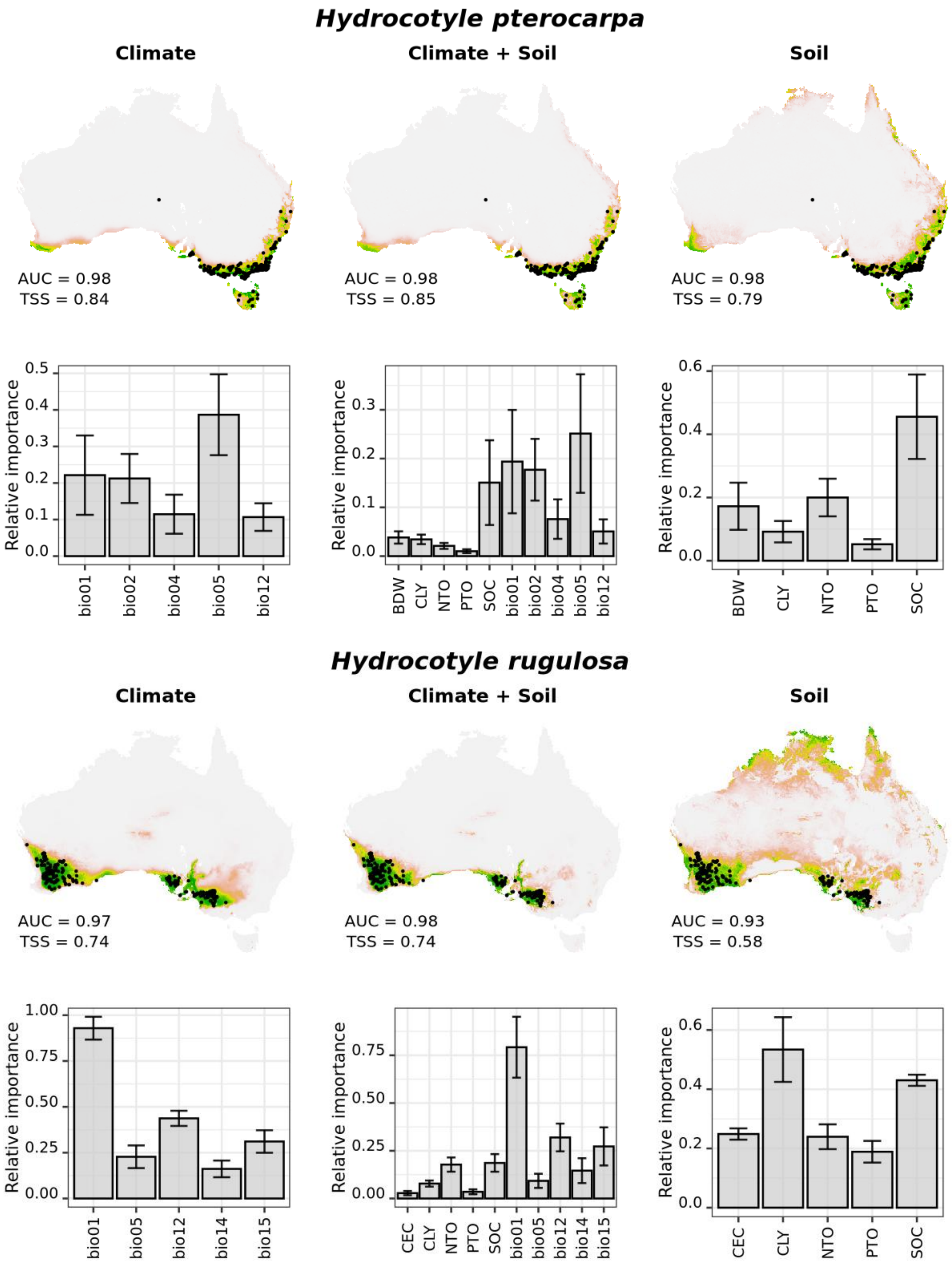

Hydrocotyle pterocarpa
Climate
Climate + Soil
Soil
AUC = 0.98
TSS = 0.84
AUC = 0.98
TSS = 0.85
AUC = 0.98
TSS = 0.79
Relative importance
bio01
bio02
bio04
bio05
bio12
BDW
CLY
NTO
PTO
SOC
Hydrocotyle rugulosa
Climate
Climate + Soil
Soil
AUC = 0.97
TSS = 0.74
AUC = 0.98
TSS = 0.74
AUC = 0.93
TSS = 0.58
Relative importance
bio01
bio05
bio12
bio14
bio15
CEC
CLY
NTO
PTO
SOC

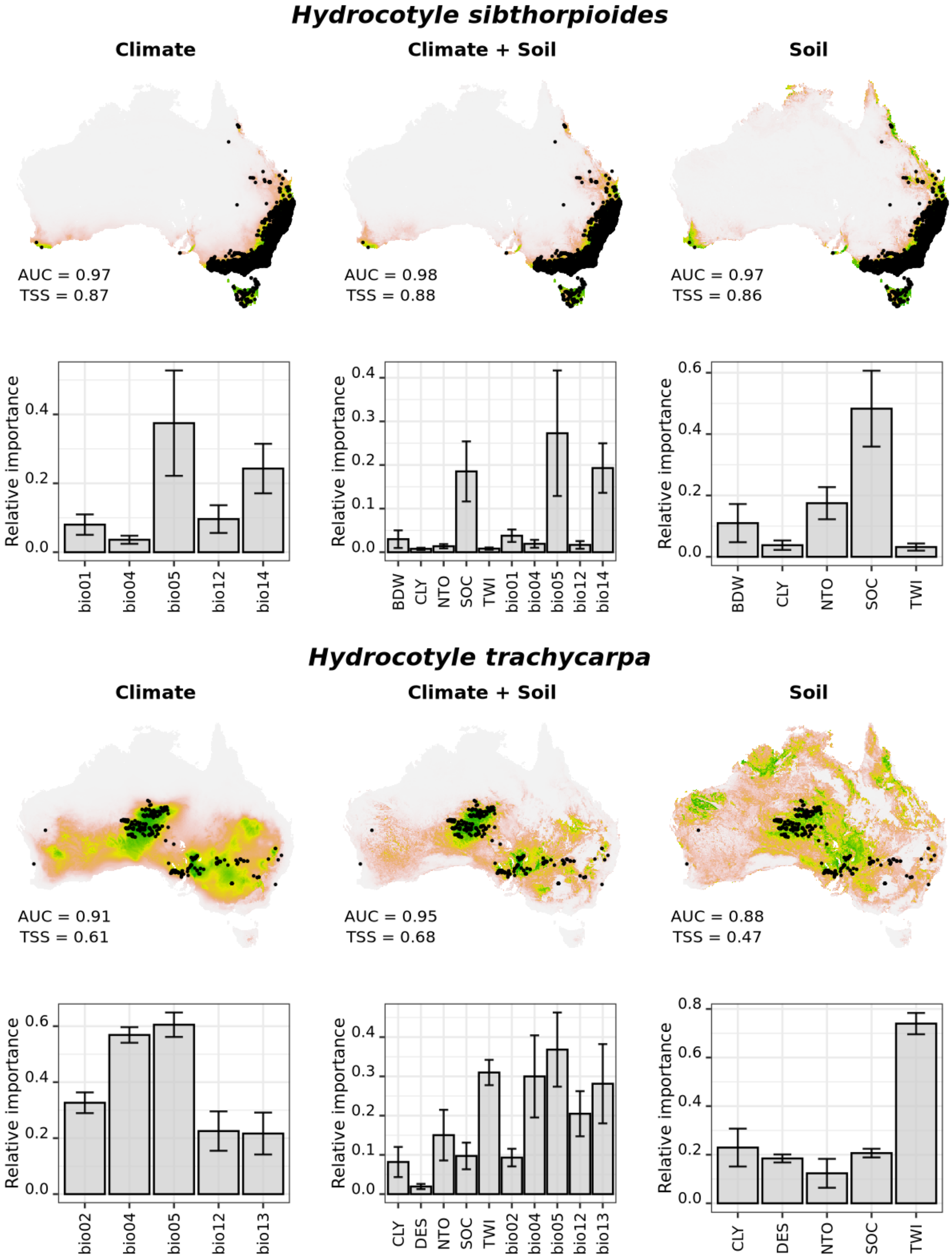
Hydrocotyle sibthorpioides
Climate
Climate + Soil
Soil
AUC = 0.97
TSS = 0.87
AUC = 0.98
TSS = 0.88
AUC = 0.97
TSS = 0.86
Relative importance
bio01
bio04
bio05
bio12
bio14
BDW
CLY
NTO
SOC
TWI
Hydrocotyle trachycarpa
Climate
Climate + Soil
Soil
AUC = 0.91
TSS = 0.61
AUC = 0.95
TSS = 0.68
AUC = 0.88
TSS = 0.47
Relative importance
bio02
bio04
bio05
bio12
bio13
CLY
DES
NTO
SOC
TWI

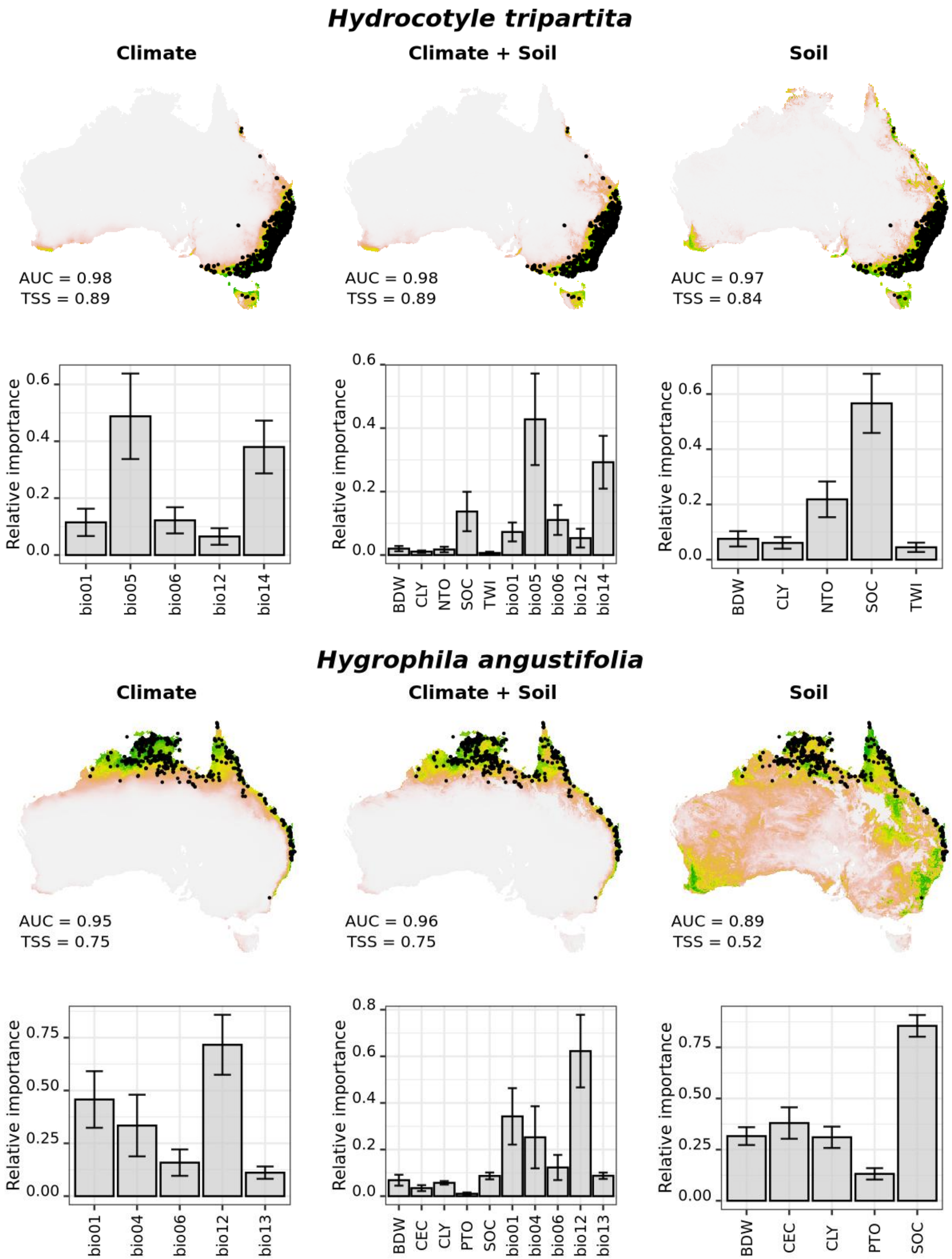
Hydrocotyle tripartita
Climate
Climate + Soil
Soil
AUC = 0.98
TSS = 0.89
AUC = 0.98
TSS = 0.89
AUC = 0.97
TSS = 0.84
Relative importance
0.0
0.2
0.4
0.6
bio01
bio05
bio06
bio12
bio14
BDW
CLY
NTO
SOC
TWI
Hygrophila angustifolia
AUC = 0.95
TSS = 0.75
AUC = 0.96
TSS = 0.75
AUC = 0.89
TSS = 0.52
0.00
0.25
0.50
0.75
0.8
bio04
bio13
CEC
PTO

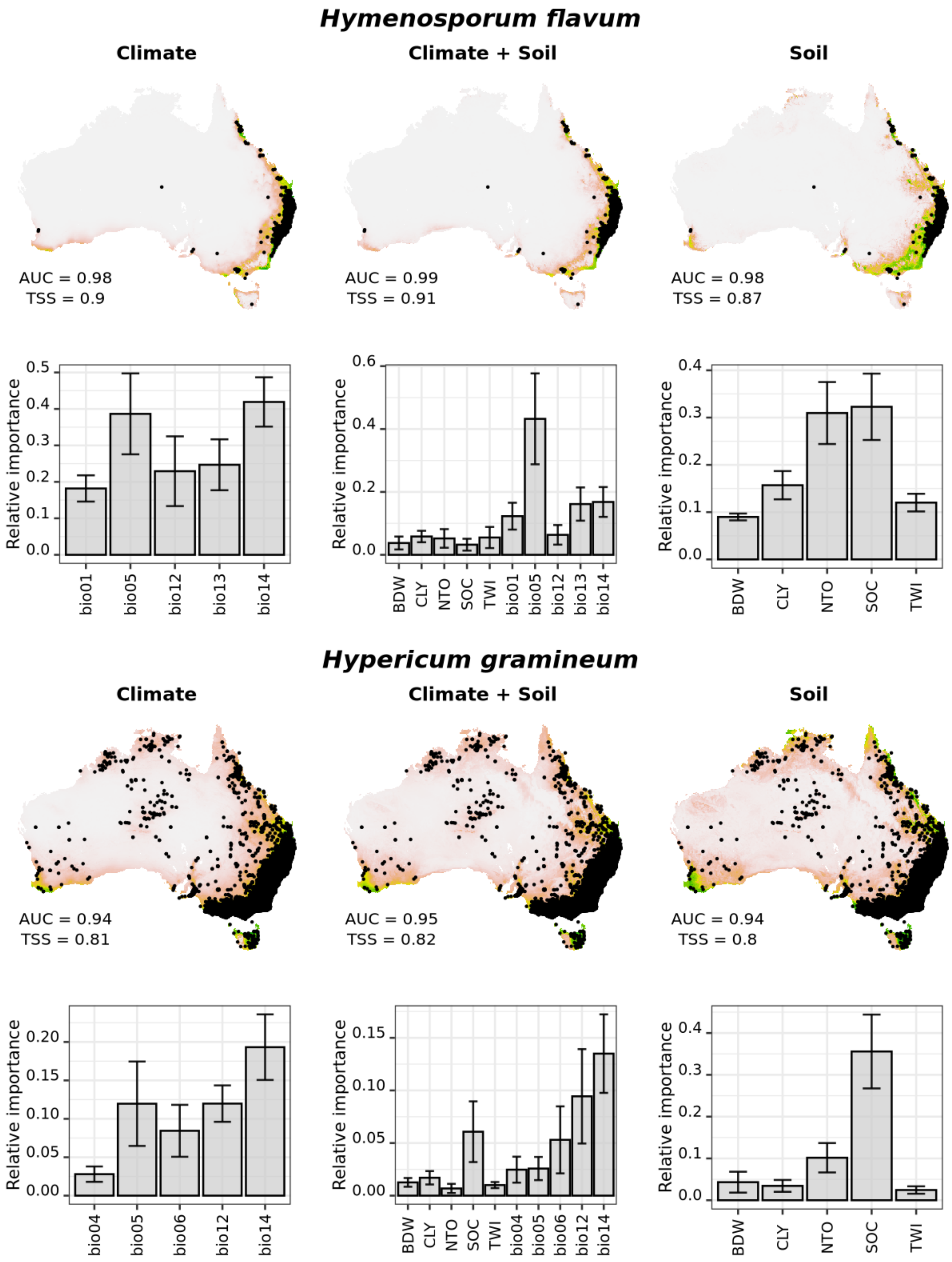
Hymenosporum flavum
Climate
Climate + Soil
Soil
AUC = 0.98
TSS = 0.9
AUC = 0.99
TSS = 0.91
AUC = 0.98
TSS = 0.87
Relative importance
bio01
bio05
bio12
bio13
bio14
BDW
CLY
NTO
SOC
TWI
Hypericum gramineum
Climate
Climate + Soil
Soil
AUC = 0.94
TSS = 0.81
AUC = 0.95
TSS = 0.82
AUC = 0.94
TSS = 0.8
Relative importance
bio04
bio05
bio06
bio12
bio14
BDW
CLY
NTO
SOC
TWI

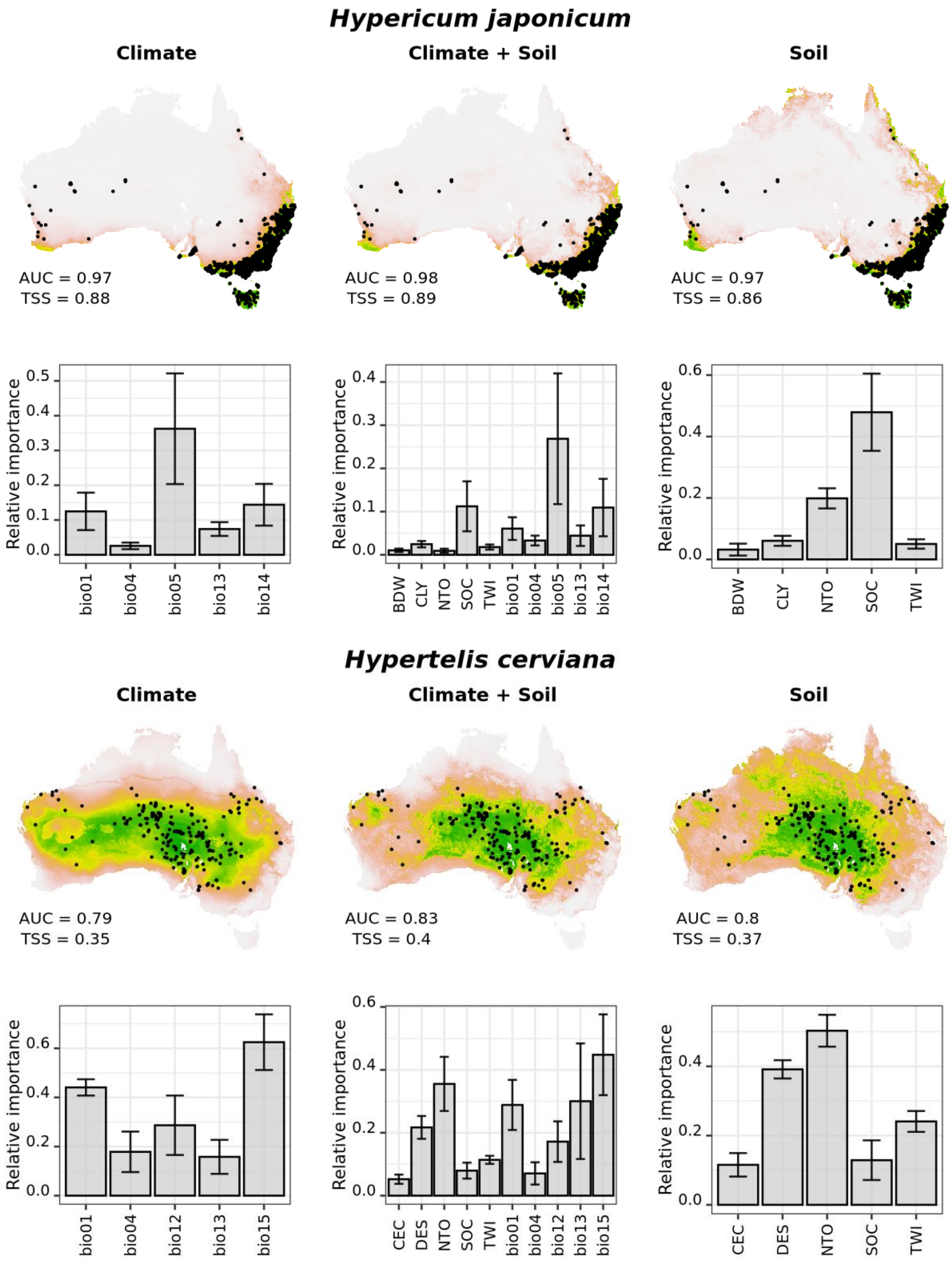
Hypericum japonicum
Climate
Climate + Soil
Soil
AUC = 0.97
TSS = 0.88
AUC = 0.98
TSS = 0.89
AUC = 0.97
TSS = 0.86
Relative importance
bio01
bio04
bio05
bio13
bio14
BDW
CLY
NTO
SOC
TWI
Hypertelis cerviana
AUC = 0.79
TSS = 0.35
AUC = 0.83
TSS = 0.4
AUC = 0.8
TSS = 0.37
bio12
bio15
CEC
DES

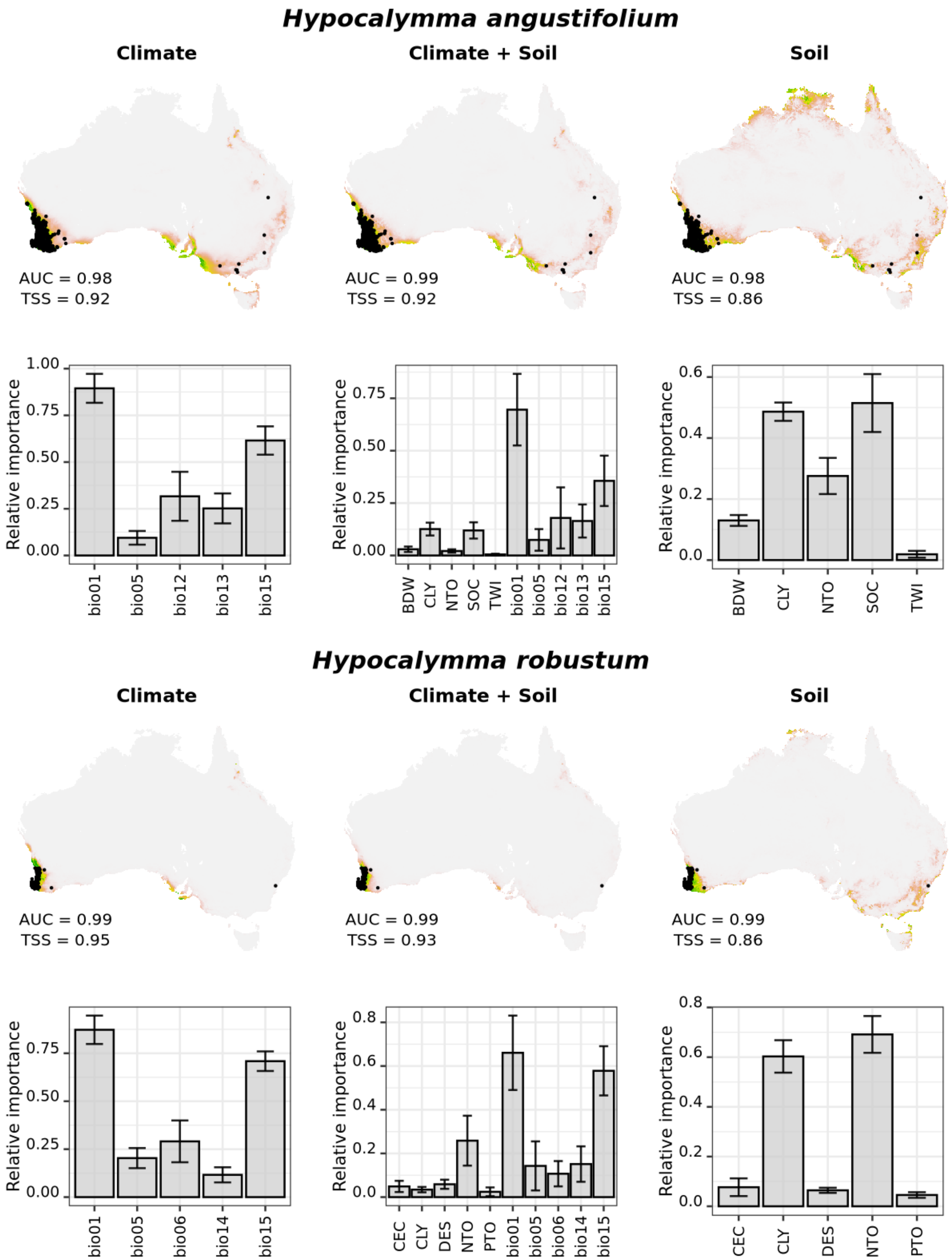

Hypocalymma angustifolium
Climate
Climate + Soil
Soil
AUC = 0.98
TSS = 0.92
AUC = 0.99
TSS = 0.92
AUC = 0.98
TSS = 0.86
Relative importance
1.00
0.75
0.50
0.25
0.00
bio01
bio05
bio12
bio13
bio15
BDW
CLY
NTO
SOC
TWI
0.6
0.4
0.2
0.0
Hypocalymma robustum
Climate
Climate + Soil
Soil
AUC = 0.99
TSS = 0.95
AUC = 0.99
TSS = 0.93
AUC = 0.99
TSS = 0.86
bio06
bio14
CEC
DES
PTO
0.8

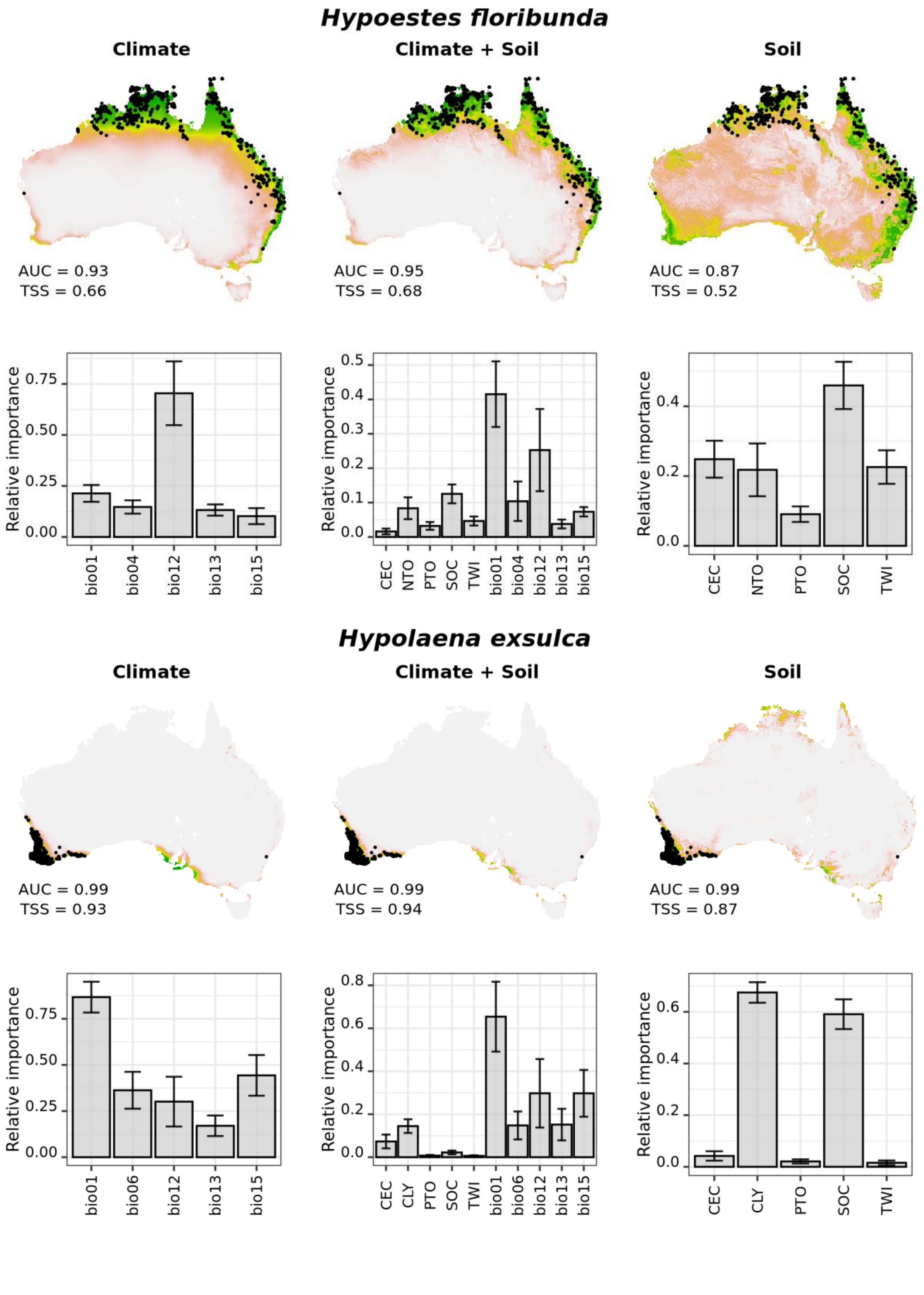

Hypoestes floribunda
Climate
Climate + Soil
Soil
AUC = 0.93
TSS = 0.66
AUC = 0.95
TSS = 0.68
AUC = 0.87
TSS = 0.52
Relative importance
bio01
bio04
bio12
bio13
bio15
CEC
NTO
PTO
SOC
TWI
Hypolaena exsulca
Climate
Climate + Soil
Soil
AUC = 0.99
TSS = 0.93
AUC = 0.99
TSS = 0.94
AUC = 0.99
TSS = 0.87
Relative importance
bio01
bio06
bio12
bio13
bio15
CEC
CLY
PTO
SOC
TWI

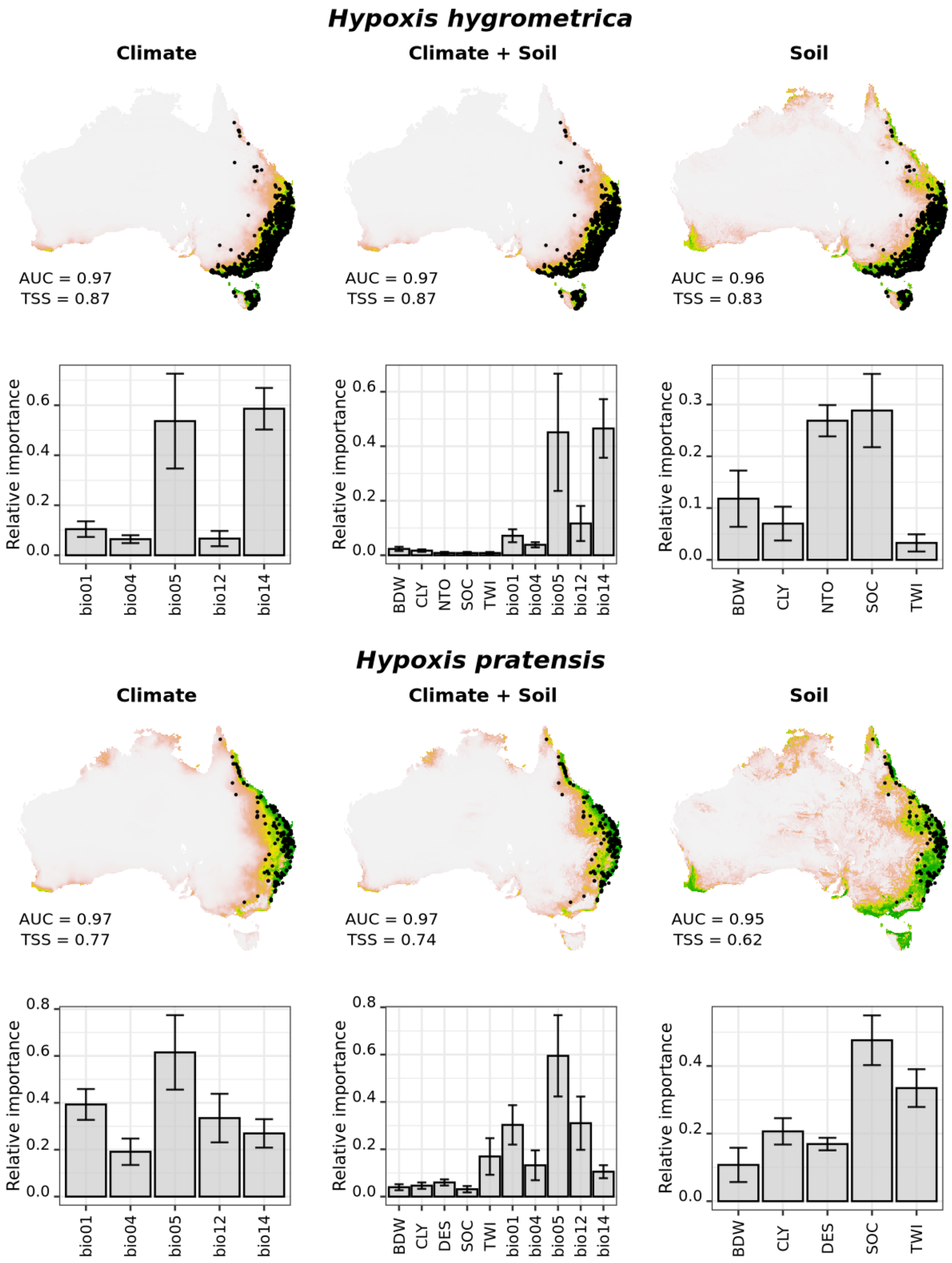
Hypoxis hygrometrica
Climate
Climate + Soil
Soil
AUC = 0.97
TSS = 0.87
AUC = 0.97
TSS = 0.87
AUC = 0.96
TSS = 0.83
Relative importance
bio01
bio04
bio05
bio12
bio14
BDW
CLY
NTO
SOC
TWI
Hypoxis pratensis
Climate
Climate + Soil
Soil
AUC = 0.97
TSS = 0.77
AUC = 0.97
TSS = 0.74
AUC = 0.95
TSS = 0.62
Relative importance
bio01
bio04
bio05
bio12
bio14
BDW
CLY
DES
SOC
TWI

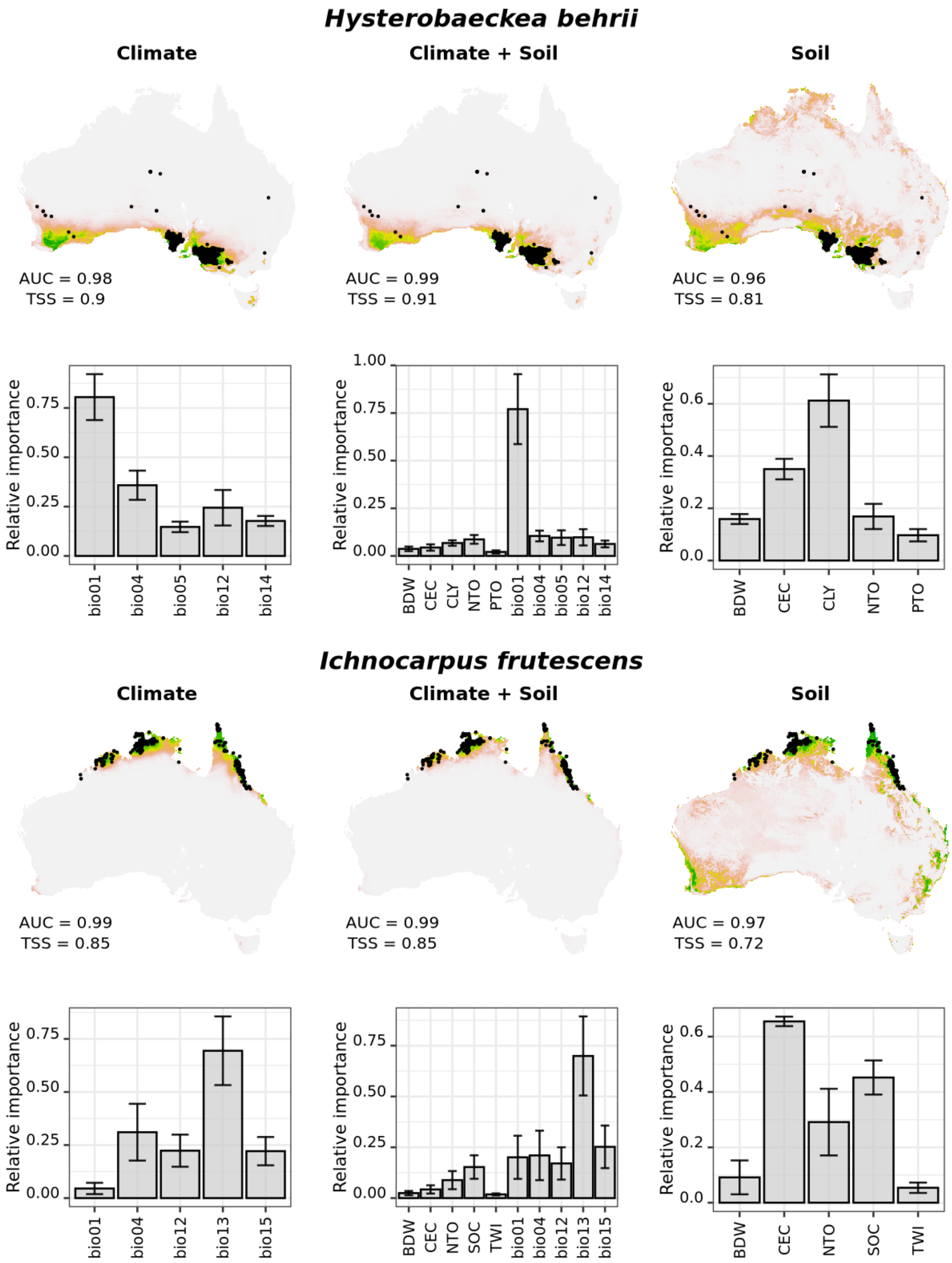
Hysterobaeckea behrii
Climate
Climate + Soil
Soil
AUC = 0.98
TSS = 0.9
AUC = 0.99
TSS = 0.91
AUC = 0.96
TSS = 0.81
Relative importance
0.00
0.25
0.50
0.75
1.00
0.0
0.2
0.4
0.6
bio01
bio04
bio05
bio12
bio14
BDW
CEC
CLY
NTO
PTO
Ichnocarpus frutescens
Climate
Climate + Soil
Soil
AUC = 0.99
TSS = 0.85
AUC = 0.99
TSS = 0.85
AUC = 0.97
TSS = 0.72
Relative importance
bio01
bio04
bio12
bio13
bio15
BDW
CEC
NTO
SOC
TWI

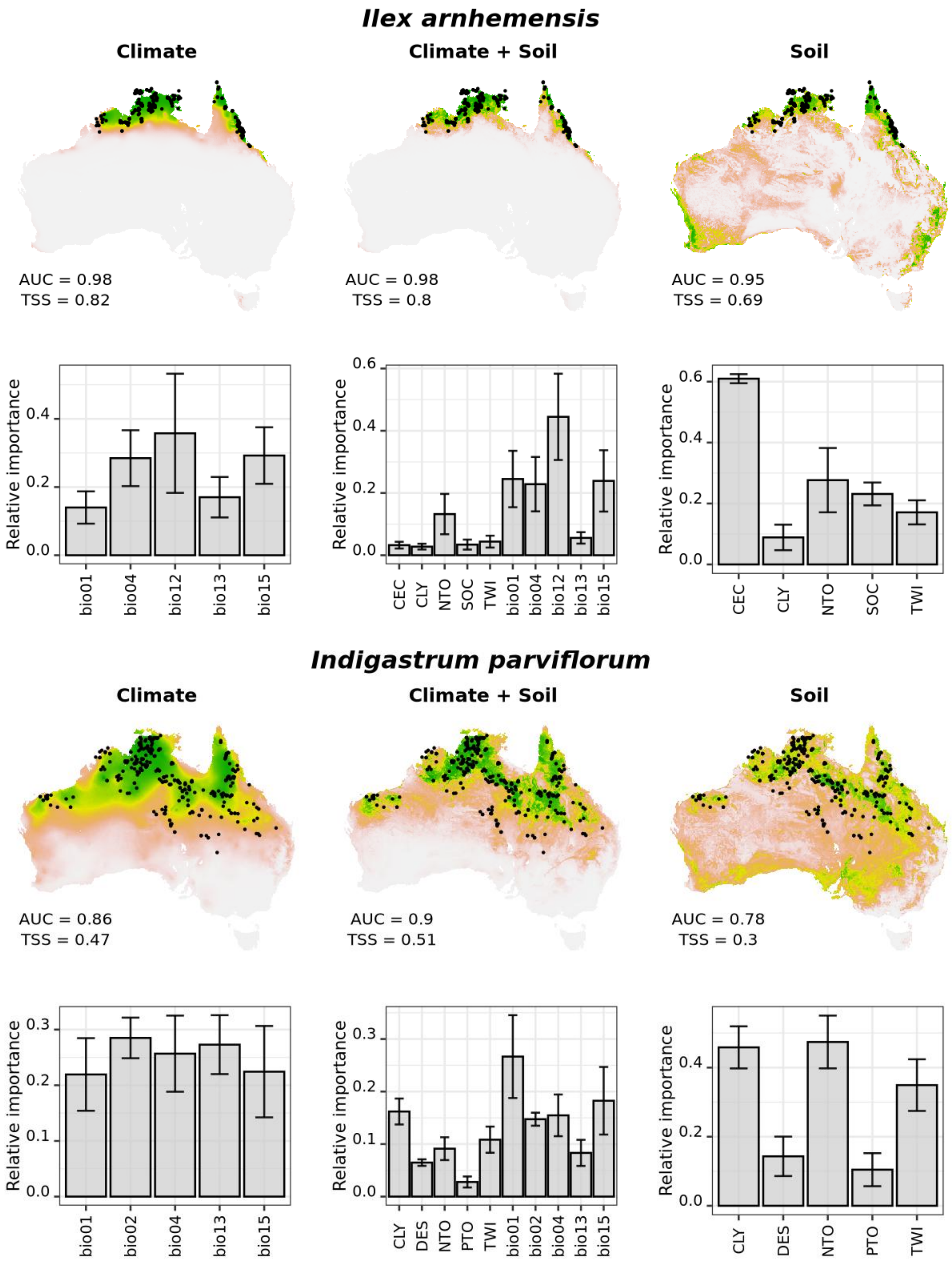

Ilex arnhemensis
Climate
Climate + Soil
Soil
AUC = 0.98
TSS = 0.82
AUC = 0.98
TSS = 0.8
AUC = 0.95
TSS = 0.69
Relative importance
0.0
0.2
0.4
0.6
bio01
bio04
bio12
bio13
bio15
CEC
CLY
NTO
SOC
TWI
Indigastrum parviflorum
Climate
Climate + Soil
Soil
AUC = 0.86
TSS = 0.47
AUC = 0.9
TSS = 0.51
AUC = 0.78
TSS = 0.3
Relative importance
0.0
0.1
0.2
0.3
0.4
bio01
bio02
bio04
bio13
bio15
CLY
DES
NTO
PTO
TWI

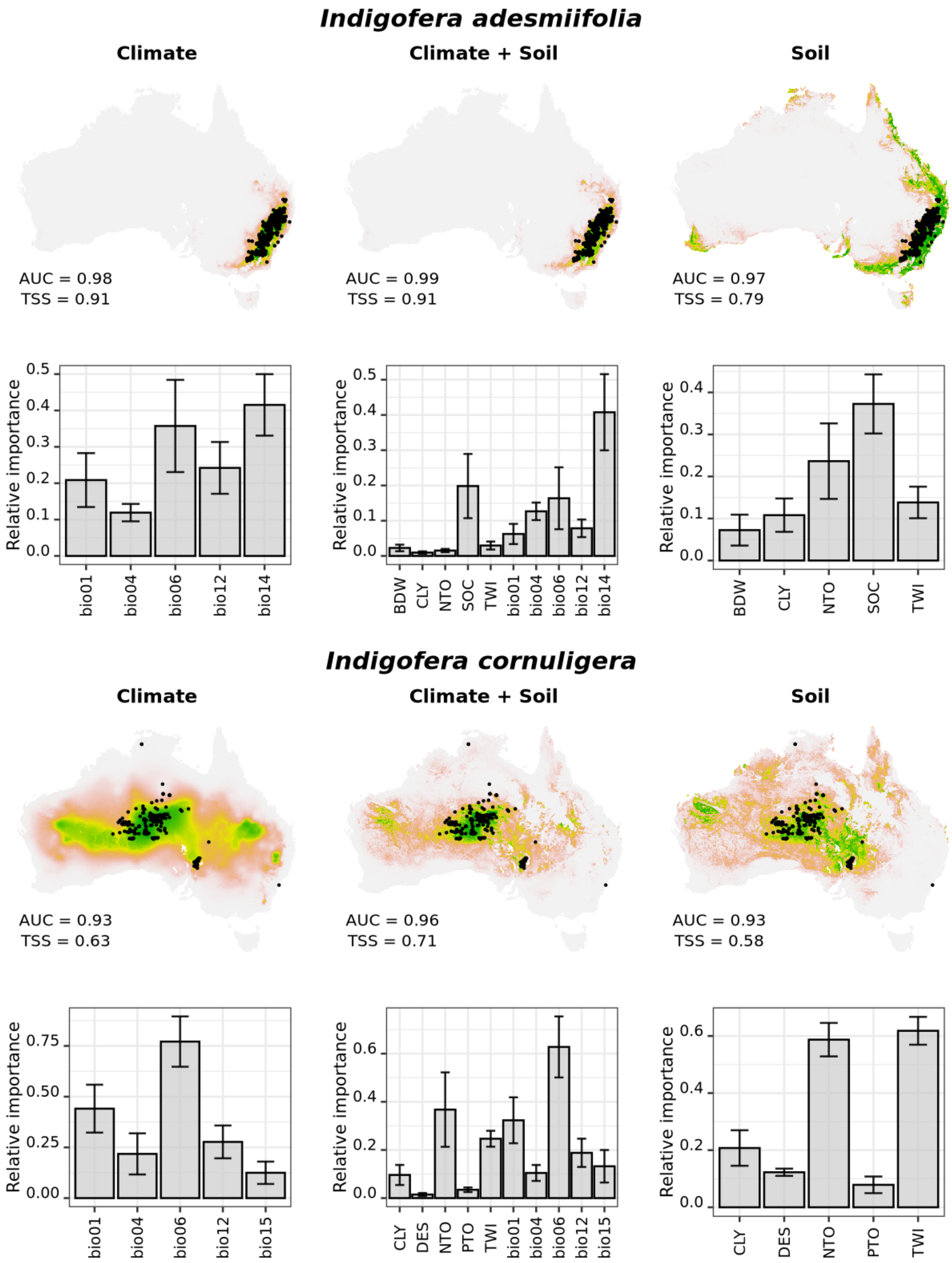

Indigofera adesmiifolia
Climate
Climate + Soil
Soil
AUC = 0.98
TSS = 0.91
AUC = 0.99
TSS = 0.91
AUC = 0.97
TSS = 0.79
Relative importance
0.0
0.1
0.2
0.3
0.4
0.5
bio01
bio04
bio06
bio12
bio14
BDW
CLY
NTO
SOC
TWI
bio01
bio04
bio06
bio12
bio14
BDW
CLY
NTO
SOC
TWI
Indigofera cornuligera
Climate
Climate + Soil
Soil
AUC = 0.93
TSS = 0.63
AUC = 0.96
TSS = 0.71
AUC = 0.93
TSS = 0.58
Relative importance
0.00
0.25
0.50
0.75
0.0
0.2
0.4
0.6
bio01
bio04
bio06
bio12
bio15
CLY
DES
NTO
PTO
TWI
bio01
bio04
bio06
bio12
bio15
CLY
DES
NTO
PTO
TWI

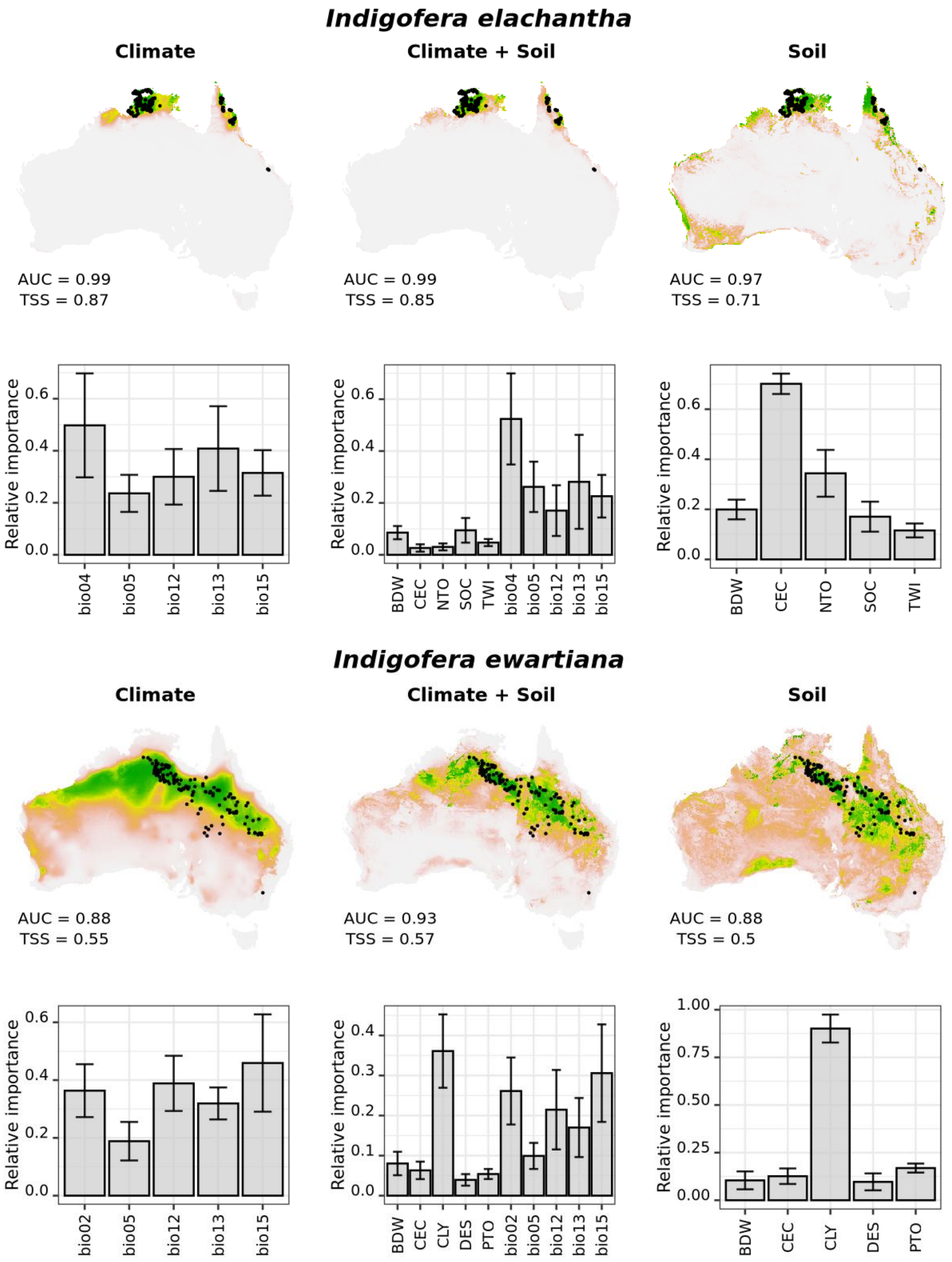
Indigofera elachantha
Climate
Climate + Soil
Soil
AUC = 0.99
TSS = 0.87
AUC = 0.99
TSS = 0.85
AUC = 0.97
TSS = 0.71
Relative importance
bio04
bio05
bio12
bio13
bio15
BDW
CEC
NTO
SOC
TWI
Indigofera ewartiana
Climate
Climate + Soil
Soil
AUC = 0.88
TSS = 0.55
AUC = 0.93
TSS = 0.57
AUC = 0.88
TSS = 0.5
Relative importance
bio02
bio05
bio12
bio13
bio15
BDW
CEC
CLY
DES
PTO

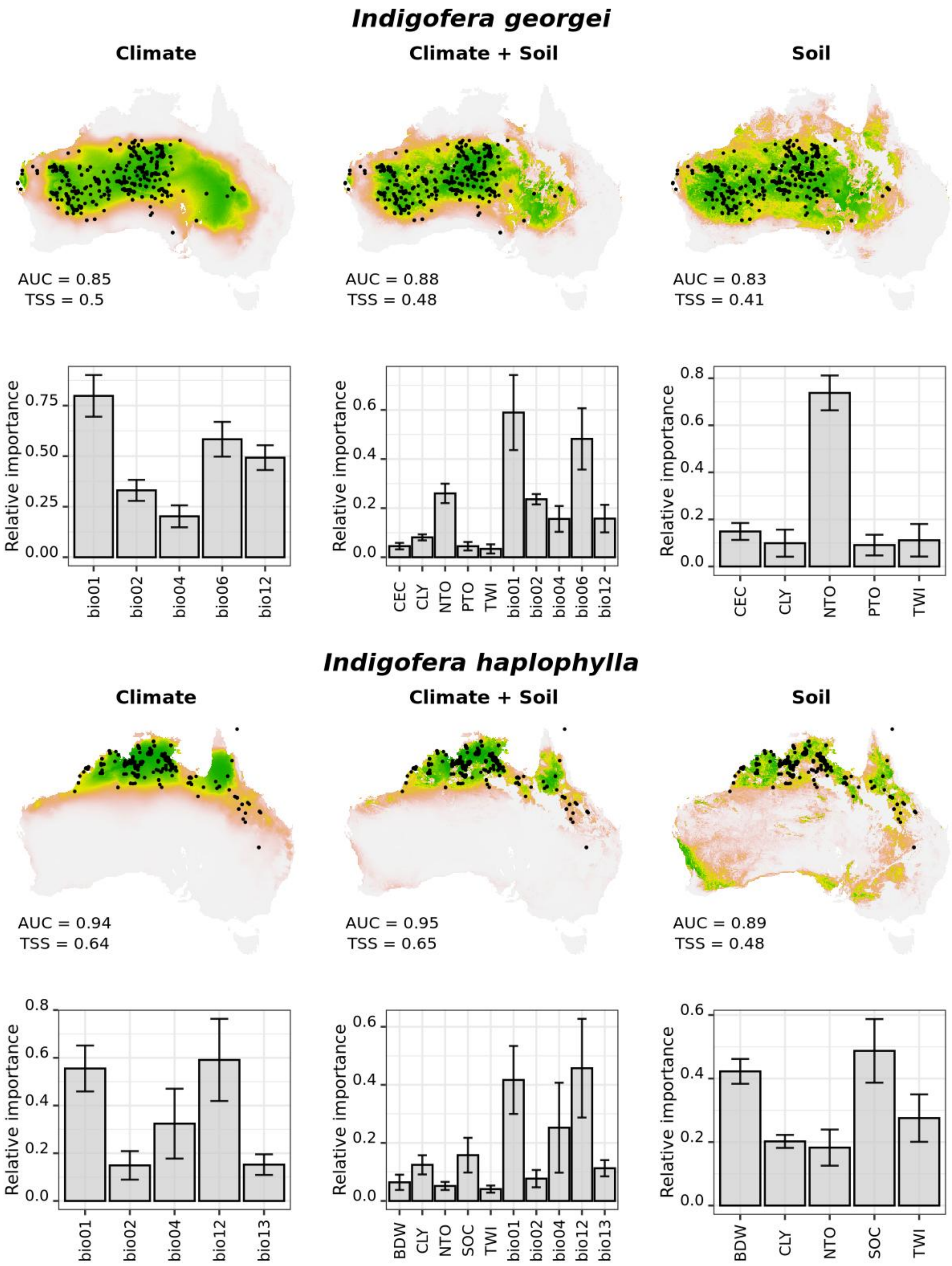
Indigofera georgei
Climate
Climate + Soil
Soil
AUC = 0.85
TSS = 0.5
AUC = 0.88
TSS = 0.48
AUC = 0.83
TSS = 0.41
Relative importance
bio01
bio02
bio04
bio06
bio12
CEC
CLY
NTO
PTO
TWI
Indigofera haplophylla
Climate
Climate + Soil
Soil
AUC = 0.94
TSS = 0.64
AUC = 0.95
TSS = 0.65
AUC = 0.89
TSS = 0.48
Relative importance
bio01
bio02
bio04
bio12
bio13
BDW
CLY
NTO
SOC
TWI

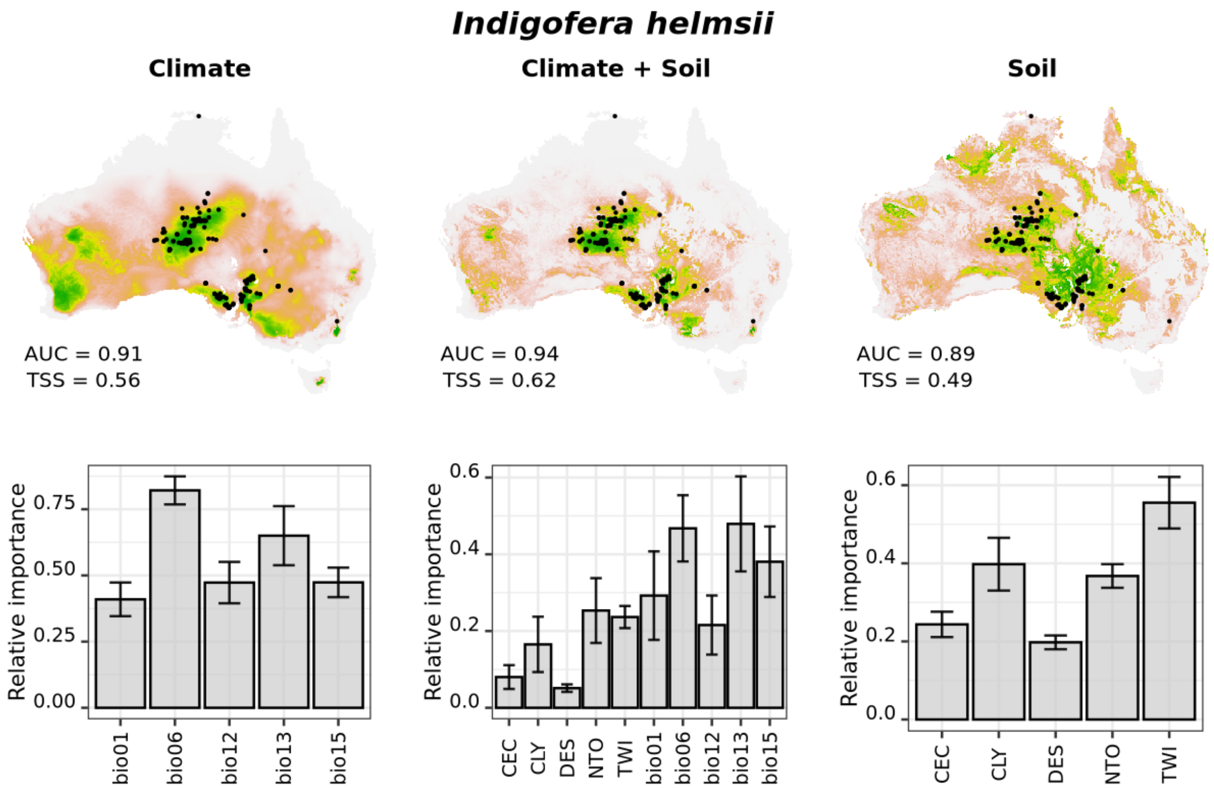

Indigofera helmsii
Climate
Climate + Soil
Soil
AUC = 0.91
TSS = 0.56
AUC = 0.94
TSS = 0.62
AUC = 0.89
TSS = 0.49
Relative importance
0.00
0.25
0.50
0.75
bio01
bio06
bio12
bio13
bio15
0.0
0.2
0.4
0.6
CEC
CLY
DES
NTO
TWI
bio01
bio06
bio12
bio13
bio15
0.0
0.2
0.4
0.6
CEC
CLY
DES
NTO
TWI

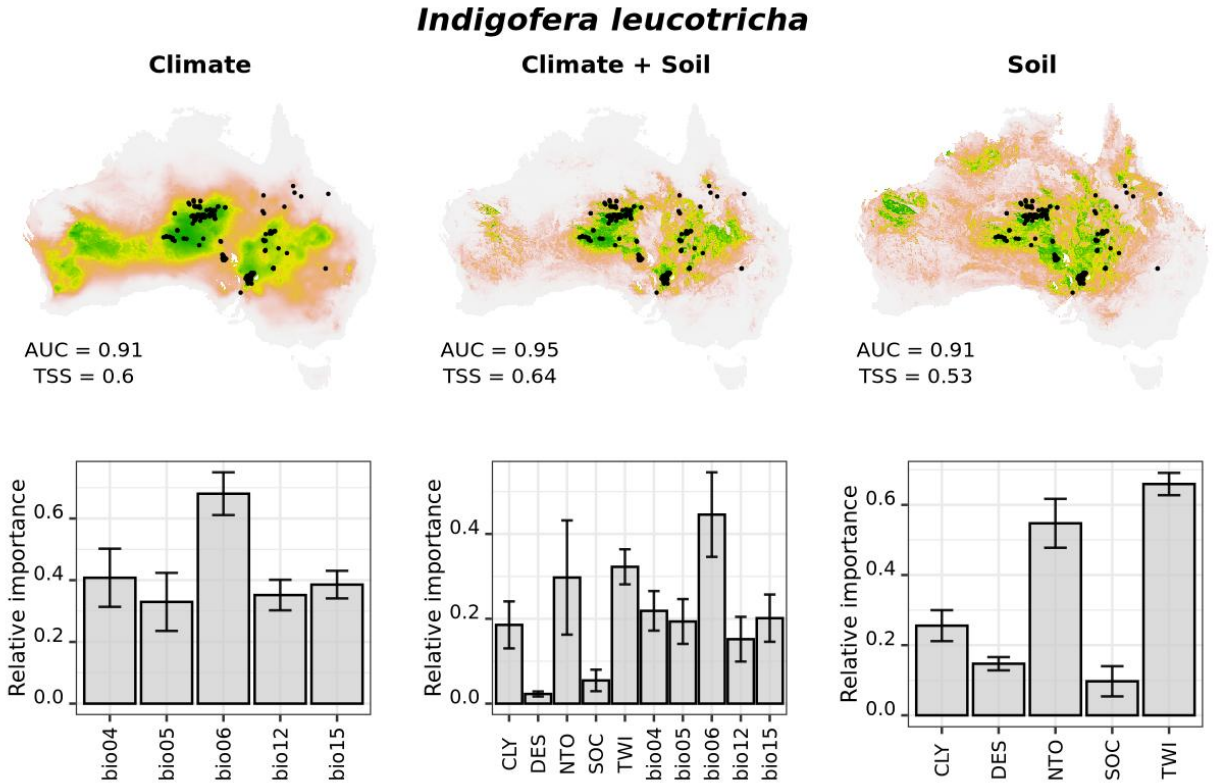

Indigofera leucotricha
Climate
Climate + Soil
Soil
AUC = 0.91
TSS = 0.6
AUC = 0.95
TSS = 0.64
AUC = 0.91
TSS = 0.53
Relative importance
0.0
0.2
0.4
0.6
bio04
bio05
bio06
bio12
bio15
0.0
0.2
0.4
CLY
DES
NTO
SOC
TWI
bio04
bio05
bio06
bio12
bio15
0.0
0.2
0.4
0.6
CLY
DES
NTO
SOC
TWI

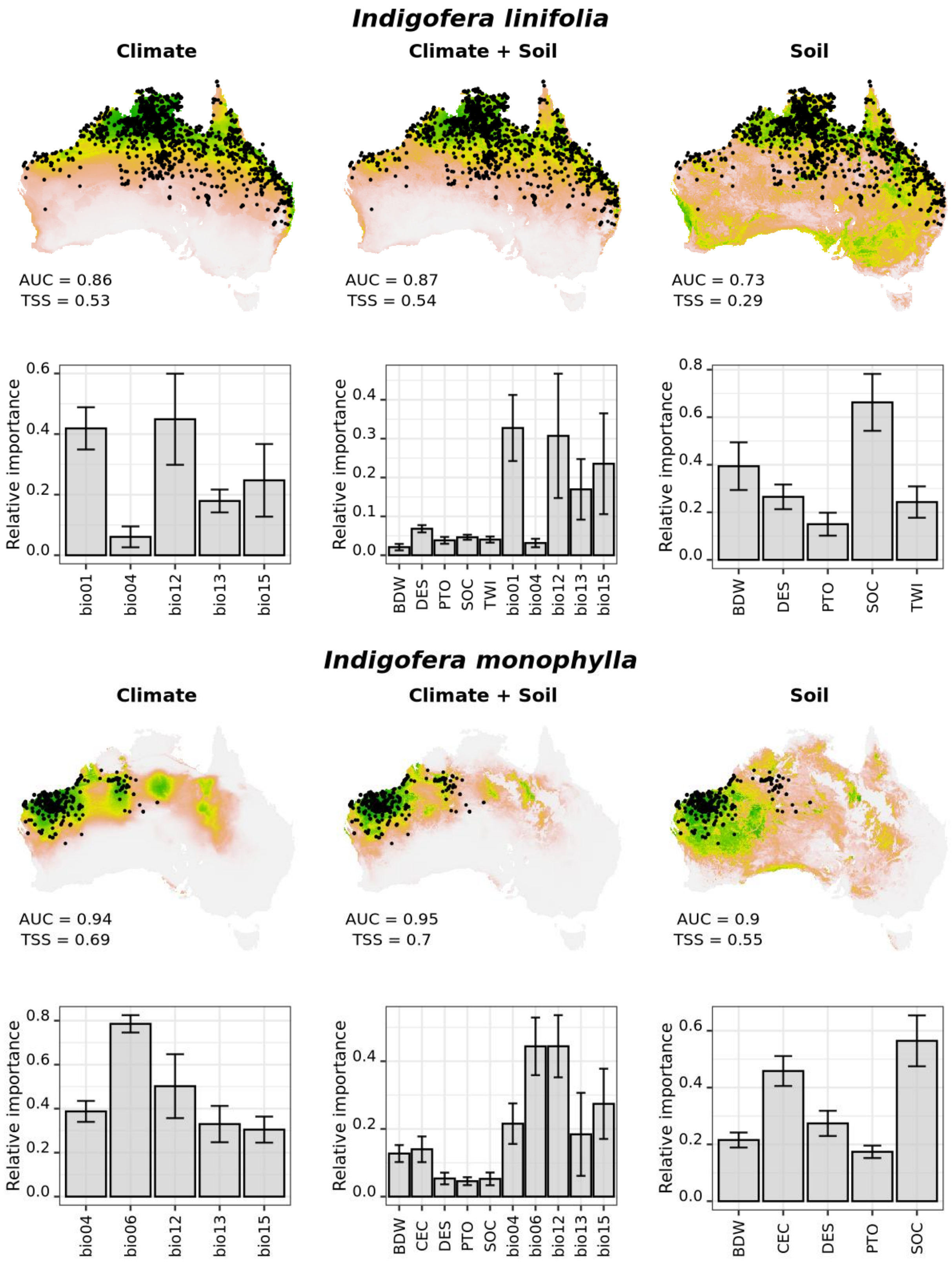

Indigofera linifolia
Climate
Climate + Soil
Soil
AUC = 0.86
TSS = 0.53
AUC = 0.87
TSS = 0.54
AUC = 0.73
TSS = 0.29
Relative importance
bio01 bio04 bio12 bio13 bio15
BDW DES PTO SOC TWI bio01 bio04 bio12 bio13 bio15
BDW DES PTO SOC TWI
Indigofera monophylla
Climate
Climate + Soil
Soil
AUC = 0.94
TSS = 0.69
AUC = 0.95
TSS = 0.7
AUC = 0.9
TSS = 0.55
Relative importance
bio04 bio06 bio12 bio13 bio15
BDW CEC DES PTO SOC bio04 bio06 bio12 bio13 bio15
BDW CEC DES PTO SOC

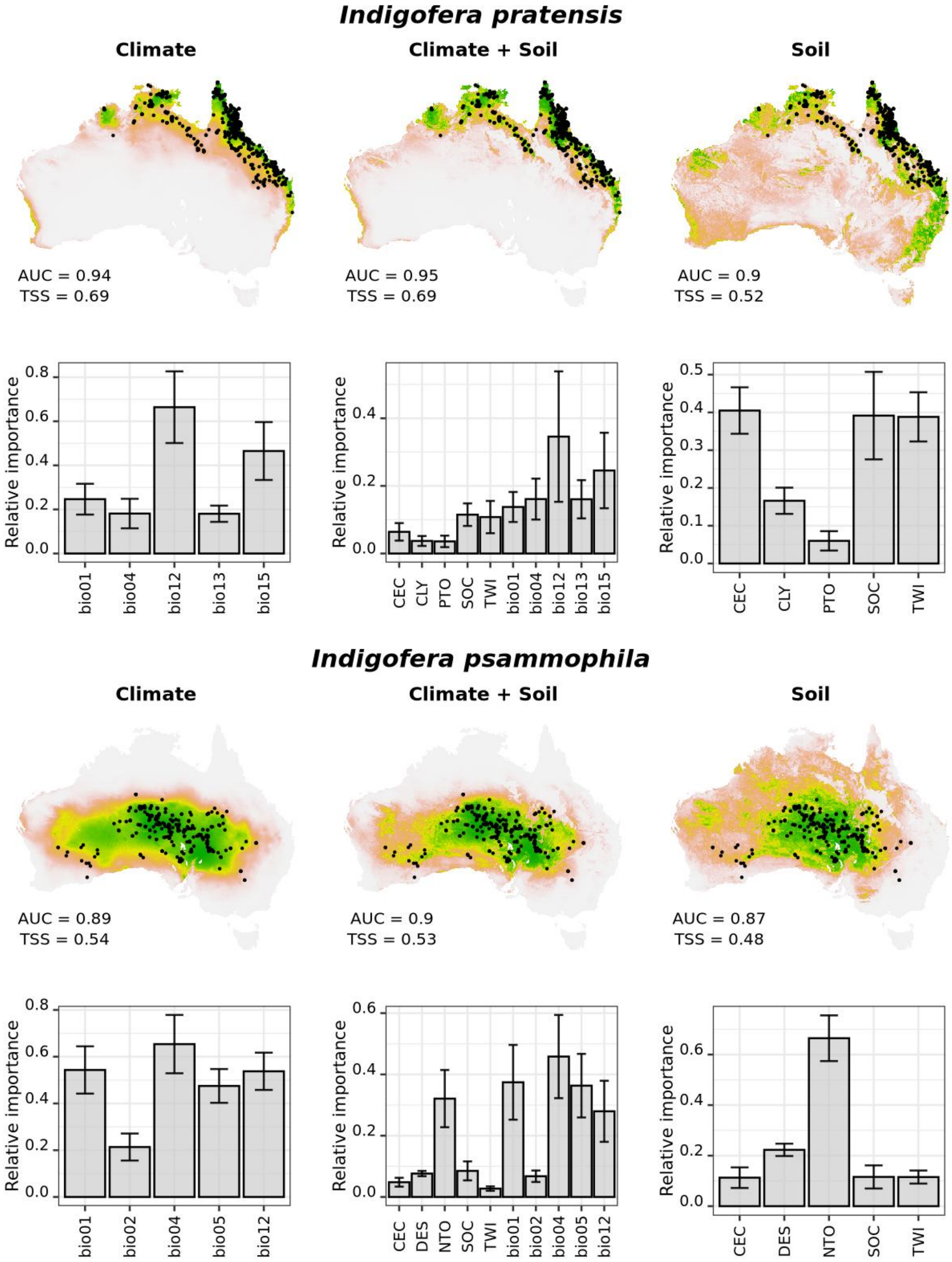
Indigofera pratensis
Climate
Climate + Soil
Soil
AUC = 0.94
TSS = 0.69
AUC = 0.95
TSS = 0.69
AUC = 0.9
TSS = 0.52
Relative importance
bio01 bio04 bio12 bio13 bio15
CEC CLY PTO SOC TWI bio01 bio04 bio12 bio13 bio15
CEC CLY PTO SOC TWI
Indigofera psammophila
Climate
Climate + Soil
Soil
AUC = 0.89
TSS = 0.54
AUC = 0.9
TSS = 0.53
AUC = 0.87
TSS = 0.48
Relative importance
bio01 bio02 bio04 bio05 bio12
CEC DES NTO SOC TWI bio01 bio02 bio04 bio05 bio12
CEC DES NTO SOC TWI

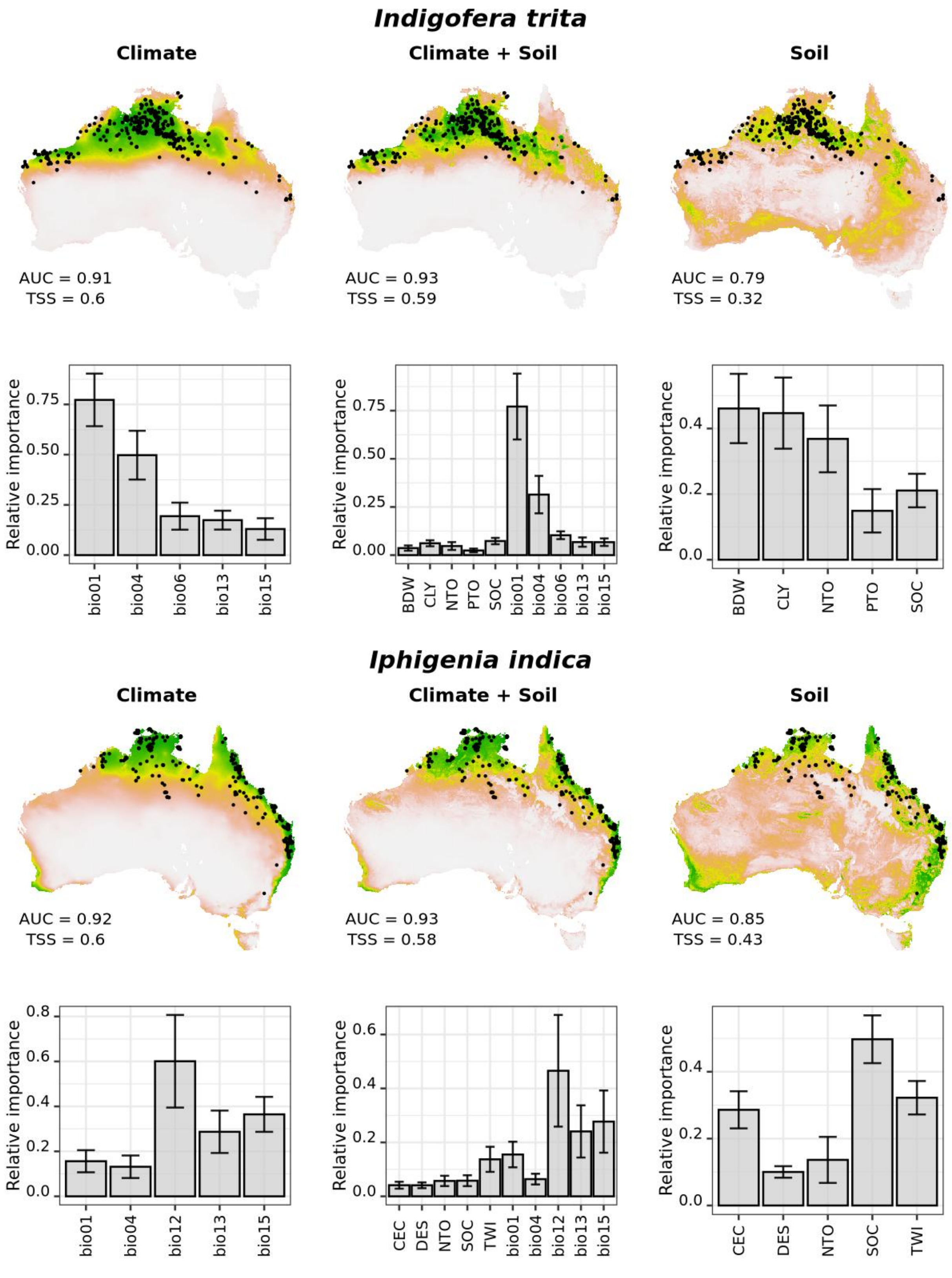
Indigofera trita
Climate
Climate + Soil
Soil
AUC = 0.91
TSS = 0.6
AUC = 0.93
TSS = 0.59
AUC = 0.79
TSS = 0.32
Relative importance
bio01 bio04 bio06 bio13 bio15
BDW CLY NTO PTO SOC bio01 bio04 bio06 bio13 bio15
BDW CLY NTO PTO SOC
Iphigenia indica
Climate
Climate + Soil
Soil
AUC = 0.92
TSS = 0.6
AUC = 0.93
TSS = 0.58
AUC = 0.85
TSS = 0.43
Relative importance
bio01 bio04 bio12 bio13 bio15
CEC DES NTO SOC TWI bio01 bio04 bio12 bio13 bio15
CEC DES NTO SOC TWI

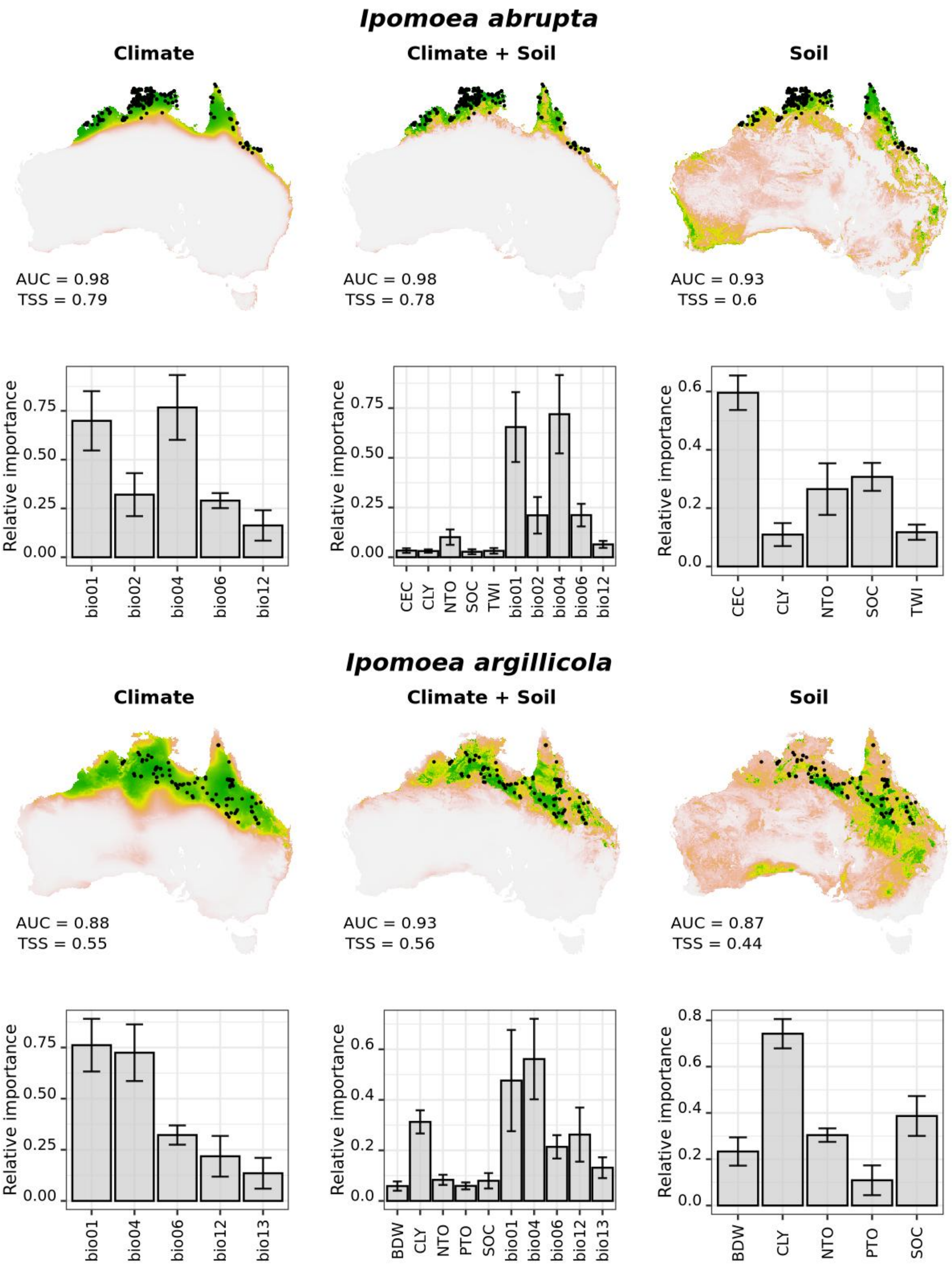
Ipomoea abrupta
Climate
Climate + Soil
Soil
AUC = 0.98
TSS = 0.79
AUC = 0.98
TSS = 0.78
AUC = 0.93
TSS = 0.6
Relative importance
0.00
0.25
0.50
0.75
bio01
bio02
bio04
bio06
bio12
CEC
CLY
NTO
SOC
TWI
0.0
0.2
0.4
0.6
Ipomoea argillicola
Climate
Climate + Soil
Soil
AUC = 0.88
TSS = 0.55
AUC = 0.93
TSS = 0.56
AUC = 0.87
TSS = 0.44
bio13
BDW
PTO
0.8

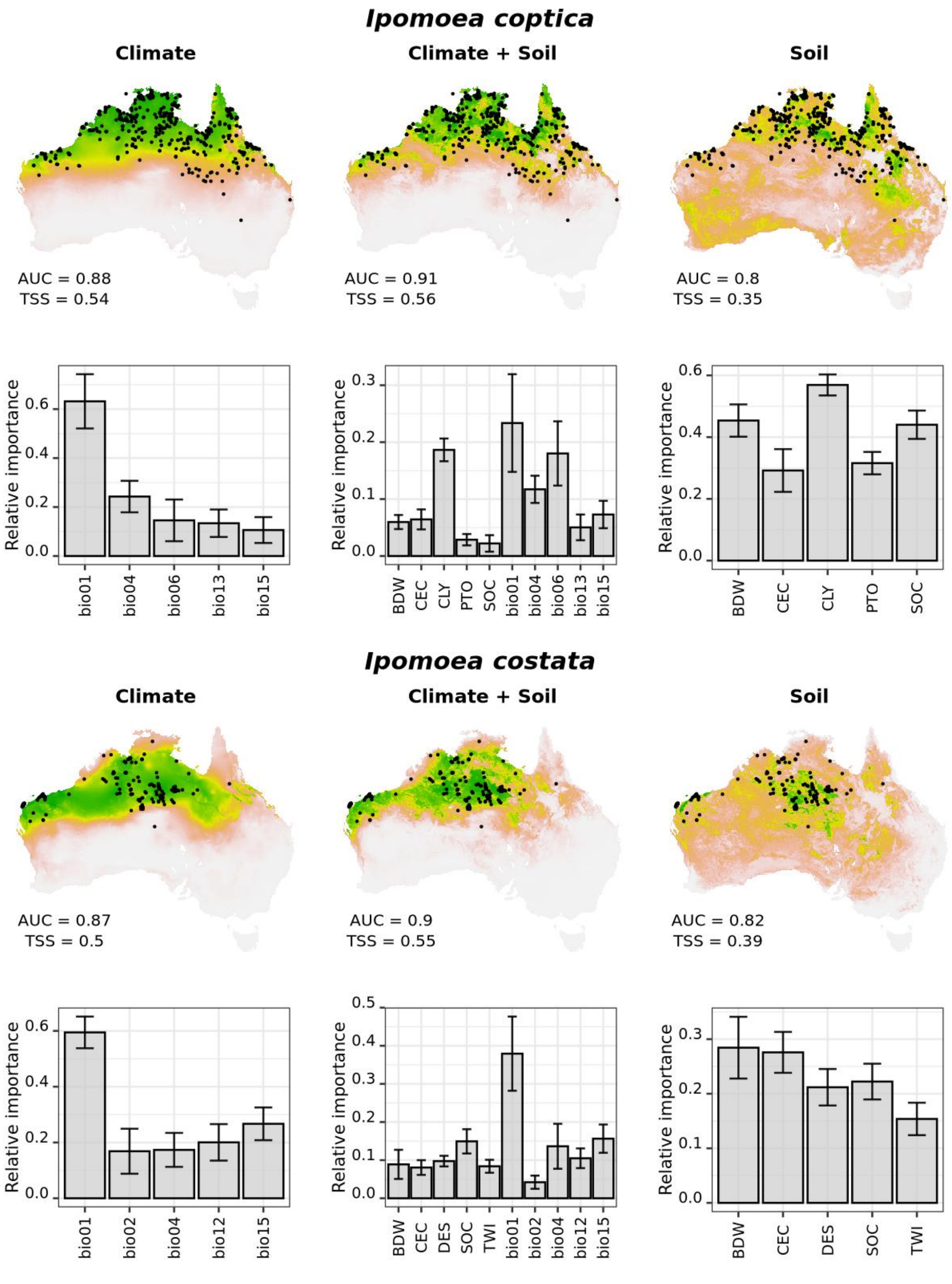

Ipomoea coptica
Climate
Climate + Soil
Soil
AUC = 0.88
TSS = 0.54
AUC = 0.91
TSS = 0.56
AUC = 0.8
TSS = 0.35
Relative importance
0.0 0.2 0.4 0.6
bio01 bio04 bio06 bio13 bio15
0.0 0.1 0.2 0.3
BDW CEC CLY PTO SOC bio01 bio04 bio06 bio13 bio15
0.0 0.2 0.4 0.6
BDW CEC CLY PTO SOC
Ipomoea costata
Climate
Climate + Soil
Soil
AUC = 0.87
TSS = 0.5
AUC = 0.9
TSS = 0.55
AUC = 0.82
TSS = 0.39
Relative importance
0.0 0.2 0.4 0.6
bio01 bio02 bio04 bio12 bio15
0.0 0.1 0.2 0.3 0.4 0.5
BDW CEC DES SOC TWI bio01 bio02 bio04 bio12 bio15
0.0 0.1 0.2 0.3
BDW CEC DES SOC TWI

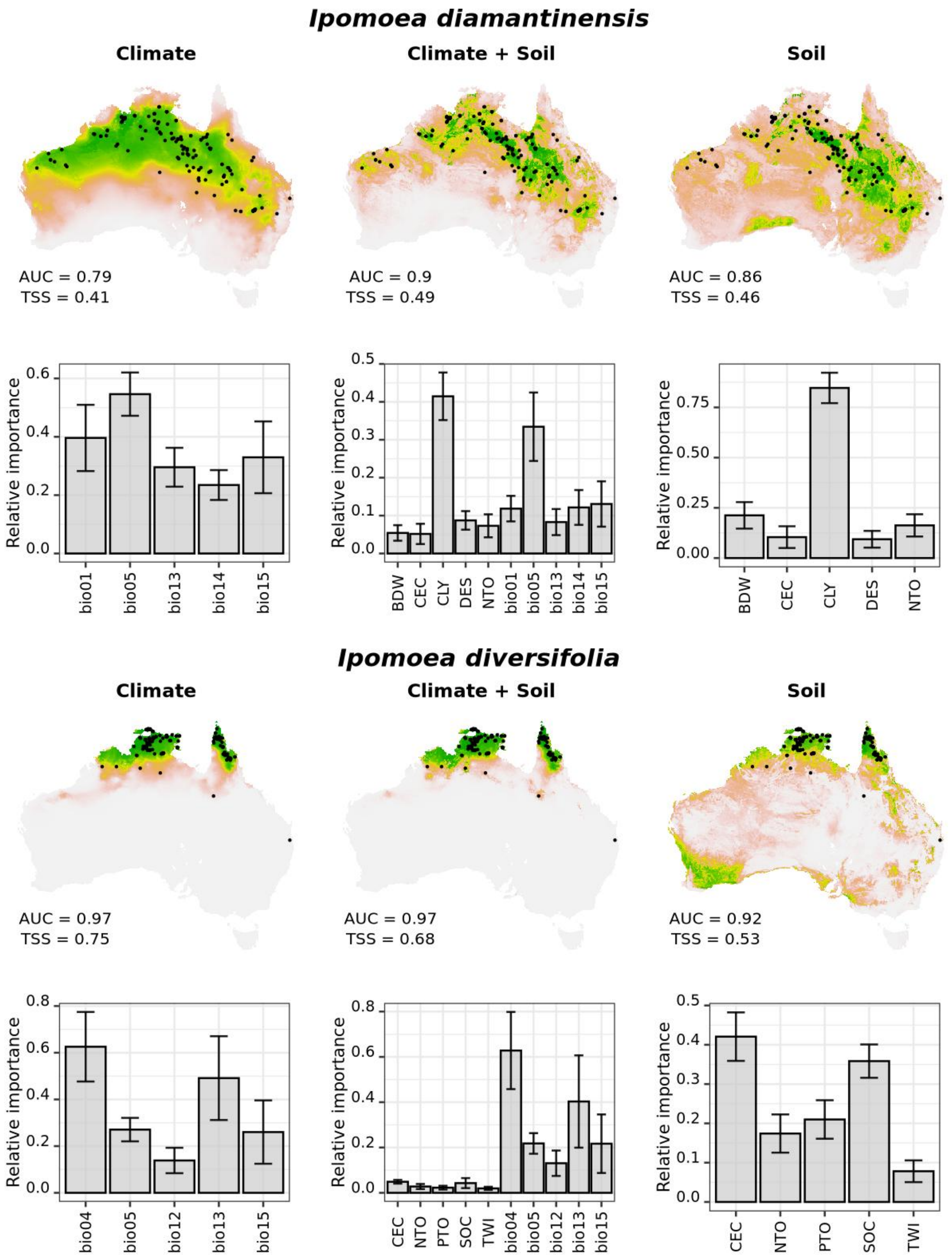

Ipomoea diamantinensis
Climate
Climate + Soil
Soil
AUC = 0.79
TSS = 0.41
AUC = 0.9
TSS = 0.49
AUC = 0.86
TSS = 0.46
Relative importance
bio01 bio05 bio13 bio14 bio15
BDW CEC CLY DES NTO bio01 bio05 bio13 bio14 bio15
BDW CEC CLY DES NTO
Ipomoea diversifolia
Climate
Climate + Soil
Soil
AUC = 0.97
TSS = 0.75
AUC = 0.97
TSS = 0.68
AUC = 0.92
TSS = 0.53
Relative importance
bio04 bio05 bio12 bio13 bio15
CEC NTO PTO SOC TWI bio04 bio05 bio12 bio13 bio15
CEC NTO PTO SOC TWI

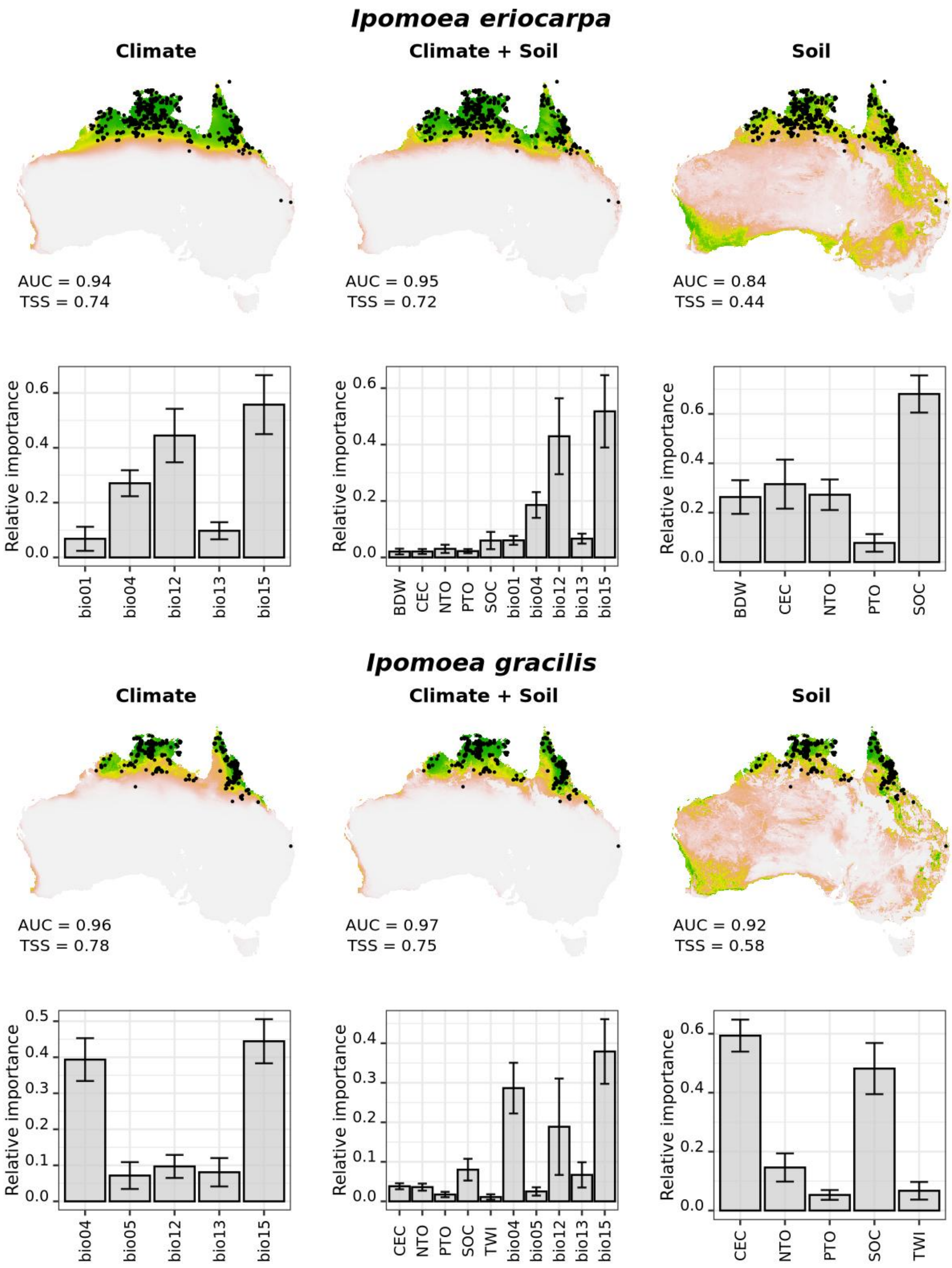
Ipomoea eriocarpa
Climate
Climate + Soil
Soil
AUC = 0.94
TSS = 0.74
AUC = 0.95
TSS = 0.72
AUC = 0.84
TSS = 0.44
Relative importance
bio01
bio04
bio12
bio13
bio15
BDW
CEC
NTO
PTO
SOC
Ipomoea gracilis
Climate
Climate + Soil
Soil
AUC = 0.96
TSS = 0.78
AUC = 0.97
TSS = 0.75
AUC = 0.92
TSS = 0.58
Relative importance
bio04
bio05
bio12
bio13
bio15
CEC
NTO
PTO
SOC
TWI

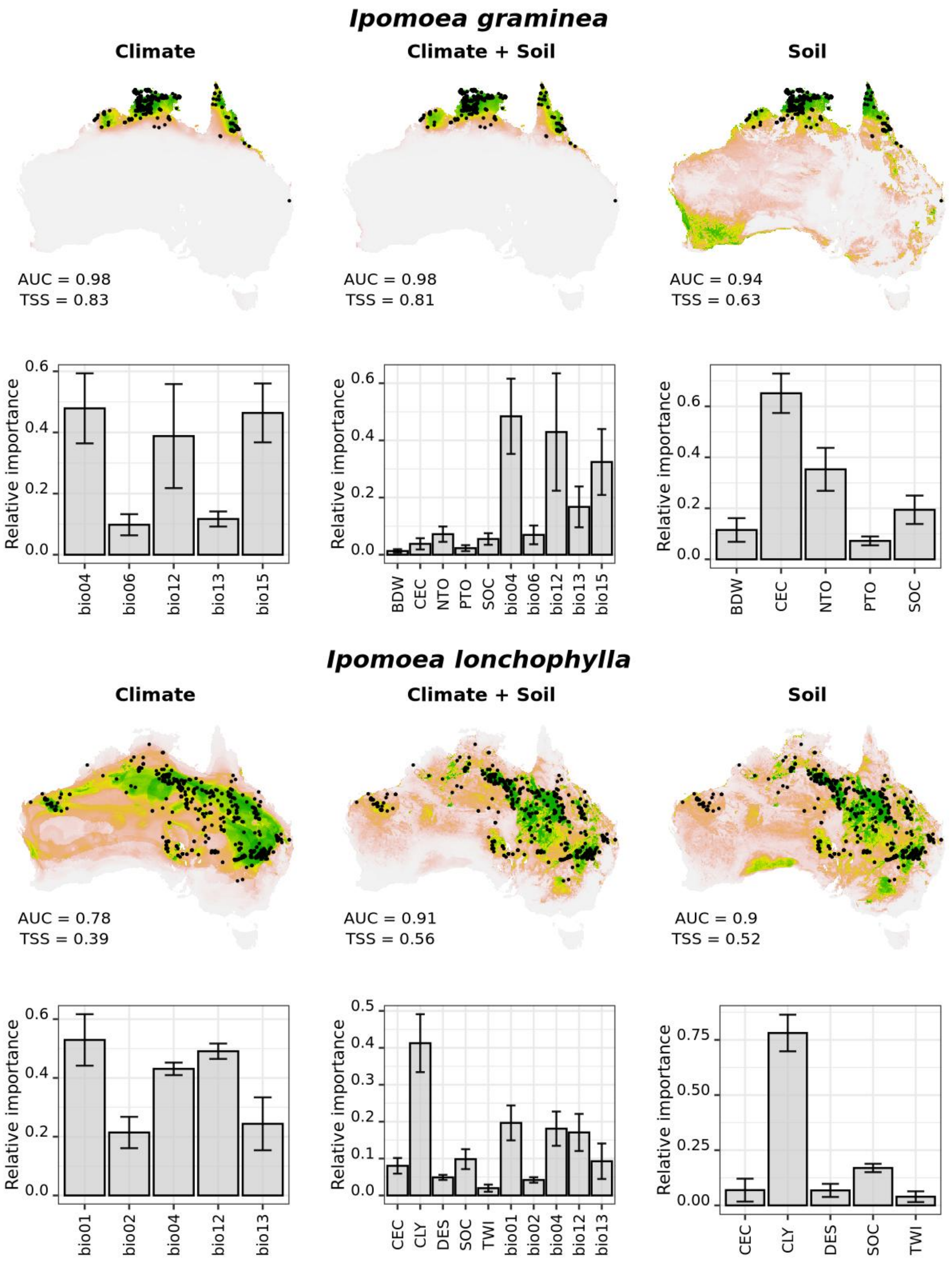
Ipomoea graminea
Climate
Climate + Soil
Soil
AUC = 0.98
TSS = 0.83
AUC = 0.98
TSS = 0.81
AUC = 0.94
TSS = 0.63
Relative importance
bio04
bio06
bio12
bio13
bio15
BDW
CEC
NTO
PTO
SOC
bio04
bio06
bio12
bio13
bio15
BDW
CEC
NTO
PTO
SOC
Ipomoea lonchophylla
Climate
Climate + Soil
Soil
AUC = 0.78
TSS = 0.39
AUC = 0.91
TSS = 0.56
AUC = 0.9
TSS = 0.52
Relative importance
bio01
bio02
bio04
bio12
bio13
CEC
CLY
DES
SOC
TWI
bio01
bio02
bio04
bio12
bio13
CEC
CLY
DES
SOC
TWI

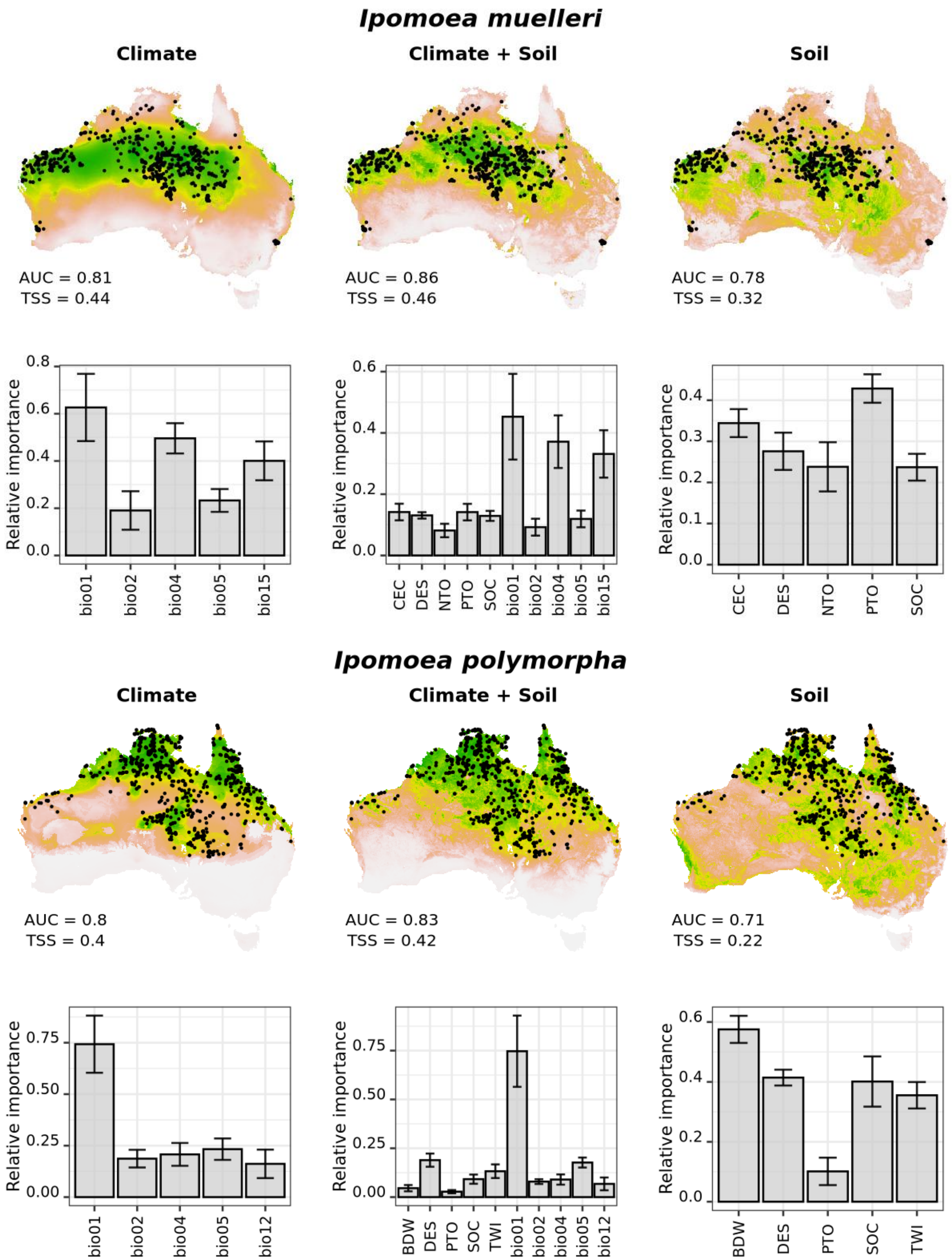
Ipomoea muelleri
Climate
Climate + Soil
Soil
AUC = 0.81
TSS = 0.44
AUC = 0.86
TSS = 0.46
AUC = 0.78
TSS = 0.32
Relative importance
bio01
bio02
bio04
bio05
bio15
CEC
DES
NTO
PTO
SOC
Ipomoea polymorpha
Climate
Climate + Soil
Soil
AUC = 0.8
TSS = 0.4
AUC = 0.83
TSS = 0.42
AUC = 0.71
TSS = 0.22
bio12
BDW
TWI

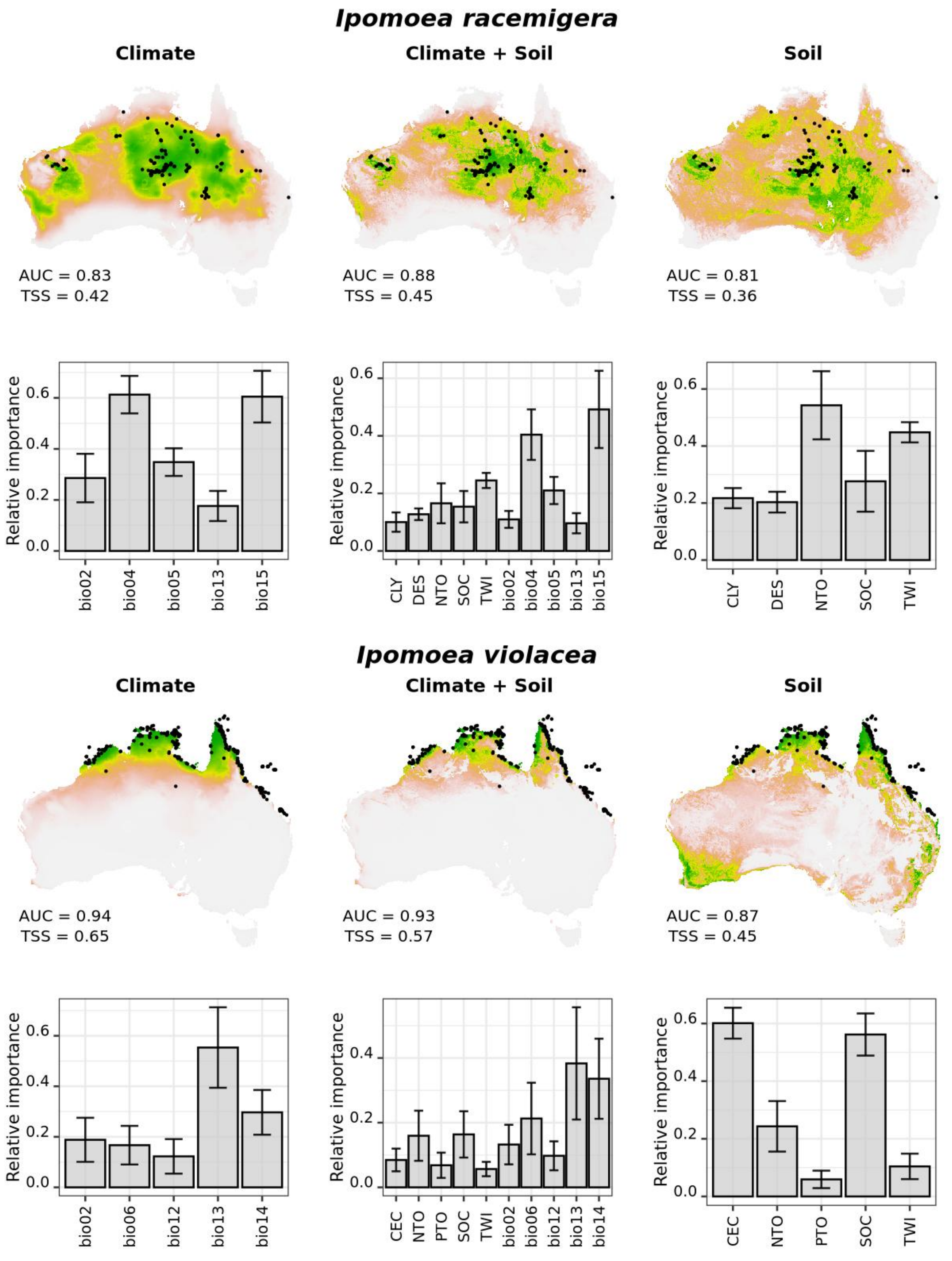

Ipomoea racemigera
Climate
Climate + Soil
Soil
AUC = 0.83
TSS = 0.42
AUC = 0.88
TSS = 0.45
AUC = 0.81
TSS = 0.36
Relative importance
bio02
bio04
bio05
bio13
bio15
CLY
DES
NTO
SOC
TWI
Ipomoea violacea
Climate
Climate + Soil
Soil
AUC = 0.94
TSS = 0.65
AUC = 0.93
TSS = 0.57
AUC = 0.87
TSS = 0.45
Relative importance
bio02
bio06
bio12
bio13
bio14
CEC
NTO
PTO
SOC
TWI

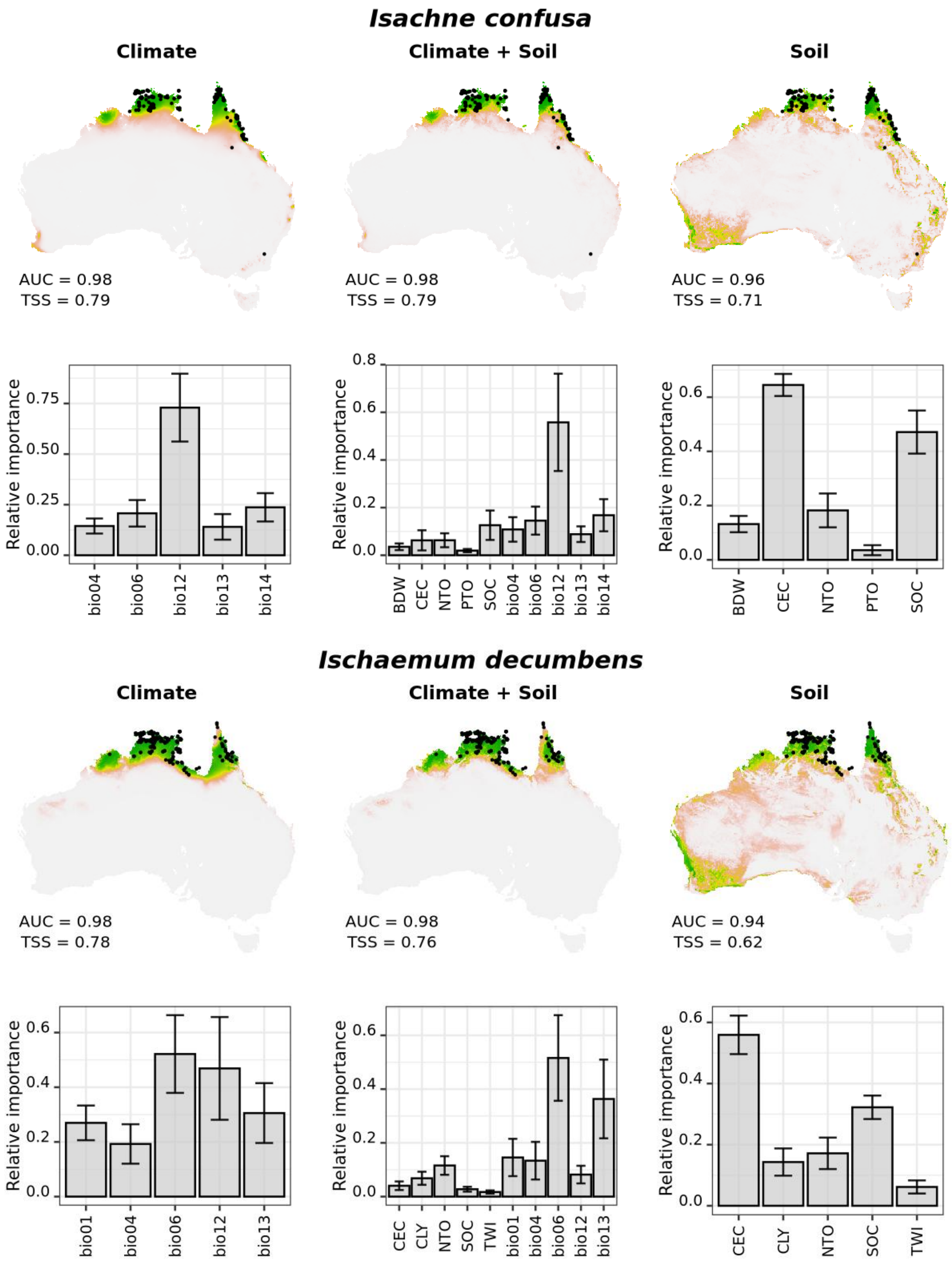
Isachne confusa
Climate
Climate + Soil
Soil
AUC = 0.98
TSS = 0.79
AUC = 0.98
TSS = 0.79
AUC = 0.96
TSS = 0.71
Relative importance
0.00
0.25
0.50
0.75
bio04
bio06
bio12
bio13
bio14
0.0
0.2
0.4
0.6
0.8
BDW
CEC
NTO
PTO
SOC
bio04
bio06
bio12
bio13
bio14
0.0
0.2
0.4
0.6
BDW
CEC
NTO
PTO
SOC
Ischaemum decumbens
Climate
Climate + Soil
Soil
AUC = 0.98
TSS = 0.78
AUC = 0.98
TSS = 0.76
AUC = 0.94
TSS = 0.62
Relative importance
0.0
0.2
0.4
0.6
bio01
bio04
bio06
bio12
bio13
CEC
CLY
NTO
SOC
TWI
bio01
bio04
bio06
bio12
bio13
CEC
CLY
NTO
SOC
TWI

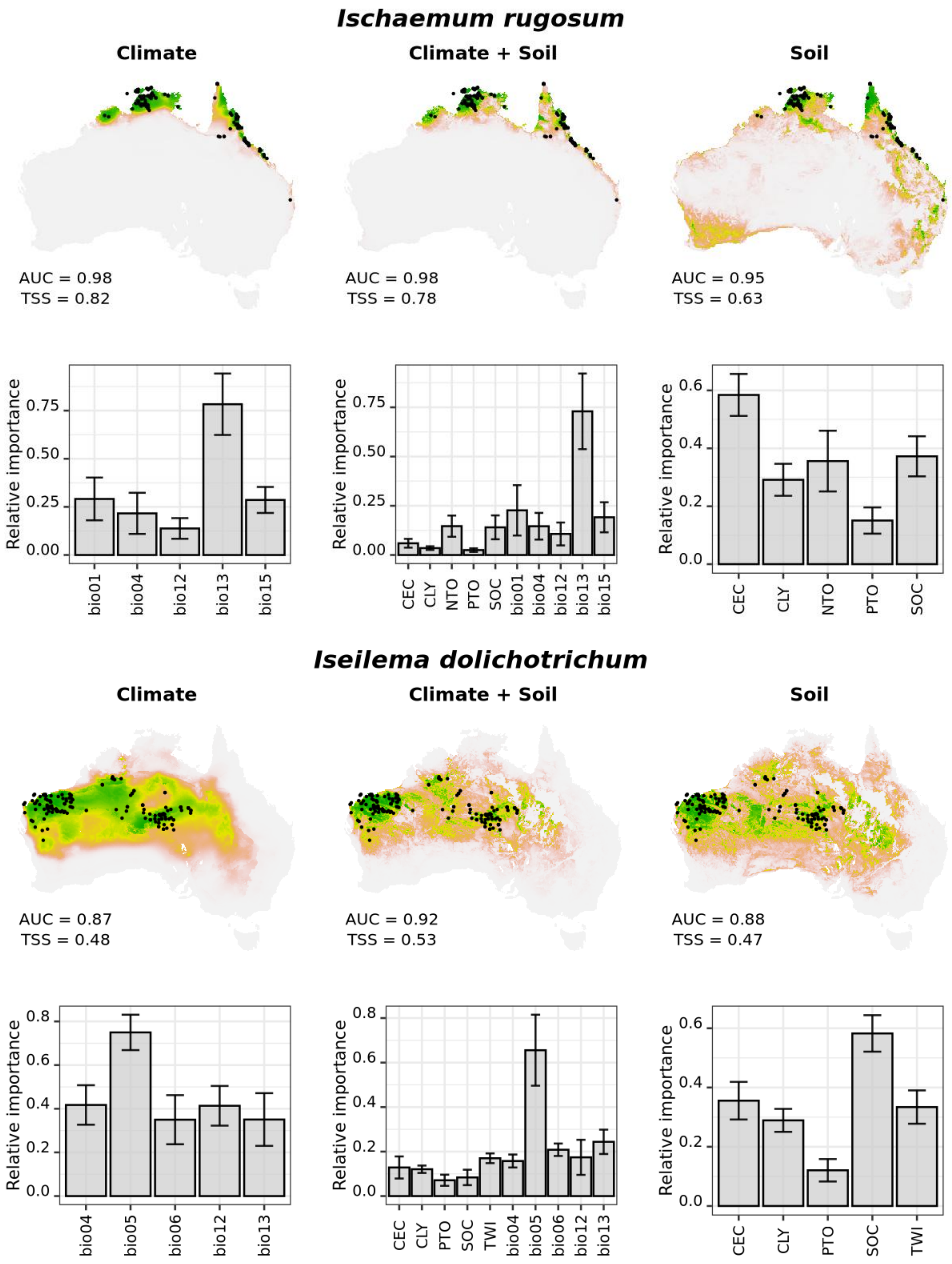
Ischaemum rugosum
Climate
Climate + Soil
Soil
AUC = 0.98
TSS = 0.82
AUC = 0.98
TSS = 0.78
AUC = 0.95
TSS = 0.63
Relative importance
0.00
0.25
0.50
0.75
bio01
bio04
bio12
bio13
bio15
CEC
CLY
NTO
PTO
SOC
0.0
0.2
0.4
0.6
Iseilema dolichotrichum
Climate
Climate + Soil
Soil
AUC = 0.87
TSS = 0.48
AUC = 0.92
TSS = 0.53
AUC = 0.88
TSS = 0.47
Relative importance
0.8
bio05
bio06
TWI

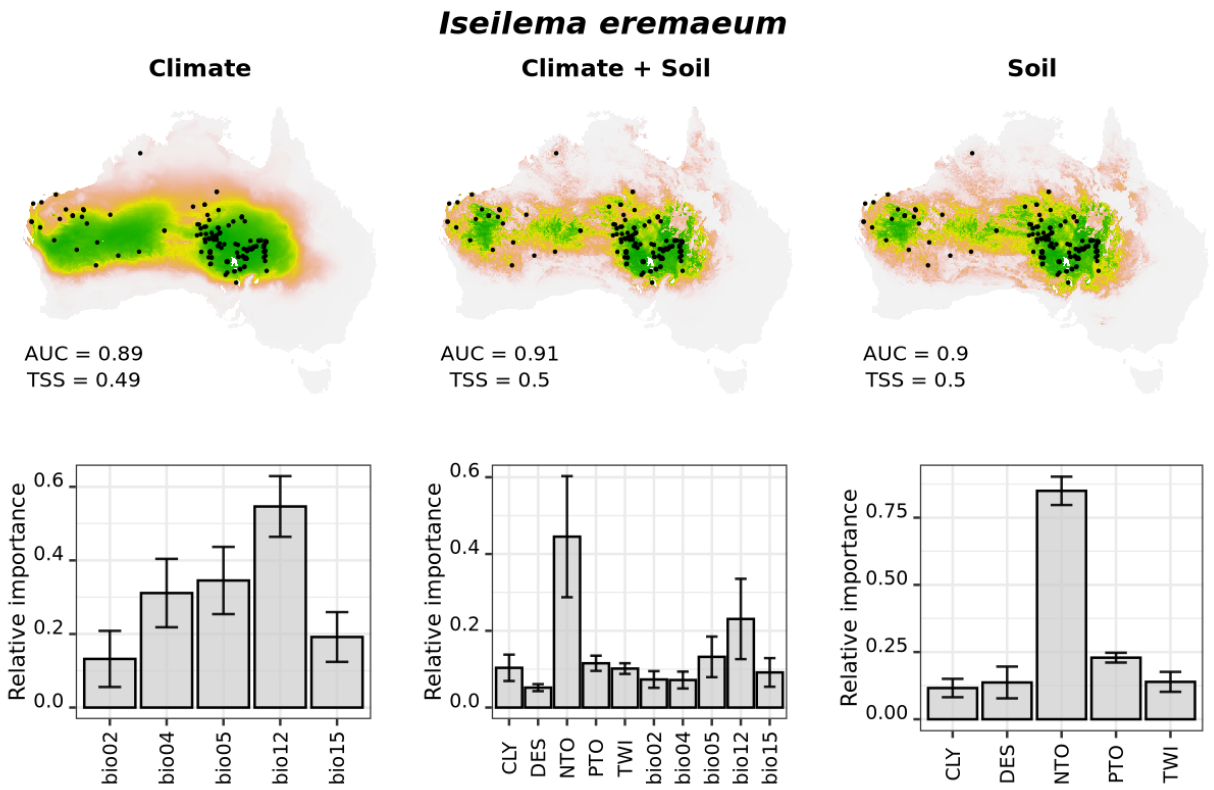

Iseilema eremaeum
Climate
Climate + Soil
Soil
AUC = 0.89
TSS = 0.49
AUC = 0.91
TSS = 0.5
AUC = 0.9
TSS = 0.5
Relative importance
0.0
0.2
0.4
0.6
bio02
bio04
bio05
bio12
bio15
CLY
DES
NTO
PTO
TWI
bio02
bio04
bio05
bio12
bio15
0.00
0.25
0.50
0.75

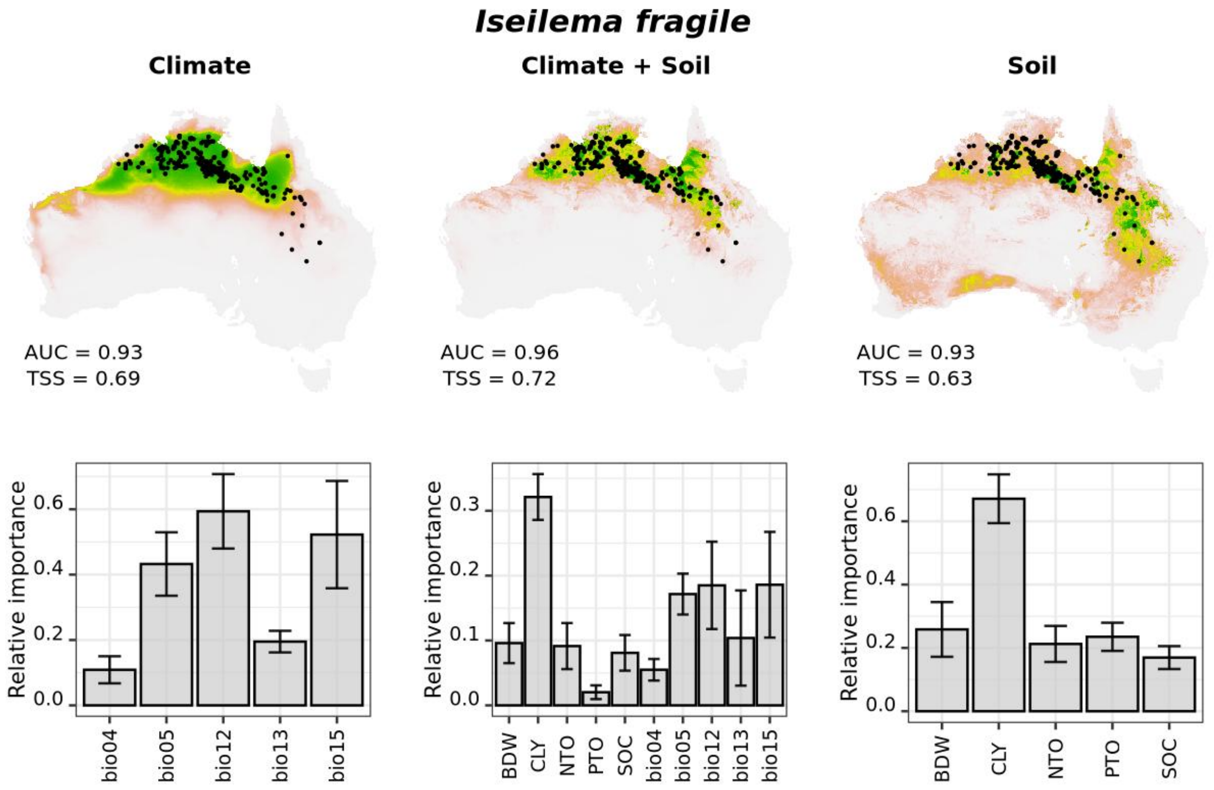

Iseilema fragile
Climate
Climate + Soil
Soil
AUC = 0.93
TSS = 0.69
AUC = 0.96
TSS = 0.72
AUC = 0.93
TSS = 0.63
Relative importance
0.0
0.2
0.4
0.6
0.1
0.3
bio04
bio05
bio12
bio13
bio15
BDW
CLY
NTO
PTO
SOC
bio04
bio05
bio12
bio13
bio15

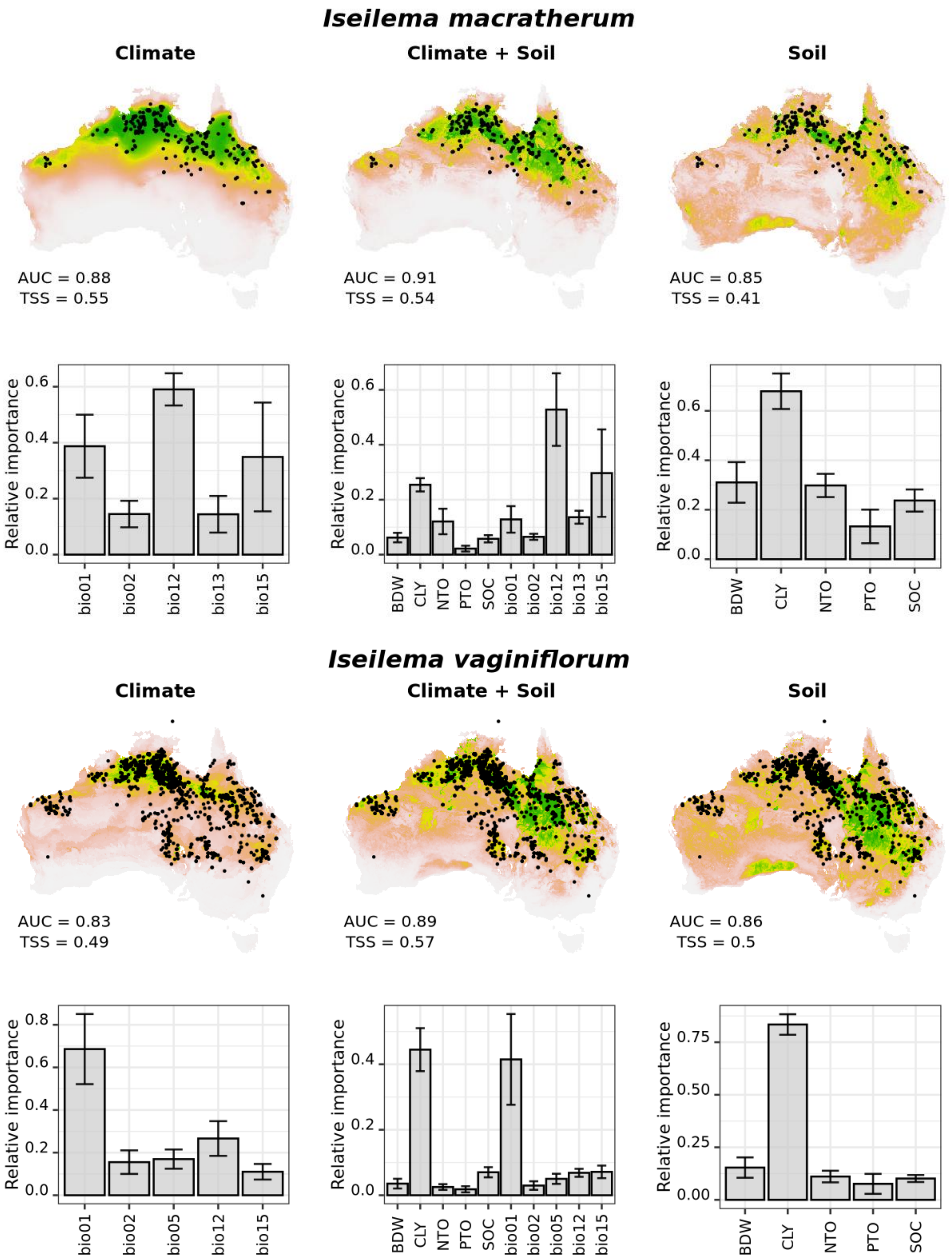

Iseilema macratherum
Climate
Climate + Soil
Soil
AUC = 0.88
TSS = 0.55
AUC = 0.91
TSS = 0.54
AUC = 0.85
TSS = 0.41
Relative importance
0.0
0.2
0.4
0.6
bio01
bio02
bio12
bio13
bio15
BDW
CLY
NTO
PTO
SOC
bio01
bio02
bio12
bio13
bio15
BDW
CLY
NTO
PTO
SOC
Iseilema vaginiflorum
Climate
Climate + Soil
Soil
AUC = 0.83
TSS = 0.49
AUC = 0.89
TSS = 0.57
AUC = 0.86
TSS = 0.5
0.8
bio01
bio02
bio05
bio12
bio15
BDW
CLY
NTO
PTO
SOC
bio01
bio02
bio05
bio12
bio15
0.00
0.25
0.50
0.75
BDW
CLY
NTO
PTO
SOC

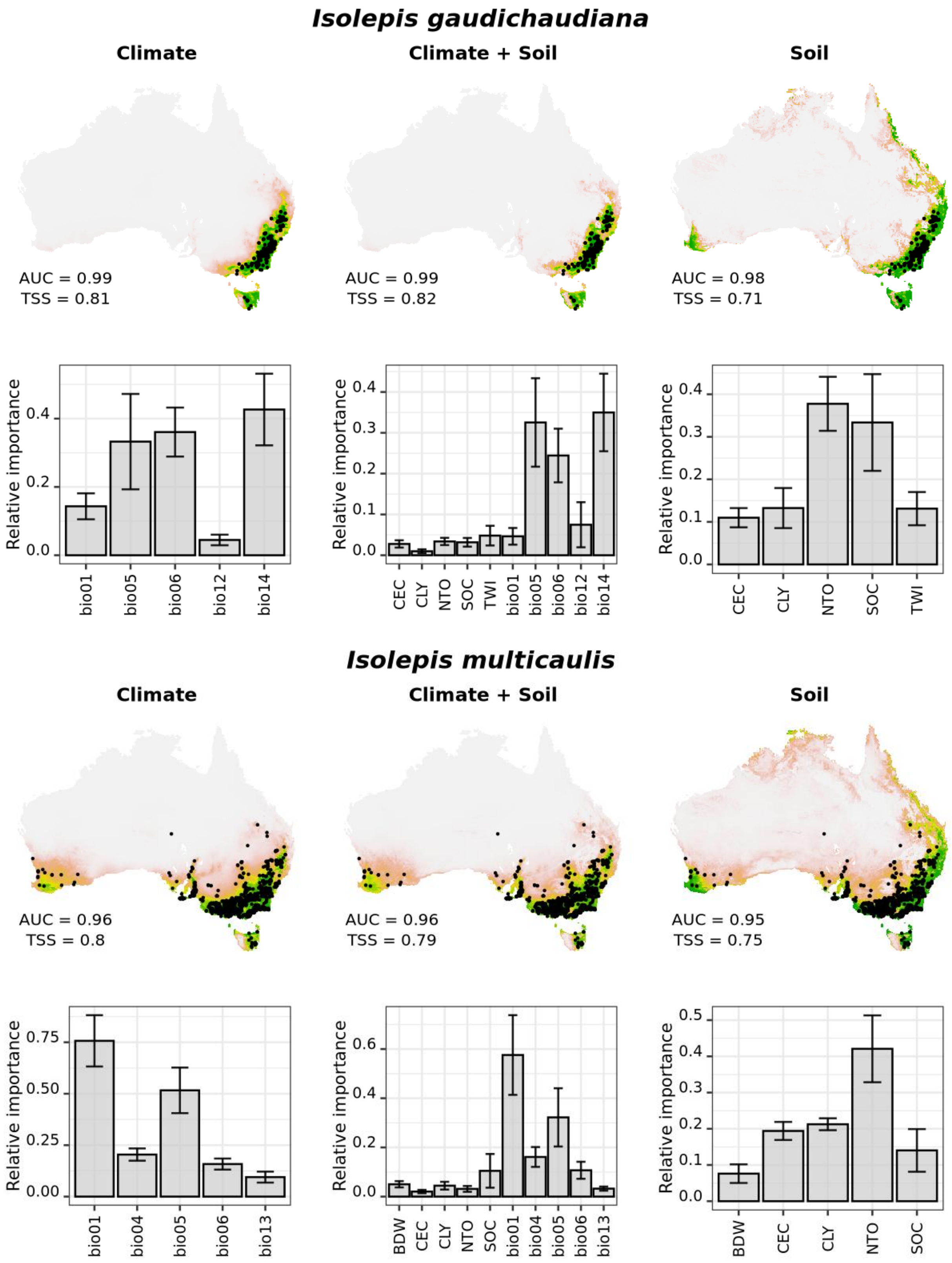

Isolepis gaudichaudiana
Climate
Climate + Soil
Soil
AUC = 0.99
TSS = 0.81
AUC = 0.99
TSS = 0.82
AUC = 0.98
TSS = 0.71
Relative importance
bio01 bio05 bio06 bio12 bio14
CEC CLY NTO SOC TWI bio01 bio05 bio06 bio12 bio14
CEC CLY NTO SOC TWI
Isolepis multicaulis
Climate
Climate + Soil
Soil
AUC = 0.96
TSS = 0.8
AUC = 0.96
TSS = 0.79
AUC = 0.95
TSS = 0.75
Relative importance
bio01 bio04 bio05 bio06 bio13
BDW CEC CLY NTO SOC bio01 bio04 bio05 bio06 bio13
BDW CEC CLY NTO SOC

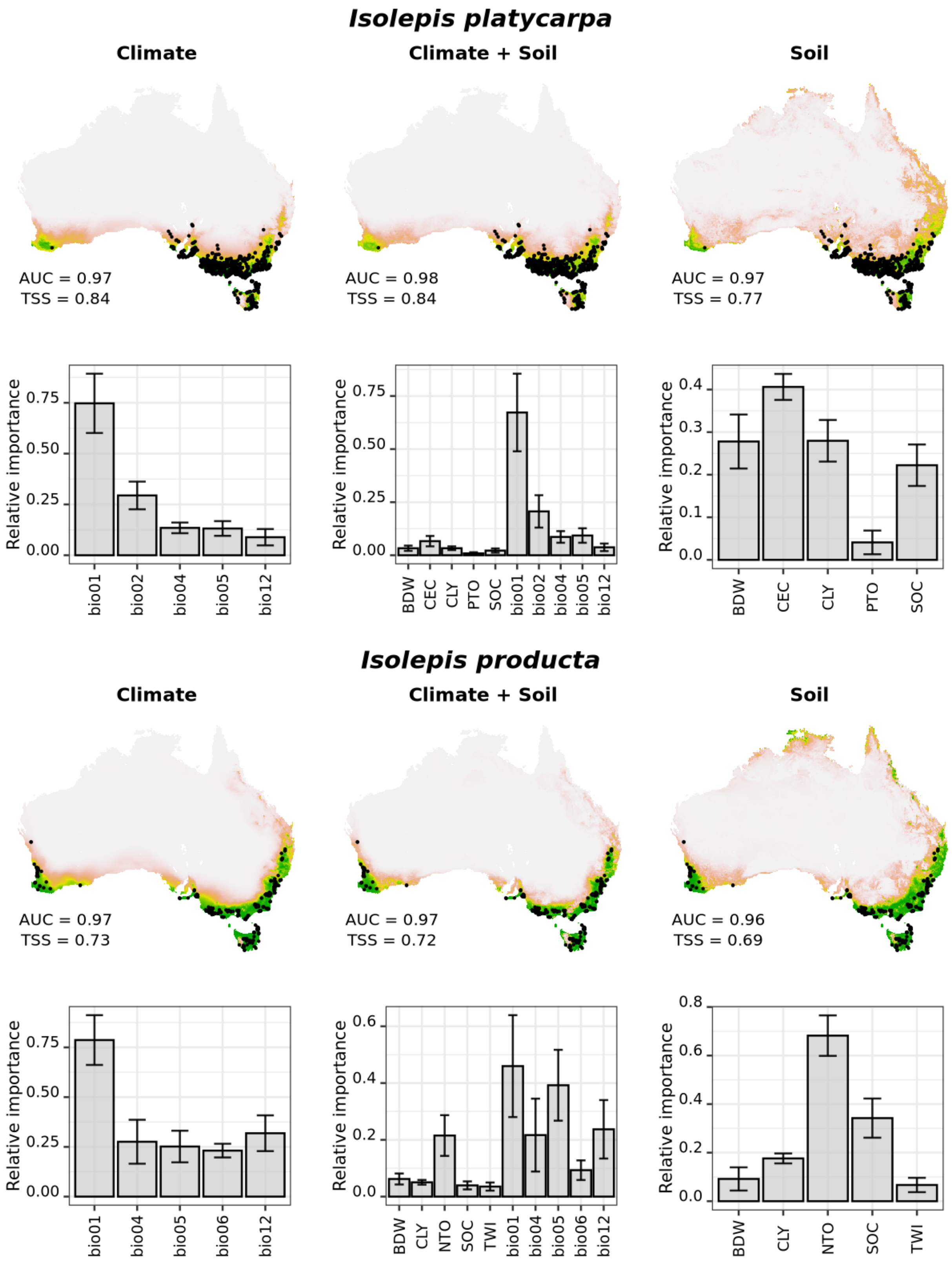
Isolepis platycarpa
Climate
Climate + Soil
Soil
AUC = 0.97
TSS = 0.84
AUC = 0.98
TSS = 0.84
AUC = 0.97
TSS = 0.77
Relative importance
bio01
bio02
bio04
bio05
bio12
BDW
CEC
CLY
PTO
SOC
Isolepis producta
AUC = 0.97
TSS = 0.73
AUC = 0.97
TSS = 0.72
AUC = 0.96
TSS = 0.69
bio06
NTO
TWI

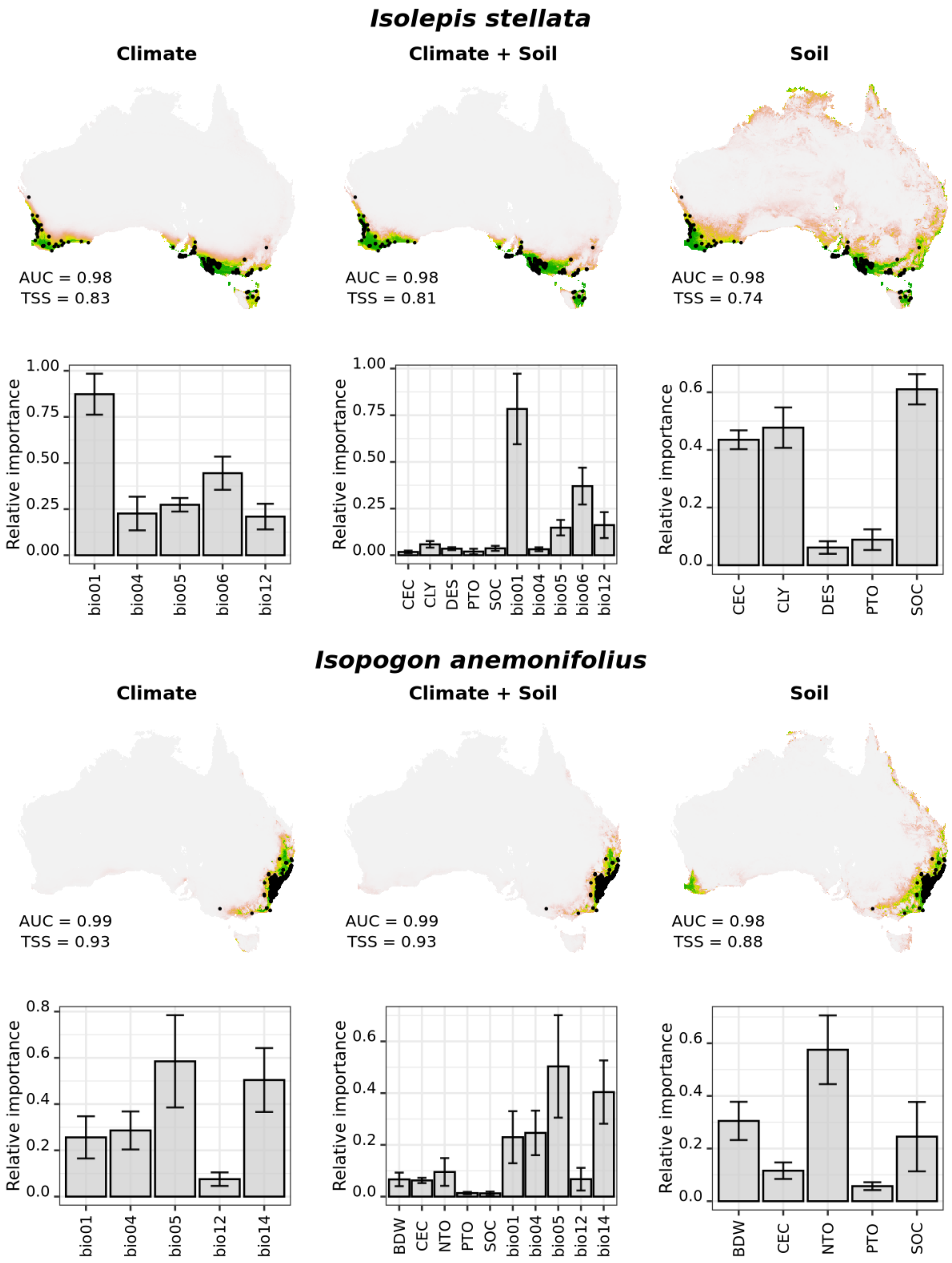
Isolepis stellata
Climate
Climate + Soil
Soil
AUC = 0.98
TSS = 0.83
AUC = 0.98
TSS = 0.81
AUC = 0.98
TSS = 0.74
Relative importance
0.00 0.25 0.50 0.75 1.00
bio01 bio04 bio05 bio06 bio12
CEC CLY DES PTO SOC bio01 bio04 bio05 bio06 bio12
0.0 0.2 0.4 0.6
CEC CLY DES PTO SOC
Isopogon anemonifolius
Climate
Climate + Soil
Soil
AUC = 0.99
TSS = 0.93
AUC = 0.99
TSS = 0.93
AUC = 0.98
TSS = 0.88
Relative importance
0.0 0.2 0.4 0.6 0.8
bio01 bio04 bio05 bio12 bio14
BDW CEC NTO PTO SOC bio01 bio04 bio05 bio12 bio14
0.0 0.2 0.4 0.6
BDW CEC NTO PTO SOC

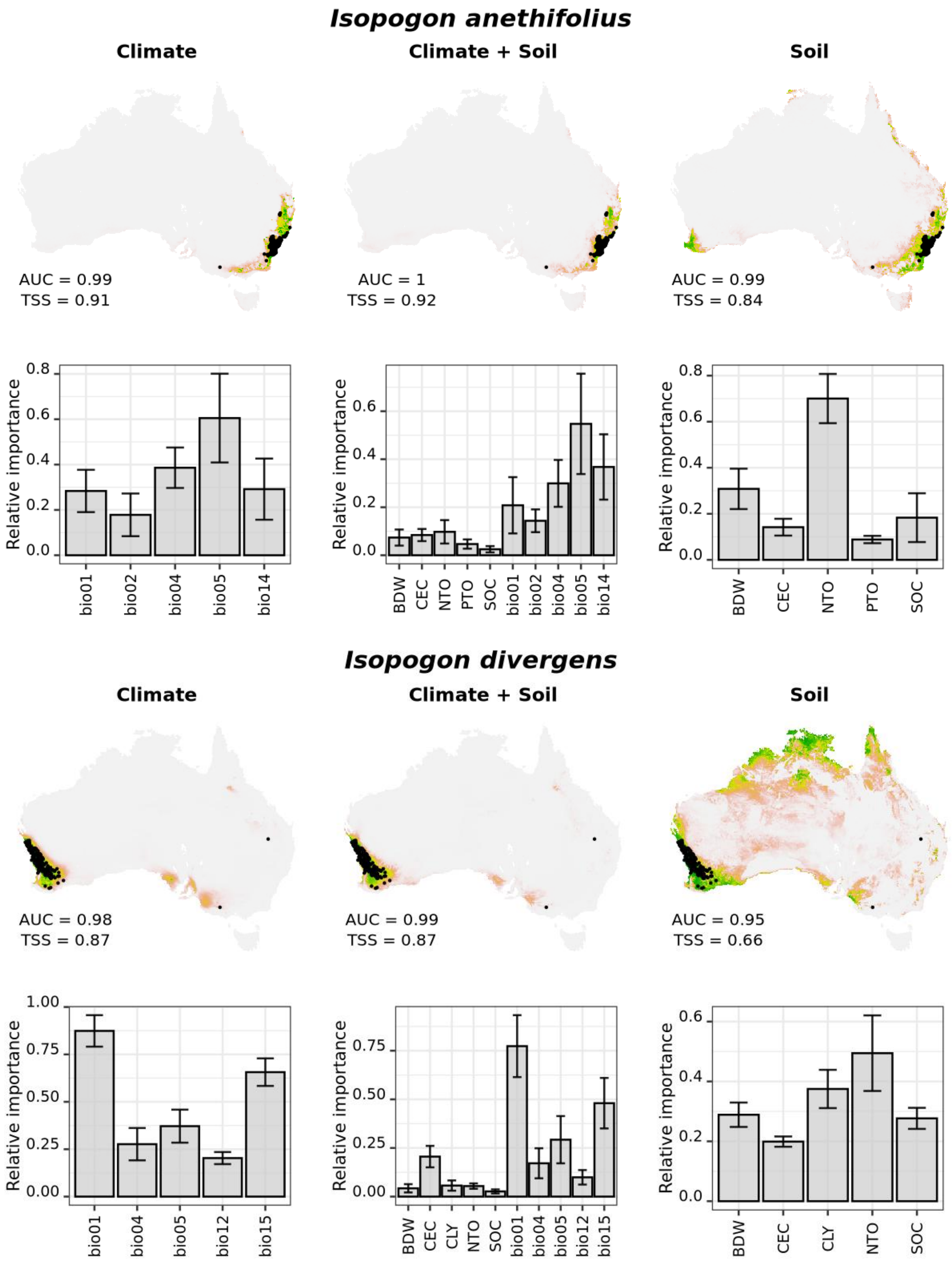
Isopogon anethifolius
Climate
Climate + Soil
Soil
AUC = 0.99
TSS = 0.91
AUC = 1
TSS = 0.92
AUC = 0.99
TSS = 0.84
Relative importance
bio01
bio02
bio04
bio05
bio14
BDW
CEC
NTO
PTO
SOC
Isopogon divergens
Climate
Climate + Soil
Soil
AUC = 0.98
TSS = 0.87
AUC = 0.99
TSS = 0.87
AUC = 0.95
TSS = 0.66
Relative importance
bio01
bio04
bio05
bio12
bio15
BDW
CEC
CLY
NTO
SOC

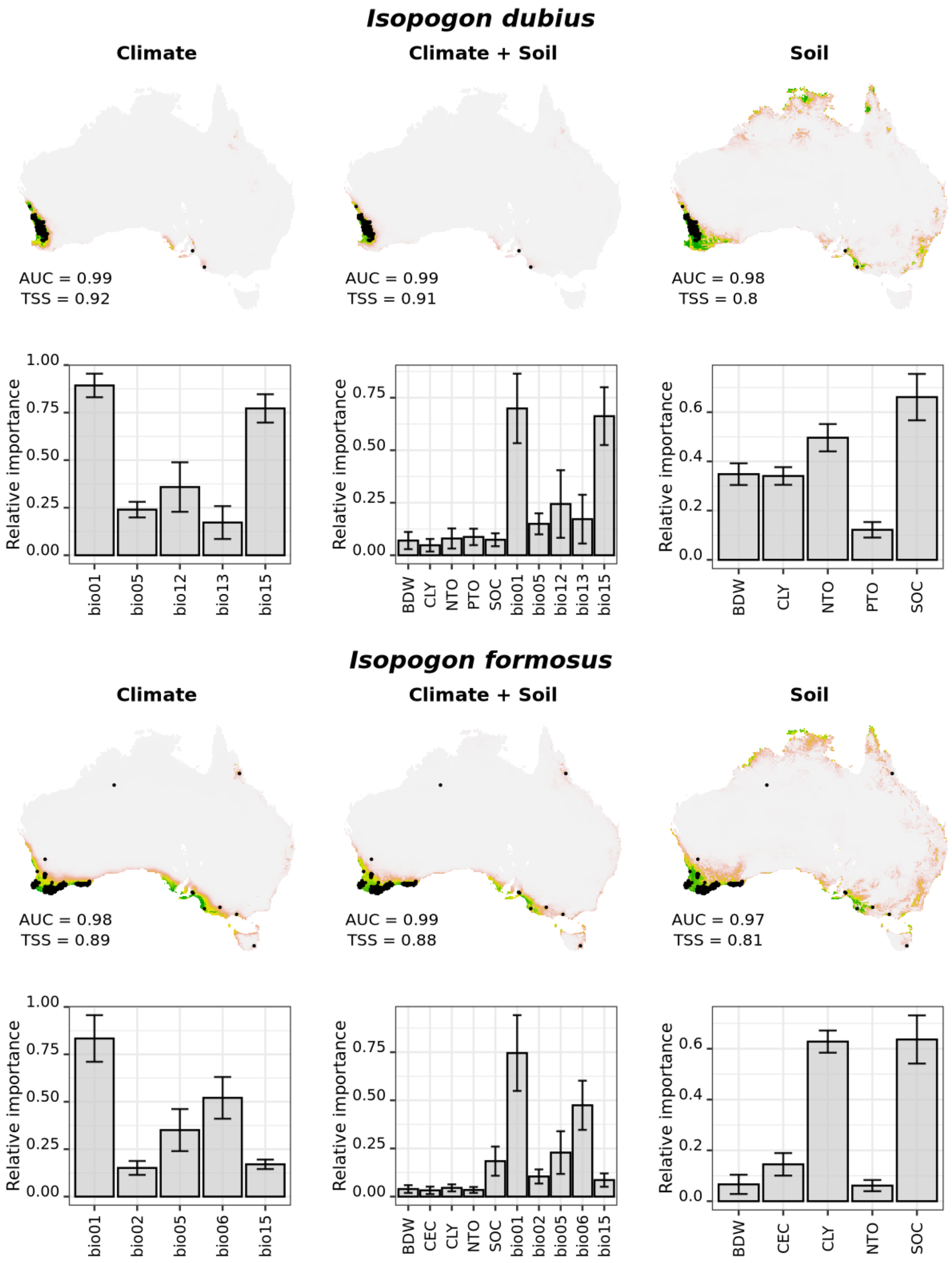
Isopogon dubius
Climate
Climate + Soil
Soil
AUC = 0.99
TSS = 0.92
AUC = 0.99
TSS = 0.91
AUC = 0.98
TSS = 0.8
Relative importance
1.00
0.75
0.50
0.25
0.00
bio01
bio05
bio12
bio13
bio15
BDW
CLY
NTO
PTO
SOC
0.6
0.4
0.2
0.0
Isopogon formosus
Climate
Climate + Soil
Soil
AUC = 0.98
TSS = 0.89
AUC = 0.99
TSS = 0.88
AUC = 0.97
TSS = 0.81
bio02
bio06
CEC

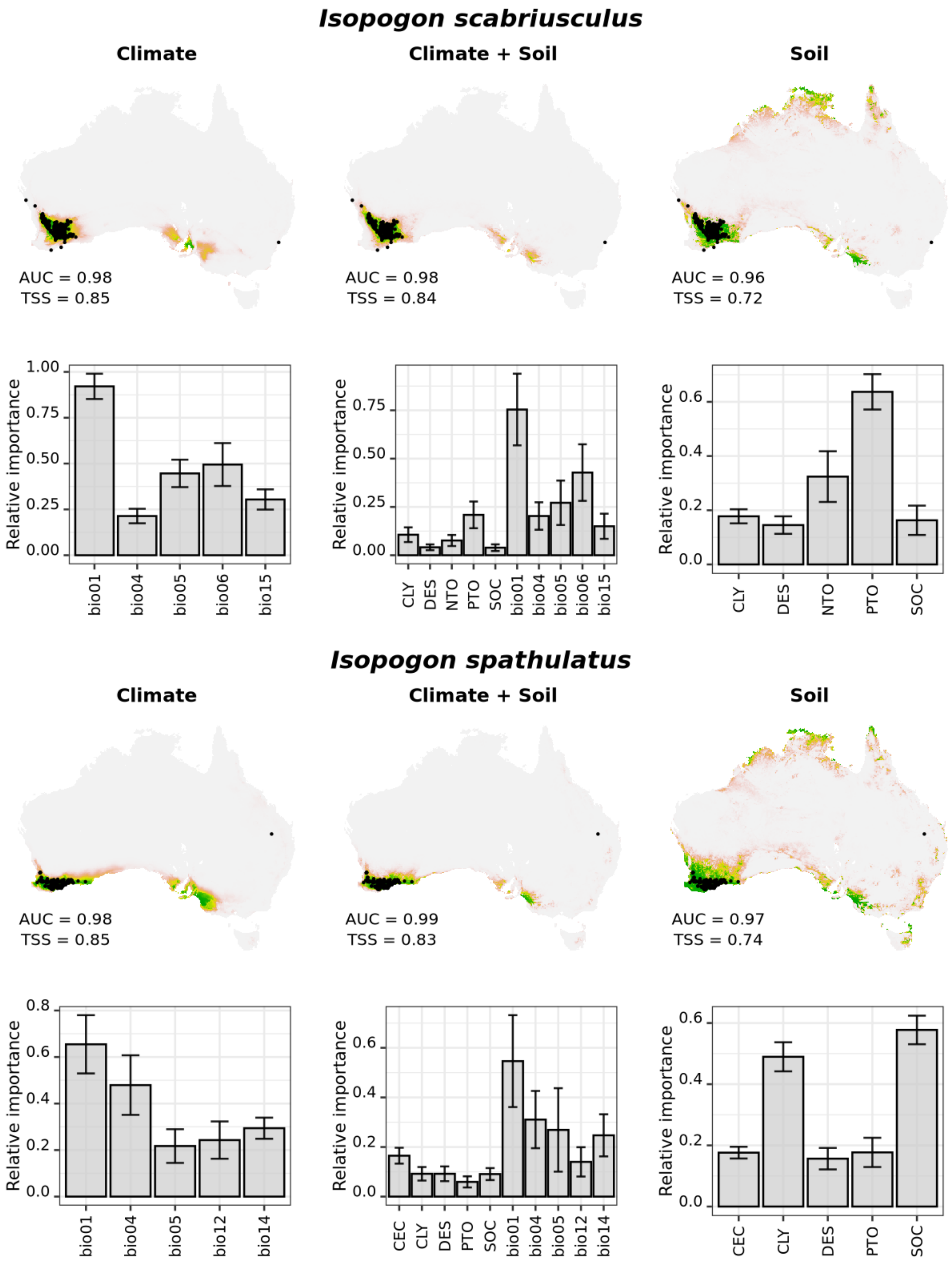
Isopogon scabriusculus
Climate
Climate + Soil
Soil
AUC = 0.98
TSS = 0.85
AUC = 0.98
TSS = 0.84
AUC = 0.96
TSS = 0.72
Relative importance
bio01
bio04
bio05
bio06
bio15
CLY
DES
NTO
PTO
SOC
Isopogon spathulatus
Climate
Climate + Soil
Soil
AUC = 0.98
TSS = 0.85
AUC = 0.99
TSS = 0.83
AUC = 0.97
TSS = 0.74
Relative importance
bio01
bio04
bio05
bio12
bio14
CEC
CLY
DES
PTO
SOC

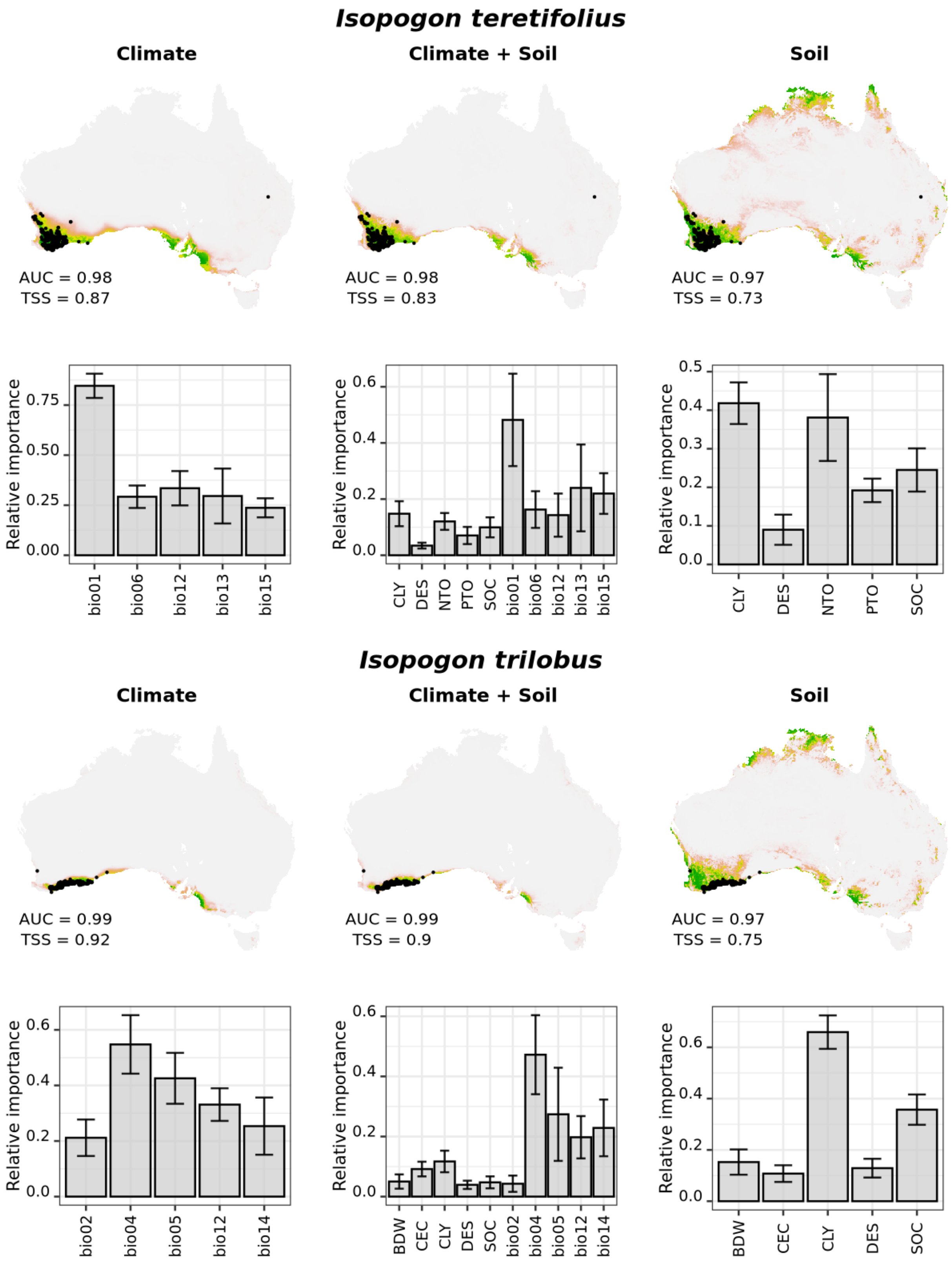
Isopogon teretifolius
Climate
Climate + Soil
Soil
AUC = 0.98
TSS = 0.87
AUC = 0.98
TSS = 0.83
AUC = 0.97
TSS = 0.73
Relative importance
0.00 0.25 0.50 0.75
bio01 bio06 bio12 bio13 bio15
0.0 0.2 0.4 0.6
CLY DES NTO PTO SOC bio01 bio06 bio12 bio13 bio15
0.0 0.1 0.2 0.3 0.4 0.5
CLY DES NTO PTO SOC
Isopogon trilobus
Climate
Climate + Soil
Soil
AUC = 0.99
TSS = 0.92
AUC = 0.99
TSS = 0.9
AUC = 0.97
TSS = 0.75
Relative importance
0.0 0.2 0.4 0.6
bio02 bio04 bio05 bio12 bio14
BDW CEC CLY DES SOC bio02 bio04 bio05 bio12 bio14
BDW CEC CLY DES SOC

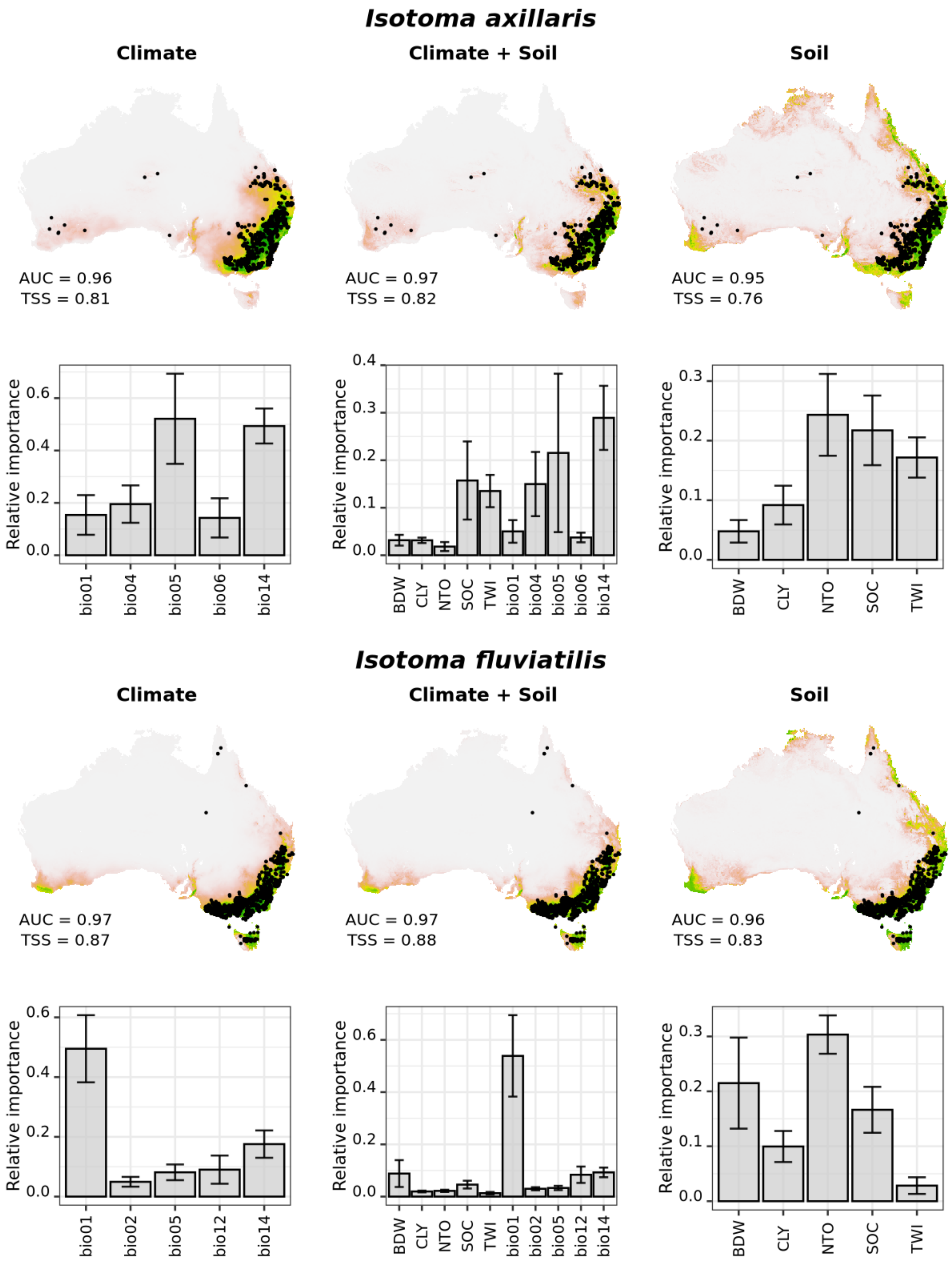

Isotoma axillaris
Climate
Climate + Soil
Soil
AUC = 0.96
TSS = 0.81
AUC = 0.97
TSS = 0.82
AUC = 0.95
TSS = 0.76
Relative importance
bio01
bio04
bio05
bio06
bio14
BDW
CLY
NTO
SOC
TWI
Isotoma fluviatilis
Climate
Climate + Soil
Soil
AUC = 0.97
TSS = 0.87
AUC = 0.97
TSS = 0.88
AUC = 0.96
TSS = 0.83
Relative importance
bio01
bio02
bio05
bio12
bio14
BDW
CLY
NTO
SOC
TWI

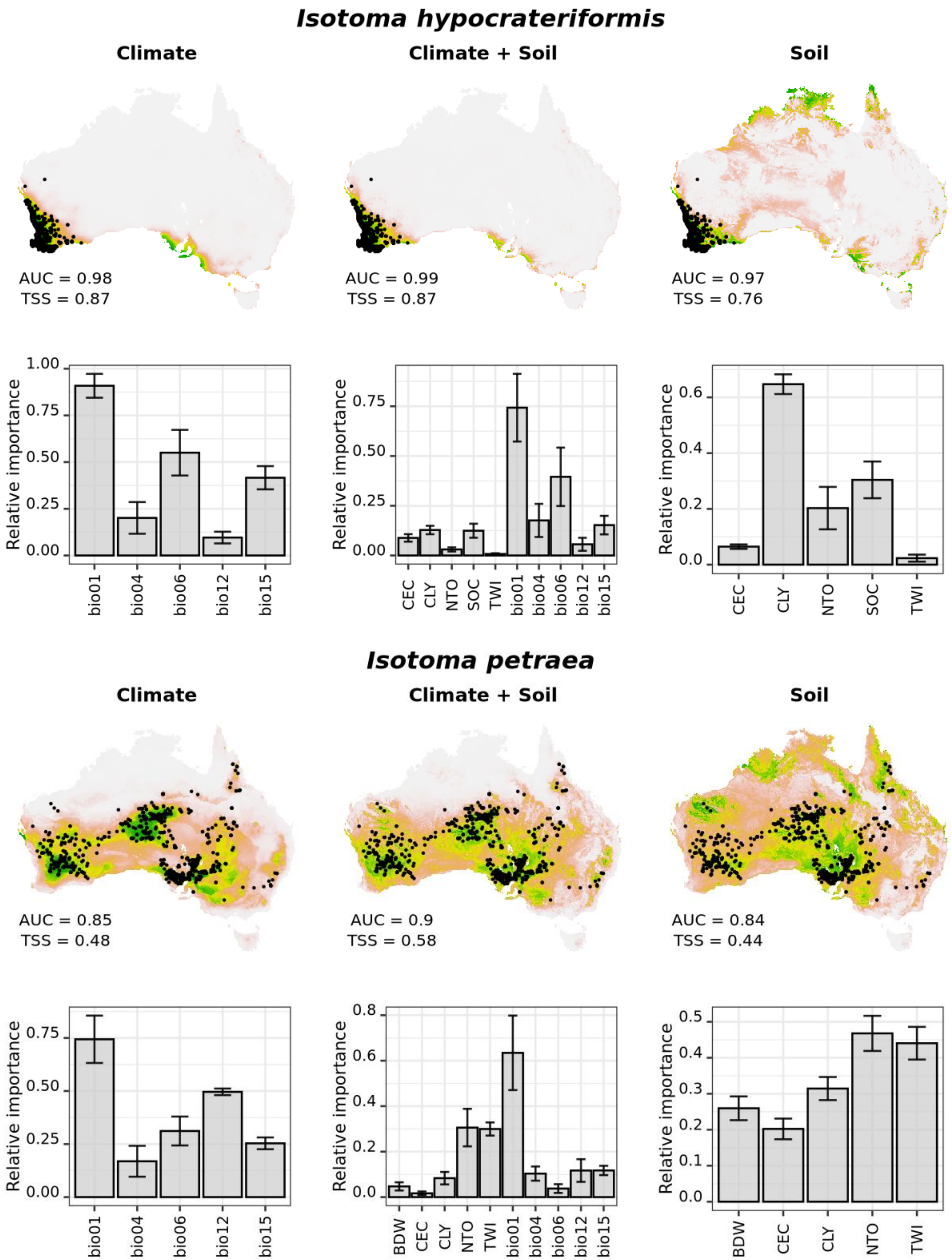

Isotoma hypocrateriformis
Climate
Climate + Soil
Soil
AUC = 0.98
TSS = 0.87
AUC = 0.99
TSS = 0.87
AUC = 0.97
TSS = 0.76
Relative importance
bio01 bio04 bio06 bio12 bio15
CEC CLY NTO SOC TWI bio01 bio04 bio06 bio12 bio15
CEC CLY NTO SOC TWI
Isotoma petraea
Climate
Climate + Soil
Soil
AUC = 0.85
TSS = 0.48
AUC = 0.9
TSS = 0.58
AUC = 0.84
TSS = 0.44
Relative importance
bio01 bio04 bio06 bio12 bio15
BDW CEC CLY NTO TWI bio01 bio04 bio06 bio12 bio15
BDW CEC CLY NTO TWI

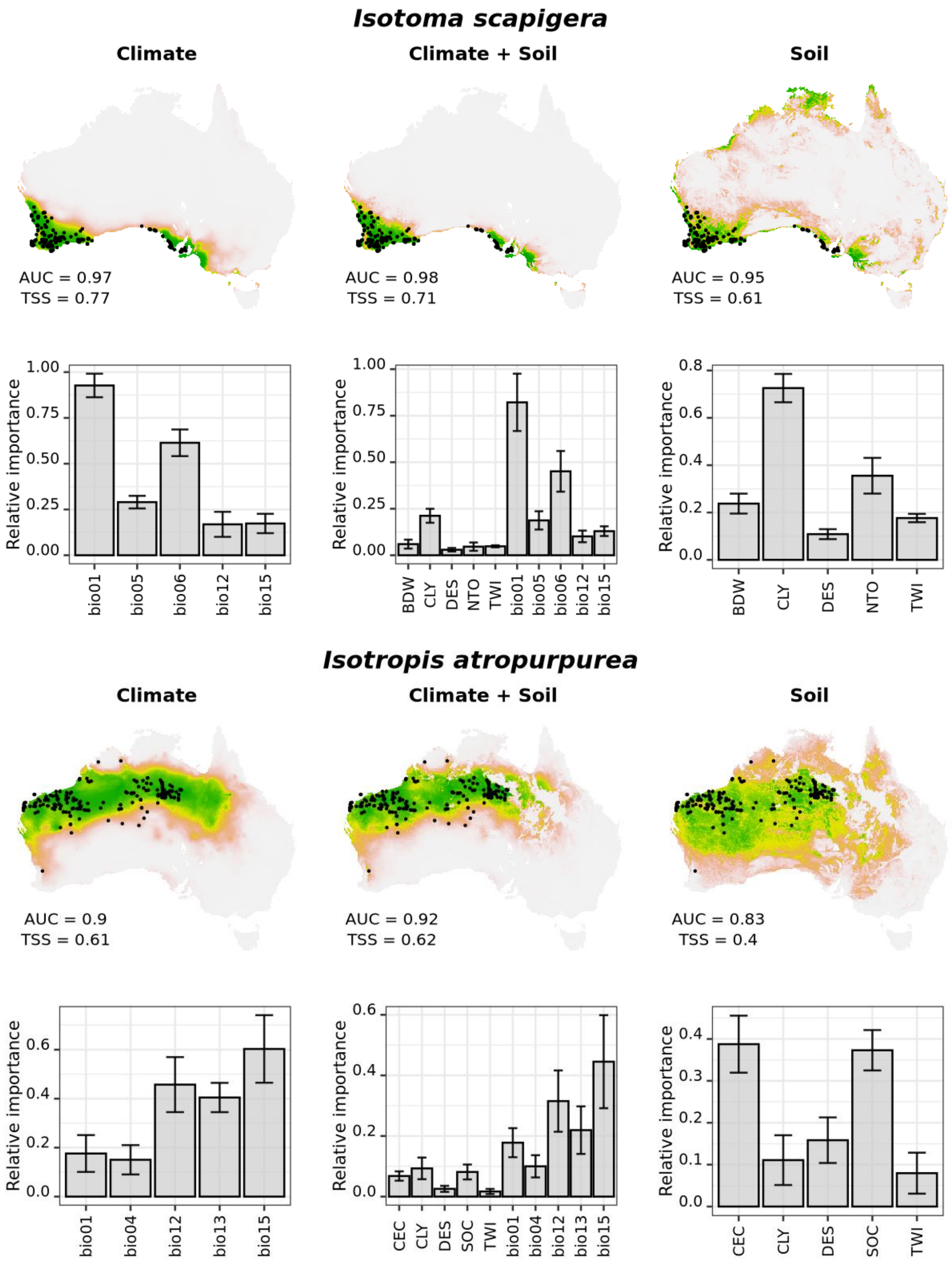
Isotoma scapigera
Climate
Climate + Soil
Soil
AUC = 0.97
TSS = 0.77
AUC = 0.98
TSS = 0.71
AUC = 0.95
TSS = 0.61
Relative importance
bio01
bio05
bio06
bio12
bio15
BDW
CLY
DES
NTO
TWI
bio01
bio05
bio06
bio12
bio15
BDW
CLY
DES
NTO
TWI
Isotropis atropurpurea
Climate
Climate + Soil
Soil
AUC = 0.9
TSS = 0.61
AUC = 0.92
TSS = 0.62
AUC = 0.83
TSS = 0.4
Relative importance
bio01
bio04
bio12
bio13
bio15
CEC
CLY
DES
SOC
TWI
bio01
bio04
bio12
bio13
bio15
CEC
CLY
DES
SOC
TWI

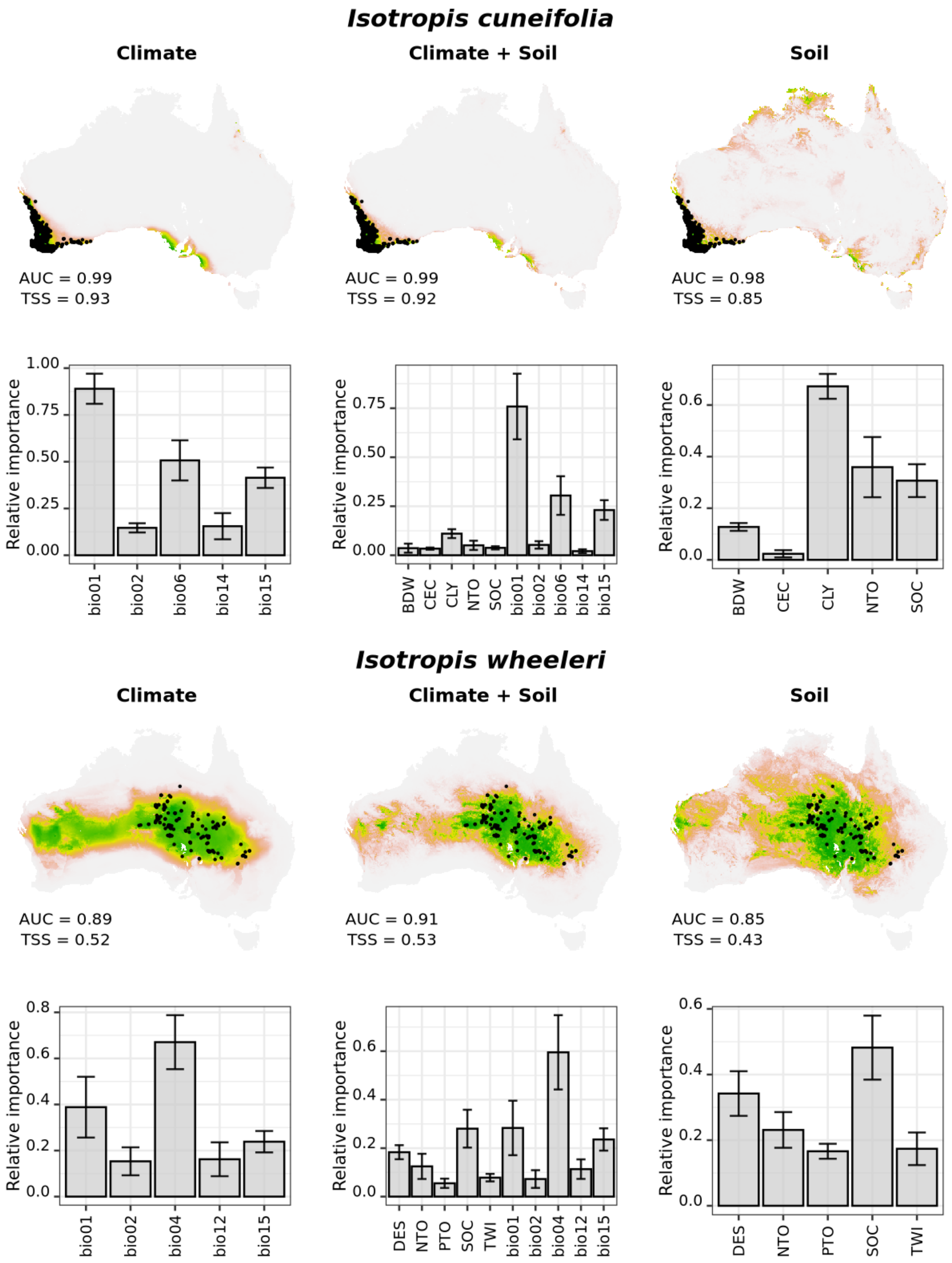
Isotropis cuneifolia
Climate
Climate + Soil
Soil
AUC = 0.99
TSS = 0.93
AUC = 0.99
TSS = 0.92
AUC = 0.98
TSS = 0.85
Relative importance
1.00
0.75
0.50
0.25
0.00
bio01
bio02
bio06
bio14
bio15
BDW
CEC
CLY
NTO
SOC
0.6
0.4
0.2
0.0
Isotropis wheeleri
Climate
Climate + Soil
Soil
AUC = 0.89
TSS = 0.52
AUC = 0.91
TSS = 0.53
AUC = 0.85
TSS = 0.43
0.8
bio04
bio12
DES
PTO
TWI

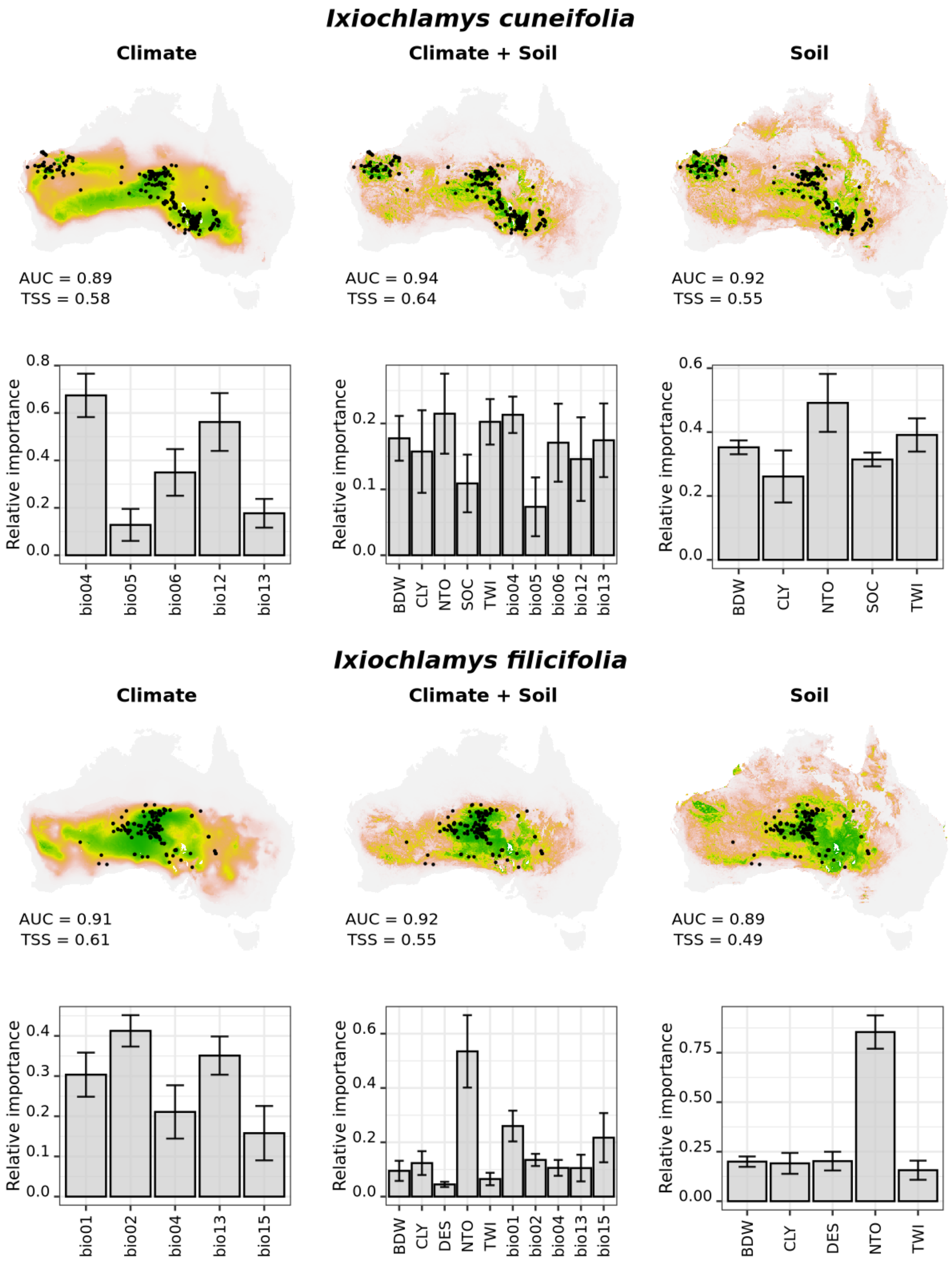
Ixiochlamys cuneifolia
Climate
Climate + Soil
Soil
AUC = 0.89
TSS = 0.58
AUC = 0.94
TSS = 0.64
AUC = 0.92
TSS = 0.55
Relative importance
bio04
bio05
bio06
bio12
bio13
BDW
CLY
NTO
SOC
TWI
bio04
bio05
bio06
bio12
bio13
BDW
CLY
NTO
SOC
TWI
Ixiochlamys filicifolia
Climate
Climate + Soil
Soil
AUC = 0.91
TSS = 0.61
AUC = 0.92
TSS = 0.55
AUC = 0.89
TSS = 0.49
Relative importance
bio01
bio02
bio04
bio13
bio15
BDW
CLY
DES
NTO
TWI
bio01
bio02
bio04
bio13
bio15
BDW
CLY
DES
NTO
TWI

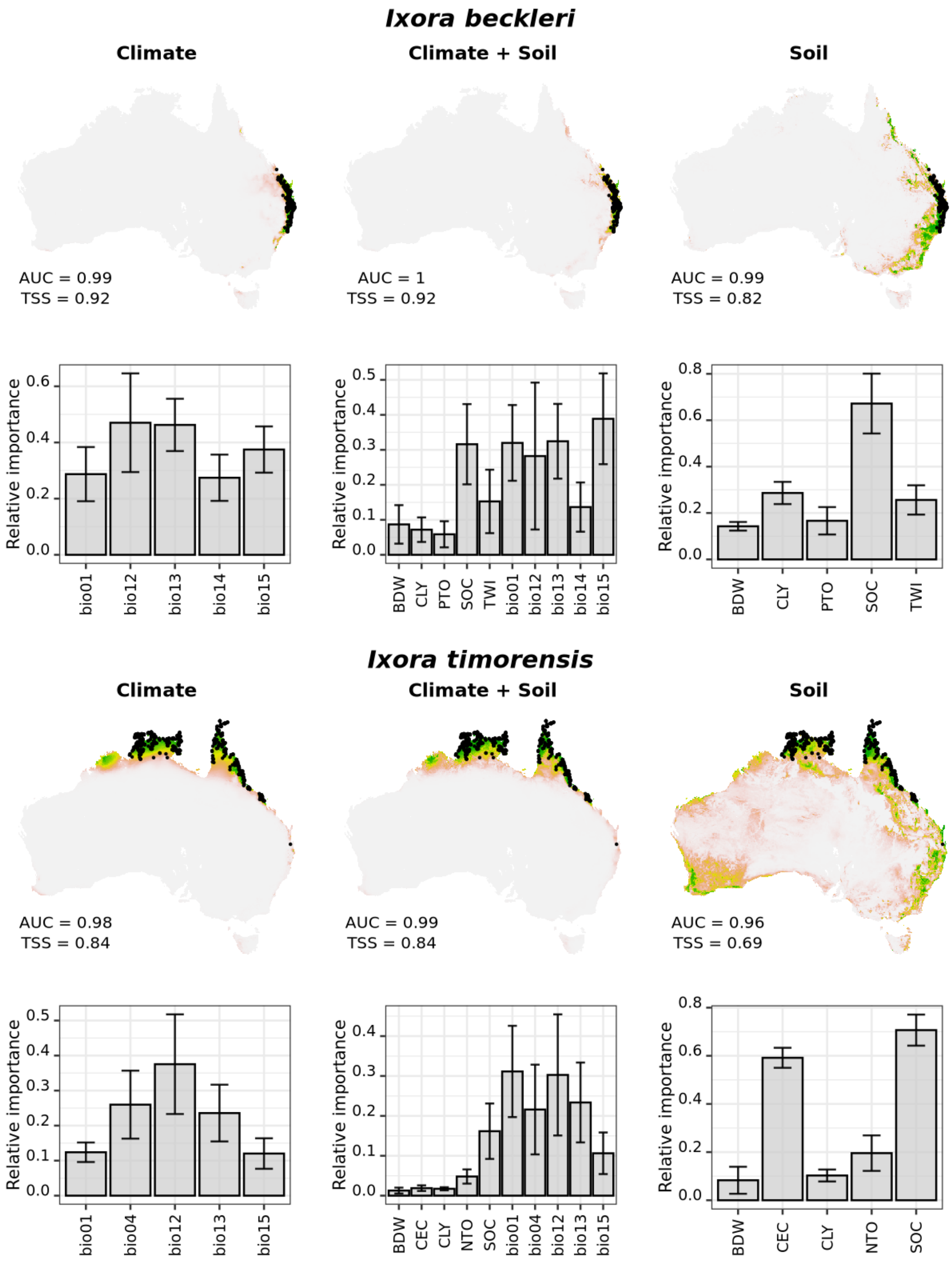
Ixora beckleri
Climate
Climate + Soil
Soil
AUC = 0.99
TSS = 0.92
AUC = 1
TSS = 0.92
AUC = 0.99
TSS = 0.82
Relative importance
0.0
0.2
0.4
0.6
bio01
bio12
bio13
bio14
bio15
0.0
0.1
0.2
0.3
0.4
0.5
BDW
CLY
PTO
SOC
TWI
bio01
bio12
bio13
bio14
bio15
0.0
0.2
0.4
0.6
0.8
BDW
CLY
PTO
SOC
TWI
Ixora timorensis
Climate
Climate + Soil
Soil
AUC = 0.98
TSS = 0.84
AUC = 0.99
TSS = 0.84
AUC = 0.96
TSS = 0.69
Relative importance
0.0
0.1
0.2
0.3
0.4
0.5
bio01
bio04
bio12
bio13
bio15
0.0
0.1
0.2
0.3
0.4
BDW
CEC
CLY
NTO
SOC
bio01
bio04
bio12
bio13
bio15
0.0
0.2
0.4
0.6
0.8
BDW
CEC
CLY
NTO
SOC

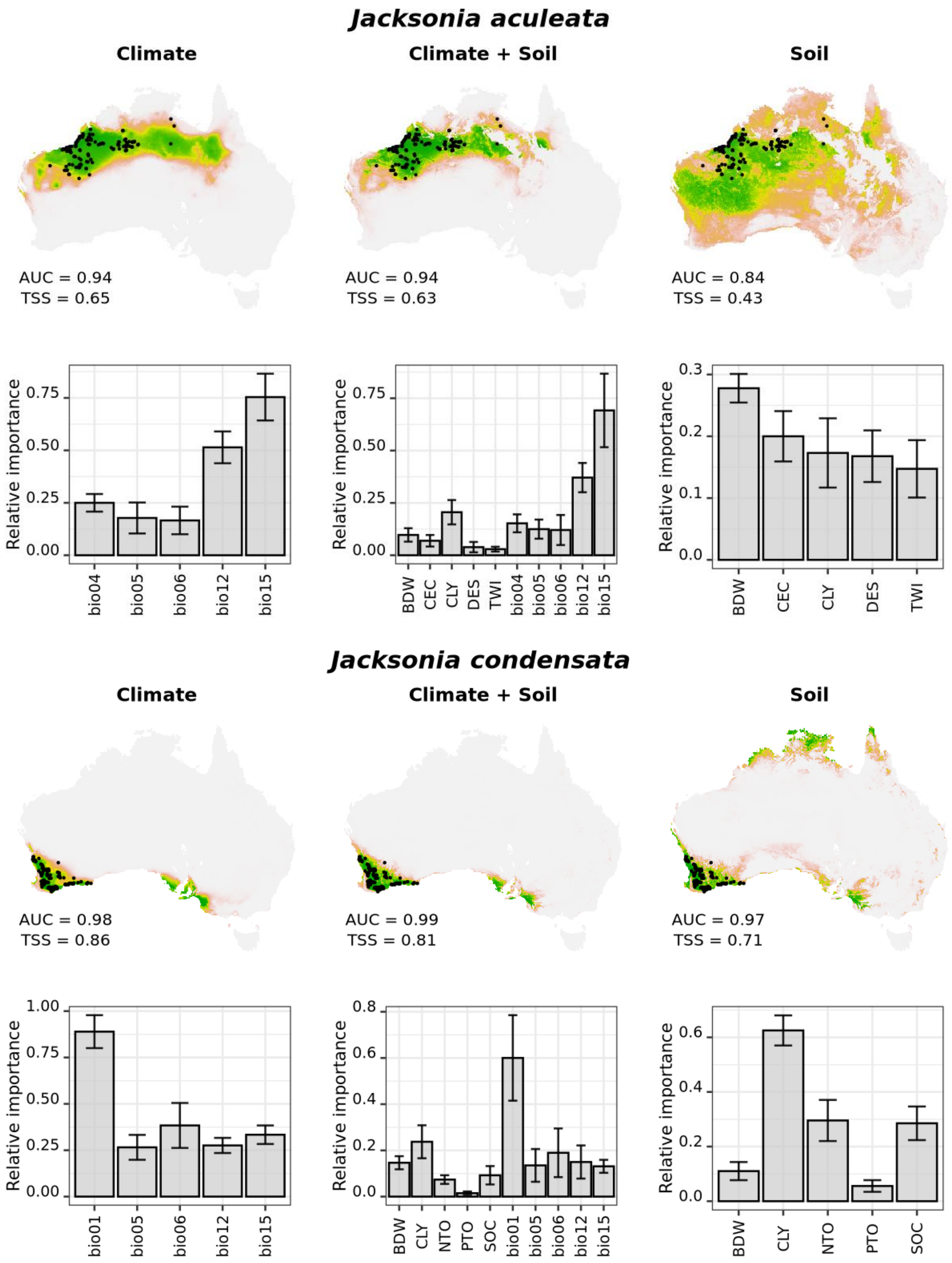

Jacksonia aculeata
Climate
Climate + Soil
Soil
AUC = 0.94
TSS = 0.65
AUC = 0.94
TSS = 0.63
AUC = 0.84
TSS = 0.43
Relative importance
bio04
bio05
bio06
bio12
bio15
BDW
CEC
CLY
DES
TWI
Jacksonia condensata
AUC = 0.98
TSS = 0.86
AUC = 0.99
TSS = 0.81
AUC = 0.97
TSS = 0.71
bio01
NTO
PTO
SOC

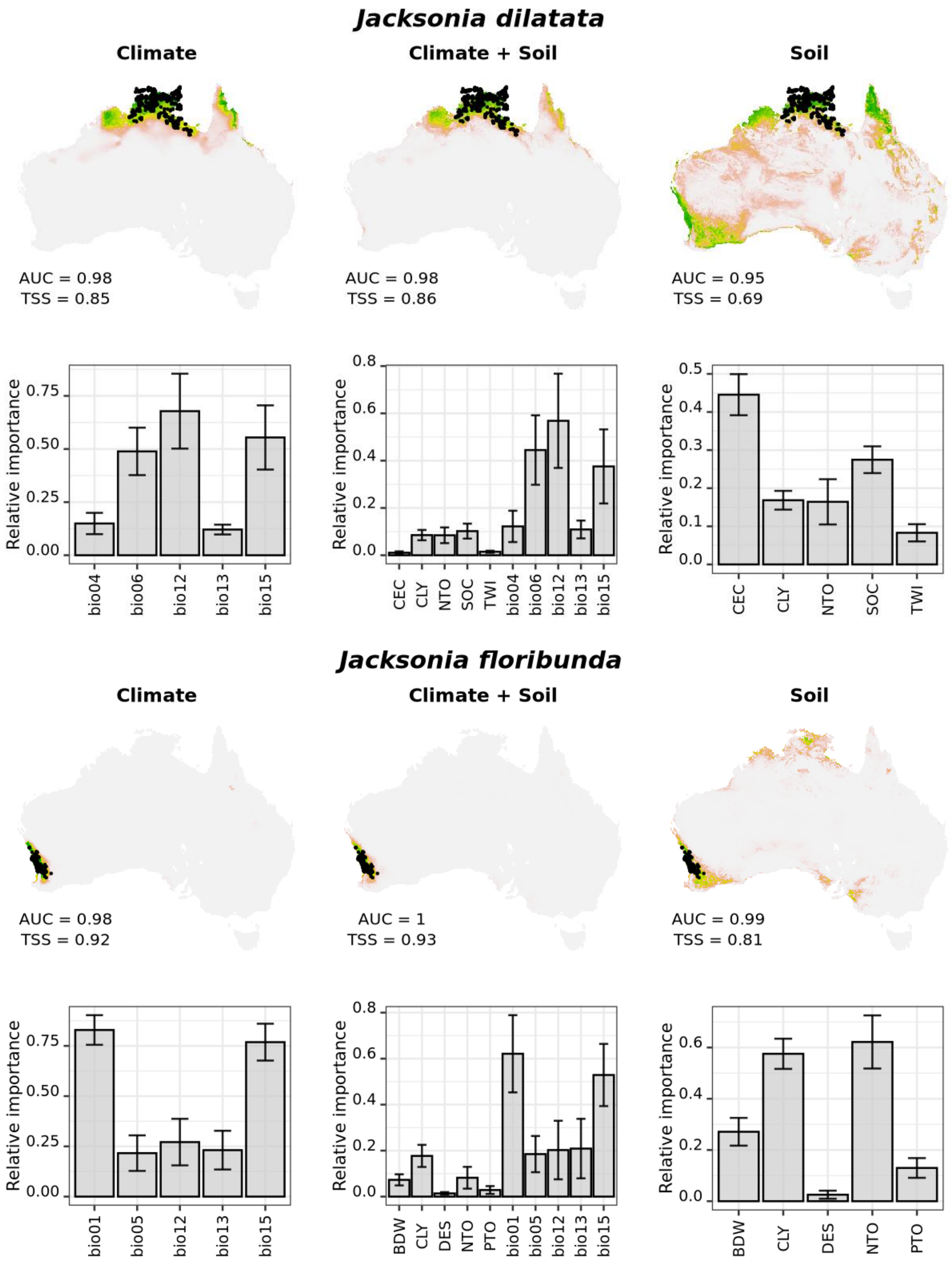
Jacksonia dilatata
Climate
Climate + Soil
Soil
AUC = 0.98
TSS = 0.85
AUC = 0.98
TSS = 0.86
AUC = 0.95
TSS = 0.69
Relative importance
bio04
bio06
bio12
bio13
bio15
CEC
CLY
NTO
SOC
TWI
Jacksonia floribunda
Climate
Climate + Soil
Soil
AUC = 0.98
TSS = 0.92
AUC = 1
TSS = 0.93
AUC = 0.99
TSS = 0.81
bio01
bio05
BDW
DES
PTO

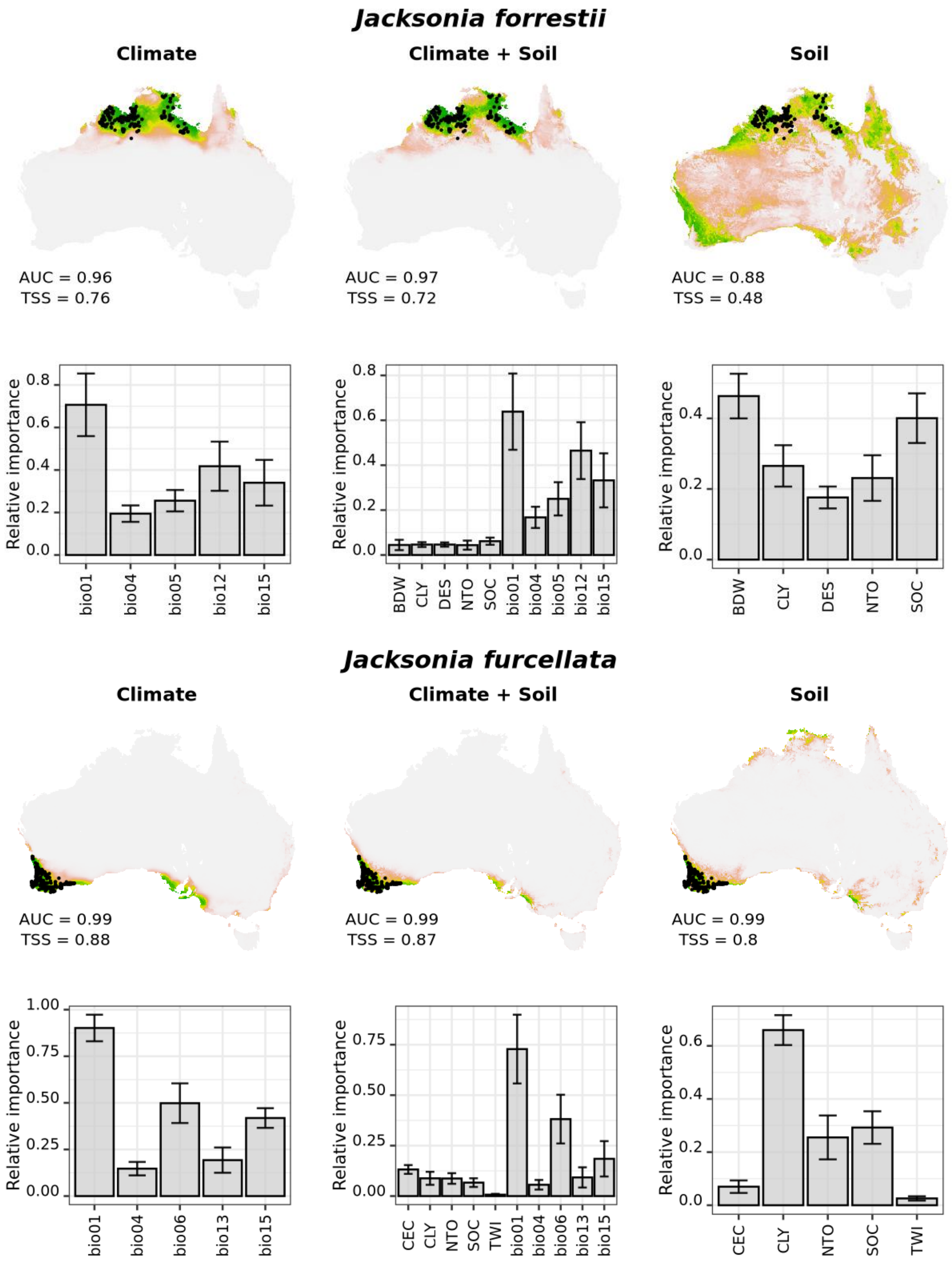

Jacksonia forrestii
Climate
Climate + Soil
Soil
AUC = 0.96
TSS = 0.76
AUC = 0.97
TSS = 0.72
AUC = 0.88
TSS = 0.48
Relative importance
bio01 bio04 bio05 bio12 bio15
BDW CLY DES NTO SOC bio01 bio04 bio05 bio12 bio15
BDW CLY DES NTO SOC
Jacksonia furcellata
Climate
Climate + Soil
Soil
AUC = 0.99
TSS = 0.88
AUC = 0.99
TSS = 0.87
AUC = 0.99
TSS = 0.8
Relative importance
bio01 bio04 bio06 bio13 bio15
CEC CLY NTO SOC TWI bio01 bio04 bio06 bio13 bio15
CEC CLY NTO SOC TWI

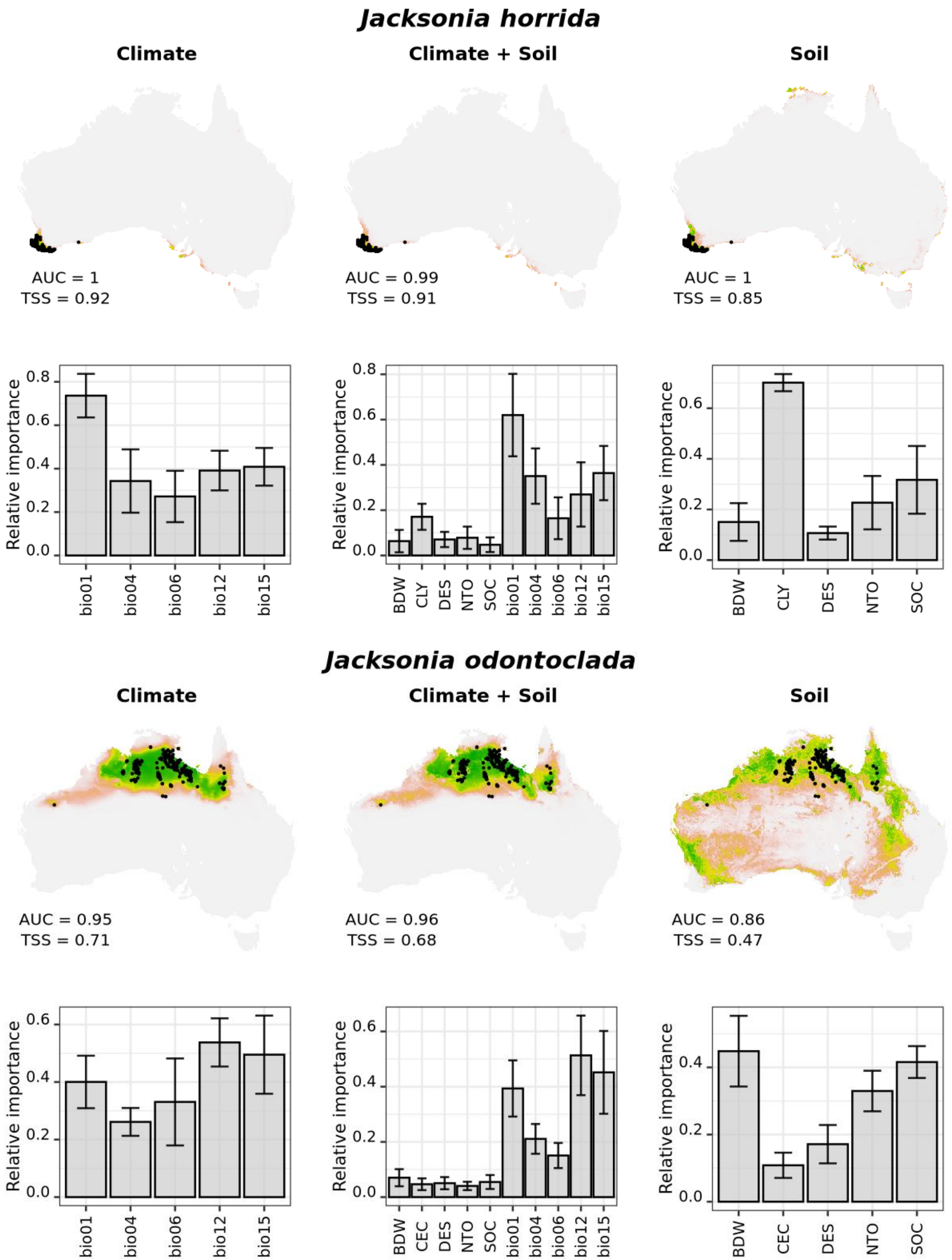
Jacksonia horrida
Climate
Climate + Soil
Soil
AUC = 1
TSS = 0.92
AUC = 0.99
TSS = 0.91
AUC = 1
TSS = 0.85
Relative importance
bio01 bio04 bio06 bio12 bio15
BDW CLY DES NTO SOC bio01 bio04 bio06 bio12 bio15
BDW CLY DES NTO SOC
Jacksonia odontoclada
Climate
Climate + Soil
Soil
AUC = 0.95
TSS = 0.71
AUC = 0.96
TSS = 0.68
AUC = 0.86
TSS = 0.47
Relative importance
bio01 bio04 bio06 bio12 bio15
BDW CEC DES NTO SOC bio01 bio04 bio06 bio12 bio15
BDW CEC DES NTO SOC

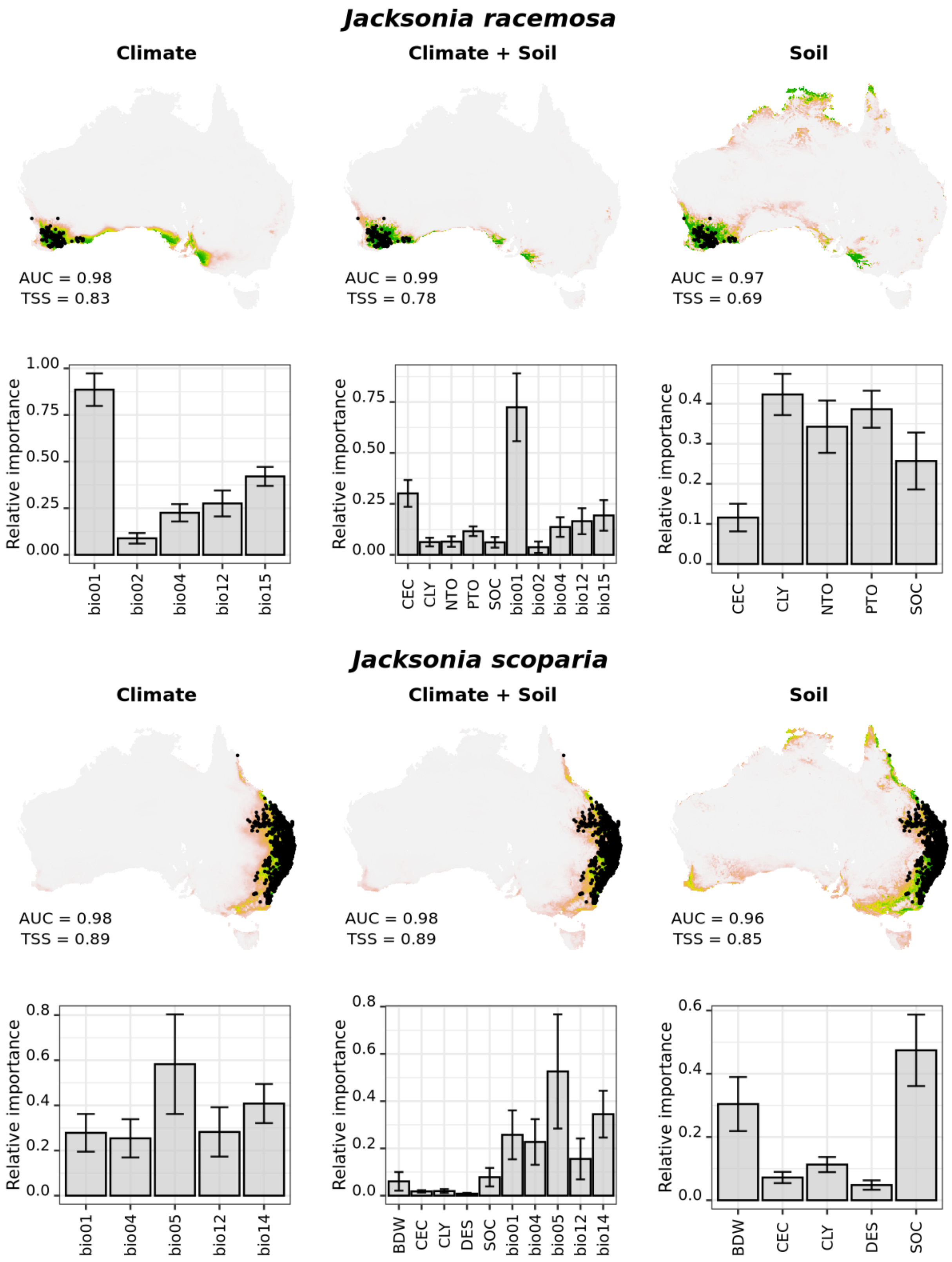
Jacksonia racemosa
Climate
Climate + Soil
Soil
AUC = 0.98
TSS = 0.83
AUC = 0.99
TSS = 0.78
AUC = 0.97
TSS = 0.69
Relative importance
1.00
0.75
0.50
0.25
0.00
bio01
bio02
bio04
bio12
bio15
CEC
CLY
NTO
PTO
SOC
0.4
0.3
0.2
0.1
0.0
Jacksonia scoparia
AUC = 0.98
TSS = 0.89
AUC = 0.96
TSS = 0.85
0.8
0.6
bio05
bio14
BDW
DES

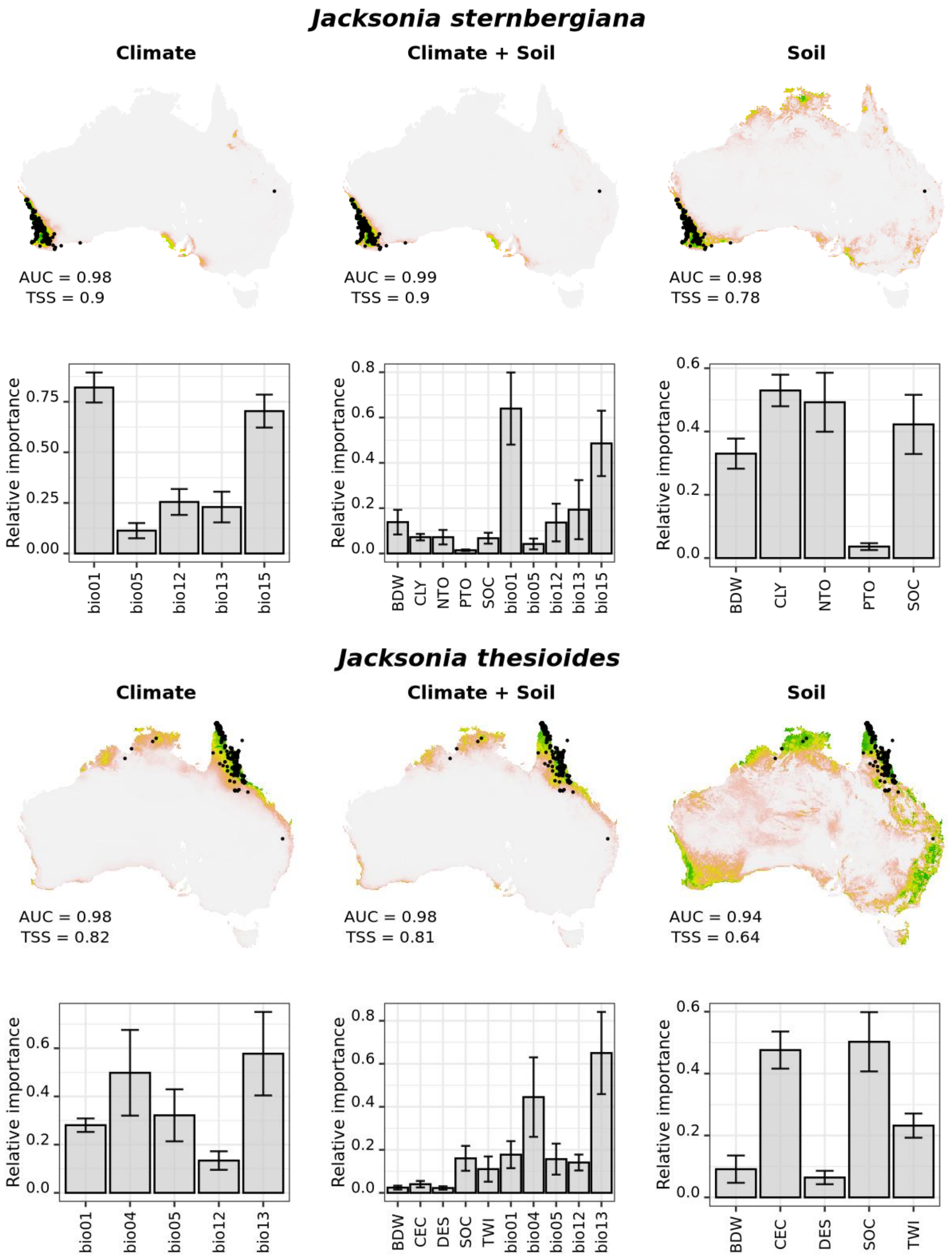

Jacksonia sternbergiana
Climate
Climate + Soil
Soil
AUC = 0.98
TSS = 0.9
AUC = 0.99
TSS = 0.9
AUC = 0.98
TSS = 0.78
Relative importance
bio01
bio05
bio12
bio13
bio15
BDW
CLY
NTO
PTO
SOC
Jacksonia thesioides
Climate
Climate + Soil
Soil
AUC = 0.98
TSS = 0.82
AUC = 0.98
TSS = 0.81
AUC = 0.94
TSS = 0.64
Relative importance
bio01
bio04
bio05
bio12
bio13
BDW
CEC
DES
SOC
TWI

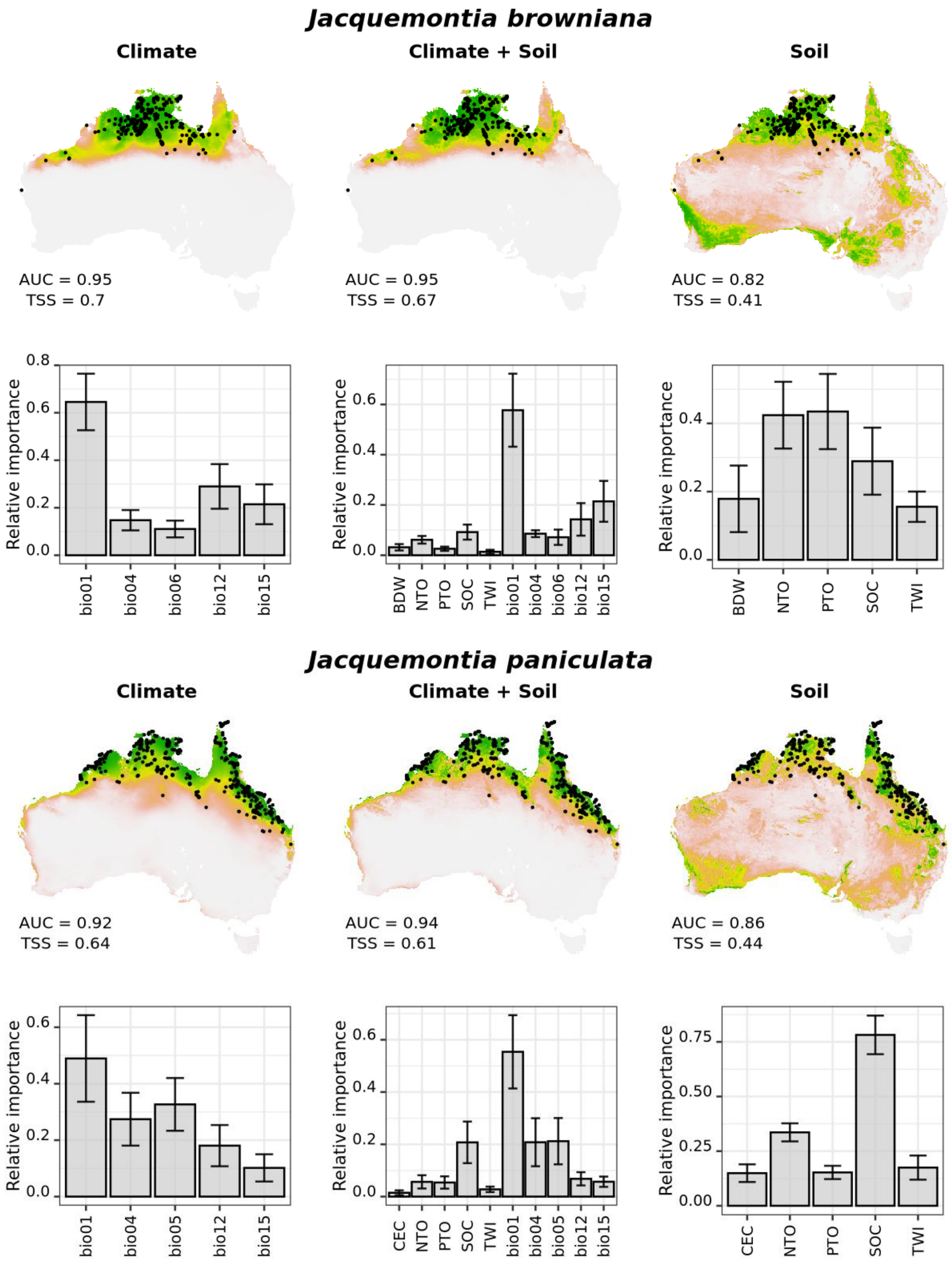
Jacquemontia browniana
Climate
Climate + Soil
Soil
AUC = 0.95
TSS = 0.7
AUC = 0.95
TSS = 0.67
AUC = 0.82
TSS = 0.41
Relative importance
bio01
bio04
bio06
bio12
bio15
BDW
NTO
PTO
SOC
TWI
Jacquemontia paniculata
AUC = 0.92
TSS = 0.64
AUC = 0.94
TSS = 0.61
AUC = 0.86
TSS = 0.44
bio05
CEC

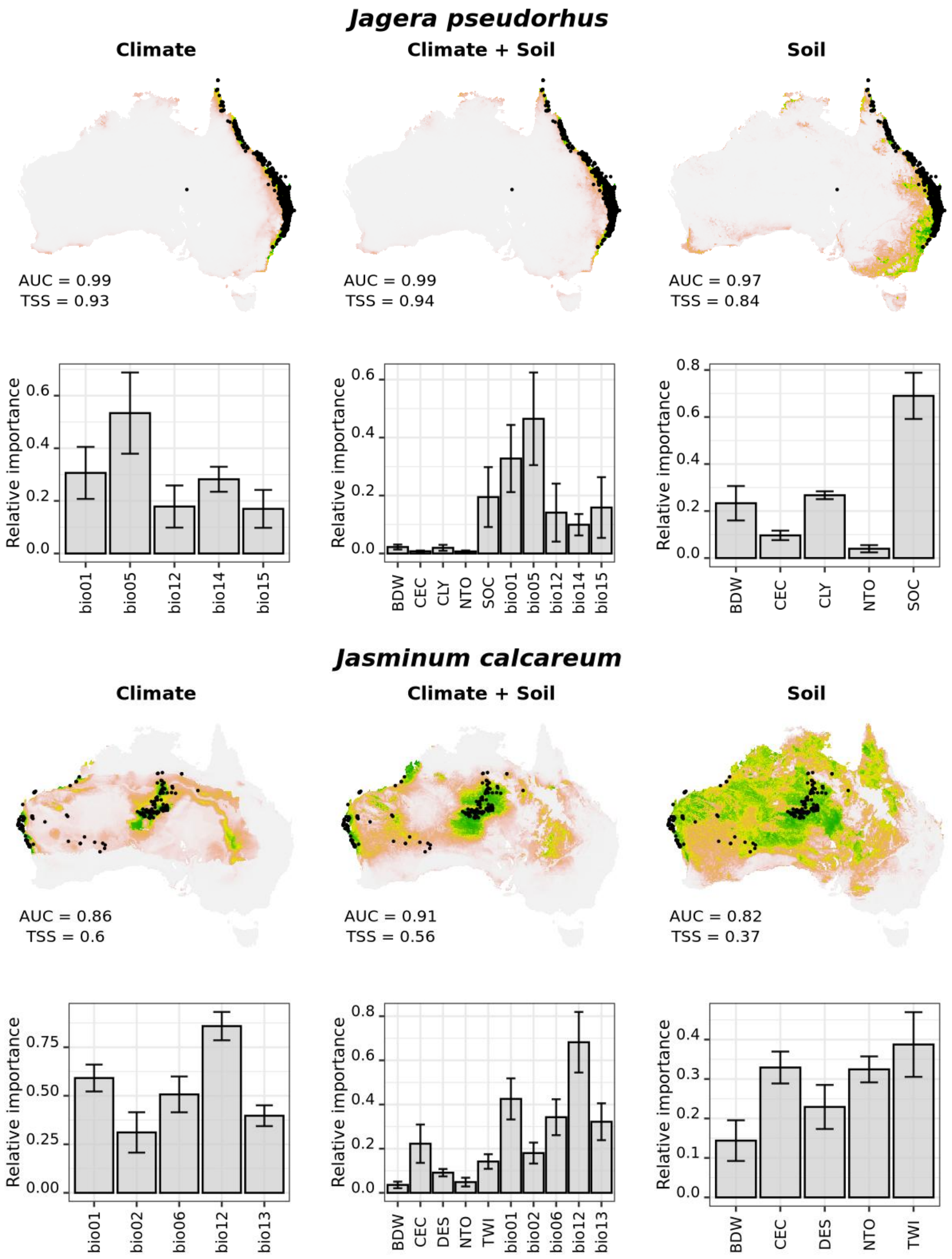
Jagera pseudorhus
Climate
Climate + Soil
Soil
AUC = 0.99
TSS = 0.93
AUC = 0.99
TSS = 0.94
AUC = 0.97
TSS = 0.84
Relative importance
bio01
bio05
bio12
bio14
bio15
BDW
CEC
CLY
NTO
SOC
Jasminum calcareum
Climate
Climate + Soil
Soil
AUC = 0.86
TSS = 0.6
AUC = 0.91
TSS = 0.56
AUC = 0.82
TSS = 0.37
Relative importance
bio01
bio02
bio06
bio12
bio13
BDW
CEC
DES
NTO
TWI

Jasminum elongatum

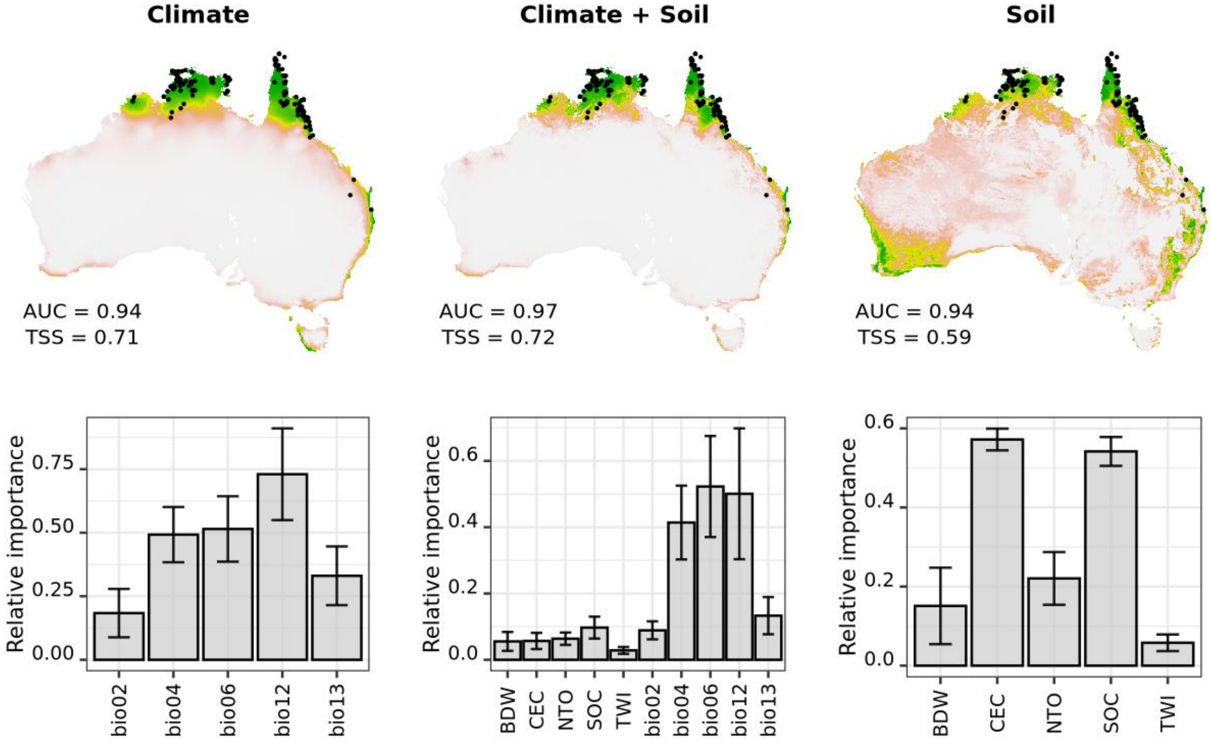

Jasminum molle

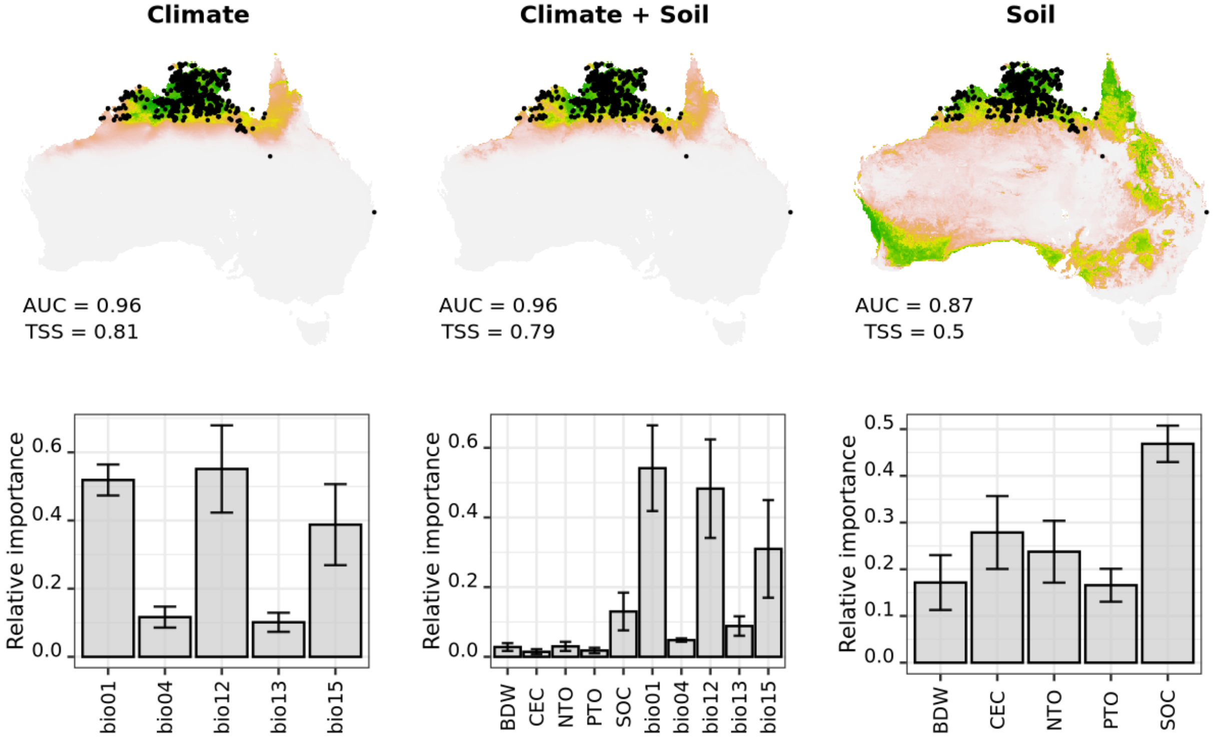

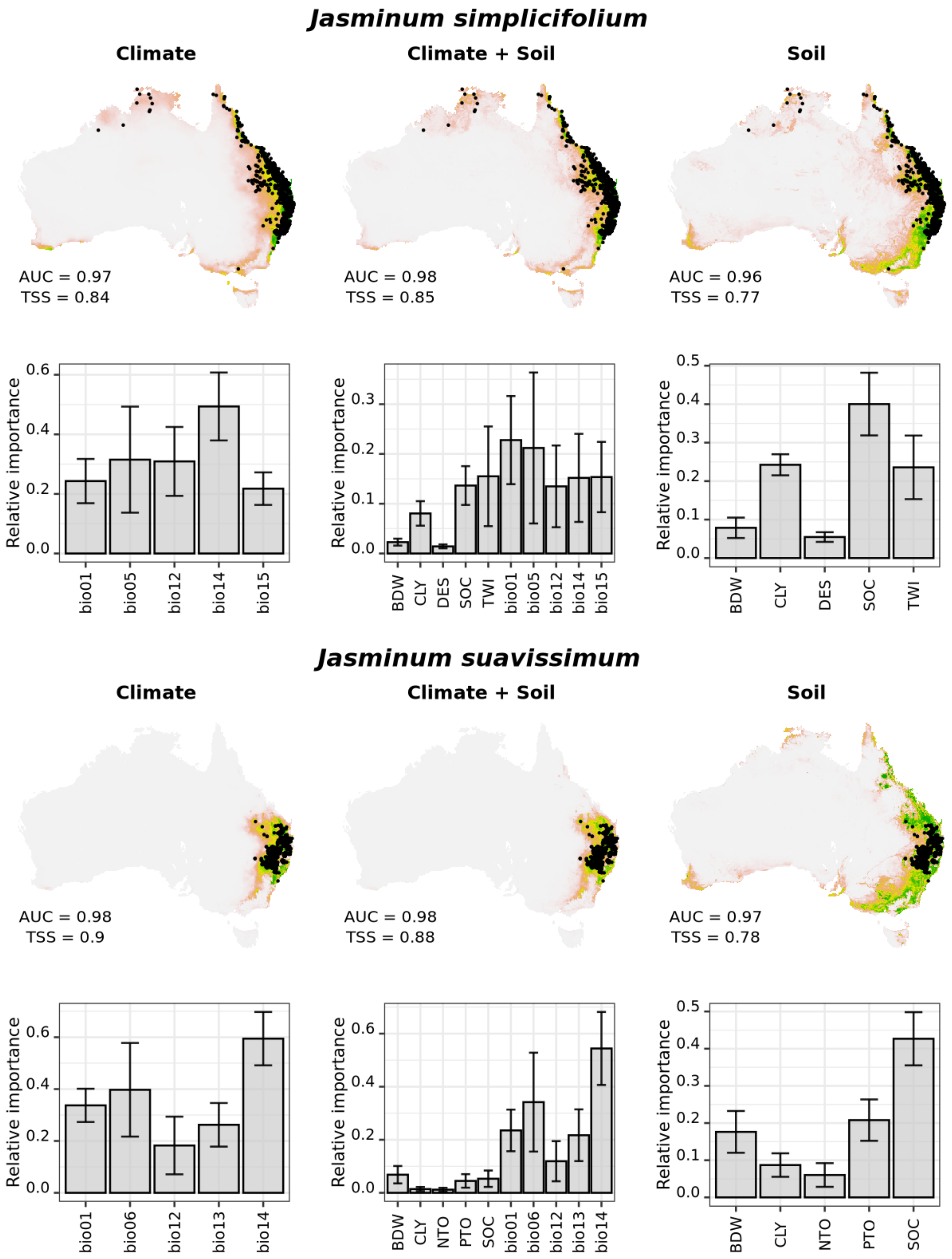

Jasminum simplicifolium
Climate
Climate + Soil
Soil
AUC = 0.97
TSS = 0.84
AUC = 0.98
TSS = 0.85
AUC = 0.96
TSS = 0.77
Relative importance
0.0
0.2
0.4
0.6
bio01
bio05
bio12
bio14
bio15
0.1
0.3
BDW
CLY
DES
SOC
TWI
0.5
Jasminum suavissimum
AUC = 0.98
TSS = 0.9
TSS = 0.88
AUC = 0.97
TSS = 0.78
bio06
bio13
NTO
PTO

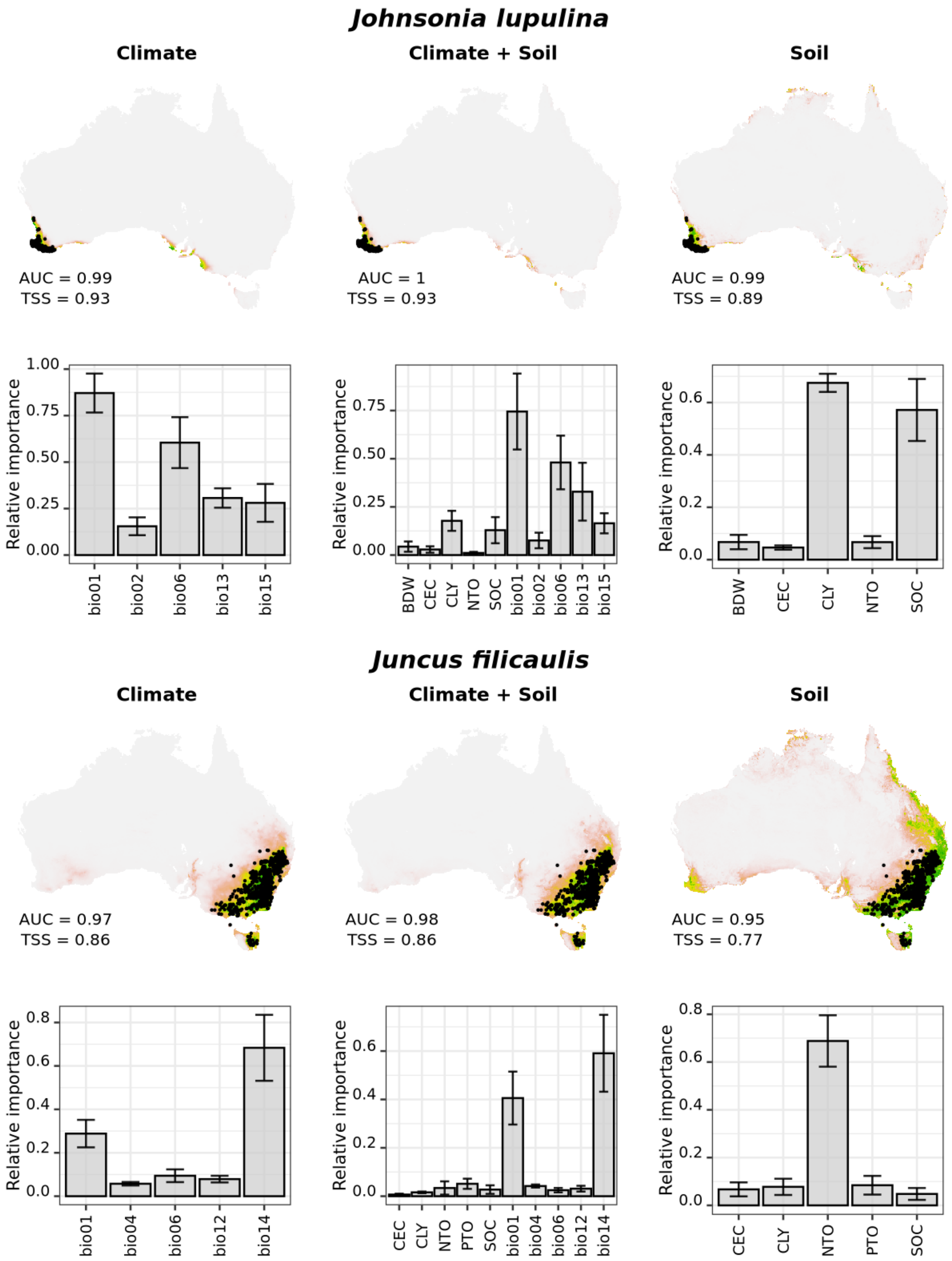
Johnsonia lupulina
Climate
Climate + Soil
Soil
AUC = 0.99
TSS = 0.93
AUC = 1
TSS = 0.93
AUC = 0.99
TSS = 0.89
Relative importance
bio01
bio02
bio06
bio13
bio15
BDW
CEC
CLY
NTO
SOC
CLY
Juncus filicaulis
Climate
Climate + Soil
Soil
AUC = 0.97
TSS = 0.86
AUC = 0.98
TSS = 0.86
AUC = 0.95
TSS = 0.77
Relative importance
bio01
bio04
bio06
bio12
bio14
CEC
CLY
NTO
PTO
SOC

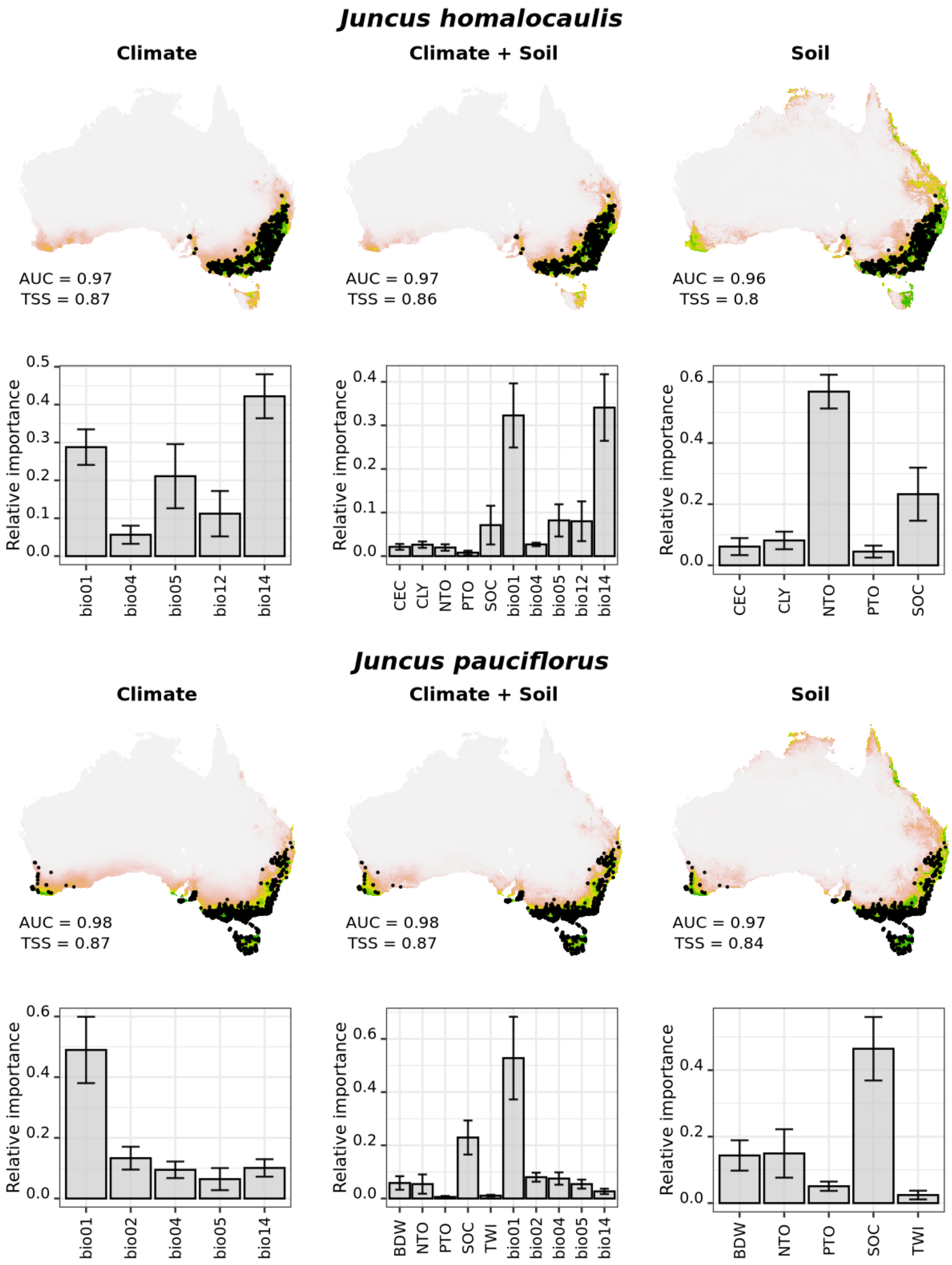

Juncus homalocaulis
Climate
Climate + Soil
Soil
AUC = 0.97
TSS = 0.87
AUC = 0.97
TSS = 0.86
AUC = 0.96
TSS = 0.8
Relative importance
bio01
bio04
bio05
bio12
bio14
CEC
CLY
NTO
PTO
SOC
Juncus pauciflorus
Climate
Climate + Soil
Soil
AUC = 0.98
TSS = 0.87
AUC = 0.98
TSS = 0.87
AUC = 0.97
TSS = 0.84
bio02
BDW
TWI

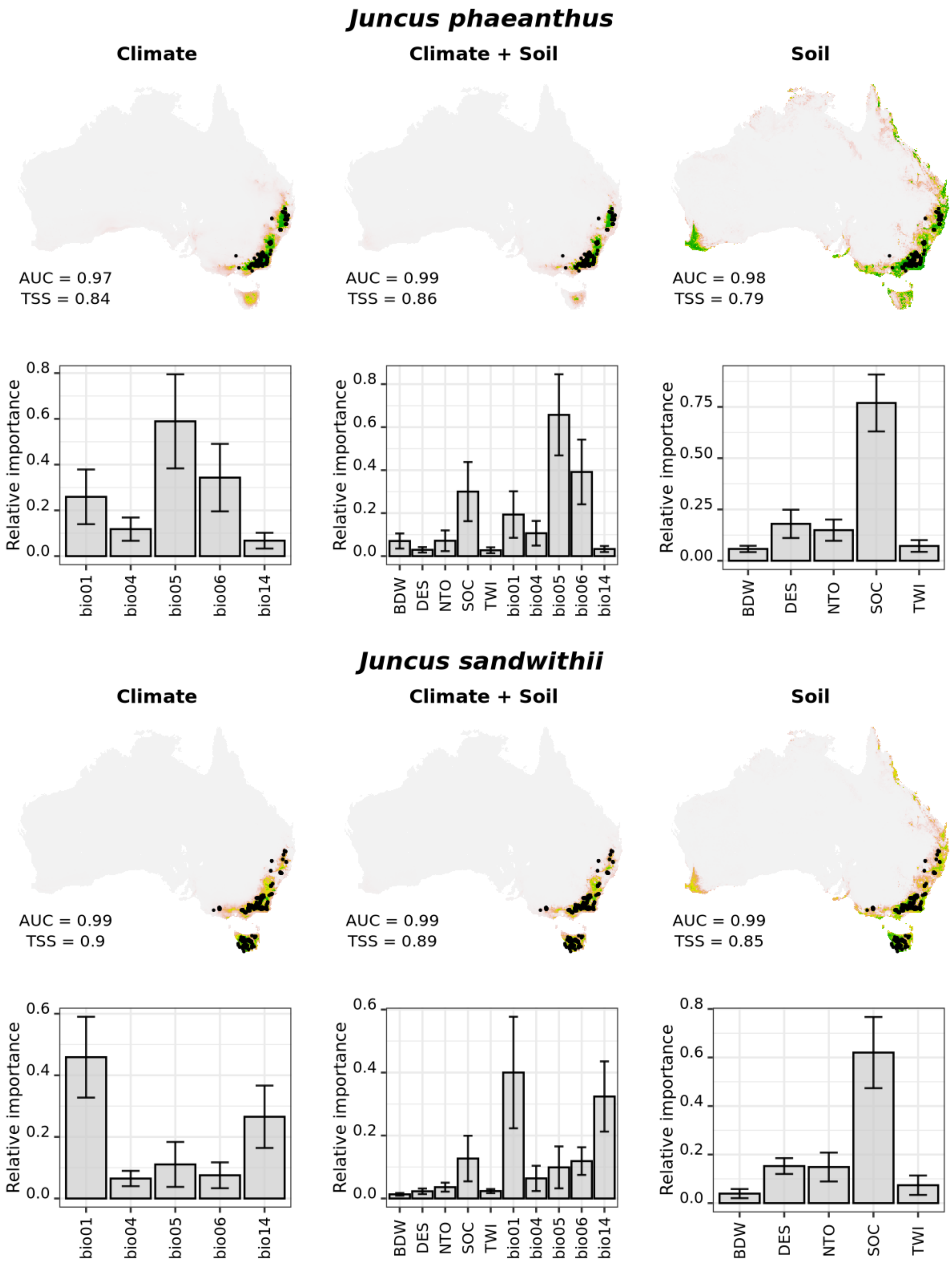
Juncus phaeanthus
Climate
Climate + Soil
Soil
AUC = 0.97
TSS = 0.84
AUC = 0.99
TSS = 0.86
AUC = 0.98
TSS = 0.79
Relative importance
bio01
bio04
bio05
bio06
bio14
BDW
DES
NTO
SOC
TWI
Juncus sandwithii
Climate
Climate + Soil
Soil
AUC = 0.99
TSS = 0.9
AUC = 0.99
TSS = 0.89
AUC = 0.99
TSS = 0.85
Relative importance
bio01
bio04
bio05
bio06
bio14
BDW
DES
NTO
SOC
TWI

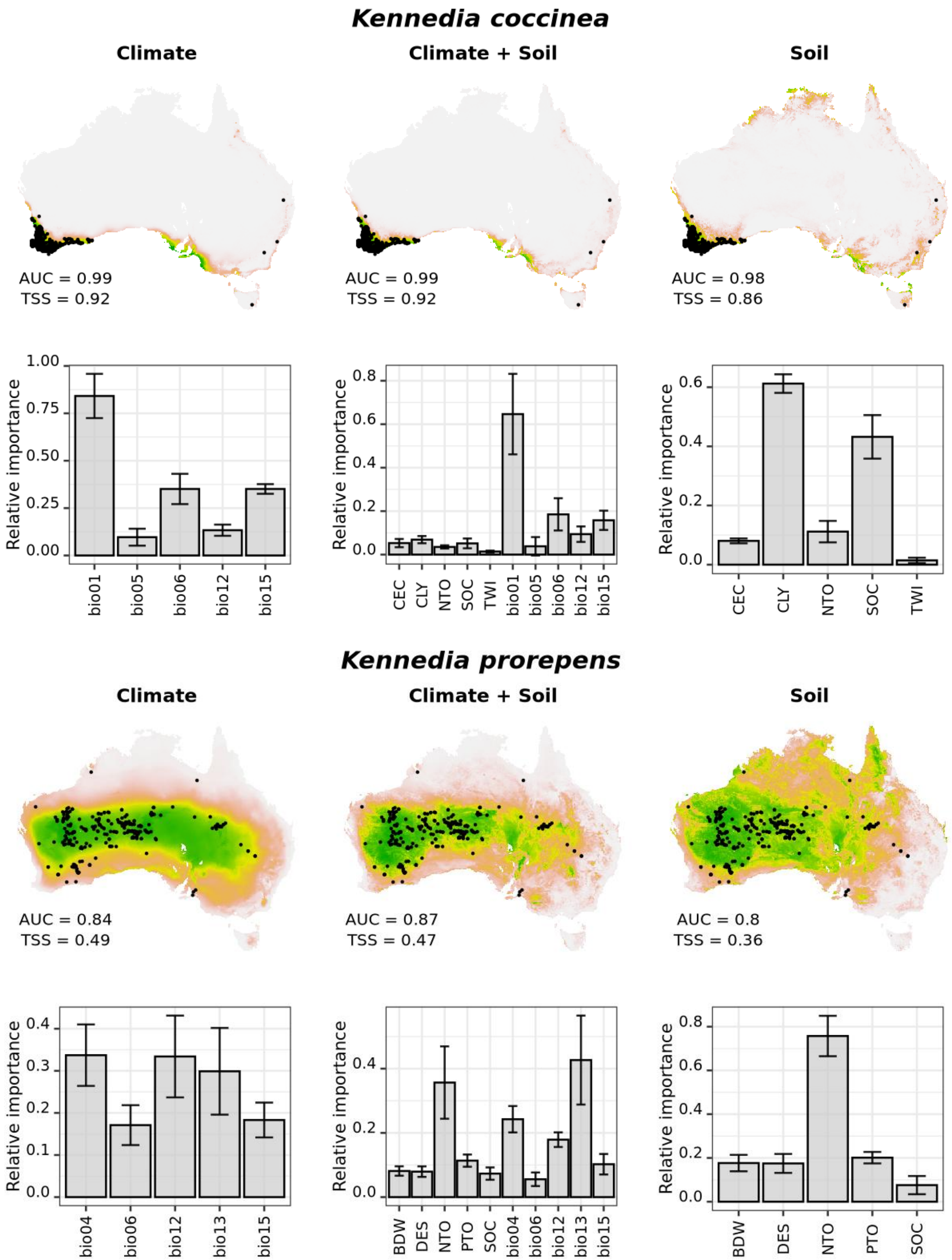
Kennedia coccinea
Climate
Climate + Soil
Soil
AUC = 0.99
TSS = 0.92
AUC = 0.99
TSS = 0.92
AUC = 0.98
TSS = 0.86
Relative importance
1.00
0.75
0.50
0.25
0.00
bio01
bio05
bio06
bio12
bio15
0.8
0.6
0.4
0.2
0.0
CEC
CLY
NTO
SOC
TWI
Kennedia prorepens
AUC = 0.84
TSS = 0.49
AUC = 0.87
TSS = 0.47
AUC = 0.8
TSS = 0.36
bio04
bio13
BDW
DES
PTO

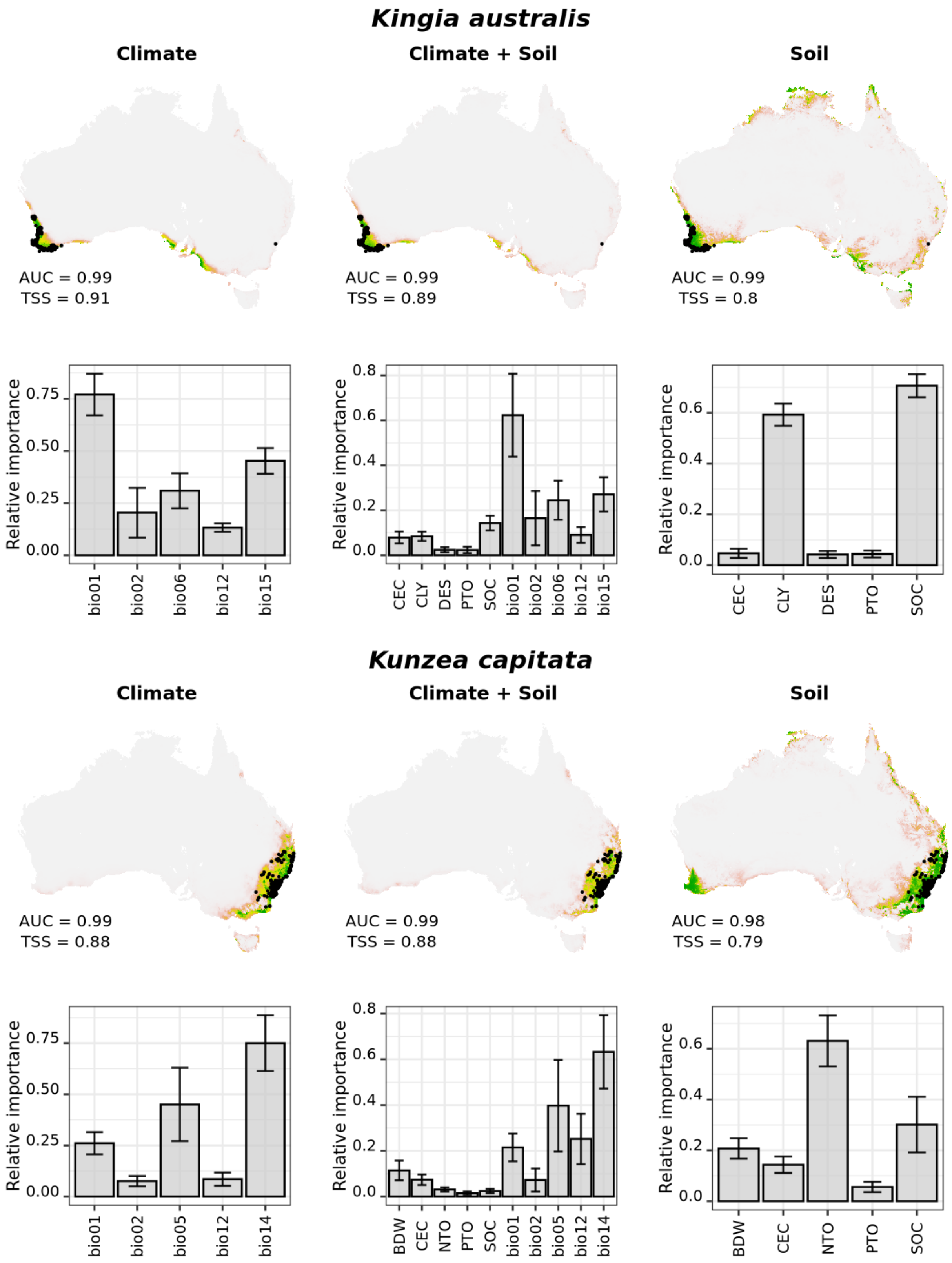

Kingia australis
Climate
Climate + Soil
Soil
AUC = 0.99
TSS = 0.91
AUC = 0.99
TSS = 0.89
AUC = 0.99
TSS = 0.8
Relative importance
0.00
0.25
0.50
0.75
bio01
bio02
bio06
bio12
bio15
0.0
0.2
0.4
0.6
0.8
CEC
CLY
DES
PTO
SOC
bio01
bio02
bio06
bio12
bio15
0.0
0.2
0.4
0.6
CEC
CLY
DES
PTO
SOC
Kunzea capitata
Climate
Climate + Soil
Soil
AUC = 0.99
TSS = 0.88
AUC = 0.99
TSS = 0.88
AUC = 0.98
TSS = 0.79
Relative importance
0.00
0.25
0.50
0.75
bio01
bio02
bio05
bio12
bio14
0.0
0.2
0.4
0.6
0.8
BDW
CEC
NTO
PTO
SOC
bio01
bio02
bio05
bio12
bio14
0.0
0.2
0.4
0.6
BDW
CEC
NTO
PTO
SOC

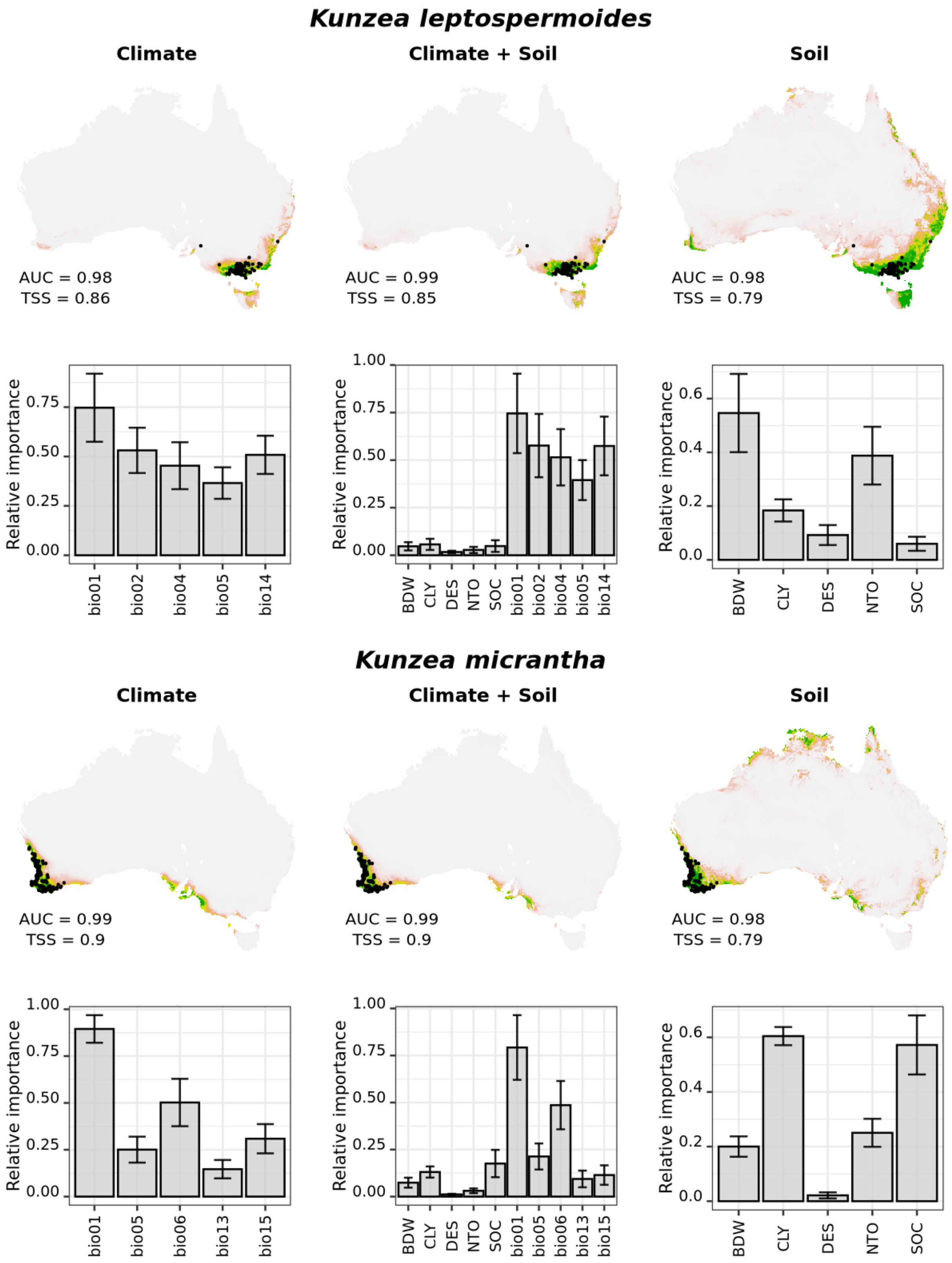
Kunzea leptospermoides
Climate
Climate + Soil
Soil
AUC = 0.98
TSS = 0.86
AUC = 0.99
TSS = 0.85
AUC = 0.98
TSS = 0.79
Relative importance
0.00
0.25
0.50
0.75
1.00
0.0
0.2
0.4
0.6
bio01
bio02
bio04
bio05
bio14
BDW
CLY
DES
NTO
SOC
Kunzea micrantha
Climate
Climate + Soil
Soil
AUC = 0.99
TSS = 0.9
AUC = 0.99
TSS = 0.9
AUC = 0.98
TSS = 0.79
bio01
bio05
bio06
bio13
bio15

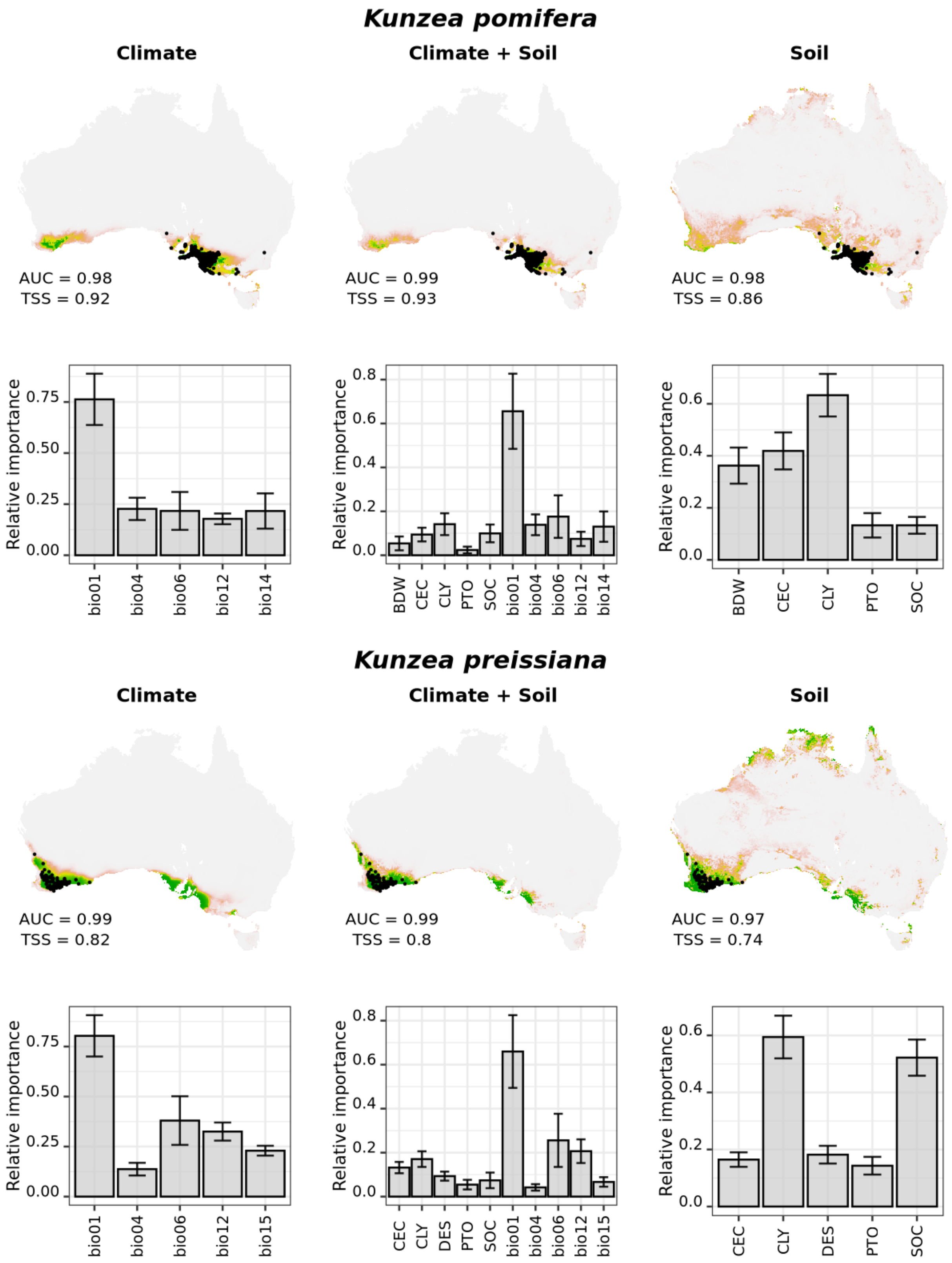
Kunzea pomifera
Climate
Climate + Soil
Soil
AUC = 0.98
TSS = 0.92
AUC = 0.99
TSS = 0.93
AUC = 0.98
TSS = 0.86
Relative importance
bio01 bio04 bio06 bio12 bio14
BDW CEC CLY PTO SOC bio01 bio04 bio06 bio12 bio14
BDW CEC CLY PTO SOC
Kunzea preissiana
Climate
Climate + Soil
Soil
AUC = 0.99
TSS = 0.82
AUC = 0.99
TSS = 0.8
AUC = 0.97
TSS = 0.74
Relative importance
bio01 bio04 bio06 bio12 bio15
CEC CLY DES PTO SOC bio01 bio04 bio06 bio12 bio15
CEC CLY DES PTO SOC

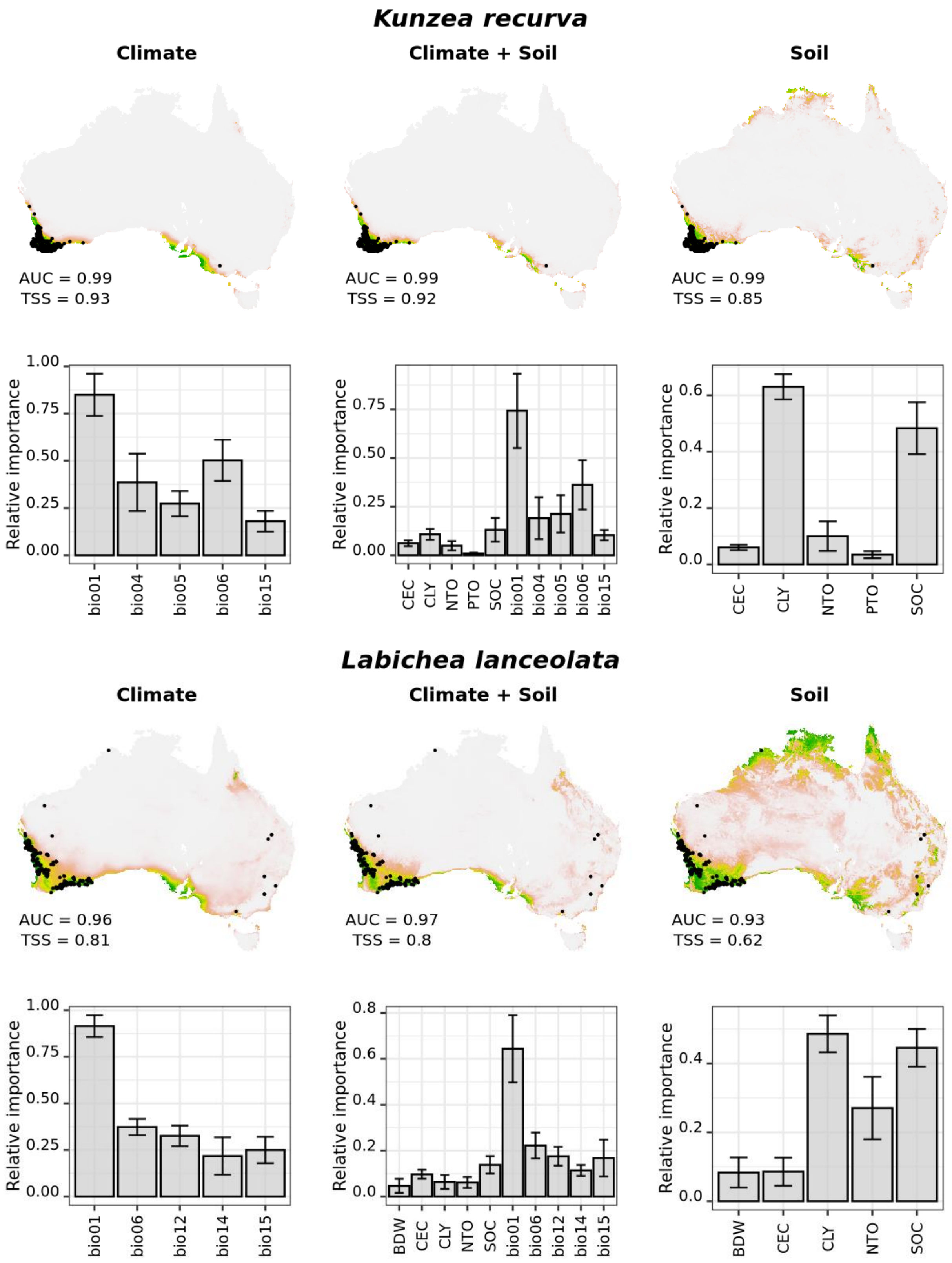

Kunzea recurva
Climate
Climate + Soil
Soil
AUC = 0.99
TSS = 0.93
AUC = 0.99
TSS = 0.92
AUC = 0.99
TSS = 0.85
Relative importance
bio01
bio04
bio05
bio06
bio15
CEC
CLY
NTO
PTO
SOC
Labichea lanceolata
AUC = 0.96
TSS = 0.81
AUC = 0.97
TSS = 0.8
AUC = 0.93
TSS = 0.62
bio12
bio14
BDW

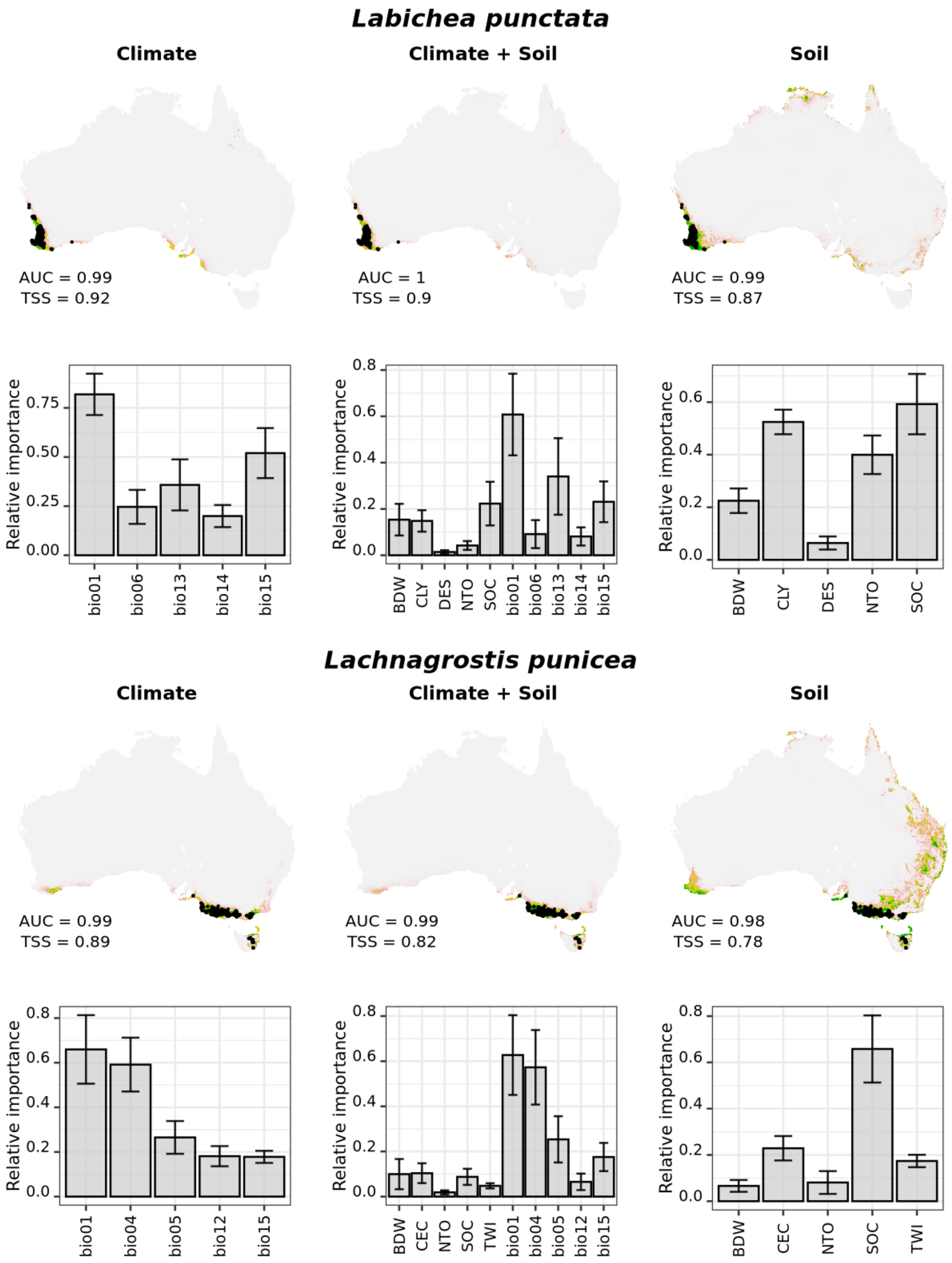
Labichea punctata
Climate
Climate + Soil
Soil
AUC = 0.99
TSS = 0.92
AUC = 1
TSS = 0.9
AUC = 0.99
TSS = 0.87
Relative importance
bio01
bio06
bio13
bio14
bio15
BDW
CLY
DES
NTO
SOC
Lachnagrostis punicea
Climate
Climate + Soil
Soil
AUC = 0.99
TSS = 0.89
AUC = 0.99
TSS = 0.82
AUC = 0.98
TSS = 0.78
Relative importance
bio01
bio04
bio05
bio12
bio15
BDW
CEC
NTO
SOC
TWI

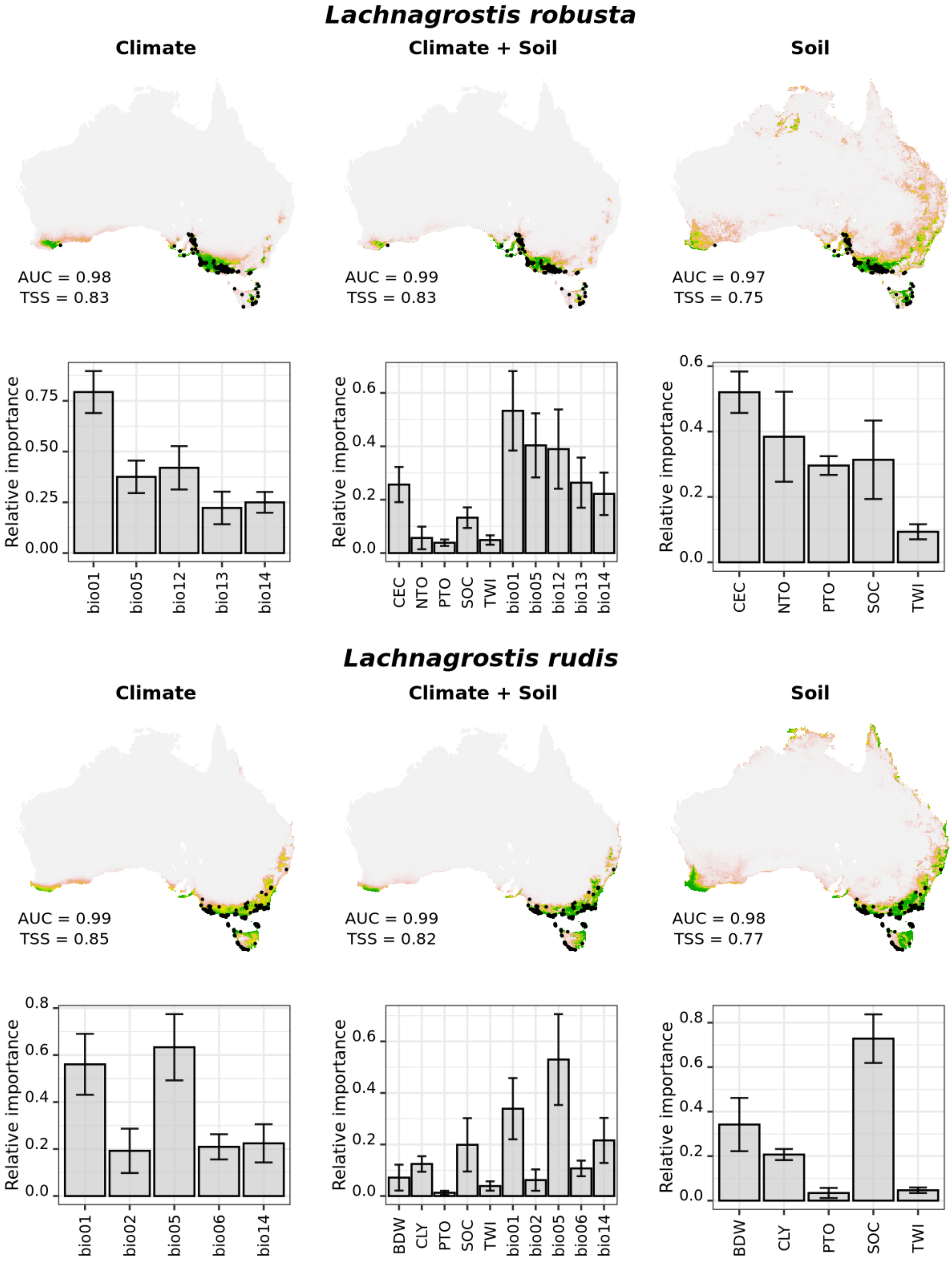

Lachnagrostis robusta
Climate
Climate + Soil
Soil
AUC = 0.98
TSS = 0.83
AUC = 0.99
TSS = 0.83
AUC = 0.97
TSS = 0.75
Relative importance
0.00
0.25
0.50
0.75
bio01
bio05
bio12
bio13
bio14
0.0
0.2
0.4
0.6
CEC
NTO
PTO
SOC
TWI
bio01
bio05
bio12
bio13
bio14
CEC
NTO
PTO
SOC
TWI
Lachnagrostis rudis
Climate
Climate + Soil
Soil
AUC = 0.99
TSS = 0.85
AUC = 0.99
TSS = 0.82
AUC = 0.98
TSS = 0.77
Relative importance
0.0
0.2
0.4
0.6
0.8
bio01
bio02
bio05
bio06
bio14
BDW
CLY
PTO
SOC
TWI
bio01
bio02
bio05
bio06
bio14
BDW
CLY
PTO
SOC
TWI

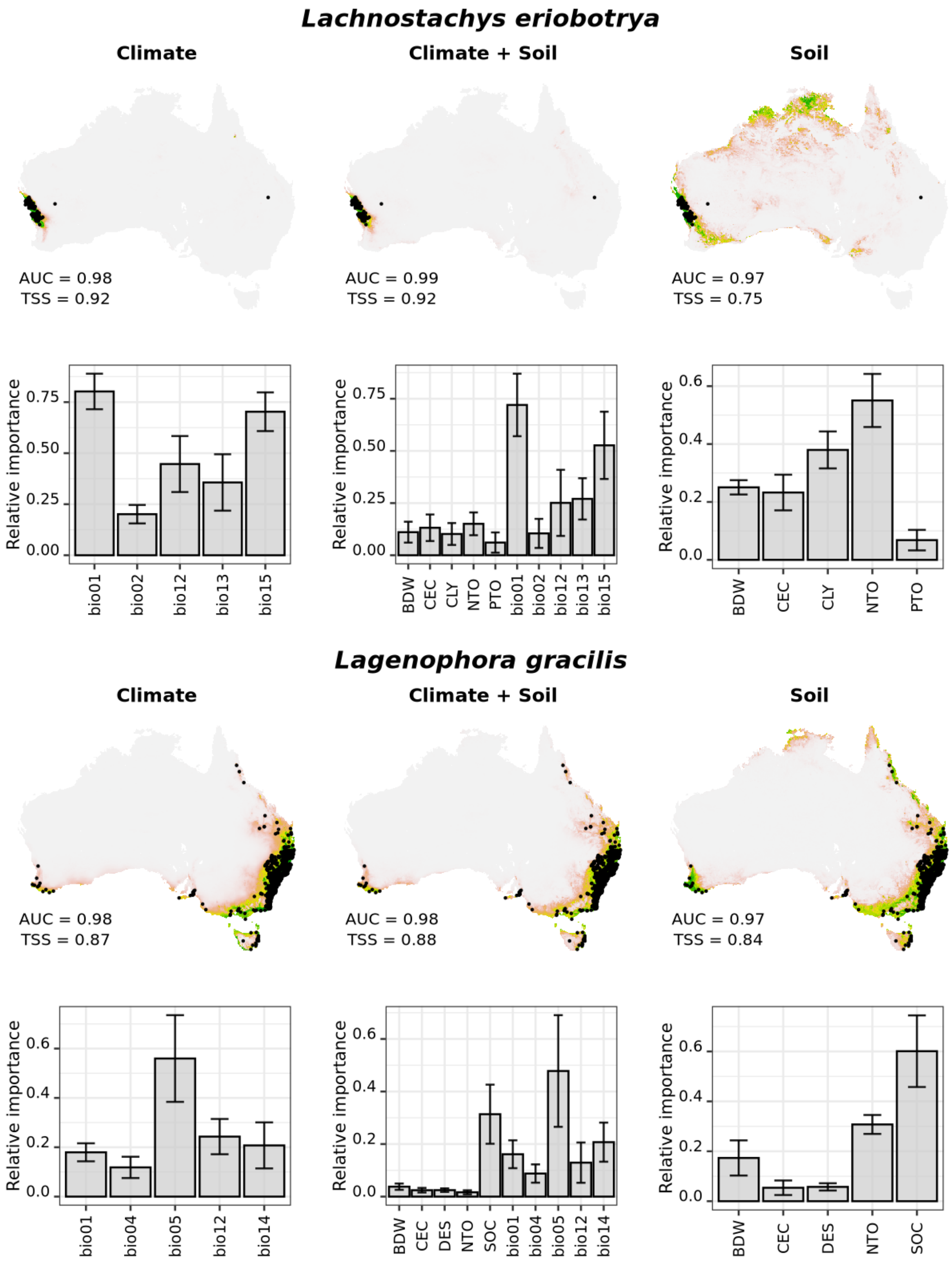
Lachnostachys eriobotrya
Climate
Climate + Soil
Soil
AUC = 0.98
TSS = 0.92
AUC = 0.99
TSS = 0.92
AUC = 0.97
TSS = 0.75
Relative importance
0.00
0.25
0.50
0.75
bio01
bio02
bio12
bio13
bio15
BDW
CEC
CLY
NTO
PTO
0.0
0.2
0.4
0.6
Lagenophora gracilis
Climate
Climate + Soil
Soil
AUC = 0.98
TSS = 0.87
AUC = 0.98
TSS = 0.88
AUC = 0.97
TSS = 0.84
Relative importance
bio01
bio04
bio05
bio12
bio14
BDW
CEC
DES
NTO
SOC

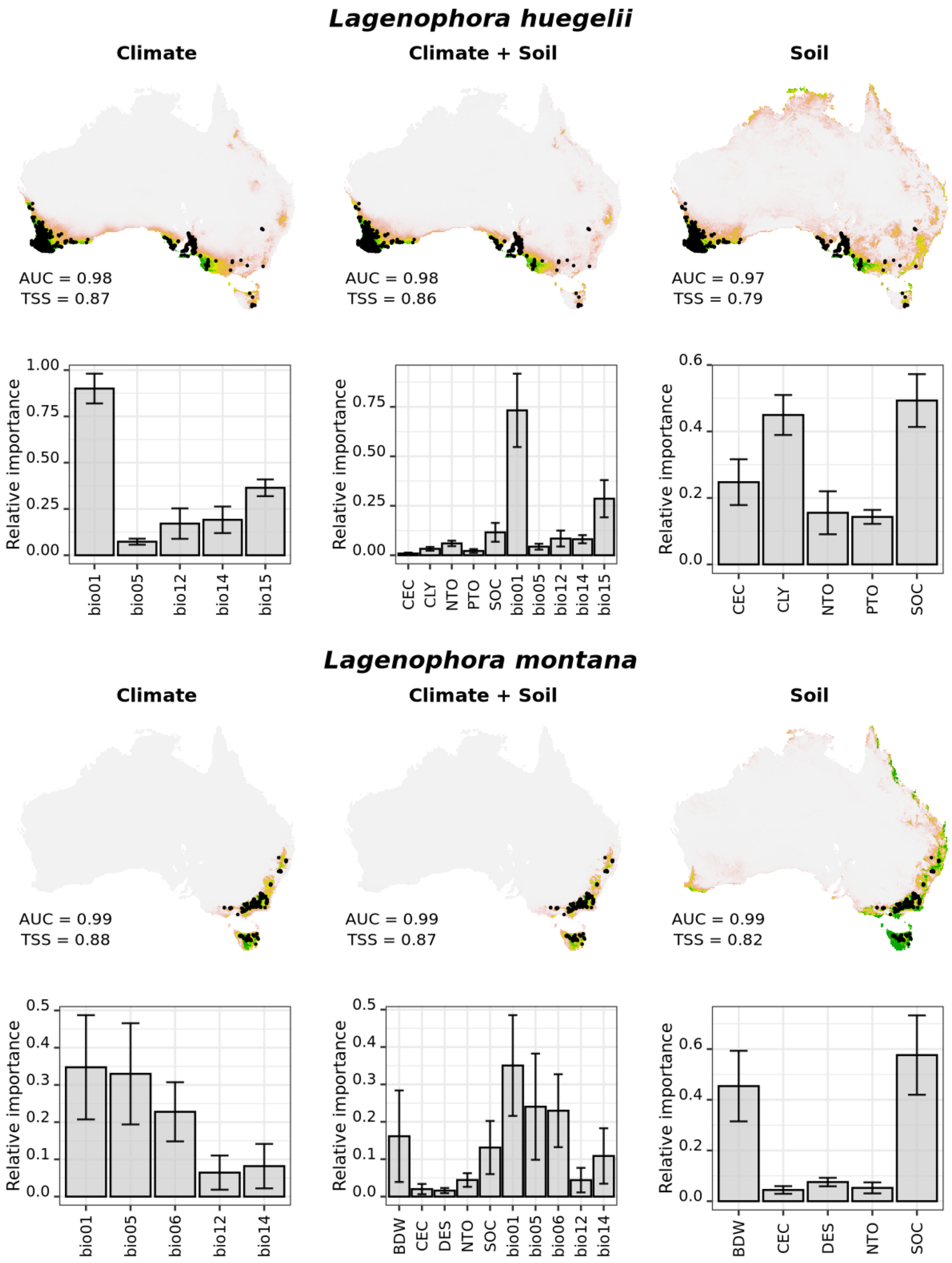

Lagenophora huegelii
Climate
Climate + Soil
Soil
AUC = 0.98
TSS = 0.87
AUC = 0.98
TSS = 0.86
AUC = 0.97
TSS = 0.79
Relative importance
bio01
bio05
bio12
bio14
bio15
CEC
CLY
NTO
PTO
SOC
Lagenophora montana
AUC = 0.99
TSS = 0.88
AUC = 0.99
TSS = 0.87
AUC = 0.99
TSS = 0.82
bio06
BDW
DES

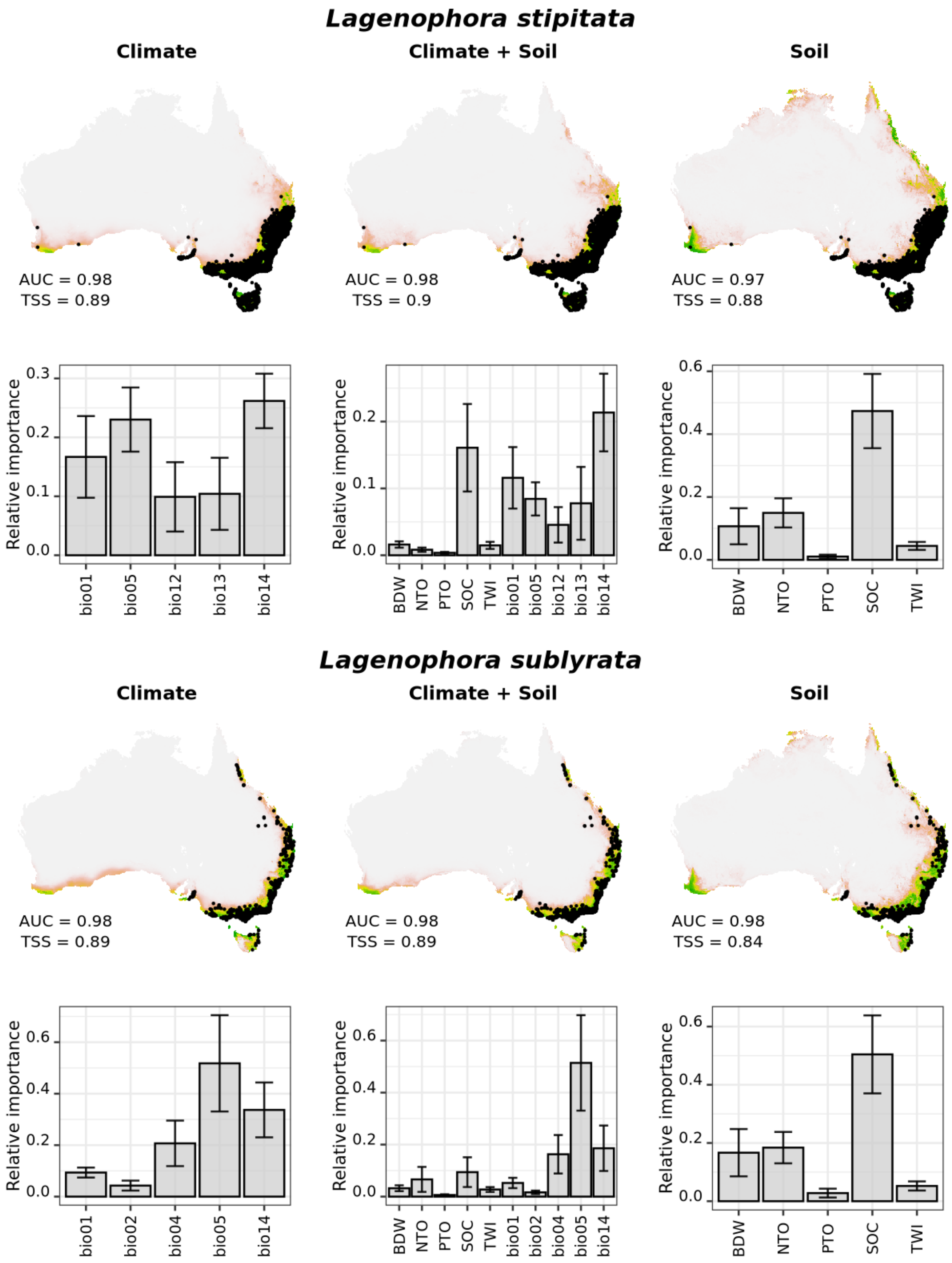
Lagenophora stipitata
Climate
Climate + Soil
Soil
AUC = 0.98
TSS = 0.89
AUC = 0.98
TSS = 0.9
AUC = 0.97
TSS = 0.88
Relative importance
bio01
bio05
bio12
bio13
bio14
BDW
NTO
PTO
SOC
TWI
Lagenophora sublyrata
Climate
Climate + Soil
Soil
AUC = 0.98
TSS = 0.89
AUC = 0.98
TSS = 0.89
AUC = 0.98
TSS = 0.84
Relative importance
bio01
bio02
bio04
bio05
bio14
BDW
NTO
PTO
SOC
TWI

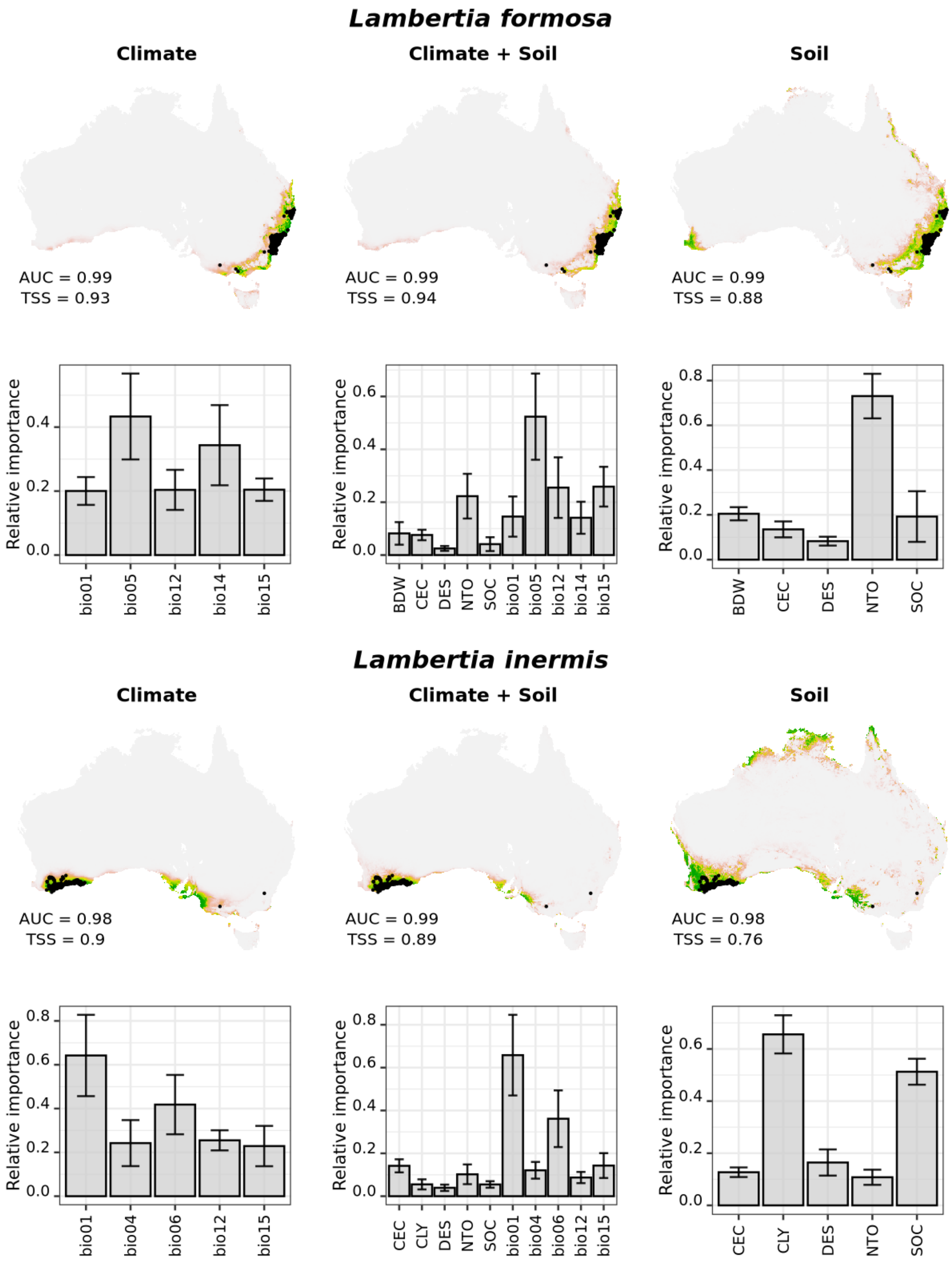
Lambertia formosa
Climate
Climate + Soil
Soil
AUC = 0.99
TSS = 0.93
AUC = 0.99
TSS = 0.94
AUC = 0.99
TSS = 0.88
Relative importance
bio01
bio05
bio12
bio14
bio15
BDW
CEC
DES
NTO
SOC
bio01
bio05
bio12
bio14
bio15
BDW
CEC
DES
NTO
SOC
Lambertia inermis
Climate
Climate + Soil
Soil
AUC = 0.98
TSS = 0.9
AUC = 0.99
TSS = 0.89
AUC = 0.98
TSS = 0.76
Relative importance
bio01
bio04
bio06
bio12
bio15
CEC
CLY
DES
NTO
SOC
bio01
bio04
bio06
bio12
bio15
CEC
CLY
DES
NTO
SOC

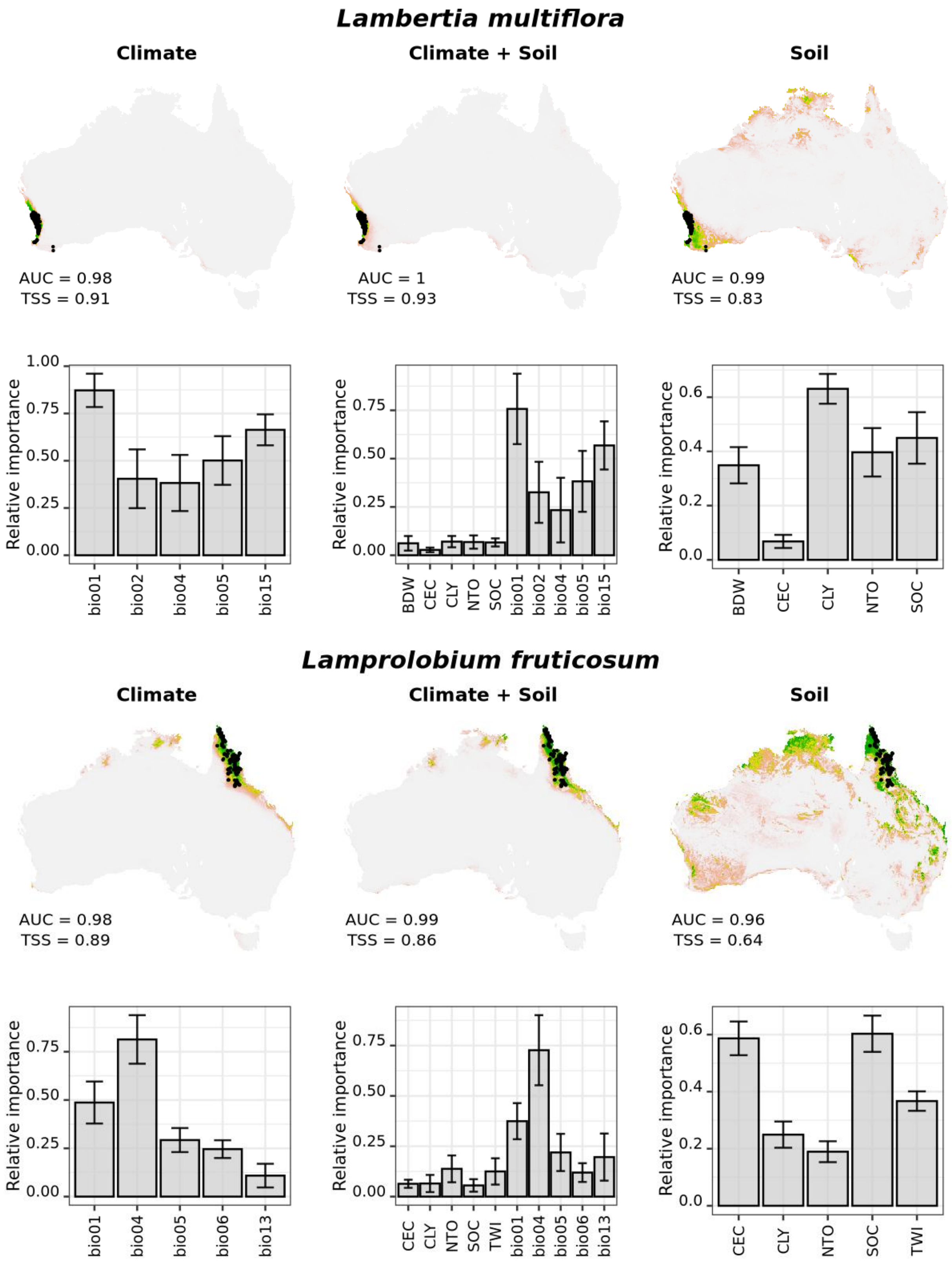

Lambertia multiflora
Climate
Climate + Soil
Soil
AUC = 0.98
TSS = 0.91
AUC = 1
TSS = 0.93
AUC = 0.99
TSS = 0.83
Relative importance
bio01
bio02
bio04
bio05
bio15
BDW
CEC
CLY
NTO
SOC
Lamprolobium fruticosum
Climate
Climate + Soil
Soil
AUC = 0.98
TSS = 0.89
AUC = 0.99
TSS = 0.86
AUC = 0.96
TSS = 0.64
Relative importance
bio01
bio04
bio05
bio06
bio13
CEC
CLY
NTO
SOC
TWI

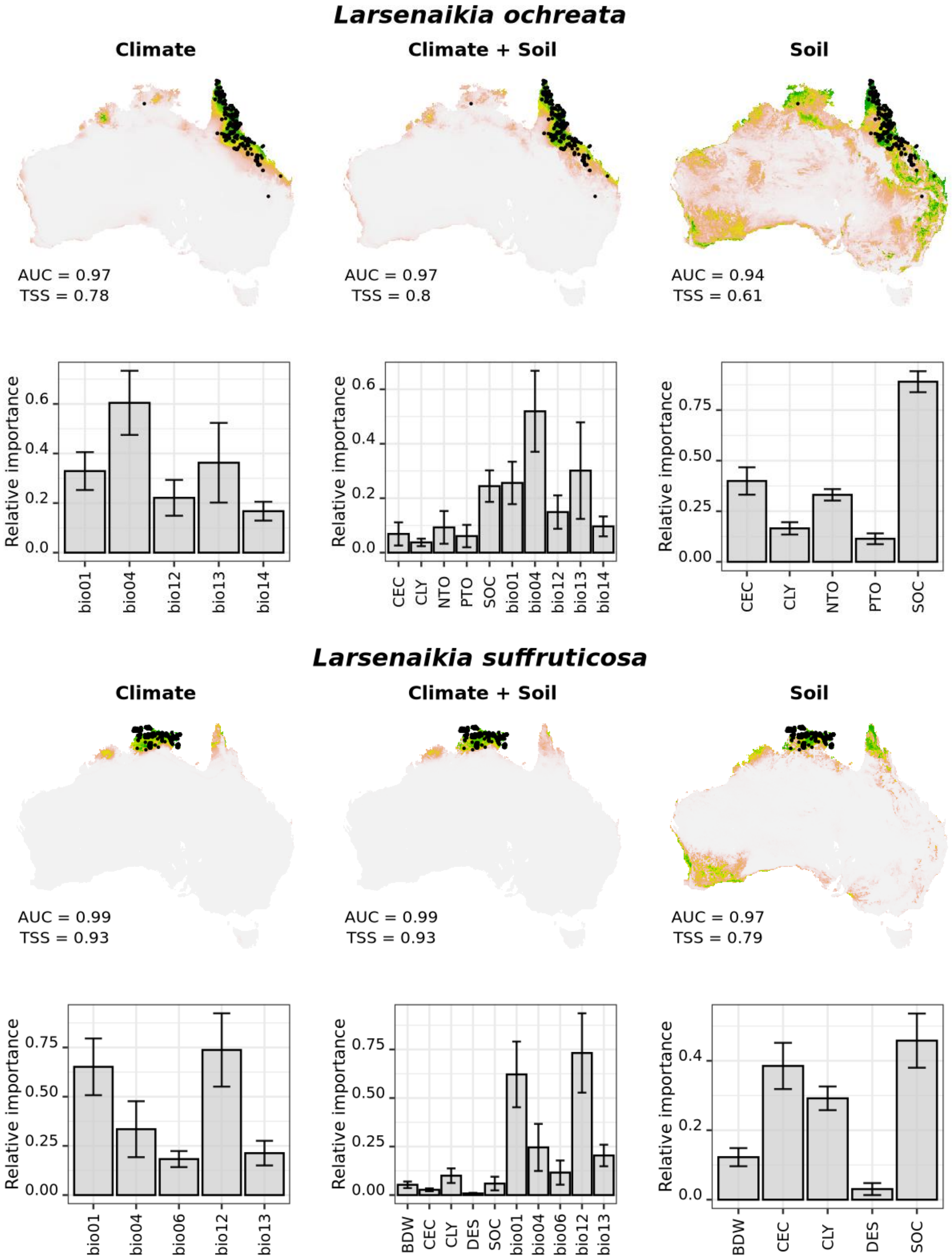

Larsenaikia ochreata
Climate
Climate + Soil
Soil
AUC = 0.97
TSS = 0.78
AUC = 0.97
TSS = 0.8
AUC = 0.94
TSS = 0.61
Relative importance
bio01
bio04
bio12
bio13
bio14
CEC
CLY
NTO
PTO
SOC
Larsenaikia suffruticosa
AUC = 0.99
TSS = 0.93
AUC = 0.99
TSS = 0.93
AUC = 0.97
TSS = 0.79
bio06
BDW
DES

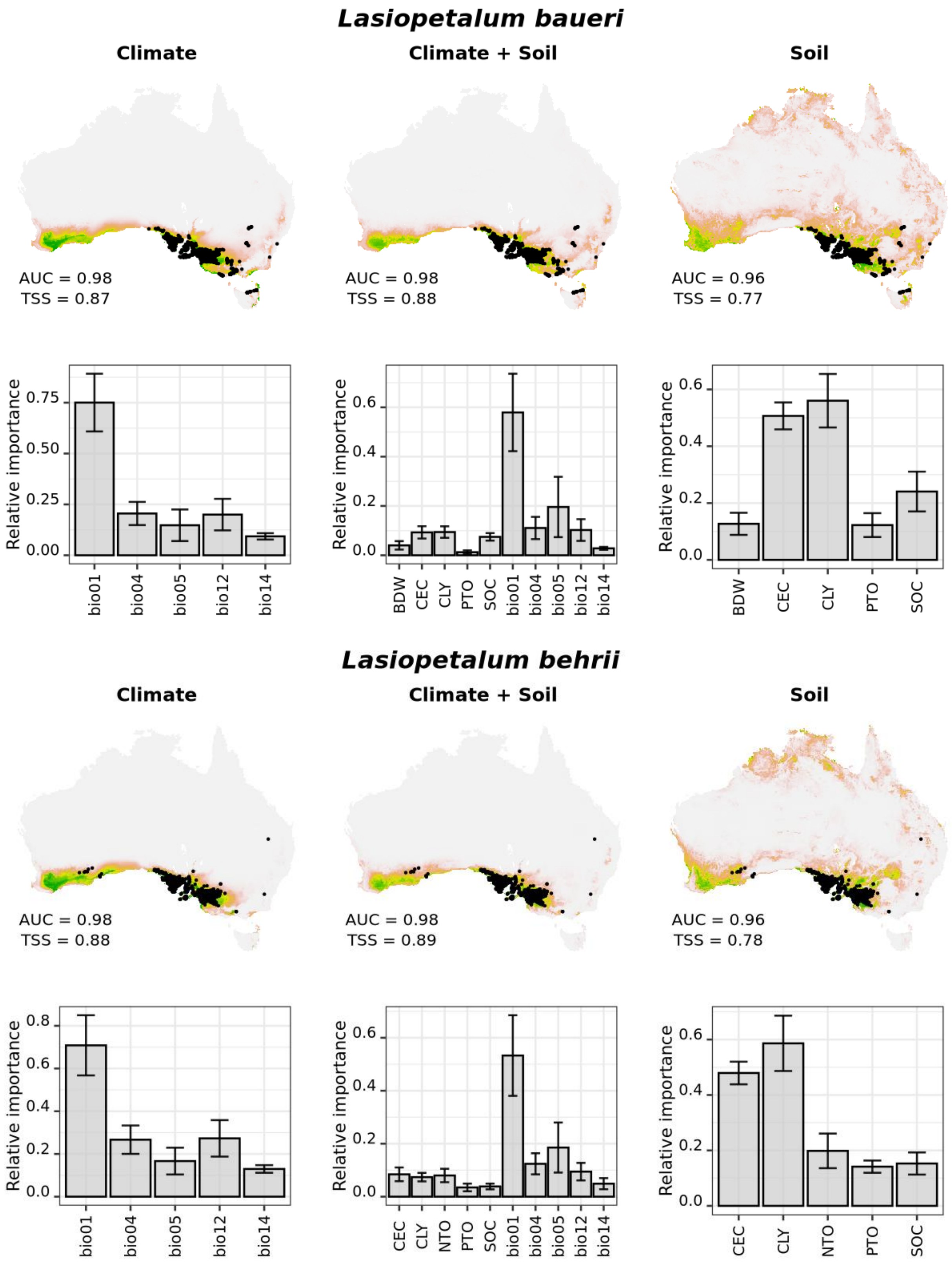
Lasiopetalum baueri
Climate
Climate + Soil
Soil
AUC = 0.98
TSS = 0.87
AUC = 0.98
TSS = 0.88
AUC = 0.96
TSS = 0.77
Relative importance
0.00
0.25
0.50
0.75
bio01
bio04
bio05
bio12
bio14
0.0
0.2
0.4
0.6
BDW
CEC
CLY
PTO
SOC
bio01
bio04
bio05
bio12
bio14
BDW
CEC
CLY
PTO
SOC
Lasiopetalum behrii
Climate
Climate + Soil
Soil
AUC = 0.98
TSS = 0.88
AUC = 0.98
TSS = 0.89
AUC = 0.96
TSS = 0.78
Relative importance
0.0
0.2
0.4
0.6
0.8
bio01
bio04
bio05
bio12
bio14
CEC
CLY
NTO
PTO
SOC
bio01
bio04
bio05
bio12
bio14
CEC
CLY
NTO
PTO
SOC

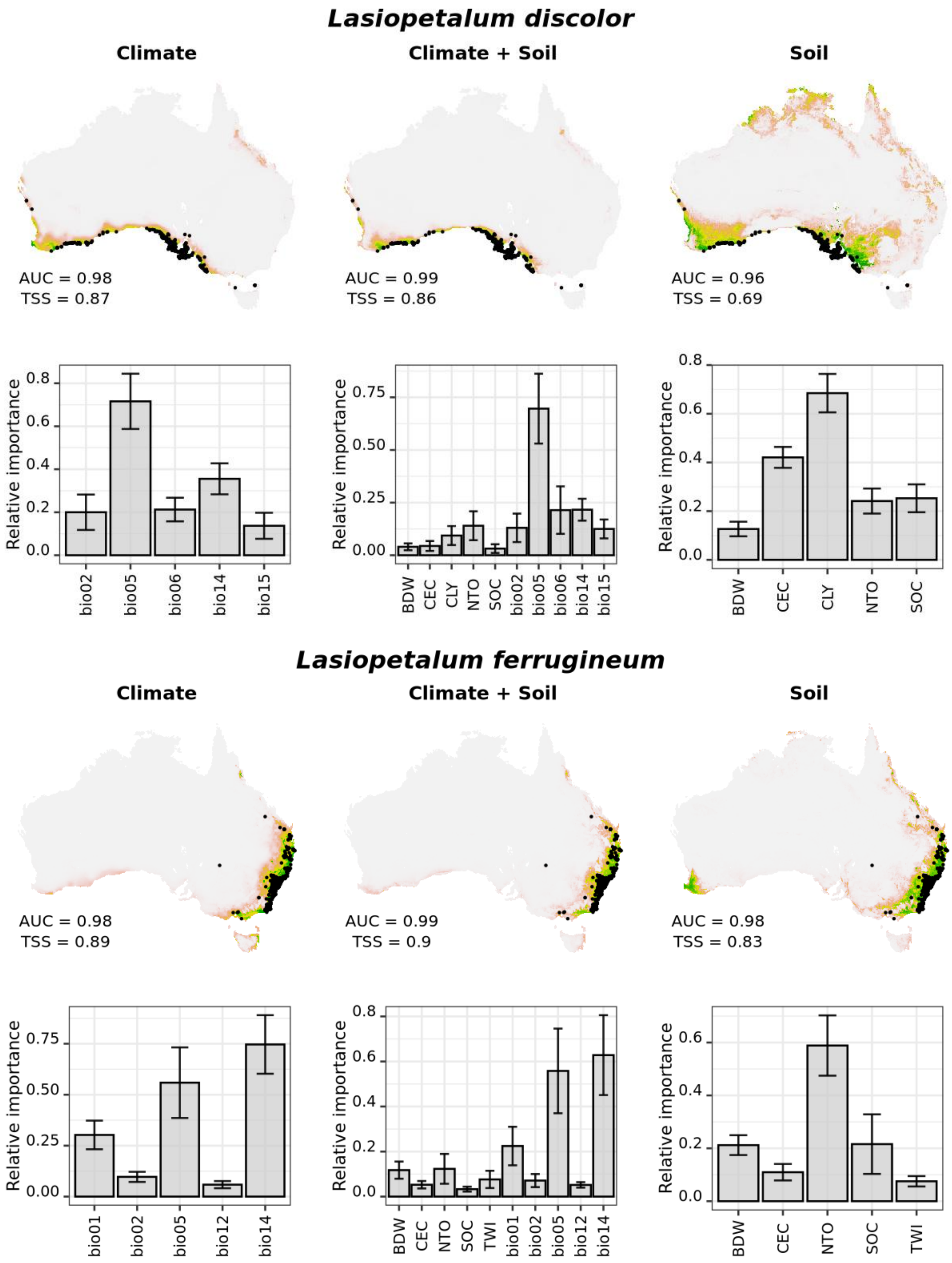

Lasiopetalum discolor
Climate
Climate + Soil
Soil
AUC = 0.98
TSS = 0.87
AUC = 0.99
TSS = 0.86
AUC = 0.96
TSS = 0.69
Relative importance
bio02
bio05
bio06
bio14
bio15
BDW
CEC
CLY
NTO
SOC
Lasiopetalum ferrugineum
AUC = 0.98
TSS = 0.89
AUC = 0.99
TSS = 0.9
AUC = 0.98
TSS = 0.83
bio01
bio12
TWI

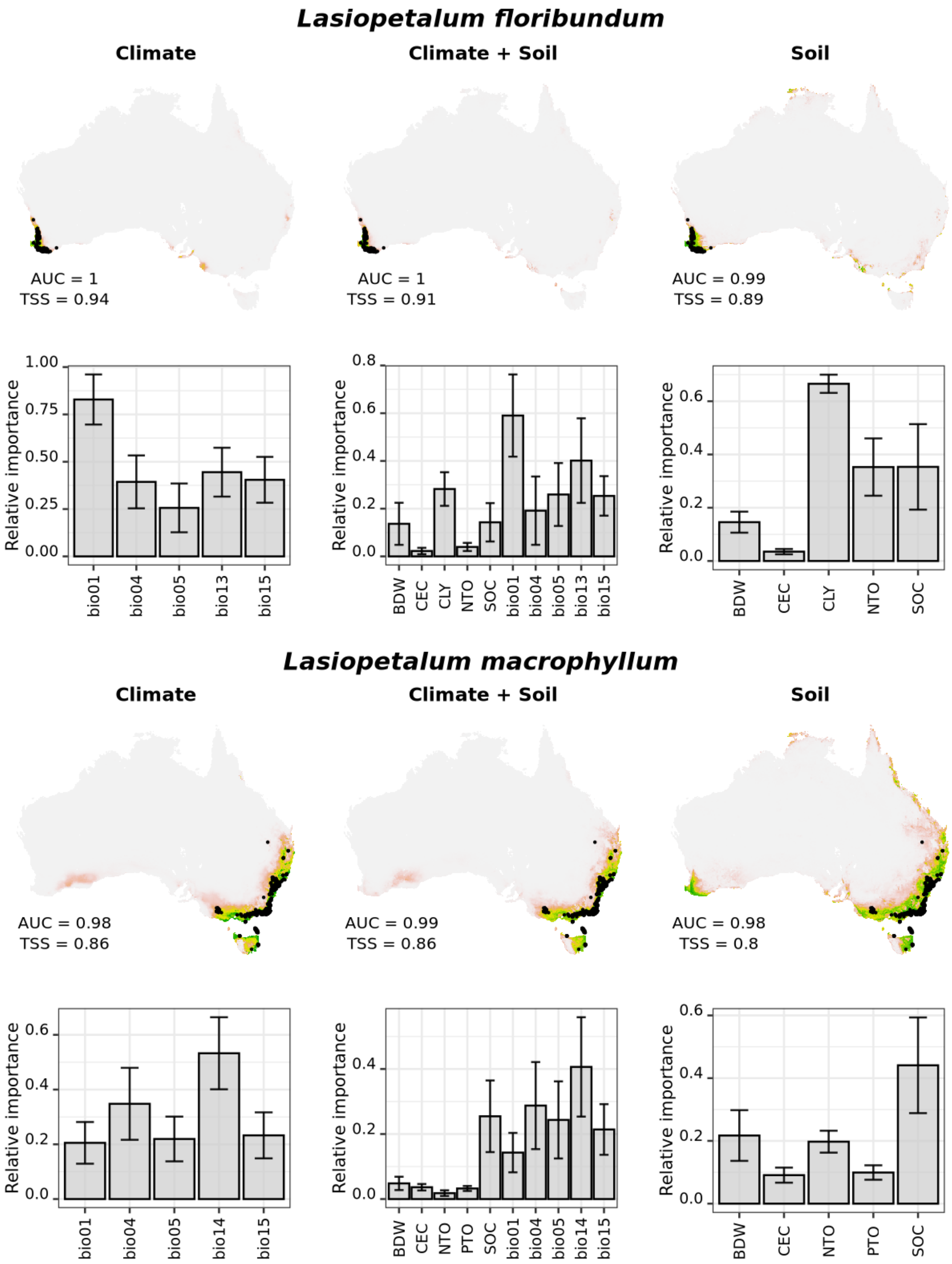
Lasiopetalum floribundum
Climate
Climate + Soil
Soil
AUC = 1
TSS = 0.94
AUC = 1
TSS = 0.91
AUC = 0.99
TSS = 0.89
Relative importance
1.00
0.75
0.50
0.25
0.00
bio01
bio04
bio05
bio13
bio15
0.8
0.6
0.4
0.2
0.0
BDW
CEC
CLY
NTO
SOC
bio01
bio04
bio05
bio13
bio15
0.6
0.4
0.2
0.0
BDW
CEC
CLY
NTO
SOC
Lasiopetalum macrophyllum
Climate
Climate + Soil
Soil
AUC = 0.98
TSS = 0.86
AUC = 0.99
TSS = 0.86
AUC = 0.98
TSS = 0.8
Relative importance
0.6
0.4
0.2
0.0
bio01
bio04
bio05
bio14
bio15
0.4
0.2
0.0
BDW
CEC
NTO
PTO
SOC
bio01
bio04
bio05
bio14
bio15
0.6
0.4
0.2
0.0
BDW
CEC
NTO
PTO
SOC

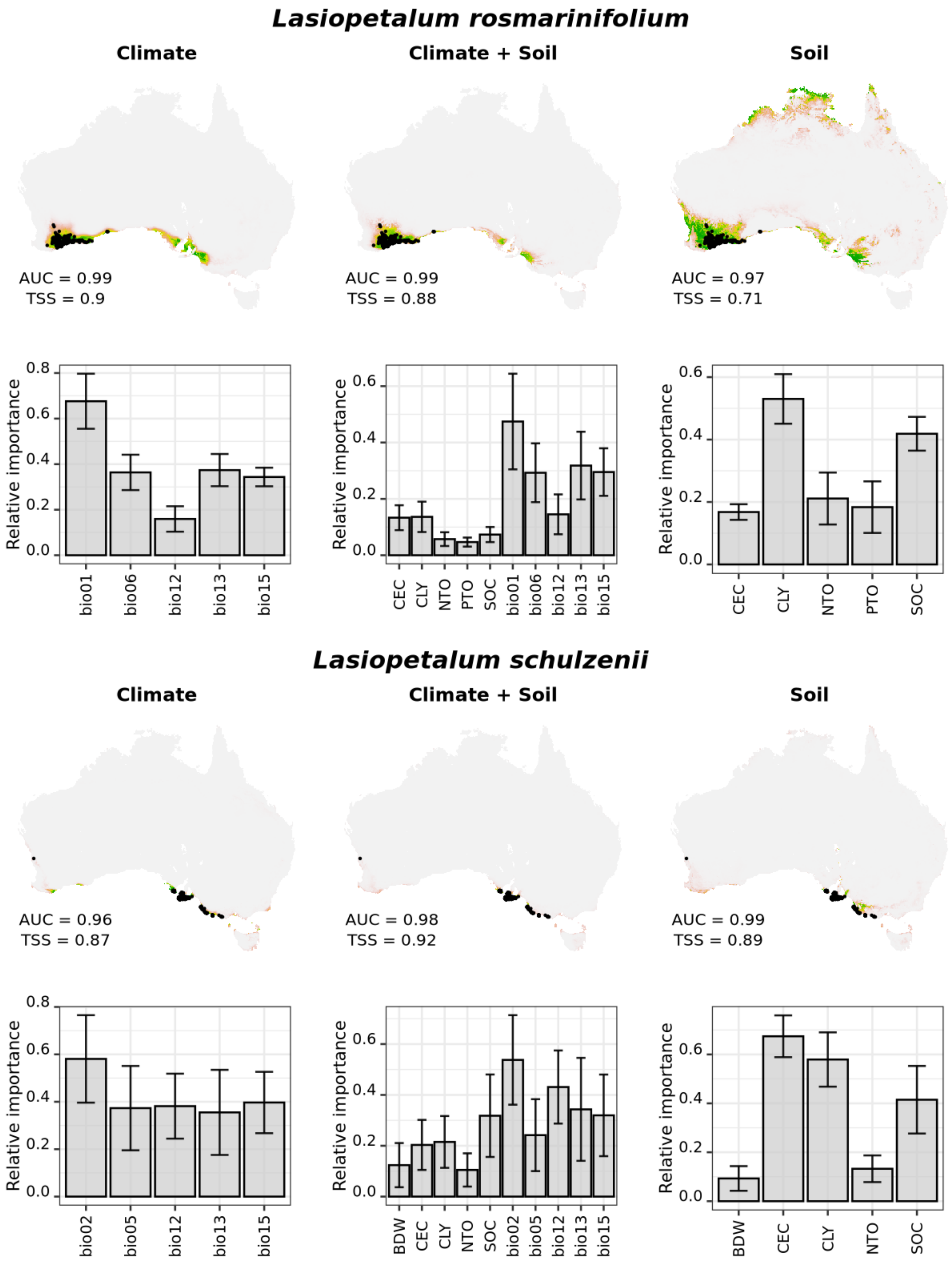

Lasiopetalum rosmarinifolium
Climate
Climate + Soil
Soil
AUC = 0.99
TSS = 0.9
AUC = 0.99
TSS = 0.88
AUC = 0.97
TSS = 0.71
Relative importance
bio01
bio06
bio12
bio13
bio15
CEC
CLY
NTO
PTO
SOC
Lasiopetalum schulzenii
AUC = 0.96
TSS = 0.87
AUC = 0.98
TSS = 0.92
AUC = 0.99
TSS = 0.89
bio02
bio05
BDW

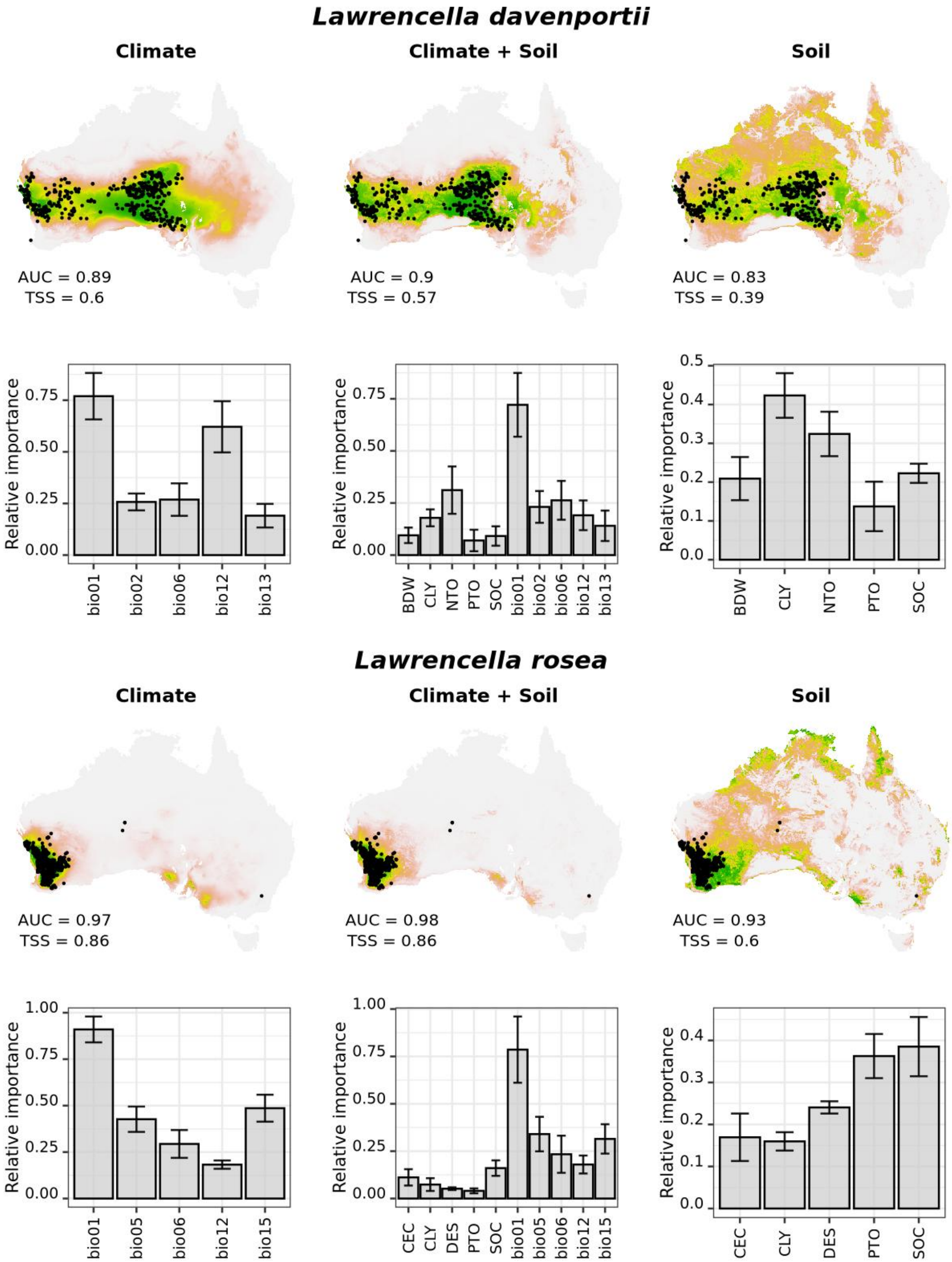

Lawrencella davenportii
Climate
Climate + Soil
Soil
AUC = 0.89
TSS = 0.6
AUC = 0.9
TSS = 0.57
AUC = 0.83
TSS = 0.39
Relative importance
0.00
0.25
0.50
0.75
bio01
bio02
bio06
bio12
bio13
BDW
CLY
NTO
PTO
SOC
bio01
bio02
bio06
bio12
bio13
0.0
0.1
0.2
0.3
0.4
0.5
BDW
CLY
NTO
PTO
SOC
Lawrencella rosea
Climate
Climate + Soil
Soil
AUC = 0.97
TSS = 0.86
AUC = 0.98
TSS = 0.86
AUC = 0.93
TSS = 0.6
Relative importance
0.00
0.25
0.50
0.75
1.00
bio01
bio05
bio06
bio12
bio15
CEC
CLY
DES
PTO
SOC
bio01
bio05
bio06
bio12
bio15
0.0
0.1
0.2
0.3
0.4
CEC
CLY
DES
PTO
SOC

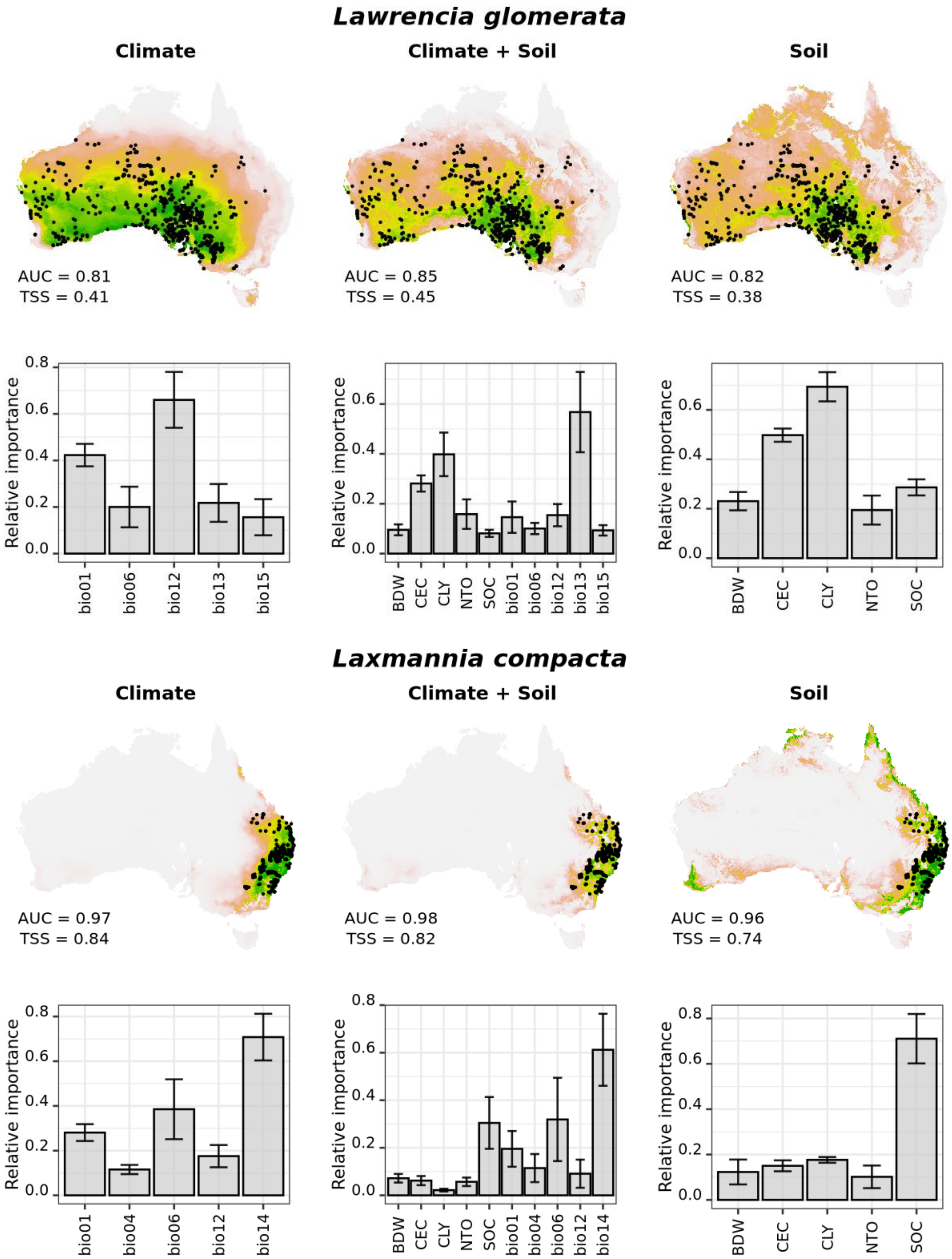

Lawrencia glomerata
Climate
Climate + Soil
Soil
AUC = 0.81
TSS = 0.41
AUC = 0.85
TSS = 0.45
AUC = 0.82
TSS = 0.38
Relative importance
bio01
bio06
bio12
bio13
bio15
BDW
CEC
CLY
NTO
SOC
Laxmannia compacta
Climate
Climate + Soil
Soil
AUC = 0.97
TSS = 0.84
AUC = 0.98
TSS = 0.82
AUC = 0.96
TSS = 0.74
Relative importance
bio01
bio04
bio06
bio12
bio14
BDW
CEC
CLY
NTO
SOC

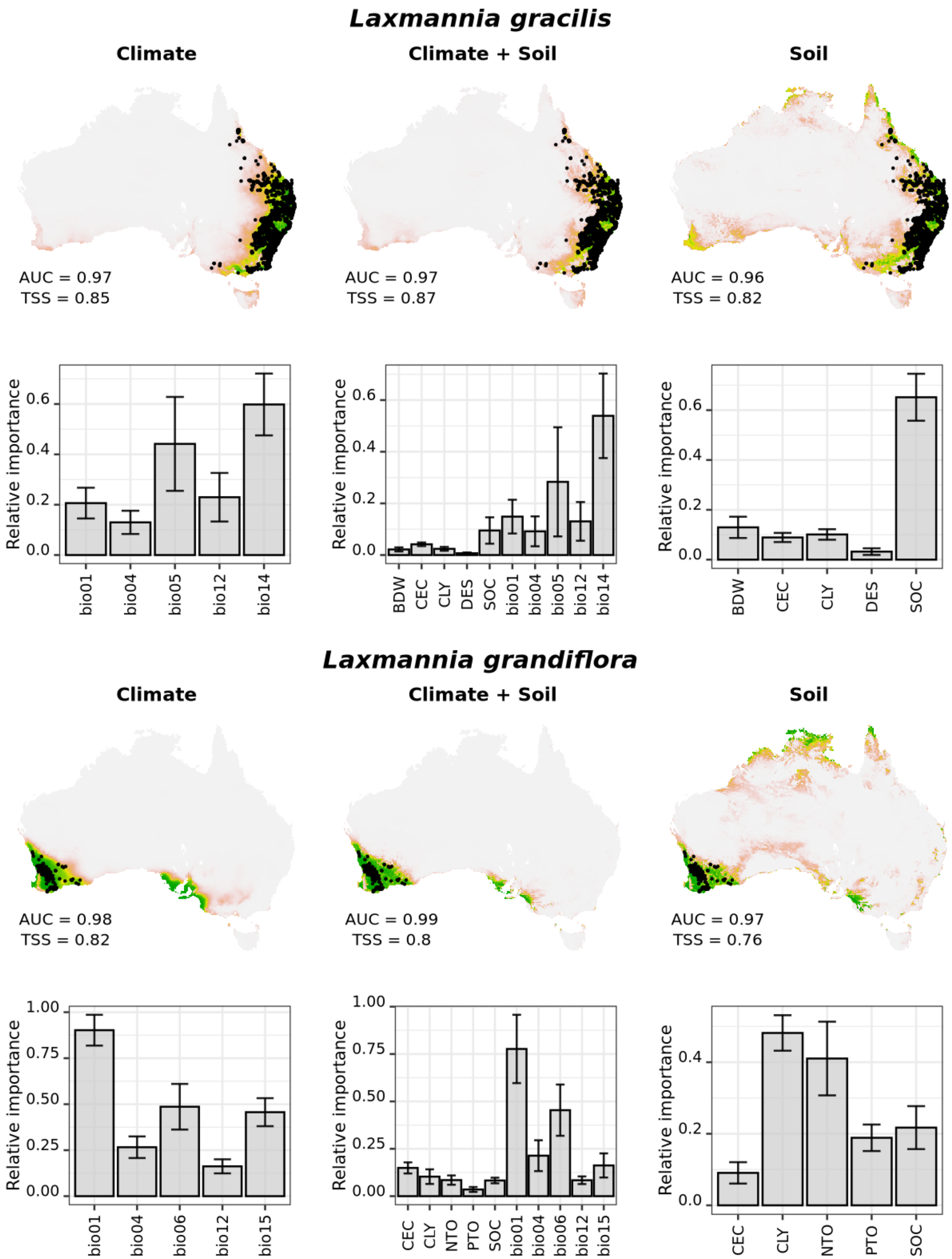
Laxmannia gracilis
Climate
Climate + Soil
Soil
AUC = 0.97
TSS = 0.85
AUC = 0.97
TSS = 0.87
AUC = 0.96
TSS = 0.82
Relative importance
bio01
bio04
bio05
bio12
bio14
BDW
CEC
CLY
DES
SOC
Laxmannia grandiflora
AUC = 0.98
TSS = 0.82
AUC = 0.99
TSS = 0.8
AUC = 0.97
TSS = 0.76
bio06
bio15
NTO
PTO

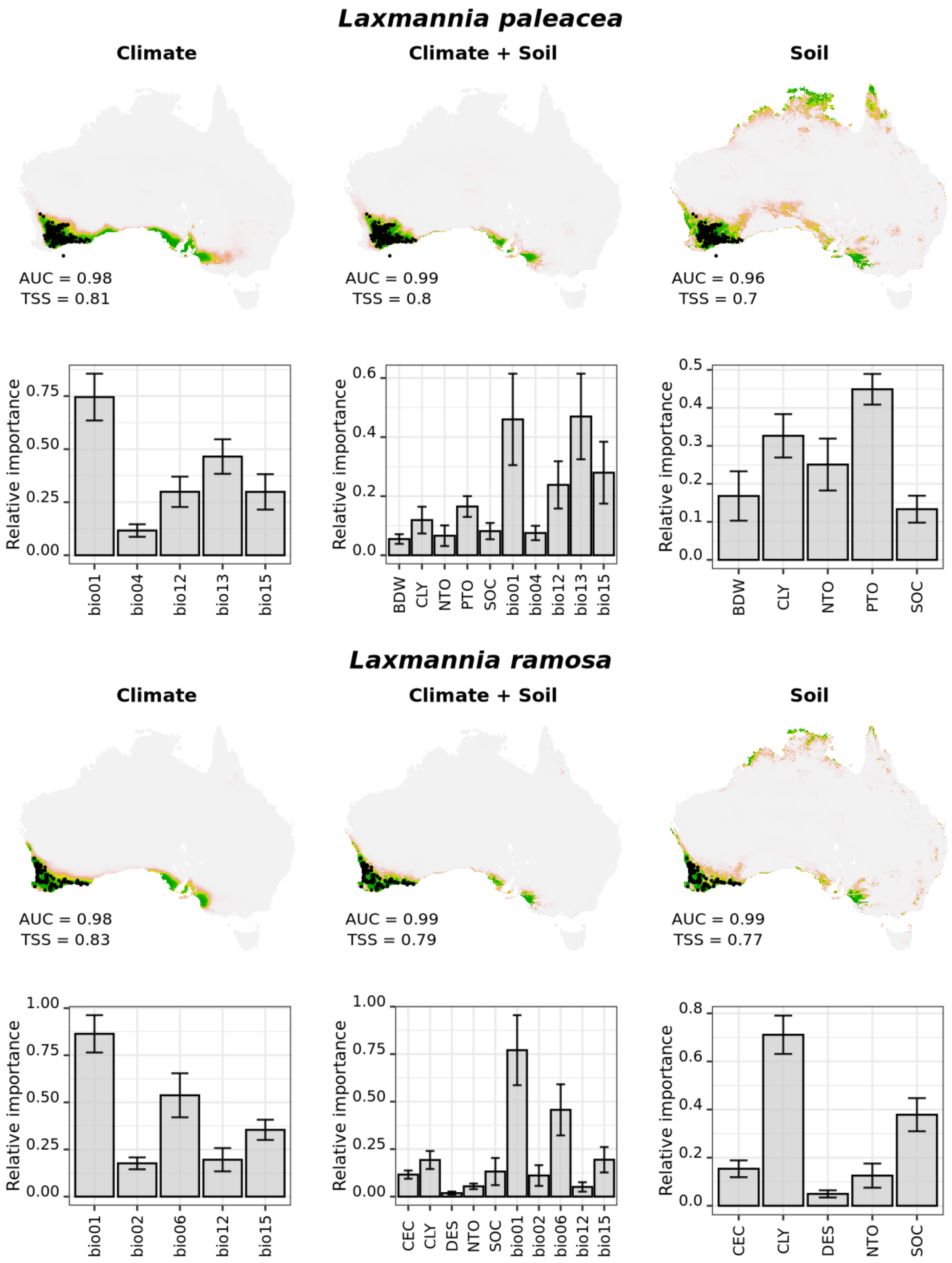

Laxmannia paleacea
Climate
Climate + Soil
Soil
AUC = 0.98
TSS = 0.81
AUC = 0.99
TSS = 0.8
AUC = 0.96
TSS = 0.7
Relative importance
bio01
bio04
bio12
bio13
bio15
BDW
CLY
NTO
PTO
SOC
Laxmannia ramosa
Climate
Climate + Soil
Soil
AUC = 0.98
TSS = 0.83
AUC = 0.99
TSS = 0.79
AUC = 0.99
TSS = 0.77
Relative importance
bio01
bio02
bio06
bio12
bio15
CEC
CLY
DES
NTO
SOC

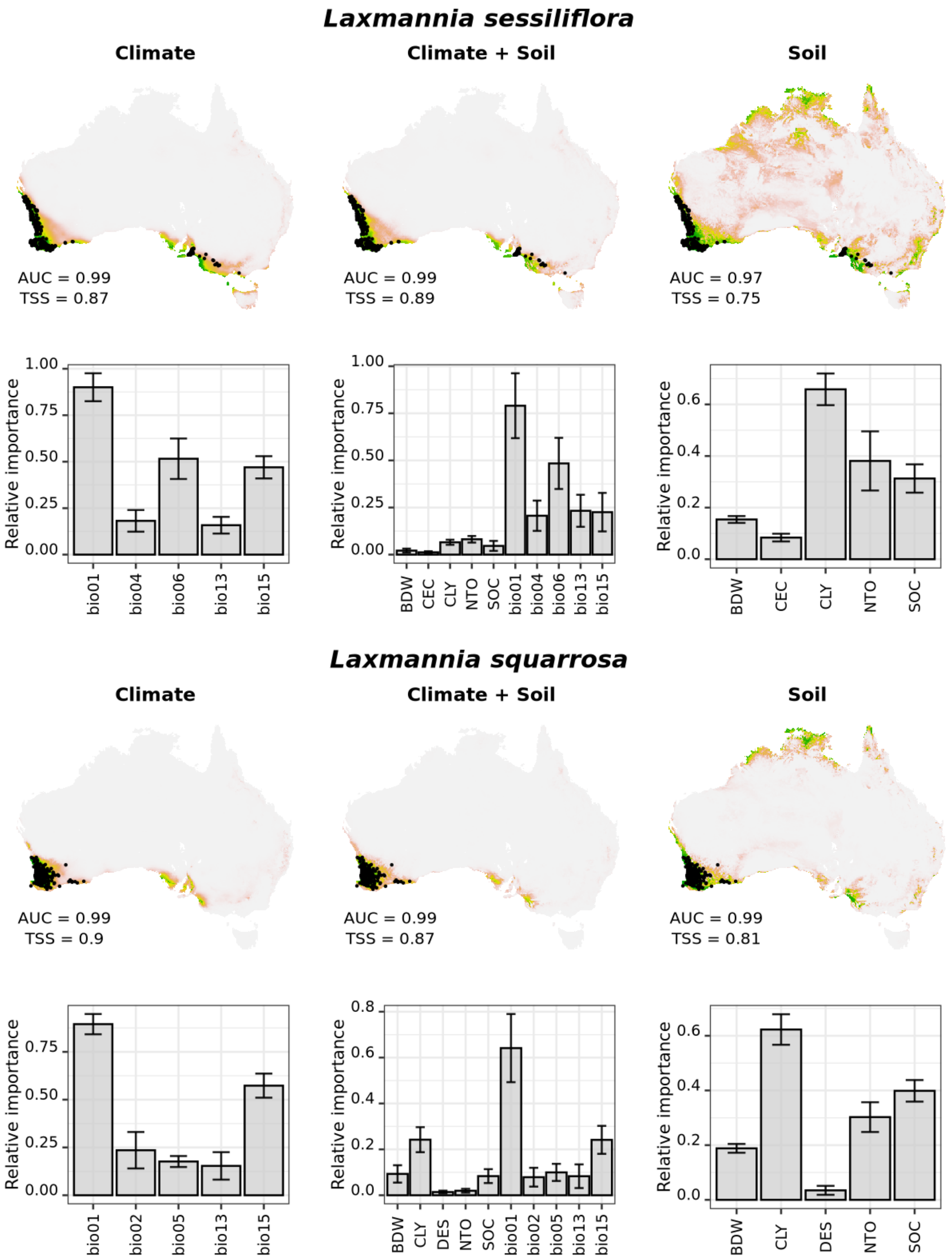

Laxmannia sessiliflora
Climate
Climate + Soil
Soil
AUC = 0.99
TSS = 0.87
AUC = 0.99
TSS = 0.89
AUC = 0.97
TSS = 0.75
Relative importance
0.00
0.25
0.50
0.75
1.00
bio01
bio04
bio06
bio13
bio15
BDW
CEC
CLY
NTO
SOC
0.0
0.2
0.4
0.6
Laxmannia squarrosa
Climate
Climate + Soil
Soil
AUC = 0.99
TSS = 0.9
AUC = 0.99
TSS = 0.87
AUC = 0.99
TSS = 0.81
Relative importance
0.00
0.25
0.50
0.75
bio01
bio02
bio05
bio13
bio15
0.0
0.2
0.4
0.6
0.8
BDW
CLY
DES
NTO
SOC

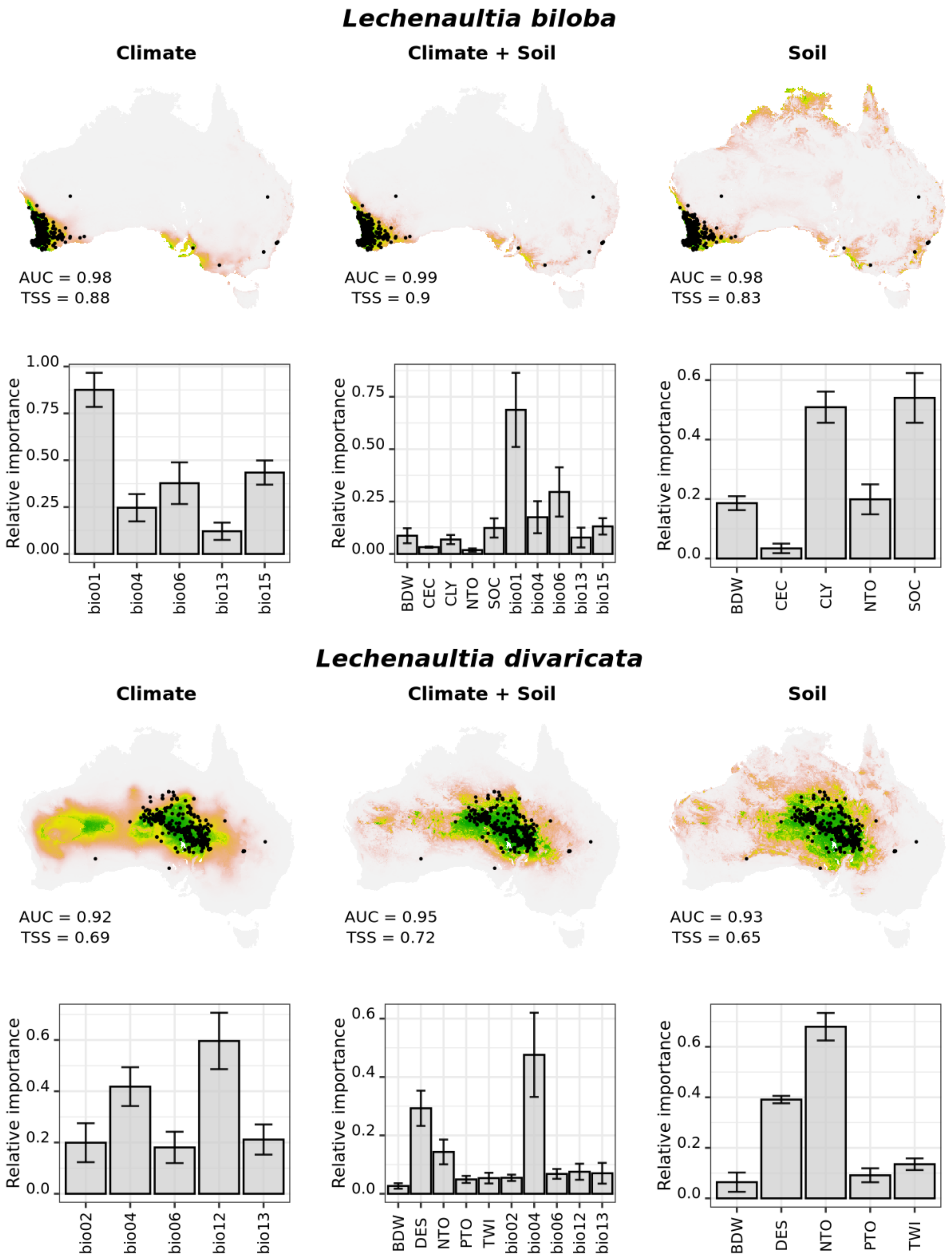
Lechenaultia biloba
Climate
Climate + Soil
Soil
AUC = 0.98
TSS = 0.88
AUC = 0.99
TSS = 0.9
AUC = 0.98
TSS = 0.83
Relative importance
1.00
0.75
0.50
0.25
0.00
bio01
bio04
bio06
bio13
bio15
BDW
CEC
CLY
NTO
SOC
0.6
0.4
0.2
0.0
Lechenaultia divaricata
AUC = 0.92
TSS = 0.69
AUC = 0.95
TSS = 0.72
AUC = 0.93
TSS = 0.65
bio02
bio12
DES
PTO
TWI

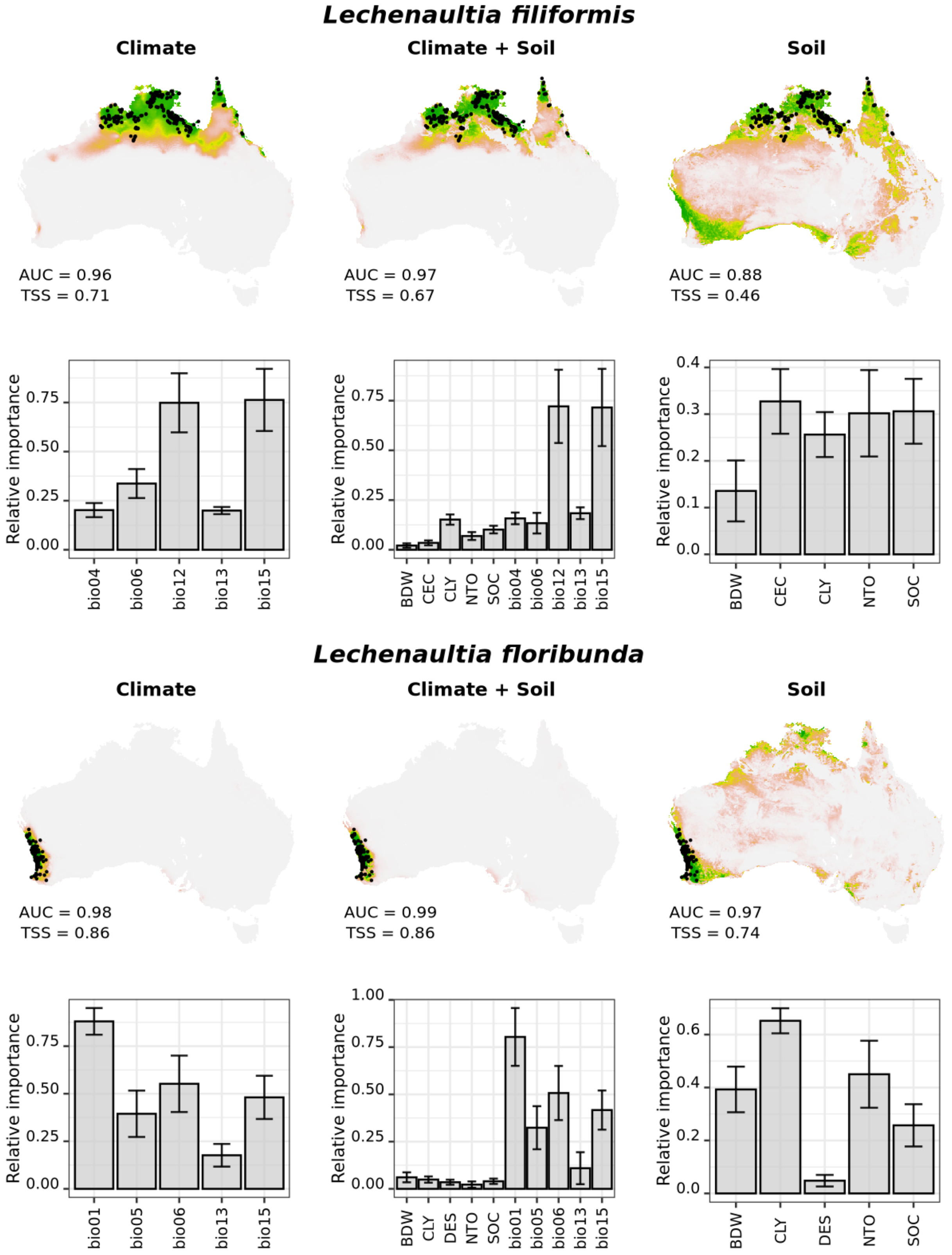

Lechenaultia filiformis
Climate
Climate + Soil
Soil
AUC = 0.96
TSS = 0.71
AUC = 0.97
TSS = 0.67
AUC = 0.88
TSS = 0.46
Relative importance
0.00
0.25
0.50
0.75
bio04
bio06
bio12
bio13
bio15
BDW
CEC
CLY
NTO
SOC
0.0
0.1
0.2
0.3
0.4
Lechenaultia floribunda
Climate
Climate + Soil
Soil
AUC = 0.98
TSS = 0.86
AUC = 0.99
TSS = 0.86
AUC = 0.97
TSS = 0.74
Relative importance
1.00
bio01
bio05
bio06
bio13
bio15
BDW
CLY
DES
NTO
SOC
0.6

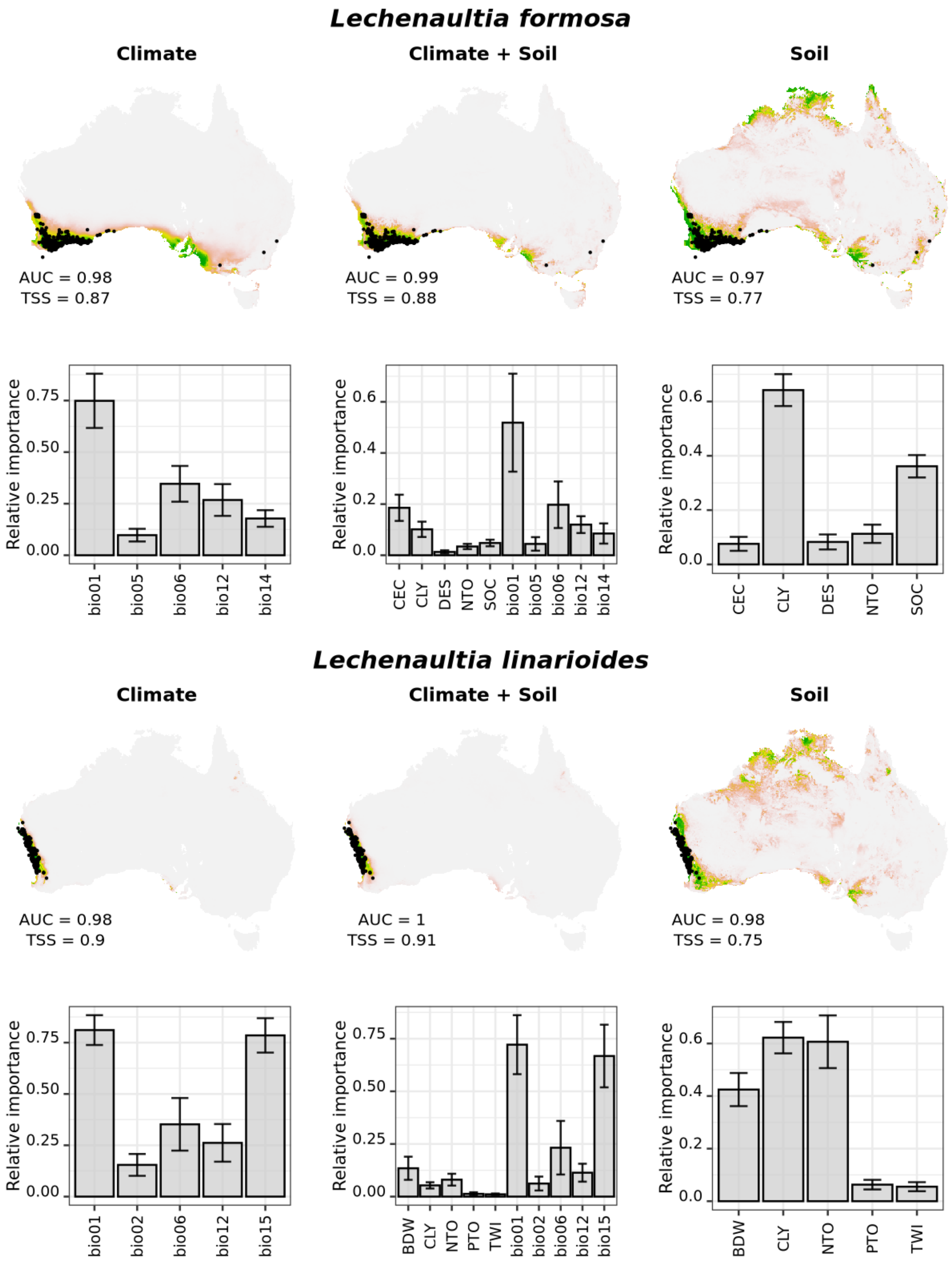
Lechenaultia formosa
Climate
Climate + Soil
Soil
AUC = 0.98
TSS = 0.87
AUC = 0.99
TSS = 0.88
AUC = 0.97
TSS = 0.77
Relative importance
bio01
bio05
bio06
bio12
bio14
CEC
CLY
DES
NTO
SOC
Lechenaultia linarioides
Climate
Climate + Soil
Soil
AUC = 0.98
TSS = 0.9
AUC = 1
TSS = 0.91
AUC = 0.98
TSS = 0.75
Relative importance
bio01
bio02
bio06
bio12
bio15
BDW
CLY
NTO
PTO
TWI

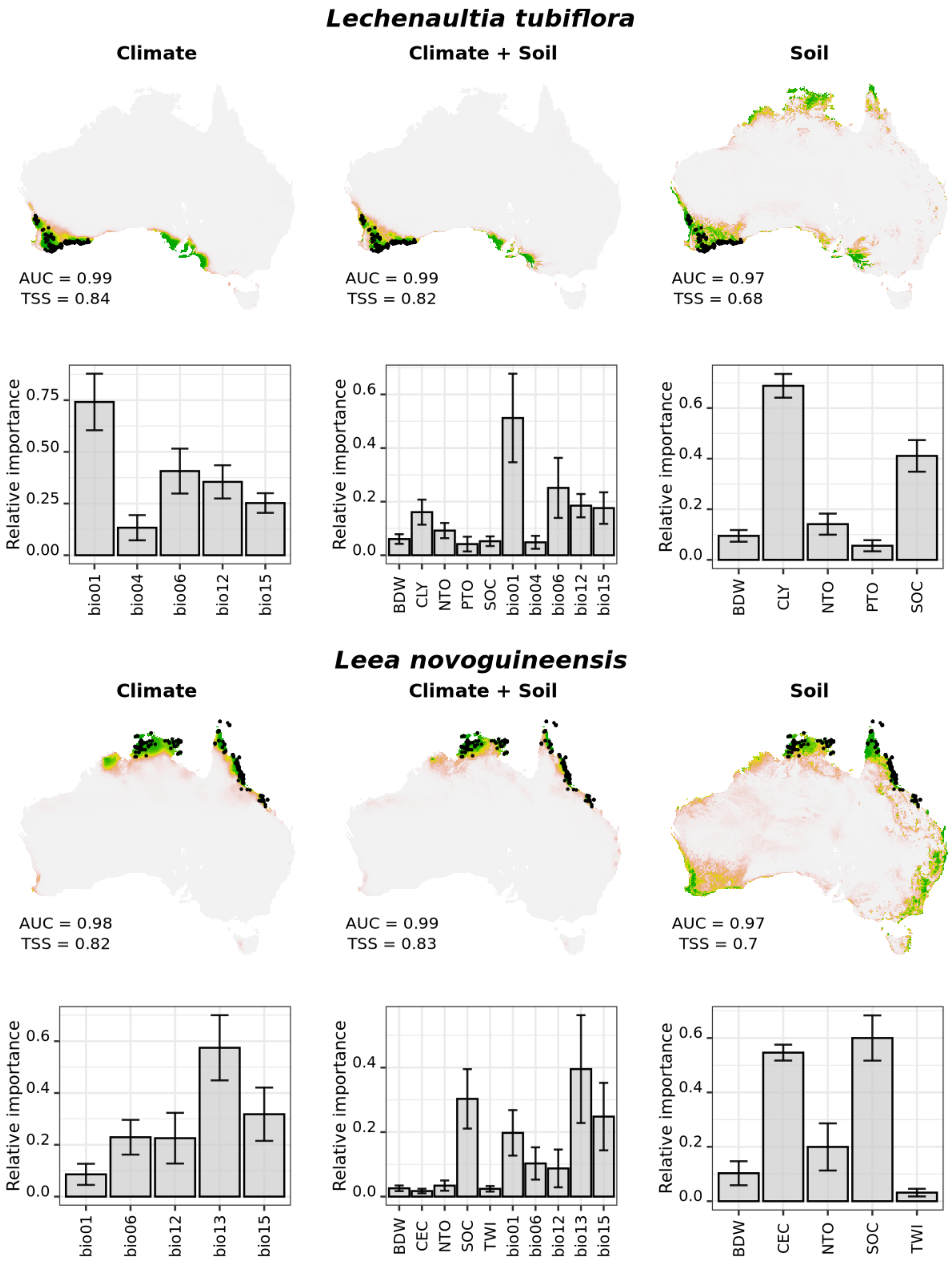
Lechenaultia tubiflora
Climate
Climate + Soil
Soil
AUC = 0.99
TSS = 0.84
AUC = 0.99
TSS = 0.82
AUC = 0.97
TSS = 0.68
Relative importance
0.00
0.25
0.50
0.75
bio01
bio04
bio06
bio12
bio15
Relative importance
0.0
0.2
0.4
0.6
BDW
CLY
NTO
PTO
SOC
bio01
bio04
bio06
bio12
bio15
Relative importance
0.0
0.2
0.4
0.6
BDW
CLY
NTO
PTO
SOC
Leea novoguineensis
Climate
Climate + Soil
Soil
AUC = 0.98
TSS = 0.82
AUC = 0.99
TSS = 0.83
AUC = 0.97
TSS = 0.7
Relative importance
0.0
0.2
0.4
0.6
bio01
bio06
bio12
bio13
bio15
Relative importance
0.0
0.2
0.4
BDW
CEC
NTO
SOC
TWI
bio01
bio06
bio12
bio13
bio15
Relative importance
0.0
0.2
0.4
0.6
BDW
CEC
NTO
SOC
TWI

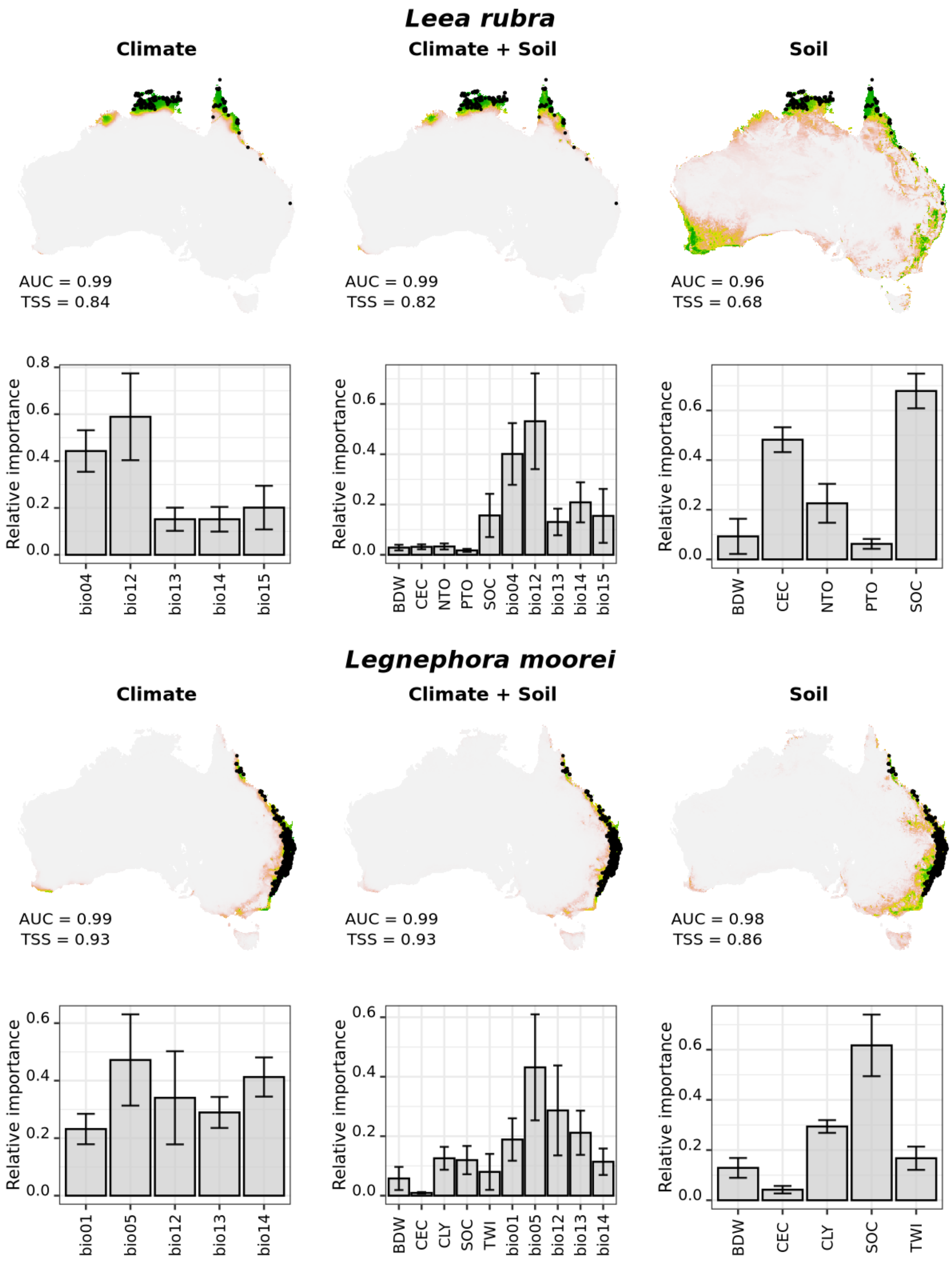
Leea rubra
Climate
Climate + Soil
Soil
AUC = 0.99
TSS = 0.84
AUC = 0.99
TSS = 0.82
AUC = 0.96
TSS = 0.68
Relative importance
bio04
bio12
bio13
bio14
bio15
BDW
CEC
NTO
PTO
SOC
Legnephora moorei
Climate
Climate + Soil
Soil
AUC = 0.99
TSS = 0.93
AUC = 0.99
TSS = 0.93
AUC = 0.98
TSS = 0.86
Relative importance
bio01
bio05
bio12
bio13
bio14
BDW
CEC
CLY
SOC
TWI

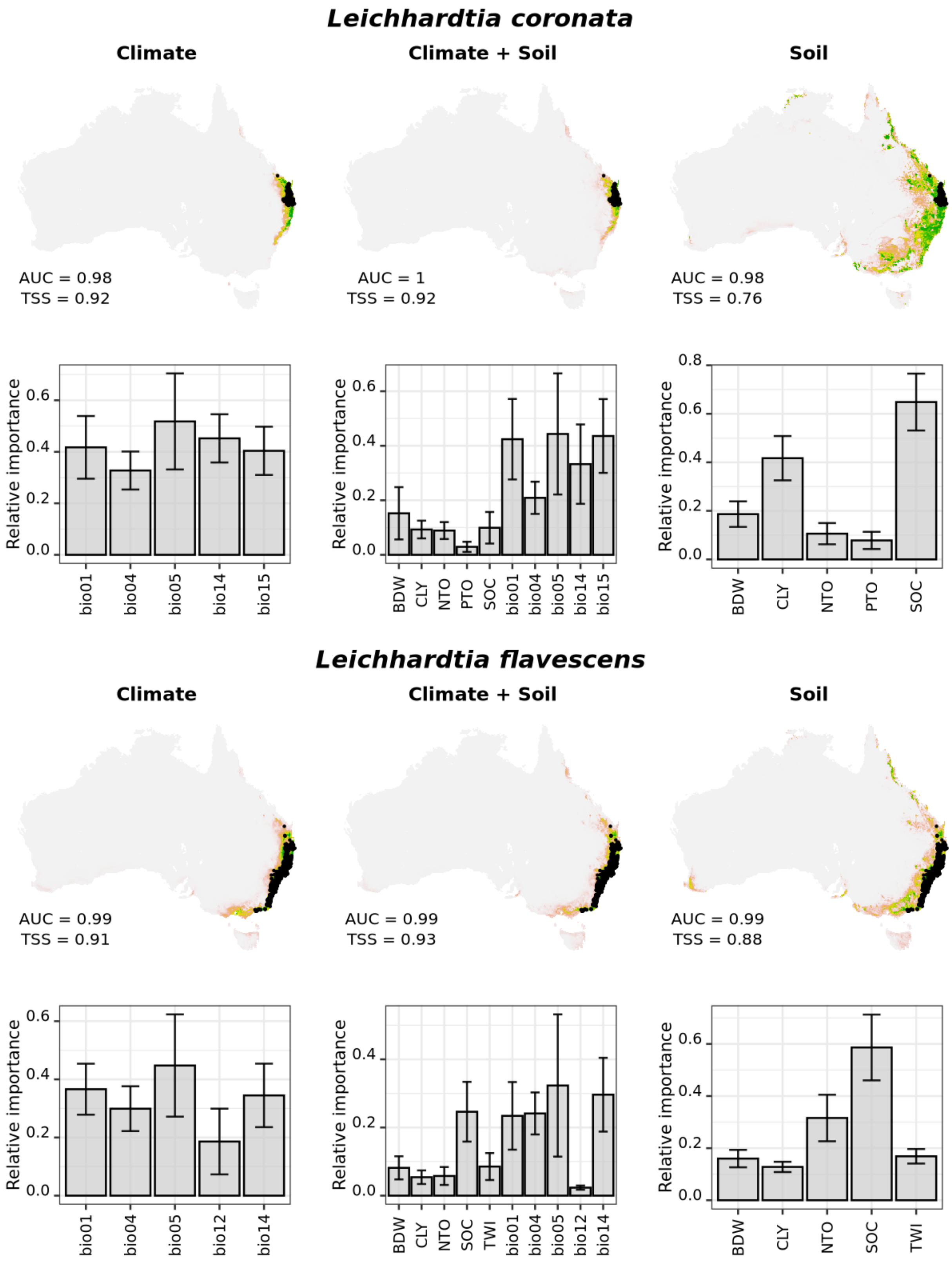

Leichhardtia coronata
Climate
Climate + Soil
Soil
AUC = 0.98
TSS = 0.92
AUC = 1
TSS = 0.92
AUC = 0.98
TSS = 0.76
Relative importance
bio01
bio04
bio05
bio14
bio15
BDW
CLY
NTO
PTO
SOC
Leichhardtia flavescens
Climate
Climate + Soil
Soil
AUC = 0.99
TSS = 0.91
AUC = 0.99
TSS = 0.93
AUC = 0.99
TSS = 0.88
Relative importance
bio01
bio04
bio05
bio12
bio14
BDW
CLY
NTO
SOC
TWI

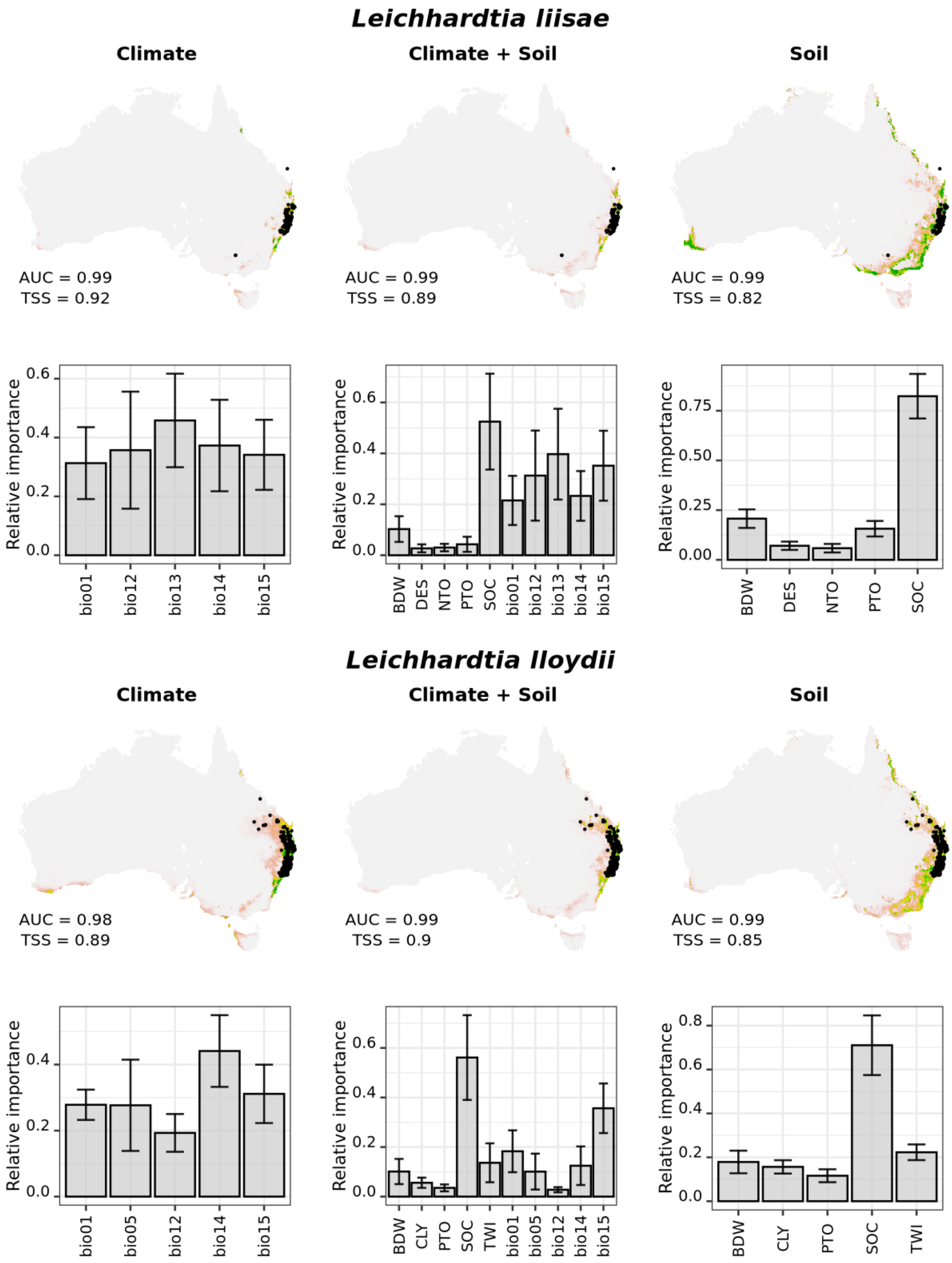

Leichhardtia liisae
Climate
Climate + Soil
Soil
AUC = 0.99
TSS = 0.92
AUC = 0.99
TSS = 0.89
AUC = 0.99
TSS = 0.82
Relative importance
0.0 0.2 0.4 0.6
bio01 bio12 bio13 bio14 bio15
Relative importance
0.0 0.2 0.4 0.6
BDW DES NTO PTO SOC bio01 bio12 bio13 bio14 bio15
Relative importance
0.00 0.25 0.50 0.75
BDW DES NTO PTO SOC
Leichhardtia lloydii
Climate
Climate + Soil
Soil
AUC = 0.98
TSS = 0.89
AUC = 0.99
TSS = 0.9
AUC = 0.99
TSS = 0.85
Relative importance
0.0 0.2 0.4
bio01 bio05 bio12 bio14 bio15
Relative importance
0.0 0.2 0.4 0.6
BDW CLY PTO SOC TWI bio01 bio05 bio12 bio14 bio15
Relative importance
0.0 0.2 0.4 0.6 0.8
BDW CLY PTO SOC TWI

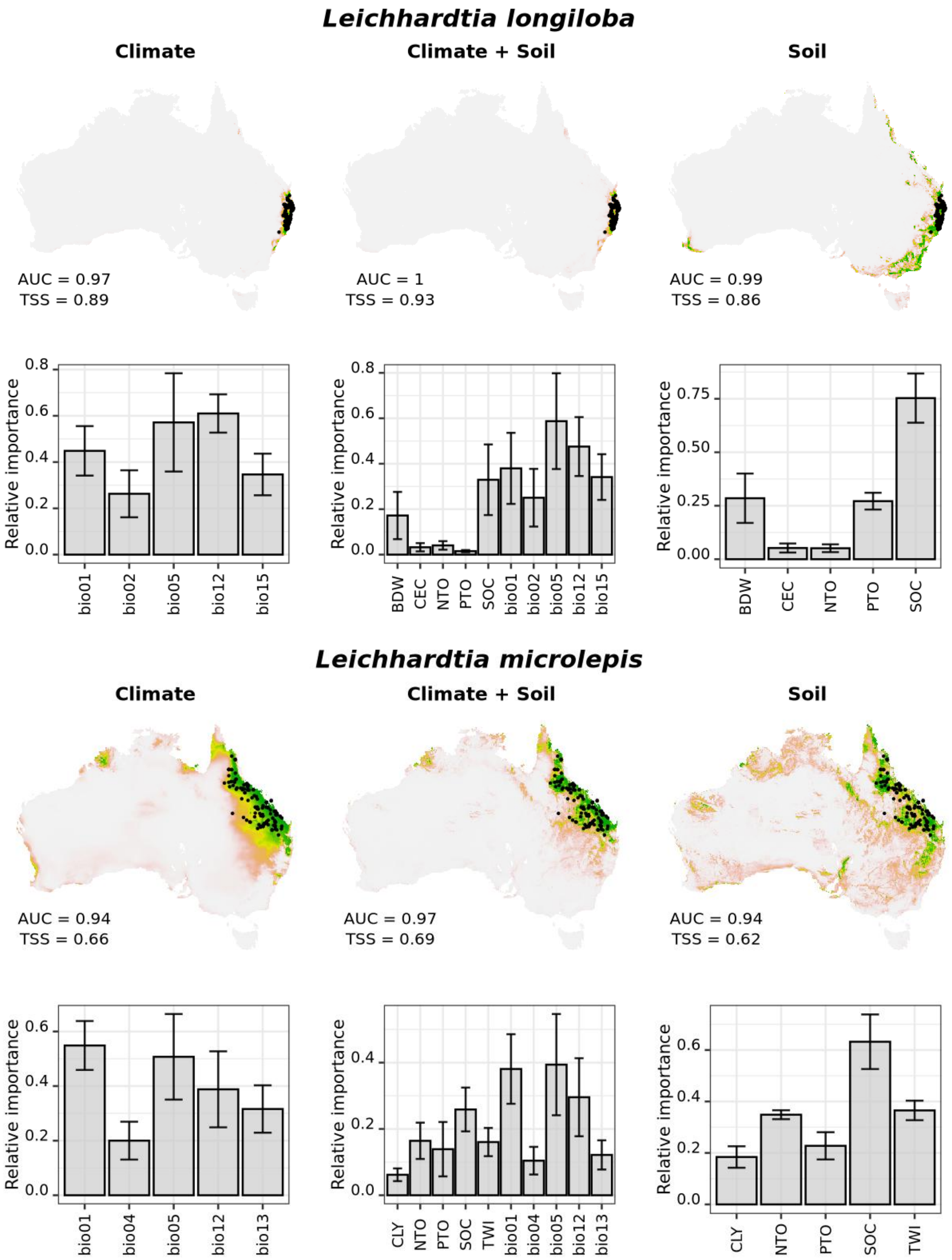
Leichhardtia longiloba
Climate
Climate + Soil
Soil
AUC = 0.97
TSS = 0.89
AUC = 1
TSS = 0.93
AUC = 0.99
TSS = 0.86
Relative importance
bio01
bio02
bio05
bio12
bio15
BDW
CEC
NTO
PTO
SOC
Leichhardtia microlepis
Climate
Climate + Soil
Soil
AUC = 0.94
TSS = 0.66
AUC = 0.97
TSS = 0.69
AUC = 0.94
TSS = 0.62
Relative importance
bio01
bio04
bio05
bio12
bio13
CLY
NTO
PTO
SOC
TWI

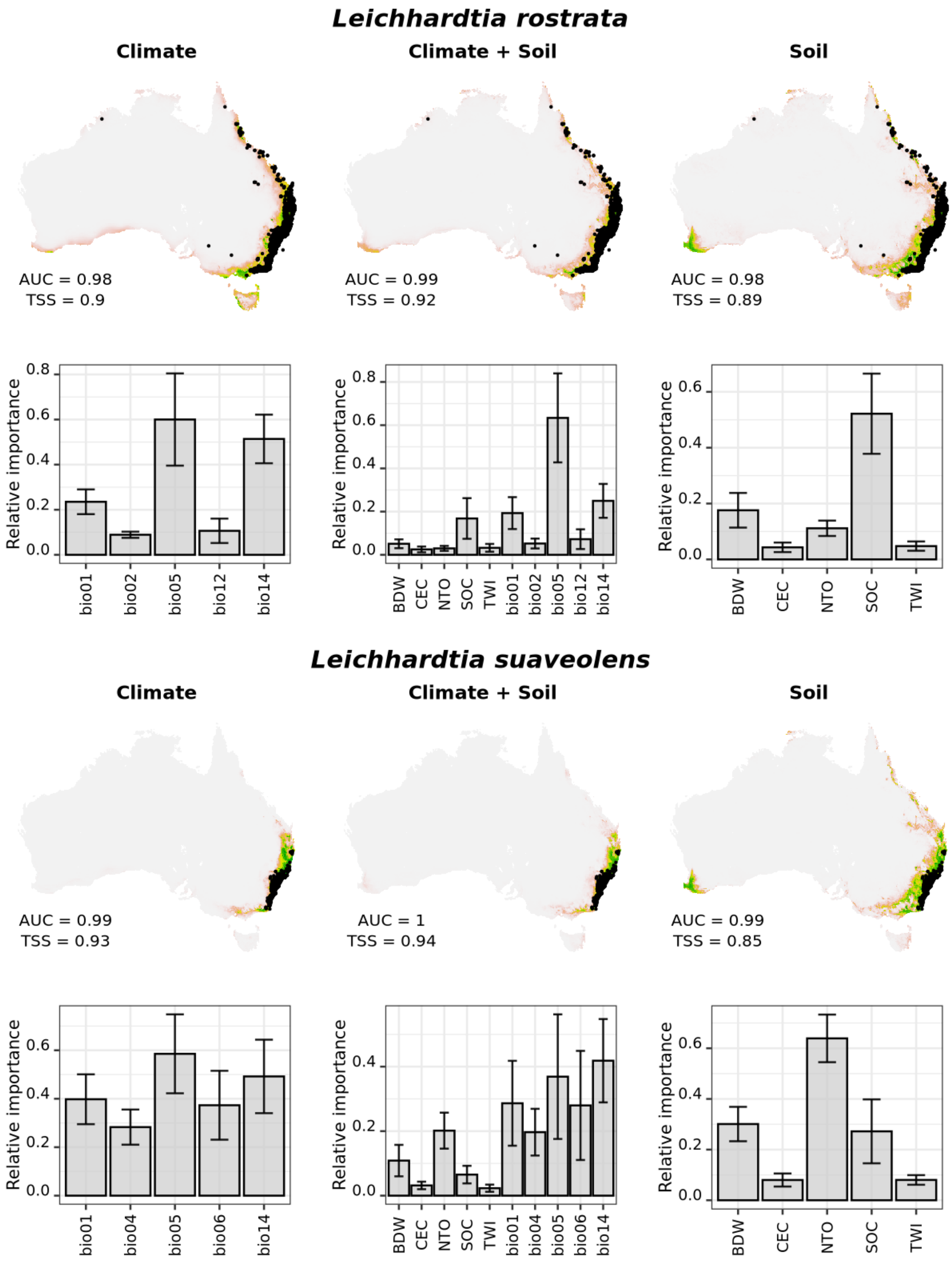
Leichhardtia rostrata
Climate
Climate + Soil
Soil
AUC = 0.98
TSS = 0.9
AUC = 0.99
TSS = 0.92
AUC = 0.98
TSS = 0.89
Relative importance
0.0
0.2
0.4
0.6
0.8
bio01
bio02
bio05
bio12
bio14
BDW
CEC
NTO
SOC
TWI
Leichhardtia suaveolens
AUC = 0.99
TSS = 0.93
AUC = 1
TSS = 0.94
AUC = 0.99
TSS = 0.85
bio04
bio06

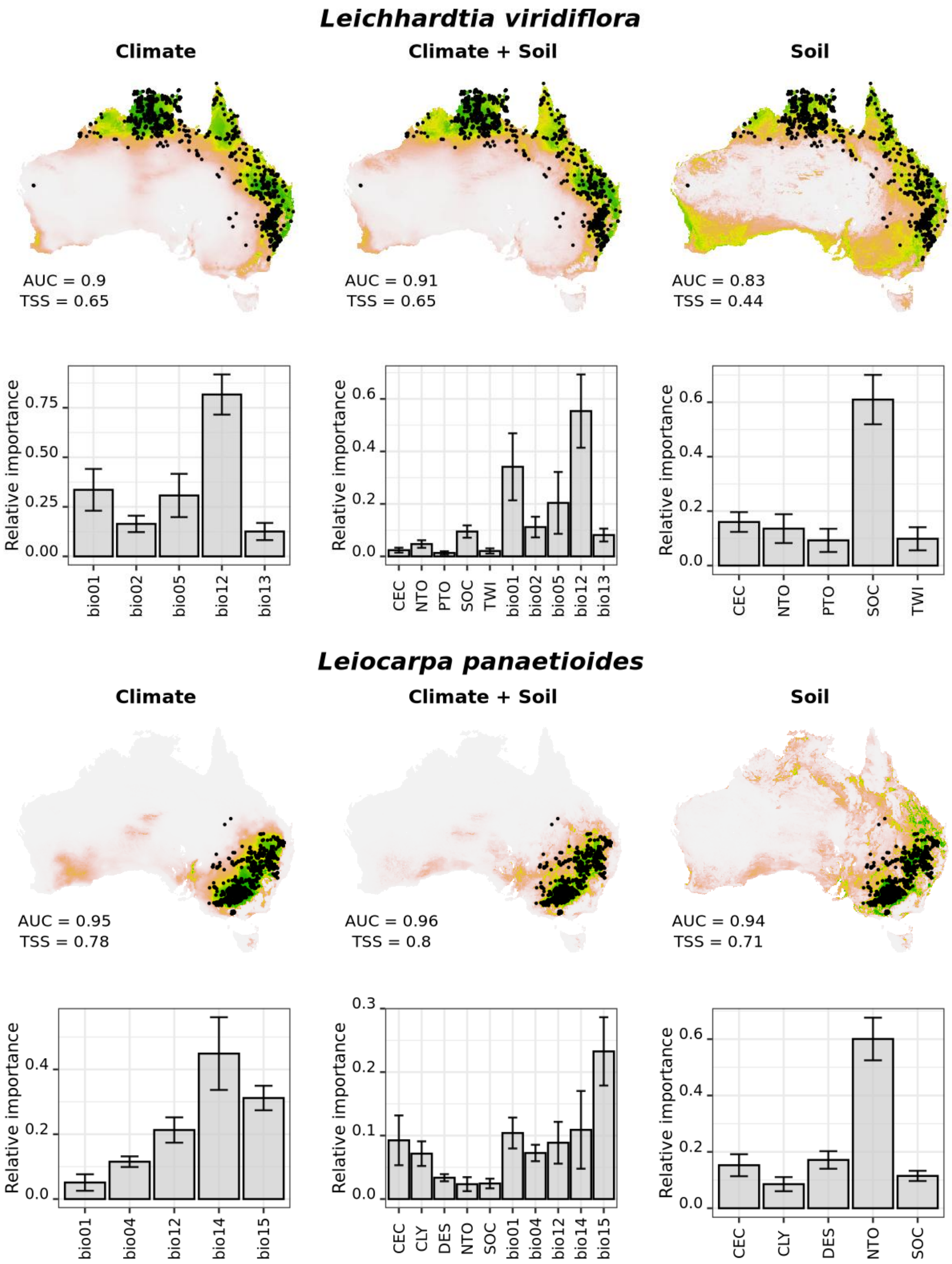
Leichhardtia viridiflora
Climate
Climate + Soil
Soil
AUC = 0.9
TSS = 0.65
AUC = 0.91
TSS = 0.65
AUC = 0.83
TSS = 0.44
Relative importance
0.00
0.25
0.50
0.75
bio01
bio02
bio05
bio12
bio13
0.0
0.2
0.4
0.6
CEC
NTO
PTO
SOC
TWI
bio01
bio02
bio05
bio12
bio13
CEC
NTO
PTO
SOC
TWI
Leiocarpa panaetioides
Climate
Climate + Soil
Soil
AUC = 0.95
TSS = 0.78
AUC = 0.96
TSS = 0.8
AUC = 0.94
TSS = 0.71
Relative importance
0.0
0.2
0.4
bio01
bio04
bio12
bio14
bio15
0.0
0.1
0.2
0.3
CEC
CLY
DES
NTO
SOC
bio01
bio04
bio12
bio14
bio15
0.0
0.2
0.4
0.6
CEC
CLY
DES
NTO
SOC

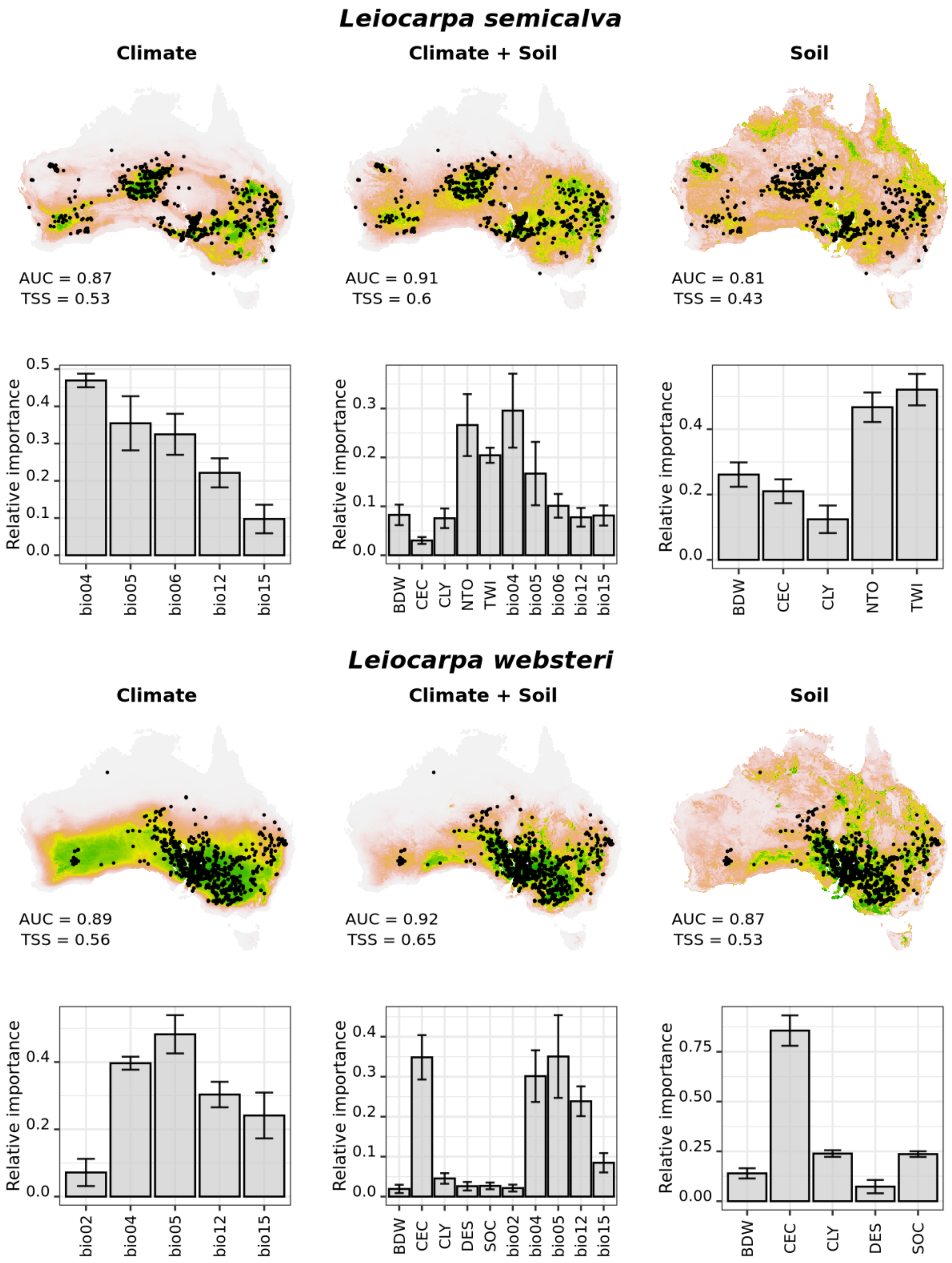
Leiocarpa semicalva
Climate
Climate + Soil
Soil
AUC = 0.87
TSS = 0.53
AUC = 0.91
TSS = 0.6
AUC = 0.81
TSS = 0.43
Relative importance
bio04
bio05
bio06
bio12
bio15
BDW
CEC
CLY
NTO
TWI
bio04
bio05
bio06
bio12
bio15
BDW
CEC
CLY
NTO
TWI
Leiocarpa websteri
Climate
Climate + Soil
Soil
AUC = 0.89
TSS = 0.56
AUC = 0.92
TSS = 0.65
AUC = 0.87
TSS = 0.53
Relative importance
bio02
bio04
bio05
bio12
bio15
BDW
CEC
CLY
DES
SOC
bio02
bio04
bio05
bio12
bio15
BDW
CEC
CLY
DES
SOC

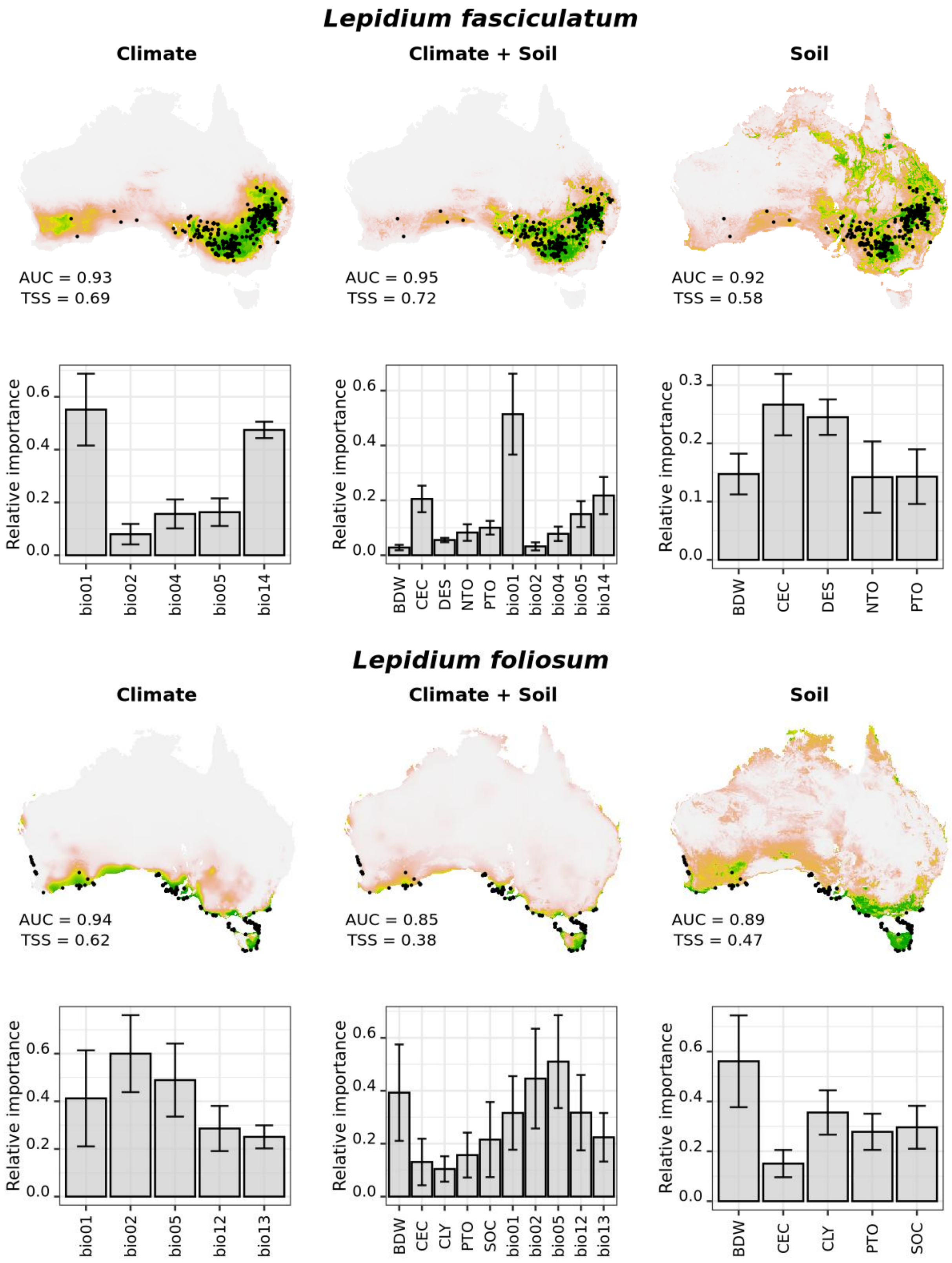

Lepidium fasciculatum
Climate
Climate + Soil
Soil
AUC = 0.93
TSS = 0.69
AUC = 0.95
TSS = 0.72
AUC = 0.92
TSS = 0.58
Relative importance
0.0
0.2
0.4
0.6
bio01
bio02
bio04
bio05
bio14
Relative importance
0.0
0.2
0.4
0.6
BDW
CEC
DES
NTO
PTO
bio01
bio02
bio04
bio05
bio14
Relative importance
0.0
0.1
0.2
0.3
BDW
CEC
DES
NTO
PTO
Lepidium foliosum
Climate
Climate + Soil
Soil
AUC = 0.94
TSS = 0.62
AUC = 0.85
TSS = 0.38
AUC = 0.89
TSS = 0.47
Relative importance
0.0
0.2
0.4
0.6
bio01
bio02
bio05
bio12
bio13
Relative importance
0.0
0.2
0.4
0.6
BDW
CEC
CLY
PTO
SOC
bio01
bio02
bio05
bio12
bio13
Relative importance
0.0
0.2
0.4
0.6
BDW
CEC
CLY
PTO
SOC

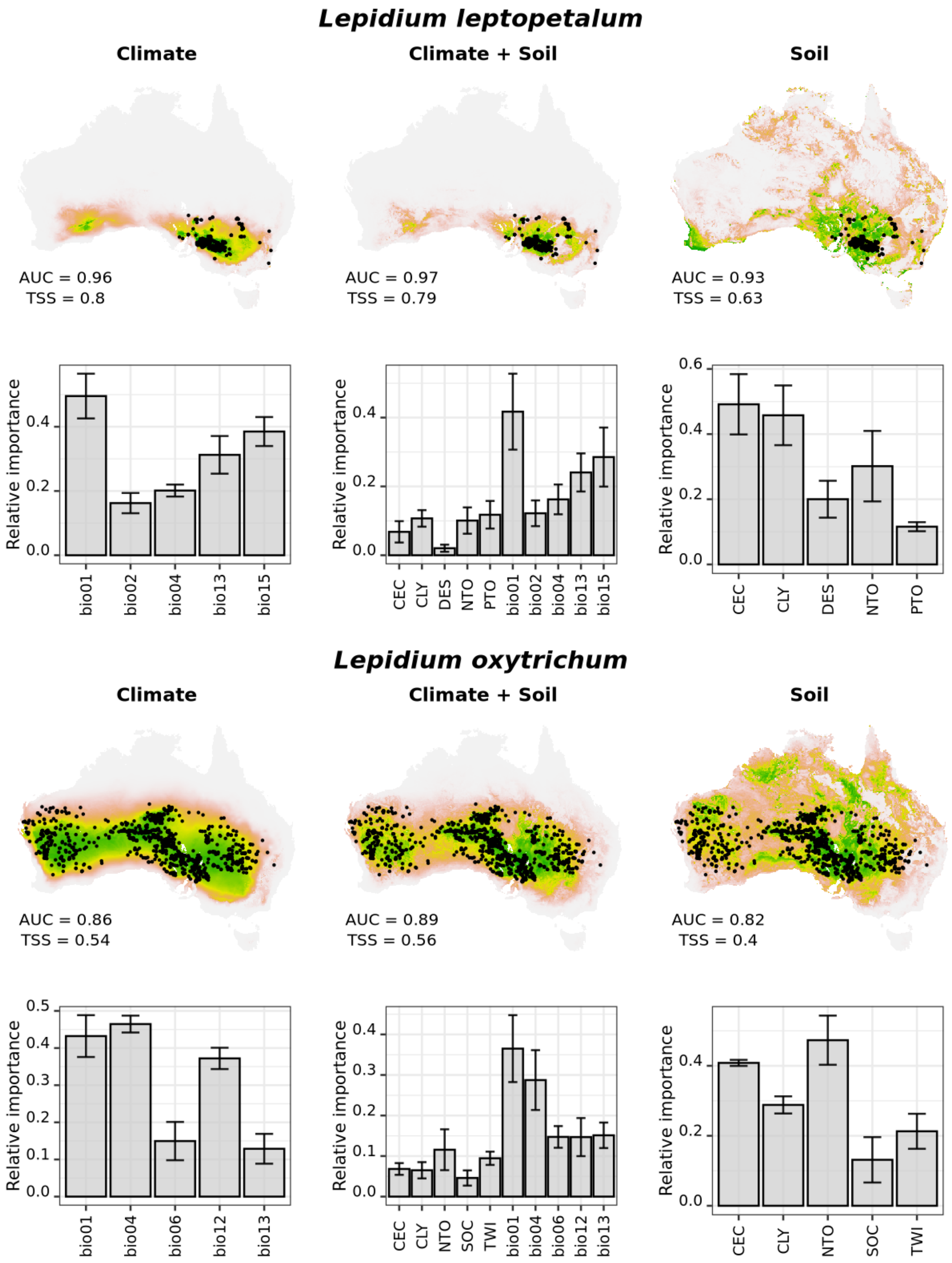
Lepidium leptopetalum
Climate
Climate + Soil
Soil
AUC = 0.96
TSS = 0.8
AUC = 0.97
TSS = 0.79
AUC = 0.93
TSS = 0.63
Relative importance
bio01
bio02
bio04
bio13
bio15
CEC
CLY
DES
NTO
PTO
Lepidium oxytrichum
AUC = 0.86
TSS = 0.54
AUC = 0.89
TSS = 0.56
AUC = 0.82
TSS = 0.4
bio01
bio04
bio06
bio12
bio13
CEC
CLY
NTO
SOC
TWI

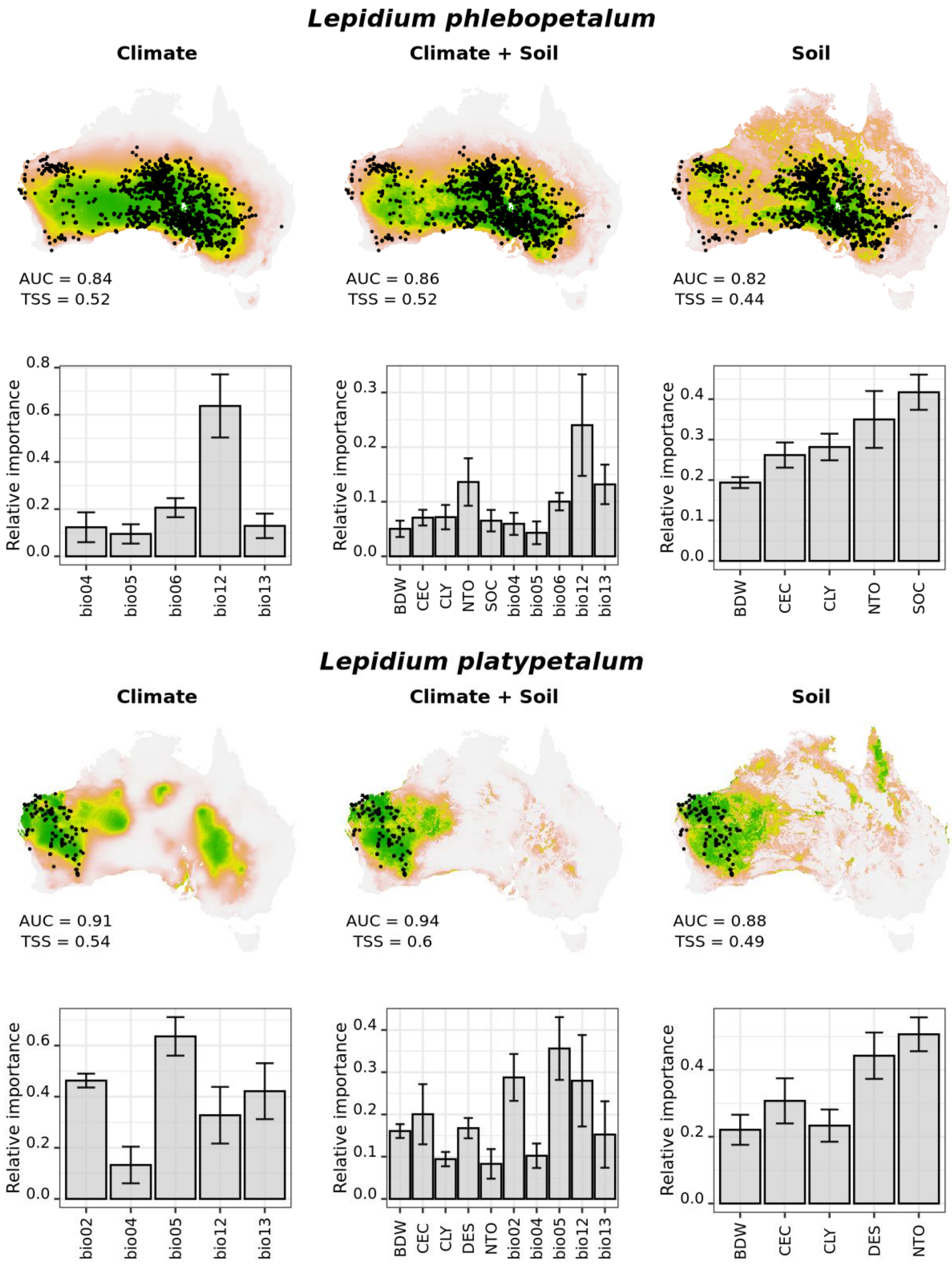

Lepidium phlebopetalum
Climate
Climate + Soil
Soil
AUC = 0.84
TSS = 0.52
AUC = 0.86
TSS = 0.52
AUC = 0.82
TSS = 0.44
Relative importance
bio04
bio05
bio06
bio12
bio13
BDW
CEC
CLY
NTO
SOC
Lepidium platypetalum
AUC = 0.91
TSS = 0.54
AUC = 0.94
TSS = 0.6
AUC = 0.88
TSS = 0.49
bio02
DES

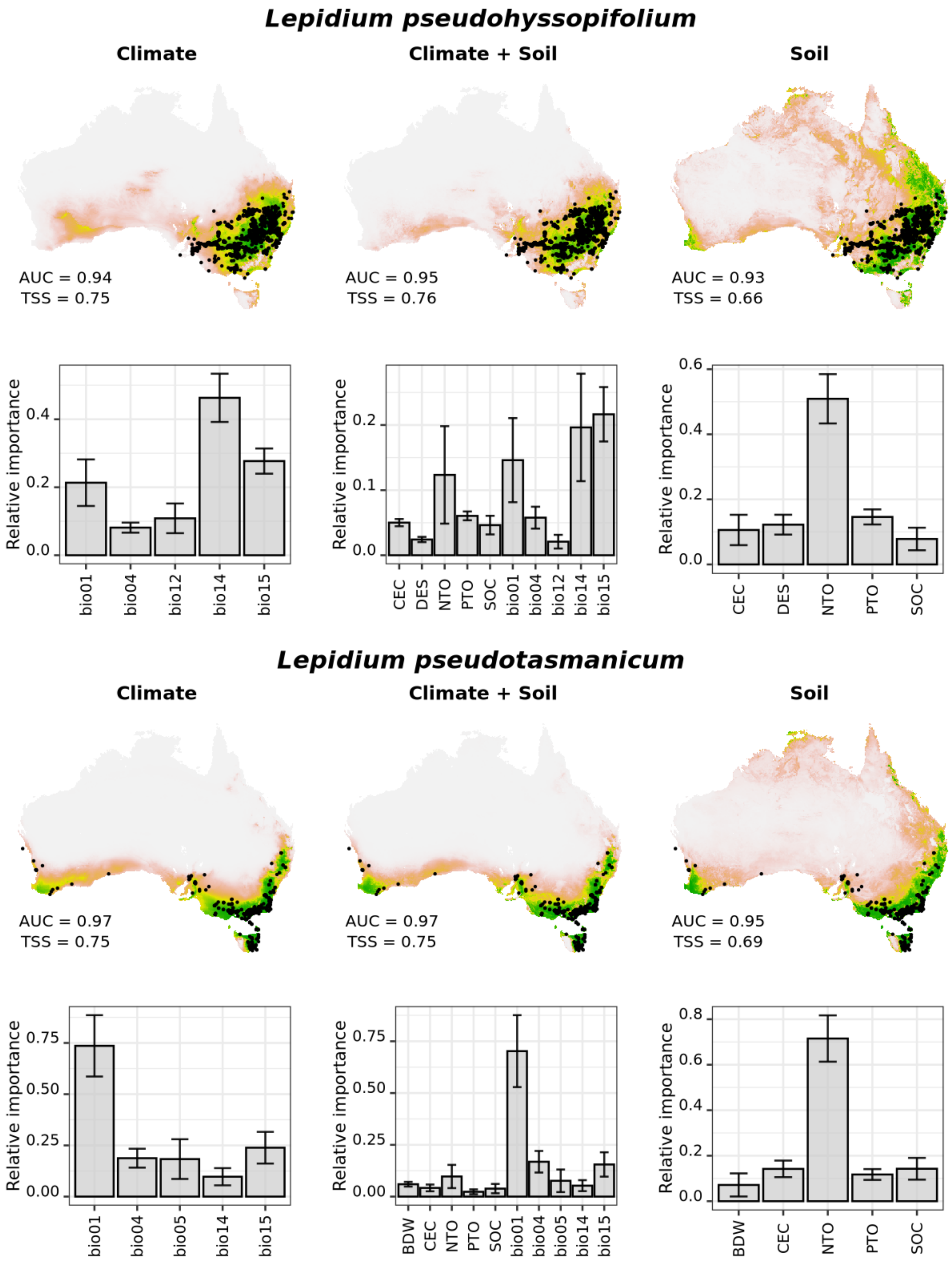

Lepidium pseudohyssopifolium
Climate
Climate + Soil
Soil
AUC = 0.94
TSS = 0.75
AUC = 0.95
TSS = 0.76
AUC = 0.93
TSS = 0.66
Relative importance
bio01
bio04
bio12
bio14
bio15
CEC
DES
NTO
PTO
SOC
Lepidium pseudotasmanicum
AUC = 0.97
TSS = 0.75
AUC = 0.97
TSS = 0.75
AUC = 0.95
TSS = 0.69
bio05
BDW

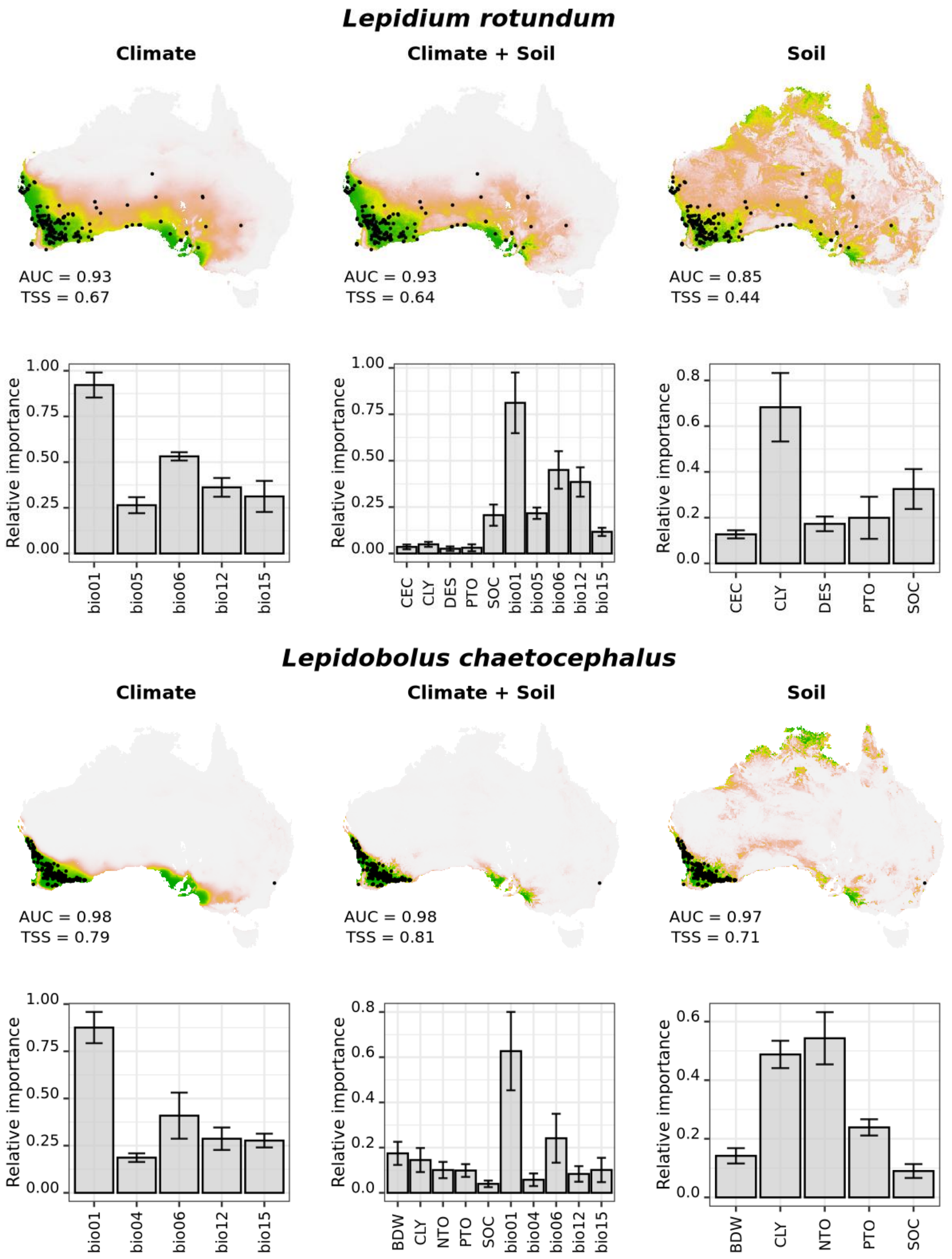
Lepidium rotundum
Climate
Climate + Soil
Soil
AUC = 0.93
TSS = 0.67
AUC = 0.93
TSS = 0.64
AUC = 0.85
TSS = 0.44
Relative importance
bio01
bio05
bio06
bio12
bio15
CEC
CLY
DES
PTO
SOC
Lepidobolus chaetocephalus
AUC = 0.98
TSS = 0.79
AUC = 0.98
TSS = 0.81
AUC = 0.97
TSS = 0.71
bio04
BDW
NTO

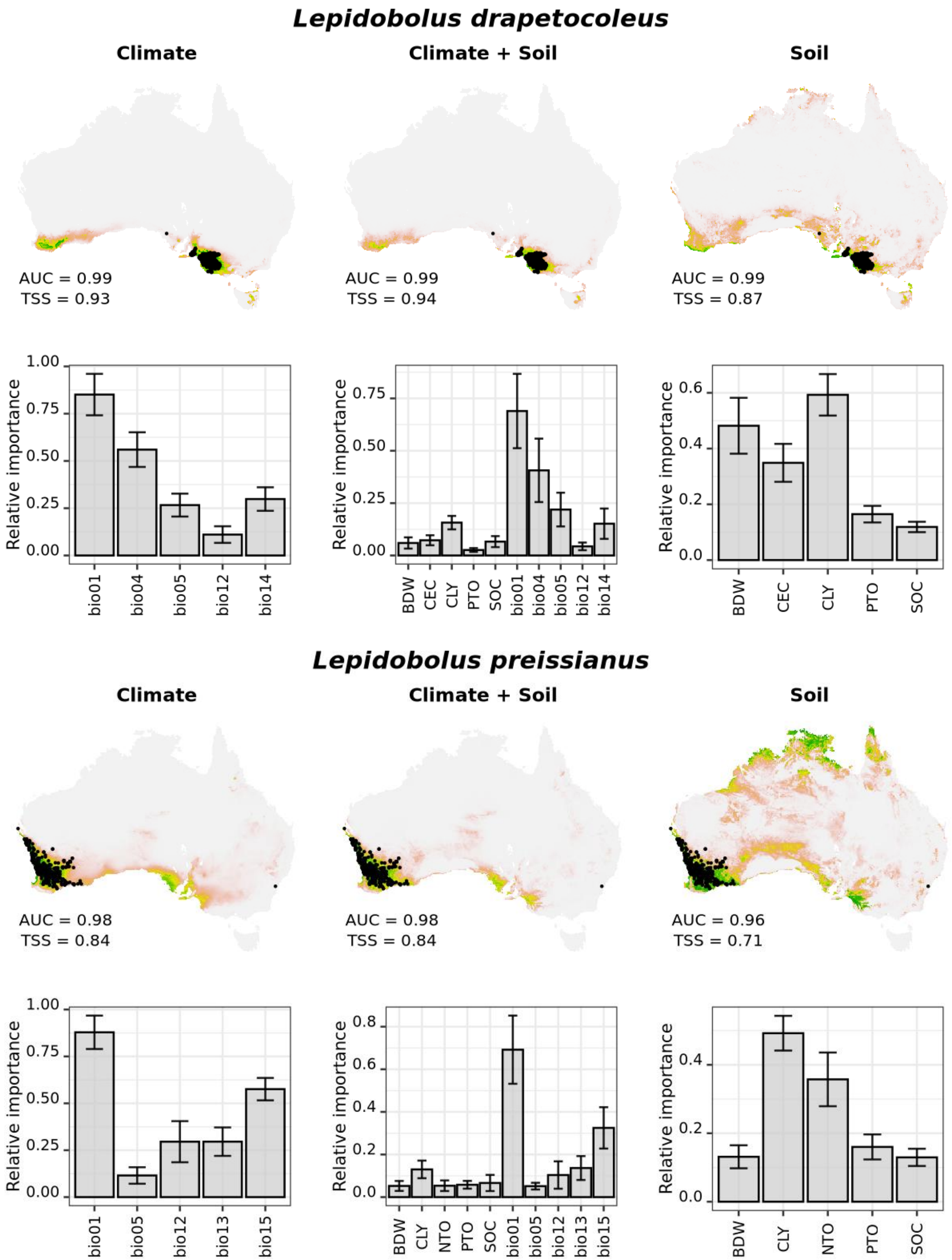
Lepidobolus drapetocoleus
Climate
Climate + Soil
Soil
AUC = 0.99
TSS = 0.93
AUC = 0.99
TSS = 0.94
AUC = 0.99
TSS = 0.87
Relative importance
0.00 0.25 0.50 0.75 1.00
bio01 bio04 bio05 bio12 bio14
BDW CEC CLY PTO SOC bio01 bio04 bio05 bio12 bio14
0.0 0.2 0.4 0.6
BDW CEC CLY PTO SOC
Lepidobolus preissianus
Climate
Climate + Soil
Soil
AUC = 0.98
TSS = 0.84
AUC = 0.98
TSS = 0.84
AUC = 0.96
TSS = 0.71
bio01 bio05 bio12 bio13 bio15
0.0 0.2 0.4 0.6 0.8
BDW CLY NTO PTO SOC bio01 bio05 bio12 bio13 bio15
BDW CLY NTO PTO SOC

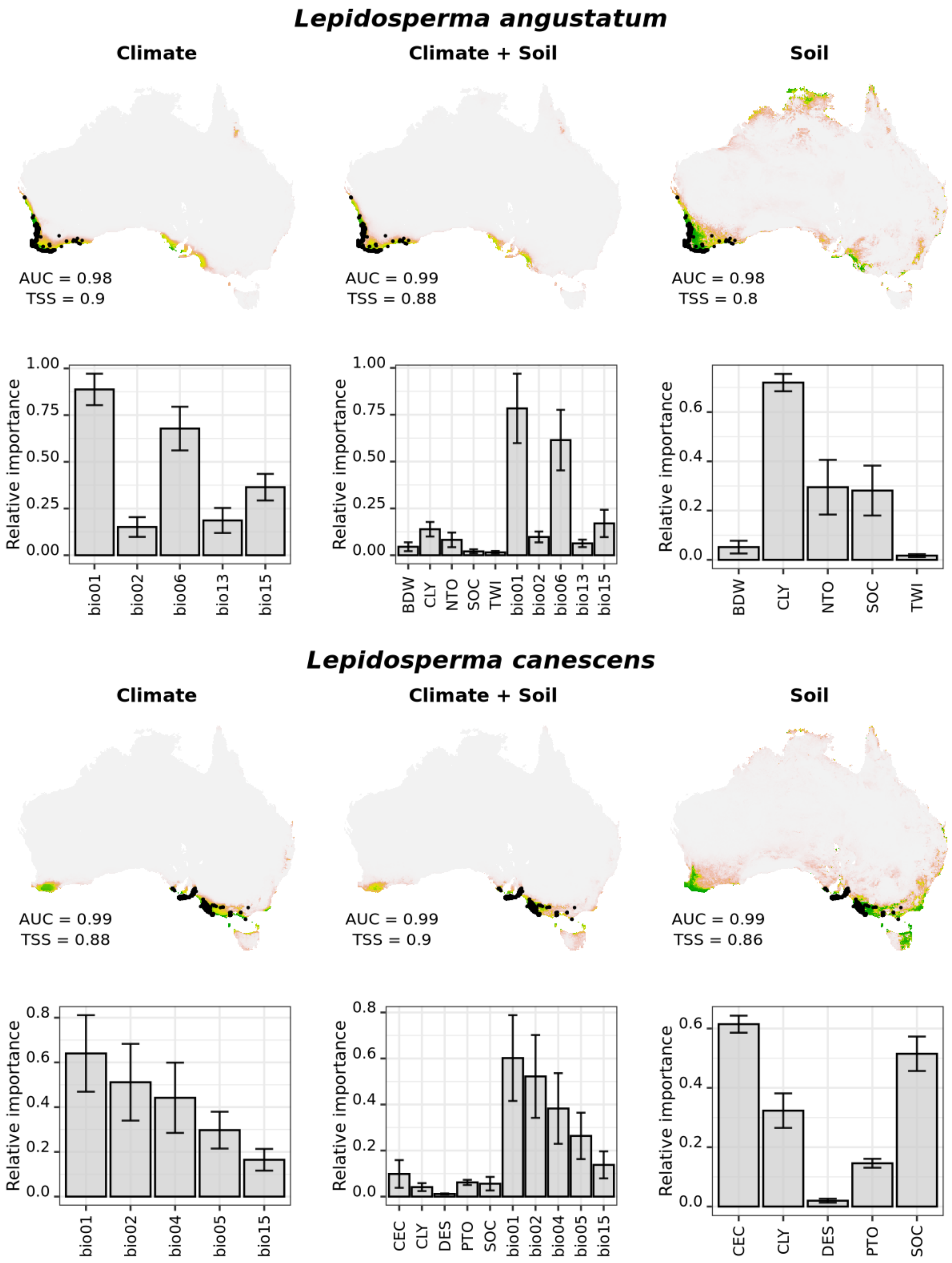

Lepidosperma angustatum
Climate
Climate + Soil
Soil
AUC = 0.98
TSS = 0.9
AUC = 0.99
TSS = 0.88
AUC = 0.98
TSS = 0.8
Relative importance
1.00
0.75
0.50
0.25
0.00
bio01
bio02
bio06
bio13
bio15
BDW
CLY
NTO
SOC
TWI
0.6
0.4
0.2
0.0
Lepidosperma canescens
Climate
Climate + Soil
Soil
AUC = 0.99
TSS = 0.88
AUC = 0.99
TSS = 0.9
AUC = 0.99
TSS = 0.86
0.8
bio04
bio05
CEC
DES
PTO

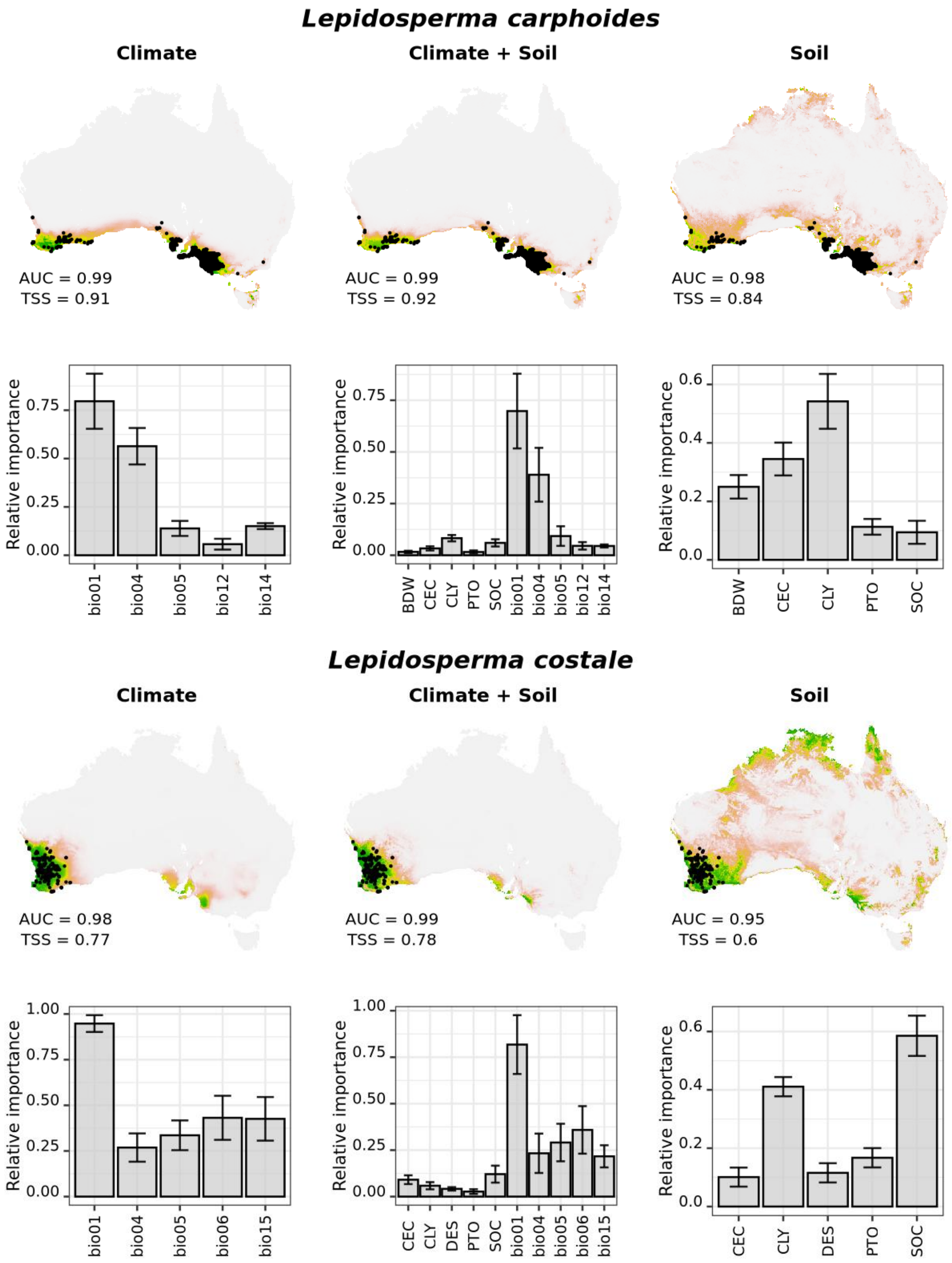
Lepidosperma carphoides
Climate
Climate + Soil
Soil
AUC = 0.99
TSS = 0.91
AUC = 0.99
TSS = 0.92
AUC = 0.98
TSS = 0.84
Relative importance
0.00 0.25 0.50 0.75
bio01 bio04 bio05 bio12 bio14
BDW CEC CLY PTO SOC bio01 bio04 bio05 bio12 bio14
0.0 0.2 0.4 0.6
BDW CEC CLY PTO SOC
Lepidosperma costale
Climate
Climate + Soil
Soil
AUC = 0.98
TSS = 0.77
AUC = 0.99
TSS = 0.78
AUC = 0.95
TSS = 0.6
Relative importance
0.00 0.25 0.50 0.75 1.00
bio01 bio04 bio05 bio06 bio15
CEC CLY DES PTO SOC bio01 bio04 bio05 bio06 bio15
0.0 0.2 0.4 0.6
CEC CLY DES PTO SOC

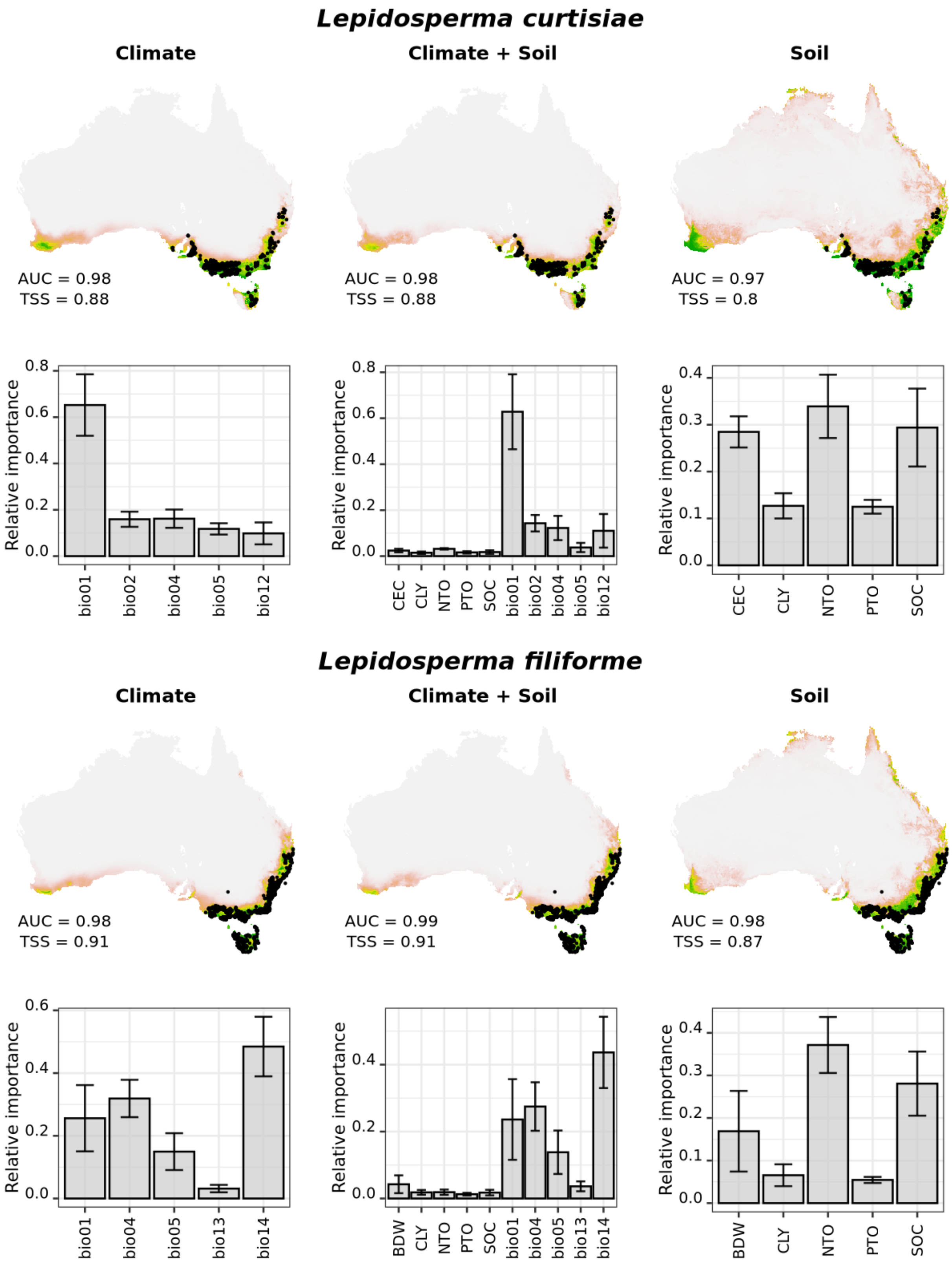

Lepidosperma curtisiae
Climate
Climate + Soil
Soil
AUC = 0.98
TSS = 0.88
AUC = 0.98
TSS = 0.88
AUC = 0.97
TSS = 0.8
Relative importance
0.0
0.2
0.4
0.6
0.8
bio01
bio02
bio04
bio05
bio12
CEC
CLY
NTO
PTO
SOC
0.1
0.3
Lepidosperma filiforme
AUC = 0.98
TSS = 0.91
AUC = 0.99
TSS = 0.91
AUC = 0.98
TSS = 0.87
bio13
bio14
BDW

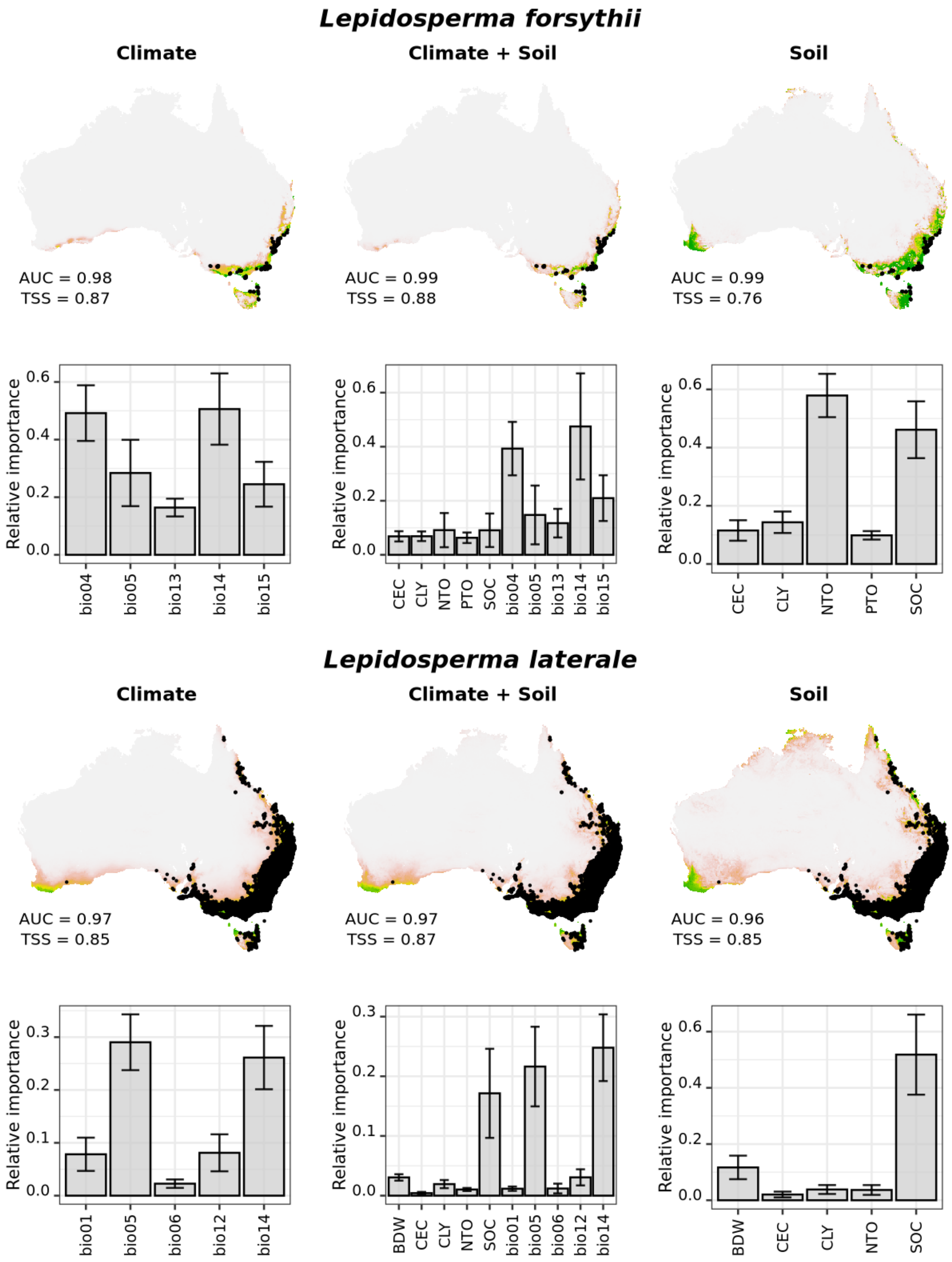
Lepidosperma forsythii
Climate
Climate + Soil
Soil
AUC = 0.98
TSS = 0.87
AUC = 0.99
TSS = 0.88
AUC = 0.99
TSS = 0.76
Relative importance
0.0
0.2
0.4
0.6
bio04
bio05
bio13
bio14
bio15
CEC
CLY
NTO
PTO
SOC
Lepidosperma laterale
Climate
Climate + Soil
Soil
AUC = 0.97
TSS = 0.85
AUC = 0.97
TSS = 0.87
AUC = 0.96
TSS = 0.85
Relative importance
0.0
0.1
0.2
0.3
bio01
bio05
bio06
bio12
bio14
BDW
CEC
CLY
NTO
SOC
0.4
0.6

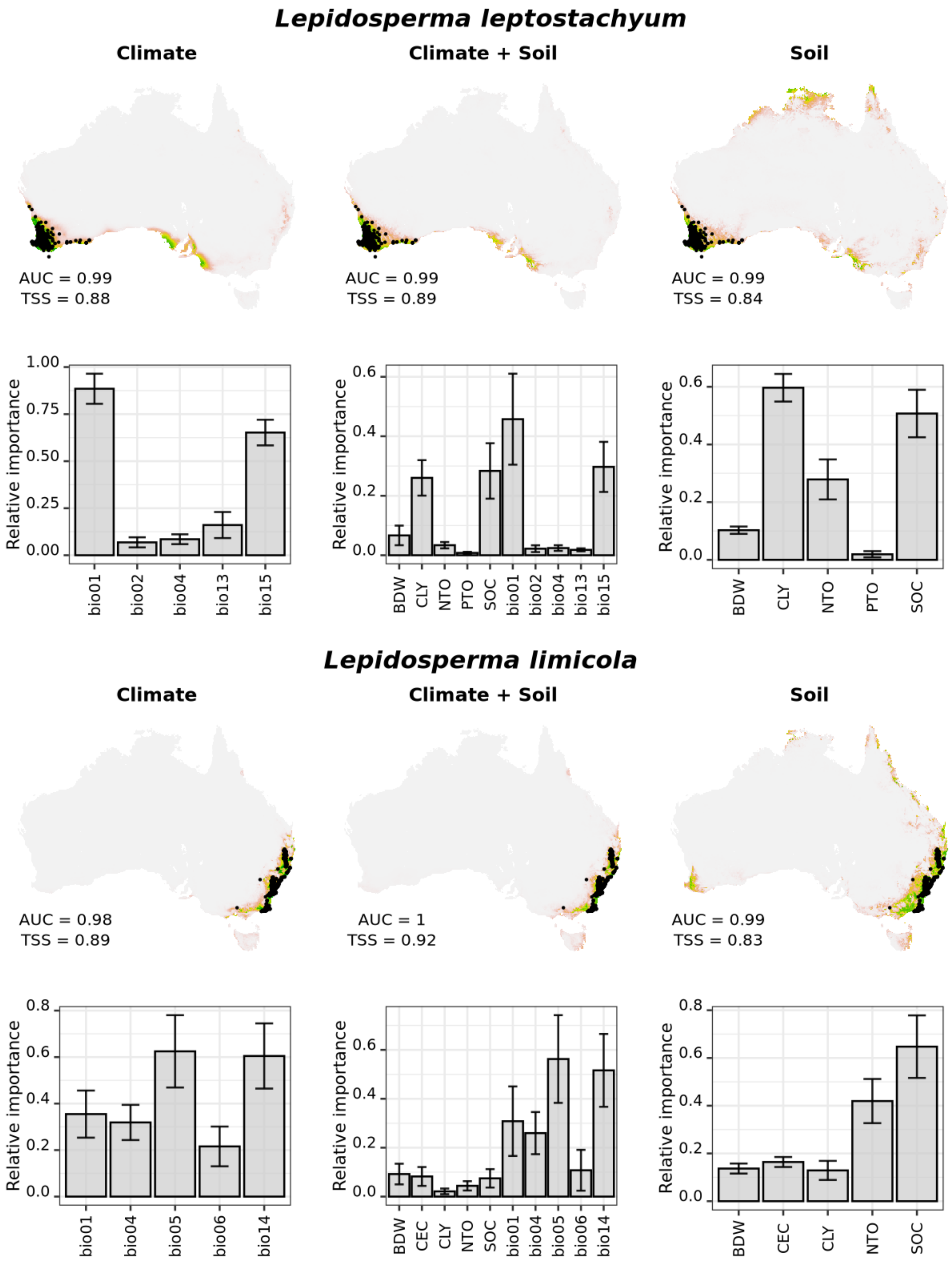
Lepidosperma leptostachyum
Climate
Climate + Soil
Soil
AUC = 0.99
TSS = 0.88
AUC = 0.99
TSS = 0.89
AUC = 0.99
TSS = 0.84
Relative importance
1.00
0.75
0.50
0.25
0.00
bio01
bio02
bio04
bio13
bio15
0.6
0.4
0.2
0.0
BDW
CLY
NTO
PTO
SOC
bio01
bio02
bio04
bio13
bio15
BDW
CLY
NTO
PTO
SOC
Lepidosperma limicola
Climate
Climate + Soil
Soil
AUC = 0.98
TSS = 0.89
AUC = 1
TSS = 0.92
AUC = 0.99
TSS = 0.83
0.8
0.6
0.4
0.2
0.0
bio01
bio04
bio05
bio06
bio14
BDW
CEC
CLY
NTO
SOC
bio01
bio04
bio05
bio06
bio14
BDW
CEC
CLY
NTO
SOC

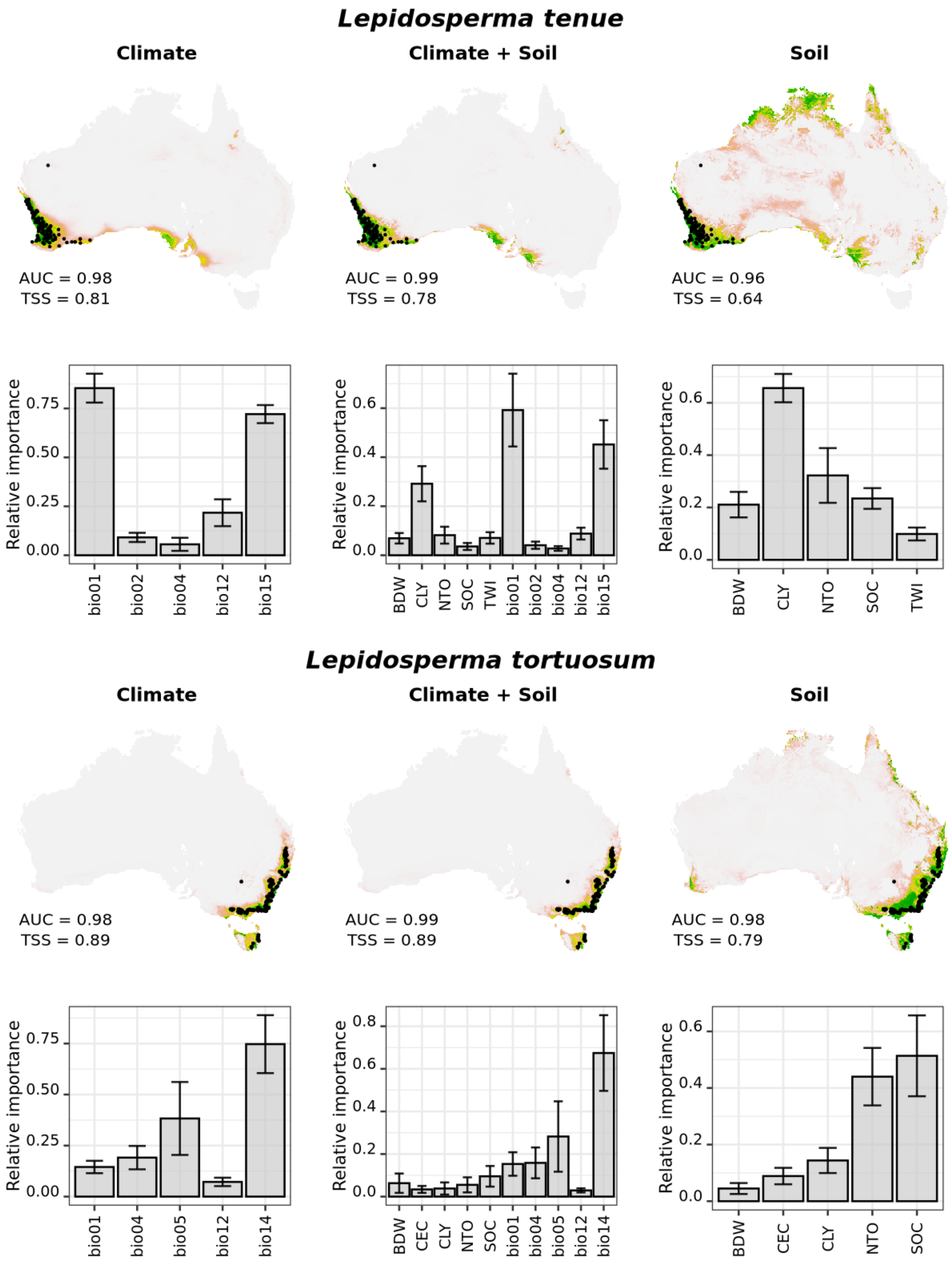
Lepidosperma tenue
Climate
Climate + Soil
Soil
AUC = 0.98
TSS = 0.81
AUC = 0.99
TSS = 0.78
AUC = 0.96
TSS = 0.64
Relative importance
bio01
bio02
bio04
bio12
bio15
BDW
CLY
NTO
SOC
TWI
Lepidosperma tortuosum
Climate
Climate + Soil
Soil
AUC = 0.98
TSS = 0.89
AUC = 0.99
TSS = 0.89
AUC = 0.98
TSS = 0.79
Relative importance
bio01
bio04
bio05
bio12
bio14
BDW
CEC
CLY
NTO
SOC

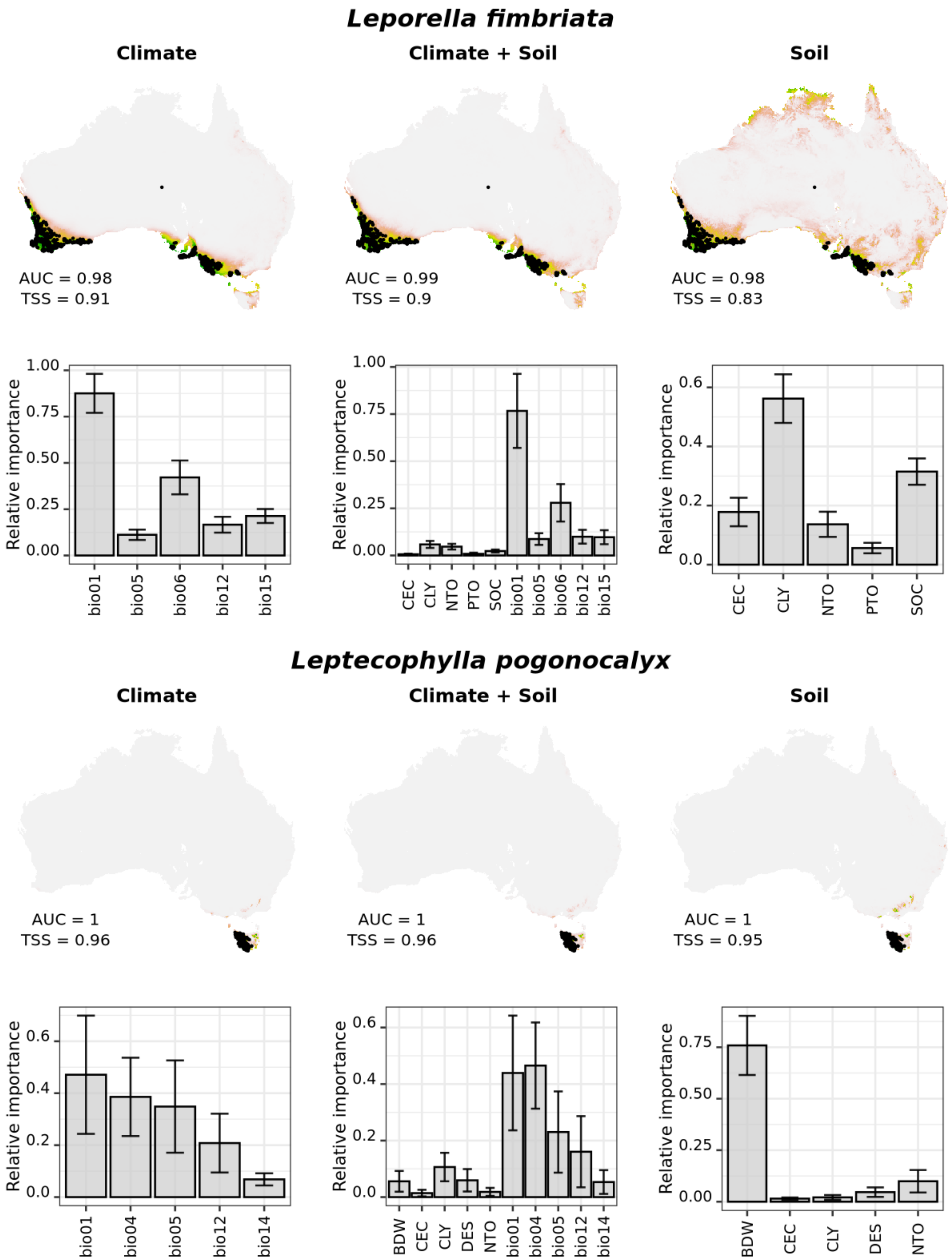
Leporella fimbriata
Climate
Climate + Soil
Soil
AUC = 0.98
TSS = 0.91
AUC = 0.99
TSS = 0.9
AUC = 0.98
TSS = 0.83
Relative importance
bio01
bio05
bio06
bio12
bio15
CEC
CLY
NTO
PTO
SOC
Leptecophylla pogonocalyx
Climate
Climate + Soil
Soil
AUC = 1
TSS = 0.96
AUC = 1
TSS = 0.96
AUC = 1
TSS = 0.95
Relative importance
bio01
bio04
bio05
bio12
bio14
BDW
CEC
CLY
DES
NTO

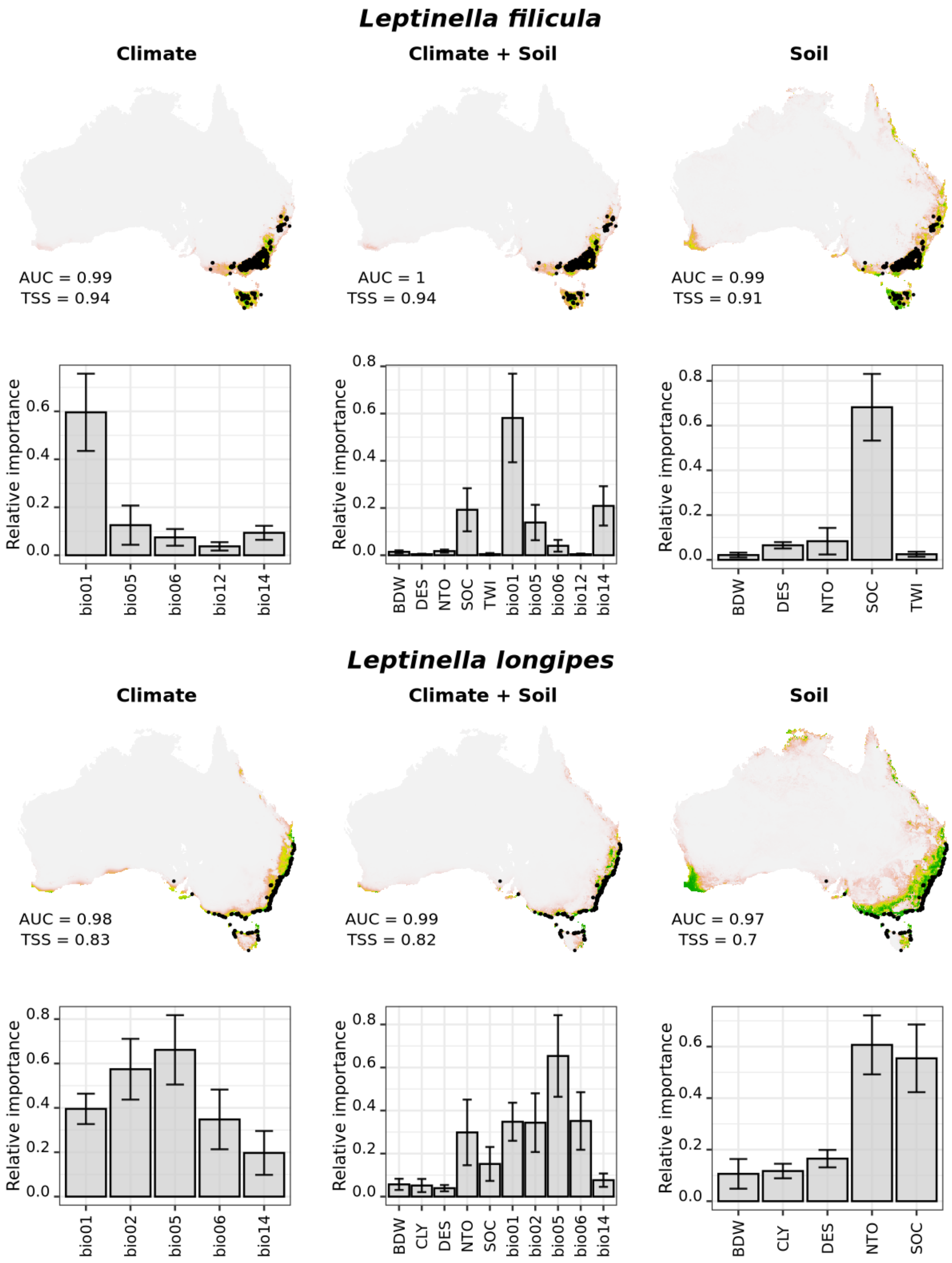
Leptinella filicula
Climate
Climate + Soil
Soil
AUC = 0.99
TSS = 0.94
AUC = 1
TSS = 0.94
AUC = 0.99
TSS = 0.91
Relative importance
bio01 bio05 bio06 bio12 bio14
BDW DES NTO SOC TWI bio01 bio05 bio06 bio12 bio14
BDW DES NTO SOC TWI
Leptinella longipes
Climate
Climate + Soil
Soil
AUC = 0.98
TSS = 0.83
AUC = 0.99
TSS = 0.82
AUC = 0.97
TSS = 0.7
Relative importance
bio01 bio02 bio05 bio06 bio14
BDW CLY DES NTO SOC bio01 bio02 bio05 bio06 bio14
BDW CLY DES NTO SOC

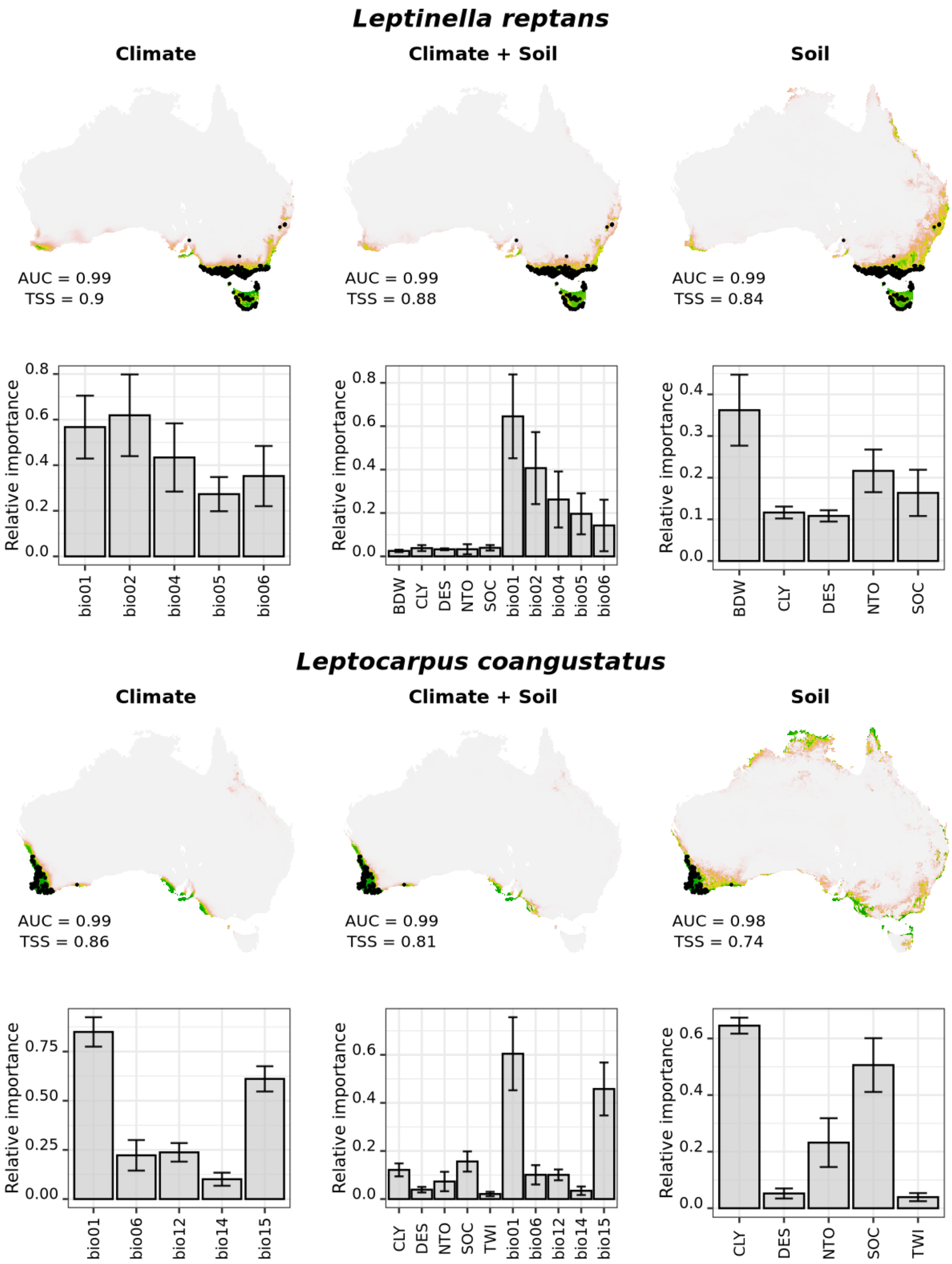
Leptinella reptans
Climate
Climate + Soil
Soil
AUC = 0.99
TSS = 0.9
AUC = 0.99
TSS = 0.88
AUC = 0.99
TSS = 0.84
Relative importance
0.0
0.2
0.4
0.6
0.8
bio01
bio02
bio04
bio05
bio06
BDW
CLY
DES
NTO
SOC
0.1
0.3
Leptocarpus coangustatus
Climate
Climate + Soil
Soil
AUC = 0.99
TSS = 0.86
AUC = 0.99
TSS = 0.81
AUC = 0.98
TSS = 0.74
0.00
0.25
0.50
0.75
bio12
bio14
bio15
TWI

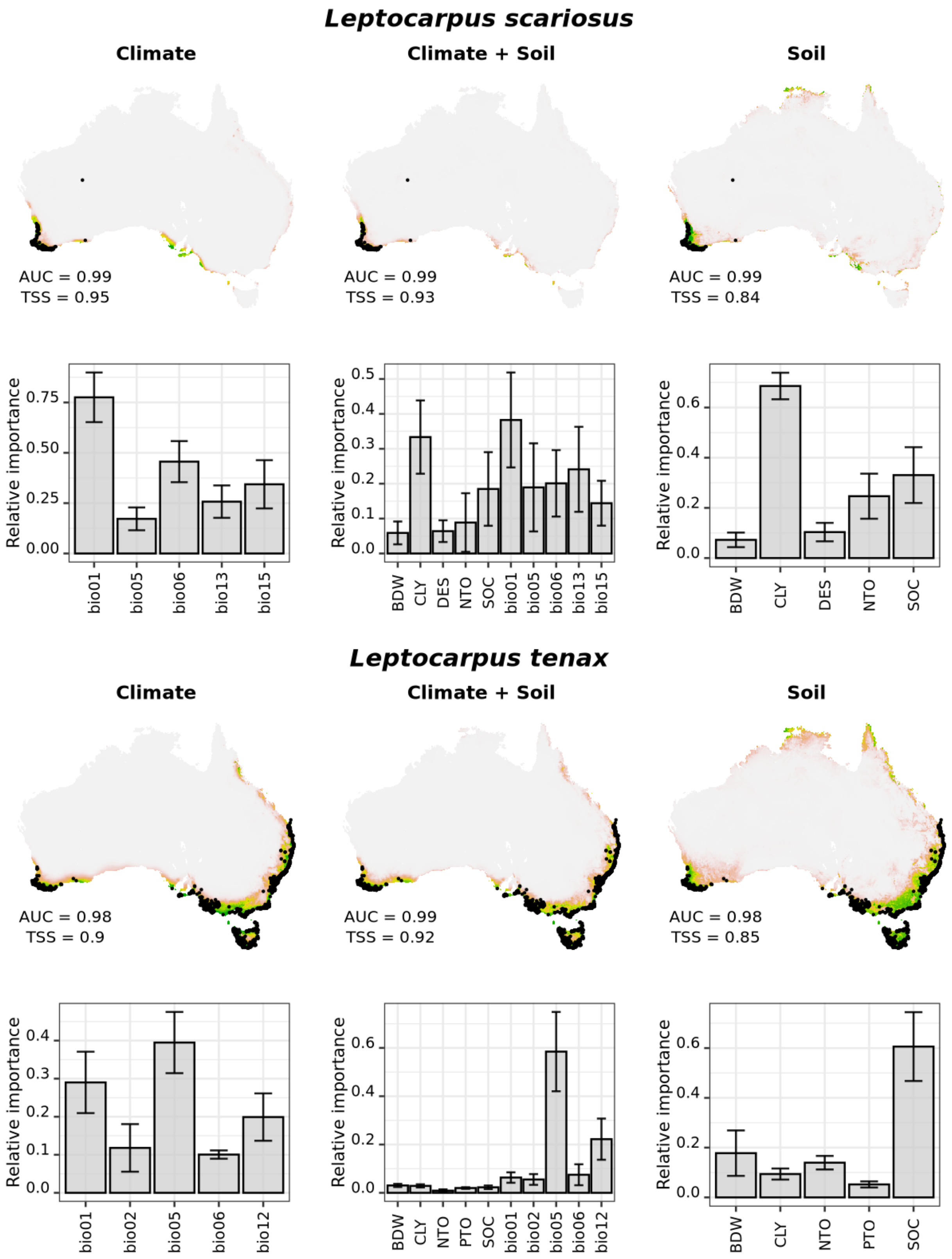
Leptocarpus scariosus
Climate
Climate + Soil
Soil
AUC = 0.99
TSS = 0.95
AUC = 0.99
TSS = 0.93
AUC = 0.99
TSS = 0.84
Relative importance
0.00
0.25
0.50
0.75
bio01
bio05
bio06
bio13
bio15
0.0
0.1
0.2
0.3
0.4
0.5
BDW
CLY
DES
NTO
SOC
bio01
bio05
bio06
bio13
bio15
0.0
0.2
0.4
0.6
BDW
CLY
DES
NTO
SOC
Leptocarpus tenax
Climate
Climate + Soil
Soil
AUC = 0.98
TSS = 0.9
AUC = 0.99
TSS = 0.92
AUC = 0.98
TSS = 0.85
Relative importance
0.0
0.1
0.2
0.3
0.4
bio01
bio02
bio05
bio06
bio12
0.0
0.2
0.4
0.6
BDW
CLY
NTO
PTO
SOC
bio01
bio02
bio05
bio06
bio12
0.0
0.2
0.4
0.6
BDW
CLY
NTO
PTO
SOC

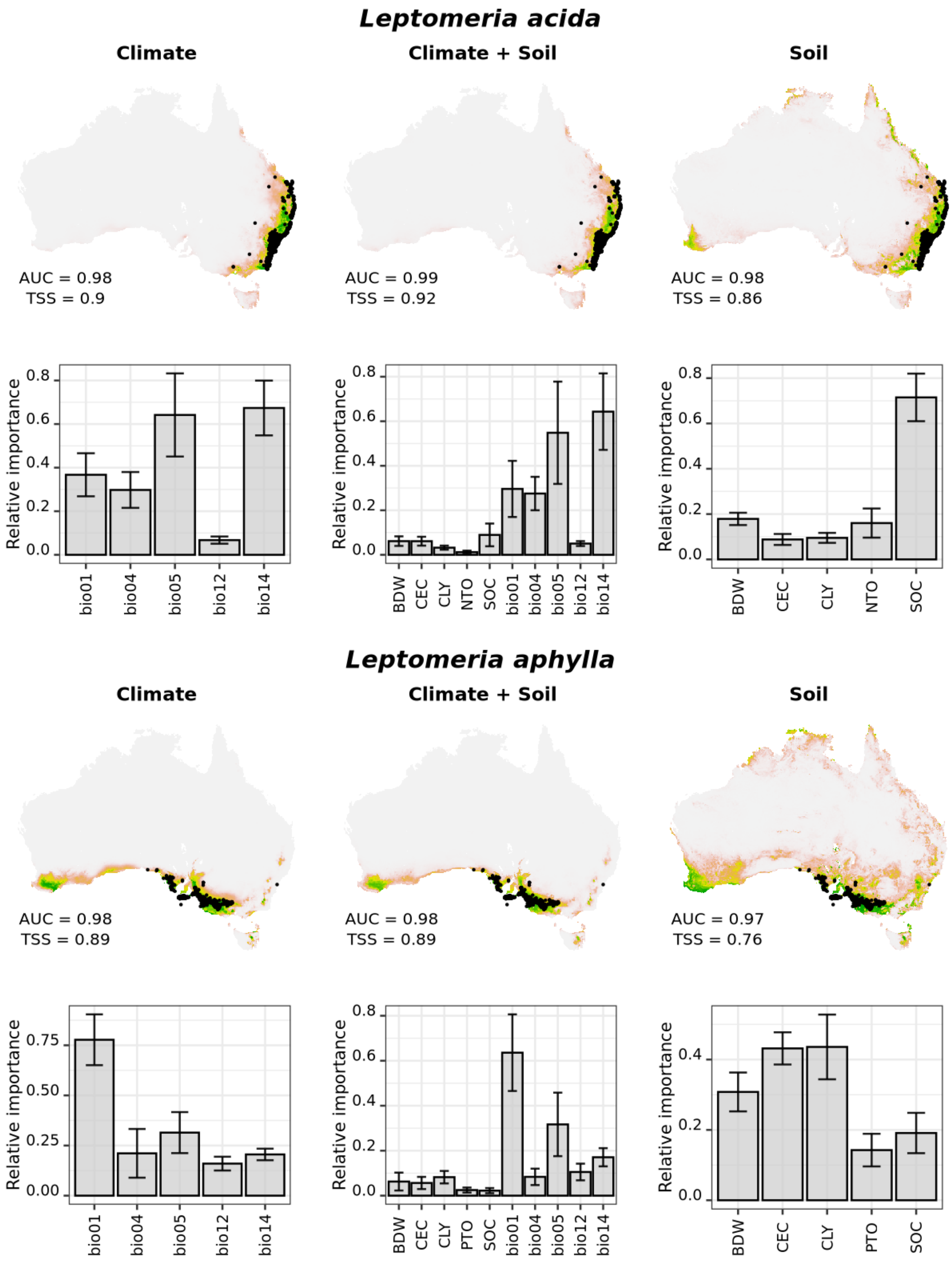

Leptomeria acida
Climate
Climate + Soil
Soil
AUC = 0.98
TSS = 0.9
AUC = 0.99
TSS = 0.92
AUC = 0.98
TSS = 0.86
Relative importance
0.0
0.2
0.4
0.6
0.8
bio01
bio04
bio05
bio12
bio14
BDW
CEC
CLY
NTO
SOC
Leptomeria aphylla
Climate
Climate + Soil
Soil
AUC = 0.98
TSS = 0.89
AUC = 0.98
TSS = 0.89
AUC = 0.97
TSS = 0.76
Relative importance
0.00
0.25
0.50
0.75
bio01
bio04
bio05
bio12
bio14
BDW
CEC
CLY
PTO
SOC

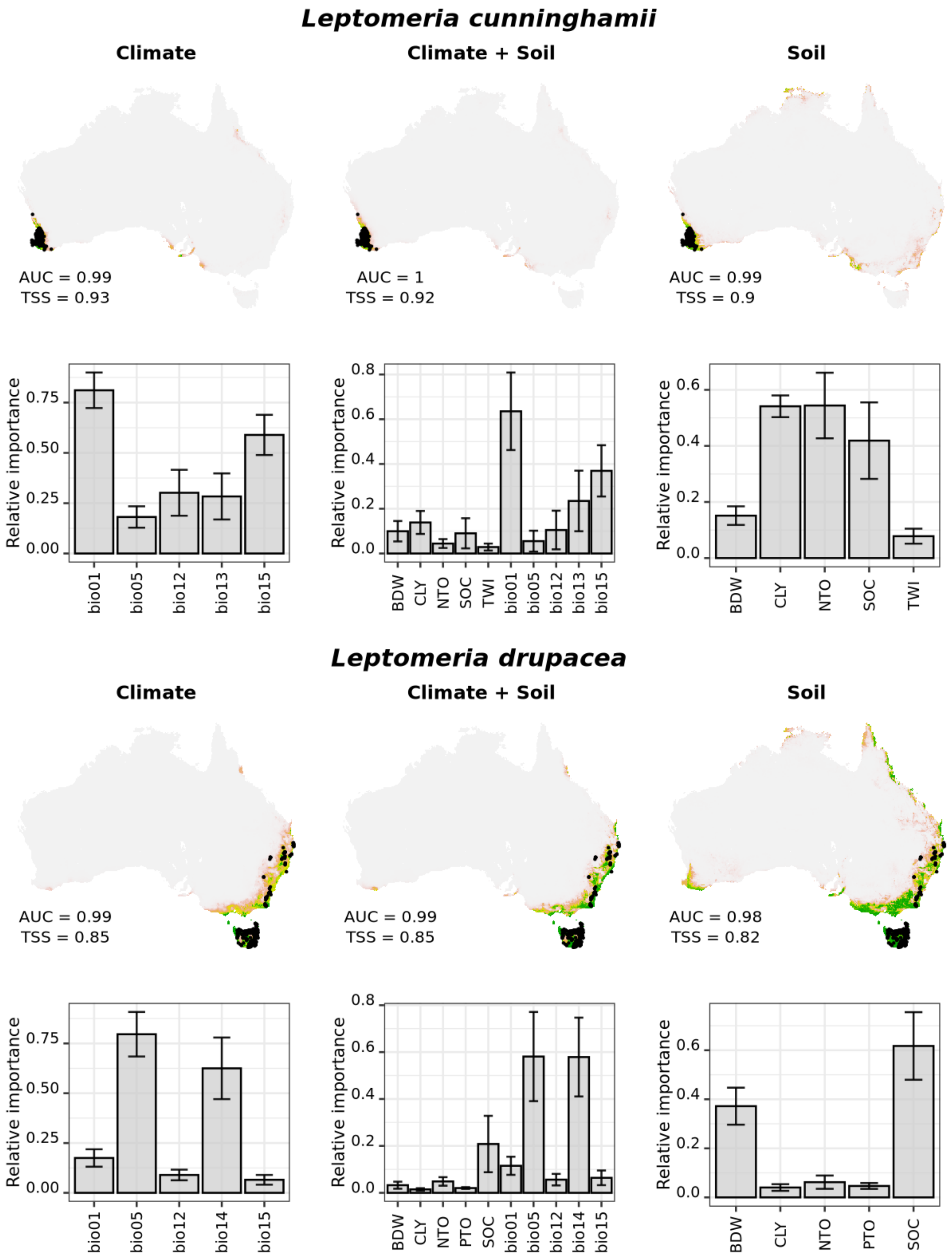
Leptomeria cunninghamii
Climate
Climate + Soil
Soil
AUC = 0.99
TSS = 0.93
AUC = 1
TSS = 0.92
AUC = 0.99
TSS = 0.9
Relative importance
bio01
bio05
bio12
bio13
bio15
BDW
CLY
NTO
SOC
TWI
Leptomeria drupacea
Climate
Climate + Soil
Soil
AUC = 0.99
TSS = 0.85
AUC = 0.99
TSS = 0.85
AUC = 0.98
TSS = 0.82
Relative importance
bio01
bio05
bio12
bio14
bio15
BDW
CLY
NTO
PTO
SOC

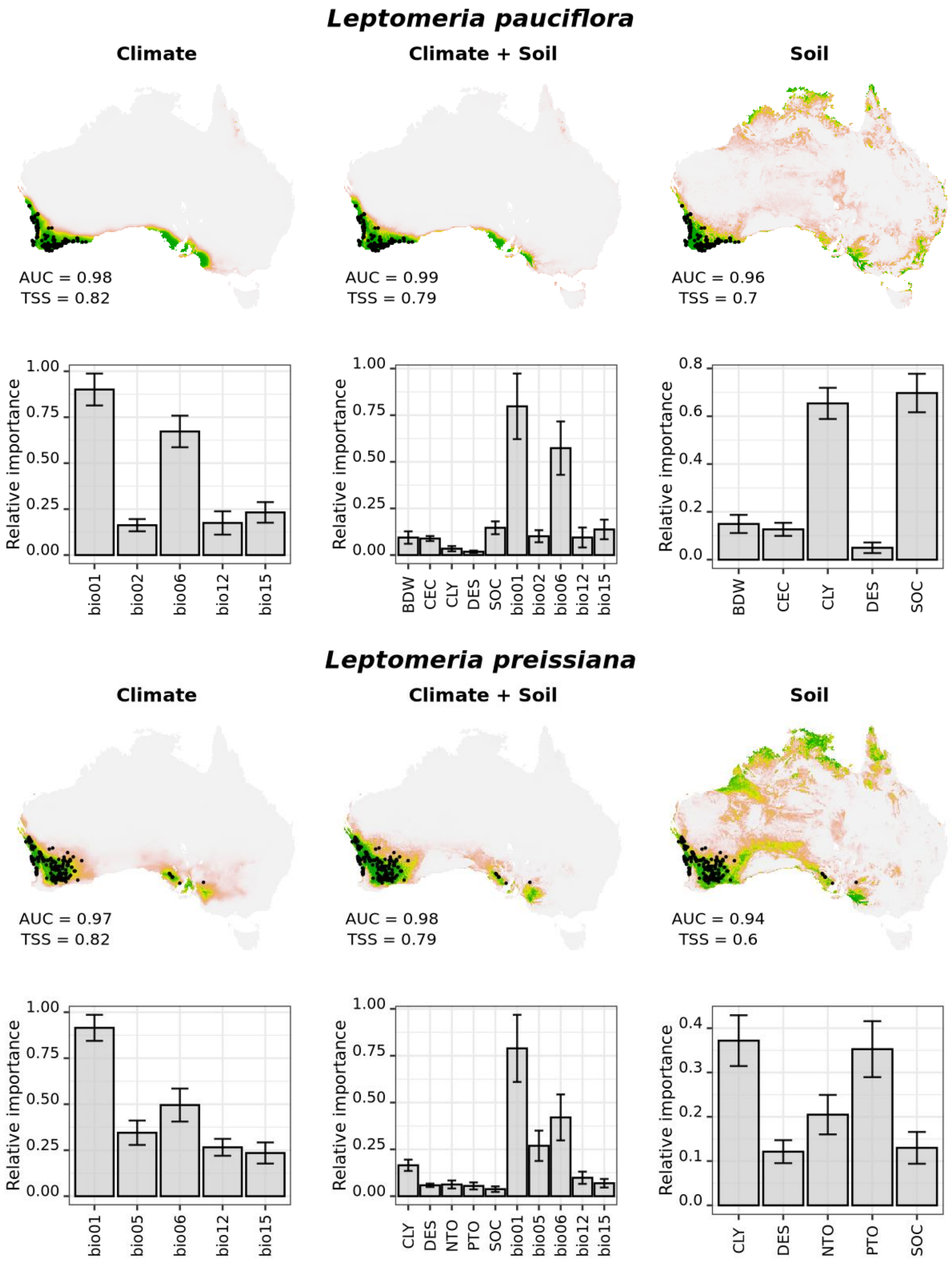

Leptomeria pauciflora
Climate
Climate + Soil
Soil
AUC = 0.98
TSS = 0.82
AUC = 0.99
TSS = 0.79
AUC = 0.96
TSS = 0.7
Relative importance
1.00
0.75
0.50
0.25
0.00
bio01
bio02
bio06
bio12
bio15
BDW
CEC
CLY
DES
SOC
0.8
0.6
0.4
0.2
0.0
Leptomeria preissiana
Climate
Climate + Soil
Soil
AUC = 0.97
TSS = 0.82
AUC = 0.98
TSS = 0.79
AUC = 0.94
TSS = 0.6
bio05
NTO
PTO
0.3
0.1

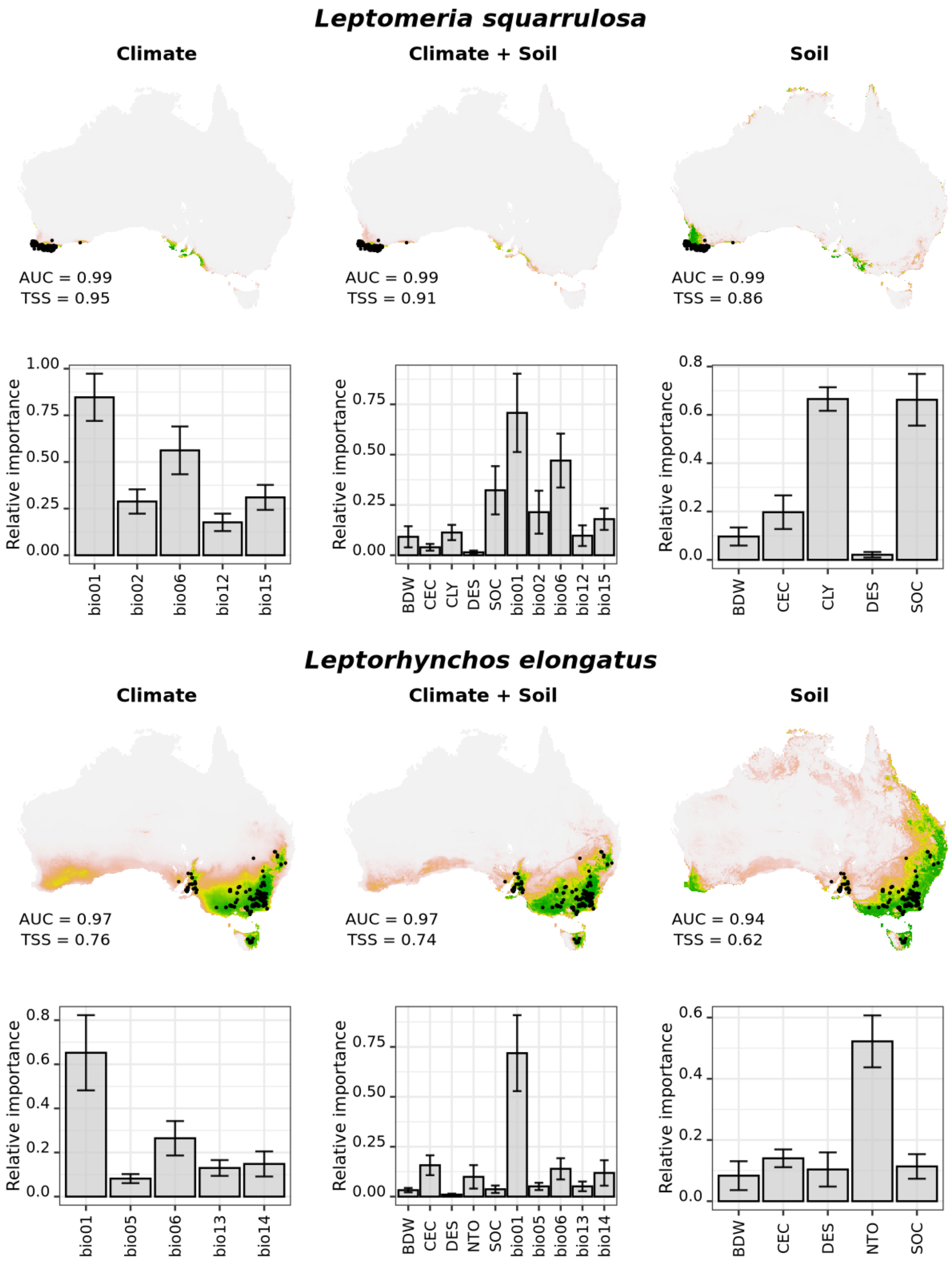

Leptomeria squarrulosa
Climate
Climate + Soil
Soil
AUC = 0.99
TSS = 0.95
AUC = 0.99
TSS = 0.91
AUC = 0.99
TSS = 0.86
Relative importance
1.00
0.75
0.50
0.25
0.00
bio01
bio02
bio06
bio12
bio15
0.75
0.50
0.25
0.00
BDW
CEC
CLY
DES
SOC
bio01
bio02
bio06
bio12
bio15
0.8
0.6
0.4
0.2
0.0
BDW
CEC
CLY
DES
SOC
Leptorhynchos elongatus
Climate
Climate + Soil
Soil
AUC = 0.97
TSS = 0.76
AUC = 0.97
TSS = 0.74
AUC = 0.94
TSS = 0.62
Relative importance
0.8
0.6
0.4
0.2
0.0
bio01
bio05
bio06
bio13
bio14
0.75
0.50
0.25
0.00
BDW
CEC
DES
NTO
SOC
bio01
bio05
bio06
bio13
bio14
0.6
0.4
0.2
0.0
BDW
CEC
DES
NTO
SOC

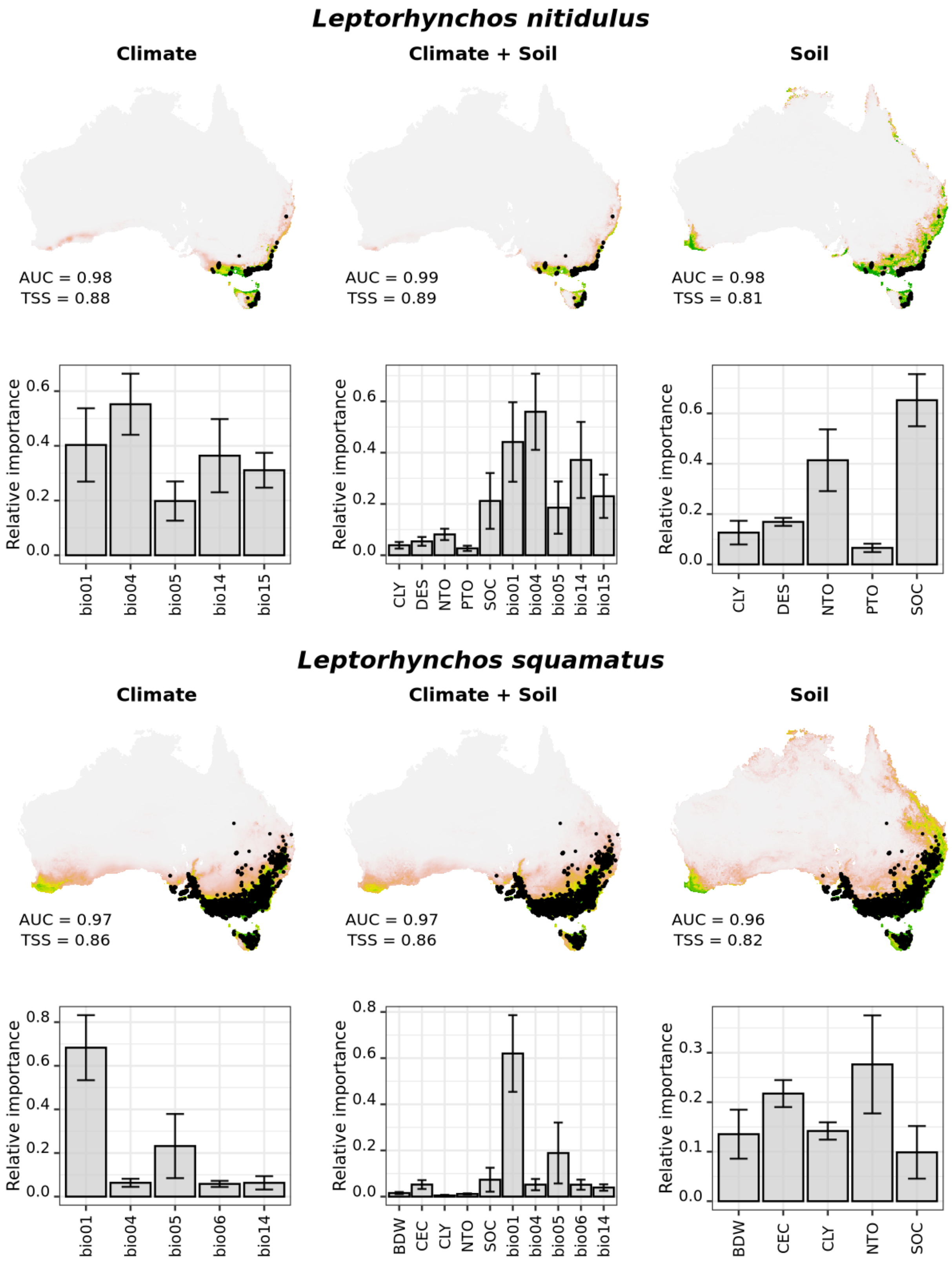
Leptorhynchos nitidulus
Climate
Climate + Soil
Soil
AUC = 0.98
TSS = 0.88
AUC = 0.99
TSS = 0.89
AUC = 0.98
TSS = 0.81
Relative importance
bio01
bio04
bio05
bio14
bio15
CLY
DES
NTO
PTO
SOC
Leptorhynchos squamatus
Climate
Climate + Soil
Soil
AUC = 0.97
TSS = 0.86
AUC = 0.97
TSS = 0.86
AUC = 0.96
TSS = 0.82
Relative importance
bio01
bio04
bio05
bio06
bio14
BDW
CEC
CLY
NTO
SOC

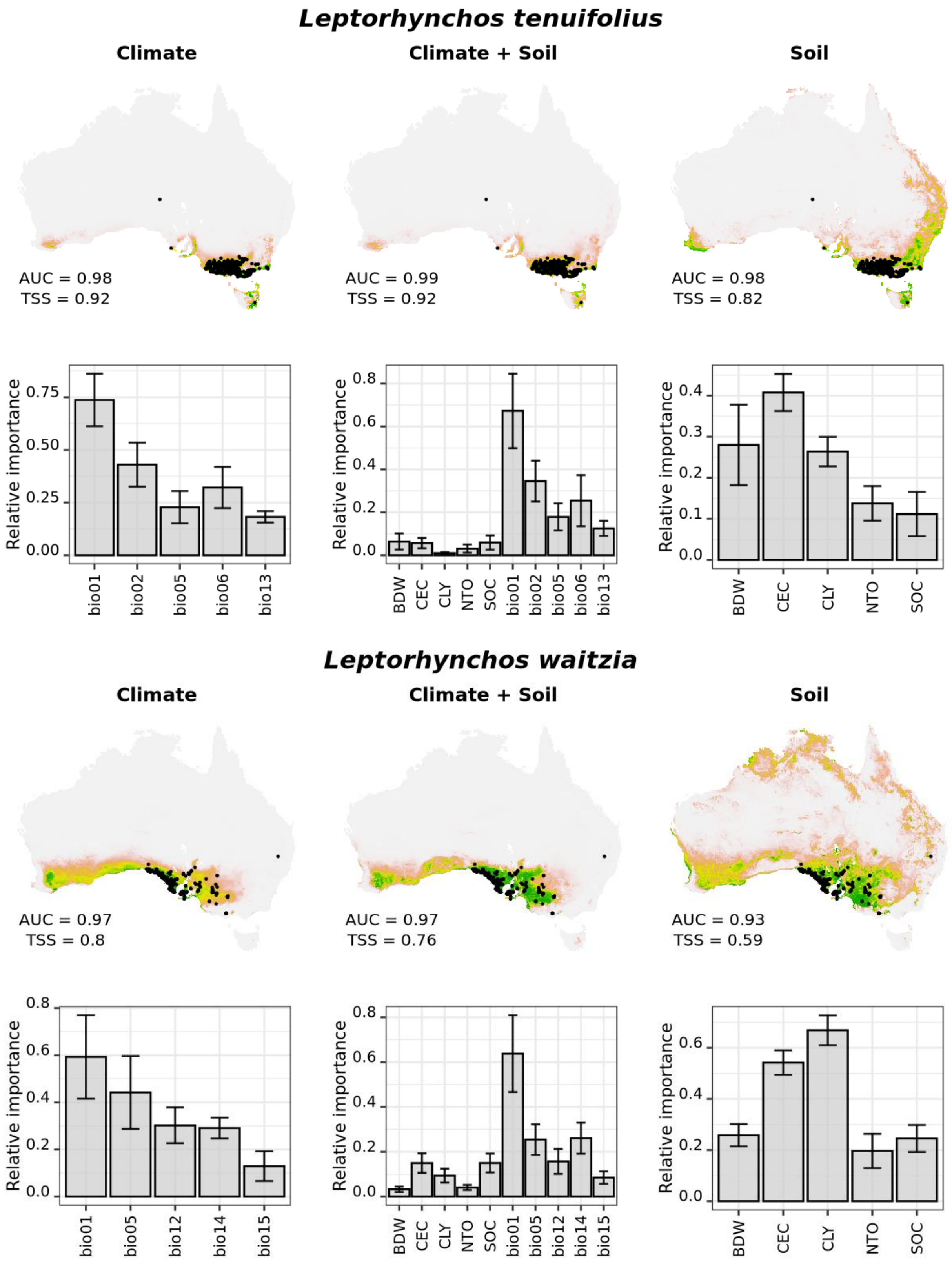

Leptorhynchos tenuifolius
Climate
Climate + Soil
Soil
AUC = 0.98
TSS = 0.92
AUC = 0.99
TSS = 0.92
AUC = 0.98
TSS = 0.82
Relative importance
0.00
0.25
0.50
0.75
bio01
bio02
bio05
bio06
bio13
0.0
0.2
0.4
0.6
0.8
BDW
CEC
CLY
NTO
SOC
bio01
bio02
bio05
bio06
bio13
0.0
0.1
0.2
0.3
0.4
BDW
CEC
CLY
NTO
SOC
Leptorhynchos waitzia
Climate
Climate + Soil
Soil
AUC = 0.97
TSS = 0.8
AUC = 0.97
TSS = 0.76
AUC = 0.93
TSS = 0.59
Relative importance
0.0
0.2
0.4
0.6
0.8
bio01
bio05
bio12
bio14
bio15
BDW
CEC
CLY
NTO
SOC
bio01
bio05
bio12
bio14
bio15
BDW
CEC
CLY
NTO
SOC

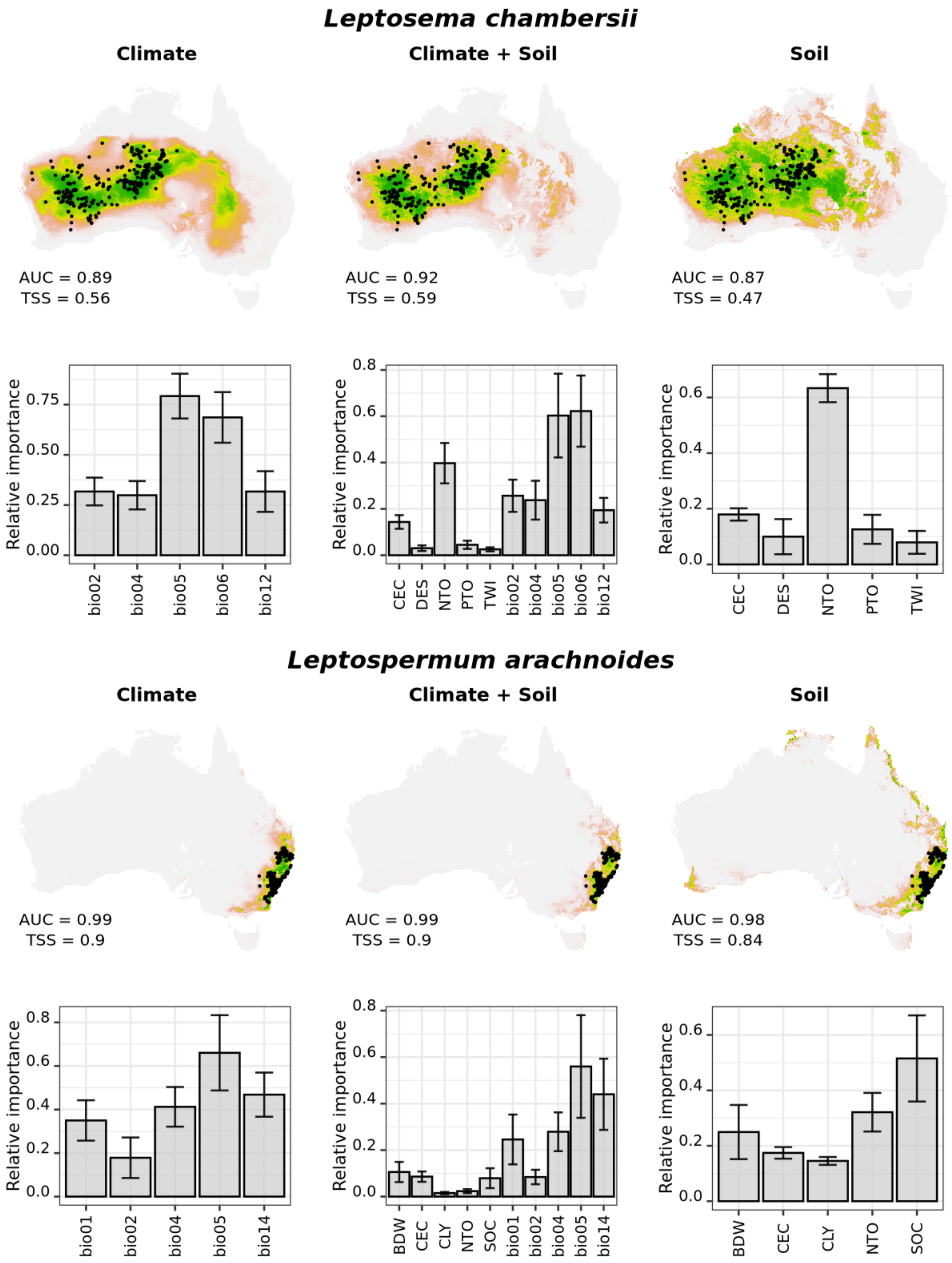

Leptosema chambersii
Climate
Climate + Soil
Soil
AUC = 0.89
TSS = 0.56
AUC = 0.92
TSS = 0.59
AUC = 0.87
TSS = 0.47
Relative importance
0.00 0.25 0.50 0.75
bio02 bio04 bio05 bio06 bio12
0.0 0.2 0.4 0.6 0.8
CEC DES NTO PTO TWI bio02 bio04 bio05 bio06 bio12
0.0 0.2 0.4 0.6
CEC DES NTO PTO TWI
Leptospermum arachnoides
Climate
Climate + Soil
Soil
AUC = 0.99
TSS = 0.9
AUC = 0.99
TSS = 0.9
AUC = 0.98
TSS = 0.84
Relative importance
0.0 0.2 0.4 0.6 0.8
bio01 bio02 bio04 bio05 bio14
0.0 0.2 0.4 0.6 0.8
BDW CEC CLY NTO SOC bio01 bio02 bio04 bio05 bio14
0.0 0.2 0.4 0.6
BDW CEC CLY NTO SOC

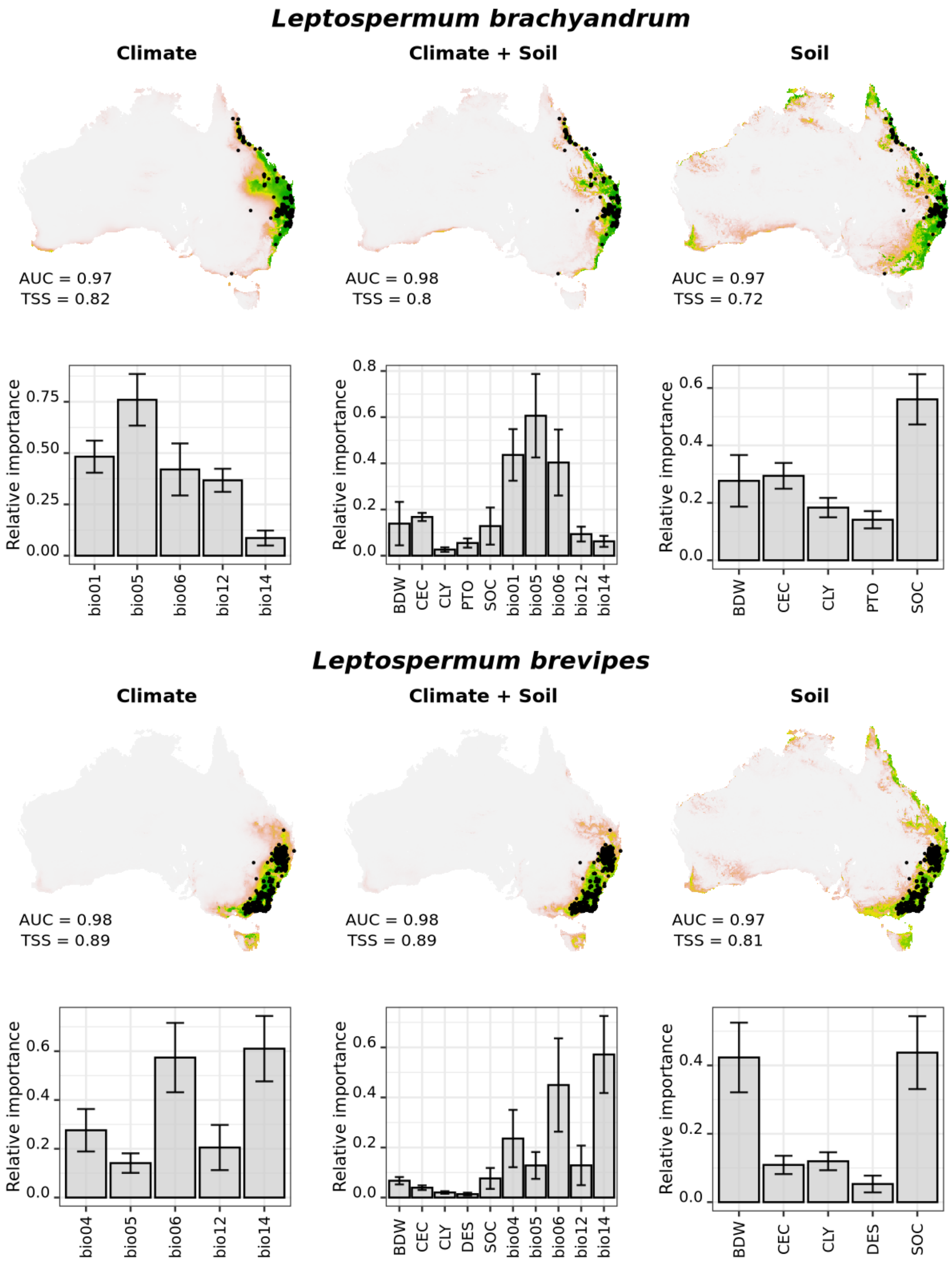

Leptospermum brachyandrum
Climate
Climate + Soil
Soil
AUC = 0.97
TSS = 0.82
AUC = 0.98
TSS = 0.8
AUC = 0.97
TSS = 0.72
Relative importance
0.00
0.25
0.50
0.75
bio01
bio05
bio06
bio12
bio14
0.0
0.2
0.4
0.6
0.8
BDW
CEC
CLY
PTO
SOC
bio01
bio05
bio06
bio12
bio14
0.0
0.2
0.4
0.6
BDW
CEC
CLY
PTO
SOC
Leptospermum brevipes
Climate
Climate + Soil
Soil
AUC = 0.98
TSS = 0.89
AUC = 0.98
TSS = 0.89
AUC = 0.97
TSS = 0.81
Relative importance
0.0
0.2
0.4
0.6
bio04
bio05
bio06
bio12
bio14
BDW
CEC
CLY
DES
SOC
bio04
bio05
bio06
bio12
bio14
BDW
CEC
CLY
DES
SOC

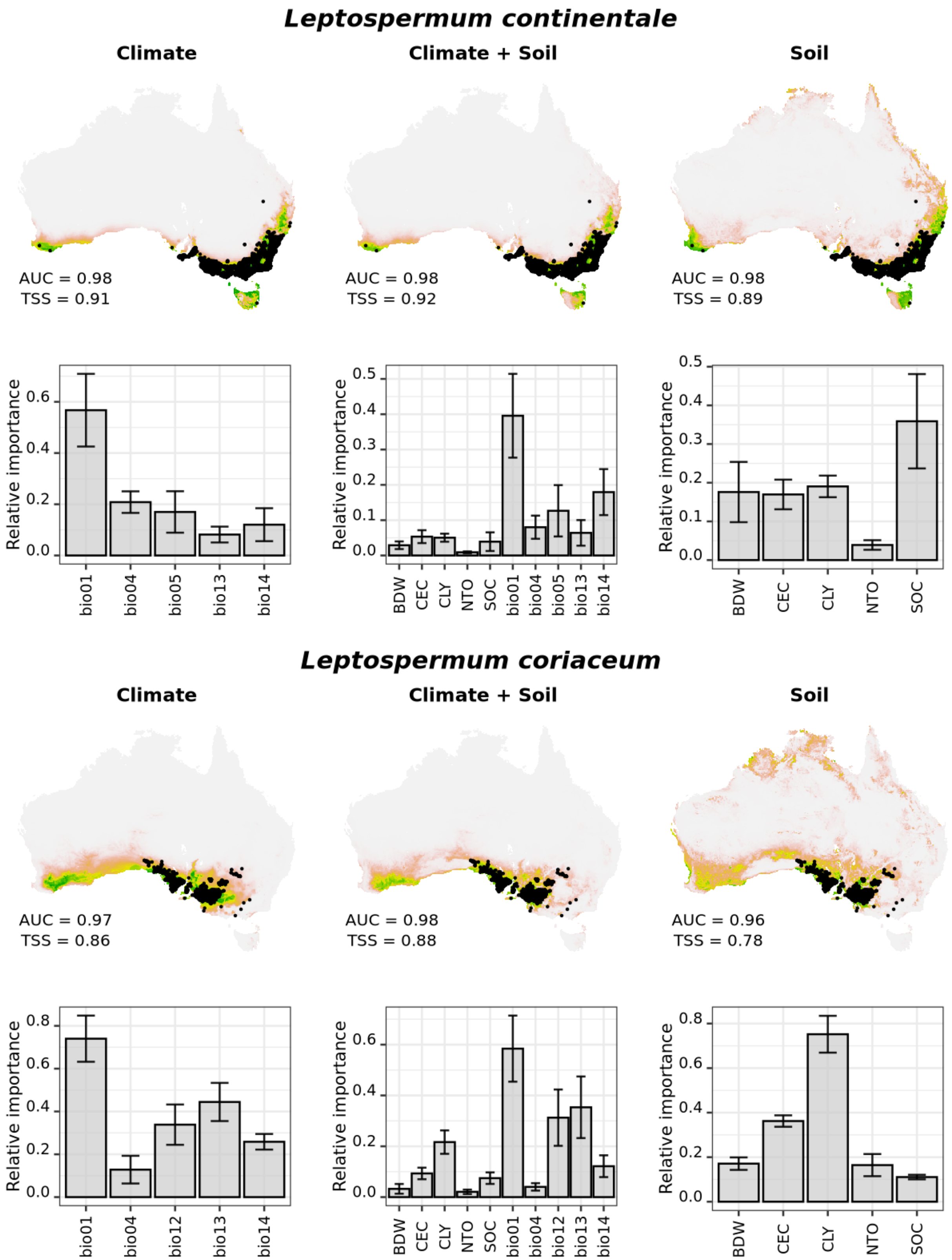
Leptospermum continentale
Climate
Climate + Soil
Soil
AUC = 0.98
TSS = 0.91
AUC = 0.98
TSS = 0.92
AUC = 0.98
TSS = 0.89
Relative importance
0.0
0.2
0.4
0.6
bio01
bio04
bio05
bio13
bio14
0.0
0.1
0.2
0.3
0.4
0.5
BDW
CEC
CLY
NTO
SOC
bio01
bio04
bio05
bio13
bio14
0.0
0.1
0.2
0.3
0.4
0.5
BDW
CEC
CLY
NTO
SOC
Leptospermum coriaceum
Climate
Climate + Soil
Soil
AUC = 0.97
TSS = 0.86
AUC = 0.98
TSS = 0.88
AUC = 0.96
TSS = 0.78
Relative importance
0.0
0.2
0.4
0.6
0.8
bio01
bio04
bio12
bio13
bio14
0.0
0.2
0.4
0.6
BDW
CEC
CLY
NTO
SOC
bio01
bio04
bio12
bio13
bio14
0.0
0.2
0.4
0.6
0.8
BDW
CEC
CLY
NTO
SOC

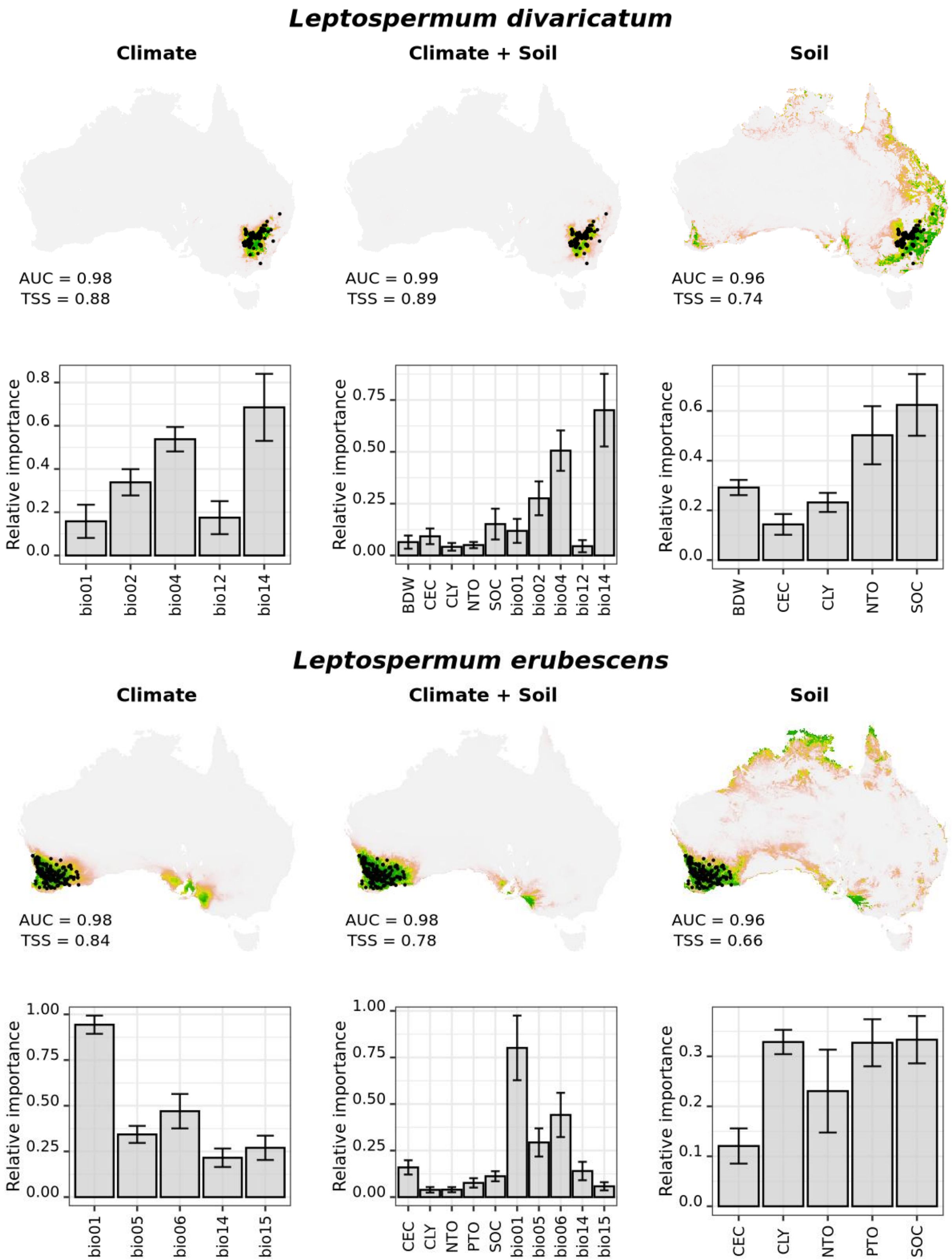
Leptospermum divaricatum
Climate
Climate + Soil
Soil
AUC = 0.98
TSS = 0.88
AUC = 0.99
TSS = 0.89
AUC = 0.96
TSS = 0.74
Relative importance
bio01
bio02
bio04
bio12
bio14
BDW
CEC
CLY
NTO
SOC
Leptospermum erubescens
Climate
Climate + Soil
Soil
AUC = 0.98
TSS = 0.84
AUC = 0.98
TSS = 0.78
AUC = 0.96
TSS = 0.66
Relative importance
bio01
bio05
bio06
bio14
bio15
CEC
CLY
NTO
PTO
SOC

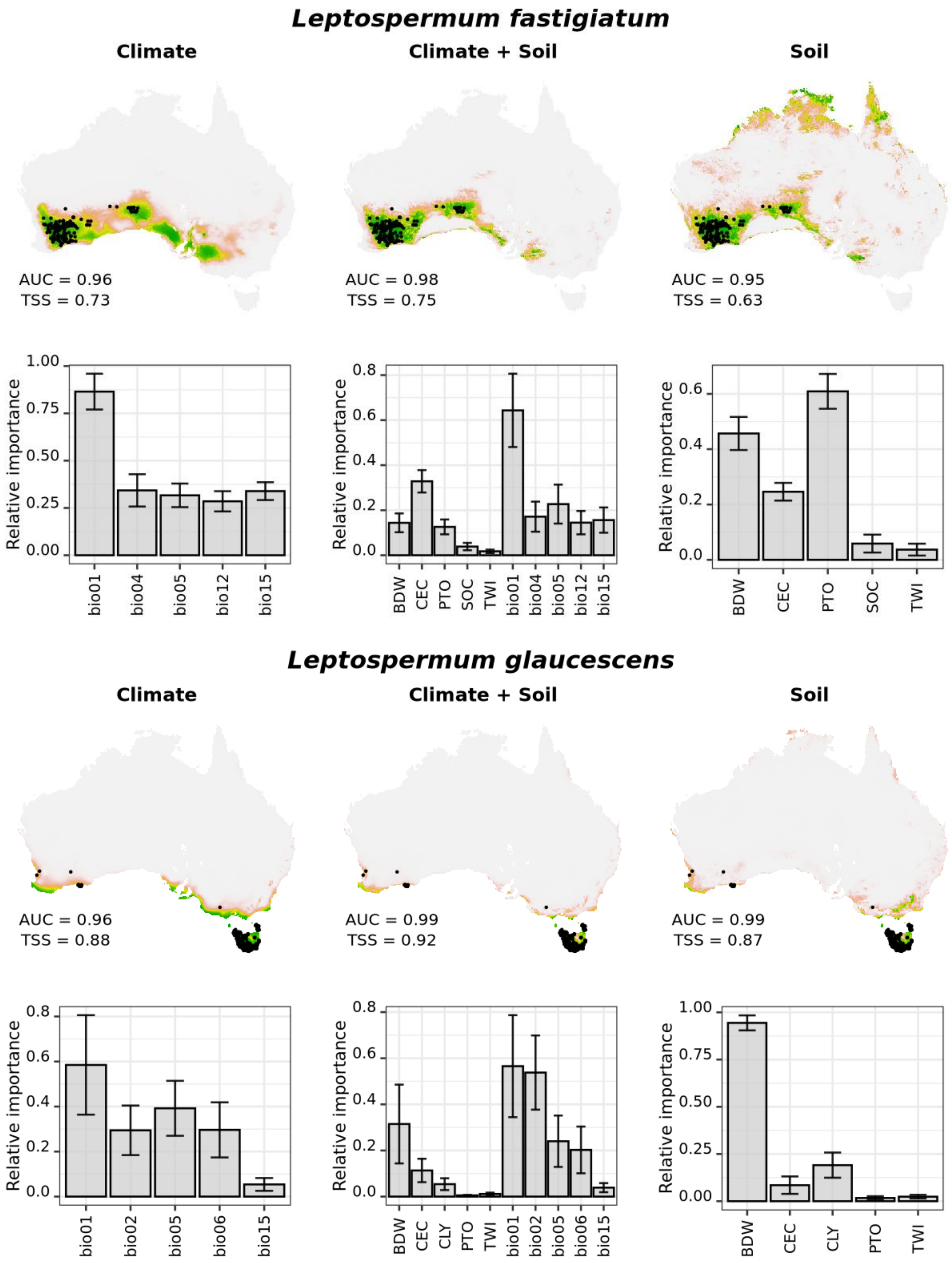

Leptospermum fastigiatum
Climate
Climate + Soil
Soil
AUC = 0.96
TSS = 0.73
AUC = 0.98
TSS = 0.75
AUC = 0.95
TSS = 0.63
Relative importance
1.00
0.75
0.50
0.25
0.00
bio01
bio04
bio05
bio12
bio15
0.8
0.6
0.4
0.2
0.0
BDW
CEC
PTO
SOC
TWI
bio01
bio04
bio05
bio12
bio15
0.6
0.4
0.2
0.0
BDW
CEC
PTO
SOC
TWI
Leptospermum glaucescens
Climate
Climate + Soil
Soil
AUC = 0.96
TSS = 0.88
AUC = 0.99
TSS = 0.92
AUC = 0.99
TSS = 0.87
Relative importance
0.8
0.6
0.4
0.2
0.0
bio01
bio02
bio05
bio06
bio15
BDW
CEC
CLY
PTO
TWI
bio01
bio02
bio05
bio06
bio15
1.00
0.75
0.50
0.25
0.00
BDW
CEC
CLY
PTO
TWI

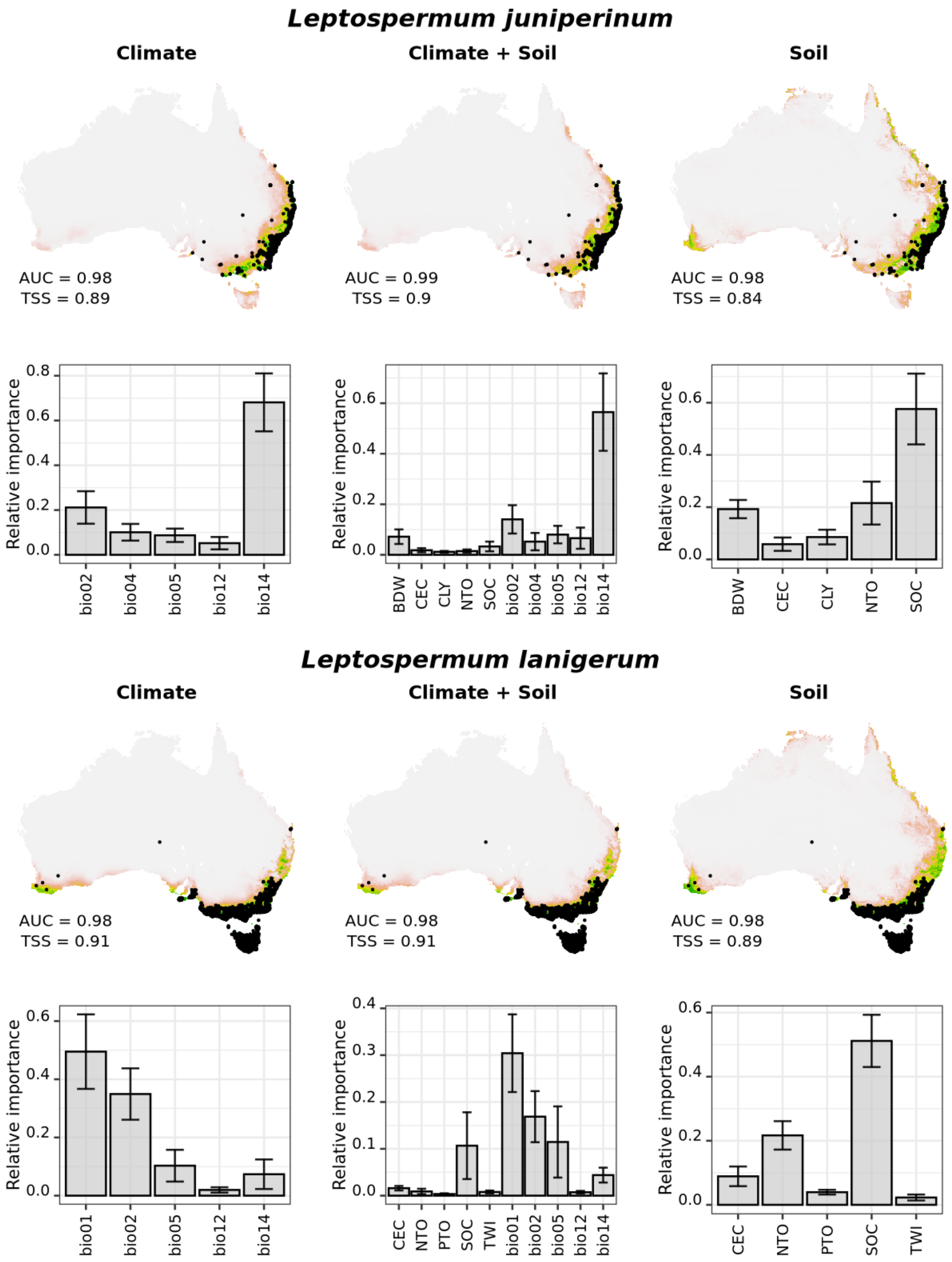
Leptospermum juniperinum
Climate
Climate + Soil
Soil
AUC = 0.98
TSS = 0.89
AUC = 0.99
TSS = 0.9
AUC = 0.98
TSS = 0.84
Relative importance
0.0
0.2
0.4
0.6
0.8
bio02
bio04
bio05
bio12
bio14
BDW
CEC
CLY
NTO
SOC
Leptospermum lanigerum
Climate
Climate + Soil
Soil
AUC = 0.98
TSS = 0.91
AUC = 0.98
TSS = 0.91
AUC = 0.98
TSS = 0.89
Relative importance
0.0
0.1
0.2
0.3
0.4
0.6
bio01
bio02
bio05
bio12
bio14
CEC
NTO
PTO
SOC
TWI

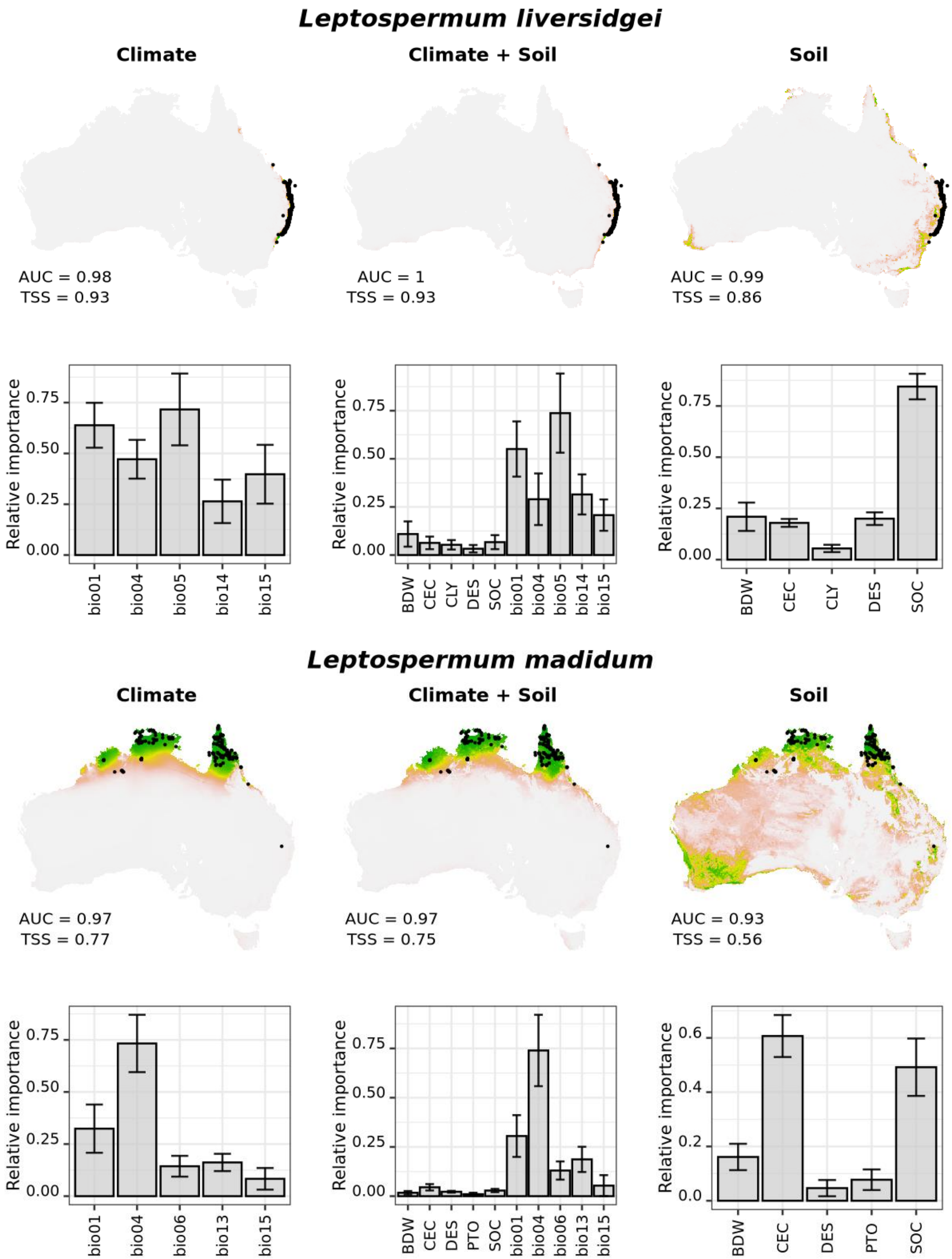
Leptospermum liversidgei
Climate
Climate + Soil
Soil
AUC = 0.98
TSS = 0.93
AUC = 1
TSS = 0.93
AUC = 0.99
TSS = 0.86
Relative importance
0.00
0.25
0.50
0.75
bio01
bio04
bio05
bio14
bio15
BDW
CEC
CLY
DES
SOC
Leptospermum madidum
Climate
Climate + Soil
Soil
AUC = 0.97
TSS = 0.77
AUC = 0.97
TSS = 0.75
AUC = 0.93
TSS = 0.56
bio06
bio13
PTO
0.0
0.2
0.4
0.6

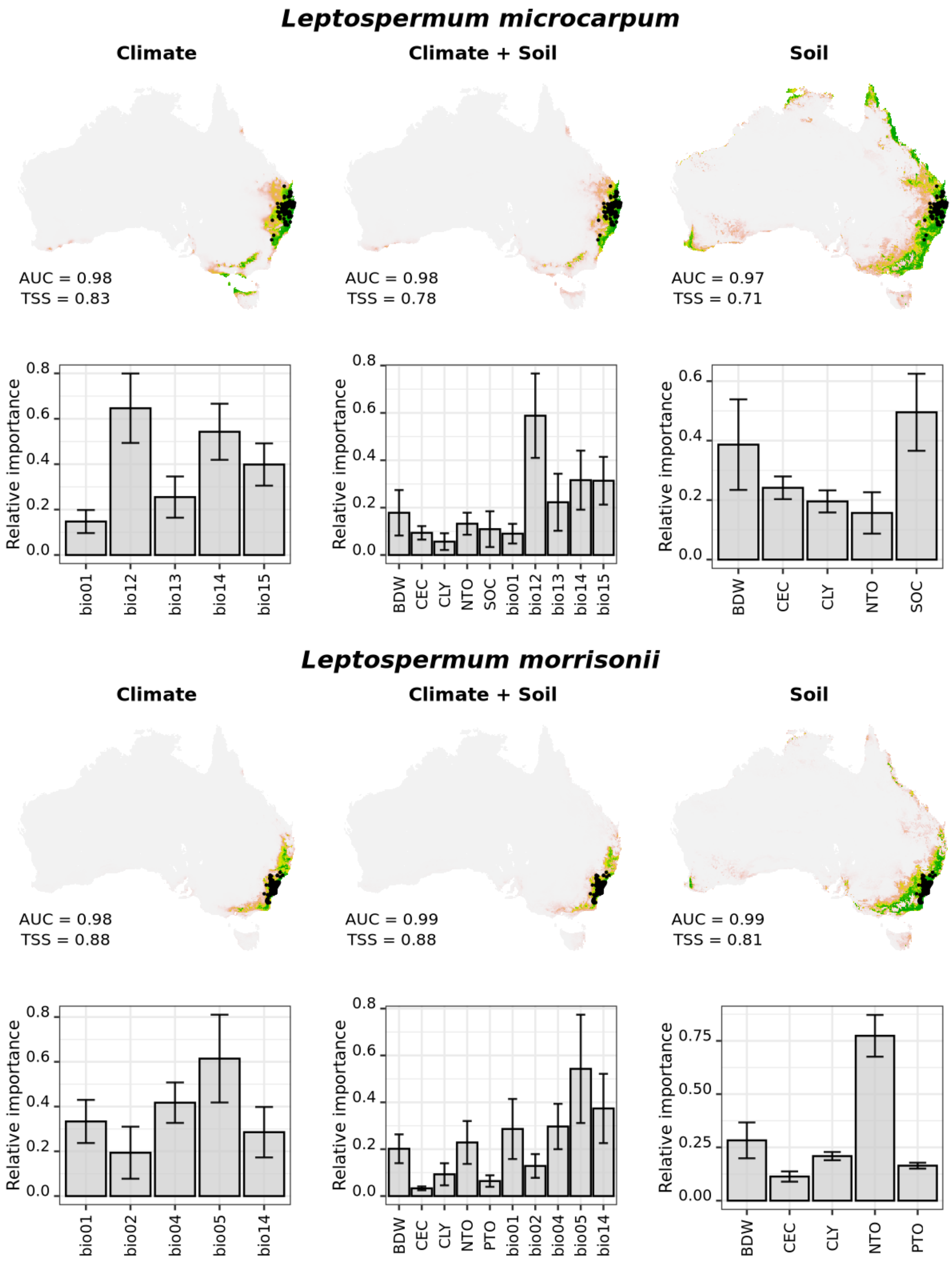

Leptospermum microcarpum
Climate
Climate + Soil
Soil
AUC = 0.98
TSS = 0.83
AUC = 0.98
TSS = 0.78
AUC = 0.97
TSS = 0.71
Relative importance
bio01
bio12
bio13
bio14
bio15
BDW
CEC
CLY
NTO
SOC
Leptospermum morrisonii
Climate
Climate + Soil
Soil
AUC = 0.98
TSS = 0.88
AUC = 0.99
TSS = 0.88
AUC = 0.99
TSS = 0.81
bio01
bio02
bio04
bio05
bio14
PTO

Leptospermum multicaule

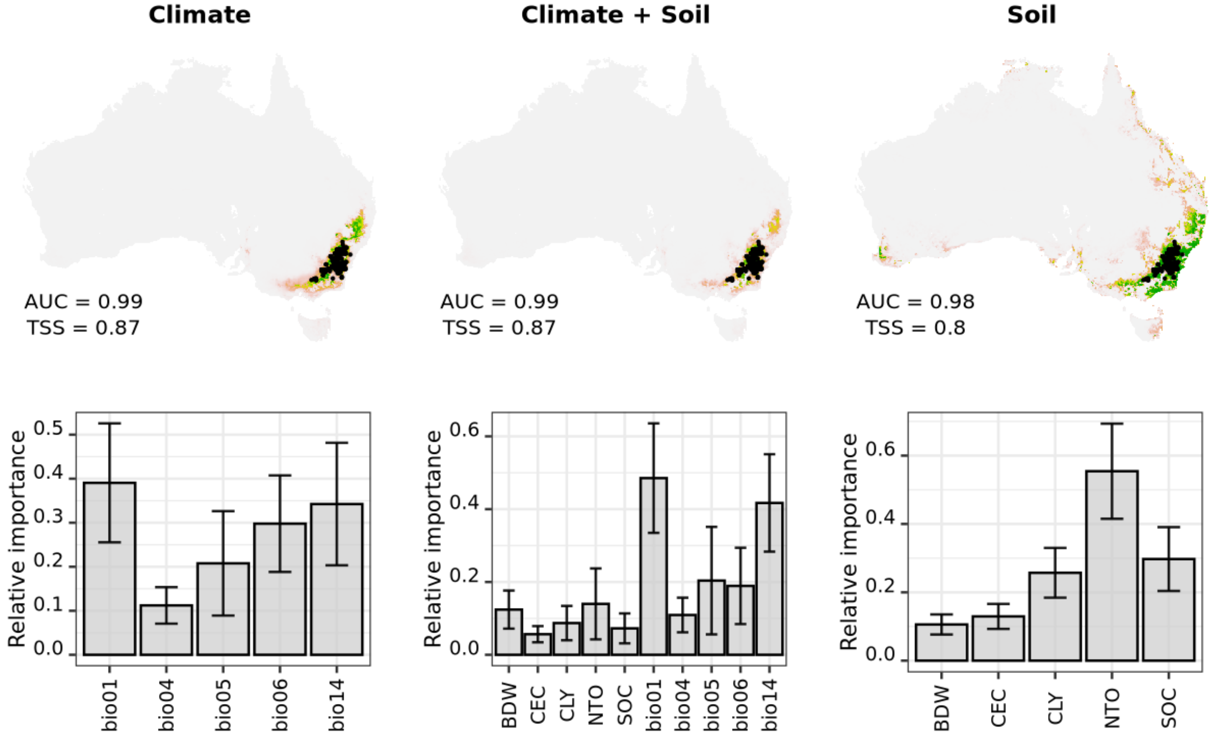

Leptospermum myrtifolium

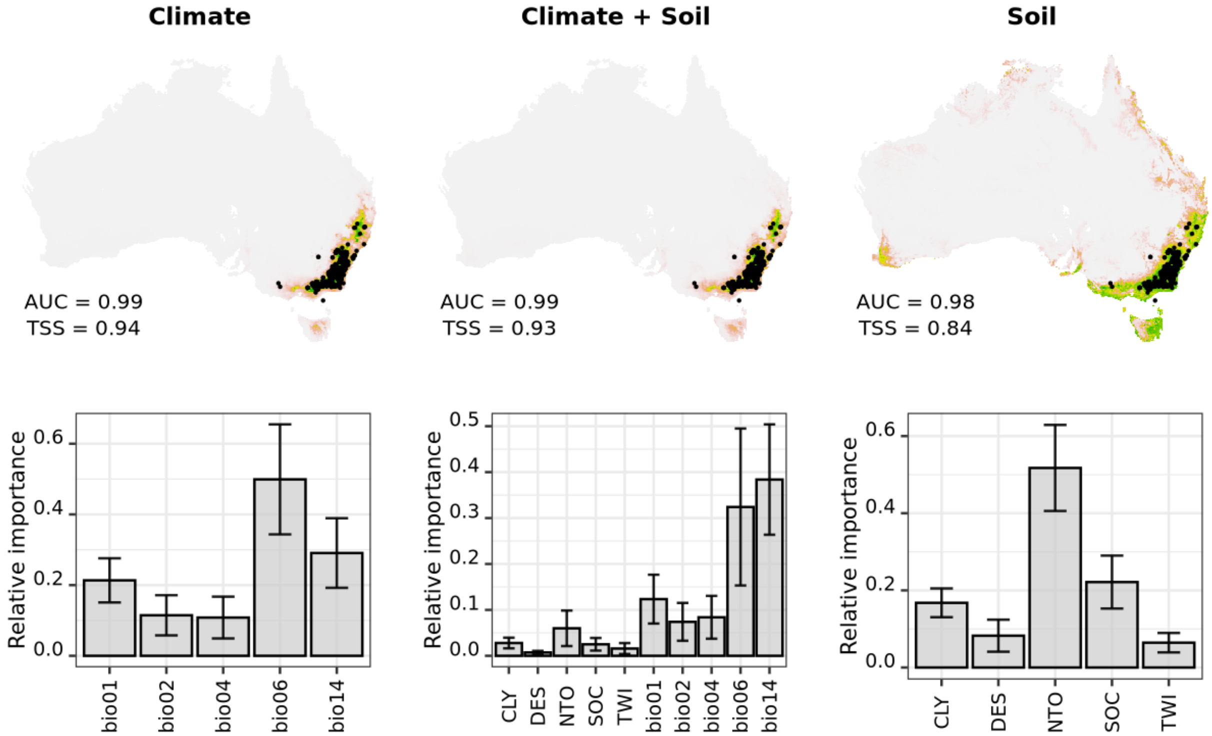

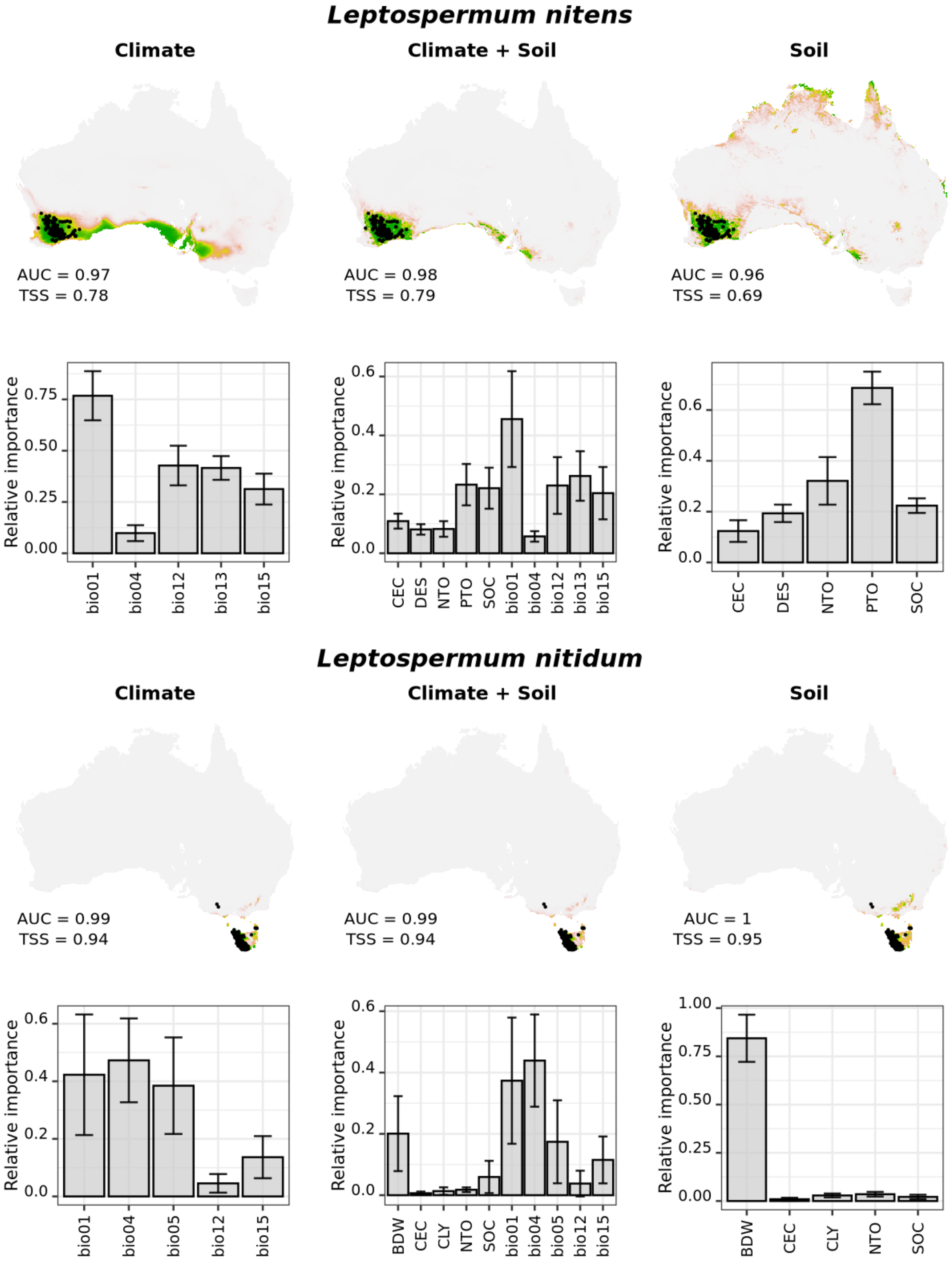
Leptospermum nitens
Climate
Climate + Soil
Soil
AUC = 0.97
TSS = 0.78
AUC = 0.98
TSS = 0.79
AUC = 0.96
TSS = 0.69
Relative importance
0.00
0.25
0.50
0.75
bio01
bio04
bio12
bio13
bio15
0.0
0.2
0.4
0.6
CEC
DES
NTO
PTO
SOC
bio01
bio04
bio12
bio13
bio15
CEC
DES
NTO
PTO
SOC
Leptospermum nitidum
Climate
Climate + Soil
Soil
AUC = 0.99
TSS = 0.94
AUC = 0.99
TSS = 0.94
AUC = 1
TSS = 0.95
bio01
bio04
bio05
bio12
bio15
BDW
CEC
CLY
NTO
SOC
bio01
bio04
bio05
bio12
bio15
1.00
BDW
CEC
CLY
NTO
SOC

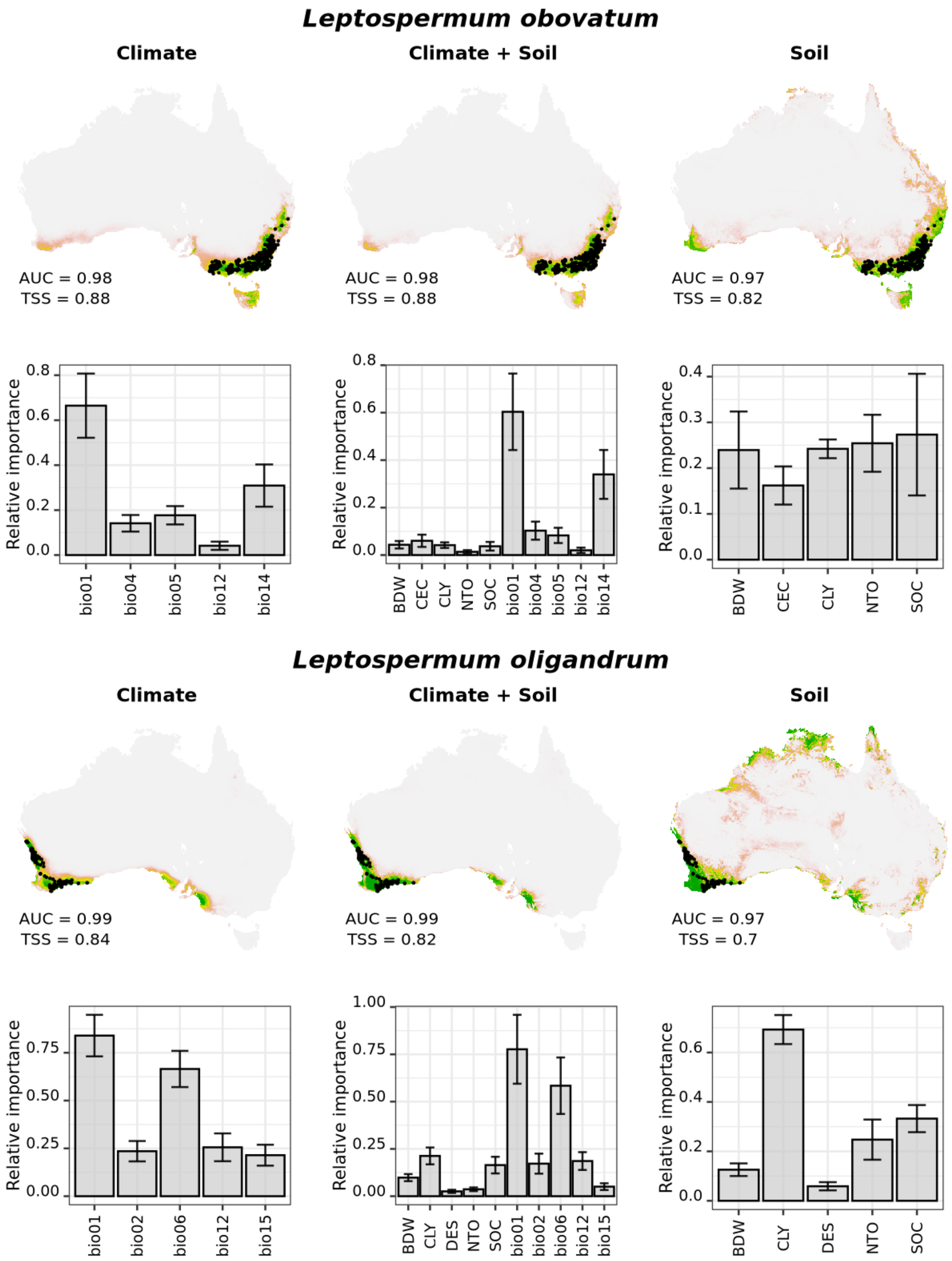
Leptospermum obovatum
Climate
Climate + Soil
Soil
AUC = 0.98
TSS = 0.88
AUC = 0.98
TSS = 0.88
AUC = 0.97
TSS = 0.82
Relative importance
bio01
bio04
bio05
bio12
bio14
BDW
CEC
CLY
NTO
SOC
Leptospermum oligandrum
Climate
Climate + Soil
Soil
AUC = 0.99
TSS = 0.84
AUC = 0.99
TSS = 0.82
AUC = 0.97
TSS = 0.7
Relative importance
bio01
bio02
bio06
bio12
bio15
BDW
CLY
DES
NTO
SOC

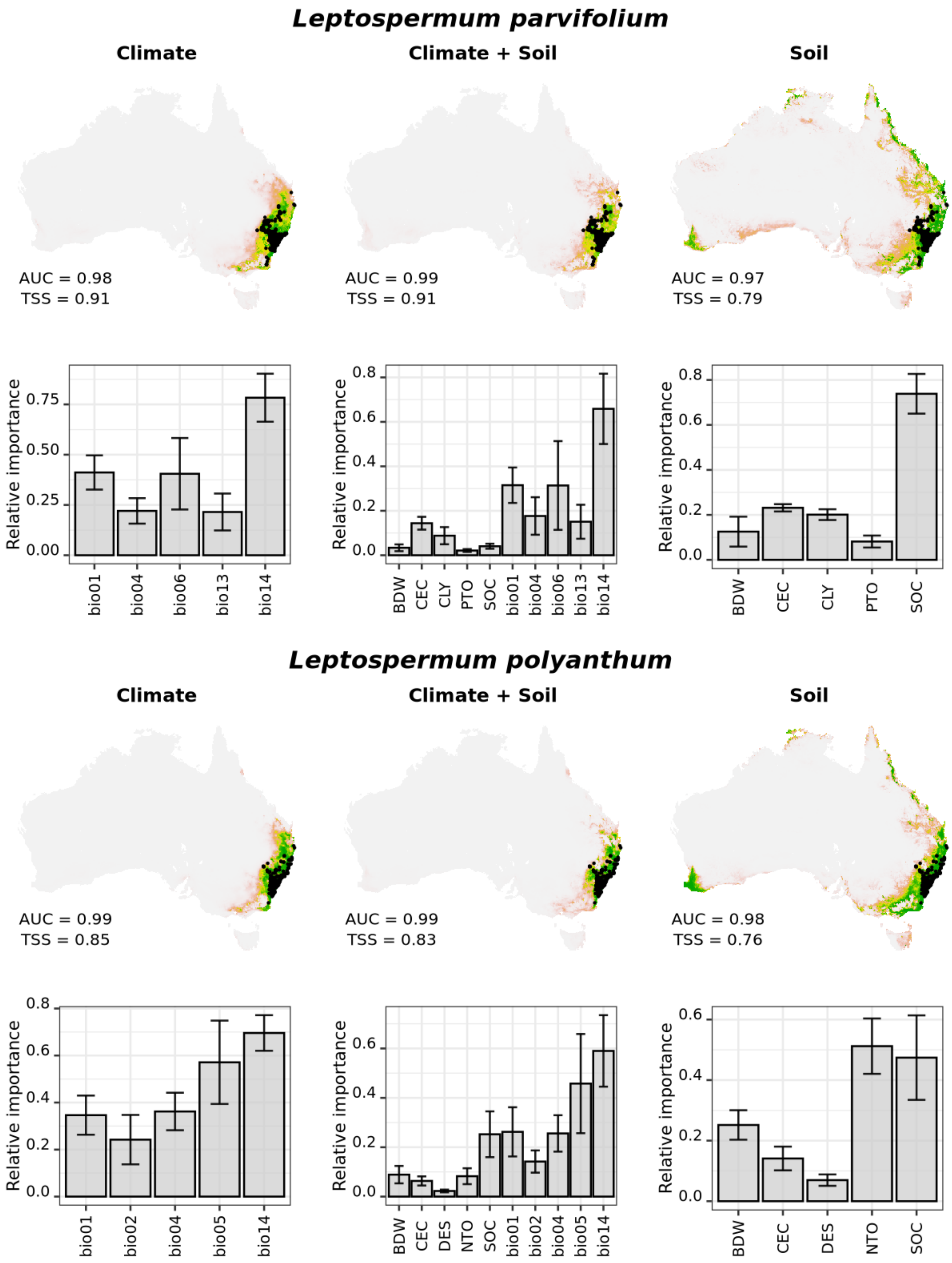
Leptospermum parvifolium
Climate
Climate + Soil
Soil
AUC = 0.98
TSS = 0.91
AUC = 0.99
TSS = 0.91
AUC = 0.97
TSS = 0.79
Relative importance
0.00
0.25
0.50
0.75
bio01
bio04
bio06
bio13
bio14
0.0
0.2
0.4
0.6
0.8
BDW
CEC
CLY
PTO
SOC
bio01
bio04
bio06
bio13
bio14
BDW
CEC
CLY
PTO
SOC
Leptospermum polyanthum
Climate
Climate + Soil
Soil
AUC = 0.99
TSS = 0.85
AUC = 0.99
TSS = 0.83
AUC = 0.98
TSS = 0.76
Relative importance
bio01
bio02
bio04
bio05
bio14
BDW
CEC
DES
NTO
SOC
bio01
bio02
bio04
bio05
bio14
BDW
CEC
DES
NTO
SOC

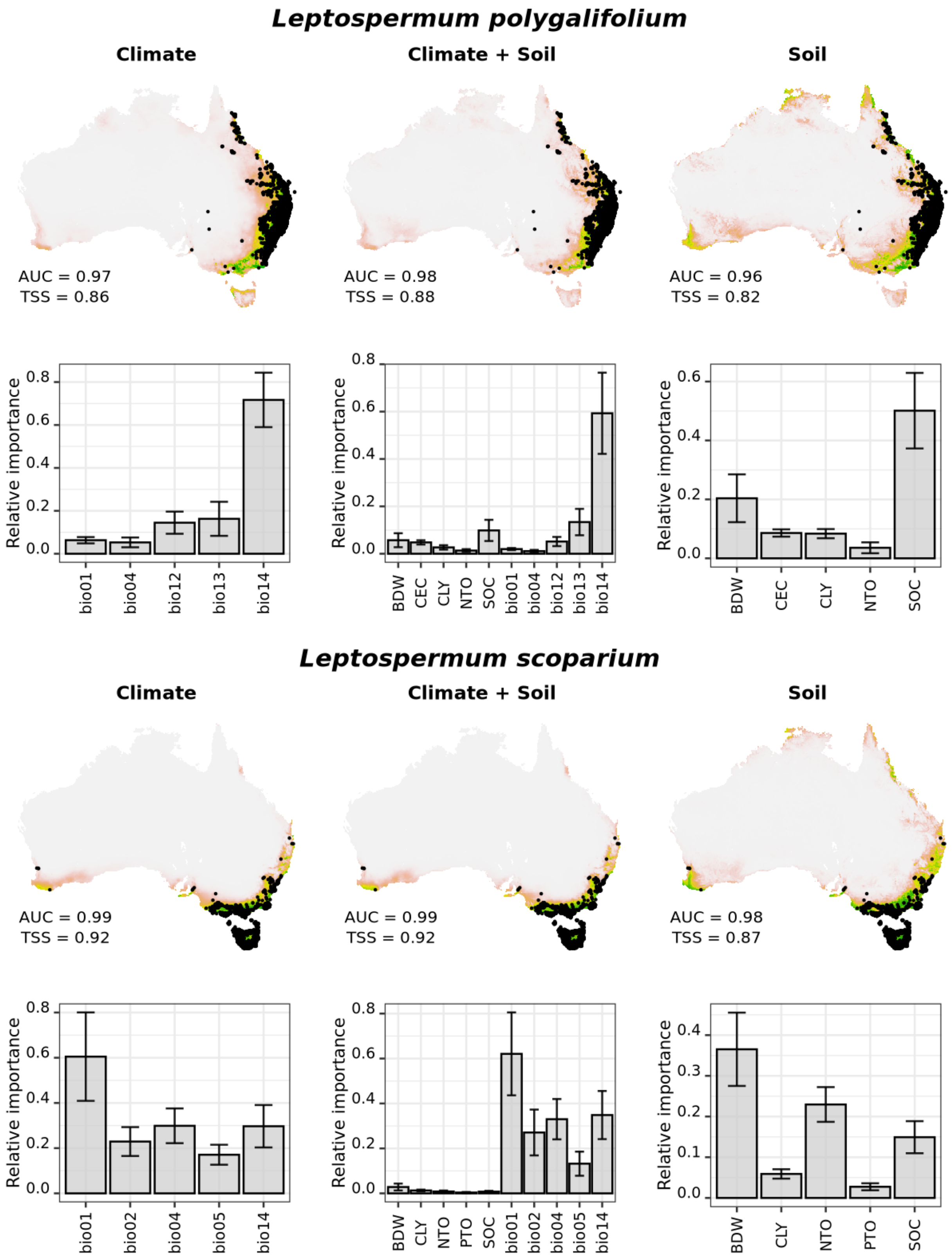
Leptospermum polygalifolium
Climate
Climate + Soil
Soil
AUC = 0.97
TSS = 0.86
AUC = 0.98
TSS = 0.88
AUC = 0.96
TSS = 0.82
Relative importance
bio01
bio04
bio12
bio13
bio14
BDW
CEC
CLY
NTO
SOC
Leptospermum scoparium
AUC = 0.99
TSS = 0.92
AUC = 0.99
TSS = 0.92
AUC = 0.98
TSS = 0.87
bio02
bio05
PTO

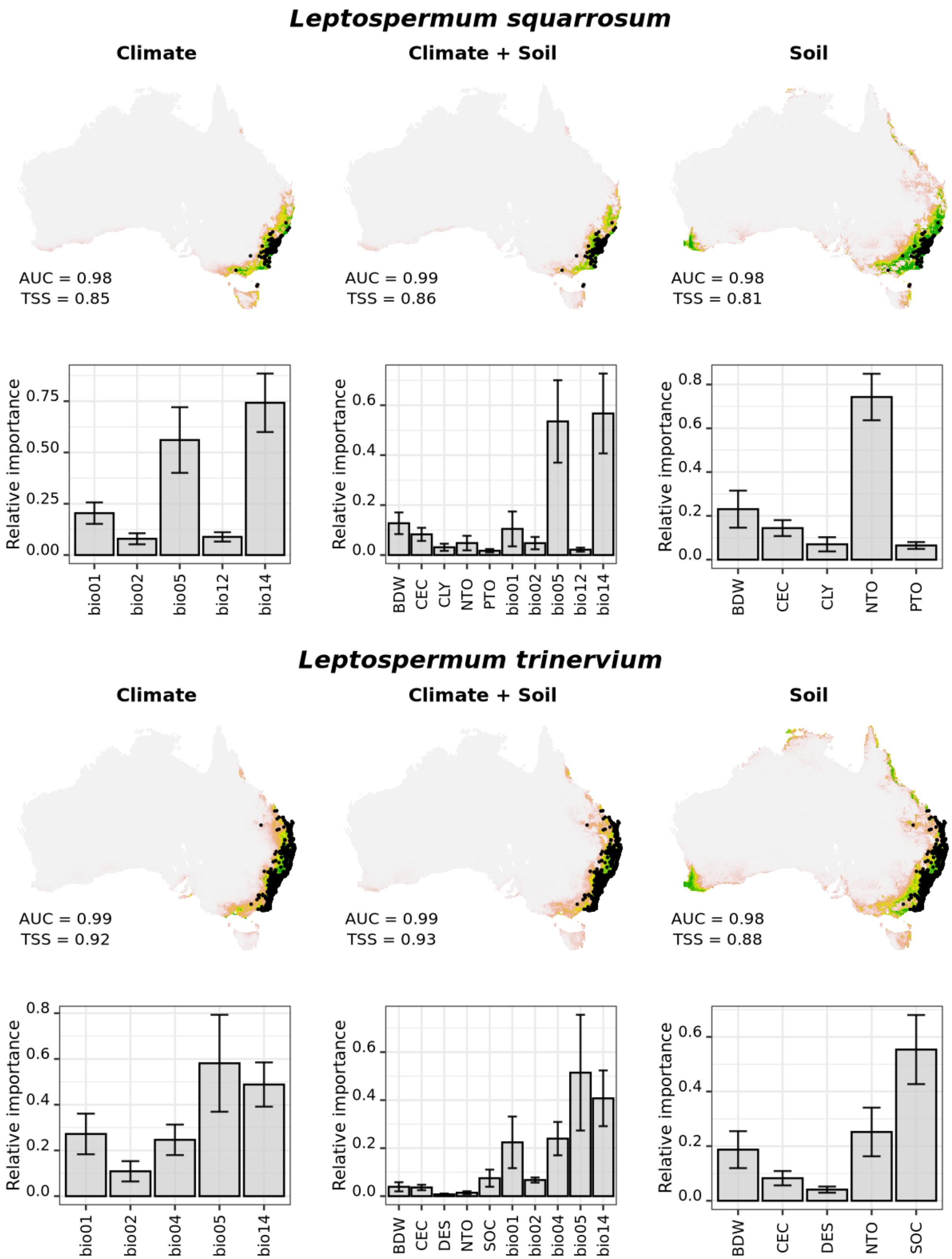

Leptospermum squarrosum
Climate
Climate + Soil
Soil
AUC = 0.98
TSS = 0.85
AUC = 0.99
TSS = 0.86
AUC = 0.98
TSS = 0.81
Relative importance
0.00
0.25
0.50
0.75
bio01
bio02
bio05
bio12
bio14
0.0
0.2
0.4
0.6
BDW
CEC
CLY
NTO
PTO
bio01
bio02
bio05
bio12
bio14
0.0
0.2
0.4
0.6
0.8
BDW
CEC
CLY
NTO
PTO
Leptospermum trinervium
Climate
Climate + Soil
Soil
AUC = 0.99
TSS = 0.92
AUC = 0.99
TSS = 0.93
AUC = 0.98
TSS = 0.88
Relative importance
0.0
0.2
0.4
0.6
0.8
bio01
bio02
bio04
bio05
bio14
0.0
0.2
0.4
0.6
BDW
CEC
DES
NTO
SOC
bio01
bio02
bio04
bio05
bio14
0.0
0.2
0.4
0.6
BDW
CEC
DES
NTO
SOC

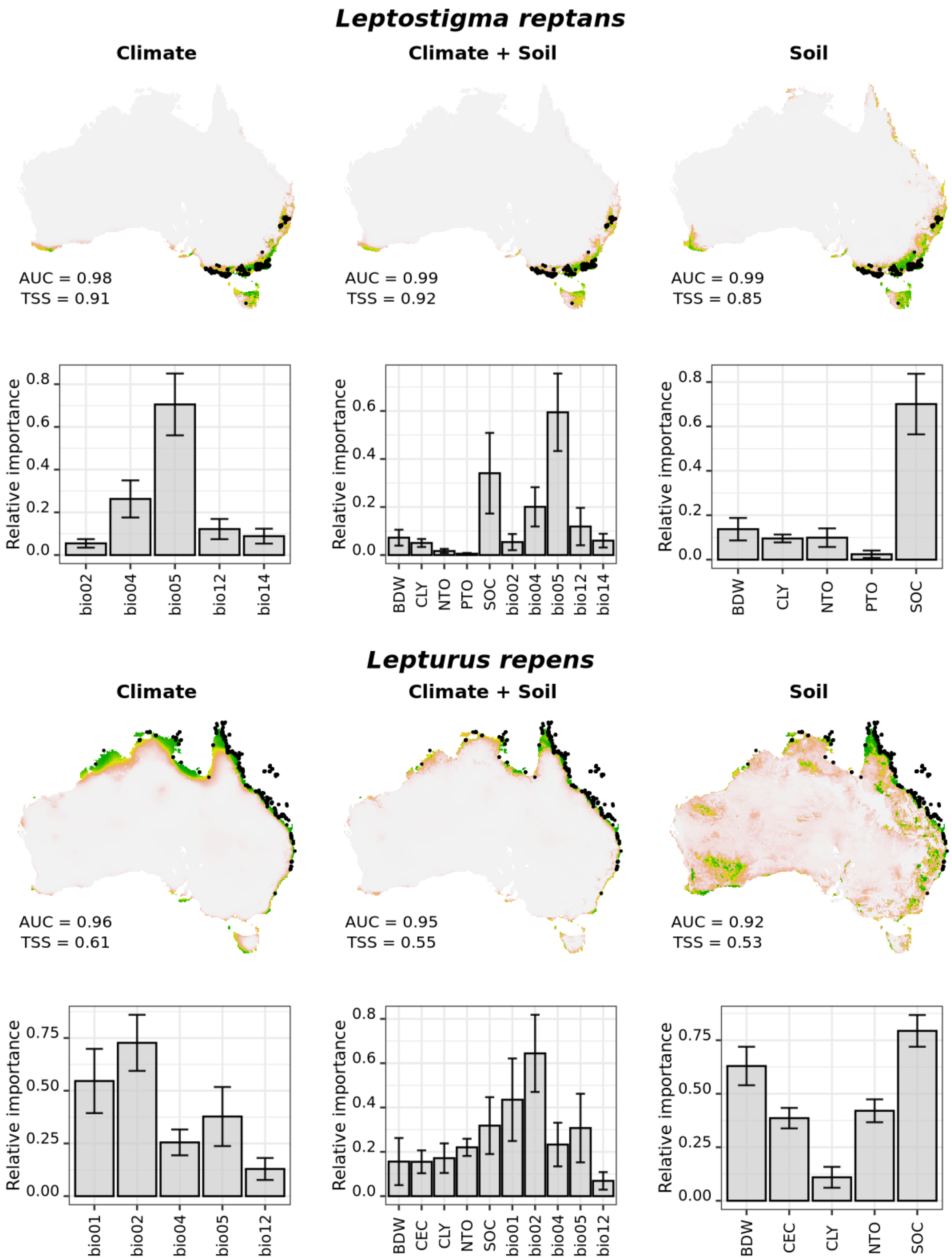

Leptostigma reptans
Climate
Climate + Soil
Soil
AUC = 0.98
TSS = 0.91
AUC = 0.99
TSS = 0.92
AUC = 0.99
TSS = 0.85
Relative importance
bio02
bio04
bio05
bio12
bio14
BDW
CLY
NTO
PTO
SOC
Lepturus repens
AUC = 0.96
TSS = 0.61
AUC = 0.95
TSS = 0.55
AUC = 0.92
TSS = 0.53
bio01
CEC

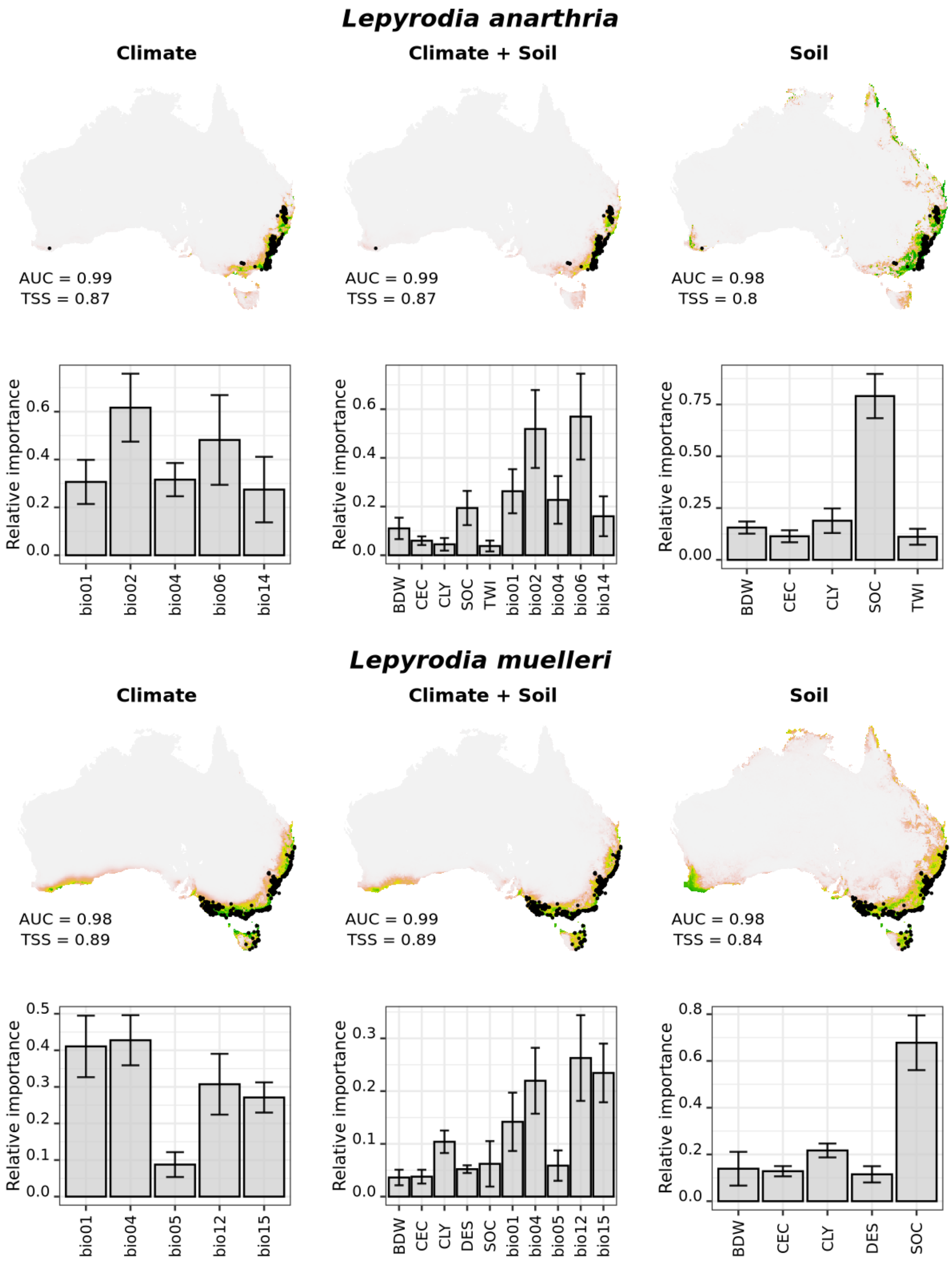
Lepyrodia anarthria
Climate
Climate + Soil
Soil
AUC = 0.99
TSS = 0.87
AUC = 0.99
TSS = 0.87
AUC = 0.98
TSS = 0.8
Relative importance
bio01 bio02 bio04 bio06 bio14
BDW CEC CLY SOC TWI bio01 bio02 bio04 bio06 bio14
BDW CEC CLY SOC TWI
Lepyrodia muelleri
Climate
Climate + Soil
Soil
AUC = 0.98
TSS = 0.89
AUC = 0.99
TSS = 0.89
AUC = 0.98
TSS = 0.84
Relative importance
bio01 bio04 bio05 bio12 bio15
BDW CEC CLY DES SOC bio01 bio04 bio05 bio12 bio15
BDW CEC CLY DES SOC

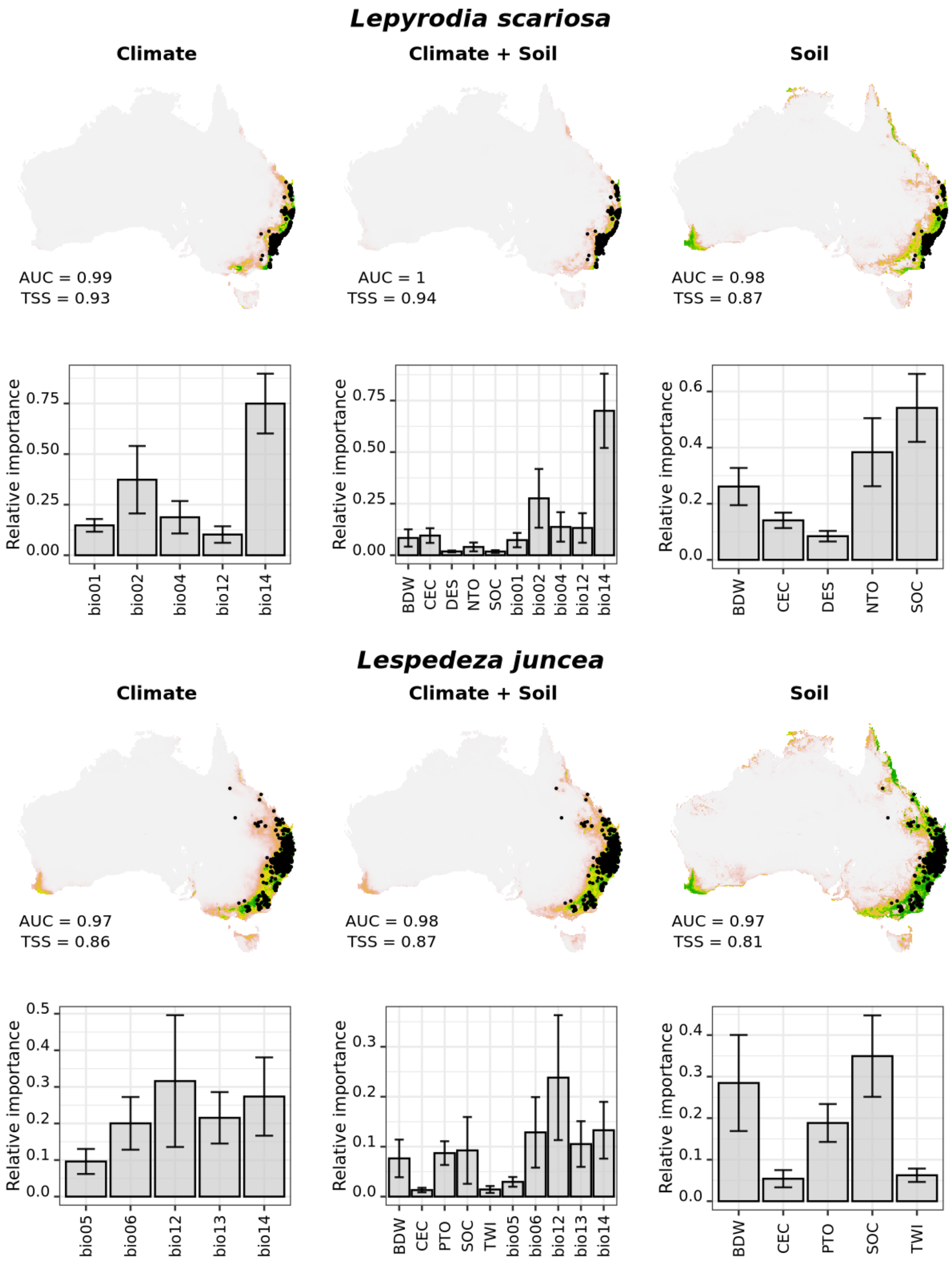

Lepyrodia scariosa
Climate
Climate + Soil
Soil
AUC = 0.99
TSS = 0.93
AUC = 1
TSS = 0.94
AUC = 0.98
TSS = 0.87
Relative importance
0.00
0.25
0.50
0.75
bio01
bio02
bio04
bio12
bio14
BDW
CEC
DES
NTO
SOC
0.0
0.2
0.4
0.6
Lespedeza juncea
Climate
Climate + Soil
Soil
AUC = 0.97
TSS = 0.86
AUC = 0.98
TSS = 0.87
AUC = 0.97
TSS = 0.81
0.1
0.3
0.5
bio05
bio06
bio13
PTO
TWI

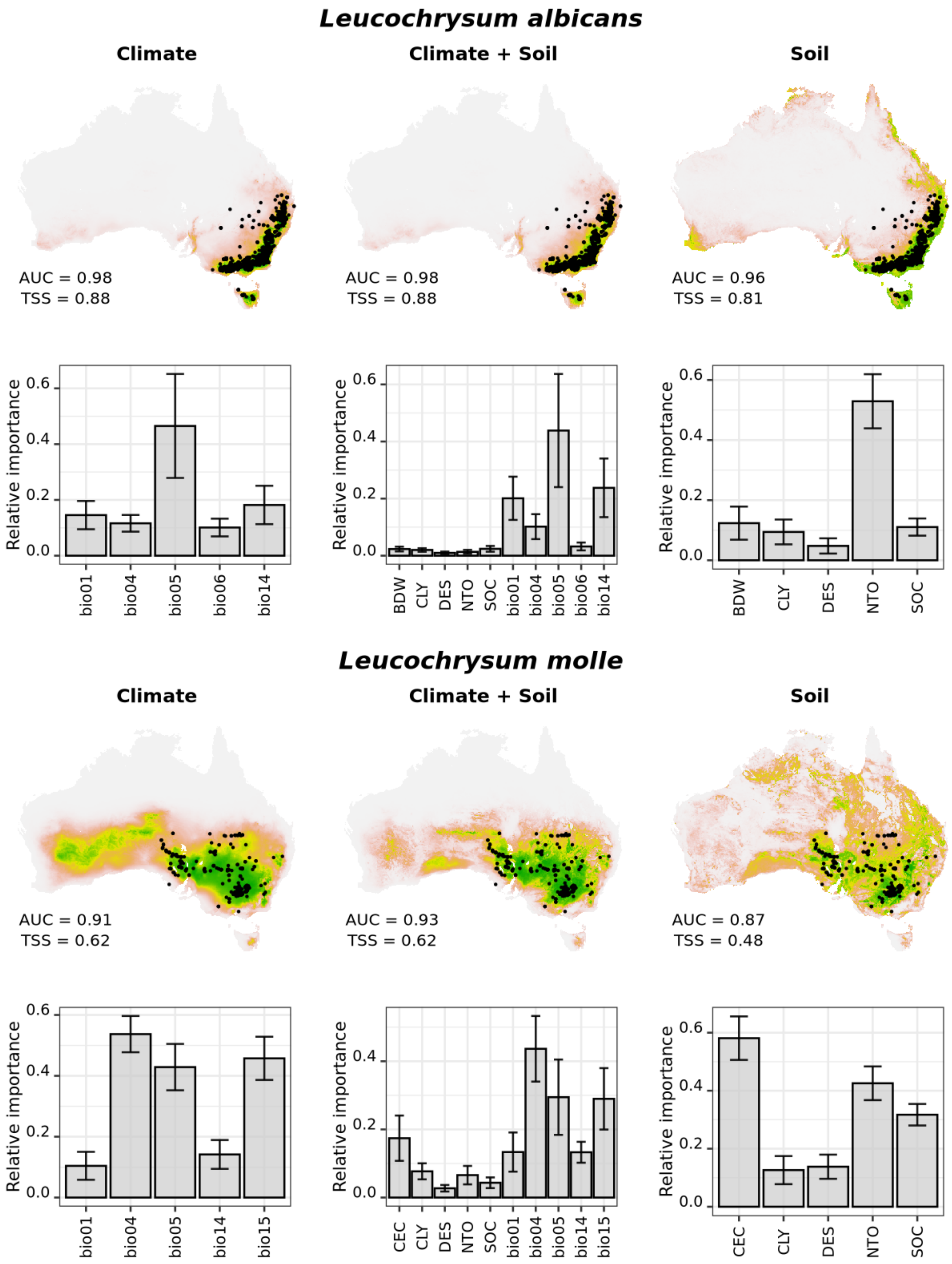

Leucochrysum albicans
Climate
Climate + Soil
Soil
AUC = 0.98
TSS = 0.88
AUC = 0.98
TSS = 0.88
AUC = 0.96
TSS = 0.81
Relative importance
bio01
bio04
bio05
bio06
bio14
BDW
CLY
DES
NTO
SOC
Leucochrysum molle
AUC = 0.91
TSS = 0.62
AUC = 0.93
TSS = 0.62
AUC = 0.87
TSS = 0.48
bio15
CEC

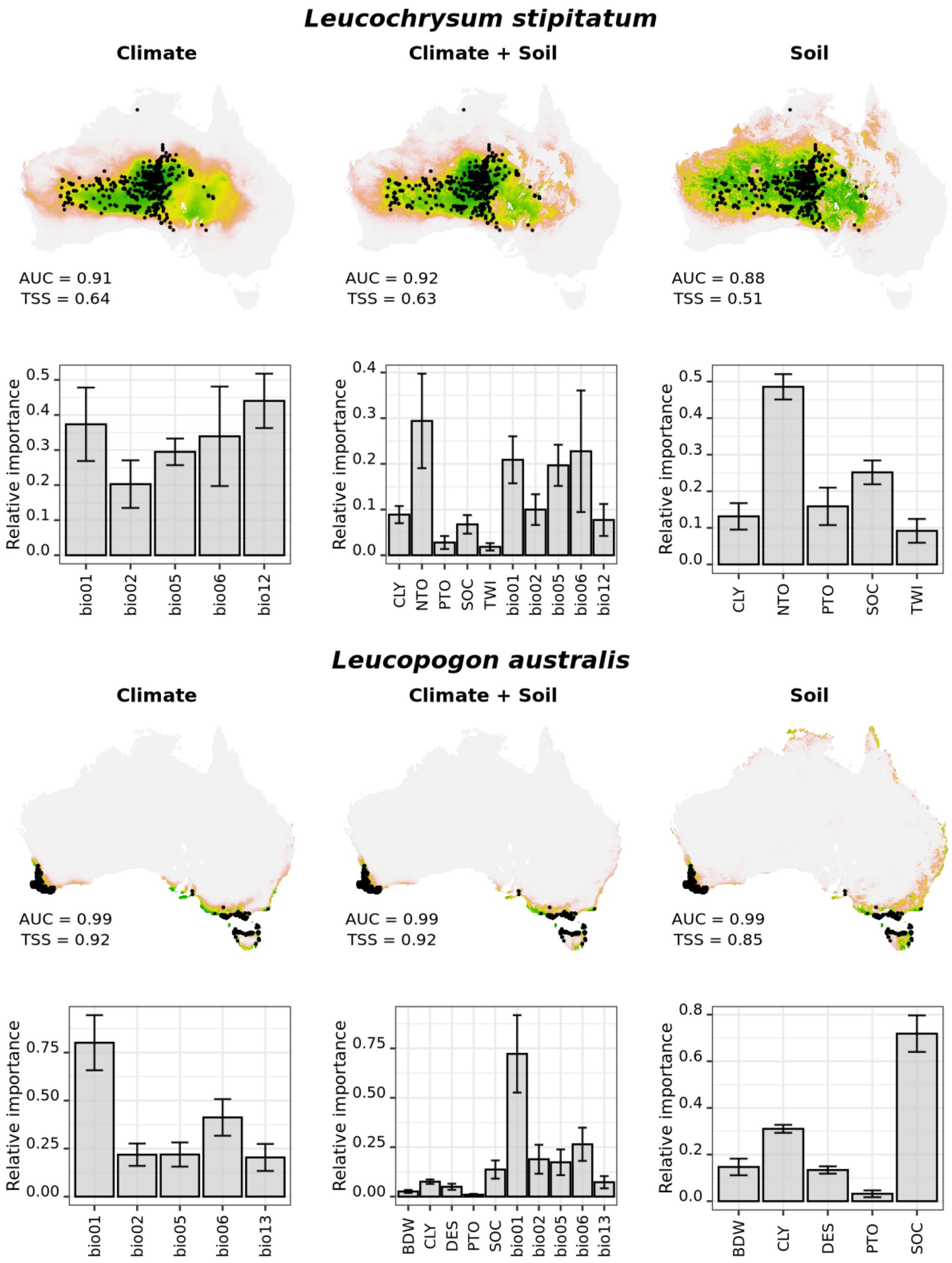

Leucochrysum stipitatum
Climate
Climate + Soil
Soil
AUC = 0.91
TSS = 0.64
AUC = 0.92
TSS = 0.63
AUC = 0.88
TSS = 0.51
Relative importance
bio01
bio02
bio05
bio06
bio12
CLY
NTO
PTO
SOC
TWI
Leucopogon australis
AUC = 0.99
TSS = 0.92
AUC = 0.99
TSS = 0.92
AUC = 0.99
TSS = 0.85
bio13
BDW
DES

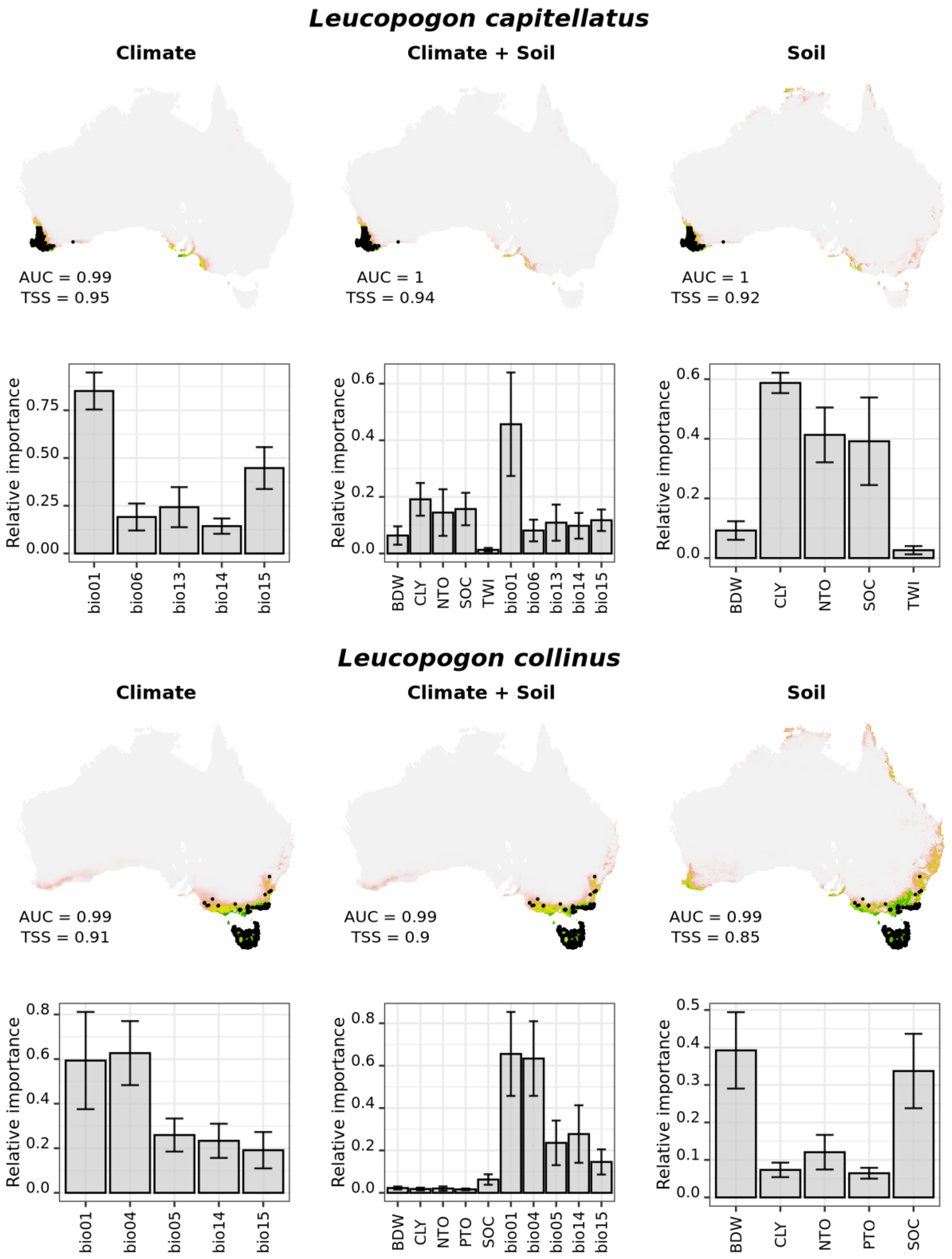
Leucopogon capitellatus
Climate
Climate + Soil
Soil
AUC = 0.99
TSS = 0.95
AUC = 1
TSS = 0.94
AUC = 1
TSS = 0.92
Relative importance
bio01
bio06
bio13
bio14
bio15
BDW
CLY
NTO
SOC
TWI
Leucopogon collinus
Climate
Climate + Soil
Soil
AUC = 0.99
TSS = 0.91
AUC = 0.99
TSS = 0.9
AUC = 0.99
TSS = 0.85
Relative importance
bio01
bio04
bio05
bio14
bio15
BDW
CLY
NTO
PTO
SOC

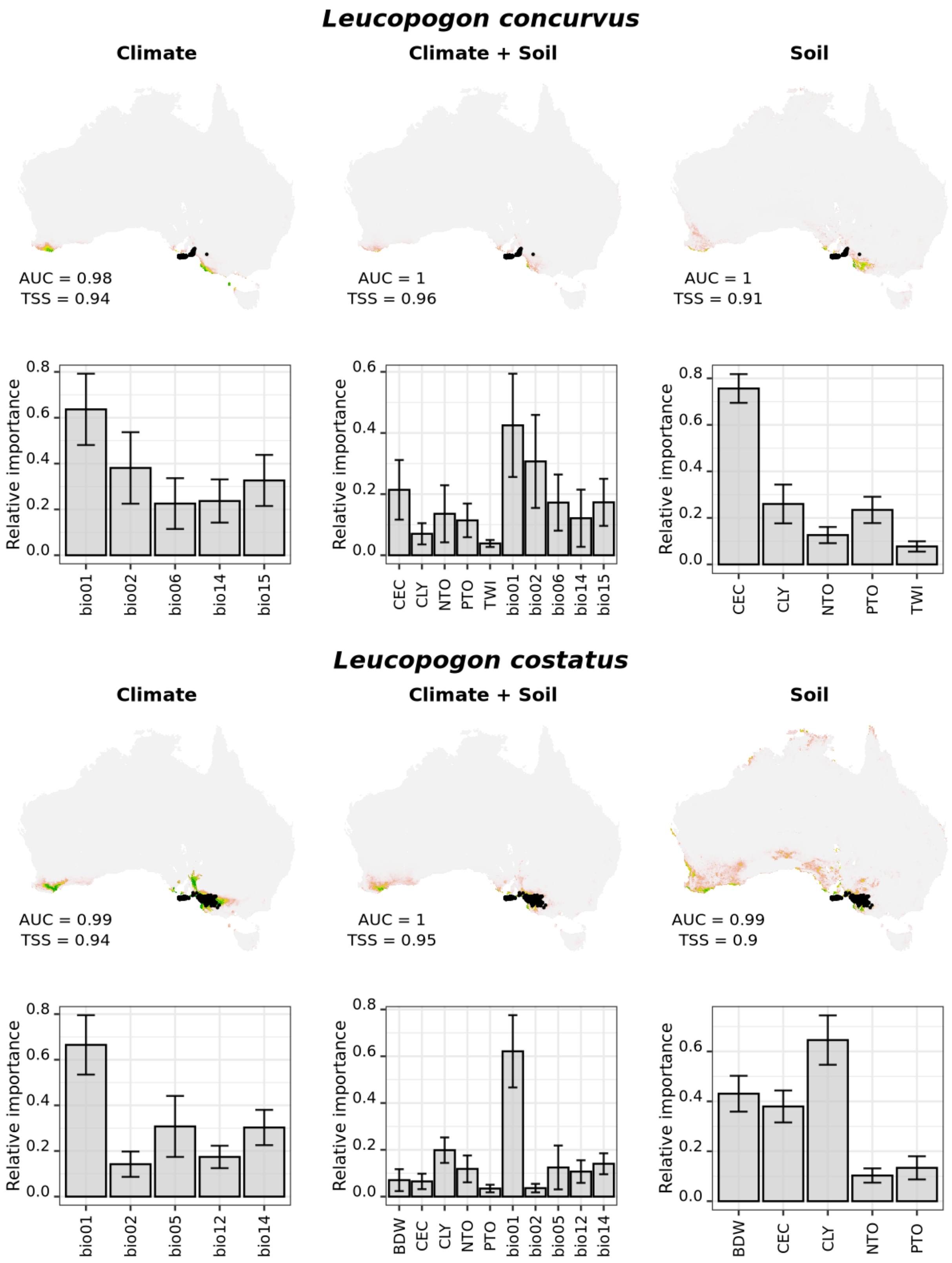

Leucopogon concurvus
Climate
Climate + Soil
Soil
AUC = 0.98
TSS = 0.94
AUC = 1
TSS = 0.96
AUC = 1
TSS = 0.91
Relative importance
0.0
0.2
0.4
0.6
0.8
bio01
bio02
bio06
bio14
bio15
CEC
CLY
NTO
PTO
TWI
Leucopogon costatus
AUC = 0.99
TSS = 0.94
AUC = 1
TSS = 0.95
AUC = 0.99
TSS = 0.9
bio05
bio12
BDW

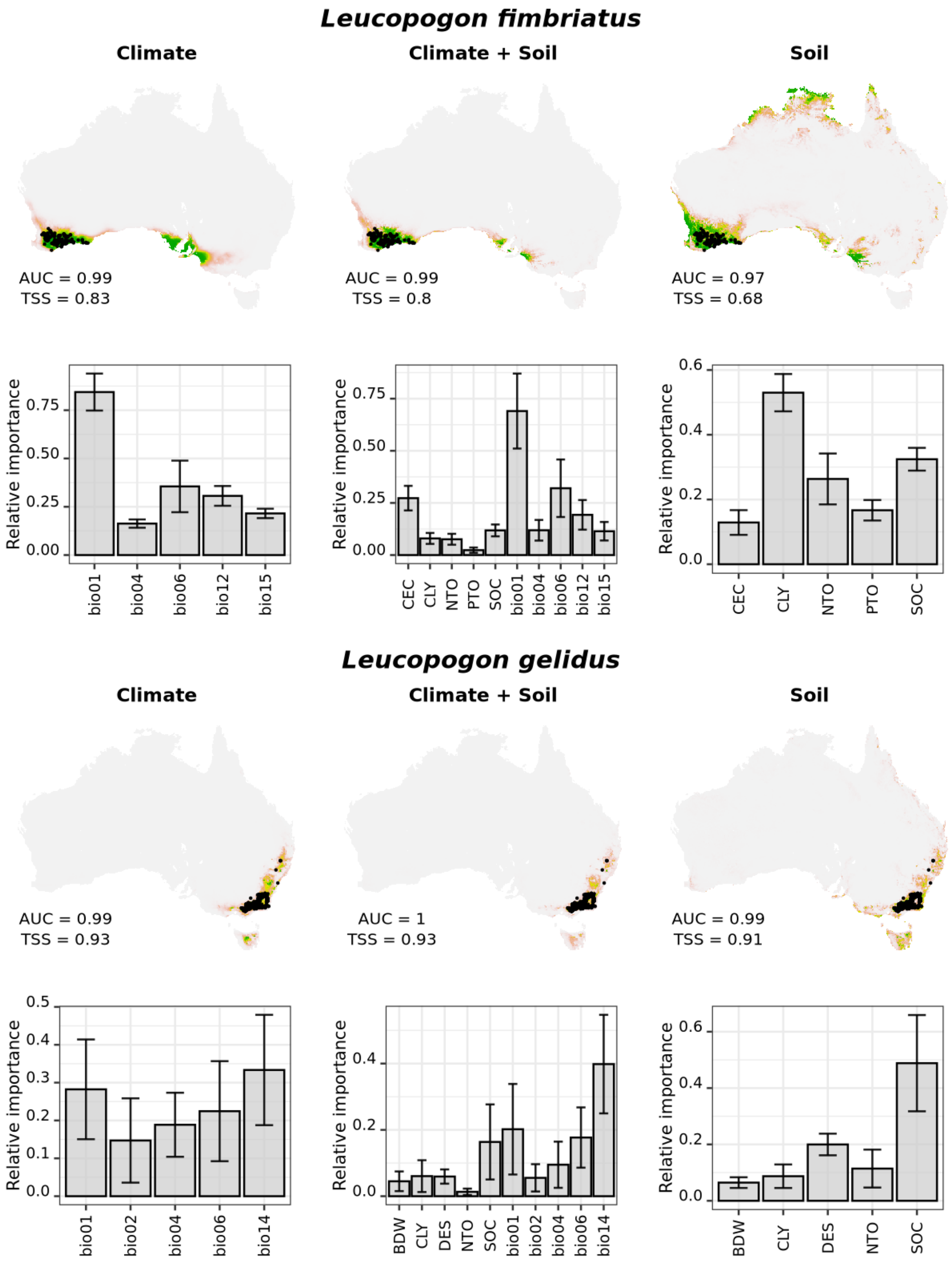
Leucopogon fimbriatus
Climate
Climate + Soil
Soil
AUC = 0.99
TSS = 0.83
AUC = 0.99
TSS = 0.8
AUC = 0.97
TSS = 0.68
Relative importance
0.00
0.25
0.50
0.75
bio01
bio04
bio06
bio12
bio15
Relative importance
0.00
0.25
0.50
0.75
CEC
CLY
NTO
PTO
SOC
bio01
bio04
bio06
bio12
bio15
Relative importance
0.0
0.2
0.4
0.6
CEC
CLY
NTO
PTO
SOC
Leucopogon gelidus
Climate
Climate + Soil
Soil
AUC = 0.99
TSS = 0.93
AUC = 1
TSS = 0.93
AUC = 0.99
TSS = 0.91
Relative importance
0.0
0.1
0.2
0.3
0.4
0.5
bio01
bio02
bio04
bio06
bio14
Relative importance
0.0
0.2
0.4
BDW
CLY
DES
NTO
SOC
bio01
bio02
bio04
bio06
bio14
Relative importance
0.0
0.2
0.4
0.6
BDW
CLY
DES
NTO
SOC

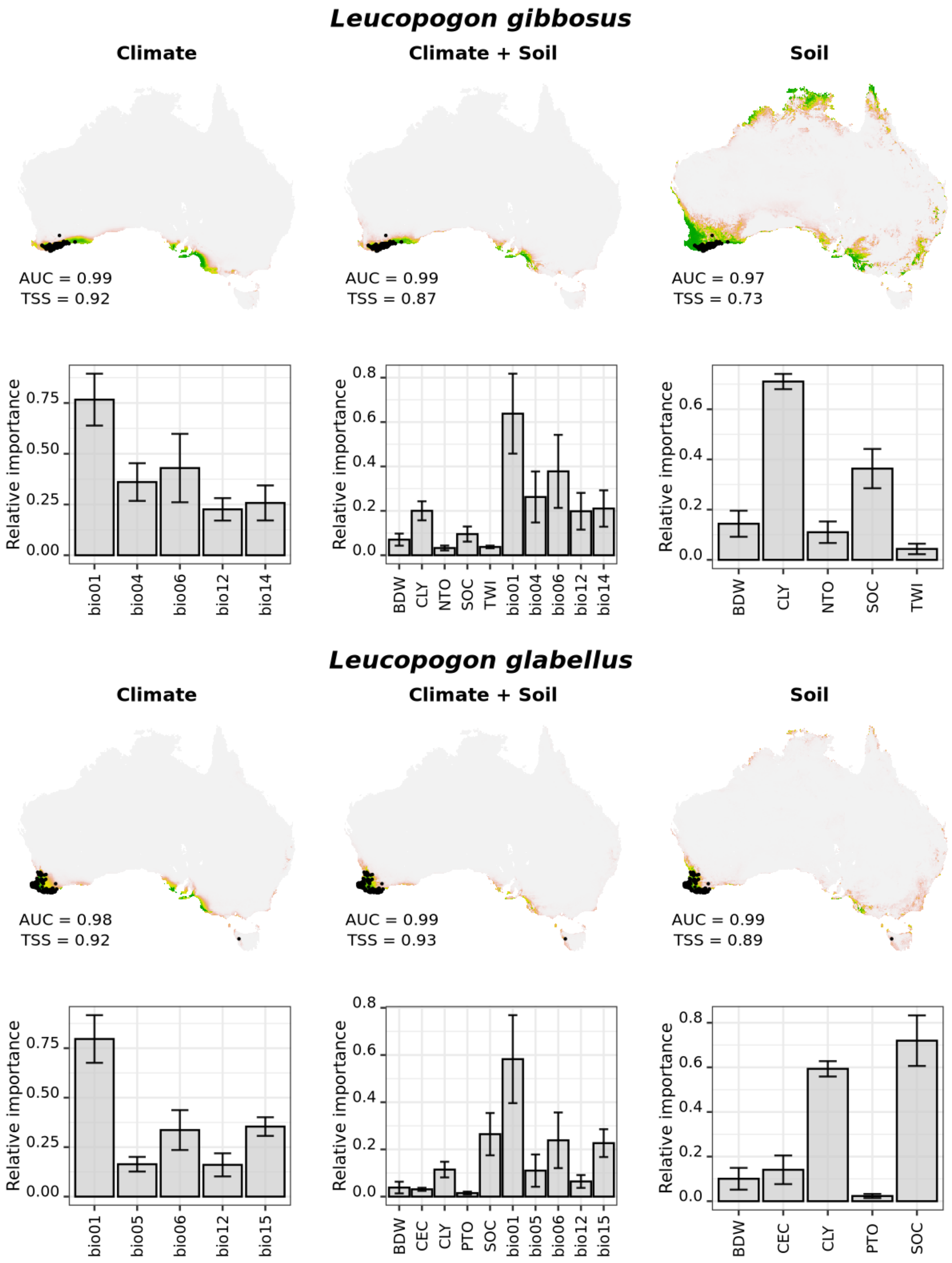

Leucopogon gibbosus
Climate
Climate + Soil
Soil
AUC = 0.99
TSS = 0.92
AUC = 0.99
TSS = 0.87
AUC = 0.97
TSS = 0.73
Relative importance
0.00
0.25
0.50
0.75
bio01
bio04
bio06
bio12
bio14
0.0
0.2
0.4
0.6
0.8
BDW
CLY
NTO
SOC
TWI
bio01
bio04
bio06
bio12
bio14
BDW
CLY
NTO
SOC
TWI
Leucopogon glabellus
Climate
Climate + Soil
Soil
AUC = 0.98
TSS = 0.92
AUC = 0.99
TSS = 0.93
AUC = 0.99
TSS = 0.89
bio01
bio05
bio06
bio12
bio15
BDW
CEC
CLY
PTO
SOC
bio01
bio05
bio06
bio12
bio15
BDW
CEC
CLY
PTO
SOC

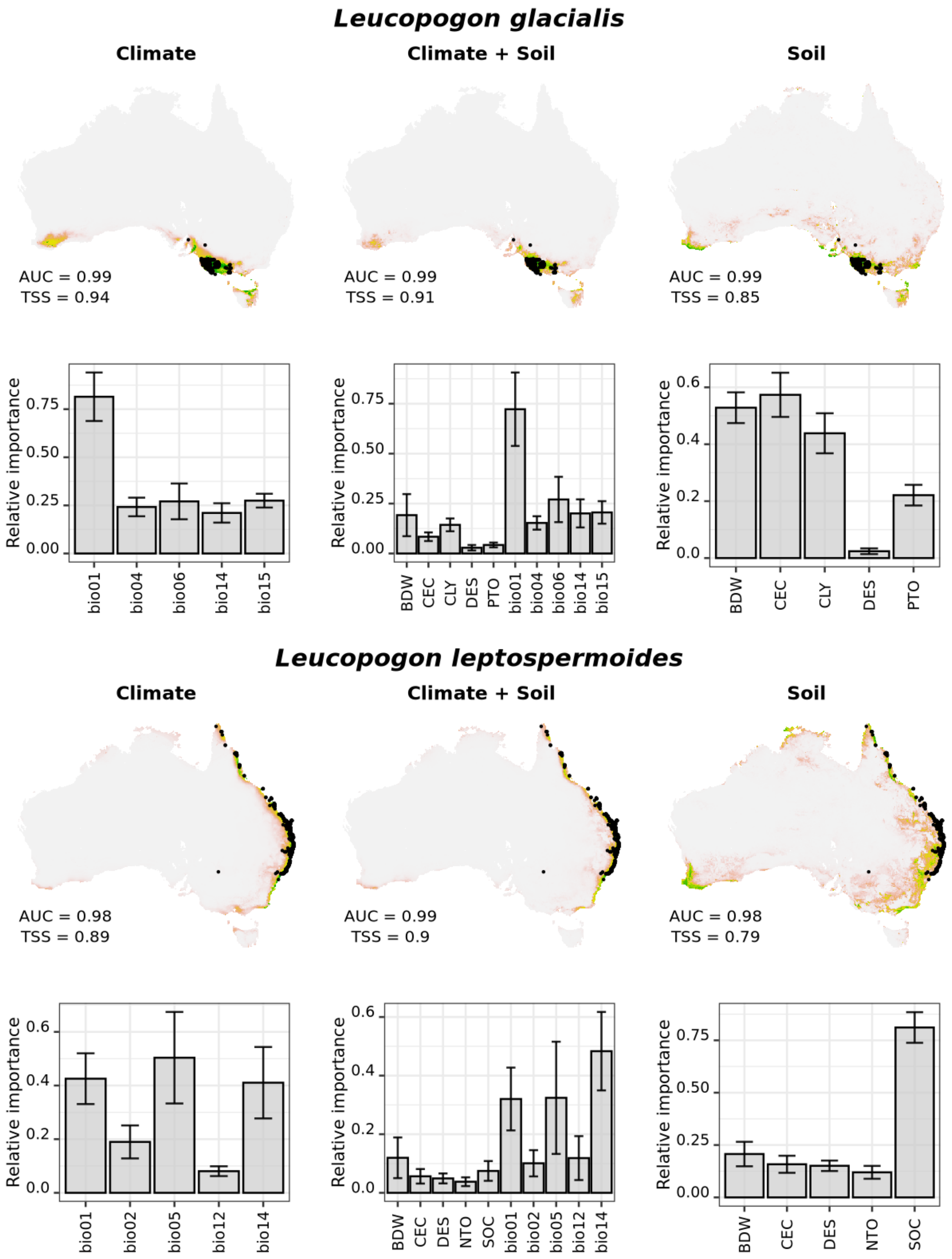
Leucopogon glacialis
Climate
Climate + Soil
Soil
AUC = 0.99
TSS = 0.94
AUC = 0.99
TSS = 0.91
AUC = 0.99
TSS = 0.85
Relative importance
bio01
bio04
bio06
bio14
bio15
BDW
CEC
CLY
DES
PTO
Leucopogon leptospermoides
Climate
Climate + Soil
Soil
AUC = 0.98
TSS = 0.89
AUC = 0.99
TSS = 0.9
AUC = 0.98
TSS = 0.79
Relative importance
bio01
bio02
bio05
bio12
bio14
BDW
CEC
DES
NTO
SOC

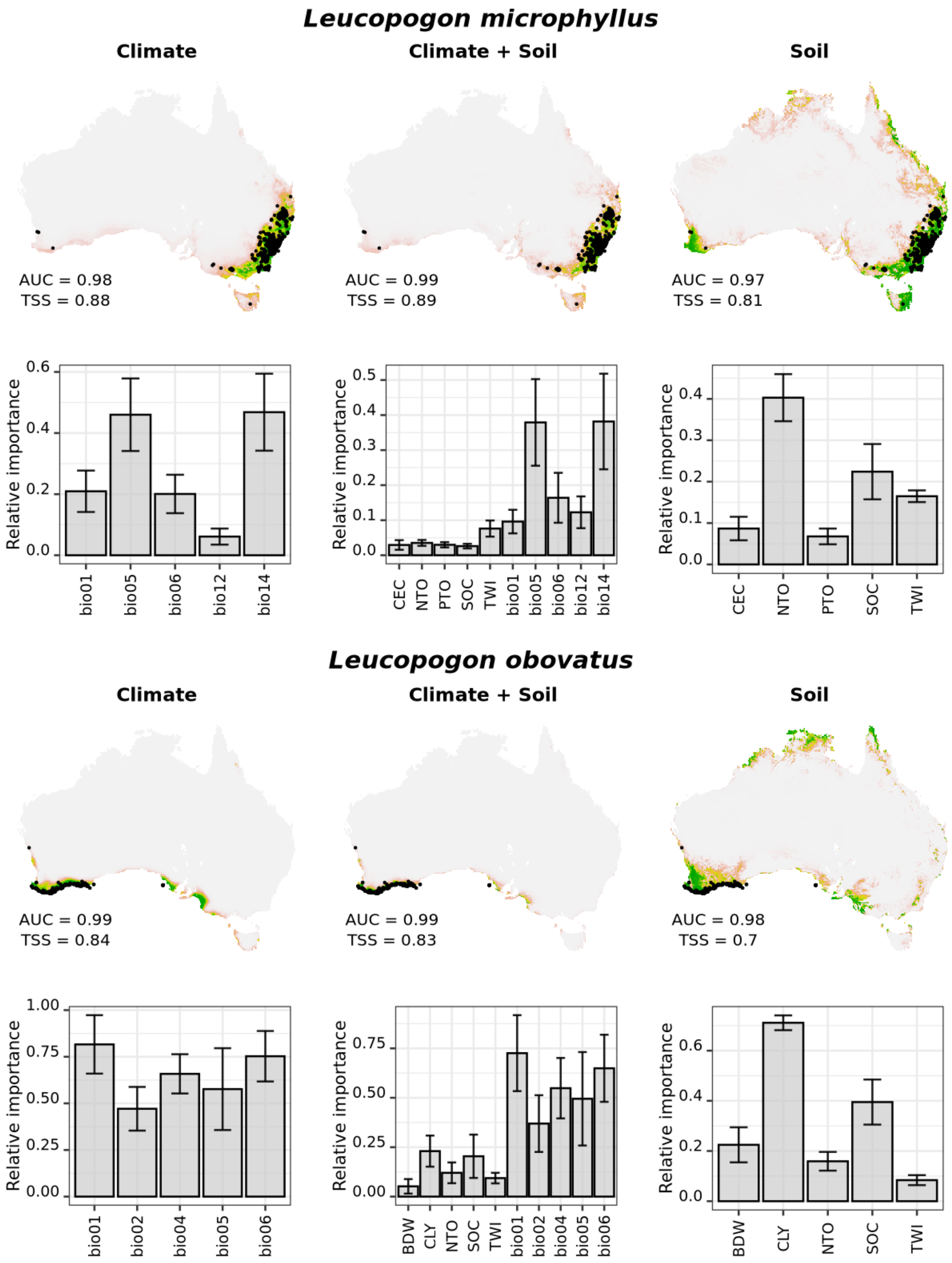

Leucopogon microphyllus
Climate
Climate + Soil
Soil
AUC = 0.98
TSS = 0.88
AUC = 0.99
TSS = 0.89
AUC = 0.97
TSS = 0.81
Relative importance
bio01
bio05
bio06
bio12
bio14
CEC
NTO
PTO
SOC
TWI
Leucopogon obovatus
Climate
Climate + Soil
Soil
AUC = 0.99
TSS = 0.84
AUC = 0.99
TSS = 0.83
AUC = 0.98
TSS = 0.7
Relative importance
bio01
bio02
bio04
bio05
bio06
BDW
CLY
NTO
SOC
TWI

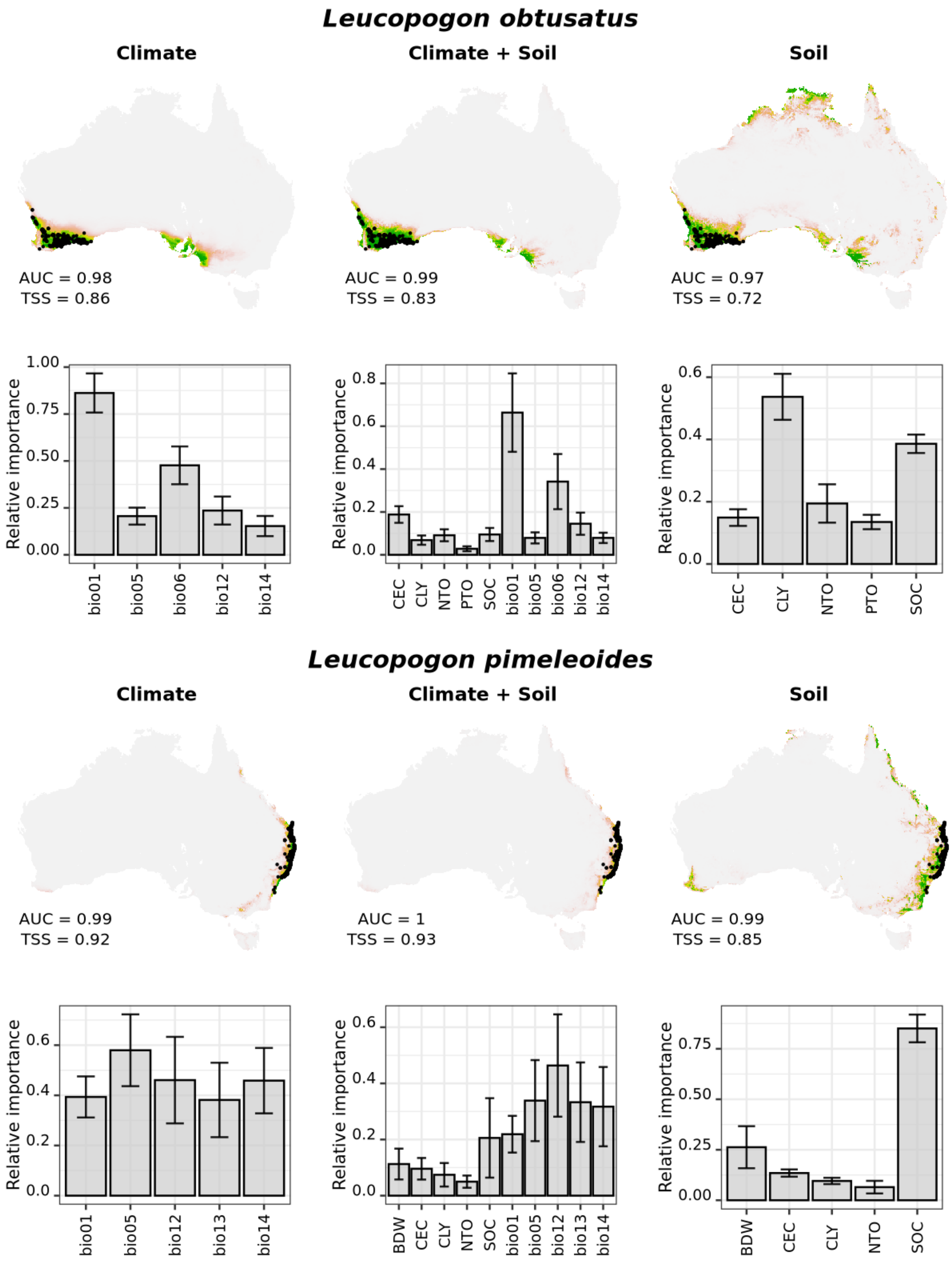

Leucopogon obtusatus
Climate
Climate + Soil
Soil
AUC = 0.98
TSS = 0.86
AUC = 0.99
TSS = 0.83
AUC = 0.97
TSS = 0.72
Relative importance
1.00
0.75
0.50
0.25
0.00
bio01
bio05
bio06
bio12
bio14
0.8
0.6
0.4
0.2
0.0
CEC
CLY
NTO
PTO
SOC
bio01
bio05
bio06
bio12
bio14
0.6
0.4
0.2
0.0
CEC
CLY
NTO
PTO
SOC
Leucopogon pimeleoides
Climate
Climate + Soil
Soil
AUC = 0.99
TSS = 0.92
AUC = 1
TSS = 0.93
AUC = 0.99
TSS = 0.85
Relative importance
0.6
0.4
0.2
0.0
bio01
bio05
bio12
bio13
bio14
0.6
0.4
0.2
0.0
BDW
CEC
CLY
NTO
SOC
bio01
bio05
bio12
bio13
bio14
0.75
0.50
0.25
0.00
BDW
CEC
CLY
NTO
SOC

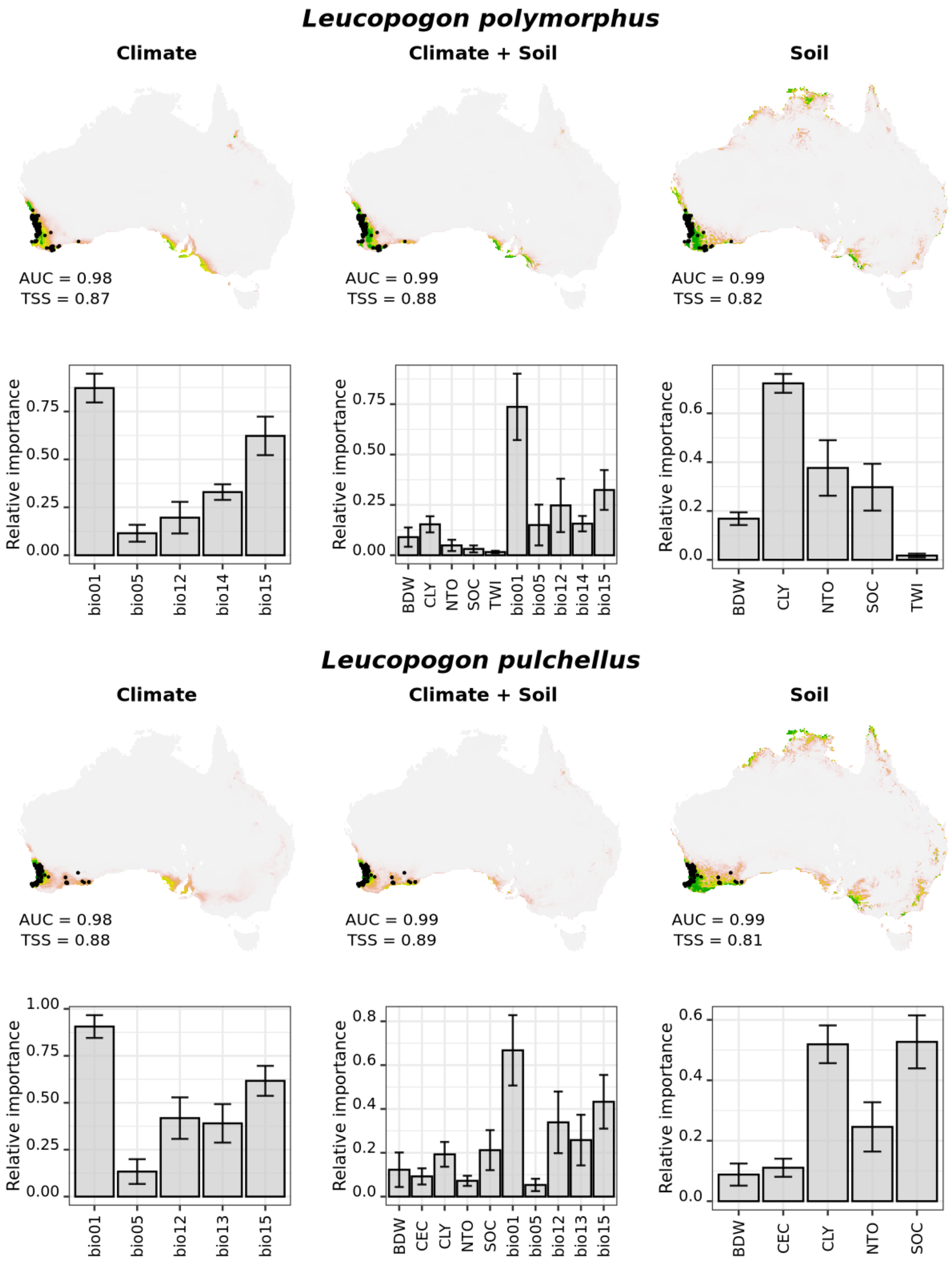

Leucopogon polymorphus
Climate
Climate + Soil
Soil
AUC = 0.98
TSS = 0.87
AUC = 0.99
TSS = 0.88
AUC = 0.99
TSS = 0.82
Relative importance
0.00
0.25
0.50
0.75
bio01
bio05
bio12
bio14
bio15
BDW
CLY
NTO
SOC
TWI
0.0
0.2
0.4
0.6
Leucopogon pulchellus
Climate
Climate + Soil
Soil
AUC = 0.98
TSS = 0.88
AUC = 0.99
TSS = 0.89
AUC = 0.99
TSS = 0.81
1.00
bio13
CEC
0.8

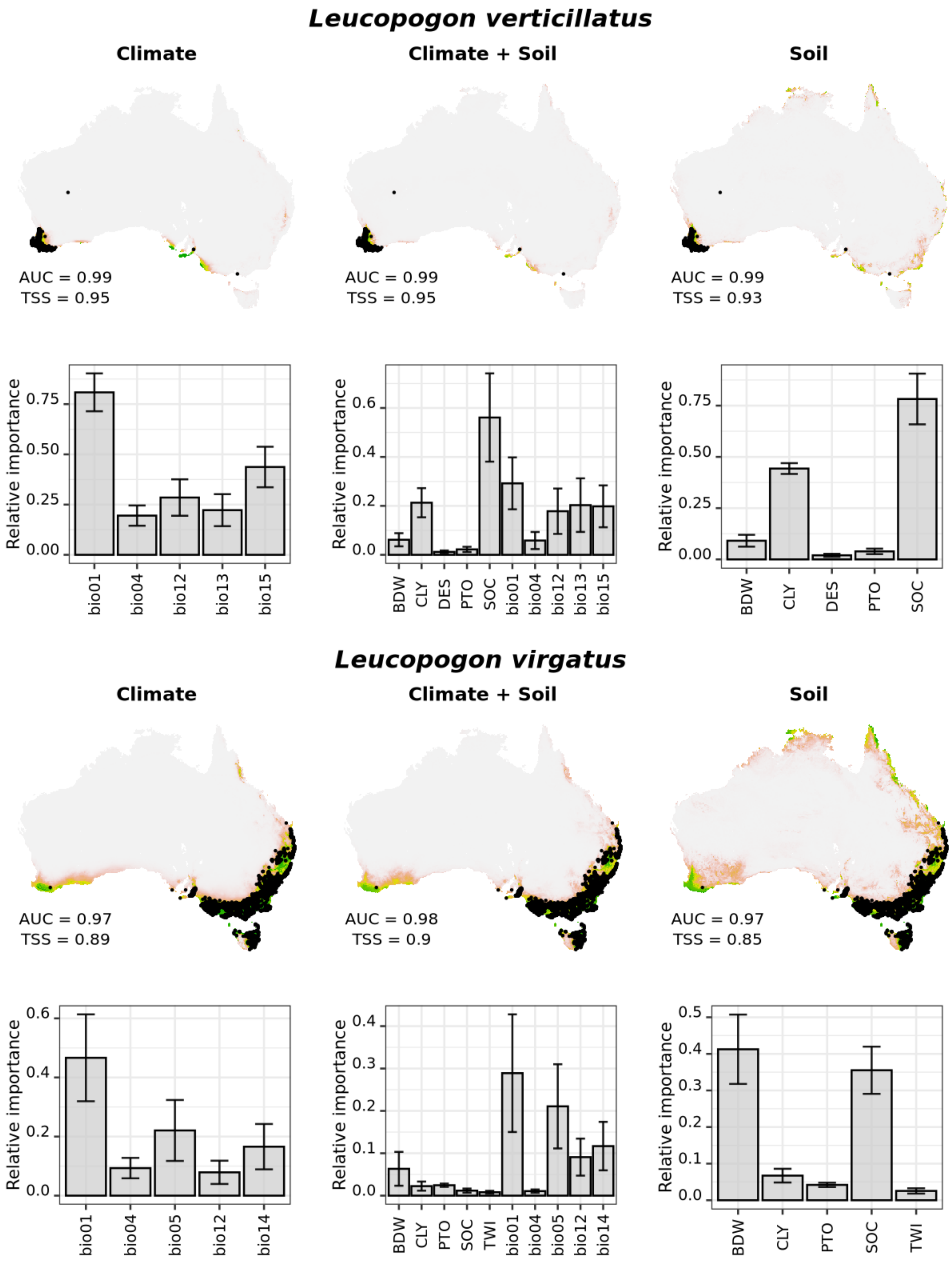

Leucopogon verticillatus
Climate
Climate + Soil
Soil
AUC = 0.99
TSS = 0.95
AUC = 0.99
TSS = 0.95
AUC = 0.99
TSS = 0.93
Relative importance
0.75
0.50
0.25
0.00
bio01
bio04
bio12
bio13
bio15
0.6
0.4
0.2
0.0
BDW
CLY
DES
PTO
SOC
bio01
bio04
bio12
bio13
bio15
BDW
CLY
DES
PTO
SOC
Leucopogon virgatus
Climate
Climate + Soil
Soil
AUC = 0.97
TSS = 0.89
AUC = 0.98
TSS = 0.9
AUC = 0.97
TSS = 0.85
Relative importance
0.6
0.4
0.2
0.0
bio01
bio04
bio05
bio12
bio14
0.4
0.3
0.2
0.1
0.0
BDW
CLY
PTO
SOC
TWI
bio01
bio04
bio05
bio12
bio14
0.5
0.4
0.3
0.2
0.1
0.0
BDW
CLY
PTO
SOC
TWI

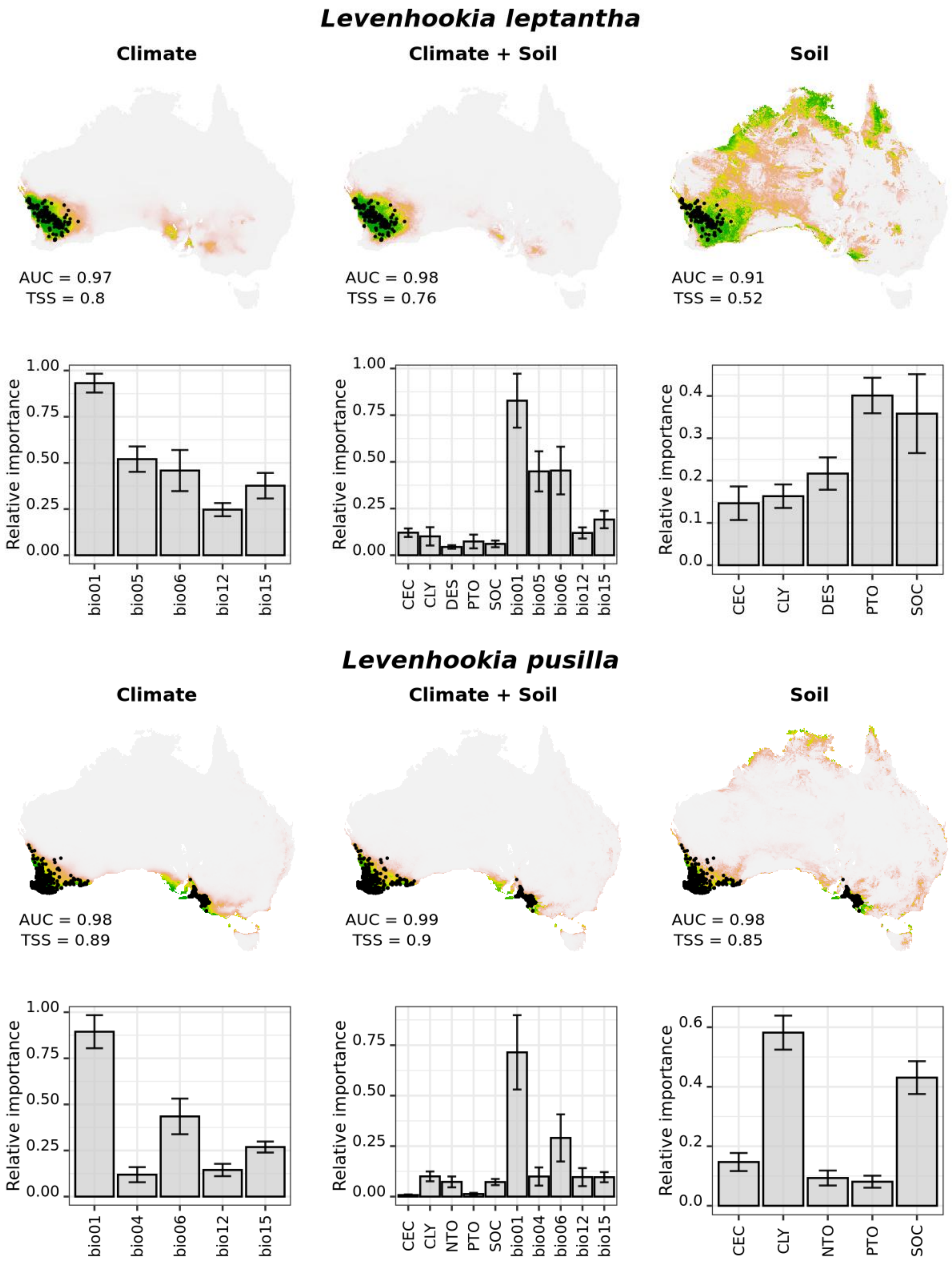

Levenhookia leptantha
Climate
Climate + Soil
Soil
AUC = 0.97
TSS = 0.8
AUC = 0.98
TSS = 0.76
AUC = 0.91
TSS = 0.52
Relative importance
bio01
bio05
bio06
bio12
bio15
CEC
CLY
DES
PTO
SOC
Levenhookia pusilla
AUC = 0.98
TSS = 0.89
AUC = 0.99
TSS = 0.9
AUC = 0.98
TSS = 0.85
bio04
NTO

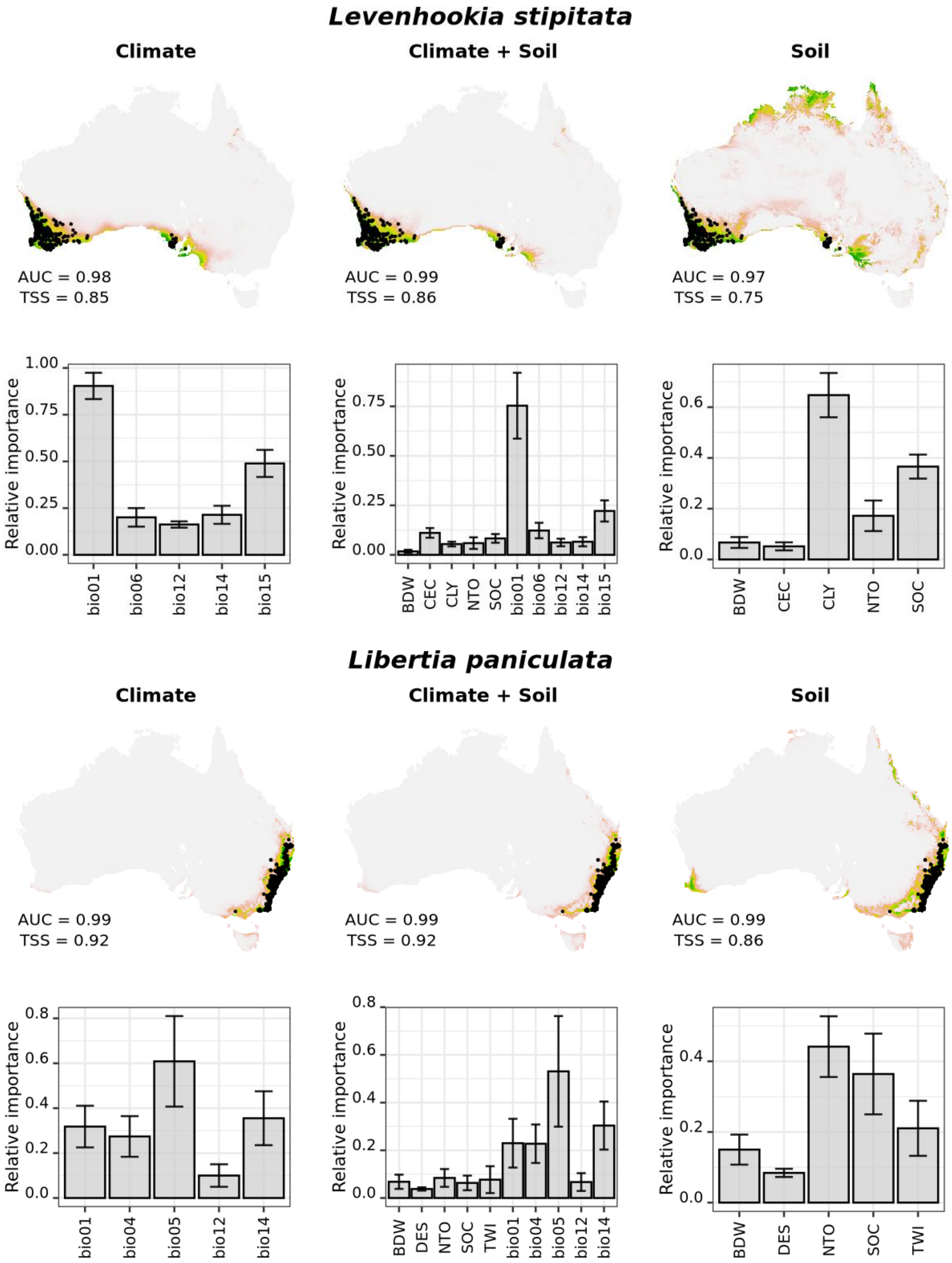
Levenhookia stipitata
Climate
Climate + Soil
Soil
AUC = 0.98
TSS = 0.85
AUC = 0.99
TSS = 0.86
AUC = 0.97
TSS = 0.75
Relative importance
1.00
0.75
0.50
0.25
0.00
bio01
bio06
bio12
bio14
bio15
BDW
CEC
CLY
NTO
SOC
0.6
0.4
0.2
0.0
Libertia paniculata
Climate
Climate + Soil
Soil
AUC = 0.99
TSS = 0.92
AUC = 0.99
TSS = 0.92
AUC = 0.99
TSS = 0.86
0.8
bio01
bio04
bio05
bio12
bio14
DES
TWI

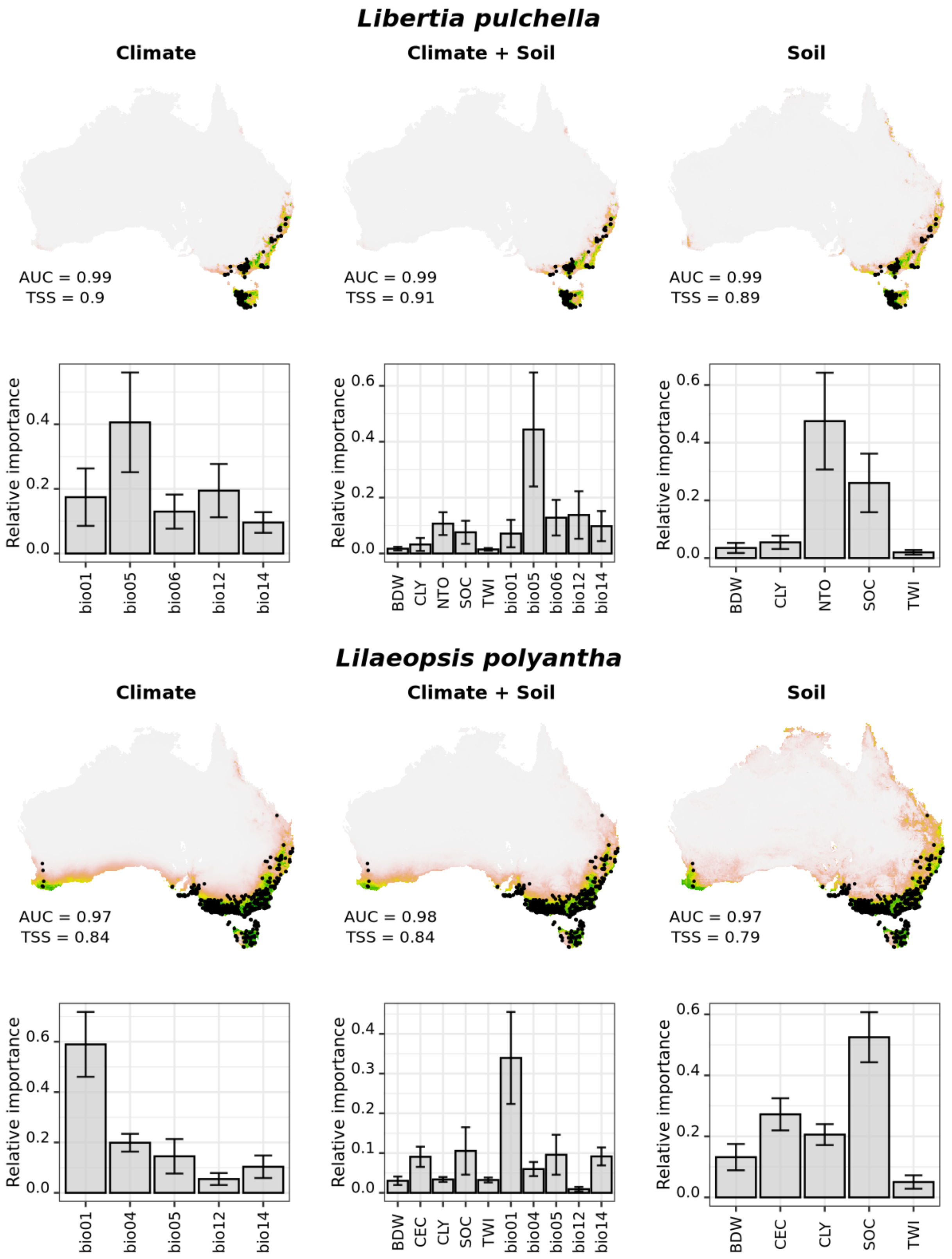
Libertia pulchella
Climate
Climate + Soil
Soil
AUC = 0.99
TSS = 0.9
AUC = 0.99
TSS = 0.91
AUC = 0.99
TSS = 0.89
Relative importance
0.0
0.2
0.4
0.6
bio01
bio05
bio06
bio12
bio14
BDW
CLY
NTO
SOC
TWI
Lilaeopsis polyantha
Climate
Climate + Soil
Soil
AUC = 0.97
TSS = 0.84
AUC = 0.98
TSS = 0.84
AUC = 0.97
TSS = 0.79
Relative importance
0.0
0.1
0.2
0.3
0.4
0.6
bio01
bio04
bio05
bio12
bio14
BDW
CEC
CLY
SOC
TWI

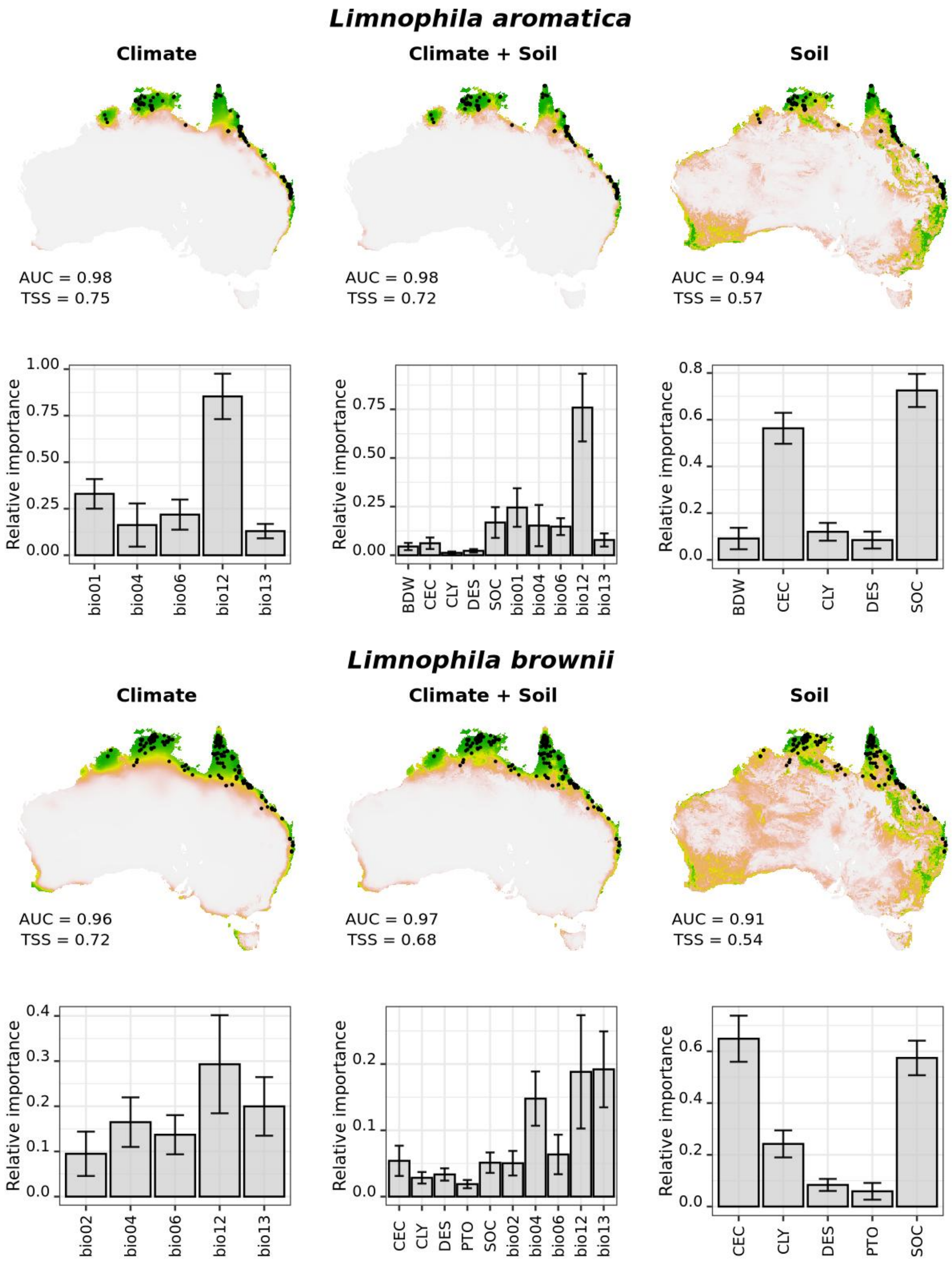
Limnophila aromatica
Climate
Climate + Soil
Soil
AUC = 0.98
TSS = 0.75
AUC = 0.98
TSS = 0.72
AUC = 0.94
TSS = 0.57
Relative importance
1.00
0.75
0.50
0.25
0.00
bio01
bio04
bio06
bio12
bio13
BDW
CEC
CLY
DES
SOC
0.8
0.6
0.4
0.2
0.0
Limnophila brownii
AUC = 0.96
TSS = 0.72
AUC = 0.97
TSS = 0.68
AUC = 0.91
TSS = 0.54
0.4
0.3
0.1
bio02
PTO

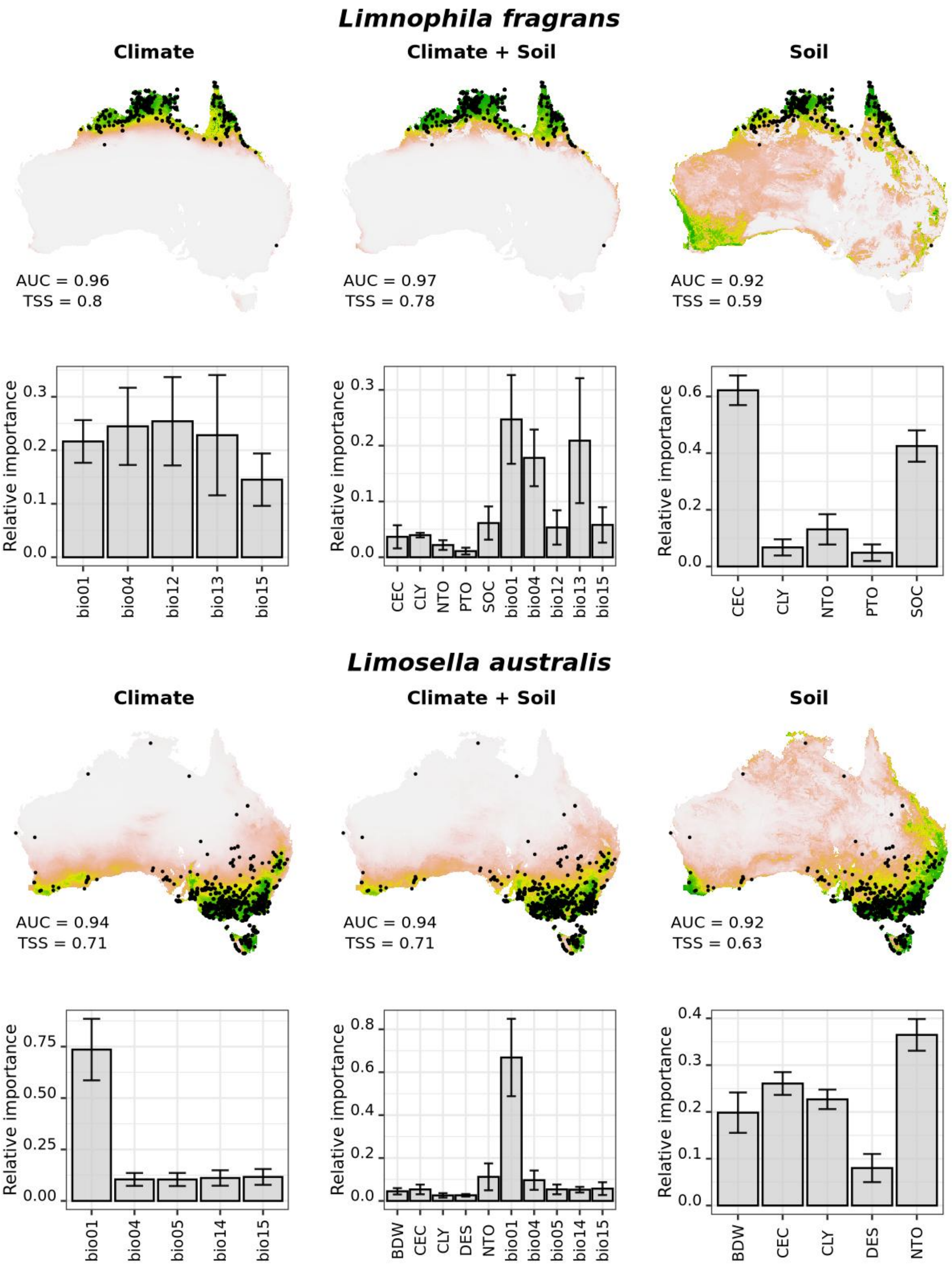

Limnophila fragrans
Climate
Climate + Soil
Soil
AUC = 0.96
TSS = 0.8
AUC = 0.97
TSS = 0.78
AUC = 0.92
TSS = 0.59
Relative importance
bio01
bio04
bio12
bio13
bio15
CEC
CLY
NTO
PTO
SOC
Limosella australis
Climate
Climate + Soil
Soil
AUC = 0.94
TSS = 0.71
AUC = 0.94
TSS = 0.71
AUC = 0.92
TSS = 0.63
Relative importance
bio01
bio04
bio05
bio14
bio15
BDW
CEC
CLY
DES
NTO

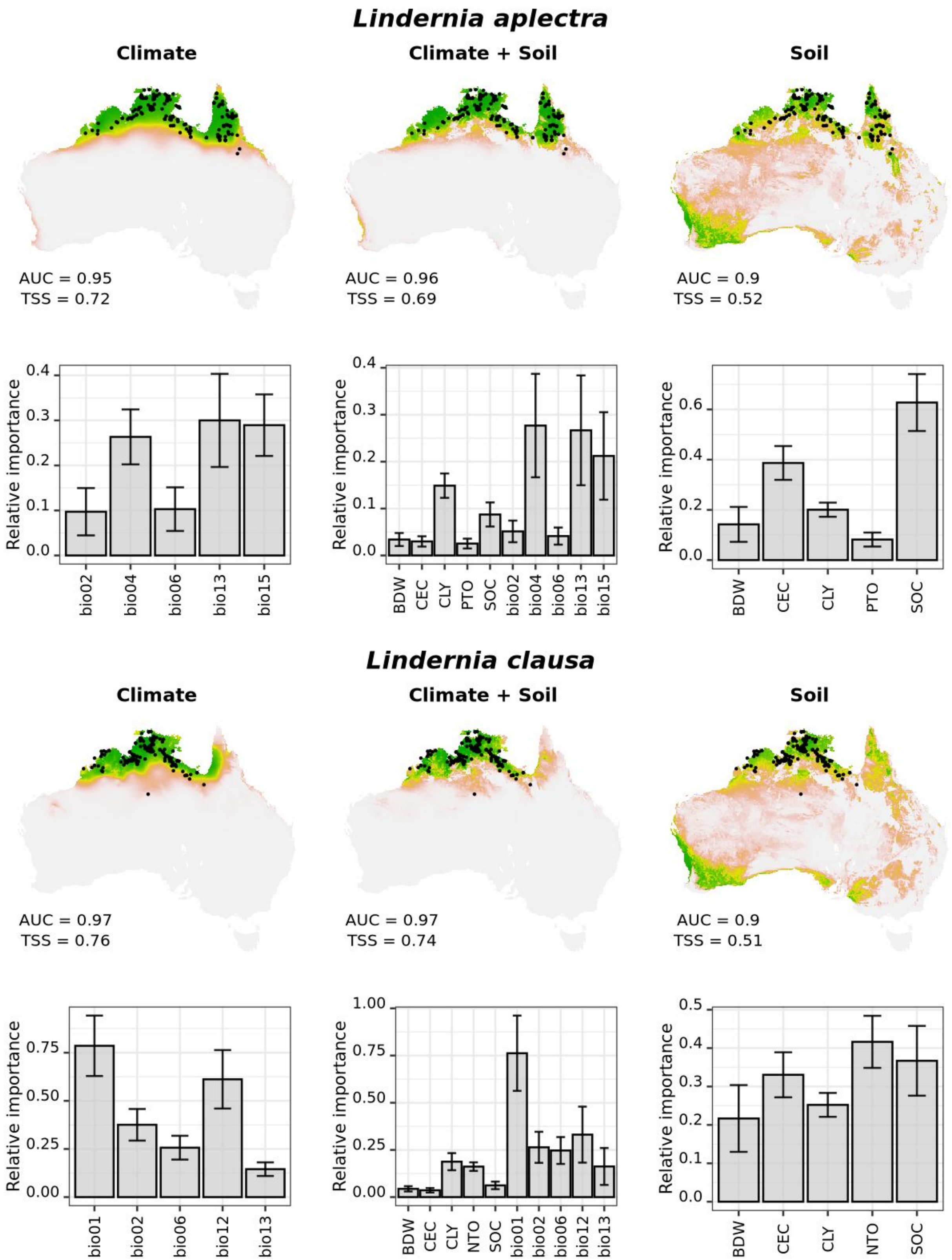
Lindernia aplectra
Climate
Climate + Soil
Soil
AUC = 0.95
TSS = 0.72
AUC = 0.96
TSS = 0.69
AUC = 0.9
TSS = 0.52
Relative importance
bio02
bio04
bio06
bio13
bio15
BDW
CEC
CLY
PTO
SOC
Lindernia clausa
Climate
Climate + Soil
Soil
AUC = 0.97
TSS = 0.76
AUC = 0.97
TSS = 0.74
AUC = 0.9
TSS = 0.51
Relative importance
bio01
bio02
bio06
bio12
bio13
BDW
CEC
CLY
NTO
SOC

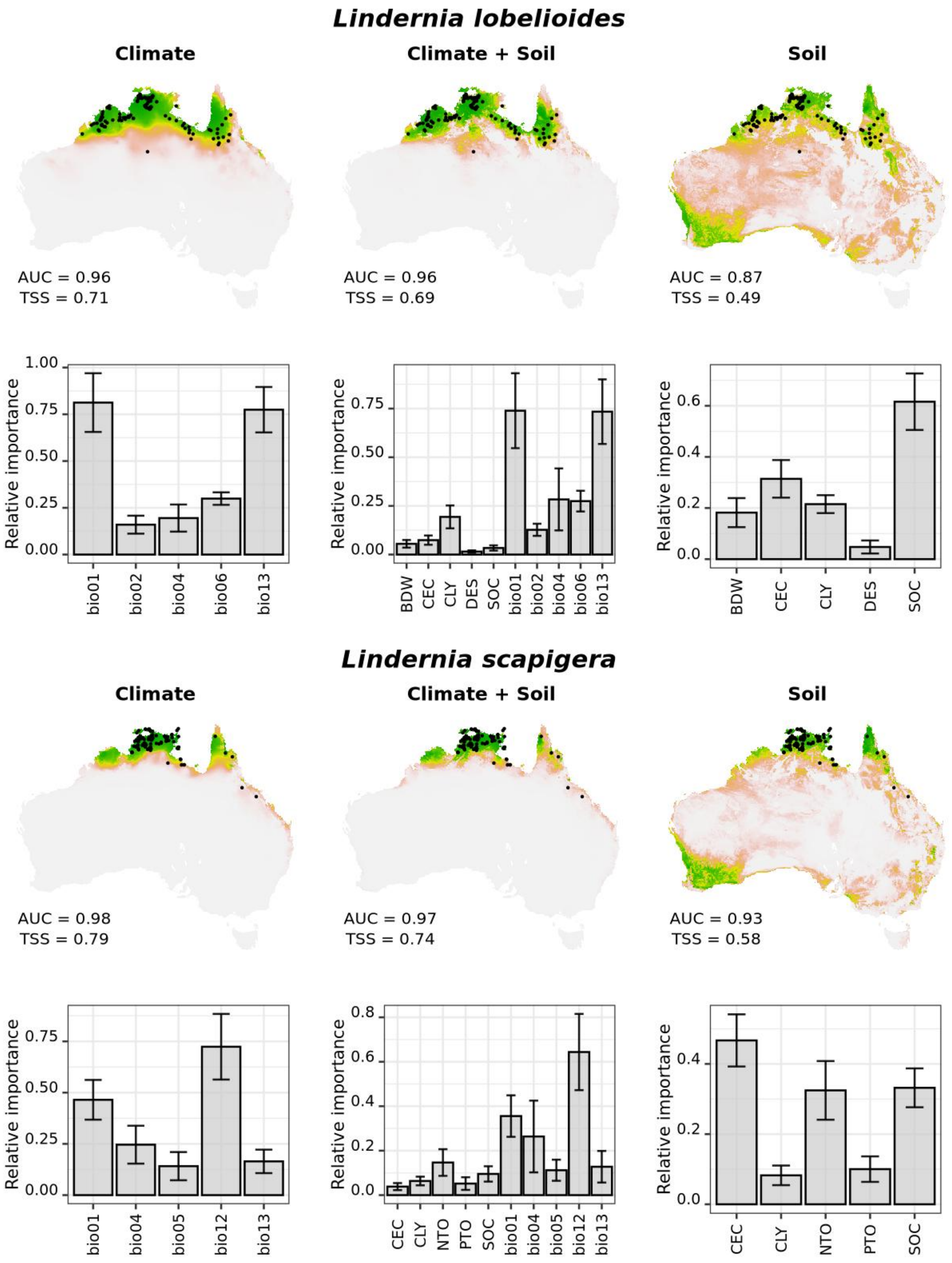
Lindernia lobelioides
Climate
Climate + Soil
Soil
AUC = 0.96
TSS = 0.71
AUC = 0.96
TSS = 0.69
AUC = 0.87
TSS = 0.49
Relative importance
bio01 bio02 bio04 bio06 bio13
BDW CEC CLY DES SOC bio01 bio02 bio04 bio06 bio13
BDW CEC CLY DES SOC
Lindernia scapigera
Climate
Climate + Soil
Soil
AUC = 0.98
TSS = 0.79
AUC = 0.97
TSS = 0.74
AUC = 0.93
TSS = 0.58
Relative importance
bio01 bio04 bio05 bio12 bio13
CEC CLY NTO PTO SOC bio01 bio04 bio05 bio12 bio13
CEC CLY NTO PTO SOC

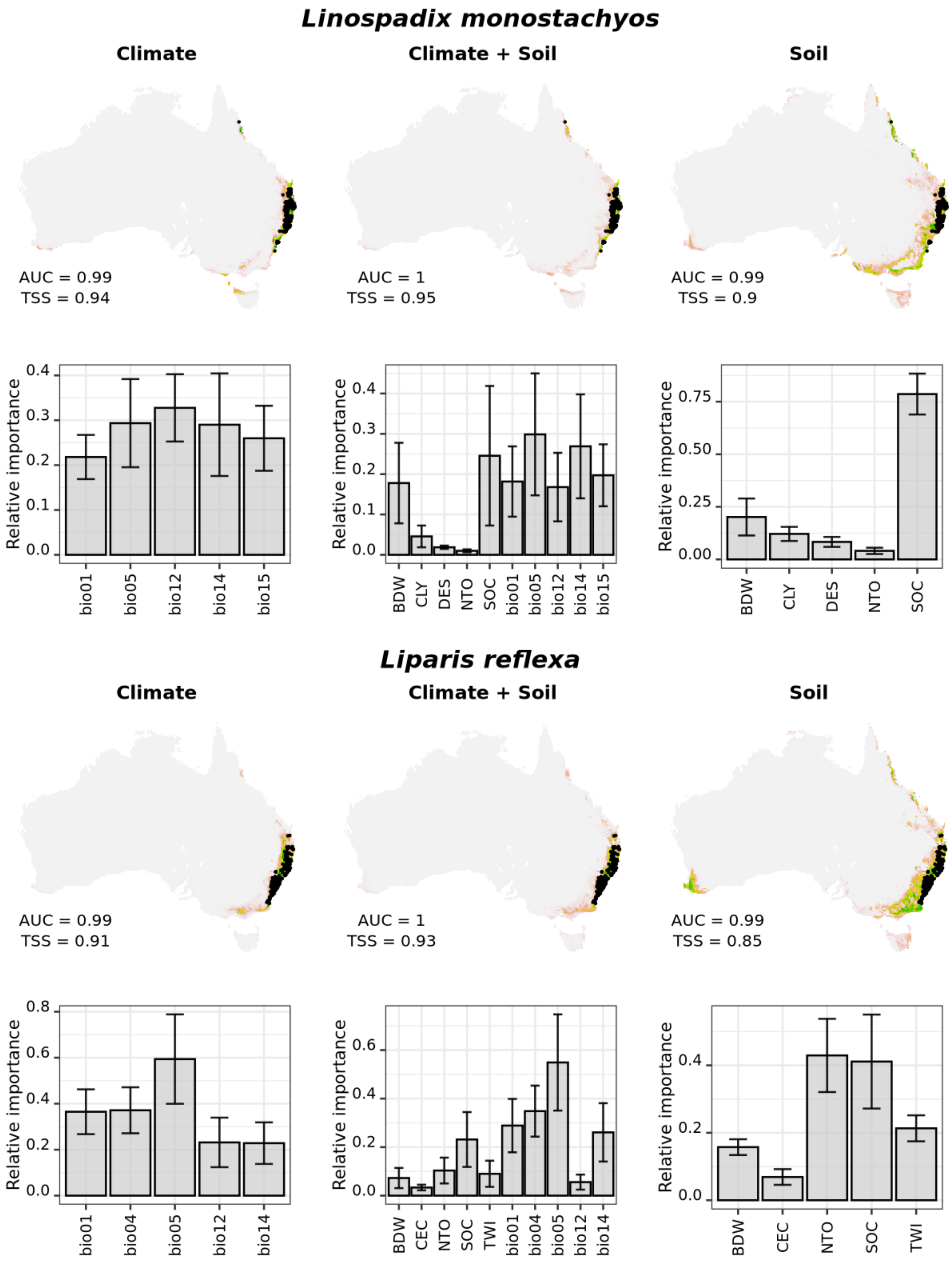

Linospadix monostachyos
Climate
Climate + Soil
Soil
AUC = 0.99
TSS = 0.94
AUC = 1
TSS = 0.95
AUC = 0.99
TSS = 0.9
Relative importance
bio01
bio05
bio12
bio14
bio15
BDW
CLY
DES
NTO
SOC
Liparis reflexa
AUC = 0.99
TSS = 0.91
AUC = 1
TSS = 0.93
AUC = 0.99
TSS = 0.85
bio04
CEC
TWI

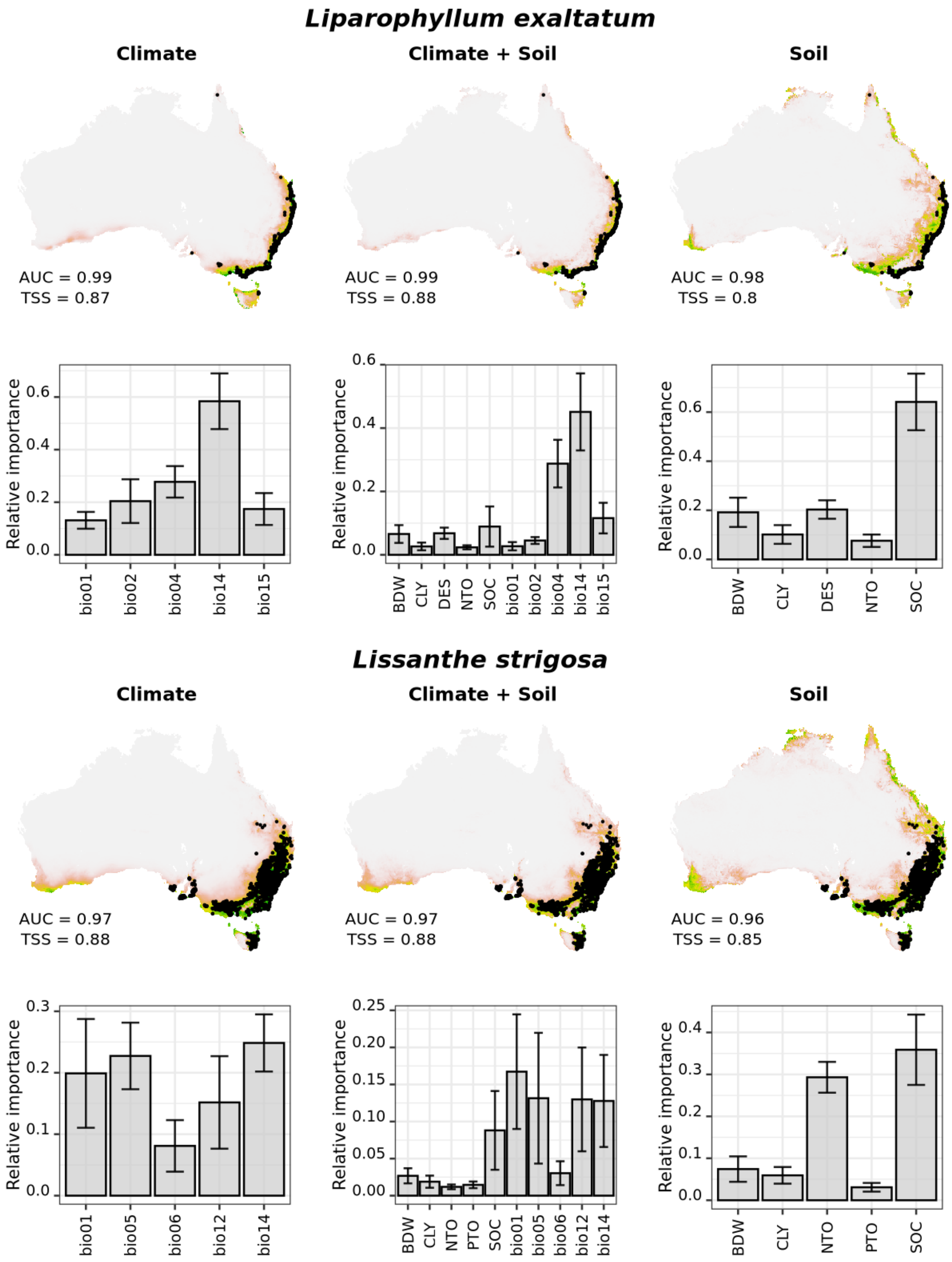

Liparophyllum exaltatum
Climate
Climate + Soil
Soil
AUC = 0.99
TSS = 0.87
AUC = 0.99
TSS = 0.88
AUC = 0.98
TSS = 0.8
Relative importance
bio01
bio02
bio04
bio14
bio15
BDW
CLY
DES
NTO
SOC
Lissanthe strigosa
AUC = 0.97
TSS = 0.88
AUC = 0.97
TSS = 0.88
AUC = 0.96
TSS = 0.85
bio05
bio06
bio12
PTO

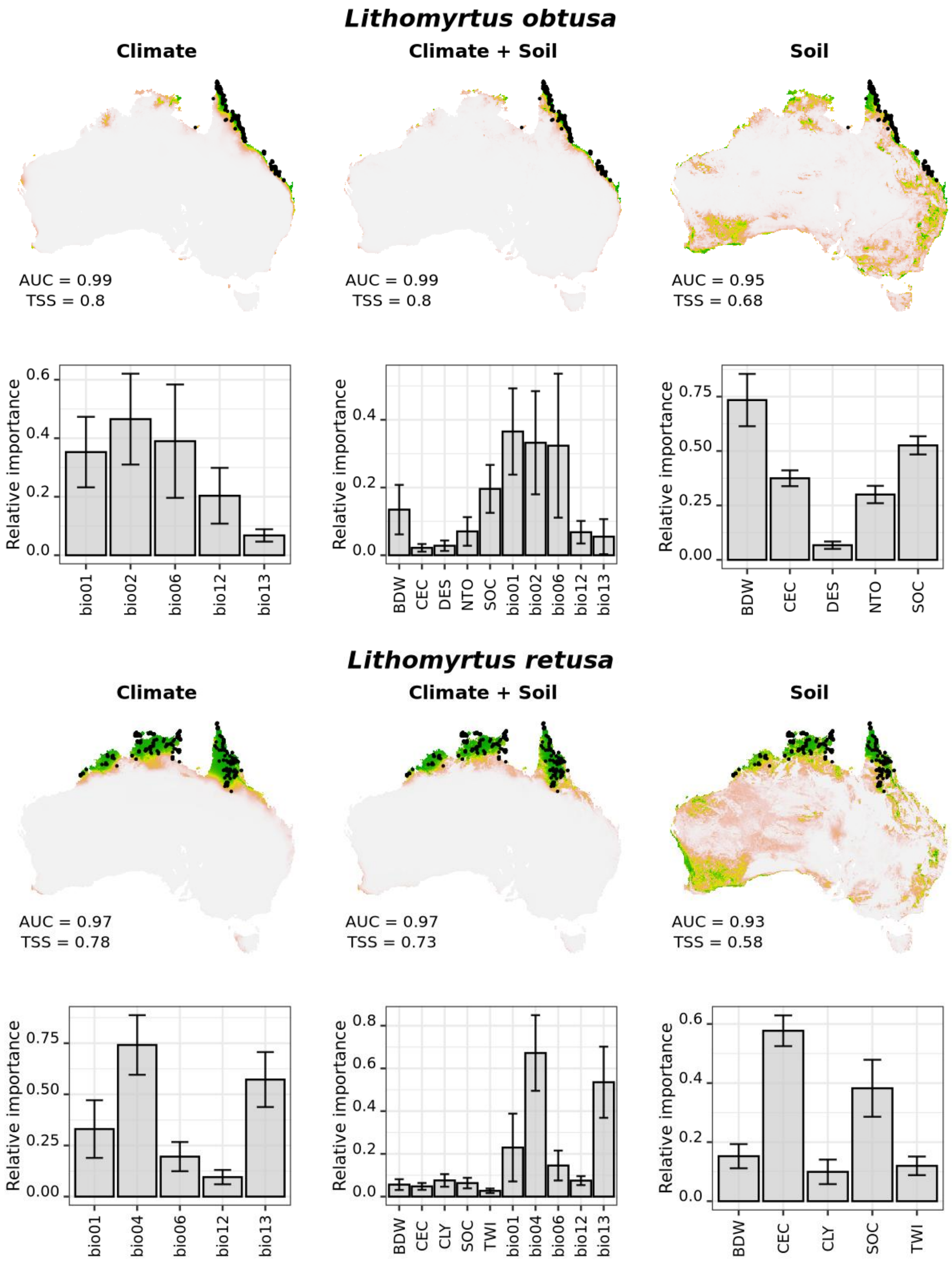
Lithomyrtus obtusa
Climate
Climate + Soil
Soil
AUC = 0.99
TSS = 0.8
AUC = 0.99
TSS = 0.8
AUC = 0.95
TSS = 0.68
Relative importance
0.0
0.2
0.4
0.6
bio01
bio02
bio06
bio12
bio13
Relative importance
0.0
0.2
0.4
BDW
CEC
DES
NTO
SOC
bio01
bio02
bio06
bio12
bio13
Relative importance
0.00
0.25
0.50
0.75
BDW
CEC
DES
NTO
SOC
Lithomyrtus retusa
Climate
Climate + Soil
Soil
AUC = 0.97
TSS = 0.78
AUC = 0.97
TSS = 0.73
AUC = 0.93
TSS = 0.58
Relative importance
0.00
0.25
0.50
0.75
bio01
bio04
bio06
bio12
bio13
Relative importance
0.0
0.2
0.4
0.6
0.8
BDW
CEC
CLY
SOC
TWI
bio01
bio04
bio06
bio12
bio13
Relative importance
0.0
0.2
0.4
0.6
BDW
CEC
CLY
SOC
TWI

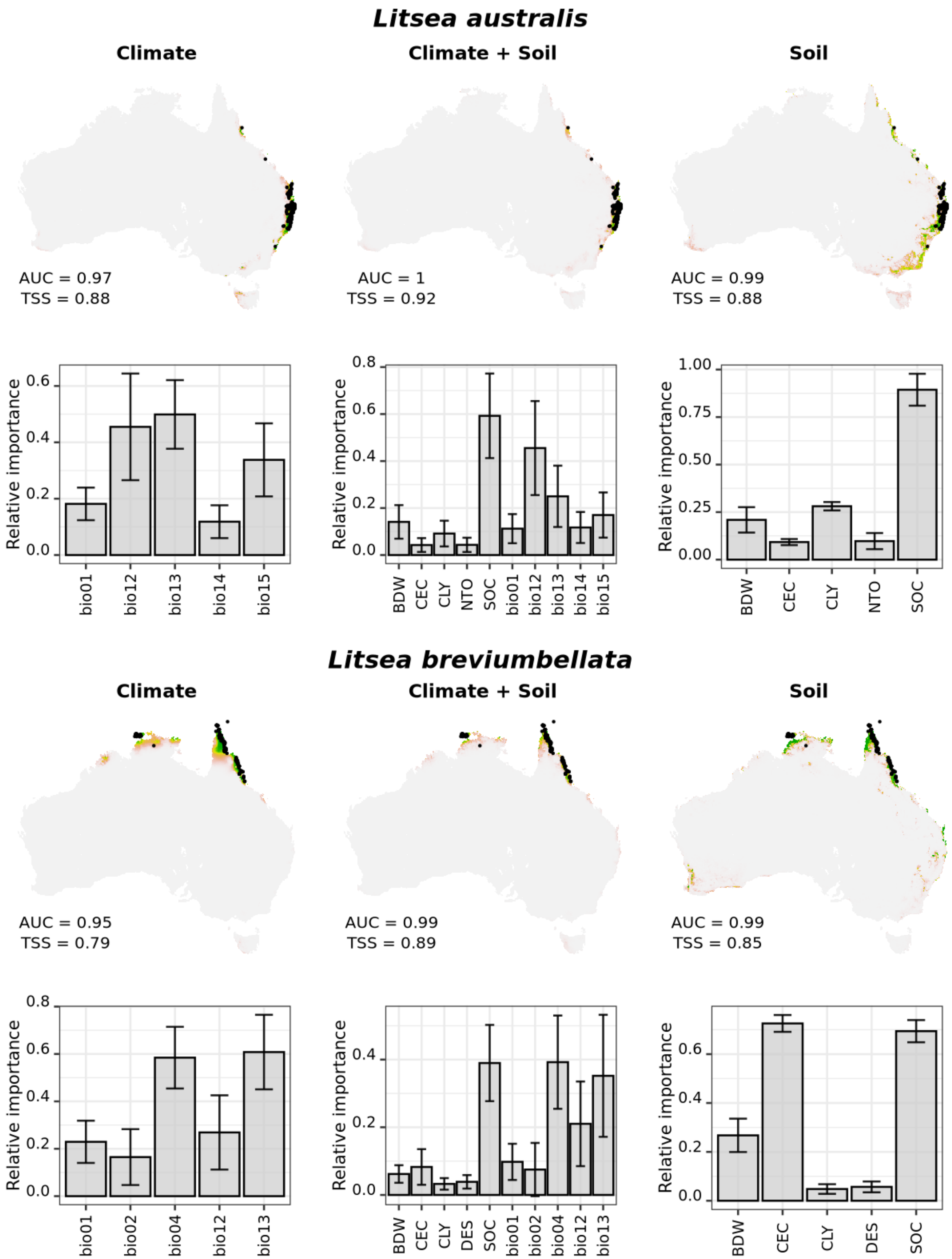
Litsea australis
Climate
Climate + Soil
Soil
AUC = 0.97
TSS = 0.88
AUC = 1
TSS = 0.92
AUC = 0.99
TSS = 0.88
Relative importance
bio01
bio12
bio13
bio14
bio15
BDW
CEC
CLY
NTO
SOC
Litsea breviumbellata
Climate
Climate + Soil
Soil
AUC = 0.95
TSS = 0.79
AUC = 0.99
TSS = 0.89
AUC = 0.99
TSS = 0.85
Relative importance
bio01
bio02
bio04
bio12
bio13
BDW
CEC
CLY
DES
SOC

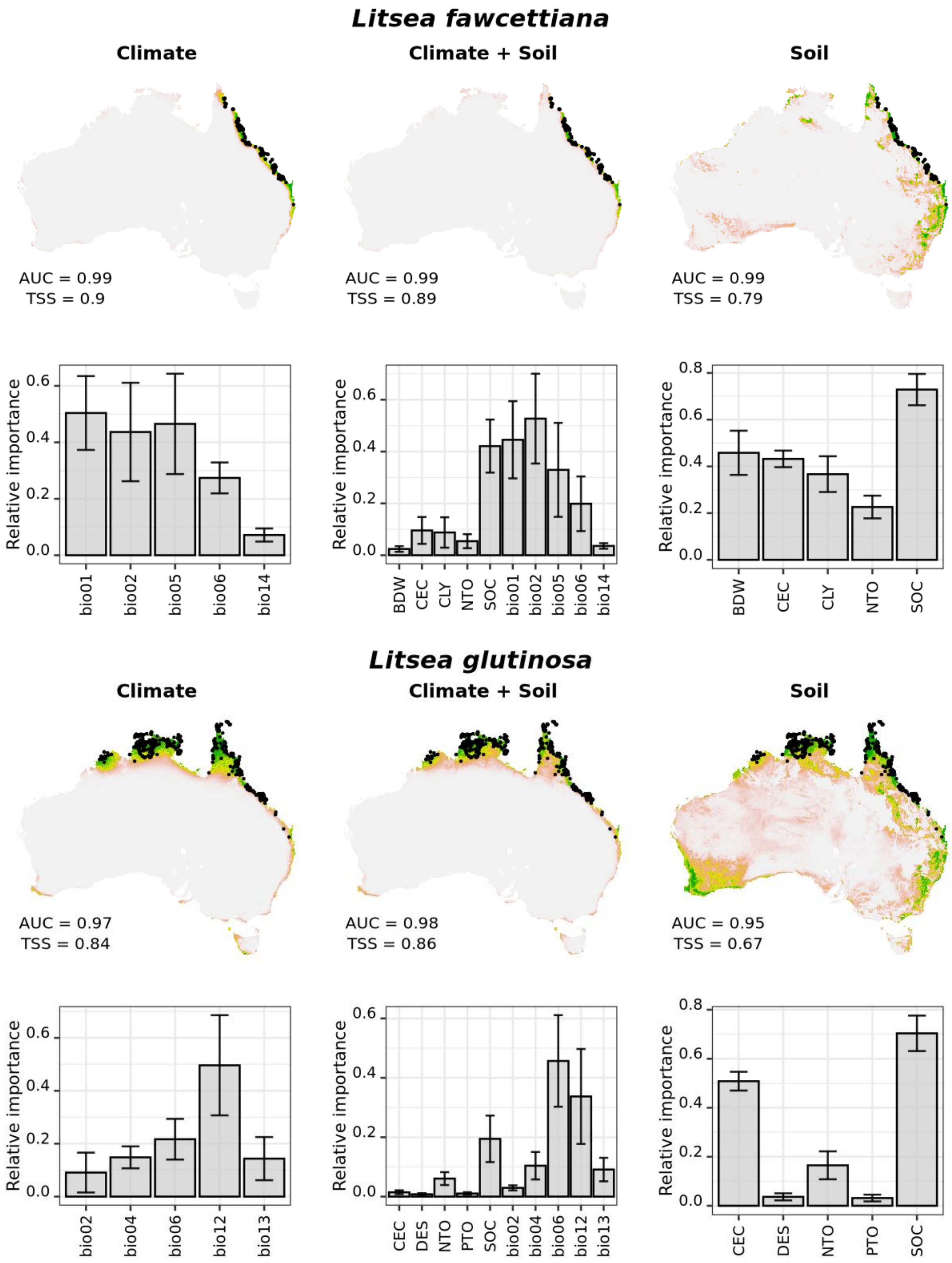

Litsea fawcettiana
Climate
Climate + Soil
Soil
AUC = 0.99
TSS = 0.9
AUC = 0.99
TSS = 0.89
AUC = 0.99
TSS = 0.79
Relative importance
bio01
bio02
bio05
bio06
bio14
BDW
CEC
CLY
NTO
SOC
Litsea glutinosa
AUC = 0.97
TSS = 0.84
AUC = 0.98
TSS = 0.86
AUC = 0.95
TSS = 0.67
bio04
bio12
bio13
DES
PTO

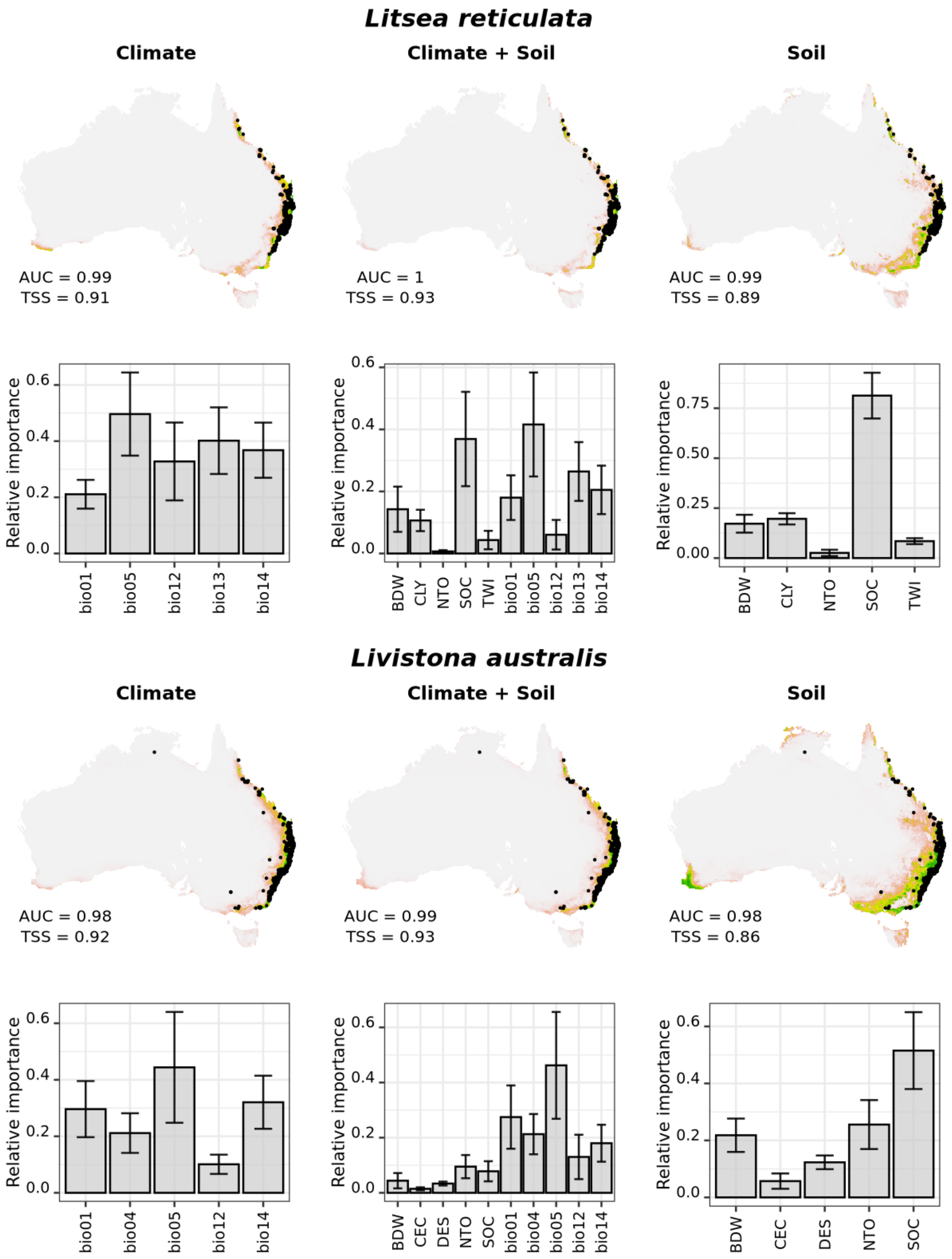
Litsea reticulata
Climate
Climate + Soil
Soil
AUC = 0.99
TSS = 0.91
AUC = 1
TSS = 0.93
AUC = 0.99
TSS = 0.89
Relative importance
bio01
bio05
bio12
bio13
bio14
BDW
CLY
NTO
SOC
TWI
Livistona australis
Climate
Climate + Soil
Soil
AUC = 0.98
TSS = 0.92
AUC = 0.99
TSS = 0.93
AUC = 0.98
TSS = 0.86
Relative importance
bio01
bio04
bio05
bio12
bio14
BDW
CEC
DES
NTO
SOC

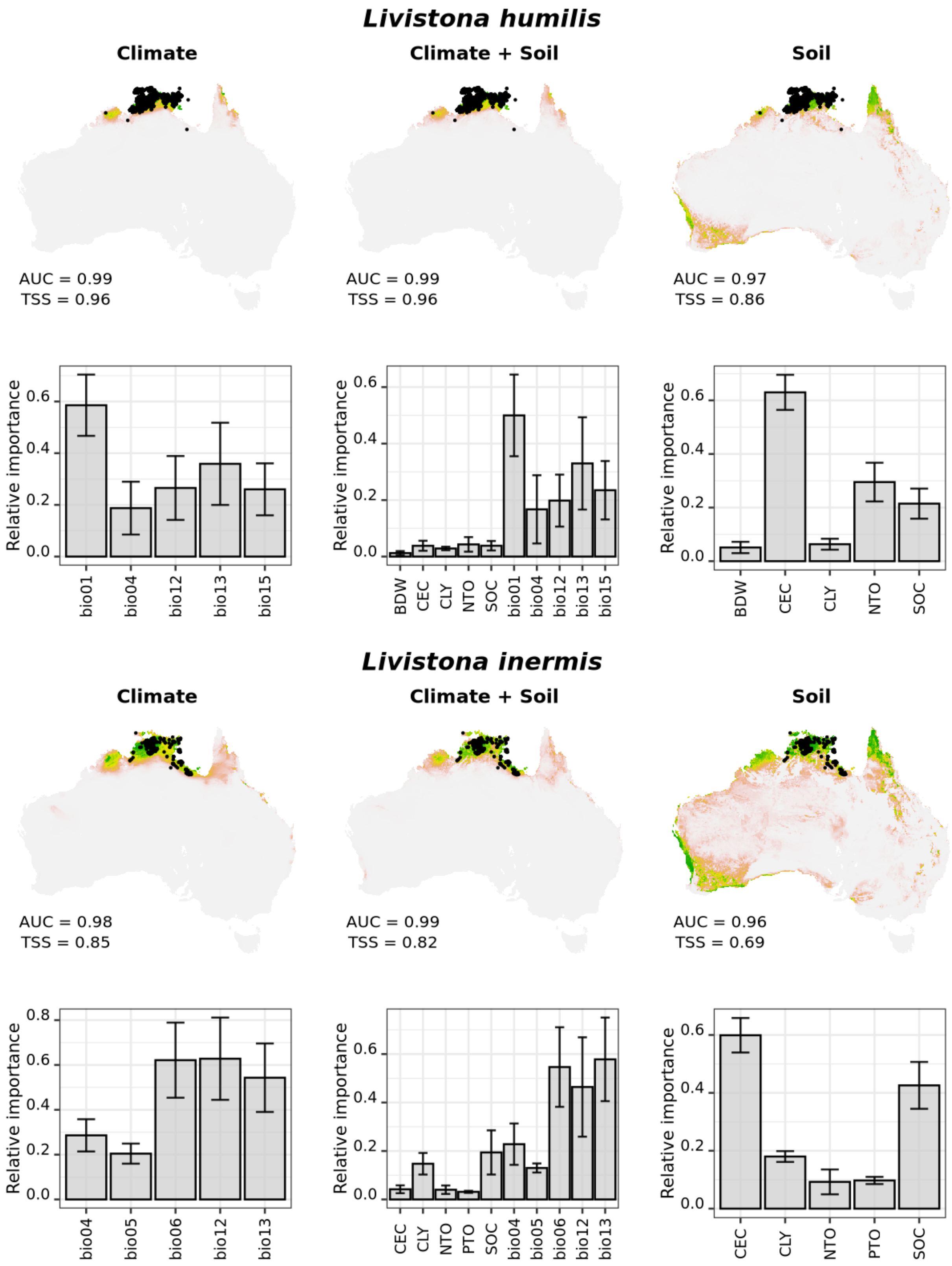
Livistona humilis
Climate
Climate + Soil
Soil
AUC = 0.99
TSS = 0.96
AUC = 0.99
TSS = 0.96
AUC = 0.97
TSS = 0.86
Relative importance
0.0
0.2
0.4
0.6
bio01
bio04
bio12
bio13
bio15
BDW
CEC
CLY
NTO
SOC
Livistona inermis
AUC = 0.98
TSS = 0.85
AUC = 0.99
TSS = 0.82
AUC = 0.96
TSS = 0.69
0.8
bio05
bio06
PTO

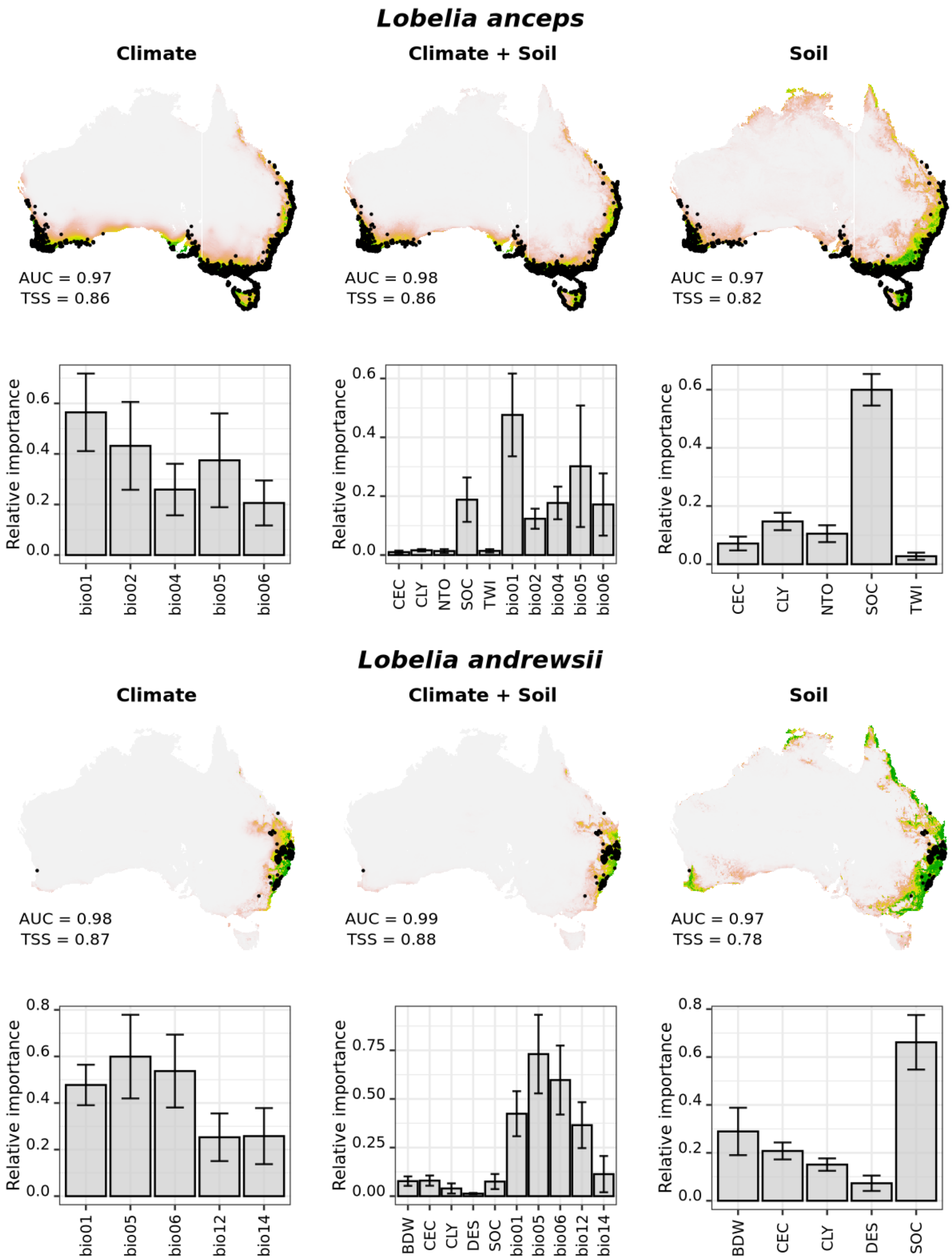
Lobelia anceps
Climate
Climate + Soil
Soil
AUC = 0.97
TSS = 0.86
AUC = 0.98
TSS = 0.86
AUC = 0.97
TSS = 0.82
Relative importance
0.0
0.2
0.4
0.6
bio01
bio02
bio04
bio05
bio06
CEC
CLY
NTO
SOC
TWI
bio01
bio02
bio04
bio05
bio06
CEC
CLY
NTO
SOC
TWI
Lobelia andrewsii
Climate
Climate + Soil
Soil
AUC = 0.98
TSS = 0.87
AUC = 0.99
TSS = 0.88
AUC = 0.97
TSS = 0.78
Relative importance
0.0
0.2
0.4
0.6
0.8
0.00
0.25
0.50
0.75
bio01
bio05
bio06
bio12
bio14
BDW
CEC
CLY
DES
SOC
bio01
bio05
bio06
bio12
bio14
BDW
CEC
CLY
DES
SOC

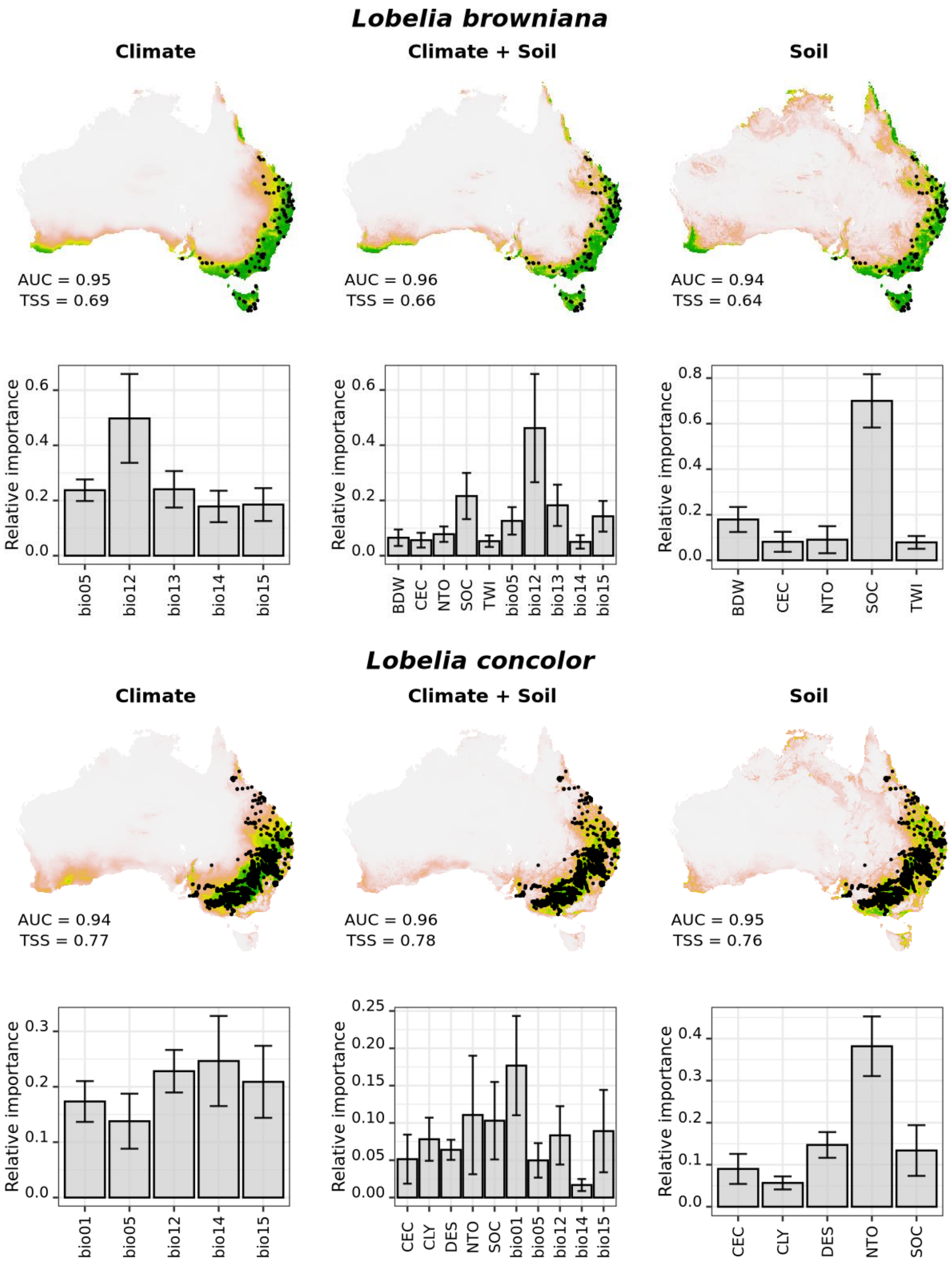
Lobelia browniana
Climate
Climate + Soil
Soil
AUC = 0.95
TSS = 0.69
AUC = 0.96
TSS = 0.66
AUC = 0.94
TSS = 0.64
Relative importance
bio05
bio12
bio13
bio14
bio15
BDW
CEC
NTO
SOC
TWI
Lobelia concolor
Climate
Climate + Soil
Soil
AUC = 0.94
TSS = 0.77
AUC = 0.96
TSS = 0.78
AUC = 0.95
TSS = 0.76
Relative importance
bio01
CLY
DES

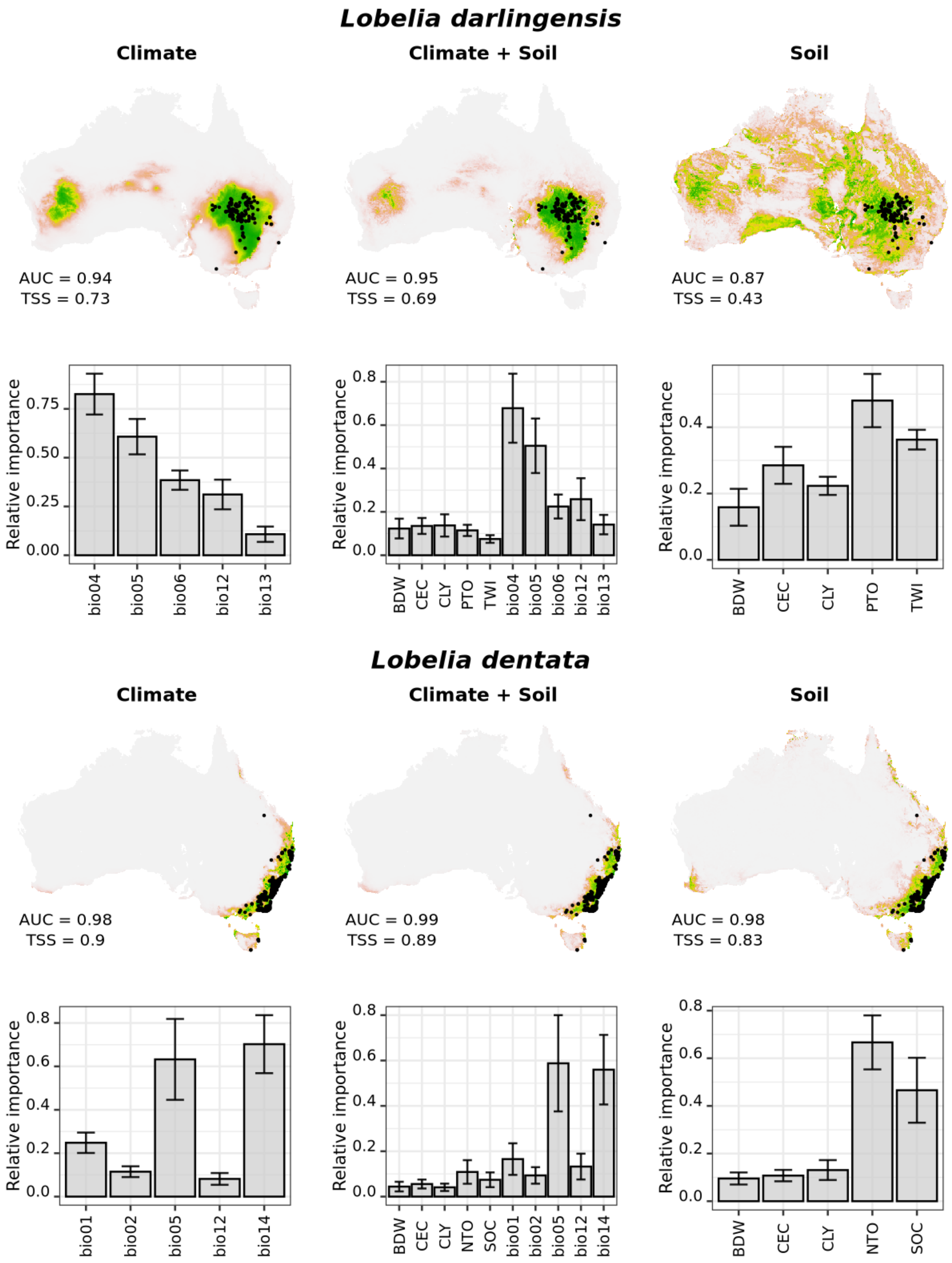
Lobelia darlingensis
Climate
Climate + Soil
Soil
AUC = 0.94
TSS = 0.73
AUC = 0.95
TSS = 0.69
AUC = 0.87
TSS = 0.43
Relative importance
0.00
0.25
0.50
0.75
bio04
bio05
bio06
bio12
bio13
0.0
0.2
0.4
0.6
0.8
BDW
CEC
CLY
PTO
TWI
bio04
bio05
bio06
bio12
bio13
0.0
0.2
0.4
BDW
CEC
CLY
PTO
TWI
Lobelia dentata
Climate
Climate + Soil
Soil
AUC = 0.98
TSS = 0.9
AUC = 0.99
TSS = 0.89
AUC = 0.98
TSS = 0.83
Relative importance
0.0
0.2
0.4
0.6
0.8
bio01
bio02
bio05
bio12
bio14
BDW
CEC
CLY
NTO
SOC
bio01
bio02
bio05
bio12
bio14
BDW
CEC
CLY
NTO
SOC

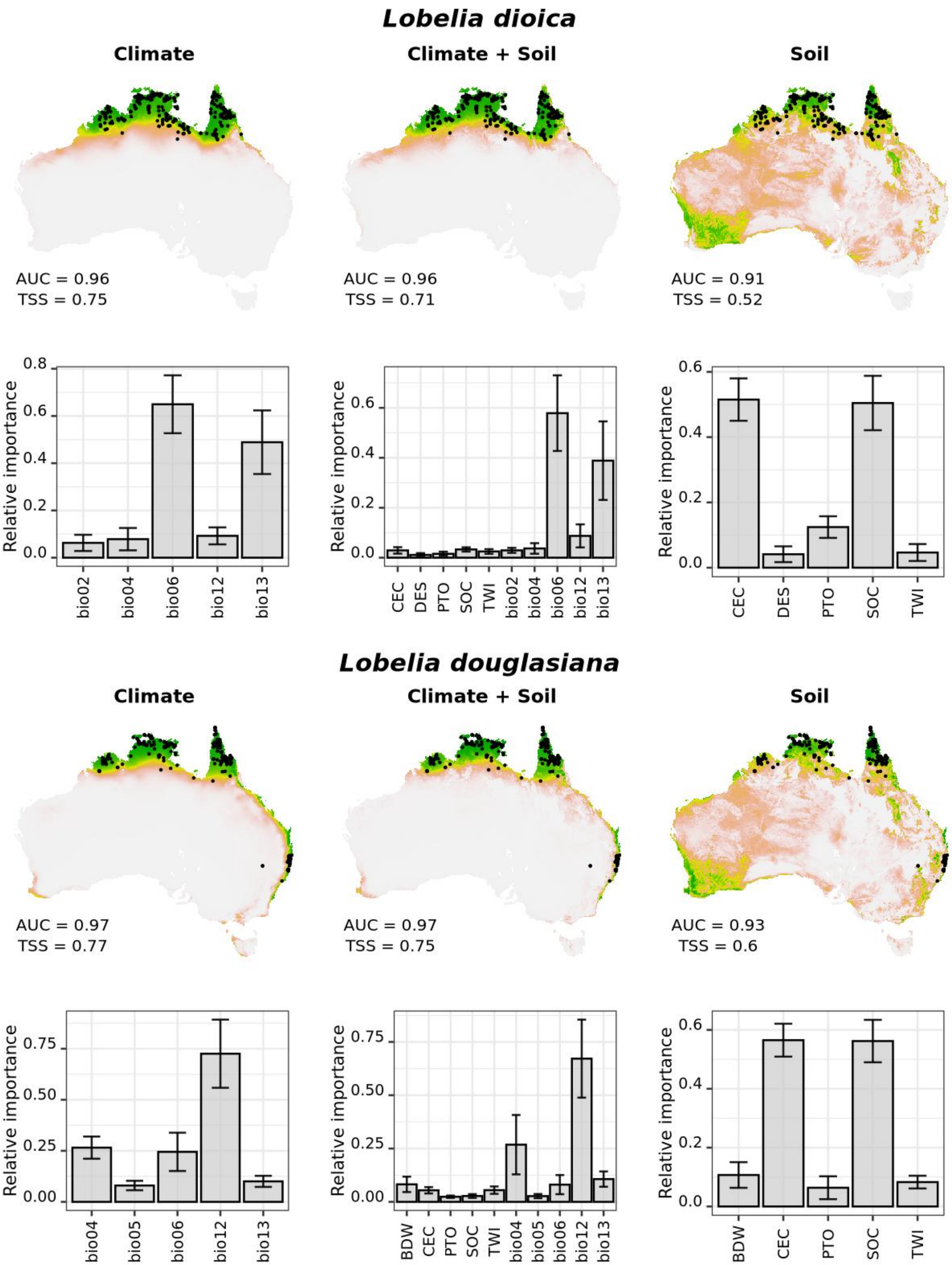
Lobelia dioica
Climate
Climate + Soil
Soil
AUC = 0.96
TSS = 0.75
AUC = 0.96
TSS = 0.71
AUC = 0.91
TSS = 0.52
Relative importance
bio02
bio04
bio06
bio12
bio13
CEC
DES
PTO
SOC
TWI
Lobelia douglasiana
Climate
Climate + Soil
Soil
AUC = 0.97
TSS = 0.77
AUC = 0.97
TSS = 0.75
AUC = 0.93
TSS = 0.6
Relative importance
bio04
bio05
bio06
bio12
bio13
BDW
CEC
PTO
SOC
TWI

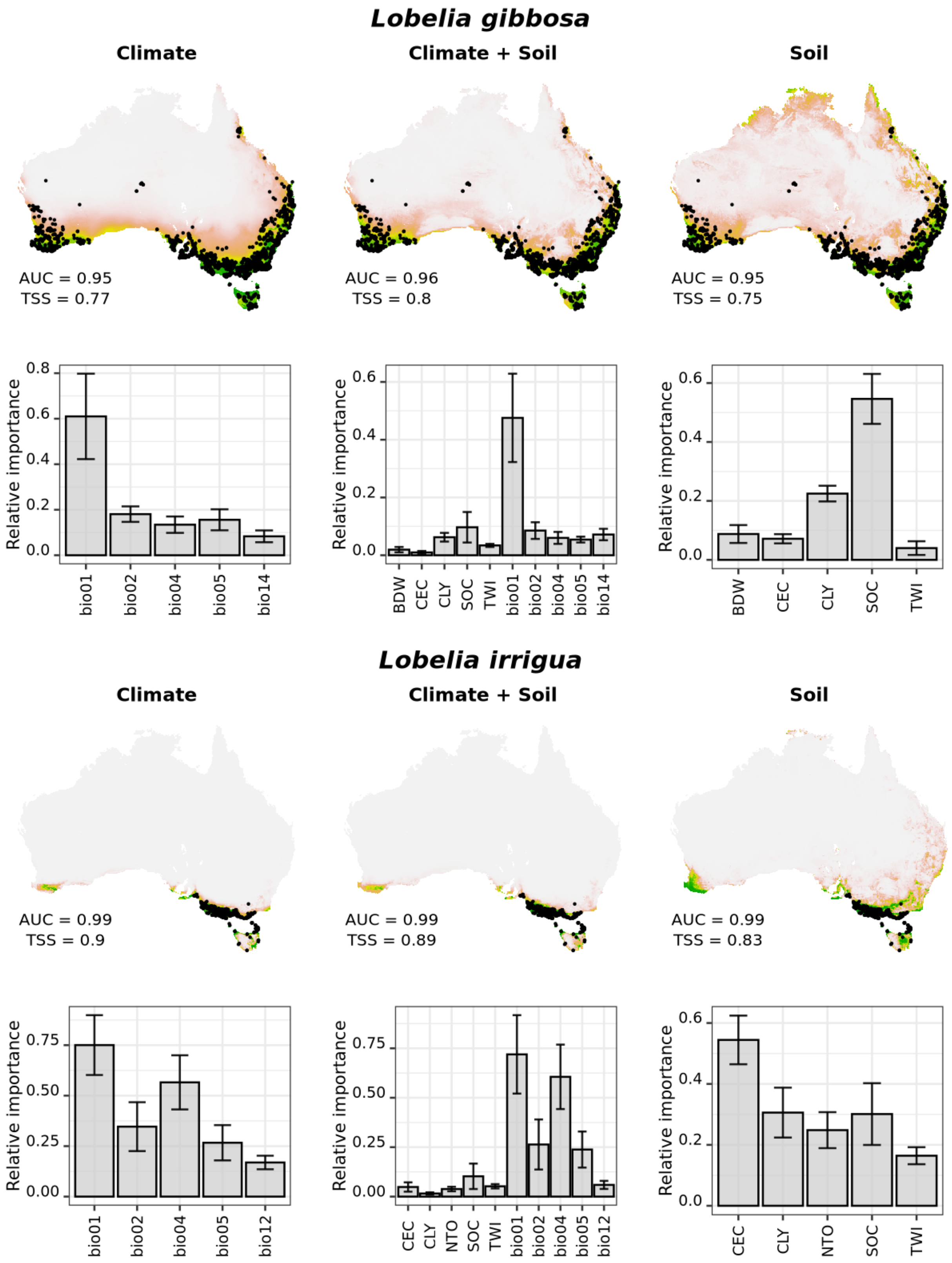

Lobelia gibbosa
Climate
Climate + Soil
Soil
AUC = 0.95
TSS = 0.77
AUC = 0.96
TSS = 0.8
AUC = 0.95
TSS = 0.75
Relative importance
0.0
0.2
0.4
0.6
0.8
bio01
bio02
bio04
bio05
bio14
BDW
CEC
CLY
SOC
TWI
Lobelia irrigua
AUC = 0.99
TSS = 0.9
AUC = 0.99
TSS = 0.89
AUC = 0.99
TSS = 0.83
0.00
0.25
0.50
0.75
bio12
NTO

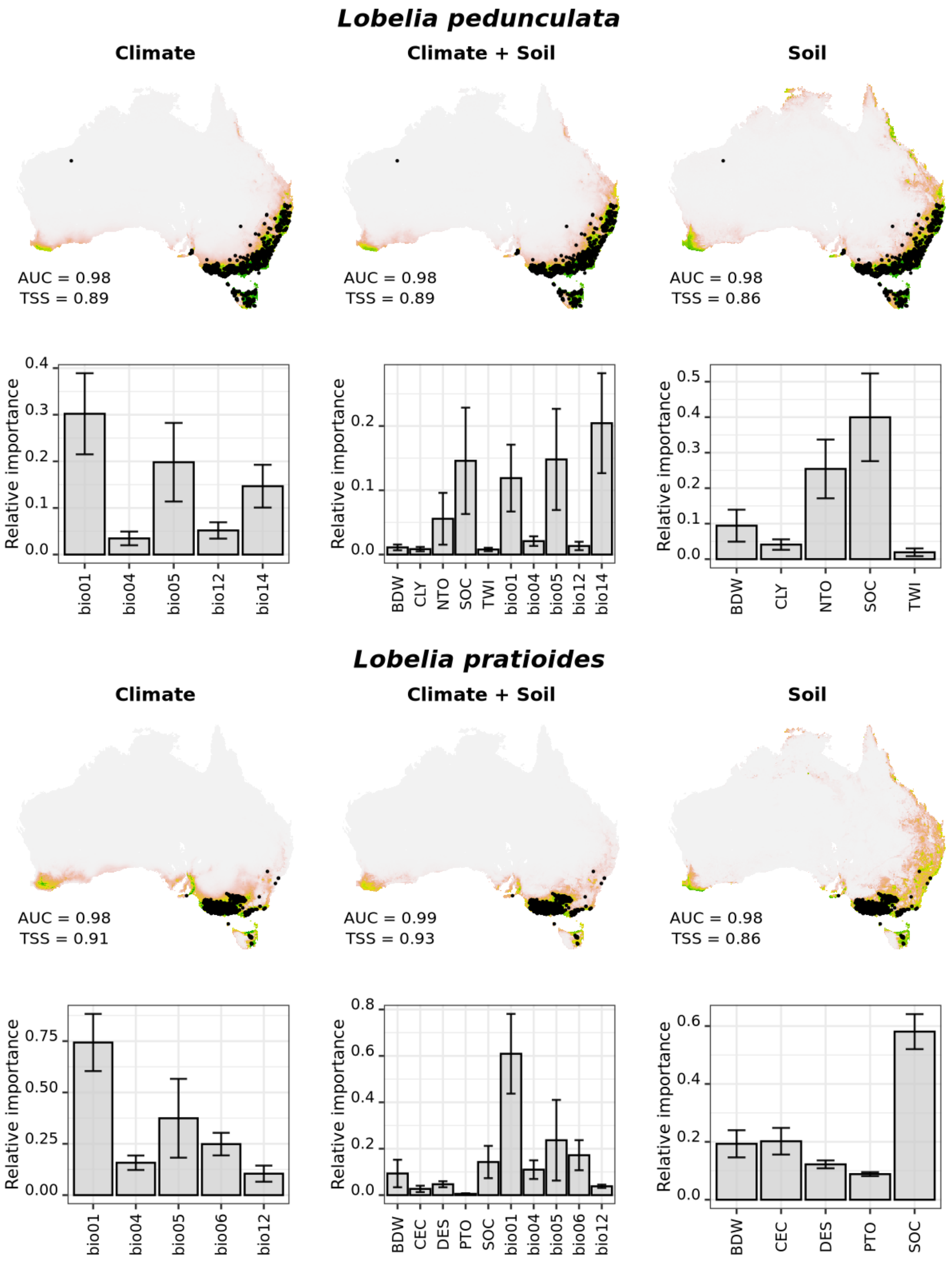
Lobelia pedunculata
Climate
Climate + Soil
Soil
AUC = 0.98
TSS = 0.89
AUC = 0.98
TSS = 0.89
AUC = 0.98
TSS = 0.86
Relative importance
bio01
bio04
bio05
bio12
bio14
BDW
CLY
NTO
SOC
TWI
Lobelia pratioides
Climate
Climate + Soil
Soil
AUC = 0.98
TSS = 0.91
AUC = 0.99
TSS = 0.93
AUC = 0.98
TSS = 0.86
bio06
CEC
DES
PTO

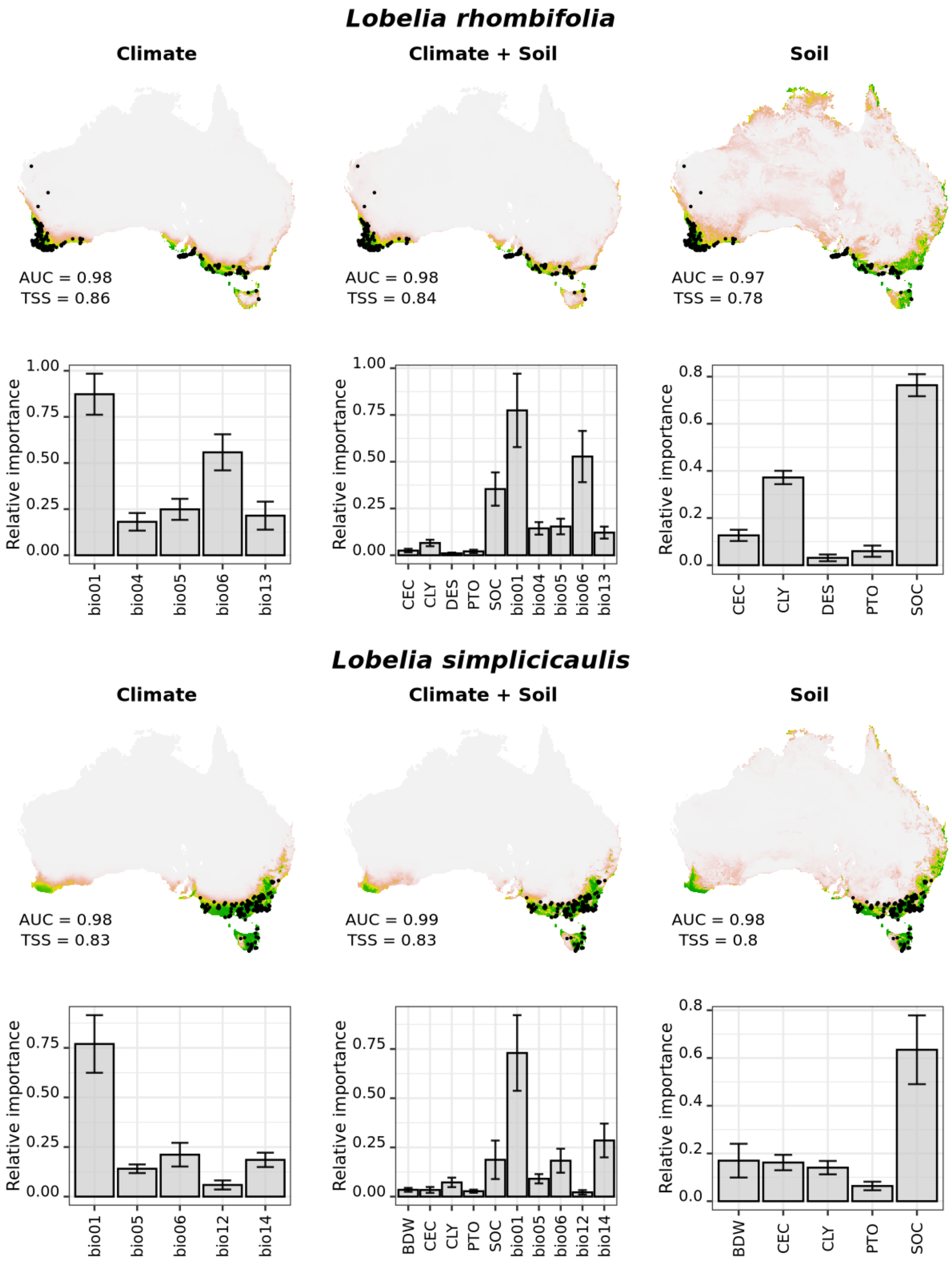
Lobelia rhombifolia
Climate
Climate + Soil
Soil
AUC = 0.98
TSS = 0.86
AUC = 0.98
TSS = 0.84
AUC = 0.97
TSS = 0.78
Relative importance
1.00
0.75
0.50
0.25
0.00
bio01
bio04
bio05
bio06
bio13
CEC
CLY
DES
PTO
SOC
0.8
0.6
0.4
0.2
0.0
Lobelia simplicicaulis
Climate
Climate + Soil
Soil
AUC = 0.98
TSS = 0.83
AUC = 0.99
TSS = 0.83
AUC = 0.98
TSS = 0.8
bio12
bio14
BDW

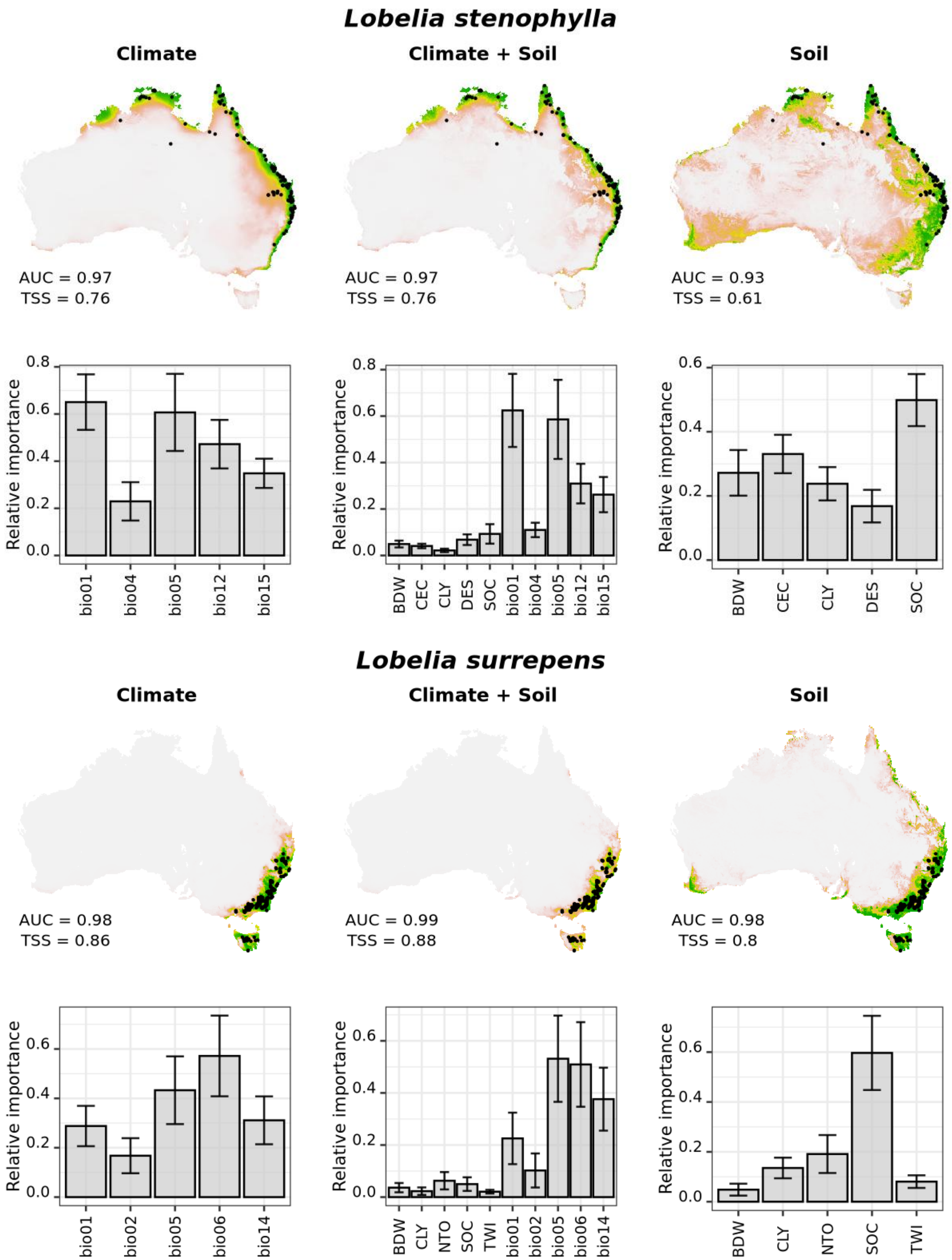
Lobelia stenophylla
Climate
Climate + Soil
Soil
AUC = 0.97
TSS = 0.76
AUC = 0.97
TSS = 0.76
AUC = 0.93
TSS = 0.61
Relative importance
bio01
bio04
bio05
bio12
bio15
BDW
CEC
CLY
DES
SOC
Lobelia surrepens
Climate
Climate + Soil
Soil
AUC = 0.98
TSS = 0.86
AUC = 0.99
TSS = 0.88
AUC = 0.98
TSS = 0.8
Relative importance
bio01
bio02
bio05
bio06
bio14
BDW
CLY
NTO
SOC
TWI

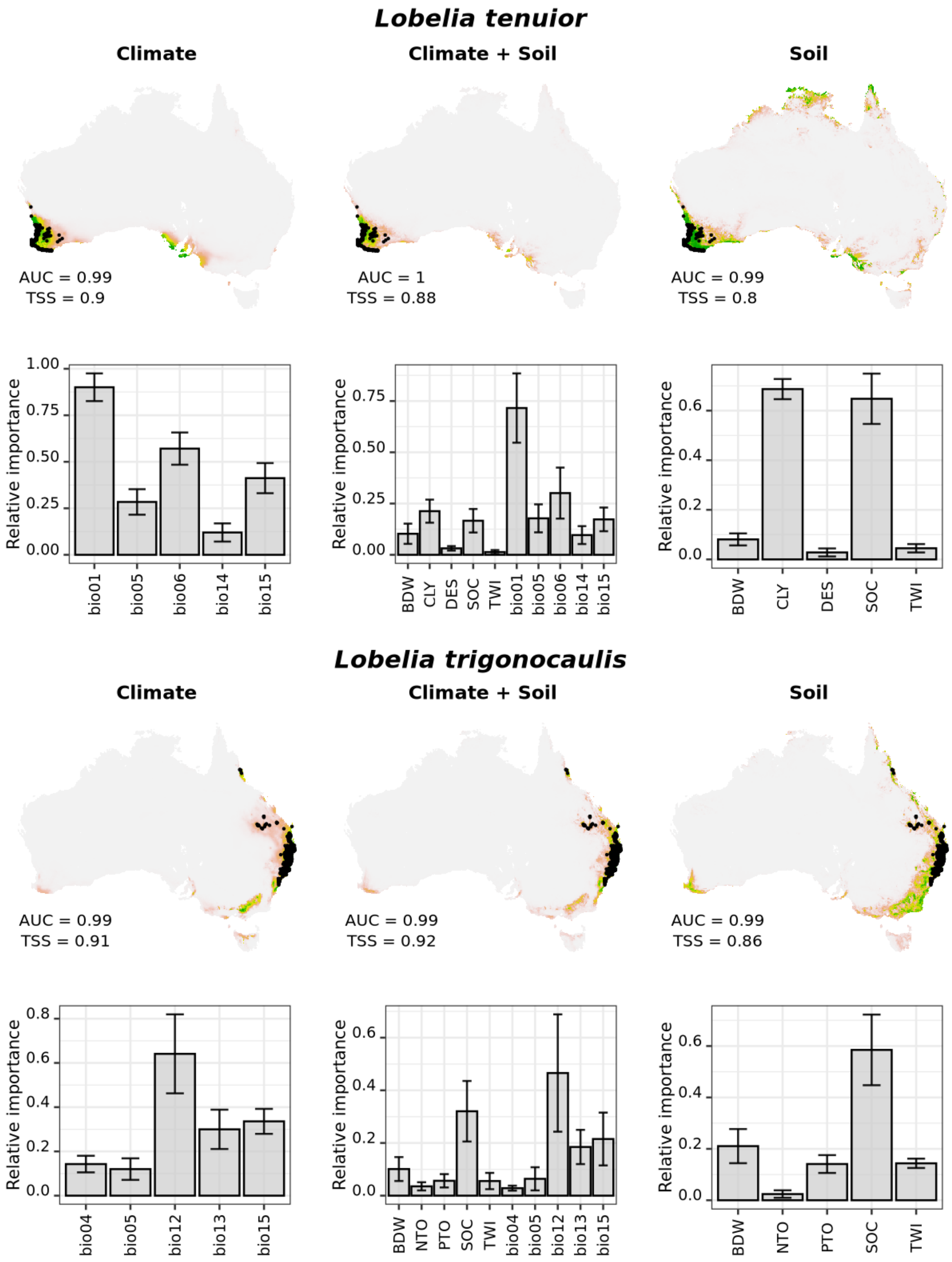

Lobelia tenuior
Climate
Climate + Soil
Soil
AUC = 0.99
TSS = 0.9
AUC = 1
TSS = 0.88
AUC = 0.99
TSS = 0.8
Relative importance
1.00
0.75
0.50
0.25
0.00
bio01
bio05
bio06
bio14
bio15
BDW
CLY
DES
SOC
TWI
0.6
0.4
0.2
0.0
Lobelia trigonocaulis
Climate
Climate + Soil
Soil
AUC = 0.99
TSS = 0.91
AUC = 0.99
TSS = 0.92
AUC = 0.99
TSS = 0.86
Relative importance
0.8
0.6
0.4
0.2
0.0
bio04
bio05
bio12
bio13
bio15
BDW
NTO
PTO
SOC
TWI

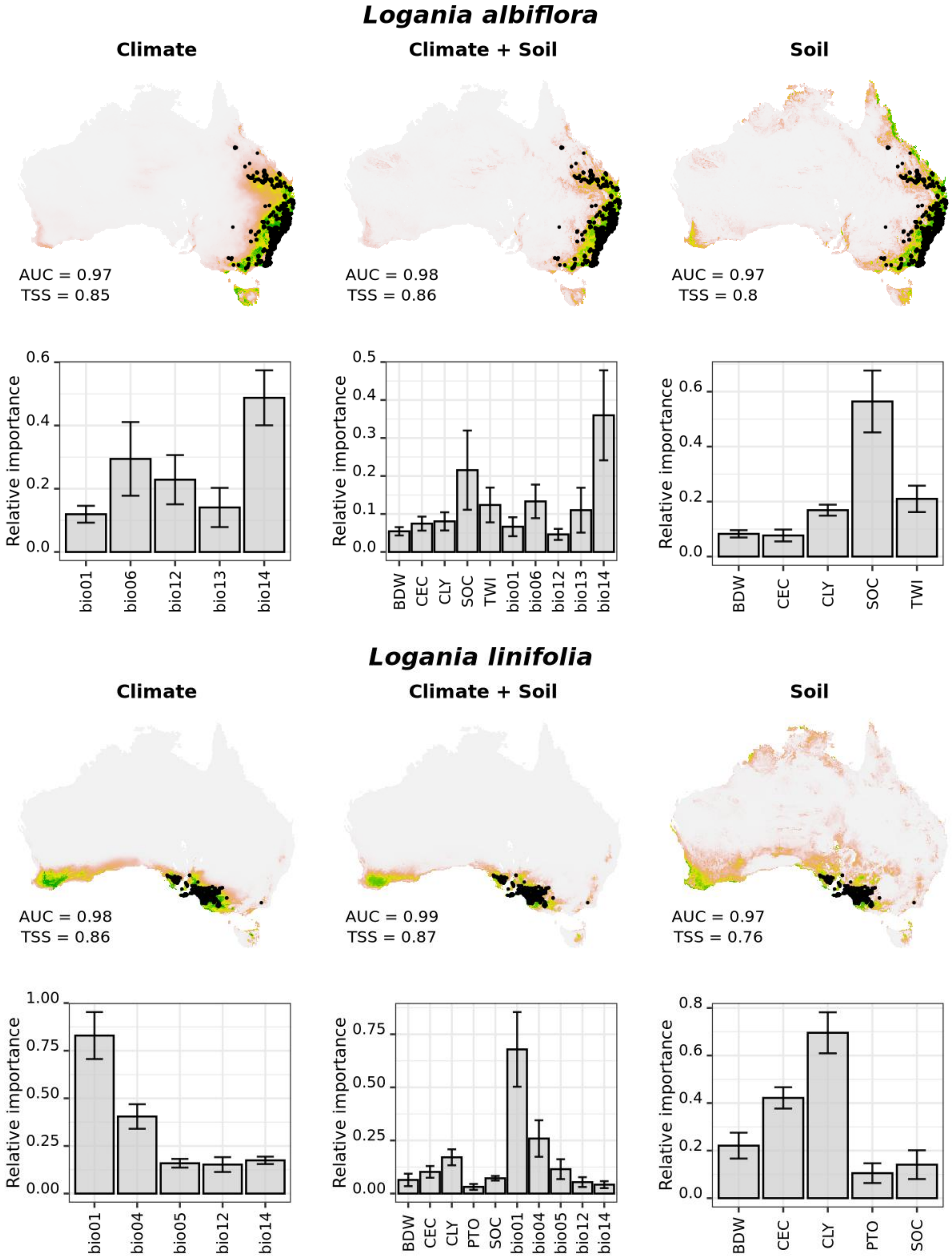
Logania albiflora
Climate
Climate + Soil
Soil
AUC = 0.97
TSS = 0.85
AUC = 0.98
TSS = 0.86
AUC = 0.97
TSS = 0.8
Relative importance
0.0
0.2
0.4
0.6
bio01
bio06
bio12
bio13
bio14
Relative importance
0.0
0.1
0.2
0.3
0.4
0.5
BDW
CEC
CLY
SOC
TWI
bio01
bio06
bio12
bio13
bio14
Relative importance
0.0
0.2
0.4
0.6
BDW
CEC
CLY
SOC
TWI
Logania linifolia
Climate
Climate + Soil
Soil
AUC = 0.98
TSS = 0.86
AUC = 0.99
TSS = 0.87
AUC = 0.97
TSS = 0.76
Relative importance
0.00
0.25
0.50
0.75
1.00
bio01
bio04
bio05
bio12
bio14
Relative importance
0.00
0.25
0.50
0.75
BDW
CEC
CLY
PTO
SOC
bio01
bio04
bio05
bio12
bio14
Relative importance
0.0
0.2
0.4
0.6
0.8
BDW
CEC
CLY
PTO
SOC

Logania ovata

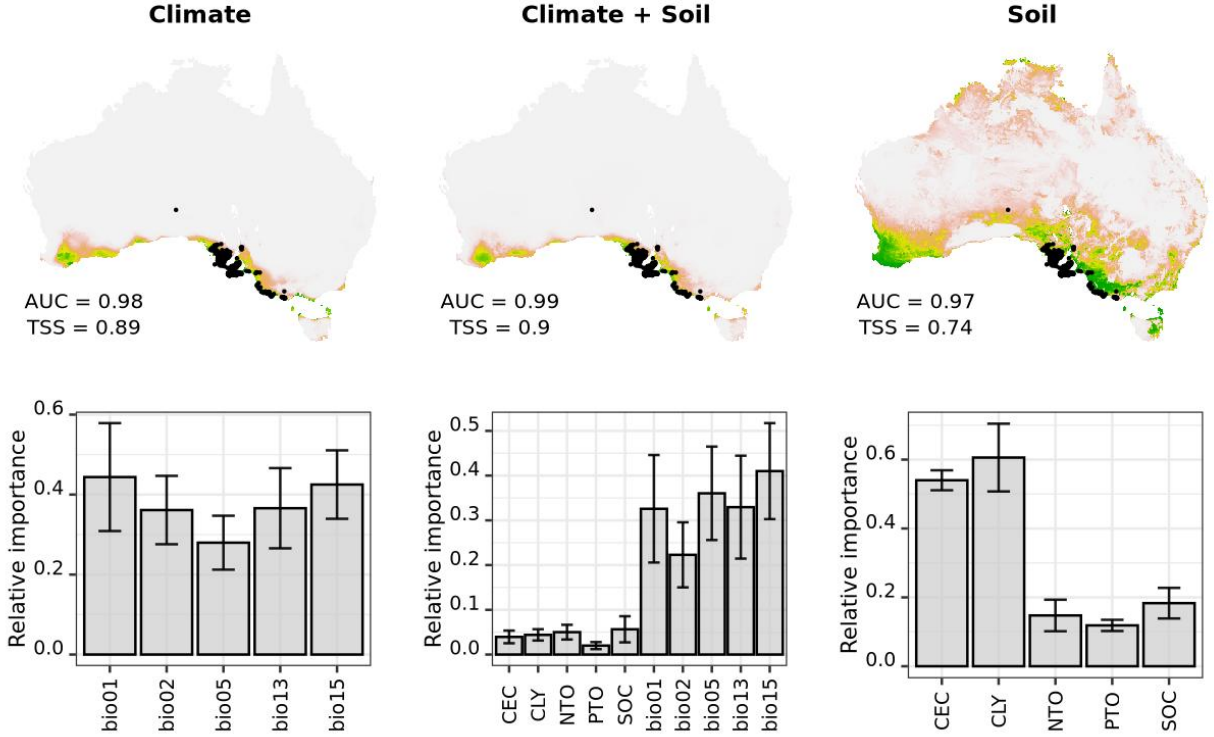

Logania vaginalis

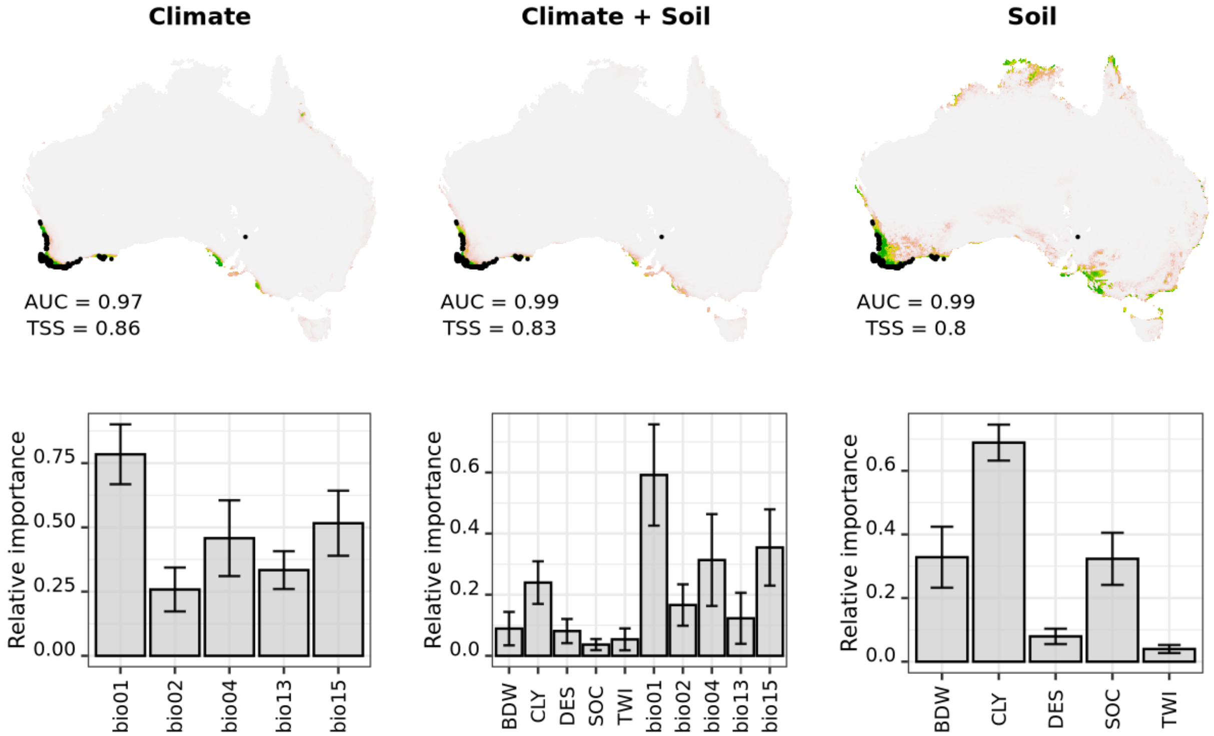

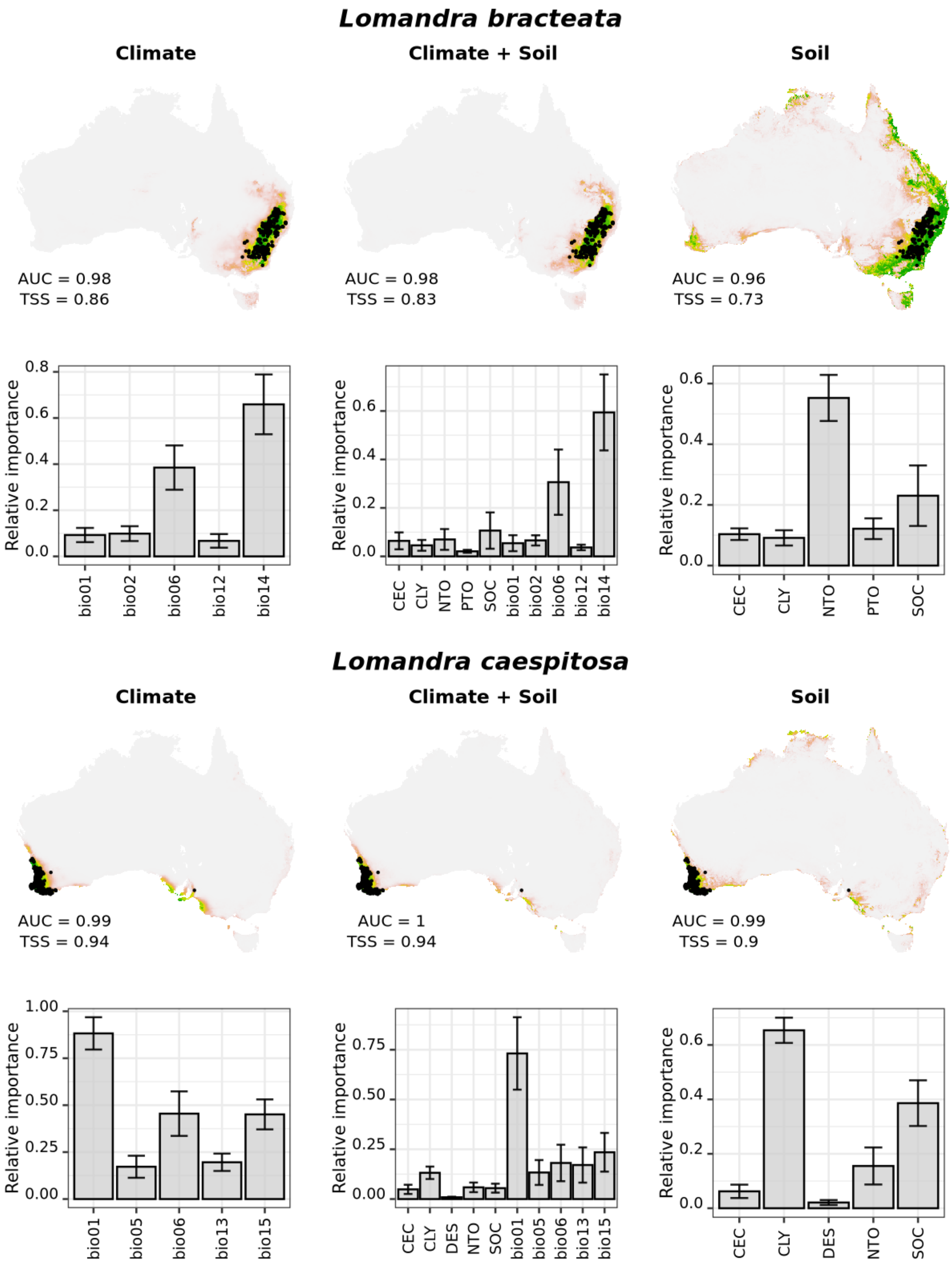

Lomandra bracteata
Climate
Climate + Soil
Soil
AUC = 0.98
TSS = 0.86
AUC = 0.98
TSS = 0.83
AUC = 0.96
TSS = 0.73
Relative importance
0.0
0.2
0.4
0.6
0.8
bio01
bio02
bio06
bio12
bio14
CEC
CLY
NTO
PTO
SOC
Lomandra caespitosa
Climate
Climate + Soil
Soil
AUC = 0.99
TSS = 0.94
AUC = 1
TSS = 0.94
AUC = 0.99
TSS = 0.9
Relative importance
0.00
0.25
0.50
0.75
1.00
bio01
bio05
bio06
bio13
bio15
CEC
CLY
DES
NTO
SOC

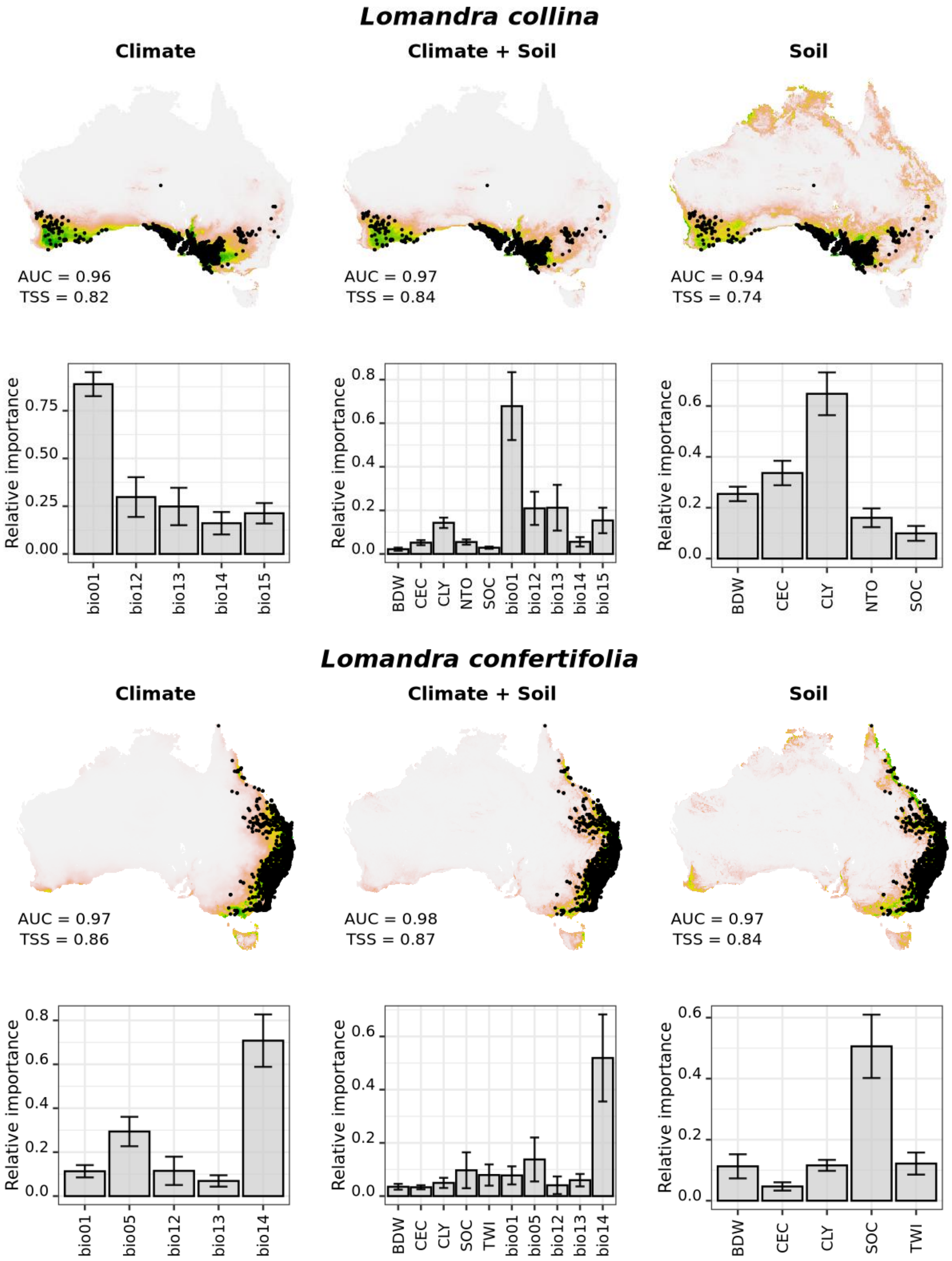
Lomandra collina
Climate
Climate + Soil
Soil
AUC = 0.96
TSS = 0.82
AUC = 0.97
TSS = 0.84
AUC = 0.94
TSS = 0.74
Relative importance
0.00
0.25
0.50
0.75
bio01
bio12
bio13
bio14
bio15
0.0
0.2
0.4
0.6
0.8
BDW
CEC
CLY
NTO
SOC
bio01
bio12
bio13
bio14
bio15
0.0
0.2
0.4
0.6
BDW
CEC
CLY
NTO
SOC
Lomandra confertifolia
Climate
Climate + Soil
Soil
AUC = 0.97
TSS = 0.86
AUC = 0.98
TSS = 0.87
AUC = 0.97
TSS = 0.84
Relative importance
0.0
0.2
0.4
0.6
0.8
bio01
bio05
bio12
bio13
bio14
0.0
0.2
0.4
0.6
BDW
CEC
CLY
SOC
TWI
bio01
bio05
bio12
bio13
bio14
0.0
0.2
0.4
0.6
BDW
CEC
CLY
SOC
TWI

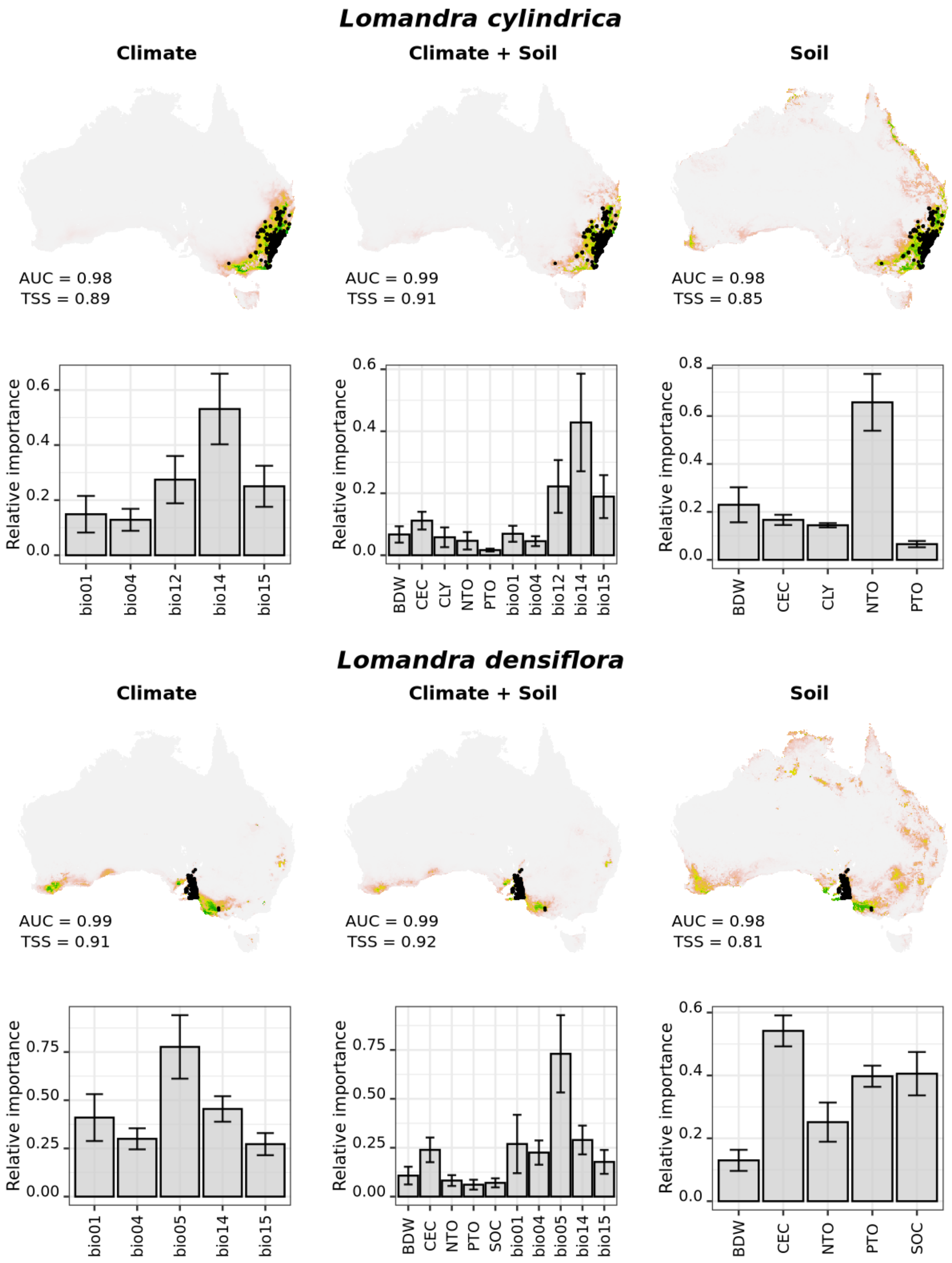

Lomandra cylindrica
Climate
Climate + Soil
Soil
AUC = 0.98
TSS = 0.89
AUC = 0.99
TSS = 0.91
AUC = 0.98
TSS = 0.85
Relative importance
bio01
bio04
bio12
bio14
bio15
BDW
CEC
CLY
NTO
PTO
Lomandra densiflora
Climate
Climate + Soil
Soil
AUC = 0.99
TSS = 0.91
AUC = 0.99
TSS = 0.92
AUC = 0.98
TSS = 0.81
Relative importance
bio01
bio04
bio05
bio14
bio15
BDW
CEC
NTO
PTO
SOC

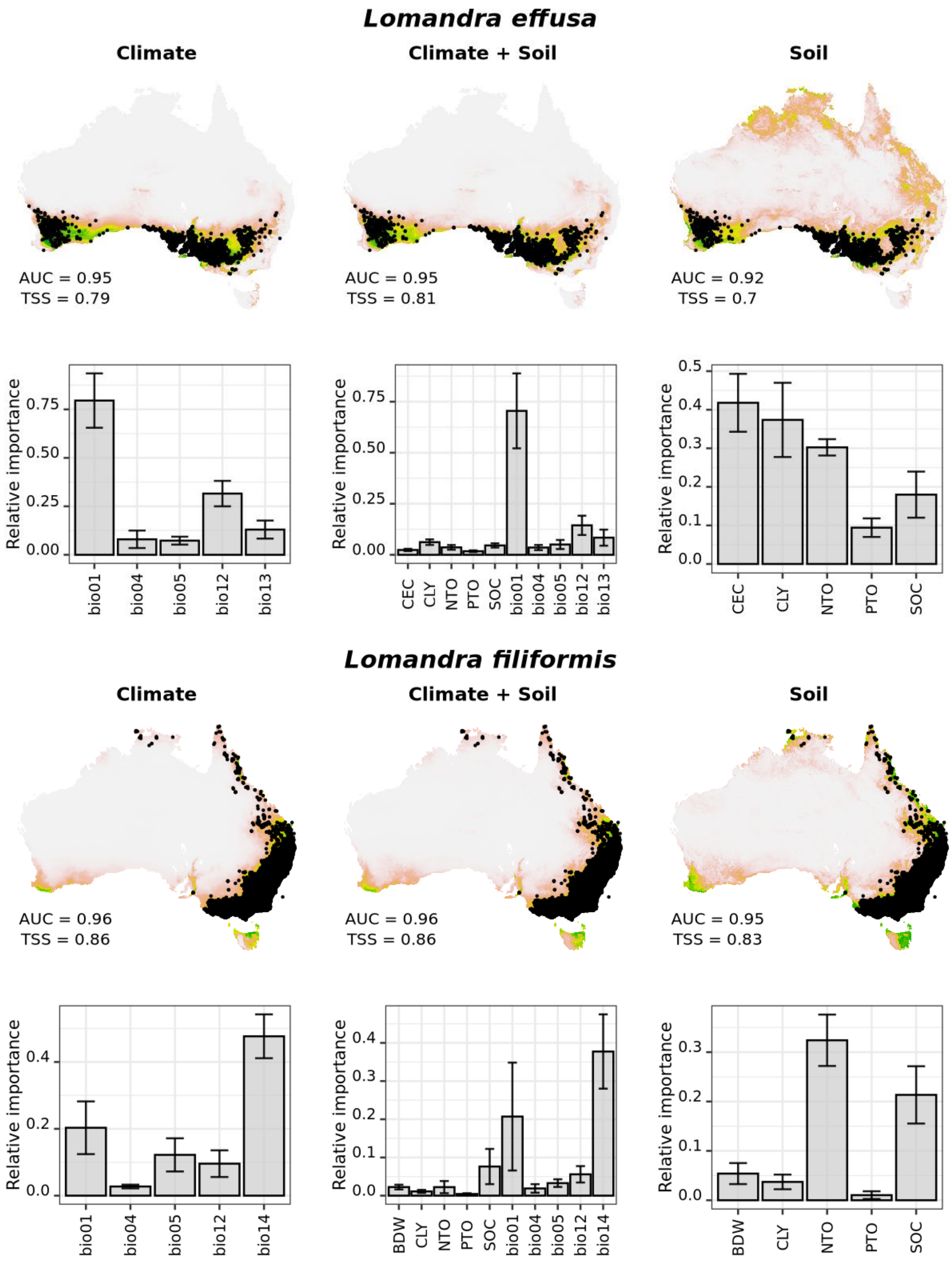

Lomandra effusa
Climate
Climate + Soil
Soil
AUC = 0.95
TSS = 0.79
AUC = 0.95
TSS = 0.81
AUC = 0.92
TSS = 0.7
Relative importance
0.00 0.25 0.50 0.75
bio01 bio04 bio05 bio12 bio13
CEC CLY NTO PTO SOC bio01 bio04 bio05 bio12 bio13
0.0 0.1 0.2 0.3 0.4 0.5
CEC CLY NTO PTO SOC
Lomandra filiformis
Climate
Climate + Soil
Soil
AUC = 0.96
TSS = 0.86
AUC = 0.96
TSS = 0.86
AUC = 0.95
TSS = 0.83
Relative importance
0.0 0.2 0.4
bio01 bio04 bio05 bio12 bio14
0.0 0.1 0.2 0.3 0.4
BDW CLY NTO PTO SOC bio01 bio04 bio05 bio12 bio14
0.0 0.1 0.2 0.3
BDW CLY NTO PTO SOC

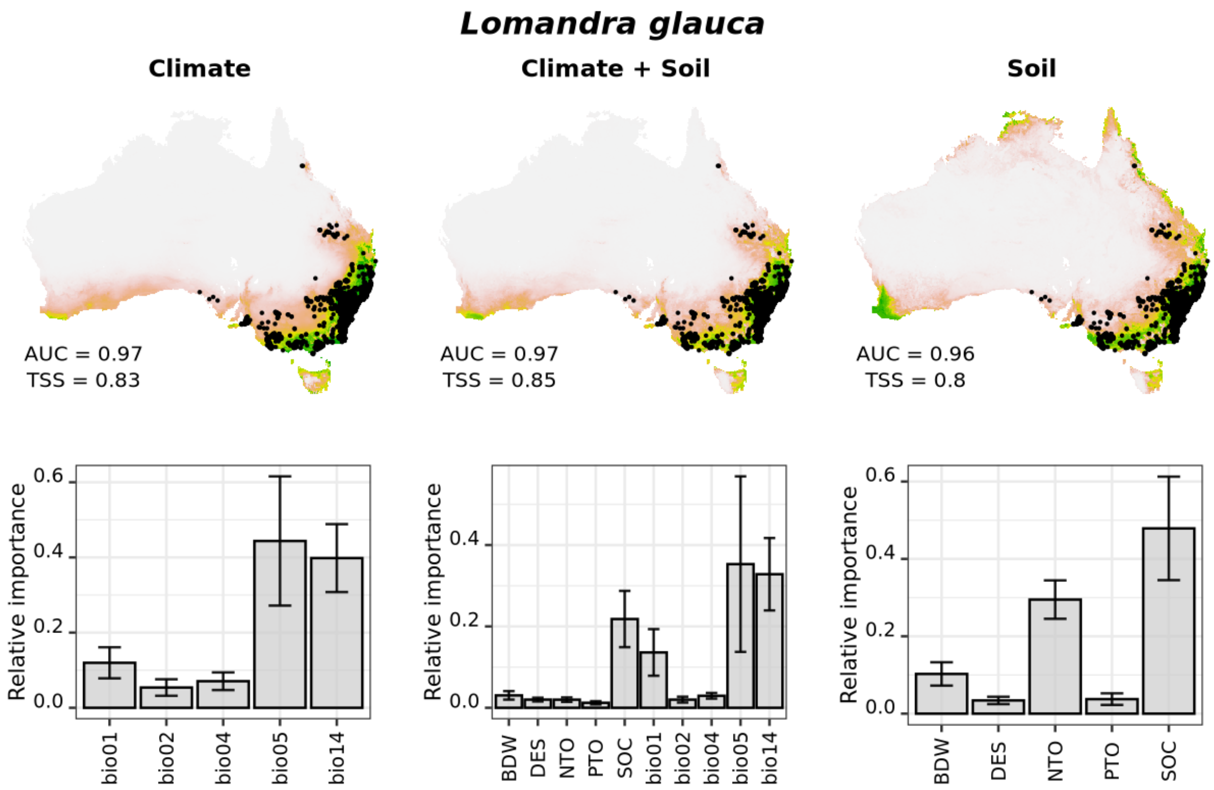
Lomandra glauca
Climate
Climate + Soil
Soil
AUC = 0.97
TSS = 0.83
AUC = 0.97
TSS = 0.85
AUC = 0.96
TSS = 0.8
Relative importance
0.0
0.2
0.4
0.6
bio01
bio02
bio04
bio05
bio14
BDW
DES
NTO
PTO
SOC
bio01
bio02
bio04
bio05
bio14
BDW
DES
NTO
PTO
SOC

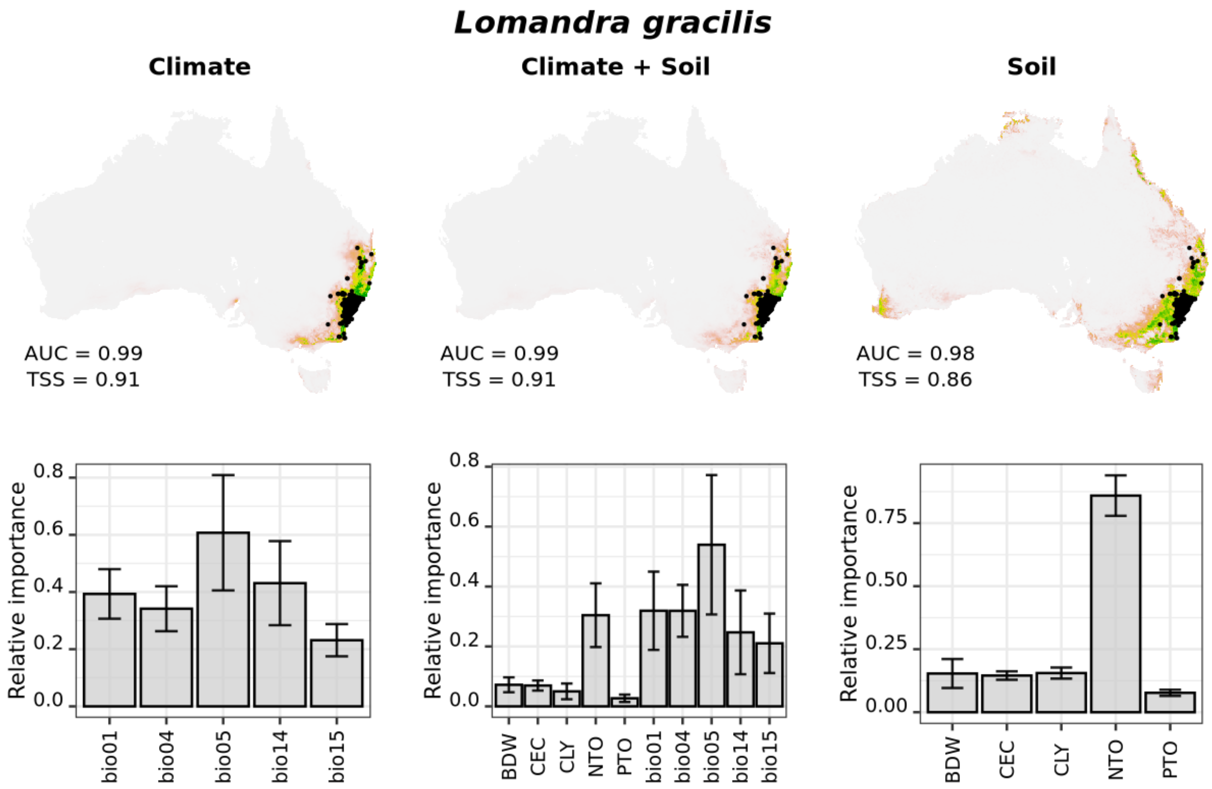
Lomandra gracilis
Climate
Climate + Soil
Soil
AUC = 0.99
TSS = 0.91
AUC = 0.99
TSS = 0.91
AUC = 0.98
TSS = 0.86
Relative importance
0.0
0.2
0.4
0.6
0.8
0.00
0.25
0.50
0.75
bio01
bio04
bio05
bio14
bio15
BDW
CEC
CLY
NTO
PTO
bio01
bio04
bio05
bio14
bio15
BDW
CEC
CLY
NTO
PTO

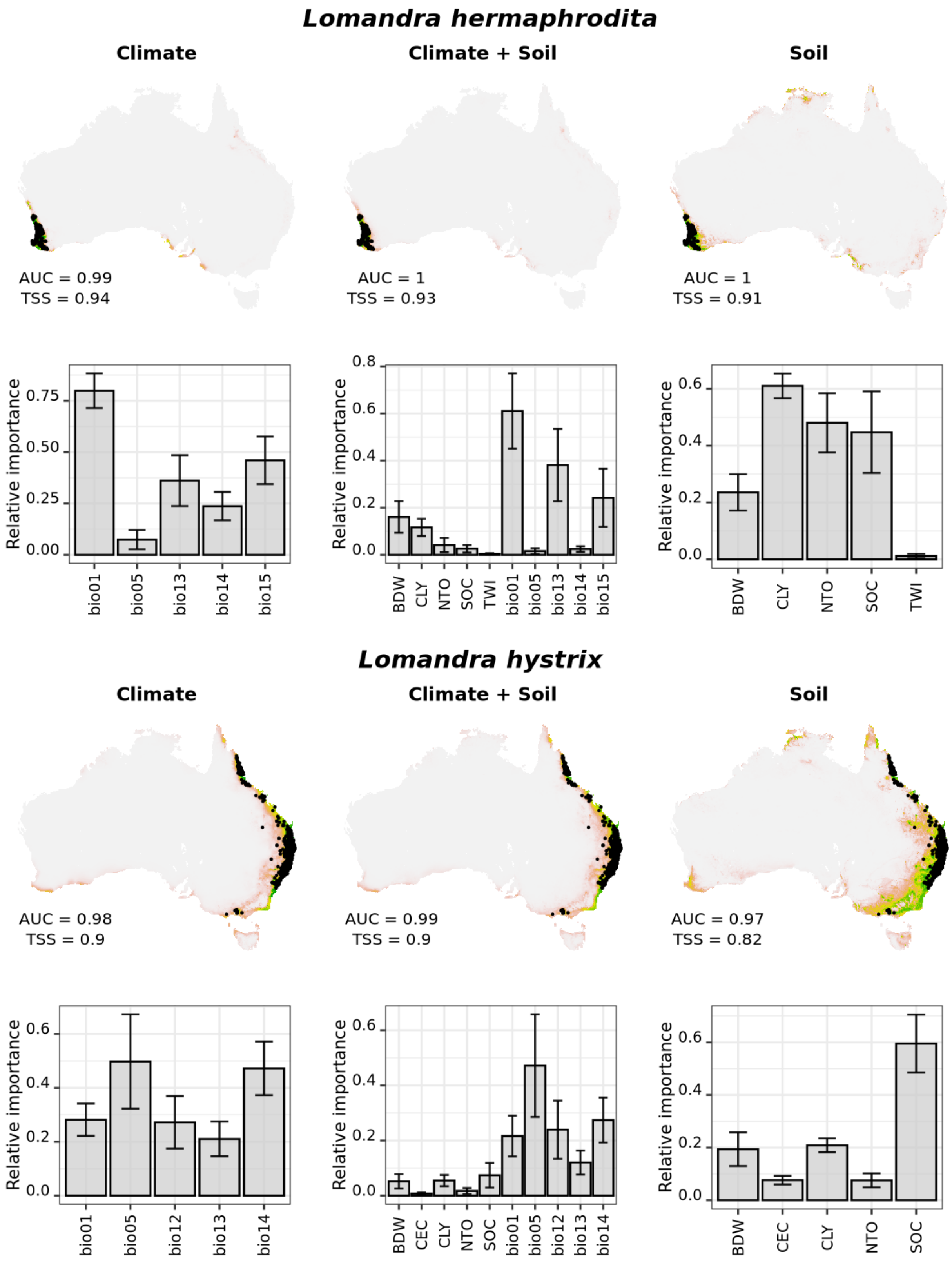

Lomandra hermaphrodita
Climate
Climate + Soil
Soil
AUC = 0.99
TSS = 0.94
AUC = 1
TSS = 0.93
AUC = 1
TSS = 0.91
Relative importance
bio01
bio05
bio13
bio14
bio15
BDW
CLY
NTO
SOC
TWI
Lomandra hystrix
Climate
Climate + Soil
Soil
AUC = 0.98
TSS = 0.9
AUC = 0.99
TSS = 0.9
AUC = 0.97
TSS = 0.82
Relative importance
bio01
bio05
bio12
bio13
bio14
BDW
CEC
CLY
NTO
SOC

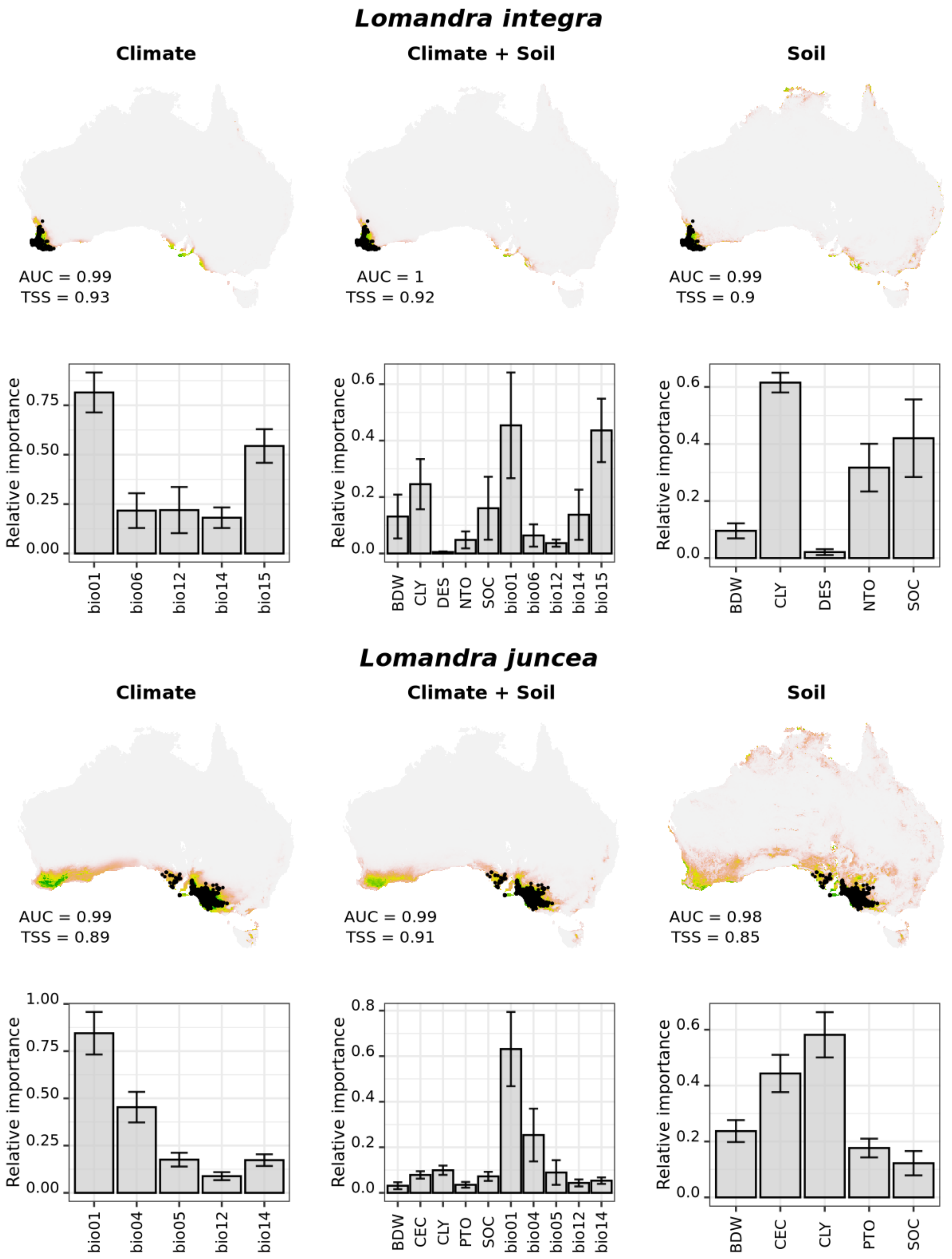
Lomandra integra
Climate
Climate + Soil
Soil
AUC = 0.99
TSS = 0.93
AUC = 1
TSS = 0.92
AUC = 0.99
TSS = 0.9
Relative importance
0.00
0.25
0.50
0.75
bio01
bio06
bio12
bio14
bio15
0.0
0.2
0.4
0.6
BDW
CLY
DES
NTO
SOC
bio01
bio06
bio12
bio14
bio15
BDW
CLY
DES
NTO
SOC
Lomandra juncea
Climate
Climate + Soil
Soil
AUC = 0.99
TSS = 0.89
AUC = 0.99
TSS = 0.91
AUC = 0.98
TSS = 0.85
Relative importance
1.00
bio01
bio04
bio05
bio12
bio14
0.8
BDW
CEC
CLY
PTO
SOC
bio01
bio04
bio05
bio12
bio14
BDW
CEC
CLY
PTO
SOC

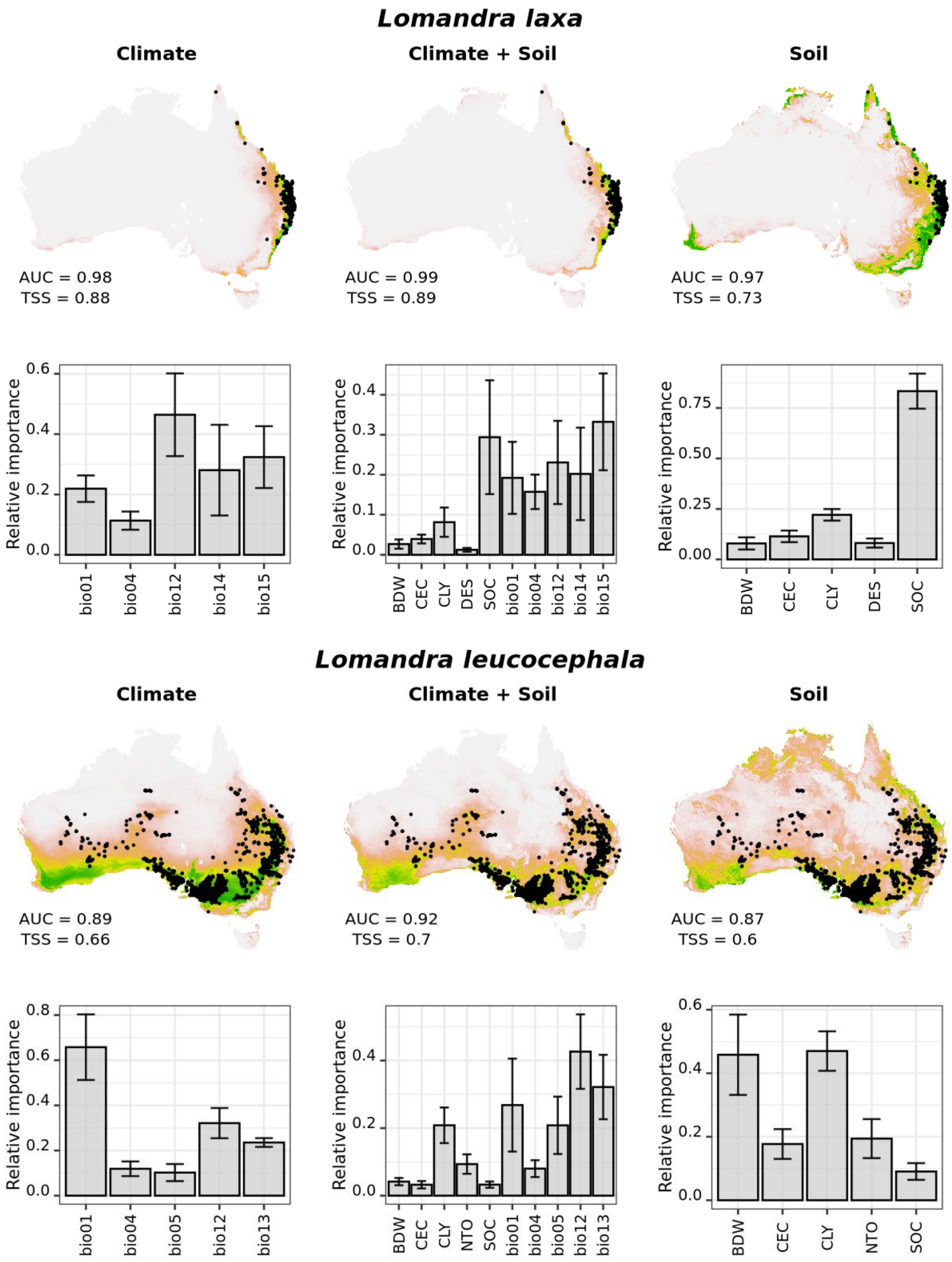
Lomandra laxa
Climate
Climate + Soil
Soil
AUC = 0.98
TSS = 0.88
AUC = 0.99
TSS = 0.89
AUC = 0.97
TSS = 0.73
Relative importance
bio01
bio04
bio12
bio14
bio15
BDW
CEC
CLY
DES
SOC
Lomandra leucocephala
Climate
Climate + Soil
Soil
AUC = 0.89
TSS = 0.66
AUC = 0.92
TSS = 0.7
AUC = 0.87
TSS = 0.6
Relative importance
bio01
bio04
bio05
bio12
bio13
BDW
CEC
CLY
NTO
SOC

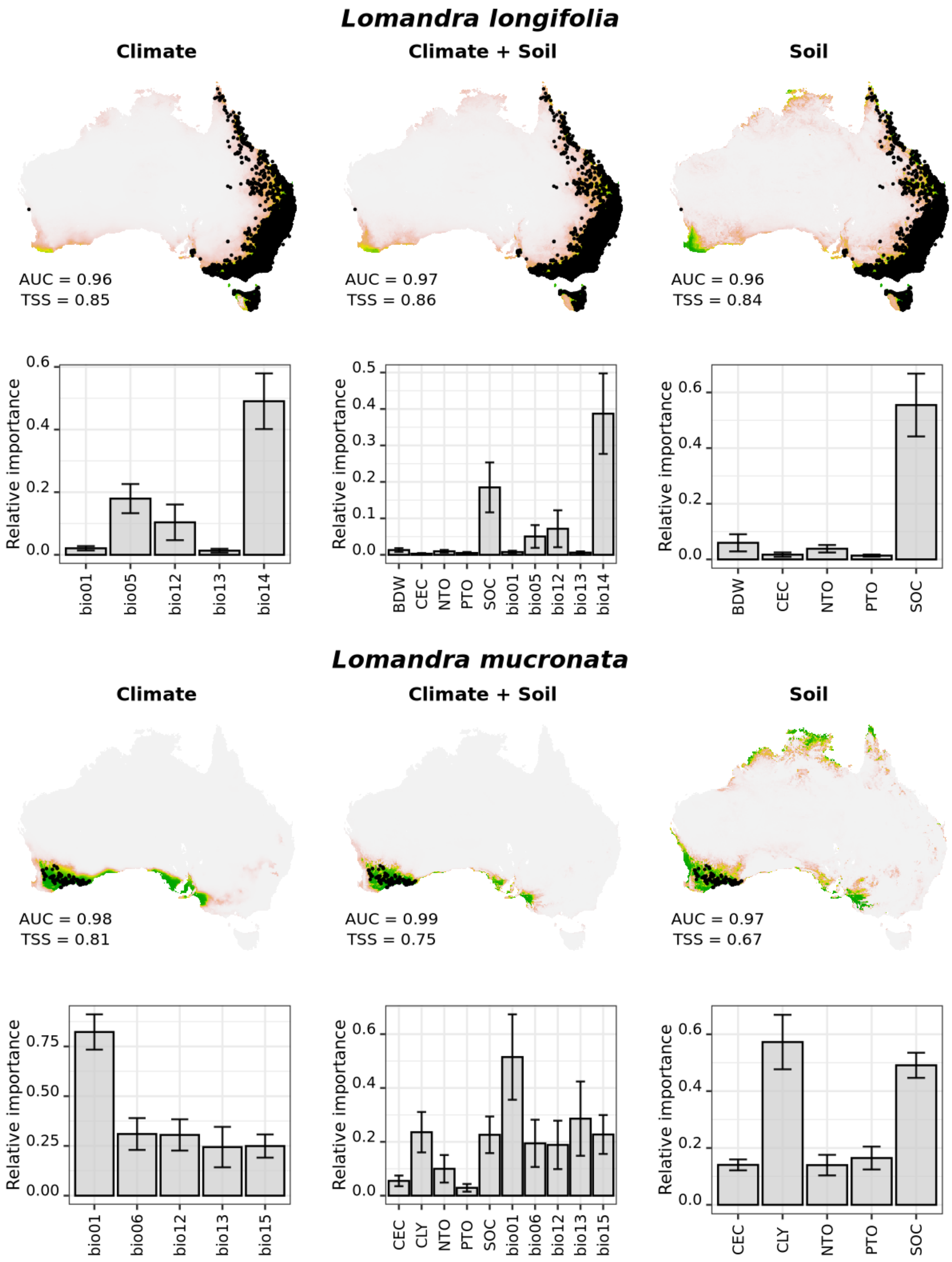
Lomandra longifolia
Climate
Climate + Soil
Soil
AUC = 0.96
TSS = 0.85
AUC = 0.97
TSS = 0.86
AUC = 0.96
TSS = 0.84
Relative importance
bio01 bio05 bio12 bio13 bio14
BDW CEC NTO PTO SOC bio01 bio05 bio12 bio13 bio14
BDW CEC NTO PTO SOC
Lomandra mucronata
Climate
Climate + Soil
Soil
AUC = 0.98
TSS = 0.81
AUC = 0.99
TSS = 0.75
AUC = 0.97
TSS = 0.67
Relative importance
bio01 bio06 bio12 bio13 bio15
CEC CLY NTO PTO SOC bio01 bio06 bio12 bio13 bio15
CEC CLY NTO PTO SOC

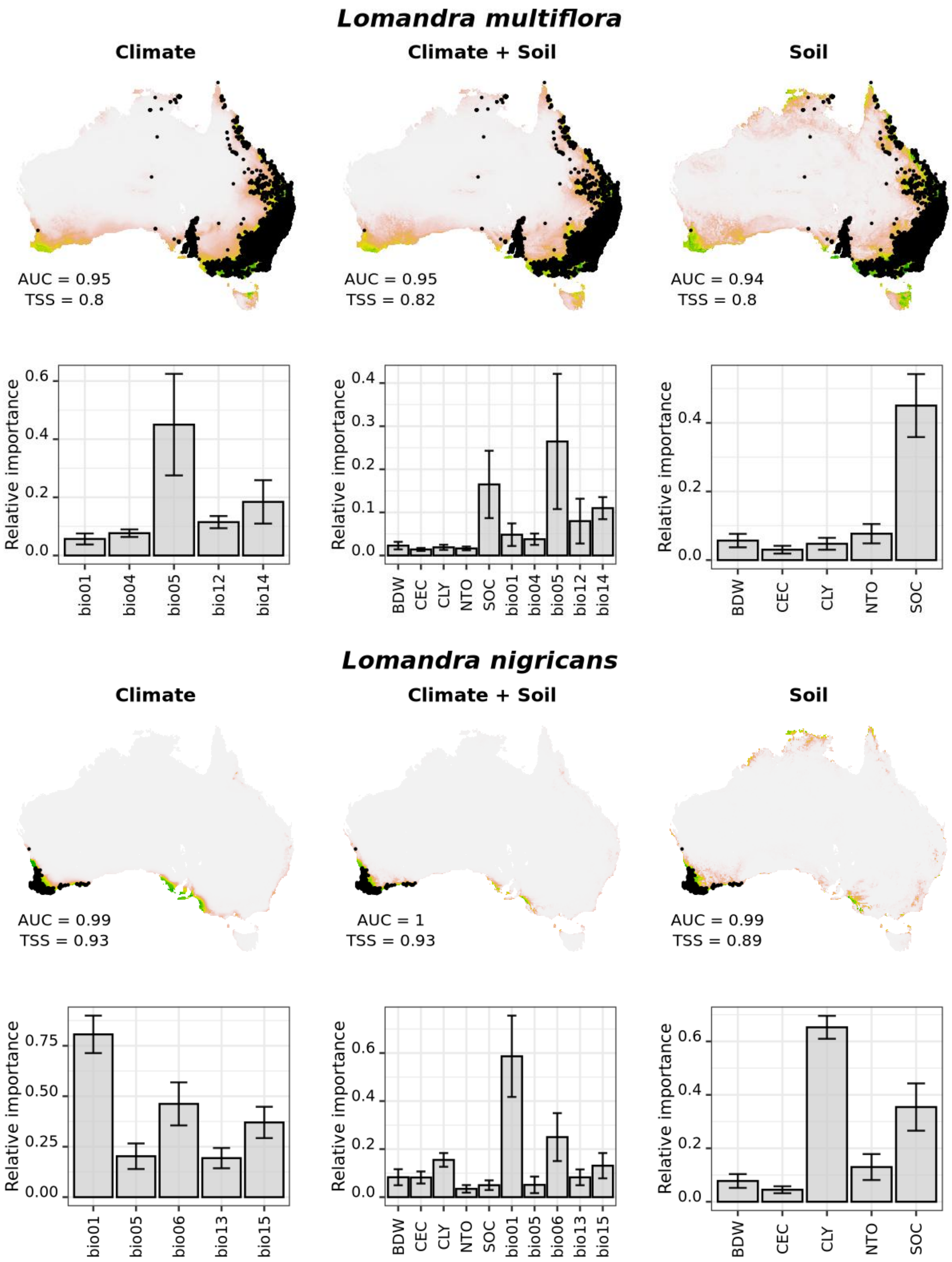

Lomandra multiflora
Climate
Climate + Soil
Soil
AUC = 0.95
TSS = 0.8
AUC = 0.95
TSS = 0.82
AUC = 0.94
TSS = 0.8
Relative importance
0.0
0.2
0.4
0.6
bio01
bio04
bio05
bio12
bio14
0.0
0.1
0.2
0.3
0.4
BDW
CEC
CLY
NTO
SOC
bio01
bio04
bio05
bio12
bio14
0.0
0.2
0.4
BDW
CEC
CLY
NTO
SOC
Lomandra nigricans
Climate
Climate + Soil
Soil
AUC = 0.99
TSS = 0.93
AUC = 1
TSS = 0.93
AUC = 0.99
TSS = 0.89
Relative importance
0.00
0.25
0.50
0.75
bio01
bio05
bio06
bio13
bio15
0.0
0.2
0.4
0.6
BDW
CEC
CLY
NTO
SOC
bio01
bio05
bio06
bio13
bio15
0.0
0.2
0.4
0.6
BDW
CEC
CLY
NTO
SOC

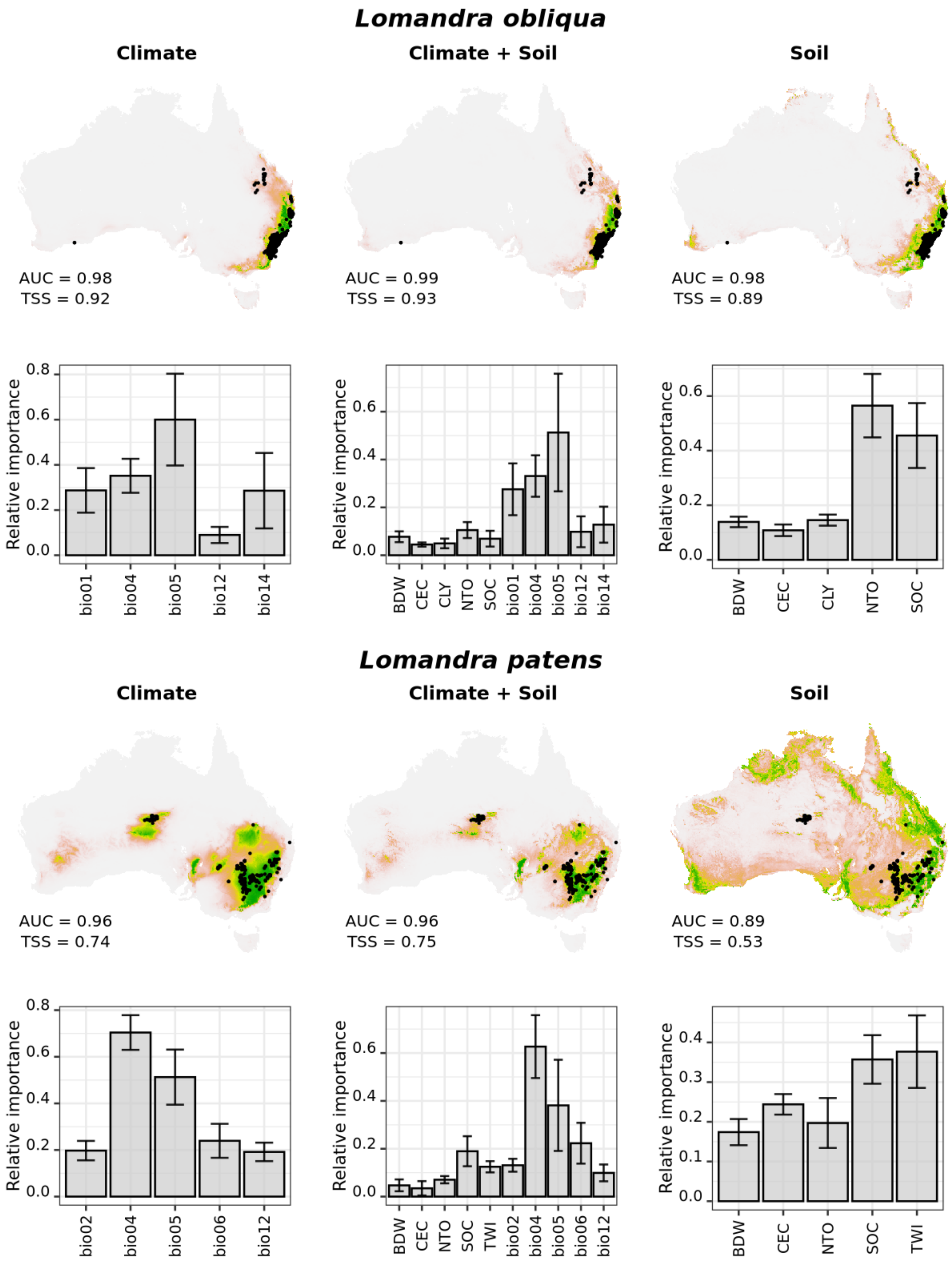

Lomandra obliqua
Climate
Climate + Soil
Soil
AUC = 0.98
TSS = 0.92
AUC = 0.99
TSS = 0.93
AUC = 0.98
TSS = 0.89
Relative importance
bio01
bio04
bio05
bio12
bio14
BDW
CEC
CLY
NTO
SOC
Lomandra patens
AUC = 0.96
TSS = 0.74
AUC = 0.96
TSS = 0.75
AUC = 0.89
TSS = 0.53
bio02
bio06
TWI

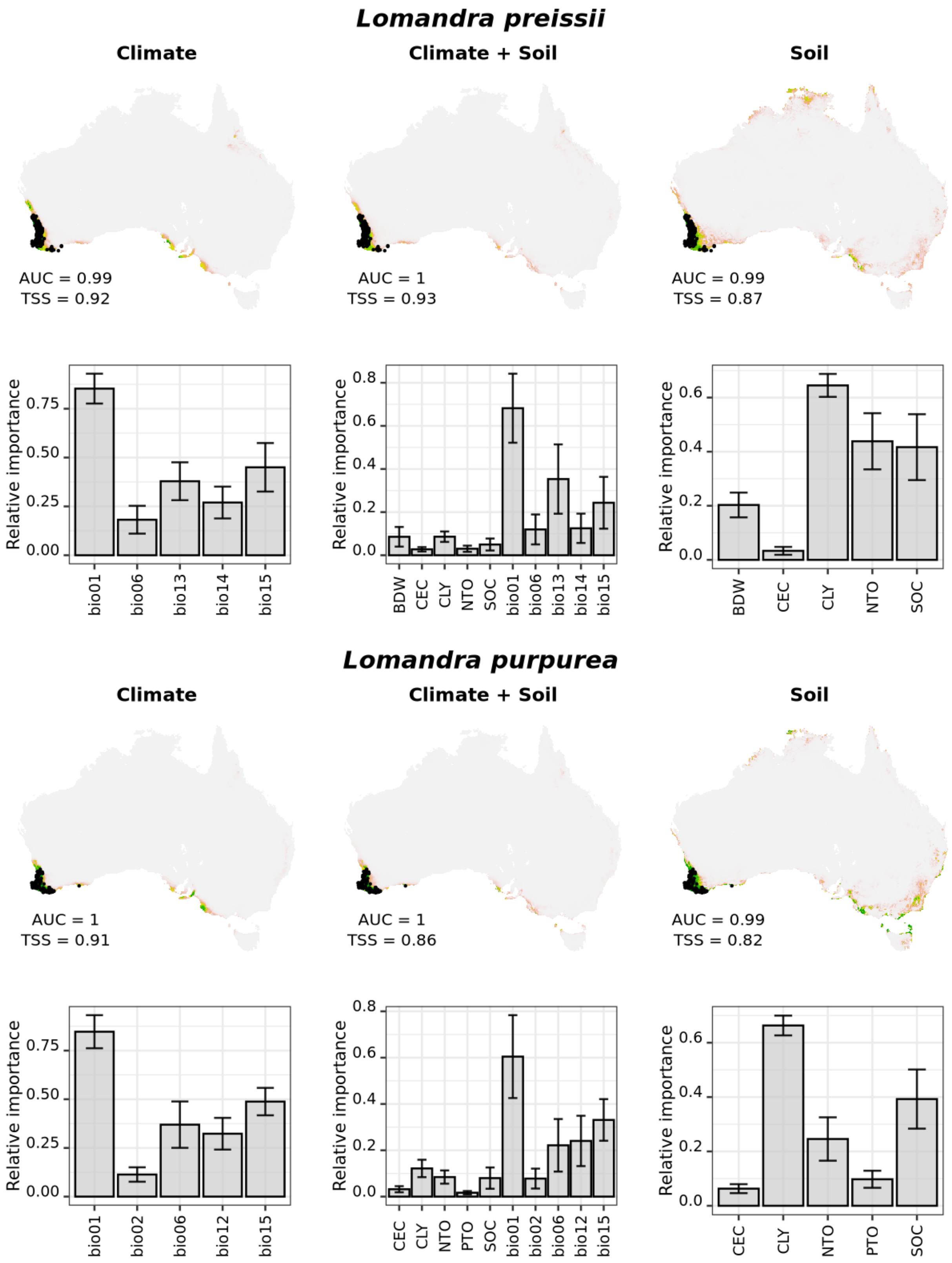
Lomandra preissii
Climate
Climate + Soil
Soil
AUC = 0.99
TSS = 0.92
AUC = 1
TSS = 0.93
AUC = 0.99
TSS = 0.87
Relative importance
0.00
0.25
0.50
0.75
bio01
bio06
bio13
bio14
bio15
0.0
0.2
0.4
0.6
0.8
BDW
CEC
CLY
NTO
SOC
bio01
bio06
bio13
bio14
bio15
0.0
0.2
0.4
0.6
BDW
CEC
CLY
NTO
SOC
Lomandra purpurea
Climate
Climate + Soil
Soil
AUC = 1
TSS = 0.91
AUC = 1
TSS = 0.86
AUC = 0.99
TSS = 0.82
Relative importance
0.00
0.25
0.50
0.75
bio01
bio02
bio06
bio12
bio15
0.0
0.2
0.4
0.6
0.8
CEC
CLY
NTO
PTO
SOC
bio01
bio02
bio06
bio12
bio15
0.0
0.2
0.4
0.6
CEC
CLY
NTO
PTO
SOC

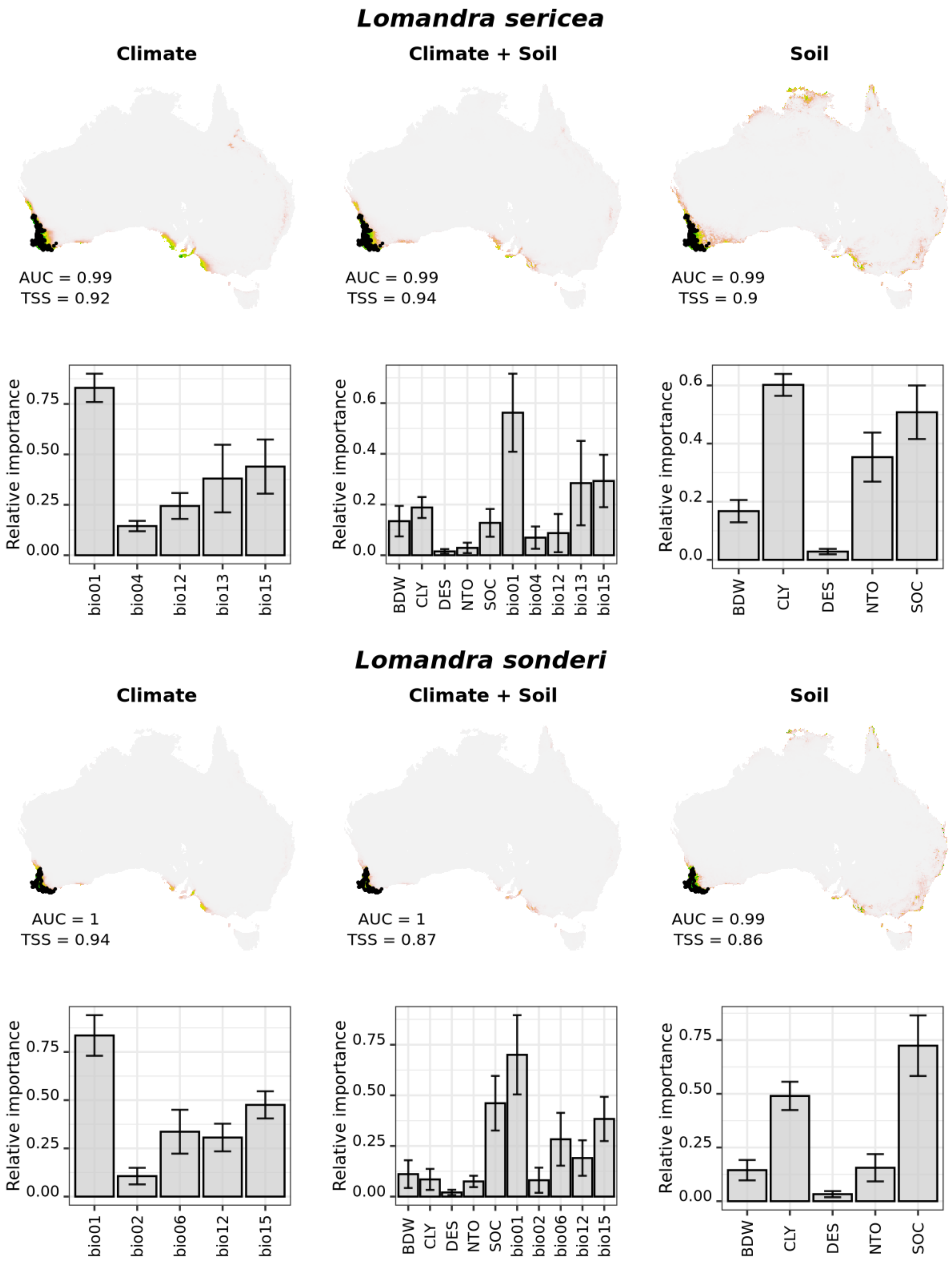
Lomandra sericea
Climate
Climate + Soil
Soil
AUC = 0.99
TSS = 0.92
AUC = 0.99
TSS = 0.94
AUC = 0.99
TSS = 0.9
Relative importance
0.00
0.25
0.50
0.75
bio01
bio04
bio12
bio13
bio15
0.0
0.2
0.4
0.6
BDW
CLY
DES
NTO
SOC
bio01
bio04
bio12
bio13
bio15
Lomandra sonderi
AUC = 1
TSS = 0.94
AUC = 1
TSS = 0.87
AUC = 0.99
TSS = 0.86
bio01
bio02
bio06
bio12
bio15
BDW
CLY
DES
NTO
SOC
bio01
bio02
bio06
bio12
bio15

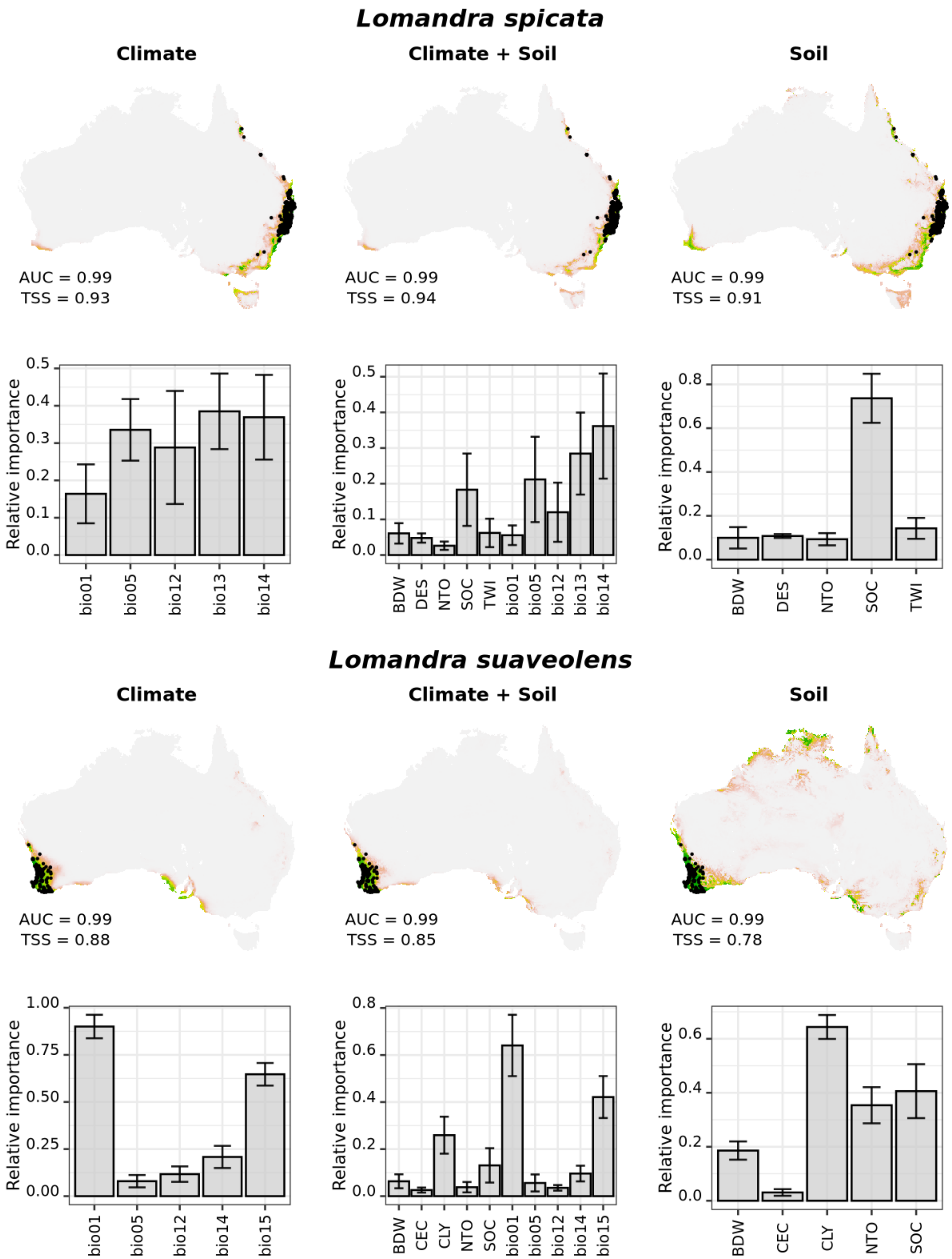

Lomandra spicata
Climate
Climate + Soil
Soil
AUC = 0.99
TSS = 0.93
AUC = 0.99
TSS = 0.94
AUC = 0.99
TSS = 0.91
Relative importance
bio01
bio05
bio12
bio13
bio14
BDW
DES
NTO
SOC
TWI
Lomandra suaveolens
Climate
Climate + Soil
Soil
AUC = 0.99
TSS = 0.88
AUC = 0.99
TSS = 0.85
AUC = 0.99
TSS = 0.78
Relative importance
bio01
bio05
bio12
bio14
bio15
BDW
CEC
CLY
NTO
SOC

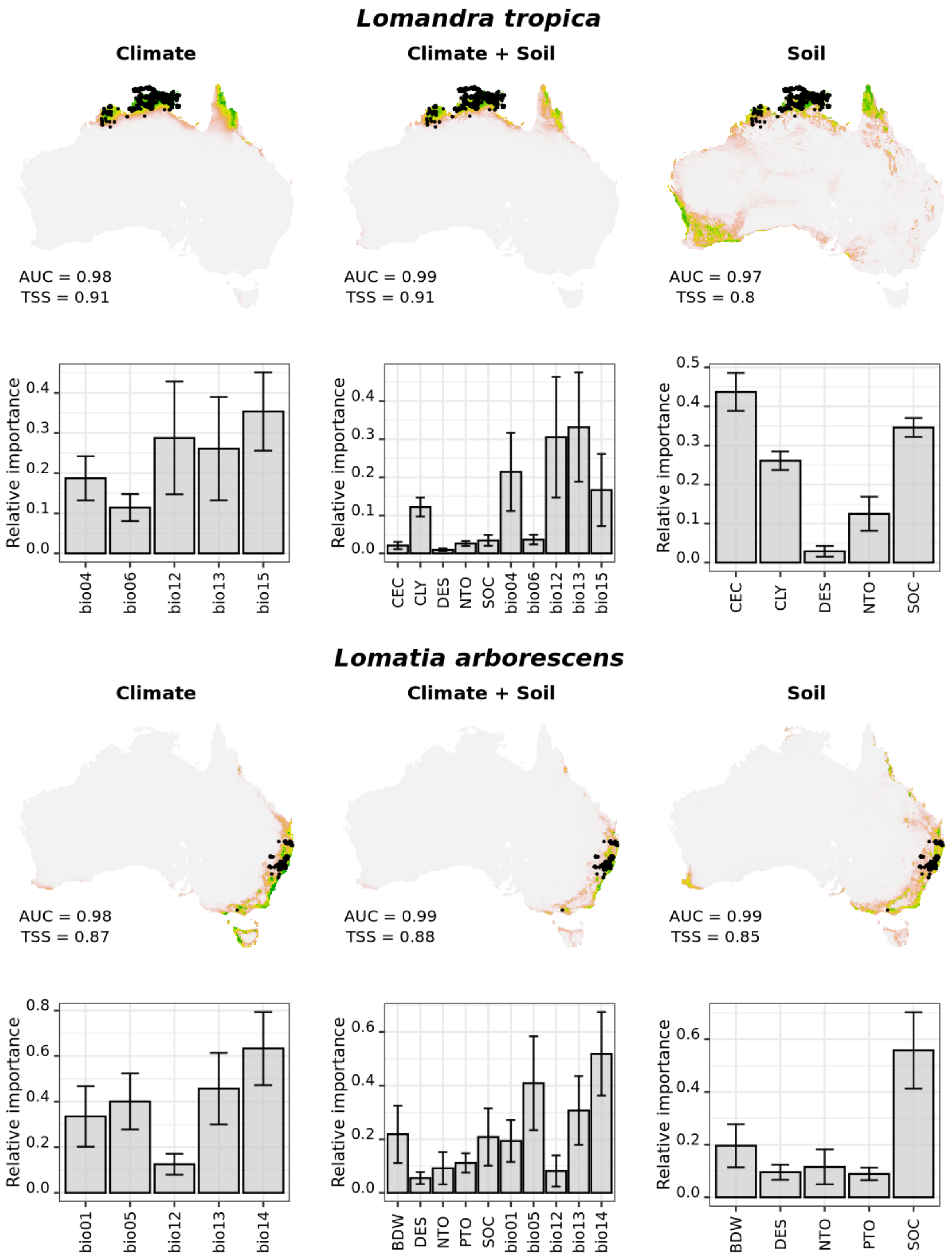

Lomandra tropica
Climate
Climate + Soil
Soil
AUC = 0.98
TSS = 0.91
AUC = 0.99
TSS = 0.91
AUC = 0.97
TSS = 0.8
Relative importance
bio04
bio06
bio12
bio13
bio15
CEC
CLY
DES
NTO
SOC
bio04
bio06
bio12
bio13
bio15
CEC
CLY
DES
NTO
SOC
Lomatia arborescens
Climate
Climate + Soil
Soil
AUC = 0.98
TSS = 0.87
AUC = 0.99
TSS = 0.88
AUC = 0.99
TSS = 0.85
Relative importance
bio01
bio05
bio12
bio13
bio14
BDW
DES
NTO
PTO
SOC
bio01
bio05
bio12
bio13
bio14
BDW
DES
NTO
PTO
SOC

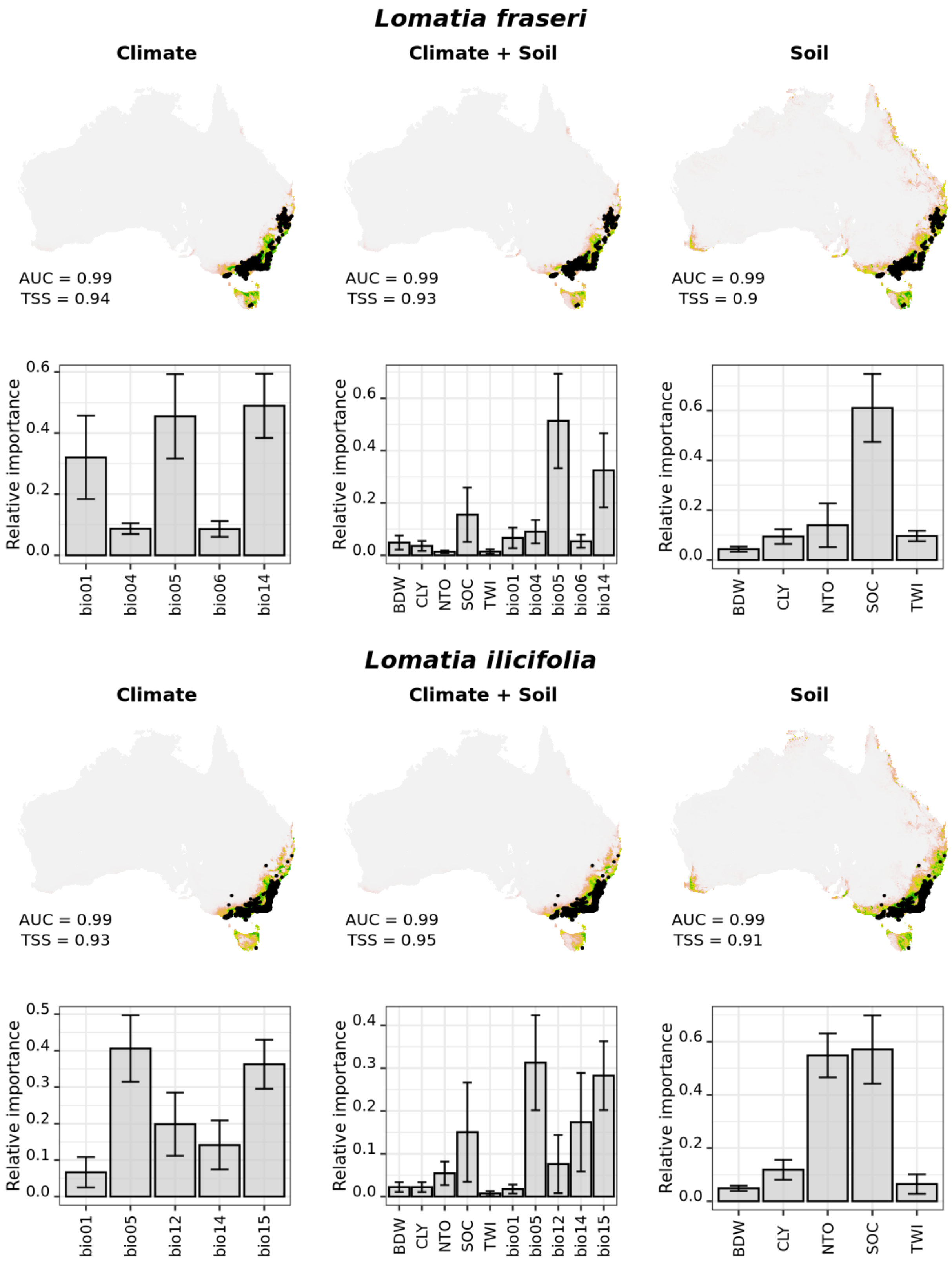
Lomatia fraseri
Climate
Climate + Soil
Soil
AUC = 0.99
TSS = 0.94
AUC = 0.99
TSS = 0.93
AUC = 0.99
TSS = 0.9
Relative importance
0.0
0.2
0.4
0.6
bio01
bio04
bio05
bio06
bio14
BDW
CLY
NTO
SOC
TWI
Lomatia ilicifolia
Climate
Climate + Soil
Soil
AUC = 0.99
TSS = 0.93
AUC = 0.99
TSS = 0.95
AUC = 0.99
TSS = 0.91
0.1
0.3
0.5
bio12
bio15

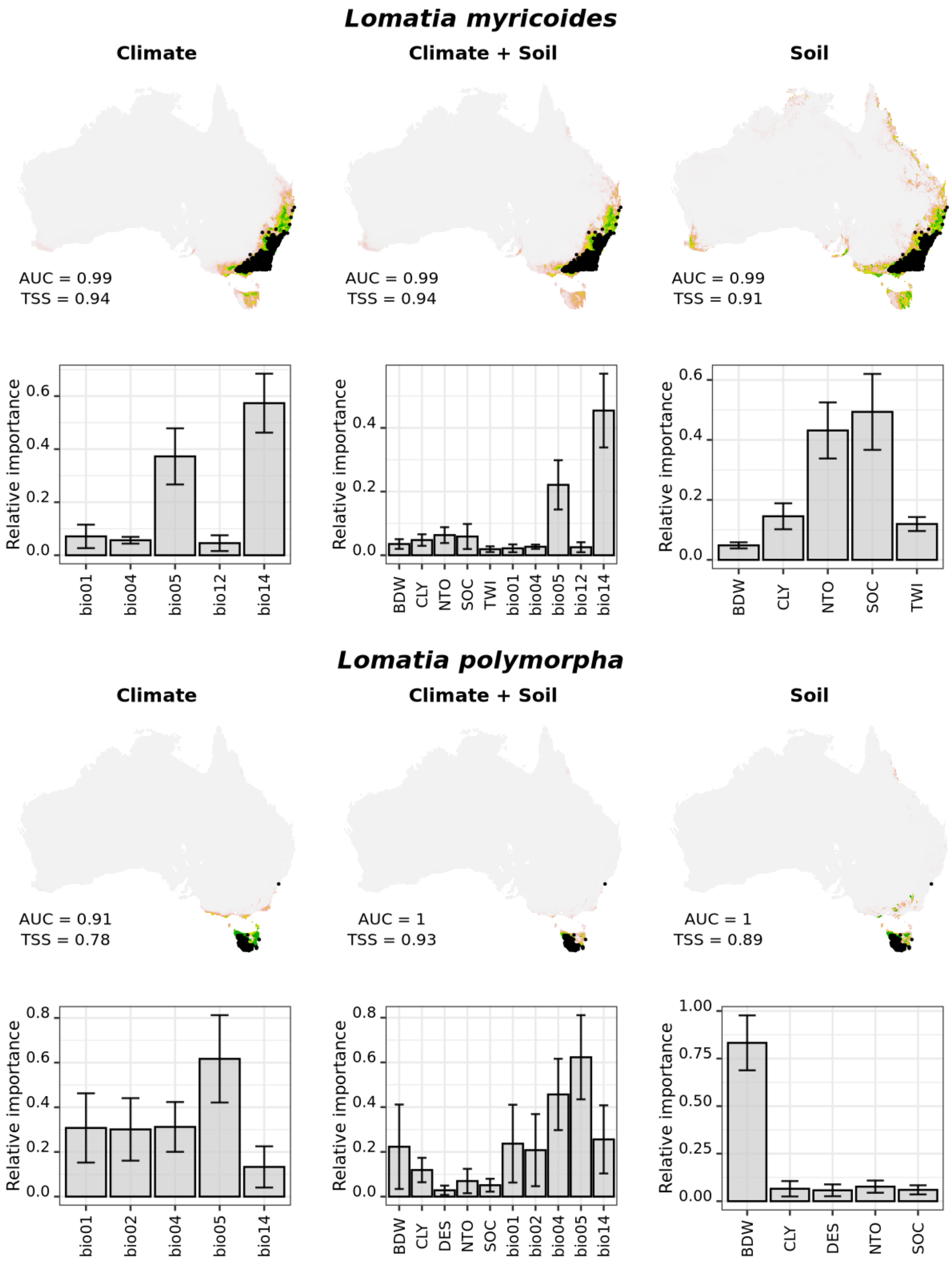
Lomatia myricoides
Climate
Climate + Soil
Soil
AUC = 0.99
TSS = 0.94
AUC = 0.99
TSS = 0.94
AUC = 0.99
TSS = 0.91
Relative importance
0.0
0.2
0.4
0.6
bio01
bio04
bio05
bio12
bio14
BDW
CLY
NTO
SOC
TWI
Lomatia polymorpha
Climate
Climate + Soil
Soil
AUC = 0.91
TSS = 0.78
AUC = 1
TSS = 0.93
AUC = 1
TSS = 0.89
0.8
bio02
DES
1.00
0.75
0.50
0.25
0.00

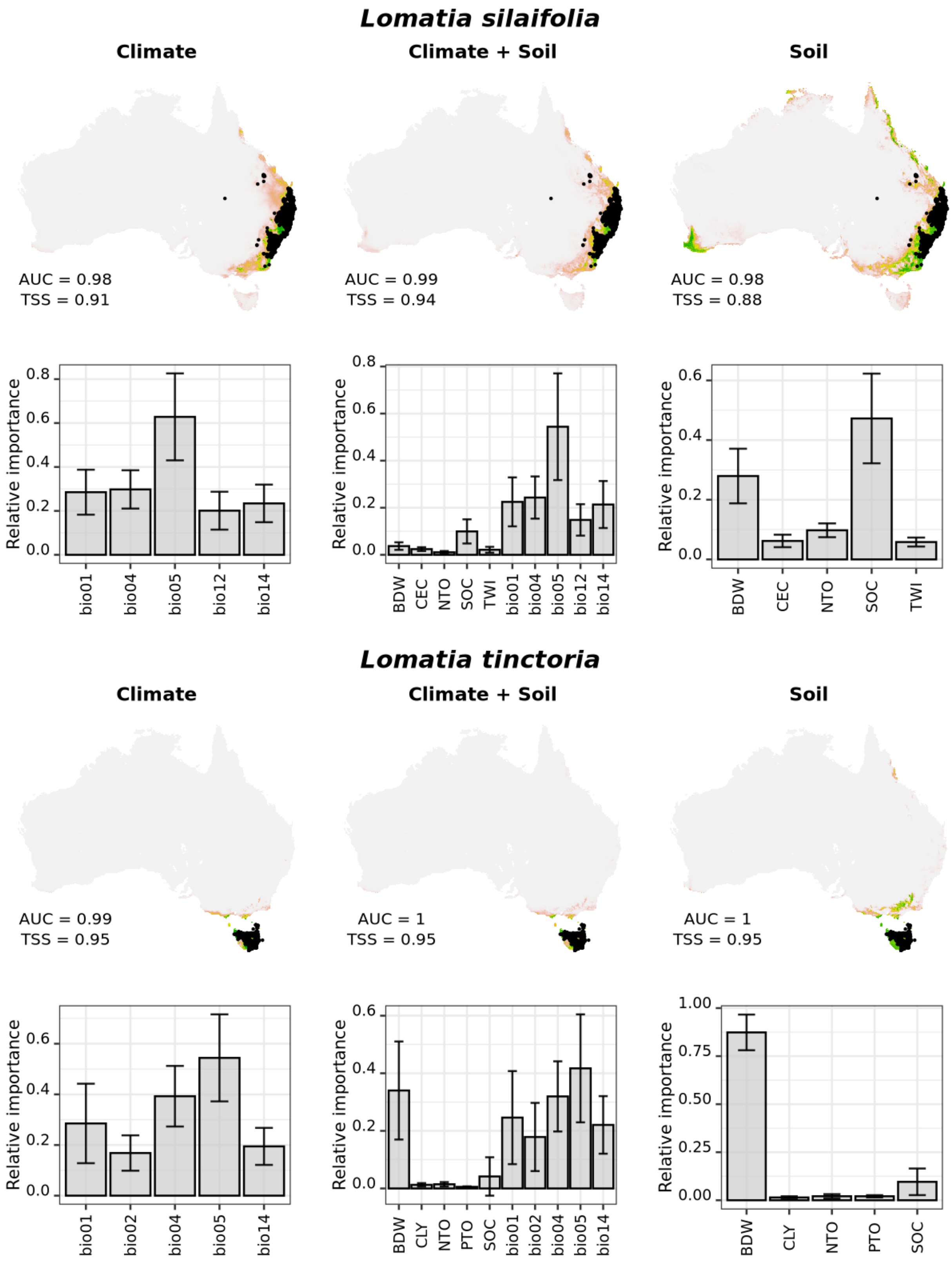
Lomatia silaifolia
Climate
Climate + Soil
Soil
AUC = 0.98
TSS = 0.91
AUC = 0.99
TSS = 0.94
AUC = 0.98
TSS = 0.88
Relative importance
bio01
bio04
bio05
bio12
bio14
BDW
CEC
NTO
SOC
TWI
Lomatia tinctoria
Climate
Climate + Soil
Soil
AUC = 0.99
TSS = 0.95
AUC = 1
TSS = 0.95
AUC = 1
TSS = 0.95
Relative importance
bio01
bio02
bio04
bio05
bio14
BDW
CLY
NTO
PTO
SOC

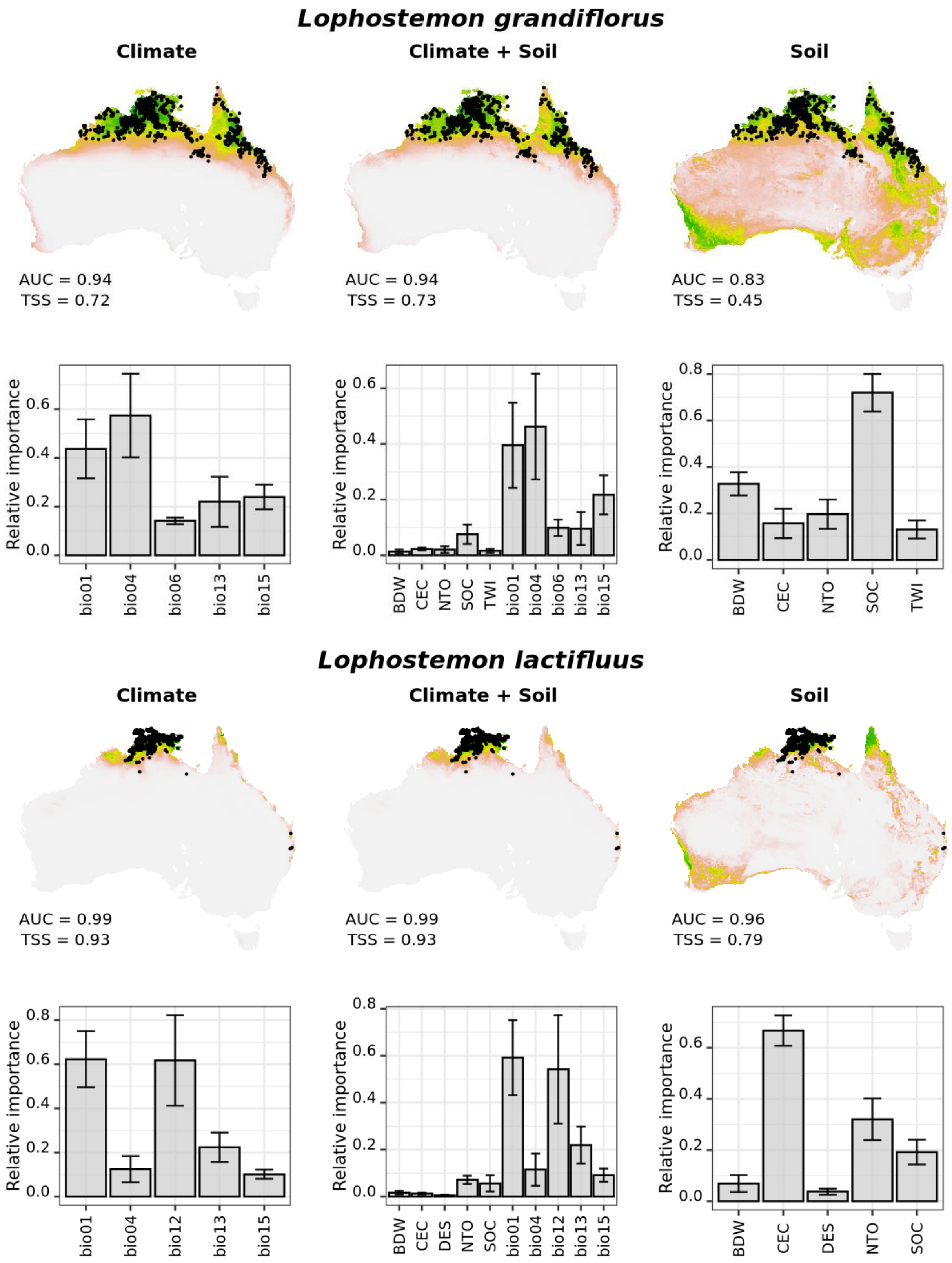

Lophostemon grandiflorus
Climate
Climate + Soil
Soil
AUC = 0.94
TSS = 0.72
AUC = 0.94
TSS = 0.73
AUC = 0.83
TSS = 0.45
Relative importance
bio01
bio04
bio06
bio13
bio15
BDW
CEC
NTO
SOC
TWI
bio01
bio04
bio06
bio13
bio15
BDW
CEC
NTO
SOC
TWI
Lophostemon lactifluus
Climate
Climate + Soil
Soil
AUC = 0.99
TSS = 0.93
AUC = 0.99
TSS = 0.93
AUC = 0.96
TSS = 0.79
Relative importance
bio01
bio04
bio12
bio13
bio15
BDW
CEC
DES
NTO
SOC
bio01
bio04
bio12
bio13
bio15
BDW
CEC
DES
NTO
SOC

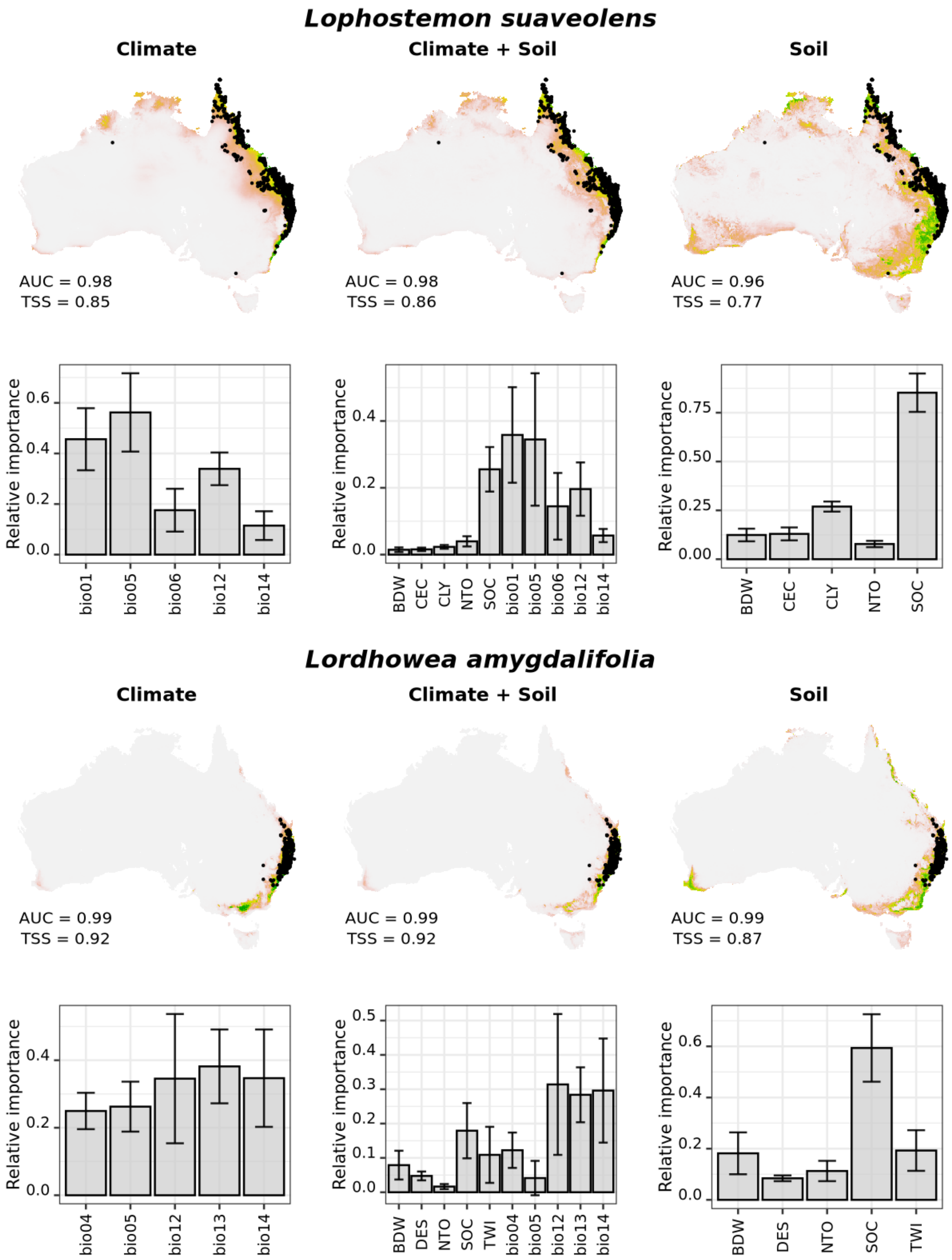
Lophostemon suaveolens
Climate
Climate + Soil
Soil
AUC = 0.98
TSS = 0.85
AUC = 0.98
TSS = 0.86
AUC = 0.96
TSS = 0.77
Relative importance
bio01 bio05 bio06 bio12 bio14
BDW CEC CLY NTO SOC bio01 bio05 bio06 bio12 bio14
BDW CEC CLY NTO SOC
Lordhowea amygdalifolia
Climate
Climate + Soil
Soil
AUC = 0.99
TSS = 0.92
AUC = 0.99
TSS = 0.92
AUC = 0.99
TSS = 0.87
Relative importance
bio04 bio05 bio12 bio13 bio14
BDW DES NTO SOC TWI bio04 bio05 bio12 bio13 bio14
BDW DES NTO SOC TWI

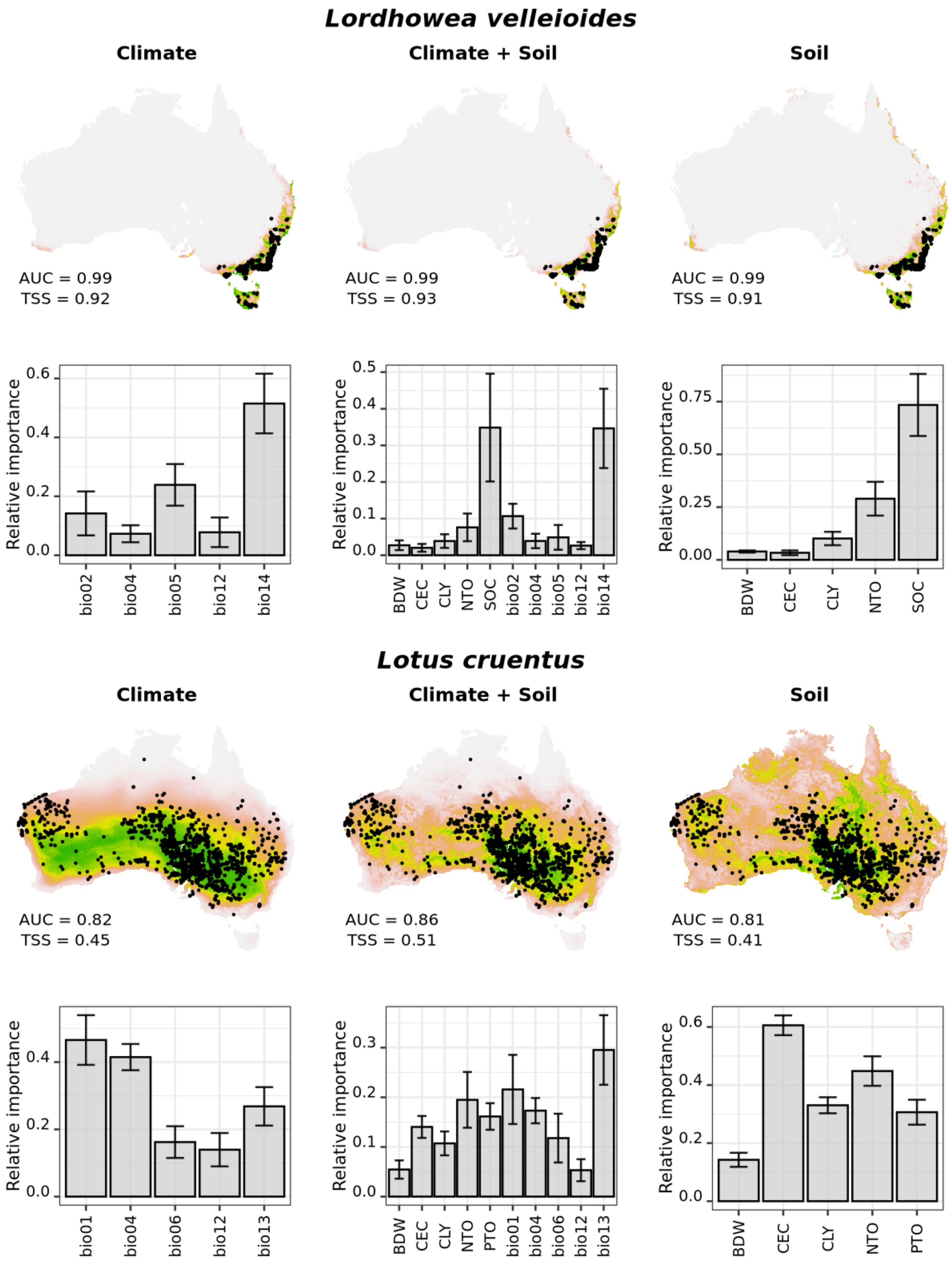
Lordhowea velleioides
Climate
Climate + Soil
Soil
AUC = 0.99
TSS = 0.92
AUC = 0.99
TSS = 0.93
AUC = 0.99
TSS = 0.91
Relative importance
bio02
bio04
bio05
bio12
bio14
BDW
CEC
CLY
NTO
SOC
Lotus cruentus
Climate
Climate + Soil
Soil
AUC = 0.82
TSS = 0.45
AUC = 0.86
TSS = 0.51
AUC = 0.81
TSS = 0.41
Relative importance
bio01
bio04
bio06
bio12
bio13
BDW
CEC
CLY
NTO
PTO

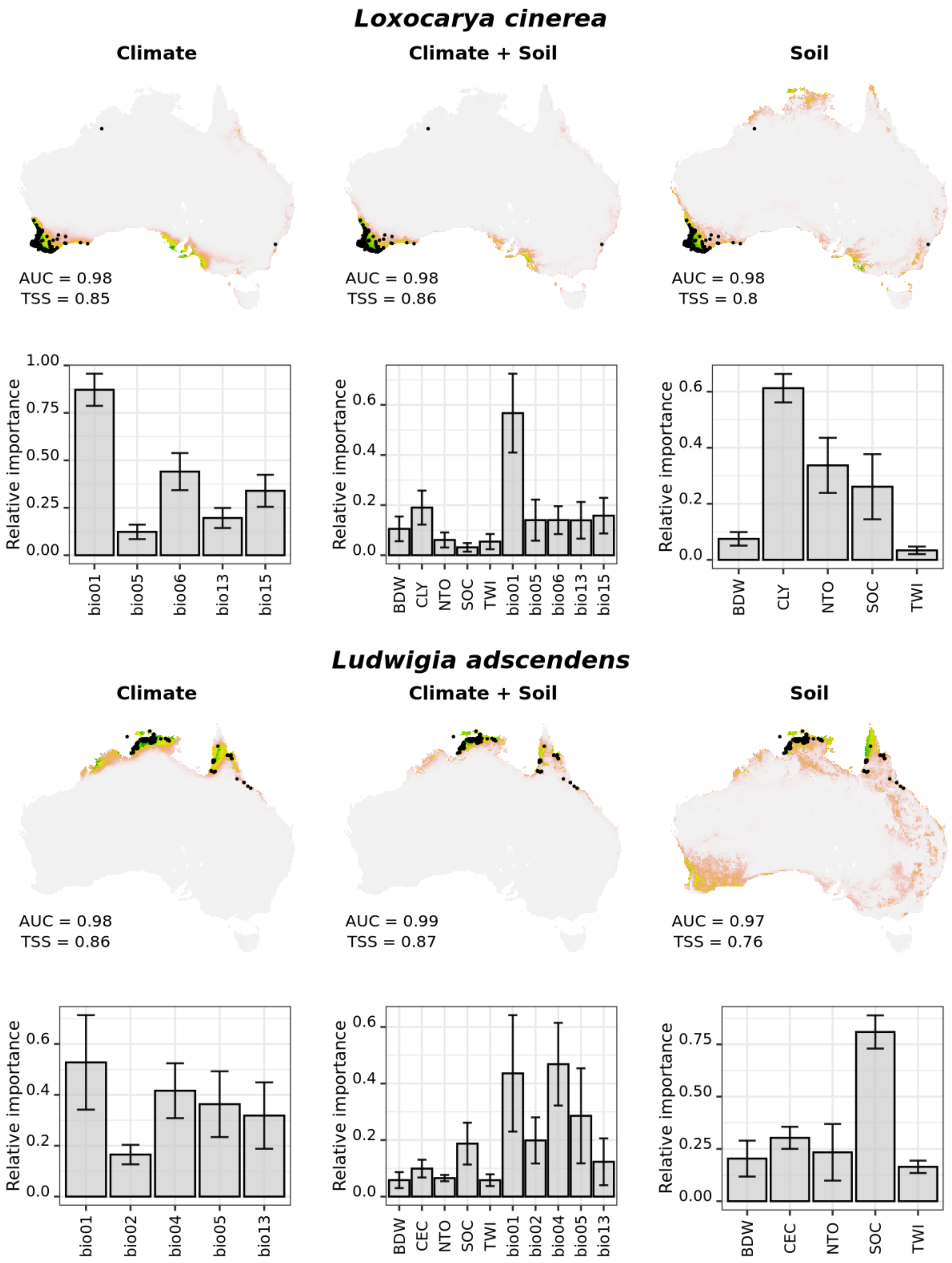

Loxocarya cinerea
Climate
Climate + Soil
Soil
AUC = 0.98
TSS = 0.85
AUC = 0.98
TSS = 0.86
AUC = 0.98
TSS = 0.8
Relative importance
bio01
bio05
bio06
bio13
bio15
BDW
CLY
NTO
SOC
TWI
Ludwigia adscendens
AUC = 0.98
TSS = 0.86
AUC = 0.99
TSS = 0.87
AUC = 0.97
TSS = 0.76
bio02
bio04
CEC

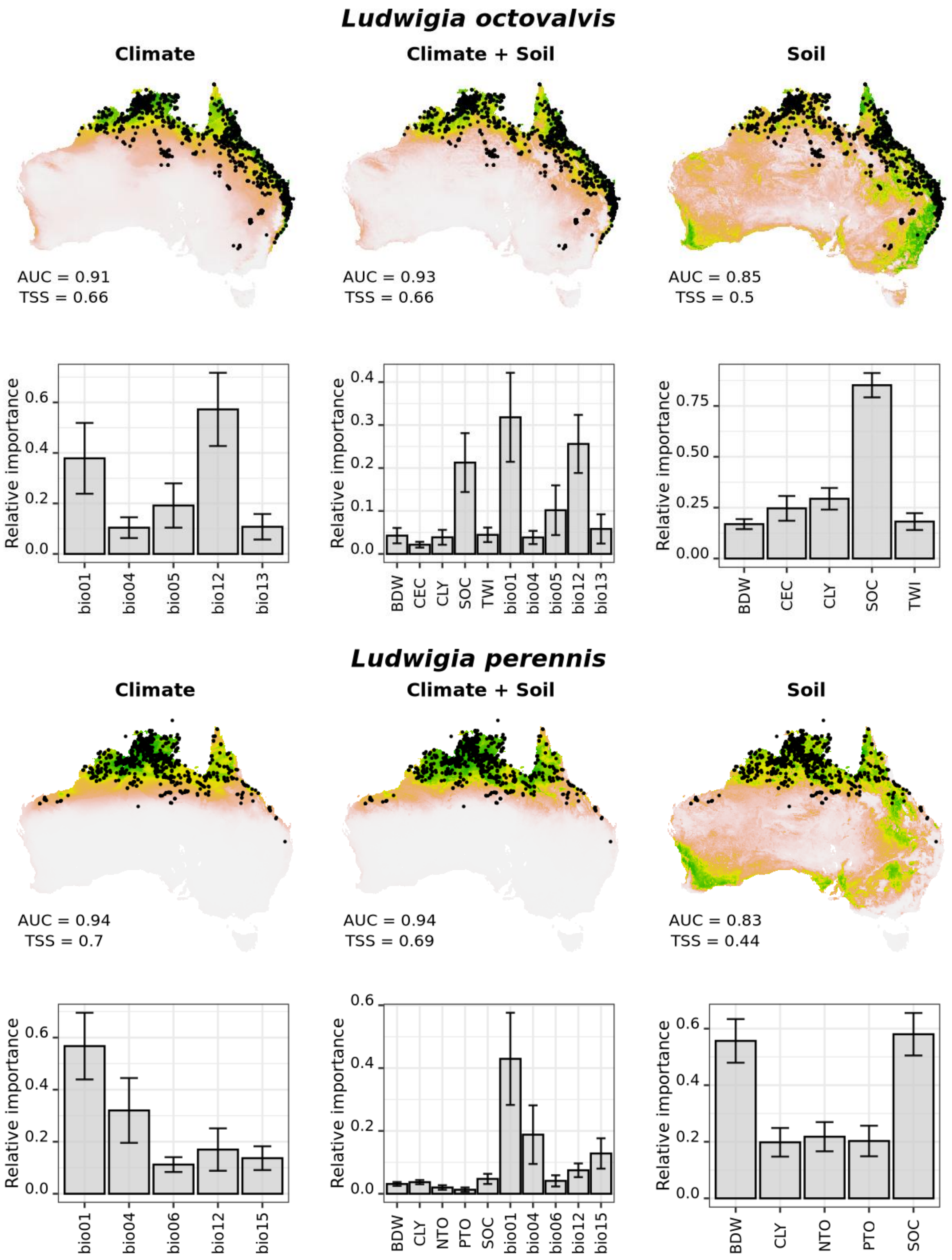

Ludwigia octovalvis
Climate
Climate + Soil
Soil
AUC = 0.91
TSS = 0.66
AUC = 0.93
TSS = 0.66
AUC = 0.85
TSS = 0.5
Relative importance
0.0
0.2
0.4
0.6
bio01
bio04
bio05
bio12
bio13
0.0
0.1
0.2
0.3
0.4
BDW
CEC
CLY
SOC
TWI
bio01
bio04
bio05
bio12
bio13
0.00
0.25
0.50
0.75
BDW
CEC
CLY
SOC
TWI
Ludwigia perennis
Climate
Climate + Soil
Soil
AUC = 0.94
TSS = 0.7
AUC = 0.94
TSS = 0.69
AUC = 0.83
TSS = 0.44
Relative importance
0.0
0.2
0.4
0.6
bio01
bio04
bio06
bio12
bio15
0.0
0.2
0.4
0.6
BDW
CLY
NTO
PTO
SOC
bio01
bio04
bio06
bio12
bio15
0.0
0.2
0.4
0.6
BDW
CLY
NTO
PTO
SOC

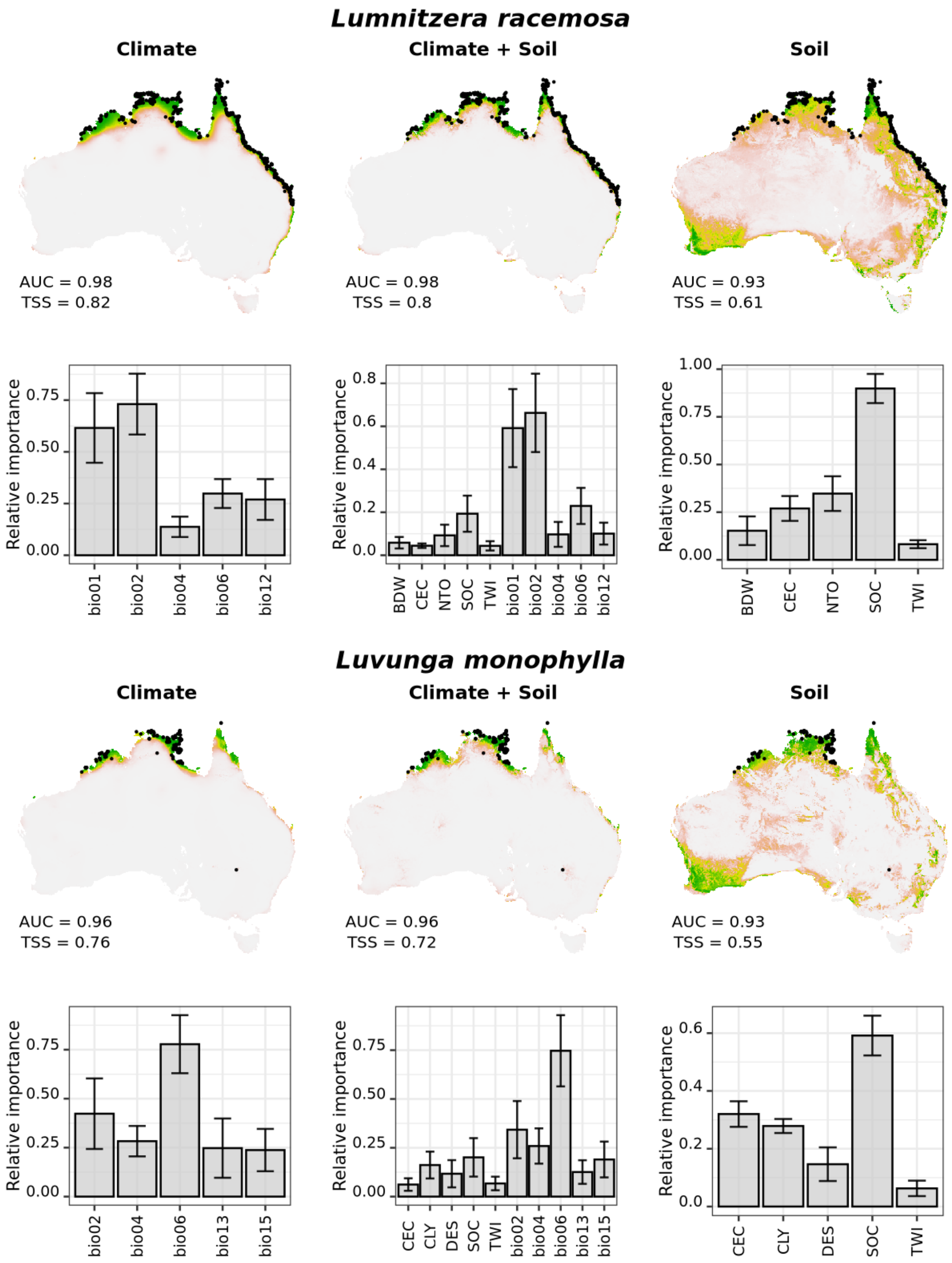
Lumnitzera racemosa
Climate
Climate + Soil
Soil
AUC = 0.98
TSS = 0.82
AUC = 0.98
TSS = 0.8
AUC = 0.93
TSS = 0.61
Relative importance
bio01
bio02
bio04
bio06
bio12
BDW
CEC
NTO
SOC
TWI
Luvunga monophylla
Climate
Climate + Soil
Soil
AUC = 0.96
TSS = 0.76
AUC = 0.96
TSS = 0.72
AUC = 0.93
TSS = 0.55
Relative importance
bio02
bio04
bio06
bio13
bio15
CEC
CLY
DES
SOC
TWI

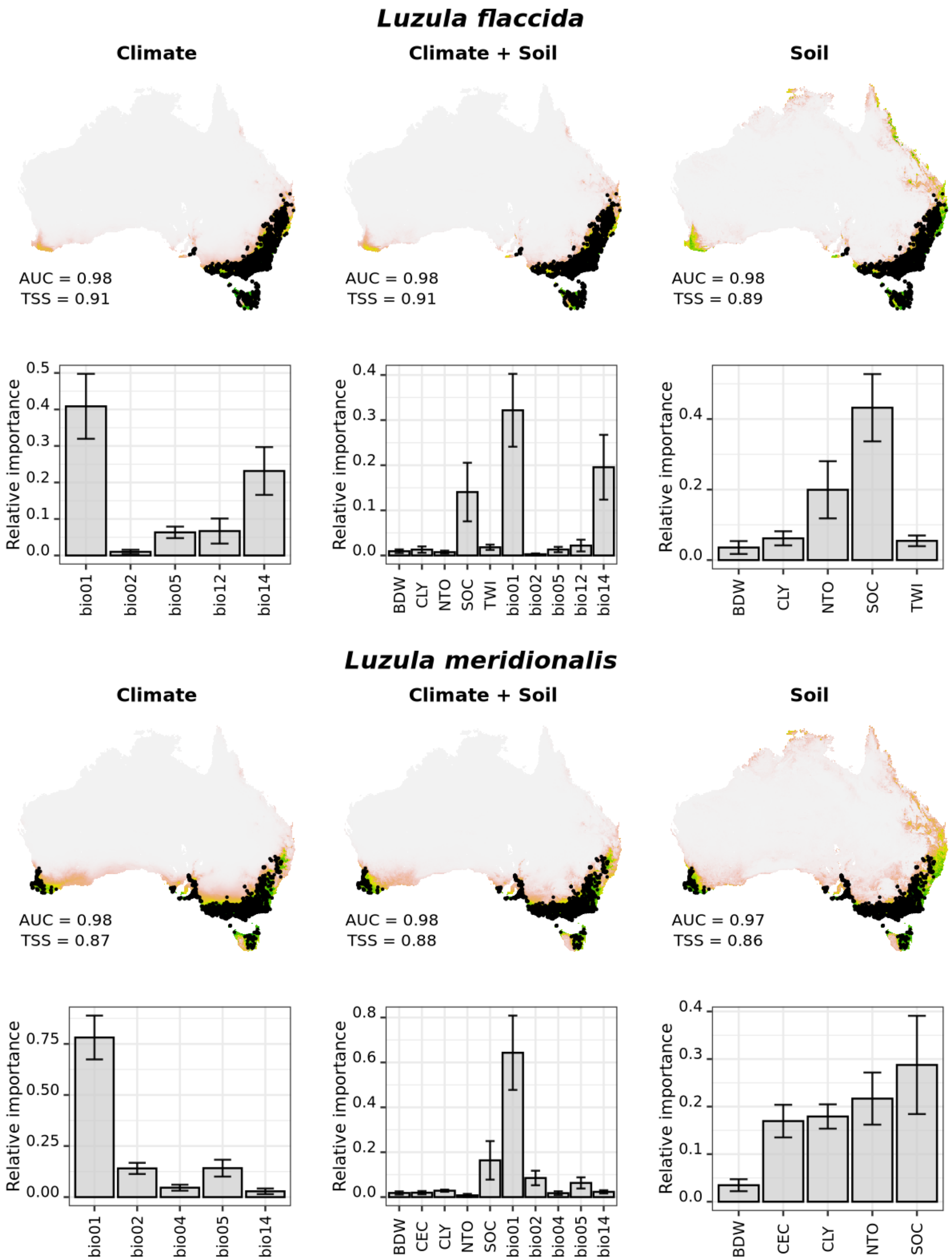
Luzula flaccida
Climate
Climate + Soil
Soil
AUC = 0.98
TSS = 0.91
AUC = 0.98
TSS = 0.91
AUC = 0.98
TSS = 0.89
Relative importance
0.5
0.4
0.3
0.2
0.1
0.0
bio01
bio02
bio05
bio12
bio14
Relative importance
0.4
0.3
0.2
0.1
0.0
BDW
CLY
NTO
SOC
TWI
bio01
bio02
bio05
bio12
bio14
Relative importance
0.4
0.2
0.0
BDW
CLY
NTO
SOC
TWI
Luzula meridionalis
Climate
Climate + Soil
Soil
AUC = 0.98
TSS = 0.87
AUC = 0.98
TSS = 0.88
AUC = 0.97
TSS = 0.86
Relative importance
0.75
0.50
0.25
0.00
bio01
bio02
bio04
bio05
bio14
Relative importance
0.8
0.6
0.4
0.2
0.0
BDW
CEC
CLY
NTO
SOC
bio01
bio02
bio04
bio05
bio14
Relative importance
0.4
0.3
0.2
0.1
0.0
BDW
CEC
CLY
NTO
SOC

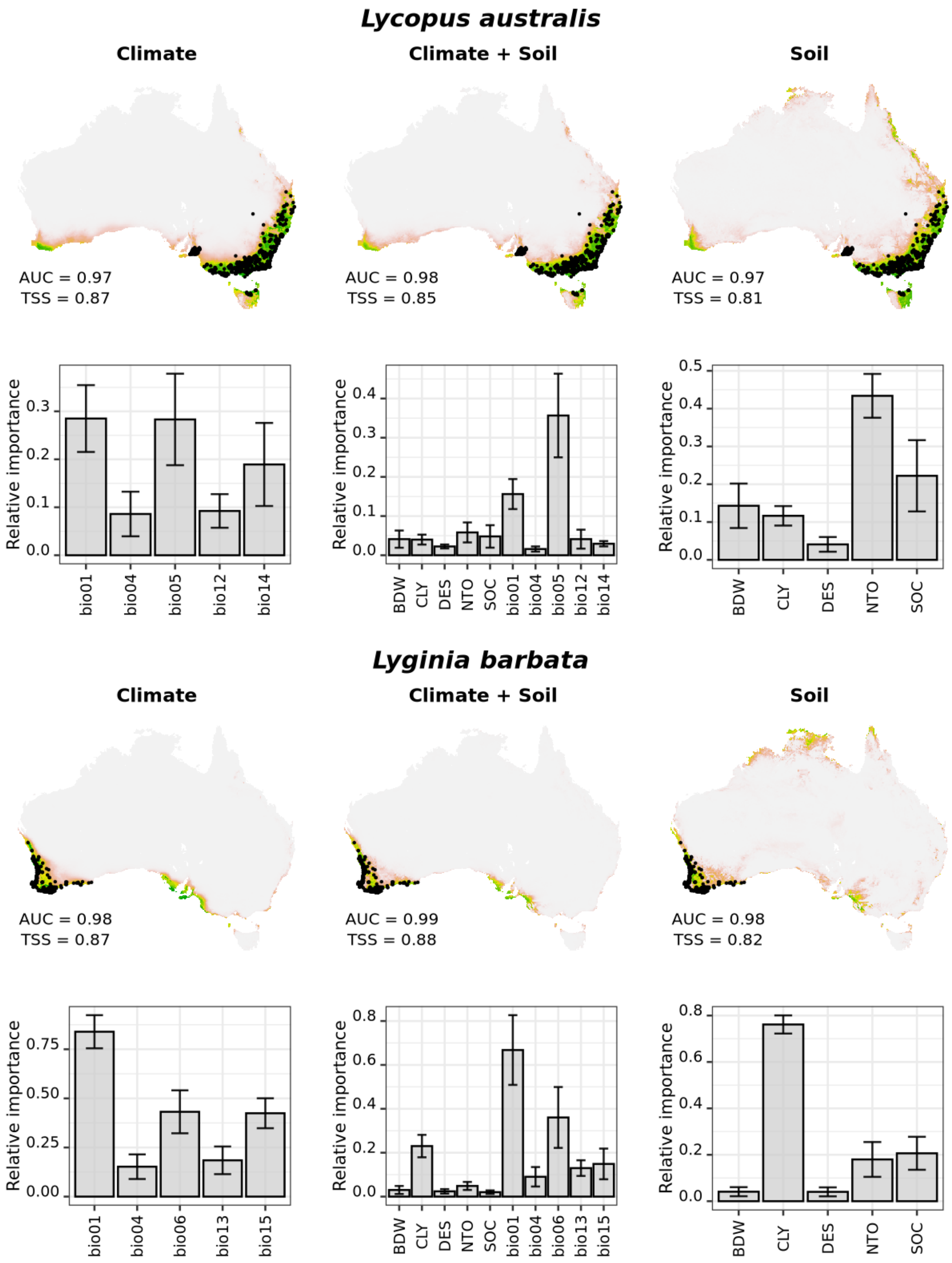
Lycopus australis
Climate
Climate + Soil
Soil
AUC = 0.97
TSS = 0.87
AUC = 0.98
TSS = 0.85
AUC = 0.97
TSS = 0.81
Relative importance
bio01
bio04
bio05
bio12
bio14
BDW
CLY
DES
NTO
SOC
Lyginia barbata
Climate
Climate + Soil
Soil
AUC = 0.98
TSS = 0.87
AUC = 0.99
TSS = 0.88
AUC = 0.98
TSS = 0.82
Relative importance
bio01
bio04
bio06
bio13
bio15
BDW
CLY
DES
NTO
SOC

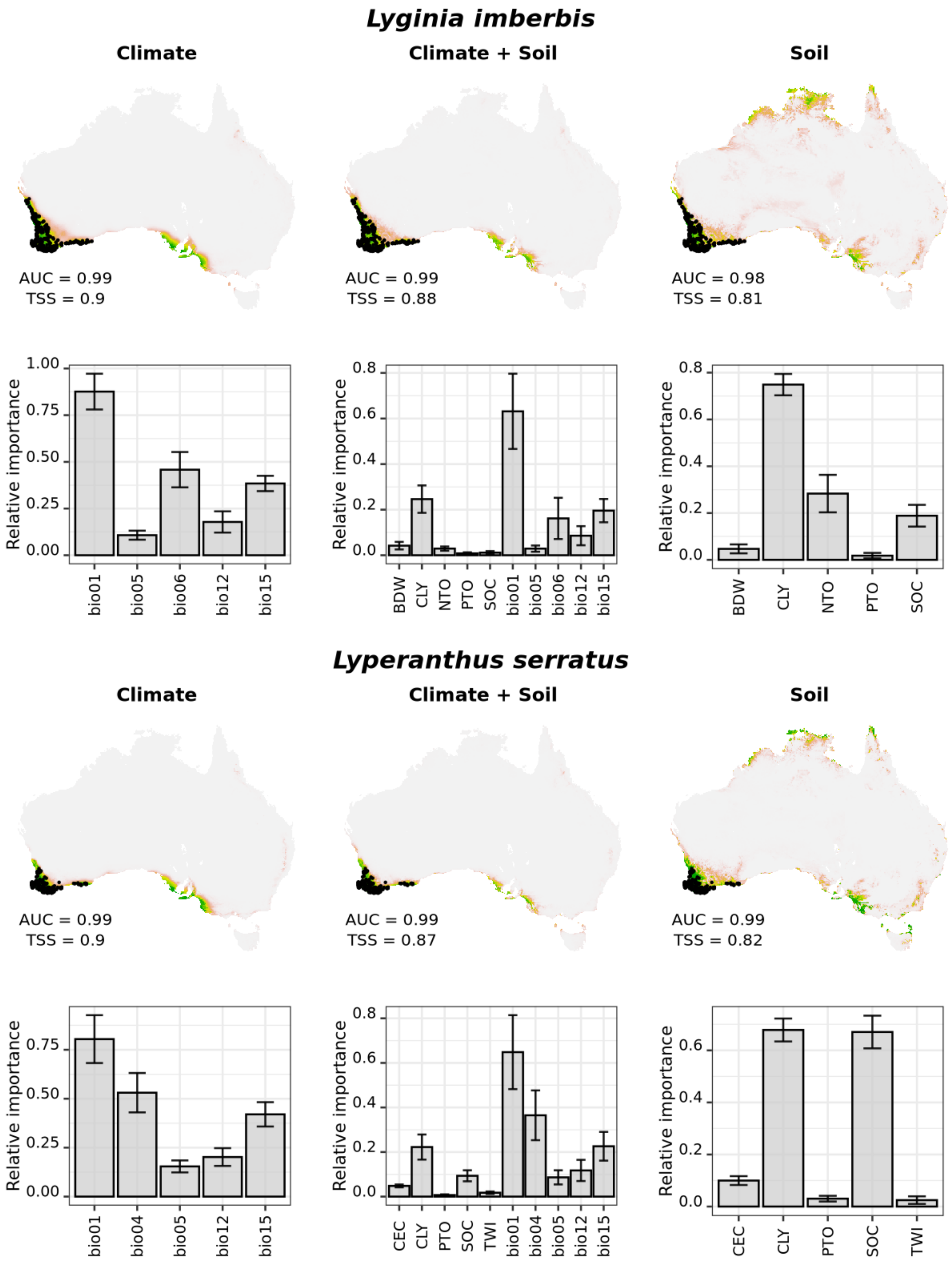
Lyginia imberbis
Climate
Climate + Soil
Soil
AUC = 0.99
TSS = 0.9
AUC = 0.99
TSS = 0.88
AUC = 0.98
TSS = 0.81
Relative importance
bio01
bio05
bio06
bio12
bio15
BDW
CLY
NTO
PTO
SOC
Lyperanthus serratus
Climate
Climate + Soil
Soil
AUC = 0.99
TSS = 0.9
AUC = 0.99
TSS = 0.87
AUC = 0.99
TSS = 0.82
Relative importance
bio01
bio04
bio05
bio12
bio15
CEC
CLY
PTO
SOC
TWI

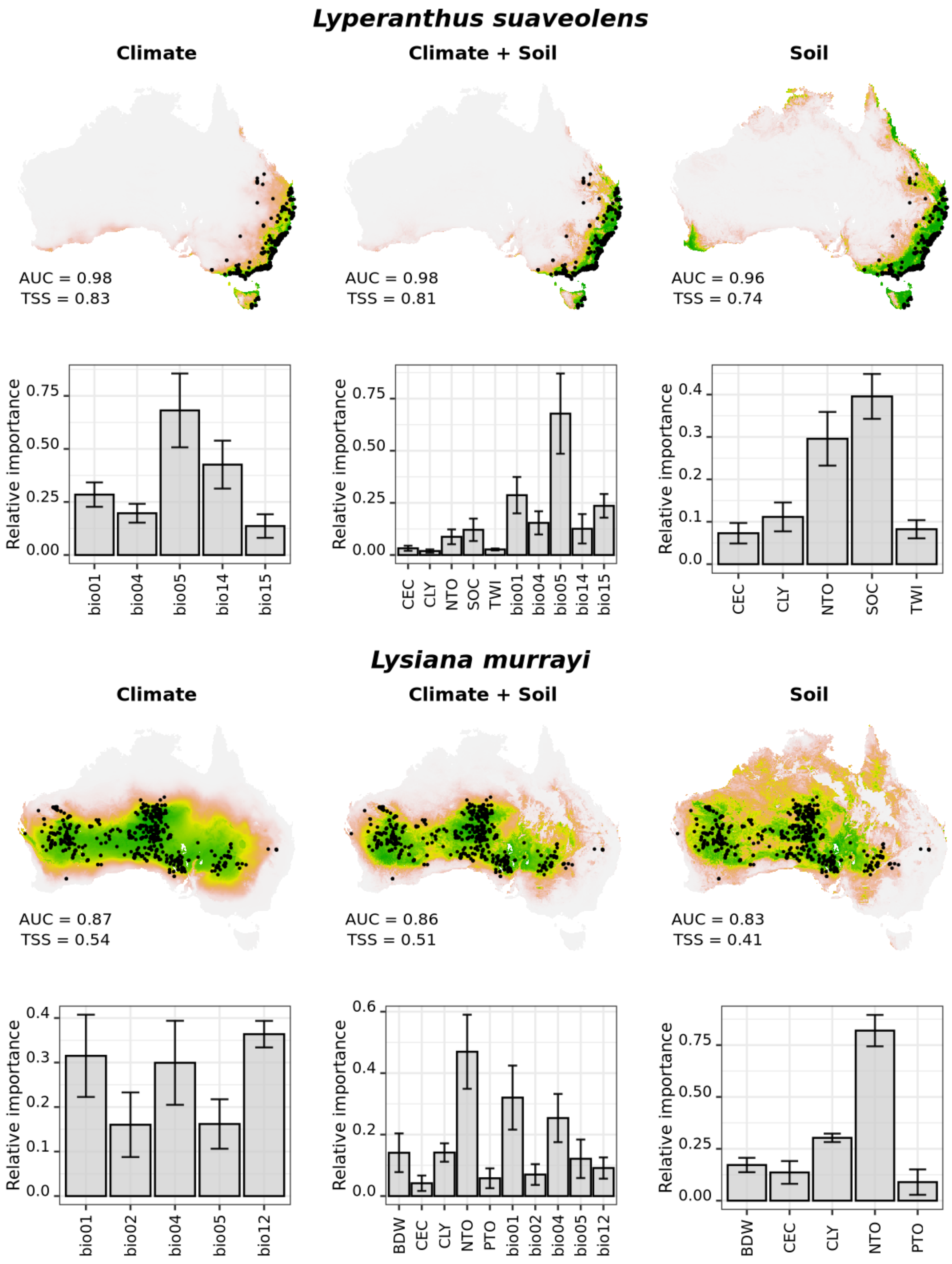

Lyperanthus suaveolens
Climate
Climate + Soil
Soil
AUC = 0.98
TSS = 0.83
AUC = 0.98
TSS = 0.81
AUC = 0.96
TSS = 0.74
Relative importance
0.00
0.25
0.50
0.75
bio01
bio04
bio05
bio14
bio15
CEC
CLY
NTO
SOC
TWI
bio01
bio04
bio05
bio14
bio15
0.0
0.1
0.2
0.3
0.4
CEC
CLY
NTO
SOC
TWI
Lysiana murrayi
Climate
Climate + Soil
Soil
AUC = 0.87
TSS = 0.54
AUC = 0.86
TSS = 0.51
AUC = 0.83
TSS = 0.41
Relative importance
0.0
0.1
0.2
0.3
0.4
bio01
bio02
bio04
bio05
bio12
0.0
0.2
0.4
0.6
BDW
CEC
CLY
NTO
PTO
bio01
bio02
bio04
bio05
bio12
0.00
0.25
0.50
0.75
BDW
CEC
CLY
NTO
PTO

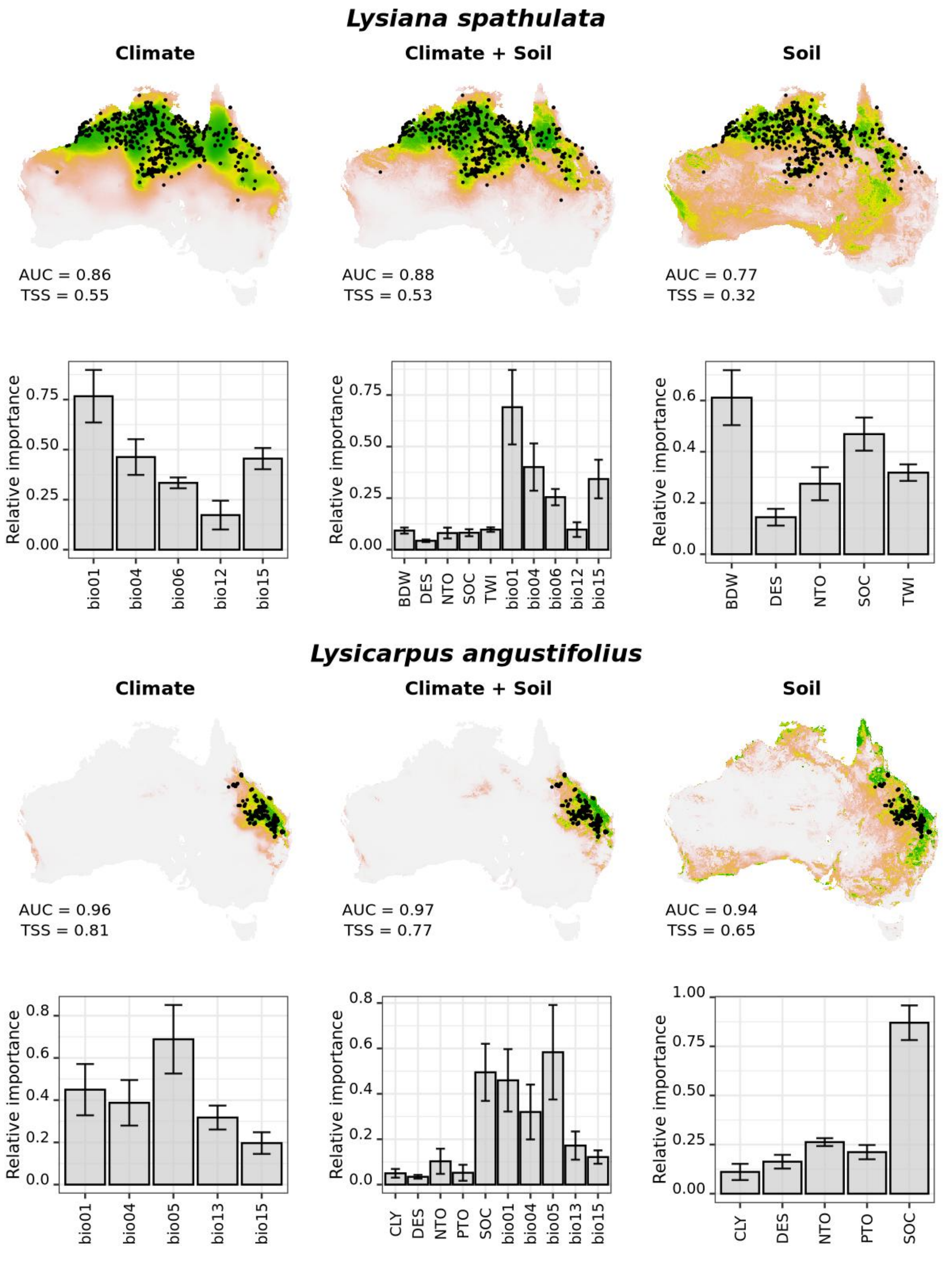
Lysiana spathulata
Climate
Climate + Soil
Soil
AUC = 0.86
TSS = 0.55
AUC = 0.88
TSS = 0.53
AUC = 0.77
TSS = 0.32
Relative importance
bio01
bio04
bio06
bio12
bio15
BDW
DES
NTO
SOC
TWI
Lysicarpus angustifolius
Climate
Climate + Soil
Soil
AUC = 0.96
TSS = 0.81
AUC = 0.97
TSS = 0.77
AUC = 0.94
TSS = 0.65
Relative importance
bio01
bio04
bio05
bio13
bio15
CLY
DES
NTO
PTO
SOC

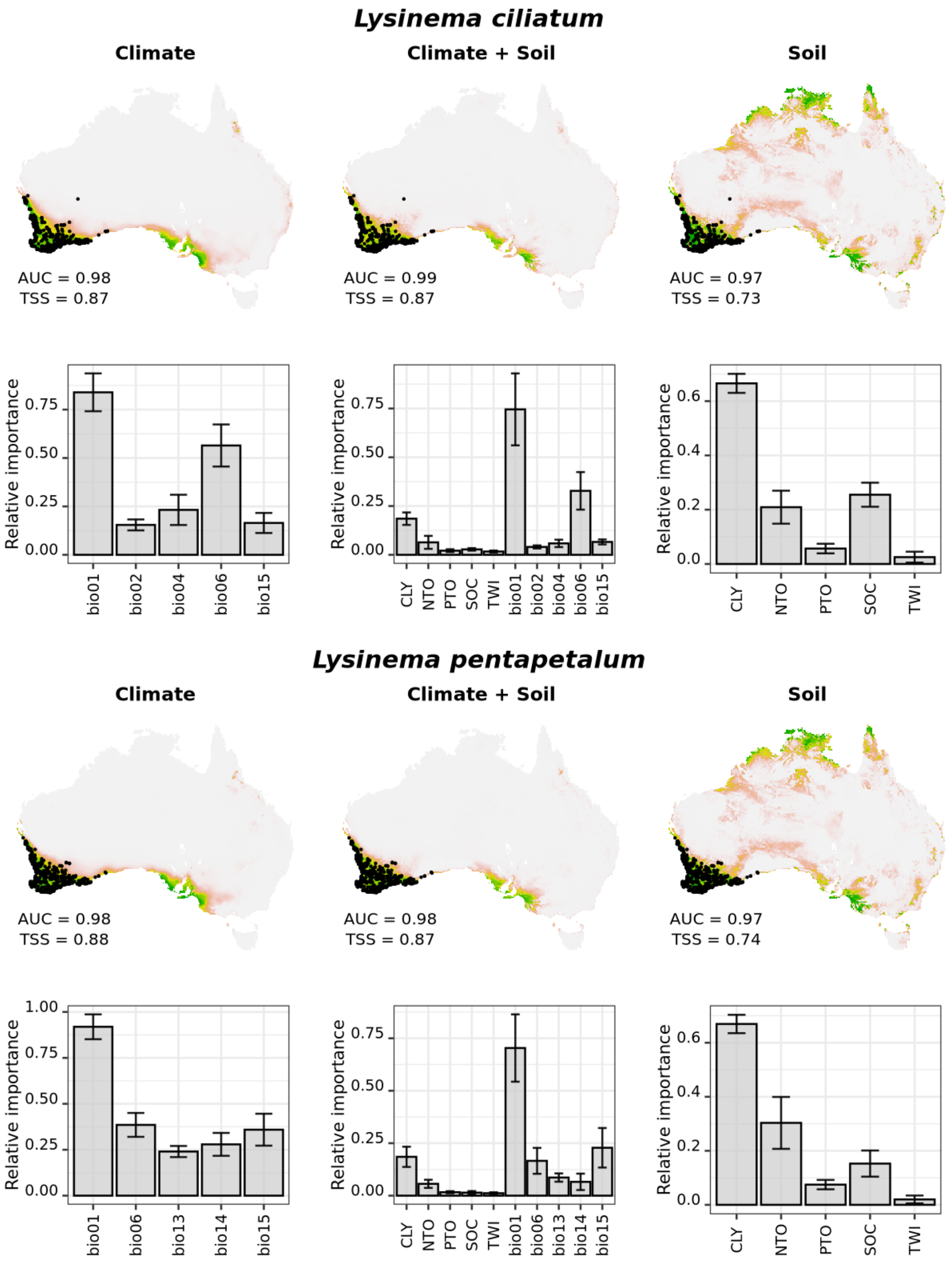
Lysinema ciliatum
Climate
Climate + Soil
Soil
AUC = 0.98
TSS = 0.87
AUC = 0.99
TSS = 0.87
AUC = 0.97
TSS = 0.73
Relative importance
bio01 bio02 bio04 bio06 bio15
CLY NTO PTO SOC TWI bio01 bio02 bio04 bio06 bio15
CLY NTO PTO SOC TWI
Lysinema pentapetalum
Climate
Climate + Soil
Soil
AUC = 0.98
TSS = 0.88
AUC = 0.98
TSS = 0.87
AUC = 0.97
TSS = 0.74
Relative importance
bio01 bio06 bio13 bio14 bio15
CLY NTO PTO SOC TWI bio01 bio06 bio13 bio14 bio15
CLY NTO PTO SOC TWI

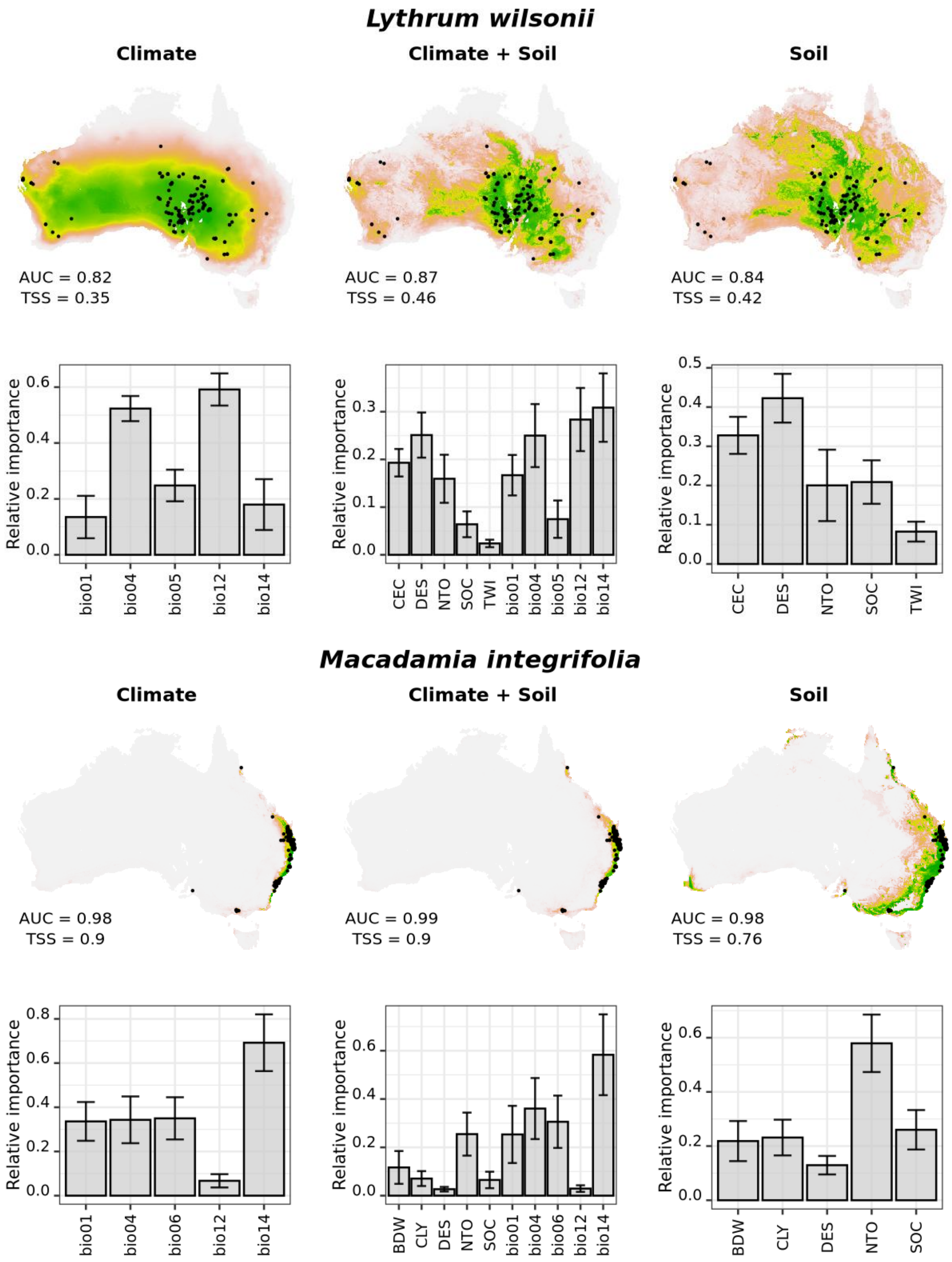

Lythrum wilsonii
Climate
Climate + Soil
Soil
AUC = 0.82
TSS = 0.35
AUC = 0.87
TSS = 0.46
AUC = 0.84
TSS = 0.42
Relative importance
bio01
bio04
bio05
bio12
bio14
CEC
DES
NTO
SOC
TWI
Macadamia integrifolia
Climate
Climate + Soil
Soil
AUC = 0.98
TSS = 0.9
AUC = 0.99
TSS = 0.9
AUC = 0.98
TSS = 0.76
Relative importance
bio01
bio04
bio06
bio12
bio14
BDW
CLY
DES
NTO
SOC

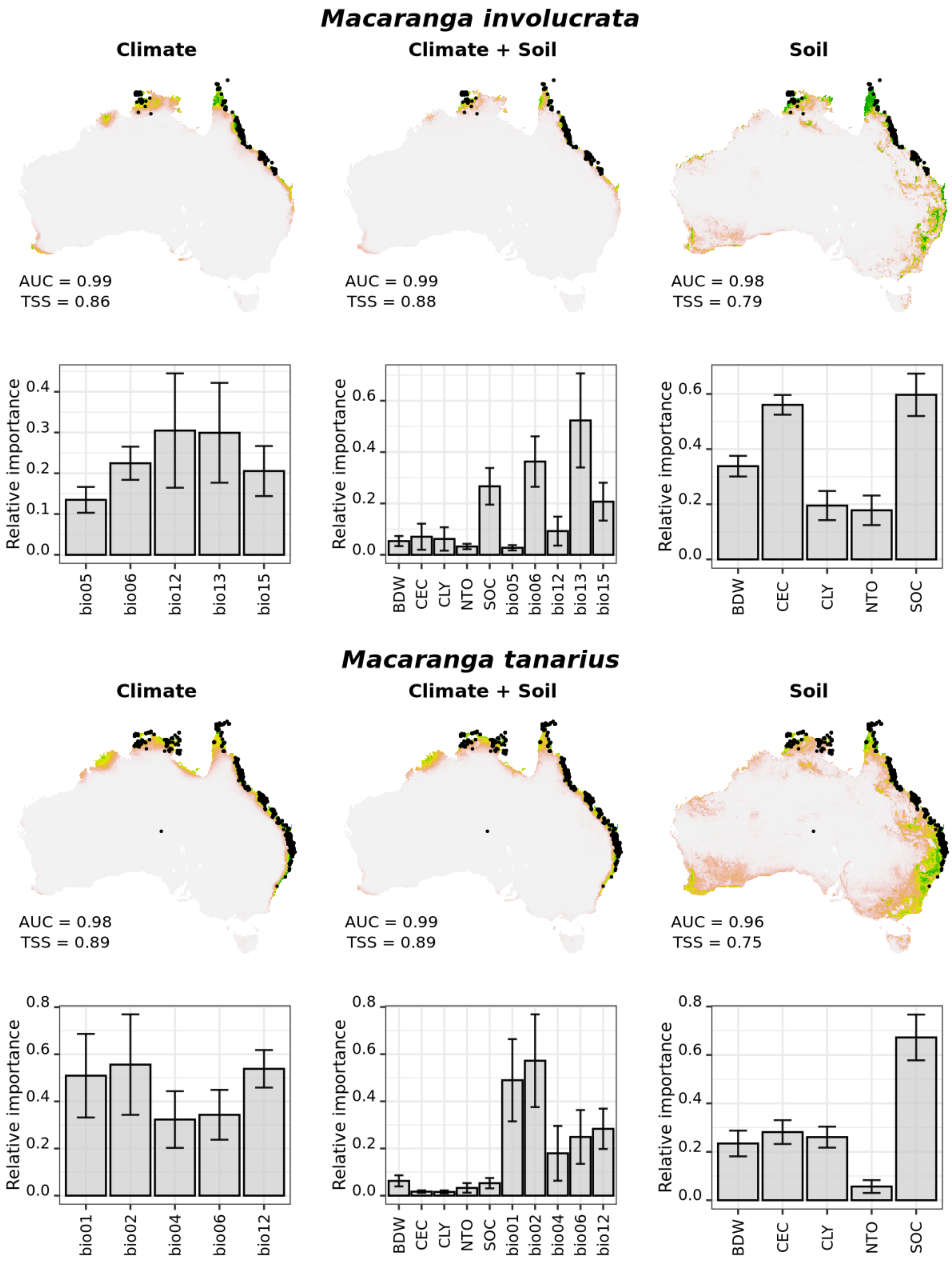
Macaranga involucrata
Climate
Climate + Soil
Soil
AUC = 0.99
TSS = 0.86
AUC = 0.99
TSS = 0.88
AUC = 0.98
TSS = 0.79
Relative importance
bio05
bio06
bio12
bio13
bio15
BDW
CEC
CLY
NTO
SOC
Macaranga tanarius
AUC = 0.98
TSS = 0.89
AUC = 0.99
TSS = 0.89
AUC = 0.96
TSS = 0.75
bio01
bio02
bio04

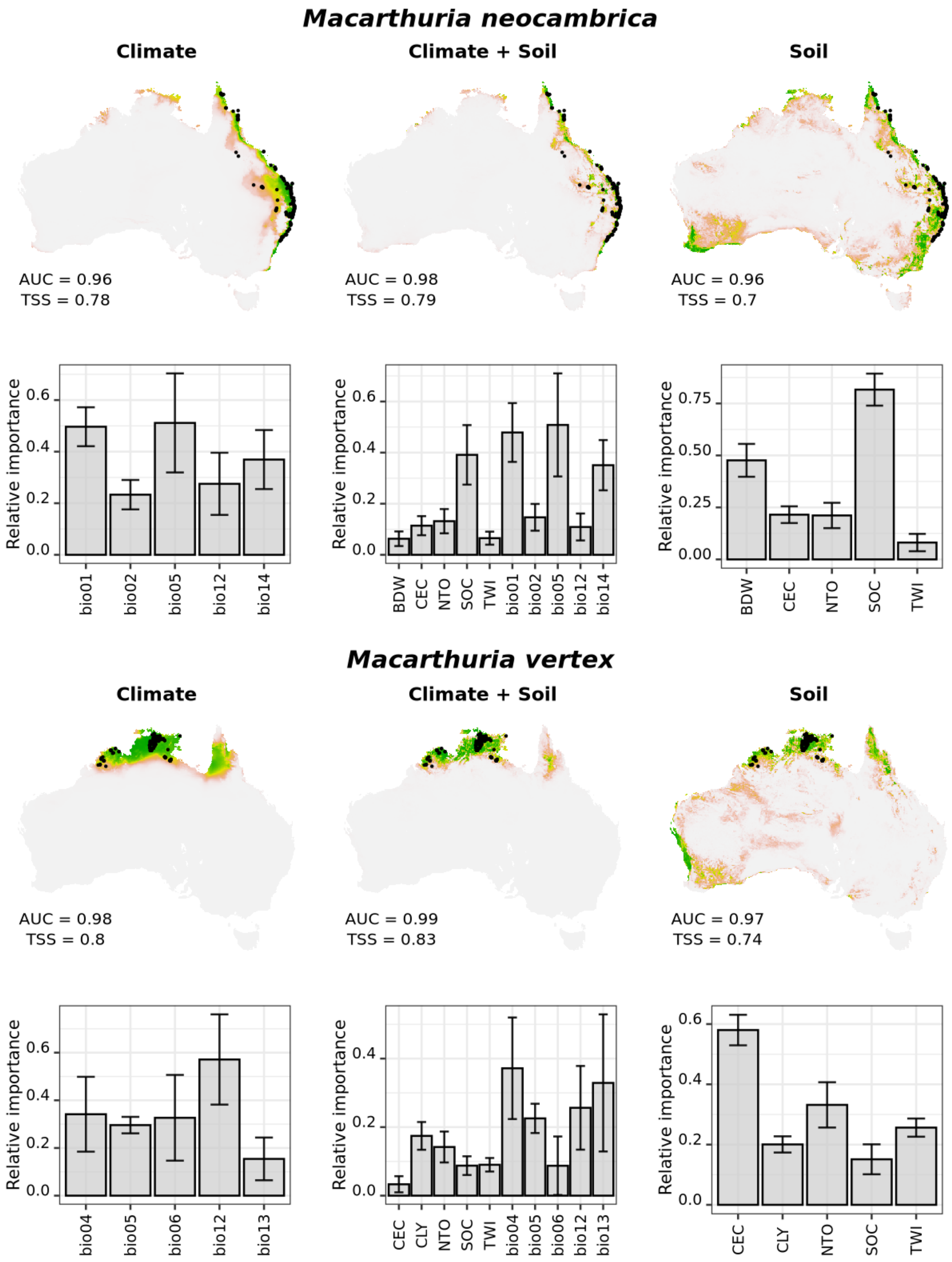
Macarthuria neocambrica
Climate
Climate + Soil
Soil
AUC = 0.96
TSS = 0.78
AUC = 0.98
TSS = 0.79
AUC = 0.96
TSS = 0.7
Relative importance
0.0
0.2
0.4
0.6
bio01
bio02
bio05
bio12
bio14
BDW
CEC
NTO
SOC
TWI
bio01
bio02
bio05
bio12
bio14
0.00
0.25
0.50
0.75
Macarthuria vertex
AUC = 0.98
TSS = 0.8
AUC = 0.99
TSS = 0.83
AUC = 0.97
TSS = 0.74
bio04
bio05
bio06
bio12
bio13
CEC
CLY
NTO
SOC
TWI
bio04
bio05
bio06
bio12
bio13

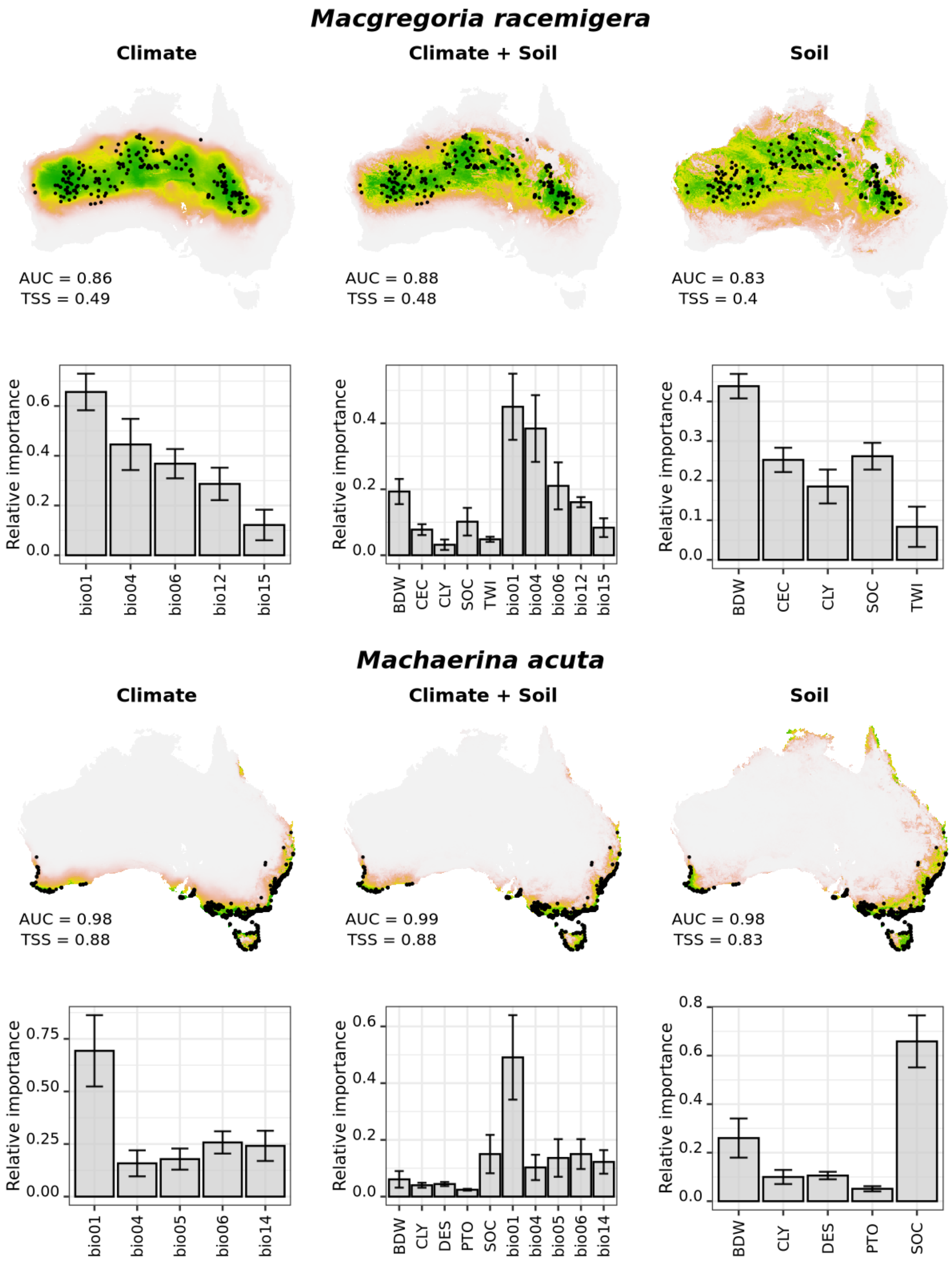
Macgregoria racemigera
Climate
Climate + Soil
Soil
AUC = 0.86
TSS = 0.49
AUC = 0.88
TSS = 0.48
AUC = 0.83
TSS = 0.4
Relative importance
bio01
bio04
bio06
bio12
bio15
BDW
CEC
CLY
SOC
TWI
Machaerina acuta
Climate
Climate + Soil
Soil
AUC = 0.98
TSS = 0.88
AUC = 0.99
TSS = 0.88
AUC = 0.98
TSS = 0.83
Relative importance
bio01
bio04
bio05
bio06
bio14
BDW
CLY
DES
PTO
SOC

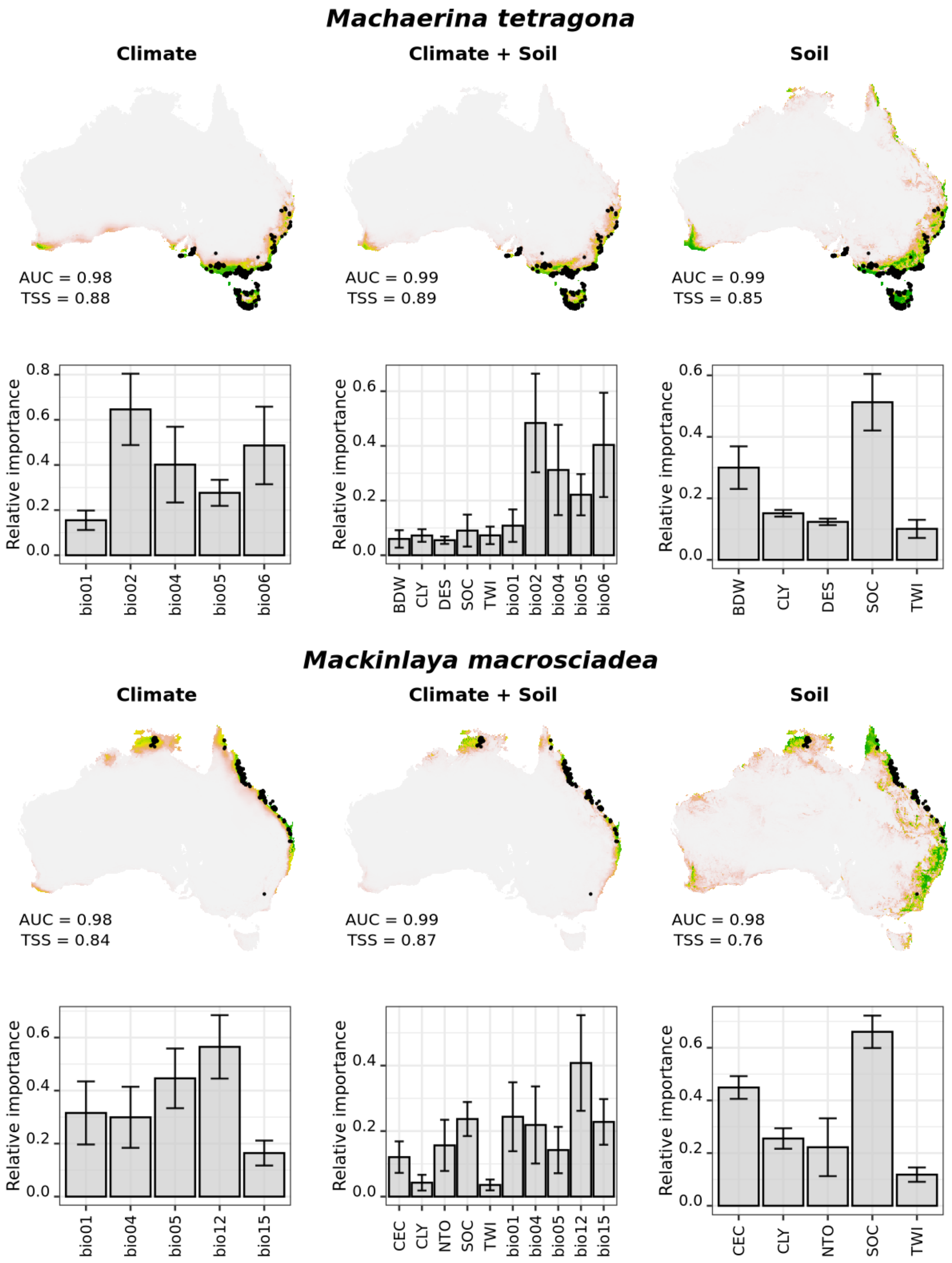
Machaerina tetragona
Climate
Climate + Soil
Soil
AUC = 0.98
TSS = 0.88
AUC = 0.99
TSS = 0.89
AUC = 0.99
TSS = 0.85
Relative importance
0.0
0.2
0.4
0.6
0.8
bio01
bio02
bio04
bio05
bio06
BDW
CLY
DES
SOC
TWI
CEC
NTO
Mackinlaya macrosciadea
AUC = 0.98
TSS = 0.84
AUC = 0.99
TSS = 0.87
AUC = 0.98
TSS = 0.76
bio12
bio15

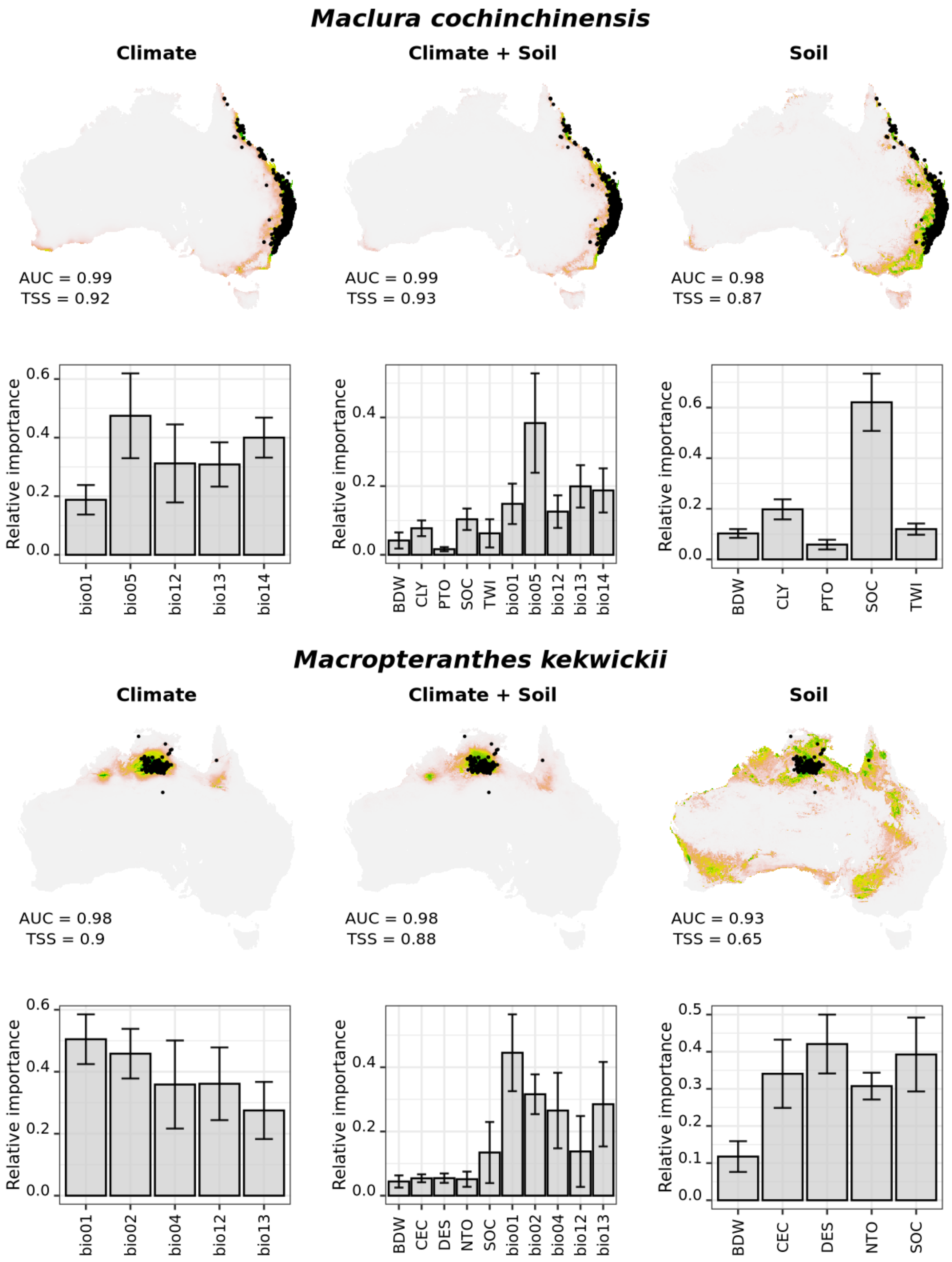
Maclura cochinchinensis
Climate
Climate + Soil
Soil
AUC = 0.99
TSS = 0.92
AUC = 0.99
TSS = 0.93
AUC = 0.98
TSS = 0.87
Relative importance
bio01
bio05
bio12
bio13
bio14
BDW
CLY
PTO
SOC
TWI
Macropteranthes kekwickii
Climate
Climate + Soil
Soil
AUC = 0.98
TSS = 0.9
AUC = 0.98
TSS = 0.88
AUC = 0.93
TSS = 0.65
Relative importance
bio01
bio02
bio04
bio12
bio13
BDW
CEC
DES
NTO
SOC

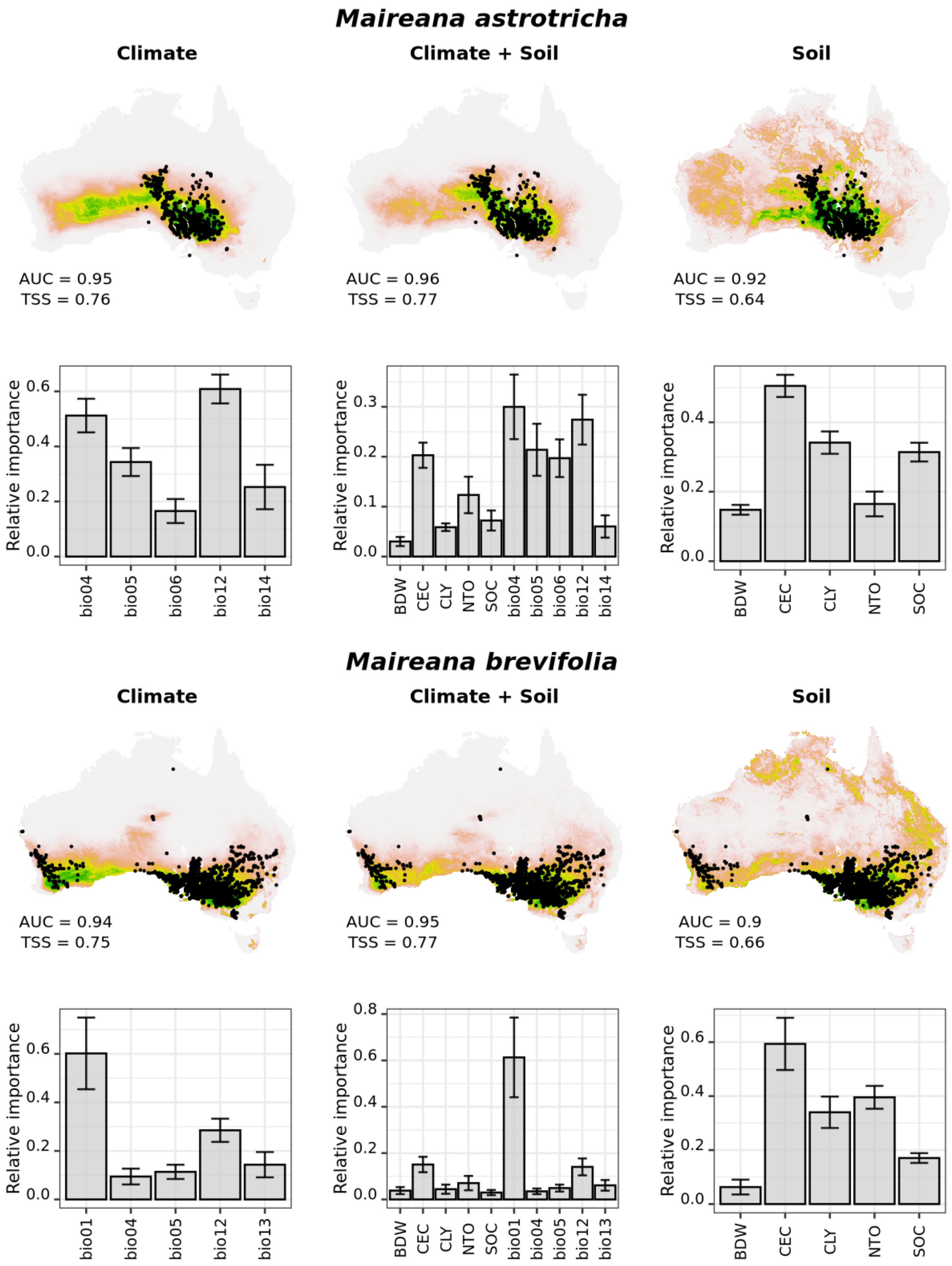

Maireana astrotricha
Climate
Climate + Soil
Soil
AUC = 0.95
TSS = 0.76
AUC = 0.96
TSS = 0.77
AUC = 0.92
TSS = 0.64
Relative importance
0.0
0.2
0.4
0.6
bio04
bio05
bio06
bio12
bio14
0.0
0.1
0.2
0.3
BDW
CEC
CLY
NTO
SOC
bio04
bio05
bio06
bio12
bio14
0.0
0.2
0.4
BDW
CEC
CLY
NTO
SOC
Maireana brevifolia
Climate
Climate + Soil
Soil
AUC = 0.94
TSS = 0.75
AUC = 0.95
TSS = 0.77
AUC = 0.9
TSS = 0.66
Relative importance
0.0
0.2
0.4
0.6
bio01
bio04
bio05
bio12
bio13
0.0
0.2
0.4
0.6
0.8
BDW
CEC
CLY
NTO
SOC
bio01
bio04
bio05
bio12
bio13
0.0
0.2
0.4
0.6
BDW
CEC
CLY
NTO
SOC

Maireana campanulata

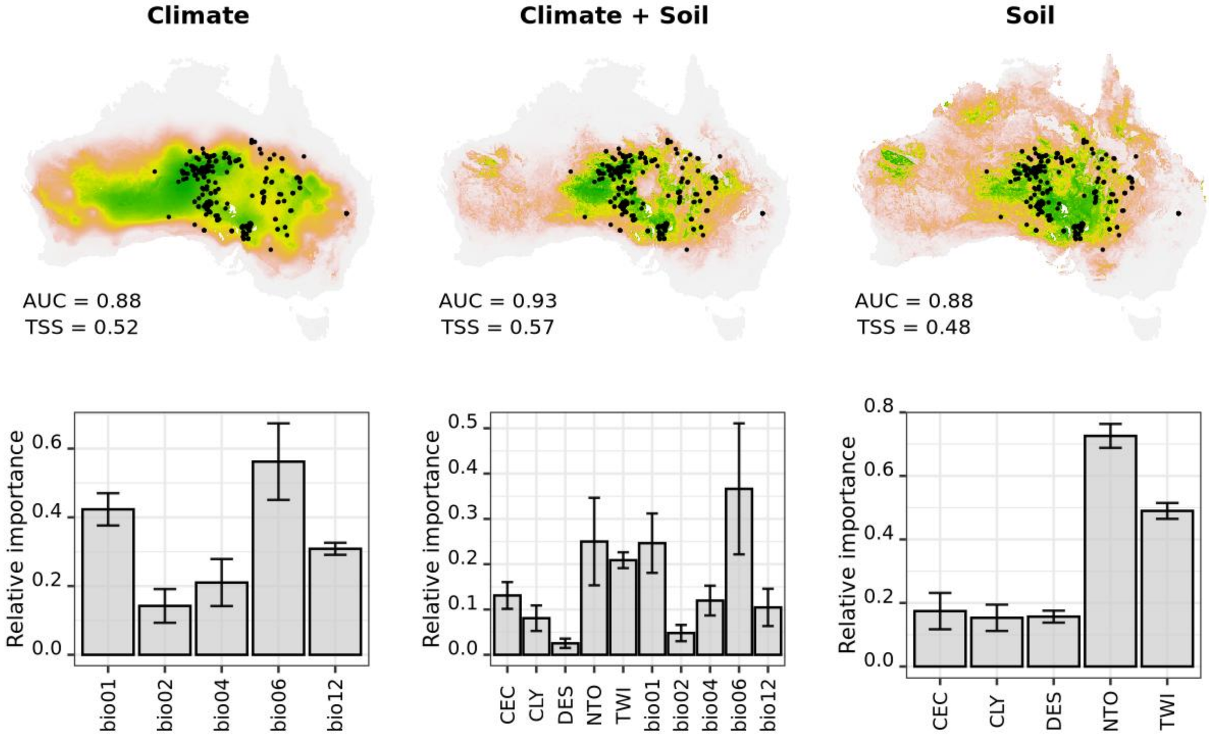

Maireana ciliata

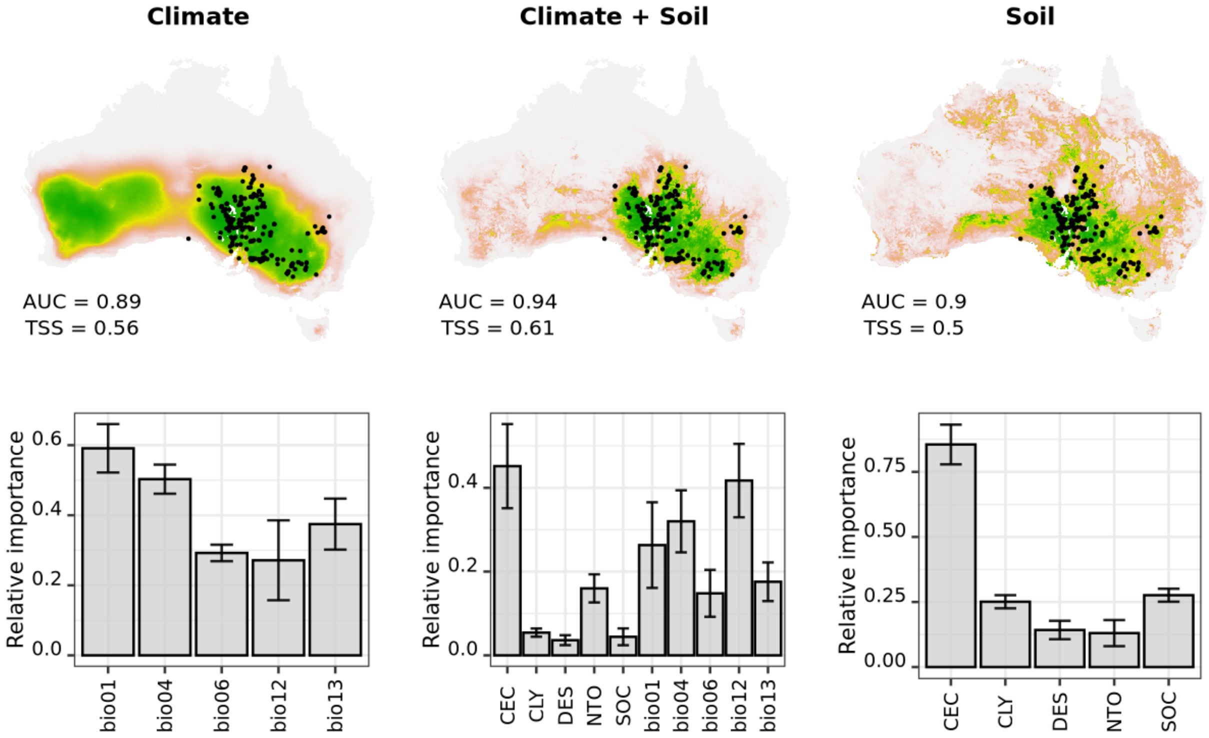

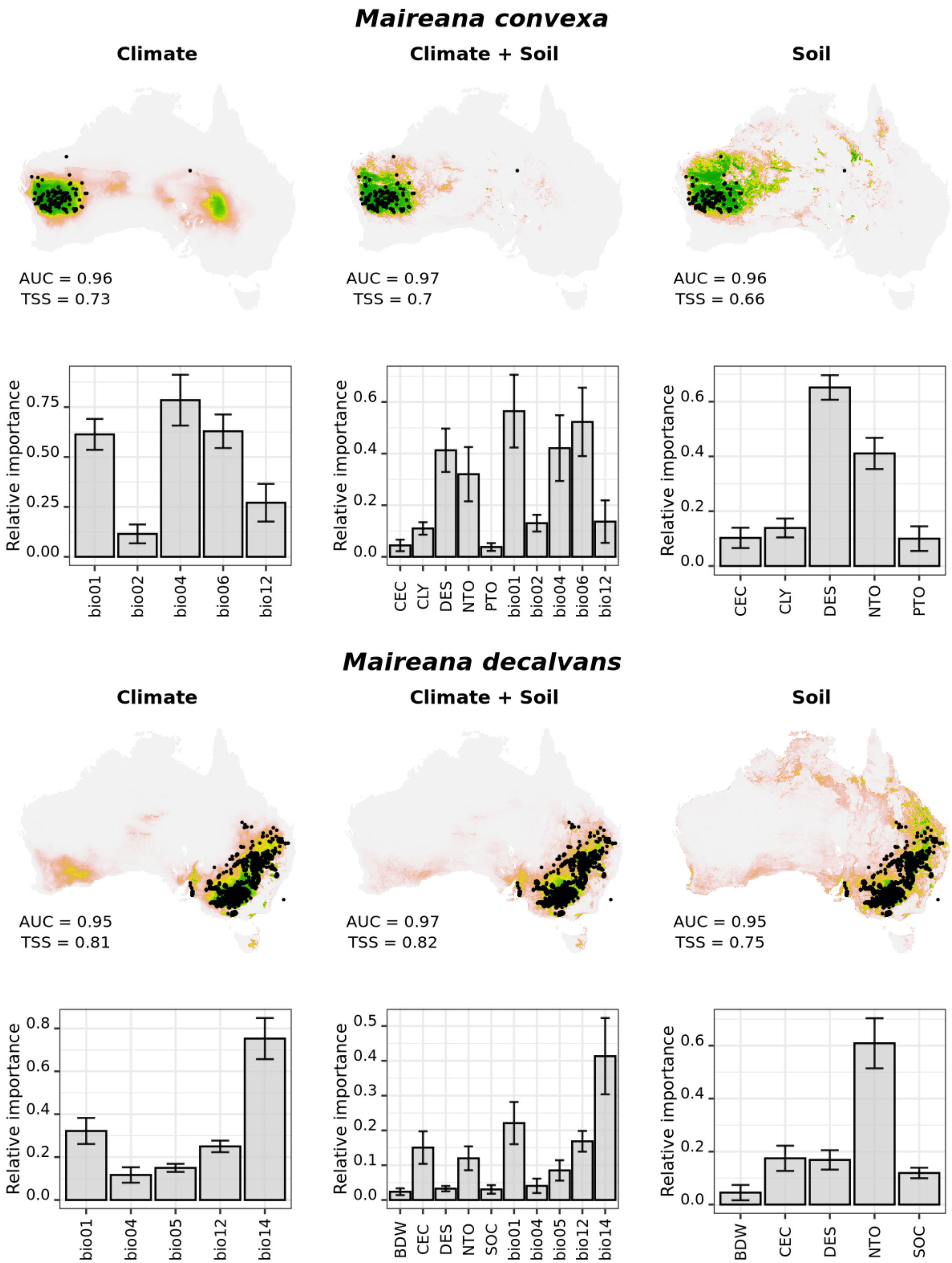
Maireana convexa
Climate
Climate + Soil
Soil
AUC = 0.96
TSS = 0.73
AUC = 0.97
TSS = 0.7
AUC = 0.96
TSS = 0.66
Relative importance
0.00
0.25
0.50
0.75
bio01
bio02
bio04
bio06
bio12
Relative importance
0.0
0.2
0.4
0.6
CEC
CLY
DES
NTO
PTO
bio01
bio02
bio04
bio06
bio12
Relative importance
0.0
0.2
0.4
0.6
CEC
CLY
DES
NTO
PTO
Maireana decalvans
Climate
Climate + Soil
Soil
AUC = 0.95
TSS = 0.81
AUC = 0.97
TSS = 0.82
AUC = 0.95
TSS = 0.75
Relative importance
0.0
0.2
0.4
0.6
0.8
bio01
bio04
bio05
bio12
bio14
Relative importance
0.0
0.1
0.2
0.3
0.4
0.5
BDW
CEC
DES
NTO
SOC
bio01
bio04
bio05
bio12
bio14
Relative importance
0.0
0.2
0.4
0.6
BDW
CEC
DES
NTO
SOC

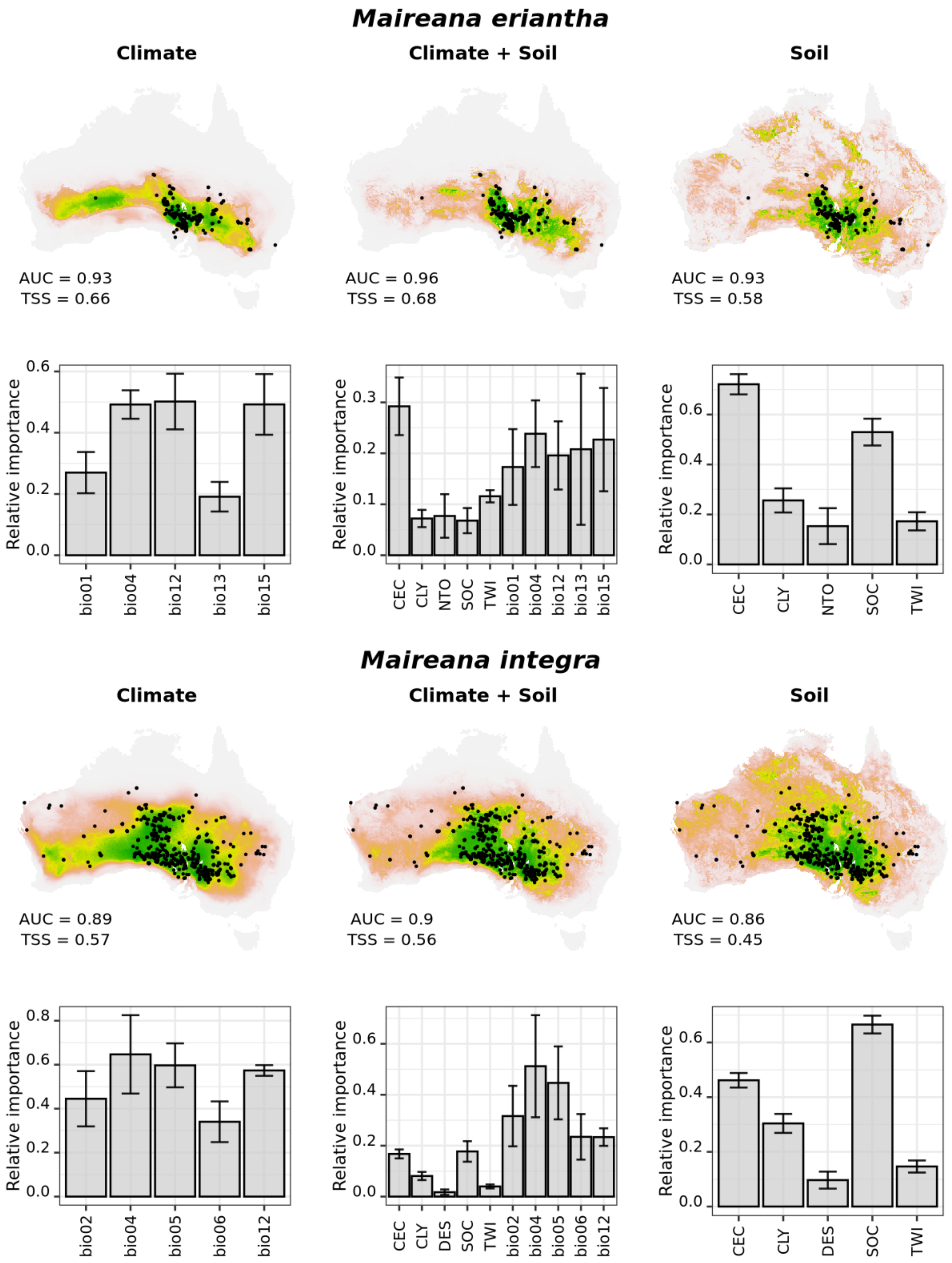

Maireana eriantha
Climate
Climate + Soil
Soil
AUC = 0.93
TSS = 0.66
AUC = 0.96
TSS = 0.68
AUC = 0.93
TSS = 0.58
Relative importance
bio01 bio04 bio12 bio13 bio15
CEC CLY NTO SOC TWI bio01 bio04 bio12 bio13 bio15
CEC CLY NTO SOC TWI
Maireana integra
Climate
Climate + Soil
Soil
AUC = 0.89
TSS = 0.57
AUC = 0.9
TSS = 0.56
AUC = 0.86
TSS = 0.45
Relative importance
bio02 bio04 bio05 bio06 bio12
CEC CLY DES SOC TWI bio02 bio04 bio05 bio06 bio12
CEC CLY DES SOC TWI

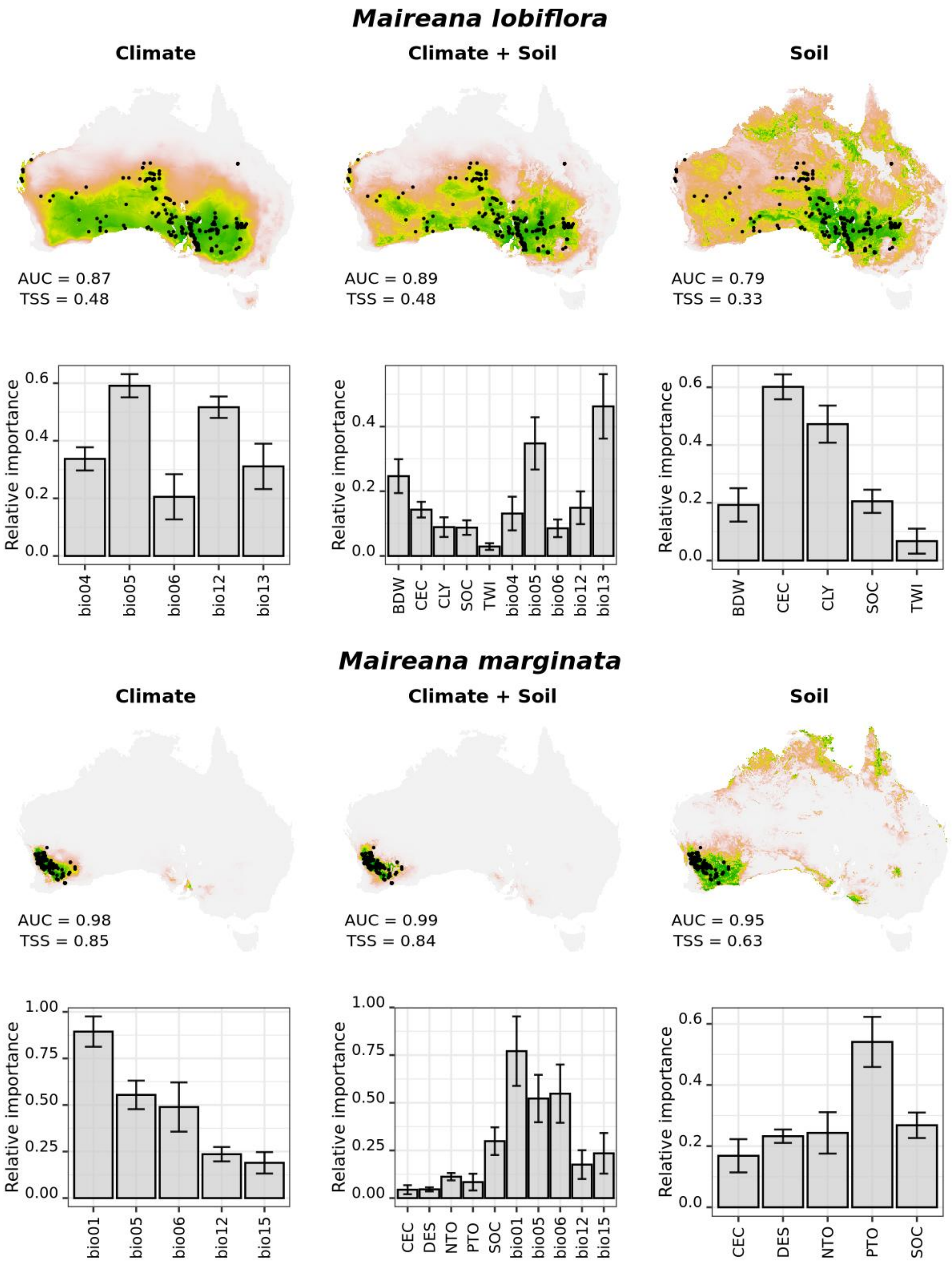
Maireana lobiflora
Climate
Climate + Soil
Soil
AUC = 0.87
TSS = 0.48
AUC = 0.89
TSS = 0.48
AUC = 0.79
TSS = 0.33
Relative importance
bio04
bio05
bio06
bio12
bio13
BDW
CEC
CLY
SOC
TWI
Maireana marginata
Climate
Climate + Soil
Soil
AUC = 0.98
TSS = 0.85
AUC = 0.99
TSS = 0.84
AUC = 0.95
TSS = 0.63
Relative importance
bio01
bio05
bio06
bio12
bio15
CEC
DES
NTO
PTO
SOC

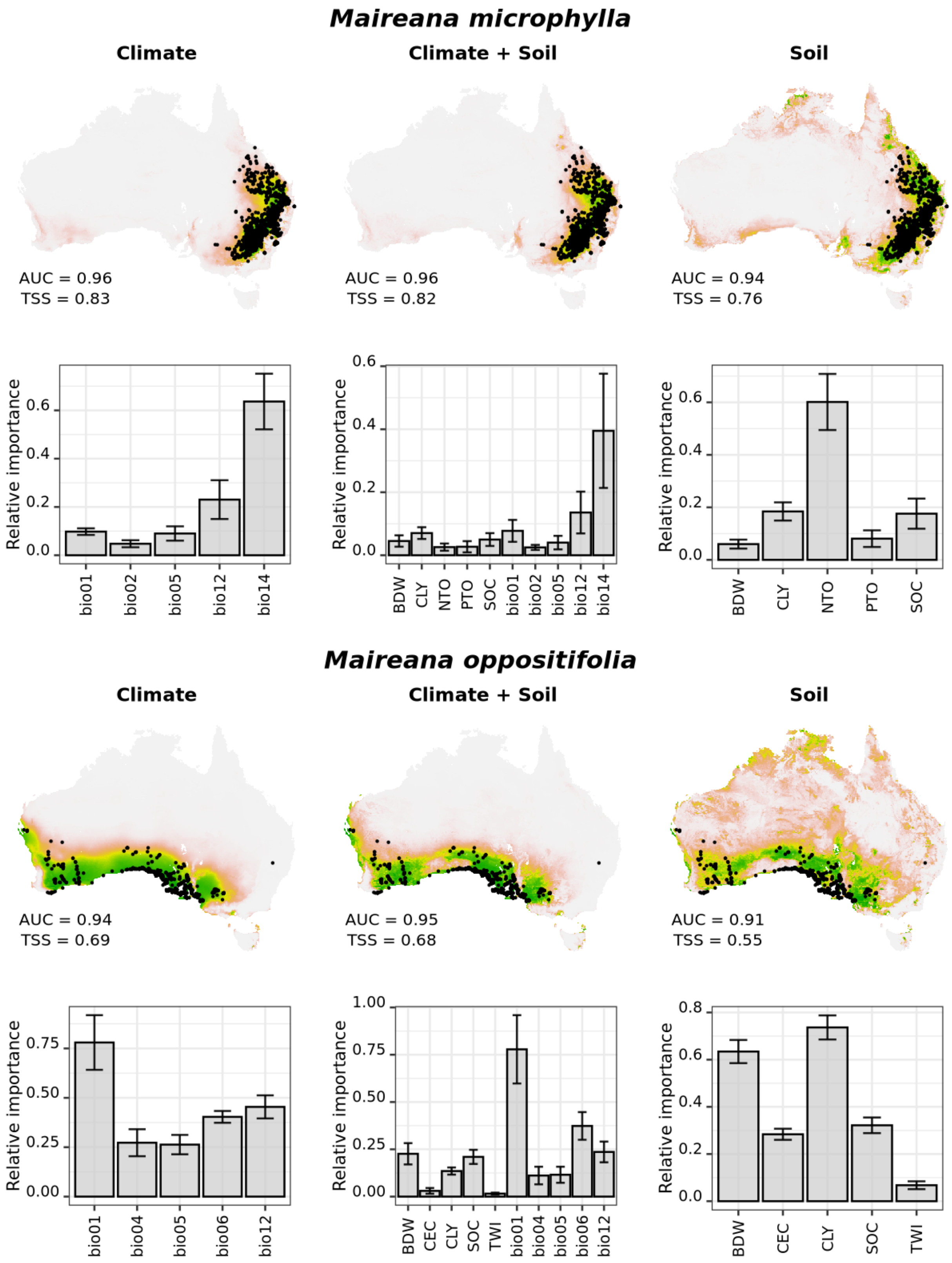
Maireana microphylla
Climate
Climate + Soil
Soil
AUC = 0.96
TSS = 0.83
AUC = 0.96
TSS = 0.82
AUC = 0.94
TSS = 0.76
Relative importance
0.0
0.2
0.4
0.6
bio01
bio02
bio05
bio12
bio14
BDW
CLY
NTO
PTO
SOC
bio01
bio02
bio05
bio12
bio14
BDW
CLY
NTO
PTO
SOC
Maireana oppositifolia
Climate
Climate + Soil
Soil
AUC = 0.94
TSS = 0.69
AUC = 0.95
TSS = 0.68
AUC = 0.91
TSS = 0.55
Relative importance
0.00
0.25
0.50
0.75
1.00
0.0
0.2
0.4
0.6
0.8
bio01
bio04
bio05
bio06
bio12
BDW
CEC
CLY
SOC
TWI
bio01
bio04
bio05
bio06
bio12
BDW
CEC
CLY
SOC
TWI

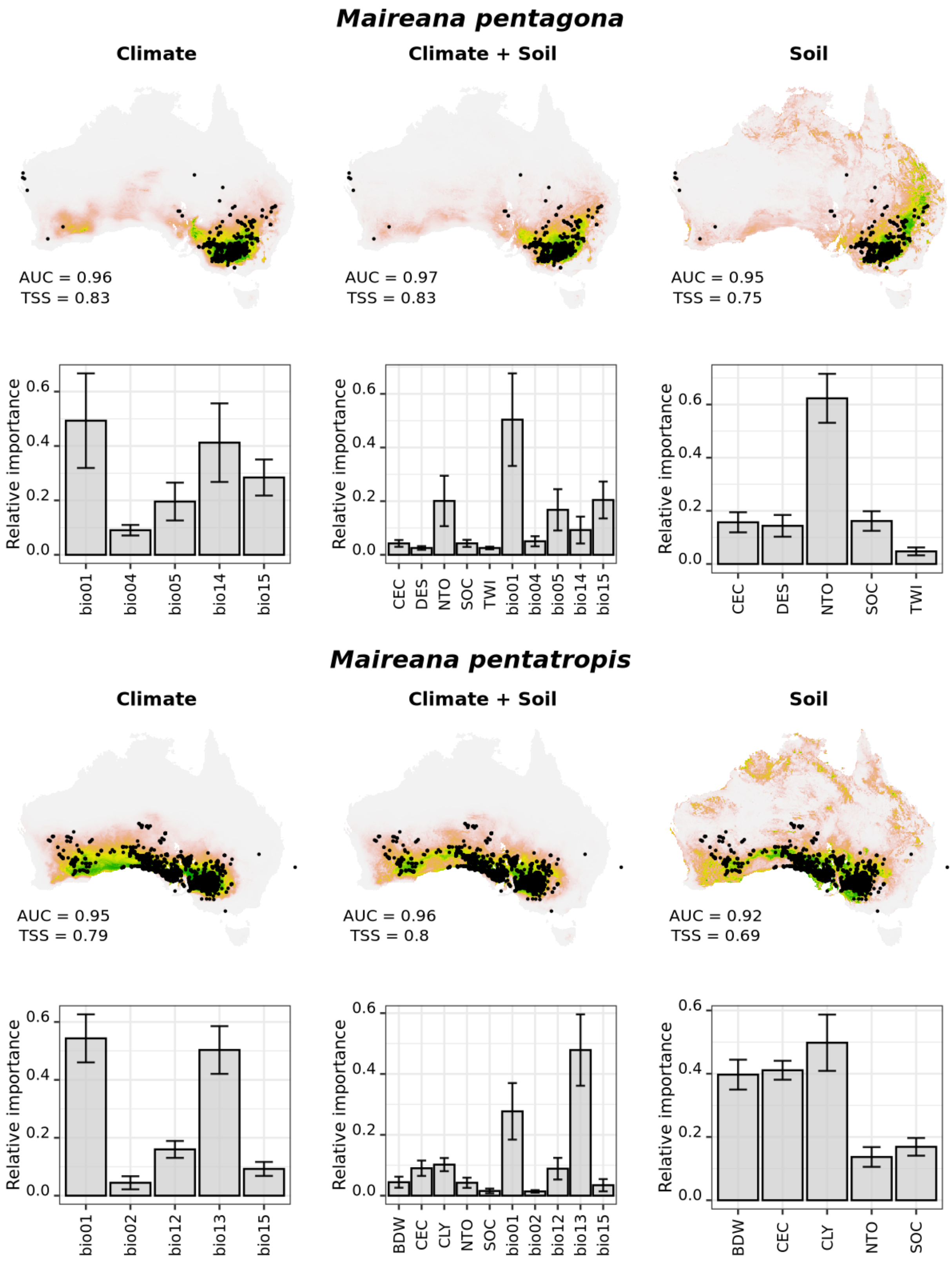
Maireana pentagona
Climate
Climate + Soil
Soil
AUC = 0.96
TSS = 0.83
AUC = 0.97
TSS = 0.83
AUC = 0.95
TSS = 0.75
Relative importance
0.0
0.2
0.4
0.6
bio01
bio04
bio05
bio14
bio15
CEC
DES
NTO
SOC
TWI
Maireana pentatropis
AUC = 0.95
TSS = 0.79
AUC = 0.96
TSS = 0.8
AUC = 0.92
TSS = 0.69
bio02
bio12
bio13
BDW
CLY

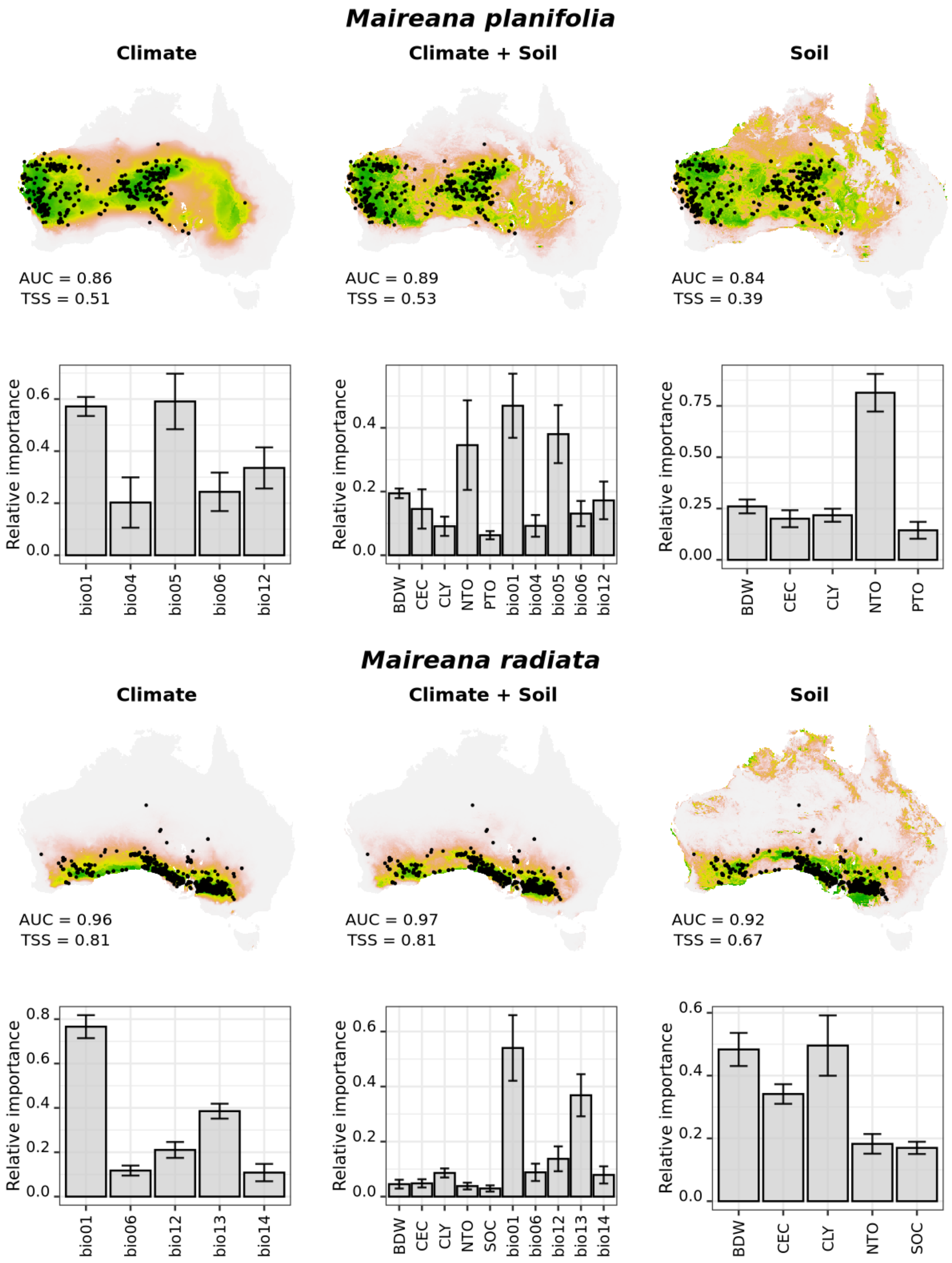
Maireana planifolia
Climate
Climate + Soil
Soil
AUC = 0.86
TSS = 0.51
AUC = 0.89
TSS = 0.53
AUC = 0.84
TSS = 0.39
Relative importance
bio01
bio04
bio05
bio06
bio12
BDW
CEC
CLY
NTO
PTO
Maireana radiata
Climate
Climate + Soil
Soil
AUC = 0.96
TSS = 0.81
AUC = 0.97
TSS = 0.81
AUC = 0.92
TSS = 0.67
Relative importance
bio01
bio06
bio12
bio13
bio14
BDW
CEC
CLY
NTO
SOC

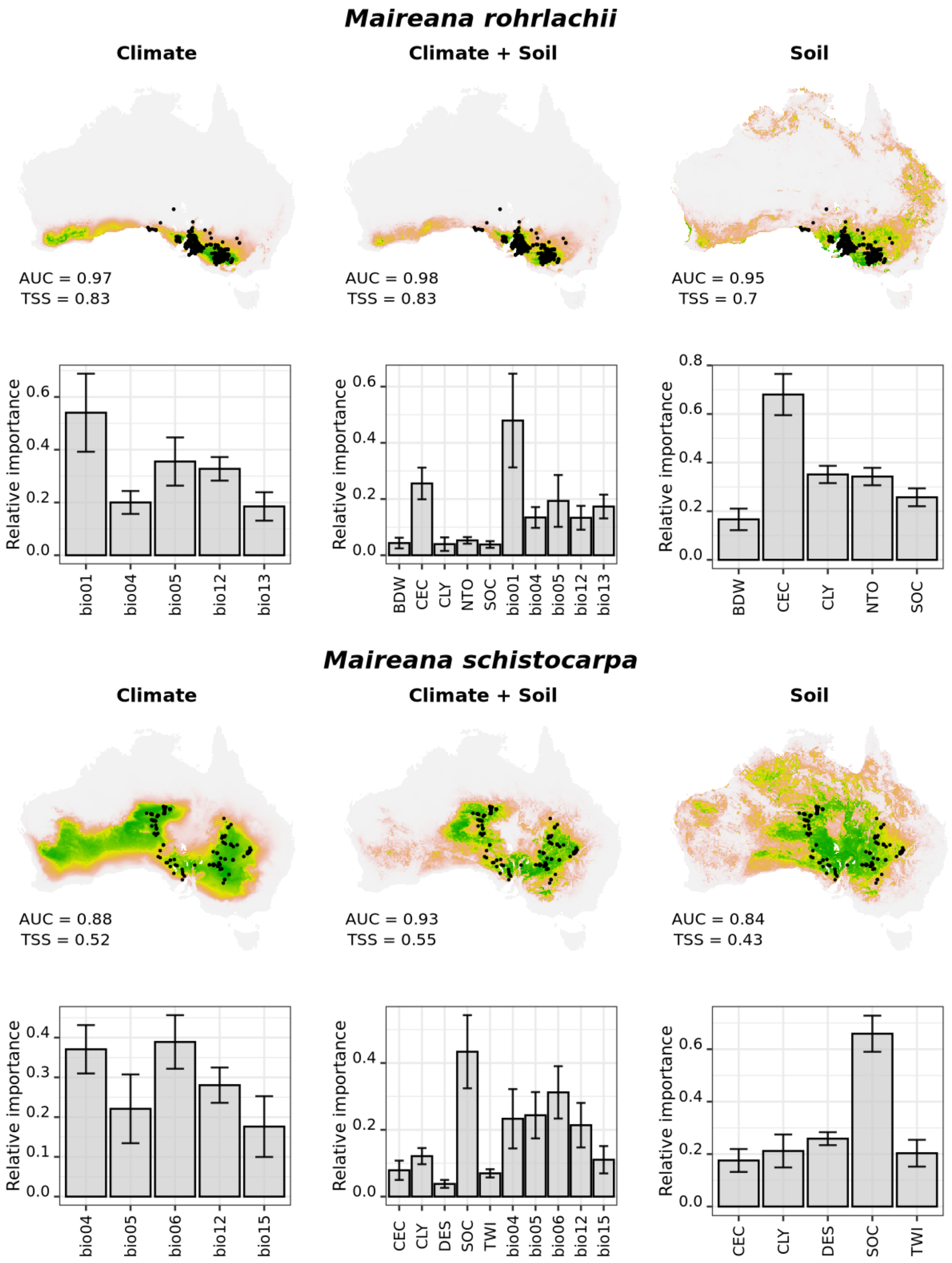
Maireana rohrlachii
Climate
Climate + Soil
Soil
AUC = 0.97
TSS = 0.83
AUC = 0.98
TSS = 0.83
AUC = 0.95
TSS = 0.7
Relative importance
bio01
bio04
bio05
bio12
bio13
BDW
CEC
CLY
NTO
SOC
bio01
bio04
bio05
bio12
bio13
BDW
CEC
CLY
NTO
SOC
Maireana schistocarpa
Climate
Climate + Soil
Soil
AUC = 0.88
TSS = 0.52
AUC = 0.93
TSS = 0.55
AUC = 0.84
TSS = 0.43
Relative importance
bio04
bio05
bio06
bio12
bio15
CEC
CLY
DES
SOC
TWI
bio04
bio05
bio06
bio12
bio15
CEC
CLY
DES
SOC
TWI

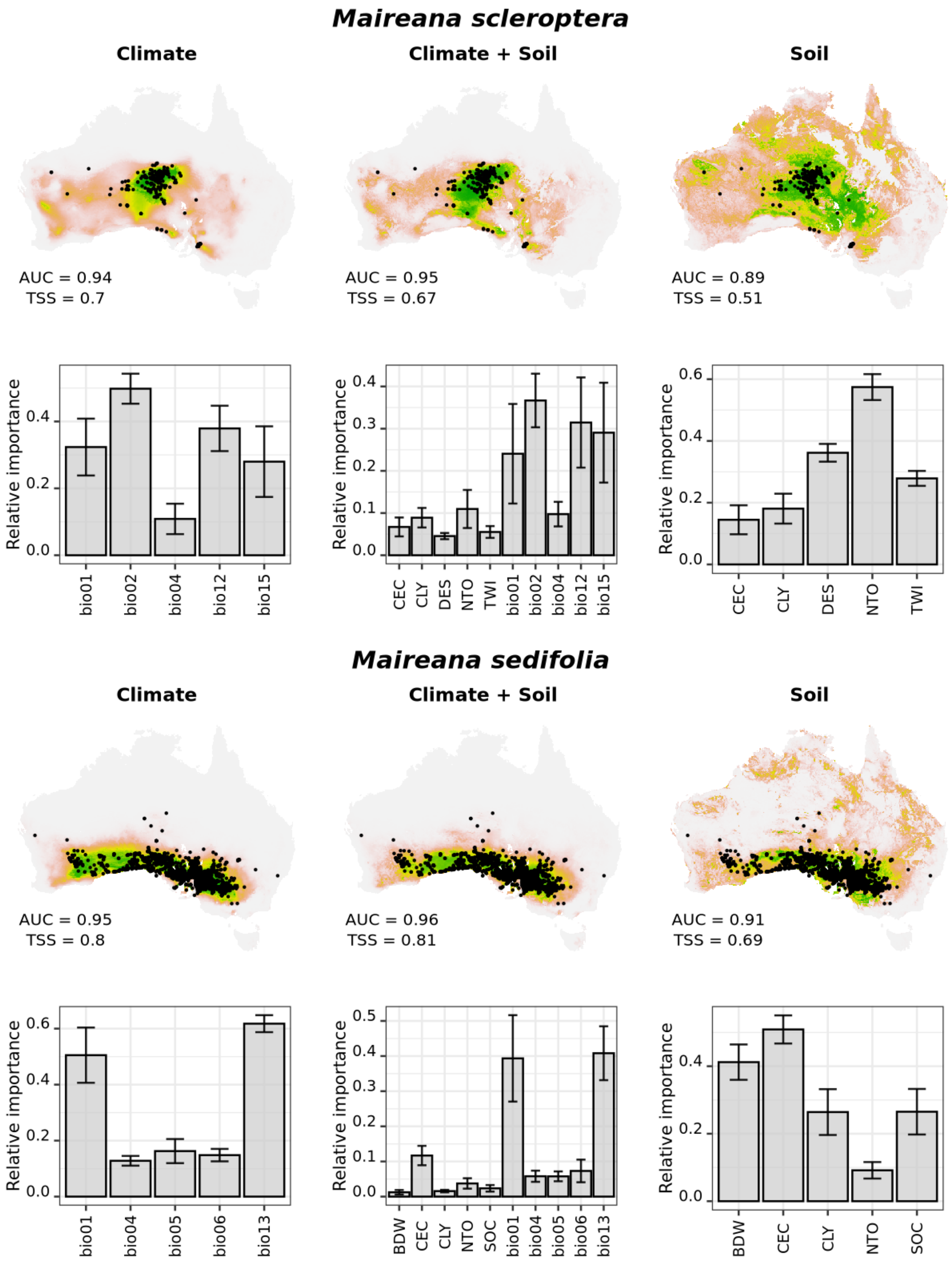

Maireana scleroptera
Climate
Climate + Soil
Soil
AUC = 0.94
TSS = 0.7
AUC = 0.95
TSS = 0.67
AUC = 0.89
TSS = 0.51
Relative importance
bio01
bio02
bio04
bio12
bio15
CEC
CLY
DES
NTO
TWI
Maireana sedifolia
Climate
Climate + Soil
Soil
AUC = 0.95
TSS = 0.8
AUC = 0.96
TSS = 0.81
AUC = 0.91
TSS = 0.69
Relative importance
bio01
bio04
bio05
bio06
bio13
BDW
CEC
CLY
NTO
SOC

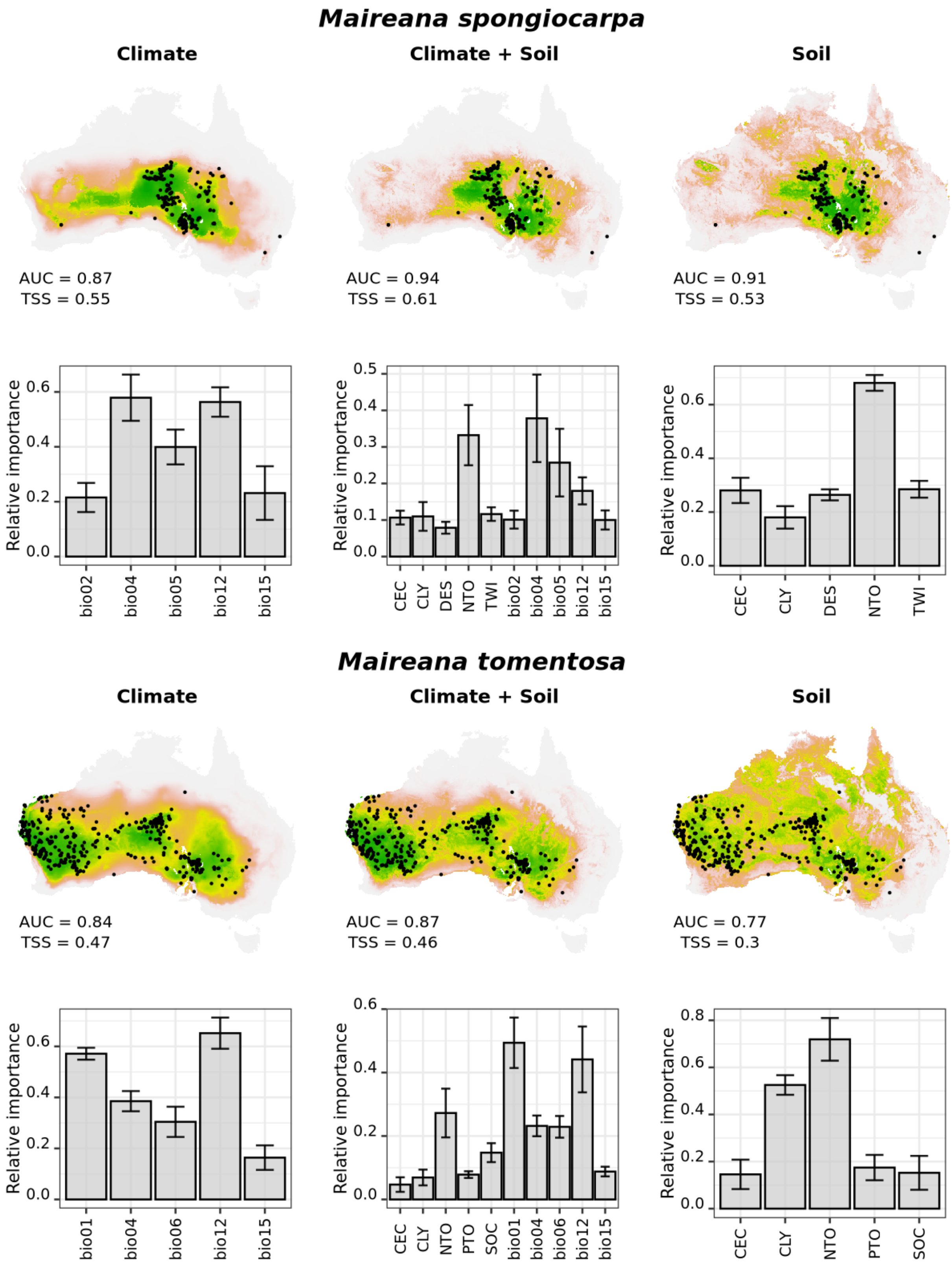

Maireana spongiocarpa
Climate
Climate + Soil
Soil
AUC = 0.87
TSS = 0.55
AUC = 0.94
TSS = 0.61
AUC = 0.91
TSS = 0.53
Relative importance
bio02
bio04
bio05
bio12
bio15
CEC
CLY
DES
NTO
TWI
Maireana tomentosa
Climate
Climate + Soil
Soil
AUC = 0.84
TSS = 0.47
AUC = 0.87
TSS = 0.46
AUC = 0.77
TSS = 0.3
Relative importance
bio01
bio04
bio06
bio12
bio15
CEC
CLY
NTO
PTO
SOC

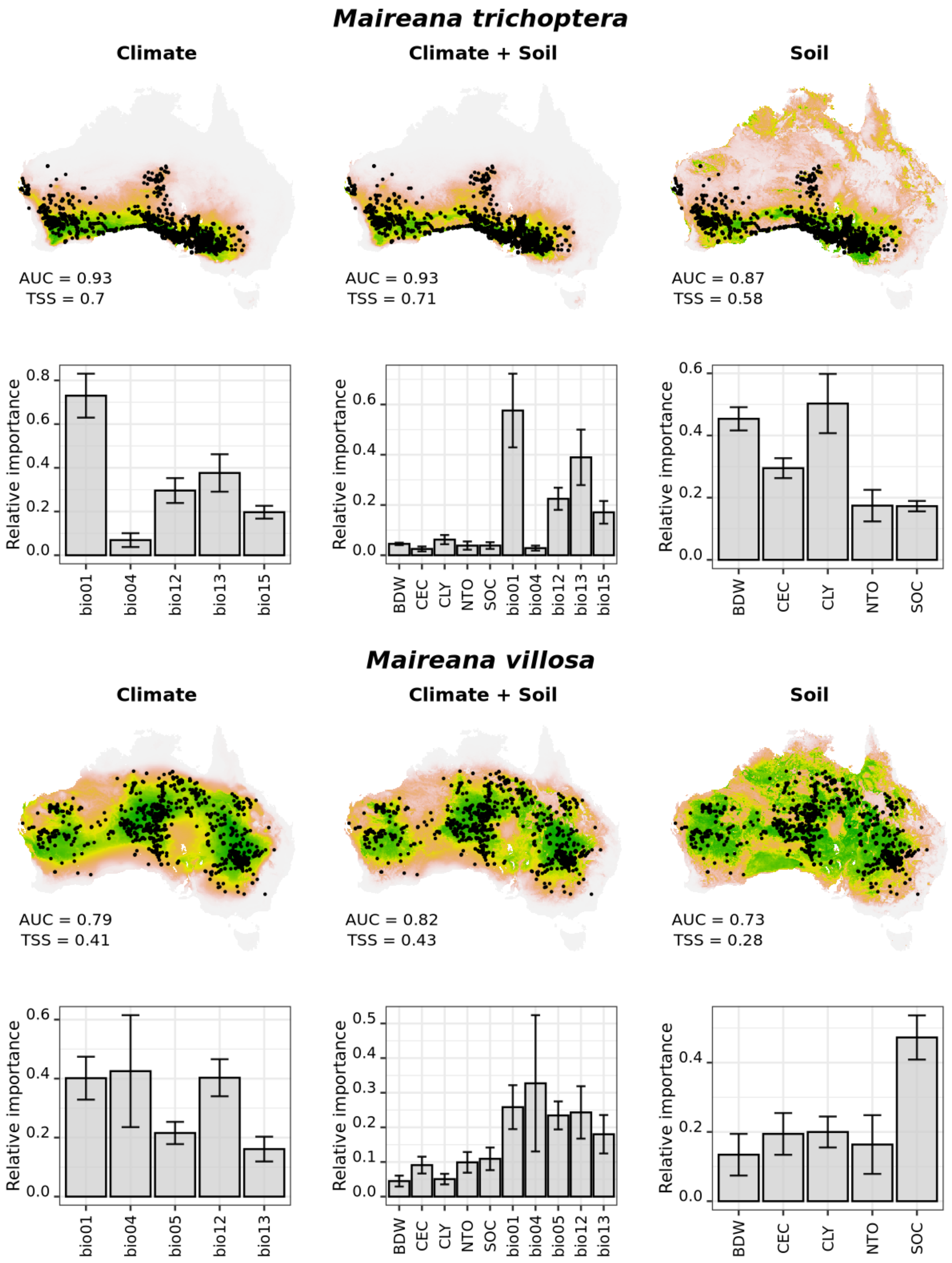
Maireana trichoptera
Climate
Climate + Soil
Soil
AUC = 0.93
TSS = 0.7
AUC = 0.93
TSS = 0.71
AUC = 0.87
TSS = 0.58
Relative importance
0.0 0.2 0.4 0.6 0.8
bio01 bio04 bio12 bio13 bio15
Relative importance
0.0 0.2 0.4 0.6
BDW CEC CLY NTO SOC bio01 bio04 bio12 bio13 bio15
Relative importance
0.0 0.2 0.4 0.6
BDW CEC CLY NTO SOC
Maireana villosa
Climate
Climate + Soil
Soil
AUC = 0.79
TSS = 0.41
AUC = 0.82
TSS = 0.43
AUC = 0.73
TSS = 0.28
Relative importance
0.0 0.2 0.4 0.6
bio01 bio04 bio05 bio12 bio13
Relative importance
0.0 0.1 0.2 0.3 0.4 0.5
BDW CEC CLY NTO SOC bio01 bio04 bio05 bio12 bio13
Relative importance
0.0 0.2 0.4
BDW CEC CLY NTO SOC

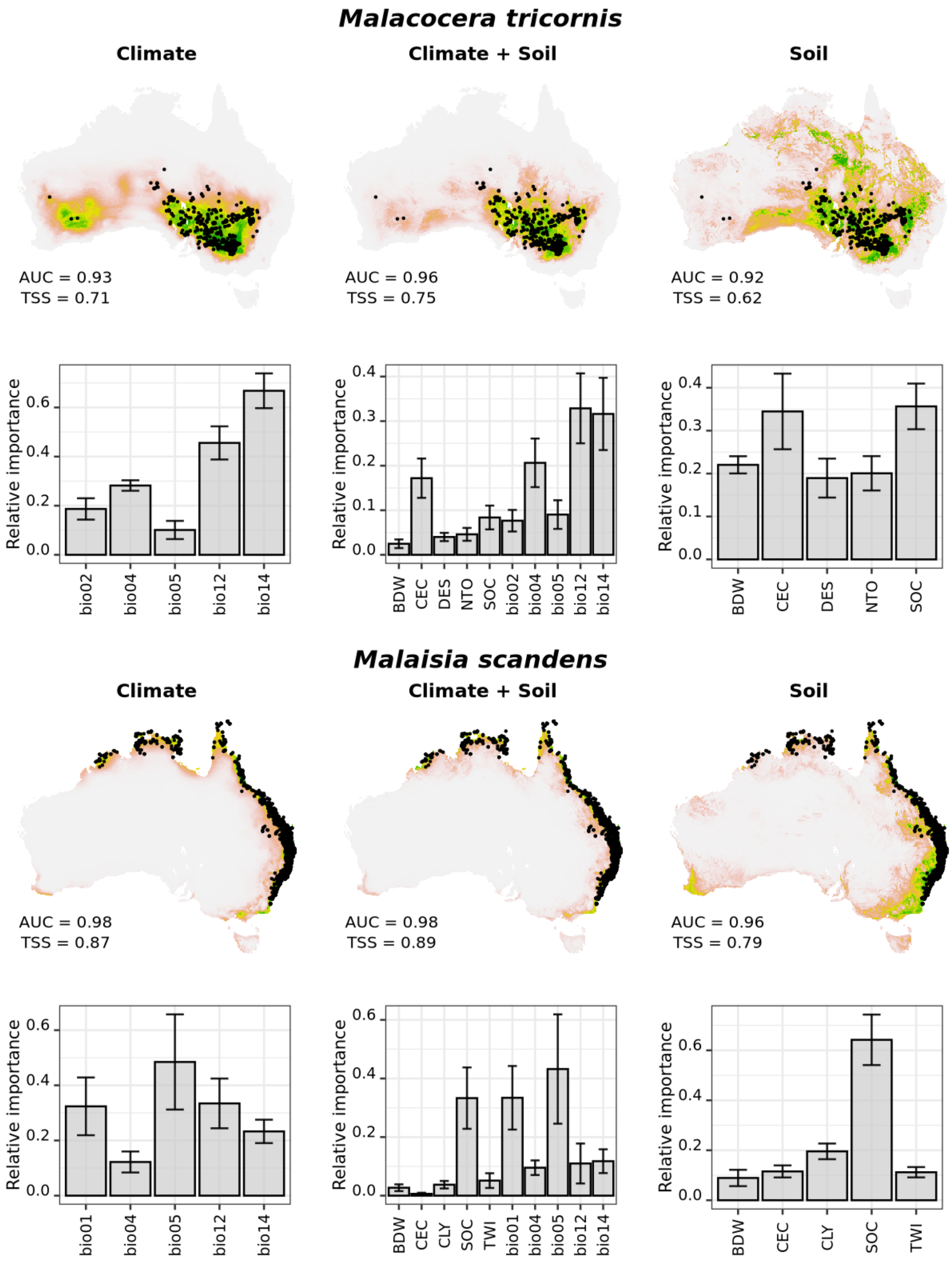

Malacocera tricornis
Climate
Climate + Soil
Soil
AUC = 0.93
TSS = 0.71
AUC = 0.96
TSS = 0.75
AUC = 0.92
TSS = 0.62
Relative importance
bio02
bio04
bio05
bio12
bio14
BDW
CEC
DES
NTO
SOC
bio02
bio04
bio05
bio12
bio14
BDW
CEC
DES
NTO
SOC
Malaisia scandens
Climate
Climate + Soil
Soil
AUC = 0.98
TSS = 0.87
AUC = 0.98
TSS = 0.89
AUC = 0.96
TSS = 0.79
Relative importance
bio01
bio04
bio05
bio12
bio14
BDW
CEC
CLY
SOC
TWI
bio01
bio04
bio05
bio12
bio14
BDW
CEC
CLY
SOC
TWI

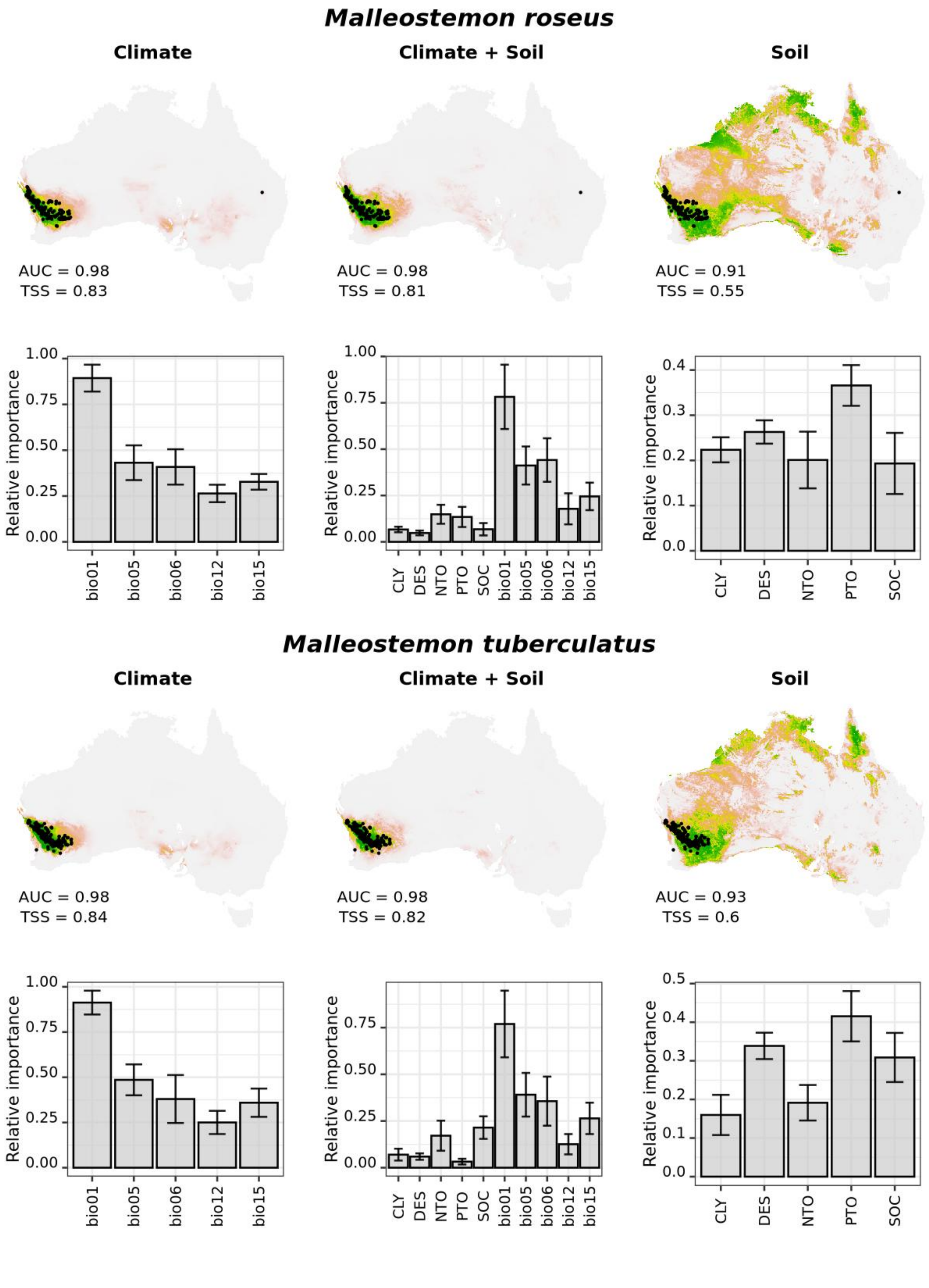

Malleostemon roseus
Climate
Climate + Soil
Soil
AUC = 0.98
TSS = 0.83
AUC = 0.98
TSS = 0.81
AUC = 0.91
TSS = 0.55
Relative importance
bio01
bio05
bio06
bio12
bio15
CLY
DES
NTO
PTO
SOC
Malleostemon tuberculatus
Climate
Climate + Soil
Soil
AUC = 0.98
TSS = 0.84
AUC = 0.98
TSS = 0.82
AUC = 0.93
TSS = 0.6

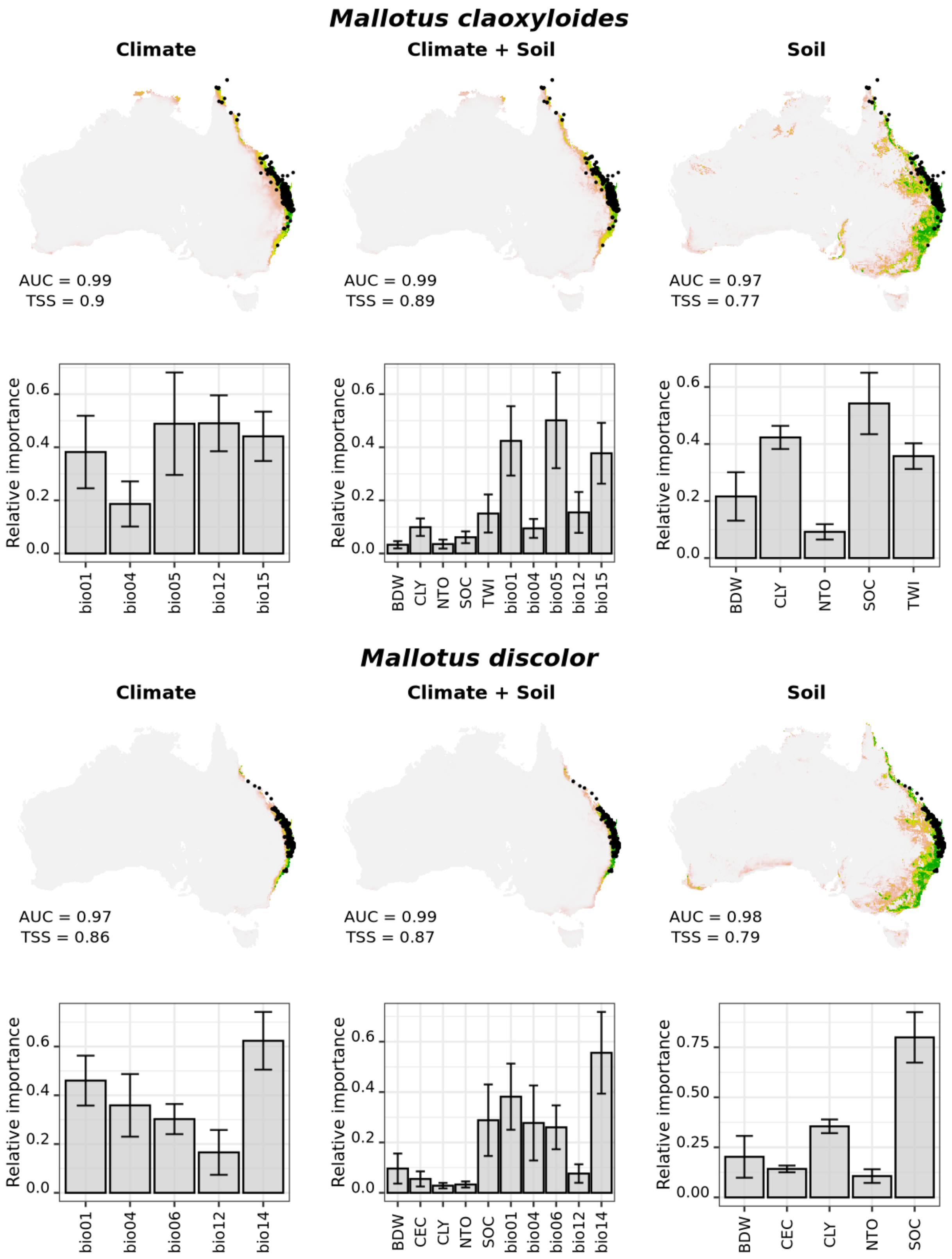

Mallotus claoxyloides
Climate
Climate + Soil
Soil
AUC = 0.99
TSS = 0.9
AUC = 0.99
TSS = 0.89
AUC = 0.97
TSS = 0.77
Relative importance
0.0
0.2
0.4
0.6
bio01
bio04
bio05
bio12
bio15
BDW
CLY
NTO
SOC
TWI
Mallotus discolor
AUC = 0.97
TSS = 0.86
AUC = 0.99
TSS = 0.87
AUC = 0.98
TSS = 0.79
bio06
bio14
CEC
0.00
0.25
0.50
0.75

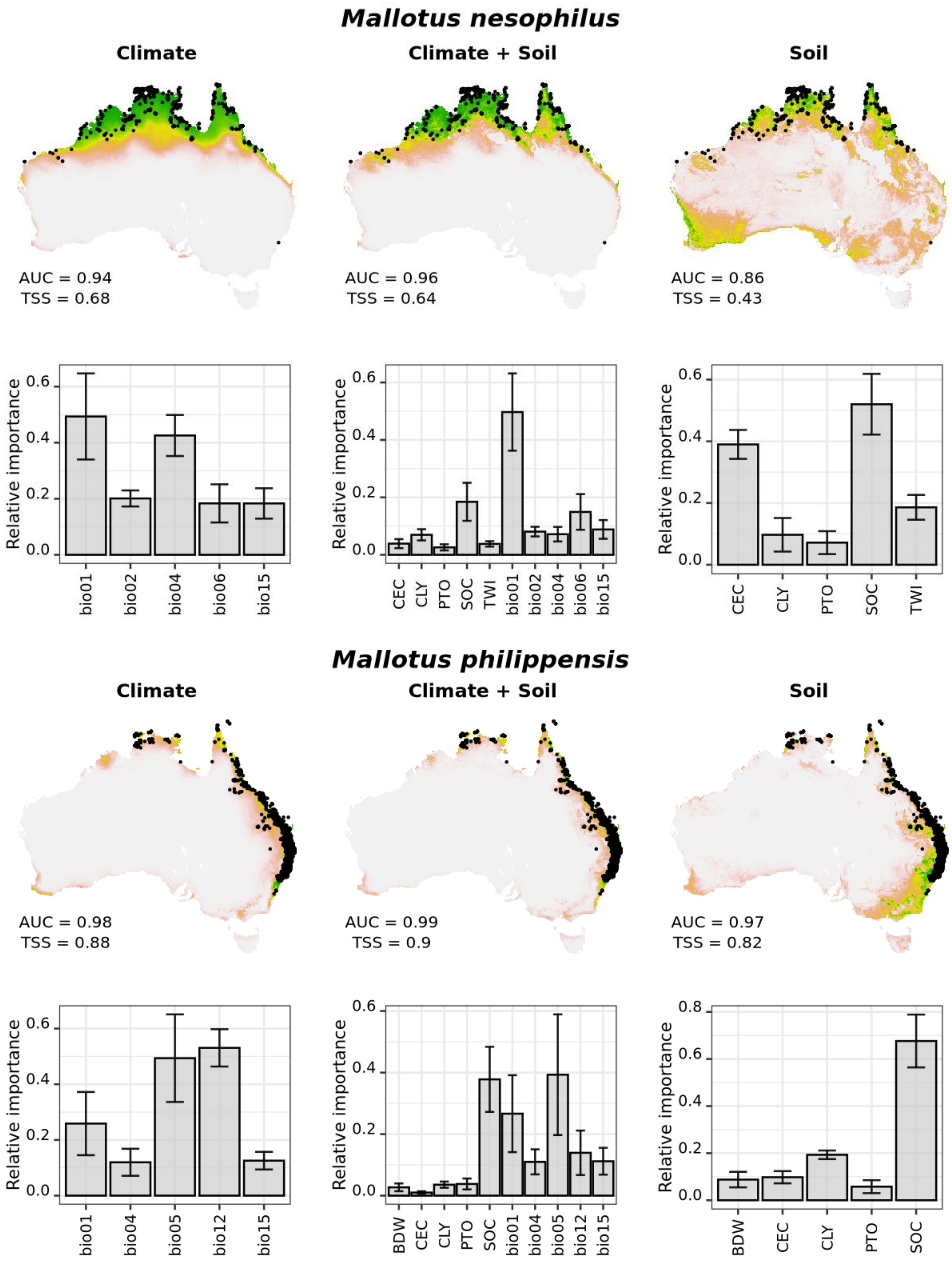

Mallotus nesophilus
Climate
Climate + Soil
Soil
AUC = 0.94
TSS = 0.68
AUC = 0.96
TSS = 0.64
AUC = 0.86
TSS = 0.43
Relative importance
bio01
bio02
bio04
bio06
bio15
CEC
CLY
PTO
SOC
TWI
Mallotus philippensis
Climate
Climate + Soil
Soil
AUC = 0.98
TSS = 0.88
AUC = 0.99
TSS = 0.9
AUC = 0.97
TSS = 0.82
Relative importance
bio01
bio04
bio05
bio12
bio15
BDW
CEC
CLY
PTO
SOC

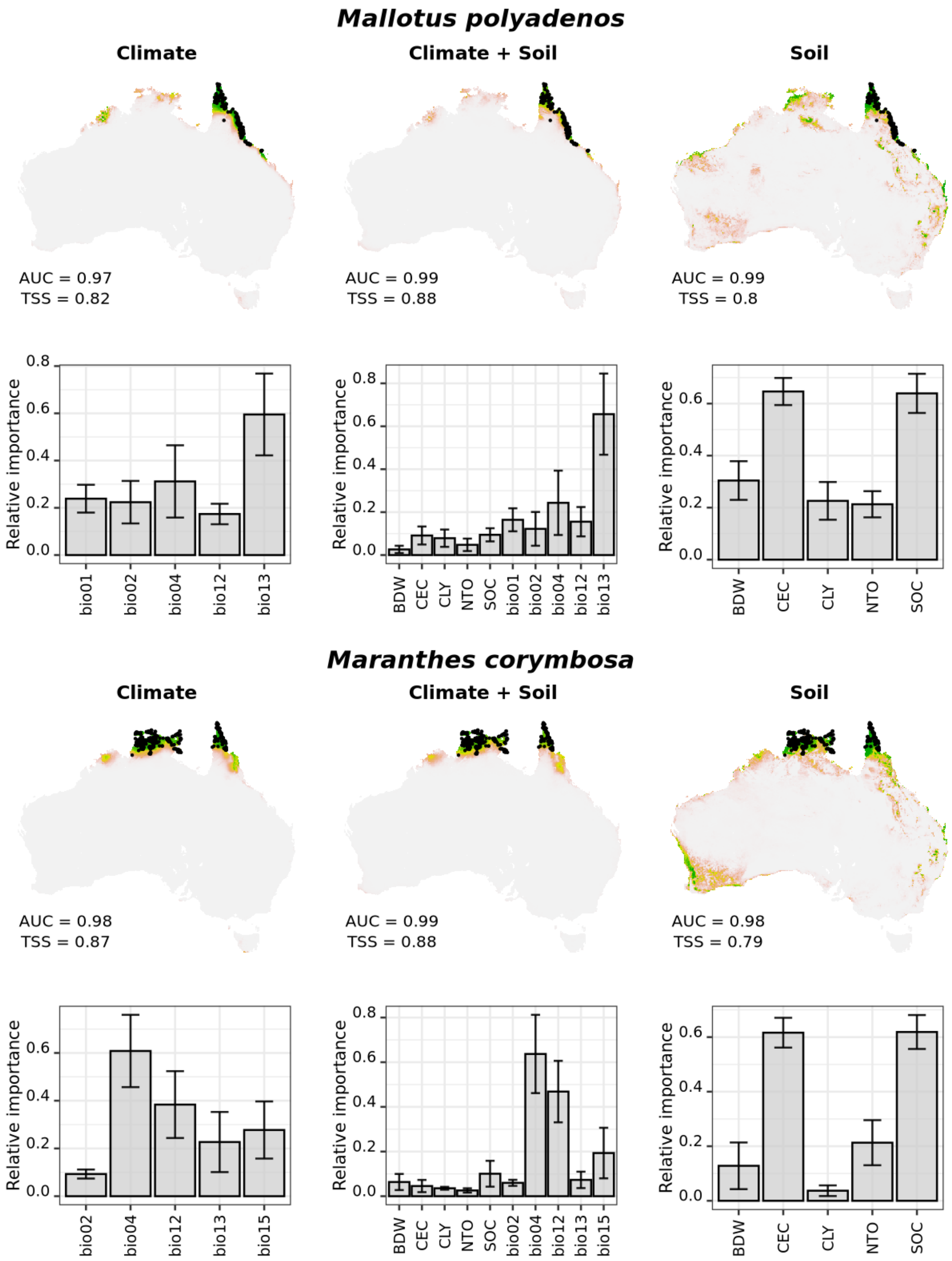

Mallotus polyadenos
Climate
Climate + Soil
Soil
AUC = 0.97
TSS = 0.82
AUC = 0.99
TSS = 0.88
AUC = 0.99
TSS = 0.8
Relative importance
0.0
0.2
0.4
0.6
0.8
bio01
bio02
bio04
bio12
bio13
BDW
CEC
CLY
NTO
SOC
Maranthes corymbosa
Climate
Climate + Soil
Soil
AUC = 0.98
TSS = 0.87
AUC = 0.99
TSS = 0.88
AUC = 0.98
TSS = 0.79
bio02
bio04
bio12
bio13
bio15

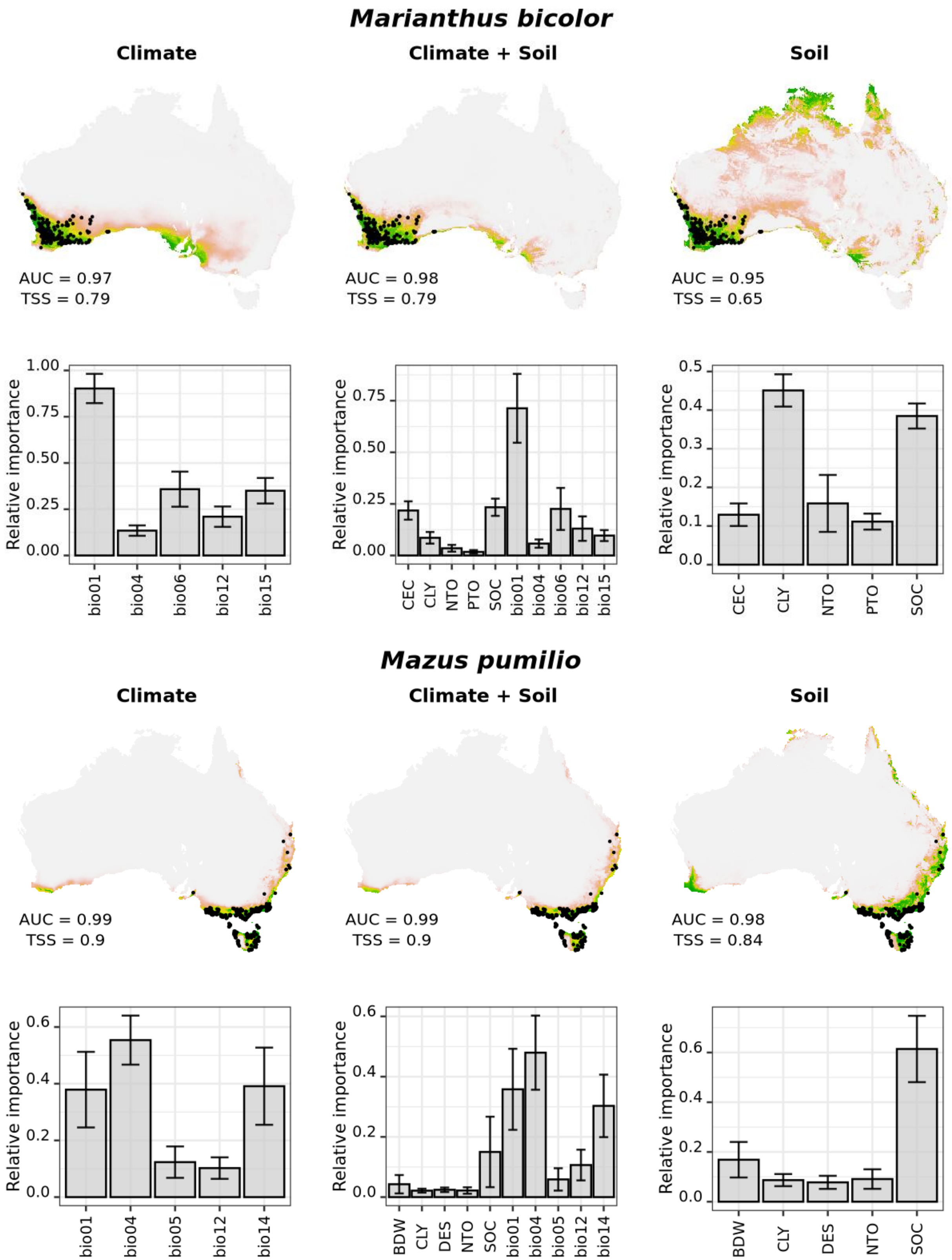
Marianthus bicolor
Climate
Climate + Soil
Soil
AUC = 0.97
TSS = 0.79
AUC = 0.98
TSS = 0.79
AUC = 0.95
TSS = 0.65
Relative importance
1.00
0.75
0.50
0.25
0.00
bio01
bio04
bio06
bio12
bio15
CEC
CLY
NTO
PTO
SOC
bio01
bio04
bio06
bio12
bio15
0.5
0.4
0.3
0.2
0.1
0.0
CEC
CLY
NTO
PTO
SOC
Mazus pumilio
Climate
Climate + Soil
Soil
AUC = 0.99
TSS = 0.9
AUC = 0.99
TSS = 0.9
AUC = 0.98
TSS = 0.84
Relative importance
0.6
0.4
0.2
0.0
bio01
bio04
bio05
bio12
bio14
BDW
CLY
DES
NTO
SOC
bio01
bio04
bio05
bio12
bio14
BDW
CLY
DES
NTO
SOC

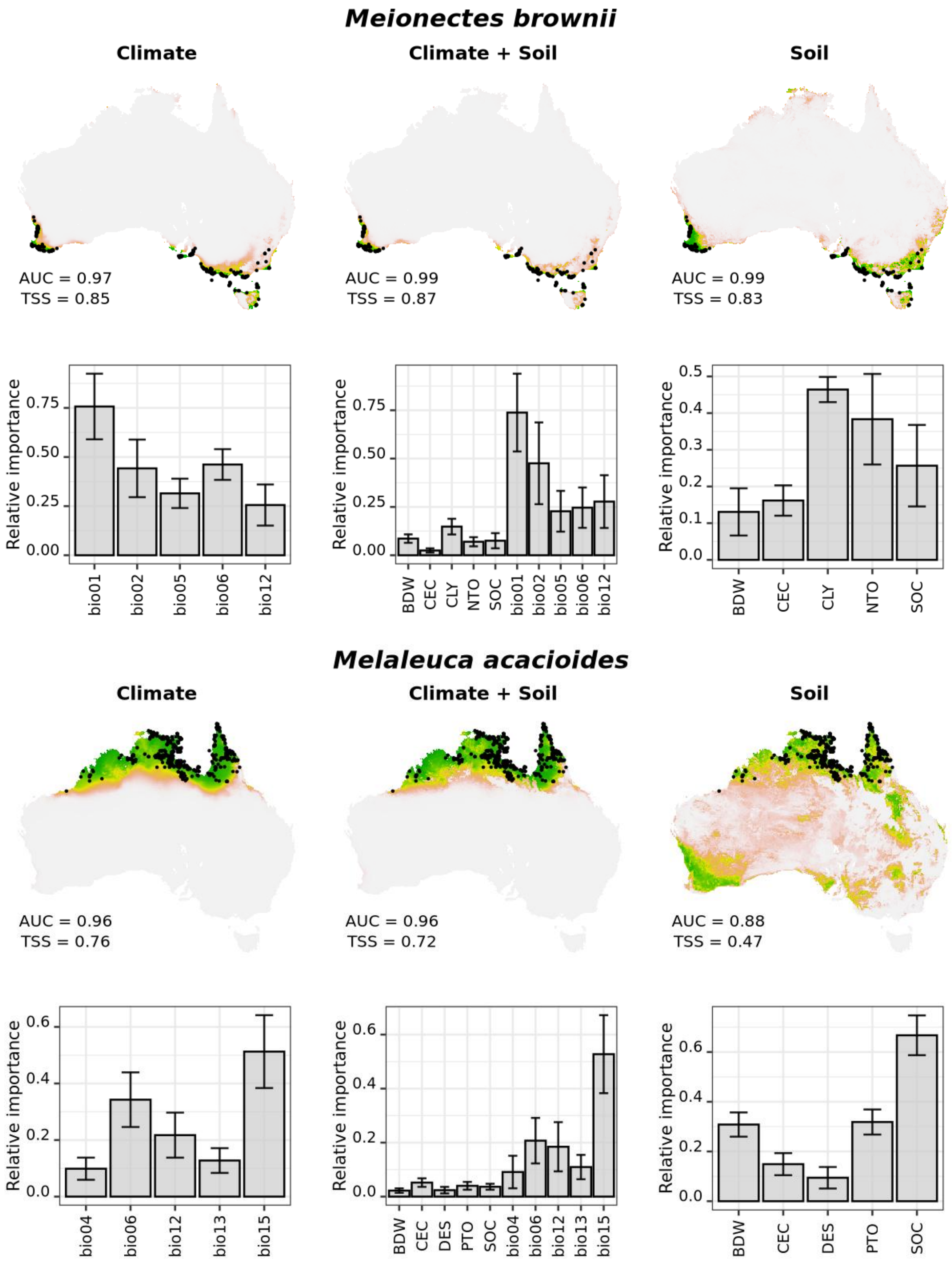
Meionectes brownii
Climate
Climate + Soil
Soil
AUC = 0.97
TSS = 0.85
AUC = 0.99
TSS = 0.87
AUC = 0.99
TSS = 0.83
Relative importance
0.00
0.25
0.50
0.75
bio01
bio02
bio05
bio06
bio12
BDW
CEC
CLY
NTO
SOC
0.0
0.1
0.2
0.3
0.4
0.5
Melaleuca acacioides
Climate
Climate + Soil
Soil
AUC = 0.96
TSS = 0.76
AUC = 0.96
TSS = 0.72
AUC = 0.88
TSS = 0.47
0.0
0.2
0.4
0.6
bio04
bio06
bio12
bio13
bio15
BDW
CEC
DES
PTO
SOC

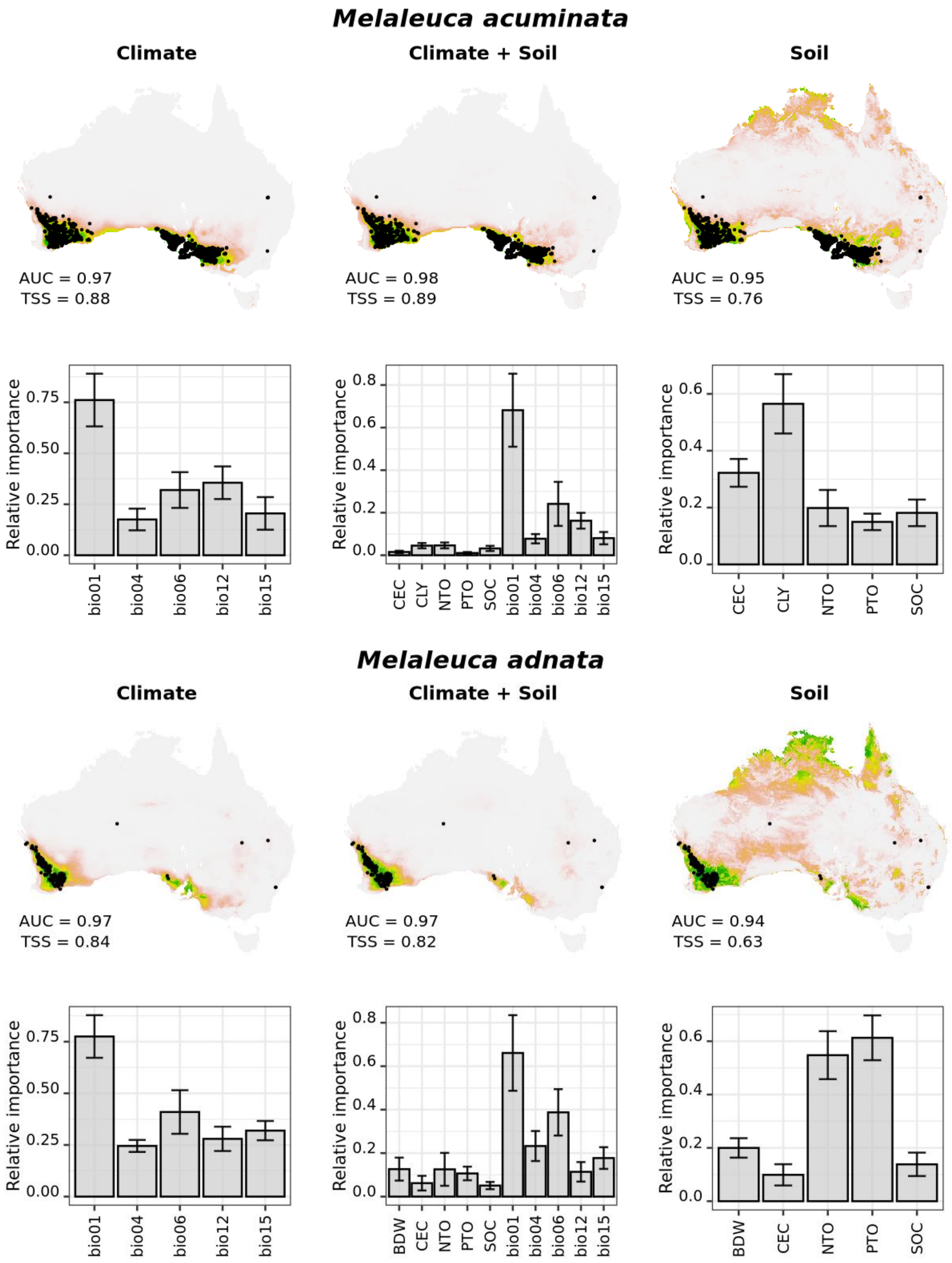
Melaleuca acuminata
Climate
Climate + Soil
Soil
AUC = 0.97
TSS = 0.88
AUC = 0.98
TSS = 0.89
AUC = 0.95
TSS = 0.76
Relative importance
0.00
0.25
0.50
0.75
bio01
bio04
bio06
bio12
bio15
0.0
0.2
0.4
0.6
0.8
CEC
CLY
NTO
PTO
SOC
Melaleuca adnata
Climate
Climate + Soil
Soil
AUC = 0.97
TSS = 0.84
AUC = 0.97
TSS = 0.82
AUC = 0.94
TSS = 0.63
BDW
CEC
NTO
PTO
SOC

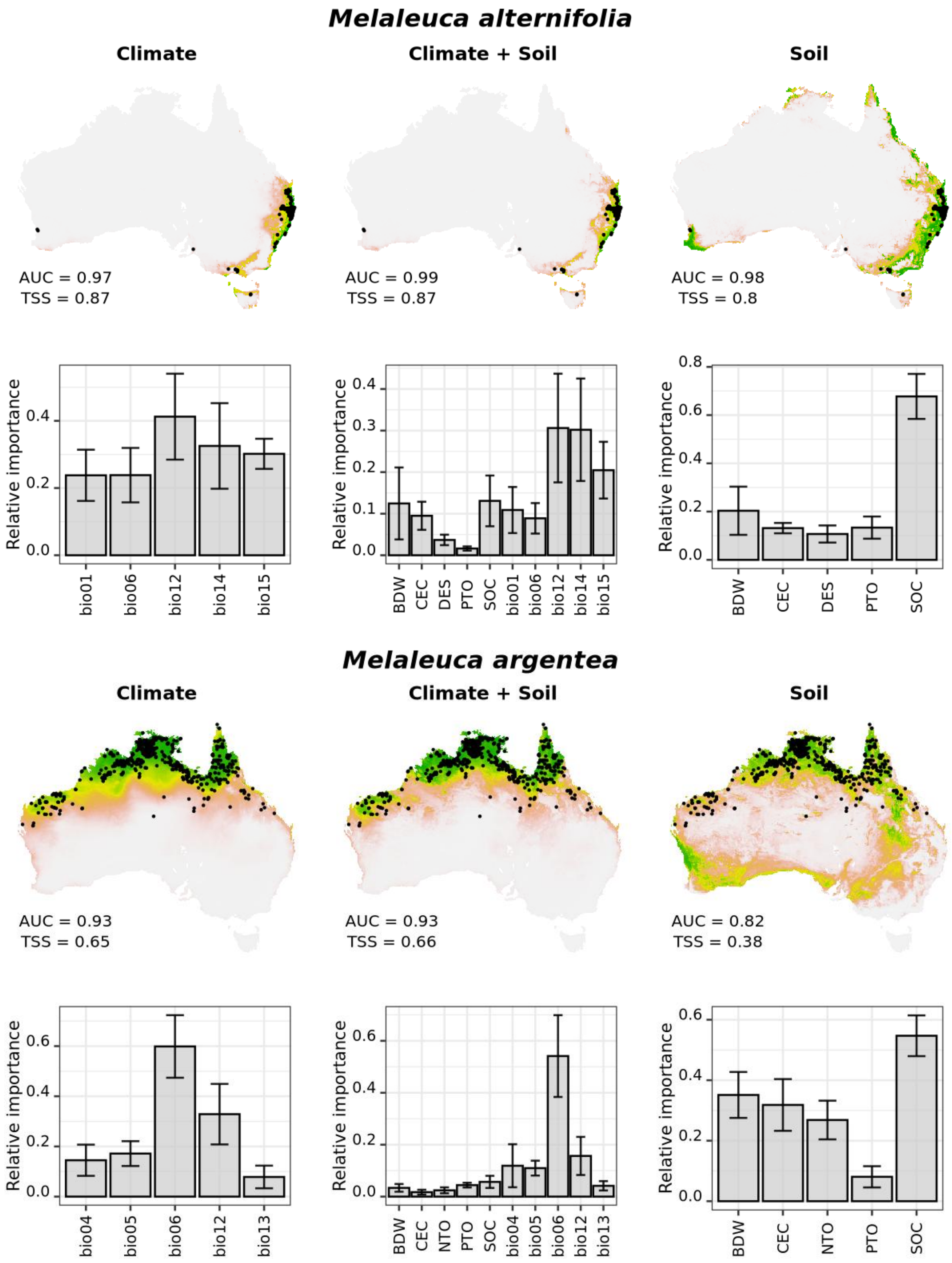
Melaleuca alternifolia
Climate
Climate + Soil
Soil
AUC = 0.97
TSS = 0.87
AUC = 0.99
TSS = 0.87
AUC = 0.98
TSS = 0.8
Relative importance
bio01
bio06
bio12
bio14
bio15
BDW
CEC
DES
PTO
SOC
Melaleuca argentea
Climate
Climate + Soil
Soil
AUC = 0.93
TSS = 0.65
AUC = 0.93
TSS = 0.66
AUC = 0.82
TSS = 0.38
Relative importance
bio04
bio05
bio06
bio12
bio13
BDW
CEC
NTO
PTO
SOC

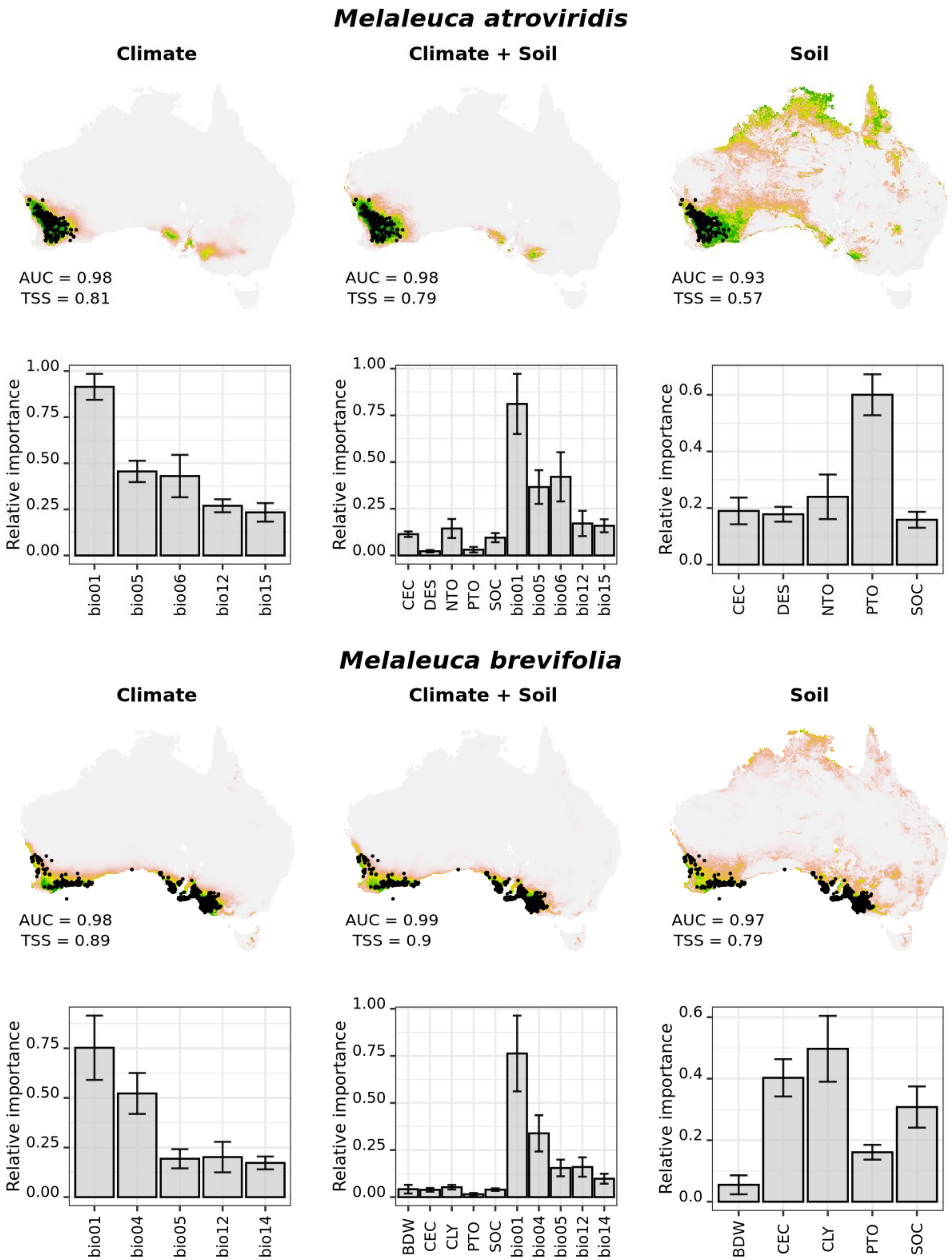

Melaleuca atroviridis
Climate
Climate + Soil
Soil
AUC = 0.98
TSS = 0.81
AUC = 0.98
TSS = 0.79
AUC = 0.93
TSS = 0.57
Relative importance
1.00
0.75
0.50
0.25
0.00
bio01
bio05
bio06
bio12
bio15
CEC
DES
NTO
PTO
SOC
0.6
0.4
0.2
0.0
Melaleuca brevifolia
AUC = 0.98
TSS = 0.89
AUC = 0.99
TSS = 0.9
AUC = 0.97
TSS = 0.79
bio04
bio14
BDW
CLY

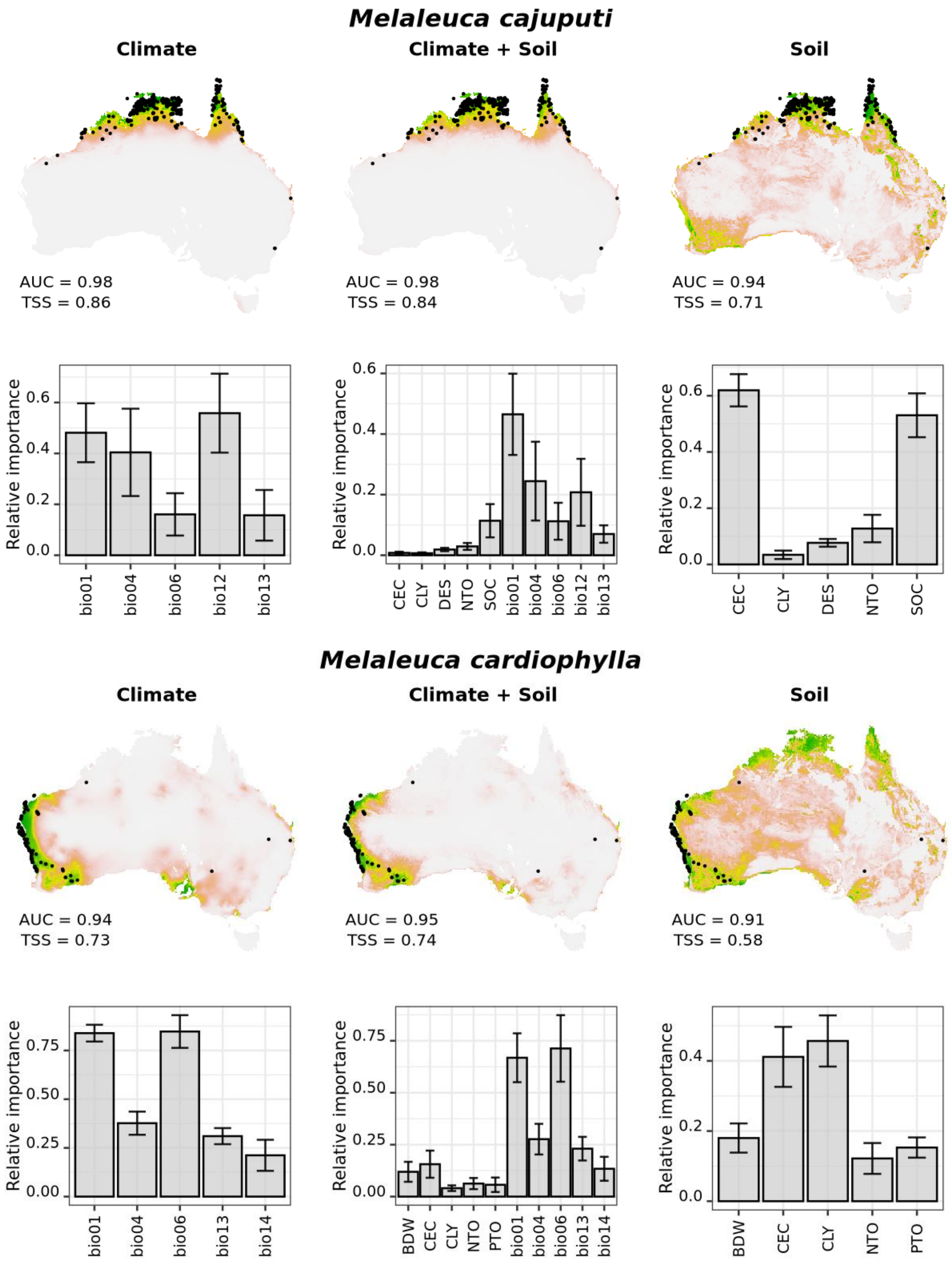
Melaleuca cajuputi
Climate
Climate + Soil
Soil
AUC = 0.98
TSS = 0.86
AUC = 0.98
TSS = 0.84
AUC = 0.94
TSS = 0.71
Relative importance
0.0
0.2
0.4
0.6
bio01
bio04
bio06
bio12
bio13
CEC
CLY
DES
NTO
SOC
Melaleuca cardiophylla
Climate
Climate + Soil
Soil
AUC = 0.94
TSS = 0.73
AUC = 0.95
TSS = 0.74
AUC = 0.91
TSS = 0.58
Relative importance
0.00
0.25
0.50
0.75
bio01
bio04
bio06
bio13
bio14
BDW
CEC
CLY
NTO
PTO

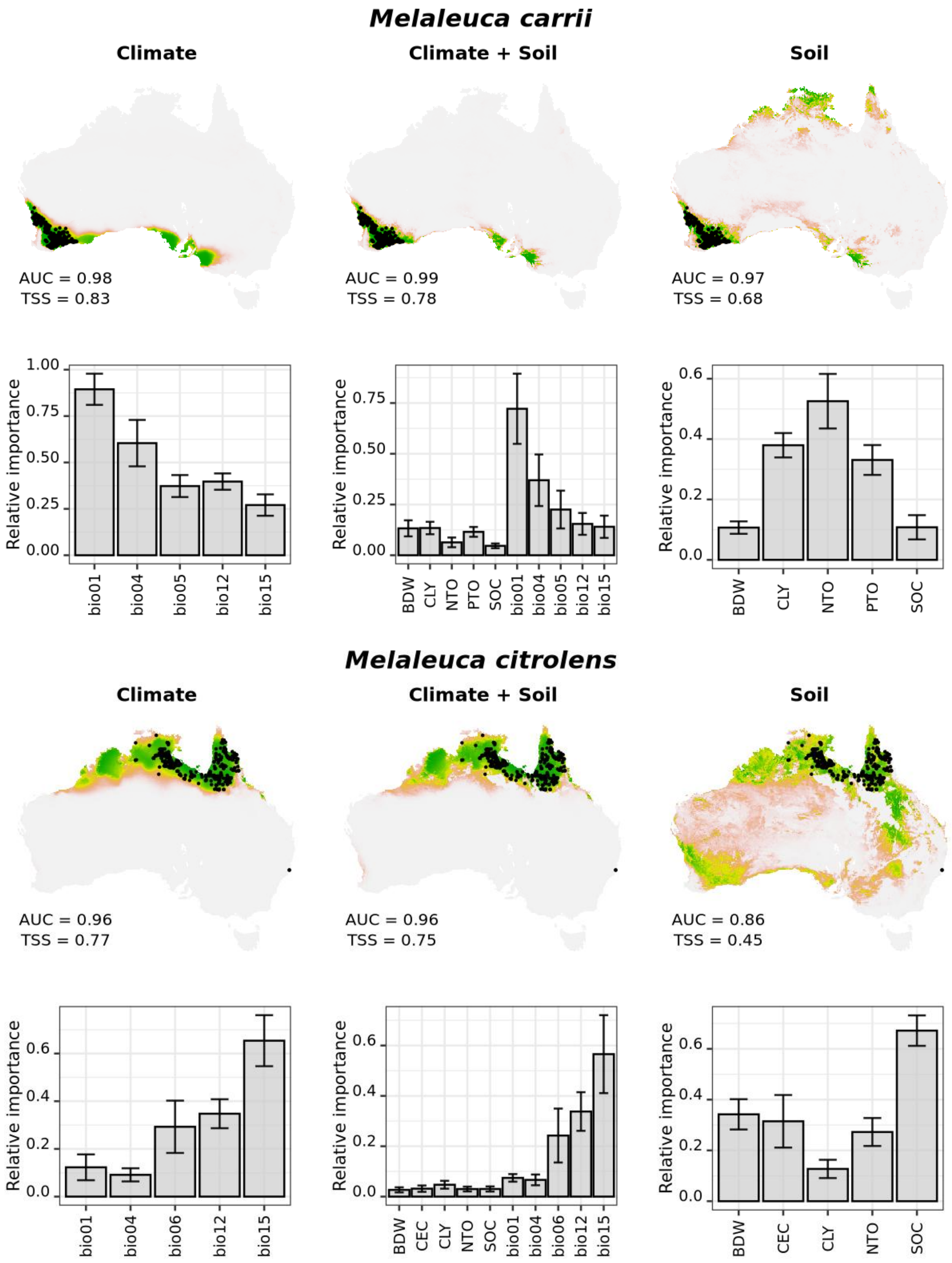

Melaleuca carrii
Climate
Climate + Soil
Soil
AUC = 0.98
TSS = 0.83
AUC = 0.99
TSS = 0.78
AUC = 0.97
TSS = 0.68
Relative importance
1.00
0.75
0.50
0.25
0.00
bio01
bio04
bio05
bio12
bio15
BDW
CLY
NTO
PTO
SOC
0.6
0.4
0.2
0.0
Melaleuca citrolens
Climate
Climate + Soil
Soil
AUC = 0.96
TSS = 0.77
AUC = 0.96
TSS = 0.75
AUC = 0.86
TSS = 0.45
bio06
CEC

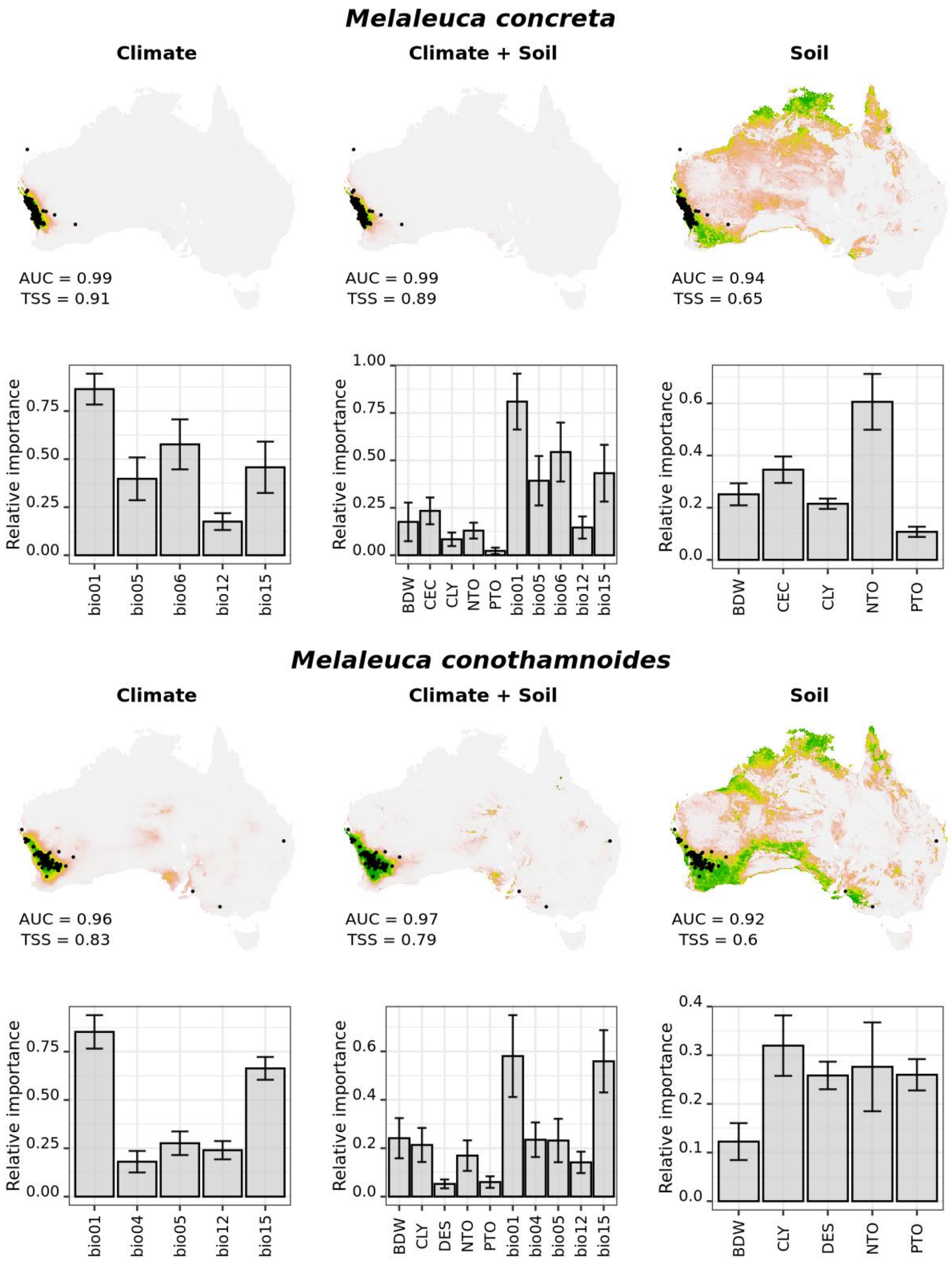
Melaleuca concreta
Climate
Climate + Soil
Soil
AUC = 0.99
TSS = 0.91
AUC = 0.99
TSS = 0.89
AUC = 0.94
TSS = 0.65
Relative importance
bio01
bio05
bio06
bio12
bio15
BDW
CEC
CLY
NTO
PTO
Melaleuca conothamnoides
Climate
Climate + Soil
Soil
AUC = 0.96
TSS = 0.83
AUC = 0.97
TSS = 0.79
AUC = 0.92
TSS = 0.6
Relative importance
bio01
bio04
bio05
bio12
bio15
BDW
CLY
DES
NTO
PTO

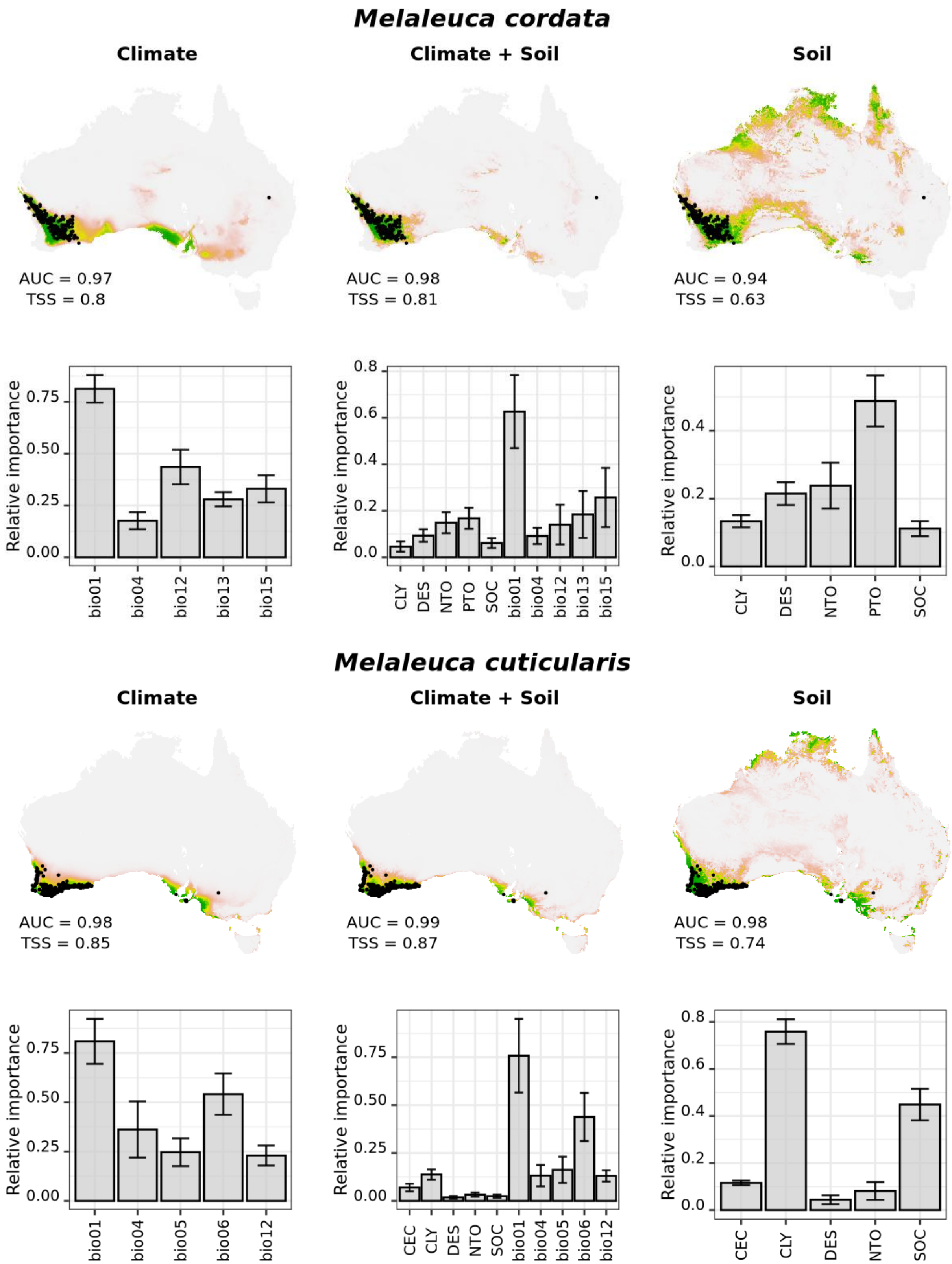
Melaleuca cordata
Climate
Climate + Soil
Soil
AUC = 0.97
TSS = 0.8
AUC = 0.98
TSS = 0.81
AUC = 0.94
TSS = 0.63
Relative importance
0.00
0.25
0.50
0.75
bio01
bio04
bio12
bio13
bio15
0.0
0.2
0.4
0.6
0.8
CLY
DES
NTO
PTO
SOC
bio01
bio04
bio12
bio13
bio15
0.0
0.2
0.4
CLY
DES
NTO
PTO
SOC
Melaleuca cuticularis
Climate
Climate + Soil
Soil
AUC = 0.98
TSS = 0.85
AUC = 0.99
TSS = 0.87
AUC = 0.98
TSS = 0.74
Relative importance
0.00
0.25
0.50
0.75
bio01
bio04
bio05
bio06
bio12
CEC
CLY
DES
NTO
SOC
bio01
bio04
bio05
bio06
bio12
0.0
0.2
0.4
0.6
0.8
CEC
CLY
DES
NTO
SOC

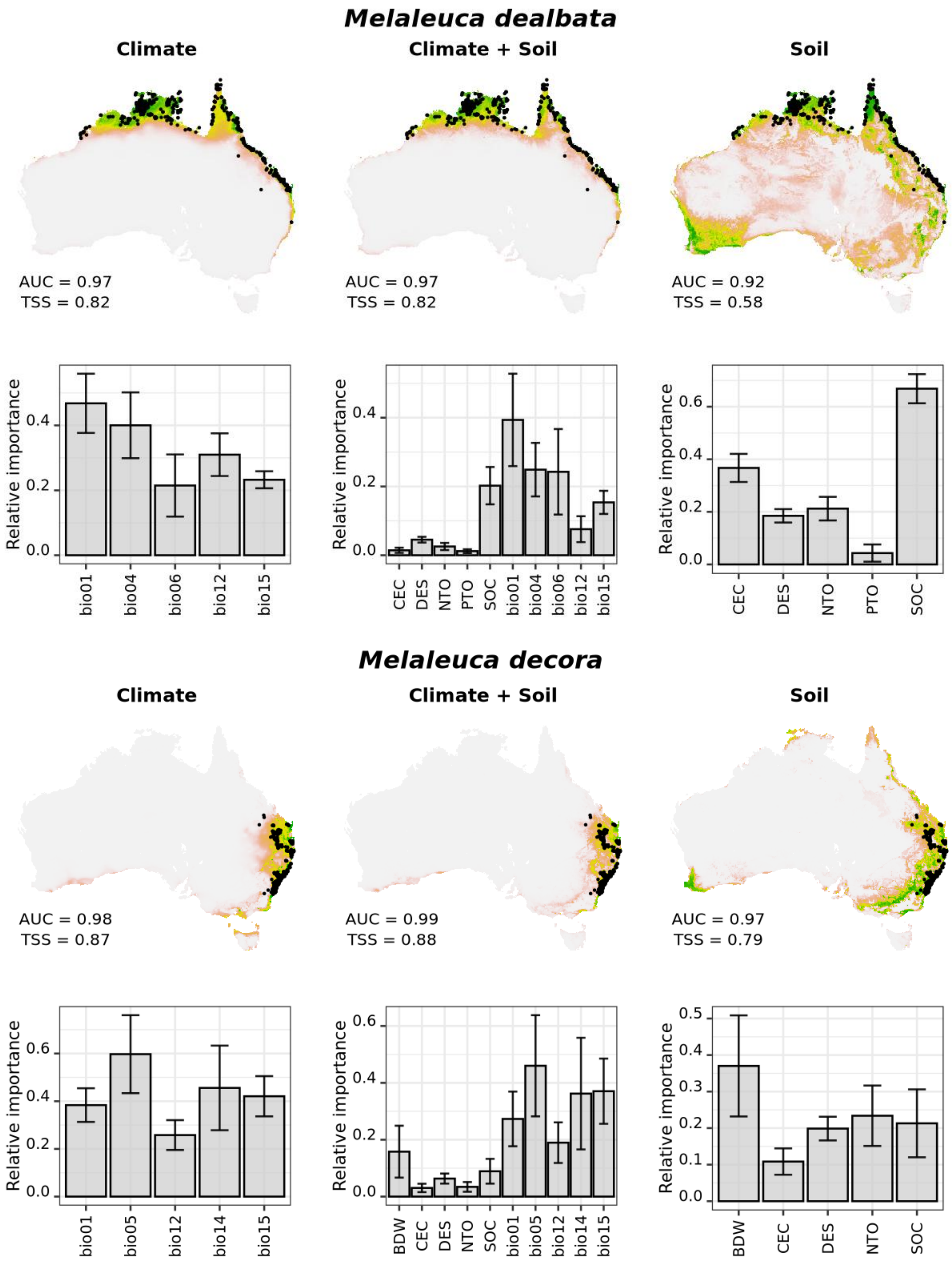

Melaleuca dealbata
Climate
Climate + Soil
Soil
AUC = 0.97
TSS = 0.82
AUC = 0.97
TSS = 0.82
AUC = 0.92
TSS = 0.58
Relative importance
bio01 bio04 bio06 bio12 bio15
CEC DES NTO PTO SOC bio01 bio04 bio06 bio12 bio15
CEC DES NTO PTO SOC
Melaleuca decora
Climate
Climate + Soil
Soil
AUC = 0.98
TSS = 0.87
AUC = 0.99
TSS = 0.88
AUC = 0.97
TSS = 0.79
bio01 bio05 bio12 bio14 bio15
BDW CEC DES NTO SOC bio01 bio05 bio12 bio14 bio15
BDW CEC DES NTO SOC

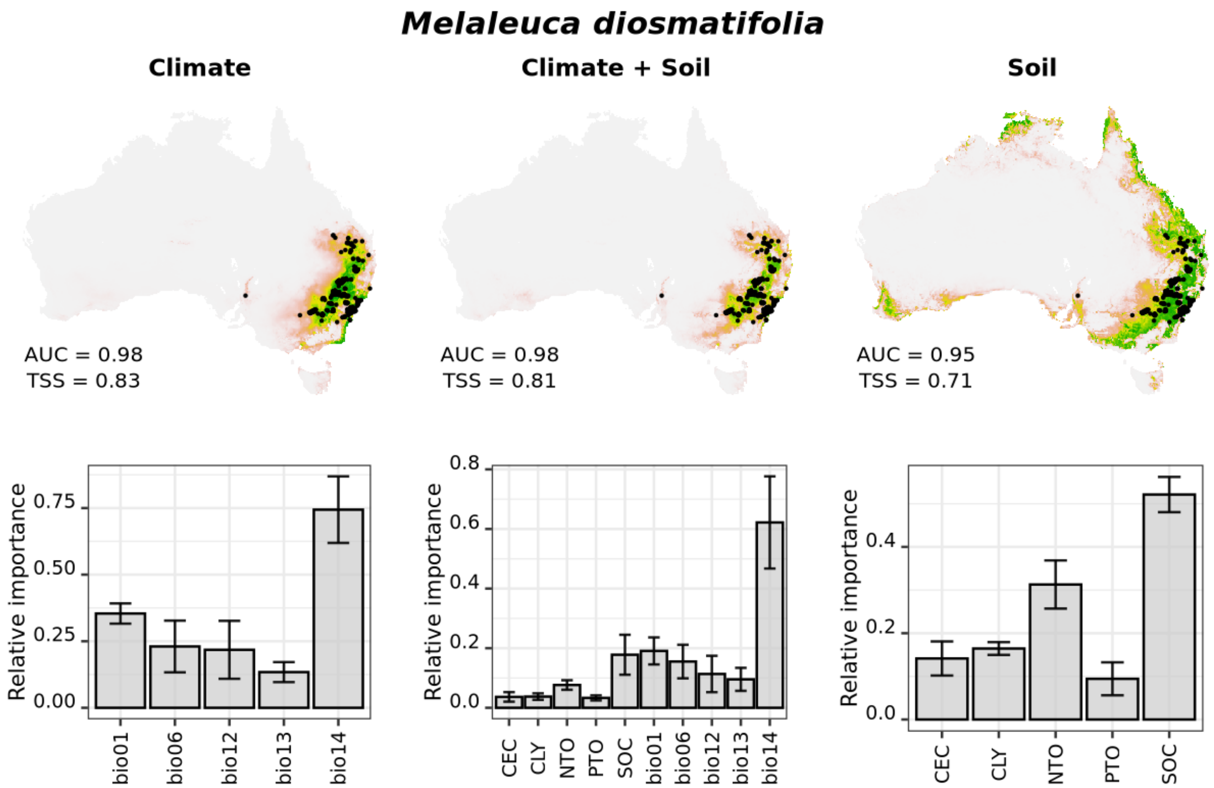
Melaleuca diosmatifolia
Climate
Climate + Soil
Soil
AUC = 0.98
TSS = 0.83
AUC = 0.98
TSS = 0.81
AUC = 0.95
TSS = 0.71
Relative importance
bio01 bio06 bio12 bio13 bio14
CEC CLY NTO PTO SOC bio01 bio06 bio12 bio13 bio14
CEC CLY NTO PTO SOC

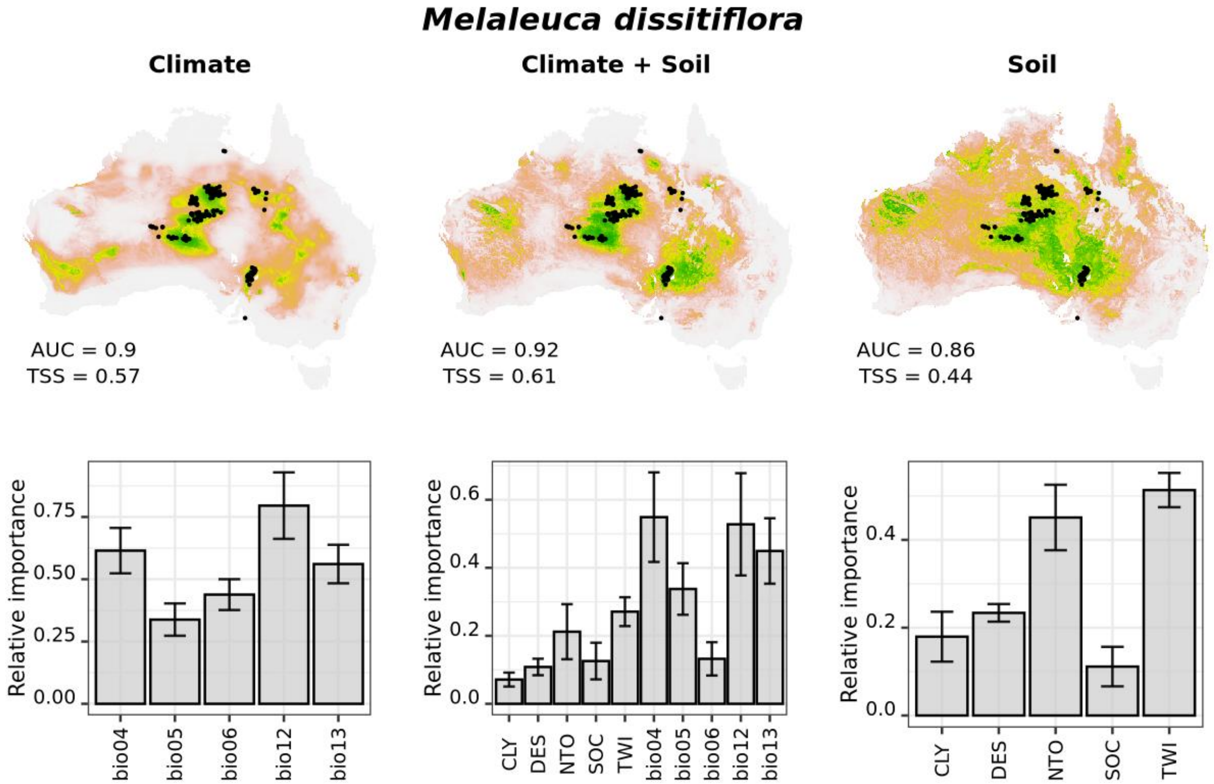
Melaleuca dissitiflora
Climate
Climate + Soil
Soil
AUC = 0.9
TSS = 0.57
AUC = 0.92
TSS = 0.61
AUC = 0.86
TSS = 0.44
Relative importance
bio04 bio05 bio06 bio12 bio13
CLY DES NTO SOC TWI bio04 bio05 bio06 bio12 bio13
CLY DES NTO SOC TWI

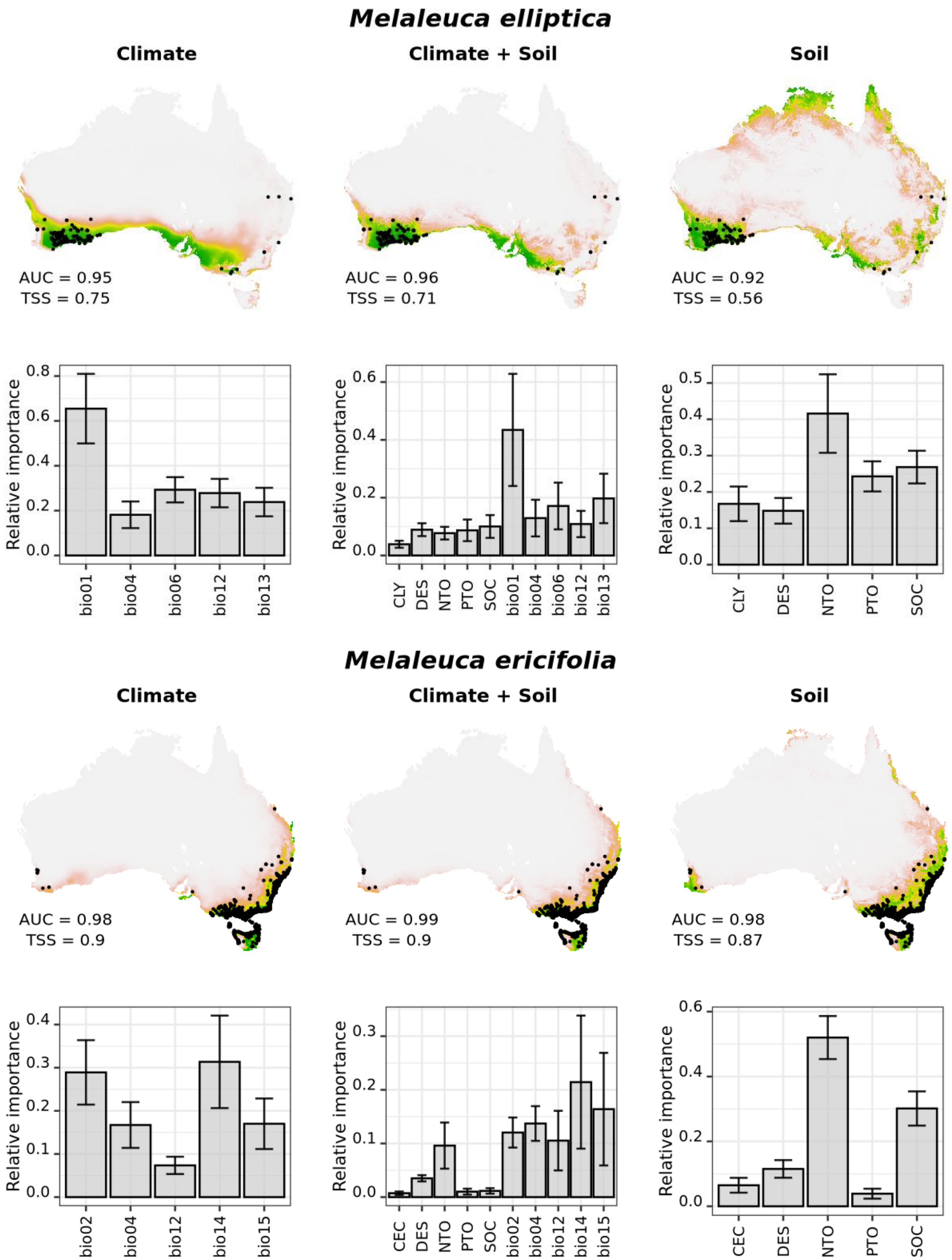
Melaleuca elliptica
Climate
Climate + Soil
Soil
AUC = 0.95
TSS = 0.75
AUC = 0.96
TSS = 0.71
AUC = 0.92
TSS = 0.56
Relative importance
bio01
bio04
bio06
bio12
bio13
CLY
DES
NTO
PTO
SOC
Melaleuca ericifolia
Climate
Climate + Soil
Soil
AUC = 0.98
TSS = 0.9
AUC = 0.99
TSS = 0.9
AUC = 0.98
TSS = 0.87
Relative importance
bio02
bio04
bio12
bio14
bio15
CEC
DES
NTO
PTO
SOC

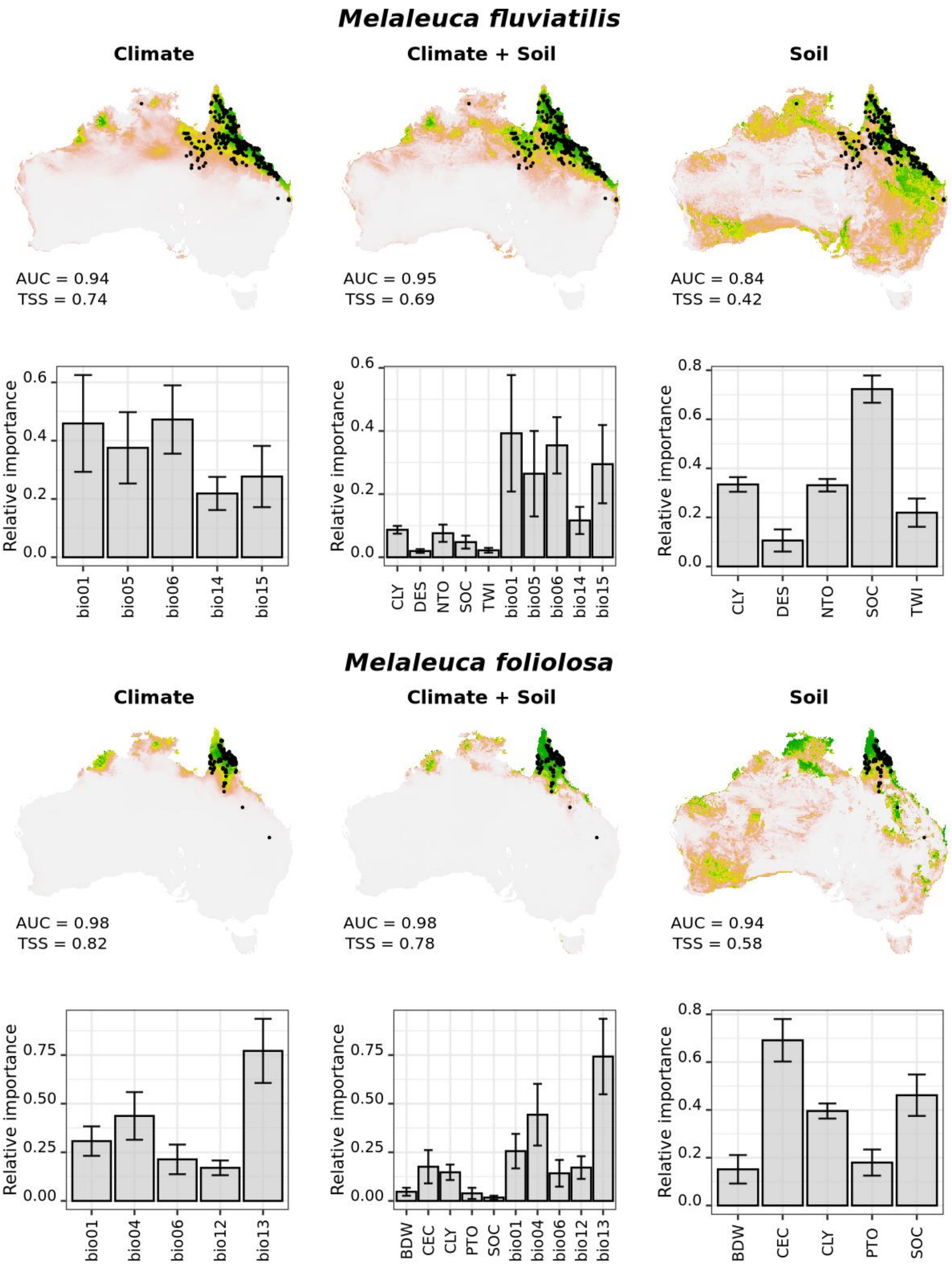

Melaleuca fluviatilis
Climate
Climate + Soil
Soil
AUC = 0.94
TSS = 0.74
AUC = 0.95
TSS = 0.69
AUC = 0.84
TSS = 0.42
Relative importance
bio01 bio05 bio06 bio14 bio15
CLY DES NTO SOC TWI bio01 bio05 bio06 bio14 bio15
CLY DES NTO SOC TWI
Melaleuca foliolosa
Climate
Climate + Soil
Soil
AUC = 0.98
TSS = 0.82
AUC = 0.98
TSS = 0.78
AUC = 0.94
TSS = 0.58
Relative importance
bio01 bio04 bio06 bio12 bio13
BDW CEC CLY PTO SOC bio01 bio04 bio06 bio12 bio13
BDW CEC CLY PTO SOC

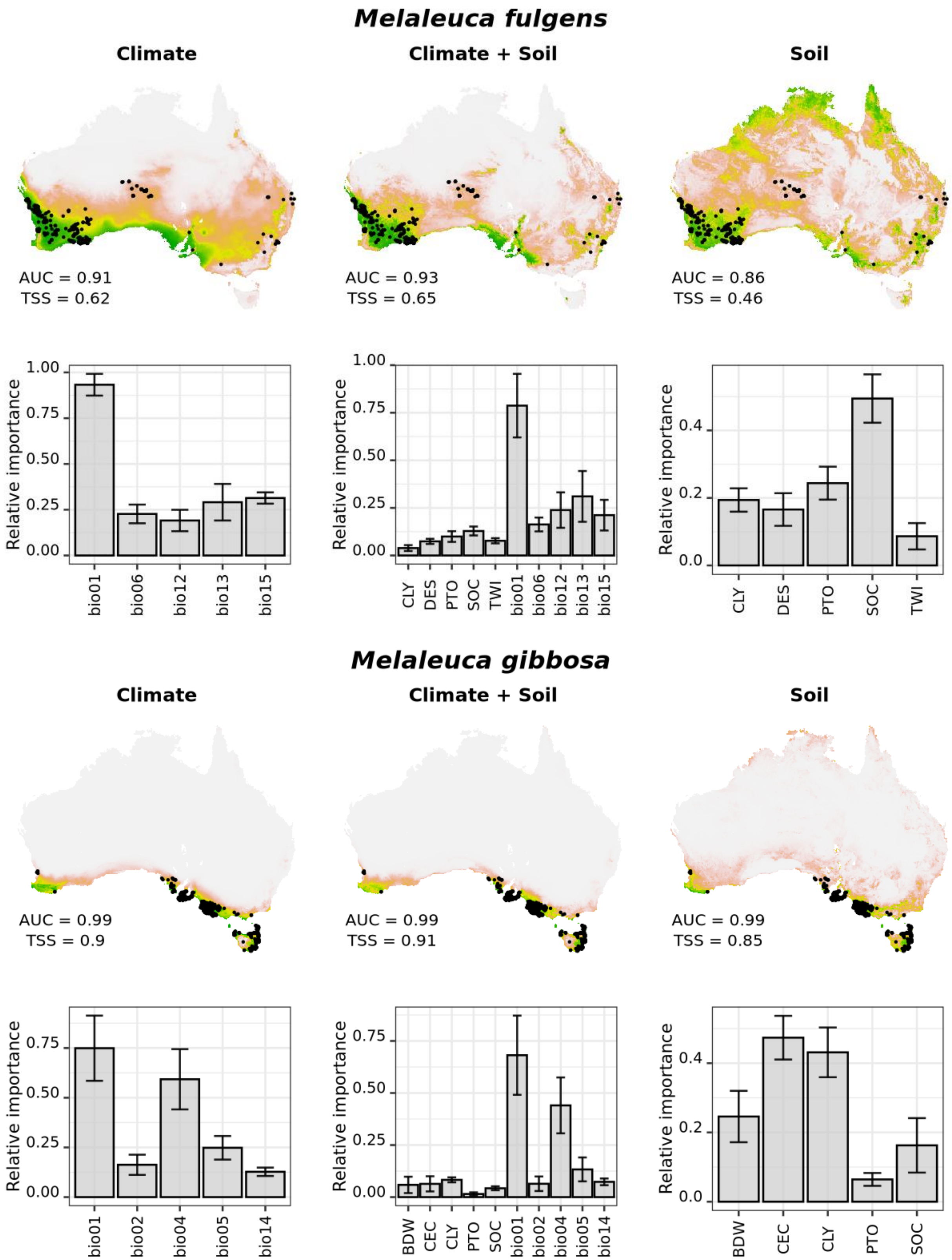

Melaleuca fulgens
Climate
Climate + Soil
Soil
AUC = 0.91
TSS = 0.62
AUC = 0.93
TSS = 0.65
AUC = 0.86
TSS = 0.46
Relative importance
1.00
0.75
0.50
0.25
0.00
bio01 bio06 bio12 bio13 bio15
CLY DES PTO SOC TWI bio01 bio06 bio12 bio13 bio15
0.4
0.2
0.0
CLY DES PTO SOC TWI
Melaleuca gibbosa
Climate
Climate + Soil
Soil
AUC = 0.99
TSS = 0.9
AUC = 0.99
TSS = 0.91
AUC = 0.99
TSS = 0.85
Relative importance
bio01 bio02 bio04 bio05 bio14
BDW CEC CLY PTO SOC bio01 bio02 bio04 bio05 bio14
BDW CEC CLY PTO SOC

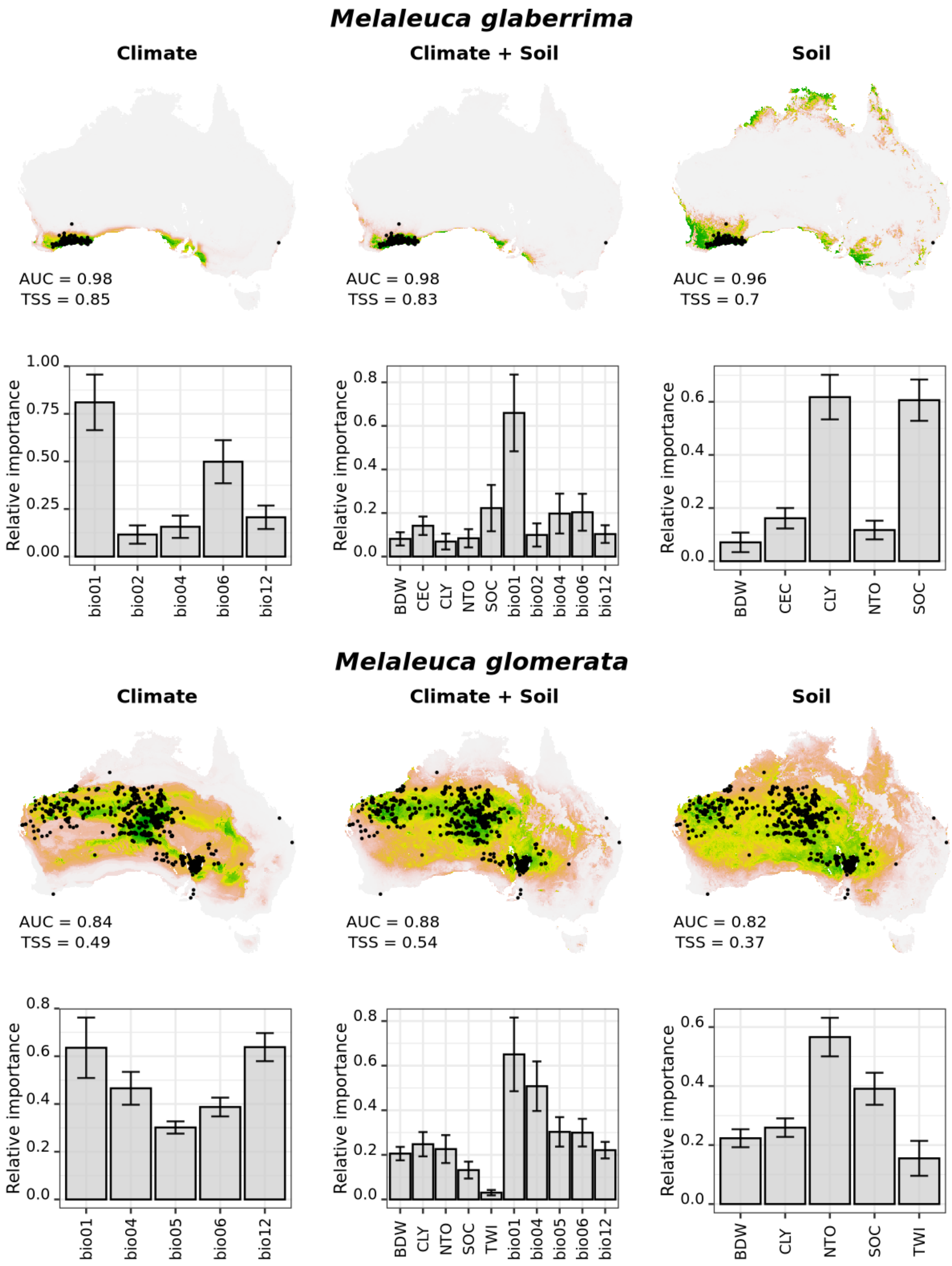
Melaleuca glaberrima
Climate
Climate + Soil
Soil
AUC = 0.98
TSS = 0.85
AUC = 0.98
TSS = 0.83
AUC = 0.96
TSS = 0.7
Relative importance
bio01
bio02
bio04
bio06
bio12
BDW
CEC
CLY
NTO
SOC
Melaleuca glomerata
AUC = 0.84
TSS = 0.49
AUC = 0.88
TSS = 0.54
AUC = 0.82
TSS = 0.37
bio05
TWI

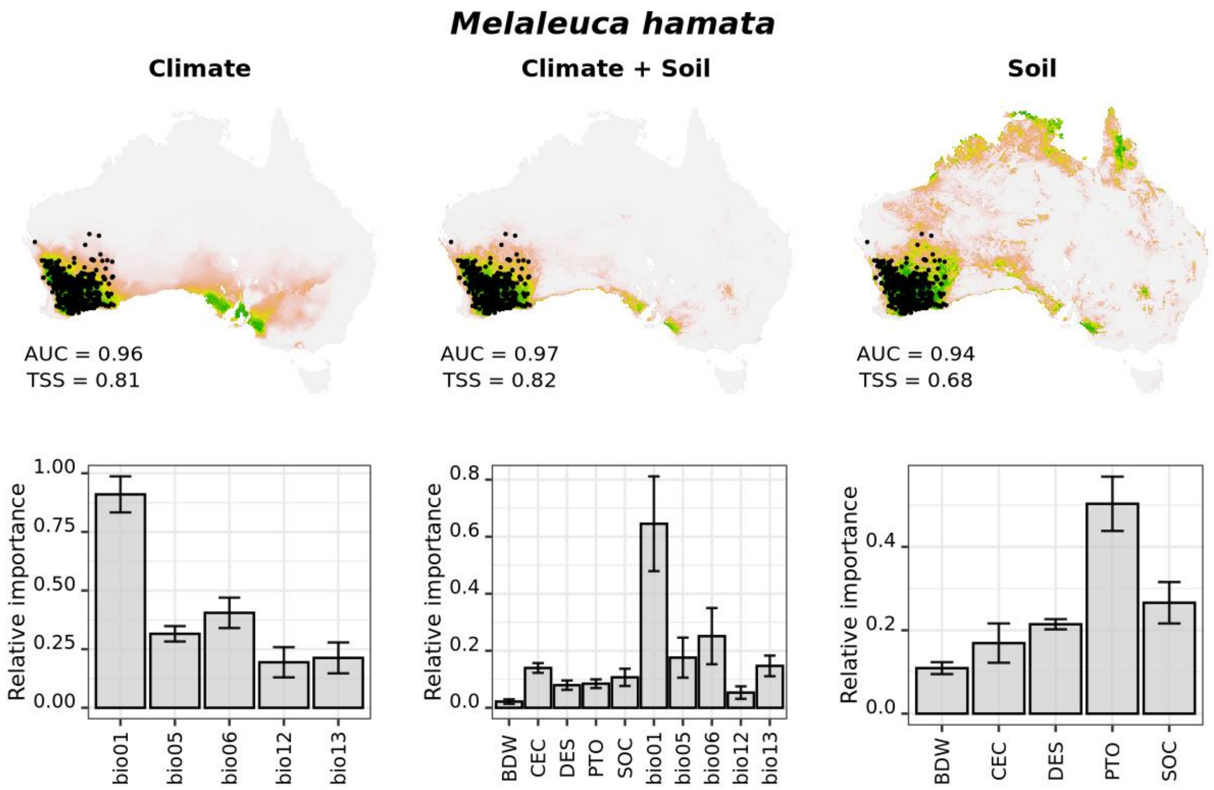
Melaleuca hamata
Climate
Climate + Soil
Soil
AUC = 0.96
TSS = 0.81
AUC = 0.97
TSS = 0.82
AUC = 0.94
TSS = 0.68
Relative importance
1.00
0.75
0.50
0.25
0.00
bio01
bio05
bio06
bio12
bio13
0.8
0.6
0.4
0.2
0.0
BDW
CEC
DES
PTO
SOC
bio01
bio05
bio06
bio12
bio13
0.4
0.2
0.0
BDW
CEC
DES
PTO
SOC

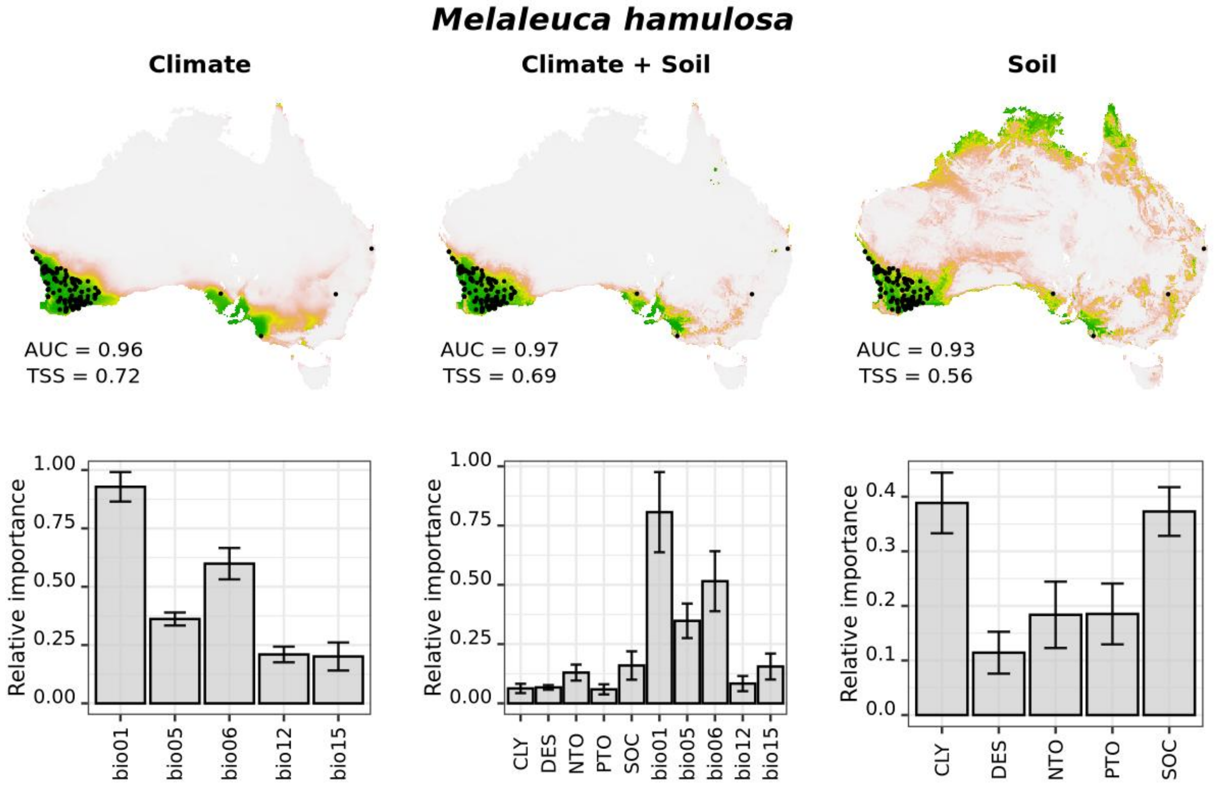
Melaleuca hamulosa
Climate
Climate + Soil
Soil
AUC = 0.96
TSS = 0.72
AUC = 0.97
TSS = 0.69
AUC = 0.93
TSS = 0.56
Relative importance
1.00
0.75
0.50
0.25
0.00
bio01
bio05
bio06
bio12
bio15
CLY
DES
NTO
PTO
SOC
bio01
bio05
bio06
bio12
bio15
0.4
0.3
0.2
0.1
0.0
CLY
DES
NTO
PTO
SOC

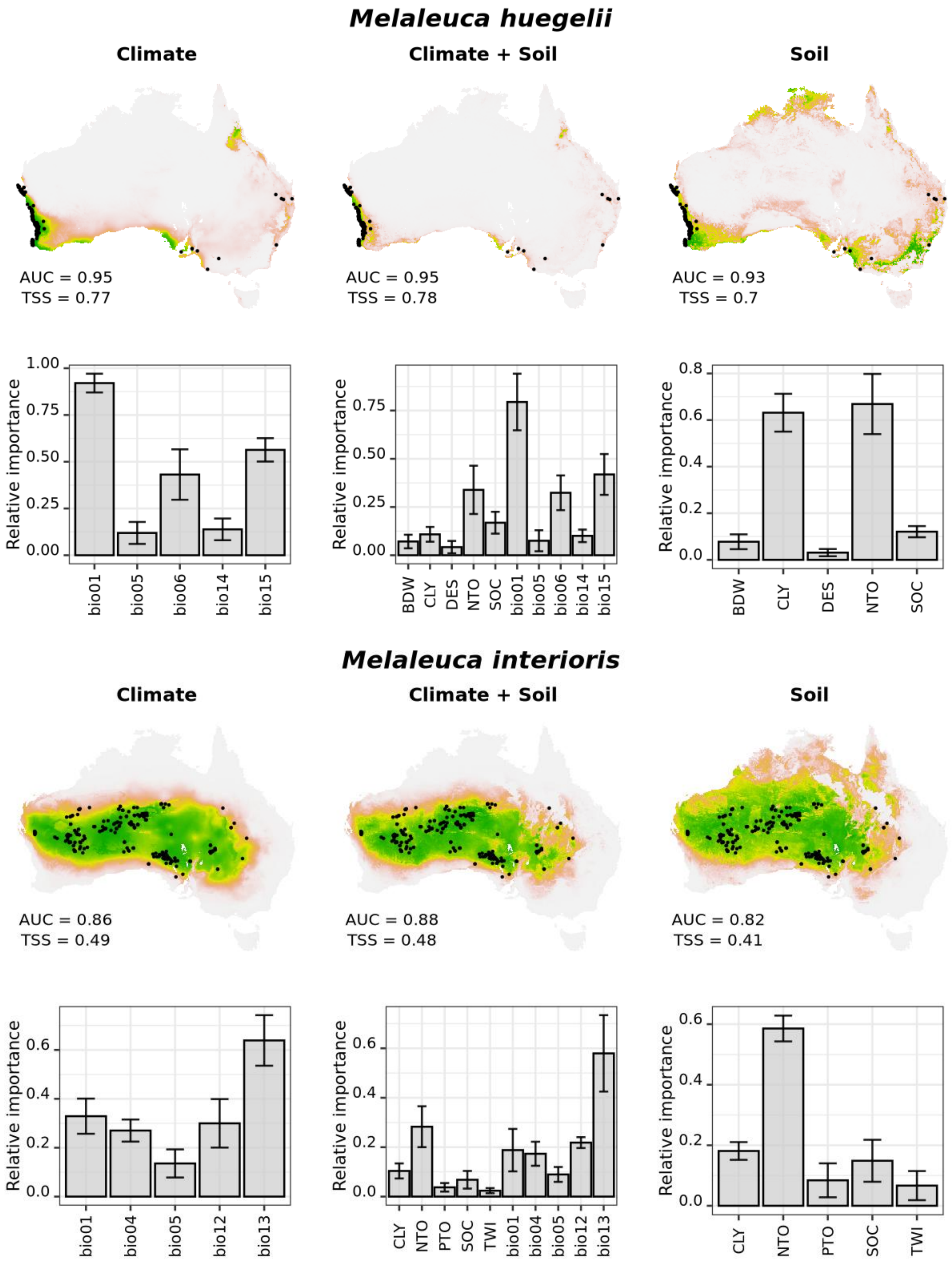

Melaleuca huegelii
Climate
Climate + Soil
Soil
AUC = 0.95
TSS = 0.77
AUC = 0.95
TSS = 0.78
AUC = 0.93
TSS = 0.7
Relative importance
0.00
0.25
0.50
0.75
1.00
bio01
bio05
bio06
bio14
bio15
BDW
CLY
DES
NTO
SOC
0.0
0.2
0.4
0.6
0.8
Melaleuca interioris
Climate
Climate + Soil
Soil
AUC = 0.86
TSS = 0.49
AUC = 0.88
TSS = 0.48
AUC = 0.82
TSS = 0.41
bio04
bio12
bio13
PTO
TWI

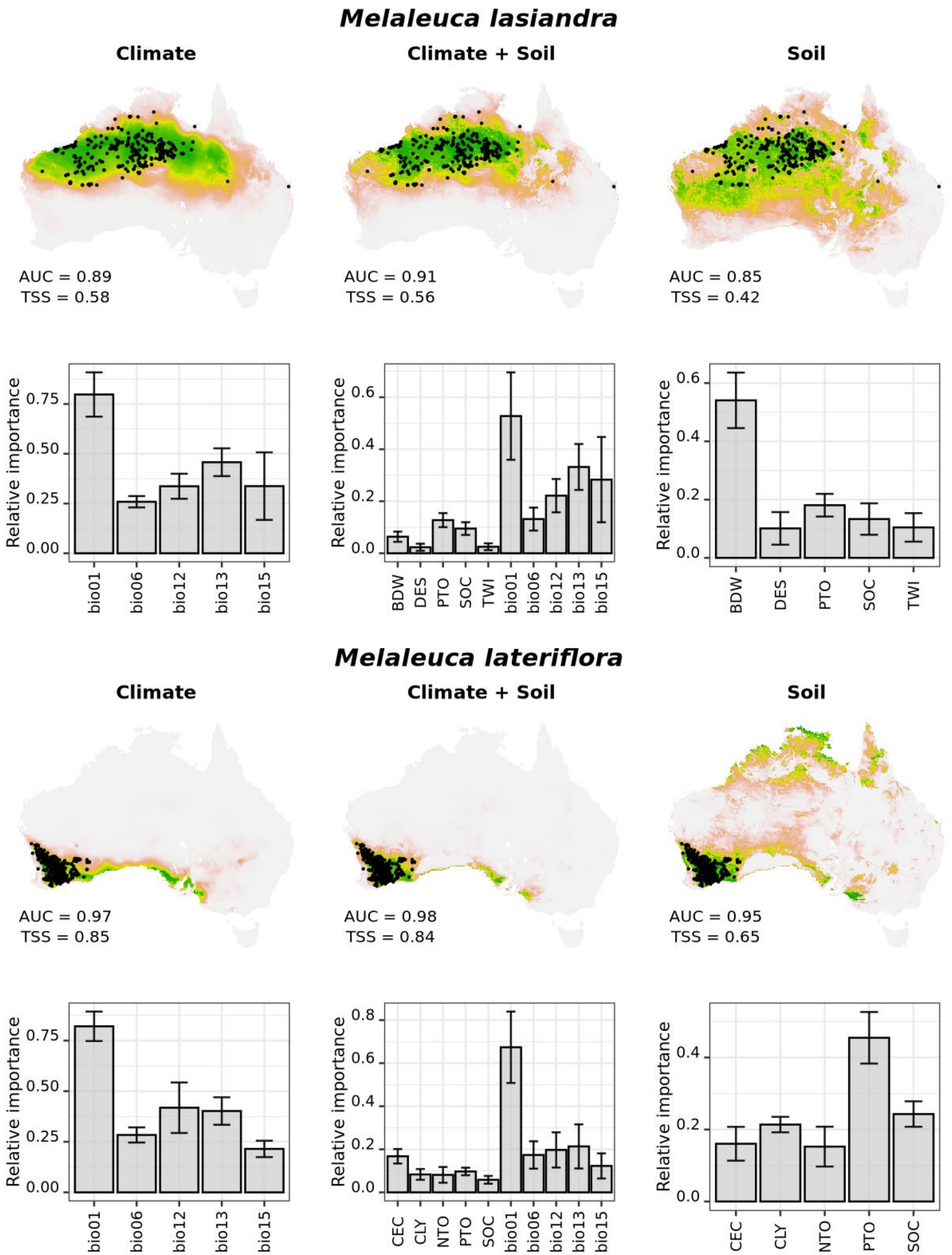

Melaleuca lasiandra
Climate
Climate + Soil
Soil
AUC = 0.89
TSS = 0.58
AUC = 0.91
TSS = 0.56
AUC = 0.85
TSS = 0.42
Relative importance
0.00
0.25
0.50
0.75
bio01
bio06
bio12
bio13
bio15
0.0
0.2
0.4
0.6
BDW
DES
PTO
SOC
TWI
bio01
bio06
bio12
bio13
bio15
BDW
DES
PTO
SOC
TWI
Melaleuca lateriflora
Climate
Climate + Soil
Soil
AUC = 0.97
TSS = 0.85
AUC = 0.98
TSS = 0.84
AUC = 0.95
TSS = 0.65
Relative importance
0.8
CEC
CLY
NTO
PTO
SOC
bio01
bio06
bio12
bio13
bio15

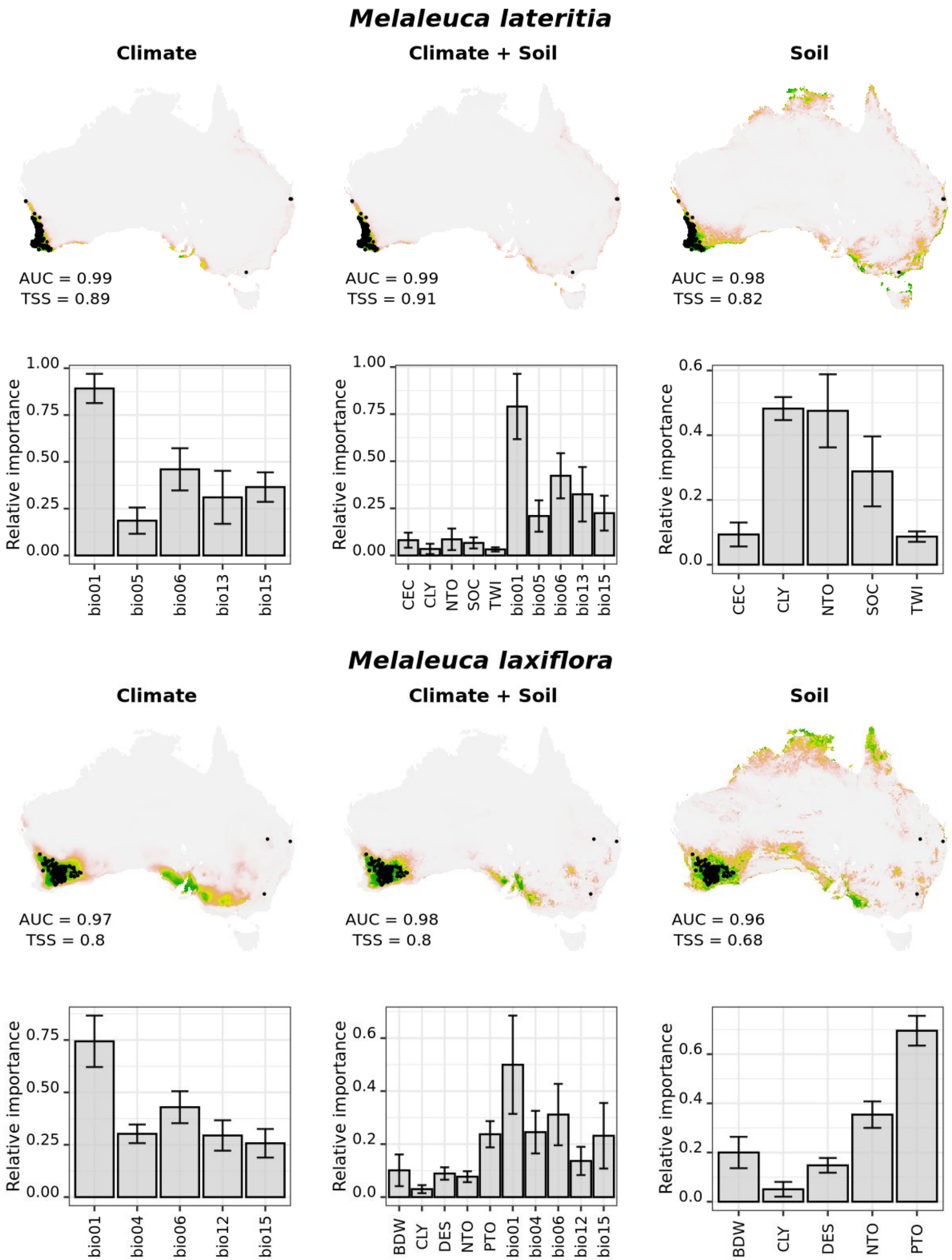

Melaleuca lateritia
Climate
Climate + Soil
Soil
AUC = 0.99
TSS = 0.89
AUC = 0.99
TSS = 0.91
AUC = 0.98
TSS = 0.82
Relative importance
1.00
0.75
0.50
0.25
0.00
bio01
bio05
bio06
bio13
bio15
CEC
CLY
NTO
SOC
TWI
0.6
0.4
0.2
0.0
Melaleuca laxiflora
Climate
Climate + Soil
Soil
AUC = 0.97
TSS = 0.8
AUC = 0.98
TSS = 0.8
AUC = 0.96
TSS = 0.68
bio01
bio04
bio06
bio12
bio15
BDW
CLY
DES
NTO
PTO

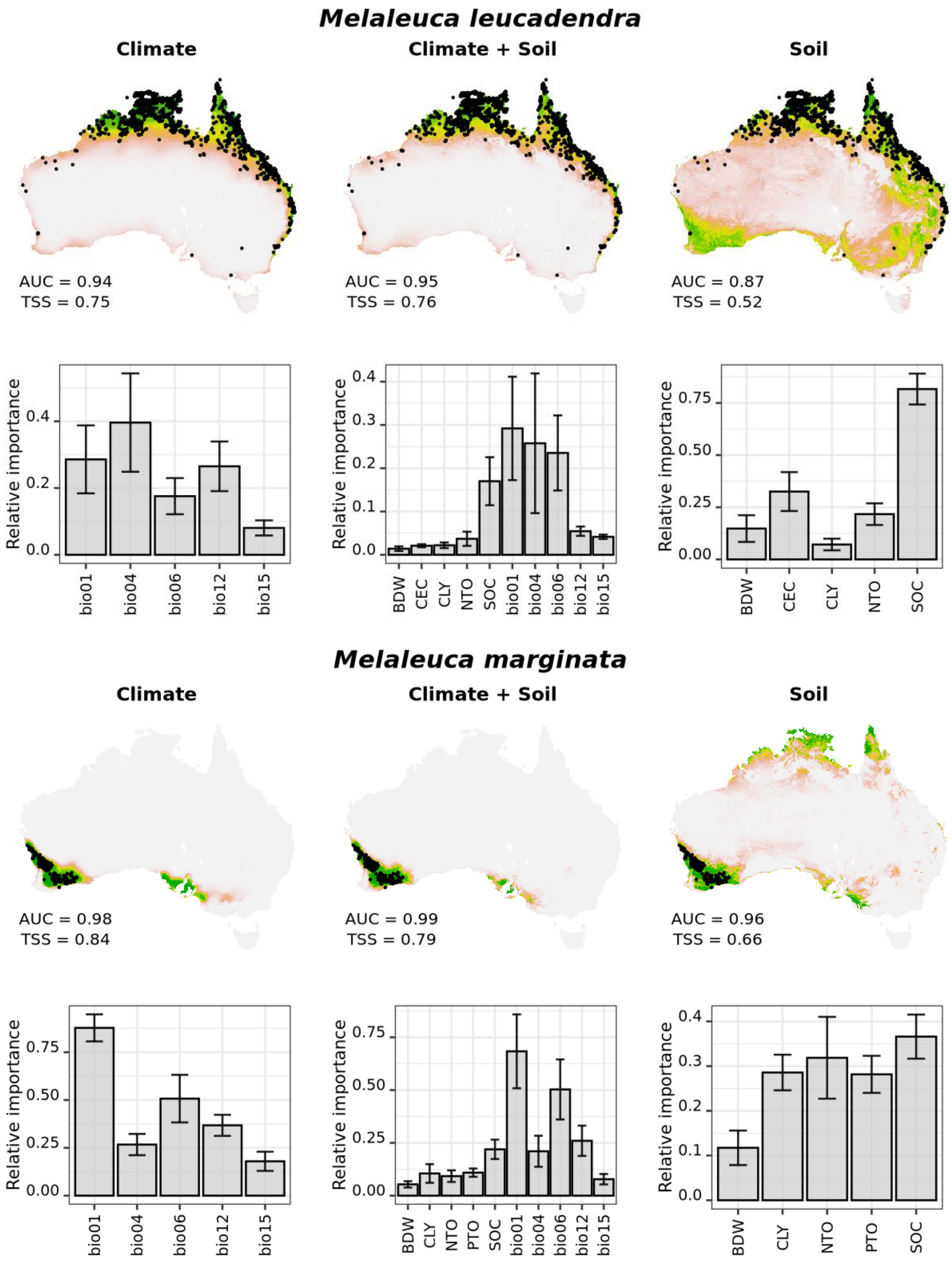

Melaleuca leucadendra
Climate
Climate + Soil
Soil
AUC = 0.94
TSS = 0.75
AUC = 0.95
TSS = 0.76
AUC = 0.87
TSS = 0.52
Relative importance
bio01
bio04
bio06
bio12
bio15
BDW
CEC
CLY
NTO
SOC
Melaleuca marginata
Climate
Climate + Soil
Soil
AUC = 0.98
TSS = 0.84
AUC = 0.99
TSS = 0.79
AUC = 0.96
TSS = 0.66
Relative importance
PTO

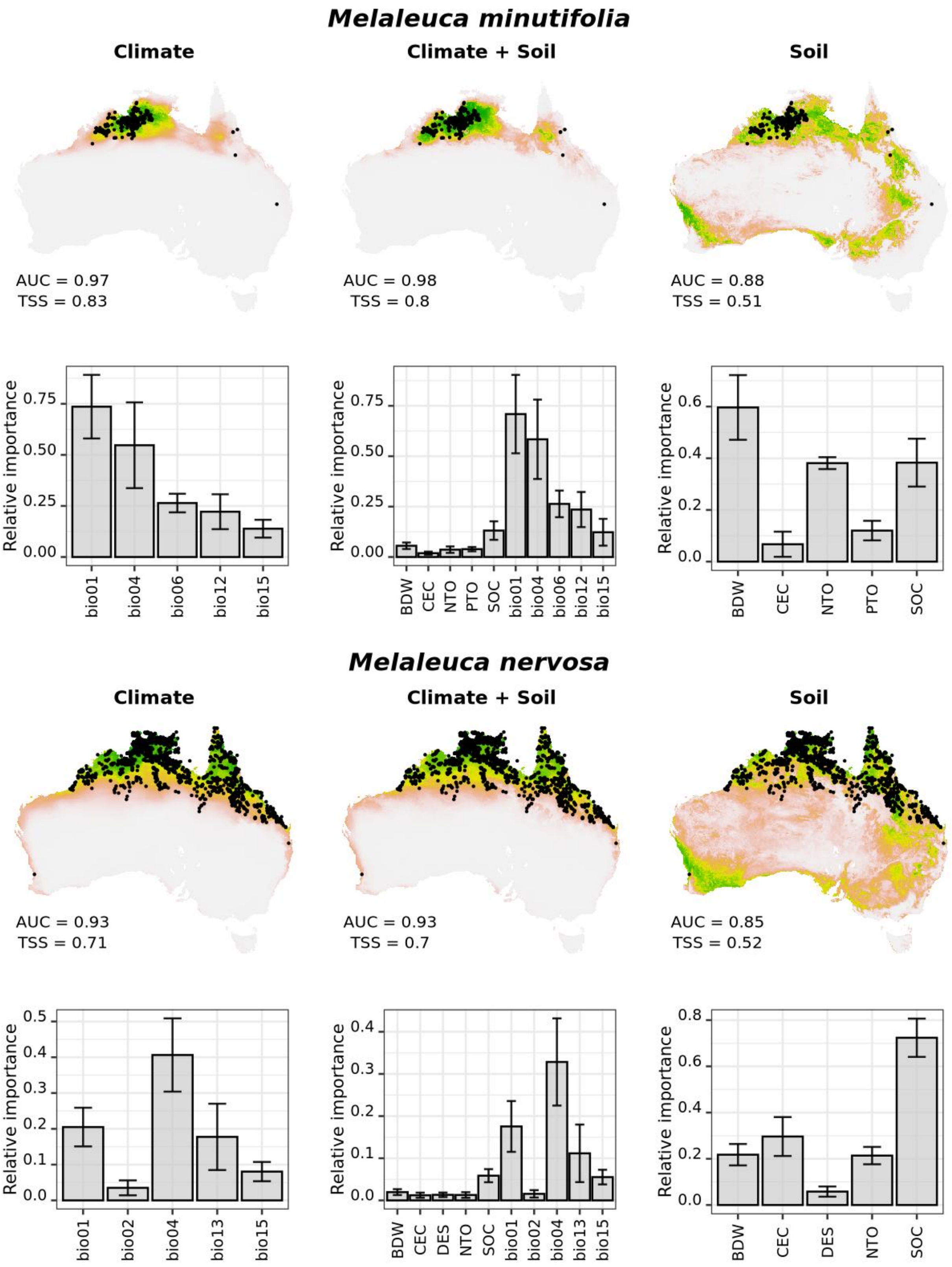
Melaleuca minutifolia
Climate
Climate + Soil
Soil
AUC = 0.97
TSS = 0.83
AUC = 0.98
TSS = 0.8
AUC = 0.88
TSS = 0.51
Relative importance
bio01
bio04
bio06
bio12
bio15
BDW
CEC
NTO
PTO
SOC
Melaleuca nervosa
Climate
Climate + Soil
Soil
AUC = 0.93
TSS = 0.71
AUC = 0.93
TSS = 0.7
AUC = 0.85
TSS = 0.52
Relative importance
bio01
bio02
bio04
bio13
bio15
BDW
CEC
DES
NTO
SOC

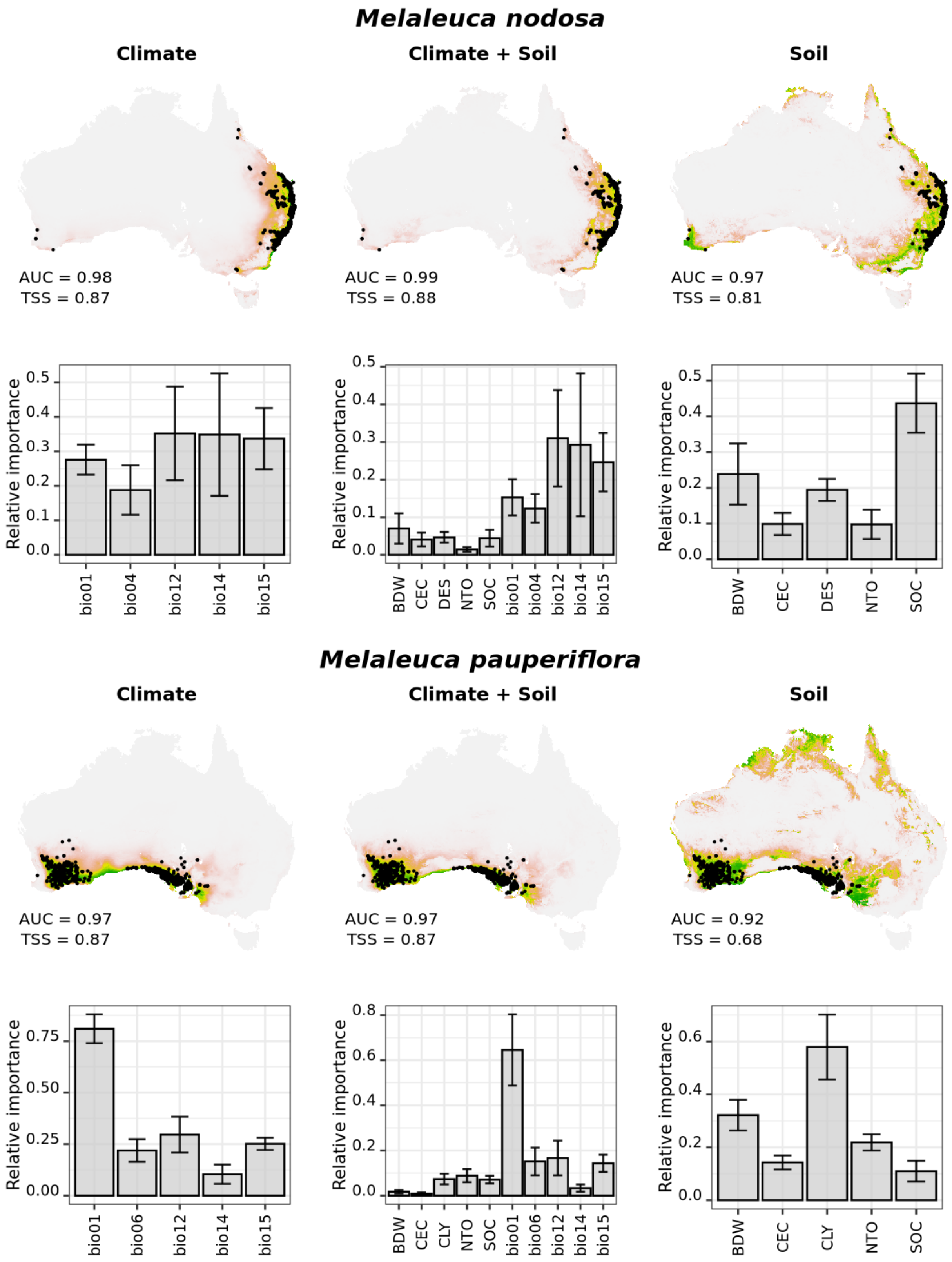
Melaleuca nodosa
Climate
Climate + Soil
Soil
AUC = 0.98
TSS = 0.87
AUC = 0.99
TSS = 0.88
AUC = 0.97
TSS = 0.81
Relative importance
0.0
0.1
0.2
0.3
0.4
0.5
bio01
bio04
bio12
bio14
bio15
BDW
CEC
DES
NTO
SOC
Melaleuca pauperiflora
Climate
Climate + Soil
Soil
AUC = 0.97
TSS = 0.87
AUC = 0.97
TSS = 0.87
AUC = 0.92
TSS = 0.68
0.00
0.25
0.50
0.75
0.6
0.8
bio06
CLY

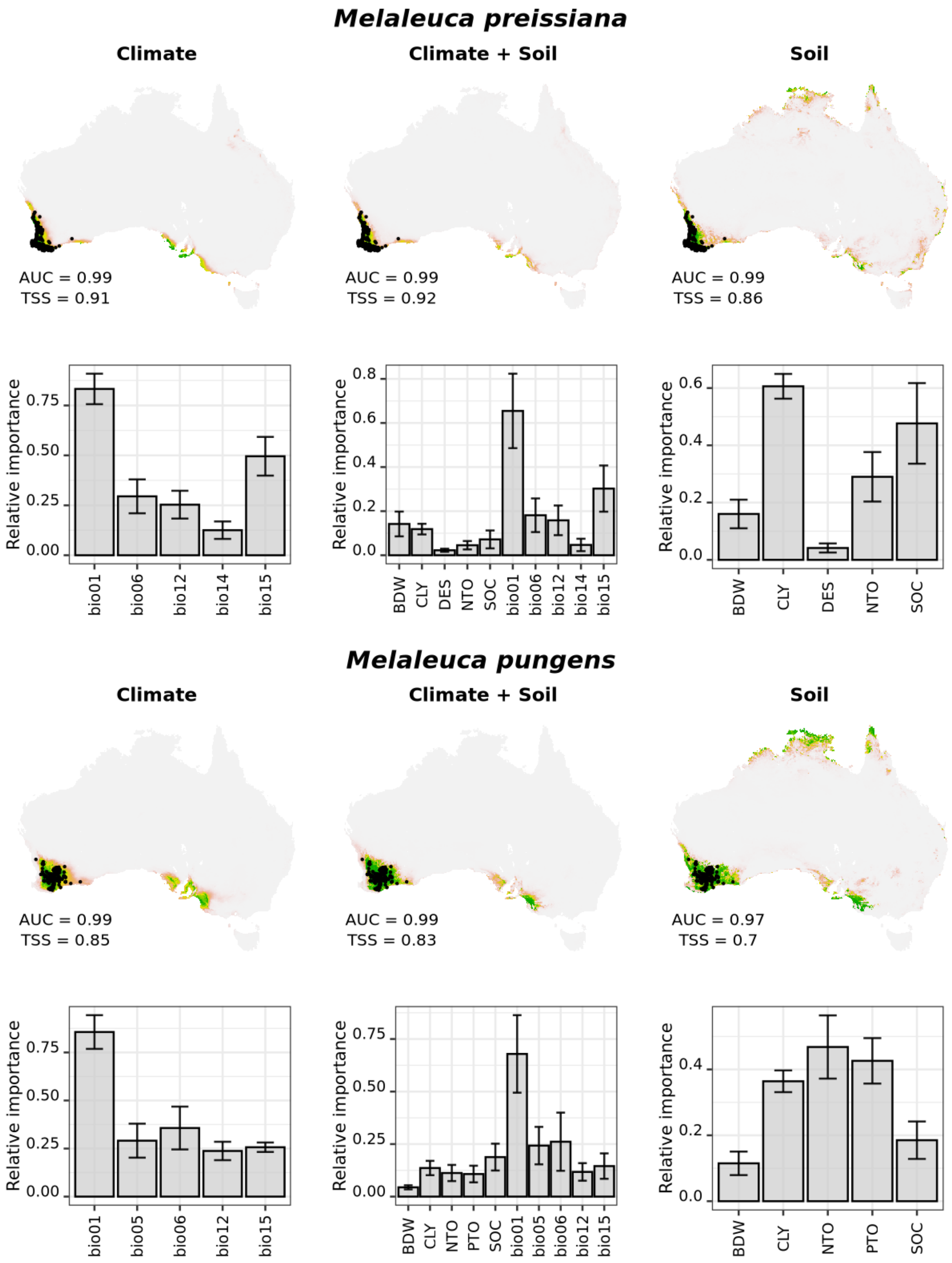

Melaleuca preissiana
Climate
Climate + Soil
Soil
AUC = 0.99
TSS = 0.91
AUC = 0.99
TSS = 0.92
AUC = 0.99
TSS = 0.86
Relative importance
0.00
0.25
0.50
0.75
bio01
bio06
bio12
bio14
bio15
0.0
0.2
0.4
0.6
0.8
BDW
CLY
DES
NTO
SOC
bio01
bio06
bio12
bio14
bio15
BDW
CLY
DES
NTO
SOC
Melaleuca pungens
Climate
Climate + Soil
Soil
AUC = 0.99
TSS = 0.85
AUC = 0.99
TSS = 0.83
AUC = 0.97
TSS = 0.7
Relative importance
bio01
bio05
bio06
bio12
bio15
BDW
CLY
NTO
PTO
SOC
bio01
bio05
bio06
bio12
bio15
BDW
CLY
NTO
PTO
SOC

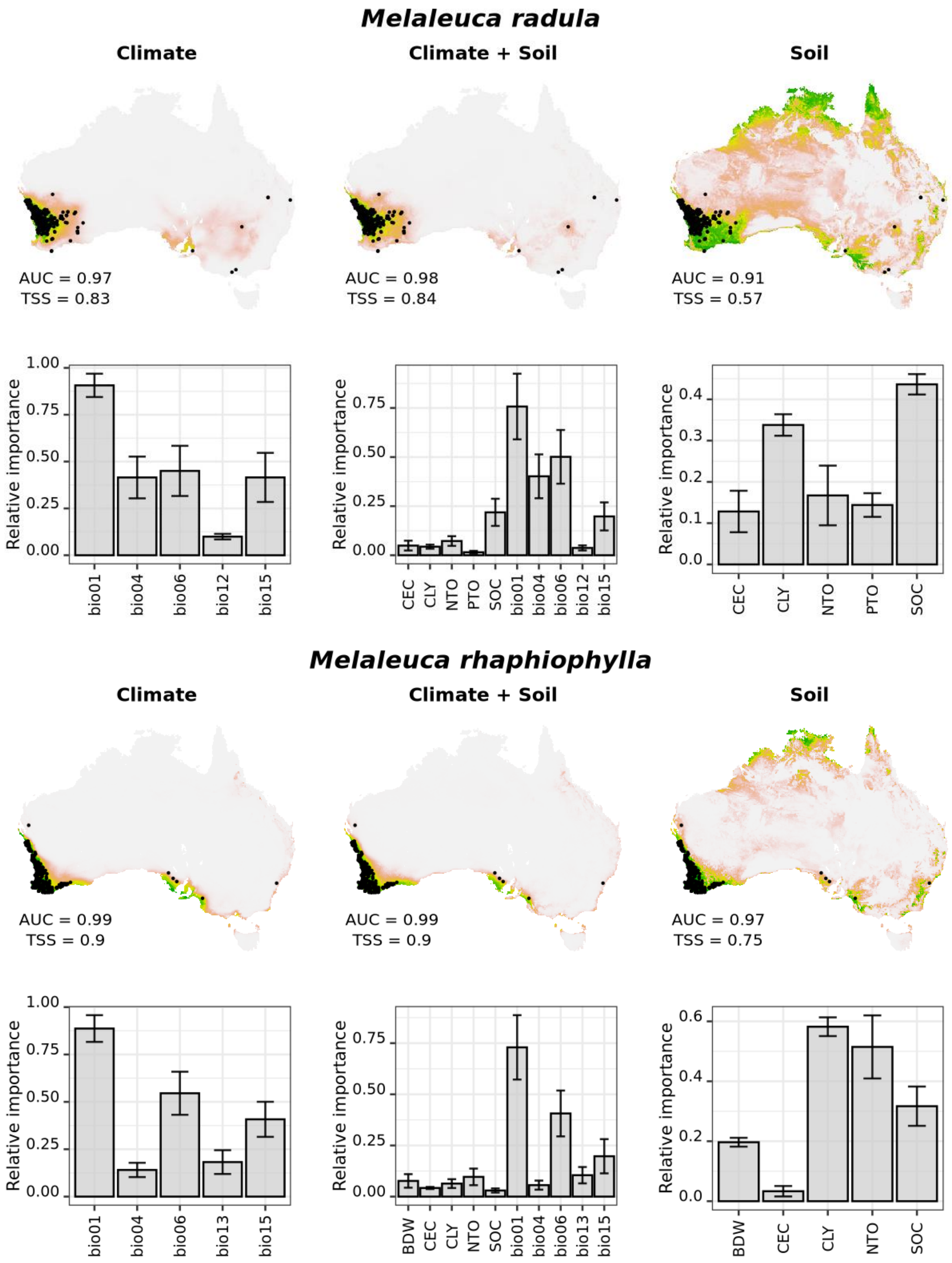

Melaleuca radula
Climate
Climate + Soil
Soil
AUC = 0.97
TSS = 0.83
AUC = 0.98
TSS = 0.84
AUC = 0.91
TSS = 0.57
Relative importance
bio01 bio04 bio06 bio12 bio15
CEC CLY NTO PTO SOC bio01 bio04 bio06 bio12 bio15
CEC CLY NTO PTO SOC
Melaleuca rhaphiophylla
Climate
Climate + Soil
Soil
AUC = 0.99
TSS = 0.9
AUC = 0.99
TSS = 0.9
AUC = 0.97
TSS = 0.75
Relative importance
bio01 bio04 bio06 bio13 bio15
BDW CEC CLY NTO SOC bio01 bio04 bio06 bio13 bio15
BDW CEC CLY NTO SOC

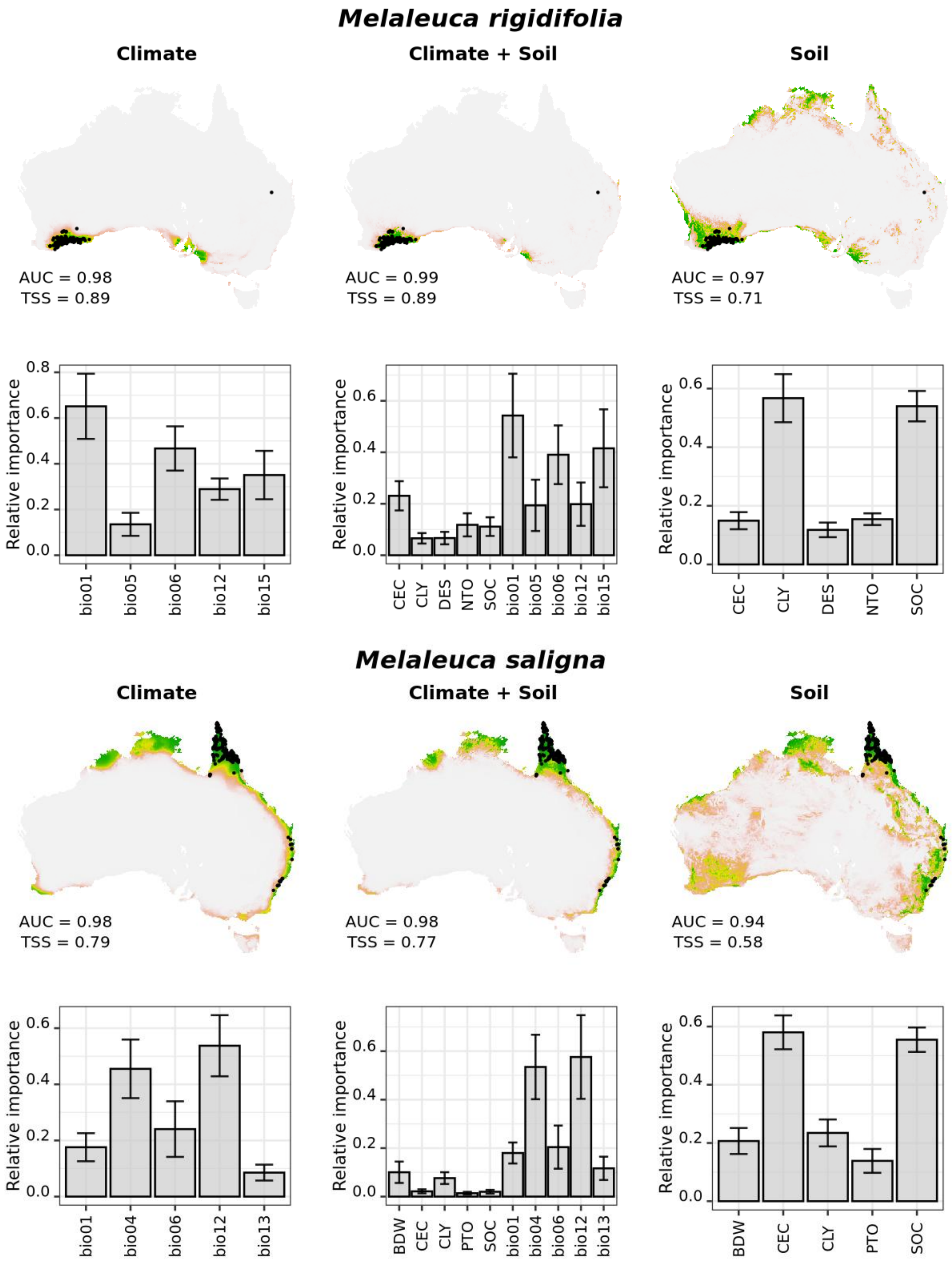

Melaleuca rigidifolia
Climate
Climate + Soil
Soil
AUC = 0.98
TSS = 0.89
AUC = 0.99
TSS = 0.89
AUC = 0.97
TSS = 0.71
Relative importance
bio01
bio05
bio06
bio12
bio15
CEC
CLY
DES
NTO
SOC
Melaleuca saligna
AUC = 0.98
TSS = 0.79
AUC = 0.98
TSS = 0.77
AUC = 0.94
TSS = 0.58
bio04
bio13
BDW
PTO

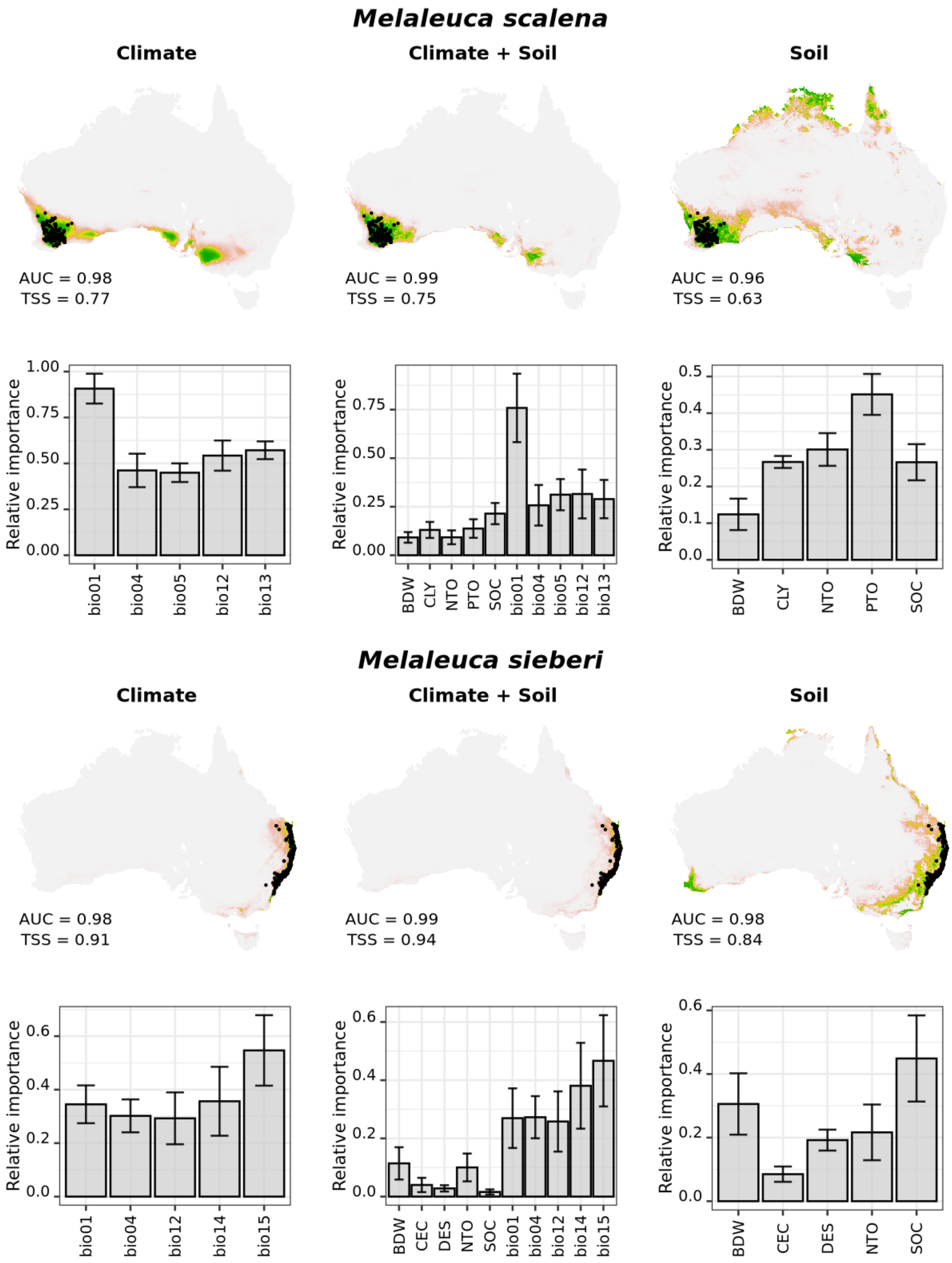
Melaleuca scalena
Climate
Climate + Soil
Soil
AUC = 0.98
TSS = 0.77
AUC = 0.99
TSS = 0.75
AUC = 0.96
TSS = 0.63
Relative importance
bio01
bio04
bio05
bio12
bio13
BDW
CLY
NTO
PTO
SOC
Melaleuca sieberi
Climate
Climate + Soil
Soil
AUC = 0.98
TSS = 0.91
AUC = 0.99
TSS = 0.94
AUC = 0.98
TSS = 0.84
bio14
bio15
CEC
DES

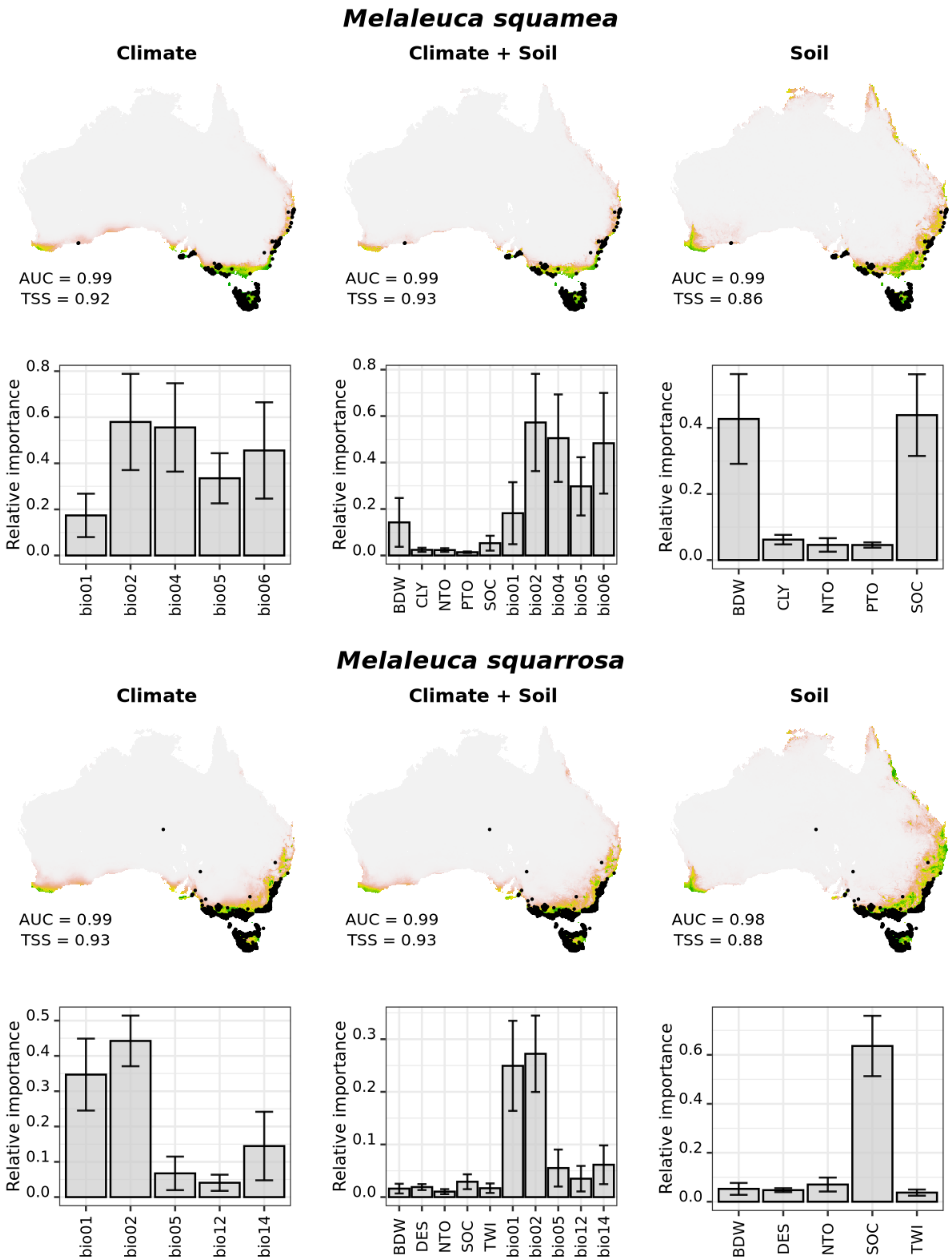
Melaleuca squamea
Climate
Climate + Soil
Soil
AUC = 0.99
TSS = 0.92
AUC = 0.99
TSS = 0.93
AUC = 0.99
TSS = 0.86
Relative importance
0.0
0.2
0.4
0.6
0.8
bio01
bio02
bio04
bio05
bio06
BDW
CLY
NTO
PTO
SOC
Melaleuca squarrosa
AUC = 0.99
TSS = 0.93
AUC = 0.99
TSS = 0.93
AUC = 0.98
TSS = 0.88
0.1
0.3
0.5
bio12
bio14
DES
TWI

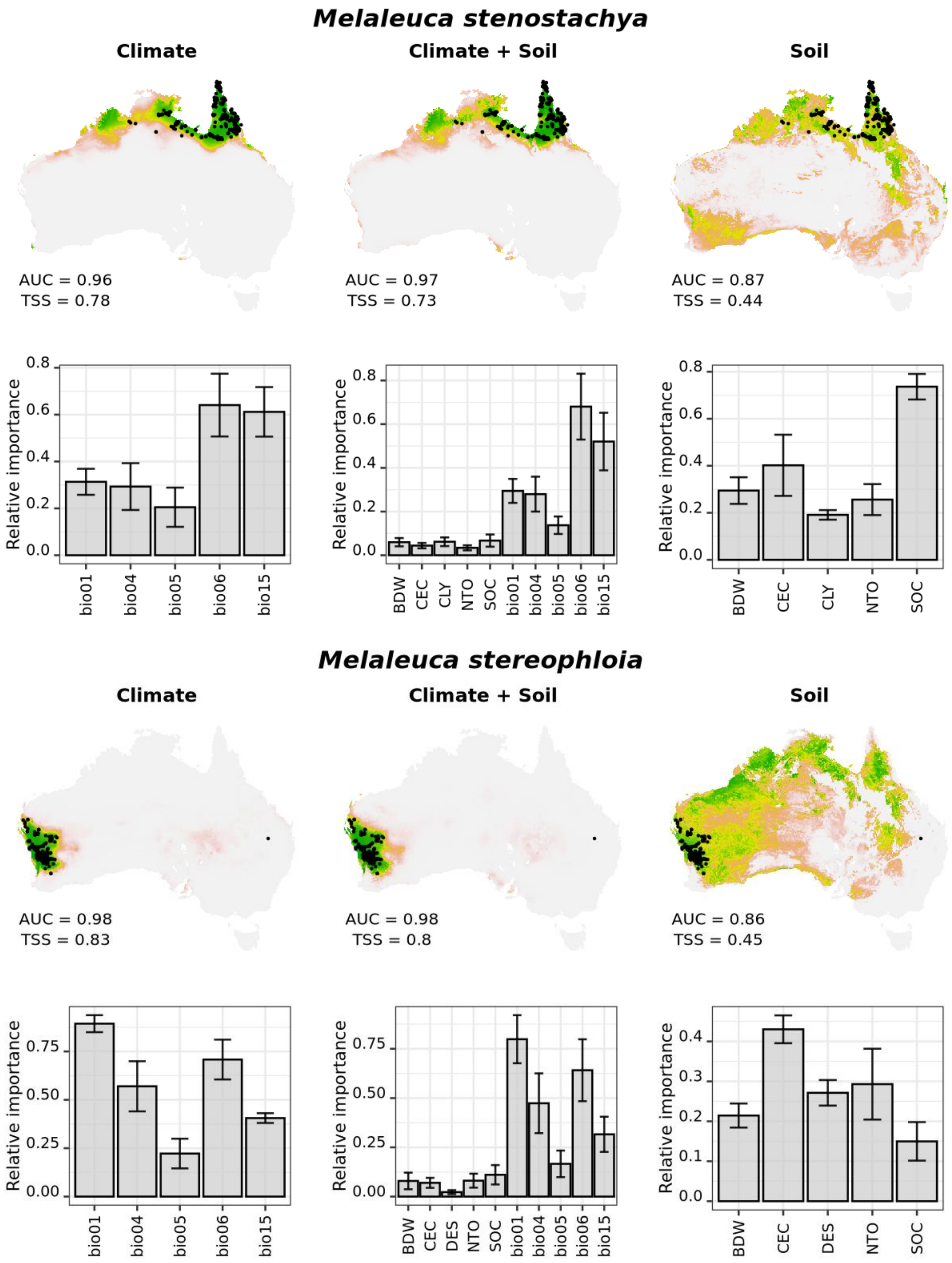
Melaleuca stenostachya
Climate
Climate + Soil
Soil
AUC = 0.96
TSS = 0.78
AUC = 0.97
TSS = 0.73
AUC = 0.87
TSS = 0.44
Relative importance
0.0
0.2
0.4
0.6
0.8
bio01
bio04
bio05
bio06
bio15
BDW
CEC
CLY
NTO
SOC
Melaleuca stereophloia
Climate
Climate + Soil
Soil
AUC = 0.98
TSS = 0.83
AUC = 0.98
TSS = 0.8
AUC = 0.86
TSS = 0.45
Relative importance
0.00
0.25
0.50
0.75
0.0
0.1
0.2
0.3
0.4
bio01
bio04
bio05
bio06
bio15
BDW
CEC
DES
NTO
SOC

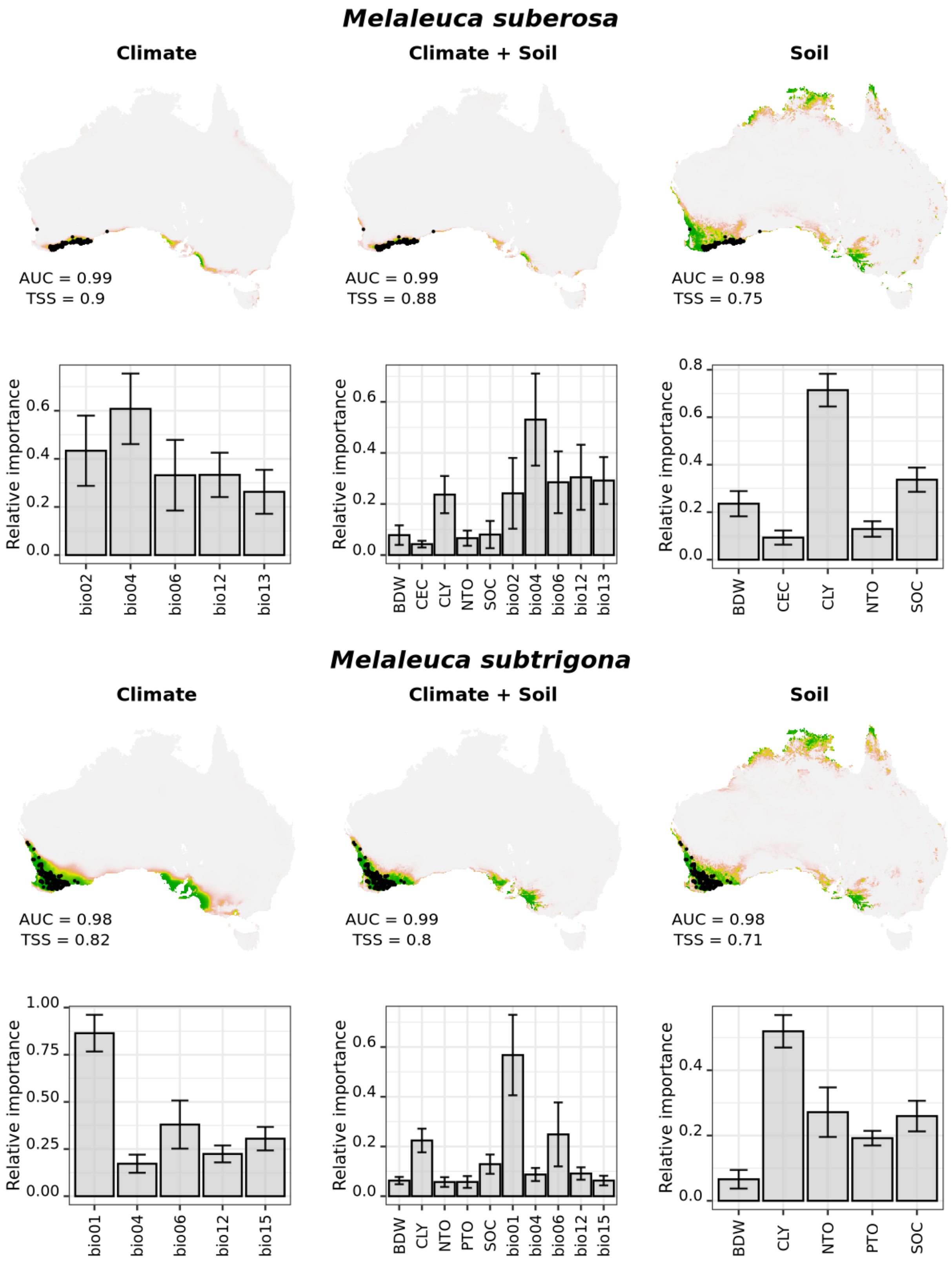

Melaleuca suberosa
Climate
Climate + Soil
Soil
AUC = 0.99
TSS = 0.9
AUC = 0.99
TSS = 0.88
AUC = 0.98
TSS = 0.75
Relative importance
0.0
0.2
0.4
0.6
0.8
bio02
bio04
bio06
bio12
bio13
BDW
CEC
CLY
NTO
SOC
Melaleuca subtrigona
Climate
Climate + Soil
Soil
AUC = 0.98
TSS = 0.82
AUC = 0.99
TSS = 0.8
AUC = 0.98
TSS = 0.71
Relative importance
0.00
0.25
0.50
0.75
1.00
bio01
bio04
bio06
bio12
bio15
BDW
CLY
NTO
PTO
SOC

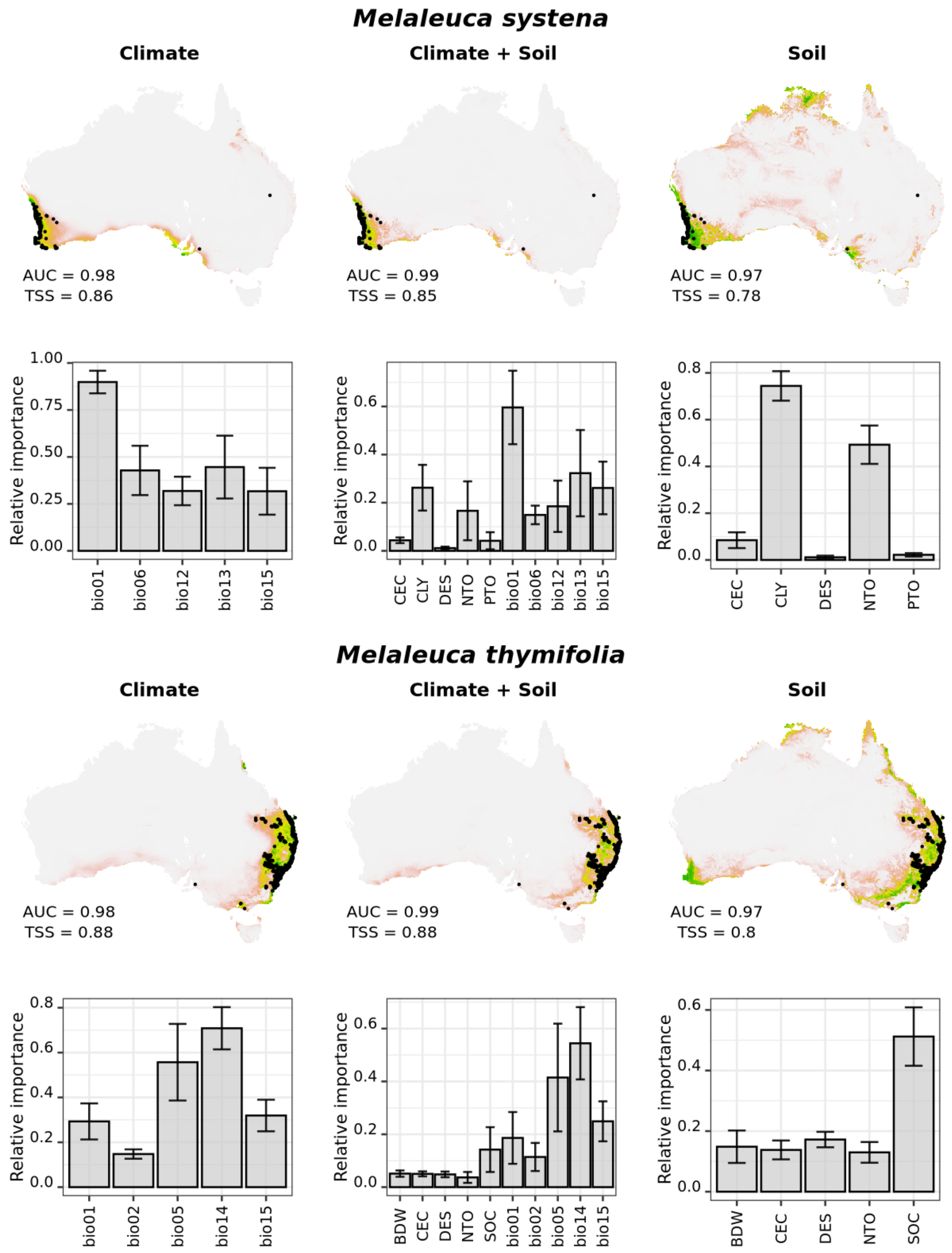

Melaleuca systena
Climate
Climate + Soil
Soil
AUC = 0.98
TSS = 0.86
AUC = 0.99
TSS = 0.85
AUC = 0.97
TSS = 0.78
Relative importance
1.00
0.75
0.50
0.25
0.00
bio01
bio06
bio12
bio13
bio15
0.6
0.4
0.2
0.0
CEC
CLY
DES
NTO
PTO
bio01
bio06
bio12
bio13
bio15
0.8
0.6
0.4
0.2
0.0
CEC
CLY
DES
NTO
PTO
Melaleuca thymifolia
Climate
Climate + Soil
Soil
AUC = 0.98
TSS = 0.88
AUC = 0.99
TSS = 0.88
AUC = 0.97
TSS = 0.8
Relative importance
0.8
0.6
0.4
0.2
0.0
bio01
bio02
bio05
bio14
bio15
0.6
0.4
0.2
0.0
BDW
CEC
DES
NTO
SOC
bio01
bio02
bio05
bio14
bio15
0.6
0.4
0.2
0.0
BDW
CEC
DES
NTO
SOC

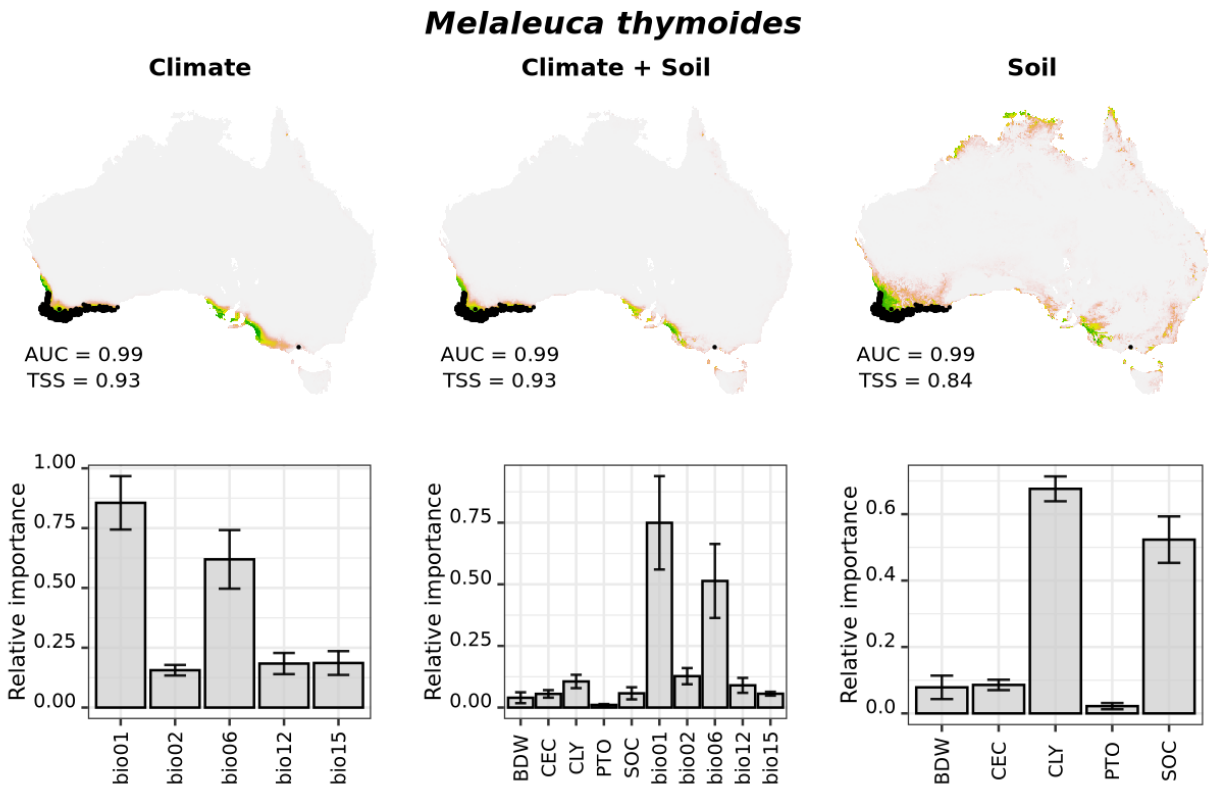
Melaleuca thymoides
Climate
Climate + Soil
Soil
AUC = 0.99
TSS = 0.93
AUC = 0.99
TSS = 0.93
AUC = 0.99
TSS = 0.84
Relative importance
1.00
0.75
0.50
0.25
0.00
bio01
bio02
bio06
bio12
bio15
BDW
CEC
CLY
PTO
SOC
0.6
0.4
0.2
0.0

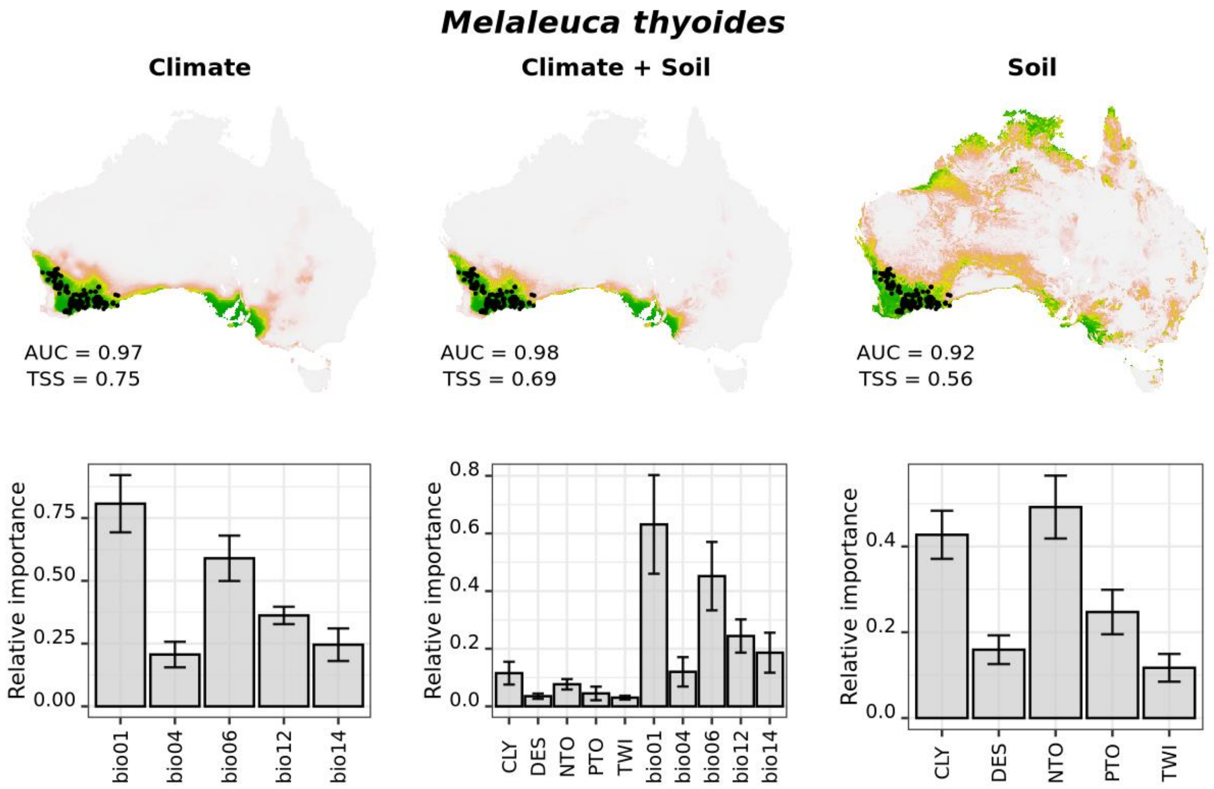
Melaleuca thyoides
Climate
Climate + Soil
Soil
AUC = 0.97
TSS = 0.75
AUC = 0.98
TSS = 0.69
AUC = 0.92
TSS = 0.56
Relative importance
0.75
0.50
0.25
0.00
0.8
0.6
0.4
0.2
0.0
bio01
bio04
bio06
bio12
bio14
CLY
DES
NTO
PTO
TWI

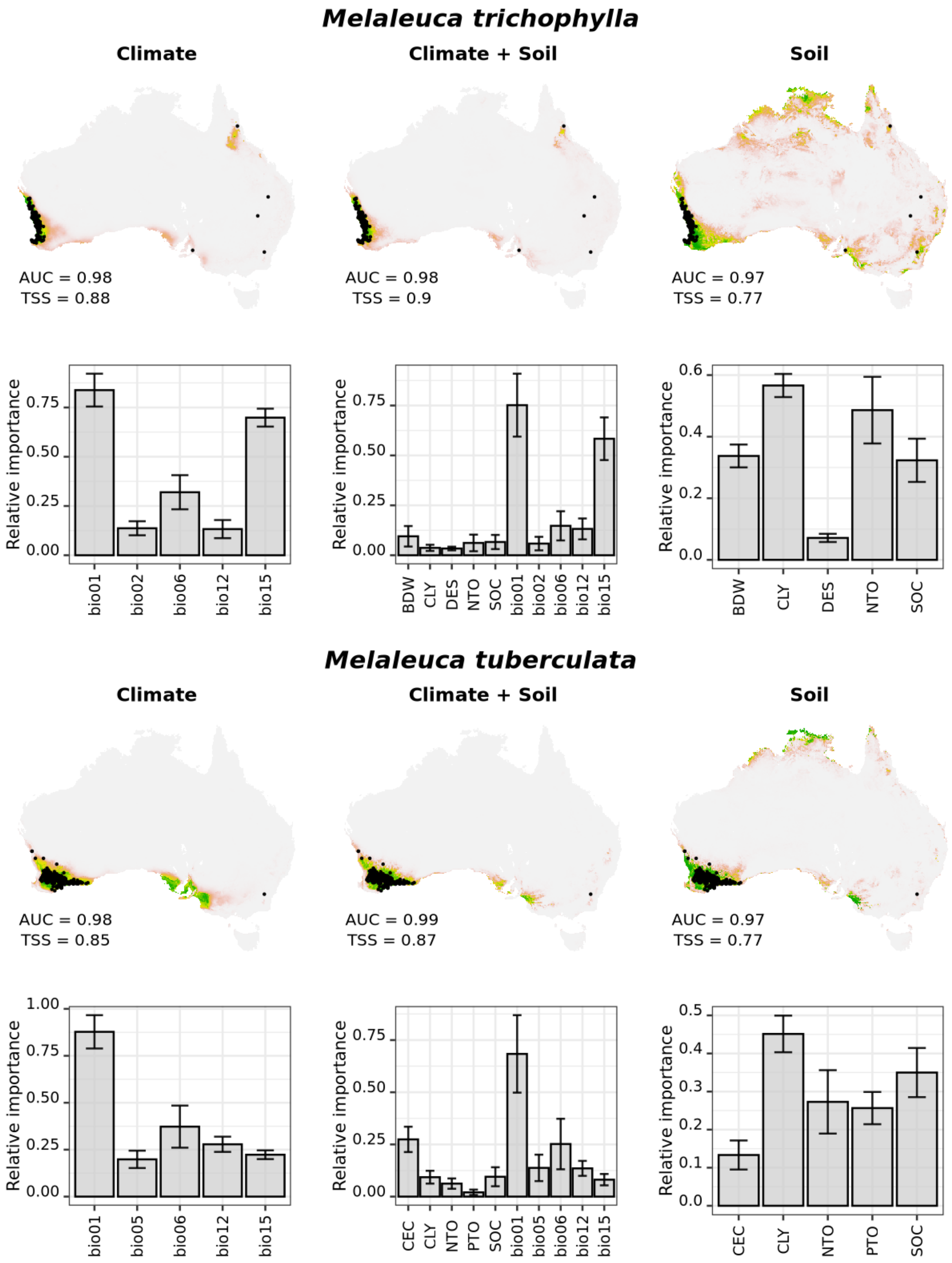

Melaleuca trichophylla
Climate
Climate + Soil
Soil
AUC = 0.98
TSS = 0.88
AUC = 0.98
TSS = 0.9
AUC = 0.97
TSS = 0.77
Relative importance
0.00
0.25
0.50
0.75
bio01
bio02
bio06
bio12
bio15
BDW
CLY
DES
NTO
SOC
0.0
0.2
0.4
0.6
Melaleuca tuberculata
AUC = 0.98
TSS = 0.85
AUC = 0.99
TSS = 0.87
AUC = 0.97
TSS = 0.77
1.00
bio05
CEC
PTO
0.1
0.3
0.5

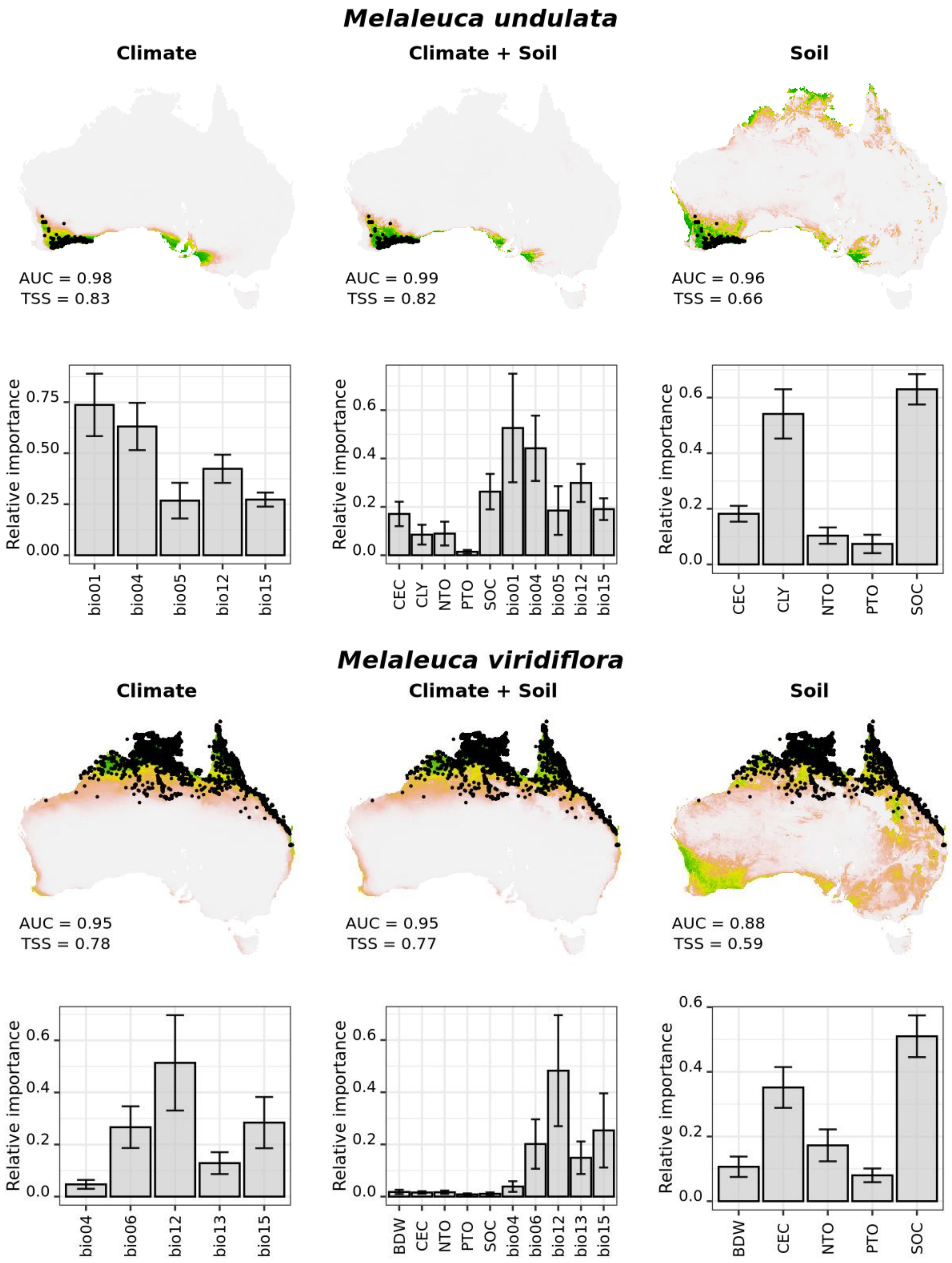
Melaleuca undulata
Climate
Climate + Soil
Soil
AUC = 0.98
TSS = 0.83
AUC = 0.99
TSS = 0.82
AUC = 0.96
TSS = 0.66
Relative importance
0.00
0.25
0.50
0.75
bio01
bio04
bio05
bio12
bio15
0.0
0.2
0.4
0.6
CEC
CLY
NTO
PTO
SOC
bio01
bio04
bio05
bio12
bio15
CEC
CLY
NTO
PTO
SOC
Melaleuca viridiflora
Climate
Climate + Soil
Soil
AUC = 0.95
TSS = 0.78
AUC = 0.95
TSS = 0.77
AUC = 0.88
TSS = 0.59
Relative importance
bio04
bio06
bio12
bio13
bio15
BDW
CEC
NTO
PTO
SOC
bio04
bio06
bio12
bio13
bio15
BDW
CEC
NTO
PTO
SOC

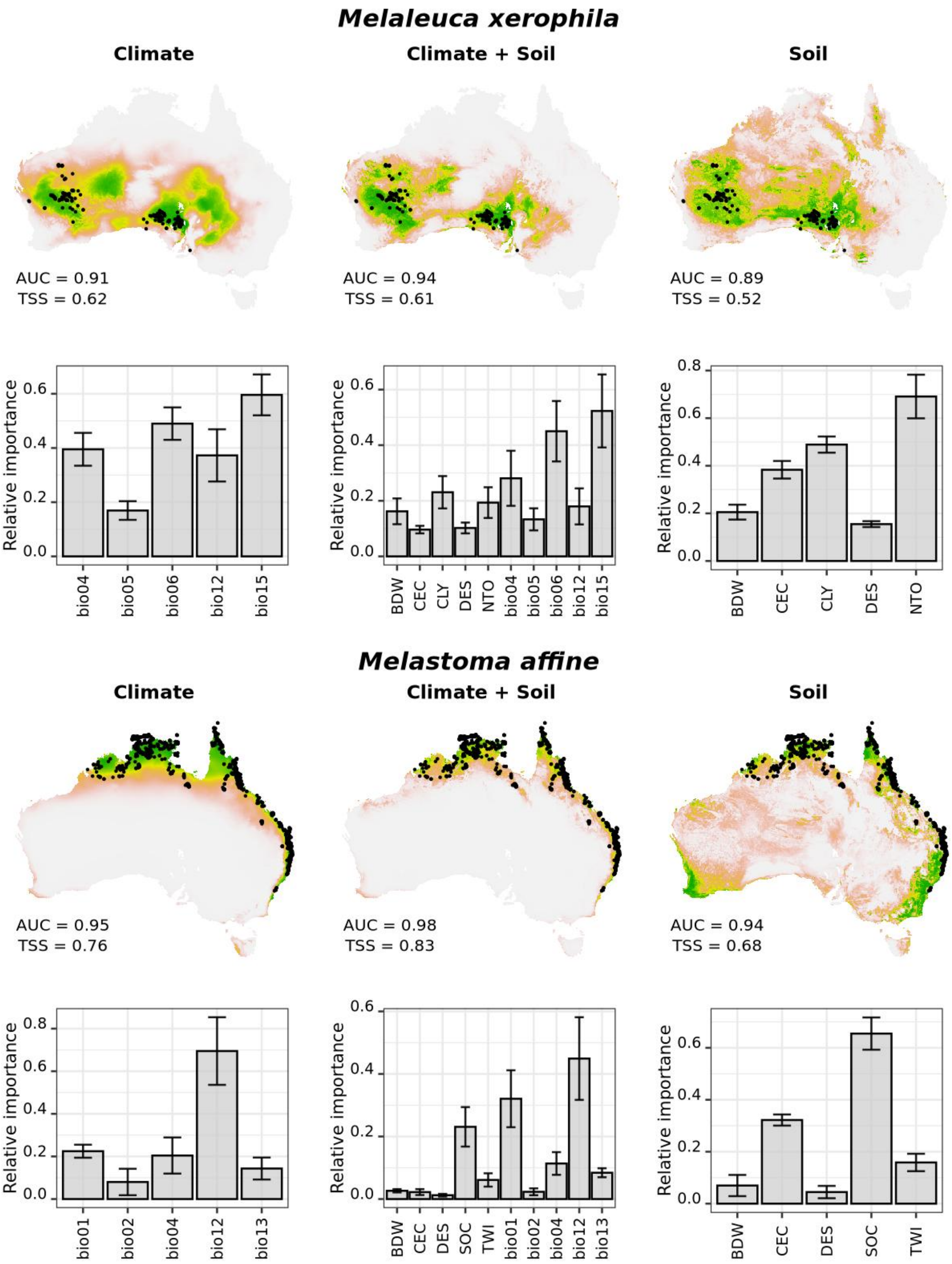

Melaleuca xerophila
Climate
Climate + Soil
Soil
AUC = 0.91
TSS = 0.62
AUC = 0.94
TSS = 0.61
AUC = 0.89
TSS = 0.52
Relative importance
0.0
0.2
0.4
0.6
0.8
bio04
bio05
bio06
bio12
bio15
BDW
CEC
CLY
DES
NTO
Melastoma affine
Climate
Climate + Soil
Soil
AUC = 0.95
TSS = 0.76
AUC = 0.98
TSS = 0.83
AUC = 0.94
TSS = 0.68
Relative importance
bio01
bio02
bio04
bio12
bio13
BDW
CEC
DES
SOC
TWI

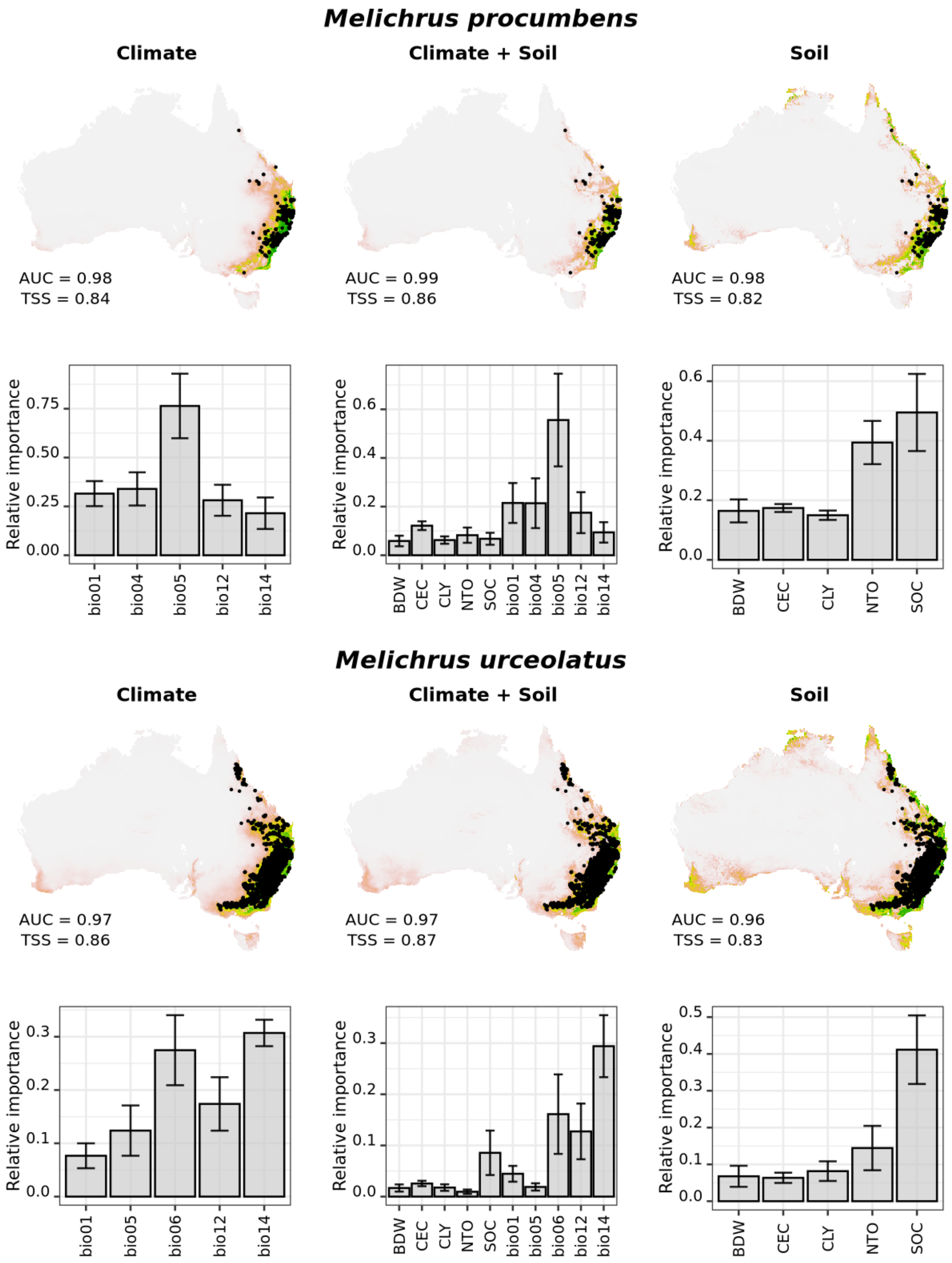
Melichrus procumbens
Climate
Climate + Soil
Soil
AUC = 0.98
TSS = 0.84
AUC = 0.99
TSS = 0.86
AUC = 0.98
TSS = 0.82
Relative importance
bio01 bio04 bio05 bio12 bio14
BDW CEC CLY NTO SOC bio01 bio04 bio05 bio12 bio14
BDW CEC CLY NTO SOC
Melichrus urceolatus
Climate
Climate + Soil
Soil
AUC = 0.97
TSS = 0.86
AUC = 0.97
TSS = 0.87
AUC = 0.96
TSS = 0.83
Relative importance
bio01 bio05 bio06 bio12 bio14
BDW CEC CLY NTO SOC bio01 bio05 bio06 bio12 bio14
BDW CEC CLY NTO SOC

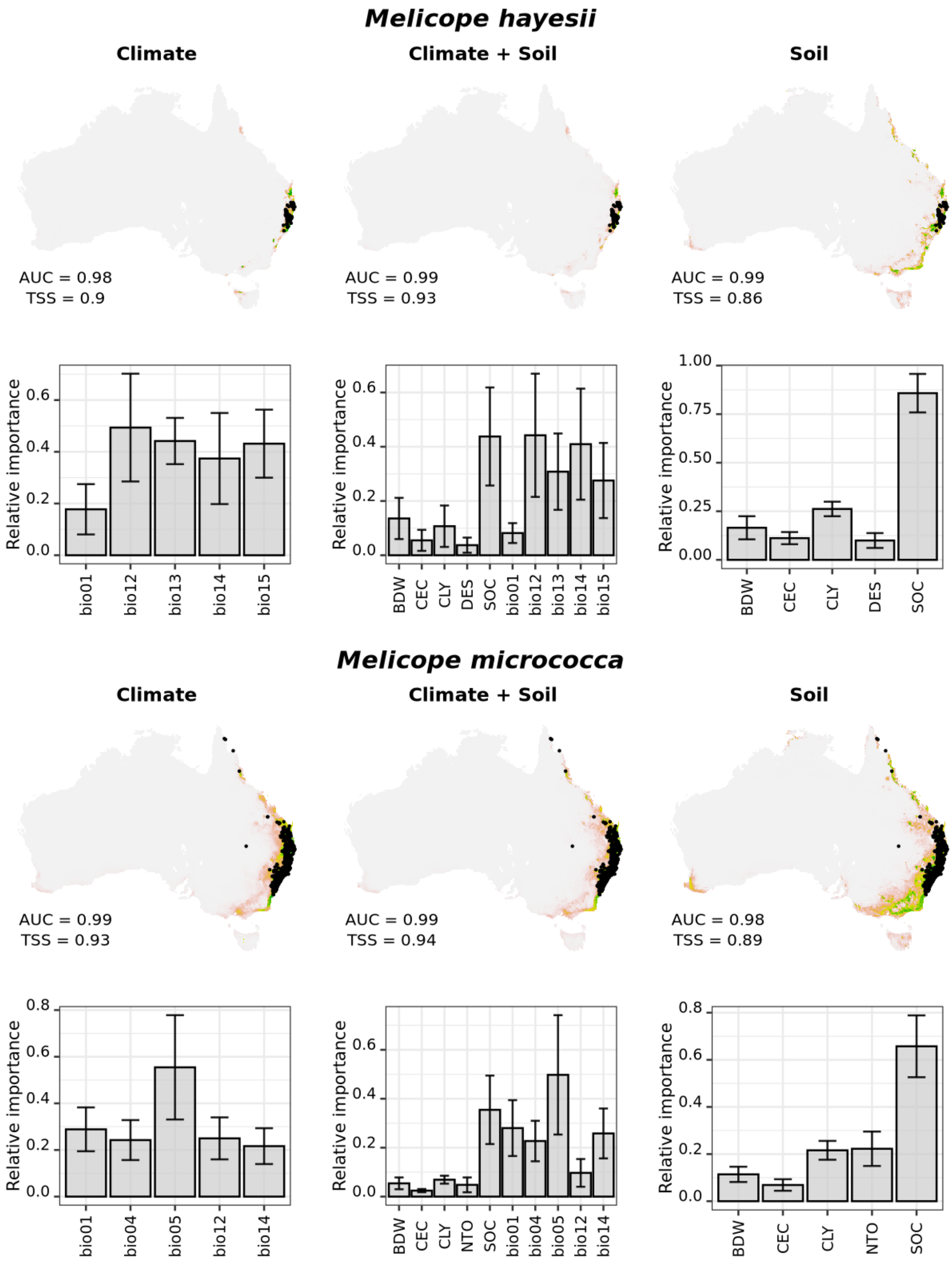
Melicope hayesii
Climate
Climate + Soil
Soil
AUC = 0.98
TSS = 0.9
AUC = 0.99
TSS = 0.93
AUC = 0.99
TSS = 0.86
Relative importance
bio01
bio12
bio13
bio14
bio15
BDW
CEC
CLY
DES
SOC
Melicope micrococca
Climate
Climate + Soil
Soil
AUC = 0.99
TSS = 0.93
AUC = 0.99
TSS = 0.94
AUC = 0.98
TSS = 0.89
Relative importance
bio01
bio04
bio05
bio12
bio14
BDW
CEC
CLY
NTO
SOC

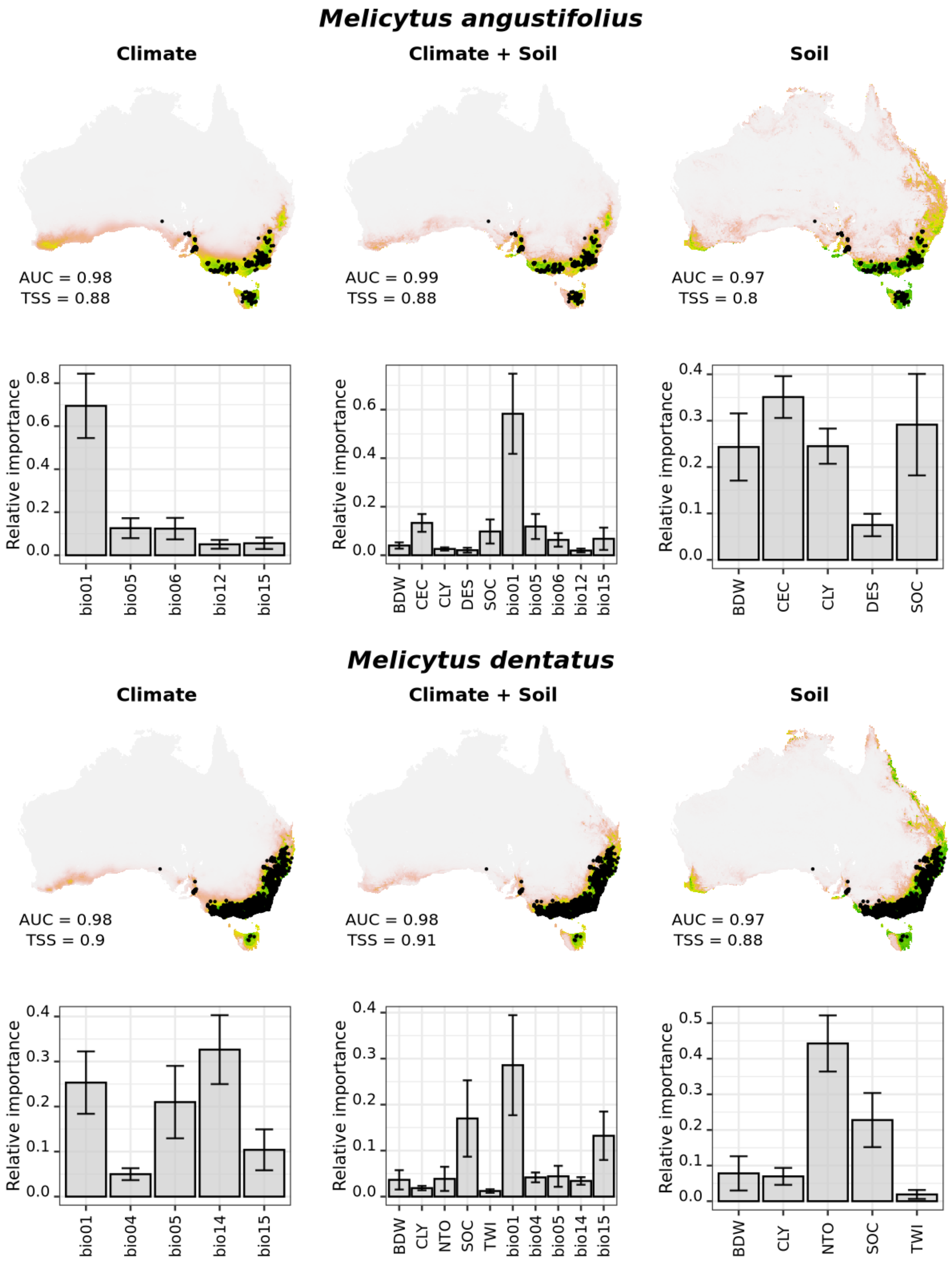

Melicytus angustifolius
Climate
Climate + Soil
Soil
AUC = 0.98
TSS = 0.88
AUC = 0.99
TSS = 0.88
AUC = 0.97
TSS = 0.8
Relative importance
bio01
bio05
bio06
bio12
bio15
BDW
CEC
CLY
DES
SOC
Melicytus dentatus
Climate
Climate + Soil
Soil
AUC = 0.98
TSS = 0.9
AUC = 0.98
TSS = 0.91
AUC = 0.97
TSS = 0.88
Relative importance
bio01
bio04
bio05
bio14
bio15
BDW
CLY
NTO
SOC
TWI

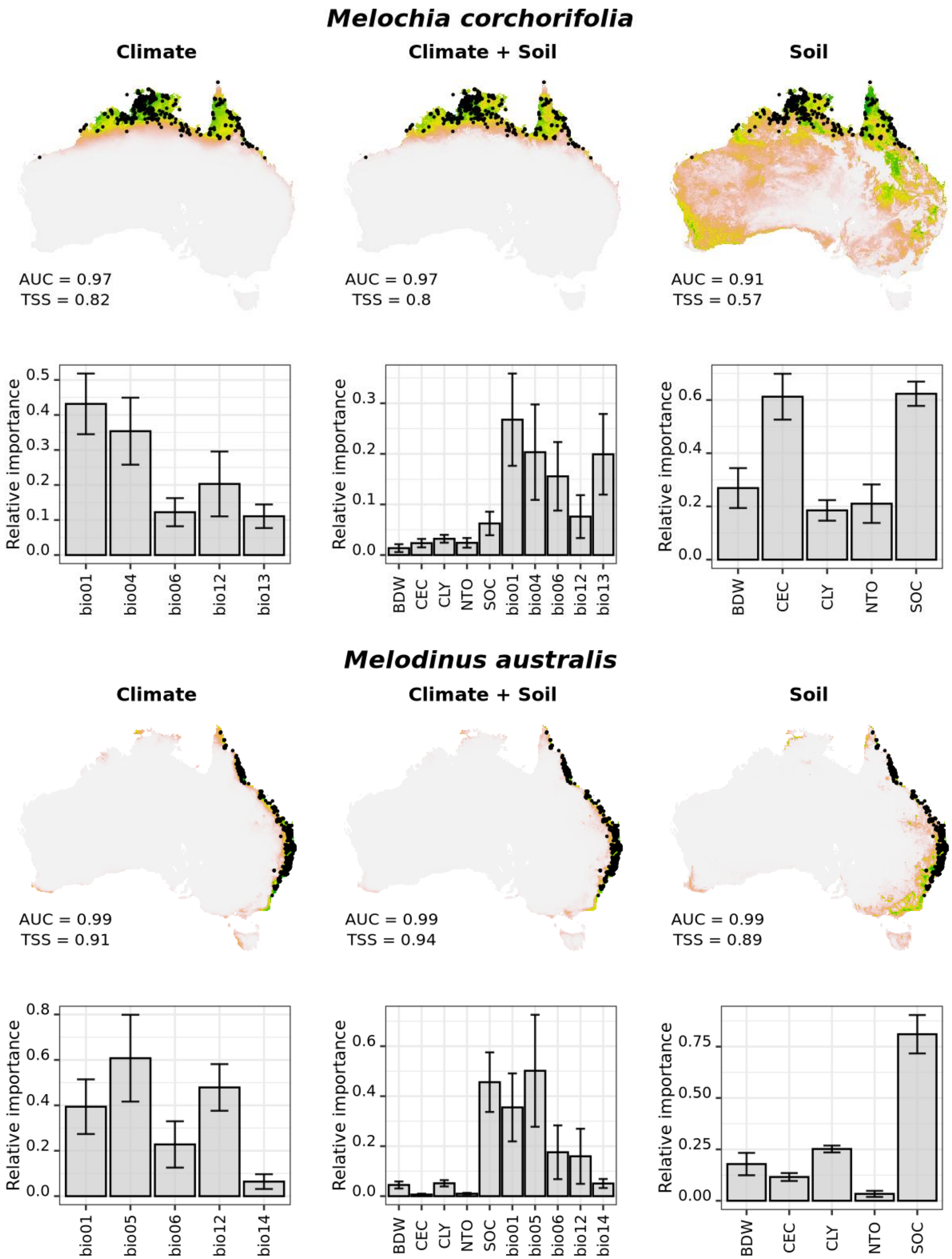
Melochia corchorifolia
Climate
Climate + Soil
Soil
AUC = 0.97
TSS = 0.82
AUC = 0.97
TSS = 0.8
AUC = 0.91
TSS = 0.57
Relative importance
0.0 0.1 0.2 0.3 0.4 0.5
bio01 bio04 bio06 bio12 bio13
Relative importance
0.0 0.1 0.2 0.3
BDW CEC CLY NTO SOC bio01 bio04 bio06 bio12 bio13
Relative importance
0.0 0.2 0.4 0.6
BDW CEC CLY NTO SOC
Melodinus australis
Climate
Climate + Soil
Soil
AUC = 0.99
TSS = 0.91
AUC = 0.99
TSS = 0.94
AUC = 0.99
TSS = 0.89
Relative importance
0.0 0.2 0.4 0.6 0.8
bio01 bio05 bio06 bio12 bio14
Relative importance
0.0 0.2 0.4 0.6
BDW CEC CLY NTO SOC bio01 bio05 bio06 bio12 bio14
Relative importance
0.00 0.25 0.50 0.75
BDW CEC CLY NTO SOC

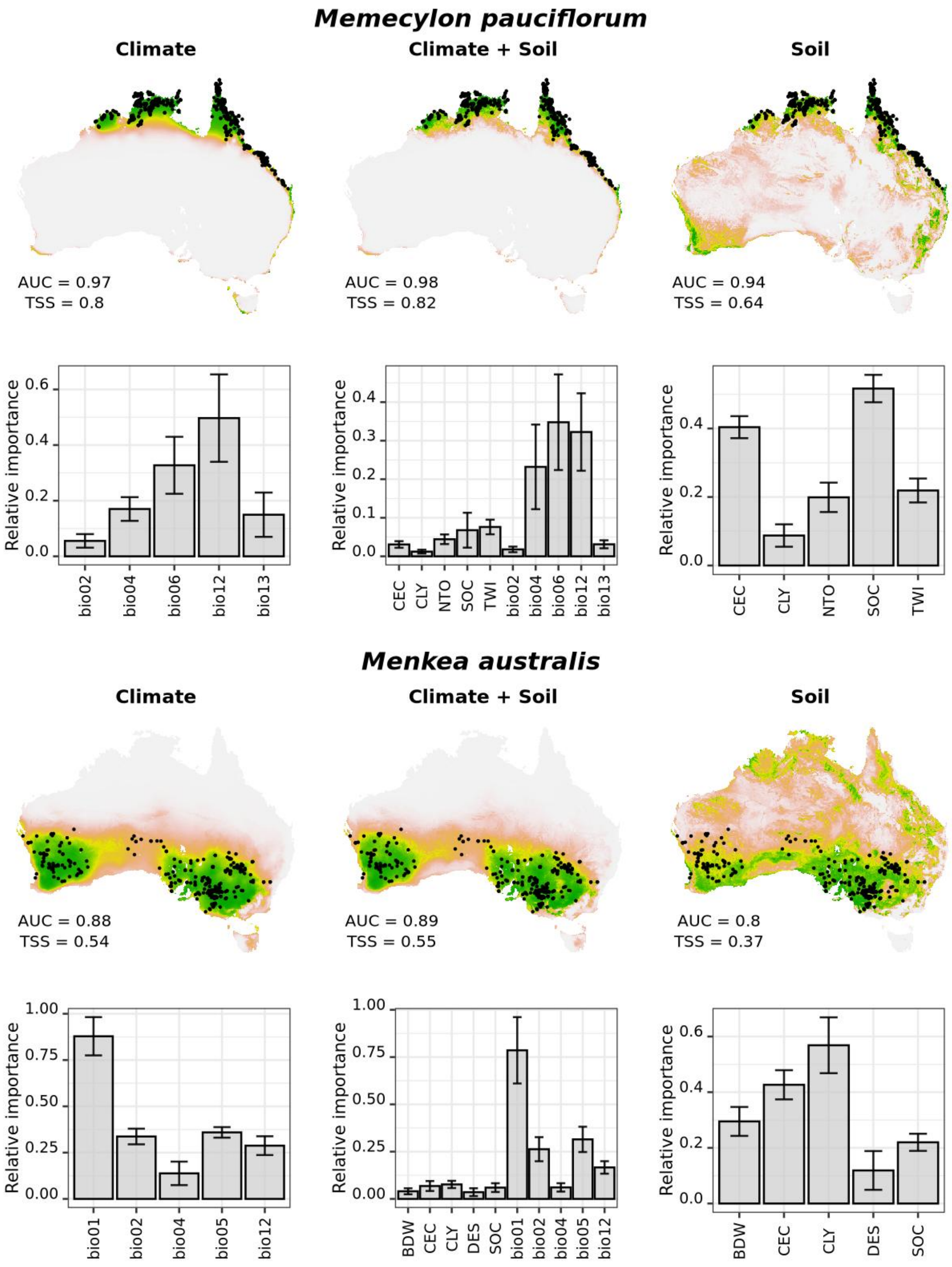
Memecylon pauciflorum
Climate
Climate + Soil
Soil
AUC = 0.97
TSS = 0.8
AUC = 0.98
TSS = 0.82
AUC = 0.94
TSS = 0.64
Relative importance
bio02
bio04
bio06
bio12
bio13
CEC
CLY
NTO
SOC
TWI
bio02
bio04
bio06
bio12
bio13
CEC
CLY
NTO
SOC
TWI
Menkea australis
Climate
Climate + Soil
Soil
AUC = 0.88
TSS = 0.54
AUC = 0.89
TSS = 0.55
AUC = 0.8
TSS = 0.37
Relative importance
bio01
bio02
bio04
bio05
bio12
BDW
CEC
CLY
DES
SOC
bio01
bio02
bio04
bio05
bio12
BDW
CEC
CLY
DES
SOC

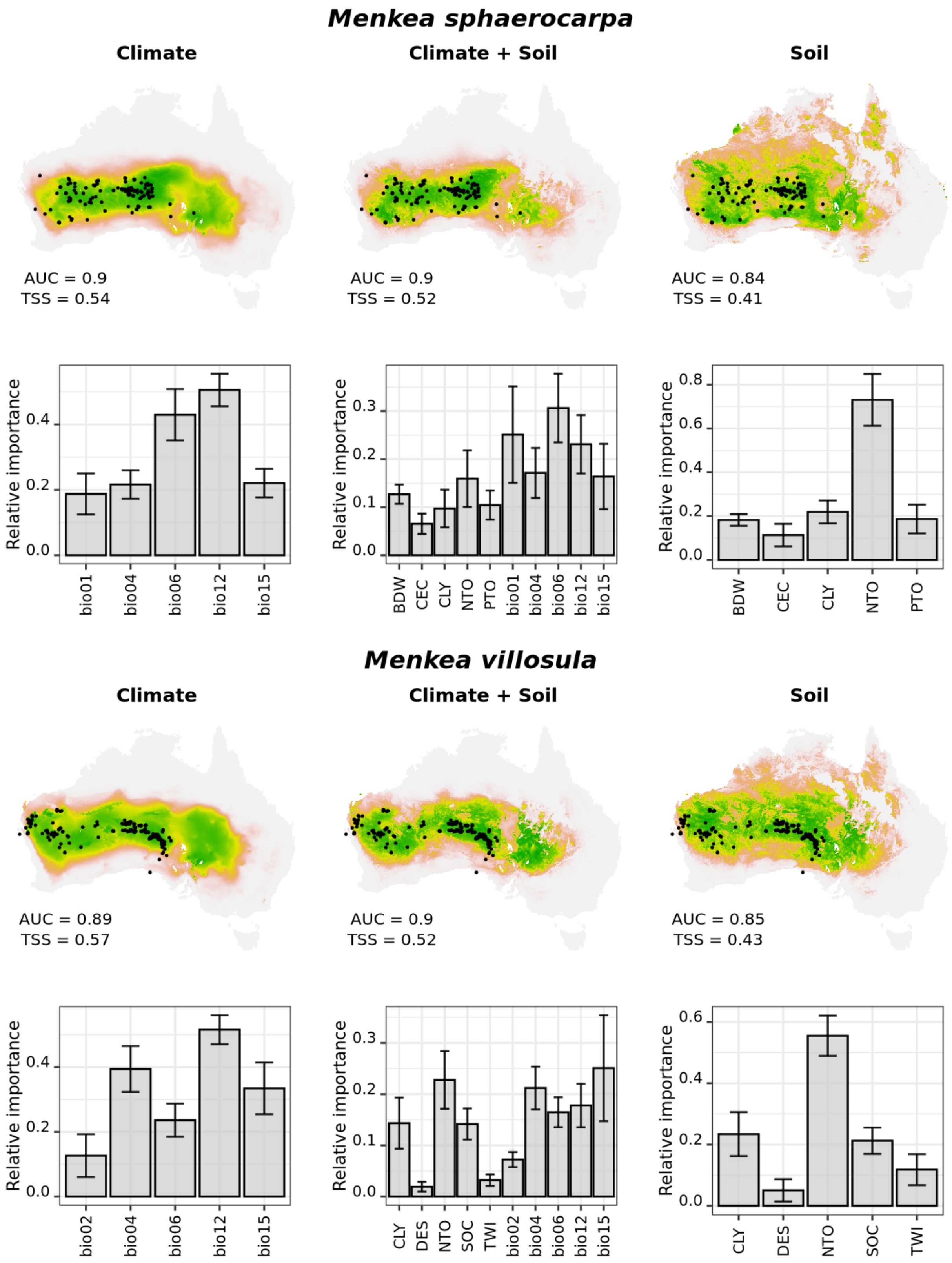

Menkea sphaerocarpa
Climate
Climate + Soil
Soil
AUC = 0.9
TSS = 0.54
AUC = 0.9
TSS = 0.52
AUC = 0.84
TSS = 0.41
Relative importance
0.0
0.2
0.4
bio01
bio04
bio06
bio12
bio15
Relative importance
0.0
0.1
0.2
0.3
BDW
CEC
CLY
NTO
PTO
bio01
bio04
bio06
bio12
bio15
Relative importance
0.0
0.2
0.4
0.6
0.8
BDW
CEC
CLY
NTO
PTO
Menkea villosula
Climate
Climate + Soil
Soil
AUC = 0.89
TSS = 0.57
AUC = 0.9
TSS = 0.52
AUC = 0.85
TSS = 0.43
Relative importance
0.0
0.2
0.4
bio02
bio04
bio06
bio12
bio15
Relative importance
0.0
0.1
0.2
0.3
CLY
DES
NTO
SOC
TWI
bio02
bio04
bio06
bio12
bio15
Relative importance
0.0
0.2
0.4
0.6
CLY
DES
NTO
SOC
TWI

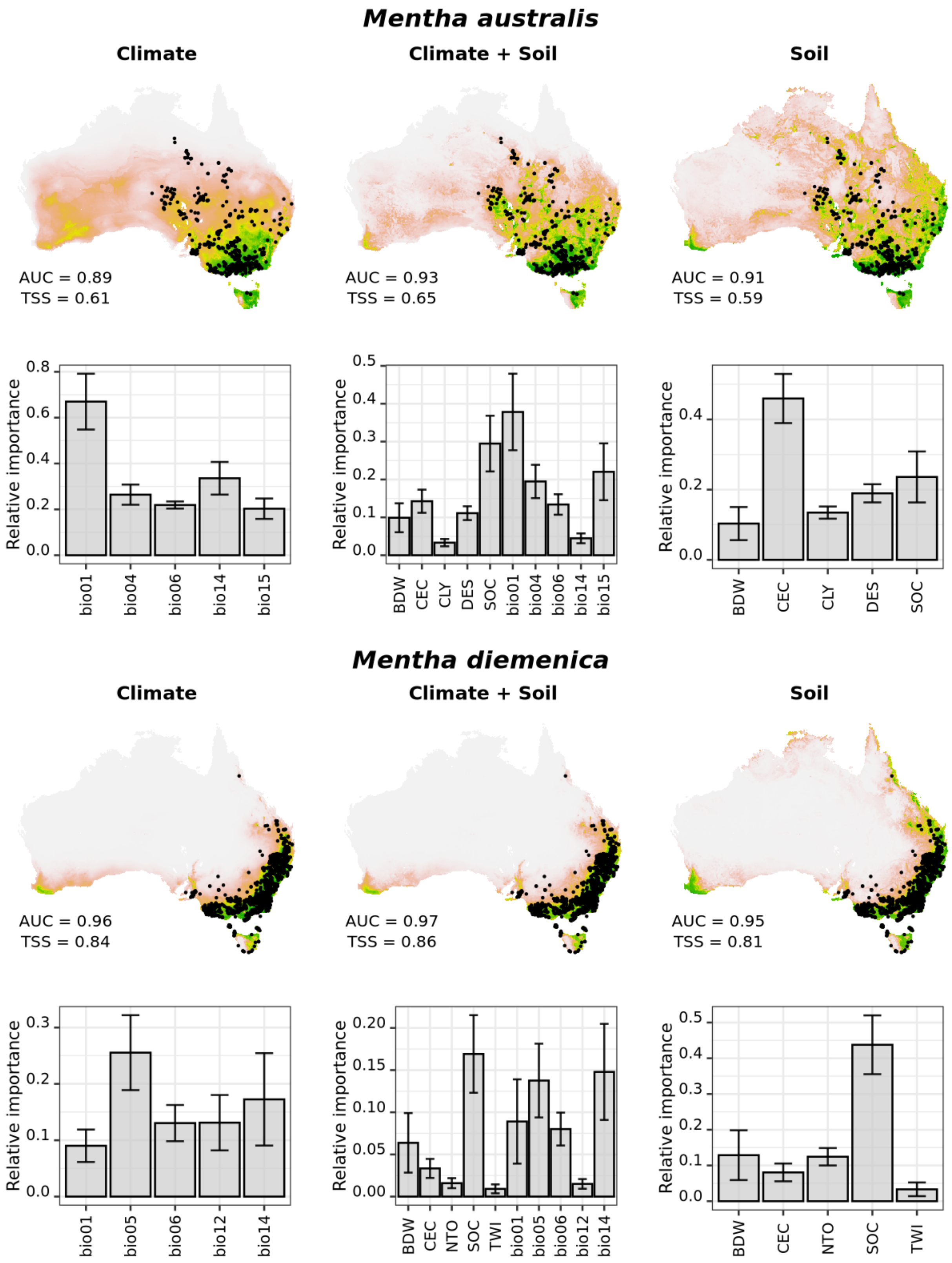

Mentha australis
Climate
Climate + Soil
Soil
AUC = 0.89
TSS = 0.61
AUC = 0.93
TSS = 0.65
AUC = 0.91
TSS = 0.59
Relative importance
bio01 bio04 bio06 bio14 bio15
BDW CEC CLY DES SOC bio01 bio04 bio06 bio14 bio15
BDW CEC CLY DES SOC
Mentha diemenica
Climate
Climate + Soil
Soil
AUC = 0.96
TSS = 0.84
AUC = 0.97
TSS = 0.86
AUC = 0.95
TSS = 0.81
Relative importance
bio01 bio05 bio06 bio12 bio14
BDW CEC NTO SOC TWI bio01 bio05 bio06 bio12 bio14
BDW CEC NTO SOC TWI

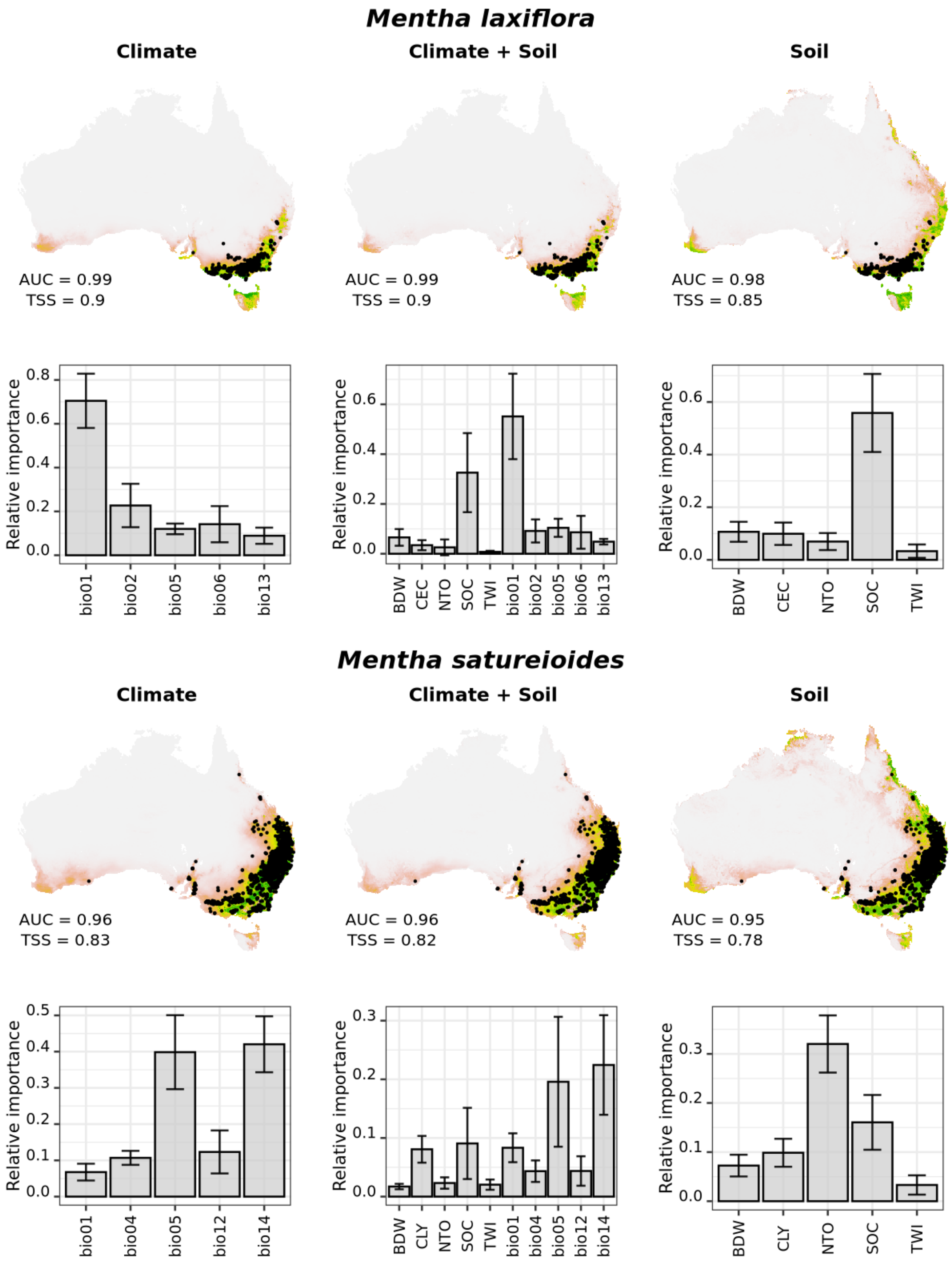
Mentha laxiflora
Climate
Climate + Soil
Soil
AUC = 0.99
TSS = 0.9
AUC = 0.99
TSS = 0.9
AUC = 0.98
TSS = 0.85
Relative importance
bio01
bio02
bio05
bio06
bio13
BDW
CEC
NTO
SOC
TWI
Mentha satureioides
Climate
Climate + Soil
Soil
AUC = 0.96
TSS = 0.83
AUC = 0.96
TSS = 0.82
AUC = 0.95
TSS = 0.78
Relative importance
bio01
bio04
bio05
bio12
bio14
BDW
CLY
NTO
SOC
TWI

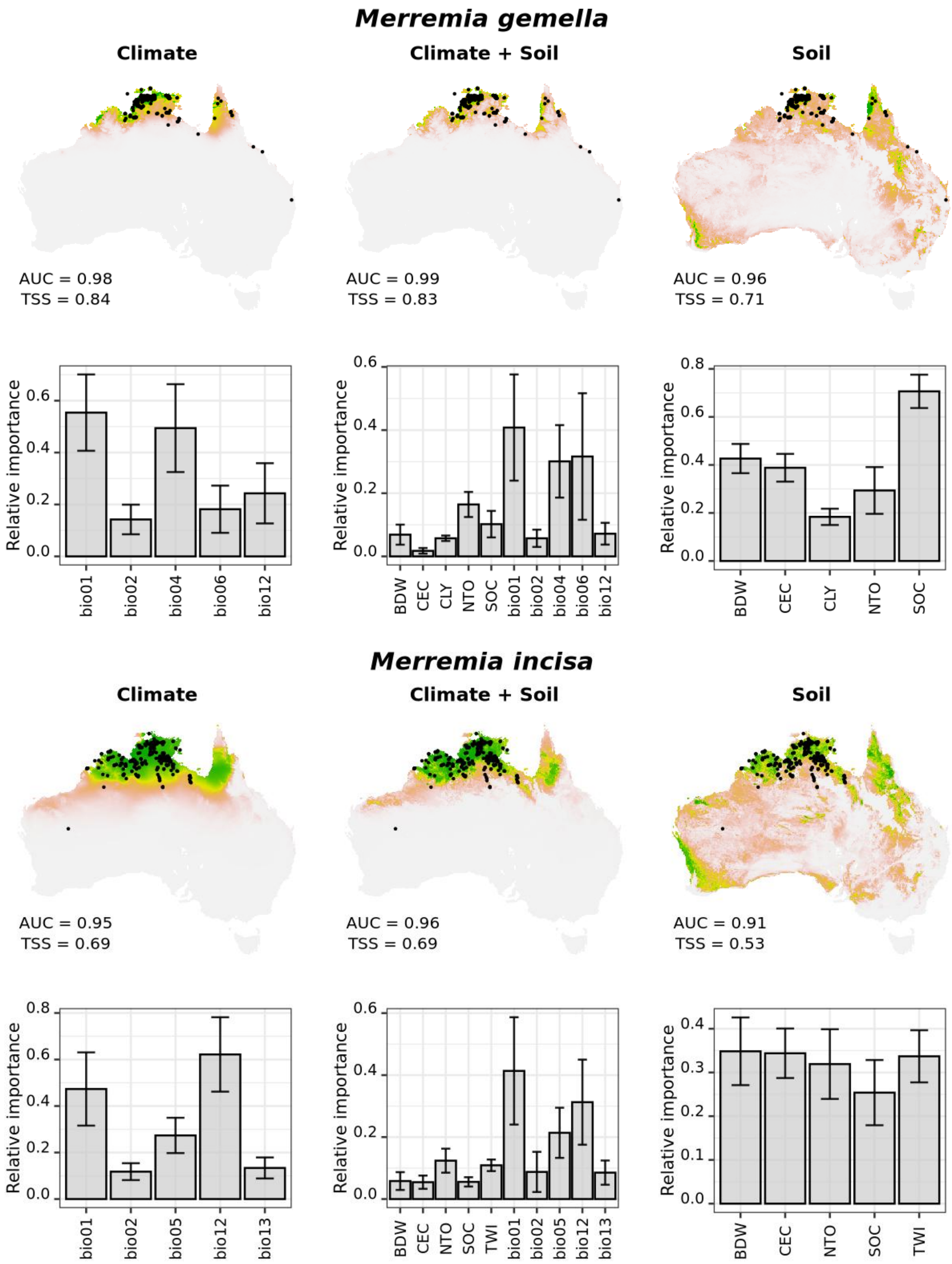
Merremia gemella
Climate
Climate + Soil
Soil
AUC = 0.98
TSS = 0.84
AUC = 0.99
TSS = 0.83
AUC = 0.96
TSS = 0.71
Relative importance
bio01
bio02
bio04
bio06
bio12
BDW
CEC
CLY
NTO
SOC
Merremia incisa
Climate
Climate + Soil
Soil
AUC = 0.95
TSS = 0.69
AUC = 0.96
TSS = 0.69
AUC = 0.91
TSS = 0.53
Relative importance
bio01
bio02
bio05
bio12
bio13
BDW
CEC
NTO
SOC
TWI

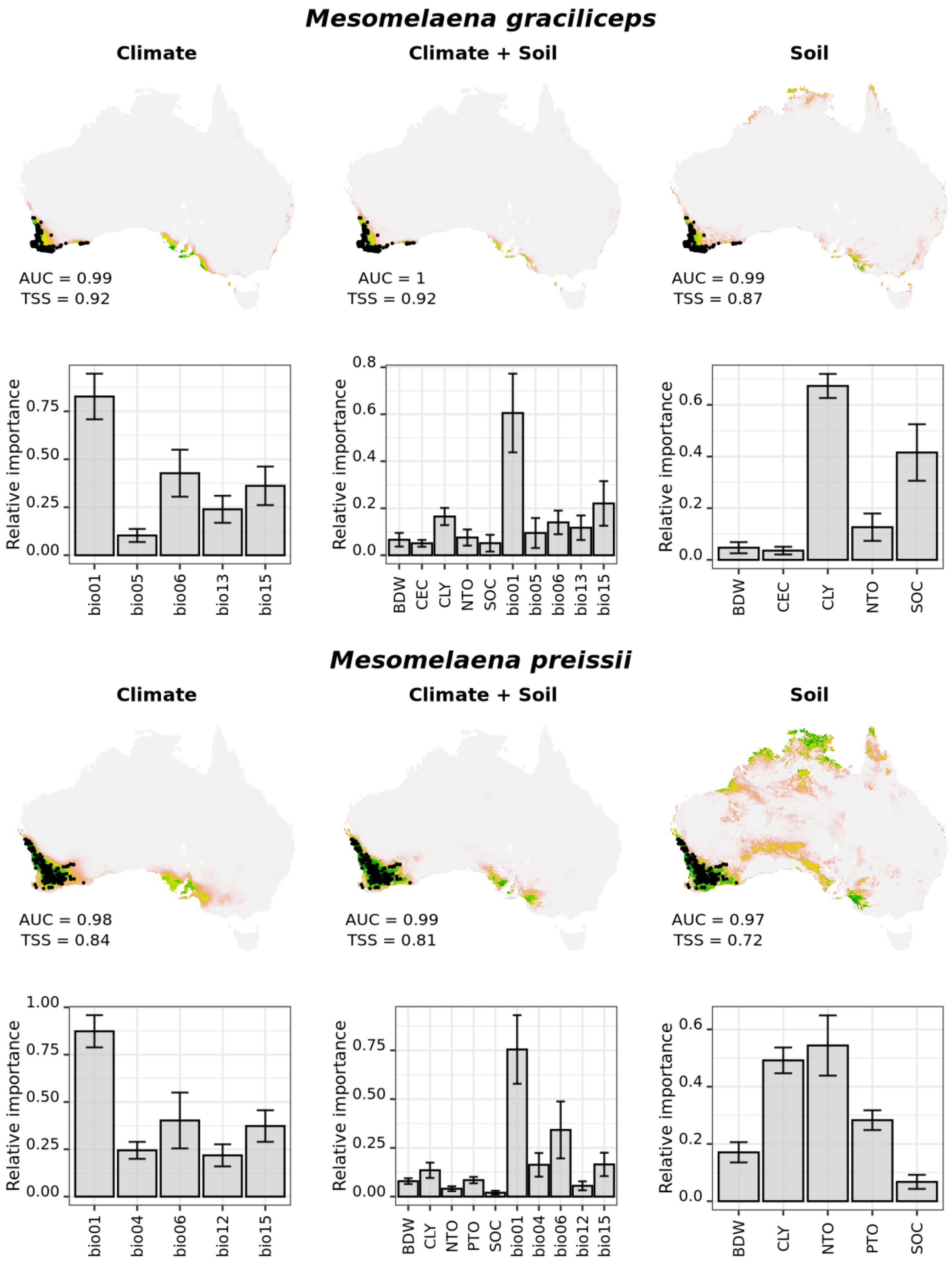

Mesomelaena graciliceps
Climate
Climate + Soil
Soil
AUC = 0.99
TSS = 0.92
AUC = 1
TSS = 0.92
AUC = 0.99
TSS = 0.87
Relative importance
bio01
bio05
bio06
bio13
bio15
BDW
CEC
CLY
NTO
SOC
Mesomelaena preissii
AUC = 0.98
TSS = 0.84
AUC = 0.99
TSS = 0.81
AUC = 0.97
TSS = 0.72
bio04
bio12
PTO

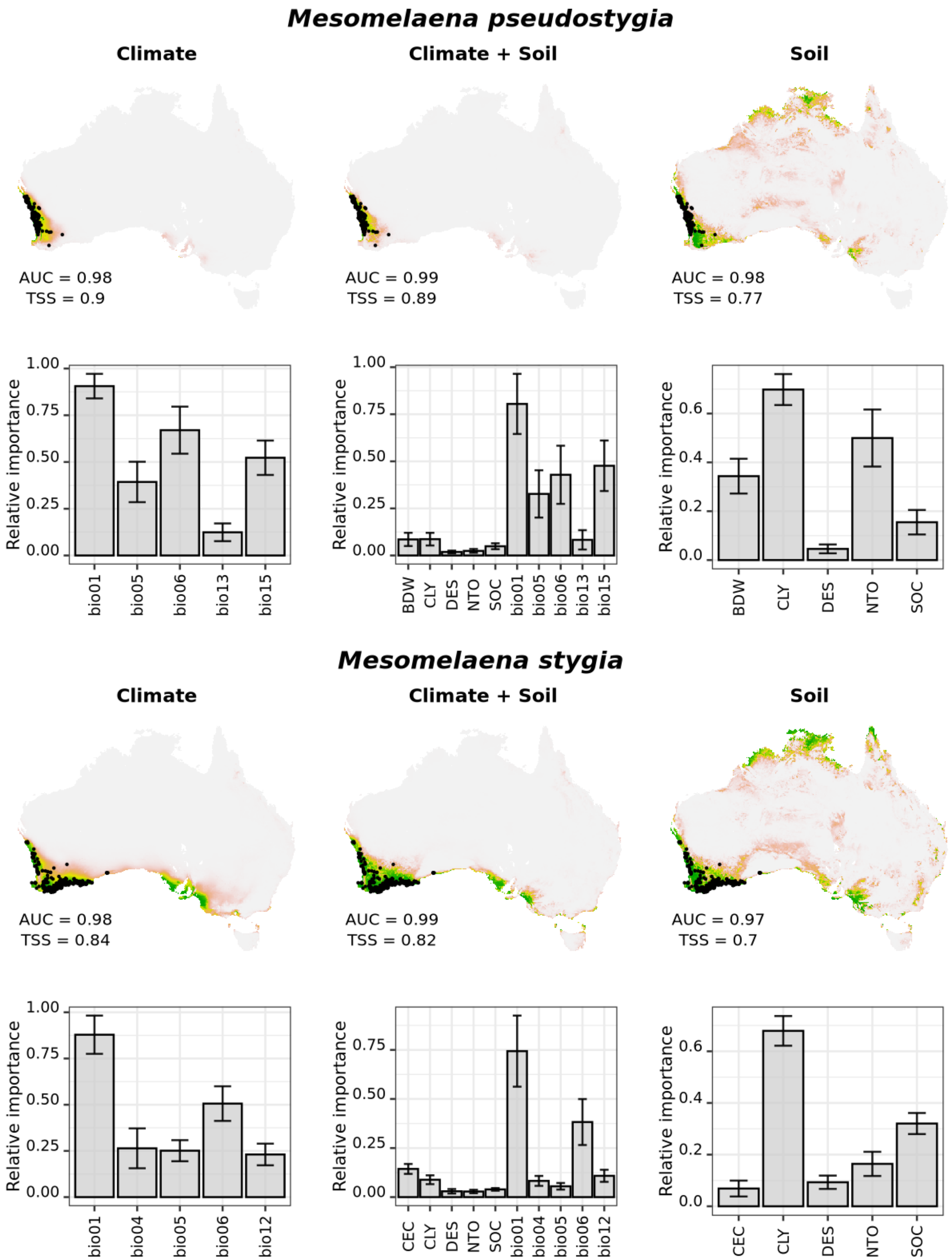

Mesomelaena pseudostygia
Climate
Climate + Soil
Soil
AUC = 0.98
TSS = 0.9
AUC = 0.99
TSS = 0.89
AUC = 0.98
TSS = 0.77
Relative importance
1.00
0.75
0.50
0.25
0.00
bio01
bio05
bio06
bio13
bio15
BDW
CLY
DES
NTO
SOC
0.6
0.4
0.2
0.0
Mesomelaena stygia
Climate
Climate + Soil
Soil
AUC = 0.98
TSS = 0.84
AUC = 0.99
TSS = 0.82
AUC = 0.97
TSS = 0.7
bio01
bio04
bio05
bio06
bio12
CEC
CLY
DES
NTO
SOC

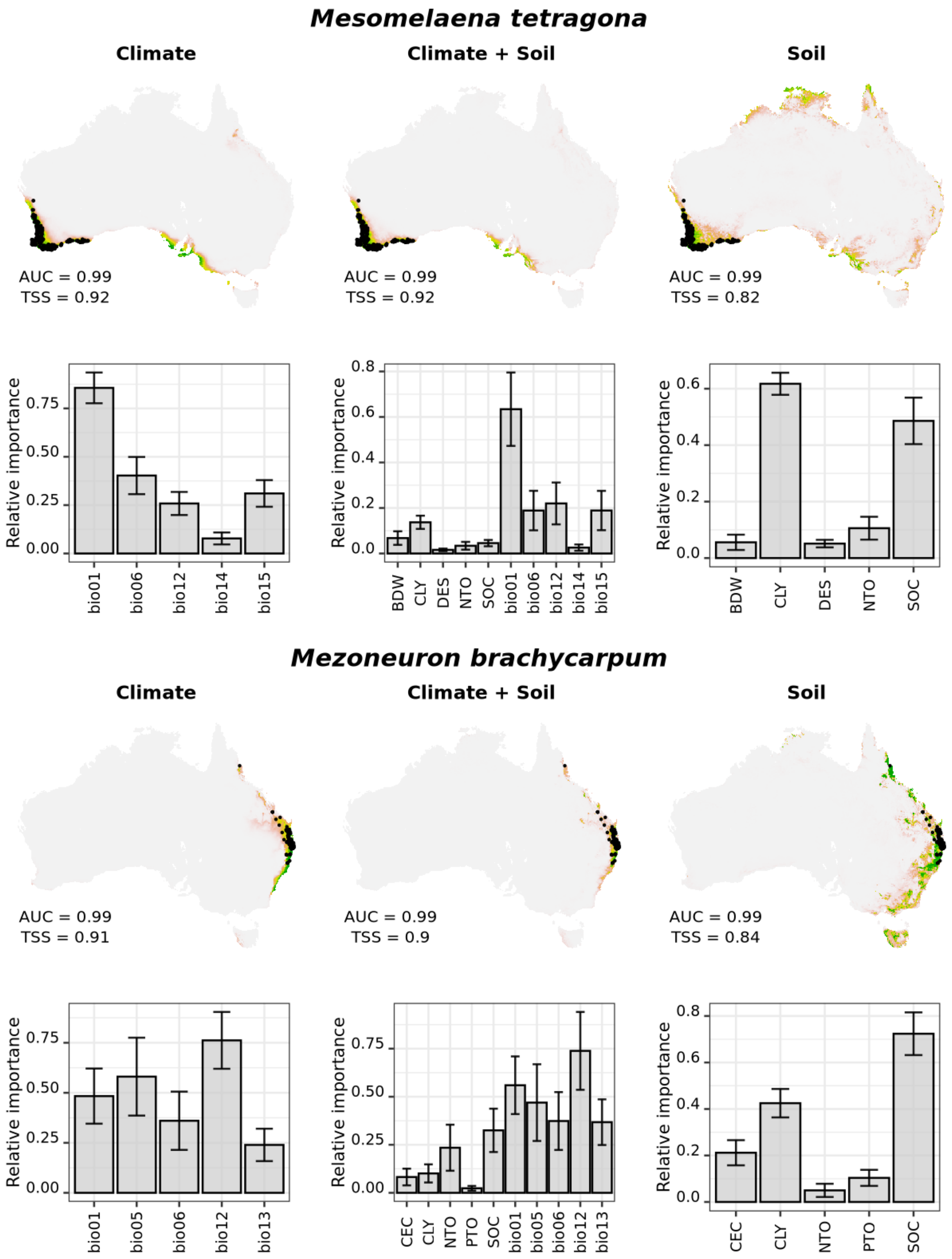
Mesomelaena tetragona
Climate
Climate + Soil
Soil
AUC = 0.99
TSS = 0.92
AUC = 0.99
TSS = 0.92
AUC = 0.99
TSS = 0.82
Relative importance
0.00
0.25
0.50
0.75
bio01
bio06
bio12
bio14
bio15
0.0
0.2
0.4
0.6
0.8
BDW
CLY
DES
NTO
SOC
bio01
bio06
bio12
bio14
bio15
0.0
0.2
0.4
0.6
BDW
CLY
DES
NTO
SOC
Mezoneuron brachycarpum
Climate
Climate + Soil
Soil
AUC = 0.99
TSS = 0.91
AUC = 0.99
TSS = 0.9
AUC = 0.99
TSS = 0.84
Relative importance
0.00
0.25
0.50
0.75
bio01
bio05
bio06
bio12
bio13
0.00
0.25
0.50
0.75
CEC
CLY
NTO
PTO
SOC
bio01
bio05
bio06
bio12
bio13
0.0
0.2
0.4
0.6
0.8
CEC
CLY
NTO
PTO
SOC

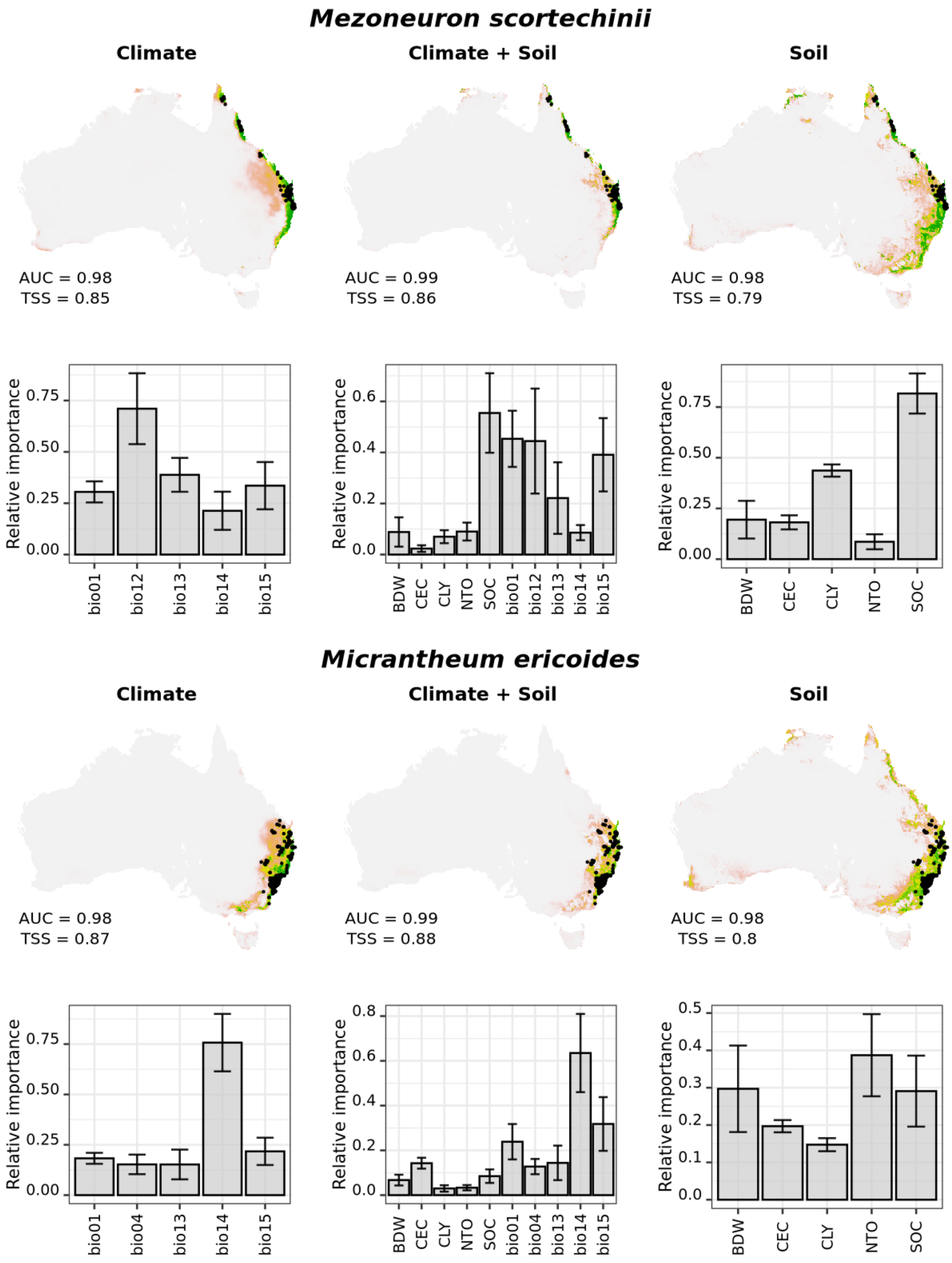
Mezoneuron scortechinii
Climate
Climate + Soil
Soil
AUC = 0.98
TSS = 0.85
AUC = 0.99
TSS = 0.86
AUC = 0.98
TSS = 0.79
Relative importance
0.00
0.25
0.50
0.75
bio01
bio12
bio13
bio14
bio15
Relative importance
0.0
0.2
0.4
0.6
BDW
CEC
CLY
NTO
SOC
bio01
bio12
bio13
bio14
bio15
Relative importance
0.00
0.25
0.50
0.75
BDW
CEC
CLY
NTO
SOC
Micrantheum ericoides
Climate
Climate + Soil
Soil
AUC = 0.98
TSS = 0.87
AUC = 0.99
TSS = 0.88
AUC = 0.98
TSS = 0.8
Relative importance
0.00
0.25
0.50
0.75
bio01
bio04
bio13
bio14
bio15
Relative importance
0.0
0.2
0.4
0.6
0.8
BDW
CEC
CLY
NTO
SOC
bio01
bio04
bio13
bio14
bio15
Relative importance
0.0
0.1
0.2
0.3
0.4
0.5
BDW
CEC
CLY
NTO
SOC

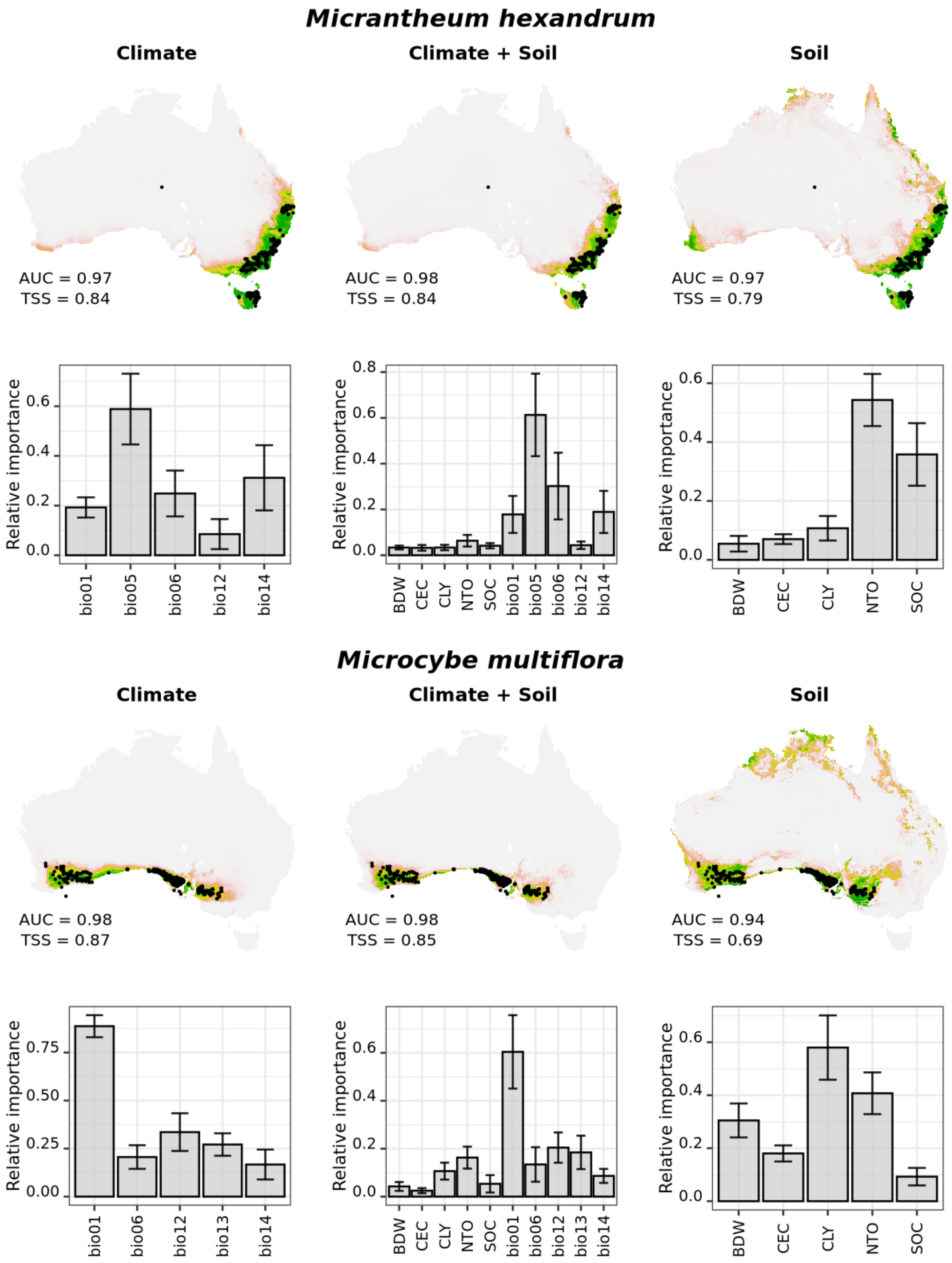
Micrantheum hexandrum
Climate
Climate + Soil
Soil
AUC = 0.97
TSS = 0.84
AUC = 0.98
TSS = 0.84
AUC = 0.97
TSS = 0.79
Relative importance
bio01
bio05
bio06
bio12
bio14
BDW
CEC
CLY
NTO
SOC
Microcybe multiflora
Climate
Climate + Soil
Soil
AUC = 0.98
TSS = 0.87
AUC = 0.98
TSS = 0.85
AUC = 0.94
TSS = 0.69
Relative importance
bio01
bio06
bio12
bio13
bio14
BDW
CEC
CLY
NTO
SOC

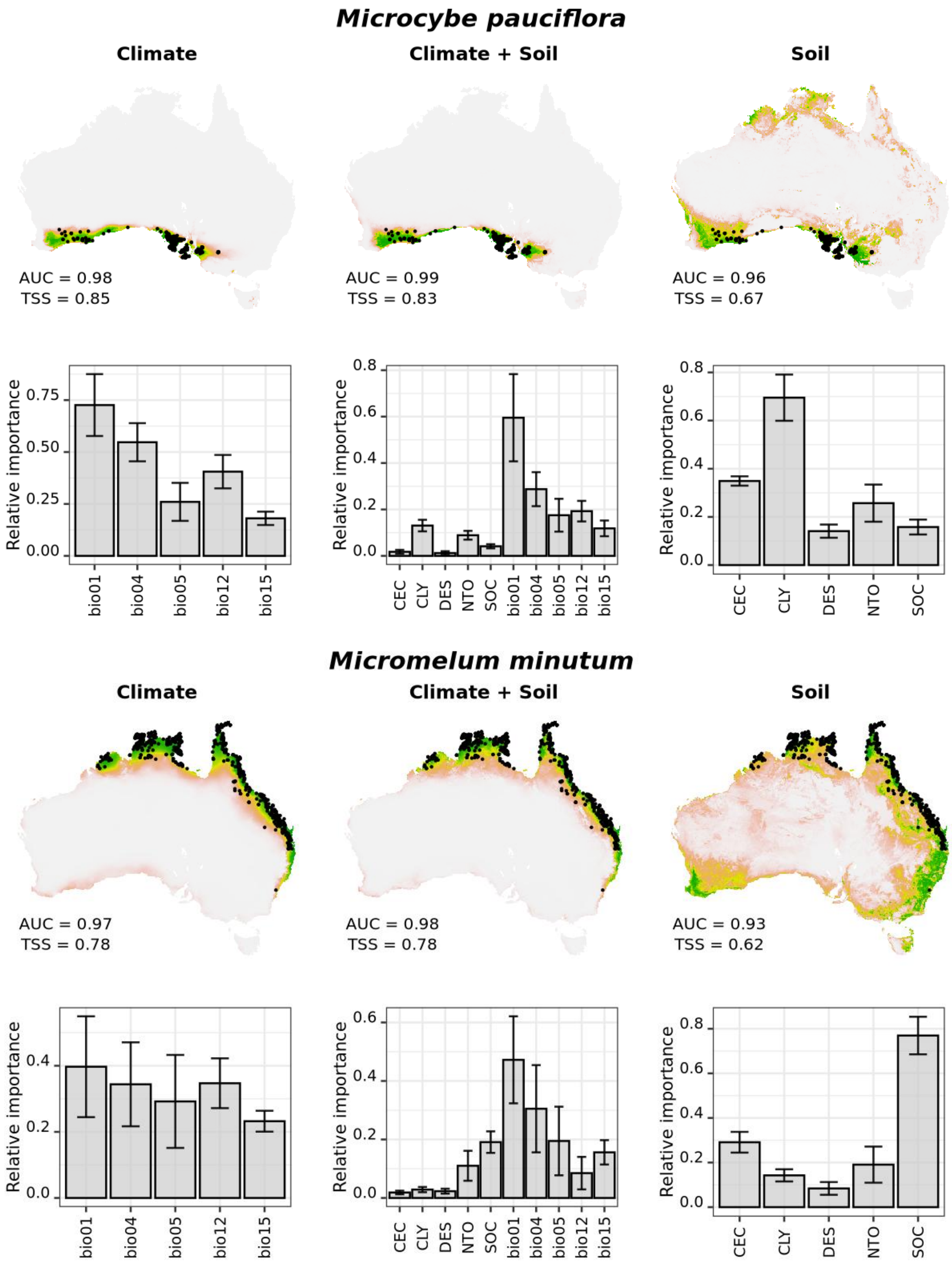

Microcybe pauciflora
Climate
Climate + Soil
Soil
AUC = 0.98
TSS = 0.85
AUC = 0.99
TSS = 0.83
AUC = 0.96
TSS = 0.67
Relative importance
bio01
bio04
bio05
bio12
bio15
CEC
CLY
DES
NTO
SOC
Micromelum minutum
Climate
Climate + Soil
Soil
AUC = 0.97
TSS = 0.78
AUC = 0.98
TSS = 0.78
AUC = 0.93
TSS = 0.62
Relative importance
bio01
bio04
bio05
bio12
bio15
CEC
CLY
DES
NTO
SOC

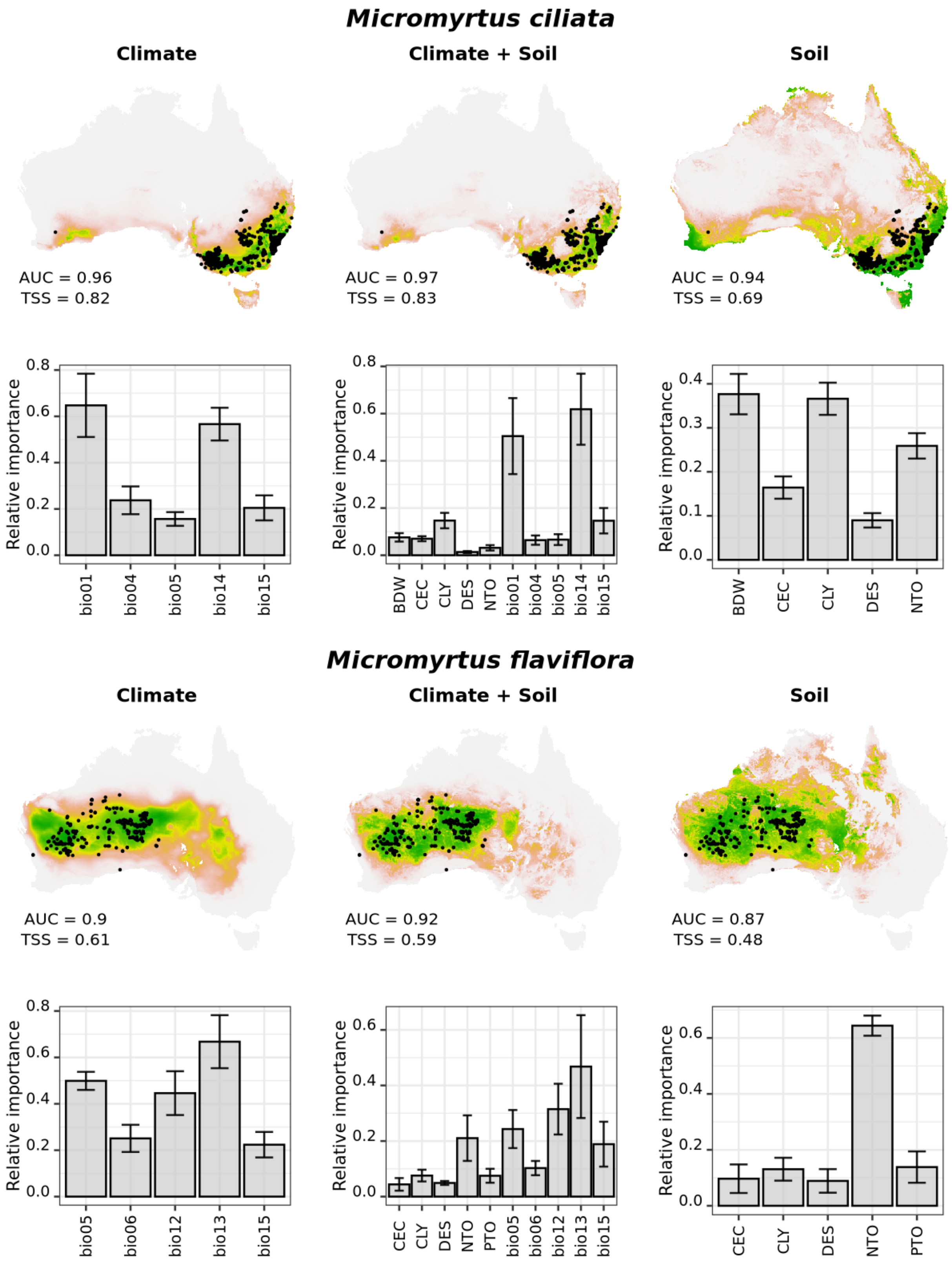

Micromyrtus ciliata
Climate
Climate + Soil
Soil
AUC = 0.96
TSS = 0.82
AUC = 0.97
TSS = 0.83
AUC = 0.94
TSS = 0.69
Relative importance
bio01
bio04
bio05
bio14
bio15
BDW
CEC
CLY
DES
NTO
Micromyrtus flaviflora
Climate
Climate + Soil
Soil
AUC = 0.9
TSS = 0.61
AUC = 0.92
TSS = 0.59
AUC = 0.87
TSS = 0.48
Relative importance
bio05
bio06
bio12
bio13
bio15
CEC
CLY
DES
NTO
PTO

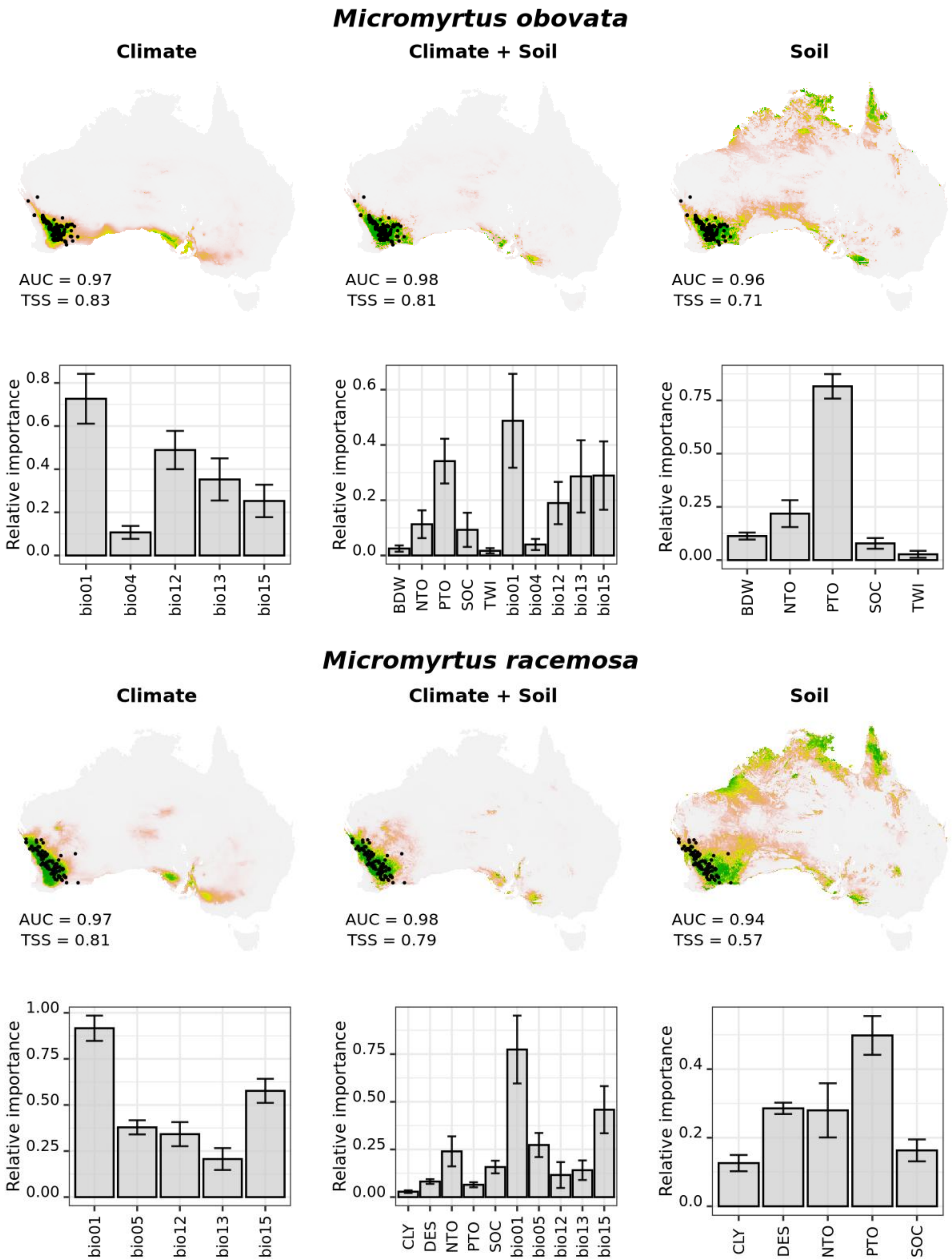

Micromyrtus obovata
Climate
Climate + Soil
Soil
AUC = 0.97
TSS = 0.83
AUC = 0.98
TSS = 0.81
AUC = 0.96
TSS = 0.71
Relative importance
bio01
bio04
bio12
bio13
bio15
BDW
NTO
PTO
SOC
TWI
Micromyrtus racemosa
Climate
Climate + Soil
Soil
AUC = 0.97
TSS = 0.81
AUC = 0.98
TSS = 0.79
AUC = 0.94
TSS = 0.57
Relative importance
bio01
bio05
bio12
bio13
bio15
CLY
DES
NTO
PTO
SOC

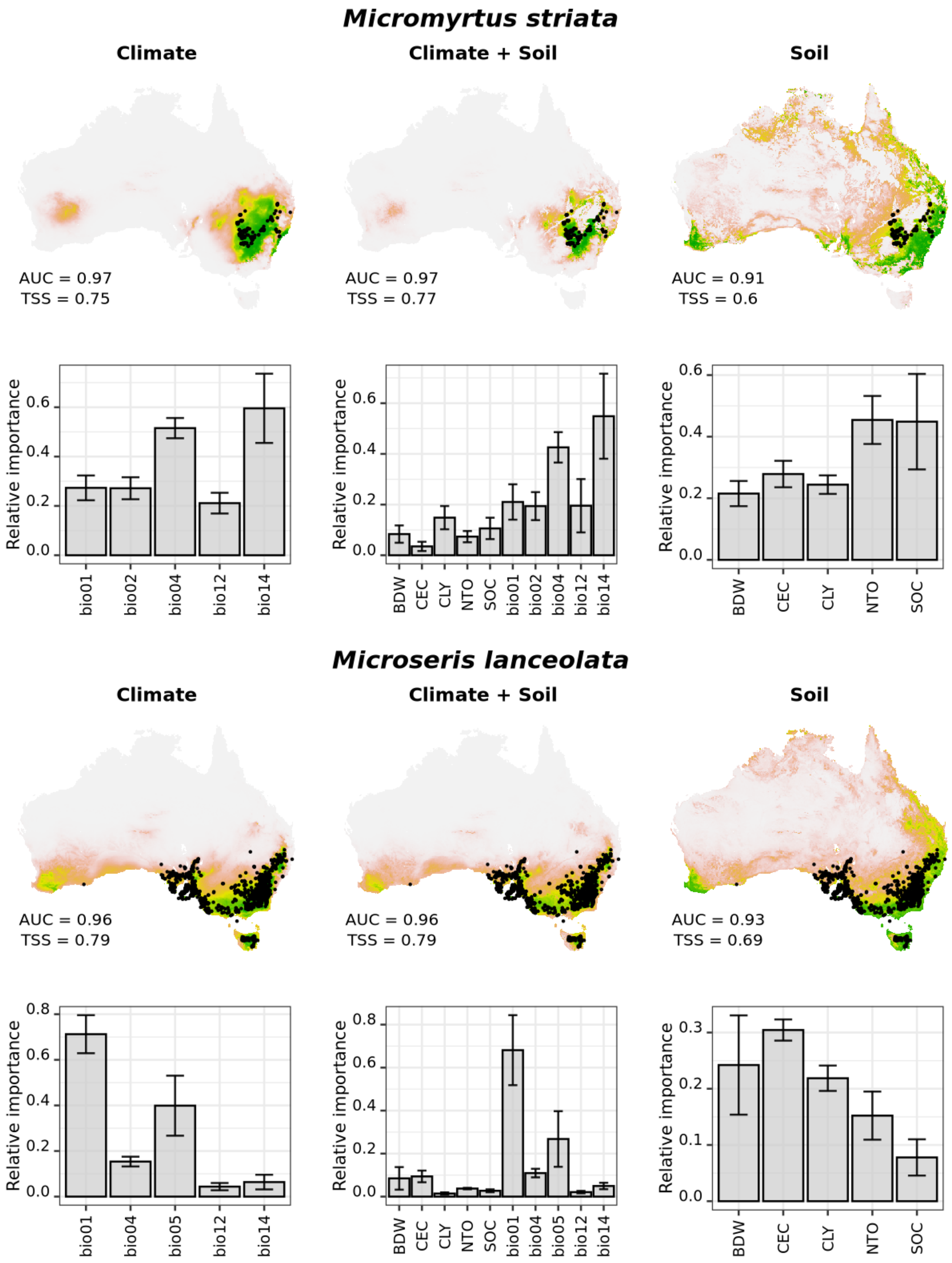

Micromyrtus striata
Climate
Climate + Soil
Soil
AUC = 0.97
TSS = 0.75
AUC = 0.97
TSS = 0.77
AUC = 0.91
TSS = 0.6
Relative importance
bio01
bio02
bio04
bio12
bio14
BDW
CEC
CLY
NTO
SOC
Microseris lanceolata
Climate
Climate + Soil
Soil
AUC = 0.96
TSS = 0.79
AUC = 0.96
TSS = 0.79
AUC = 0.93
TSS = 0.69
Relative importance
bio01
bio04
bio05
bio12
bio14
BDW
CEC
CLY
NTO
SOC

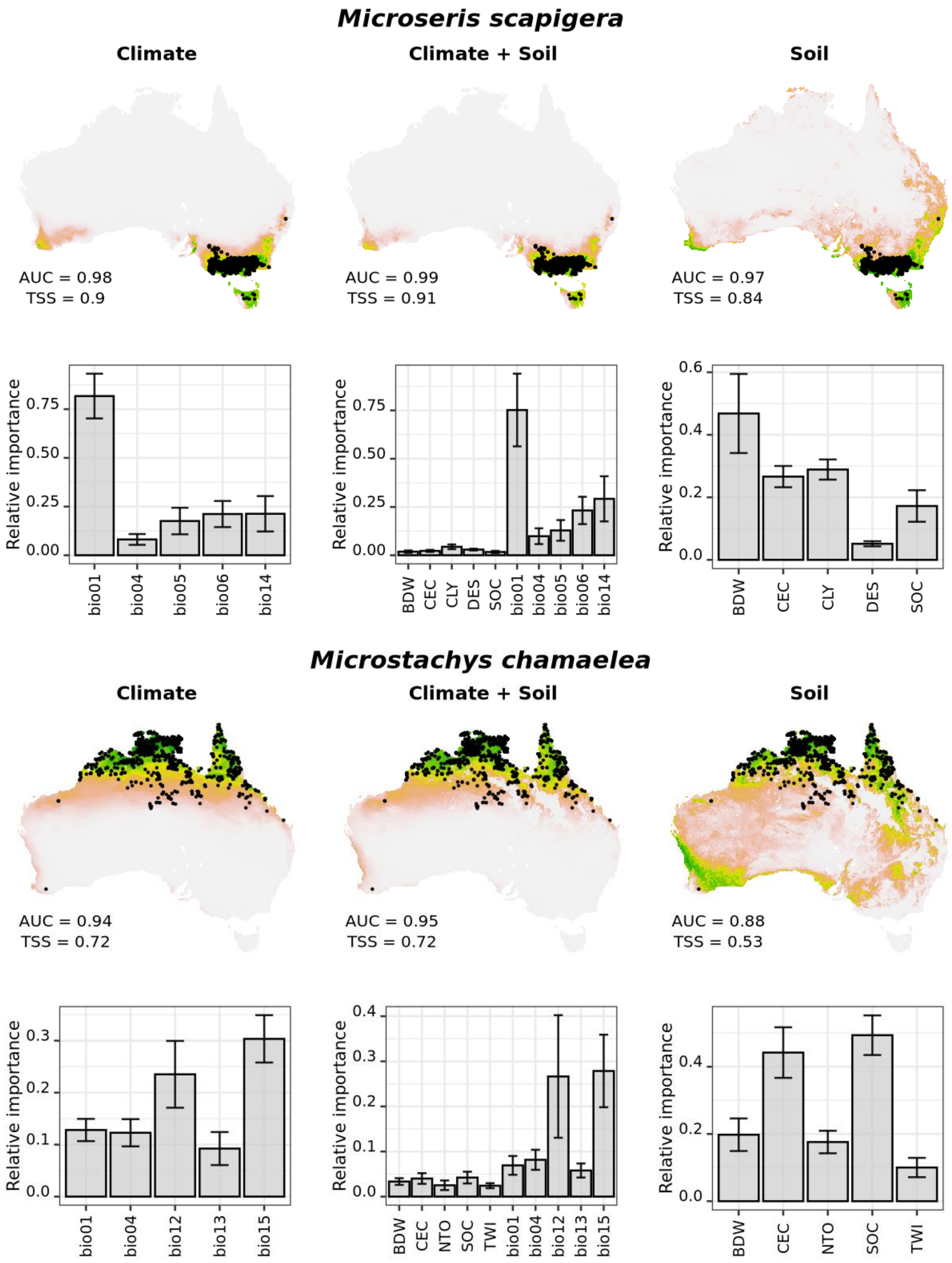
Microseris scapigera
Climate
Climate + Soil
Soil
AUC = 0.98
TSS = 0.9
AUC = 0.99
TSS = 0.91
AUC = 0.97
TSS = 0.84
Relative importance
bio01 bio04 bio05 bio06 bio14
BDW CEC CLY DES SOC bio01 bio04 bio05 bio06 bio14
BDW CEC CLY DES SOC
Microstachys chamaelea
Climate
Climate + Soil
Soil
AUC = 0.94
TSS = 0.72
AUC = 0.95
TSS = 0.72
AUC = 0.88
TSS = 0.53
Relative importance
bio01 bio04 bio12 bio13 bio15
BDW CEC NTO SOC TWI bio01 bio04 bio12 bio13 bio15
BDW CEC NTO SOC TWI

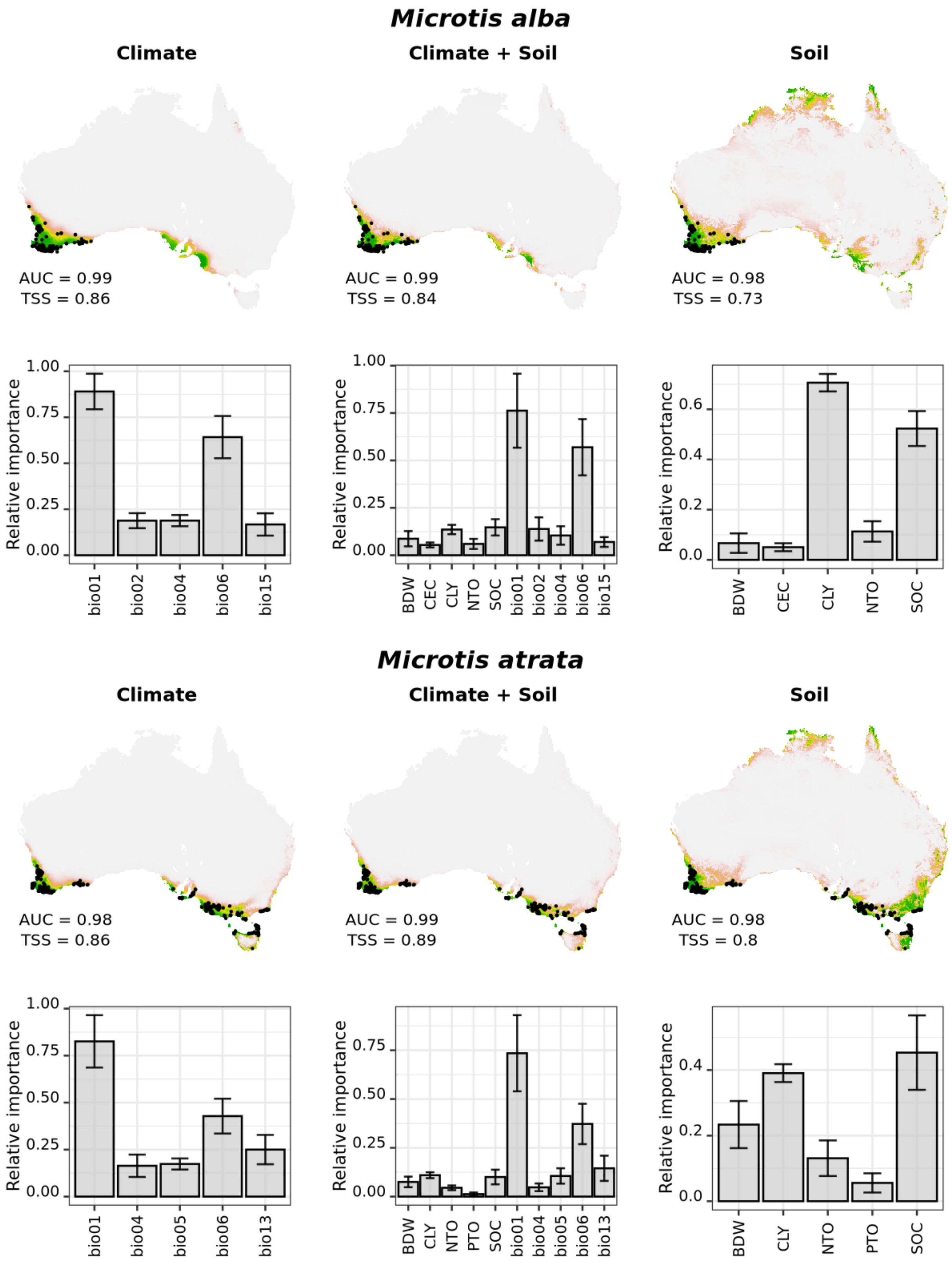

Microtis alba
Climate
Climate + Soil
Soil
AUC = 0.99
TSS = 0.86
AUC = 0.99
TSS = 0.84
AUC = 0.98
TSS = 0.73
Relative importance
bio01 bio02 bio04 bio06 bio15
BDW CEC CLY NTO SOC bio01 bio02 bio04 bio06 bio15
BDW CEC CLY NTO SOC
Microtis atrata
Climate
Climate + Soil
Soil
AUC = 0.98
TSS = 0.86
AUC = 0.99
TSS = 0.89
AUC = 0.98
TSS = 0.8
Relative importance
bio01 bio04 bio05 bio06 bio13
BDW CLY NTO PTO SOC bio01 bio04 bio05 bio06 bio13
BDW CLY NTO PTO SOC

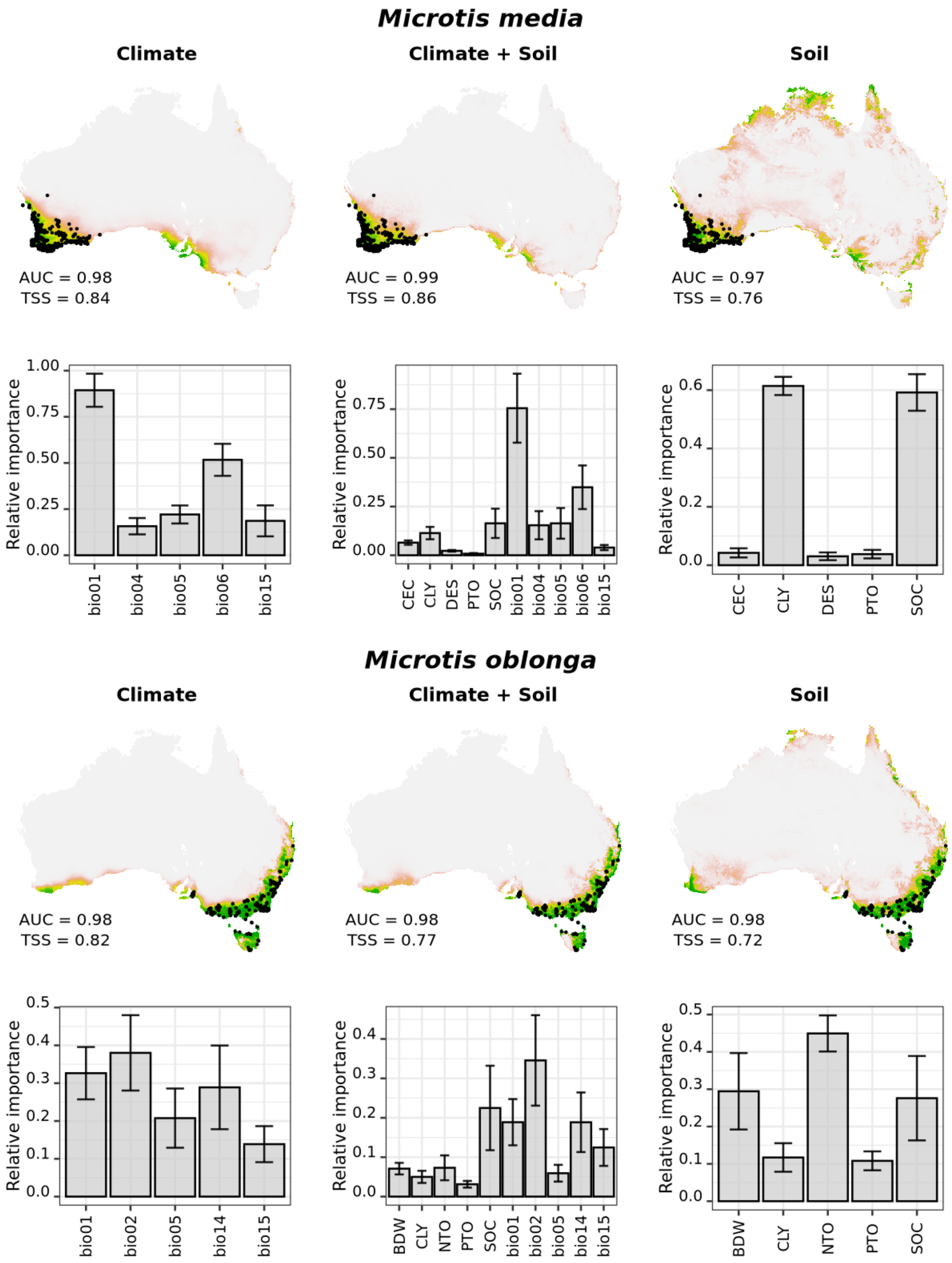

Microtis media
Climate
Climate + Soil
Soil
AUC = 0.98
TSS = 0.84
AUC = 0.99
TSS = 0.86
AUC = 0.97
TSS = 0.76
Relative importance
bio01
bio04
bio05
bio06
bio15
CEC
CLY
DES
PTO
SOC
Microtis oblonga
Climate
Climate + Soil
Soil
AUC = 0.98
TSS = 0.82
AUC = 0.98
TSS = 0.77
AUC = 0.98
TSS = 0.72
Relative importance
bio01
bio02
bio05
bio14
bio15
BDW
CLY
NTO
PTO
SOC

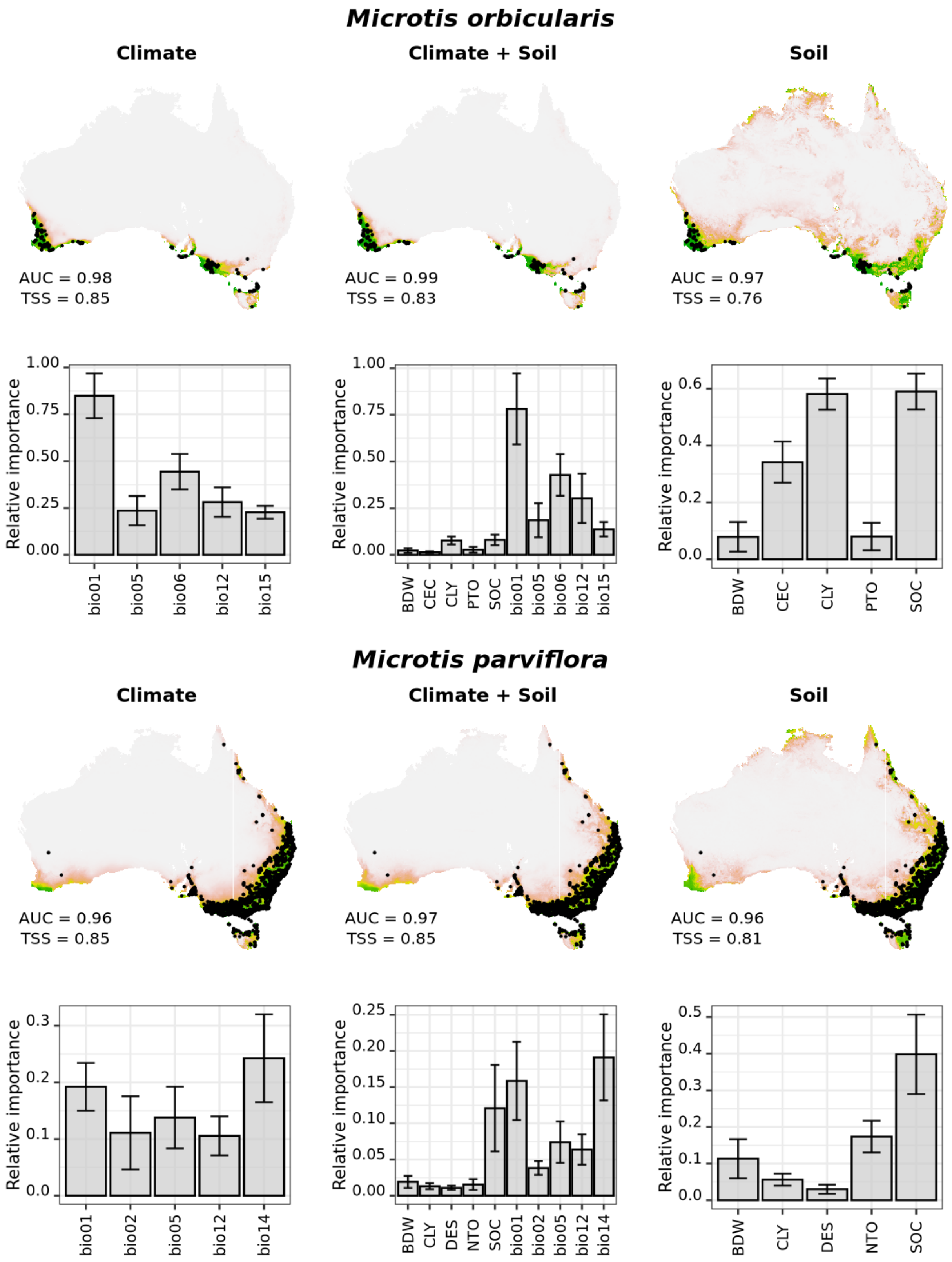

Microtis orbicularis
Climate
Climate + Soil
Soil
AUC = 0.98
TSS = 0.85
AUC = 0.99
TSS = 0.83
AUC = 0.97
TSS = 0.76
Relative importance
bio01
bio05
bio06
bio12
bio15
BDW
CEC
CLY
PTO
SOC
Microtis parviflora
Climate
Climate + Soil
Soil
AUC = 0.96
TSS = 0.85
AUC = 0.97
TSS = 0.85
AUC = 0.96
TSS = 0.81
Relative importance
bio01
bio02
bio05
bio12
bio14
BDW
CLY
DES
NTO
SOC

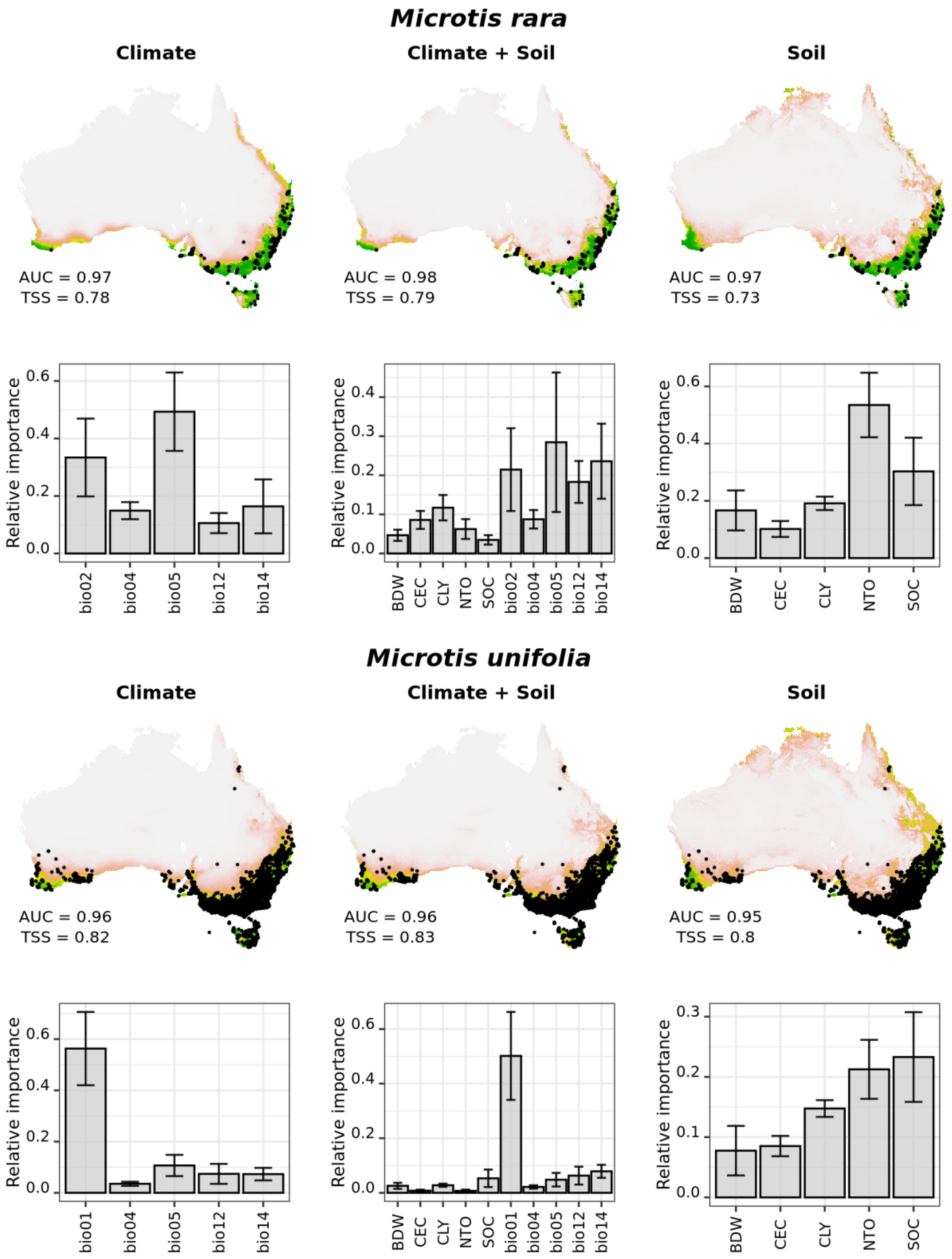
Microtis rara
Climate
Climate + Soil
Soil
AUC = 0.97
TSS = 0.78
AUC = 0.98
TSS = 0.79
AUC = 0.97
TSS = 0.73
Relative importance
bio02
bio04
bio05
bio12
bio14
BDW
CEC
CLY
NTO
SOC
Microtis unifolia
Climate
Climate + Soil
Soil
AUC = 0.96
TSS = 0.82
AUC = 0.96
TSS = 0.83
AUC = 0.95
TSS = 0.8
Relative importance
bio01
bio04
bio05
bio12
bio14
BDW
CEC
CLY
NTO
SOC

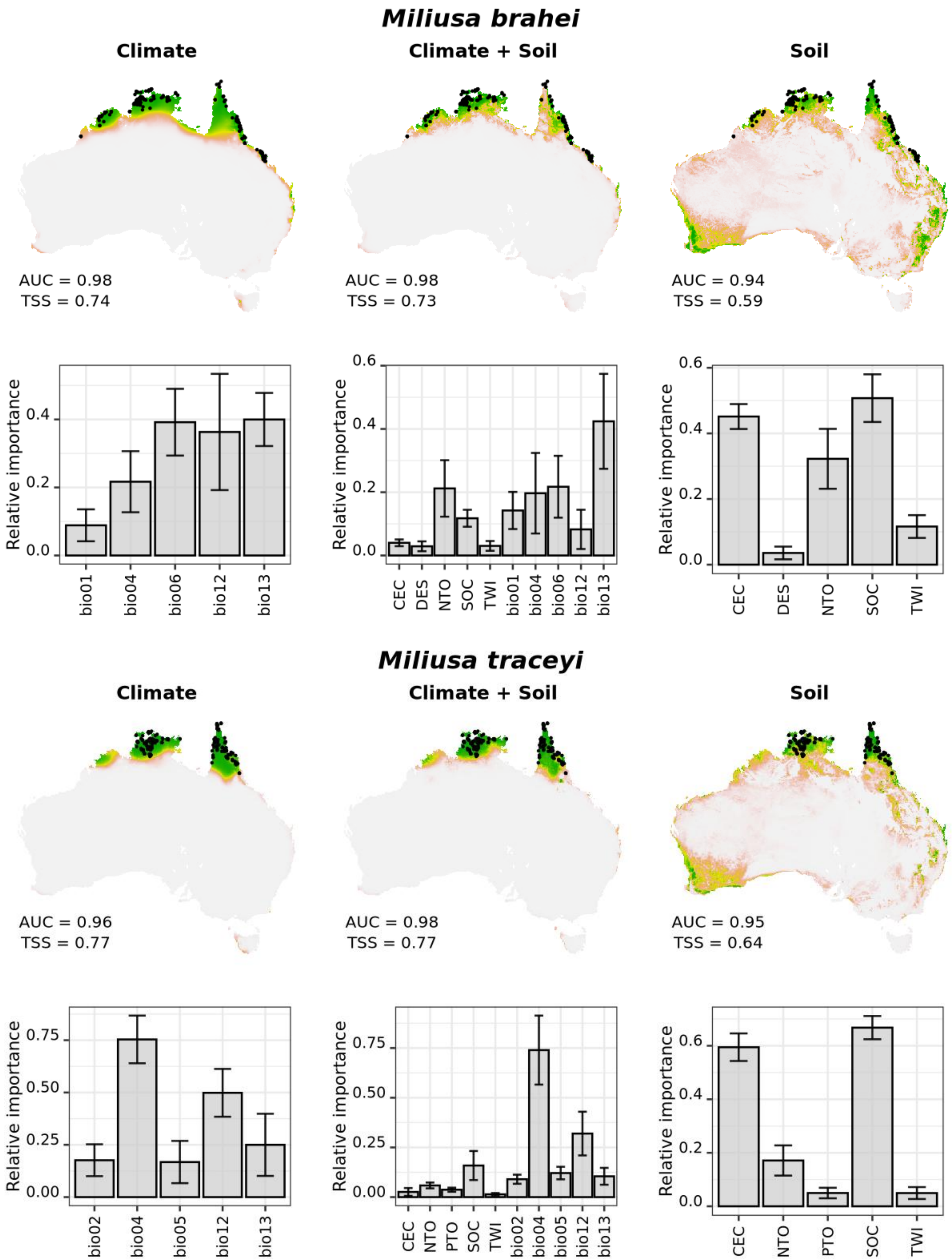

Miliusa brahei
Climate
Climate + Soil
Soil
AUC = 0.98
TSS = 0.74
AUC = 0.98
TSS = 0.73
AUC = 0.94
TSS = 0.59
Relative importance
bio01
bio04
bio06
bio12
bio13
CEC
DES
NTO
SOC
TWI
Miliusa traceyi
Climate
Climate + Soil
Soil
AUC = 0.96
TSS = 0.77
AUC = 0.98
TSS = 0.77
AUC = 0.95
TSS = 0.64
Relative importance
bio02
bio04
bio05
bio12
bio13
CEC
NTO
PTO
SOC
TWI

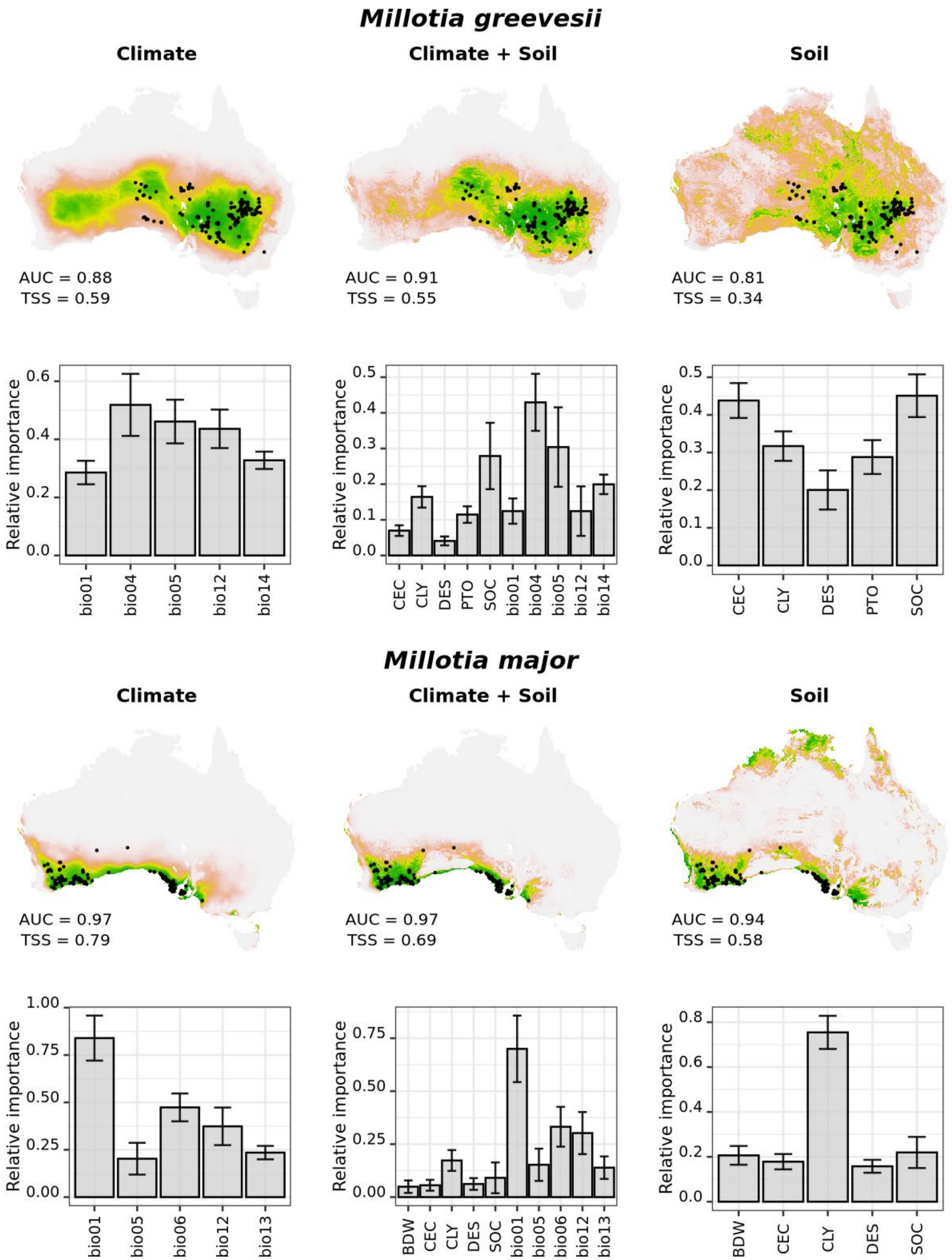
Millotia greevesii
Climate
Climate + Soil
Soil
AUC = 0.88
TSS = 0.59
AUC = 0.91
TSS = 0.55
AUC = 0.81
TSS = 0.34
Relative importance
bio01 bio04 bio05 bio12 bio14
CEC CLY DES PTO SOC bio01 bio04 bio05 bio12 bio14
CEC CLY DES PTO SOC
Millotia major
Climate
Climate + Soil
Soil
AUC = 0.97
TSS = 0.79
AUC = 0.97
TSS = 0.69
AUC = 0.94
TSS = 0.58
Relative importance
bio01 bio05 bio06 bio12 bio13
BDW CEC CLY DES SOC bio01 bio05 bio06 bio12 bio13
BDW CEC CLY DES SOC

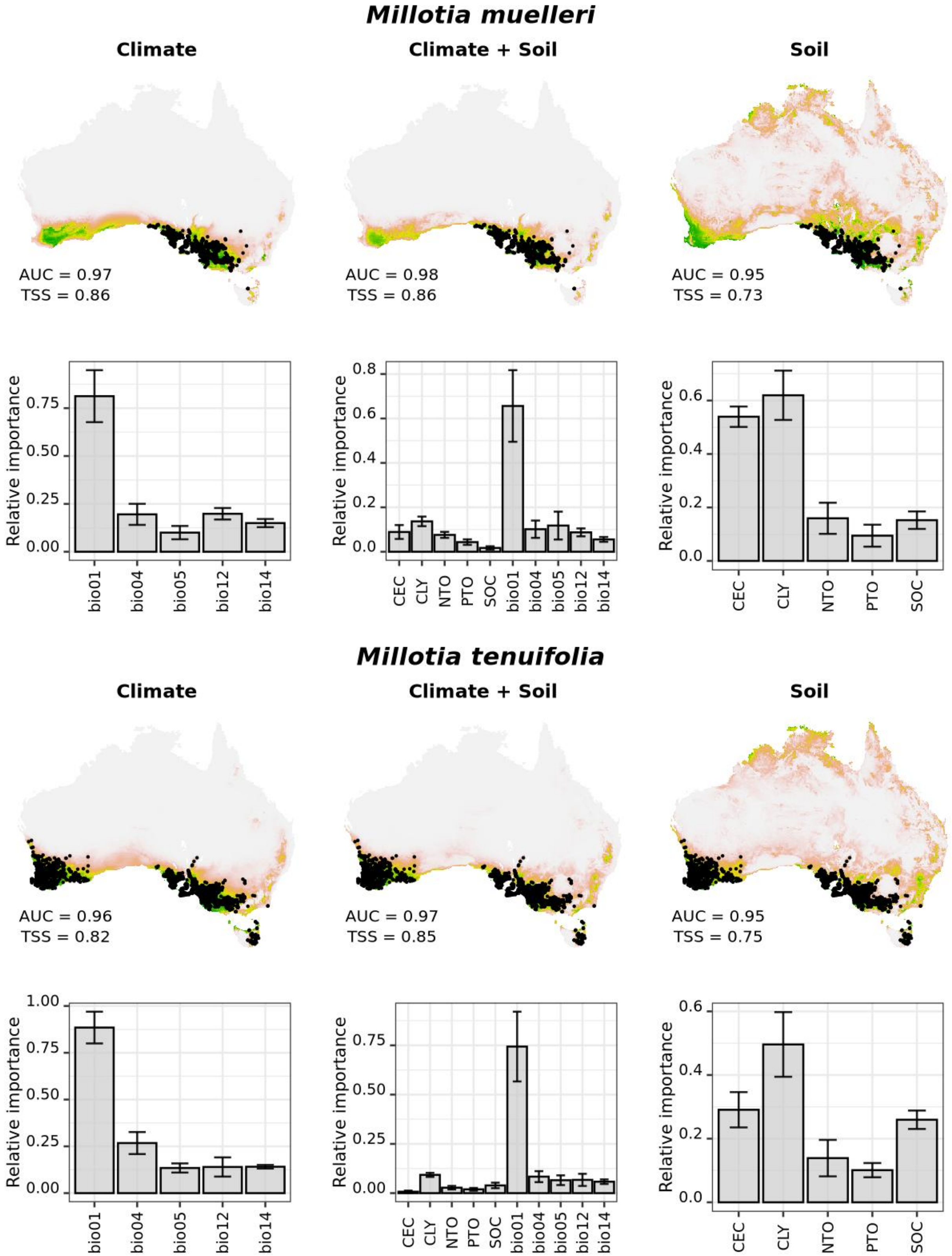
Millotia muelleri
Climate
Climate + Soil
Soil
AUC = 0.97
TSS = 0.86
AUC = 0.98
TSS = 0.86
AUC = 0.95
TSS = 0.73
Relative importance
bio01 bio04 bio05 bio12 bio14
CEC CLY NTO PTO SOC bio01 bio04 bio05 bio12 bio14
CEC CLY NTO PTO SOC
Millotia tenuifolia
Climate
Climate + Soil
Soil
AUC = 0.96
TSS = 0.82
AUC = 0.97
TSS = 0.85
AUC = 0.95
TSS = 0.75
Relative importance
bio01 bio04 bio05 bio12 bio14
CEC CLY NTO PTO SOC bio01 bio04 bio05 bio12 bio14
CEC CLY NTO PTO SOC

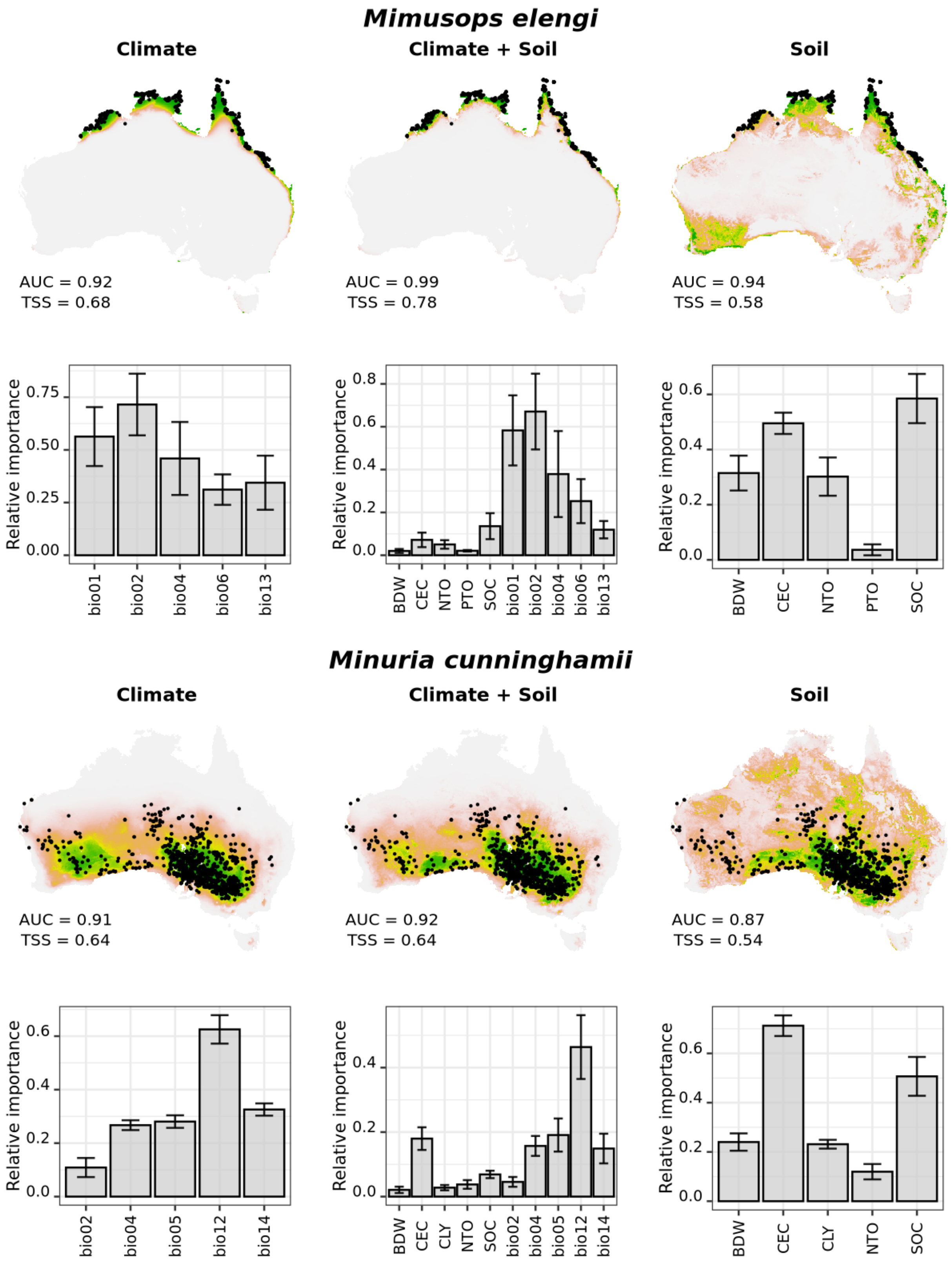

Mimusops elengi
Climate
Climate + Soil
Soil
AUC = 0.92
TSS = 0.68
AUC = 0.99
TSS = 0.78
AUC = 0.94
TSS = 0.58
Relative importance
bio01 bio02 bio04 bio06 bio13
BDW CEC NTO PTO SOC bio01 bio02 bio04 bio06 bio13
BDW CEC NTO PTO SOC
Minuria cunninghamii
Climate
Climate + Soil
Soil
AUC = 0.91
TSS = 0.64
AUC = 0.92
TSS = 0.64
AUC = 0.87
TSS = 0.54
Relative importance
bio02 bio04 bio05 bio12 bio14
BDW CEC CLY NTO SOC bio02 bio04 bio05 bio12 bio14
BDW CEC CLY NTO SOC

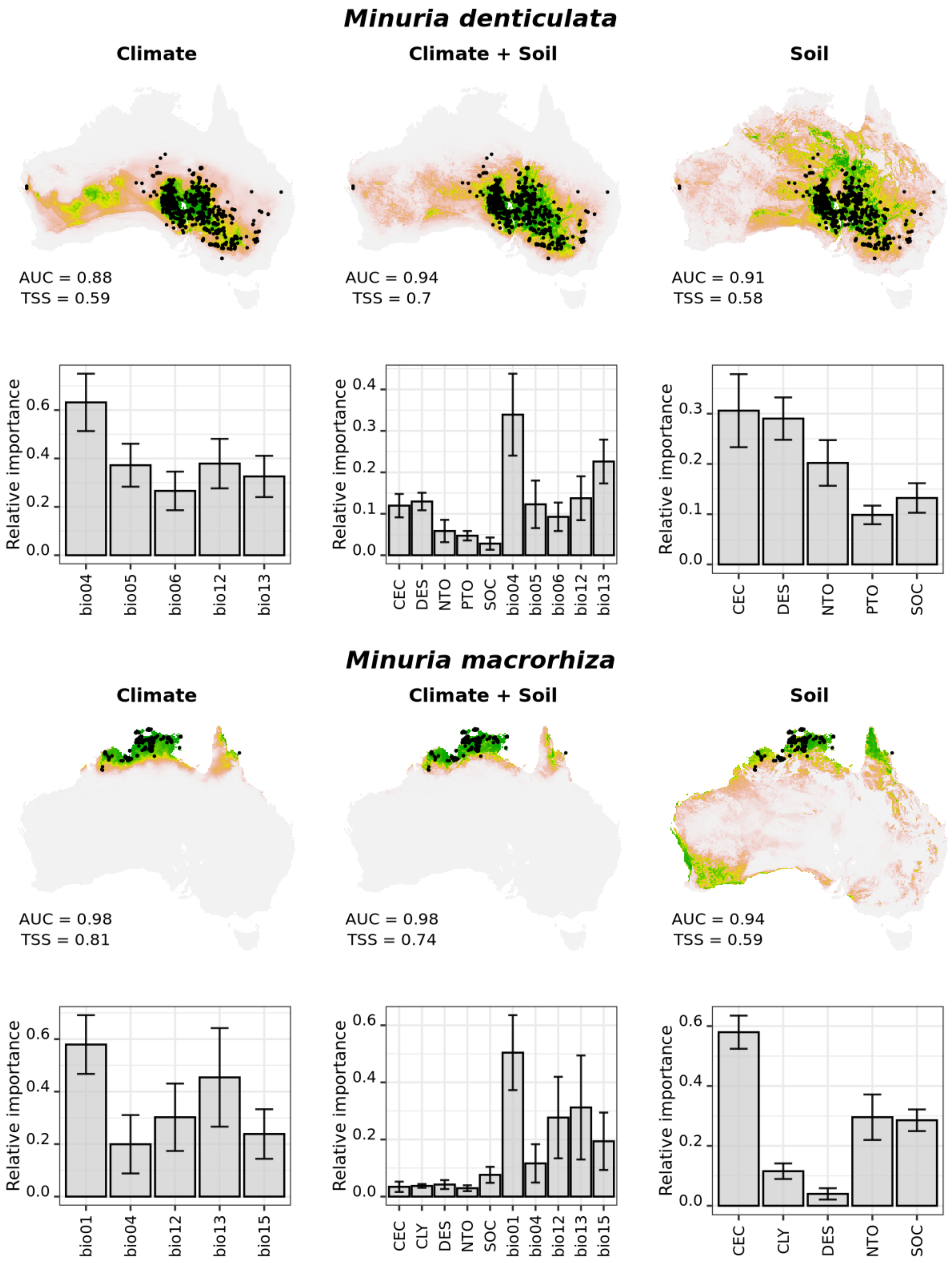
Minuria denticulata
Climate
Climate + Soil
Soil
AUC = 0.88
TSS = 0.59
AUC = 0.94
TSS = 0.7
AUC = 0.91
TSS = 0.58
Relative importance
0.0
0.2
0.4
0.6
bio04
bio05
bio06
bio12
bio13
0.0
0.1
0.2
0.3
0.4
CEC
DES
NTO
PTO
SOC
bio04
bio05
bio06
bio12
bio13
0.0
0.1
0.2
0.3
CEC
DES
NTO
PTO
SOC
Minuria macrorhiza
Climate
Climate + Soil
Soil
AUC = 0.98
TSS = 0.81
AUC = 0.98
TSS = 0.74
AUC = 0.94
TSS = 0.59
Relative importance
0.0
0.2
0.4
0.6
bio01
bio04
bio12
bio13
bio15
CEC
CLY
DES
NTO
SOC
bio01
bio04
bio12
bio13
bio15
CEC
CLY
DES
NTO
SOC

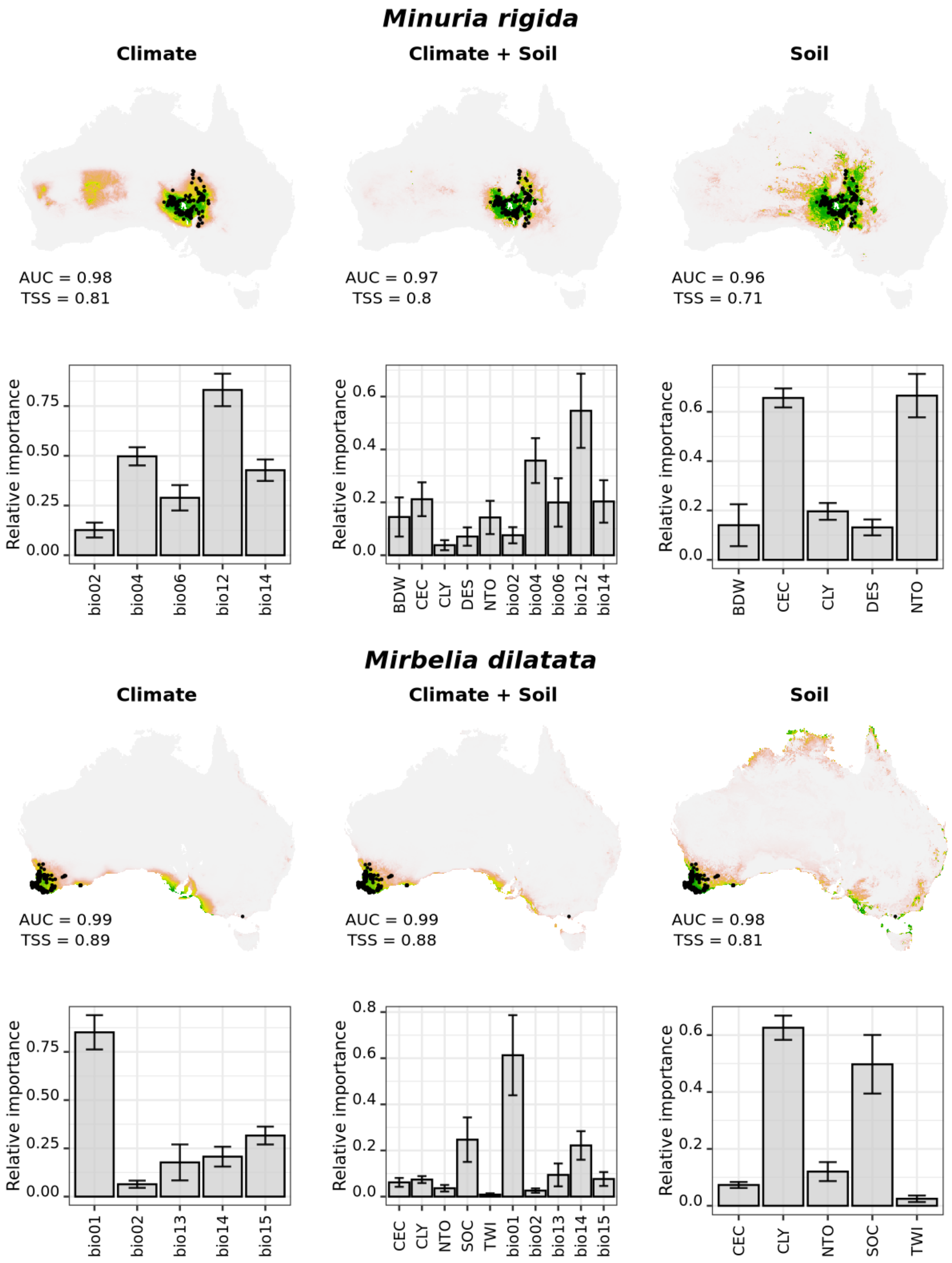
Minuria rigida
Climate
Climate + Soil
Soil
AUC = 0.98
TSS = 0.81
AUC = 0.97
TSS = 0.8
AUC = 0.96
TSS = 0.71
Relative importance
0.00
0.25
0.50
0.75
bio02
bio04
bio06
bio12
bio14
0.0
0.2
0.4
0.6
BDW
CEC
CLY
DES
NTO
Mirbelia dilatata
AUC = 0.99
TSS = 0.89
AUC = 0.99
TSS = 0.88
AUC = 0.98
TSS = 0.81
bio01
bio13
bio15
0.8
SOC
TWI

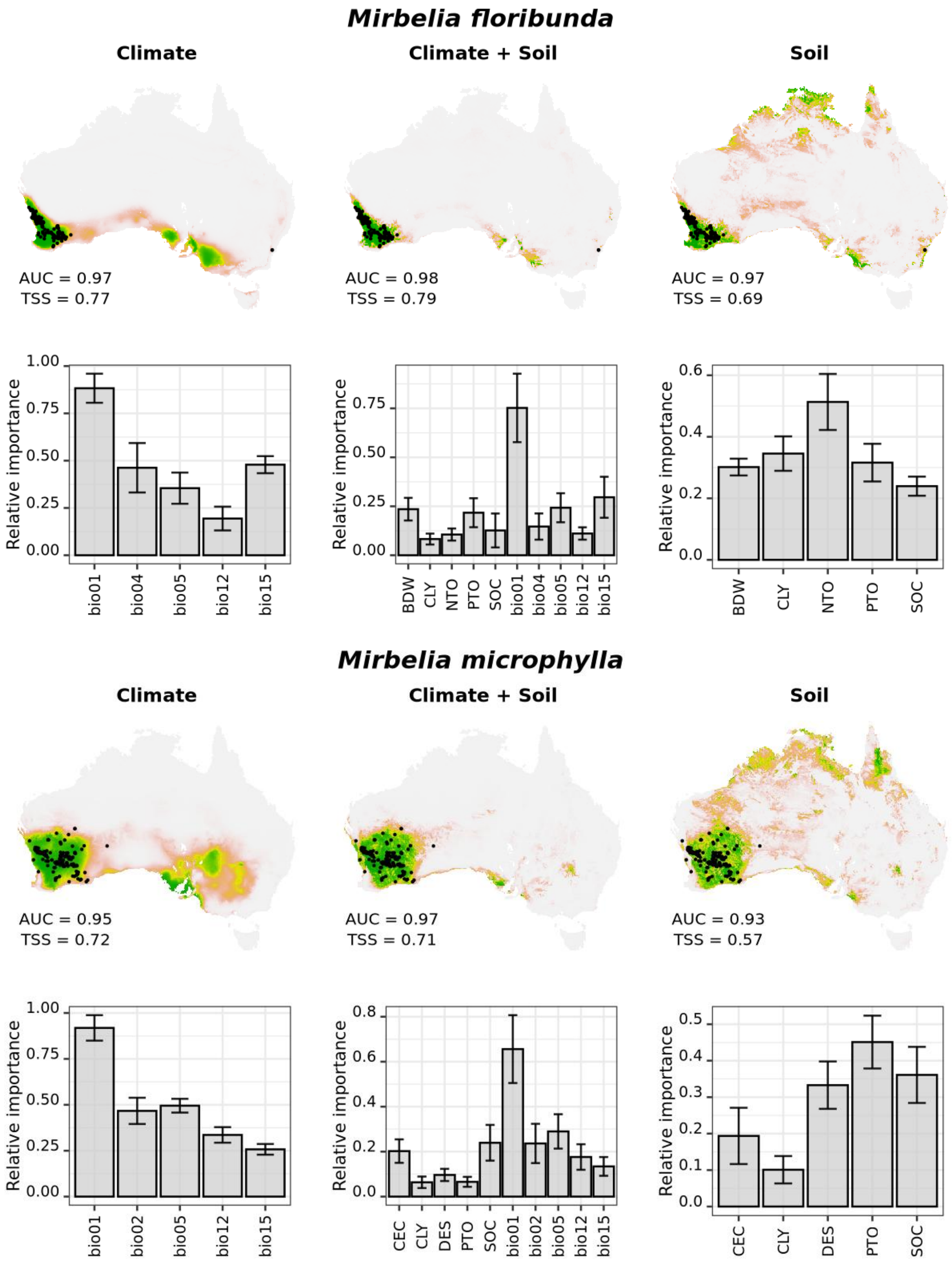

Mirbelia floribunda
Climate
Climate + Soil
Soil
AUC = 0.97
TSS = 0.77
AUC = 0.98
TSS = 0.79
AUC = 0.97
TSS = 0.69
Relative importance
1.00
0.75
0.50
0.25
0.00
bio01
bio04
bio05
bio12
bio15
Relative importance
0.75
0.50
0.25
0.00
BDW
CLY
NTO
PTO
SOC
bio01
bio04
bio05
bio12
bio15
Relative importance
0.6
0.4
0.2
0.0
BDW
CLY
NTO
PTO
SOC
Mirbelia microphylla
Climate
Climate + Soil
Soil
AUC = 0.95
TSS = 0.72
AUC = 0.97
TSS = 0.71
AUC = 0.93
TSS = 0.57
Relative importance
1.00
0.75
0.50
0.25
0.00
bio01
bio02
bio05
bio12
bio15
Relative importance
0.8
0.6
0.4
0.2
0.0
CEC
CLY
DES
PTO
SOC
bio01
bio02
bio05
bio12
bio15
Relative importance
0.5
0.4
0.3
0.2
0.1
0.0
CEC
CLY
DES
PTO
SOC

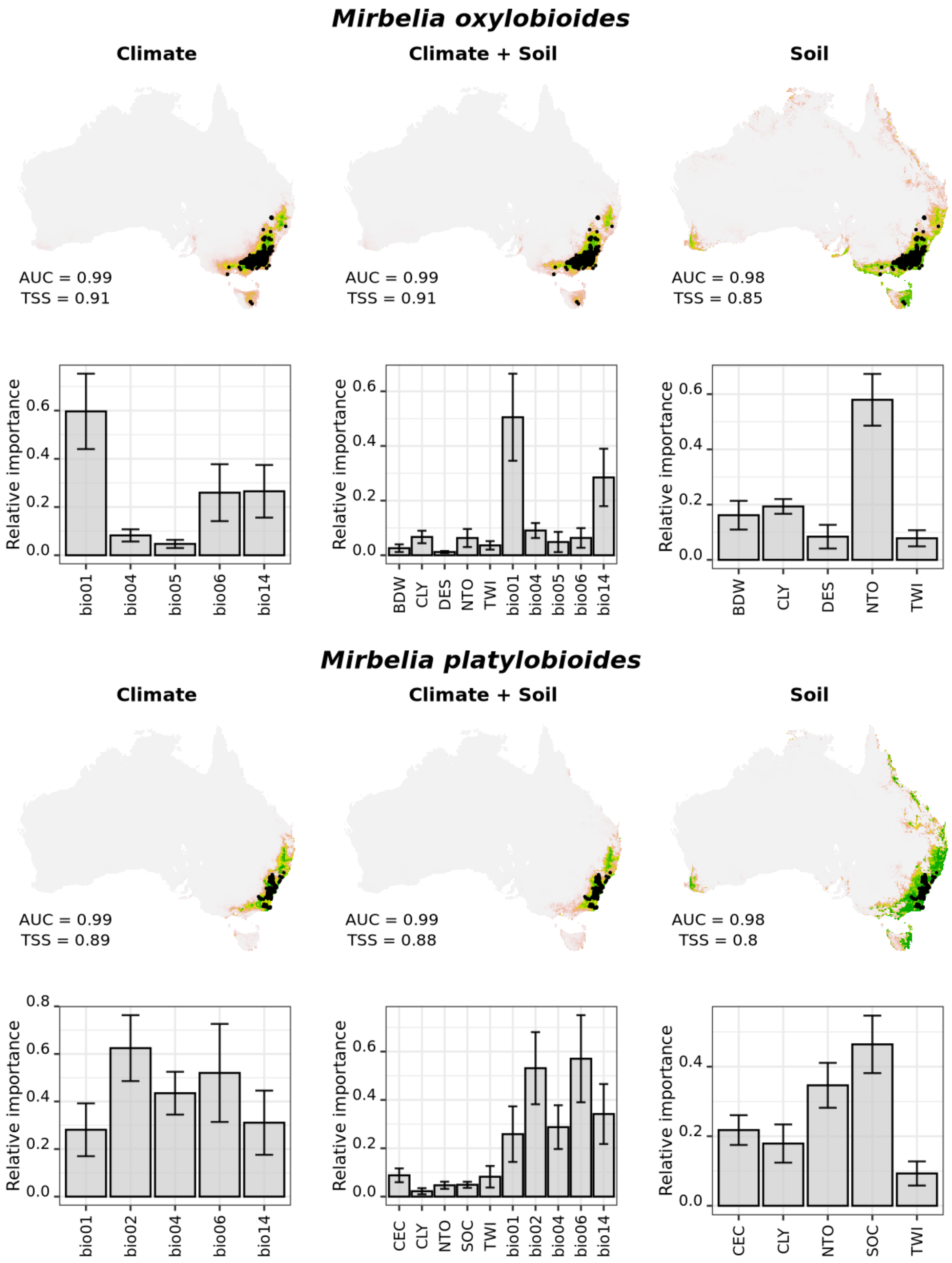

Mirbelia oxylobioides
Climate
Climate + Soil
Soil
AUC = 0.99
TSS = 0.91
AUC = 0.99
TSS = 0.91
AUC = 0.98
TSS = 0.85
Relative importance
bio01
bio04
bio05
bio06
bio14
BDW
CLY
DES
NTO
TWI
Mirbelia platylobioides
Climate
Climate + Soil
Soil
AUC = 0.99
TSS = 0.89
AUC = 0.99
TSS = 0.88
AUC = 0.98
TSS = 0.8
Relative importance
bio01
bio02
bio04
bio06
bio14
CEC
CLY
NTO
SOC
TWI

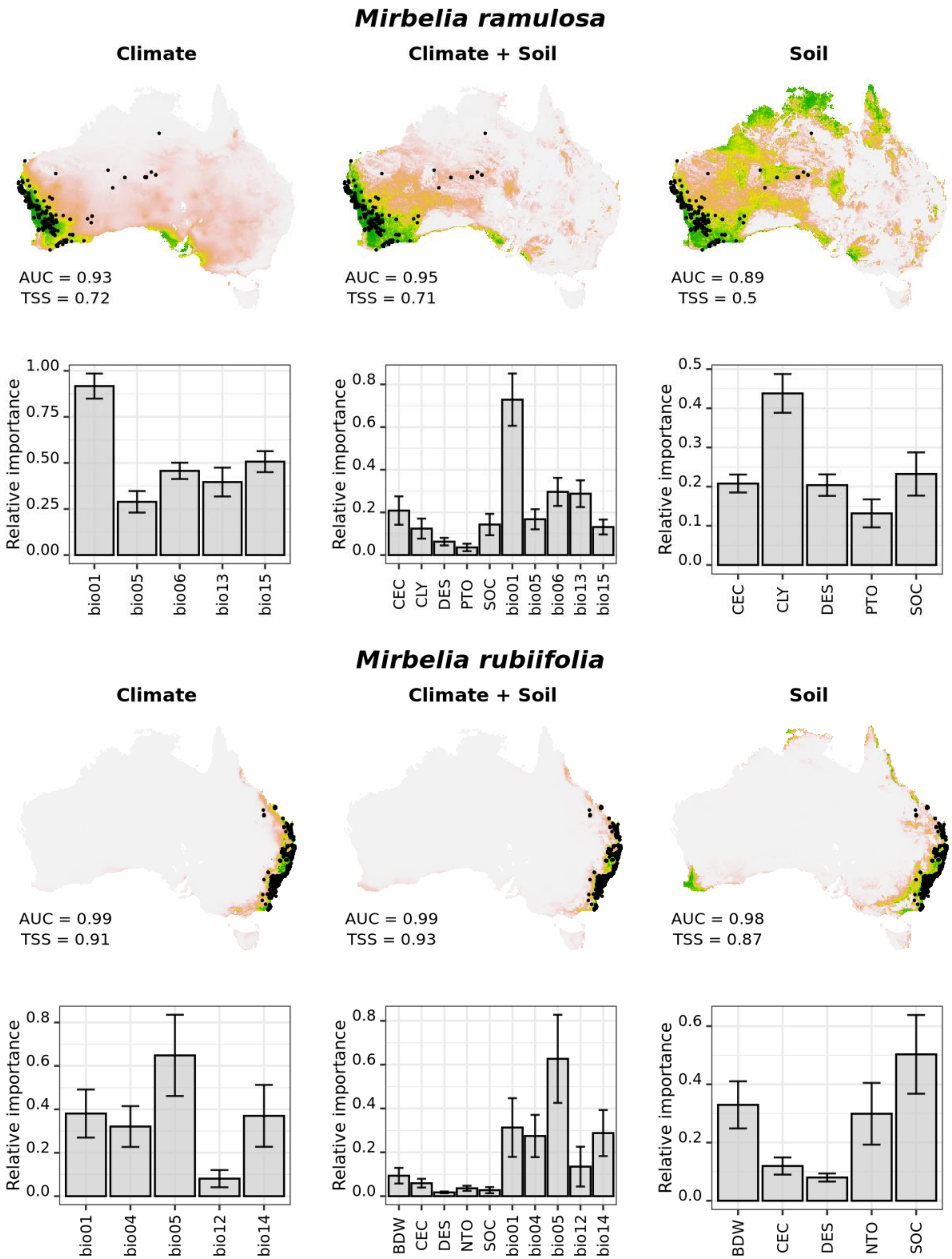
Mirbelia ramulosa
Climate
Climate + Soil
Soil
AUC = 0.93
TSS = 0.72
AUC = 0.95
TSS = 0.71
AUC = 0.89
TSS = 0.5
Relative importance
bio01
bio05
bio06
bio13
bio15
CEC
CLY
DES
PTO
SOC
Mirbelia rubiifolia
Climate
Climate + Soil
Soil
AUC = 0.99
TSS = 0.91
AUC = 0.99
TSS = 0.93
AUC = 0.98
TSS = 0.87
Relative importance
bio01
bio04
bio05
bio12
bio14
BDW
CEC
DES
NTO
SOC

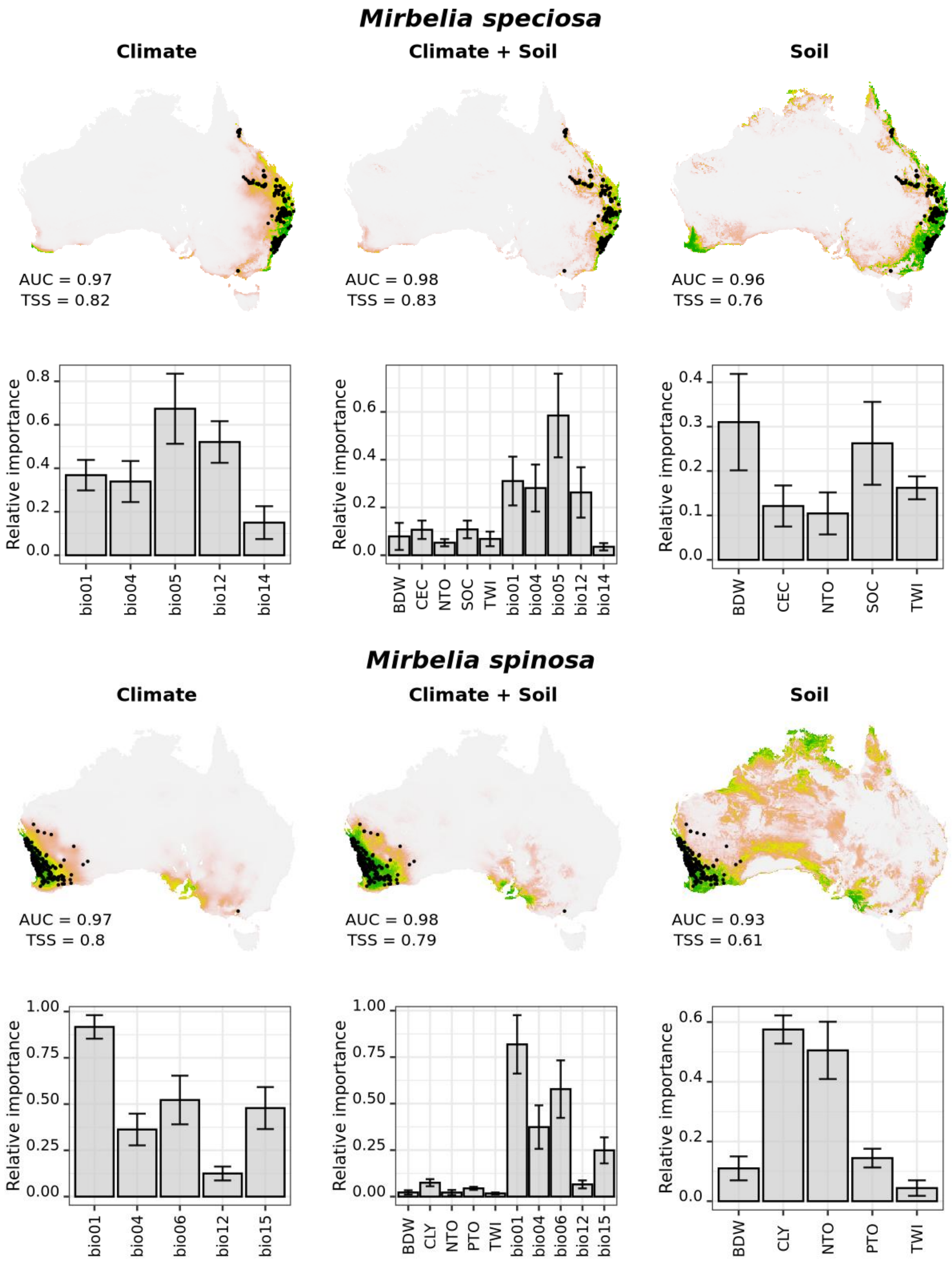
Mirbelia speciosa
Climate
Climate + Soil
Soil
AUC = 0.97
TSS = 0.82
AUC = 0.98
TSS = 0.83
AUC = 0.96
TSS = 0.76
Relative importance
bio01
bio04
bio05
bio12
bio14
BDW
CEC
NTO
SOC
TWI
Mirbelia spinosa
Climate
Climate + Soil
Soil
AUC = 0.97
TSS = 0.8
AUC = 0.98
TSS = 0.79
AUC = 0.93
TSS = 0.61
Relative importance
bio01
bio04
bio06
bio12
bio15
BDW
CLY
NTO
PTO
TWI

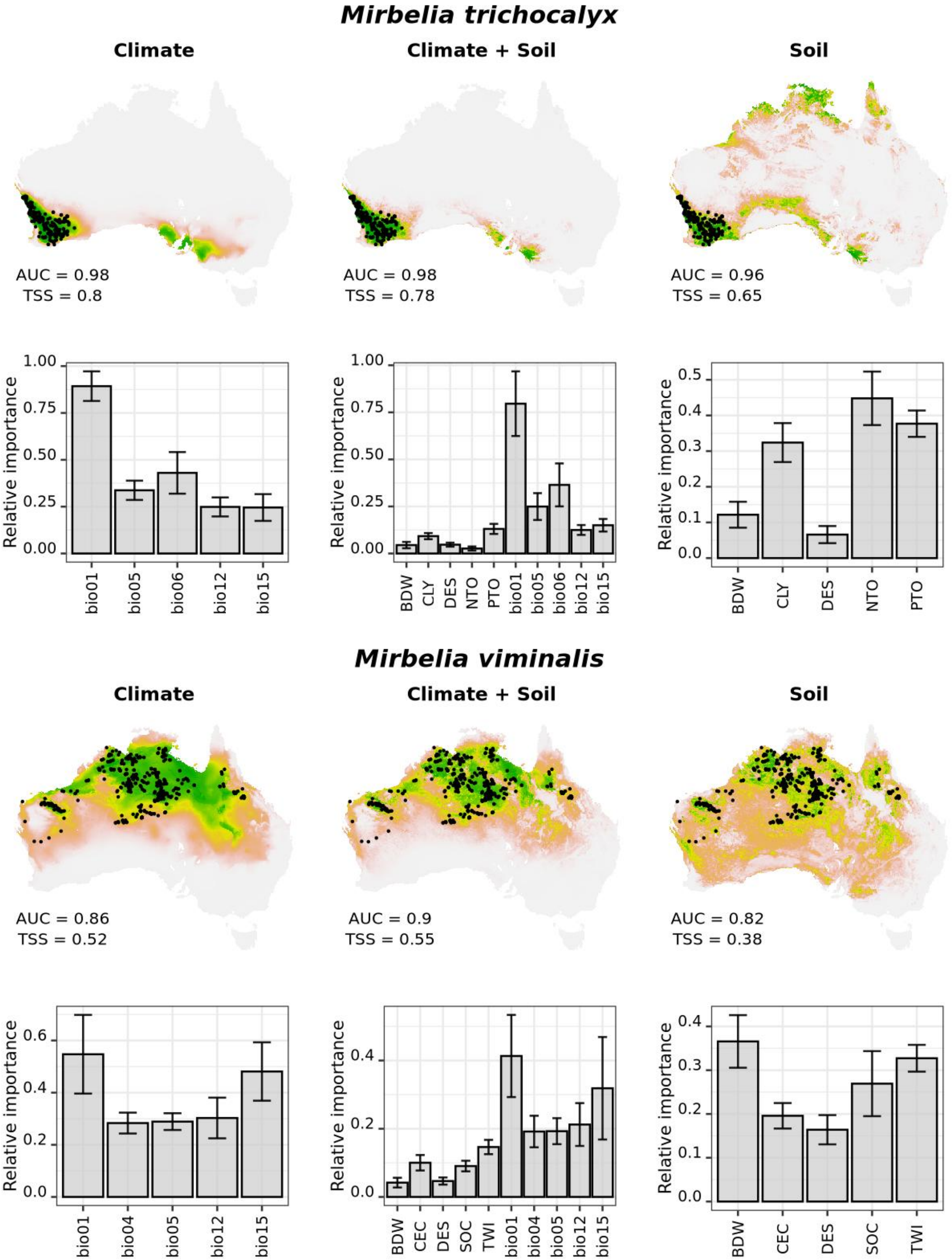

Mirbelia trichocalyx
Climate
Climate + Soil
Soil
AUC = 0.98
TSS = 0.8
AUC = 0.98
TSS = 0.78
AUC = 0.96
TSS = 0.65
Relative importance
1.00
0.75
0.50
0.25
0.00
bio01
bio05
bio06
bio12
bio15
BDW
CLY
DES
NTO
PTO
0.5
0.4
0.3
0.2
0.1
0.0
Mirbelia viminalis
Climate
Climate + Soil
Soil
AUC = 0.86
TSS = 0.52
AUC = 0.9
TSS = 0.55
AUC = 0.82
TSS = 0.38
0.6
0.4
0.2
0.0
bio01
bio04
bio05
bio12
bio15
BDW
CEC
DES
SOC
TWI

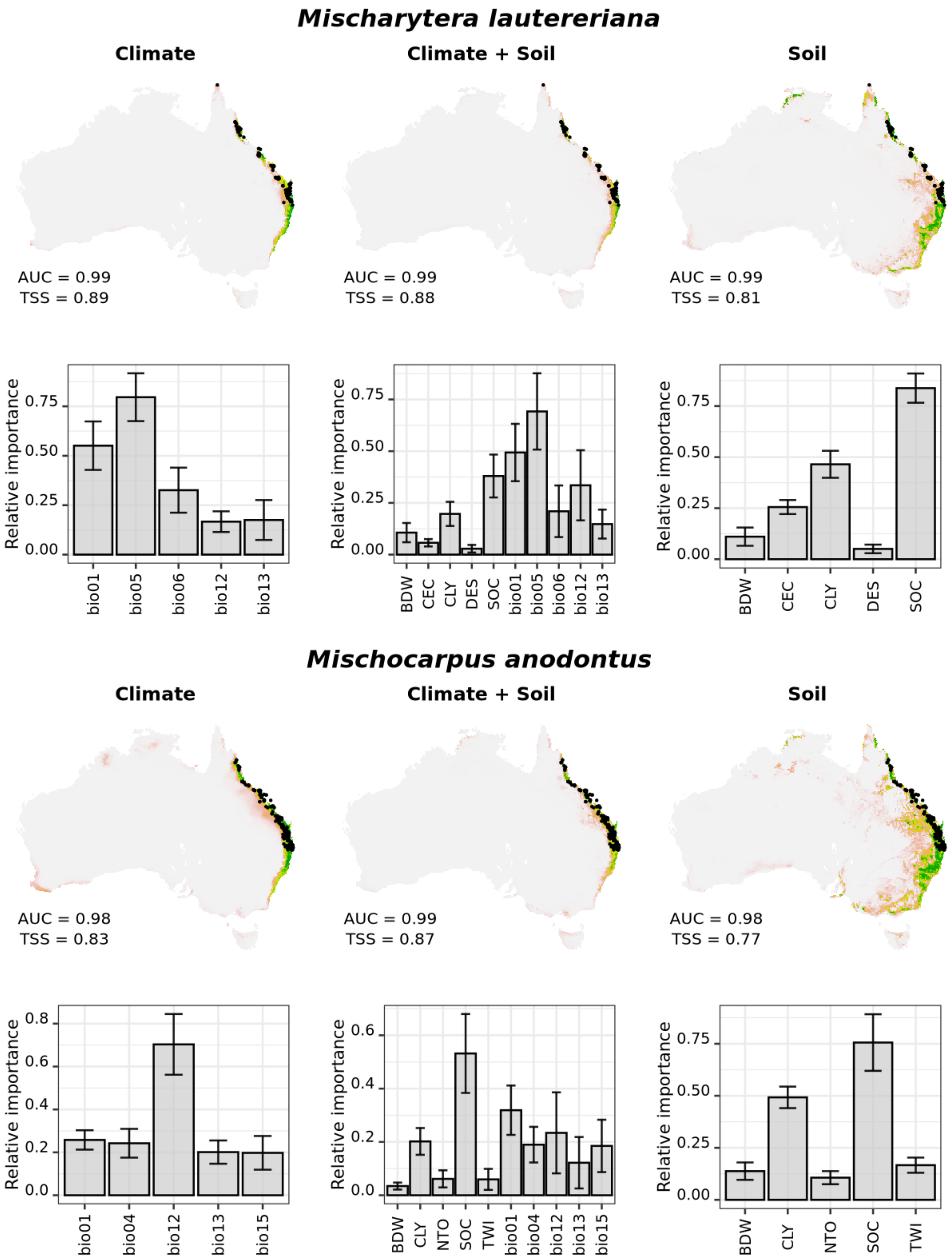
Mischarytera lautereriana
Climate
Climate + Soil
Soil
AUC = 0.99
TSS = 0.89
AUC = 0.99
TSS = 0.88
AUC = 0.99
TSS = 0.81
Relative importance
0.00
0.25
0.50
0.75
bio01
bio05
bio06
bio12
bio13
BDW
CEC
CLY
DES
SOC
bio01
bio05
bio06
bio12
bio13
BDW
CEC
CLY
DES
SOC
Mischocarpus anodontus
Climate
Climate + Soil
Soil
AUC = 0.98
TSS = 0.83
AUC = 0.99
TSS = 0.87
AUC = 0.98
TSS = 0.77
0.0
0.2
0.4
0.6
0.8
bio01
bio04
bio12
bio13
bio15
BDW
CLY
NTO
SOC
TWI
bio01
bio04
bio12
bio13
bio15
BDW
CLY
NTO
SOC
TWI

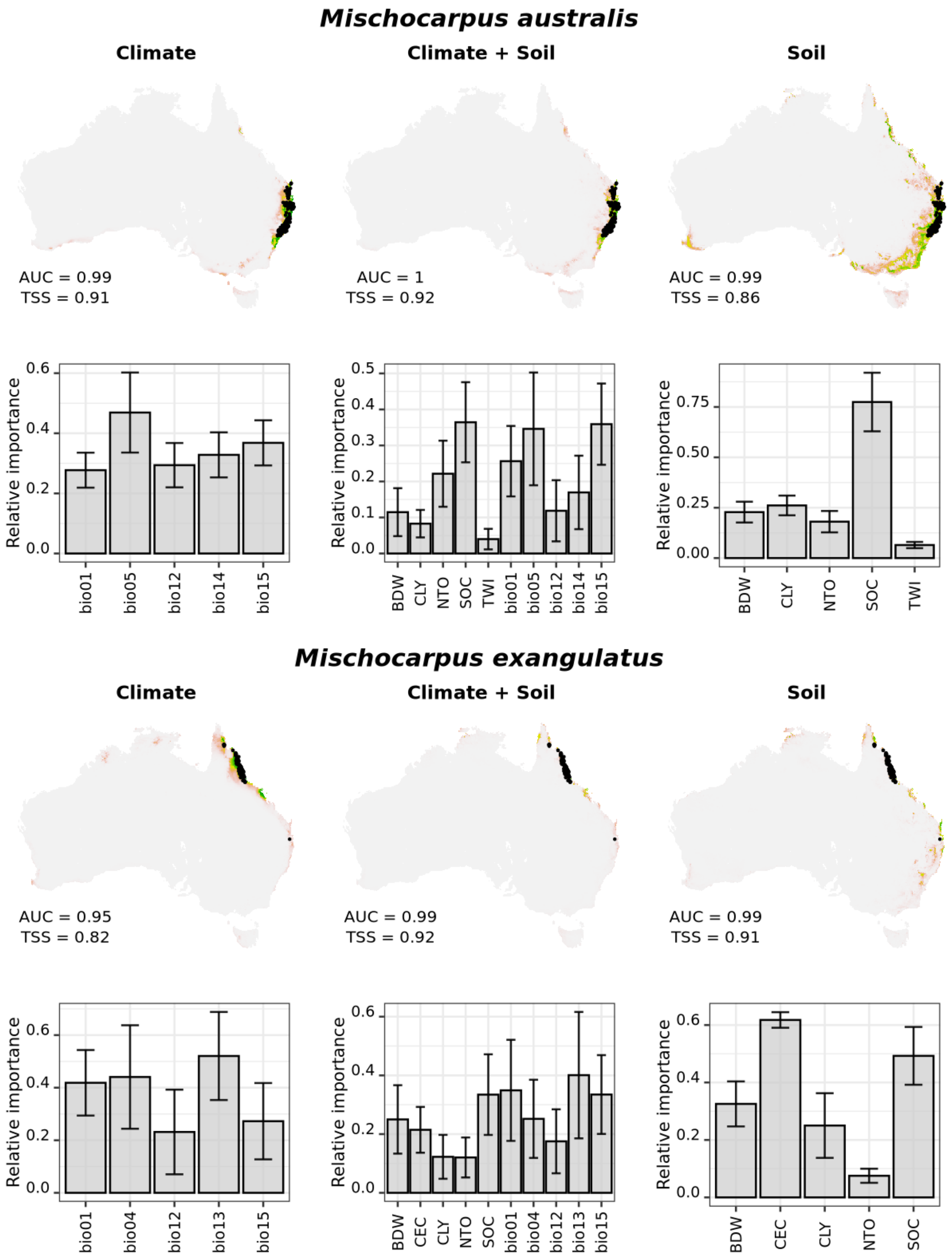
Mischocarpus australis
Climate
Climate + Soil
Soil
AUC = 0.99
TSS = 0.91
AUC = 1
TSS = 0.92
AUC = 0.99
TSS = 0.86
Relative importance
bio01
bio05
bio12
bio14
bio15
BDW
CLY
NTO
SOC
TWI
Mischocarpus exangulatus
Climate
Climate + Soil
Soil
AUC = 0.95
TSS = 0.82
AUC = 0.99
TSS = 0.92
AUC = 0.99
TSS = 0.91
Relative importance
bio01
bio04
bio12
bio13
bio15
BDW
CEC
CLY
NTO
SOC

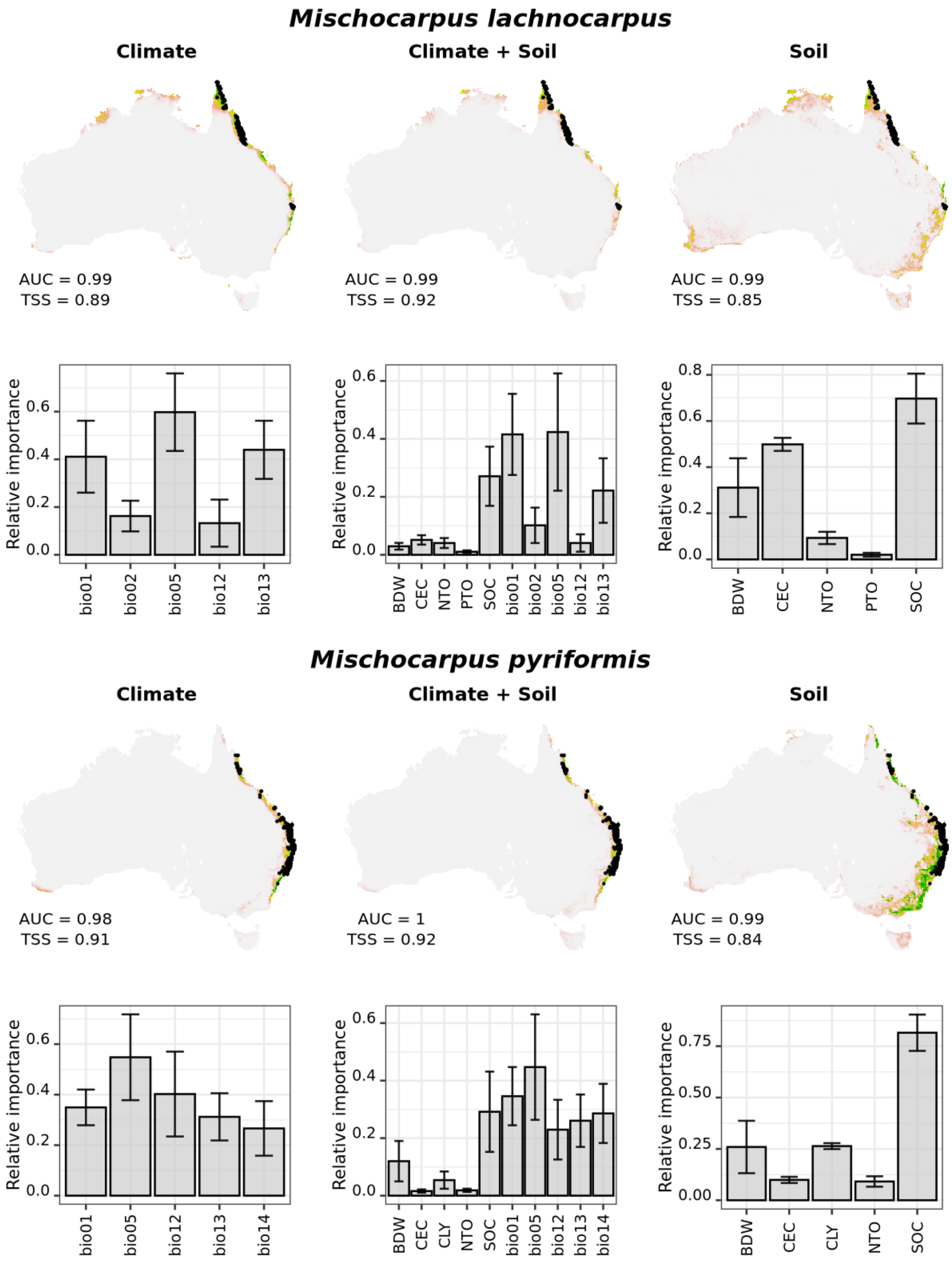

Mischocarpus lachnocarpus
Climate
Climate + Soil
Soil
AUC = 0.99
TSS = 0.89
AUC = 0.99
TSS = 0.92
AUC = 0.99
TSS = 0.85
Relative importance
bio01
bio02
bio05
bio12
bio13
BDW
CEC
NTO
PTO
SOC
Mischocarpus pyriformis
Climate
Climate + Soil
Soil
AUC = 0.98
TSS = 0.91
AUC = 1
TSS = 0.92
AUC = 0.99
TSS = 0.84
Relative importance
bio01
bio05
bio12
bio13
bio14
BDW
CEC
CLY
NTO
SOC

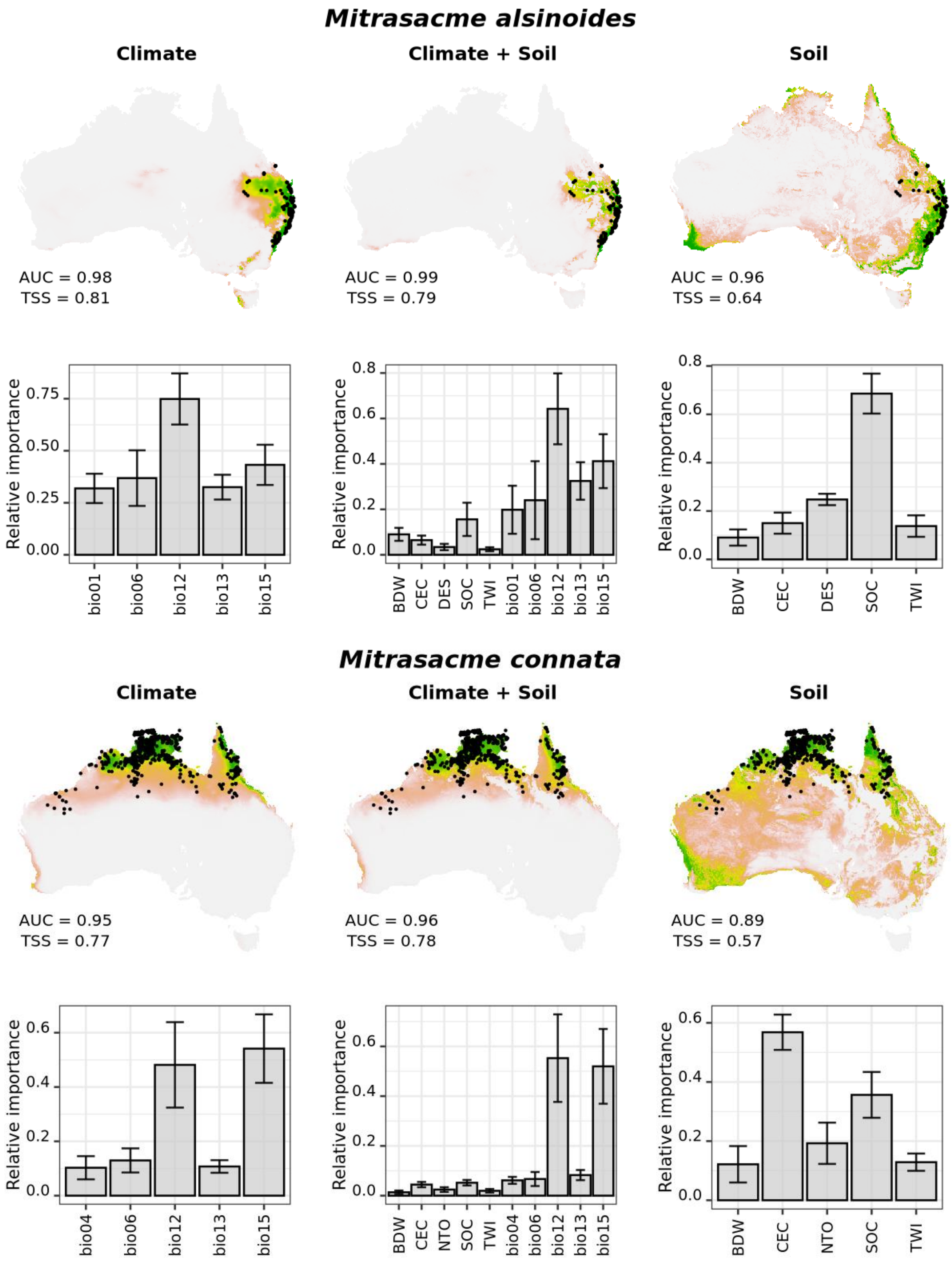

Mitrasacme alsinoides
Climate
Climate + Soil
Soil
AUC = 0.98
TSS = 0.81
AUC = 0.99
TSS = 0.79
AUC = 0.96
TSS = 0.64
Relative importance
0.00
0.25
0.50
0.75
bio01
bio06
bio12
bio13
bio15
0.0
0.2
0.4
0.6
0.8
BDW
CEC
DES
SOC
TWI
bio01
bio06
bio12
bio13
bio15
BDW
CEC
DES
SOC
TWI
Mitrasacme connata
Climate
Climate + Soil
Soil
AUC = 0.95
TSS = 0.77
AUC = 0.96
TSS = 0.78
AUC = 0.89
TSS = 0.57
bio04
bio06
bio12
bio13
bio15
BDW
CEC
NTO
SOC
TWI
bio04
bio06
bio12
bio13
bio15
BDW
CEC
NTO
SOC
TWI

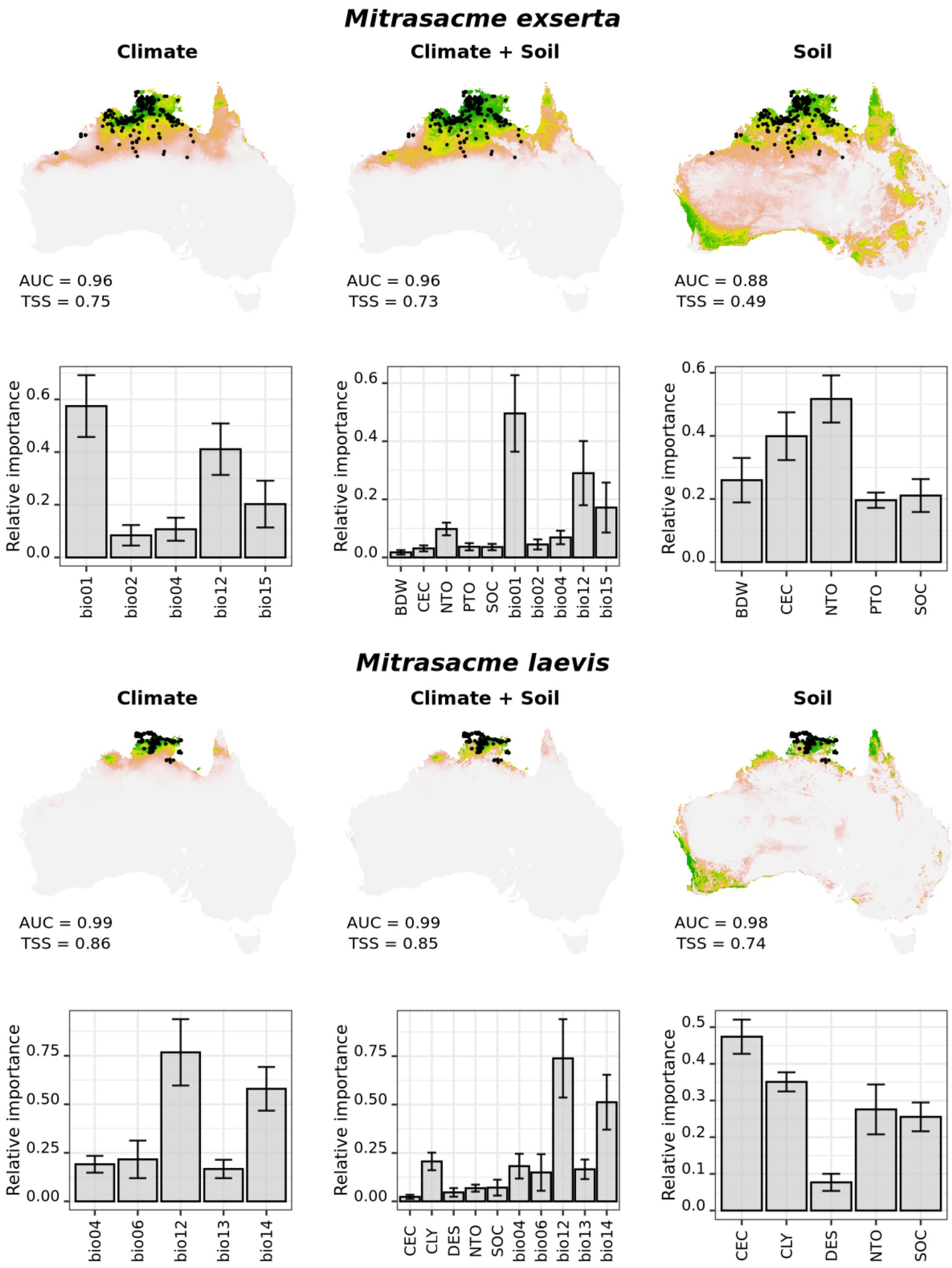
Mitrasacme exserta
Climate
Climate + Soil
Soil
AUC = 0.96
TSS = 0.75
AUC = 0.96
TSS = 0.73
AUC = 0.88
TSS = 0.49
Relative importance
0.0
0.2
0.4
0.6
bio01
bio02
bio04
bio12
bio15
BDW
CEC
NTO
PTO
SOC
bio01
bio02
bio04
bio12
bio15
BDW
CEC
NTO
PTO
SOC
Mitrasacme laevis
Climate
Climate + Soil
Soil
AUC = 0.99
TSS = 0.86
AUC = 0.99
TSS = 0.85
AUC = 0.98
TSS = 0.74
0.00
0.25
0.50
0.75
0.1
0.3
0.5
bio04
bio06
bio12
bio13
bio14
CEC
CLY
DES
NTO
SOC
bio04
bio06
bio12
bio13
bio14
CEC
CLY
DES
NTO
SOC

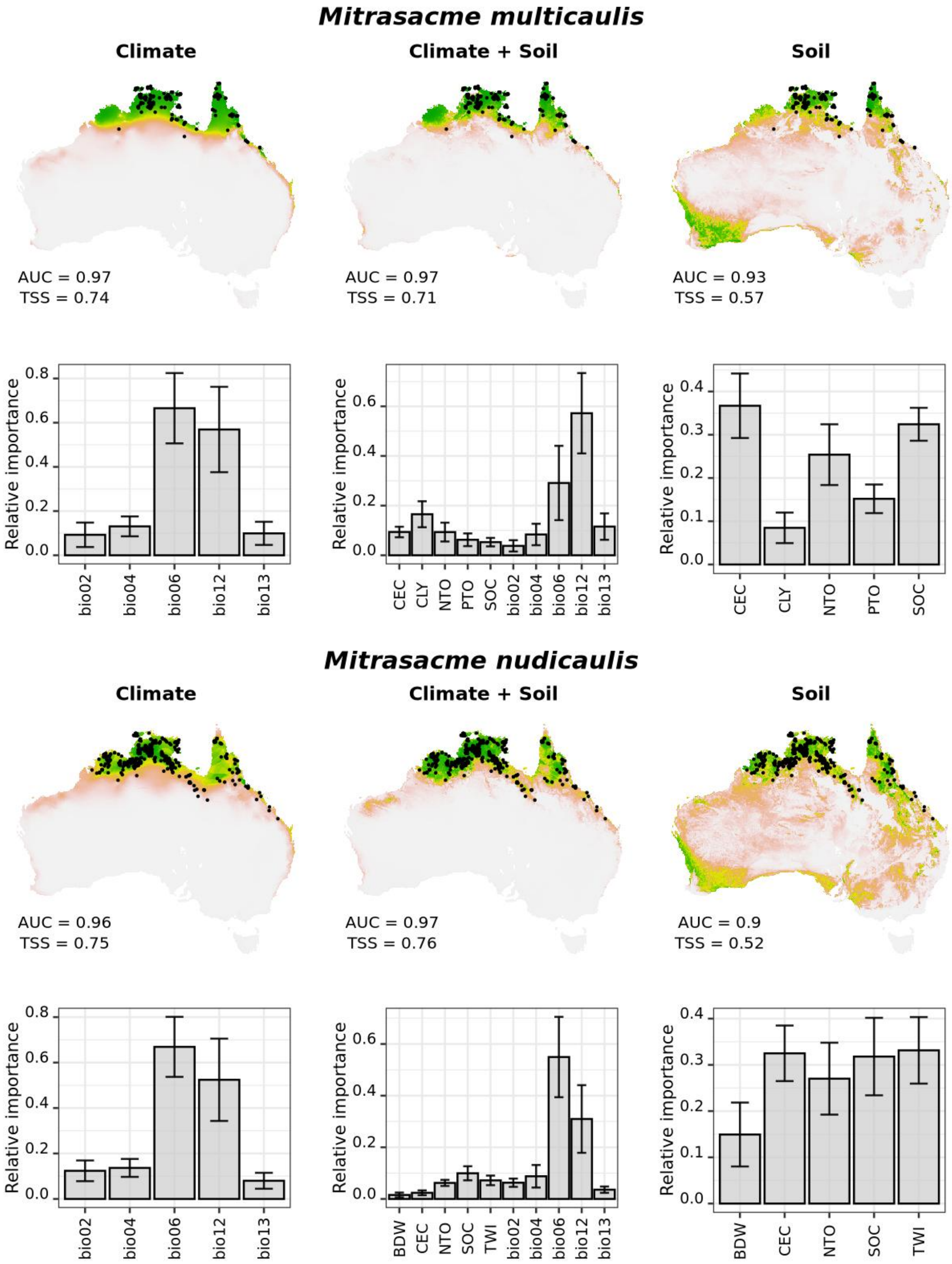
Mitrasacme multicaulis
Climate
Climate + Soil
Soil
AUC = 0.97
TSS = 0.74
AUC = 0.97
TSS = 0.71
AUC = 0.93
TSS = 0.57
Relative importance
bio02
bio04
bio06
bio12
bio13
CEC
CLY
NTO
PTO
SOC
Mitrasacme nudicaulis
Climate
Climate + Soil
Soil
AUC = 0.96
TSS = 0.75
AUC = 0.97
TSS = 0.76
AUC = 0.9
TSS = 0.52
Relative importance
BDW
CEC
NTO
SOC
TWI
bio02
bio04
bio06
bio12
bio13

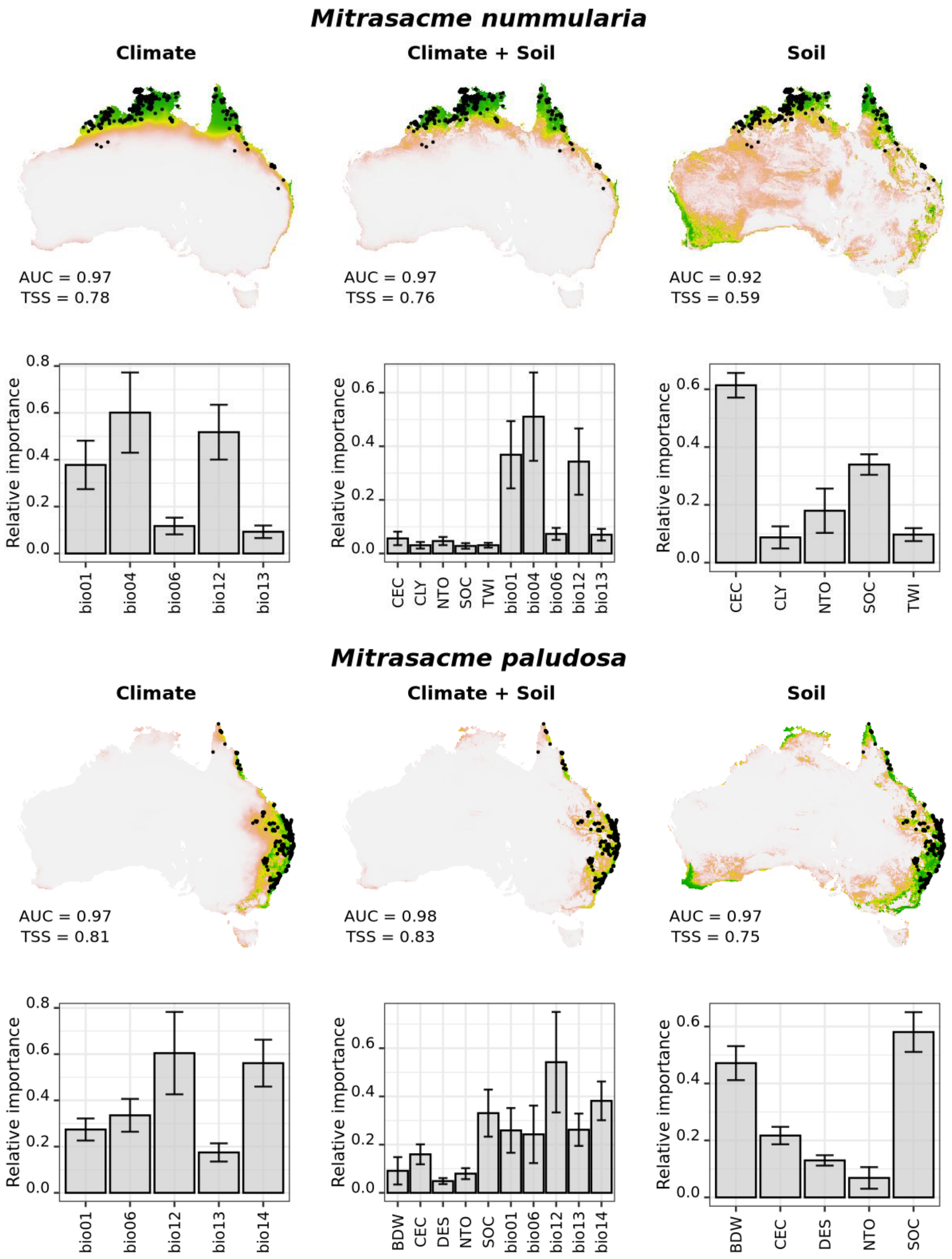
Mitrasacme nummularia
Climate
Climate + Soil
Soil
AUC = 0.97
TSS = 0.78
AUC = 0.97
TSS = 0.76
AUC = 0.92
TSS = 0.59
Relative importance
bio01
bio04
bio06
bio12
bio13
CEC
CLY
NTO
SOC
TWI
Mitrasacme paludosa
Climate
Climate + Soil
Soil
AUC = 0.97
TSS = 0.81
AUC = 0.98
TSS = 0.83
AUC = 0.97
TSS = 0.75
Relative importance
bio01
bio06
bio12
bio13
bio14
BDW
CEC
DES
NTO
SOC

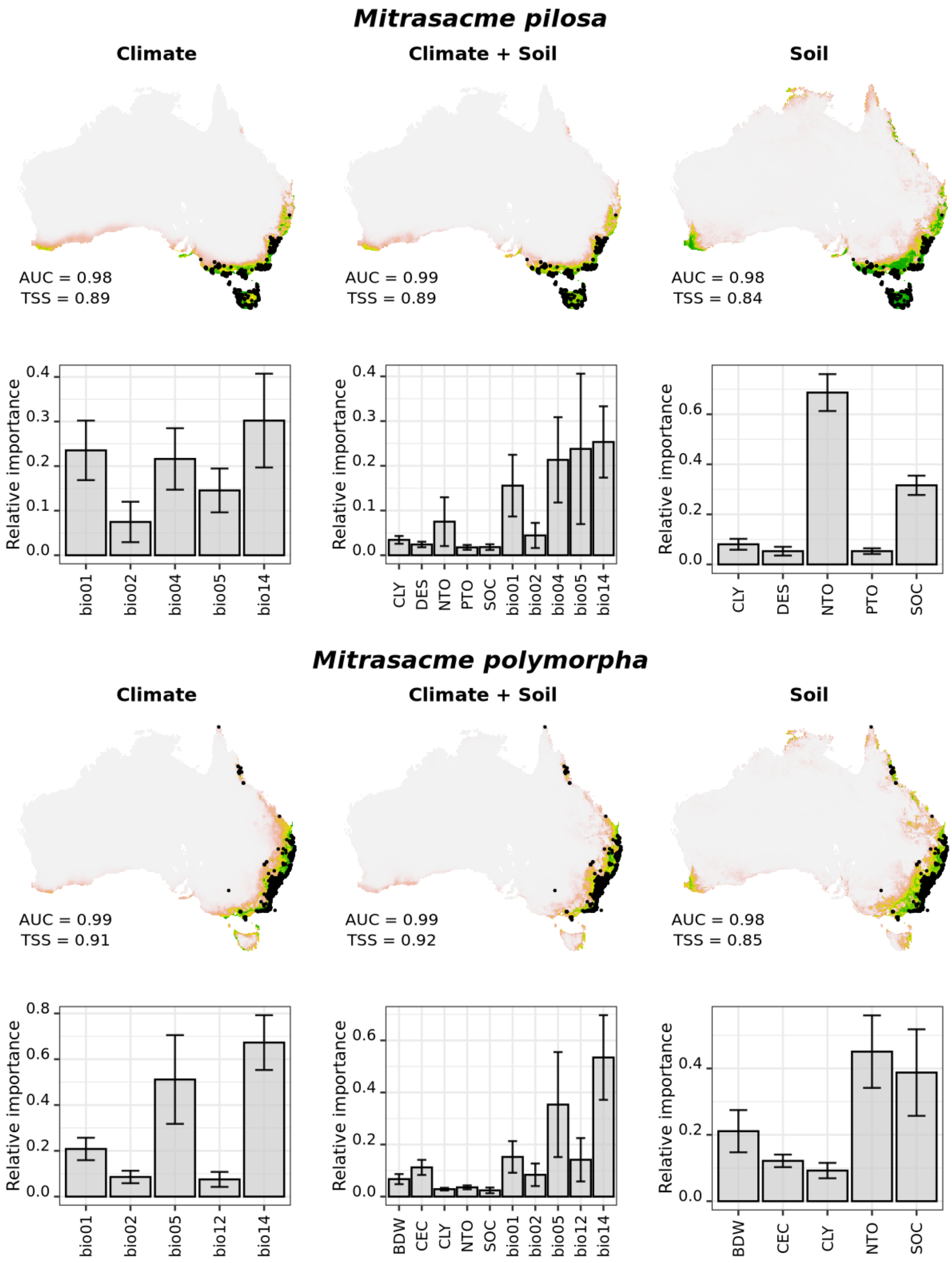
Mitrasacme pilosa
Climate
Climate + Soil
Soil
AUC = 0.98
TSS = 0.89
AUC = 0.99
TSS = 0.89
AUC = 0.98
TSS = 0.84
Relative importance
bio01
bio02
bio04
bio05
bio14
CLY
DES
NTO
PTO
SOC
Mitrasacme polymorpha
Climate
Climate + Soil
Soil
AUC = 0.99
TSS = 0.91
AUC = 0.99
TSS = 0.92
AUC = 0.98
TSS = 0.85
Relative importance
bio01
bio02
bio05
bio12
bio14
BDW
CEC
CLY
NTO
SOC

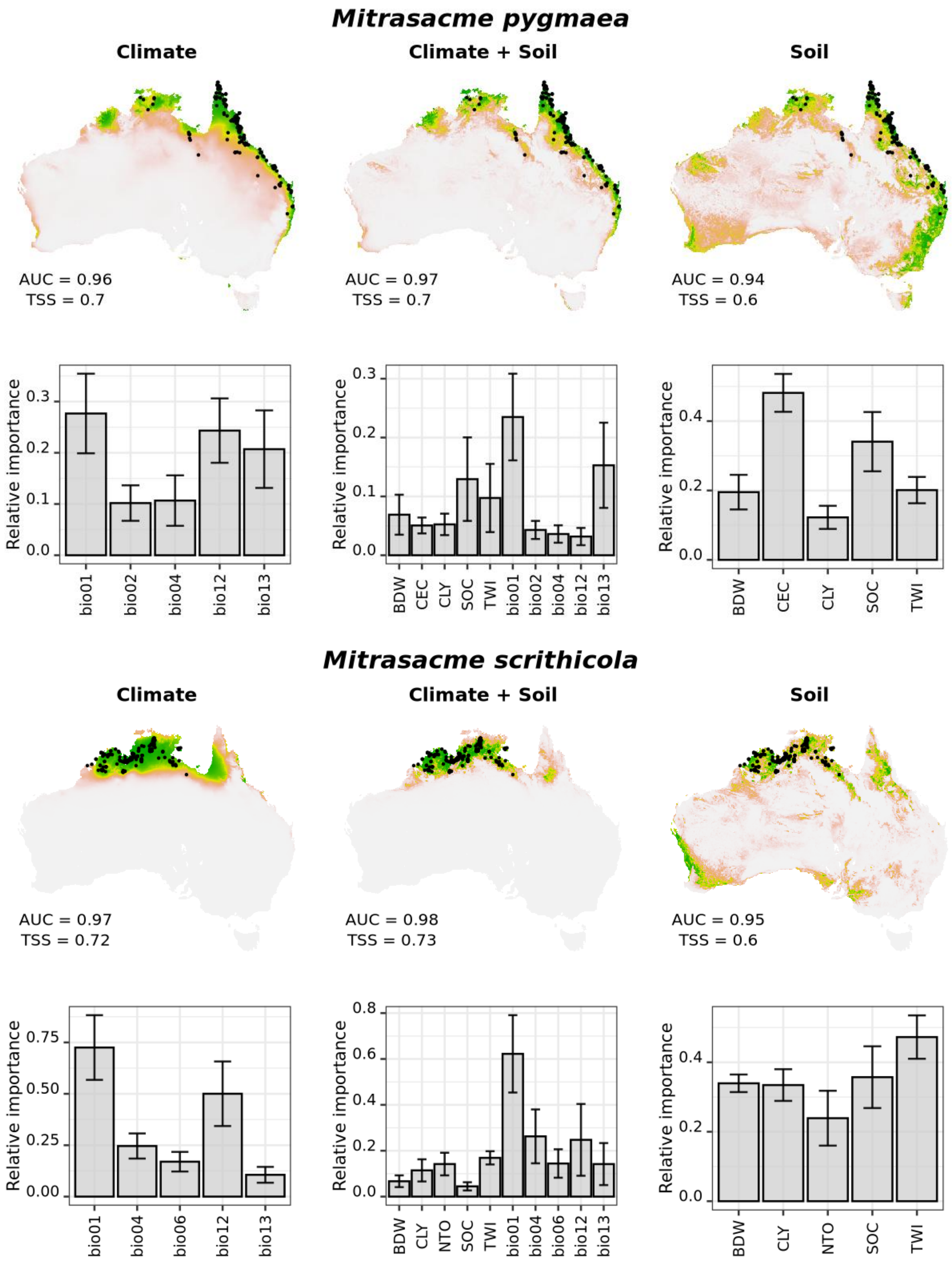
Mitrasacme pygmaea
Climate
Climate + Soil
Soil
AUC = 0.96
TSS = 0.7
AUC = 0.97
TSS = 0.7
AUC = 0.94
TSS = 0.6
Relative importance
bio01 bio02 bio04 bio12 bio13
BDW CEC CLY SOC TWI bio01 bio02 bio04 bio12 bio13
BDW CEC CLY SOC TWI
Mitrasacme scrithicola
Climate
Climate + Soil
Soil
AUC = 0.97
TSS = 0.72
AUC = 0.98
TSS = 0.73
AUC = 0.95
TSS = 0.6
Relative importance
bio01 bio04 bio06 bio12 bio13
BDW CLY NTO SOC TWI bio01 bio04 bio06 bio12 bio13
BDW CLY NTO SOC TWI

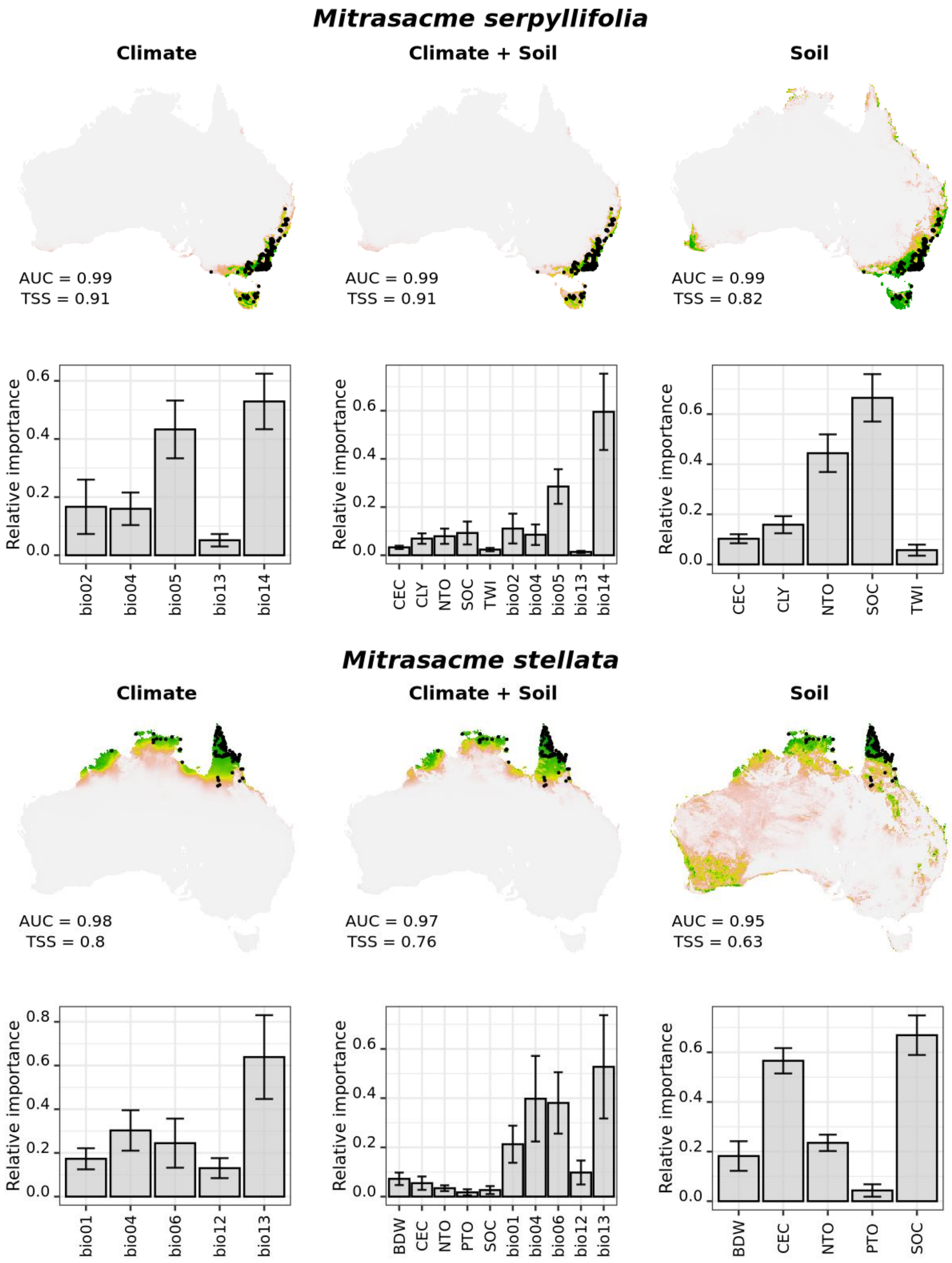
Mitrasacme serpyllifolia
Climate
Climate + Soil
Soil
AUC = 0.99
TSS = 0.91
AUC = 0.99
TSS = 0.91
AUC = 0.99
TSS = 0.82
Relative importance
bio02
bio04
bio05
bio13
bio14
CEC
CLY
NTO
SOC
TWI
Mitrasacme stellata
AUC = 0.98
TSS = 0.8
AUC = 0.97
TSS = 0.76
AUC = 0.95
TSS = 0.63
bio01
bio06
bio12
BDW
PTO

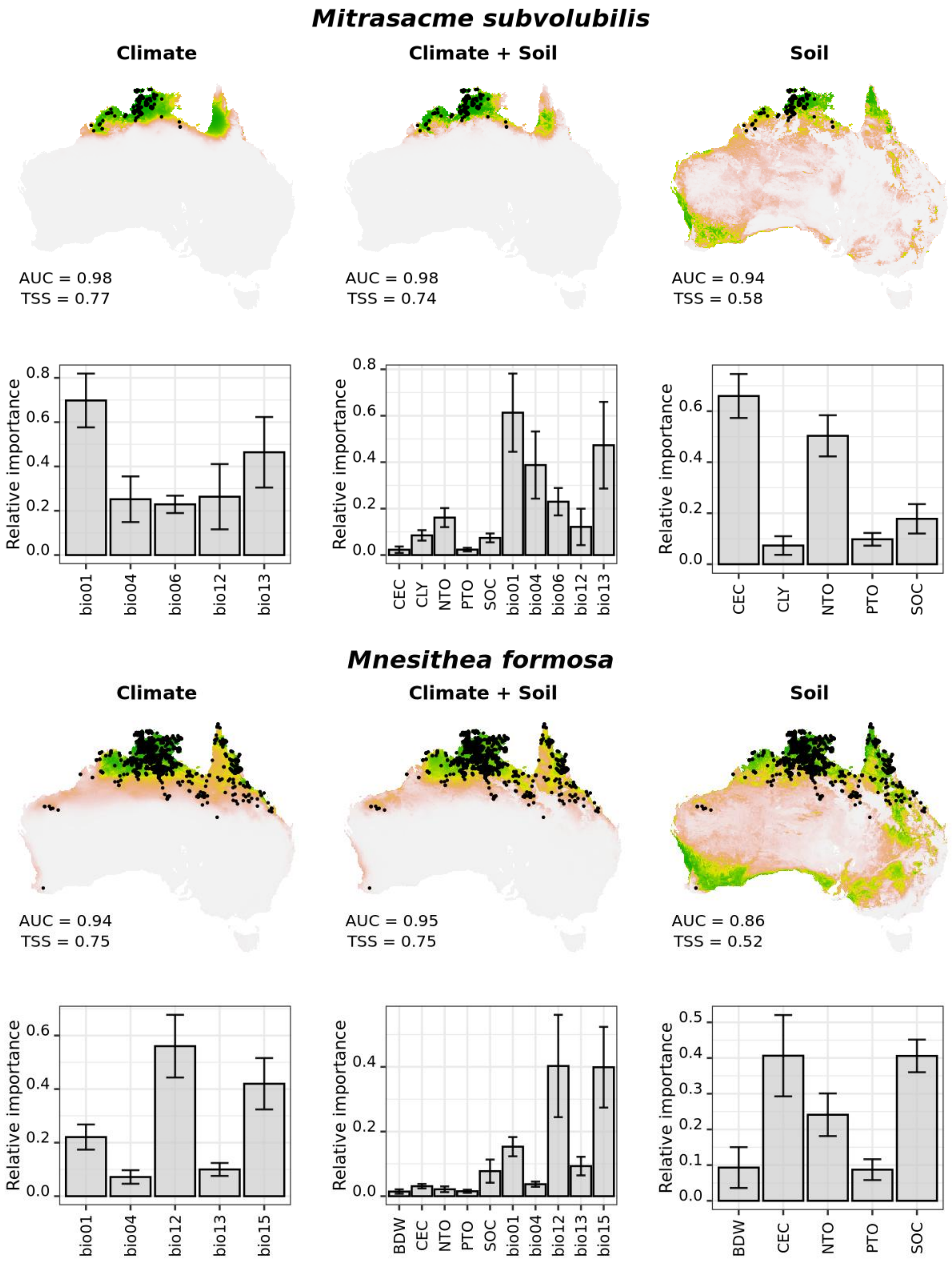

Mitrasacme subvolubilis
Climate
Climate + Soil
Soil
AUC = 0.98
TSS = 0.77
AUC = 0.98
TSS = 0.74
AUC = 0.94
TSS = 0.58
Relative importance
bio01
bio04
bio06
bio12
bio13
CEC
CLY
NTO
PTO
SOC
Mnesithea formosa
Climate
Climate + Soil
Soil
AUC = 0.94
TSS = 0.75
AUC = 0.95
TSS = 0.75
AUC = 0.86
TSS = 0.52
Relative importance
bio01
bio04
bio12
bio13
bio15
BDW
CEC
NTO
PTO
SOC

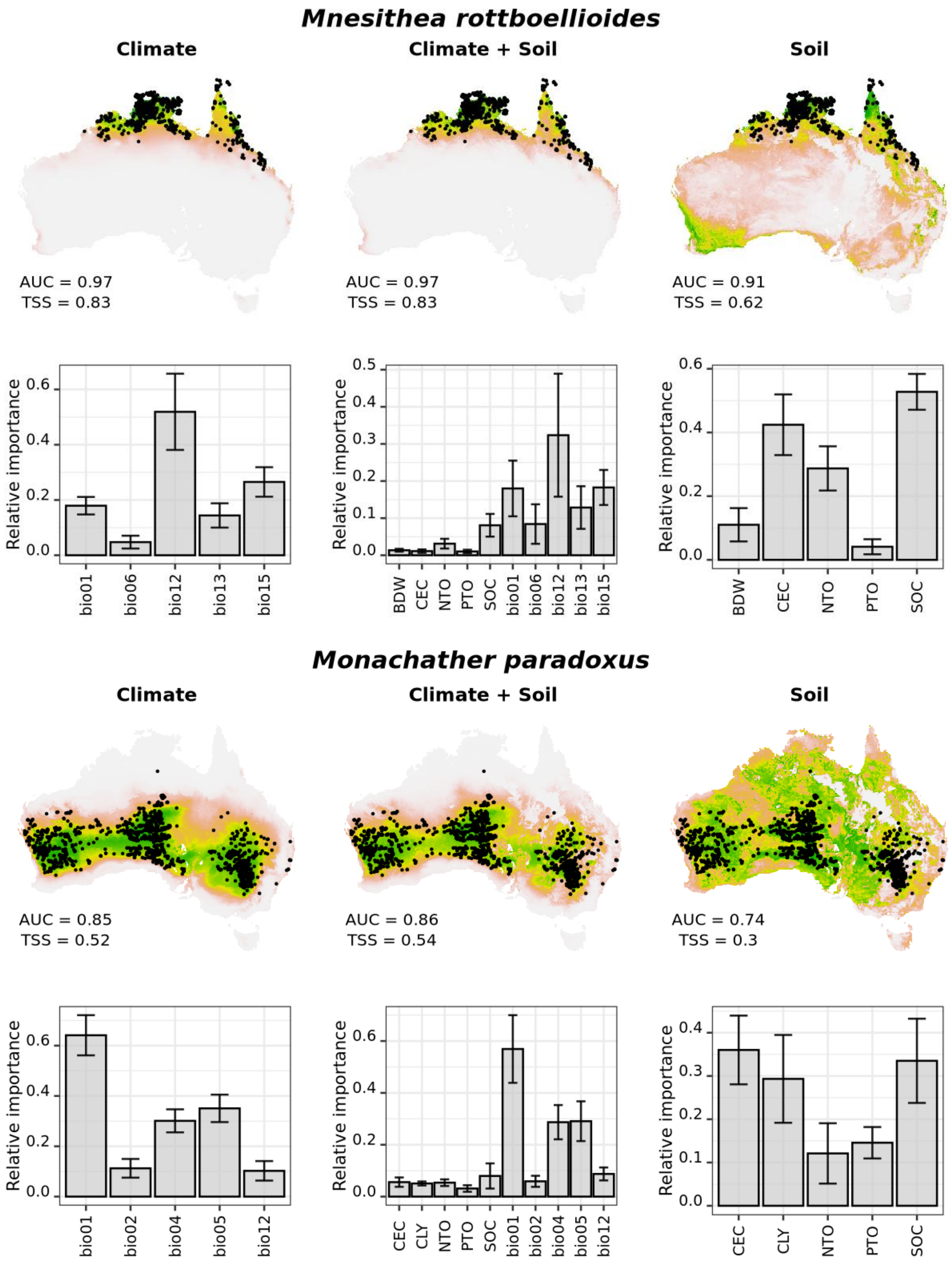
Mnesithea rottboellioides
Climate
Climate + Soil
Soil
AUC = 0.97
TSS = 0.83
AUC = 0.97
TSS = 0.83
AUC = 0.91
TSS = 0.62
Relative importance
bio01
bio06
bio12
bio13
bio15
BDW
CEC
NTO
PTO
SOC
Monachather paradoxus
Climate
Climate + Soil
Soil
AUC = 0.85
TSS = 0.52
AUC = 0.86
TSS = 0.54
AUC = 0.74
TSS = 0.3
Relative importance
bio01
bio02
bio04
bio05
bio12
CEC
CLY
NTO
PTO
SOC

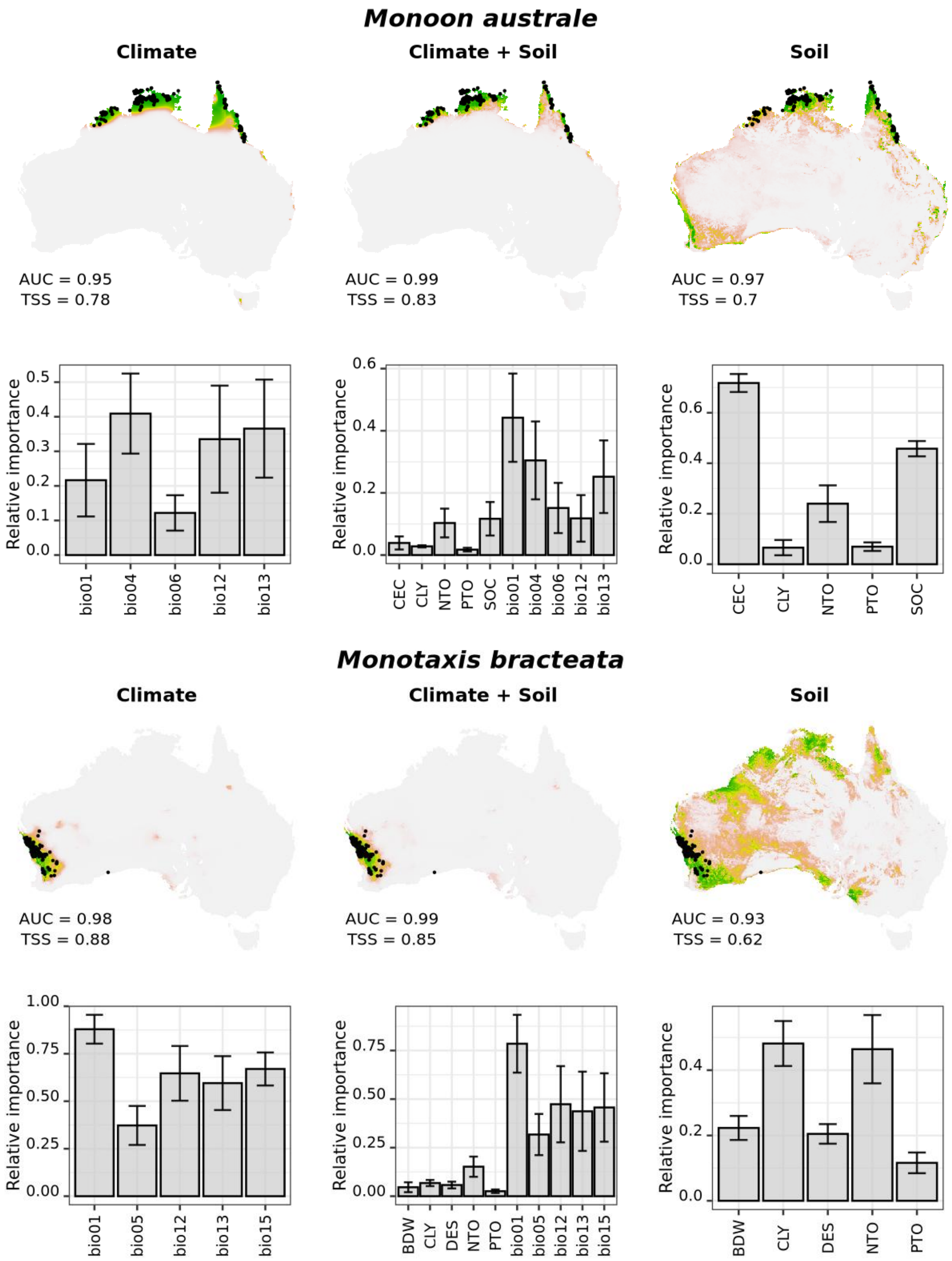

Monoon australe
Climate
Climate + Soil
Soil
AUC = 0.95
TSS = 0.78
AUC = 0.99
TSS = 0.83
AUC = 0.97
TSS = 0.7
Relative importance
bio01
bio04
bio06
bio12
bio13
CEC
CLY
NTO
PTO
SOC
Monotaxis bracteata
Climate
Climate + Soil
Soil
AUC = 0.98
TSS = 0.88
AUC = 0.99
TSS = 0.85
AUC = 0.93
TSS = 0.62
Relative importance
bio01
bio05
bio12
bio13
bio15
BDW
CLY
DES
NTO
PTO

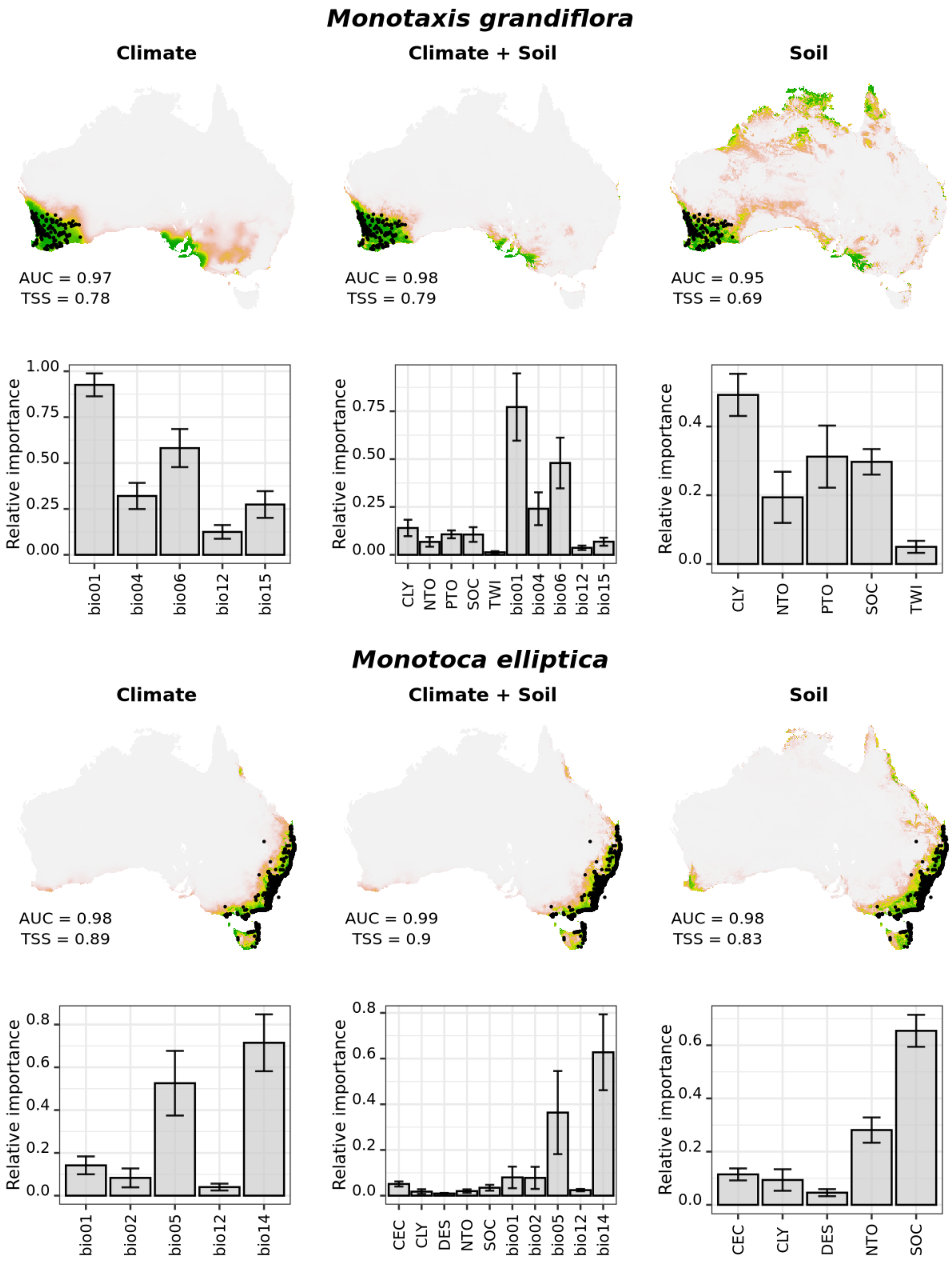

Monotaxis grandiflora
Climate
Climate + Soil
Soil
AUC = 0.97
TSS = 0.78
AUC = 0.98
TSS = 0.79
AUC = 0.95
TSS = 0.69
Relative importance
1.00
0.75
0.50
0.25
0.00
bio01
bio04
bio06
bio12
bio15
0.75
0.50
0.25
0.00
CLY
NTO
PTO
SOC
TWI
bio01
bio04
bio06
bio12
bio15
0.4
0.2
0.0
CLY
NTO
PTO
SOC
TWI
Monotoca elliptica
Climate
Climate + Soil
Soil
AUC = 0.98
TSS = 0.89
AUC = 0.99
TSS = 0.9
AUC = 0.98
TSS = 0.83
0.8
0.6
0.4
0.2
0.0
bio01
bio02
bio05
bio12
bio14
0.8
0.6
0.4
0.2
0.0
CEC
CLY
DES
NTO
SOC
bio01
bio02
bio05
bio12
bio14
0.6
0.4
0.2
0.0
CEC
CLY
DES
NTO
SOC

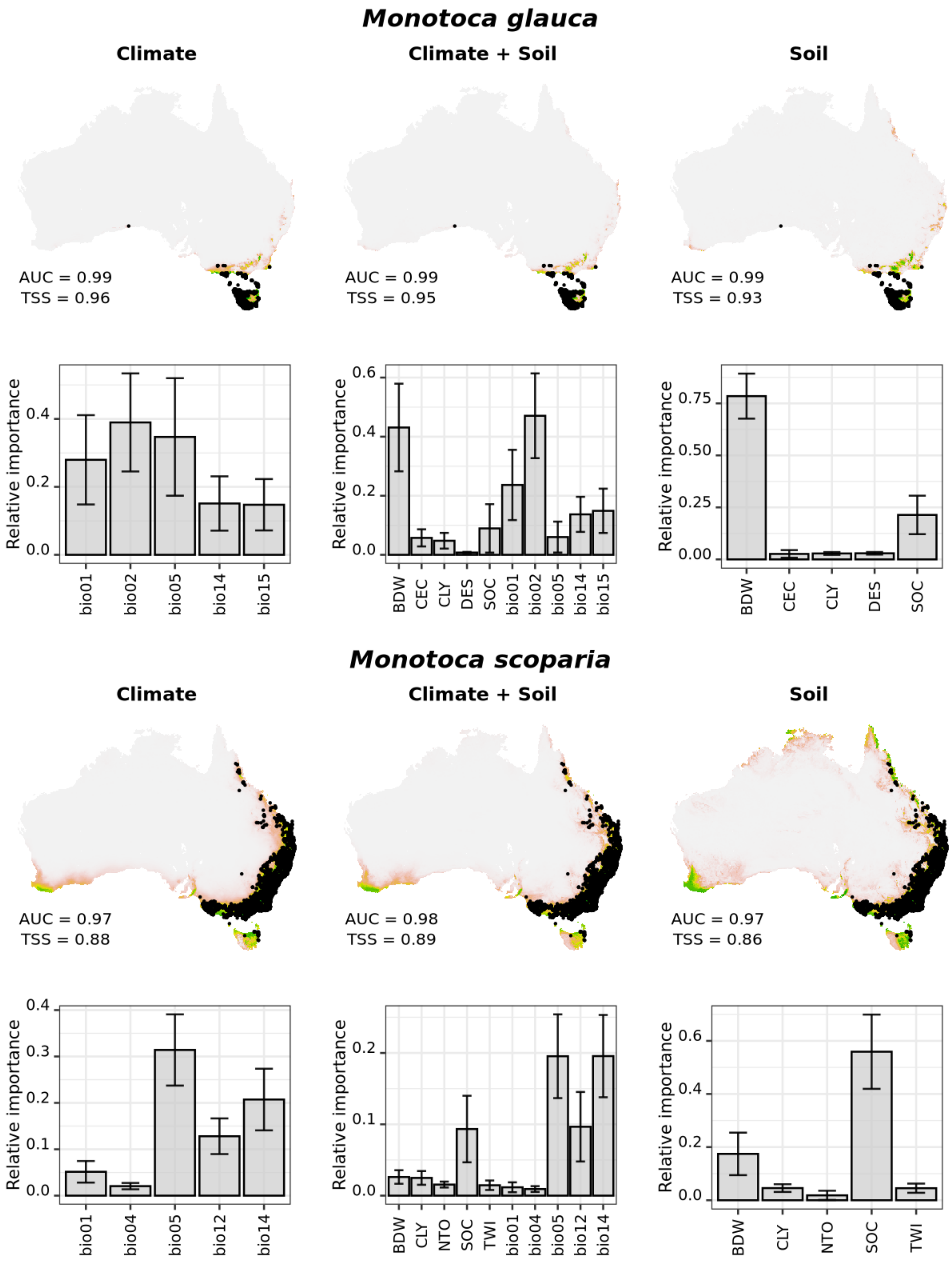
Monotoca glauca
Climate
Climate + Soil
Soil
AUC = 0.99
TSS = 0.96
AUC = 0.99
TSS = 0.95
AUC = 0.99
TSS = 0.93
Relative importance
0.0
0.2
0.4
bio01
bio02
bio05
bio14
bio15
0.6
BDW
CEC
CLY
DES
SOC
0.00
0.25
0.50
0.75
Monotoca scoparia
Climate
Climate + Soil
Soil
AUC = 0.97
TSS = 0.88
AUC = 0.98
TSS = 0.89
AUC = 0.97
TSS = 0.86
0.1
0.3
bio04
bio12
NTO
TWI

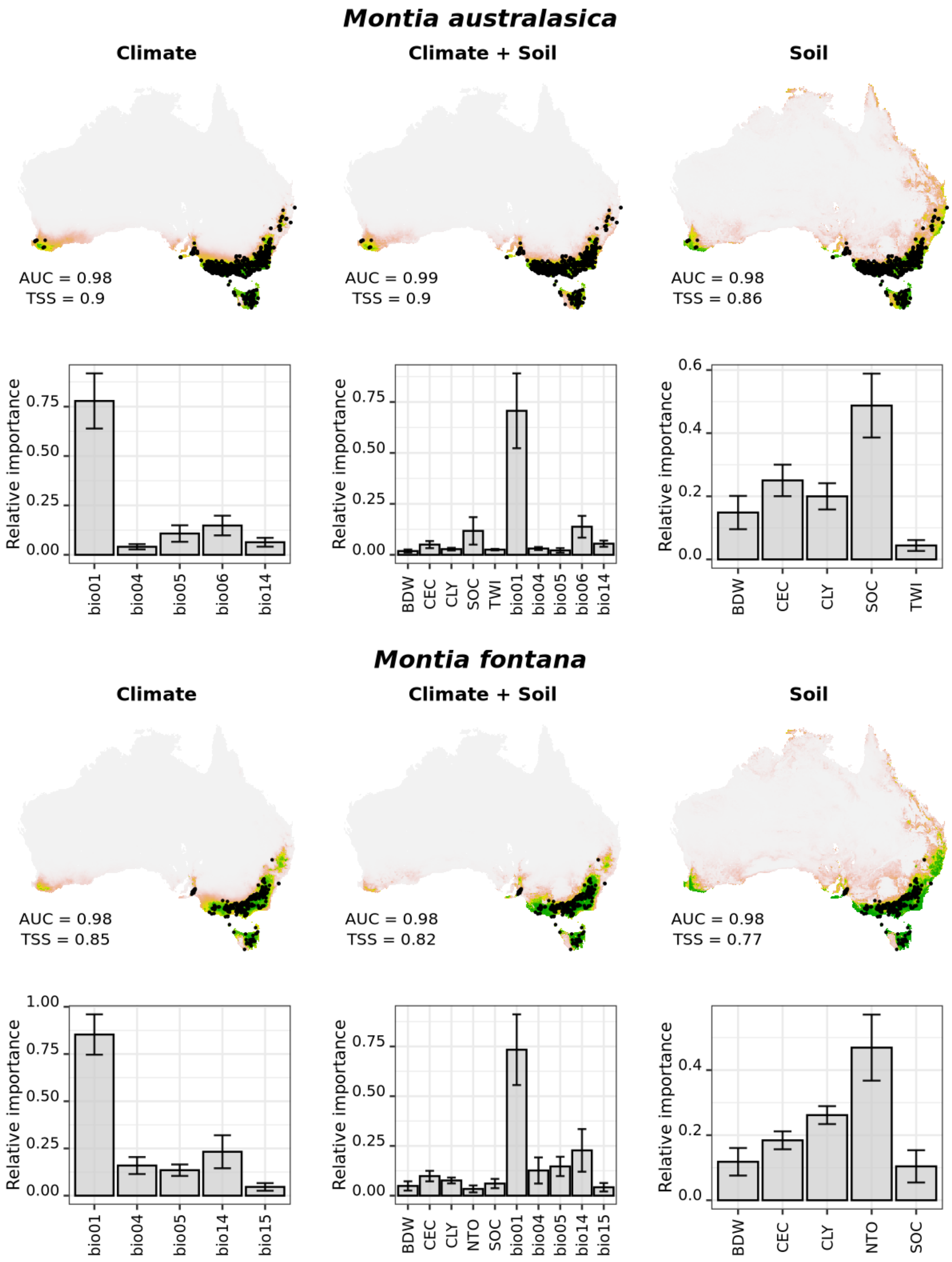
Montia australasica
Climate
Climate + Soil
Soil
AUC = 0.98
TSS = 0.9
AUC = 0.99
TSS = 0.9
AUC = 0.98
TSS = 0.86
Relative importance
bio01
bio04
bio05
bio06
bio14
BDW
CEC
CLY
SOC
TWI
Montia fontana
Climate
Climate + Soil
Soil
AUC = 0.98
TSS = 0.85
AUC = 0.98
TSS = 0.82
AUC = 0.98
TSS = 0.77
Relative importance
bio01
bio04
bio05
bio14
bio15
BDW
CEC
CLY
NTO
SOC

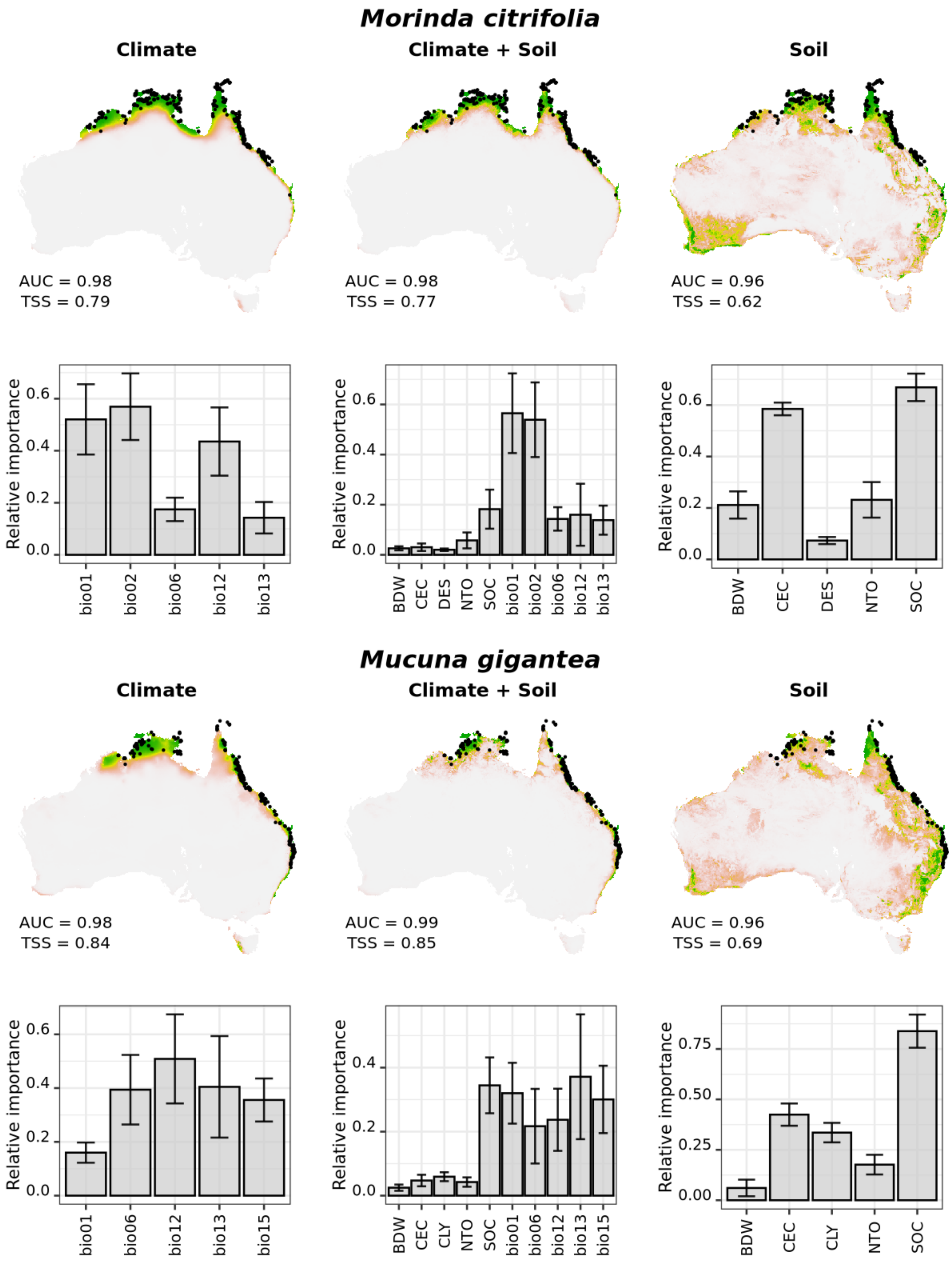
Morinda citrifolia
Climate
Climate + Soil
Soil
AUC = 0.98
TSS = 0.79
AUC = 0.98
TSS = 0.77
AUC = 0.96
TSS = 0.62
Relative importance
bio01
bio02
bio06
bio12
bio13
BDW
CEC
DES
NTO
SOC
Mucuna gigantea
Climate
Climate + Soil
Soil
AUC = 0.98
TSS = 0.84
AUC = 0.99
TSS = 0.85
AUC = 0.96
TSS = 0.69
Relative importance
bio01
bio06
bio12
bio13
bio15
BDW
CEC
CLY
NTO
SOC

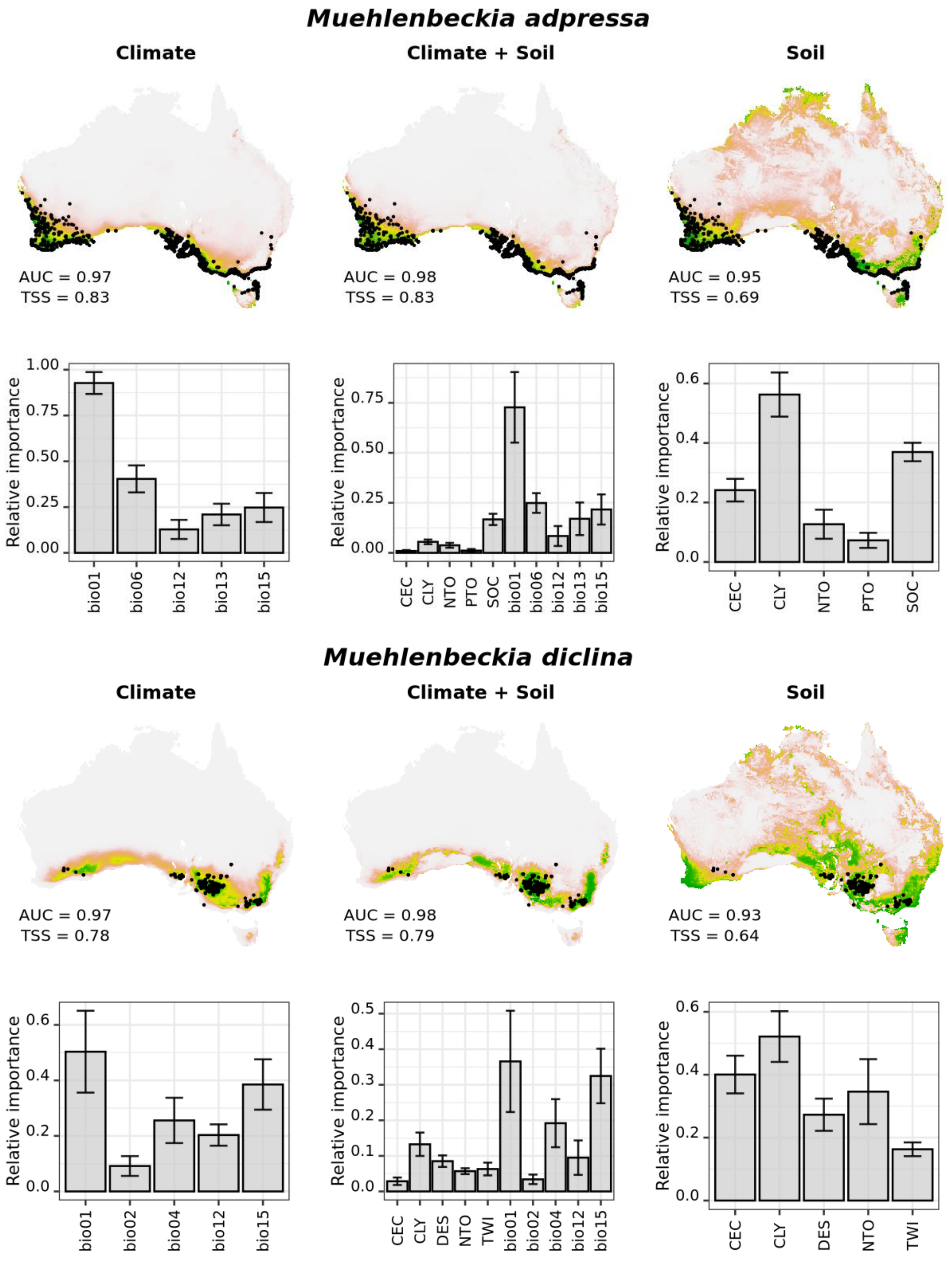

Muehlenbeckia adpressa
Climate
Climate + Soil
Soil
AUC = 0.97
TSS = 0.83
AUC = 0.98
TSS = 0.83
AUC = 0.95
TSS = 0.69
Relative importance
bio01 bio06 bio12 bio13 bio15
CEC CLY NTO PTO SOC bio01 bio06 bio12 bio13 bio15
CEC CLY NTO PTO SOC
Muehlenbeckia diclina
Climate
Climate + Soil
Soil
AUC = 0.97
TSS = 0.78
AUC = 0.98
TSS = 0.79
AUC = 0.93
TSS = 0.64
Relative importance
bio01 bio02 bio04 bio12 bio15
CEC CLY DES NTO TWI bio01 bio02 bio04 bio12 bio15
CEC CLY DES NTO TWI

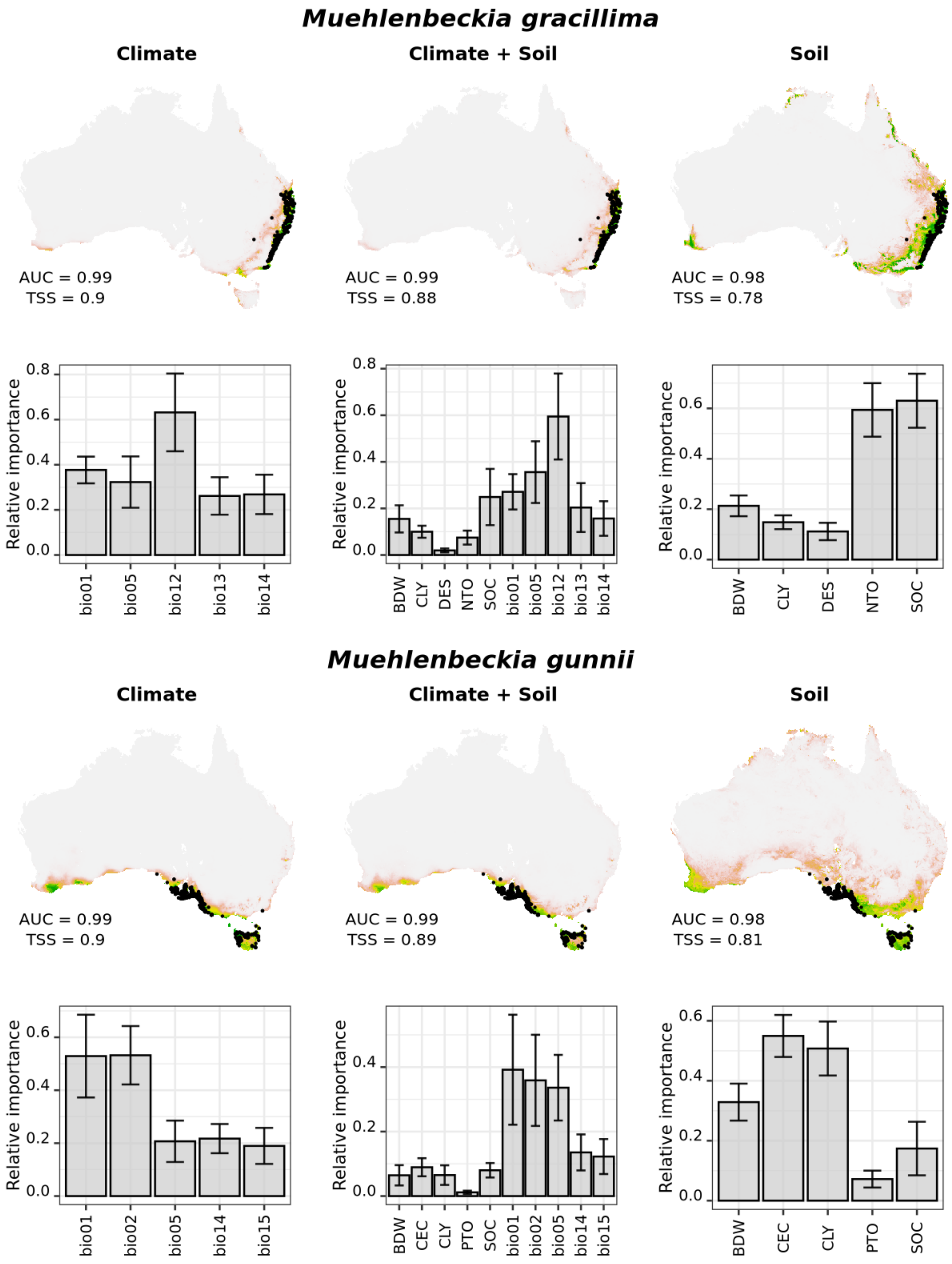
Muehlenbeckia gracillima
Climate
Climate + Soil
Soil
AUC = 0.99
TSS = 0.9
AUC = 0.99
TSS = 0.88
AUC = 0.98
TSS = 0.78
Relative importance
bio01
bio05
bio12
bio13
bio14
BDW
CLY
DES
NTO
SOC
Muehlenbeckia gunnii
Climate
Climate + Soil
Soil
AUC = 0.99
TSS = 0.9
AUC = 0.99
TSS = 0.89
AUC = 0.98
TSS = 0.81
Relative importance
bio01
bio02
bio05
bio14
bio15
BDW
CEC
CLY
PTO
SOC

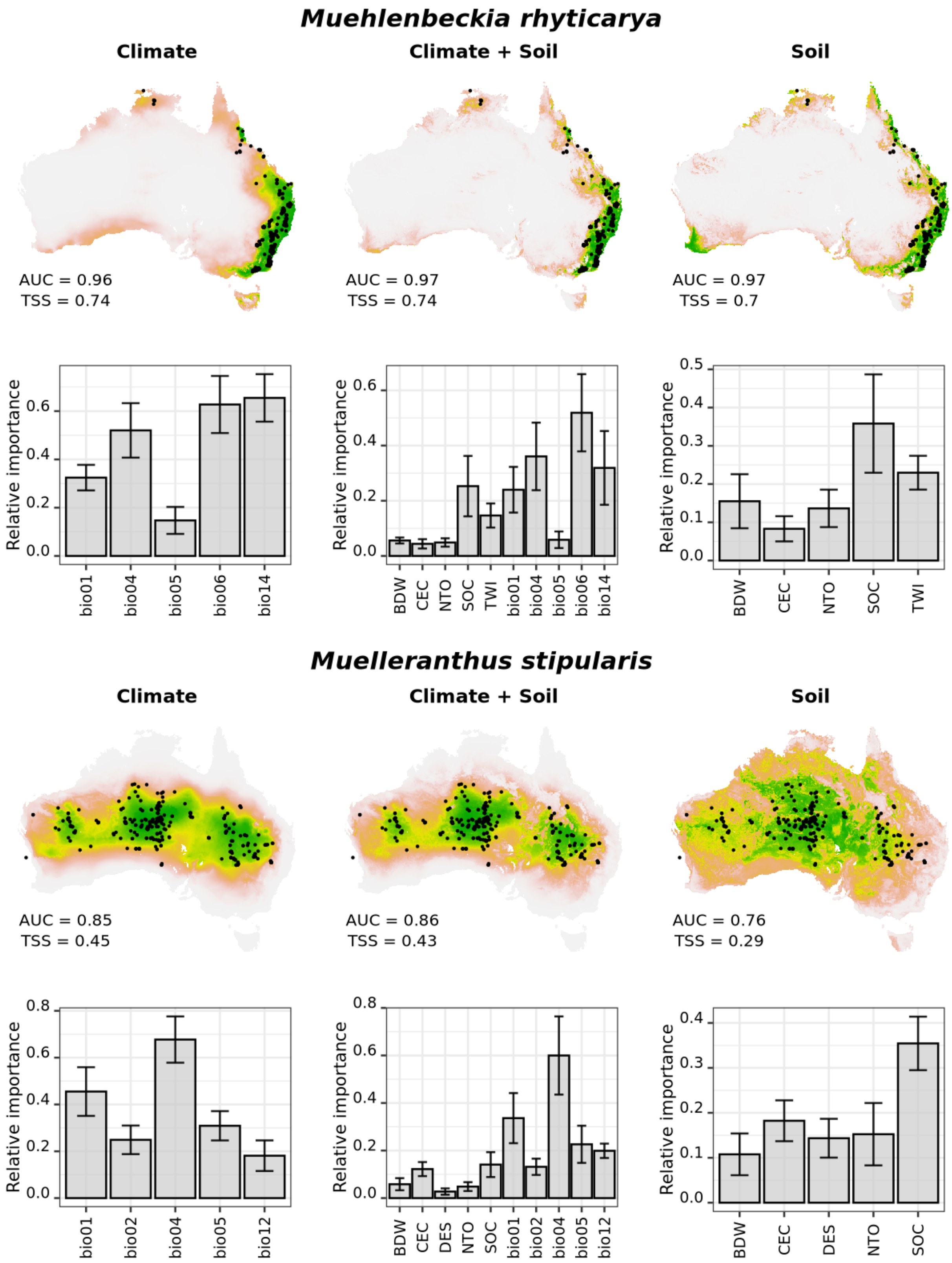

Muehlenbeckia rhyticarya
Climate
Climate + Soil
Soil
AUC = 0.96
TSS = 0.74
AUC = 0.97
TSS = 0.74
AUC = 0.97
TSS = 0.7
Relative importance
bio01
bio04
bio05
bio06
bio14
BDW
CEC
NTO
SOC
TWI
bio01
bio04
bio05
bio06
bio14
BDW
CEC
NTO
SOC
TWI
Muelleranthus stipularis
Climate
Climate + Soil
Soil
AUC = 0.85
TSS = 0.45
AUC = 0.86
TSS = 0.43
AUC = 0.76
TSS = 0.29
Relative importance
bio01
bio02
bio04
bio05
bio12
BDW
CEC
DES
NTO
SOC
bio01
bio02
bio04
bio05
bio12
BDW
CEC
DES
NTO
SOC

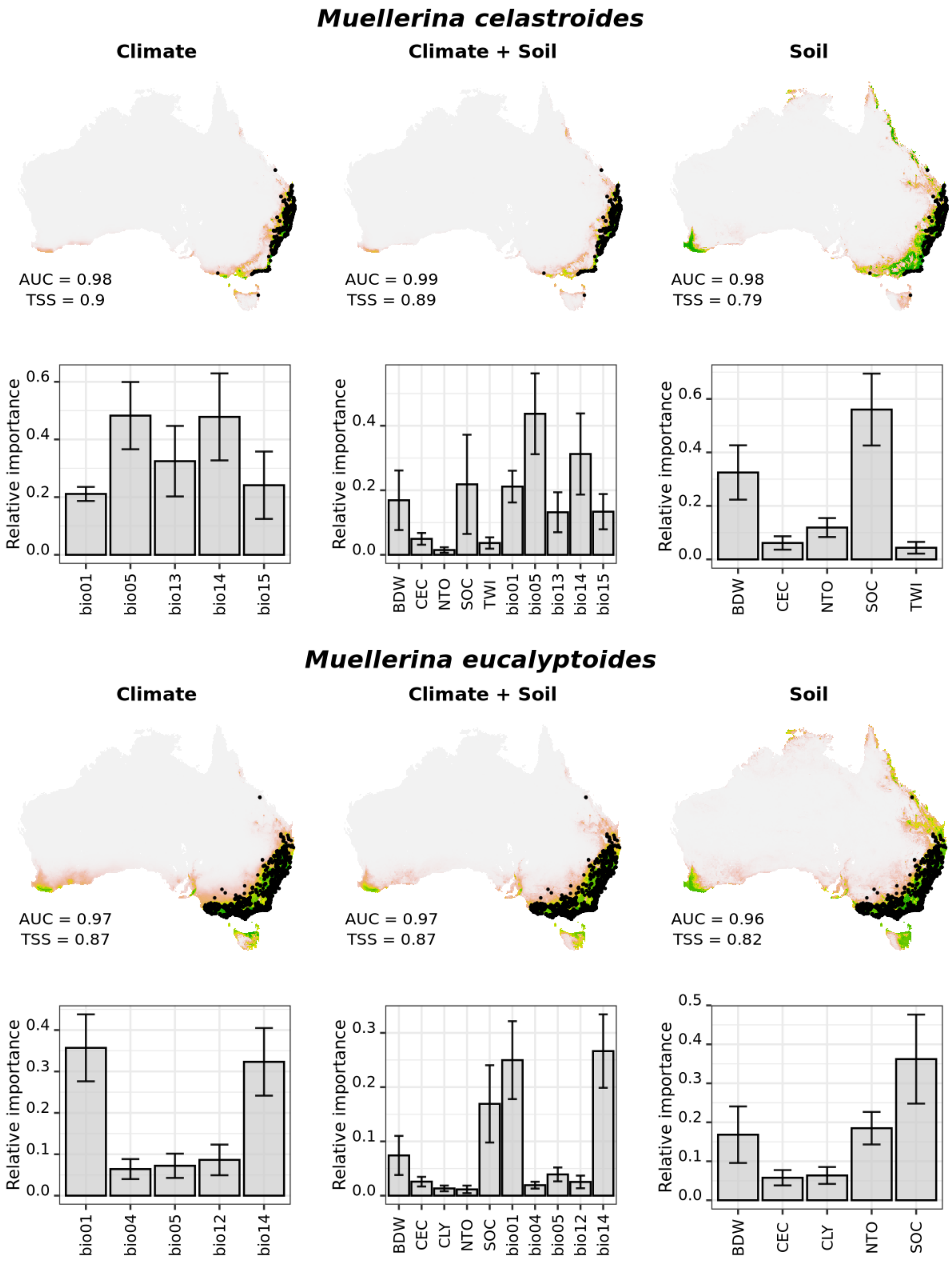
Muellerina celastroides
Climate
Climate + Soil
Soil
AUC = 0.98
TSS = 0.9
AUC = 0.99
TSS = 0.89
AUC = 0.98
TSS = 0.79
Relative importance
bio01
bio05
bio13
bio14
bio15
BDW
CEC
NTO
SOC
TWI
Muellerina eucalyptoides
Climate
Climate + Soil
Soil
AUC = 0.97
TSS = 0.87
AUC = 0.97
TSS = 0.87
AUC = 0.96
TSS = 0.82
bio04
bio12
CLY

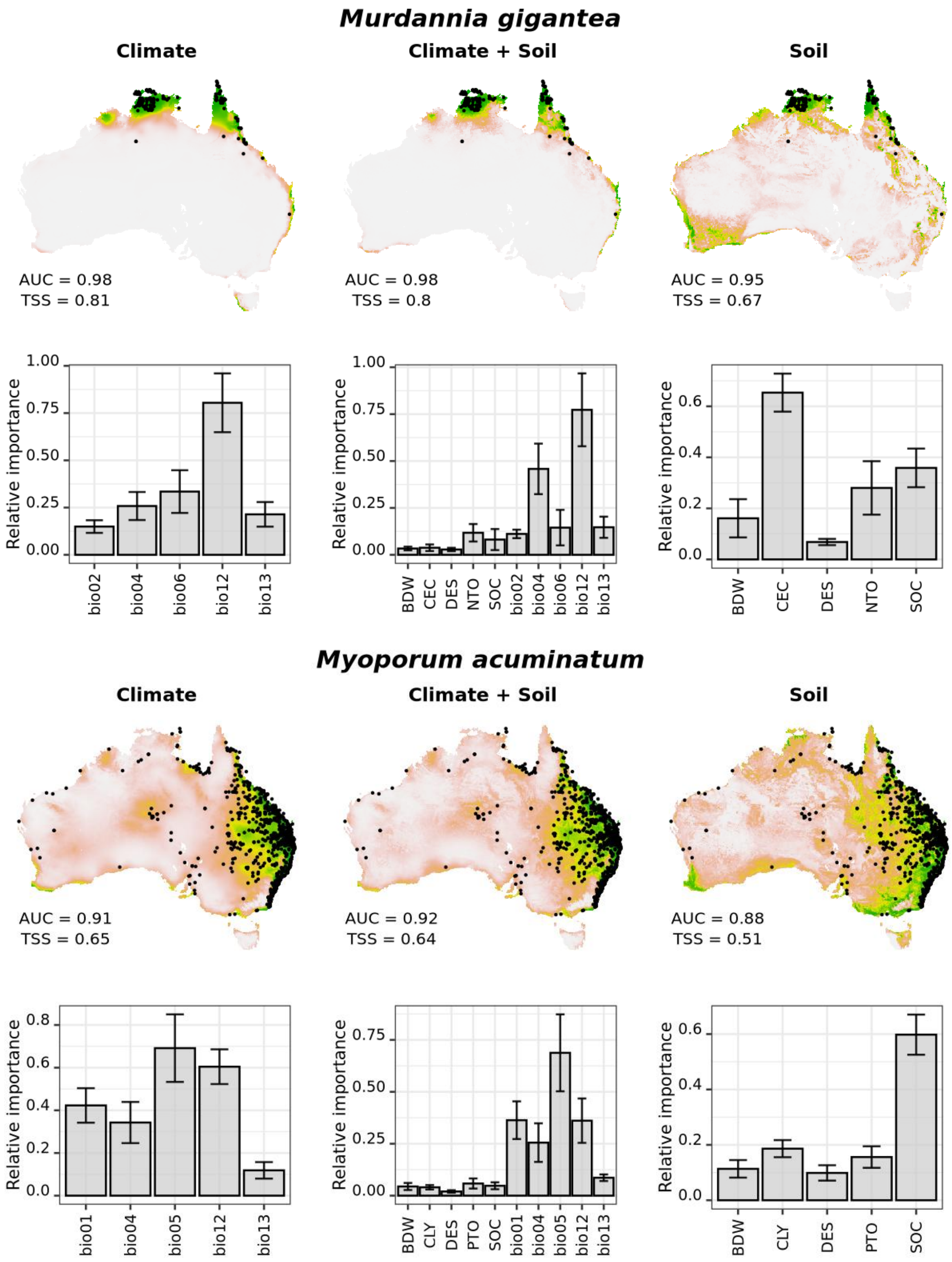
Murdannia gigantea
Climate
Climate + Soil
Soil
AUC = 0.98
TSS = 0.81
AUC = 0.98
TSS = 0.8
AUC = 0.95
TSS = 0.67
Relative importance
bio02
bio04
bio06
bio12
bio13
BDW
CEC
DES
NTO
SOC
Myoporum acuminatum
Climate
Climate + Soil
Soil
AUC = 0.91
TSS = 0.65
AUC = 0.92
TSS = 0.64
AUC = 0.88
TSS = 0.51
Relative importance
bio01
bio04
bio05
bio12
bio13
BDW
CLY
DES
PTO
SOC

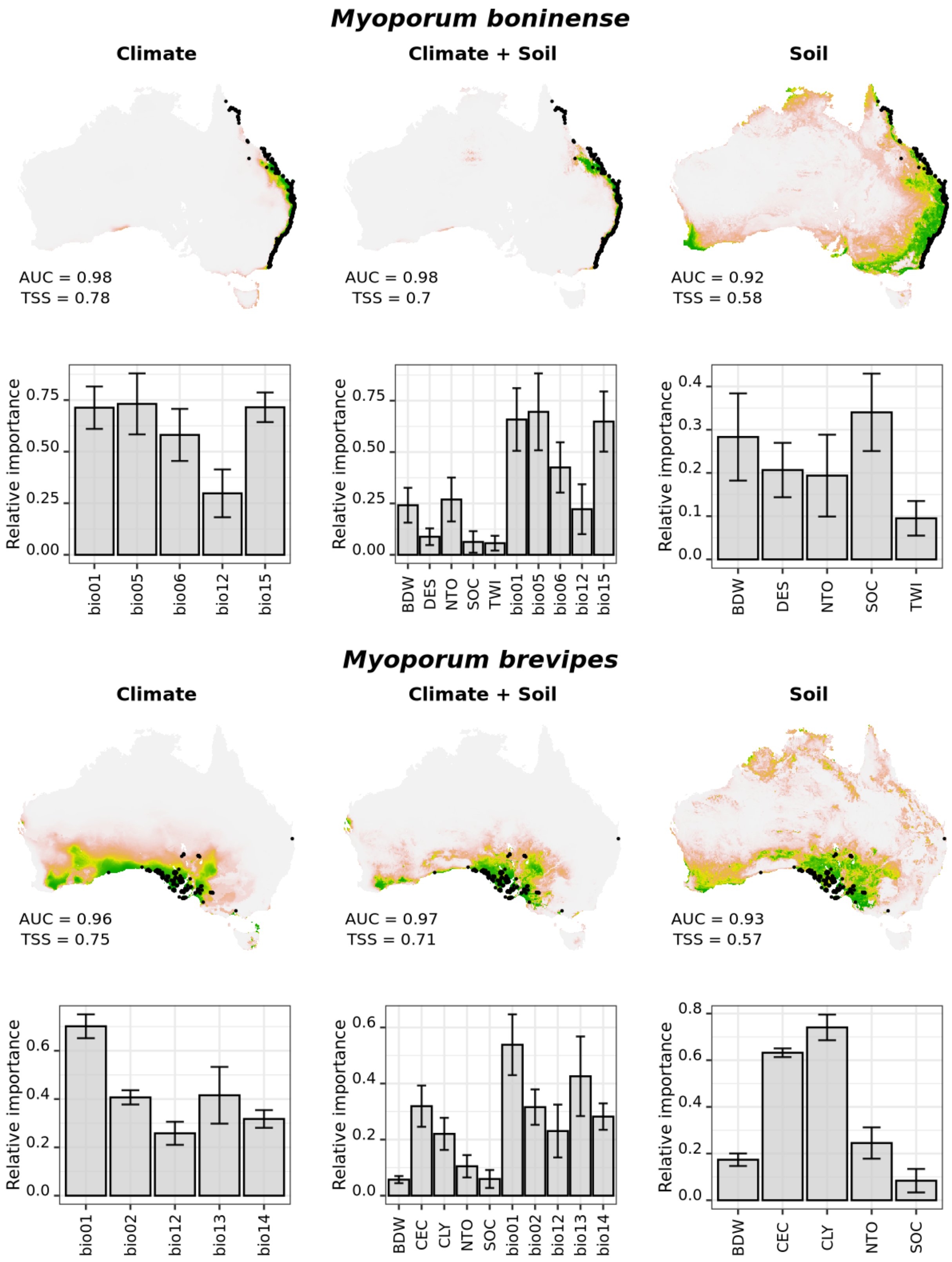
Myoporum boninense
Climate
Climate + Soil
Soil
AUC = 0.98
TSS = 0.78
AUC = 0.98
TSS = 0.7
AUC = 0.92
TSS = 0.58
Relative importance
0.00
0.25
0.50
0.75
bio01
bio05
bio06
bio12
bio15
BDW
DES
NTO
SOC
TWI
bio01
bio05
bio06
bio12
bio15
0.0
0.1
0.2
0.3
0.4
BDW
DES
NTO
SOC
TWI
Myoporum brevipes
Climate
Climate + Soil
Soil
AUC = 0.96
TSS = 0.75
AUC = 0.97
TSS = 0.71
AUC = 0.93
TSS = 0.57
Relative importance
0.0
0.2
0.4
0.6
bio01
bio02
bio12
bio13
bio14
BDW
CEC
CLY
NTO
SOC
bio01
bio02
bio12
bio13
bio14
0.8
BDW
CEC
CLY
NTO
SOC

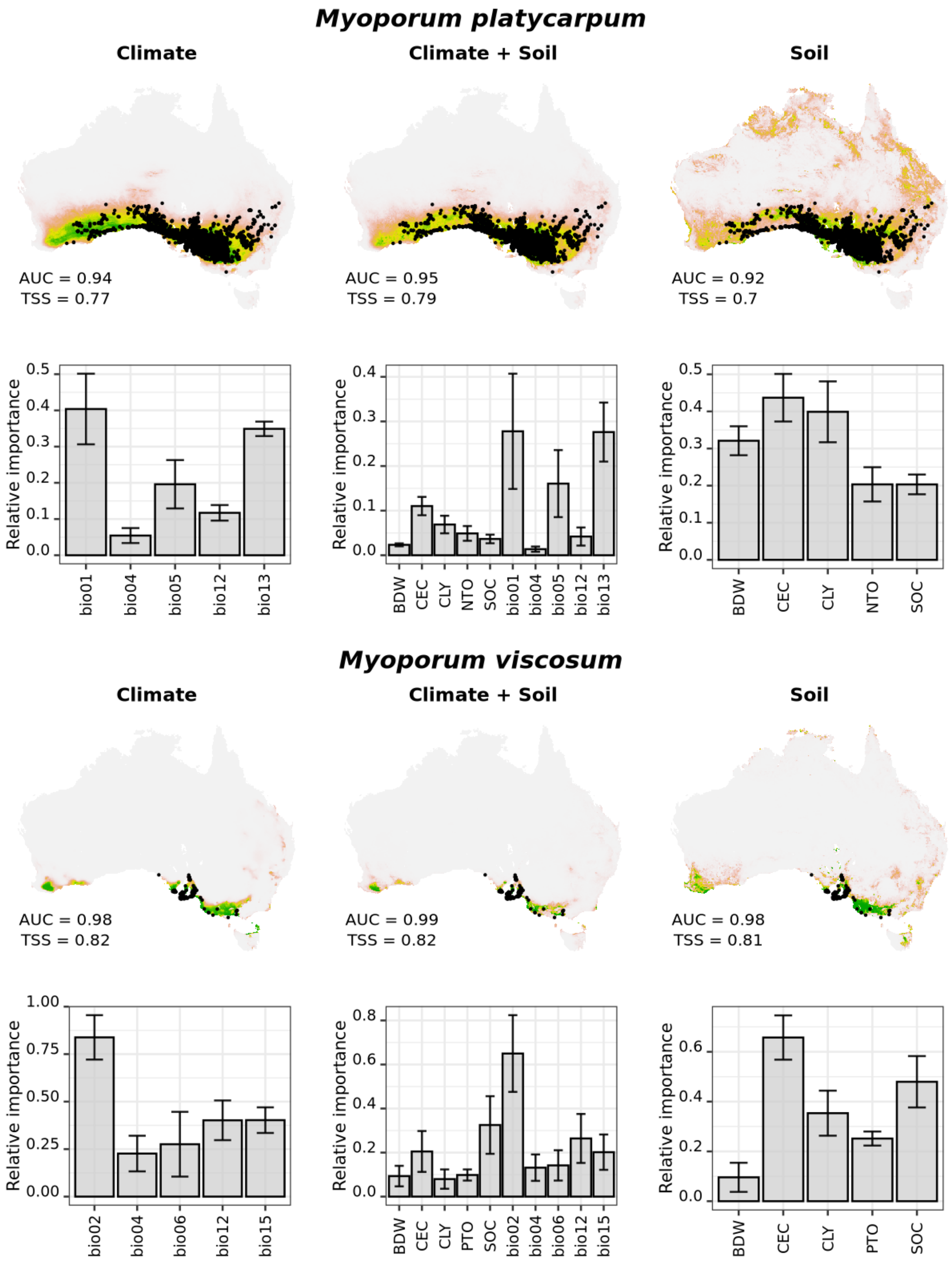

Myoporum platycarpum
Climate
Climate + Soil
Soil
AUC = 0.94
TSS = 0.77
AUC = 0.95
TSS = 0.79
AUC = 0.92
TSS = 0.7
Relative importance
bio01
bio04
bio05
bio12
bio13
BDW
CEC
CLY
NTO
SOC
Myoporum viscosum
Climate
Climate + Soil
Soil
AUC = 0.98
TSS = 0.82
AUC = 0.99
TSS = 0.82
AUC = 0.98
TSS = 0.81
Relative importance
bio02
bio04
bio06
bio12
bio15
BDW
CEC
CLY
PTO
SOC

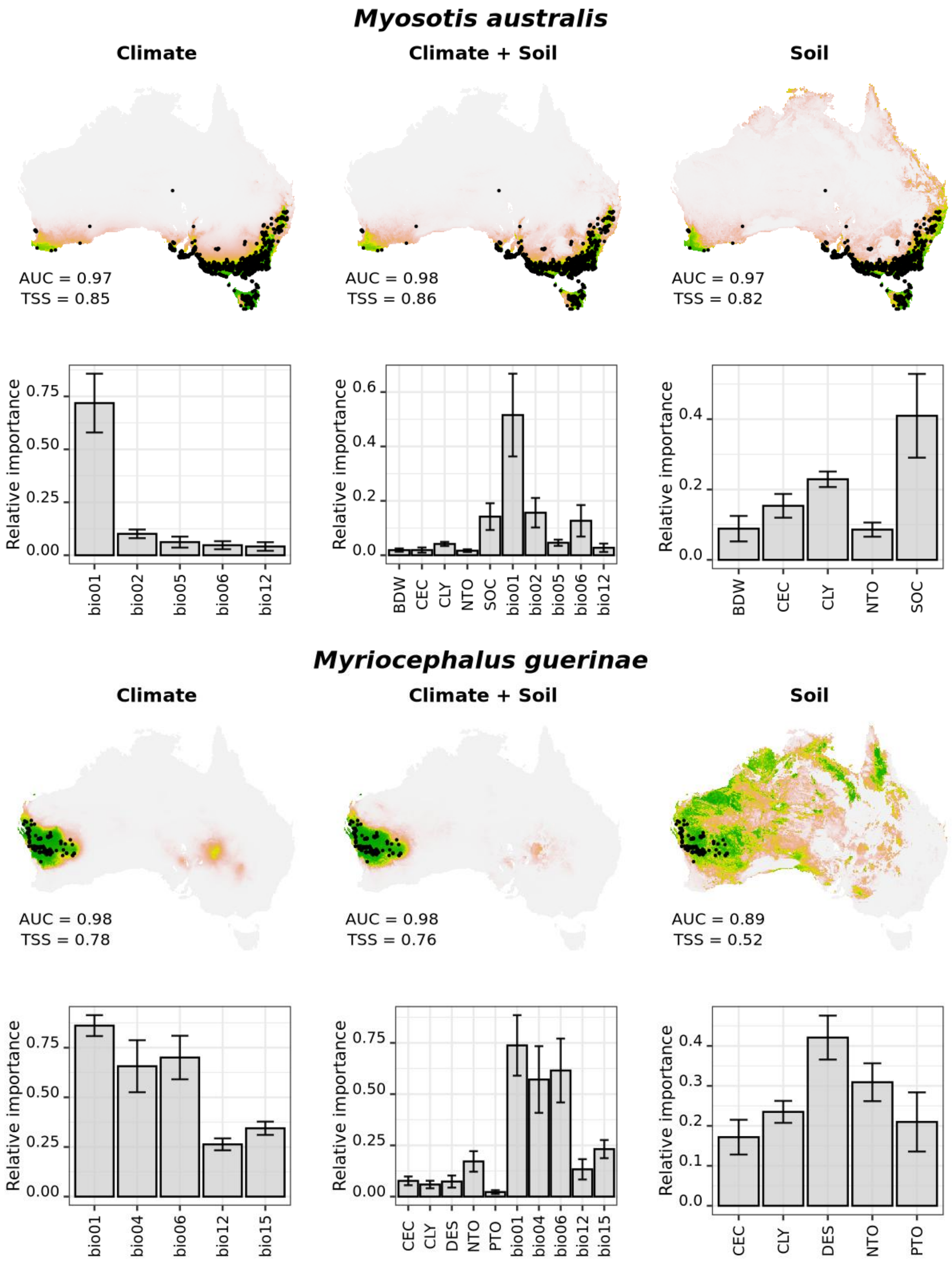

Myosotis australis
Climate
Climate + Soil
Soil
AUC = 0.97
TSS = 0.85
AUC = 0.98
TSS = 0.86
AUC = 0.97
TSS = 0.82
Relative importance
bio01
bio02
bio05
bio06
bio12
BDW
CEC
CLY
NTO
SOC
Myriocephalus guerinae
Climate
Climate + Soil
Soil
AUC = 0.98
TSS = 0.78
AUC = 0.98
TSS = 0.76
AUC = 0.89
TSS = 0.52
Relative importance
bio01
bio04
bio06
bio12
bio15
CEC
CLY
DES
NTO
PTO

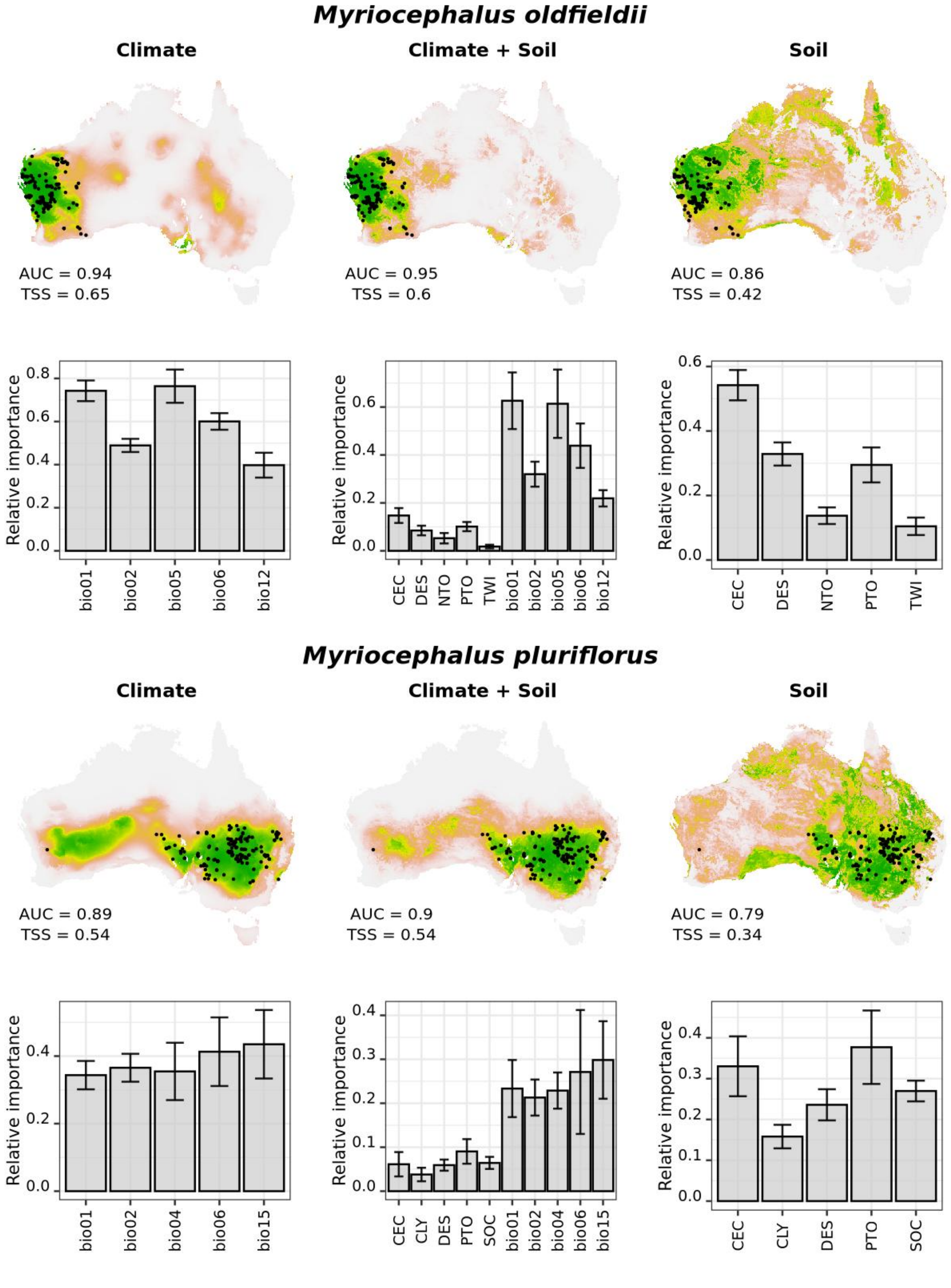
Myriocephalus oldfieldii
Climate
Climate + Soil
Soil
AUC = 0.94
TSS = 0.65
AUC = 0.95
TSS = 0.6
AUC = 0.86
TSS = 0.42
Relative importance
bio01
bio02
bio05
bio06
bio12
CEC
DES
NTO
PTO
TWI
Myriocephalus pluriflorus
Climate
Climate + Soil
Soil
AUC = 0.89
TSS = 0.54
AUC = 0.9
TSS = 0.54
AUC = 0.79
TSS = 0.34
Relative importance
bio01
bio02
bio04
bio06
bio15
CEC
CLY
DES
PTO
SOC

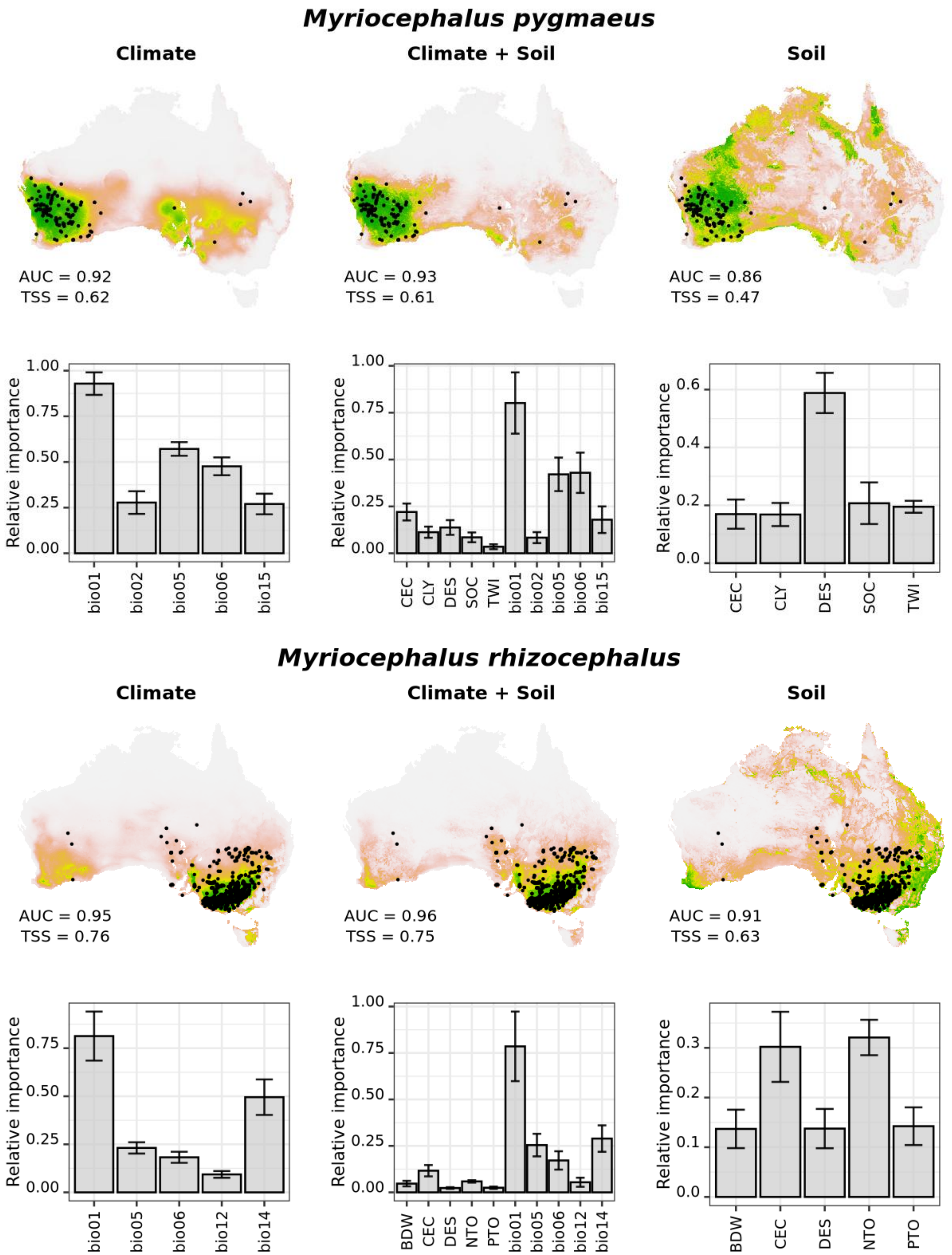
Myriocephalus pygmaeus
Climate
Climate + Soil
Soil
AUC = 0.92
TSS = 0.62
AUC = 0.93
TSS = 0.61
AUC = 0.86
TSS = 0.47
Relative importance
bio01
bio02
bio05
bio06
bio15
CEC
CLY
DES
SOC
TWI
Myriocephalus rhizocephalus
Climate
Climate + Soil
Soil
AUC = 0.95
TSS = 0.76
AUC = 0.96
TSS = 0.75
AUC = 0.91
TSS = 0.63
Relative importance
bio01
bio05
bio06
bio12
bio14
BDW
CEC
DES
NTO
PTO

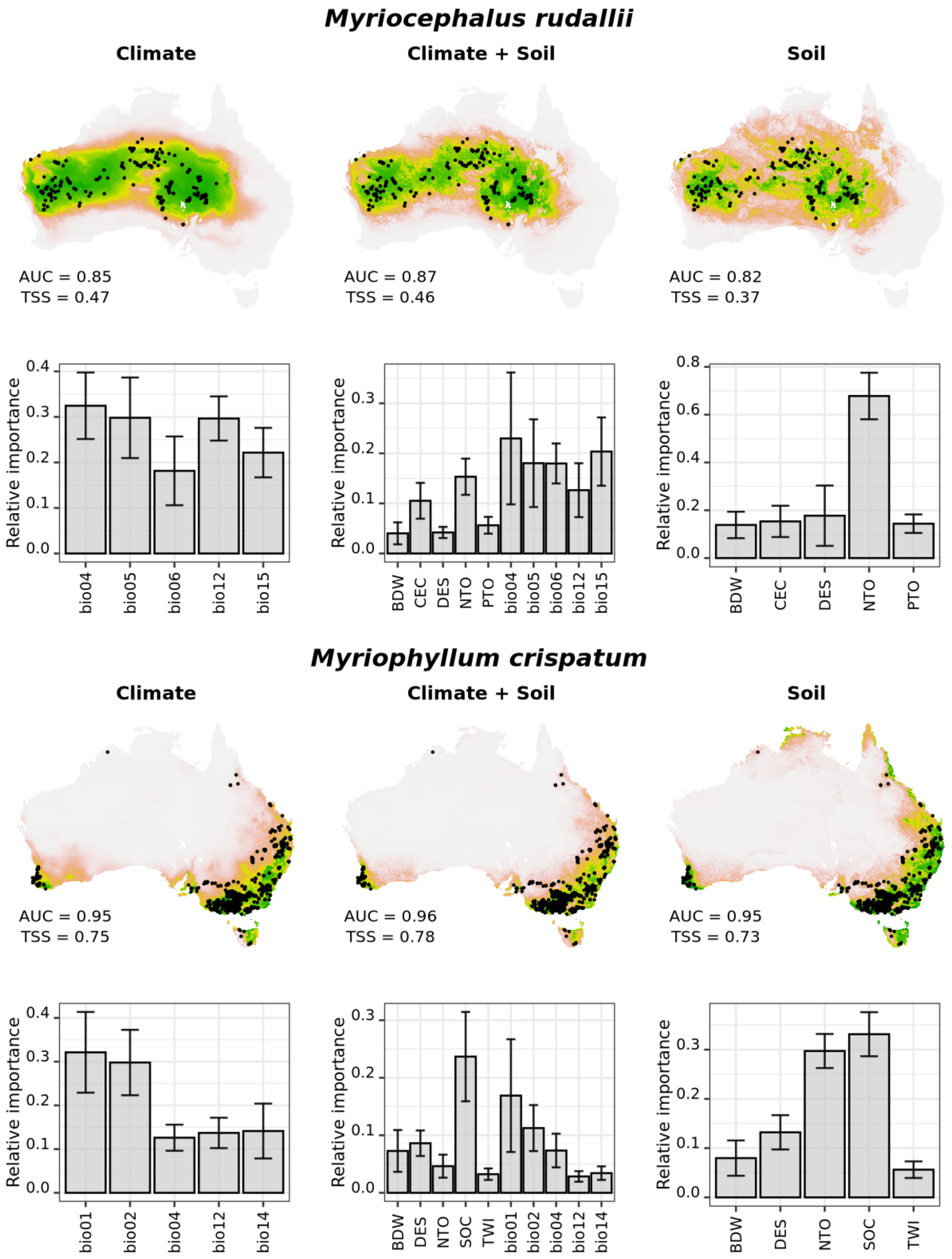

Myriocephalus rudallii
Climate
Climate + Soil
Soil
AUC = 0.85
TSS = 0.47
AUC = 0.87
TSS = 0.46
AUC = 0.82
TSS = 0.37
Relative importance
bio04 bio05 bio06 bio12 bio15
BDW CEC DES NTO PTO bio04 bio05 bio06 bio12 bio15
BDW CEC DES NTO PTO
Myriophyllum crispatum
Climate
Climate + Soil
Soil
AUC = 0.95
TSS = 0.75
AUC = 0.96
TSS = 0.78
AUC = 0.95
TSS = 0.73
Relative importance
bio01 bio02 bio04 bio12 bio14
BDW DES NTO SOC TWI bio01 bio02 bio04 bio12 bio14
BDW DES NTO SOC TWI

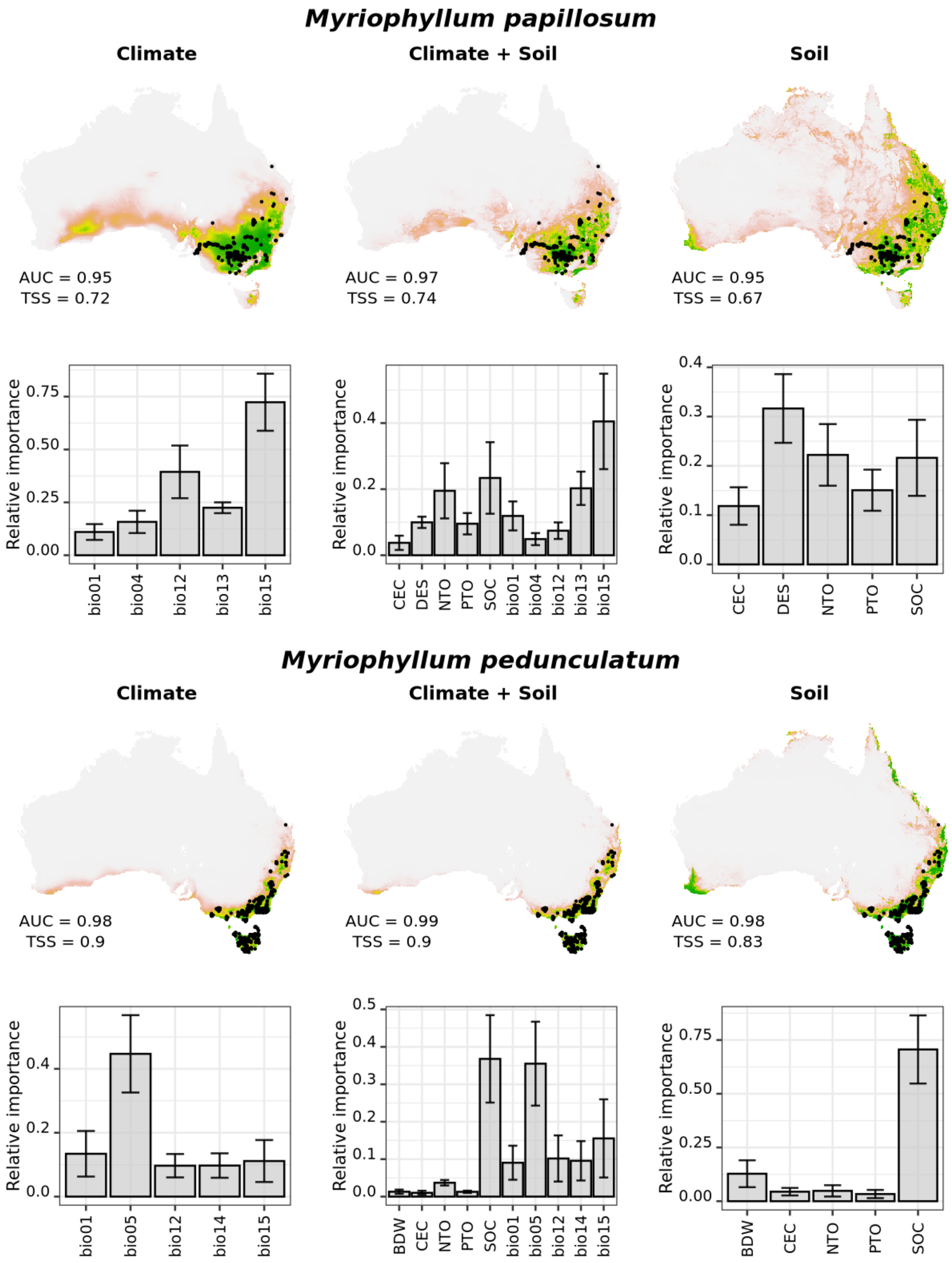
Myriophyllum papillosum
Climate
Climate + Soil
Soil
AUC = 0.95
TSS = 0.72
AUC = 0.97
TSS = 0.74
AUC = 0.95
TSS = 0.67
Relative importance
0.00
0.25
0.50
0.75
bio01
bio04
bio12
bio13
bio15
Relative importance
0.0
0.2
0.4
CEC
DES
NTO
PTO
SOC
bio01
bio04
bio12
bio13
bio15
Relative importance
0.0
0.1
0.2
0.3
0.4
CEC
DES
NTO
PTO
SOC
Myriophyllum pedunculatum
Climate
Climate + Soil
Soil
AUC = 0.98
TSS = 0.9
AUC = 0.99
TSS = 0.9
AUC = 0.98
TSS = 0.83
Relative importance
0.0
0.2
0.4
bio01
bio05
bio12
bio14
bio15
Relative importance
0.0
0.1
0.2
0.3
0.4
0.5
BDW
CEC
NTO
PTO
SOC
bio01
bio05
bio12
bio14
bio15
Relative importance
0.00
0.25
0.50
0.75
BDW
CEC
NTO
PTO
SOC

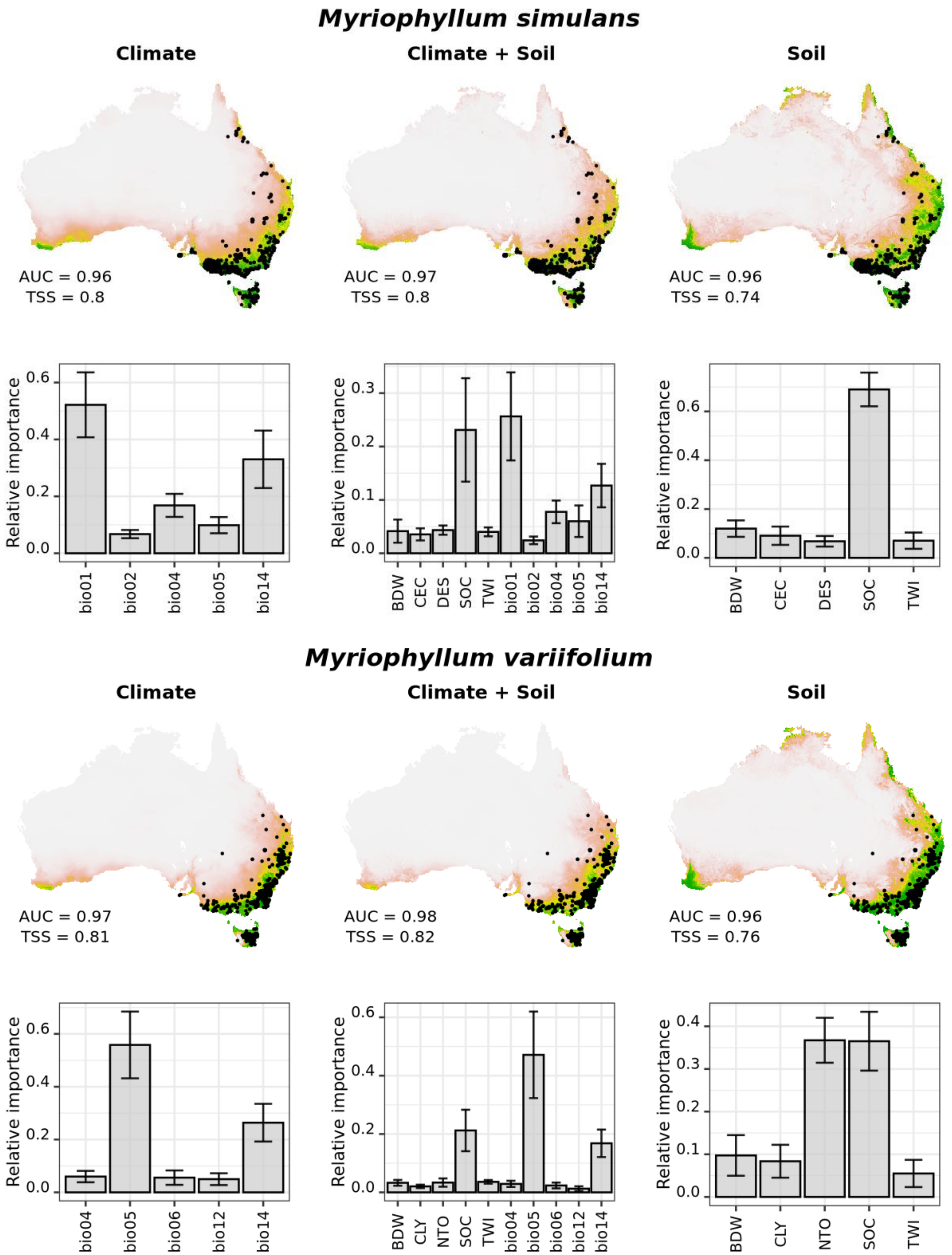

Myriophyllum simulans
Climate
Climate + Soil
Soil
AUC = 0.96
TSS = 0.8
AUC = 0.97
TSS = 0.8
AUC = 0.96
TSS = 0.74
Relative importance
bio01
bio02
bio04
bio05
bio14
BDW
CEC
DES
SOC
TWI
Myriophyllum variifolium
Climate
Climate + Soil
Soil
AUC = 0.97
TSS = 0.81
AUC = 0.98
TSS = 0.82
AUC = 0.96
TSS = 0.76
Relative importance
bio04
bio05
bio06
bio12
bio14
BDW
CLY
NTO
SOC
TWI

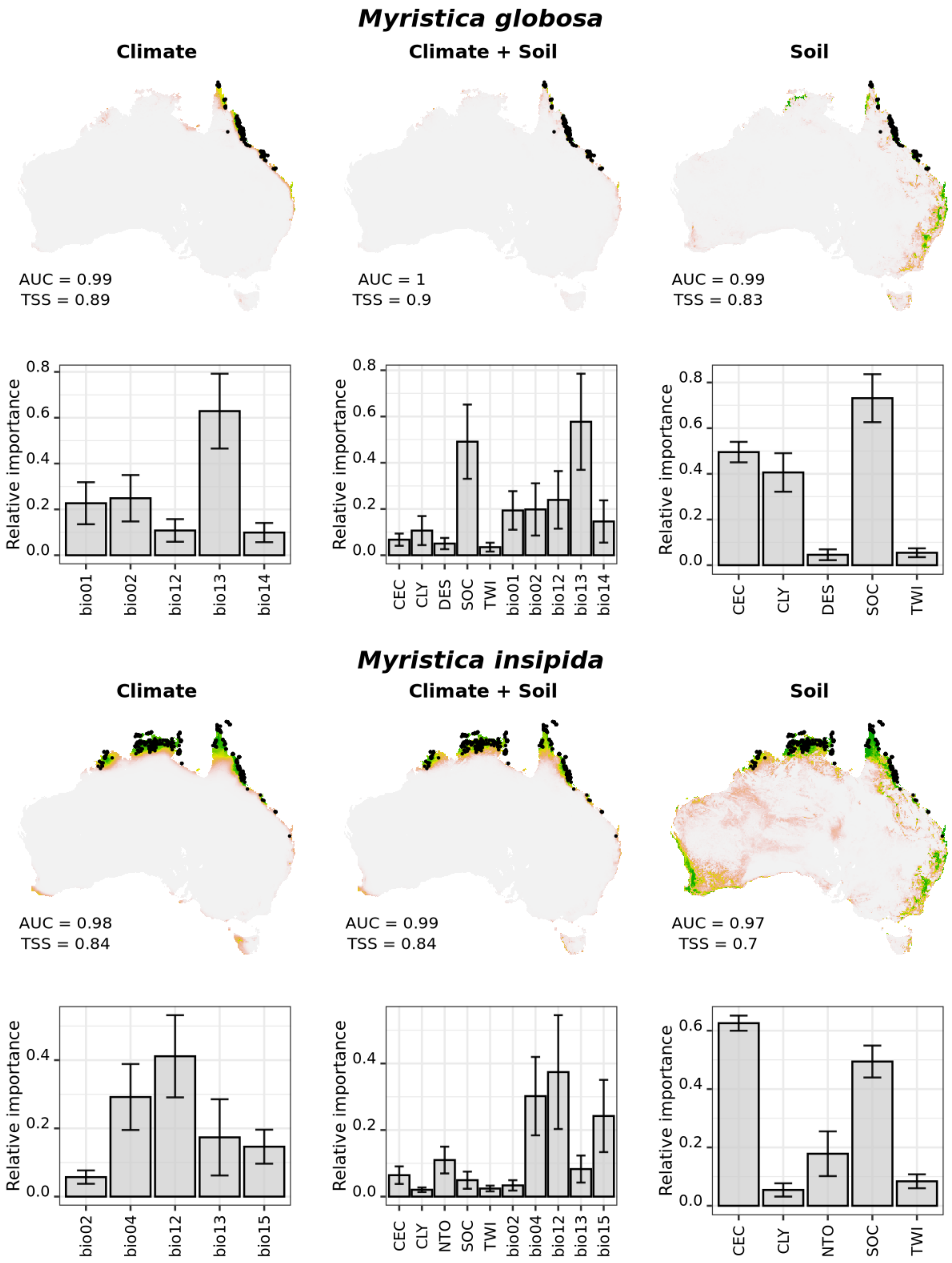
Myristica globosa
Climate
Climate + Soil
Soil
AUC = 0.99
TSS = 0.89
AUC = 1
TSS = 0.9
AUC = 0.99
TSS = 0.83
Relative importance
0.0
0.2
0.4
0.6
0.8
bio01
bio02
bio12
bio13
bio14
CEC
CLY
DES
SOC
TWI
Myristica insipida
Climate
Climate + Soil
Soil
AUC = 0.98
TSS = 0.84
AUC = 0.99
TSS = 0.84
AUC = 0.97
TSS = 0.7
bio02
bio04
bio15
NTO

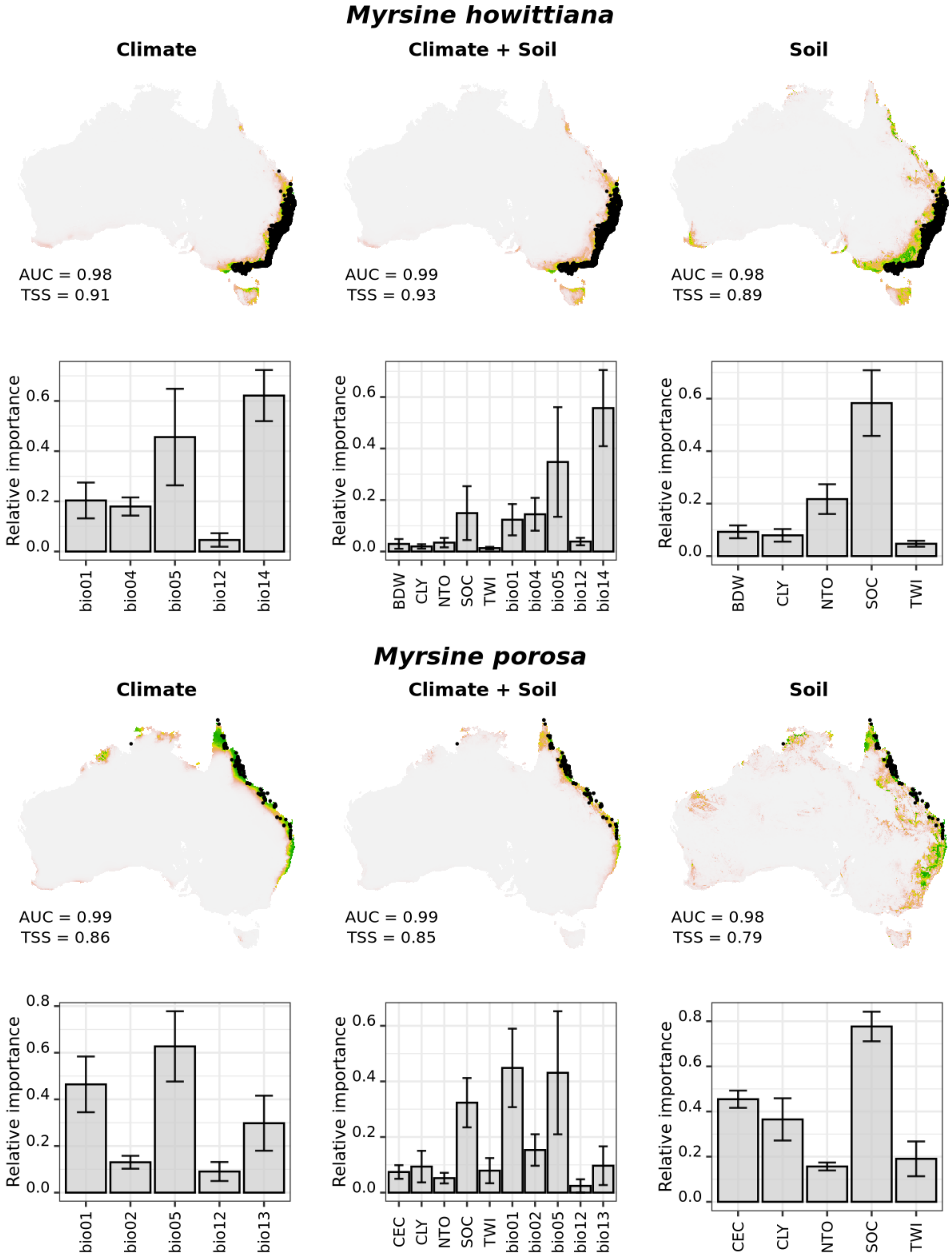

Myrsine howittiana
Climate
Climate + Soil
Soil
AUC = 0.98
TSS = 0.91
AUC = 0.99
TSS = 0.93
AUC = 0.98
TSS = 0.89
Relative importance
bio01
bio04
bio05
bio12
bio14
BDW
CLY
NTO
SOC
TWI
Myrsine porosa
Climate
Climate + Soil
Soil
AUC = 0.99
TSS = 0.86
AUC = 0.99
TSS = 0.85
AUC = 0.98
TSS = 0.79
Relative importance
bio01
bio02
bio05
bio12
bio13
CEC
CLY
NTO
SOC
TWI

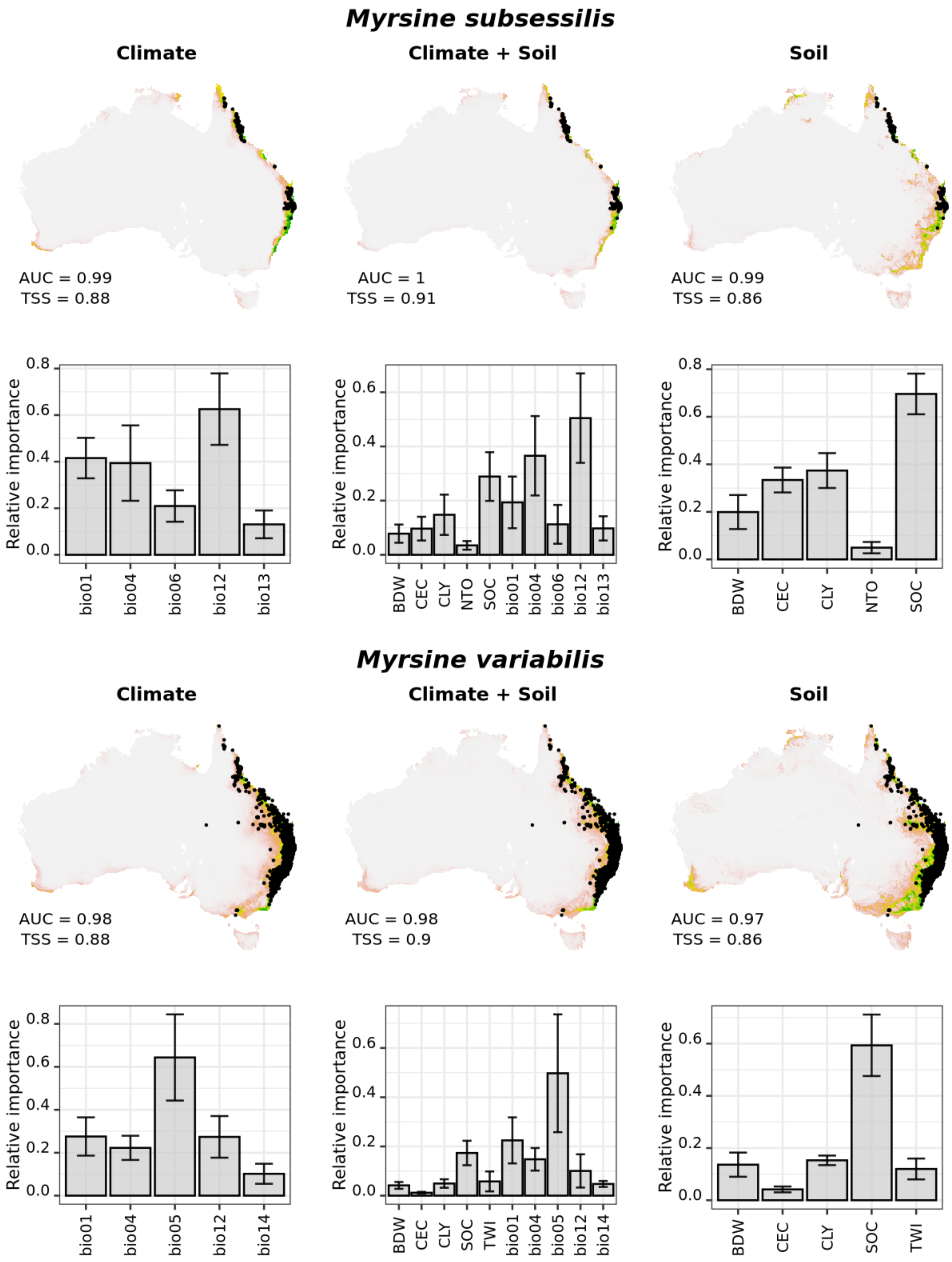
Myrsine subsessilis
Climate
Climate + Soil
Soil
AUC = 0.99
TSS = 0.88
AUC = 1
TSS = 0.91
AUC = 0.99
TSS = 0.86
Relative importance
0.0
0.2
0.4
0.6
0.8
bio01
bio04
bio06
bio12
bio13
BDW
CEC
CLY
NTO
SOC
Myrsine variabilis
Climate
Climate + Soil
Soil
AUC = 0.98
TSS = 0.88
AUC = 0.98
TSS = 0.9
AUC = 0.97
TSS = 0.86
bio05
bio14
TWI

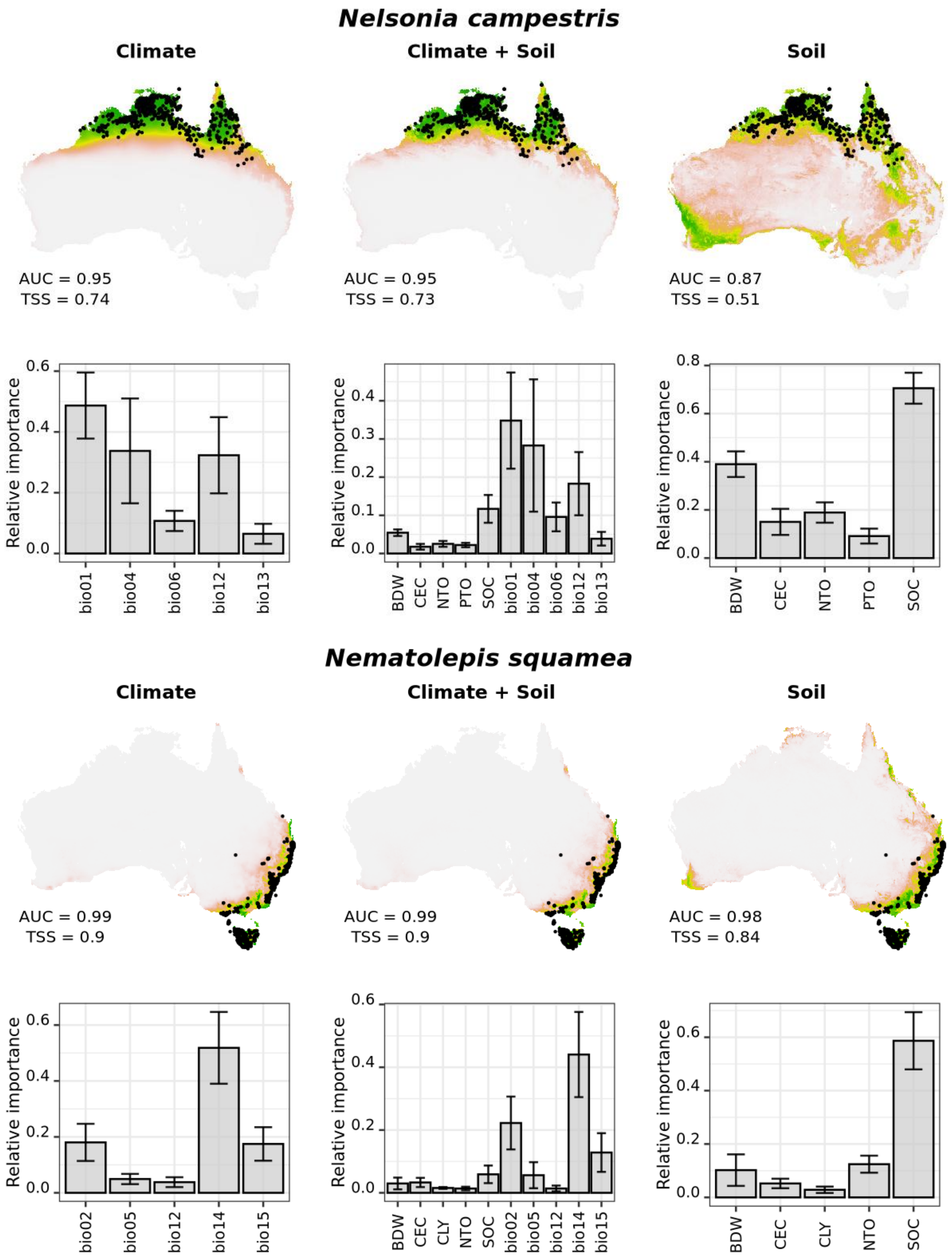
Nelsonia campestris
Climate
Climate + Soil
Soil
AUC = 0.95
TSS = 0.74
AUC = 0.95
TSS = 0.73
AUC = 0.87
TSS = 0.51
Relative importance
bio01 bio04 bio06 bio12 bio13
BDW CEC NTO PTO SOC bio01 bio04 bio06 bio12 bio13
BDW CEC NTO PTO SOC
Nematolepis squamea
Climate
Climate + Soil
Soil
AUC = 0.99
TSS = 0.9
AUC = 0.99
TSS = 0.9
AUC = 0.98
TSS = 0.84
Relative importance
bio02 bio05 bio12 bio14 bio15
BDW CEC CLY NTO SOC bio02 bio05 bio12 bio14 bio15
BDW CEC CLY NTO SOC

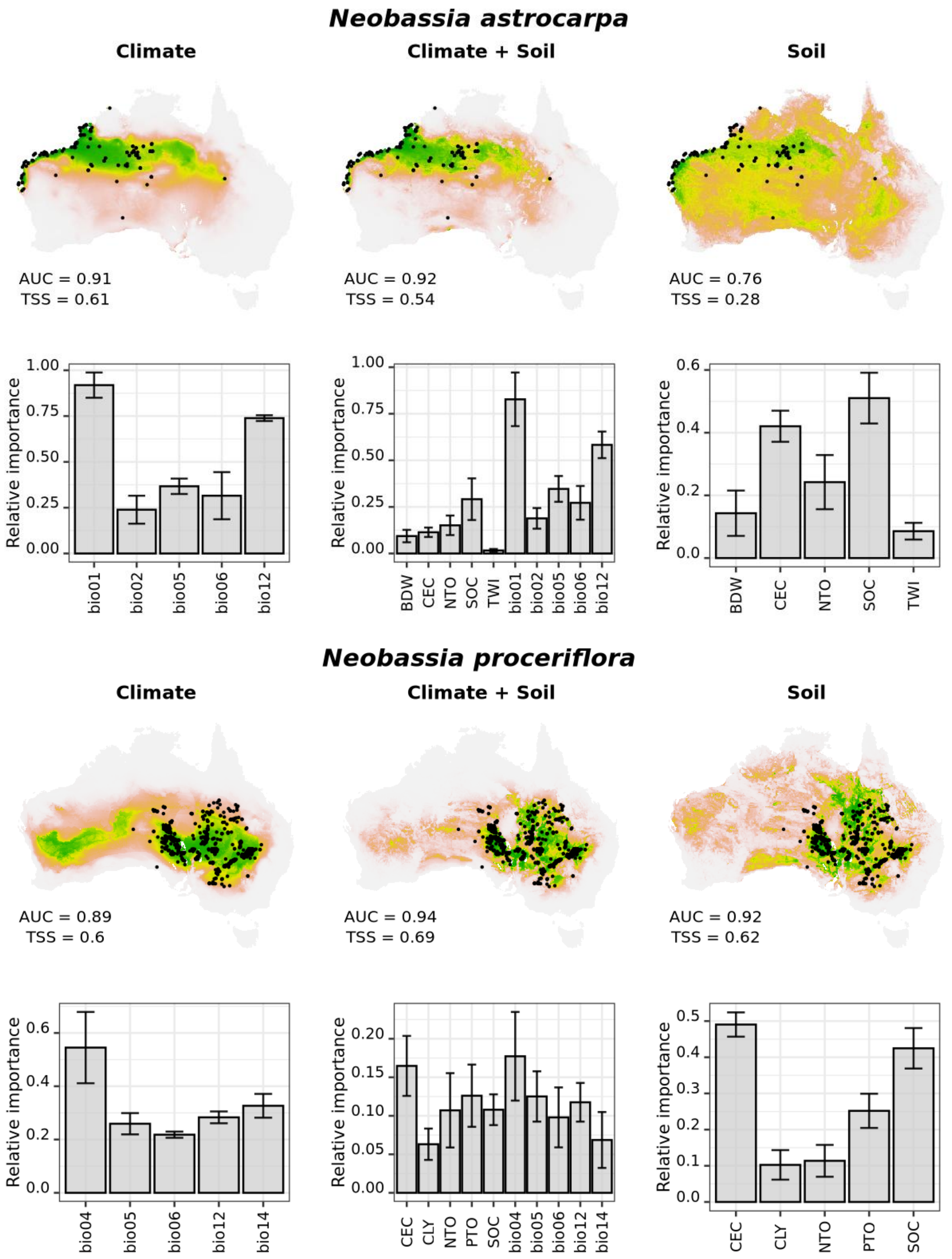

Neobassia astrocarpa
Climate
Climate + Soil
Soil
AUC = 0.91
TSS = 0.61
AUC = 0.92
TSS = 0.54
AUC = 0.76
TSS = 0.28
Relative importance
bio01
bio02
bio05
bio06
bio12
BDW
CEC
NTO
SOC
TWI
Neobassia proceriflora
Climate
Climate + Soil
Soil
AUC = 0.89
TSS = 0.6
AUC = 0.94
TSS = 0.69
AUC = 0.92
TSS = 0.62
Relative importance
bio04
bio05
bio06
bio12
bio14
CEC
CLY
NTO
PTO
SOC

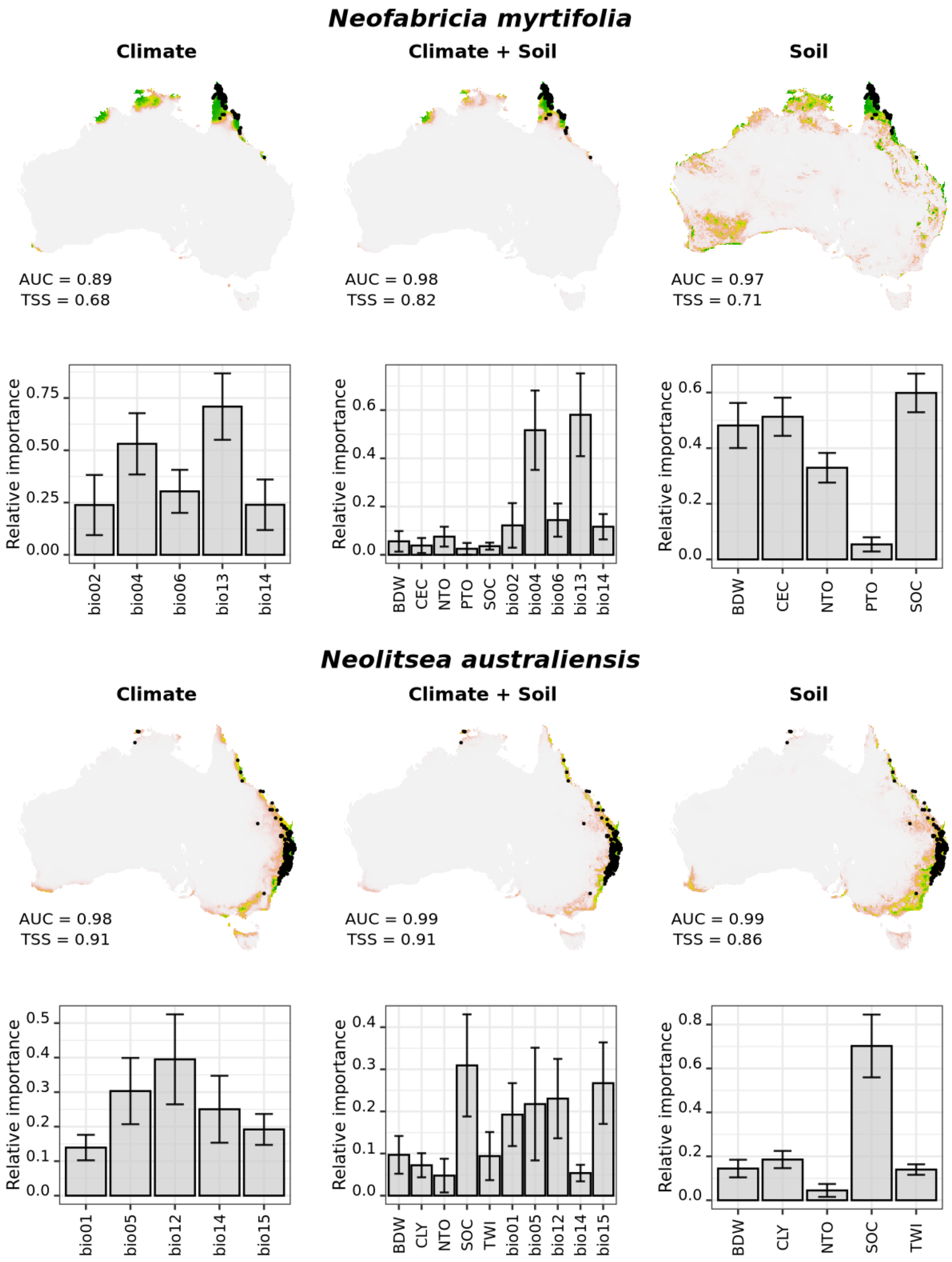

Neofabricia myrtifolia
Climate
Climate + Soil
Soil
AUC = 0.89
TSS = 0.68
AUC = 0.98
TSS = 0.82
AUC = 0.97
TSS = 0.71
Relative importance
bio02
bio04
bio06
bio13
bio14
BDW
CEC
NTO
PTO
SOC
Neolitsea australiensis
AUC = 0.98
TSS = 0.91
AUC = 0.99
TSS = 0.91
AUC = 0.99
TSS = 0.86
bio01
bio05
bio12
bio15
CLY
TWI

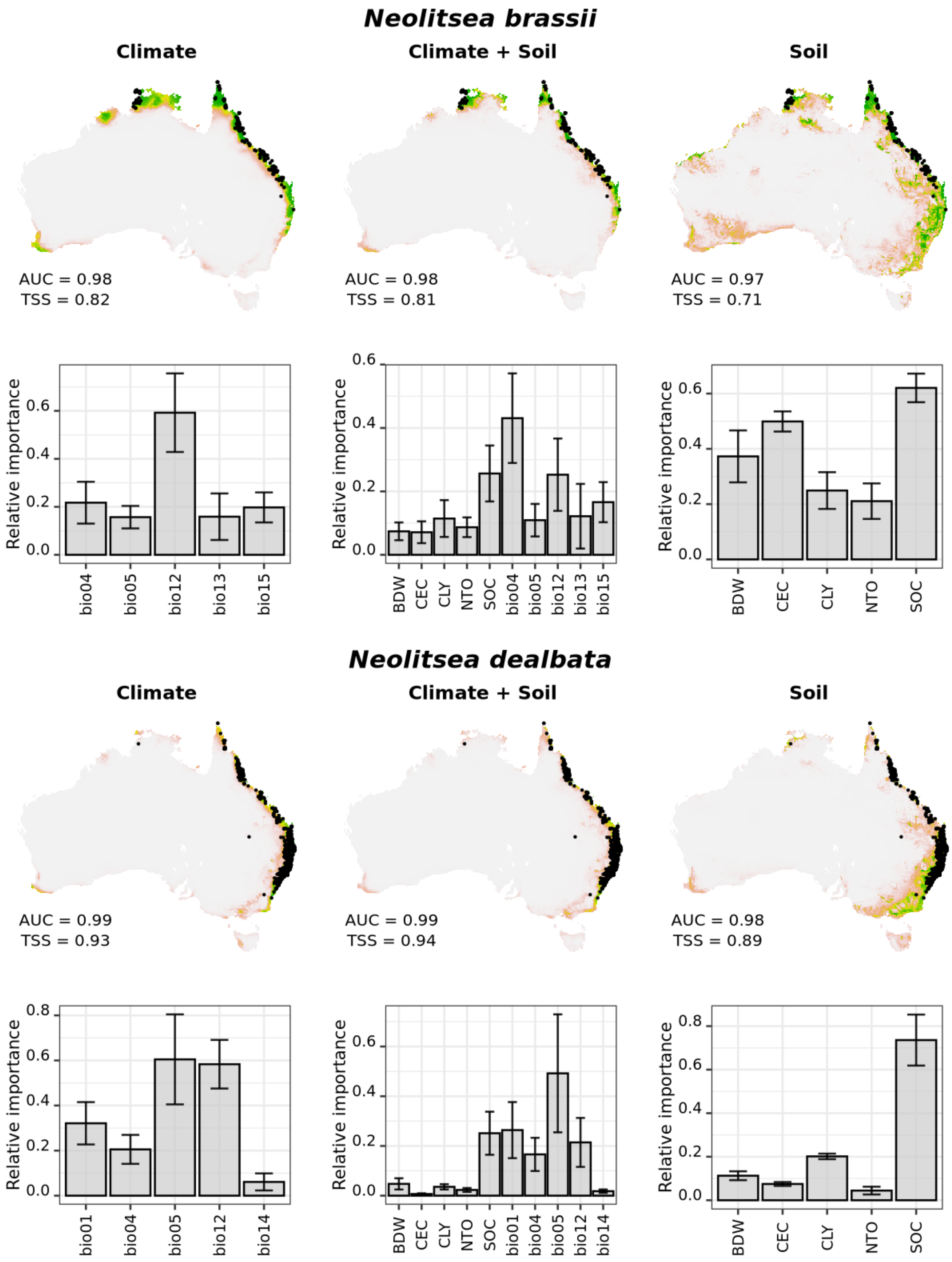

Neolitsea brassii
Climate
Climate + Soil
Soil
AUC = 0.98
TSS = 0.82
AUC = 0.98
TSS = 0.81
AUC = 0.97
TSS = 0.71
Relative importance
0.0
0.2
0.4
0.6
bio04
bio05
bio12
bio13
bio15
BDW
CEC
CLY
NTO
SOC
Neolitsea dealbata
Climate
Climate + Soil
Soil
AUC = 0.99
TSS = 0.93
AUC = 0.99
TSS = 0.94
AUC = 0.98
TSS = 0.89
Relative importance
0.8
bio01
bio14

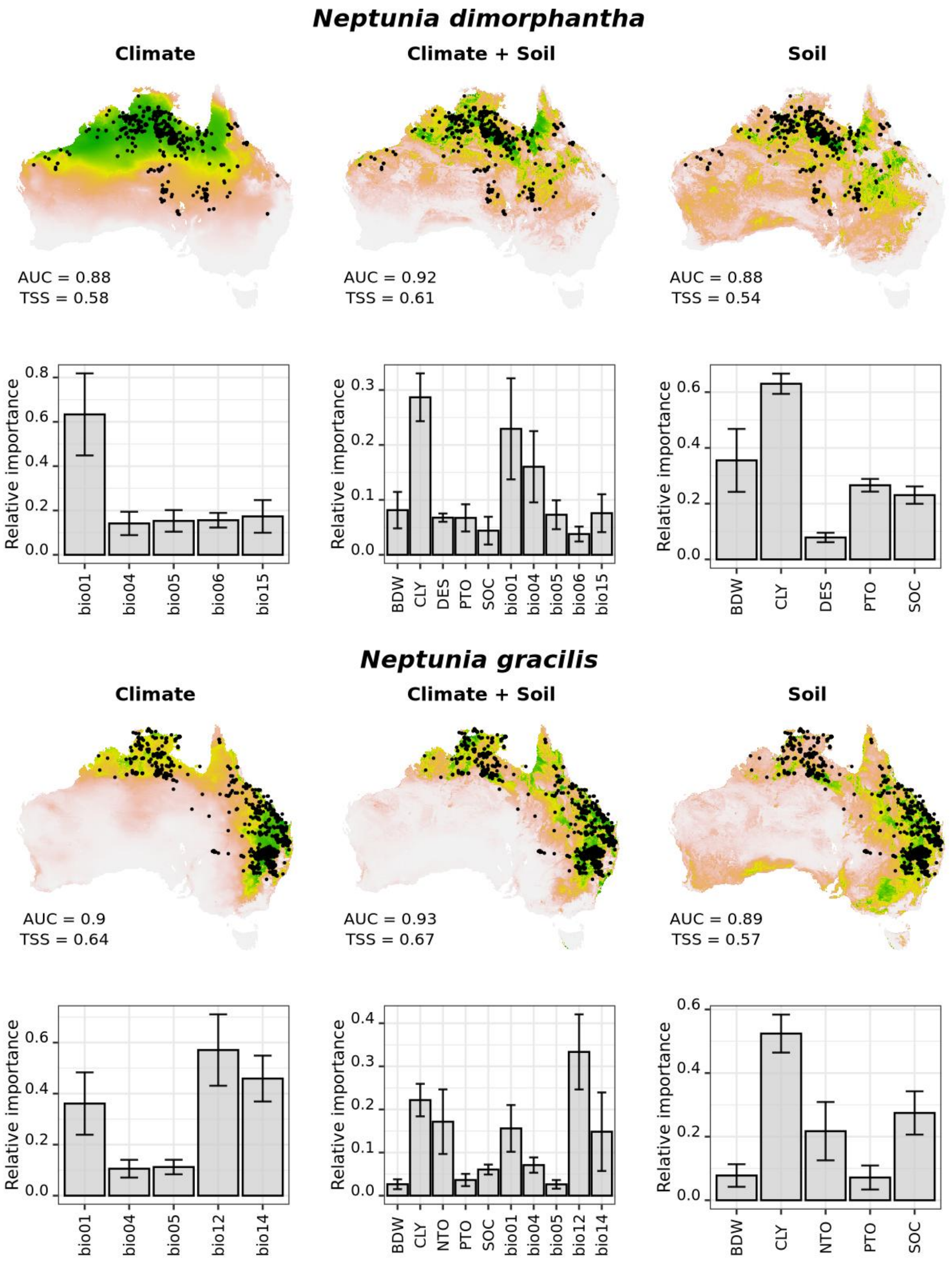
Neptunia dimorphantha
Climate
Climate + Soil
Soil
AUC = 0.88
TSS = 0.58
AUC = 0.92
TSS = 0.61
AUC = 0.88
TSS = 0.54
Relative importance
bio01 bio04 bio05 bio06 bio15
BDW CLY DES PTO SOC bio01 bio04 bio05 bio06 bio15
BDW CLY DES PTO SOC
Neptunia gracilis
Climate
Climate + Soil
Soil
AUC = 0.9
TSS = 0.64
AUC = 0.93
TSS = 0.67
AUC = 0.89
TSS = 0.57
Relative importance
bio01 bio04 bio05 bio12 bio14
BDW CLY NTO PTO SOC bio01 bio04 bio05 bio12 bio14
BDW CLY NTO PTO SOC

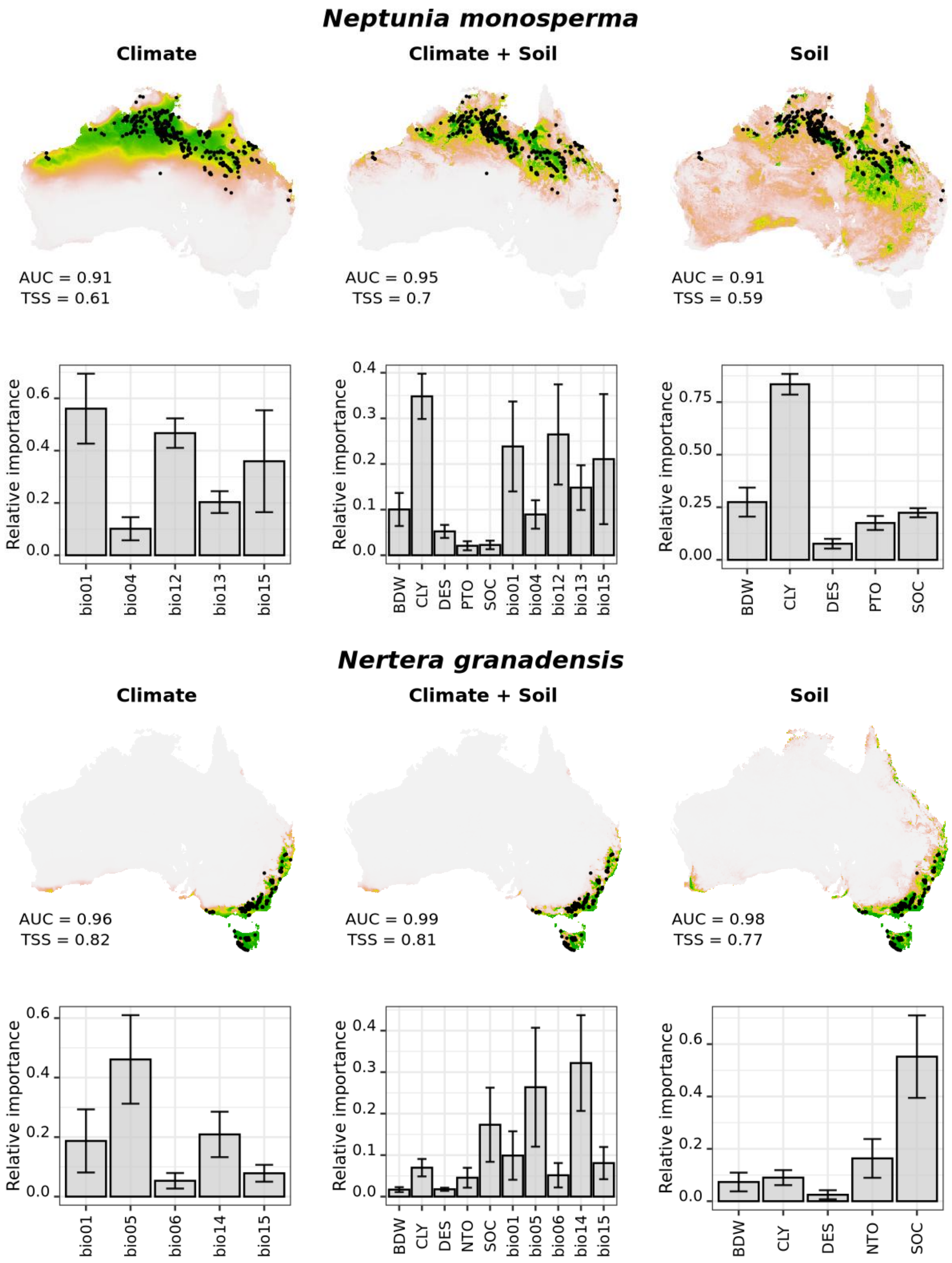

Neptunia monosperma
Climate
Climate + Soil
Soil
AUC = 0.91
TSS = 0.61
AUC = 0.95
TSS = 0.7
AUC = 0.91
TSS = 0.59
Relative importance
bio01
bio04
bio12
bio13
bio15
BDW
CLY
DES
PTO
SOC
Nertera granadensis
Climate
Climate + Soil
Soil
AUC = 0.96
TSS = 0.82
AUC = 0.99
TSS = 0.81
AUC = 0.98
TSS = 0.77
Relative importance
bio01
bio05
bio06
bio14
bio15
BDW
CLY
DES
NTO
SOC

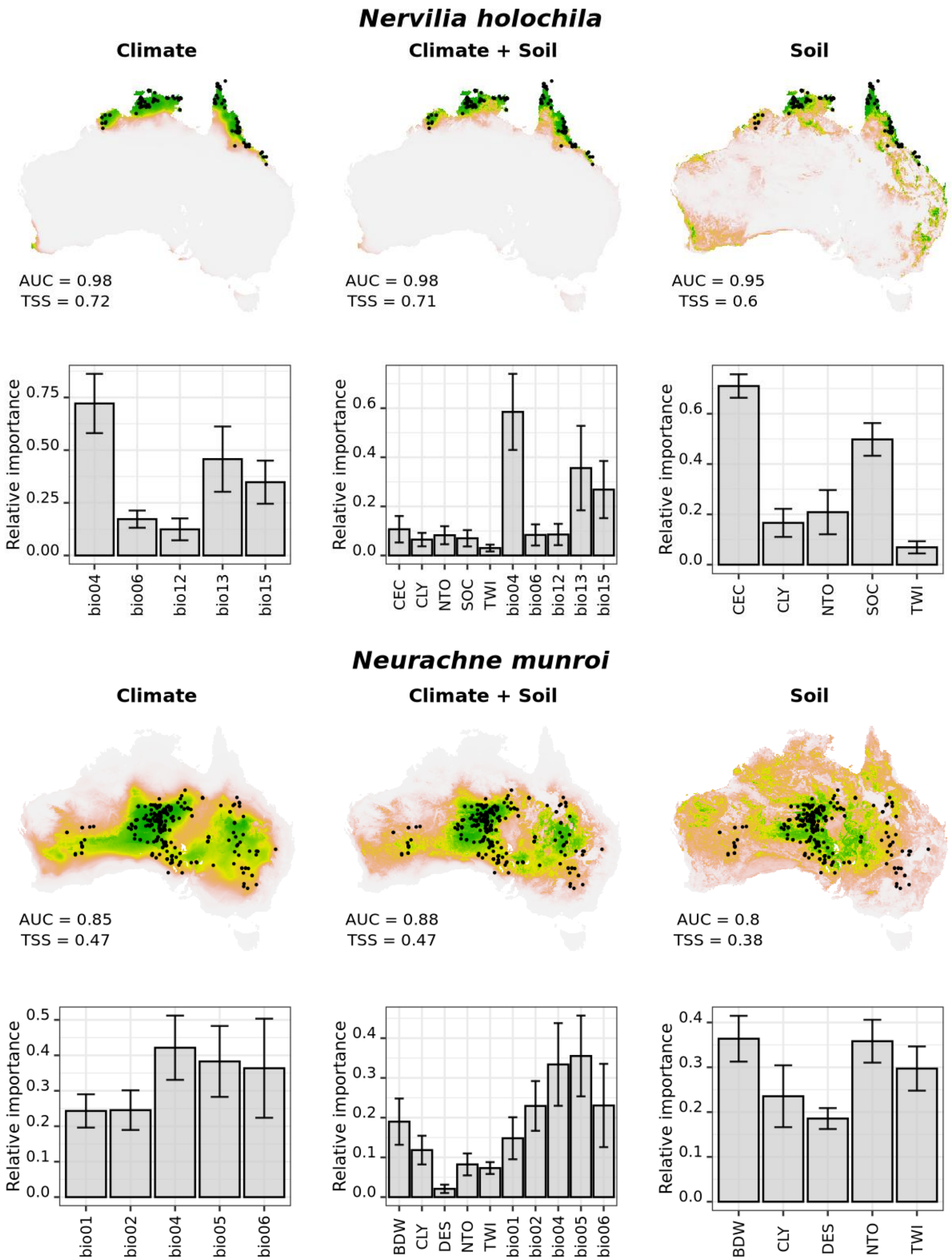

Nervilia holochila
Climate
Climate + Soil
Soil
AUC = 0.98
TSS = 0.72
AUC = 0.98
TSS = 0.71
AUC = 0.95
TSS = 0.6
Relative importance
0.00
0.25
0.50
0.75
bio04
bio06
bio12
bio13
bio15
0.0
0.2
0.4
0.6
CEC
CLY
NTO
SOC
TWI
bio04
bio06
bio12
bio13
bio15
CEC
CLY
NTO
SOC
TWI
Neurachne munroi
Climate
Climate + Soil
Soil
AUC = 0.85
TSS = 0.47
AUC = 0.88
TSS = 0.47
AUC = 0.8
TSS = 0.38
0.0
0.1
0.2
0.3
0.4
0.5
bio01
bio02
bio04
bio05
bio06
BDW
CLY
DES
NTO
TWI
bio01
bio02
bio04
bio05
bio06
BDW
CLY
DES
NTO
TWI

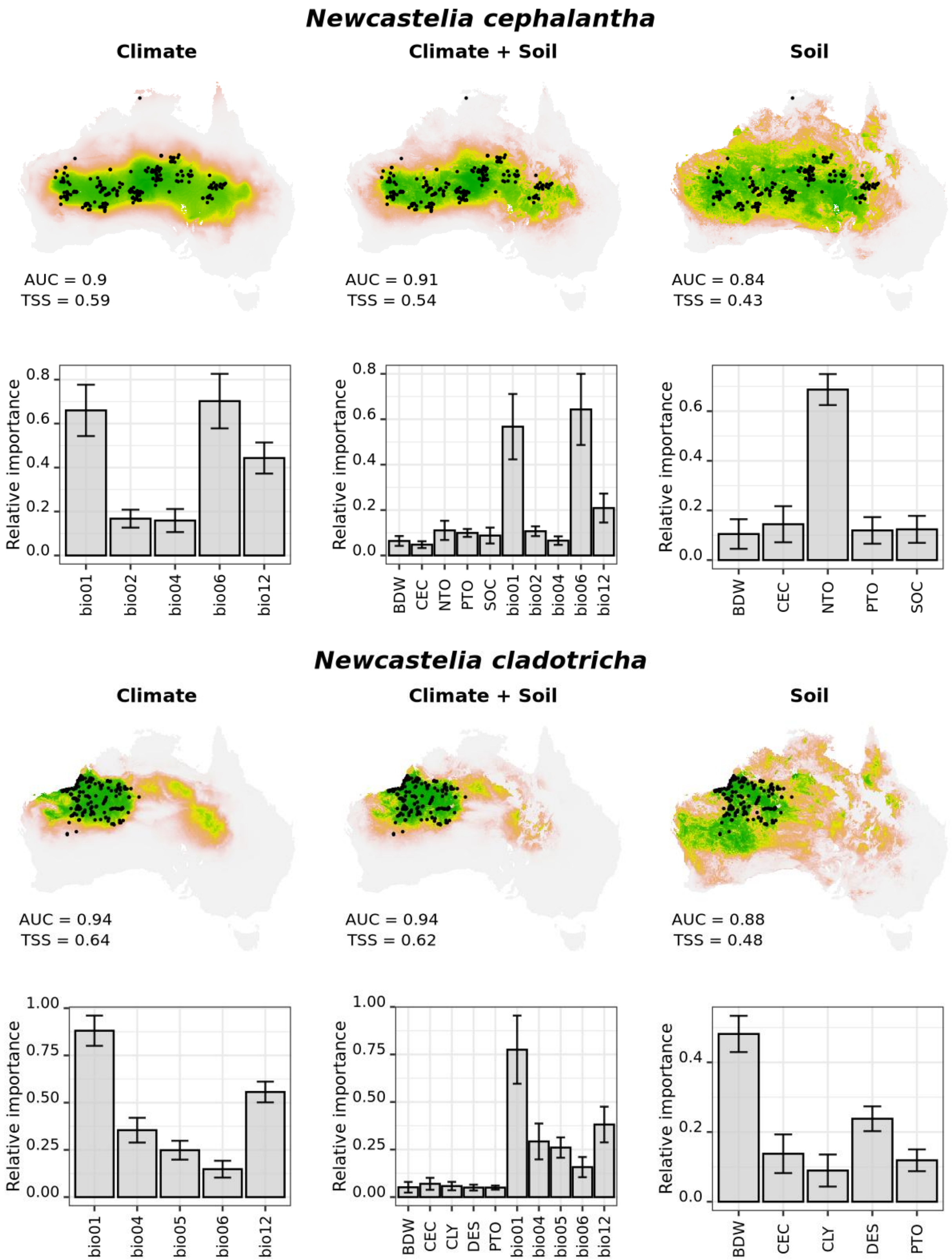
Newcastelia cephalantha
Climate
Climate + Soil
Soil
AUC = 0.9
TSS = 0.59
AUC = 0.91
TSS = 0.54
AUC = 0.84
TSS = 0.43
Relative importance
bio01
bio02
bio04
bio06
bio12
BDW
CEC
NTO
PTO
SOC
Newcastelia cladotricha
Climate
Climate + Soil
Soil
AUC = 0.94
TSS = 0.64
AUC = 0.94
TSS = 0.62
AUC = 0.88
TSS = 0.48
Relative importance
bio01
bio04
bio05
bio06
bio12
BDW
CEC
CLY
DES
PTO

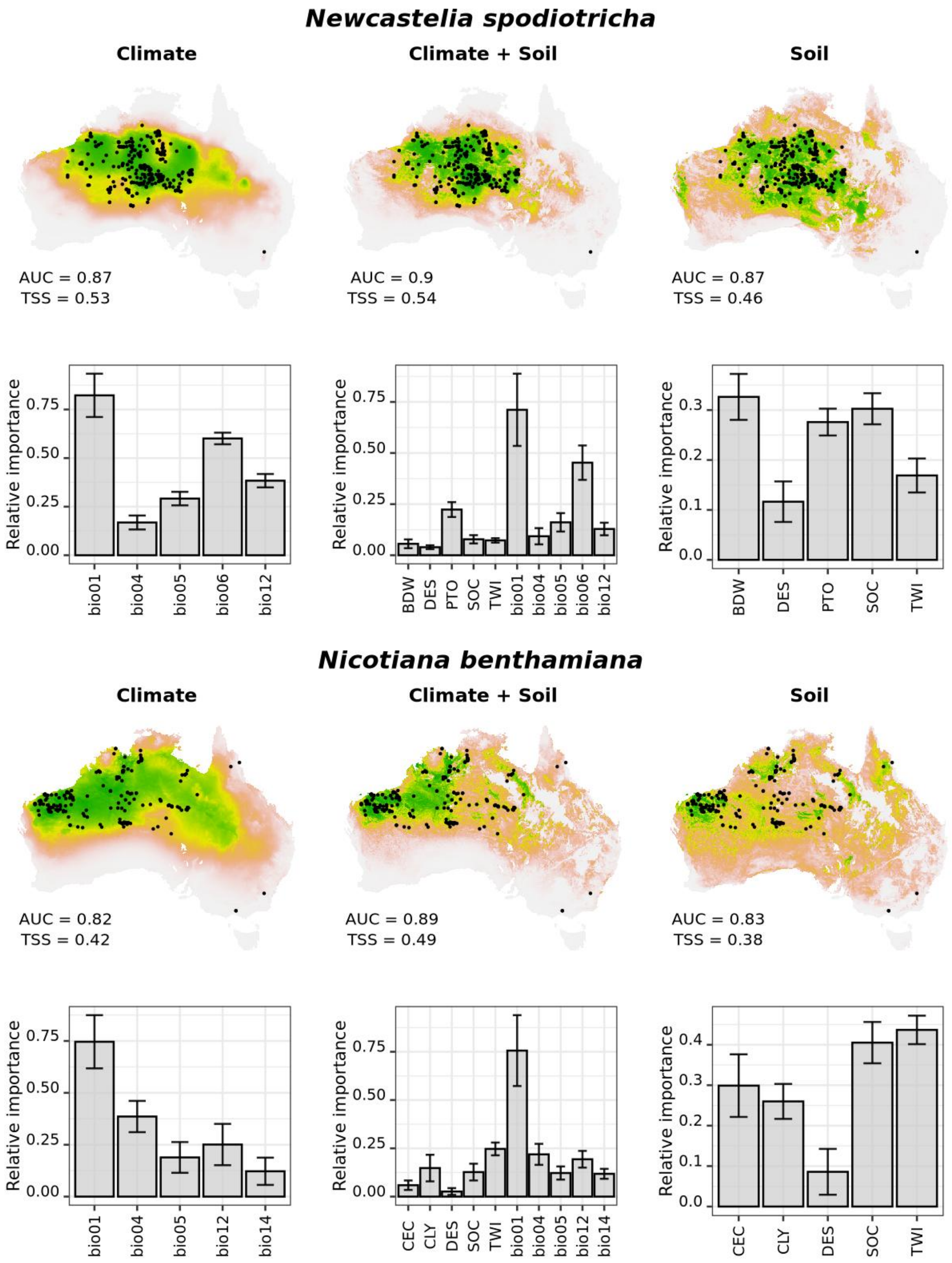
Newcastelia spodiotricha
Climate
Climate + Soil
Soil
AUC = 0.87
TSS = 0.53
AUC = 0.9
TSS = 0.54
AUC = 0.87
TSS = 0.46
Relative importance
0.00
0.25
0.50
0.75
bio01
bio04
bio05
bio06
bio12
BDW
DES
PTO
SOC
TWI
0.0
0.1
0.2
0.3
Nicotiana benthamiana
Climate
Climate + Soil
Soil
AUC = 0.82
TSS = 0.42
AUC = 0.89
TSS = 0.49
AUC = 0.83
TSS = 0.38
Relative importance
0.00
0.25
0.50
0.75
bio01
bio04
bio05
bio12
bio14
CEC
CLY
DES
SOC
TWI
0.0
0.1
0.2
0.3
0.4

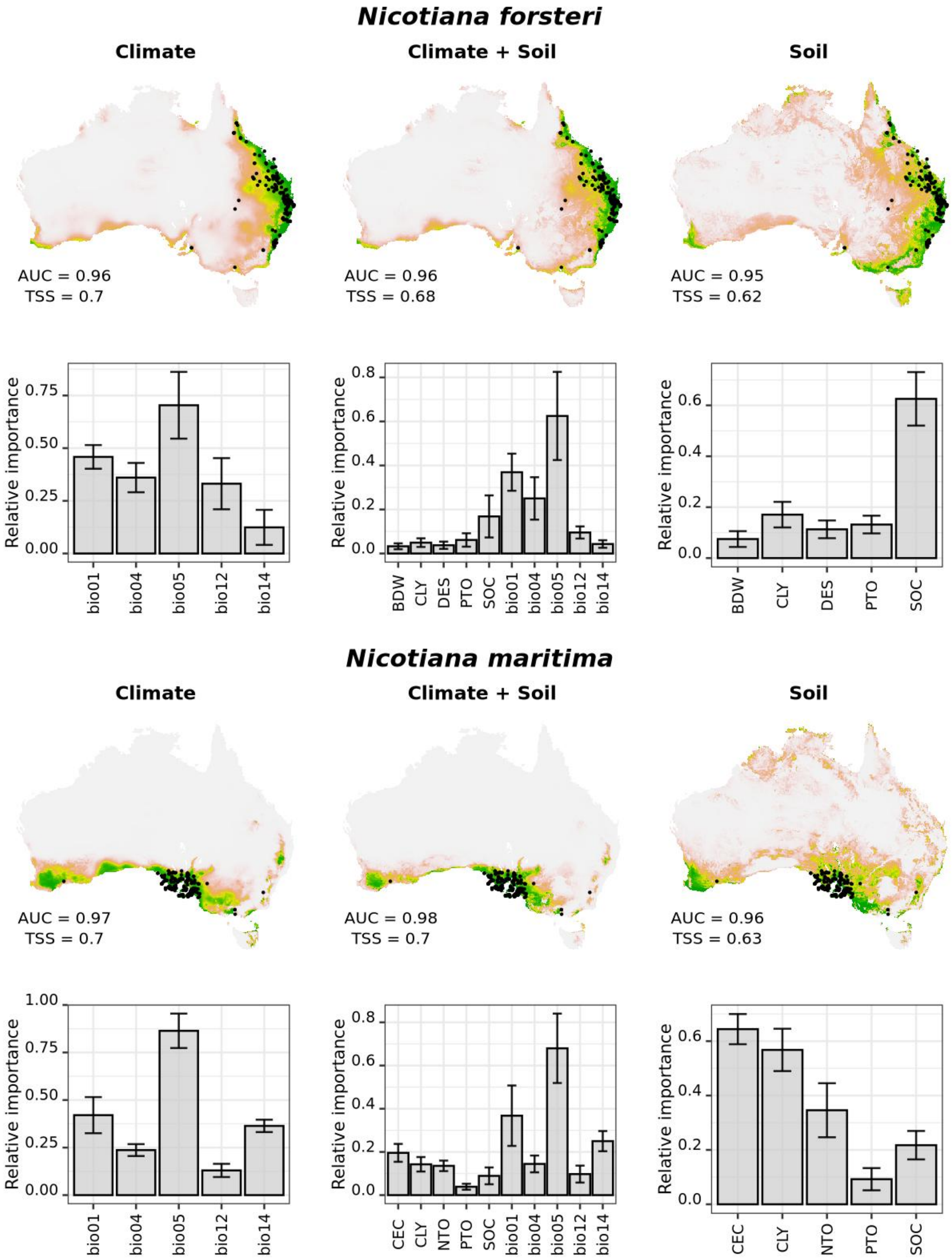

Nicotiana forsteri
Climate
Climate + Soil
Soil
AUC = 0.96
TSS = 0.7
AUC = 0.96
TSS = 0.68
AUC = 0.95
TSS = 0.62
Relative importance
0.00
0.25
0.50
0.75
bio01
bio04
bio05
bio12
bio14
0.0
0.2
0.4
0.6
0.8
BDW
CLY
DES
PTO
SOC
bio01
bio04
bio05
bio12
bio14
BDW
CLY
DES
PTO
SOC
Nicotiana maritima
Climate
Climate + Soil
Soil
AUC = 0.97
TSS = 0.7
AUC = 0.98
TSS = 0.7
AUC = 0.96
TSS = 0.63
Relative importance
0.00
0.25
0.50
0.75
1.00
bio01
bio04
bio05
bio12
bio14
CEC
CLY
NTO
PTO
SOC
bio01
bio04
bio05
bio12
bio14
CEC
CLY
NTO
PTO
SOC

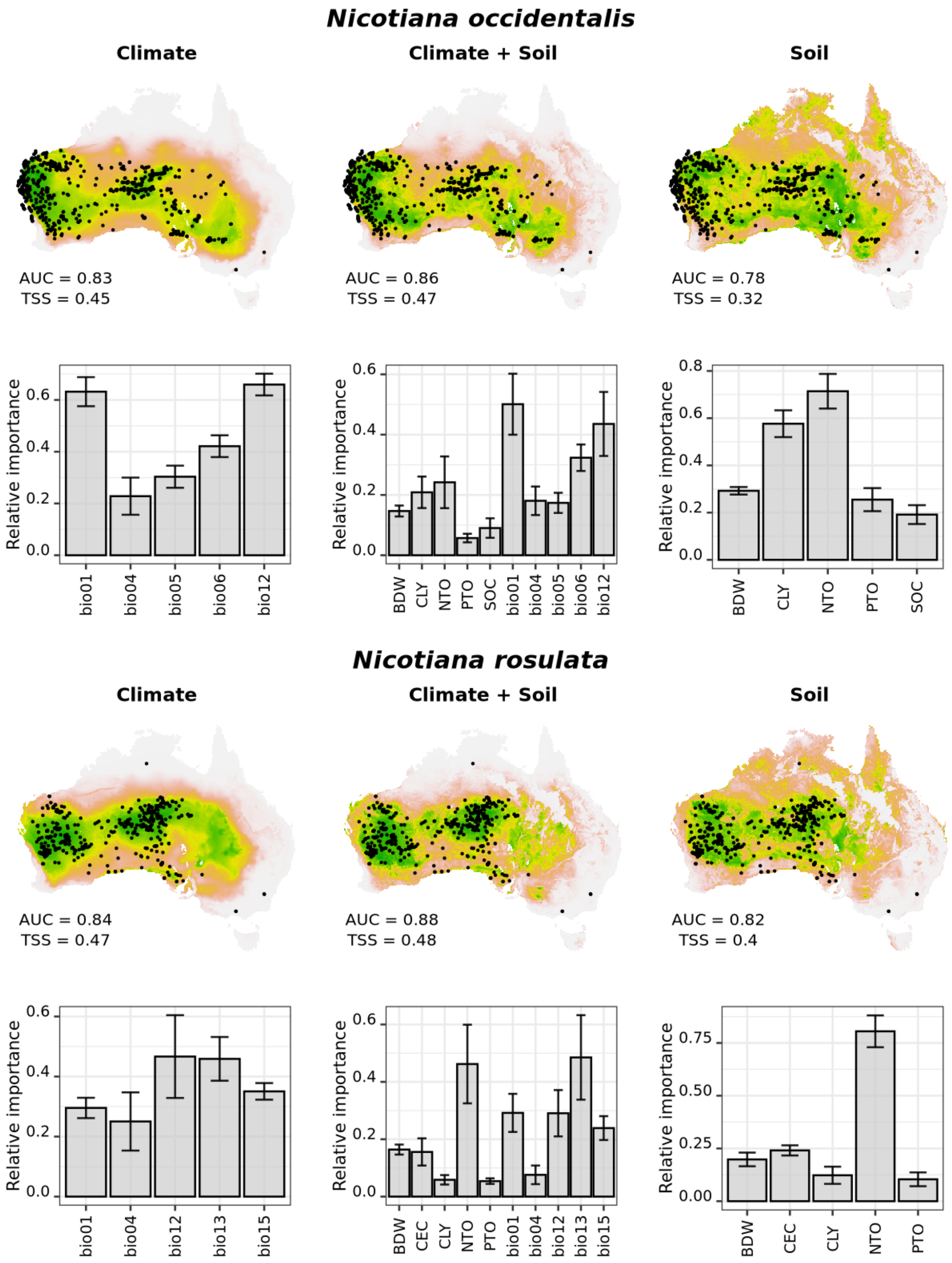
Nicotiana occidentalis
Climate
Climate + Soil
Soil
AUC = 0.83
TSS = 0.45
AUC = 0.86
TSS = 0.47
AUC = 0.78
TSS = 0.32
Relative importance
bio01
bio04
bio05
bio06
bio12
BDW
CLY
NTO
PTO
SOC
Nicotiana rosulata
AUC = 0.84
TSS = 0.47
AUC = 0.88
TSS = 0.48
AUC = 0.82
TSS = 0.4
bio13
bio15
CEC

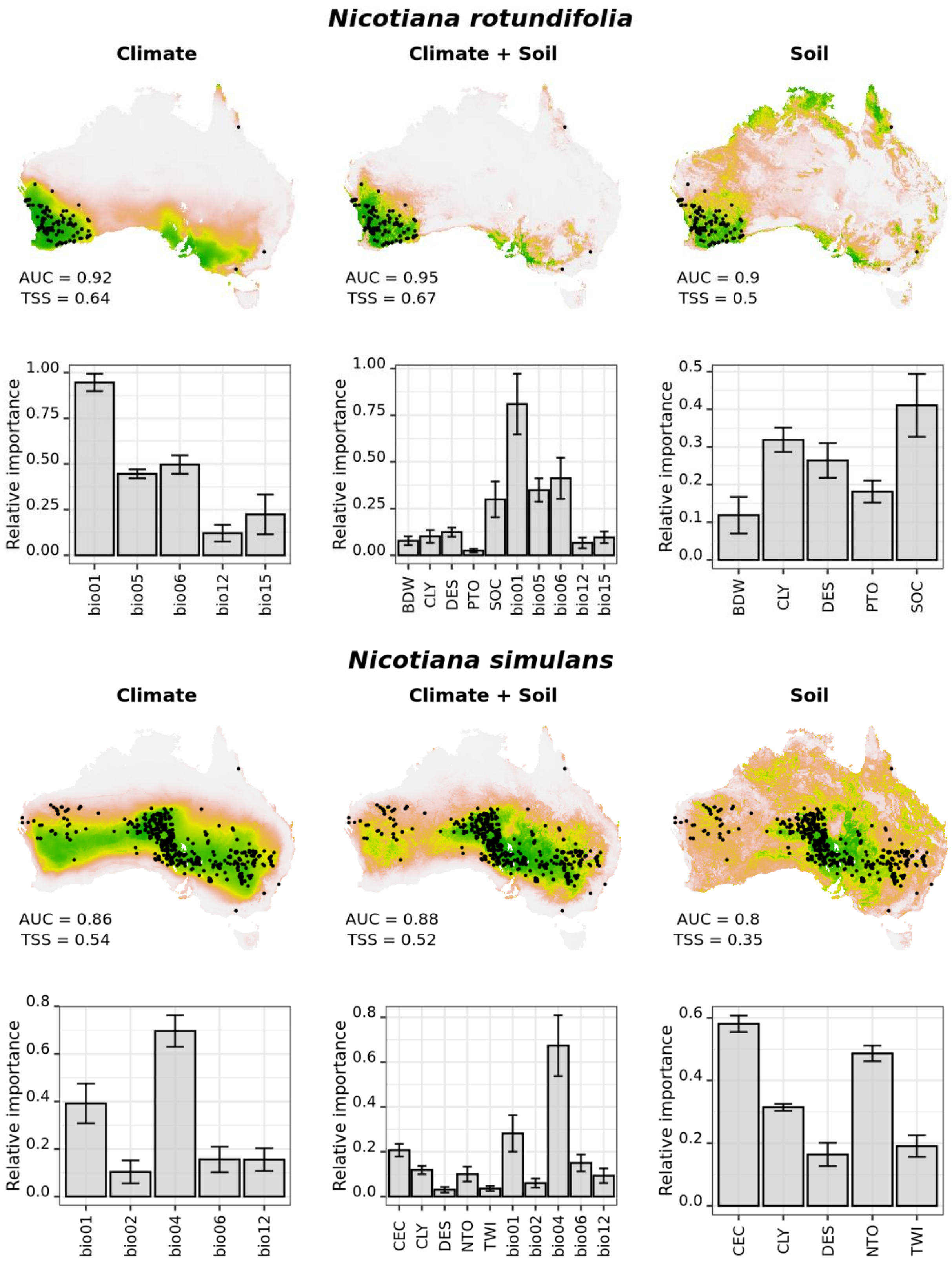

Nicotiana rotundifolia
Climate
Climate + Soil
Soil
AUC = 0.92
TSS = 0.64
AUC = 0.95
TSS = 0.67
AUC = 0.9
TSS = 0.5
Relative importance
1.00
0.75
0.50
0.25
0.00
bio01
bio05
bio06
bio12
bio15
BDW
CLY
DES
PTO
SOC
0.5
0.4
0.3
0.2
0.1
0.0
Nicotiana simulans
Climate
Climate + Soil
Soil
AUC = 0.86
TSS = 0.54
AUC = 0.88
TSS = 0.52
AUC = 0.8
TSS = 0.35
Relative importance
0.8
0.6
0.4
0.2
0.0
bio01
bio02
bio04
bio06
bio12
CEC
CLY
DES
NTO
TWI

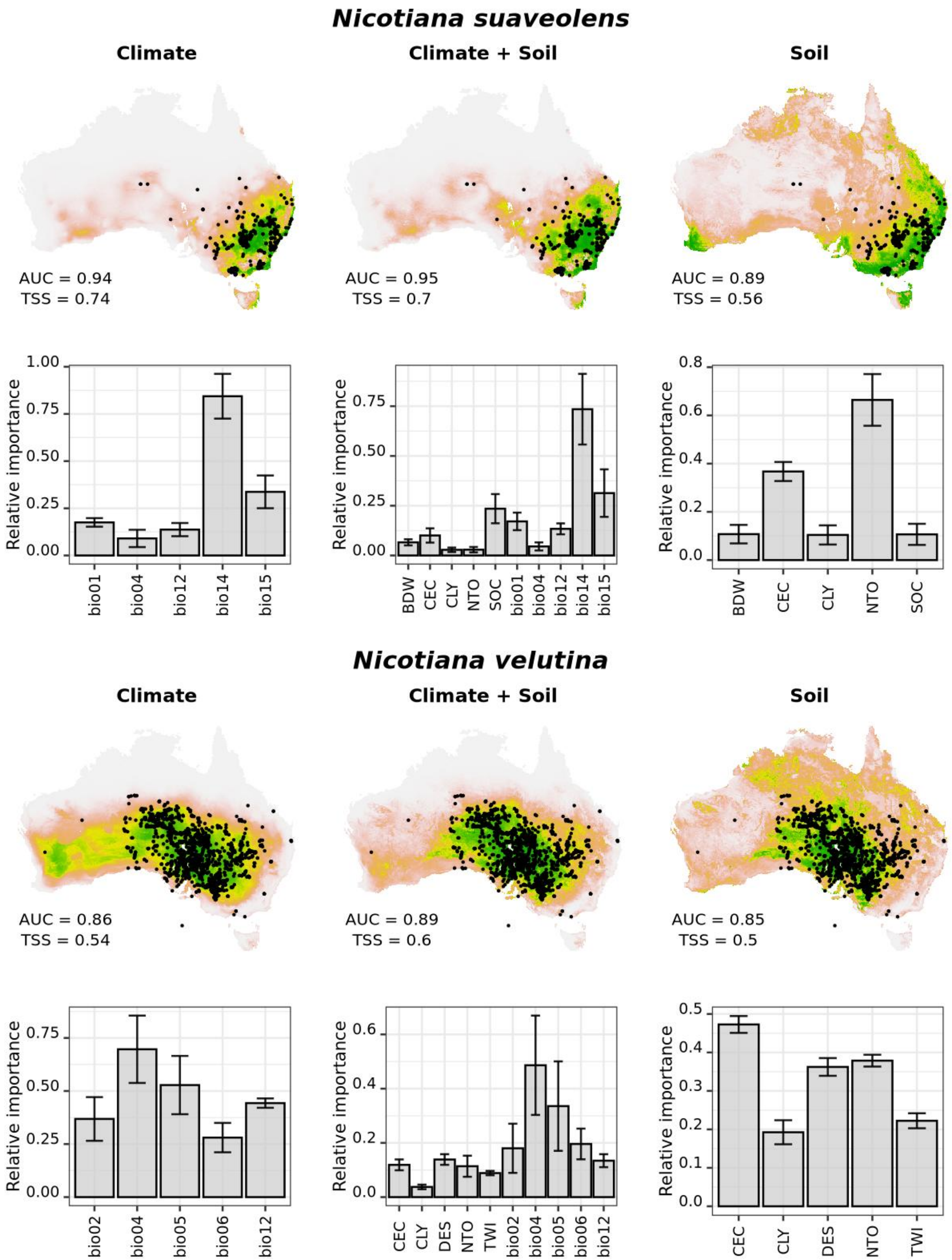
Nicotiana suaveolens
Climate
Climate + Soil
Soil
AUC = 0.94
TSS = 0.74
AUC = 0.95
TSS = 0.7
AUC = 0.89
TSS = 0.56
Relative importance
bio01 bio04 bio12 bio14 bio15
BDW CEC CLY NTO SOC bio01 bio04 bio12 bio14 bio15
BDW CEC CLY NTO SOC
Nicotiana velutina
Climate
Climate + Soil
Soil
AUC = 0.86
TSS = 0.54
AUC = 0.89
TSS = 0.6
AUC = 0.85
TSS = 0.5
Relative importance
bio02 bio04 bio05 bio06 bio12
CEC CLY DES NTO TWI bio02 bio04 bio05 bio06 bio12
CEC CLY DES NTO TWI

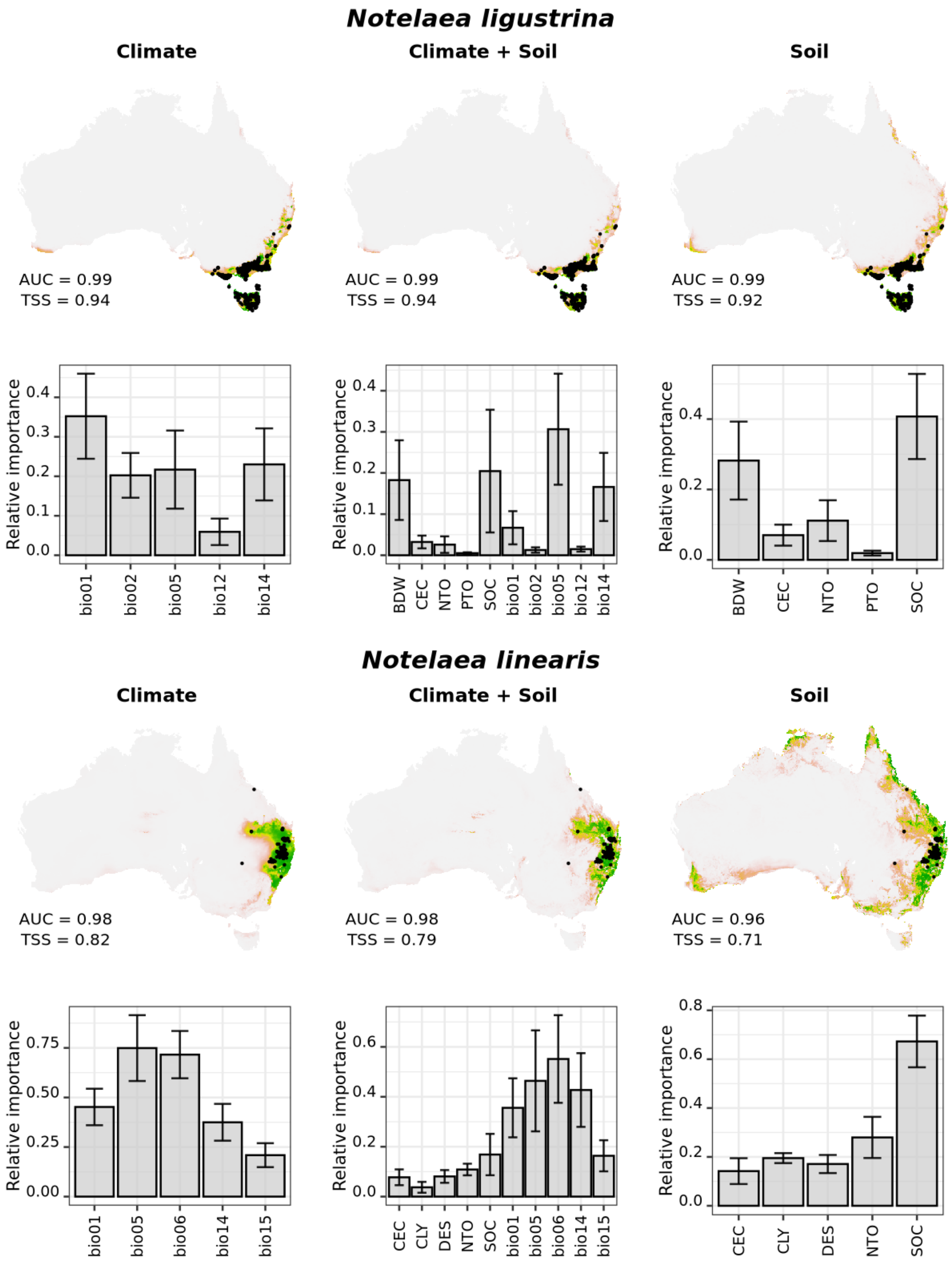

Notelaea ligustrina
Climate
Climate + Soil
Soil
AUC = 0.99
TSS = 0.94
AUC = 0.99
TSS = 0.94
AUC = 0.99
TSS = 0.92
Relative importance
bio01
bio02
bio05
bio12
bio14
BDW
CEC
NTO
PTO
SOC
Notelaea linearis
AUC = 0.98
TSS = 0.82
AUC = 0.98
TSS = 0.79
AUC = 0.96
TSS = 0.71
bio06
bio15
CLY
DES

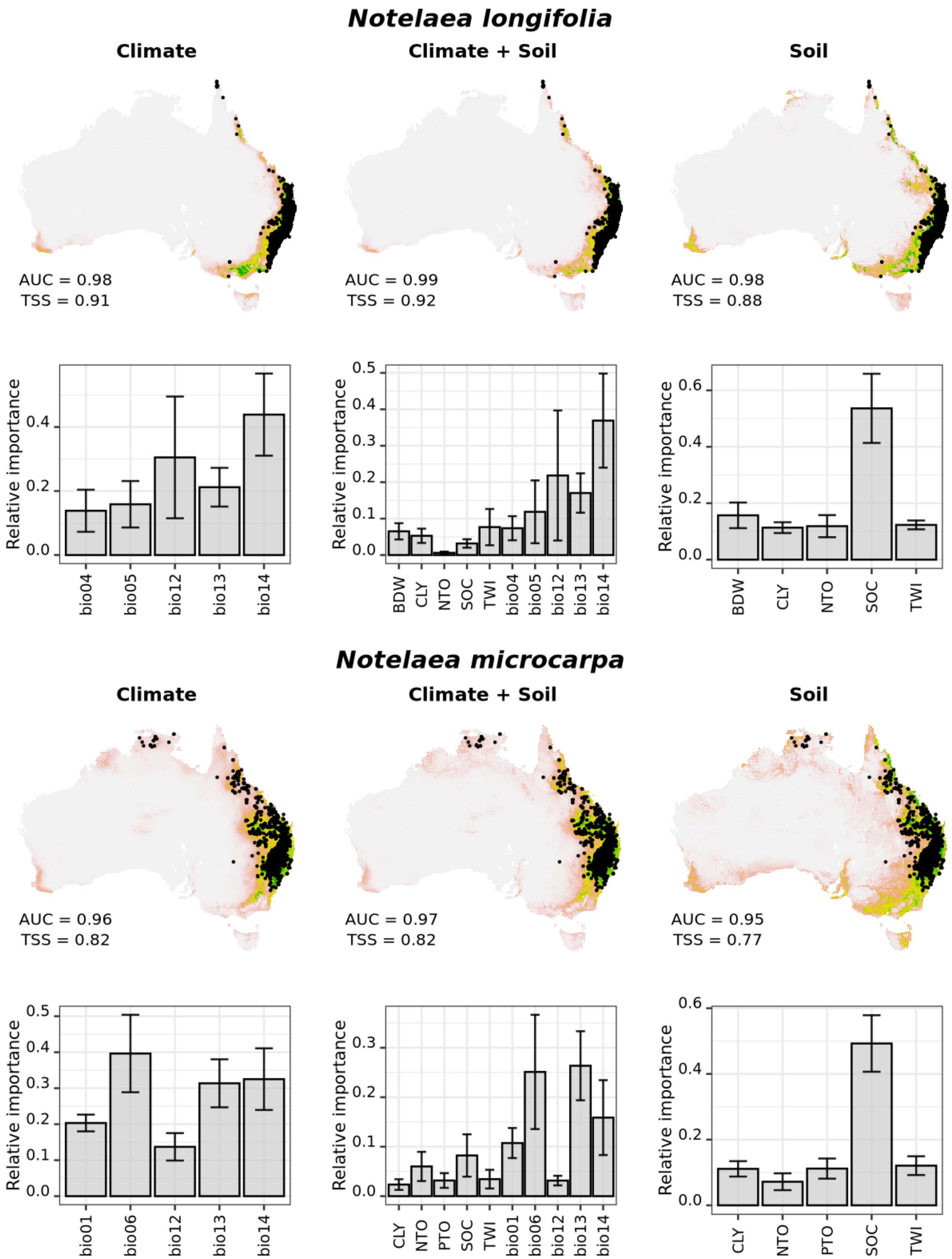

Notelaea longifolia
Climate
Climate + Soil
Soil
AUC = 0.98
TSS = 0.91
AUC = 0.99
TSS = 0.92
AUC = 0.98
TSS = 0.88
Relative importance
bio04
bio05
bio12
bio13
bio14
BDW
CLY
NTO
SOC
TWI
Notelaea microcarpa
Climate
Climate + Soil
Soil
AUC = 0.96
TSS = 0.82
AUC = 0.97
TSS = 0.82
AUC = 0.95
TSS = 0.77
bio01
bio06
PTO

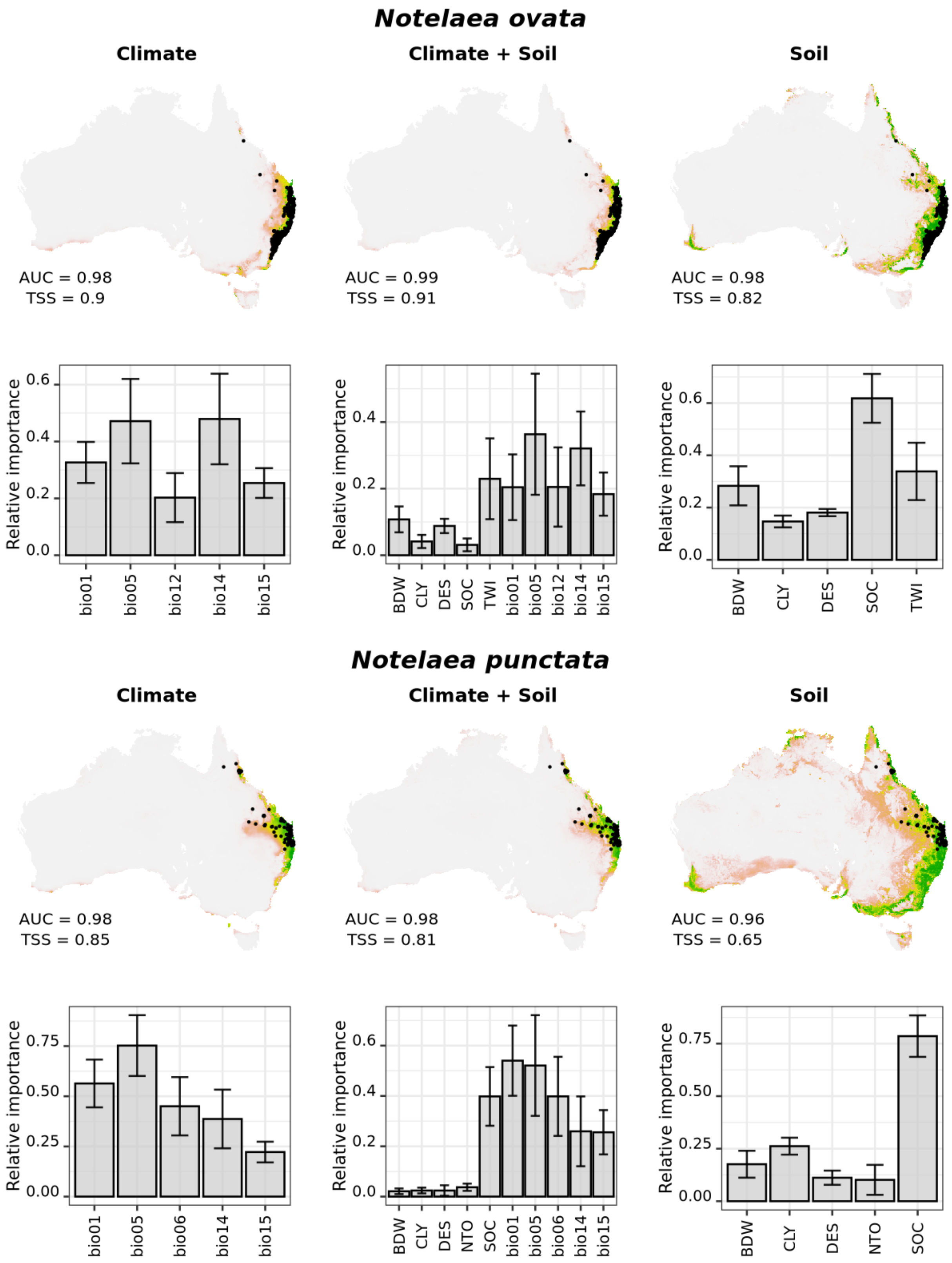

Notelaea ovata
Climate
Climate + Soil
Soil
AUC = 0.98
TSS = 0.9
AUC = 0.99
TSS = 0.91
AUC = 0.98
TSS = 0.82
Relative importance
bio01
bio05
bio12
bio14
bio15
BDW
CLY
DES
SOC
TWI
Notelaea punctata
AUC = 0.98
TSS = 0.85
AUC = 0.98
TSS = 0.81
AUC = 0.96
TSS = 0.65
bio06
NTO

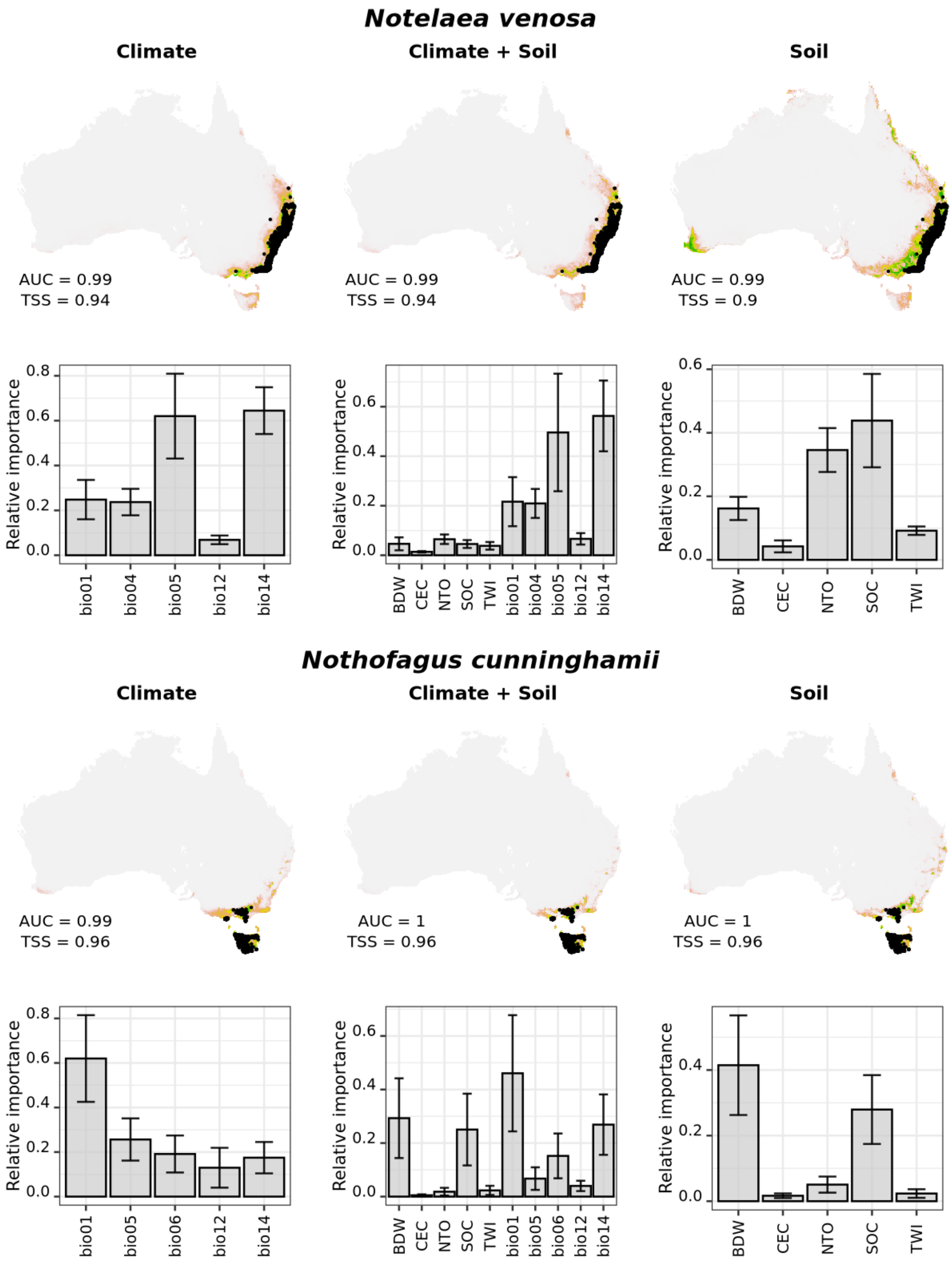
Notelaea venosa
Climate
Climate + Soil
Soil
AUC = 0.99
TSS = 0.94
AUC = 0.99
TSS = 0.94
AUC = 0.99
TSS = 0.9
Relative importance
bio01
bio04
bio05
bio12
bio14
BDW
CEC
NTO
SOC
TWI
Nothofagus cunninghamii
Climate
Climate + Soil
Soil
AUC = 0.99
TSS = 0.96
AUC = 1
TSS = 0.96
AUC = 1
TSS = 0.96
Relative importance
bio01
bio05
bio06
bio12
bio14
BDW
CEC
NTO
SOC
TWI

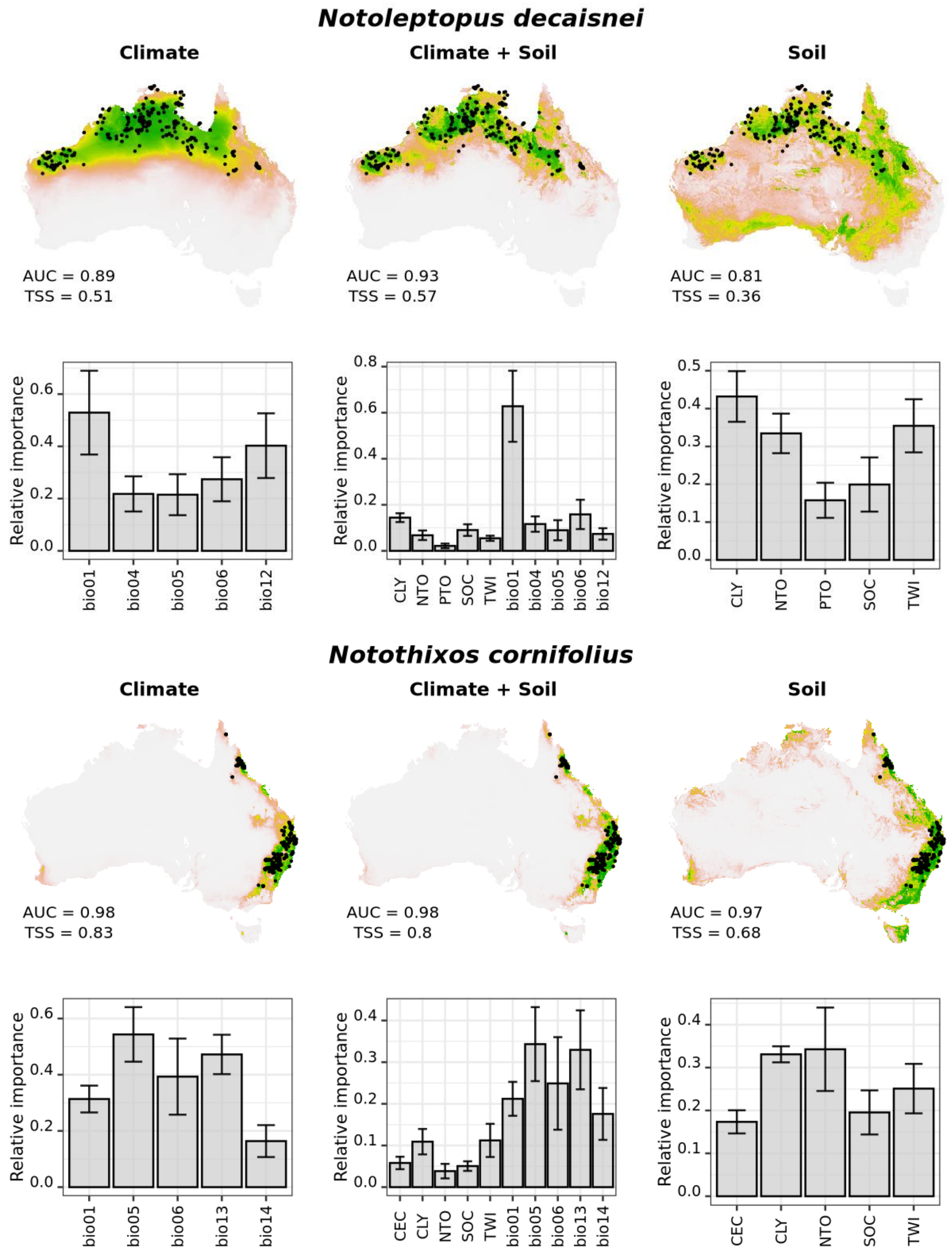
Notoleptopus decaisnei
Climate
Climate + Soil
Soil
AUC = 0.89
TSS = 0.51
AUC = 0.93
TSS = 0.57
AUC = 0.81
TSS = 0.36
Relative importance
0.0
0.2
0.4
0.6
bio01
bio04
bio05
bio06
bio12
Relative importance
0.0
0.2
0.4
0.6
0.8
CLY
NTO
PTO
SOC
TWI
bio01
bio04
bio05
bio06
bio12
Relative importance
0.0
0.1
0.2
0.3
0.4
0.5
CLY
NTO
PTO
SOC
TWI
Notothixos cornifolius
Climate
Climate + Soil
Soil
AUC = 0.98
TSS = 0.83
AUC = 0.98
TSS = 0.8
AUC = 0.97
TSS = 0.68
Relative importance
0.0
0.2
0.4
0.6
bio01
bio05
bio06
bio13
bio14
Relative importance
0.0
0.1
0.2
0.3
0.4
CEC
CLY
NTO
SOC
TWI
bio01
bio05
bio06
bio13
bio14
Relative importance
0.0
0.1
0.2
0.3
0.4
CEC
CLY
NTO
SOC
TWI

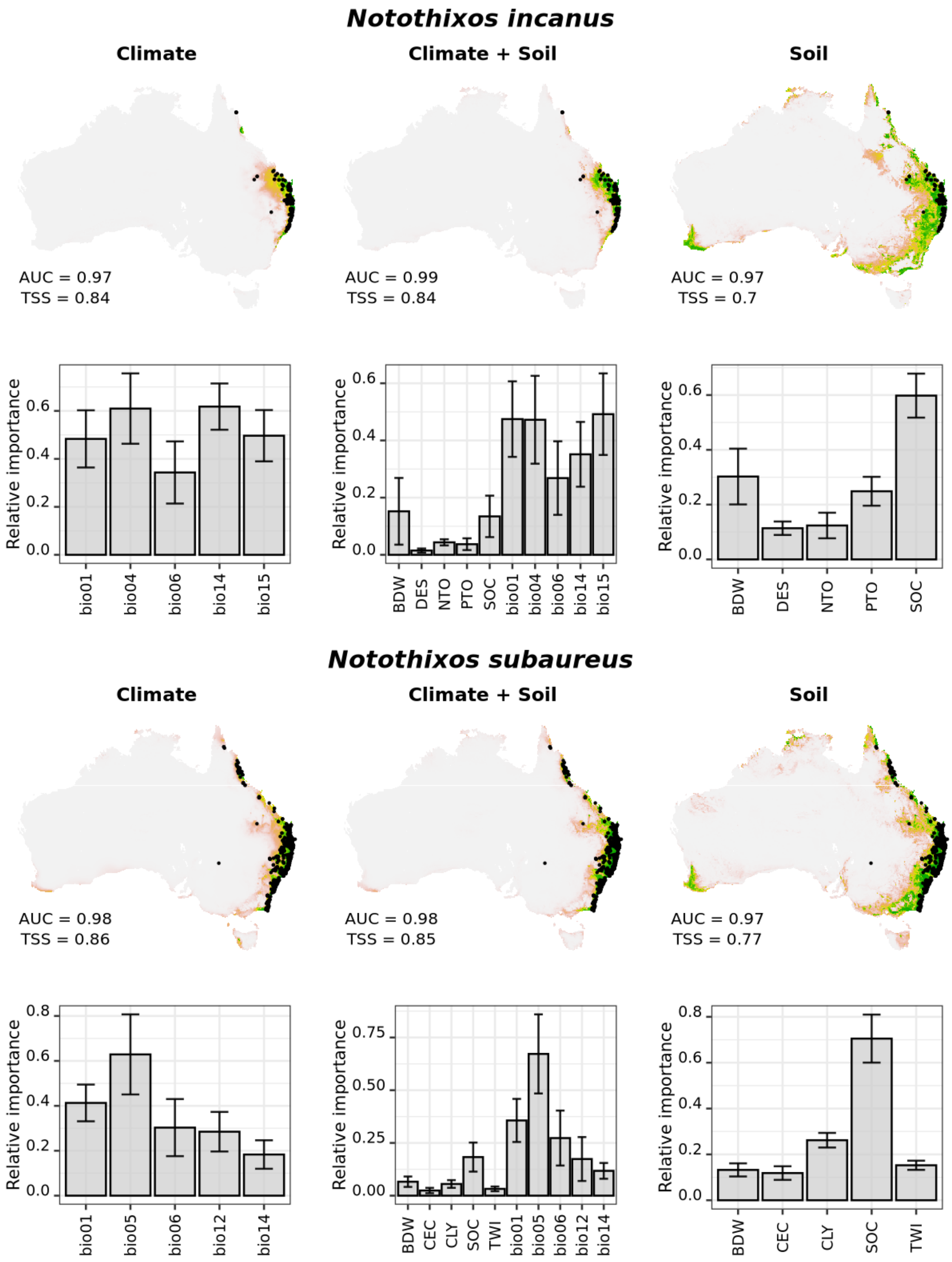
Notothixos incanus
Climate
Climate + Soil
Soil
AUC = 0.97
TSS = 0.84
AUC = 0.99
TSS = 0.84
AUC = 0.97
TSS = 0.7
Relative importance
0.0
0.2
0.4
0.6
bio01
bio04
bio06
bio14
bio15
BDW
DES
NTO
PTO
SOC
Notothixos subaureus
Climate
Climate + Soil
Soil
AUC = 0.98
TSS = 0.86
AUC = 0.98
TSS = 0.85
AUC = 0.97
TSS = 0.77
Relative importance
0.0
0.2
0.4
0.6
0.8
0.00
0.25
0.50
0.75
bio01
bio05
bio06
bio12
bio14
BDW
CEC
CLY
SOC
TWI

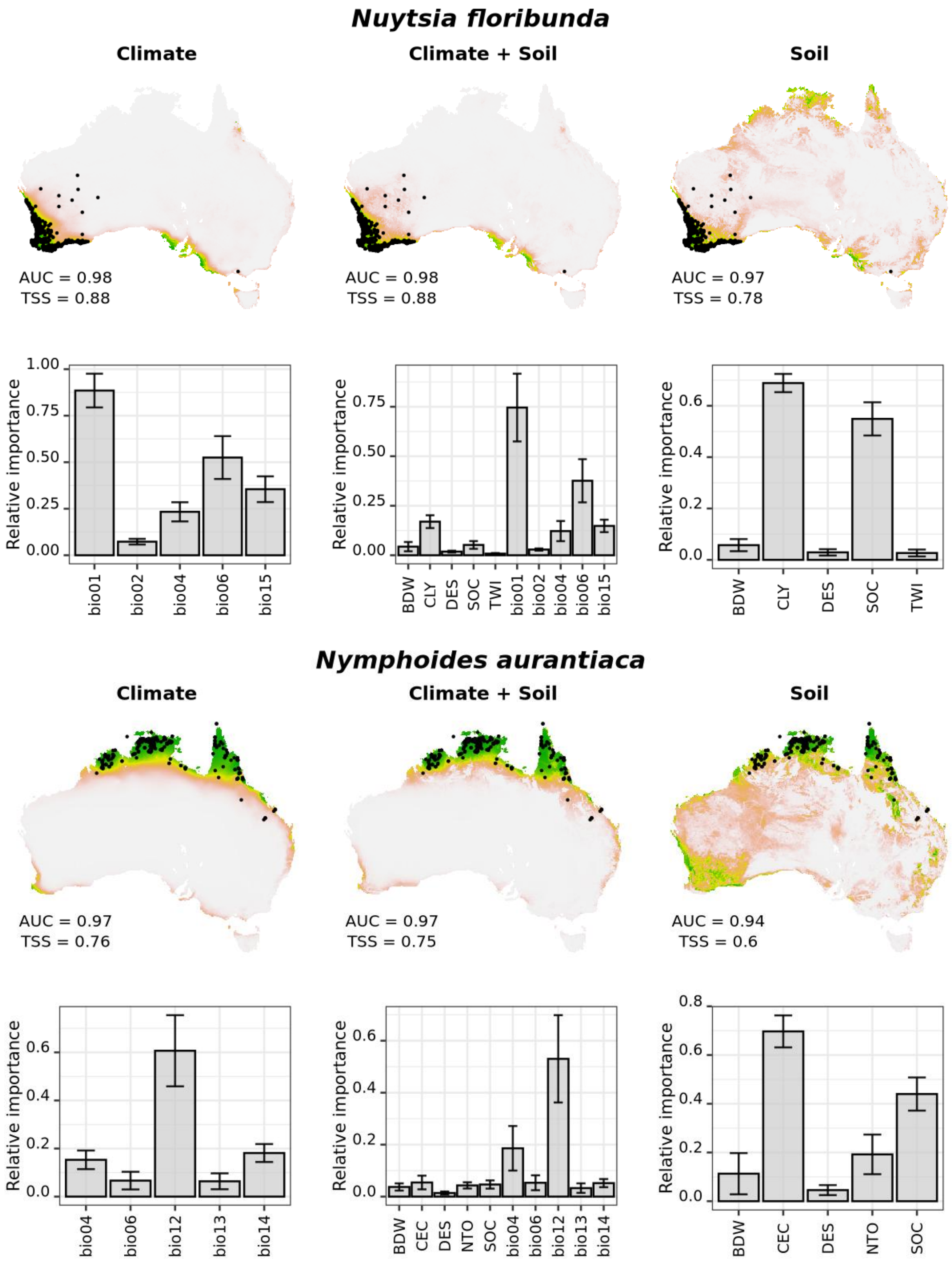
Nuytsia floribunda
Climate
Climate + Soil
Soil
AUC = 0.98
TSS = 0.88
AUC = 0.98
TSS = 0.88
AUC = 0.97
TSS = 0.78
Relative importance
bio01
bio02
bio04
bio06
bio15
BDW
CLY
DES
SOC
TWI
Nymphoides aurantiaca
Climate
Climate + Soil
Soil
AUC = 0.97
TSS = 0.76
AUC = 0.97
TSS = 0.75
AUC = 0.94
TSS = 0.6
Relative importance
bio04
bio06
bio12
bio13
bio14
BDW
CEC
DES
NTO
SOC

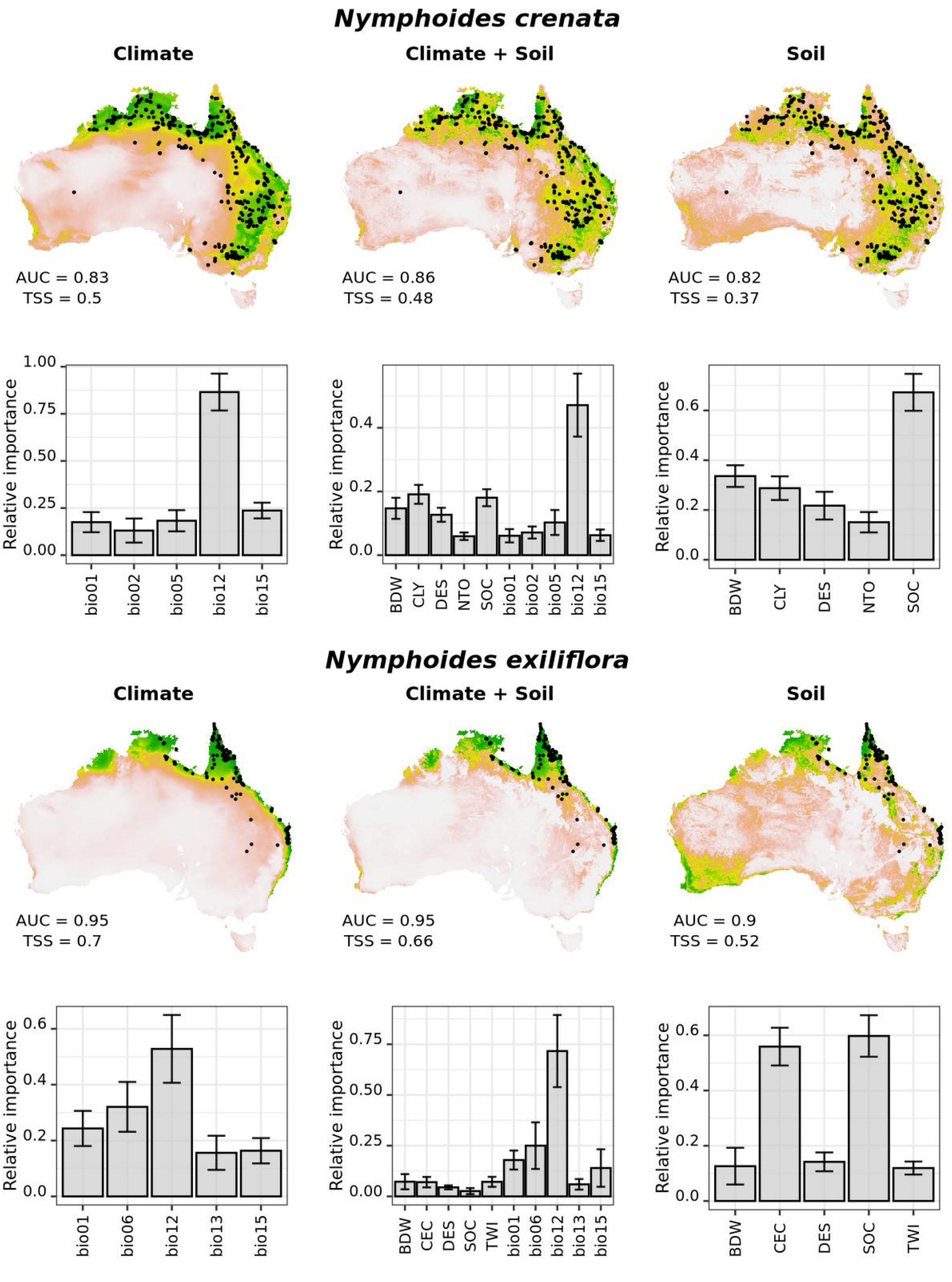
Nymphoides crenata
Climate
Climate + Soil
Soil
AUC = 0.83
TSS = 0.5
AUC = 0.86
TSS = 0.48
AUC = 0.82
TSS = 0.37
Relative importance
1.00
0.75
0.50
0.25
0.00
bio01
bio02
bio05
bio12
bio15
0.4
0.2
0.0
BDW
CLY
DES
NTO
SOC
bio01
bio02
bio05
bio12
bio15
0.6
0.4
0.2
0.0
BDW
CLY
DES
NTO
SOC
Nymphoides exiliflora
Climate
Climate + Soil
Soil
AUC = 0.95
TSS = 0.7
AUC = 0.95
TSS = 0.66
AUC = 0.9
TSS = 0.52
Relative importance
0.6
0.4
0.2
0.0
bio01
bio06
bio12
bio13
bio15
0.75
0.50
0.25
0.00
BDW
CEC
DES
SOC
TWI
bio01
bio06
bio12
bio13
bio15
0.6
0.4
0.2
0.0
BDW
CEC
DES
SOC
TWI

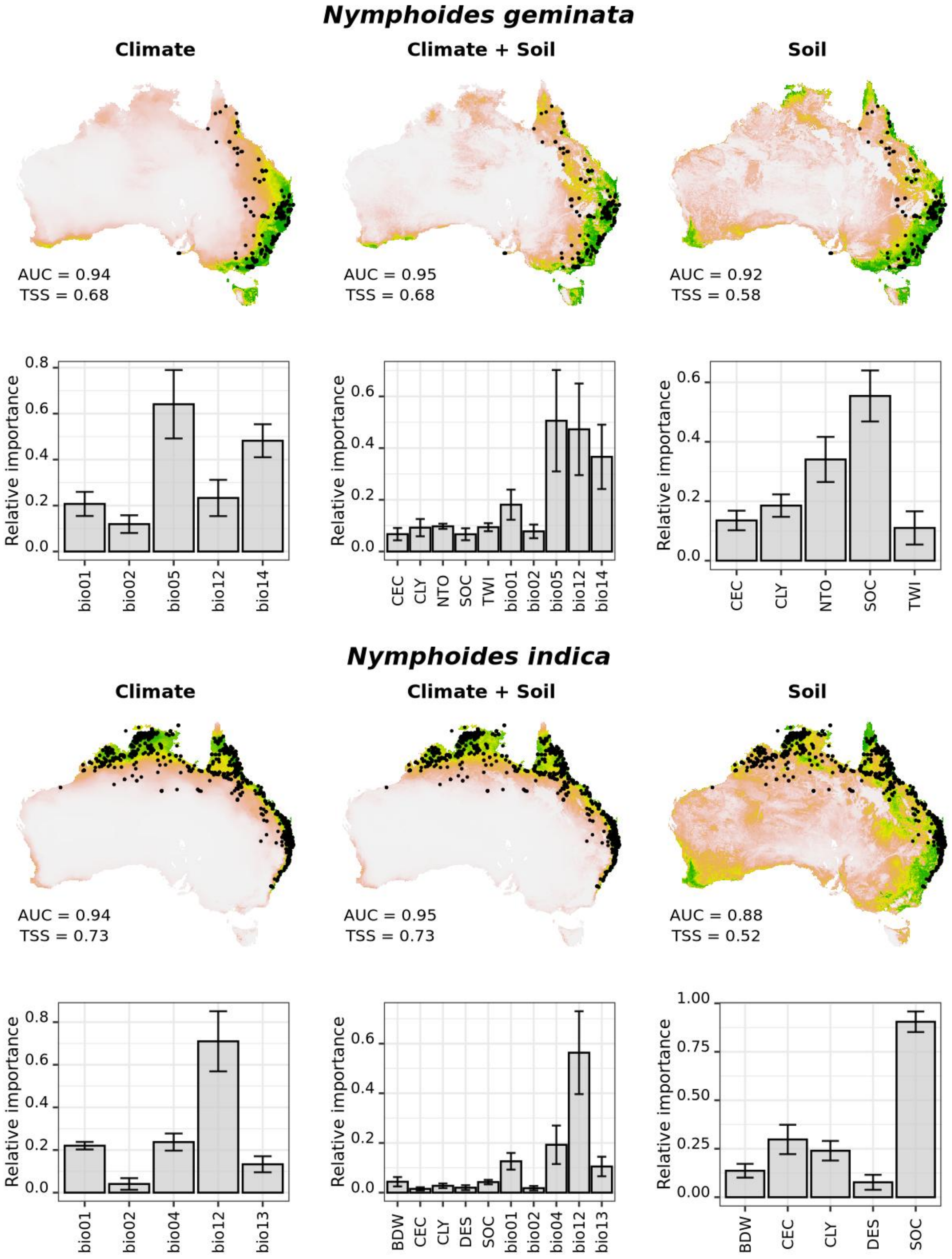

Nymphoides geminata
Climate
Climate + Soil
Soil
AUC = 0.94
TSS = 0.68
AUC = 0.95
TSS = 0.68
AUC = 0.92
TSS = 0.58
Relative importance
0.0
0.2
0.4
0.6
0.8
bio01
bio02
bio05
bio12
bio14
CEC
CLY
NTO
SOC
TWI
bio01
bio02
bio05
bio12
bio14
CEC
CLY
NTO
SOC
TWI
Nymphoides indica
Climate
Climate + Soil
Soil
AUC = 0.94
TSS = 0.73
AUC = 0.95
TSS = 0.73
AUC = 0.88
TSS = 0.52
0.00
0.25
0.50
0.75
1.00
bio01
bio02
bio04
bio12
bio13
BDW
CEC
CLY
DES
SOC
bio01
bio02
bio04
bio12
bio13
BDW
CEC
CLY
DES
SOC

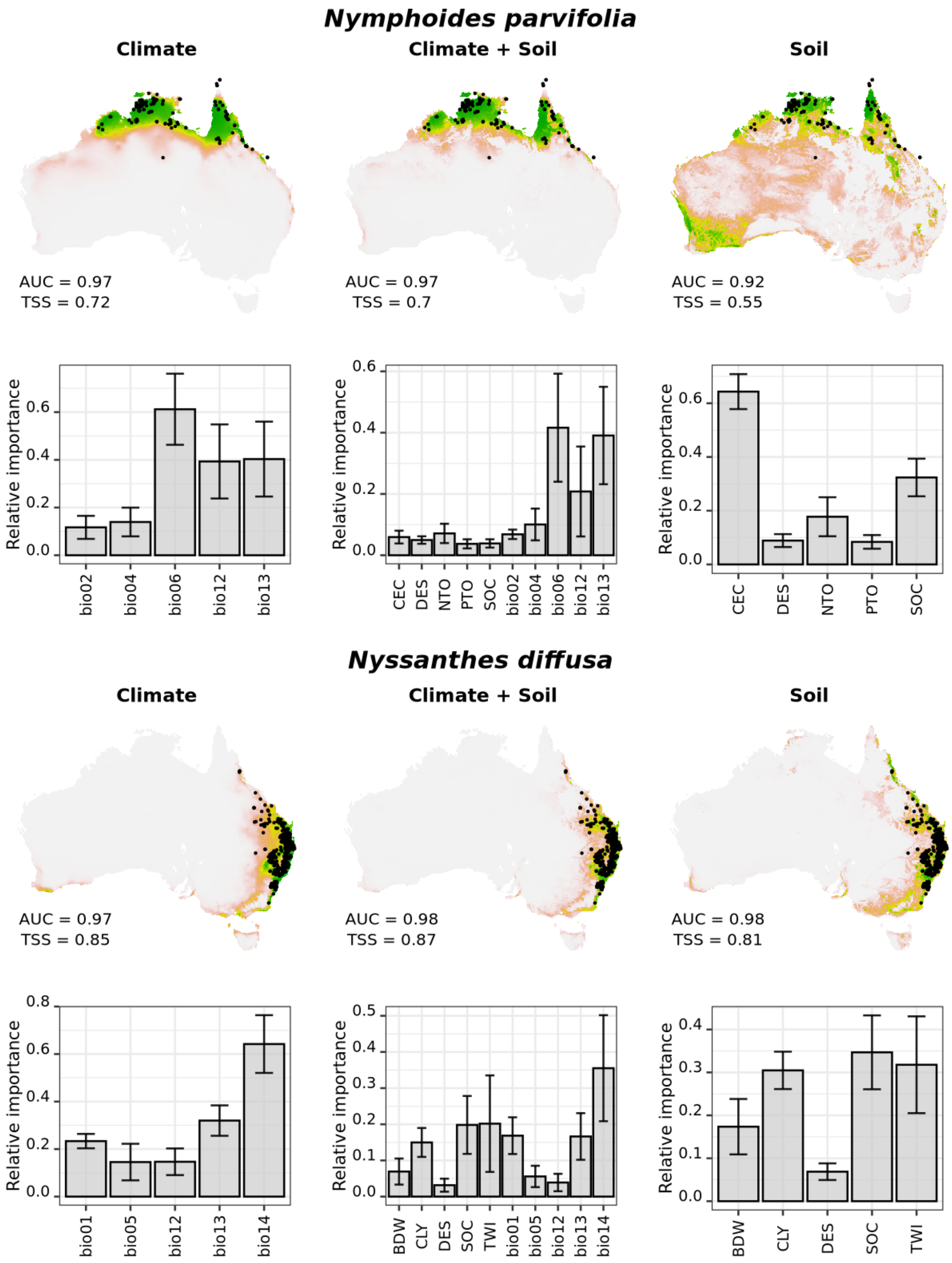
Nymphoides parvifolia
Climate
Climate + Soil
Soil
AUC = 0.97
TSS = 0.72
AUC = 0.97
TSS = 0.7
AUC = 0.92
TSS = 0.55
Relative importance
bio02
bio04
bio06
bio12
bio13
CEC
DES
NTO
PTO
SOC
Nyssanthes diffusa
Climate
Climate + Soil
Soil
AUC = 0.97
TSS = 0.85
AUC = 0.98
TSS = 0.87
AUC = 0.98
TSS = 0.81
Relative importance
bio01
bio05
bio12
bio13
bio14
BDW
CLY
DES
SOC
TWI

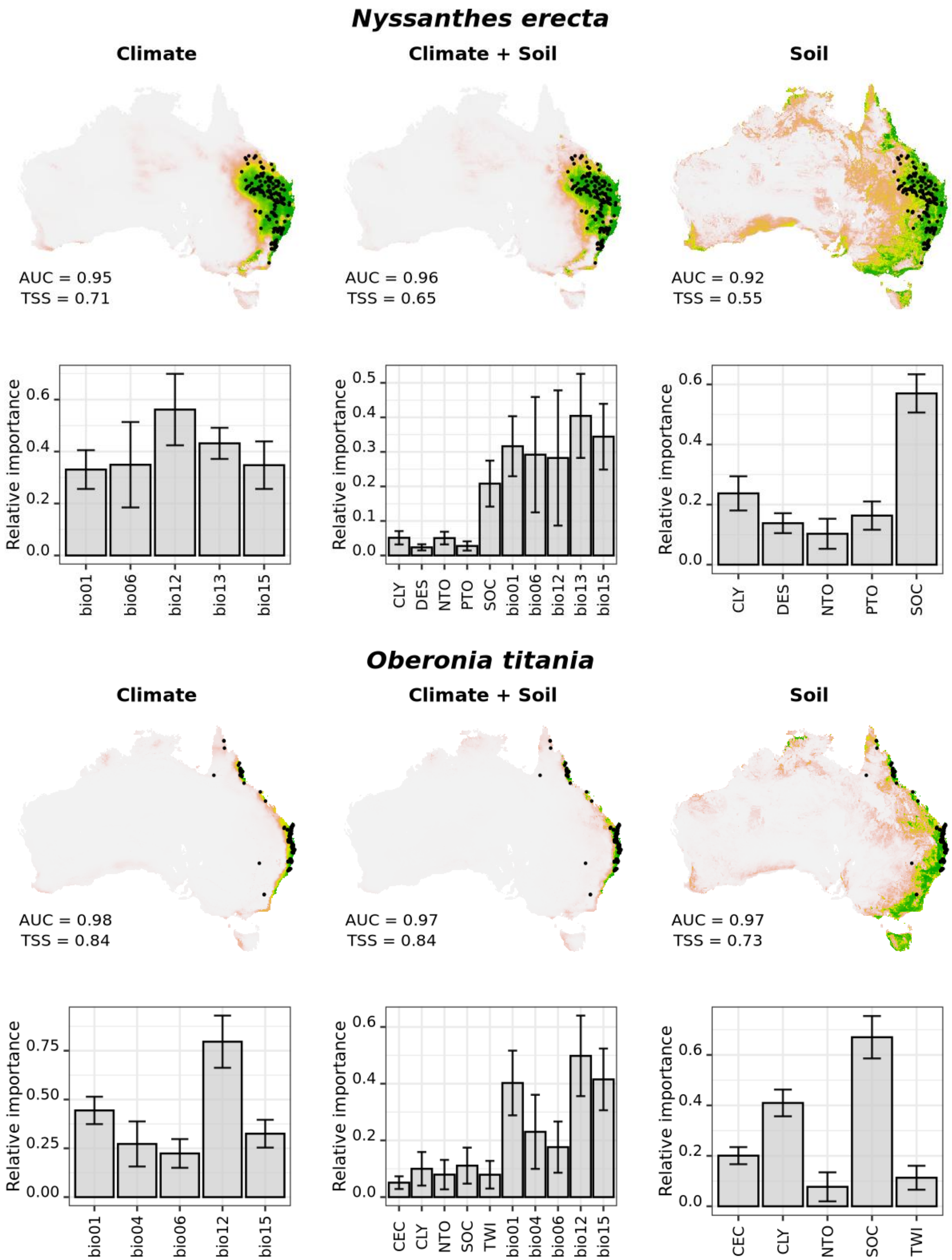
Nyssanthes erecta
Climate
Climate + Soil
Soil
AUC = 0.95
TSS = 0.71
AUC = 0.96
TSS = 0.65
AUC = 0.92
TSS = 0.55
Relative importance
bio01 bio06 bio12 bio13 bio15
CLY DES NTO PTO SOC bio01 bio06 bio12 bio13 bio15
CLY DES NTO PTO SOC
Oberonia titania
Climate
Climate + Soil
Soil
AUC = 0.98
TSS = 0.84
AUC = 0.97
TSS = 0.84
AUC = 0.97
TSS = 0.73
Relative importance
bio01 bio04 bio06 bio12 bio15
CEC CLY NTO SOC TWI bio01 bio04 bio06 bio12 bio15
CEC CLY NTO SOC TWI

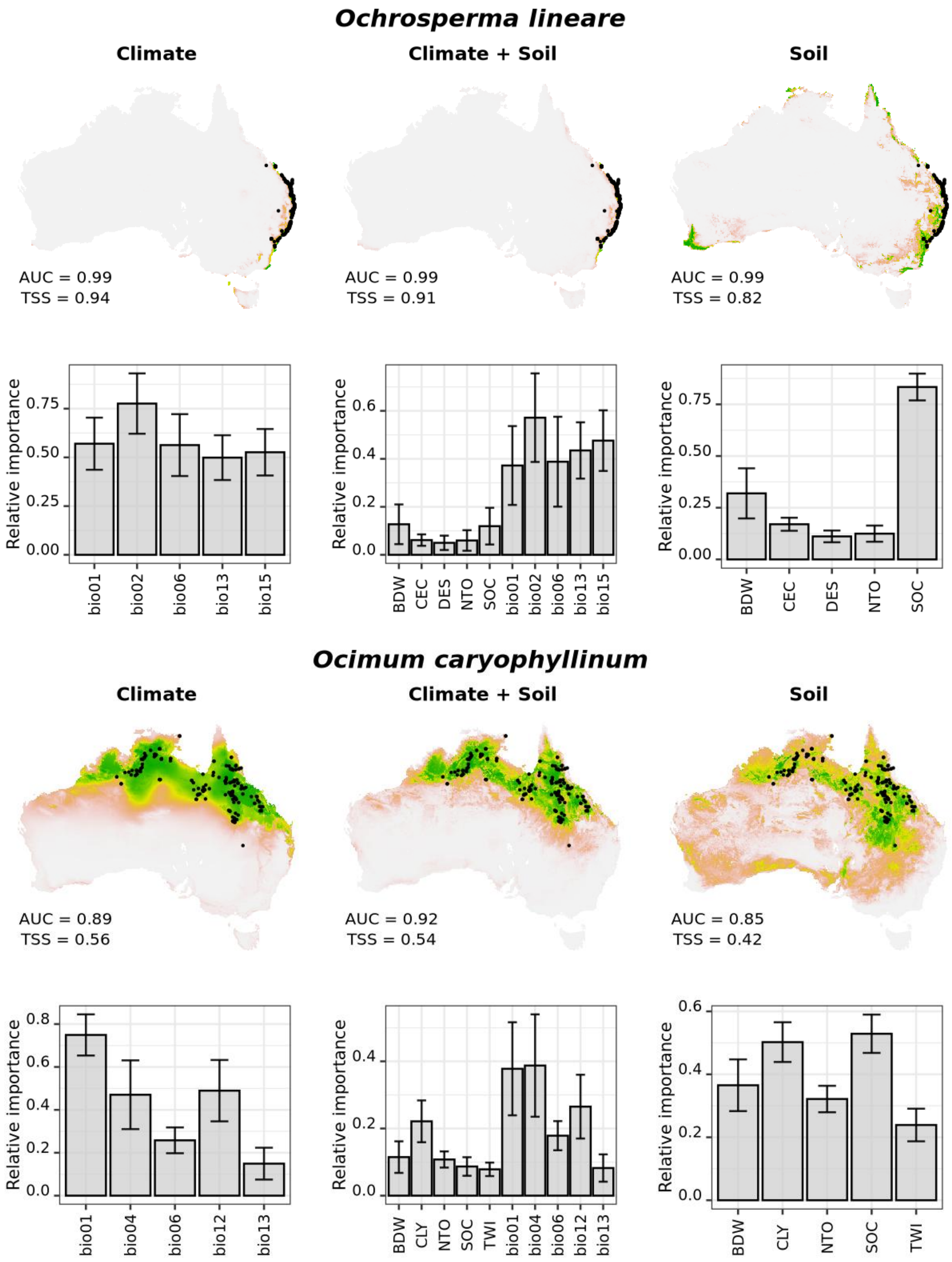
Ochrosperma lineare
Climate
Climate + Soil
Soil
AUC = 0.99
TSS = 0.94
AUC = 0.99
TSS = 0.91
AUC = 0.99
TSS = 0.82
Relative importance
bio01
bio02
bio06
bio13
bio15
BDW
CEC
DES
NTO
SOC
Ocimum caryophyllinum
Climate
Climate + Soil
Soil
AUC = 0.89
TSS = 0.56
AUC = 0.92
TSS = 0.54
AUC = 0.85
TSS = 0.42
bio04
bio12
CLY
TWI

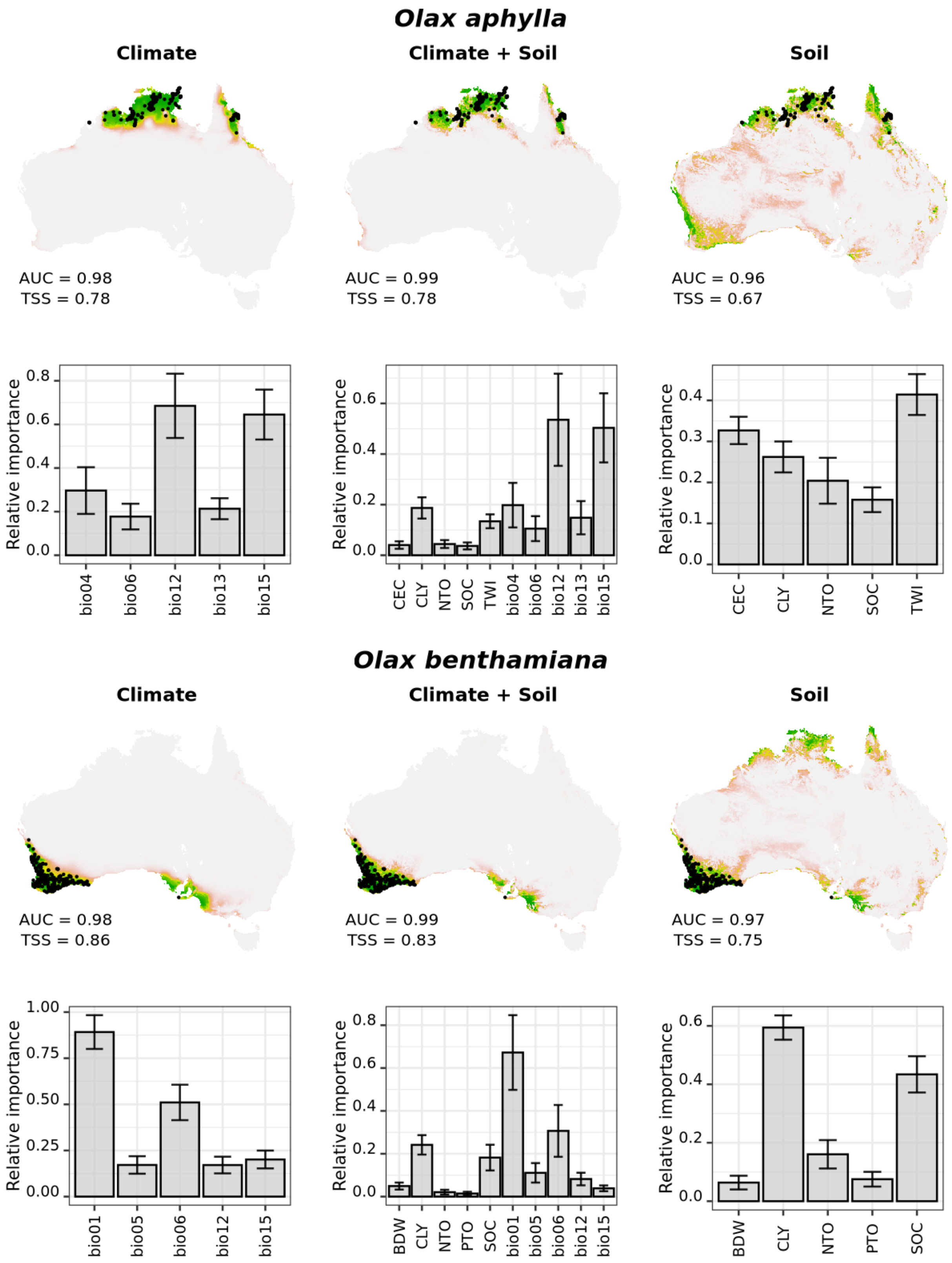

Olax aphylla
Climate
Climate + Soil
Soil
AUC = 0.98
TSS = 0.78
AUC = 0.99
TSS = 0.78
AUC = 0.96
TSS = 0.67
Relative importance
bio04 bio06 bio12 bio13 bio15
CEC CLY NTO SOC TWI bio04 bio06 bio12 bio13 bio15
CEC CLY NTO SOC TWI
Olax benthamiana
Climate
Climate + Soil
Soil
AUC = 0.98
TSS = 0.86
AUC = 0.99
TSS = 0.83
AUC = 0.97
TSS = 0.75
Relative importance
bio01 bio05 bio06 bio12 bio15
BDW CLY NTO PTO SOC bio01 bio05 bio06 bio12 bio15
BDW CLY NTO PTO SOC

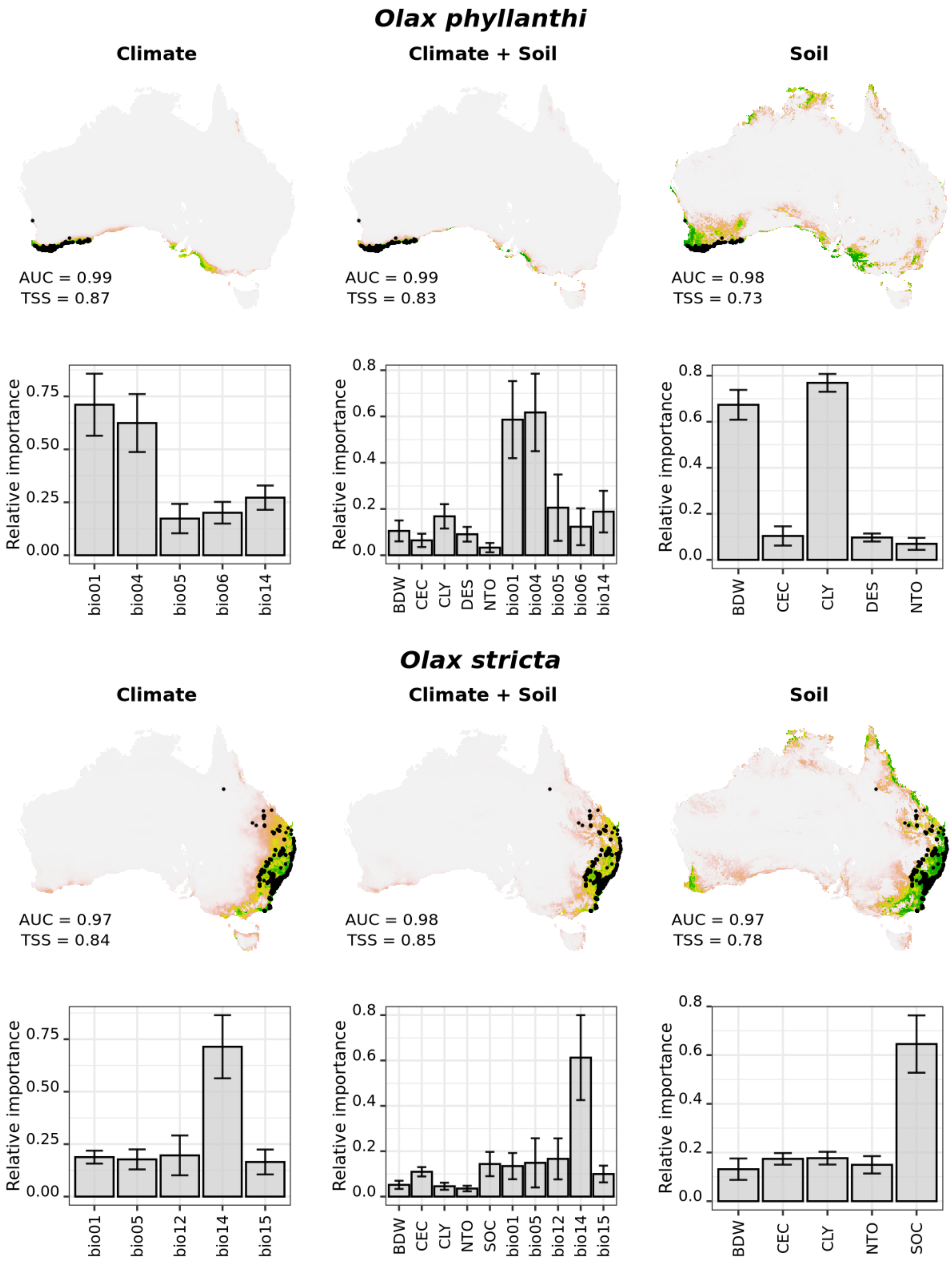

Olax phyllanthi
Climate
Climate + Soil
Soil
AUC = 0.99
TSS = 0.87
AUC = 0.99
TSS = 0.83
AUC = 0.98
TSS = 0.73
Relative importance
0.00
0.25
0.50
0.75
0.0
0.2
0.4
0.6
0.8
bio01
bio04
bio05
bio06
bio14
BDW
CEC
CLY
DES
NTO
Olax stricta
AUC = 0.97
TSS = 0.84
AUC = 0.98
TSS = 0.85
AUC = 0.97
TSS = 0.78
bio12
bio15
SOC

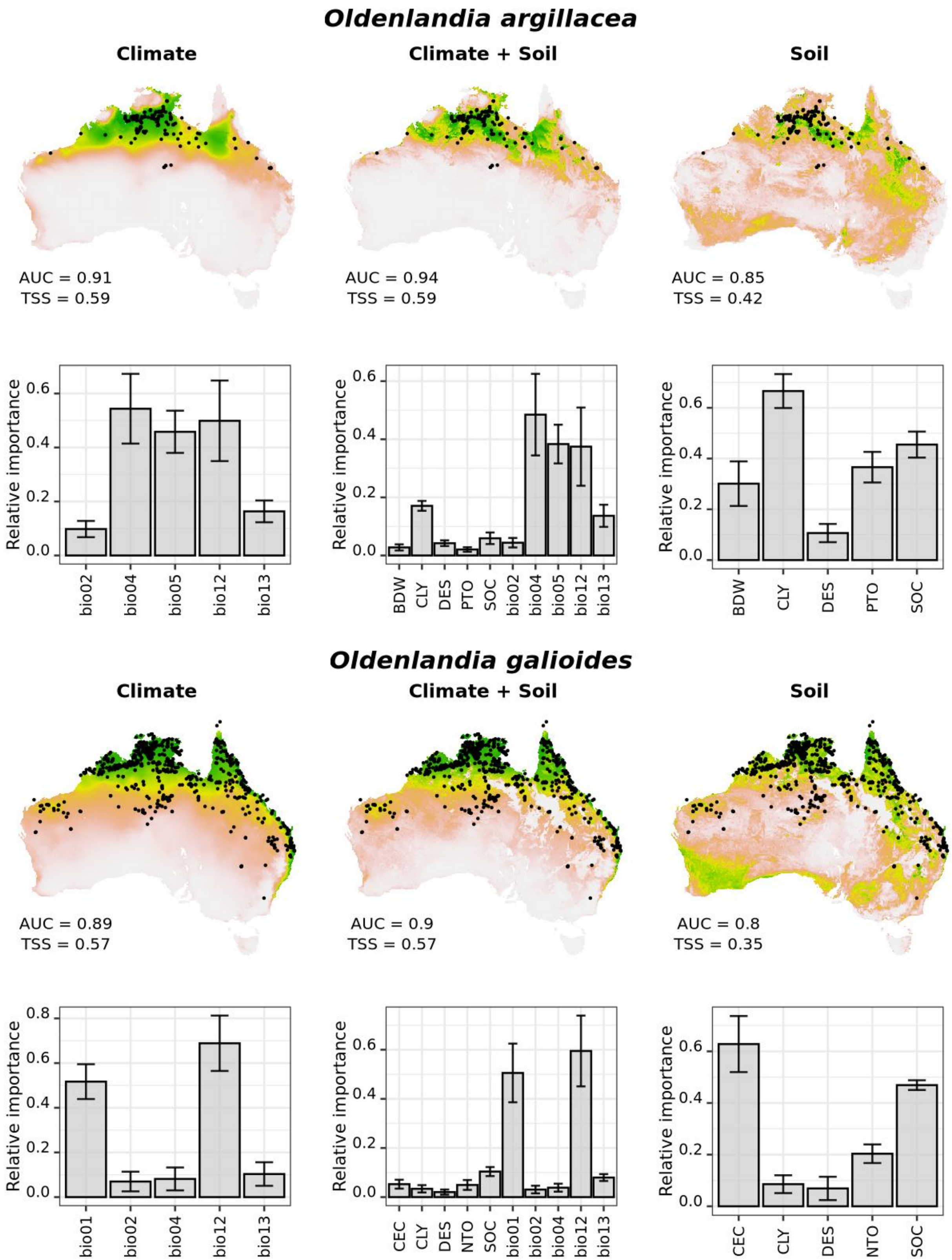

Oldenlandia argillacea
Climate
Climate + Soil
Soil
AUC = 0.91
TSS = 0.59
AUC = 0.94
TSS = 0.59
AUC = 0.85
TSS = 0.42
Relative importance
bio02
bio04
bio05
bio12
bio13
BDW
CLY
DES
PTO
SOC
Oldenlandia galioides
Climate
Climate + Soil
Soil
AUC = 0.89
TSS = 0.57
AUC = 0.9
TSS = 0.57
AUC = 0.8
TSS = 0.35
Relative importance
bio01
bio02
bio04
bio12
bio13
CEC
CLY
DES
NTO
SOC

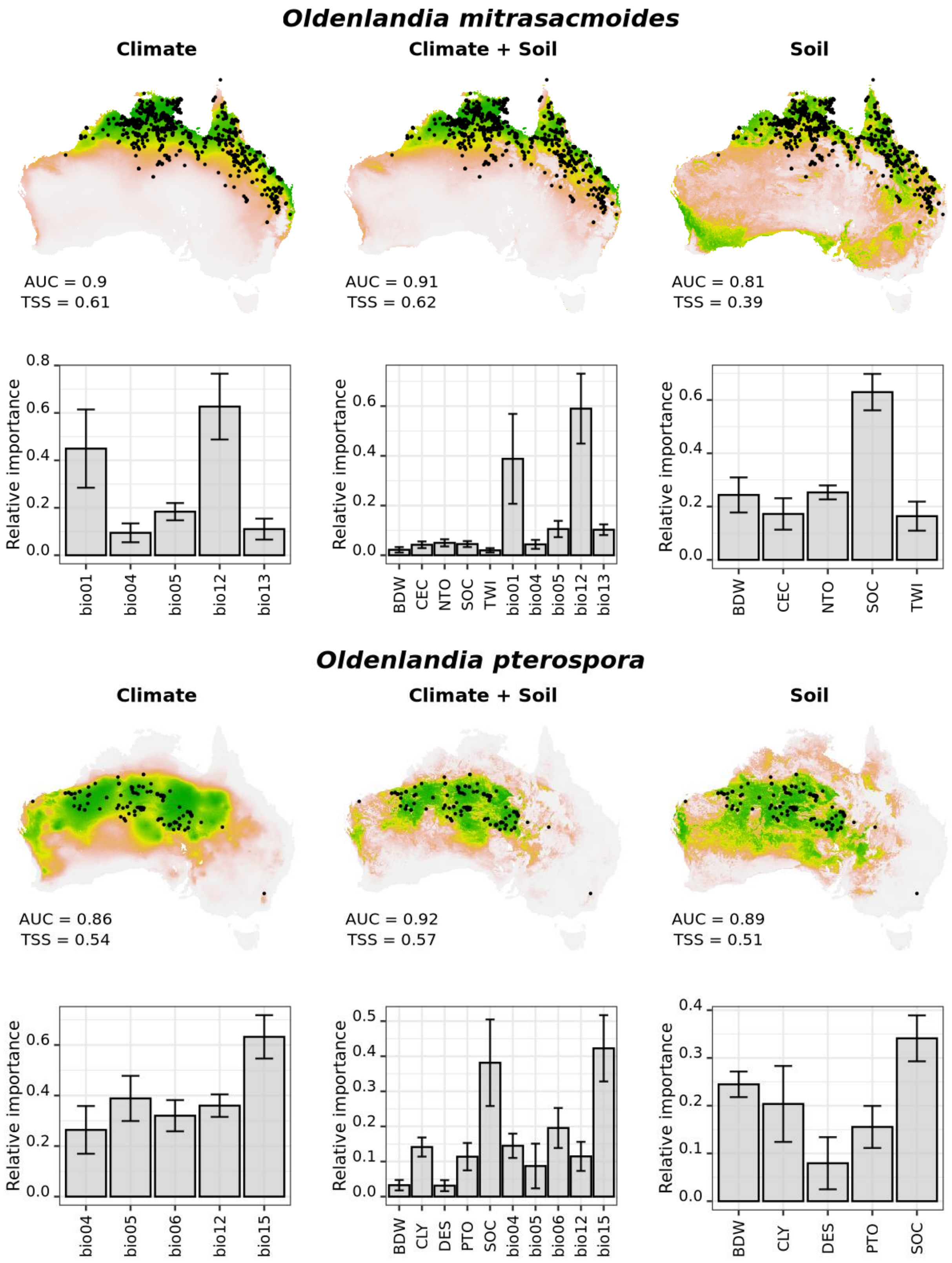
Oldenlandia mitrasacmoides
Climate
Climate + Soil
Soil
AUC = 0.9
TSS = 0.61
AUC = 0.91
TSS = 0.62
AUC = 0.81
TSS = 0.39
Relative importance
bio01
bio04
bio05
bio12
bio13
BDW
CEC
NTO
SOC
TWI
Oldenlandia pterospora
AUC = 0.86
TSS = 0.54
AUC = 0.92
TSS = 0.57
AUC = 0.89
TSS = 0.51
bio06
bio15
CLY
DES
PTO

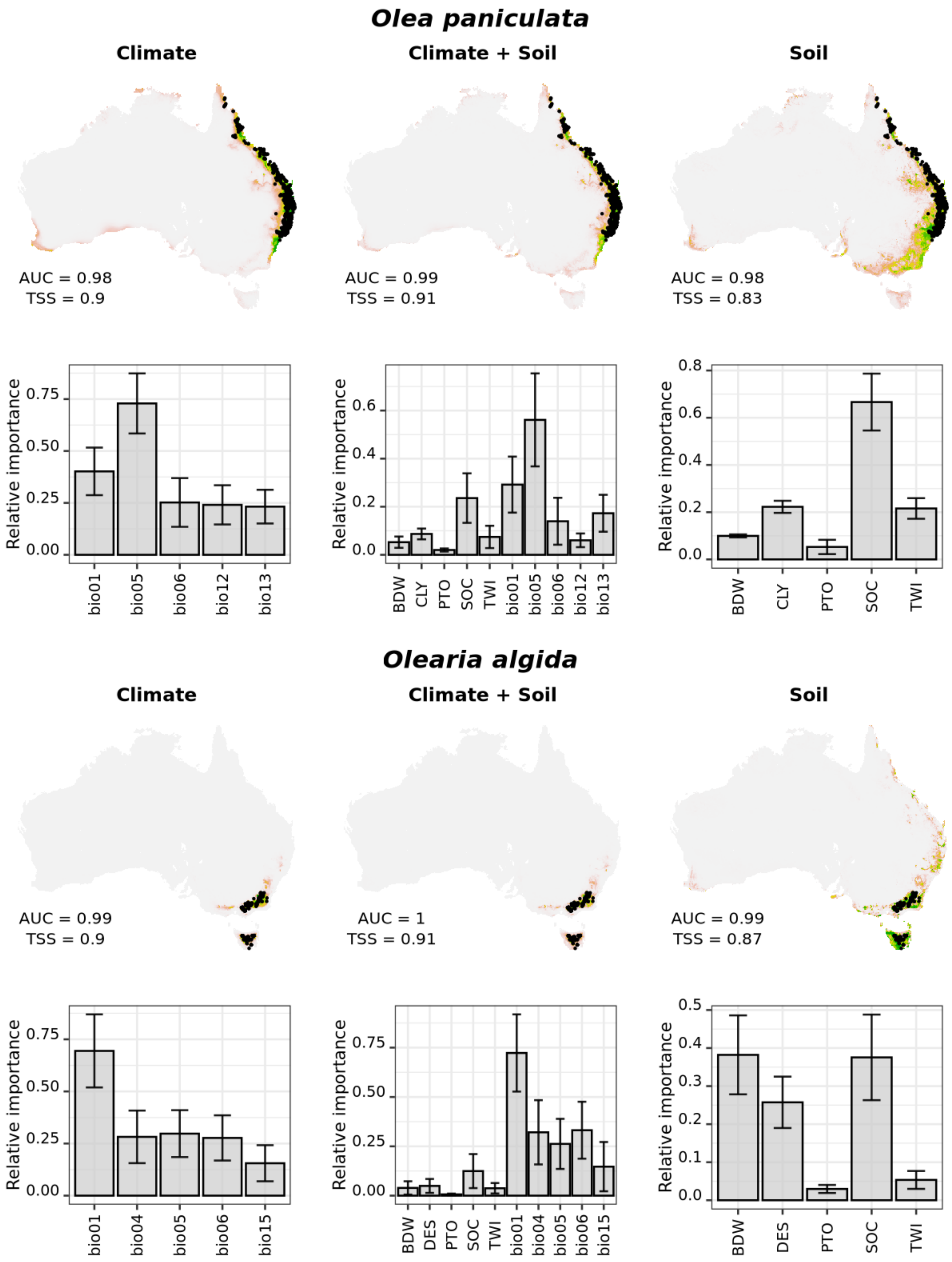
Olea paniculata
Climate
Climate + Soil
Soil
AUC = 0.98
TSS = 0.9
AUC = 0.99
TSS = 0.91
AUC = 0.98
TSS = 0.83
Relative importance
0.00
0.25
0.50
0.75
bio01
bio05
bio06
bio12
bio13
0.0
0.2
0.4
0.6
BDW
CLY
PTO
SOC
TWI
bio01
bio05
bio06
bio12
bio13
0.8
Olearia algida
Climate
Climate + Soil
Soil
AUC = 0.99
TSS = 0.9
AUC = 1
TSS = 0.91
AUC = 0.99
TSS = 0.87
bio01
bio04
bio05
bio06
bio15
BDW
DES
PTO
SOC
TWI
0.1
0.3
0.5

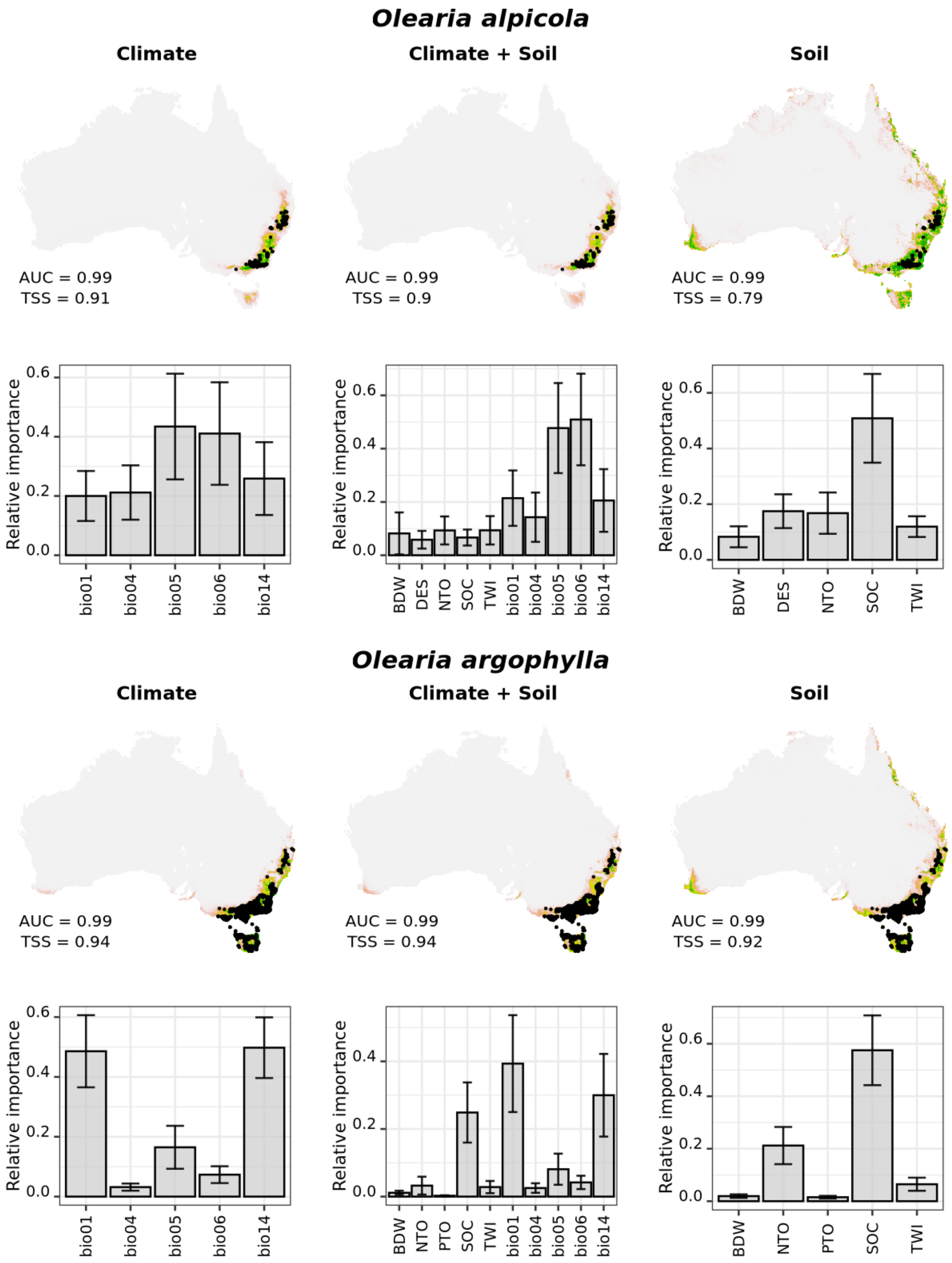

Olearia alpicola
Climate
Climate + Soil
Soil
AUC = 0.99
TSS = 0.91
AUC = 0.99
TSS = 0.9
AUC = 0.99
TSS = 0.79
Relative importance
0.0
0.2
0.4
0.6
bio01
bio04
bio05
bio06
bio14
BDW
DES
NTO
SOC
TWI
Olearia argophylla
AUC = 0.99
TSS = 0.94
AUC = 0.99
TSS = 0.94
AUC = 0.99
TSS = 0.92
PTO

Olearia axillaris

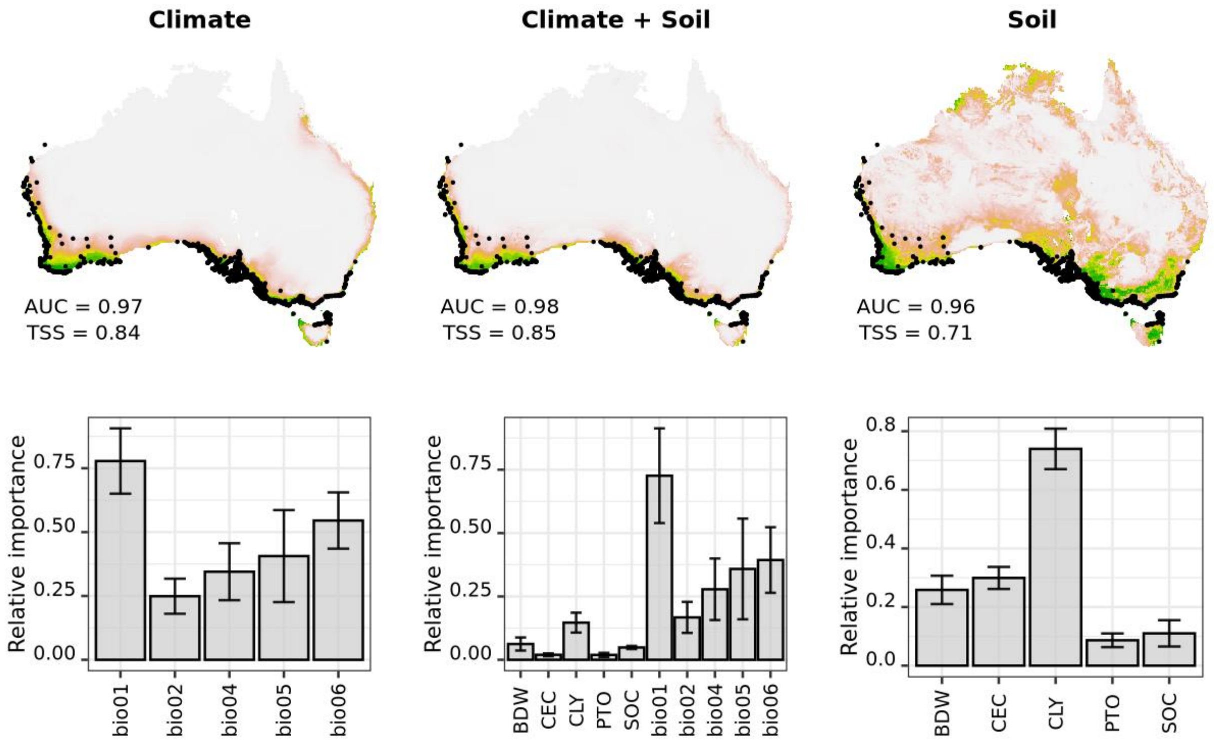

Olearia brachyphylla

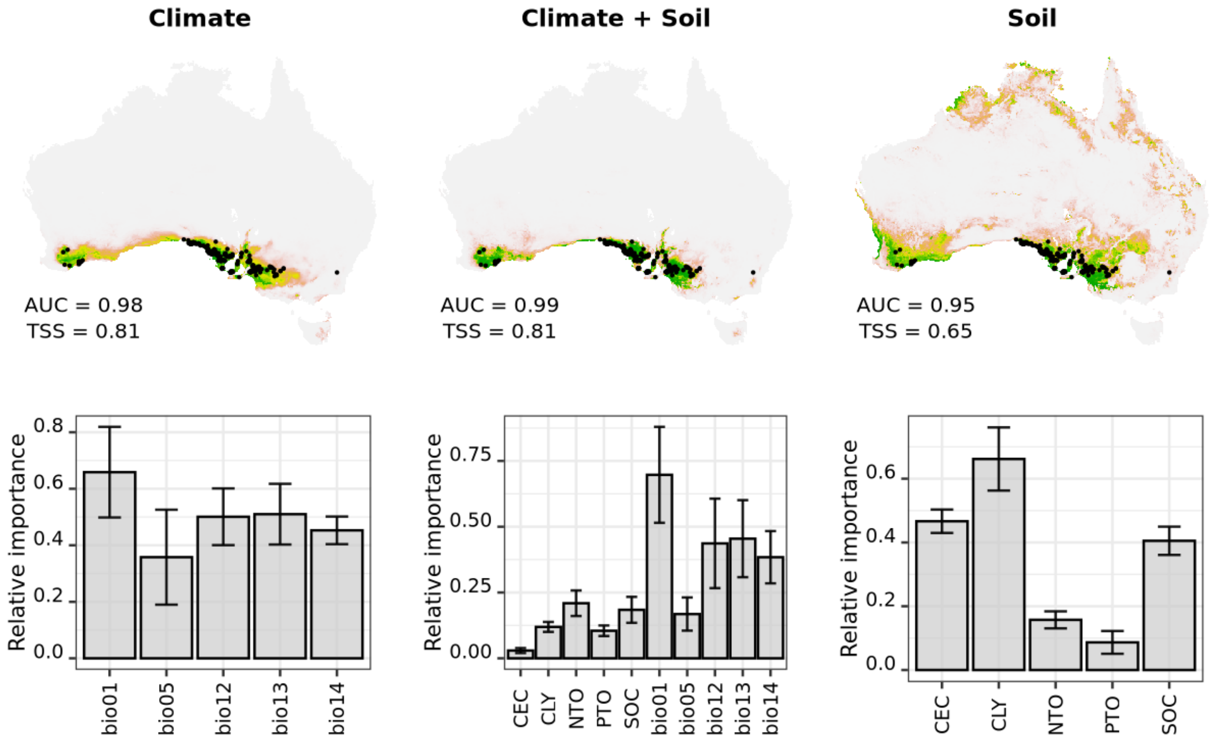

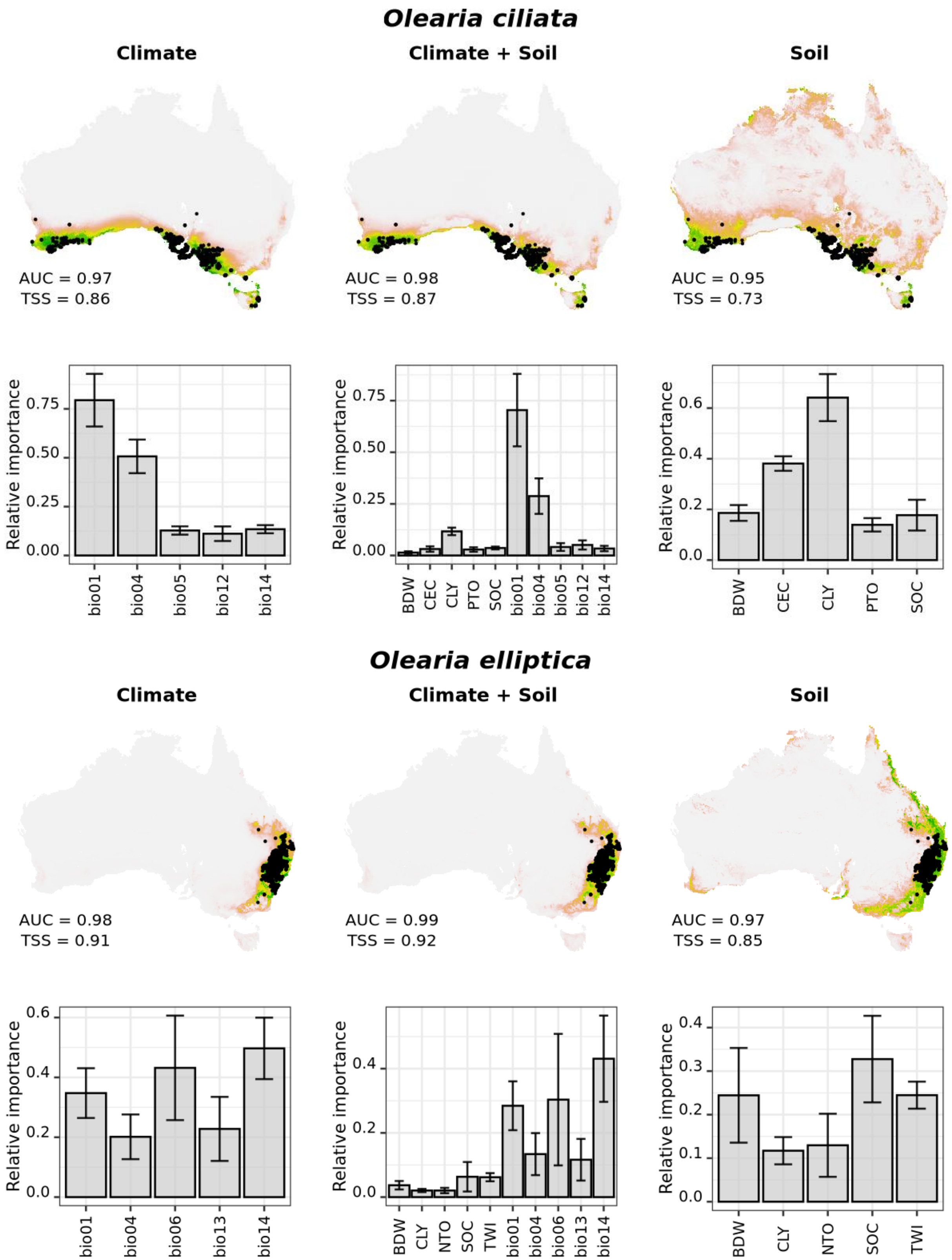
Olearia ciliata
Climate
Climate + Soil
Soil
AUC = 0.97
TSS = 0.86
AUC = 0.98
TSS = 0.87
AUC = 0.95
TSS = 0.73
Relative importance
0.00
0.25
0.50
0.75
bio01
bio04
bio05
bio12
bio14
BDW
CEC
CLY
PTO
SOC
0.0
0.2
0.4
0.6
Olearia elliptica
AUC = 0.98
TSS = 0.91
AUC = 0.99
TSS = 0.92
AUC = 0.97
TSS = 0.85
bio06
bio13
NTO
TWI
0.1
0.3

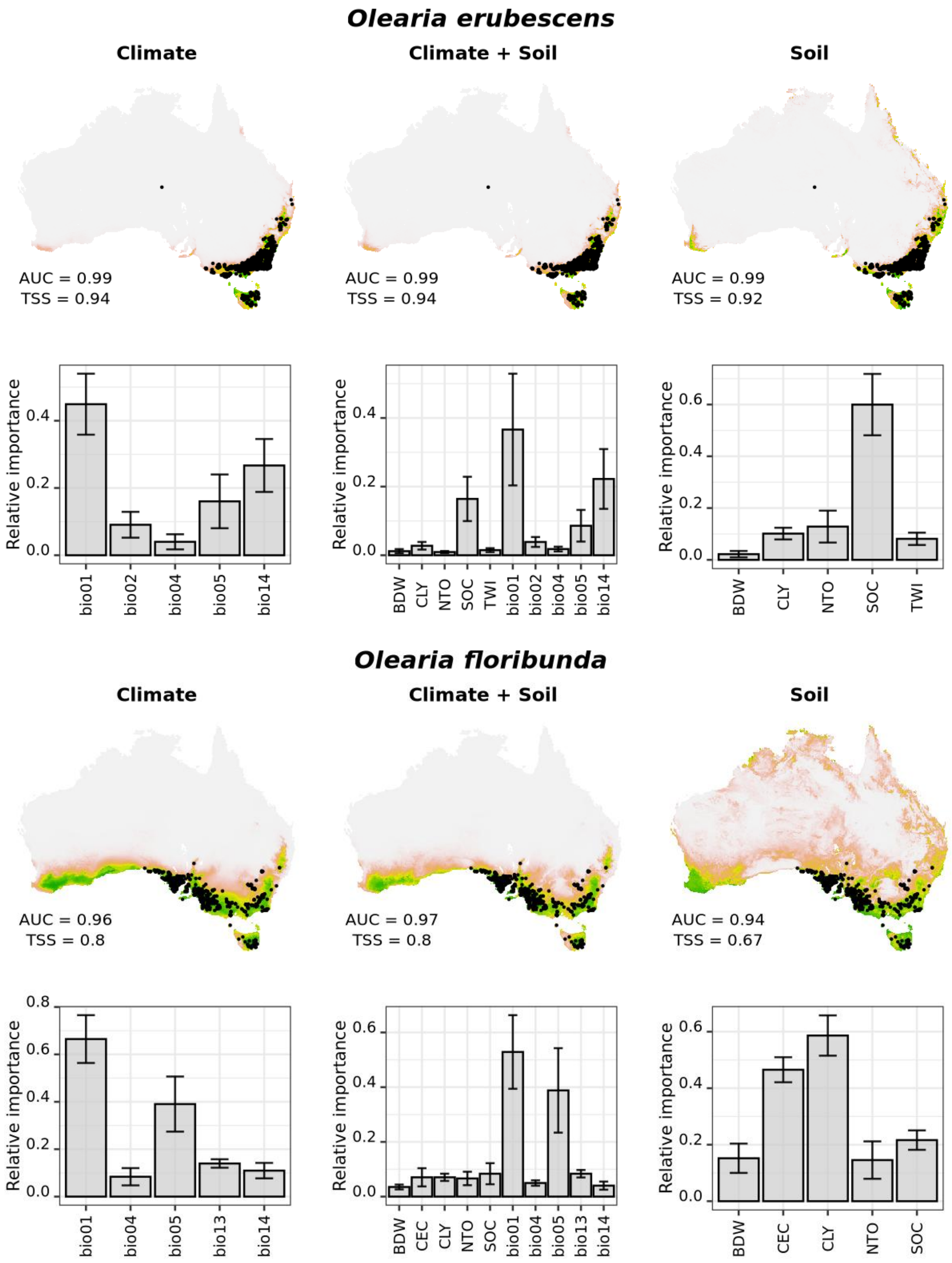

Olearia erubescens
Climate
Climate + Soil
Soil
AUC = 0.99
TSS = 0.94
AUC = 0.99
TSS = 0.94
AUC = 0.99
TSS = 0.92
Relative importance
bio01 bio02 bio04 bio05 bio14
BDW CLY NTO SOC TWI bio01 bio02 bio04 bio05 bio14
BDW CLY NTO SOC TWI
Olearia floribunda
Climate
Climate + Soil
Soil
AUC = 0.96
TSS = 0.8
AUC = 0.97
TSS = 0.8
AUC = 0.94
TSS = 0.67
Relative importance
bio01 bio04 bio05 bio13 bio14
BDW CEC CLY NTO SOC bio01 bio04 bio05 bio13 bio14
BDW CEC CLY NTO SOC

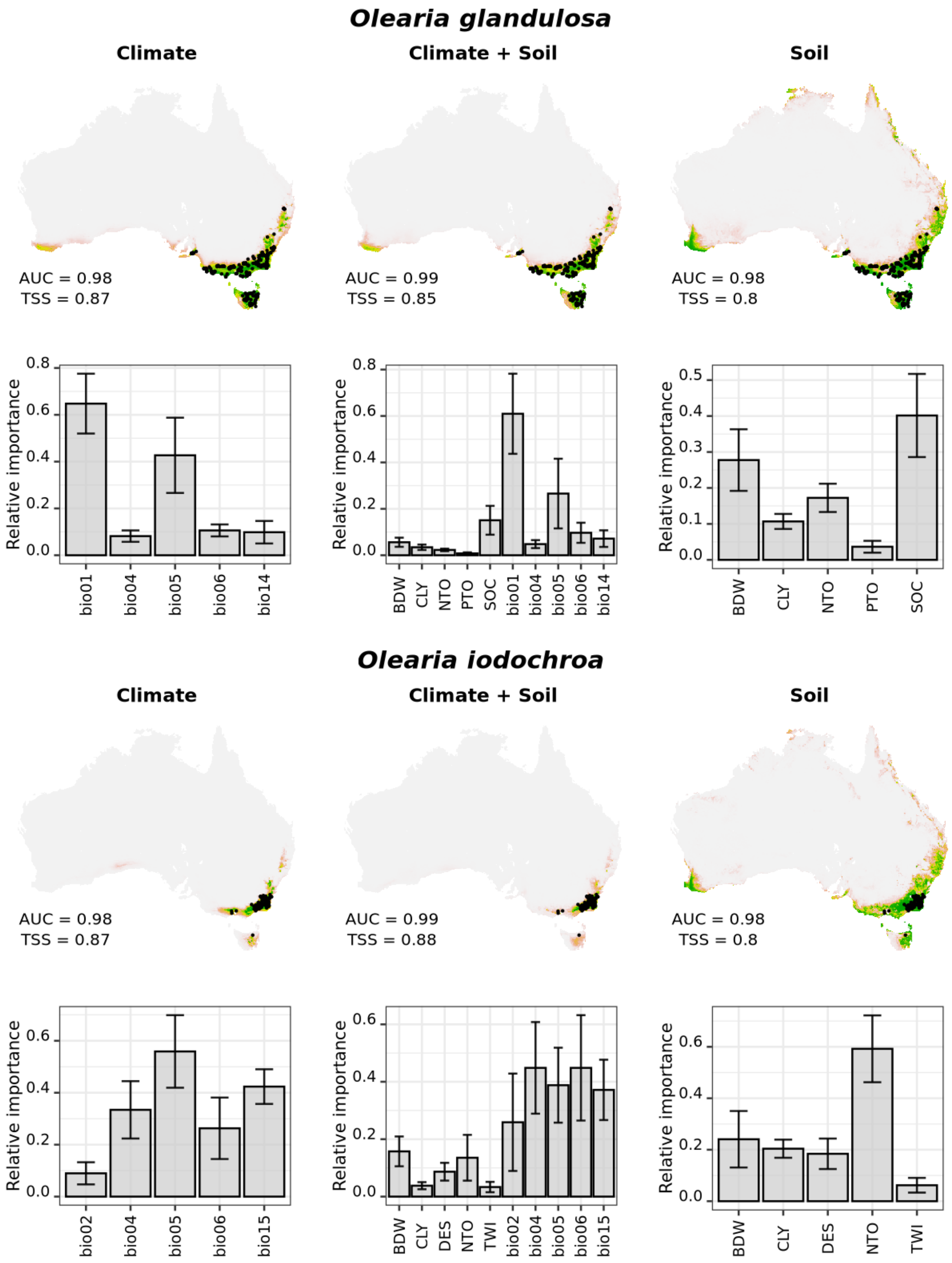
Olearia glandulosa
Climate
Climate + Soil
Soil
AUC = 0.98
TSS = 0.87
AUC = 0.99
TSS = 0.85
AUC = 0.98
TSS = 0.8
Relative importance
0.0
0.2
0.4
0.6
0.8
bio01
bio04
bio05
bio06
bio14
BDW
CLY
NTO
PTO
SOC
0.1
0.3
0.5
Olearia iodochroa
Climate
Climate + Soil
Soil
AUC = 0.98
TSS = 0.87
AUC = 0.99
TSS = 0.88
AUC = 0.98
TSS = 0.8
bio02
bio15
DES
TWI

Olearia lanuginosa

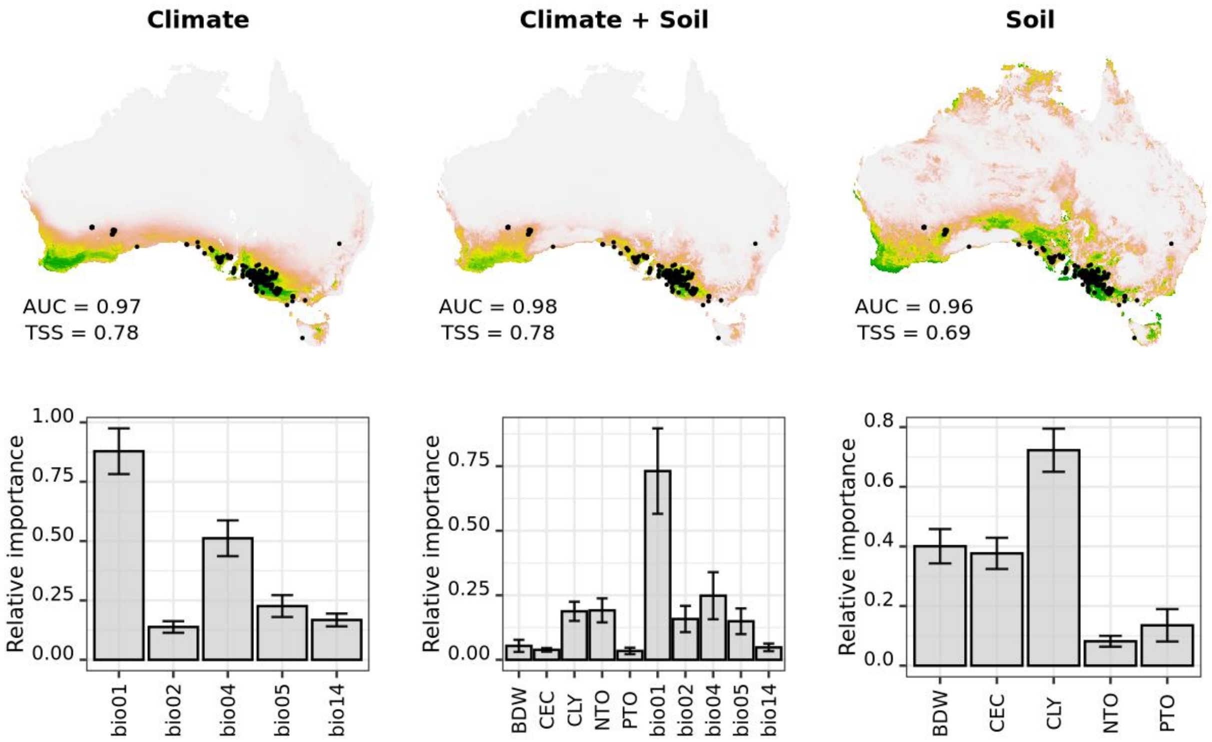

Olearia lepidophylla

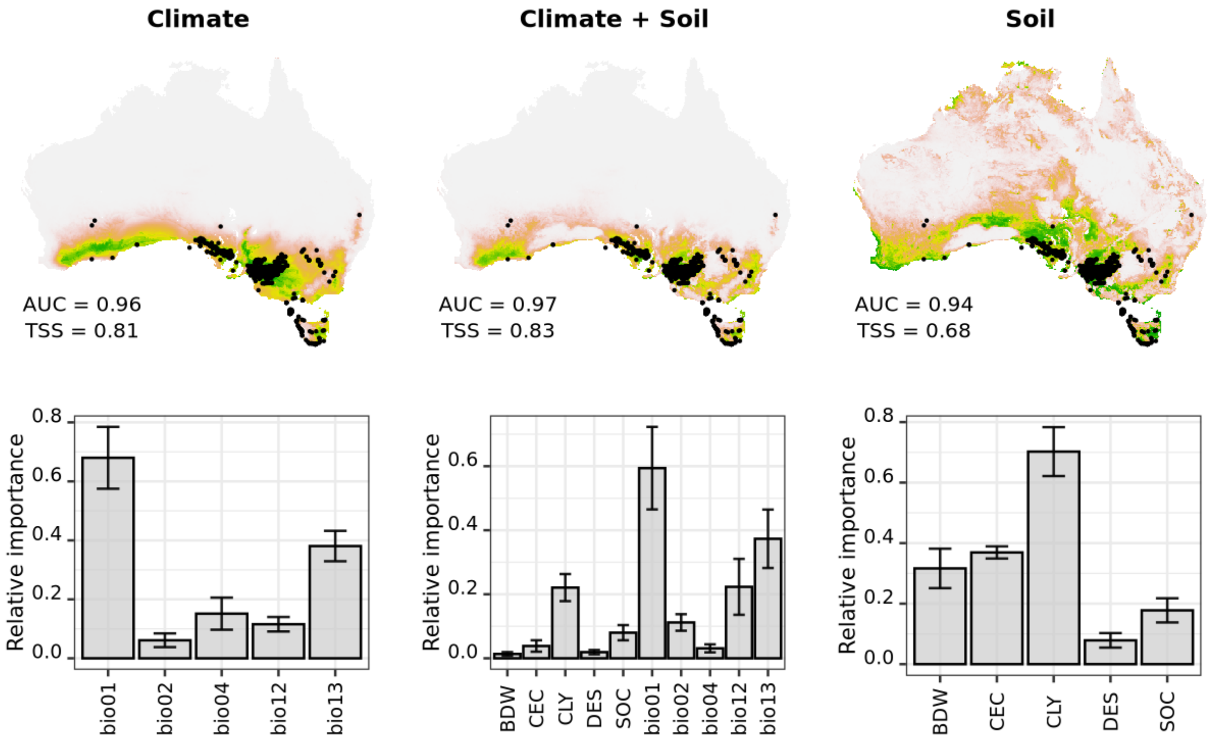

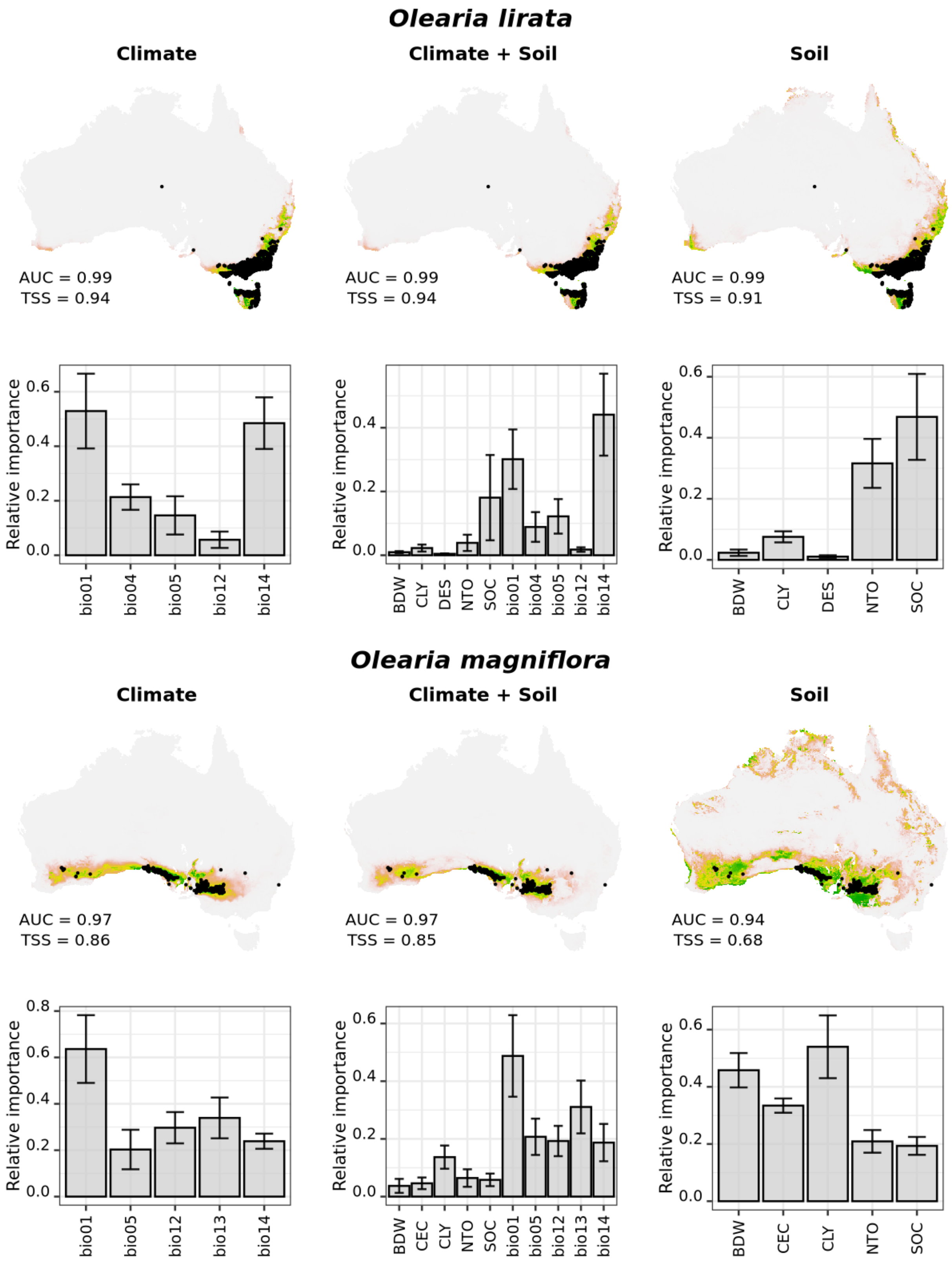

Olearia lirata
Climate
Climate + Soil
Soil
AUC = 0.99
TSS = 0.94
AUC = 0.99
TSS = 0.94
AUC = 0.99
TSS = 0.91
Relative importance
bio01
bio04
bio05
bio12
bio14
BDW
CLY
DES
NTO
SOC
Olearia magniflora
Climate
Climate + Soil
Soil
AUC = 0.97
TSS = 0.86
AUC = 0.97
TSS = 0.85
AUC = 0.94
TSS = 0.68
Relative importance
bio01
bio05
bio12
bio13
bio14
BDW
CEC
CLY
NTO
SOC

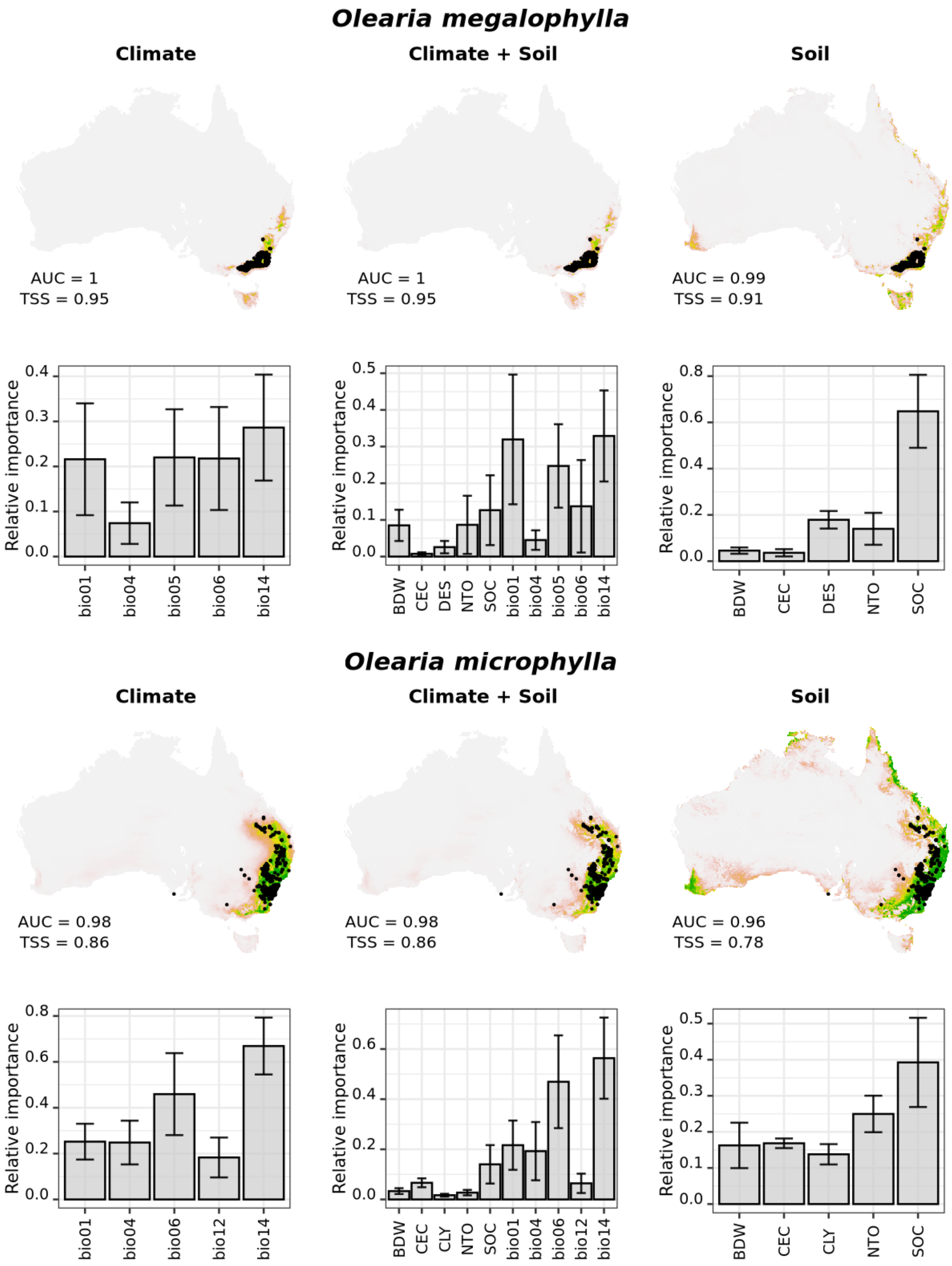
Olearia megalophylla
Climate
Climate + Soil
Soil
AUC = 1
TSS = 0.95
AUC = 1
TSS = 0.95
AUC = 0.99
TSS = 0.91
Relative importance
bio01
bio04
bio05
bio06
bio14
BDW
CEC
DES
NTO
SOC
bio01
bio04
bio05
bio06
bio14
BDW
CEC
DES
NTO
SOC
Olearia microphylla
Climate
Climate + Soil
Soil
AUC = 0.98
TSS = 0.86
AUC = 0.98
TSS = 0.86
AUC = 0.96
TSS = 0.78
Relative importance
bio01
bio04
bio06
bio12
bio14
BDW
CEC
CLY
NTO
SOC
bio01
bio04
bio06
bio12
bio14
BDW
CEC
CLY
NTO
SOC

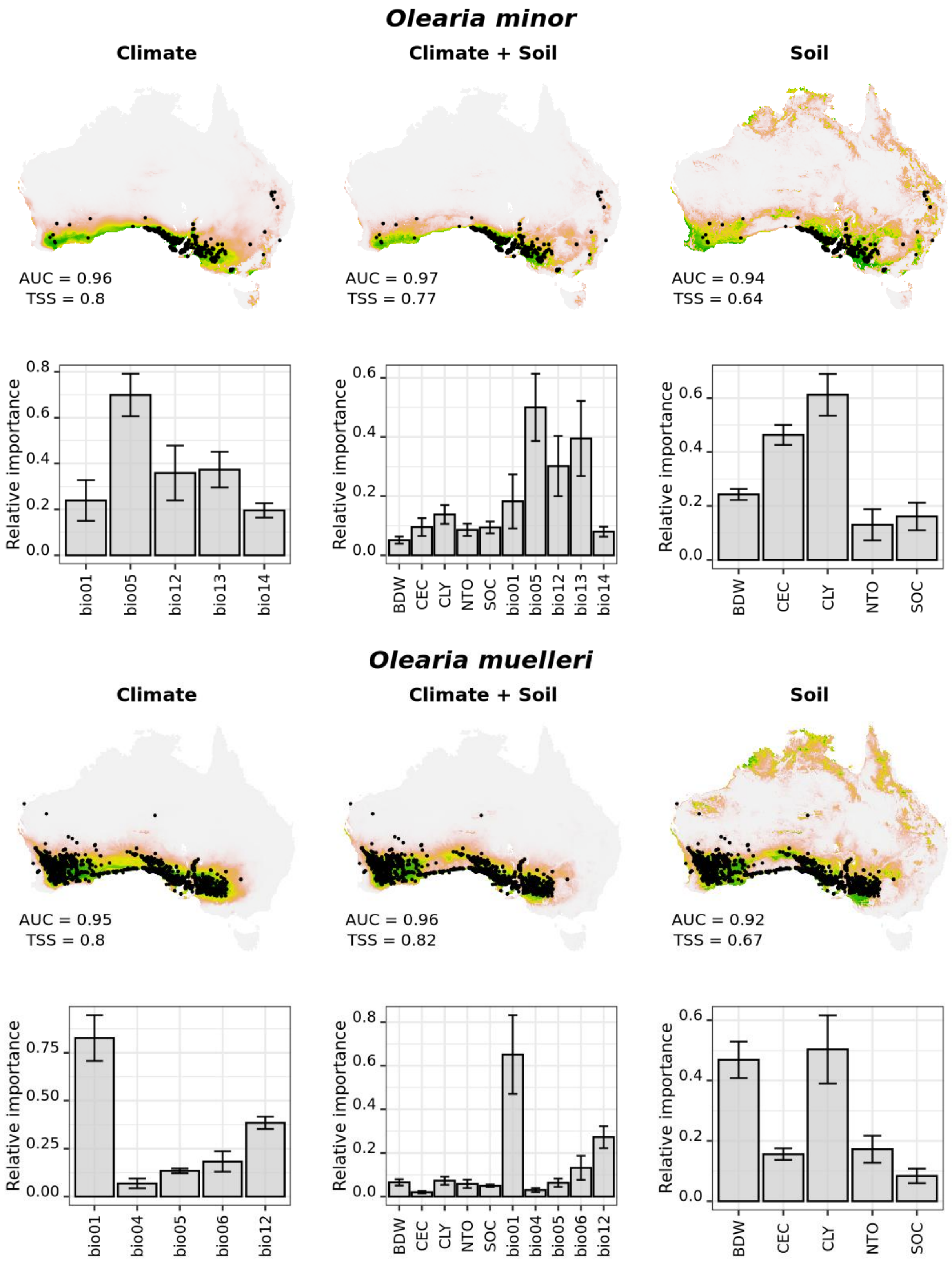
Olearia minor
Climate
Climate + Soil
Soil
AUC = 0.96
TSS = 0.8
AUC = 0.97
TSS = 0.77
AUC = 0.94
TSS = 0.64
Relative importance
bio01
bio05
bio12
bio13
bio14
BDW
CEC
CLY
NTO
SOC
Olearia muelleri
Climate
Climate + Soil
Soil
AUC = 0.95
TSS = 0.8
AUC = 0.96
TSS = 0.82
AUC = 0.92
TSS = 0.67
Relative importance
bio01
bio04
bio05
bio06
bio12
BDW
CEC
CLY
NTO
SOC

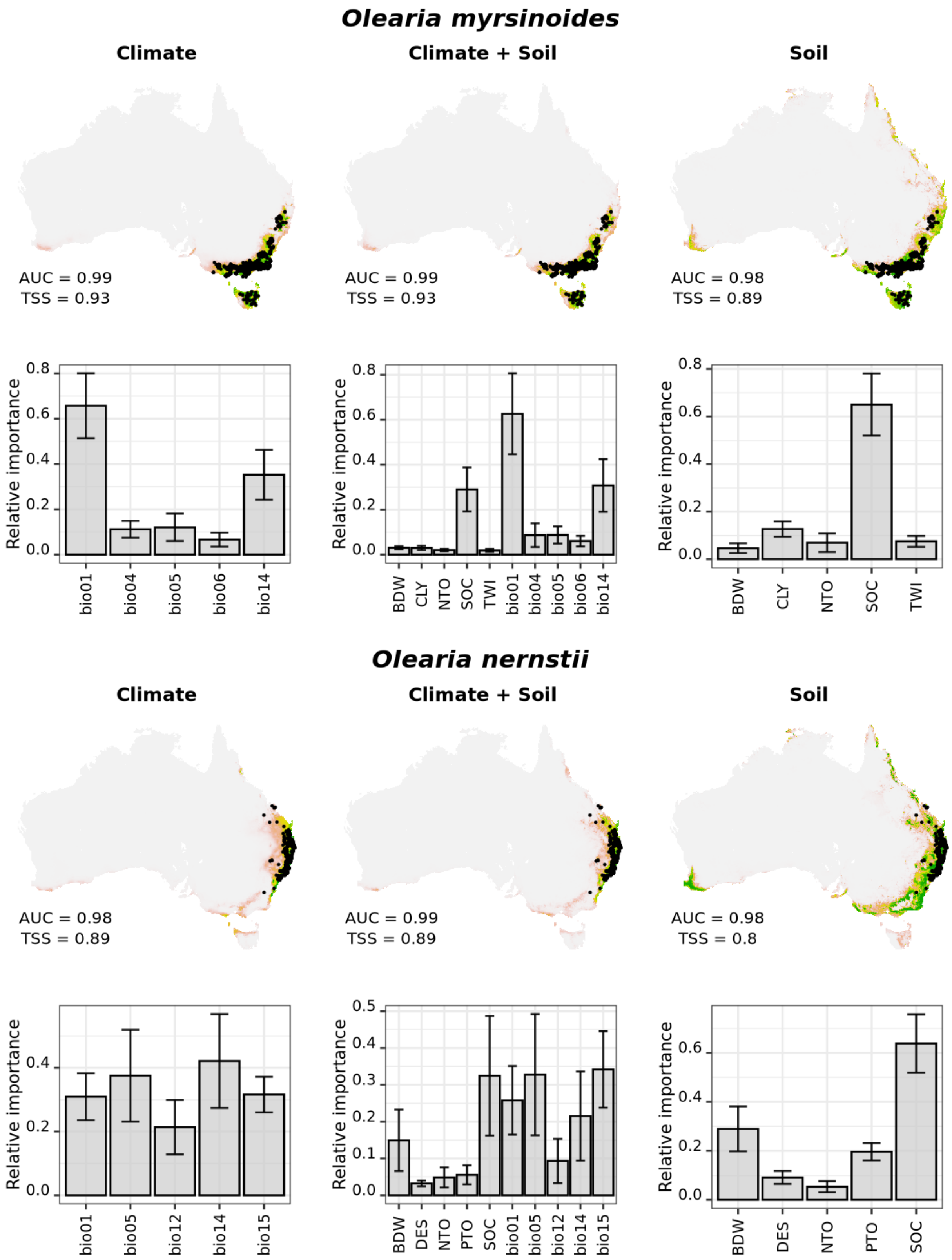

Olearia myrsinoides
Climate
Climate + Soil
Soil
AUC = 0.99
TSS = 0.93
AUC = 0.99
TSS = 0.93
AUC = 0.98
TSS = 0.89
Relative importance
bio01 bio04 bio05 bio06 bio14
BDW CLY NTO SOC TWI bio01 bio04 bio05 bio06 bio14
BDW CLY NTO SOC TWI
Olearia nernstii
Climate
Climate + Soil
Soil
AUC = 0.98
TSS = 0.89
AUC = 0.99
TSS = 0.89
AUC = 0.98
TSS = 0.8
Relative importance
bio01 bio05 bio12 bio14 bio15
BDW DES NTO PTO SOC bio01 bio05 bio12 bio14 bio15
BDW DES NTO PTO SOC

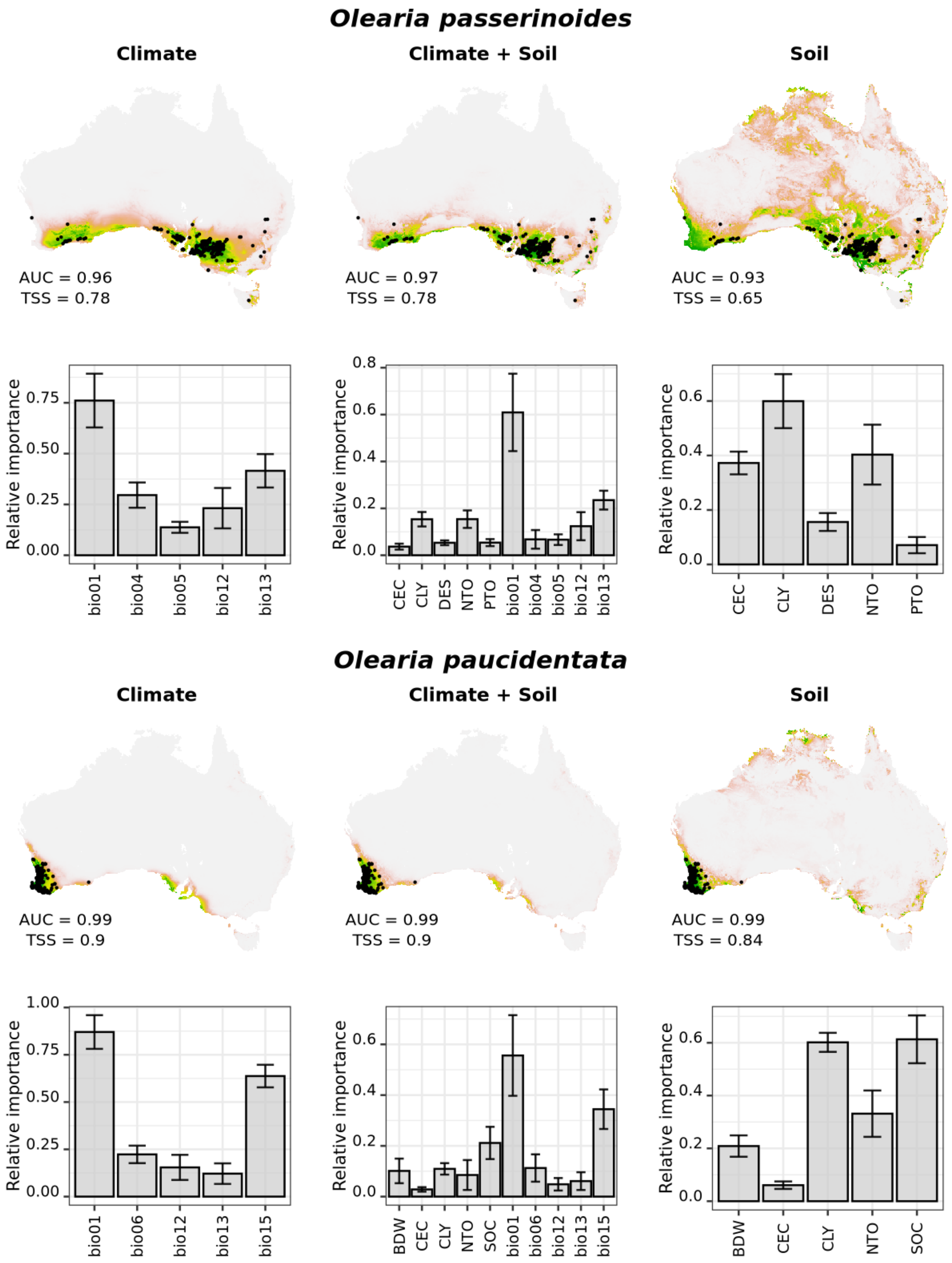

Olearia passerinoides
Climate
Climate + Soil
Soil
AUC = 0.96
TSS = 0.78
AUC = 0.97
TSS = 0.78
AUC = 0.93
TSS = 0.65
Relative importance
bio01
bio04
bio05
bio12
bio13
CEC
CLY
DES
NTO
PTO
Olearia paucidentata
Climate
Climate + Soil
Soil
AUC = 0.99
TSS = 0.9
AUC = 0.99
TSS = 0.9
AUC = 0.99
TSS = 0.84
Relative importance
bio01
bio06
bio12
bio13
bio15
BDW
CEC
CLY
NTO
SOC

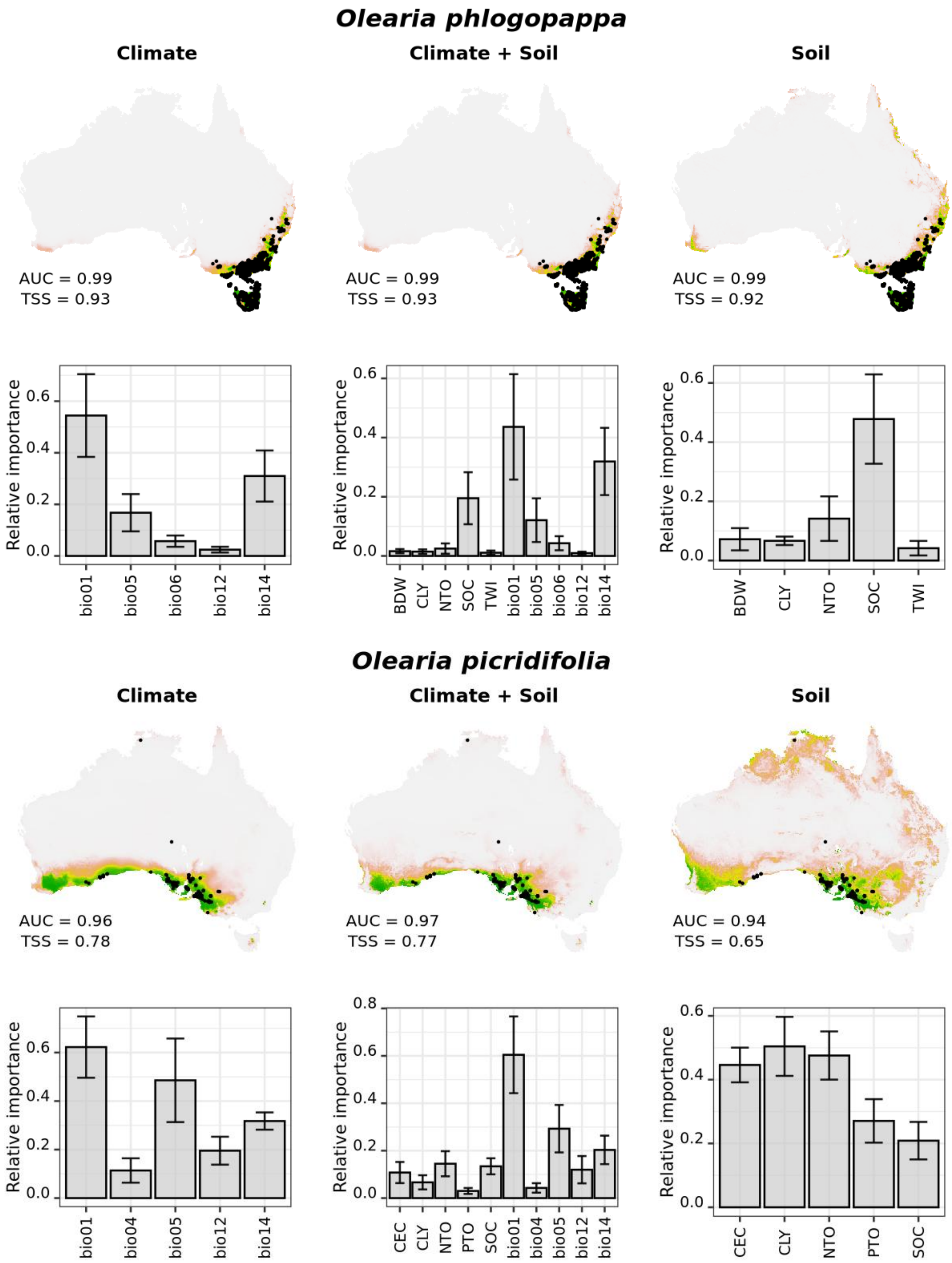
Olearia phlogopappa
Climate
Climate + Soil
Soil
AUC = 0.99
TSS = 0.93
AUC = 0.99
TSS = 0.93
AUC = 0.99
TSS = 0.92
Relative importance
bio01
bio05
bio06
bio12
bio14
BDW
CLY
NTO
SOC
TWI
Olearia picridifolia
Climate
Climate + Soil
Soil
AUC = 0.96
TSS = 0.78
AUC = 0.97
TSS = 0.77
AUC = 0.94
TSS = 0.65
Relative importance
bio01
bio04
bio05
bio12
bio14
CEC
CLY
NTO
PTO
SOC

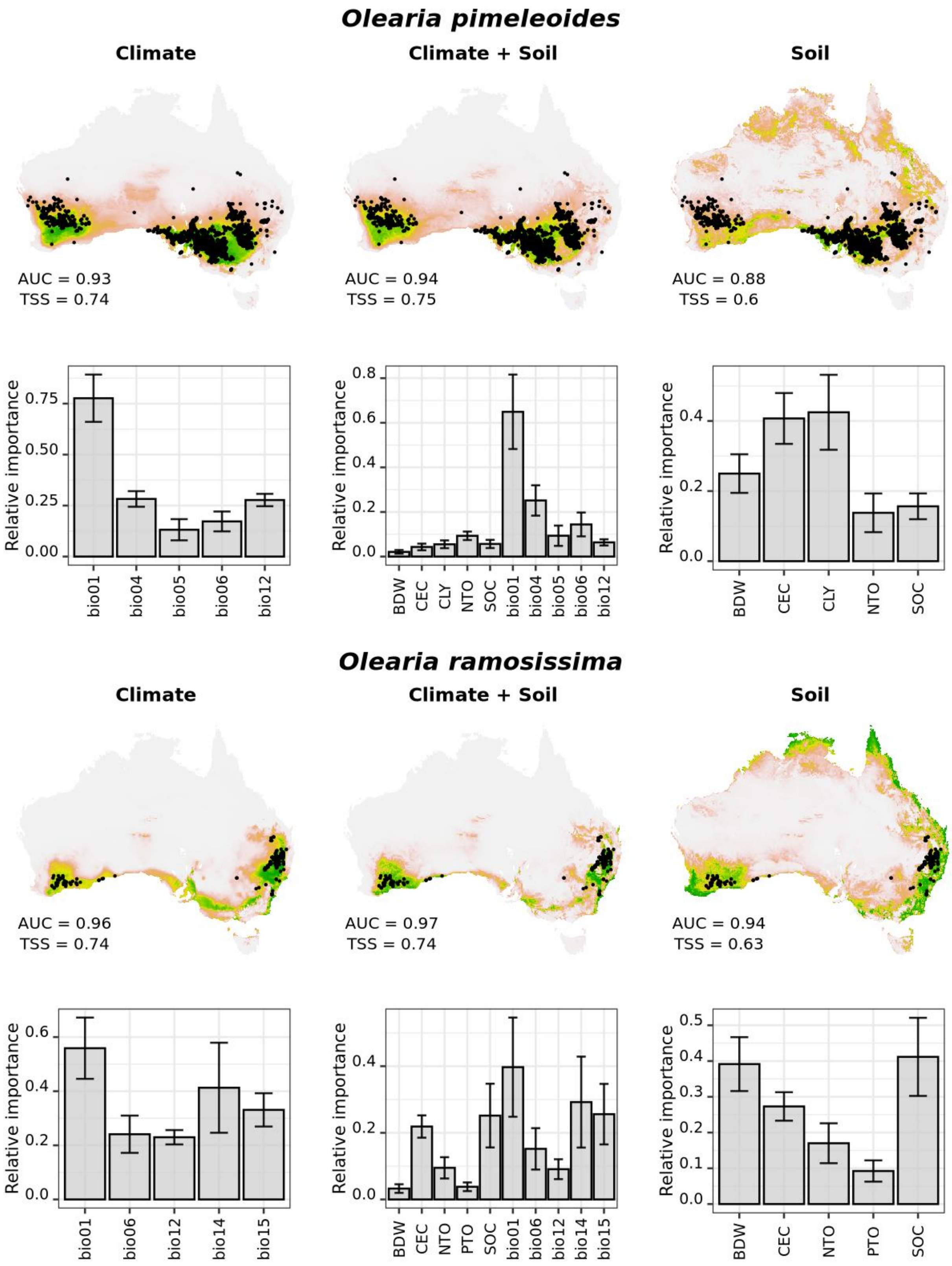
Olearia pimeleoides
Climate
Climate + Soil
Soil
AUC = 0.93
TSS = 0.74
AUC = 0.94
TSS = 0.75
AUC = 0.88
TSS = 0.6
Relative importance
bio01
bio04
bio05
bio06
bio12
BDW
CEC
CLY
NTO
SOC
Olearia ramosissima
Climate
Climate + Soil
Soil
AUC = 0.96
TSS = 0.74
AUC = 0.97
TSS = 0.74
AUC = 0.94
TSS = 0.63
Relative importance
bio01
bio06
bio12
bio14
bio15
BDW
CEC
NTO
PTO
SOC

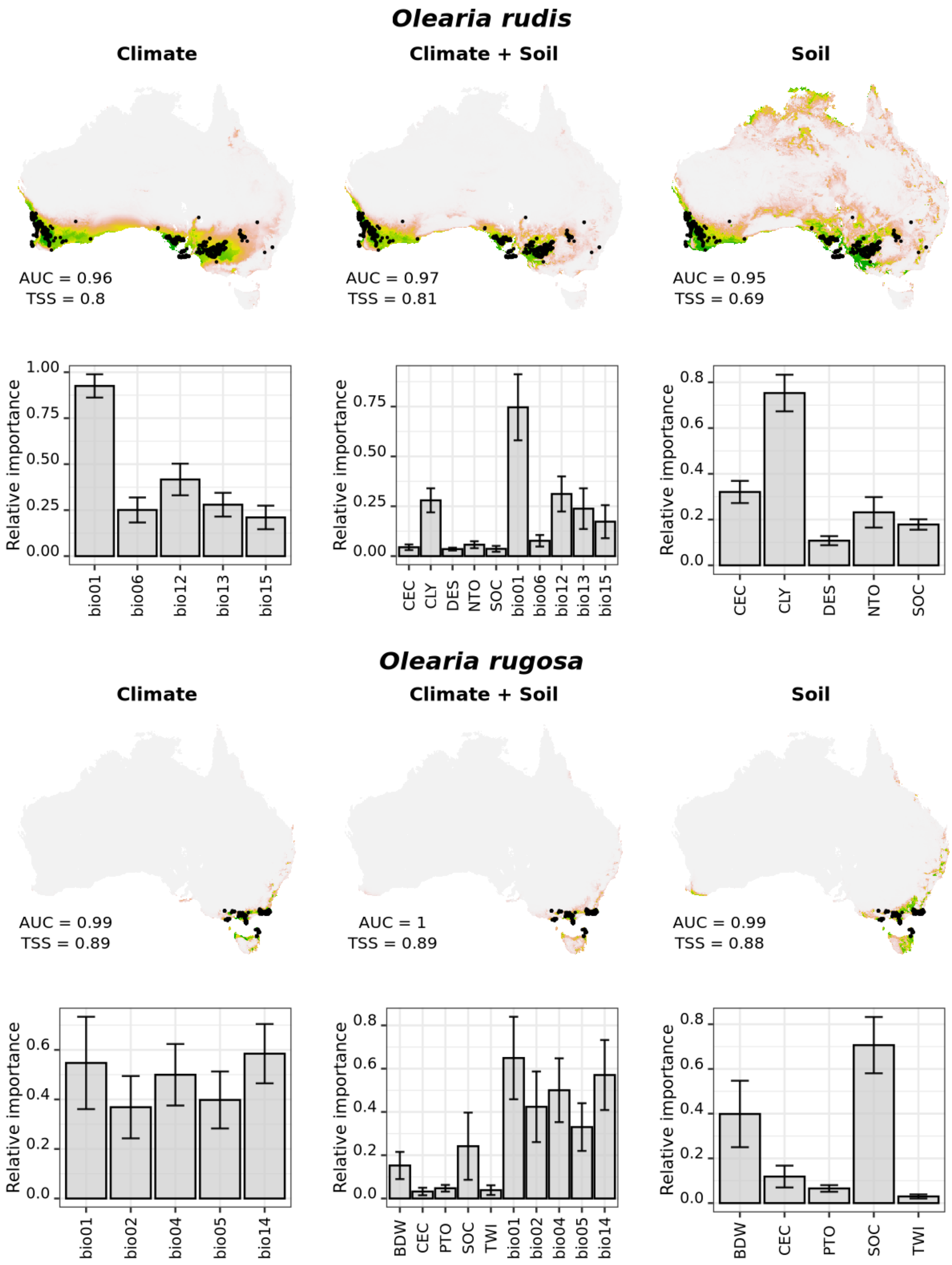

Olearia rudis
Climate
Climate + Soil
Soil
AUC = 0.96
TSS = 0.8
AUC = 0.97
TSS = 0.81
AUC = 0.95
TSS = 0.69
Relative importance
1.00
0.75
0.50
0.25
0.00
bio01
bio06
bio12
bio13
bio15
CEC
CLY
DES
NTO
SOC
0.8
0.6
0.4
0.2
0.0
Olearia rugosa
Climate
Climate + Soil
Soil
AUC = 0.99
TSS = 0.89
AUC = 1
TSS = 0.89
AUC = 0.99
TSS = 0.88
Relative importance
bio01
bio02
bio04
bio05
bio14
BDW
CEC
PTO
SOC
TWI

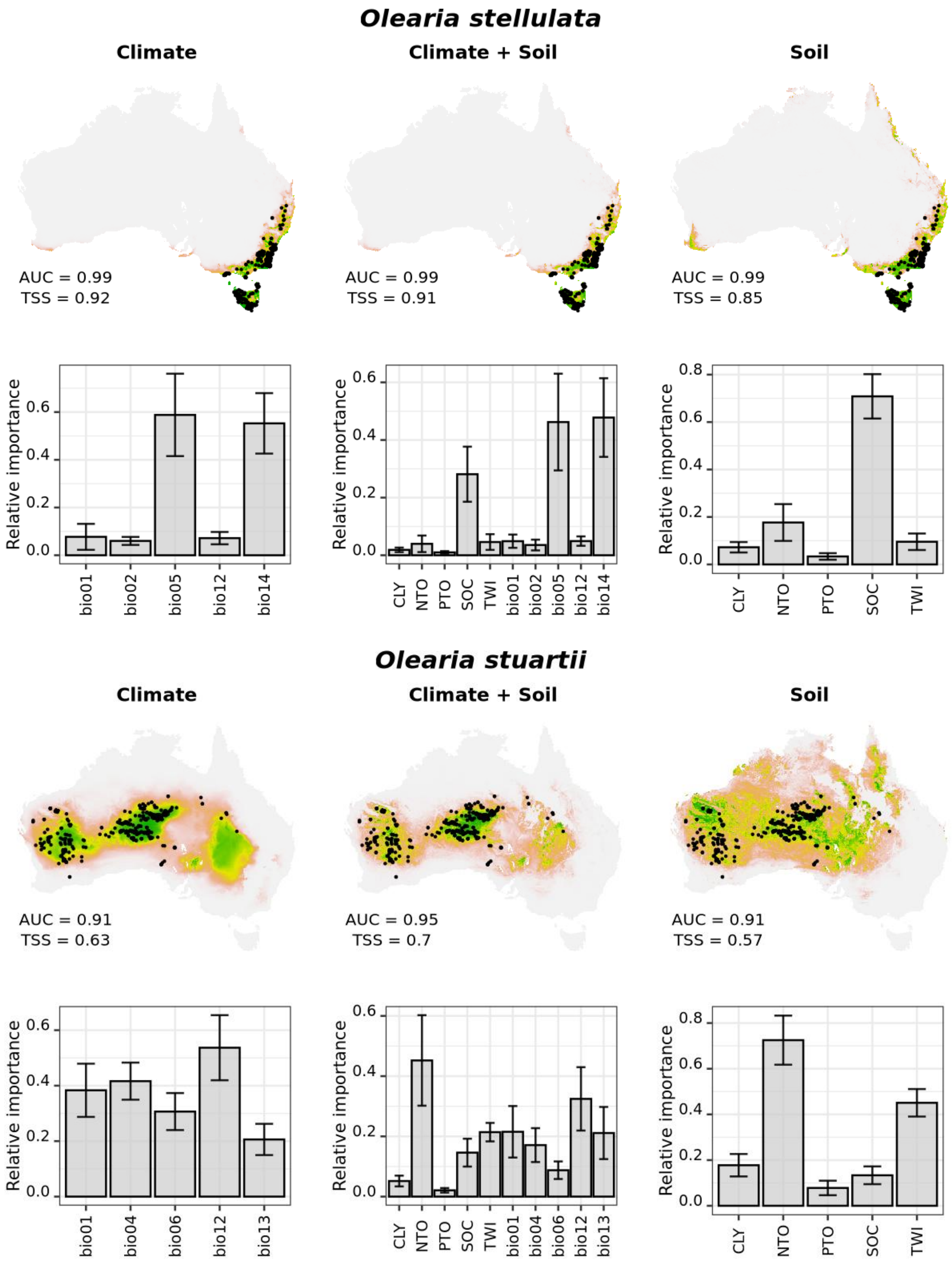

Olearia stellulata
Climate
Climate + Soil
Soil
AUC = 0.99
TSS = 0.92
AUC = 0.99
TSS = 0.91
AUC = 0.99
TSS = 0.85
Relative importance
bio01
bio02
bio05
bio12
bio14
CLY
NTO
PTO
SOC
TWI
bio01
bio02
bio05
bio12
bio14
Olearia stuartii
Climate
Climate + Soil
Soil
AUC = 0.91
TSS = 0.63
AUC = 0.95
TSS = 0.7
AUC = 0.91
TSS = 0.57
Relative importance
bio01
bio04
bio06
bio12
bio13
CLY
NTO
PTO
SOC
TWI
bio01
bio04
bio06
bio12
bio13

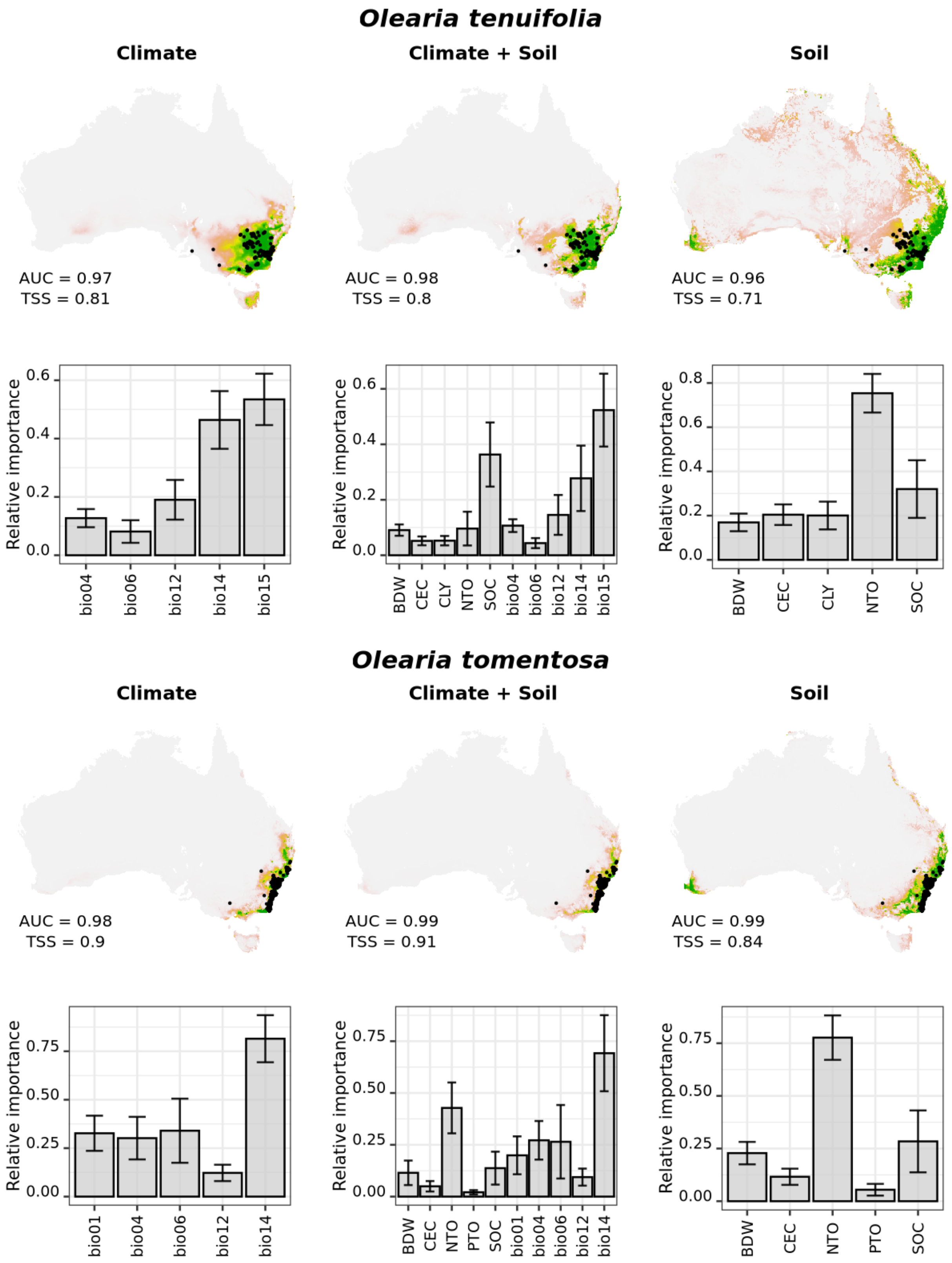
Olearia tenuifolia
Climate
Climate + Soil
Soil
AUC = 0.97
TSS = 0.81
AUC = 0.98
TSS = 0.8
AUC = 0.96
TSS = 0.71
Relative importance
0.0
0.2
0.4
0.6
0.8
bio04
bio06
bio12
bio14
bio15
BDW
CEC
CLY
NTO
SOC
Olearia tomentosa
Climate
Climate + Soil
Soil
AUC = 0.98
TSS = 0.9
AUC = 0.99
TSS = 0.91
AUC = 0.99
TSS = 0.84
Relative importance
0.00
0.25
0.50
0.75
bio01
bio04
bio06
bio12
bio14
BDW
CEC
NTO
PTO
SOC

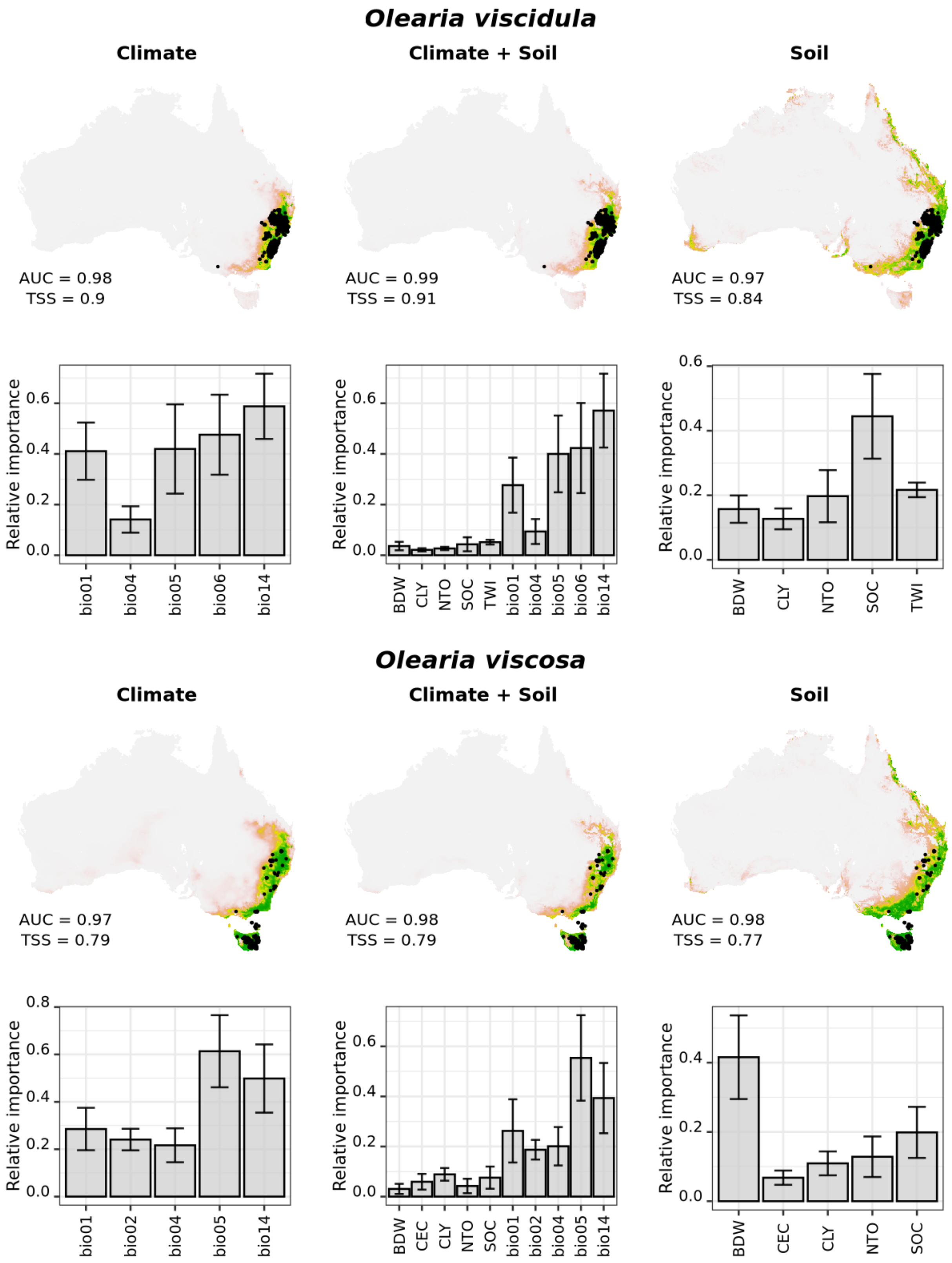
Olearia viscidula
Climate
Climate + Soil
Soil
AUC = 0.98
TSS = 0.9
AUC = 0.99
TSS = 0.91
AUC = 0.97
TSS = 0.84
Relative importance
bio01
bio04
bio05
bio06
bio14
BDW
CLY
NTO
SOC
TWI
Olearia viscosa
Climate
Climate + Soil
Soil
AUC = 0.97
TSS = 0.79
AUC = 0.98
TSS = 0.79
AUC = 0.98
TSS = 0.77
Relative importance
bio01
bio02
bio04
bio05
bio14
BDW
CEC
CLY
NTO
SOC

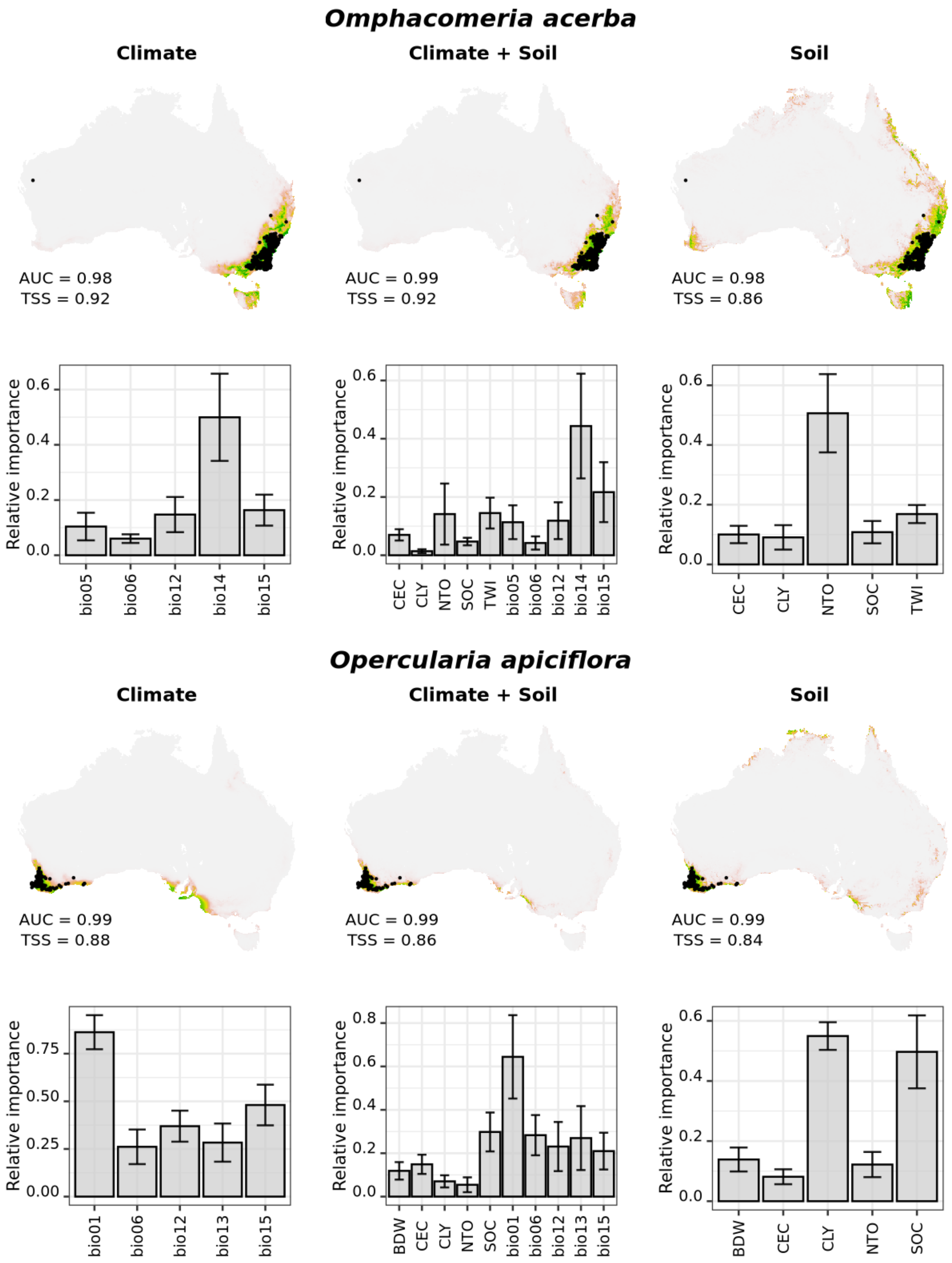

Omphacomeria acerba
Climate
Climate + Soil
Soil
AUC = 0.98
TSS = 0.92
AUC = 0.99
TSS = 0.92
AUC = 0.98
TSS = 0.86
Relative importance
bio05 bio06 bio12 bio14 bio15
CEC CLY NTO SOC TWI bio05 bio06 bio12 bio14 bio15
CEC CLY NTO SOC TWI
Opercularia apiciflora
Climate
Climate + Soil
Soil
AUC = 0.99
TSS = 0.88
AUC = 0.99
TSS = 0.86
AUC = 0.99
TSS = 0.84
Relative importance
bio01 bio06 bio12 bio13 bio15
BDW CEC CLY NTO SOC bio01 bio06 bio12 bio13 bio15
BDW CEC CLY NTO SOC

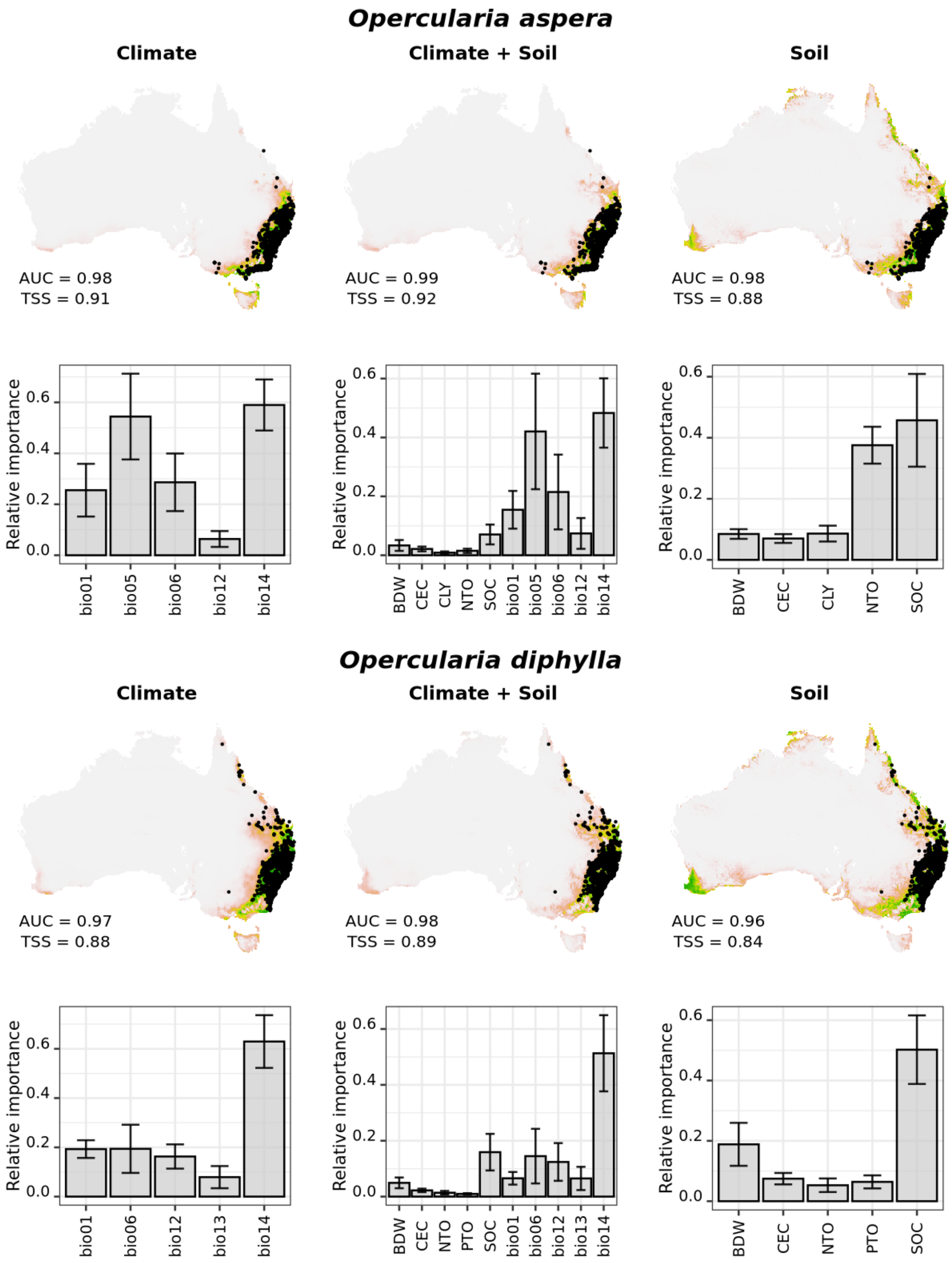

Opercularia aspera
Climate
Climate + Soil
Soil
AUC = 0.98
TSS = 0.91
AUC = 0.99
TSS = 0.92
AUC = 0.98
TSS = 0.88
Relative importance
0.0
0.2
0.4
0.6
bio01
bio05
bio06
bio12
bio14
BDW
CEC
CLY
NTO
SOC
bio01
bio05
bio06
bio12
bio14
BDW
CEC
CLY
NTO
SOC
Opercularia diphylla
Climate
Climate + Soil
Soil
AUC = 0.97
TSS = 0.88
AUC = 0.98
TSS = 0.89
AUC = 0.96
TSS = 0.84
Relative importance
0.0
0.2
0.4
0.6
bio01
bio06
bio12
bio13
bio14
BDW
CEC
NTO
PTO
SOC
bio01
bio06
bio12
bio13
bio14
BDW
CEC
NTO
PTO
SOC

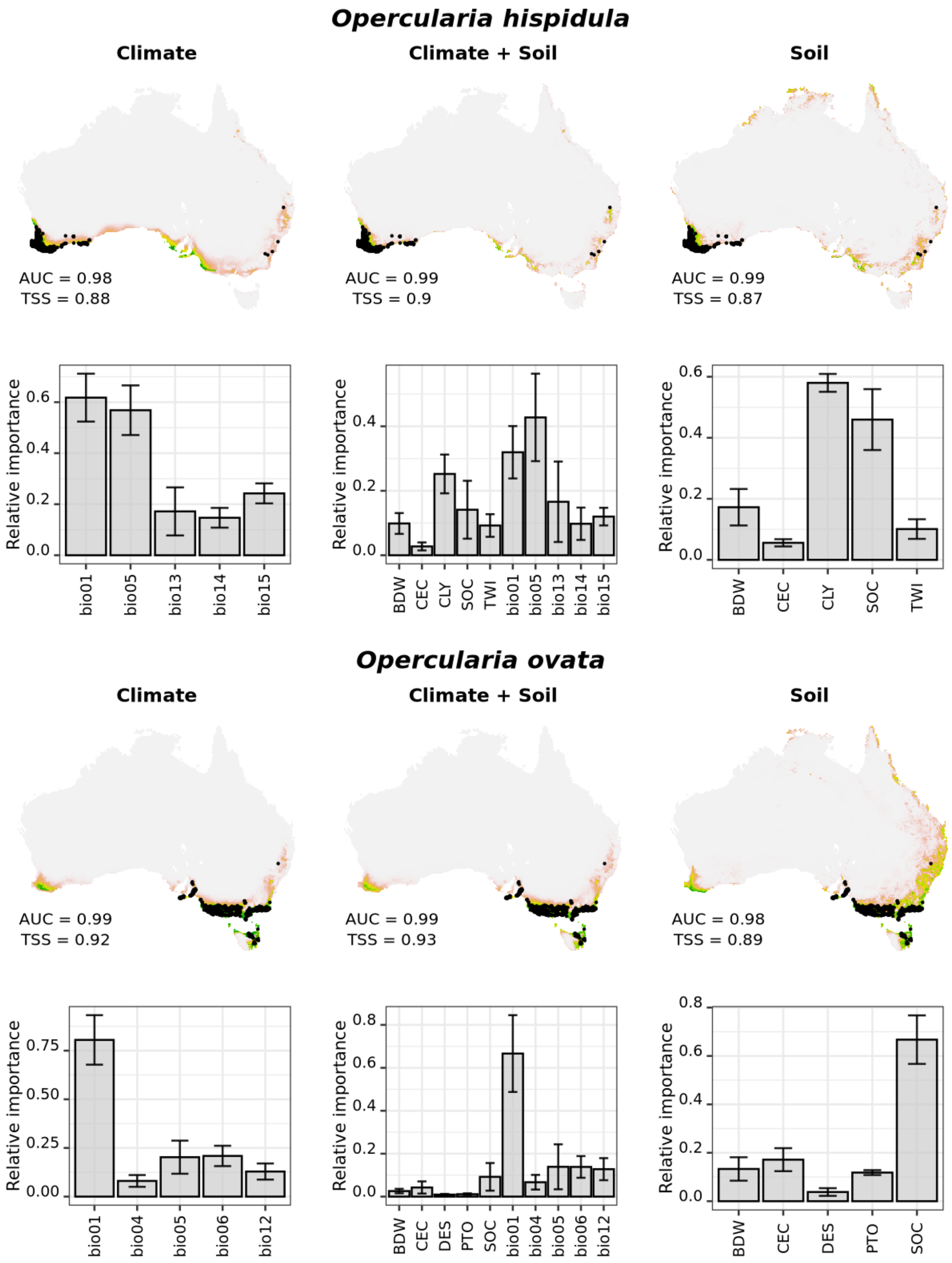
Opercularia hispidula
Climate
Climate + Soil
Soil
AUC = 0.98
TSS = 0.88
AUC = 0.99
TSS = 0.9
AUC = 0.99
TSS = 0.87
Relative importance
bio01
bio05
bio13
bio14
bio15
BDW
CEC
CLY
SOC
TWI
Opercularia ovata
AUC = 0.99
TSS = 0.92
AUC = 0.99
TSS = 0.93
AUC = 0.98
TSS = 0.89
bio04
bio06
bio12
DES
PTO

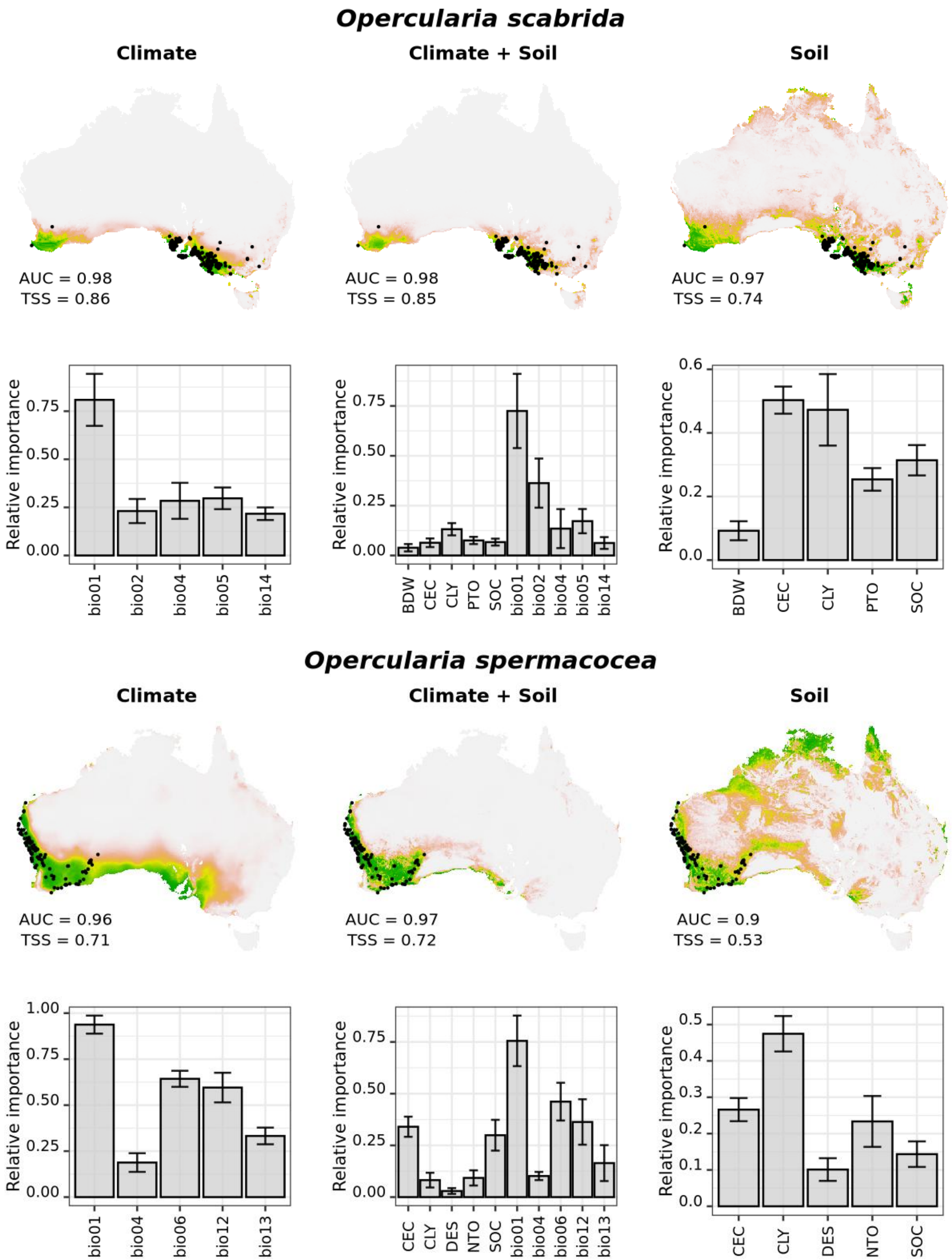

Opercularia scabrida
Climate
Climate + Soil
Soil
AUC = 0.98
TSS = 0.86
AUC = 0.98
TSS = 0.85
AUC = 0.97
TSS = 0.74
Relative importance
0.00
0.25
0.50
0.75
bio01
bio02
bio04
bio05
bio14
BDW
CEC
CLY
PTO
SOC
bio01
bio02
bio04
bio05
bio14
0.0
0.2
0.4
0.6
BDW
CEC
CLY
PTO
SOC
Opercularia spermacocea
Climate
Climate + Soil
Soil
AUC = 0.96
TSS = 0.71
AUC = 0.97
TSS = 0.72
AUC = 0.9
TSS = 0.53
Relative importance
0.00
0.25
0.50
0.75
1.00
bio01
bio04
bio06
bio12
bio13
CEC
CLY
DES
NTO
SOC
bio01
bio04
bio06
bio12
bio13
0.0
0.1
0.2
0.3
0.4
0.5
CEC
CLY
DES
NTO
SOC

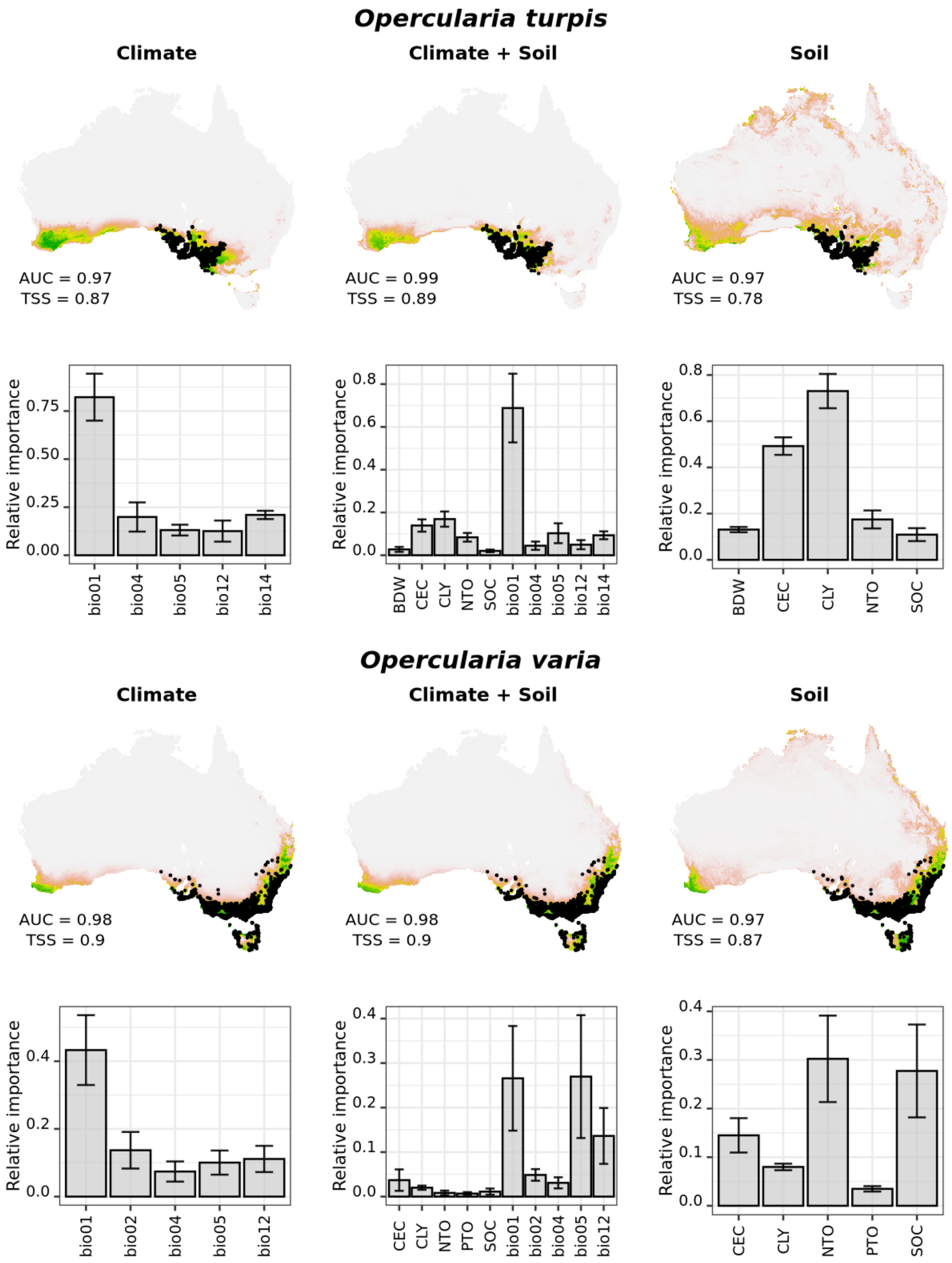

Opercularia turpis
Climate
Climate + Soil
Soil
AUC = 0.97
TSS = 0.87
AUC = 0.99
TSS = 0.89
AUC = 0.97
TSS = 0.78
Relative importance
0.00
0.25
0.50
0.75
bio01
bio04
bio05
bio12
bio14
0.0
0.2
0.4
0.6
0.8
BDW
CEC
CLY
NTO
SOC
bio01
bio04
bio05
bio12
bio14
BDW
CEC
CLY
NTO
SOC
Opercularia varia
Climate
Climate + Soil
Soil
AUC = 0.98
TSS = 0.9
AUC = 0.98
TSS = 0.9
AUC = 0.97
TSS = 0.87
Relative importance
0.0
0.2
0.4
bio01
bio02
bio04
bio05
bio12
0.0
0.1
0.2
0.3
0.4
CEC
CLY
NTO
PTO
SOC
bio01
bio02
bio04
bio05
bio12
CEC
CLY
NTO
PTO
SOC

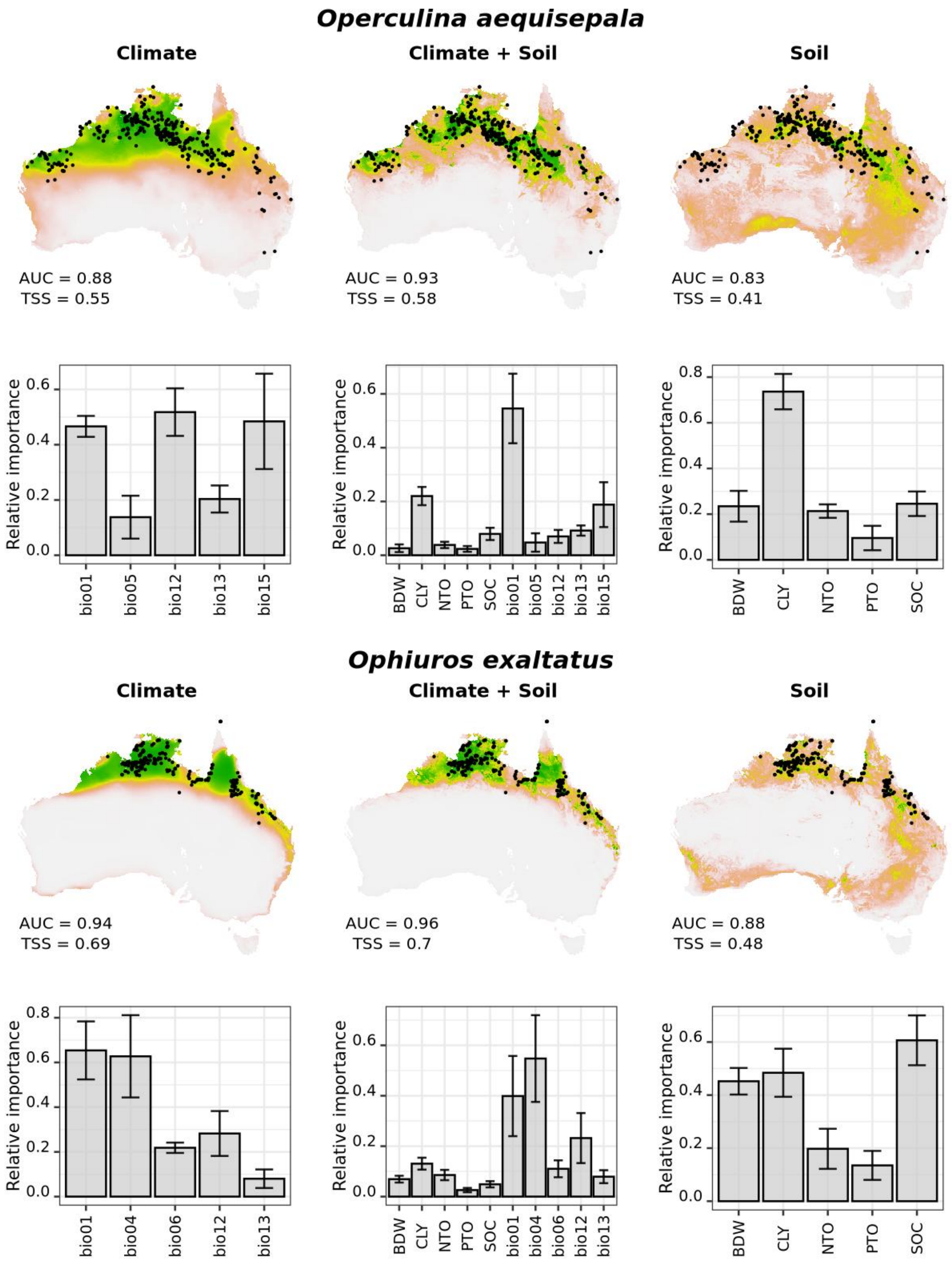

Operculina aequisepala
Climate
Climate + Soil
Soil
AUC = 0.88
TSS = 0.55
AUC = 0.93
TSS = 0.58
AUC = 0.83
TSS = 0.41
Relative importance
bio01
bio05
bio12
bio13
bio15
BDW
CLY
NTO
PTO
SOC
Ophiuros exaltatus
Climate
Climate + Soil
Soil
AUC = 0.94
TSS = 0.69
AUC = 0.96
TSS = 0.7
AUC = 0.88
TSS = 0.48
Relative importance
bio01
bio04
bio06
bio12
bio13
BDW
CLY
NTO
PTO
SOC

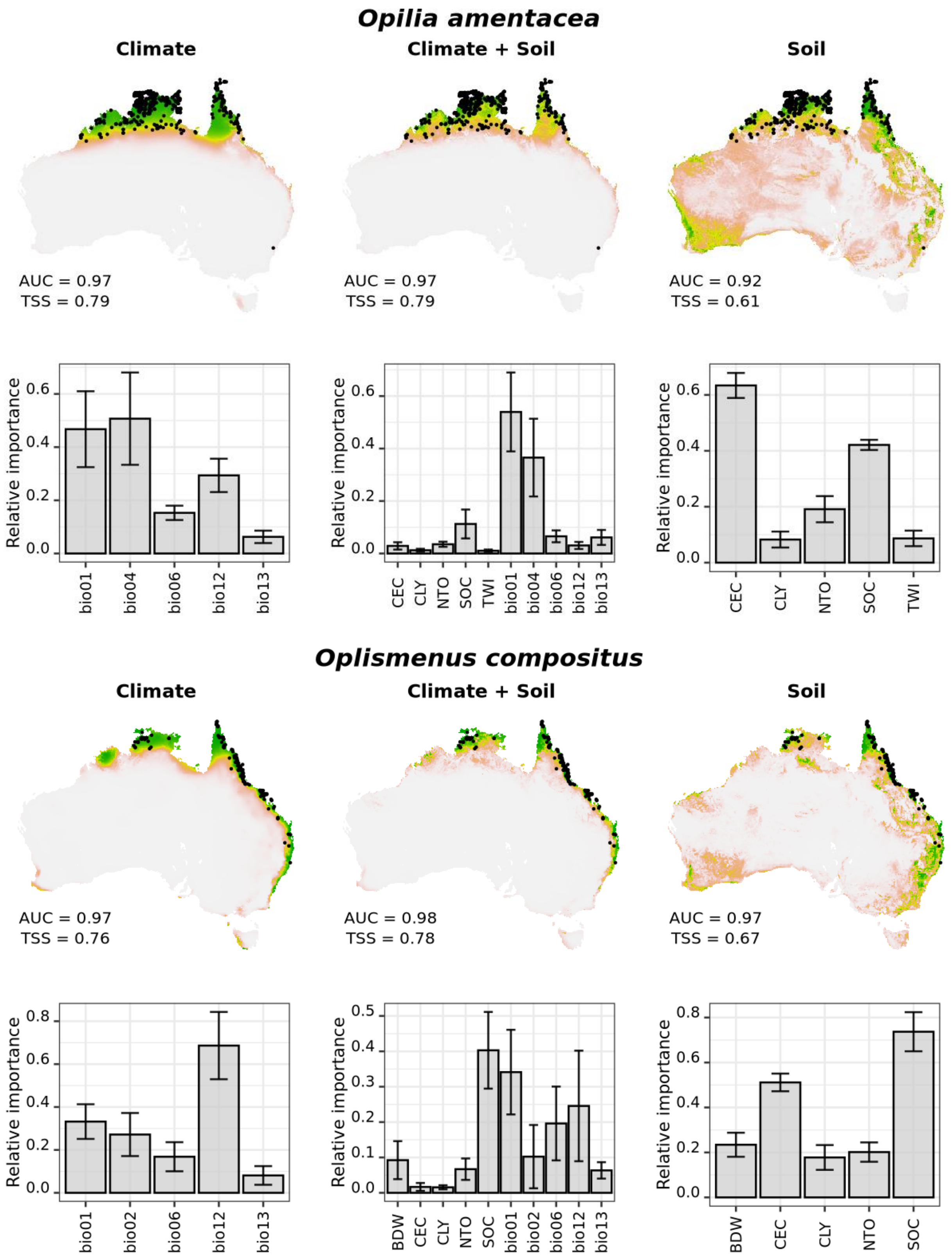
Opilia amentacea
Climate
Climate + Soil
Soil
AUC = 0.97
TSS = 0.79
AUC = 0.97
TSS = 0.79
AUC = 0.92
TSS = 0.61
Relative importance
bio01
bio04
bio06
bio12
bio13
CEC
CLY
NTO
SOC
TWI
Oplismenus compositus
AUC = 0.97
TSS = 0.76
AUC = 0.98
TSS = 0.78
AUC = 0.97
TSS = 0.67
bio02
BDW

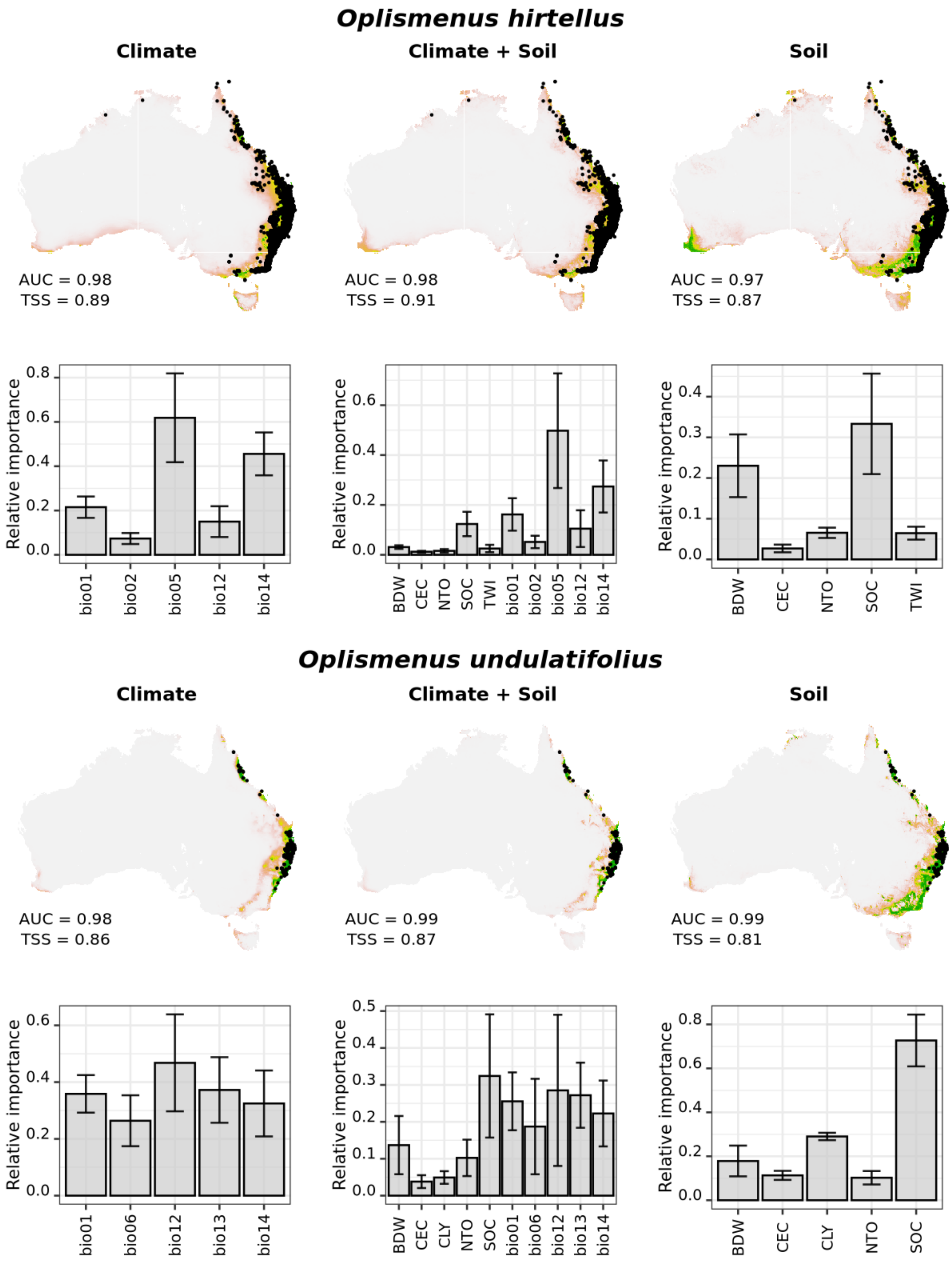

Oplismenus hirtellus
Climate
Climate + Soil
Soil
AUC = 0.98
TSS = 0.89
AUC = 0.98
TSS = 0.91
AUC = 0.97
TSS = 0.87
Relative importance
bio01
bio02
bio05
bio12
bio14
BDW
CEC
NTO
SOC
TWI
Oplismenus undulatifolius
AUC = 0.98
TSS = 0.86
AUC = 0.99
TSS = 0.87
AUC = 0.99
TSS = 0.81
bio06
bio13
CLY

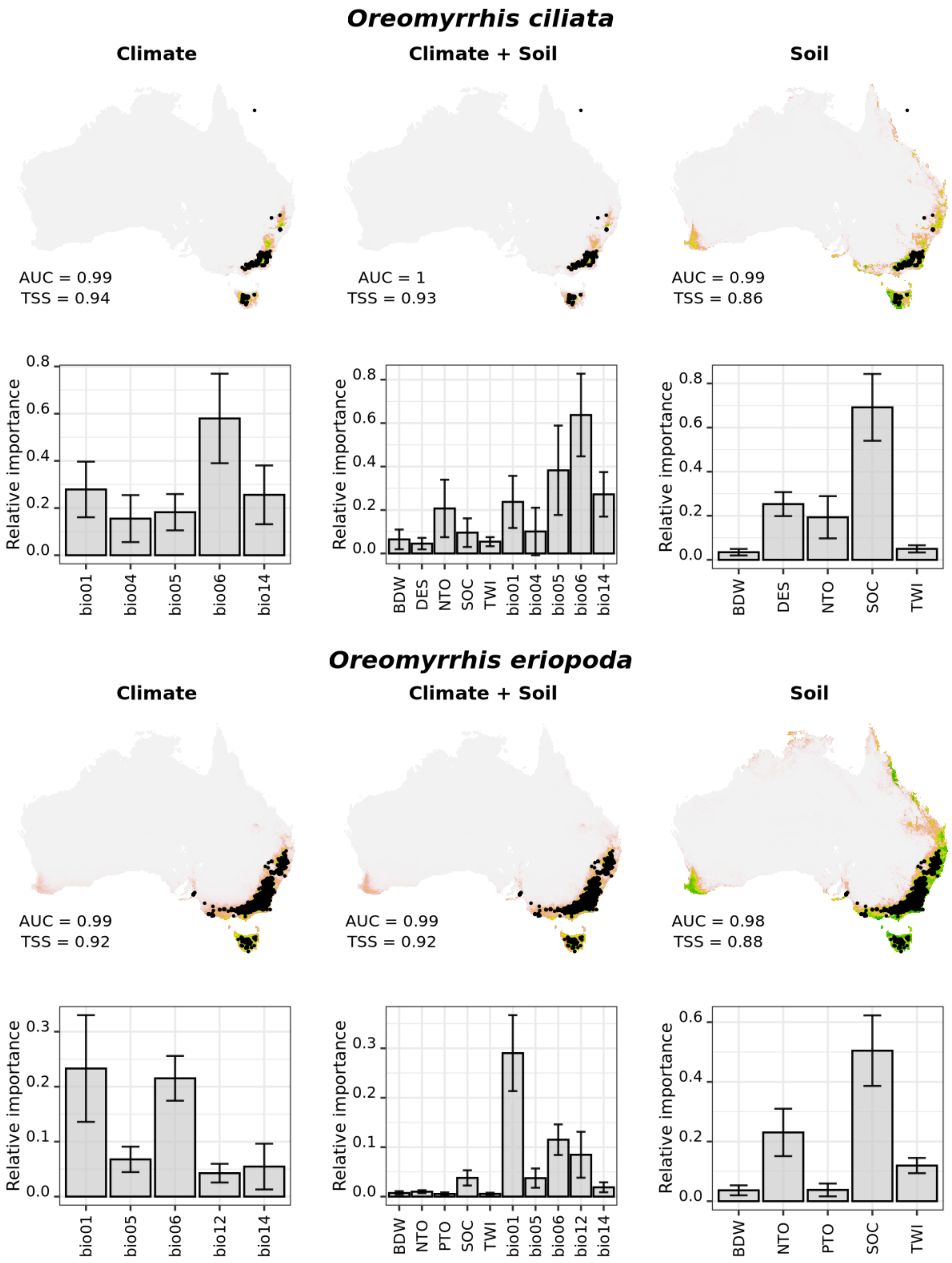
Oreomyrrhis ciliata
Climate
Climate + Soil
Soil
AUC = 0.99
TSS = 0.94
AUC = 1
TSS = 0.93
AUC = 0.99
TSS = 0.86
Relative importance
bio01
bio04
bio05
bio06
bio14
BDW
DES
NTO
SOC
TWI
Oreomyrrhis eriopoda
Climate
Climate + Soil
Soil
AUC = 0.99
TSS = 0.92
AUC = 0.99
TSS = 0.92
AUC = 0.98
TSS = 0.88
Relative importance
bio01
bio05
bio06
bio12
bio14
BDW
NTO
PTO
SOC
TWI

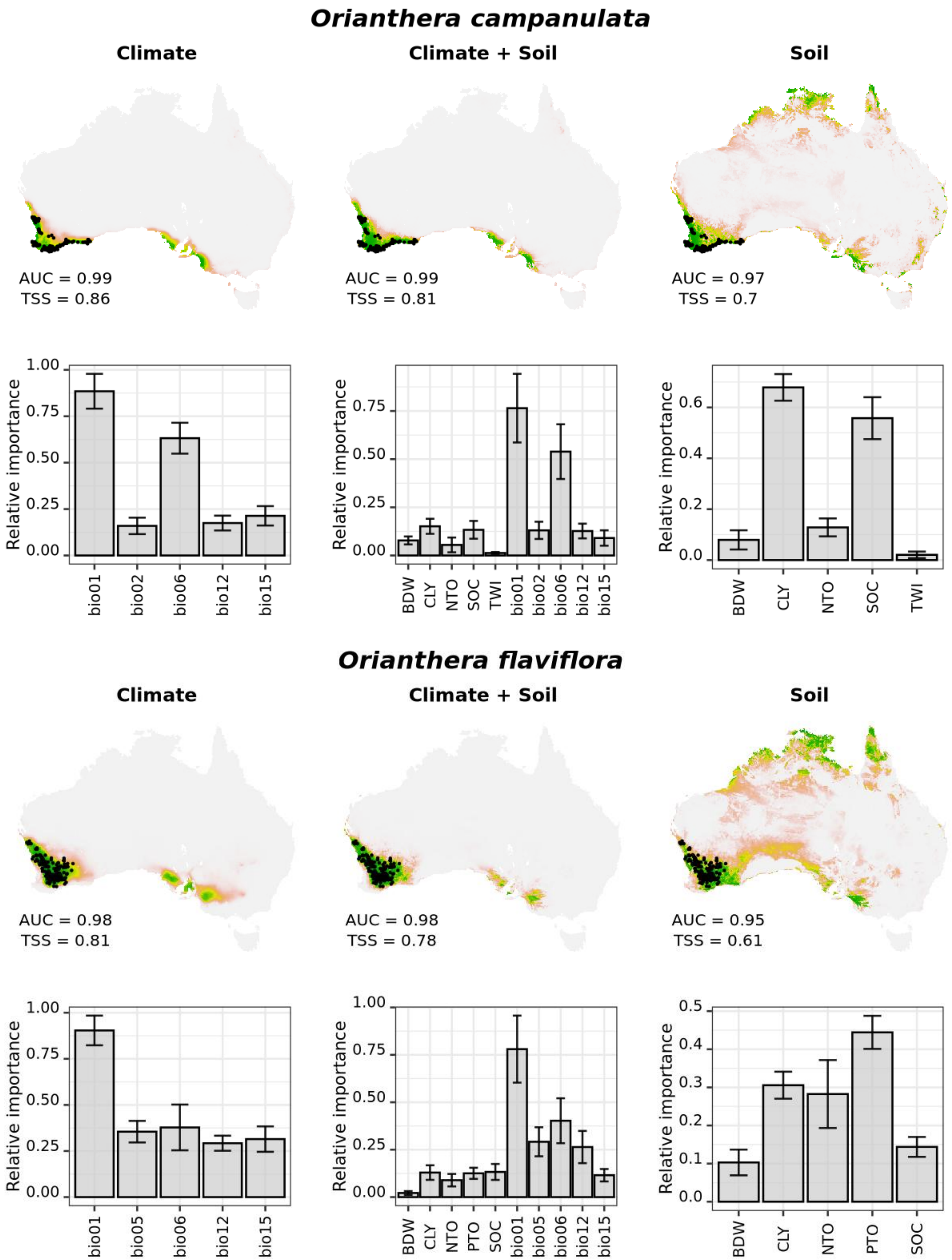

Orianthera campanulata
Climate
Climate + Soil
Soil
AUC = 0.99
TSS = 0.86
AUC = 0.99
TSS = 0.81
AUC = 0.97
TSS = 0.7
Relative importance
bio01
bio02
bio06
bio12
bio15
BDW
CLY
NTO
SOC
TWI
Orianthera flaviflora
Climate
Climate + Soil
Soil
AUC = 0.98
TSS = 0.81
AUC = 0.98
TSS = 0.78
AUC = 0.95
TSS = 0.61
Relative importance
bio01
bio05
bio06
bio12
bio15
BDW
CLY
NTO
PTO
SOC

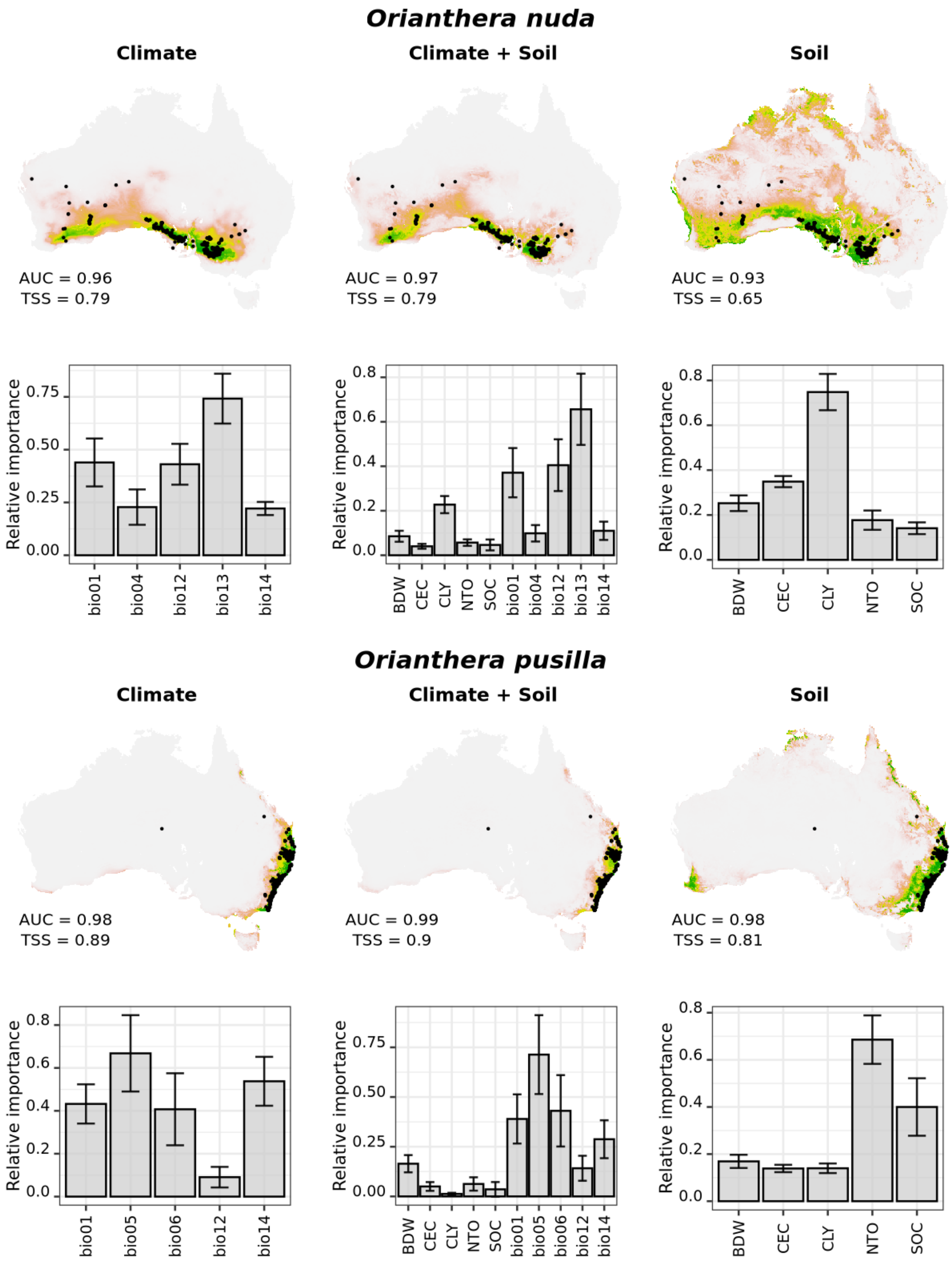

Orianthera nuda
Climate
Climate + Soil
Soil
AUC = 0.96
TSS = 0.79
AUC = 0.97
TSS = 0.79
AUC = 0.93
TSS = 0.65
Relative importance
0.00
0.25
0.50
0.75
bio01
bio04
bio12
bio13
bio14
0.0
0.2
0.4
0.6
0.8
BDW
CEC
CLY
NTO
SOC
Orianthera pusilla
AUC = 0.98
TSS = 0.89
AUC = 0.99
TSS = 0.9
AUC = 0.98
TSS = 0.81
bio05
bio06

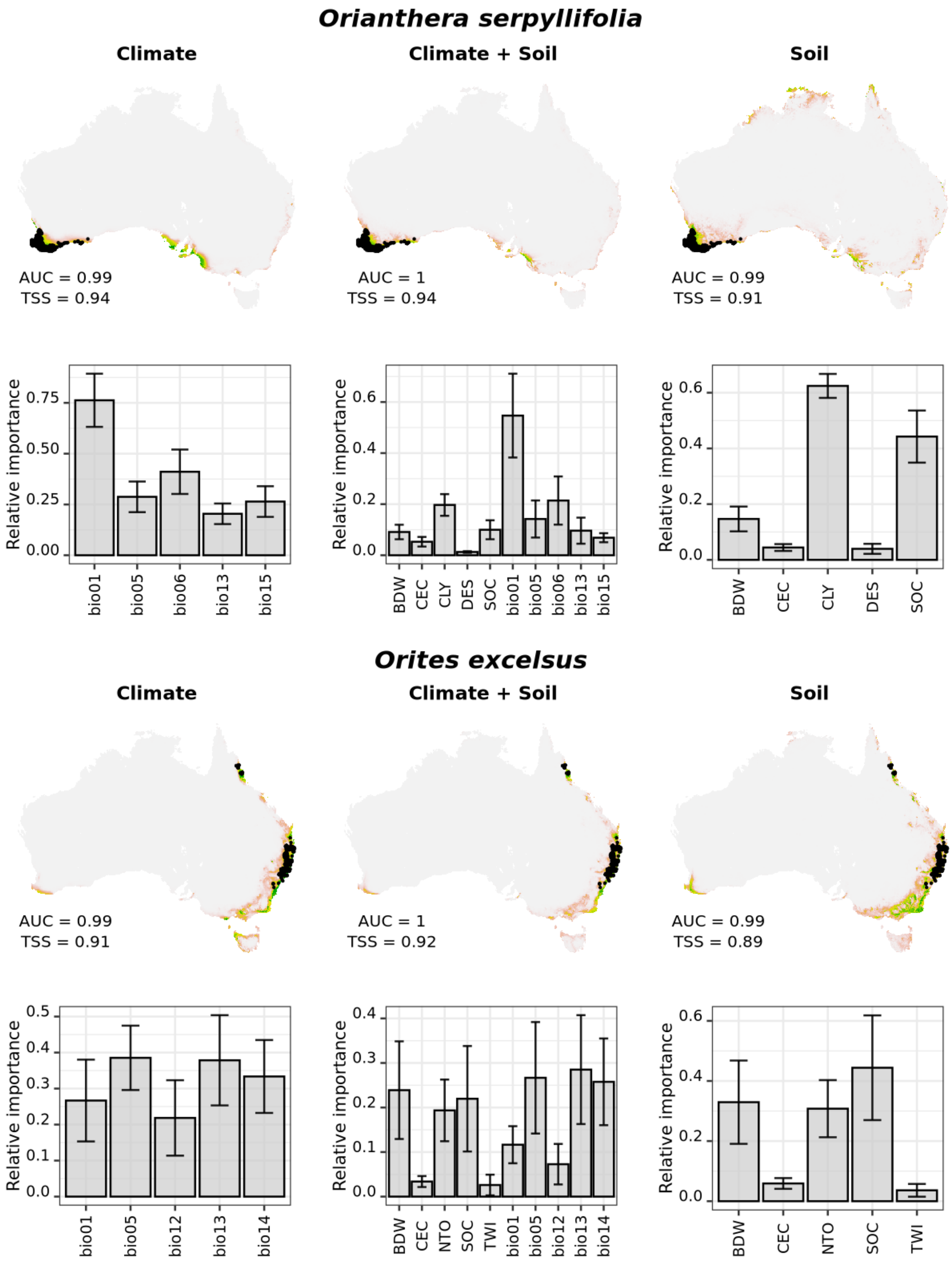

Orianthera serpyllifolia
Climate
Climate + Soil
Soil
AUC = 0.99
TSS = 0.94
AUC = 1
TSS = 0.94
AUC = 0.99
TSS = 0.91
Relative importance
0.00
0.25
0.50
0.75
bio01
bio05
bio06
bio13
bio15
Relative importance
0.0
0.2
0.4
0.6
BDW
CEC
CLY
DES
SOC
bio01
bio05
bio06
bio13
bio15
Relative importance
0.0
0.2
0.4
0.6
BDW
CEC
CLY
DES
SOC
Orites excelsus
Climate
Climate + Soil
Soil
AUC = 0.99
TSS = 0.91
AUC = 1
TSS = 0.92
AUC = 0.99
TSS = 0.89
Relative importance
0.0
0.1
0.2
0.3
0.4
0.5
bio01
bio05
bio12
bio13
bio14
Relative importance
0.0
0.1
0.2
0.3
0.4
BDW
CEC
NTO
SOC
TWI
bio01
bio05
bio12
bio13
bio14
Relative importance
0.0
0.2
0.4
0.6
BDW
CEC
NTO
SOC
TWI

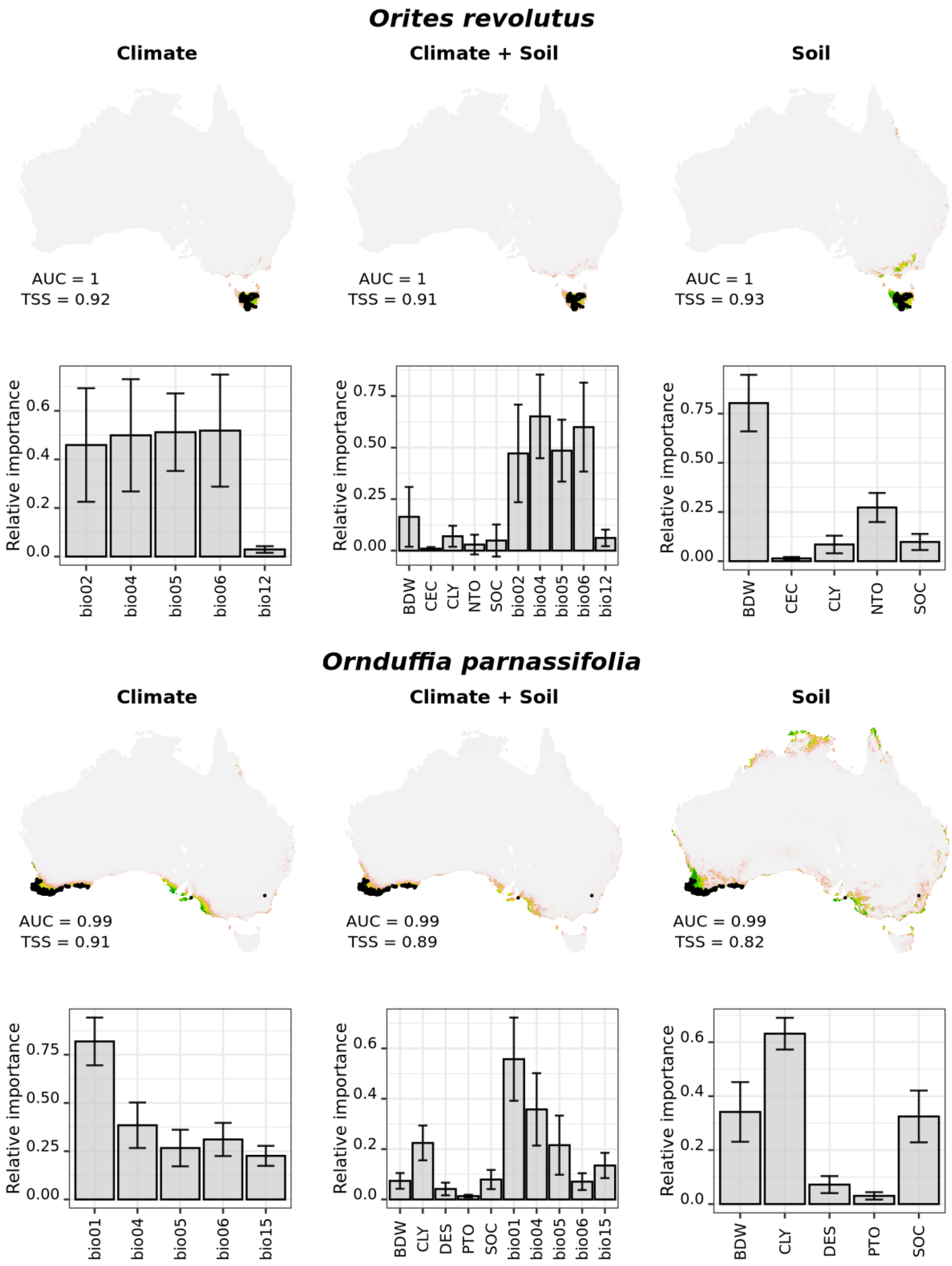
Orites revolutus
Climate
Climate + Soil
Soil
AUC = 1
TSS = 0.92
AUC = 1
TSS = 0.91
AUC = 1
TSS = 0.93
Relative importance
bio02
bio04
bio05
bio06
bio12
BDW
CEC
CLY
NTO
SOC
Ornduffia parnassifolia
Climate
Climate + Soil
Soil
AUC = 0.99
TSS = 0.91
AUC = 0.99
TSS = 0.89
AUC = 0.99
TSS = 0.82
Relative importance
bio01
bio04
bio05
bio06
bio15
BDW
CLY
DES
PTO
SOC

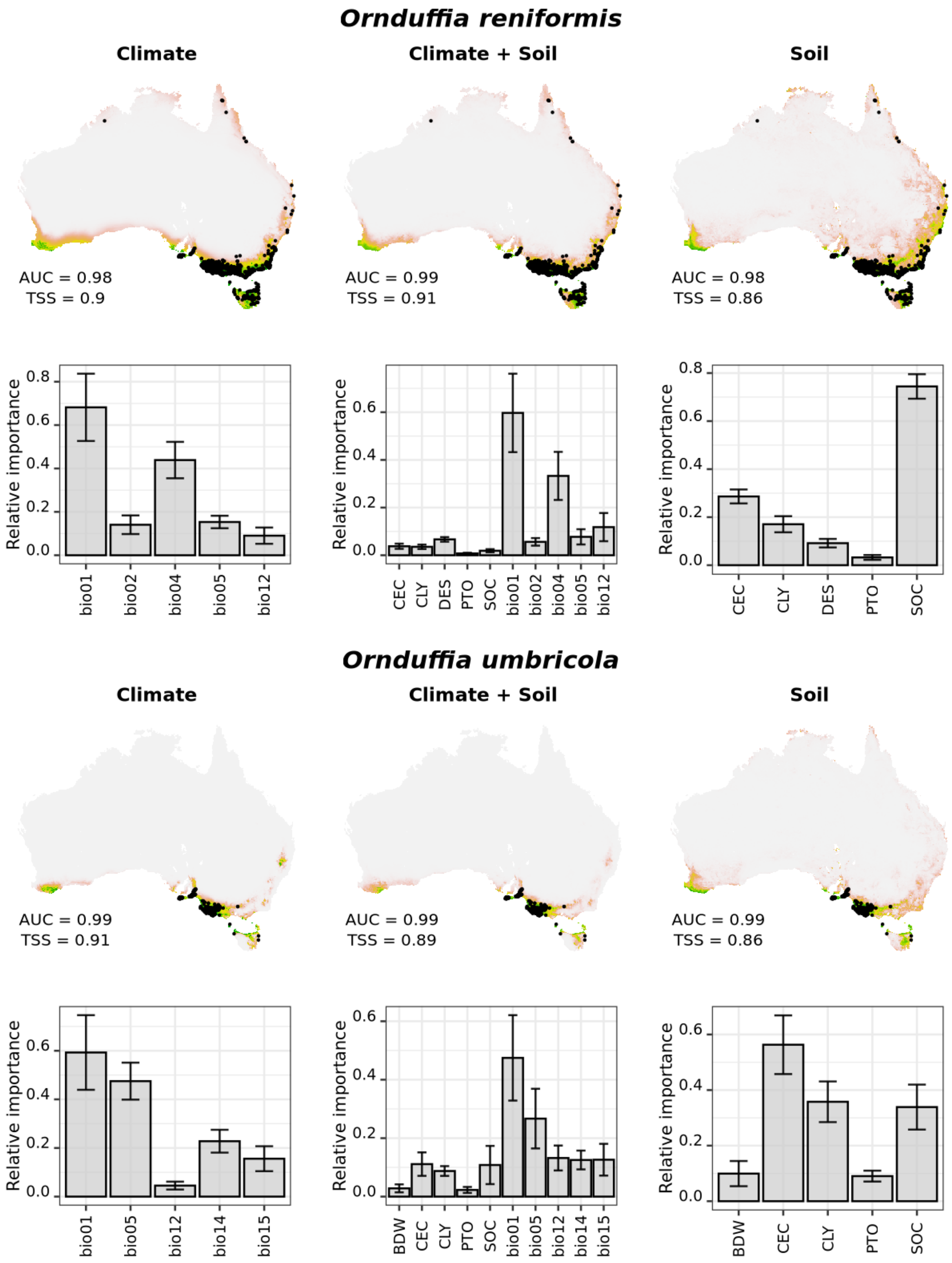

Ornduffia reniformis
Climate
Climate + Soil
Soil
AUC = 0.98
TSS = 0.9
AUC = 0.99
TSS = 0.91
AUC = 0.98
TSS = 0.86
Relative importance
bio01
bio02
bio04
bio05
bio12
CEC
CLY
DES
PTO
SOC
Ornduffia umbricola
Climate
Climate + Soil
Soil
AUC = 0.99
TSS = 0.91
AUC = 0.99
TSS = 0.89
AUC = 0.99
TSS = 0.86
Relative importance
bio01
bio05
bio12
bio14
bio15
BDW
CEC
CLY
PTO
SOC

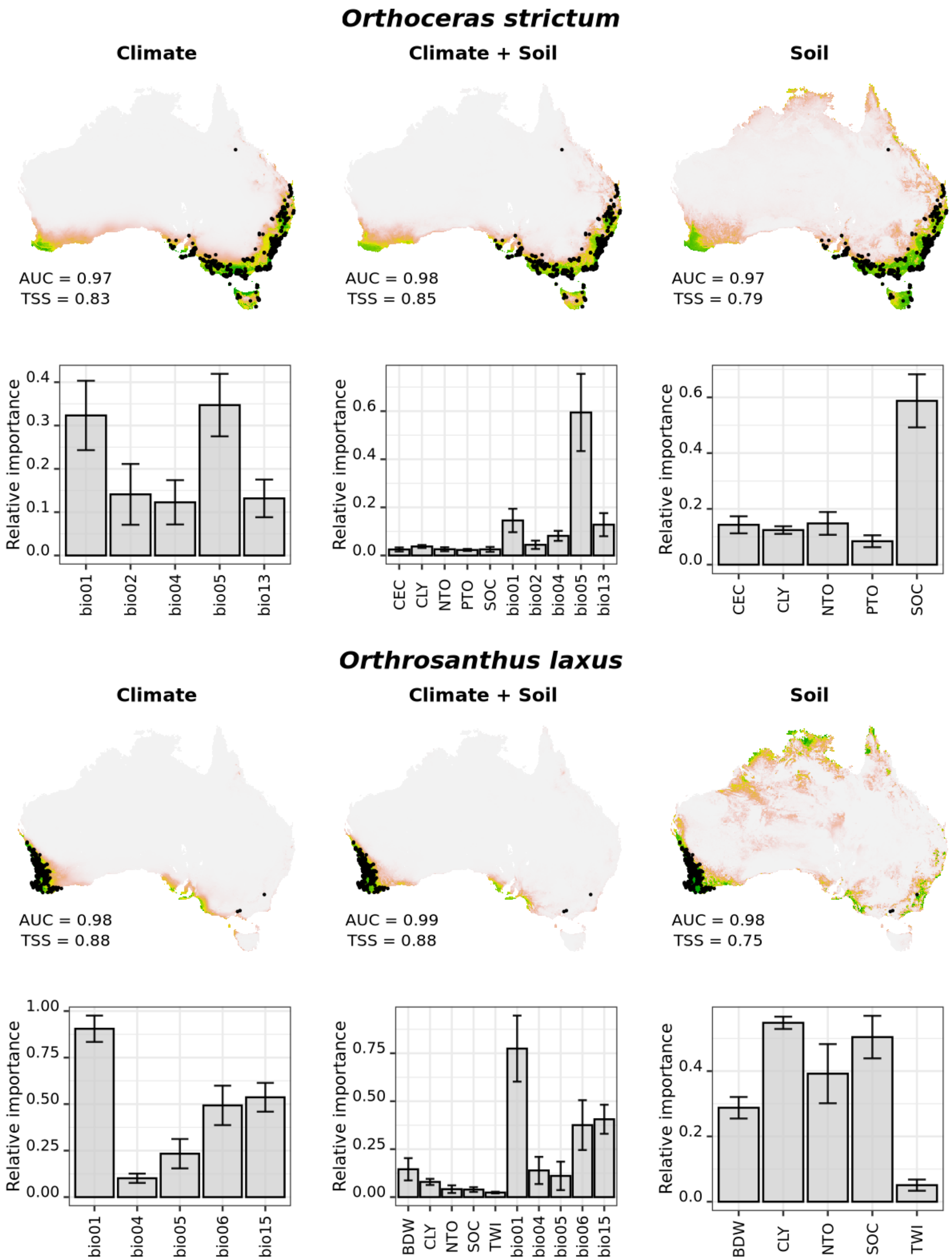
Orthoceras strictum
Climate
Climate + Soil
Soil
AUC = 0.97
TSS = 0.83
AUC = 0.98
TSS = 0.85
AUC = 0.97
TSS = 0.79
Relative importance
0.0
0.1
0.2
0.3
0.4
bio01
bio02
bio04
bio05
bio13
Relative importance
0.0
0.2
0.4
0.6
CEC
CLY
NTO
PTO
SOC
bio01
bio02
bio04
bio05
bio13
Relative importance
0.0
0.2
0.4
0.6
CEC
CLY
NTO
PTO
SOC
Orthrosanthus laxus
Climate
Climate + Soil
Soil
AUC = 0.98
TSS = 0.88
AUC = 0.99
TSS = 0.88
AUC = 0.98
TSS = 0.75
Relative importance
0.00
0.25
0.50
0.75
1.00
bio01
bio04
bio05
bio06
bio15
Relative importance
0.00
0.25
0.50
0.75
BDW
CLY
NTO
SOC
TWI
bio01
bio04
bio05
bio06
bio15
Relative importance
0.0
0.2
0.4
BDW
CLY
NTO
SOC
TWI

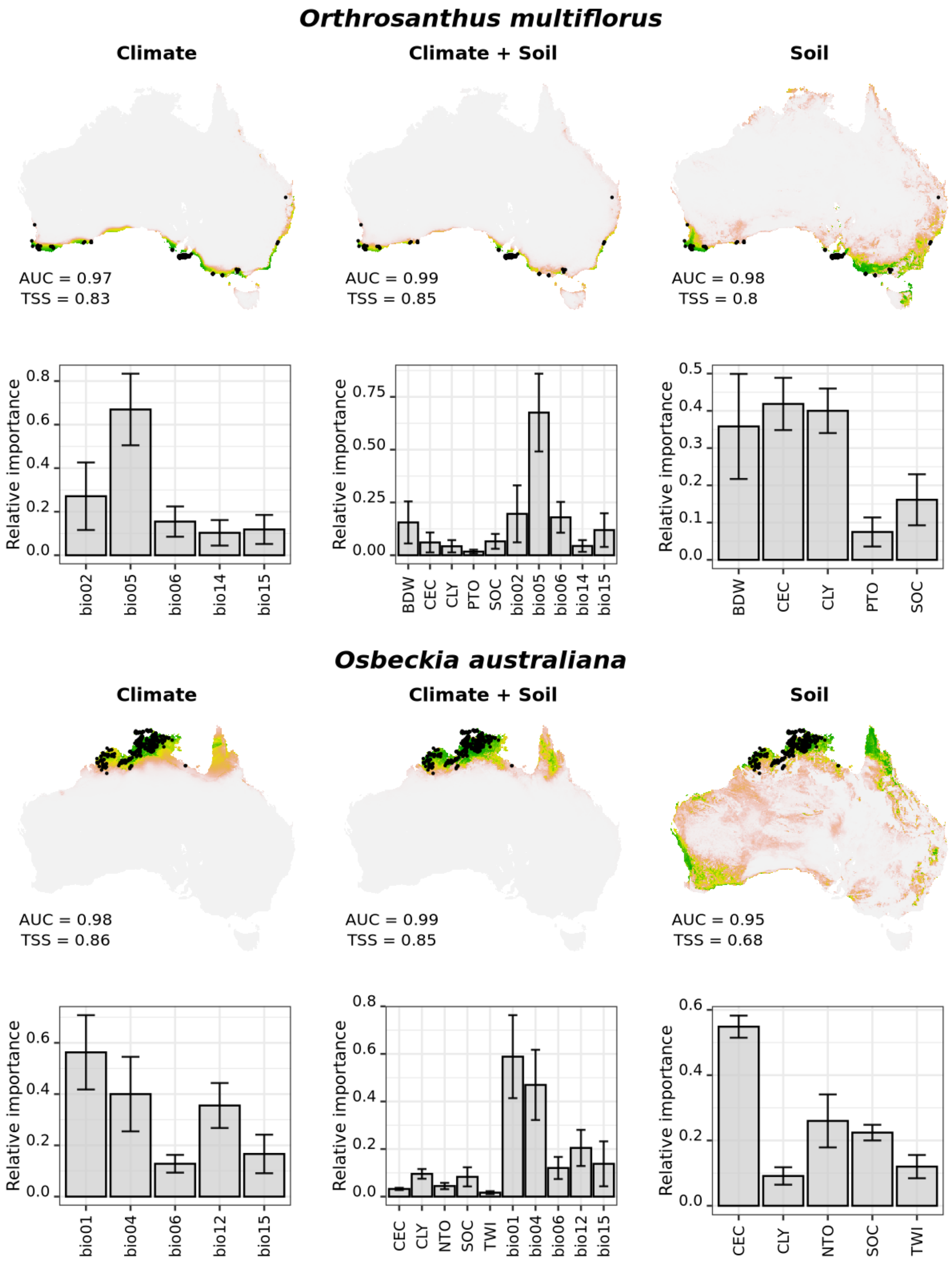
Orthrosanthus multiflorus
Climate
Climate + Soil
Soil
AUC = 0.97
TSS = 0.83
AUC = 0.99
TSS = 0.85
AUC = 0.98
TSS = 0.8
Relative importance
bio02
bio05
bio06
bio14
bio15
BDW
CEC
CLY
PTO
SOC
Osbeckia australiana
AUC = 0.98
TSS = 0.86
AUC = 0.99
TSS = 0.85
AUC = 0.95
TSS = 0.68
bio01
bio04
bio12
NTO
TWI

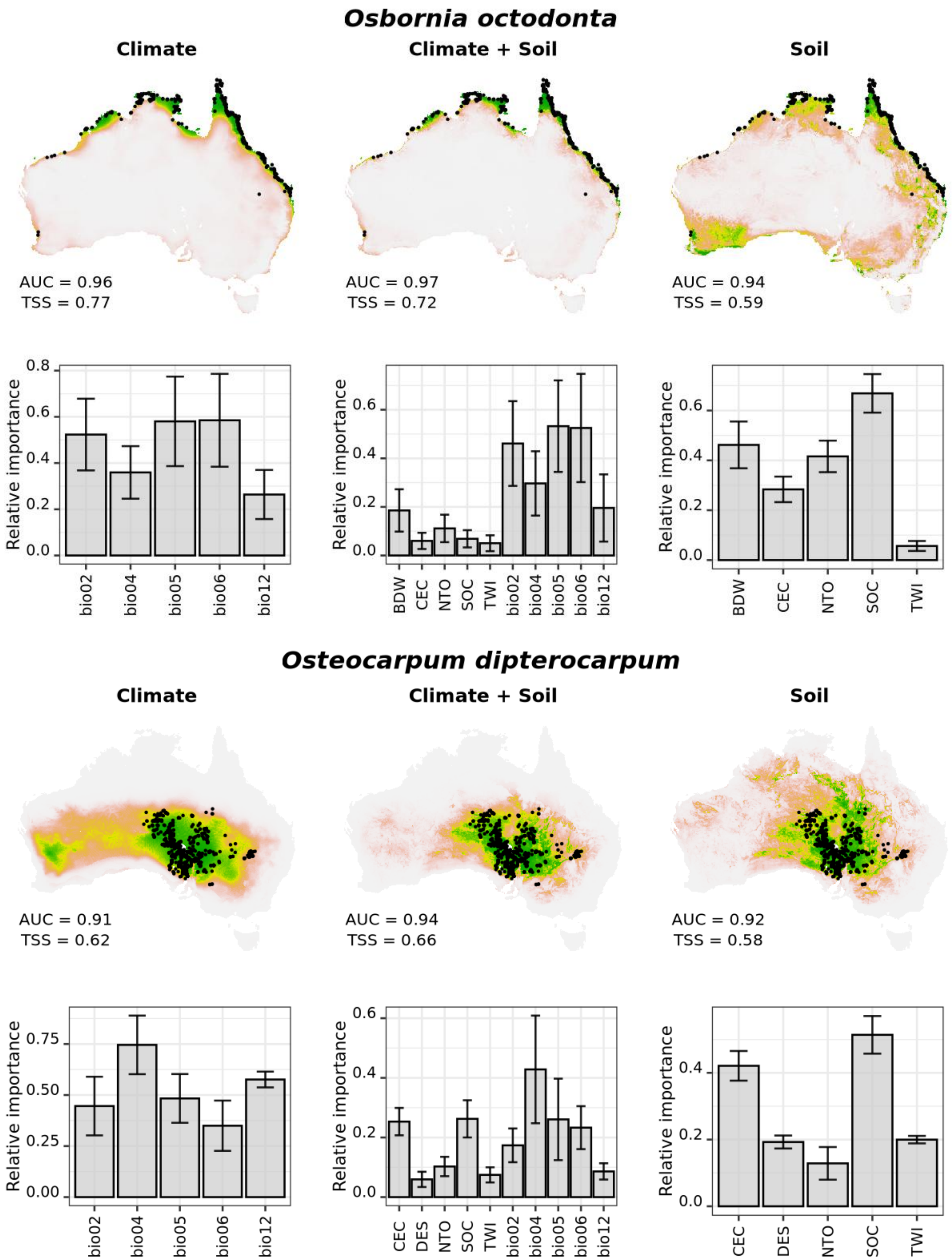
Osbornia octodonta
Climate
Climate + Soil
Soil
AUC = 0.96
TSS = 0.77
AUC = 0.97
TSS = 0.72
AUC = 0.94
TSS = 0.59
Relative importance
bio02
bio04
bio05
bio06
bio12
BDW
CEC
NTO
SOC
TWI
Osteocarpum dipterocarpum
Climate
Climate + Soil
Soil
AUC = 0.91
TSS = 0.62
AUC = 0.94
TSS = 0.66
AUC = 0.92
TSS = 0.58
Relative importance
bio02
bio04
bio05
bio06
bio12
CEC
DES
NTO
SOC
TWI

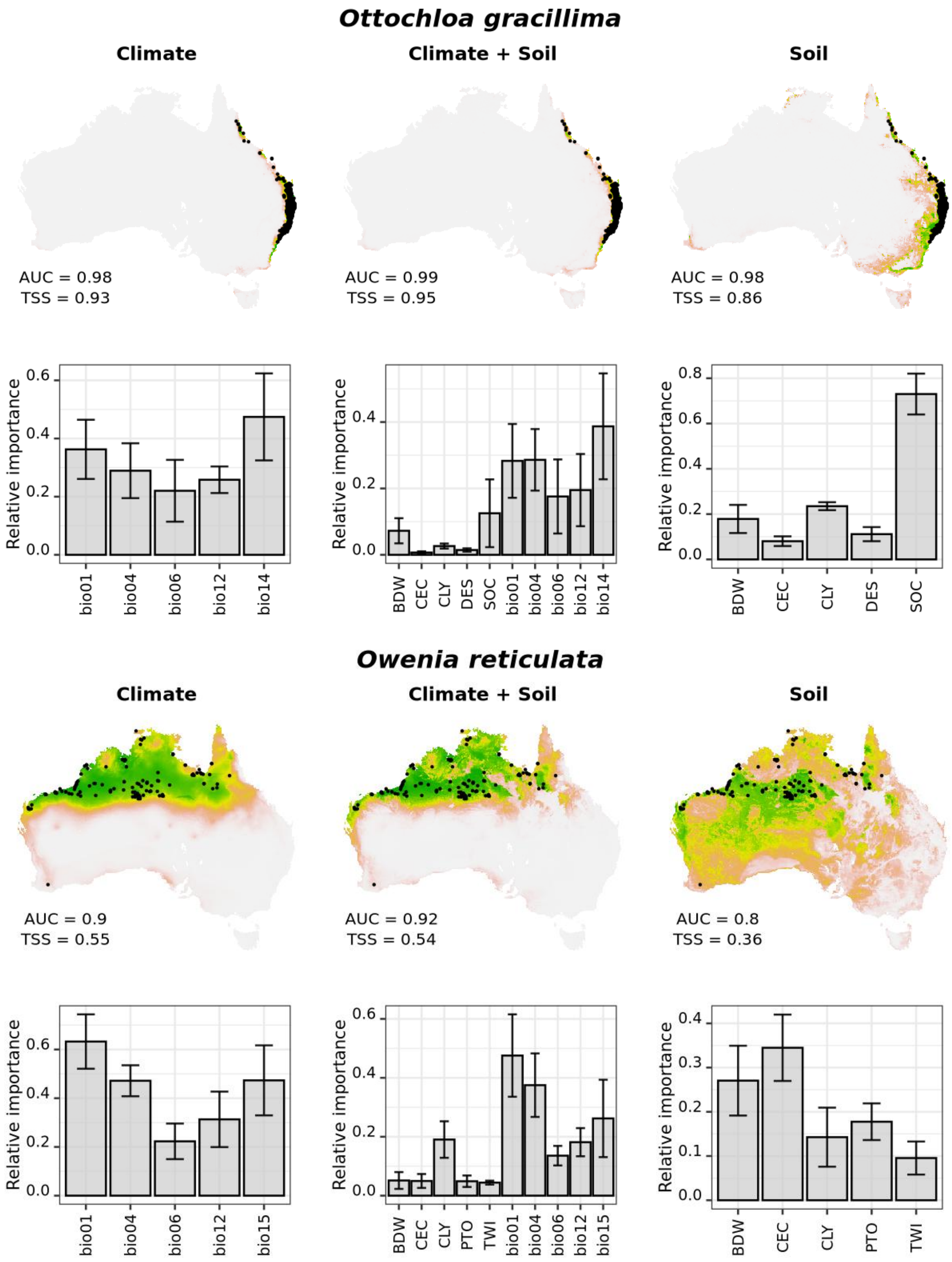
Ottochloa gracillima
Climate
Climate + Soil
Soil
AUC = 0.98
TSS = 0.93
AUC = 0.99
TSS = 0.95
AUC = 0.98
TSS = 0.86
Relative importance
bio01
bio04
bio06
bio12
bio14
BDW
CEC
CLY
DES
SOC
Owenia reticulata
Climate
Climate + Soil
Soil
AUC = 0.9
TSS = 0.55
AUC = 0.92
TSS = 0.54
AUC = 0.8
TSS = 0.36
Relative importance
bio01
bio04
bio06
bio12
bio15
BDW
CEC
CLY
PTO
TWI

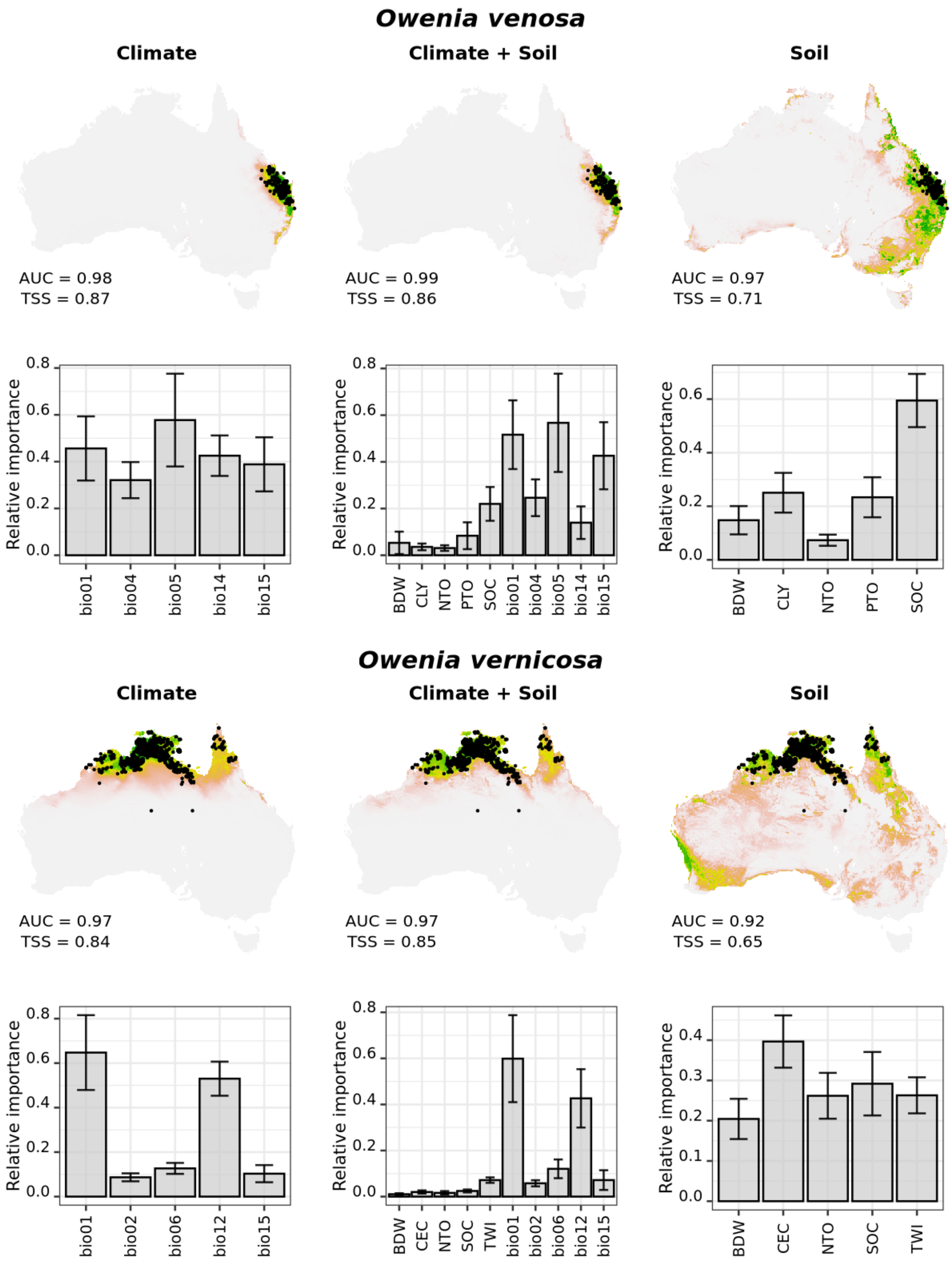
Owenia venosa
Climate
Climate + Soil
Soil
AUC = 0.98
TSS = 0.87
AUC = 0.99
TSS = 0.86
AUC = 0.97
TSS = 0.71
Relative importance
bio01
bio04
bio05
bio14
bio15
BDW
CLY
NTO
PTO
SOC
Owenia vernicosa
Climate
Climate + Soil
Soil
AUC = 0.97
TSS = 0.84
AUC = 0.97
TSS = 0.85
AUC = 0.92
TSS = 0.65
bio02
bio06
bio12
CEC
TWI

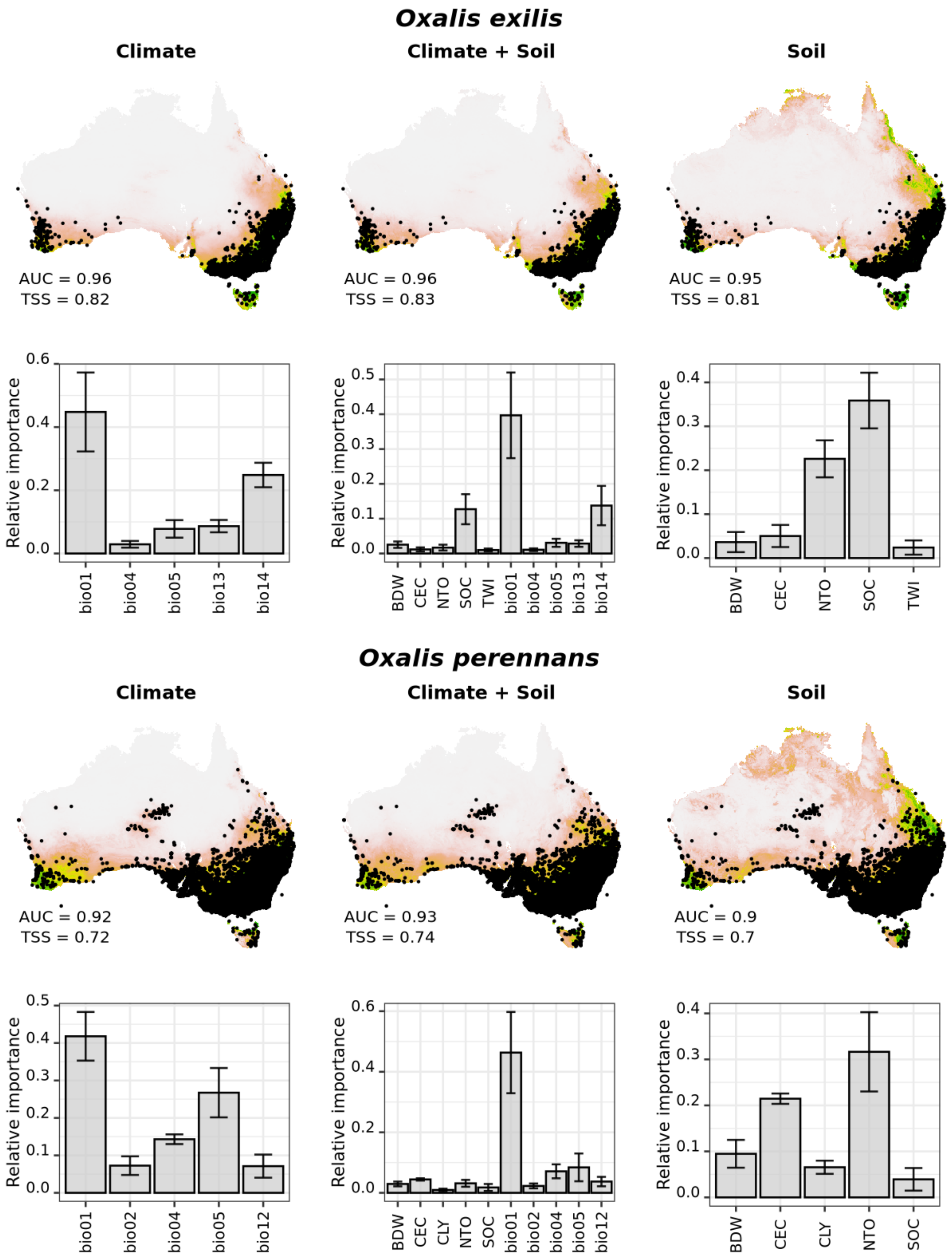

Oxalis exilis
Climate
Climate + Soil
Soil
AUC = 0.96
TSS = 0.82
AUC = 0.96
TSS = 0.83
AUC = 0.95
TSS = 0.81
Relative importance
bio01
bio04
bio05
bio13
bio14
BDW
CEC
NTO
SOC
TWI
Oxalis perennans
Climate
Climate + Soil
Soil
AUC = 0.92
TSS = 0.72
AUC = 0.93
TSS = 0.74
AUC = 0.9
TSS = 0.7
Relative importance
bio01
bio02
bio04
bio05
bio12
BDW
CEC
CLY
NTO
SOC

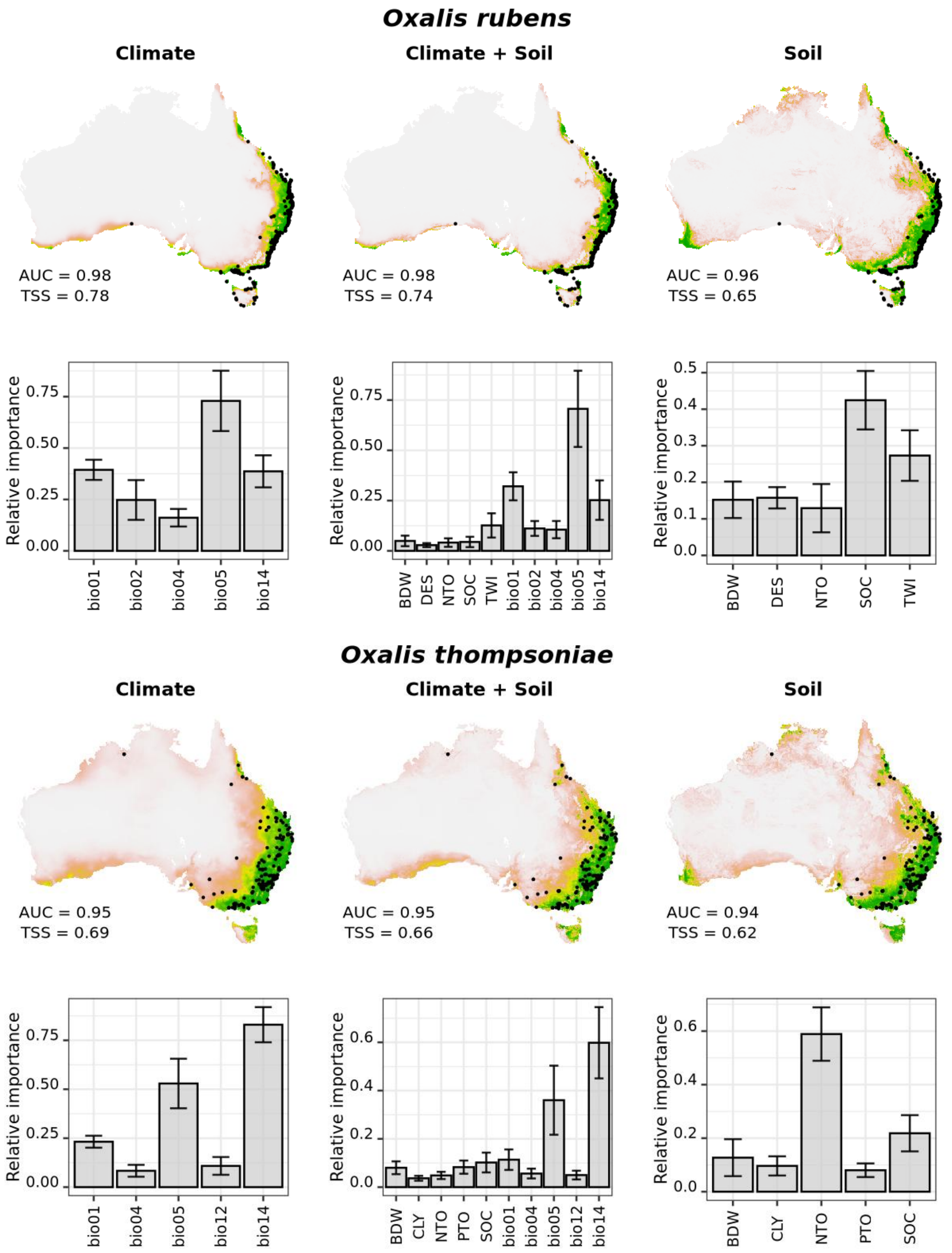
Oxalis rubens
Climate
Climate + Soil
Soil
AUC = 0.98
TSS = 0.78
AUC = 0.98
TSS = 0.74
AUC = 0.96
TSS = 0.65
Relative importance
0.00
0.25
0.50
0.75
bio01
bio02
bio04
bio05
bio14
BDW
DES
NTO
SOC
TWI
0.0
0.1
0.2
0.3
0.4
0.5
Oxalis thompsoniae
Climate
Climate + Soil
Soil
AUC = 0.95
TSS = 0.69
AUC = 0.95
TSS = 0.66
AUC = 0.94
TSS = 0.62
bio12
CLY
PTO
0.6

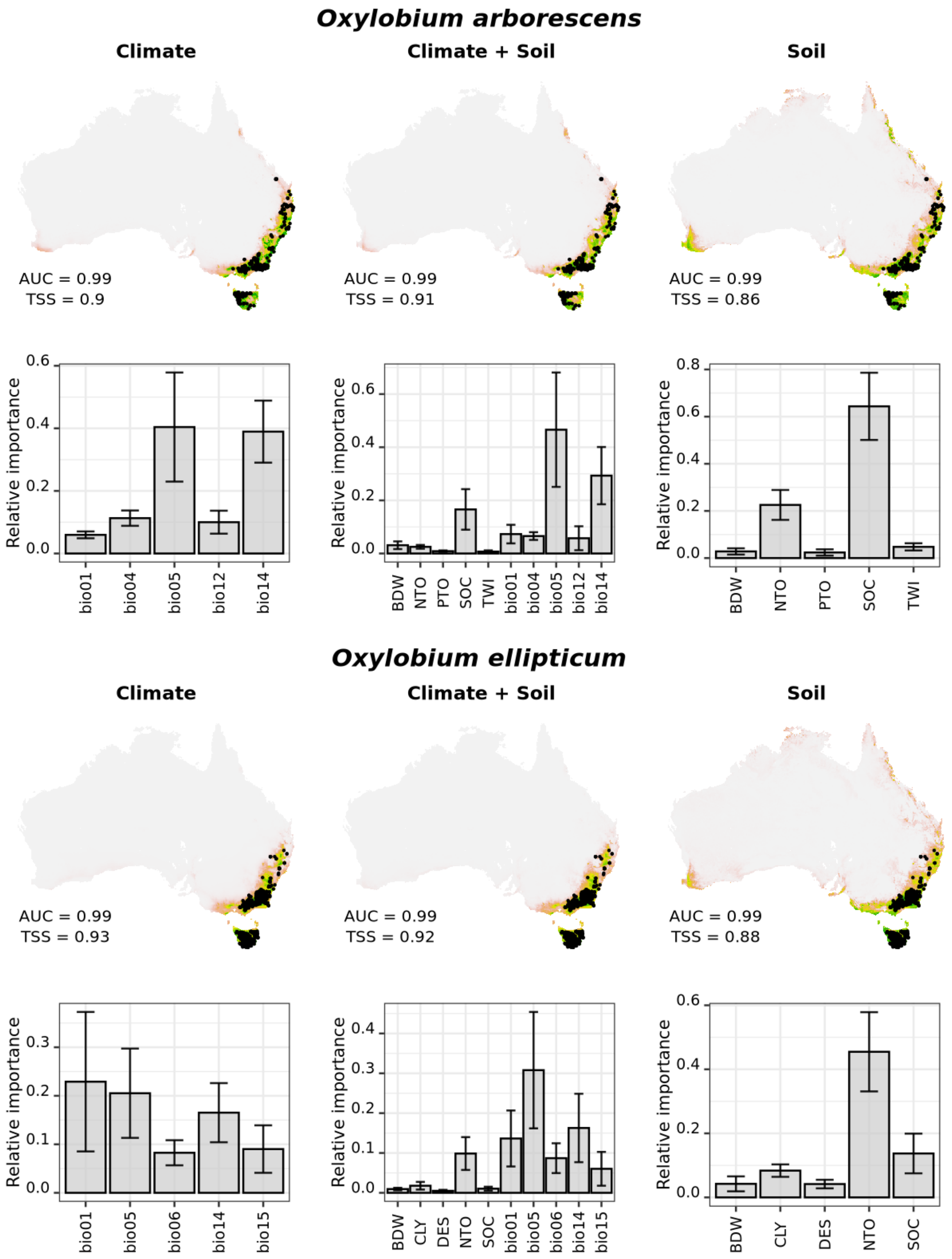

Oxylobium arborescens
Climate
Climate + Soil
Soil
AUC = 0.99
TSS = 0.9
AUC = 0.99
TSS = 0.91
AUC = 0.99
TSS = 0.86
Relative importance
bio01 bio04 bio05 bio12 bio14
BDW NTO PTO SOC TWI bio01 bio04 bio05 bio12 bio14
BDW NTO PTO SOC TWI
Oxylobium ellipticum
Climate
Climate + Soil
Soil
AUC = 0.99
TSS = 0.93
AUC = 0.99
TSS = 0.92
AUC = 0.99
TSS = 0.88
Relative importance
bio01 bio05 bio06 bio14 bio15
BDW CLY DES NTO SOC bio01 bio05 bio06 bio14 bio15
BDW CLY DES NTO SOC

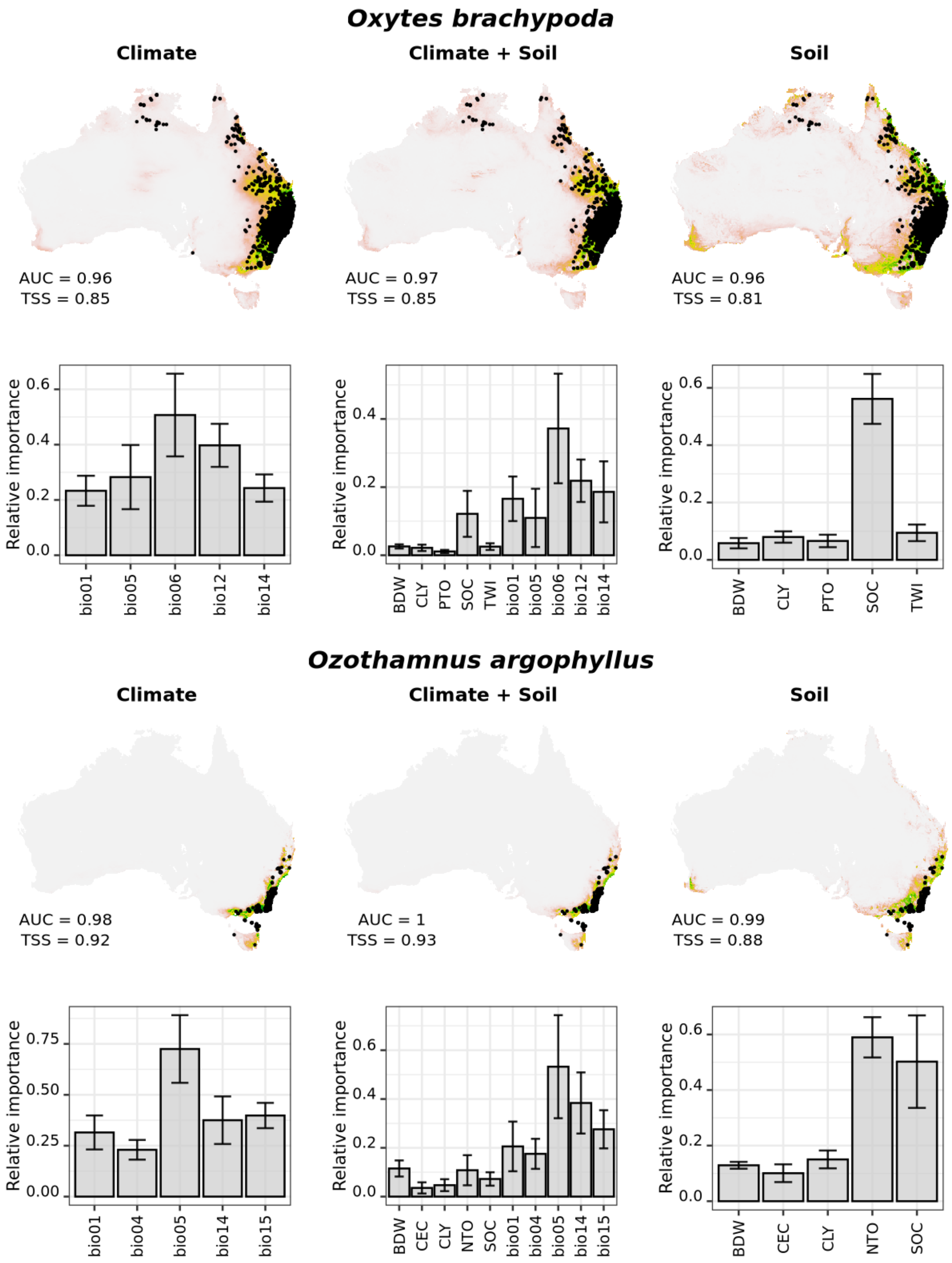
Oxytes brachypoda
Climate
Climate + Soil
Soil
AUC = 0.96
TSS = 0.85
AUC = 0.97
TSS = 0.85
AUC = 0.96
TSS = 0.81
Relative importance
0.0
0.2
0.4
0.6
bio01
bio05
bio06
bio12
bio14
BDW
CLY
PTO
SOC
TWI
Ozothamnus argophyllus
AUC = 0.98
TSS = 0.92
AUC = 1
TSS = 0.93
AUC = 0.99
TSS = 0.88
0.00
0.25
0.50
0.75
bio04
bio15
CEC
NTO

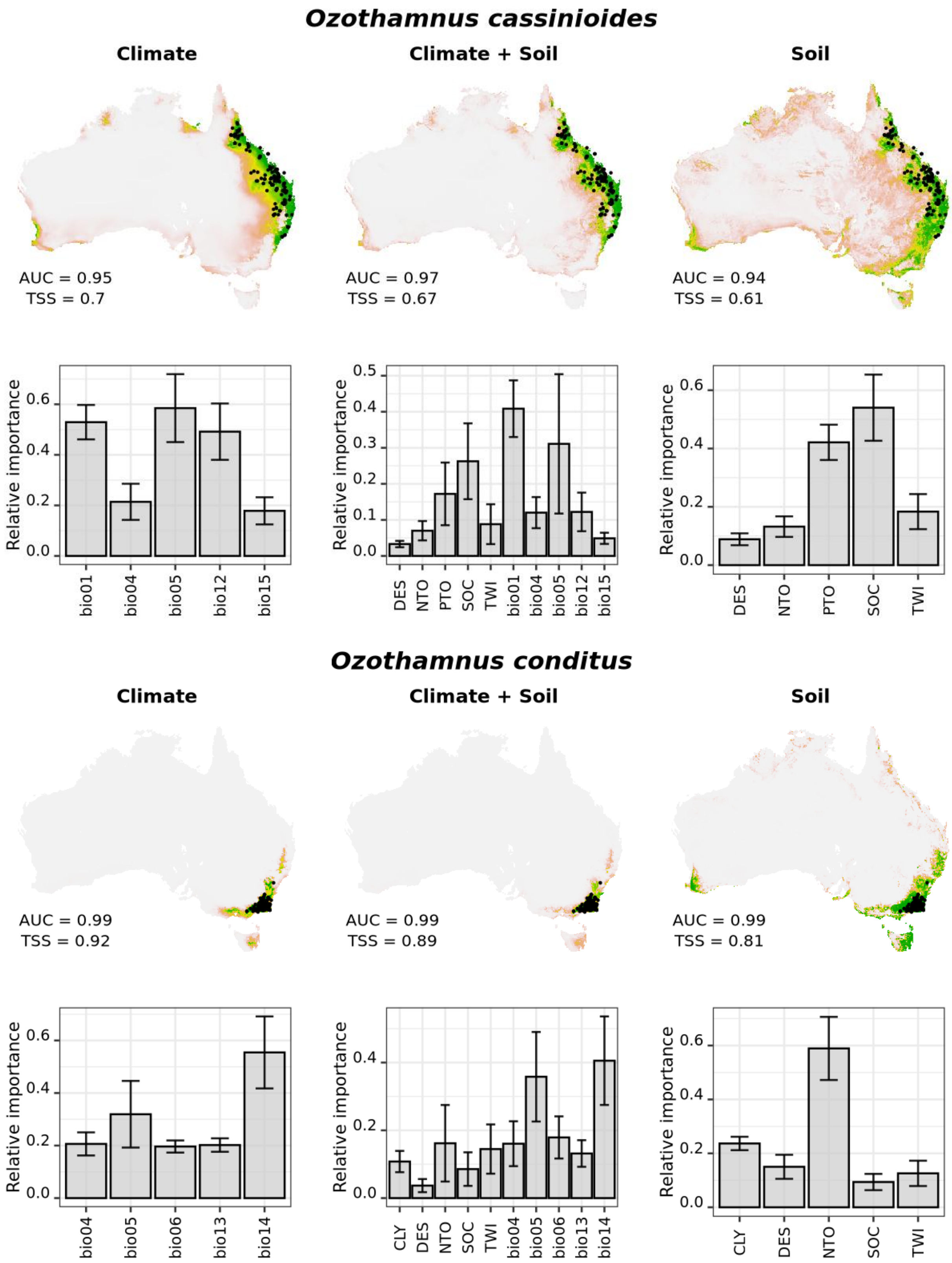
Ozothamnus cassinioides
Climate
Climate + Soil
Soil
AUC = 0.95
TSS = 0.7
AUC = 0.97
TSS = 0.67
AUC = 0.94
TSS = 0.61
Relative importance
bio01
bio04
bio05
bio12
bio15
DES
NTO
PTO
SOC
TWI
Ozothamnus conditus
Climate
Climate + Soil
Soil
AUC = 0.99
TSS = 0.92
AUC = 0.99
TSS = 0.89
AUC = 0.99
TSS = 0.81
Relative importance
bio04
bio05
bio06
bio13
bio14
CLY
DES
NTO
SOC
TWI

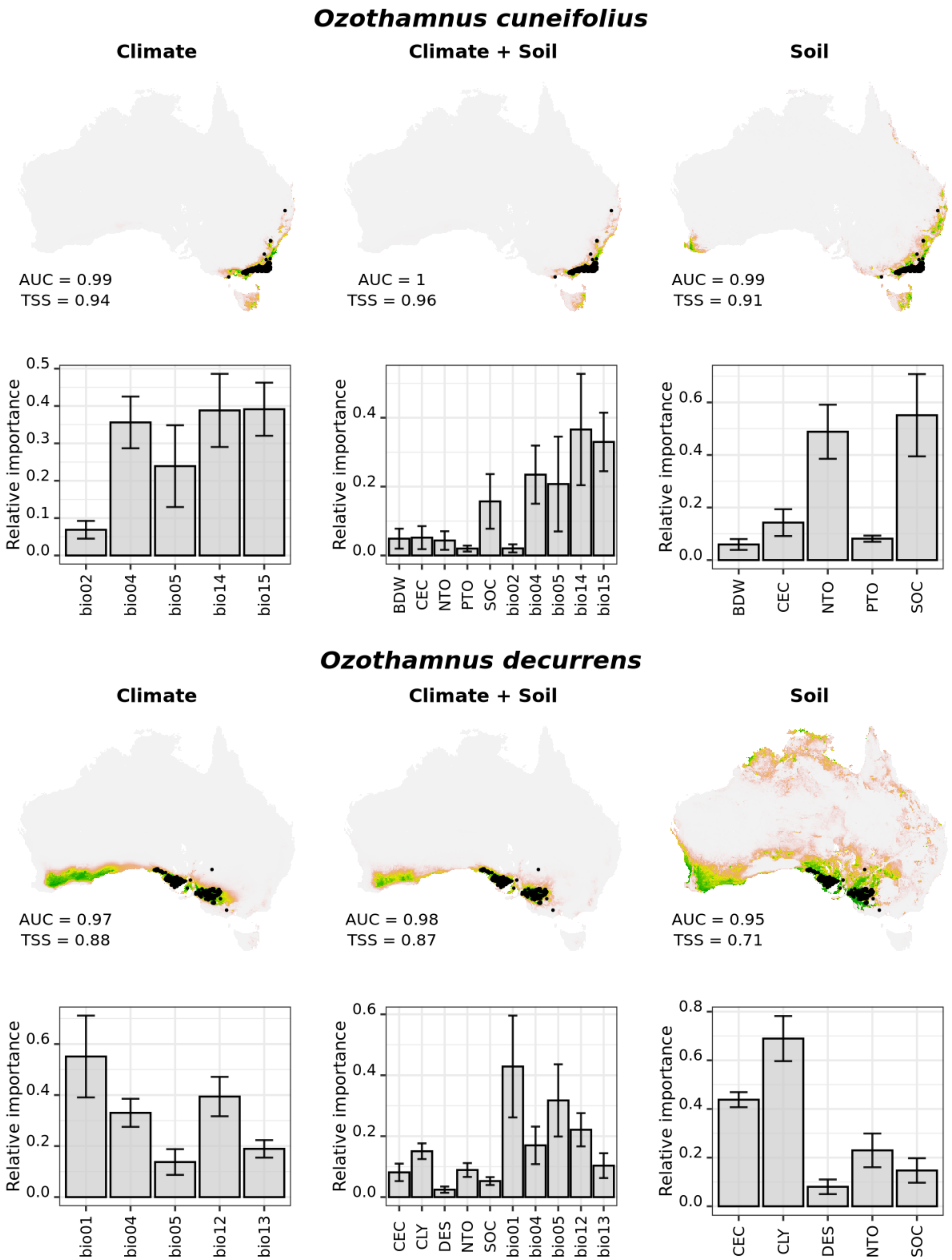
Ozothamnus cuneifolius
Climate
Climate + Soil
Soil
AUC = 0.99
TSS = 0.94
AUC = 1
TSS = 0.96
AUC = 0.99
TSS = 0.91
Relative importance
bio02
bio04
bio05
bio14
bio15
BDW
CEC
NTO
PTO
SOC
Ozothamnus decurrens
AUC = 0.97
TSS = 0.88
AUC = 0.98
TSS = 0.87
AUC = 0.95
TSS = 0.71
bio01
bio12
bio13
CLY
DES

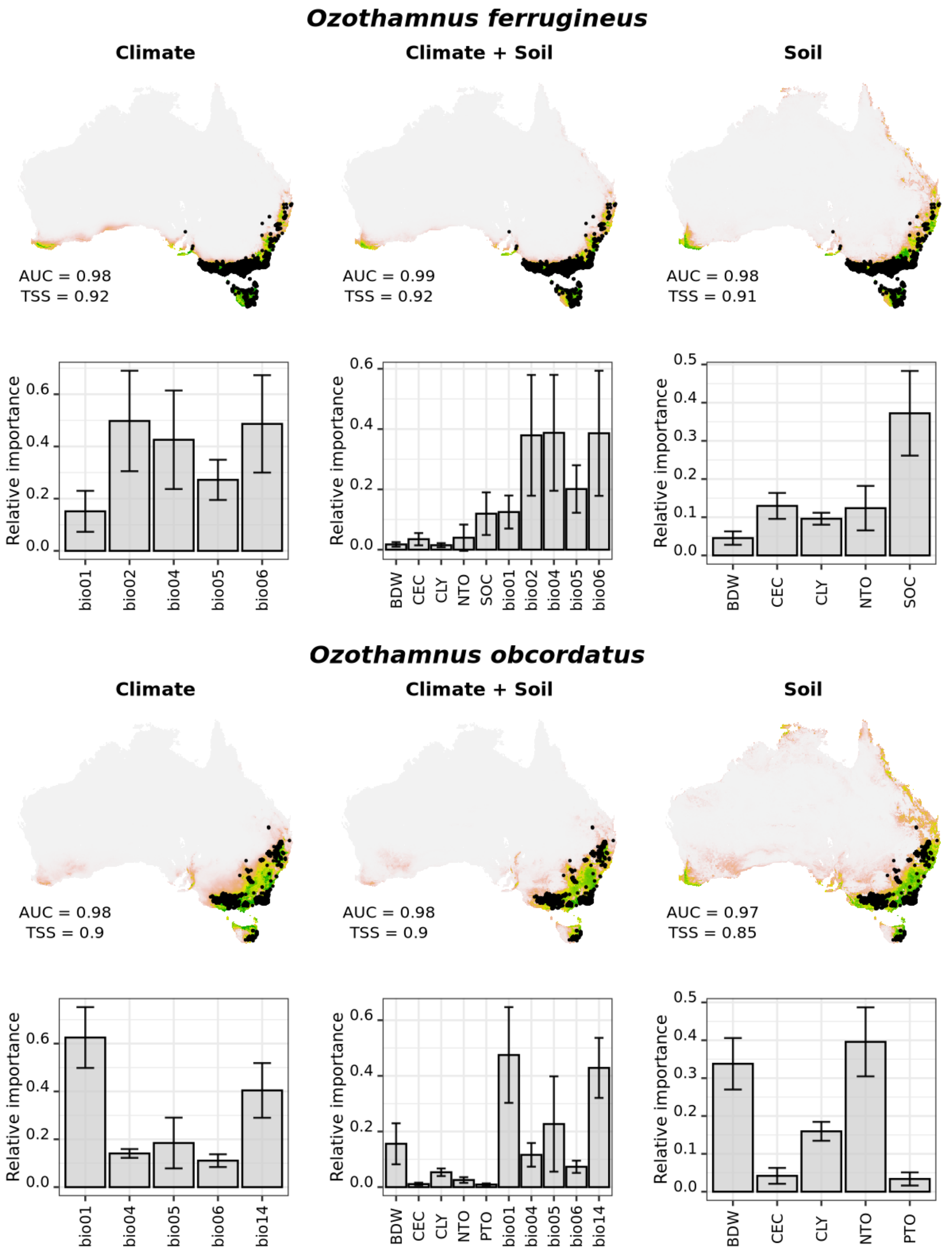
Ozothamnus ferrugineus
Climate
Climate + Soil
Soil
AUC = 0.98
TSS = 0.92
AUC = 0.99
TSS = 0.92
AUC = 0.98
TSS = 0.91
Relative importance
bio01
bio02
bio04
bio05
bio06
BDW
CEC
CLY
NTO
SOC
Ozothamnus obcordatus
Climate
Climate + Soil
Soil
AUC = 0.98
TSS = 0.9
AUC = 0.98
TSS = 0.9
AUC = 0.97
TSS = 0.85
Relative importance
bio01
bio04
bio05
bio06
bio14
BDW
CEC
CLY
NTO
PTO

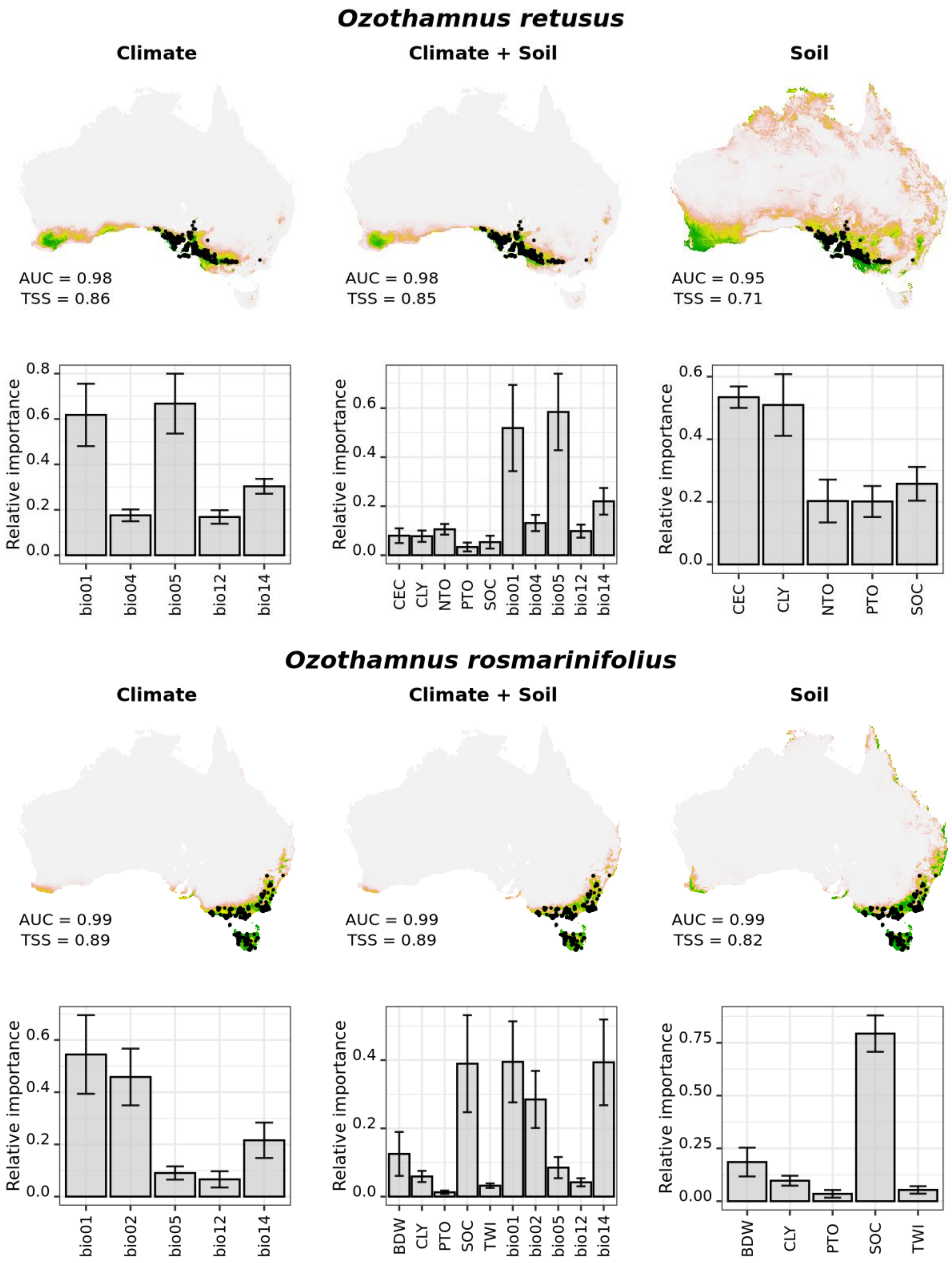

Ozothamnus retusus
Climate
Climate + Soil
Soil
AUC = 0.98
TSS = 0.86
AUC = 0.98
TSS = 0.85
AUC = 0.95
TSS = 0.71
Relative importance
bio01
bio04
bio05
bio12
bio14
CEC
CLY
NTO
PTO
SOC
Ozothamnus rosmarinifolius
Climate
Climate + Soil
Soil
AUC = 0.99
TSS = 0.89
AUC = 0.99
TSS = 0.89
AUC = 0.99
TSS = 0.82
Relative importance
bio01
bio02
bio05
bio12
bio14
BDW
CLY
PTO
SOC
TWI

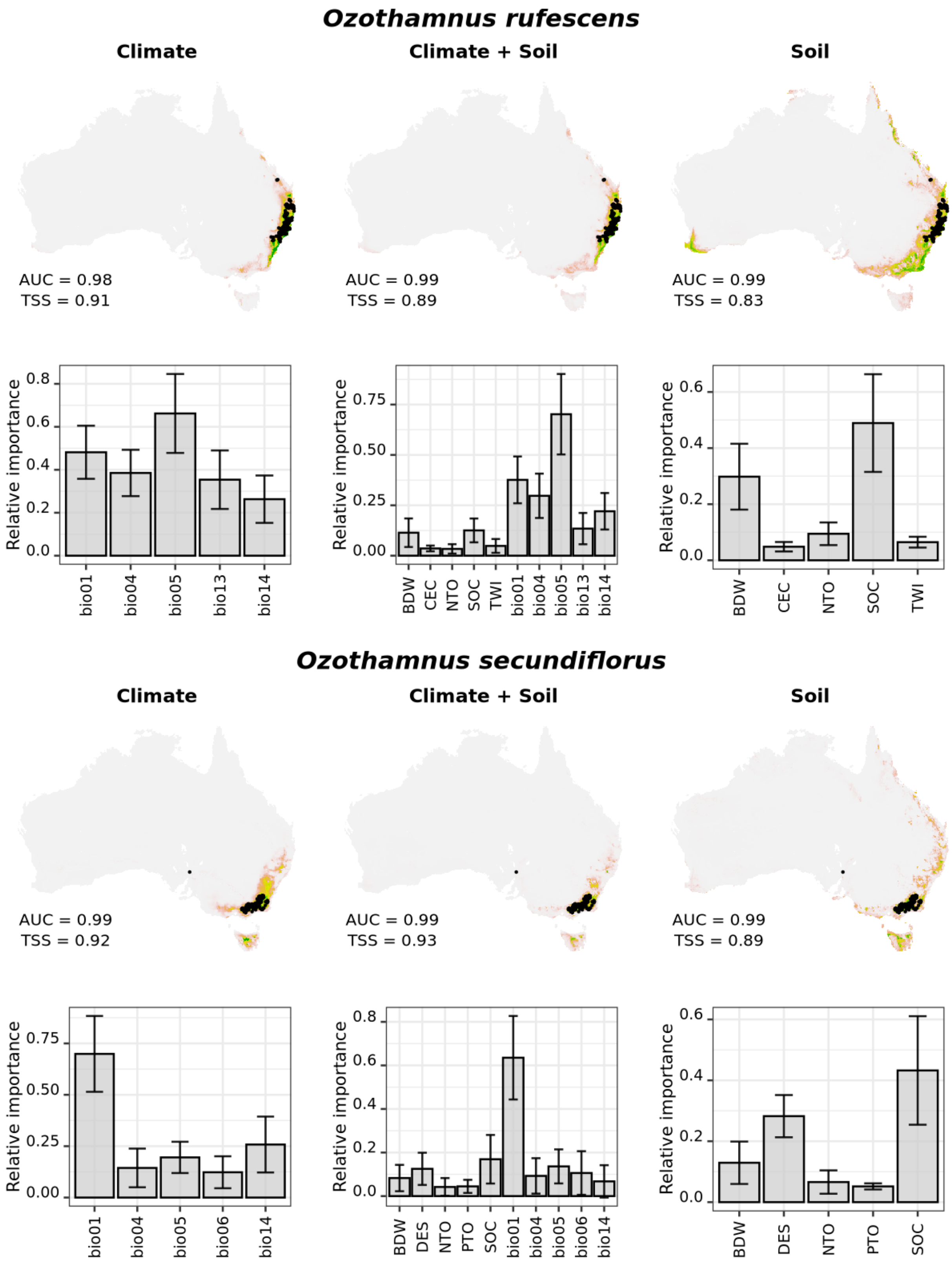
Ozothamnus rufescens
Climate
Climate + Soil
Soil
AUC = 0.98
TSS = 0.91
AUC = 0.99
TSS = 0.89
AUC = 0.99
TSS = 0.83
Relative importance
bio01 bio04 bio05 bio13 bio14
BDW CEC NTO SOC TWI bio01 bio04 bio05 bio13 bio14
BDW CEC NTO SOC TWI
Ozothamnus secundiflorus
Climate
Climate + Soil
Soil
AUC = 0.99
TSS = 0.92
AUC = 0.99
TSS = 0.93
AUC = 0.99
TSS = 0.89
Relative importance
bio01 bio04 bio05 bio06 bio14
BDW DES NTO PTO SOC bio01 bio04 bio05 bio06 bio14
BDW DES NTO PTO SOC

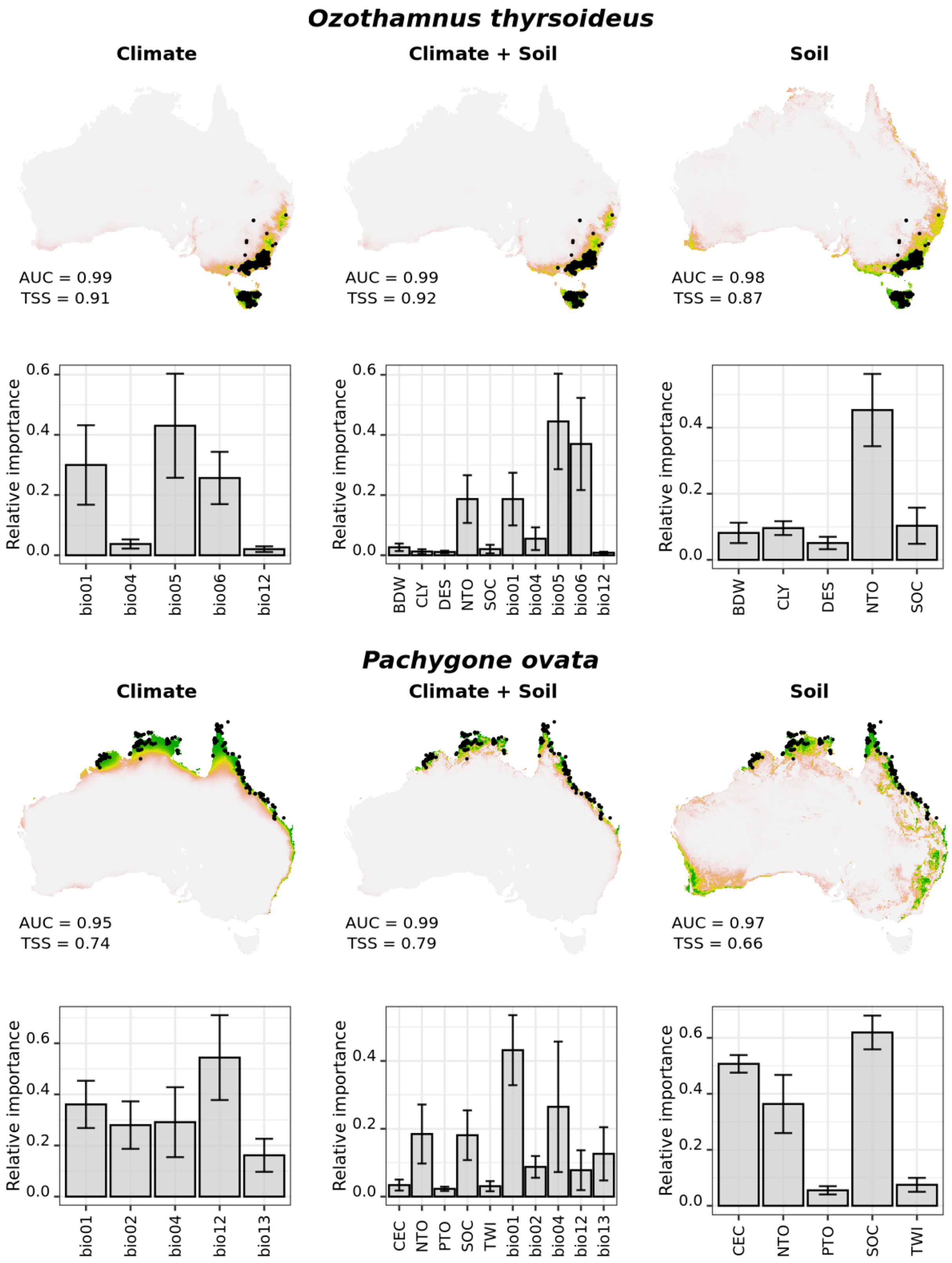

Ozothamnus thyrsoideus
Climate
Climate + Soil
Soil
AUC = 0.99
TSS = 0.91
AUC = 0.99
TSS = 0.92
AUC = 0.98
TSS = 0.87
Relative importance
bio01
bio04
bio05
bio06
bio12
BDW
CLY
DES
NTO
SOC
Pachygone ovata
AUC = 0.95
TSS = 0.74
AUC = 0.99
TSS = 0.79
AUC = 0.97
TSS = 0.66
bio02
bio13
CEC
PTO
TWI

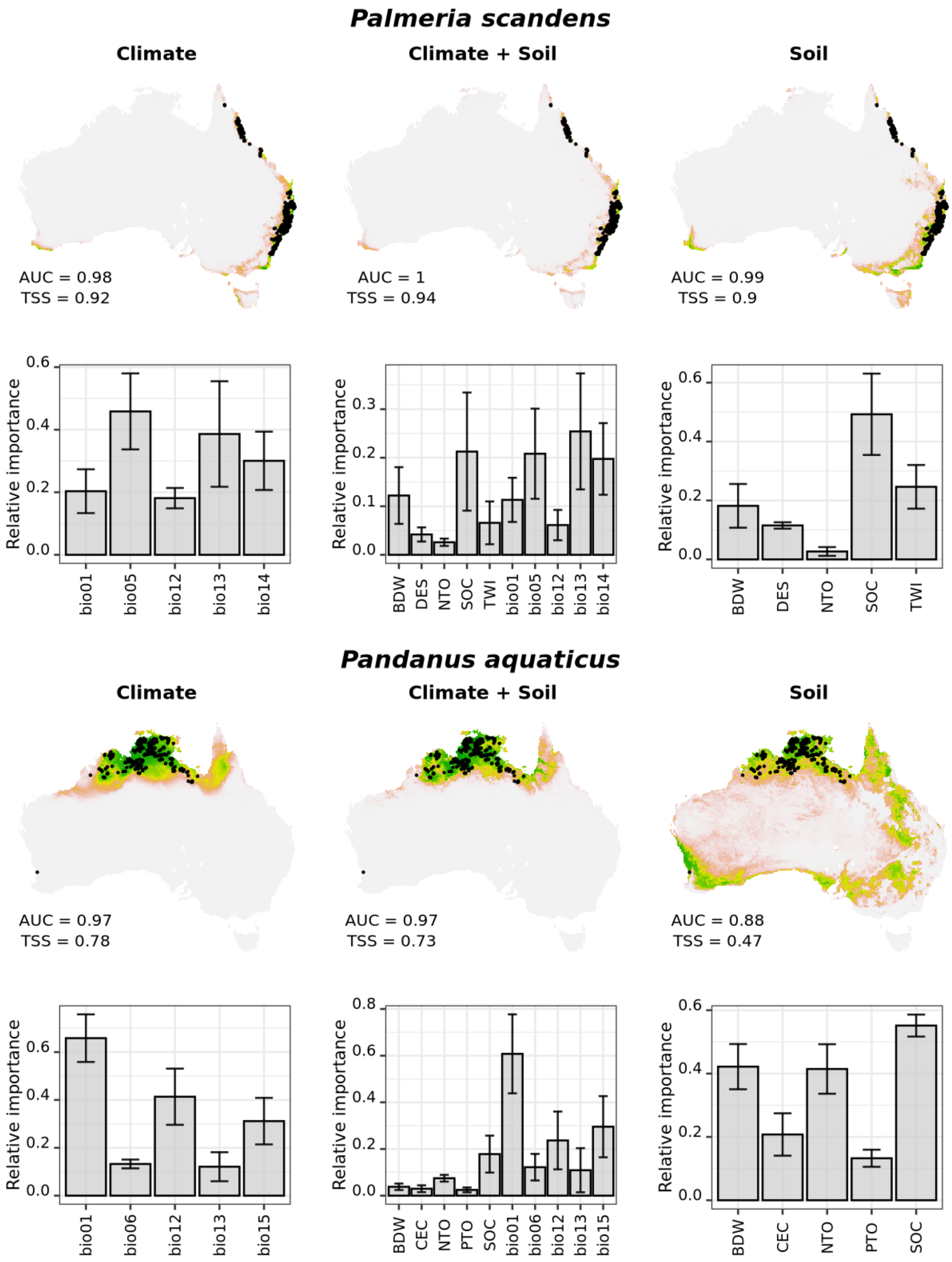
Palmeria scandens
Climate
Climate + Soil
Soil
AUC = 0.98
TSS = 0.92
AUC = 1
TSS = 0.94
AUC = 0.99
TSS = 0.9
Relative importance
0.0
0.2
0.4
0.6
bio01
bio05
bio12
bio13
bio14
0.0
0.1
0.2
0.3
BDW
DES
NTO
SOC
TWI
bio01
bio05
bio12
bio13
bio14
BDW
DES
NTO
SOC
TWI
Pandanus aquaticus
Climate
Climate + Soil
Soil
AUC = 0.97
TSS = 0.78
AUC = 0.97
TSS = 0.73
AUC = 0.88
TSS = 0.47
bio01
bio06
bio12
bio13
bio15
0.8
BDW
CEC
NTO
PTO
SOC
bio01
bio06
bio12
bio13
bio15
BDW
CEC
NTO
PTO
SOC

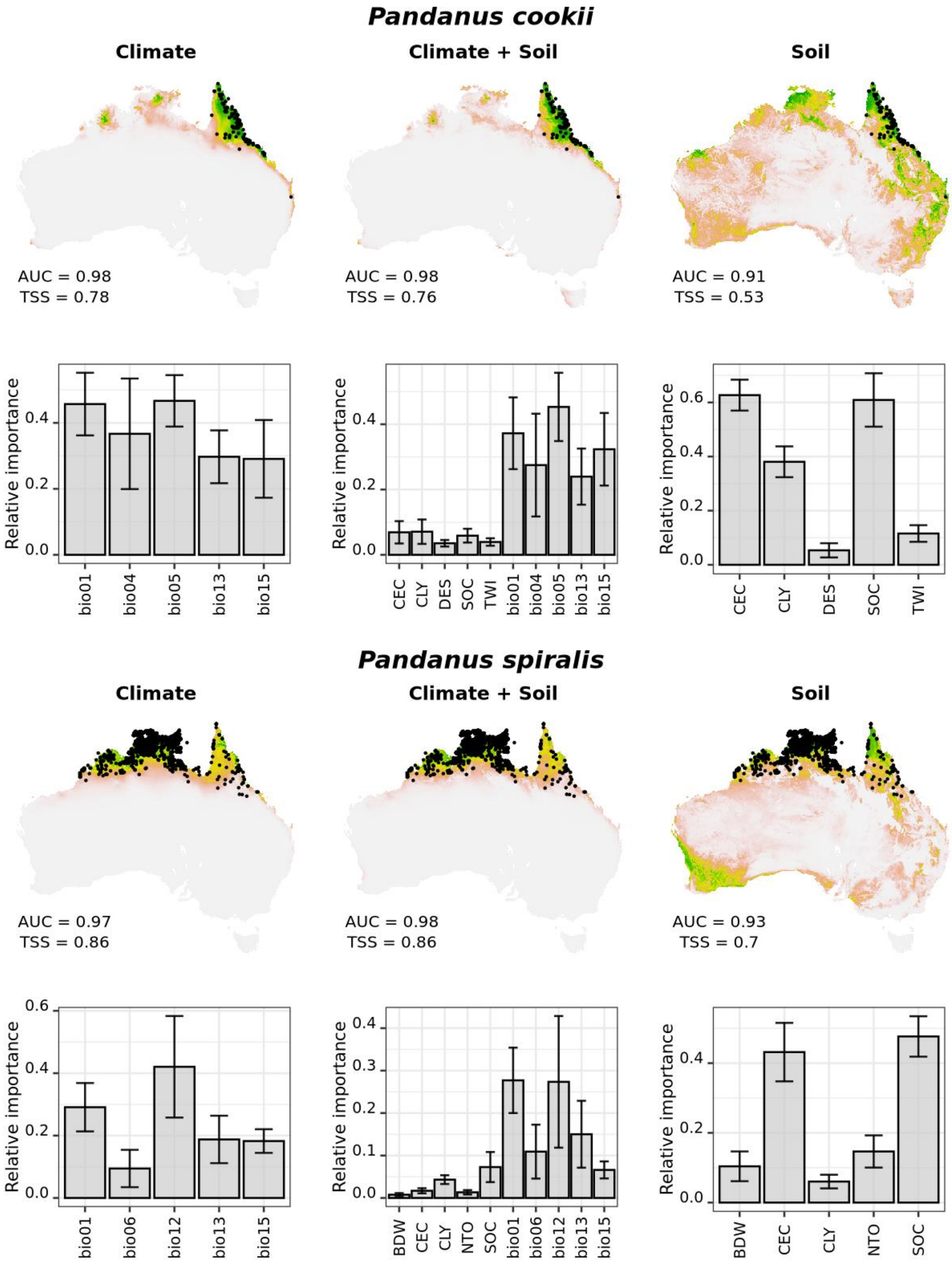

Pandanus cookii
Climate
Climate + Soil
Soil
AUC = 0.98
TSS = 0.78
AUC = 0.98
TSS = 0.76
AUC = 0.91
TSS = 0.53
Relative importance
bio01
bio04
bio05
bio13
bio15
CEC
CLY
DES
SOC
TWI
Pandanus spiralis
Climate
Climate + Soil
Soil
AUC = 0.97
TSS = 0.86
AUC = 0.98
TSS = 0.86
AUC = 0.93
TSS = 0.7
Relative importance
bio01
bio06
bio12
bio13
bio15
BDW
CEC
CLY
NTO
SOC

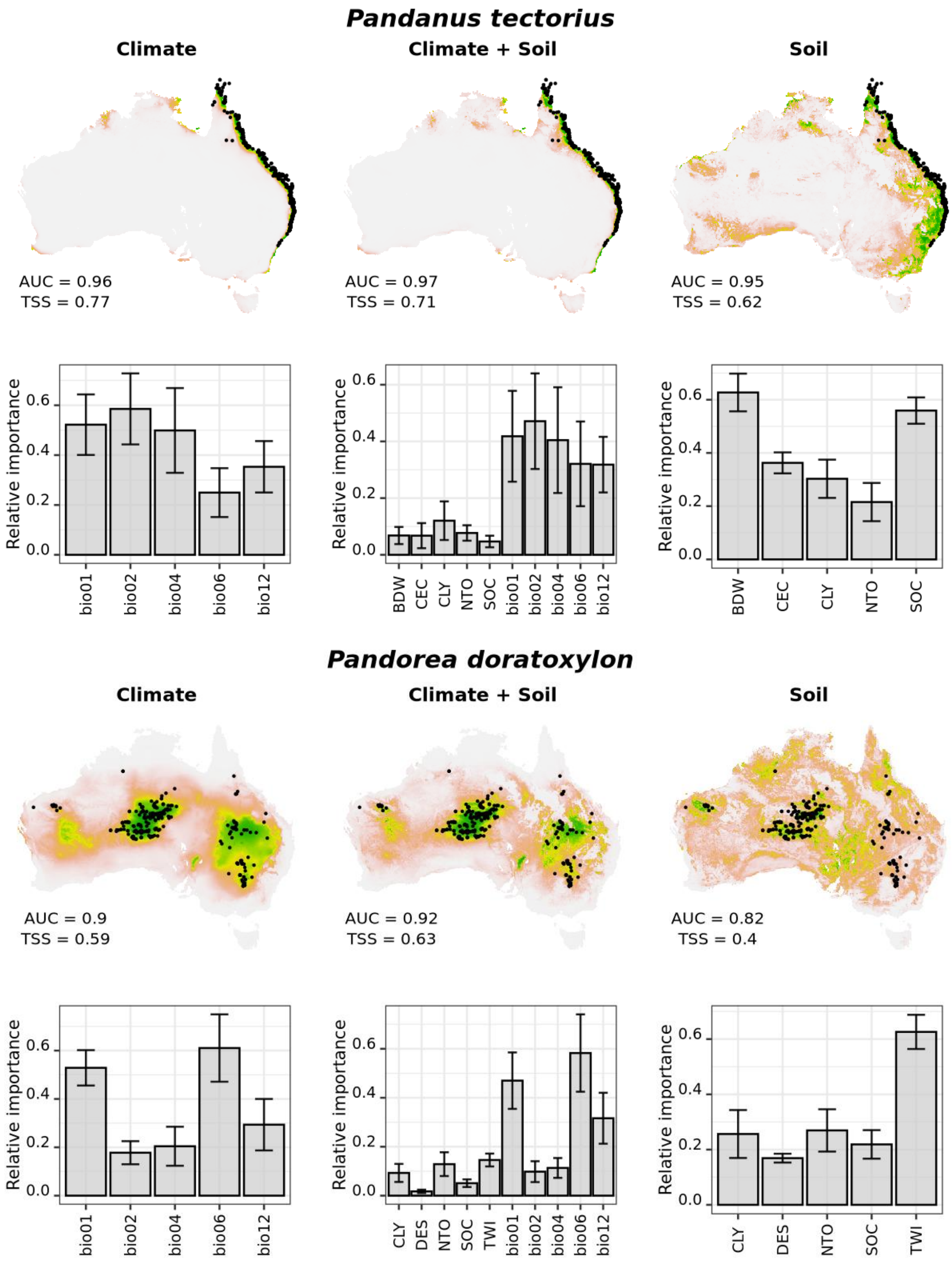

Pandanus tectorius
Climate
Climate + Soil
Soil
AUC = 0.96
TSS = 0.77
AUC = 0.97
TSS = 0.71
AUC = 0.95
TSS = 0.62
Relative importance
0.0
0.2
0.4
0.6
bio01
bio02
bio04
bio06
bio12
BDW
CEC
CLY
NTO
SOC
Pandorea doratoxylon
Climate
Climate + Soil
Soil
AUC = 0.9
TSS = 0.59
AUC = 0.92
TSS = 0.63
AUC = 0.82
TSS = 0.4
Relative importance
0.0
0.2
0.4
0.6
bio01
bio02
bio04
bio06
bio12
CLY
DES
NTO
SOC
TWI

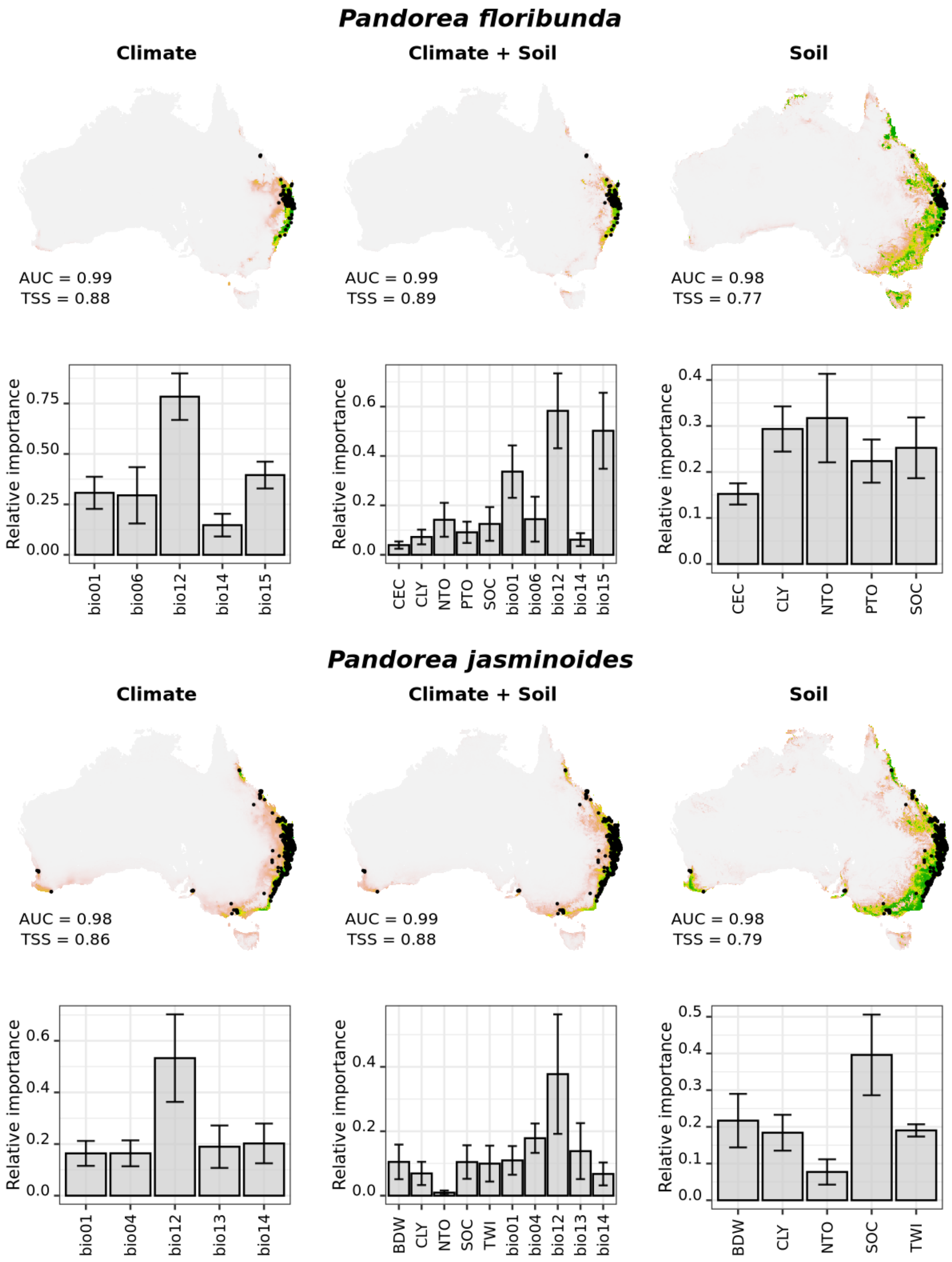

Pandorea floribunda
Climate
Climate + Soil
Soil
AUC = 0.99
TSS = 0.88
AUC = 0.99
TSS = 0.89
AUC = 0.98
TSS = 0.77
Relative importance
bio01
bio06
bio12
bio14
bio15
CEC
CLY
NTO
PTO
SOC
Pandorea jasminoides
Climate
Climate + Soil
Soil
AUC = 0.98
TSS = 0.86
AUC = 0.99
TSS = 0.88
AUC = 0.98
TSS = 0.79
Relative importance
bio01
bio04
bio12
bio13
bio14
BDW
CLY
NTO
SOC
TWI

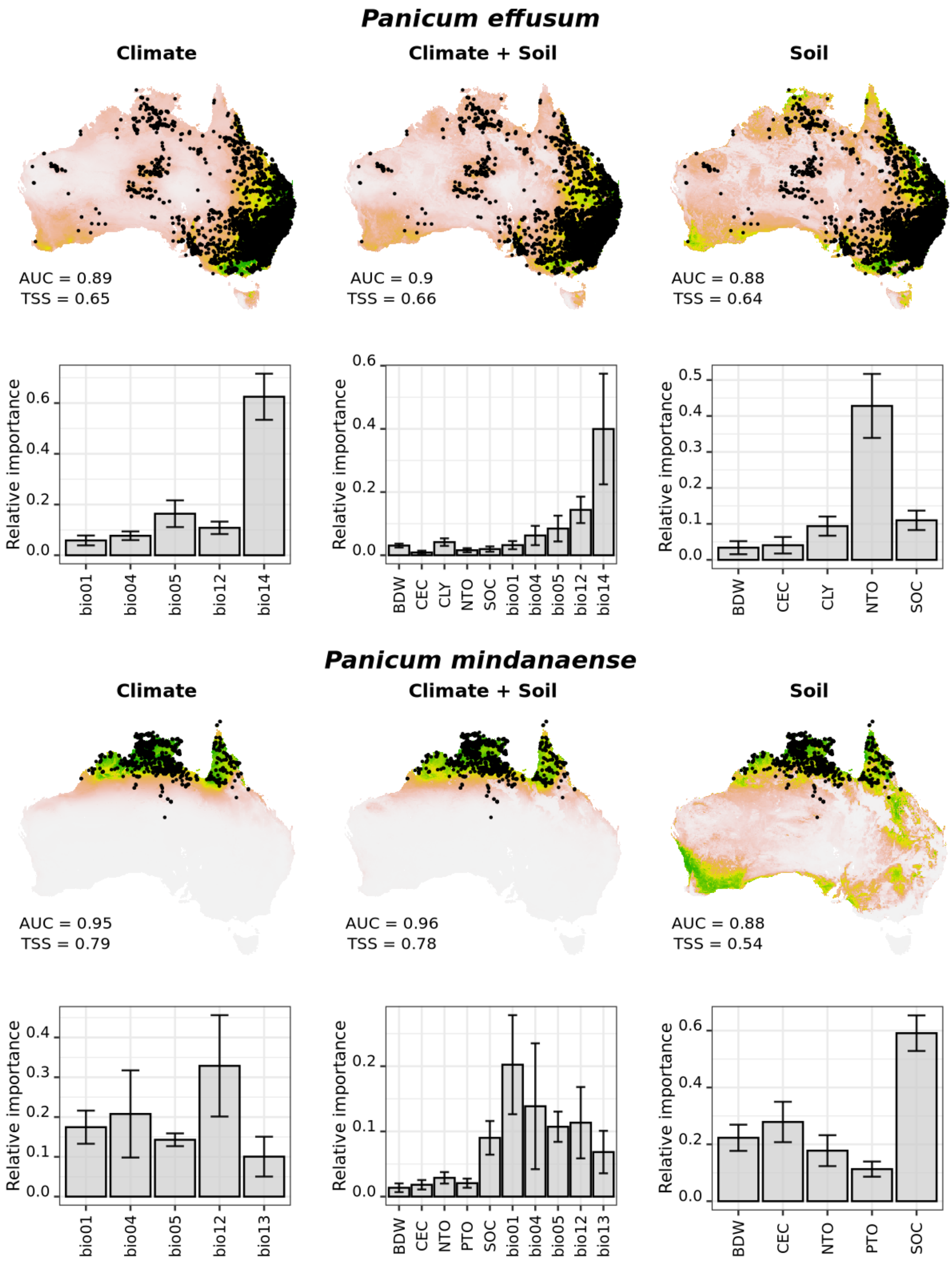

Panicum effusum
Climate
Climate + Soil
Soil
AUC = 0.89
TSS = 0.65
AUC = 0.9
TSS = 0.66
AUC = 0.88
TSS = 0.64
Relative importance
bio01
bio04
bio05
bio12
bio14
BDW
CEC
CLY
NTO
SOC
Panicum mindanaense
Climate
Climate + Soil
Soil
AUC = 0.95
TSS = 0.79
AUC = 0.96
TSS = 0.78
AUC = 0.88
TSS = 0.54
Relative importance
bio13
PTO

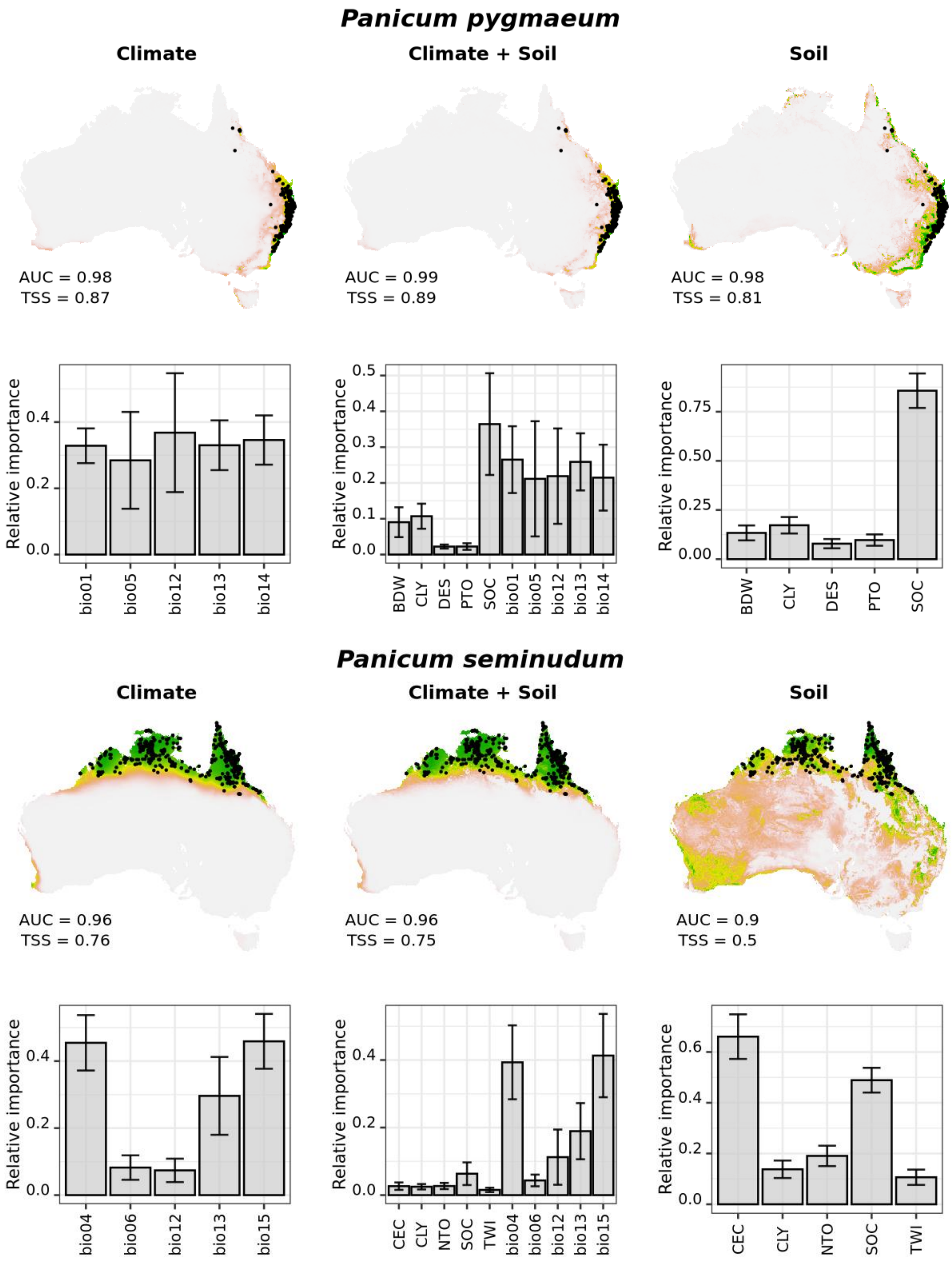

Panicum pygmaeum
Climate
Climate + Soil
Soil
AUC = 0.98
TSS = 0.87
AUC = 0.99
TSS = 0.89
AUC = 0.98
TSS = 0.81
Relative importance
0.0
0.2
0.4
bio01
bio05
bio12
bio13
bio14
0.1
0.3
0.5
BDW
CLY
DES
PTO
SOC
0.00
0.25
0.50
0.75
Panicum seminudum
AUC = 0.96
TSS = 0.76
AUC = 0.96
TSS = 0.75
AUC = 0.9
TSS = 0.5
bio04
bio06
bio15
CEC
NTO
TWI
0.6

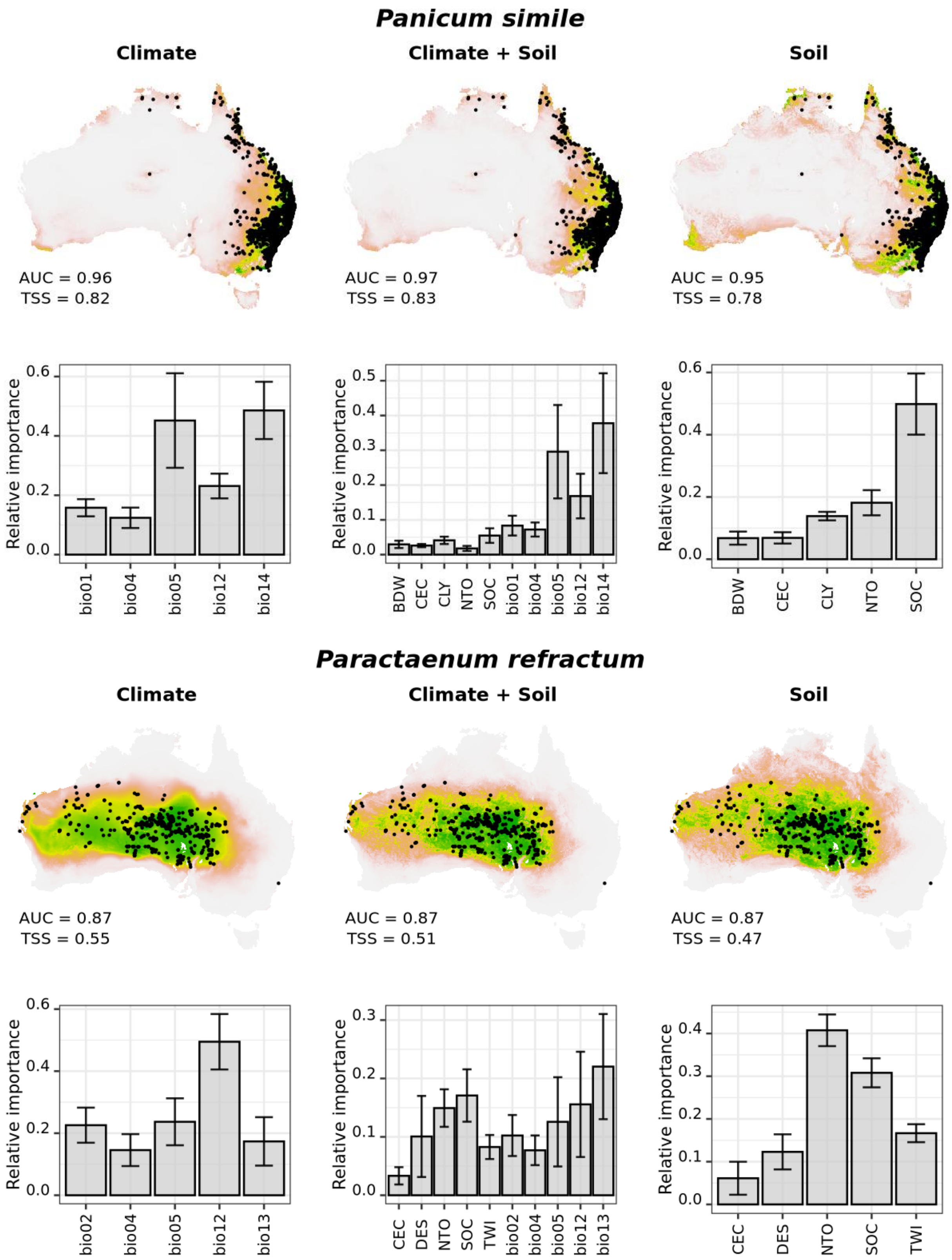

Panicum simile
Climate
Climate + Soil
Soil
AUC = 0.96
TSS = 0.82
AUC = 0.97
TSS = 0.83
AUC = 0.95
TSS = 0.78
Relative importance
bio01
bio04
bio05
bio12
bio14
BDW
CEC
CLY
NTO
SOC
Paractaenum refractum
AUC = 0.87
TSS = 0.55
AUC = 0.87
TSS = 0.51
AUC = 0.87
TSS = 0.47
bio02
bio13
DES
TWI

Paraneurachne muelleri

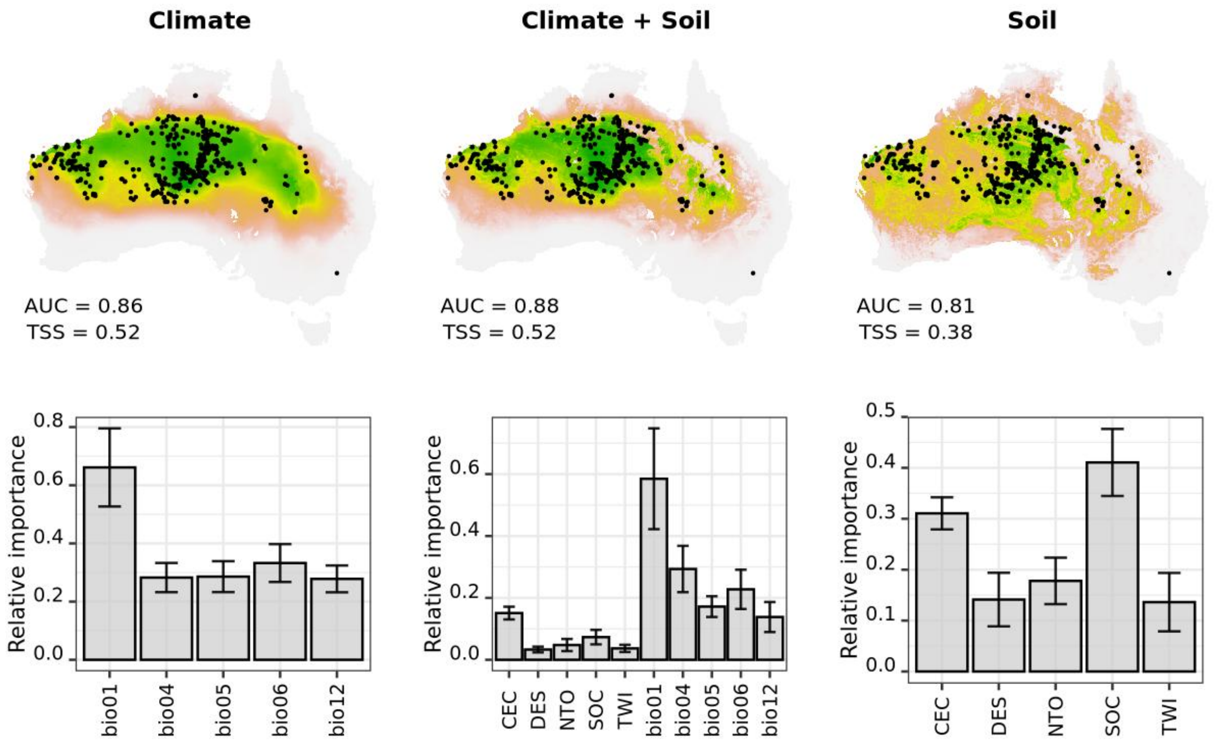

Pararchidendron pruinosum

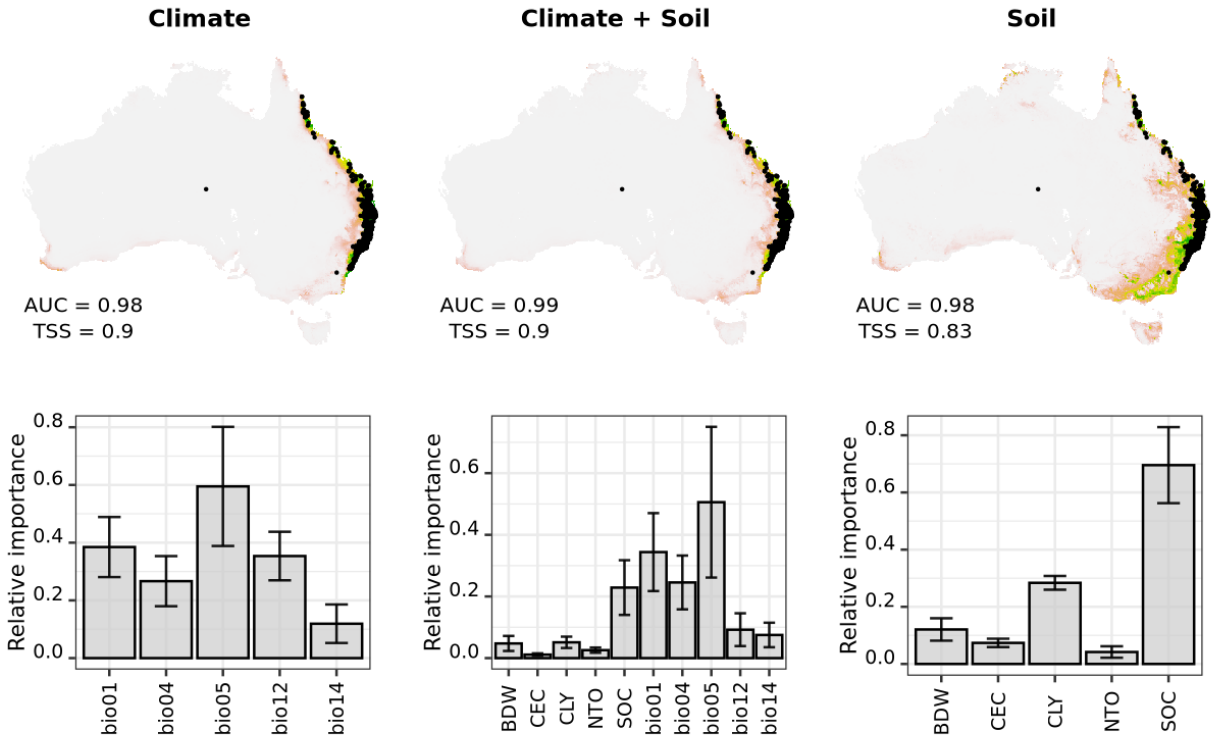

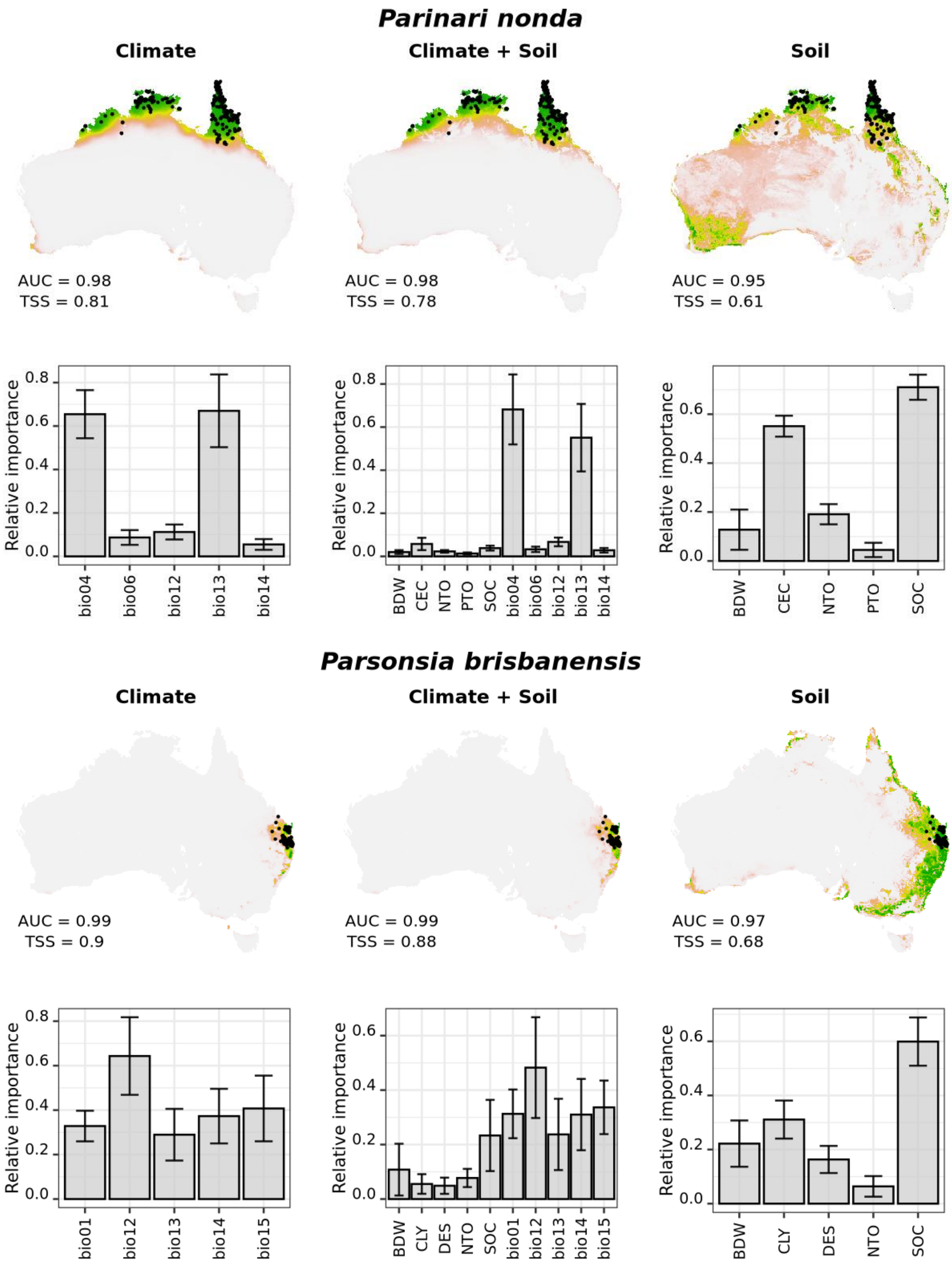

Parinari nonda
Climate
Climate + Soil
Soil
AUC = 0.98
TSS = 0.81
AUC = 0.98
TSS = 0.78
AUC = 0.95
TSS = 0.61
Relative importance
bio04
bio06
bio12
bio13
bio14
BDW
CEC
NTO
PTO
SOC
Parsonsia brisbanensis
Climate
Climate + Soil
Soil
AUC = 0.99
TSS = 0.9
AUC = 0.99
TSS = 0.88
AUC = 0.97
TSS = 0.68
Relative importance
bio01
bio12
bio13
bio14
bio15
BDW
CLY
DES
NTO
SOC

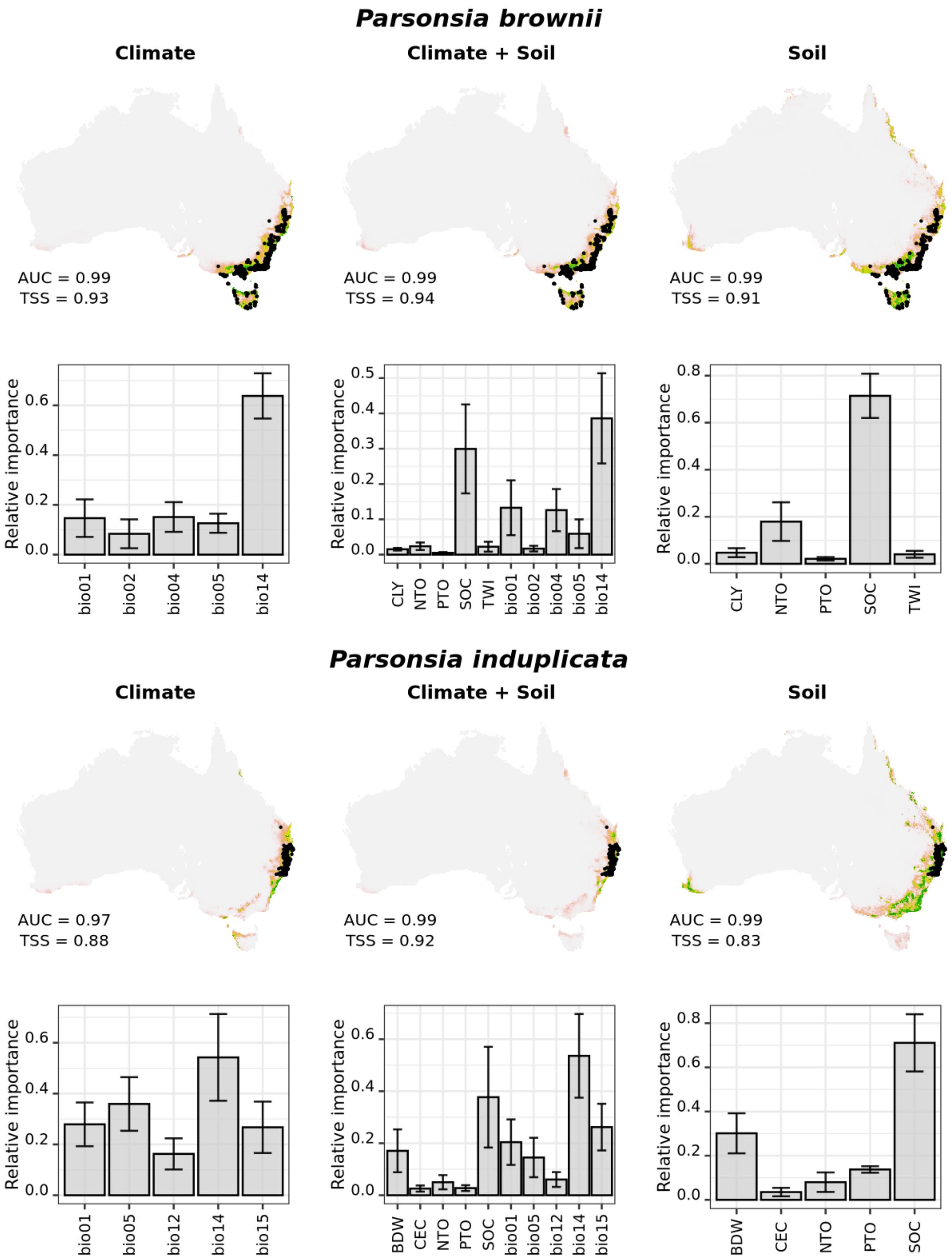

Parsonsia brownii
Climate
Climate + Soil
Soil
AUC = 0.99
TSS = 0.93
AUC = 0.99
TSS = 0.94
AUC = 0.99
TSS = 0.91
Relative importance
bio01 bio02 bio04 bio05 bio14
CLY NTO PTO SOC TWI bio01 bio02 bio04 bio05 bio14
CLY NTO PTO SOC TWI
Parsonsia induplicata
Climate
Climate + Soil
Soil
AUC = 0.97
TSS = 0.88
AUC = 0.99
TSS = 0.92
AUC = 0.99
TSS = 0.83
Relative importance
bio01 bio05 bio12 bio14 bio15
BDW CEC NTO PTO SOC bio01 bio05 bio12 bio14 bio15
BDW CEC NTO PTO SOC

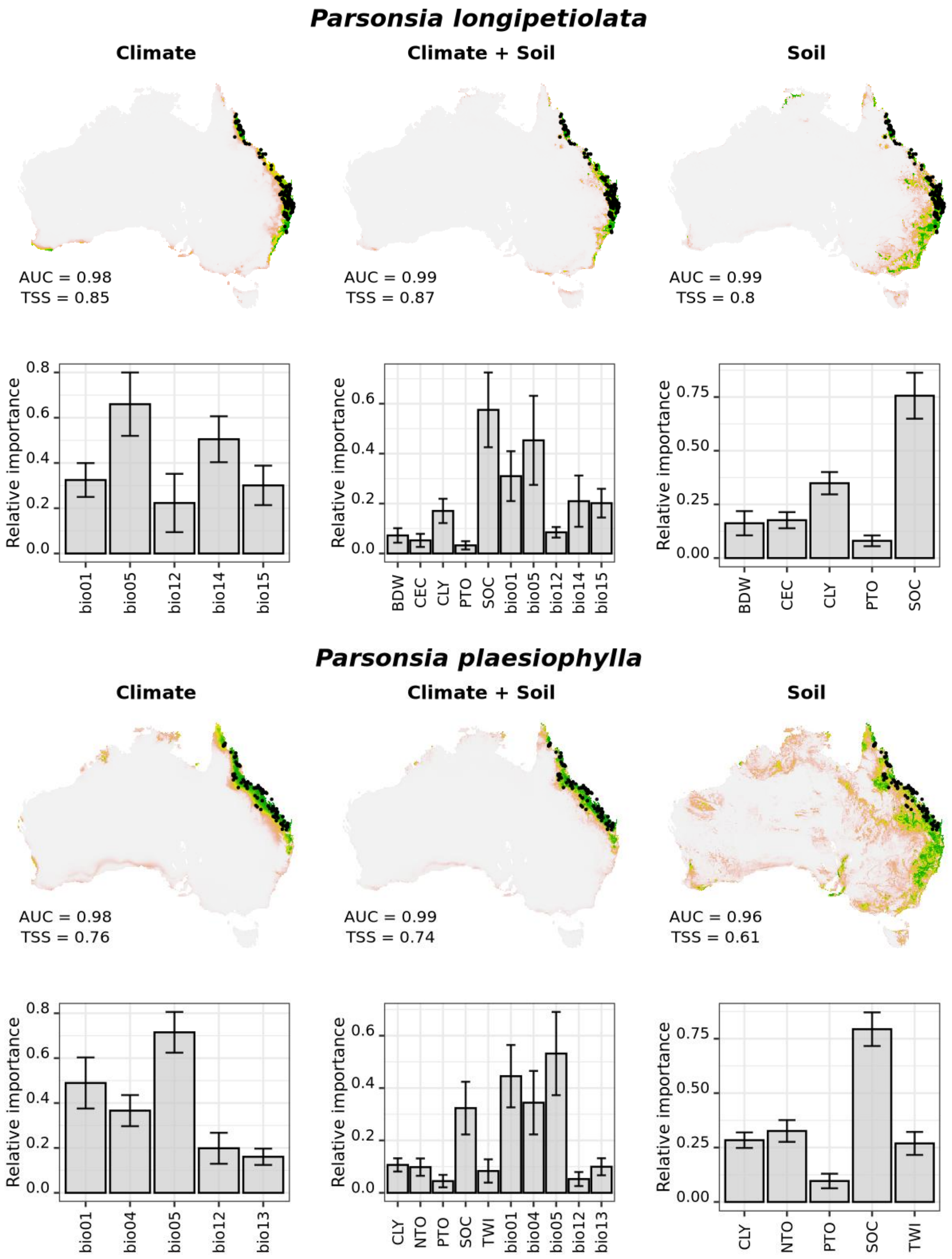
Parsonsia longipetiolata
Climate
Climate + Soil
Soil
AUC = 0.98
TSS = 0.85
AUC = 0.99
TSS = 0.87
AUC = 0.99
TSS = 0.8
Relative importance
bio01
bio05
bio12
bio14
bio15
BDW
CEC
CLY
PTO
SOC
Parsonsia plaesiophylla
AUC = 0.98
TSS = 0.76
AUC = 0.99
TSS = 0.74
AUC = 0.96
TSS = 0.61
bio04
bio13
NTO
TWI

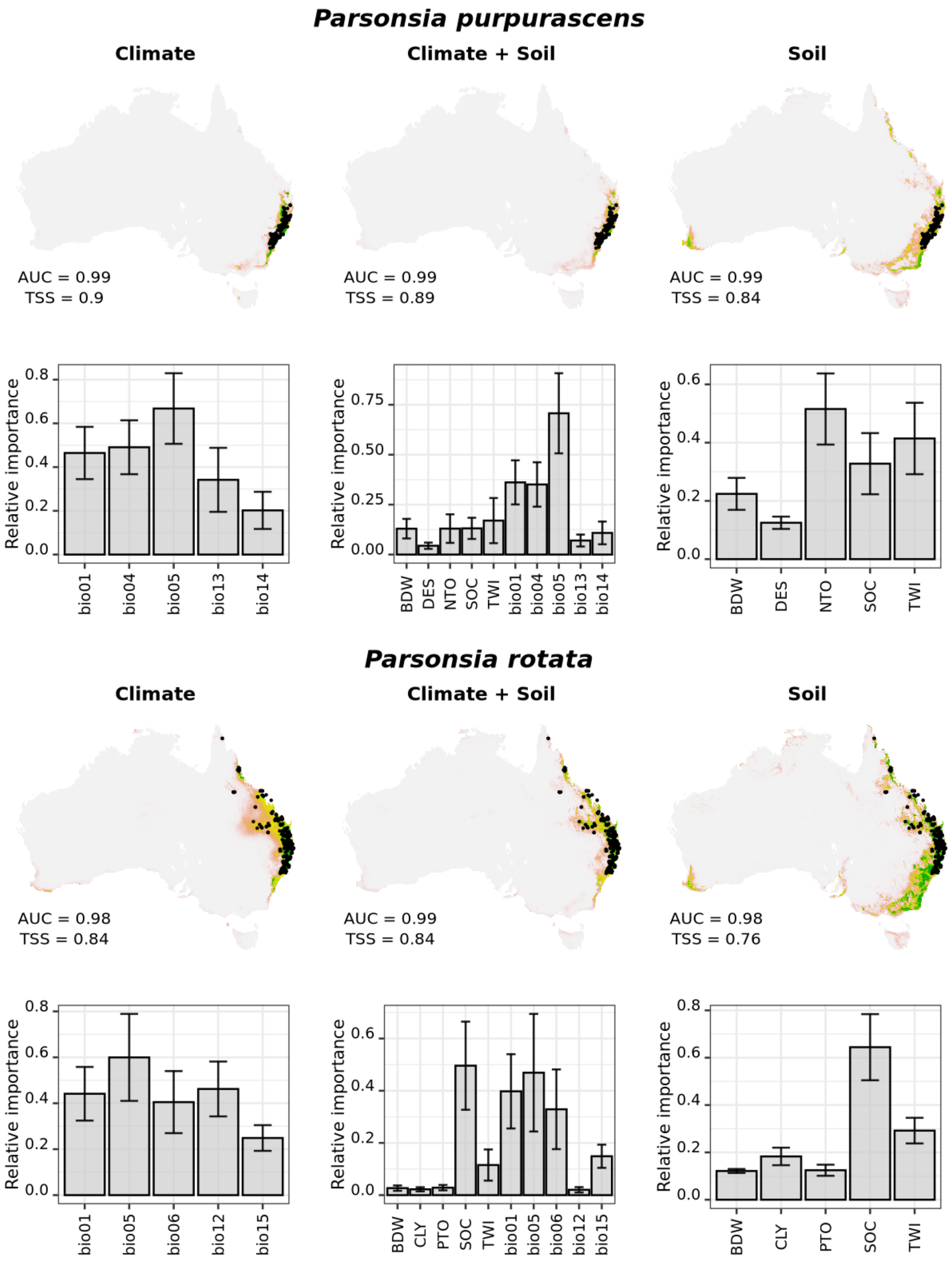
Parsonsia purpurascens
Climate
Climate + Soil
Soil
AUC = 0.99
TSS = 0.9
AUC = 0.99
TSS = 0.89
AUC = 0.99
TSS = 0.84
Relative importance
bio01
bio04
bio05
bio13
bio14
BDW
DES
NTO
SOC
TWI
bio01
bio04
bio05
bio13
bio14
BDW
DES
NTO
SOC
TWI
Parsonsia rotata
Climate
Climate + Soil
Soil
AUC = 0.98
TSS = 0.84
AUC = 0.99
TSS = 0.84
AUC = 0.98
TSS = 0.76
Relative importance
bio01
bio05
bio06
bio12
bio15
BDW
CLY
PTO
SOC
TWI
bio01
bio05
bio06
bio12
bio15
BDW
CLY
PTO
SOC
TWI

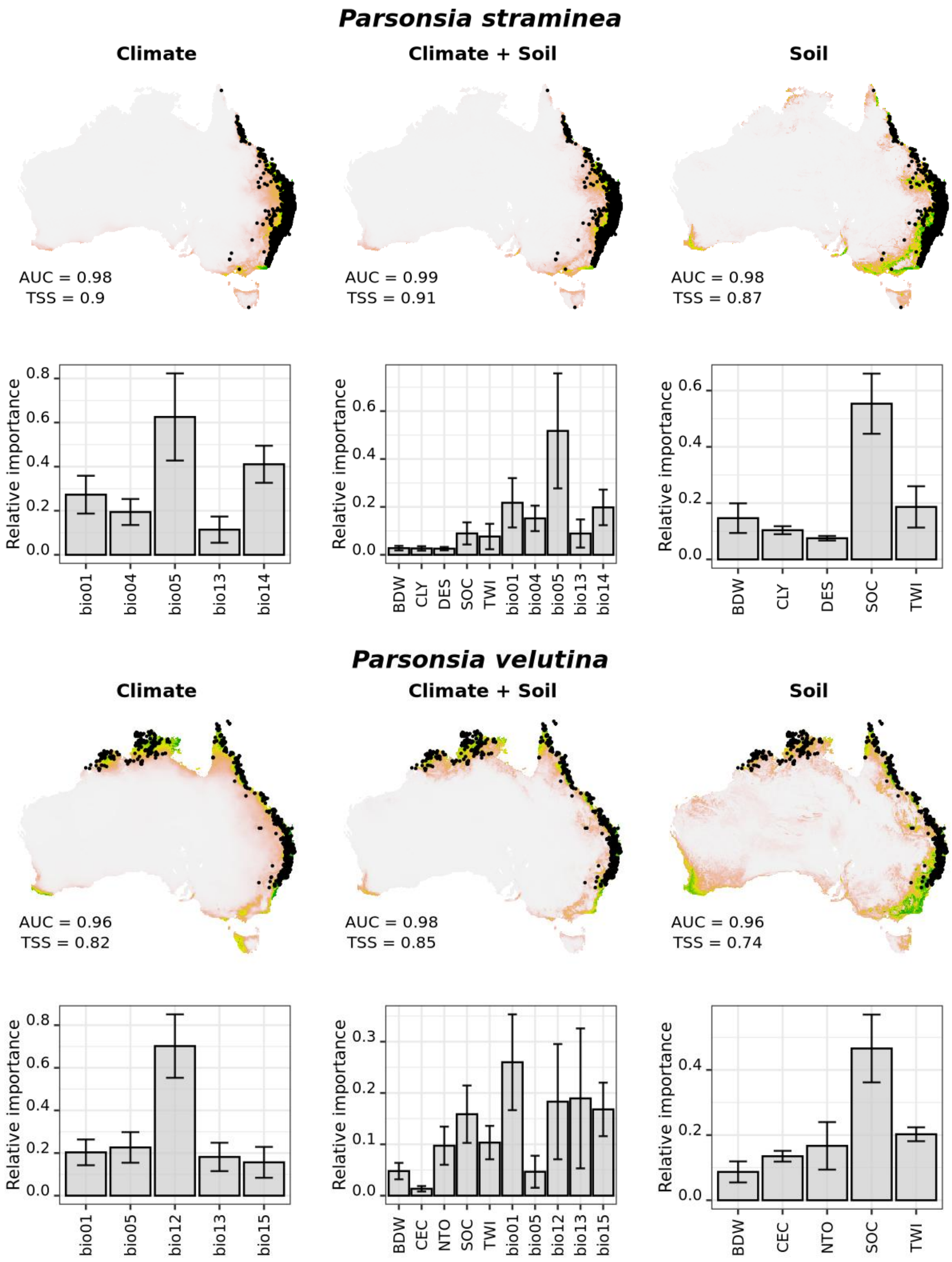

Parsonsia straminea
Climate
Climate + Soil
Soil
AUC = 0.98
TSS = 0.9
AUC = 0.99
TSS = 0.91
AUC = 0.98
TSS = 0.87
Relative importance
bio01
bio04
bio05
bio13
bio14
BDW
CLY
DES
SOC
TWI
Parsonsia velutina
Climate
Climate + Soil
Soil
AUC = 0.96
TSS = 0.82
AUC = 0.98
TSS = 0.85
AUC = 0.96
TSS = 0.74
Relative importance
bio01
bio05
bio12
bio13
bio15
BDW
CEC
NTO
SOC
TWI

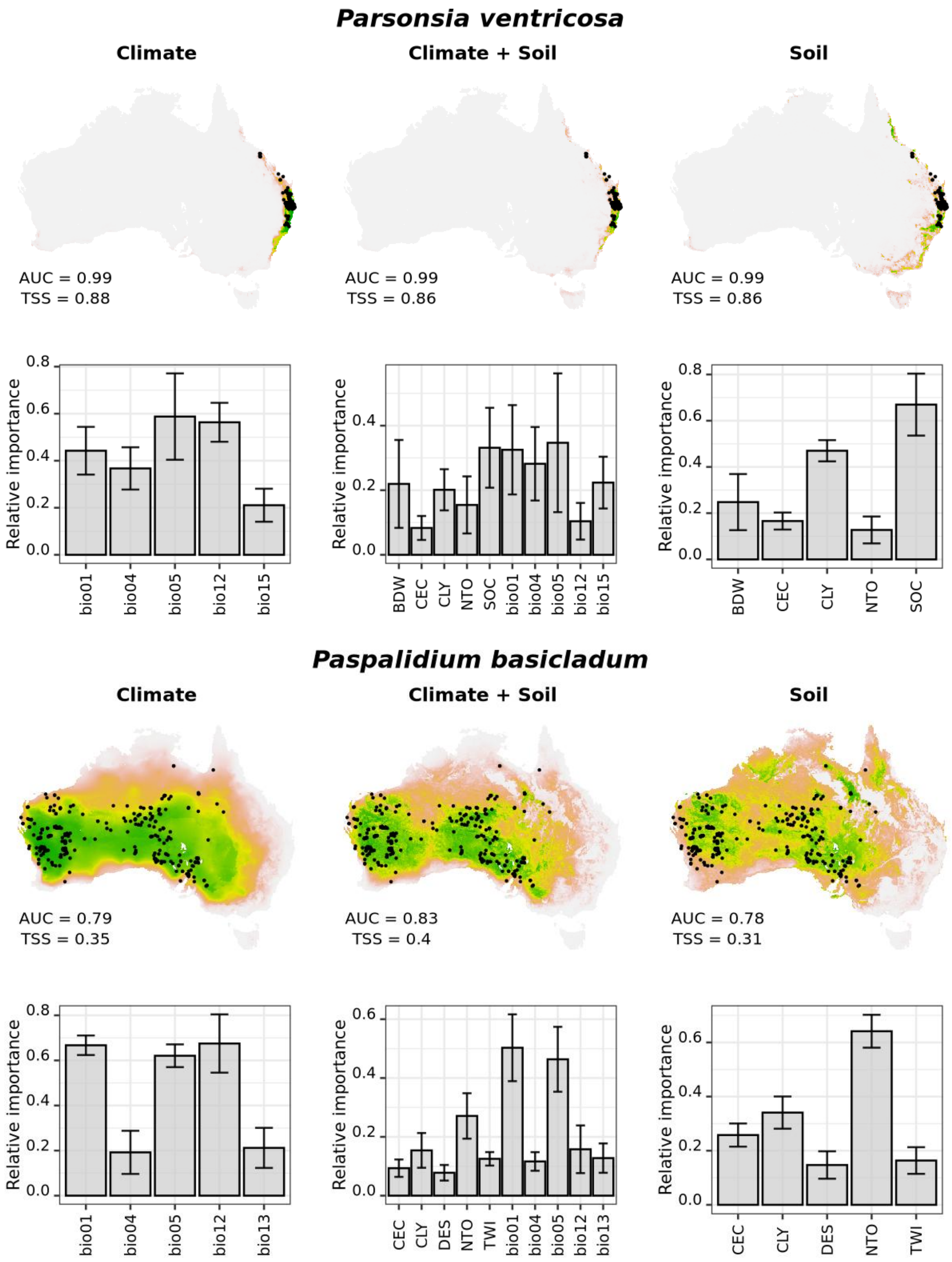

Parsonsia ventricosa
Climate
Climate + Soil
Soil
AUC = 0.99
TSS = 0.88
AUC = 0.99
TSS = 0.86
AUC = 0.99
TSS = 0.86
Relative importance
bio01
bio04
bio05
bio12
bio15
BDW
CEC
CLY
NTO
SOC
Paspalidium basicladum
AUC = 0.79
TSS = 0.35
AUC = 0.83
TSS = 0.4
AUC = 0.78
TSS = 0.31
bio13
DES
TWI

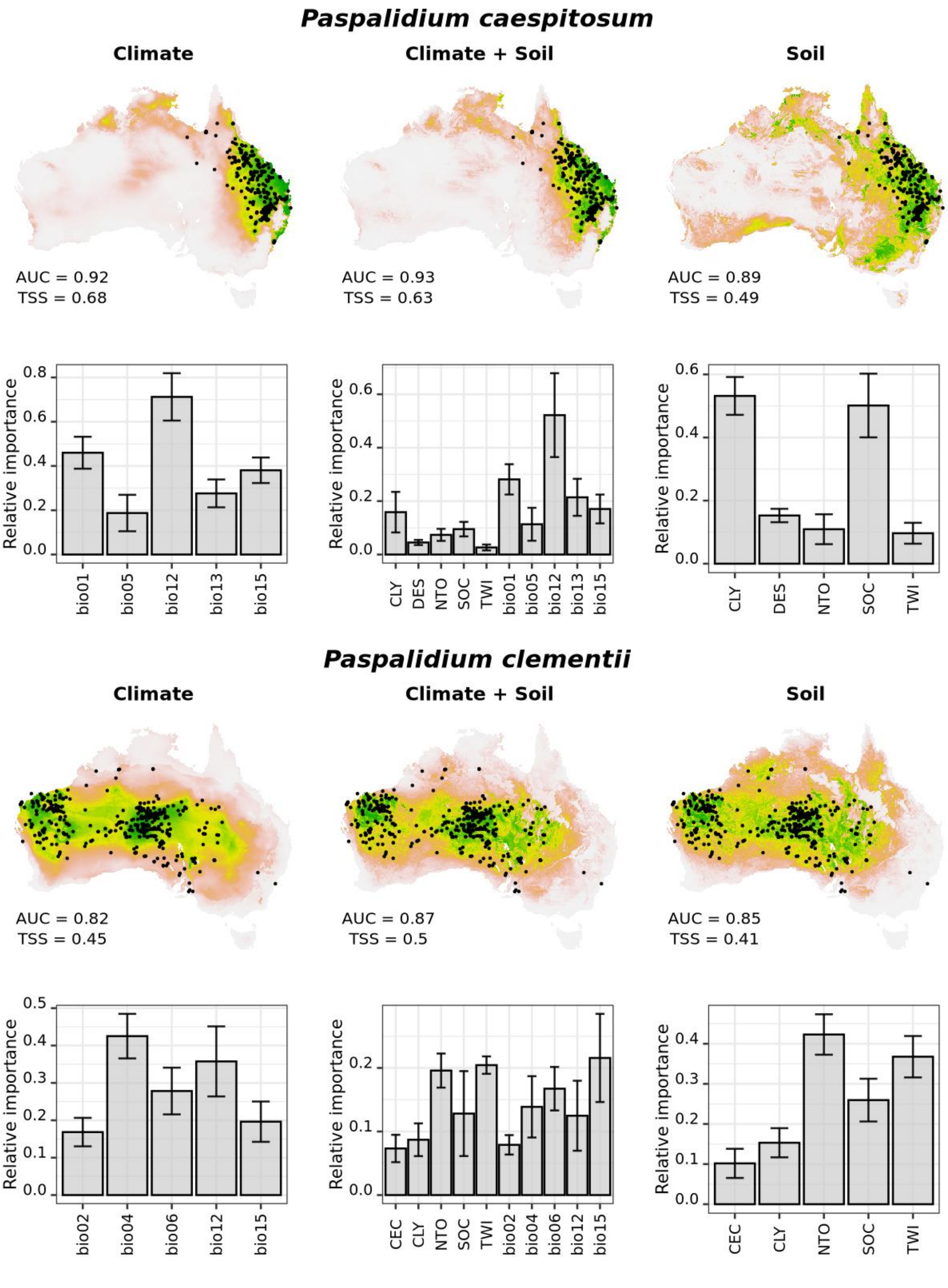
Paspalidium caespitosum
Climate
Climate + Soil
Soil
AUC = 0.92
TSS = 0.68
AUC = 0.93
TSS = 0.63
AUC = 0.89
TSS = 0.49
Relative importance
bio01
bio05
bio12
bio13
bio15
CLY
DES
NTO
SOC
TWI
Paspalidium clementii
Climate
Climate + Soil
Soil
AUC = 0.82
TSS = 0.45
AUC = 0.87
TSS = 0.5
AUC = 0.85
TSS = 0.41
Relative importance
bio02
bio04
bio06
bio12
bio15
CEC
CLY
NTO
SOC
TWI

Paspalidium criniforme

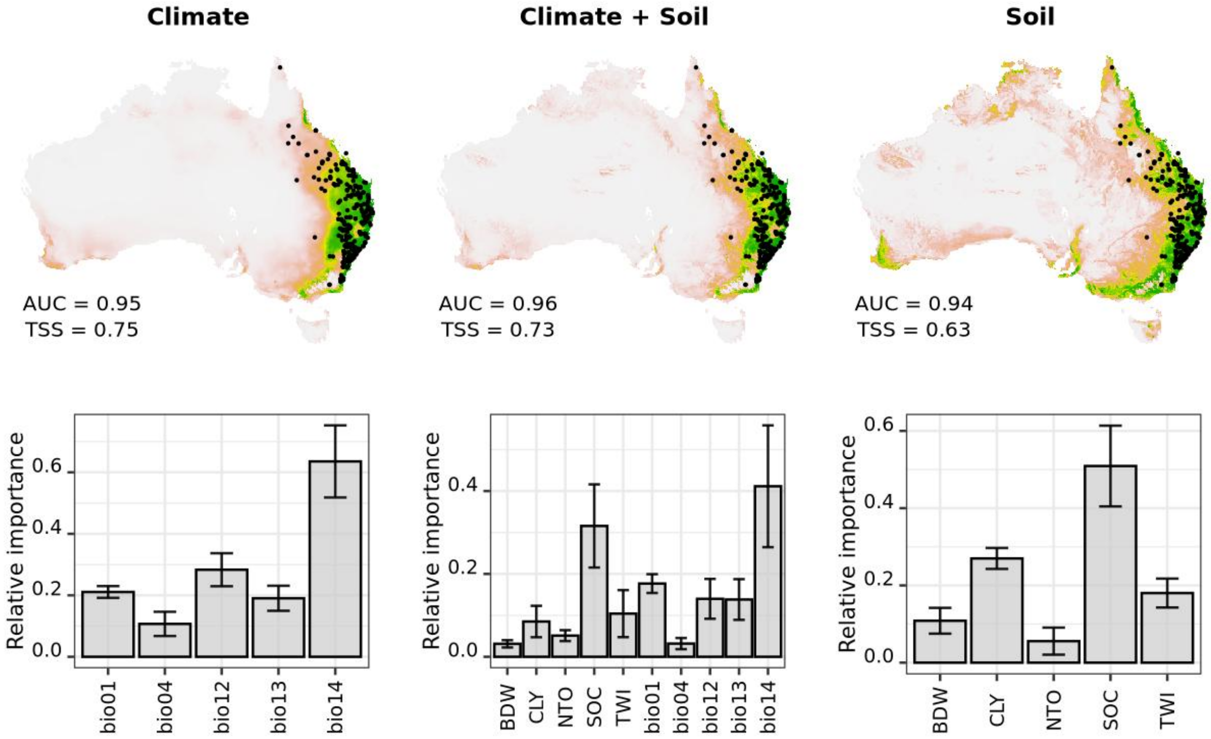

Paspalidium gausum

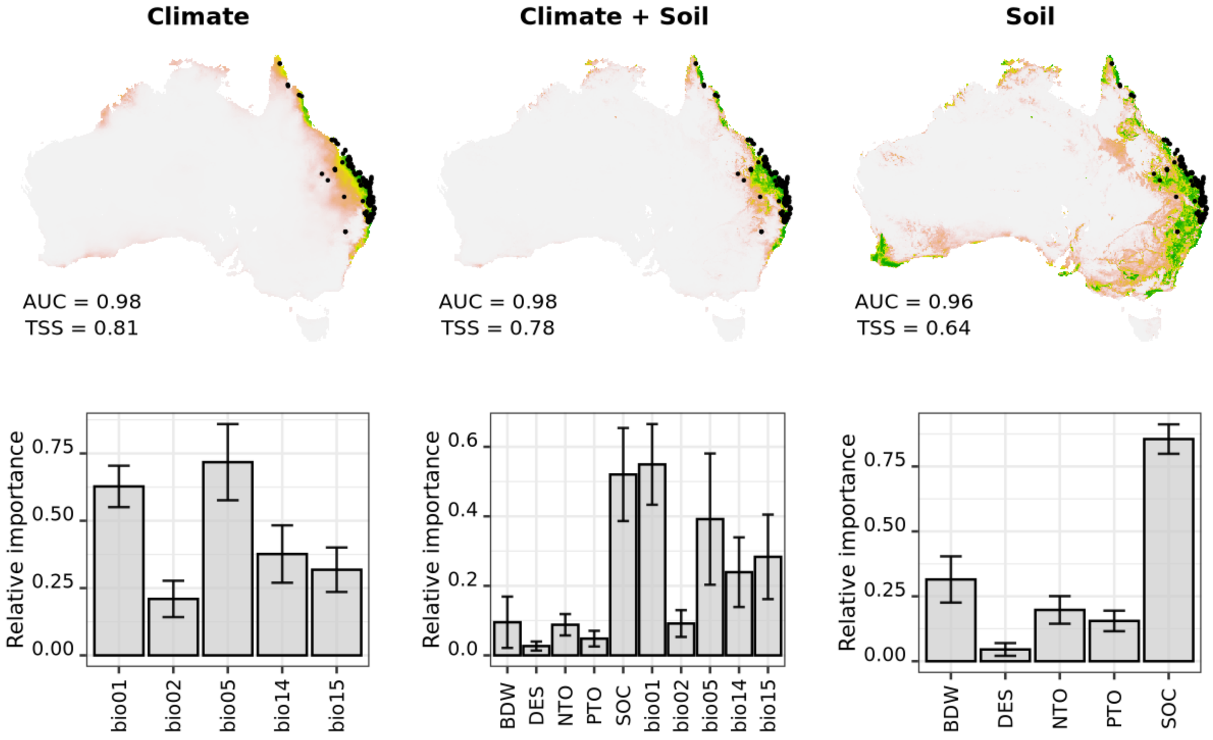

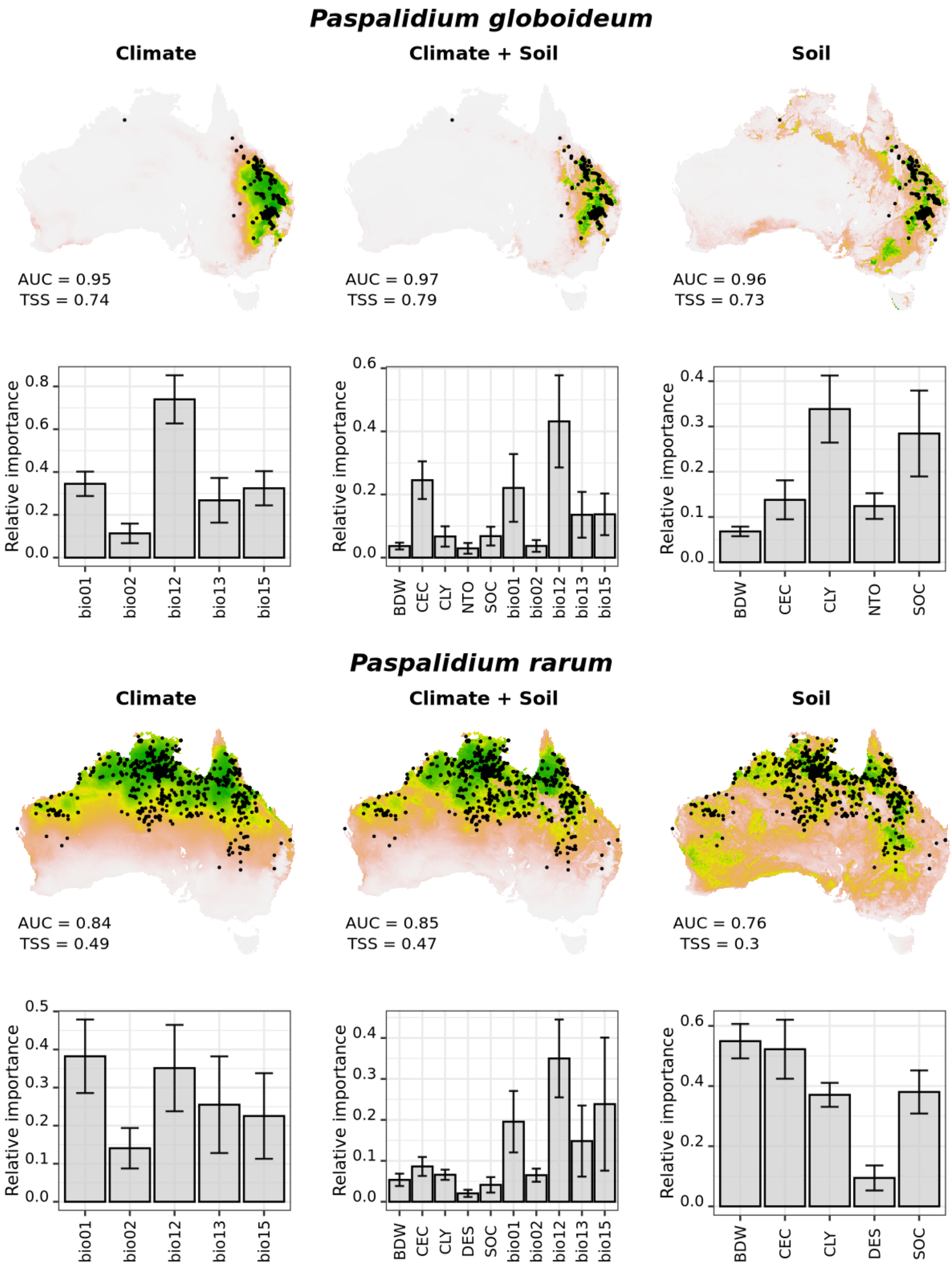
Paspalidium globoideum
Climate
Climate + Soil
Soil
AUC = 0.95
TSS = 0.74
AUC = 0.97
TSS = 0.79
AUC = 0.96
TSS = 0.73
Relative importance
bio01
bio02
bio12
bio13
bio15
BDW
CEC
CLY
NTO
SOC
Paspalidium rarum
Climate
Climate + Soil
Soil
AUC = 0.84
TSS = 0.49
AUC = 0.85
TSS = 0.47
AUC = 0.76
TSS = 0.3
Relative importance
bio01
bio02
bio12
bio13
bio15
BDW
CEC
CLY
DES
SOC

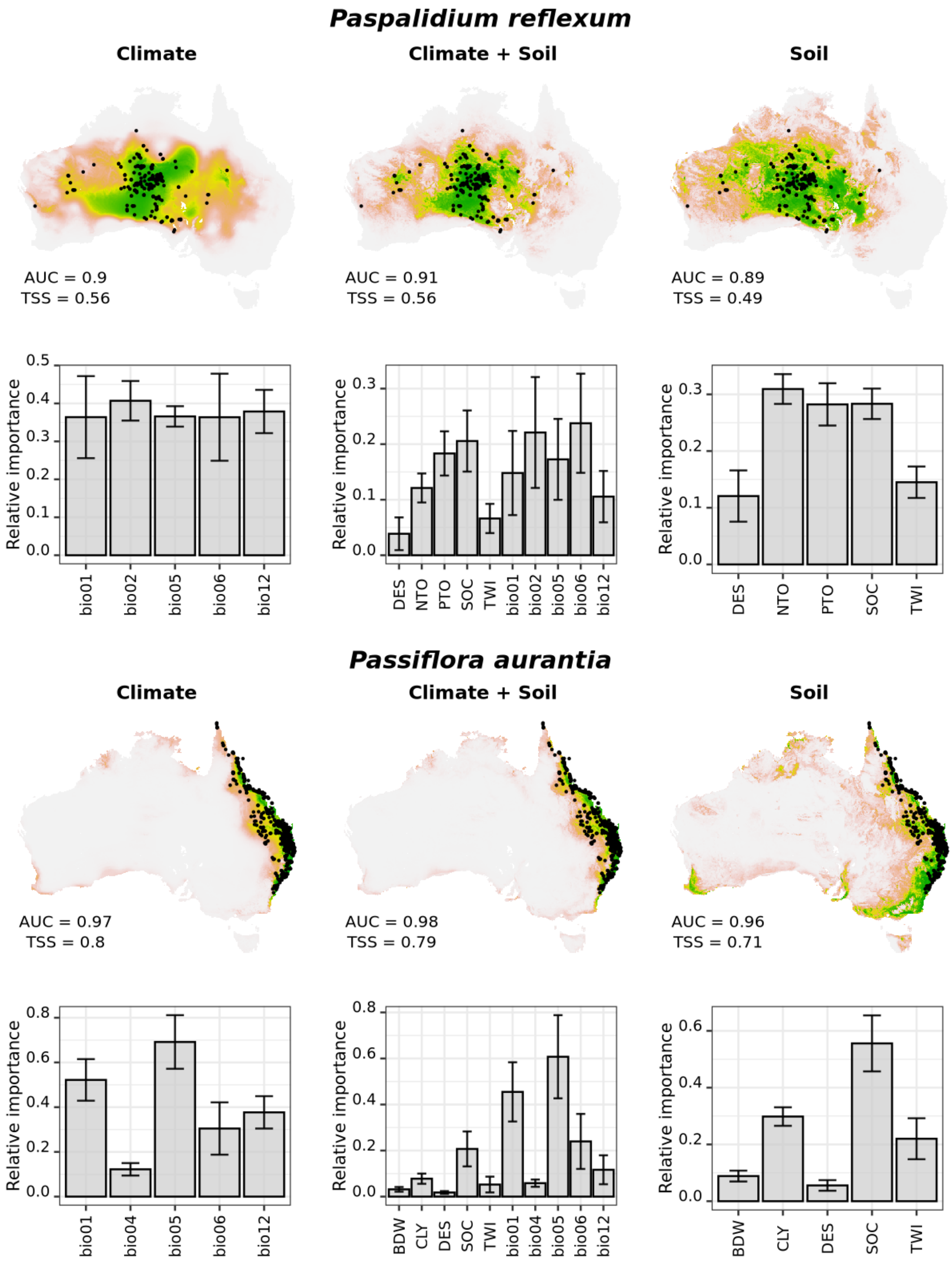
Paspalidium reflexum
Climate
Climate + Soil
Soil
AUC = 0.9
TSS = 0.56
AUC = 0.91
TSS = 0.56
AUC = 0.89
TSS = 0.49
Relative importance
bio01 bio02 bio05 bio06 bio12
DES NTO PTO SOC TWI bio01 bio02 bio05 bio06 bio12
DES NTO PTO SOC TWI
Passiflora aurantia
Climate
Climate + Soil
Soil
AUC = 0.97
TSS = 0.8
AUC = 0.98
TSS = 0.79
AUC = 0.96
TSS = 0.71
Relative importance
bio01 bio04 bio05 bio06 bio12
BDW CLY DES SOC TWI bio01 bio04 bio05 bio06 bio12
BDW CLY DES SOC TWI

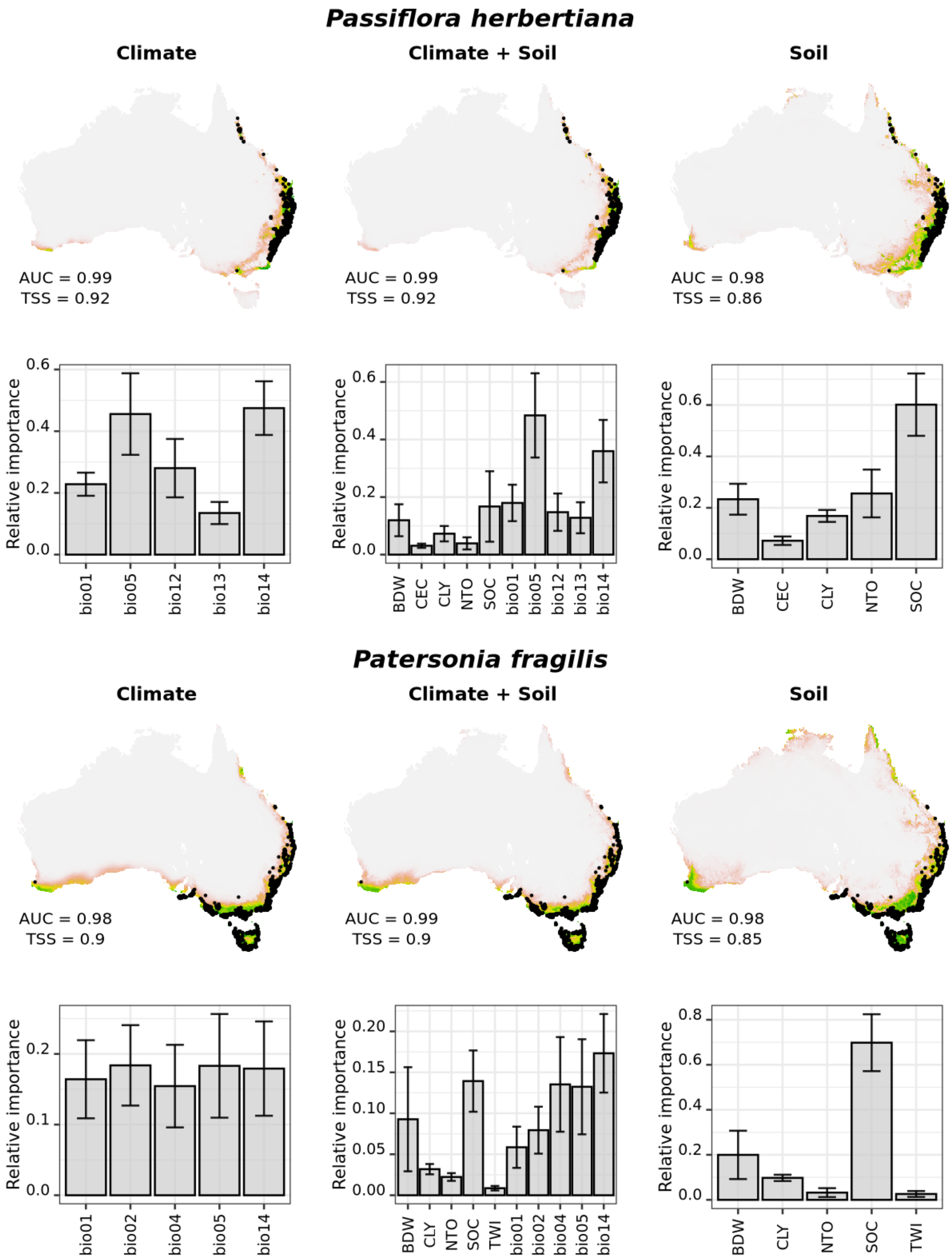

Passiflora herbertiana
Climate
Climate + Soil
Soil
AUC = 0.99
TSS = 0.92
AUC = 0.99
TSS = 0.92
AUC = 0.98
TSS = 0.86
Relative importance
0.0
0.2
0.4
0.6
bio01
bio05
bio12
bio13
bio14
BDW
CEC
CLY
NTO
SOC
bio01
bio05
bio12
bio13
bio14
BDW
CEC
CLY
NTO
SOC
Patersonia fragilis
Climate
Climate + Soil
Soil
AUC = 0.98
TSS = 0.9
AUC = 0.99
TSS = 0.9
AUC = 0.98
TSS = 0.85
Relative importance
0.0
0.1
0.2
0.00
0.05
0.10
0.15
0.20
0.0
0.2
0.4
0.6
0.8
bio01
bio02
bio04
bio05
bio14
BDW
CLY
NTO
SOC
TWI
bio01
bio02
bio04
bio05
bio14
BDW
CLY
NTO
SOC
TWI

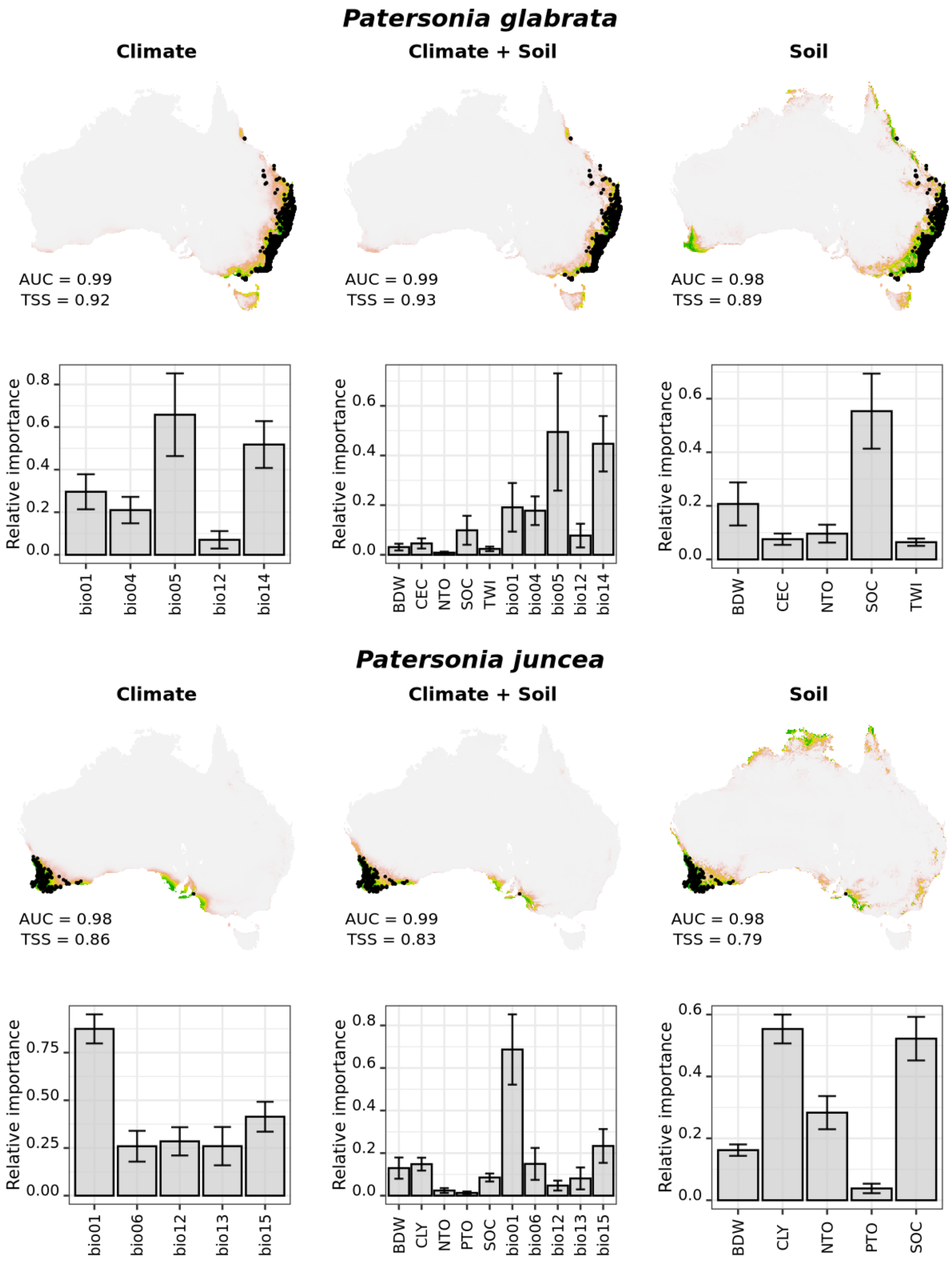
Patersonia glabrata
Climate
Climate + Soil
Soil
AUC = 0.99
TSS = 0.92
AUC = 0.99
TSS = 0.93
AUC = 0.98
TSS = 0.89
Relative importance
bio01
bio04
bio05
bio12
bio14
BDW
CEC
NTO
SOC
TWI
Patersonia juncea
Climate
Climate + Soil
Soil
AUC = 0.98
TSS = 0.86
AUC = 0.99
TSS = 0.83
AUC = 0.98
TSS = 0.79
Relative importance
bio01
bio06
bio12
bio13
bio15
BDW
CLY
NTO
PTO
SOC

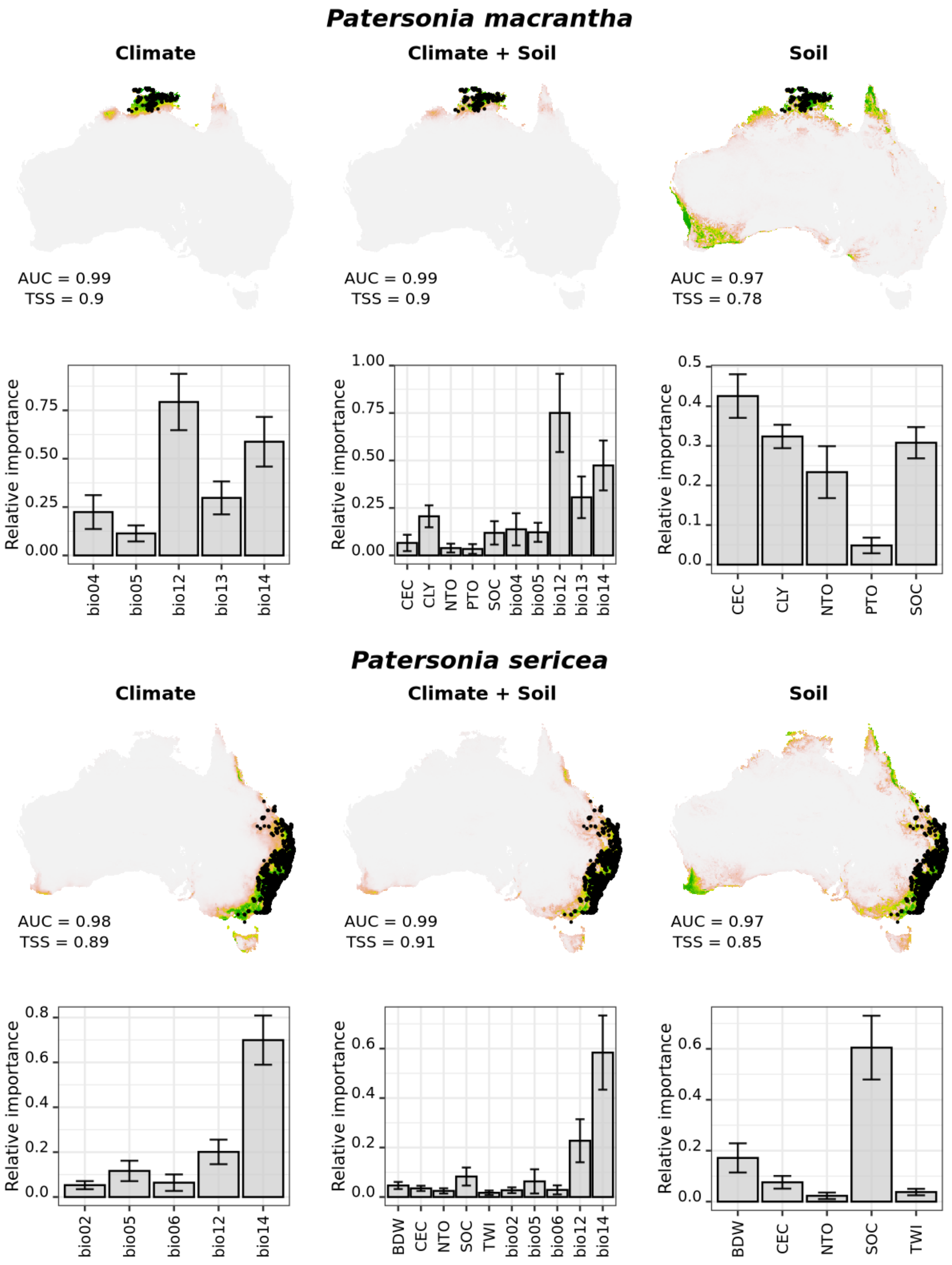

Patersonia macrantha
Climate
Climate + Soil
Soil
AUC = 0.99
TSS = 0.9
AUC = 0.99
TSS = 0.9
AUC = 0.97
TSS = 0.78
Relative importance
0.00
0.25
0.50
0.75
bio04
bio05
bio12
bio13
bio14
Relative importance
0.00
0.25
0.50
0.75
1.00
CEC
CLY
NTO
PTO
SOC
bio04
bio05
bio12
bio13
bio14
Relative importance
0.0
0.1
0.2
0.3
0.4
0.5
CEC
CLY
NTO
PTO
SOC
Patersonia sericea
Climate
Climate + Soil
Soil
AUC = 0.98
TSS = 0.89
AUC = 0.99
TSS = 0.91
AUC = 0.97
TSS = 0.85
Relative importance
0.0
0.2
0.4
0.6
0.8
bio02
bio05
bio06
bio12
bio14
Relative importance
0.0
0.2
0.4
0.6
BDW
CEC
NTO
SOC
TWI
bio02
bio05
bio06
bio12
bio14
Relative importance
0.0
0.2
0.4
0.6
BDW
CEC
NTO
SOC
TWI

Patersonia umbrosa

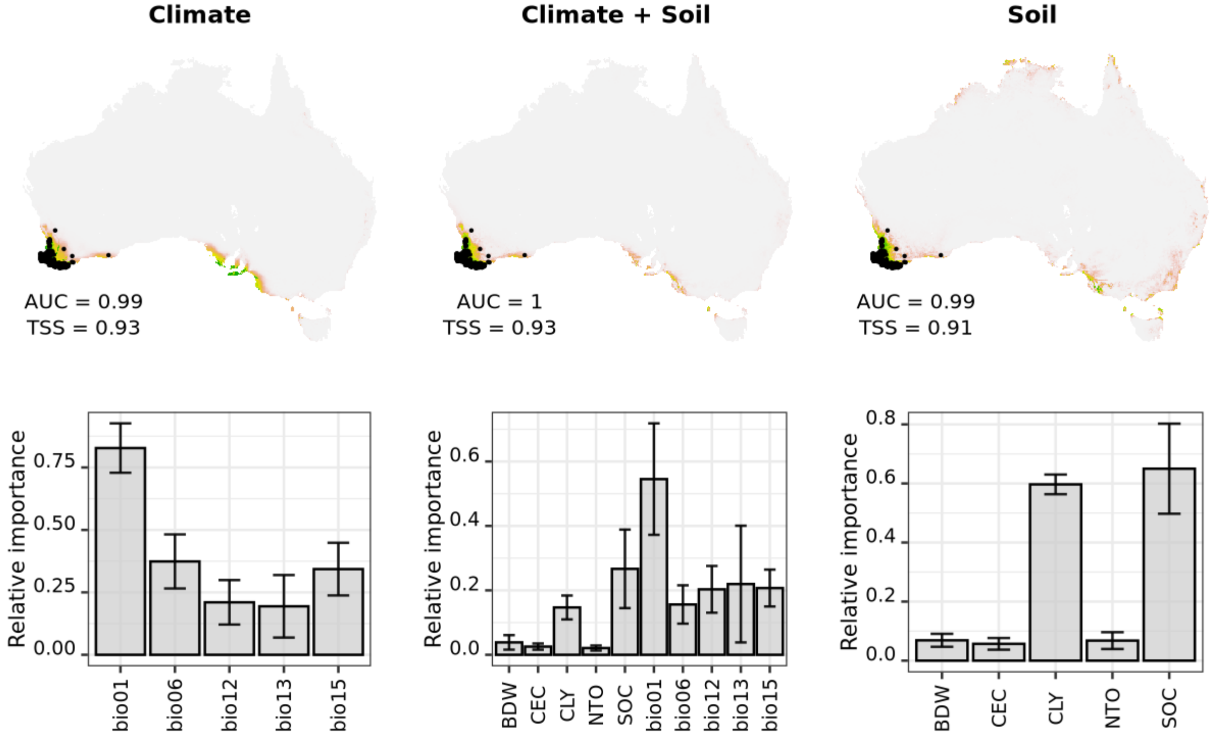

Pauridia occidentalis

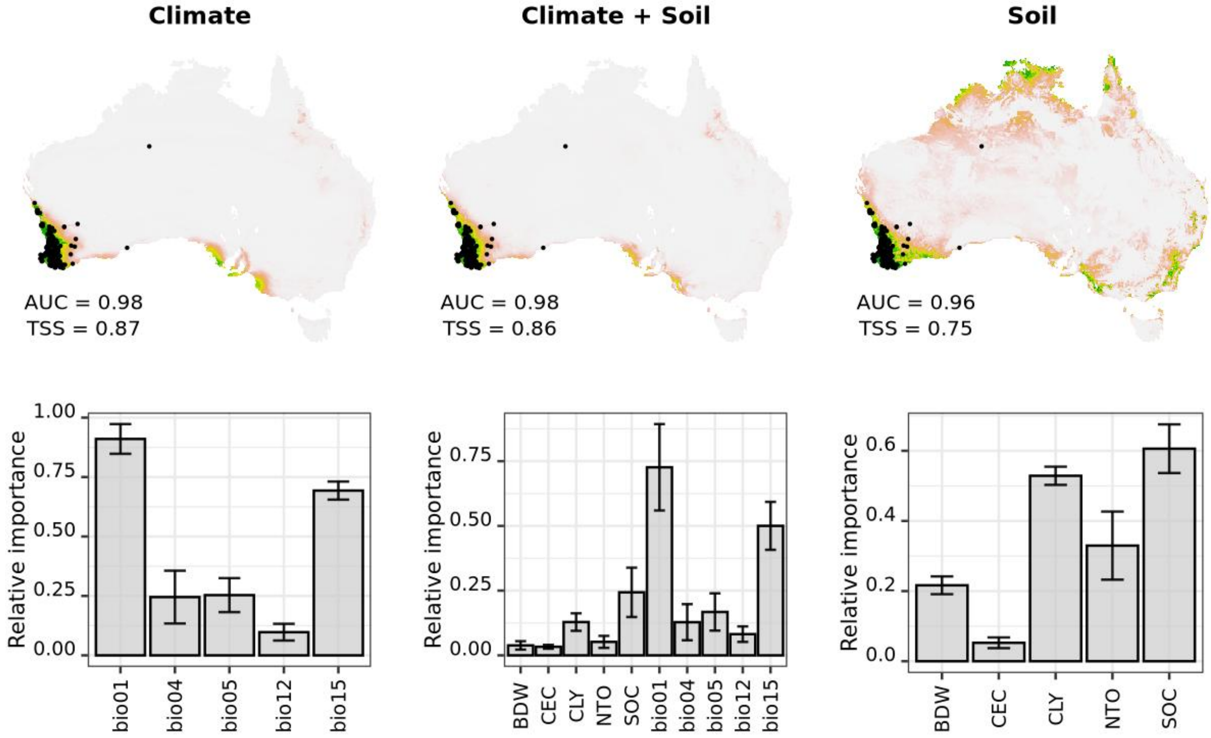

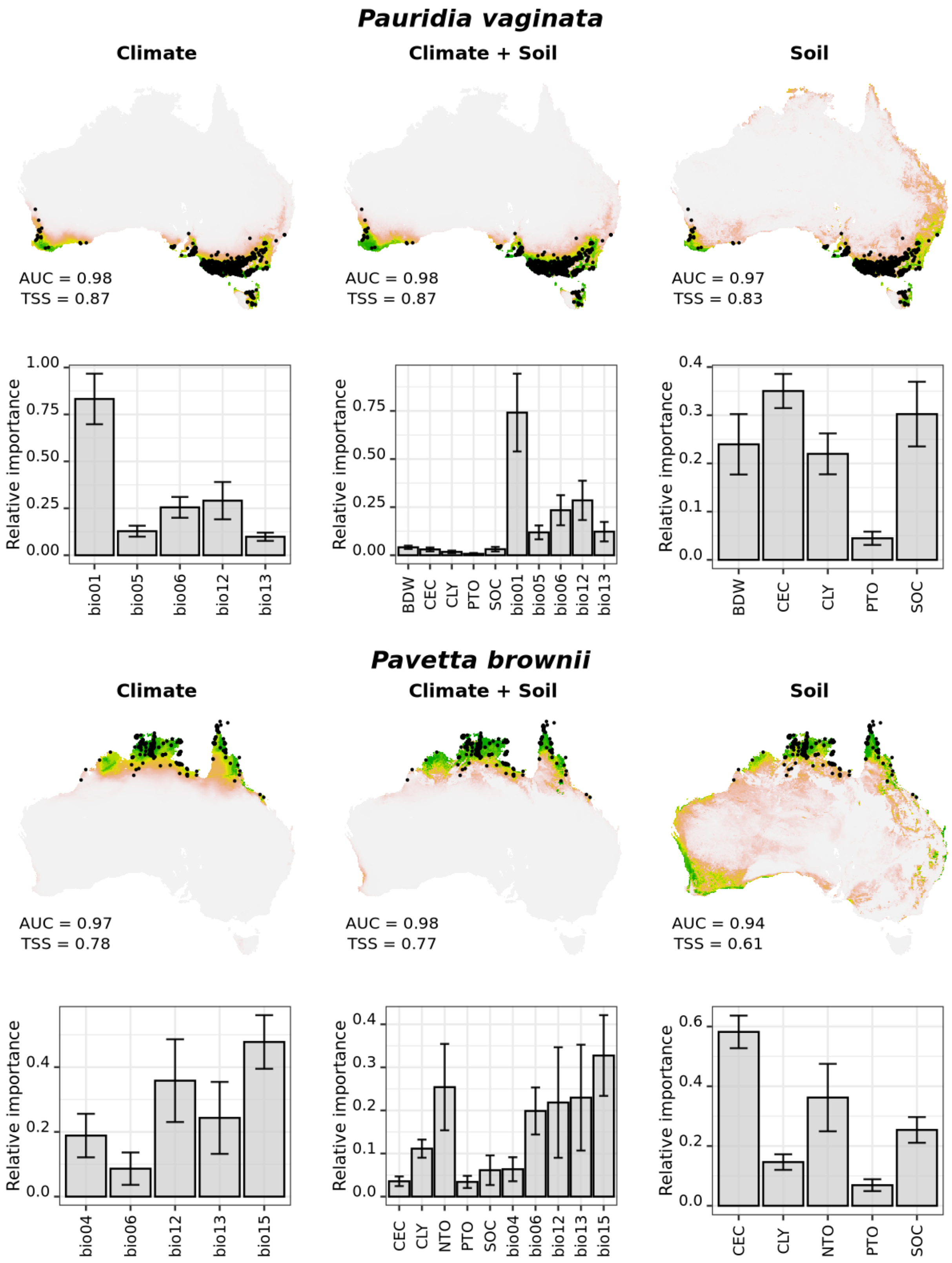

Pauridia vaginata
Climate
Climate + Soil
Soil
AUC = 0.98
TSS = 0.87
AUC = 0.98
TSS = 0.87
AUC = 0.97
TSS = 0.83
Relative importance
1.00
0.75
0.50
0.25
0.00
bio01
bio05
bio06
bio12
bio13
Relative importance
0.75
0.50
0.25
0.00
BDW
CEC
CLY
PTO
SOC
bio01
bio05
bio06
bio12
bio13
Relative importance
0.4
0.3
0.2
0.1
0.0
BDW
CEC
CLY
PTO
SOC
Pavetta brownii
Climate
Climate + Soil
Soil
AUC = 0.97
TSS = 0.78
AUC = 0.98
TSS = 0.77
AUC = 0.94
TSS = 0.61
Relative importance
0.4
0.2
0.0
bio04
bio06
bio12
bio13
bio15
Relative importance
0.4
0.3
0.2
0.1
0.0
CEC
CLY
NTO
PTO
SOC
bio04
bio06
bio12
bio13
bio15
Relative importance
0.6
0.4
0.2
0.0
CEC
CLY
NTO
PTO
SOC

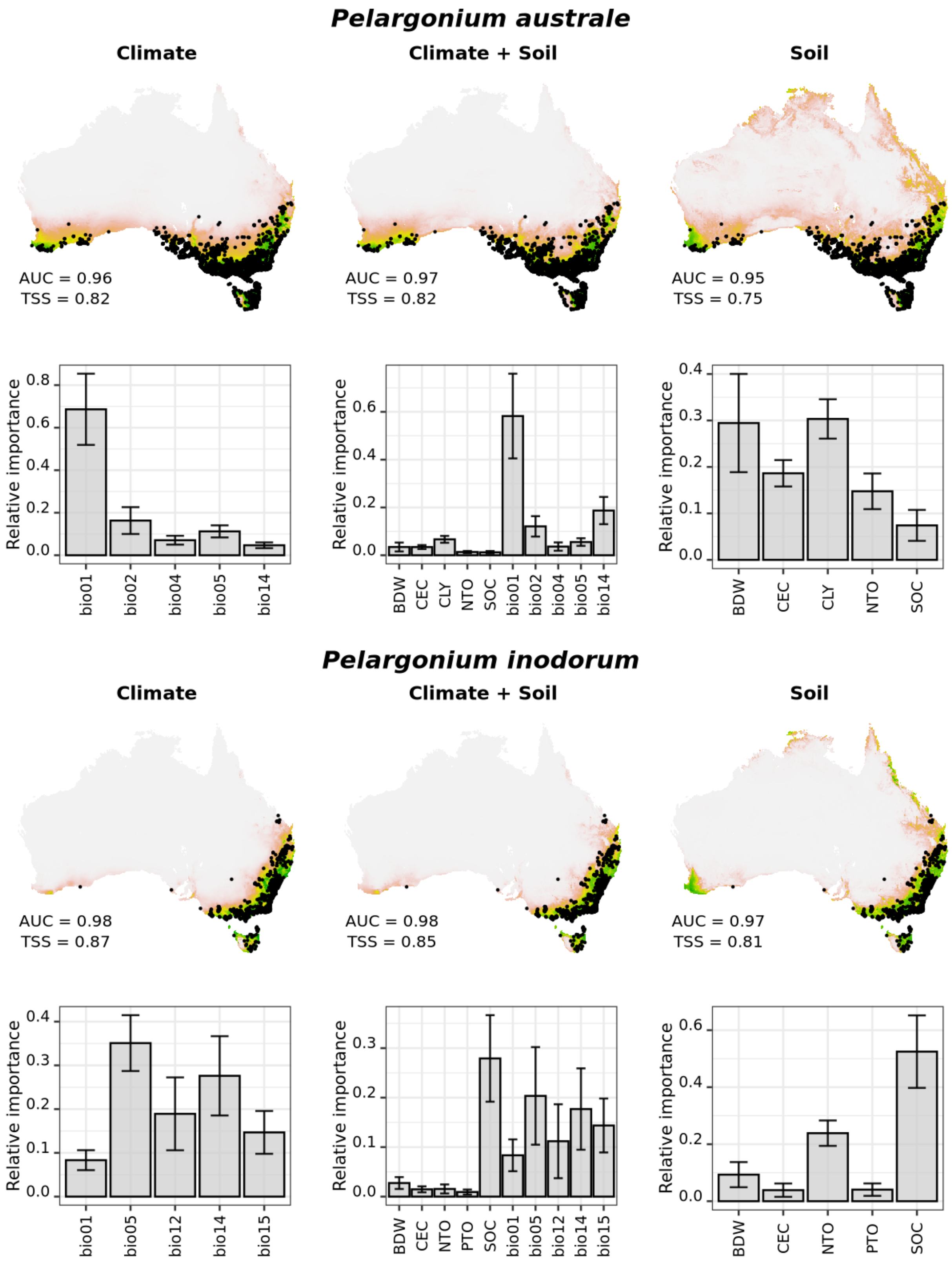

Pelargonium australe
Climate
Climate + Soil
Soil
AUC = 0.96
TSS = 0.82
AUC = 0.97
TSS = 0.82
AUC = 0.95
TSS = 0.75
Relative importance
bio01 bio02 bio04 bio05 bio14
BDW CEC CLY NTO SOC bio01 bio02 bio04 bio05 bio14
BDW CEC CLY NTO SOC
Pelargonium inodorum
Climate
Climate + Soil
Soil
AUC = 0.98
TSS = 0.87
AUC = 0.98
TSS = 0.85
AUC = 0.97
TSS = 0.81
Relative importance
bio01 bio05 bio12 bio14 bio15
BDW CEC NTO PTO SOC bio01 bio05 bio12 bio14 bio15
BDW CEC NTO PTO SOC

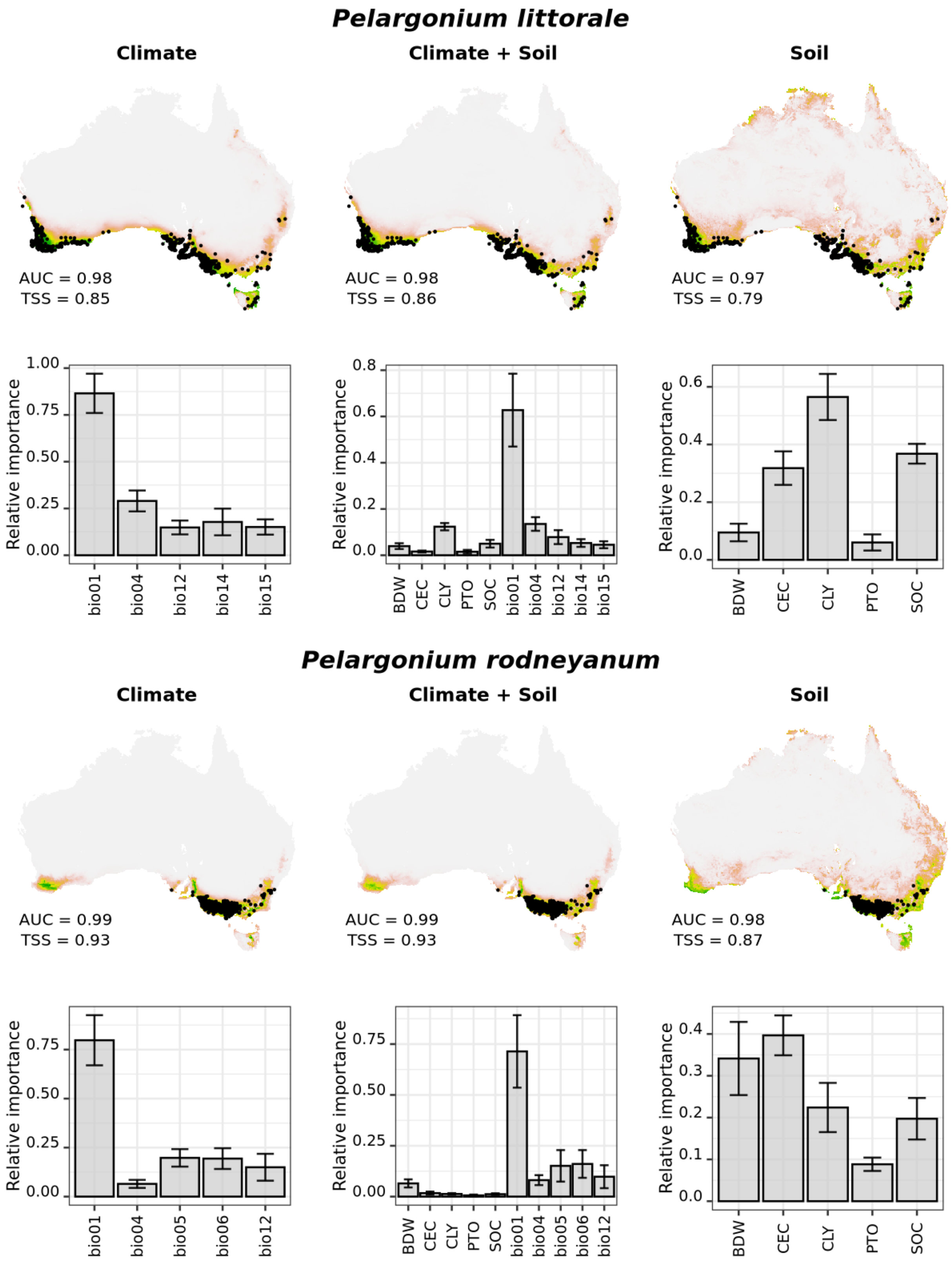
Pelargonium littorale
Climate
Climate + Soil
Soil
AUC = 0.98
TSS = 0.85
AUC = 0.98
TSS = 0.86
AUC = 0.97
TSS = 0.79
Relative importance
bio01
bio04
bio12
bio14
bio15
BDW
CEC
CLY
PTO
SOC
Pelargonium rodneyanum
Climate
Climate + Soil
Soil
AUC = 0.99
TSS = 0.93
AUC = 0.99
TSS = 0.93
AUC = 0.98
TSS = 0.87
Relative importance
bio01
bio04
bio05
bio06
bio12
BDW
CEC
CLY
PTO
SOC

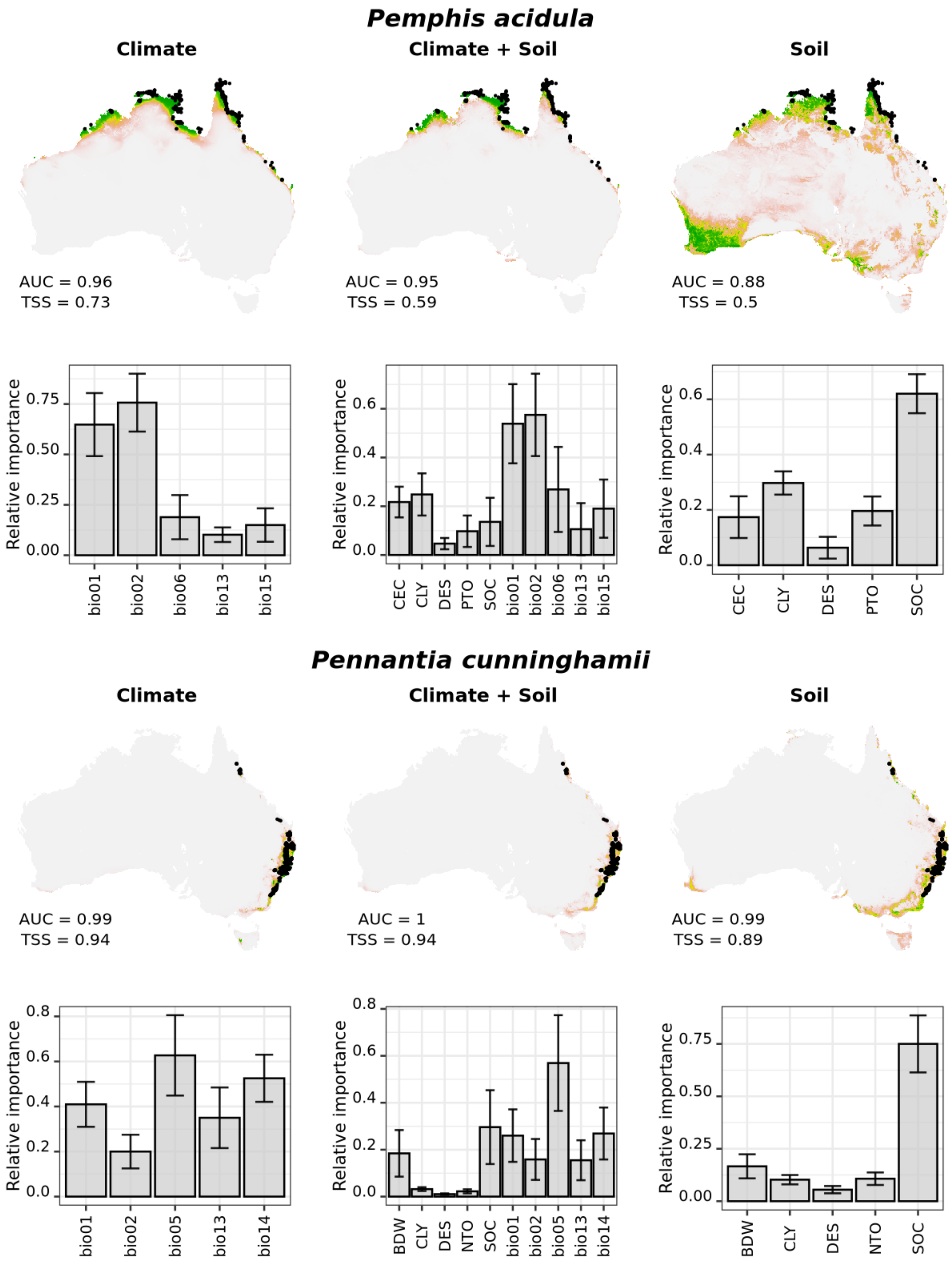

Pemphis acidula
Climate
Climate + Soil
Soil
AUC = 0.96
TSS = 0.73
AUC = 0.95
TSS = 0.59
AUC = 0.88
TSS = 0.5
Relative importance
bio01
bio02
bio06
bio13
bio15
CEC
CLY
DES
PTO
SOC
bio01
bio02
bio06
bio13
bio15
CEC
CLY
DES
PTO
SOC
Pennantia cunninghamii
Climate
Climate + Soil
Soil
AUC = 0.99
TSS = 0.94
AUC = 1
TSS = 0.94
AUC = 0.99
TSS = 0.89
Relative importance
bio01
bio02
bio05
bio13
bio14
BDW
CLY
DES
NTO
SOC
bio01
bio02
bio05
bio13
bio14
BDW
CLY
DES
NTO
SOC

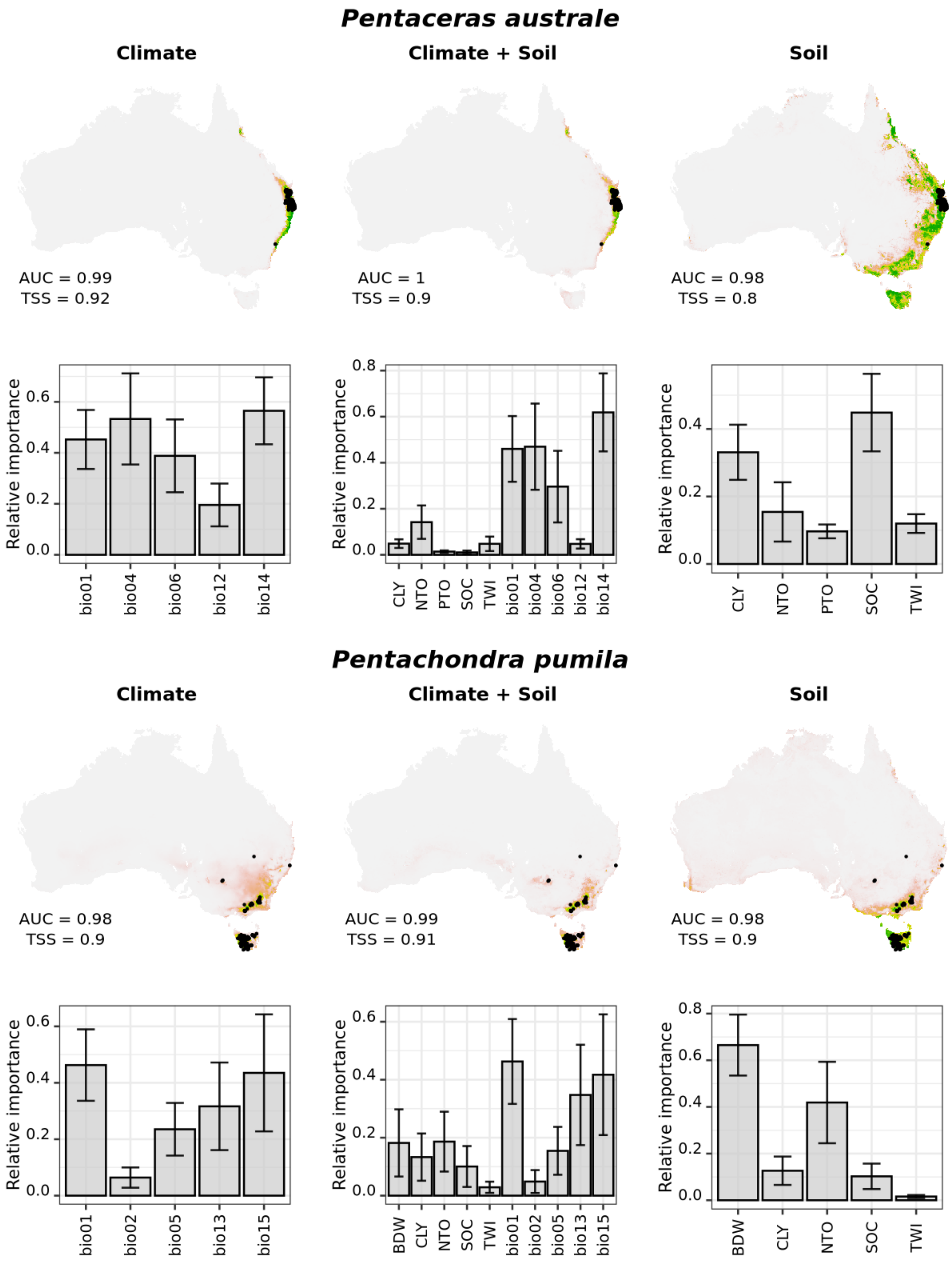
Pentaceras australe
Climate
Climate + Soil
Soil
AUC = 0.99
TSS = 0.92
AUC = 1
TSS = 0.9
AUC = 0.98
TSS = 0.8
Relative importance
bio01
bio04
bio06
bio12
bio14
CLY
NTO
PTO
SOC
TWI
Pentachondra pumila
Climate
Climate + Soil
Soil
AUC = 0.98
TSS = 0.9
AUC = 0.99
TSS = 0.91
AUC = 0.98
TSS = 0.9
Relative importance
bio01
bio02
bio05
bio13
bio15
BDW
CLY
NTO
SOC
TWI

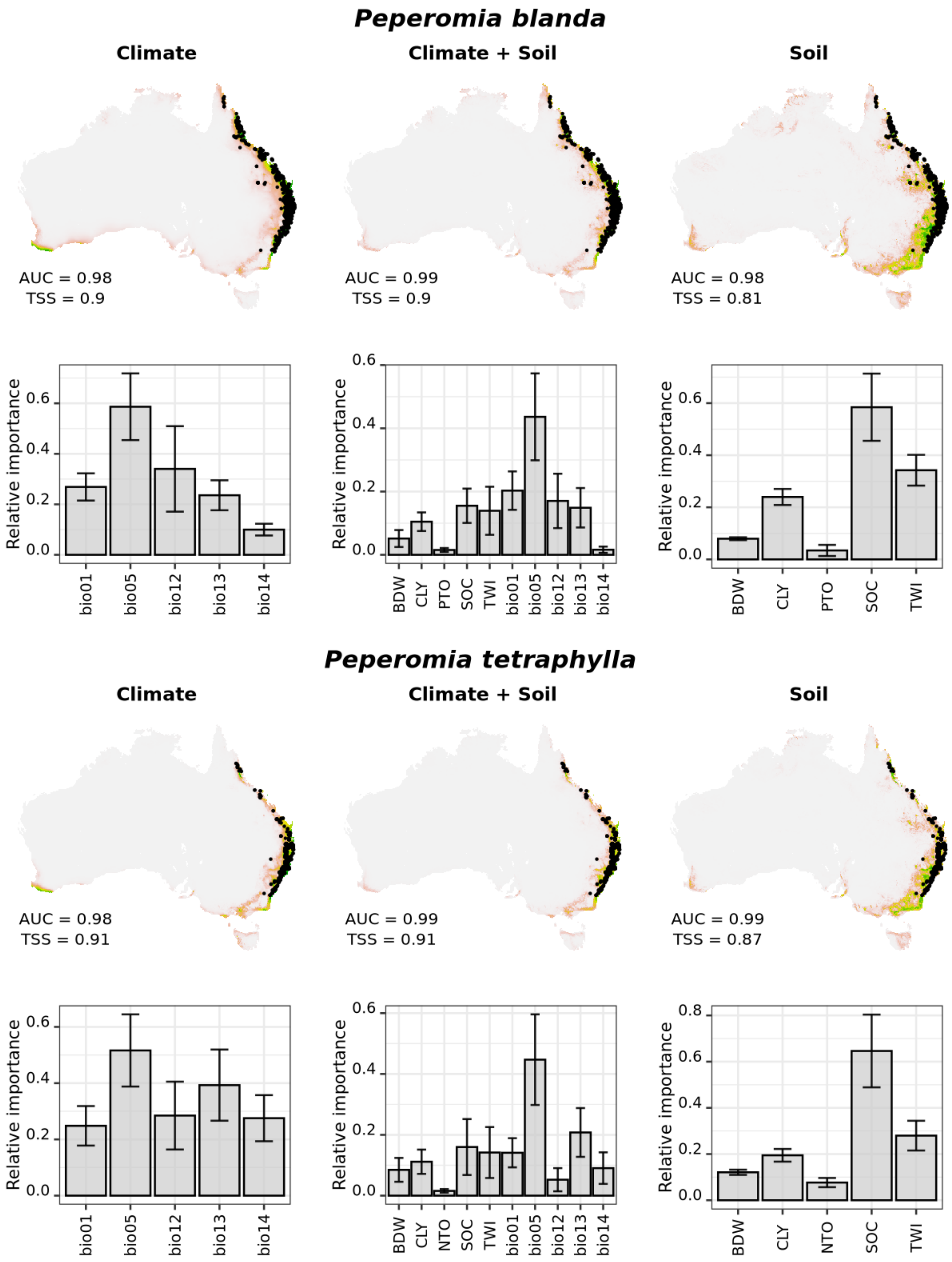
Peperomia blanda
Climate
Climate + Soil
Soil
AUC = 0.98
TSS = 0.9
AUC = 0.99
TSS = 0.9
AUC = 0.98
TSS = 0.81
Relative importance
0.0
0.2
0.4
0.6
bio01
bio05
bio12
bio13
bio14
BDW
CLY
PTO
SOC
TWI
Peperomia tetraphylla
AUC = 0.98
TSS = 0.91
AUC = 0.99
TSS = 0.91
AUC = 0.99
TSS = 0.87
0.8
NTO

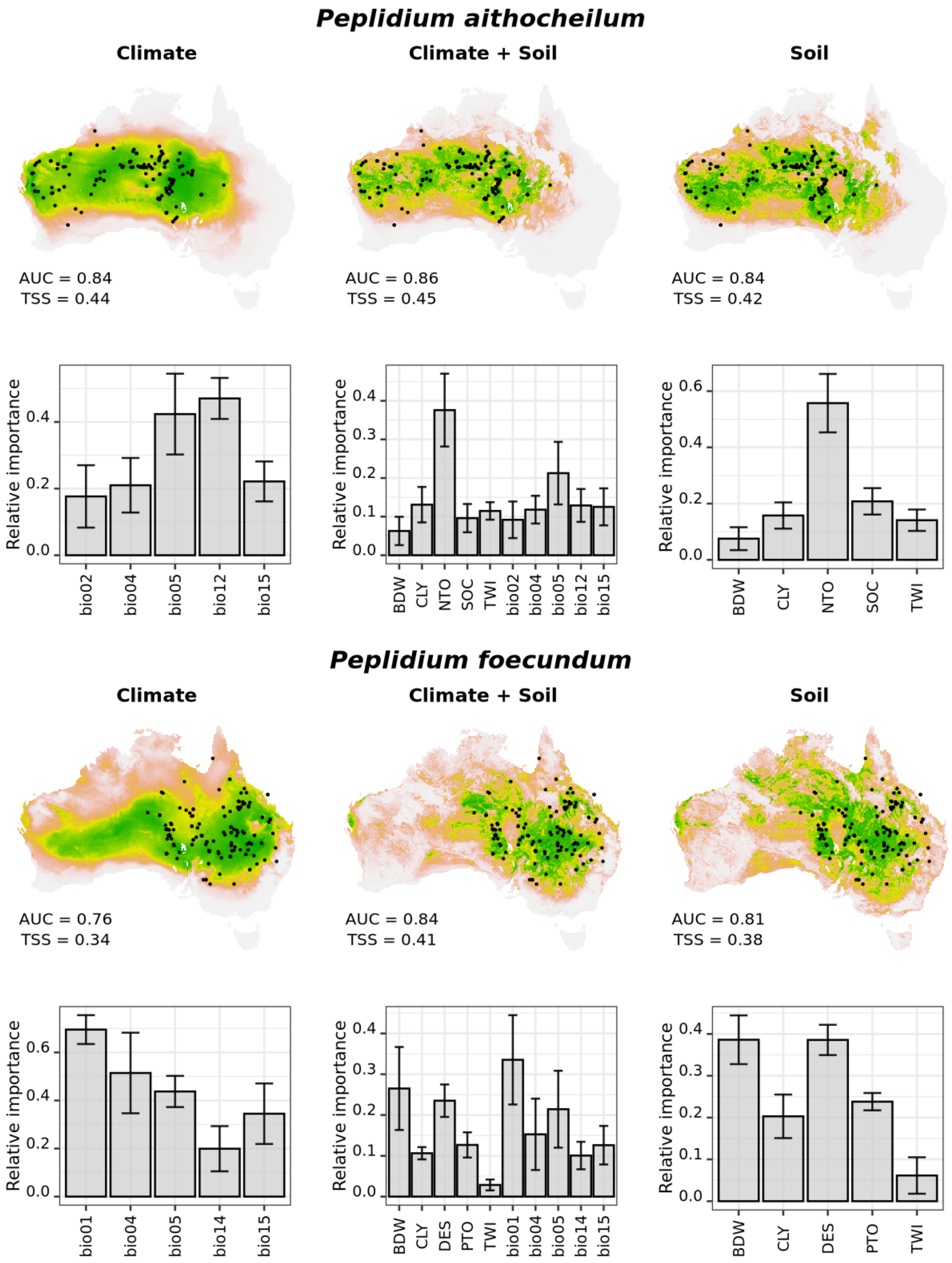
Peplidium aithocheilum
Climate
Climate + Soil
Soil
AUC = 0.84
TSS = 0.44
AUC = 0.86
TSS = 0.45
AUC = 0.84
TSS = 0.42
Relative importance
bio02
bio04
bio05
bio12
bio15
BDW
CLY
NTO
SOC
TWI
Peplidium foecundum
Climate
Climate + Soil
Soil
AUC = 0.76
TSS = 0.34
AUC = 0.84
TSS = 0.41
AUC = 0.81
TSS = 0.38
Relative importance
bio01
bio04
bio05
bio14
bio15
BDW
CLY
DES
PTO
TWI

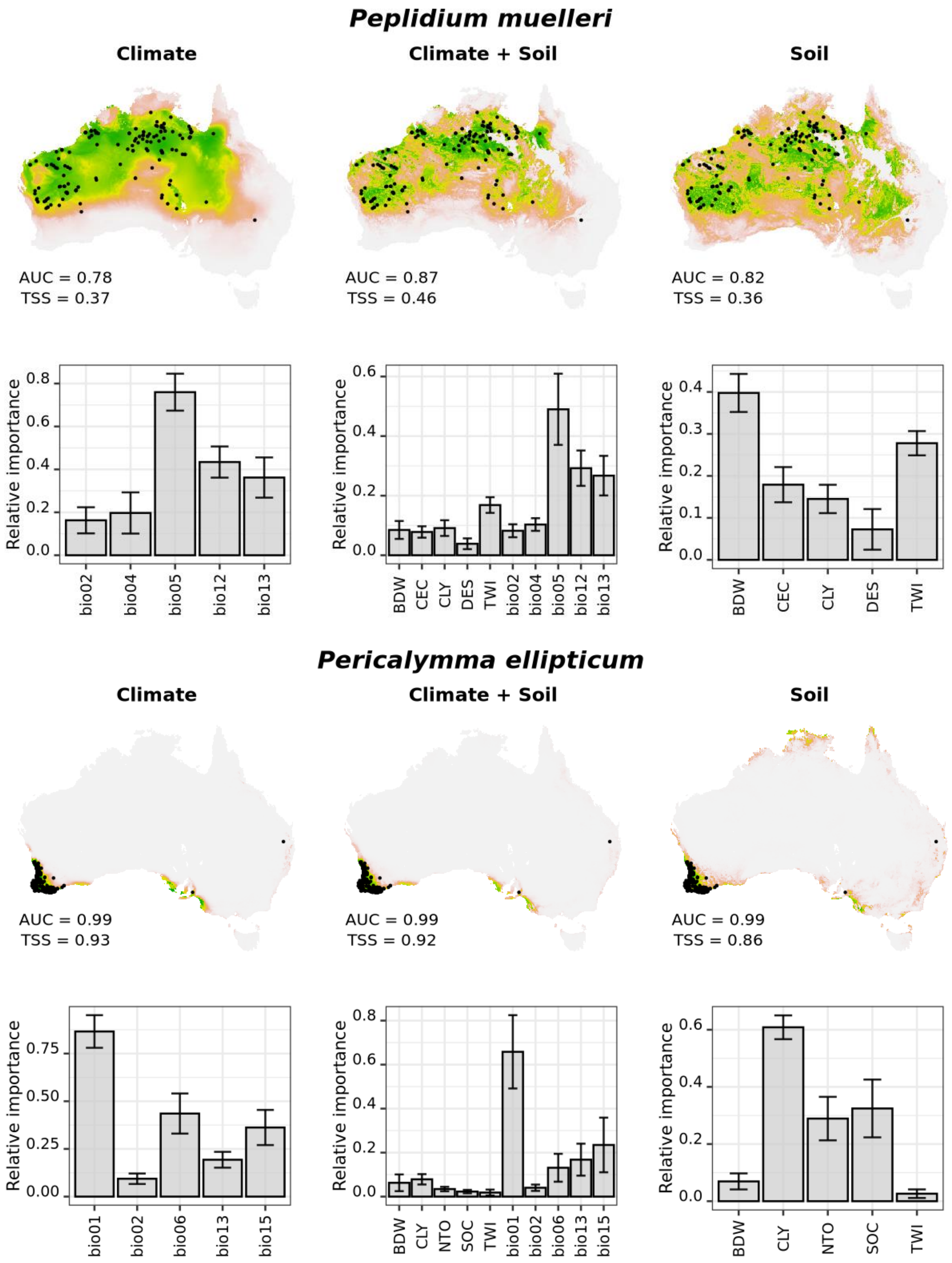
Peplidium muelleri
Climate
Climate + Soil
Soil
AUC = 0.78
TSS = 0.37
AUC = 0.87
TSS = 0.46
AUC = 0.82
TSS = 0.36
Relative importance
bio02 bio04 bio05 bio12 bio13
BDW CEC CLY DES TWI bio02 bio04 bio05 bio12 bio13
BDW CEC CLY DES TWI
Pericalymma ellipticum
Climate
Climate + Soil
Soil
AUC = 0.99
TSS = 0.93
AUC = 0.99
TSS = 0.92
AUC = 0.99
TSS = 0.86
Relative importance
bio01 bio02 bio06 bio13 bio15
BDW CLY NTO SOC TWI bio01 bio02 bio06 bio13 bio15
BDW CLY NTO SOC TWI

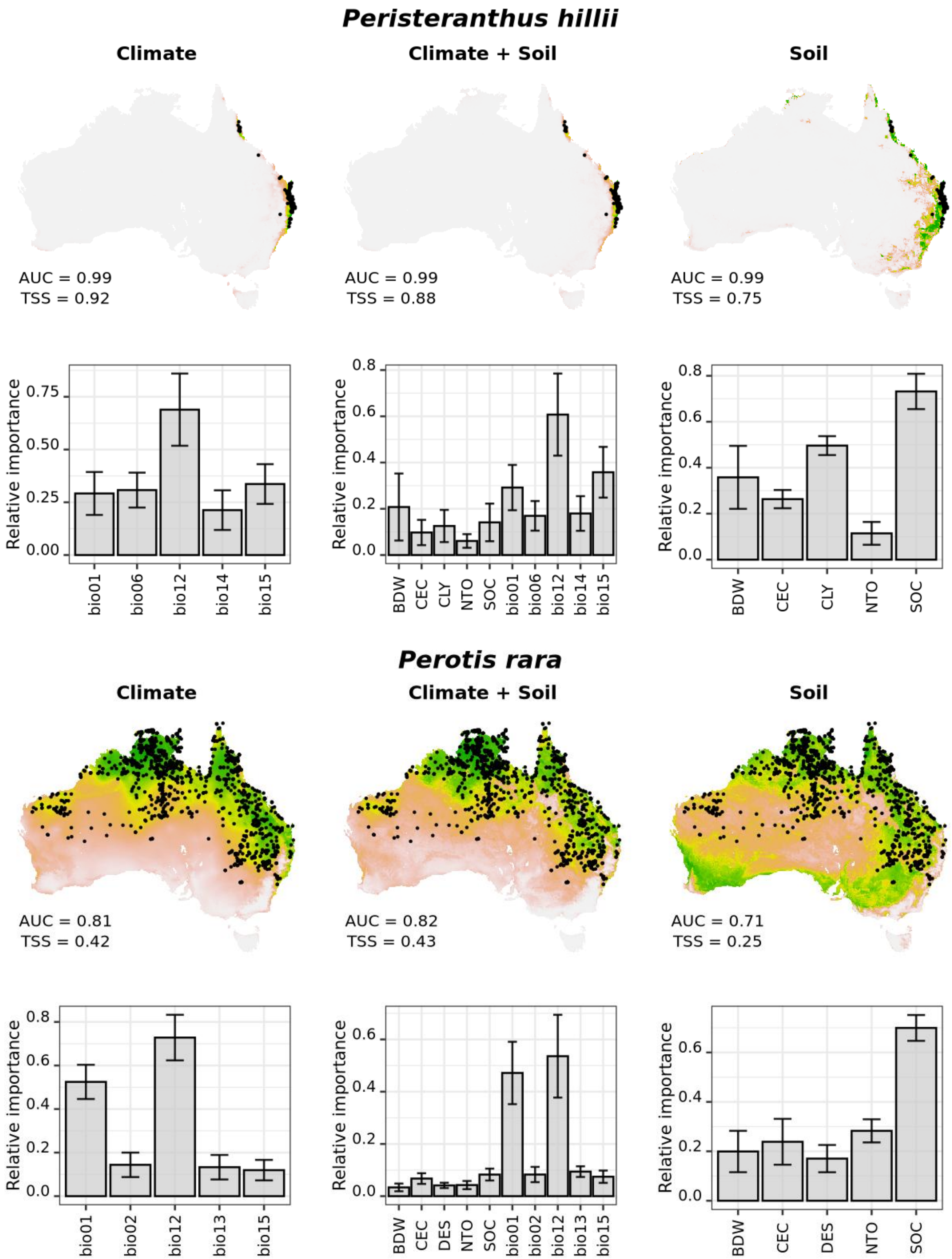
Peristeranthus hillii
Climate
Climate + Soil
Soil
AUC = 0.99
TSS = 0.92
AUC = 0.99
TSS = 0.88
AUC = 0.99
TSS = 0.75
Relative importance
bio01
bio06
bio12
bio14
bio15
BDW
CEC
CLY
NTO
SOC
Perotis rara
Climate
Climate + Soil
Soil
AUC = 0.81
TSS = 0.42
AUC = 0.82
TSS = 0.43
AUC = 0.71
TSS = 0.25
Relative importance
bio01
bio02
bio12
bio13
bio15
BDW
CEC
DES
NTO
SOC

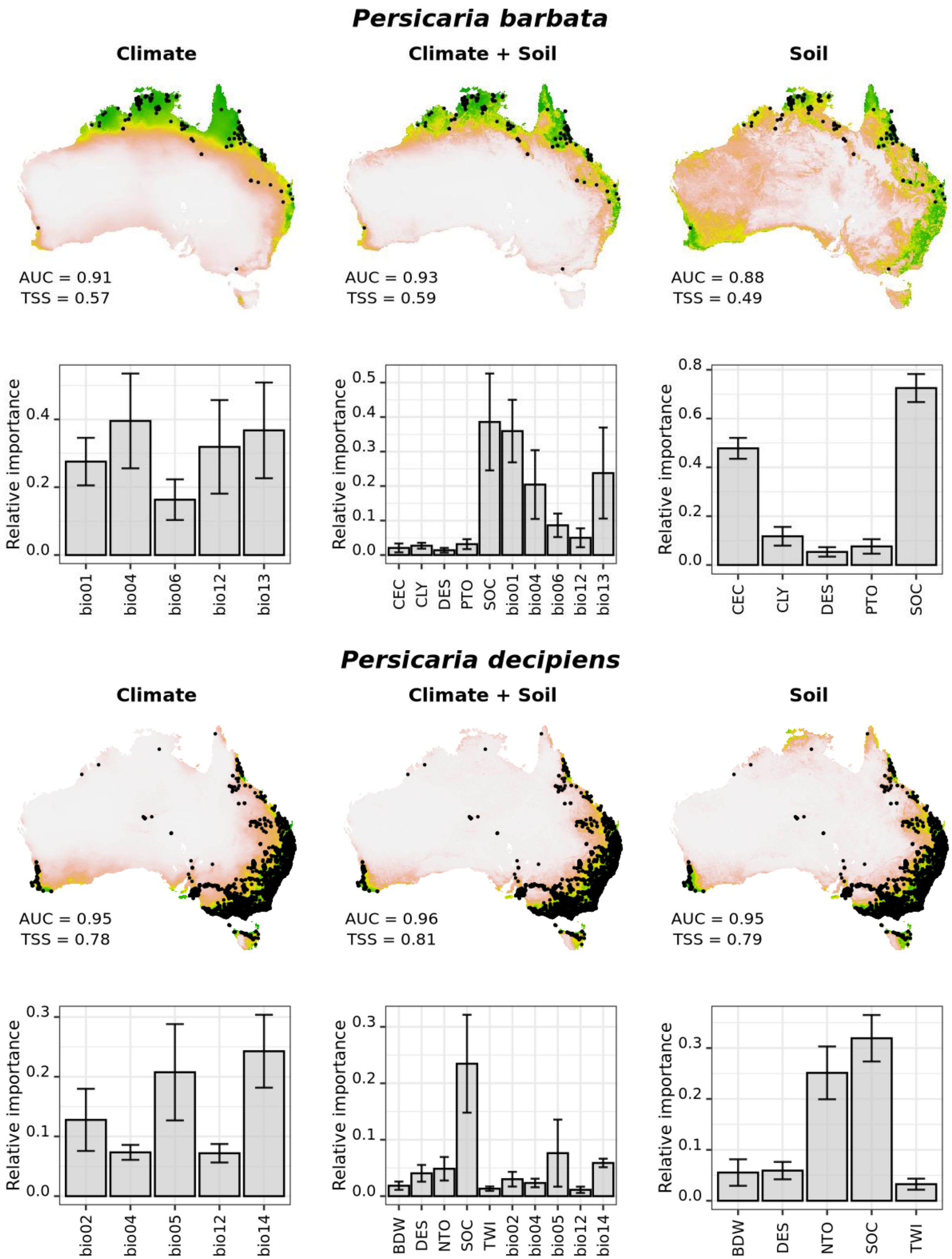
Persicaria barbata
Climate
Climate + Soil
Soil
AUC = 0.91
TSS = 0.57
AUC = 0.93
TSS = 0.59
AUC = 0.88
TSS = 0.49
Relative importance
bio01
bio04
bio06
bio12
bio13
CEC
CLY
DES
PTO
SOC
bio01
bio04
bio06
bio12
bio13
CEC
CLY
DES
PTO
SOC
Persicaria decipiens
Climate
Climate + Soil
Soil
AUC = 0.95
TSS = 0.78
AUC = 0.96
TSS = 0.81
AUC = 0.95
TSS = 0.79
Relative importance
bio02
bio04
bio05
bio12
bio14
BDW
DES
NTO
SOC
TWI
bio02
bio04
bio05
bio12
bio14
BDW
DES
NTO
SOC
TWI

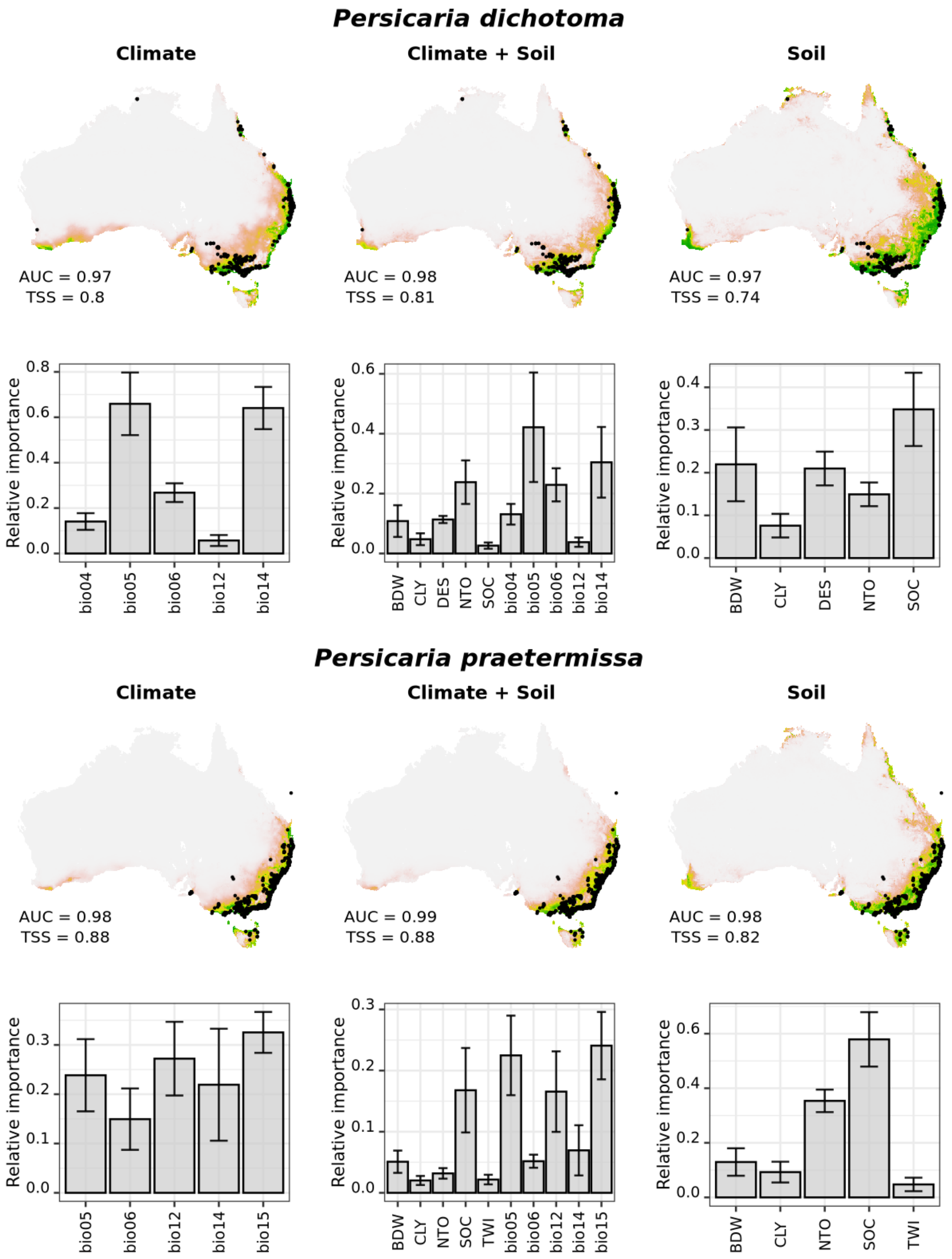

Persicaria dichotoma
Climate
Climate + Soil
Soil
AUC = 0.97
TSS = 0.8
AUC = 0.98
TSS = 0.81
AUC = 0.97
TSS = 0.74
Relative importance
bio04
bio05
bio06
bio12
bio14
BDW
CLY
DES
NTO
SOC
Persicaria praetermissa
AUC = 0.98
TSS = 0.88
AUC = 0.99
TSS = 0.88
AUC = 0.98
TSS = 0.82
bio15
TWI

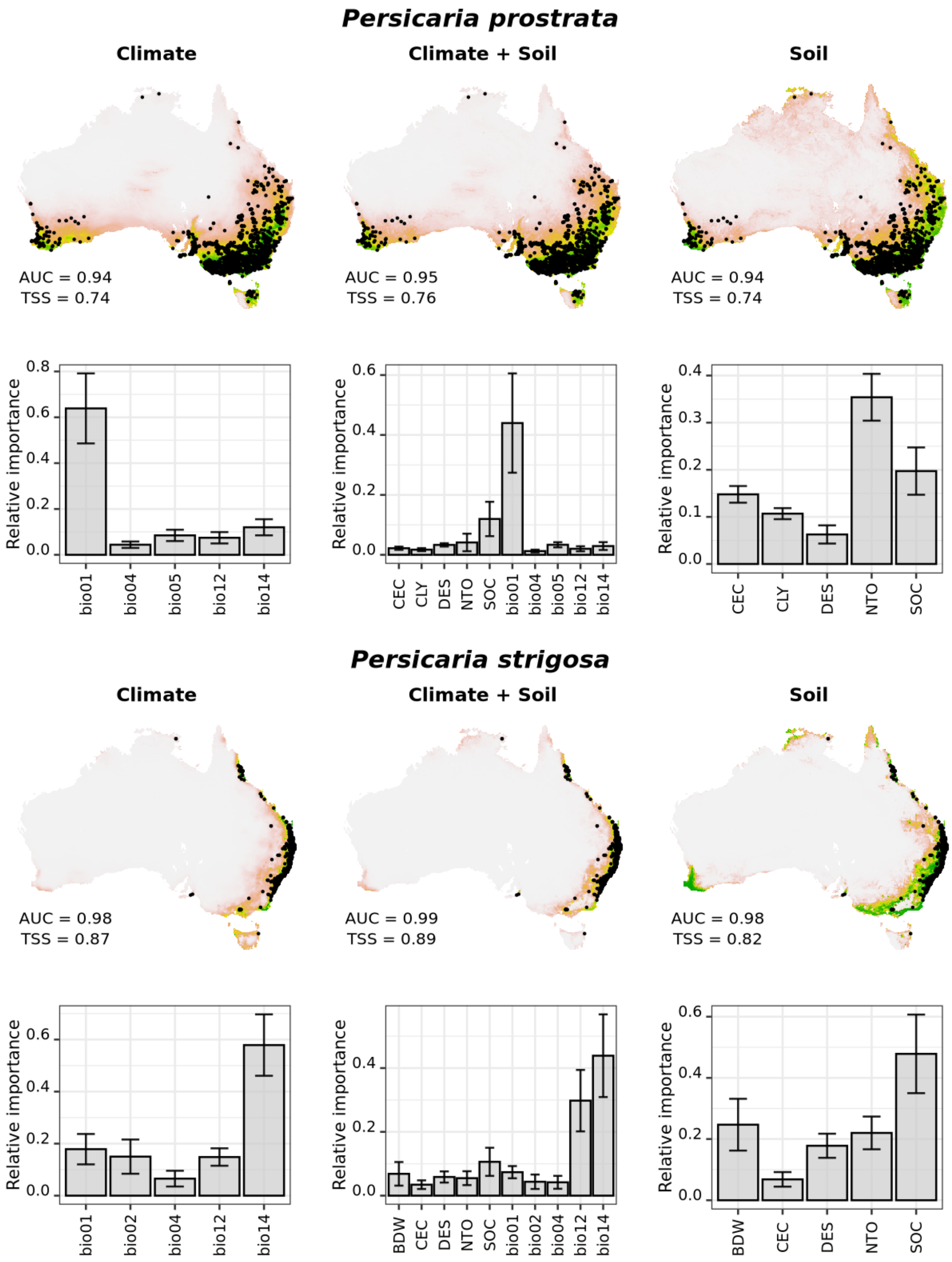

Persicaria prostrata
Climate
Climate + Soil
Soil
AUC = 0.94
TSS = 0.74
AUC = 0.95
TSS = 0.76
AUC = 0.94
TSS = 0.74
Relative importance
bio01
bio04
bio05
bio12
bio14
CEC
CLY
DES
NTO
SOC
Persicaria strigosa
Climate
Climate + Soil
Soil
AUC = 0.98
TSS = 0.87
AUC = 0.99
TSS = 0.89
AUC = 0.98
TSS = 0.82
Relative importance
bio01
bio02
bio04
bio12
bio14
BDW
CEC
DES
NTO
SOC

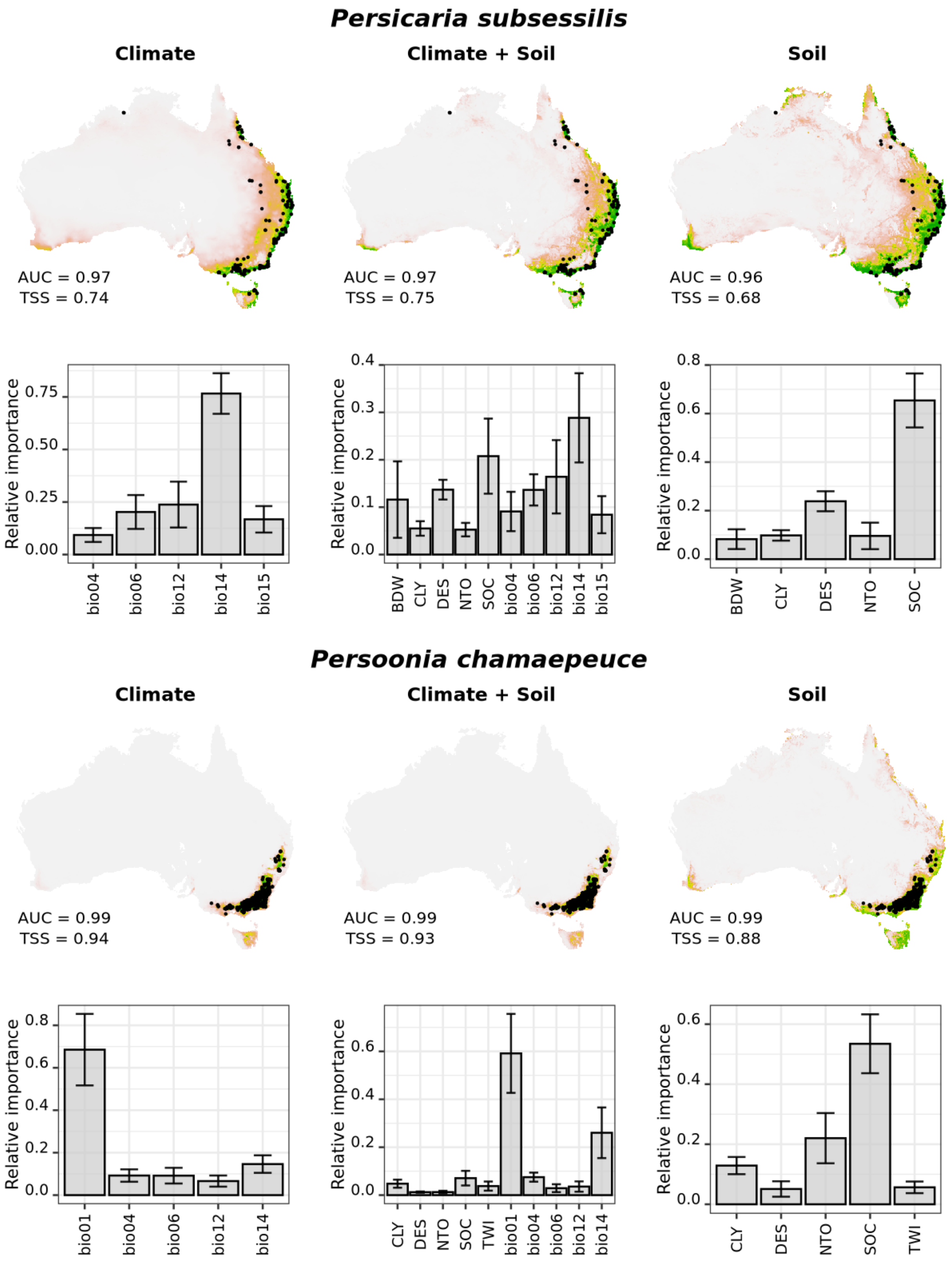
Persicaria subsessilis
Climate
Climate + Soil
Soil
AUC = 0.97
TSS = 0.74
AUC = 0.97
TSS = 0.75
AUC = 0.96
TSS = 0.68
Relative importance
0.00
0.25
0.50
0.75
bio04
bio06
bio12
bio14
bio15
0.0
0.1
0.2
0.3
0.4
BDW
CLY
DES
NTO
SOC
bio04
bio06
bio12
bio14
bio15
0.0
0.2
0.4
0.6
0.8
BDW
CLY
DES
NTO
SOC
Persoonia chamaepeuce
Climate
Climate + Soil
Soil
AUC = 0.99
TSS = 0.94
AUC = 0.99
TSS = 0.93
AUC = 0.99
TSS = 0.88
Relative importance
0.0
0.2
0.4
0.6
0.8
bio01
bio04
bio06
bio12
bio14
0.0
0.2
0.4
0.6
CLY
DES
NTO
SOC
TWI
bio01
bio04
bio06
bio12
bio14
0.0
0.2
0.4
0.6
CLY
DES
NTO
SOC
TWI

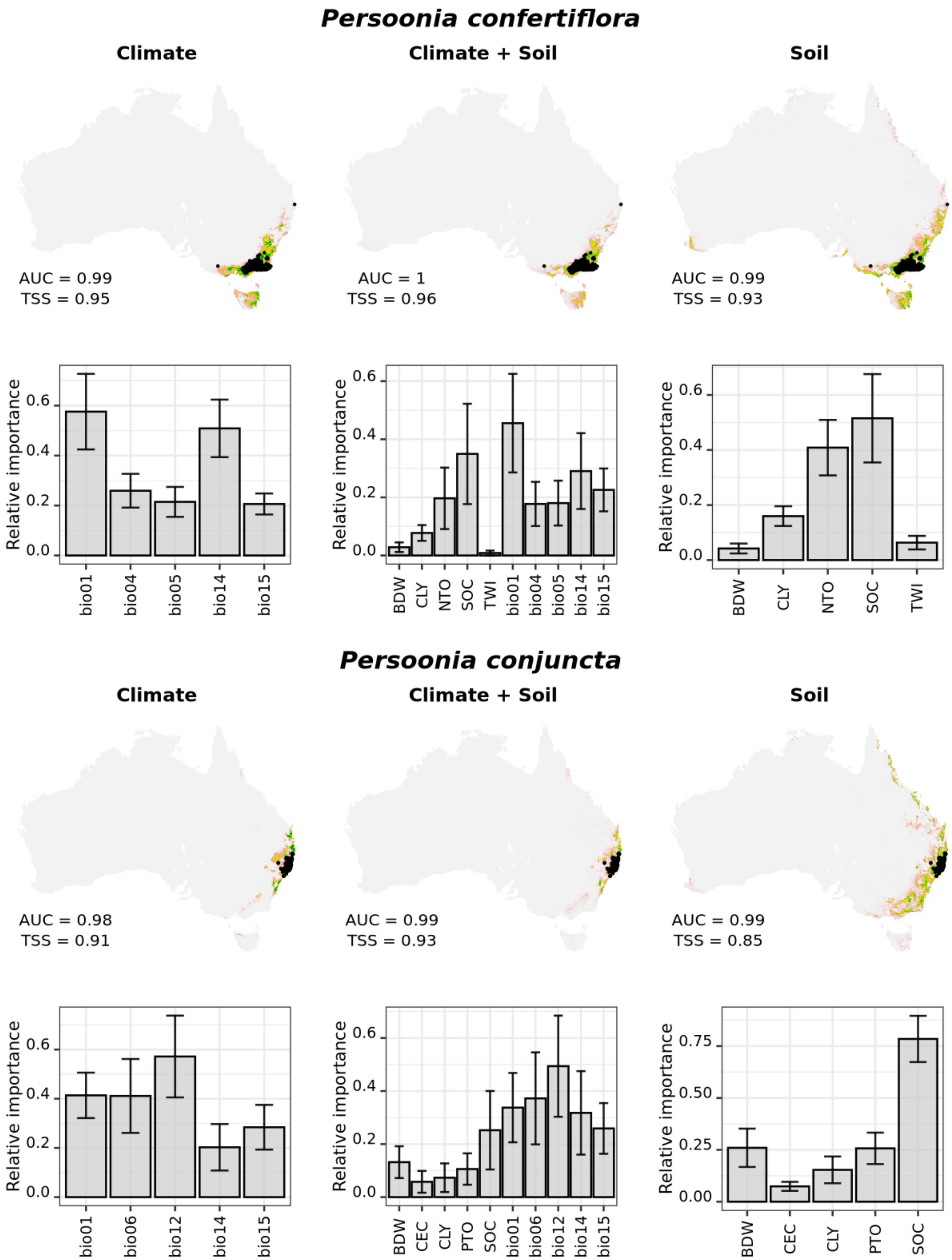

Persoonia confertiflora
Climate
Climate + Soil
Soil
AUC = 0.99
TSS = 0.95
AUC = 1
TSS = 0.96
AUC = 0.99
TSS = 0.93
Relative importance
0.0
0.2
0.4
0.6
bio01
bio04
bio05
bio14
bio15
BDW
CLY
NTO
SOC
TWI
Persoonia conjuncta
AUC = 0.98
TSS = 0.91
AUC = 0.99
TSS = 0.93
AUC = 0.99
TSS = 0.85
bio06
bio12
CEC
PTO
0.00
0.25
0.50
0.75

Persoonia coriacea

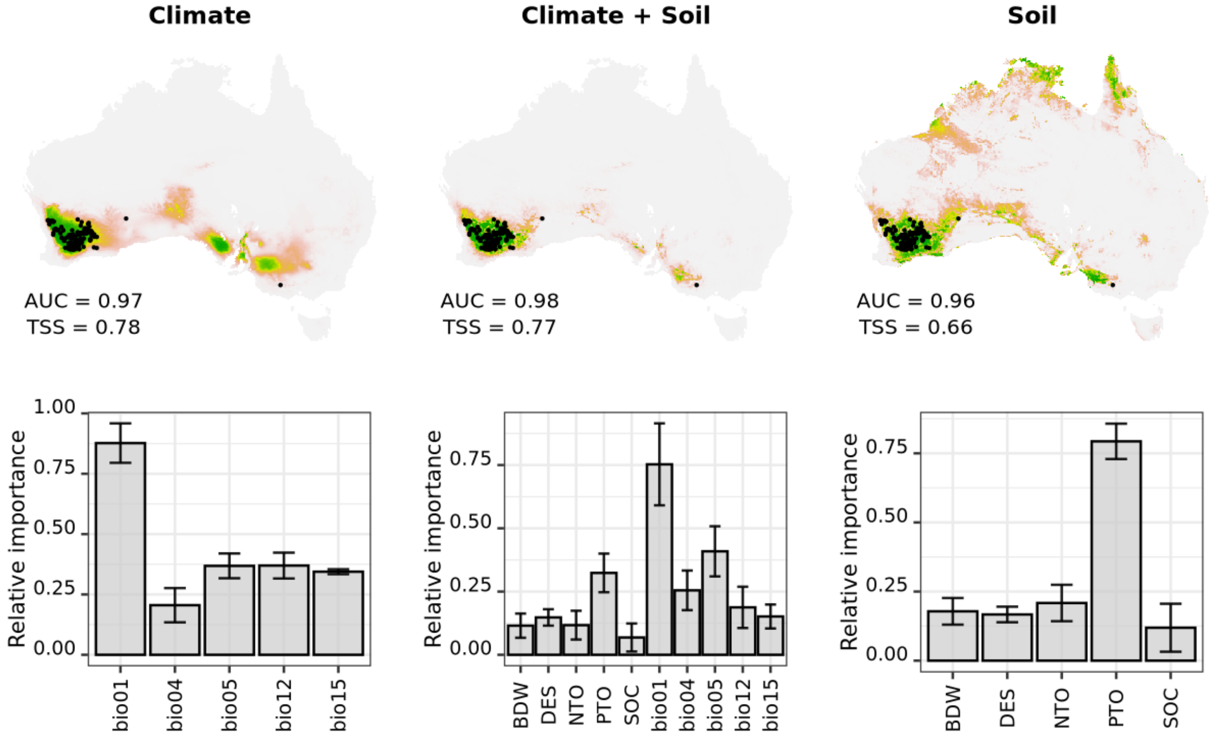

Persoonia cornifolia

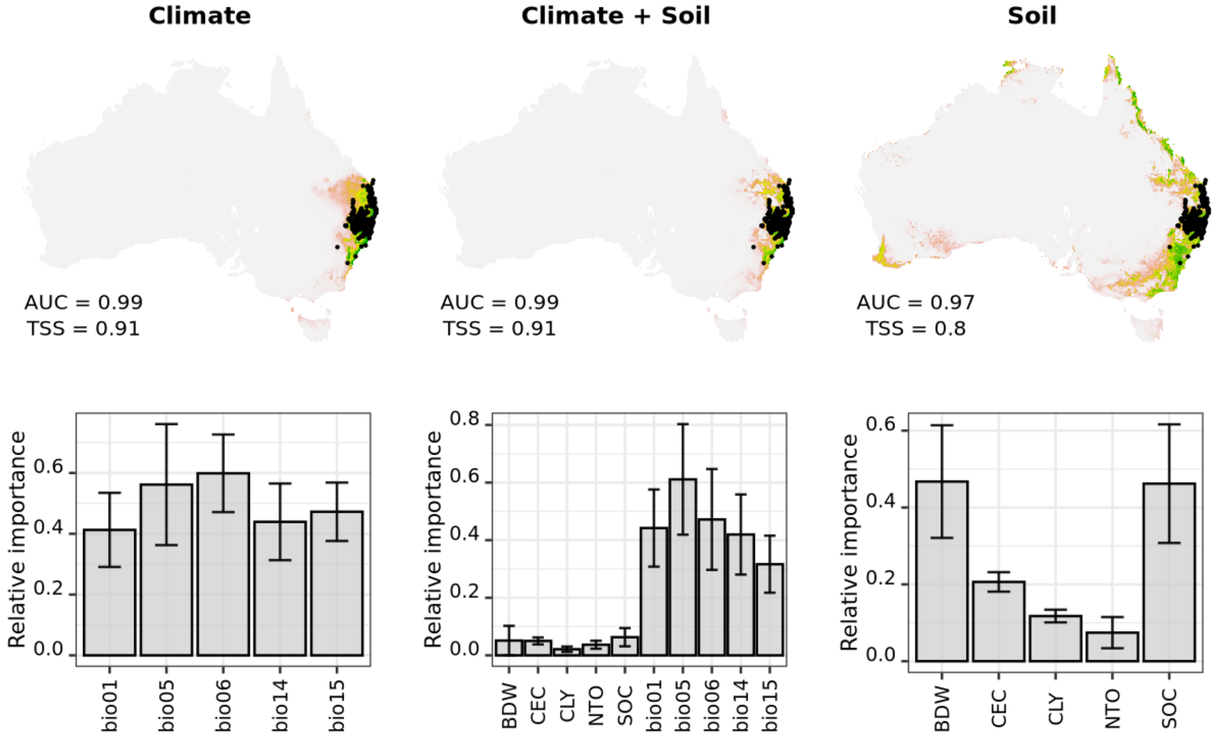

Persoonia curvifolia

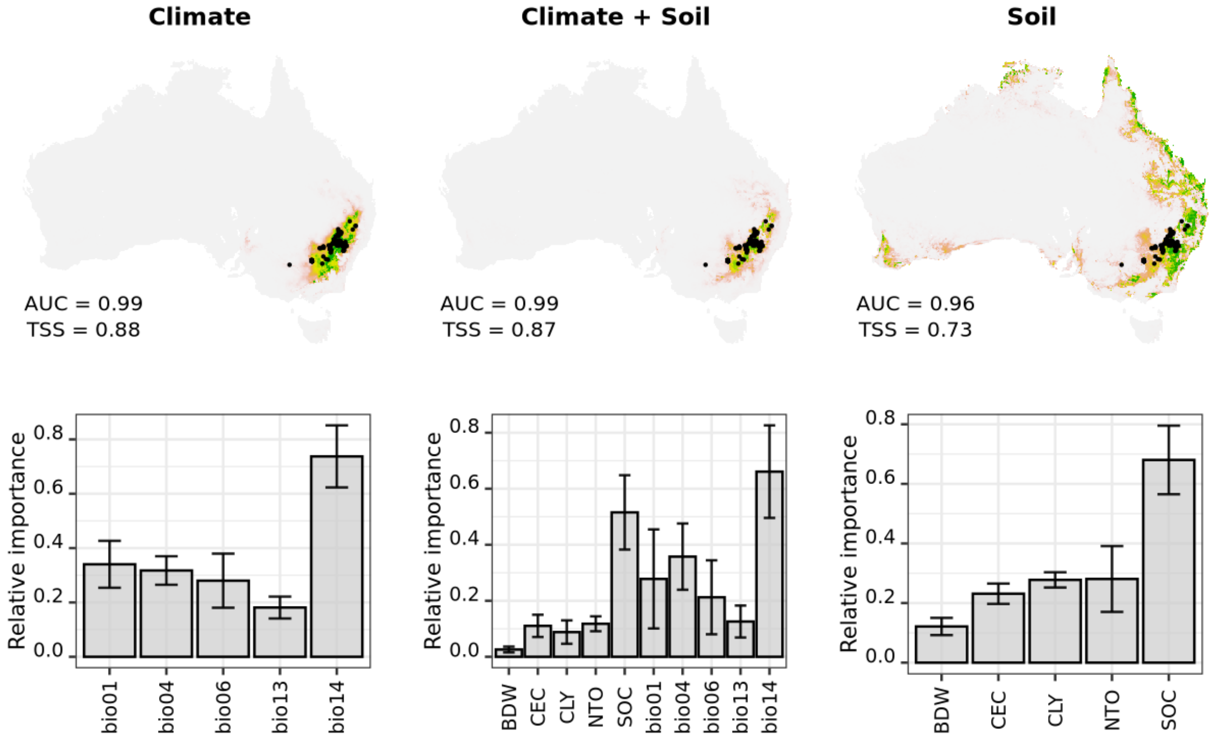

Persoonia falcata

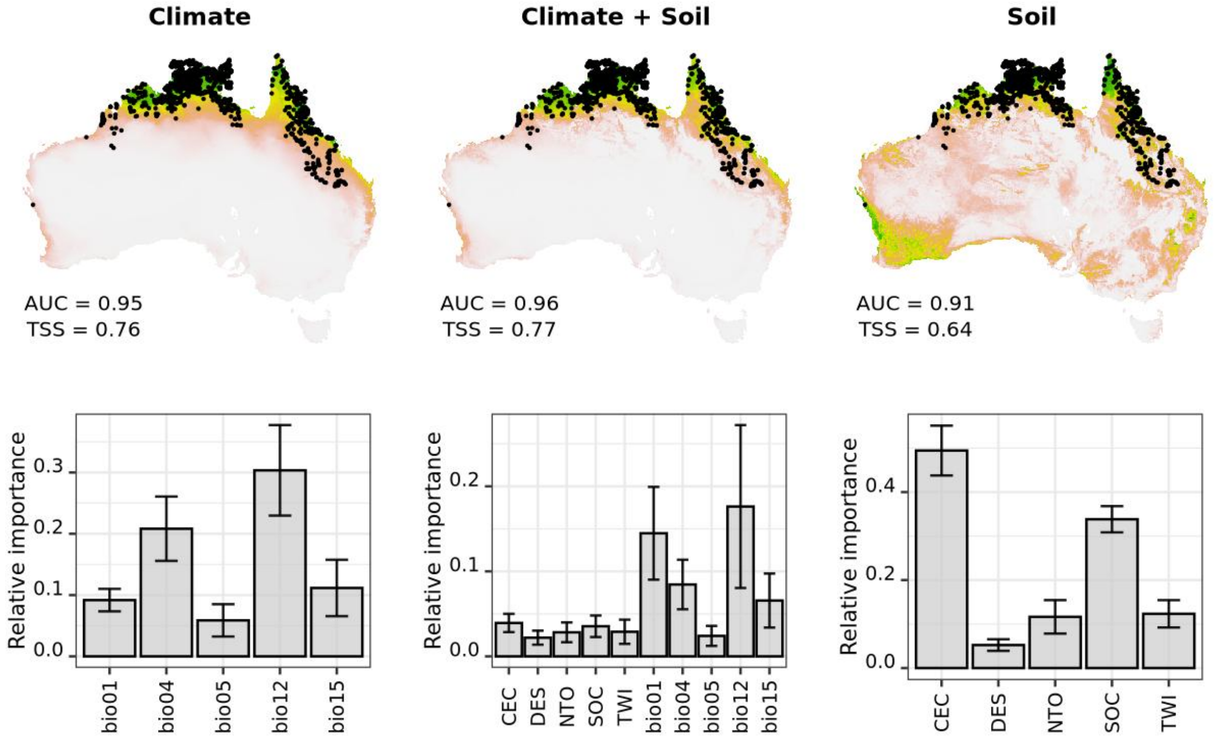

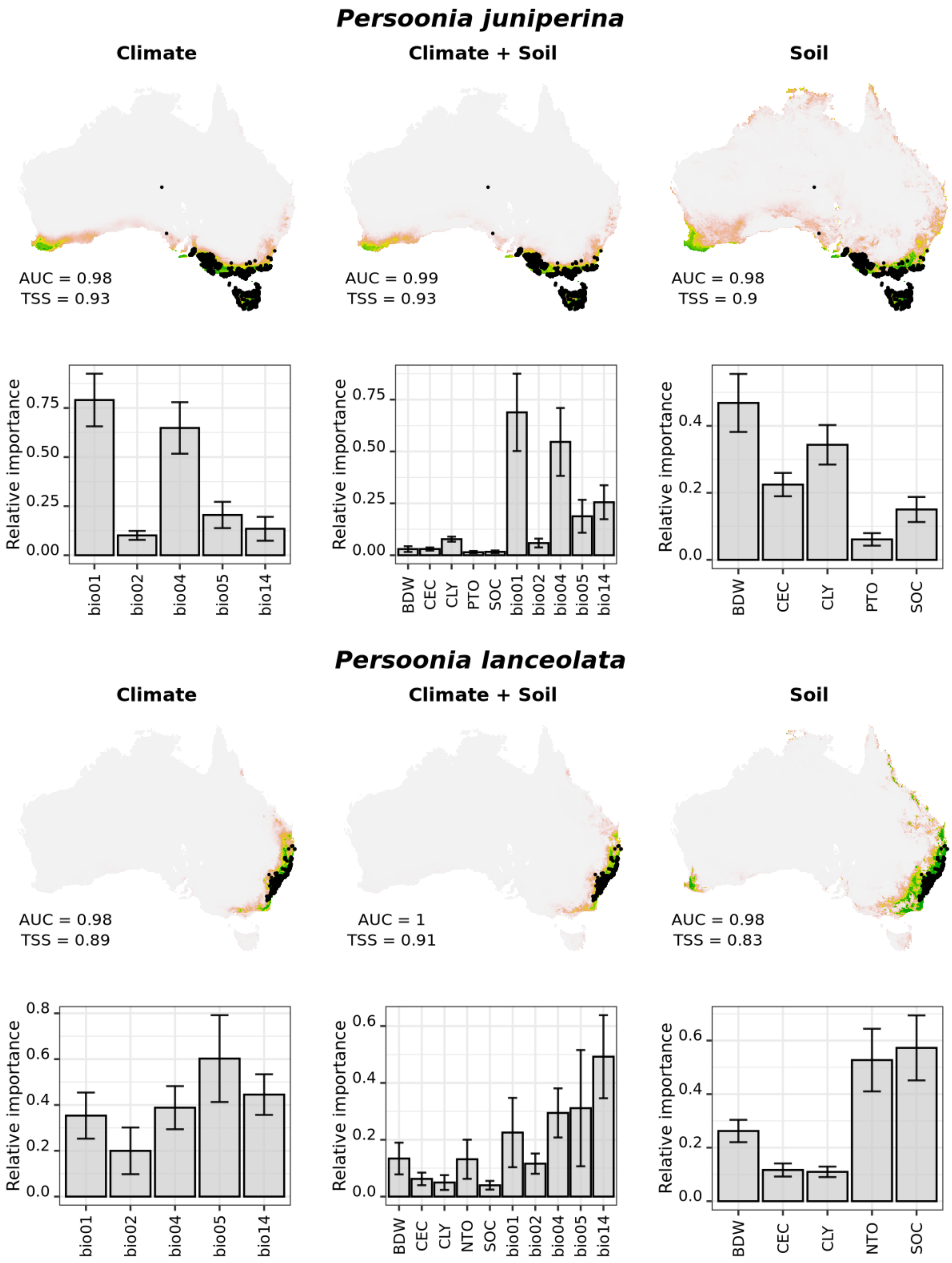

Persoonia juniperina
Climate
Climate + Soil
Soil
AUC = 0.98
TSS = 0.93
AUC = 0.99
TSS = 0.93
AUC = 0.98
TSS = 0.9
Relative importance
bio01
bio02
bio04
bio05
bio14
BDW
CEC
CLY
PTO
SOC
Persoonia lanceolata
Climate
Climate + Soil
Soil
AUC = 0.98
TSS = 0.89
AUC = 1
TSS = 0.91
AUC = 0.98
TSS = 0.83
Relative importance
bio01
bio02
bio04
bio05
bio14
BDW
CEC
CLY
NTO
SOC

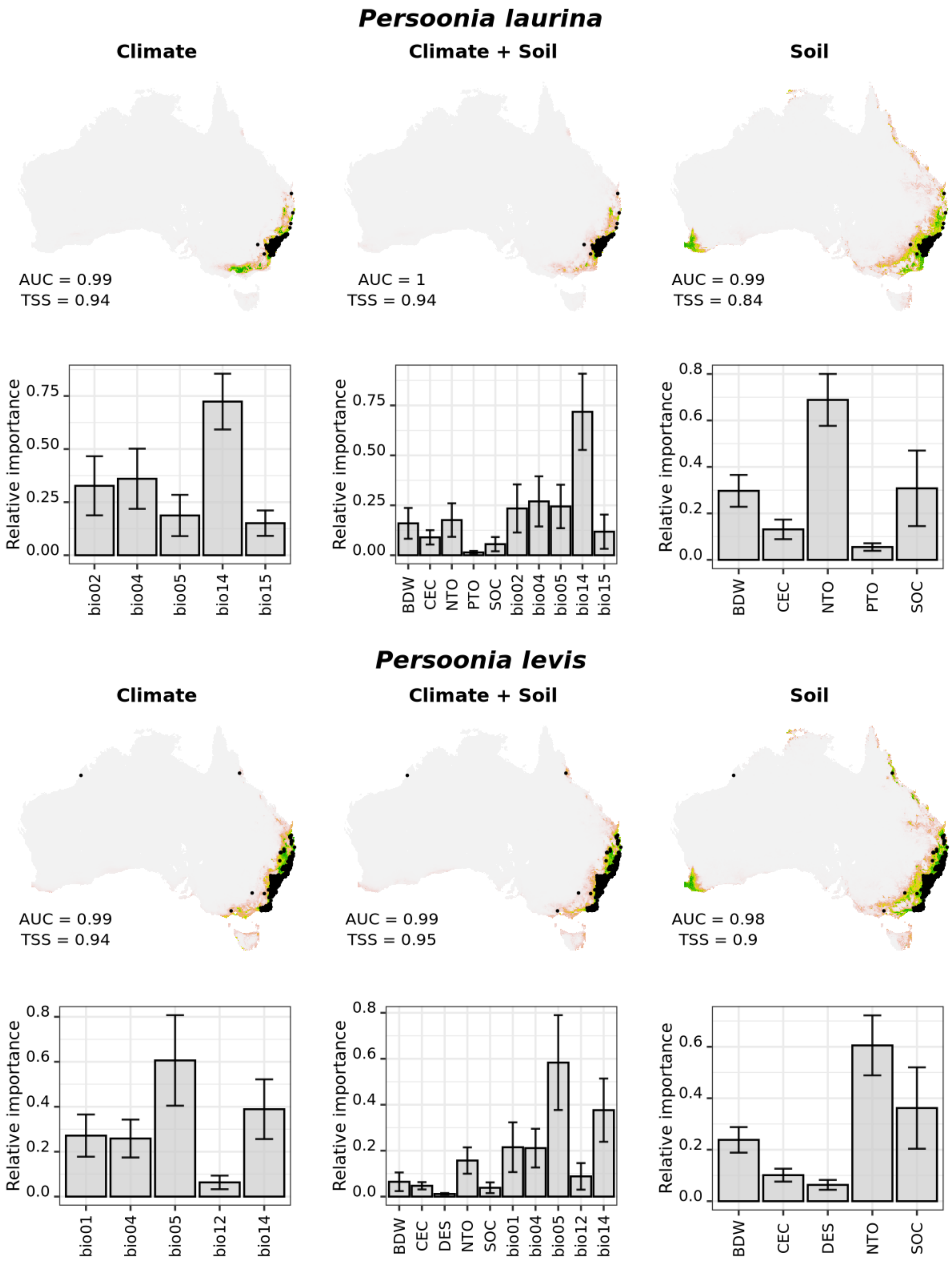

Persoonia laurina
Climate
Climate + Soil
Soil
AUC = 0.99
TSS = 0.94
AUC = 1
TSS = 0.94
AUC = 0.99
TSS = 0.84
Relative importance
bio02
bio04
bio05
bio14
bio15
BDW
CEC
NTO
PTO
SOC
Persoonia levis
Climate
Climate + Soil
Soil
AUC = 0.99
TSS = 0.94
AUC = 0.99
TSS = 0.95
AUC = 0.98
TSS = 0.9
Relative importance
bio01
bio04
bio05
bio12
bio14
BDW
CEC
DES
NTO
SOC

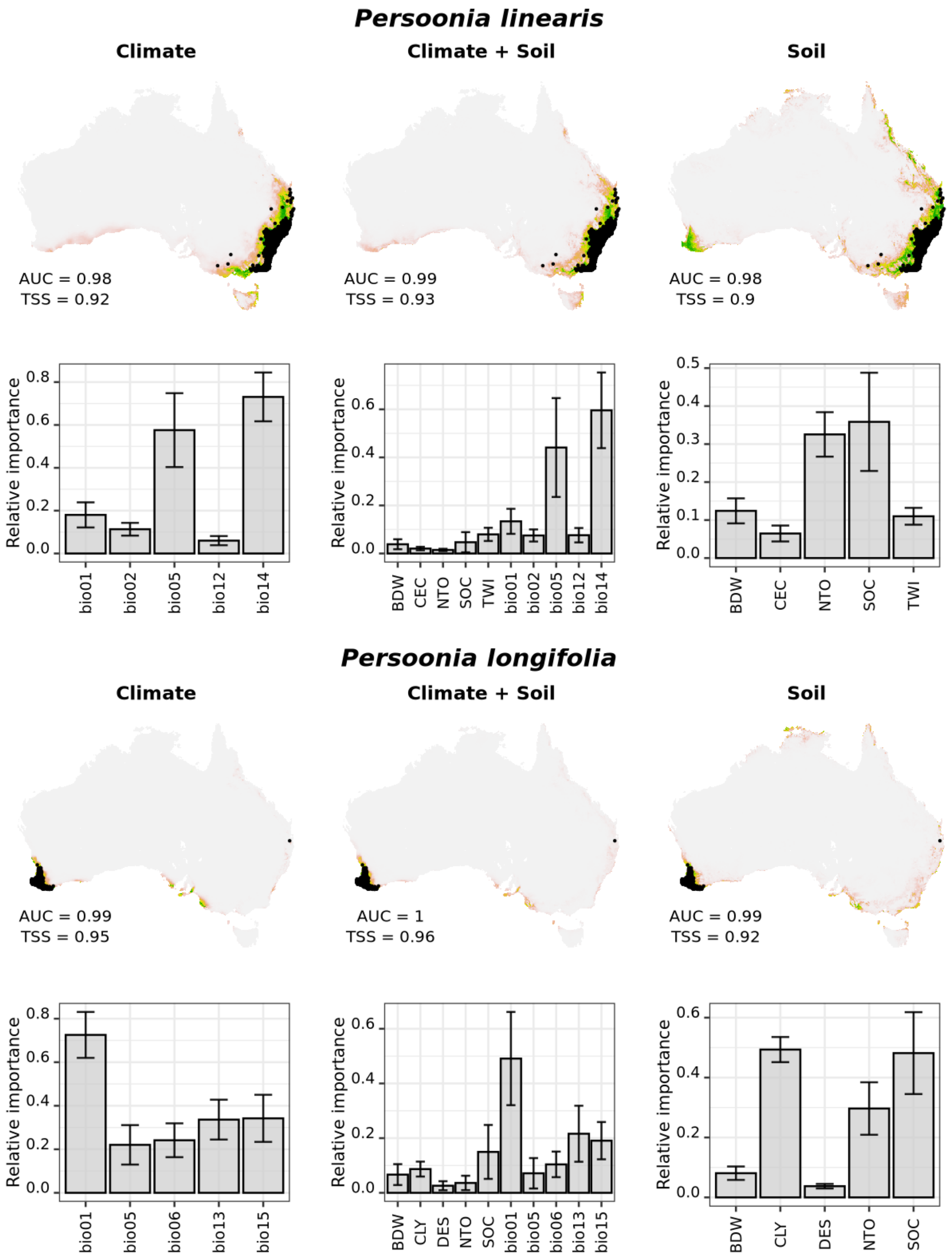
Persoonia linearis
Climate
Climate + Soil
Soil
AUC = 0.98
TSS = 0.92
AUC = 0.99
TSS = 0.93
AUC = 0.98
TSS = 0.9
Relative importance
0.0
0.2
0.4
0.6
0.8
bio01
bio02
bio05
bio12
bio14
Relative importance
0.0
0.2
0.4
0.6
BDW
CEC
NTO
SOC
TWI
bio01
bio02
bio05
bio12
bio14
Relative importance
0.0
0.1
0.2
0.3
0.4
0.5
BDW
CEC
NTO
SOC
TWI
Persoonia longifolia
Climate
Climate + Soil
Soil
AUC = 0.99
TSS = 0.95
AUC = 1
TSS = 0.96
AUC = 0.99
TSS = 0.92
Relative importance
0.0
0.2
0.4
0.6
0.8
bio01
bio05
bio06
bio13
bio15
Relative importance
0.0
0.2
0.4
0.6
BDW
CLY
DES
NTO
SOC
bio01
bio05
bio06
bio13
bio15
Relative importance
0.0
0.2
0.4
0.6
BDW
CLY
DES
NTO
SOC

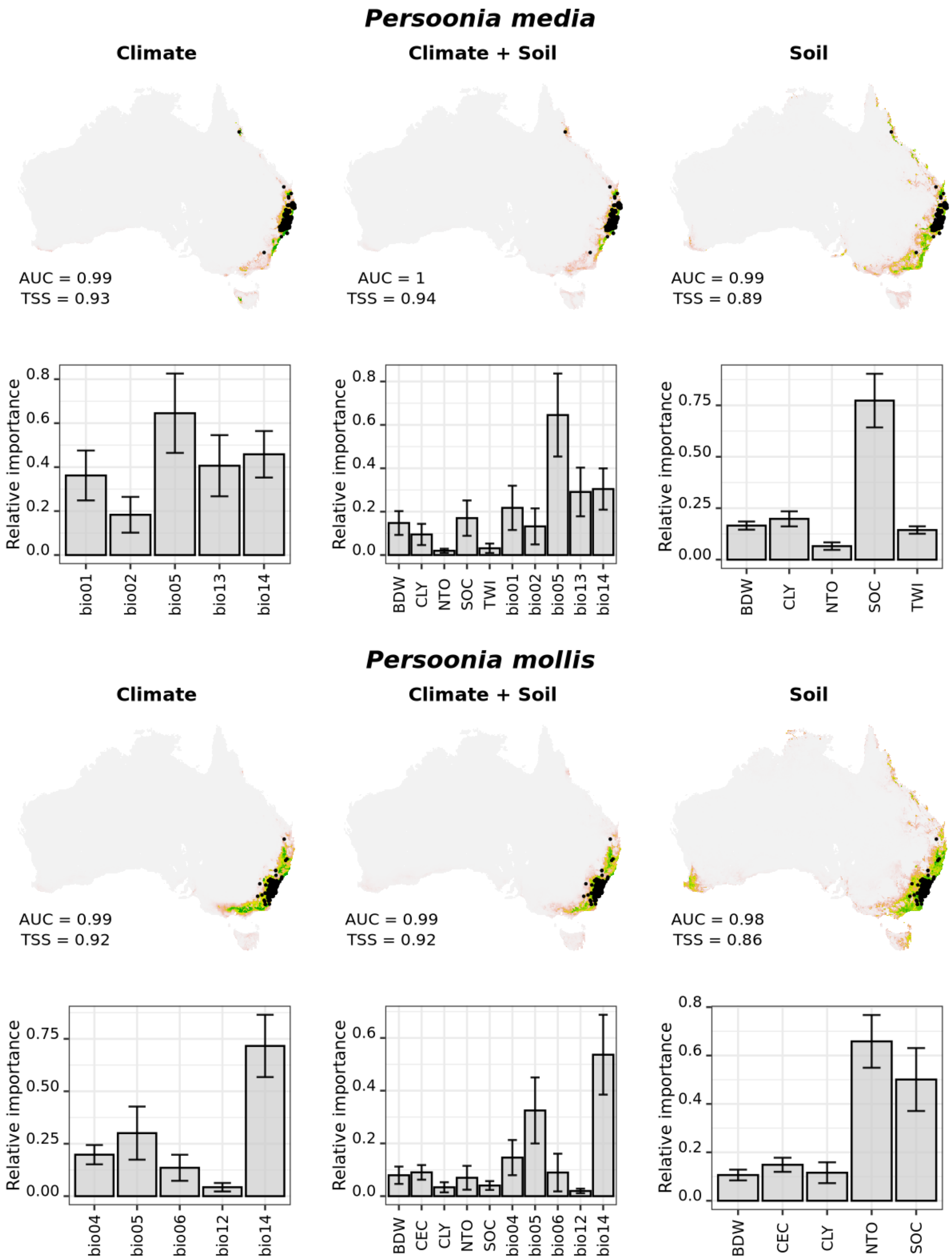
Persoonia media
Climate
Climate + Soil
Soil
AUC = 0.99
TSS = 0.93
AUC = 1
TSS = 0.94
AUC = 0.99
TSS = 0.89
Relative importance
bio01
bio02
bio05
bio13
bio14
BDW
CLY
NTO
SOC
TWI
Persoonia mollis
Climate
Climate + Soil
Soil
AUC = 0.99
TSS = 0.92
AUC = 0.99
TSS = 0.92
AUC = 0.98
TSS = 0.86
Relative importance
bio04
bio05
bio06
bio12
bio14
BDW
CEC
CLY
NTO
SOC

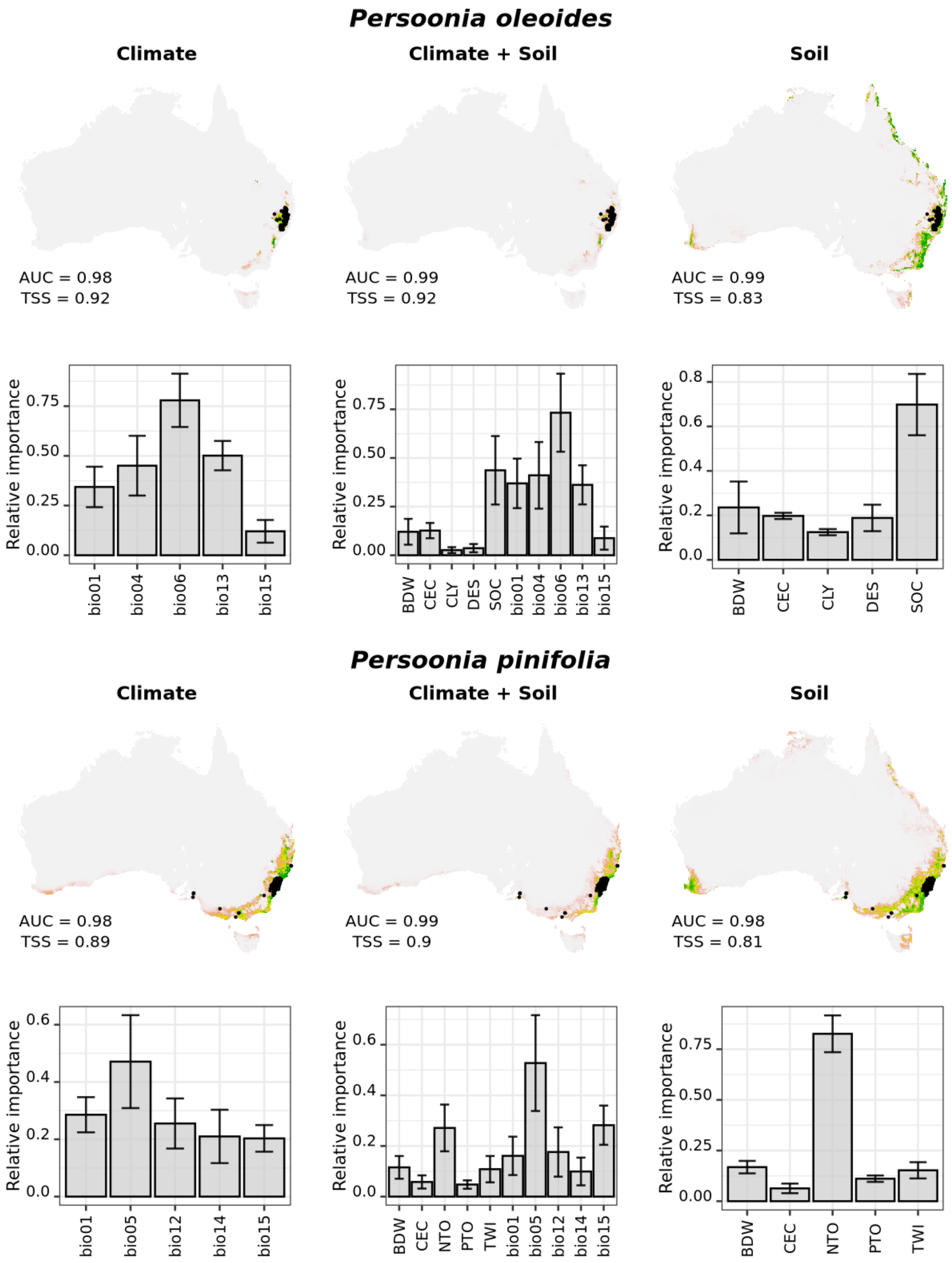

Persoonia oleoides
Climate
Climate + Soil
Soil
AUC = 0.98
TSS = 0.92
AUC = 0.99
TSS = 0.92
AUC = 0.99
TSS = 0.83
Relative importance
0.00
0.25
0.50
0.75
bio01
bio04
bio06
bio13
bio15
Relative importance
0.00
0.25
0.50
0.75
BDW
CEC
CLY
DES
SOC
bio01
bio04
bio06
bio13
bio15
Relative importance
0.0
0.2
0.4
0.6
0.8
BDW
CEC
CLY
DES
SOC
Persoonia pinifolia
Climate
Climate + Soil
Soil
AUC = 0.98
TSS = 0.89
AUC = 0.99
TSS = 0.9
AUC = 0.98
TSS = 0.81
Relative importance
0.0
0.2
0.4
0.6
bio01
bio05
bio12
bio14
bio15
Relative importance
0.0
0.2
0.4
0.6
BDW
CEC
NTO
PTO
TWI
bio01
bio05
bio12
bio14
bio15
Relative importance
0.00
0.25
0.50
0.75
BDW
CEC
NTO
PTO
TWI

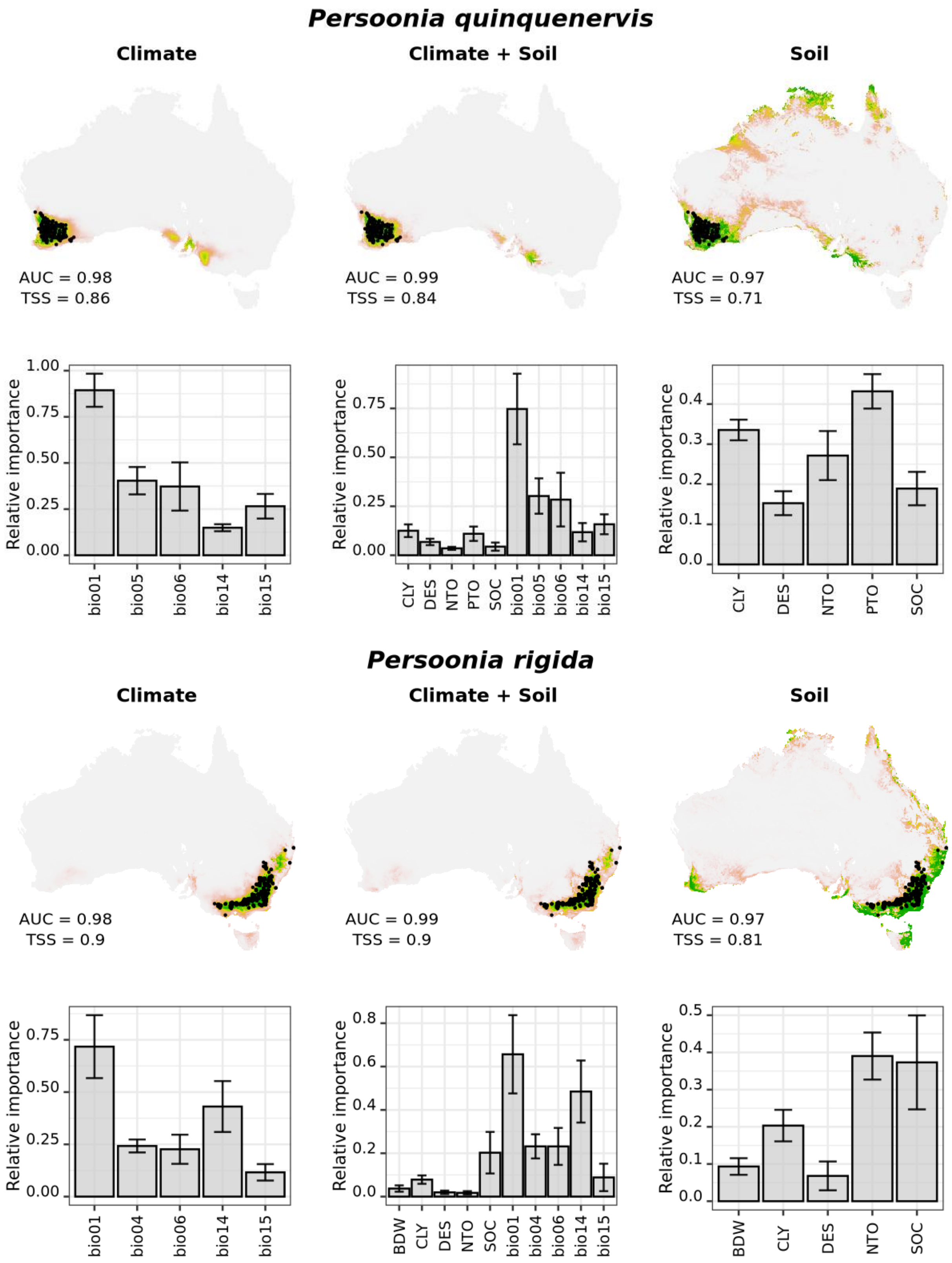

Persoonia quinquenervis
Climate
Climate + Soil
Soil
AUC = 0.98
TSS = 0.86
AUC = 0.99
TSS = 0.84
AUC = 0.97
TSS = 0.71
Relative importance
bio01 bio05 bio06 bio14 bio15
CLY DES NTO PTO SOC bio01 bio05 bio06 bio14 bio15
CLY DES NTO PTO SOC
Persoonia rigida
Climate
Climate + Soil
Soil
AUC = 0.98
TSS = 0.9
AUC = 0.99
TSS = 0.9
AUC = 0.97
TSS = 0.81
Relative importance
bio01 bio04 bio06 bio14 bio15
BDW CLY DES NTO SOC bio01 bio04 bio06 bio14 bio15
BDW CLY DES NTO SOC

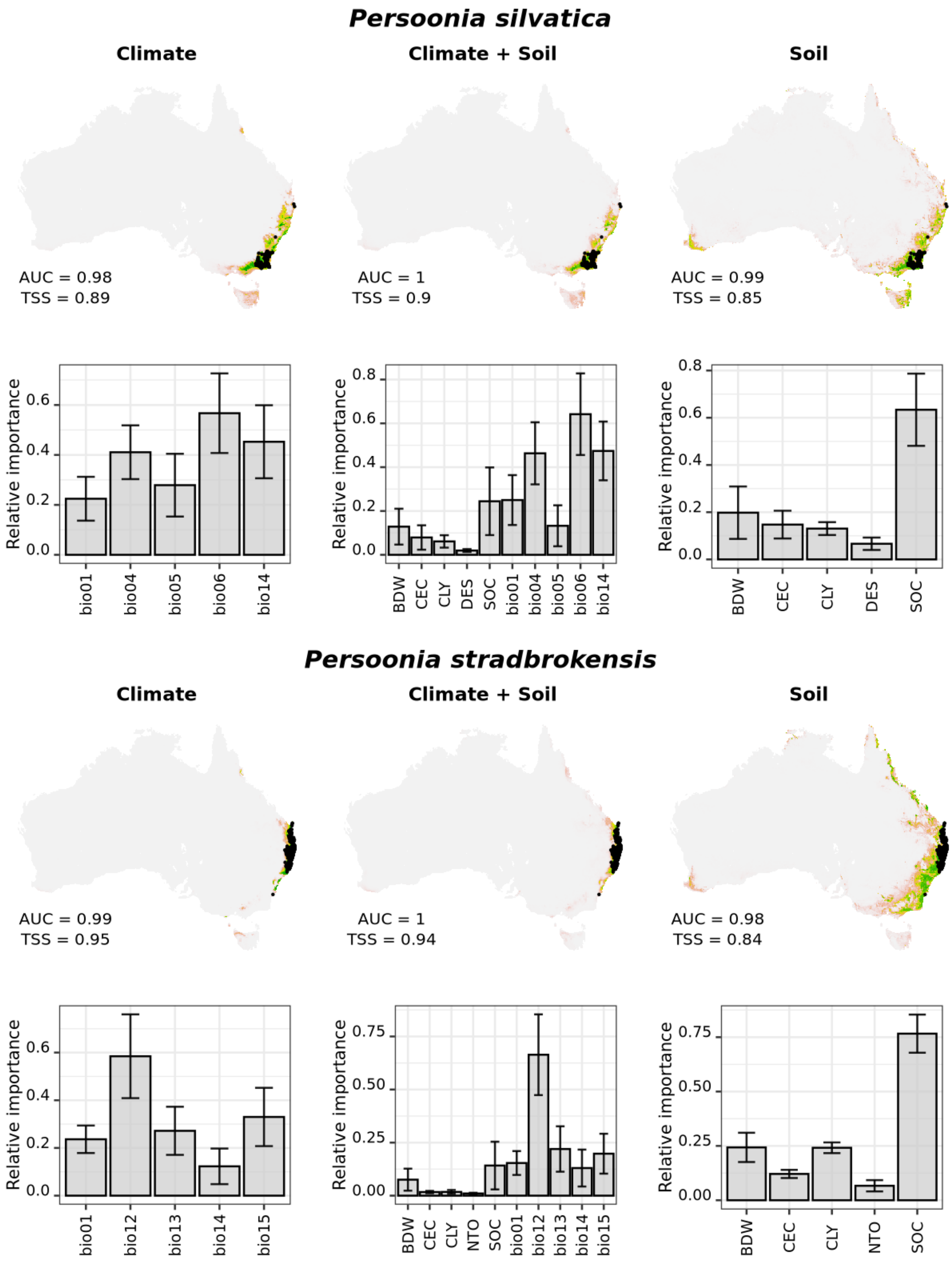
Persoonia silvatica
Climate
Climate + Soil
Soil
AUC = 0.98
TSS = 0.89
AUC = 1
TSS = 0.9
AUC = 0.99
TSS = 0.85
Relative importance
bio01
bio04
bio05
bio06
bio14
BDW
CEC
CLY
DES
SOC
Persoonia stradbrokensis
Climate
Climate + Soil
Soil
AUC = 0.99
TSS = 0.95
AUC = 1
TSS = 0.94
AUC = 0.98
TSS = 0.84
Relative importance
bio01
bio12
bio13
bio14
bio15
BDW
CEC
CLY
NTO
SOC

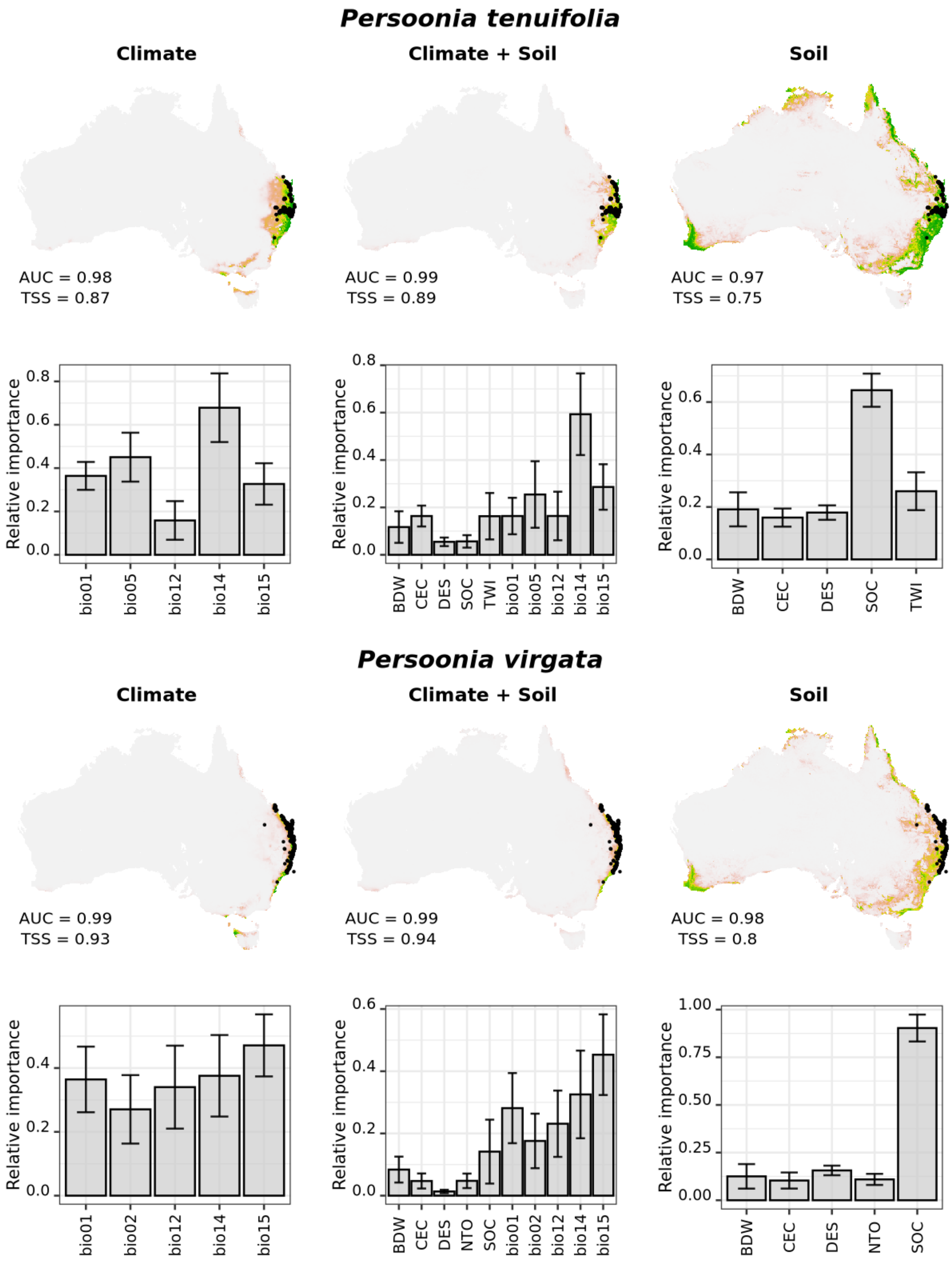
Persoonia tenuifolia
Climate
Climate + Soil
Soil
AUC = 0.98
TSS = 0.87
AUC = 0.99
TSS = 0.89
AUC = 0.97
TSS = 0.75
Relative importance
bio01
bio05
bio12
bio14
bio15
BDW
CEC
DES
SOC
TWI
bio01
bio05
bio12
bio14
bio15
BDW
CEC
DES
SOC
TWI
Persoonia virgata
Climate
Climate + Soil
Soil
AUC = 0.99
TSS = 0.93
AUC = 0.99
TSS = 0.94
AUC = 0.98
TSS = 0.8
Relative importance
bio01
bio02
bio12
bio14
bio15
BDW
CEC
DES
NTO
SOC
bio01
bio02
bio12
bio14
bio15
BDW
CEC
DES
NTO
SOC

Petalostigma banksii

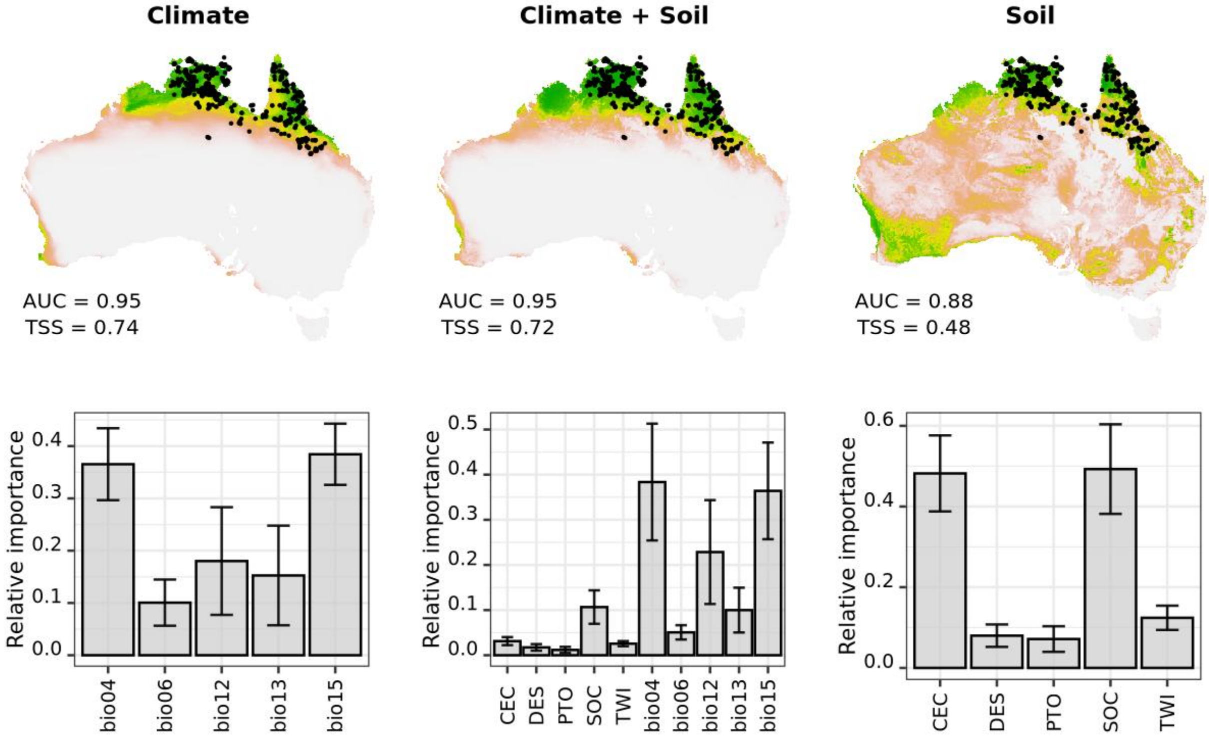

Petalostigma pubescens

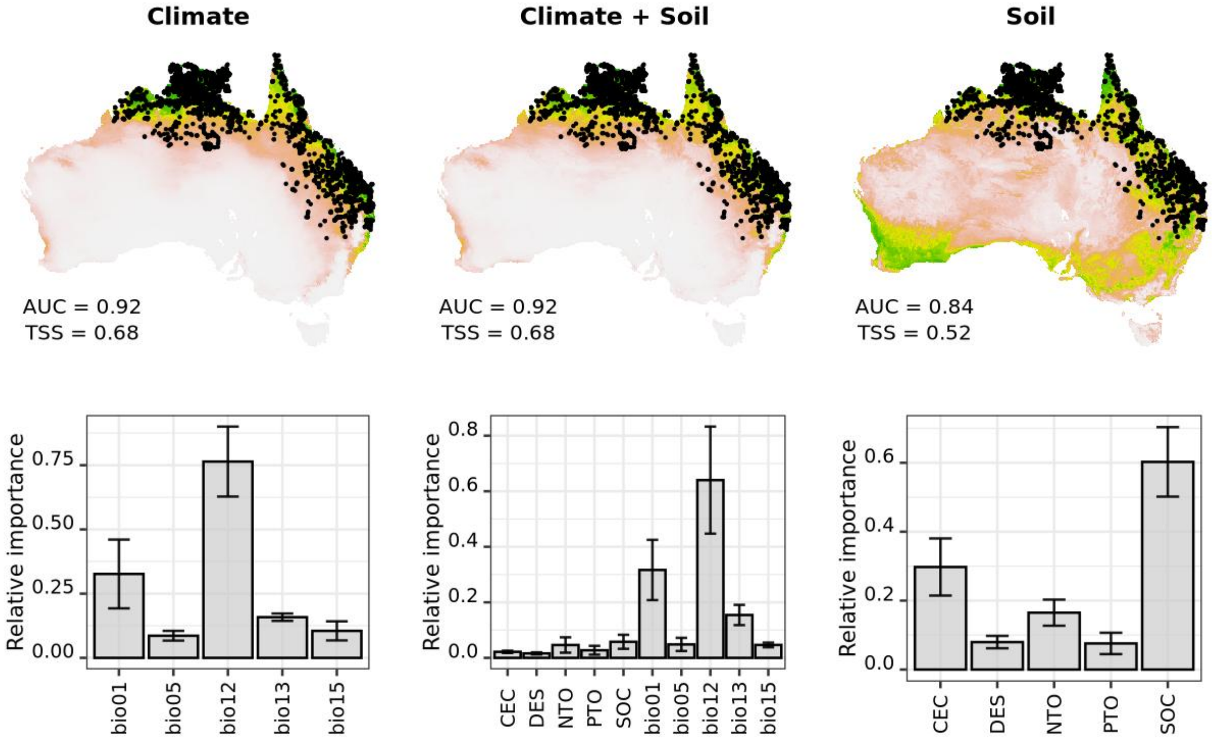

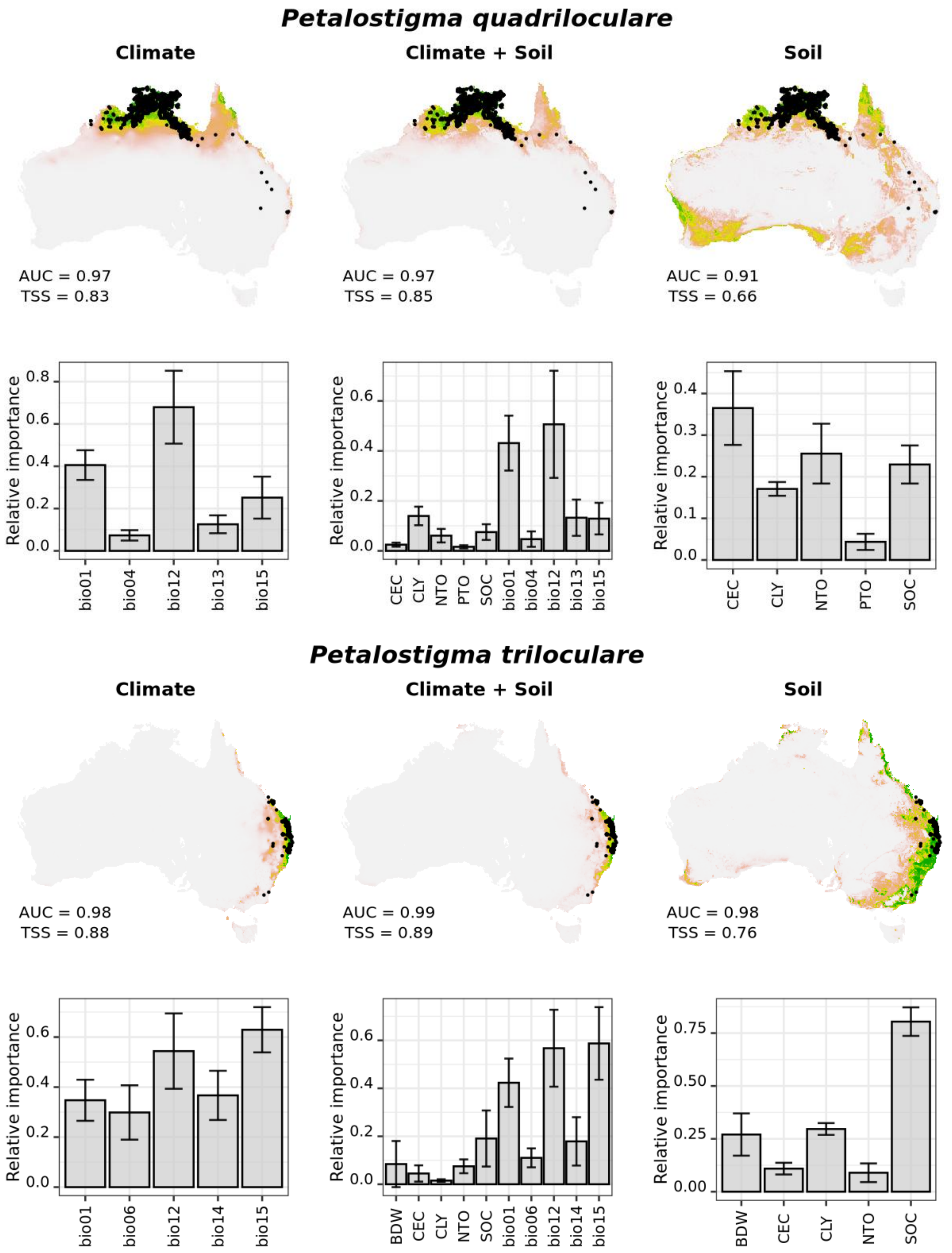
Petalostigma quadriloculare
Climate
Climate + Soil
Soil
AUC = 0.97
TSS = 0.83
AUC = 0.97
TSS = 0.85
AUC = 0.91
TSS = 0.66
Relative importance
0.0
0.2
0.4
0.6
0.8
bio01
bio04
bio12
bio13
bio15
CEC
CLY
NTO
PTO
SOC
bio01
bio04
bio12
bio13
bio15
0.1
0.3
CEC
CLY
NTO
PTO
SOC
Petalostigma triloculare
Climate
Climate + Soil
Soil
AUC = 0.98
TSS = 0.88
AUC = 0.99
TSS = 0.89
AUC = 0.98
TSS = 0.76
bio01
bio06
bio12
bio14
bio15
BDW
CEC
CLY
NTO
SOC
bio01
bio06
bio12
bio14
bio15
0.00
0.25
0.50
0.75
BDW
CEC
CLY
NTO
SOC

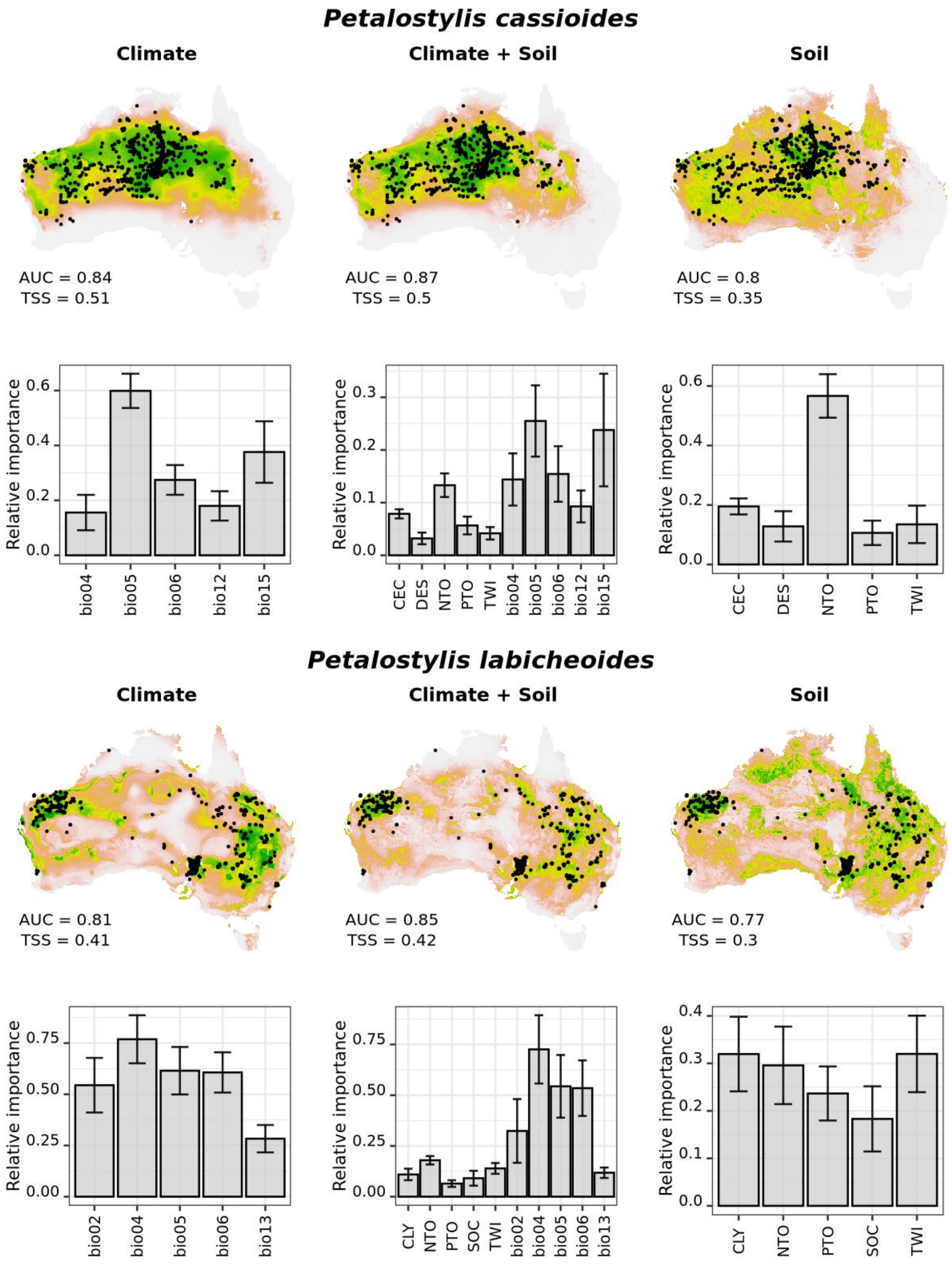

Petalostylis cassioides
Climate
Climate + Soil
Soil
AUC = 0.84
TSS = 0.51
AUC = 0.87
TSS = 0.5
AUC = 0.8
TSS = 0.35
Relative importance
0.0
0.2
0.4
0.6
bio04
bio05
bio06
bio12
bio15
0.0
0.1
0.2
0.3
CEC
DES
NTO
PTO
TWI
bio04
bio05
bio06
bio12
bio15
0.0
0.2
0.4
0.6
CEC
DES
NTO
PTO
TWI
Petalostylis labicheoides
Climate
Climate + Soil
Soil
AUC = 0.81
TSS = 0.41
AUC = 0.85
TSS = 0.42
AUC = 0.77
TSS = 0.3
Relative importance
0.00
0.25
0.50
0.75
bio02
bio04
bio05
bio06
bio13
0.00
0.25
0.50
0.75
CLY
NTO
PTO
SOC
TWI
bio02
bio04
bio05
bio06
bio13
0.0
0.1
0.2
0.3
0.4
CLY
NTO
PTO
SOC
TWI

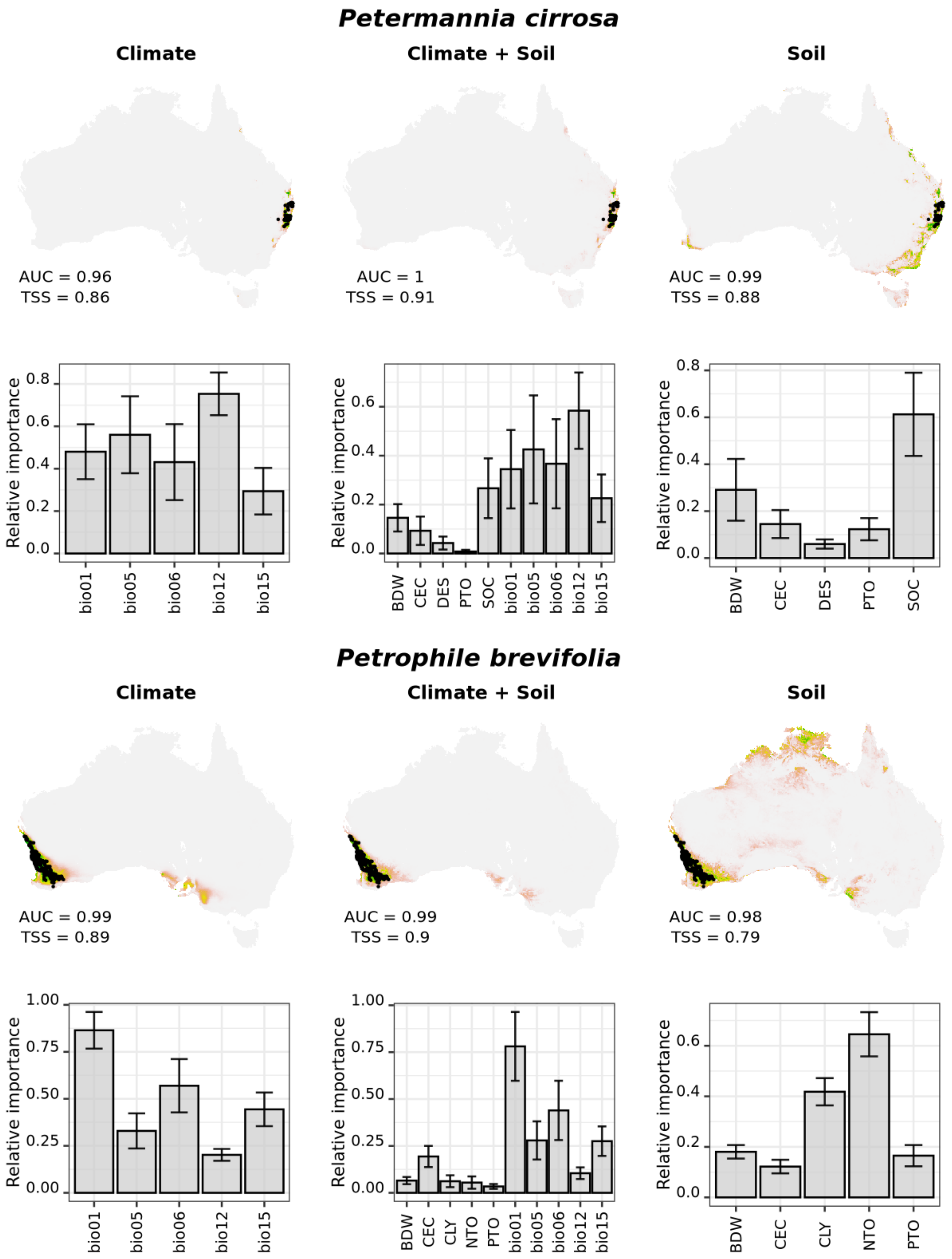
Petermannia cirrosa
Climate
Climate + Soil
Soil
AUC = 0.96
TSS = 0.86
AUC = 1
TSS = 0.91
AUC = 0.99
TSS = 0.88
Relative importance
bio01 bio05 bio06 bio12 bio15
BDW CEC DES PTO SOC bio01 bio05 bio06 bio12 bio15
BDW CEC DES PTO SOC
Petrophile brevifolia
Climate
Climate + Soil
Soil
AUC = 0.99
TSS = 0.89
AUC = 0.99
TSS = 0.9
AUC = 0.98
TSS = 0.79
Relative importance
bio01 bio05 bio06 bio12 bio15
BDW CEC CLY NTO PTO bio01 bio05 bio06 bio12 bio15
BDW CEC CLY NTO PTO

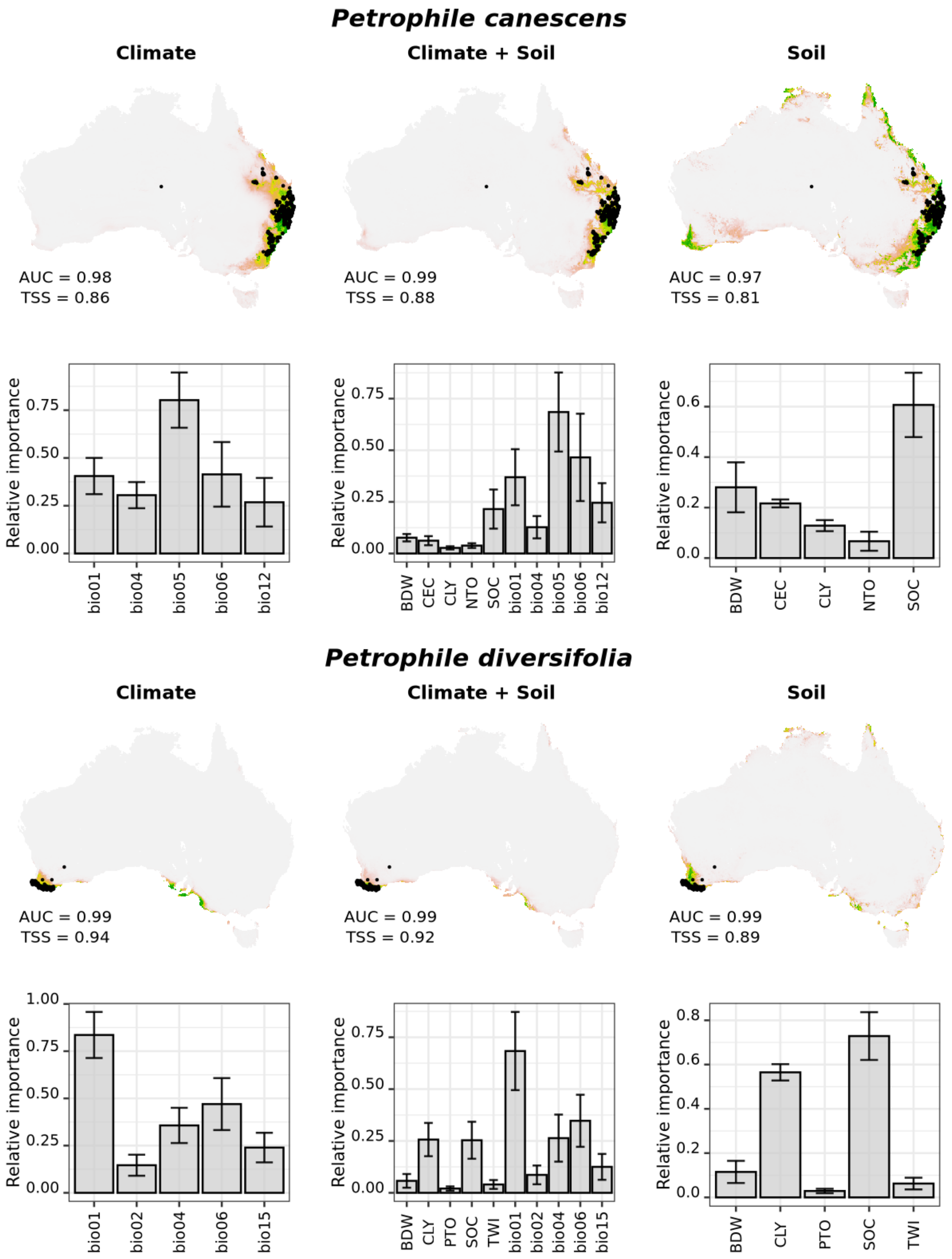
Petrophile canescens
Climate
Climate + Soil
Soil
AUC = 0.98
TSS = 0.86
AUC = 0.99
TSS = 0.88
AUC = 0.97
TSS = 0.81
Relative importance
0.00
0.25
0.50
0.75
bio01
bio04
bio05
bio06
bio12
BDW
CEC
CLY
NTO
SOC
0.0
0.2
0.4
0.6
Petrophile diversifolia
Climate
Climate + Soil
Soil
AUC = 0.99
TSS = 0.94
AUC = 0.99
TSS = 0.92
AUC = 0.99
TSS = 0.89
1.00
bio02
bio15
PTO
TWI
0.8

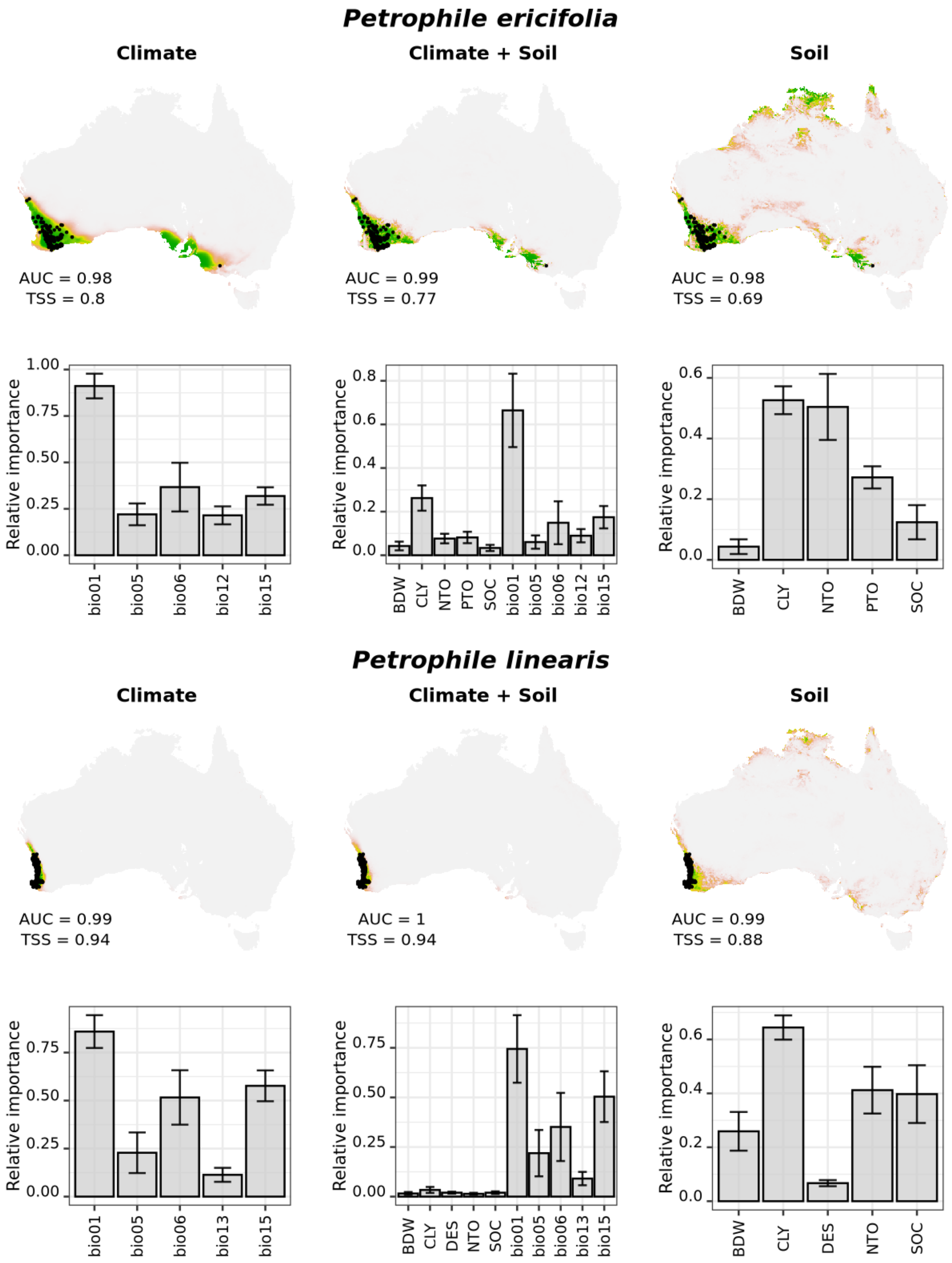
Petrophile ericifolia
Climate
Climate + Soil
Soil
AUC = 0.98
TSS = 0.8
AUC = 0.99
TSS = 0.77
AUC = 0.98
TSS = 0.69
Relative importance
bio01
bio05
bio06
bio12
bio15
BDW
CLY
NTO
PTO
SOC
Petrophile linearis
Climate
Climate + Soil
Soil
AUC = 0.99
TSS = 0.94
AUC = 1
TSS = 0.94
AUC = 0.99
TSS = 0.88
Relative importance
bio01
bio05
bio06
bio13
bio15
BDW
CLY
DES
NTO
SOC

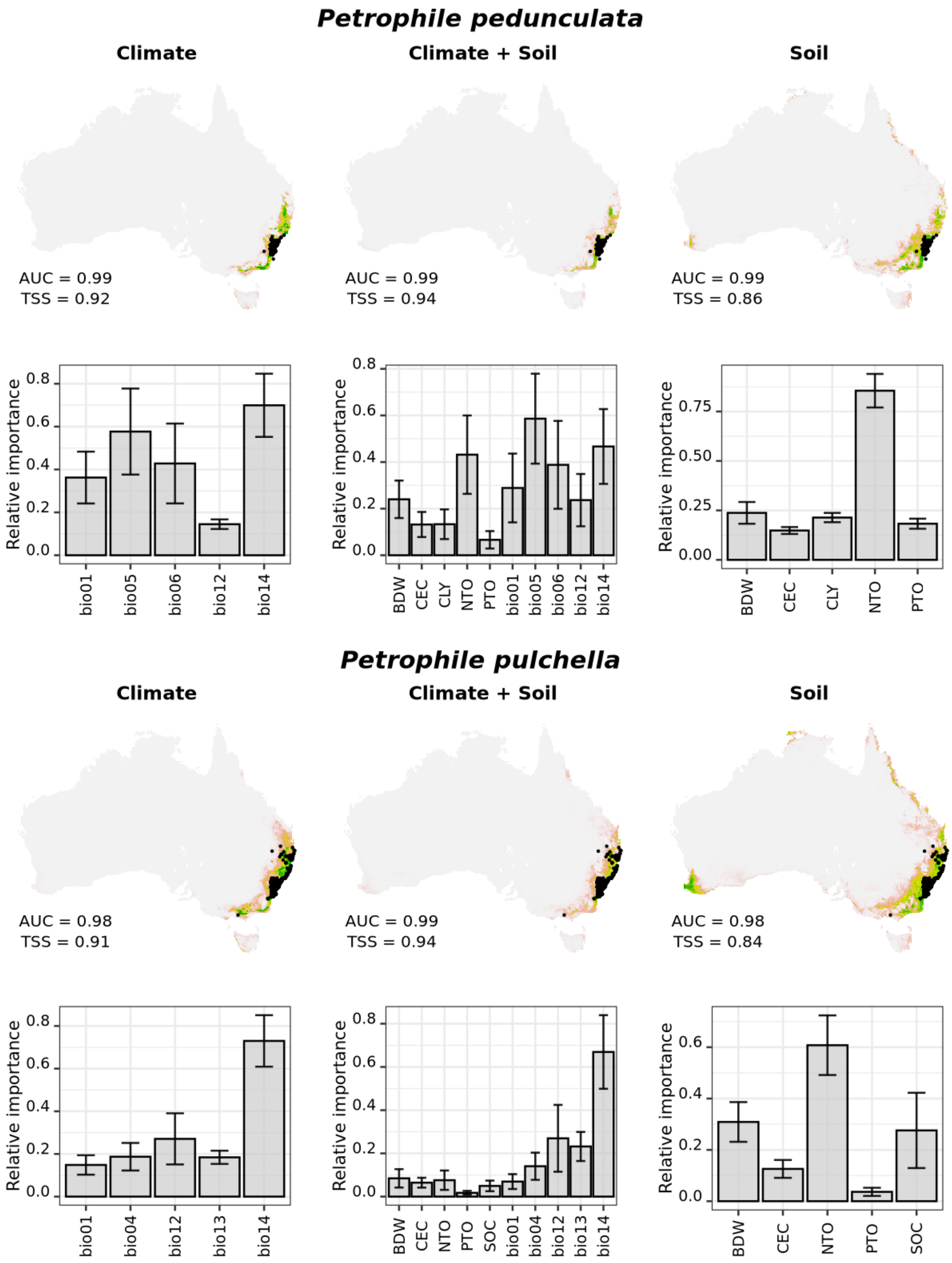

Petrophile pedunculata
Climate
Climate + Soil
Soil
AUC = 0.99
TSS = 0.92
AUC = 0.99
TSS = 0.94
AUC = 0.99
TSS = 0.86
Relative importance
bio01
bio05
bio06
bio12
bio14
BDW
CEC
CLY
NTO
PTO
bio01
bio05
bio06
bio12
bio14
BDW
CEC
CLY
NTO
PTO
Petrophile pulchella
Climate
Climate + Soil
Soil
AUC = 0.98
TSS = 0.91
AUC = 0.99
TSS = 0.94
AUC = 0.98
TSS = 0.84
Relative importance
bio01
bio04
bio12
bio13
bio14
BDW
CEC
NTO
PTO
SOC
bio01
bio04
bio12
bio13
bio14
BDW
CEC
NTO
PTO
SOC

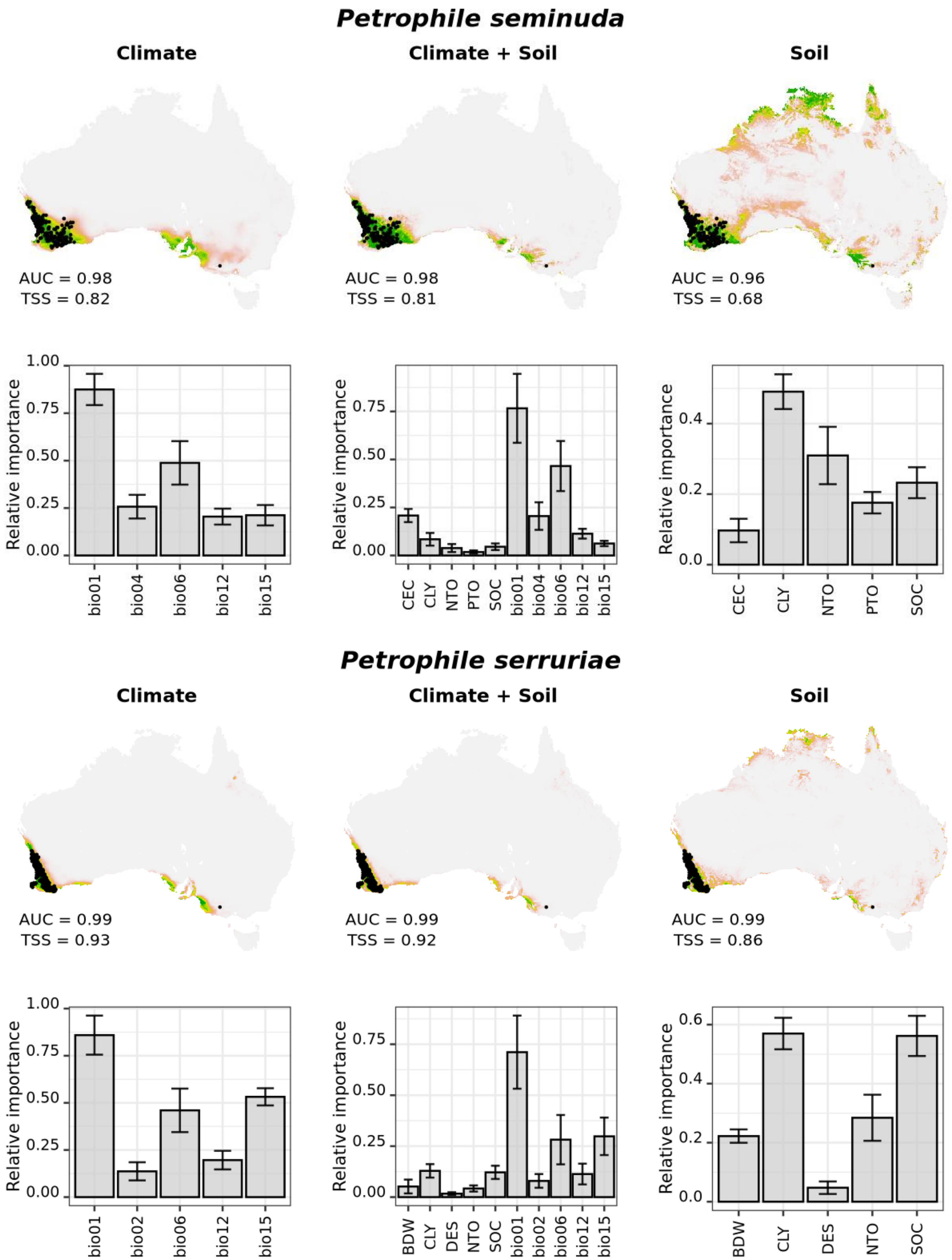

Petrophile seminuda
Climate
Climate + Soil
Soil
AUC = 0.98
TSS = 0.82
AUC = 0.98
TSS = 0.81
AUC = 0.96
TSS = 0.68
Relative importance
0.00 0.25 0.50 0.75 1.00
bio01 bio04 bio06 bio12 bio15
0.00 0.25 0.50 0.75
CEC CLY NTO PTO SOC bio01 bio04 bio06 bio12 bio15
0.0 0.2 0.4
CEC CLY NTO PTO SOC
Petrophile serruriae
Climate
Climate + Soil
Soil
AUC = 0.99
TSS = 0.93
AUC = 0.99
TSS = 0.92
AUC = 0.99
TSS = 0.86
Relative importance
0.00 0.25 0.50 0.75 1.00
bio01 bio02 bio06 bio12 bio15
0.00 0.25 0.50 0.75
BDW CLY DES NTO SOC bio01 bio02 bio06 bio12 bio15
0.0 0.2 0.4 0.6
BDW CLY DES NTO SOC

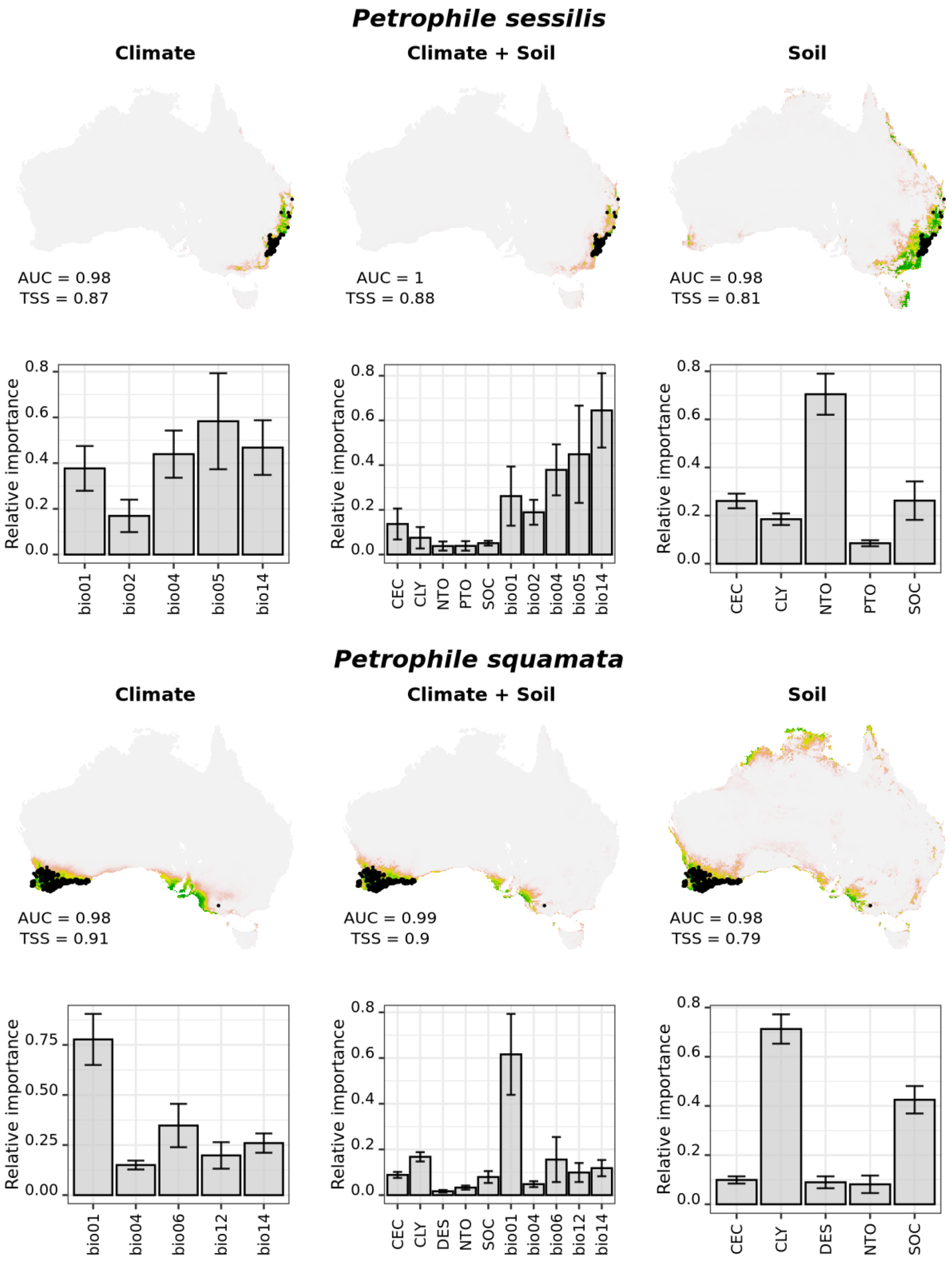
Petrophile sessilis
Climate
Climate + Soil
Soil
AUC = 0.98
TSS = 0.87
AUC = 1
TSS = 0.88
AUC = 0.98
TSS = 0.81
Relative importance
bio01
bio02
bio04
bio05
bio14
CEC
CLY
NTO
PTO
SOC
Petrophile squamata
Climate
Climate + Soil
Soil
AUC = 0.98
TSS = 0.91
AUC = 0.99
TSS = 0.9
AUC = 0.98
TSS = 0.79
Relative importance
bio01
bio04
bio06
bio12
bio14
CEC
CLY
DES
NTO
SOC

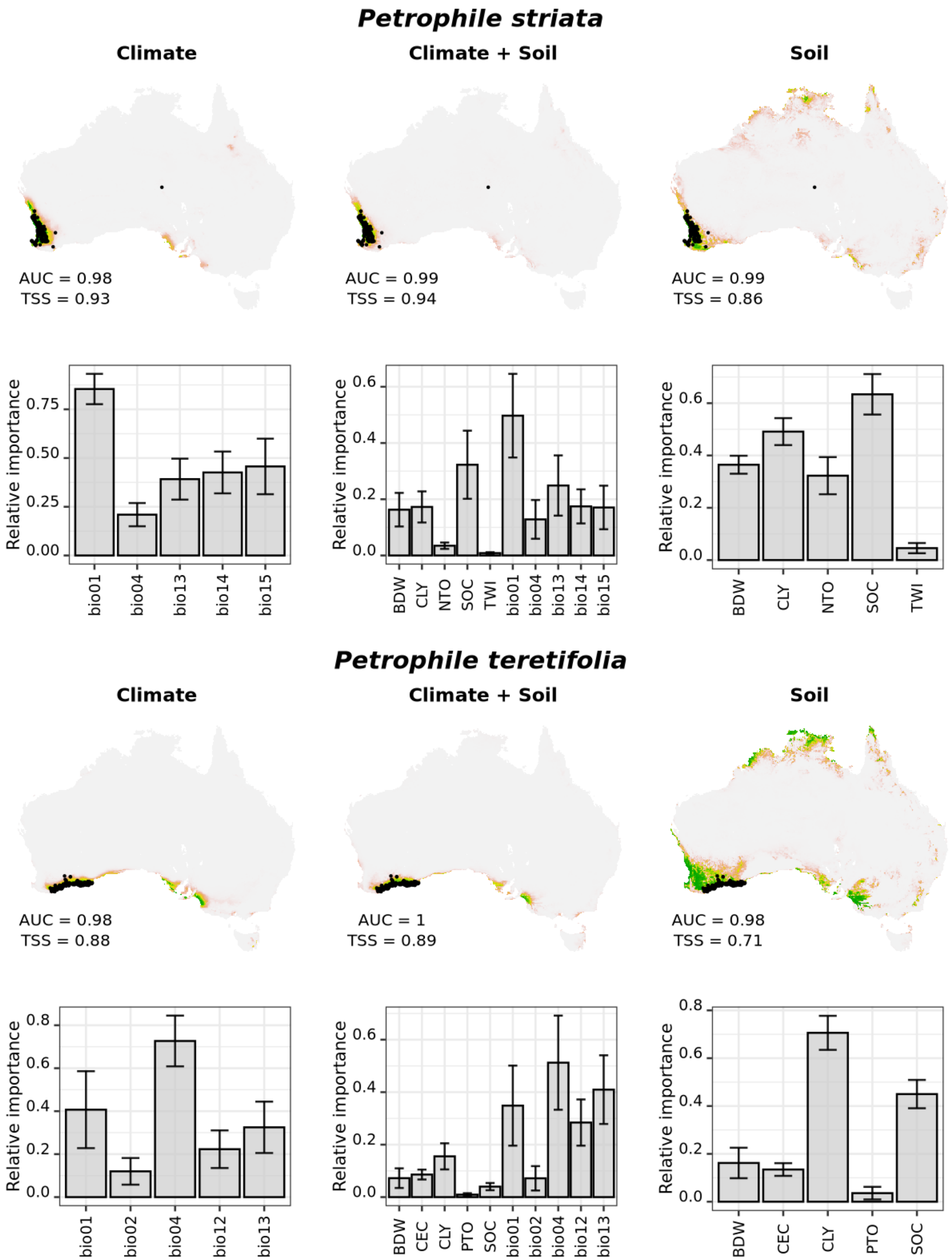
Petrophile striata
Climate
Climate + Soil
Soil
AUC = 0.98
TSS = 0.93
AUC = 0.99
TSS = 0.94
AUC = 0.99
TSS = 0.86
Relative importance
0.00
0.25
0.50
0.75
bio01
bio04
bio13
bio14
bio15
0.0
0.2
0.4
0.6
BDW
CLY
NTO
SOC
TWI
bio01
bio04
bio13
bio14
bio15
BDW
CLY
NTO
SOC
TWI
Petrophile teretifolia
Climate
Climate + Soil
Soil
AUC = 0.98
TSS = 0.88
AUC = 1
TSS = 0.89
AUC = 0.98
TSS = 0.71
0.8
bio01
bio02
bio04
bio12
bio13
BDW
CEC
CLY
PTO
SOC
bio01
bio02
bio04
bio12
bio13
BDW
CEC
CLY
PTO
SOC

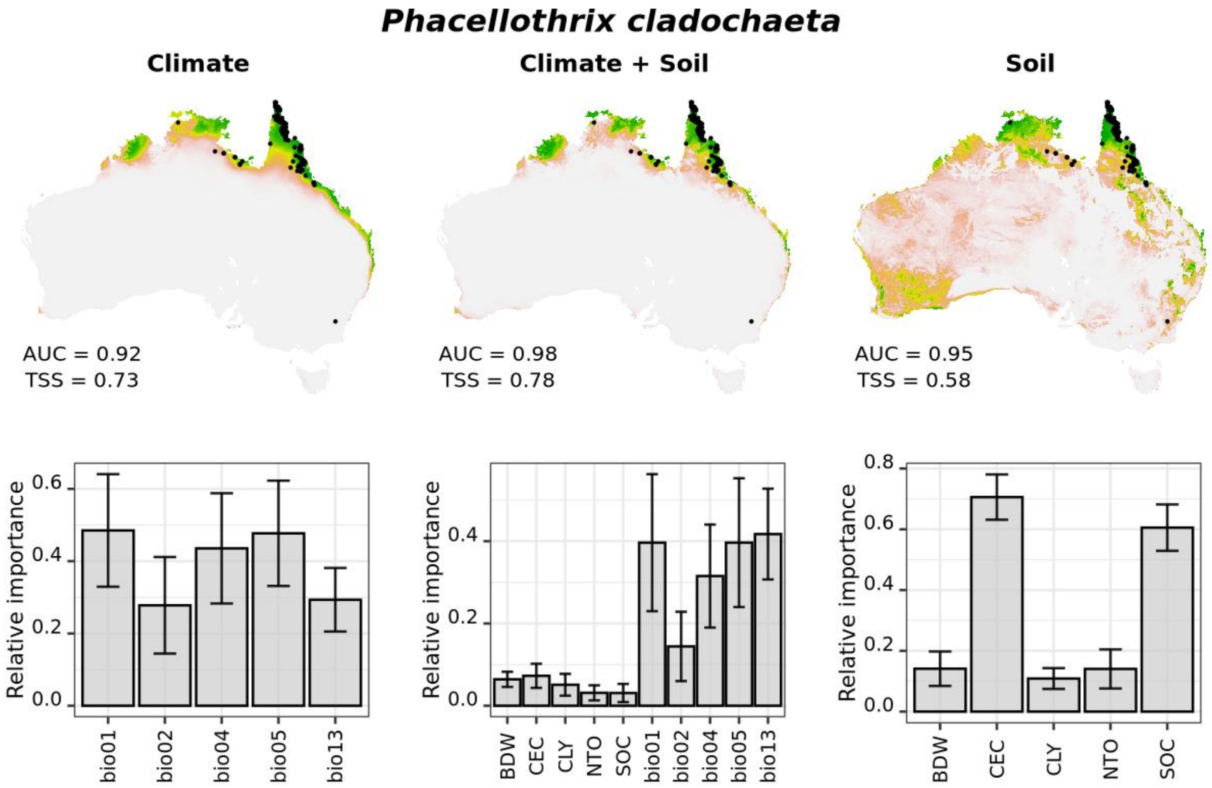
Phacellothrix cladochaeta
Climate
Climate + Soil
Soil
AUC = 0.92
TSS = 0.73
AUC = 0.98
TSS = 0.78
AUC = 0.95
TSS = 0.58
Relative importance
0.0
0.2
0.4
0.6
0.8
bio01
bio02
bio04
bio05
bio13
BDW
CEC
CLY
NTO
SOC

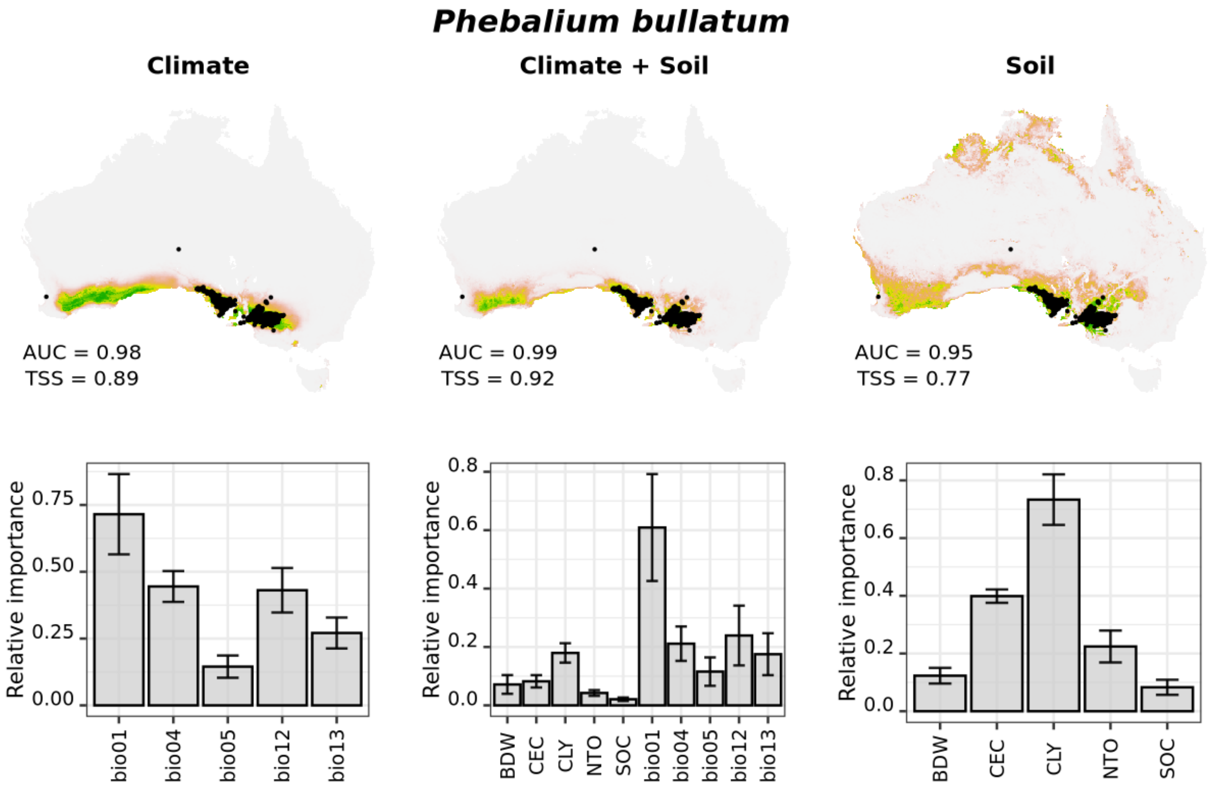
Phebalium bullatum
Climate
Climate + Soil
Soil
AUC = 0.98
TSS = 0.89
AUC = 0.99
TSS = 0.92
AUC = 0.95
TSS = 0.77
Relative importance
0.00
0.25
0.50
0.75
0.0
0.2
0.4
0.6
0.8
bio01
bio04
bio05
bio12
bio13
BDW
CEC
CLY
NTO
SOC

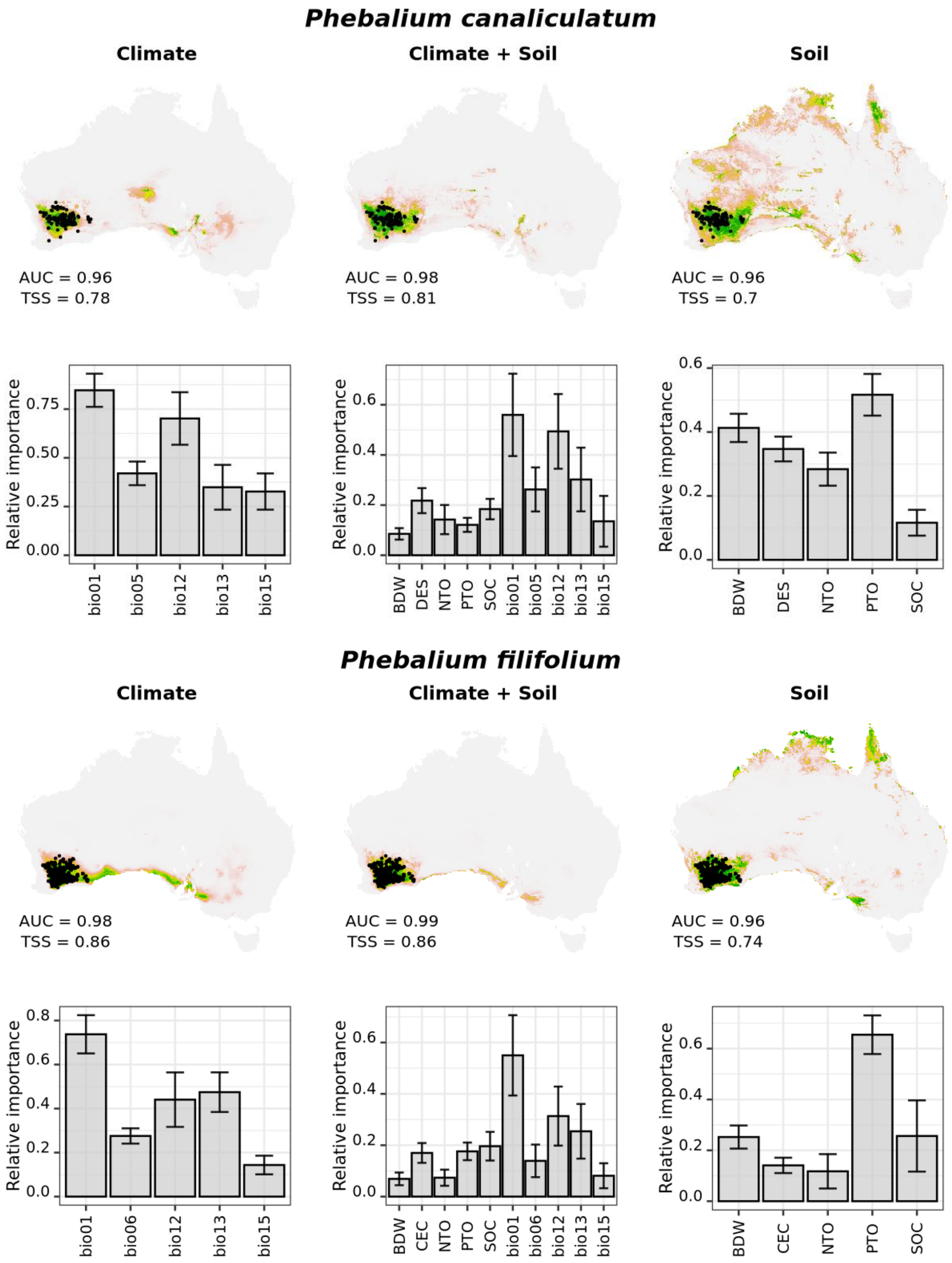

Phebalium canaliculatum
Climate
Climate + Soil
Soil
AUC = 0.96
TSS = 0.78
AUC = 0.98
TSS = 0.81
AUC = 0.96
TSS = 0.7
Relative importance
0.00
0.25
0.50
0.75
bio01
bio05
bio12
bio13
bio15
0.0
0.2
0.4
0.6
BDW
DES
NTO
PTO
SOC
bio01
bio05
bio12
bio13
bio15
BDW
DES
NTO
PTO
SOC
Phebalium filifolium
Climate
Climate + Soil
Soil
AUC = 0.98
TSS = 0.86
AUC = 0.99
TSS = 0.86
AUC = 0.96
TSS = 0.74
Relative importance
0.0
0.2
0.4
0.6
0.8
bio01
bio06
bio12
bio13
bio15
BDW
CEC
NTO
PTO
SOC
bio01
bio06
bio12
bio13
bio15
BDW
CEC
NTO
PTO
SOC

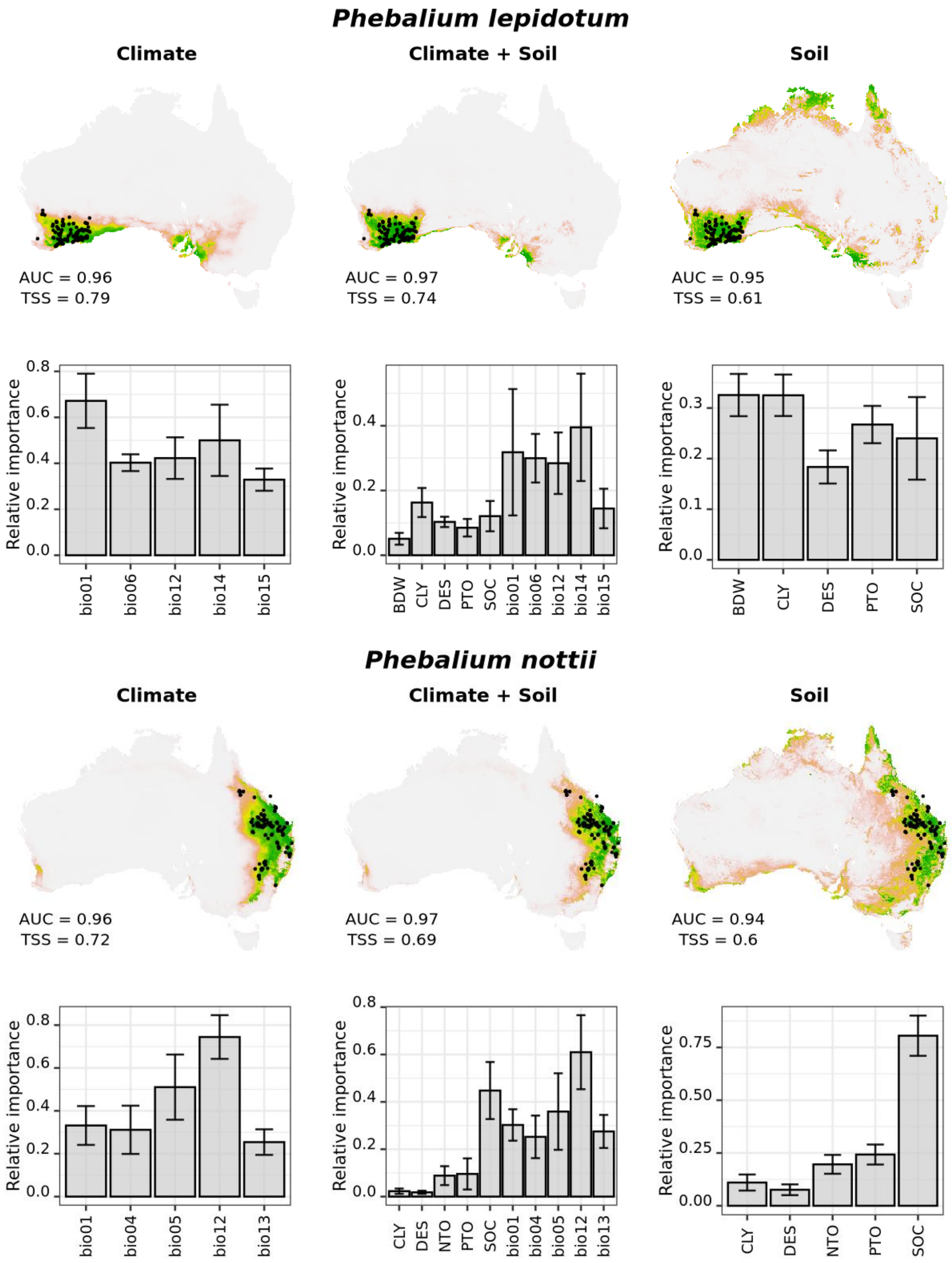
Phebalium lepidotum
Climate
Climate + Soil
Soil
AUC = 0.96
TSS = 0.79
AUC = 0.97
TSS = 0.74
AUC = 0.95
TSS = 0.61
Relative importance
0.0
0.2
0.4
0.6
0.8
bio01
bio06
bio12
bio14
bio15
Relative importance
0.0
0.2
0.4
BDW
CLY
DES
PTO
SOC
bio01
bio06
bio12
bio14
bio15
Relative importance
0.0
0.1
0.2
0.3
BDW
CLY
DES
PTO
SOC
Phebalium nottii
Climate
Climate + Soil
Soil
AUC = 0.96
TSS = 0.72
AUC = 0.97
TSS = 0.69
AUC = 0.94
TSS = 0.6
Relative importance
0.0
0.2
0.4
0.6
0.8
bio01
bio04
bio05
bio12
bio13
Relative importance
0.0
0.2
0.4
0.6
0.8
CLY
DES
NTO
PTO
SOC
bio01
bio04
bio05
bio12
bio13
Relative importance
0.00
0.25
0.50
0.75
CLY
DES
NTO
PTO
SOC

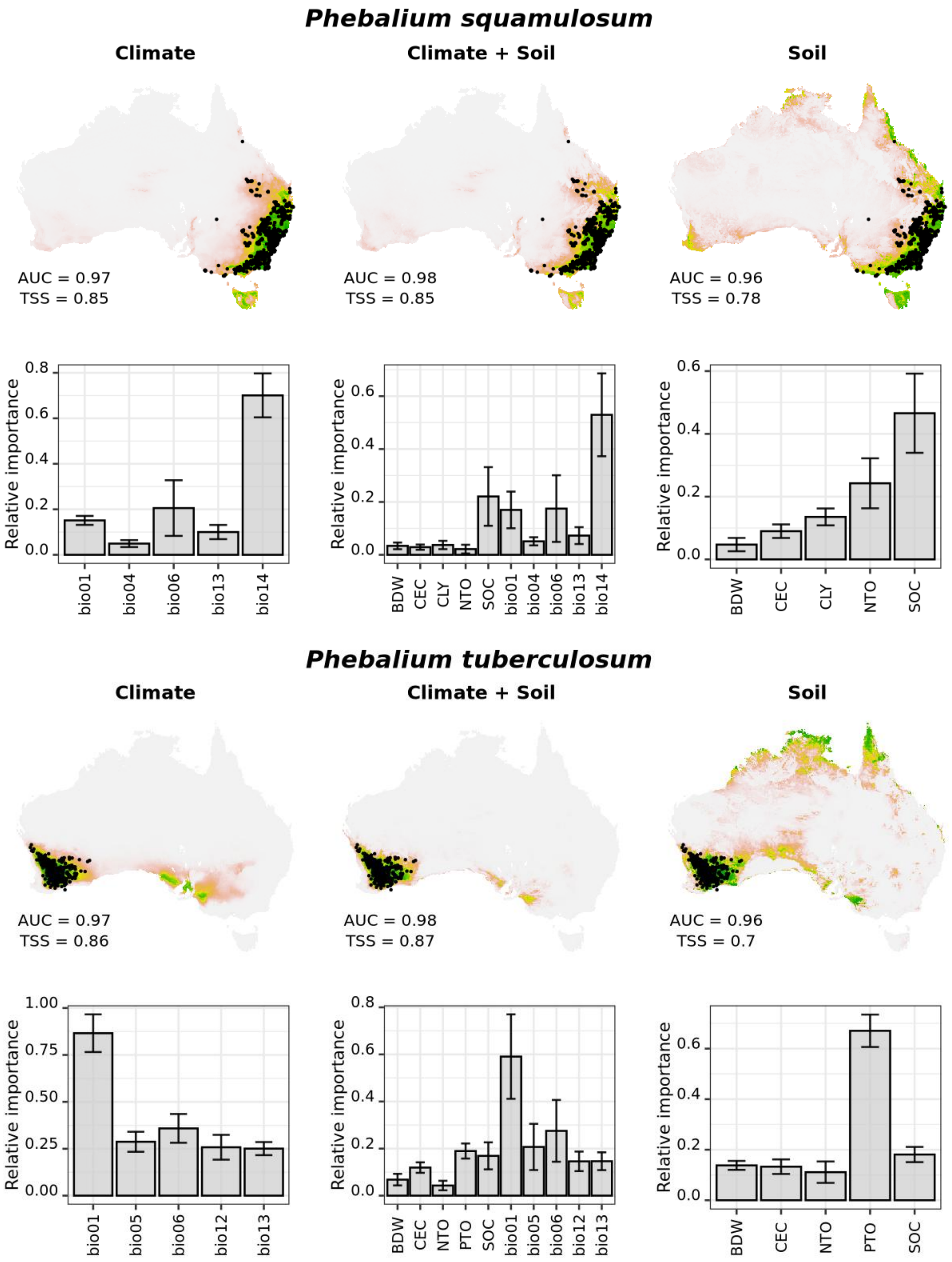
Phebalium squamulosum
Climate
Climate + Soil
Soil
AUC = 0.97
TSS = 0.85
AUC = 0.98
TSS = 0.85
AUC = 0.96
TSS = 0.78
Relative importance
0.0
0.2
0.4
0.6
0.8
bio01
bio04
bio06
bio13
bio14
BDW
CEC
CLY
NTO
SOC
Phebalium tuberculosum
AUC = 0.97
TSS = 0.86
AUC = 0.98
TSS = 0.87
AUC = 0.96
TSS = 0.7
0.00
0.25
0.50
0.75
1.00
bio05
bio12
PTO

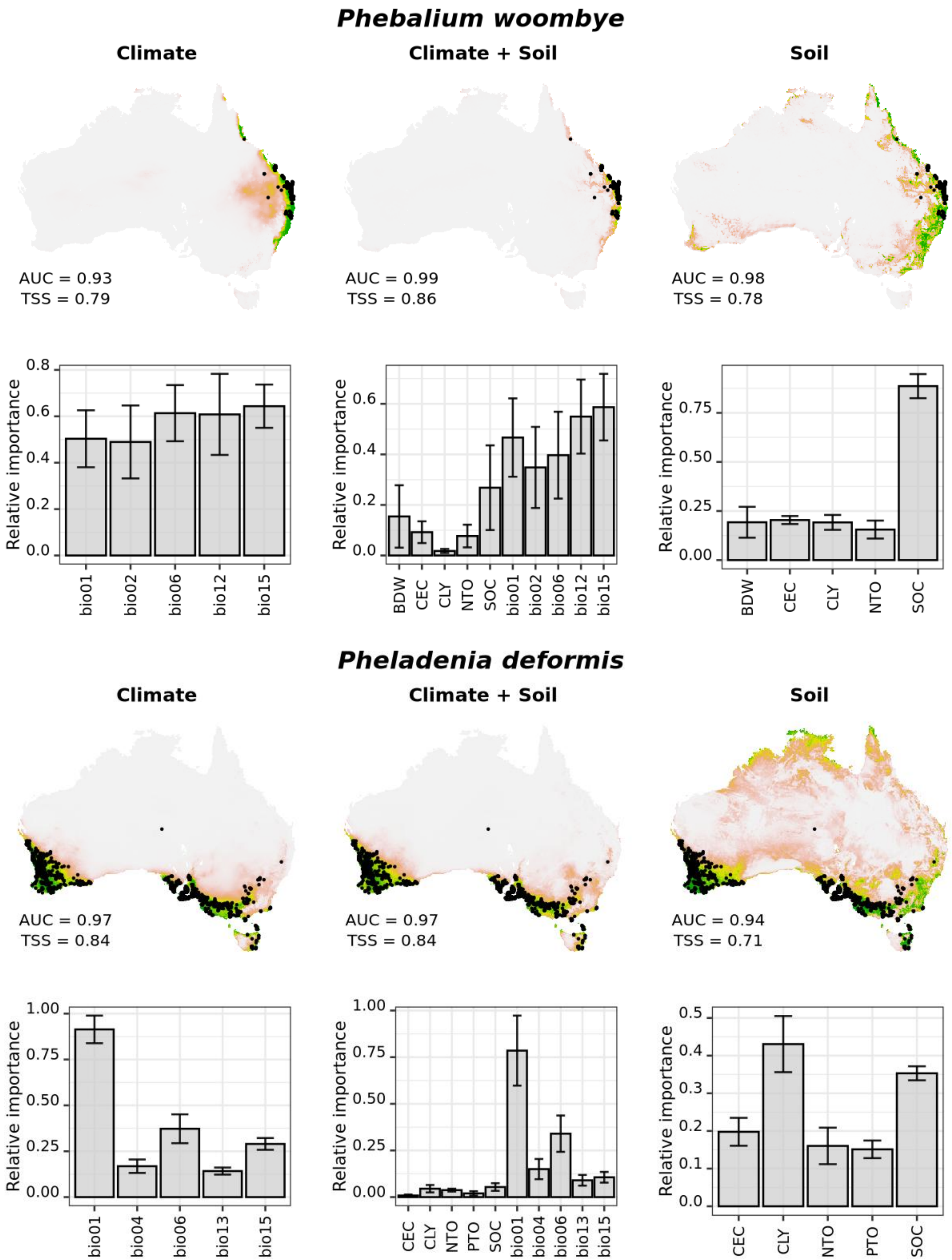

Phebalium woombye
Climate
Climate + Soil
Soil
AUC = 0.93
TSS = 0.79
AUC = 0.99
TSS = 0.86
AUC = 0.98
TSS = 0.78
Relative importance
bio01 bio02 bio06 bio12 bio15
BDW CEC CLY NTO SOC bio01 bio02 bio06 bio12 bio15
BDW CEC CLY NTO SOC
Pheladenia deformis
Climate
Climate + Soil
Soil
AUC = 0.97
TSS = 0.84
AUC = 0.97
TSS = 0.84
AUC = 0.94
TSS = 0.71
Relative importance
bio01 bio04 bio06 bio13 bio15
CEC CLY NTO PTO SOC bio01 bio04 bio06 bio13 bio15
CEC CLY NTO PTO SOC

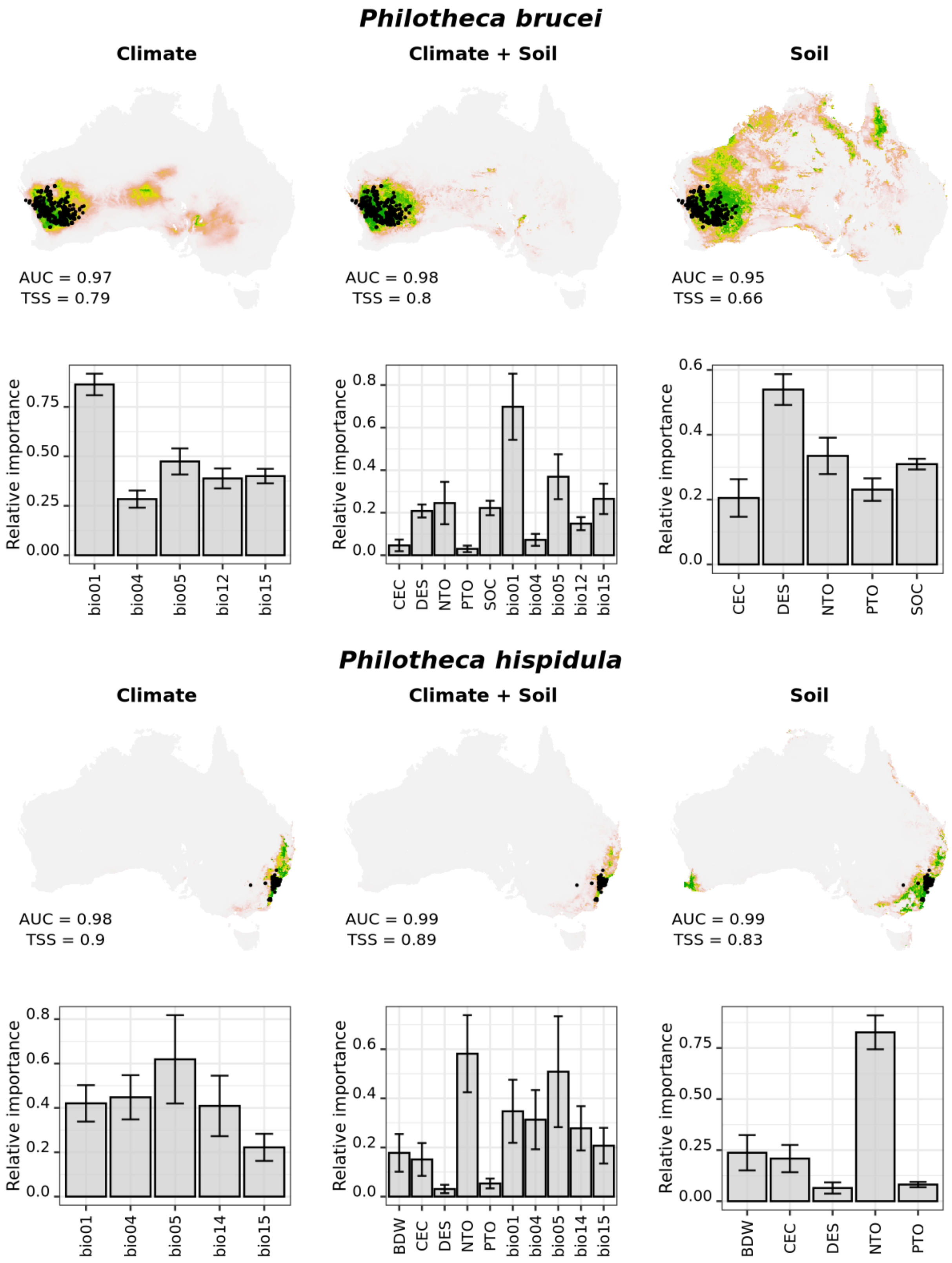

Philotheca brucei
Climate
Climate + Soil
Soil
AUC = 0.97
TSS = 0.79
AUC = 0.98
TSS = 0.8
AUC = 0.95
TSS = 0.66
Relative importance
bio01
bio04
bio05
bio12
bio15
CEC
DES
NTO
PTO
SOC
Philotheca hispidula
Climate
Climate + Soil
Soil
AUC = 0.98
TSS = 0.9
AUC = 0.99
TSS = 0.89
AUC = 0.99
TSS = 0.83
Relative importance
bio01
bio04
bio05
bio14
bio15
BDW
CEC
DES
NTO
PTO

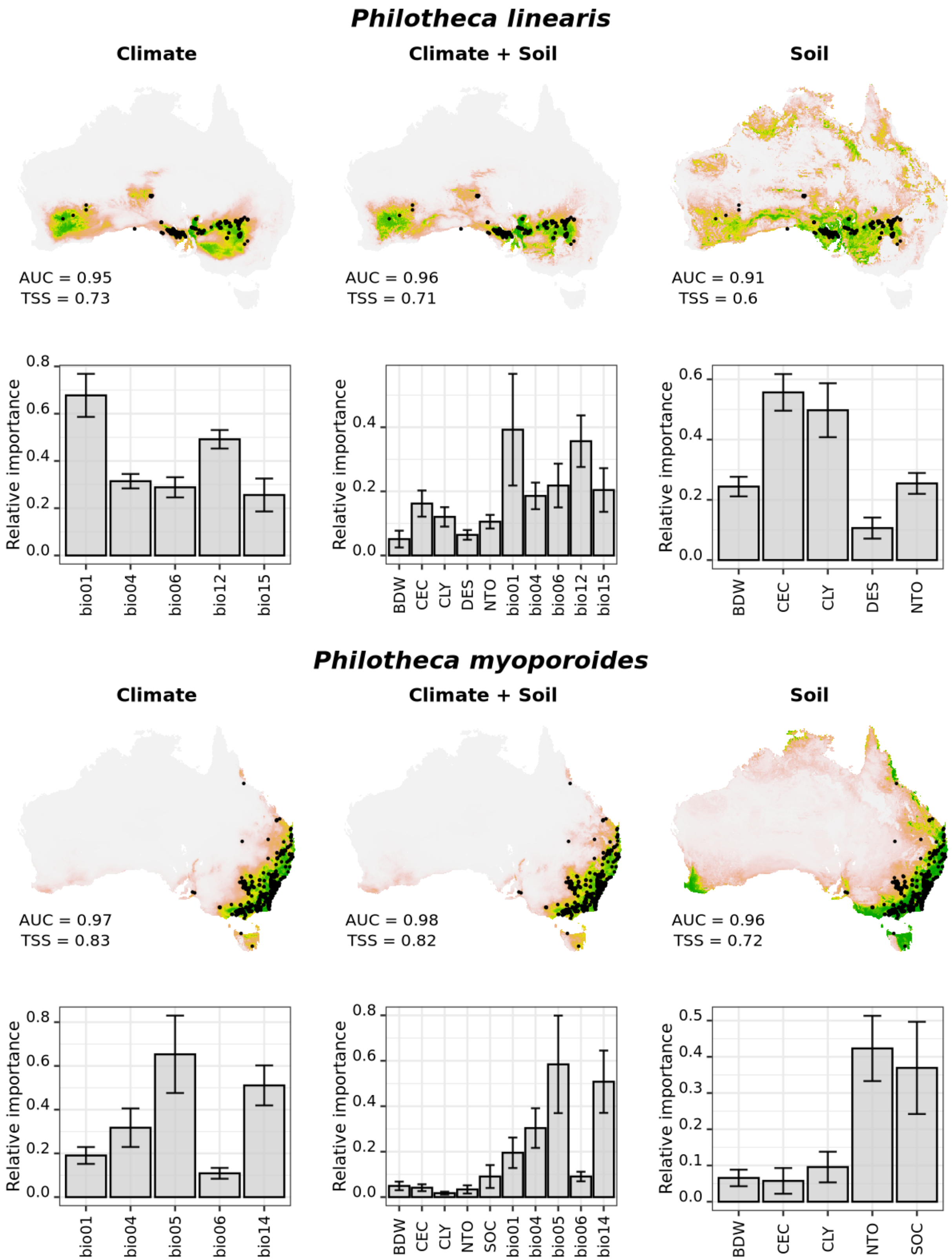
Philotheca linearis
Climate
Climate + Soil
Soil
AUC = 0.95
TSS = 0.73
AUC = 0.96
TSS = 0.71
AUC = 0.91
TSS = 0.6
Relative importance
bio01 bio04 bio06 bio12 bio15
BDW CEC CLY DES NTO bio01 bio04 bio06 bio12 bio15
BDW CEC CLY DES NTO
Philotheca myoporoides
Climate
Climate + Soil
Soil
AUC = 0.97
TSS = 0.83
AUC = 0.98
TSS = 0.82
AUC = 0.96
TSS = 0.72
Relative importance
bio01 bio04 bio05 bio06 bio14
BDW CEC CLY NTO SOC bio01 bio04 bio05 bio06 bio14
BDW CEC CLY NTO SOC

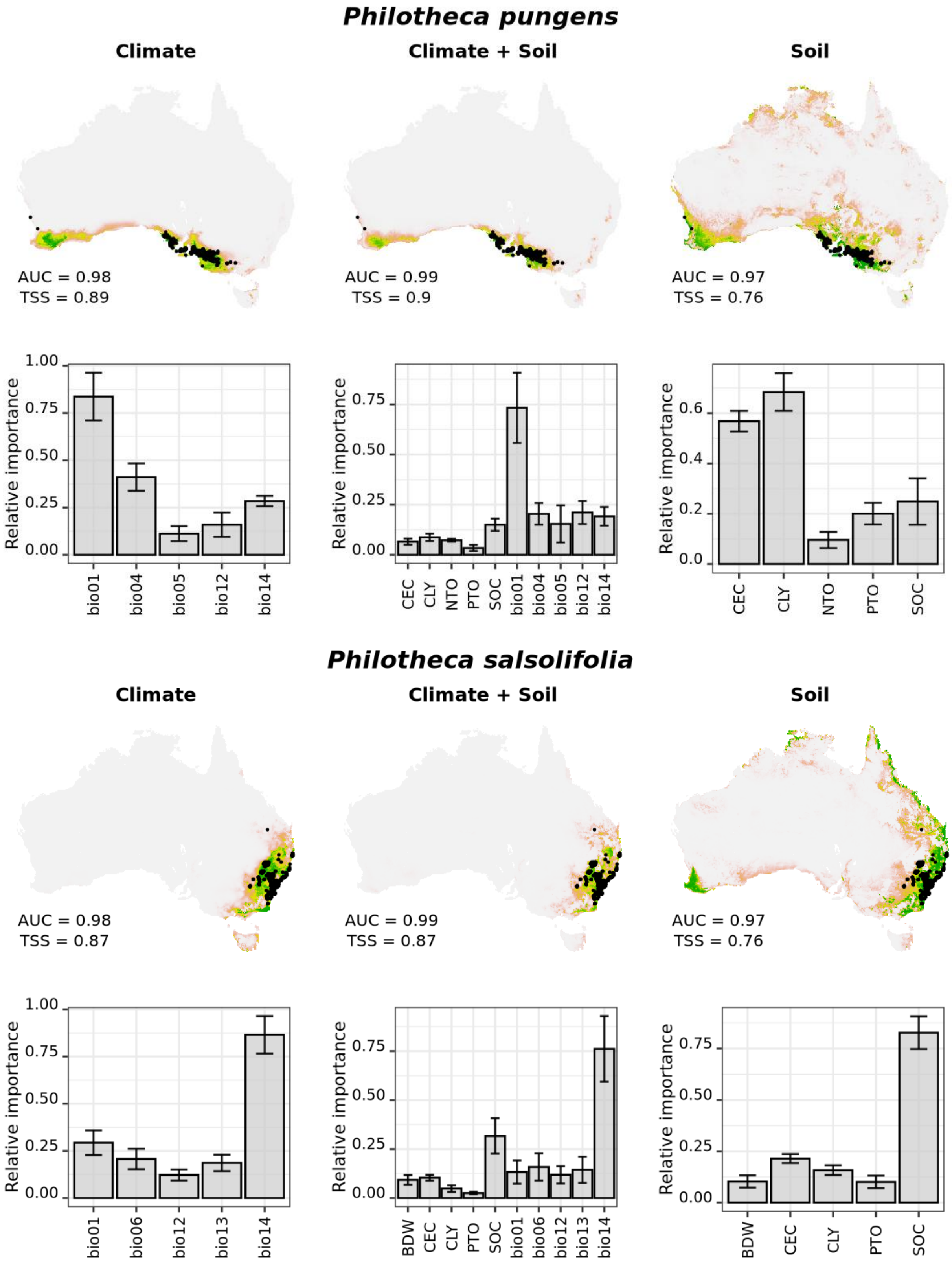
Philotheca pungens
Climate
Climate + Soil
Soil
AUC = 0.98
TSS = 0.89
AUC = 0.99
TSS = 0.9
AUC = 0.97
TSS = 0.76
Relative importance
1.00
0.75
0.50
0.25
0.00
bio01
bio04
bio05
bio12
bio14
CEC
CLY
NTO
PTO
SOC
0.6
0.4
0.2
0.0
Philotheca salsolifolia
Climate
Climate + Soil
Soil
AUC = 0.98
TSS = 0.87
AUC = 0.99
TSS = 0.87
AUC = 0.97
TSS = 0.76
bio06
bio13
BDW

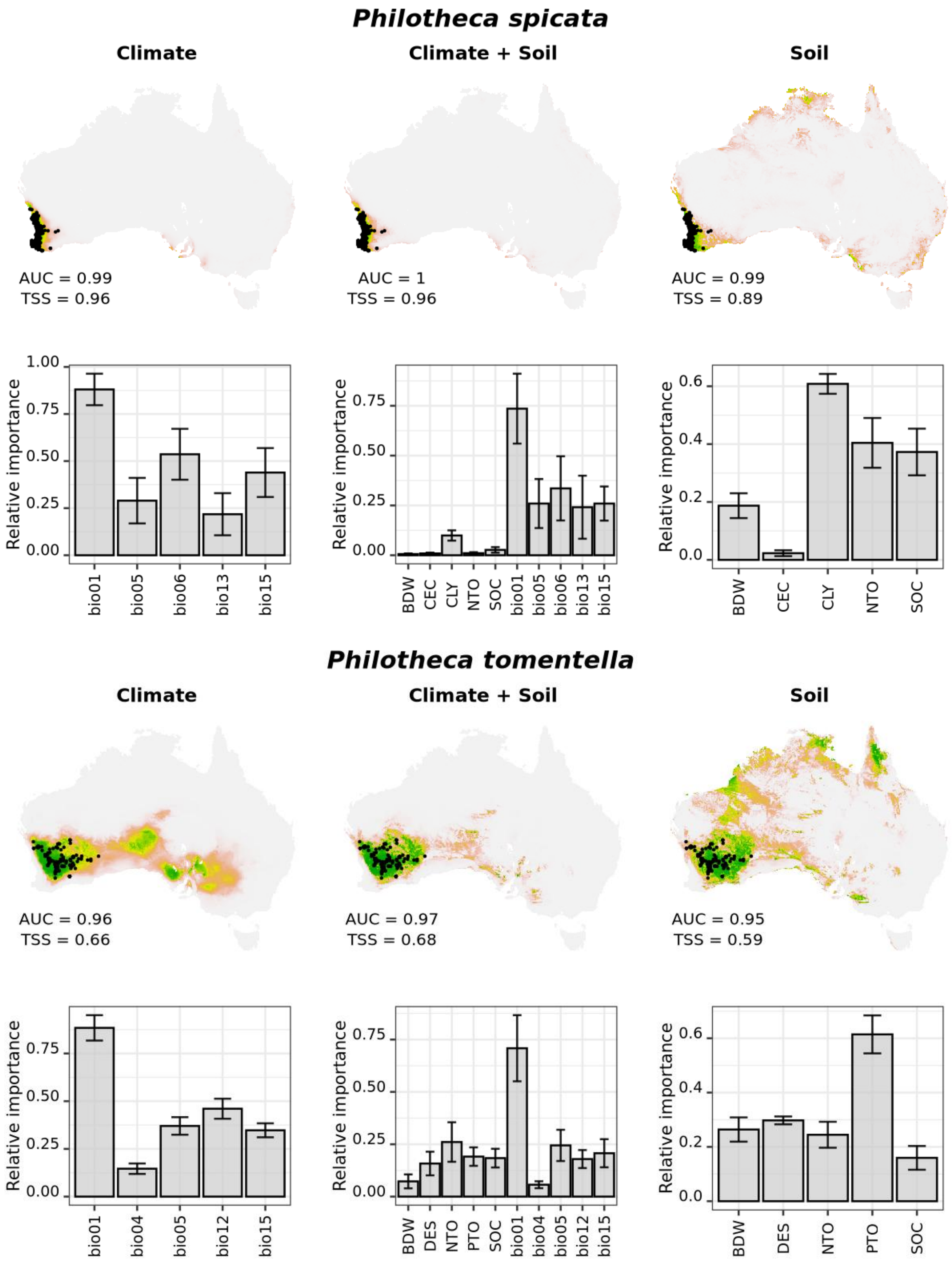
Philotheca spicata
Climate
Climate + Soil
Soil
AUC = 0.99
TSS = 0.96
AUC = 1
TSS = 0.96
AUC = 0.99
TSS = 0.89
Relative importance
bio01
bio05
bio06
bio13
bio15
BDW
CEC
CLY
NTO
SOC
Philotheca tomentella
Climate
Climate + Soil
Soil
AUC = 0.96
TSS = 0.66
AUC = 0.97
TSS = 0.68
AUC = 0.95
TSS = 0.59
Relative importance
bio01
bio04
bio05
bio12
bio15
BDW
DES
NTO
PTO
SOC

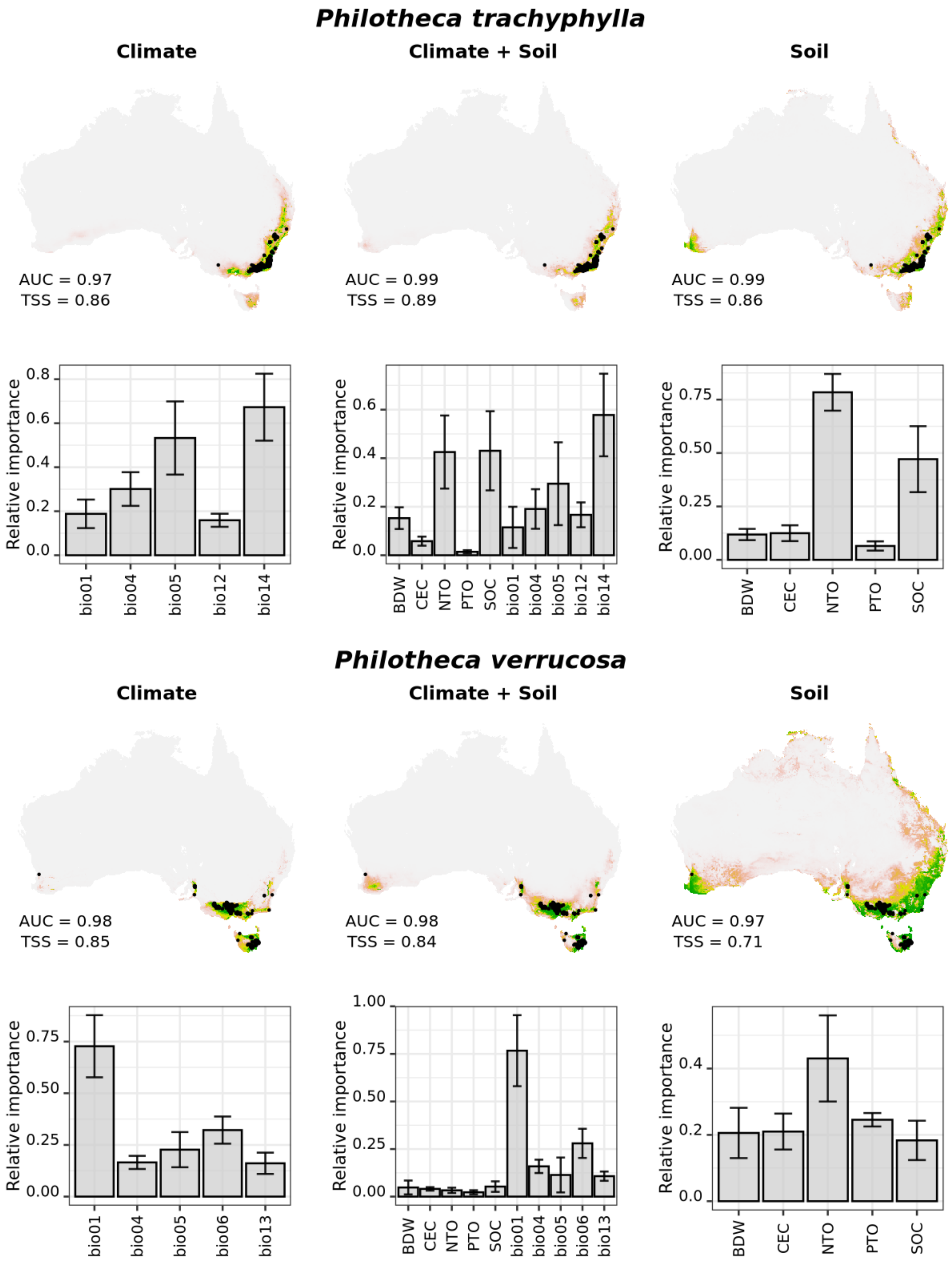
Philotheca trachyphylla
Climate
Climate + Soil
Soil
AUC = 0.97
TSS = 0.86
AUC = 0.99
TSS = 0.89
AUC = 0.99
TSS = 0.86
Relative importance
0.0
0.2
0.4
0.6
0.8
bio01
bio04
bio05
bio12
bio14
BDW
CEC
NTO
PTO
SOC
bio01
bio04
bio05
bio12
bio14
0.00
0.25
0.50
0.75
Philotheca verrucosa
Climate
Climate + Soil
Soil
AUC = 0.98
TSS = 0.85
AUC = 0.98
TSS = 0.84
AUC = 0.97
TSS = 0.71
1.00
bio06
bio13

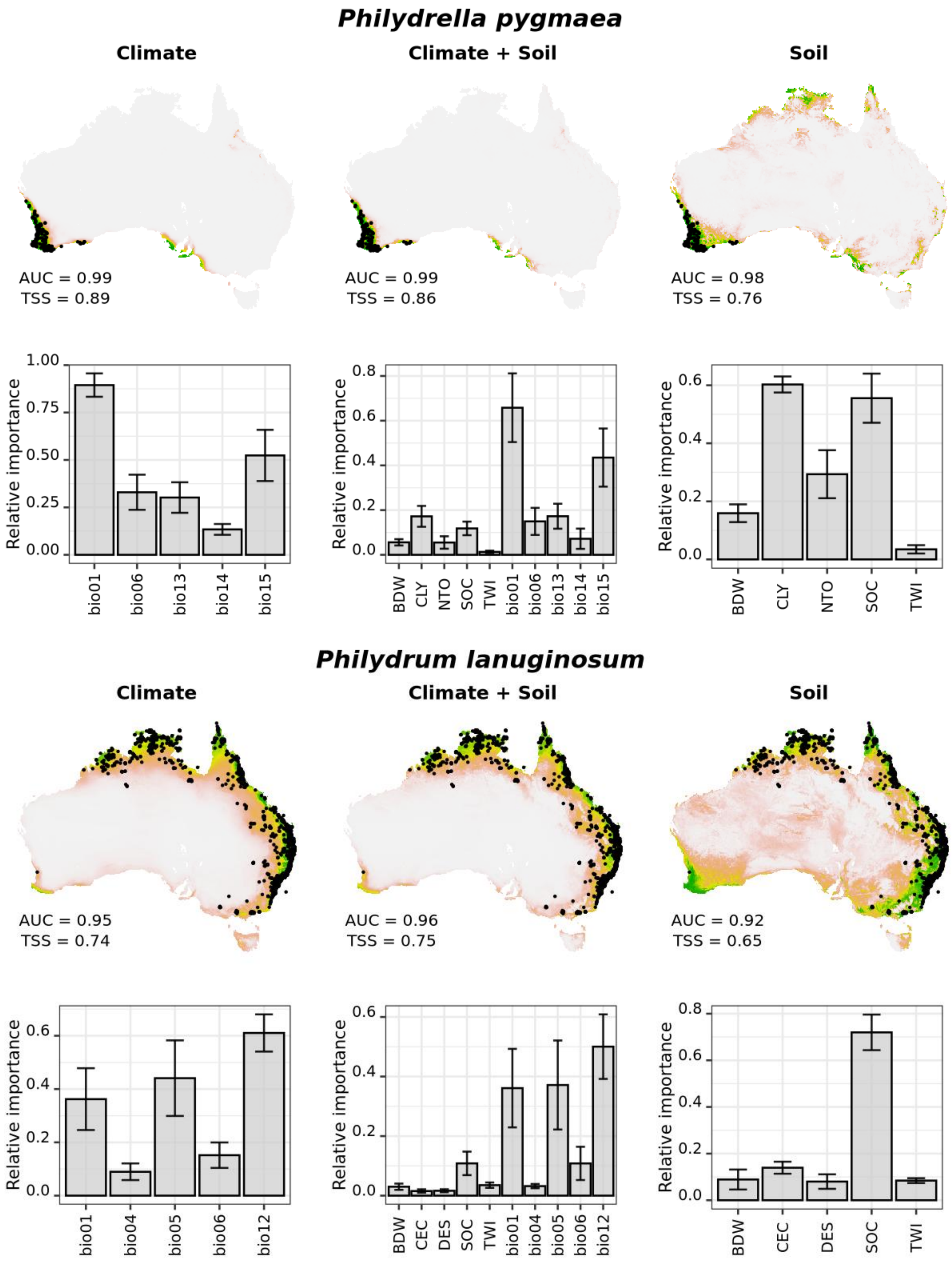

Philydrella pygmaea
Climate
Climate + Soil
Soil
AUC = 0.99
TSS = 0.89
AUC = 0.99
TSS = 0.86
AUC = 0.98
TSS = 0.76
Relative importance
bio01
bio06
bio13
bio14
bio15
BDW
CLY
NTO
SOC
TWI
Philydrum lanuginosum
AUC = 0.95
TSS = 0.74
AUC = 0.96
TSS = 0.75
AUC = 0.92
TSS = 0.65
bio04
bio05
bio12
CEC
DES

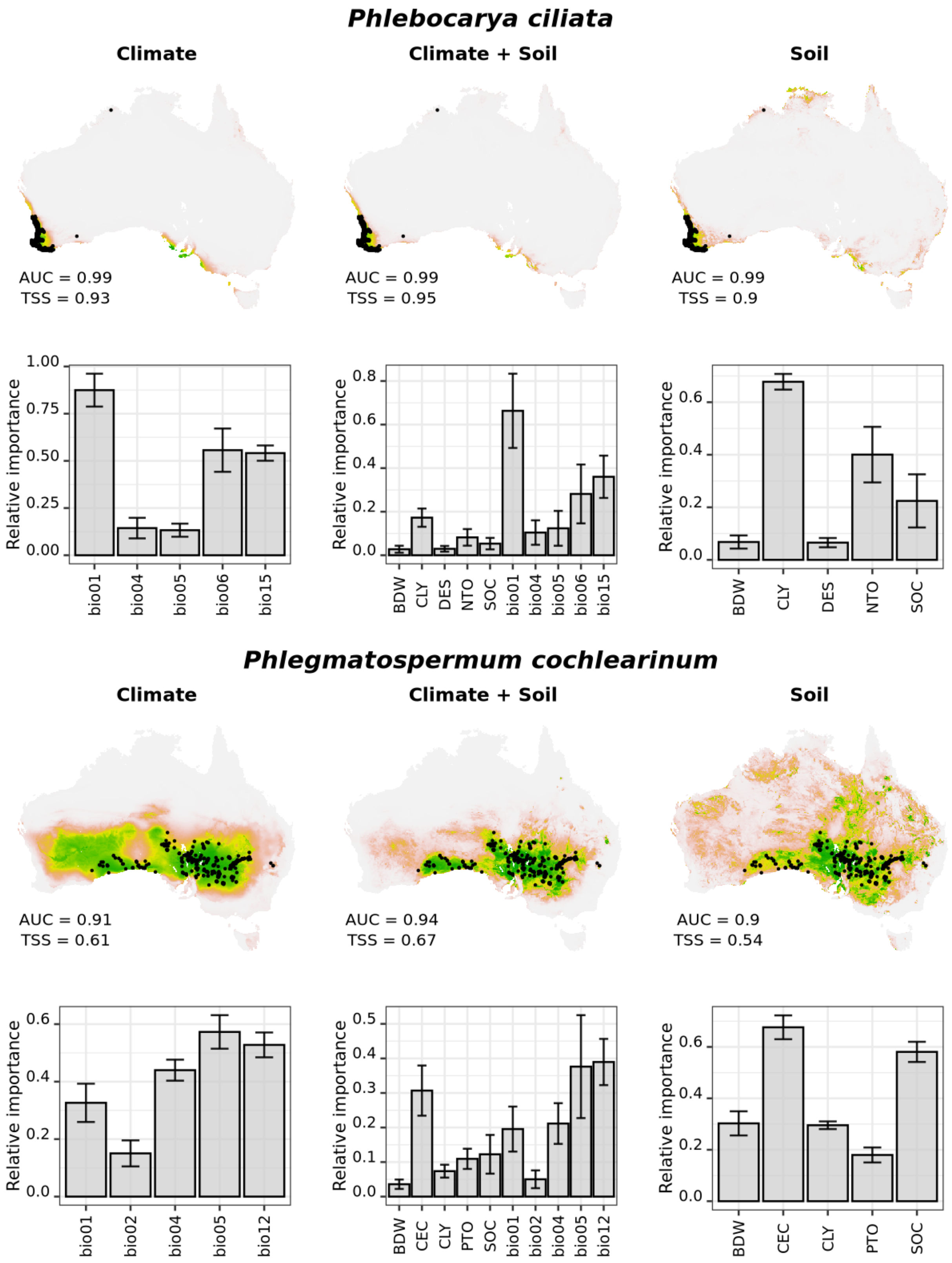
Phlebocarya ciliata
Climate
Climate + Soil
Soil
AUC = 0.99
TSS = 0.93
AUC = 0.99
TSS = 0.95
AUC = 0.99
TSS = 0.9
Relative importance
bio01
bio04
bio05
bio06
bio15
BDW
CLY
DES
NTO
SOC
Phlegmatospermum cochlearinum
AUC = 0.91
TSS = 0.61
AUC = 0.94
TSS = 0.67
AUC = 0.9
TSS = 0.54
bio02
bio12
CEC
PTO

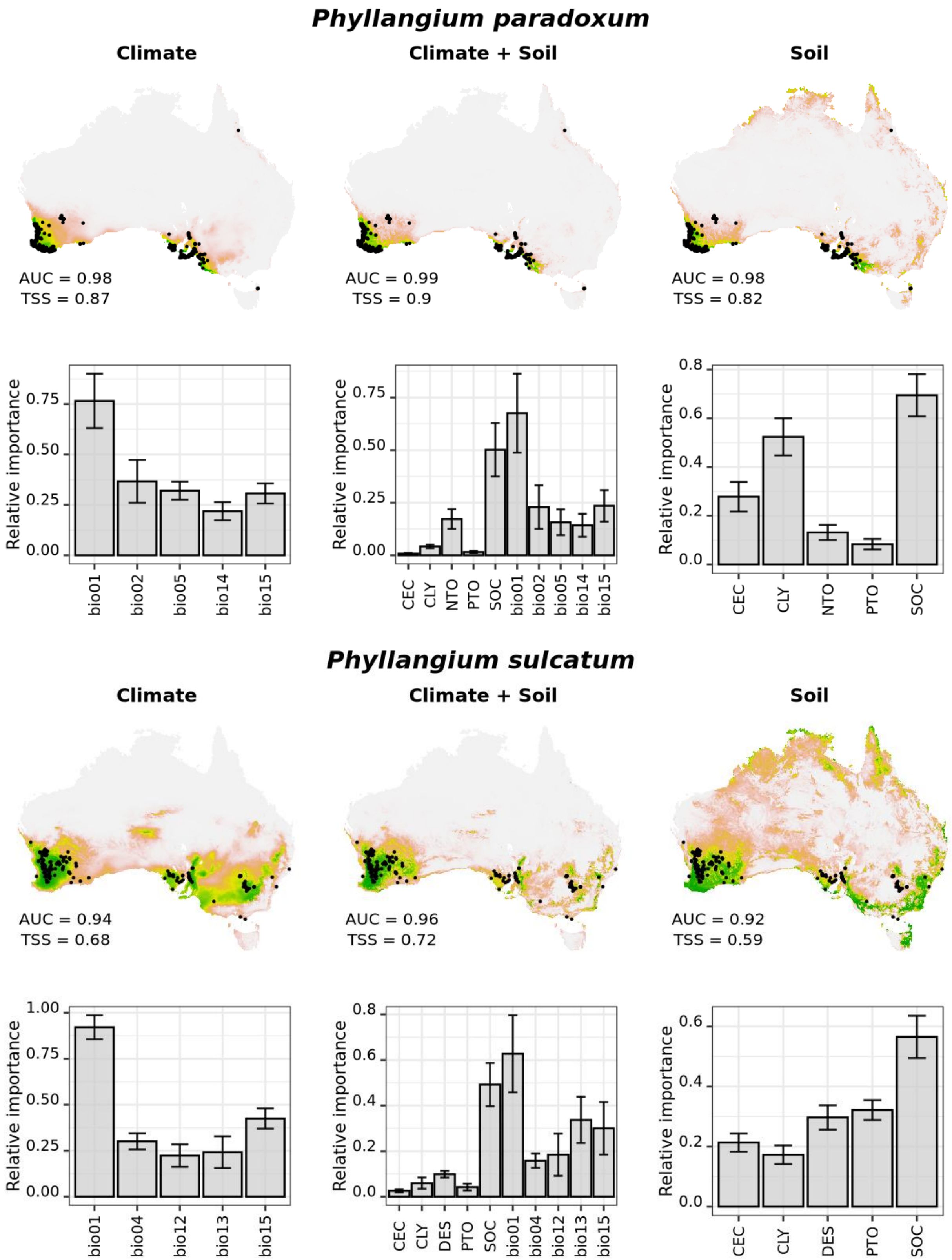
Phyllangium paradoxum
Climate
Climate + Soil
Soil
AUC = 0.98
TSS = 0.87
AUC = 0.99
TSS = 0.9
AUC = 0.98
TSS = 0.82
Relative importance
bio01
bio02
bio05
bio14
bio15
CEC
CLY
NTO
PTO
SOC
Phyllangium sulcatum
Climate
Climate + Soil
Soil
AUC = 0.94
TSS = 0.68
AUC = 0.96
TSS = 0.72
AUC = 0.92
TSS = 0.59
Relative importance
bio01
bio04
bio12
bio13
bio15
CEC
CLY
DES
PTO
SOC

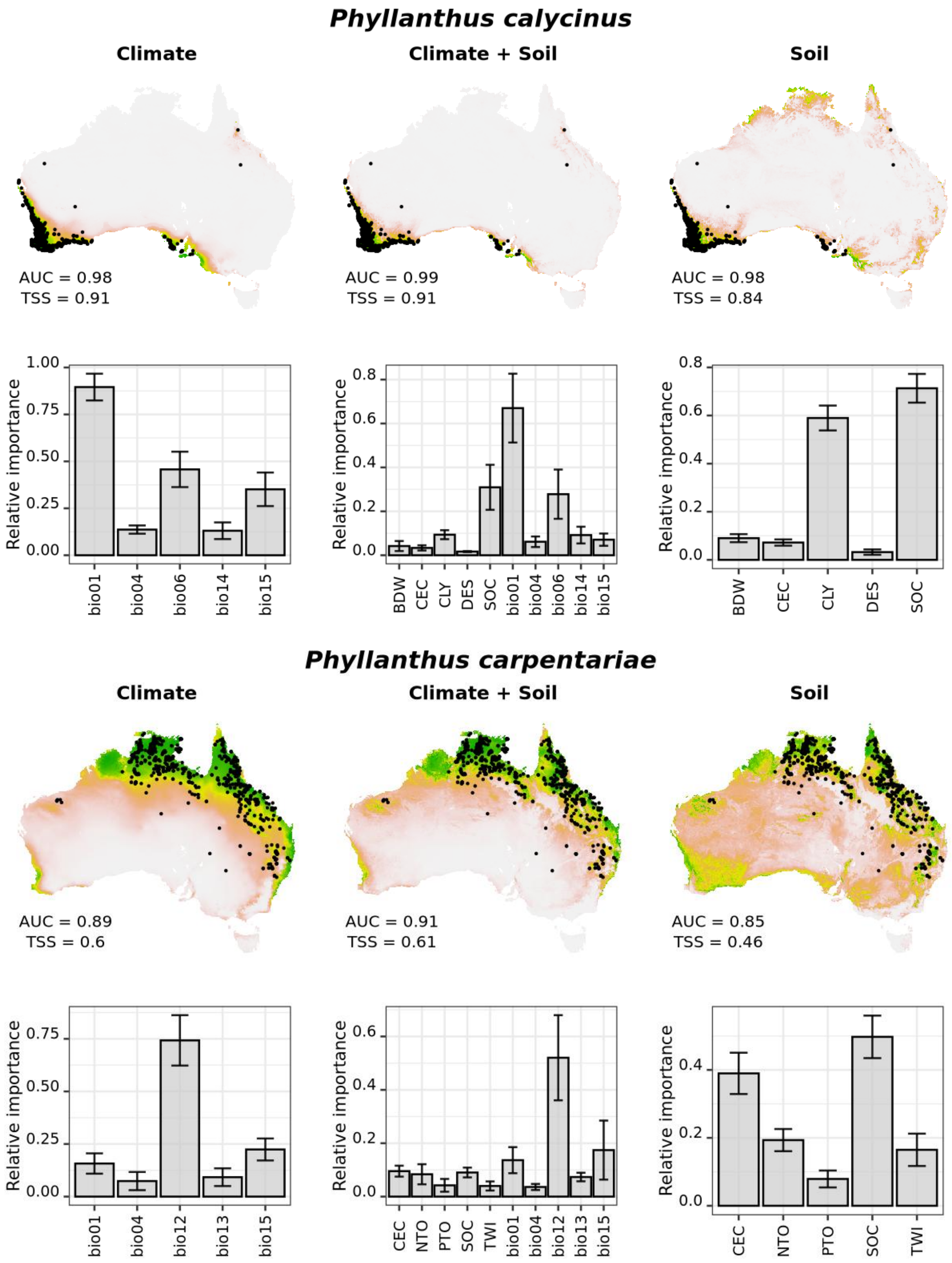
Phyllanthus calycinus
Climate
Climate + Soil
Soil
AUC = 0.98
TSS = 0.91
AUC = 0.99
TSS = 0.91
AUC = 0.98
TSS = 0.84
Relative importance
bio01
bio04
bio06
bio14
bio15
BDW
CEC
CLY
DES
SOC
Phyllanthus carpentariae
Climate
Climate + Soil
Soil
AUC = 0.89
TSS = 0.6
AUC = 0.91
TSS = 0.61
AUC = 0.85
TSS = 0.46
Relative importance
bio01
bio04
bio12
bio13
bio15
CEC
NTO
PTO
SOC
TWI

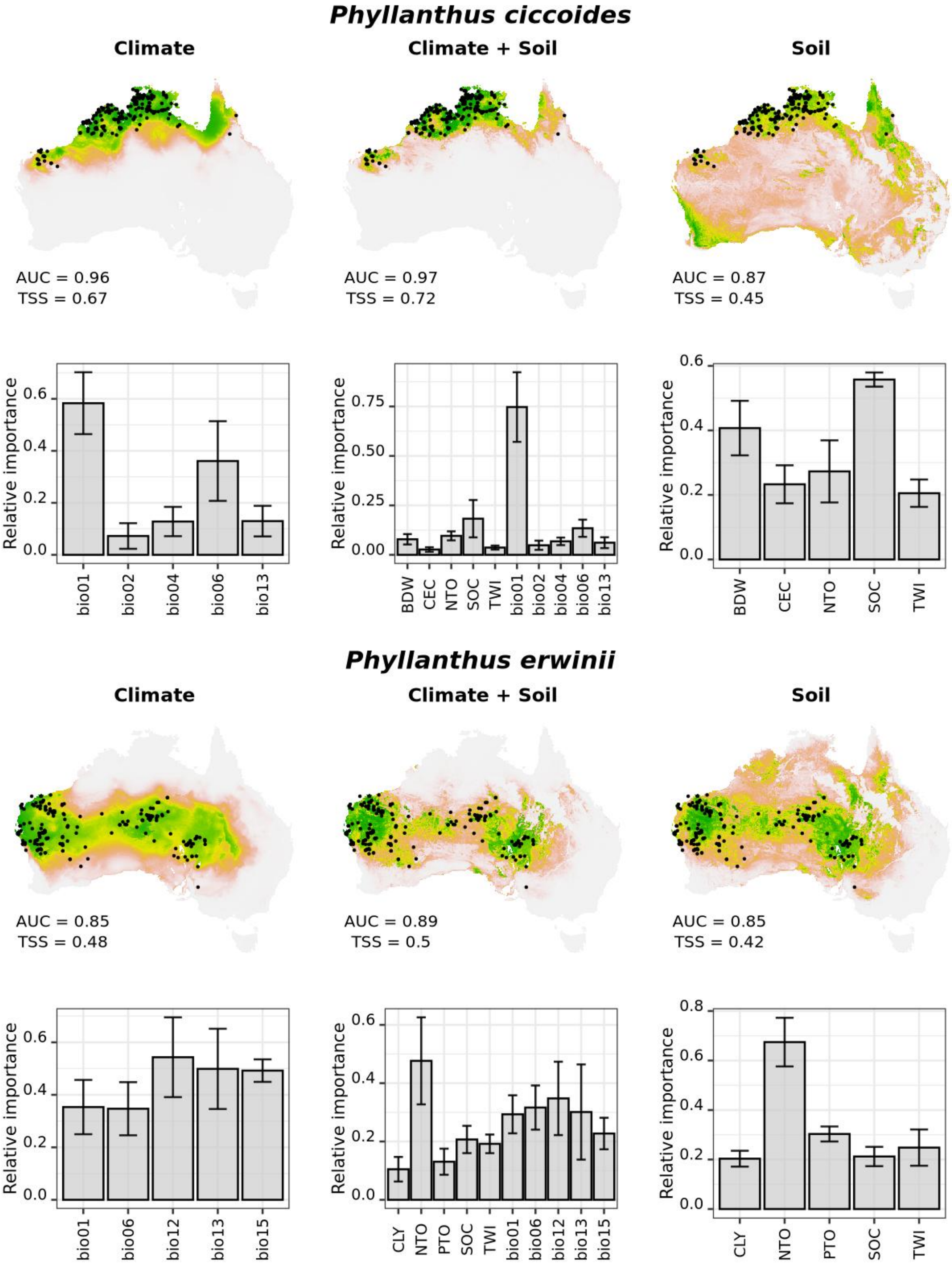
Phyllanthus ciccoides
Climate
Climate + Soil
Soil
AUC = 0.96
TSS = 0.67
AUC = 0.97
TSS = 0.72
AUC = 0.87
TSS = 0.45
Relative importance
bio01
bio02
bio04
bio06
bio13
BDW
CEC
NTO
SOC
TWI
Phyllanthus erwinii
Climate
Climate + Soil
Soil
AUC = 0.85
TSS = 0.48
AUC = 0.89
TSS = 0.5
AUC = 0.85
TSS = 0.42
Relative importance
bio01
bio06
bio12
bio13
bio15
CLY
NTO
PTO
SOC
TWI

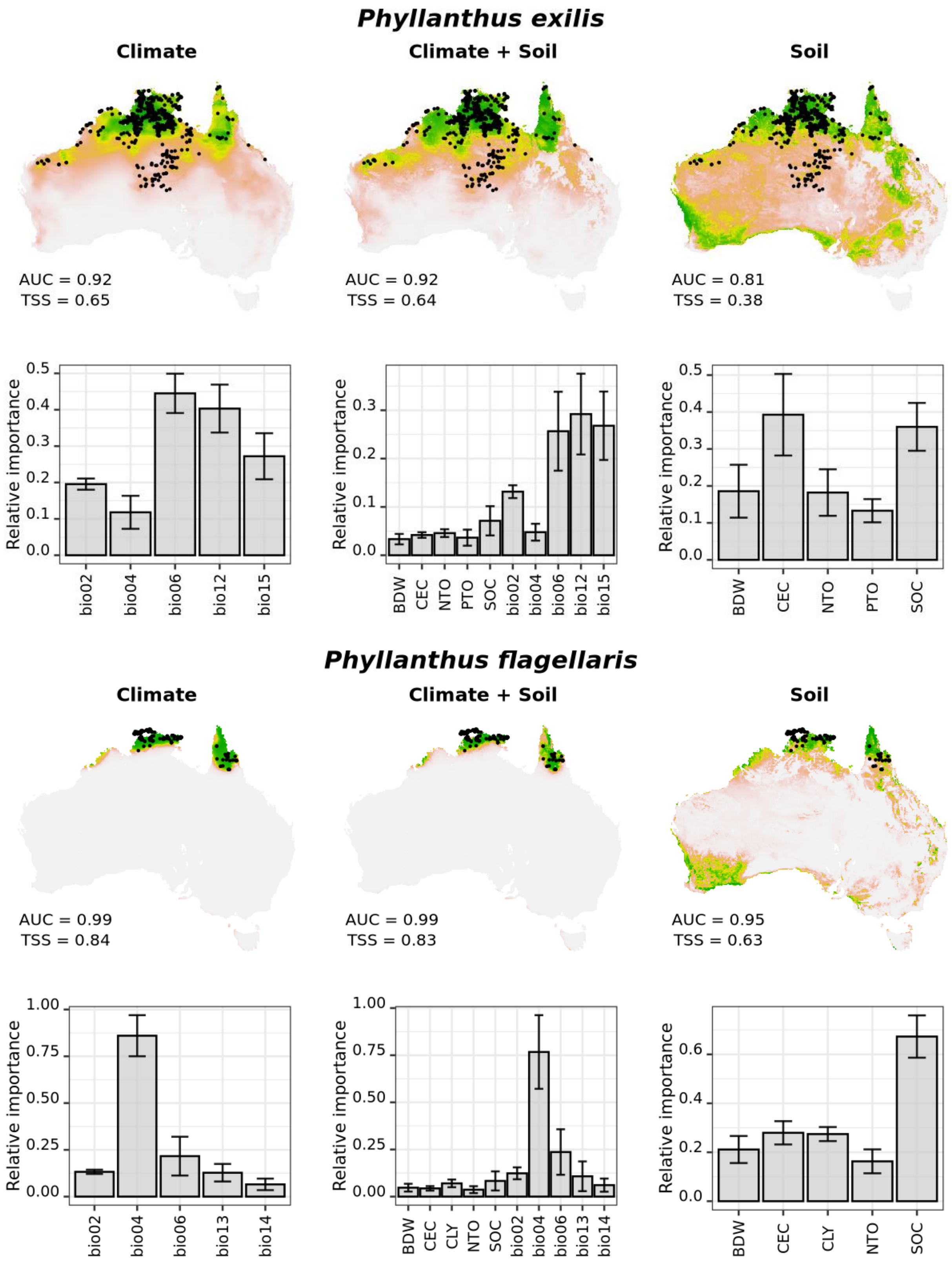

Phyllanthus exilis
Climate
Climate + Soil
Soil
AUC = 0.92
TSS = 0.65
AUC = 0.92
TSS = 0.64
AUC = 0.81
TSS = 0.38
Relative importance
bio02
bio04
bio06
bio12
bio15
BDW
CEC
NTO
PTO
SOC
Phyllanthus flagellaris
Climate
Climate + Soil
Soil
AUC = 0.99
TSS = 0.84
AUC = 0.99
TSS = 0.83
AUC = 0.95
TSS = 0.63
Relative importance
bio02
bio04
bio06
bio13
bio14
BDW
CEC
CLY
NTO
SOC

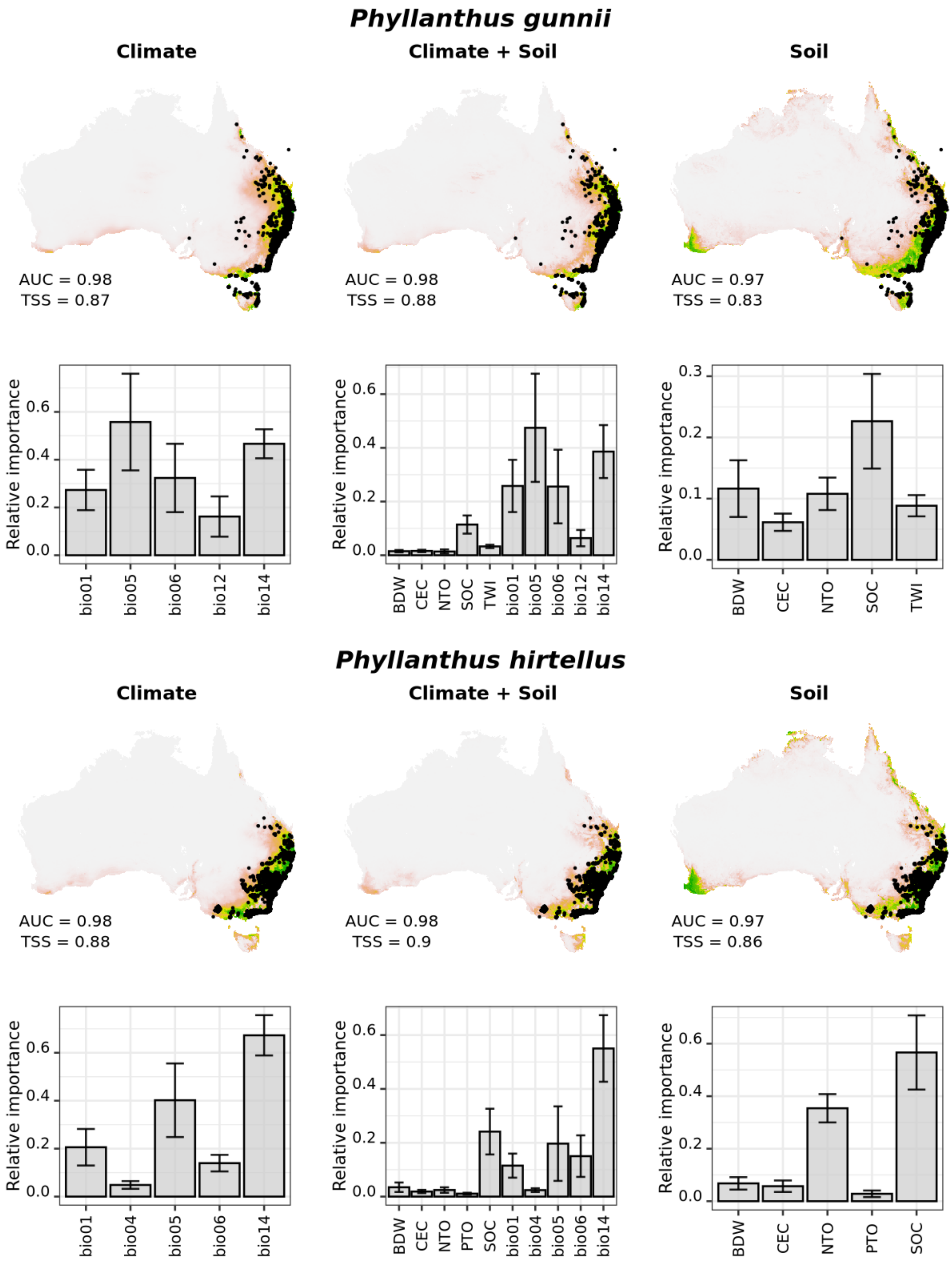
Phyllanthus gunnii
Climate
Climate + Soil
Soil
AUC = 0.98
TSS = 0.87
AUC = 0.98
TSS = 0.88
AUC = 0.97
TSS = 0.83
Relative importance
bio01
bio05
bio06
bio12
bio14
BDW
CEC
NTO
SOC
TWI
Phyllanthus hirtellus
Climate
Climate + Soil
Soil
AUC = 0.98
TSS = 0.88
AUC = 0.98
TSS = 0.9
AUC = 0.97
TSS = 0.86
Relative importance
bio01
bio04
bio05
bio06
bio14
BDW
CEC
NTO
PTO
SOC

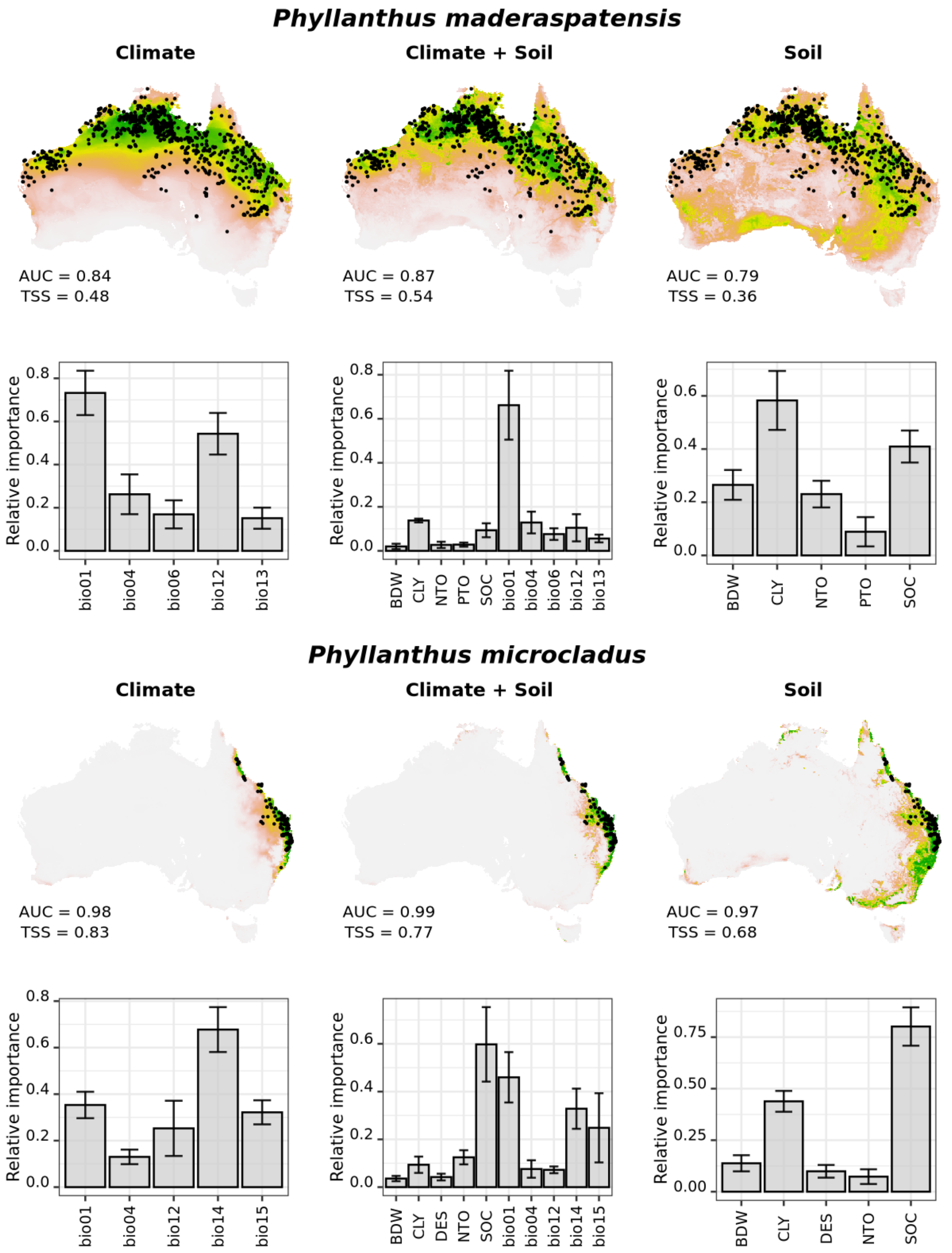
Phyllanthus maderaspatensis
Climate
Climate + Soil
Soil
AUC = 0.84
TSS = 0.48
AUC = 0.87
TSS = 0.54
AUC = 0.79
TSS = 0.36
Relative importance
bio01
bio04
bio06
bio12
bio13
BDW
CLY
NTO
PTO
SOC
Phyllanthus microcladus
AUC = 0.98
TSS = 0.83
AUC = 0.99
TSS = 0.77
AUC = 0.97
TSS = 0.68
bio14
bio15
DES

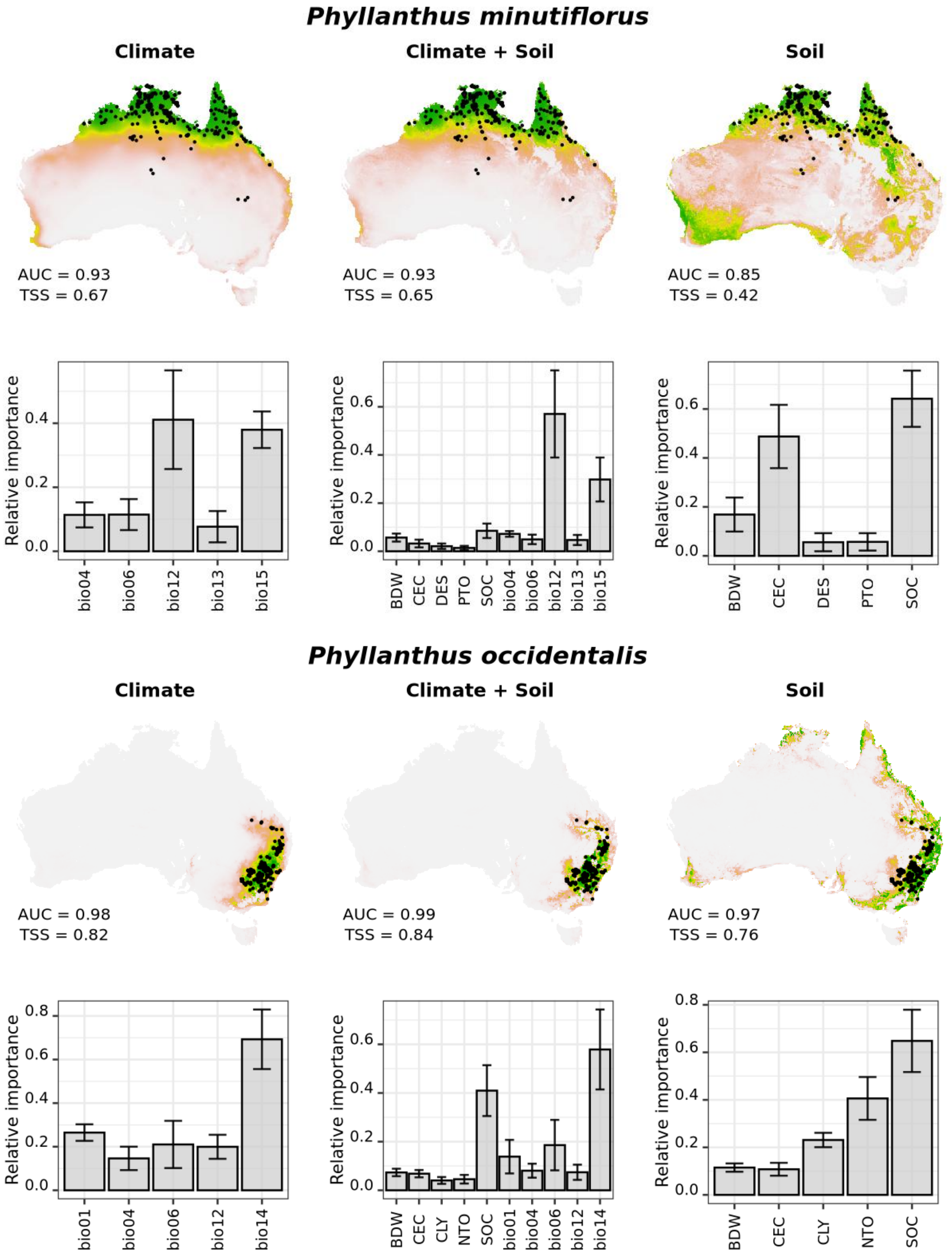
Phyllanthus minutiflorus
Climate
Climate + Soil
Soil
AUC = 0.93
TSS = 0.67
AUC = 0.93
TSS = 0.65
AUC = 0.85
TSS = 0.42
Relative importance
bio04
bio06
bio12
bio13
bio15
BDW
CEC
DES
PTO
SOC
Phyllanthus occidentalis
Climate
Climate + Soil
Soil
AUC = 0.98
TSS = 0.82
AUC = 0.99
TSS = 0.84
AUC = 0.97
TSS = 0.76
Relative importance
bio01
bio04
bio06
bio12
bio14
BDW
CEC
CLY
NTO
SOC

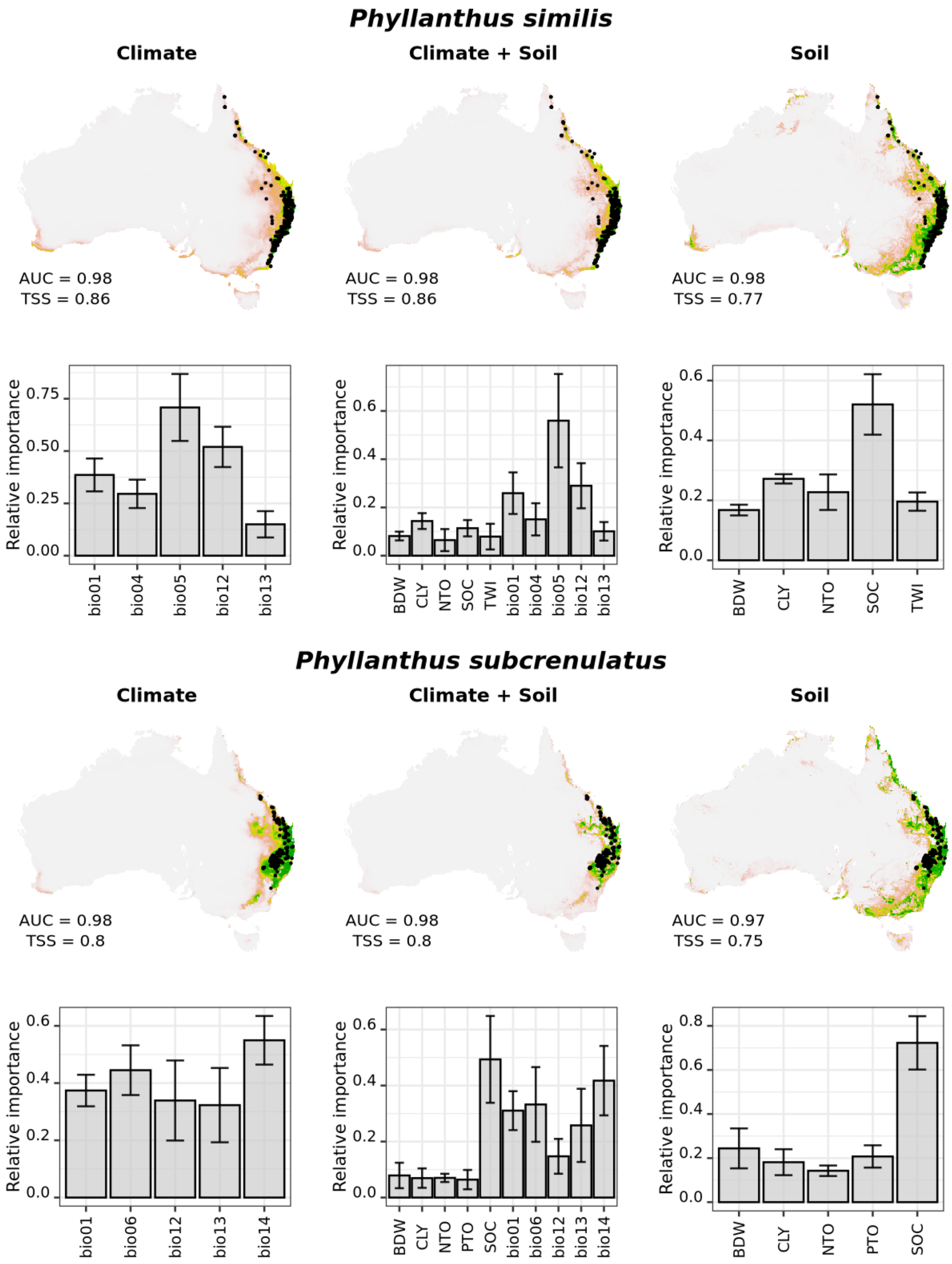
Phyllanthus similis
Climate
Climate + Soil
Soil
AUC = 0.98
TSS = 0.86
AUC = 0.98
TSS = 0.86
AUC = 0.98
TSS = 0.77
Relative importance
0.00
0.25
0.50
0.75
bio01
bio04
bio05
bio12
bio13
0.0
0.2
0.4
0.6
BDW
CLY
NTO
SOC
TWI
bio01
bio04
bio05
bio12
bio13
BDW
CLY
NTO
SOC
TWI
Phyllanthus subcrenulatus
Climate
Climate + Soil
Soil
AUC = 0.98
TSS = 0.8
AUC = 0.98
TSS = 0.8
AUC = 0.97
TSS = 0.75
Relative importance
0.0
0.2
0.4
0.6
0.8
bio01
bio06
bio12
bio13
bio14
BDW
CLY
NTO
PTO
SOC
bio01
bio06
bio12
bio13
bio14
BDW
CLY
NTO
PTO
SOC

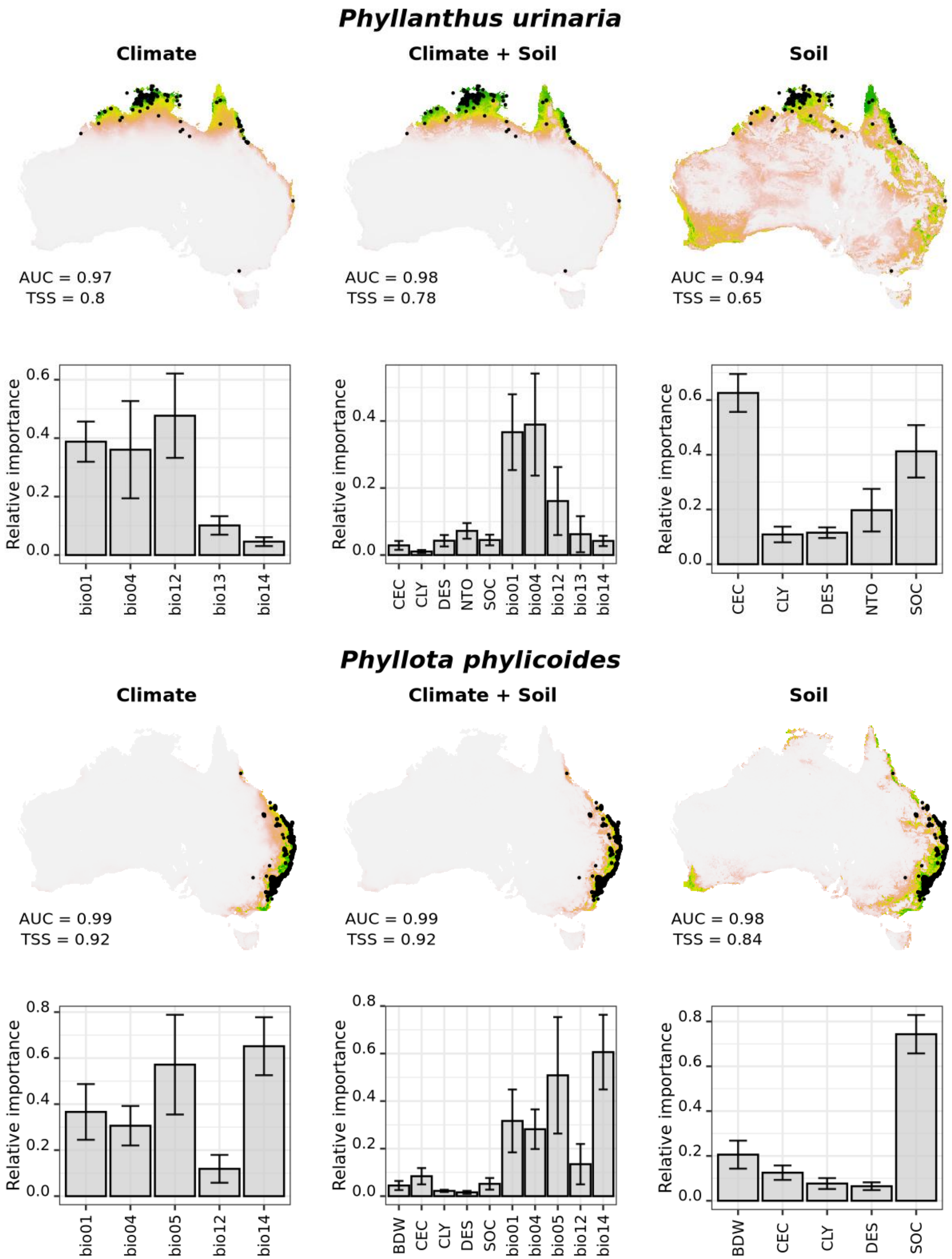
Phyllanthus urinaria
Climate
Climate + Soil
Soil
AUC = 0.97
TSS = 0.8
AUC = 0.98
TSS = 0.78
AUC = 0.94
TSS = 0.65
Relative importance
0.0
0.2
0.4
0.6
bio01
bio04
bio12
bio13
bio14
CEC
CLY
DES
NTO
SOC
Phyllota phylicoides
AUC = 0.99
TSS = 0.92
AUC = 0.99
TSS = 0.92
AUC = 0.98
TSS = 0.84
0.8
bio05
BDW

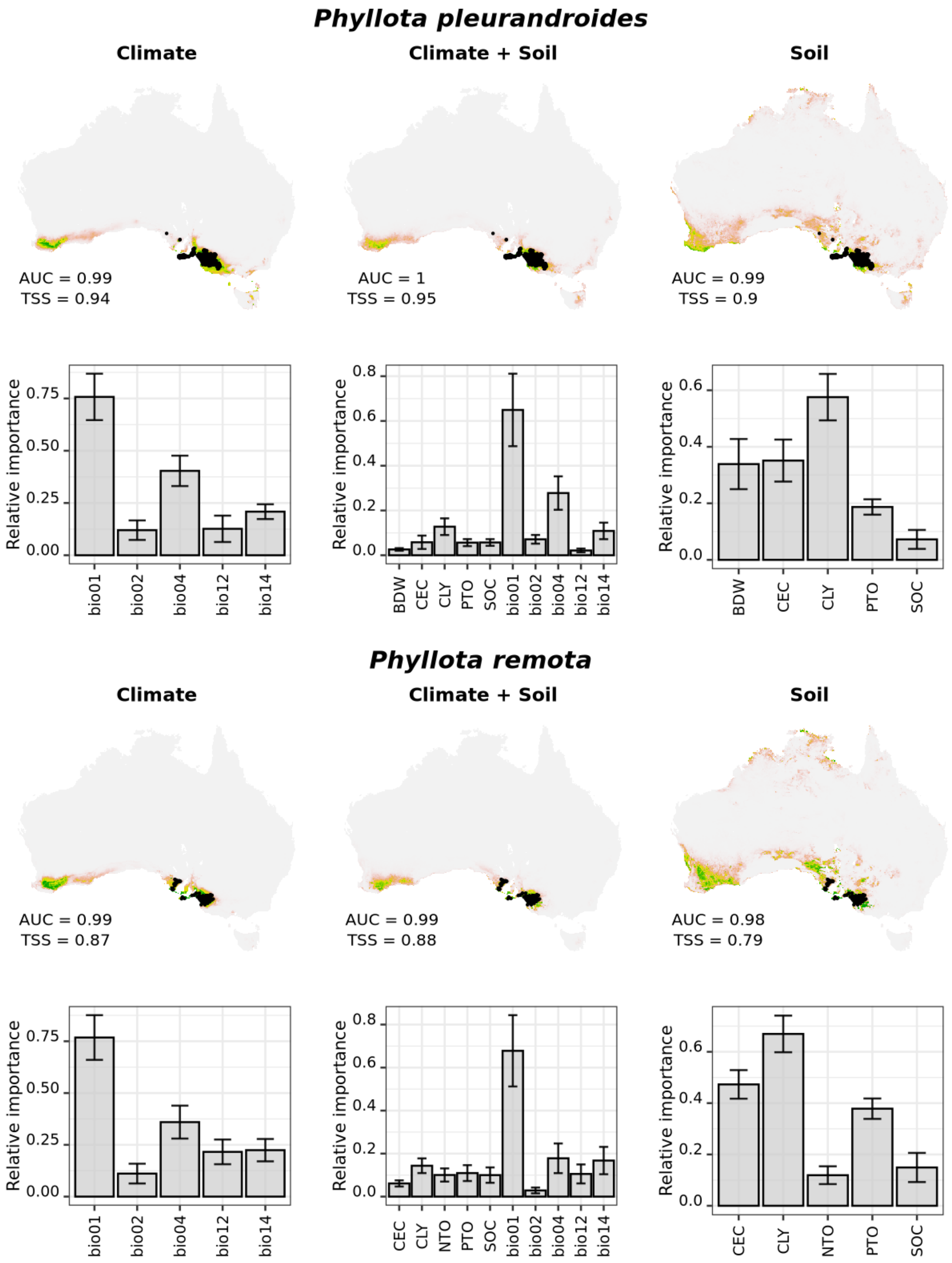
Phyllota pleurandroides
Climate
Climate + Soil
Soil
AUC = 0.99
TSS = 0.94
AUC = 1
TSS = 0.95
AUC = 0.99
TSS = 0.9
Relative importance
0.00
0.25
0.50
0.75
bio01
bio02
bio04
bio12
bio14
0.0
0.2
0.4
0.6
0.8
BDW
CEC
CLY
PTO
SOC
bio01
bio02
bio04
bio12
bio14
0.0
0.2
0.4
0.6
BDW
CEC
CLY
PTO
SOC
Phyllota remota
Climate
Climate + Soil
Soil
AUC = 0.99
TSS = 0.87
AUC = 0.99
TSS = 0.88
AUC = 0.98
TSS = 0.79
Relative importance
0.00
0.25
0.50
0.75
bio01
bio02
bio04
bio12
bio14
0.0
0.2
0.4
0.6
0.8
CEC
CLY
NTO
PTO
SOC
bio01
bio02
bio04
bio12
bio14
0.0
0.2
0.4
0.6
CEC
CLY
NTO
PTO
SOC

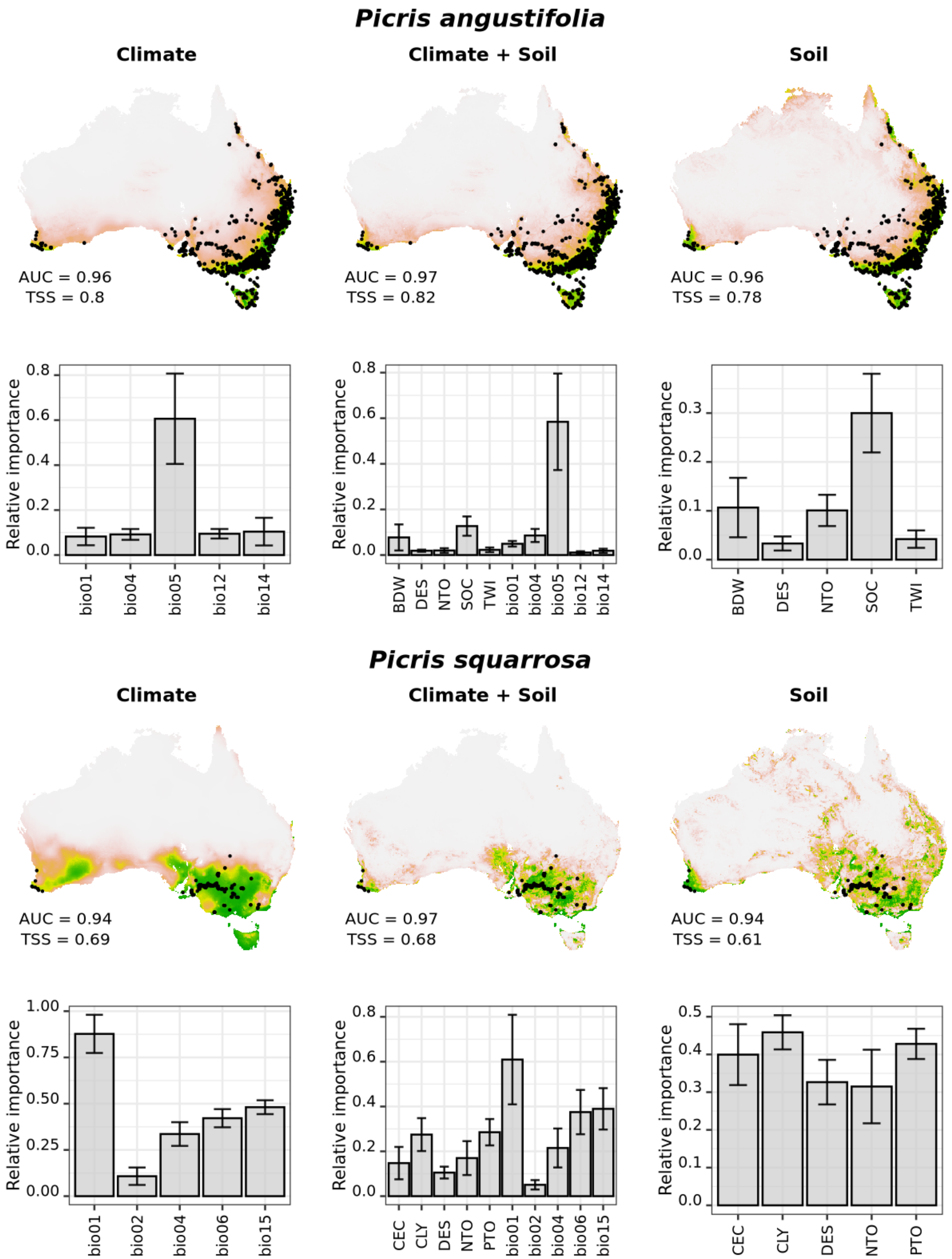

Picris angustifolia
Climate
Climate + Soil
Soil
AUC = 0.96
TSS = 0.8
AUC = 0.97
TSS = 0.82
AUC = 0.96
TSS = 0.78
Relative importance
bio01 bio04 bio05 bio12 bio14
BDW DES NTO SOC TWI bio01 bio04 bio05 bio12 bio14
BDW DES NTO SOC TWI
Picris squarrosa
Climate
Climate + Soil
Soil
AUC = 0.94
TSS = 0.69
AUC = 0.97
TSS = 0.68
AUC = 0.94
TSS = 0.61
Relative importance
bio01 bio02 bio04 bio06 bio15
CEC CLY DES NTO PTO bio01 bio02 bio04 bio06 bio15
CEC CLY DES NTO PTO

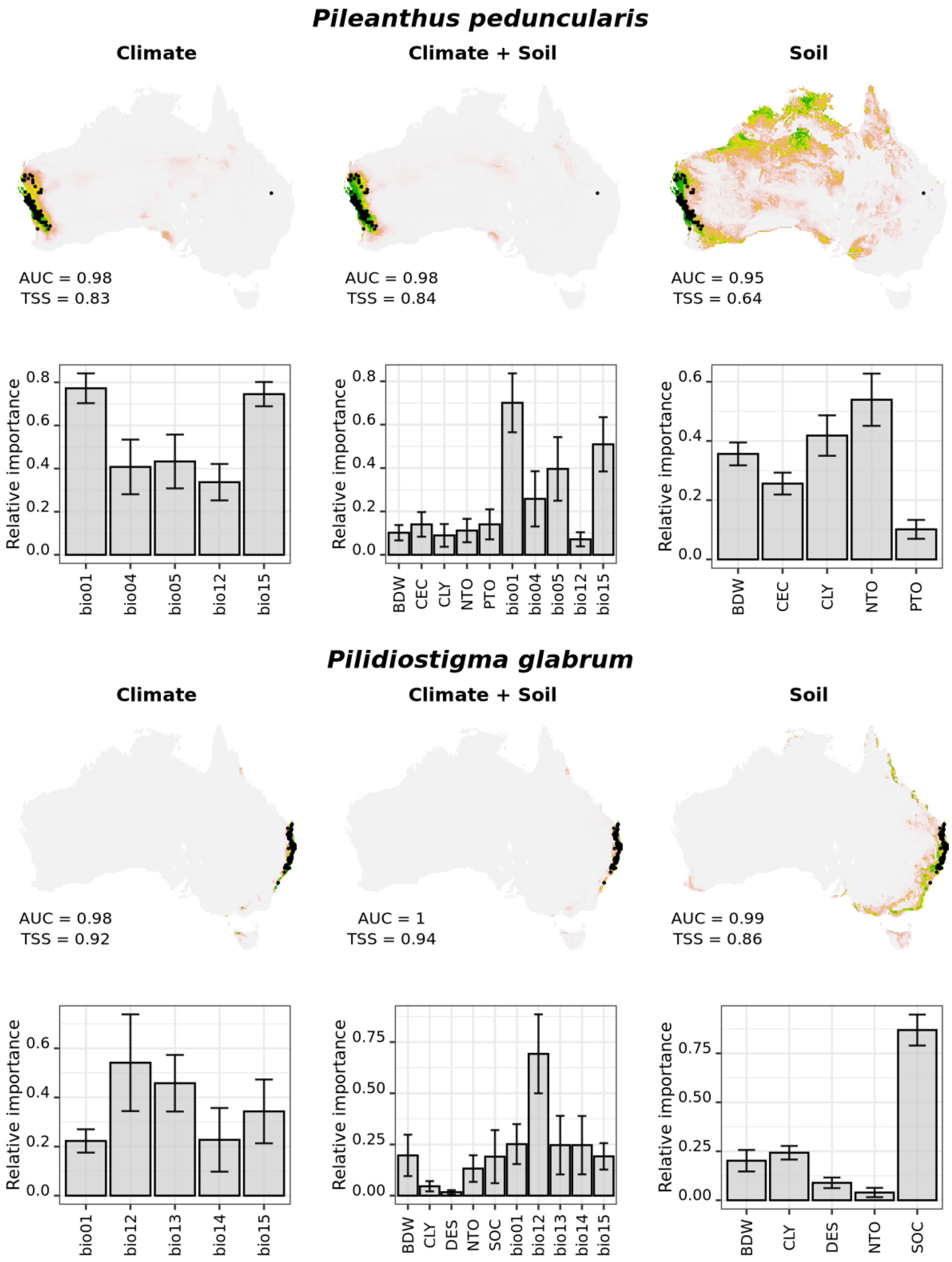
Pileanthus peduncularis
Climate
Climate + Soil
Soil
AUC = 0.98
TSS = 0.83
AUC = 0.98
TSS = 0.84
AUC = 0.95
TSS = 0.64
Relative importance
bio01 bio04 bio05 bio12 bio15
BDW CEC CLY NTO PTO bio01 bio04 bio05 bio12 bio15
BDW CEC CLY NTO PTO
Pilidiostigma glabrum
Climate
Climate + Soil
Soil
AUC = 0.98
TSS = 0.92
AUC = 1
TSS = 0.94
AUC = 0.99
TSS = 0.86
Relative importance
bio01 bio12 bio13 bio14 bio15
BDW CLY DES NTO SOC bio01 bio12 bio13 bio14 bio15
BDW CLY DES NTO SOC

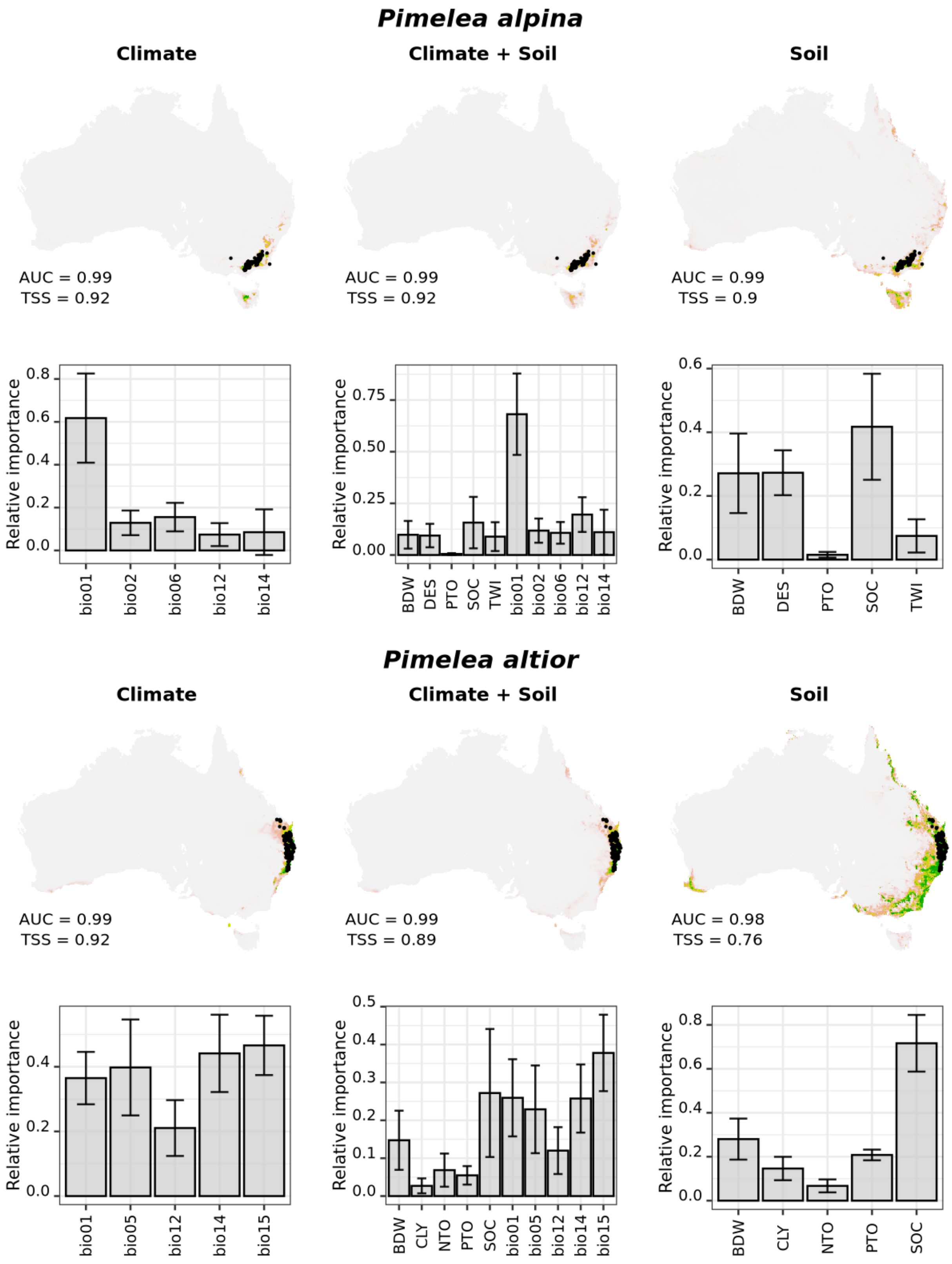

Pimelea alpina
Climate
Climate + Soil
Soil
AUC = 0.99
TSS = 0.92
AUC = 0.99
TSS = 0.92
AUC = 0.99
TSS = 0.9
Relative importance
bio01
bio02
bio06
bio12
bio14
BDW
DES
PTO
SOC
TWI
Pimelea altior
Climate
Climate + Soil
Soil
AUC = 0.99
TSS = 0.92
AUC = 0.99
TSS = 0.89
AUC = 0.98
TSS = 0.76
Relative importance
bio01
bio05
bio12
bio14
bio15
BDW
CLY
NTO
PTO
SOC

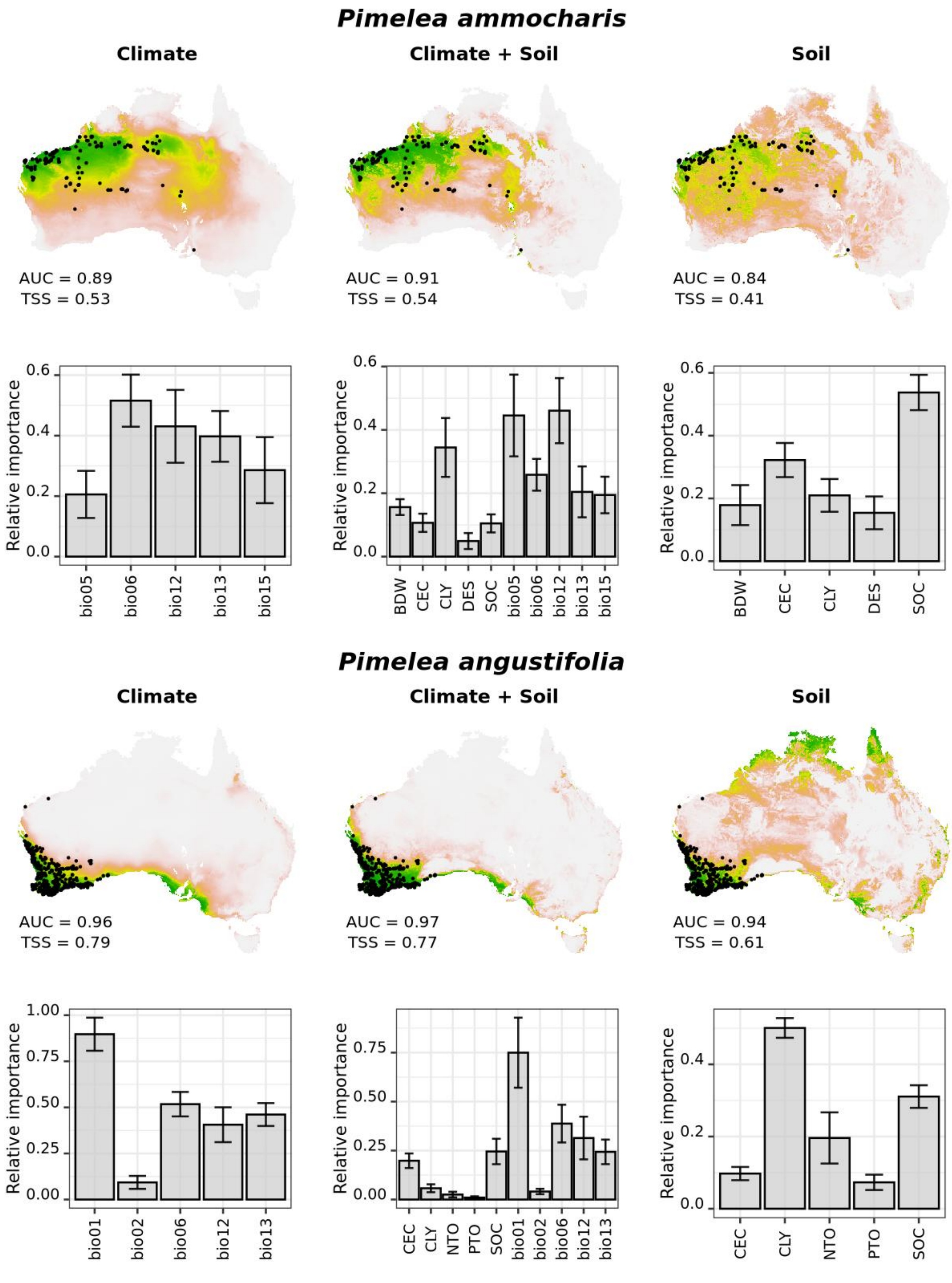
Pimelea ammocharis
Climate
Climate + Soil
Soil
AUC = 0.89
TSS = 0.53
AUC = 0.91
TSS = 0.54
AUC = 0.84
TSS = 0.41
Relative importance
bio05
bio06
bio12
bio13
bio15
BDW
CEC
CLY
DES
SOC
Pimelea angustifolia
Climate
Climate + Soil
Soil
AUC = 0.96
TSS = 0.79
AUC = 0.97
TSS = 0.77
AUC = 0.94
TSS = 0.61
Relative importance
bio01
bio02
bio06
bio12
bio13
CEC
CLY
NTO
PTO
SOC

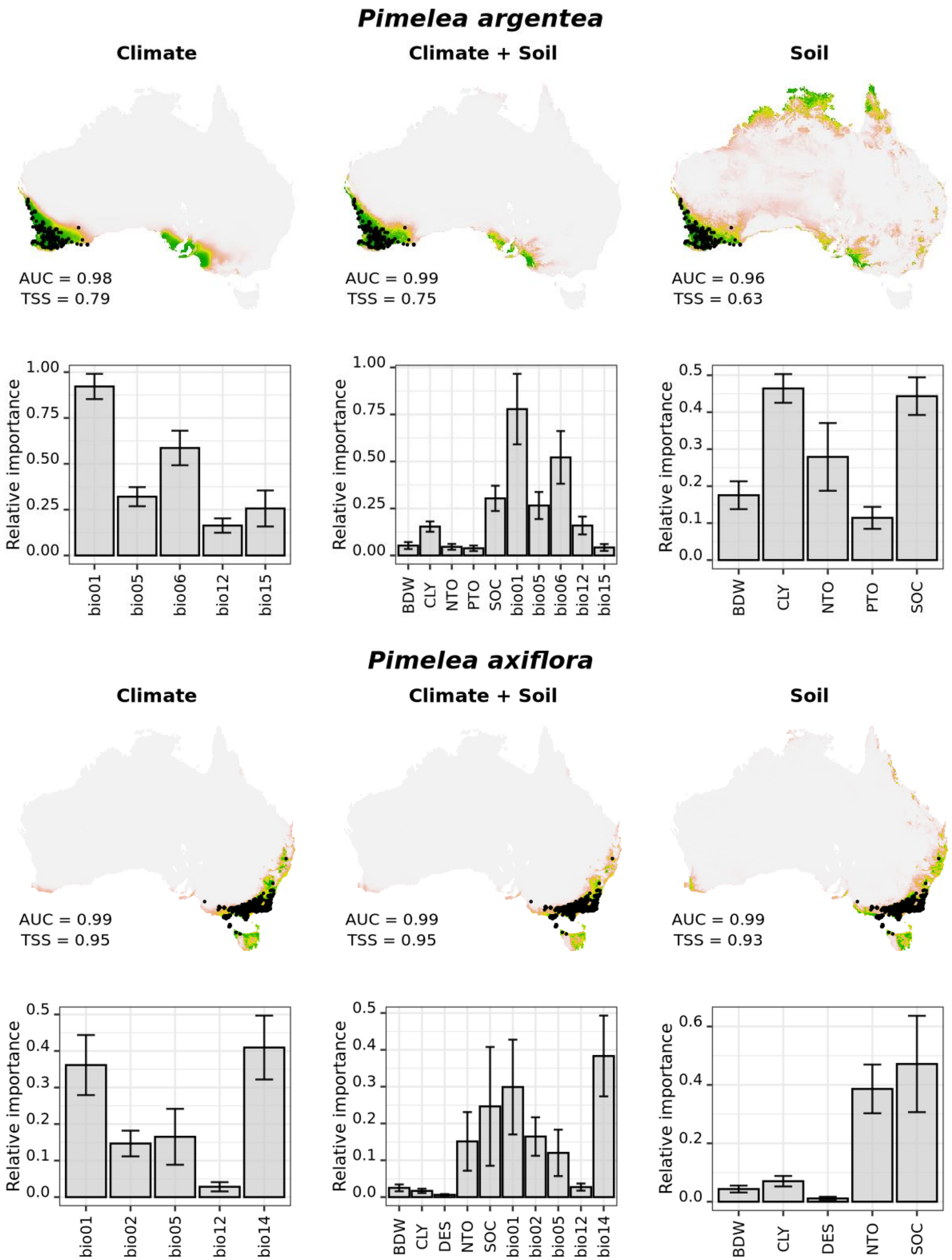
Pimelea argentea
Climate
Climate + Soil
Soil
AUC = 0.98
TSS = 0.79
AUC = 0.99
TSS = 0.75
AUC = 0.96
TSS = 0.63
Relative importance
1.00
0.75
0.50
0.25
0.00
bio01
bio05
bio06
bio12
bio15
BDW
CLY
NTO
PTO
SOC
bio01
bio05
bio06
bio12
bio15
0.5
0.4
0.3
0.2
0.1
0.0
BDW
CLY
NTO
PTO
SOC
Pimelea axiflora
Climate
Climate + Soil
Soil
AUC = 0.99
TSS = 0.95
AUC = 0.99
TSS = 0.95
AUC = 0.99
TSS = 0.93
Relative importance
0.5
0.4
0.3
0.2
0.1
0.0
bio01
bio02
bio05
bio12
bio14
BDW
CLY
DES
NTO
SOC
bio01
bio02
bio05
bio12
bio14
0.6
0.4
0.2
0.0
BDW
CLY
DES
NTO
SOC

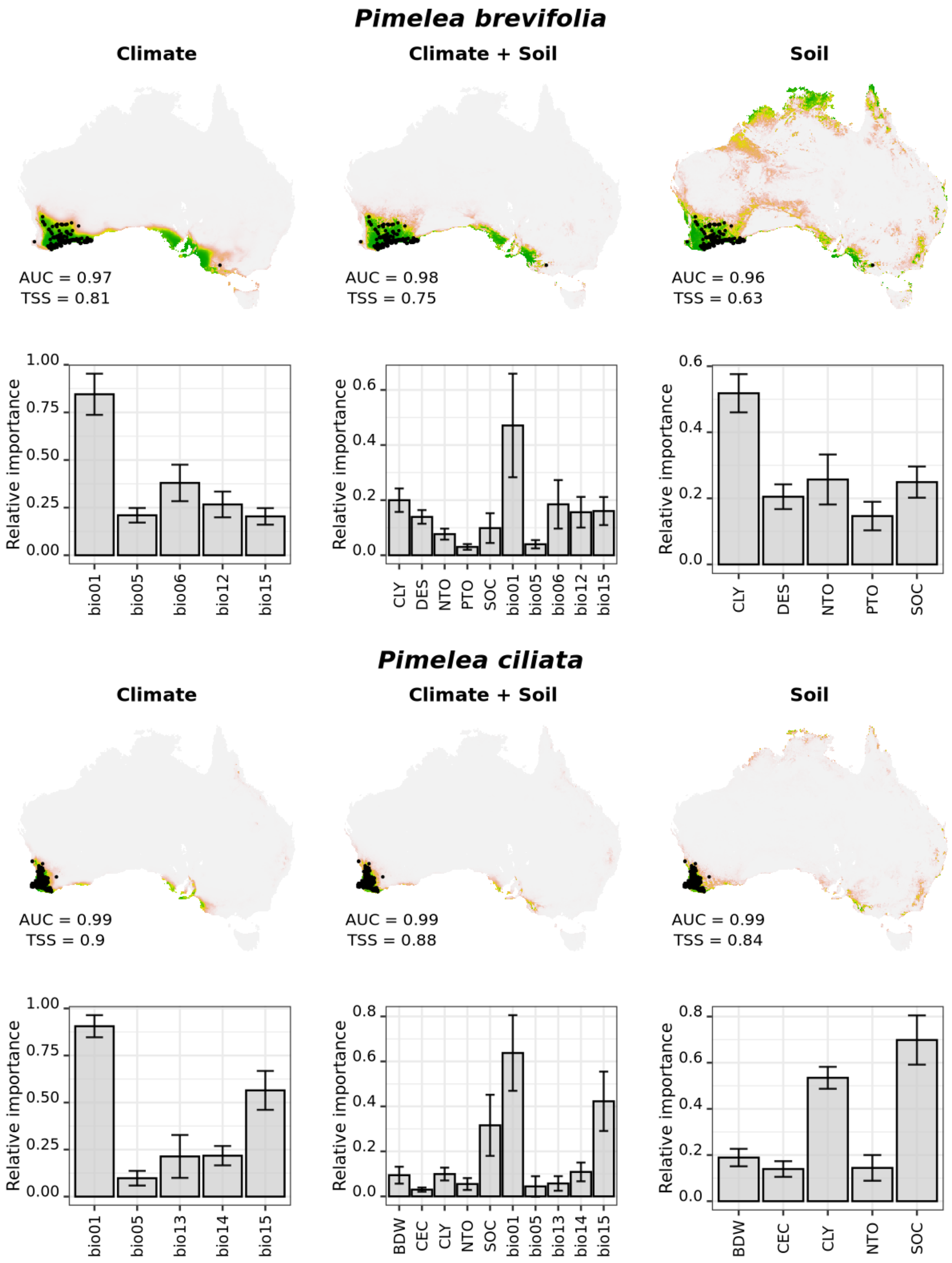
Pimelea brevifolia
Climate
Climate + Soil
Soil
AUC = 0.97
TSS = 0.81
AUC = 0.98
TSS = 0.75
AUC = 0.96
TSS = 0.63
Relative importance
bio01
bio05
bio06
bio12
bio15
CLY
DES
NTO
PTO
SOC
Pimelea ciliata
Climate
Climate + Soil
Soil
AUC = 0.99
TSS = 0.9
AUC = 0.99
TSS = 0.88
AUC = 0.99
TSS = 0.84
Relative importance
bio01
bio05
bio13
bio14
bio15
BDW
CEC
CLY
NTO
SOC

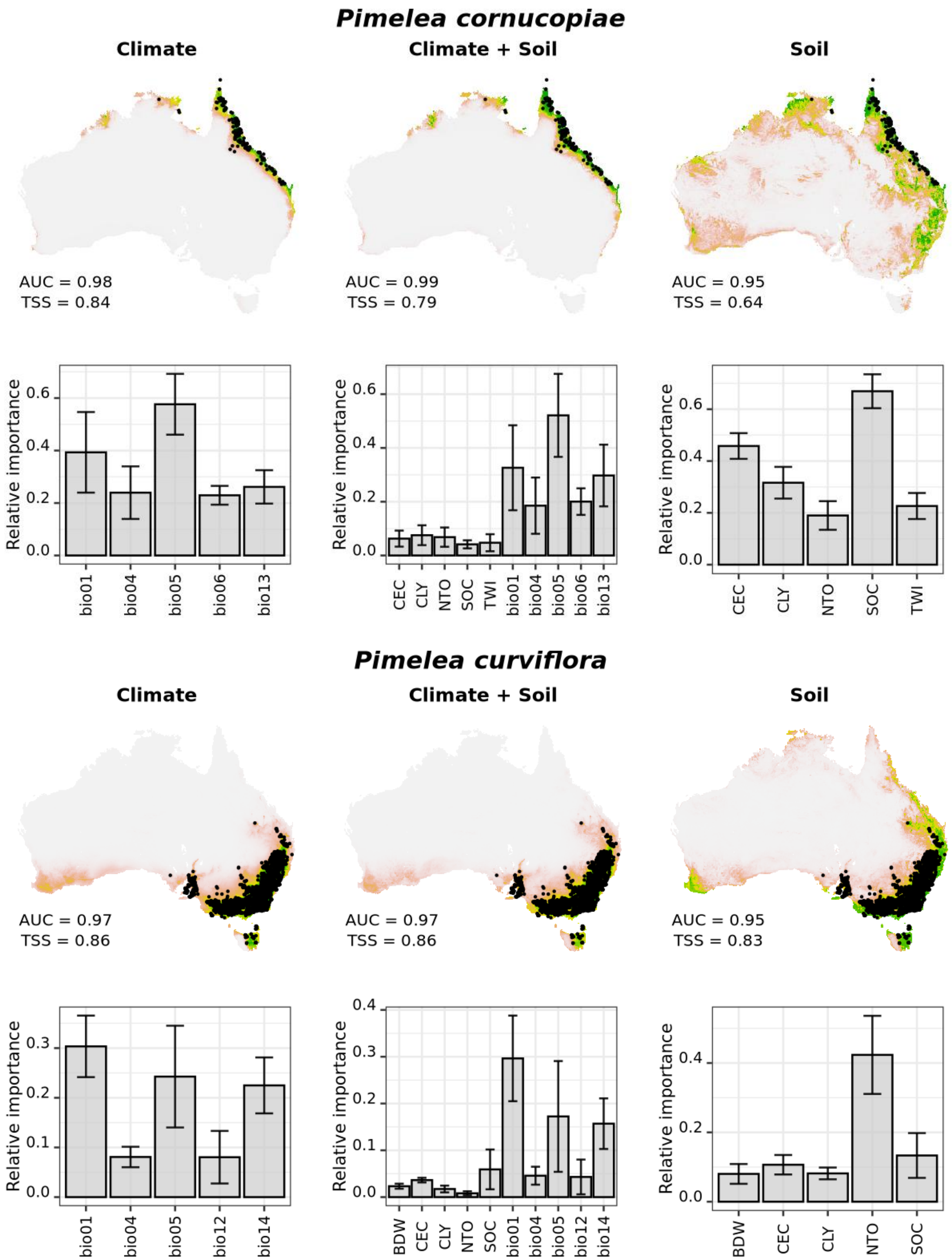
Pimelea cornucopiae
Climate
Climate + Soil
Soil
AUC = 0.98
TSS = 0.84
AUC = 0.99
TSS = 0.79
AUC = 0.95
TSS = 0.64
Relative importance
bio01
bio04
bio05
bio06
bio13
CEC
CLY
NTO
SOC
TWI
Pimelea curviflora
Climate
Climate + Soil
Soil
AUC = 0.97
TSS = 0.86
AUC = 0.97
TSS = 0.86
AUC = 0.95
TSS = 0.83
bio12
bio14
BDW

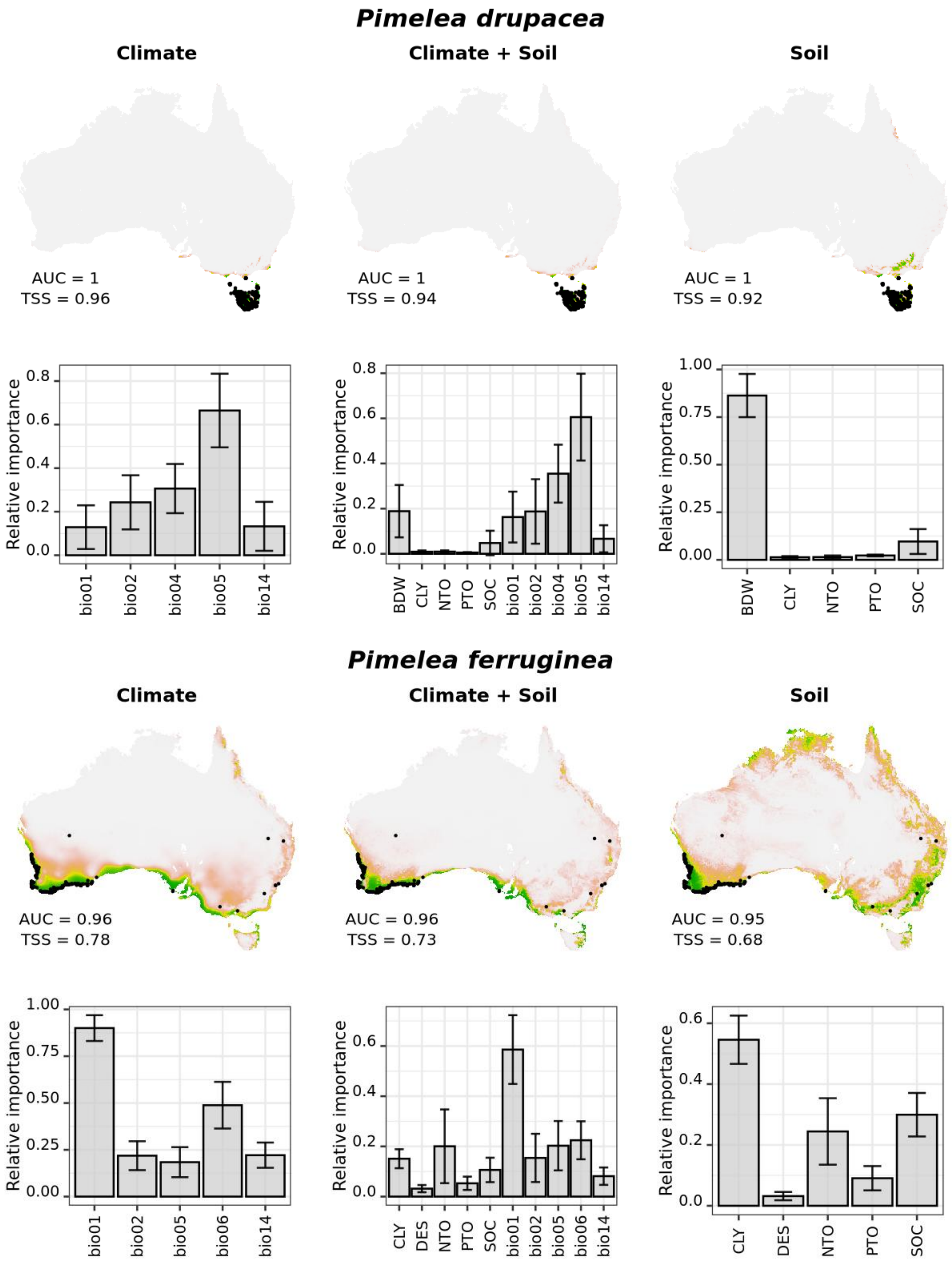

Pimelea drupacea
Climate
Climate + Soil
Soil
AUC = 1
TSS = 0.96
AUC = 1
TSS = 0.94
AUC = 1
TSS = 0.92
Relative importance
bio01
bio02
bio04
bio05
bio14
BDW
CLY
NTO
PTO
SOC
Pimelea ferruginea
AUC = 0.96
TSS = 0.78
AUC = 0.96
TSS = 0.73
AUC = 0.95
TSS = 0.68
bio06
DES

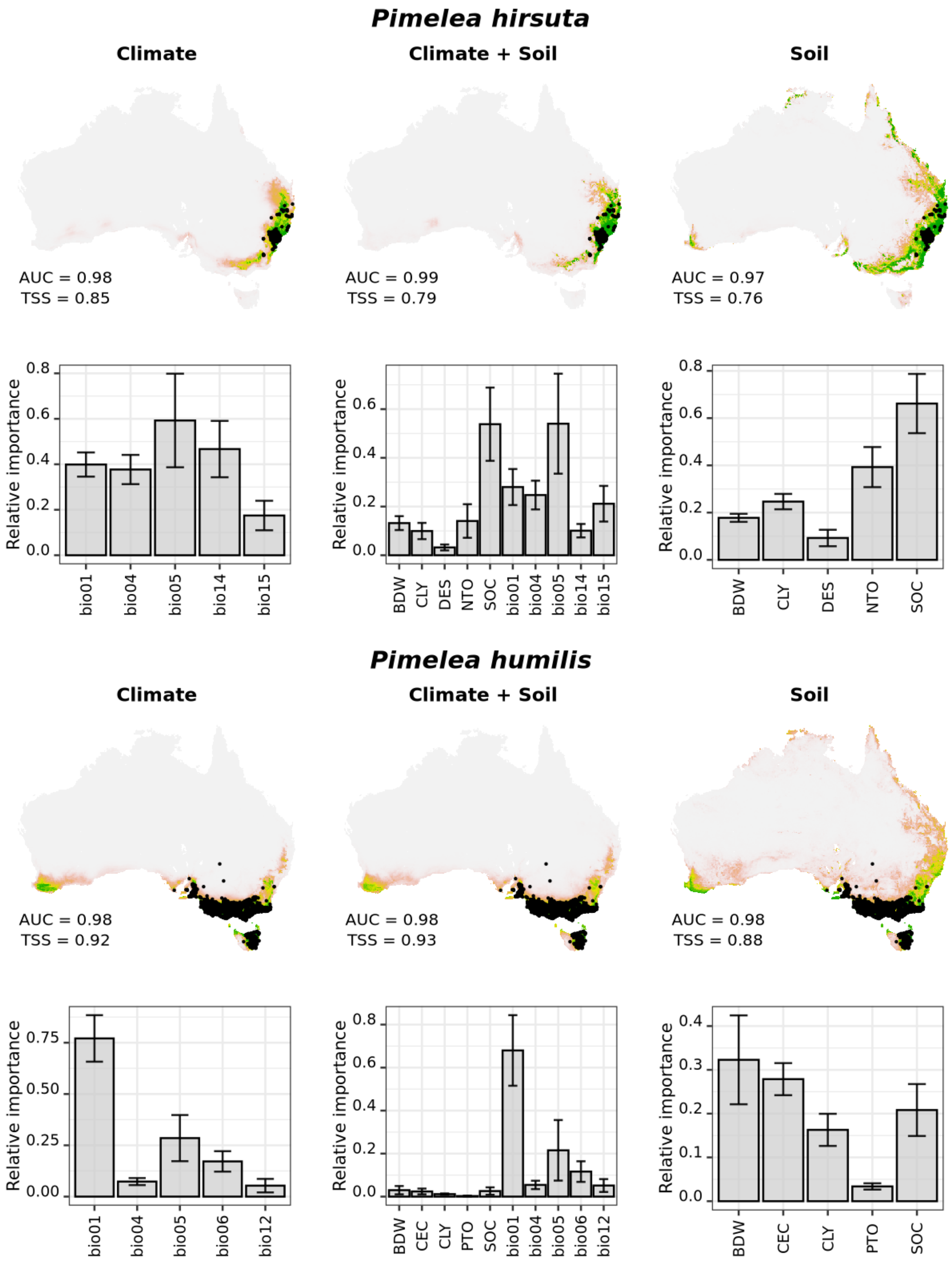
Pimelea hirsuta
Climate
Climate + Soil
Soil
AUC = 0.98
TSS = 0.85
AUC = 0.99
TSS = 0.79
AUC = 0.97
TSS = 0.76
Relative importance
bio01 bio04 bio05 bio14 bio15
BDW CLY DES NTO SOC bio01 bio04 bio05 bio14 bio15
BDW CLY DES NTO SOC
Pimelea humilis
Climate
Climate + Soil
Soil
AUC = 0.98
TSS = 0.92
AUC = 0.98
TSS = 0.93
AUC = 0.98
TSS = 0.88
Relative importance
bio01 bio04 bio05 bio06 bio12
BDW CEC CLY PTO SOC bio01 bio04 bio05 bio06 bio12
BDW CEC CLY PTO SOC

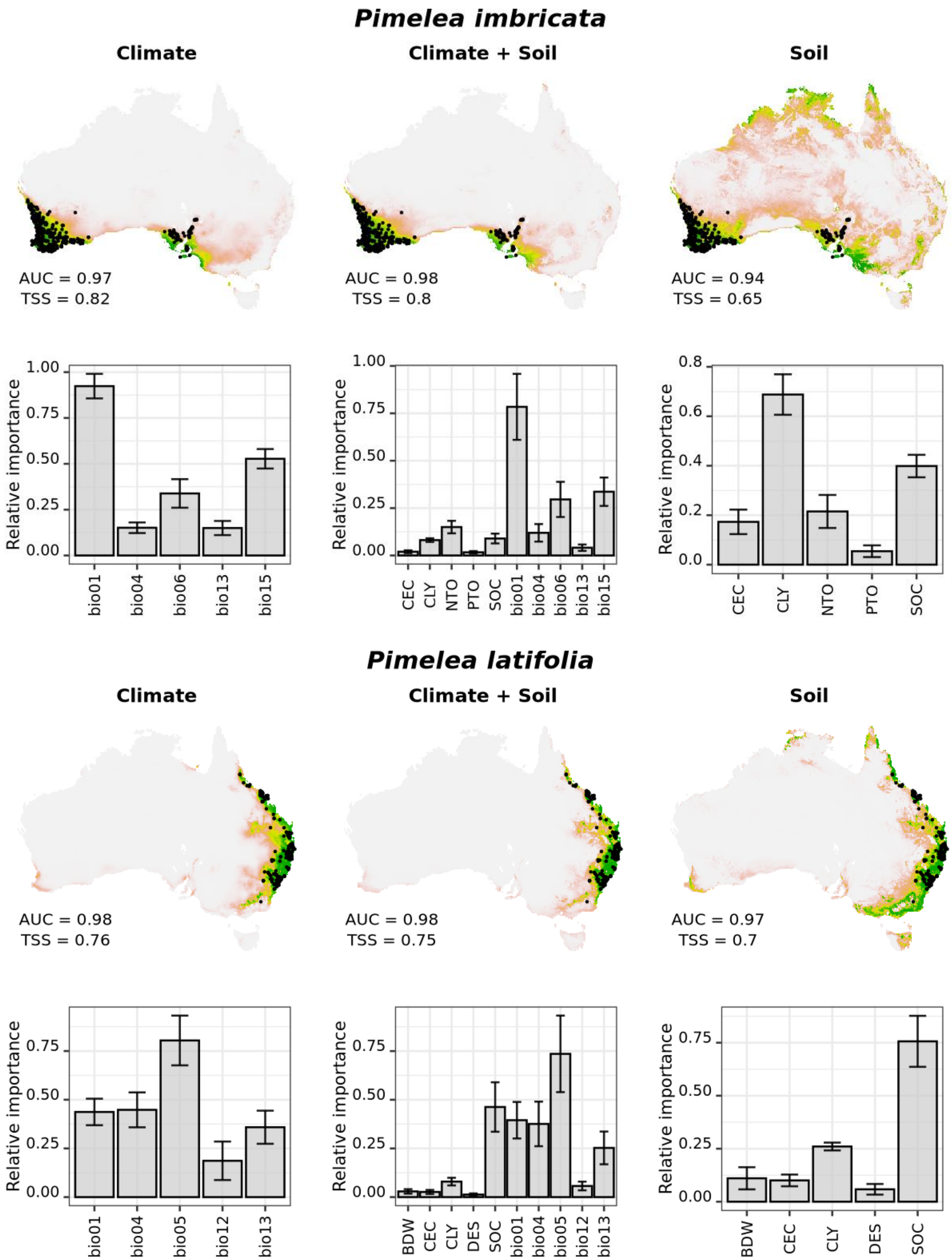
Pimelea imbricata
Climate
Climate + Soil
Soil
AUC = 0.97
TSS = 0.82
AUC = 0.98
TSS = 0.8
AUC = 0.94
TSS = 0.65
Relative importance
1.00
0.75
0.50
0.25
0.00
bio01
bio04
bio06
bio13
bio15
CEC
CLY
NTO
PTO
SOC
0.8
0.6
0.4
0.2
0.0
Pimelea latifolia
Climate
Climate + Soil
Soil
AUC = 0.98
TSS = 0.76
AUC = 0.98
TSS = 0.75
AUC = 0.97
TSS = 0.7
bio05
bio12
BDW
DES

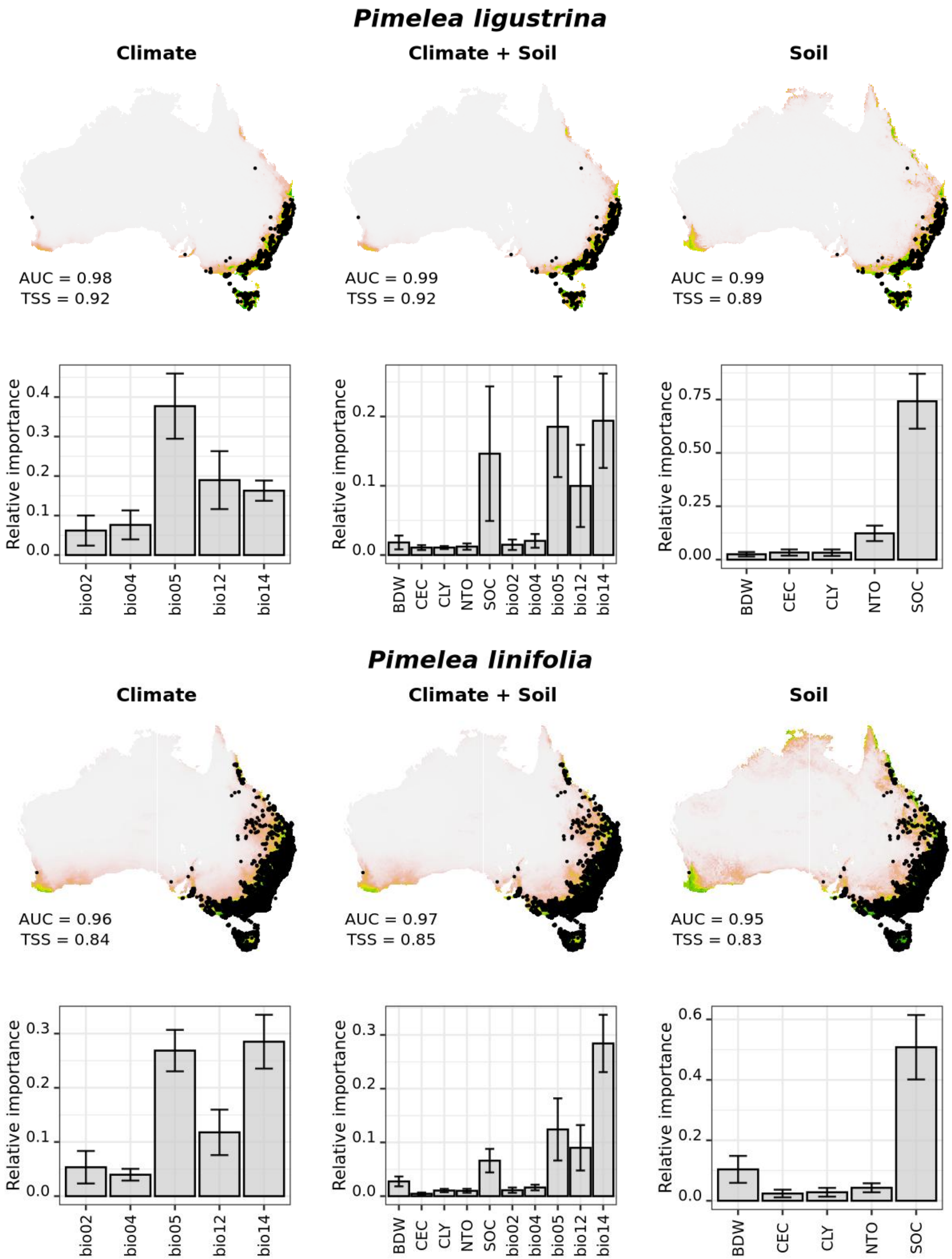

Pimelea ligustrina
Climate
Climate + Soil
Soil
AUC = 0.98
TSS = 0.92
AUC = 0.99
TSS = 0.92
AUC = 0.99
TSS = 0.89
Relative importance
bio02
bio04
bio05
bio12
bio14
BDW
CEC
CLY
NTO
SOC
Pimelea linifolia
Climate
Climate + Soil
Soil
AUC = 0.96
TSS = 0.84
AUC = 0.97
TSS = 0.85
AUC = 0.95
TSS = 0.83
Relative importance
bio02
bio04
bio05
bio12
bio14
BDW
CEC
CLY
NTO
SOC

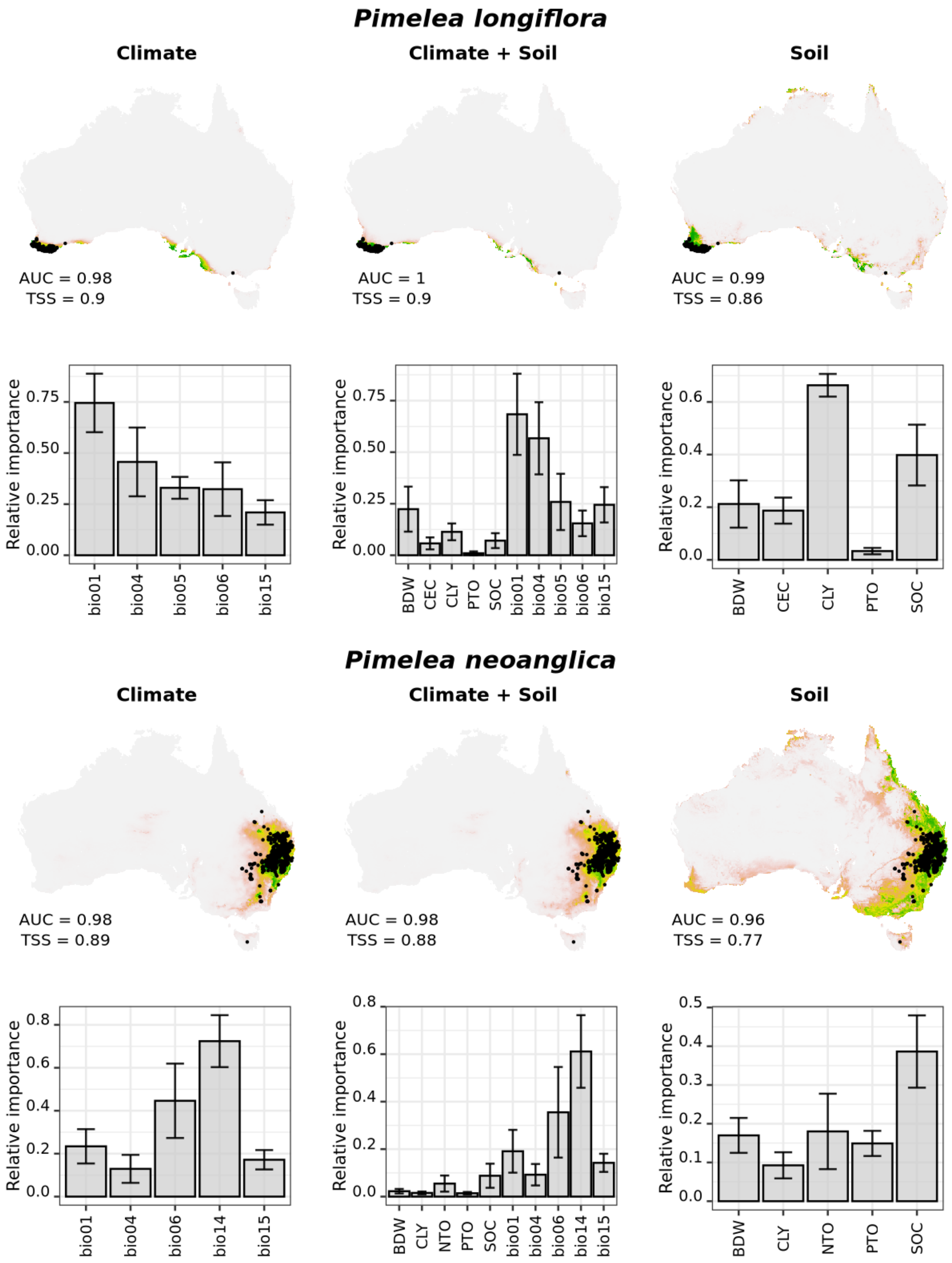
Pimelea longiflora
Climate
Climate + Soil
Soil
AUC = 0.98
TSS = 0.9
AUC = 1
TSS = 0.9
AUC = 0.99
TSS = 0.86
Relative importance
0.00
0.25
0.50
0.75
bio01
bio04
bio05
bio06
bio15
BDW
CEC
CLY
PTO
SOC
0.0
0.2
0.4
0.6
Pimelea neoanglica
Climate
Climate + Soil
Soil
AUC = 0.98
TSS = 0.89
AUC = 0.98
TSS = 0.88
AUC = 0.96
TSS = 0.77
0.0
0.2
0.4
0.6
0.8
bio01
bio04
bio06
bio14
bio15
BDW
CLY
NTO
PTO
SOC
0.1
0.3
0.5

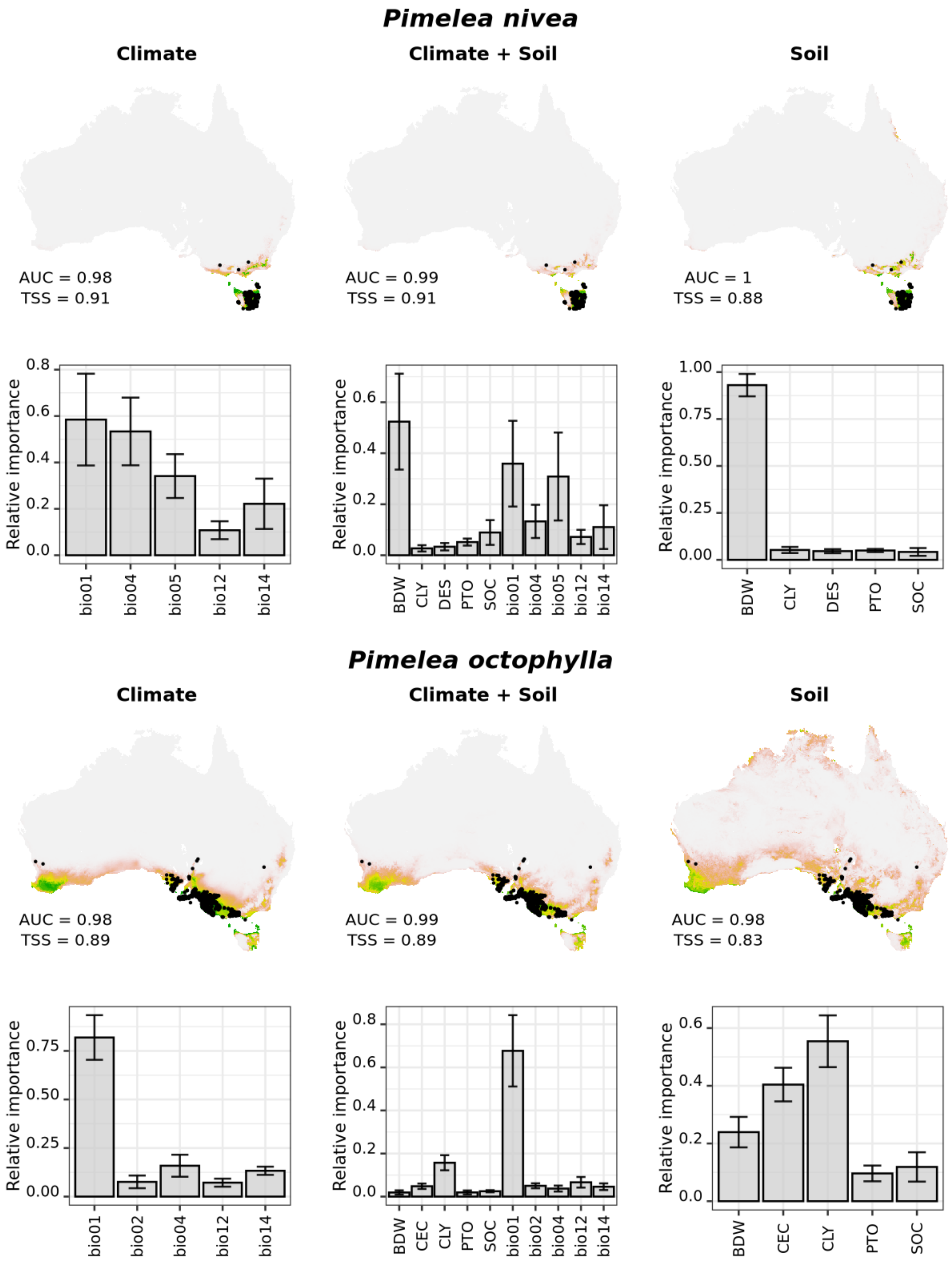
Pimelea nivea
Climate
Climate + Soil
Soil
AUC = 0.98
TSS = 0.91
AUC = 0.99
TSS = 0.91
AUC = 1
TSS = 0.88
Relative importance
bio01
bio04
bio05
bio12
bio14
BDW
CLY
DES
PTO
SOC
Pimelea octophylla
Climate
Climate + Soil
Soil
AUC = 0.98
TSS = 0.89
AUC = 0.99
TSS = 0.89
AUC = 0.98
TSS = 0.83
Relative importance
bio01
bio02
bio04
bio12
bio14
BDW
CEC
CLY
PTO
SOC

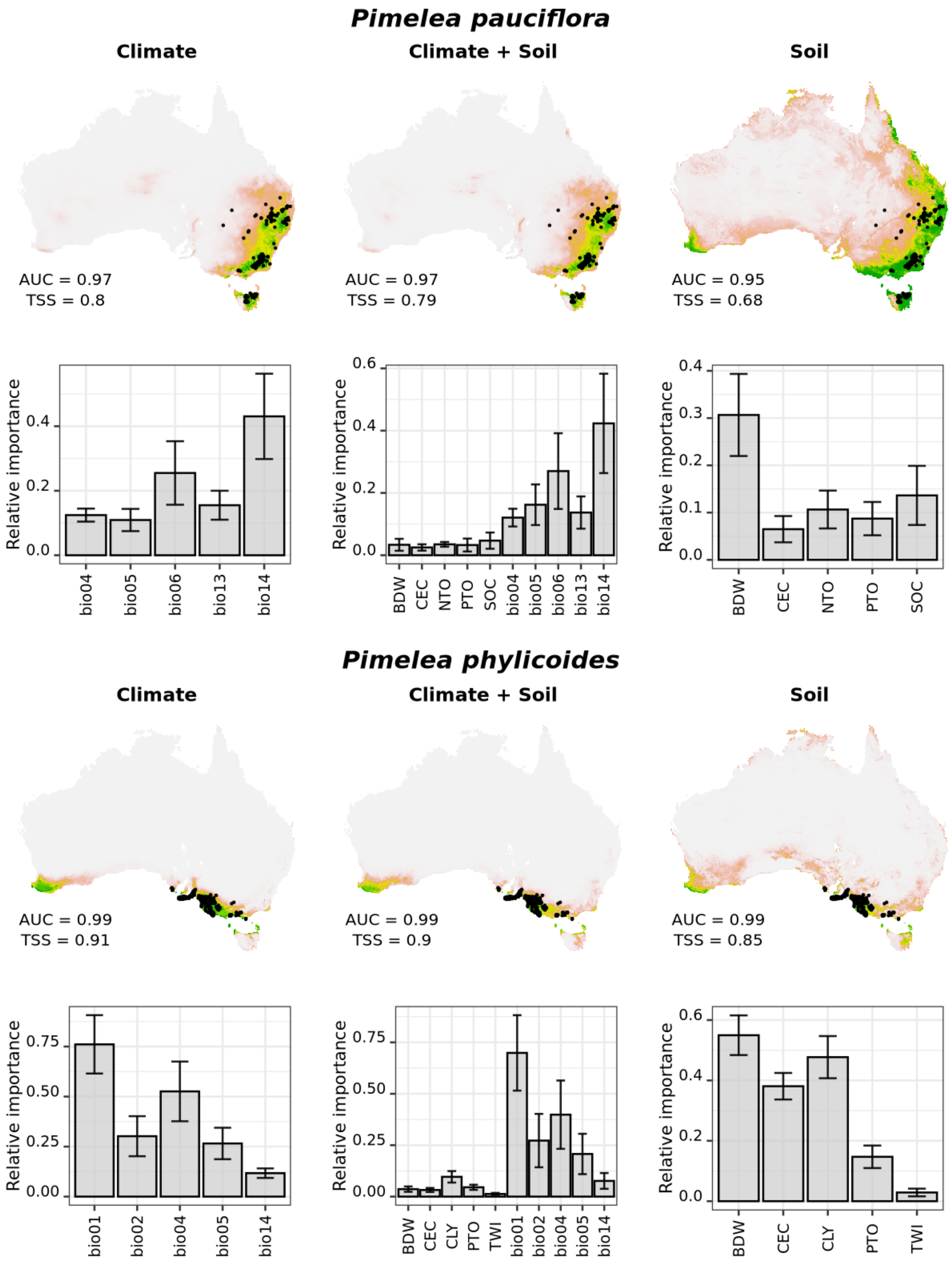

Pimelea pauciflora
Climate
Climate + Soil
Soil
AUC = 0.97
TSS = 0.8
AUC = 0.97
TSS = 0.79
AUC = 0.95
TSS = 0.68
Relative importance
bio04
bio05
bio06
bio13
bio14
BDW
CEC
NTO
PTO
SOC
Pimelea phylicoides
Climate
Climate + Soil
Soil
AUC = 0.99
TSS = 0.91
AUC = 0.99
TSS = 0.9
AUC = 0.99
TSS = 0.85
Relative importance
bio01
bio02
bio04
bio05
bio14
BDW
CEC
CLY
PTO
TWI

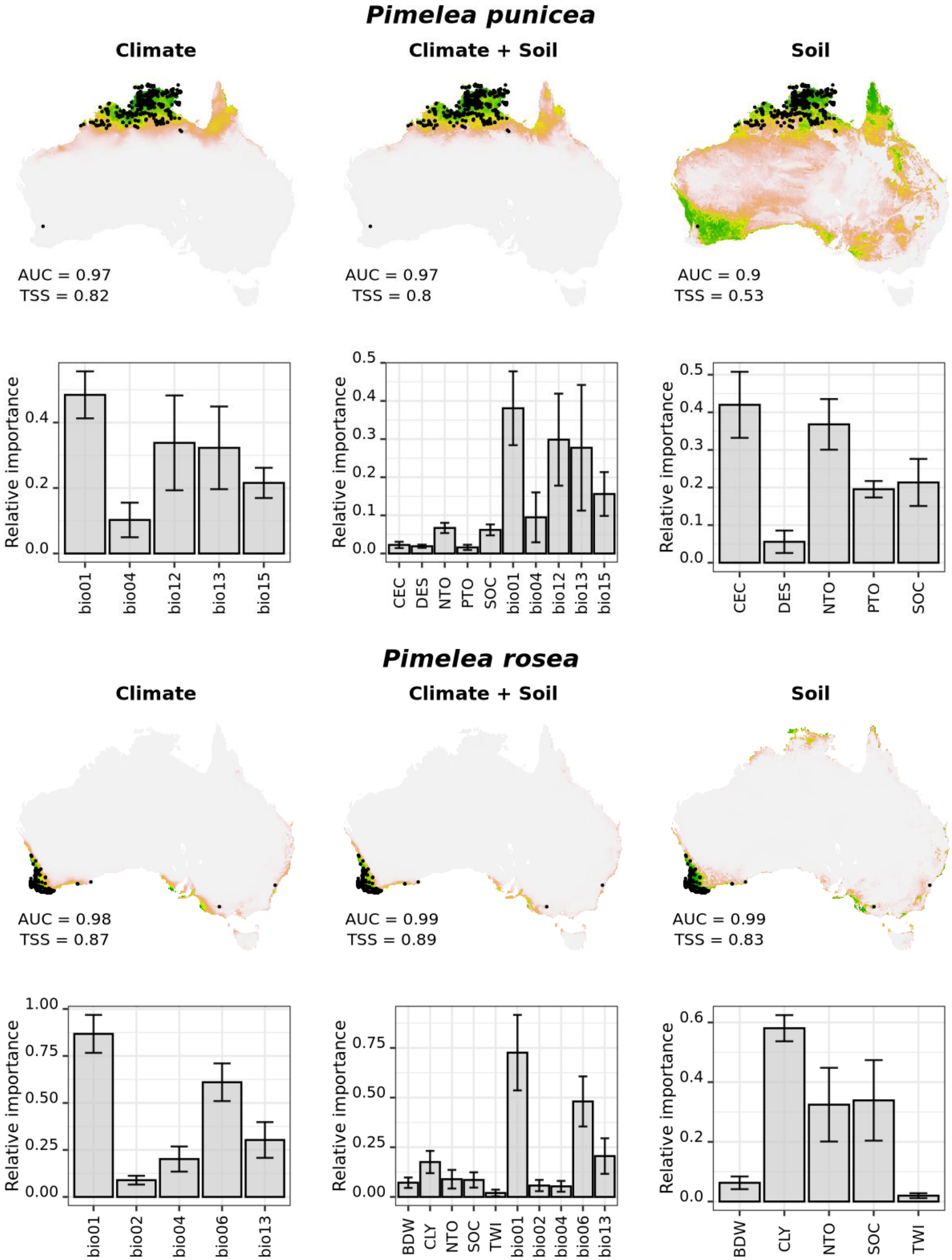

Pimelea punicea
Climate
Climate + Soil
Soil
AUC = 0.97
TSS = 0.82
AUC = 0.97
TSS = 0.8
AUC = 0.9
TSS = 0.53
Relative importance
bio01 bio04 bio12 bio13 bio15
CEC DES NTO PTO SOC bio01 bio04 bio12 bio13 bio15
CEC DES NTO PTO SOC
Pimelea rosea
Climate
Climate + Soil
Soil
AUC = 0.98
TSS = 0.87
AUC = 0.99
TSS = 0.89
AUC = 0.99
TSS = 0.83
Relative importance
bio01 bio02 bio04 bio06 bio13
BDW CLY NTO SOC TWI bio01 bio02 bio04 bio06 bio13
BDW CLY NTO SOC TWI

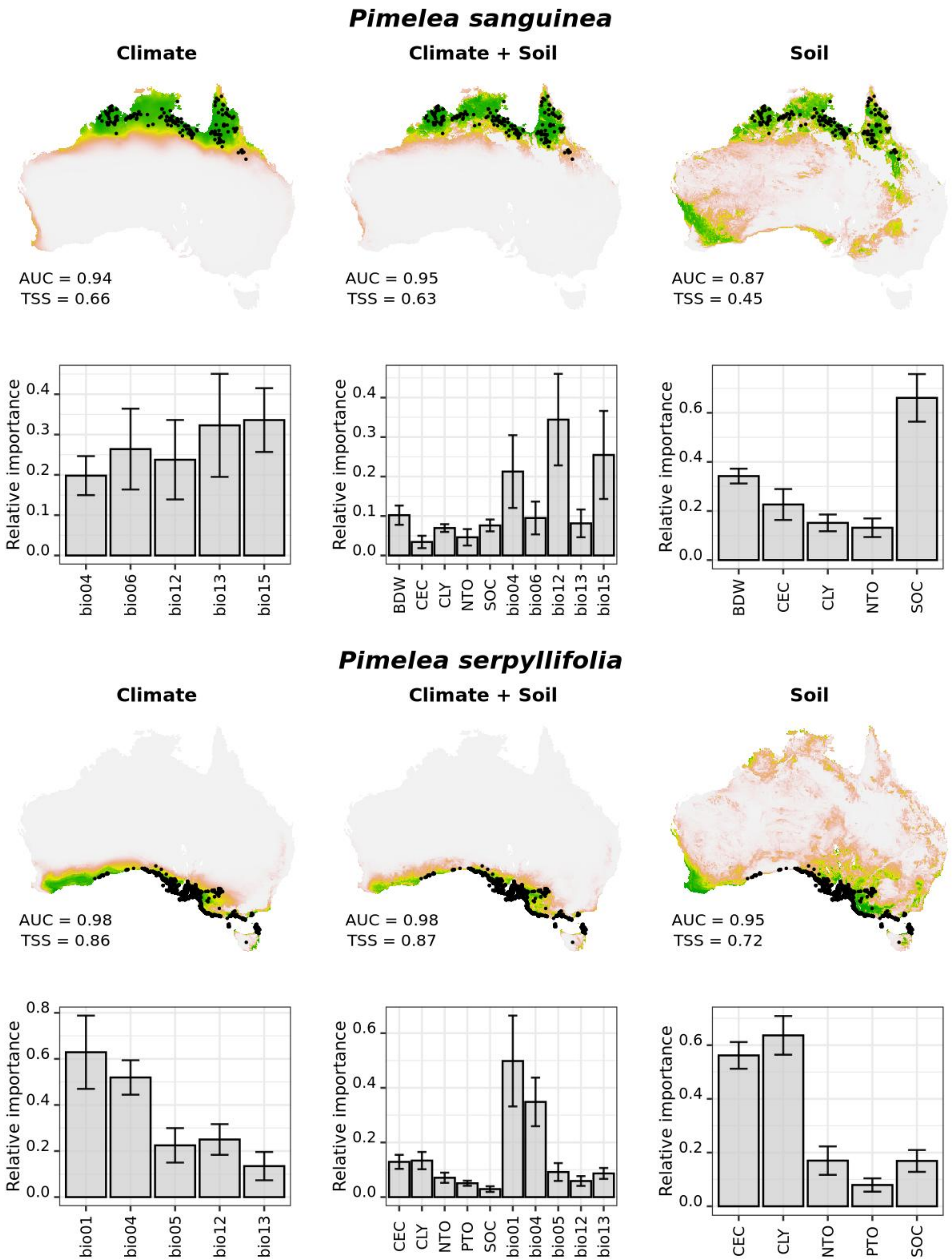
Pimelea sanguinea
Climate
Climate + Soil
Soil
AUC = 0.94
TSS = 0.66
AUC = 0.95
TSS = 0.63
AUC = 0.87
TSS = 0.45
Relative importance
0.0 0.1 0.2 0.3 0.4
bio04 bio06 bio12 bio13 bio15
Relative importance
0.0 0.1 0.2 0.3 0.4
BDW CEC CLY NTO SOC bio04 bio06 bio12 bio13 bio15
Relative importance
0.0 0.2 0.4 0.6
BDW CEC CLY NTO SOC
Pimelea serpyllifolia
Climate
Climate + Soil
Soil
AUC = 0.98
TSS = 0.86
AUC = 0.98
TSS = 0.87
AUC = 0.95
TSS = 0.72
Relative importance
0.0 0.2 0.4 0.6 0.8
bio01 bio04 bio05 bio12 bio13
Relative importance
0.0 0.2 0.4 0.6
CEC CLY NTO PTO SOC bio01 bio04 bio05 bio12 bio13
Relative importance
0.0 0.2 0.4 0.6
CEC CLY NTO PTO SOC

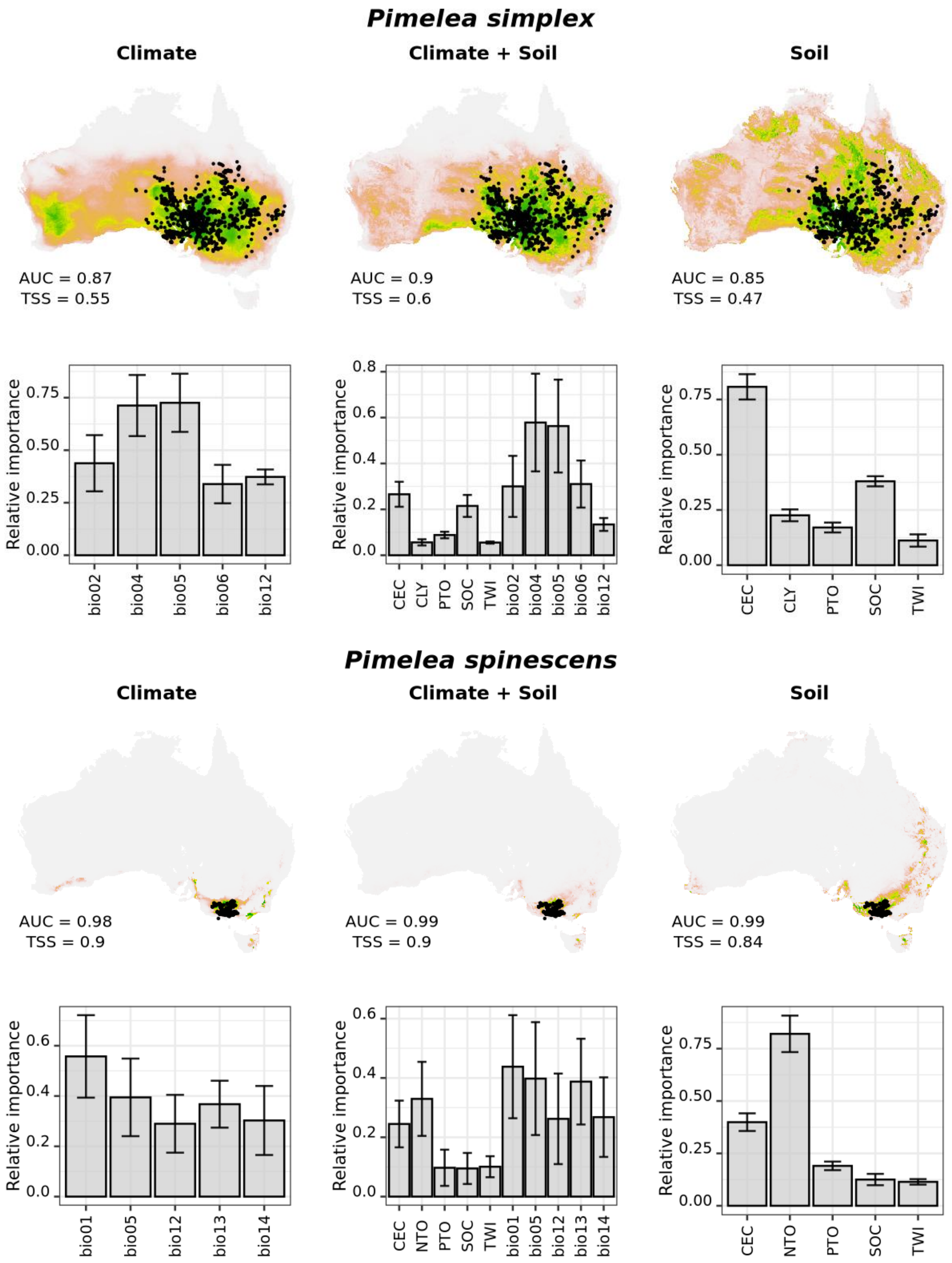
Pimelea simplex
Climate
Climate + Soil
Soil
AUC = 0.87
TSS = 0.55
AUC = 0.9
TSS = 0.6
AUC = 0.85
TSS = 0.47
Relative importance
bio02 bio04 bio05 bio06 bio12
CEC CLY PTO SOC TWI bio02 bio04 bio05 bio06 bio12
CEC CLY PTO SOC TWI
Pimelea spinescens
Climate
Climate + Soil
Soil
AUC = 0.98
TSS = 0.9
AUC = 0.99
TSS = 0.9
AUC = 0.99
TSS = 0.84
Relative importance
bio01 bio05 bio12 bio13 bio14
CEC NTO PTO SOC TWI bio01 bio05 bio12 bio13 bio14
CEC NTO PTO SOC TWI

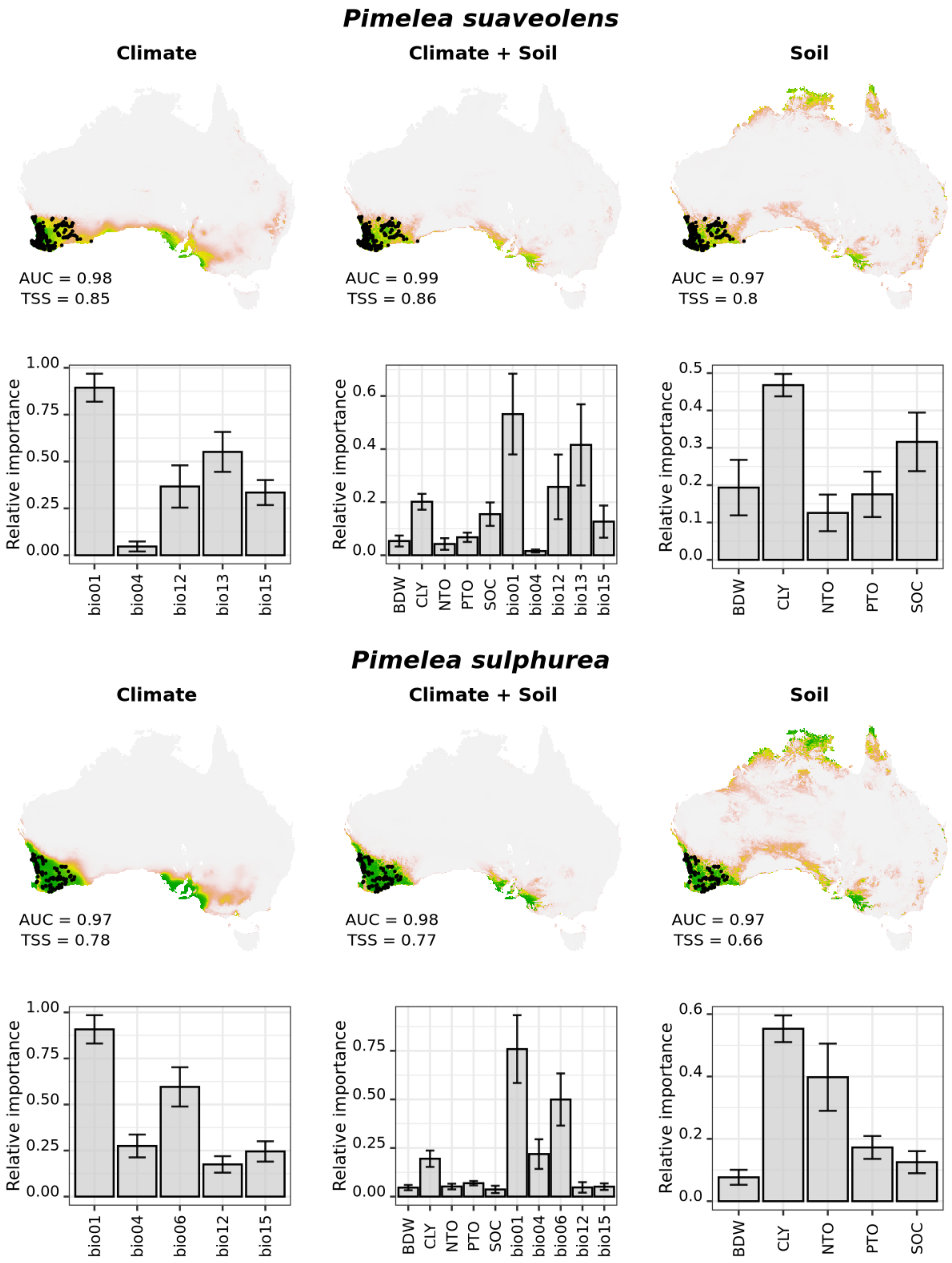
Pimelea suaveolens
Climate
Climate + Soil
Soil
AUC = 0.98
TSS = 0.85
AUC = 0.99
TSS = 0.86
AUC = 0.97
TSS = 0.8
Relative importance
bio01
bio04
bio12
bio13
bio15
BDW
CLY
NTO
PTO
SOC
Pimelea sulphurea
Climate
Climate + Soil
Soil
AUC = 0.97
TSS = 0.78
AUC = 0.98
TSS = 0.77
AUC = 0.97
TSS = 0.66
Relative importance
bio01
bio04
bio06
bio12
bio15
BDW
CLY
NTO
PTO
SOC

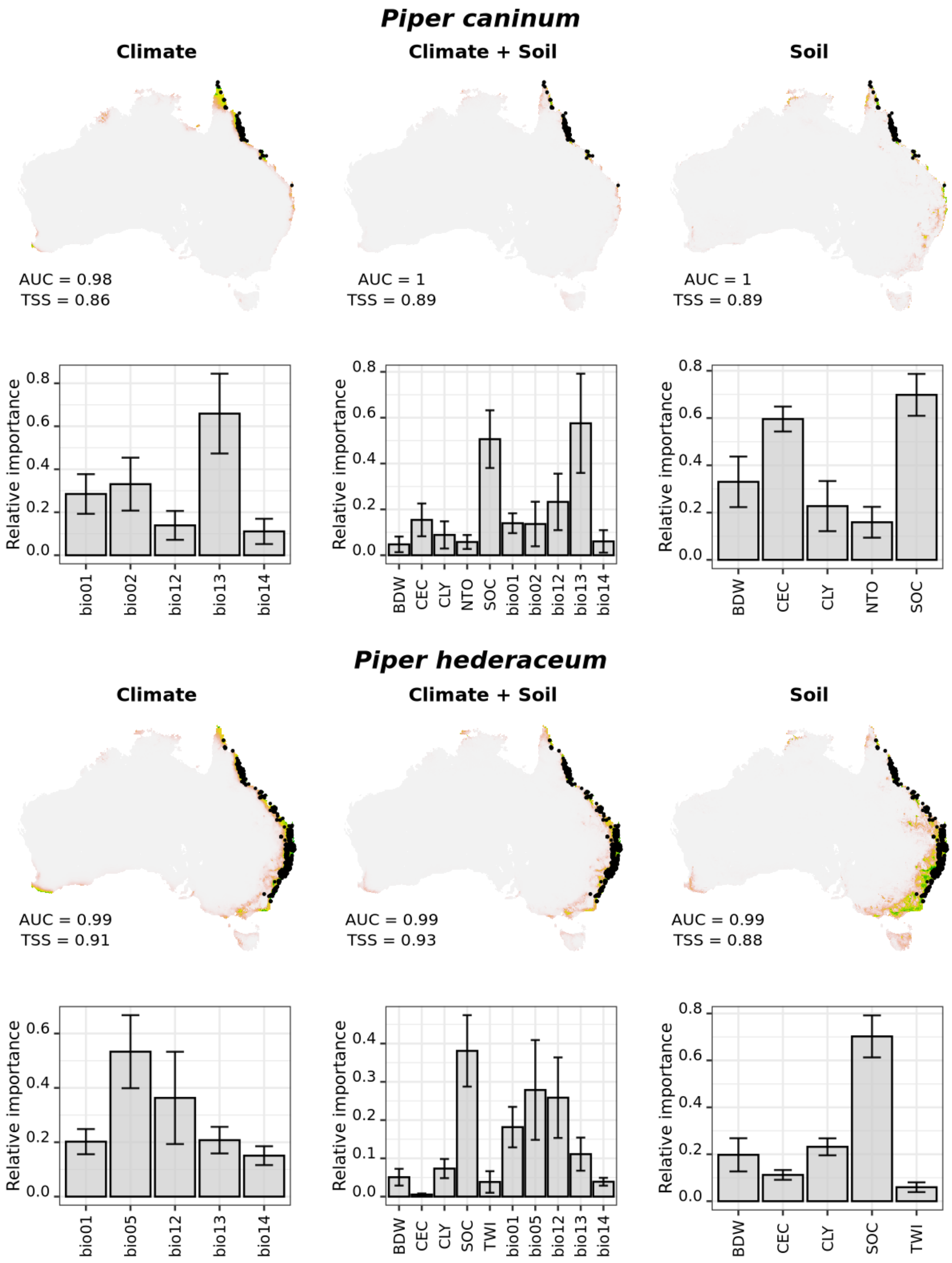
Piper caninum
Climate
Climate + Soil
Soil
AUC = 0.98
TSS = 0.86
AUC = 1
TSS = 0.89
AUC = 1
TSS = 0.89
Relative importance
bio01
bio02
bio12
bio13
bio14
BDW
CEC
CLY
NTO
SOC
Piper hederaceum
Climate
Climate + Soil
Soil
AUC = 0.99
TSS = 0.91
AUC = 0.99
TSS = 0.93
AUC = 0.99
TSS = 0.88
Relative importance
bio01
bio05
bio12
bio13
bio14
BDW
CEC
CLY
SOC
TWI

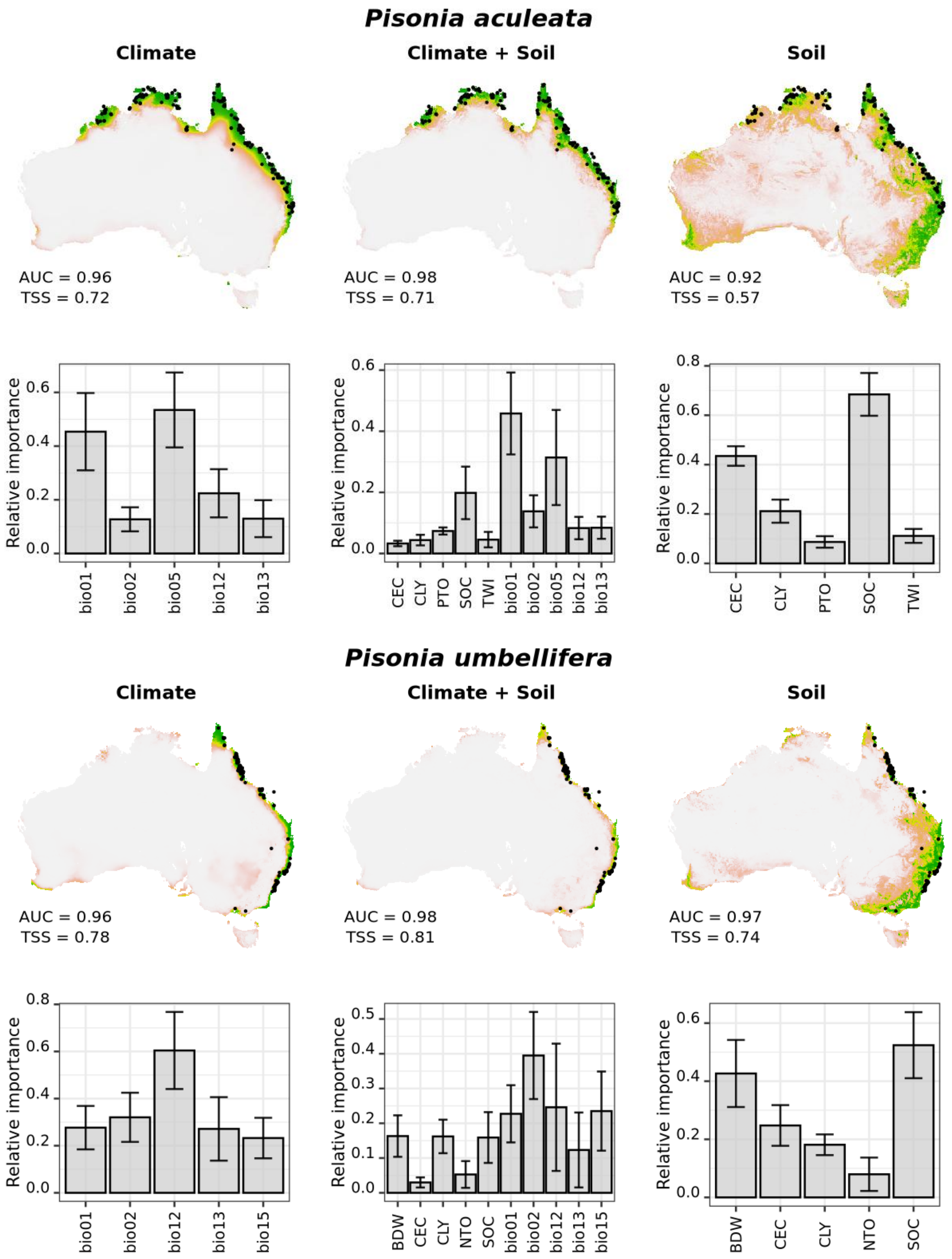

Pisonia aculeata
Climate
Climate + Soil
Soil
AUC = 0.96
TSS = 0.72
AUC = 0.98
TSS = 0.71
AUC = 0.92
TSS = 0.57
Relative importance
bio01
bio02
bio05
bio12
bio13
CEC
CLY
PTO
SOC
TWI
Pisonia umbellifera
Climate
Climate + Soil
Soil
AUC = 0.96
TSS = 0.78
AUC = 0.98
TSS = 0.81
AUC = 0.97
TSS = 0.74
Relative importance
bio01
bio02
bio12
bio13
bio15
BDW
CEC
CLY
NTO
SOC

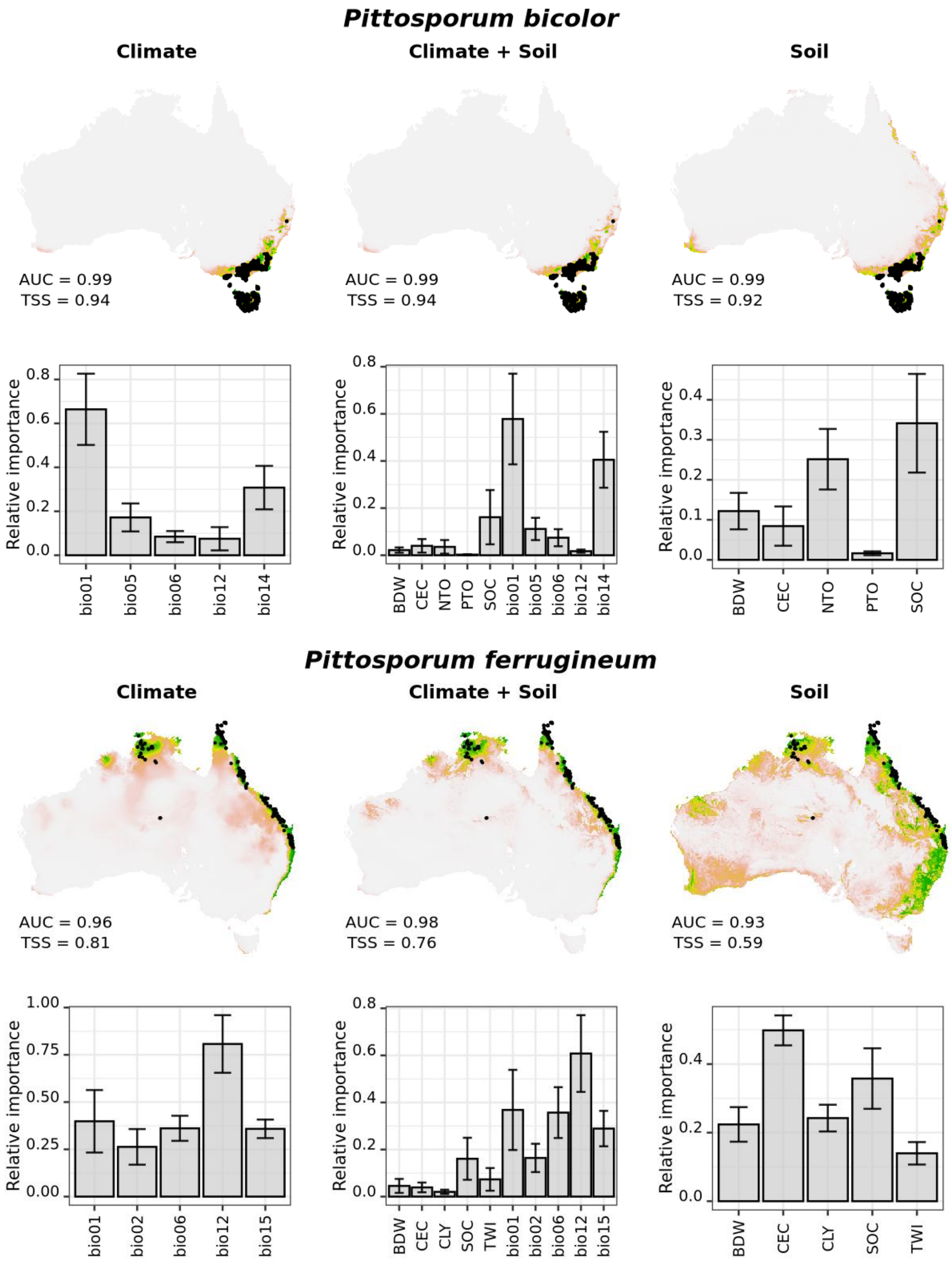
Pittosporum bicolor
Climate
Climate + Soil
Soil
AUC = 0.99
TSS = 0.94
AUC = 0.99
TSS = 0.94
AUC = 0.99
TSS = 0.92
Relative importance
bio01
bio05
bio06
bio12
bio14
BDW
CEC
NTO
PTO
SOC
Pittosporum ferrugineum
Climate
Climate + Soil
Soil
AUC = 0.96
TSS = 0.81
AUC = 0.98
TSS = 0.76
AUC = 0.93
TSS = 0.59
Relative importance
bio01
bio02
bio06
bio12
bio15
BDW
CEC
CLY
SOC
TWI

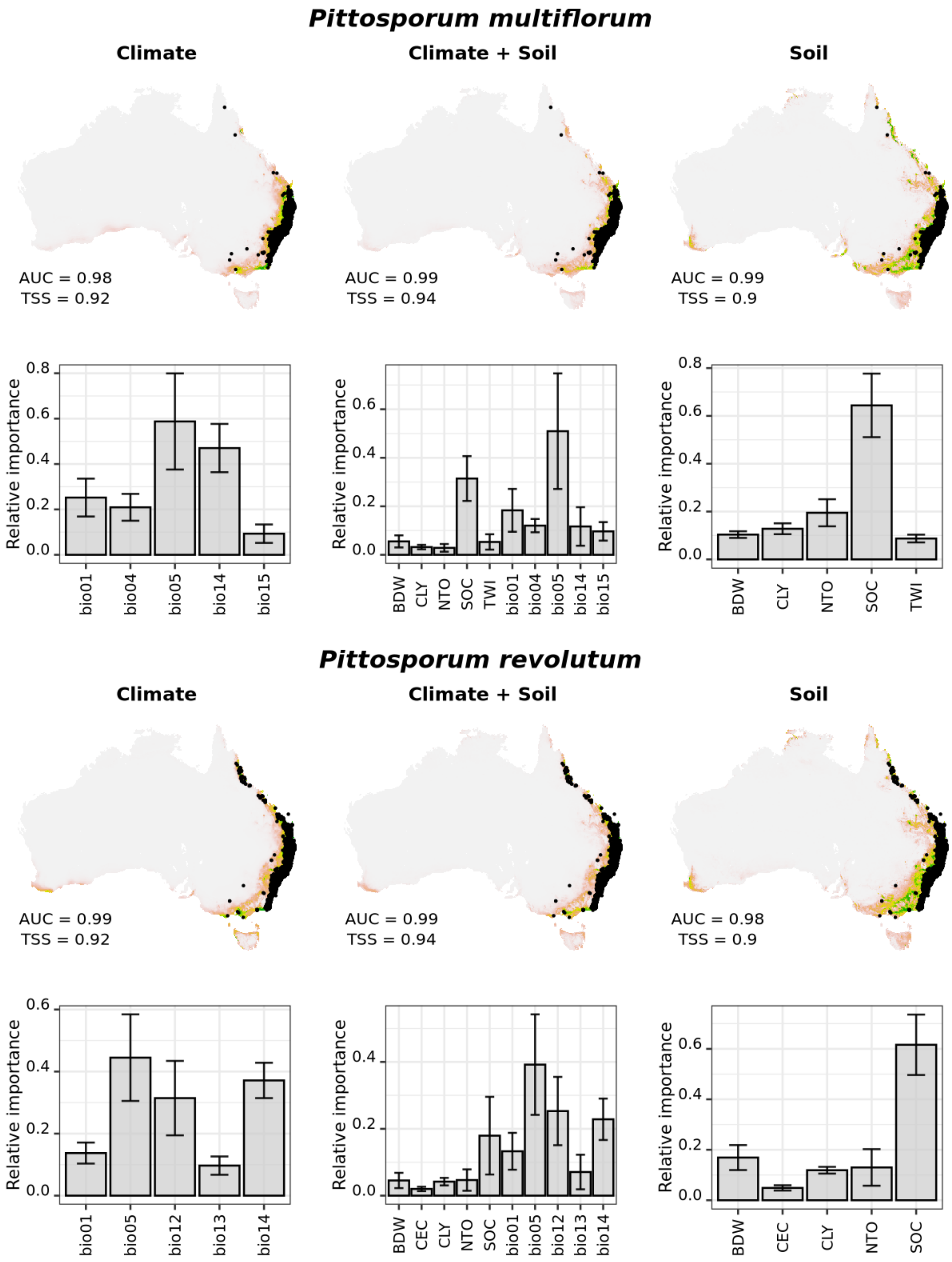

Pittosporum multiflorum
Climate
Climate + Soil
Soil
AUC = 0.98
TSS = 0.92
AUC = 0.99
TSS = 0.94
AUC = 0.99
TSS = 0.9
Relative importance
0.0
0.2
0.4
0.6
0.8
bio01
bio04
bio05
bio14
bio15
BDW
CLY
NTO
SOC
TWI
bio01
bio04
bio05
bio14
bio15
BDW
CLY
NTO
SOC
TWI
Pittosporum revolutum
Climate
Climate + Soil
Soil
AUC = 0.99
TSS = 0.92
AUC = 0.99
TSS = 0.94
AUC = 0.98
TSS = 0.9
Relative importance
0.0
0.2
0.4
0.6
bio01
bio05
bio12
bio13
bio14
BDW
CEC
CLY
NTO
SOC
bio01
bio05
bio12
bio13
bio14
BDW
CEC
CLY
NTO
SOC

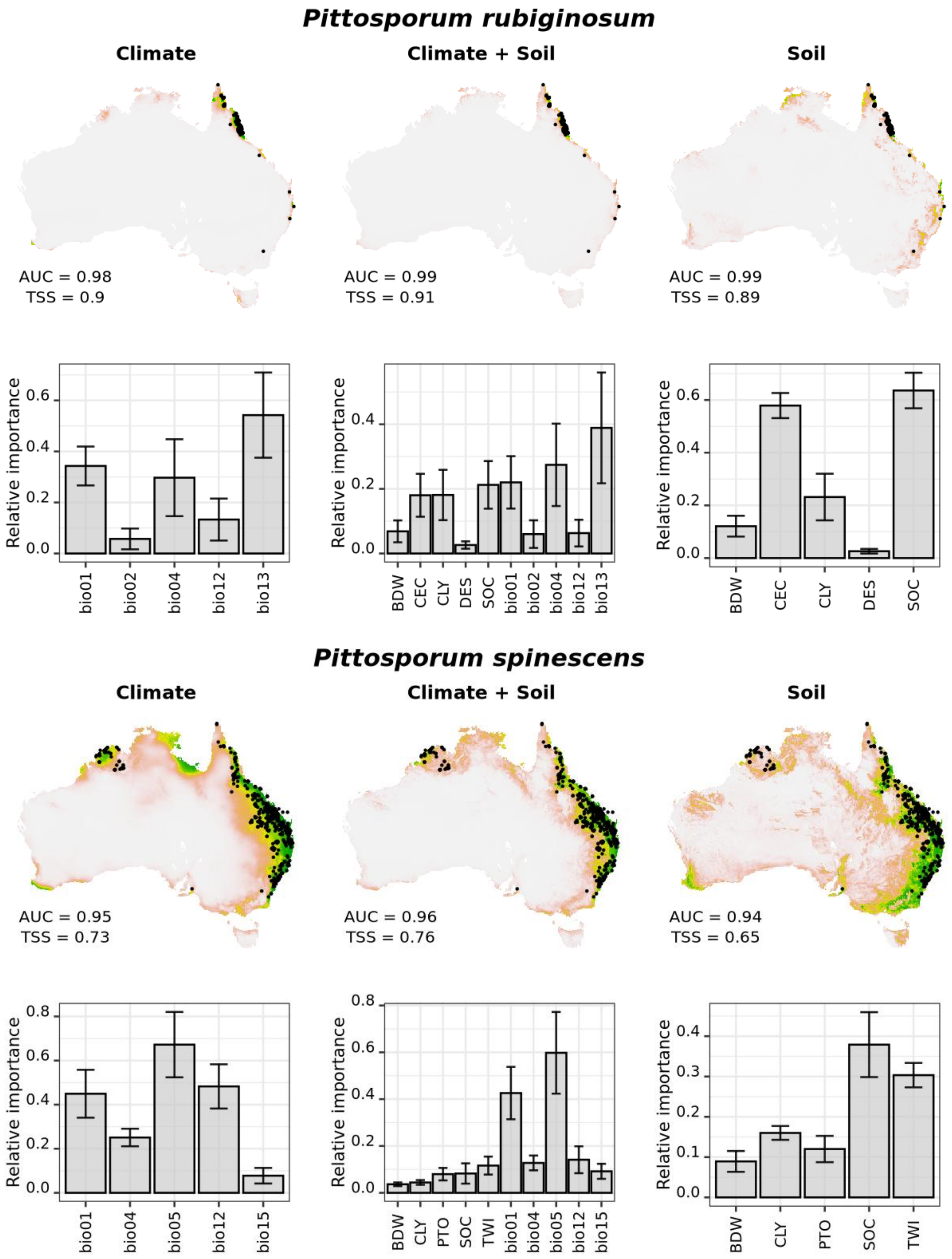
Pittosporum rubiginosum
Climate
Climate + Soil
Soil
AUC = 0.98
TSS = 0.9
AUC = 0.99
TSS = 0.91
AUC = 0.99
TSS = 0.89
Relative importance
bio01
bio02
bio04
bio12
bio13
BDW
CEC
CLY
DES
SOC
bio01
bio02
bio04
bio12
bio13
BDW
CEC
CLY
DES
SOC
Pittosporum spinescens
Climate
Climate + Soil
Soil
AUC = 0.95
TSS = 0.73
AUC = 0.96
TSS = 0.76
AUC = 0.94
TSS = 0.65
Relative importance
bio01
bio04
bio05
bio12
bio15
BDW
CLY
PTO
SOC
TWI
bio01
bio04
bio05
bio12
bio15
BDW
CLY
PTO
SOC
TWI

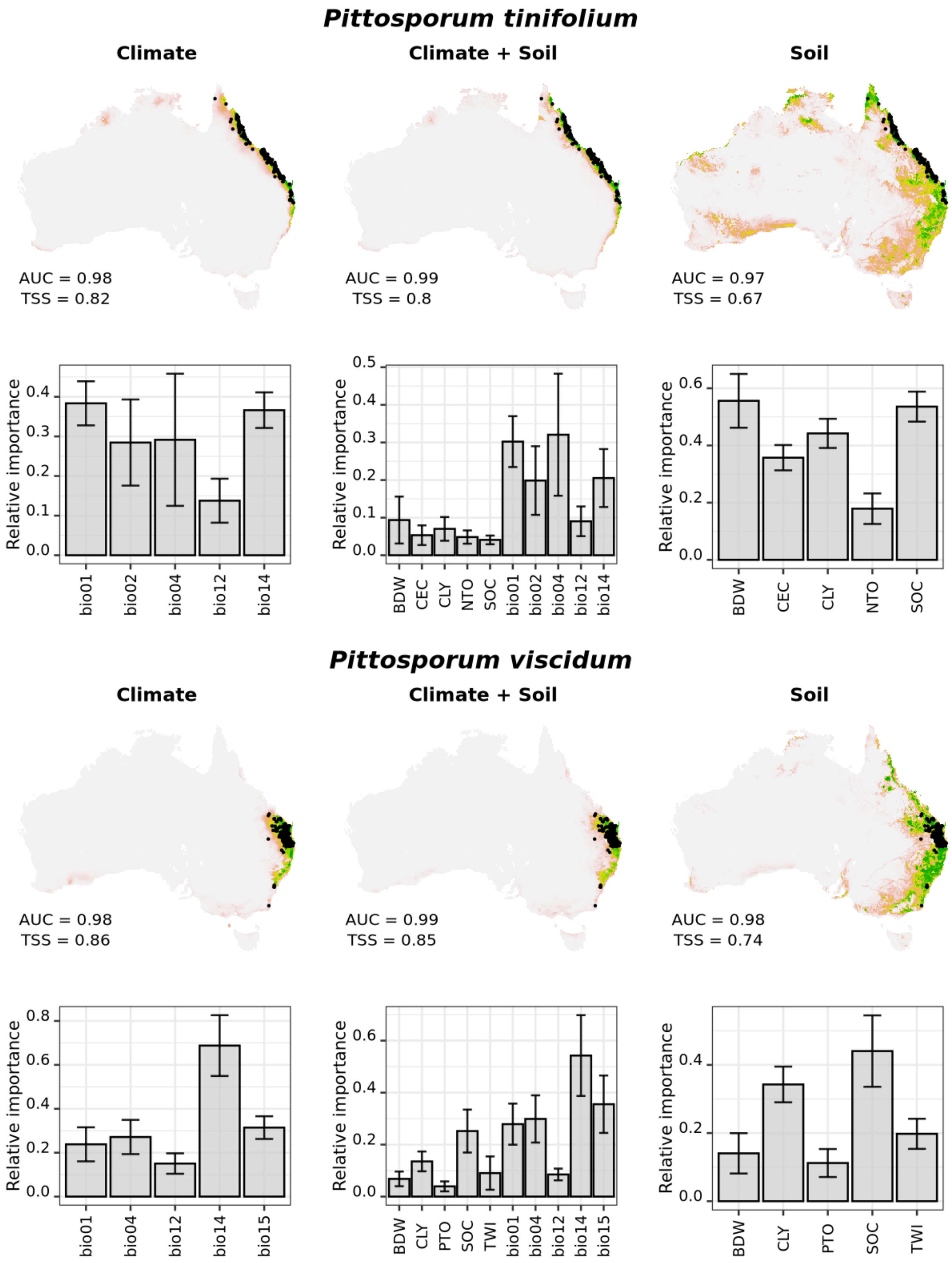
Pittosporum tinifolium
Climate
Climate + Soil
Soil
AUC = 0.98
TSS = 0.82
AUC = 0.99
TSS = 0.8
AUC = 0.97
TSS = 0.67
Relative importance
bio01
bio02
bio04
bio12
bio14
BDW
CEC
CLY
NTO
SOC
Pittosporum viscidum
Climate
Climate + Soil
Soil
AUC = 0.98
TSS = 0.86
AUC = 0.99
TSS = 0.85
AUC = 0.98
TSS = 0.74
Relative importance
bio01
bio04
bio12
bio14
bio15
BDW
CLY
PTO
SOC
TWI

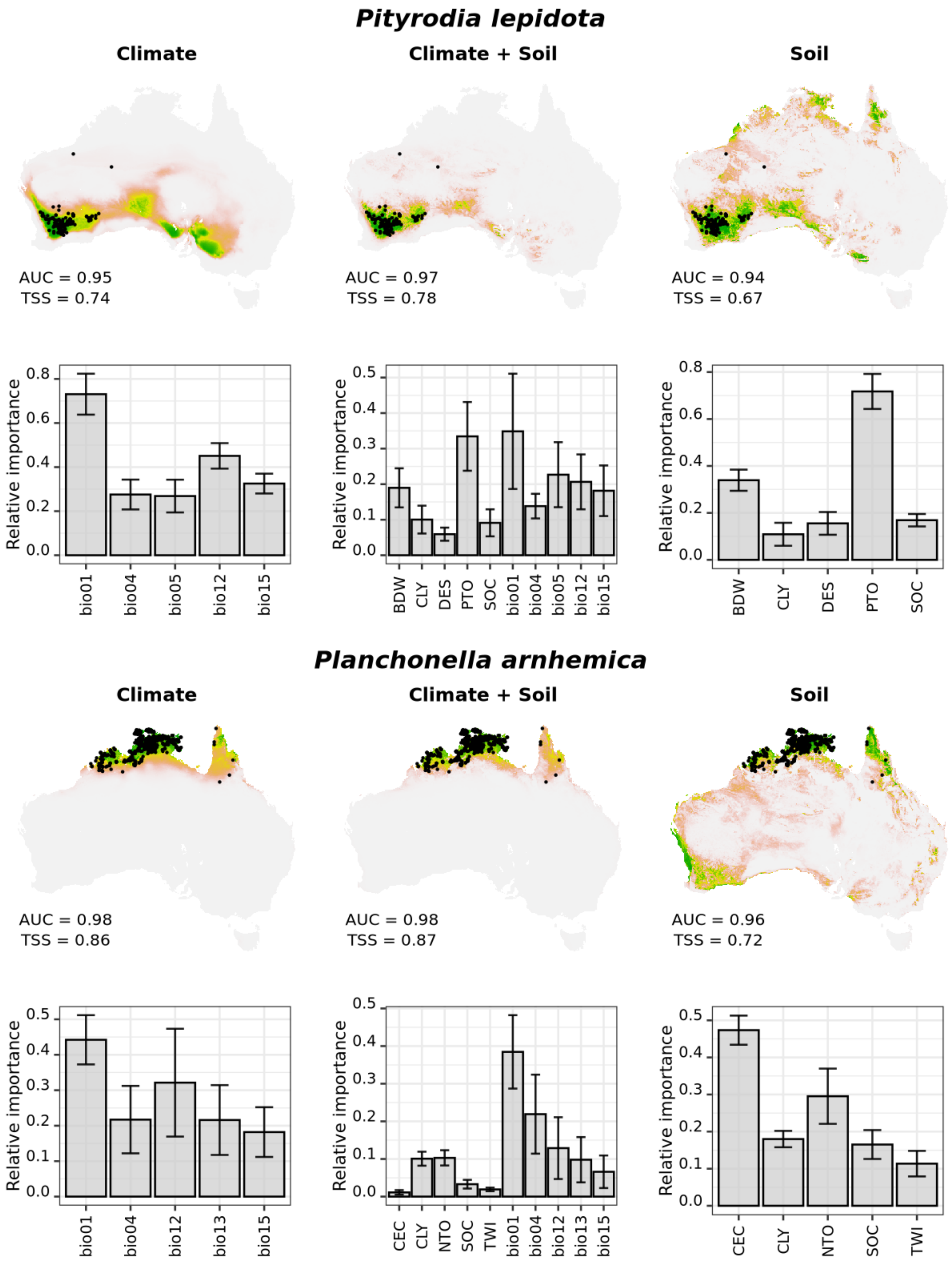
Pityrodia lepidota
Climate
Climate + Soil
Soil
AUC = 0.95
TSS = 0.74
AUC = 0.97
TSS = 0.78
AUC = 0.94
TSS = 0.67
Relative importance
bio01
bio04
bio05
bio12
bio15
BDW
CLY
DES
PTO
SOC
Planchonella arnhemica
AUC = 0.98
TSS = 0.86
AUC = 0.98
TSS = 0.87
AUC = 0.96
TSS = 0.72
bio13
CEC
NTO
TWI

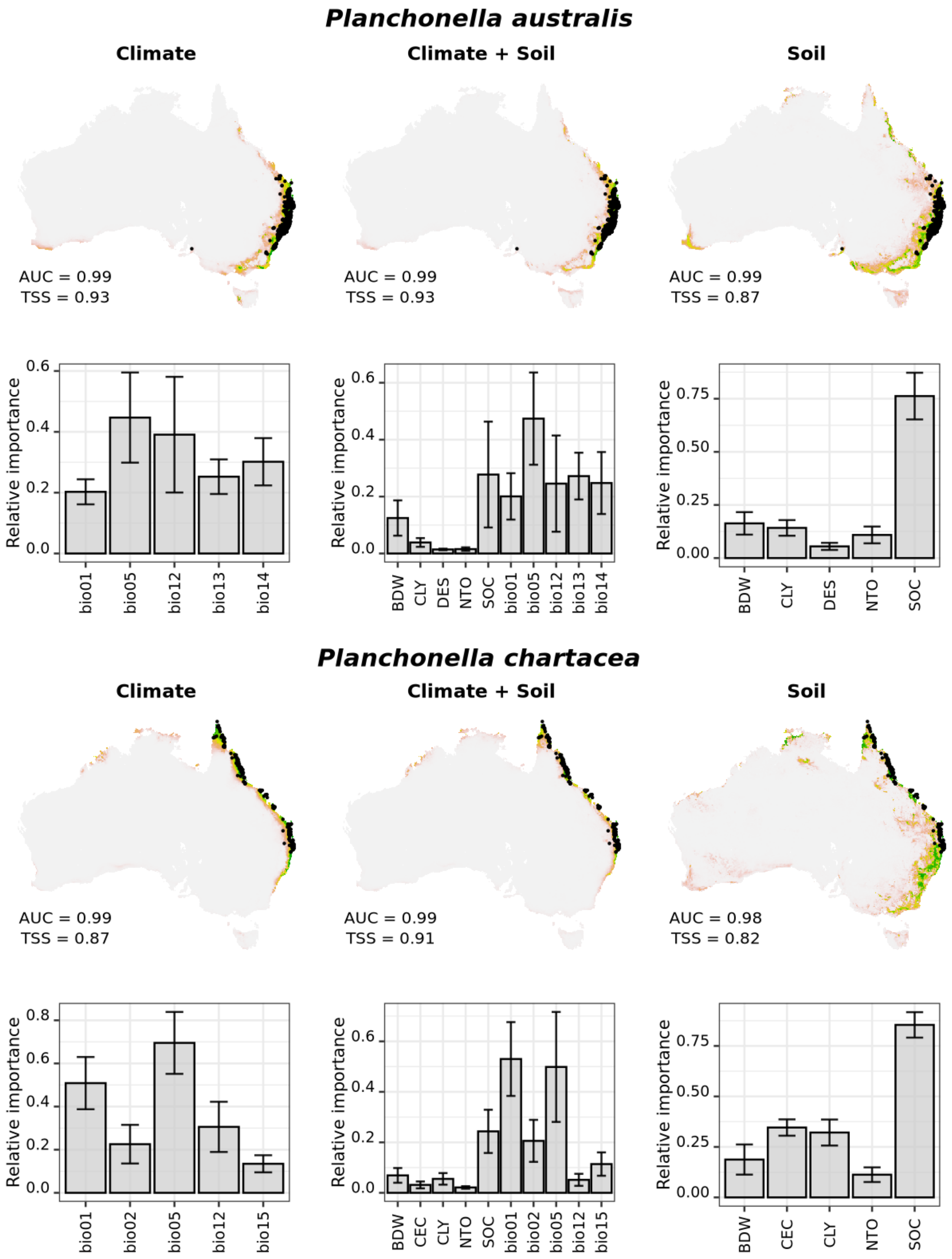

Planchonella australis
Climate
Climate + Soil
Soil
AUC = 0.99
TSS = 0.93
AUC = 0.99
TSS = 0.93
AUC = 0.99
TSS = 0.87
Relative importance
0.0
0.2
0.4
0.6
bio01
bio05
bio12
bio13
bio14
BDW
CLY
DES
NTO
SOC
0.00
0.25
0.50
0.75
Planchonella chartacea
Climate
Climate + Soil
Soil
AUC = 0.99
TSS = 0.87
AUC = 0.99
TSS = 0.91
AUC = 0.98
TSS = 0.82
0.8
bio02
bio15
CEC

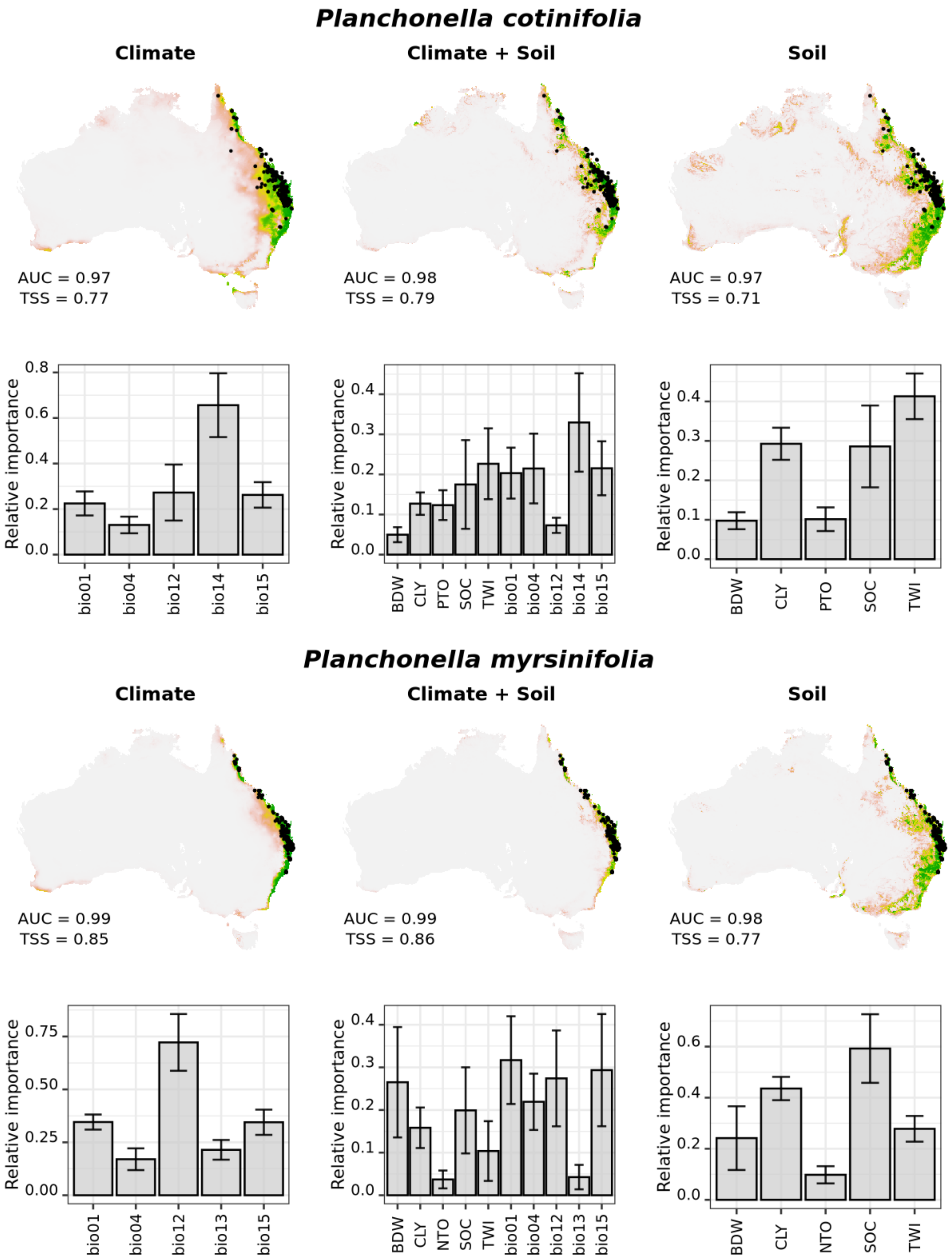
Planchonella cotinifolia
Climate
Climate + Soil
Soil
AUC = 0.97
TSS = 0.77
AUC = 0.98
TSS = 0.79
AUC = 0.97
TSS = 0.71
Relative importance
bio01 bio04 bio12 bio14 bio15
BDW CLY PTO SOC TWI bio01 bio04 bio12 bio14 bio15
BDW CLY PTO SOC TWI
Planchonella myrsinifolia
Climate
Climate + Soil
Soil
AUC = 0.99
TSS = 0.85
AUC = 0.99
TSS = 0.86
AUC = 0.98
TSS = 0.77
Relative importance
bio01 bio04 bio12 bio13 bio15
BDW CLY NTO SOC TWI bio01 bio04 bio12 bio13 bio15
BDW CLY NTO SOC TWI

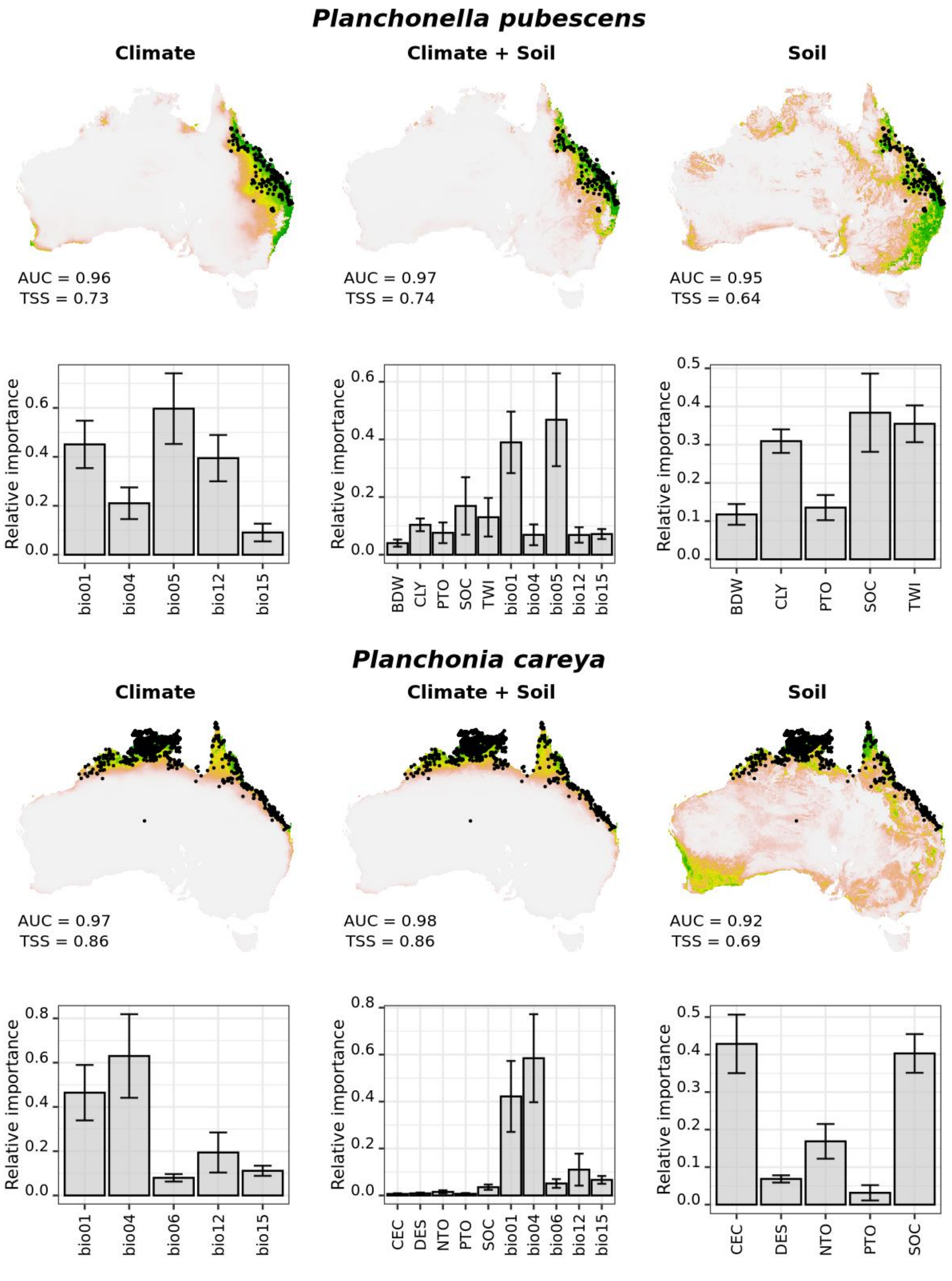
Planchonella pubescens
Climate
Climate + Soil
Soil
AUC = 0.96
TSS = 0.73
AUC = 0.97
TSS = 0.74
AUC = 0.95
TSS = 0.64
Relative importance
bio01 bio04 bio05 bio12 bio15
BDW CLY PTO SOC TWI bio01 bio04 bio05 bio12 bio15
BDW CLY PTO SOC TWI
Planchonia careya
Climate
Climate + Soil
Soil
AUC = 0.97
TSS = 0.86
AUC = 0.98
TSS = 0.86
AUC = 0.92
TSS = 0.69
Relative importance
bio01 bio04 bio06 bio12 bio15
CEC DES NTO PTO SOC bio01 bio04 bio06 bio12 bio15
CEC DES NTO PTO SOC

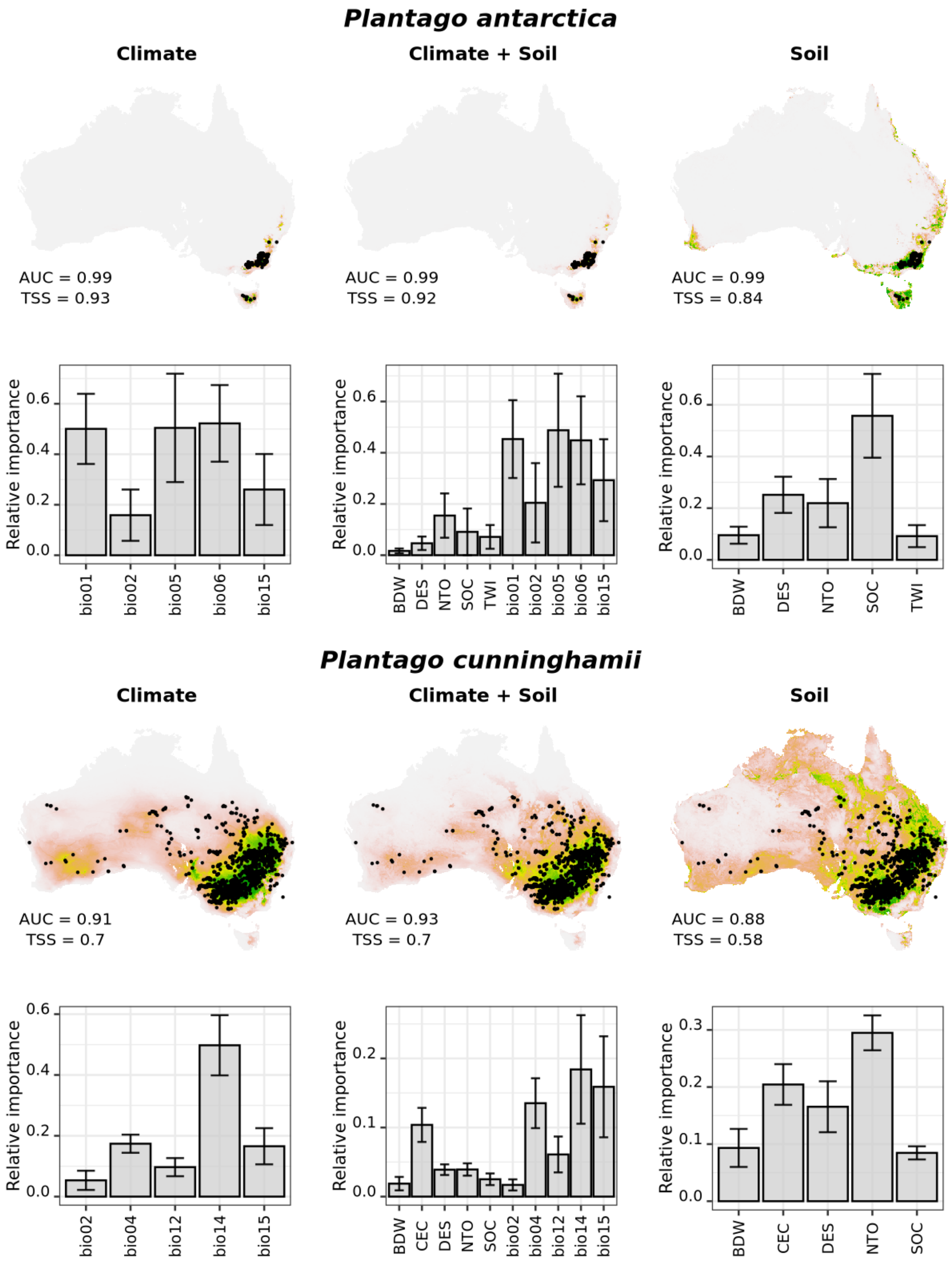
Plantago antarctica
Climate
Climate + Soil
Soil
AUC = 0.99
TSS = 0.93
AUC = 0.99
TSS = 0.92
AUC = 0.99
TSS = 0.84
Relative importance
bio01
bio02
bio05
bio06
bio15
BDW
DES
NTO
SOC
TWI
Plantago cunninghamii
Climate
Climate + Soil
Soil
AUC = 0.91
TSS = 0.7
AUC = 0.93
TSS = 0.7
AUC = 0.88
TSS = 0.58
bio02
bio04
bio12
bio14
bio15
BDW
CEC
DES
NTO
SOC

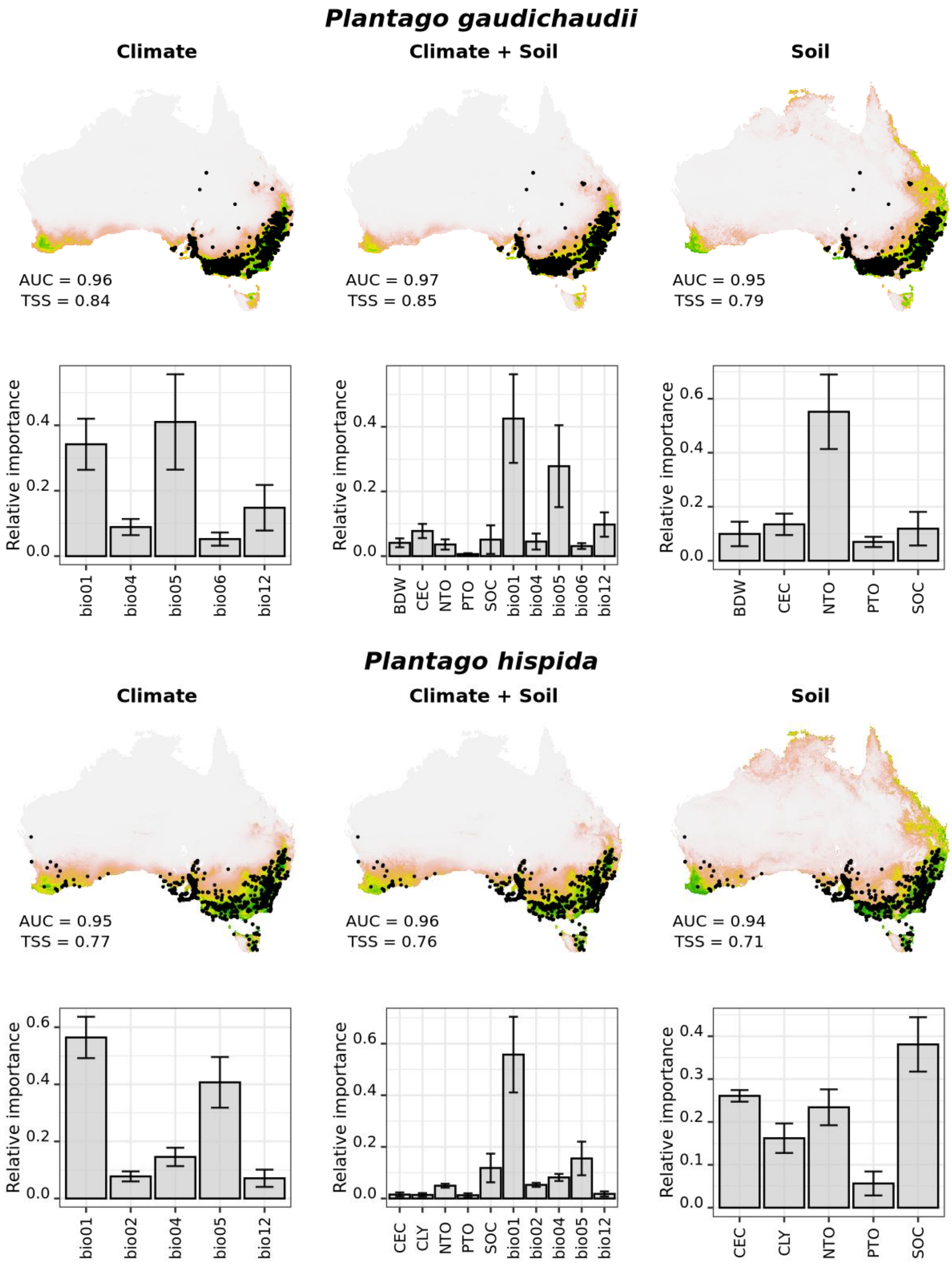

Plantago gaudichaudii
Climate
Climate + Soil
Soil
AUC = 0.96
TSS = 0.84
AUC = 0.97
TSS = 0.85
AUC = 0.95
TSS = 0.79
Relative importance
0.0
0.2
0.4
0.6
bio01
bio04
bio05
bio06
bio12
BDW
CEC
NTO
PTO
SOC
Plantago hispida
AUC = 0.95
TSS = 0.77
AUC = 0.96
TSS = 0.76
AUC = 0.94
TSS = 0.71
0.1
0.3
bio02
CLY

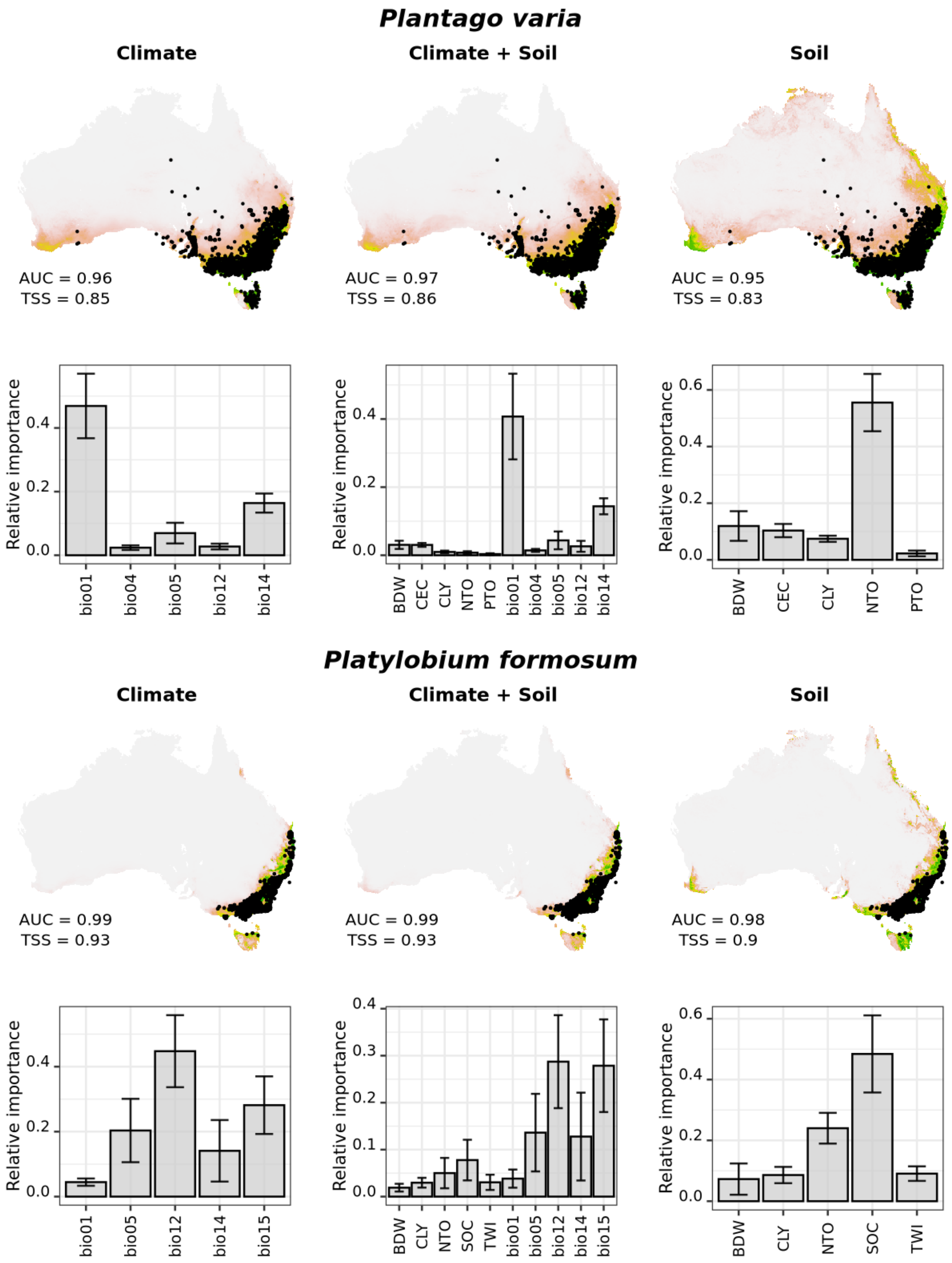
Plantago varia
Climate
Climate + Soil
Soil
AUC = 0.96
TSS = 0.85
AUC = 0.97
TSS = 0.86
AUC = 0.95
TSS = 0.83
Relative importance
bio01
bio04
bio05
bio12
bio14
BDW
CEC
CLY
NTO
PTO
Platylobium formosum
Climate
Climate + Soil
Soil
AUC = 0.99
TSS = 0.93
AUC = 0.99
TSS = 0.93
AUC = 0.98
TSS = 0.9
Relative importance
bio01
bio05
bio12
bio14
bio15
BDW
CLY
NTO
SOC
TWI

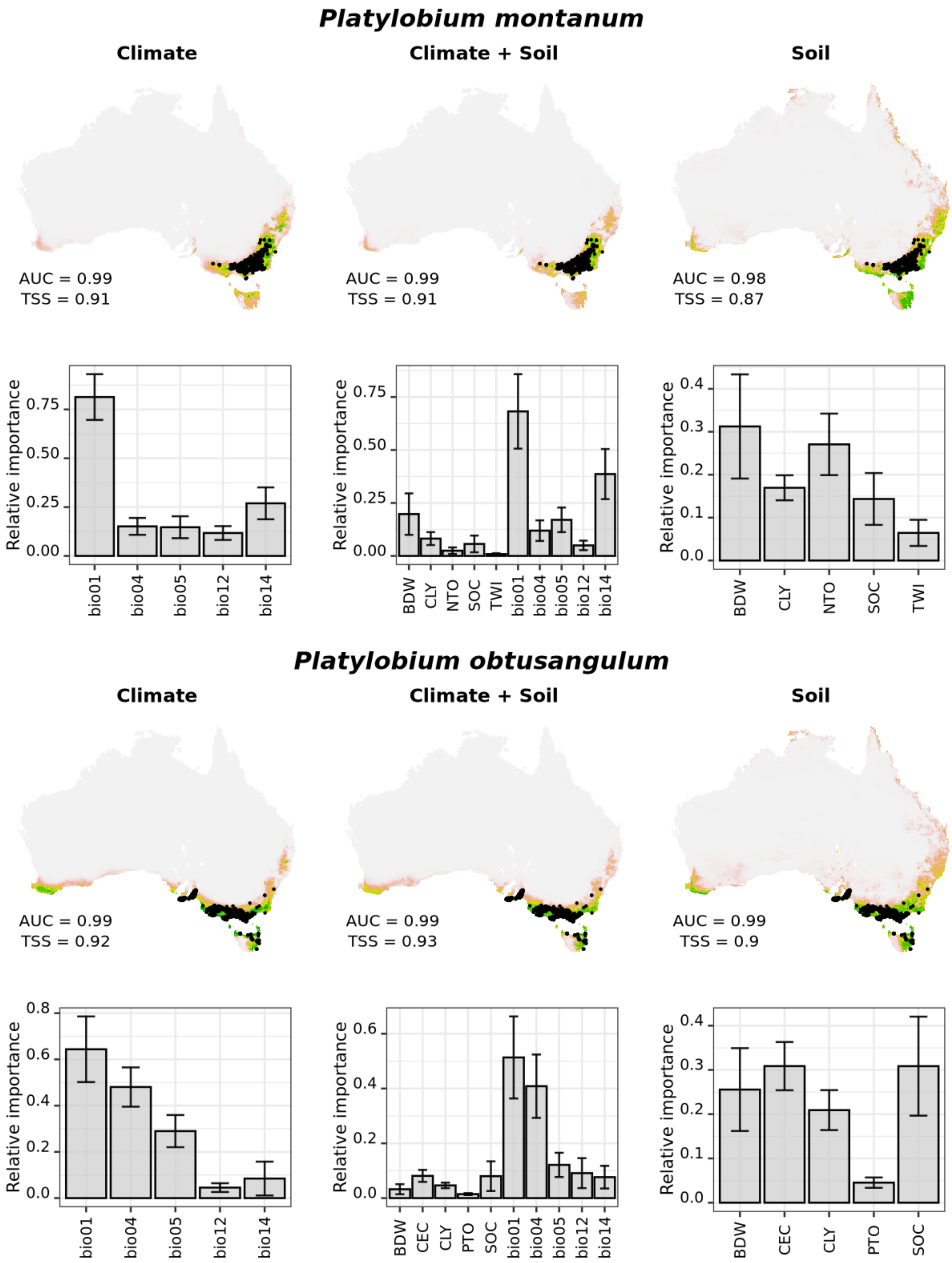
Platylobium montanum
Climate
Climate + Soil
Soil
AUC = 0.99
TSS = 0.91
AUC = 0.99
TSS = 0.91
AUC = 0.98
TSS = 0.87
Relative importance
0.00
0.25
0.50
0.75
bio01
bio04
bio05
bio12
bio14
BDW
CLY
NTO
SOC
TWI
0.0
0.1
0.2
0.3
0.4
Platylobium obtusangulum
Climate
Climate + Soil
Soil
AUC = 0.99
TSS = 0.92
AUC = 0.99
TSS = 0.93
AUC = 0.99
TSS = 0.9
Relative importance
0.0
0.2
0.4
0.6
0.8
BDW
CEC
CLY
PTO
SOC
bio01
bio04
bio05
bio12
bio14

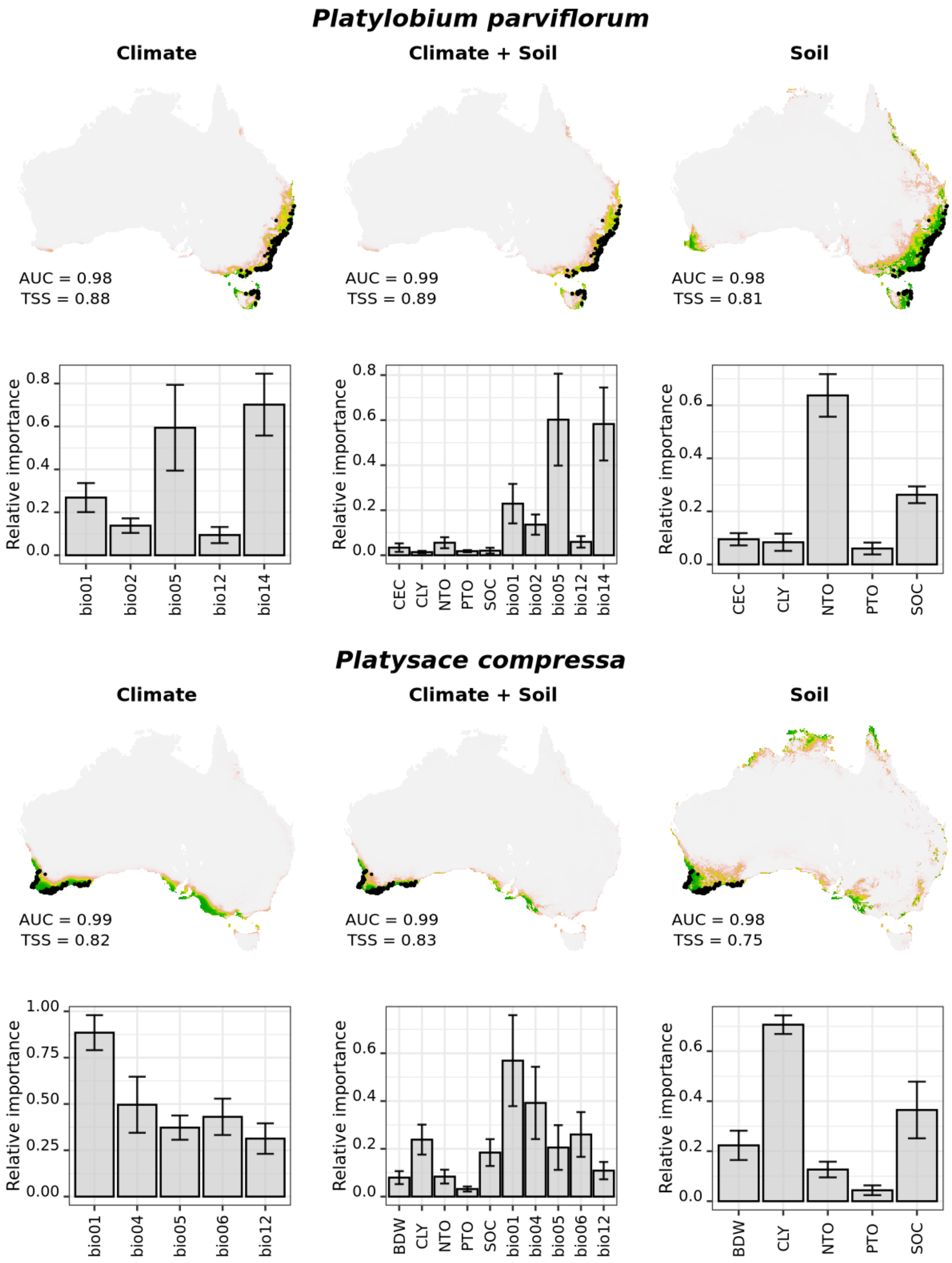

Platylobium parviflorum
Climate
Climate + Soil
Soil
AUC = 0.98
TSS = 0.88
AUC = 0.99
TSS = 0.89
AUC = 0.98
TSS = 0.81
Relative importance
bio01 bio02 bio05 bio12 bio14
CEC CLY NTO PTO SOC bio01 bio02 bio05 bio12 bio14
CEC CLY NTO PTO SOC
Platysace compressa
Climate
Climate + Soil
Soil
AUC = 0.99
TSS = 0.82
AUC = 0.99
TSS = 0.83
AUC = 0.98
TSS = 0.75
Relative importance
bio01 bio04 bio05 bio06 bio12
BDW CLY NTO PTO SOC bio01 bio04 bio05 bio06 bio12
BDW CLY NTO PTO SOC

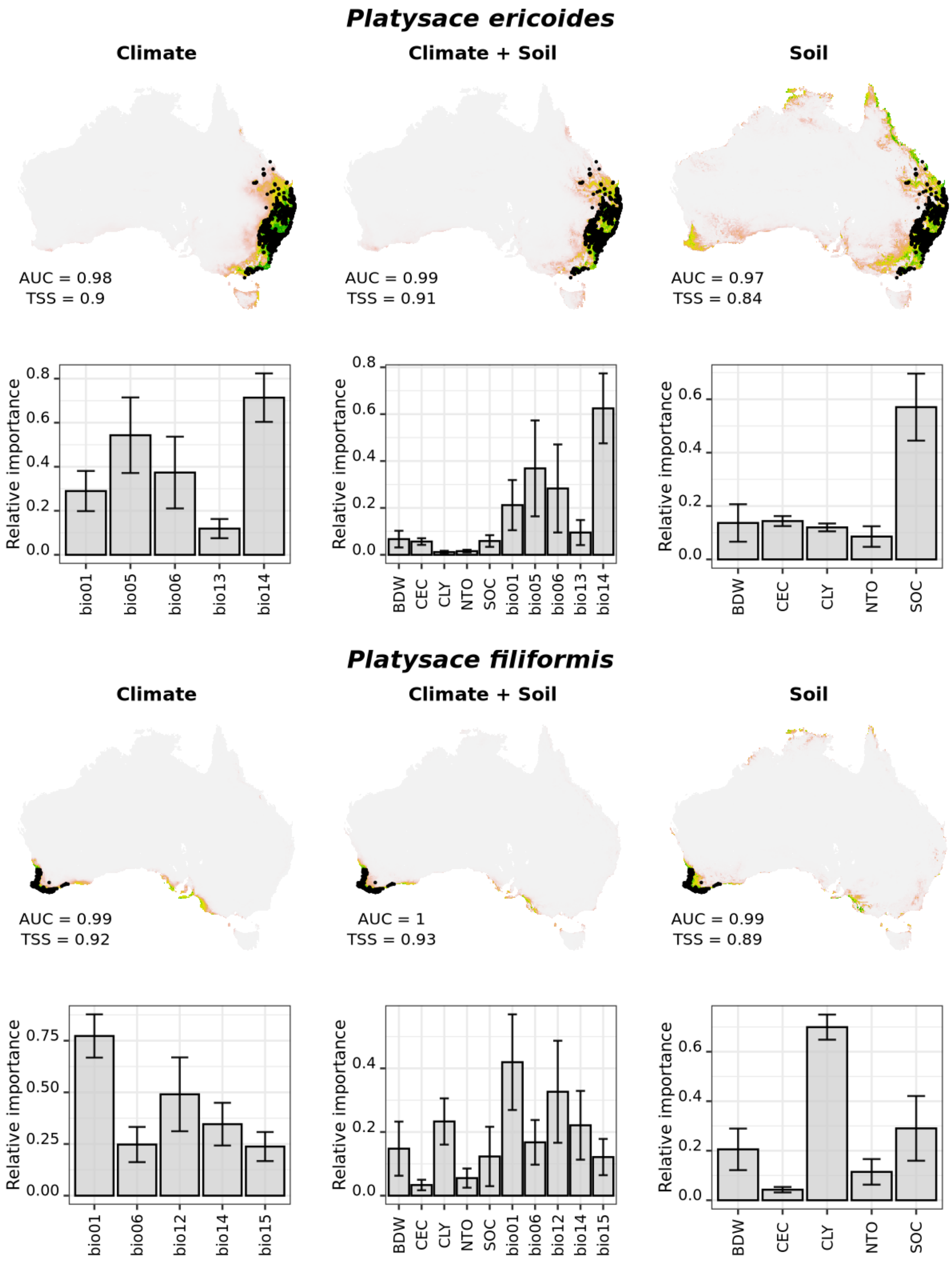
Platysace ericoides
Climate
Climate + Soil
Soil
AUC = 0.98
TSS = 0.9
AUC = 0.99
TSS = 0.91
AUC = 0.97
TSS = 0.84
Relative importance
bio01
bio05
bio06
bio13
bio14
BDW
CEC
CLY
NTO
SOC
Platysace filiformis
Climate
Climate + Soil
Soil
AUC = 0.99
TSS = 0.92
AUC = 1
TSS = 0.93
AUC = 0.99
TSS = 0.89
Relative importance
bio01
bio06
bio12
bio14
bio15
BDW
CEC
CLY
NTO
SOC

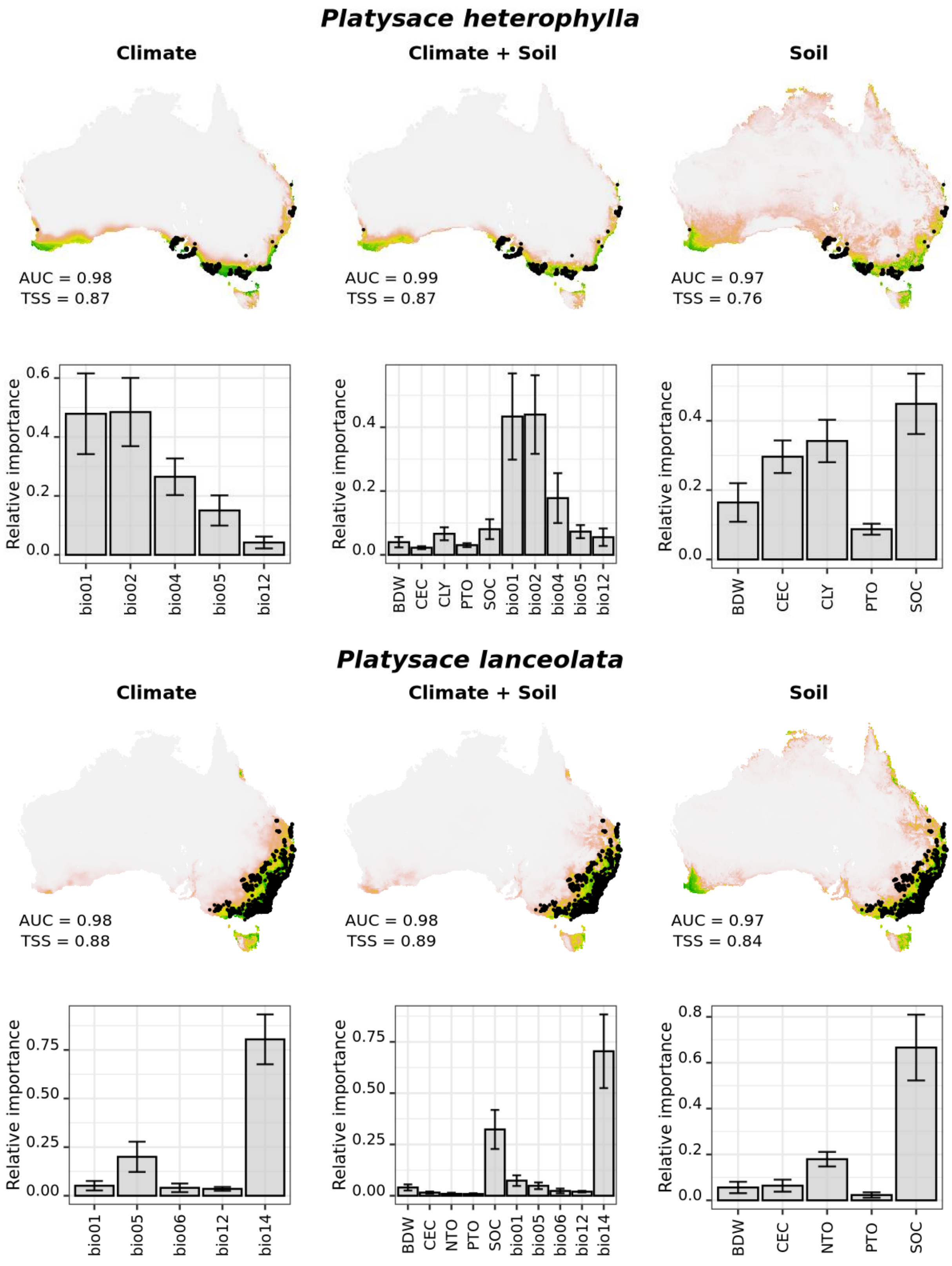
Platysace heterophylla
Climate
Climate + Soil
Soil
AUC = 0.98
TSS = 0.87
AUC = 0.99
TSS = 0.87
AUC = 0.97
TSS = 0.76
Relative importance
0.0
0.2
0.4
0.6
bio01
bio02
bio04
bio05
bio12
BDW
CEC
CLY
PTO
SOC
Platysace lanceolata
Climate
Climate + Soil
Soil
AUC = 0.98
TSS = 0.88
AUC = 0.98
TSS = 0.89
AUC = 0.97
TSS = 0.84
Relative importance
0.00
0.25
0.50
0.75
0.8
bio01
bio05
bio06
bio12
bio14
BDW
CEC
NTO
PTO
SOC

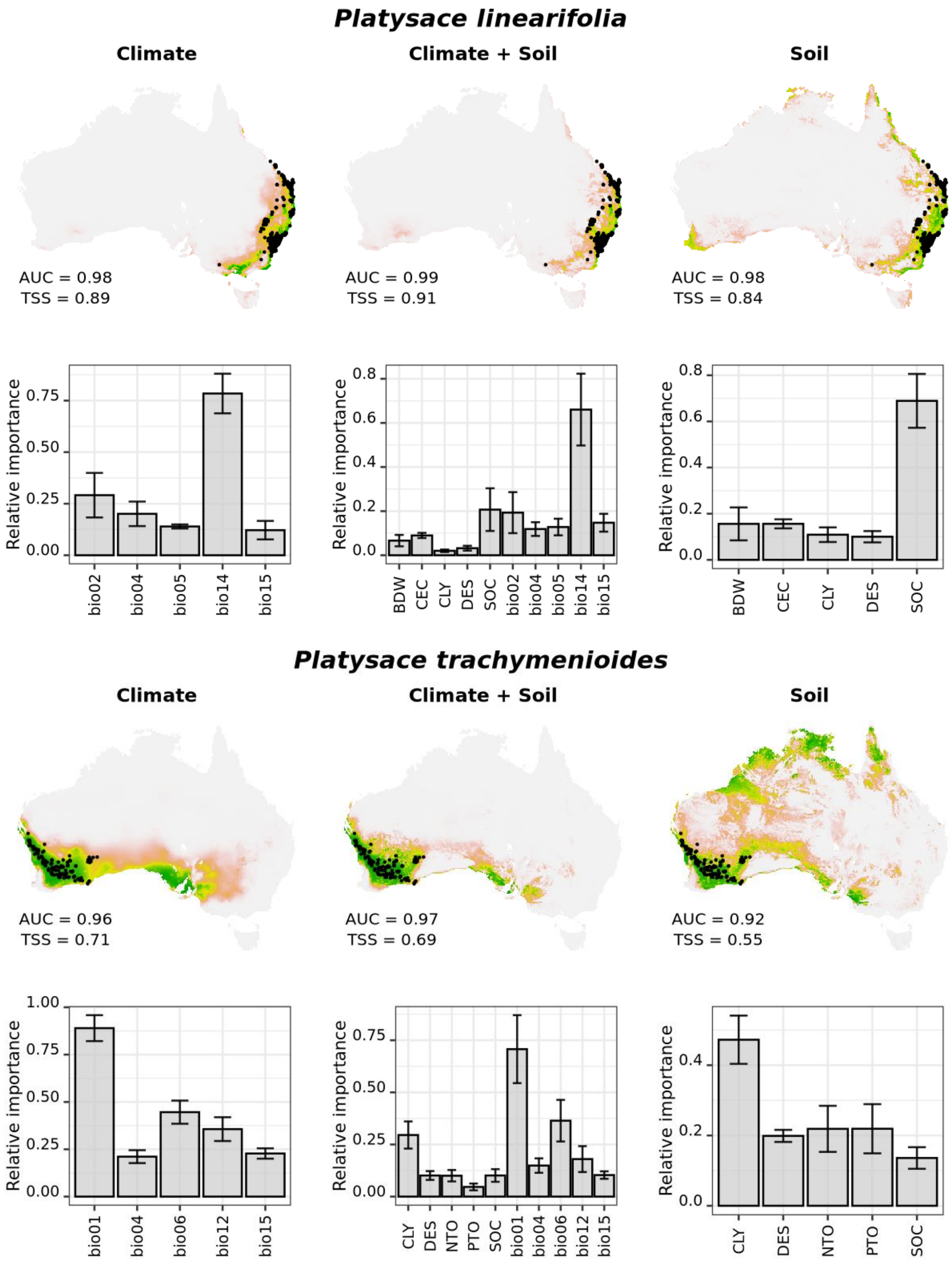

Platysace linearifolia
Climate
Climate + Soil
Soil
AUC = 0.98
TSS = 0.89
AUC = 0.99
TSS = 0.91
AUC = 0.98
TSS = 0.84
Relative importance
bio02
bio04
bio05
bio14
bio15
BDW
CEC
CLY
DES
SOC
Platysace trachymenioides
AUC = 0.96
TSS = 0.71
AUC = 0.97
TSS = 0.69
AUC = 0.92
TSS = 0.55
bio01
bio06
bio12
NTO
PTO

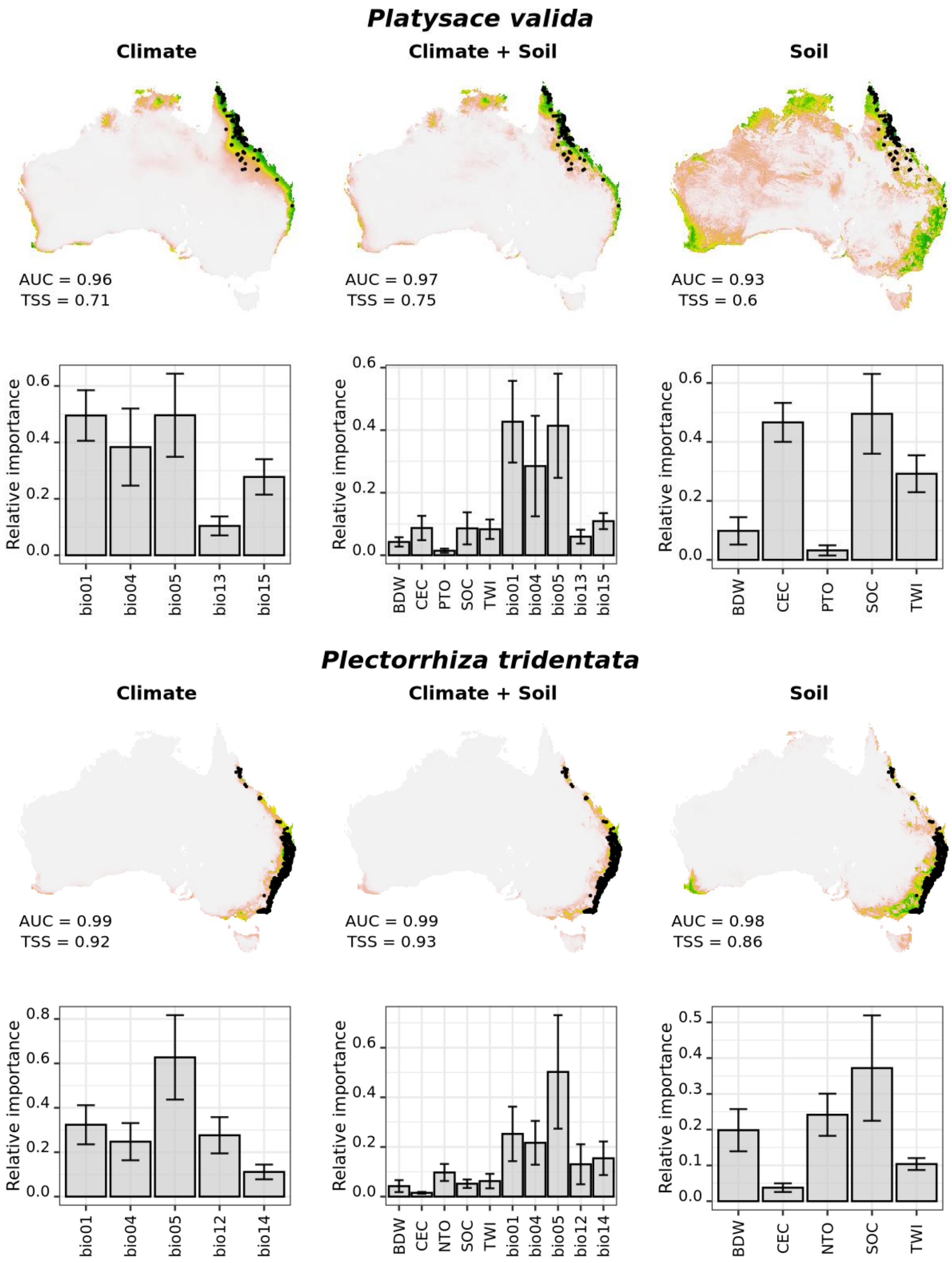

Platysace valida
Climate
Climate + Soil
Soil
AUC = 0.96
TSS = 0.71
AUC = 0.97
TSS = 0.75
AUC = 0.93
TSS = 0.6
Relative importance
bio01
bio04
bio05
bio13
bio15
BDW
CEC
PTO
SOC
TWI
Plectorrhiza tridentata
Climate
Climate + Soil
Soil
AUC = 0.99
TSS = 0.92
AUC = 0.99
TSS = 0.93
AUC = 0.98
TSS = 0.86
bio01
bio04
bio05
bio12
bio14
BDW
CEC
NTO
SOC
TWI

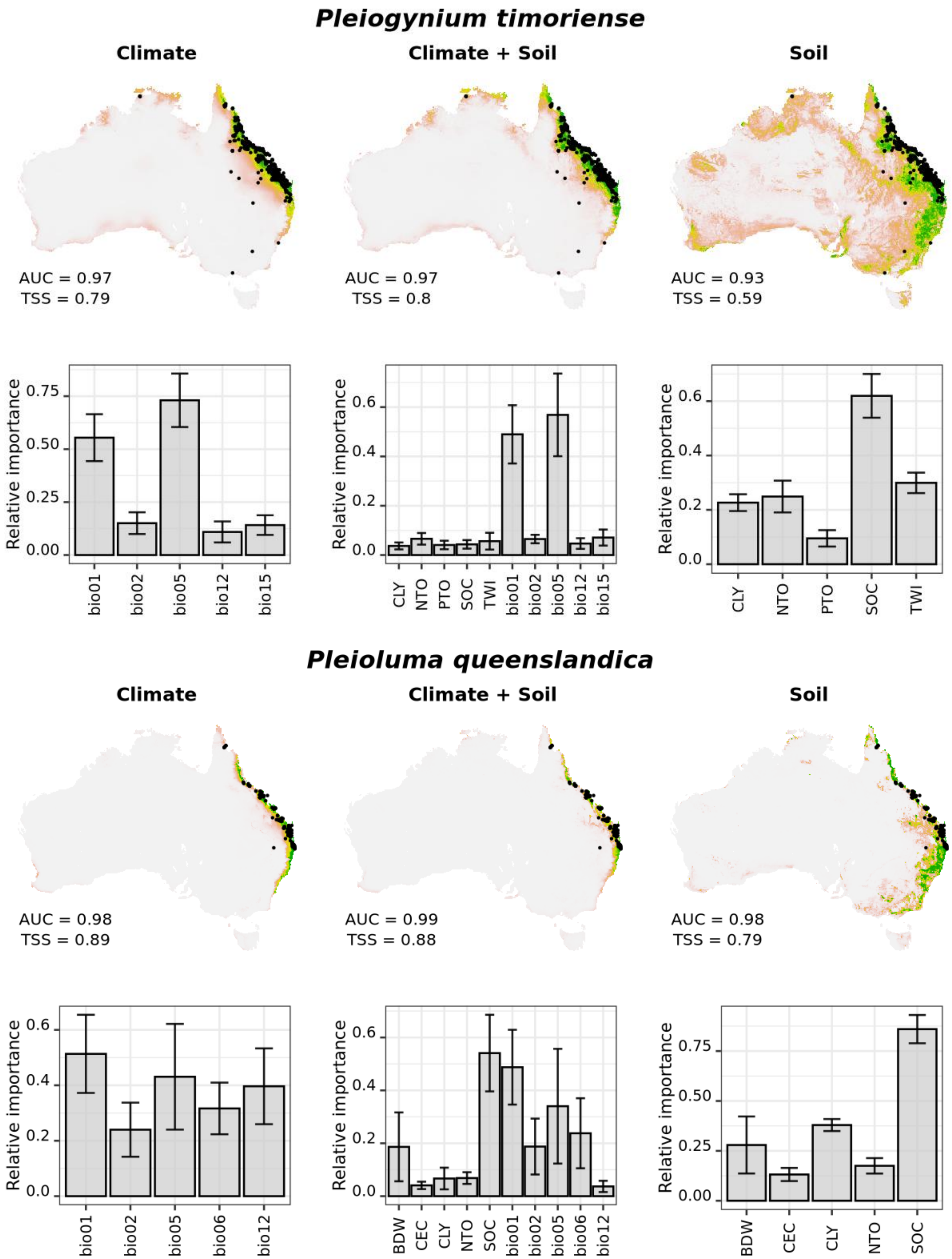
Pleiogynium timoriense
Climate
Climate + Soil
Soil
AUC = 0.97
TSS = 0.79
AUC = 0.97
TSS = 0.8
AUC = 0.93
TSS = 0.59
Relative importance
bio01
bio02
bio05
bio12
bio15
CLY
NTO
PTO
SOC
TWI
Pleioluma queenslandica
Climate
Climate + Soil
Soil
AUC = 0.98
TSS = 0.89
AUC = 0.99
TSS = 0.88
AUC = 0.98
TSS = 0.79
Relative importance
bio01
bio02
bio05
bio06
bio12
BDW
CEC
CLY
NTO
SOC

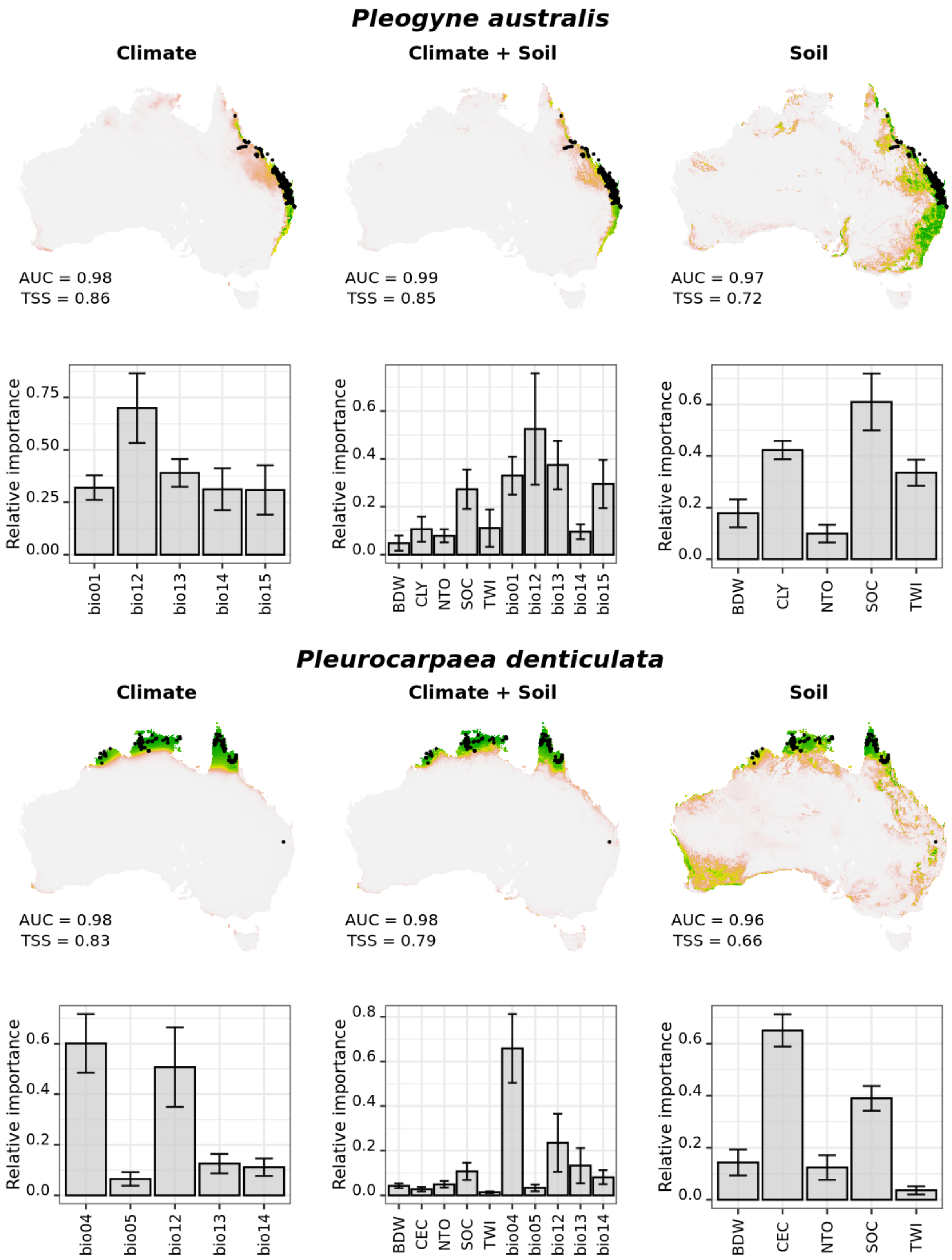
Pleogyne australis
Climate
Climate + Soil
Soil
AUC = 0.98
TSS = 0.86
AUC = 0.99
TSS = 0.85
AUC = 0.97
TSS = 0.72
Relative importance
0.00
0.25
0.50
0.75
bio01
bio12
bio13
bio14
bio15
0.0
0.2
0.4
0.6
BDW
CLY
NTO
SOC
TWI
Pleurocarpaea denticulata
AUC = 0.98
TSS = 0.83
AUC = 0.98
TSS = 0.79
AUC = 0.96
TSS = 0.66
0.8
bio04
bio05
CEC

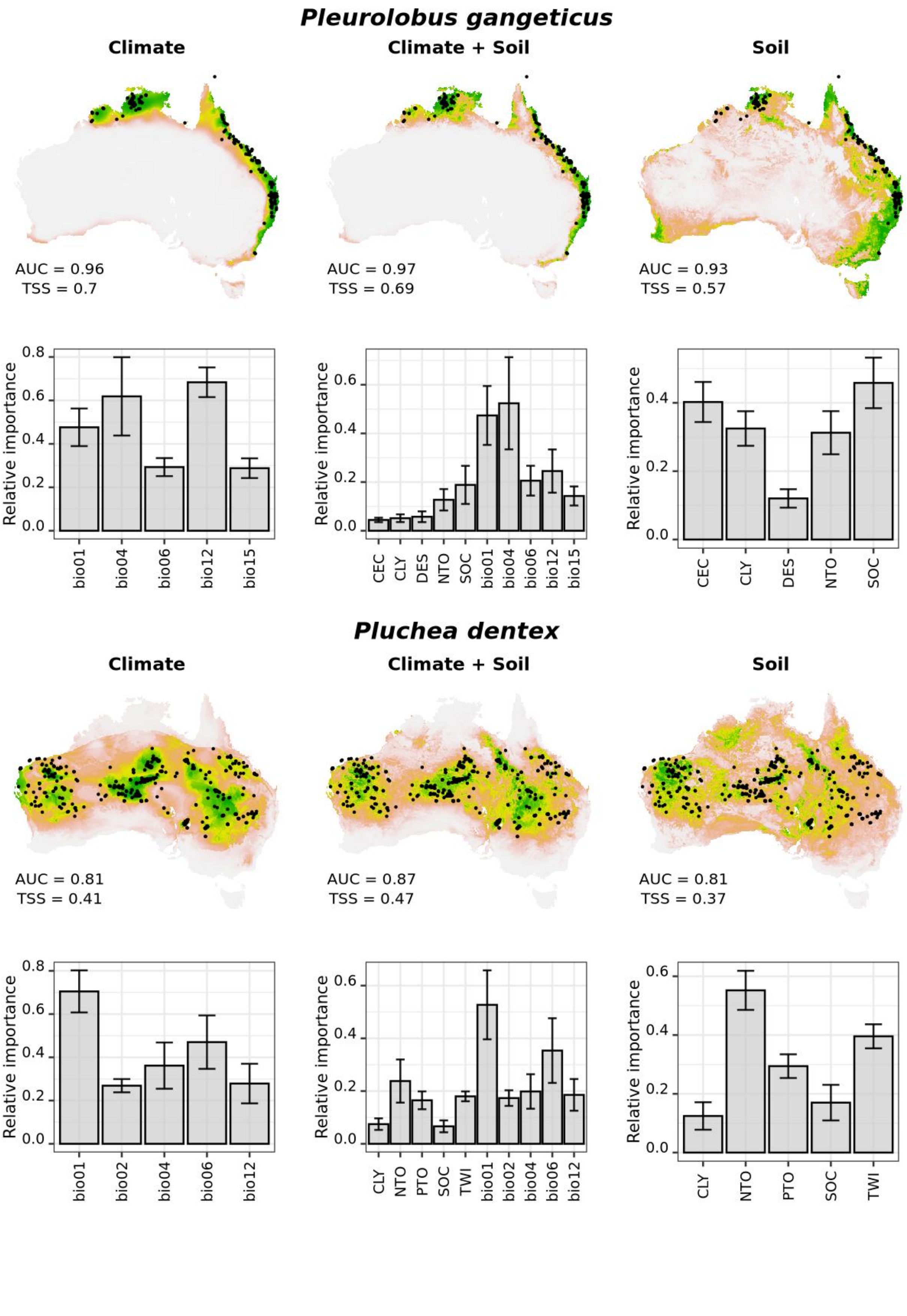
Pleurolobus gangeticus
Climate
Climate + Soil
Soil
AUC = 0.96
TSS = 0.7
AUC = 0.97
TSS = 0.69
AUC = 0.93
TSS = 0.57
Relative importance
bio01
bio04
bio06
bio12
bio15
CEC
CLY
DES
NTO
SOC
Pluchea dentex
Climate
Climate + Soil
Soil
AUC = 0.81
TSS = 0.41
AUC = 0.87
TSS = 0.47
AUC = 0.81
TSS = 0.37
Relative importance
bio01
bio02
bio04
bio06
bio12
CLY
NTO
PTO
SOC
TWI

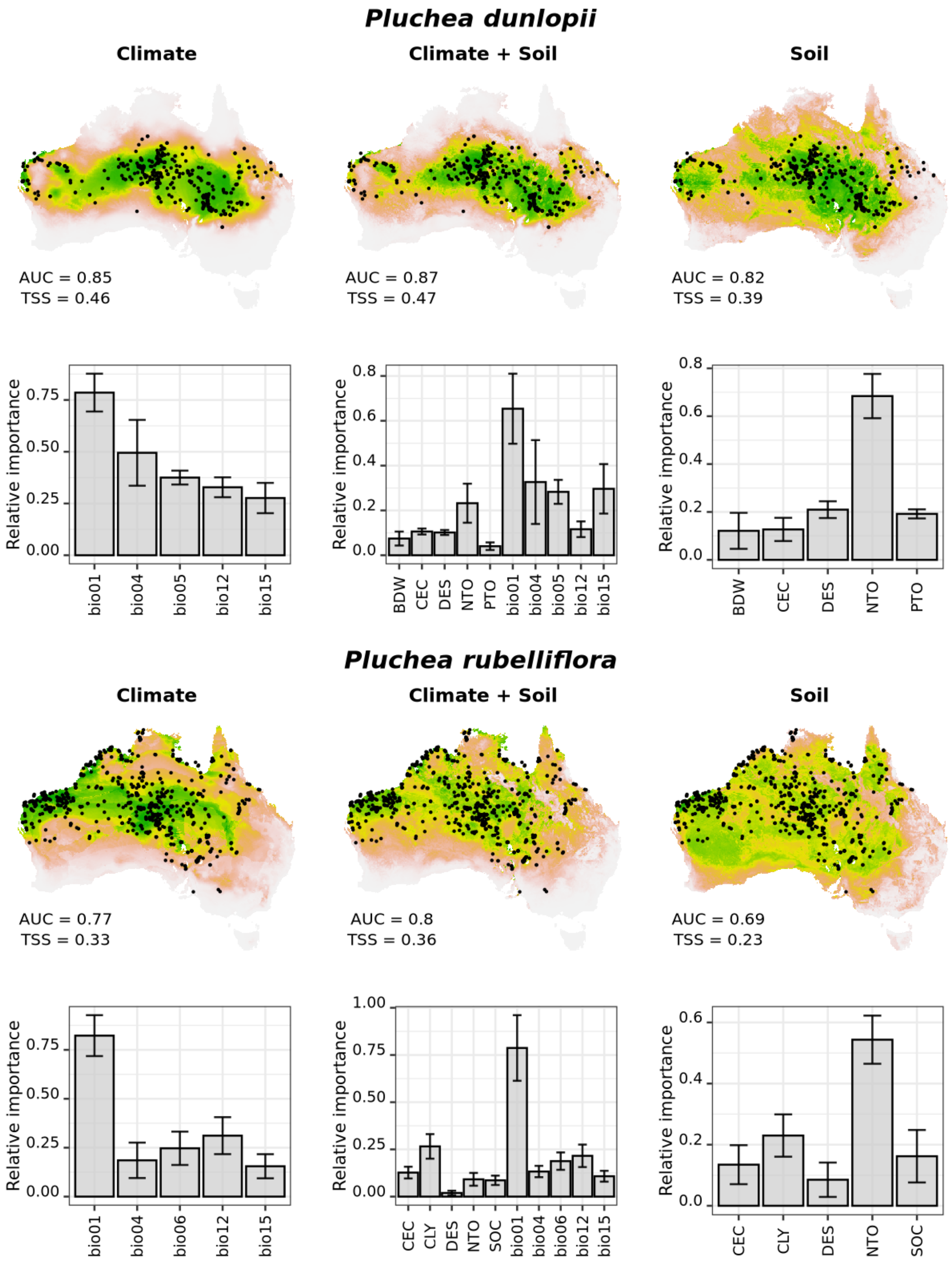

Pluchea dunlopii
Climate
Climate + Soil
Soil
AUC = 0.85
TSS = 0.46
AUC = 0.87
TSS = 0.47
AUC = 0.82
TSS = 0.39
Relative importance
bio01
bio04
bio05
bio12
bio15
BDW
CEC
DES
NTO
PTO
Pluchea rubelliflora
Climate
Climate + Soil
Soil
AUC = 0.77
TSS = 0.33
AUC = 0.8
TSS = 0.36
AUC = 0.69
TSS = 0.23
Relative importance
bio01
bio04
bio06
bio12
bio15
CEC
CLY
DES
NTO
SOC

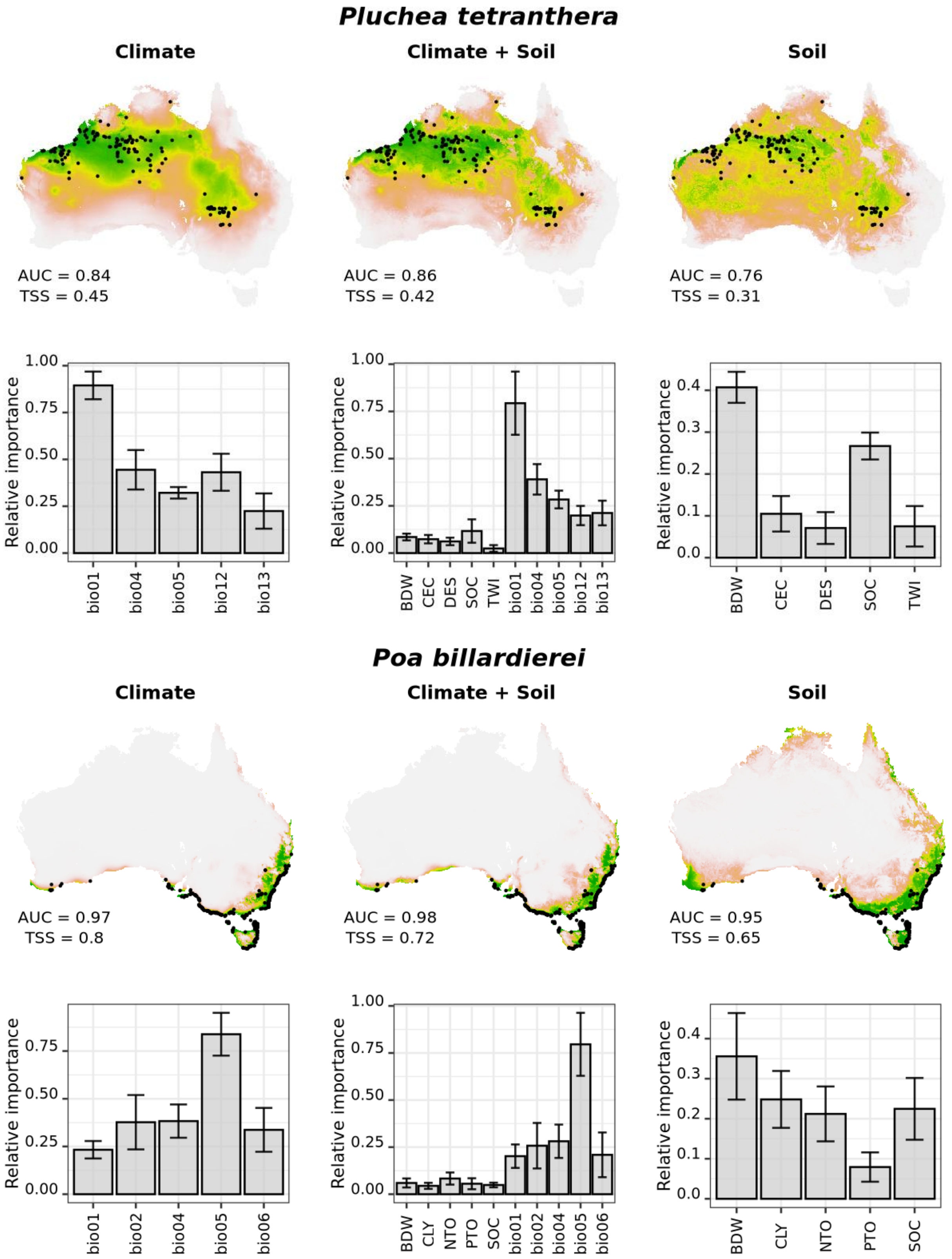

Pluchea tetranthera
Climate
Climate + Soil
Soil
AUC = 0.84
TSS = 0.45
AUC = 0.86
TSS = 0.42
AUC = 0.76
TSS = 0.31
Relative importance
1.00
0.75
0.50
0.25
0.00
bio01
bio04
bio05
bio12
bio13
BDW
CEC
DES
SOC
TWI
0.4
0.3
0.2
0.1
0.0
Poa billardierei
Climate
Climate + Soil
Soil
AUC = 0.97
TSS = 0.8
AUC = 0.98
TSS = 0.72
AUC = 0.95
TSS = 0.65
bio01
bio02
bio04
bio05
bio06
BDW
CLY
NTO
PTO
SOC

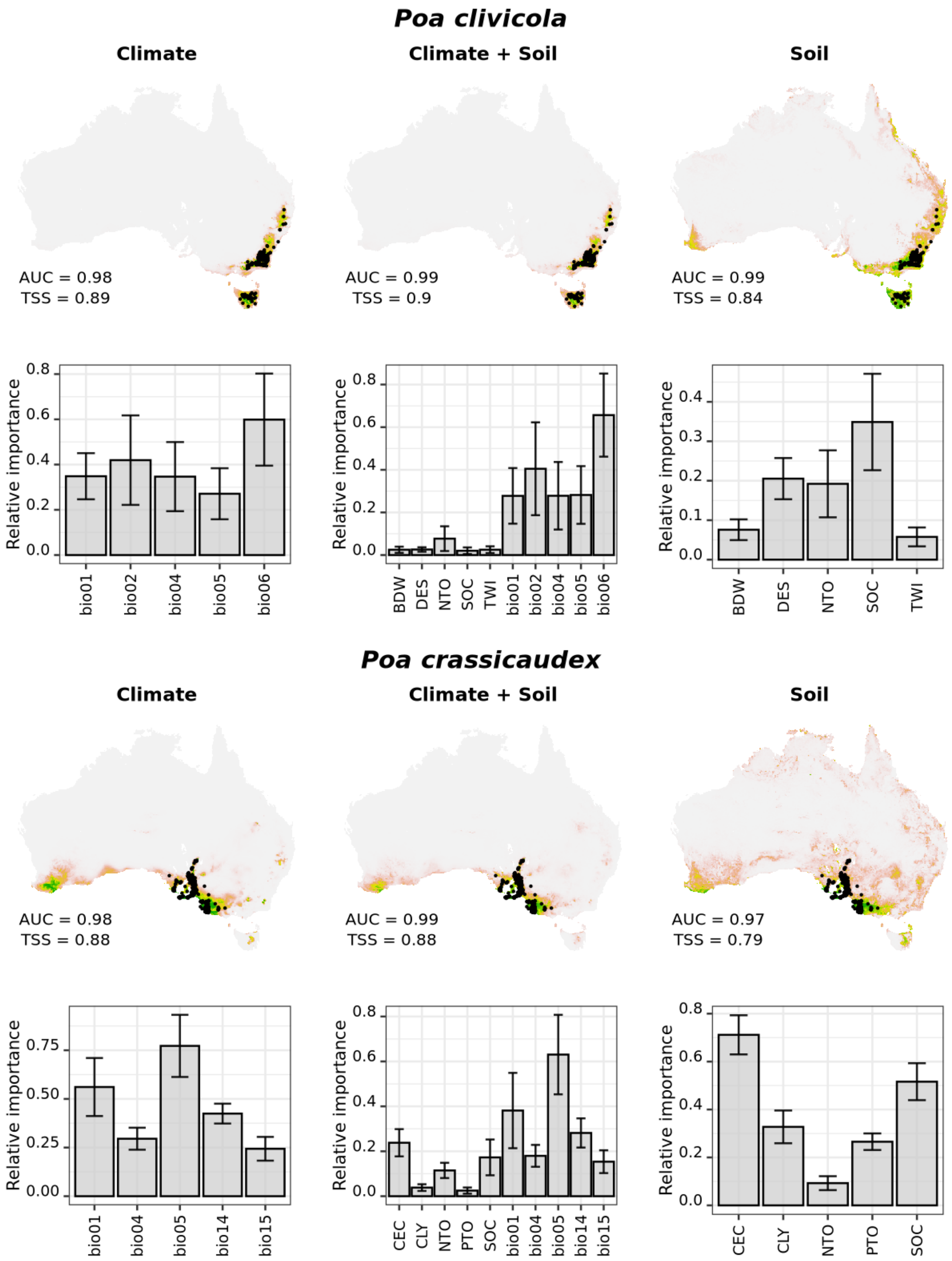

Poa clivicola
Climate
Climate + Soil
Soil
AUC = 0.98
TSS = 0.89
AUC = 0.99
TSS = 0.9
AUC = 0.99
TSS = 0.84
Relative importance
0.0
0.2
0.4
0.6
0.8
bio01
bio02
bio04
bio05
bio06
BDW
DES
NTO
SOC
TWI
0.1
0.3
Poa crassicaudex
Climate
Climate + Soil
Soil
AUC = 0.98
TSS = 0.88
AUC = 0.99
TSS = 0.88
AUC = 0.97
TSS = 0.79
0.00
0.25
0.50
0.75
bio14
bio15
CEC
CLY
PTO

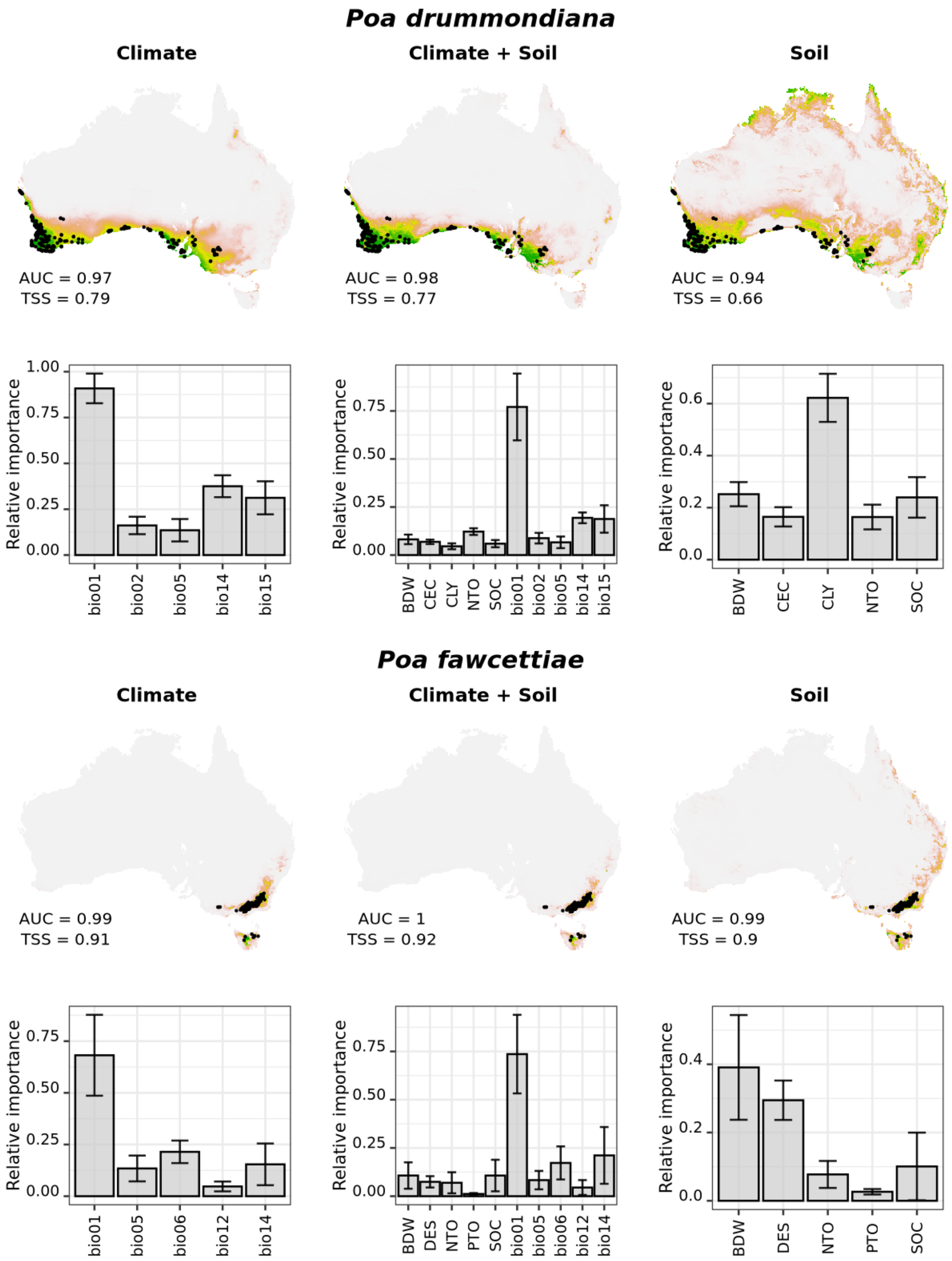

Poa drummondiana
Climate
Climate + Soil
Soil
AUC = 0.97
TSS = 0.79
AUC = 0.98
TSS = 0.77
AUC = 0.94
TSS = 0.66
Relative importance
1.00
0.75
0.50
0.25
0.00
bio01
bio02
bio05
bio14
bio15
BDW
CEC
CLY
NTO
SOC
0.6
0.4
0.2
0.0
Poa fawcettiae
Climate
Climate + Soil
Soil
AUC = 0.99
TSS = 0.91
AUC = 1
TSS = 0.92
AUC = 0.99
TSS = 0.9
bio06
bio12
DES
PTO

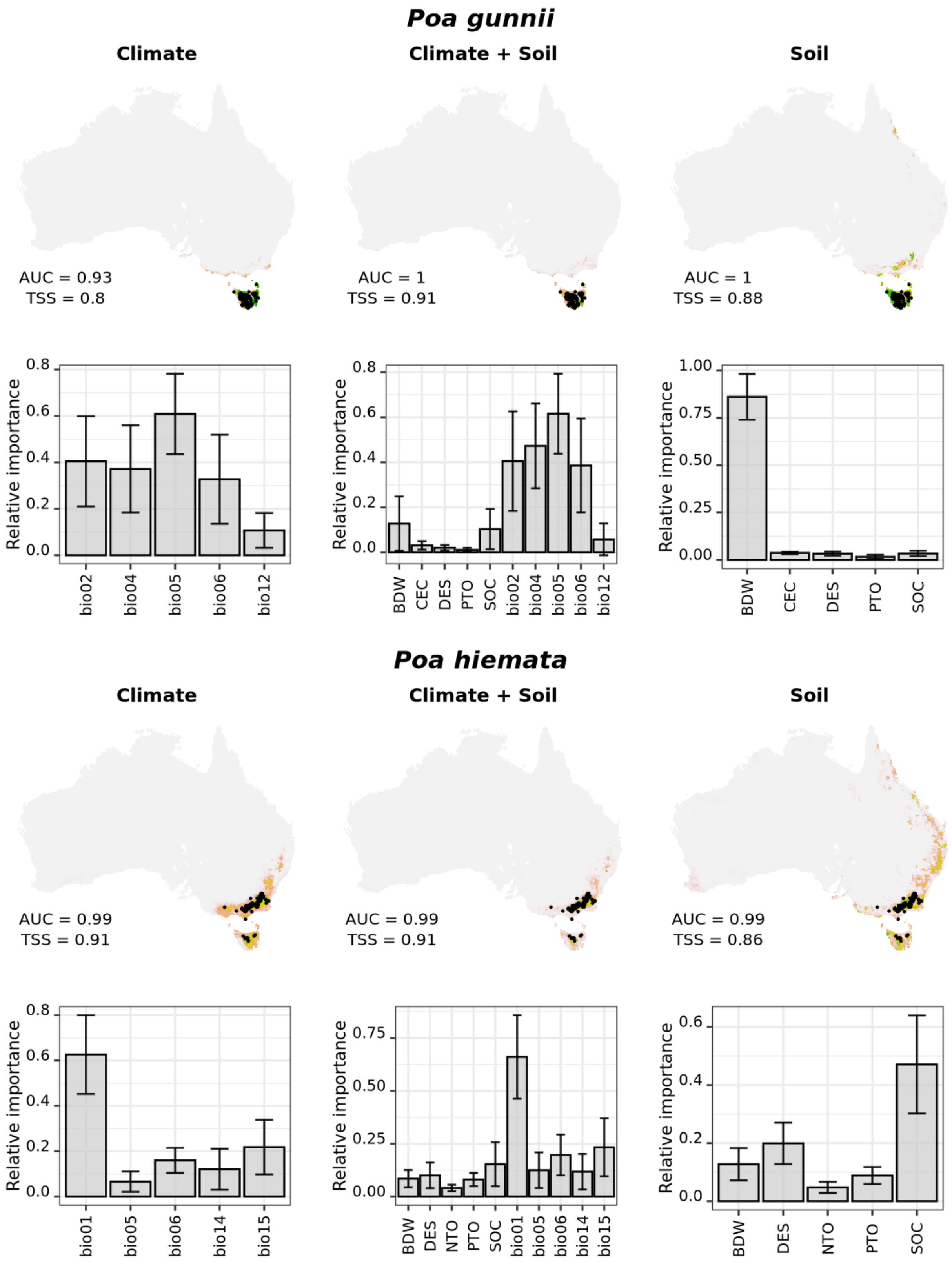

Poa gunnii
Climate
Climate + Soil
Soil
AUC = 0.93
TSS = 0.8
AUC = 1
TSS = 0.91
AUC = 1
TSS = 0.88
Relative importance
bio02
bio04
bio05
bio06
bio12
BDW
CEC
DES
PTO
SOC
Poa hiemata
Climate
Climate + Soil
Soil
AUC = 0.99
TSS = 0.91
AUC = 0.99
TSS = 0.91
AUC = 0.99
TSS = 0.86
Relative importance
bio01
bio05
bio06
bio14
bio15
BDW
DES
NTO
PTO
SOC

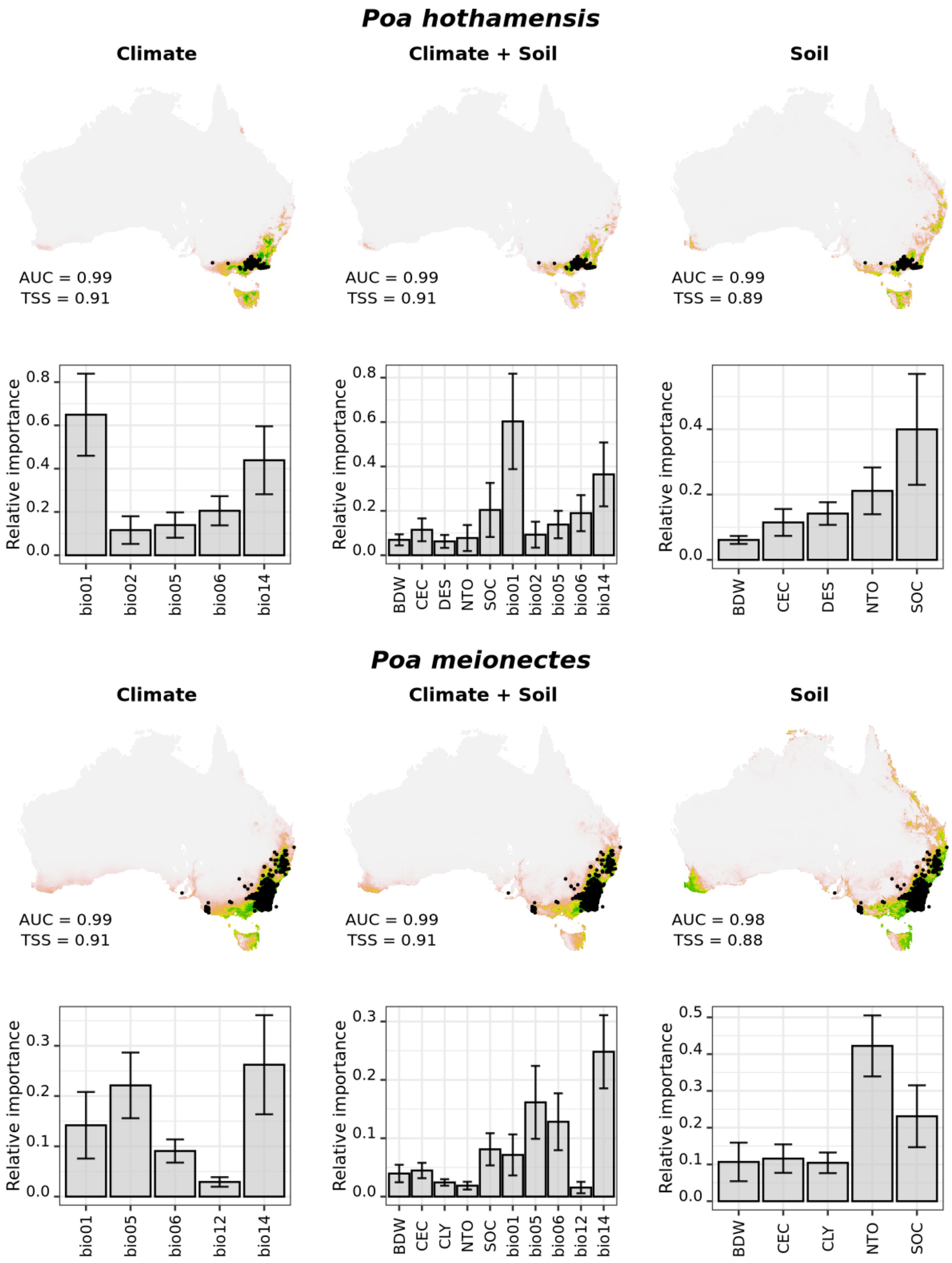
Poa hothamensis
Climate
Climate + Soil
Soil
AUC = 0.99
TSS = 0.91
AUC = 0.99
TSS = 0.91
AUC = 0.99
TSS = 0.89
Relative importance
bio01
bio02
bio05
bio06
bio14
BDW
CEC
DES
NTO
SOC
Poa meionectes
Climate
Climate + Soil
Soil
AUC = 0.99
TSS = 0.91
AUC = 0.99
TSS = 0.91
AUC = 0.98
TSS = 0.88
Relative importance
bio01
bio05
bio06
bio12
bio14
BDW
CEC
CLY
NTO
SOC

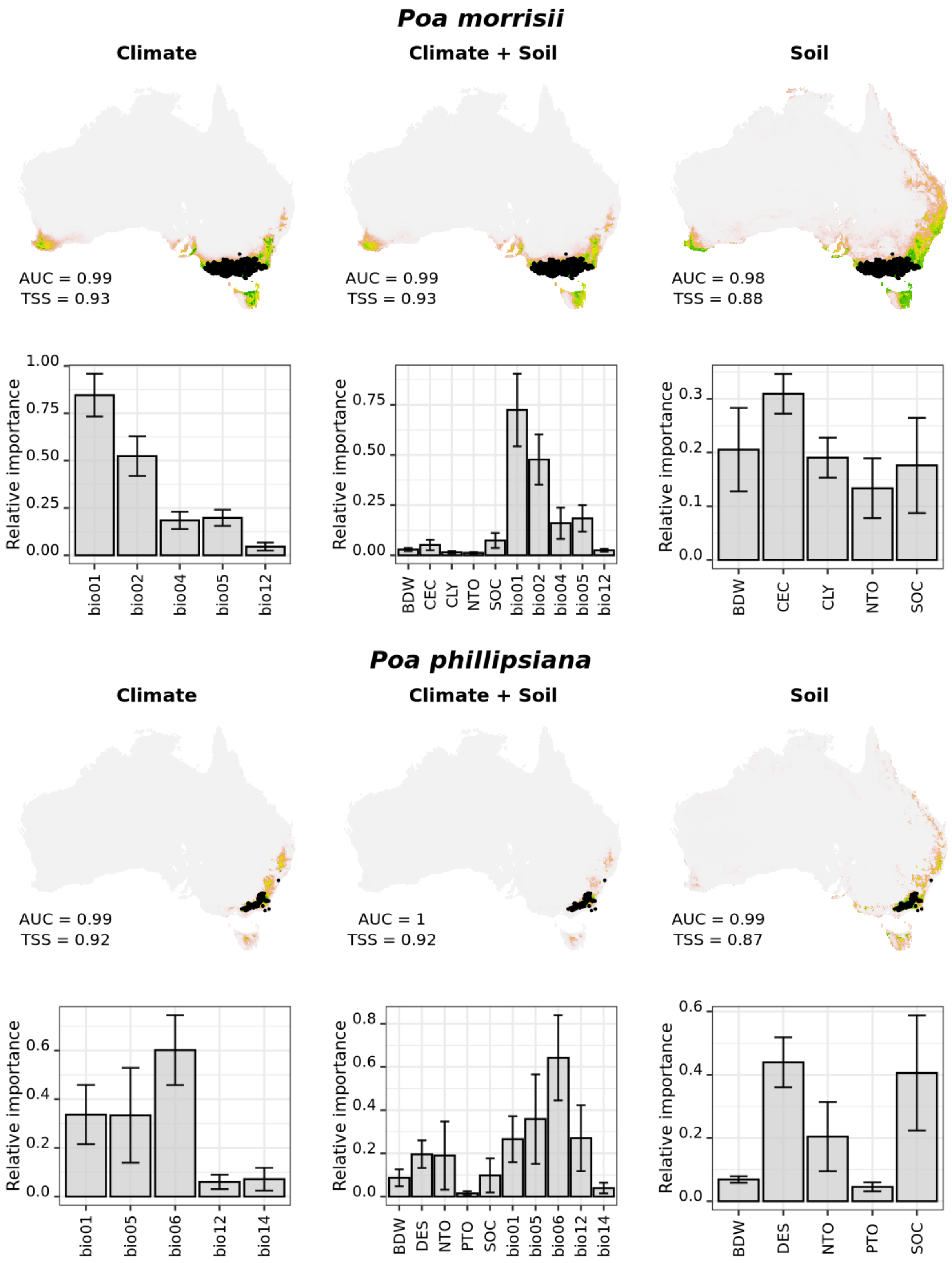

Poa morrisii
Climate
Climate + Soil
Soil
AUC = 0.99
TSS = 0.93
AUC = 0.99
TSS = 0.93
AUC = 0.98
TSS = 0.88
Relative importance
bio01
bio02
bio04
bio05
bio12
BDW
CEC
CLY
NTO
SOC
Poa phillipsiana
Climate
Climate + Soil
Soil
AUC = 0.99
TSS = 0.92
AUC = 1
TSS = 0.92
AUC = 0.99
TSS = 0.87
Relative importance
bio01
bio05
bio06
bio12
bio14
BDW
DES
NTO
PTO
SOC

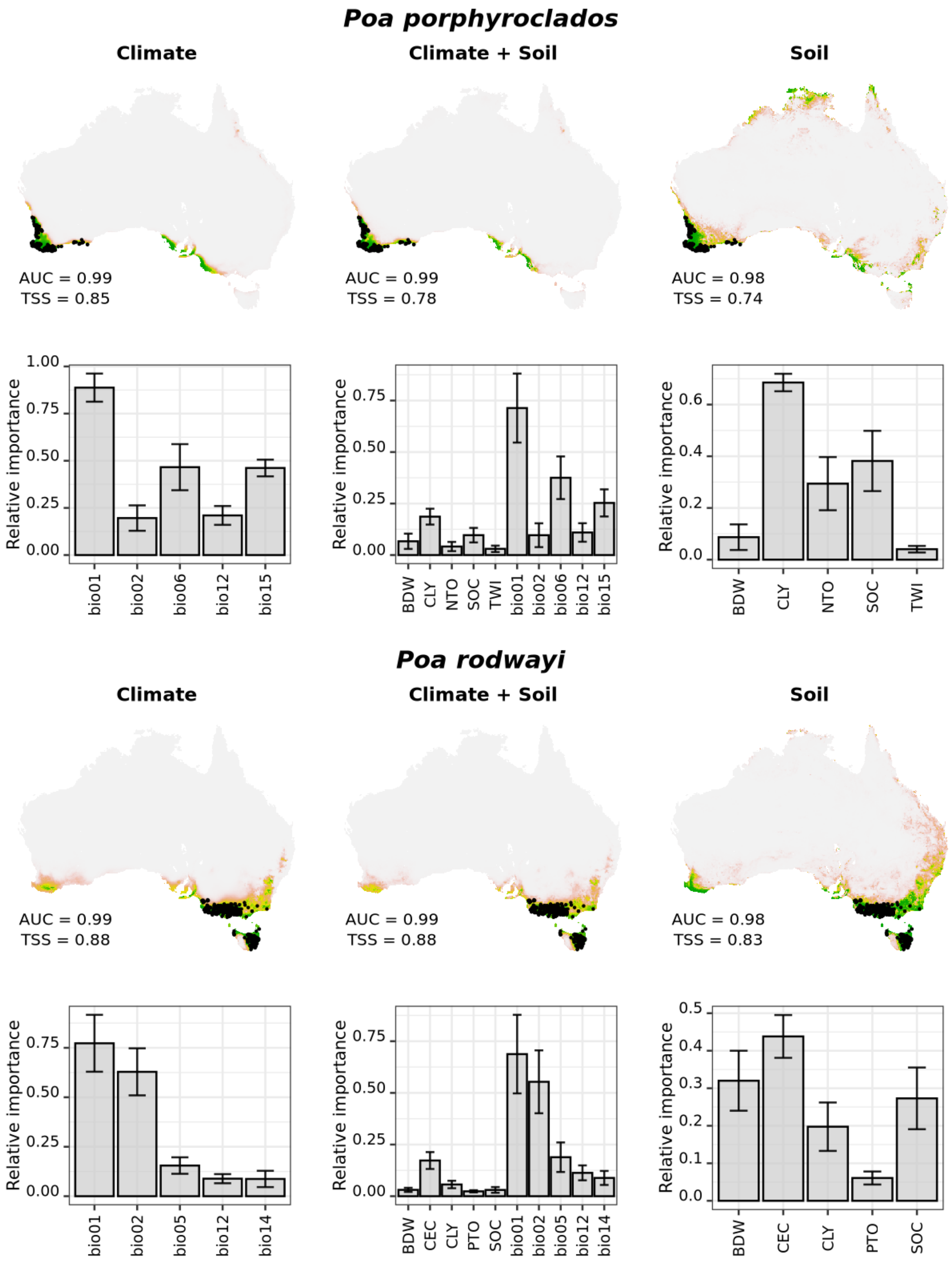
Poa porphyroclados
Climate
Climate + Soil
Soil
AUC = 0.99
TSS = 0.85
AUC = 0.99
TSS = 0.78
AUC = 0.98
TSS = 0.74
Relative importance
0.00
0.25
0.50
0.75
1.00
bio01
bio02
bio06
bio12
bio15
BDW
CLY
NTO
SOC
TWI
0.0
0.2
0.4
0.6
Poa rodwayi
Climate
Climate + Soil
Soil
AUC = 0.99
TSS = 0.88
AUC = 0.99
TSS = 0.88
AUC = 0.98
TSS = 0.83
bio05
bio14
CEC
PTO
0.1
0.3
0.5

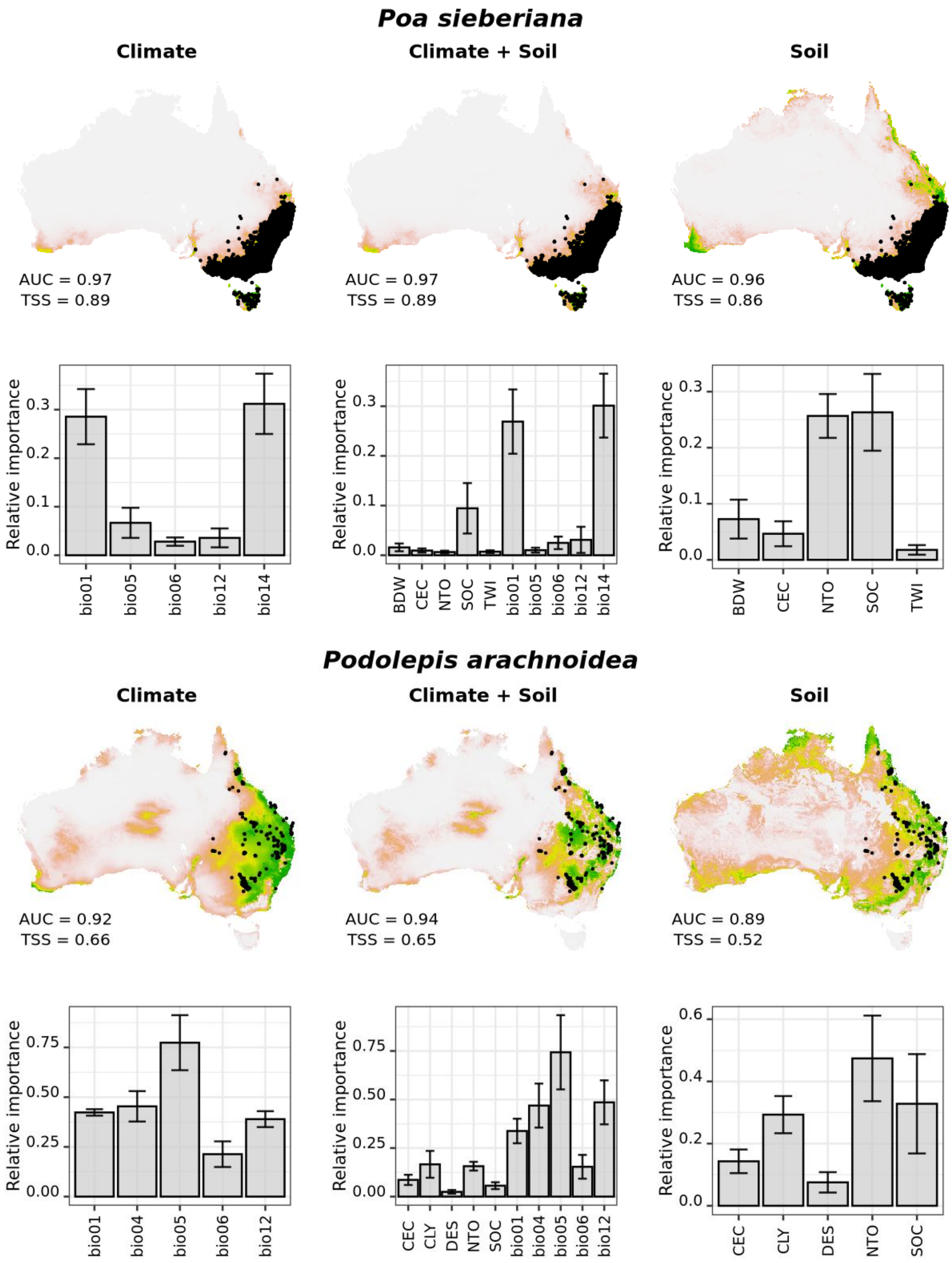

Poa sieberiana
Climate
Climate + Soil
Soil
AUC = 0.97
TSS = 0.89
AUC = 0.97
TSS = 0.89
AUC = 0.96
TSS = 0.86
Relative importance
0.0
0.1
0.2
0.3
bio01
bio05
bio06
bio12
bio14
BDW
CEC
NTO
SOC
TWI
Podolepis arachnoidea
Climate
Climate + Soil
Soil
AUC = 0.92
TSS = 0.66
AUC = 0.94
TSS = 0.65
AUC = 0.89
TSS = 0.52
Relative importance
0.00
0.25
0.50
0.75
0.0
0.2
0.4
0.6
bio01
bio04
bio05
bio06
bio12
CEC
CLY
DES
NTO
SOC

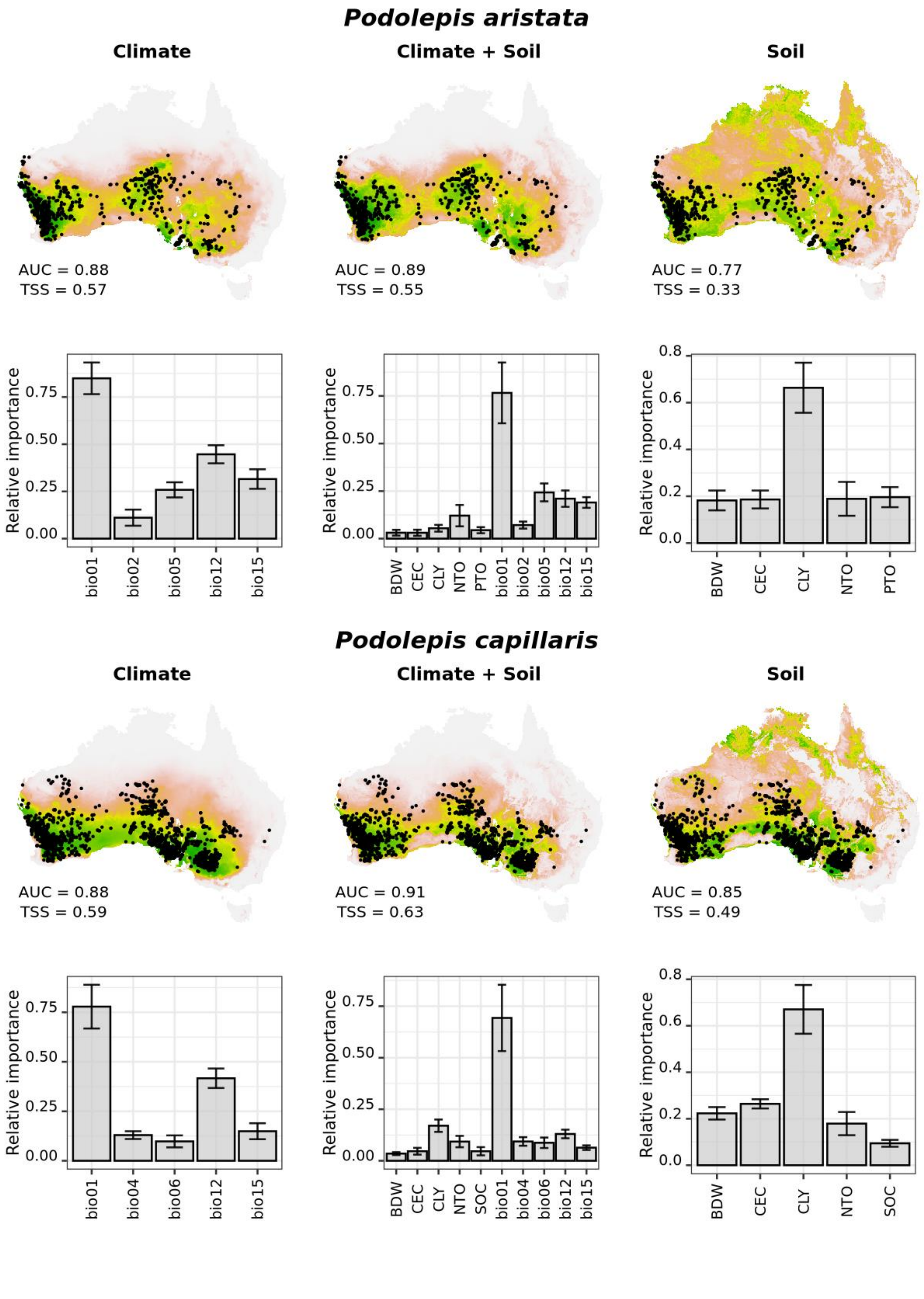

Podolepis aristata
Climate
Climate + Soil
Soil
AUC = 0.88
TSS = 0.57
AUC = 0.89
TSS = 0.55
AUC = 0.77
TSS = 0.33
Relative importance
0.00
0.25
0.50
0.75
bio01
bio02
bio05
bio12
bio15
BDW
CEC
CLY
NTO
PTO
bio01
bio02
bio05
bio12
bio15
0.0
0.2
0.4
0.6
0.8
BDW
CEC
CLY
NTO
PTO
Podolepis capillaris
Climate
Climate + Soil
Soil
AUC = 0.88
TSS = 0.59
AUC = 0.91
TSS = 0.63
AUC = 0.85
TSS = 0.49
Relative importance
bio01
bio04
bio06
bio12
bio15
BDW
CEC
CLY
NTO
SOC
bio01
bio04
bio06
bio12
bio15
BDW
CEC
CLY
NTO
SOC

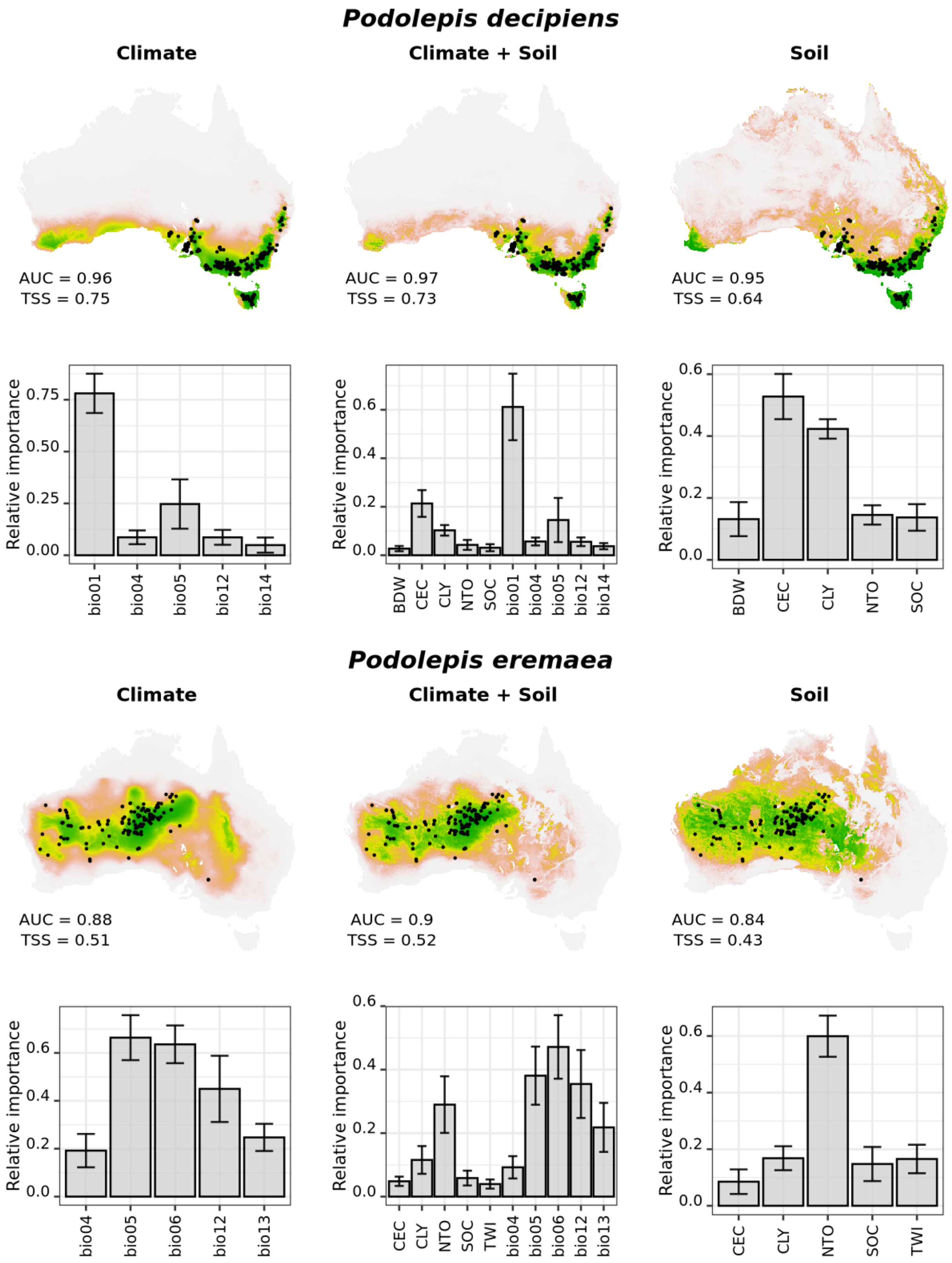
Podolepis decipiens
Climate
Climate + Soil
Soil
AUC = 0.96
TSS = 0.75
AUC = 0.97
TSS = 0.73
AUC = 0.95
TSS = 0.64
Relative importance
0.00
0.25
0.50
0.75
bio01
bio04
bio05
bio12
bio14
0.0
0.2
0.4
0.6
BDW
CEC
CLY
NTO
SOC
bio01
bio04
bio05
bio12
bio14
BDW
CEC
CLY
NTO
SOC
Podolepis eremaea
Climate
Climate + Soil
Soil
AUC = 0.88
TSS = 0.51
AUC = 0.9
TSS = 0.52
AUC = 0.84
TSS = 0.43
Relative importance
0.0
0.2
0.4
0.6
bio04
bio05
bio06
bio12
bio13
CEC
CLY
NTO
SOC
TWI
bio04
bio05
bio06
bio12
bio13
CEC
CLY
NTO
SOC
TWI

Podolepis gracilis

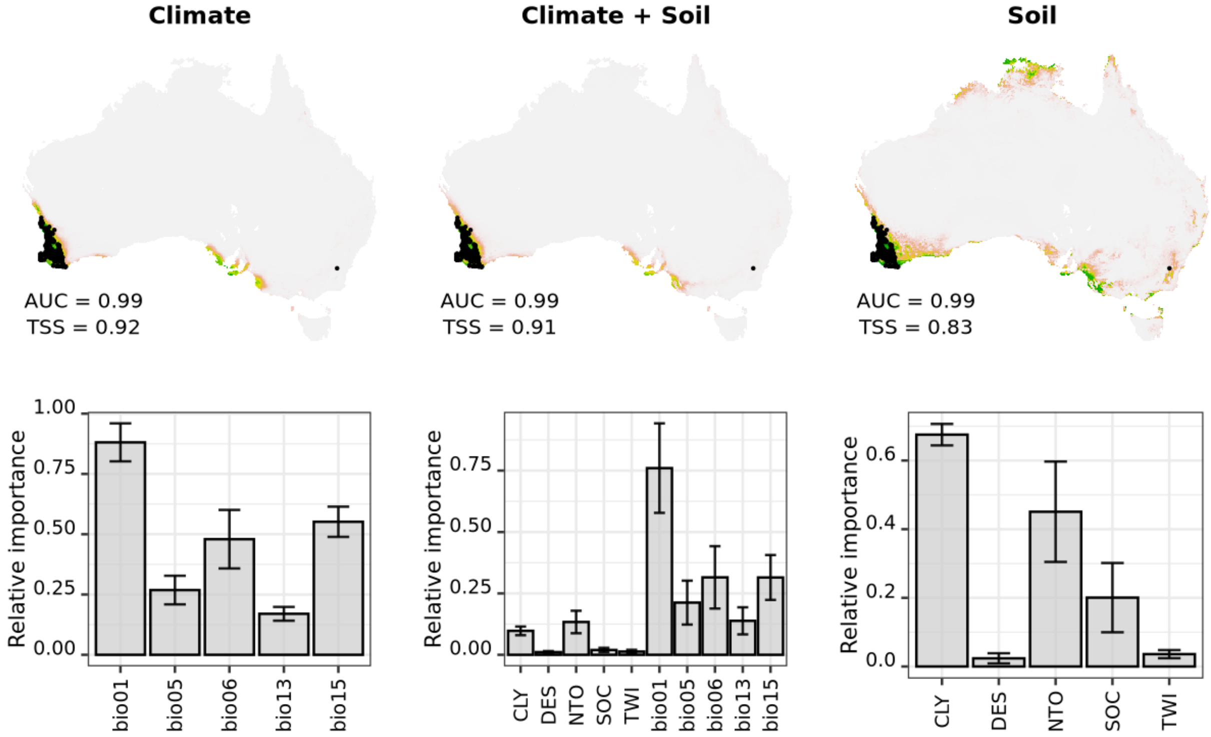

Podolepis hieracioides

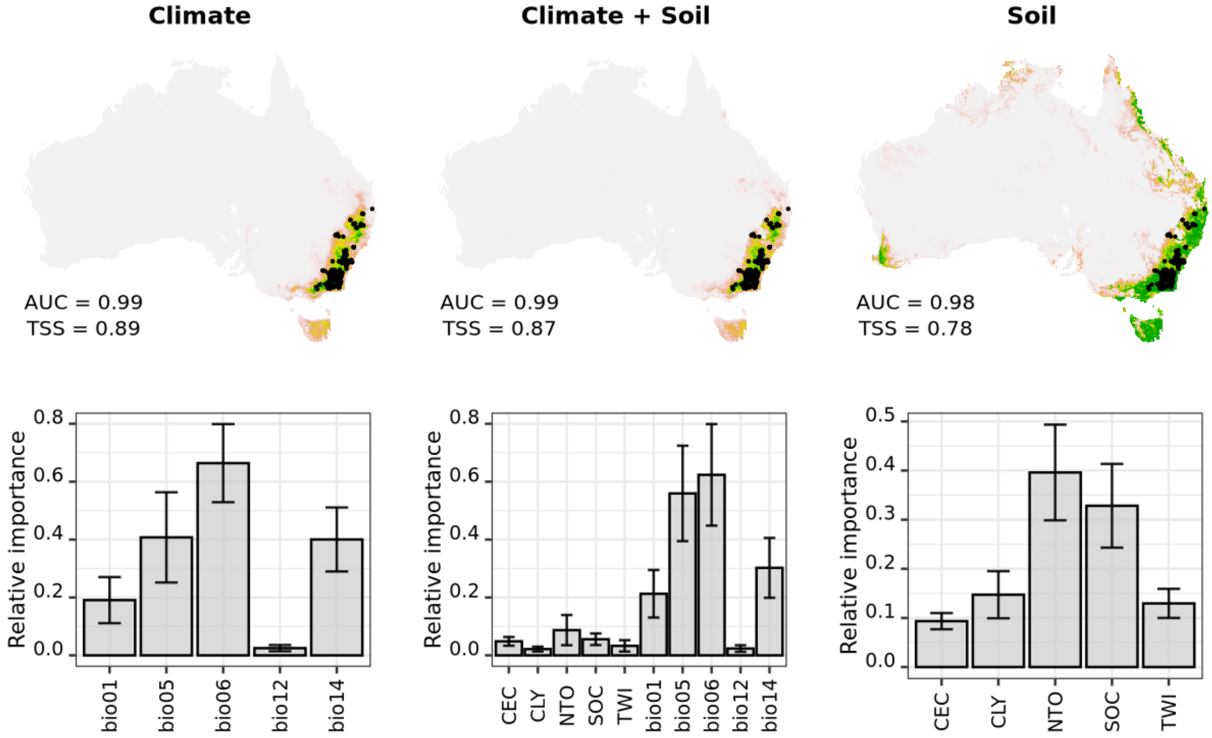

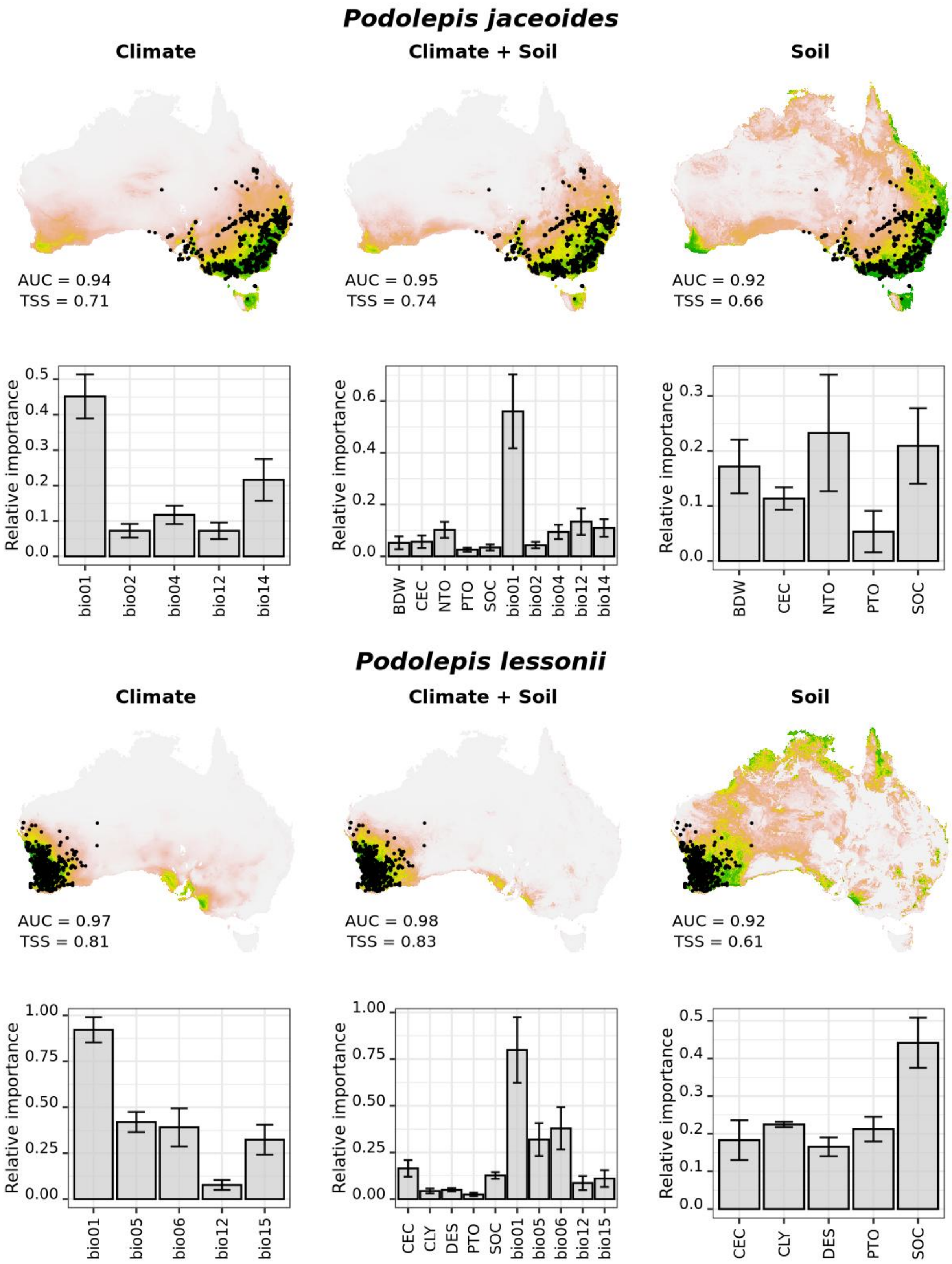
Podolepis jaceoides
Climate
Climate + Soil
Soil
AUC = 0.94
TSS = 0.71
AUC = 0.95
TSS = 0.74
AUC = 0.92
TSS = 0.66
Relative importance
bio01
bio02
bio04
bio12
bio14
BDW
CEC
NTO
PTO
SOC
Podolepis lessonii
Climate
Climate + Soil
Soil
AUC = 0.97
TSS = 0.81
AUC = 0.98
TSS = 0.83
AUC = 0.92
TSS = 0.61
Relative importance
bio01
bio05
bio06
bio12
bio15
CEC
CLY
DES
PTO
SOC

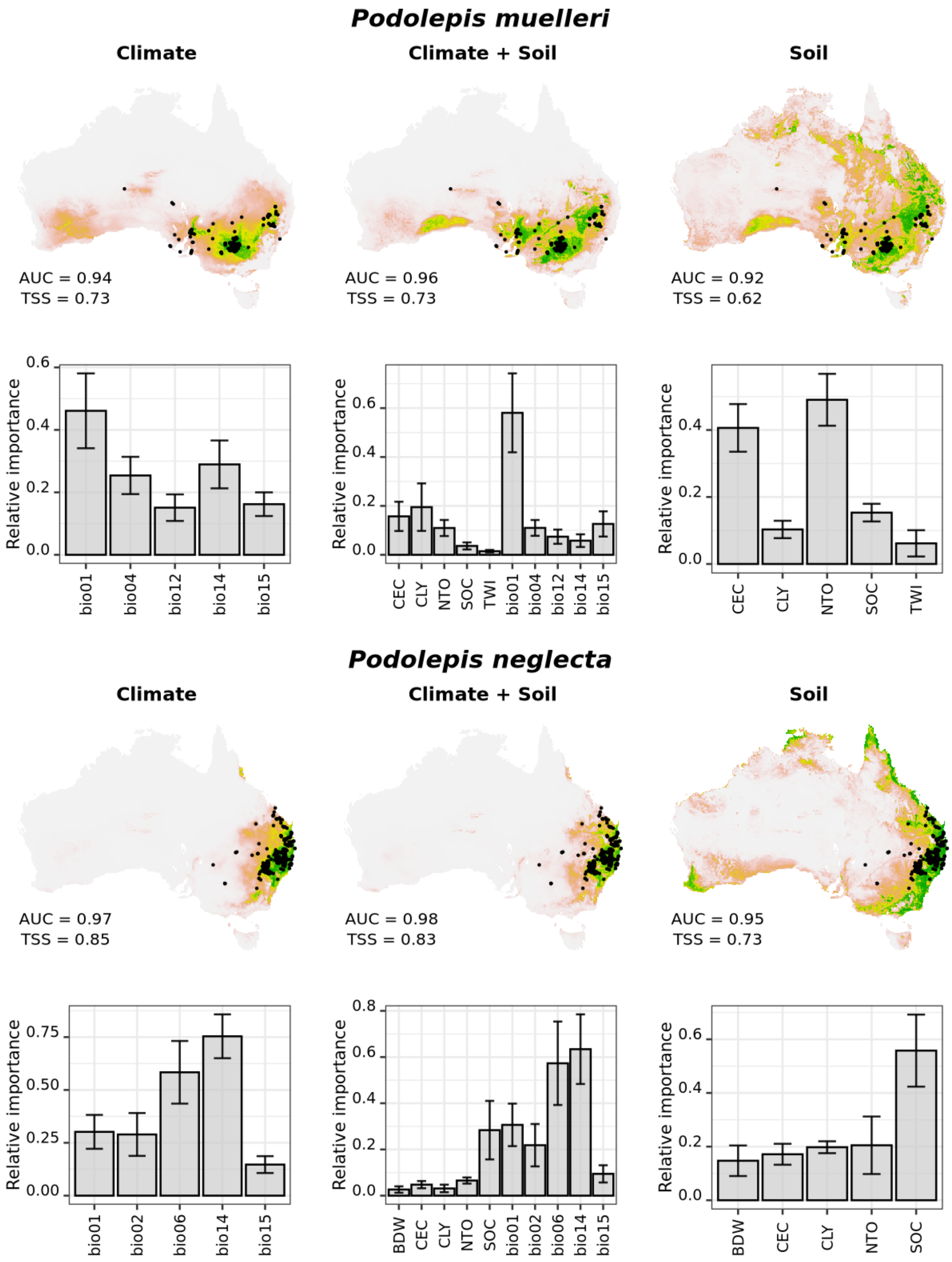

Podolepis muelleri
Climate
Climate + Soil
Soil
AUC = 0.94
TSS = 0.73
AUC = 0.96
TSS = 0.73
AUC = 0.92
TSS = 0.62
Relative importance
bio01
bio04
bio12
bio14
bio15
CEC
CLY
NTO
SOC
TWI
Podolepis neglecta
AUC = 0.97
TSS = 0.85
AUC = 0.98
TSS = 0.83
AUC = 0.95
TSS = 0.73
bio02
bio06
BDW

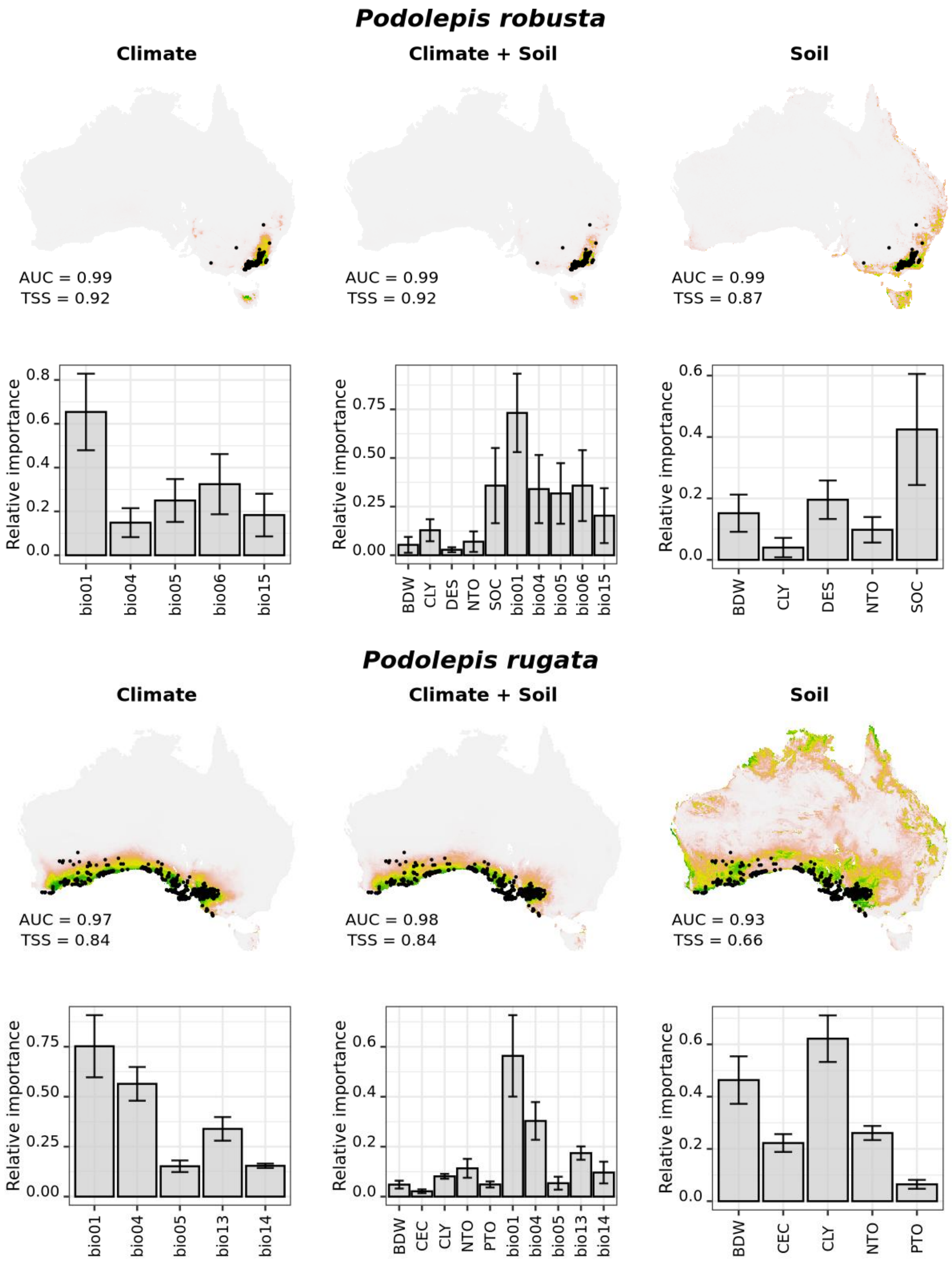
Podolepis robusta
Climate
Climate + Soil
Soil
AUC = 0.99
TSS = 0.92
AUC = 0.99
TSS = 0.92
AUC = 0.99
TSS = 0.87
Relative importance
0.0 0.2 0.4 0.6 0.8
bio01 bio04 bio05 bio06 bio15
0.00 0.25 0.50 0.75
BDW CLY DES NTO SOC bio01 bio04 bio05 bio06 bio15
0.0 0.2 0.4 0.6
BDW CLY DES NTO SOC
Podolepis rugata
Climate
Climate + Soil
Soil
AUC = 0.97
TSS = 0.84
AUC = 0.98
TSS = 0.84
AUC = 0.93
TSS = 0.66
Relative importance
0.00 0.25 0.50 0.75
bio01 bio04 bio05 bio13 bio14
0.0 0.2 0.4 0.6
BDW CEC CLY NTO PTO bio01 bio04 bio05 bio13 bio14
0.0 0.2 0.4 0.6
BDW CEC CLY NTO PTO

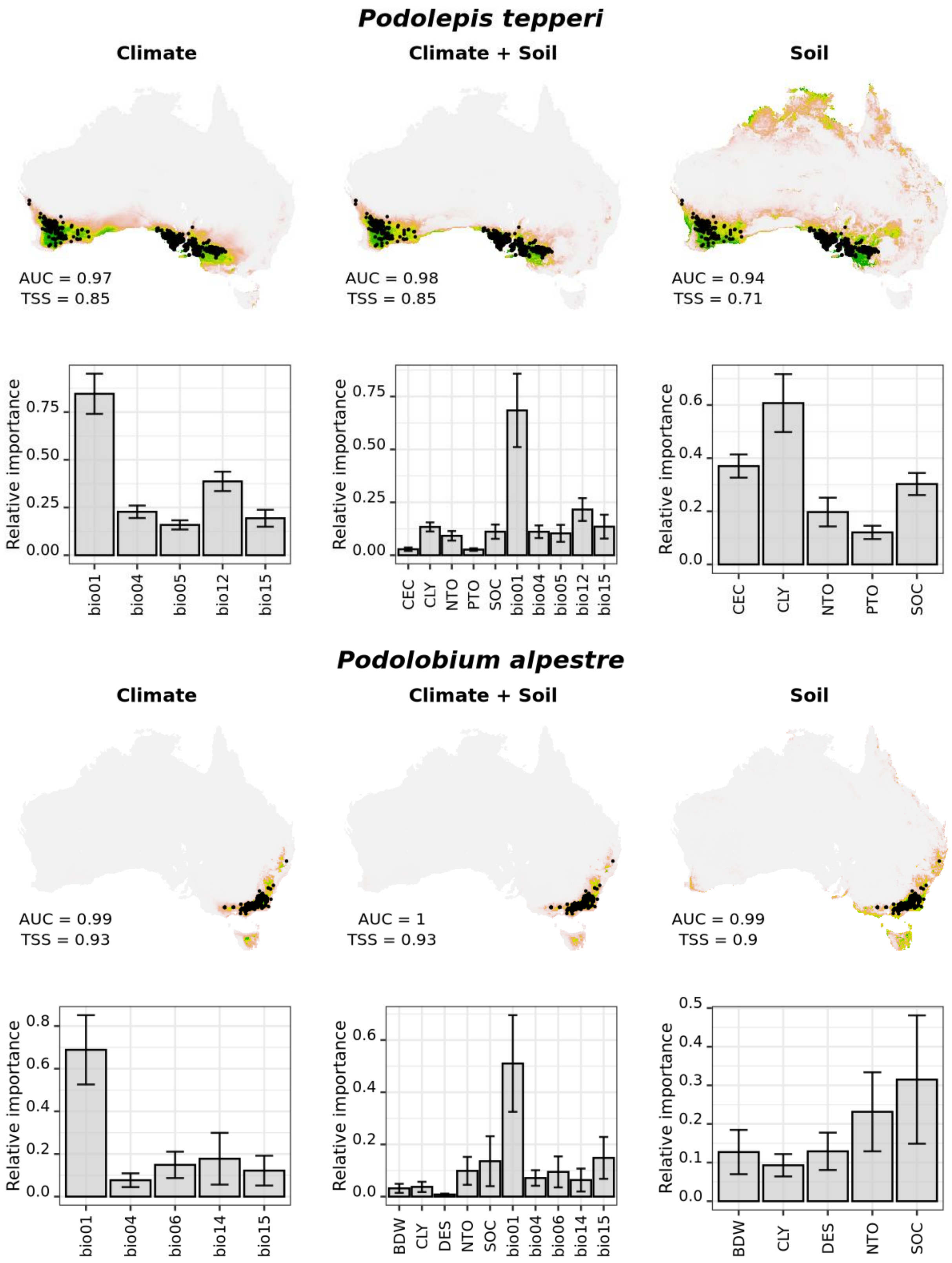
Podolepis tepperi
Climate
Climate + Soil
Soil
AUC = 0.97
TSS = 0.85
AUC = 0.98
TSS = 0.85
AUC = 0.94
TSS = 0.71
Relative importance
0.00
0.25
0.50
0.75
bio01
bio04
bio05
bio12
bio15
CEC
CLY
NTO
PTO
SOC
0.0
0.2
0.4
0.6
Podolobium alpestre
AUC = 0.99
TSS = 0.93
AUC = 1
TSS = 0.93
AUC = 0.99
TSS = 0.9
0.8
bio06
bio14
BDW
DES
0.1
0.3
0.5

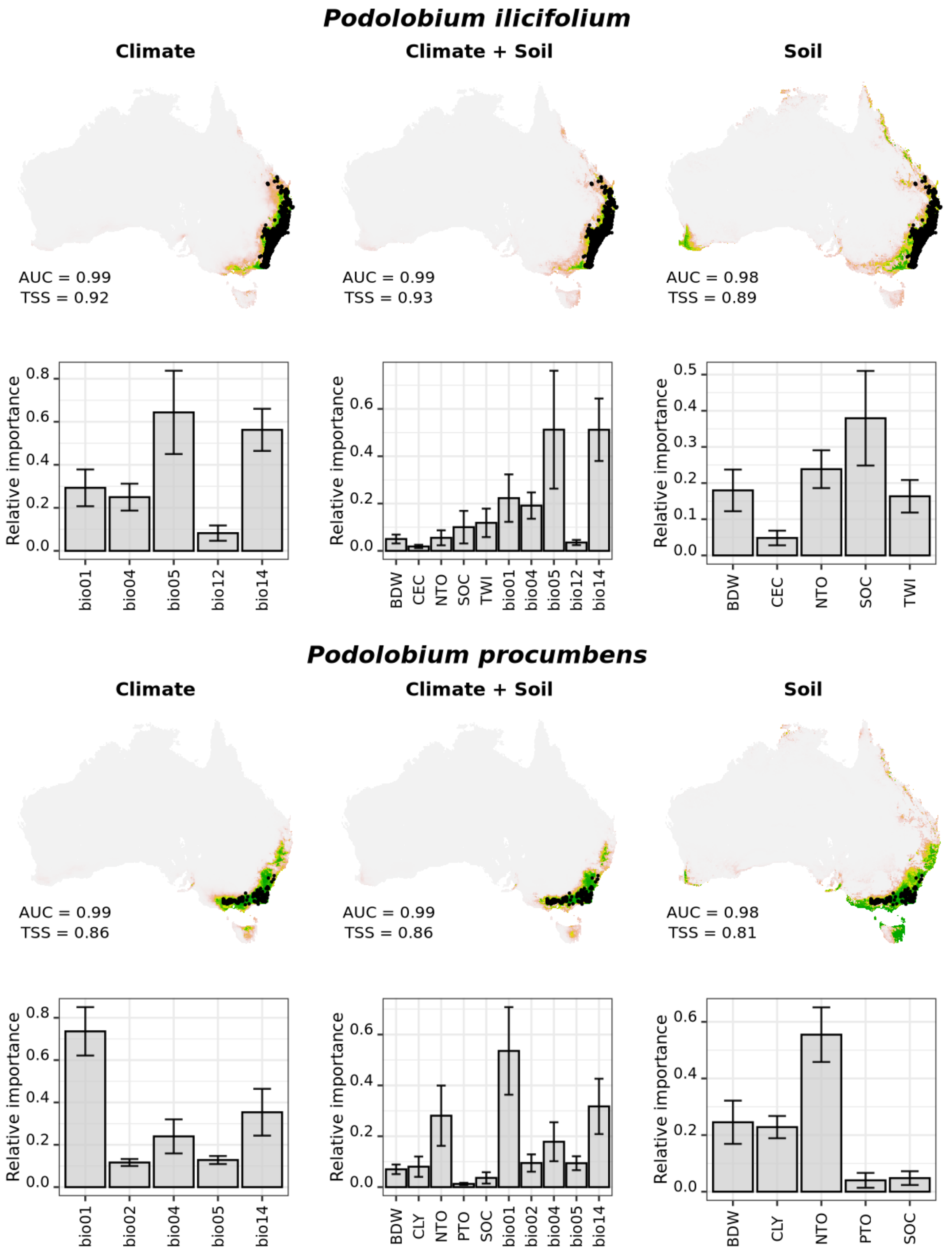
Podolobium ilicifolium
Climate
Climate + Soil
Soil
AUC = 0.99
TSS = 0.92
AUC = 0.99
TSS = 0.93
AUC = 0.98
TSS = 0.89
Relative importance
0.0 0.2 0.4 0.6 0.8
bio01 bio04 bio05 bio12 bio14
0.0 0.2 0.4 0.6
BDW CEC NTO SOC TWI bio01 bio04 bio05 bio12 bio14
0.0 0.1 0.2 0.3 0.4 0.5
BDW CEC NTO SOC TWI
Podolobium procumbens
Climate
Climate + Soil
Soil
AUC = 0.99
TSS = 0.86
AUC = 0.99
TSS = 0.86
AUC = 0.98
TSS = 0.81
Relative importance
0.0 0.2 0.4 0.6 0.8
bio01 bio02 bio04 bio05 bio14
0.0 0.2 0.4 0.6
BDW CLY NTO PTO SOC bio01 bio02 bio04 bio05 bio14
0.0 0.2 0.4 0.6
BDW CLY NTO PTO SOC

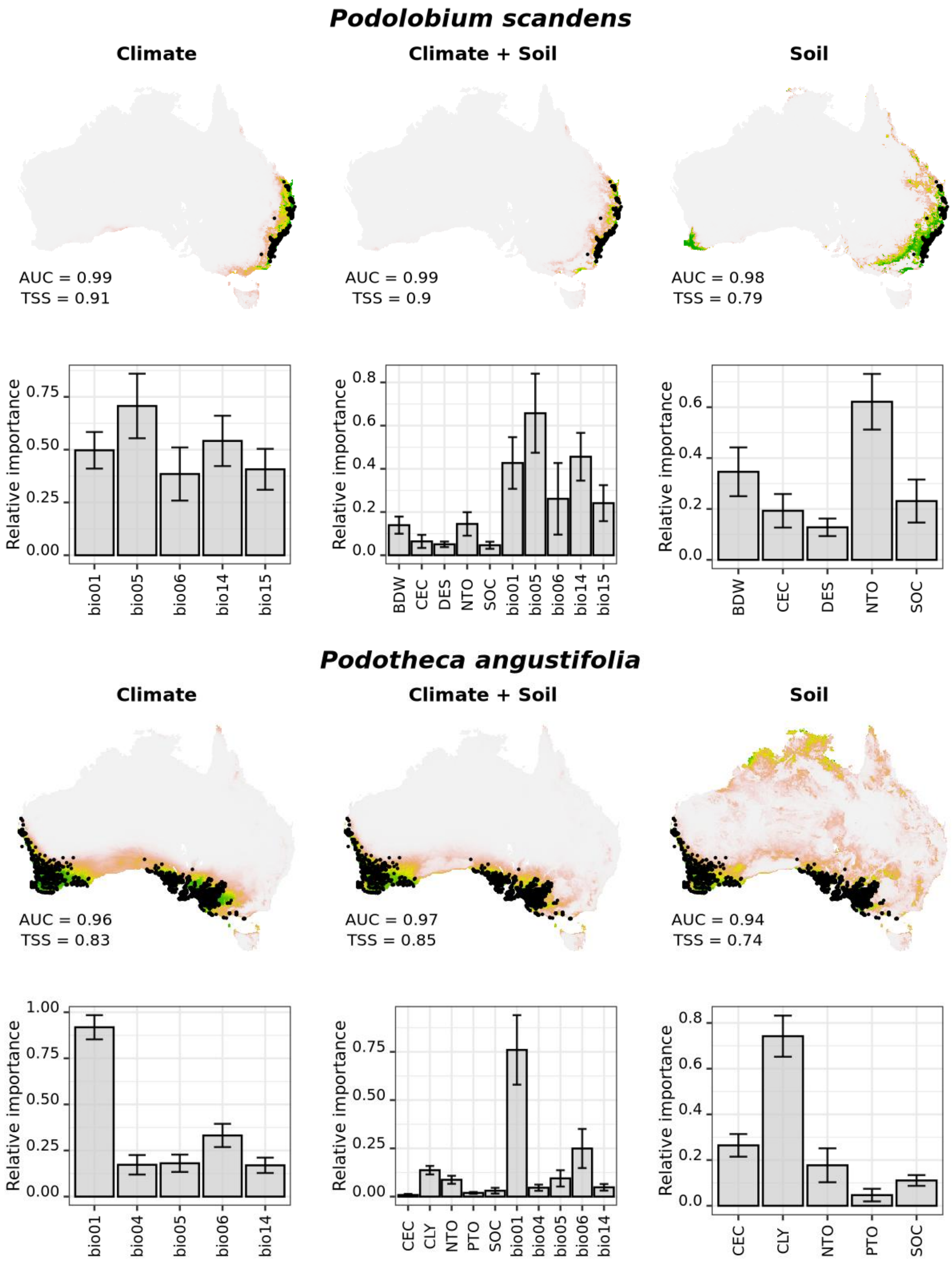

Podolobium scandens
Climate
Climate + Soil
Soil
AUC = 0.99
TSS = 0.91
AUC = 0.99
TSS = 0.9
AUC = 0.98
TSS = 0.79
Relative importance
bio01 bio05 bio06 bio14 bio15
BDW CEC DES NTO SOC bio01 bio05 bio06 bio14 bio15
BDW CEC DES NTO SOC
Podotheca angustifolia
Climate
Climate + Soil
Soil
AUC = 0.96
TSS = 0.83
AUC = 0.97
TSS = 0.85
AUC = 0.94
TSS = 0.74
Relative importance
bio01 bio04 bio05 bio06 bio14
CEC CLY NTO PTO SOC bio01 bio04 bio05 bio06 bio14
CEC CLY NTO PTO SOC

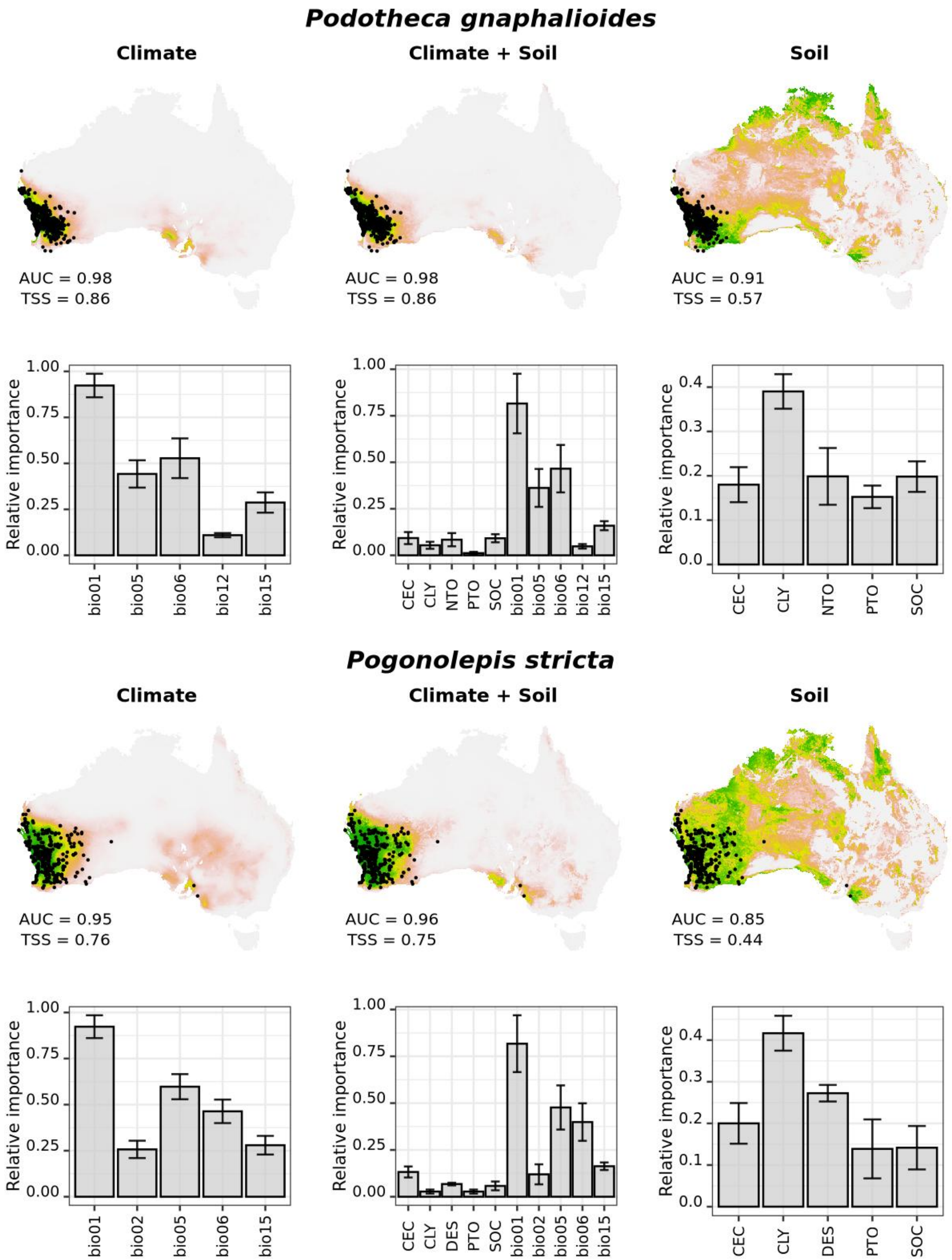
Podotheca gnaphalioides
Climate
Climate + Soil
Soil
AUC = 0.98
TSS = 0.86
AUC = 0.98
TSS = 0.86
AUC = 0.91
TSS = 0.57
Relative importance
bio01
bio05
bio06
bio12
bio15
CEC
CLY
NTO
PTO
SOC
Pogonolepis stricta
Climate
Climate + Soil
Soil
AUC = 0.95
TSS = 0.76
AUC = 0.96
TSS = 0.75
AUC = 0.85
TSS = 0.44
Relative importance
bio01
bio02
bio05
bio06
bio15
CEC
CLY
DES
PTO
SOC

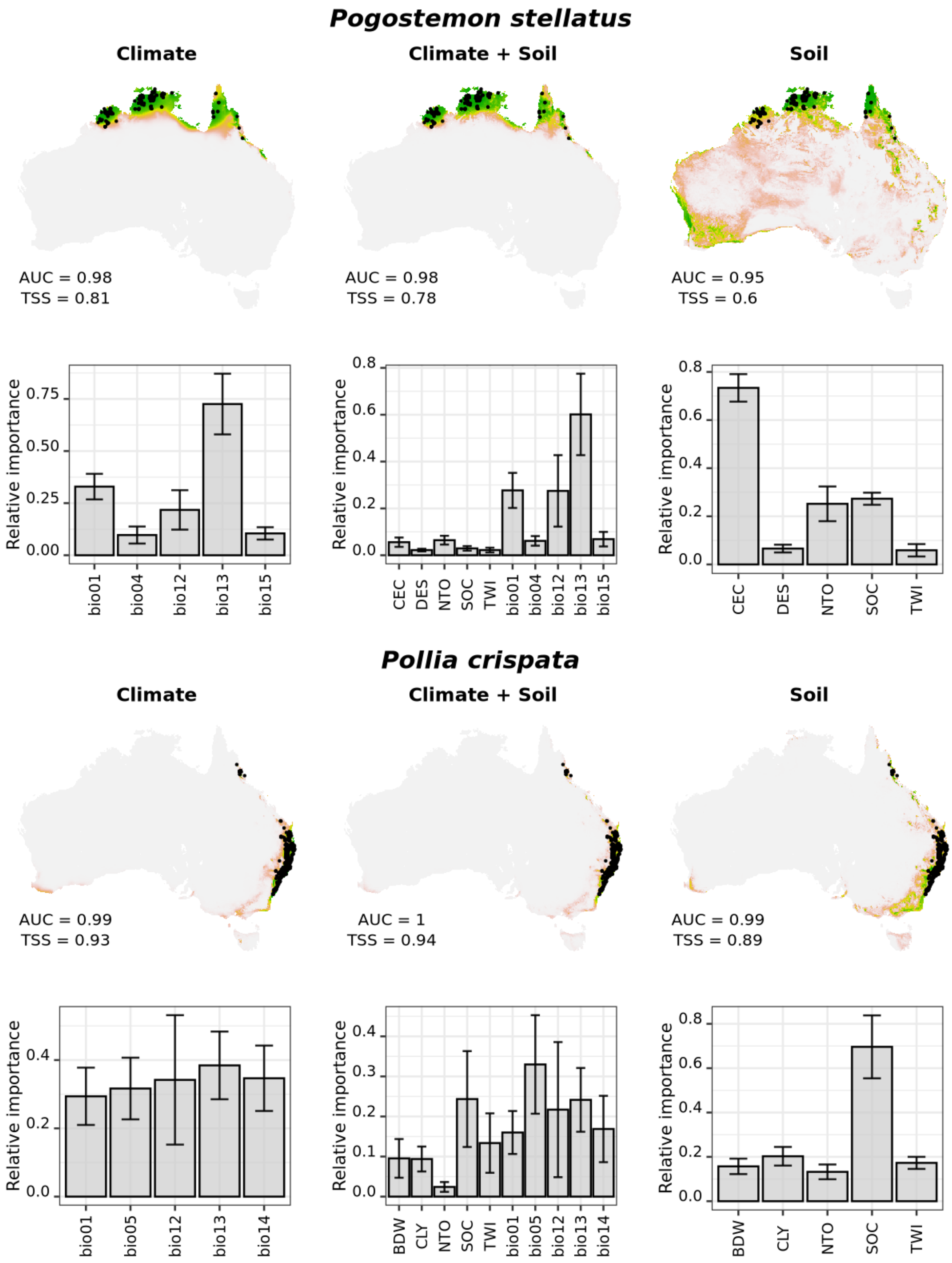
Pogostemon stellatus
Climate
Climate + Soil
Soil
AUC = 0.98
TSS = 0.81
AUC = 0.98
TSS = 0.78
AUC = 0.95
TSS = 0.6
Relative importance
0.00
0.25
0.50
0.75
bio01
bio04
bio12
bio13
bio15
0.0
0.2
0.4
0.6
0.8
CEC
DES
NTO
SOC
TWI
bio01
bio04
bio12
bio13
bio15
Pollia crispata
Climate
Climate + Soil
Soil
AUC = 0.99
TSS = 0.93
AUC = 1
TSS = 0.94
AUC = 0.99
TSS = 0.89
0.0
0.1
0.2
0.3
0.4
bio01
bio05
bio12
bio13
bio14
BDW
CLY
NTO
SOC
TWI

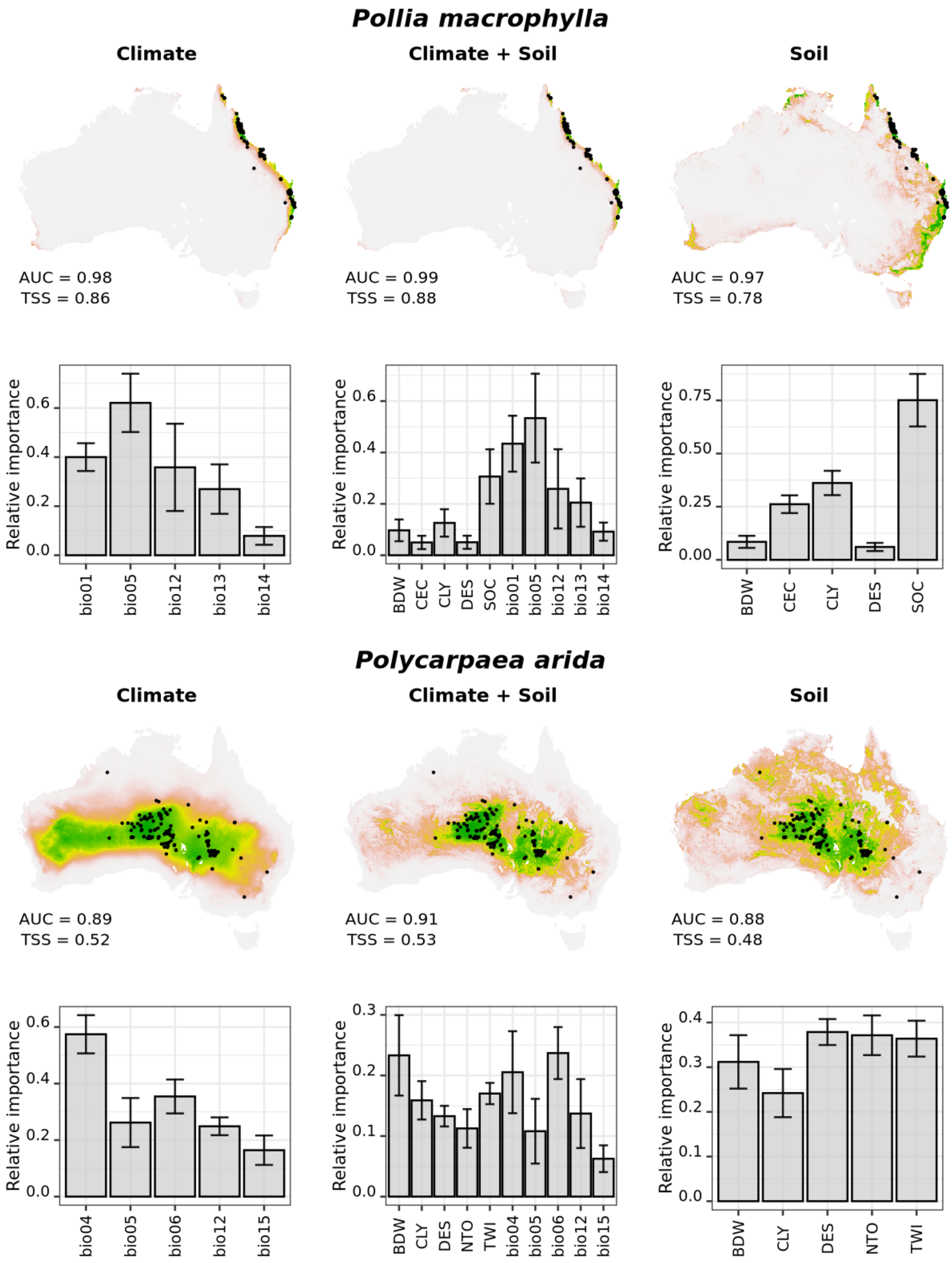

Pollia macrophylla
Climate
Climate + Soil
Soil
AUC = 0.98
TSS = 0.86
AUC = 0.99
TSS = 0.88
AUC = 0.97
TSS = 0.78
Relative importance
bio01
bio05
bio12
bio13
bio14
BDW
CEC
CLY
DES
SOC
Polycarpaea arida
Climate
Climate + Soil
Soil
AUC = 0.89
TSS = 0.52
AUC = 0.91
TSS = 0.53
AUC = 0.88
TSS = 0.48
Relative importance
bio04
bio05
bio06
bio12
bio15
BDW
CLY
DES
NTO
TWI

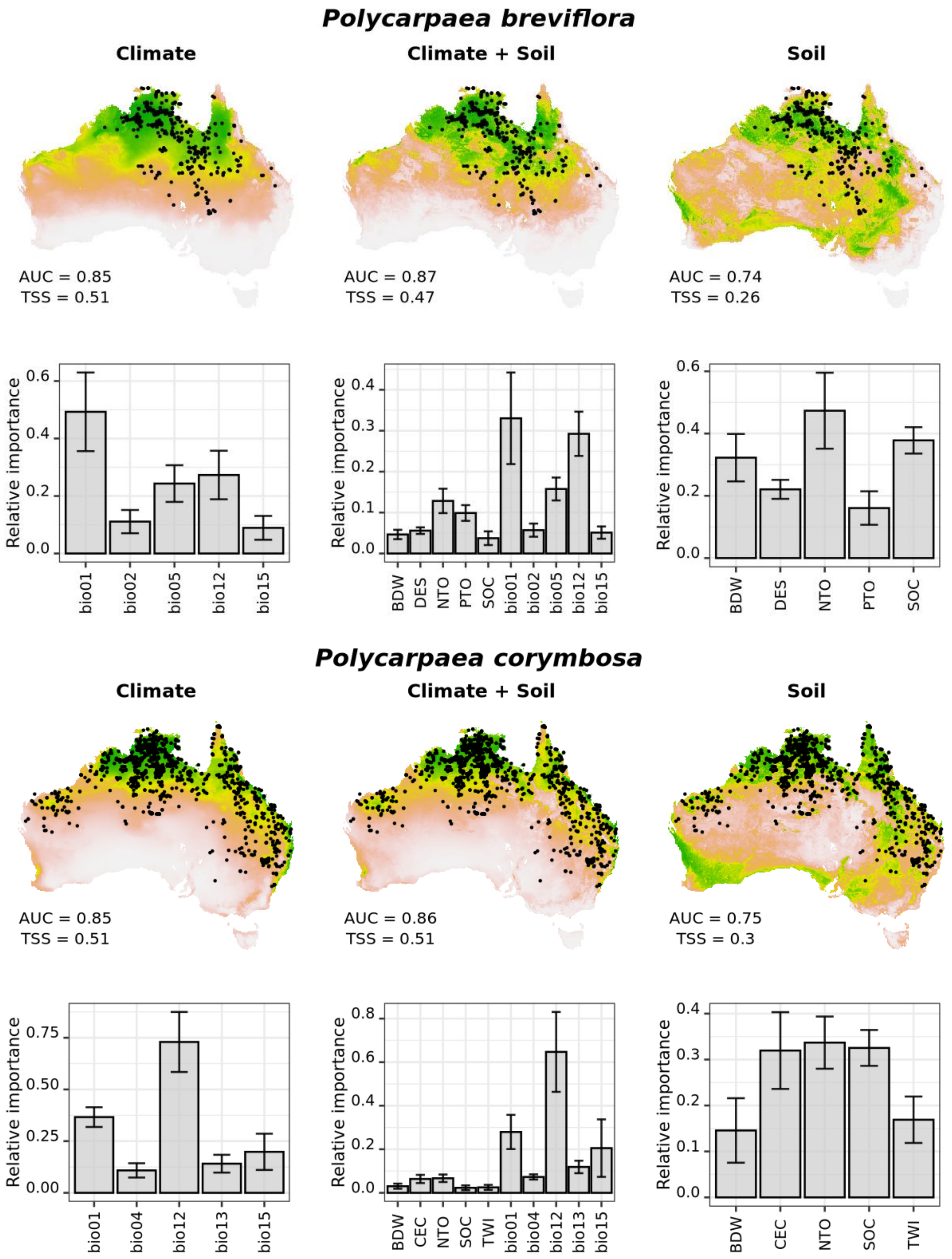
Polycarpaea breviflora
Climate
Climate + Soil
Soil
AUC = 0.85
TSS = 0.51
AUC = 0.87
TSS = 0.47
AUC = 0.74
TSS = 0.26
Relative importance
0.6
0.4
0.2
0.0
bio01
bio02
bio05
bio12
bio15
0.4
0.3
0.2
0.1
0.0
BDW
DES
NTO
PTO
SOC
bio01
bio02
bio05
bio12
bio15
0.6
0.4
0.2
0.0
BDW
DES
NTO
PTO
SOC
Polycarpaea corymbosa
Climate
Climate + Soil
Soil
AUC = 0.85
TSS = 0.51
AUC = 0.86
TSS = 0.51
AUC = 0.75
TSS = 0.3
Relative importance
0.75
0.50
0.25
0.00
bio01
bio04
bio12
bio13
bio15
0.8
0.6
0.4
0.2
0.0
BDW
CEC
NTO
SOC
TWI
bio01
bio04
bio12
bio13
bio15
0.4
0.3
0.2
0.1
0.0
BDW
CEC
NTO
SOC
TWI

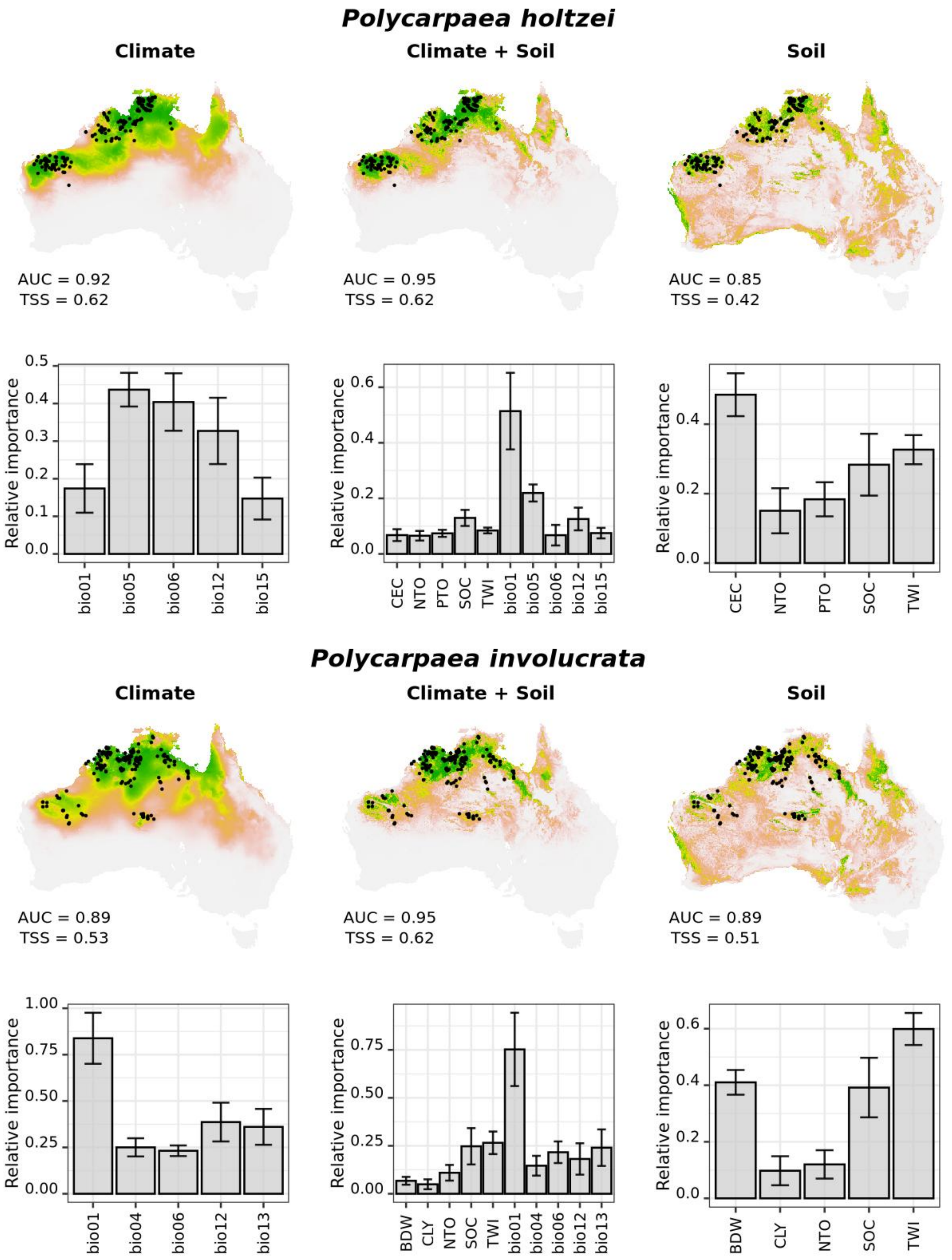
Polycarpaea holtzei
Climate
Climate + Soil
Soil
AUC = 0.92
TSS = 0.62
AUC = 0.95
TSS = 0.62
AUC = 0.85
TSS = 0.42
Relative importance
bio01
bio05
bio06
bio12
bio15
CEC
NTO
PTO
SOC
TWI
Polycarpaea involucrata
Climate
Climate + Soil
Soil
AUC = 0.89
TSS = 0.53
AUC = 0.95
TSS = 0.62
AUC = 0.89
TSS = 0.51
Relative importance
bio01
bio04
bio06
bio12
bio13
BDW
CLY
NTO
SOC
TWI

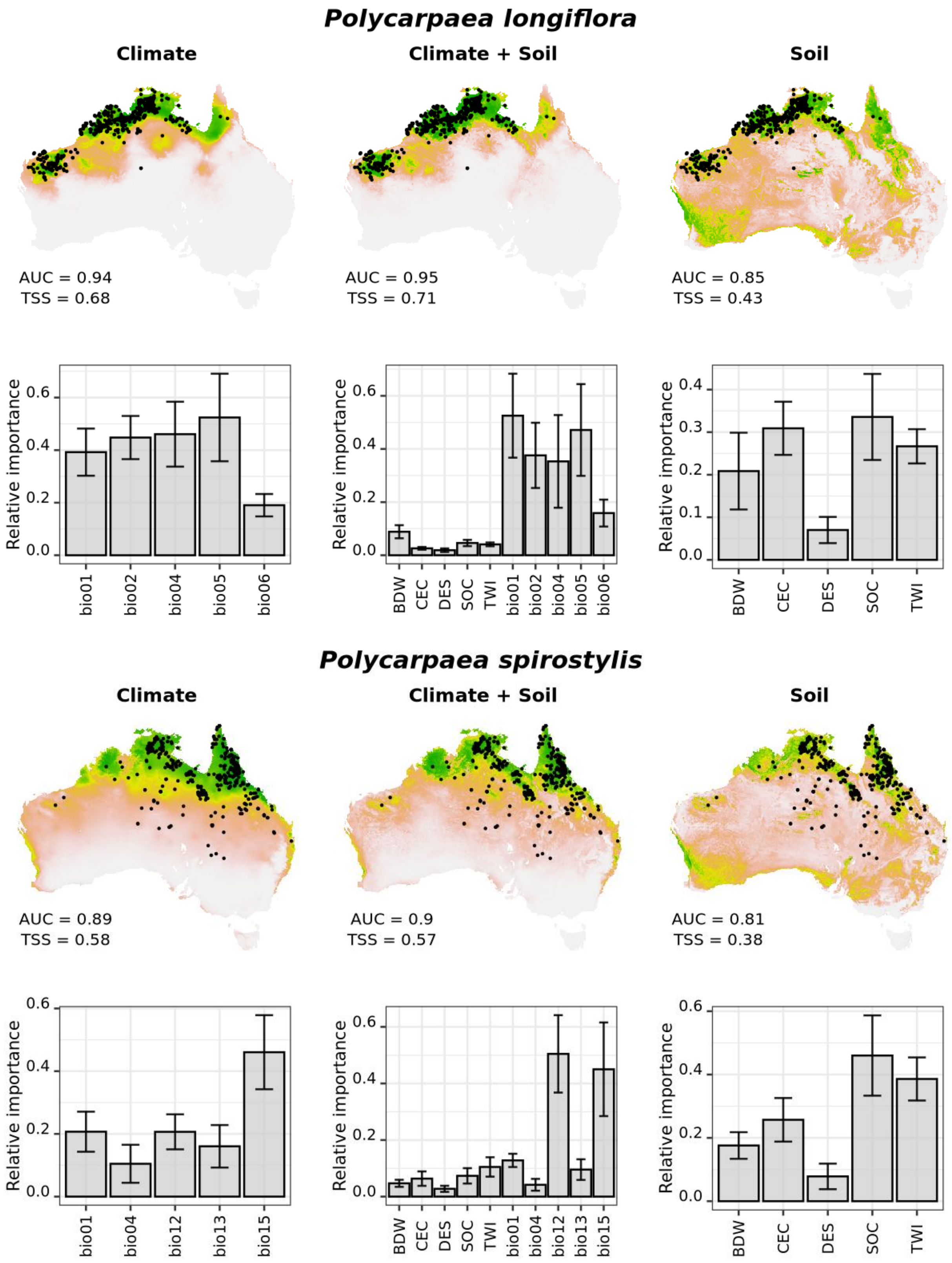

Polycarpaea longiflora
Climate
Climate + Soil
Soil
AUC = 0.94
TSS = 0.68
AUC = 0.95
TSS = 0.71
AUC = 0.85
TSS = 0.43
Relative importance
bio01
bio02
bio04
bio05
bio06
BDW
CEC
DES
SOC
TWI
Polycarpaea spirostylis
Climate
Climate + Soil
Soil
AUC = 0.89
TSS = 0.58
AUC = 0.9
TSS = 0.57
AUC = 0.81
TSS = 0.38
Relative importance
bio01
bio04
bio12
bio13
bio15
BDW
CEC
DES
SOC
TWI

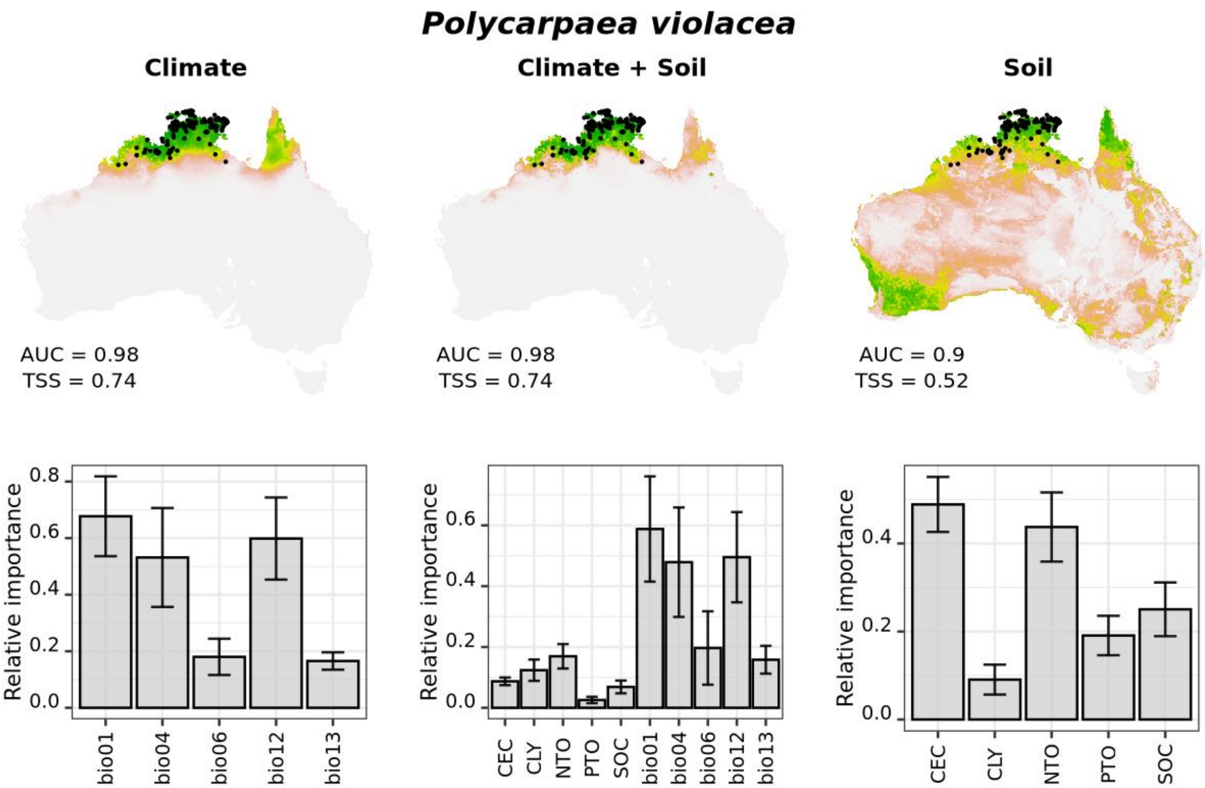
Polycarpaea violacea
Climate
Climate + Soil
Soil
AUC = 0.98
TSS = 0.74
AUC = 0.98
TSS = 0.74
AUC = 0.9
TSS = 0.52
Relative importance
0.0
0.2
0.4
0.6
0.8
bio01
bio04
bio06
bio12
bio13
CEC
CLY
NTO
PTO
SOC
bio01
bio04
bio06
bio12
bio13
CEC
CLY
NTO
PTO
SOC

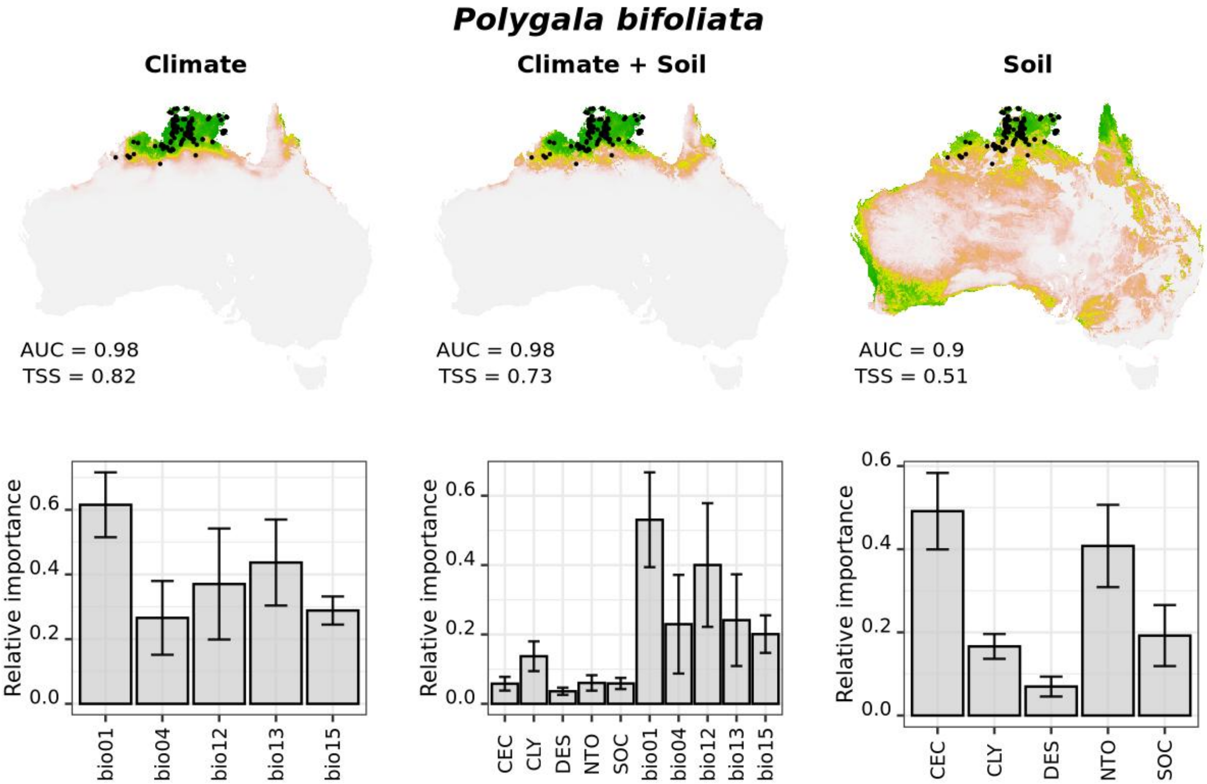
Polygala bifoliata
Climate
Climate + Soil
Soil
AUC = 0.98
TSS = 0.82
AUC = 0.98
TSS = 0.73
AUC = 0.9
TSS = 0.51
Relative importance
0.0
0.2
0.4
0.6
bio01
bio04
bio12
bio13
bio15
CEC
CLY
DES
NTO
SOC
bio01
bio04
bio12
bio13
bio15
CEC
CLY
DES
NTO
SOC

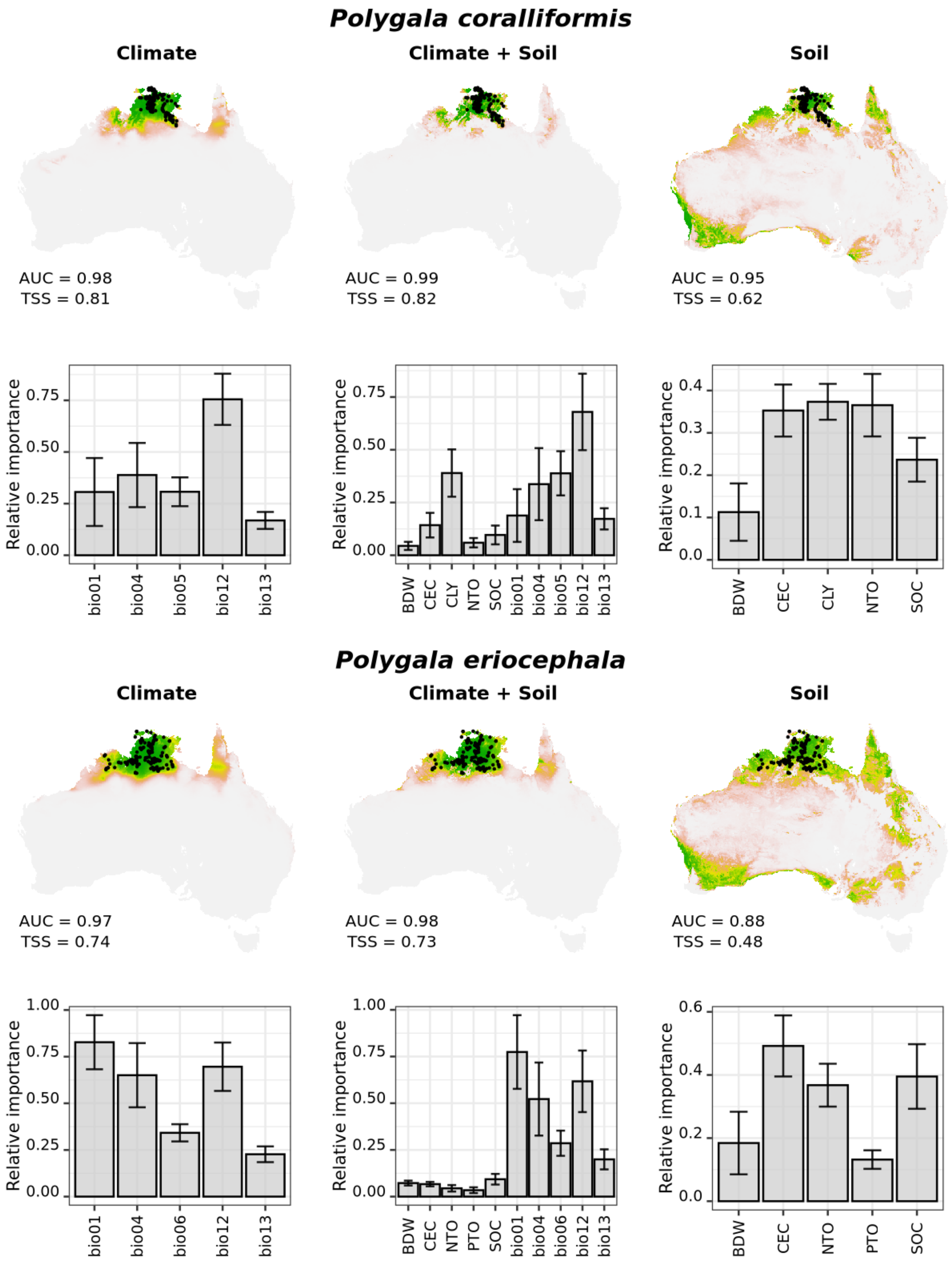
Polygala coralliformis
Climate
Climate + Soil
Soil
AUC = 0.98
TSS = 0.81
AUC = 0.99
TSS = 0.82
AUC = 0.95
TSS = 0.62
Relative importance
0.00
0.25
0.50
0.75
bio01
bio04
bio05
bio12
bio13
BDW
CEC
CLY
NTO
SOC
0.0
0.1
0.2
0.3
0.4
Polygala eriocephala
Climate
Climate + Soil
Soil
AUC = 0.97
TSS = 0.74
AUC = 0.98
TSS = 0.73
AUC = 0.88
TSS = 0.48
1.00
bio06
PTO
0.6

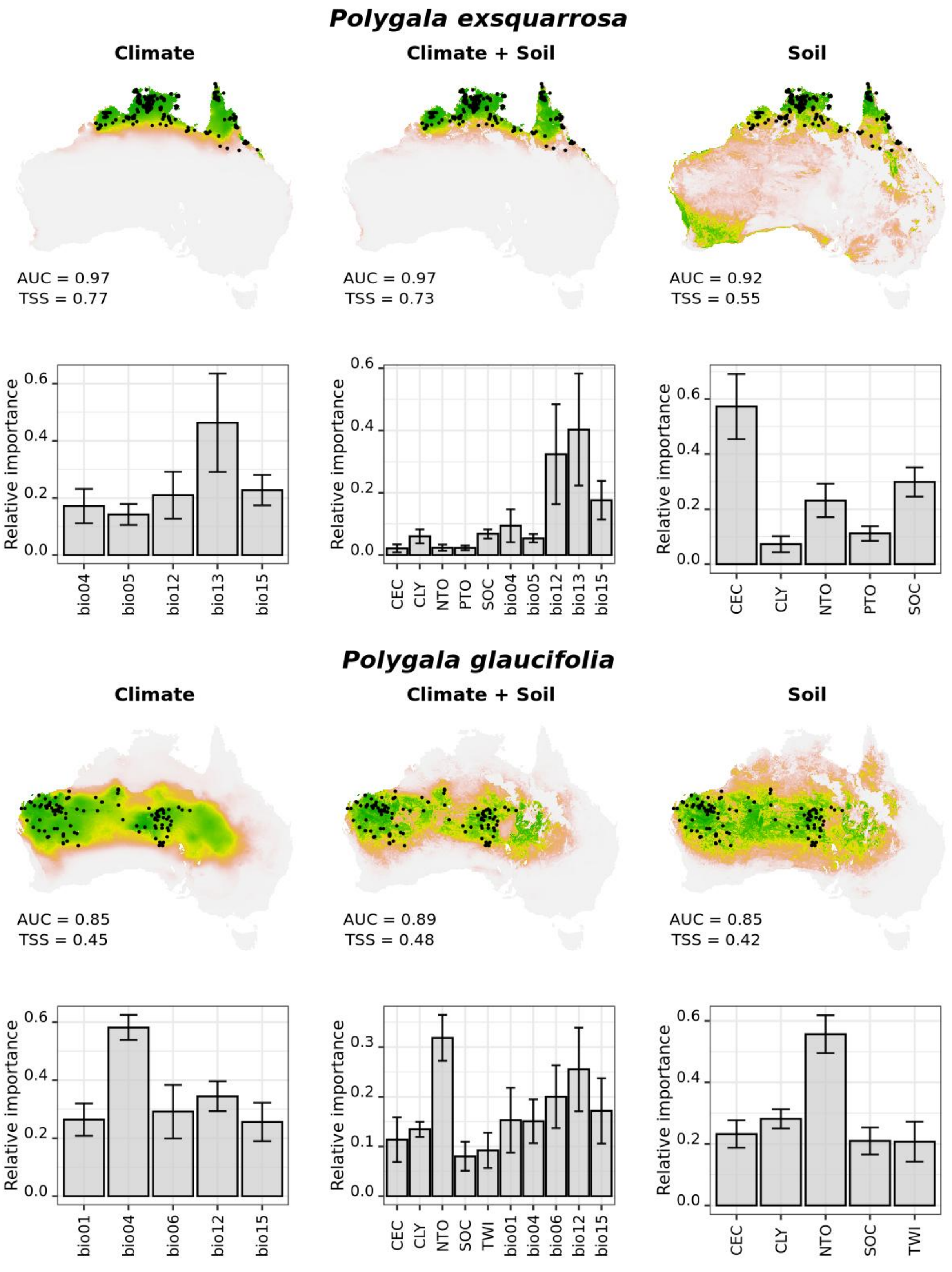

Polygala exsquarrosa
Climate
Climate + Soil
Soil
AUC = 0.97
TSS = 0.77
AUC = 0.97
TSS = 0.73
AUC = 0.92
TSS = 0.55
Relative importance
bio04
bio05
bio12
bio13
bio15
CEC
CLY
NTO
PTO
SOC
Polygala glaucifolia
Climate
Climate + Soil
Soil
AUC = 0.85
TSS = 0.45
AUC = 0.89
TSS = 0.48
AUC = 0.85
TSS = 0.42
Relative importance
bio01
bio04
bio06
bio12
bio15
CEC
CLY
NTO
SOC
TWI

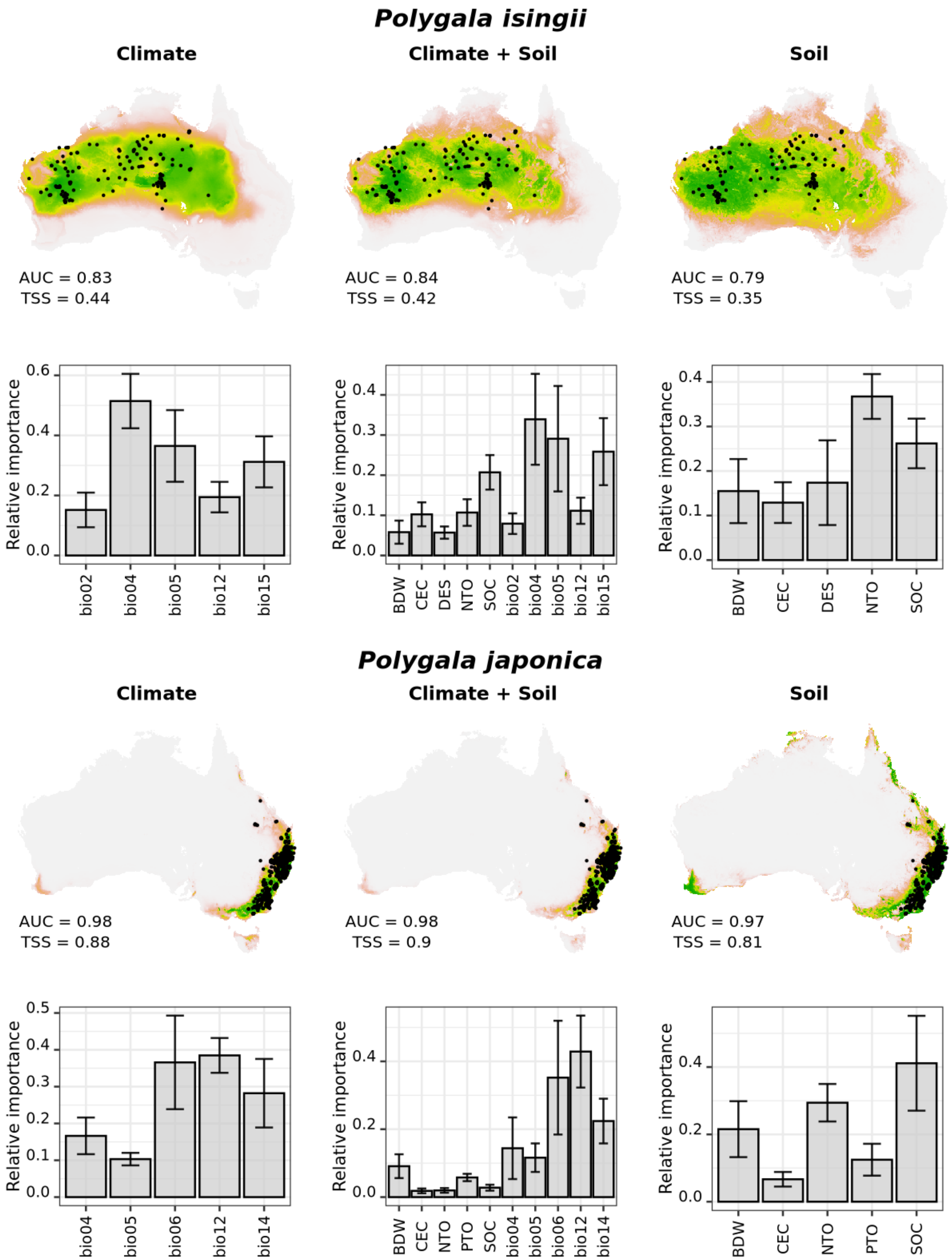

Polygala isingii
Climate
Climate + Soil
Soil
AUC = 0.83
TSS = 0.44
AUC = 0.84
TSS = 0.42
AUC = 0.79
TSS = 0.35
Relative importance
0.0
0.2
0.4
0.6
bio02
bio04
bio05
bio12
bio15
0.0
0.1
0.2
0.3
0.4
BDW
CEC
DES
NTO
SOC
bio02
bio04
bio05
bio12
bio15
0.0
0.1
0.2
0.3
0.4
BDW
CEC
DES
NTO
SOC
Polygala japonica
Climate
Climate + Soil
Soil
AUC = 0.98
TSS = 0.88
AUC = 0.98
TSS = 0.9
AUC = 0.97
TSS = 0.81
Relative importance
0.0
0.1
0.2
0.3
0.4
0.5
bio04
bio05
bio06
bio12
bio14
0.0
0.2
0.4
BDW
CEC
NTO
PTO
SOC
bio04
bio05
bio06
bio12
bio14
0.0
0.2
0.4
BDW
CEC
NTO
PTO
SOC

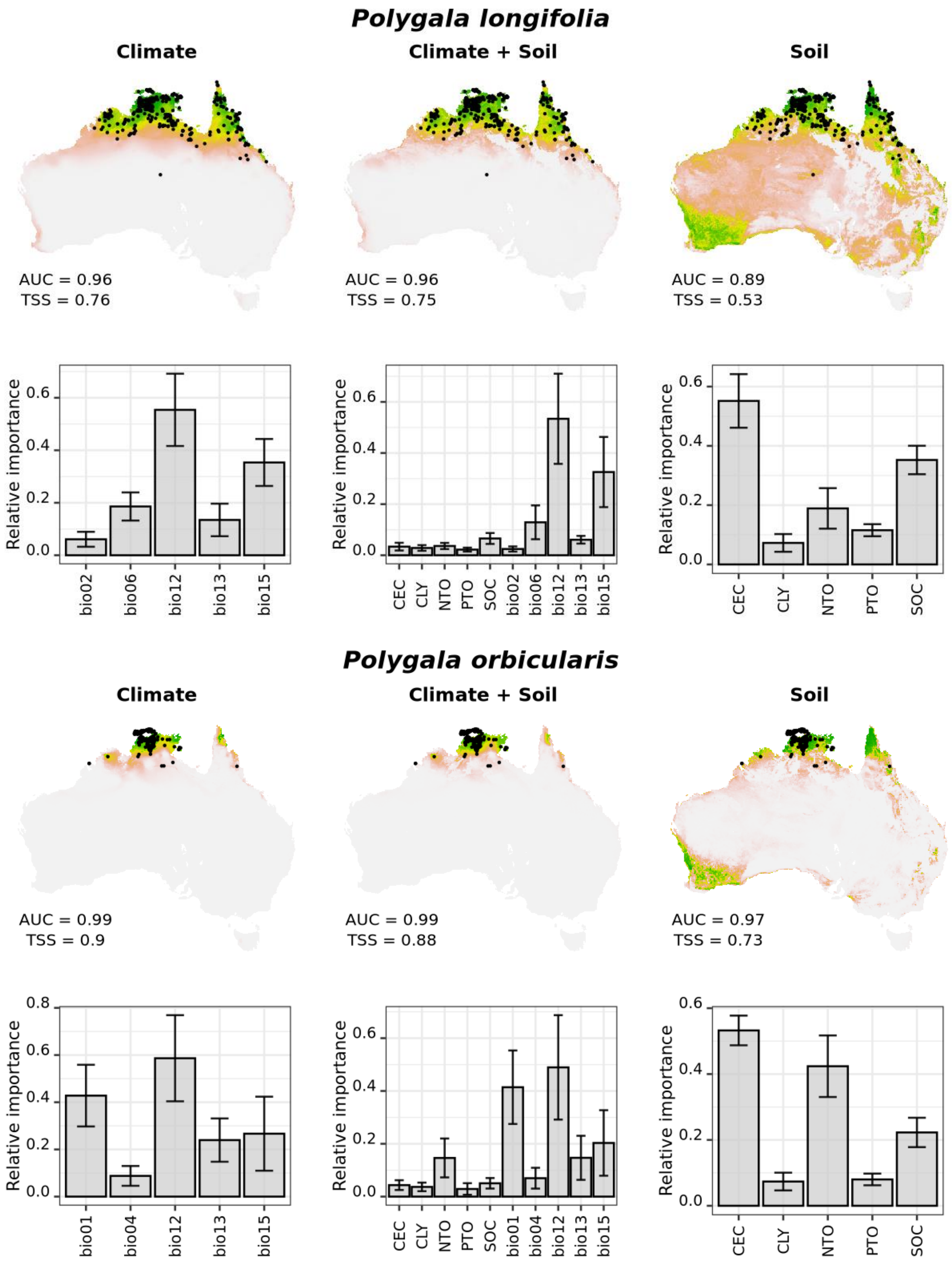
Polygala longifolia
Climate
Climate + Soil
Soil
AUC = 0.96
TSS = 0.76
AUC = 0.96
TSS = 0.75
AUC = 0.89
TSS = 0.53
Relative importance
0.0
0.2
0.4
0.6
bio02
bio06
bio12
bio13
bio15
CEC
CLY
NTO
PTO
SOC
Polygala orbicularis
Climate
Climate + Soil
Soil
AUC = 0.99
TSS = 0.9
AUC = 0.99
TSS = 0.88
AUC = 0.97
TSS = 0.73
Relative importance
0.0
0.2
0.4
0.6
0.8
bio01
bio04
bio12
bio13
bio15
CEC
CLY
NTO
PTO
SOC

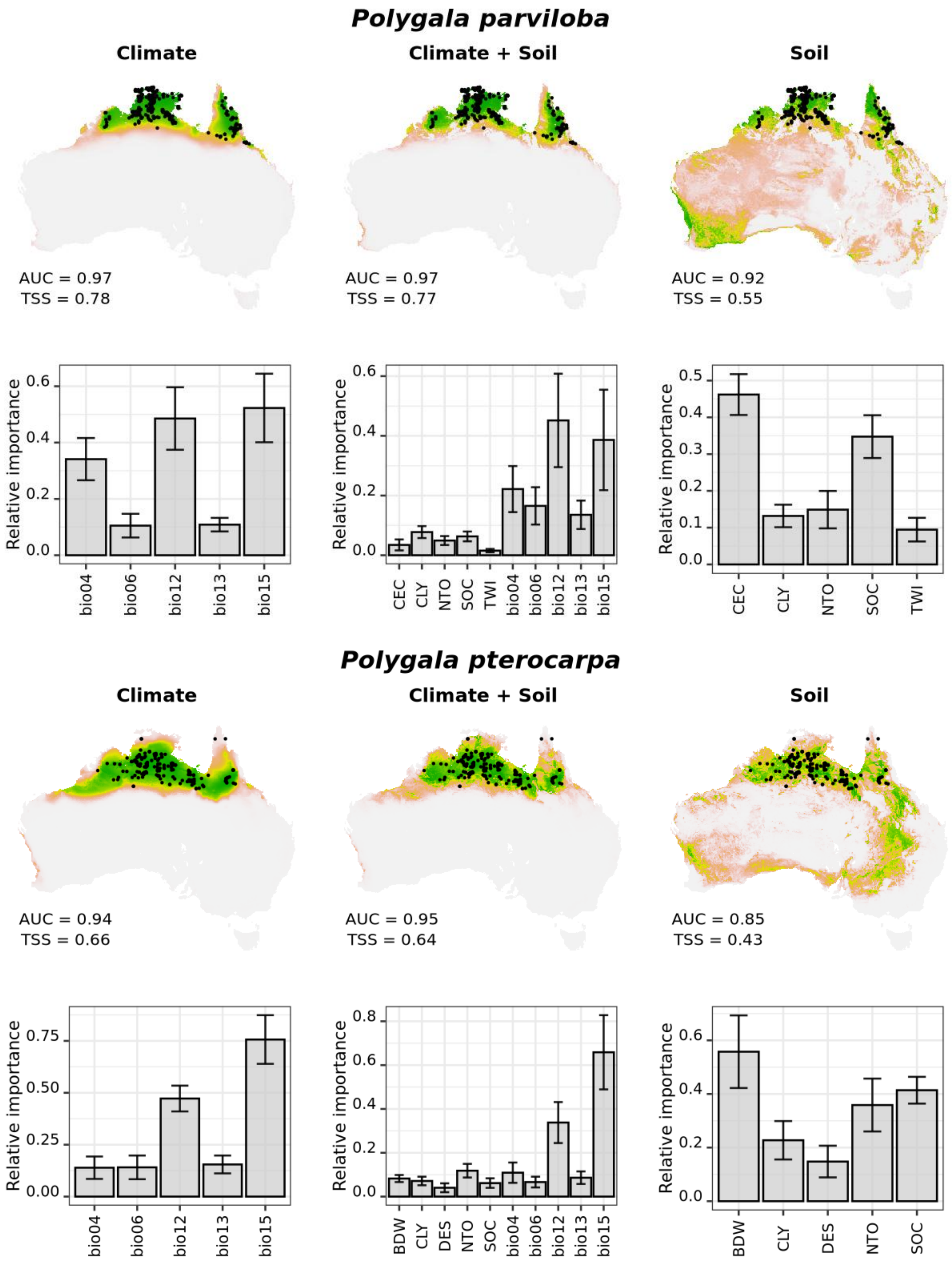
Polygala parviloba
Climate
Climate + Soil
Soil
AUC = 0.97
TSS = 0.78
AUC = 0.97
TSS = 0.77
AUC = 0.92
TSS = 0.55
Relative importance
bio04
bio06
bio12
bio13
bio15
CEC
CLY
NTO
SOC
TWI
Polygala pterocarpa
Climate
Climate + Soil
Soil
AUC = 0.94
TSS = 0.66
AUC = 0.95
TSS = 0.64
AUC = 0.85
TSS = 0.43
Relative importance
bio04
bio06
bio12
bio13
bio15
BDW
CLY
DES
NTO
SOC

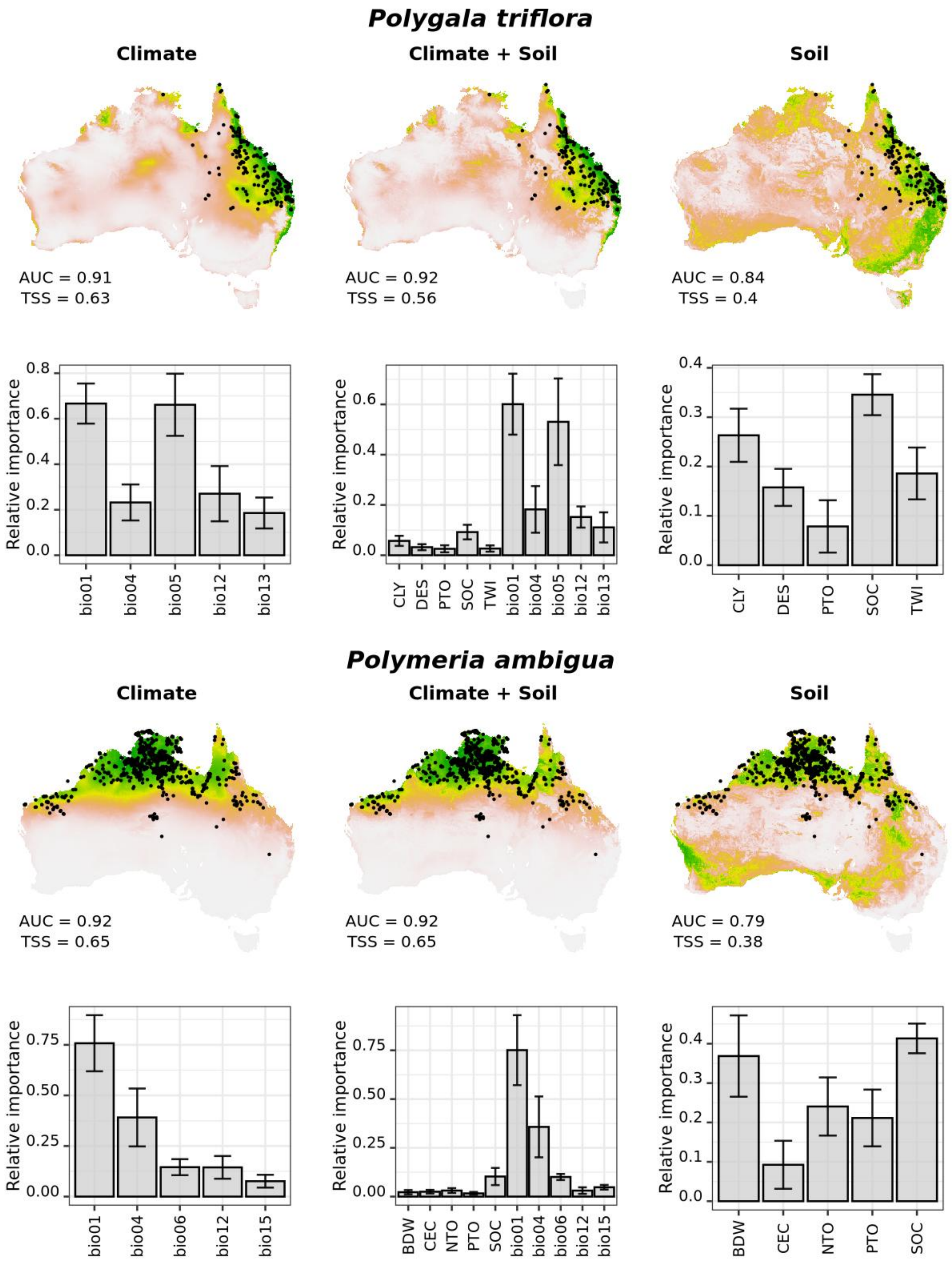
Polygala triflora
Climate
Climate + Soil
Soil
AUC = 0.91
TSS = 0.63
AUC = 0.92
TSS = 0.56
AUC = 0.84
TSS = 0.4
Relative importance
bio01
bio04
bio05
bio12
bio13
CLY
DES
PTO
SOC
TWI
Polymeria ambigua
Climate
Climate + Soil
Soil
AUC = 0.92
TSS = 0.65
AUC = 0.92
TSS = 0.65
AUC = 0.79
TSS = 0.38
Relative importance
bio01
bio04
bio06
bio12
bio15
BDW
CEC
NTO
PTO
SOC

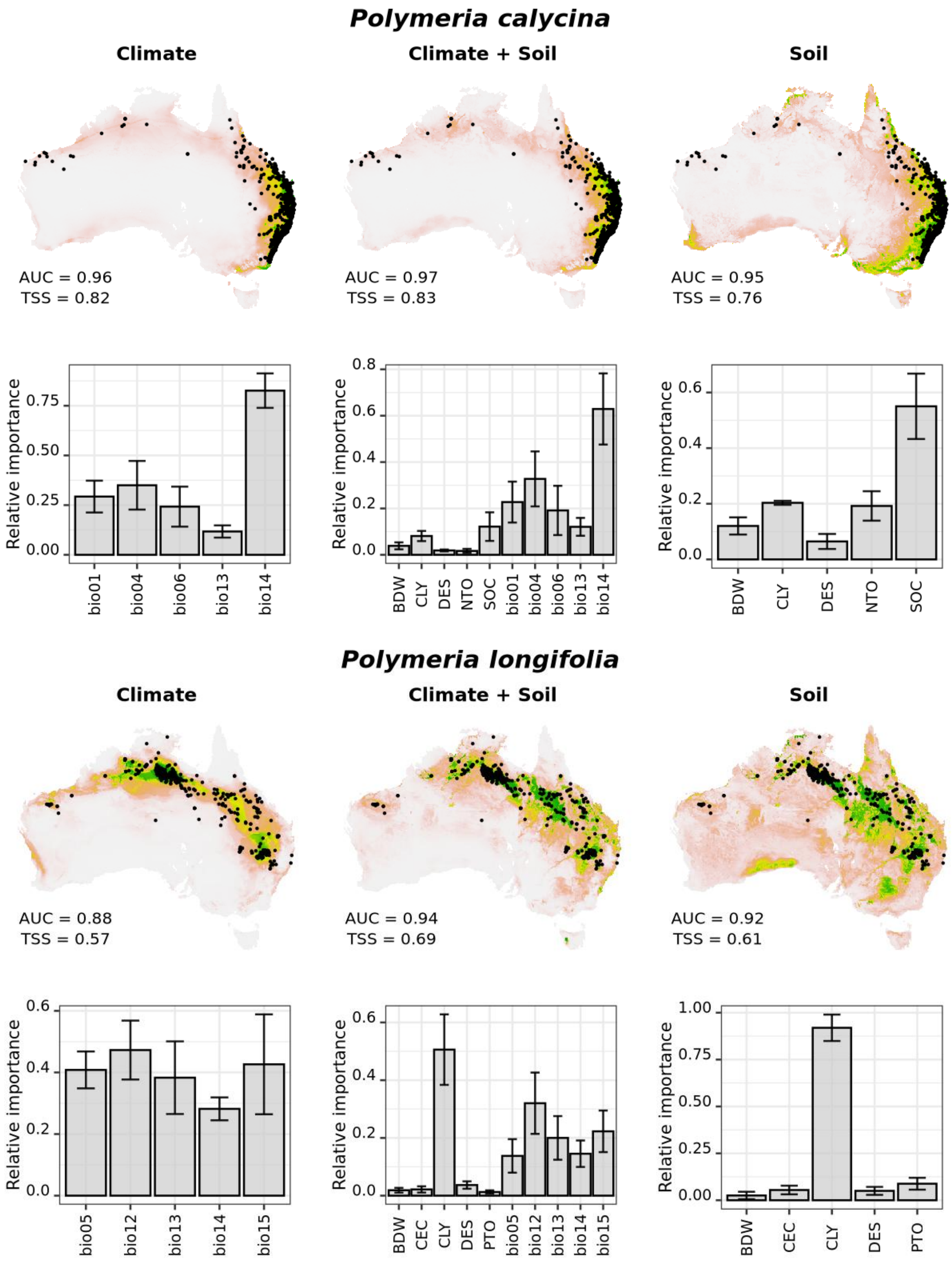

Polymeria calycina
Climate
Climate + Soil
Soil
AUC = 0.96
TSS = 0.82
AUC = 0.97
TSS = 0.83
AUC = 0.95
TSS = 0.76
Relative importance
0.00
0.25
0.50
0.75
bio01
bio04
bio06
bio13
bio14
0.0
0.2
0.4
0.6
0.8
BDW
CLY
DES
NTO
SOC
bio01
bio04
bio06
bio13
bio14
0.0
0.2
0.4
0.6
BDW
CLY
DES
NTO
SOC
Polymeria longifolia
Climate
Climate + Soil
Soil
AUC = 0.88
TSS = 0.57
AUC = 0.94
TSS = 0.69
AUC = 0.92
TSS = 0.61
Relative importance
0.0
0.2
0.4
0.6
bio05
bio12
bio13
bio14
bio15
BDW
CEC
CLY
DES
PTO
bio05
bio12
bio13
bio14
bio15
0.00
0.25
0.50
0.75
1.00
BDW
CEC
CLY
DES
PTO

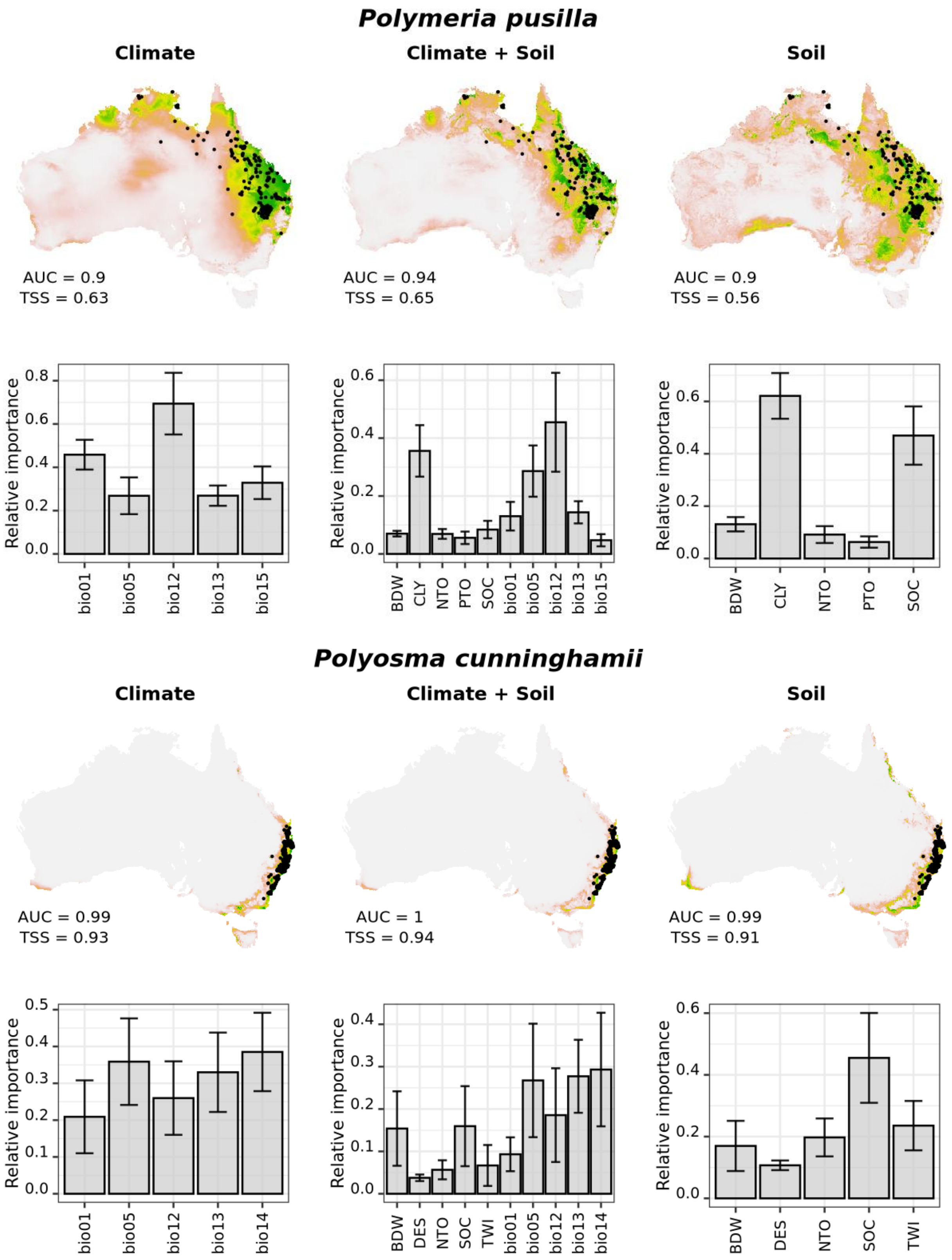
Polymeria pusilla
Climate
Climate + Soil
Soil
AUC = 0.9
TSS = 0.63
AUC = 0.94
TSS = 0.65
AUC = 0.9
TSS = 0.56
Relative importance
bio01 bio05 bio12 bio13 bio15
BDW CLY NTO PTO SOC bio01 bio05 bio12 bio13 bio15
BDW CLY NTO PTO SOC
Polyosma cunninghamii
Climate
Climate + Soil
Soil
AUC = 0.99
TSS = 0.93
AUC = 1
TSS = 0.94
AUC = 0.99
TSS = 0.91
Relative importance
bio01 bio05 bio12 bio13 bio14
BDW DES NTO SOC TWI bio01 bio05 bio12 bio13 bio14
BDW DES NTO SOC TWI

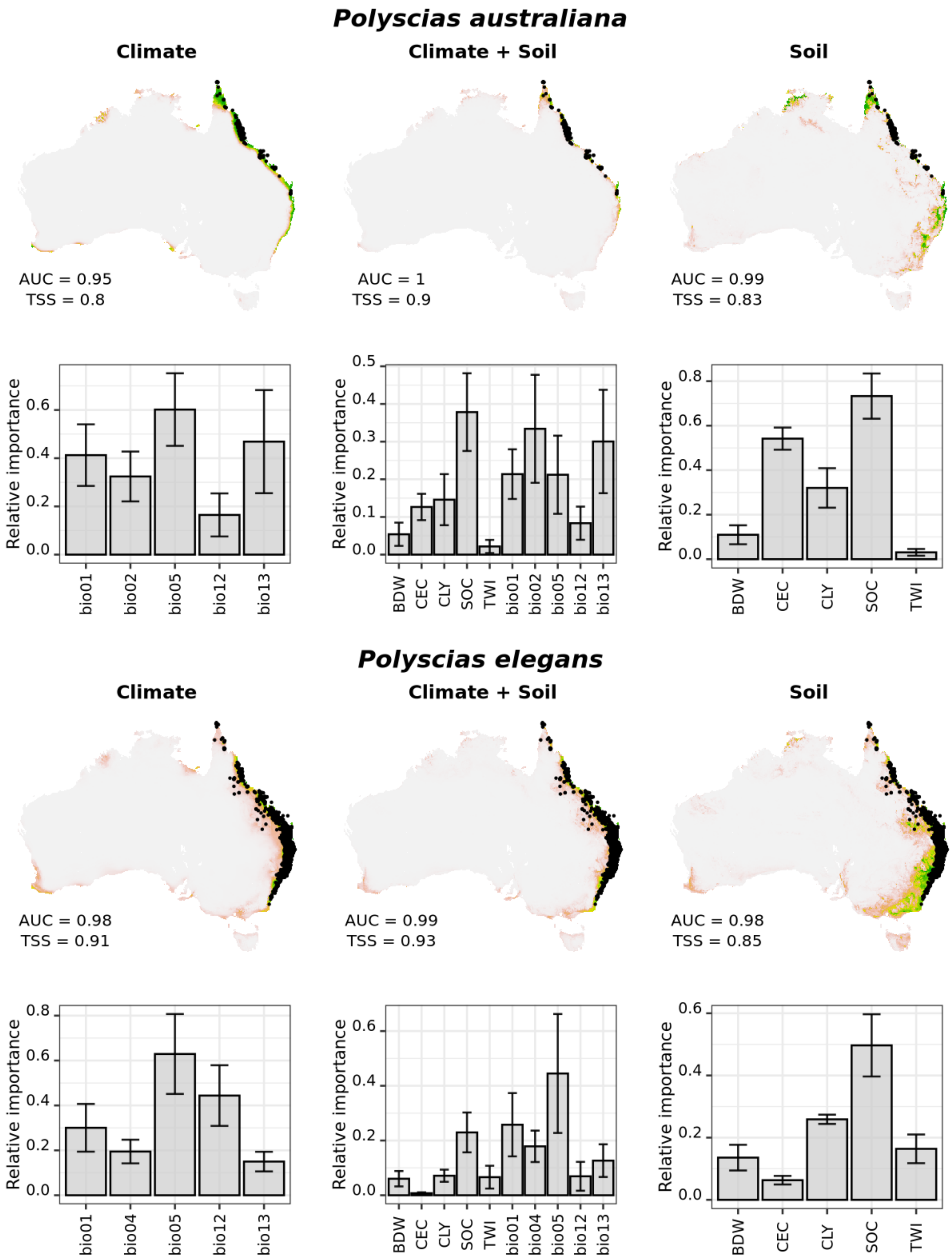

Polyscias australiana
Climate
Climate + Soil
Soil
AUC = 0.95
TSS = 0.8
AUC = 1
TSS = 0.9
AUC = 0.99
TSS = 0.83
Relative importance
bio01
bio02
bio05
bio12
bio13
BDW
CEC
CLY
SOC
TWI
Polyscias elegans
Climate
Climate + Soil
Soil
AUC = 0.98
TSS = 0.91
AUC = 0.99
TSS = 0.93
AUC = 0.98
TSS = 0.85
Relative importance
bio01
bio04
bio05
bio12
bio13
BDW
CEC
CLY
SOC
TWI

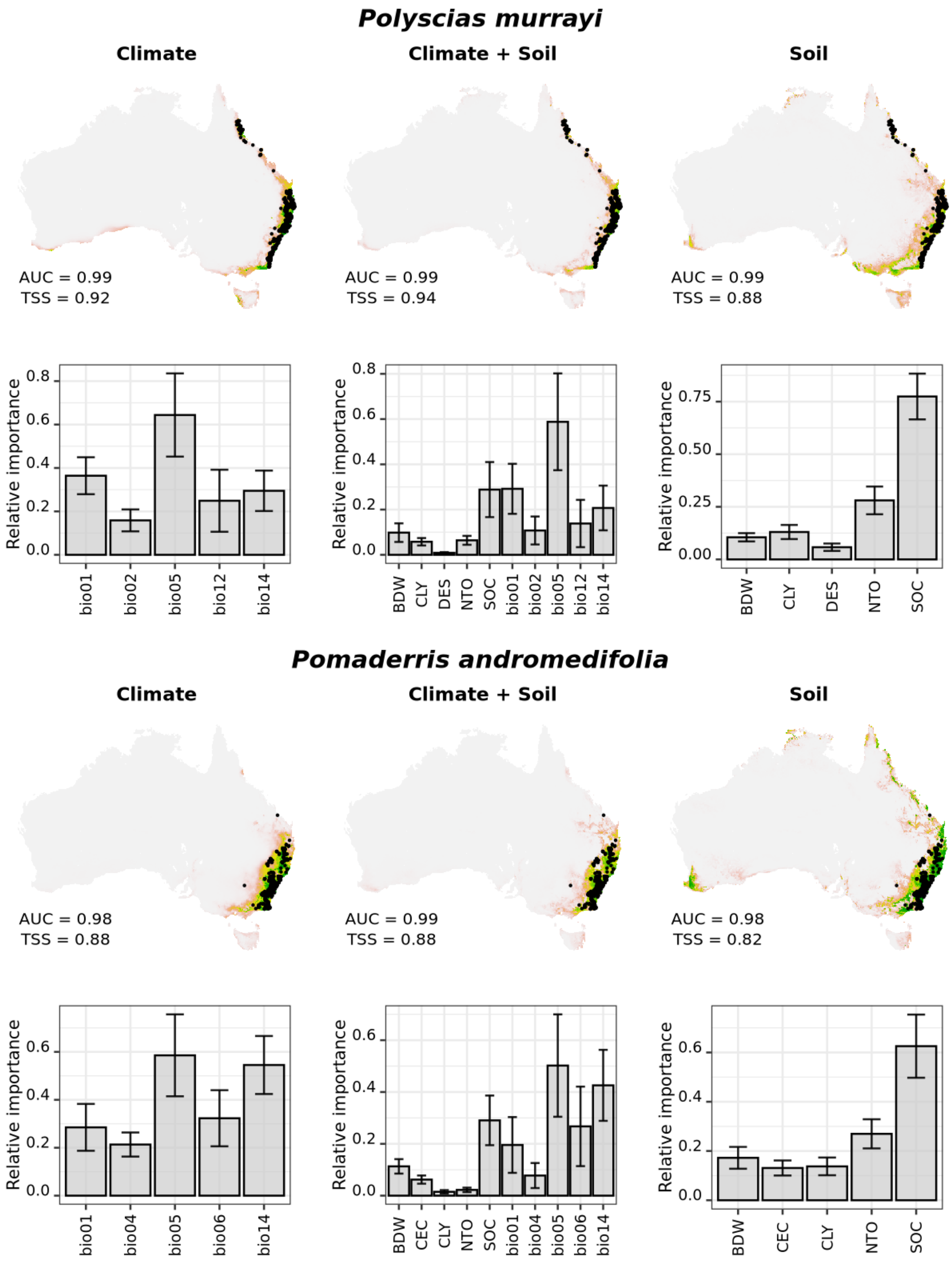
Polyscias murrayi
Climate
Climate + Soil
Soil
AUC = 0.99
TSS = 0.92
AUC = 0.99
TSS = 0.94
AUC = 0.99
TSS = 0.88
Relative importance
bio01
bio02
bio05
bio12
bio14
BDW
CLY
DES
NTO
SOC
Pomaderris andromedifolia
Climate
Climate + Soil
Soil
AUC = 0.98
TSS = 0.88
AUC = 0.99
TSS = 0.88
AUC = 0.98
TSS = 0.82
Relative importance
bio01
bio04
bio05
bio06
bio14
BDW
CEC
CLY
NTO
SOC

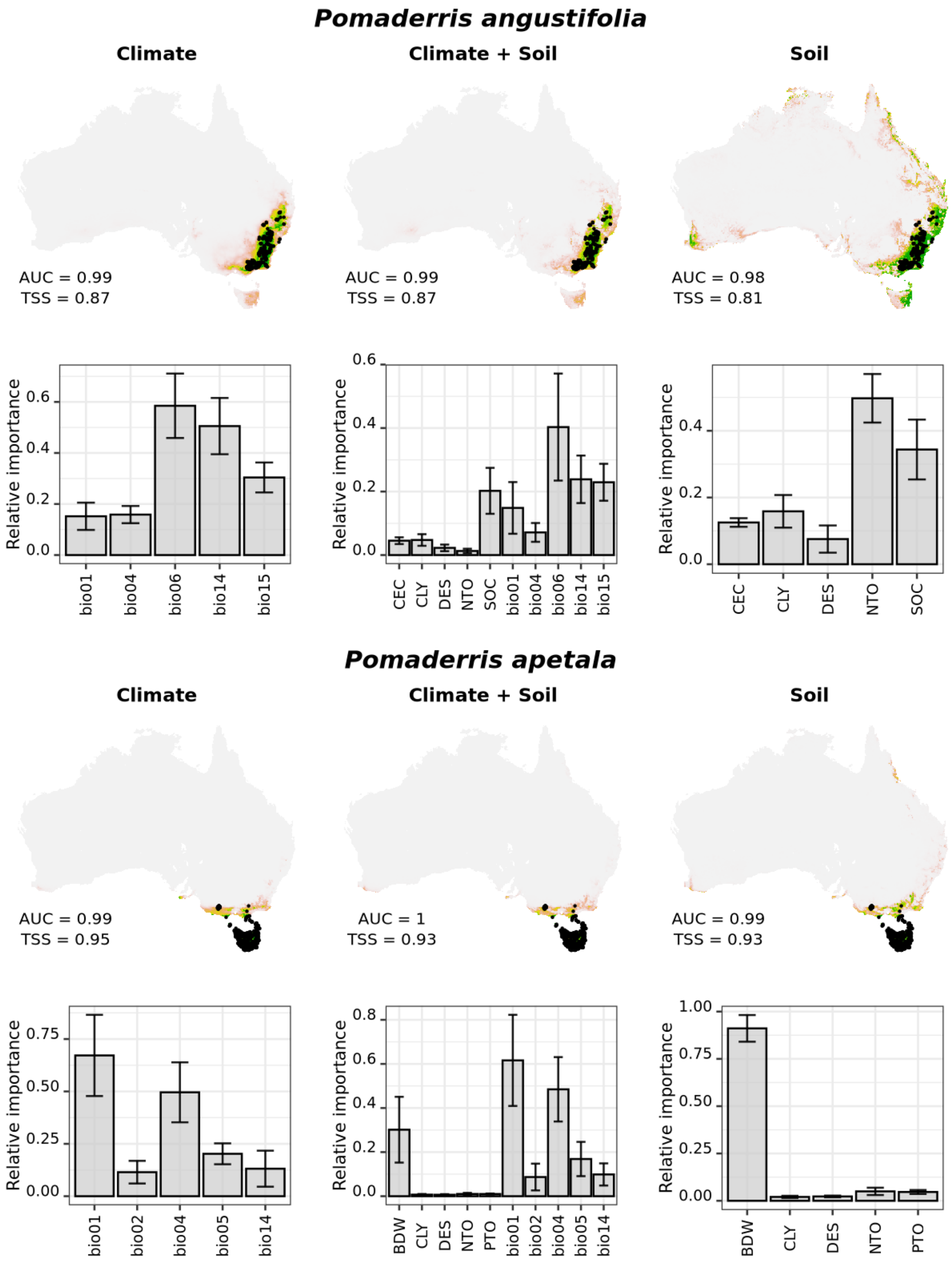
Pomaderris angustifolia
Climate
Climate + Soil
Soil
AUC = 0.99
TSS = 0.87
AUC = 0.99
TSS = 0.87
AUC = 0.98
TSS = 0.81
Relative importance
bio01
bio04
bio06
bio14
bio15
CEC
CLY
DES
NTO
SOC
Pomaderris apetala
Climate
Climate + Soil
Soil
AUC = 0.99
TSS = 0.95
AUC = 1
TSS = 0.93
AUC = 0.99
TSS = 0.93
bio01
bio02
bio04
bio05
bio14
BDW
CLY
DES
NTO
PTO

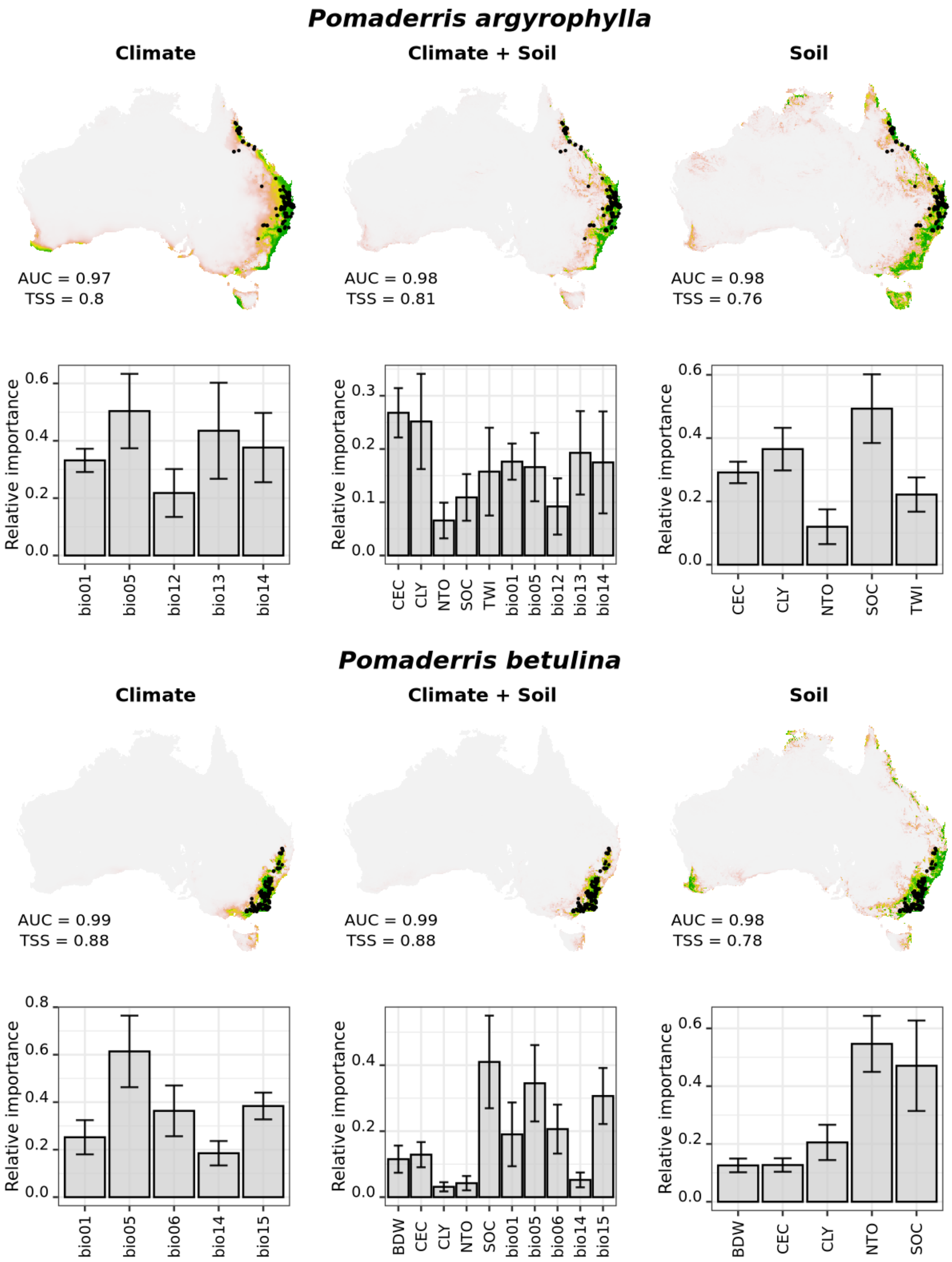

Pomaderris argyrophylla
Climate
Climate + Soil
Soil
AUC = 0.97
TSS = 0.8
AUC = 0.98
TSS = 0.81
AUC = 0.98
TSS = 0.76
Relative importance
0.0
0.2
0.4
0.6
bio01
bio05
bio12
bio13
bio14
Relative importance
0.0
0.1
0.2
0.3
CEC
CLY
NTO
SOC
TWI
bio01
bio05
bio12
bio13
bio14
Relative importance
0.0
0.2
0.4
0.6
CEC
CLY
NTO
SOC
TWI
Pomaderris betulina
Climate
Climate + Soil
Soil
AUC = 0.99
TSS = 0.88
AUC = 0.99
TSS = 0.88
AUC = 0.98
TSS = 0.78
Relative importance
0.0
0.2
0.4
0.6
0.8
bio01
bio05
bio06
bio14
bio15
Relative importance
0.0
0.2
0.4
BDW
CEC
CLY
NTO
SOC
bio01
bio05
bio06
bio14
bio15
Relative importance
0.0
0.2
0.4
0.6
BDW
CEC
CLY
NTO
SOC

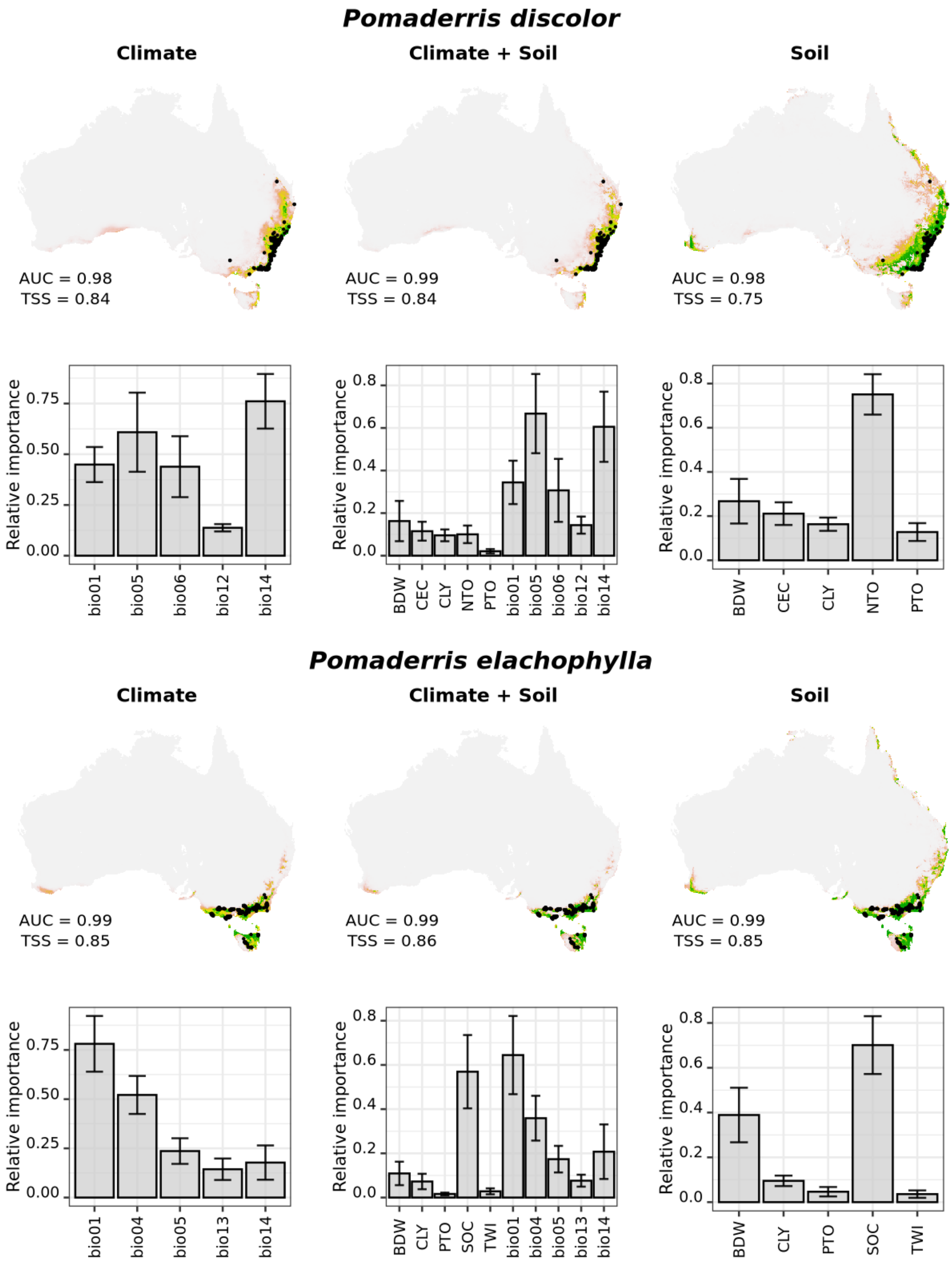

Pomaderris discolor
Climate
Climate + Soil
Soil
AUC = 0.98
TSS = 0.84
AUC = 0.99
TSS = 0.84
AUC = 0.98
TSS = 0.75
Relative importance
0.00
0.25
0.50
0.75
bio01
bio05
bio06
bio12
bio14
0.0
0.2
0.4
0.6
0.8
BDW
CEC
CLY
NTO
PTO
bio01
bio05
bio06
bio12
bio14
BDW
CEC
CLY
NTO
PTO
Pomaderris elachophylla
Climate
Climate + Soil
Soil
AUC = 0.99
TSS = 0.85
AUC = 0.99
TSS = 0.86
AUC = 0.99
TSS = 0.85
bio01
bio04
bio05
bio13
bio14
BDW
CLY
PTO
SOC
TWI
bio01
bio04
bio05
bio13
bio14
BDW
CLY
PTO
SOC
TWI

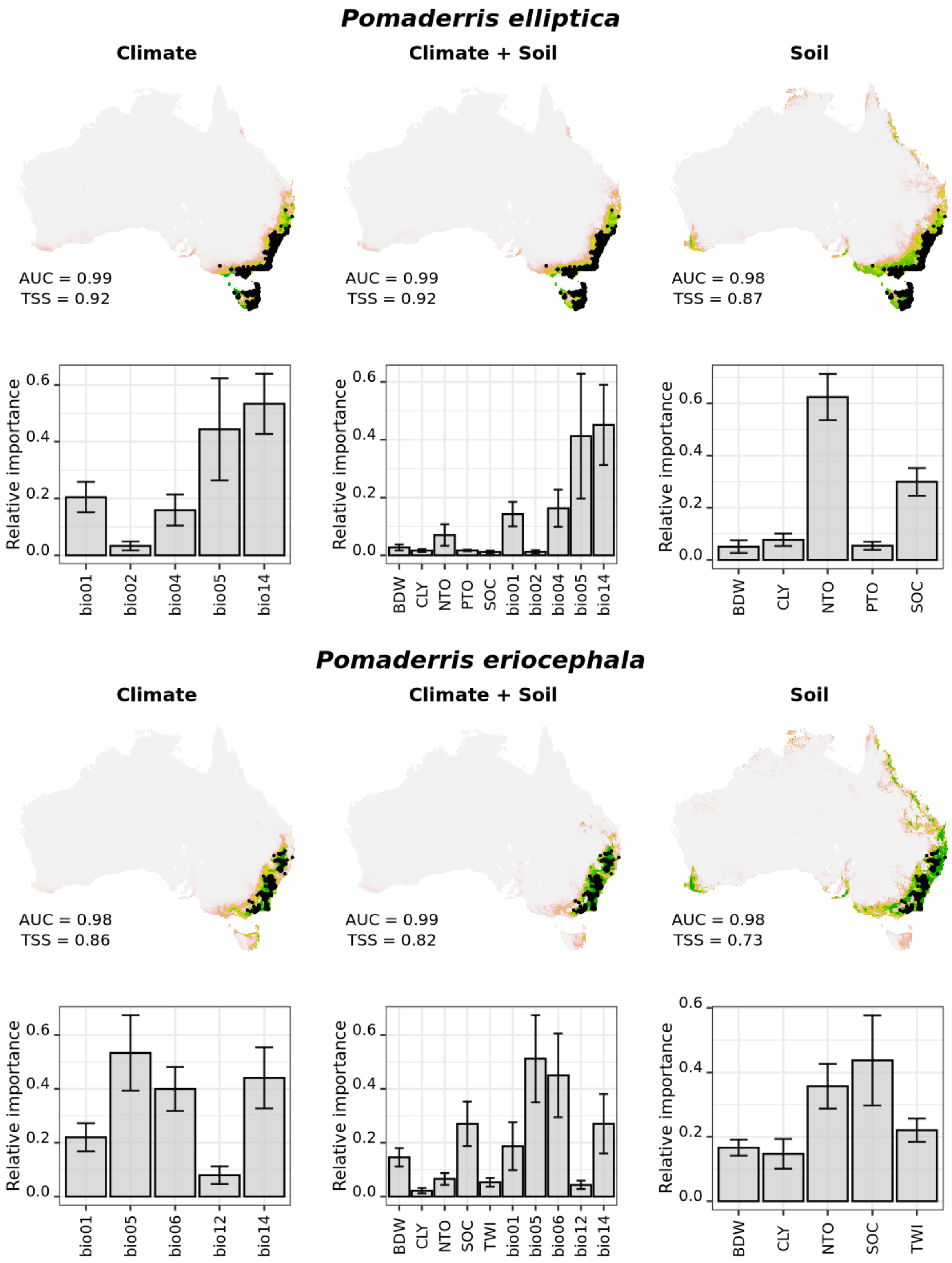

Pomaderris elliptica
Climate
Climate + Soil
Soil
AUC = 0.99
TSS = 0.92
AUC = 0.99
TSS = 0.92
AUC = 0.98
TSS = 0.87
Relative importance
0.0
0.2
0.4
0.6
bio01
bio02
bio04
bio05
bio14
BDW
CLY
NTO
PTO
SOC
bio01
bio02
bio04
bio05
bio14
BDW
CLY
NTO
PTO
SOC
Pomaderris eriocephala
Climate
Climate + Soil
Soil
AUC = 0.98
TSS = 0.86
AUC = 0.99
TSS = 0.82
AUC = 0.98
TSS = 0.73
bio01
bio05
bio06
bio12
bio14
BDW
CLY
NTO
SOC
TWI
bio01
bio05
bio06
bio12
bio14
BDW
CLY
NTO
SOC
TWI

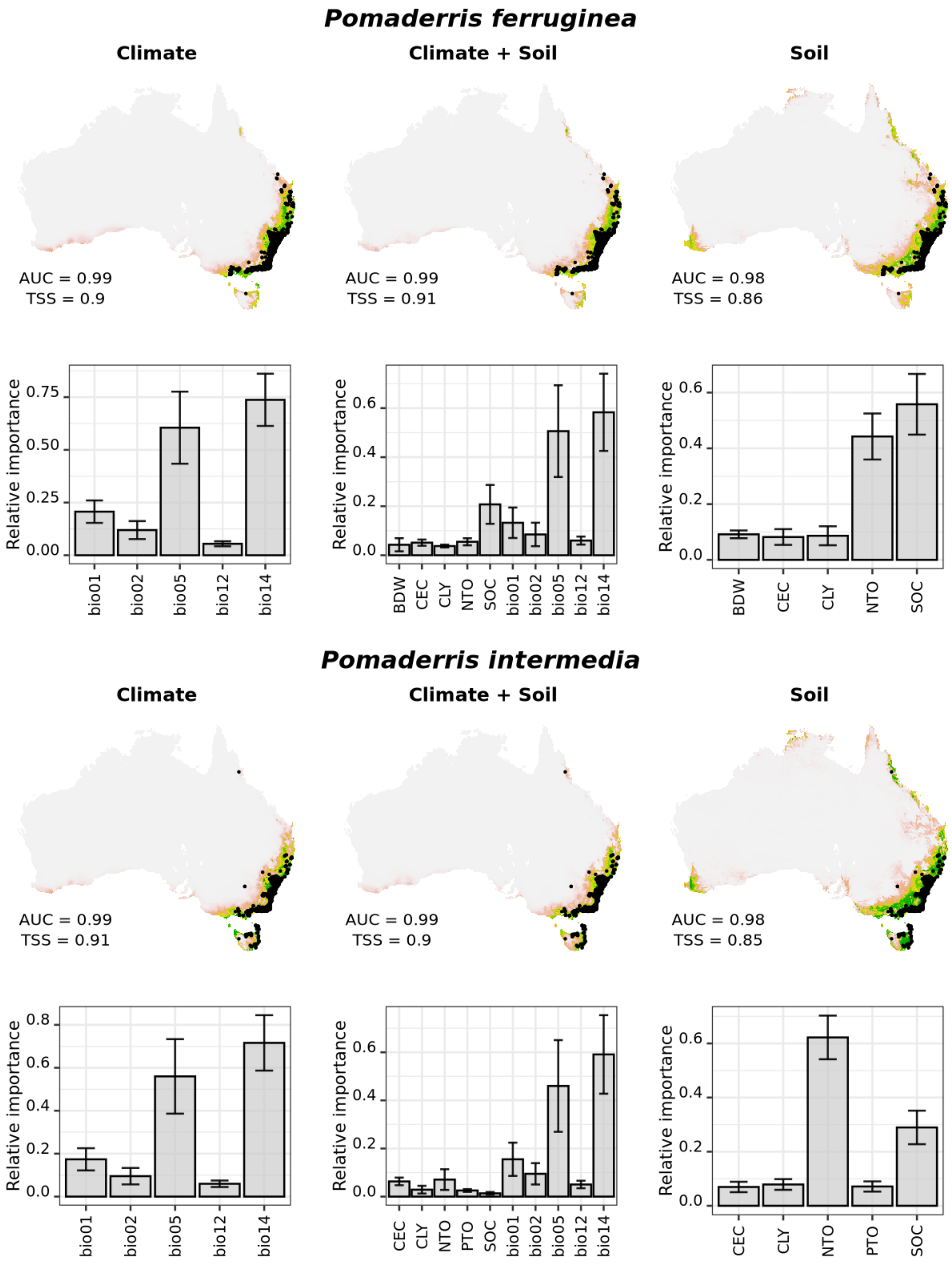
Pomaderris ferruginea
Climate
Climate + Soil
Soil
AUC = 0.99
TSS = 0.9
AUC = 0.99
TSS = 0.91
AUC = 0.98
TSS = 0.86
Relative importance
0.00
0.25
0.50
0.75
bio01
bio02
bio05
bio12
bio14
0.0
0.2
0.4
0.6
BDW
CEC
CLY
NTO
SOC
bio01
bio02
bio05
bio12
bio14
BDW
CEC
CLY
NTO
SOC
Pomaderris intermedia
Climate
Climate + Soil
Soil
AUC = 0.99
TSS = 0.91
AUC = 0.99
TSS = 0.9
AUC = 0.98
TSS = 0.85
Relative importance
0.0
0.2
0.4
0.6
0.8
bio01
bio02
bio05
bio12
bio14
CEC
CLY
NTO
PTO
SOC
bio01
bio02
bio05
bio12
bio14
CEC
CLY
NTO
PTO
SOC

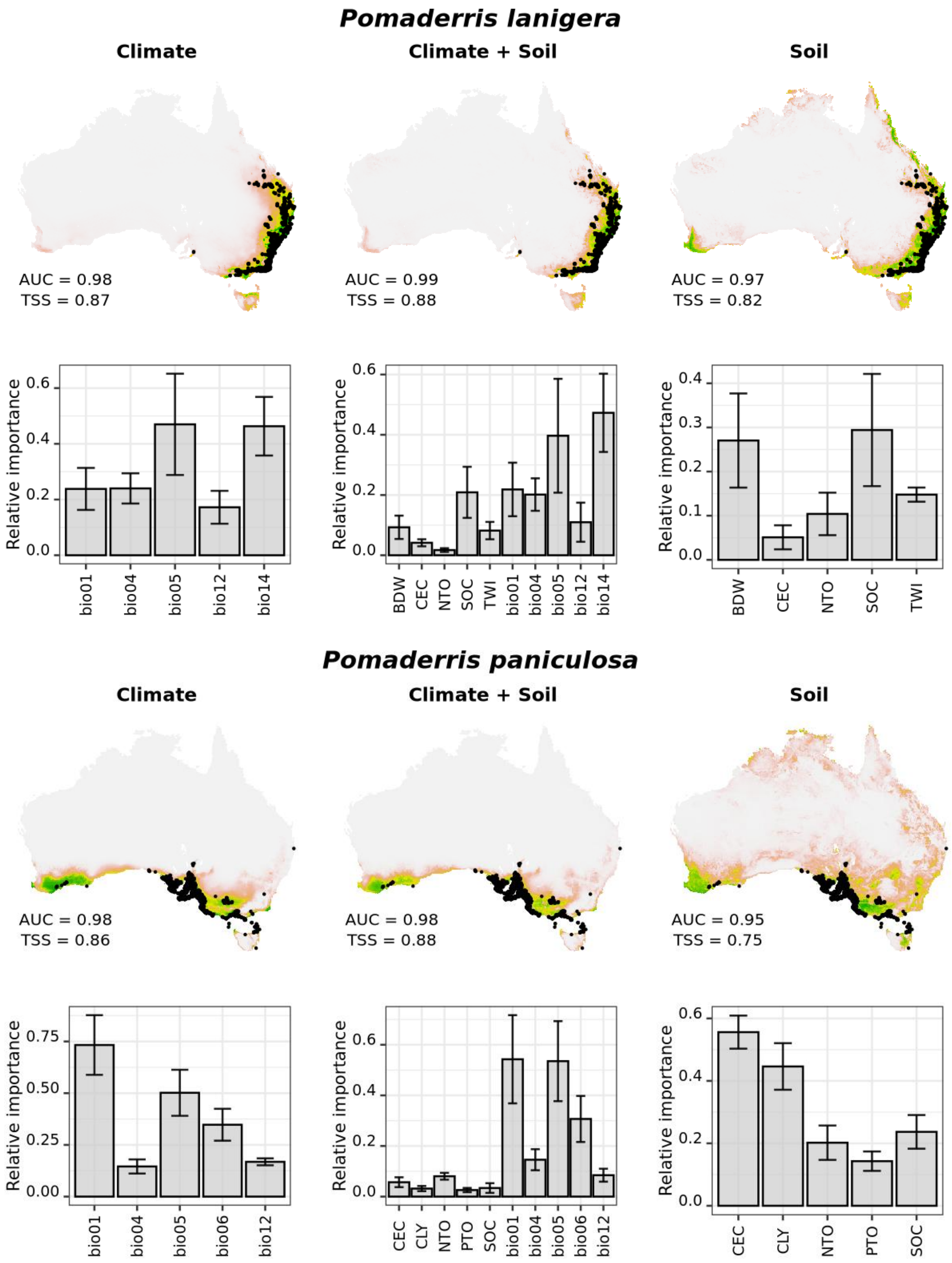

Pomaderris lanigera
Climate
Climate + Soil
Soil
AUC = 0.98
TSS = 0.87
AUC = 0.99
TSS = 0.88
AUC = 0.97
TSS = 0.82
Relative importance
bio01 bio04 bio05 bio12 bio14
BDW CEC NTO SOC TWI bio01 bio04 bio05 bio12 bio14
BDW CEC NTO SOC TWI
Pomaderris paniculosa
Climate
Climate + Soil
Soil
AUC = 0.98
TSS = 0.86
AUC = 0.98
TSS = 0.88
AUC = 0.95
TSS = 0.75
Relative importance
bio01 bio04 bio05 bio06 bio12
CEC CLY NTO PTO SOC bio01 bio04 bio05 bio06 bio12
CEC CLY NTO PTO SOC

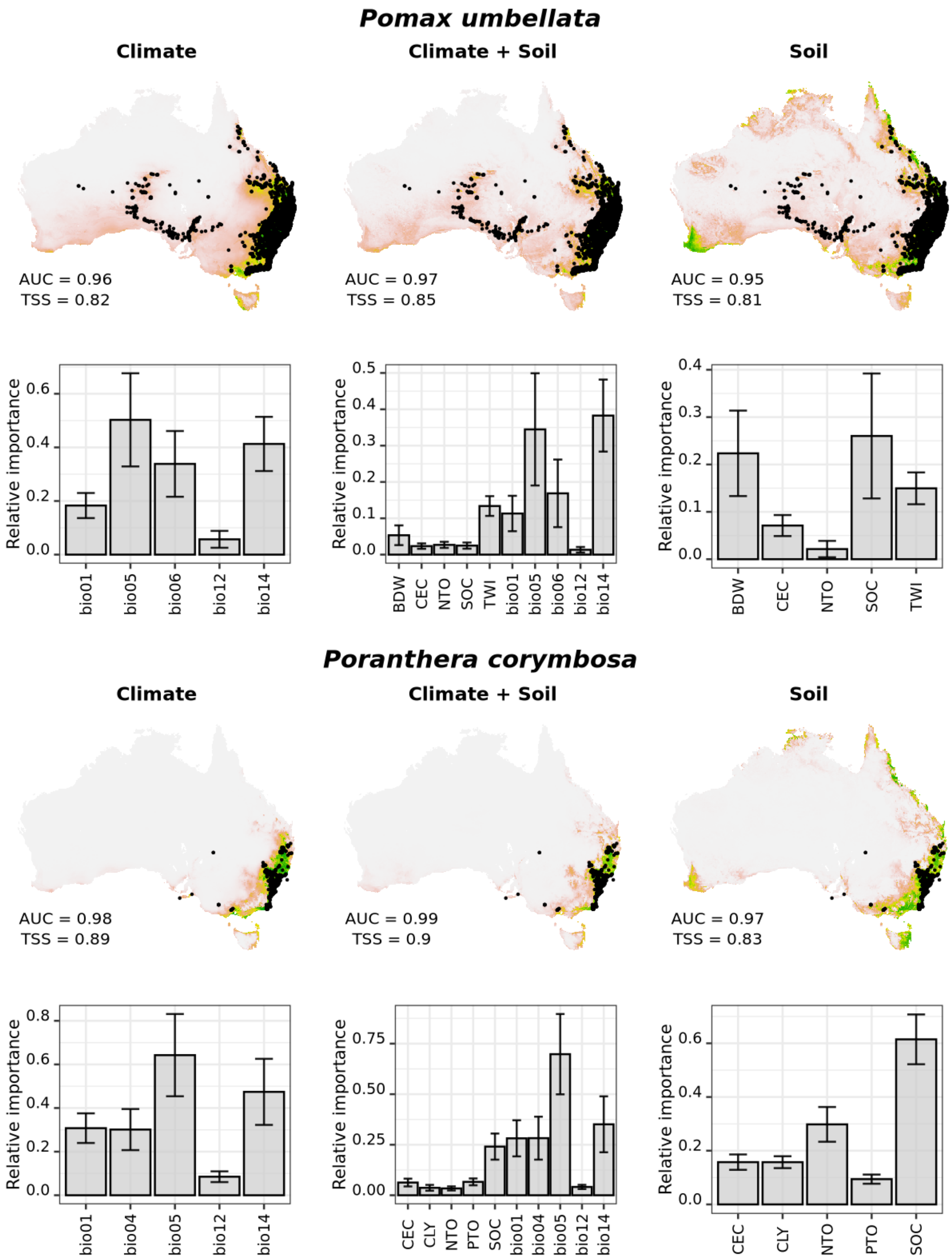
Pomax umbellata
Climate
Climate + Soil
Soil
AUC = 0.96
TSS = 0.82
AUC = 0.97
TSS = 0.85
AUC = 0.95
TSS = 0.81
Relative importance
bio01 bio05 bio06 bio12 bio14
BDW CEC NTO SOC TWI bio01 bio05 bio06 bio12 bio14
BDW CEC NTO SOC TWI
Poranthera corymbosa
Climate
Climate + Soil
Soil
AUC = 0.98
TSS = 0.89
AUC = 0.99
TSS = 0.9
AUC = 0.97
TSS = 0.83
Relative importance
bio01 bio04 bio05 bio12 bio14
CEC CLY NTO PTO SOC bio01 bio04 bio05 bio12 bio14
CEC CLY NTO PTO SOC

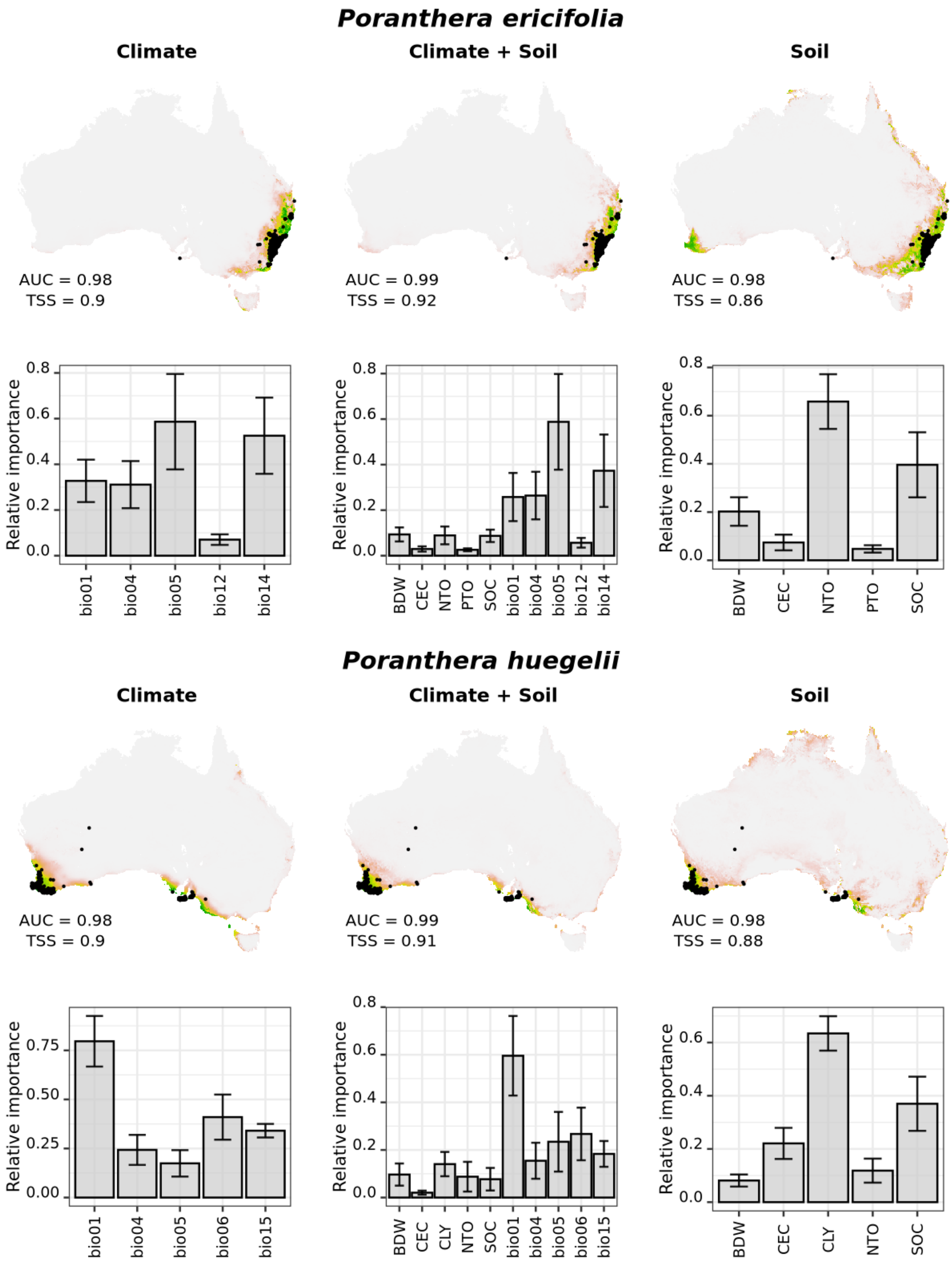
Poranthera ericifolia
Climate
Climate + Soil
Soil
AUC = 0.98
TSS = 0.9
AUC = 0.99
TSS = 0.92
AUC = 0.98
TSS = 0.86
Relative importance
bio01 bio04 bio05 bio12 bio14
BDW CEC NTO PTO SOC bio01 bio04 bio05 bio12 bio14
BDW CEC NTO PTO SOC
Poranthera huegelii
Climate
Climate + Soil
Soil
AUC = 0.98
TSS = 0.9
AUC = 0.99
TSS = 0.91
AUC = 0.98
TSS = 0.88
Relative importance
bio01 bio04 bio05 bio06 bio15
BDW CEC CLY NTO SOC bio01 bio04 bio05 bio06 bio15
BDW CEC CLY NTO SOC

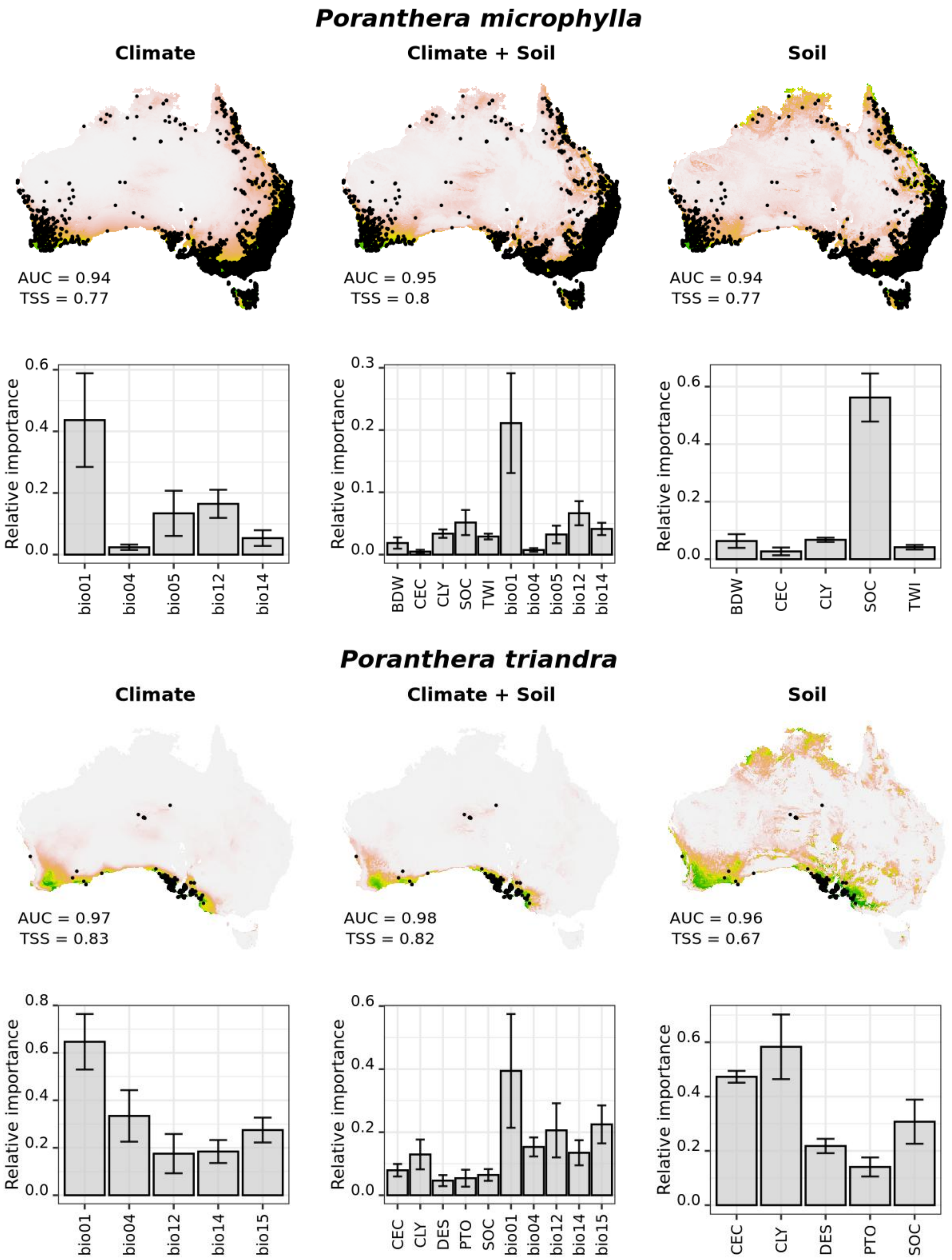
Poranthera microphylla
Climate
Climate + Soil
Soil
AUC = 0.94
TSS = 0.77
AUC = 0.95
TSS = 0.8
AUC = 0.94
TSS = 0.77
Relative importance
0.0
0.2
0.4
0.6
bio01
bio04
bio05
bio12
bio14
0.0
0.1
0.2
0.3
BDW
CEC
CLY
SOC
TWI
bio01
bio04
bio05
bio12
bio14
0.0
0.2
0.4
0.6
BDW
CEC
CLY
SOC
TWI
Poranthera triandra
Climate
Climate + Soil
Soil
AUC = 0.97
TSS = 0.83
AUC = 0.98
TSS = 0.82
AUC = 0.96
TSS = 0.67
Relative importance
0.0
0.2
0.4
0.6
0.8
bio01
bio04
bio12
bio14
bio15
0.0
0.2
0.4
0.6
CEC
CLY
DES
PTO
SOC
bio01
bio04
bio12
bio14
bio15
0.0
0.2
0.4
0.6
CEC
CLY
DES
PTO
SOC

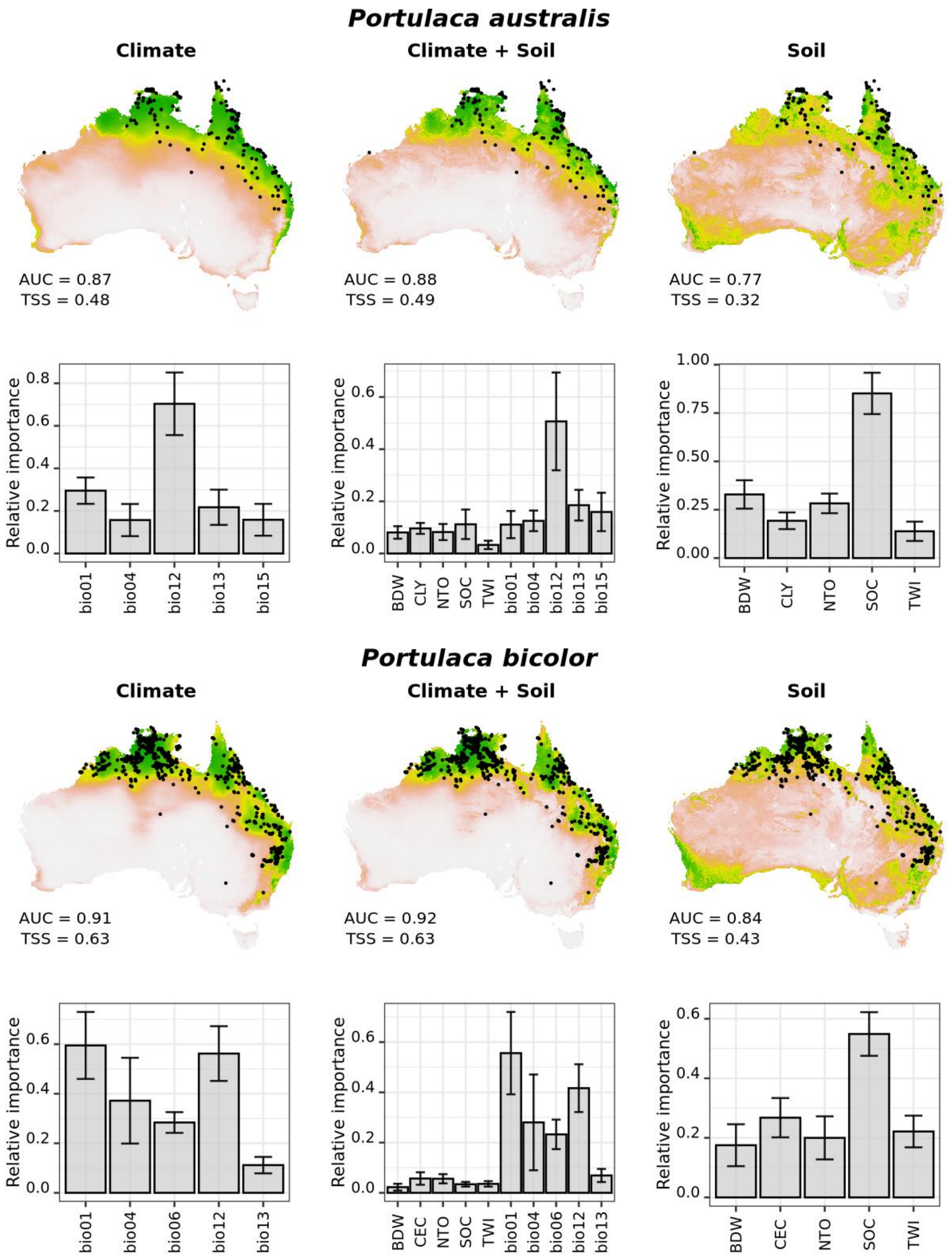
Portulaca australis
Climate
Climate + Soil
Soil
AUC = 0.87
TSS = 0.48
AUC = 0.88
TSS = 0.49
AUC = 0.77
TSS = 0.32
Relative importance
bio01
bio04
bio12
bio13
bio15
BDW
CLY
NTO
SOC
TWI
Portulaca bicolor
Climate
Climate + Soil
Soil
AUC = 0.91
TSS = 0.63
AUC = 0.92
TSS = 0.63
AUC = 0.84
TSS = 0.43
Relative importance
bio01
bio04
bio06
bio12
bio13
BDW
CEC
NTO
SOC
TWI

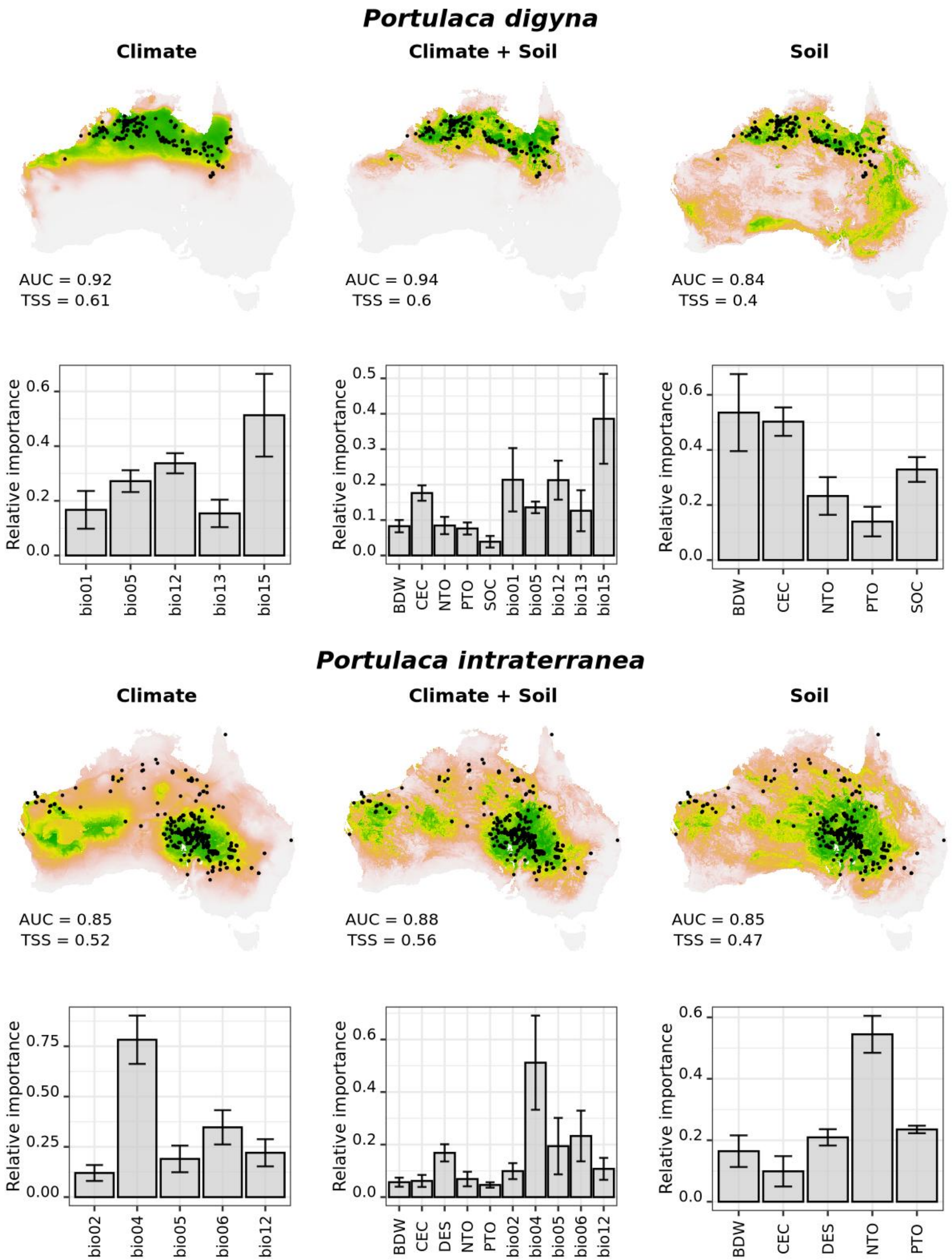
Portulaca digyna
Climate
Climate + Soil
Soil
AUC = 0.92
TSS = 0.61
AUC = 0.94
TSS = 0.6
AUC = 0.84
TSS = 0.4
Relative importance
bio01
bio05
bio12
bio13
bio15
BDW
CEC
NTO
PTO
SOC
Portulaca intraterranea
AUC = 0.85
TSS = 0.52
AUC = 0.88
TSS = 0.56
AUC = 0.85
TSS = 0.47
bio02
bio04
bio05
bio06
bio12
DES

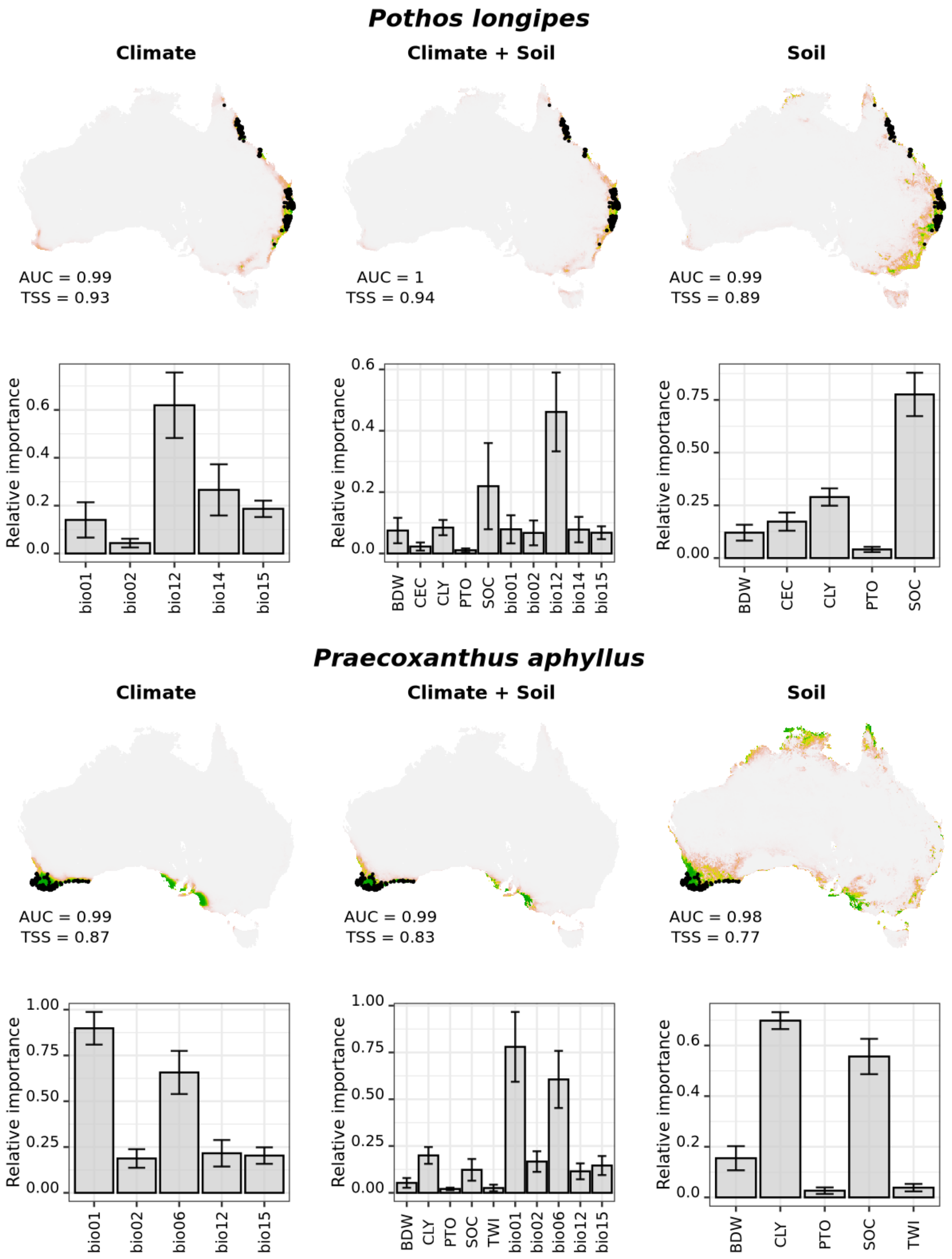

Pothos longipes
Climate
Climate + Soil
Soil
AUC = 0.99
TSS = 0.93
AUC = 1
TSS = 0.94
AUC = 0.99
TSS = 0.89
Relative importance
0.0
0.2
0.4
0.6
bio01
bio02
bio12
bio14
bio15
0.0
0.2
0.4
0.6
BDW
CEC
CLY
PTO
SOC
bio01
bio02
bio12
bio14
bio15
0.00
0.25
0.50
0.75
BDW
CEC
CLY
PTO
SOC
Praecoxanthus aphyllus
Climate
Climate + Soil
Soil
AUC = 0.99
TSS = 0.87
AUC = 0.99
TSS = 0.83
AUC = 0.98
TSS = 0.77
Relative importance
0.00
0.25
0.50
0.75
1.00
bio01
bio02
bio06
bio12
bio15
0.00
0.25
0.50
0.75
1.00
BDW
CLY
PTO
SOC
TWI
bio01
bio02
bio06
bio12
bio15
0.0
0.2
0.4
0.6
BDW
CLY
PTO
SOC
TWI

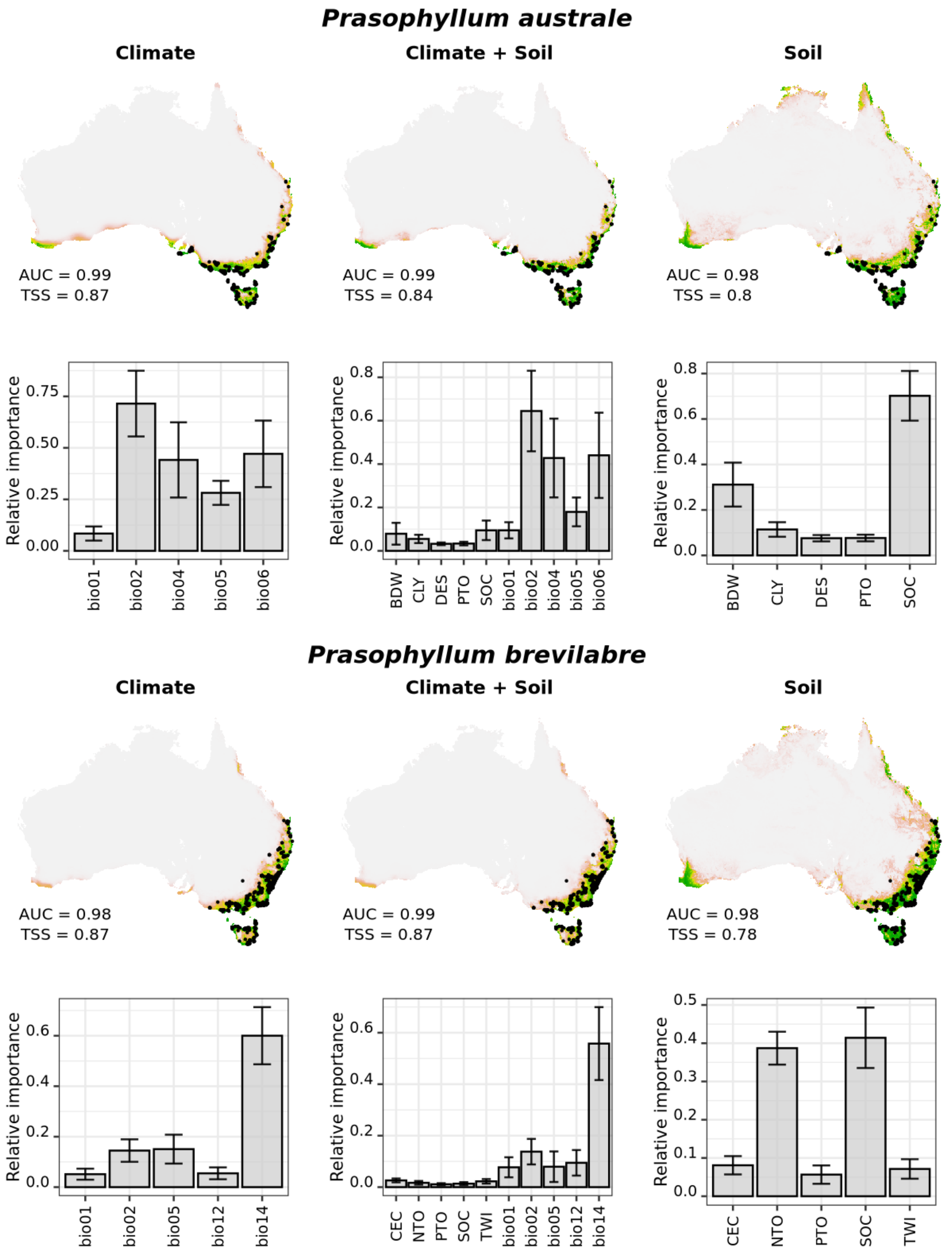

Prasophyllum australe
Climate
Climate + Soil
Soil
AUC = 0.99
TSS = 0.87
AUC = 0.99
TSS = 0.84
AUC = 0.98
TSS = 0.8
Relative importance
0.00
0.25
0.50
0.75
bio01
bio02
bio04
bio05
bio06
0.0
0.2
0.4
0.6
0.8
BDW
CLY
DES
PTO
SOC
bio01
bio02
bio04
bio05
bio06
BDW
CLY
DES
PTO
SOC
Prasophyllum brevilabre
Climate
Climate + Soil
Soil
AUC = 0.98
TSS = 0.87
AUC = 0.99
TSS = 0.87
AUC = 0.98
TSS = 0.78
Relative importance
0.0
0.2
0.4
0.6
bio01
bio02
bio05
bio12
bio14
CEC
NTO
PTO
SOC
TWI
bio01
bio02
bio05
bio12
bio14
0.0
0.1
0.2
0.3
0.4
0.5
CEC
NTO
PTO
SOC
TWI

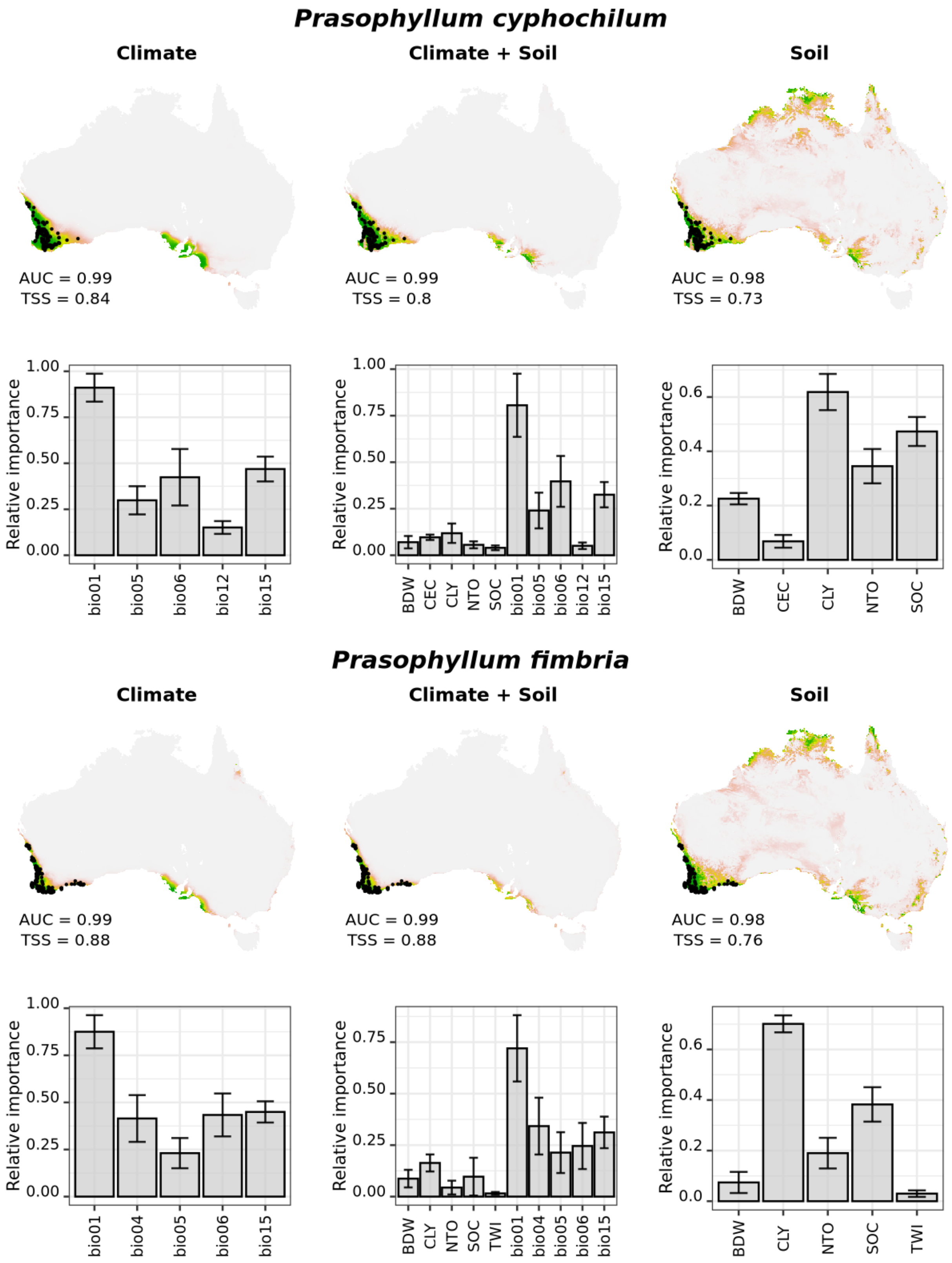
Prasophyllum cyphochilum
Climate
Climate + Soil
Soil
AUC = 0.99
TSS = 0.84
AUC = 0.99
TSS = 0.8
AUC = 0.98
TSS = 0.73
Relative importance
0.00
0.25
0.50
0.75
1.00
bio01
bio05
bio06
bio12
bio15
BDW
CEC
CLY
NTO
SOC
0.0
0.2
0.4
0.6
Prasophyllum fimbria
Climate
Climate + Soil
Soil
AUC = 0.99
TSS = 0.88
AUC = 0.99
TSS = 0.88
AUC = 0.98
TSS = 0.76
bio04
TWI

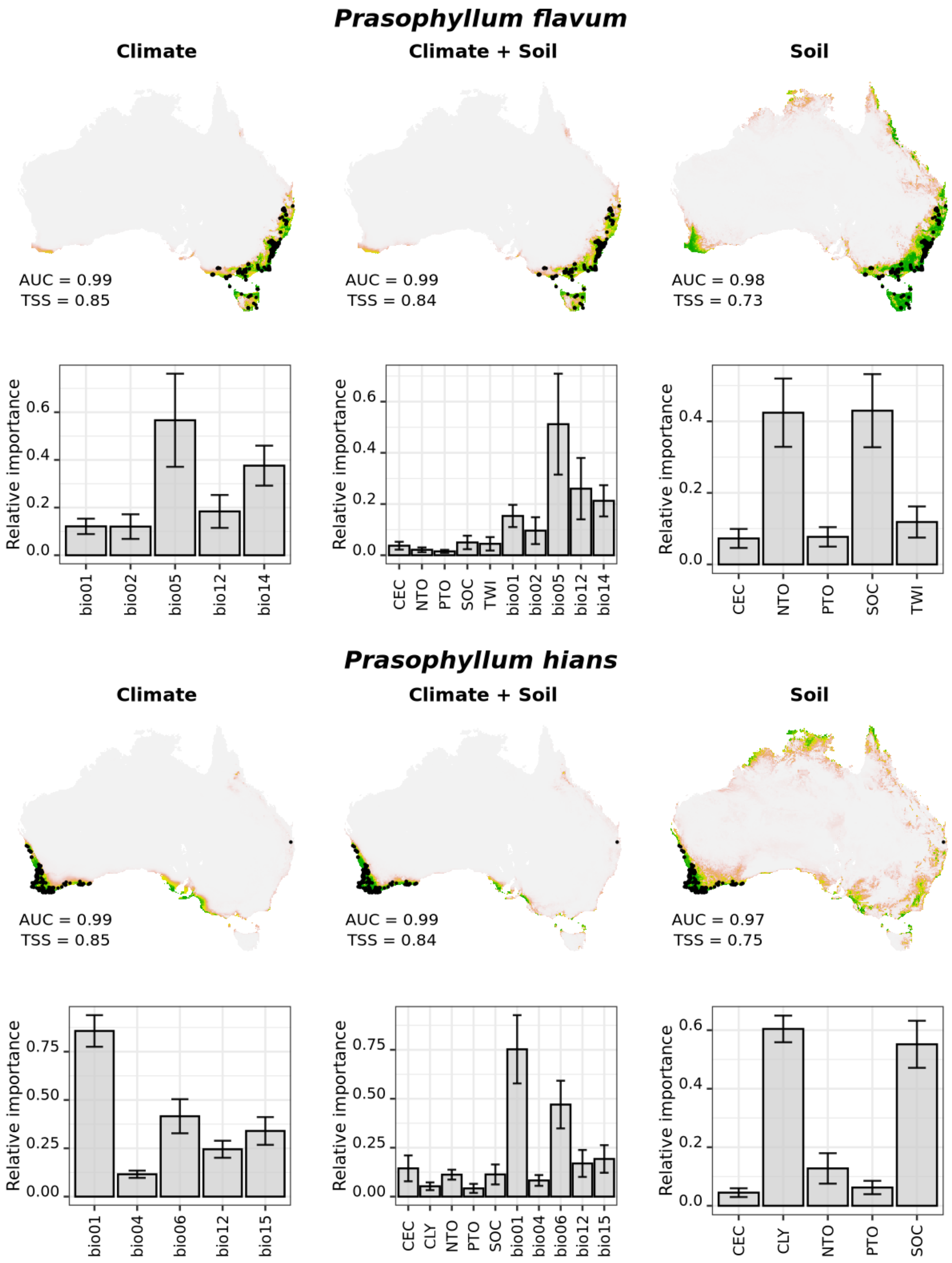
Prasophyllum flavum
Climate
Climate + Soil
Soil
AUC = 0.99
TSS = 0.85
AUC = 0.99
TSS = 0.84
AUC = 0.98
TSS = 0.73
Relative importance
bio01
bio02
bio05
bio12
bio14
CEC
NTO
PTO
SOC
TWI
Prasophyllum hians
Climate
Climate + Soil
Soil
AUC = 0.99
TSS = 0.85
AUC = 0.99
TSS = 0.84
AUC = 0.97
TSS = 0.75
Relative importance
bio01
bio04
bio06
bio12
bio15
CEC
CLY
NTO
PTO
SOC

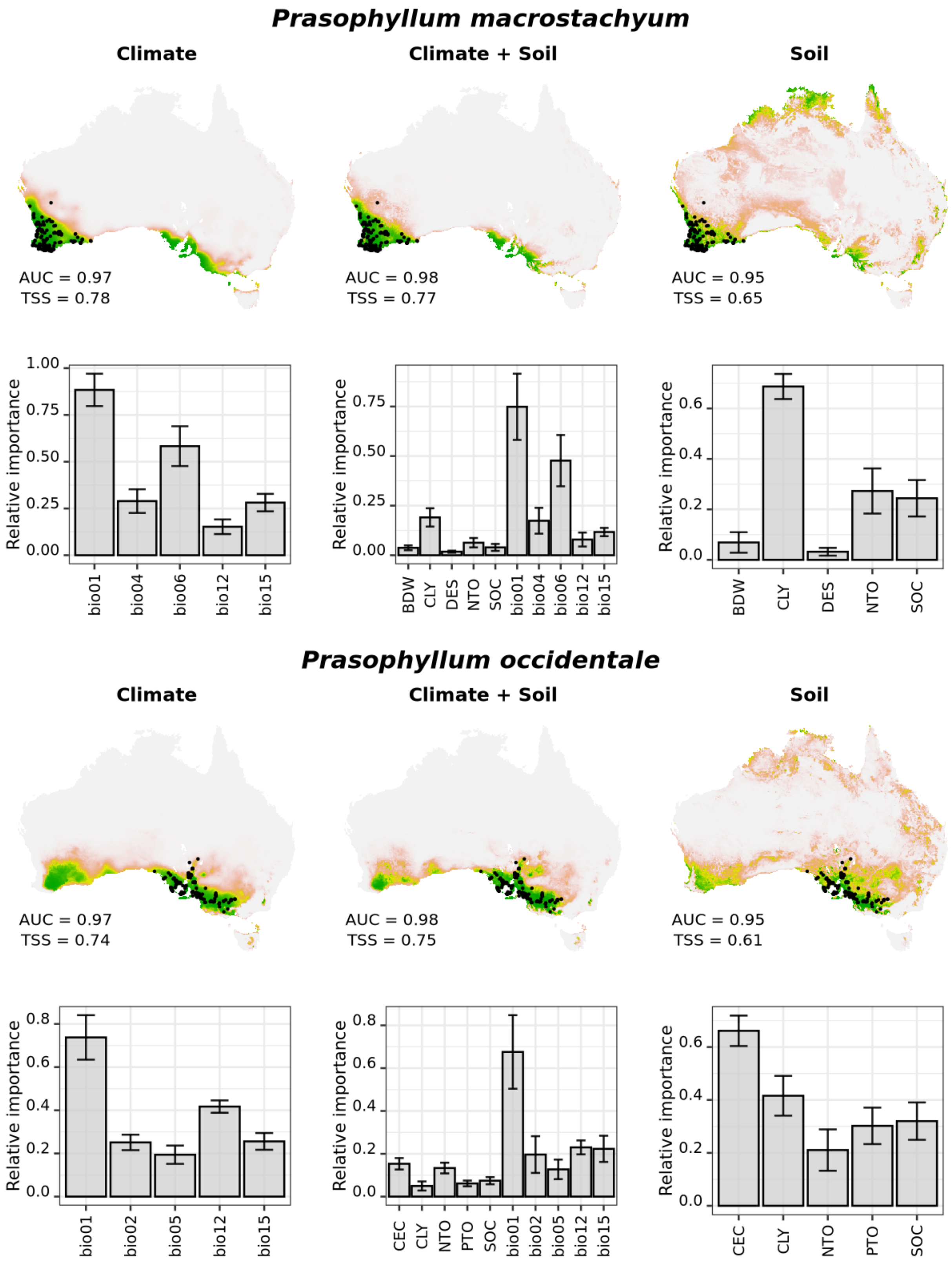

Prasophyllum macrostachyum
Climate
Climate + Soil
Soil
AUC = 0.97
TSS = 0.78
AUC = 0.98
TSS = 0.77
AUC = 0.95
TSS = 0.65
Relative importance
bio01
bio04
bio06
bio12
bio15
BDW
CLY
DES
NTO
SOC
Prasophyllum occidentale
AUC = 0.97
TSS = 0.74
AUC = 0.98
TSS = 0.75
AUC = 0.95
TSS = 0.61
bio02
bio05
CEC
PTO

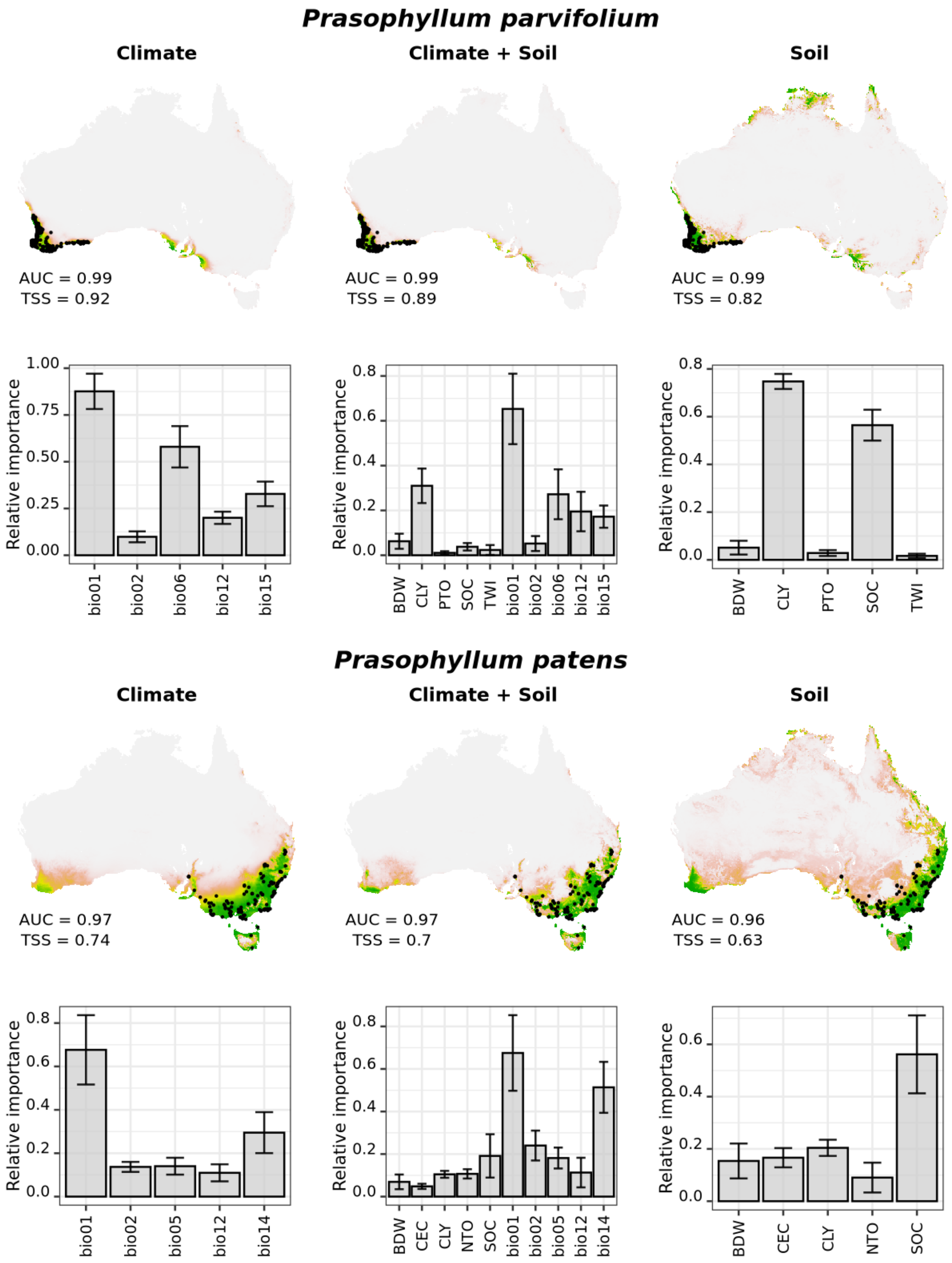

Prasophyllum parvifolium
Climate
Climate + Soil
Soil
AUC = 0.99
TSS = 0.92
AUC = 0.99
TSS = 0.89
AUC = 0.99
TSS = 0.82
Relative importance
1.00
0.75
0.50
0.25
0.00
bio01
bio02
bio06
bio12
bio15
0.8
0.6
0.4
0.2
0.0
BDW
CLY
PTO
SOC
TWI
bio01
bio02
bio06
bio12
bio15
BDW
CLY
PTO
SOC
TWI
Prasophyllum patens
Climate
Climate + Soil
Soil
AUC = 0.97
TSS = 0.74
AUC = 0.97
TSS = 0.7
AUC = 0.96
TSS = 0.63
bio01
bio02
bio05
bio12
bio14
BDW
CEC
CLY
NTO
SOC
bio01
bio02
bio05
bio12
bio14
BDW
CEC
CLY
NTO
SOC

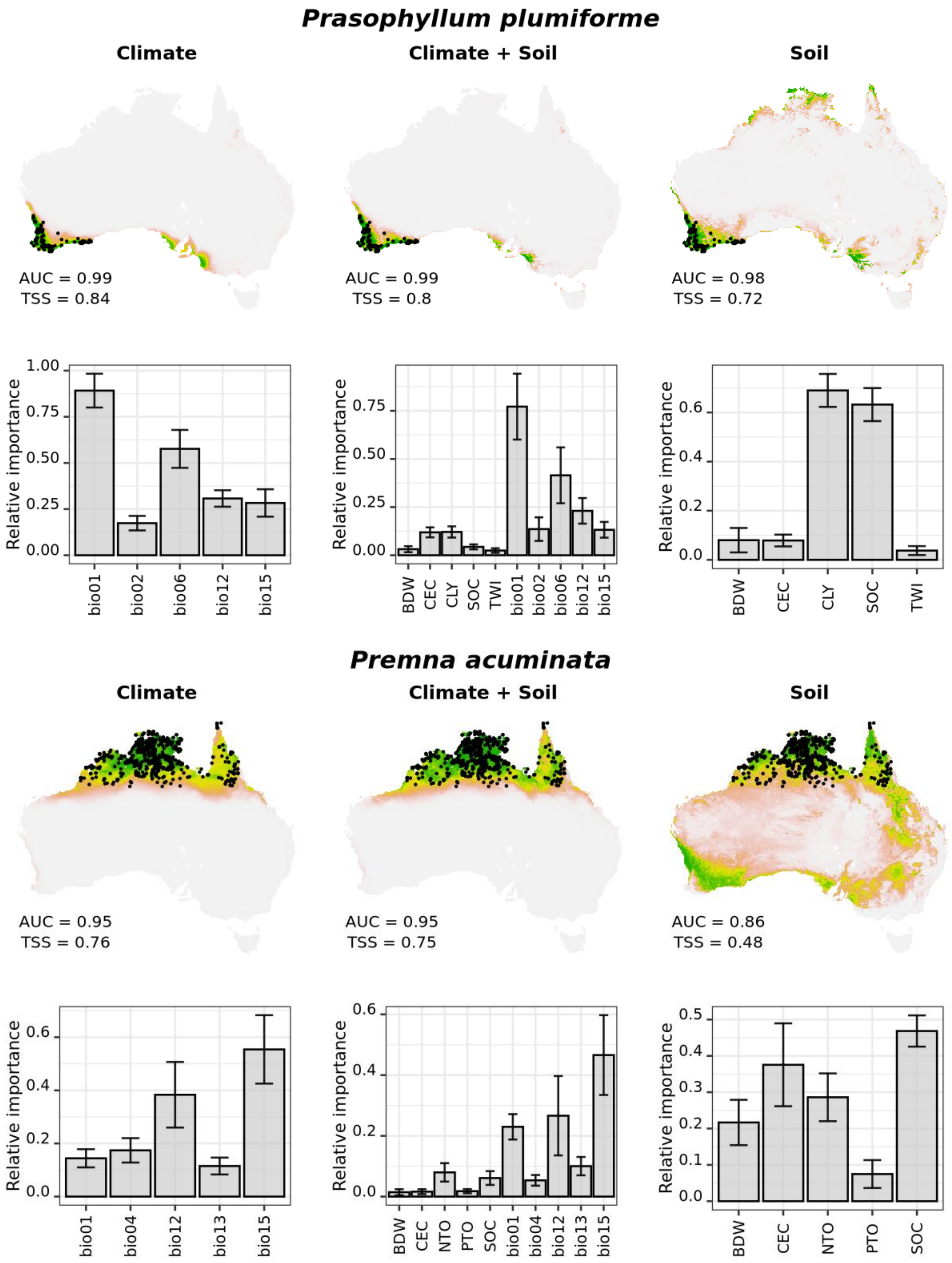

Prasophyllum plumiforme
Climate
Climate + Soil
Soil
AUC = 0.99
TSS = 0.84
AUC = 0.99
TSS = 0.8
AUC = 0.98
TSS = 0.72
Relative importance
bio01
bio02
bio06
bio12
bio15
BDW
CEC
CLY
SOC
TWI
Premna acuminata
Climate
Climate + Soil
Soil
AUC = 0.95
TSS = 0.76
AUC = 0.95
TSS = 0.75
AUC = 0.86
TSS = 0.48
Relative importance
bio01
bio04
bio12
bio13
bio15
BDW
CEC
NTO
PTO
SOC

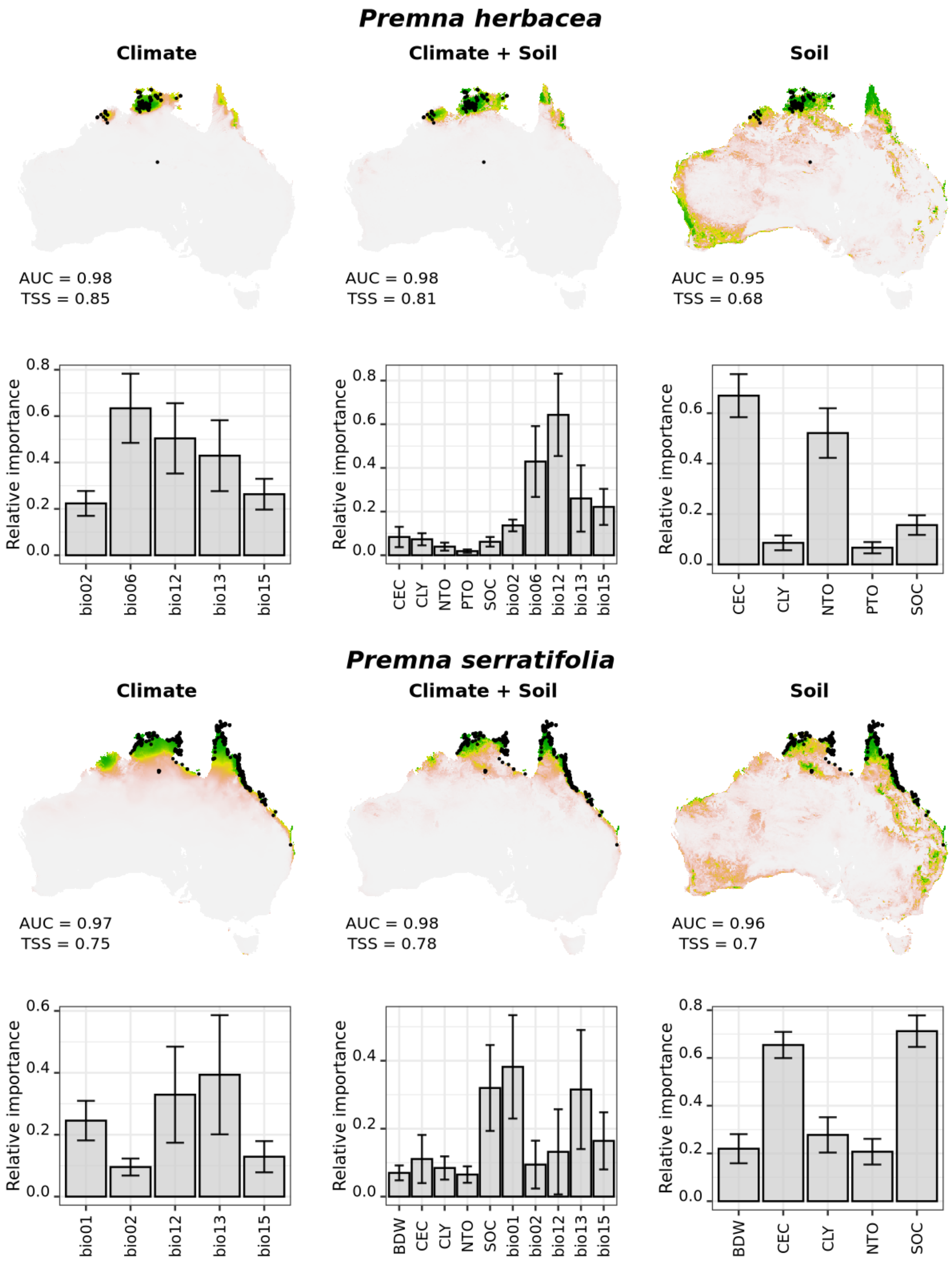

Premna herbacea
Climate
Climate + Soil
Soil
AUC = 0.98
TSS = 0.85
AUC = 0.98
TSS = 0.81
AUC = 0.95
TSS = 0.68
Relative importance
0.0
0.2
0.4
0.6
0.8
bio02
bio06
bio12
bio13
bio15
CEC
CLY
NTO
PTO
SOC
Premna serratifolia
Climate
Climate + Soil
Soil
AUC = 0.97
TSS = 0.75
AUC = 0.98
TSS = 0.78
AUC = 0.96
TSS = 0.7
bio01
BDW

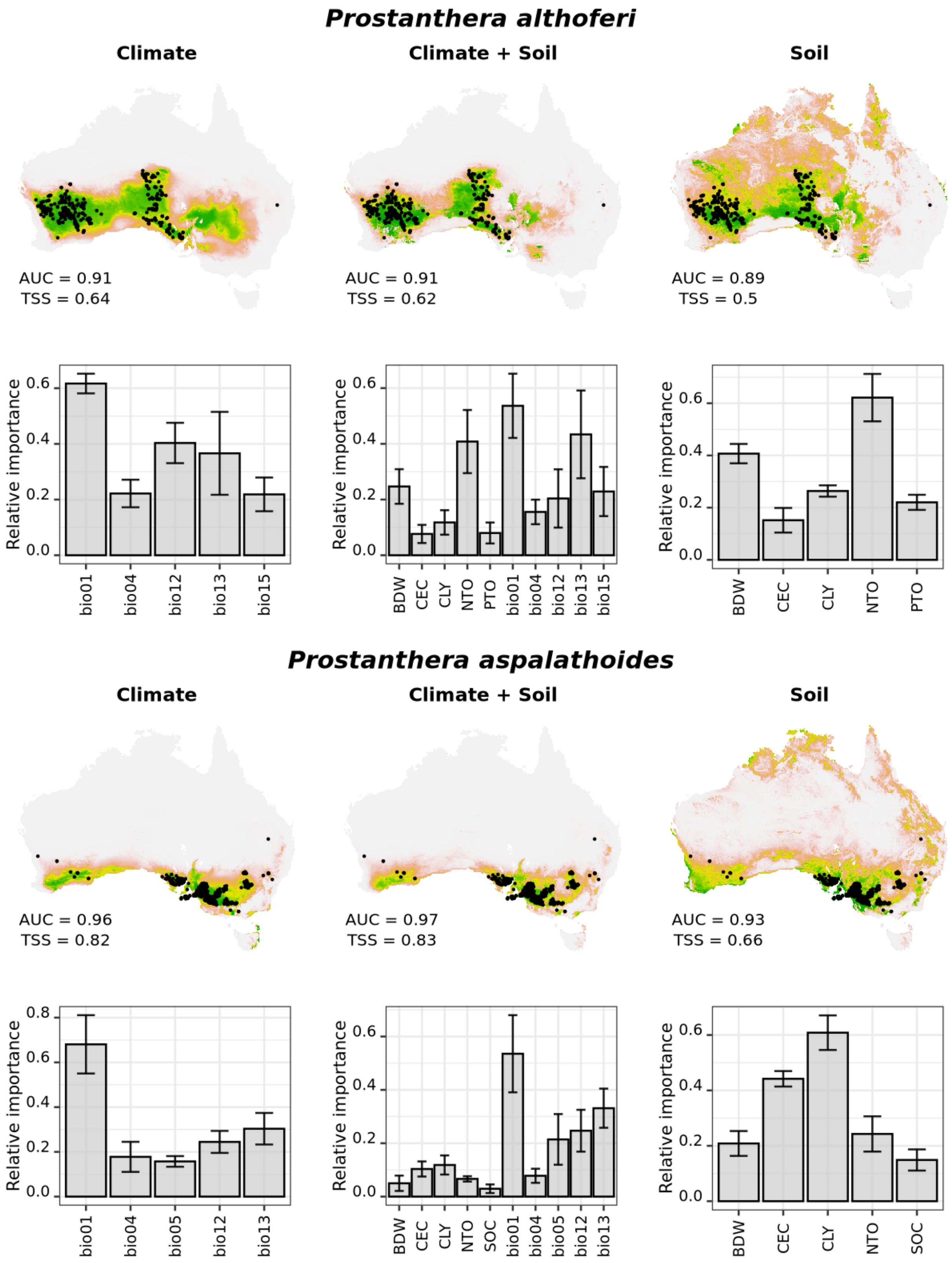

Prostanthera althoferi
Climate
Climate + Soil
Soil
AUC = 0.91
TSS = 0.64
AUC = 0.91
TSS = 0.62
AUC = 0.89
TSS = 0.5
Relative importance
bio01 bio04 bio12 bio13 bio15
BDW CEC CLY NTO PTO bio01 bio04 bio12 bio13 bio15
BDW CEC CLY NTO PTO
Prostanthera aspalathoides
Climate
Climate + Soil
Soil
AUC = 0.96
TSS = 0.82
AUC = 0.97
TSS = 0.83
AUC = 0.93
TSS = 0.66
Relative importance
bio01 bio04 bio05 bio12 bio13
BDW CEC CLY NTO SOC bio01 bio04 bio05 bio12 bio13
BDW CEC CLY NTO SOC

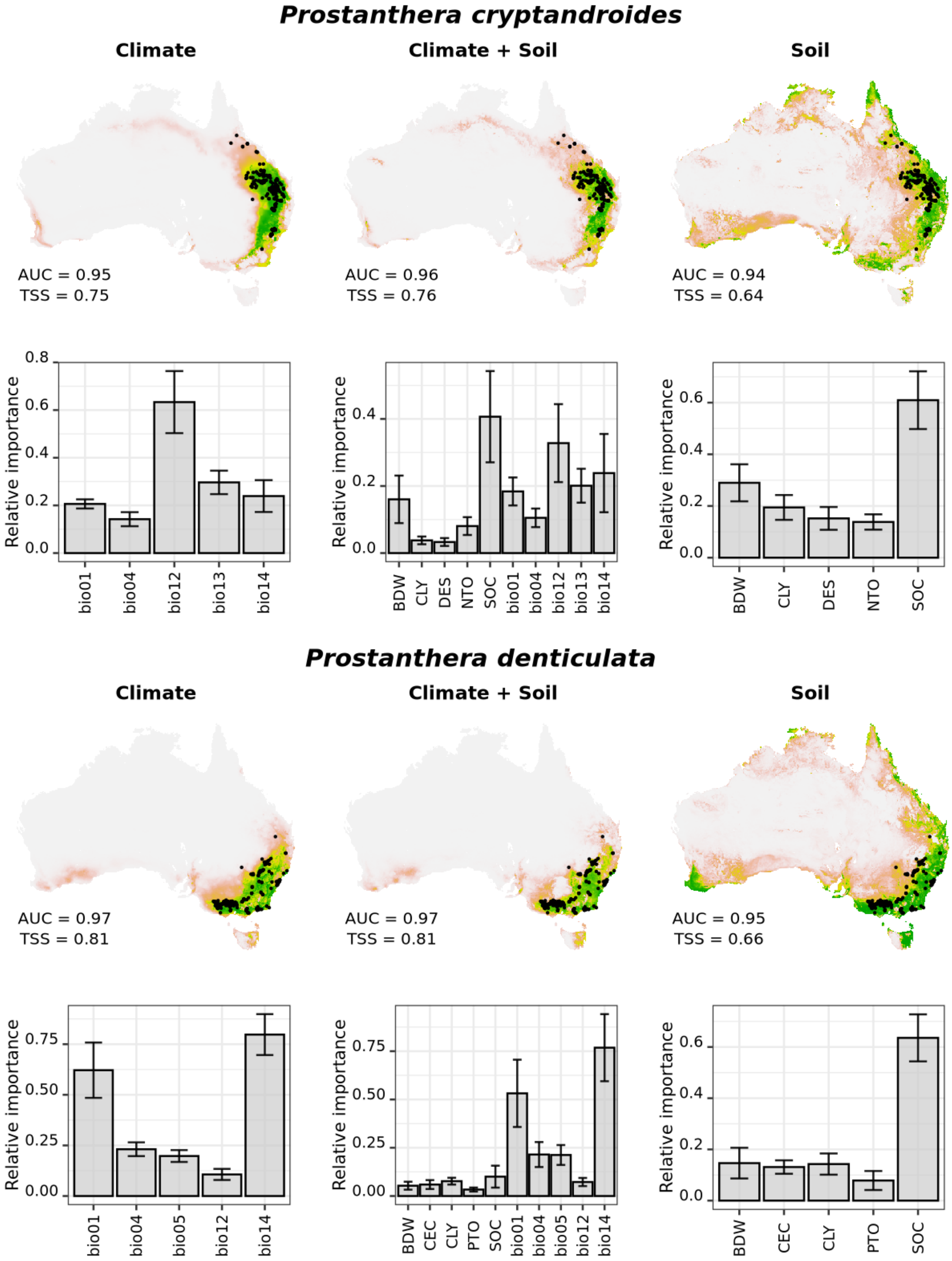
Prostanthera cryptandroides
Climate
Climate + Soil
Soil
AUC = 0.95
TSS = 0.75
AUC = 0.96
TSS = 0.76
AUC = 0.94
TSS = 0.64
Relative importance
0.0
0.2
0.4
0.6
0.8
bio01
bio04
bio12
bio13
bio14
BDW
CLY
DES
NTO
SOC
Prostanthera denticulata
AUC = 0.97
TSS = 0.81
AUC = 0.97
TSS = 0.81
AUC = 0.95
TSS = 0.66
0.00
0.25
0.50
0.75
bio05
CEC
PTO

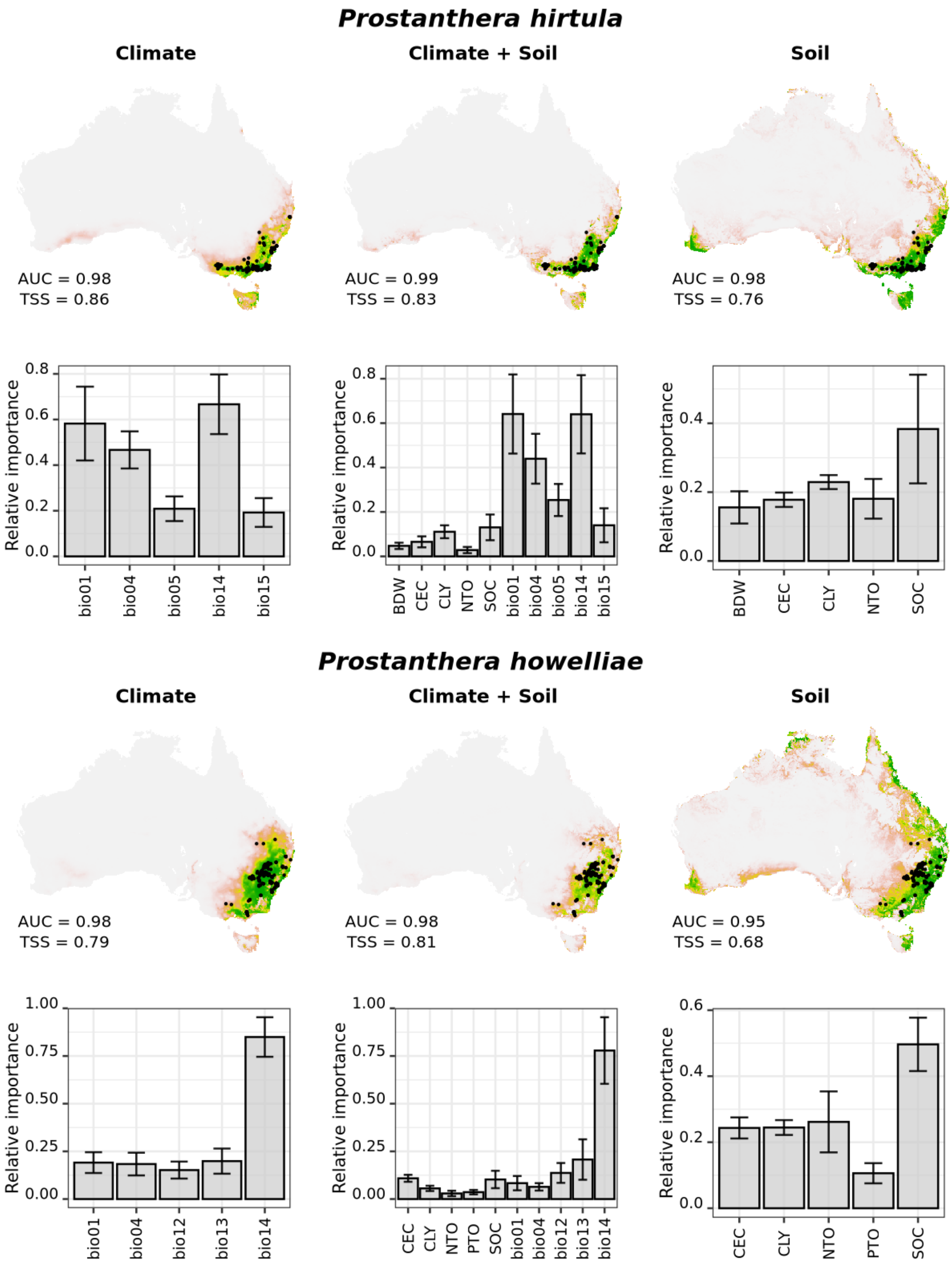
Prostanthera hirtula
Climate
Climate + Soil
Soil
AUC = 0.98
TSS = 0.86
AUC = 0.99
TSS = 0.83
AUC = 0.98
TSS = 0.76
Relative importance
bio01 bio04 bio05 bio14 bio15
BDW CEC CLY NTO SOC bio01 bio04 bio05 bio14 bio15
BDW CEC CLY NTO SOC
Prostanthera howelliae
Climate
Climate + Soil
Soil
AUC = 0.98
TSS = 0.79
AUC = 0.98
TSS = 0.81
AUC = 0.95
TSS = 0.68
Relative importance
bio01 bio04 bio12 bio13 bio14
CEC CLY NTO PTO SOC bio01 bio04 bio12 bio13 bio14
CEC CLY NTO PTO SOC

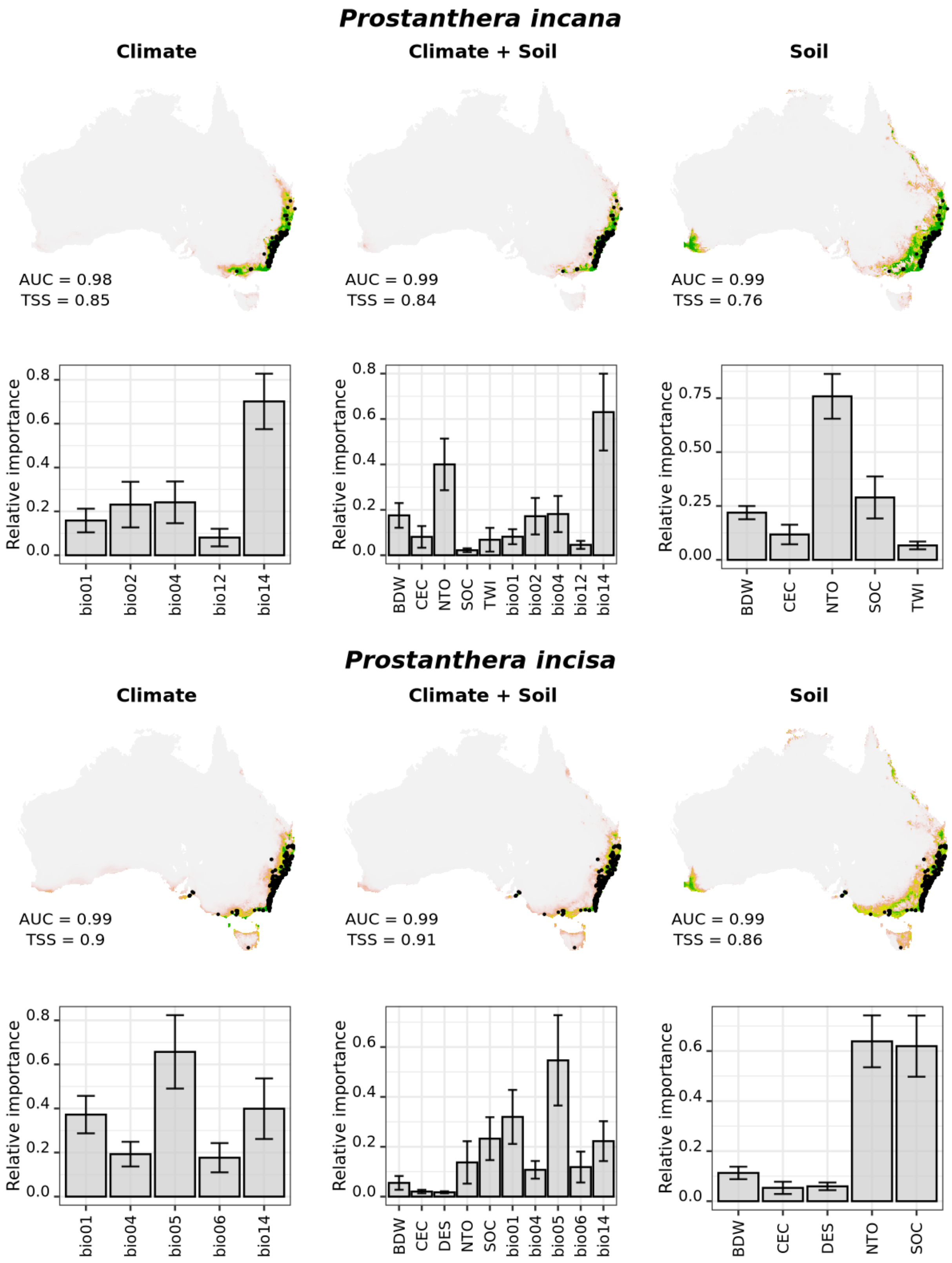
Prostanthera incana
Climate
Climate + Soil
Soil
AUC = 0.98
TSS = 0.85
AUC = 0.99
TSS = 0.84
AUC = 0.99
TSS = 0.76
Relative importance
0.0
0.2
0.4
0.6
0.8
bio01
bio02
bio04
bio12
bio14
BDW
CEC
NTO
SOC
TWI
bio01
bio02
bio04
bio12
bio14
0.00
0.25
0.50
0.75
BDW
CEC
NTO
SOC
TWI
Prostanthera incisa
Climate
Climate + Soil
Soil
AUC = 0.99
TSS = 0.9
AUC = 0.99
TSS = 0.91
AUC = 0.99
TSS = 0.86
Relative importance
bio01
bio04
bio05
bio06
bio14
BDW
CEC
DES
NTO
SOC
bio01
bio04
bio05
bio06
bio14
BDW
CEC
DES
NTO
SOC

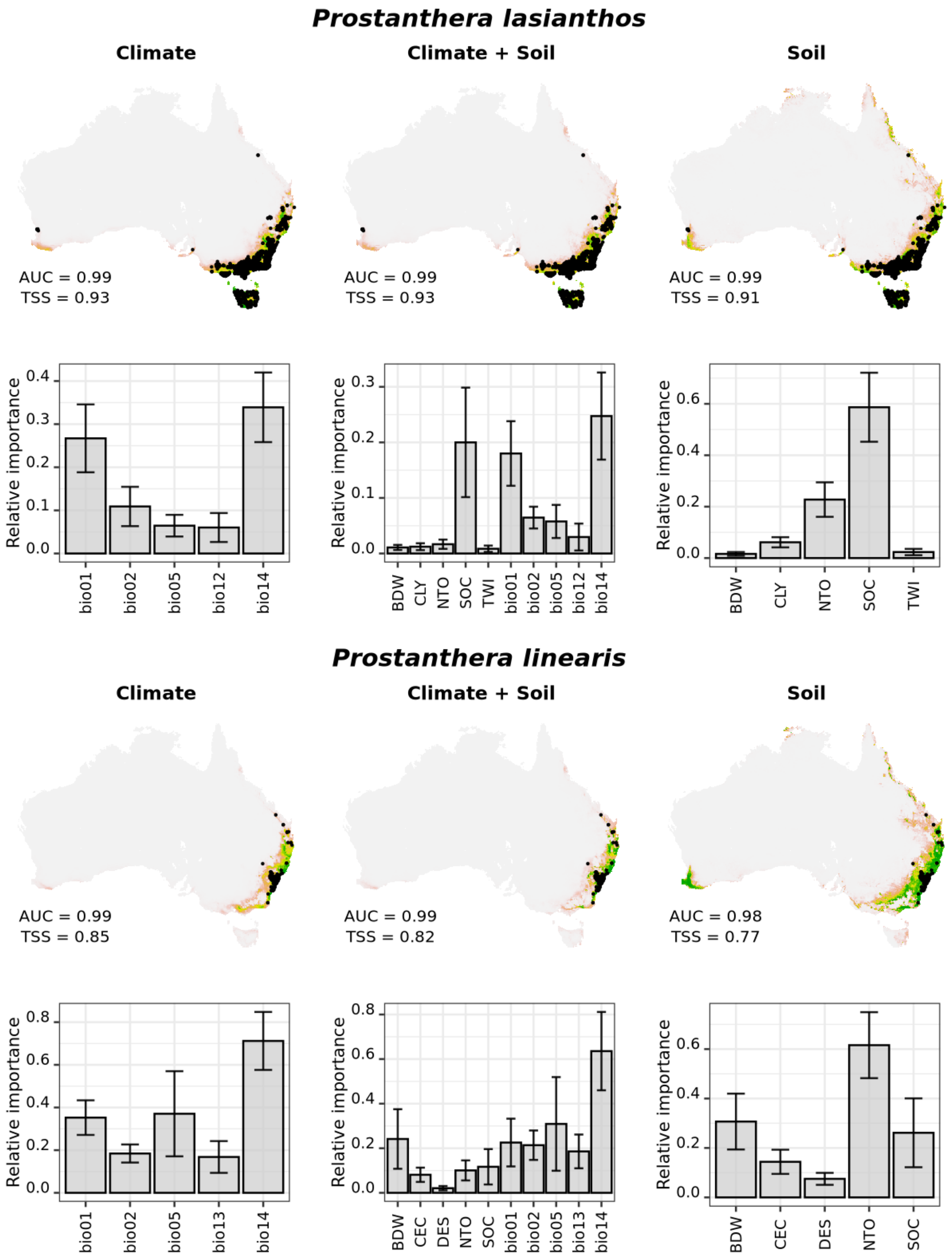

Prostanthera lasianthos
Climate
Climate + Soil
Soil
AUC = 0.99
TSS = 0.93
AUC = 0.99
TSS = 0.93
AUC = 0.99
TSS = 0.91
Relative importance
bio01
bio02
bio05
bio12
bio14
BDW
CLY
NTO
SOC
TWI
Prostanthera linearis
Climate
Climate + Soil
Soil
AUC = 0.99
TSS = 0.85
AUC = 0.99
TSS = 0.82
AUC = 0.98
TSS = 0.77
Relative importance
bio01
bio02
bio05
bio13
bio14
BDW
CEC
DES
NTO
SOC

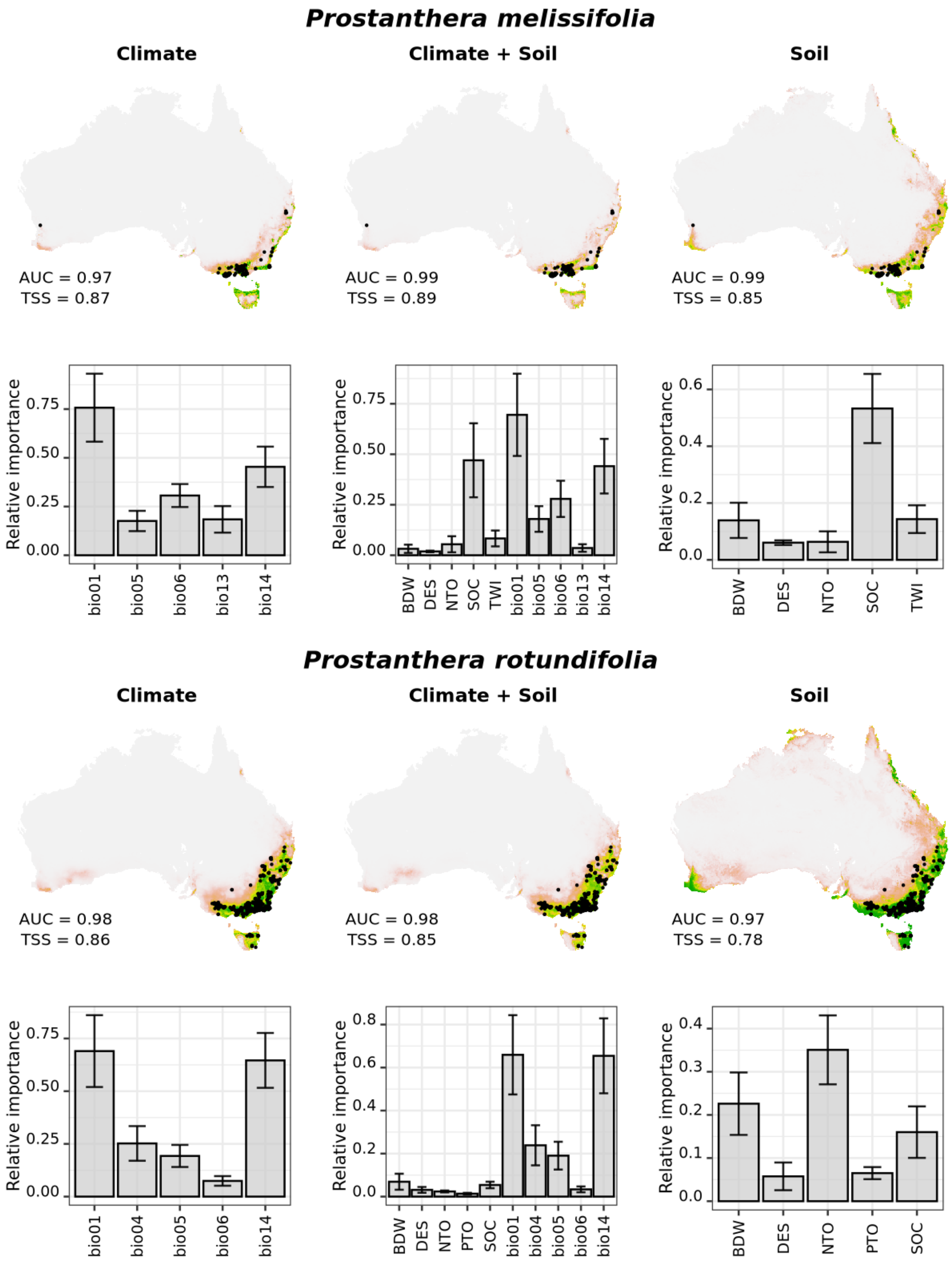
Prostanthera melissifolia
Climate
Climate + Soil
Soil
AUC = 0.97
TSS = 0.87
AUC = 0.99
TSS = 0.89
AUC = 0.99
TSS = 0.85
Relative importance
bio01
bio05
bio06
bio13
bio14
BDW
DES
NTO
SOC
TWI
Prostanthera rotundifolia
Climate
Climate + Soil
Soil
AUC = 0.98
TSS = 0.86
AUC = 0.98
TSS = 0.85
AUC = 0.97
TSS = 0.78
bio04
PTO

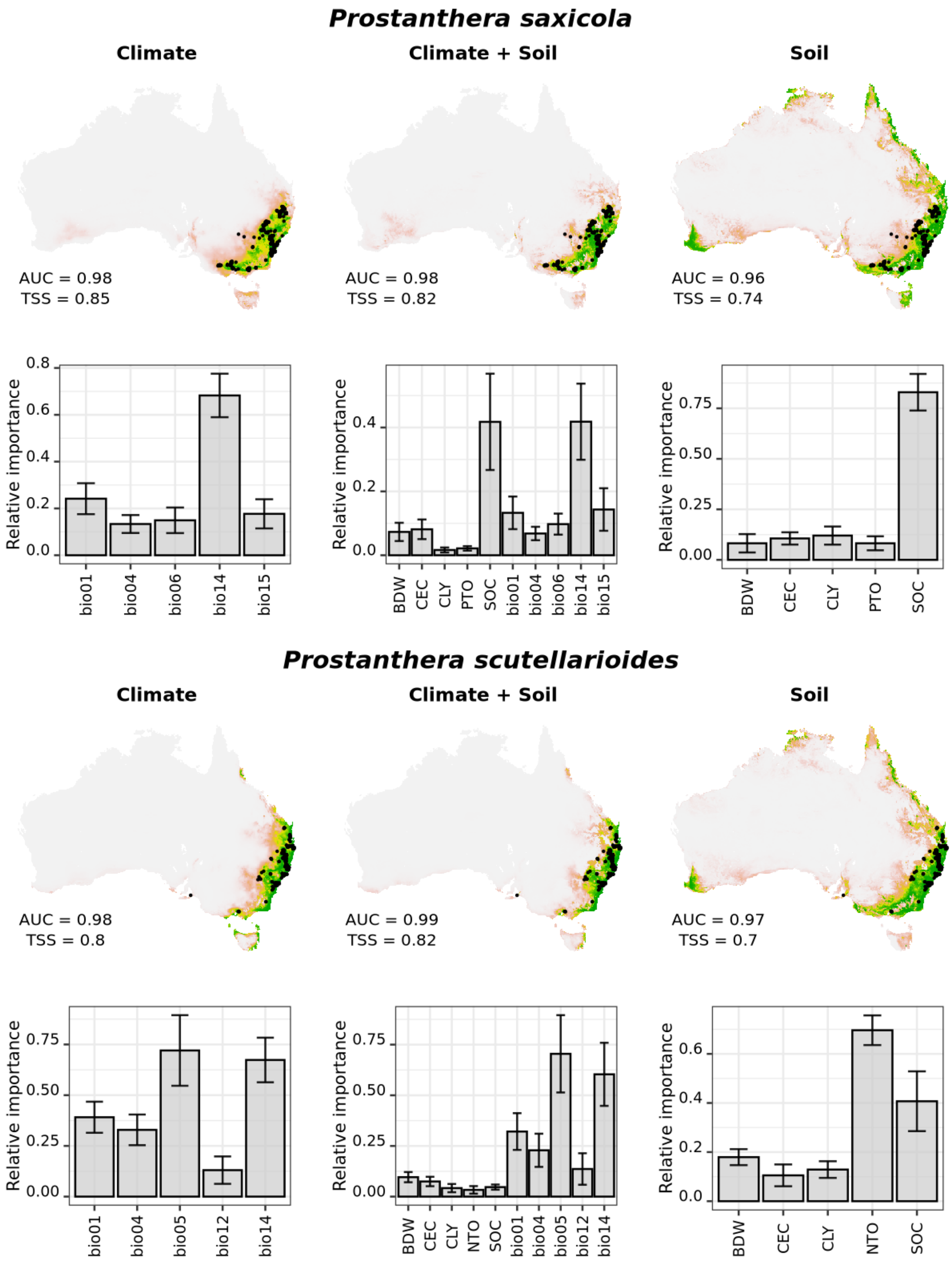

Prostanthera saxicola
Climate
Climate + Soil
Soil
AUC = 0.98
TSS = 0.85
AUC = 0.98
TSS = 0.82
AUC = 0.96
TSS = 0.74
Relative importance
bio01
bio04
bio06
bio14
bio15
BDW
CEC
CLY
PTO
SOC
Prostanthera scutellarioides
Climate
Climate + Soil
Soil
AUC = 0.98
TSS = 0.8
AUC = 0.99
TSS = 0.82
AUC = 0.97
TSS = 0.7
Relative importance
bio01
bio04
bio05
bio12
bio14
BDW
CEC
CLY
NTO
SOC

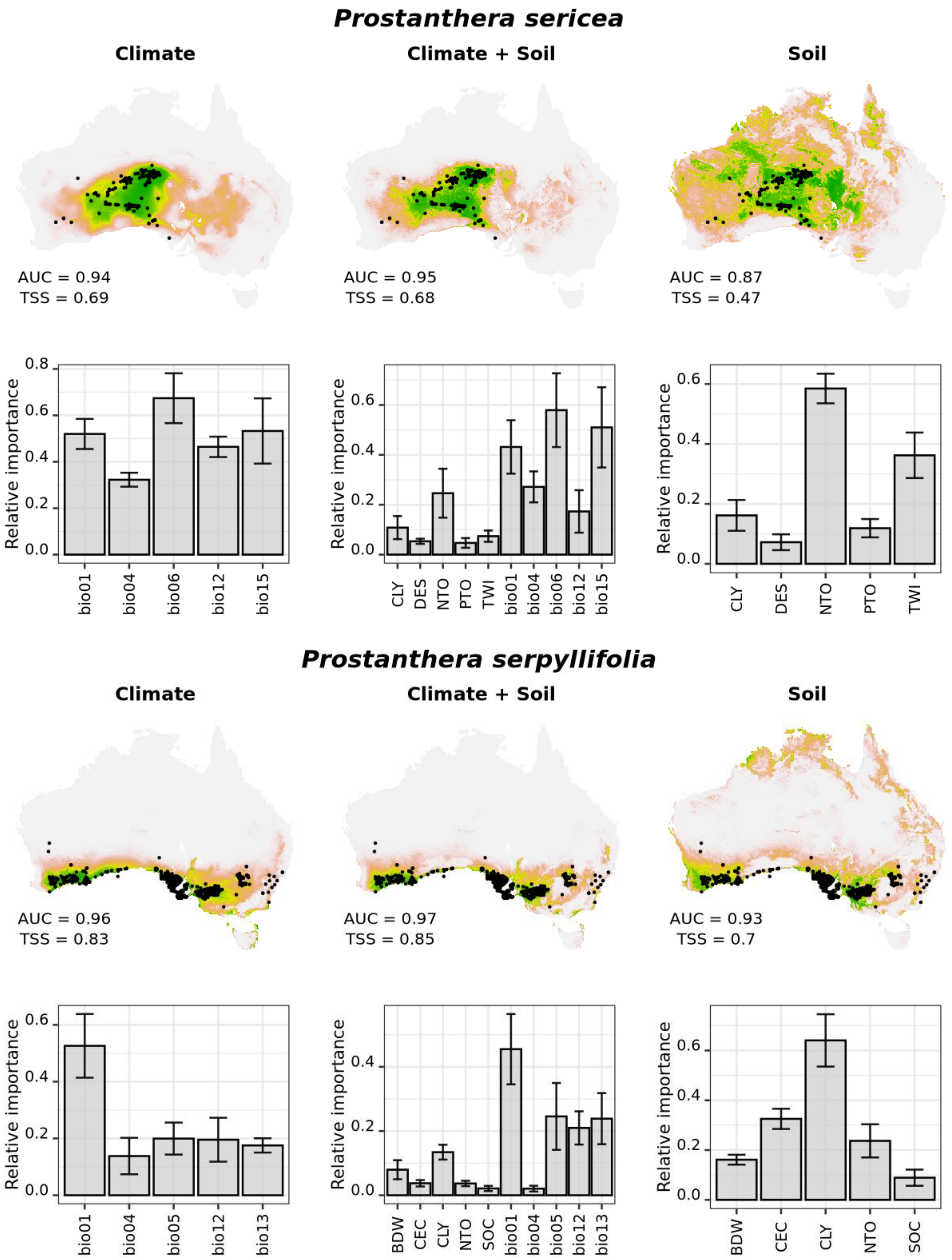
Prostanthera sericea
Climate
Climate + Soil
Soil
AUC = 0.94
TSS = 0.69
AUC = 0.95
TSS = 0.68
AUC = 0.87
TSS = 0.47
Relative importance
bio01
bio04
bio06
bio12
bio15
CLY
DES
NTO
PTO
TWI
Prostanthera serpyllifolia
AUC = 0.96
TSS = 0.83
AUC = 0.97
TSS = 0.85
AUC = 0.93
TSS = 0.7
bio05
bio13
BDW
CEC
SOC

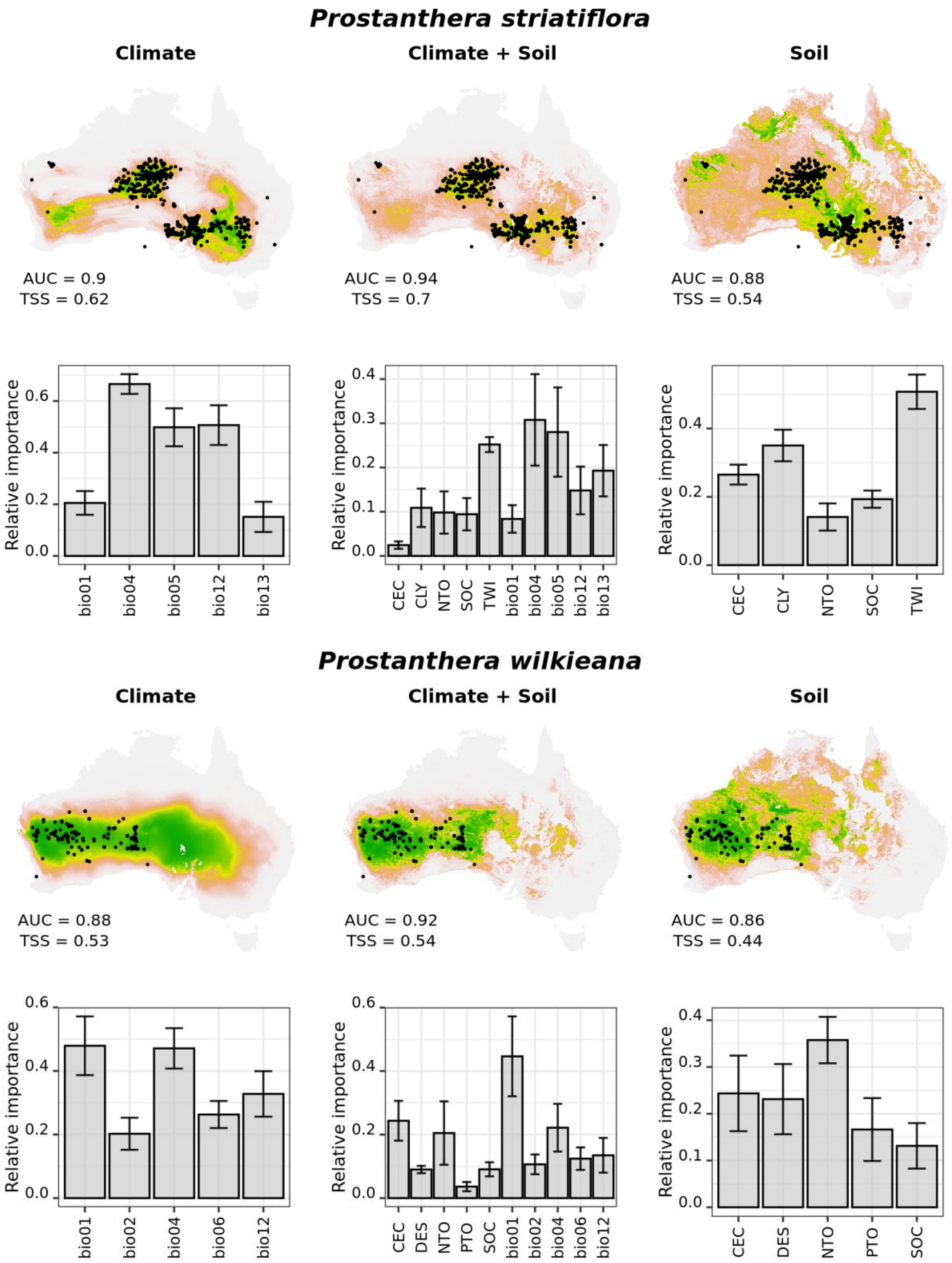
Prostanthera striatiflora
Climate
Climate + Soil
Soil
AUC = 0.9
TSS = 0.62
AUC = 0.94
TSS = 0.7
AUC = 0.88
TSS = 0.54
Relative importance
bio01 bio04 bio05 bio12 bio13
CEC CLY NTO SOC TWI bio01 bio04 bio05 bio12 bio13
CEC CLY NTO SOC TWI
Prostanthera wilkieana
Climate
Climate + Soil
Soil
AUC = 0.88
TSS = 0.53
AUC = 0.92
TSS = 0.54
AUC = 0.86
TSS = 0.44
Relative importance
bio01 bio02 bio04 bio06 bio12
CEC DES NTO PTO SOC bio01 bio02 bio04 bio06 bio12
CEC DES NTO PTO SOC

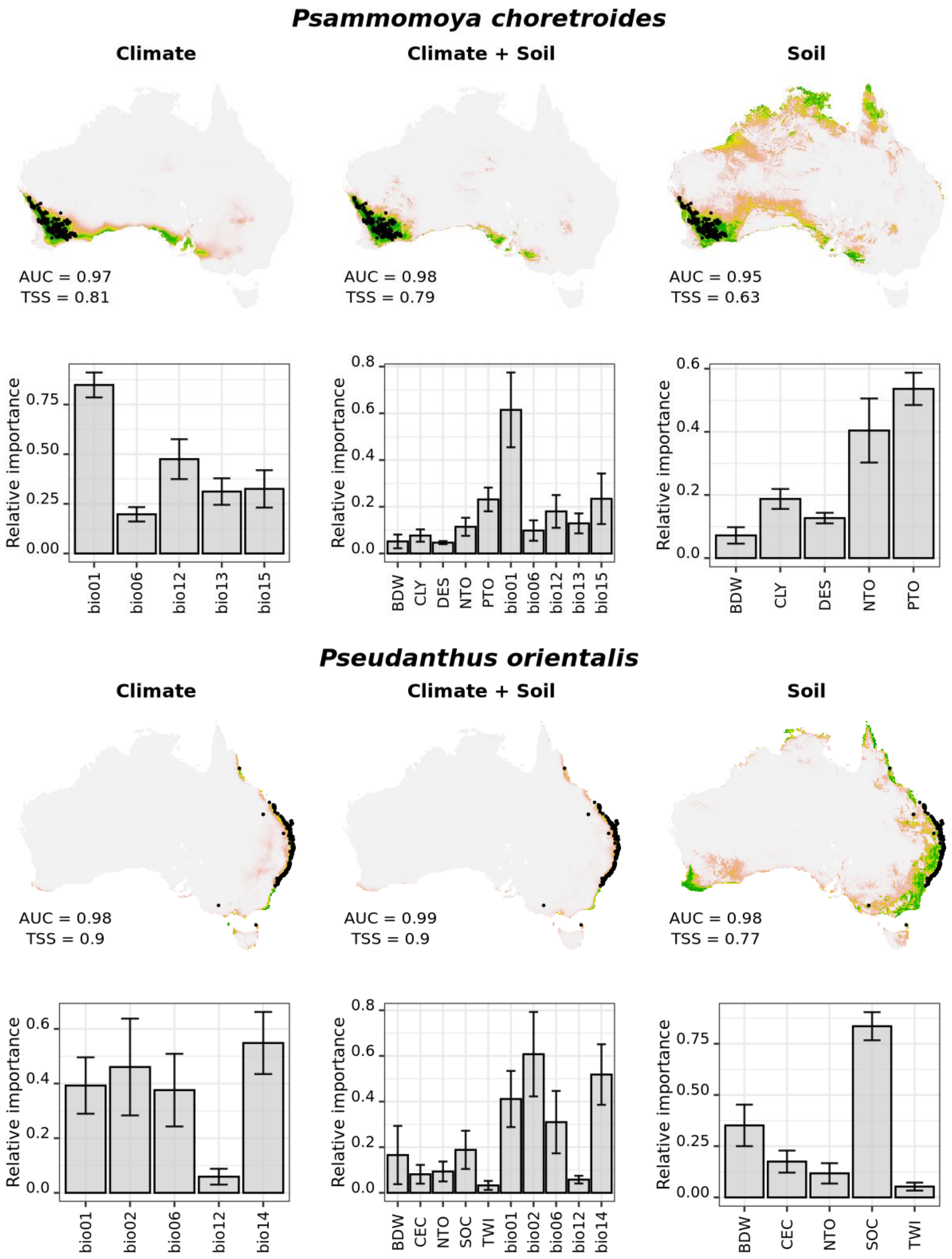

Psammomoya choretroides
Climate
Climate + Soil
Soil
AUC = 0.97
TSS = 0.81
AUC = 0.98
TSS = 0.79
AUC = 0.95
TSS = 0.63
Relative importance
0.00
0.25
0.50
0.75
bio01
bio06
bio12
bio13
bio15
0.0
0.2
0.4
0.6
0.8
BDW
CLY
DES
NTO
PTO
bio01
bio06
bio12
bio13
bio15
0.0
0.2
0.4
0.6
BDW
CLY
DES
NTO
PTO
Pseudanthus orientalis
Climate
Climate + Soil
Soil
AUC = 0.98
TSS = 0.9
AUC = 0.99
TSS = 0.9
AUC = 0.98
TSS = 0.77
Relative importance
0.0
0.2
0.4
0.6
bio01
bio02
bio06
bio12
bio14
0.0
0.2
0.4
0.6
0.8
BDW
CEC
NTO
SOC
TWI
bio01
bio02
bio06
bio12
bio14
0.00
0.25
0.50
0.75
BDW
CEC
NTO
SOC
TWI

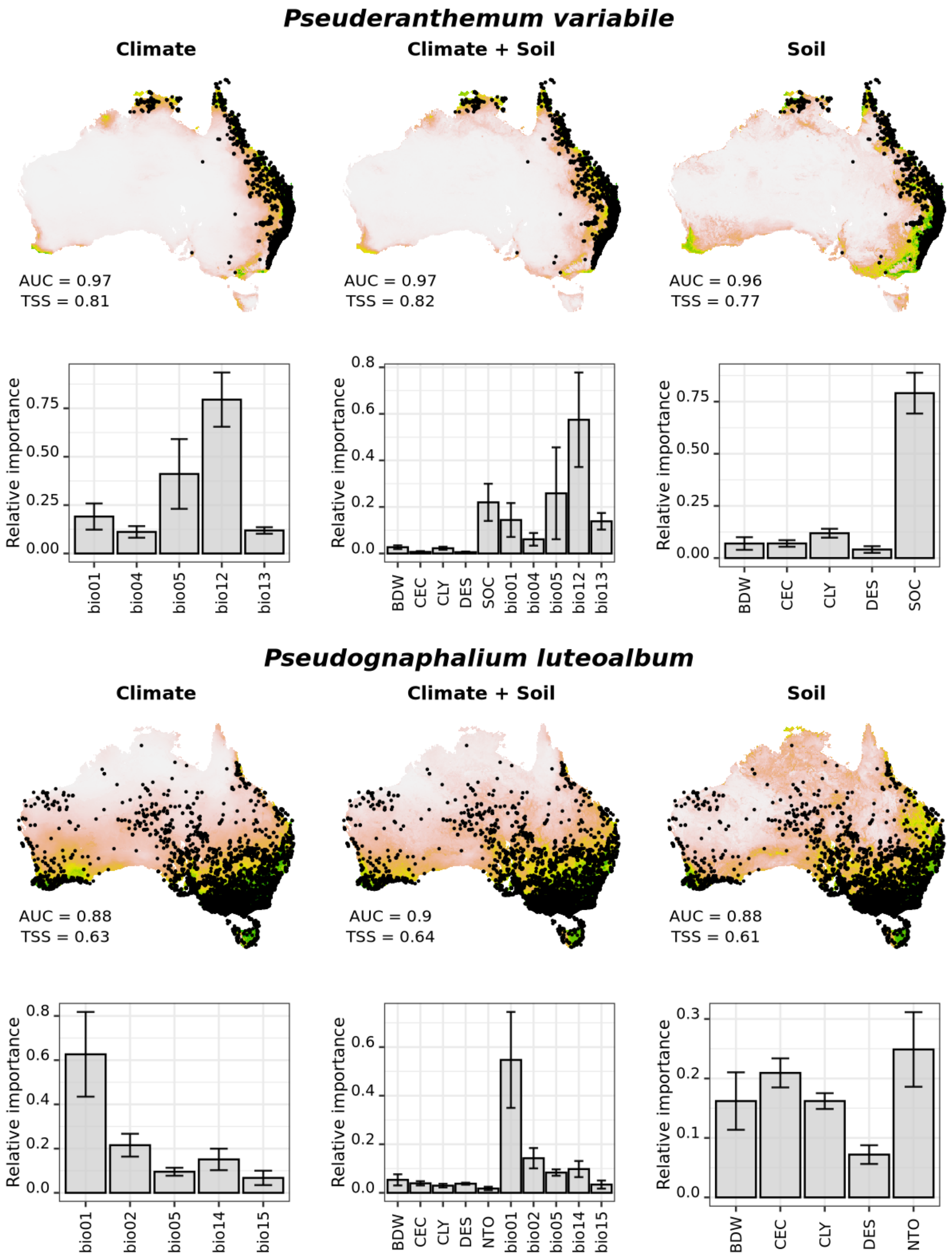

Pseuderanthemum variabile
Climate
Climate + Soil
Soil
AUC = 0.97
TSS = 0.81
AUC = 0.97
TSS = 0.82
AUC = 0.96
TSS = 0.77
Relative importance
0.00
0.25
0.50
0.75
bio01
bio04
bio05
bio12
bio13
0.0
0.2
0.4
0.6
0.8
BDW
CEC
CLY
DES
SOC
bio01
bio04
bio05
bio12
bio13
BDW
CEC
CLY
DES
SOC
Pseudognaphalium luteoalbum
Climate
Climate + Soil
Soil
AUC = 0.88
TSS = 0.63
AUC = 0.9
TSS = 0.64
AUC = 0.88
TSS = 0.61
bio01
bio02
bio05
bio14
bio15
BDW
CEC
CLY
DES
NTO
bio01
bio02
bio05
bio14
bio15
0.0
0.1
0.2
0.3
BDW
CEC
CLY
DES
NTO

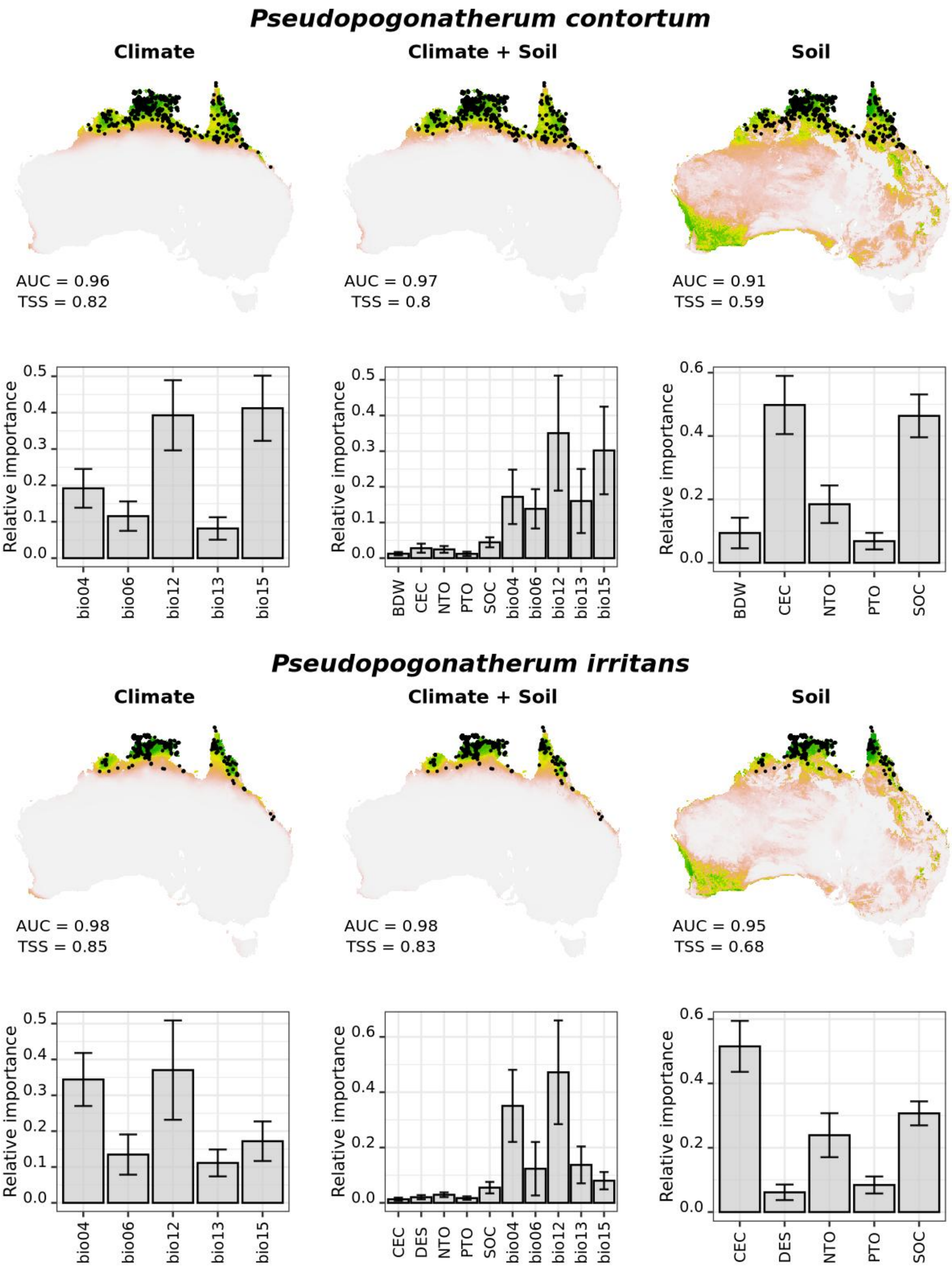

Pseudopogonatherum contortum
Climate
Climate + Soil
Soil
AUC = 0.96
TSS = 0.82
AUC = 0.97
TSS = 0.8
AUC = 0.91
TSS = 0.59
Relative importance
bio04
bio06
bio12
bio13
bio15
BDW
CEC
NTO
PTO
SOC
Pseudopogonatherum irritans
Climate
Climate + Soil
Soil
AUC = 0.98
TSS = 0.85
AUC = 0.98
TSS = 0.83
AUC = 0.95
TSS = 0.68
Relative importance
bio04
bio06
bio12
bio13
bio15
CEC
DES
NTO
PTO
SOC

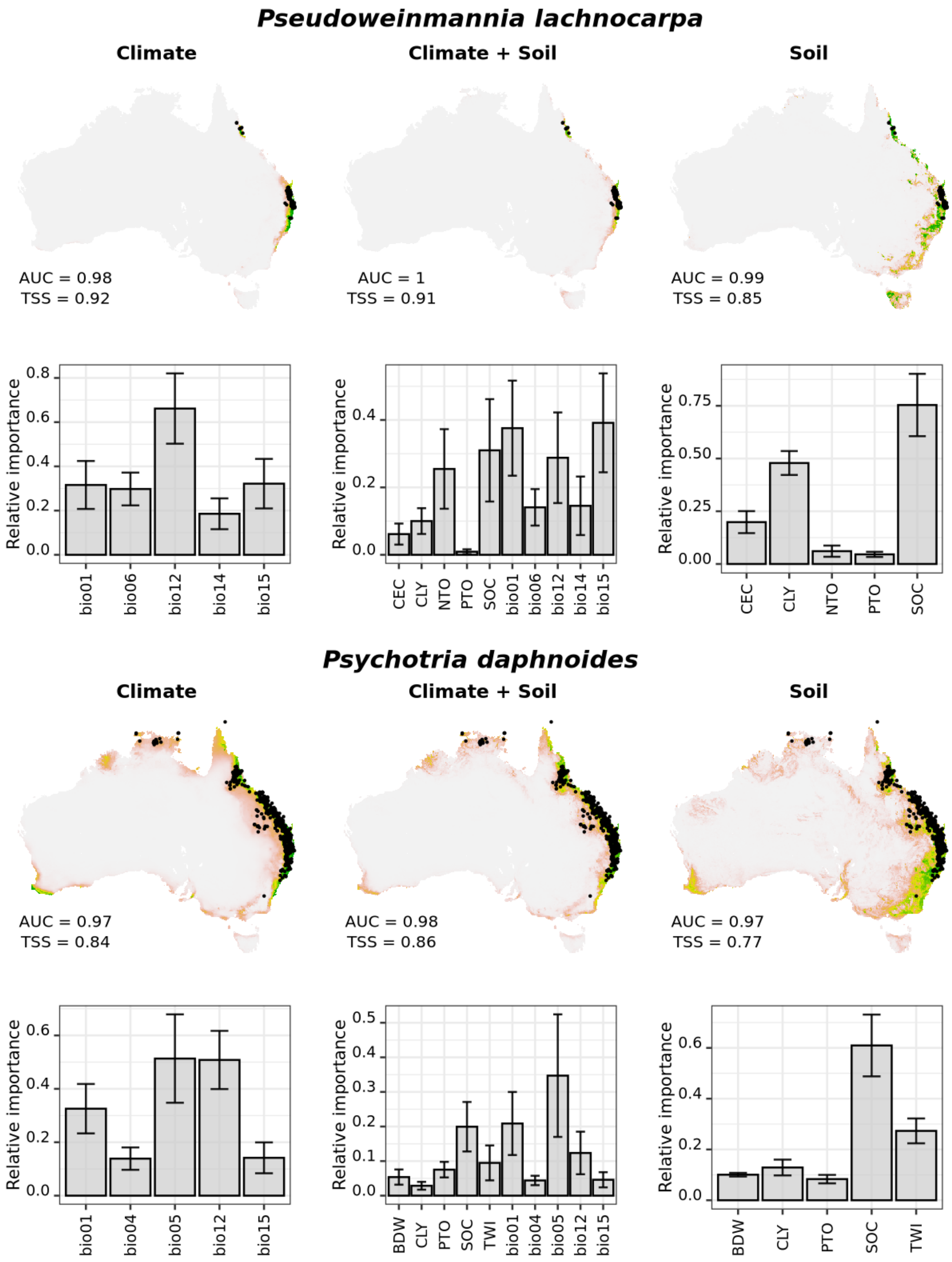
Pseudoweinmannia lachnocarpa
Climate
Climate + Soil
Soil
AUC = 0.98
TSS = 0.92
AUC = 1
TSS = 0.91
AUC = 0.99
TSS = 0.85
Relative importance
bio01
bio06
bio12
bio14
bio15
CEC
CLY
NTO
PTO
SOC
Psychotria daphnoides
AUC = 0.97
TSS = 0.84
AUC = 0.98
TSS = 0.86
AUC = 0.97
TSS = 0.77
bio04
bio05
BDW
TWI

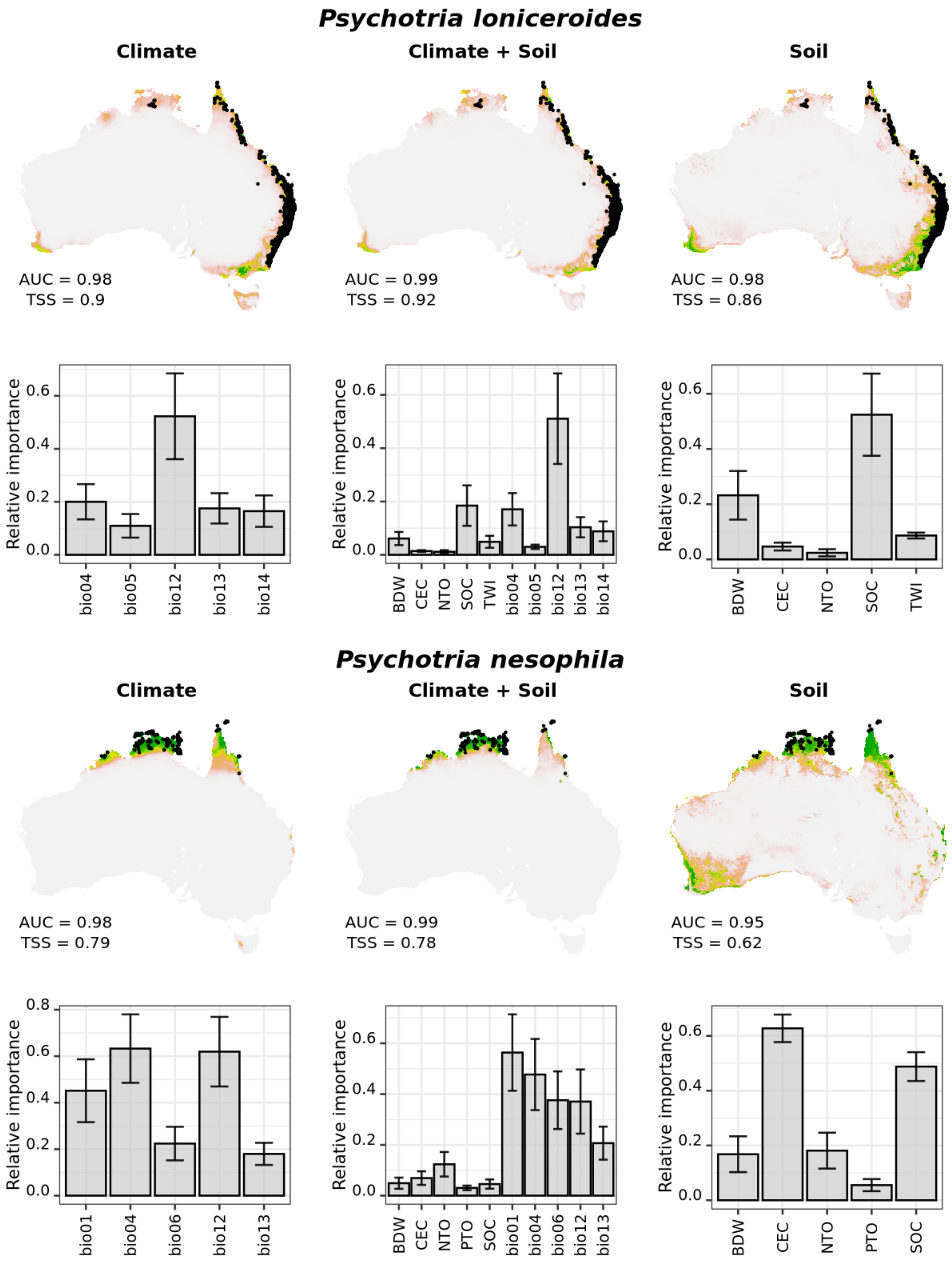

Psychotria loniceroides
Climate
Climate + Soil
Soil
AUC = 0.98
TSS = 0.9
AUC = 0.99
TSS = 0.92
AUC = 0.98
TSS = 0.86
Relative importance
0.0
0.2
0.4
0.6
bio04
bio05
bio12
bio13
bio14
BDW
CEC
NTO
SOC
TWI
bio04
bio05
bio12
bio13
bio14
BDW
CEC
NTO
SOC
TWI
Psychotria nesophila
Climate
Climate + Soil
Soil
AUC = 0.98
TSS = 0.79
AUC = 0.99
TSS = 0.78
AUC = 0.95
TSS = 0.62
Relative importance
0.0
0.2
0.4
0.6
0.8
bio01
bio04
bio06
bio12
bio13
BDW
CEC
NTO
PTO
SOC
bio01
bio04
bio06
bio12
bio13
BDW
CEC
NTO
PTO
SOC

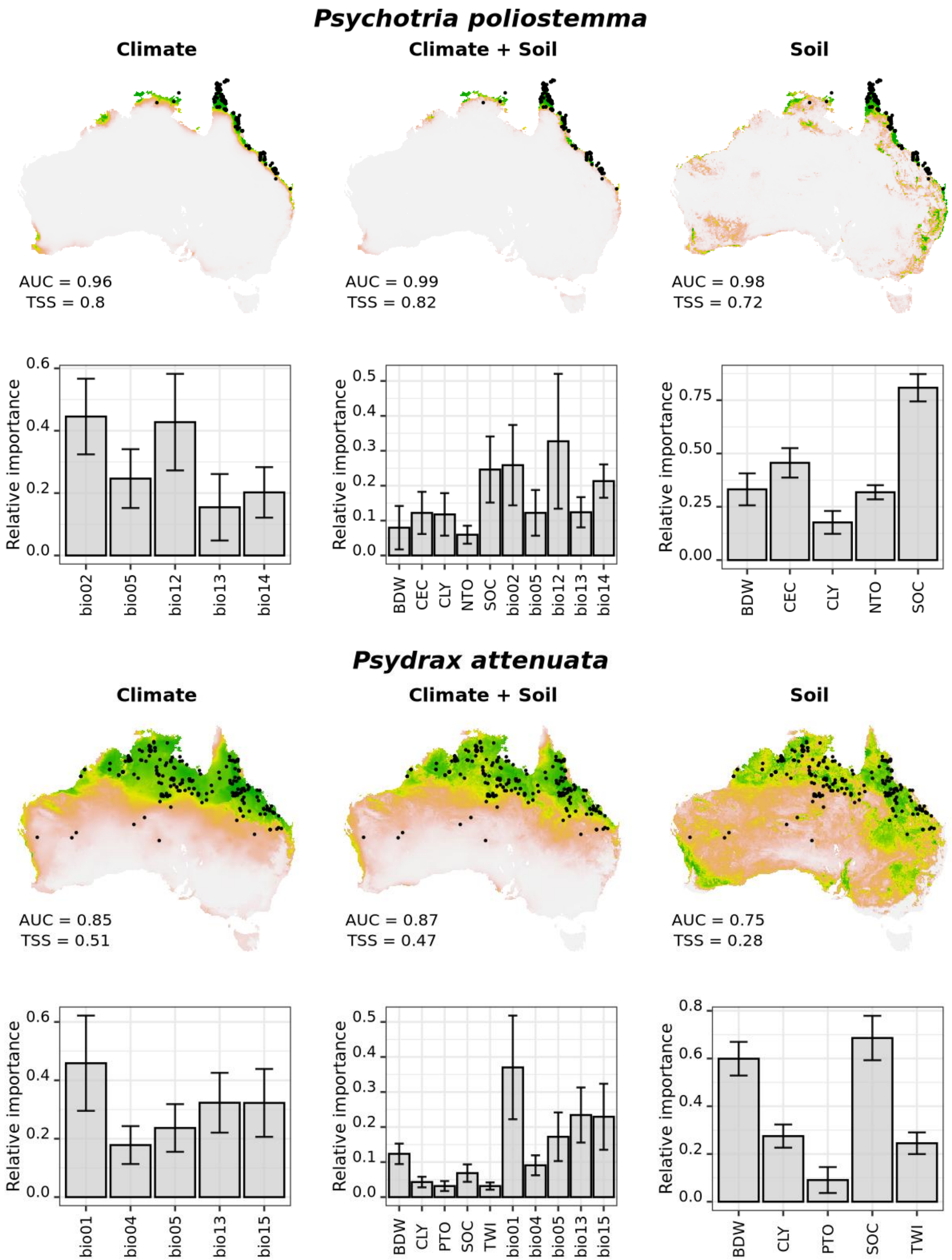
Psychotria poliostemma
Climate
Climate + Soil
Soil
AUC = 0.96
TSS = 0.8
AUC = 0.99
TSS = 0.82
AUC = 0.98
TSS = 0.72
Relative importance
bio02 bio05 bio12 bio13 bio14
BDW CEC CLY NTO SOC bio02 bio05 bio12 bio13 bio14
BDW CEC CLY NTO SOC
Psydrax attenuata
Climate
Climate + Soil
Soil
AUC = 0.85
TSS = 0.51
AUC = 0.87
TSS = 0.47
AUC = 0.75
TSS = 0.28
bio01 bio04 bio05 bio13 bio15
BDW CLY PTO SOC TWI bio01 bio04 bio05 bio13 bio15
BDW CLY PTO SOC TWI

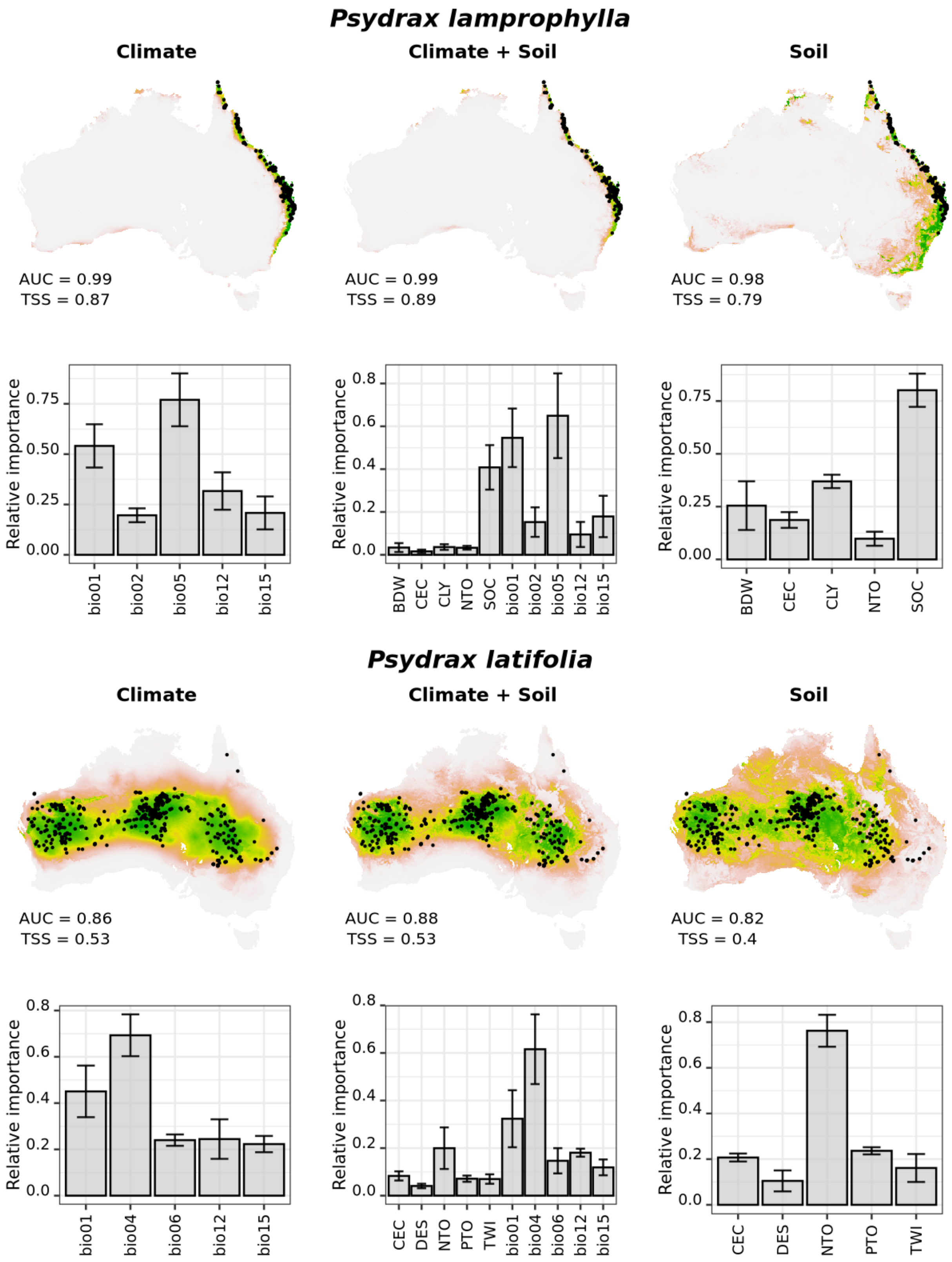
Psydrax lamprophylla
Climate
Climate + Soil
Soil
AUC = 0.99
TSS = 0.87
AUC = 0.99
TSS = 0.89
AUC = 0.98
TSS = 0.79
Relative importance
bio01
bio02
bio05
bio12
bio15
BDW
CEC
CLY
NTO
SOC
Psydrax latifolia
Climate
Climate + Soil
Soil
AUC = 0.86
TSS = 0.53
AUC = 0.88
TSS = 0.53
AUC = 0.82
TSS = 0.4
bio04
bio06
DES
PTO
TWI

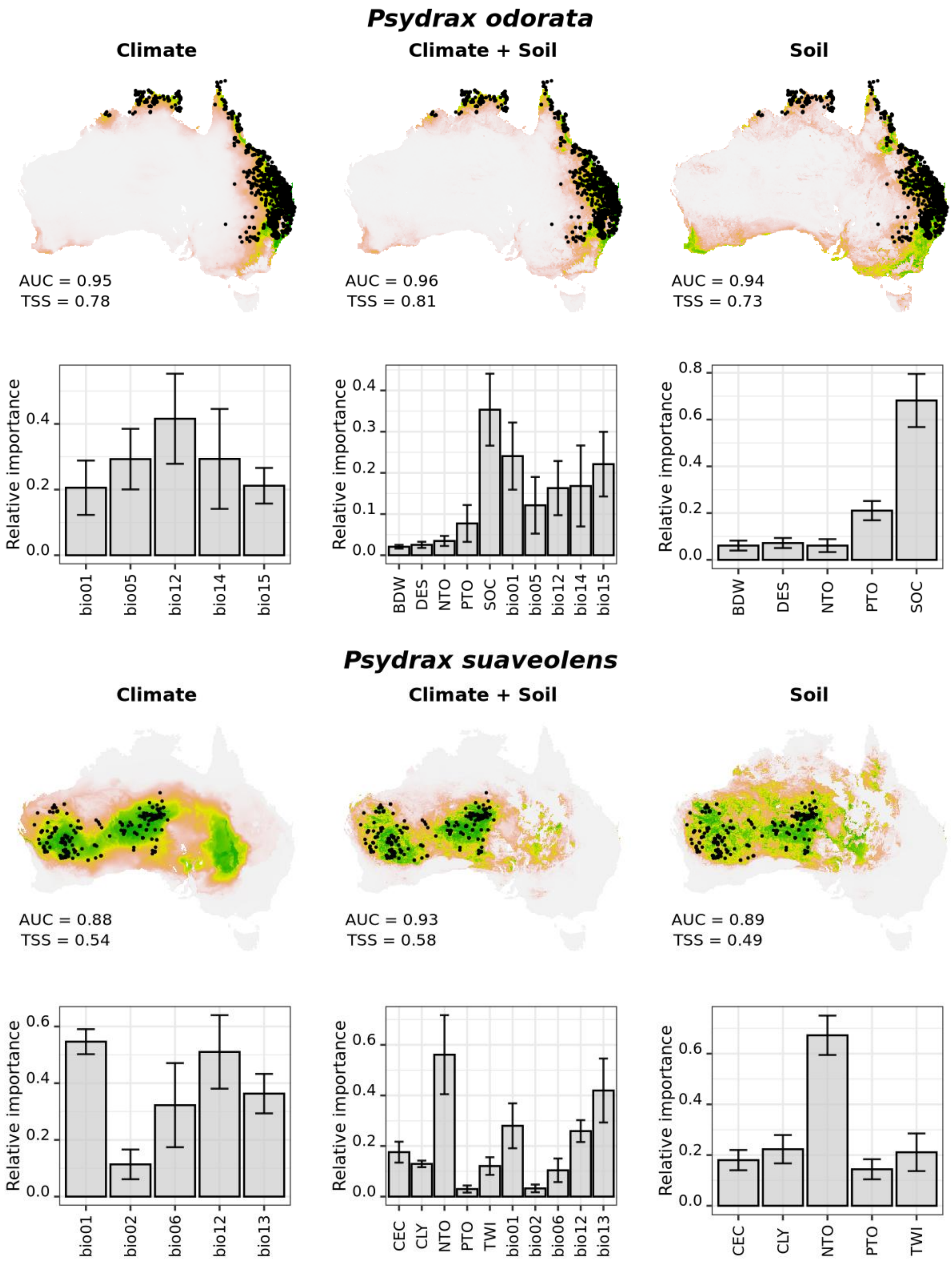

Psydrax odorata
Climate
Climate + Soil
Soil
AUC = 0.95
TSS = 0.78
AUC = 0.96
TSS = 0.81
AUC = 0.94
TSS = 0.73
Relative importance
0.0
0.2
0.4
bio01
bio05
bio12
bio14
bio15
Relative importance
0.0
0.1
0.2
0.3
0.4
BDW
DES
NTO
PTO
SOC
bio01
bio05
bio12
bio14
bio15
Relative importance
0.0
0.2
0.4
0.6
0.8
BDW
DES
NTO
PTO
SOC
Psydrax suaveolens
Climate
Climate + Soil
Soil
AUC = 0.88
TSS = 0.54
AUC = 0.93
TSS = 0.58
AUC = 0.89
TSS = 0.49
Relative importance
0.0
0.2
0.4
0.6
bio01
bio02
bio06
bio12
bio13
Relative importance
0.0
0.2
0.4
0.6
CEC
CLY
NTO
PTO
TWI
bio01
bio02
bio06
bio12
bio13
Relative importance
0.0
0.2
0.4
0.6
CEC
CLY
NTO
PTO
TWI

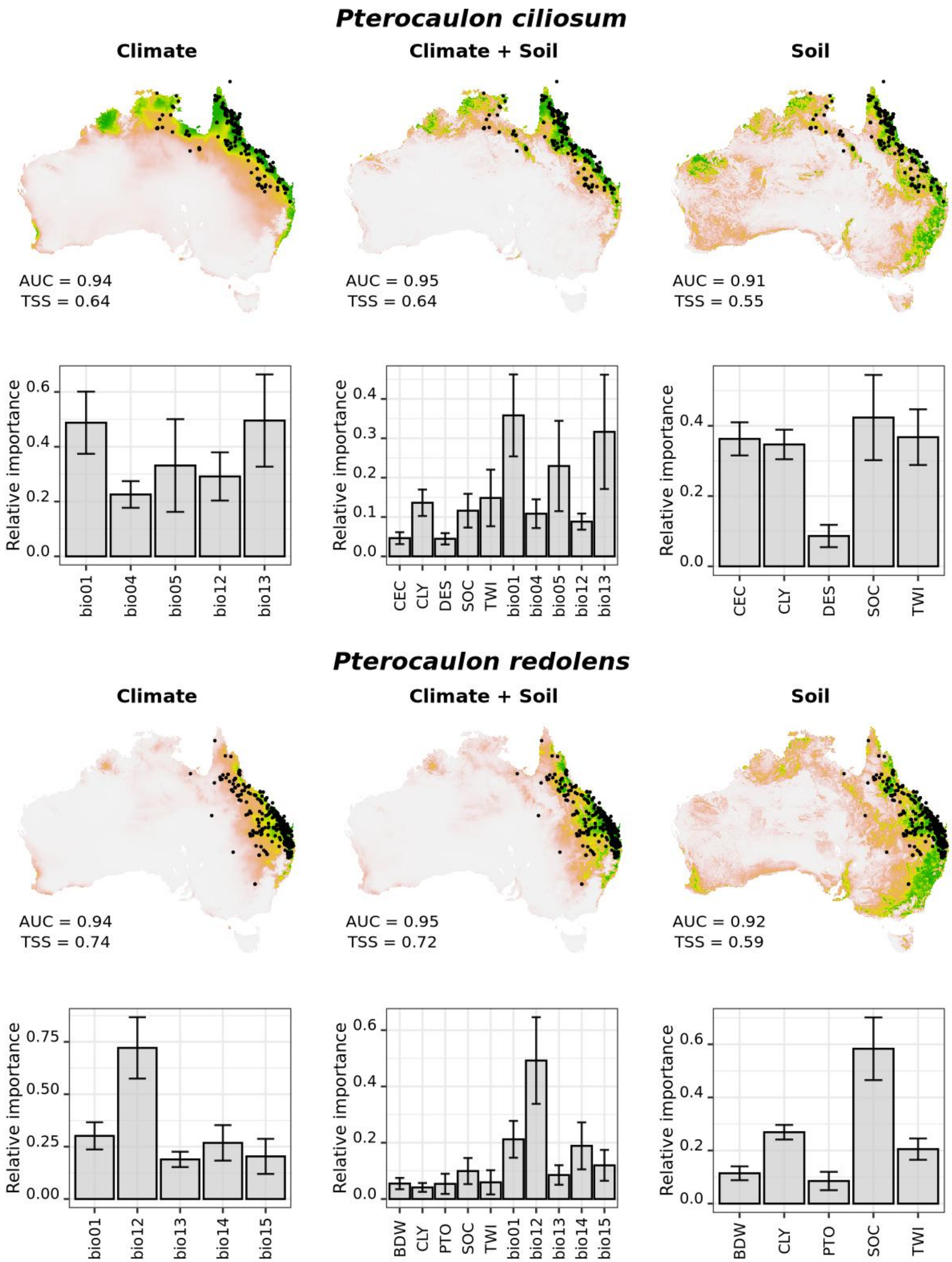
Pterocaulon ciliosum
Climate
Climate + Soil
Soil
AUC = 0.94
TSS = 0.64
AUC = 0.95
TSS = 0.64
AUC = 0.91
TSS = 0.55
Relative importance
0.0
0.2
0.4
0.6
bio01
bio04
bio05
bio12
bio13
Relative importance
0.0
0.1
0.2
0.3
0.4
CEC
CLY
DES
SOC
TWI
bio01
bio04
bio05
bio12
bio13
Relative importance
0.0
0.2
0.4
CEC
CLY
DES
SOC
TWI
Pterocaulon redolens
Climate
Climate + Soil
Soil
AUC = 0.94
TSS = 0.74
AUC = 0.95
TSS = 0.72
AUC = 0.92
TSS = 0.59
Relative importance
0.00
0.25
0.50
0.75
bio01
bio12
bio13
bio14
bio15
Relative importance
0.0
0.2
0.4
0.6
BDW
CLY
PTO
SOC
TWI
bio01
bio12
bio13
bio14
bio15
Relative importance
0.0
0.2
0.4
0.6
BDW
CLY
PTO
SOC
TWI

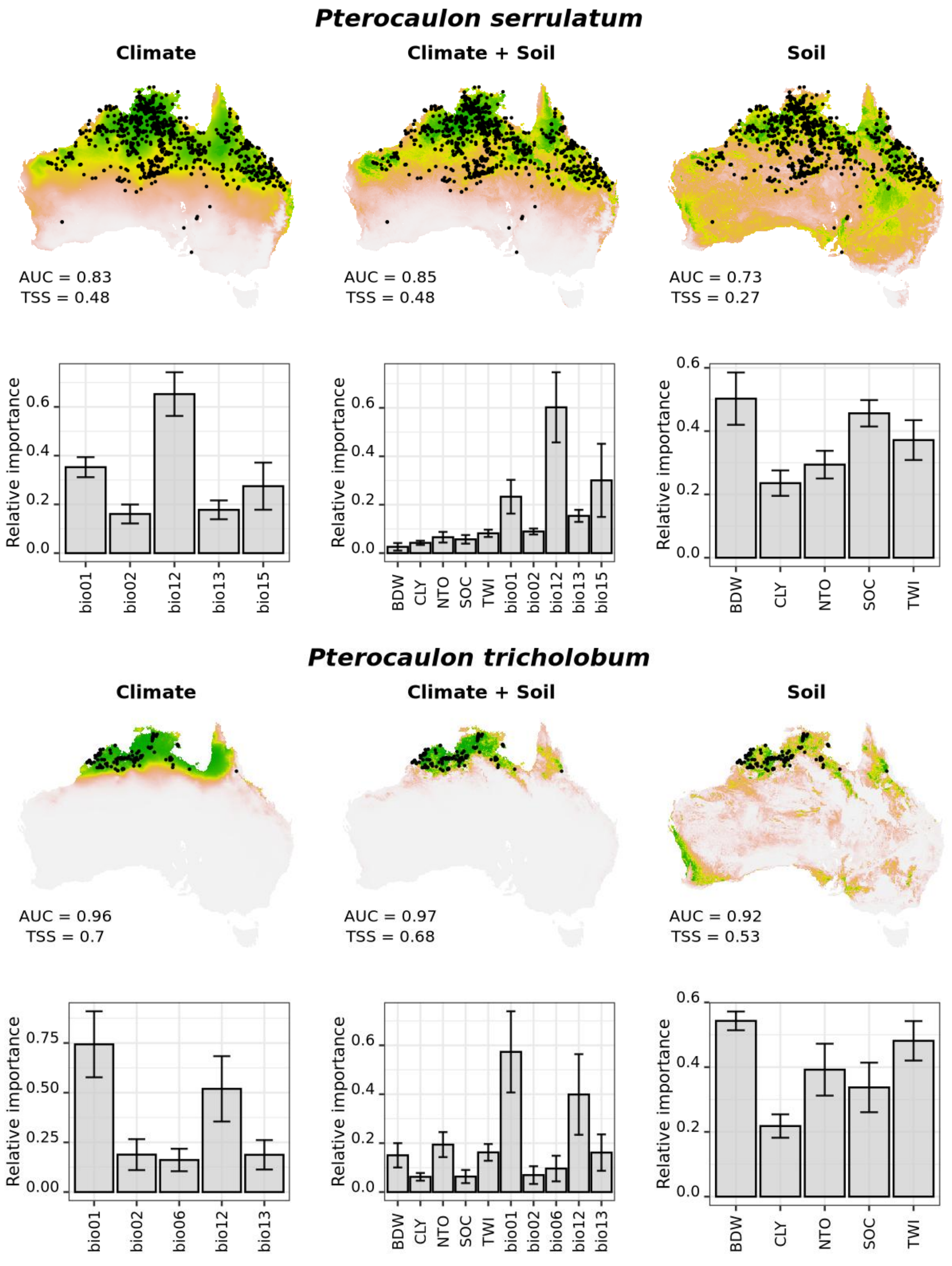

Pterocaulon serrulatum
Climate
Climate + Soil
Soil
AUC = 0.83
TSS = 0.48
AUC = 0.85
TSS = 0.48
AUC = 0.73
TSS = 0.27
Relative importance
bio01
bio02
bio12
bio13
bio15
BDW
CLY
NTO
SOC
TWI
Pterocaulon tricholobum
Climate
Climate + Soil
Soil
AUC = 0.96
TSS = 0.7
AUC = 0.97
TSS = 0.68
AUC = 0.92
TSS = 0.53
Relative importance
bio01
bio02
bio06
bio12
bio13
BDW
CLY
NTO
SOC
TWI

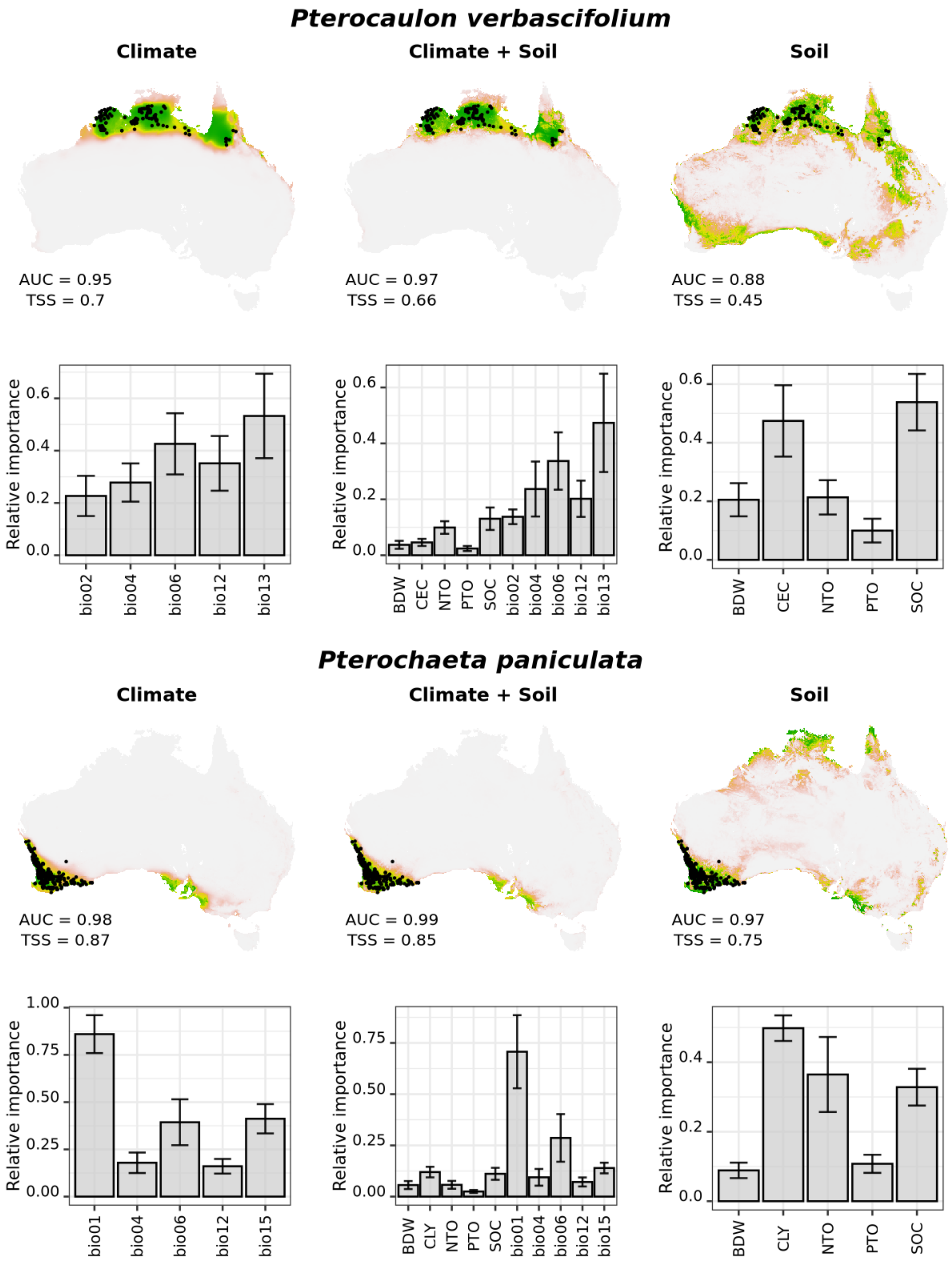

Pterocaulon verbascifolium
Climate
Climate + Soil
Soil
AUC = 0.95
TSS = 0.7
AUC = 0.97
TSS = 0.66
AUC = 0.88
TSS = 0.45
Relative importance
bio02
bio04
bio06
bio12
bio13
BDW
CEC
NTO
PTO
SOC
Pterochaeta paniculata
Climate
Climate + Soil
Soil
AUC = 0.98
TSS = 0.87
AUC = 0.99
TSS = 0.85
AUC = 0.97
TSS = 0.75
Relative importance
bio01
bio04
bio06
bio12
bio15
BDW
CLY
NTO
PTO
SOC

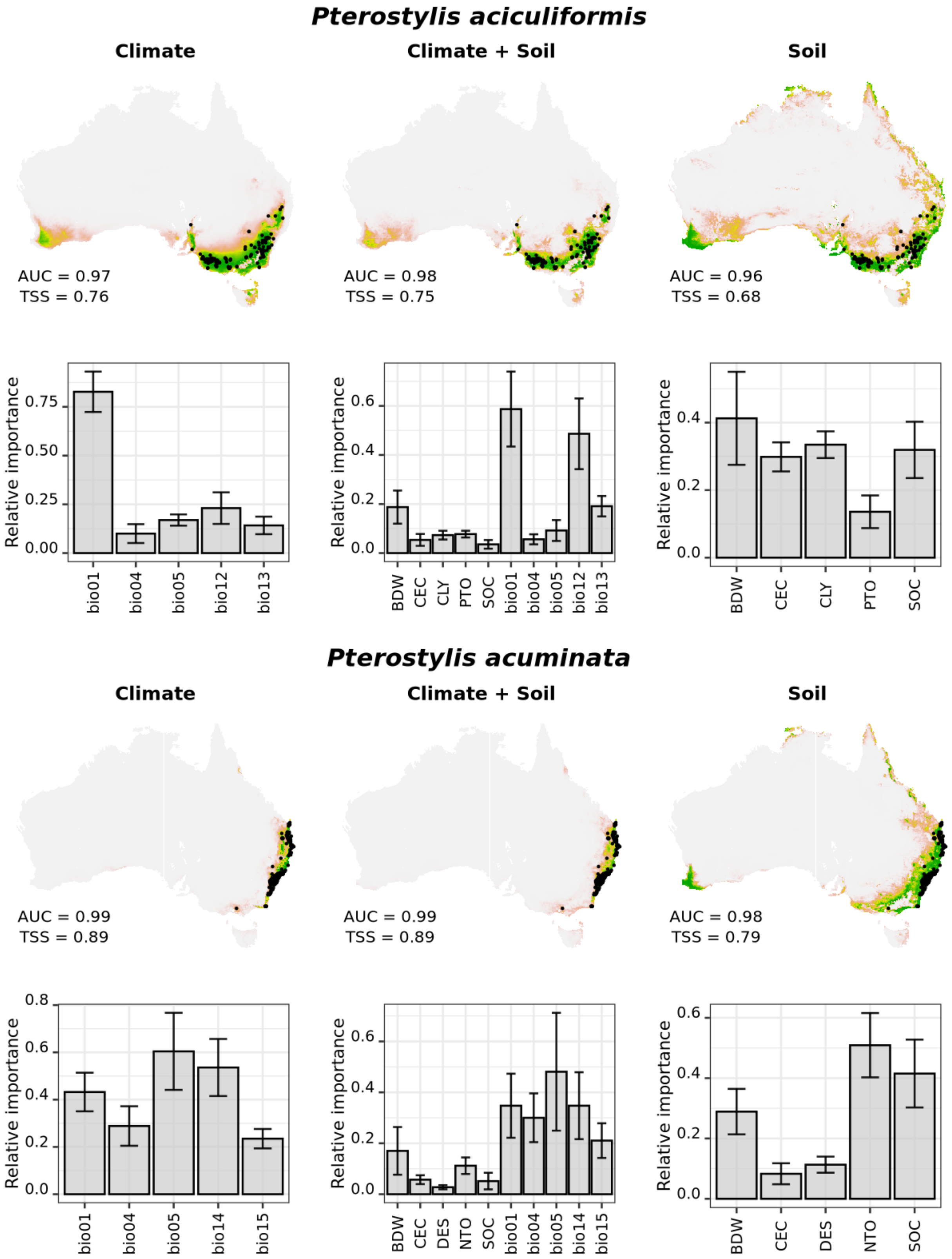

Pterostylis aciculiformis
Climate
Climate + Soil
Soil
AUC = 0.97
TSS = 0.76
AUC = 0.98
TSS = 0.75
AUC = 0.96
TSS = 0.68
Relative importance
0.00
0.25
0.50
0.75
bio01
bio04
bio05
bio12
bio13
Relative importance
0.0
0.2
0.4
0.6
BDW
CEC
CLY
PTO
SOC
bio01
bio04
bio05
bio12
bio13
Relative importance
0.0
0.2
0.4
BDW
CEC
CLY
PTO
SOC
Pterostylis acuminata
Climate
Climate + Soil
Soil
AUC = 0.99
TSS = 0.89
AUC = 0.99
TSS = 0.89
AUC = 0.98
TSS = 0.79
Relative importance
0.0
0.2
0.4
0.6
0.8
bio01
bio04
bio05
bio14
bio15
Relative importance
0.0
0.2
0.4
0.6
BDW
CEC
DES
NTO
SOC
bio01
bio04
bio05
bio14
bio15
Relative importance
0.0
0.2
0.4
0.6
BDW
CEC
DES
NTO
SOC

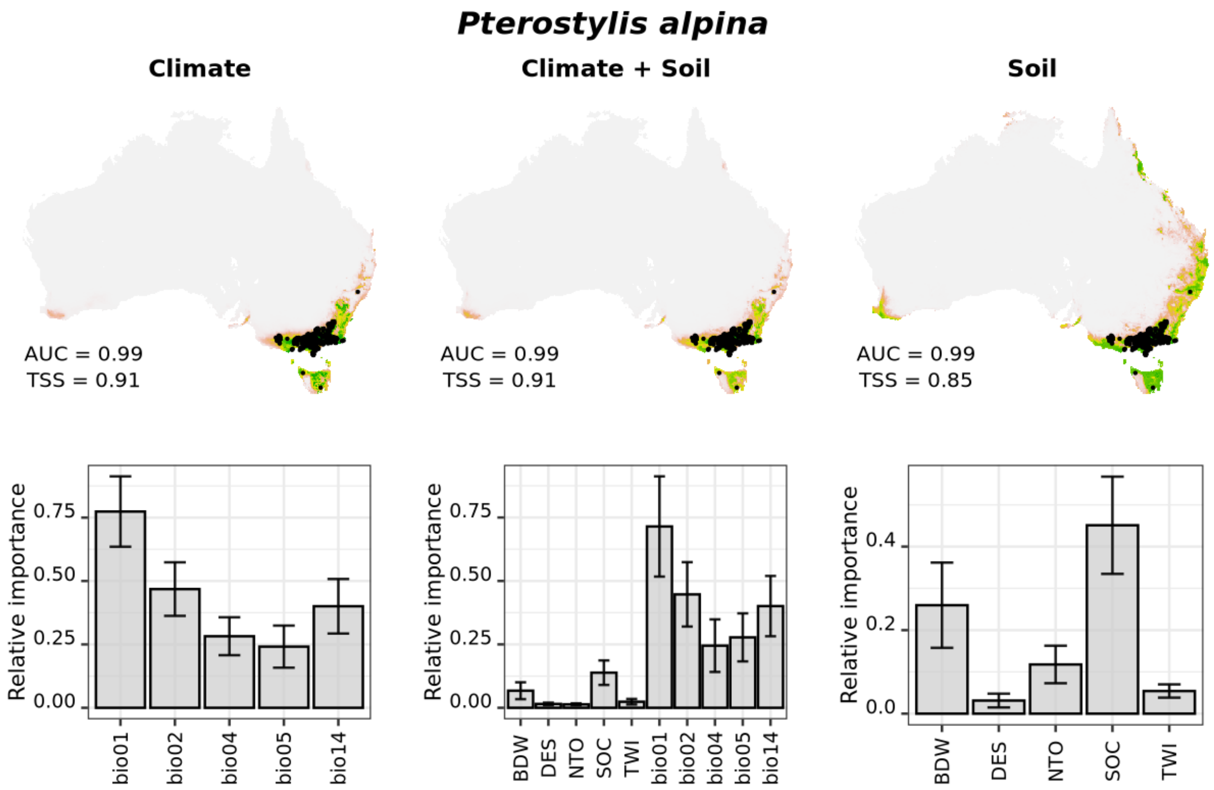
Pterostylis alpina
Climate
Climate + Soil
Soil
AUC = 0.99
TSS = 0.91
AUC = 0.99
TSS = 0.91
AUC = 0.99
TSS = 0.85
Relative importance
0.00
0.25
0.50
0.75
bio01
bio02
bio04
bio05
bio14
BDW
DES
NTO
SOC
TWI
0.0
0.2
0.4

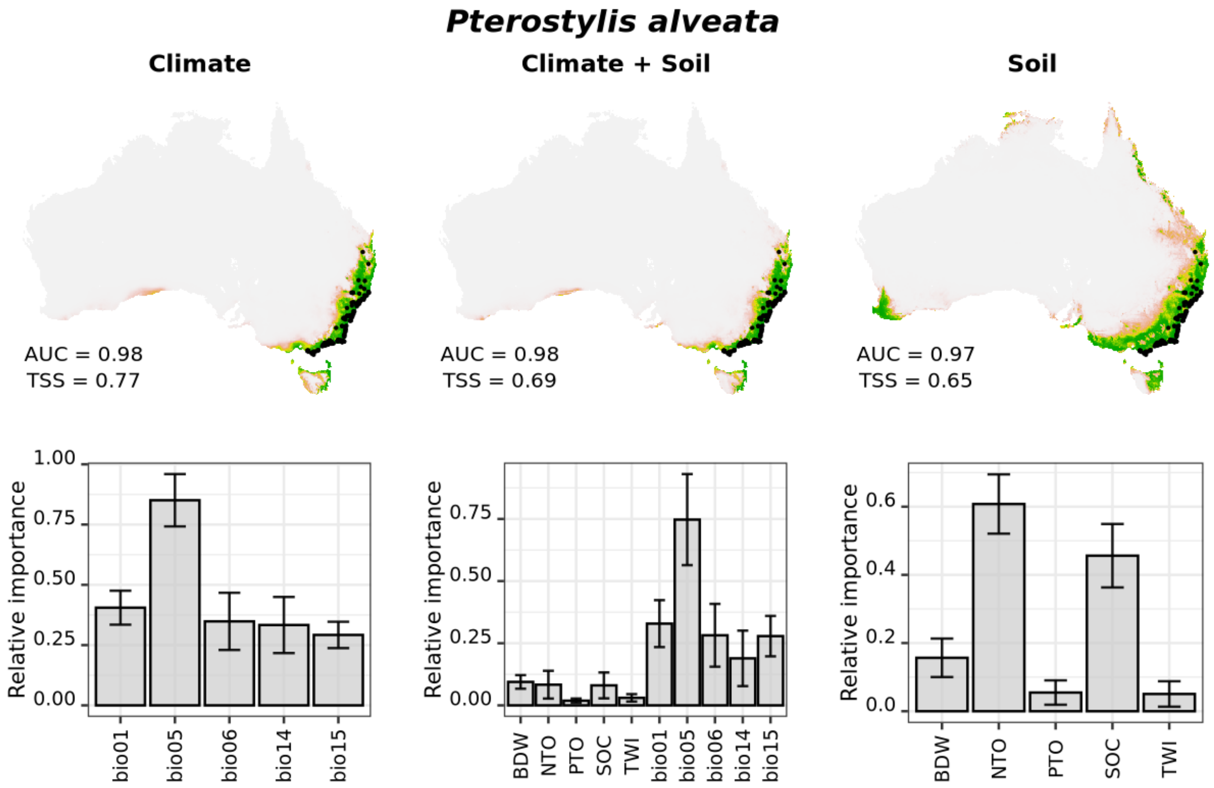
Pterostylis alveata
Climate
Climate + Soil
Soil
AUC = 0.98
TSS = 0.77
AUC = 0.98
TSS = 0.69
AUC = 0.97
TSS = 0.65
Relative importance
0.00
0.25
0.50
0.75
1.00
bio01
bio05
bio06
bio14
bio15
BDW
NTO
PTO
SOC
TWI
0.0
0.2
0.4
0.6

Pterostylis arbuscula

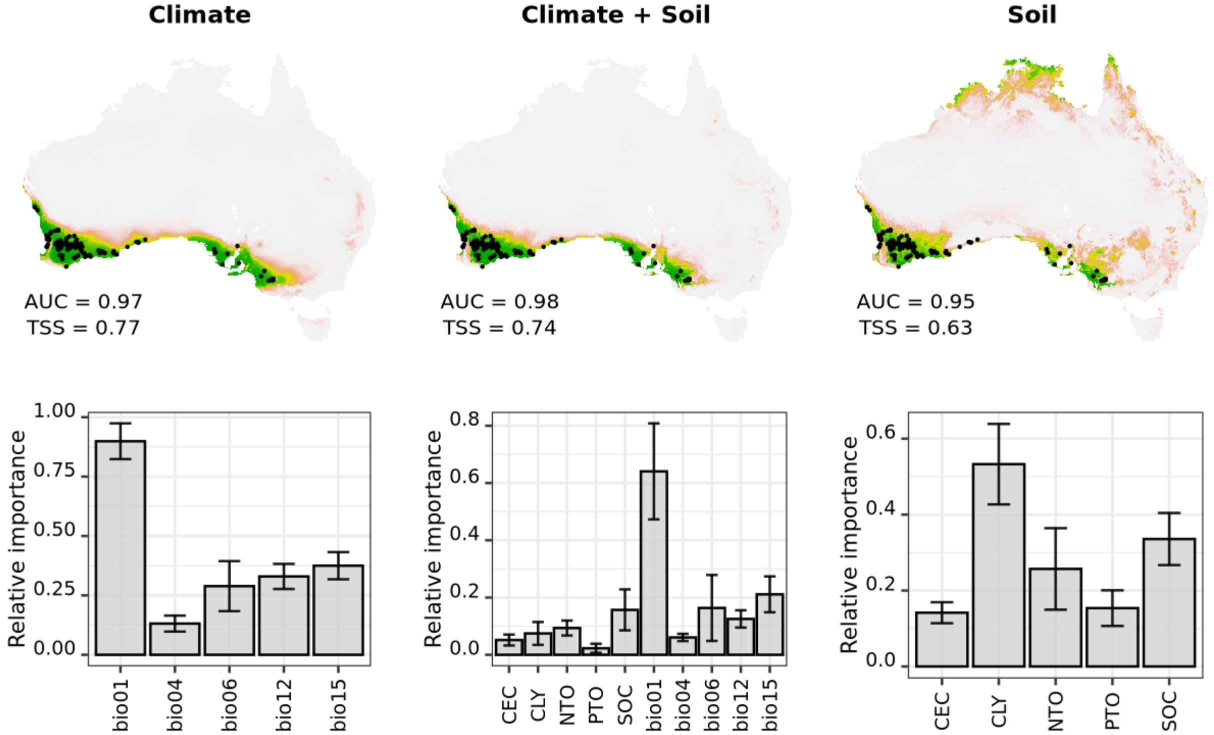

Pterostylis atrans

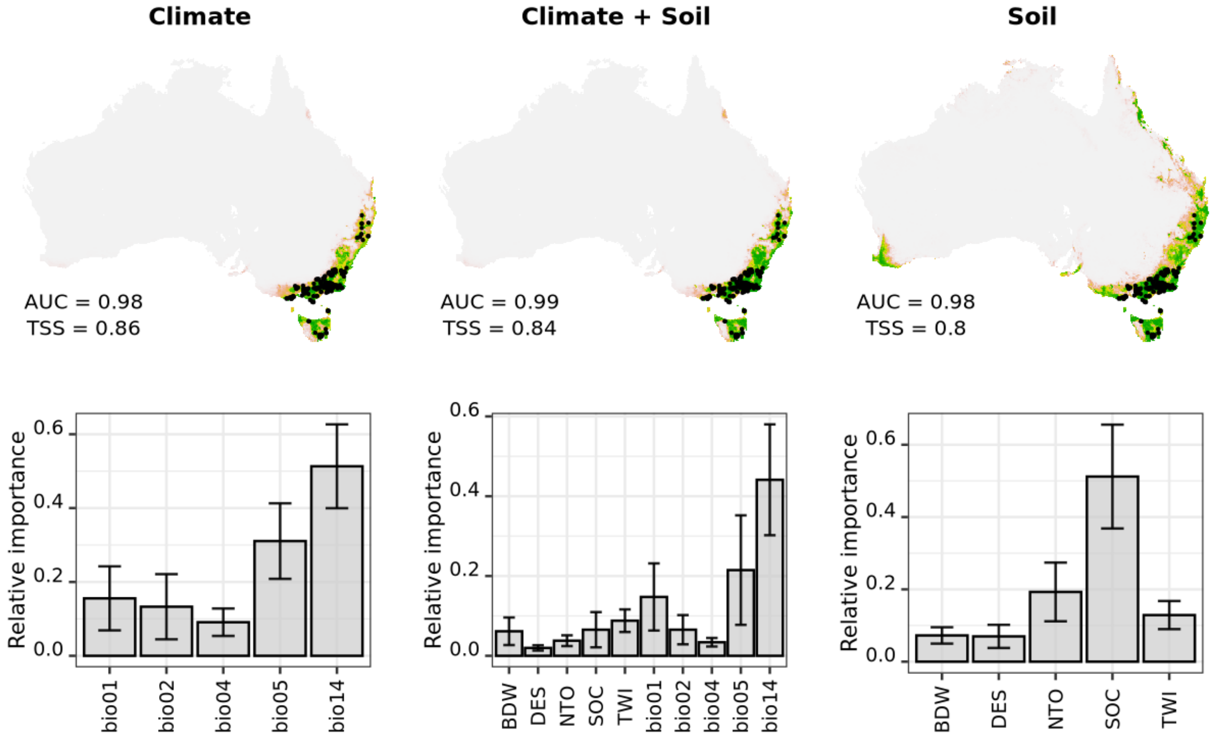

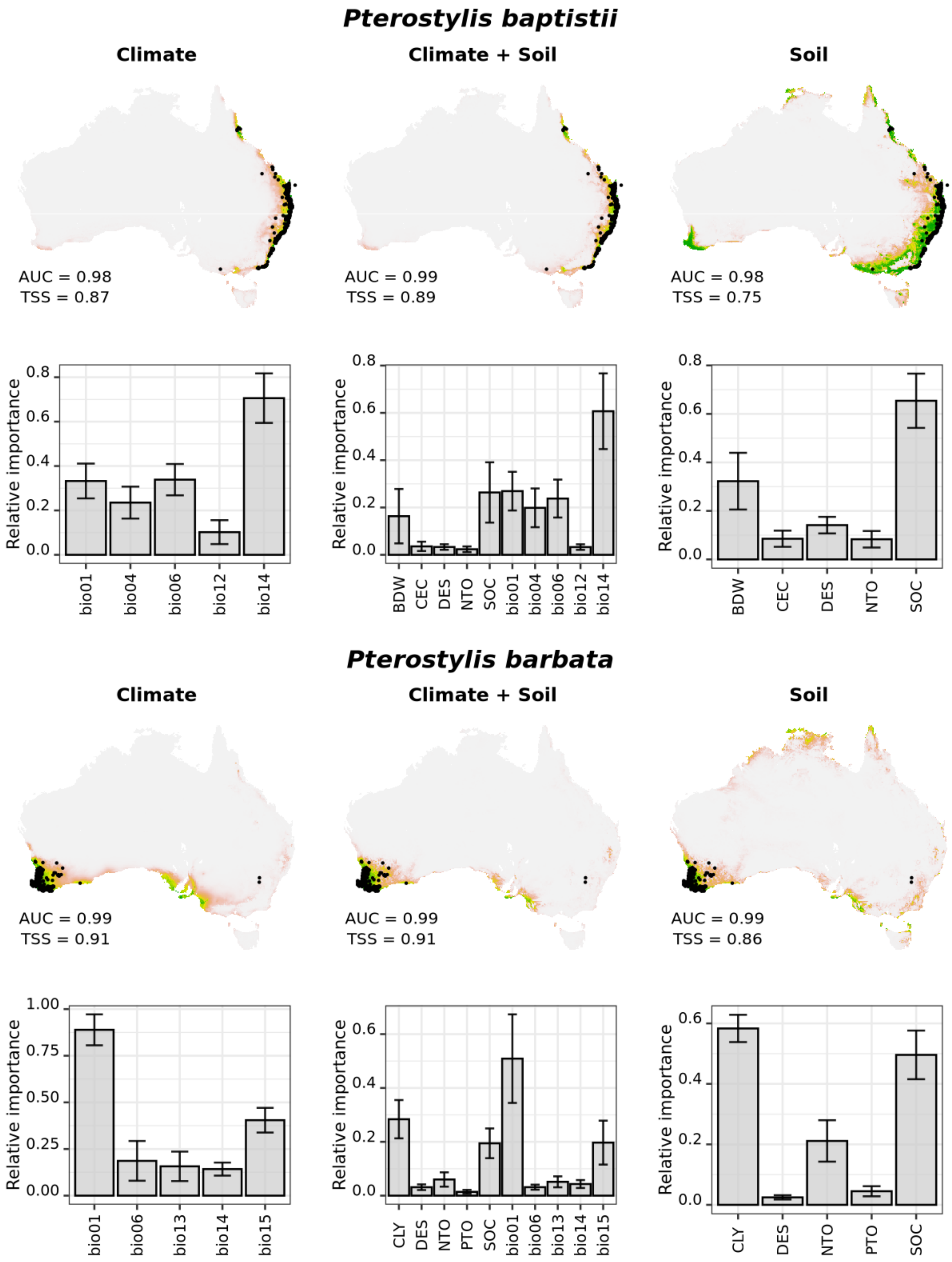
Pterostylis baptistii
Climate
Climate + Soil
Soil
AUC = 0.98
TSS = 0.87
AUC = 0.99
TSS = 0.89
AUC = 0.98
TSS = 0.75
Relative importance
bio01
bio04
bio06
bio12
bio14
BDW
CEC
DES
NTO
SOC
Pterostylis barbata
AUC = 0.99
TSS = 0.91
AUC = 0.99
TSS = 0.91
AUC = 0.99
TSS = 0.86
bio13
bio15
CLY
PTO

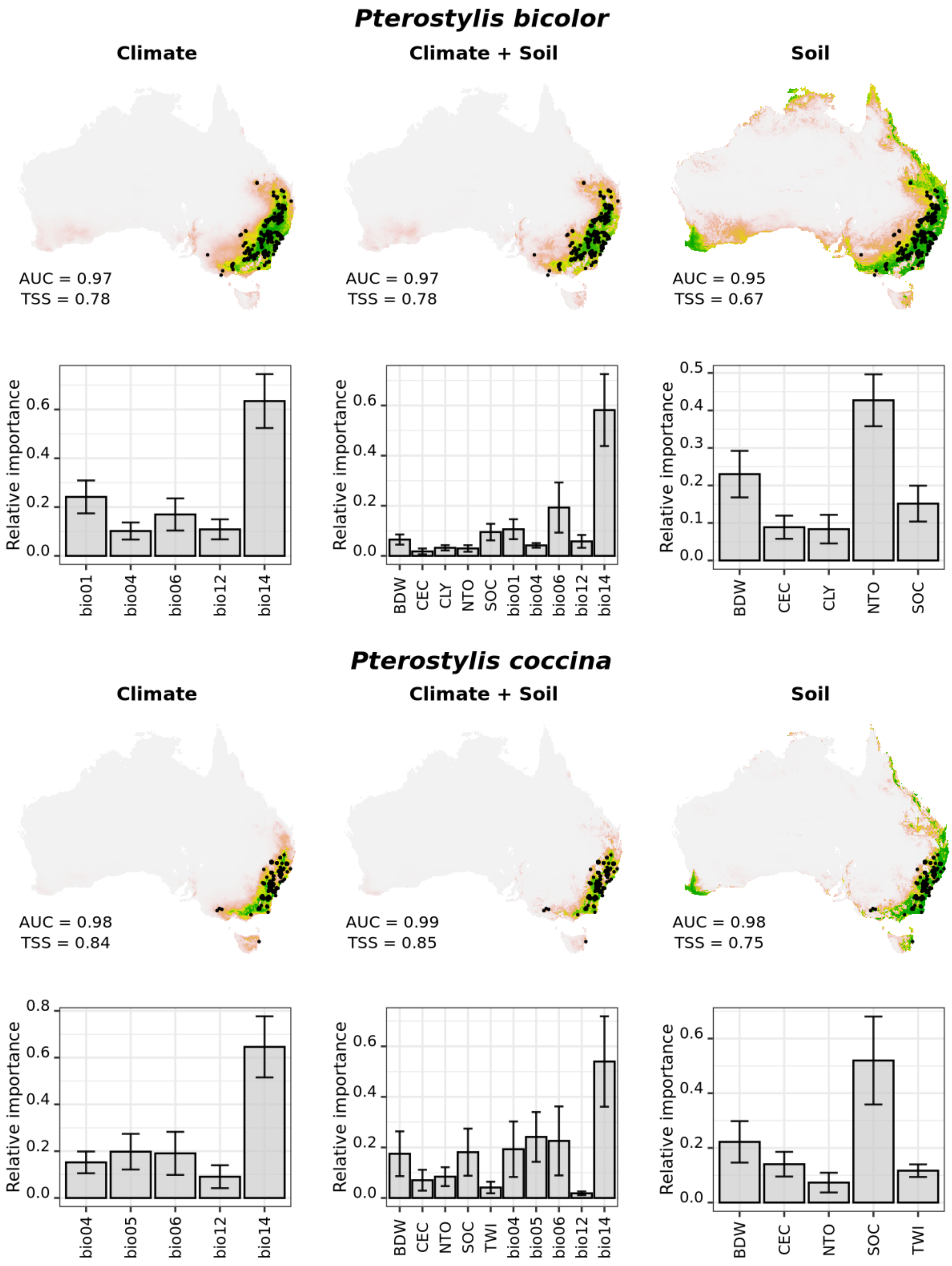

Pterostylis bicolor
Climate
Climate + Soil
Soil
AUC = 0.97
TSS = 0.78
AUC = 0.97
TSS = 0.78
AUC = 0.95
TSS = 0.67
Relative importance
bio01
bio04
bio06
bio12
bio14
BDW
CEC
CLY
NTO
SOC
bio01
bio04
bio06
bio12
bio14
BDW
CEC
CLY
NTO
SOC
Pterostylis coccina
Climate
Climate + Soil
Soil
AUC = 0.98
TSS = 0.84
AUC = 0.99
TSS = 0.85
AUC = 0.98
TSS = 0.75
Relative importance
bio04
bio05
bio06
bio12
bio14
BDW
CEC
NTO
SOC
TWI
bio04
bio05
bio06
bio12
bio14
BDW
CEC
NTO
SOC
TWI

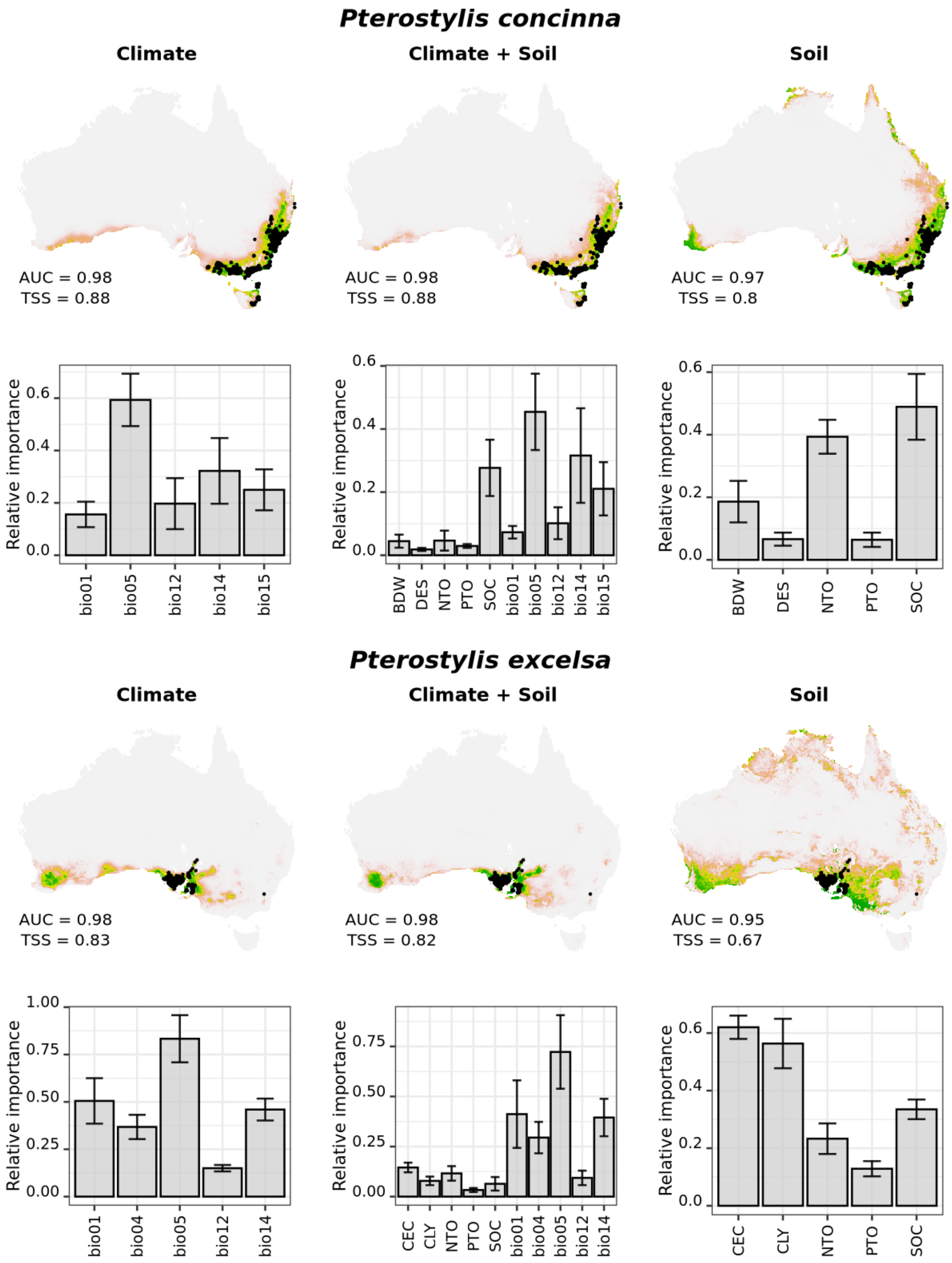
Pterostylis concinna
Climate
Climate + Soil
Soil
AUC = 0.98
TSS = 0.88
AUC = 0.98
TSS = 0.88
AUC = 0.97
TSS = 0.8
Relative importance
bio01
bio05
bio12
bio14
bio15
BDW
DES
NTO
PTO
SOC
Pterostylis excelsa
AUC = 0.98
TSS = 0.83
AUC = 0.98
TSS = 0.82
AUC = 0.95
TSS = 0.67
bio04
CEC
CLY

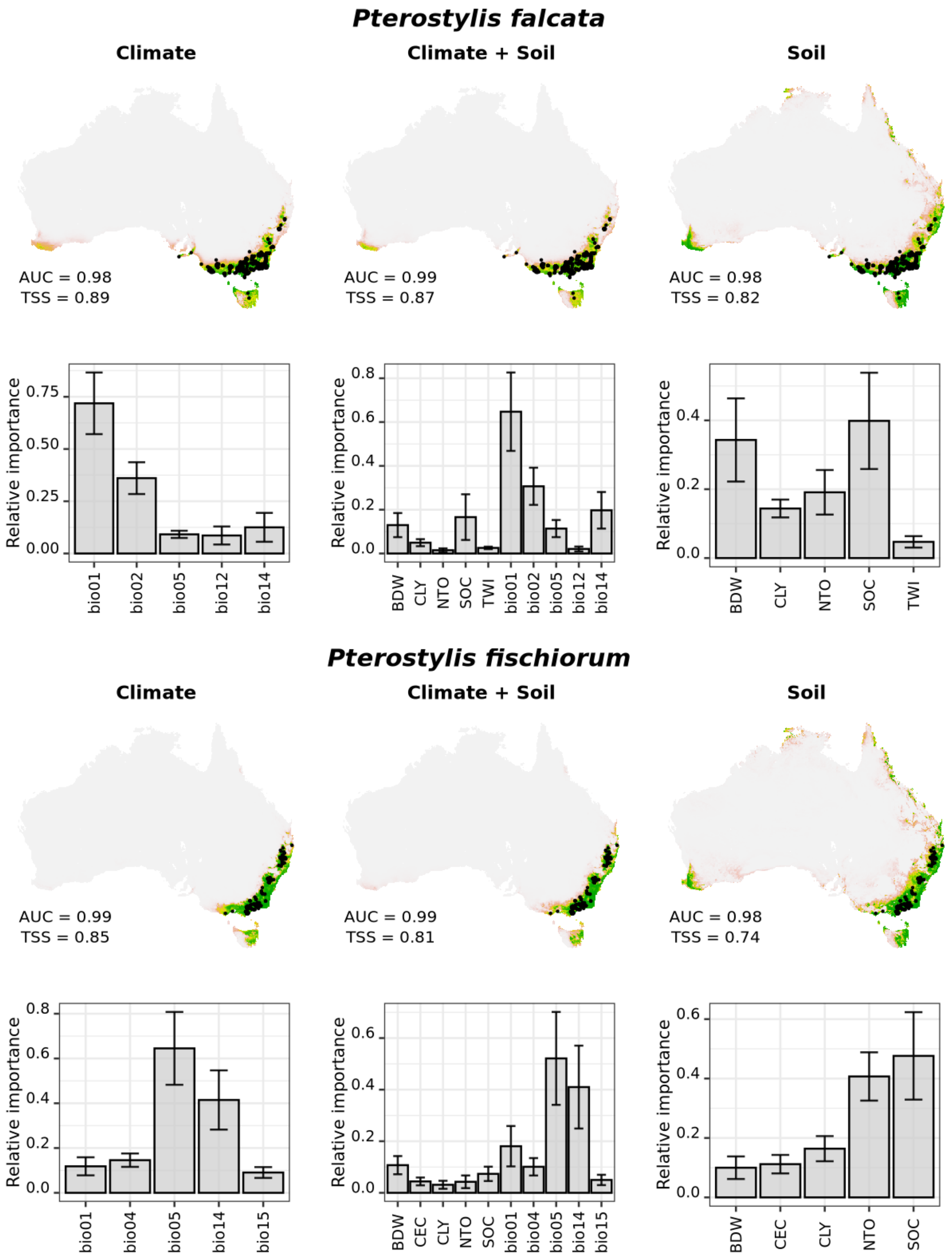

Pterostylis falcata
Climate
Climate + Soil
Soil
AUC = 0.98
TSS = 0.89
AUC = 0.99
TSS = 0.87
AUC = 0.98
TSS = 0.82
Relative importance
0.00
0.25
0.50
0.75
bio01
bio02
bio05
bio12
bio14
0.0
0.2
0.4
0.6
0.8
BDW
CLY
NTO
SOC
TWI
bio01
bio02
bio05
bio12
bio14
0.0
0.2
0.4
BDW
CLY
NTO
SOC
TWI
Pterostylis fischiorum
Climate
Climate + Soil
Soil
AUC = 0.99
TSS = 0.85
AUC = 0.99
TSS = 0.81
AUC = 0.98
TSS = 0.74
Relative importance
0.0
0.2
0.4
0.6
0.8
bio01
bio04
bio05
bio14
bio15
0.0
0.2
0.4
0.6
BDW
CEC
CLY
NTO
SOC
bio01
bio04
bio05
bio14
bio15
0.0
0.2
0.4
0.6
BDW
CEC
CLY
NTO
SOC

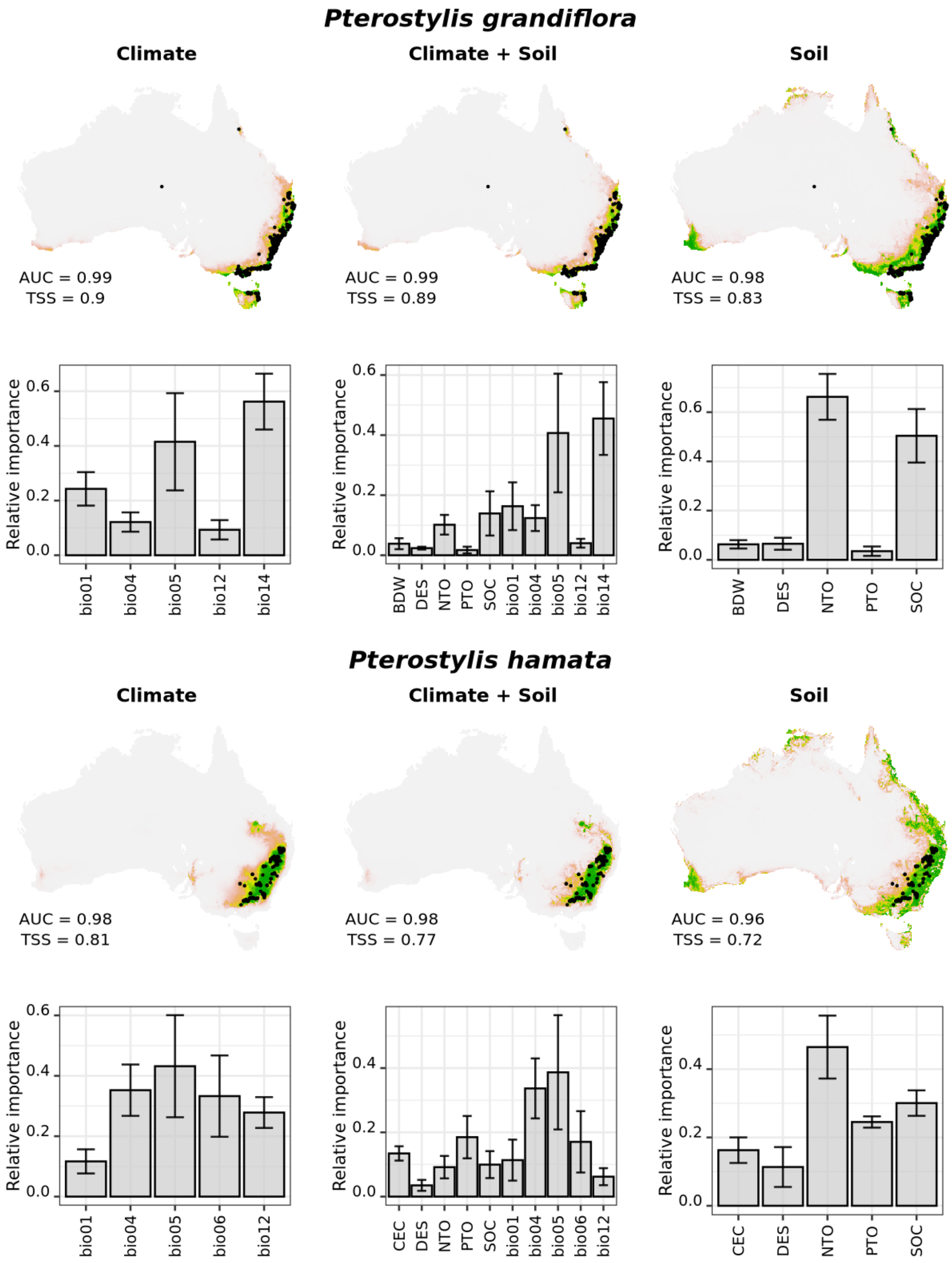
Pterostylis grandiflora
Climate
Climate + Soil
Soil
AUC = 0.99
TSS = 0.9
AUC = 0.99
TSS = 0.89
AUC = 0.98
TSS = 0.83
Relative importance
0.0
0.2
0.4
0.6
bio01
bio04
bio05
bio12
bio14
BDW
DES
NTO
PTO
SOC
Pterostylis hamata
Climate
Climate + Soil
Soil
AUC = 0.98
TSS = 0.81
AUC = 0.98
TSS = 0.77
AUC = 0.96
TSS = 0.72
bio06
CEC

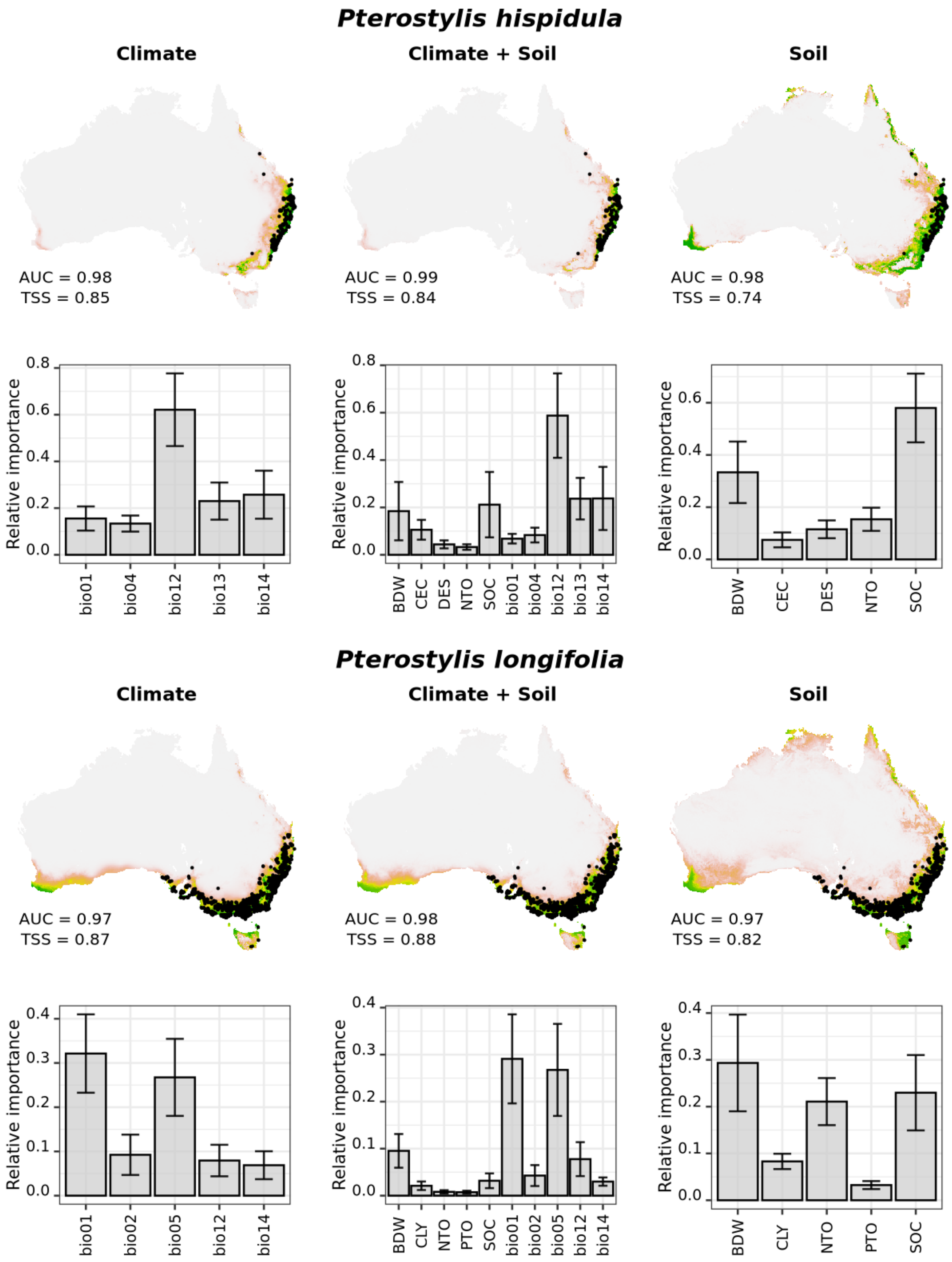
Pterostylis hispidula
Climate
Climate + Soil
Soil
AUC = 0.98
TSS = 0.85
AUC = 0.99
TSS = 0.84
AUC = 0.98
TSS = 0.74
Relative importance
bio01
bio04
bio12
bio13
bio14
BDW
CEC
DES
NTO
SOC
Pterostylis longifolia
AUC = 0.97
TSS = 0.87
AUC = 0.98
TSS = 0.88
AUC = 0.97
TSS = 0.82
bio02
bio05
CLY
PTO

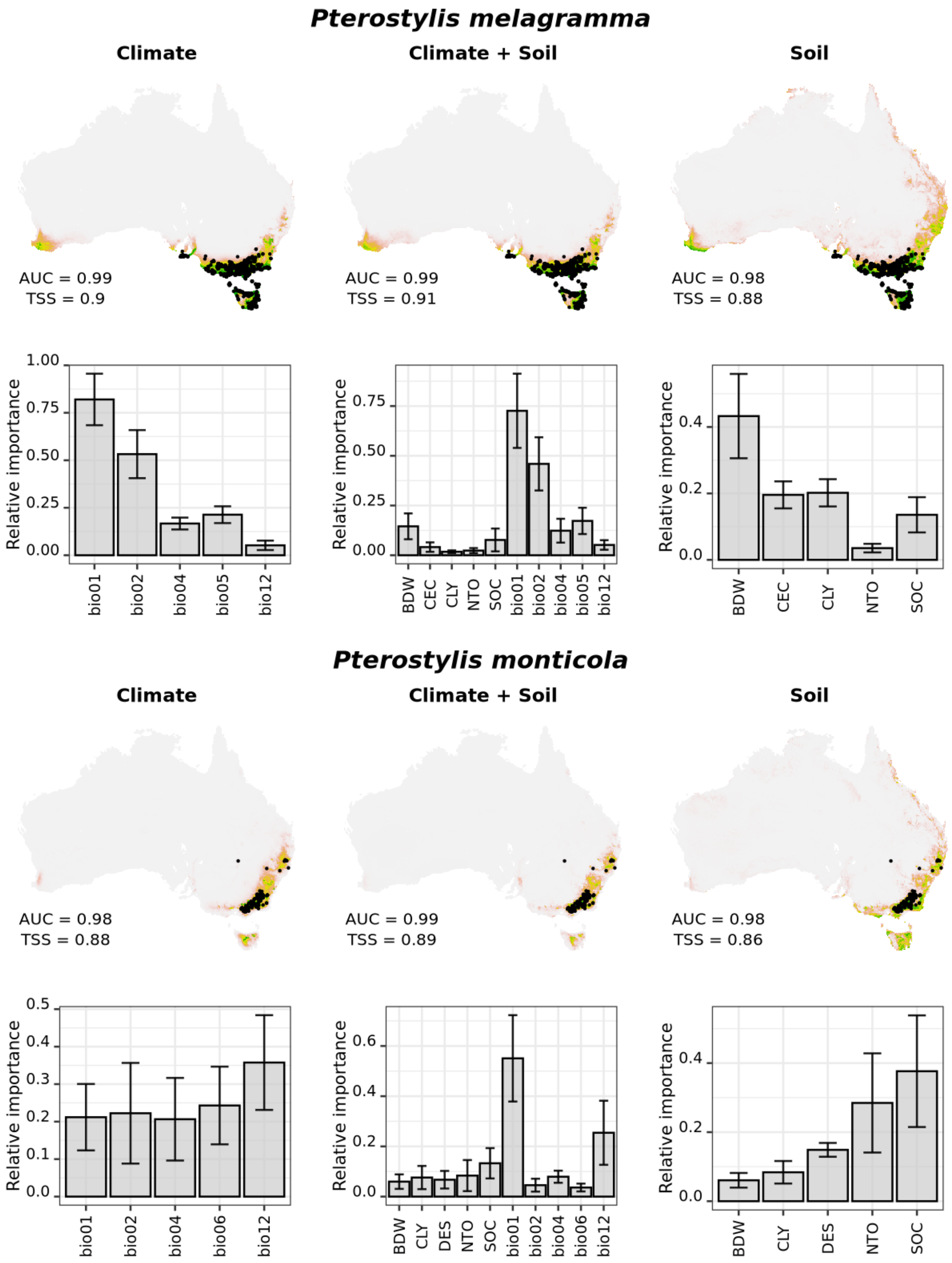

Pterostylis melagramma
Climate
Climate + Soil
Soil
AUC = 0.99
TSS = 0.9
AUC = 0.99
TSS = 0.91
AUC = 0.98
TSS = 0.88
Relative importance
bio01
bio02
bio04
bio05
bio12
BDW
CEC
CLY
NTO
SOC
Pterostylis monticola
AUC = 0.98
TSS = 0.88
AUC = 0.99
TSS = 0.89
AUC = 0.98
TSS = 0.86
bio06
DES

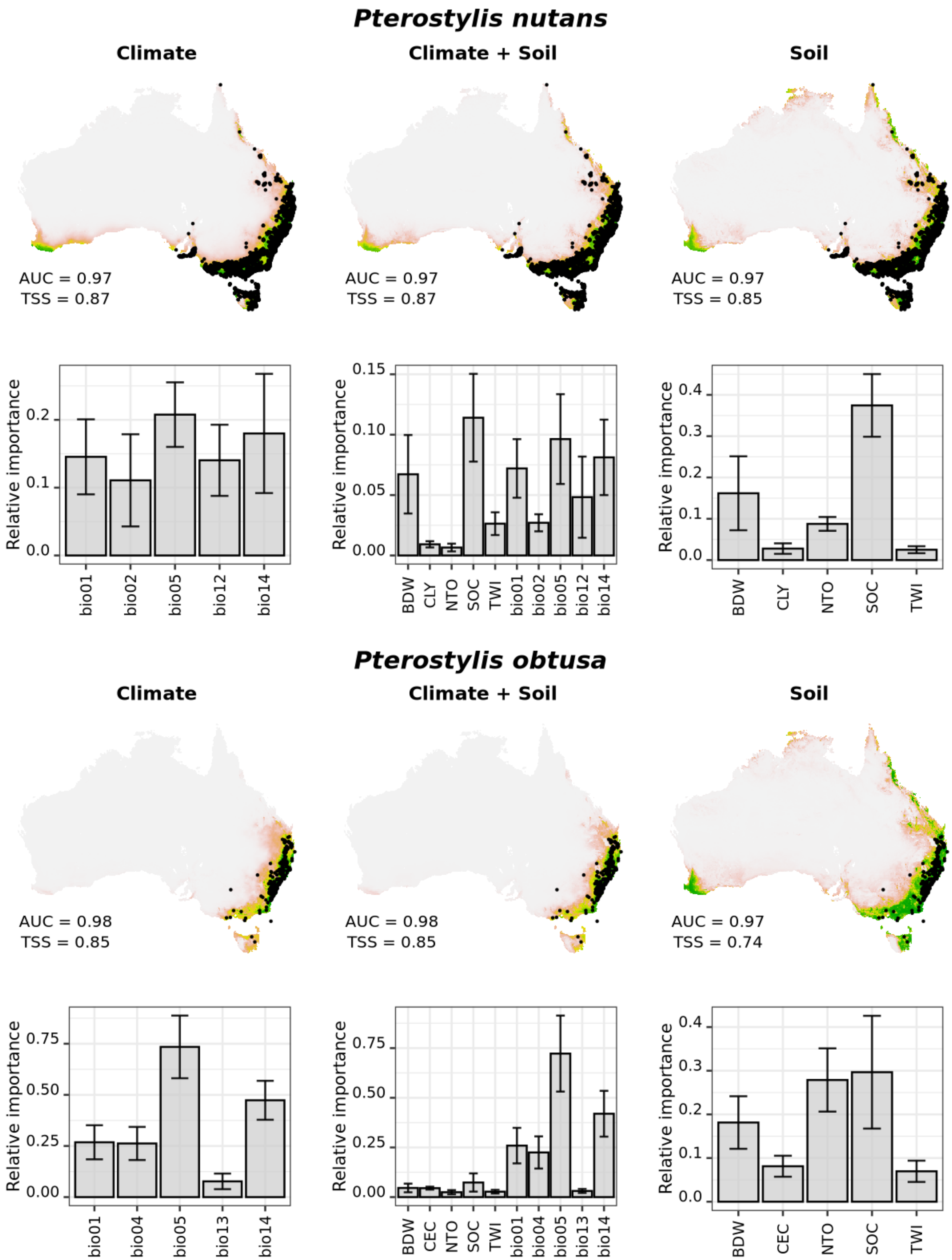
Pterostylis nutans
Climate
Climate + Soil
Soil
AUC = 0.97
TSS = 0.87
AUC = 0.97
TSS = 0.87
AUC = 0.97
TSS = 0.85
Relative importance
0.0
0.1
0.2
bio01
bio02
bio05
bio12
bio14
Relative importance
0.00
0.05
0.10
0.15
BDW
CLY
NTO
SOC
TWI
bio01
bio02
bio05
bio12
bio14
Relative importance
0.0
0.1
0.2
0.3
0.4
BDW
CLY
NTO
SOC
TWI
Pterostylis obtusa
Climate
Climate + Soil
Soil
AUC = 0.98
TSS = 0.85
AUC = 0.98
TSS = 0.85
AUC = 0.97
TSS = 0.74
Relative importance
0.00
0.25
0.50
0.75
bio01
bio04
bio05
bio13
bio14
Relative importance
0.00
0.25
0.50
0.75
BDW
CEC
NTO
SOC
TWI
bio01
bio04
bio05
bio13
bio14
Relative importance
0.0
0.1
0.2
0.3
0.4
BDW
CEC
NTO
SOC
TWI

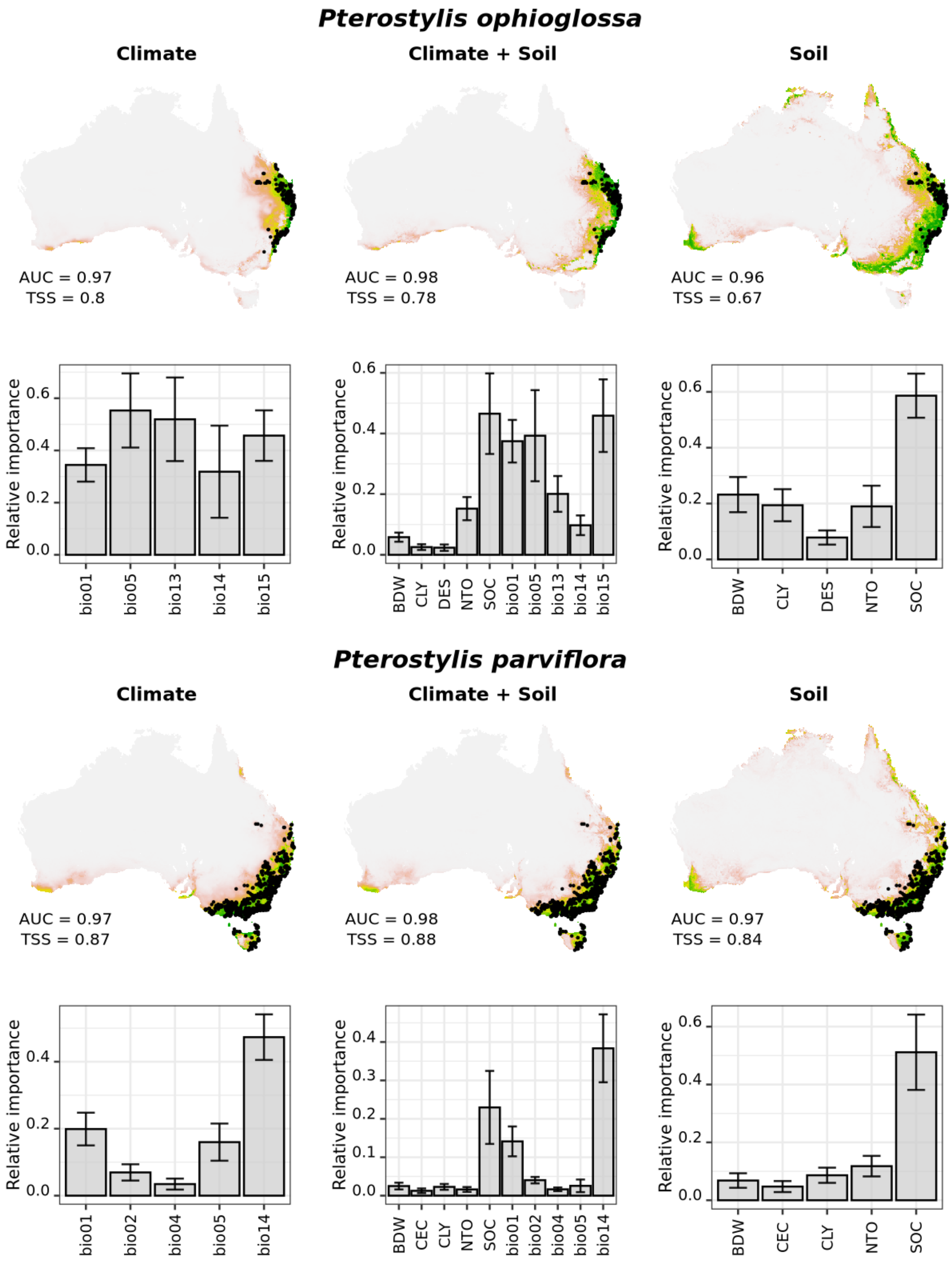
Pterostylis ophioglossa
Climate
Climate + Soil
Soil
AUC = 0.97
TSS = 0.8
AUC = 0.98
TSS = 0.78
AUC = 0.96
TSS = 0.67
Relative importance
bio01
bio05
bio13
bio14
bio15
BDW
CLY
DES
NTO
SOC
Pterostylis parviflora
AUC = 0.97
TSS = 0.87
AUC = 0.98
TSS = 0.88
AUC = 0.97
TSS = 0.84
bio02
bio04
CEC

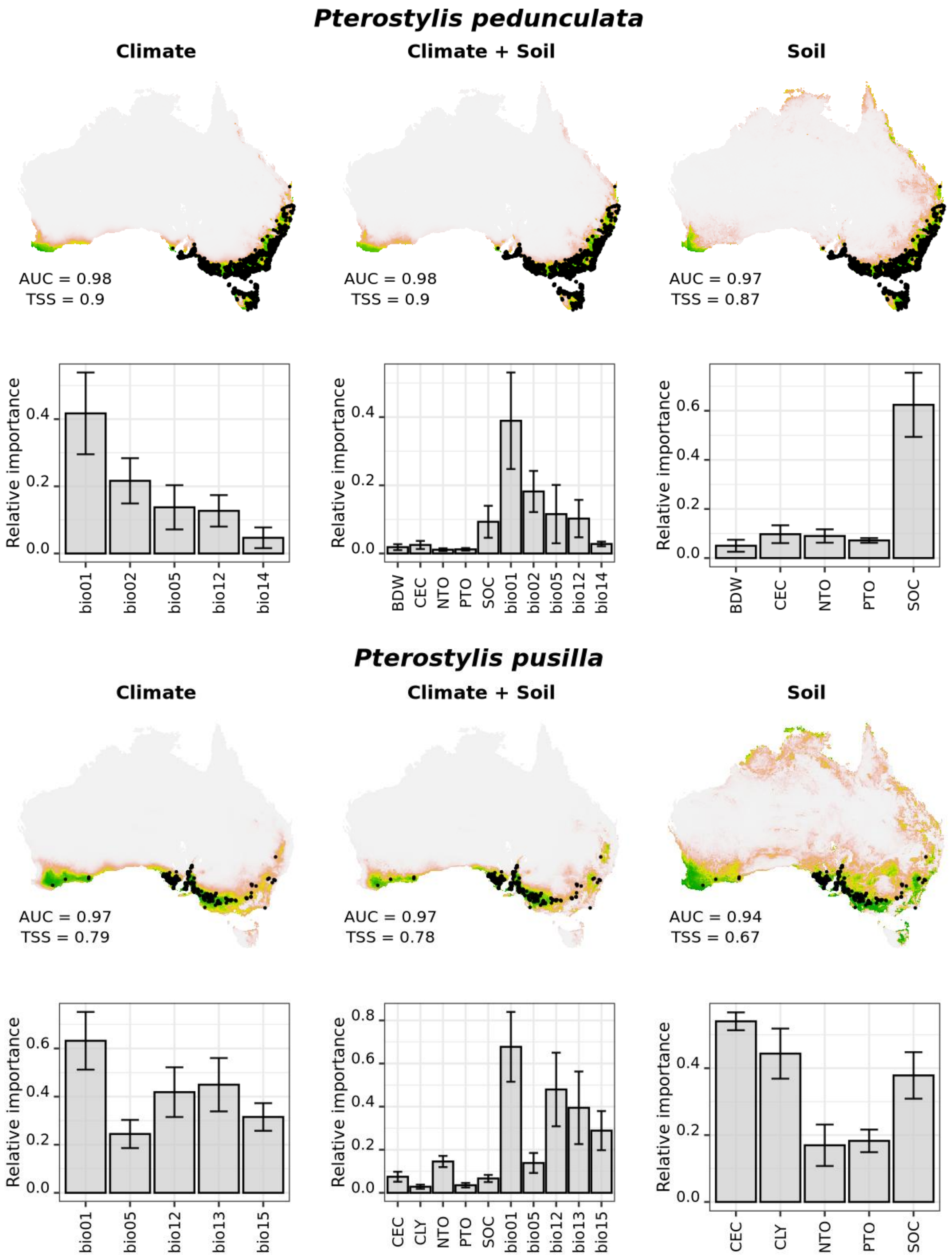
Pterostylis pedunculata
Climate
Climate + Soil
Soil
AUC = 0.98
TSS = 0.9
AUC = 0.98
TSS = 0.9
AUC = 0.97
TSS = 0.87
Relative importance
0.0
0.2
0.4
bio01
bio02
bio05
bio12
bio14
BDW
CEC
NTO
PTO
SOC
bio01
bio02
bio05
bio12
bio14
0.6
BDW
CEC
NTO
PTO
SOC
Pterostylis pusilla
Climate
Climate + Soil
Soil
AUC = 0.97
TSS = 0.79
AUC = 0.97
TSS = 0.78
AUC = 0.94
TSS = 0.67
bio01
bio05
bio12
bio13
bio15
0.8
CEC
CLY
NTO
PTO
SOC
bio01
bio05
bio12
bio13
bio15
CEC
CLY
NTO
PTO
SOC

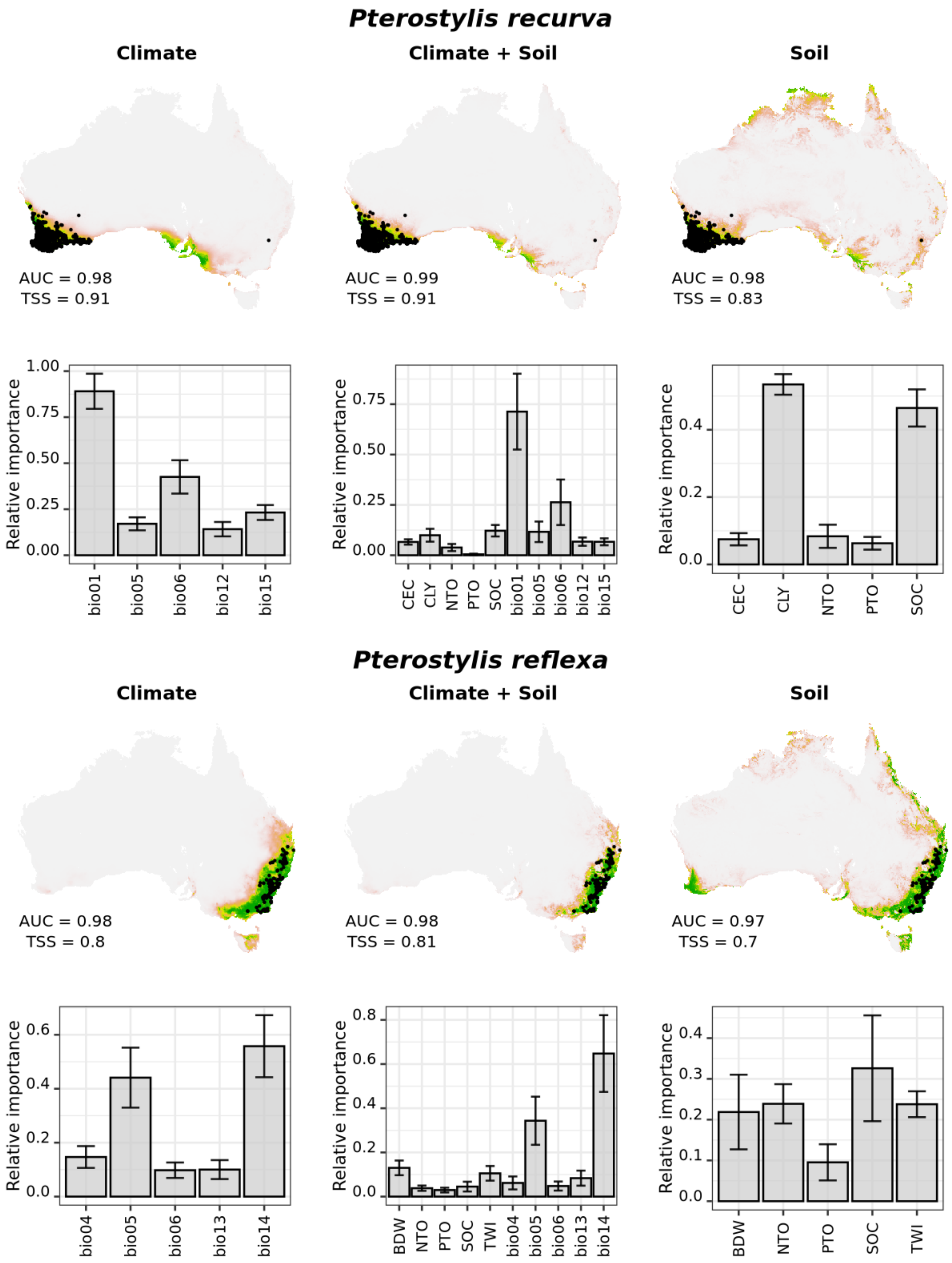

Pterostylis recurva
Climate
Climate + Soil
Soil
AUC = 0.98
TSS = 0.91
AUC = 0.99
TSS = 0.91
AUC = 0.98
TSS = 0.83
Relative importance
bio01 bio05 bio06 bio12 bio15
CEC CLY NTO PTO SOC bio01 bio05 bio06 bio12 bio15
CEC CLY NTO PTO SOC
Pterostylis reflexa
Climate
Climate + Soil
Soil
AUC = 0.98
TSS = 0.8
AUC = 0.98
TSS = 0.81
AUC = 0.97
TSS = 0.7
Relative importance
bio04 bio05 bio06 bio13 bio14
BDW NTO PTO SOC TWI bio04 bio05 bio06 bio13 bio14
BDW NTO PTO SOC TWI

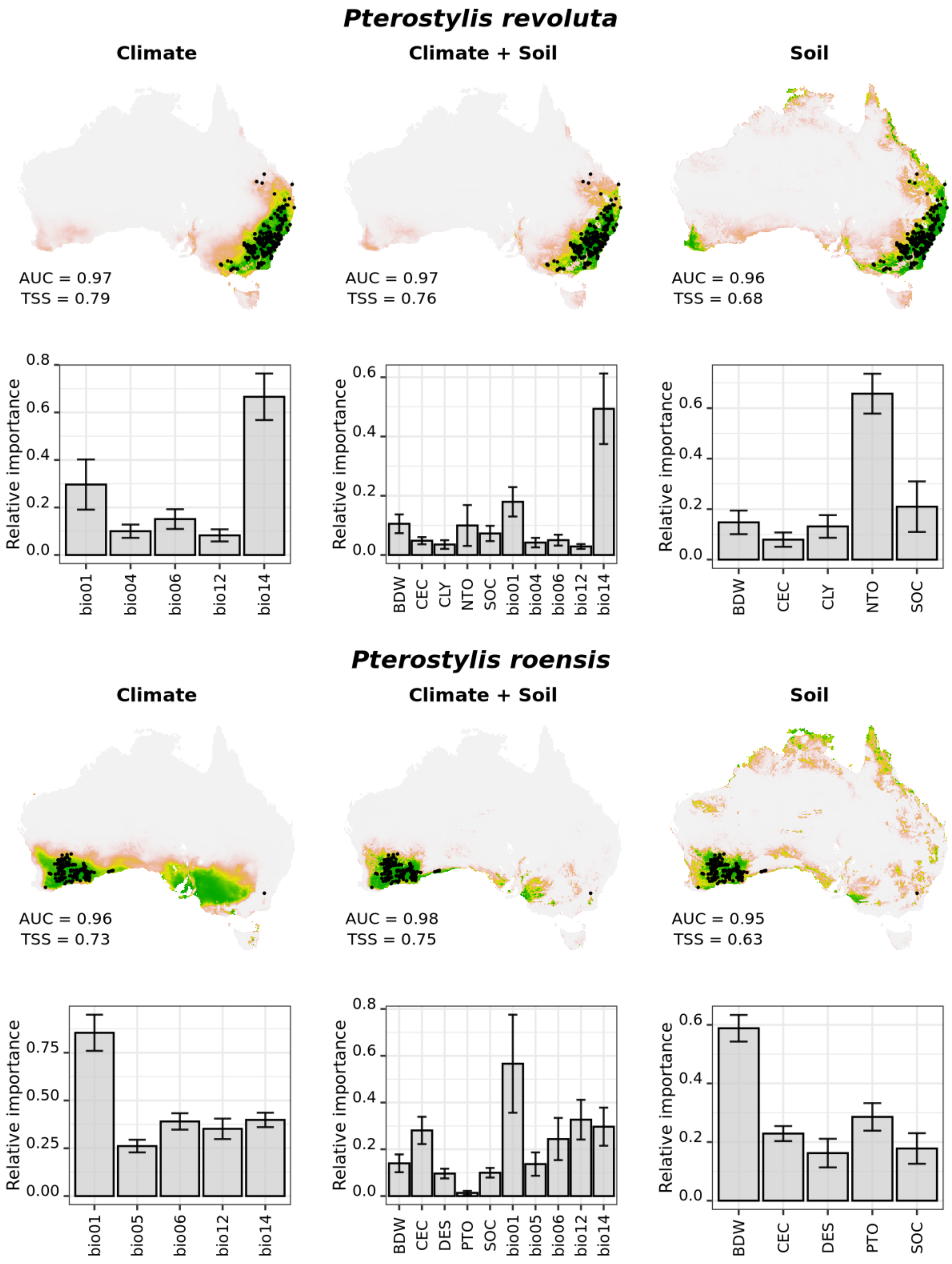
Pterostylis revoluta
Climate
Climate + Soil
Soil
AUC = 0.97
TSS = 0.79
AUC = 0.97
TSS = 0.76
AUC = 0.96
TSS = 0.68
Relative importance
bio01
bio04
bio06
bio12
bio14
BDW
CEC
CLY
NTO
SOC
Pterostylis roensis
AUC = 0.96
TSS = 0.73
AUC = 0.98
TSS = 0.75
AUC = 0.95
TSS = 0.63
bio05
DES
PTO

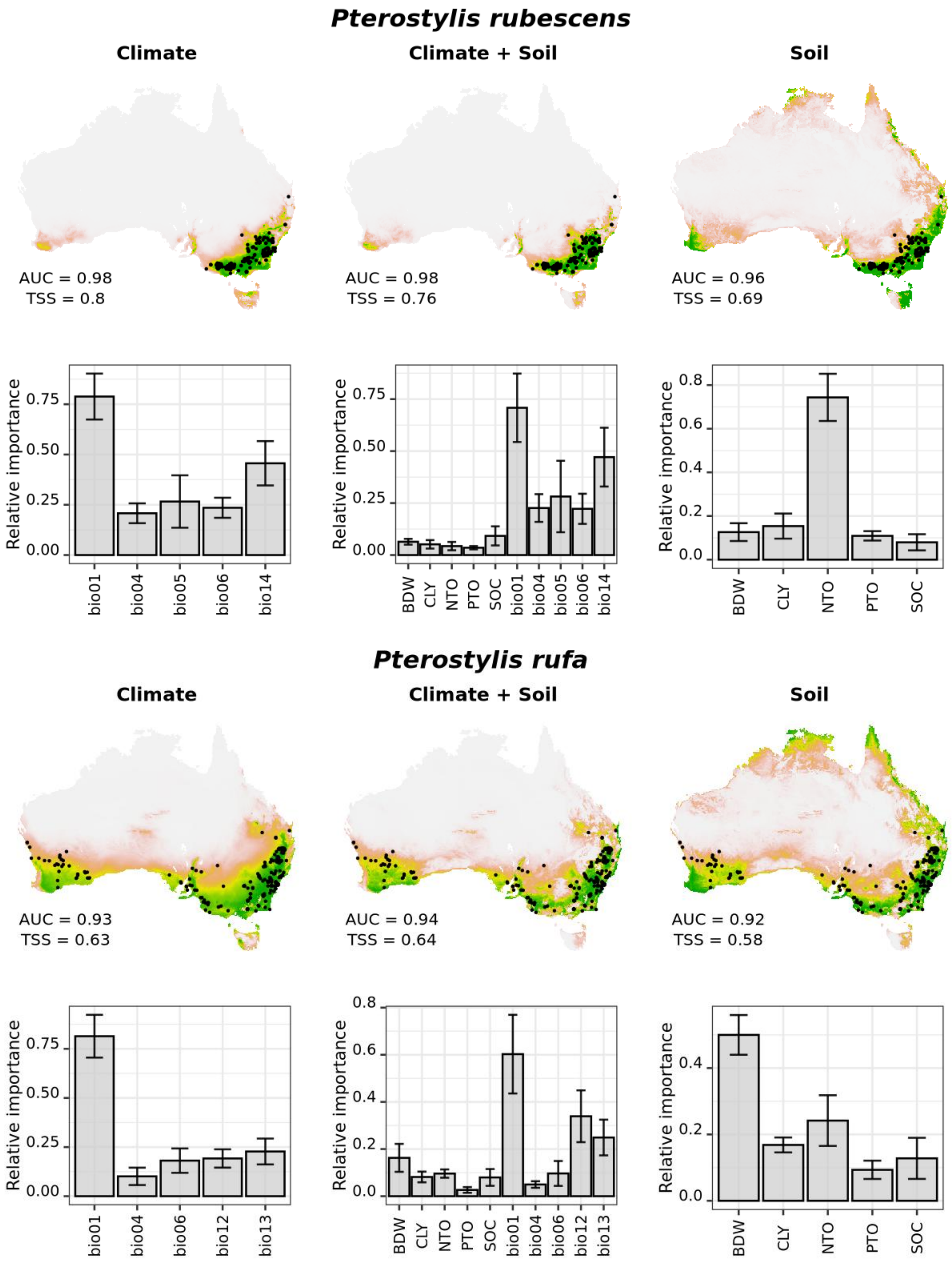
Pterostylis rubescens
Climate
Climate + Soil
Soil
AUC = 0.98
TSS = 0.8
AUC = 0.98
TSS = 0.76
AUC = 0.96
TSS = 0.69
Relative importance
0.00
0.25
0.50
0.75
bio01
bio04
bio05
bio06
bio14
BDW
CLY
NTO
PTO
SOC
0.0
0.2
0.4
0.6
0.8
Pterostylis rufa
AUC = 0.93
TSS = 0.63
AUC = 0.94
TSS = 0.64
AUC = 0.92
TSS = 0.58
bio12
bio13

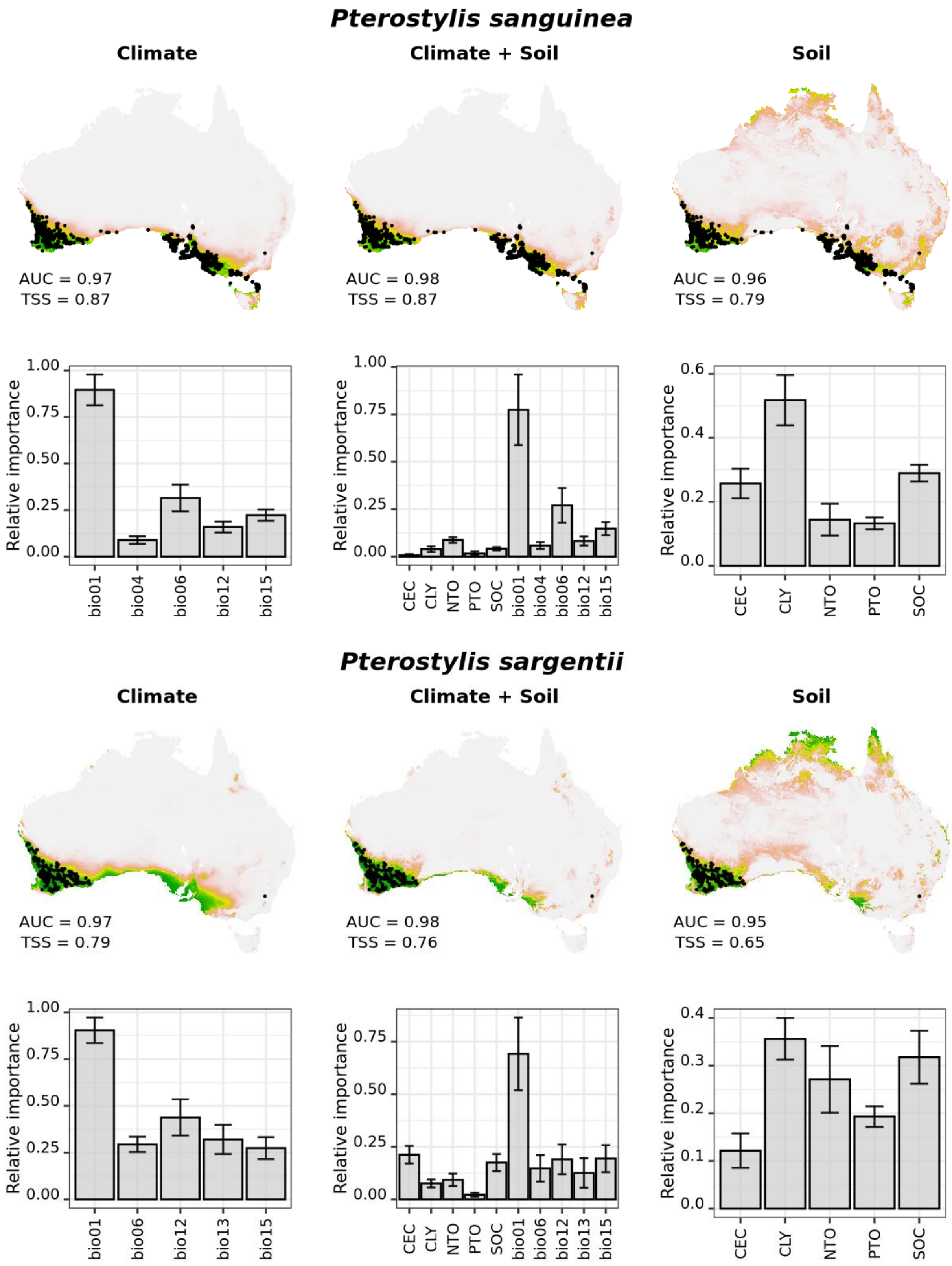
Pterostylis sanguinea
Climate
Climate + Soil
Soil
AUC = 0.97
TSS = 0.87
AUC = 0.98
TSS = 0.87
AUC = 0.96
TSS = 0.79
Relative importance
1.00
0.75
0.50
0.25
0.00
bio01
bio04
bio06
bio12
bio15
CEC
CLY
NTO
PTO
SOC
0.6
0.4
0.2
0.0
Pterostylis sargentii
Climate
Climate + Soil
Soil
AUC = 0.97
TSS = 0.79
AUC = 0.98
TSS = 0.76
AUC = 0.95
TSS = 0.65
Relative importance
bio01
bio06
bio12
bio13
bio15
CEC
CLY
NTO
PTO
SOC
0.4
0.3
0.2
0.1
0.0

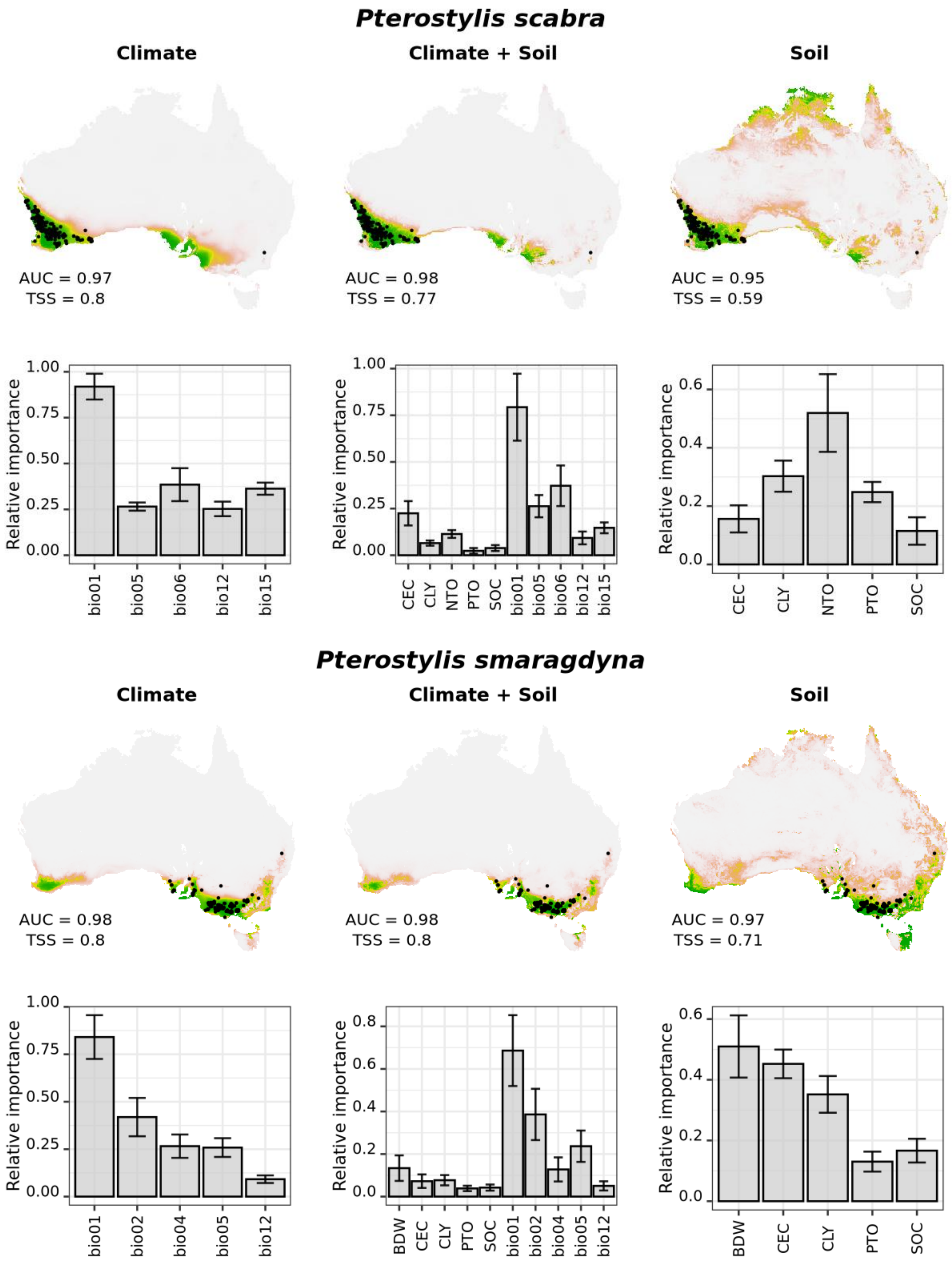

Pterostylis scabra
Climate
Climate + Soil
Soil
AUC = 0.97
TSS = 0.8
AUC = 0.98
TSS = 0.77
AUC = 0.95
TSS = 0.59
Relative importance
bio01
bio05
bio06
bio12
bio15
CEC
CLY
NTO
PTO
SOC
Pterostylis smaragdyna
AUC = 0.98
TSS = 0.8
AUC = 0.98
TSS = 0.8
AUC = 0.97
TSS = 0.71
bio02
bio04
BDW

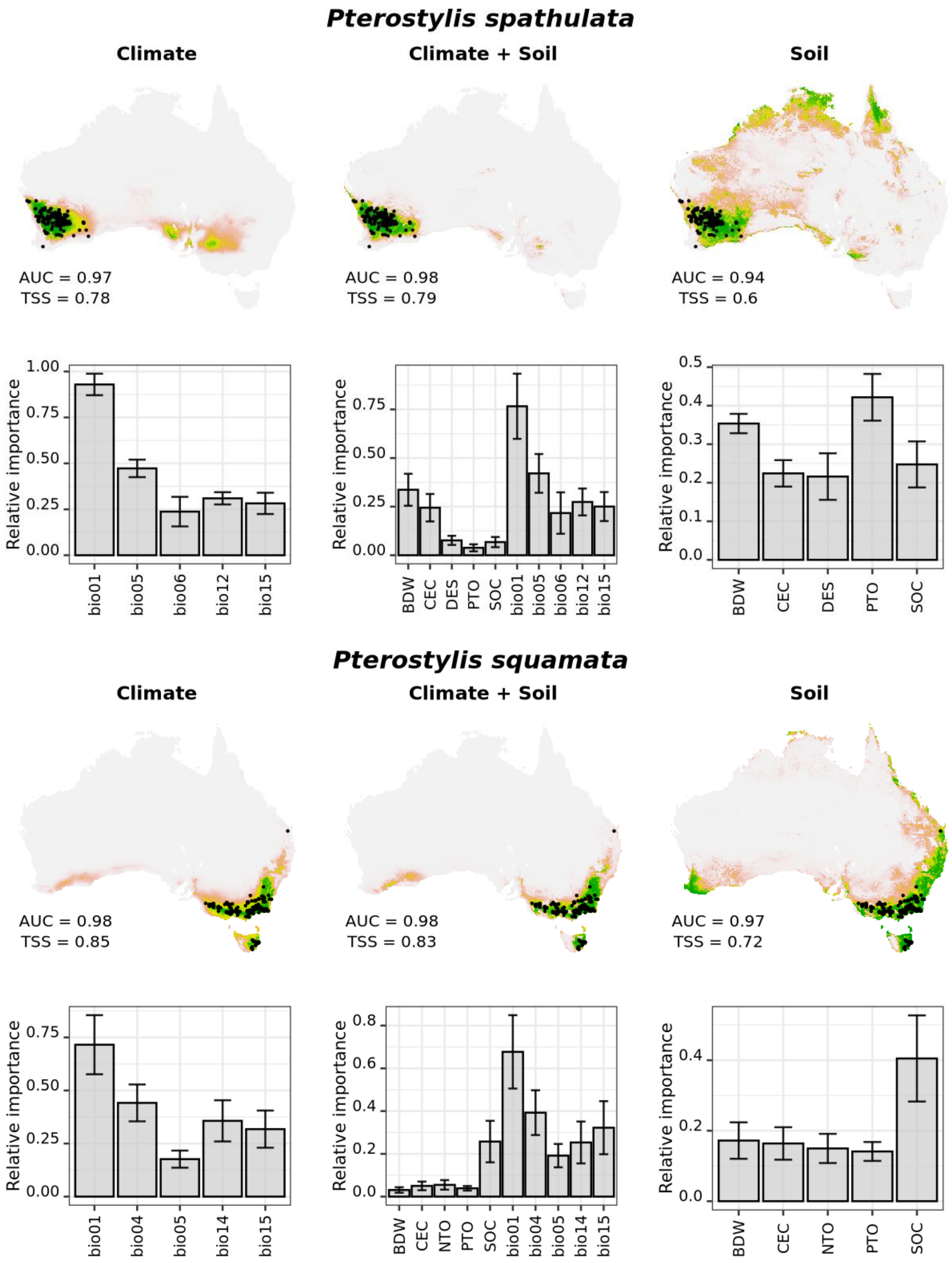

Pterostylis spathulata
Climate
Climate + Soil
Soil
AUC = 0.97
TSS = 0.78
AUC = 0.98
TSS = 0.79
AUC = 0.94
TSS = 0.6
Relative importance
1.00
0.75
0.50
0.25
0.00
bio01
bio05
bio06
bio12
bio15
BDW
CEC
DES
PTO
SOC
0.5
0.4
0.3
0.2
0.1
0.0
Pterostylis squamata
AUC = 0.98
TSS = 0.85
AUC = 0.98
TSS = 0.83
AUC = 0.97
TSS = 0.72
bio04
bio14
NTO
0.8
0.6

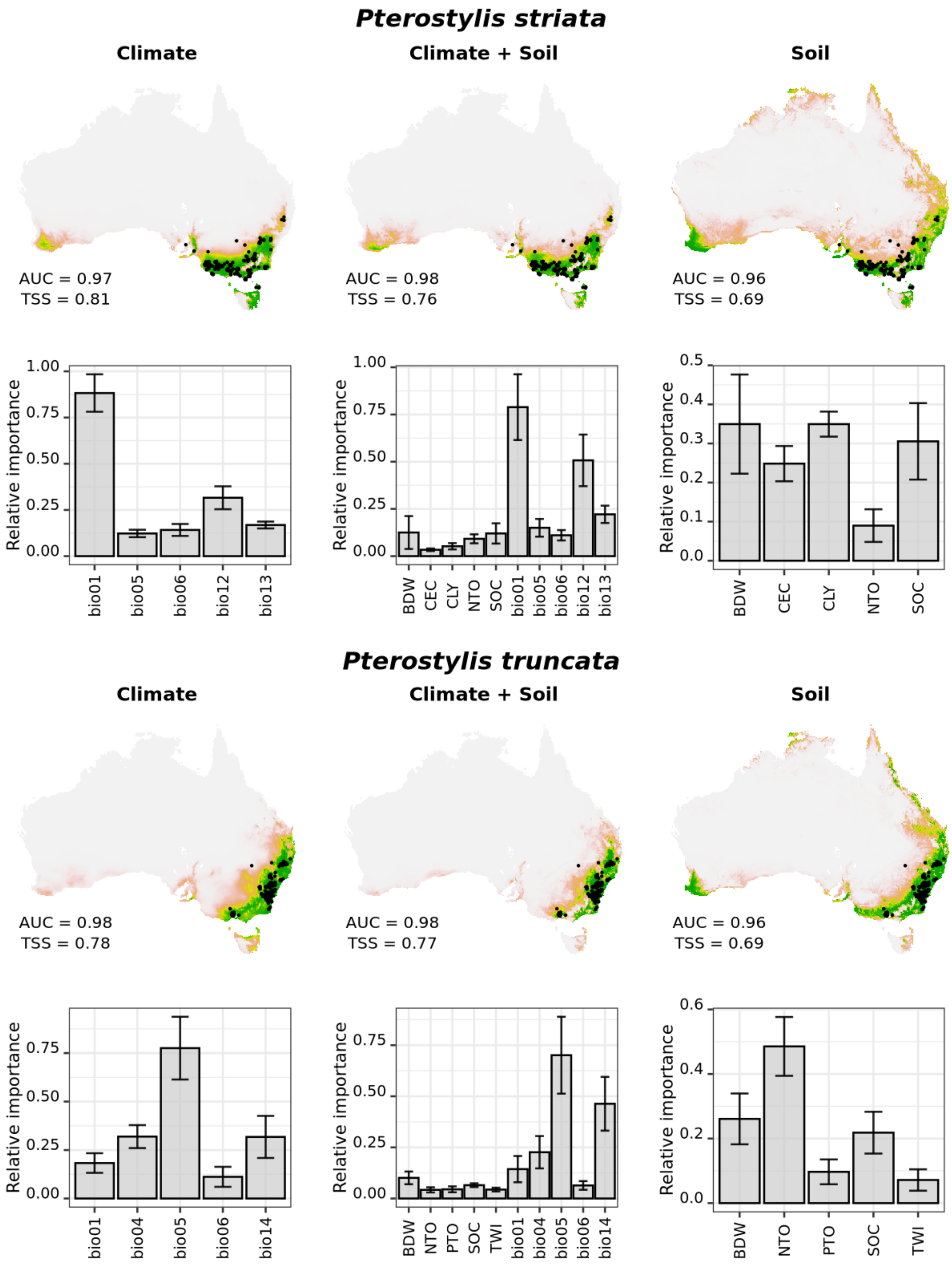
Pterostylis striata
Climate
Climate + Soil
Soil
AUC = 0.97
TSS = 0.81
AUC = 0.98
TSS = 0.76
AUC = 0.96
TSS = 0.69
Relative importance
bio01
bio05
bio06
bio12
bio13
BDW
CEC
CLY
NTO
SOC
Pterostylis truncata
AUC = 0.98
TSS = 0.78
AUC = 0.98
TSS = 0.77
AUC = 0.96
TSS = 0.69
bio04
bio14
PTO
TWI

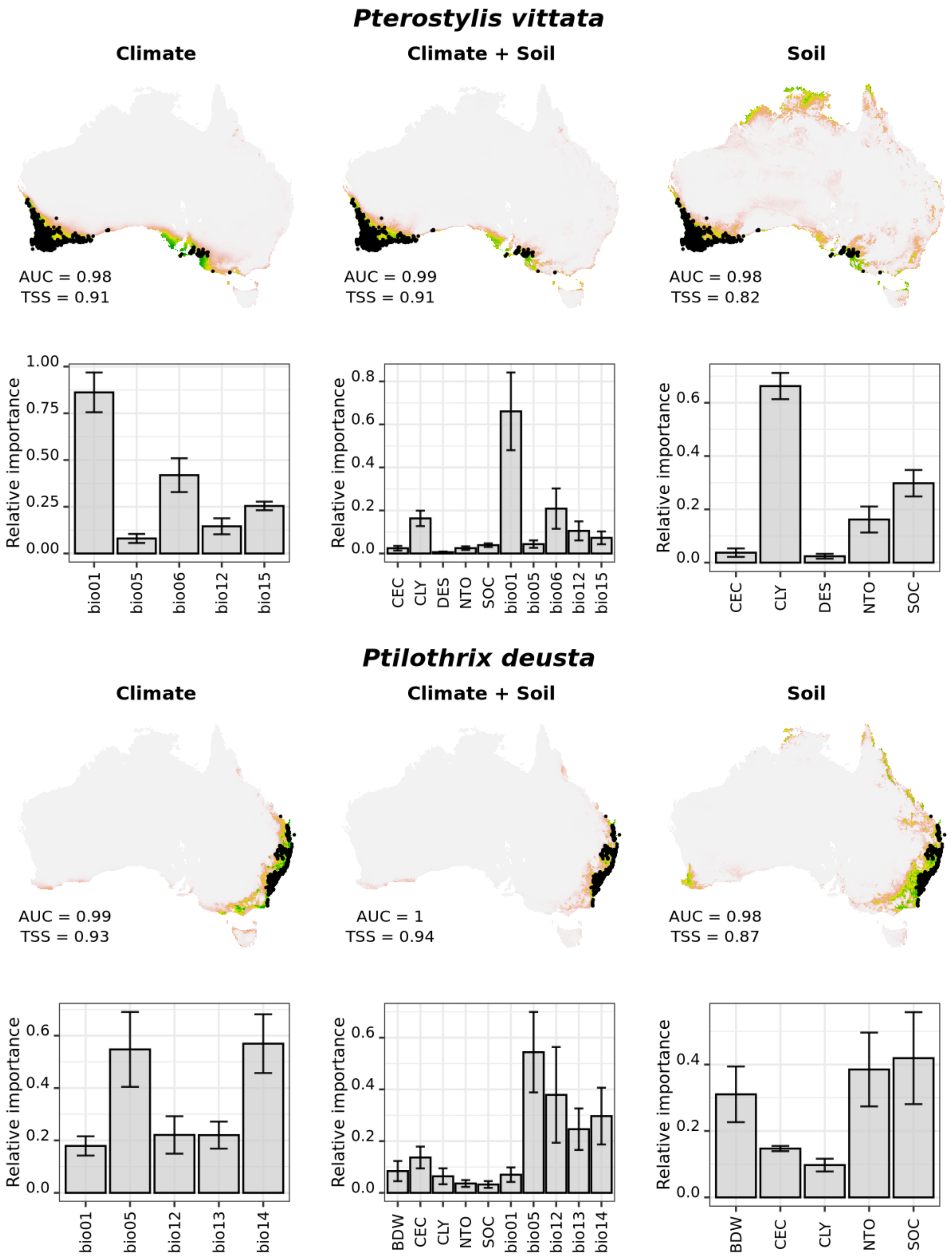
Pterostylis vittata
Climate
Climate + Soil
Soil
AUC = 0.98
TSS = 0.91
AUC = 0.99
TSS = 0.91
AUC = 0.98
TSS = 0.82
Relative importance
bio01
bio05
bio06
bio12
bio15
CEC
CLY
DES
NTO
SOC
Ptilothrix deusta
AUC = 0.99
TSS = 0.93
AUC = 1
TSS = 0.94
AUC = 0.98
TSS = 0.87
bio13
bio14
BDW

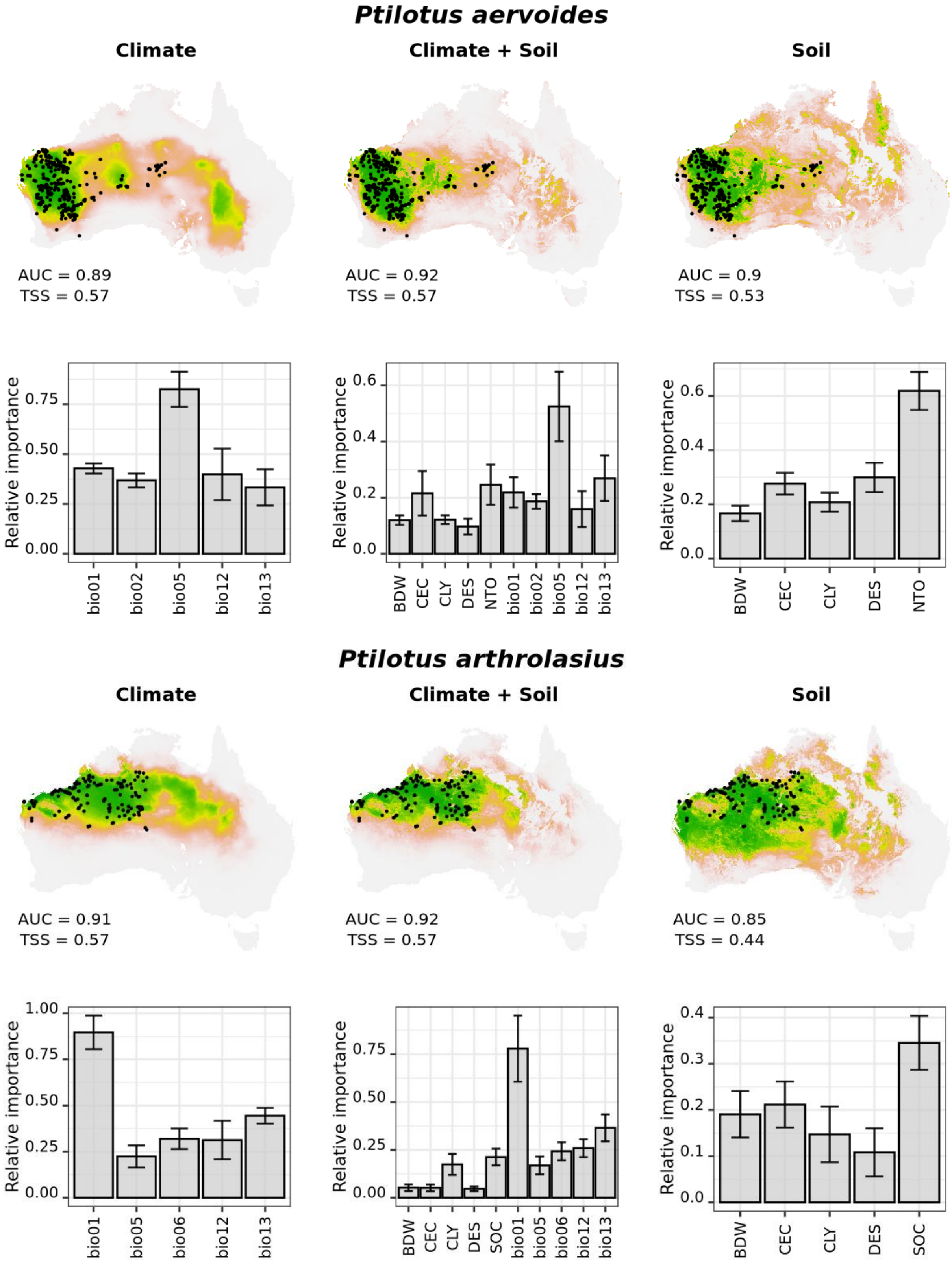

Ptilotus aervoides
Climate
Climate + Soil
Soil
AUC = 0.89
TSS = 0.57
AUC = 0.92
TSS = 0.57
AUC = 0.9
TSS = 0.53
Relative importance
bio01
bio02
bio05
bio12
bio13
BDW
CEC
CLY
DES
NTO
Ptilotus arthrolasius
Climate
Climate + Soil
Soil
AUC = 0.91
TSS = 0.57
AUC = 0.92
TSS = 0.57
AUC = 0.85
TSS = 0.44
Relative importance
bio01
bio05
bio06
bio12
bio13
BDW
CEC
CLY
DES
SOC

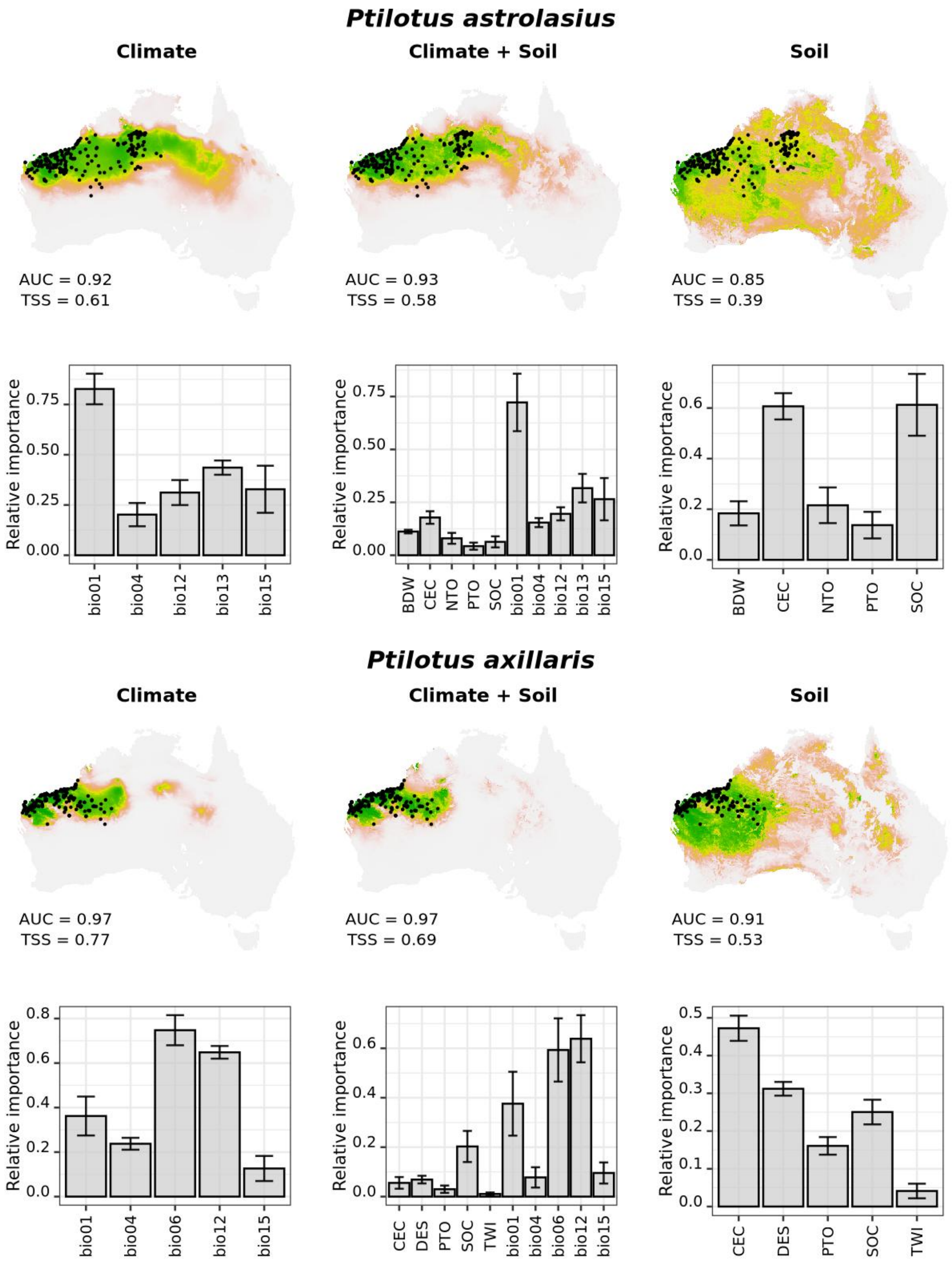

Ptilotus astrolasius
Climate
Climate + Soil
Soil
AUC = 0.92
TSS = 0.61
AUC = 0.93
TSS = 0.58
AUC = 0.85
TSS = 0.39
Relative importance
bio01
bio04
bio12
bio13
bio15
BDW
CEC
NTO
PTO
SOC
Ptilotus axillaris
Climate
Climate + Soil
Soil
AUC = 0.97
TSS = 0.77
AUC = 0.97
TSS = 0.69
AUC = 0.91
TSS = 0.53
Relative importance
bio01
bio04
bio06
bio12
bio15
CEC
DES
PTO
SOC
TWI

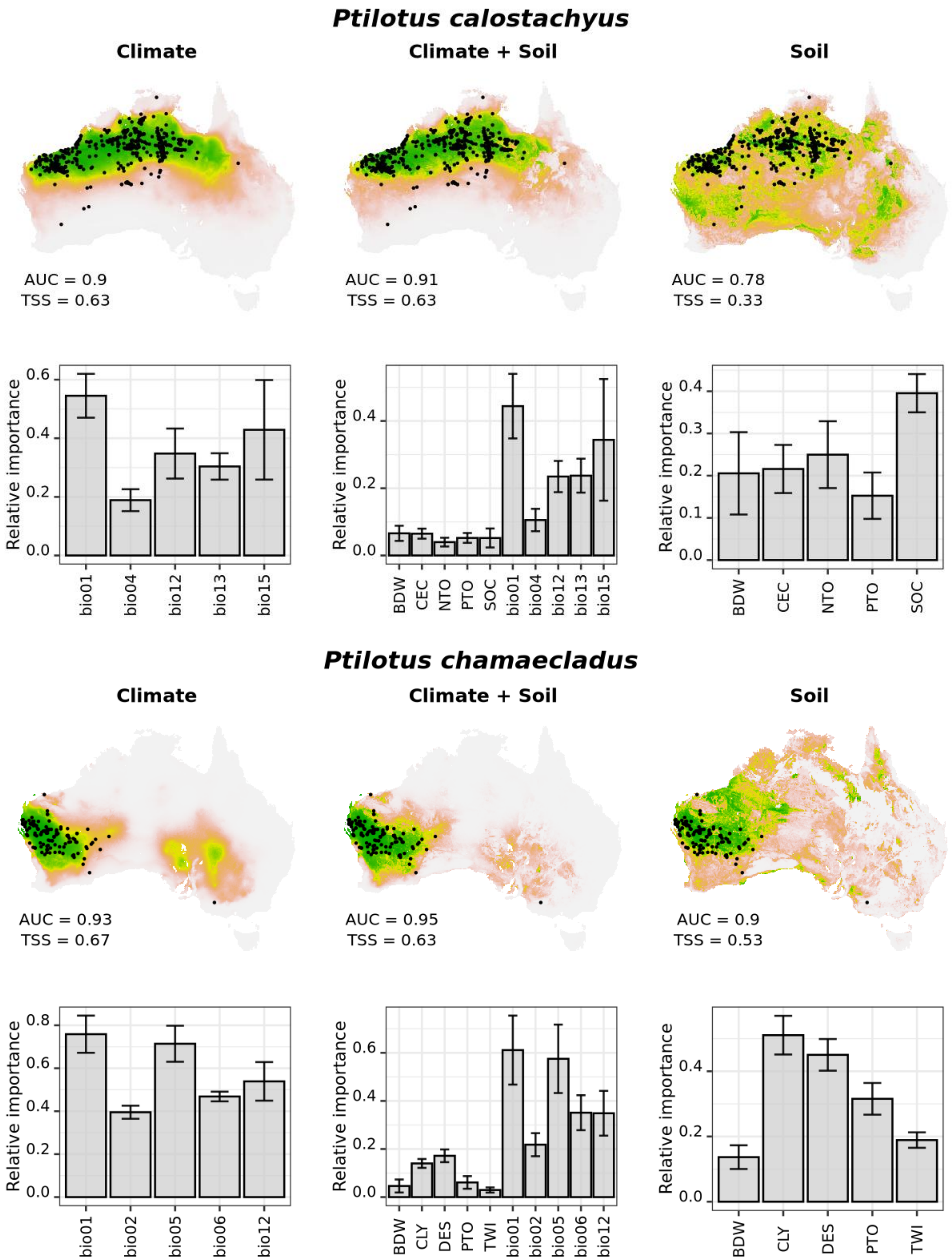
Ptilotus calostachyus
Climate
Climate + Soil
Soil
AUC = 0.9
TSS = 0.63
AUC = 0.91
TSS = 0.63
AUC = 0.78
TSS = 0.33
Relative importance
bio01
bio04
bio12
bio13
bio15
BDW
CEC
NTO
PTO
SOC
Ptilotus chamaecladus
Climate
Climate + Soil
Soil
AUC = 0.93
TSS = 0.67
AUC = 0.95
TSS = 0.63
AUC = 0.9
TSS = 0.53
Relative importance
bio01
bio02
bio05
bio06
bio12
BDW
CLY
DES
PTO
TWI

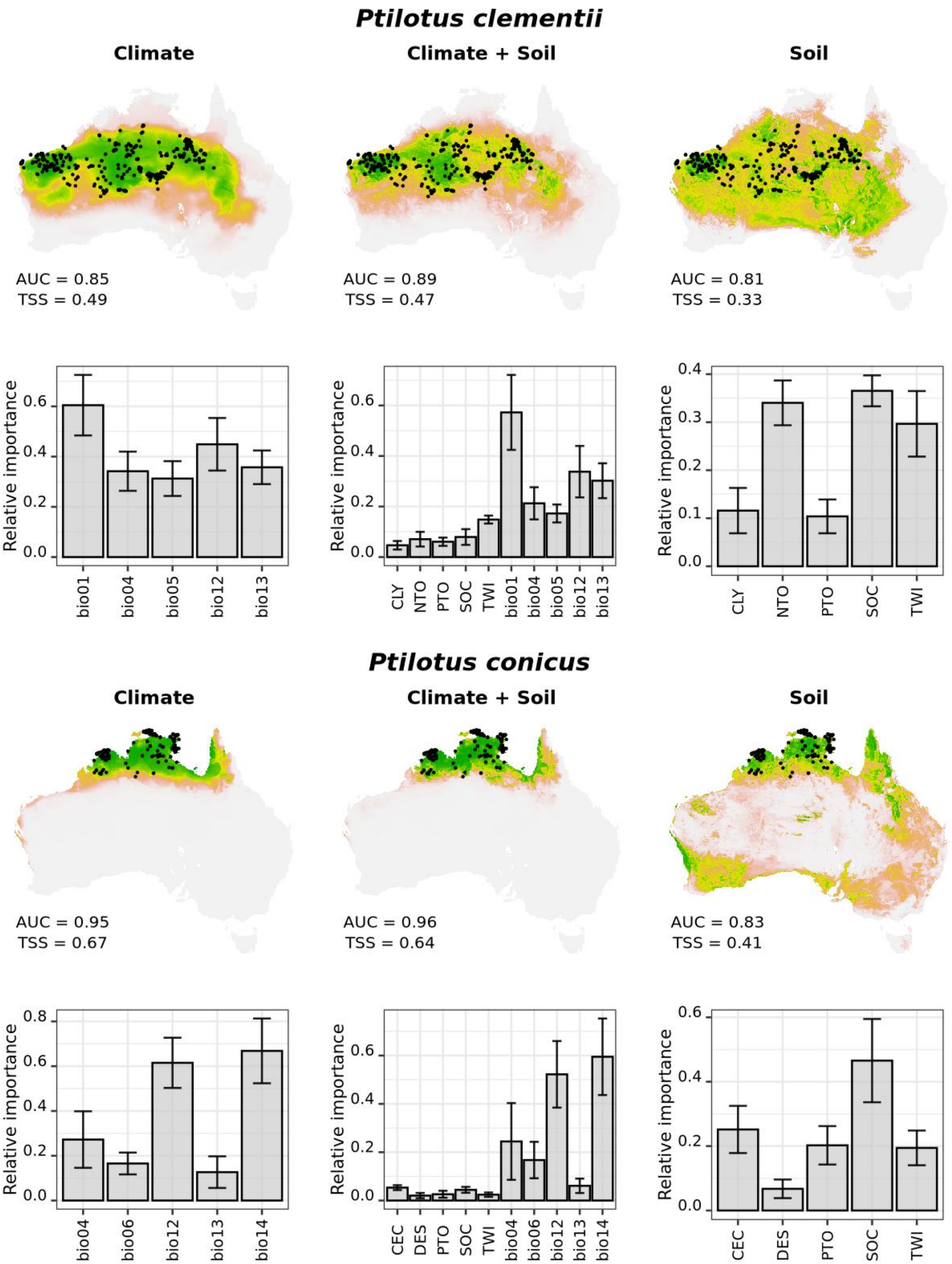

Ptilotus clementii
Climate
Climate + Soil
Soil
AUC = 0.85
TSS = 0.49
AUC = 0.89
TSS = 0.47
AUC = 0.81
TSS = 0.33
Relative importance
0.0
0.2
0.4
0.6
bio01
bio04
bio05
bio12
bio13
CLY
NTO
PTO
SOC
TWI
bio01
bio04
bio05
bio12
bio13
0.0
0.1
0.2
0.3
0.4
CLY
NTO
PTO
SOC
TWI
Ptilotus conicus
Climate
Climate + Soil
Soil
AUC = 0.95
TSS = 0.67
AUC = 0.96
TSS = 0.64
AUC = 0.83
TSS = 0.41
Relative importance
0.0
0.2
0.4
0.6
0.8
bio04
bio06
bio12
bio13
bio14
CEC
DES
PTO
SOC
TWI
bio04
bio06
bio12
bio13
bio14
CEC
DES
PTO
SOC
TWI

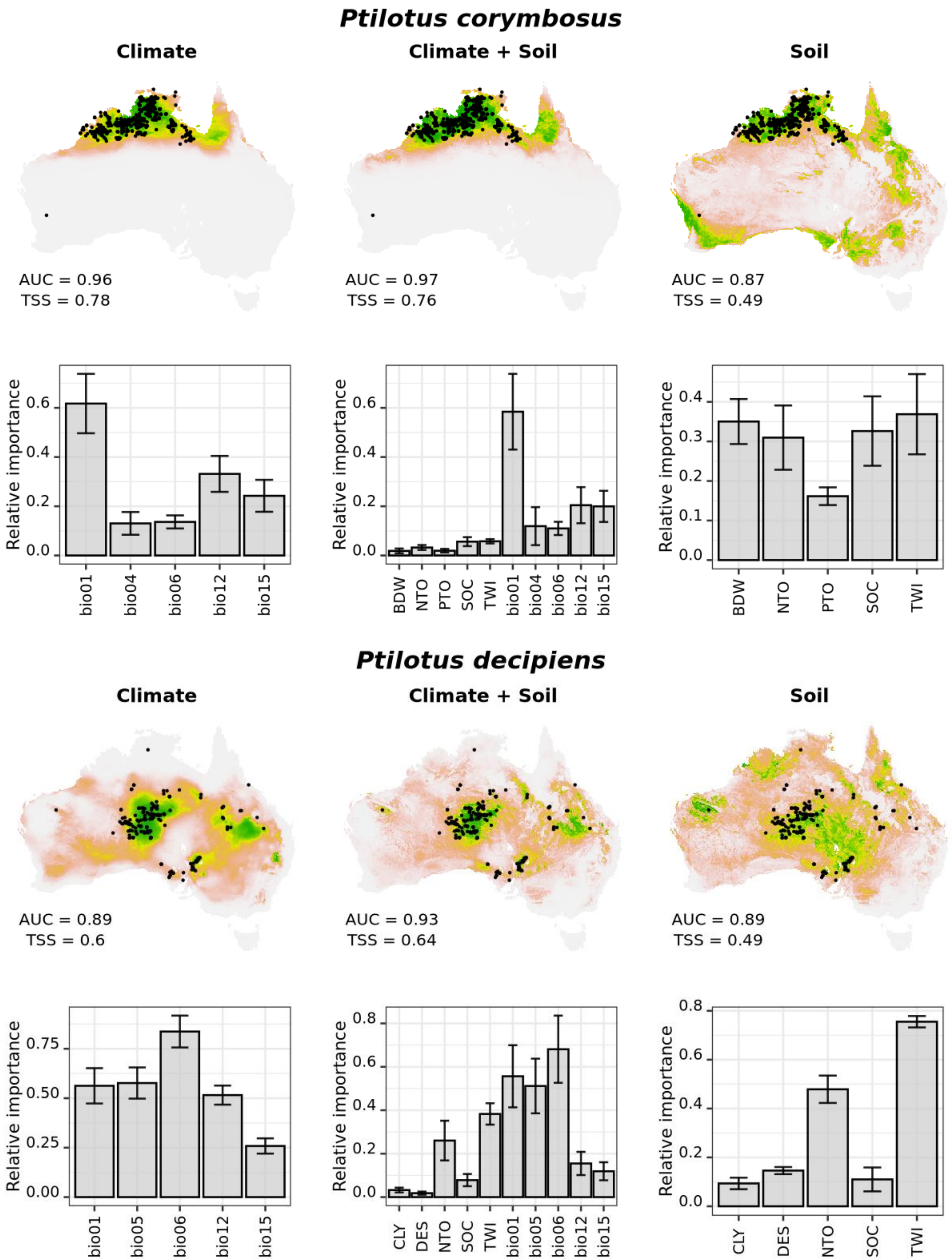

Ptilotus corymbosus
Climate
Climate + Soil
Soil
AUC = 0.96
TSS = 0.78
AUC = 0.97
TSS = 0.76
AUC = 0.87
TSS = 0.49
Relative importance
0.0
0.2
0.4
0.6
bio01
bio04
bio06
bio12
bio15
Relative importance
0.0
0.2
0.4
0.6
BDW
NTO
PTO
SOC
TWI
bio01
bio04
bio06
bio12
bio15
Relative importance
0.0
0.1
0.2
0.3
0.4
BDW
NTO
PTO
SOC
TWI
Ptilotus decipiens
Climate
Climate + Soil
Soil
AUC = 0.89
TSS = 0.6
AUC = 0.93
TSS = 0.64
AUC = 0.89
TSS = 0.49
Relative importance
0.00
0.25
0.50
0.75
bio01
bio05
bio06
bio12
bio15
Relative importance
0.0
0.2
0.4
0.6
0.8
CLY
DES
NTO
SOC
TWI
bio01
bio05
bio06
bio12
bio15
Relative importance
0.0
0.2
0.4
0.6
0.8
CLY
DES
NTO
SOC
TWI

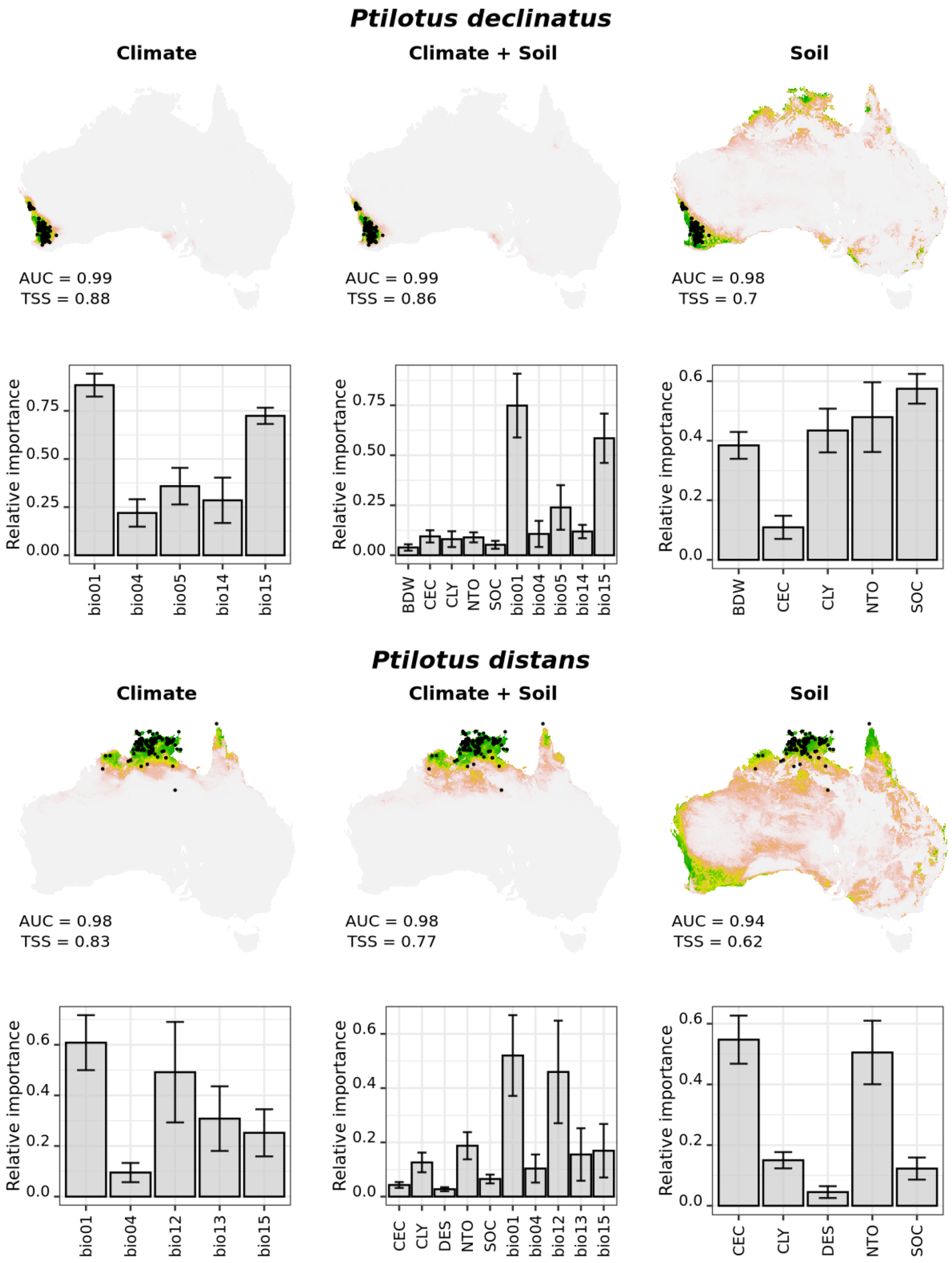

Ptilotus declinatus
Climate
Climate + Soil
Soil
AUC = 0.99
TSS = 0.88
AUC = 0.99
TSS = 0.86
AUC = 0.98
TSS = 0.7
Relative importance
bio01
bio04
bio05
bio14
bio15
BDW
CEC
CLY
NTO
SOC
Ptilotus distans
Climate
Climate + Soil
Soil
AUC = 0.98
TSS = 0.83
AUC = 0.98
TSS = 0.77
AUC = 0.94
TSS = 0.62
Relative importance
bio01
bio04
bio12
bio13
bio15
CEC
CLY
DES
NTO
SOC

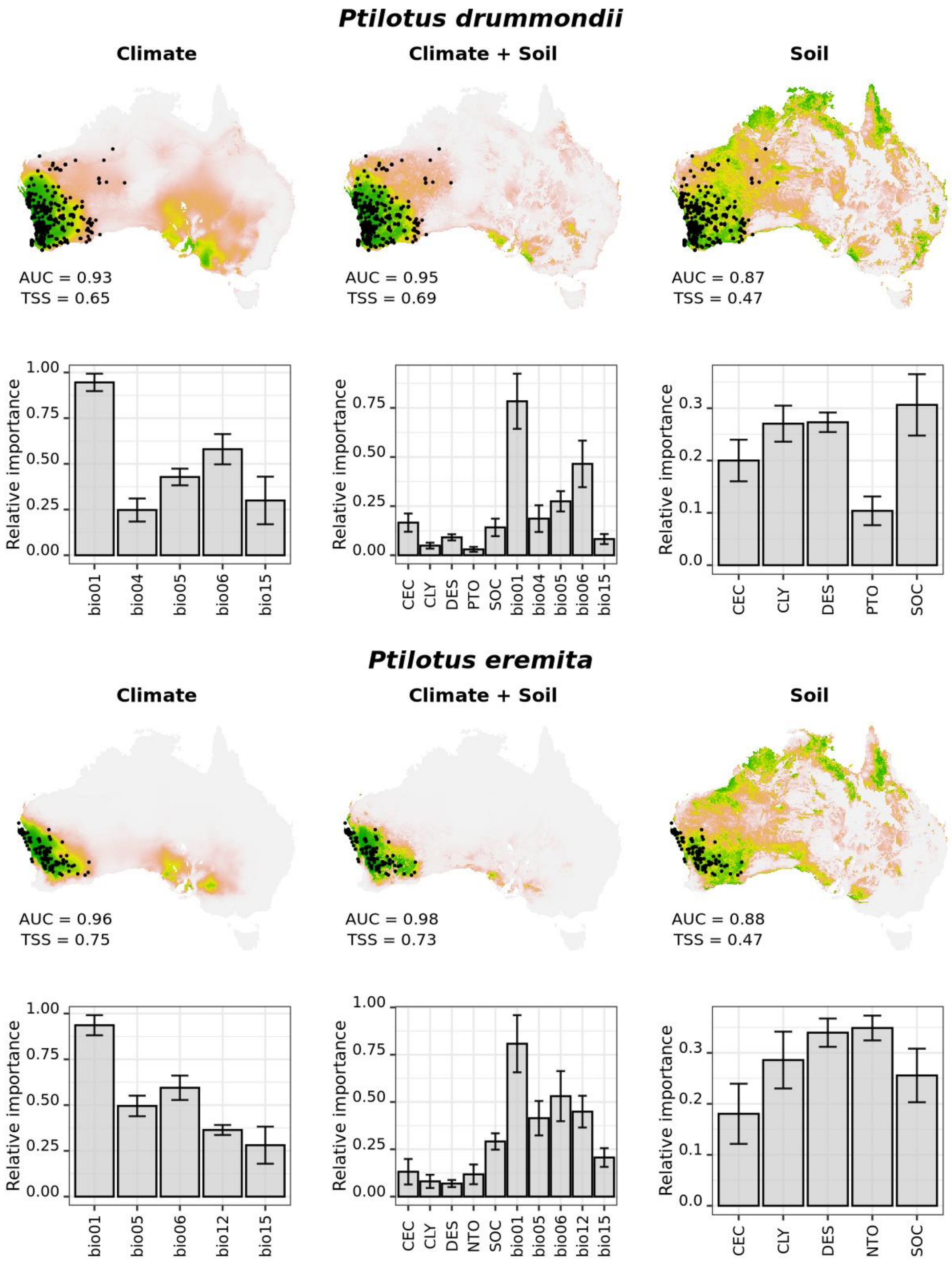
Ptilotus drummondii
Climate
Climate + Soil
Soil
AUC = 0.93
TSS = 0.65
AUC = 0.95
TSS = 0.69
AUC = 0.87
TSS = 0.47
Relative importance
bio01 bio04 bio05 bio06 bio15
CEC CLY DES PTO SOC bio01 bio04 bio05 bio06 bio15
CEC CLY DES PTO SOC
Ptilotus eremita
Climate
Climate + Soil
Soil
AUC = 0.96
TSS = 0.75
AUC = 0.98
TSS = 0.73
AUC = 0.88
TSS = 0.47
Relative importance
bio01 bio05 bio06 bio12 bio15
CEC CLY DES NTO SOC bio01 bio05 bio06 bio12 bio15
CEC CLY DES NTO SOC

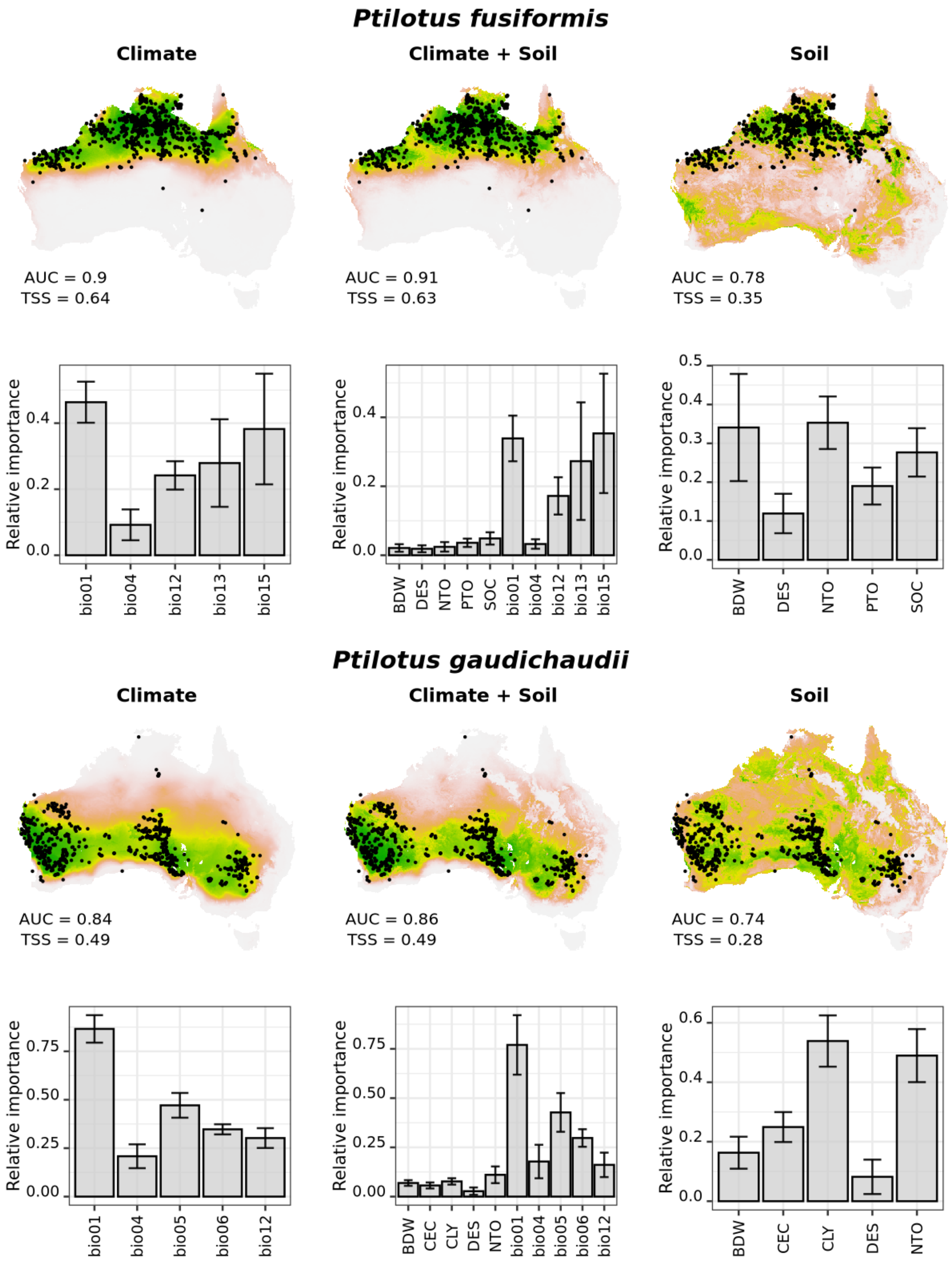
Ptilotus fusiformis
Climate
Climate + Soil
Soil
AUC = 0.9
TSS = 0.64
AUC = 0.91
TSS = 0.63
AUC = 0.78
TSS = 0.35
Relative importance
bio01
bio04
bio12
bio13
bio15
BDW
DES
NTO
PTO
SOC
Ptilotus gaudichaudii
Climate
Climate + Soil
Soil
AUC = 0.84
TSS = 0.49
AUC = 0.86
TSS = 0.49
AUC = 0.74
TSS = 0.28
Relative importance
bio01
bio04
bio05
bio06
bio12
BDW
CEC
CLY
DES
NTO

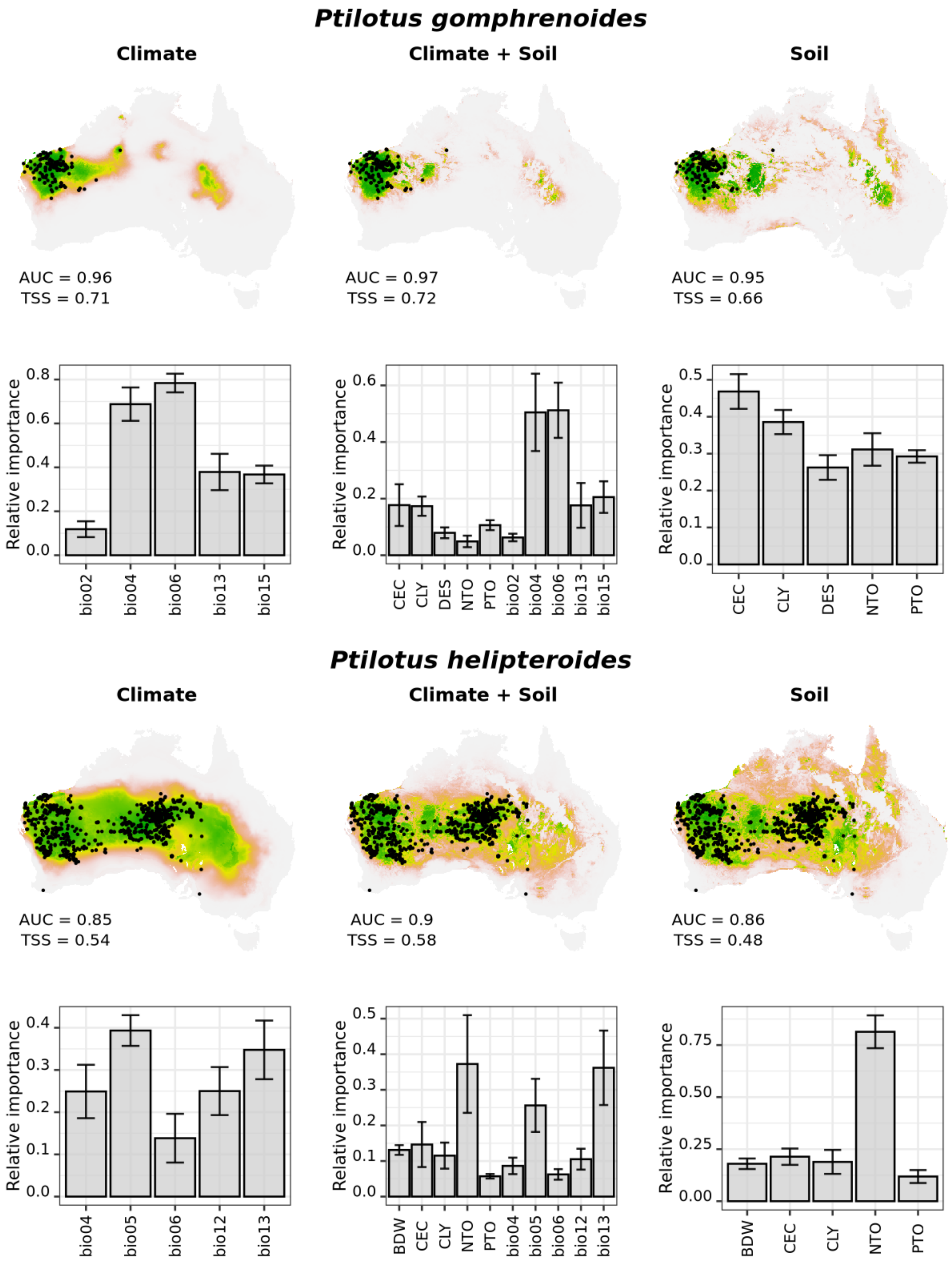
Ptilotus gomphrenoides
Climate
Climate + Soil
Soil
AUC = 0.96
TSS = 0.71
AUC = 0.97
TSS = 0.72
AUC = 0.95
TSS = 0.66
Relative importance
bio02
bio04
bio06
bio13
bio15
CEC
CLY
DES
NTO
PTO
Ptilotus helipteroides
Climate
Climate + Soil
Soil
AUC = 0.85
TSS = 0.54
AUC = 0.9
TSS = 0.58
AUC = 0.86
TSS = 0.48
Relative importance
bio04
bio05
bio06
bio12
bio13
BDW
CEC
CLY
NTO
PTO

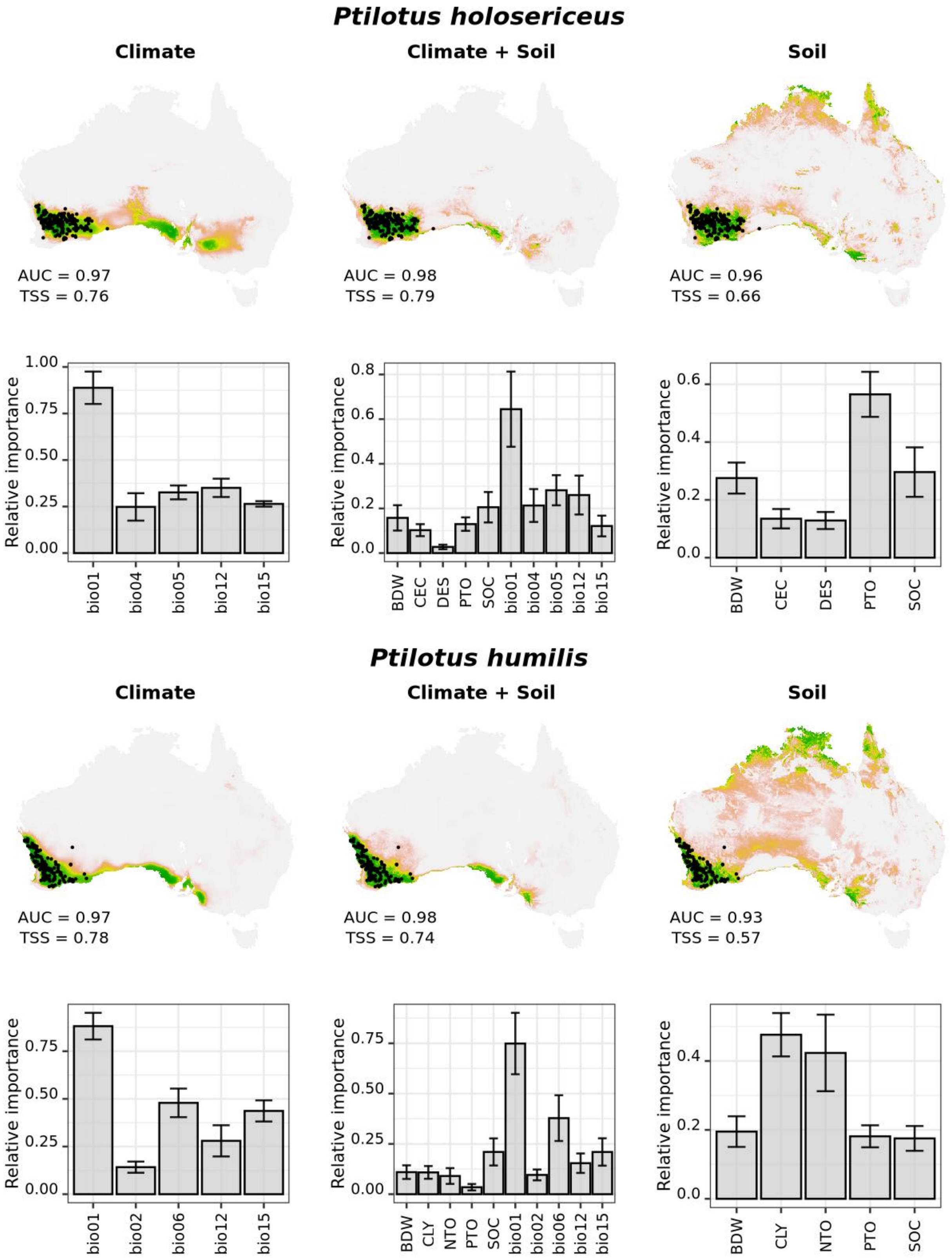

Ptilotus holosericeus
Climate
Climate + Soil
Soil
AUC = 0.97
TSS = 0.76
AUC = 0.98
TSS = 0.79
AUC = 0.96
TSS = 0.66
Relative importance
bio01
bio04
bio05
bio12
bio15
BDW
CEC
DES
PTO
SOC
Ptilotus humilis
Climate
Climate + Soil
Soil
AUC = 0.97
TSS = 0.78
AUC = 0.98
TSS = 0.74
AUC = 0.93
TSS = 0.57
Relative importance
bio01
bio02
bio06
bio12
bio15
BDW
CLY
NTO
PTO
SOC

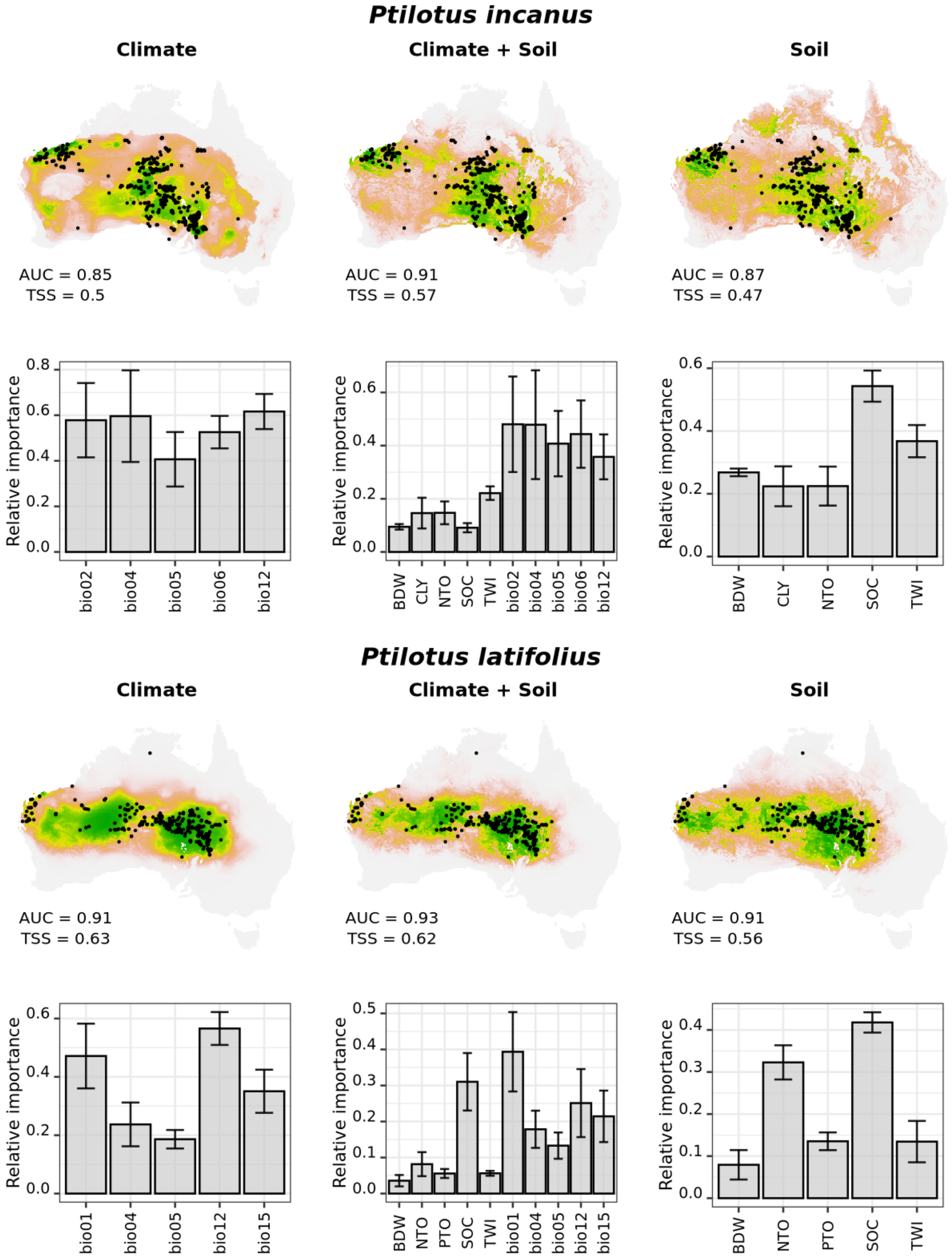
Ptilotus incanus
Climate
Climate + Soil
Soil
AUC = 0.85
TSS = 0.5
AUC = 0.91
TSS = 0.57
AUC = 0.87
TSS = 0.47
Relative importance
bio02
bio04
bio05
bio06
bio12
BDW
CLY
NTO
SOC
TWI
Ptilotus latifolius
Climate
Climate + Soil
Soil
AUC = 0.91
TSS = 0.63
AUC = 0.93
TSS = 0.62
AUC = 0.91
TSS = 0.56
bio01
bio04
bio05
bio12
bio15
BDW
NTO
PTO
SOC
TWI

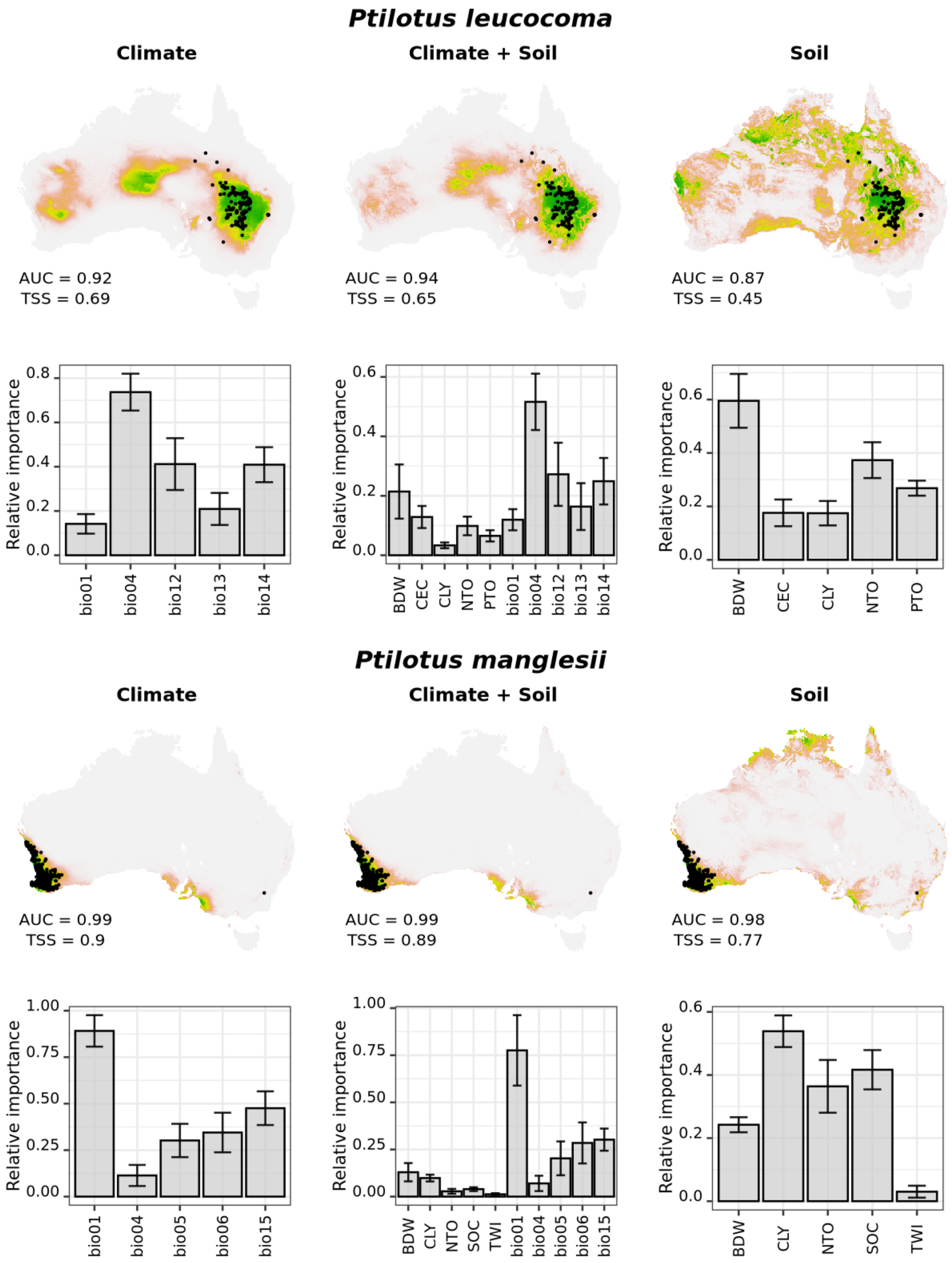
Ptilotus leucocoma
Climate
Climate + Soil
Soil
AUC = 0.92
TSS = 0.69
AUC = 0.94
TSS = 0.65
AUC = 0.87
TSS = 0.45
Relative importance
bio01
bio04
bio12
bio13
bio14
BDW
CEC
CLY
NTO
PTO
Ptilotus manglesii
AUC = 0.99
TSS = 0.9
AUC = 0.99
TSS = 0.89
AUC = 0.98
TSS = 0.77
bio05
bio06
bio15
SOC
TWI

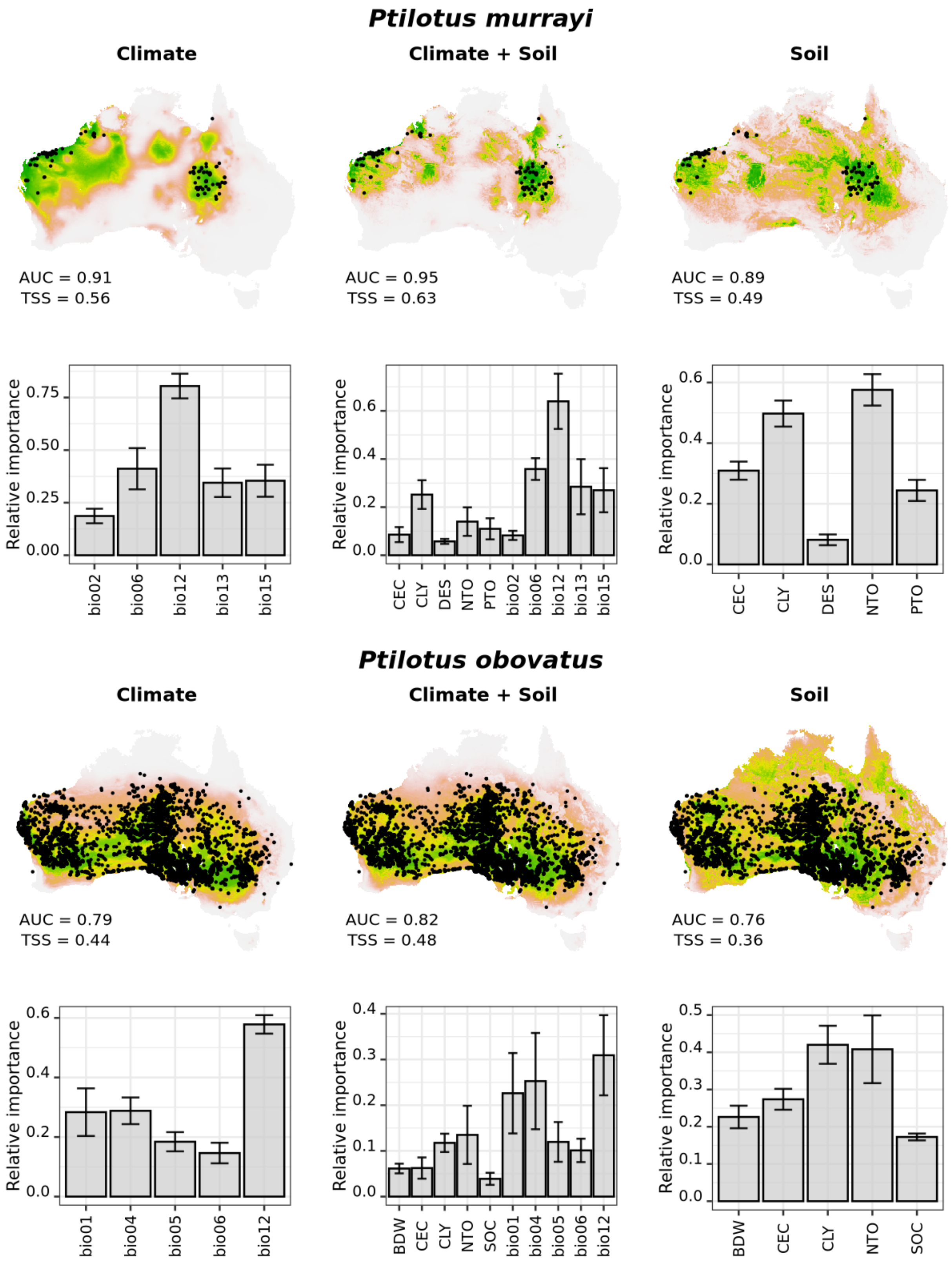

Ptilotus murrayi
Climate
Climate + Soil
Soil
AUC = 0.91
TSS = 0.56
AUC = 0.95
TSS = 0.63
AUC = 0.89
TSS = 0.49
Relative importance
0.00
0.25
0.50
0.75
bio02
bio06
bio12
bio13
bio15
0.0
0.2
0.4
0.6
CEC
CLY
DES
NTO
PTO
bio02
bio06
bio12
bio13
bio15
CEC
CLY
DES
NTO
PTO
Ptilotus obovatus
Climate
Climate + Soil
Soil
AUC = 0.79
TSS = 0.44
AUC = 0.82
TSS = 0.48
AUC = 0.76
TSS = 0.36
Relative importance
0.0
0.2
0.4
0.6
bio01
bio04
bio05
bio06
bio12
0.0
0.1
0.2
0.3
0.4
BDW
CEC
CLY
NTO
SOC
bio01
bio04
bio05
bio06
bio12
0.5
BDW
CEC
CLY
NTO
SOC

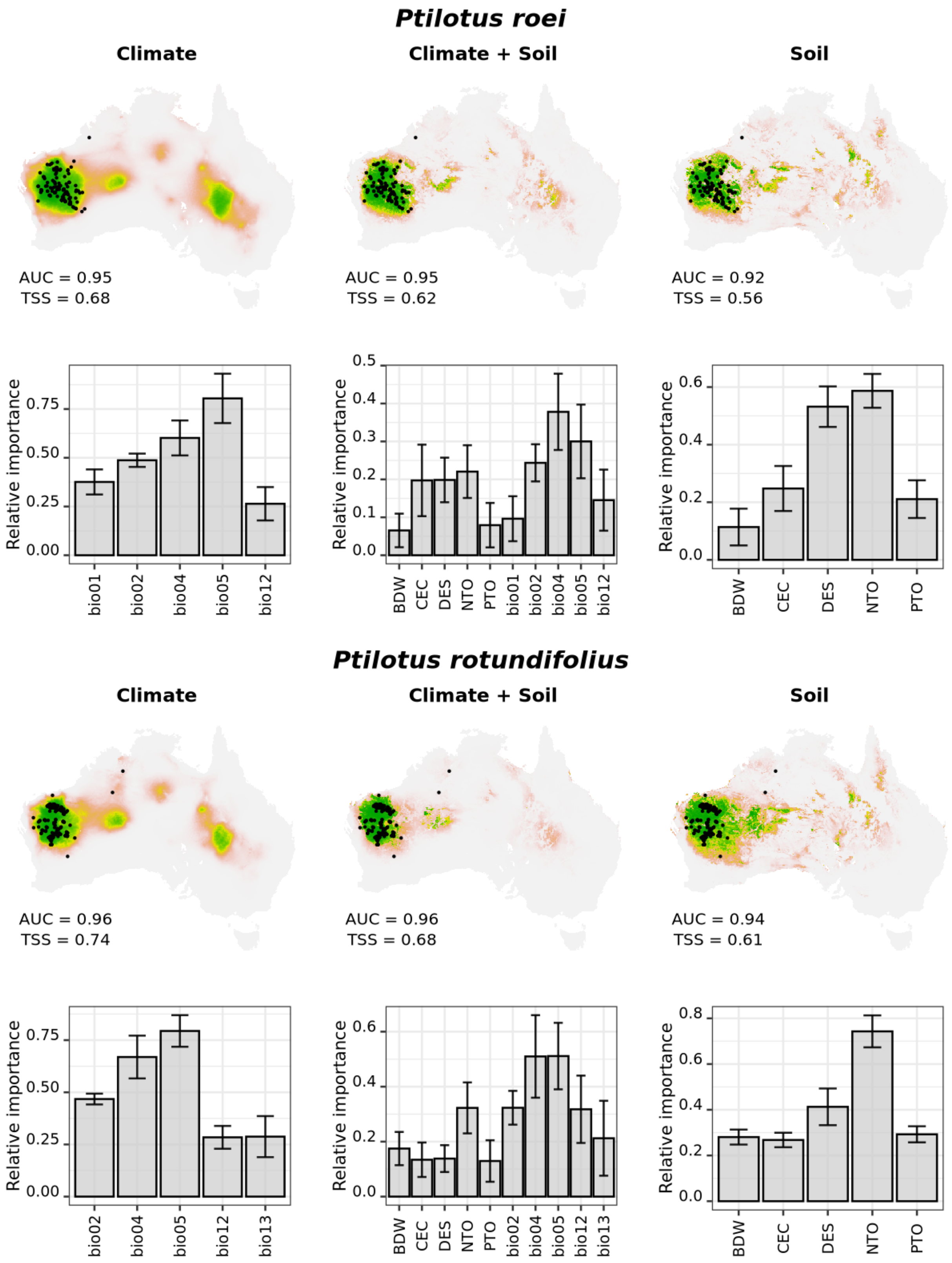
Ptilotus roei
Climate
Climate + Soil
Soil
AUC = 0.95
TSS = 0.68
AUC = 0.95
TSS = 0.62
AUC = 0.92
TSS = 0.56
Relative importance
0.00
0.25
0.50
0.75
bio01
bio02
bio04
bio05
bio12
0.0
0.1
0.2
0.3
0.4
0.5
BDW
CEC
DES
NTO
PTO
bio01
bio02
bio04
bio05
bio12
0.0
0.2
0.4
0.6
BDW
CEC
DES
NTO
PTO
Ptilotus rotundifolius
Climate
Climate + Soil
Soil
AUC = 0.96
TSS = 0.74
AUC = 0.96
TSS = 0.68
AUC = 0.94
TSS = 0.61
Relative importance
0.00
0.25
0.50
0.75
bio02
bio04
bio05
bio12
bio13
0.0
0.2
0.4
0.6
BDW
CEC
DES
NTO
PTO
bio02
bio04
bio05
bio12
bio13
0.0
0.2
0.4
0.6
0.8
BDW
CEC
DES
NTO
PTO

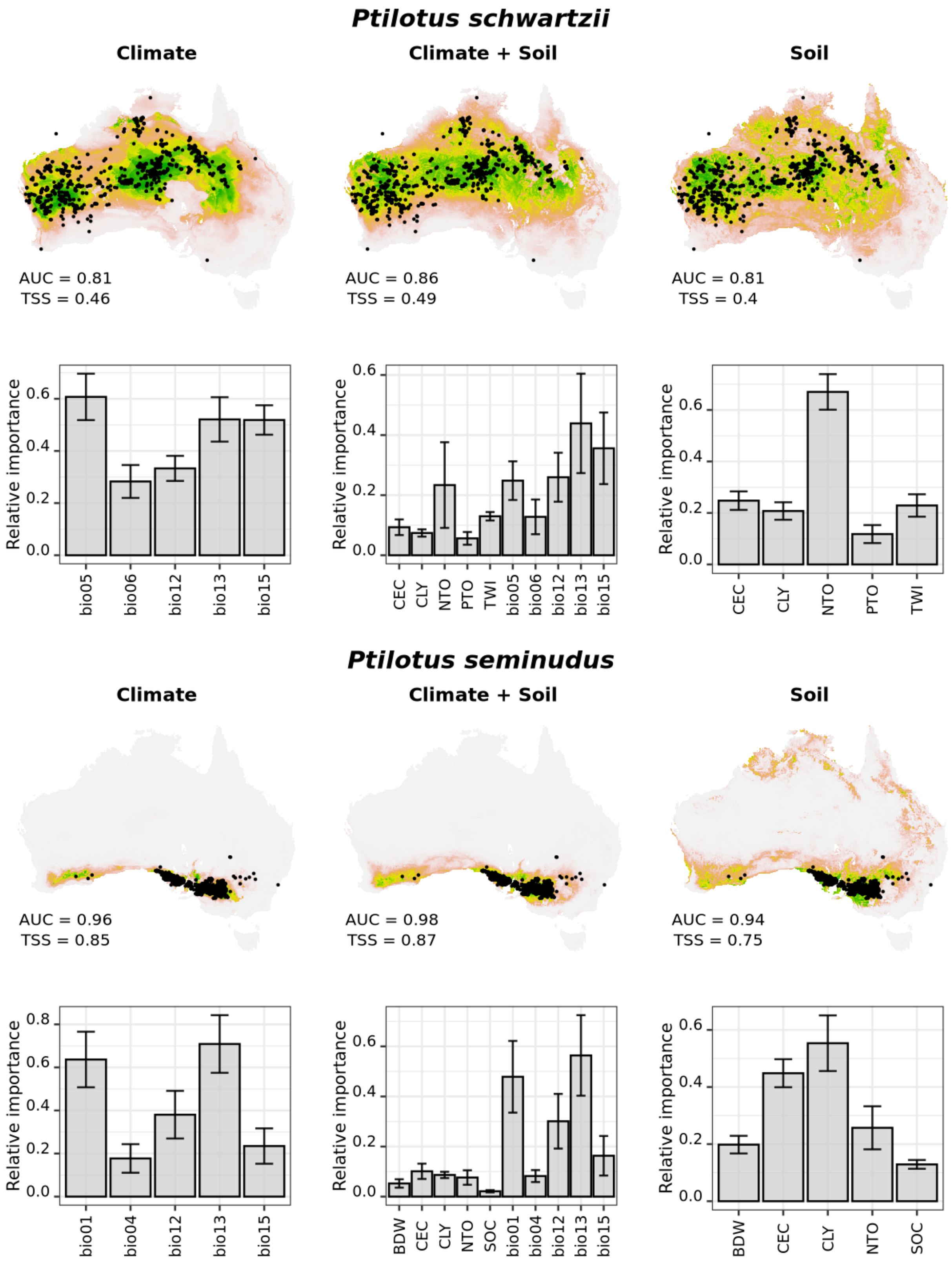
Ptilotus schwartzii
Climate
Climate + Soil
Soil
AUC = 0.81
TSS = 0.46
AUC = 0.86
TSS = 0.49
AUC = 0.81
TSS = 0.4
Relative importance
bio05 bio06 bio12 bio13 bio15
CEC CLY NTO PTO TWI bio05 bio06 bio12 bio13 bio15
CEC CLY NTO PTO TWI
Ptilotus seminudus
Climate
Climate + Soil
Soil
AUC = 0.96
TSS = 0.85
AUC = 0.98
TSS = 0.87
AUC = 0.94
TSS = 0.75
Relative importance
bio01 bio04 bio12 bio13 bio15
BDW CEC CLY NTO SOC bio01 bio04 bio12 bio13 bio15
BDW CEC CLY NTO SOC

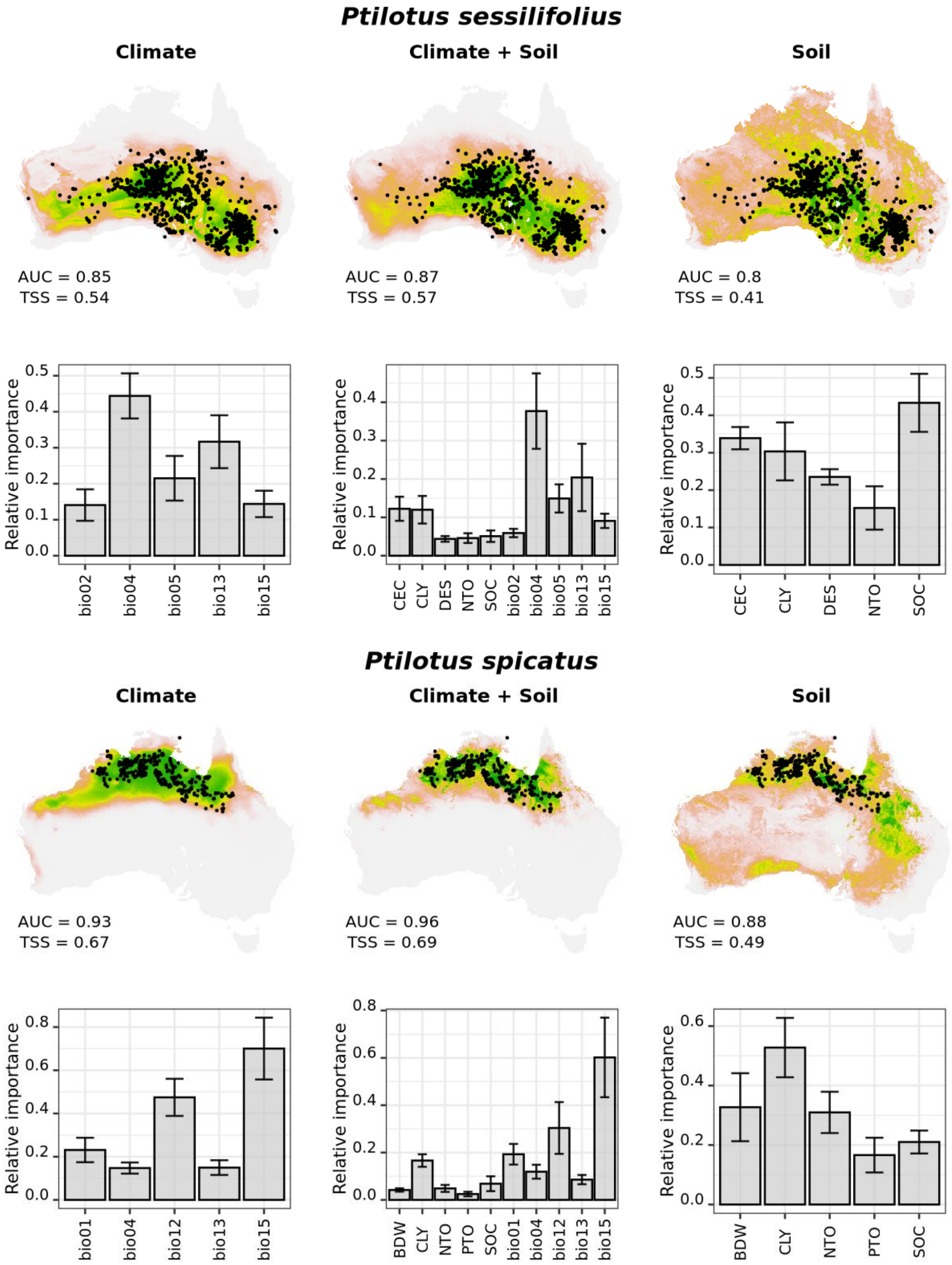

Ptilotus sessilifolius
Climate
Climate + Soil
Soil
AUC = 0.85
TSS = 0.54
AUC = 0.87
TSS = 0.57
AUC = 0.8
TSS = 0.41
Relative importance
0.0
0.1
0.2
0.3
0.4
0.5
bio02
bio04
bio05
bio13
bio15
CEC
CLY
DES
NTO
SOC
bio02
bio04
bio05
bio13
bio15
CEC
CLY
DES
NTO
SOC
Ptilotus spicatus
Climate
Climate + Soil
Soil
AUC = 0.93
TSS = 0.67
AUC = 0.96
TSS = 0.69
AUC = 0.88
TSS = 0.49
Relative importance
0.0
0.2
0.4
0.6
0.8
bio01
bio04
bio12
bio13
bio15
BDW
CLY
NTO
PTO
SOC
bio01
bio04
bio12
bio13
bio15
BDW
CLY
NTO
PTO
SOC

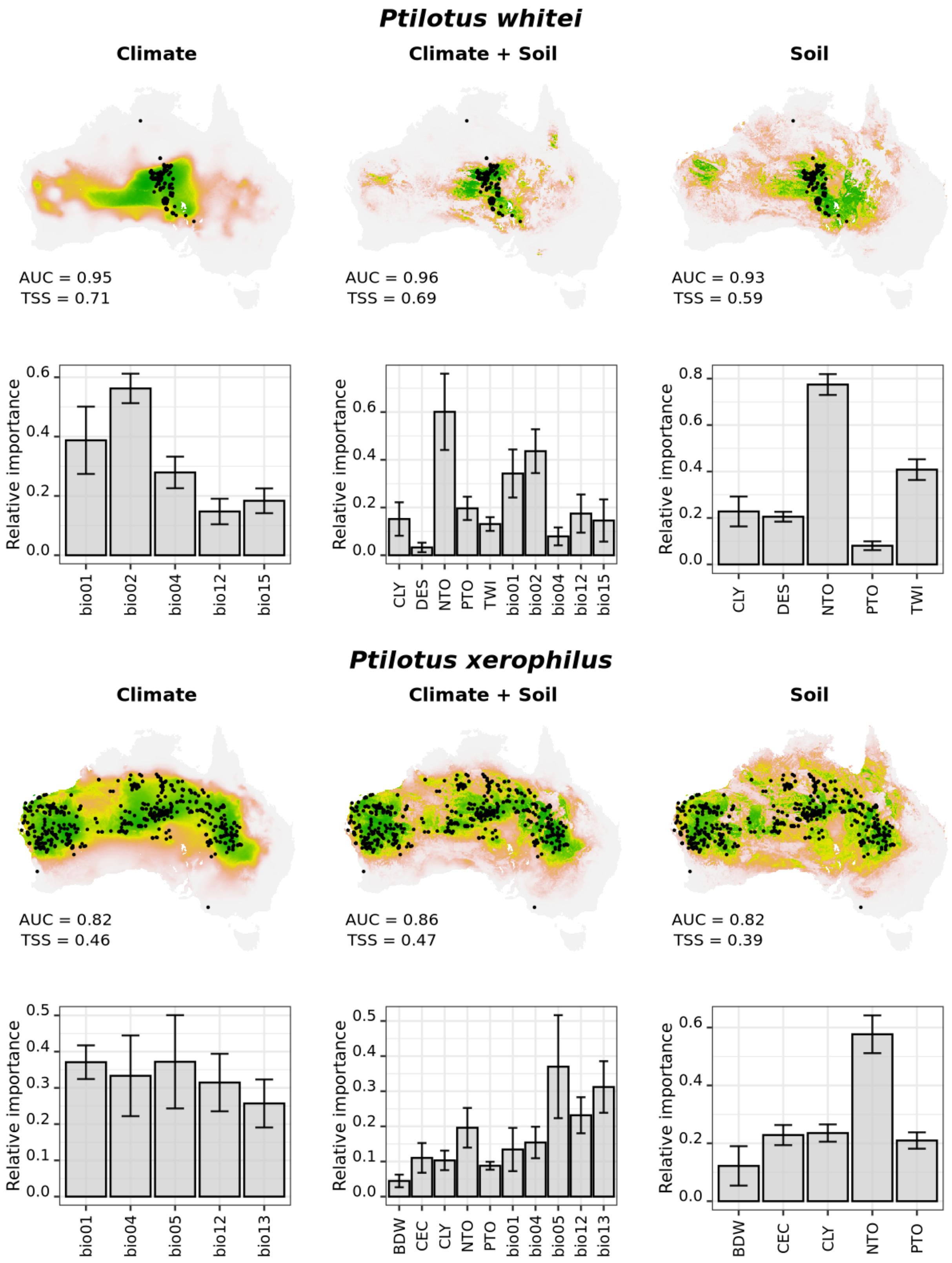
Ptilotus whitei
Climate
Climate + Soil
Soil
AUC = 0.95
TSS = 0.71
AUC = 0.96
TSS = 0.69
AUC = 0.93
TSS = 0.59
Relative importance
bio01 bio02 bio04 bio12 bio15
CLY DES NTO PTO TWI bio01 bio02 bio04 bio12 bio15
CLY DES NTO PTO TWI
Ptilotus xerophilus
Climate
Climate + Soil
Soil
AUC = 0.82
TSS = 0.46
AUC = 0.86
TSS = 0.47
AUC = 0.82
TSS = 0.39
Relative importance
bio01 bio04 bio05 bio12 bio13
BDW CEC CLY NTO PTO bio01 bio04 bio05 bio12 bio13
BDW CEC CLY NTO PTO

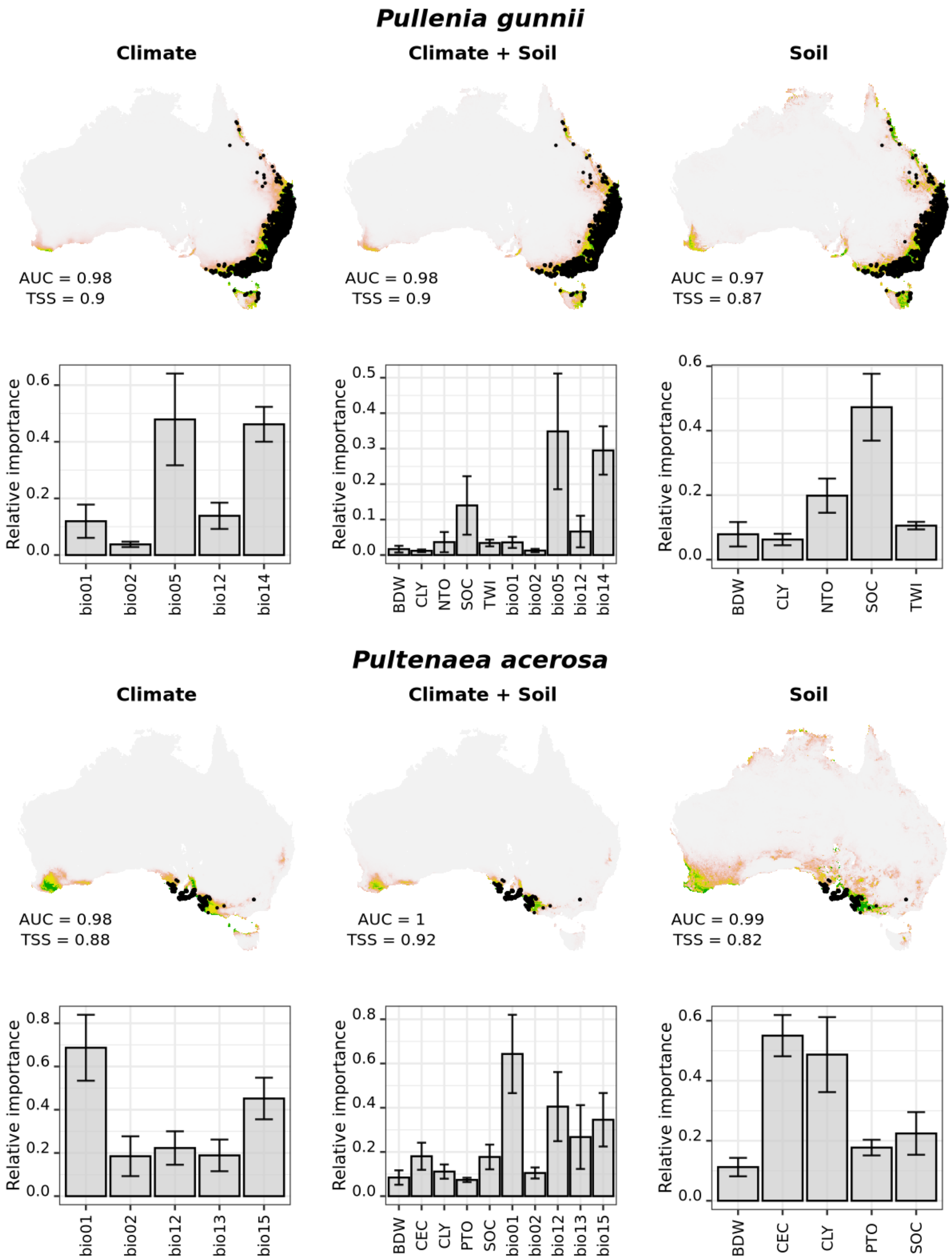
Pullenia gunnii
Climate
Climate + Soil
Soil
AUC = 0.98
TSS = 0.9
AUC = 0.98
TSS = 0.9
AUC = 0.97
TSS = 0.87
Relative importance
bio01
bio02
bio05
bio12
bio14
BDW
CLY
NTO
SOC
TWI
Pultenaea acerosa
AUC = 0.98
TSS = 0.88
AUC = 1
TSS = 0.92
AUC = 0.99
TSS = 0.82
bio13
bio15
CEC
PTO

Pultenaea blakelyi

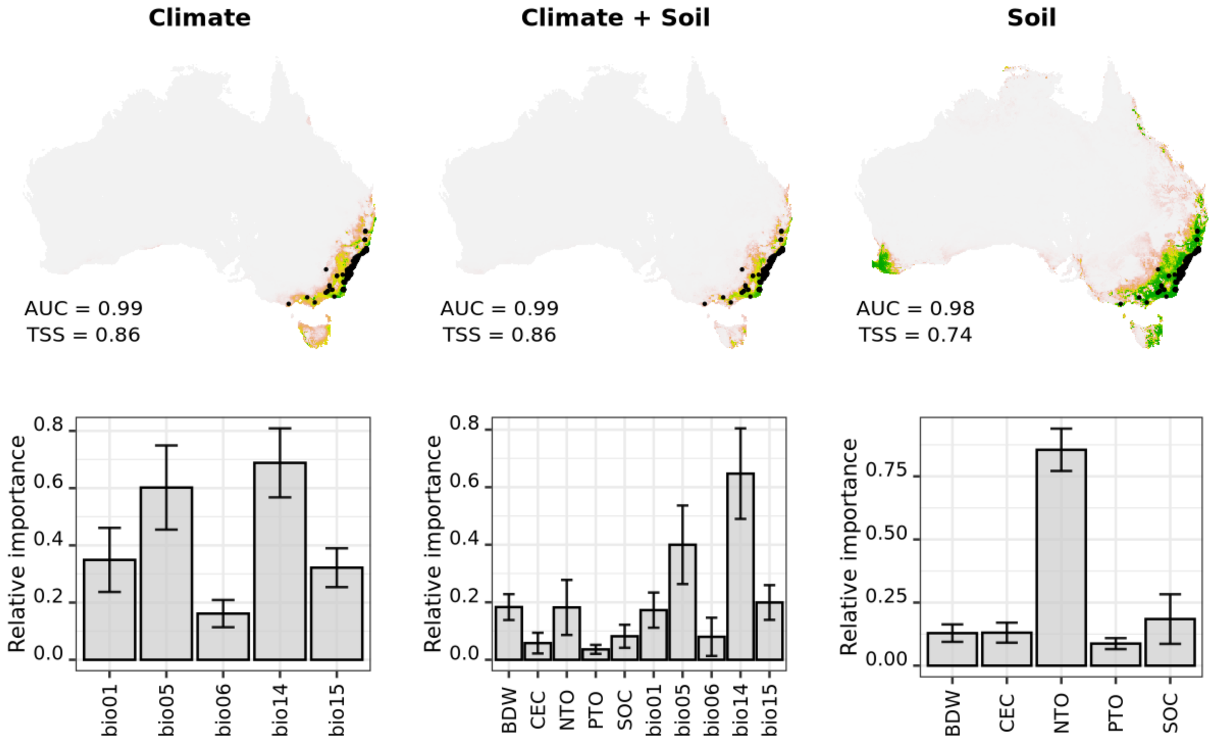

Pultenaea canaliculata

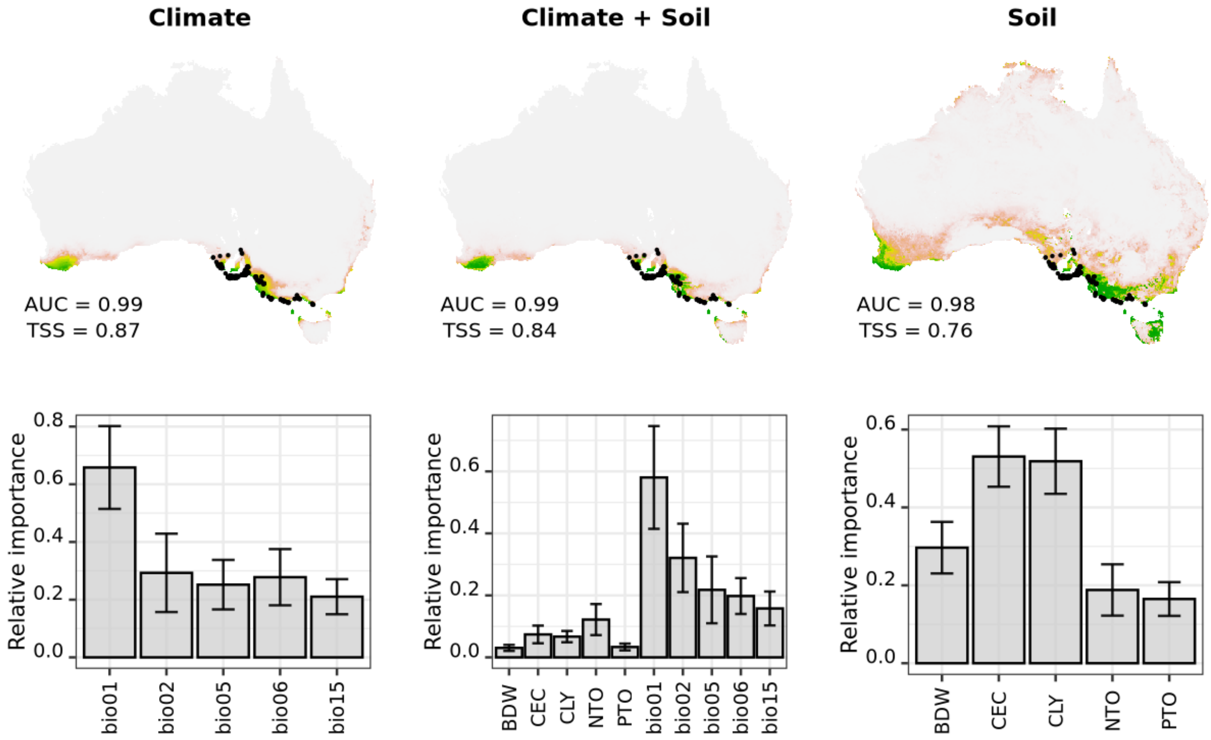

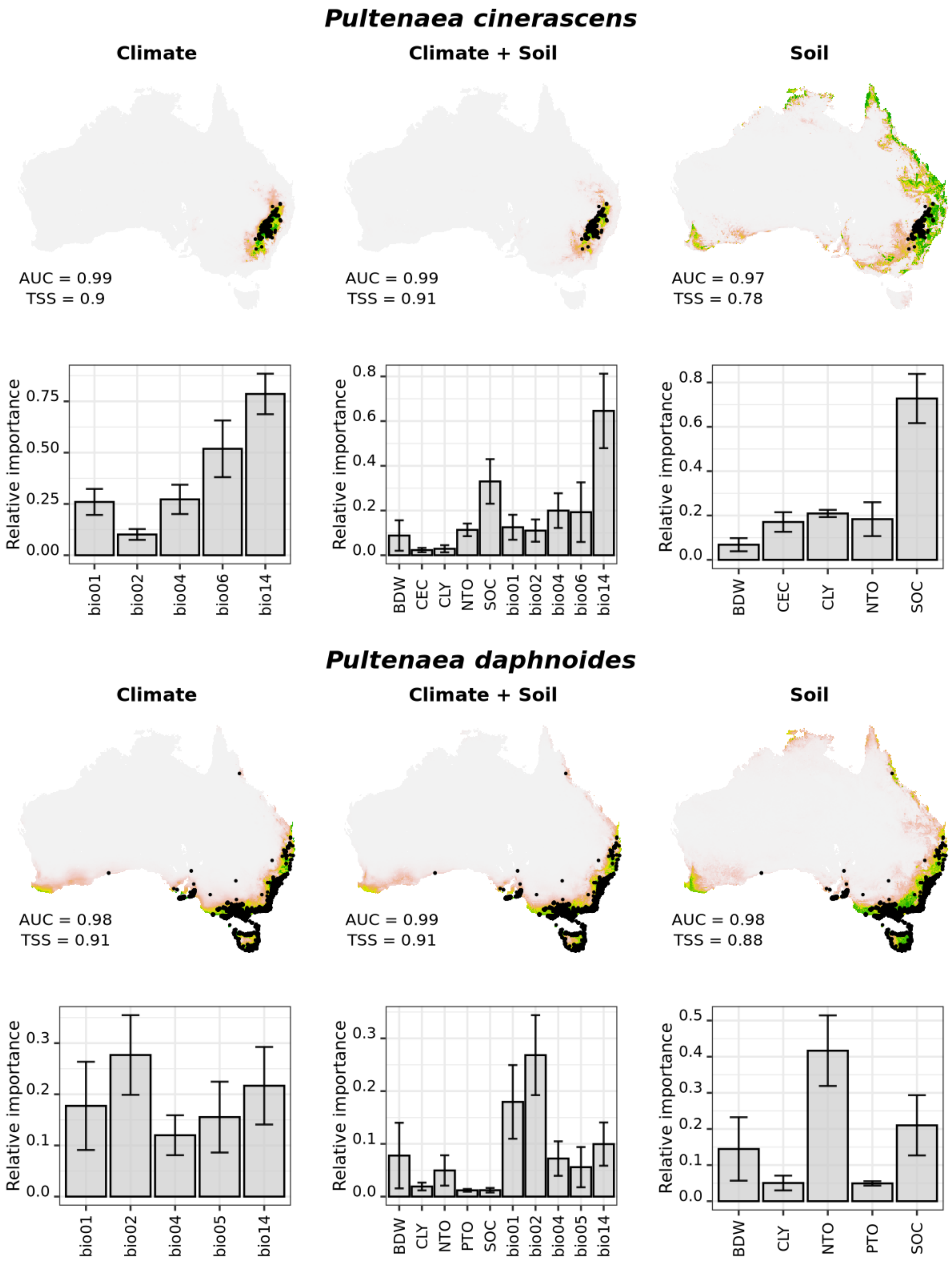
Pultenaea cinerascens
Climate
Climate + Soil
Soil
AUC = 0.99
TSS = 0.9
AUC = 0.99
TSS = 0.91
AUC = 0.97
TSS = 0.78
Relative importance
0.00
0.25
0.50
0.75
bio01
bio02
bio04
bio06
bio14
0.0
0.2
0.4
0.6
0.8
BDW
CEC
CLY
NTO
SOC
bio01
bio02
bio04
bio06
bio14
BDW
CEC
CLY
NTO
SOC
Pultenaea daphnoides
Climate
Climate + Soil
Soil
AUC = 0.98
TSS = 0.91
AUC = 0.99
TSS = 0.91
AUC = 0.98
TSS = 0.88
Relative importance
0.0
0.1
0.2
0.3
0.4
0.5
bio01
bio02
bio04
bio05
bio14
BDW
CLY
NTO
PTO
SOC
bio01
bio02
bio04
bio05
bio14
BDW
CLY
NTO
PTO
SOC

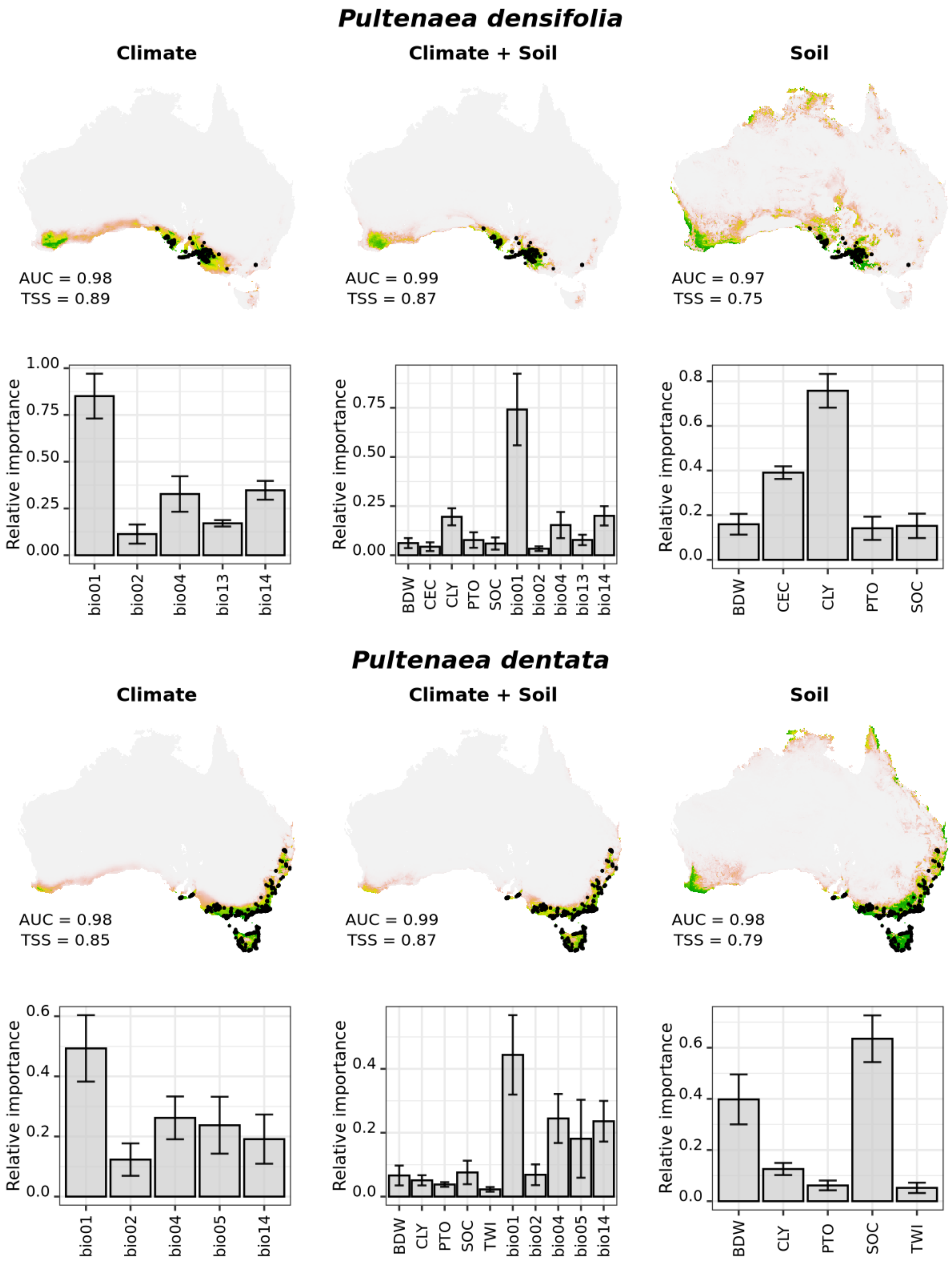
Pultenaea densifolia
Climate
Climate + Soil
Soil
AUC = 0.98
TSS = 0.89
AUC = 0.99
TSS = 0.87
AUC = 0.97
TSS = 0.75
Relative importance
1.00
0.75
0.50
0.25
0.00
bio01
bio02
bio04
bio13
bio14
Relative importance
0.75
0.50
0.25
0.00
BDW
CEC
CLY
PTO
SOC
bio01
bio02
bio04
bio13
bio14
Relative importance
0.8
0.6
0.4
0.2
0.0
BDW
CEC
CLY
PTO
SOC
Pultenaea dentata
Climate
Climate + Soil
Soil
AUC = 0.98
TSS = 0.85
AUC = 0.99
TSS = 0.87
AUC = 0.98
TSS = 0.79
Relative importance
0.6
0.4
0.2
0.0
bio01
bio02
bio04
bio05
bio14
Relative importance
0.4
0.2
0.0
BDW
CLY
PTO
SOC
TWI
bio01
bio02
bio04
bio05
bio14
Relative importance
0.6
0.4
0.2
0.0
BDW
CLY
PTO
SOC
TWI

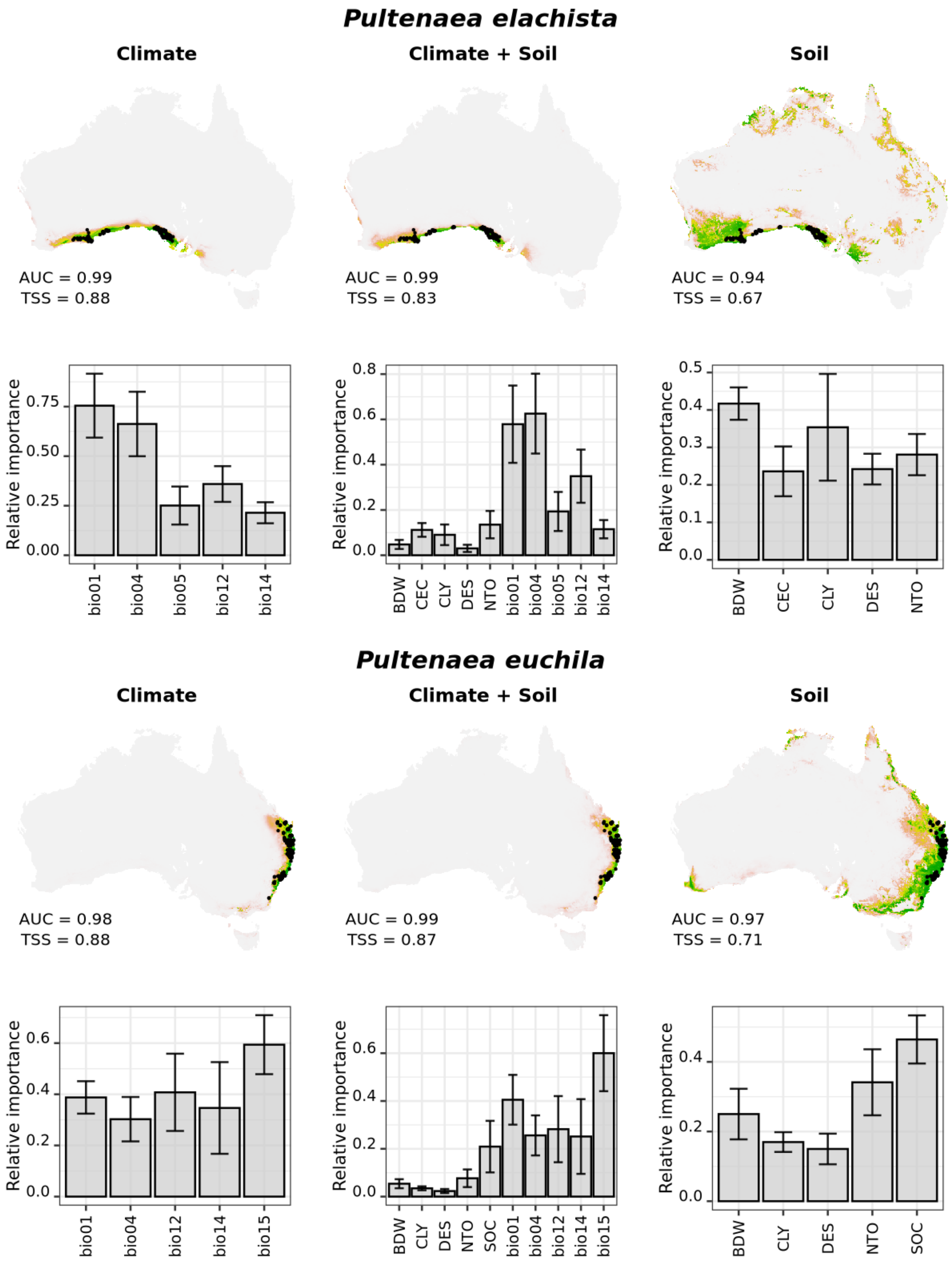
Pultenaea elachista
Climate
Climate + Soil
Soil
AUC = 0.99
TSS = 0.88
AUC = 0.99
TSS = 0.83
AUC = 0.94
TSS = 0.67
Relative importance
0.00
0.25
0.50
0.75
bio01
bio04
bio05
bio12
bio14
0.0
0.2
0.4
0.6
0.8
BDW
CEC
CLY
DES
NTO
bio01
bio04
bio05
bio12
bio14
0.0
0.1
0.2
0.3
0.4
0.5
BDW
CEC
CLY
DES
NTO
Pultenaea euchila
Climate
Climate + Soil
Soil
AUC = 0.98
TSS = 0.88
AUC = 0.99
TSS = 0.87
AUC = 0.97
TSS = 0.71
Relative importance
0.0
0.2
0.4
0.6
bio01
bio04
bio12
bio14
bio15
BDW
CLY
DES
NTO
SOC
bio01
bio04
bio12
bio14
bio15
0.0
0.2
0.4
BDW
CLY
DES
NTO
SOC

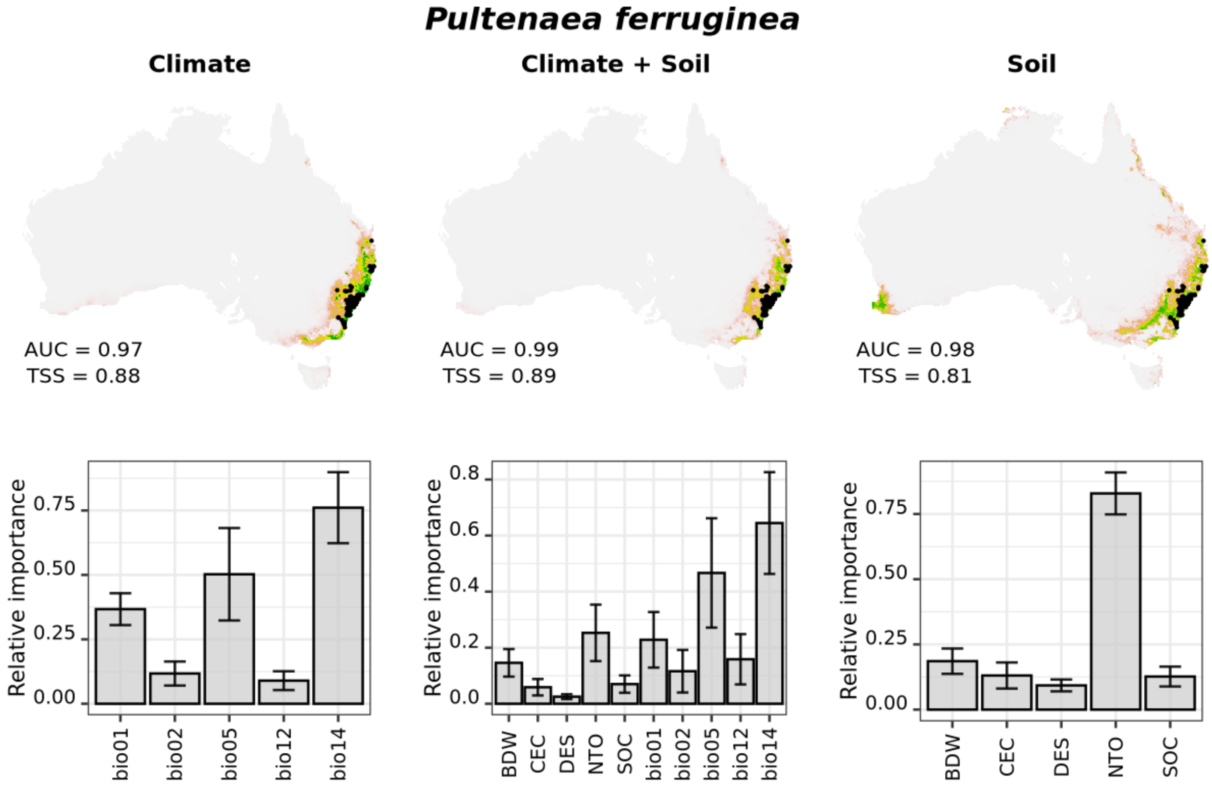
Pultenaea ferruginea
Climate
Climate + Soil
Soil
AUC = 0.97
TSS = 0.88
AUC = 0.99
TSS = 0.89
AUC = 0.98
TSS = 0.81
Relative importance
0.00
0.25
0.50
0.75
bio01
bio02
bio05
bio12
bio14
0.0
0.2
0.4
0.6
0.8
BDW
CEC
DES
NTO
SOC
bio01
bio02
bio05
bio12
bio14
BDW
CEC
DES
NTO
SOC

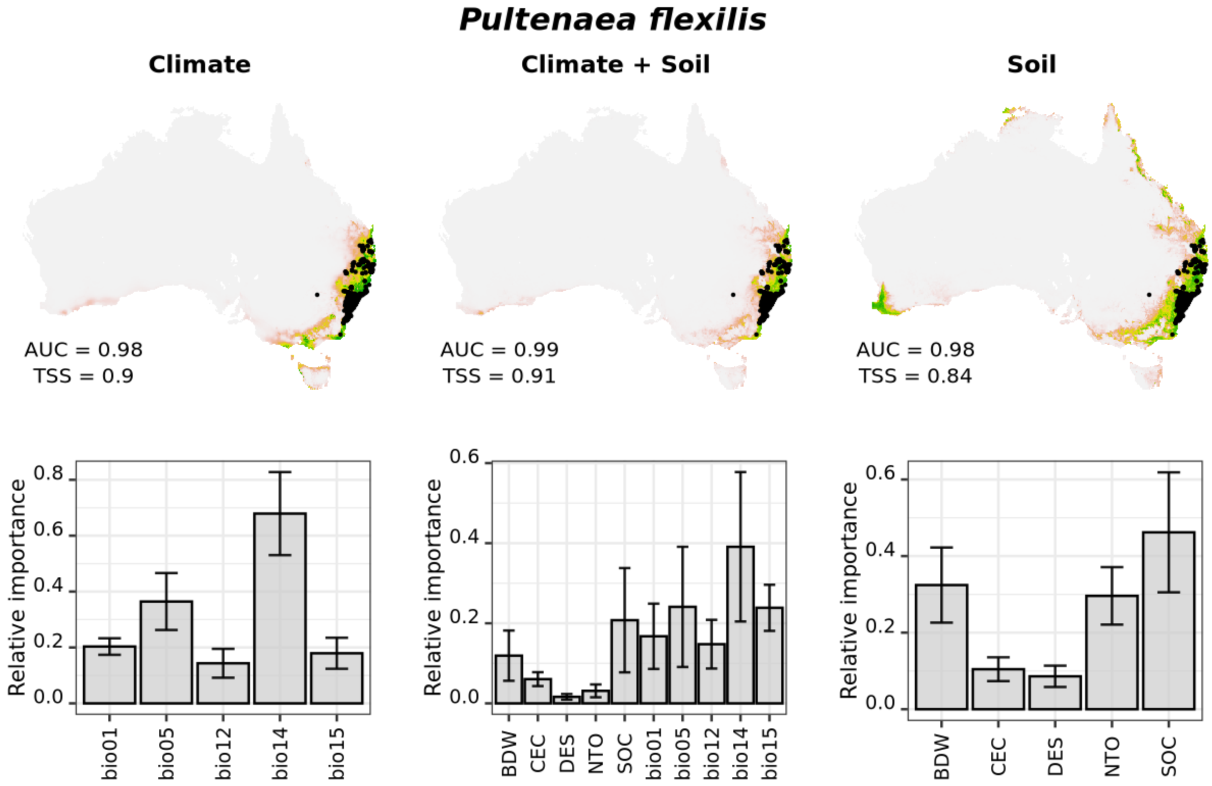
Pultenaea flexilis
Climate
Climate + Soil
Soil
AUC = 0.98
TSS = 0.9
AUC = 0.99
TSS = 0.91
AUC = 0.98
TSS = 0.84
Relative importance
0.0
0.2
0.4
0.6
0.8
bio01
bio05
bio12
bio14
bio15
BDW
CEC
DES
NTO
SOC
bio01
bio05
bio12
bio14
bio15
BDW
CEC
DES
NTO
SOC

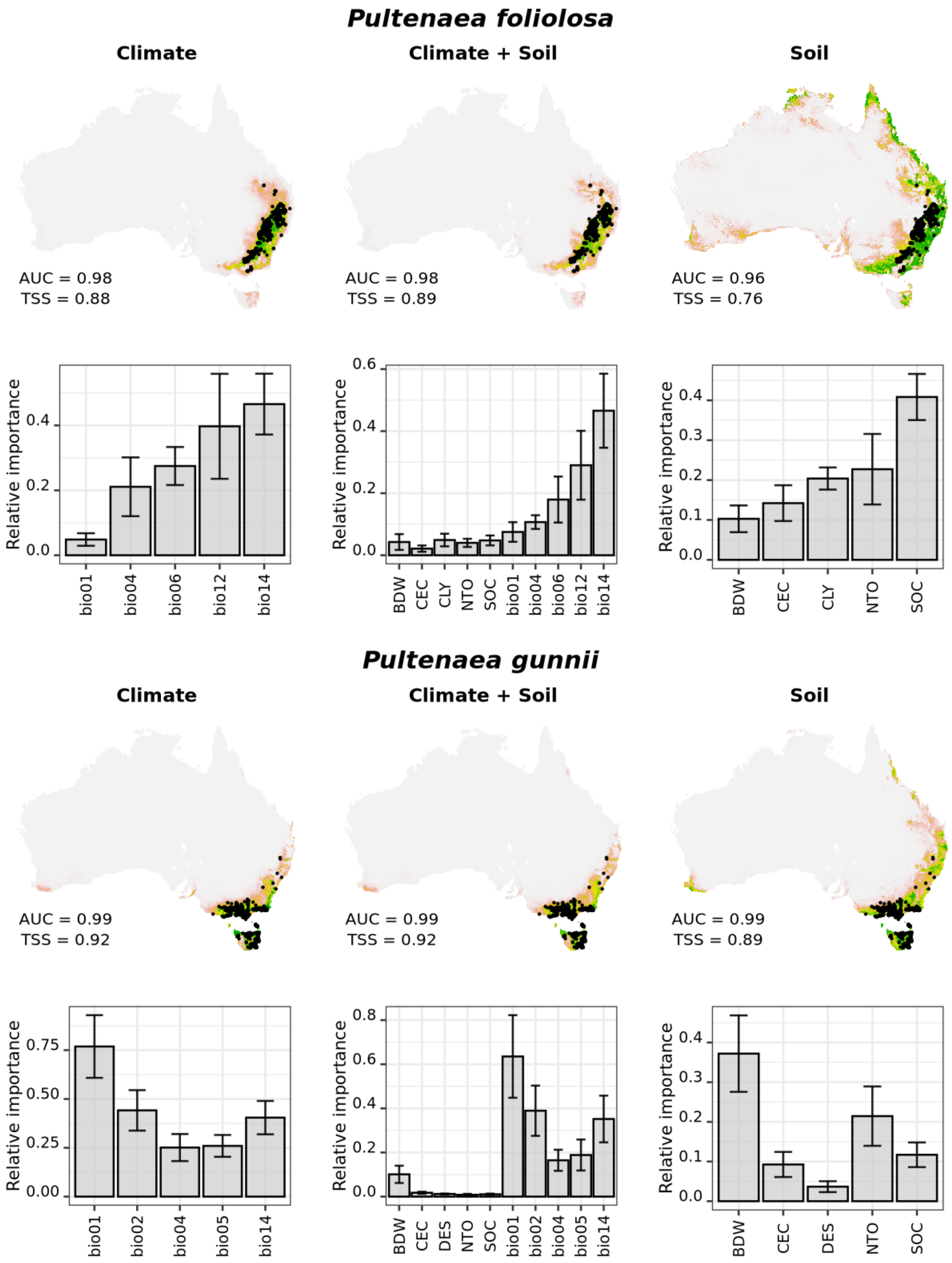

Pultenaea foliolosa
Climate
Climate + Soil
Soil
AUC = 0.98
TSS = 0.88
AUC = 0.98
TSS = 0.89
AUC = 0.96
TSS = 0.76
Relative importance
0.0
0.2
0.4
0.6
0.1
0.3
bio01
bio04
bio06
bio12
bio14
BDW
CEC
CLY
NTO
SOC
Pultenaea gunnii
Climate
Climate + Soil
Soil
AUC = 0.99
TSS = 0.92
AUC = 0.99
TSS = 0.92
AUC = 0.99
TSS = 0.89
Relative importance
0.00
0.25
0.50
0.75
0.8
bio01
bio02
bio04
bio05
bio14
BDW
CEC
DES
NTO
SOC

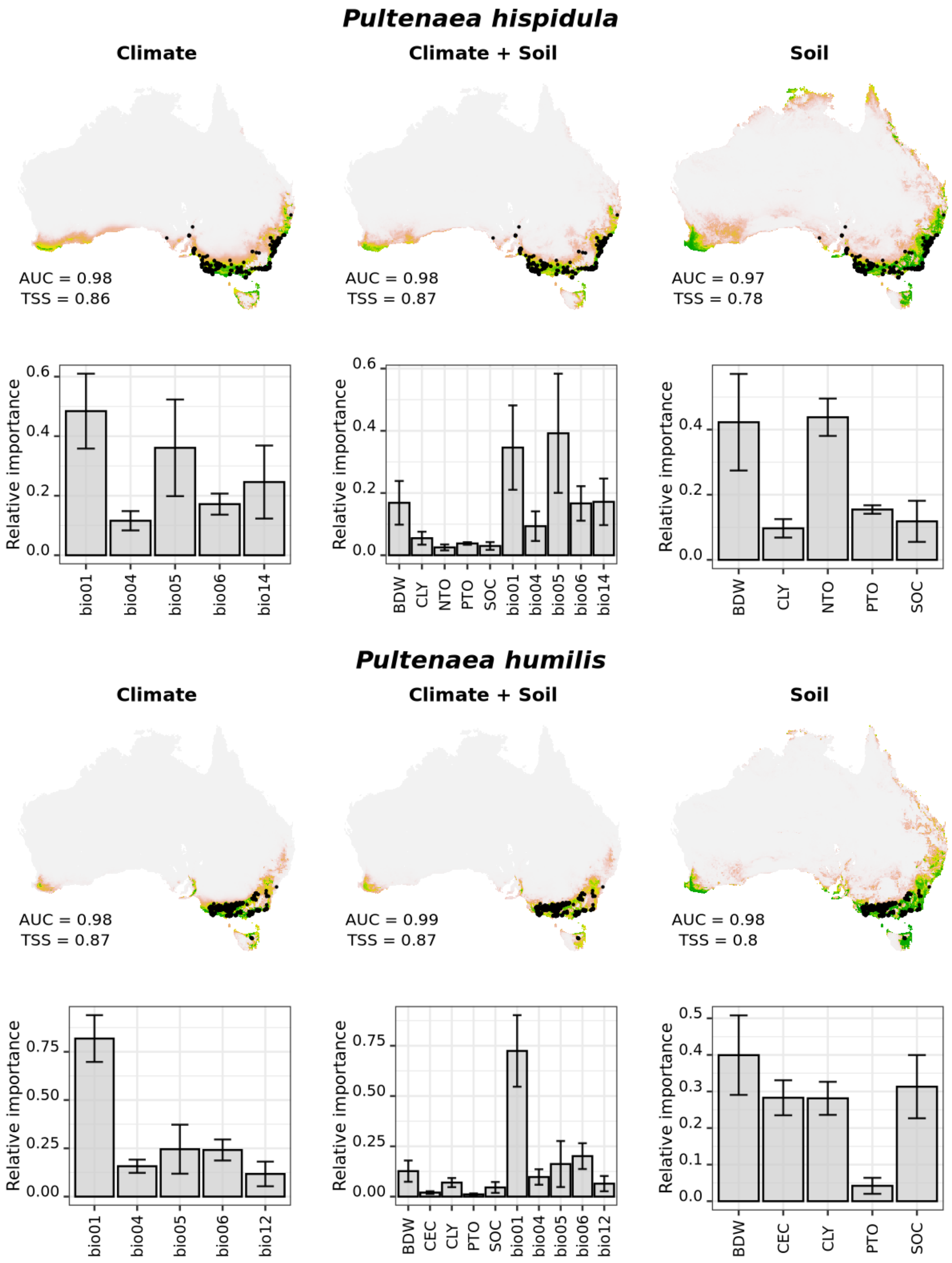
Pultenaea hispidula
Climate
Climate + Soil
Soil
AUC = 0.98
TSS = 0.86
AUC = 0.98
TSS = 0.87
AUC = 0.97
TSS = 0.78
Relative importance
bio01 bio04 bio05 bio06 bio14
BDW CLY NTO PTO SOC bio01 bio04 bio05 bio06 bio14
BDW CLY NTO PTO SOC
Pultenaea humilis
Climate
Climate + Soil
Soil
AUC = 0.98
TSS = 0.87
AUC = 0.99
TSS = 0.87
AUC = 0.98
TSS = 0.8
Relative importance
bio01 bio04 bio05 bio06 bio12
BDW CEC CLY PTO SOC bio01 bio04 bio05 bio06 bio12
BDW CEC CLY PTO SOC

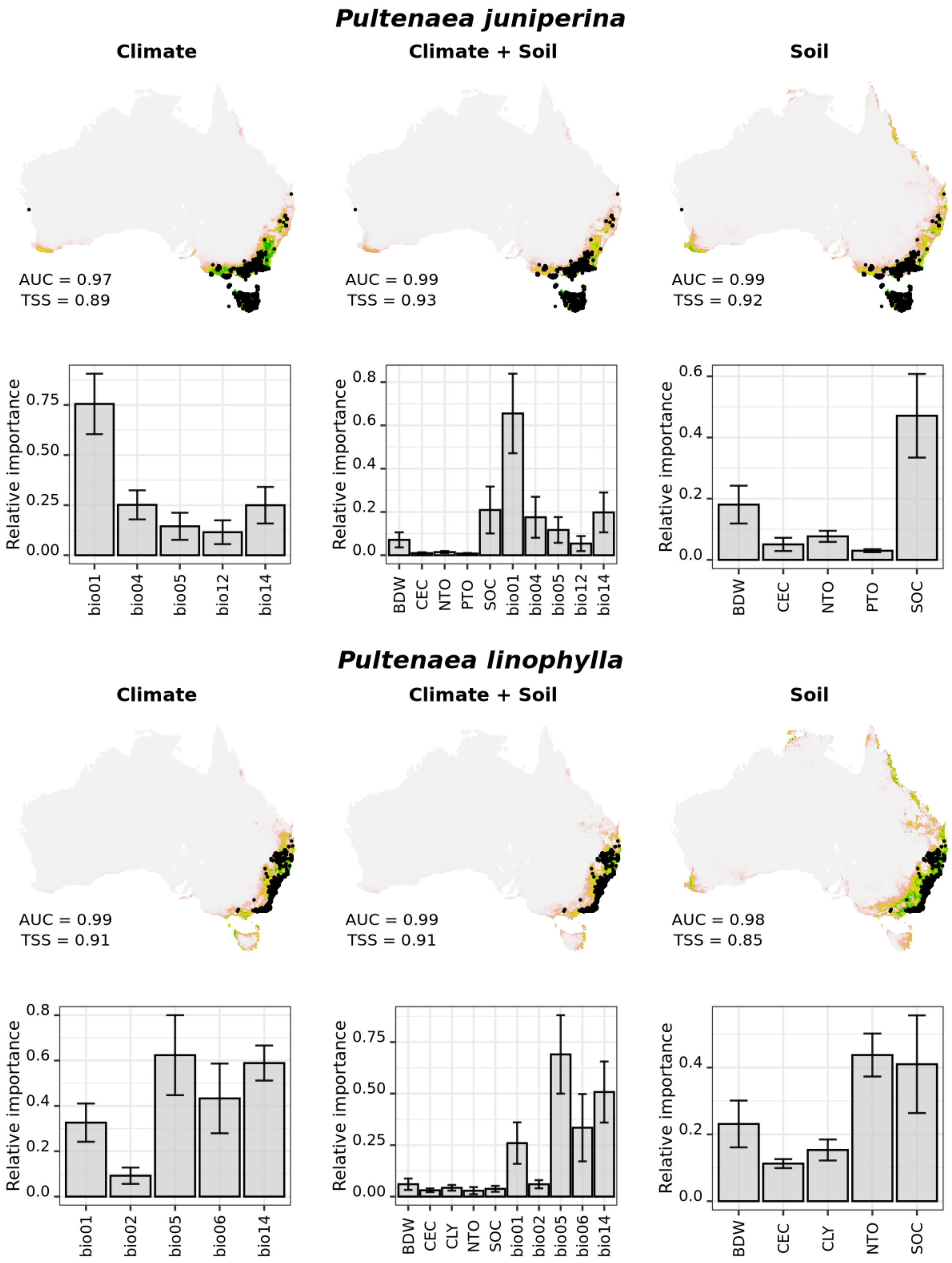

Pultenaea juniperina
Climate
Climate + Soil
Soil
AUC = 0.97
TSS = 0.89
AUC = 0.99
TSS = 0.93
AUC = 0.99
TSS = 0.92
Relative importance
bio01 bio04 bio05 bio12 bio14
BDW CEC NTO PTO SOC bio01 bio04 bio05 bio12 bio14
BDW CEC NTO PTO SOC
Pultenaea linophylla
Climate
Climate + Soil
Soil
AUC = 0.99
TSS = 0.91
AUC = 0.99
TSS = 0.91
AUC = 0.98
TSS = 0.85
Relative importance
bio01 bio02 bio05 bio06 bio14
BDW CEC CLY NTO SOC bio01 bio02 bio05 bio06 bio14
BDW CEC CLY NTO SOC

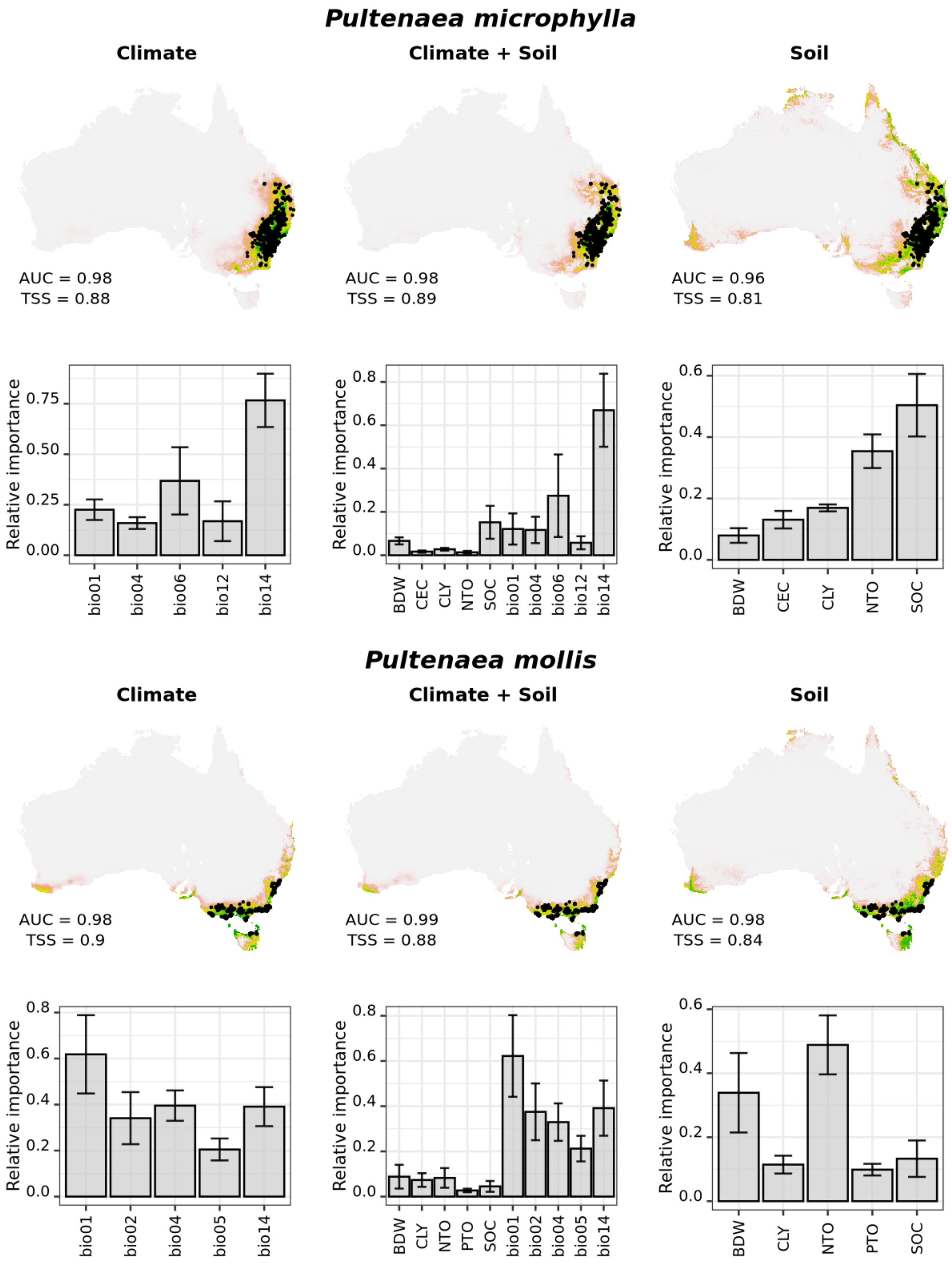

Pultenaea microphylla
Climate
Climate + Soil
Soil
AUC = 0.98
TSS = 0.88
AUC = 0.98
TSS = 0.89
AUC = 0.96
TSS = 0.81
Relative importance
0.00
0.25
0.50
0.75
bio01
bio04
bio06
bio12
bio14
0.0
0.2
0.4
0.6
0.8
BDW
CEC
CLY
NTO
SOC
bio01
bio04
bio06
bio12
bio14
0.0
0.2
0.4
0.6
BDW
CEC
CLY
NTO
SOC
Pultenaea mollis
Climate
Climate + Soil
Soil
AUC = 0.98
TSS = 0.9
AUC = 0.99
TSS = 0.88
AUC = 0.98
TSS = 0.84
Relative importance
0.0
0.2
0.4
0.6
0.8
bio01
bio02
bio04
bio05
bio14
BDW
CLY
NTO
PTO
SOC
bio01
bio02
bio04
bio05
bio14
0.0
0.2
0.4
0.6
BDW
CLY
NTO
PTO
SOC

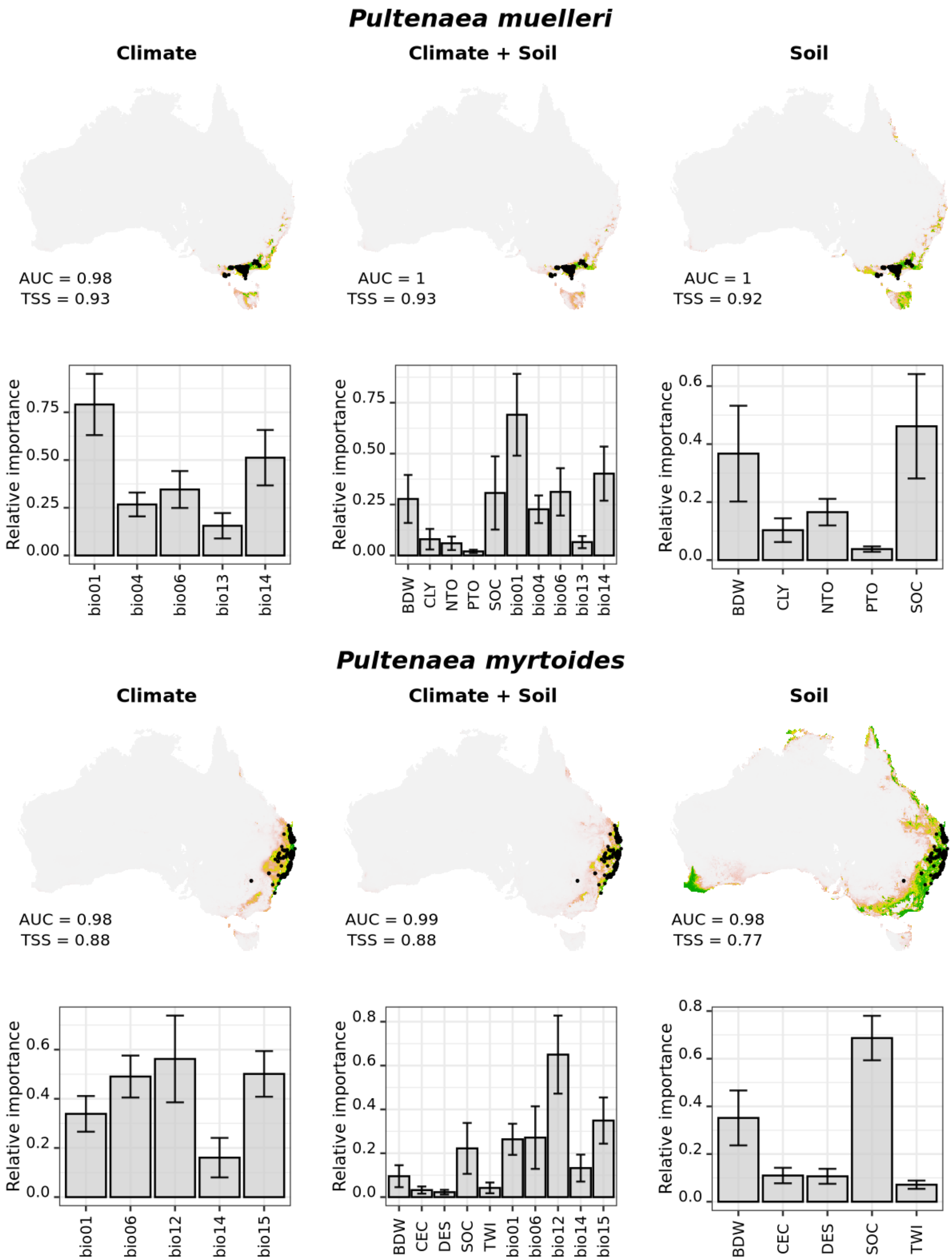
Pultenaea muelleri
Climate
Climate + Soil
Soil
AUC = 0.98
TSS = 0.93
AUC = 1
TSS = 0.93
AUC = 1
TSS = 0.92
Relative importance
0.00
0.25
0.50
0.75
bio01
bio04
bio06
bio13
bio14
BDW
CLY
NTO
PTO
SOC
bio01
bio04
bio06
bio13
bio14
0.0
0.2
0.4
0.6
BDW
CLY
NTO
PTO
SOC
Pultenaea myrtoides
Climate
Climate + Soil
Soil
AUC = 0.98
TSS = 0.88
AUC = 0.99
TSS = 0.88
AUC = 0.98
TSS = 0.77
Relative importance
0.0
0.2
0.4
0.6
0.8
bio01
bio06
bio12
bio14
bio15
BDW
CEC
DES
SOC
TWI
bio01
bio06
bio12
bio14
bio15
BDW
CEC
DES
SOC
TWI

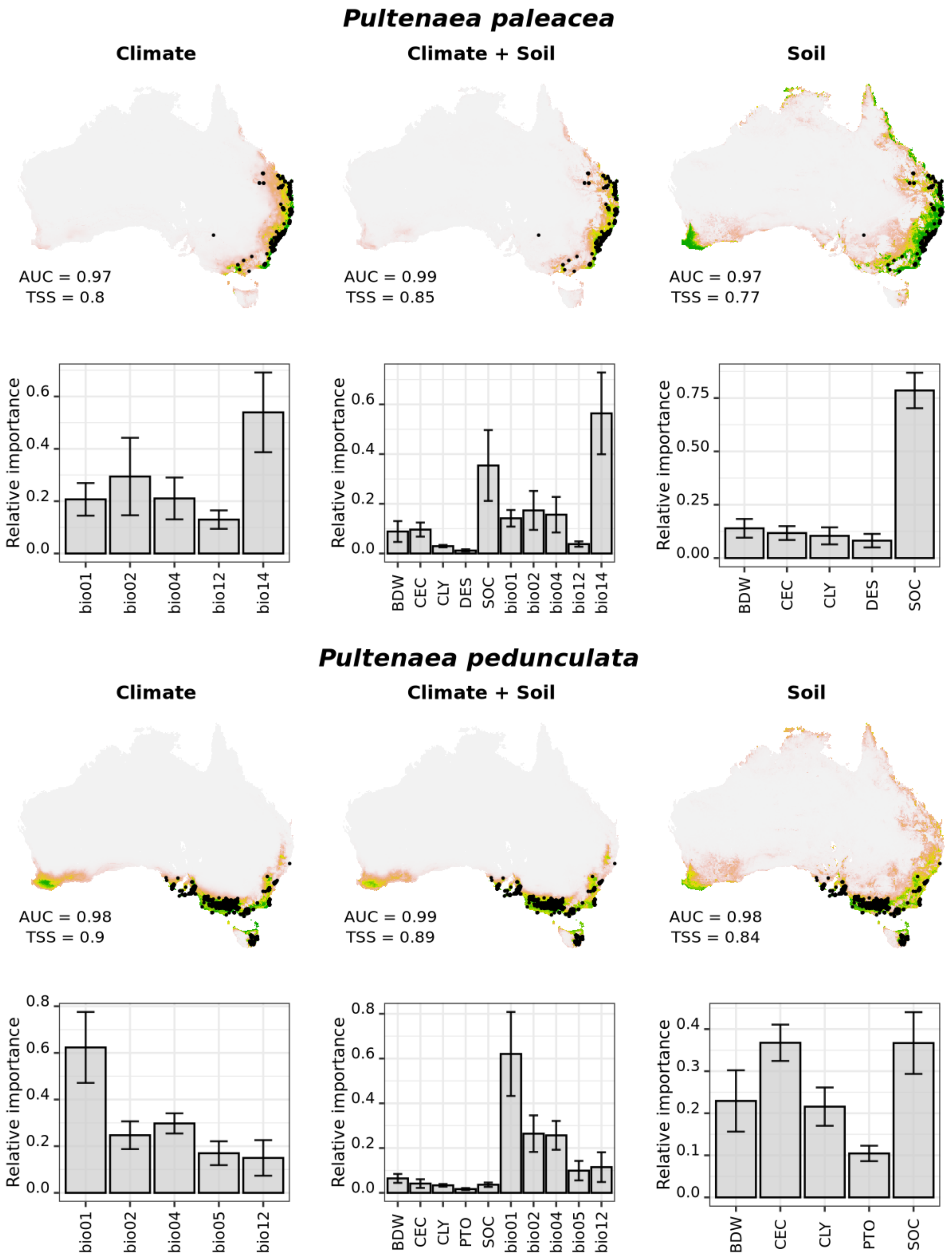

Pultenaea paleacea
Climate
Climate + Soil
Soil
AUC = 0.97
TSS = 0.8
AUC = 0.99
TSS = 0.85
AUC = 0.97
TSS = 0.77
Relative importance
0.0
0.2
0.4
0.6
bio01
bio02
bio04
bio12
bio14
Relative importance
0.0
0.2
0.4
0.6
BDW
CEC
CLY
DES
SOC
bio01
bio02
bio04
bio12
bio14
Relative importance
0.00
0.25
0.50
0.75
BDW
CEC
CLY
DES
SOC
Pultenaea pedunculata
Climate
Climate + Soil
Soil
AUC = 0.98
TSS = 0.9
AUC = 0.99
TSS = 0.89
AUC = 0.98
TSS = 0.84
Relative importance
0.0
0.2
0.4
0.6
0.8
bio01
bio02
bio04
bio05
bio12
Relative importance
0.0
0.2
0.4
0.6
0.8
BDW
CEC
CLY
PTO
SOC
bio01
bio02
bio04
bio05
bio12
Relative importance
0.0
0.1
0.2
0.3
0.4
BDW
CEC
CLY
PTO
SOC

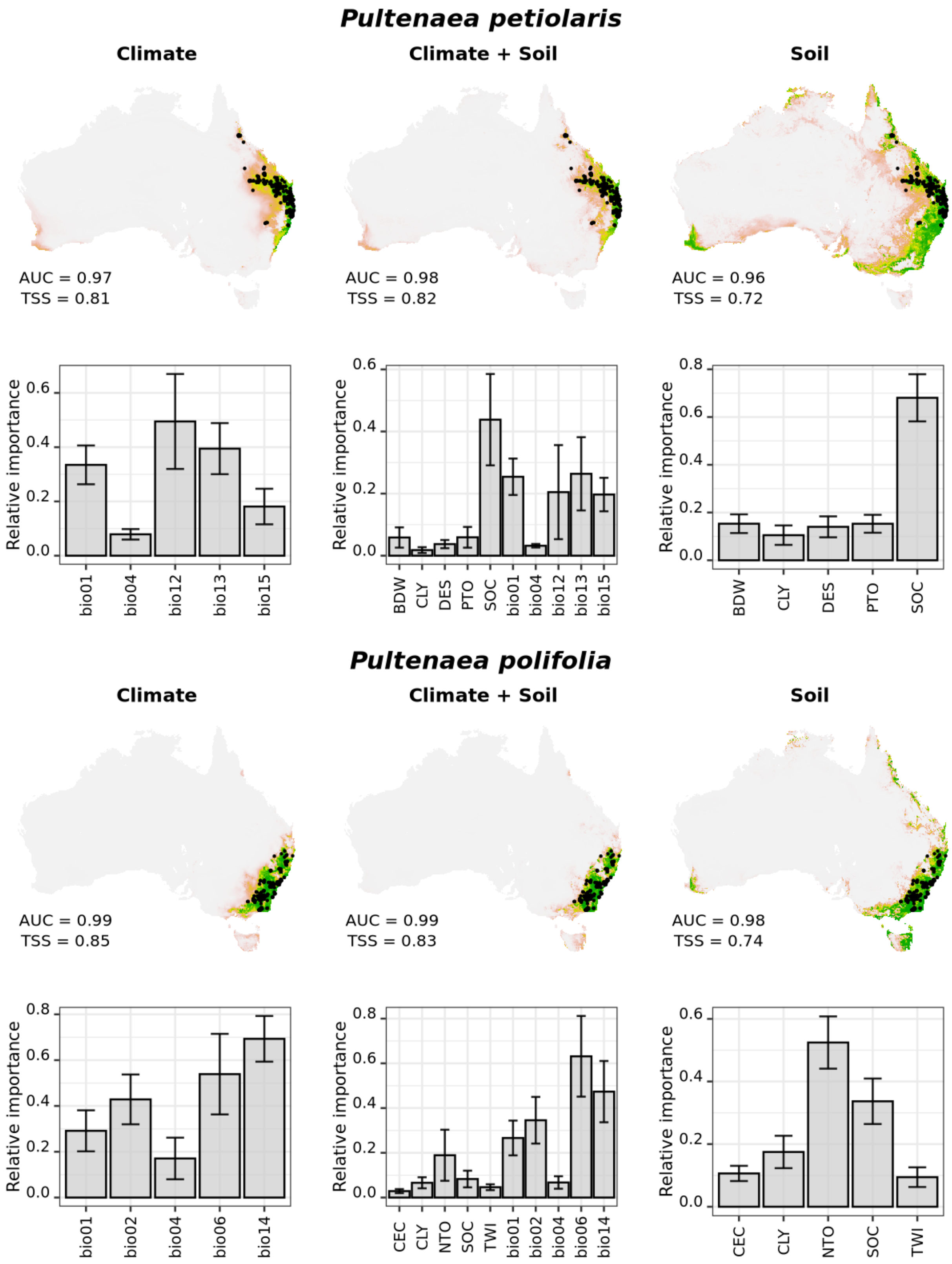
Pultenaea petiolaris
Climate
Climate + Soil
Soil
AUC = 0.97
TSS = 0.81
AUC = 0.98
TSS = 0.82
AUC = 0.96
TSS = 0.72
Relative importance
bio01 bio04 bio12 bio13 bio15
BDW CLY DES PTO SOC bio01 bio04 bio12 bio13 bio15
BDW CLY DES PTO SOC
Pultenaea polifolia
Climate
Climate + Soil
Soil
AUC = 0.99
TSS = 0.85
AUC = 0.99
TSS = 0.83
AUC = 0.98
TSS = 0.74
Relative importance
bio01 bio02 bio04 bio06 bio14
CEC CLY NTO SOC TWI bio01 bio02 bio04 bio06 bio14
CEC CLY NTO SOC TWI

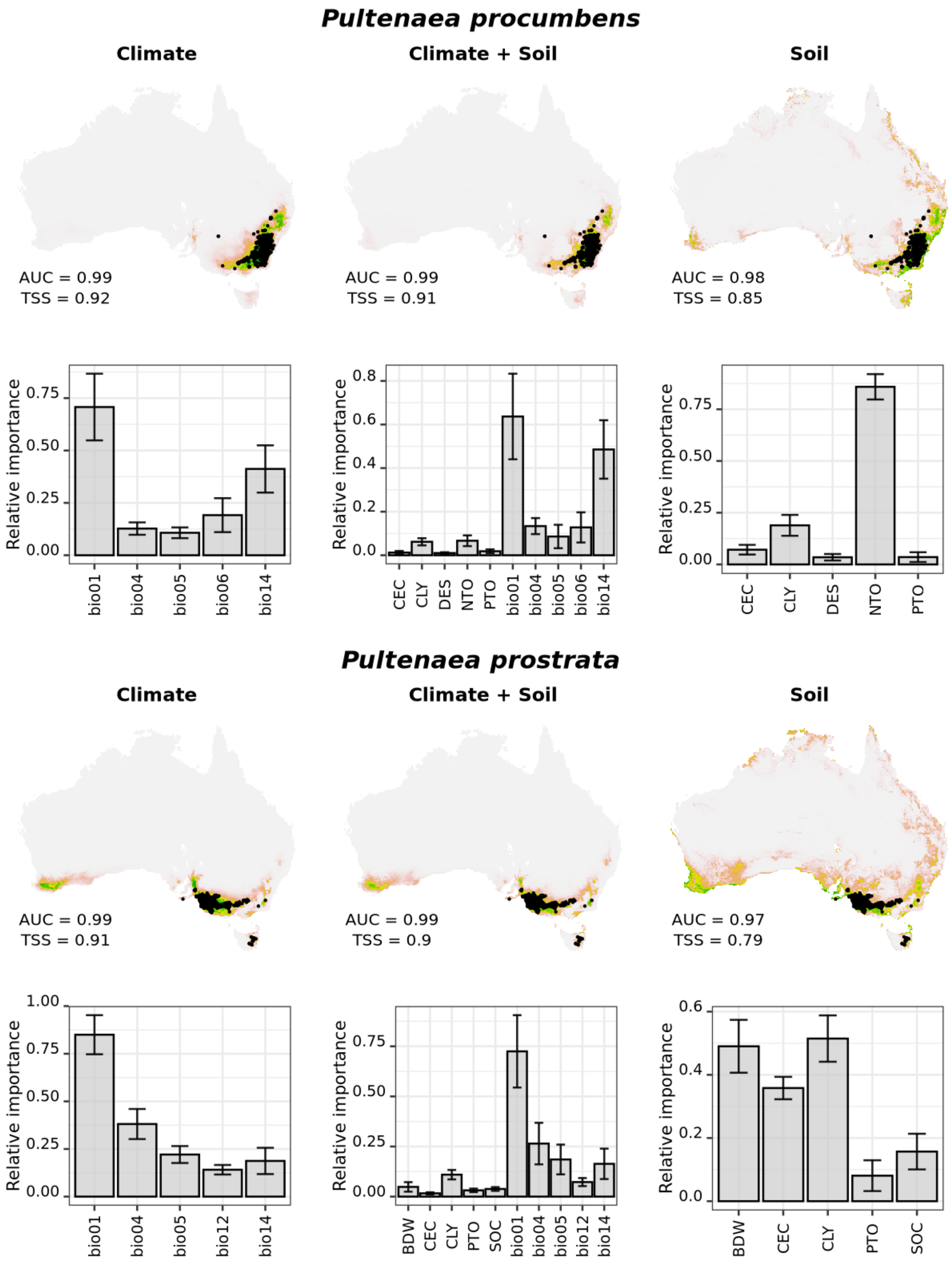

Pultenaea procumbens
Climate
Climate + Soil
Soil
AUC = 0.99
TSS = 0.92
AUC = 0.99
TSS = 0.91
AUC = 0.98
TSS = 0.85
Relative importance
0.00
0.25
0.50
0.75
bio01
bio04
bio05
bio06
bio14
0.0
0.2
0.4
0.6
0.8
CEC
CLY
DES
NTO
PTO
bio01
bio04
bio05
bio06
bio14
CEC
CLY
DES
NTO
PTO
Pultenaea prostrata
Climate
Climate + Soil
Soil
AUC = 0.99
TSS = 0.91
AUC = 0.99
TSS = 0.9
AUC = 0.97
TSS = 0.79
Relative importance
1.00
bio01
bio04
bio05
bio12
bio14
BDW
CEC
CLY
PTO
SOC
bio01
bio04
bio05
bio12
bio14
0.6
BDW
CEC
CLY
PTO
SOC

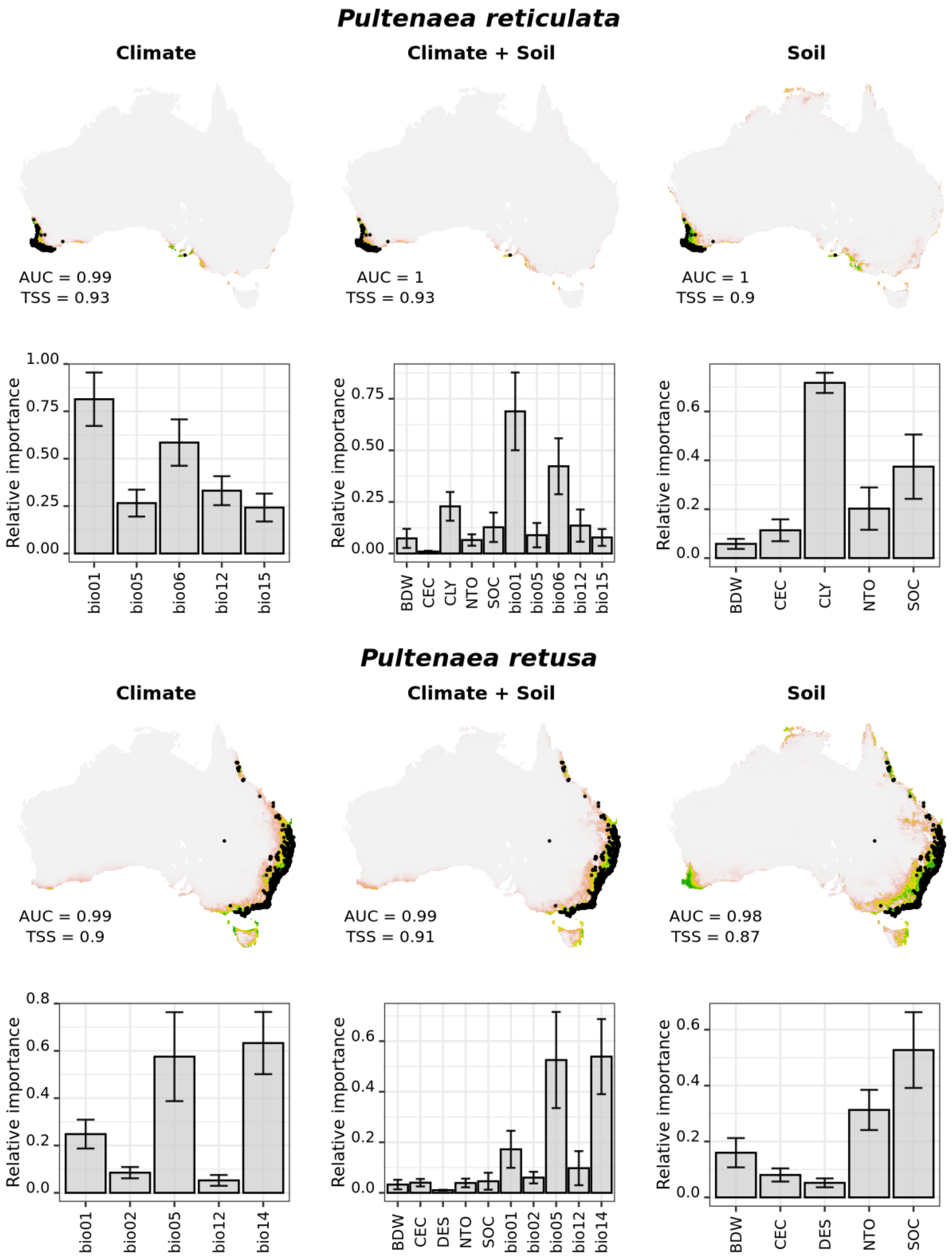
Pultenaea reticulata
Climate
Climate + Soil
Soil
AUC = 0.99
TSS = 0.93
AUC = 1
TSS = 0.93
AUC = 1
TSS = 0.9
Relative importance
bio01
bio05
bio06
bio12
bio15
BDW
CEC
CLY
NTO
SOC
Pultenaea retusa
Climate
Climate + Soil
Soil
AUC = 0.99
TSS = 0.9
AUC = 0.99
TSS = 0.91
AUC = 0.98
TSS = 0.87
Relative importance
bio01
bio02
bio05
bio12
bio14
BDW
CEC
DES
NTO
SOC

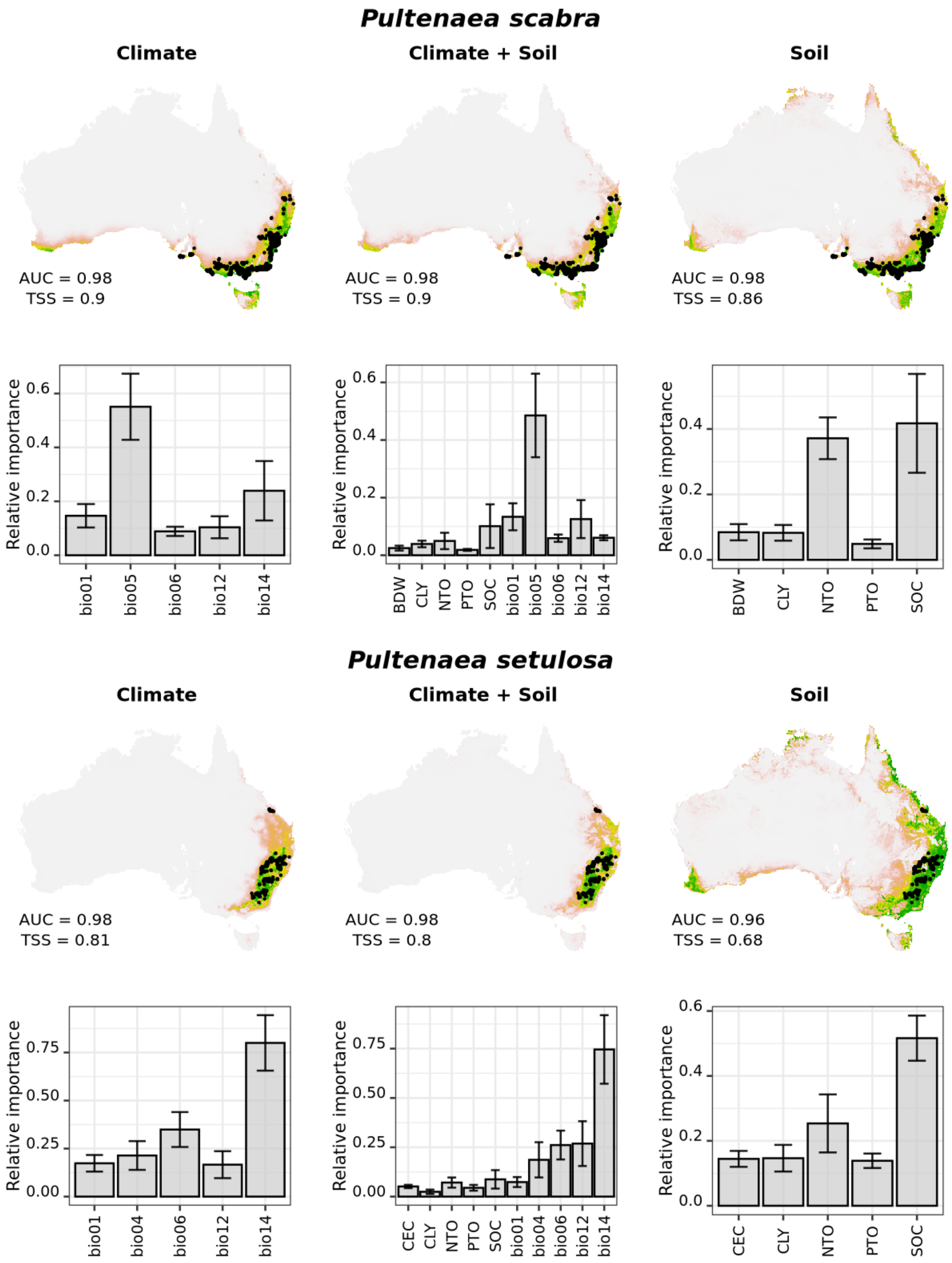
Pultenaea scabra
Climate
Climate + Soil
Soil
AUC = 0.98
TSS = 0.9
AUC = 0.98
TSS = 0.9
AUC = 0.98
TSS = 0.86
Relative importance
0.0
0.2
0.4
0.6
bio01
bio05
bio06
bio12
bio14
BDW
CLY
NTO
PTO
SOC
Pultenaea setulosa
Climate
Climate + Soil
Soil
AUC = 0.98
TSS = 0.81
AUC = 0.98
TSS = 0.8
AUC = 0.96
TSS = 0.68
Relative importance
0.00
0.25
0.50
0.75
bio01
bio04
bio06
bio12
bio14
CEC
CLY
NTO
PTO
SOC

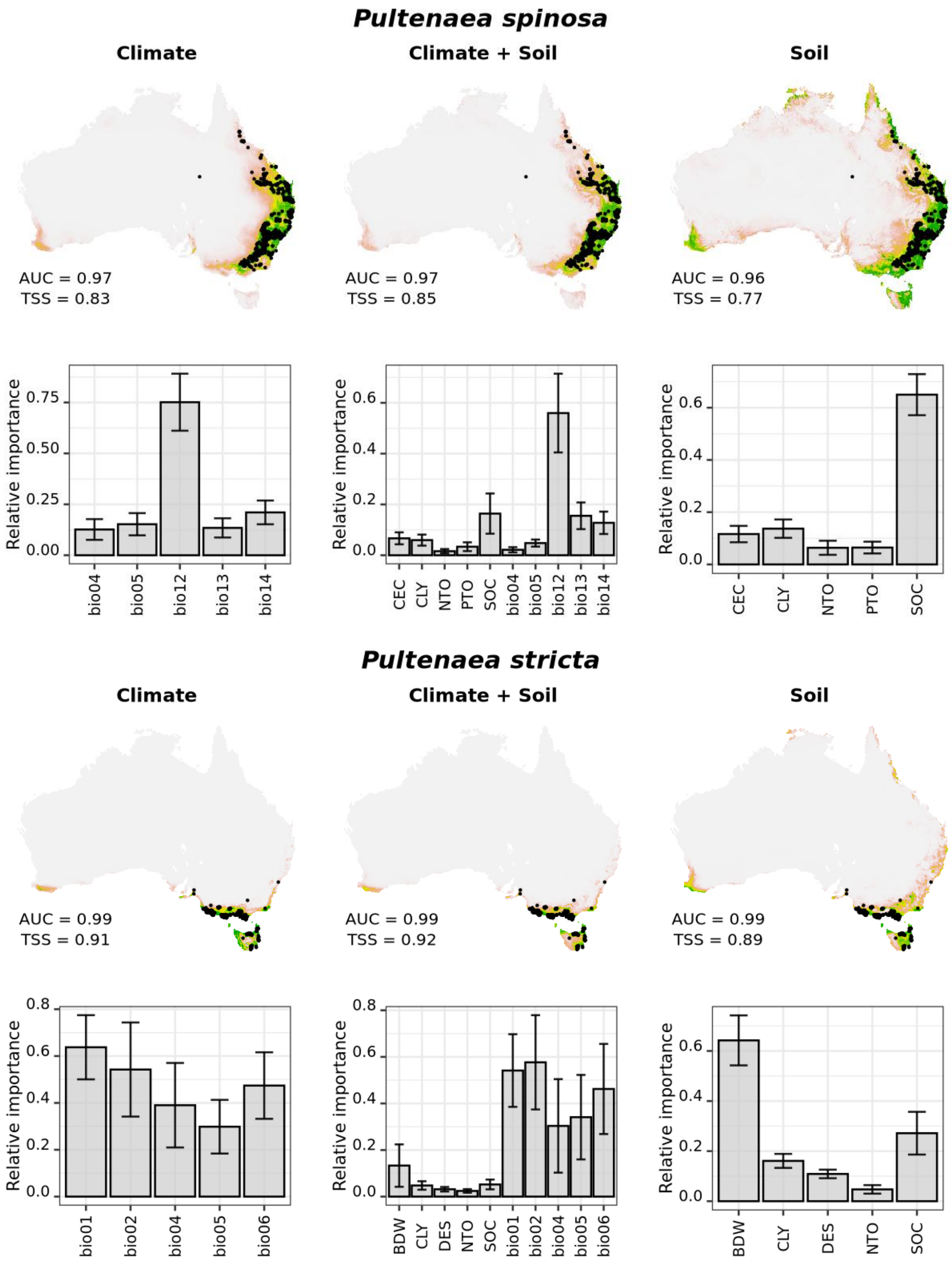

Pultenaea spinosa
Climate
Climate + Soil
Soil
AUC = 0.97
TSS = 0.83
AUC = 0.97
TSS = 0.85
AUC = 0.96
TSS = 0.77
Relative importance
bio04
bio05
bio12
bio13
bio14
CEC
CLY
NTO
PTO
SOC
bio04
bio05
bio12
bio13
bio14
CEC
CLY
NTO
PTO
SOC
Pultenaea stricta
Climate
Climate + Soil
Soil
AUC = 0.99
TSS = 0.91
AUC = 0.99
TSS = 0.92
AUC = 0.99
TSS = 0.89
Relative importance
bio01
bio02
bio04
bio05
bio06
BDW
CLY
DES
NTO
SOC
bio01
bio02
bio04
bio05
bio06
BDW
CLY
DES
NTO
SOC

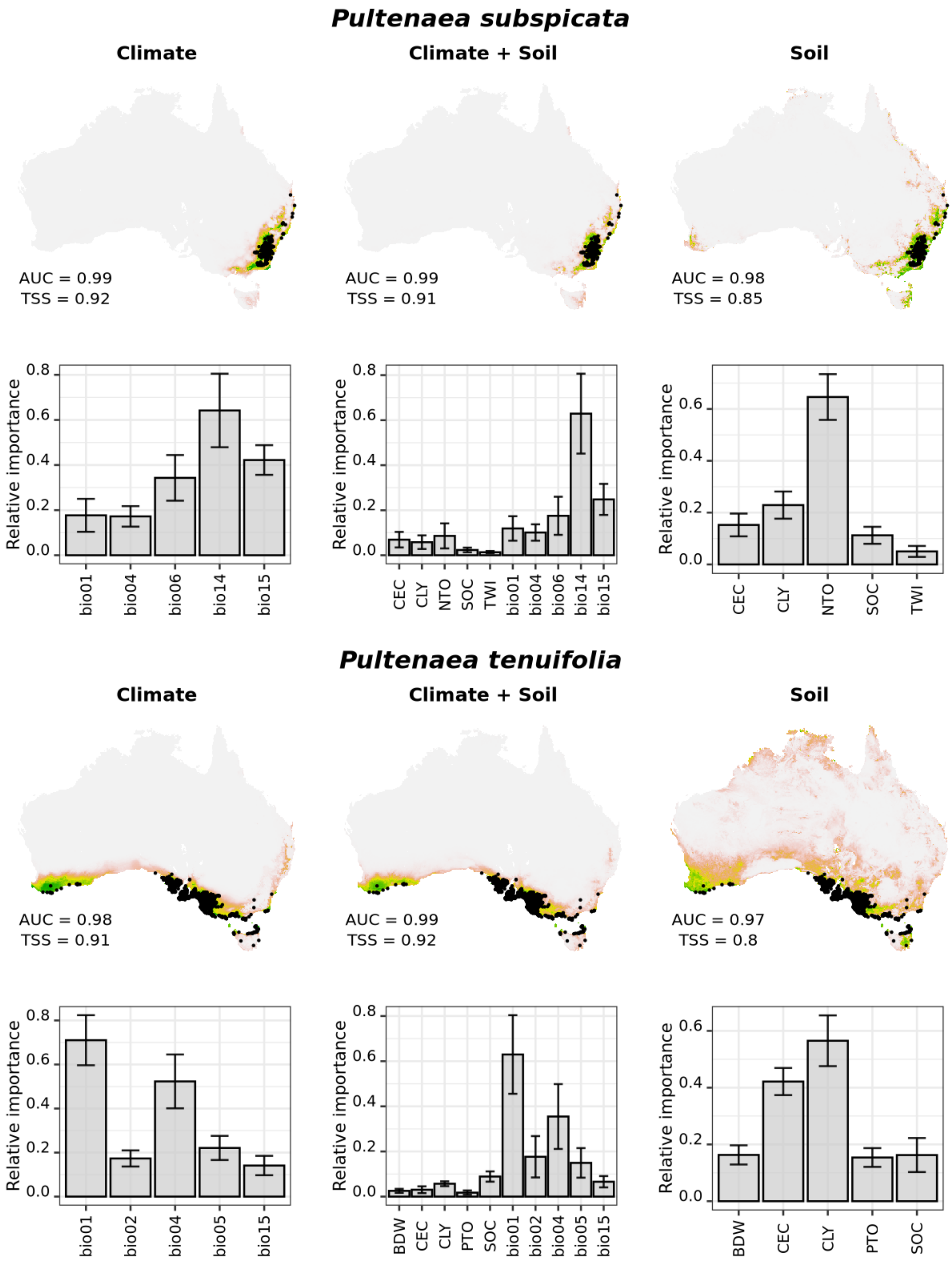
Pultenaea subspicata
Climate
Climate + Soil
Soil
AUC = 0.99
TSS = 0.92
AUC = 0.99
TSS = 0.91
AUC = 0.98
TSS = 0.85
Relative importance
bio01
bio04
bio06
bio14
bio15
CEC
CLY
NTO
SOC
TWI
Pultenaea tenuifolia
Climate
Climate + Soil
Soil
AUC = 0.98
TSS = 0.91
AUC = 0.99
TSS = 0.92
AUC = 0.97
TSS = 0.8
Relative importance
bio01
bio02
bio04
bio05
bio15
BDW
CEC
CLY
PTO
SOC

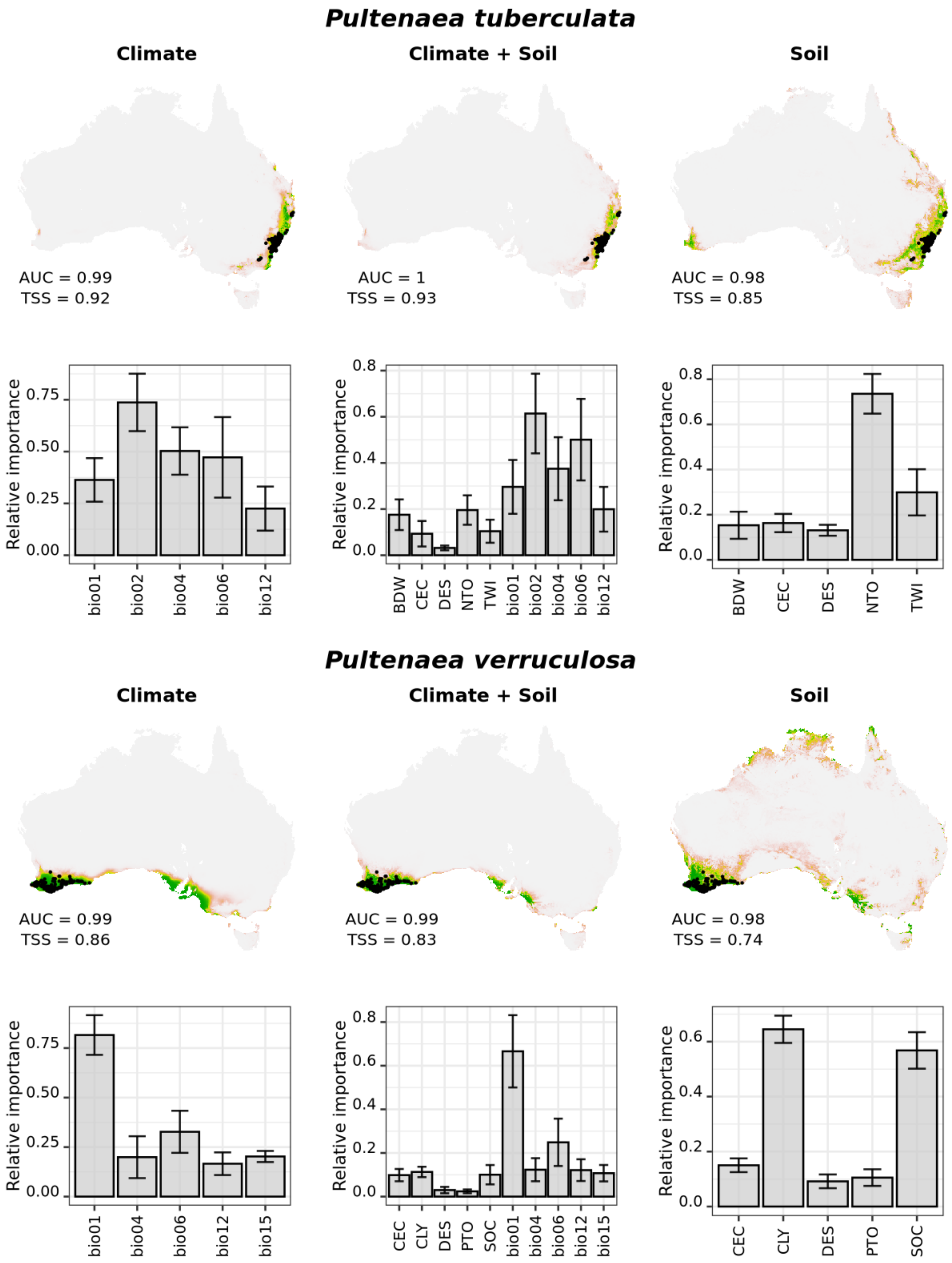
Pultenaea tuberculata
Climate
Climate + Soil
Soil
AUC = 0.99
TSS = 0.92
AUC = 1
TSS = 0.93
AUC = 0.98
TSS = 0.85
Relative importance
0.00
0.25
0.50
0.75
bio01
bio02
bio04
bio06
bio12
0.0
0.2
0.4
0.6
0.8
BDW
CEC
DES
NTO
TWI
bio01
bio02
bio04
bio06
bio12
BDW
CEC
DES
NTO
TWI
Pultenaea verruculosa
Climate
Climate + Soil
Soil
AUC = 0.99
TSS = 0.86
AUC = 0.99
TSS = 0.83
AUC = 0.98
TSS = 0.74
bio01
bio04
bio06
bio12
bio15
CEC
CLY
DES
PTO
SOC
bio01
bio04
bio06
bio12
bio15
CEC
CLY
DES
PTO
SOC

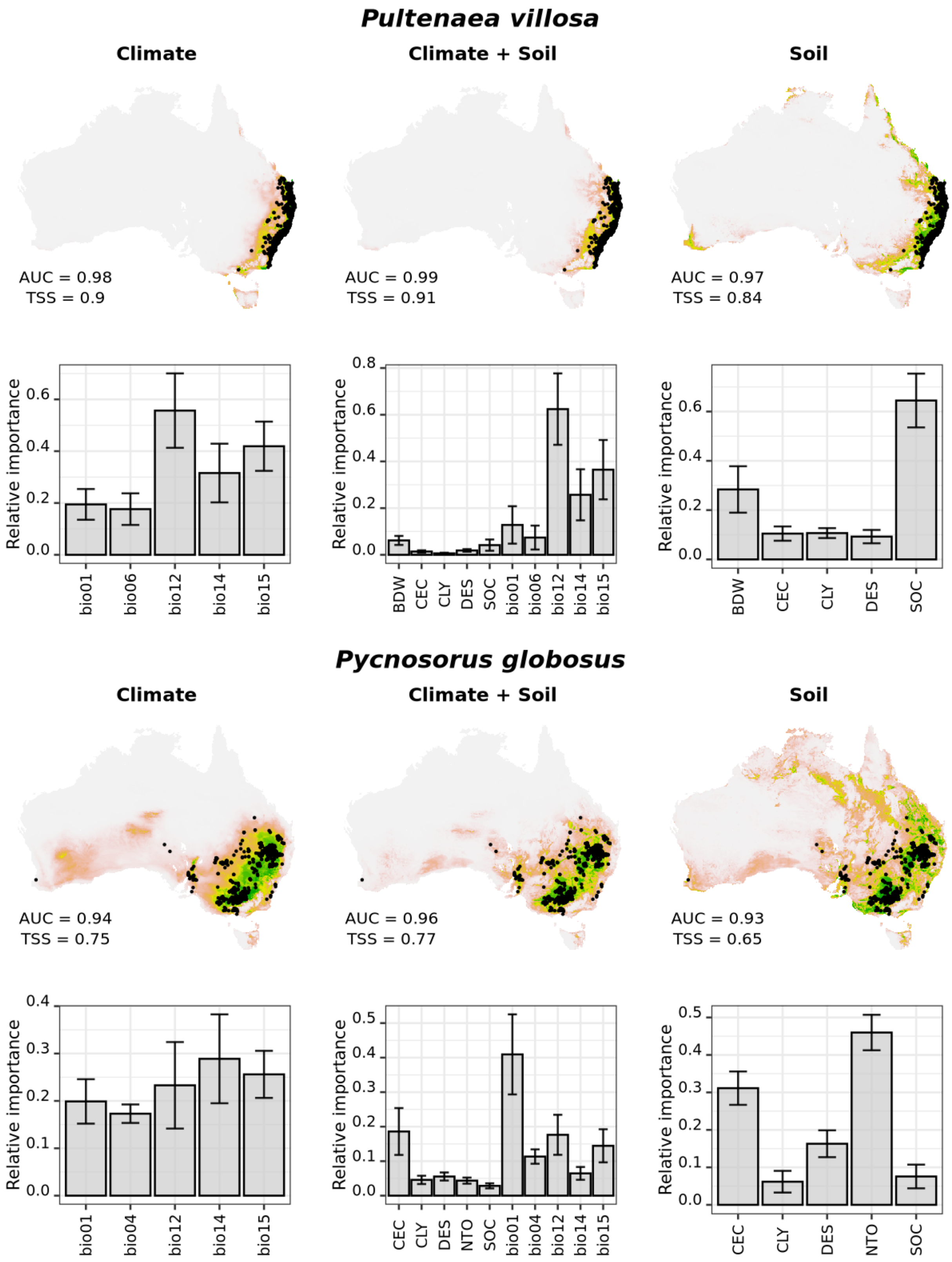
Pultenaea villosa
Climate
Climate + Soil
Soil
AUC = 0.98
TSS = 0.9
AUC = 0.99
TSS = 0.91
AUC = 0.97
TSS = 0.84
Relative importance
0.0
0.2
0.4
0.6
0.8
bio01
bio06
bio12
bio14
bio15
BDW
CEC
CLY
DES
SOC
Pycnosorus globosus
Climate
Climate + Soil
Soil
AUC = 0.94
TSS = 0.75
AUC = 0.96
TSS = 0.77
AUC = 0.93
TSS = 0.65
Relative importance
0.0
0.1
0.2
0.3
0.4
0.5
bio01
bio04
bio12
bio14
bio15
CEC
CLY
DES
NTO
SOC

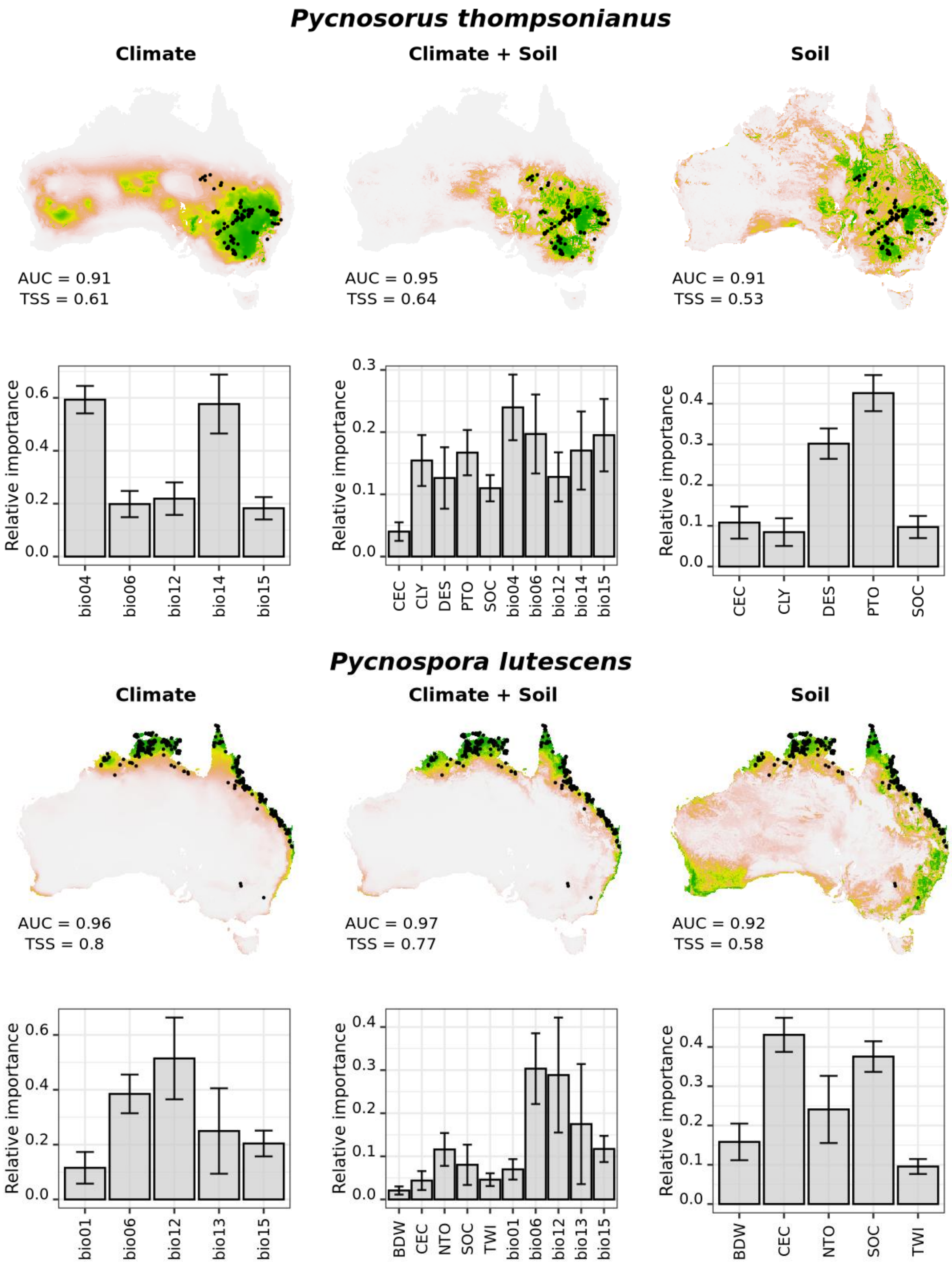
Pycnosorus thompsonianus
Climate
Climate + Soil
Soil
AUC = 0.91
TSS = 0.61
AUC = 0.95
TSS = 0.64
AUC = 0.91
TSS = 0.53
Relative importance
0.0
0.2
0.4
0.6
bio04
bio06
bio12
bio14
bio15
Relative importance
0.0
0.1
0.2
0.3
CEC
CLY
DES
PTO
SOC
bio04
bio06
bio12
bio14
bio15
Relative importance
0.0
0.1
0.2
0.3
0.4
CEC
CLY
DES
PTO
SOC
Pycnospora lutescens
Climate
Climate + Soil
Soil
AUC = 0.96
TSS = 0.8
AUC = 0.97
TSS = 0.77
AUC = 0.92
TSS = 0.58
Relative importance
0.0
0.2
0.4
0.6
bio01
bio06
bio12
bio13
bio15
Relative importance
0.0
0.1
0.2
0.3
0.4
BDW
CEC
NTO
SOC
TWI
bio01
bio06
bio12
bio13
bio15
Relative importance
0.0
0.1
0.2
0.3
0.4
BDW
CEC
NTO
SOC
TWI

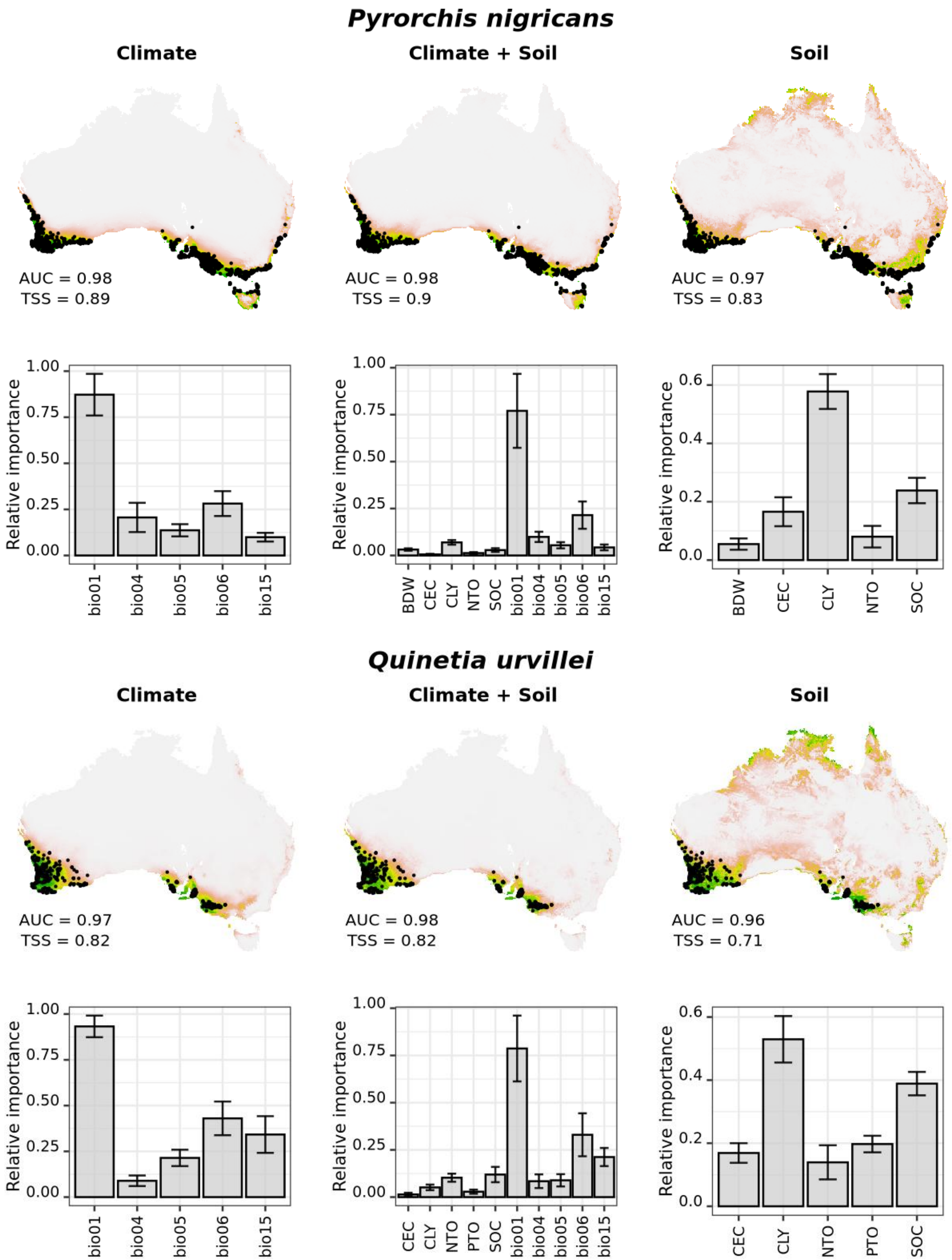

Pyrorchis nigricans
Climate
Climate + Soil
Soil
AUC = 0.98
TSS = 0.89
AUC = 0.98
TSS = 0.9
AUC = 0.97
TSS = 0.83
Relative importance
1.00
0.75
0.50
0.25
0.00
bio01
bio04
bio05
bio06
bio15
BDW
CEC
CLY
NTO
SOC
0.6
0.4
0.2
0.0
Quinetia urvillei
Climate
Climate + Soil
Soil
AUC = 0.97
TSS = 0.82
AUC = 0.98
TSS = 0.82
AUC = 0.96
TSS = 0.71
PTO

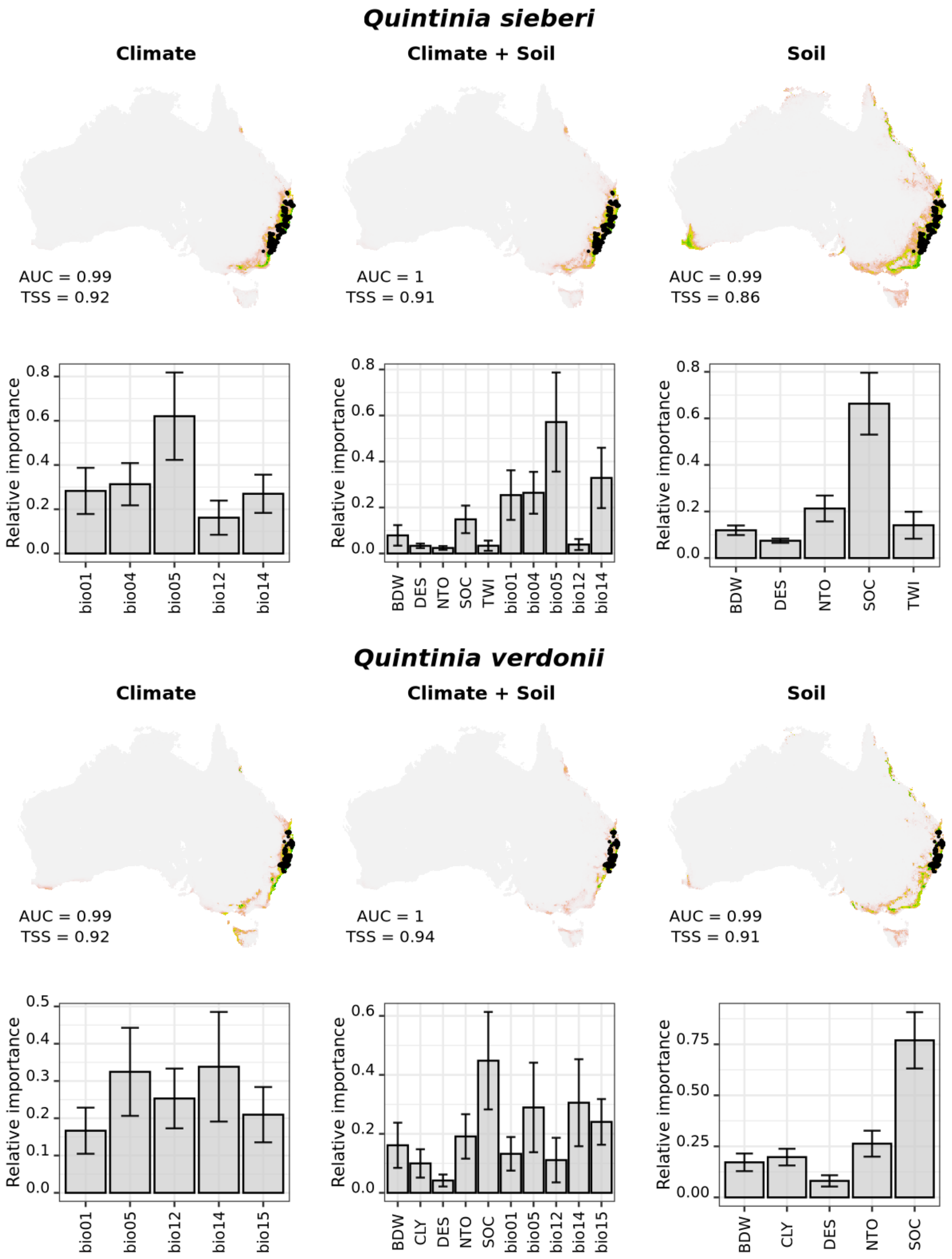

Quintinia sieberi
Climate
Climate + Soil
Soil
AUC = 0.99
TSS = 0.92
AUC = 1
TSS = 0.91
AUC = 0.99
TSS = 0.86
Relative importance
bio01
bio04
bio05
bio12
bio14
BDW
DES
NTO
SOC
TWI
Quintinia verdonii
Climate
Climate + Soil
Soil
AUC = 0.99
TSS = 0.92
AUC = 1
TSS = 0.94
AUC = 0.99
TSS = 0.91
Relative importance
bio01
bio05
bio12
bio14
bio15
BDW
CLY
DES
NTO
SOC

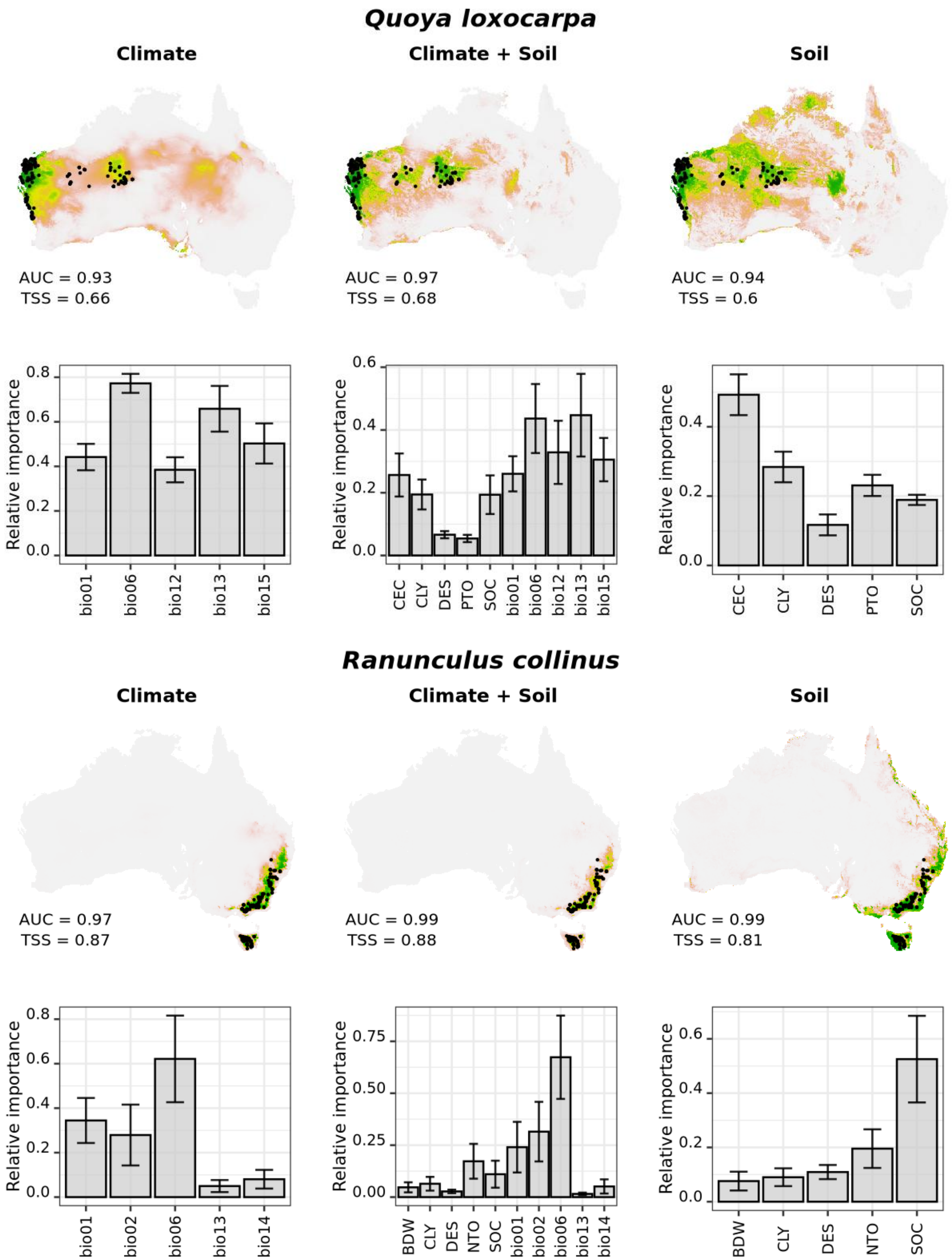
Quoya loxocarpa
Climate
Climate + Soil
Soil
AUC = 0.93
TSS = 0.66
AUC = 0.97
TSS = 0.68
AUC = 0.94
TSS = 0.6
Relative importance
bio01
bio06
bio12
bio13
bio15
CEC
CLY
DES
PTO
SOC
Ranunculus collinus
Climate
Climate + Soil
Soil
AUC = 0.97
TSS = 0.87
AUC = 0.99
TSS = 0.88
AUC = 0.99
TSS = 0.81
Relative importance
bio01
bio02
bio06
bio13
bio14
BDW
CLY
DES
NTO
SOC

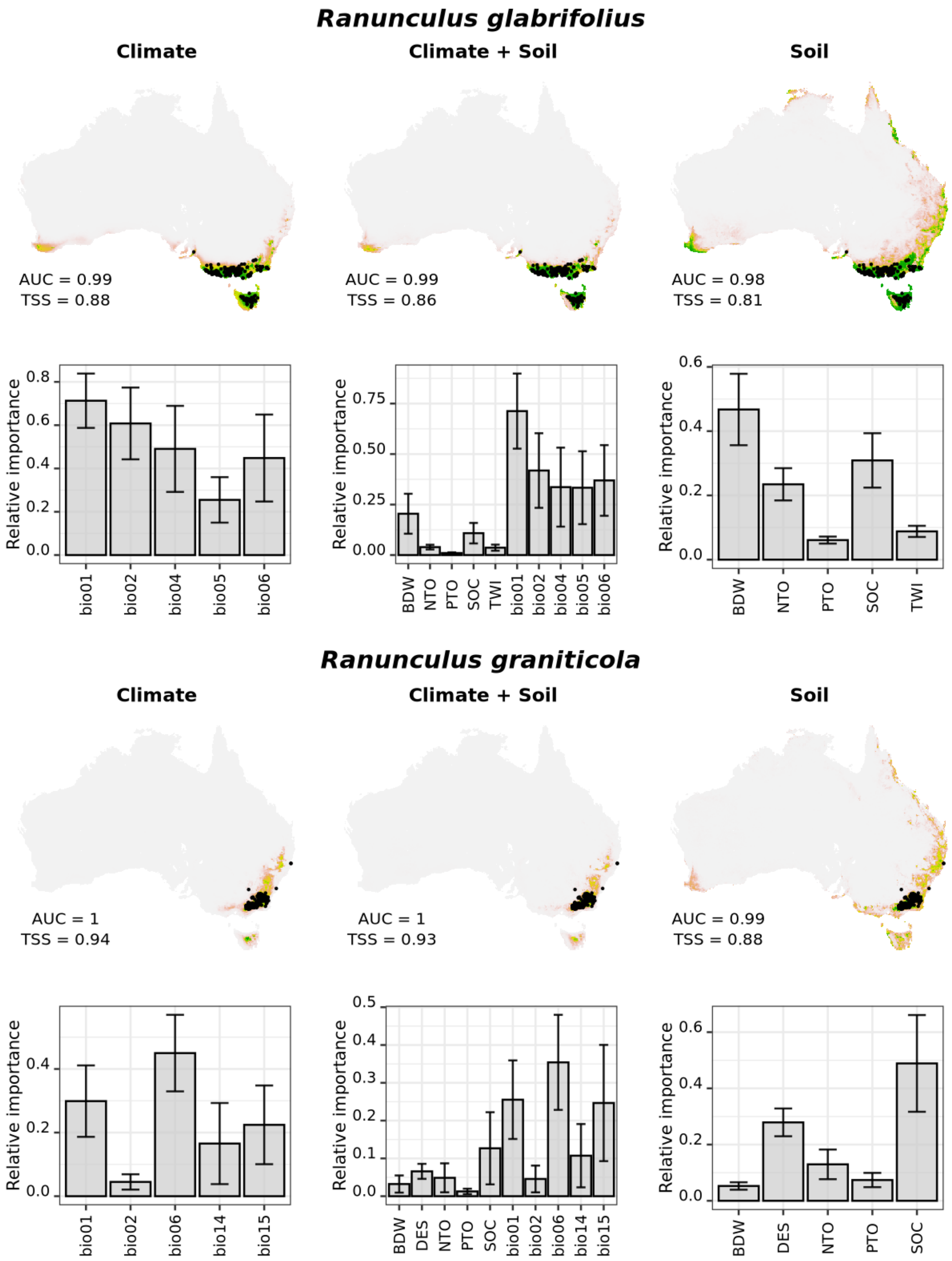
Ranunculus glabrifolius
Climate
Climate + Soil
Soil
AUC = 0.99
TSS = 0.88
AUC = 0.99
TSS = 0.86
AUC = 0.98
TSS = 0.81
Relative importance
0.0
0.2
0.4
0.6
0.8
bio01
bio02
bio04
bio05
bio06
0.00
0.25
0.50
0.75
BDW
NTO
PTO
SOC
TWI
bio01
bio02
bio04
bio05
bio06
0.0
0.2
0.4
0.6
BDW
NTO
PTO
SOC
TWI
Ranunculus graniticola
Climate
Climate + Soil
Soil
AUC = 1
TSS = 0.94
AUC = 1
TSS = 0.93
AUC = 0.99
TSS = 0.88
Relative importance
0.0
0.2
0.4
bio01
bio02
bio06
bio14
bio15
0.0
0.1
0.2
0.3
0.4
0.5
BDW
DES
NTO
PTO
SOC
bio01
bio02
bio06
bio14
bio15
0.0
0.2
0.4
0.6
BDW
DES
NTO
PTO
SOC

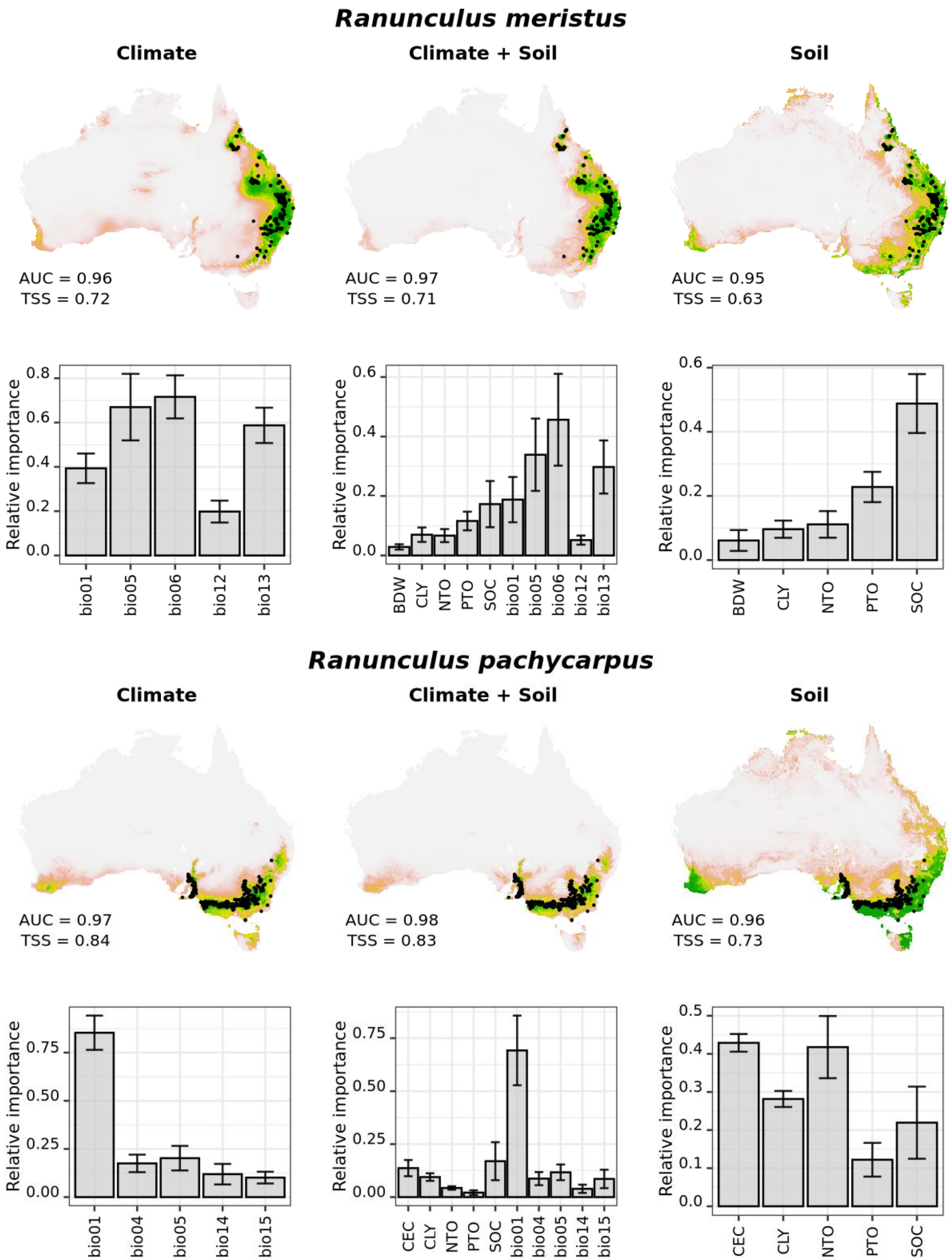
Ranunculus meristus
Climate
Climate + Soil
Soil
AUC = 0.96
TSS = 0.72
AUC = 0.97
TSS = 0.71
AUC = 0.95
TSS = 0.63
Relative importance
bio01
bio05
bio06
bio12
bio13
BDW
CLY
NTO
PTO
SOC
Ranunculus pachycarpus
Climate
Climate + Soil
Soil
AUC = 0.97
TSS = 0.84
AUC = 0.98
TSS = 0.83
AUC = 0.96
TSS = 0.73
Relative importance
bio01
bio04
bio05
bio14
bio15
CEC
CLY
NTO
PTO
SOC

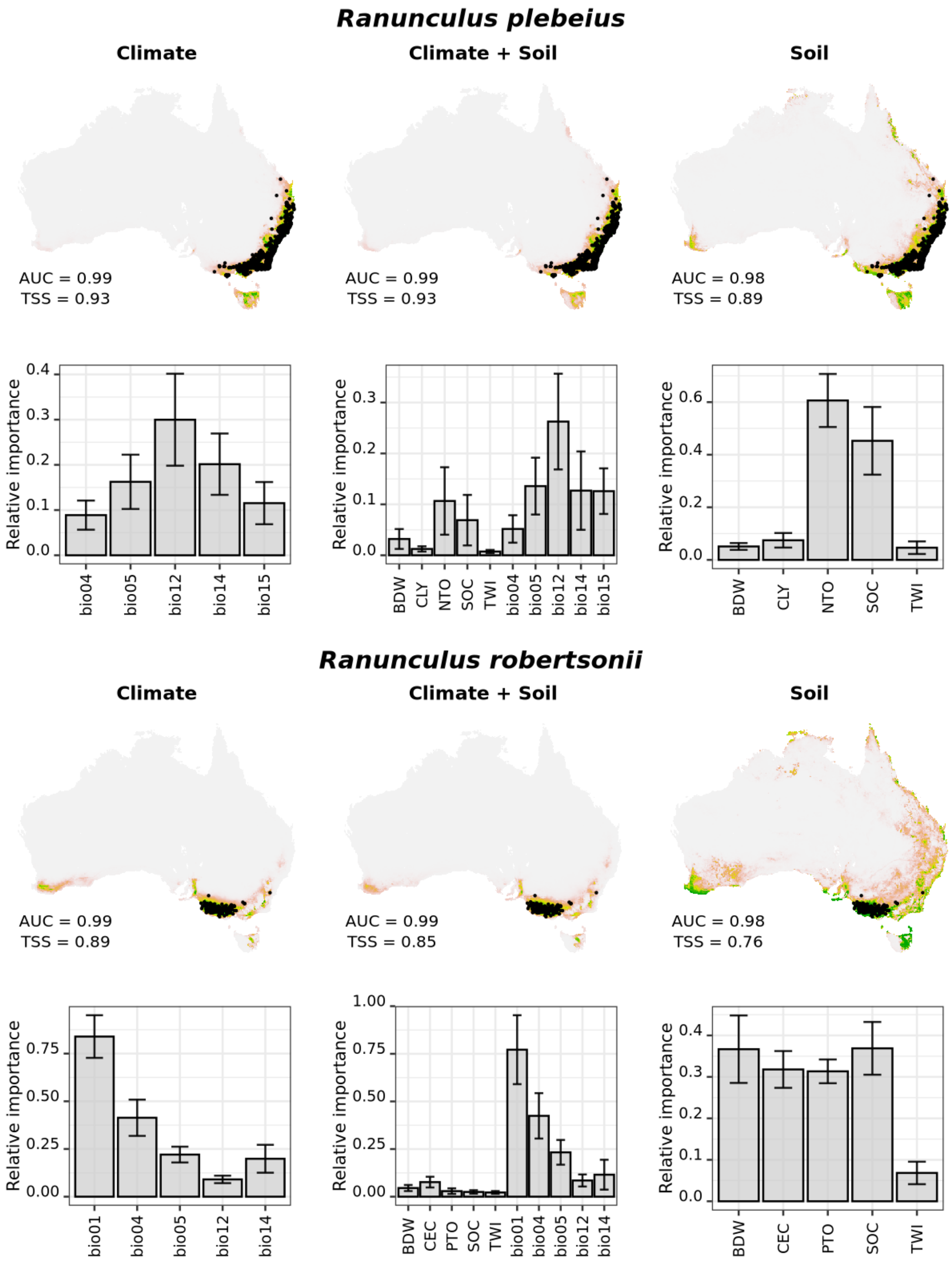

Ranunculus plebeius
Climate
Climate + Soil
Soil
AUC = 0.99
TSS = 0.93
AUC = 0.99
TSS = 0.93
AUC = 0.98
TSS = 0.89
Relative importance
bio04 bio05 bio12 bio14 bio15
BDW CLY NTO SOC TWI bio04 bio05 bio12 bio14 bio15
BDW CLY NTO SOC TWI
Ranunculus robertsonii
Climate
Climate + Soil
Soil
AUC = 0.99
TSS = 0.89
AUC = 0.99
TSS = 0.85
AUC = 0.98
TSS = 0.76
Relative importance
bio01 bio04 bio05 bio12 bio14
BDW CEC PTO SOC TWI bio01 bio04 bio05 bio12 bio14
BDW CEC PTO SOC TWI

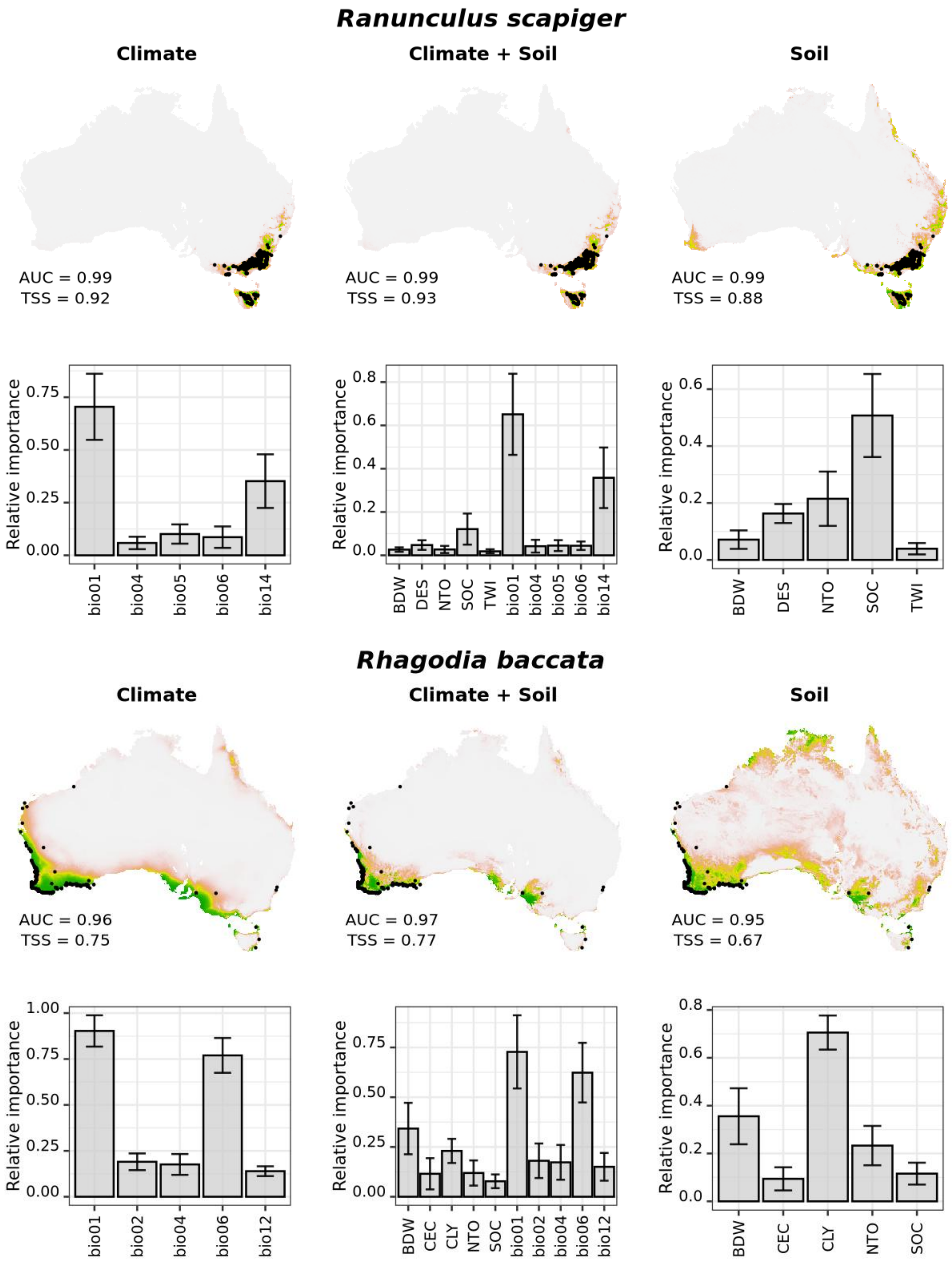
Ranunculus scapiger
Climate
Climate + Soil
Soil
AUC = 0.99
TSS = 0.92
AUC = 0.99
TSS = 0.93
AUC = 0.99
TSS = 0.88
Relative importance
bio01
bio04
bio05
bio06
bio14
BDW
DES
NTO
SOC
TWI
Rhagodia baccata
AUC = 0.96
TSS = 0.75
AUC = 0.97
TSS = 0.77
AUC = 0.95
TSS = 0.67
bio02
bio12
CEC
CLY

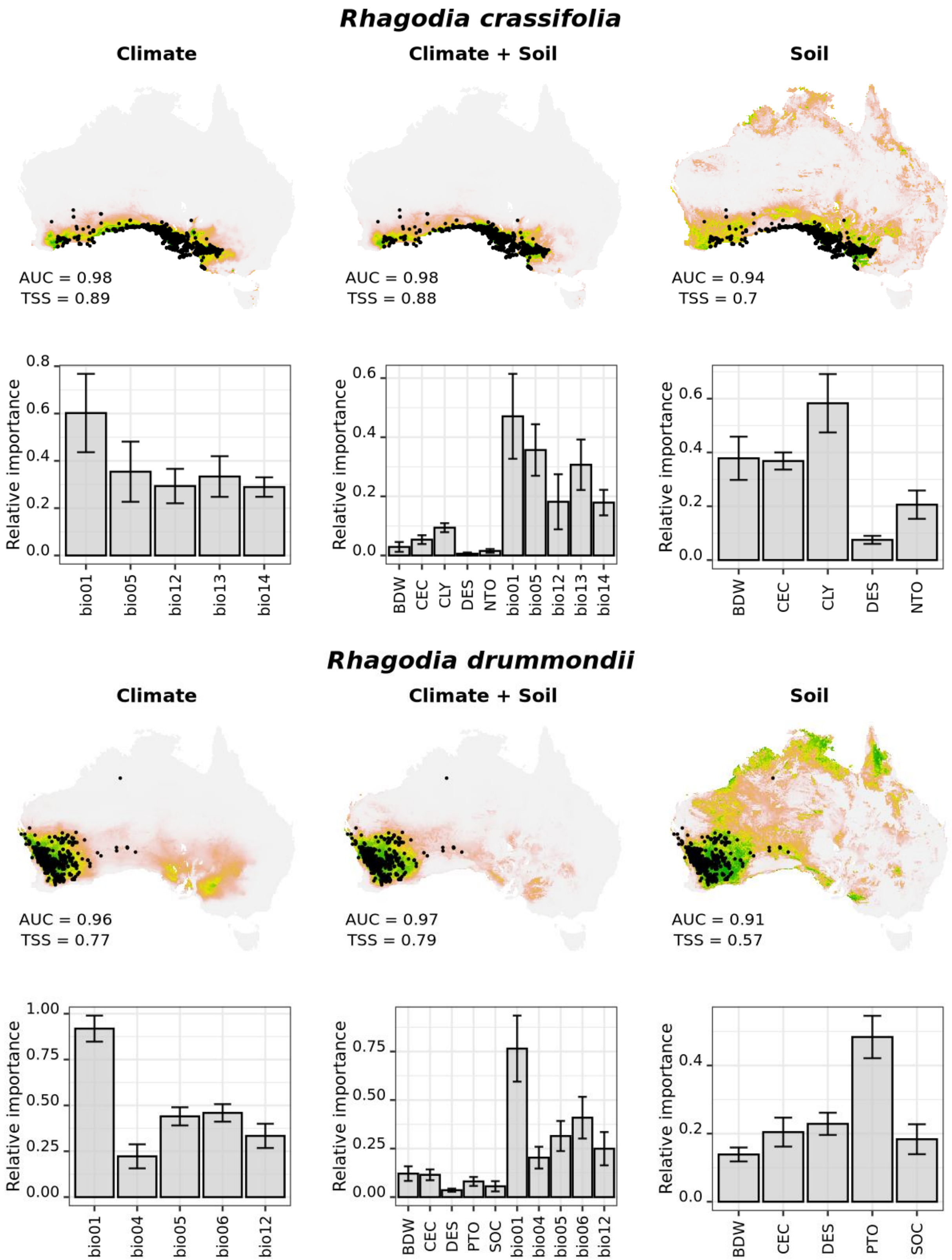

Rhagodia crassifolia
Climate
Climate + Soil
Soil
AUC = 0.98
TSS = 0.89
AUC = 0.98
TSS = 0.88
AUC = 0.94
TSS = 0.7
Relative importance
0.0
0.2
0.4
0.6
0.8
bio01
bio05
bio12
bio13
bio14
BDW
CEC
CLY
DES
NTO
Rhagodia drummondii
Climate
Climate + Soil
Soil
AUC = 0.96
TSS = 0.77
AUC = 0.97
TSS = 0.79
AUC = 0.91
TSS = 0.57
Relative importance
0.00
0.25
0.50
0.75
1.00
bio01
bio04
bio05
bio06
bio12
BDW
CEC
DES
PTO
SOC

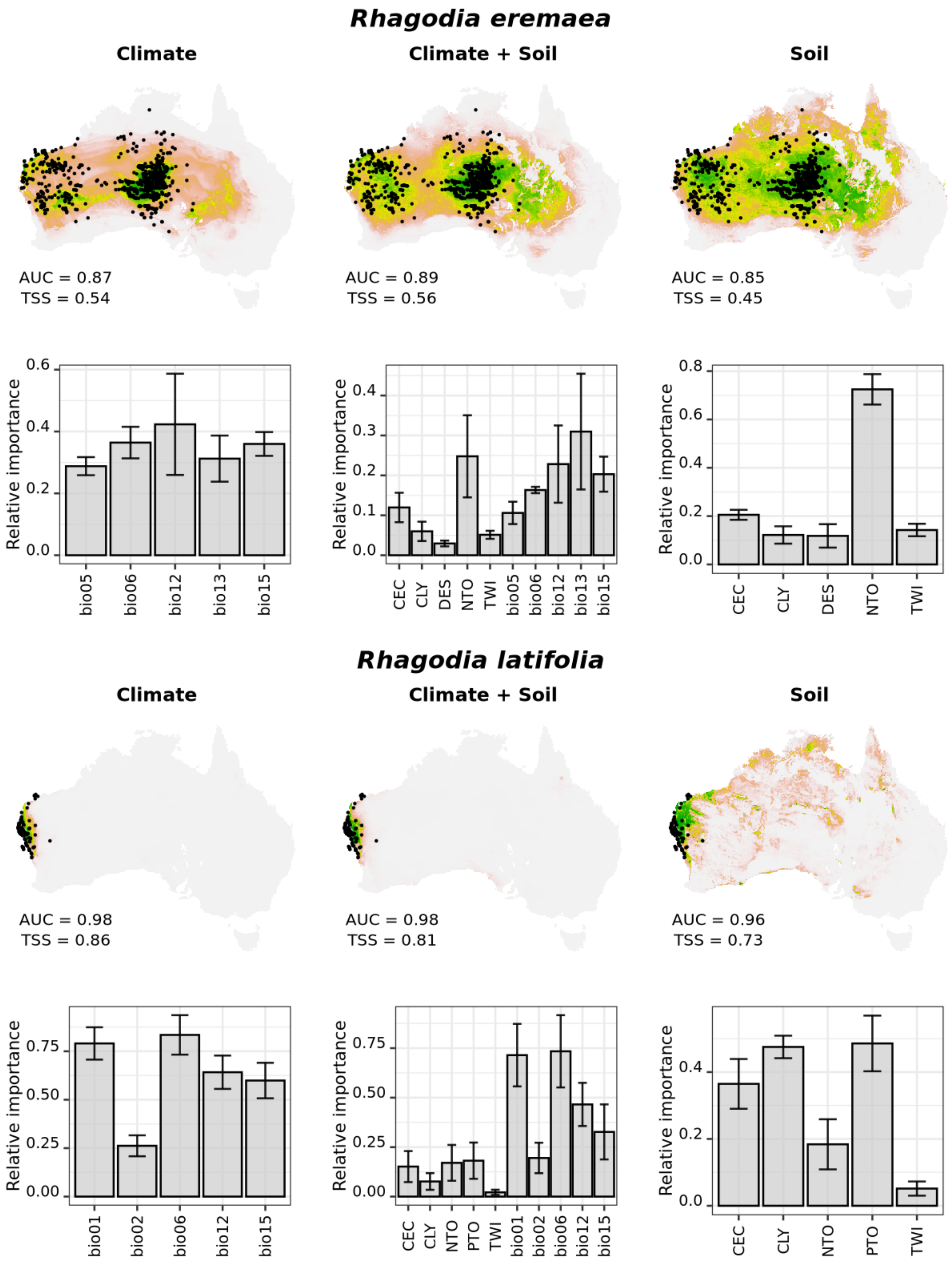
Rhagodia eremaea
Climate
Climate + Soil
Soil
AUC = 0.87
TSS = 0.54
AUC = 0.89
TSS = 0.56
AUC = 0.85
TSS = 0.45
Relative importance
bio05
bio06
bio12
bio13
bio15
CEC
CLY
DES
NTO
TWI
Rhagodia latifolia
AUC = 0.98
TSS = 0.86
AUC = 0.98
TSS = 0.81
AUC = 0.96
TSS = 0.73
bio01
bio02
PTO

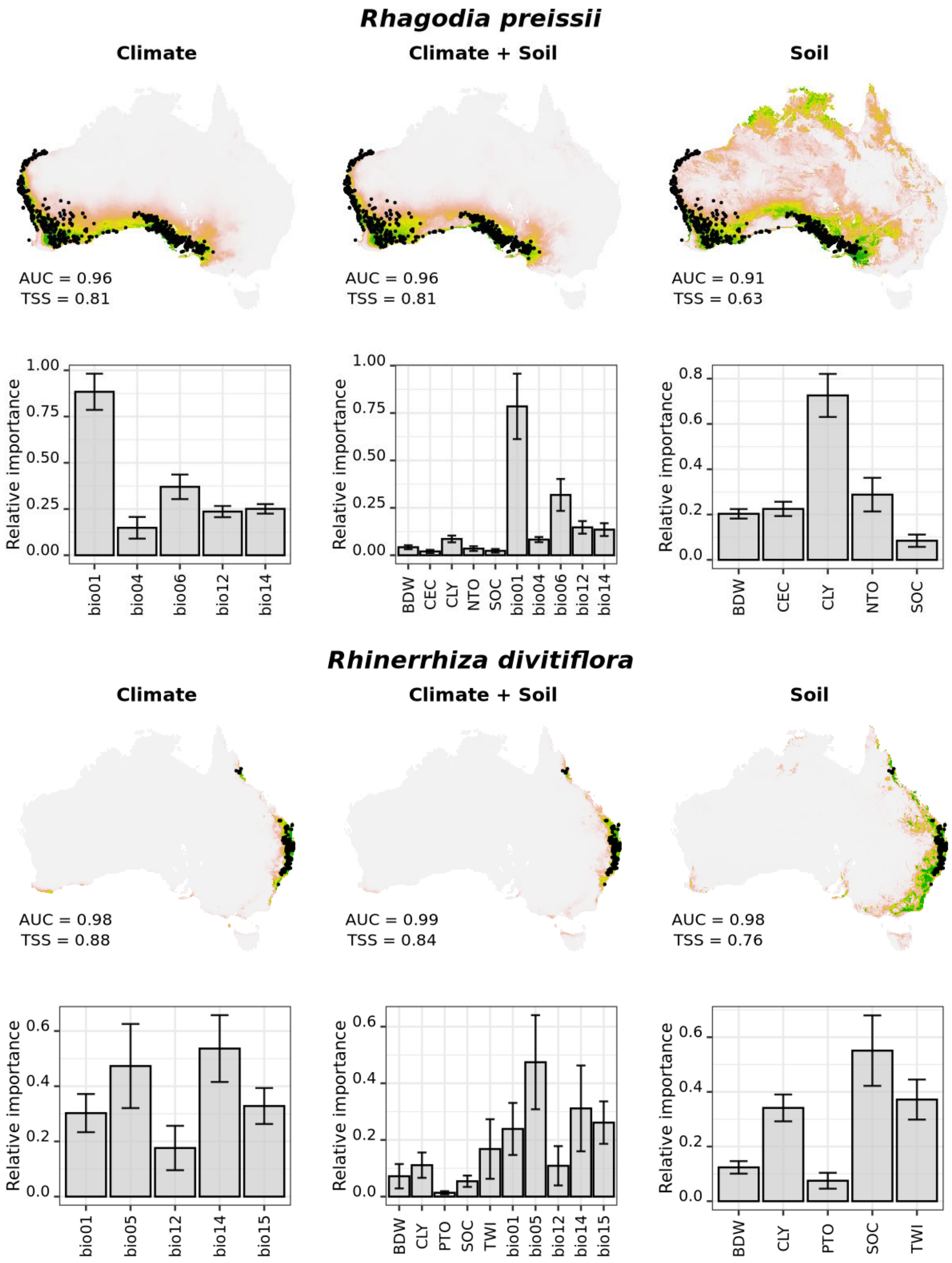
Rhagodia preissii
Climate
Climate + Soil
Soil
AUC = 0.96
TSS = 0.81
AUC = 0.96
TSS = 0.81
AUC = 0.91
TSS = 0.63
Relative importance
1.00
0.75
0.50
0.25
0.00
bio01
bio04
bio06
bio12
bio14
BDW
CEC
CLY
NTO
SOC
0.8
0.6
0.4
0.2
0.0
Rhinerrhiza divitiflora
Climate
Climate + Soil
Soil
AUC = 0.98
TSS = 0.88
AUC = 0.99
TSS = 0.84
AUC = 0.98
TSS = 0.76
bio01
bio05
bio12
bio14
bio15
BDW
CLY
PTO
SOC
TWI

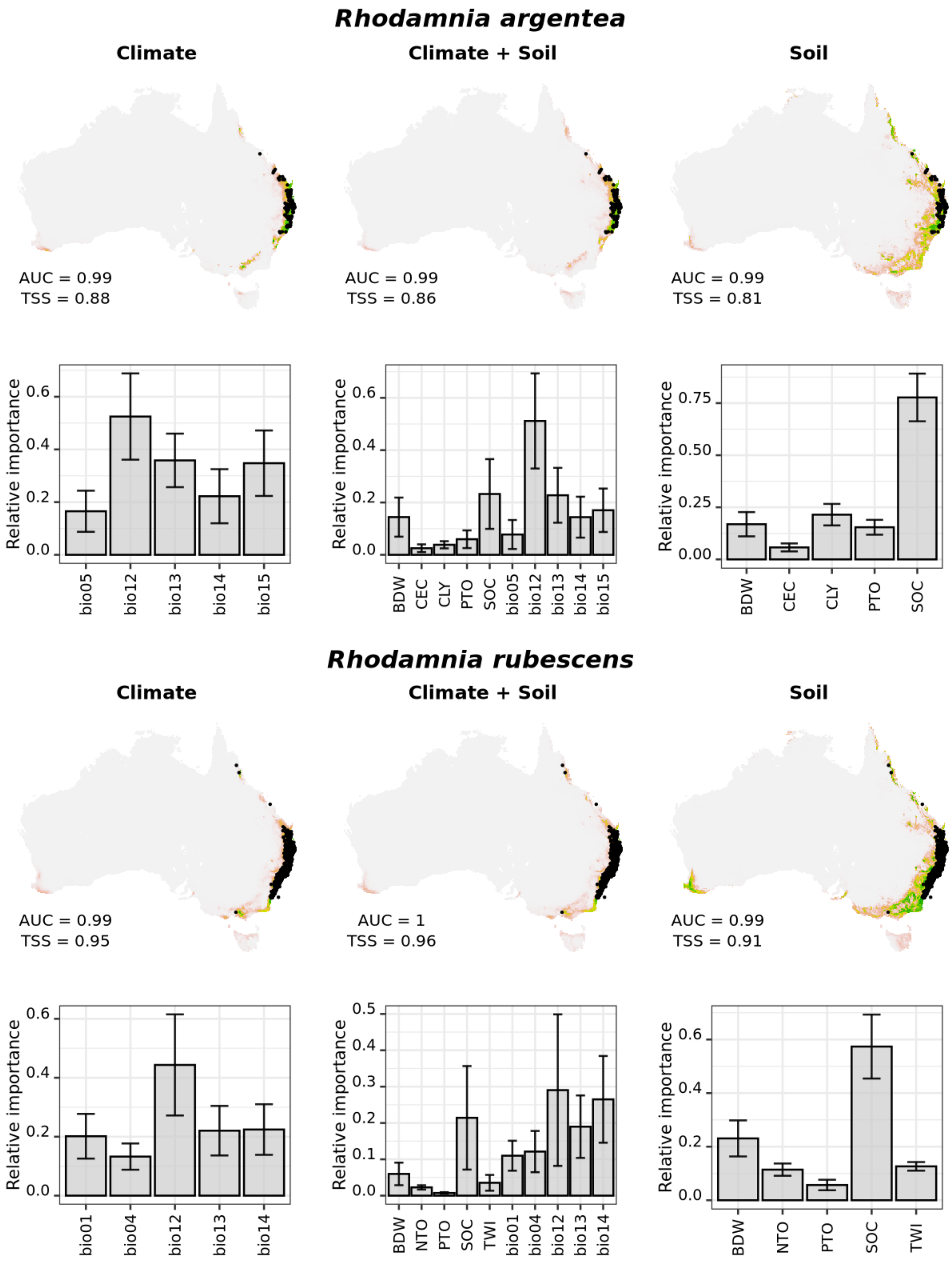

Rhodamnia argentea
Climate
Climate + Soil
Soil
AUC = 0.99
TSS = 0.88
AUC = 0.99
TSS = 0.86
AUC = 0.99
TSS = 0.81
Relative importance
0.0
0.2
0.4
0.6
bio05
bio12
bio13
bio14
bio15
BDW
CEC
CLY
PTO
SOC
0.00
0.25
0.50
0.75
Rhodamnia rubescens
Climate
Climate + Soil
Soil
AUC = 0.99
TSS = 0.95
AUC = 1
TSS = 0.96
AUC = 0.99
TSS = 0.91
0.1
0.3
0.5
bio01
bio04
NTO
TWI

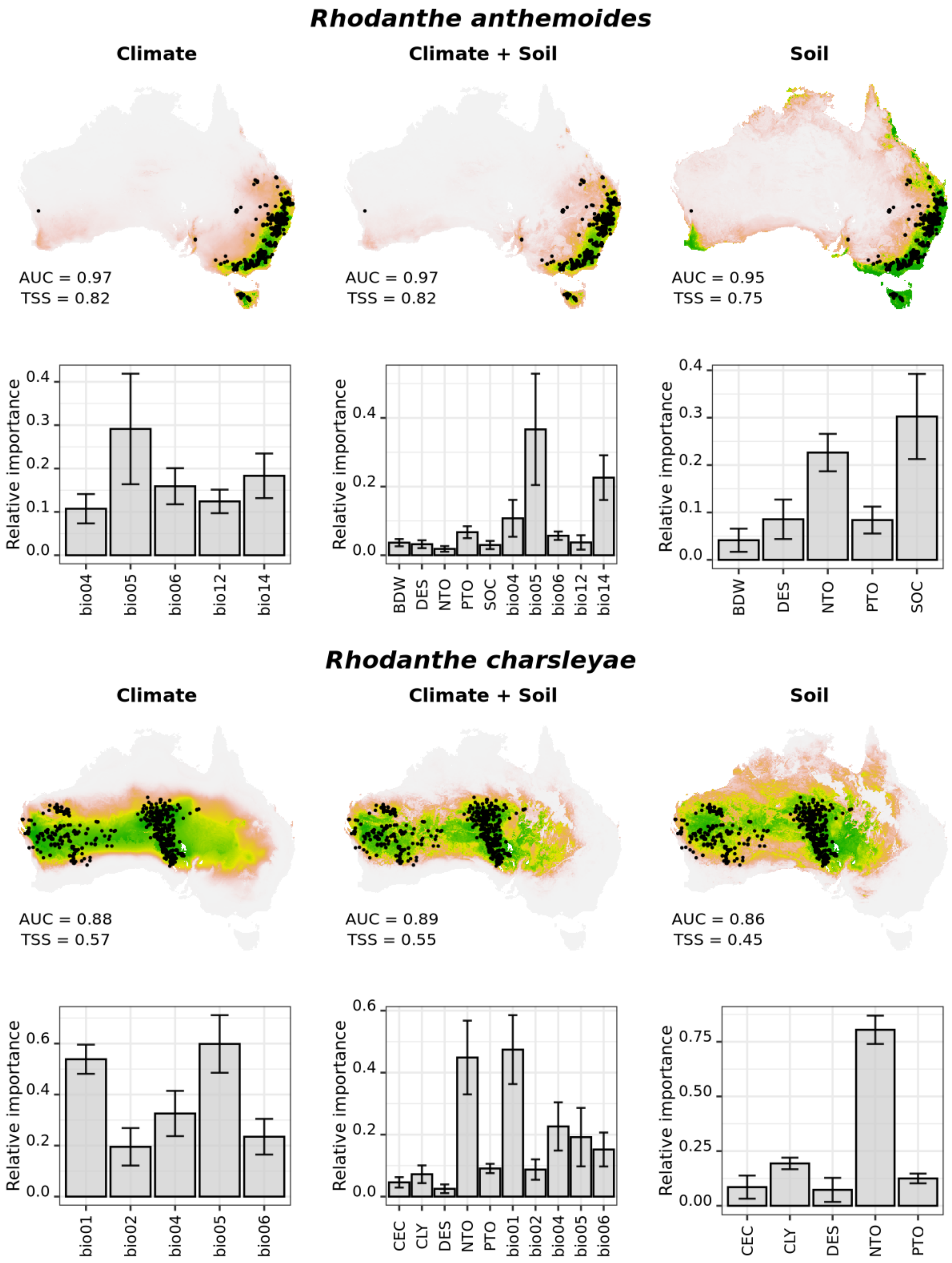

Rhodanthe anthemoides
Climate
Climate + Soil
Soil
AUC = 0.97
TSS = 0.82
AUC = 0.97
TSS = 0.82
AUC = 0.95
TSS = 0.75
Relative importance
bio04
bio05
bio06
bio12
bio14
BDW
DES
NTO
PTO
SOC
Rhodanthe charsleyae
Climate
Climate + Soil
Soil
AUC = 0.88
TSS = 0.57
AUC = 0.89
TSS = 0.55
AUC = 0.86
TSS = 0.45
Relative importance
bio01
bio02
bio04
bio05
bio06
CEC
CLY
DES
NTO
PTO

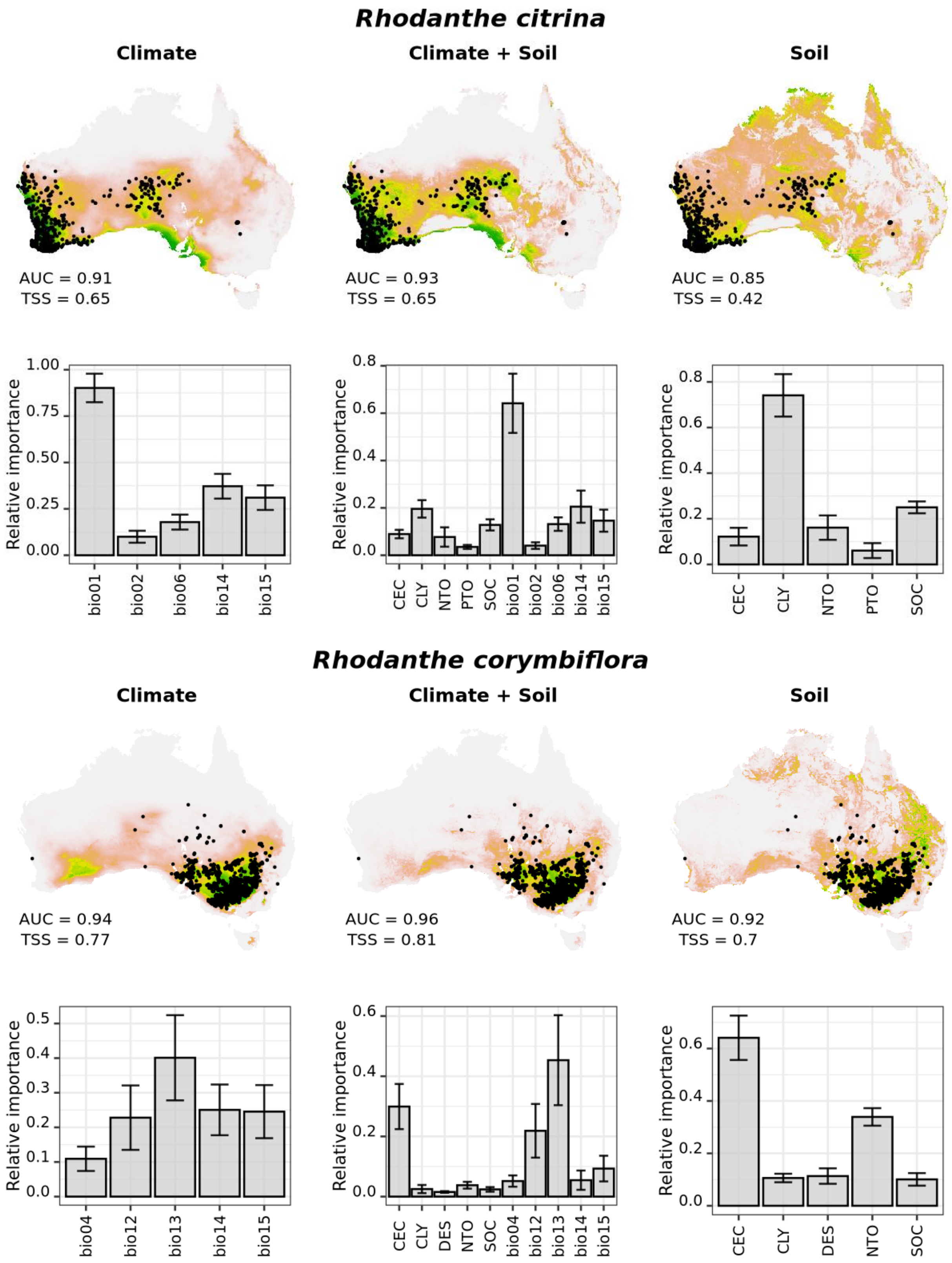

Rhodanthe citrina
Climate
Climate + Soil
Soil
AUC = 0.91
TSS = 0.65
AUC = 0.93
TSS = 0.65
AUC = 0.85
TSS = 0.42
Relative importance
bio01
bio02
bio06
bio14
bio15
CEC
CLY
NTO
PTO
SOC
Rhodanthe corymbiflora
Climate
Climate + Soil
Soil
AUC = 0.94
TSS = 0.77
AUC = 0.96
TSS = 0.81
AUC = 0.92
TSS = 0.7
Relative importance
bio04
bio12
bio13
bio14
bio15
CEC
CLY
DES
NTO
SOC

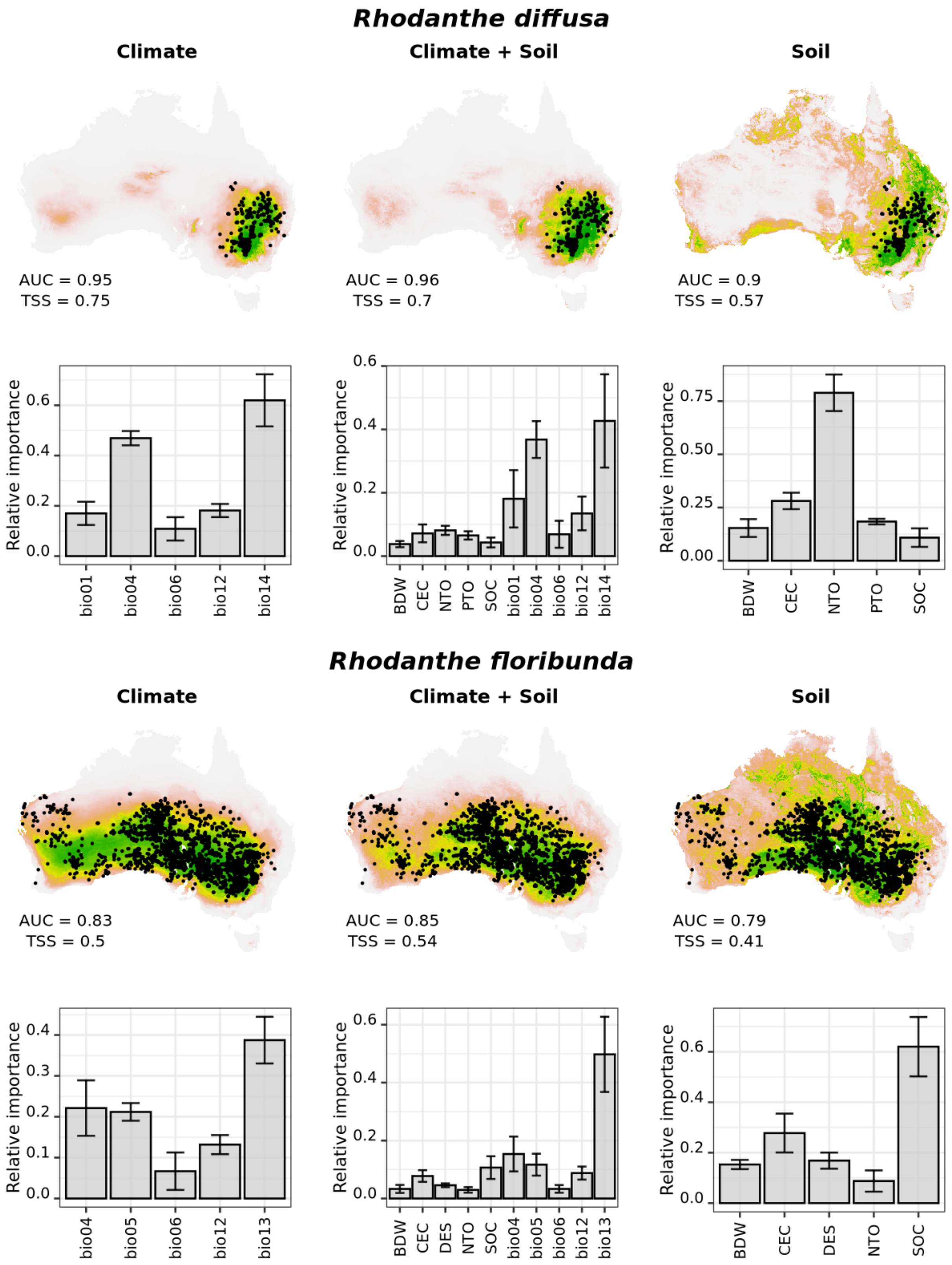

Rhodanthe diffusa
Climate
Climate + Soil
Soil
AUC = 0.95
TSS = 0.75
AUC = 0.96
TSS = 0.7
AUC = 0.9
TSS = 0.57
Relative importance
0.0
0.2
0.4
0.6
bio01
bio04
bio06
bio12
bio14
BDW
CEC
NTO
PTO
SOC
0.00
0.25
0.50
0.75
Rhodanthe floribunda
AUC = 0.83
TSS = 0.5
AUC = 0.85
TSS = 0.54
AUC = 0.79
TSS = 0.41
0.1
0.3
bio05
bio13
DES

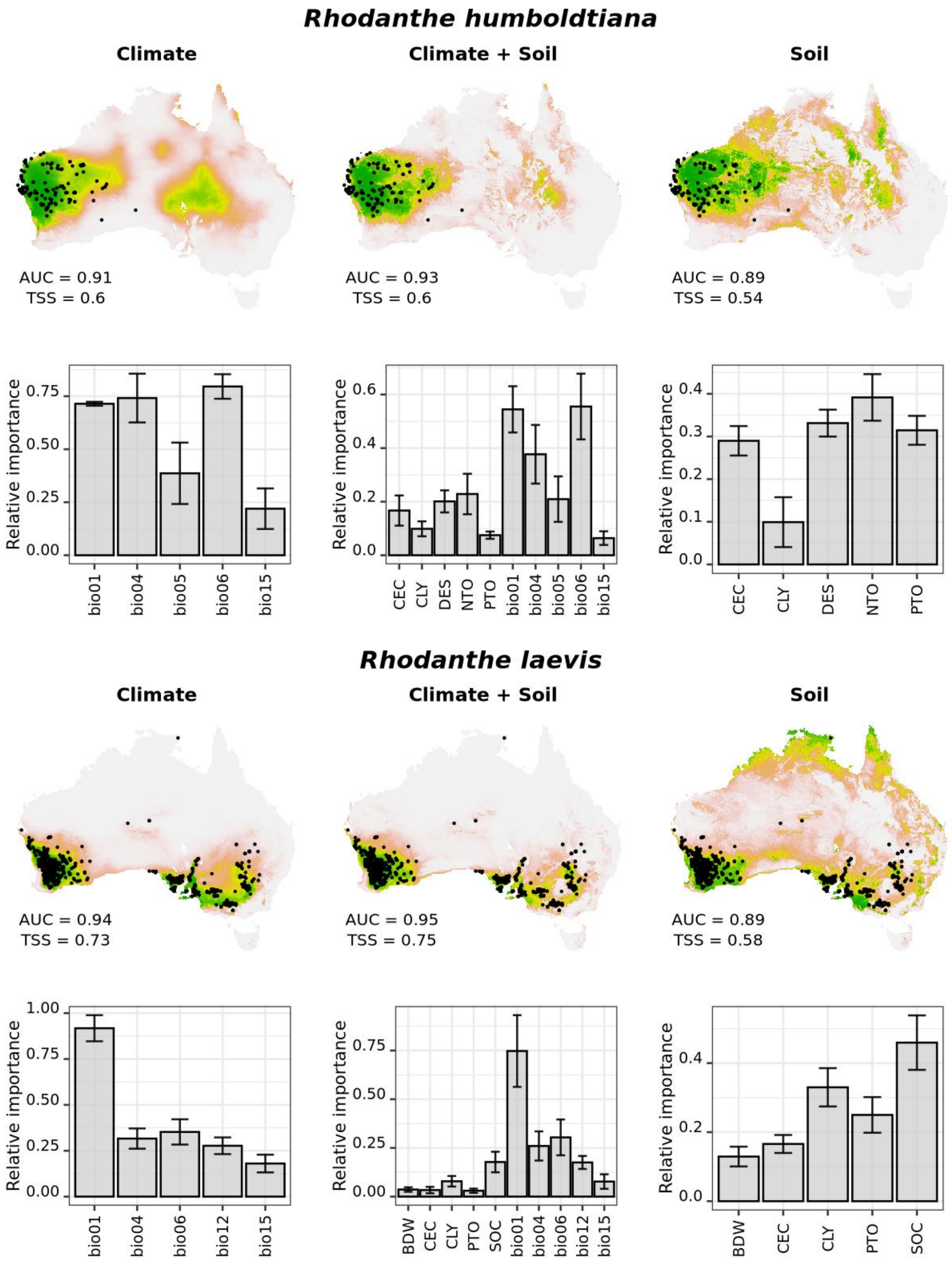
Rhodanthe humboldtiana
Climate
Climate + Soil
Soil
AUC = 0.91
TSS = 0.6
AUC = 0.93
TSS = 0.6
AUC = 0.89
TSS = 0.54
Relative importance
0.00
0.25
0.50
0.75
bio01
bio04
bio05
bio06
bio15
0.0
0.2
0.4
0.6
CEC
CLY
DES
NTO
PTO
bio01
bio04
bio05
bio06
bio15
0.0
0.1
0.2
0.3
0.4
CEC
CLY
DES
NTO
PTO
Rhodanthe laevis
Climate
Climate + Soil
Soil
AUC = 0.94
TSS = 0.73
AUC = 0.95
TSS = 0.75
AUC = 0.89
TSS = 0.58
Relative importance
0.00
0.25
0.50
0.75
1.00
bio01
bio04
bio06
bio12
bio15
0.00
0.25
0.50
0.75
BDW
CEC
CLY
PTO
SOC
bio01
bio04
bio06
bio12
bio15
0.0
0.2
0.4
BDW
CEC
CLY
PTO
SOC

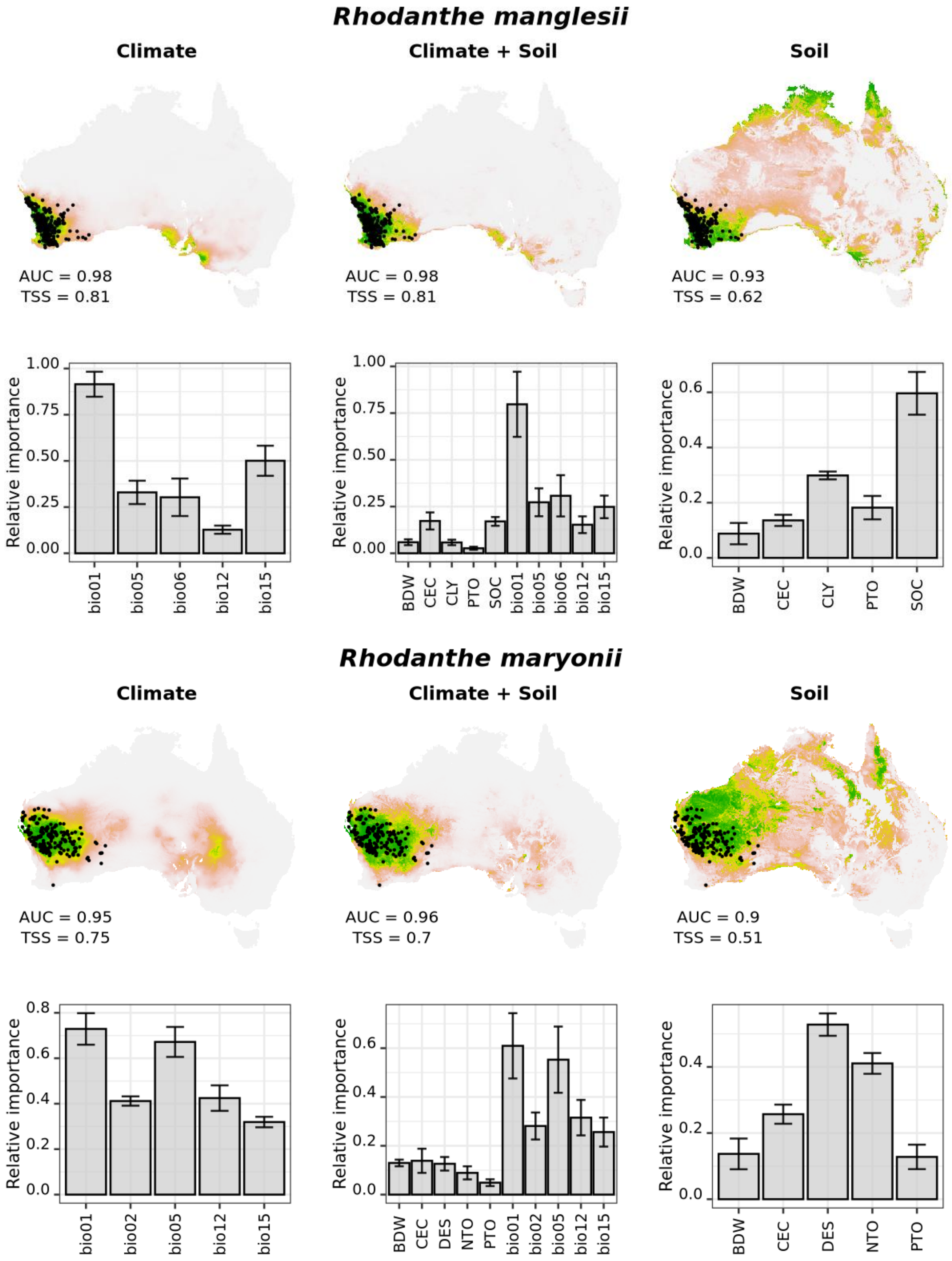
Rhodanthe manglesii
Climate
Climate + Soil
Soil
AUC = 0.98
TSS = 0.81
AUC = 0.98
TSS = 0.81
AUC = 0.93
TSS = 0.62
Relative importance
1.00
0.75
0.50
0.25
0.00
bio01
bio05
bio06
bio12
bio15
BDW
CEC
CLY
PTO
SOC
0.6
0.4
0.2
0.0
Rhodanthe maryonii
Climate
Climate + Soil
Soil
AUC = 0.95
TSS = 0.75
AUC = 0.96
TSS = 0.7
AUC = 0.9
TSS = 0.51
0.8
bio02
DES
NTO

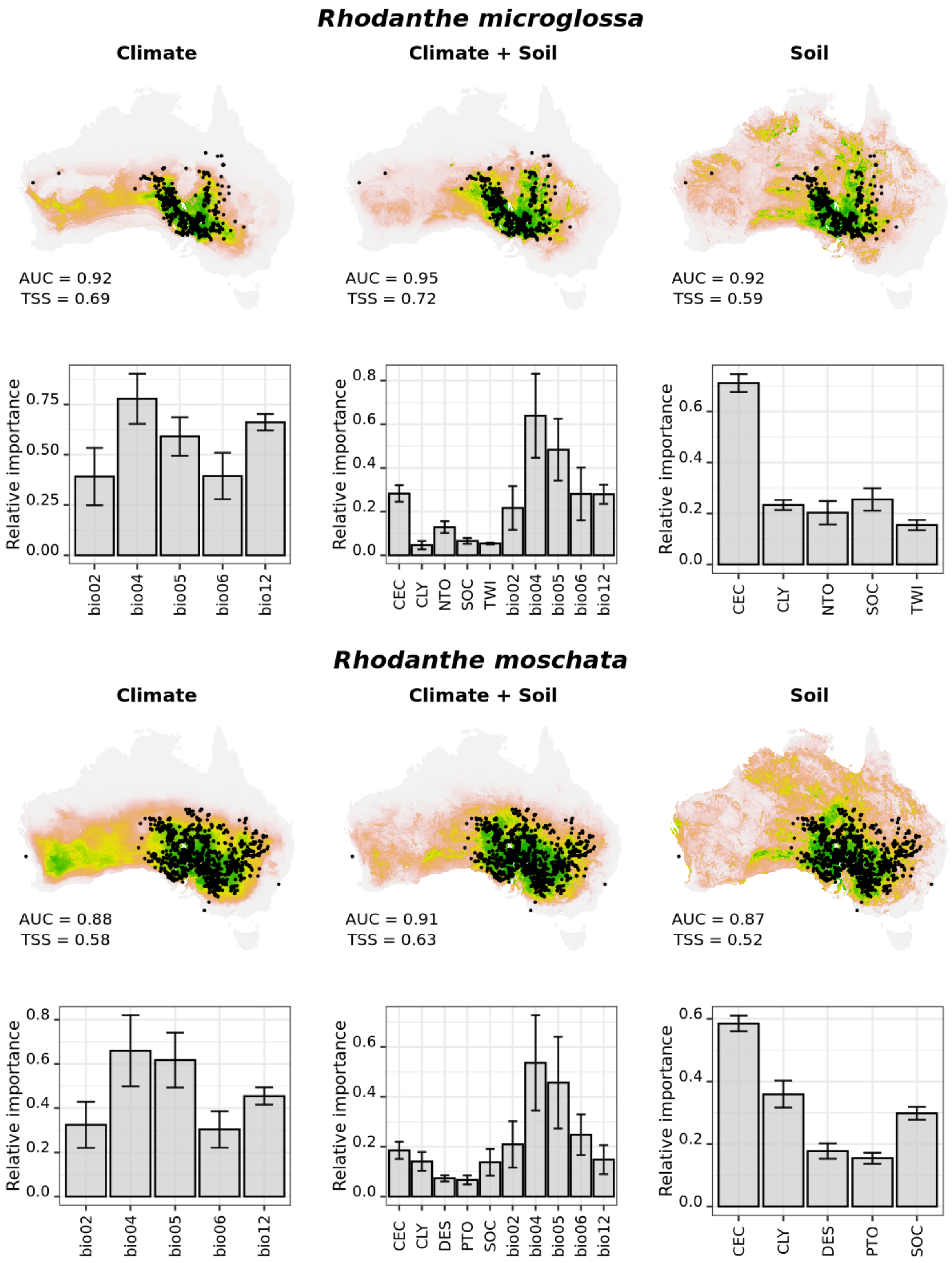
Rhodanthe microglossa
Climate
Climate + Soil
Soil
AUC = 0.92
TSS = 0.69
AUC = 0.95
TSS = 0.72
AUC = 0.92
TSS = 0.59
Relative importance
bio02
bio04
bio05
bio06
bio12
CEC
CLY
NTO
SOC
TWI
Rhodanthe moschata
Climate
Climate + Soil
Soil
AUC = 0.88
TSS = 0.58
AUC = 0.91
TSS = 0.63
AUC = 0.87
TSS = 0.52
Relative importance
bio02
bio04
bio05
bio06
bio12
CEC
CLY
DES
PTO
SOC

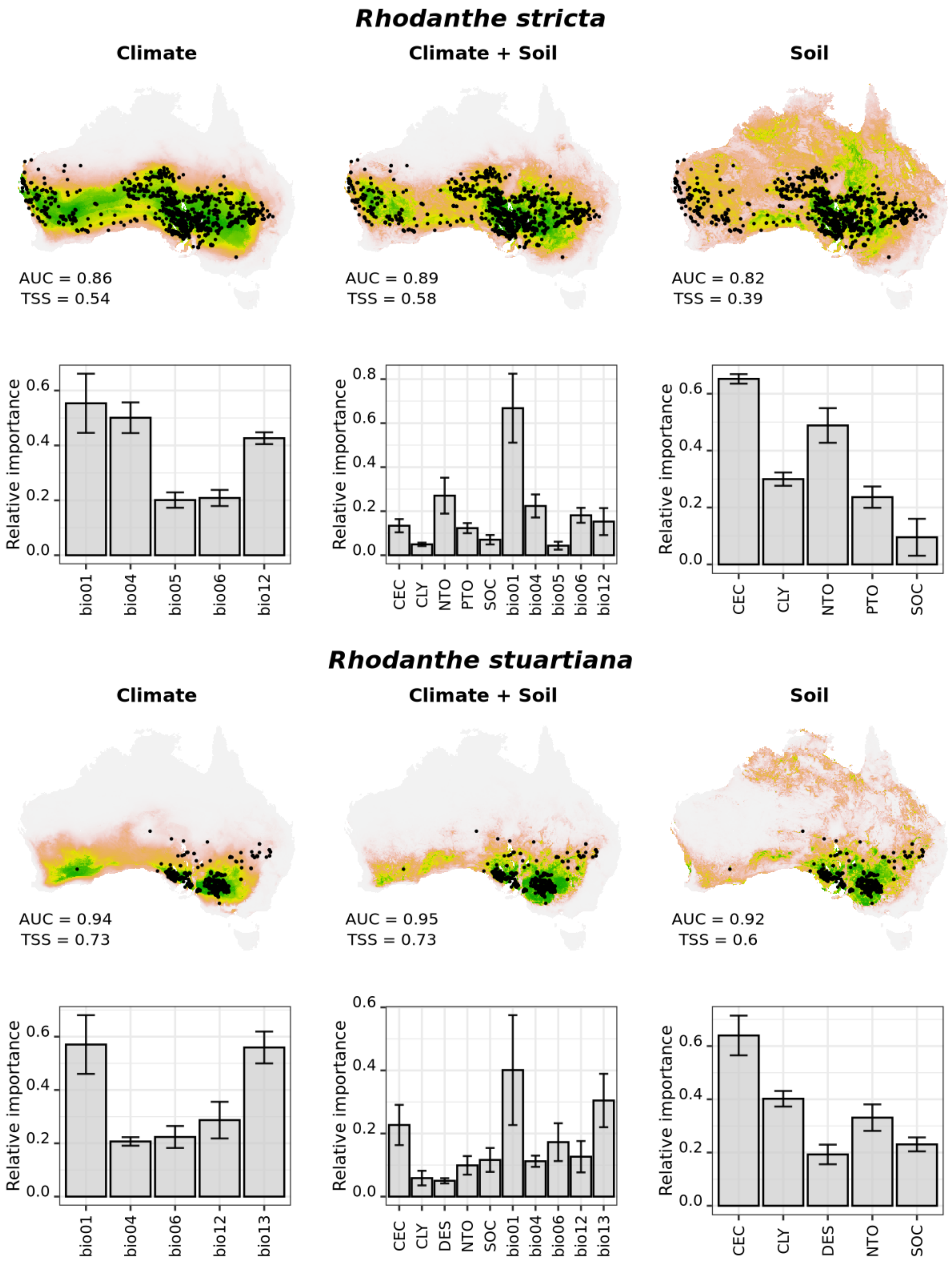
Rhodanthe stricta
Climate
Climate + Soil
Soil
AUC = 0.86
TSS = 0.54
AUC = 0.89
TSS = 0.58
AUC = 0.82
TSS = 0.39
Relative importance
bio01
bio04
bio05
bio06
bio12
CEC
CLY
NTO
PTO
SOC
Rhodanthe stuartiana
Climate
Climate + Soil
Soil
AUC = 0.94
TSS = 0.73
AUC = 0.95
TSS = 0.73
AUC = 0.92
TSS = 0.6
Relative importance
bio01
bio04
bio06
bio12
bio13
CEC
CLY
DES
NTO
SOC

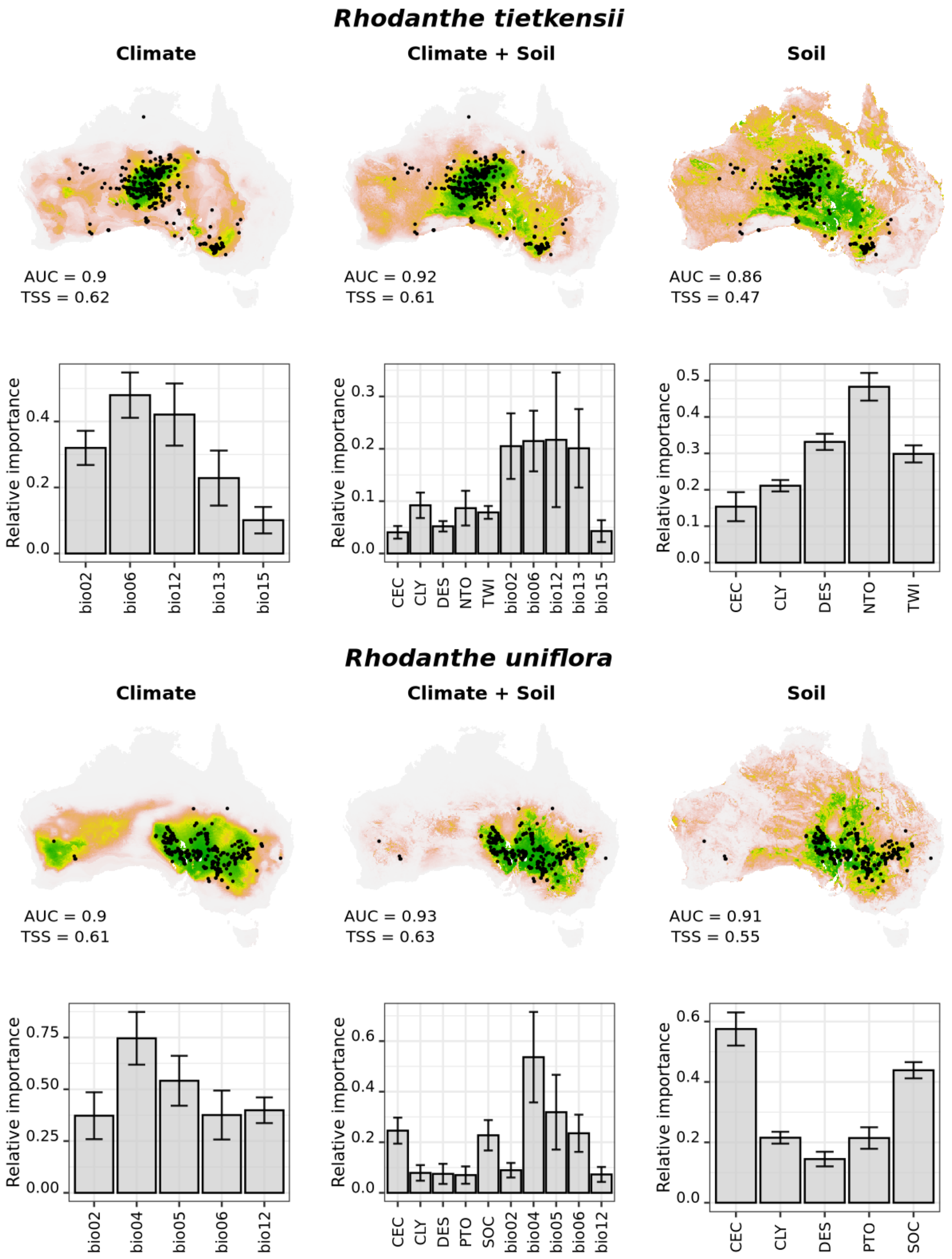

Rhodanthe tietkensii
Climate
Climate + Soil
Soil
AUC = 0.9
TSS = 0.62
AUC = 0.92
TSS = 0.61
AUC = 0.86
TSS = 0.47
Relative importance
bio02
bio06
bio12
bio13
bio15
CEC
CLY
DES
NTO
TWI
Rhodanthe uniflora
Climate
Climate + Soil
Soil
AUC = 0.9
TSS = 0.61
AUC = 0.93
TSS = 0.63
AUC = 0.91
TSS = 0.55
bio04
bio05
PTO
SOC

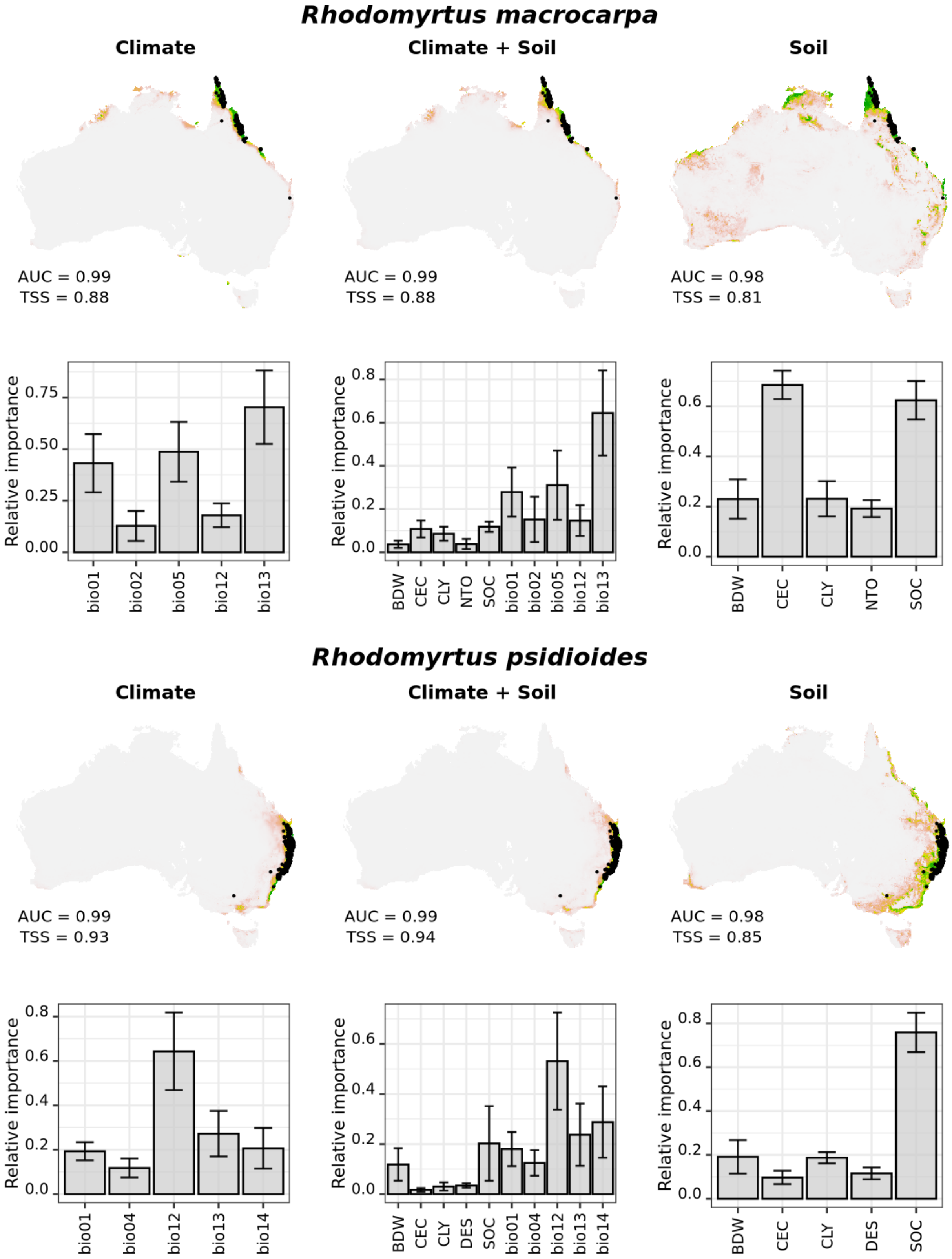

Rhodomyrtus macrocarpa
Climate
Climate + Soil
Soil
AUC = 0.99
TSS = 0.88
AUC = 0.99
TSS = 0.88
AUC = 0.98
TSS = 0.81
Relative importance
0.00
0.25
0.50
0.75
bio01
bio02
bio05
bio12
bio13
0.0
0.2
0.4
0.6
0.8
BDW
CEC
CLY
NTO
SOC
bio01
bio02
bio05
bio12
bio13
0.0
0.2
0.4
0.6
BDW
CEC
CLY
NTO
SOC
Rhodomyrtus psidioides
Climate
Climate + Soil
Soil
AUC = 0.99
TSS = 0.93
AUC = 0.99
TSS = 0.94
AUC = 0.98
TSS = 0.85
Relative importance
0.0
0.2
0.4
0.6
0.8
bio01
bio04
bio12
bio13
bio14
0.0
0.2
0.4
0.6
BDW
CEC
CLY
DES
SOC
bio01
bio04
bio12
bio13
bio14
0.0
0.2
0.4
0.6
0.8
BDW
CEC
CLY
DES
SOC

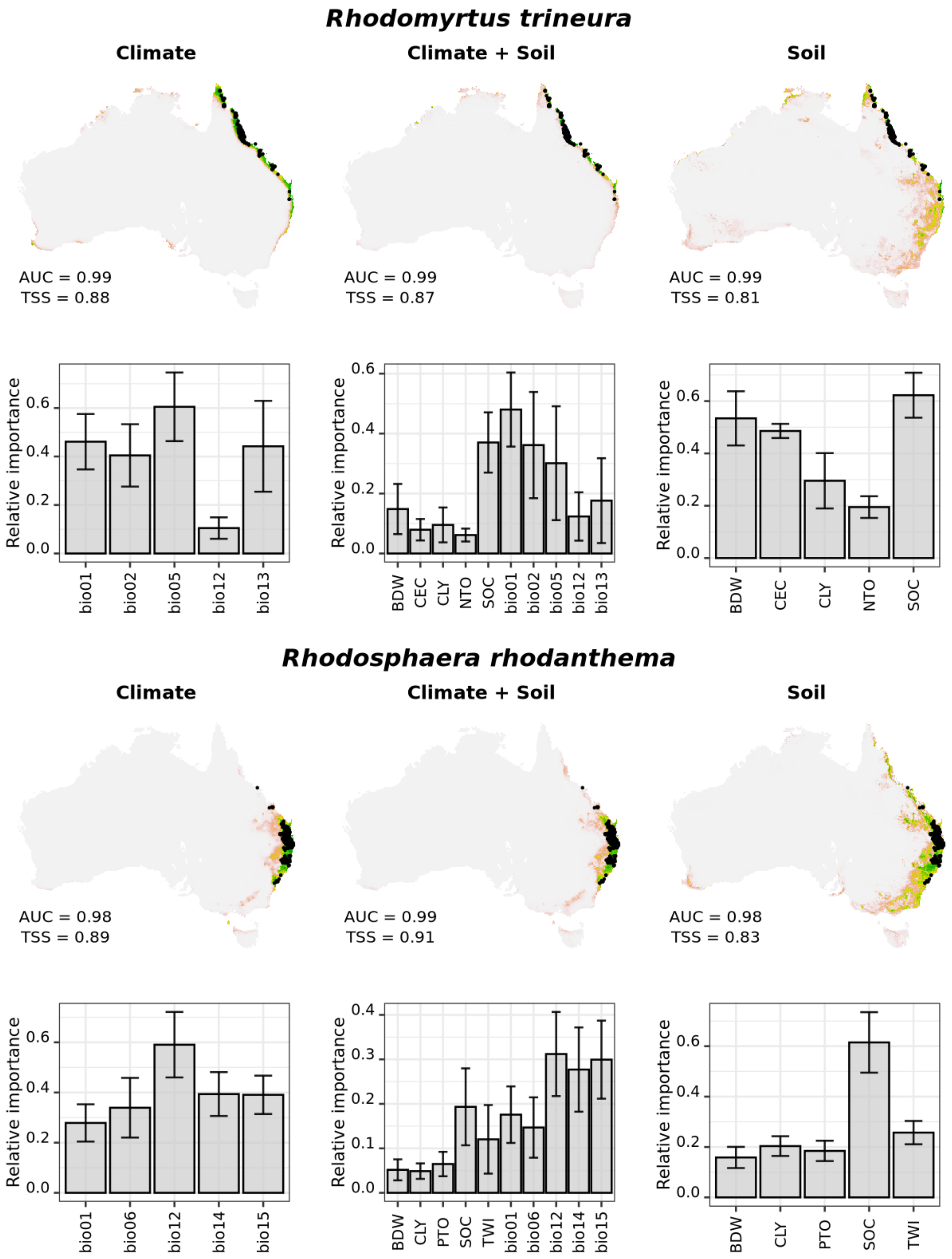
Rhodomyrtus trineura
Climate
Climate + Soil
Soil
AUC = 0.99
TSS = 0.88
AUC = 0.99
TSS = 0.87
AUC = 0.99
TSS = 0.81
Relative importance
0.0
0.2
0.4
0.6
bio01
bio02
bio05
bio12
bio13
BDW
CEC
CLY
NTO
SOC
bio01
bio02
bio05
bio12
bio13
BDW
CEC
CLY
NTO
SOC
Rhodosphaera rhodanthema
Climate
Climate + Soil
Soil
AUC = 0.98
TSS = 0.89
AUC = 0.99
TSS = 0.91
AUC = 0.98
TSS = 0.83
Relative importance
0.0
0.1
0.2
0.3
0.4
0.6
bio01
bio06
bio12
bio14
bio15
BDW
CLY
PTO
SOC
TWI
bio01
bio06
bio12
bio14
bio15
BDW
CLY
PTO
SOC
TWI

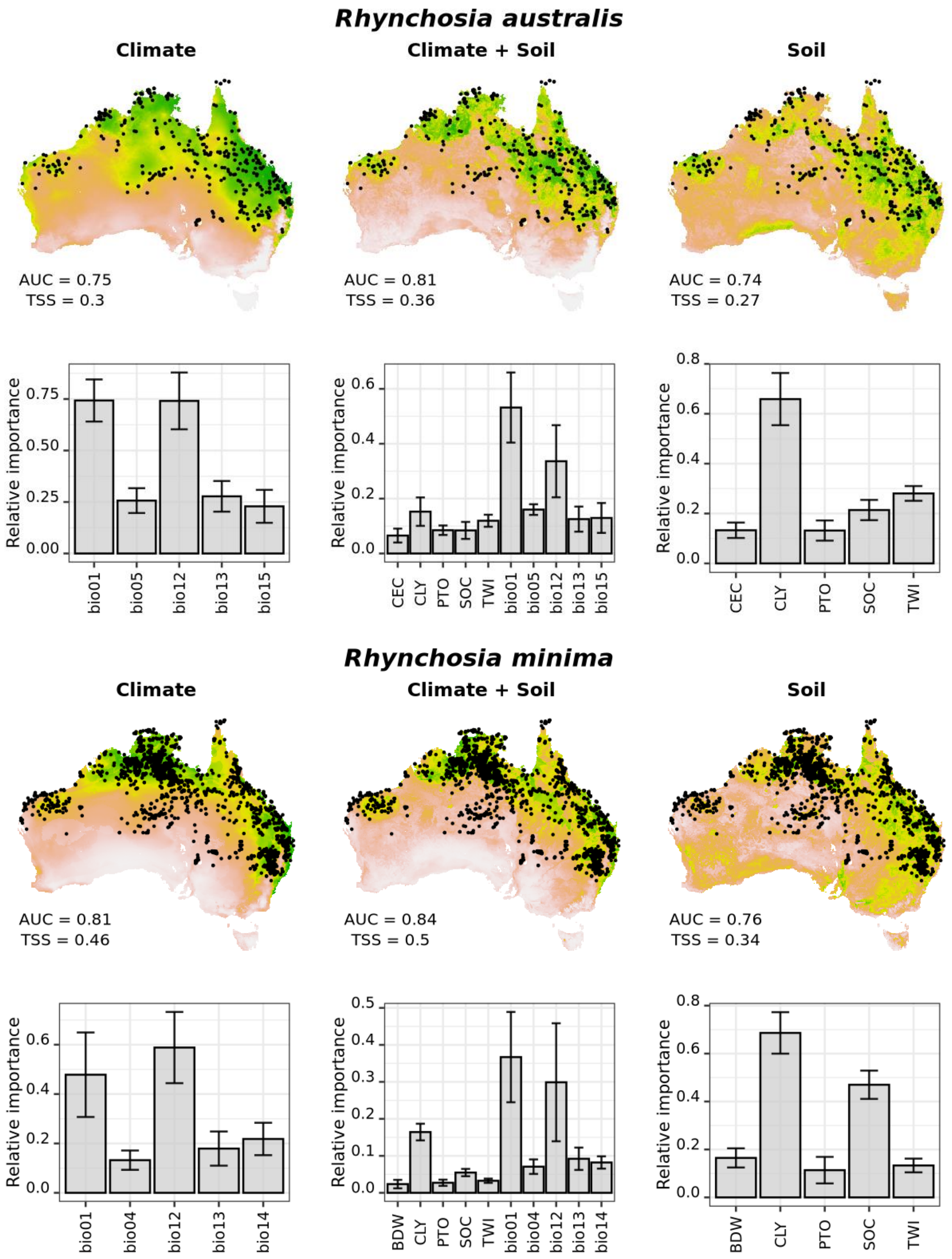

Rhynchosia australis
Climate
Climate + Soil
Soil
AUC = 0.75
TSS = 0.3
AUC = 0.81
TSS = 0.36
AUC = 0.74
TSS = 0.27
Relative importance
0.00
0.25
0.50
0.75
bio01
bio05
bio12
bio13
bio15
Relative importance
0.0
0.2
0.4
0.6
CEC
CLY
PTO
SOC
TWI
bio01
bio05
bio12
bio13
bio15
Relative importance
0.0
0.2
0.4
0.6
0.8
CEC
CLY
PTO
SOC
TWI
Rhynchosia minima
Climate
Climate + Soil
Soil
AUC = 0.81
TSS = 0.46
AUC = 0.84
TSS = 0.5
AUC = 0.76
TSS = 0.34
Relative importance
0.0
0.2
0.4
0.6
bio01
bio04
bio12
bio13
bio14
Relative importance
0.0
0.1
0.2
0.3
0.4
0.5
BDW
CLY
PTO
SOC
TWI
bio01
bio04
bio12
bio13
bio14
Relative importance
0.0
0.2
0.4
0.6
0.8
BDW
CLY
PTO
SOC
TWI

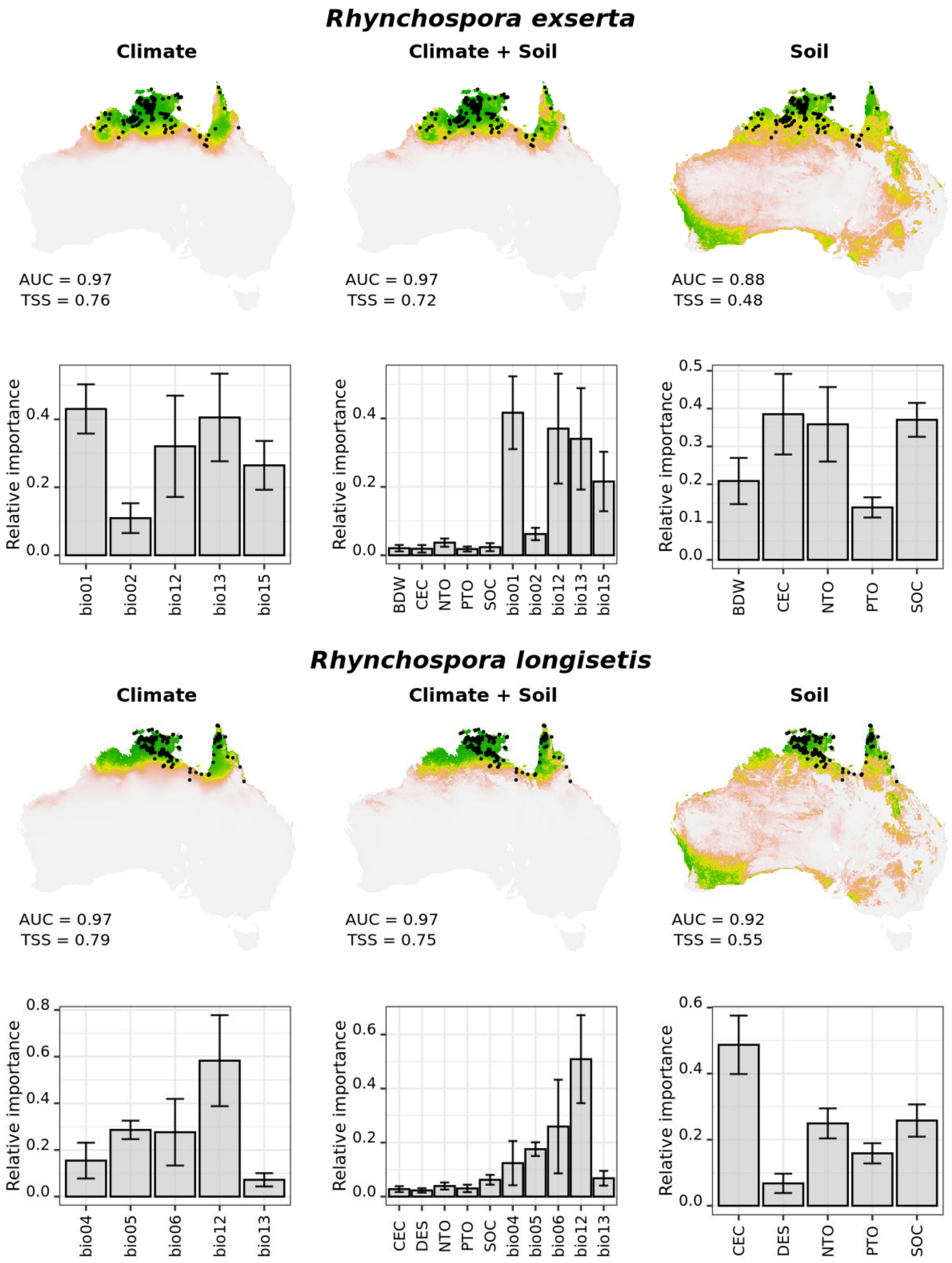
Rhynchospora exserta
Climate
Climate + Soil
Soil
AUC = 0.97
TSS = 0.76
AUC = 0.97
TSS = 0.72
AUC = 0.88
TSS = 0.48
Relative importance
0.0
0.2
0.4
bio01
bio02
bio12
bio13
bio15
Relative importance
0.0
0.2
0.4
BDW
CEC
NTO
PTO
SOC
bio01
bio02
bio12
bio13
bio15
Relative importance
0.0
0.1
0.2
0.3
0.4
0.5
BDW
CEC
NTO
PTO
SOC
Rhynchospora longisetis
Climate
Climate + Soil
Soil
AUC = 0.97
TSS = 0.79
AUC = 0.97
TSS = 0.75
AUC = 0.92
TSS = 0.55
Relative importance
0.0
0.2
0.4
0.6
0.8
bio04
bio05
bio06
bio12
bio13
Relative importance
0.0
0.2
0.4
0.6
CEC
DES
NTO
PTO
SOC
bio04
bio05
bio06
bio12
bio13
Relative importance
0.0
0.2
0.4
0.6
CEC
DES
NTO
PTO
SOC

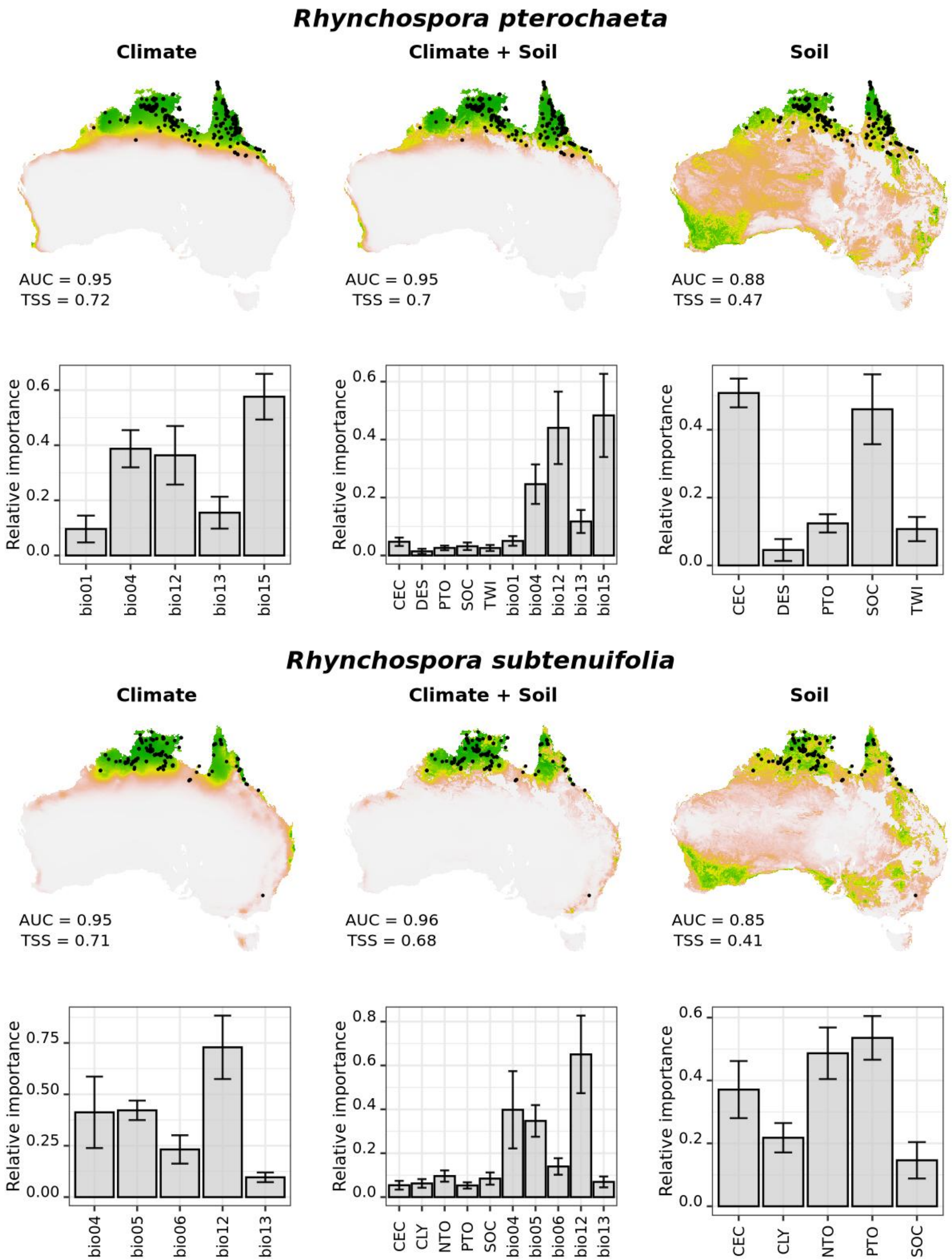
Rhynchospora pterochaeta
Climate
Climate + Soil
Soil
AUC = 0.95
TSS = 0.72
AUC = 0.95
TSS = 0.7
AUC = 0.88
TSS = 0.47
Relative importance
bio01
bio04
bio12
bio13
bio15
CEC
DES
PTO
SOC
TWI
Rhynchospora subtenuifolia
AUC = 0.95
TSS = 0.71
AUC = 0.96
TSS = 0.68
AUC = 0.85
TSS = 0.41
bio05
bio06
CLY
NTO

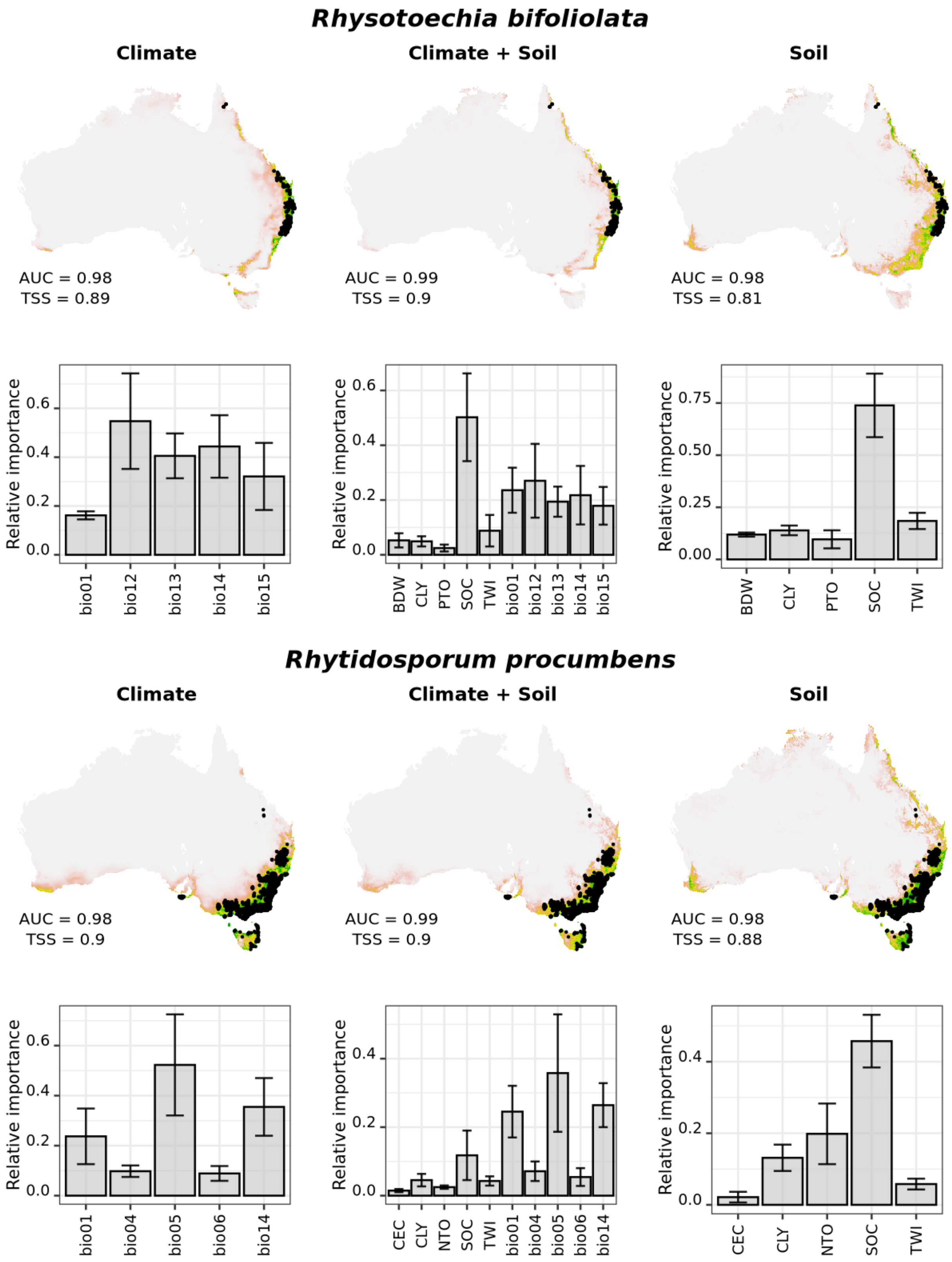
Rhysotoechia bifoliolata
Climate
Climate + Soil
Soil
AUC = 0.98
TSS = 0.89
AUC = 0.99
TSS = 0.9
AUC = 0.98
TSS = 0.81
Relative importance
0.0
0.2
0.4
0.6
bio01
bio12
bio13
bio14
bio15
BDW
CLY
PTO
SOC
TWI
bio01
bio12
bio13
bio14
bio15
0.00
0.25
0.50
0.75
BDW
CLY
PTO
SOC
TWI
Rhytidosporum procumbens
Climate
Climate + Soil
Soil
AUC = 0.98
TSS = 0.9
AUC = 0.99
TSS = 0.9
AUC = 0.98
TSS = 0.88
Relative importance
bio01
bio04
bio05
bio06
bio14
CEC
CLY
NTO
SOC
TWI
bio01
bio04
bio05
bio06
bio14
CEC
CLY
NTO
SOC
TWI

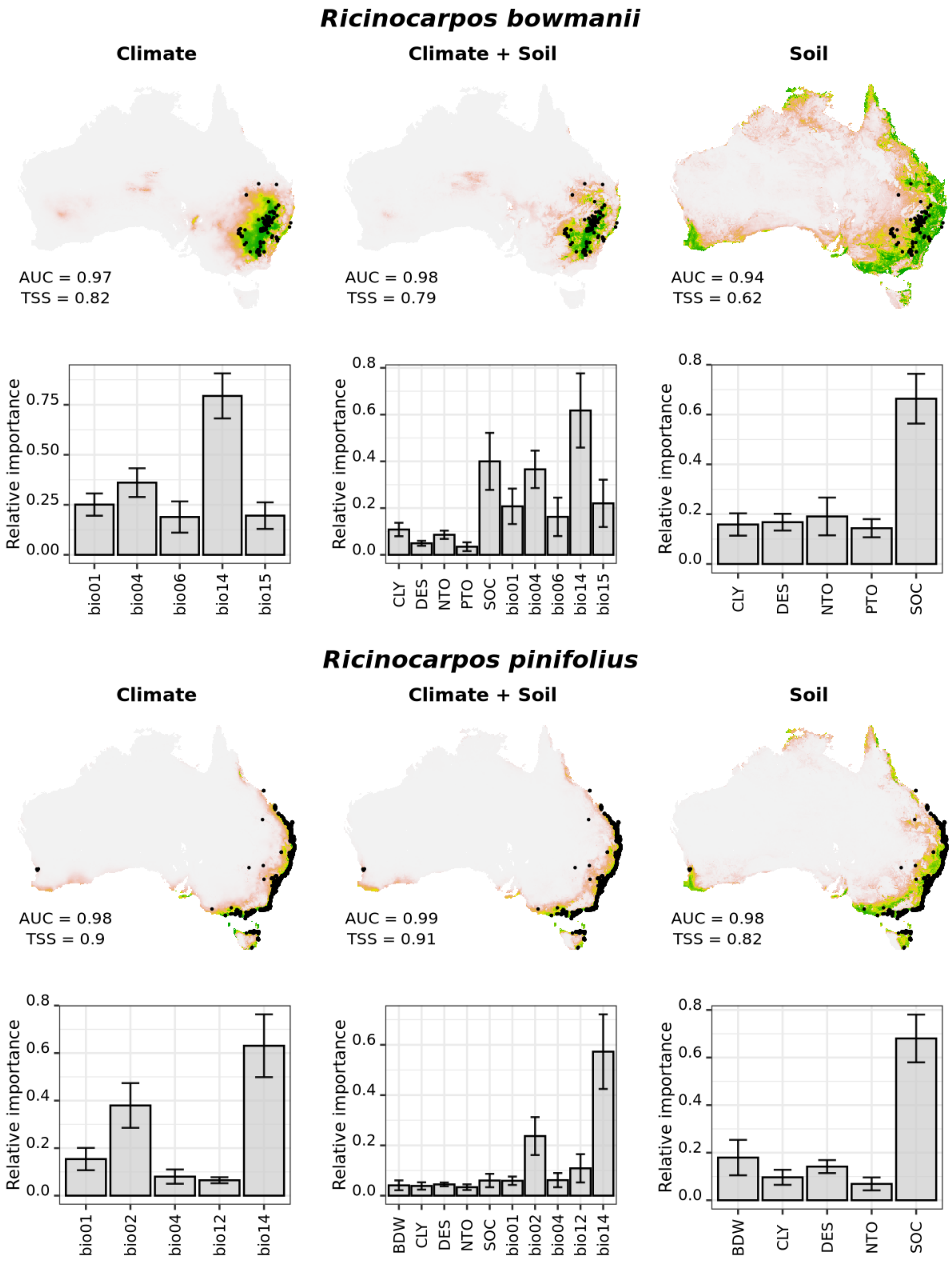

Ricinocarpos bowmanii
Climate
Climate + Soil
Soil
AUC = 0.97
TSS = 0.82
AUC = 0.98
TSS = 0.79
AUC = 0.94
TSS = 0.62
Relative importance
0.00
0.25
0.50
0.75
bio01
bio04
bio06
bio14
bio15
0.0
0.2
0.4
0.6
0.8
CLY
DES
NTO
PTO
SOC
bio01
bio04
bio06
bio14
bio15
CLY
DES
NTO
PTO
SOC
Ricinocarpos pinifolius
Climate
Climate + Soil
Soil
AUC = 0.98
TSS = 0.9
AUC = 0.99
TSS = 0.91
AUC = 0.98
TSS = 0.82
Relative importance
bio01
bio02
bio04
bio12
bio14
BDW
CLY
DES
NTO
SOC
bio01
bio02
bio04
bio12
bio14
BDW
CLY
DES
NTO
SOC

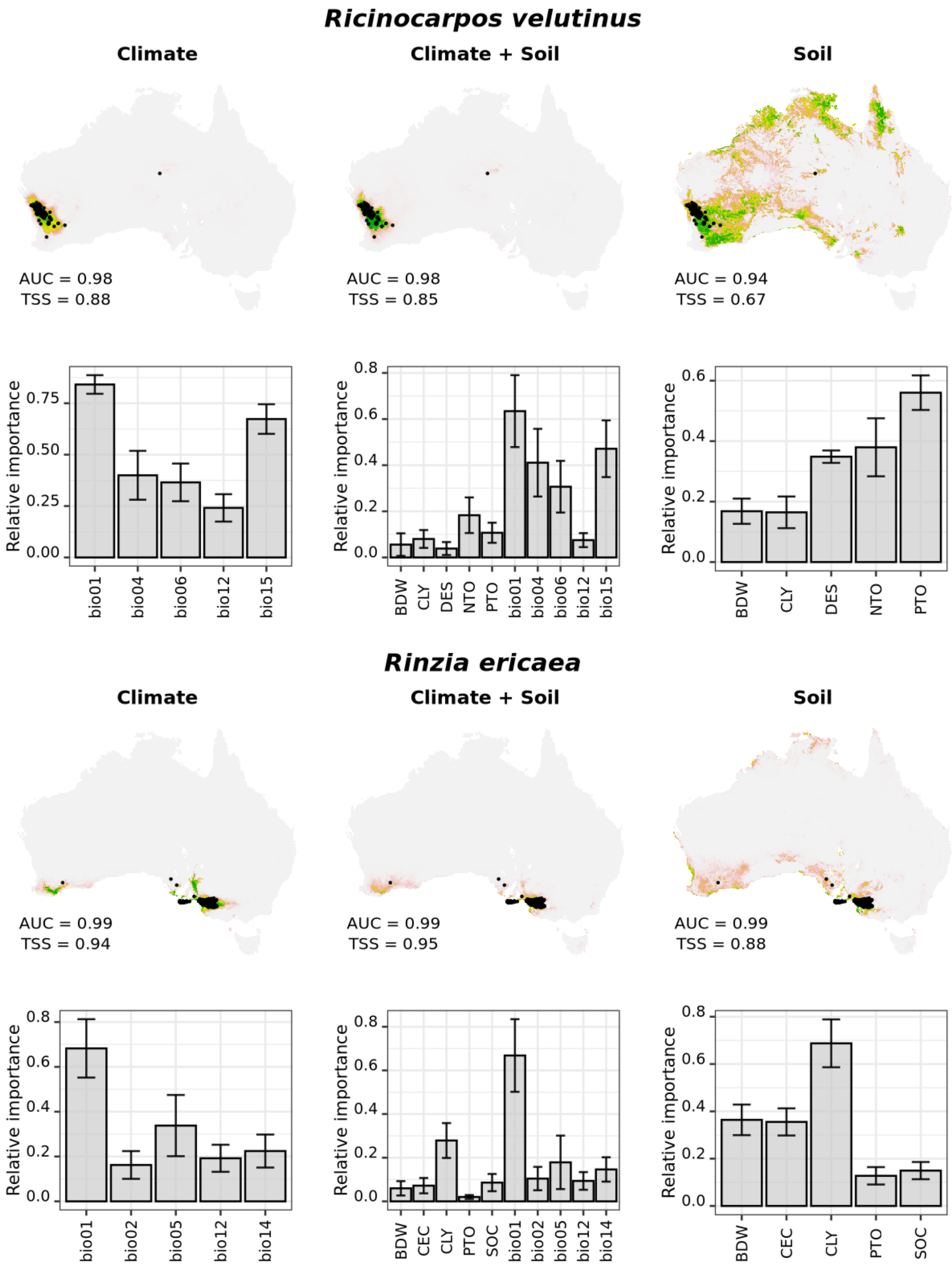

Ricinocarpos velutinus
Climate
Climate + Soil
Soil
AUC = 0.98
TSS = 0.88
AUC = 0.98
TSS = 0.85
AUC = 0.94
TSS = 0.67
Relative importance
0.00
0.25
0.50
0.75
bio01
bio04
bio06
bio12
bio15
0.0
0.2
0.4
0.6
0.8
BDW
CLY
DES
NTO
PTO
bio01
bio04
bio06
bio12
bio15
BDW
CLY
DES
NTO
PTO
Rinzia ericaea
Climate
Climate + Soil
Soil
AUC = 0.99
TSS = 0.94
AUC = 0.99
TSS = 0.95
AUC = 0.99
TSS = 0.88
bio01
bio02
bio05
bio12
bio14
BDW
CEC
CLY
PTO
SOC
bio01
bio02
bio05
bio12
bio14
BDW
CEC
CLY
PTO
SOC

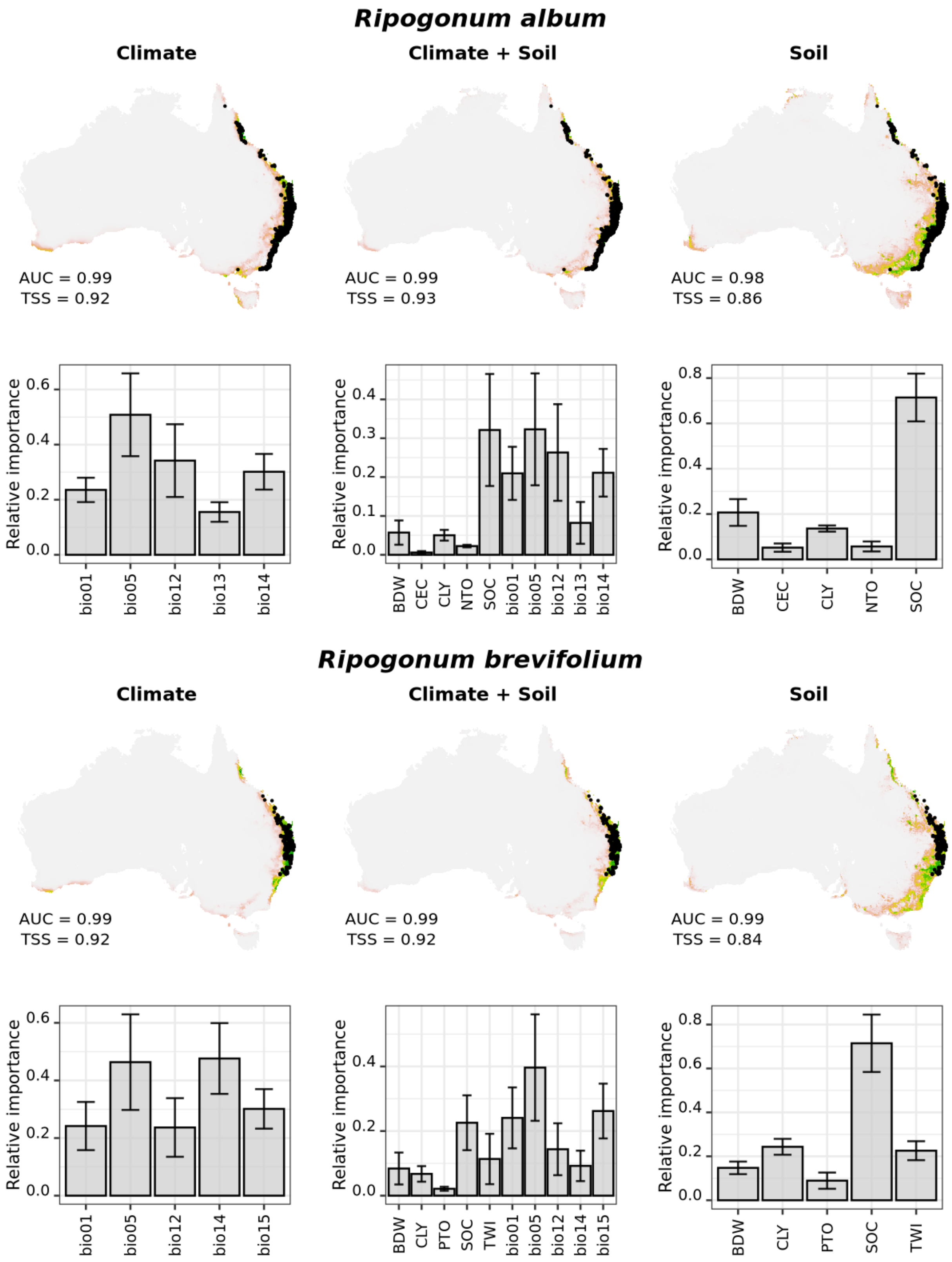

Ripogonum album
Climate
Climate + Soil
Soil
AUC = 0.99
TSS = 0.92
AUC = 0.99
TSS = 0.93
AUC = 0.98
TSS = 0.86
Relative importance
bio01 bio05 bio12 bio13 bio14
BDW CEC CLY NTO SOC bio01 bio05 bio12 bio13 bio14
BDW CEC CLY NTO SOC
Ripogonum brevifolium
Climate
Climate + Soil
Soil
AUC = 0.99
TSS = 0.92
AUC = 0.99
TSS = 0.92
AUC = 0.99
TSS = 0.84
Relative importance
bio01 bio05 bio12 bio14 bio15
BDW CLY PTO SOC TWI bio01 bio05 bio12 bio14 bio15
BDW CLY PTO SOC TWI

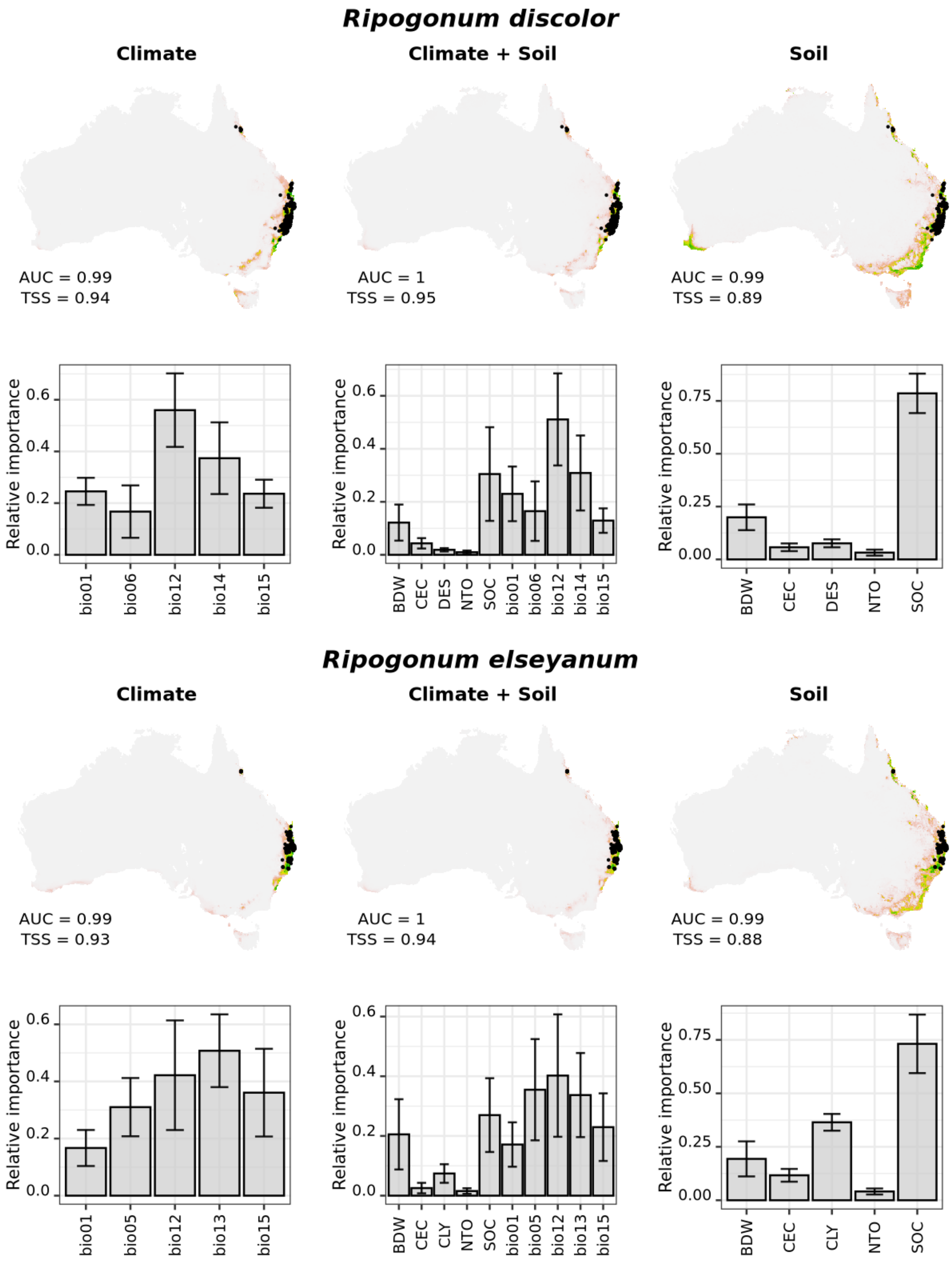

Ripogonum discolor
Climate
Climate + Soil
Soil
AUC = 0.99
TSS = 0.94
AUC = 1
TSS = 0.95
AUC = 0.99
TSS = 0.89
Relative importance
bio01
bio06
bio12
bio14
bio15
BDW
CEC
DES
NTO
SOC
Ripogonum elseyanum
Climate
Climate + Soil
Soil
AUC = 0.99
TSS = 0.93
AUC = 1
TSS = 0.94
AUC = 0.99
TSS = 0.88
Relative importance
bio01
bio05
bio12
bio13
bio15
BDW
CEC
CLY
NTO
SOC

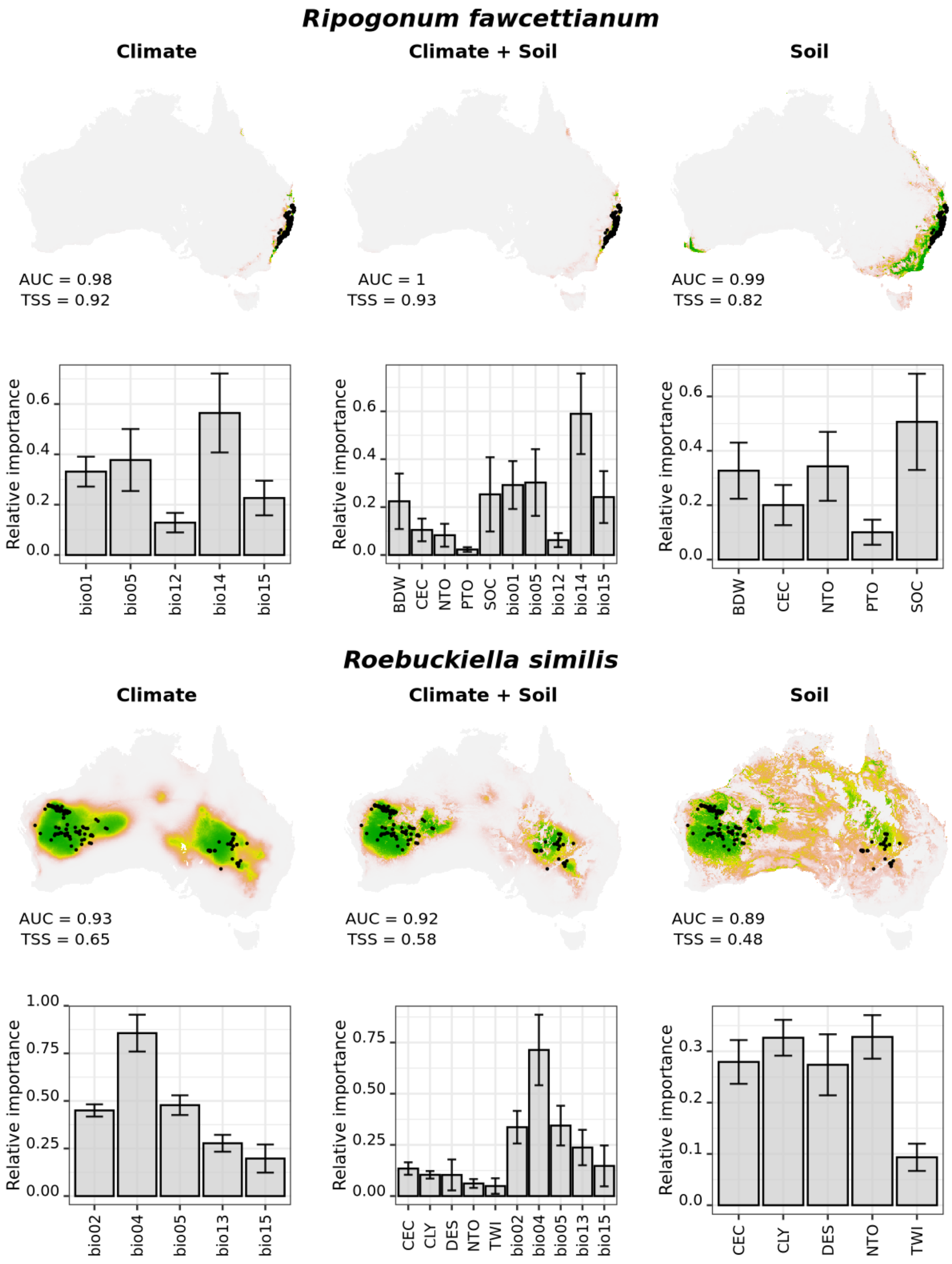
Ripogonum fawcettianum
Climate
Climate + Soil
Soil
AUC = 0.98
TSS = 0.92
AUC = 1
TSS = 0.93
AUC = 0.99
TSS = 0.82
Relative importance
bio01 bio05 bio12 bio14 bio15
BDW CEC NTO PTO SOC bio01 bio05 bio12 bio14 bio15
BDW CEC NTO PTO SOC
Roebuckiella similis
Climate
Climate + Soil
Soil
AUC = 0.93
TSS = 0.65
AUC = 0.92
TSS = 0.58
AUC = 0.89
TSS = 0.48
Relative importance
bio02 bio04 bio05 bio13 bio15
CEC CLY DES NTO TWI bio02 bio04 bio05 bio13 bio15
CEC CLY DES NTO TWI

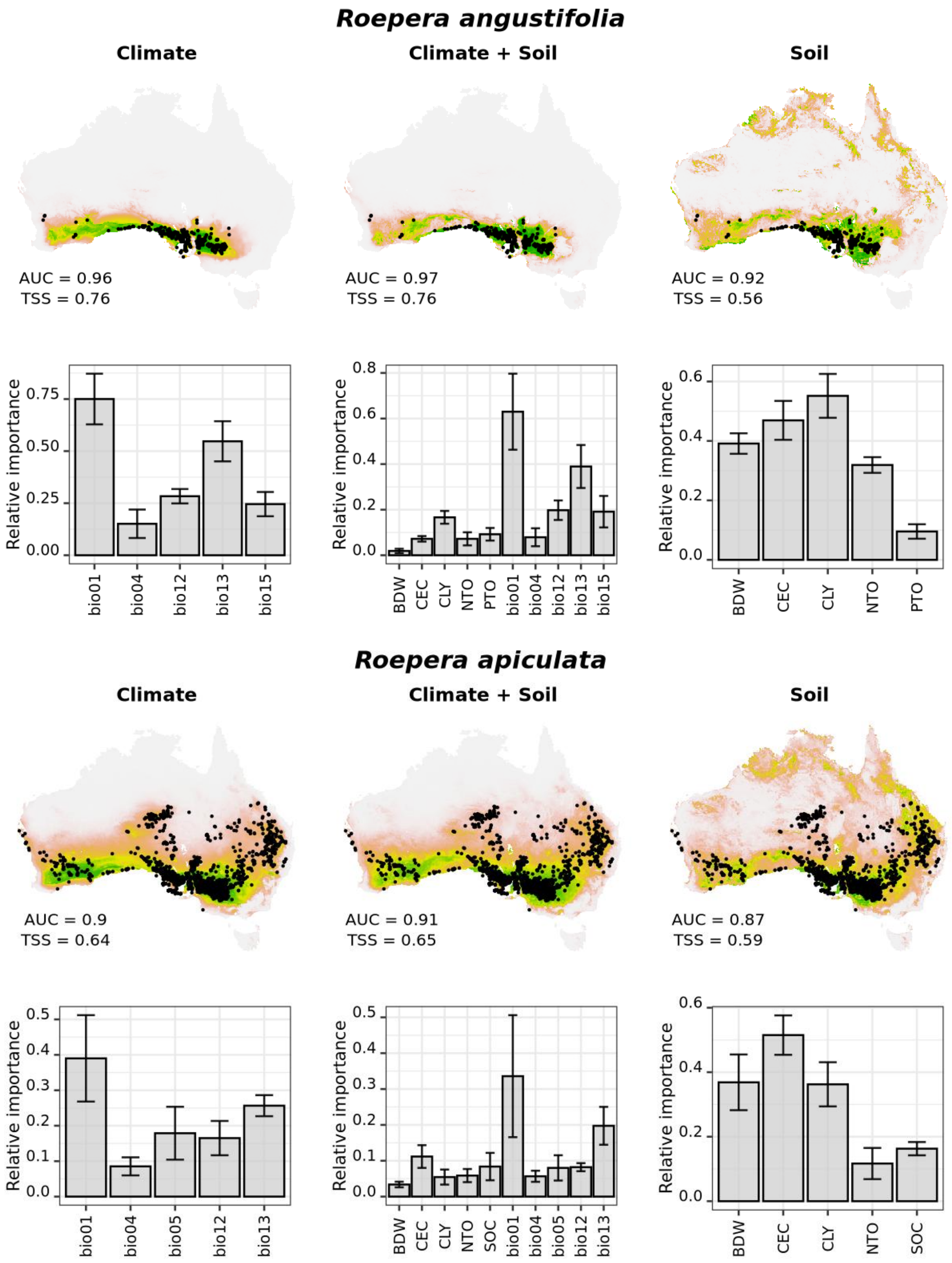

Roepera angustifolia
Climate
Climate + Soil
Soil
AUC = 0.96
TSS = 0.76
AUC = 0.97
TSS = 0.76
AUC = 0.92
TSS = 0.56
Relative importance
bio01
bio04
bio12
bio13
bio15
BDW
CEC
CLY
NTO
PTO
Roepera apiculata
Climate
Climate + Soil
Soil
AUC = 0.9
TSS = 0.64
AUC = 0.91
TSS = 0.65
AUC = 0.87
TSS = 0.59
Relative importance
bio05
SOC

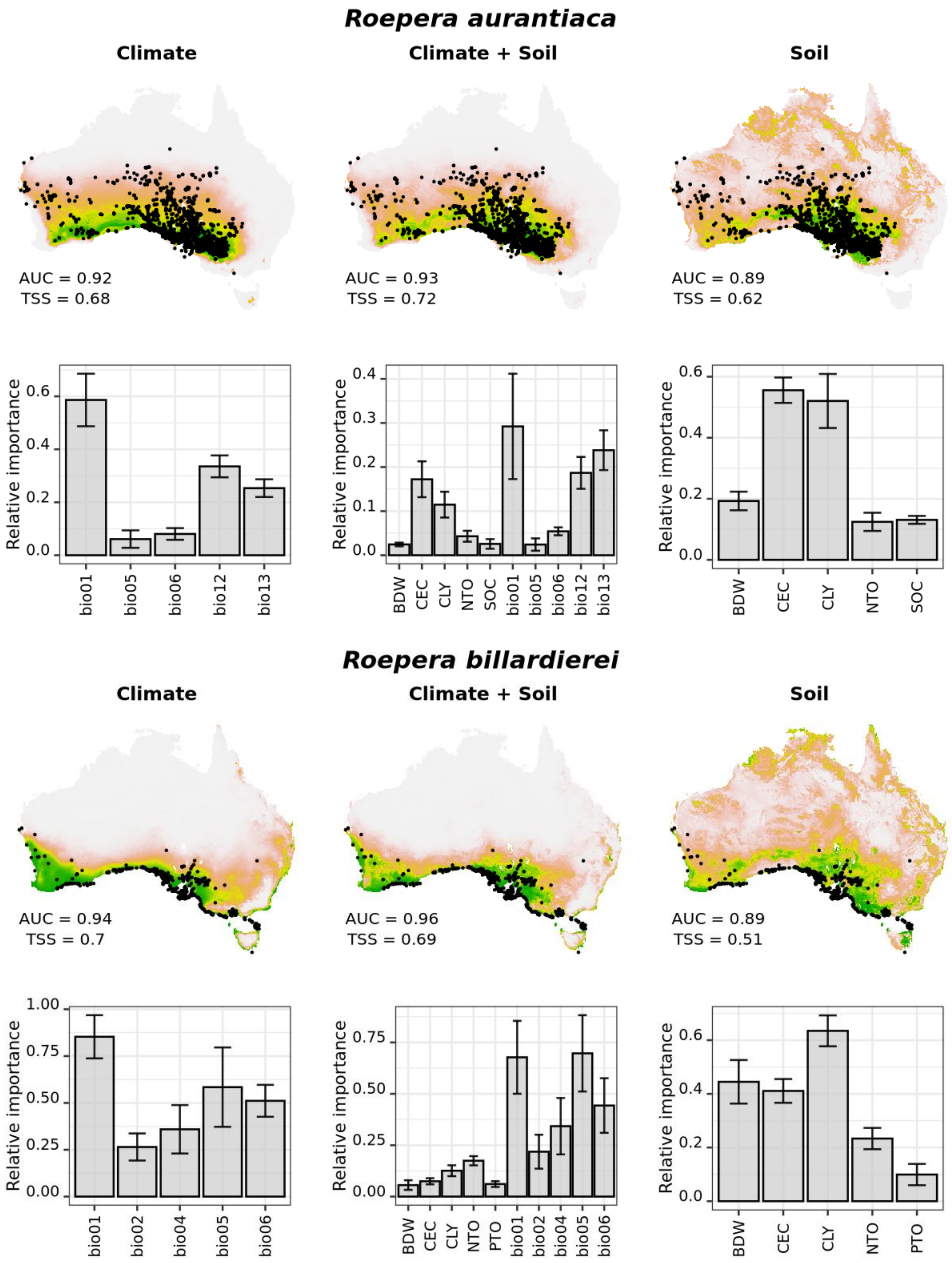

Roepera aurantiaca
Climate
Climate + Soil
Soil
AUC = 0.92
TSS = 0.68
AUC = 0.93
TSS = 0.72
AUC = 0.89
TSS = 0.62
Relative importance
bio01
bio05
bio06
bio12
bio13
BDW
CEC
CLY
NTO
SOC
Roepera billardierei
AUC = 0.94
TSS = 0.7
AUC = 0.96
TSS = 0.69
AUC = 0.89
TSS = 0.51
bio02
bio04
PTO

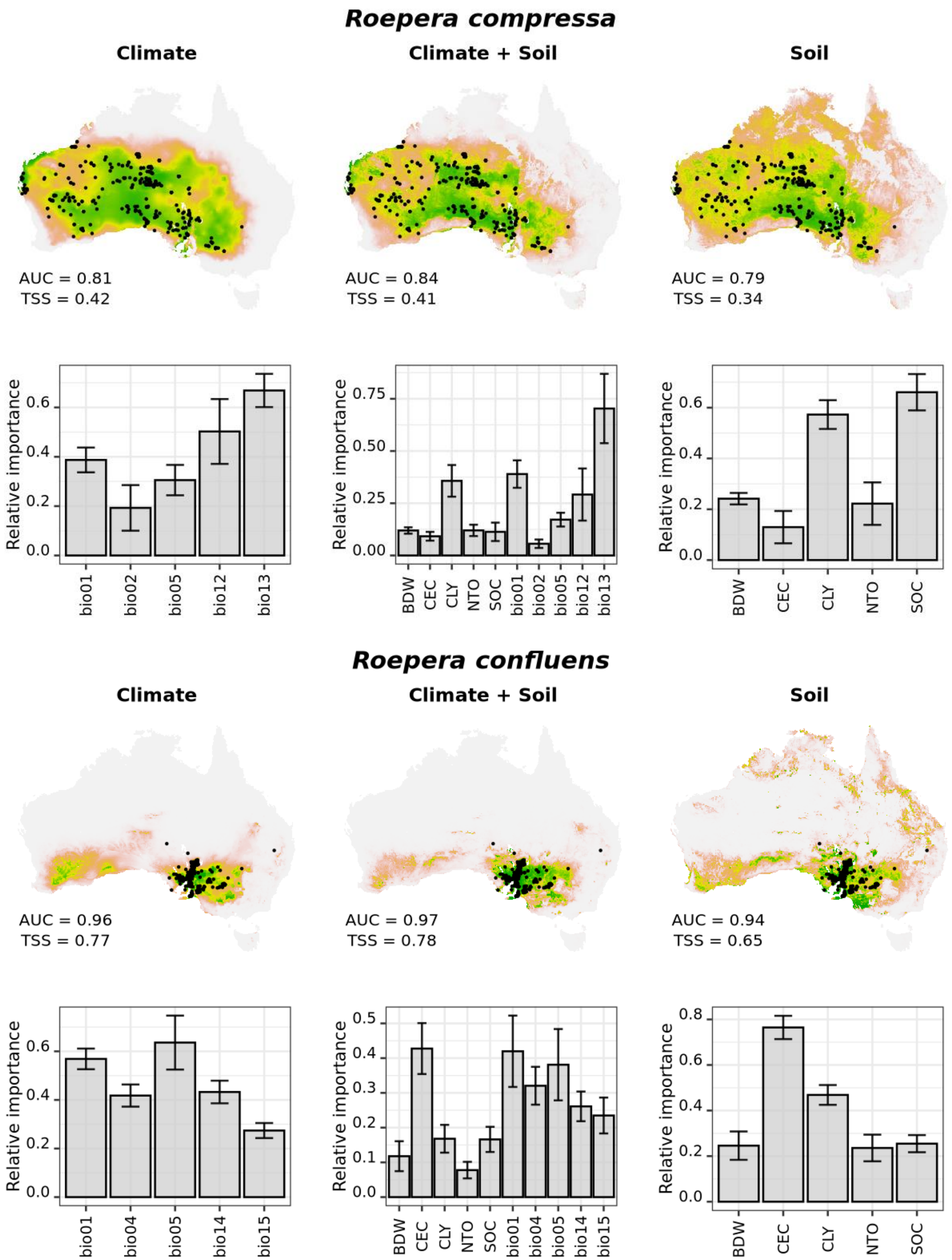
Roepera compressa
Climate
Climate + Soil
Soil
AUC = 0.81
TSS = 0.42
AUC = 0.84
TSS = 0.41
AUC = 0.79
TSS = 0.34
Relative importance
bio01
bio02
bio05
bio12
bio13
BDW
CEC
CLY
NTO
SOC
Roepera confluens
Climate
Climate + Soil
Soil
AUC = 0.96
TSS = 0.77
AUC = 0.97
TSS = 0.78
AUC = 0.94
TSS = 0.65
Relative importance
bio01
bio04
bio05
bio14
bio15
BDW
CEC
CLY
NTO
SOC

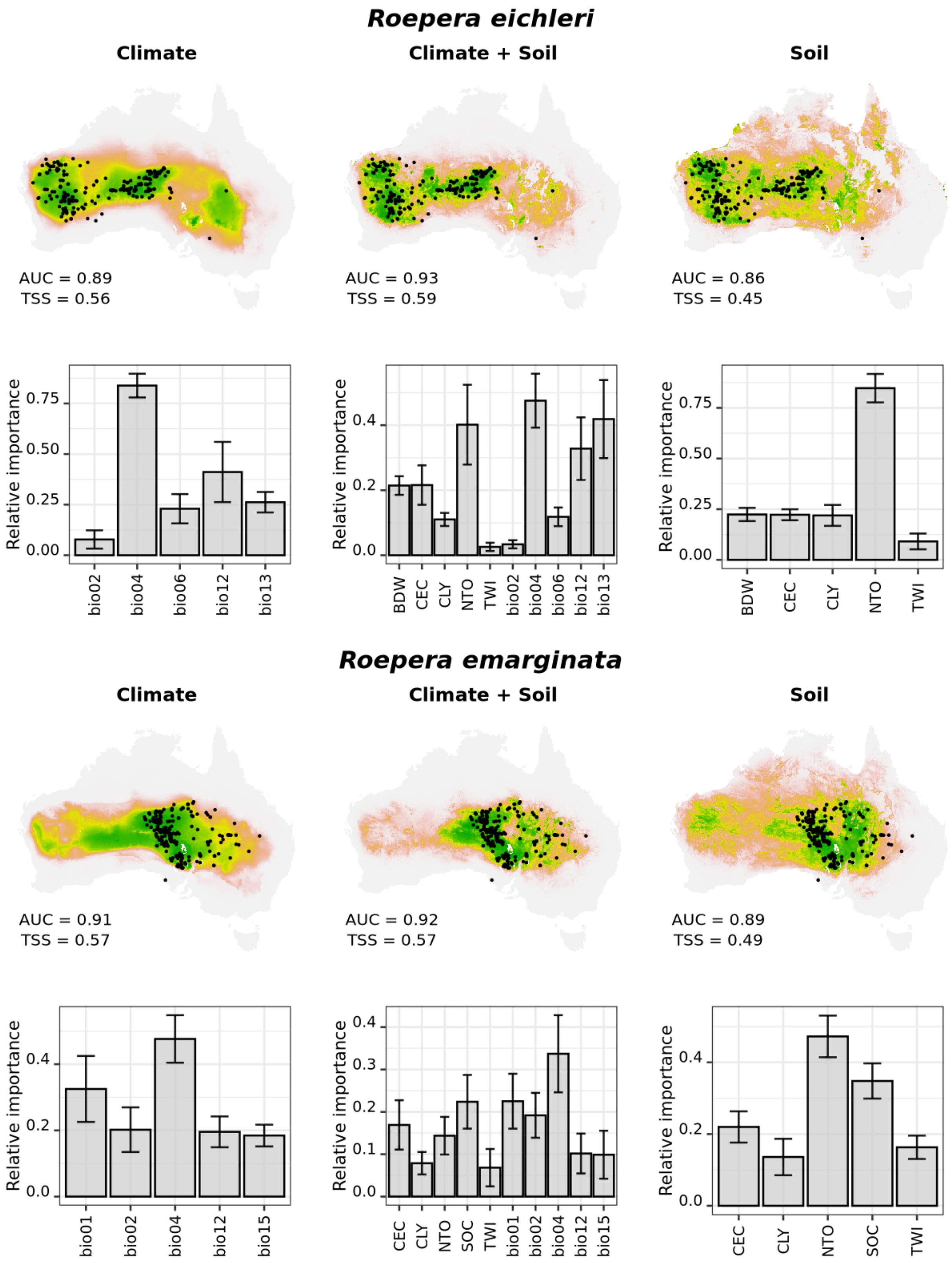
Roepera eichleri
Climate
Climate + Soil
Soil
AUC = 0.89
TSS = 0.56
AUC = 0.93
TSS = 0.59
AUC = 0.86
TSS = 0.45
Relative importance
0.00
0.25
0.50
0.75
bio02
bio04
bio06
bio12
bio13
Relative importance
0.0
0.2
0.4
BDW
CEC
CLY
NTO
TWI
bio02
bio04
bio06
bio12
bio13
Relative importance
0.00
0.25
0.50
0.75
BDW
CEC
CLY
NTO
TWI
Roepera emarginata
Climate
Climate + Soil
Soil
AUC = 0.91
TSS = 0.57
AUC = 0.92
TSS = 0.57
AUC = 0.89
TSS = 0.49
Relative importance
0.0
0.2
0.4
bio01
bio02
bio04
bio12
bio15
Relative importance
0.0
0.1
0.2
0.3
0.4
CEC
CLY
NTO
SOC
TWI
bio01
bio02
bio04
bio12
bio15
Relative importance
0.0
0.2
0.4
CEC
CLY
NTO
SOC
TWI

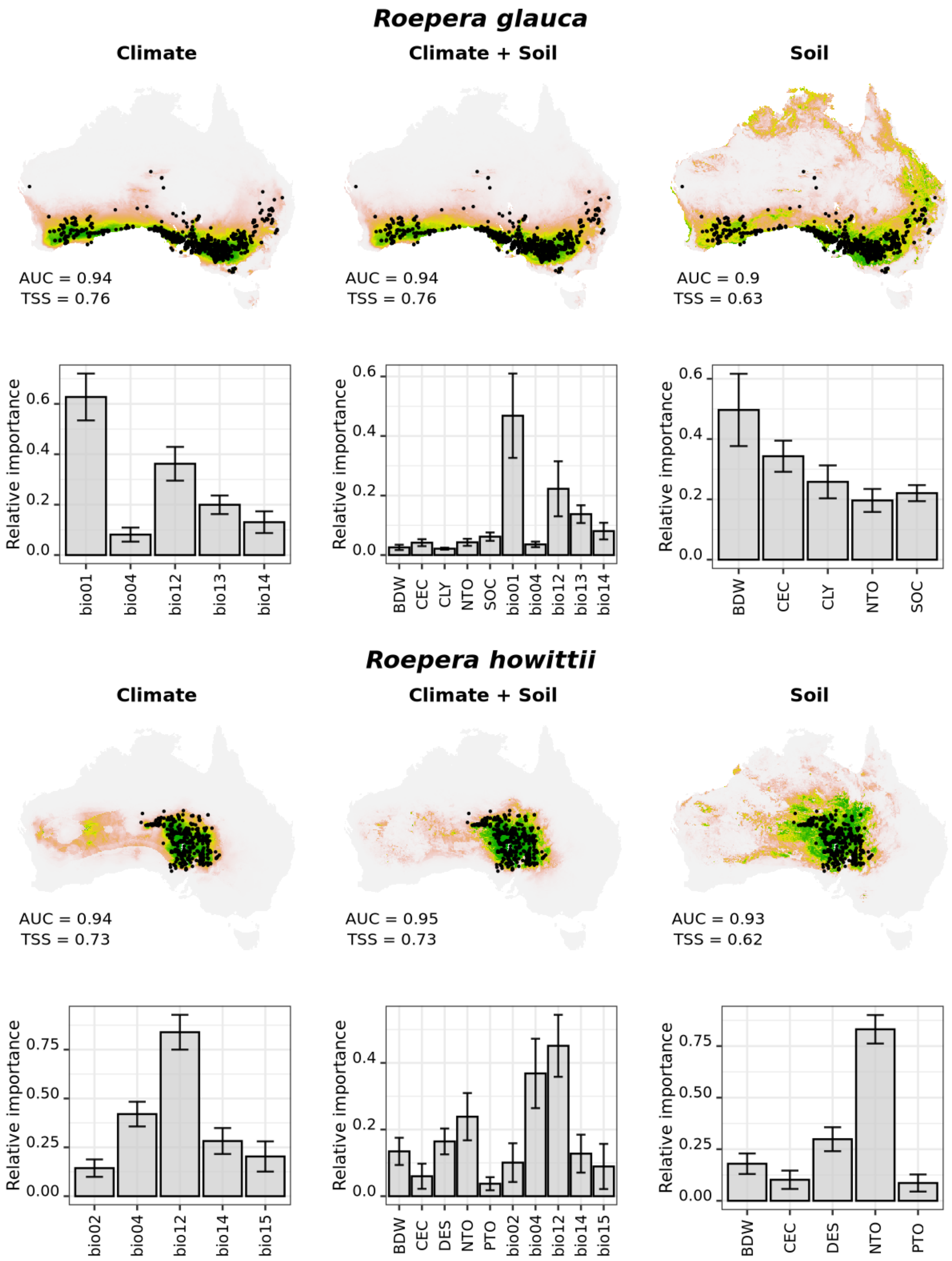
Roepera glauca
Climate
Climate + Soil
Soil
AUC = 0.94
TSS = 0.76
AUC = 0.94
TSS = 0.76
AUC = 0.9
TSS = 0.63
Relative importance
0.0
0.2
0.4
0.6
bio01
bio04
bio12
bio13
bio14
BDW
CEC
CLY
NTO
SOC
bio01
bio04
bio12
bio13
bio14
BDW
CEC
CLY
NTO
SOC
Roepera howittii
Climate
Climate + Soil
Soil
AUC = 0.94
TSS = 0.73
AUC = 0.95
TSS = 0.73
AUC = 0.93
TSS = 0.62
Relative importance
0.00
0.25
0.50
0.75
bio02
bio04
bio12
bio14
bio15
BDW
CEC
DES
NTO
PTO
bio02
bio04
bio12
bio14
bio15
BDW
CEC
DES
NTO
PTO

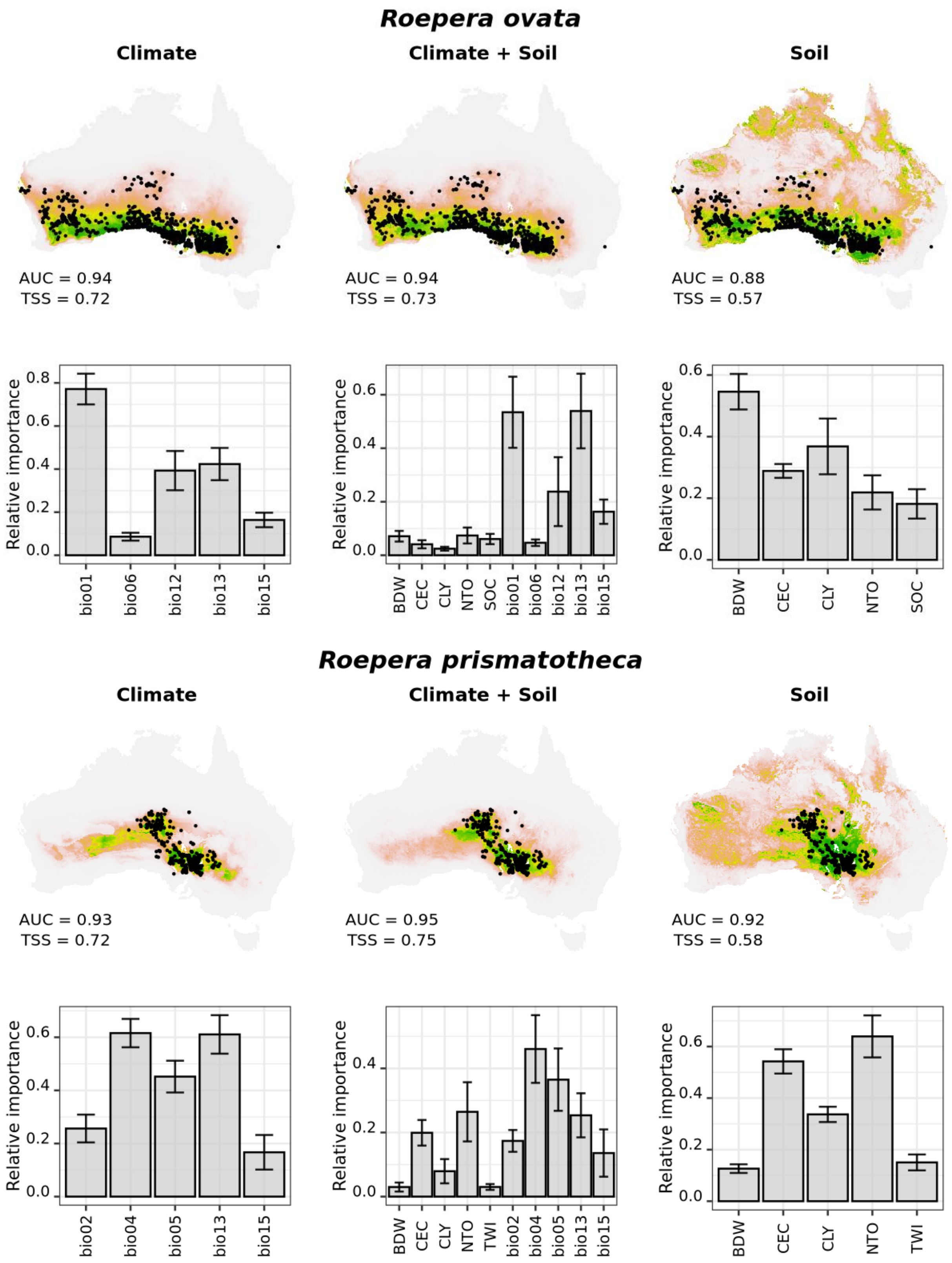
Roepera ovata
Climate
Climate + Soil
Soil
AUC = 0.94
TSS = 0.72
AUC = 0.94
TSS = 0.73
AUC = 0.88
TSS = 0.57
Relative importance
0.0
0.2
0.4
0.6
0.8
bio01
bio06
bio12
bio13
bio15
BDW
CEC
CLY
NTO
SOC
bio01
bio06
bio12
bio13
bio15
BDW
CEC
CLY
NTO
SOC
Roepera prismatotheca
Climate
Climate + Soil
Soil
AUC = 0.93
TSS = 0.72
AUC = 0.95
TSS = 0.75
AUC = 0.92
TSS = 0.58
Relative importance
bio02
bio04
bio05
bio13
bio15
BDW
CEC
CLY
NTO
TWI
bio02
bio04
bio05
bio13
bio15
BDW
CEC
CLY
NTO
TWI

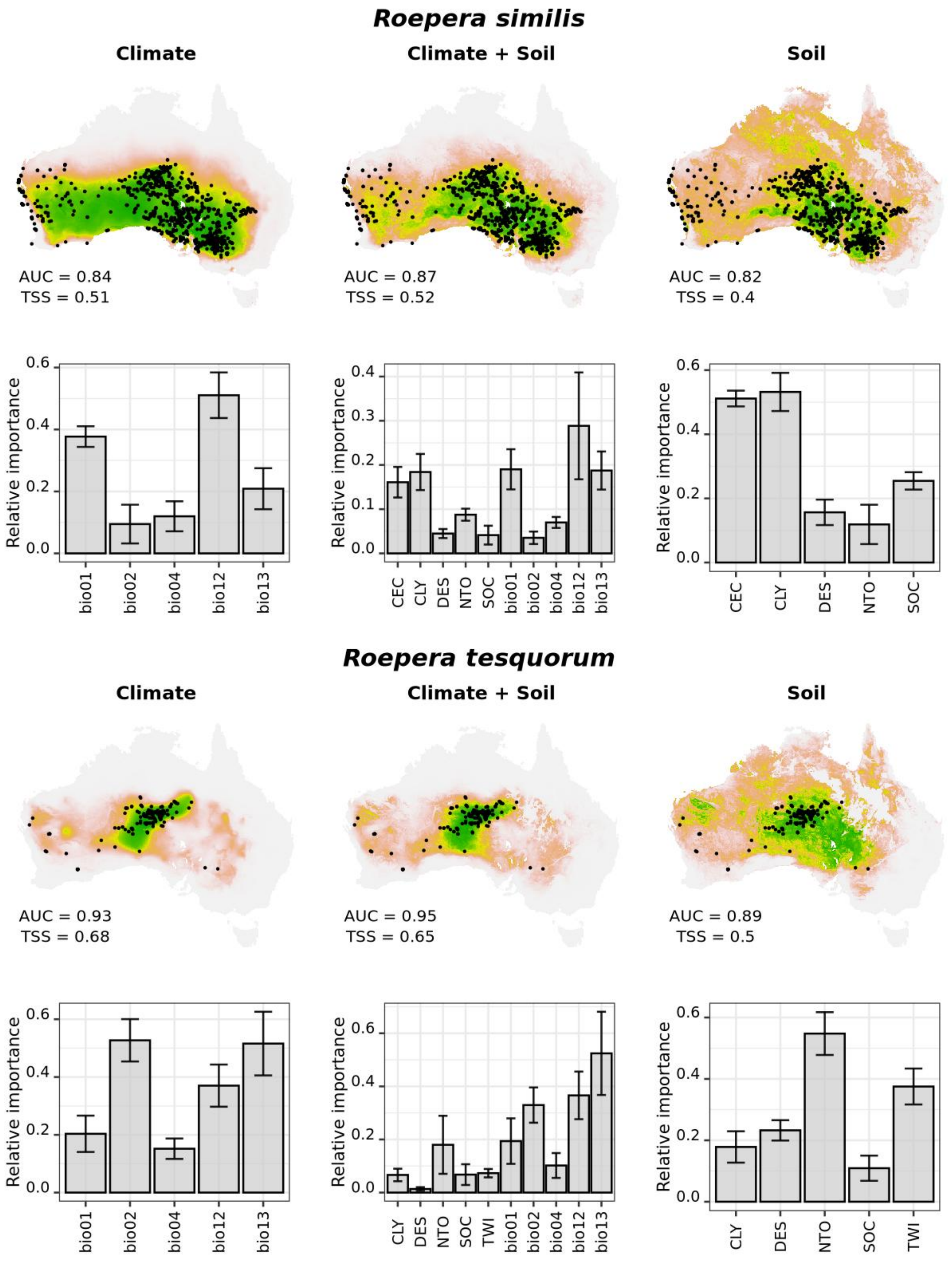

Roepera similis
Climate
Climate + Soil
Soil
AUC = 0.84
TSS = 0.51
AUC = 0.87
TSS = 0.52
AUC = 0.82
TSS = 0.4
Relative importance
bio01
bio02
bio04
bio12
bio13
CEC
CLY
DES
NTO
SOC
Roepera tesquorum
Climate
Climate + Soil
Soil
AUC = 0.93
TSS = 0.68
AUC = 0.95
TSS = 0.65
AUC = 0.89
TSS = 0.5
Relative importance
TWI

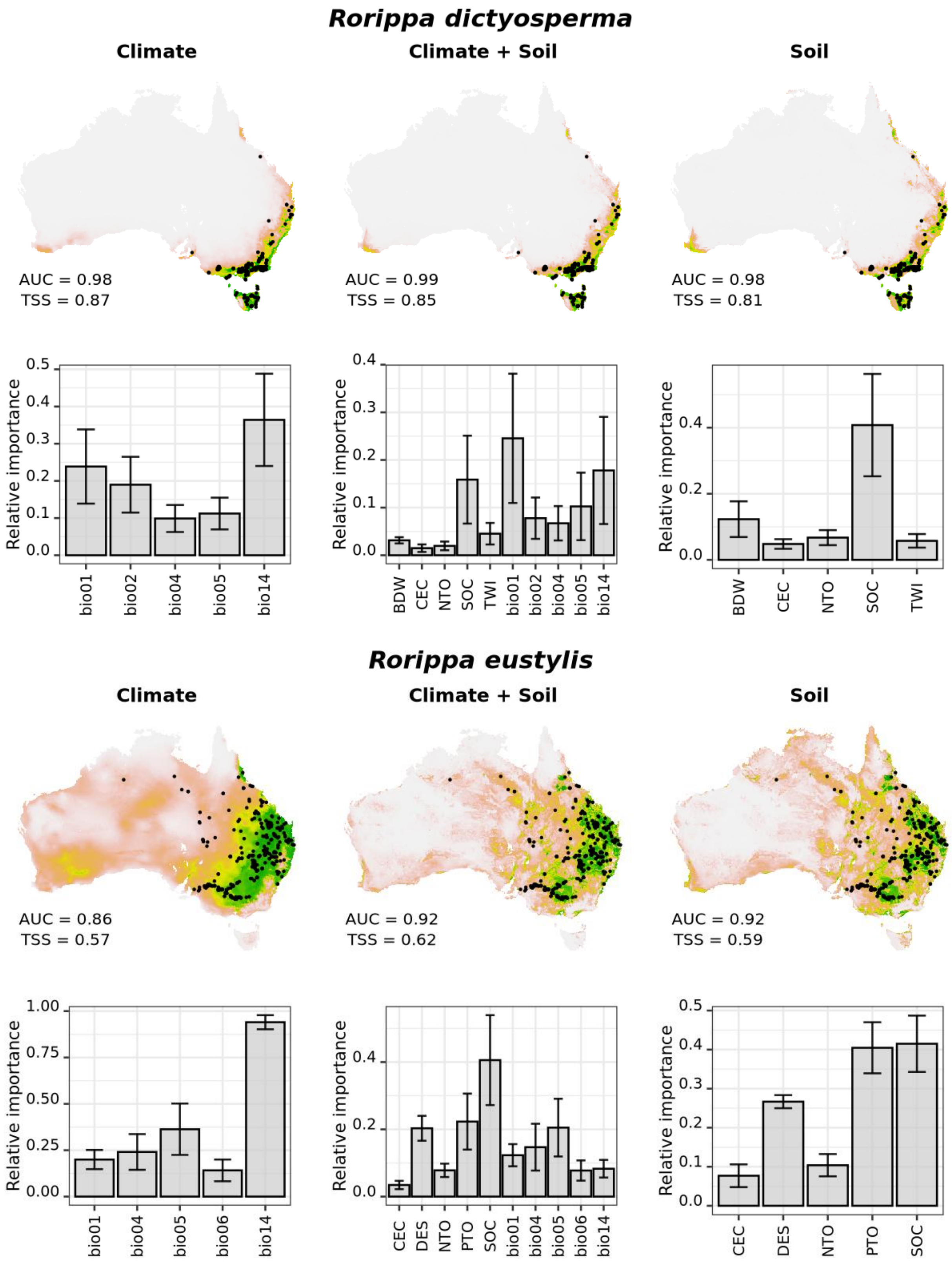
Rorippa dictyosperma
Climate
Climate + Soil
Soil
AUC = 0.98
TSS = 0.87
AUC = 0.99
TSS = 0.85
AUC = 0.98
TSS = 0.81
Relative importance
bio01
bio02
bio04
bio05
bio14
BDW
CEC
NTO
SOC
TWI
Rorippa eustylis
AUC = 0.86
TSS = 0.57
AUC = 0.92
TSS = 0.62
AUC = 0.92
TSS = 0.59
bio06
DES
PTO

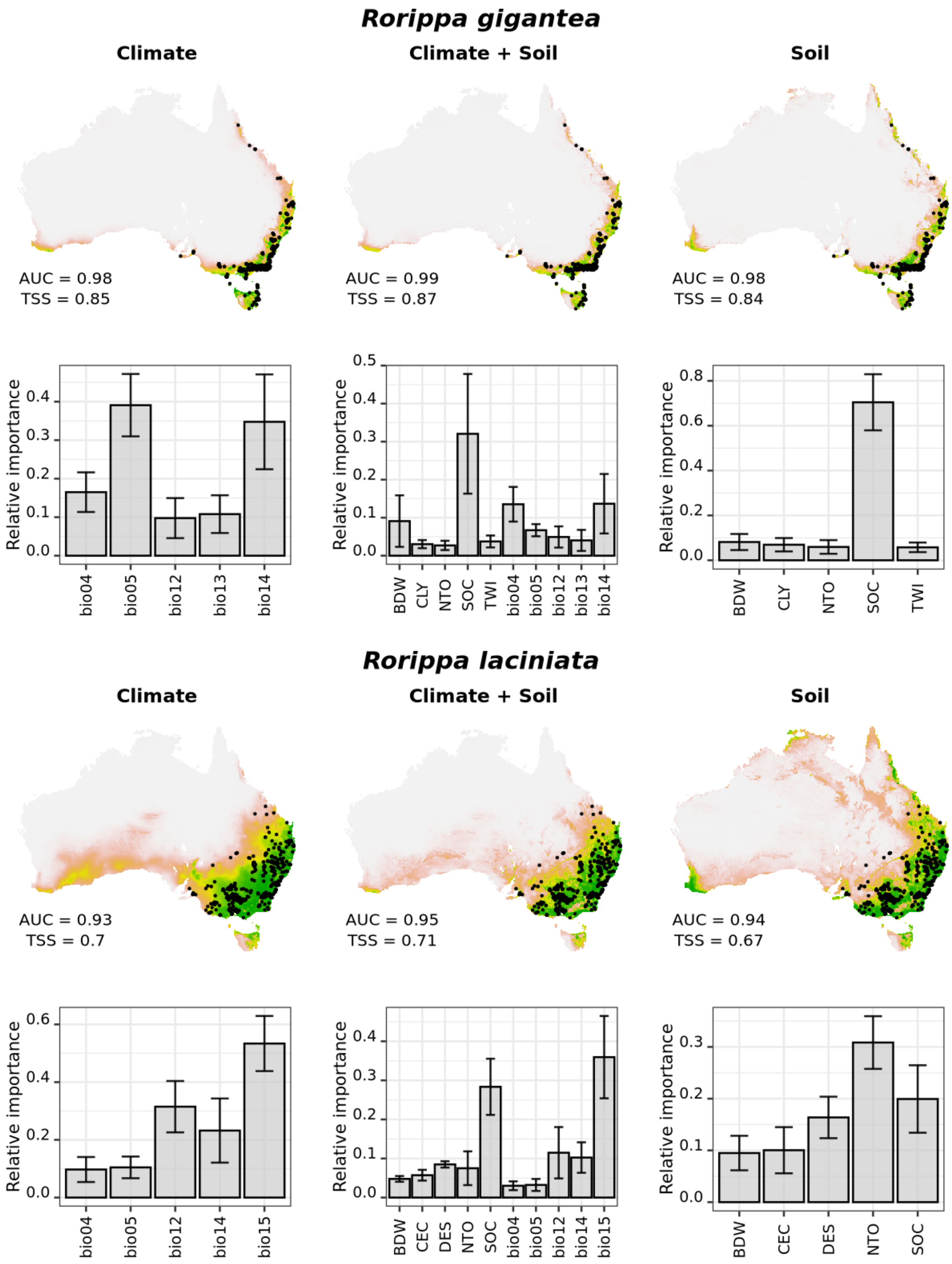

Rorippa gigantea
Climate
Climate + Soil
Soil
AUC = 0.98
TSS = 0.85
AUC = 0.99
TSS = 0.87
AUC = 0.98
TSS = 0.84
Relative importance
bio04 bio05 bio12 bio13 bio14
BDW CLY NTO SOC TWI bio04 bio05 bio12 bio13 bio14
BDW CLY NTO SOC TWI
Rorippa laciniata
Climate
Climate + Soil
Soil
AUC = 0.93
TSS = 0.7
AUC = 0.95
TSS = 0.71
AUC = 0.94
TSS = 0.67
Relative importance
bio04 bio05 bio12 bio14 bio15
BDW CEC DES NTO SOC bio04 bio05 bio12 bio14 bio15
BDW CEC DES NTO SOC

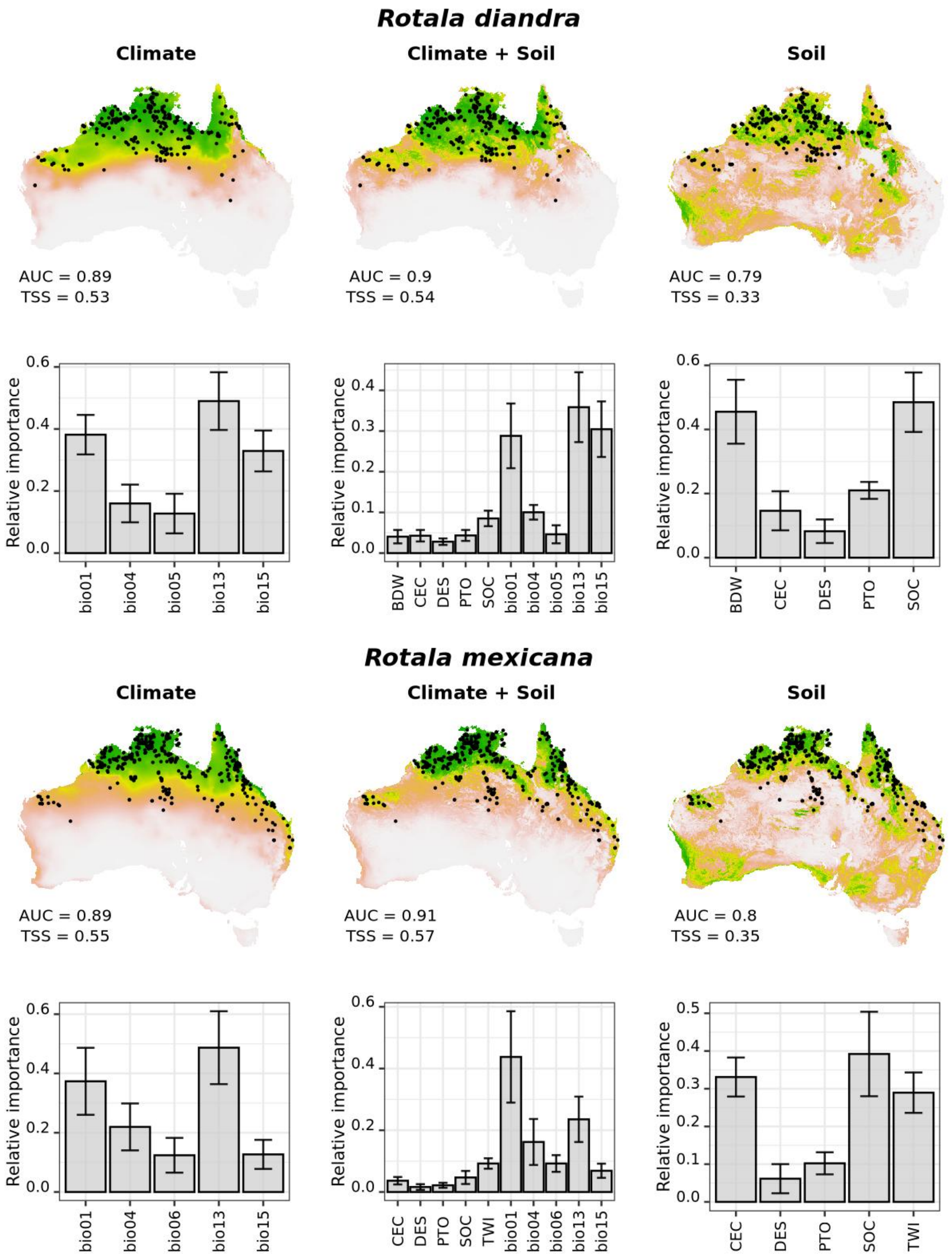

Rotala diandra
Climate
Climate + Soil
Soil
AUC = 0.89
TSS = 0.53
AUC = 0.9
TSS = 0.54
AUC = 0.79
TSS = 0.33
Relative importance
bio01
bio04
bio05
bio13
bio15
BDW
CEC
DES
PTO
SOC
Rotala mexicana
Climate
Climate + Soil
Soil
AUC = 0.89
TSS = 0.55
AUC = 0.91
TSS = 0.57
AUC = 0.8
TSS = 0.35
Relative importance
bio01
bio04
bio06
bio13
bio15
CEC
DES
PTO
SOC
TWI

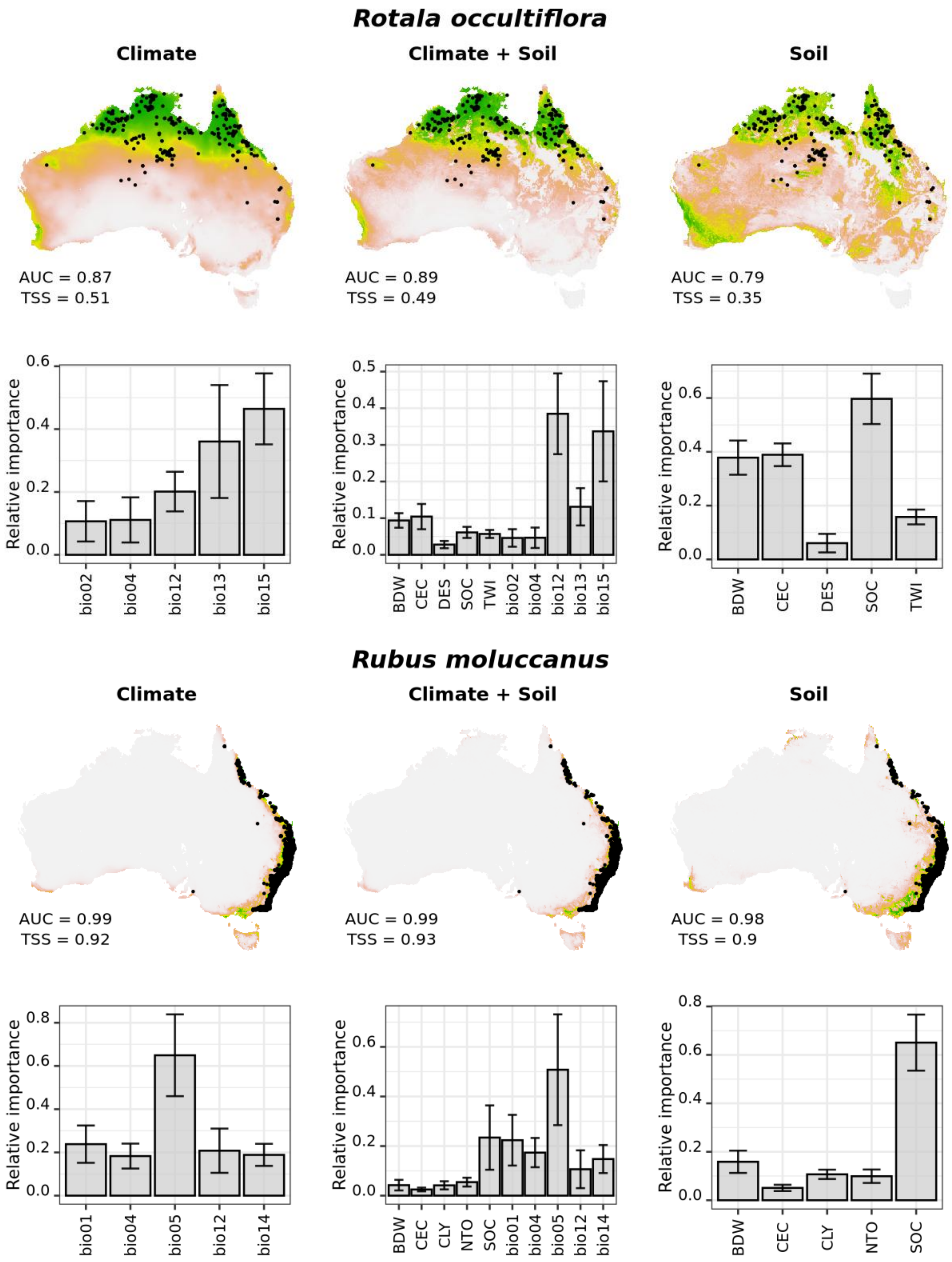
Rotala occultiflora
Climate
Climate + Soil
Soil
AUC = 0.87
TSS = 0.51
AUC = 0.89
TSS = 0.49
AUC = 0.79
TSS = 0.35
Relative importance
bio02
bio04
bio12
bio13
bio15
BDW
CEC
DES
SOC
TWI
Rubus moluccanus
AUC = 0.99
TSS = 0.92
AUC = 0.99
TSS = 0.93
AUC = 0.98
TSS = 0.9
bio01
bio05
bio14
CLY
NTO

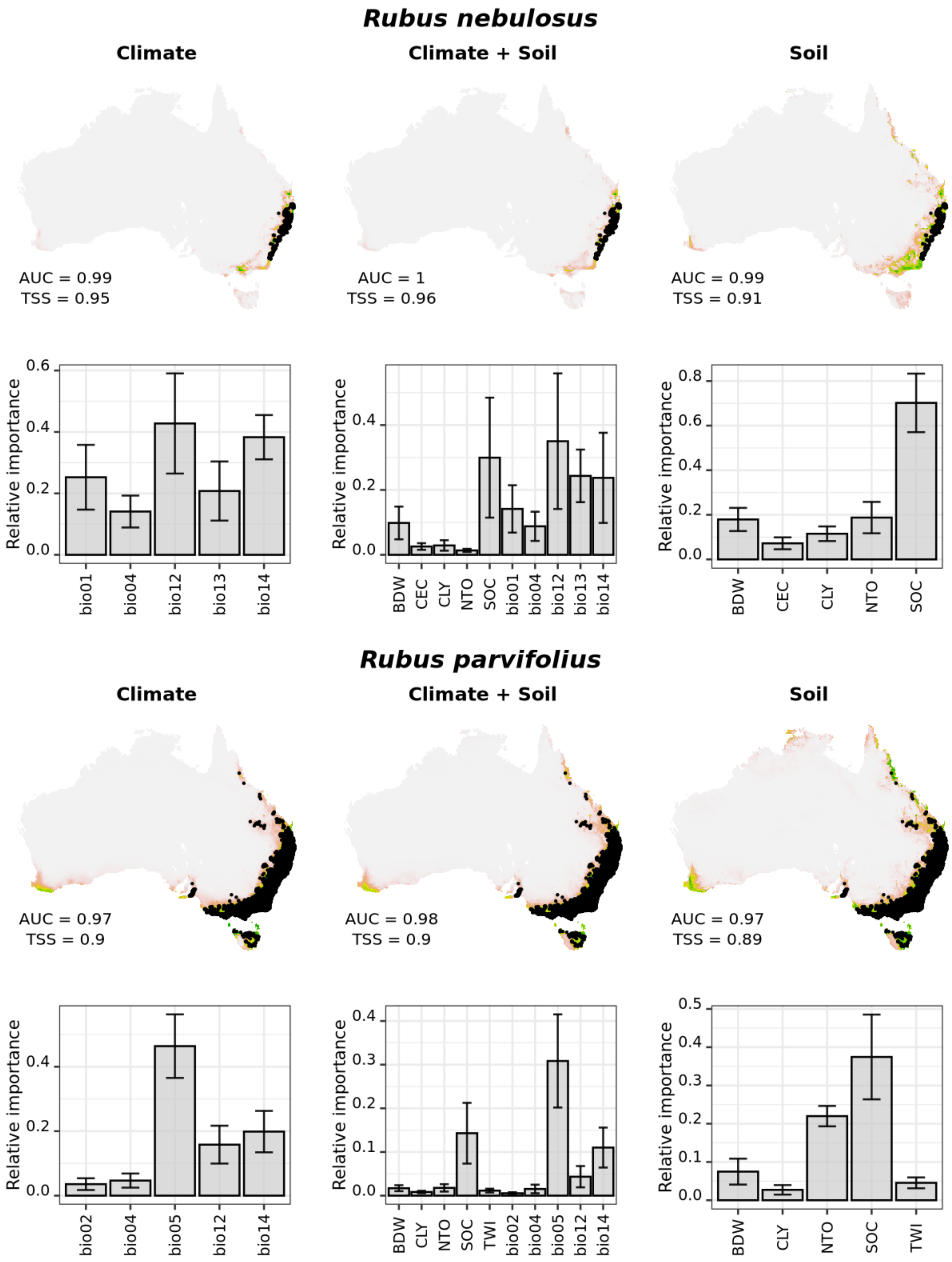
Rubus nebulosus
Climate
Climate + Soil
Soil
AUC = 0.99
TSS = 0.95
AUC = 1
TSS = 0.96
AUC = 0.99
TSS = 0.91
Relative importance
0.0
0.2
0.4
0.6
bio01
bio04
bio12
bio13
bio14
BDW
CEC
CLY
NTO
SOC
0.8
Rubus parvifolius
AUC = 0.97
TSS = 0.9
AUC = 0.98
TSS = 0.9
AUC = 0.97
TSS = 0.89
0.1
0.3
0.5
bio02
bio05
TWI

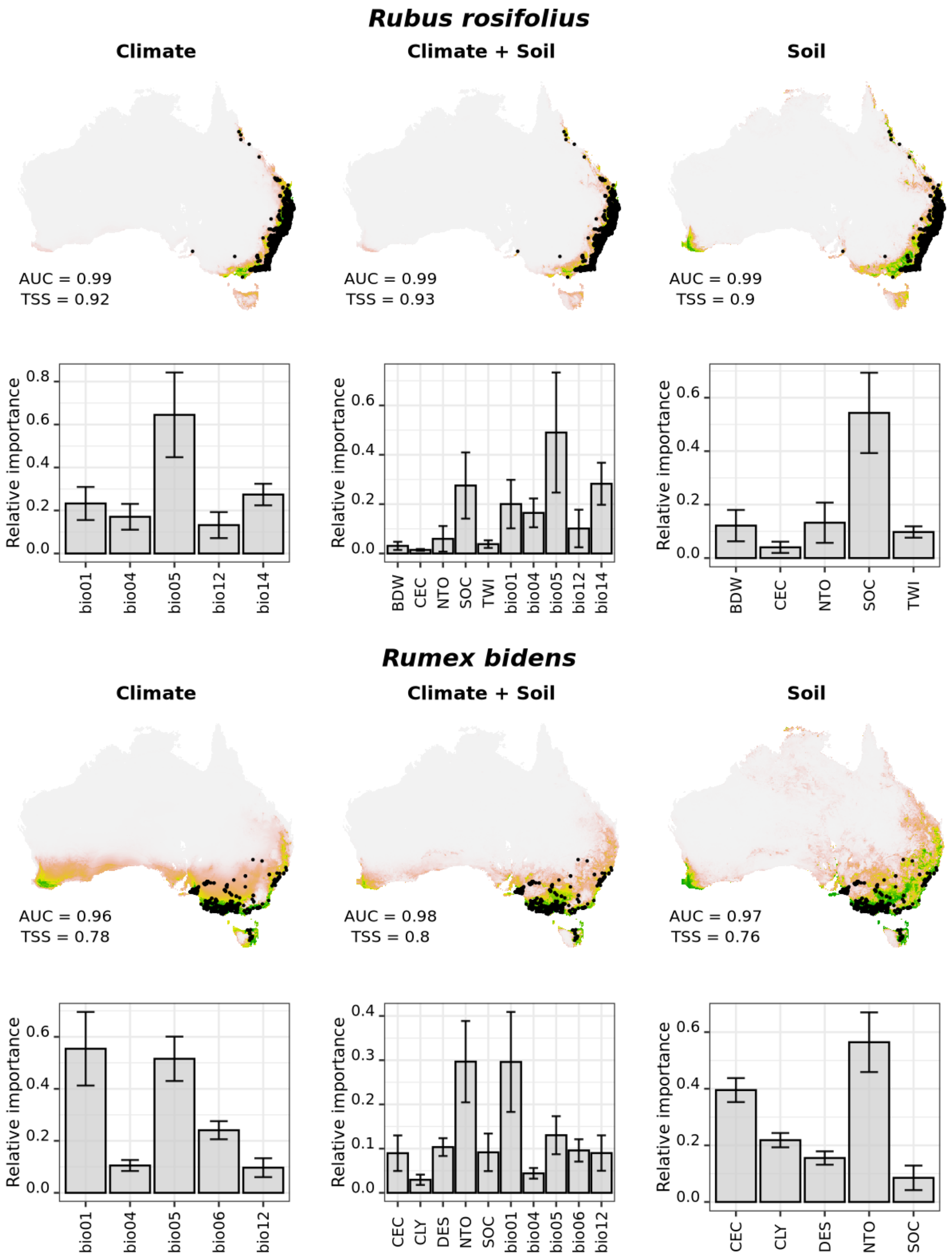
Rubus rosifolius
Climate
Climate + Soil
Soil
AUC = 0.99
TSS = 0.92
AUC = 0.99
TSS = 0.93
AUC = 0.99
TSS = 0.9
Relative importance
bio01
bio04
bio05
bio12
bio14
BDW
CEC
NTO
SOC
TWI
Rumex bidens
Climate
Climate + Soil
Soil
AUC = 0.96
TSS = 0.78
AUC = 0.98
TSS = 0.8
AUC = 0.97
TSS = 0.76
Relative importance
bio01
bio04
bio05
bio06
bio12
CEC
CLY
DES
NTO
SOC

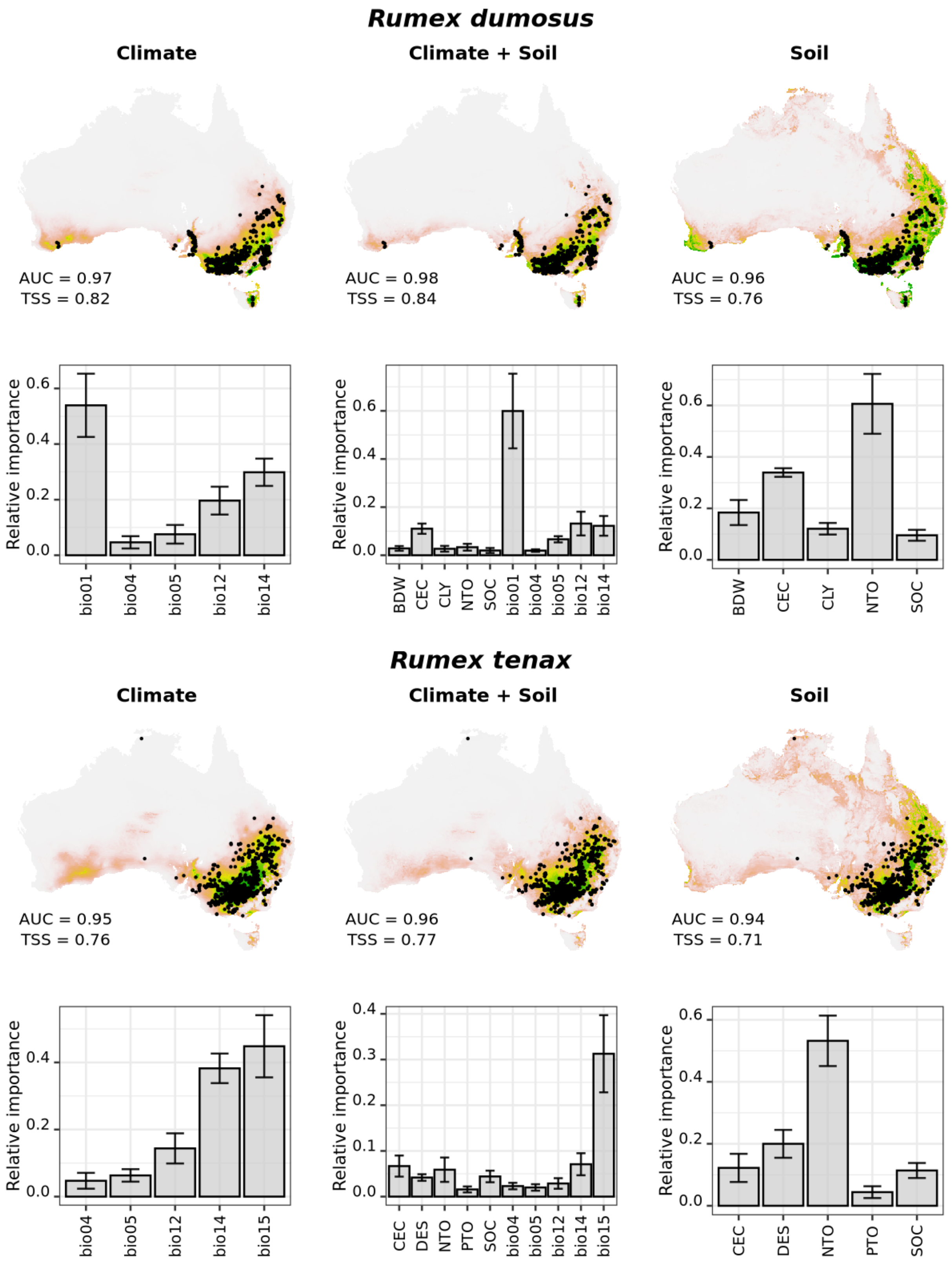
Rumex dumosus
Climate
Climate + Soil
Soil
AUC = 0.97
TSS = 0.82
AUC = 0.98
TSS = 0.84
AUC = 0.96
TSS = 0.76
Relative importance
bio01 bio04 bio05 bio12 bio14
BDW CEC CLY NTO SOC bio01 bio04 bio05 bio12 bio14
BDW CEC CLY NTO SOC
Rumex tenax
Climate
Climate + Soil
Soil
AUC = 0.95
TSS = 0.76
AUC = 0.96
TSS = 0.77
AUC = 0.94
TSS = 0.71
Relative importance
bio04 bio05 bio12 bio14 bio15
CEC DES NTO PTO SOC bio04 bio05 bio12 bio14 bio15
CEC DES NTO PTO SOC

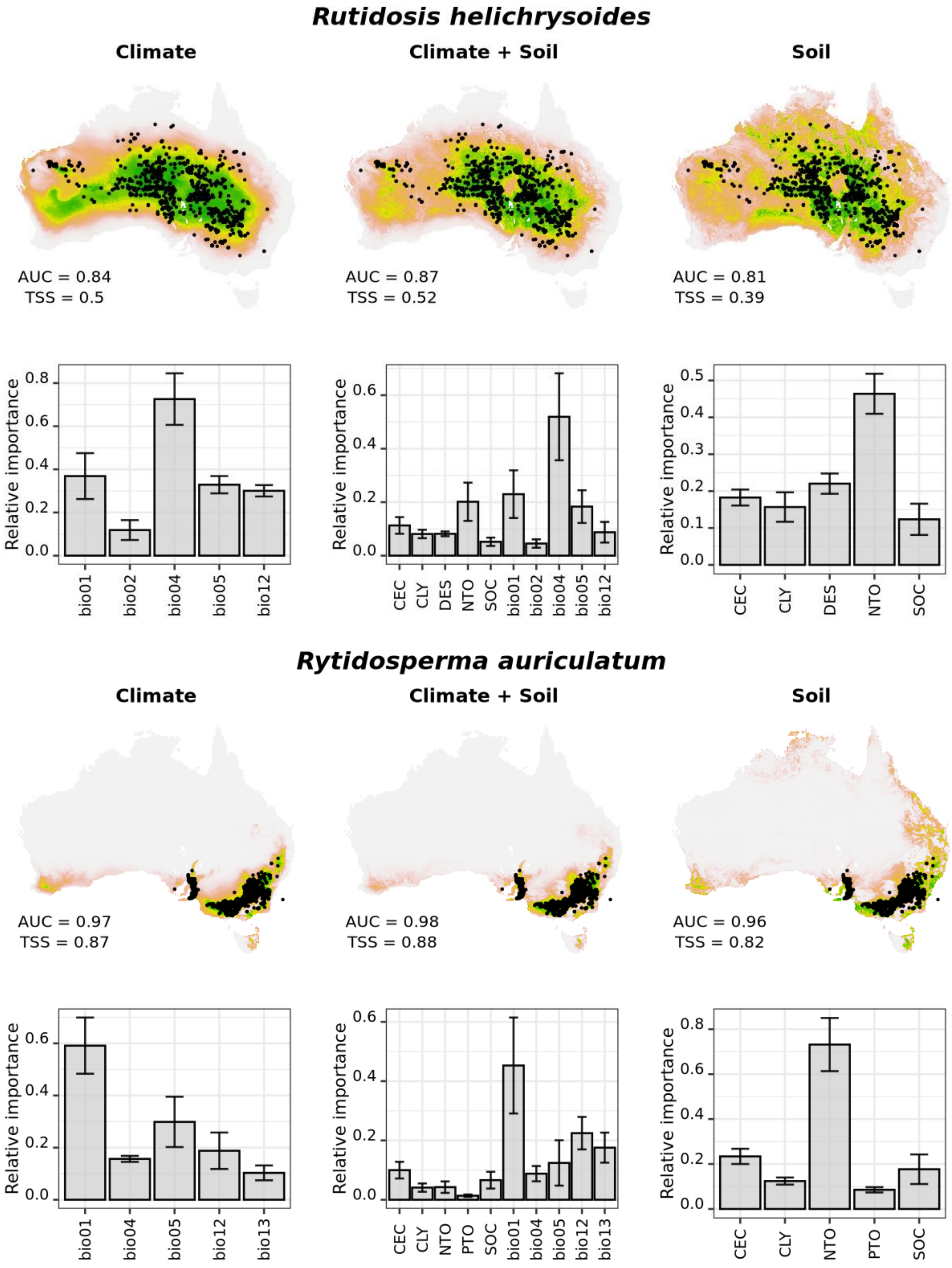
Rutidosis helichrysoides
Climate
Climate + Soil
Soil
AUC = 0.84
TSS = 0.5
AUC = 0.87
TSS = 0.52
AUC = 0.81
TSS = 0.39
Relative importance
bio01
bio02
bio04
bio05
bio12
CEC
CLY
DES
NTO
SOC
Rytidosperma auriculatum
Climate
Climate + Soil
Soil
AUC = 0.97
TSS = 0.87
AUC = 0.98
TSS = 0.88
AUC = 0.96
TSS = 0.82
Relative importance
bio01
bio04
bio05
bio12
bio13
CEC
CLY
NTO
PTO
SOC

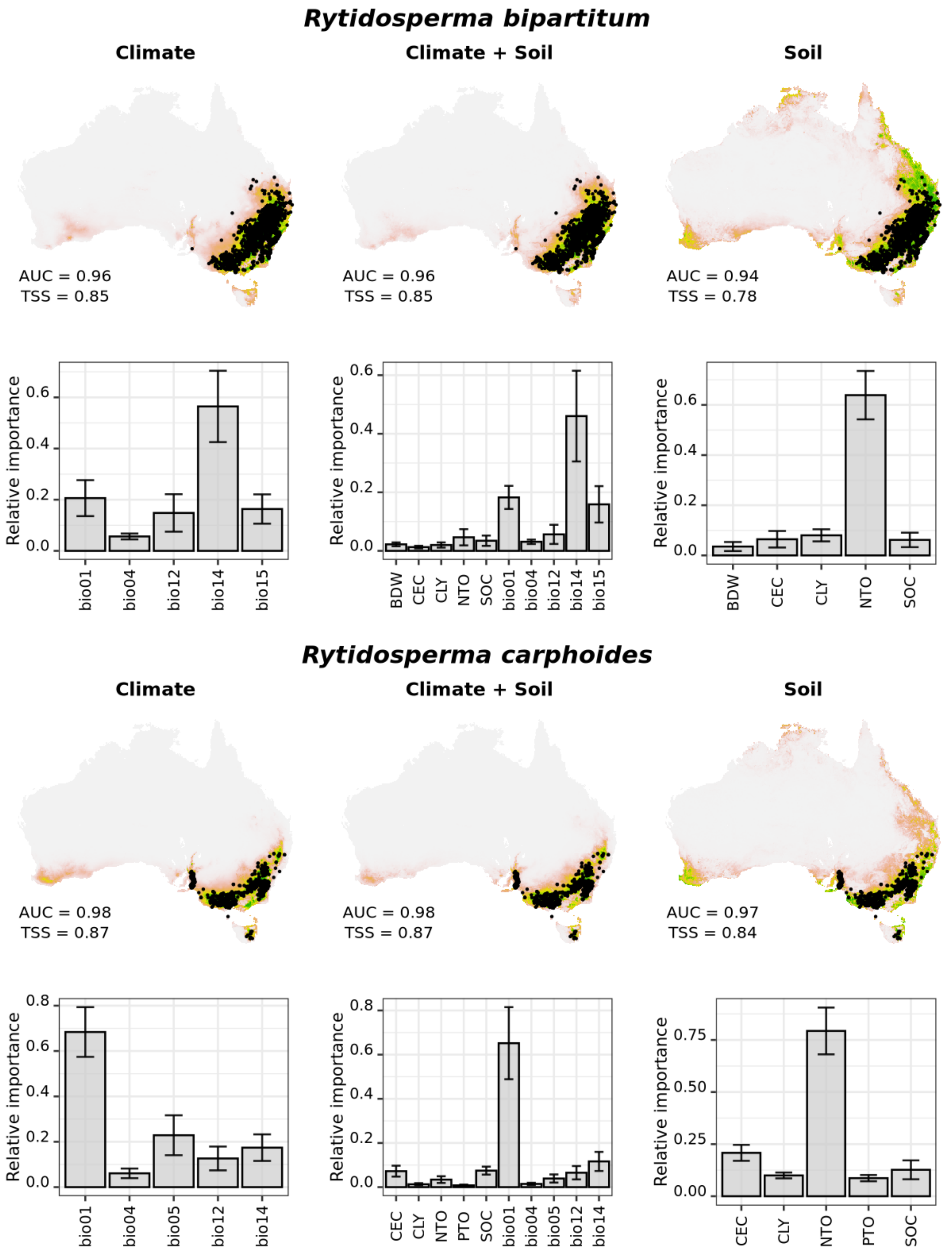

Rytidosperma bipartitum
Climate
Climate + Soil
Soil
AUC = 0.96
TSS = 0.85
AUC = 0.96
TSS = 0.85
AUC = 0.94
TSS = 0.78
Relative importance
0.0 0.2 0.4 0.6
bio01 bio04 bio12 bio14 bio15
Relative importance
0.0 0.2 0.4 0.6
BDW CEC CLY NTO SOC bio01 bio04 bio12 bio14 bio15
Relative importance
0.0 0.2 0.4 0.6
BDW CEC CLY NTO SOC
Rytidosperma carphoides
Climate
Climate + Soil
Soil
AUC = 0.98
TSS = 0.87
AUC = 0.98
TSS = 0.87
AUC = 0.97
TSS = 0.84
Relative importance
0.0 0.2 0.4 0.6 0.8
bio01 bio04 bio05 bio12 bio14
Relative importance
0.0 0.2 0.4 0.6 0.8
CEC CLY NTO PTO SOC bio01 bio04 bio05 bio12 bio14
Relative importance
0.00 0.25 0.50 0.75
CEC CLY NTO PTO SOC

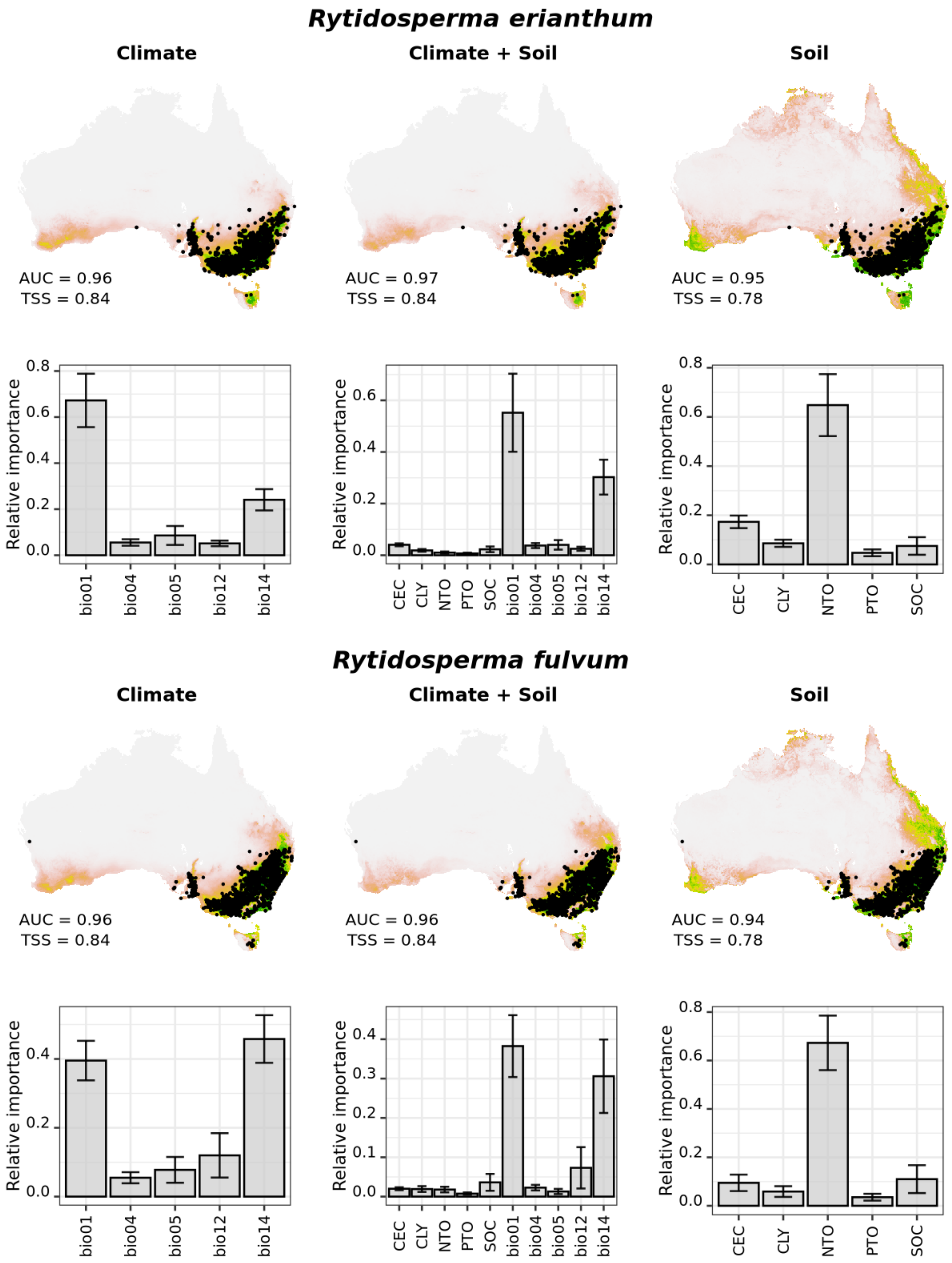

Rytidosperma erianthum
Climate
Climate + Soil
Soil
AUC = 0.96
TSS = 0.84
AUC = 0.97
TSS = 0.84
AUC = 0.95
TSS = 0.78
Relative importance
0.0
0.2
0.4
0.6
0.8
bio01
bio04
bio05
bio12
bio14
CEC
CLY
NTO
PTO
SOC
Rytidosperma fulvum
Climate
Climate + Soil
Soil
AUC = 0.96
TSS = 0.84
AUC = 0.96
TSS = 0.84
AUC = 0.94
TSS = 0.78
0.1
0.3

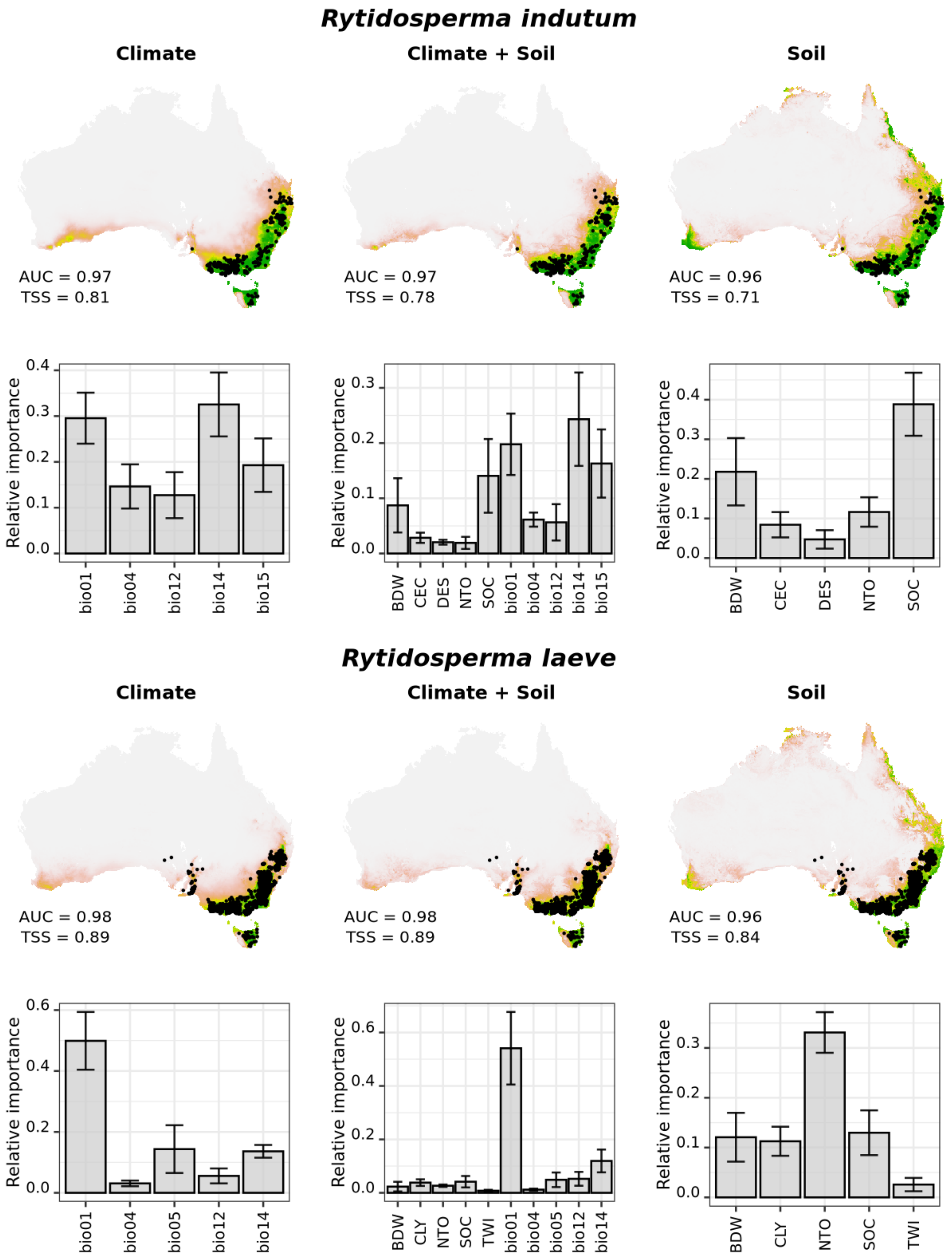

Rytidosperma indutum
Climate
Climate + Soil
Soil
AUC = 0.97
TSS = 0.81
AUC = 0.97
TSS = 0.78
AUC = 0.96
TSS = 0.71
Relative importance
0.0
0.1
0.2
0.3
0.4
bio01
bio04
bio12
bio14
bio15
BDW
CEC
DES
NTO
SOC
Rytidosperma laeve
AUC = 0.98
TSS = 0.89
AUC = 0.98
TSS = 0.89
AUC = 0.96
TSS = 0.84
0.6
bio05
CLY
TWI

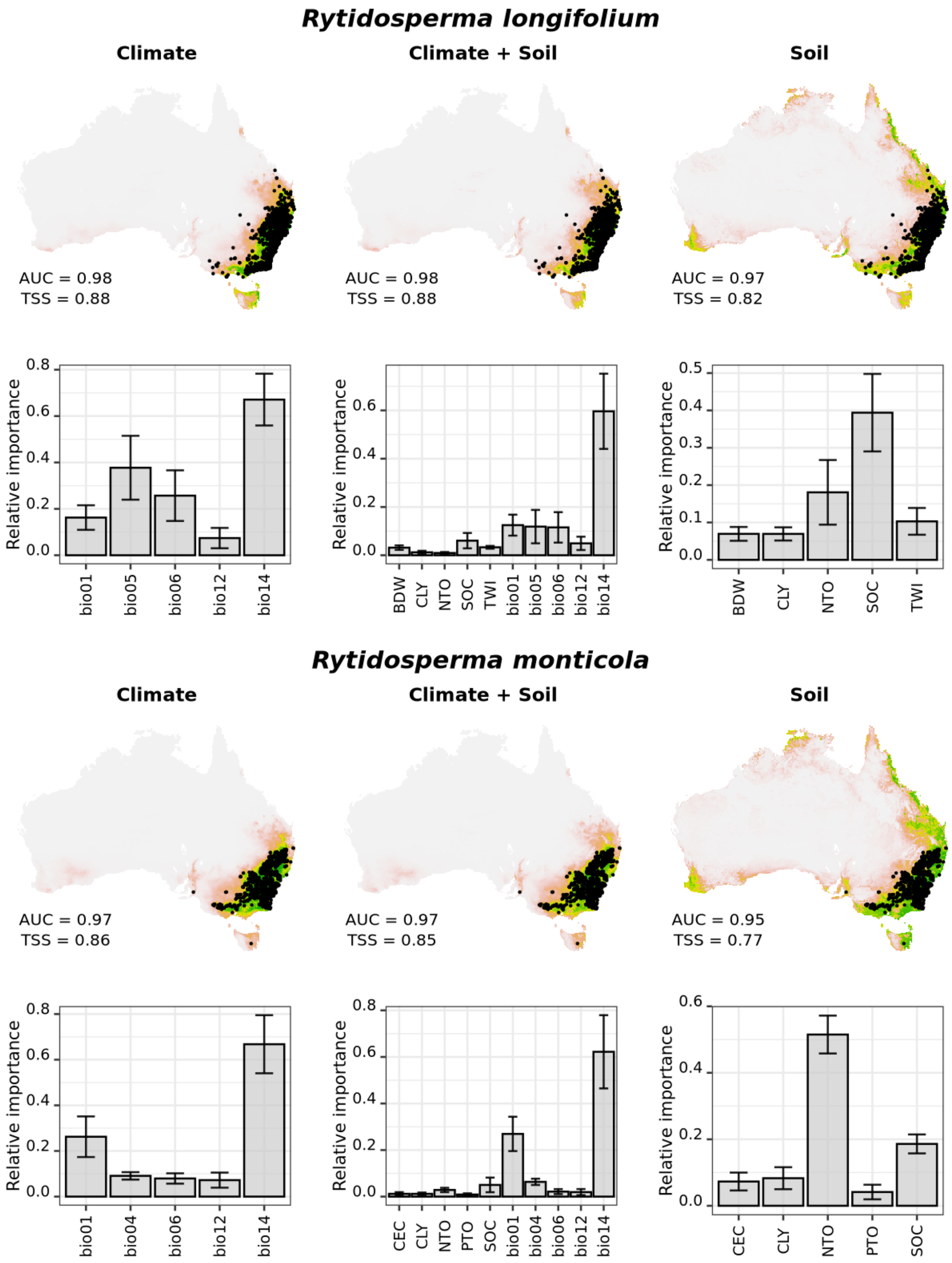
Rytidosperma longifolium
Climate
Climate + Soil
Soil
AUC = 0.98
TSS = 0.88
AUC = 0.98
TSS = 0.88
AUC = 0.97
TSS = 0.82
Relative importance
bio01
bio05
bio06
bio12
bio14
BDW
CLY
NTO
SOC
TWI
Rytidosperma monticola
AUC = 0.97
TSS = 0.86
AUC = 0.97
TSS = 0.85
AUC = 0.95
TSS = 0.77
bio04
CEC
PTO

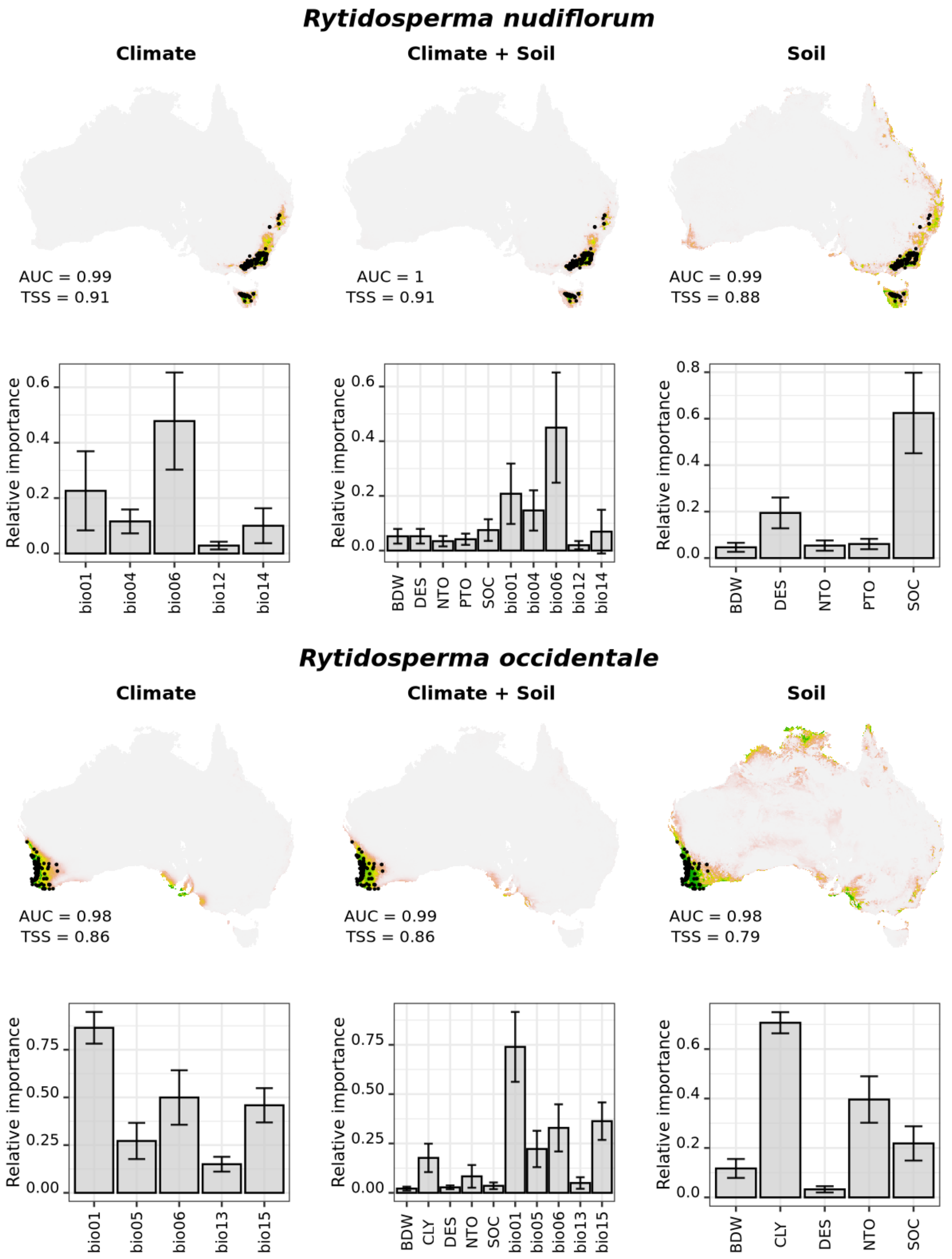
Rytidosperma nudiflorum
Climate
Climate + Soil
Soil
AUC = 0.99
TSS = 0.91
AUC = 1
TSS = 0.91
AUC = 0.99
TSS = 0.88
Relative importance
0.0
0.2
0.4
0.6
0.8
bio01
bio04
bio06
bio12
bio14
BDW
DES
NTO
PTO
SOC
Rytidosperma occidentale
Climate
Climate + Soil
Soil
AUC = 0.98
TSS = 0.86
AUC = 0.99
TSS = 0.86
AUC = 0.98
TSS = 0.79
Relative importance
0.00
0.25
0.50
0.75
bio01
bio05
bio06
bio13
bio15
BDW
CLY
DES
NTO
SOC

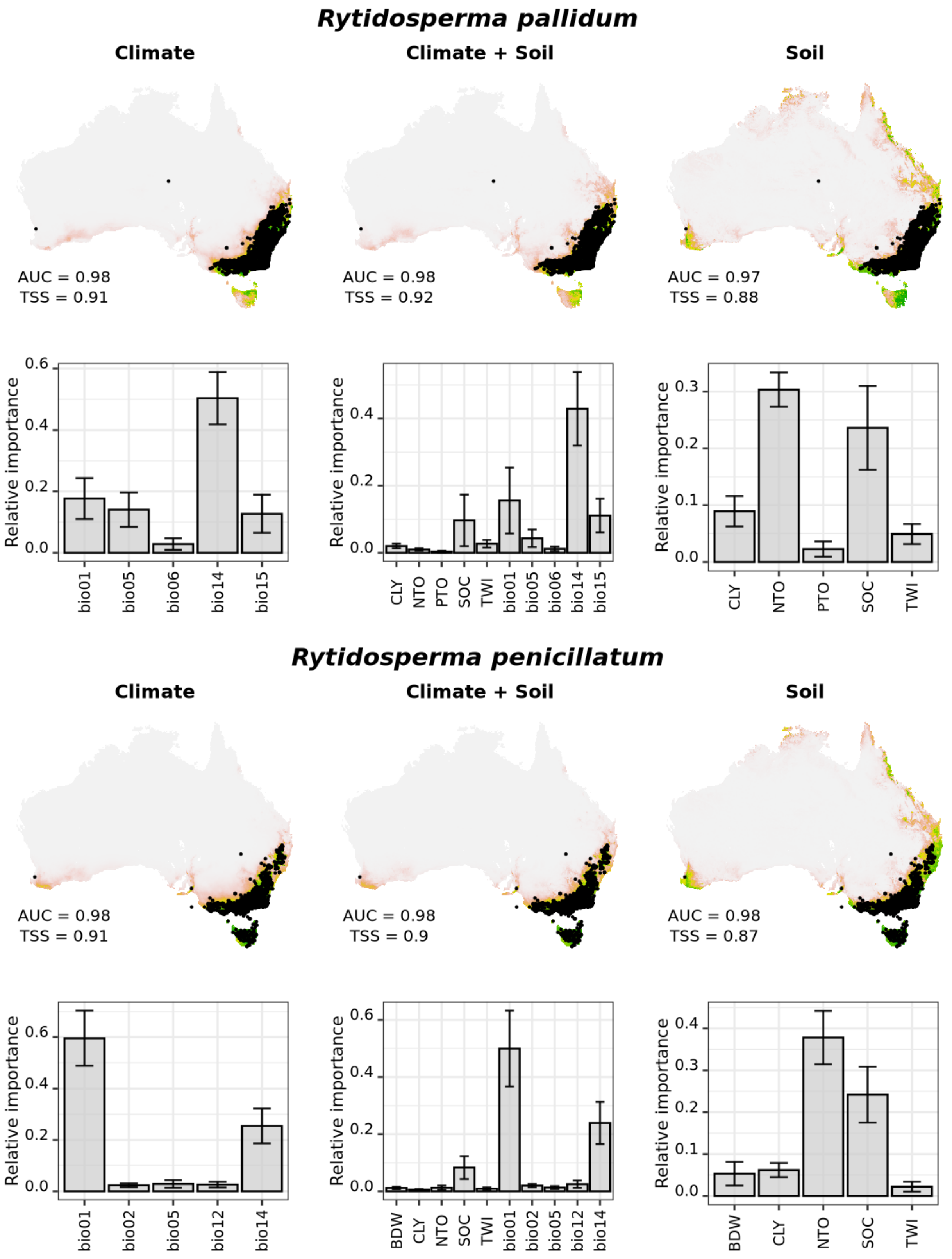

Rytidosperma pallidum
Climate
Climate + Soil
Soil
AUC = 0.98
TSS = 0.91
AUC = 0.98
TSS = 0.92
AUC = 0.97
TSS = 0.88
Relative importance
bio01
bio05
bio06
bio14
bio15
CLY
NTO
PTO
SOC
TWI
Rytidosperma penicillatum
Climate
Climate + Soil
Soil
AUC = 0.98
TSS = 0.91
AUC = 0.98
TSS = 0.9
AUC = 0.98
TSS = 0.87
Relative importance
bio01
bio02
bio05
bio12
bio14
BDW
CLY
NTO
SOC
TWI

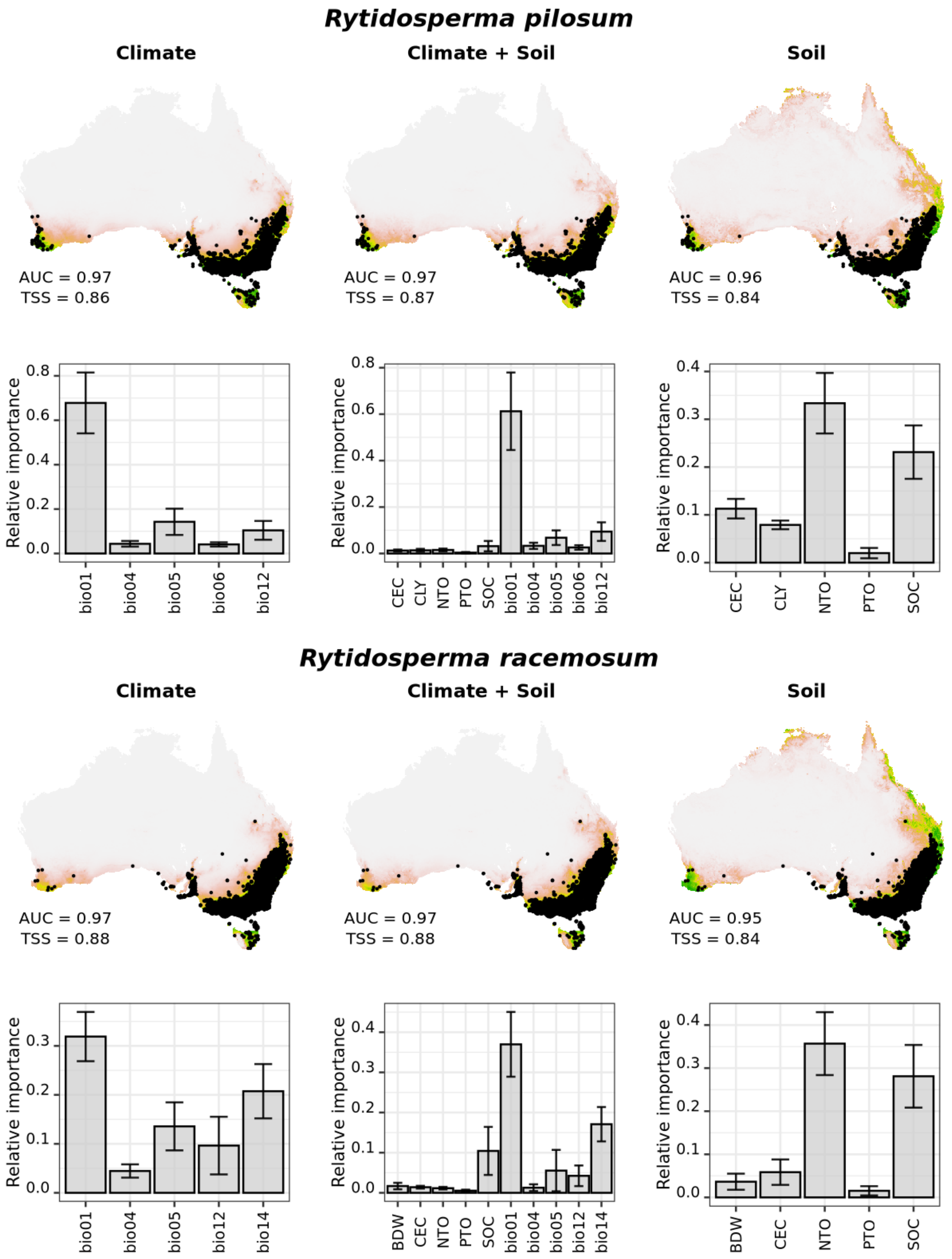
Rytidosperma pilosum
Climate
Climate + Soil
Soil
AUC = 0.97
TSS = 0.86
AUC = 0.97
TSS = 0.87
AUC = 0.96
TSS = 0.84
Relative importance
bio01
bio04
bio05
bio06
bio12
CEC
CLY
NTO
PTO
SOC
Rytidosperma racemosum
Climate
Climate + Soil
Soil
AUC = 0.97
TSS = 0.88
AUC = 0.97
TSS = 0.88
AUC = 0.95
TSS = 0.84
Relative importance
bio01
bio04
bio05
bio12
bio14
BDW
CEC
NTO
PTO
SOC

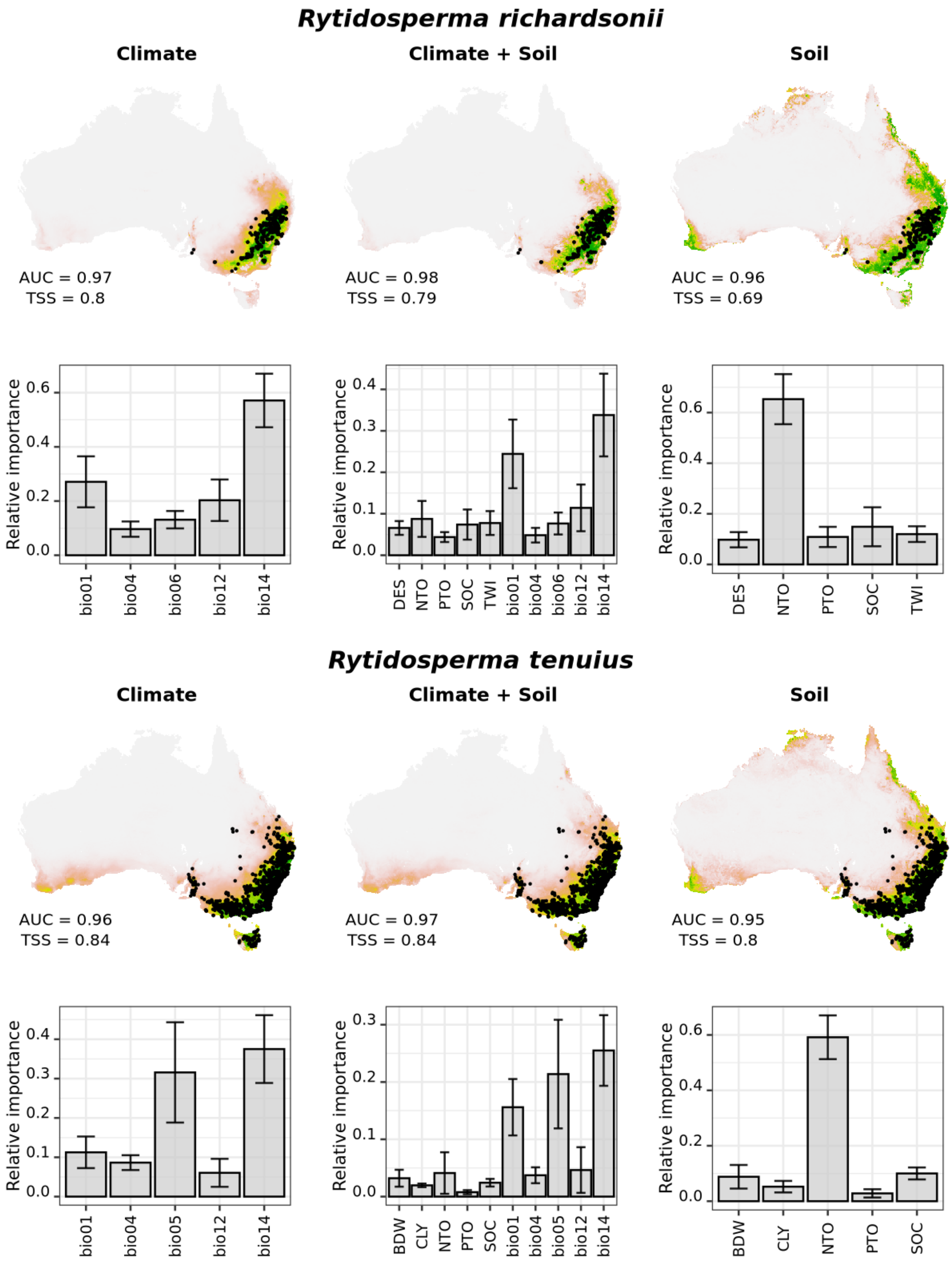
Rytidosperma richardsonii
Climate
Climate + Soil
Soil
AUC = 0.97
TSS = 0.8
AUC = 0.98
TSS = 0.79
AUC = 0.96
TSS = 0.69
Relative importance
bio01
bio04
bio06
bio12
bio14
DES
NTO
PTO
SOC
TWI
Rytidosperma tenuius
Climate
Climate + Soil
Soil
AUC = 0.96
TSS = 0.84
AUC = 0.97
TSS = 0.84
AUC = 0.95
TSS = 0.8
Relative importance
bio01
bio04
bio05
bio12
bio14
BDW
CLY
NTO
PTO
SOC

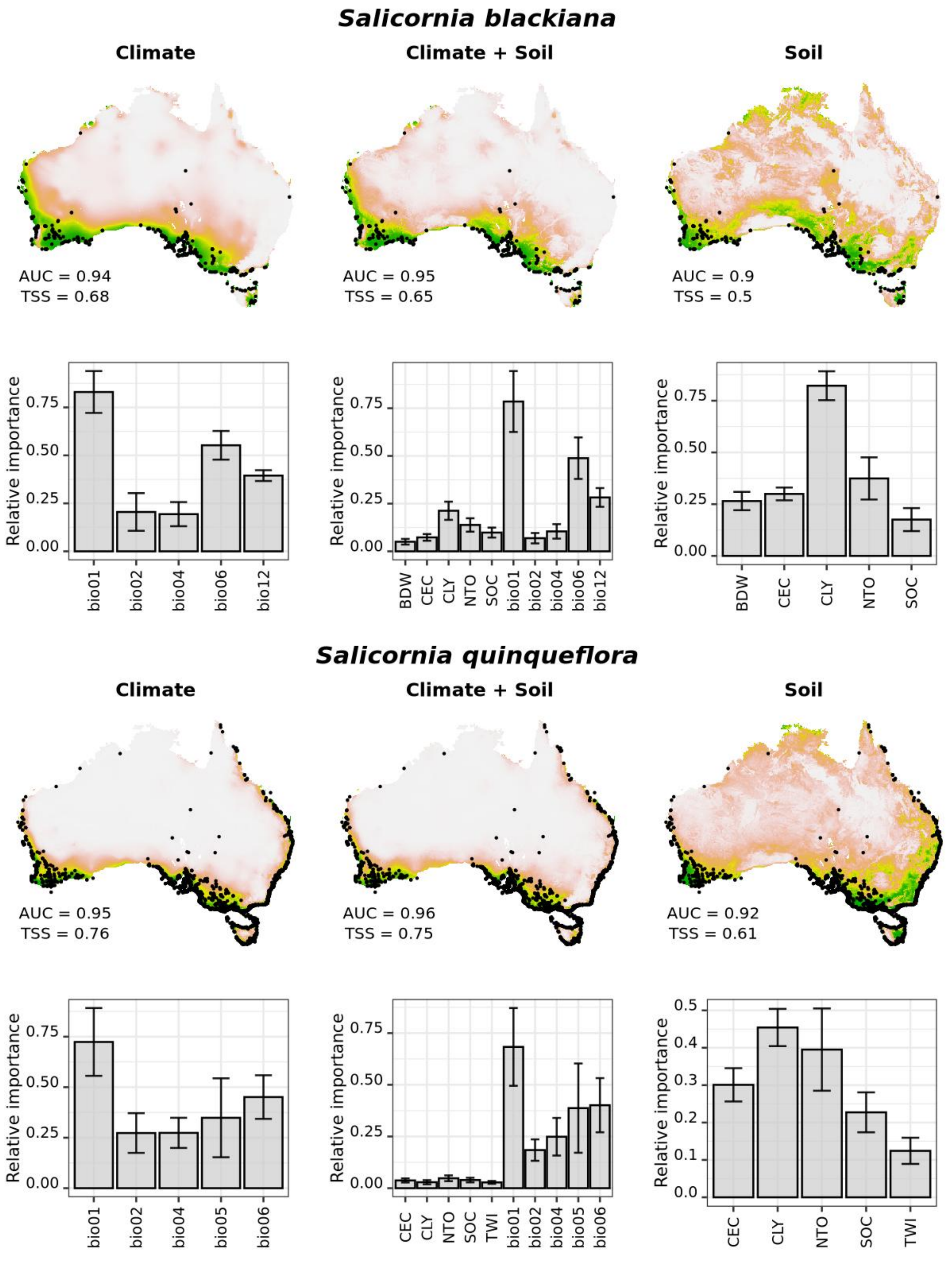
Salicornia blackiana
Climate
Climate + Soil
Soil
AUC = 0.94
TSS = 0.68
AUC = 0.95
TSS = 0.65
AUC = 0.9
TSS = 0.5
Relative importance
bio01
bio02
bio04
bio06
bio12
BDW
CEC
CLY
NTO
SOC
Salicornia quinqueflora
AUC = 0.95
TSS = 0.76
AUC = 0.96
TSS = 0.75
AUC = 0.92
TSS = 0.61
bio05
TWI

Salomonia ciliata

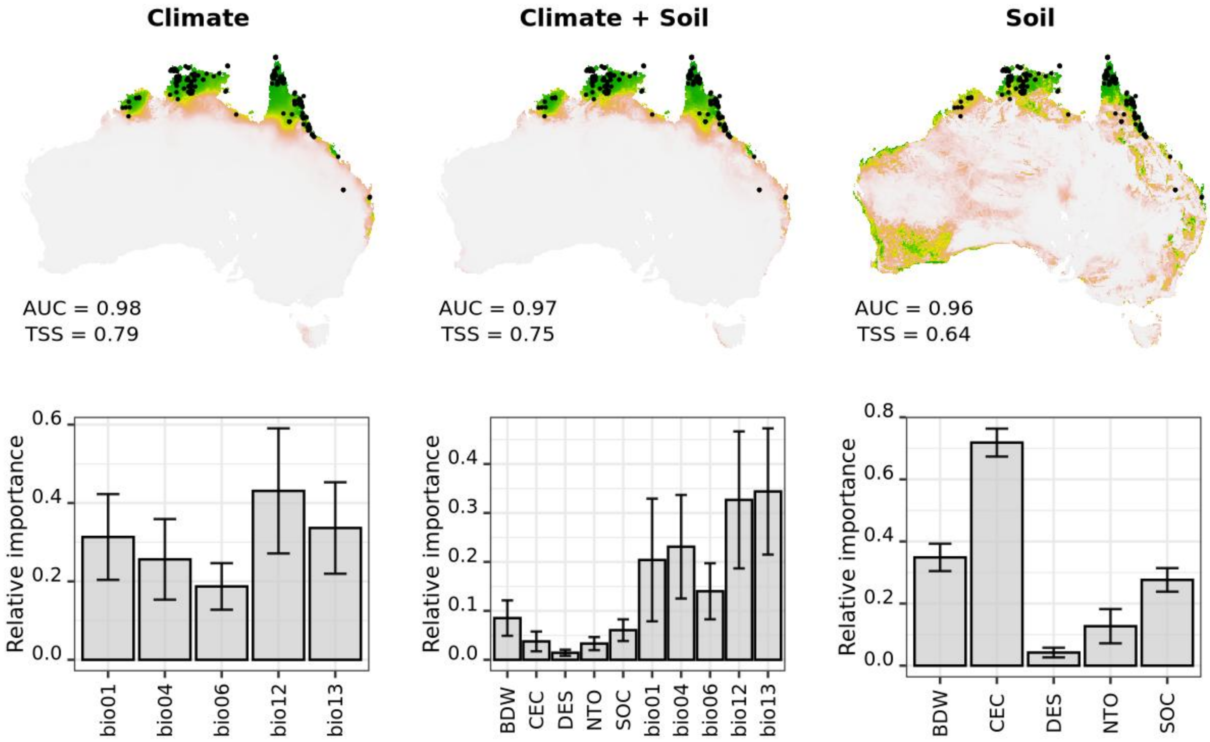

Sambucus australasica

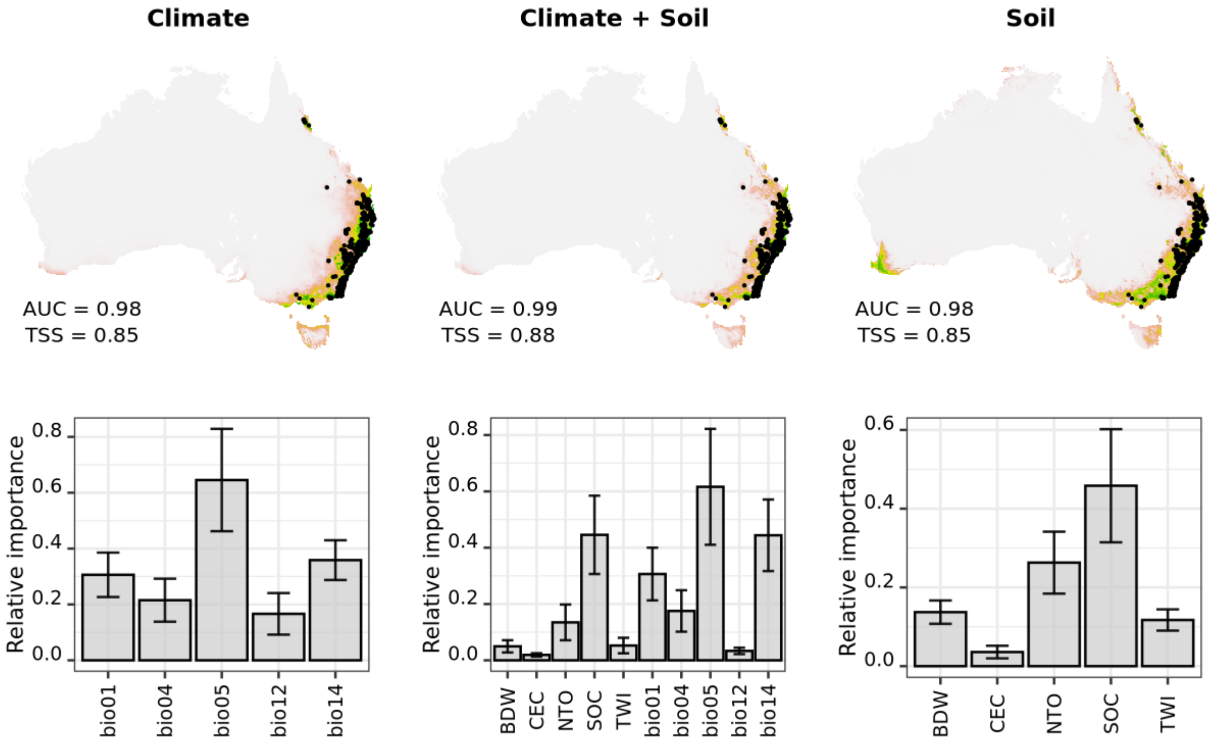

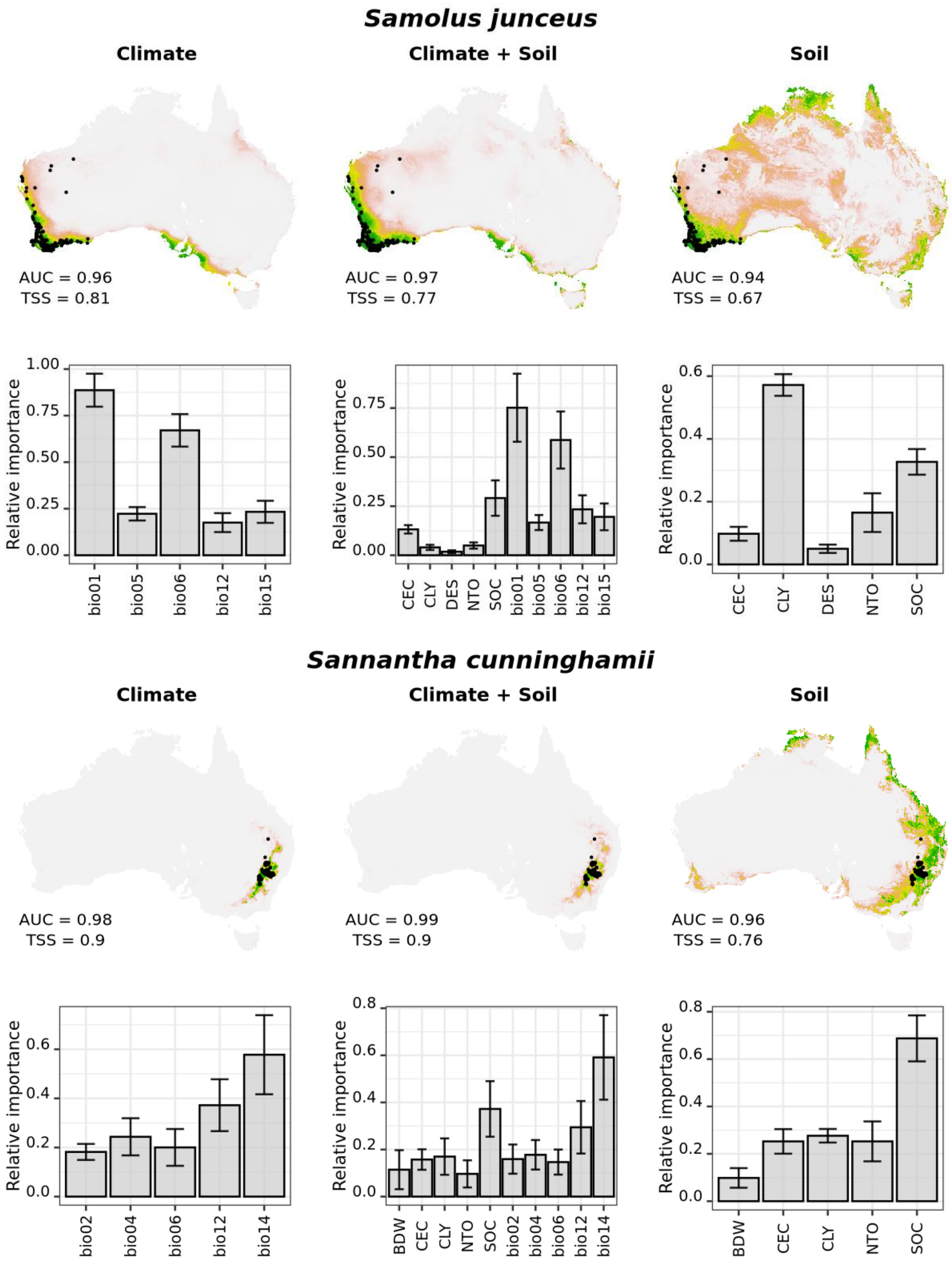
Samolus junceus
Climate
Climate + Soil
Soil
AUC = 0.96
TSS = 0.81
AUC = 0.97
TSS = 0.77
AUC = 0.94
TSS = 0.67
Relative importance
bio01
bio05
bio06
bio12
bio15
CEC
CLY
DES
NTO
SOC
Sannantha cunninghamii
Climate
Climate + Soil
Soil
AUC = 0.98
TSS = 0.9
AUC = 0.99
TSS = 0.9
AUC = 0.96
TSS = 0.76
Relative importance
bio02
bio04
bio06
bio12
bio14
BDW
CEC
CLY
NTO
SOC

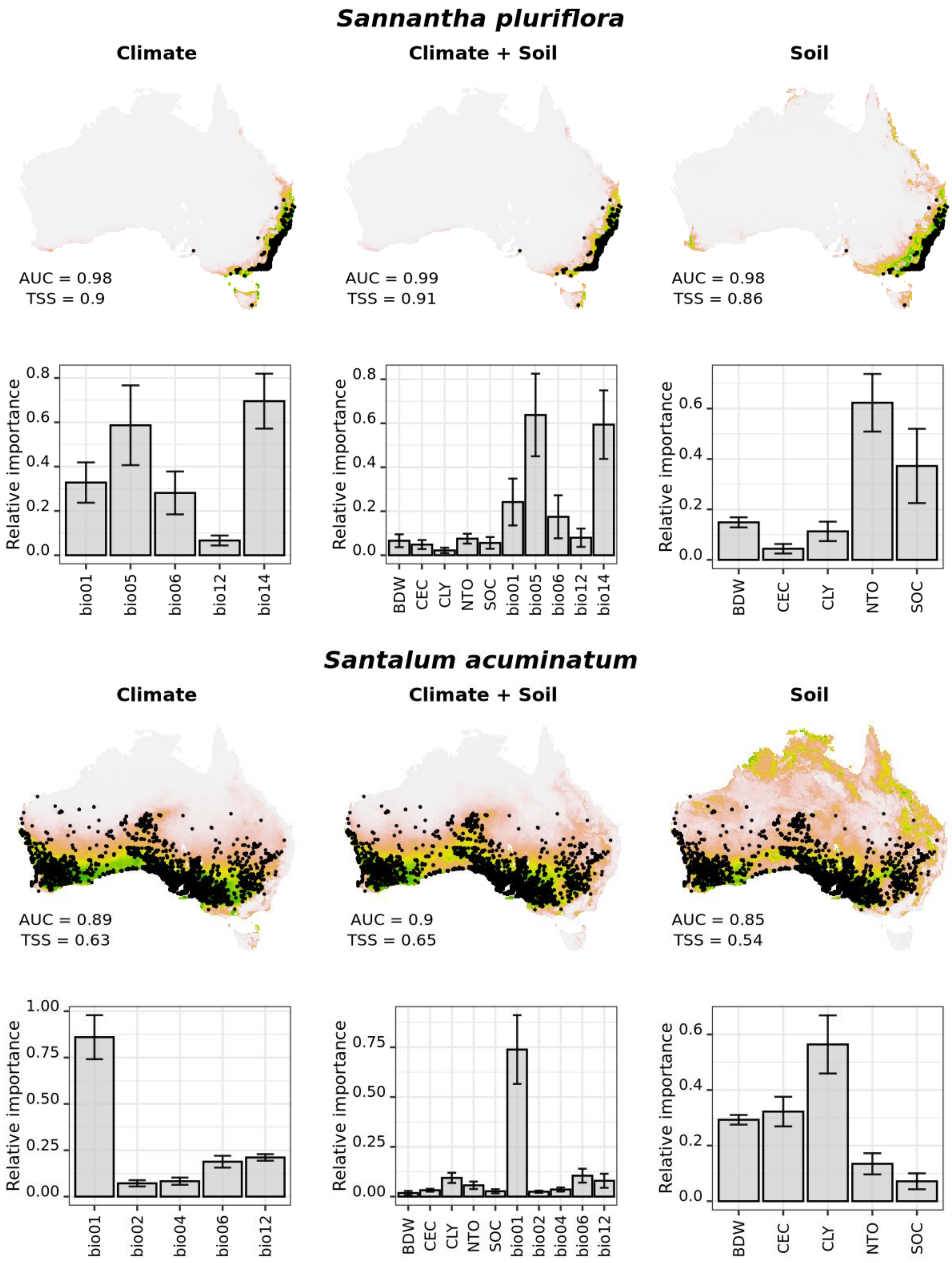
Sannantha pluriflora
Climate
Climate + Soil
Soil
AUC = 0.98
TSS = 0.9
AUC = 0.99
TSS = 0.91
AUC = 0.98
TSS = 0.86
Relative importance
bio01
bio05
bio06
bio12
bio14
BDW
CEC
CLY
NTO
SOC
Santalum acuminatum
Climate
Climate + Soil
Soil
AUC = 0.89
TSS = 0.63
AUC = 0.9
TSS = 0.65
AUC = 0.85
TSS = 0.54
Relative importance
bio01
bio02
bio04
bio06
bio12
BDW
CEC
CLY
NTO
SOC

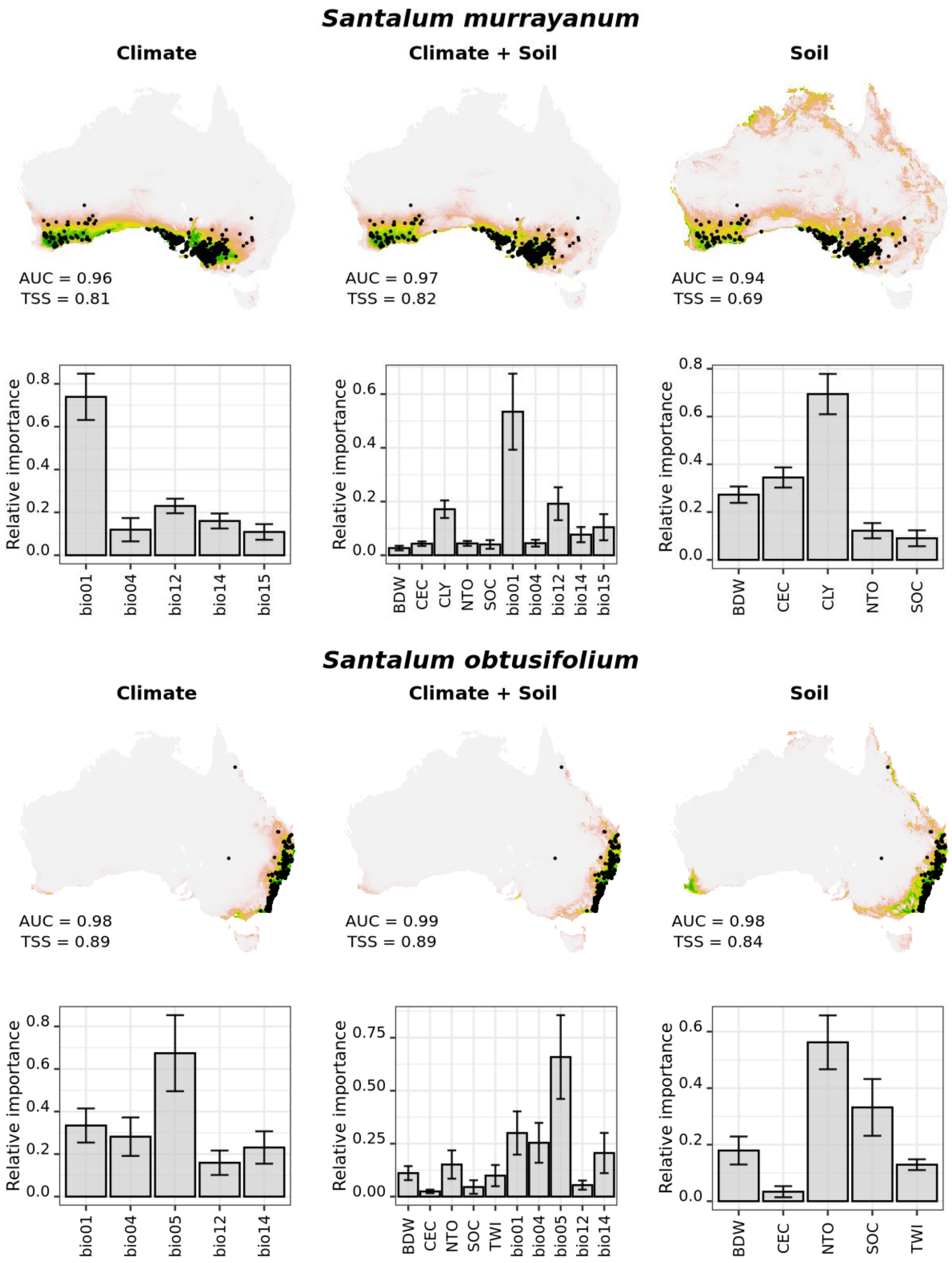

Santalum murrayanum
Climate
Climate + Soil
Soil
AUC = 0.96
TSS = 0.81
AUC = 0.97
TSS = 0.82
AUC = 0.94
TSS = 0.69
Relative importance
bio01
bio04
bio12
bio14
bio15
BDW
CEC
CLY
NTO
SOC
Santalum obtusifolium
Climate
Climate + Soil
Soil
AUC = 0.98
TSS = 0.89
AUC = 0.99
TSS = 0.89
AUC = 0.98
TSS = 0.84
Relative importance
bio01
bio04
bio05
bio12
bio14
BDW
CEC
NTO
SOC
TWI

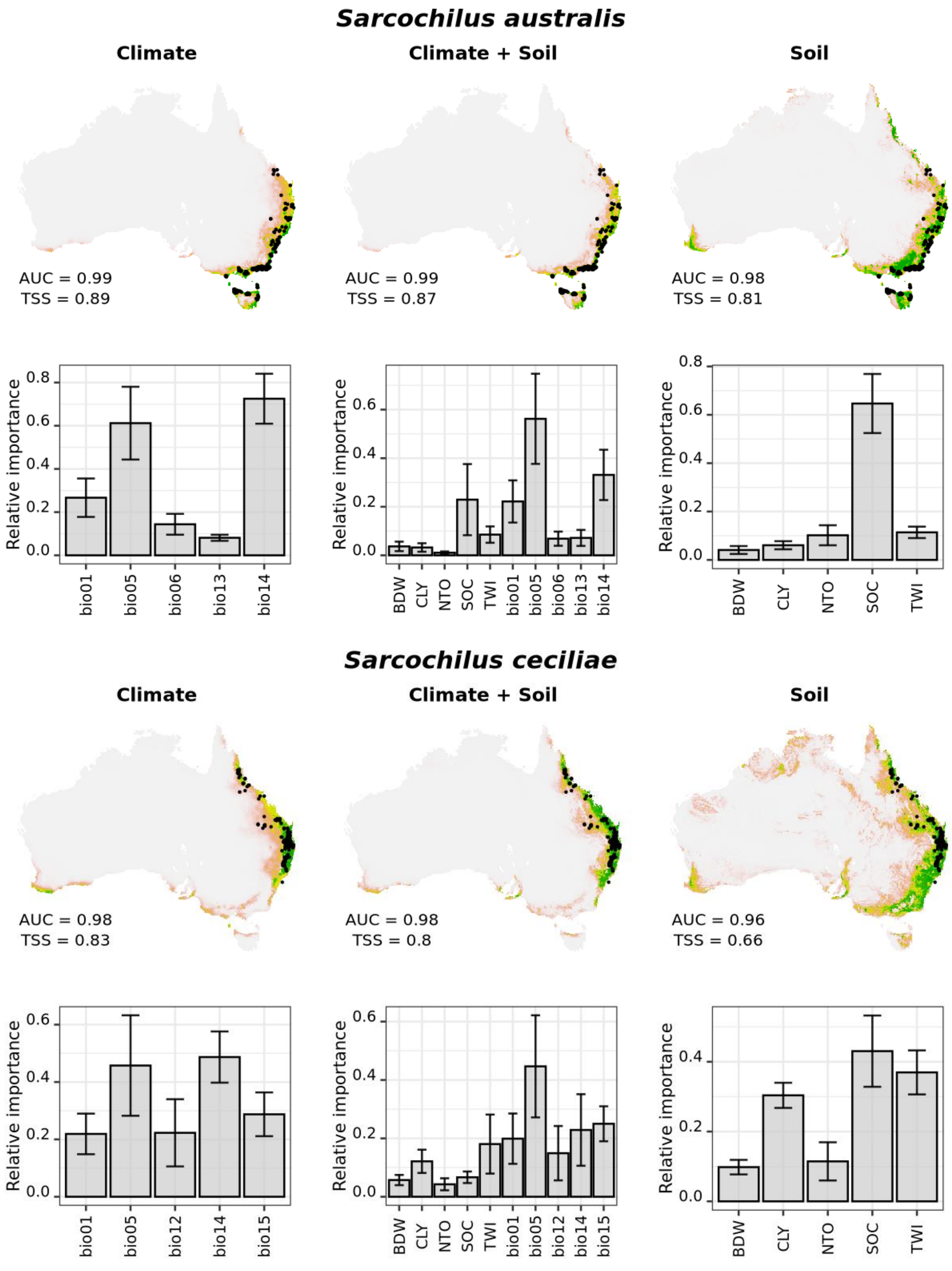
Sarcochilus australis
Climate
Climate + Soil
Soil
AUC = 0.99
TSS = 0.89
AUC = 0.99
TSS = 0.87
AUC = 0.98
TSS = 0.81
Relative importance
bio01
bio05
bio06
bio13
bio14
BDW
CLY
NTO
SOC
TWI
Sarcochilus ceciliae
Climate
Climate + Soil
Soil
AUC = 0.98
TSS = 0.83
AUC = 0.98
TSS = 0.8
AUC = 0.96
TSS = 0.66
Relative importance
bio01
bio05
bio12
bio14
bio15
BDW
CLY
NTO
SOC
TWI

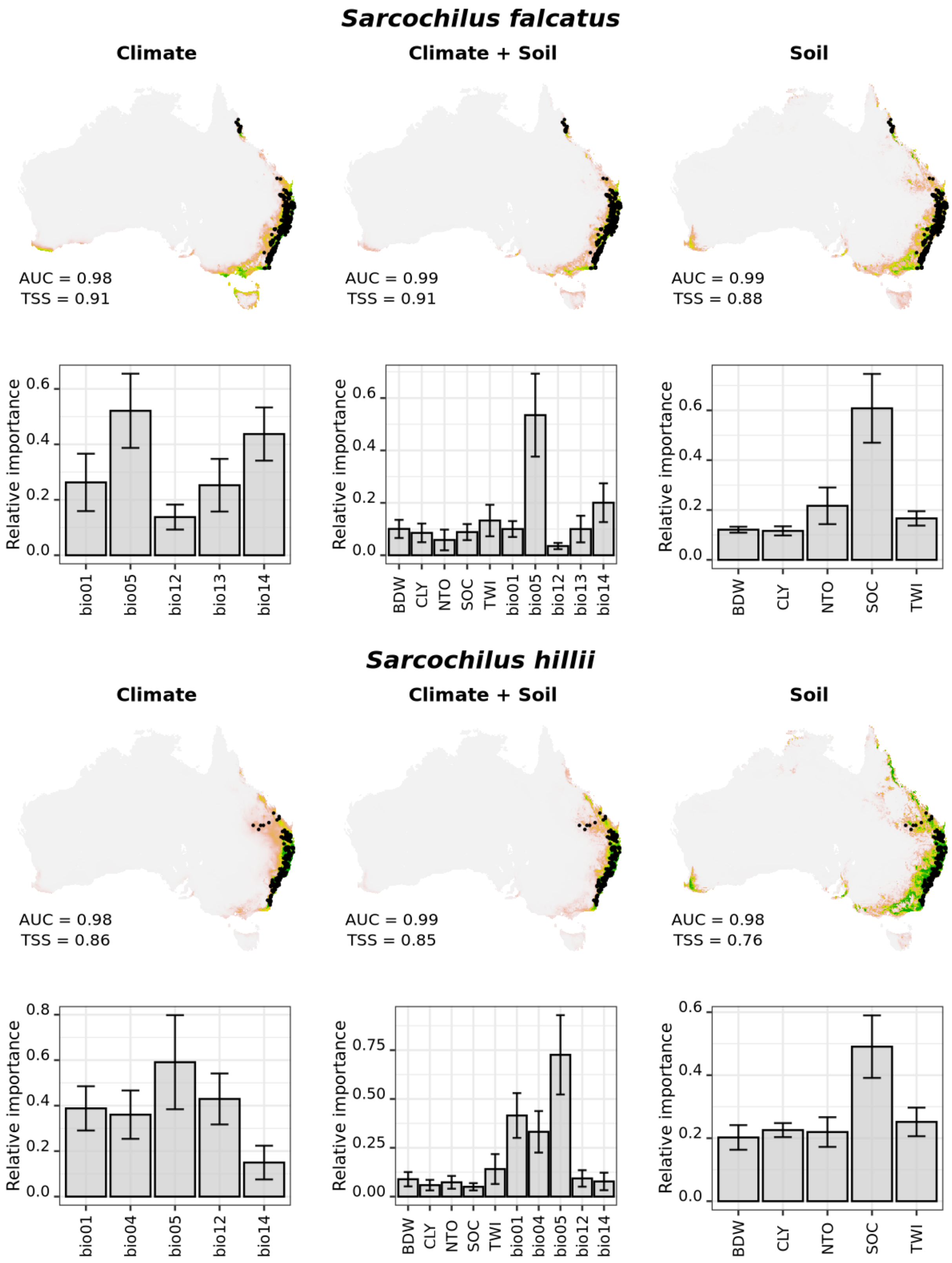

Sarcochilus falcatus
Climate
Climate + Soil
Soil
AUC = 0.98
TSS = 0.91
AUC = 0.99
TSS = 0.91
AUC = 0.99
TSS = 0.88
Relative importance
bio01
bio05
bio12
bio13
bio14
BDW
CLY
NTO
SOC
TWI
Sarcochilus hillii
Climate
Climate + Soil
Soil
AUC = 0.98
TSS = 0.86
AUC = 0.99
TSS = 0.85
AUC = 0.98
TSS = 0.76
Relative importance
bio01
bio04
bio05
bio12
bio14

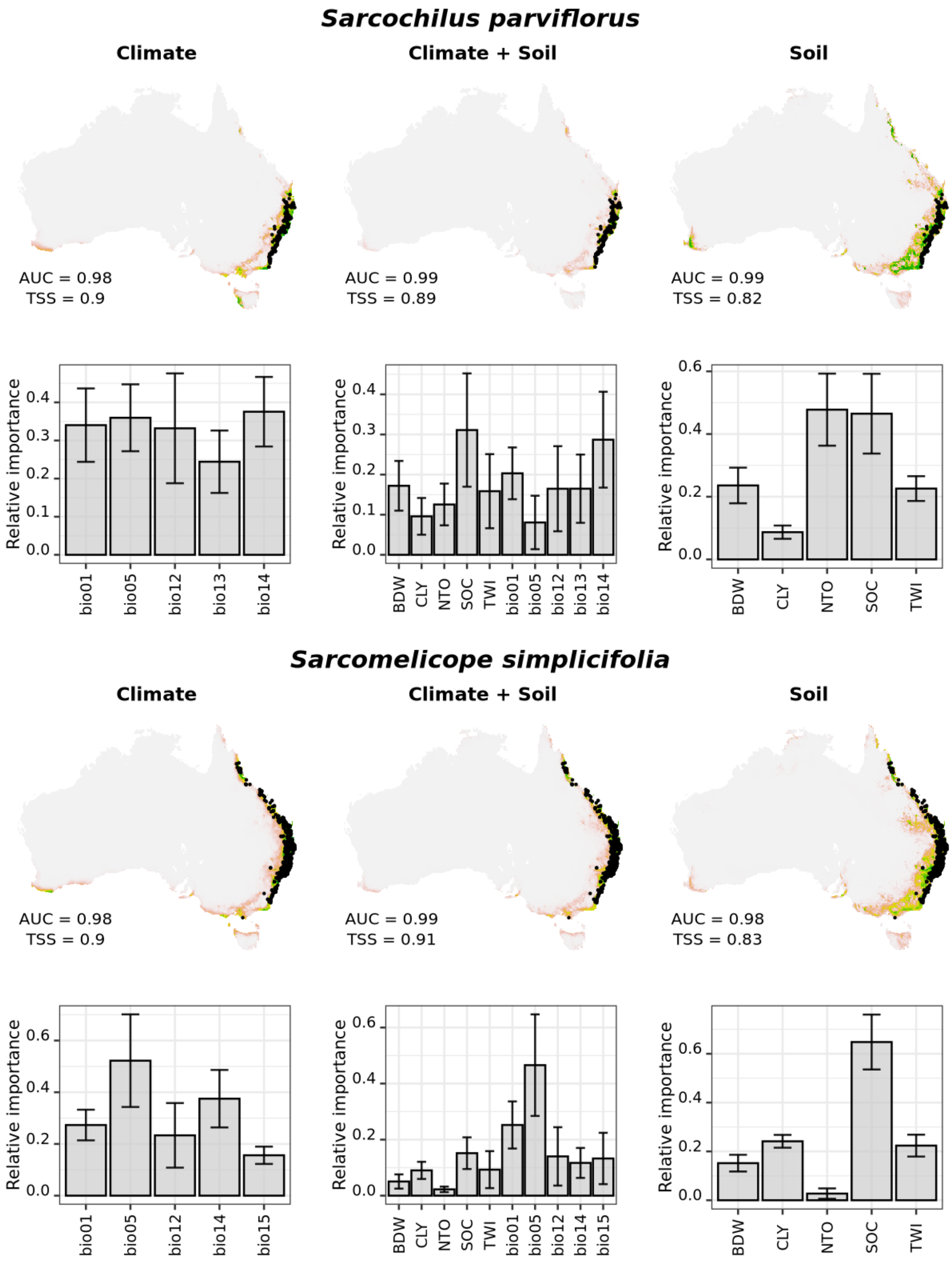
Sarcochilus parviflorus
Climate
Climate + Soil
Soil
AUC = 0.98
TSS = 0.9
AUC = 0.99
TSS = 0.89
AUC = 0.99
TSS = 0.82
Relative importance
bio01
bio05
bio12
bio13
bio14
BDW
CLY
NTO
SOC
TWI
Sarcomelicope simplicifolia
Climate
Climate + Soil
Soil
AUC = 0.98
TSS = 0.9
AUC = 0.99
TSS = 0.91
AUC = 0.98
TSS = 0.83
Relative importance
bio01
bio05
bio12
bio14
bio15
BDW
CLY
NTO
SOC
TWI

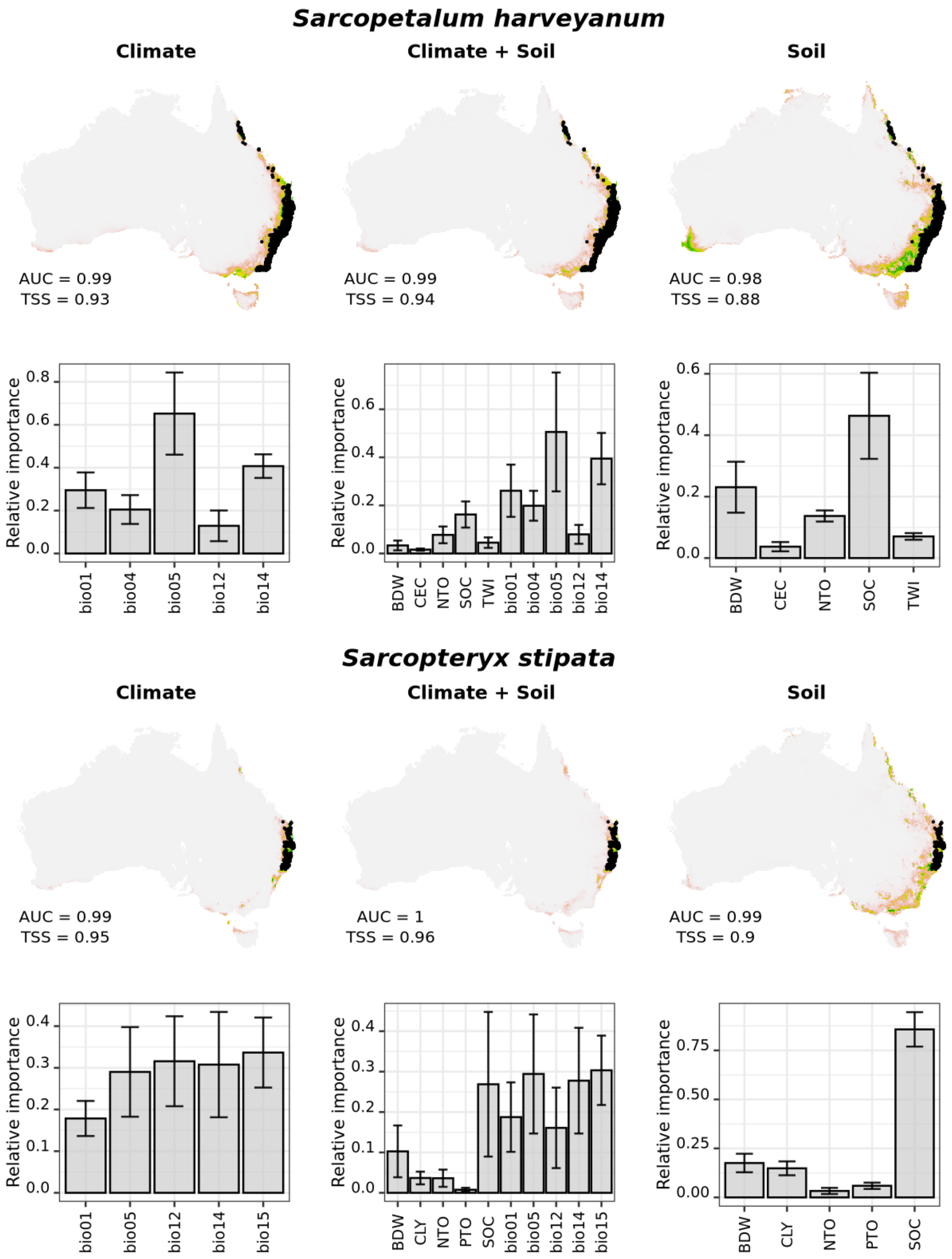

Sarcopetalum harveyanum
Climate
Climate + Soil
Soil
AUC = 0.99
TSS = 0.93
AUC = 0.99
TSS = 0.94
AUC = 0.98
TSS = 0.88
Relative importance
bio01
bio04
bio05
bio12
bio14
BDW
CEC
NTO
SOC
TWI
Sarcopteryx stipata
Climate
Climate + Soil
Soil
AUC = 0.99
TSS = 0.95
AUC = 1
TSS = 0.96
AUC = 0.99
TSS = 0.9
Relative importance
bio01
bio05
bio12
bio14
bio15
BDW
CLY
NTO
PTO
SOC

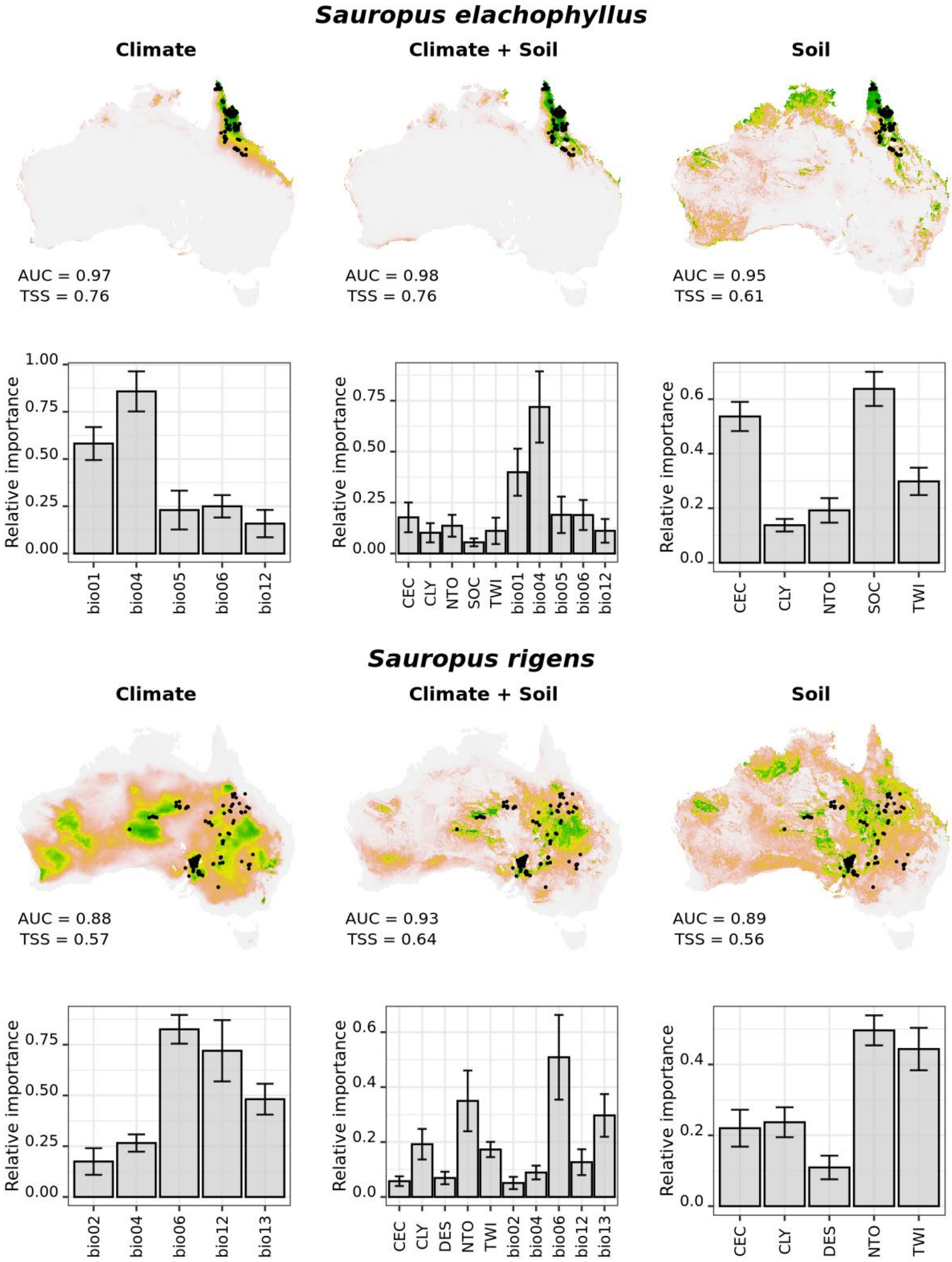
Sauropus elachophyllus
Climate
Climate + Soil
Soil
AUC = 0.97
TSS = 0.76
AUC = 0.98
TSS = 0.76
AUC = 0.95
TSS = 0.61
Relative importance
1.00
0.75
0.50
0.25
0.00
bio01
bio04
bio05
bio06
bio12
CEC
CLY
NTO
SOC
TWI
0.6
0.4
0.2
0.0
Sauropus rigens
AUC = 0.88
TSS = 0.57
AUC = 0.93
TSS = 0.64
AUC = 0.89
TSS = 0.56
bio02
bio13
DES

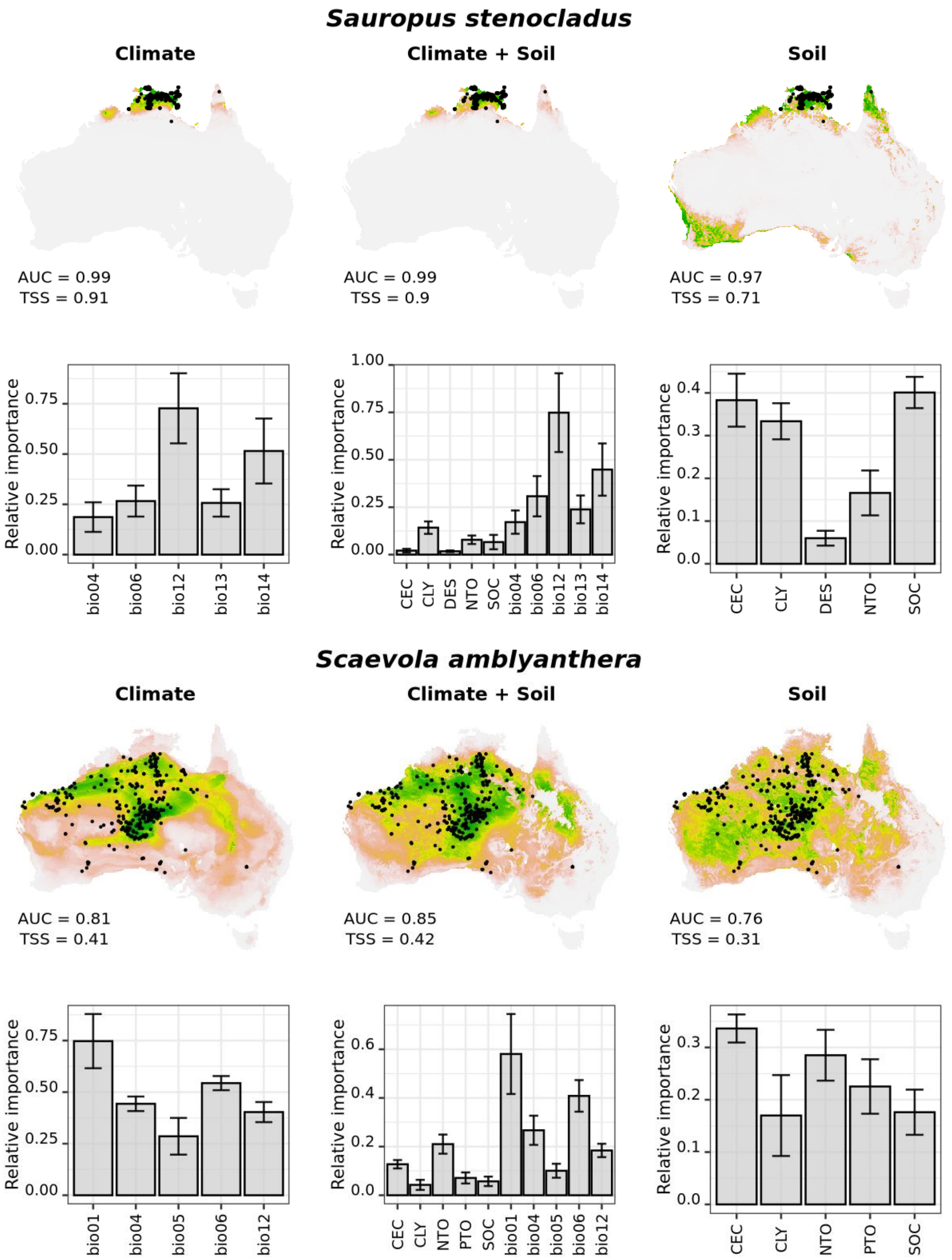
Sauropus stenocladus
Climate
Climate + Soil
Soil
AUC = 0.99
TSS = 0.91
AUC = 0.99
TSS = 0.9
AUC = 0.97
TSS = 0.71
Relative importance
0.00 0.25 0.50 0.75
bio04 bio06 bio12 bio13 bio14
0.00 0.25 0.50 0.75 1.00
CEC CLY DES NTO SOC bio04 bio06 bio12 bio13 bio14
0.0 0.1 0.2 0.3 0.4
CEC CLY DES NTO SOC
Scaevola amblyanthera
Climate
Climate + Soil
Soil
AUC = 0.81
TSS = 0.41
AUC = 0.85
TSS = 0.42
AUC = 0.76
TSS = 0.31
Relative importance
0.00 0.25 0.50 0.75
bio01 bio04 bio05 bio06 bio12
0.0 0.2 0.4 0.6
CEC CLY NTO PTO SOC bio01 bio04 bio05 bio06 bio12
0.0 0.1 0.2 0.3
CEC CLY NTO PTO SOC

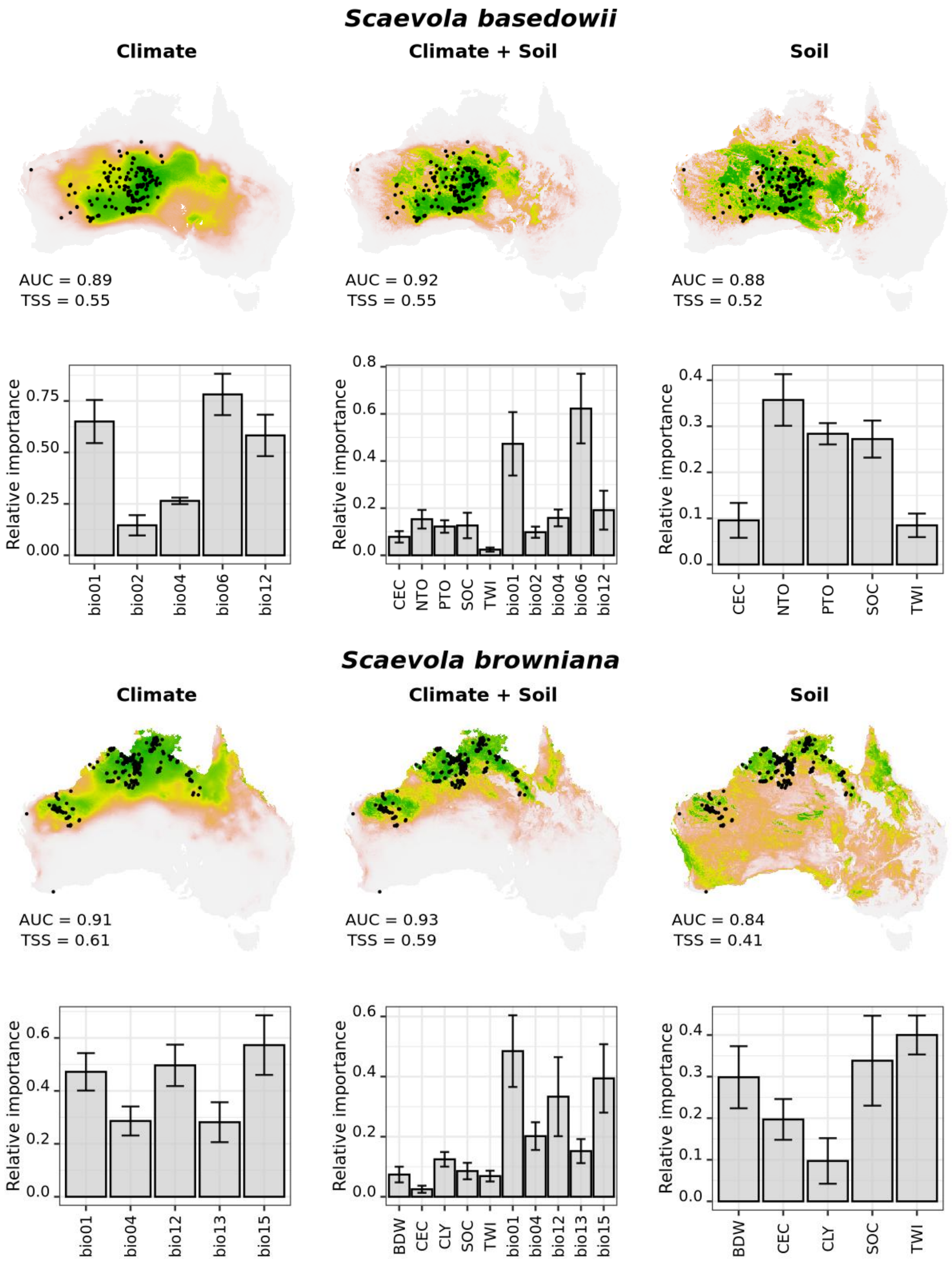

Scaevola basedowii
Climate
Climate + Soil
Soil
AUC = 0.89
TSS = 0.55
AUC = 0.92
TSS = 0.55
AUC = 0.88
TSS = 0.52
Relative importance
bio01
bio02
bio04
bio06
bio12
CEC
NTO
PTO
SOC
TWI
Scaevola browniana
Climate
Climate + Soil
Soil
AUC = 0.91
TSS = 0.61
AUC = 0.93
TSS = 0.59
AUC = 0.84
TSS = 0.41
Relative importance
bio01
bio04
bio12
bio13
bio15
BDW
CEC
CLY
SOC
TWI

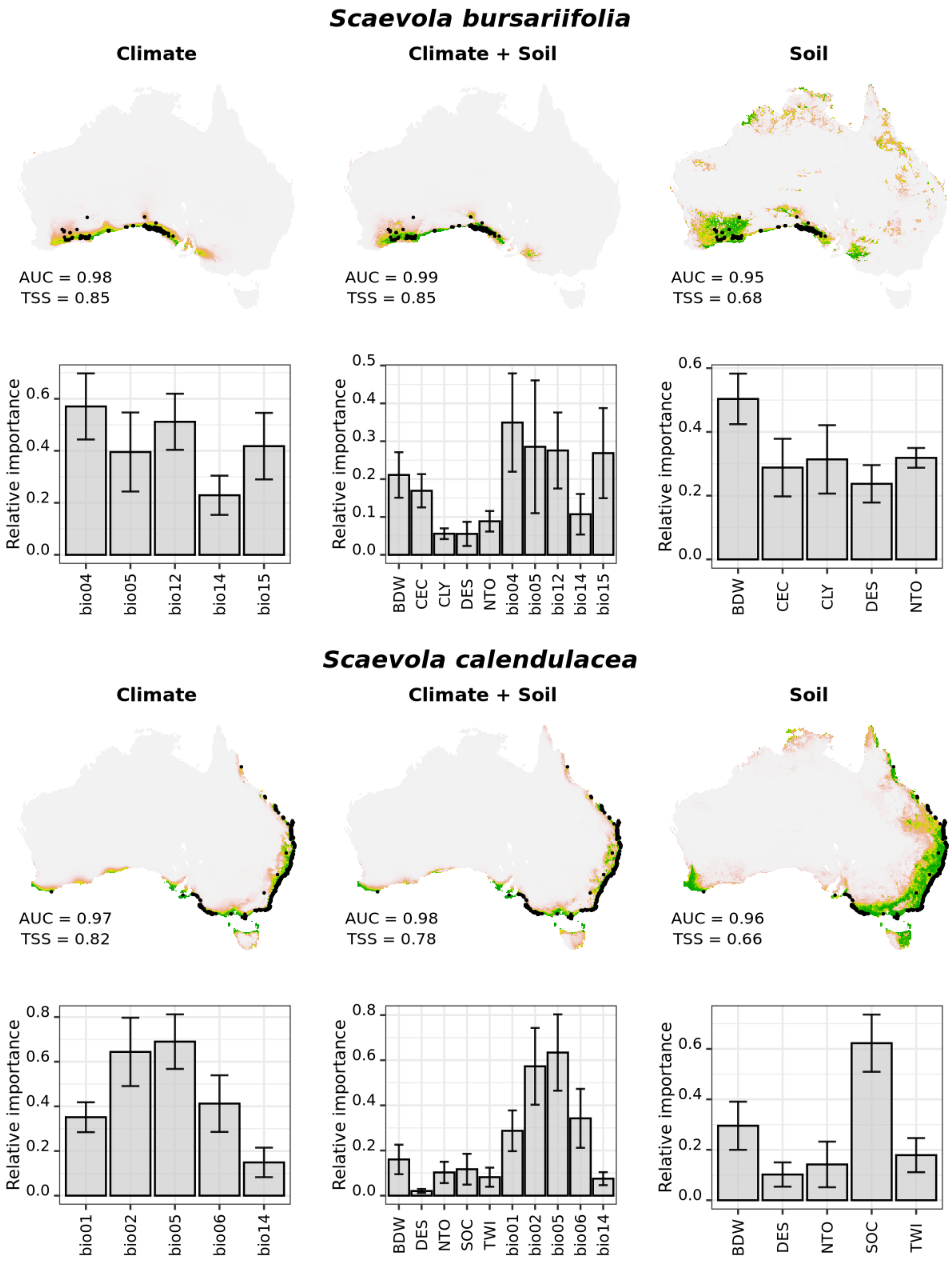
Scaevola bursariifolia
Climate
Climate + Soil
Soil
AUC = 0.98
TSS = 0.85
AUC = 0.99
TSS = 0.85
AUC = 0.95
TSS = 0.68
Relative importance
bio04
bio05
bio12
bio14
bio15
BDW
CEC
CLY
DES
NTO
Scaevola calendulacea
Climate
Climate + Soil
Soil
AUC = 0.97
TSS = 0.82
AUC = 0.98
TSS = 0.78
AUC = 0.96
TSS = 0.66
Relative importance
bio01
bio02
bio05
bio06
bio14
BDW
DES
NTO
SOC
TWI

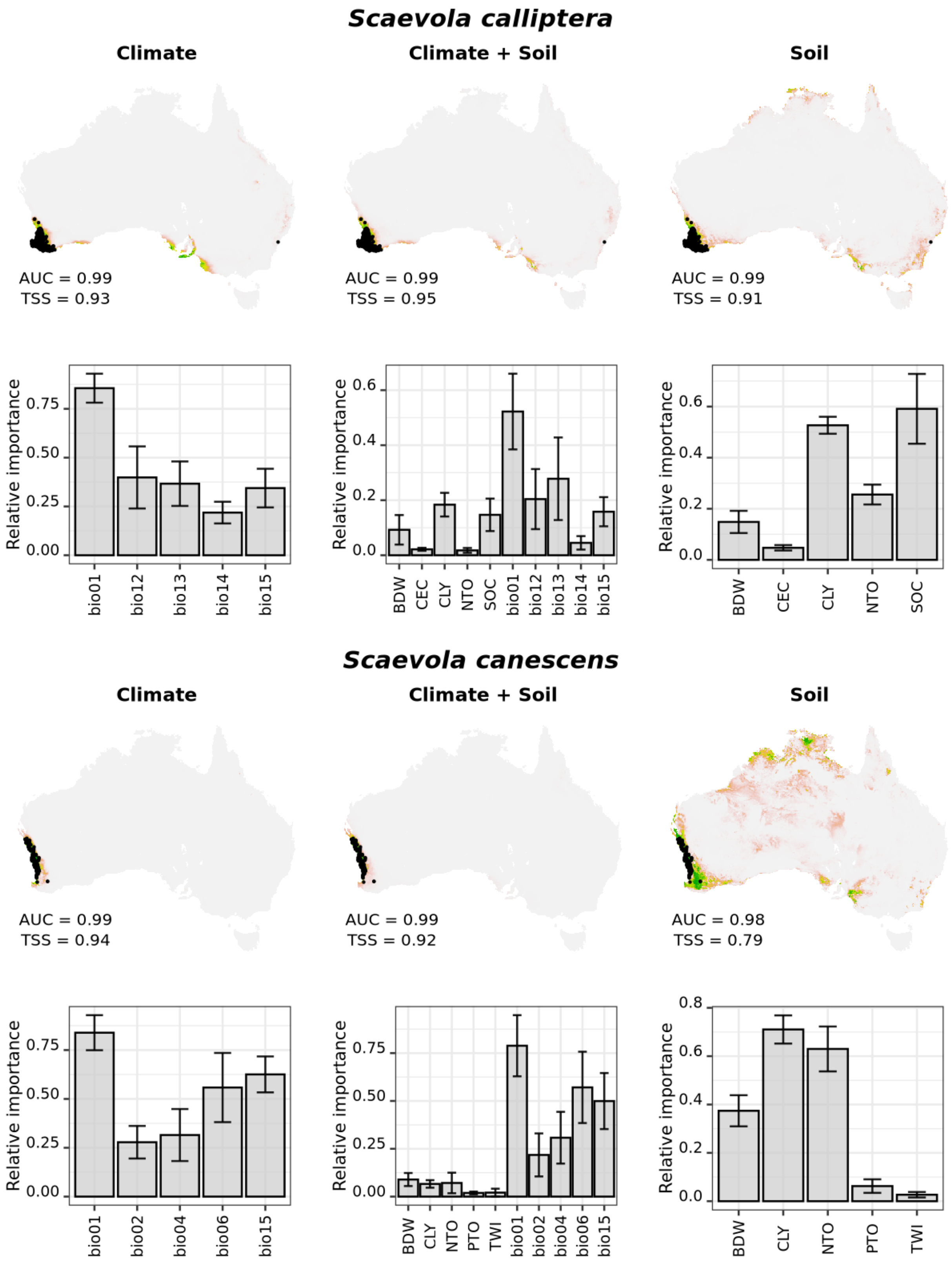
Scaevola calliptera
Climate
Climate + Soil
Soil
AUC = 0.99
TSS = 0.93
AUC = 0.99
TSS = 0.95
AUC = 0.99
TSS = 0.91
Relative importance
bio01
bio12
bio13
bio14
bio15
BDW
CEC
CLY
NTO
SOC
Scaevola canescens
Climate
Climate + Soil
Soil
AUC = 0.99
TSS = 0.94
AUC = 0.99
TSS = 0.92
AUC = 0.98
TSS = 0.79
Relative importance
bio01
bio02
bio04
bio06
bio15
BDW
CLY
NTO
PTO
TWI

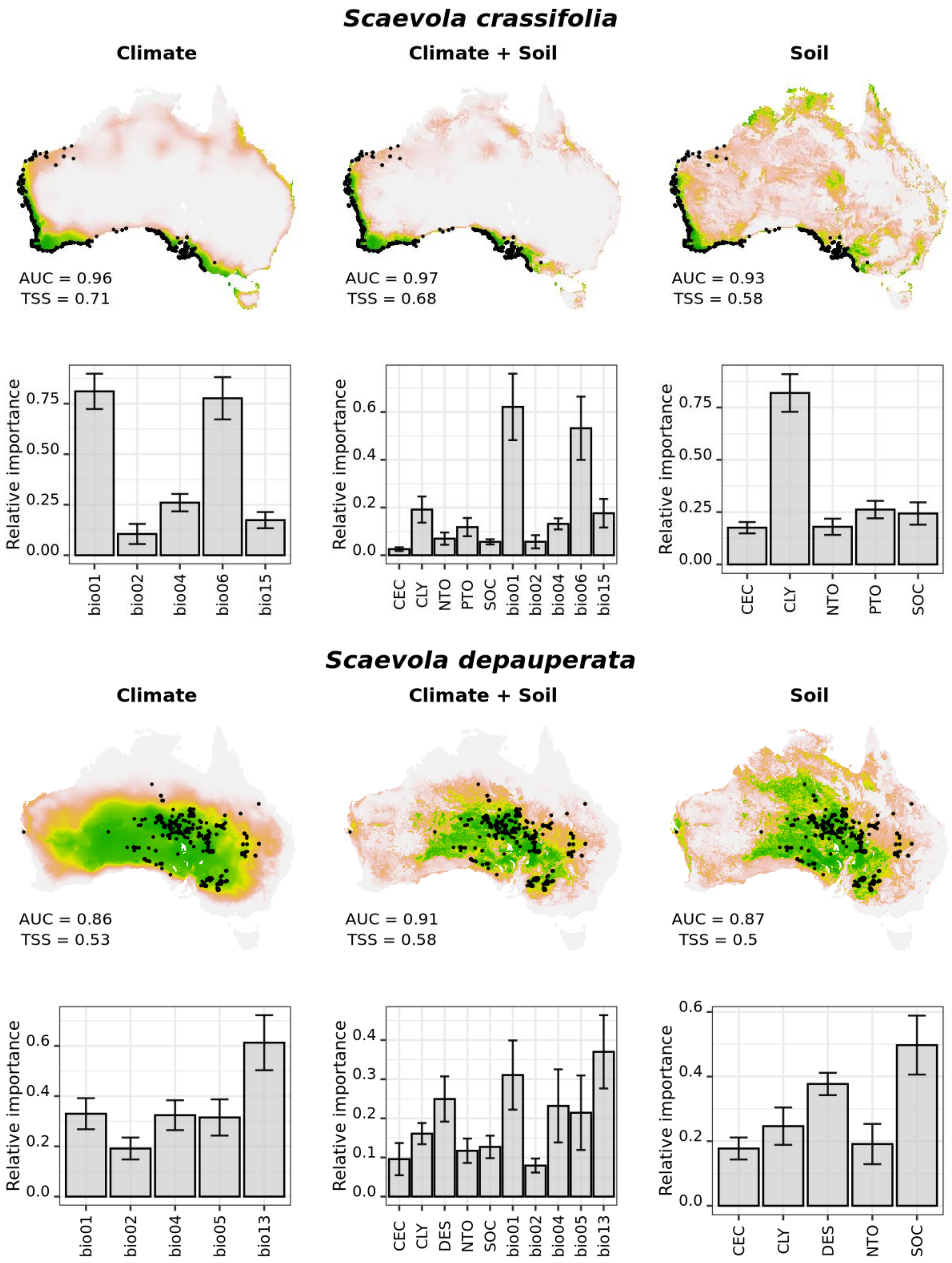
Scaevola crassifolia
Climate
Climate + Soil
Soil
AUC = 0.96
TSS = 0.71
AUC = 0.97
TSS = 0.68
AUC = 0.93
TSS = 0.58
Relative importance
0.00
0.25
0.50
0.75
bio01
bio02
bio04
bio06
bio15
0.0
0.2
0.4
0.6
CEC
CLY
NTO
PTO
SOC
bio01
bio02
bio04
bio06
bio15
CEC
CLY
NTO
PTO
SOC
Scaevola depauperata
Climate
Climate + Soil
Soil
AUC = 0.86
TSS = 0.53
AUC = 0.91
TSS = 0.58
AUC = 0.87
TSS = 0.5
Relative importance
0.0
0.2
0.4
0.6
bio01
bio02
bio04
bio05
bio13
0.0
0.1
0.2
0.3
0.4
CEC
CLY
DES
NTO
SOC
bio01
bio02
bio04
bio05
bio13
CEC
CLY
DES
NTO
SOC

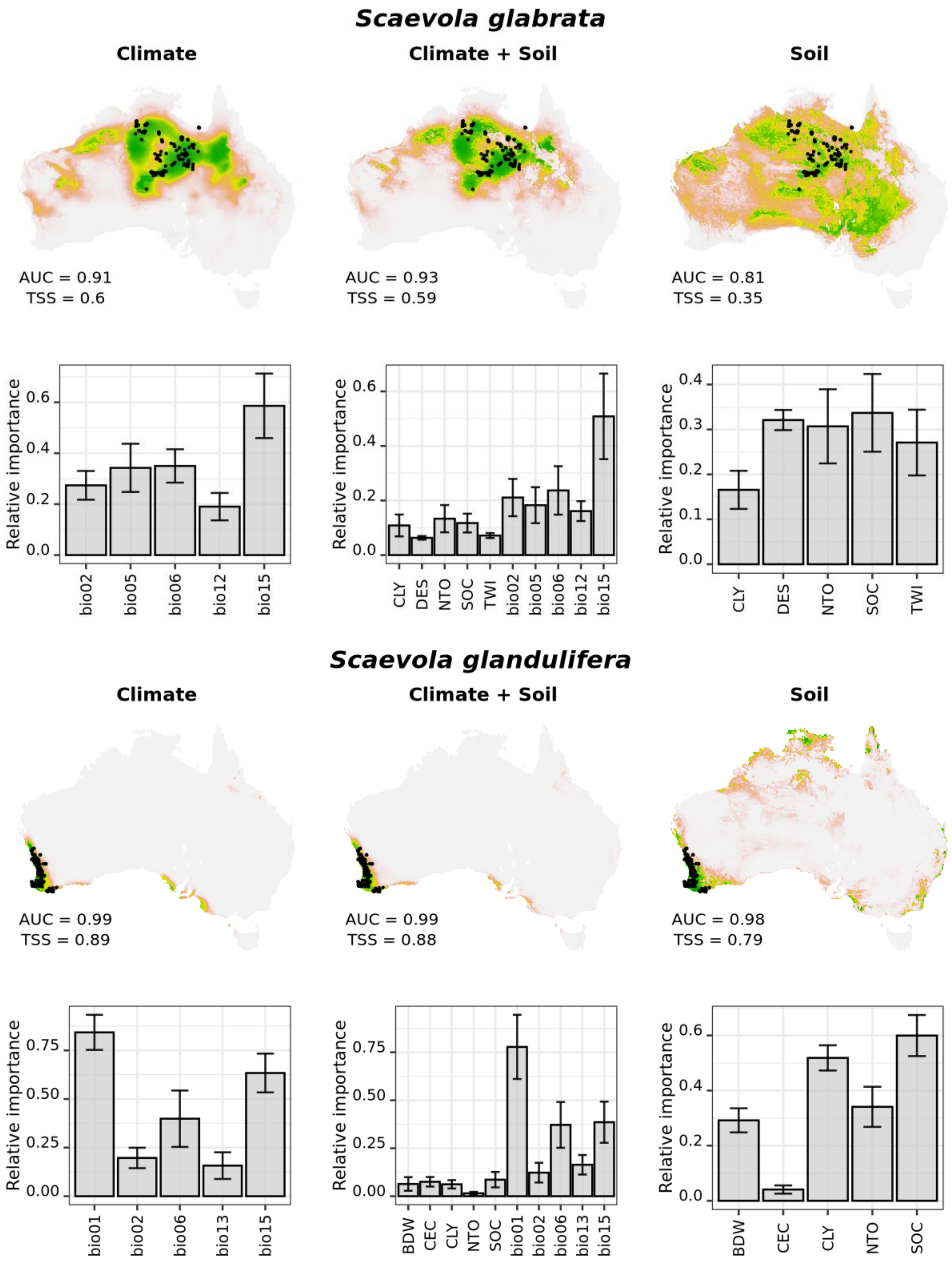

Scaevola glabrata
Climate
Climate + Soil
Soil
AUC = 0.91
TSS = 0.6
AUC = 0.93
TSS = 0.59
AUC = 0.81
TSS = 0.35
Relative importance
bio02
bio05
bio06
bio12
bio15
CLY
DES
NTO
SOC
TWI
Scaevola glandulifera
Climate
Climate + Soil
Soil
AUC = 0.99
TSS = 0.89
AUC = 0.99
TSS = 0.88
AUC = 0.98
TSS = 0.79
Relative importance
bio01
bio02
bio06
bio13
bio15
BDW
CEC
CLY
NTO
SOC

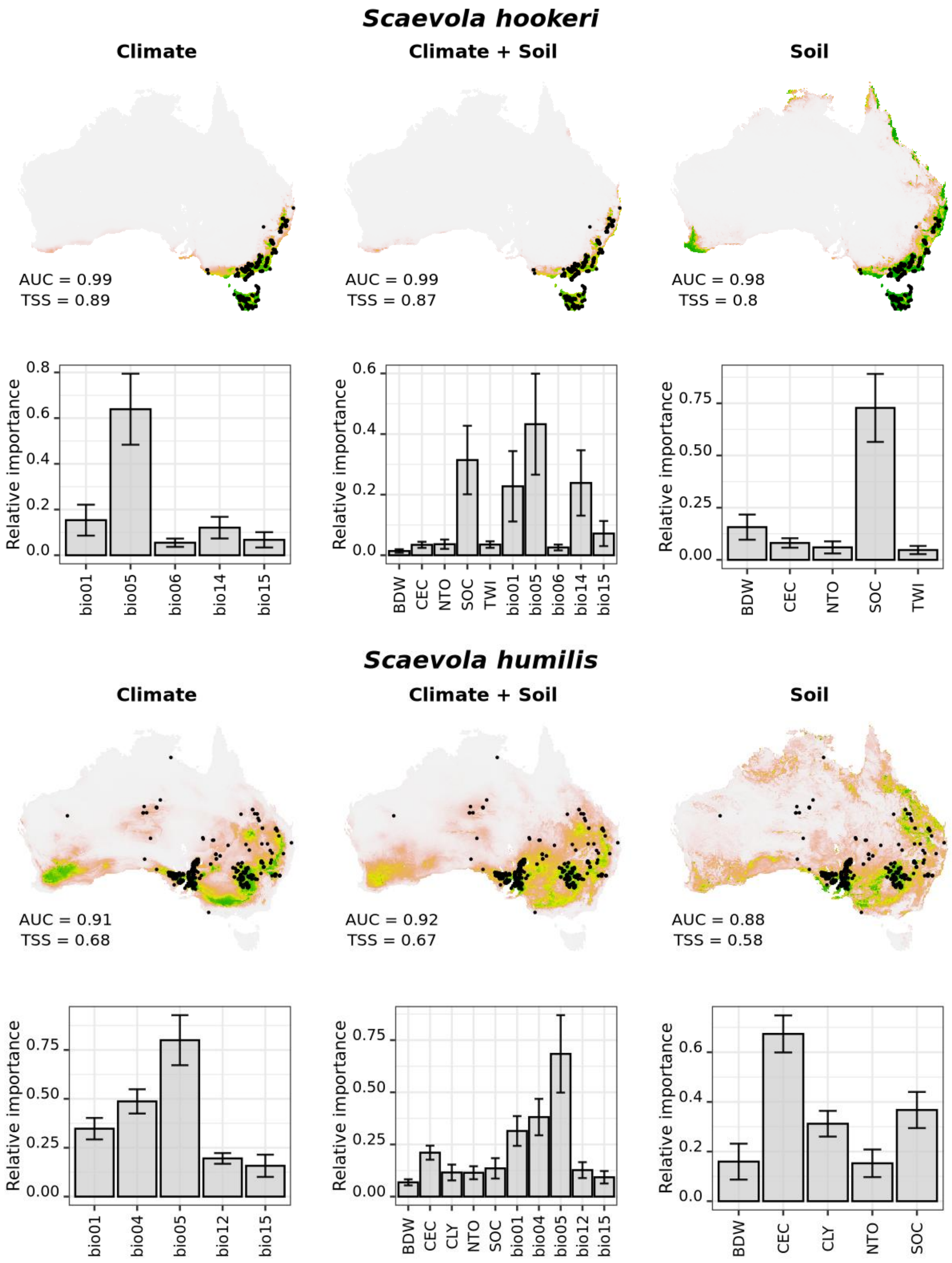

Scaevola hookeri
Climate
Climate + Soil
Soil
AUC = 0.99
TSS = 0.89
AUC = 0.99
TSS = 0.87
AUC = 0.98
TSS = 0.8
Relative importance
bio01
bio05
bio06
bio14
bio15
BDW
CEC
NTO
SOC
TWI
Scaevola humilis
Climate
Climate + Soil
Soil
AUC = 0.91
TSS = 0.68
AUC = 0.92
TSS = 0.67
AUC = 0.88
TSS = 0.58
Relative importance
bio01
bio04
bio05
bio12
bio15
BDW
CEC
CLY
NTO
SOC

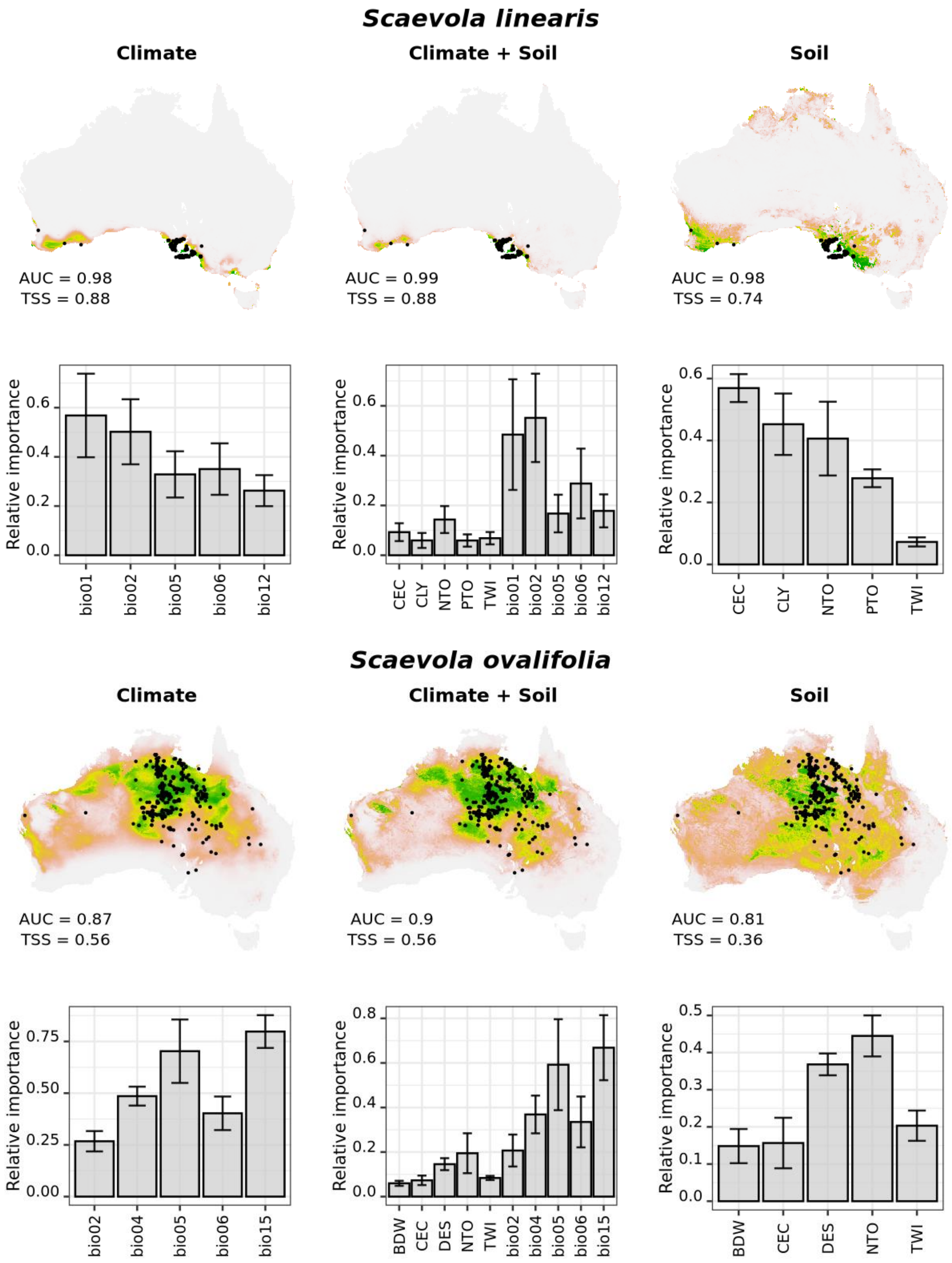
Scaevola linearis
Climate
Climate + Soil
Soil
AUC = 0.98
TSS = 0.88
AUC = 0.99
TSS = 0.88
AUC = 0.98
TSS = 0.74
Relative importance
bio01
bio02
bio05
bio06
bio12
CEC
CLY
NTO
PTO
TWI
Scaevola ovalifolia
AUC = 0.87
TSS = 0.56
AUC = 0.9
TSS = 0.56
AUC = 0.81
TSS = 0.36
bio04
bio15
BDW
DES

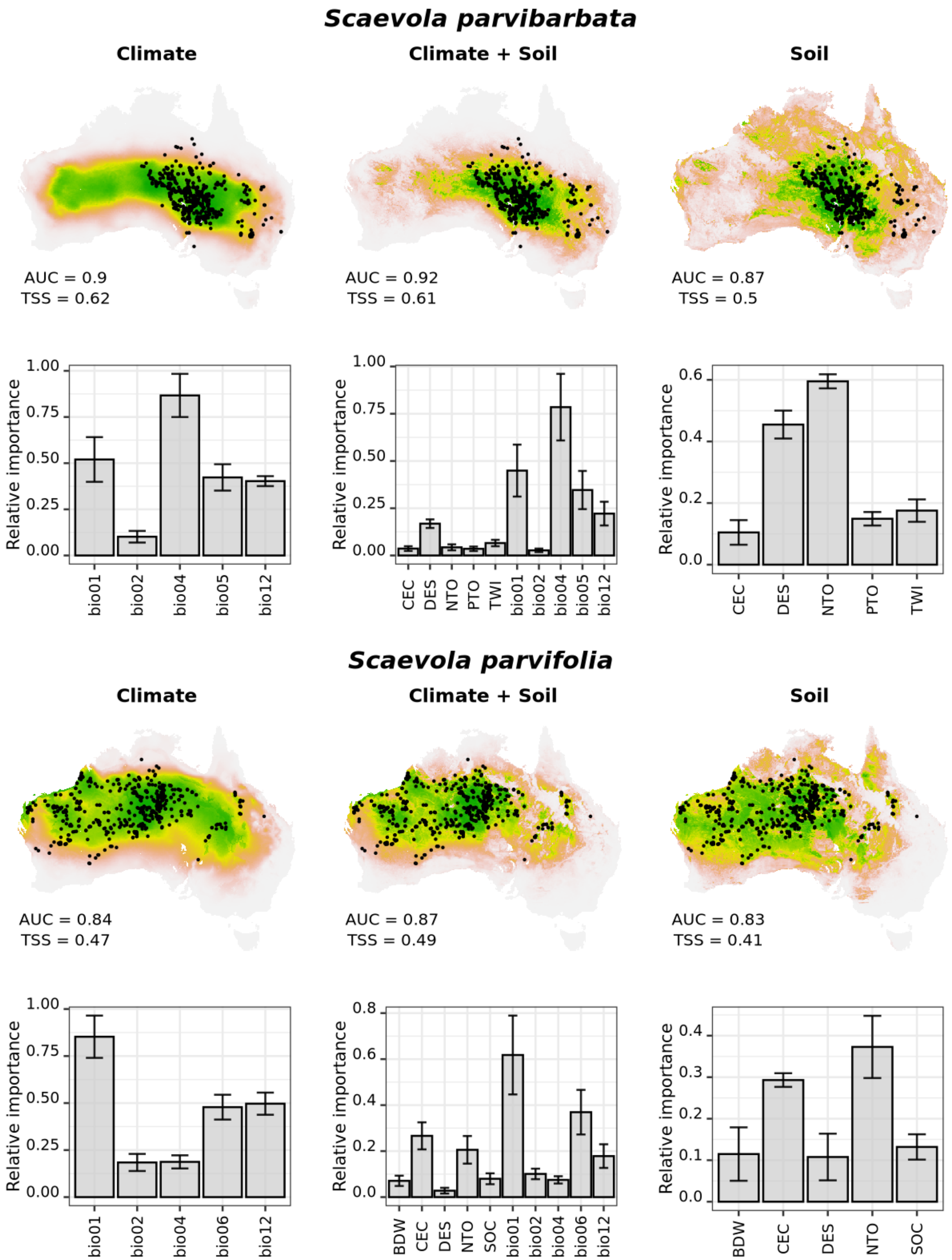
Scaevola parvibarbata
Climate
Climate + Soil
Soil
AUC = 0.9
TSS = 0.62
AUC = 0.92
TSS = 0.61
AUC = 0.87
TSS = 0.5
Relative importance
bio01
bio02
bio04
bio05
bio12
CEC
DES
NTO
PTO
TWI
Scaevola parvifolia
AUC = 0.84
TSS = 0.47
AUC = 0.87
TSS = 0.49
AUC = 0.83
TSS = 0.41
bio06
BDW
SOC

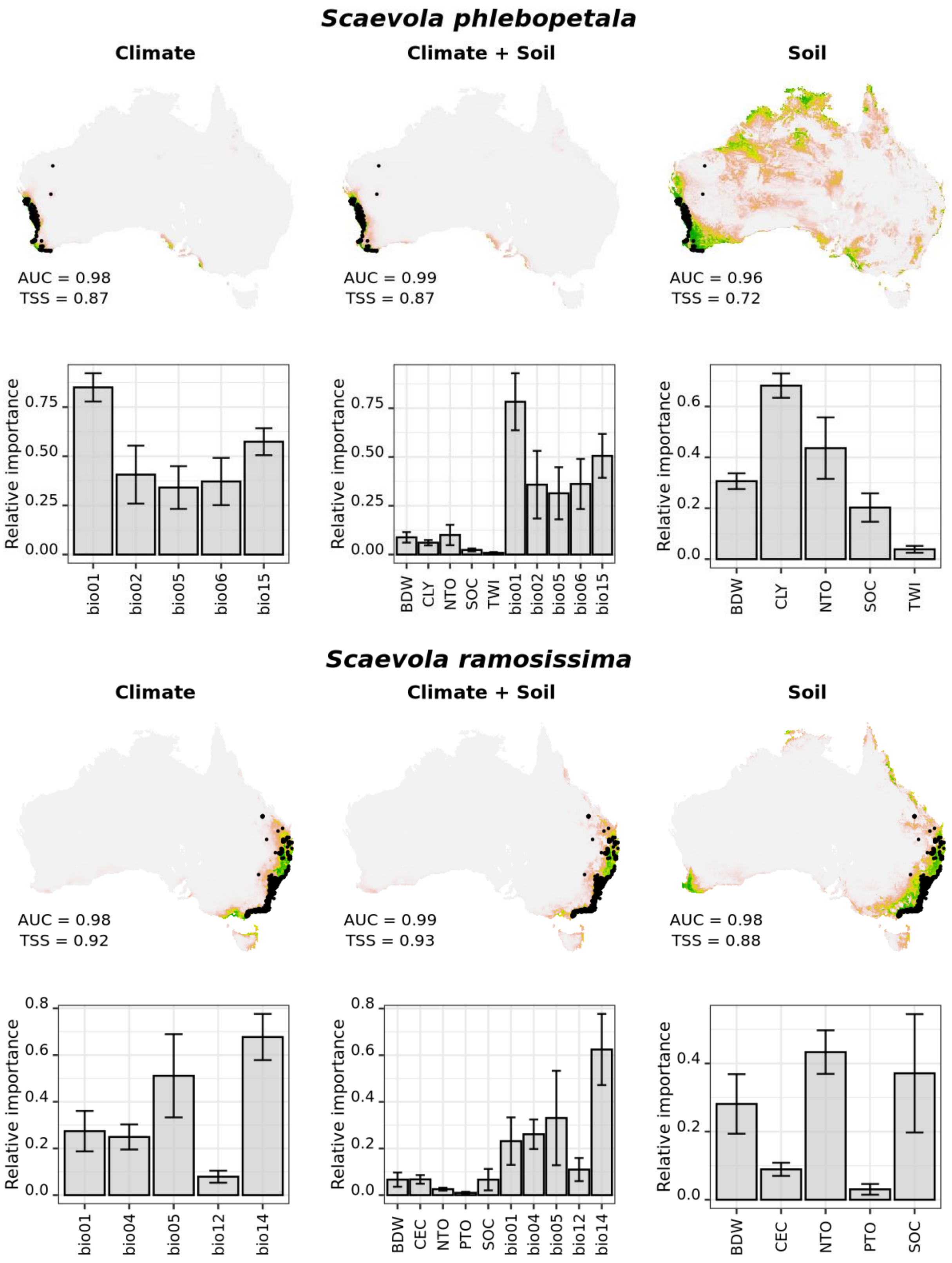
Scaevola phlebopetala
Climate
Climate + Soil
Soil
AUC = 0.98
TSS = 0.87
AUC = 0.99
TSS = 0.87
AUC = 0.96
TSS = 0.72
Relative importance
0.00
0.25
0.50
0.75
bio01
bio02
bio05
bio06
bio15
BDW
CLY
NTO
SOC
TWI
0.0
0.2
0.4
0.6
Scaevola ramosissima
Climate
Climate + Soil
Soil
AUC = 0.98
TSS = 0.92
AUC = 0.99
TSS = 0.93
AUC = 0.98
TSS = 0.88
Relative importance
0.0
0.2
0.4
0.6
0.8
bio01
bio04
bio05
bio12
bio14
BDW
CEC
NTO
PTO
SOC

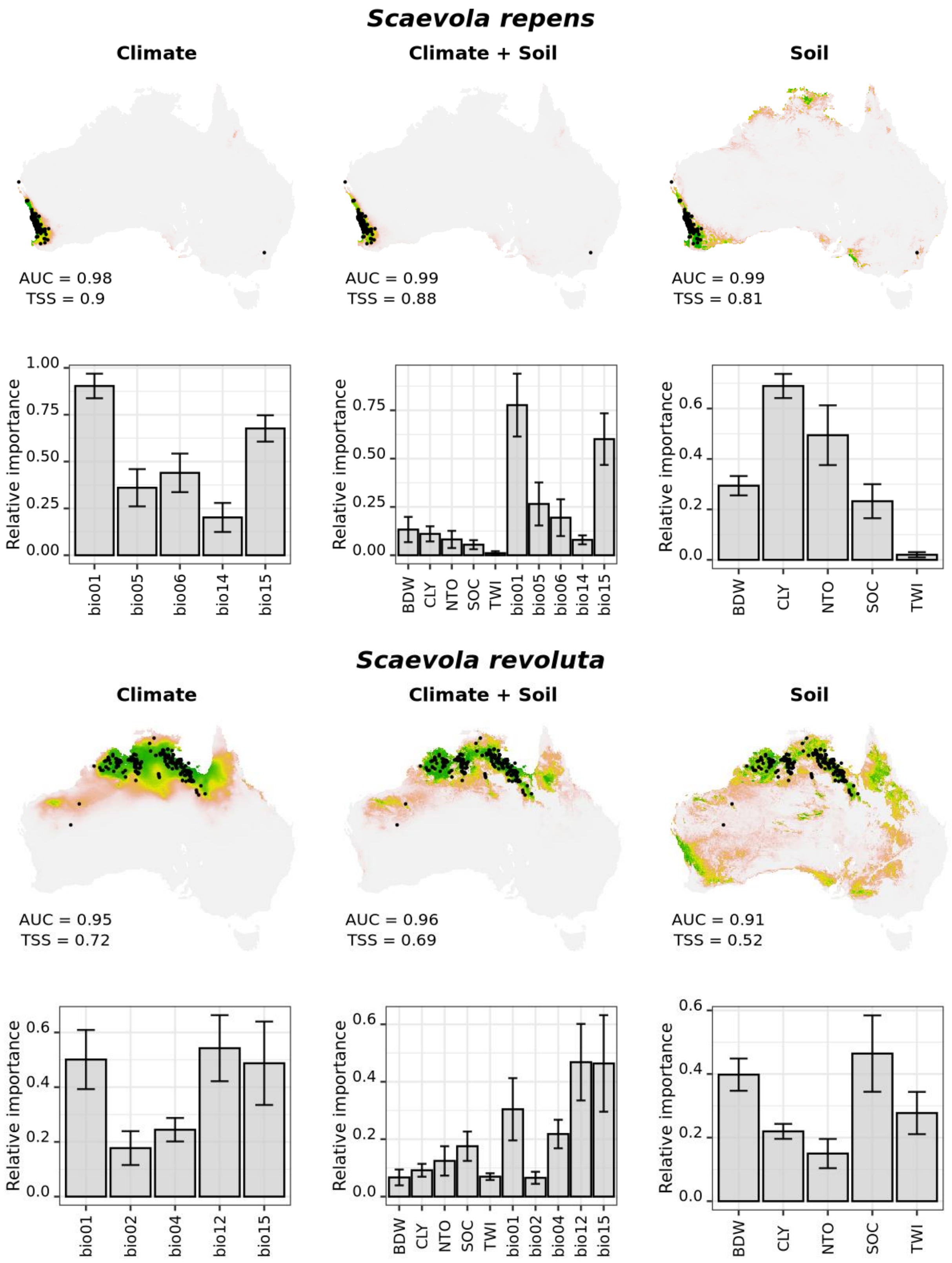

Scaevola repens
Climate
Climate + Soil
Soil
AUC = 0.98
TSS = 0.9
AUC = 0.99
TSS = 0.88
AUC = 0.99
TSS = 0.81
Relative importance
bio01
bio05
bio06
bio14
bio15
BDW
CLY
NTO
SOC
TWI
Scaevola revoluta
Climate
Climate + Soil
Soil
AUC = 0.95
TSS = 0.72
AUC = 0.96
TSS = 0.69
AUC = 0.91
TSS = 0.52
Relative importance
bio01
bio02
bio04
bio12
bio15
BDW
CLY
NTO
SOC
TWI

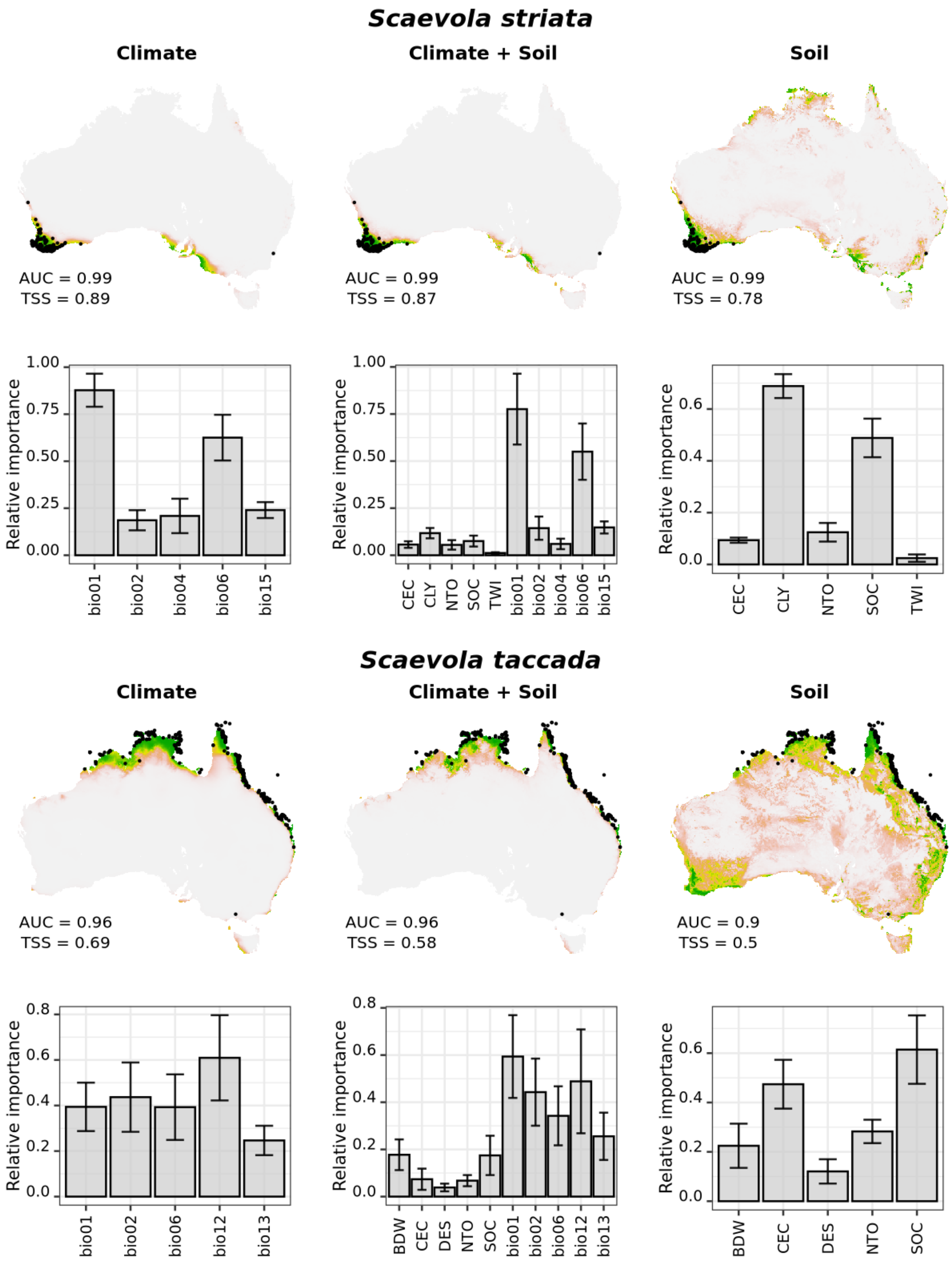

Scaevola striata
Climate
Climate + Soil
Soil
AUC = 0.99
TSS = 0.89
AUC = 0.99
TSS = 0.87
AUC = 0.99
TSS = 0.78
Relative importance
bio01
bio02
bio04
bio06
bio15
CEC
CLY
NTO
SOC
TWI
Scaevola taccada
AUC = 0.96
TSS = 0.69
AUC = 0.96
TSS = 0.58
AUC = 0.9
TSS = 0.5
bio12
bio13
BDW
DES

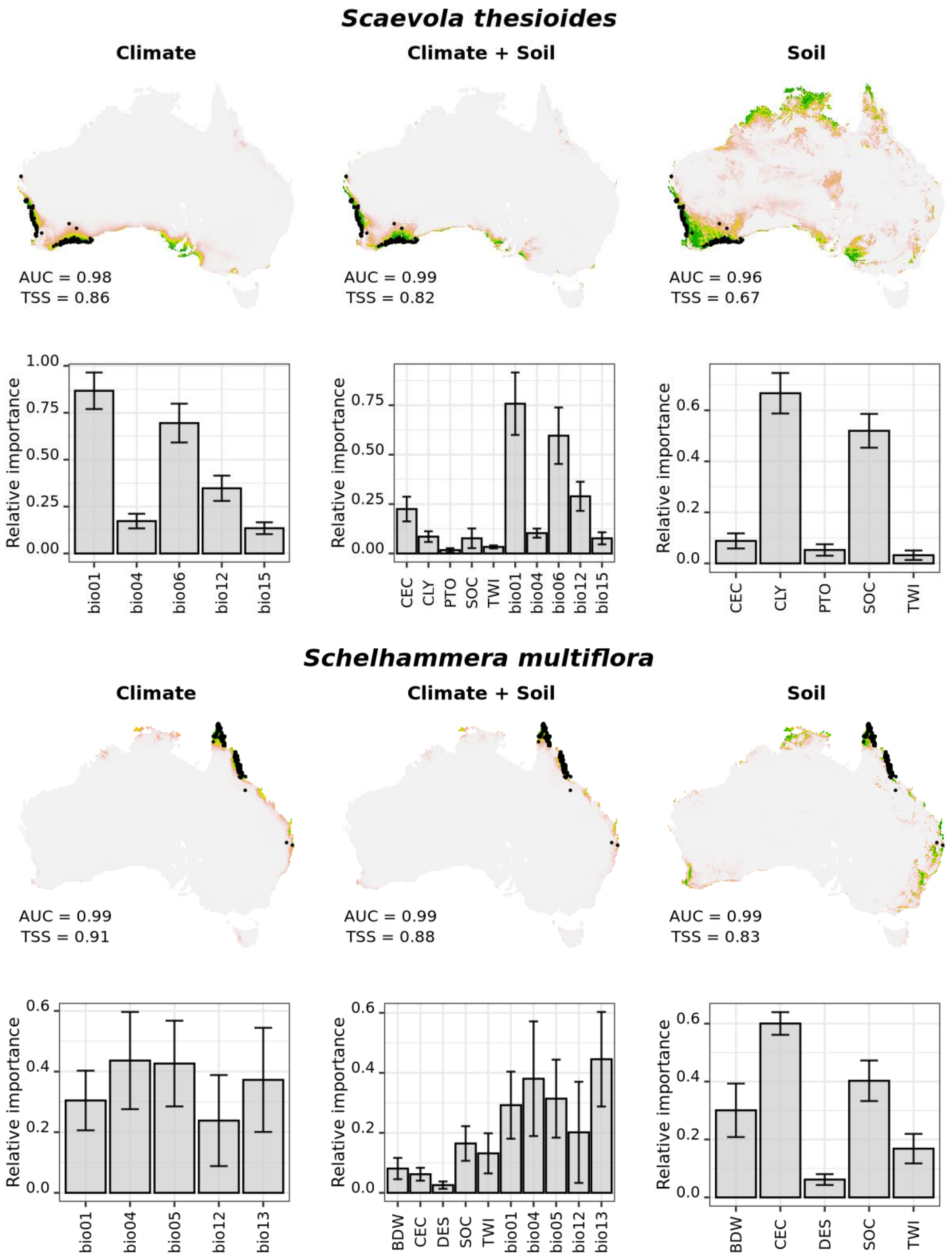
Scaevola thesioides
Climate
Climate + Soil
Soil
AUC = 0.98
TSS = 0.86
AUC = 0.99
TSS = 0.82
AUC = 0.96
TSS = 0.67
Relative importance
bio01
bio04
bio06
bio12
bio15
CEC
CLY
PTO
SOC
TWI
Schelhammera multiflora
Climate
Climate + Soil
Soil
AUC = 0.99
TSS = 0.91
AUC = 0.99
TSS = 0.88
AUC = 0.99
TSS = 0.83
Relative importance
bio01
bio04
bio05
bio12
bio13
BDW
CEC
DES
SOC
TWI

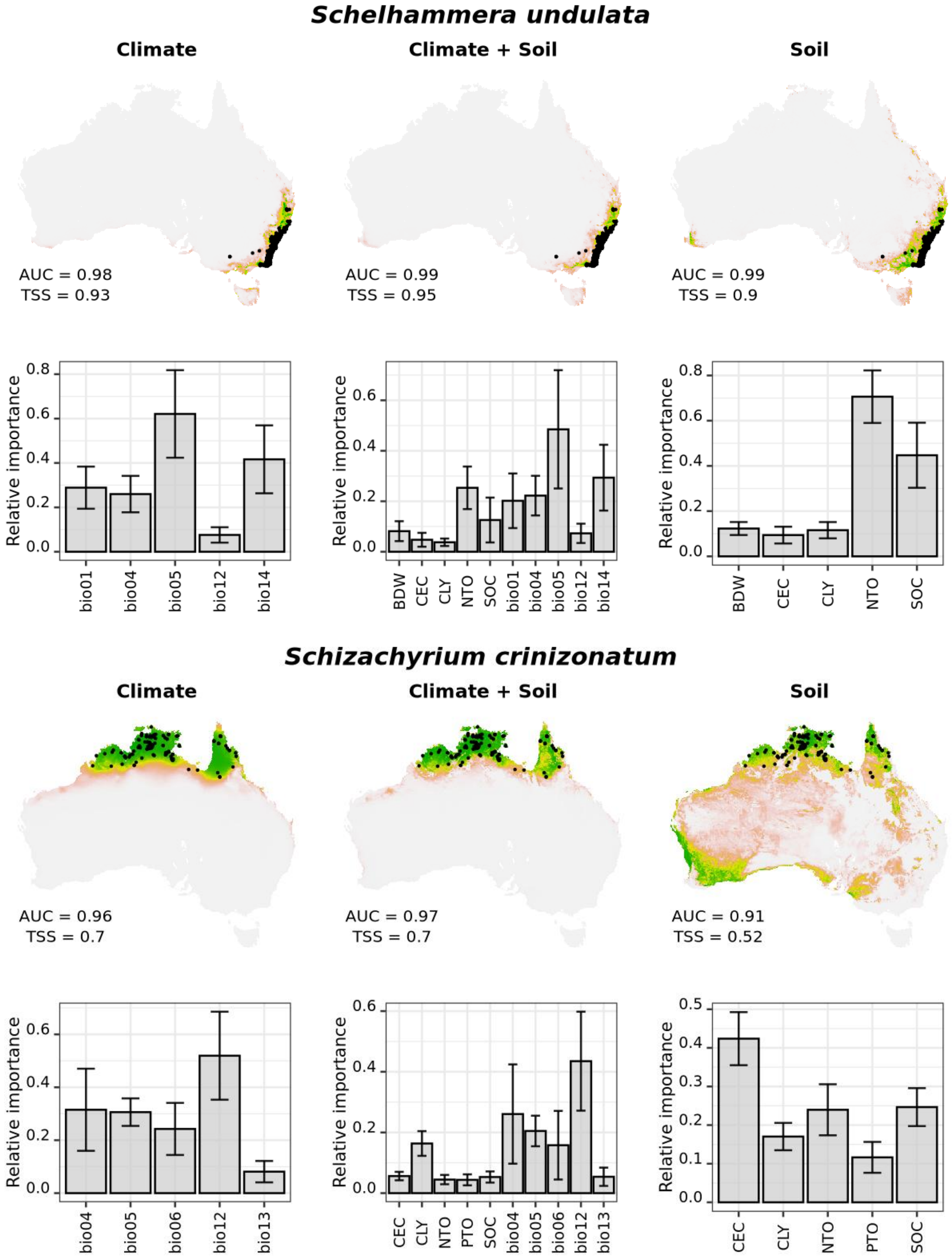

Schelhammera undulata
Climate
Climate + Soil
Soil
AUC = 0.98
TSS = 0.93
AUC = 0.99
TSS = 0.95
AUC = 0.99
TSS = 0.9
Relative importance
bio01
bio04
bio05
bio12
bio14
BDW
CEC
CLY
NTO
SOC
Schizachyrium crinizonatum
Climate
Climate + Soil
Soil
AUC = 0.96
TSS = 0.7
AUC = 0.97
TSS = 0.7
AUC = 0.91
TSS = 0.52
Relative importance
bio04
bio05
bio06
bio12
bio13
CEC
CLY
NTO
PTO
SOC

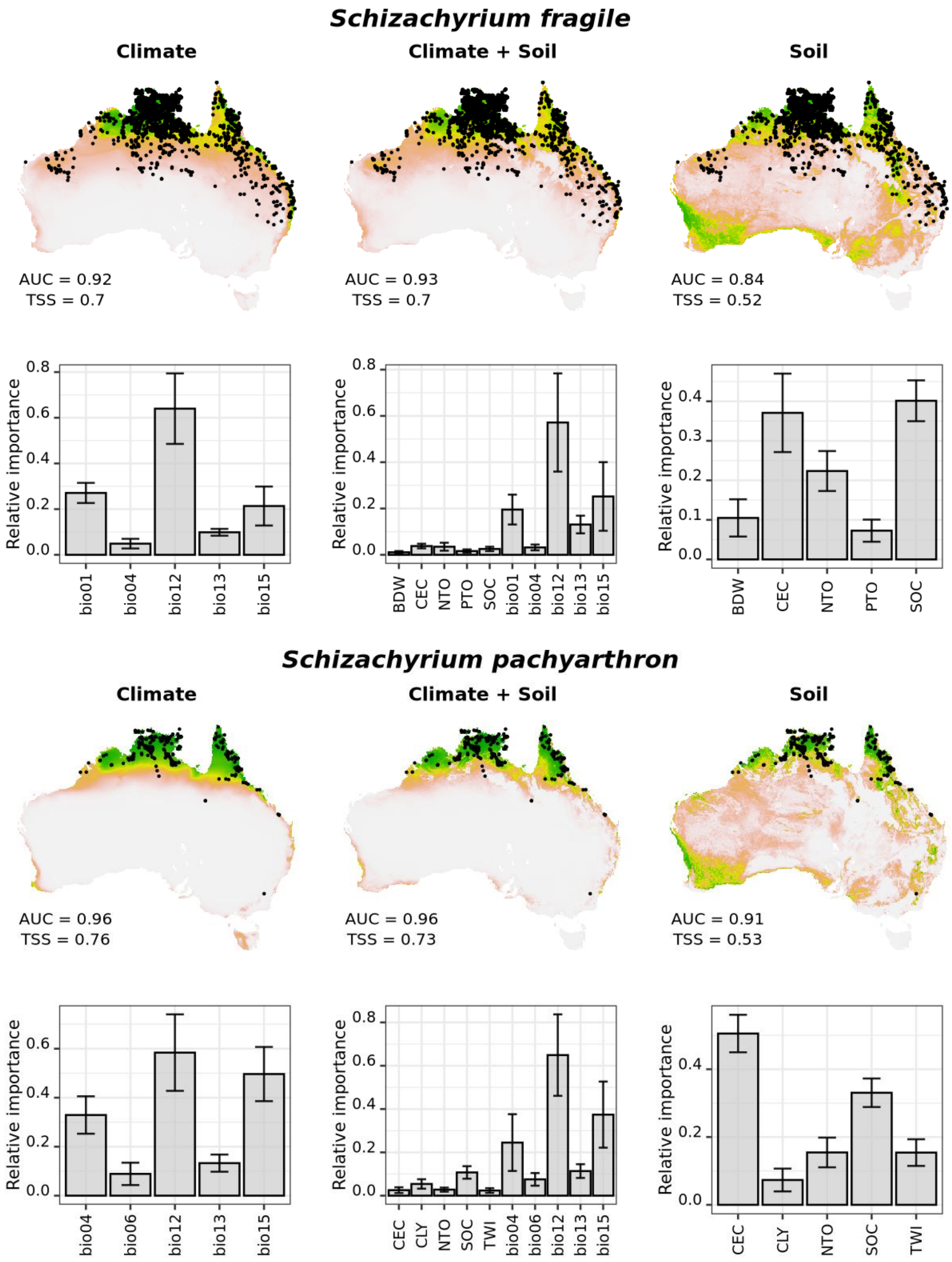
Schizachyrium fragile
Climate
Climate + Soil
Soil
AUC = 0.92
TSS = 0.7
AUC = 0.93
TSS = 0.7
AUC = 0.84
TSS = 0.52
Relative importance
0.0
0.2
0.4
0.6
0.8
bio01
bio04
bio12
bio13
bio15
BDW
CEC
NTO
PTO
SOC
0.1
0.3
Schizachyrium pachyarthron
Climate
Climate + Soil
Soil
AUC = 0.96
TSS = 0.76
AUC = 0.96
TSS = 0.73
AUC = 0.91
TSS = 0.53
Relative importance
bio04
bio06
bio12
bio13
bio15
CEC
CLY
NTO
SOC
TWI

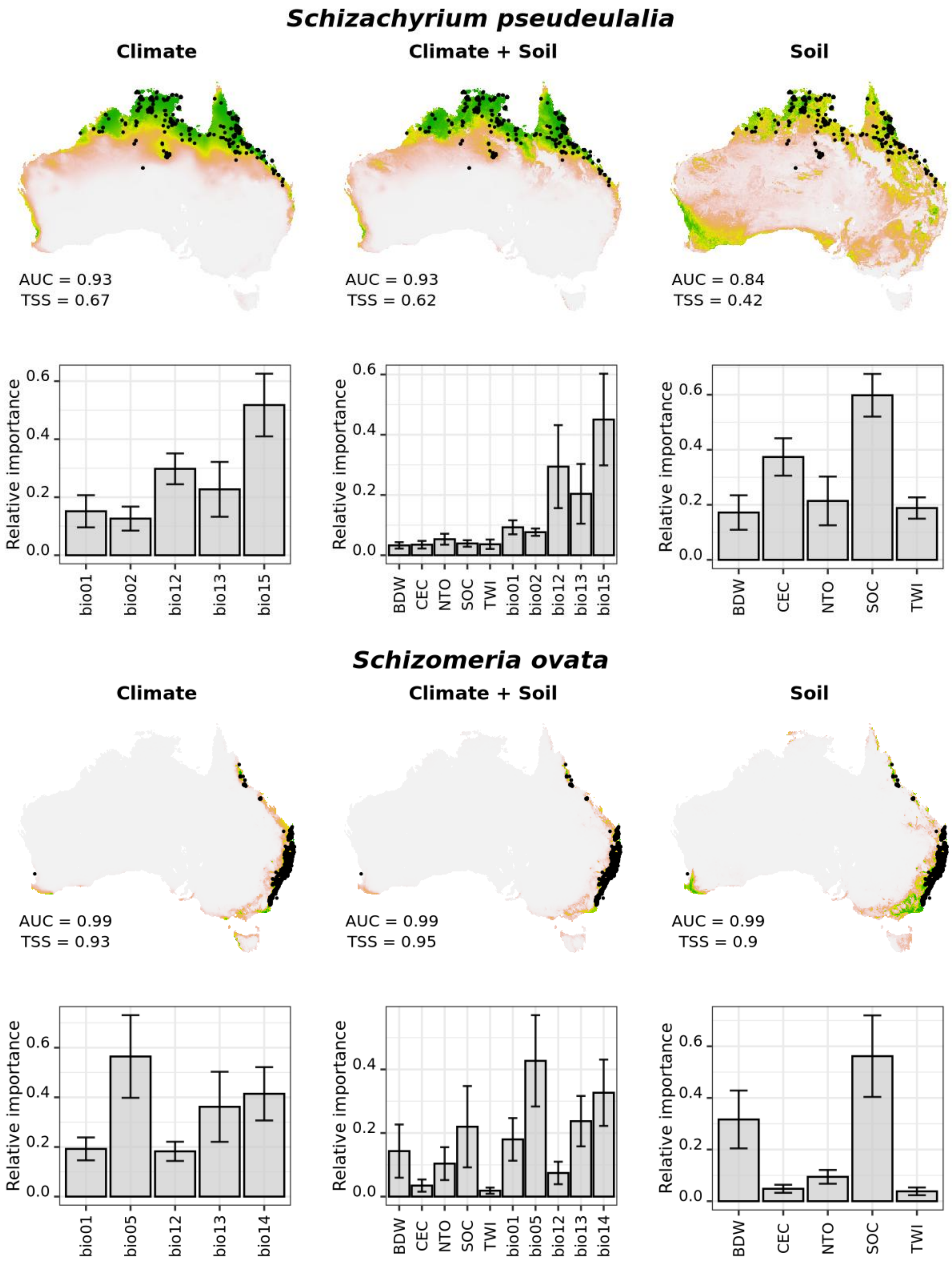

Schizachyrium pseudeulalia
Climate
Climate + Soil
Soil
AUC = 0.93
TSS = 0.67
AUC = 0.93
TSS = 0.62
AUC = 0.84
TSS = 0.42
Relative importance
bio01
bio02
bio12
bio13
bio15
BDW
CEC
NTO
SOC
TWI
Schizomeria ovata
Climate
Climate + Soil
Soil
AUC = 0.99
TSS = 0.93
AUC = 0.99
TSS = 0.95
AUC = 0.99
TSS = 0.9
bio05
bio14

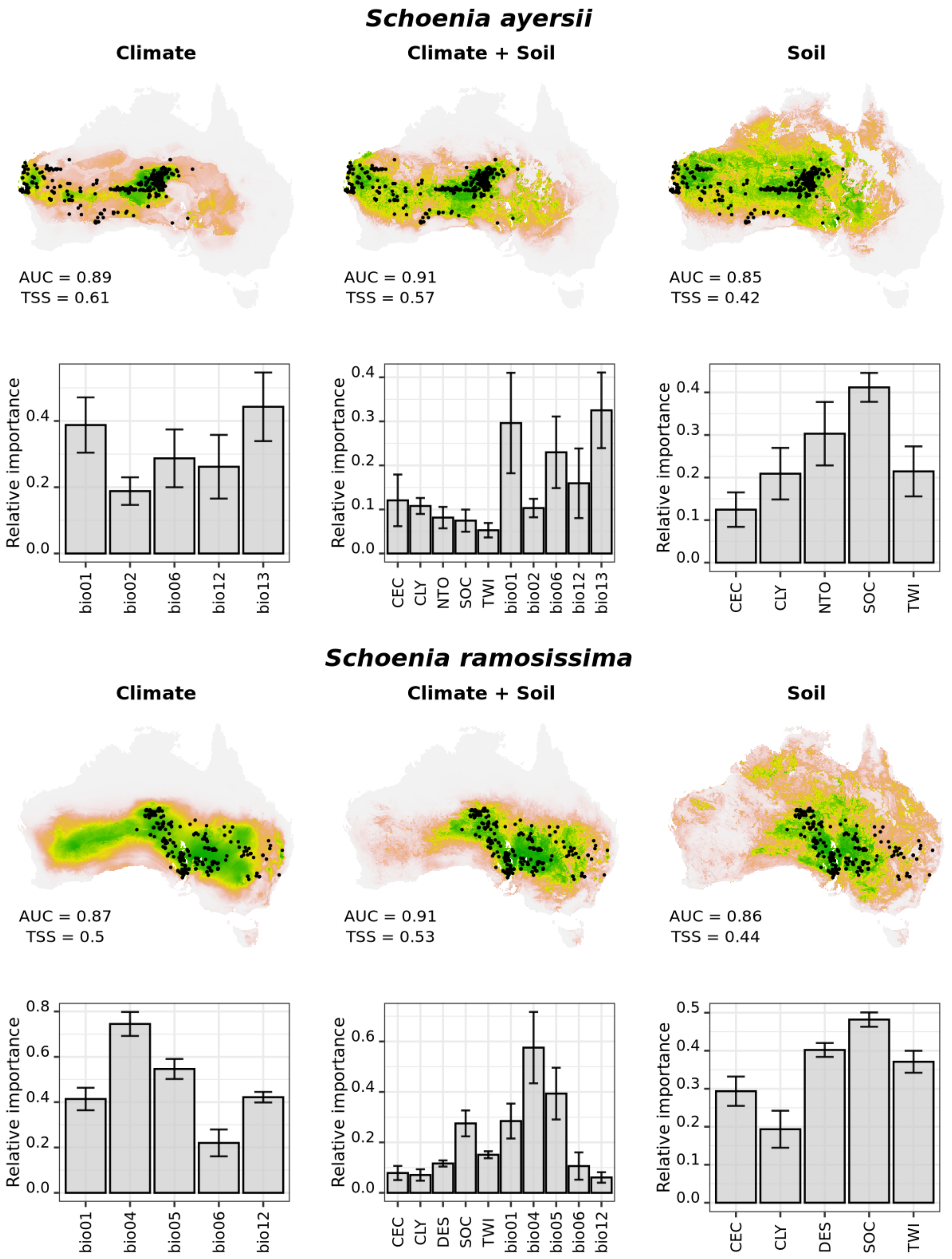
Schoenia ayersii
Climate
Climate + Soil
Soil
AUC = 0.89
TSS = 0.61
AUC = 0.91
TSS = 0.57
AUC = 0.85
TSS = 0.42
Relative importance
bio01
bio02
bio06
bio12
bio13
CEC
CLY
NTO
SOC
TWI
Schoenia ramosissima
Climate
Climate + Soil
Soil
AUC = 0.87
TSS = 0.5
AUC = 0.91
TSS = 0.53
AUC = 0.86
TSS = 0.44
Relative importance
bio01
bio04
bio05
bio06
bio12
CEC
CLY
DES
SOC
TWI

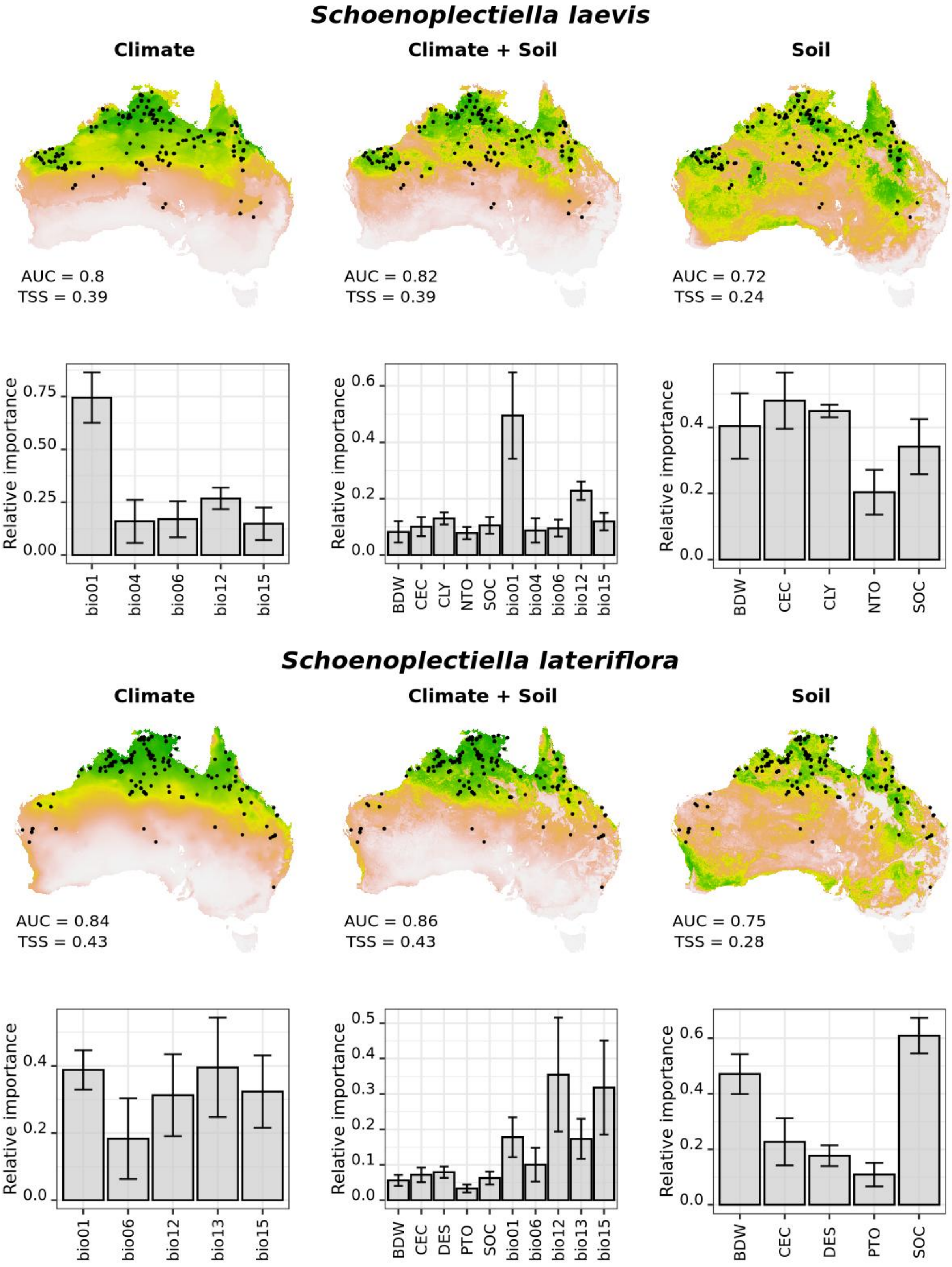

Schoenoplectiella laevis
Climate
Climate + Soil
Soil
AUC = 0.8
TSS = 0.39
AUC = 0.82
TSS = 0.39
AUC = 0.72
TSS = 0.24
Relative importance
bio01
bio04
bio06
bio12
bio15
BDW
CEC
CLY
NTO
SOC
Schoenoplectiella lateriflora
Climate
Climate + Soil
Soil
AUC = 0.84
TSS = 0.43
AUC = 0.86
TSS = 0.43
AUC = 0.75
TSS = 0.28
Relative importance
bio01
bio06
bio12
bio13
bio15
BDW
CEC
DES
PTO
SOC

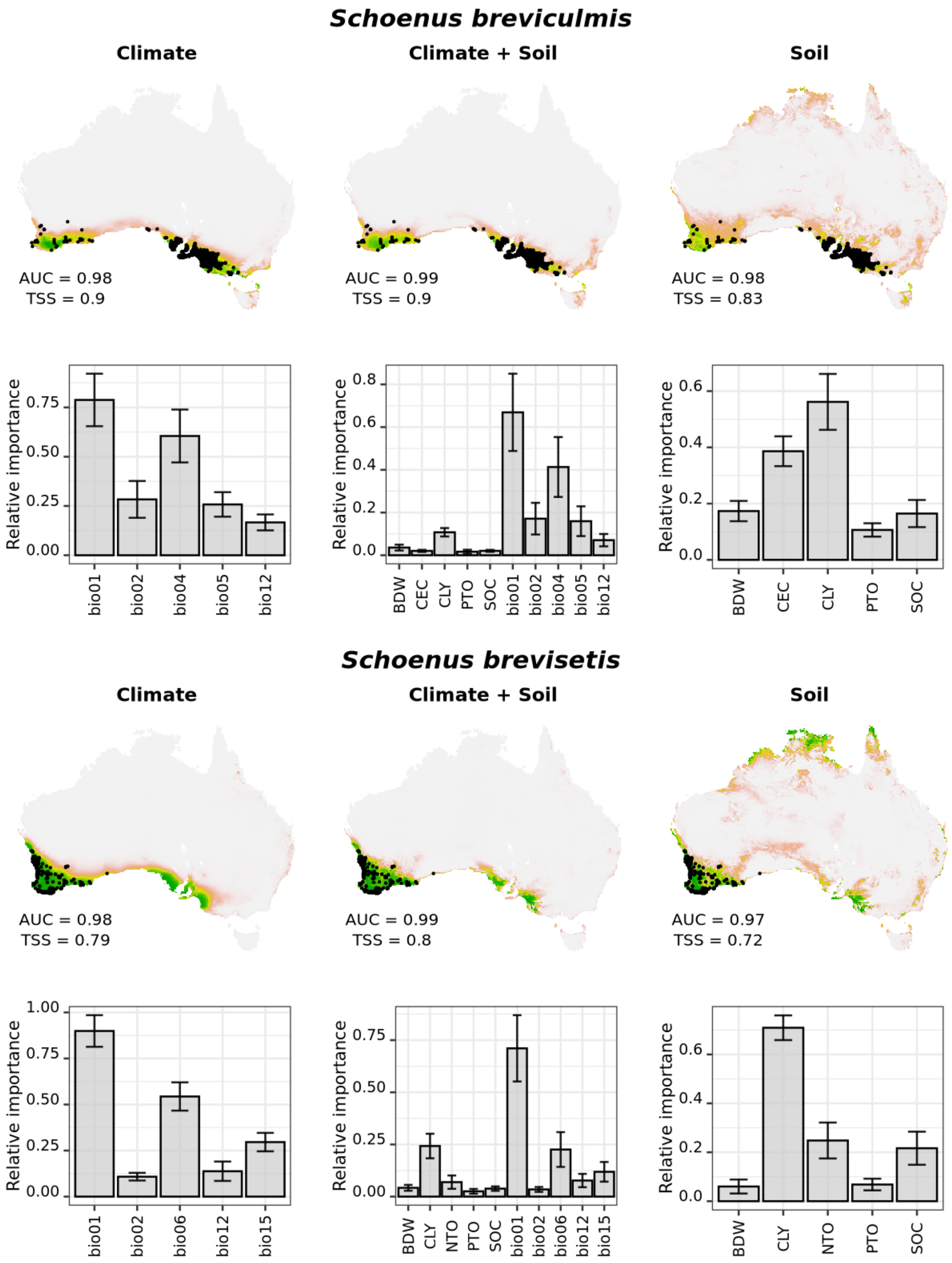

Schoenus breviculmis
Climate
Climate + Soil
Soil
AUC = 0.98
TSS = 0.9
AUC = 0.99
TSS = 0.9
AUC = 0.98
TSS = 0.83
Relative importance
0.00
0.25
0.50
0.75
bio01
bio02
bio04
bio05
bio12
0.0
0.2
0.4
0.6
0.8
BDW
CEC
CLY
PTO
SOC
bio01
bio02
bio04
bio05
bio12
0.0
0.2
0.4
0.6
BDW
CEC
CLY
PTO
SOC
Schoenus brevisetis
Climate
Climate + Soil
Soil
AUC = 0.98
TSS = 0.79
AUC = 0.99
TSS = 0.8
AUC = 0.97
TSS = 0.72
Relative importance
0.00
0.25
0.50
0.75
1.00
bio01
bio02
bio06
bio12
bio15
0.00
0.25
0.50
0.75
BDW
CLY
NTO
PTO
SOC
bio01
bio02
bio06
bio12
bio15
0.0
0.2
0.4
0.6
BDW
CLY
NTO
PTO
SOC

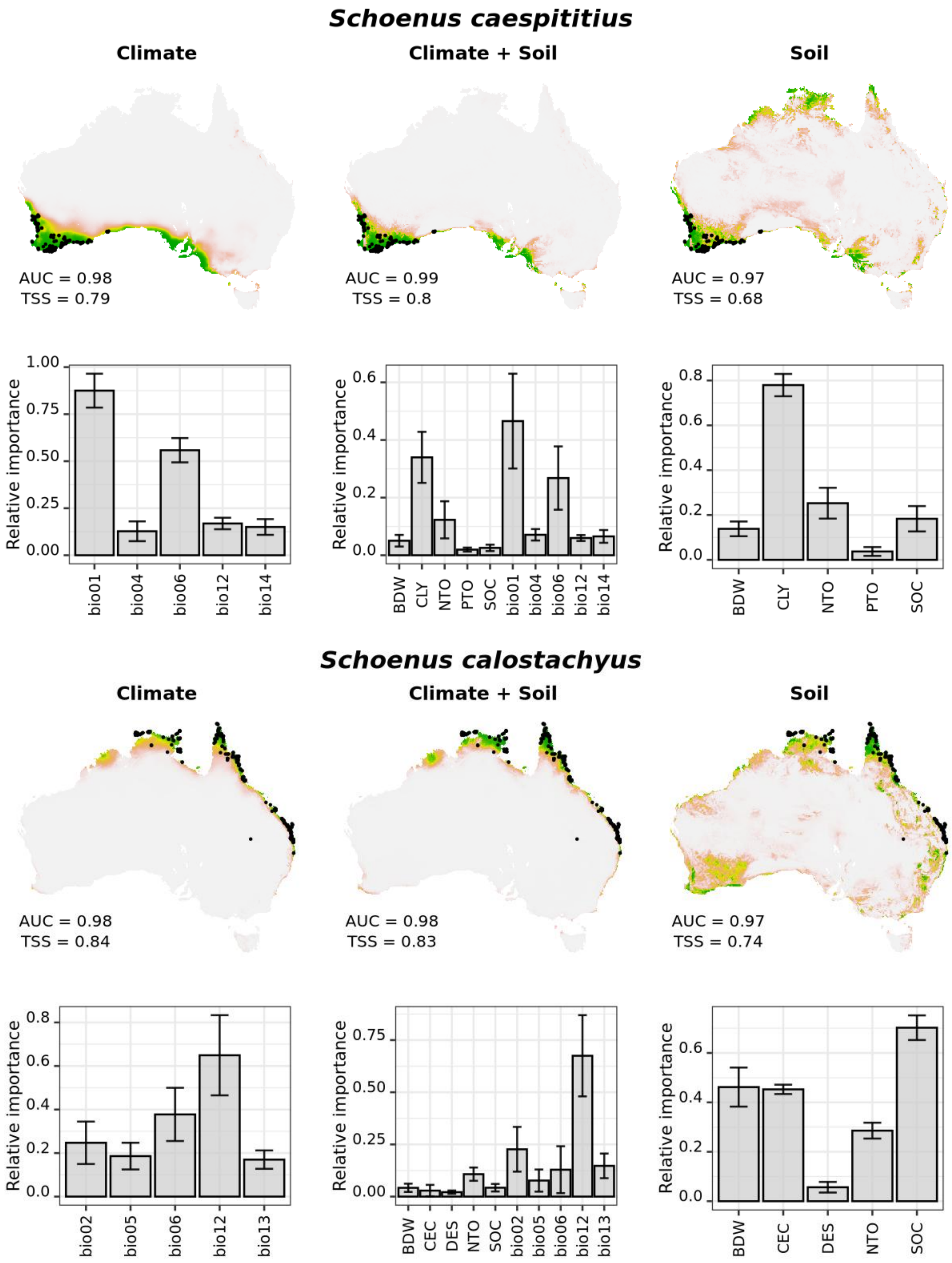

Schoenus caespititius
Climate
Climate + Soil
Soil
AUC = 0.98
TSS = 0.79
AUC = 0.99
TSS = 0.8
AUC = 0.97
TSS = 0.68
Relative importance
bio01
bio04
bio06
bio12
bio14
BDW
CLY
NTO
PTO
SOC
Schoenus calostachyus
Climate
Climate + Soil
Soil
AUC = 0.98
TSS = 0.84
AUC = 0.98
TSS = 0.83
AUC = 0.97
TSS = 0.74
Relative importance
bio02
bio05
bio06
bio12
bio13
BDW
CEC
DES
NTO
SOC

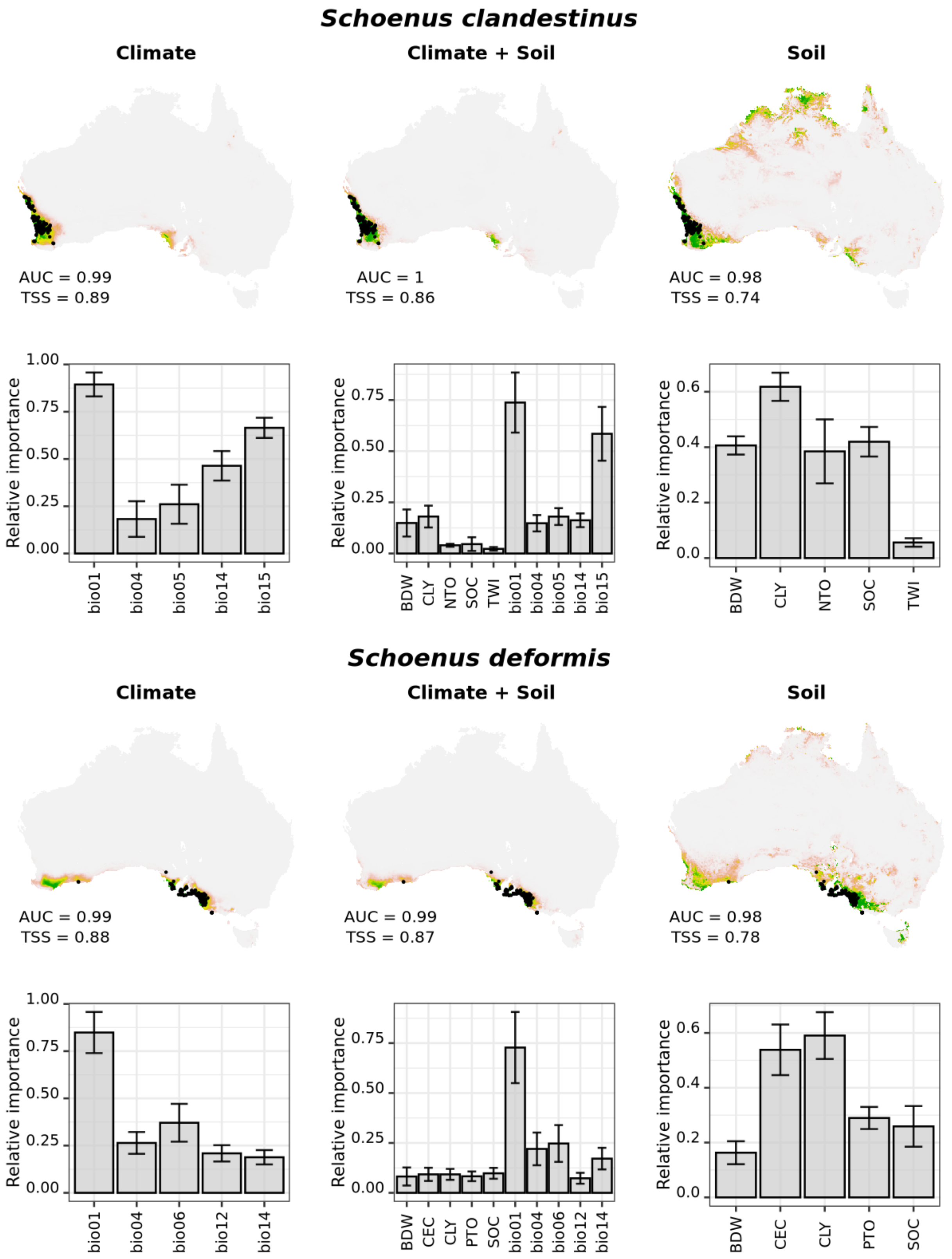
Schoenus clandestinus
Climate
Climate + Soil
Soil
AUC = 0.99
TSS = 0.89
AUC = 1
TSS = 0.86
AUC = 0.98
TSS = 0.74
Relative importance
bio01 bio04 bio05 bio14 bio15
BDW CLY NTO SOC TWI bio01 bio04 bio05 bio14 bio15
BDW CLY NTO SOC TWI
Schoenus deformis
Climate
Climate + Soil
Soil
AUC = 0.99
TSS = 0.88
AUC = 0.99
TSS = 0.87
AUC = 0.98
TSS = 0.78
Relative importance
bio01 bio04 bio06 bio12 bio14
BDW CEC CLY PTO SOC bio01 bio04 bio06 bio12 bio14
BDW CEC CLY PTO SOC

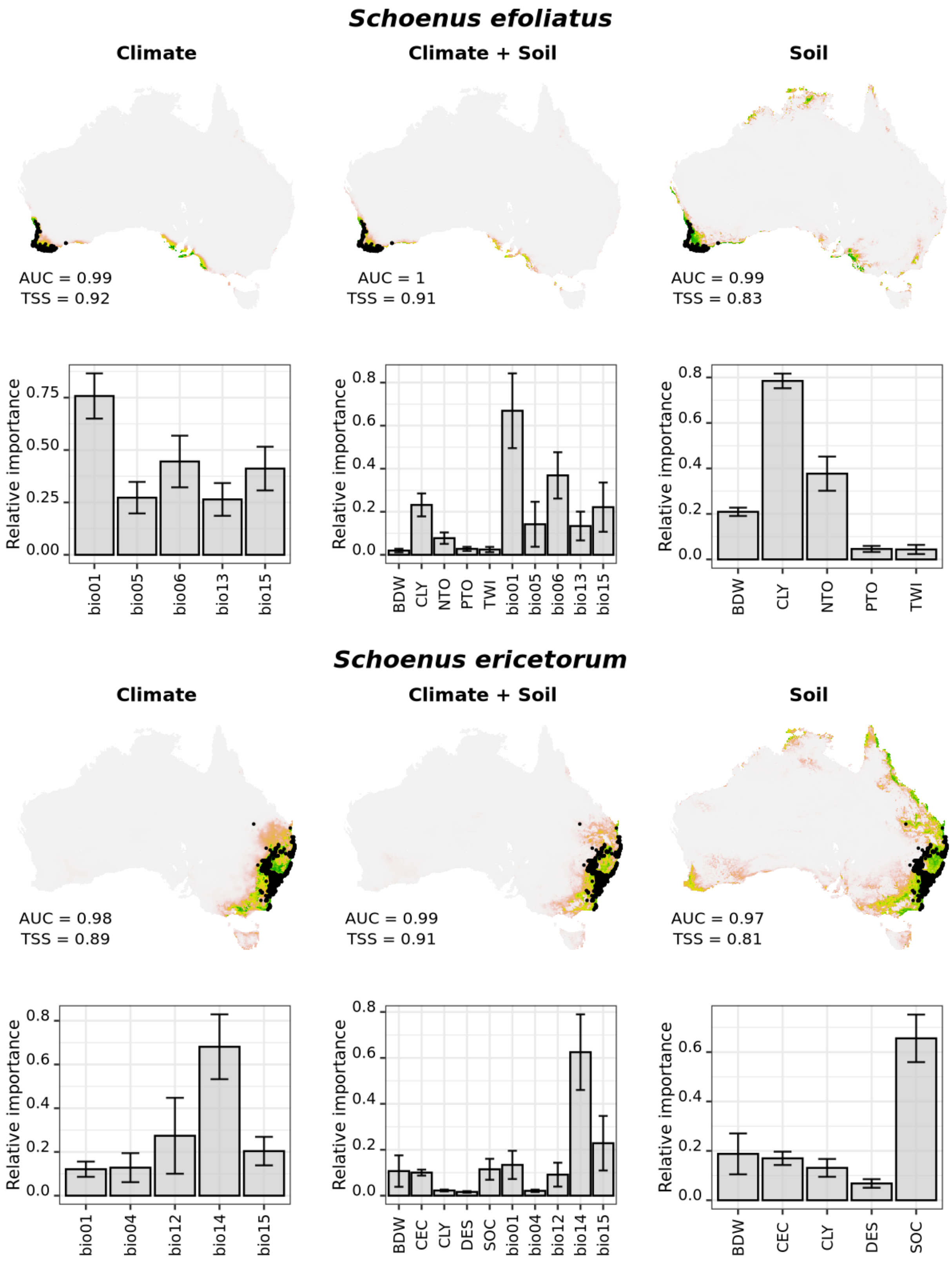
Schoenus efoliatus
Climate
Climate + Soil
Soil
AUC = 0.99
TSS = 0.92
AUC = 1
TSS = 0.91
AUC = 0.99
TSS = 0.83
Relative importance
0.00
0.25
0.50
0.75
bio01
bio05
bio06
bio13
bio15
0.0
0.2
0.4
0.6
0.8
BDW
CLY
NTO
PTO
TWI
Schoenus ericetorum
AUC = 0.98
TSS = 0.89
AUC = 0.99
TSS = 0.91
AUC = 0.97
TSS = 0.81
bio01
bio04
bio12
bio14
bio15
BDW
CEC
CLY
DES
SOC

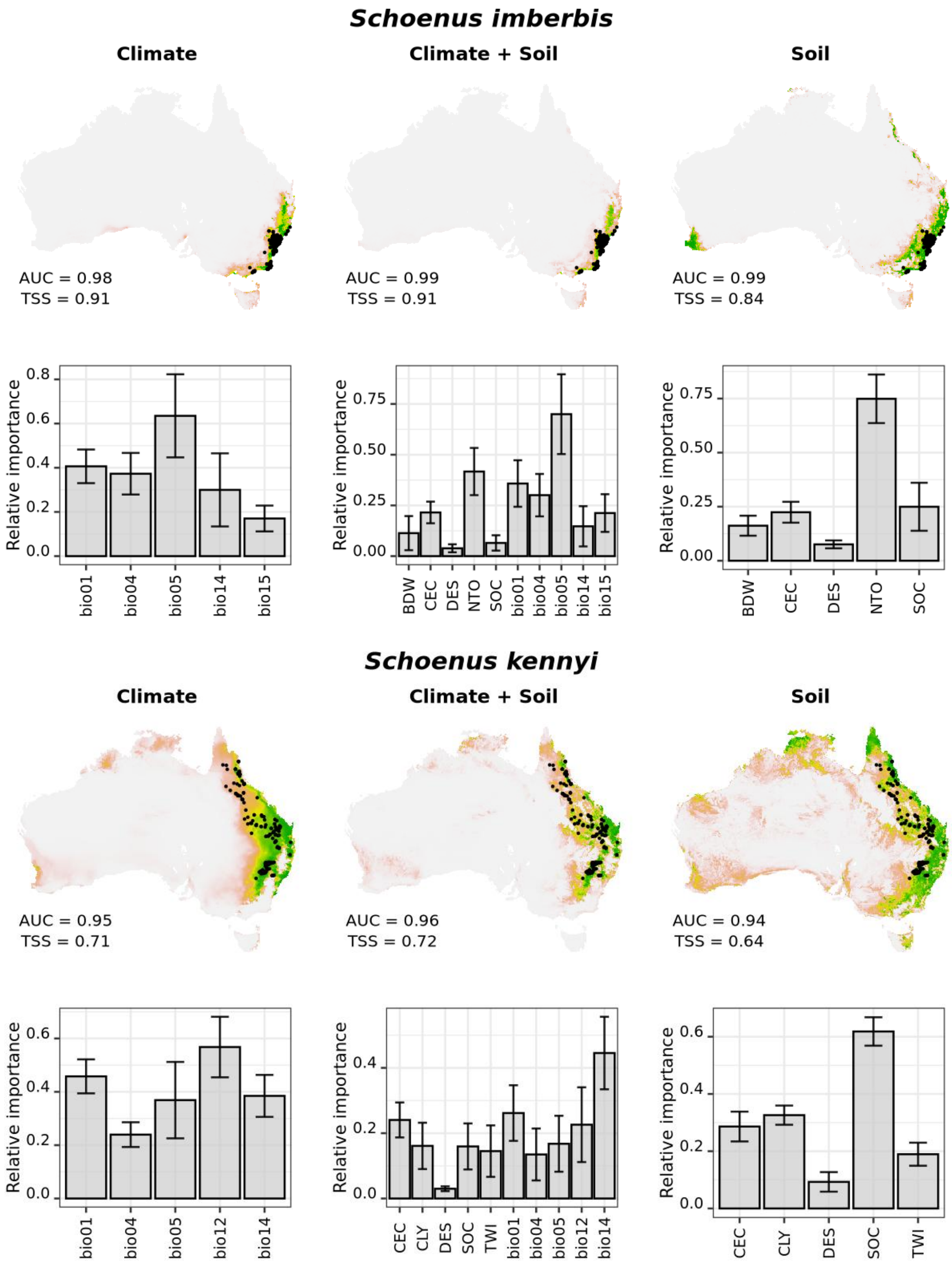
Schoenus imberbis
Climate
Climate + Soil
Soil
AUC = 0.98
TSS = 0.91
AUC = 0.99
TSS = 0.91
AUC = 0.99
TSS = 0.84
Relative importance
bio01
bio04
bio05
bio14
bio15
BDW
CEC
DES
NTO
SOC
Schoenus kennyi
AUC = 0.95
TSS = 0.71
AUC = 0.96
TSS = 0.72
AUC = 0.94
TSS = 0.64
bio12
CLY
TWI

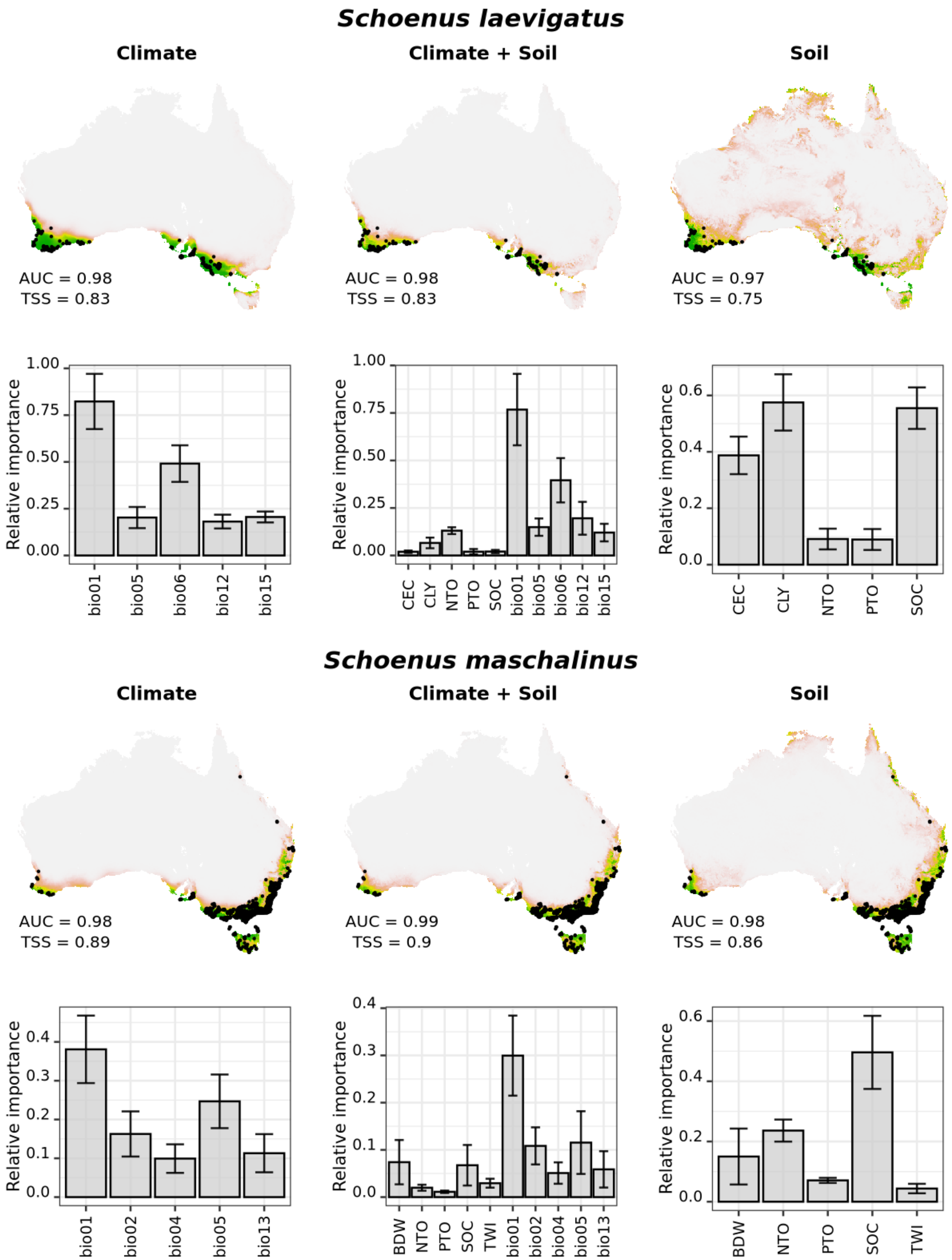

Schoenus laevigatus
Climate
Climate + Soil
Soil
AUC = 0.98
TSS = 0.83
AUC = 0.98
TSS = 0.83
AUC = 0.97
TSS = 0.75
Relative importance
1.00
0.75
0.50
0.25
0.00
bio01
bio05
bio06
bio12
bio15
CEC
CLY
NTO
PTO
SOC
0.6
0.4
0.2
0.0
Schoenus maschalinus
Climate
Climate + Soil
Soil
AUC = 0.98
TSS = 0.89
AUC = 0.99
TSS = 0.9
AUC = 0.98
TSS = 0.86
0.4
0.3
0.2
0.1
0.0
bio01
bio02
bio04
bio05
bio13
BDW
NTO
PTO
SOC
TWI

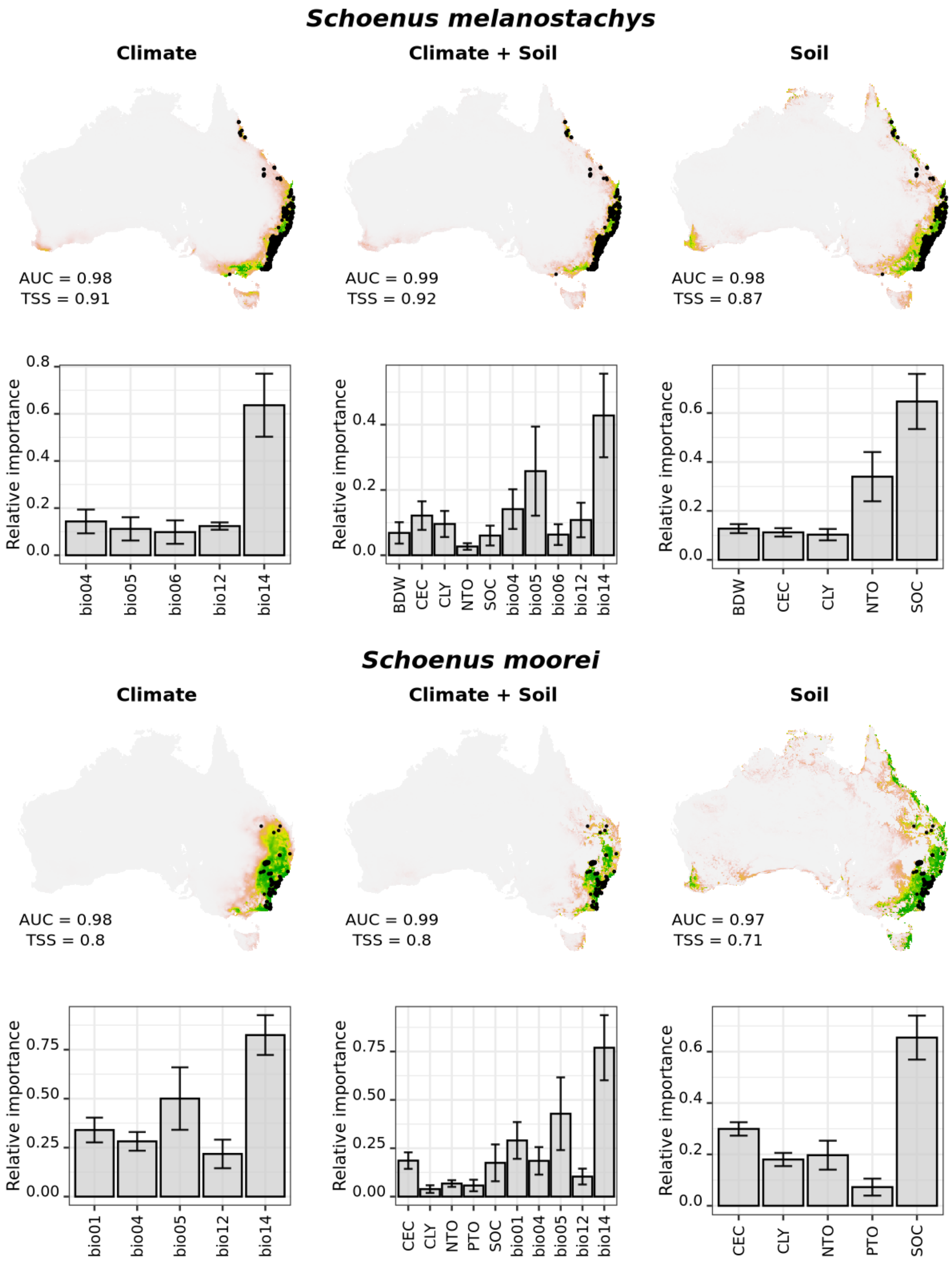
Schoenus melanostachys
Climate
Climate + Soil
Soil
AUC = 0.98
TSS = 0.91
AUC = 0.99
TSS = 0.92
AUC = 0.98
TSS = 0.87
Relative importance
0.0
0.2
0.4
0.6
0.8
bio04
bio05
bio06
bio12
bio14
BDW
CEC
CLY
NTO
SOC
bio04
bio05
bio06
bio12
bio14
BDW
CEC
CLY
NTO
SOC
Schoenus moorei
Climate
Climate + Soil
Soil
AUC = 0.98
TSS = 0.8
AUC = 0.99
TSS = 0.8
AUC = 0.97
TSS = 0.71
Relative importance
0.00
0.25
0.50
0.75
bio01
bio04
bio05
bio12
bio14
CEC
CLY
NTO
PTO
SOC
bio01
bio04
bio05
bio12
bio14
CEC
CLY
NTO
PTO
SOC

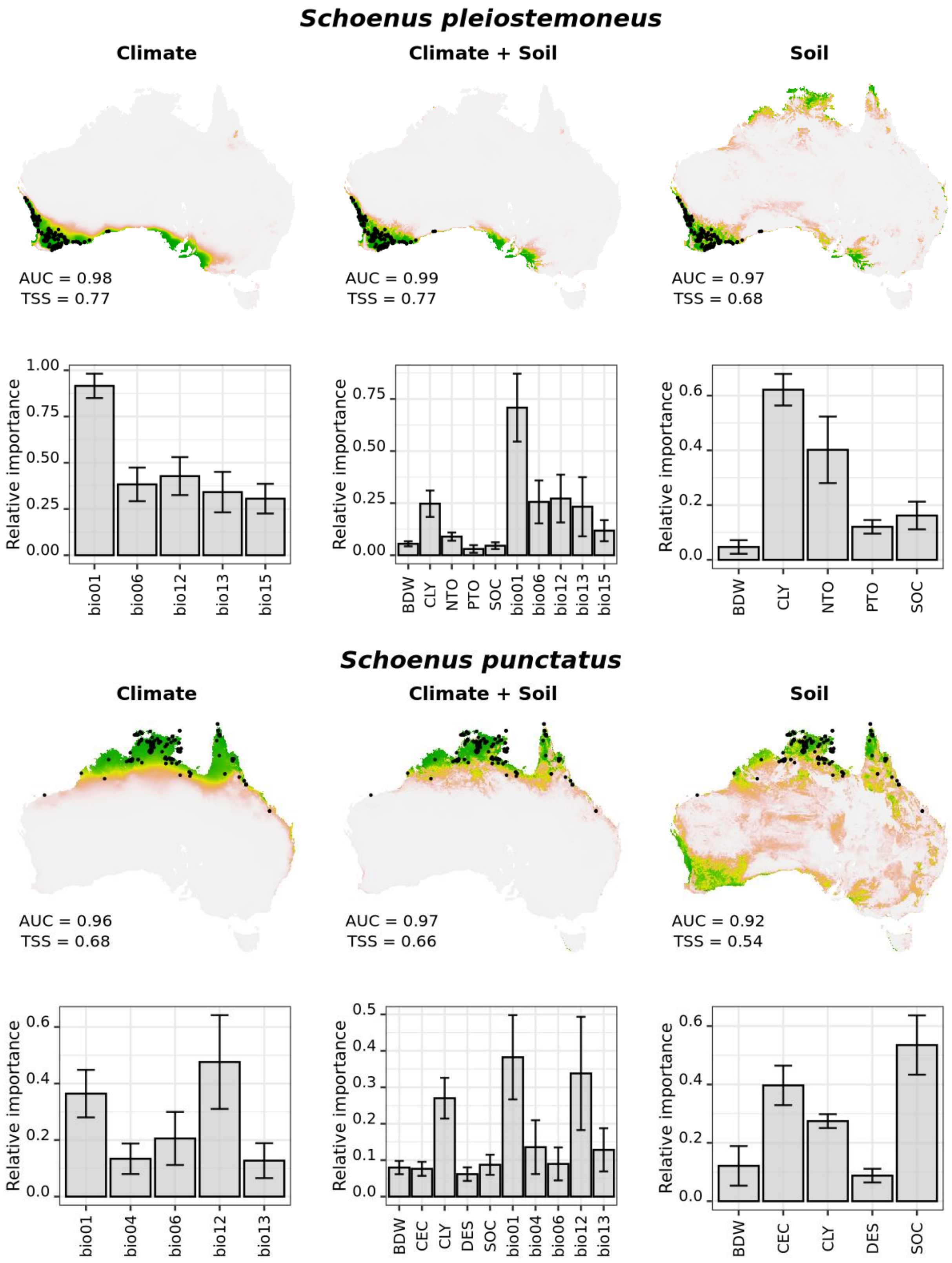

Schoenus pleiostemoneus
Climate
Climate + Soil
Soil
AUC = 0.98
TSS = 0.77
AUC = 0.99
TSS = 0.77
AUC = 0.97
TSS = 0.68
Relative importance
bio01
bio06
bio12
bio13
bio15
BDW
CLY
NTO
PTO
SOC
Schoenus punctatus
Climate
Climate + Soil
Soil
AUC = 0.96
TSS = 0.68
AUC = 0.97
TSS = 0.66
AUC = 0.92
TSS = 0.54
Relative importance
bio01
bio04
bio06
bio12
bio13
BDW
CEC
CLY
DES
SOC

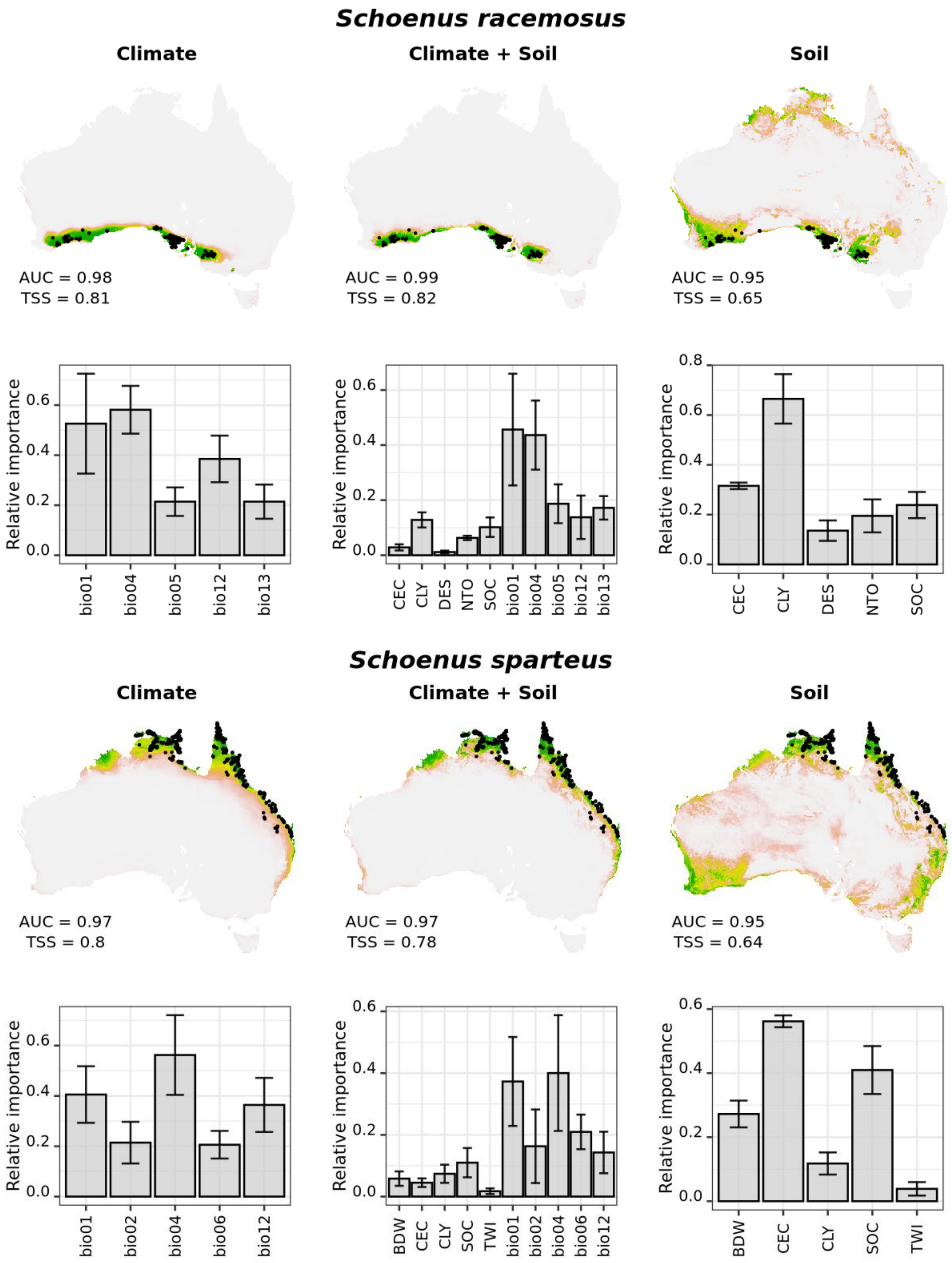

Schoenus racemosus
Climate
Climate + Soil
Soil
AUC = 0.98
TSS = 0.81
AUC = 0.99
TSS = 0.82
AUC = 0.95
TSS = 0.65
Relative importance
bio01
bio04
bio05
bio12
bio13
CEC
CLY
DES
NTO
SOC
Schoenus sparteus
Climate
Climate + Soil
Soil
AUC = 0.97
TSS = 0.8
AUC = 0.97
TSS = 0.78
AUC = 0.95
TSS = 0.64
bio01
bio02
bio04
bio06
bio12
BDW
CEC
CLY
SOC
TWI

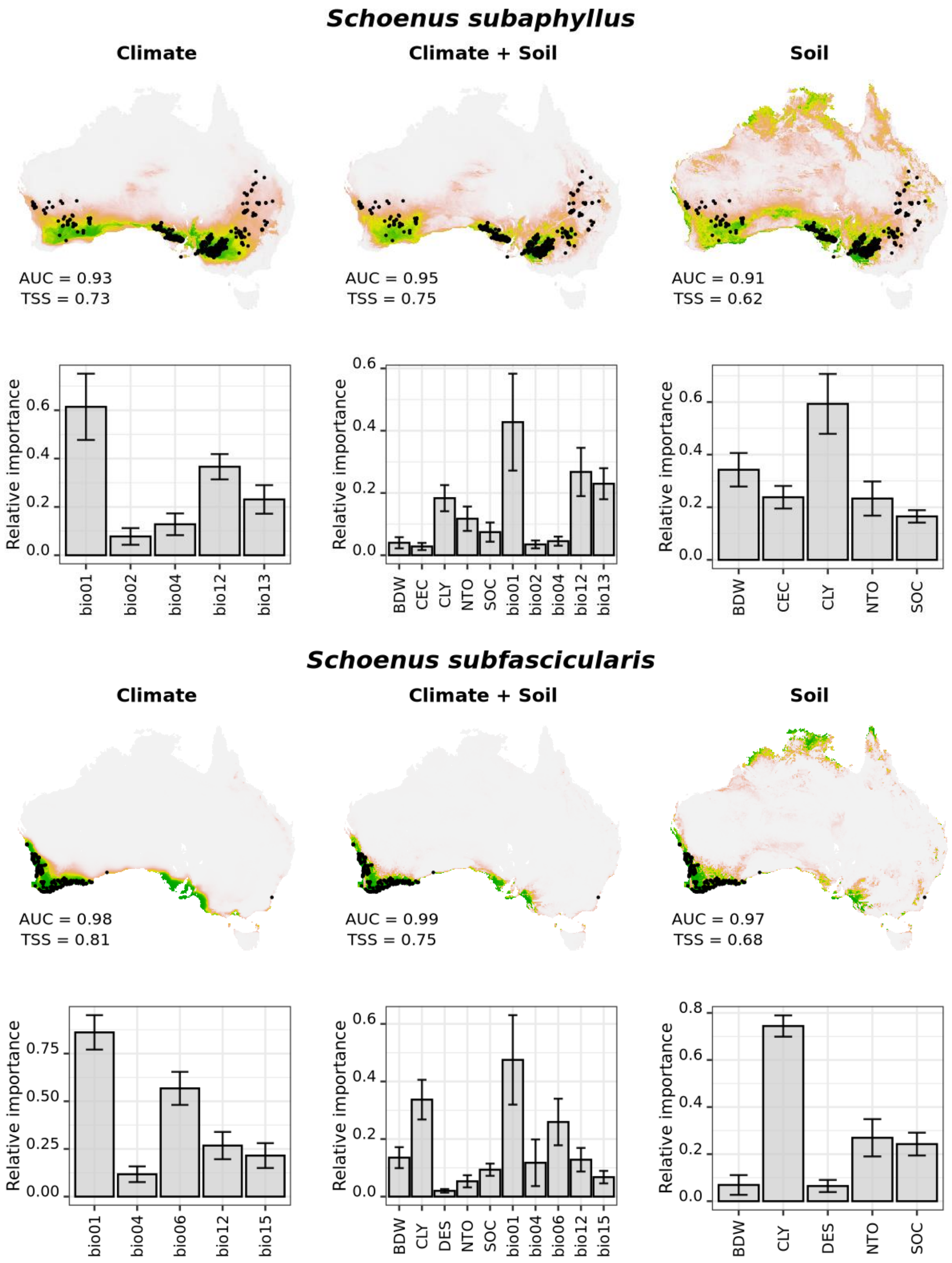
Schoenus subaphyllus
Climate
Climate + Soil
Soil
AUC = 0.93
TSS = 0.73
AUC = 0.95
TSS = 0.75
AUC = 0.91
TSS = 0.62
Relative importance
bio01
bio02
bio04
bio12
bio13
BDW
CEC
CLY
NTO
SOC
Schoenus subfascicularis
AUC = 0.98
TSS = 0.81
AUC = 0.99
TSS = 0.75
AUC = 0.97
TSS = 0.68
bio06
bio15
DES

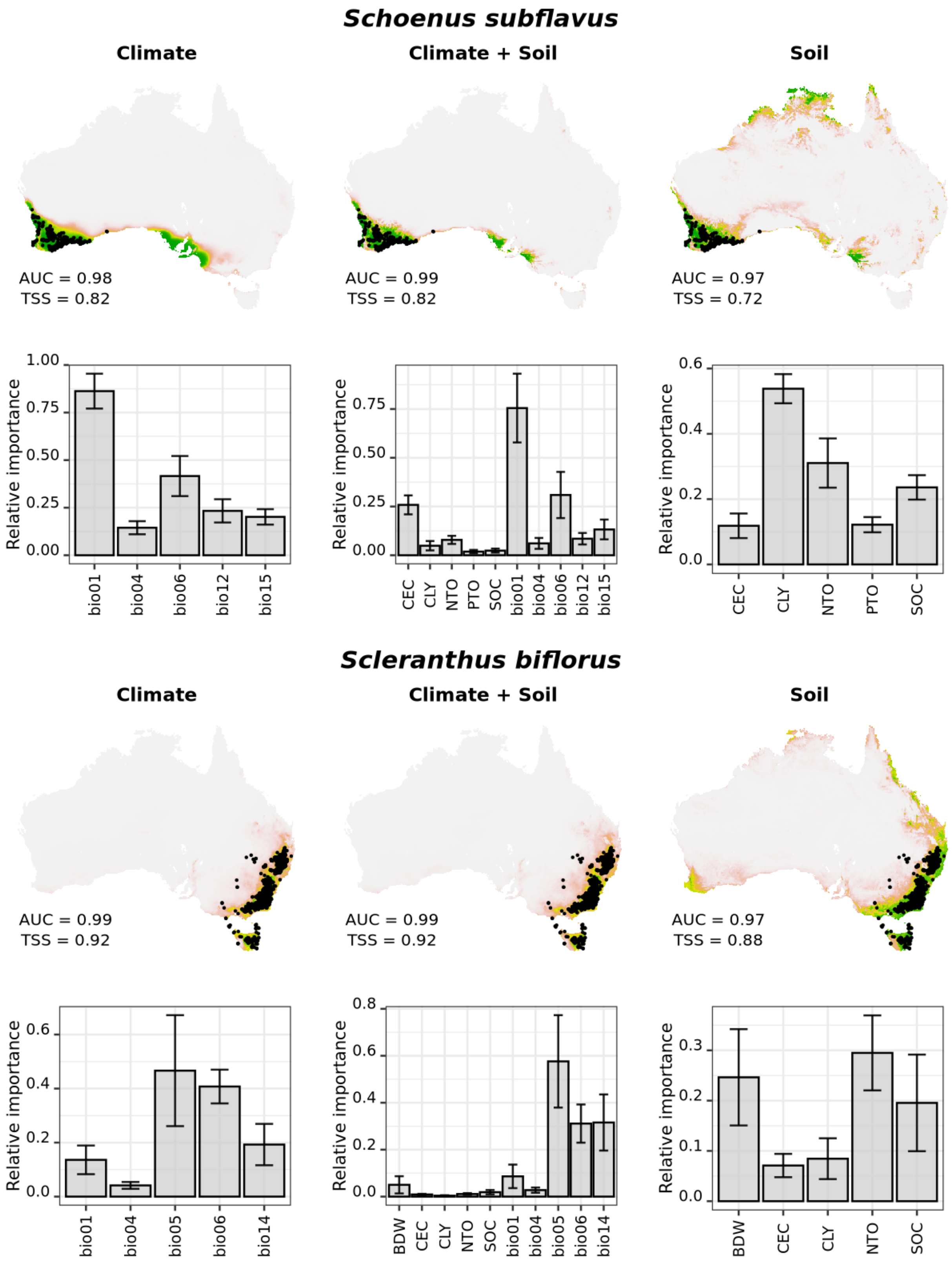

Schoenus subflavus
Climate
Climate + Soil
Soil
AUC = 0.98
TSS = 0.82
AUC = 0.99
TSS = 0.82
AUC = 0.97
TSS = 0.72
Relative importance
1.00
0.75
0.50
0.25
0.00
bio01
bio04
bio06
bio12
bio15
CEC
CLY
NTO
PTO
SOC
0.6
0.4
0.2
0.0
Scleranthus biflorus
Climate
Climate + Soil
Soil
AUC = 0.99
TSS = 0.92
AUC = 0.99
TSS = 0.92
AUC = 0.97
TSS = 0.88
bio05
bio14
BDW
0.8
0.3
0.1

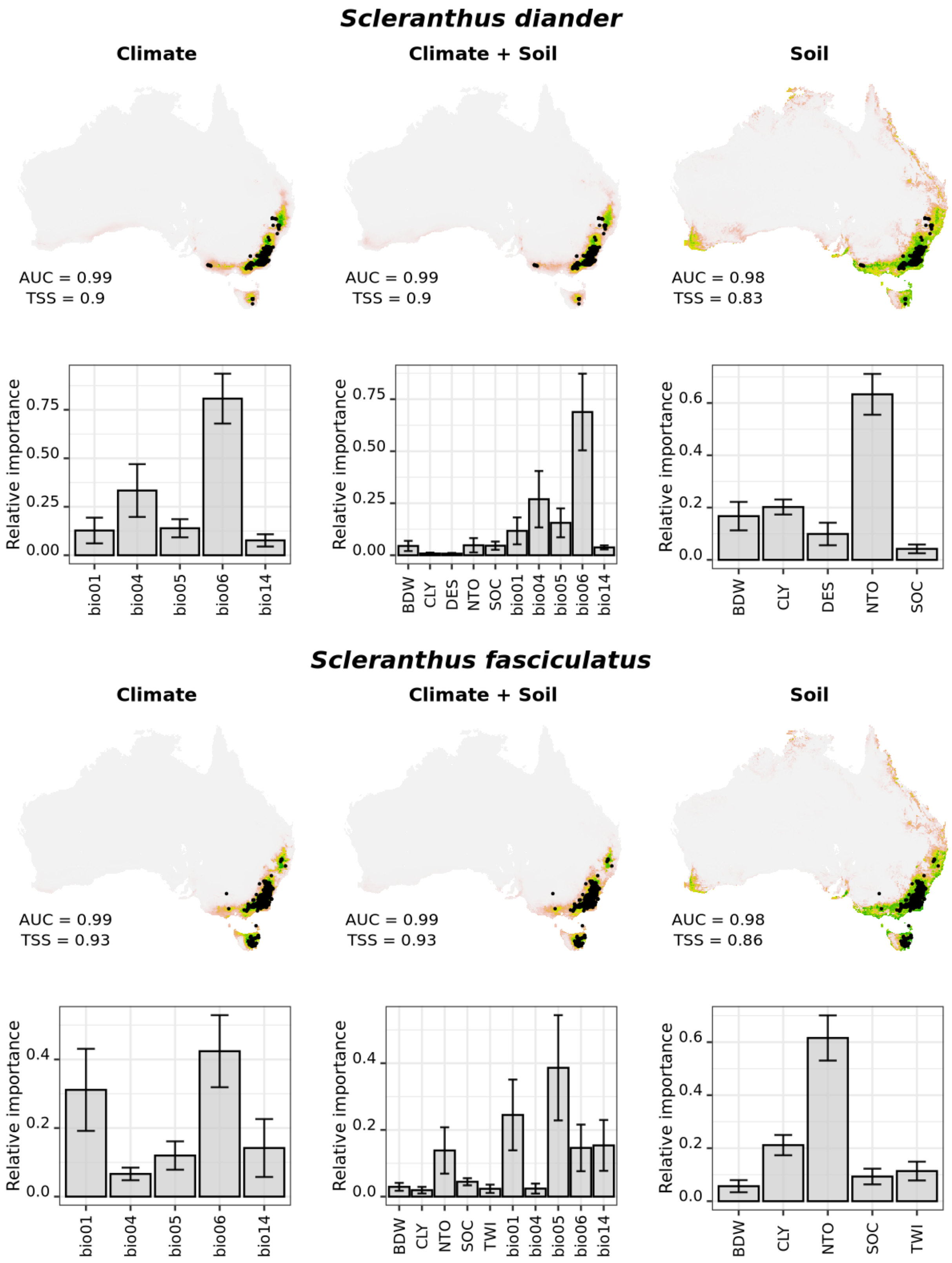
Scleranthus diander
Climate
Climate + Soil
Soil
AUC = 0.99
TSS = 0.9
AUC = 0.99
TSS = 0.9
AUC = 0.98
TSS = 0.83
Relative importance
bio01
bio04
bio05
bio06
bio14
BDW
CLY
DES
NTO
SOC
Scleranthus fasciculatus
AUC = 0.99
TSS = 0.93
AUC = 0.99
TSS = 0.93
AUC = 0.98
TSS = 0.86
TWI

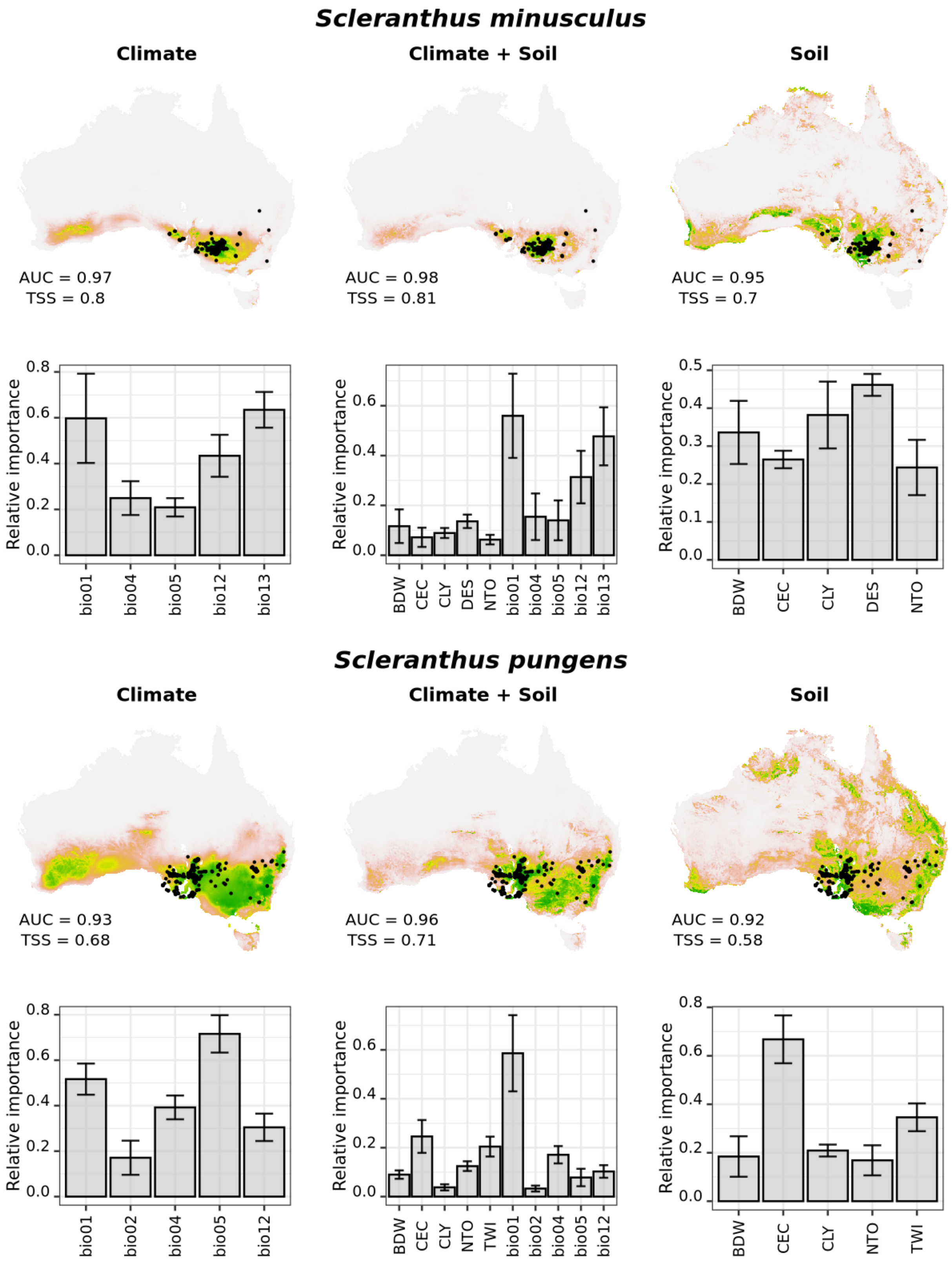
Scleranthus minusculus
Climate
Climate + Soil
Soil
AUC = 0.97
TSS = 0.8
AUC = 0.98
TSS = 0.81
AUC = 0.95
TSS = 0.7
Relative importance
0.0
0.2
0.4
0.6
0.8
bio01
bio04
bio05
bio12
bio13
BDW
CEC
CLY
DES
NTO
0.1
0.3
0.5
Scleranthus pungens
Climate
Climate + Soil
Soil
AUC = 0.93
TSS = 0.68
AUC = 0.96
TSS = 0.71
AUC = 0.92
TSS = 0.58
bio02
TWI

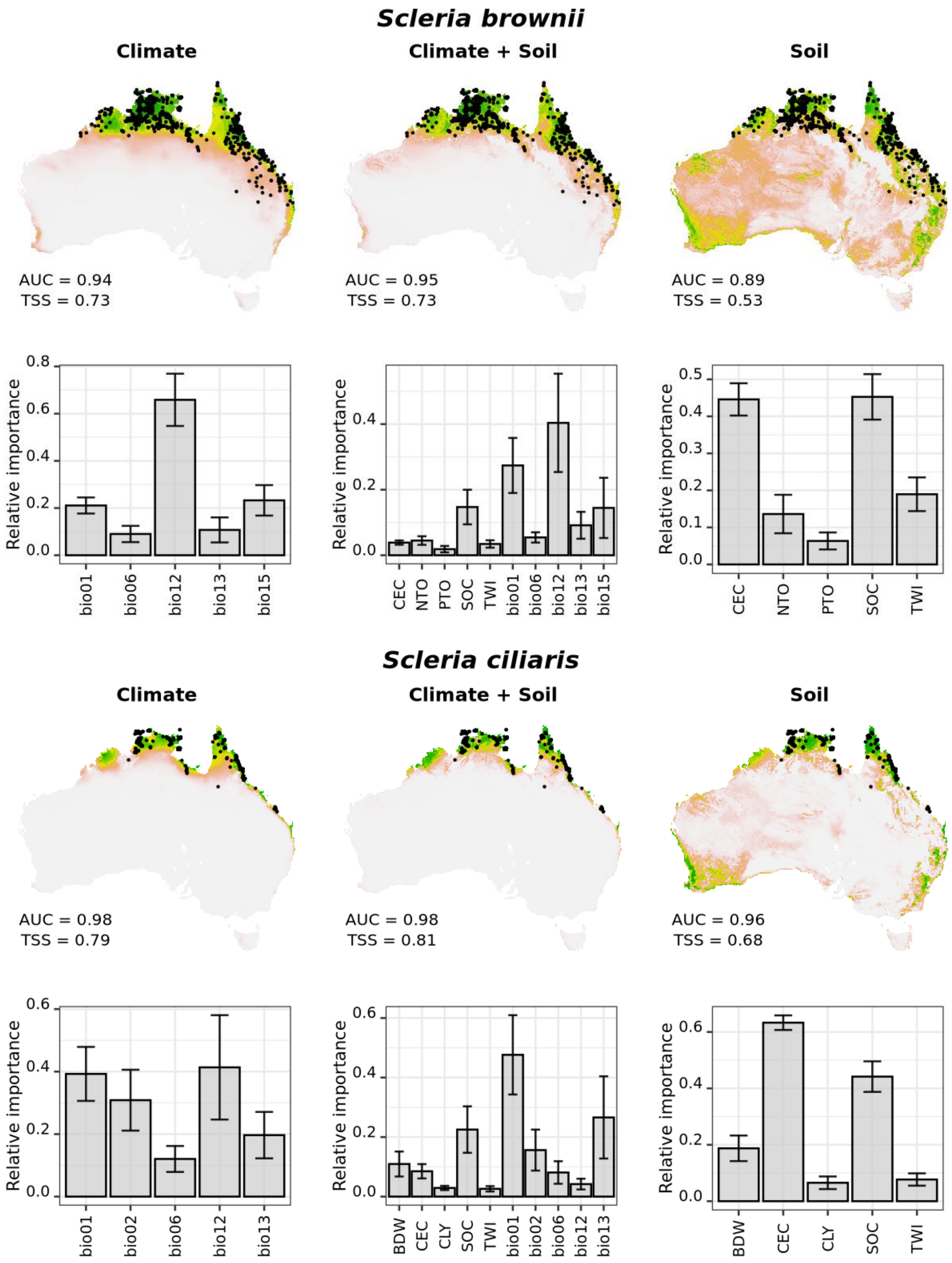

Scleria brownii
Climate
Climate + Soil
Soil
AUC = 0.94
TSS = 0.73
AUC = 0.95
TSS = 0.73
AUC = 0.89
TSS = 0.53
Relative importance
bio01
bio06
bio12
bio13
bio15
CEC
NTO
PTO
SOC
TWI
Scleria ciliaris
AUC = 0.98
TSS = 0.79
AUC = 0.98
TSS = 0.81
AUC = 0.96
TSS = 0.68
BDW
CLY
bio02

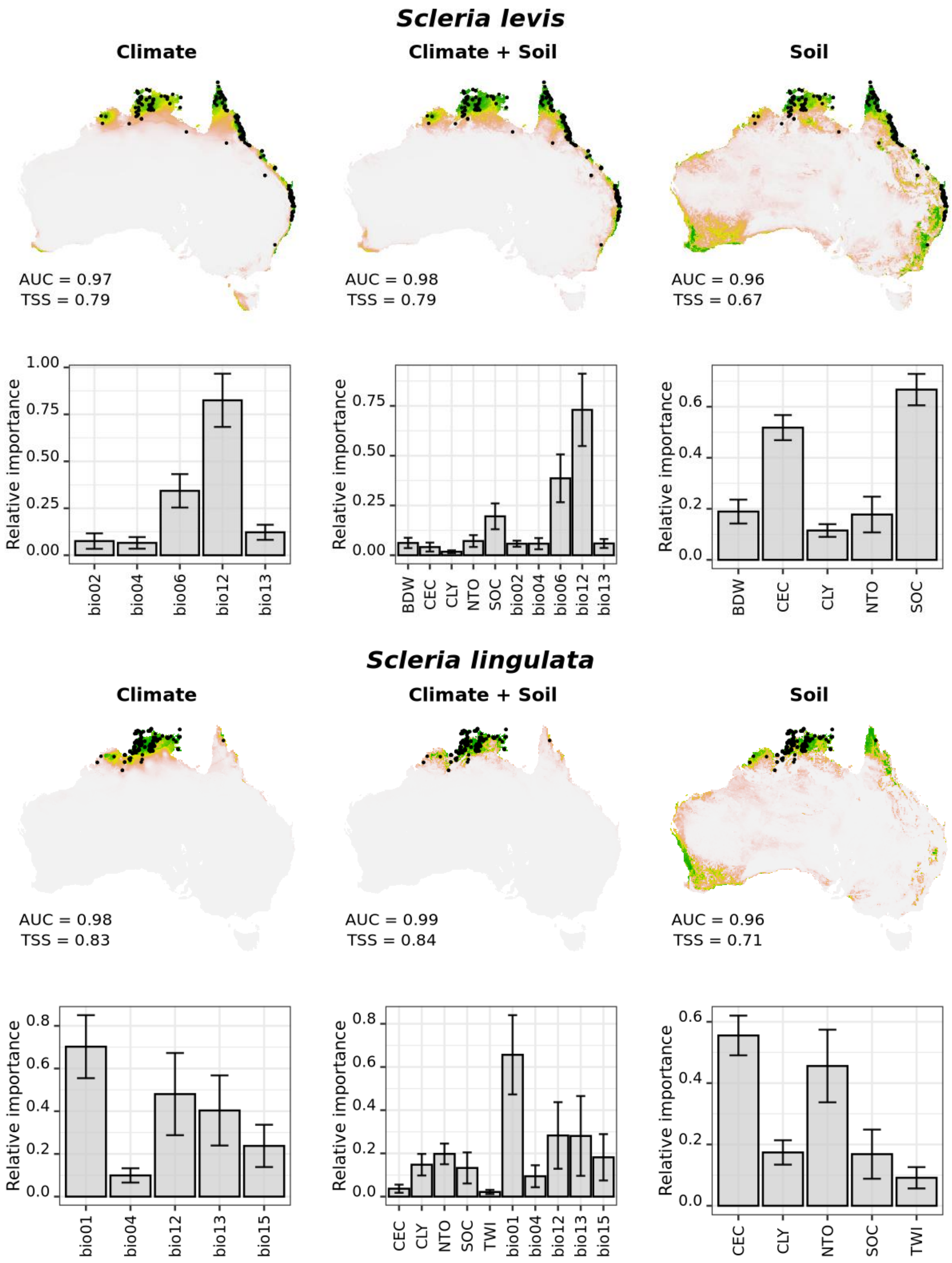
Scleria levis
Climate
Climate + Soil
Soil
AUC = 0.97
TSS = 0.79
AUC = 0.98
TSS = 0.79
AUC = 0.96
TSS = 0.67
Relative importance
bio02 bio04 bio06 bio12 bio13
BDW CEC CLY NTO SOC bio02 bio04 bio06 bio12 bio13
BDW CEC CLY NTO SOC
Scleria lingulata
Climate
Climate + Soil
Soil
AUC = 0.98
TSS = 0.83
AUC = 0.99
TSS = 0.84
AUC = 0.96
TSS = 0.71
Relative importance
bio01 bio04 bio12 bio13 bio15
CEC CLY NTO SOC TWI bio01 bio04 bio12 bio13 bio15
CEC CLY NTO SOC TWI

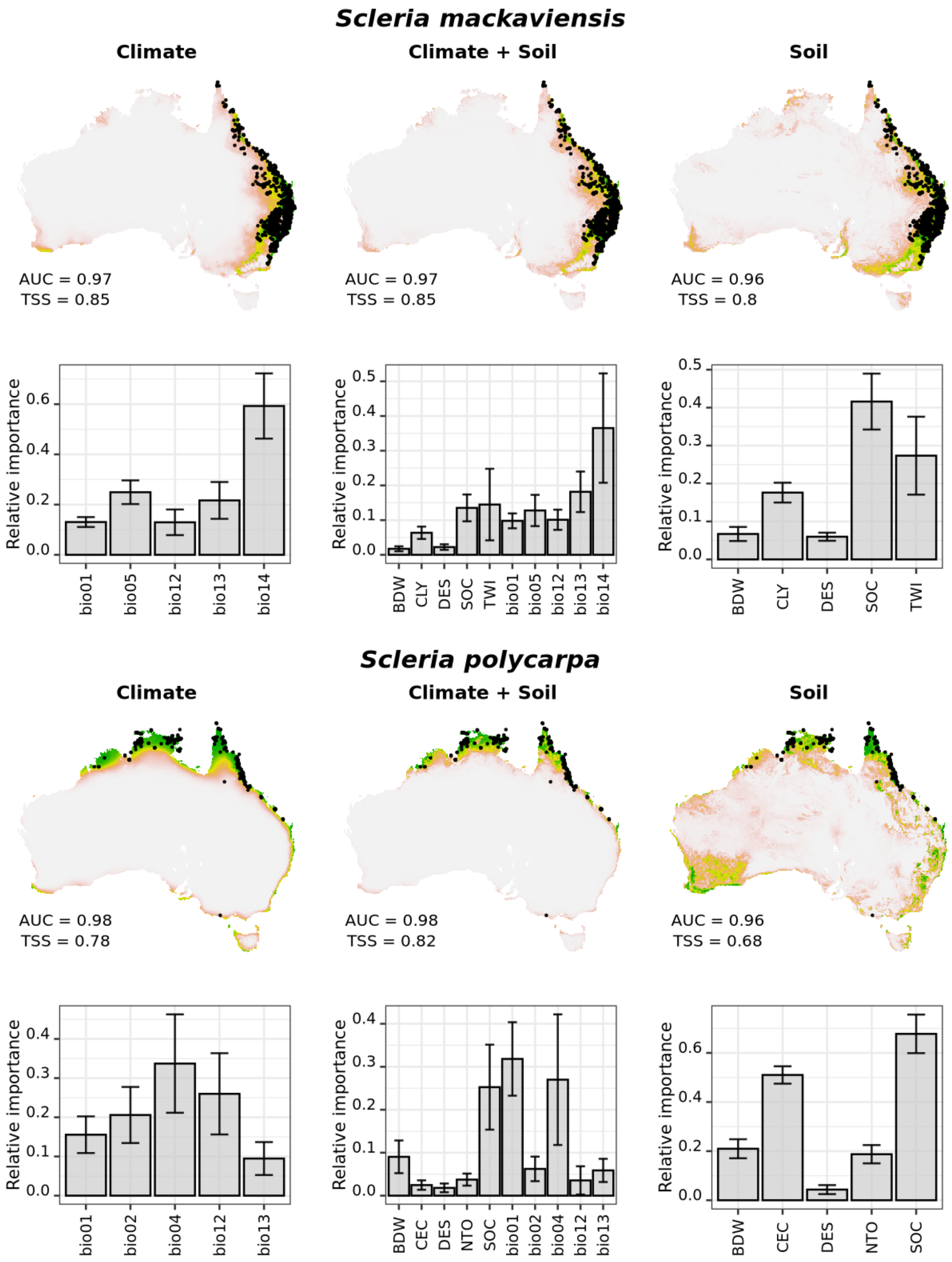

Scleria mackaviensis
Climate
Climate + Soil
Soil
AUC = 0.97
TSS = 0.85
AUC = 0.97
TSS = 0.85
AUC = 0.96
TSS = 0.8
Relative importance
bio01
bio05
bio12
bio13
bio14
BDW
CLY
DES
SOC
TWI
Scleria polycarpa
Climate
Climate + Soil
Soil
AUC = 0.98
TSS = 0.78
AUC = 0.98
TSS = 0.82
AUC = 0.96
TSS = 0.68
Relative importance
bio01
bio02
bio04
bio12
bio13
BDW
CEC
DES
NTO
SOC

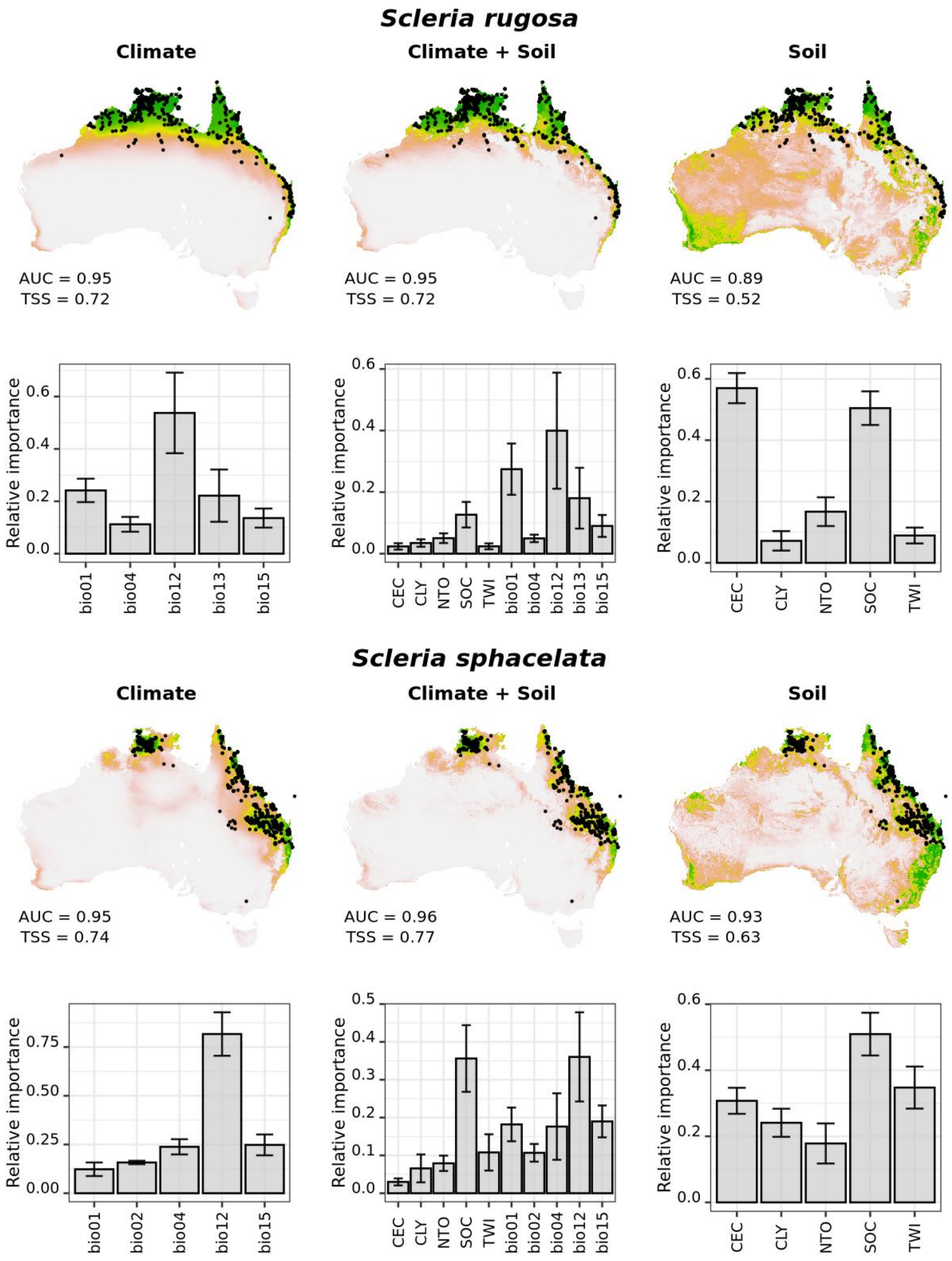
Scleria rugosa
Climate
Climate + Soil
Soil
AUC = 0.95
TSS = 0.72
AUC = 0.95
TSS = 0.72
AUC = 0.89
TSS = 0.52
Relative importance
0.0
0.2
0.4
0.6
bio01
bio04
bio12
bio13
bio15
CEC
CLY
NTO
SOC
TWI
Scleria sphacelata
Climate
Climate + Soil
Soil
AUC = 0.95
TSS = 0.74
AUC = 0.96
TSS = 0.77
AUC = 0.93
TSS = 0.63
Relative importance
0.00
0.25
0.50
0.75
0.0
0.1
0.2
0.3
0.4
0.5
bio01
bio02
bio04
bio12
bio15

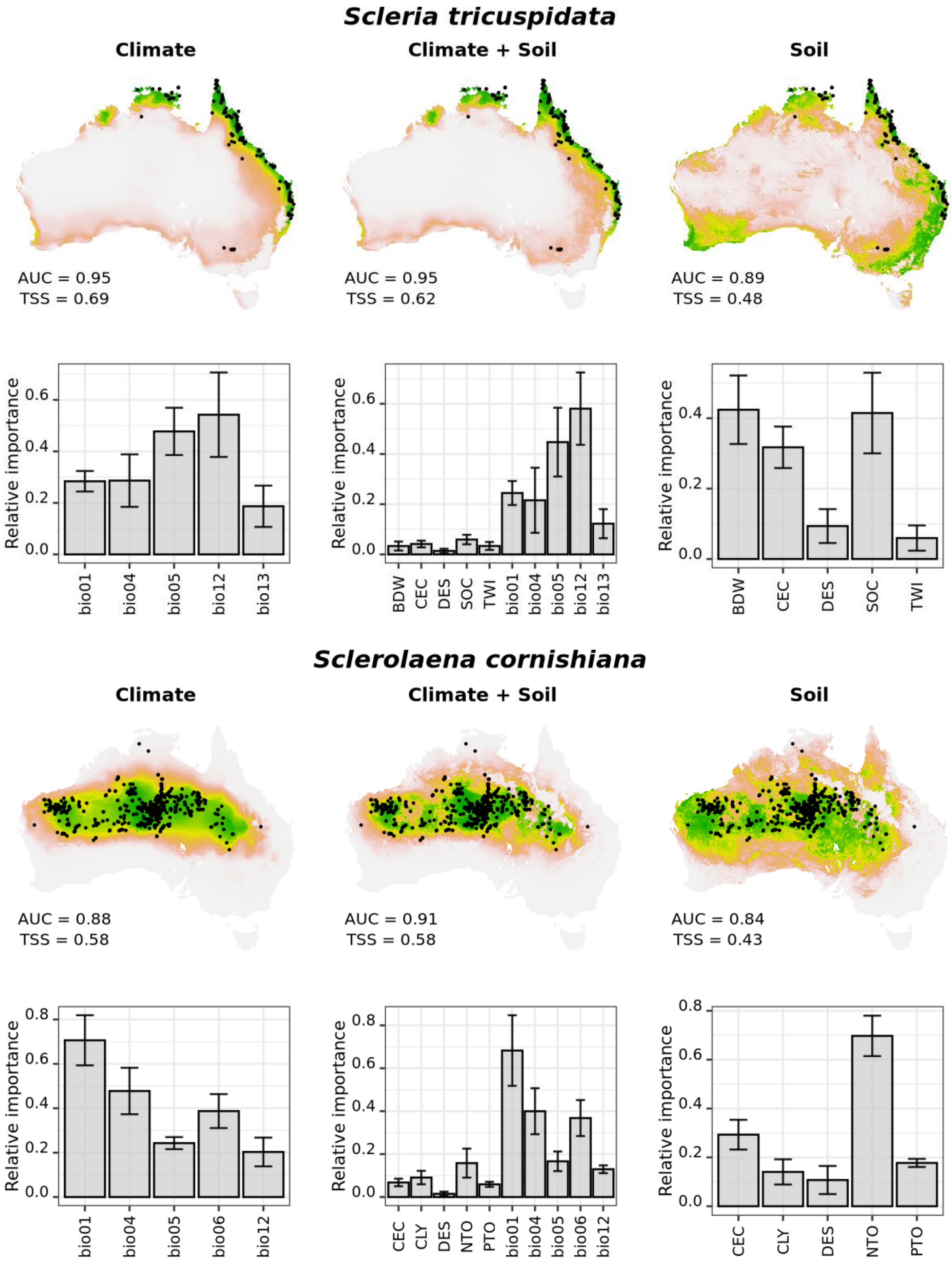

Scleria tricuspidata
Climate
Climate + Soil
Soil
AUC = 0.95
TSS = 0.69
AUC = 0.95
TSS = 0.62
AUC = 0.89
TSS = 0.48
Relative importance
0.0
0.2
0.4
0.6
bio01
bio04
bio05
bio12
bio13
BDW
CEC
DES
SOC
TWI
Sclerolaena cornishiana
AUC = 0.88
TSS = 0.58
AUC = 0.91
TSS = 0.58
AUC = 0.84
TSS = 0.43
0.8
bio06
CLY
NTO
PTO

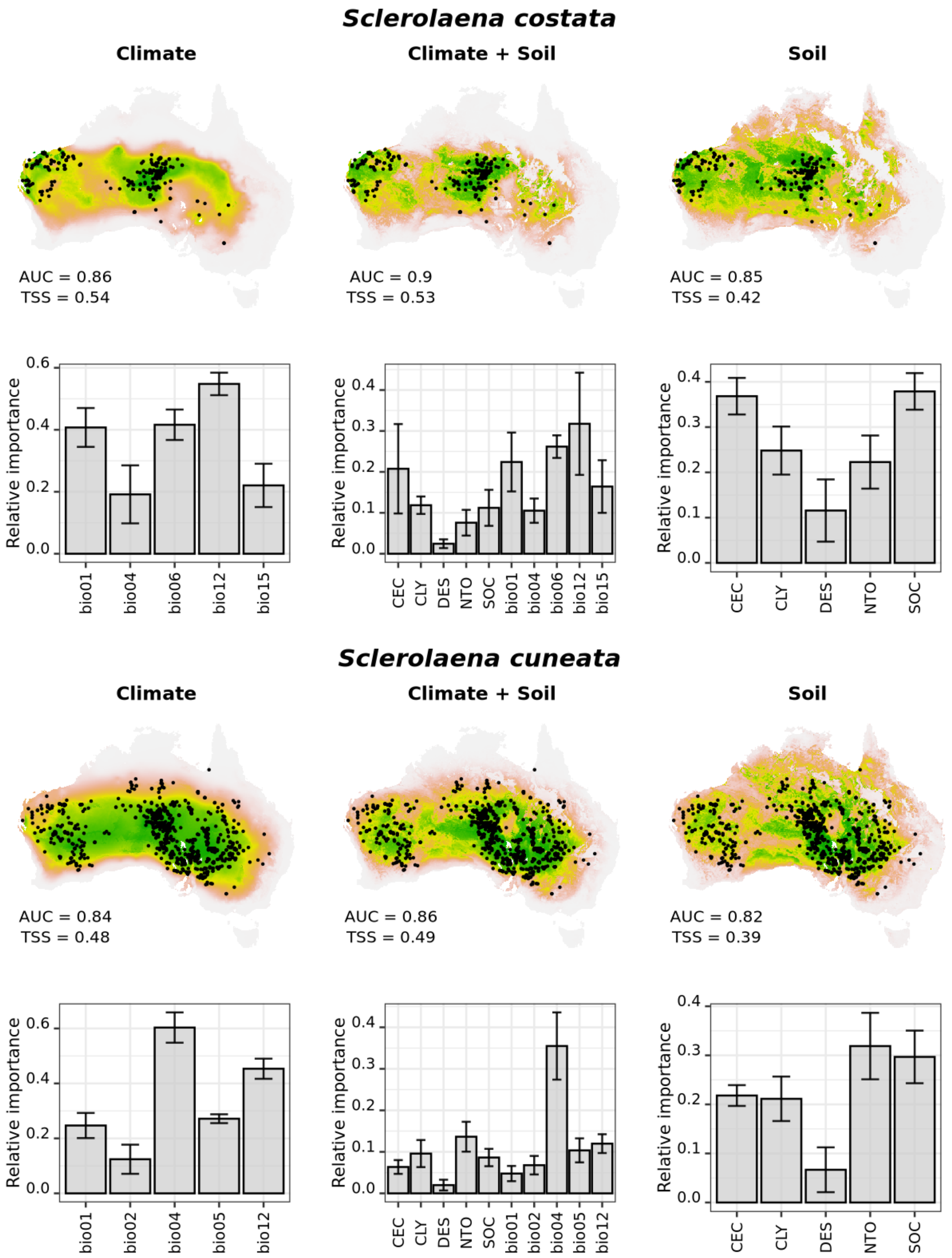
Sclerolaena costata
Climate
Climate + Soil
Soil
AUC = 0.86
TSS = 0.54
AUC = 0.9
TSS = 0.53
AUC = 0.85
TSS = 0.42
Relative importance
bio01 bio04 bio06 bio12 bio15
CEC CLY DES NTO SOC bio01 bio04 bio06 bio12 bio15
CEC CLY DES NTO SOC
Sclerolaena cuneata
Climate
Climate + Soil
Soil
AUC = 0.84
TSS = 0.48
AUC = 0.86
TSS = 0.49
AUC = 0.82
TSS = 0.39
Relative importance
bio01 bio02 bio04 bio05 bio12
CEC CLY DES NTO SOC bio01 bio02 bio04 bio05 bio12
CEC CLY DES NTO SOC

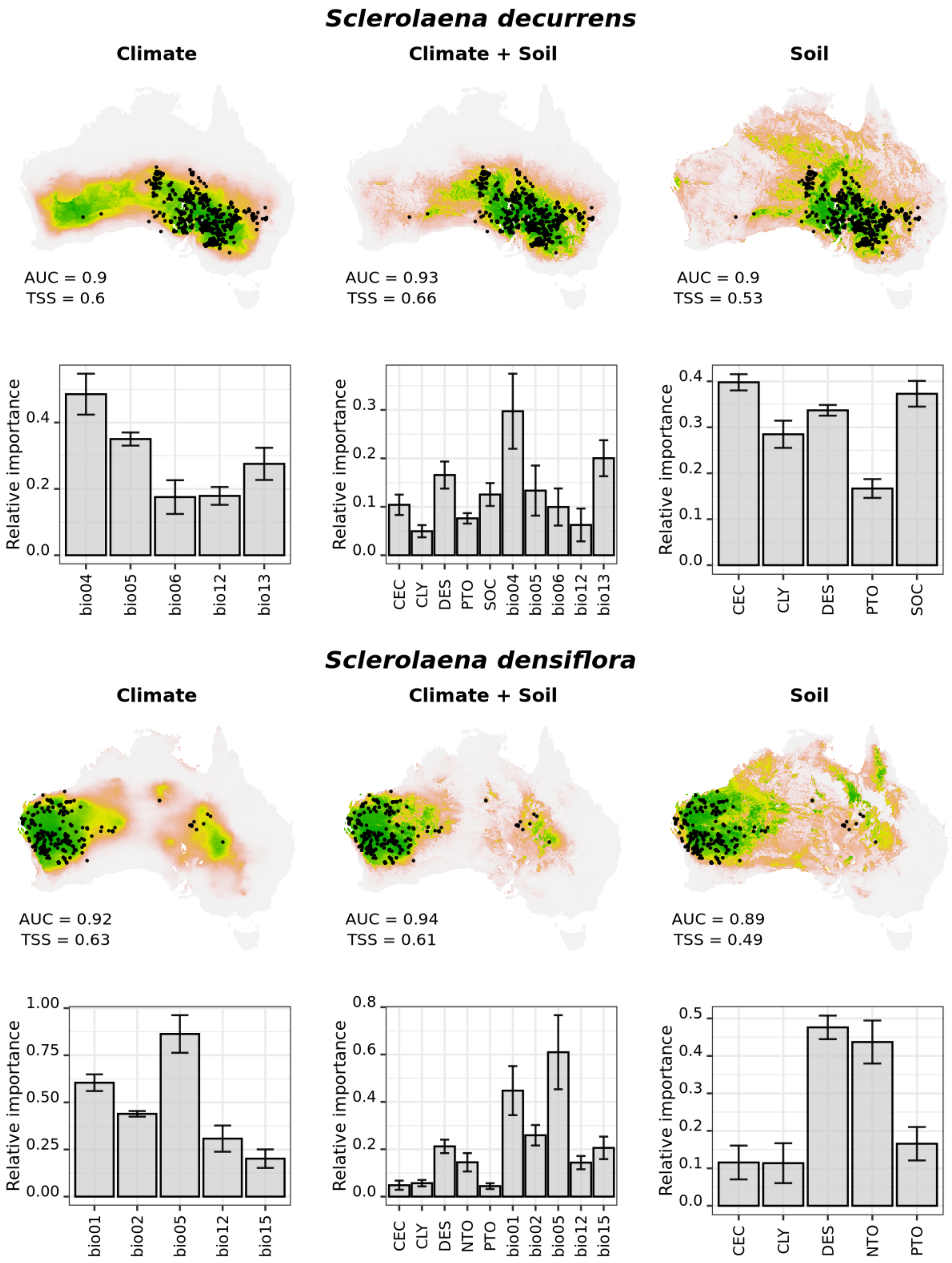
Sclerolaena decurrens
Climate
Climate + Soil
Soil
AUC = 0.9
TSS = 0.6
AUC = 0.93
TSS = 0.66
AUC = 0.9
TSS = 0.53
Relative importance
0.0
0.2
0.4
bio04
bio05
bio06
bio12
bio13
Relative importance
0.0
0.1
0.2
0.3
CEC
CLY
DES
PTO
SOC
bio04
bio05
bio06
bio12
bio13
Relative importance
0.0
0.1
0.2
0.3
0.4
CEC
CLY
DES
PTO
SOC
Sclerolaena densiflora
Climate
Climate + Soil
Soil
AUC = 0.92
TSS = 0.63
AUC = 0.94
TSS = 0.61
AUC = 0.89
TSS = 0.49
Relative importance
0.00
0.25
0.50
0.75
1.00
bio01
bio02
bio05
bio12
bio15
Relative importance
0.0
0.2
0.4
0.6
0.8
CEC
CLY
DES
NTO
PTO
bio01
bio02
bio05
bio12
bio15
Relative importance
0.0
0.1
0.2
0.3
0.4
0.5
CEC
CLY
DES
NTO
PTO

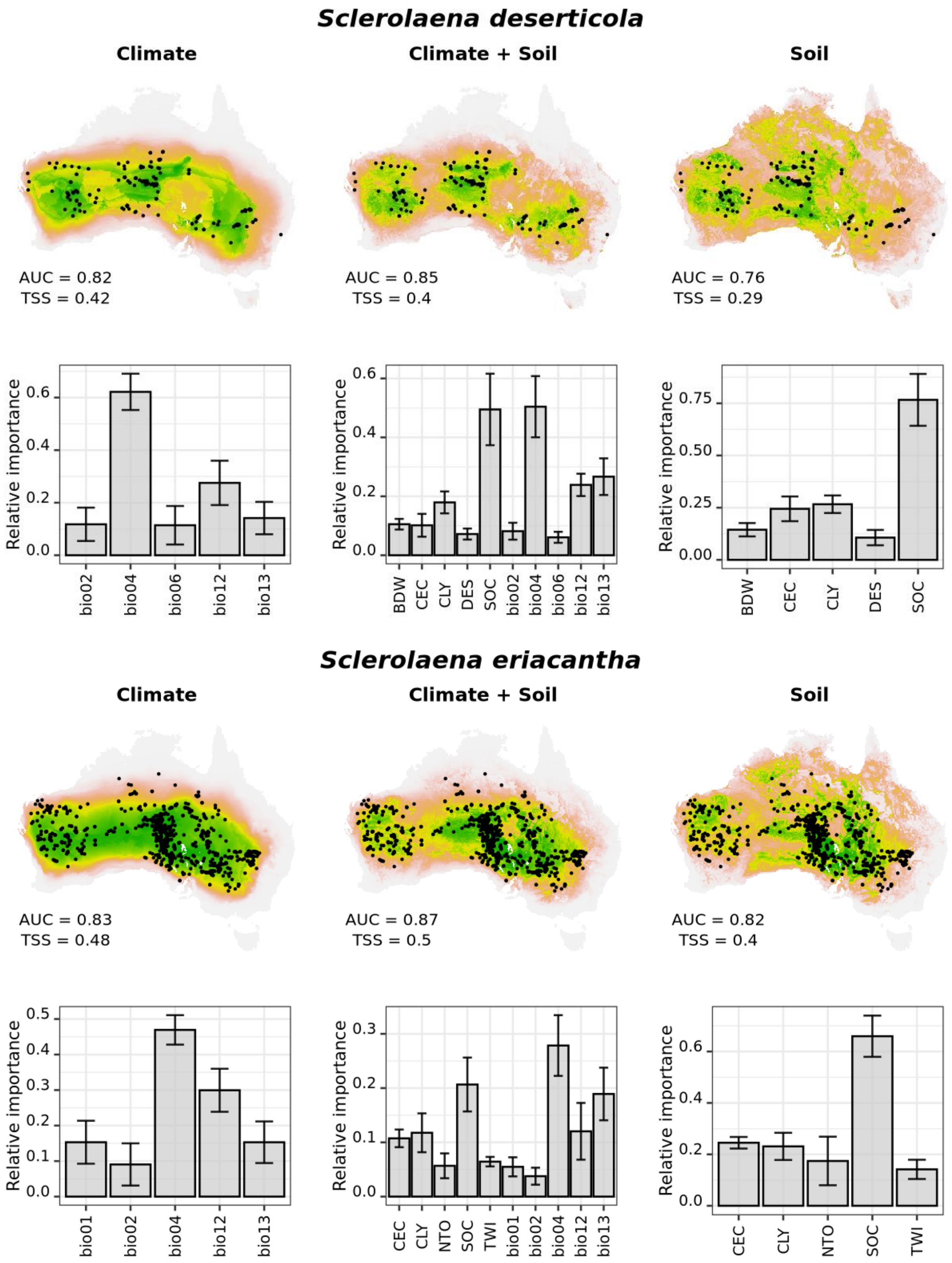
Sclerolaena deserticola
Climate
Climate + Soil
Soil
AUC = 0.82
TSS = 0.42
AUC = 0.85
TSS = 0.4
AUC = 0.76
TSS = 0.29
Relative importance
bio02
bio04
bio06
bio12
bio13
BDW
CEC
CLY
DES
SOC
bio02
bio04
bio06
bio12
bio13
BDW
CEC
CLY
DES
SOC
Sclerolaena eriacantha
Climate
Climate + Soil
Soil
AUC = 0.83
TSS = 0.48
AUC = 0.87
TSS = 0.5
AUC = 0.82
TSS = 0.4
Relative importance
bio01
bio02
bio04
bio12
bio13
CEC
CLY
NTO
SOC
TWI
bio01
bio02
bio04
bio12
bio13
CEC
CLY
NTO
SOC
TWI

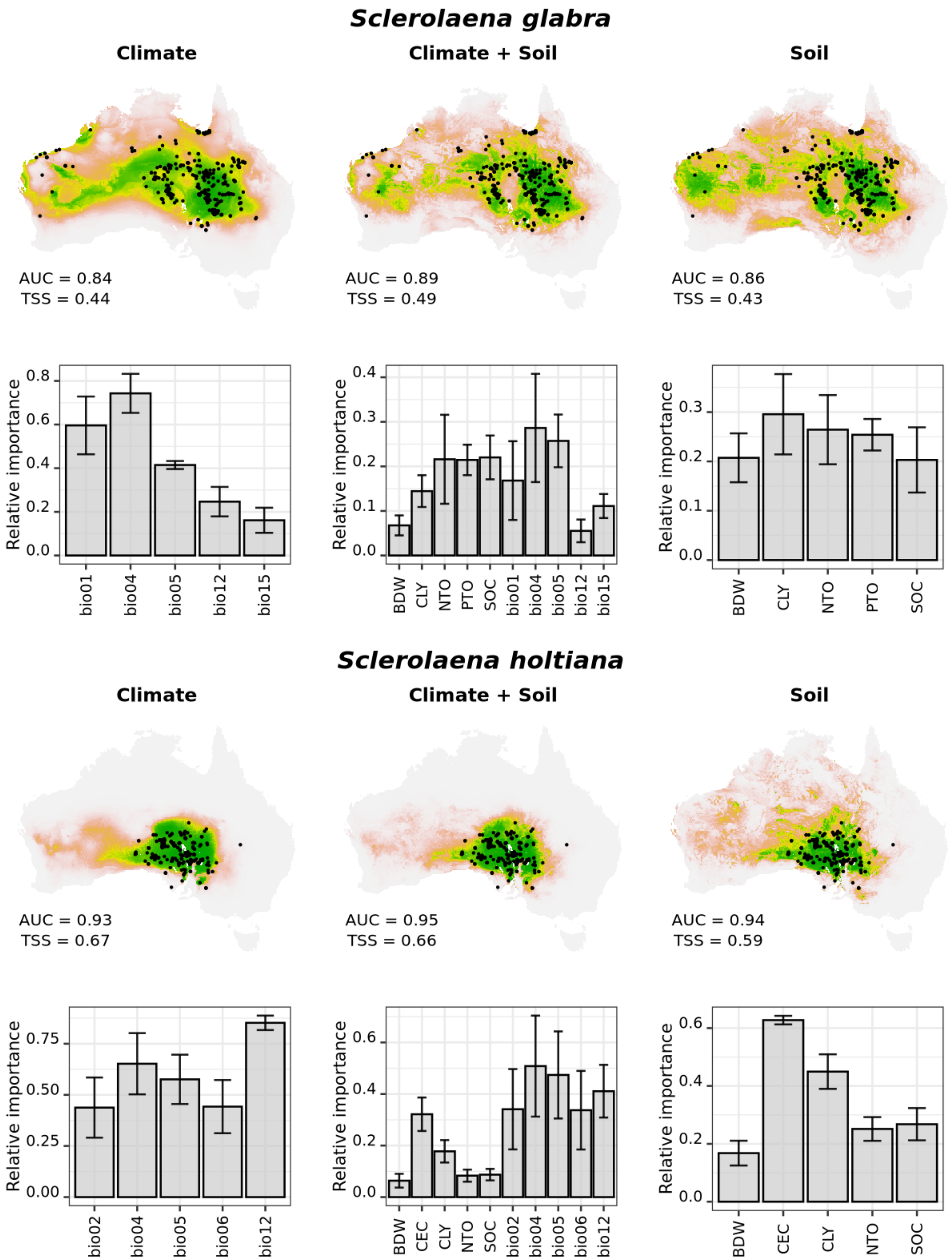
Sclerolaena glabra
Climate
Climate + Soil
Soil
AUC = 0.84
TSS = 0.44
AUC = 0.89
TSS = 0.49
AUC = 0.86
TSS = 0.43
Relative importance
bio01
bio04
bio05
bio12
bio15
BDW
CLY
NTO
PTO
SOC
bio01
bio04
bio05
bio12
bio15
BDW
CLY
NTO
PTO
SOC
Sclerolaena holtiana
Climate
Climate + Soil
Soil
AUC = 0.93
TSS = 0.67
AUC = 0.95
TSS = 0.66
AUC = 0.94
TSS = 0.59
Relative importance
bio02
bio04
bio05
bio06
bio12
BDW
CEC
CLY
NTO
SOC
bio02
bio04
bio05
bio06
bio12
BDW
CEC
CLY
NTO
SOC

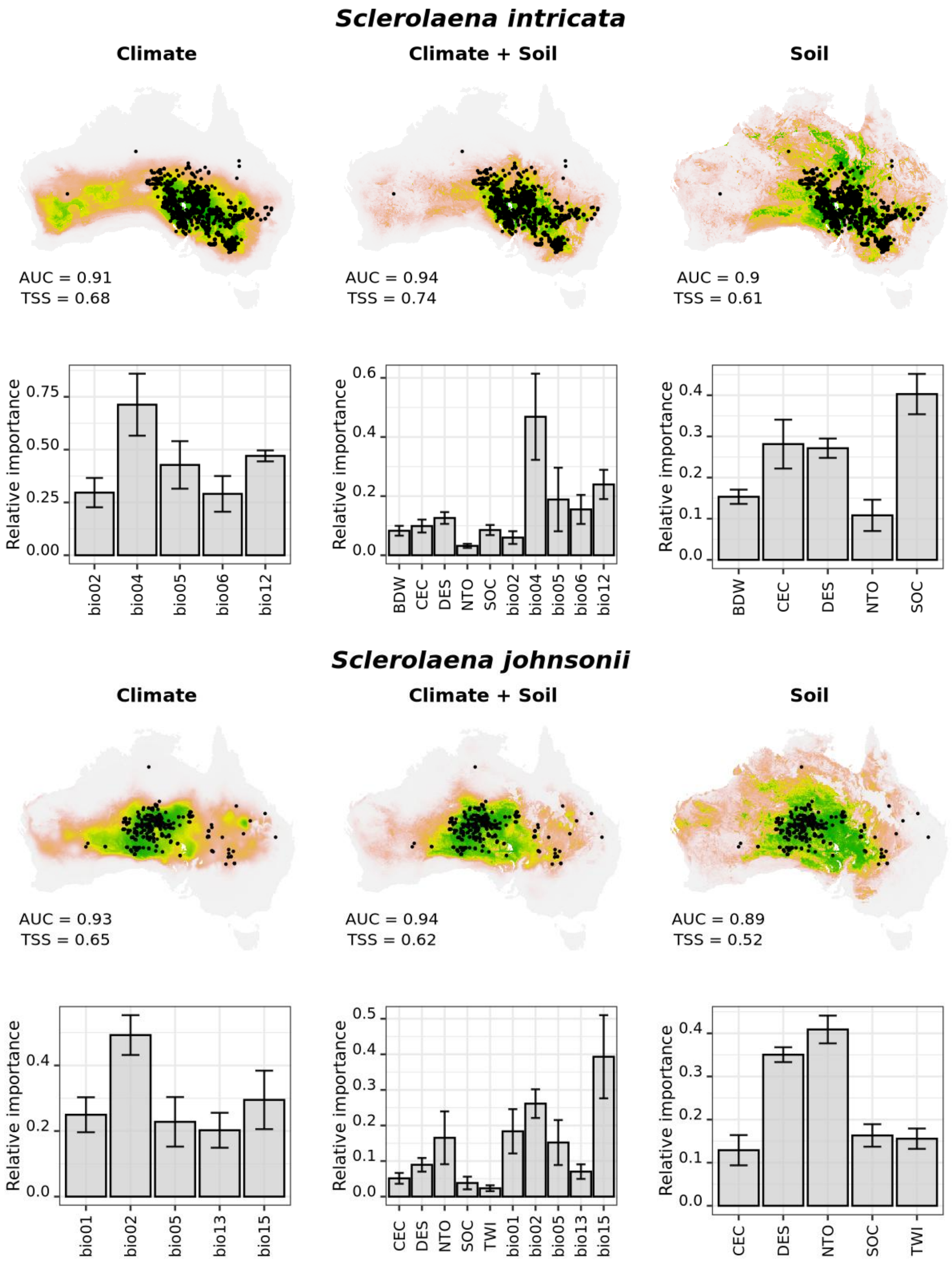

Sclerolaena intricata
Climate
Climate + Soil
Soil
AUC = 0.91
TSS = 0.68
AUC = 0.94
TSS = 0.74
AUC = 0.9
TSS = 0.61
Relative importance
0.00
0.25
0.50
0.75
bio02
bio04
bio05
bio06
bio12
0.0
0.2
0.4
0.6
BDW
CEC
DES
NTO
SOC
bio02
bio04
bio05
bio06
bio12
0.0
0.1
0.2
0.3
0.4
BDW
CEC
DES
NTO
SOC
Sclerolaena johnsonii
Climate
Climate + Soil
Soil
AUC = 0.93
TSS = 0.65
AUC = 0.94
TSS = 0.62
AUC = 0.89
TSS = 0.52
Relative importance
0.0
0.2
0.4
bio01
bio02
bio05
bio13
bio15
0.0
0.1
0.2
0.3
0.4
0.5
CEC
DES
NTO
SOC
TWI
bio01
bio02
bio05
bio13
bio15
0.0
0.1
0.2
0.3
0.4
CEC
DES
NTO
SOC
TWI

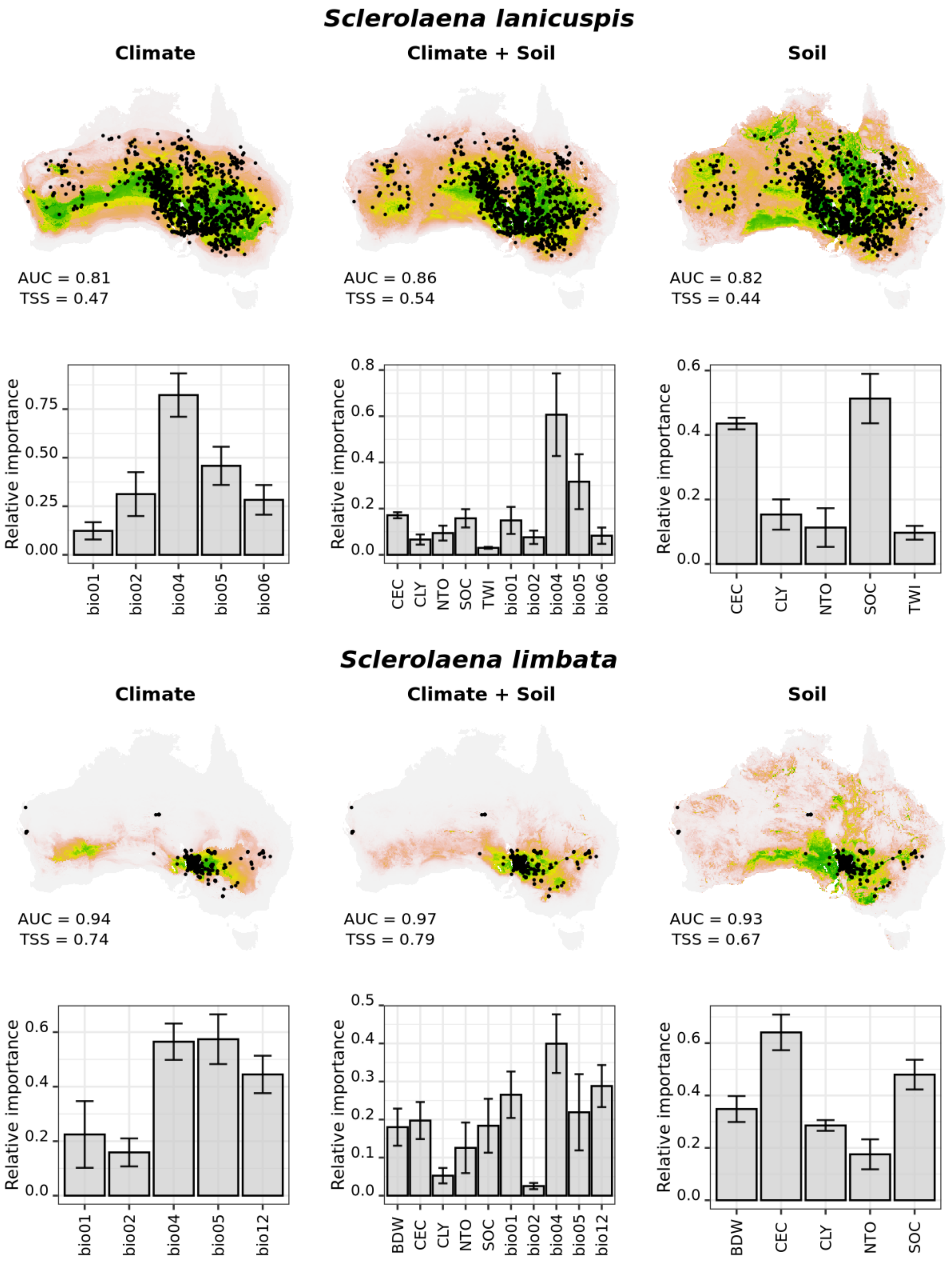
Sclerolaena lanicuspis
Climate
Climate + Soil
Soil
AUC = 0.81
TSS = 0.47
AUC = 0.86
TSS = 0.54
AUC = 0.82
TSS = 0.44
Relative importance
0.00
0.25
0.50
0.75
bio01
bio02
bio04
bio05
bio06
0.0
0.2
0.4
0.6
0.8
CEC
CLY
NTO
SOC
TWI
bio01
bio02
bio04
bio05
bio06
0.0
0.2
0.4
0.6
CEC
CLY
NTO
SOC
TWI
Sclerolaena limbata
Climate
Climate + Soil
Soil
AUC = 0.94
TSS = 0.74
AUC = 0.97
TSS = 0.79
AUC = 0.93
TSS = 0.67
Relative importance
0.0
0.2
0.4
0.6
bio01
bio02
bio04
bio05
bio12
0.0
0.1
0.2
0.3
0.4
0.5
BDW
CEC
CLY
NTO
SOC
bio01
bio02
bio04
bio05
bio12
0.0
0.2
0.4
0.6
BDW
CEC
CLY
NTO
SOC

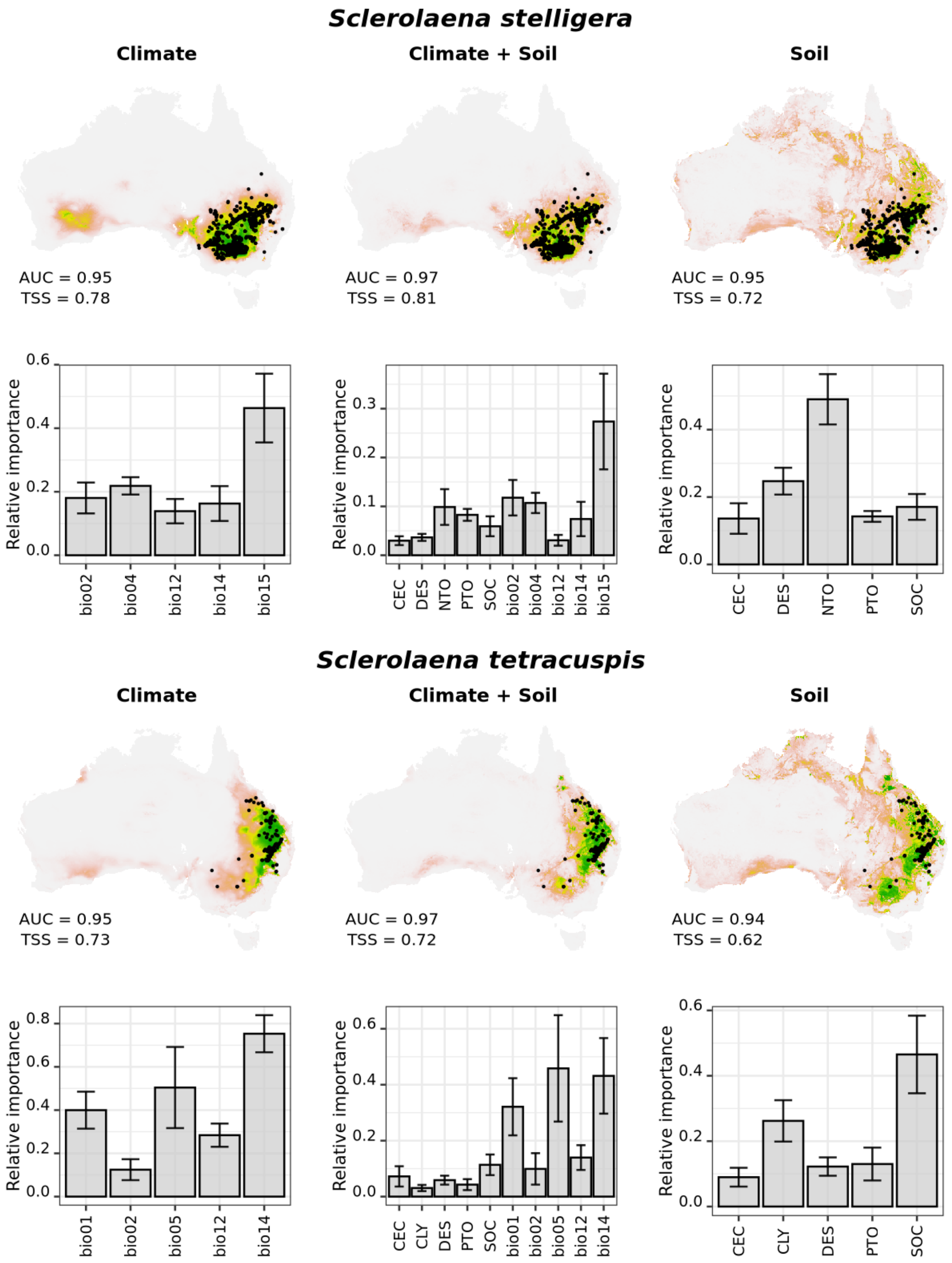

Sclerolaena stelligera
Climate
Climate + Soil
Soil
AUC = 0.95
TSS = 0.78
AUC = 0.97
TSS = 0.81
AUC = 0.95
TSS = 0.72
Relative importance
bio02
bio04
bio12
bio14
bio15
CEC
DES
NTO
PTO
SOC
Sclerolaena tetracuspis
Climate
Climate + Soil
Soil
AUC = 0.95
TSS = 0.73
AUC = 0.97
TSS = 0.72
AUC = 0.94
TSS = 0.62
Relative importance
bio01
bio02
bio05
bio12
bio14
CEC
CLY
DES
PTO
SOC

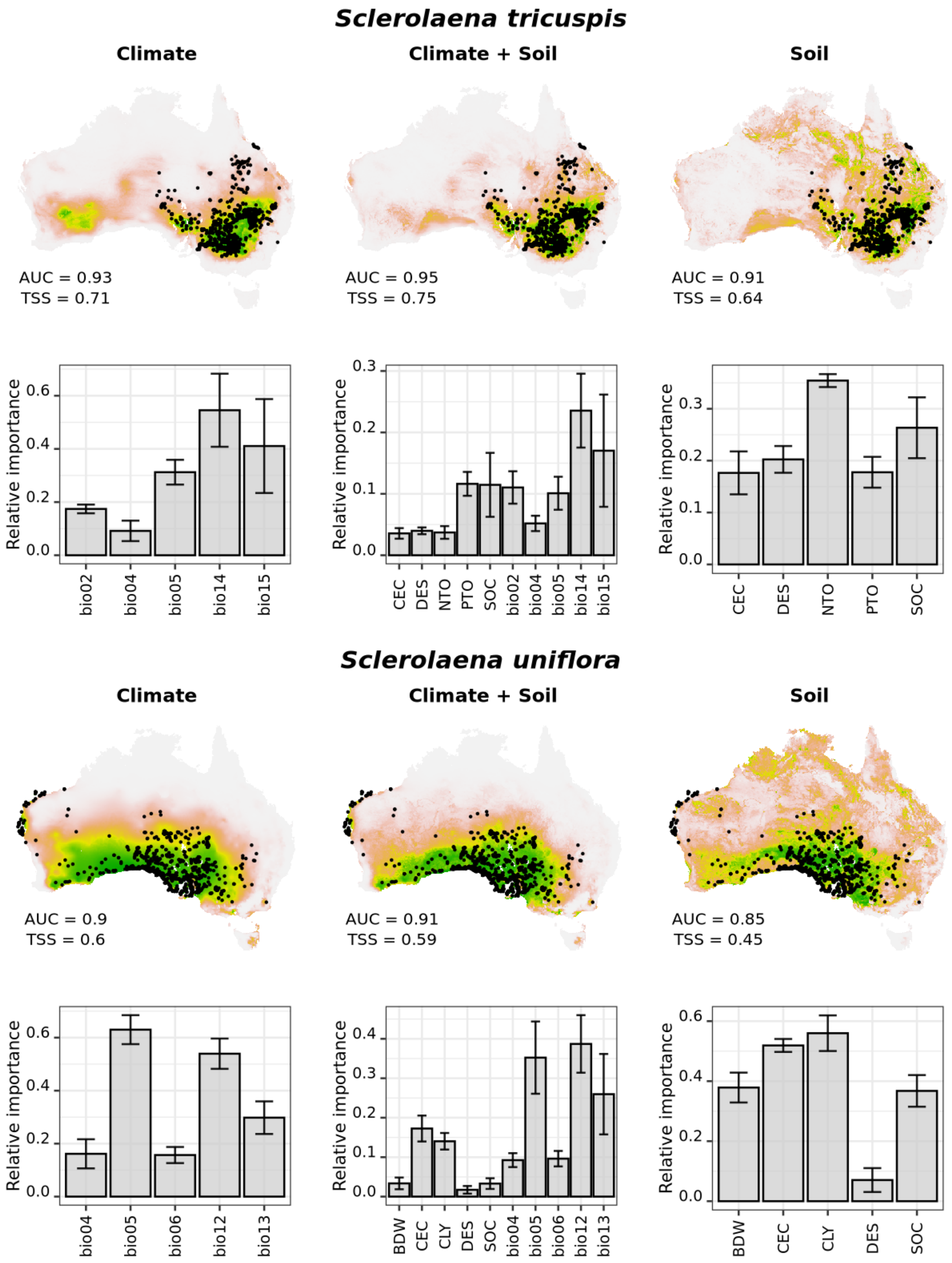
Sclerolaena tricuspis
Climate
Climate + Soil
Soil
AUC = 0.93
TSS = 0.71
AUC = 0.95
TSS = 0.75
AUC = 0.91
TSS = 0.64
Relative importance
0.0
0.2
0.4
0.6
bio02
bio04
bio05
bio14
bio15
0.0
0.1
0.2
0.3
CEC
DES
NTO
PTO
SOC
bio02
bio04
bio05
bio14
bio15
0.0
0.1
0.2
0.3
CEC
DES
NTO
PTO
SOC
Sclerolaena uniflora
Climate
Climate + Soil
Soil
AUC = 0.9
TSS = 0.6
AUC = 0.91
TSS = 0.59
AUC = 0.85
TSS = 0.45
Relative importance
0.0
0.2
0.4
0.6
bio04
bio05
bio06
bio12
bio13
0.0
0.1
0.2
0.3
0.4
BDW
CEC
CLY
DES
SOC
bio04
bio05
bio06
bio12
bio13
0.0
0.2
0.4
0.6
BDW
CEC
CLY
DES
SOC

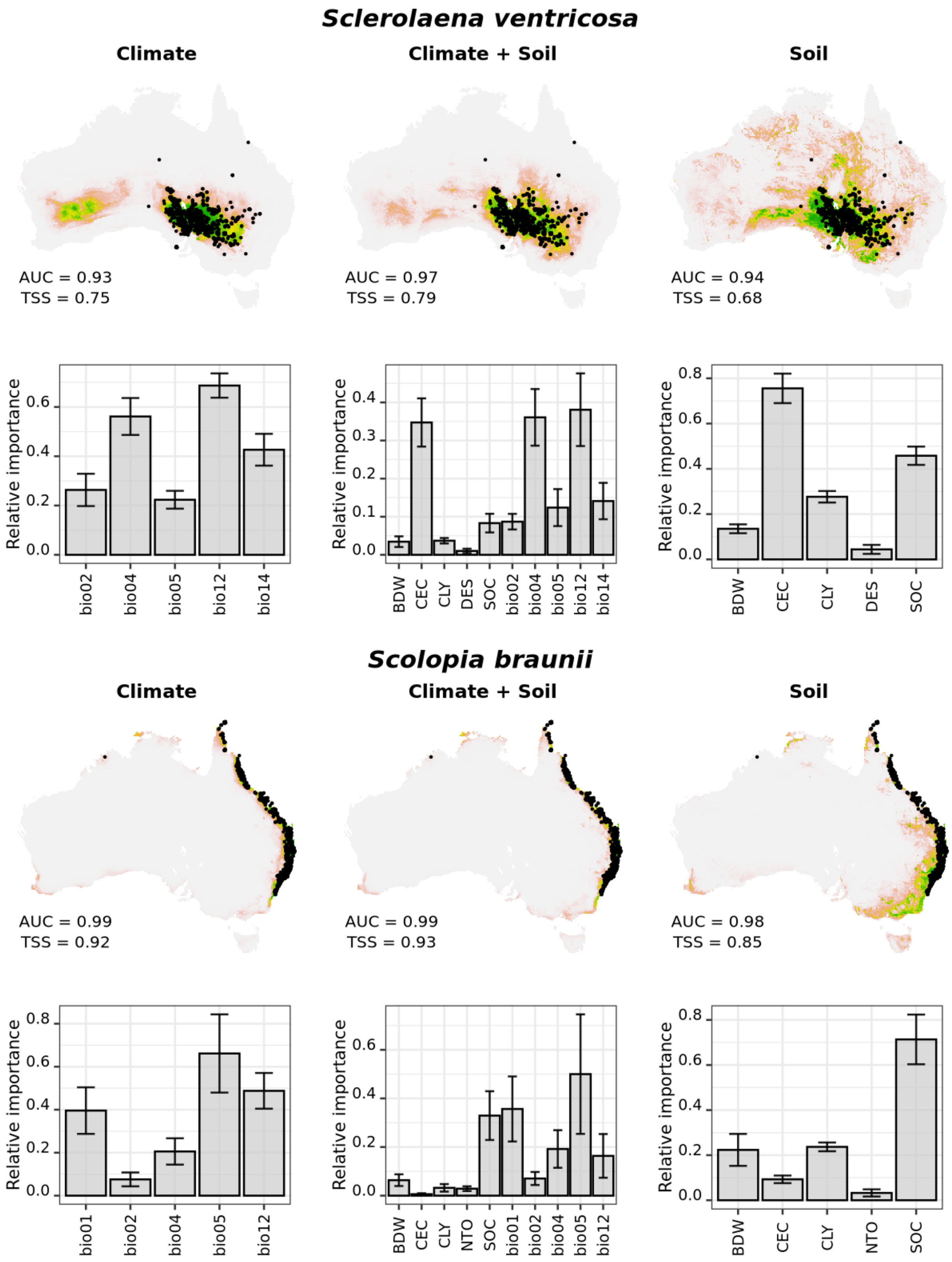
Sclerolaena ventricosa
Climate
Climate + Soil
Soil
AUC = 0.93
TSS = 0.75
AUC = 0.97
TSS = 0.79
AUC = 0.94
TSS = 0.68
Relative importance
bio02 bio04 bio05 bio12 bio14
BDW CEC CLY DES SOC bio02 bio04 bio05 bio12 bio14
BDW CEC CLY DES SOC
Scolopia braunii
Climate
Climate + Soil
Soil
AUC = 0.99
TSS = 0.92
AUC = 0.99
TSS = 0.93
AUC = 0.98
TSS = 0.85
Relative importance
bio01 bio02 bio04 bio05 bio12
BDW CEC CLY NTO SOC bio01 bio02 bio04 bio05 bio12
BDW CEC CLY NTO SOC

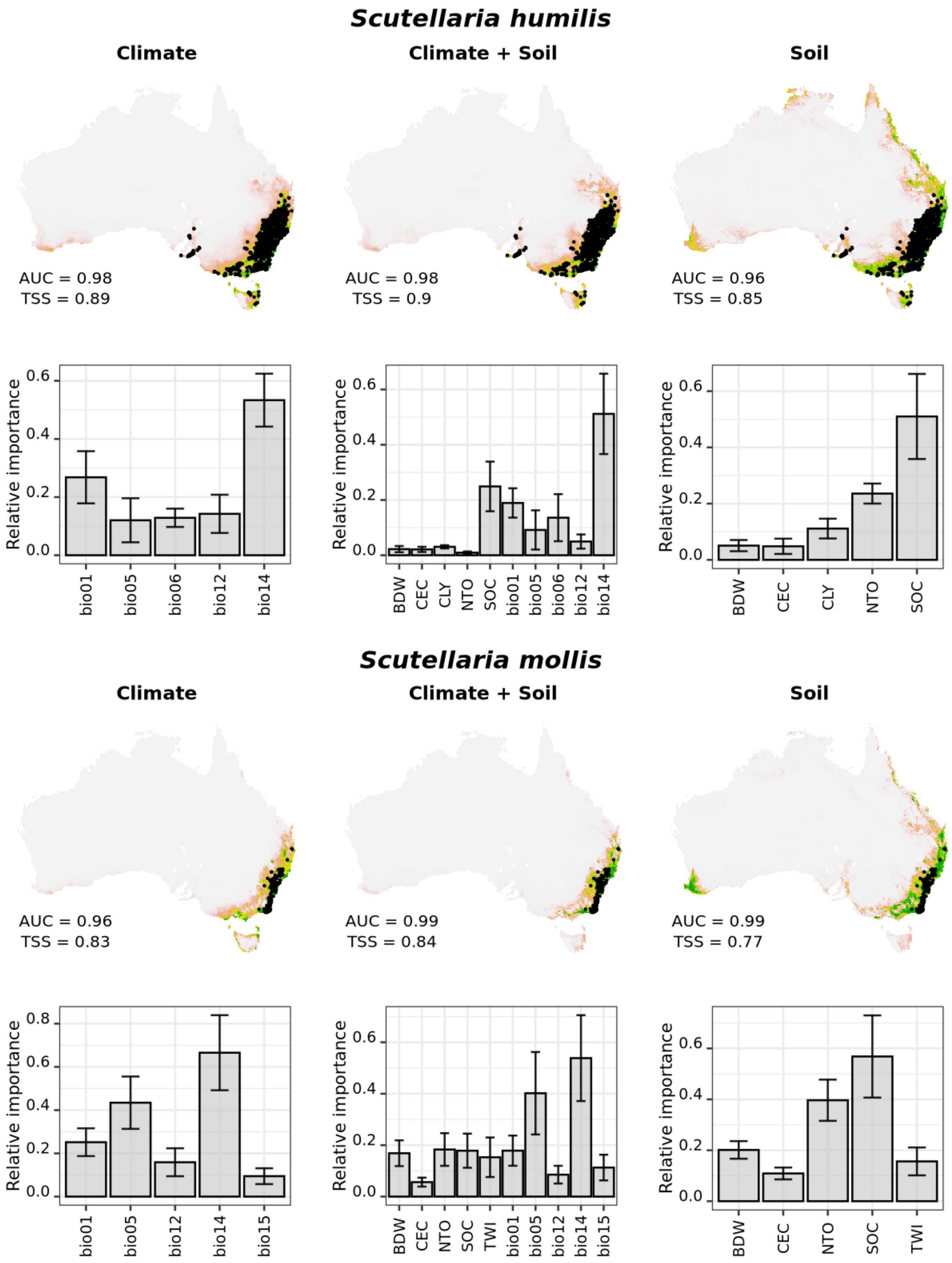
Scutellaria humilis
Climate
Climate + Soil
Soil
AUC = 0.98
TSS = 0.89
AUC = 0.98
TSS = 0.9
AUC = 0.96
TSS = 0.85
Relative importance
0.0
0.2
0.4
0.6
bio01
bio05
bio06
bio12
bio14
BDW
CEC
CLY
NTO
SOC
bio01
bio05
bio06
bio12
bio14
BDW
CEC
CLY
NTO
SOC
Scutellaria mollis
Climate
Climate + Soil
Soil
AUC = 0.96
TSS = 0.83
AUC = 0.99
TSS = 0.84
AUC = 0.99
TSS = 0.77
Relative importance
0.0
0.2
0.4
0.6
0.8
bio01
bio05
bio12
bio14
bio15
BDW
CEC
NTO
SOC
TWI
bio01
bio05
bio12
bio14
bio15
BDW
CEC
NTO
SOC
TWI

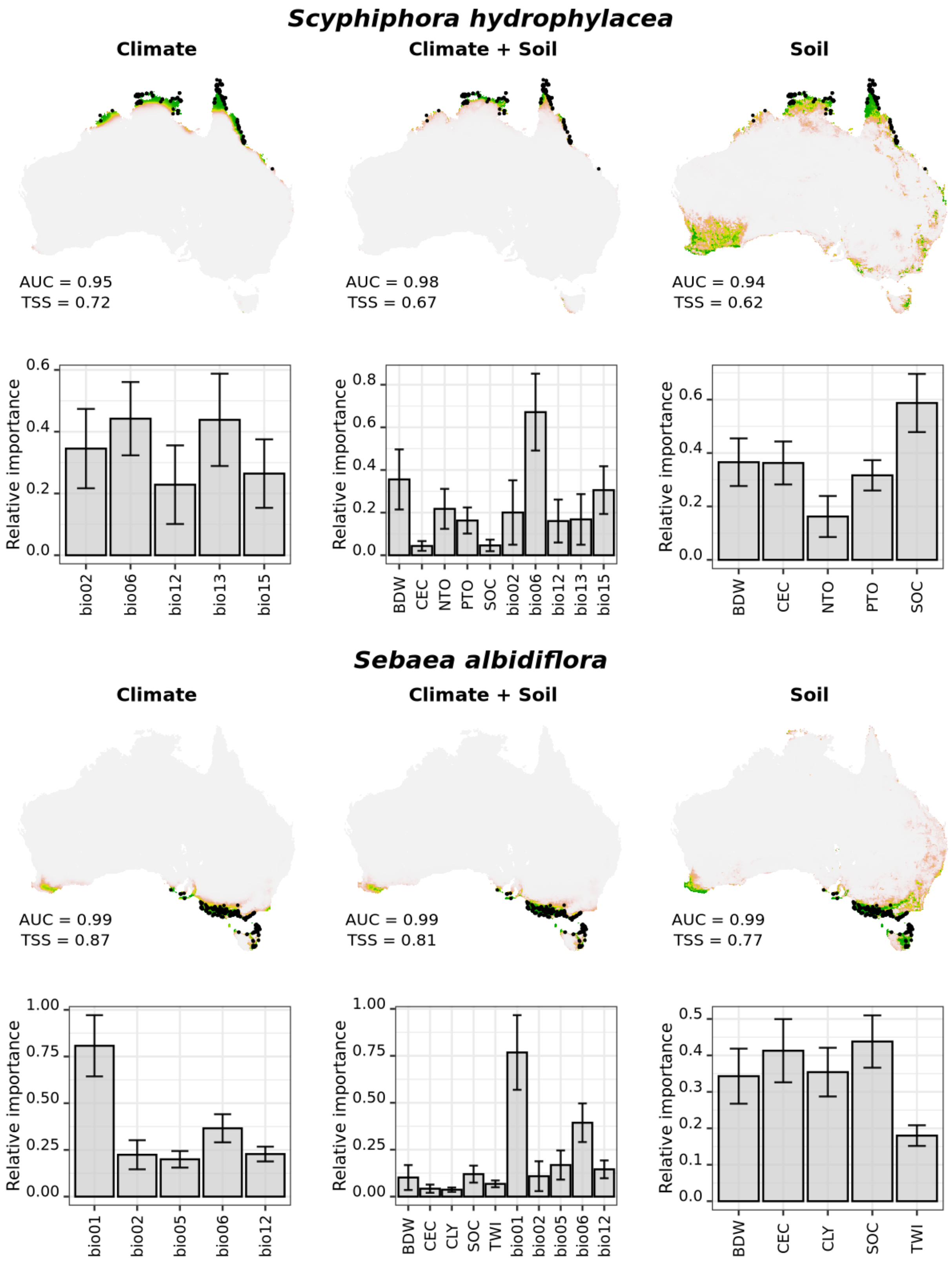
Scyphiphora hydrophylacea
Climate
Climate + Soil
Soil
AUC = 0.95
TSS = 0.72
AUC = 0.98
TSS = 0.67
AUC = 0.94
TSS = 0.62
Relative importance
bio02
bio06
bio12
bio13
bio15
BDW
CEC
NTO
PTO
SOC
Sebaea albidiflora
AUC = 0.99
TSS = 0.87
AUC = 0.99
TSS = 0.81
AUC = 0.99
TSS = 0.77
bio01
bio05
CLY
TWI

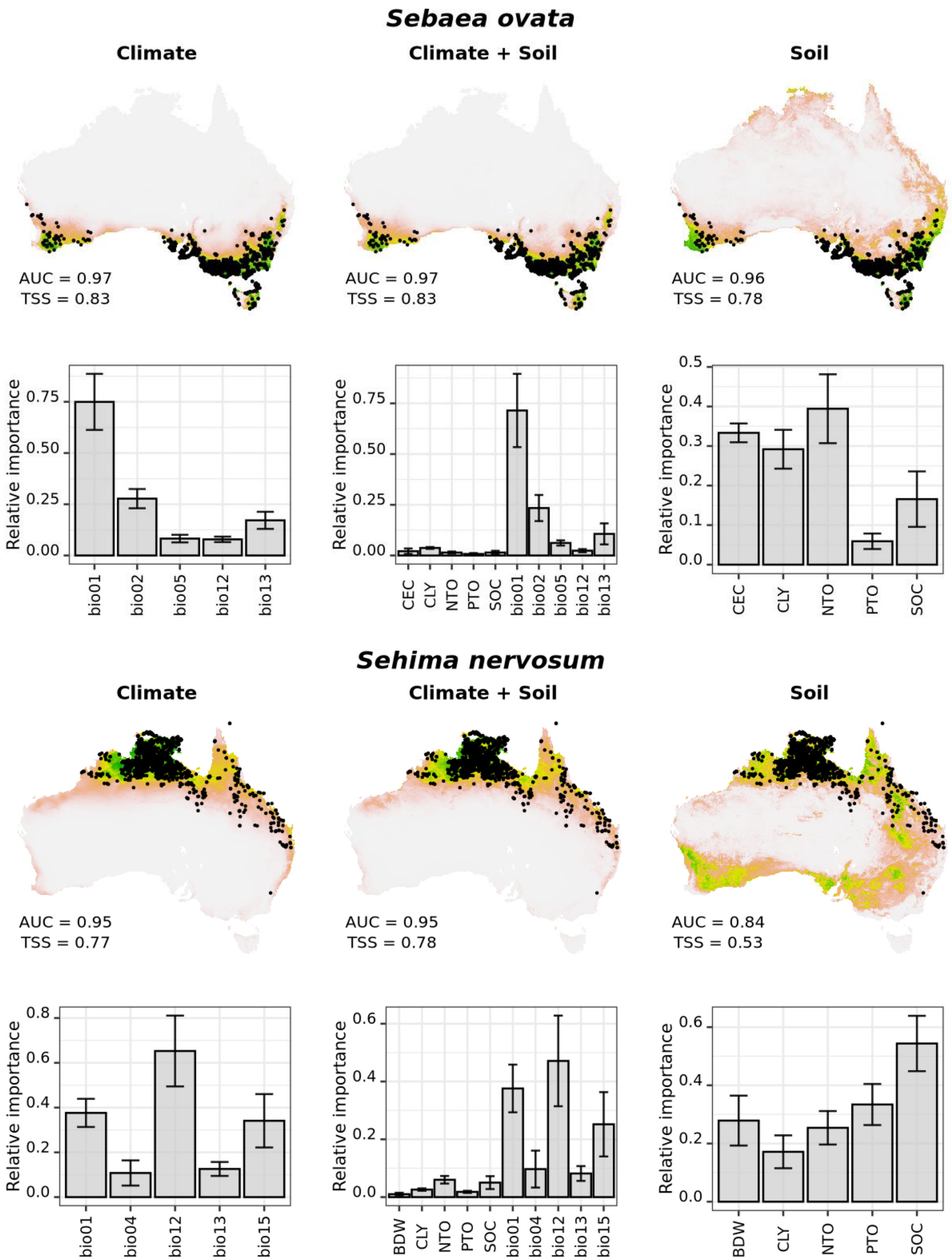
Sebaea ovata
Climate
Climate + Soil
Soil
AUC = 0.97
TSS = 0.83
AUC = 0.97
TSS = 0.83
AUC = 0.96
TSS = 0.78
Relative importance
0.00
0.25
0.50
0.75
bio01
bio02
bio05
bio12
bio13
CEC
CLY
NTO
PTO
SOC
0.0
0.1
0.2
0.3
0.4
0.5
Sehima nervosum
Climate
Climate + Soil
Soil
AUC = 0.95
TSS = 0.77
AUC = 0.95
TSS = 0.78
AUC = 0.84
TSS = 0.53
0.0
0.2
0.4
0.6
0.8
bio01
bio04
bio12
bio13
bio15
BDW
CLY
NTO
PTO
SOC

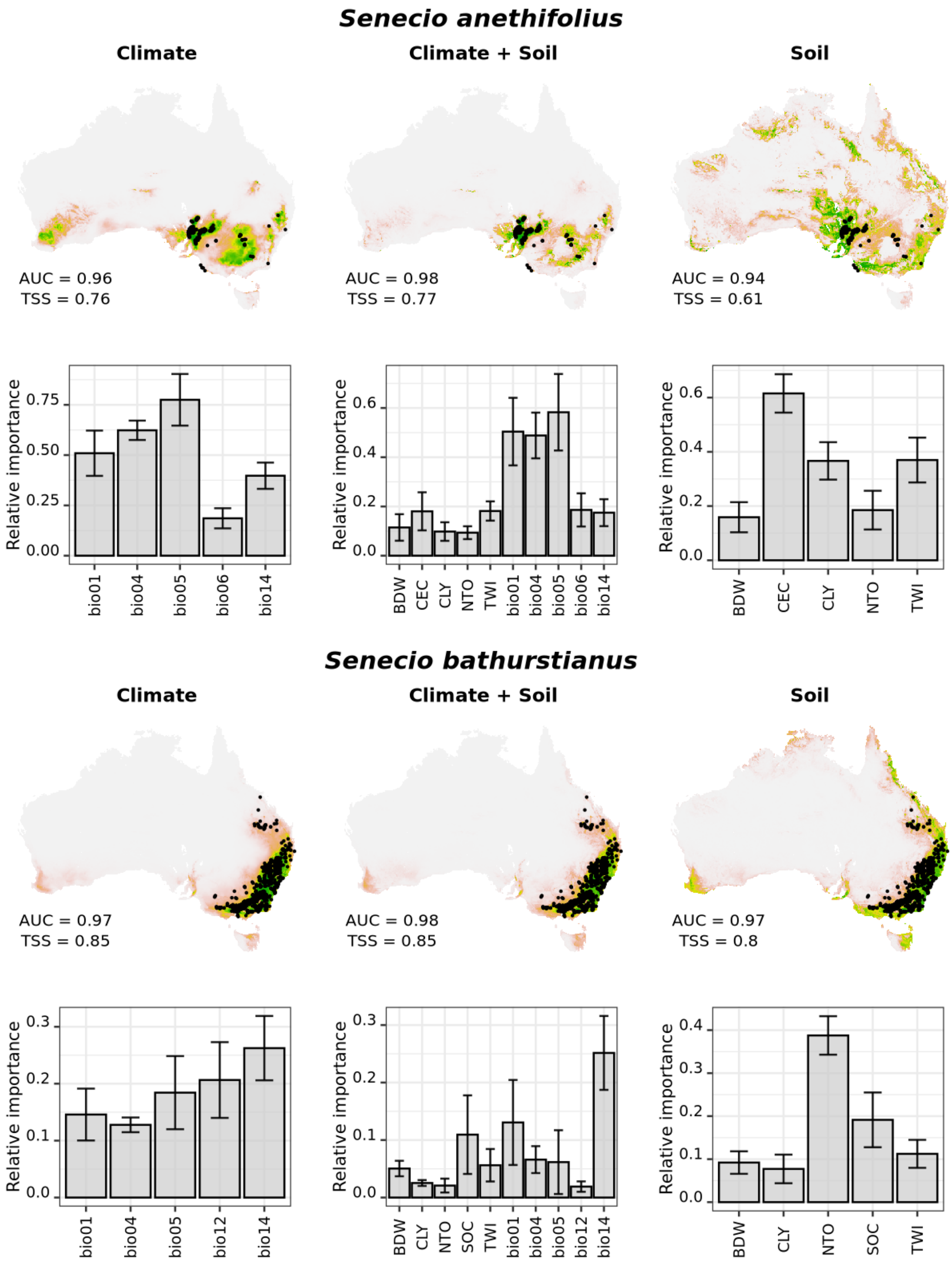

Senecio anethifolius
Climate
Climate + Soil
Soil
AUC = 0.96
TSS = 0.76
AUC = 0.98
TSS = 0.77
AUC = 0.94
TSS = 0.61
Relative importance
bio01
bio04
bio05
bio06
bio14
BDW
CEC
CLY
NTO
TWI
Senecio bathurstianus
AUC = 0.97
TSS = 0.85
AUC = 0.98
TSS = 0.85
AUC = 0.97
TSS = 0.8
bio12
SOC

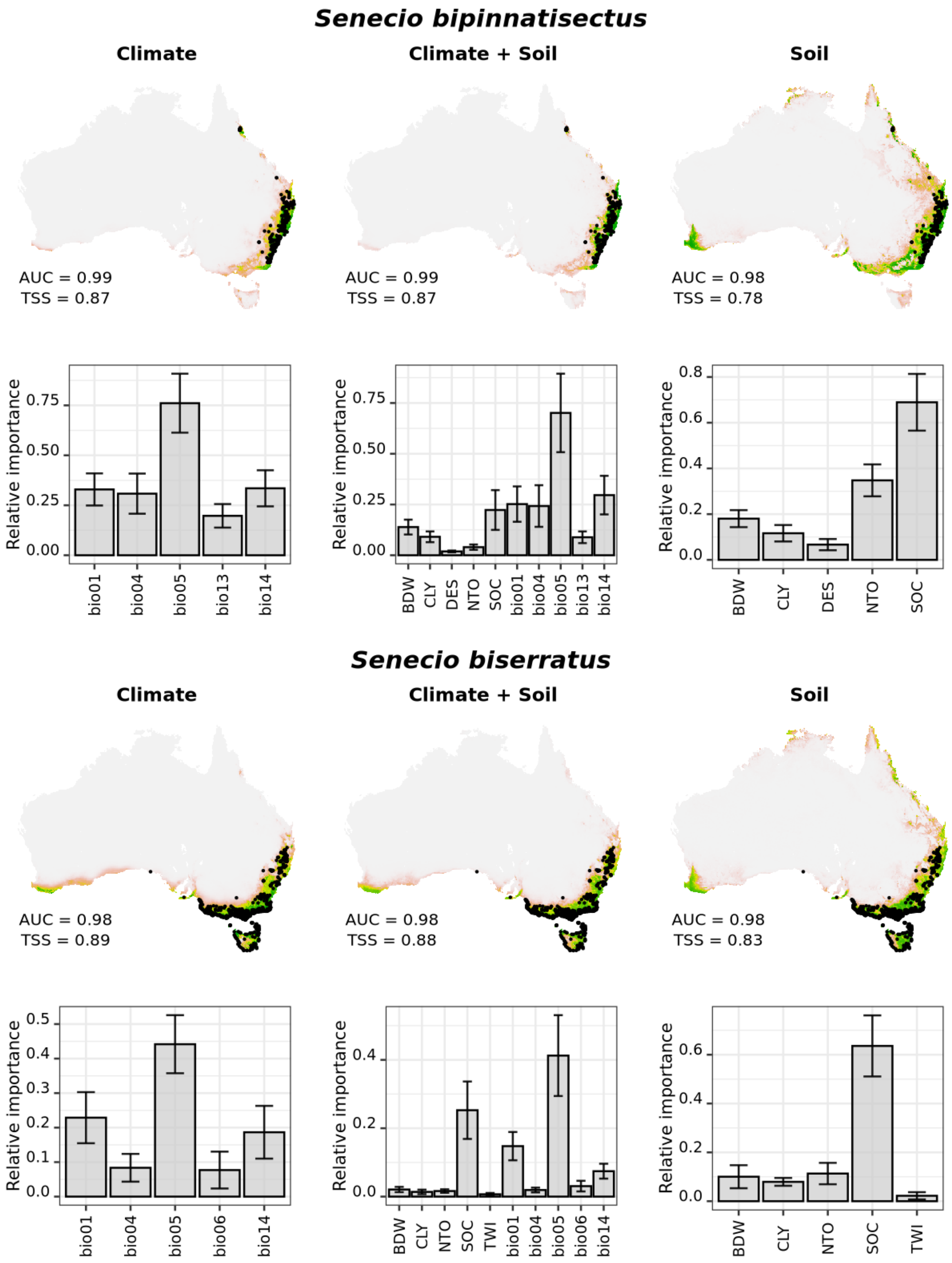
Senecio bipinnatisectus
Climate
Climate + Soil
Soil
AUC = 0.99
TSS = 0.87
AUC = 0.99
TSS = 0.87
AUC = 0.98
TSS = 0.78
Relative importance
0.00
0.25
0.50
0.75
bio01
bio04
bio05
bio13
bio14
Relative importance
0.00
0.25
0.50
0.75
BDW
CLY
DES
NTO
SOC
bio01
bio04
bio05
bio13
bio14
Relative importance
0.0
0.2
0.4
0.6
0.8
BDW
CLY
DES
NTO
SOC
Senecio biserratus
Climate
Climate + Soil
Soil
AUC = 0.98
TSS = 0.89
AUC = 0.98
TSS = 0.88
AUC = 0.98
TSS = 0.83
Relative importance
0.0
0.1
0.2
0.3
0.4
0.5
bio01
bio04
bio05
bio06
bio14
Relative importance
0.0
0.2
0.4
BDW
CLY
NTO
SOC
TWI
bio01
bio04
bio05
bio06
bio14
Relative importance
0.0
0.2
0.4
0.6
BDW
CLY
NTO
SOC
TWI

Senecio brigalowensis

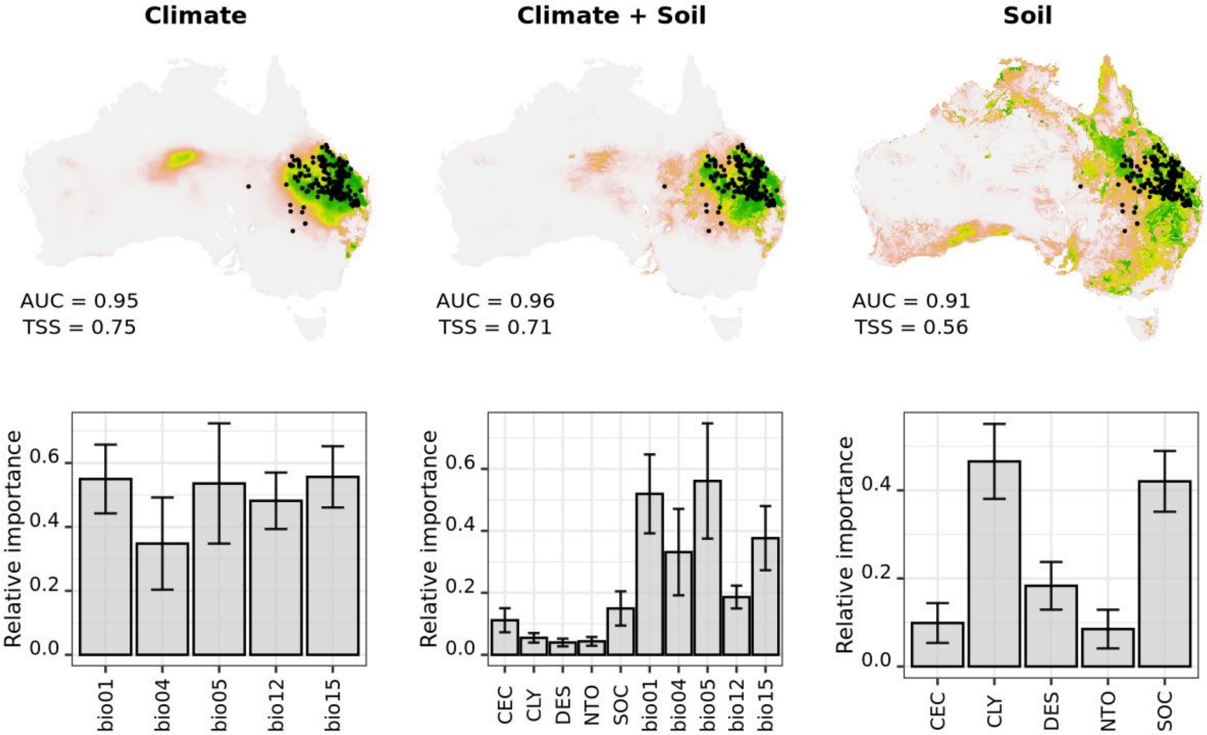

Senecio cunninghamii

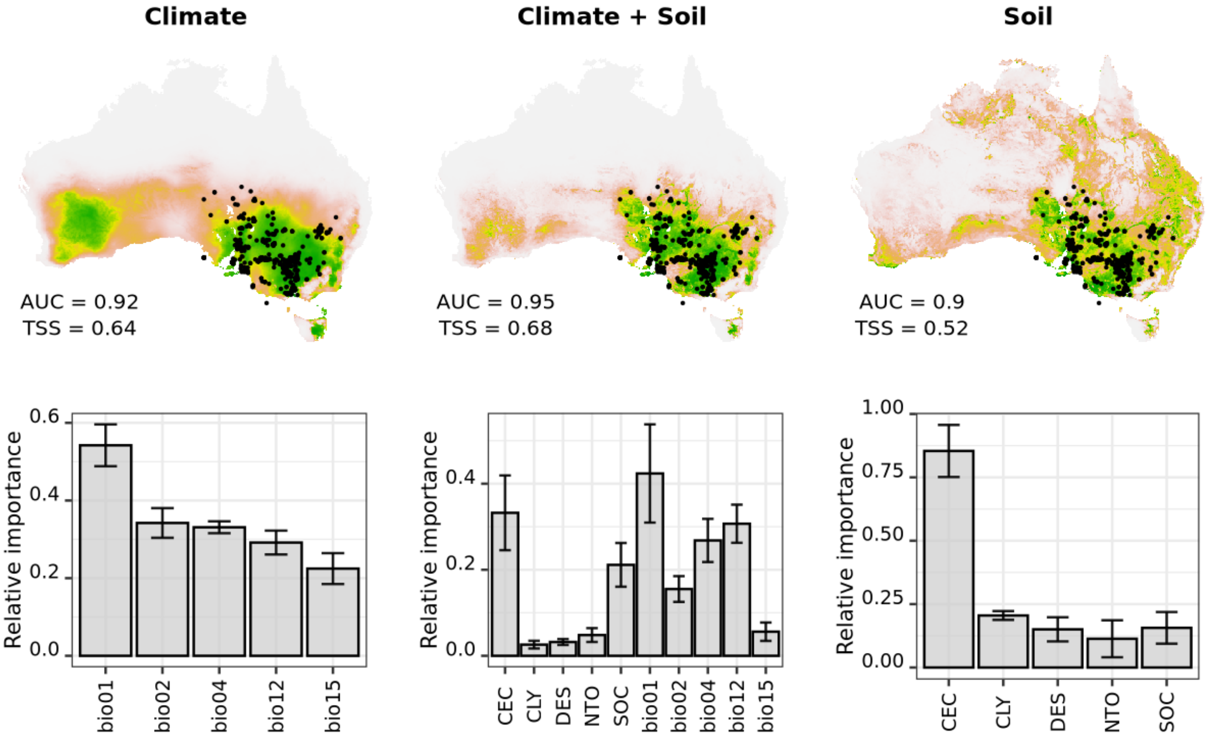

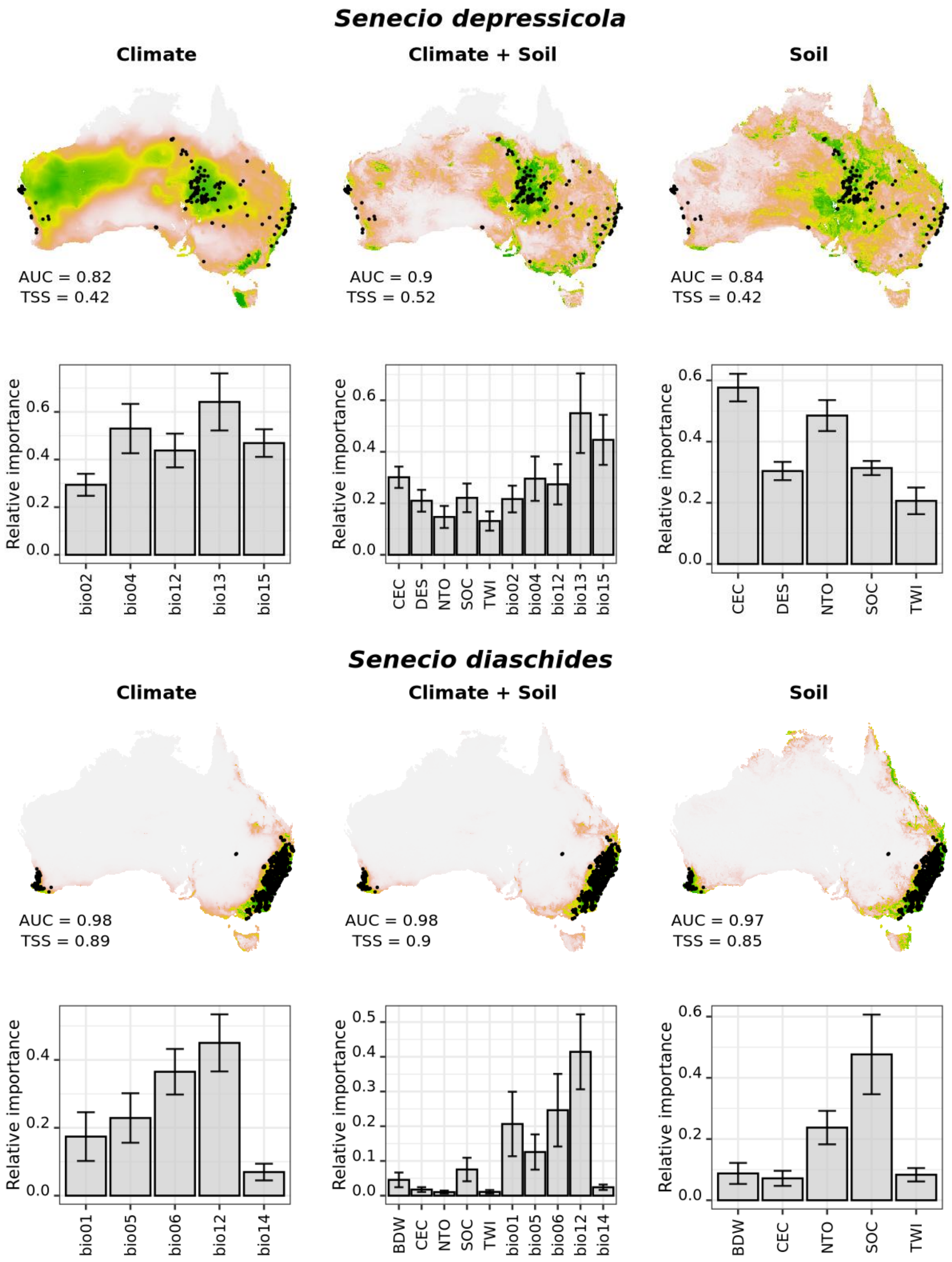

Senecio depressicola
Climate
Climate + Soil
Soil
AUC = 0.82
TSS = 0.42
AUC = 0.9
TSS = 0.52
AUC = 0.84
TSS = 0.42
Relative importance
0.0
0.2
0.4
0.6
bio02
bio04
bio12
bio13
bio15
CEC
DES
NTO
SOC
TWI
Senecio diaschides
AUC = 0.98
TSS = 0.89
AUC = 0.98
TSS = 0.9
AUC = 0.97
TSS = 0.85
0.1
0.3
0.5
bio01
bio05
bio06
bio14
BDW

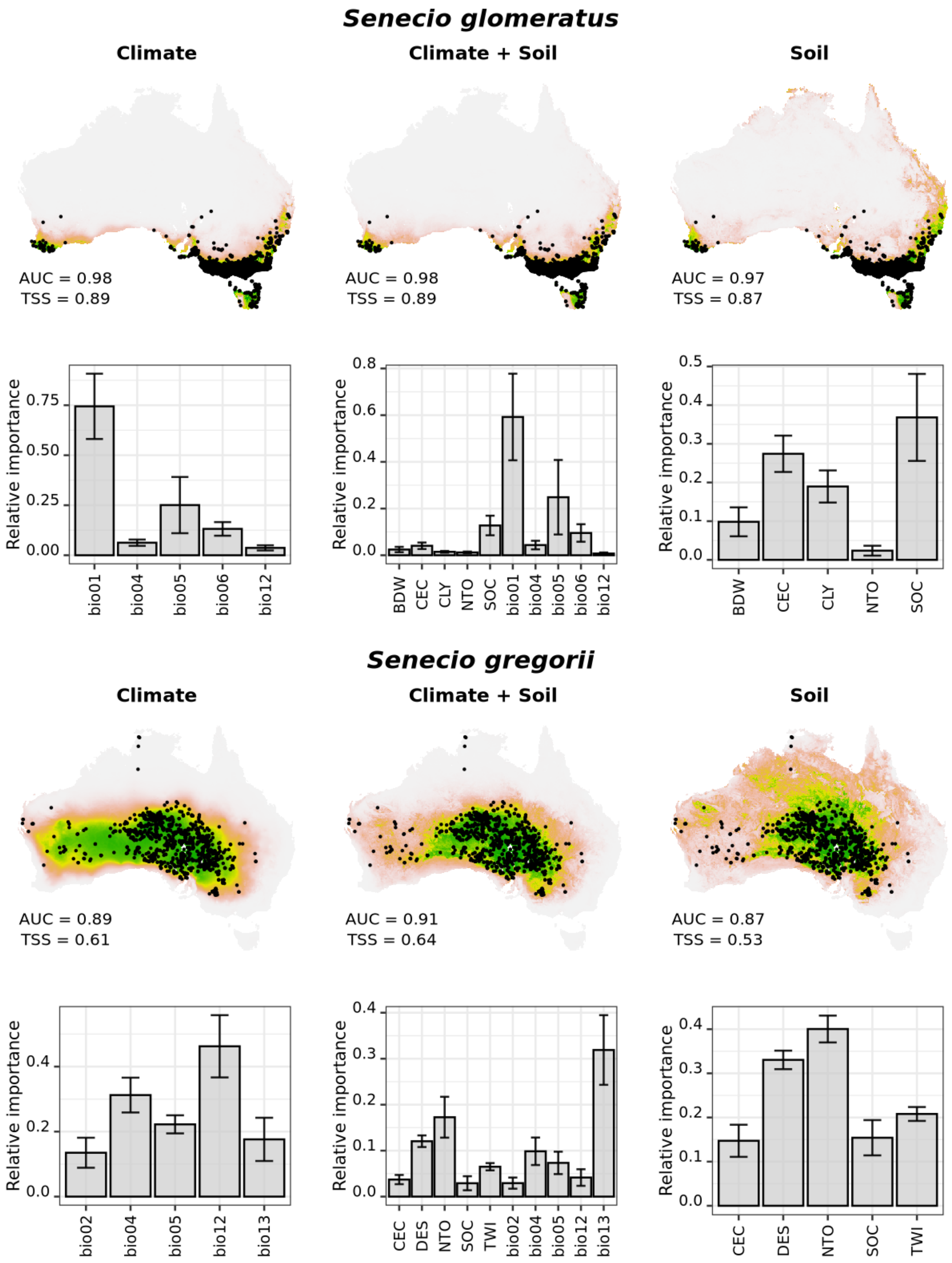
Senecio glomeratus
Climate
Climate + Soil
Soil
AUC = 0.98
TSS = 0.89
AUC = 0.98
TSS = 0.89
AUC = 0.97
TSS = 0.87
Relative importance
0.00
0.25
0.50
0.75
bio01
bio04
bio05
bio06
bio12
0.0
0.2
0.4
0.6
0.8
BDW
CEC
CLY
NTO
SOC
bio01
bio04
bio05
bio06
bio12
0.0
0.1
0.2
0.3
0.4
0.5
BDW
CEC
CLY
NTO
SOC
Senecio gregorii
Climate
Climate + Soil
Soil
AUC = 0.89
TSS = 0.61
AUC = 0.91
TSS = 0.64
AUC = 0.87
TSS = 0.53
Relative importance
0.0
0.2
0.4
bio02
bio04
bio05
bio12
bio13
0.0
0.1
0.2
0.3
0.4
CEC
DES
NTO
SOC
TWI
bio02
bio04
bio05
bio12
bio13
0.0
0.1
0.2
0.3
0.4
CEC
DES
NTO
SOC
TWI

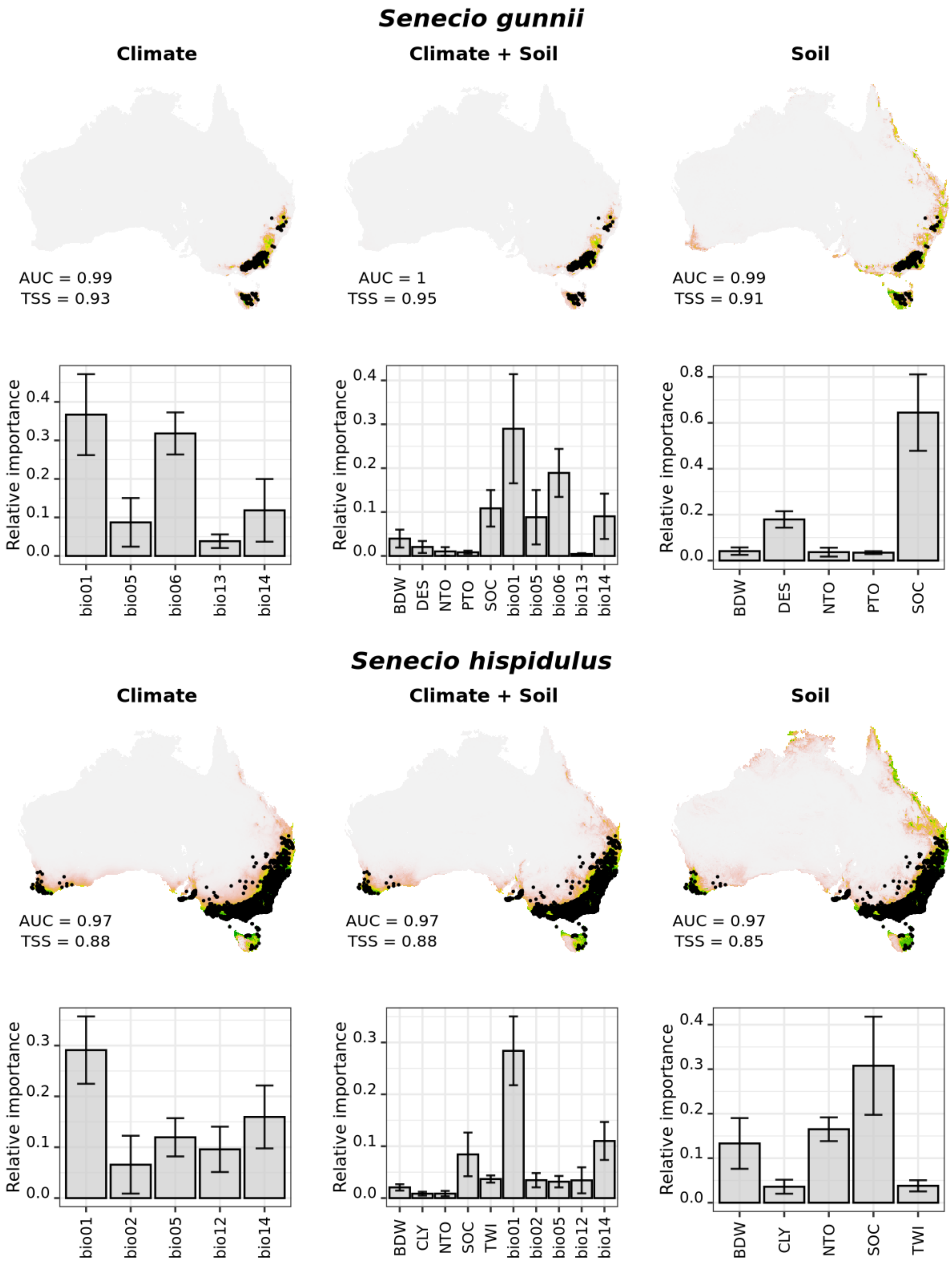

Senecio gunnii
Climate
Climate + Soil
Soil
AUC = 0.99
TSS = 0.93
AUC = 1
TSS = 0.95
AUC = 0.99
TSS = 0.91
Relative importance
bio01
bio05
bio06
bio13
bio14
BDW
DES
NTO
PTO
SOC
Senecio hispidulus
Climate
Climate + Soil
Soil
AUC = 0.97
TSS = 0.88
AUC = 0.97
TSS = 0.88
AUC = 0.97
TSS = 0.85
Relative importance
bio01
bio02
bio05
bio12
bio14
BDW
CLY
NTO
SOC
TWI

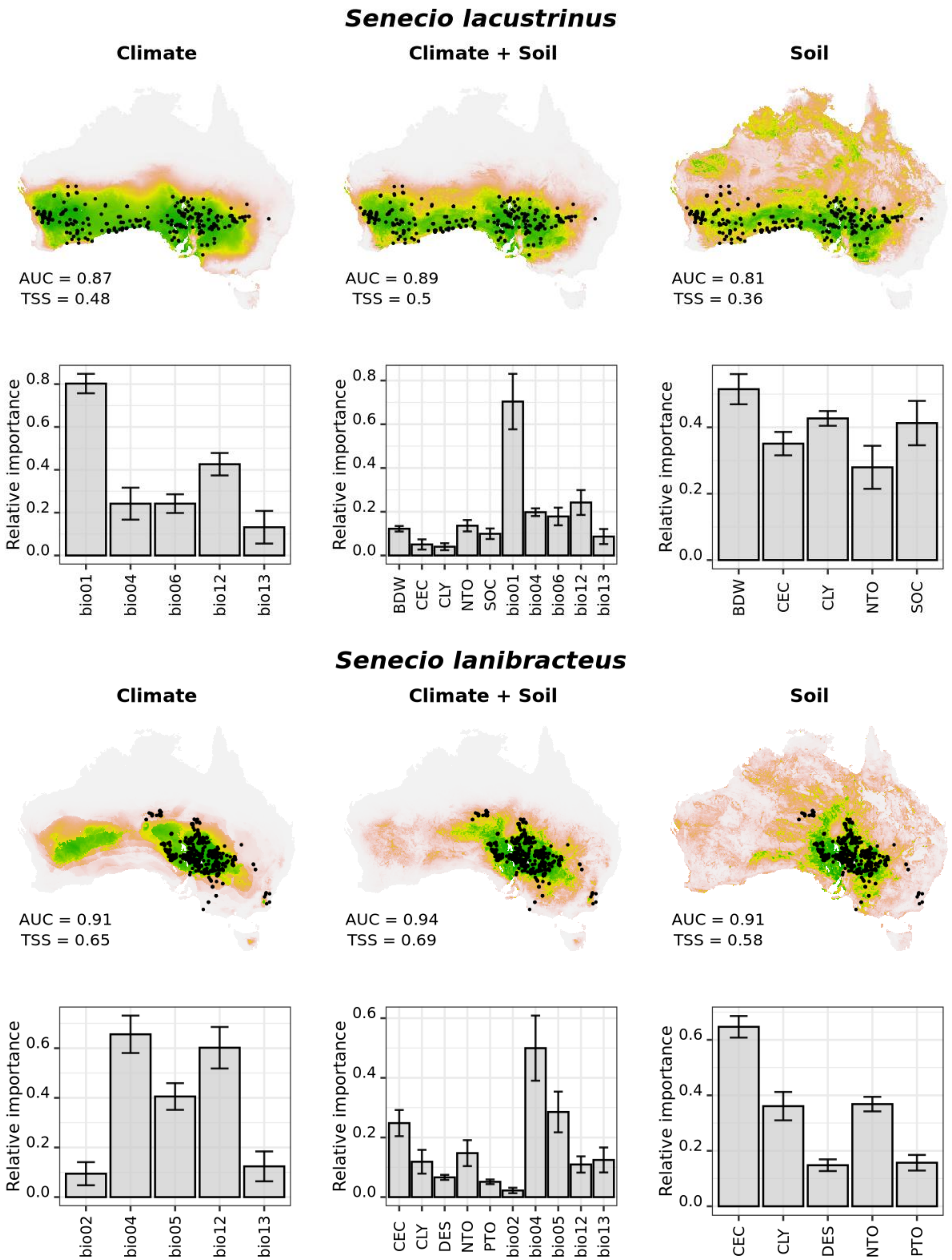
Senecio lacustrinus
Climate
Climate + Soil
Soil
AUC = 0.87
TSS = 0.48
AUC = 0.89
TSS = 0.5
AUC = 0.81
TSS = 0.36
Relative importance
0.0
0.2
0.4
0.6
0.8
bio01
bio04
bio06
bio12
bio13
BDW
CEC
CLY
NTO
SOC
Senecio lanibracteus
Climate
Climate + Soil
Soil
AUC = 0.91
TSS = 0.65
AUC = 0.94
TSS = 0.69
AUC = 0.91
TSS = 0.58
Relative importance
bio02
bio04
bio05
bio12
bio13
CEC
CLY
DES
NTO
PTO

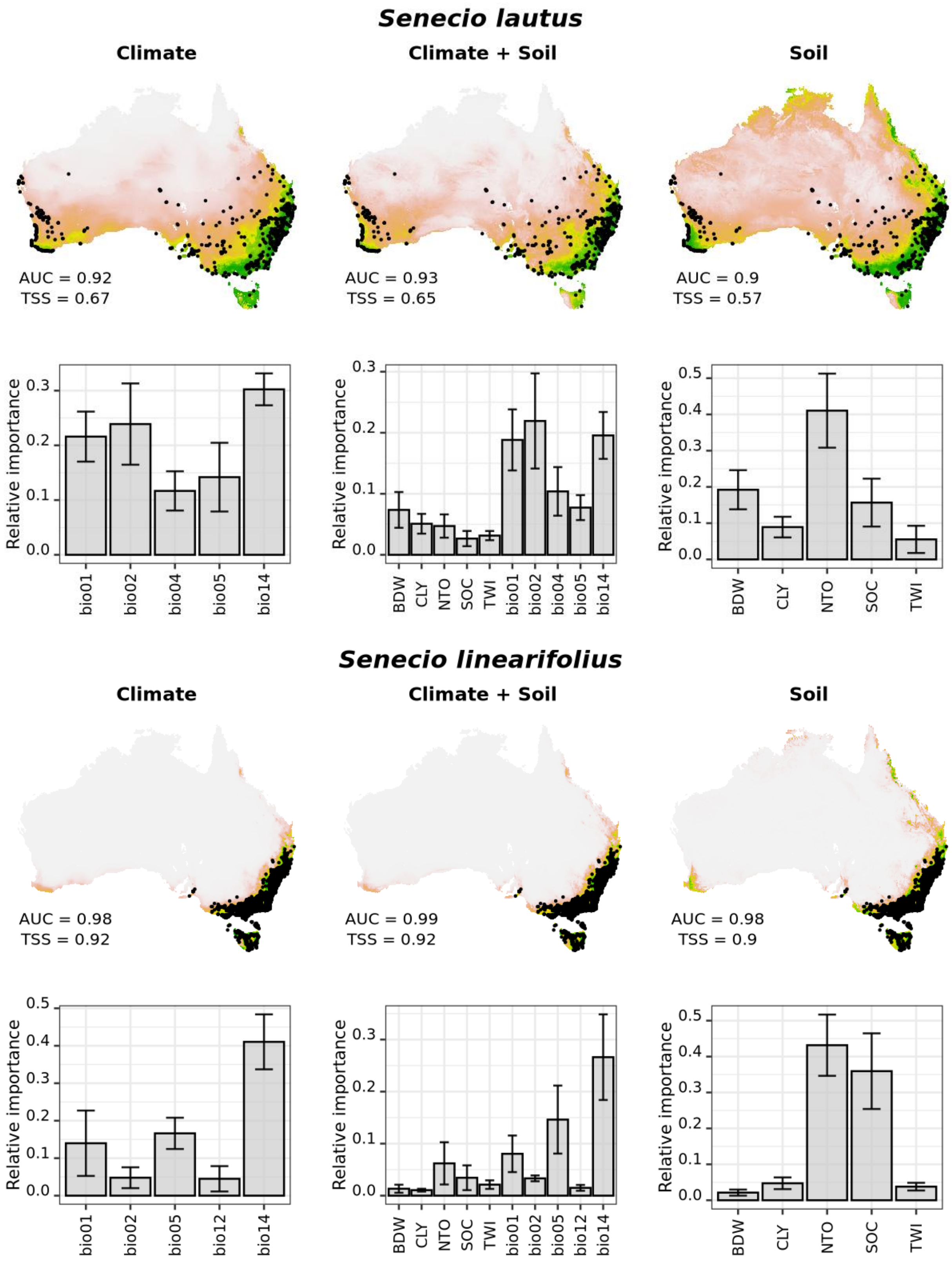
Senecio lautus
Climate
Climate + Soil
Soil
AUC = 0.92
TSS = 0.67
AUC = 0.93
TSS = 0.65
AUC = 0.9
TSS = 0.57
Relative importance
bio01
bio02
bio04
bio05
bio14
BDW
CLY
NTO
SOC
TWI
Senecio linearifolius
Climate
Climate + Soil
Soil
AUC = 0.98
TSS = 0.92
AUC = 0.99
TSS = 0.92
AUC = 0.98
TSS = 0.9
Relative importance
bio01
bio02
bio05
bio12
bio14
BDW
CLY
NTO
SOC
TWI

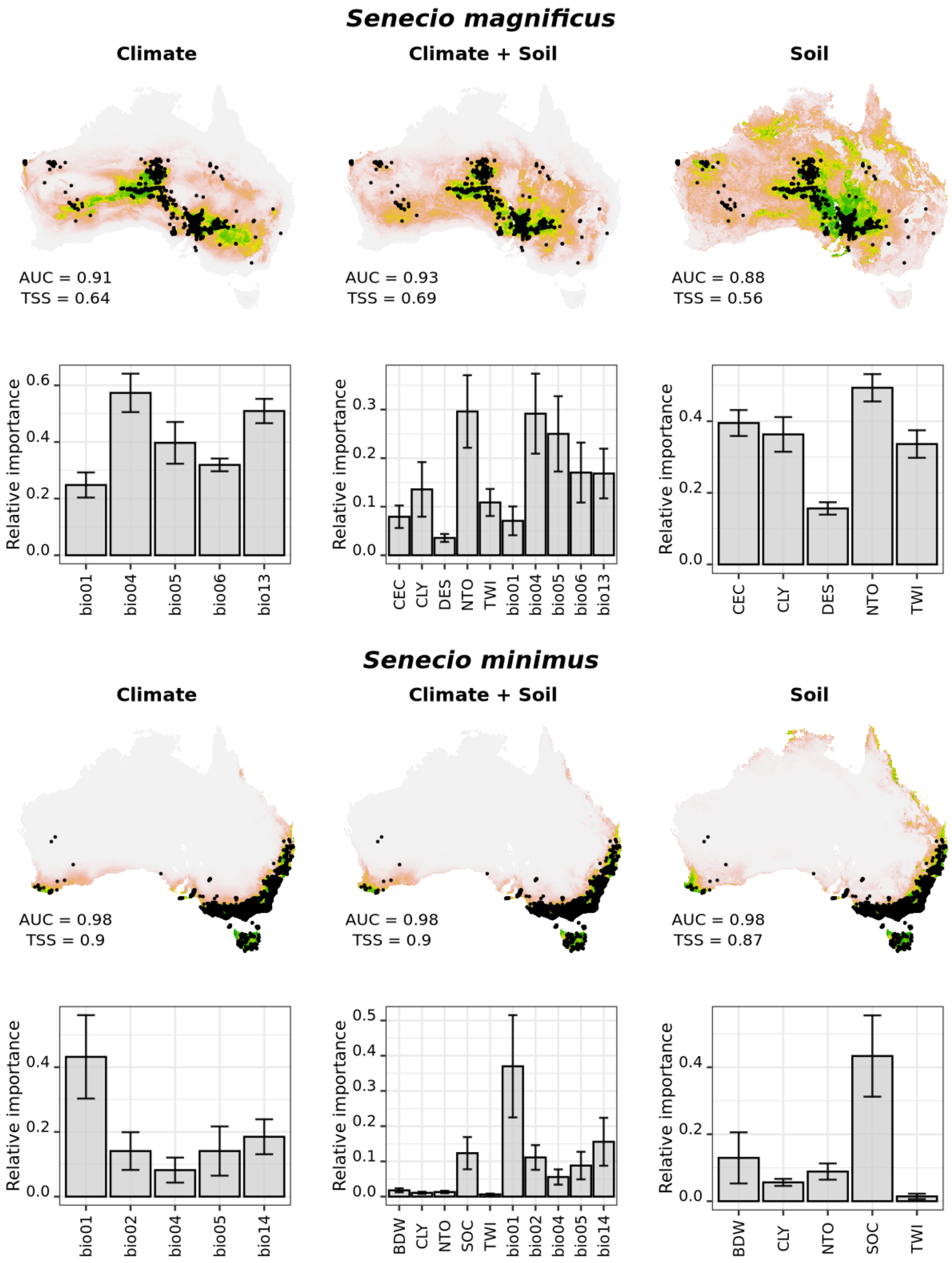

Senecio magnificus
Climate
Climate + Soil
Soil
AUC = 0.91
TSS = 0.64
AUC = 0.93
TSS = 0.69
AUC = 0.88
TSS = 0.56
Relative importance
0.0
0.2
0.4
0.6
bio01
bio04
bio05
bio06
bio13
0.0
0.1
0.2
0.3
CEC
CLY
DES
NTO
TWI
bio01
bio04
bio05
bio06
bio13
0.0
0.2
0.4
CEC
CLY
DES
NTO
TWI
Senecio minimus
Climate
Climate + Soil
Soil
AUC = 0.98
TSS = 0.9
AUC = 0.98
TSS = 0.9
AUC = 0.98
TSS = 0.87
Relative importance
0.0
0.2
0.4
bio01
bio02
bio04
bio05
bio14
0.0
0.1
0.2
0.3
0.4
0.5
BDW
CLY
NTO
SOC
TWI
bio01
bio02
bio04
bio05
bio14
0.0
0.2
0.4
BDW
CLY
NTO
SOC
TWI

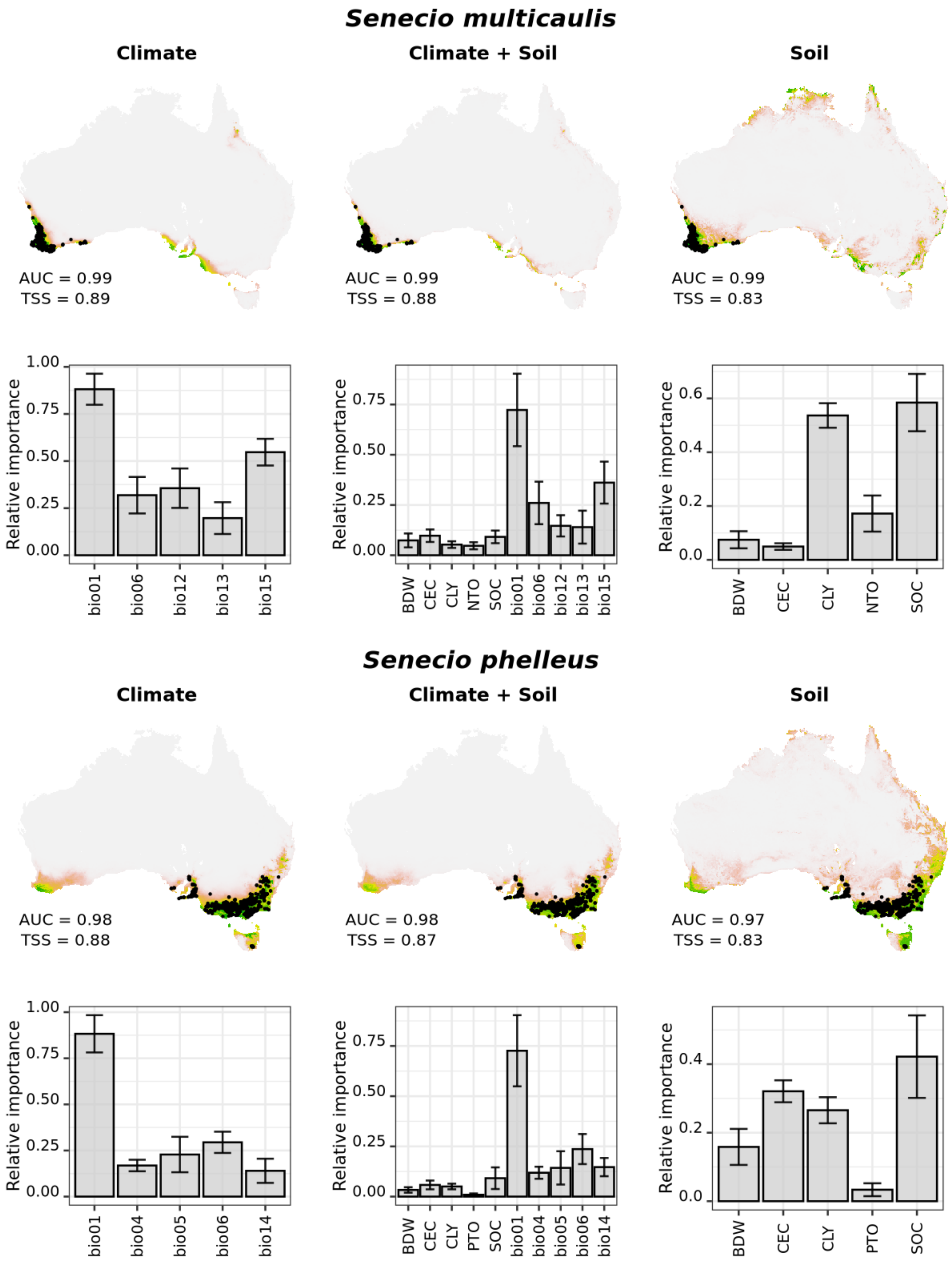
Senecio multicaulis
Climate
Climate + Soil
Soil
AUC = 0.99
TSS = 0.89
AUC = 0.99
TSS = 0.88
AUC = 0.99
TSS = 0.83
Relative importance
1.00
0.75
0.50
0.25
0.00
bio01
bio06
bio12
bio13
bio15
0.75
0.50
0.25
0.00
BDW
CEC
CLY
NTO
SOC
bio01
bio06
bio12
bio13
bio15
0.6
0.4
0.2
0.0
BDW
CEC
CLY
NTO
SOC
Senecio phelleus
Climate
Climate + Soil
Soil
AUC = 0.98
TSS = 0.88
AUC = 0.98
TSS = 0.87
AUC = 0.97
TSS = 0.83
Relative importance
1.00
0.75
0.50
0.25
0.00
bio01
bio04
bio05
bio06
bio14
0.75
0.50
0.25
0.00
BDW
CEC
CLY
PTO
SOC
bio01
bio04
bio05
bio06
bio14
0.4
0.2
0.0
BDW
CEC
CLY
PTO
SOC

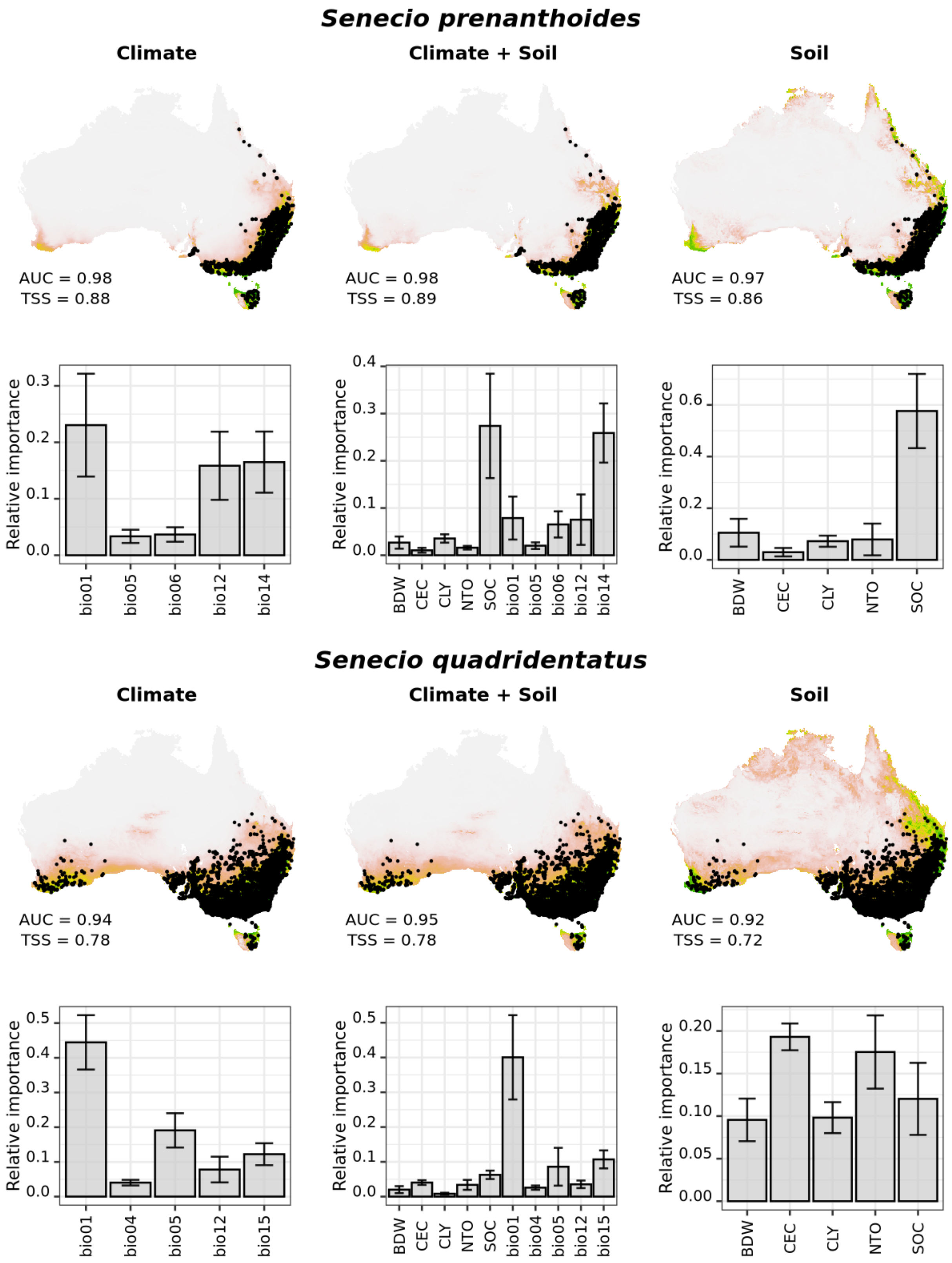
Senecio prenanthoides
Climate
Climate + Soil
Soil
AUC = 0.98
TSS = 0.88
AUC = 0.98
TSS = 0.89
AUC = 0.97
TSS = 0.86
Relative importance
bio01
bio05
bio06
bio12
bio14
BDW
CEC
CLY
NTO
SOC
Senecio quadridentatus
Climate
Climate + Soil
Soil
AUC = 0.94
TSS = 0.78
AUC = 0.95
TSS = 0.78
AUC = 0.92
TSS = 0.72
Relative importance
bio01
bio04
bio05
bio12
bio15
BDW
CEC
CLY
NTO
SOC

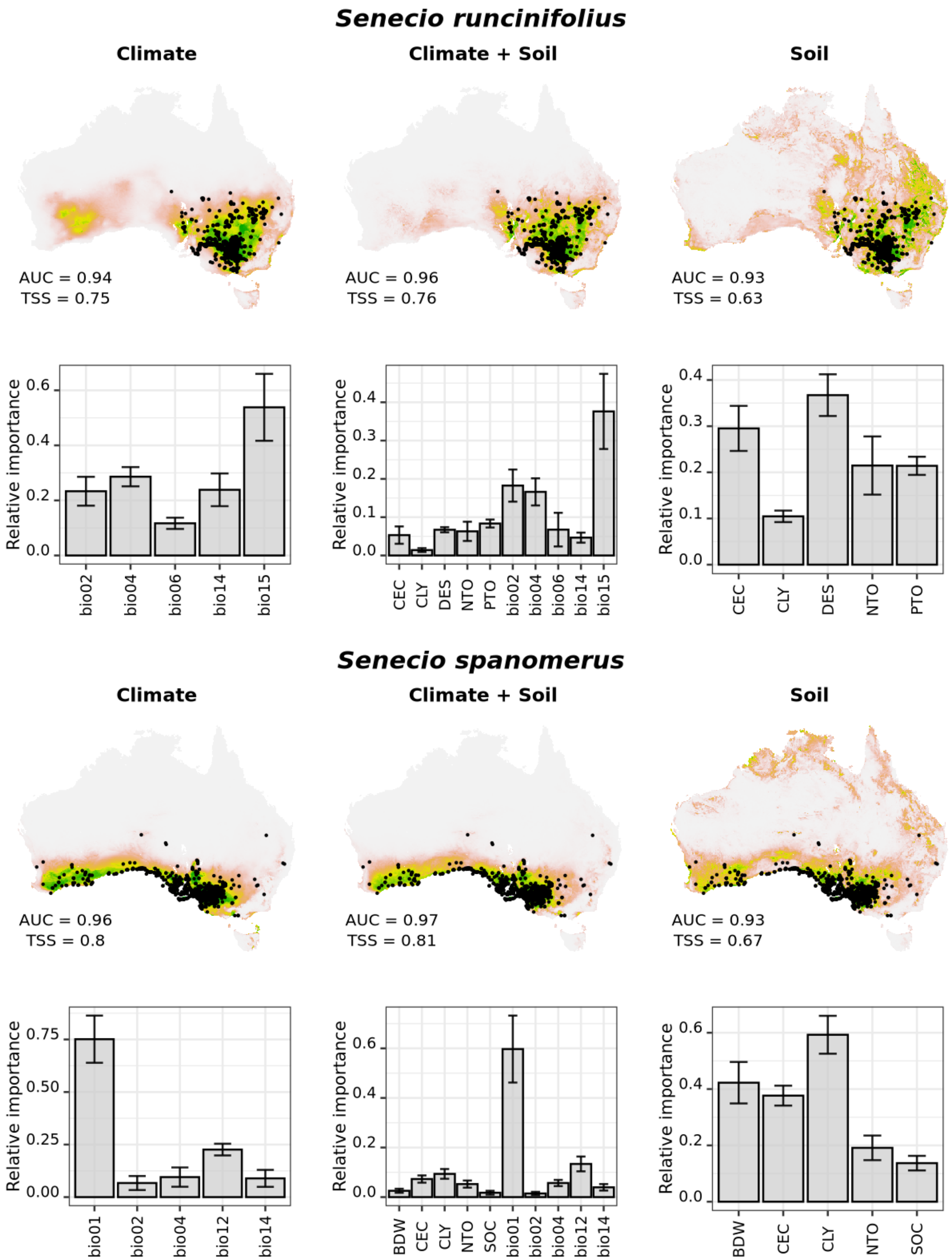
Senecio runcinifolius
Climate
Climate + Soil
Soil
AUC = 0.94
TSS = 0.75
AUC = 0.96
TSS = 0.76
AUC = 0.93
TSS = 0.63
Relative importance
bio02
bio04
bio06
bio14
bio15
CEC
CLY
DES
NTO
PTO
bio02
bio04
bio06
bio14
bio15
CEC
CLY
DES
NTO
PTO
Senecio spanomerus
Climate
Climate + Soil
Soil
AUC = 0.96
TSS = 0.8
AUC = 0.97
TSS = 0.81
AUC = 0.93
TSS = 0.67
Relative importance
bio01
bio02
bio04
bio12
bio14
BDW
CEC
CLY
NTO
SOC
bio01
bio02
bio04
bio12
bio14
BDW
CEC
CLY
NTO
SOC

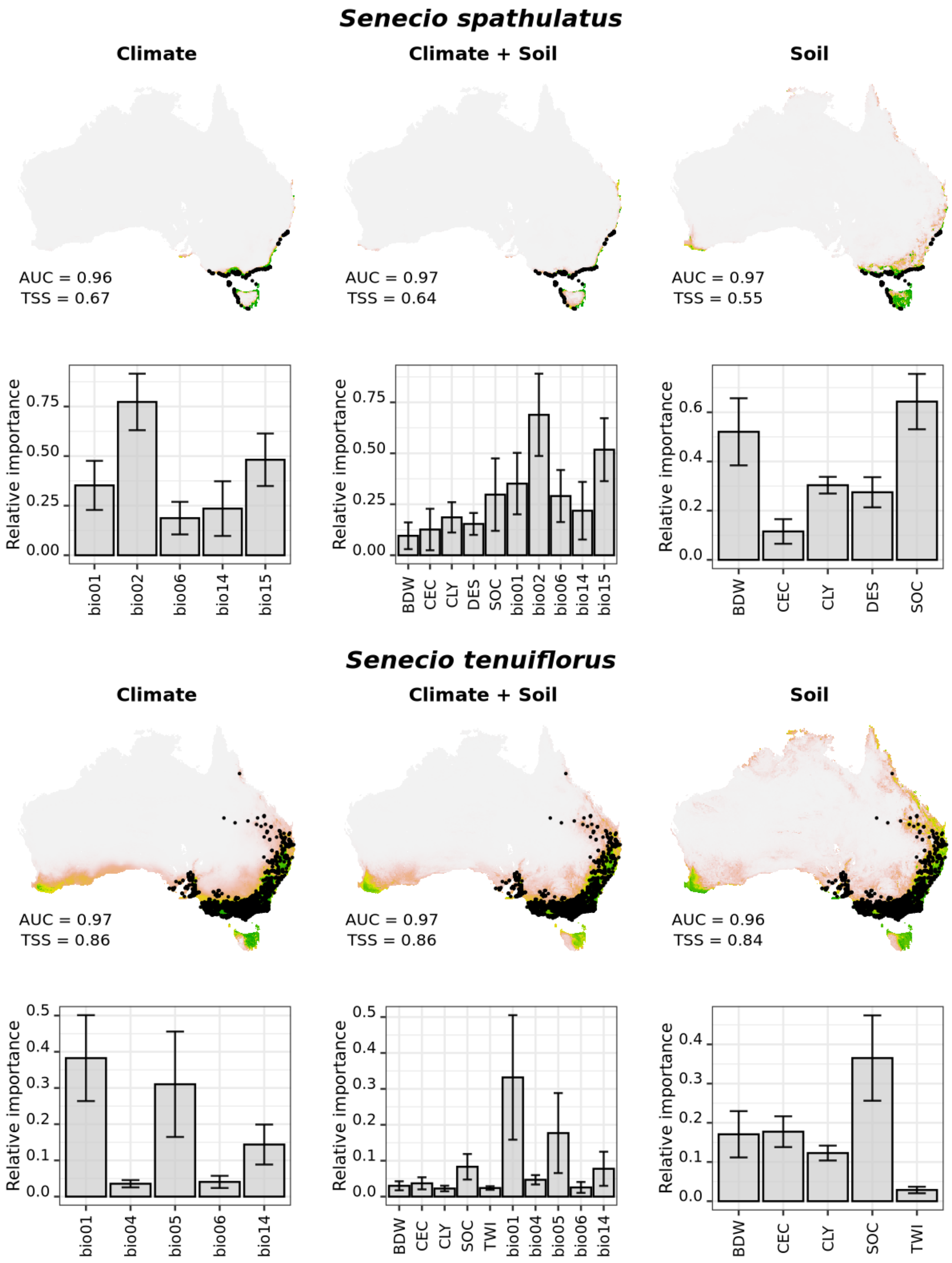
Senecio spathulatus
Climate
Climate + Soil
Soil
AUC = 0.96
TSS = 0.67
AUC = 0.97
TSS = 0.64
AUC = 0.97
TSS = 0.55
Relative importance
bio01
bio02
bio06
bio14
bio15
BDW
CEC
CLY
DES
SOC
Senecio tenuiflorus
Climate
Climate + Soil
Soil
AUC = 0.97
TSS = 0.86
AUC = 0.97
TSS = 0.86
AUC = 0.96
TSS = 0.84
Relative importance
bio01
bio04
bio05
bio06
bio14
BDW
CEC
CLY
SOC
TWI

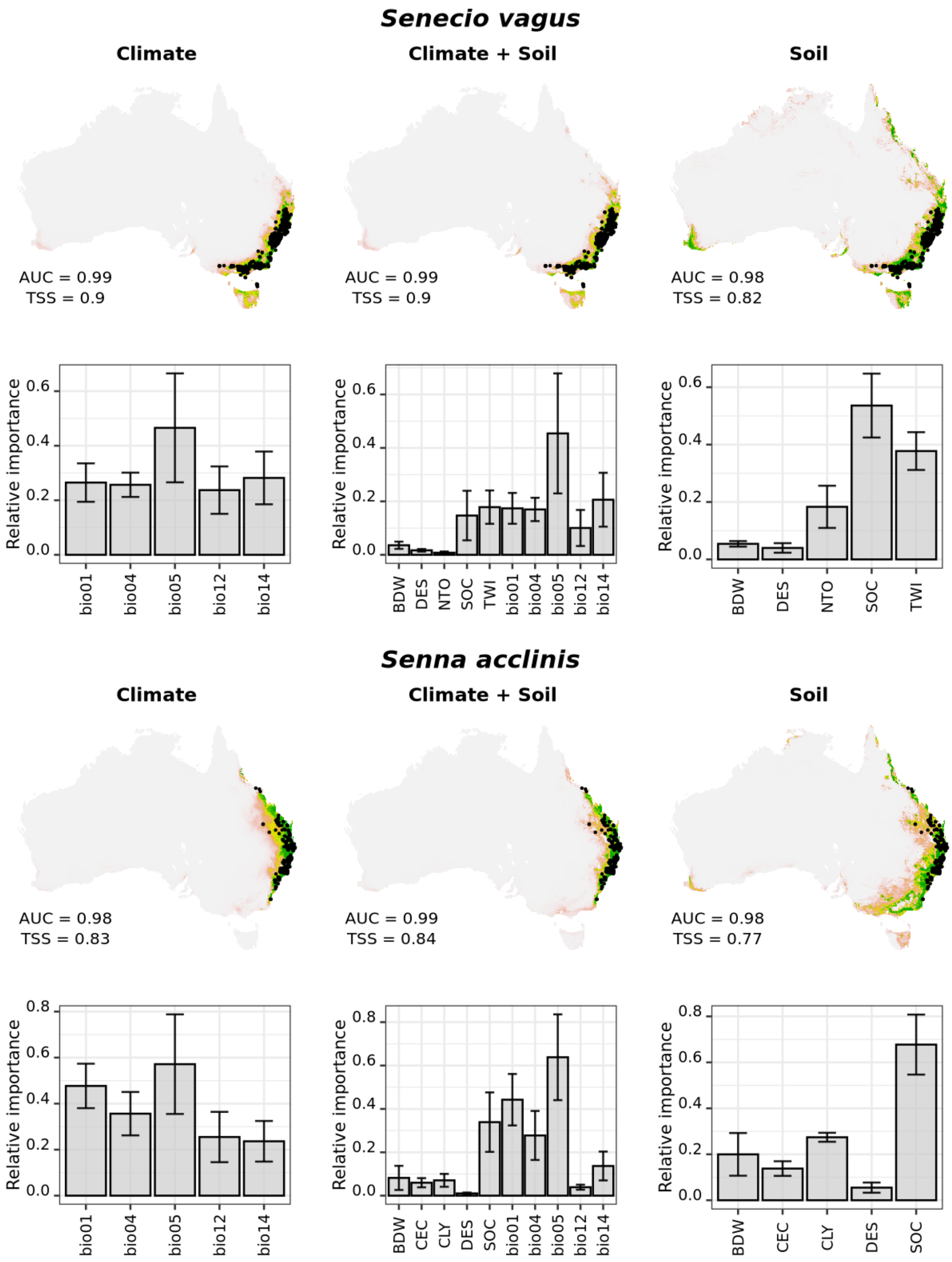
Senecio vagus
Climate
Climate + Soil
Soil
AUC = 0.99
TSS = 0.9
AUC = 0.99
TSS = 0.9
AUC = 0.98
TSS = 0.82
Relative importance
bio01 bio04 bio05 bio12 bio14
BDW DES NTO SOC TWI bio01 bio04 bio05 bio12 bio14
BDW DES NTO SOC TWI
Senna acclinis
Climate
Climate + Soil
Soil
AUC = 0.98
TSS = 0.83
AUC = 0.99
TSS = 0.84
AUC = 0.98
TSS = 0.77
Relative importance
bio01 bio04 bio05 bio12 bio14
BDW CEC CLY DES SOC bio01 bio04 bio05 bio12 bio14
BDW CEC CLY DES SOC

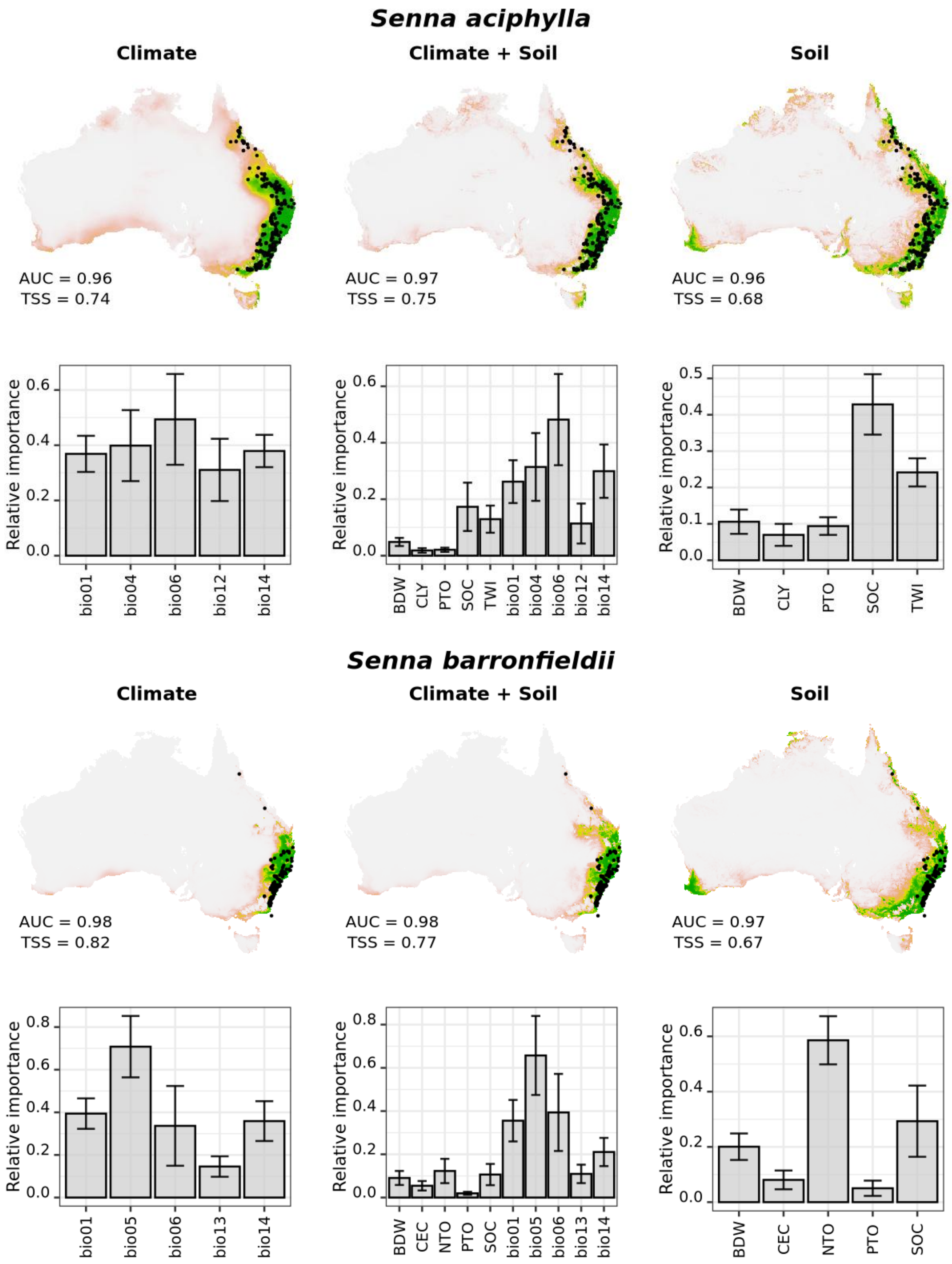

Senna aciphylla
Climate
Climate + Soil
Soil
AUC = 0.96
TSS = 0.74
AUC = 0.97
TSS = 0.75
AUC = 0.96
TSS = 0.68
Relative importance
bio01
bio04
bio06
bio12
bio14
BDW
CLY
PTO
SOC
TWI
Senna barronfieldii
Climate
Climate + Soil
Soil
AUC = 0.98
TSS = 0.82
AUC = 0.98
TSS = 0.77
AUC = 0.97
TSS = 0.67
Relative importance
bio01
bio05
bio06
bio13
bio14
BDW
CEC
NTO
PTO
SOC

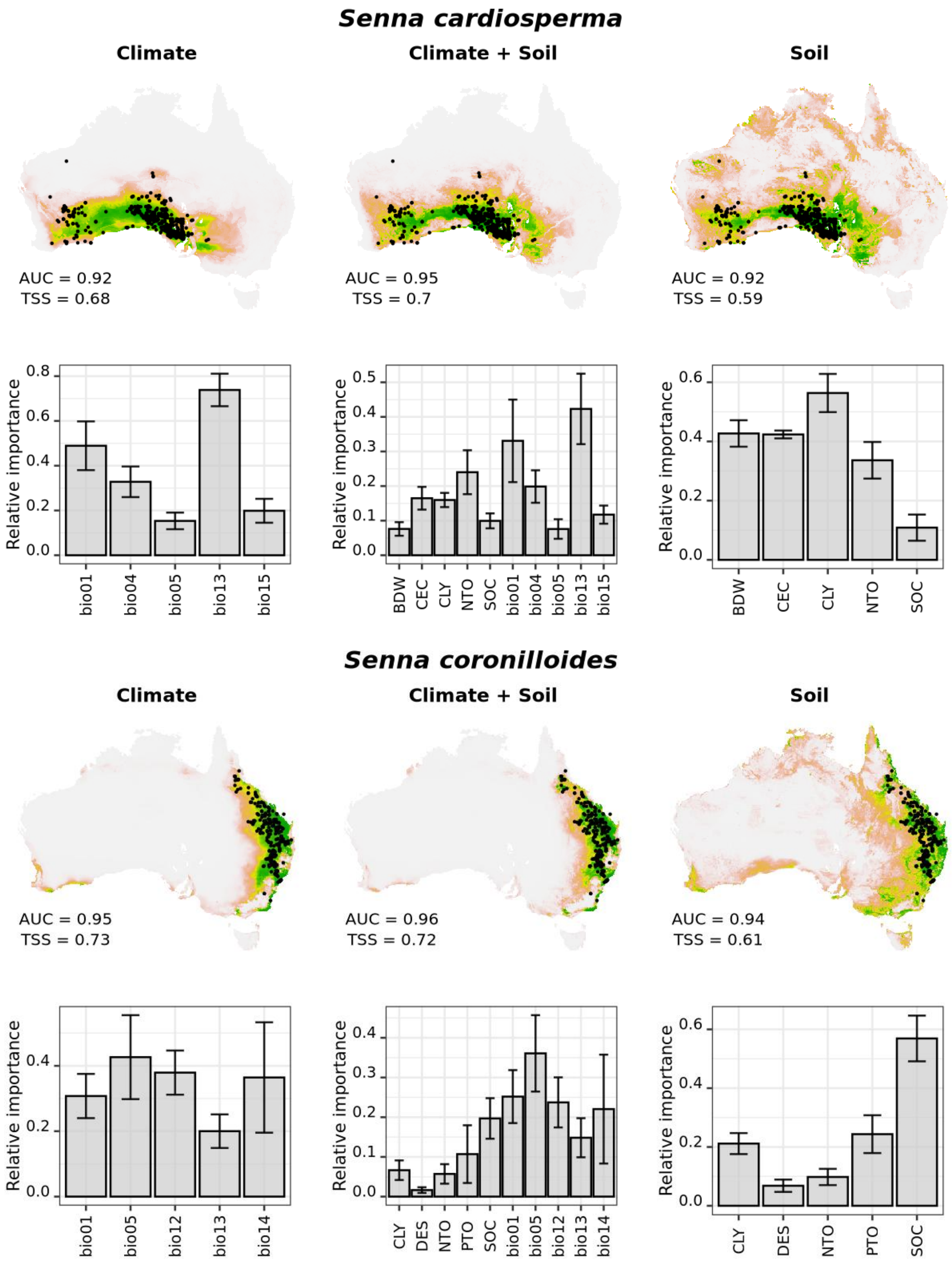

Senna cardiosperma
Climate
Climate + Soil
Soil
AUC = 0.92
TSS = 0.68
AUC = 0.95
TSS = 0.7
AUC = 0.92
TSS = 0.59
Relative importance
bio01
bio04
bio05
bio13
bio15
BDW
CEC
CLY
NTO
SOC
Senna coronilloides
Climate
Climate + Soil
Soil
AUC = 0.95
TSS = 0.73
AUC = 0.96
TSS = 0.72
AUC = 0.94
TSS = 0.61
Relative importance
bio01
bio05
bio12
bio13
bio14
CLY
DES
NTO
PTO
SOC

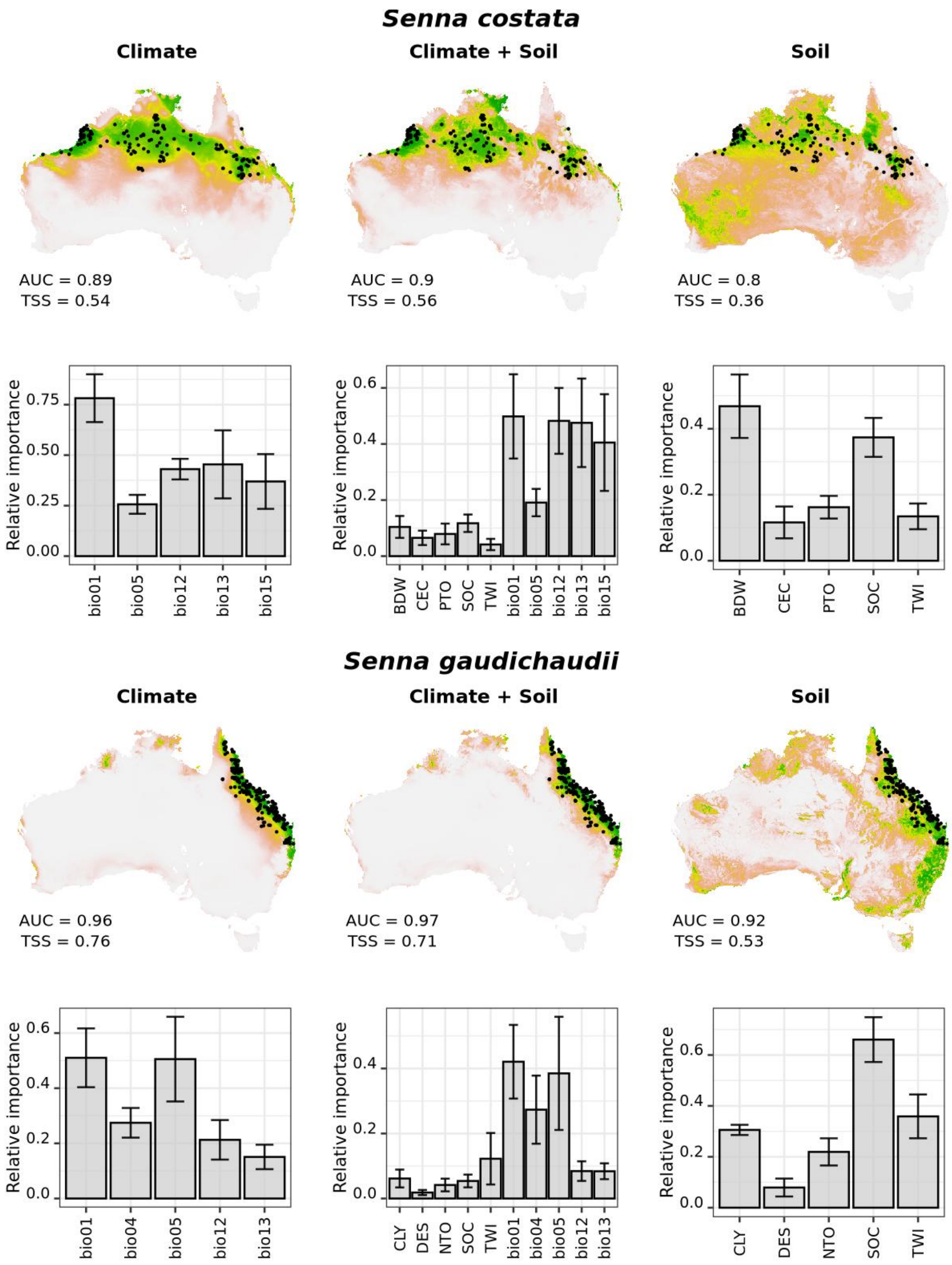
Senna costata
Climate
Climate + Soil
Soil
AUC = 0.89
TSS = 0.54
AUC = 0.9
TSS = 0.56
AUC = 0.8
TSS = 0.36
Relative importance
0.00
0.25
0.50
0.75
bio01
bio05
bio12
bio13
bio15
0.0
0.2
0.4
0.6
BDW
CEC
PTO
SOC
TWI
bio01
bio05
bio12
bio13
bio15
0.0
0.2
0.4
BDW
CEC
PTO
SOC
TWI
Senna gaudichaudii
Climate
Climate + Soil
Soil
AUC = 0.96
TSS = 0.76
AUC = 0.97
TSS = 0.71
AUC = 0.92
TSS = 0.53
Relative importance
0.0
0.2
0.4
0.6
bio01
bio04
bio05
bio12
bio13
0.0
0.2
0.4
CLY
DES
NTO
SOC
TWI
bio01
bio04
bio05
bio12
bio13
0.0
0.2
0.4
0.6
CLY
DES
NTO
SOC
TWI

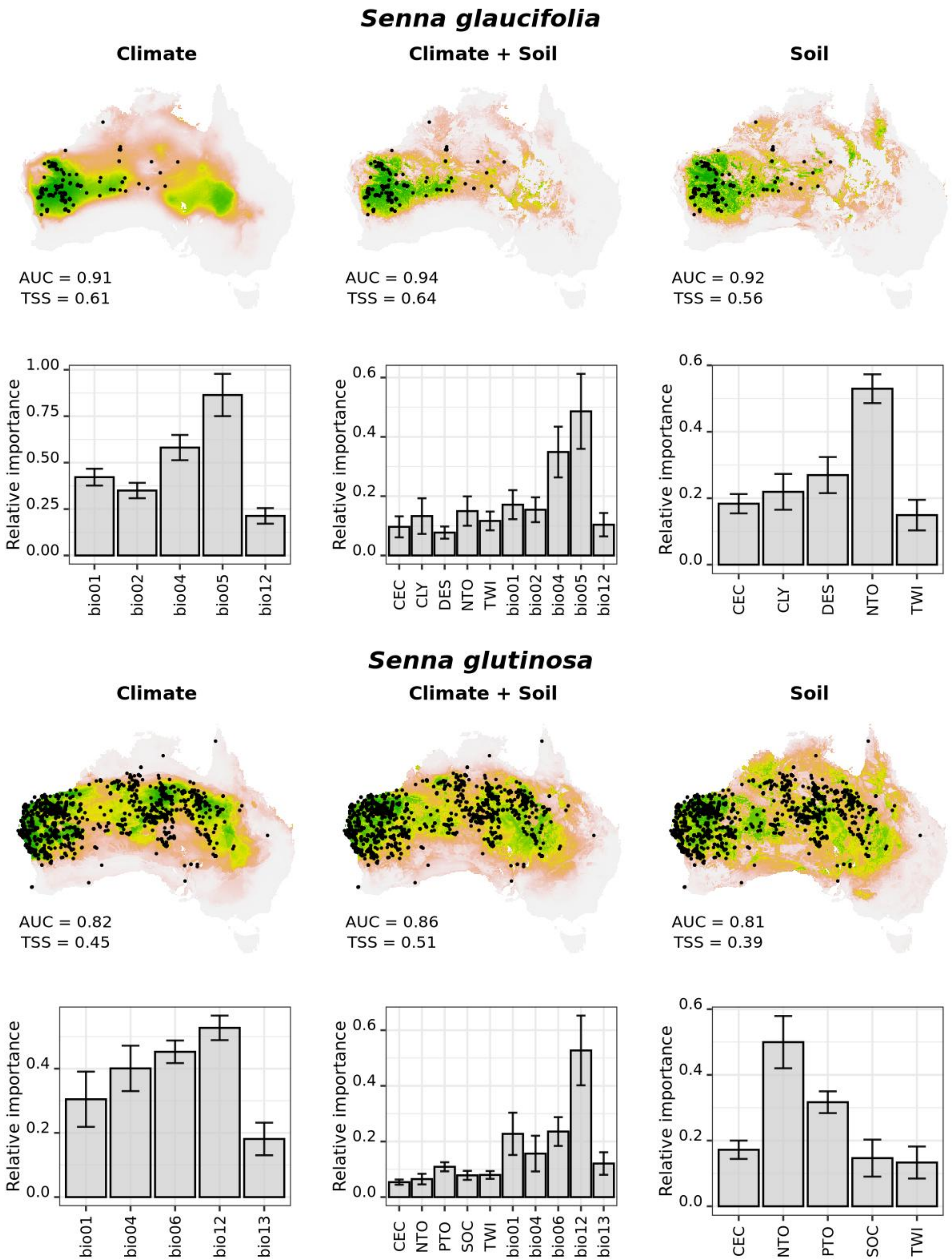

Senna glaucifolia
Climate
Climate + Soil
Soil
AUC = 0.91
TSS = 0.61
AUC = 0.94
TSS = 0.64
AUC = 0.92
TSS = 0.56
Relative importance
0.00
0.25
0.50
0.75
1.00
bio01
bio02
bio04
bio05
bio12
Relative importance
0.0
0.2
0.4
0.6
CEC
CLY
DES
NTO
TWI
bio01
bio02
bio04
bio05
bio12
Relative importance
0.0
0.2
0.4
0.6
CEC
CLY
DES
NTO
TWI
Senna glutinosa
Climate
Climate + Soil
Soil
AUC = 0.82
TSS = 0.45
AUC = 0.86
TSS = 0.51
AUC = 0.81
TSS = 0.39
Relative importance
0.0
0.2
0.4
bio01
bio04
bio06
bio12
bio13
Relative importance
0.0
0.2
0.4
0.6
CEC
NTO
PTO
SOC
TWI
bio01
bio04
bio06
bio12
bio13
Relative importance
0.0
0.2
0.4
0.6
CEC
NTO
PTO
SOC
TWI

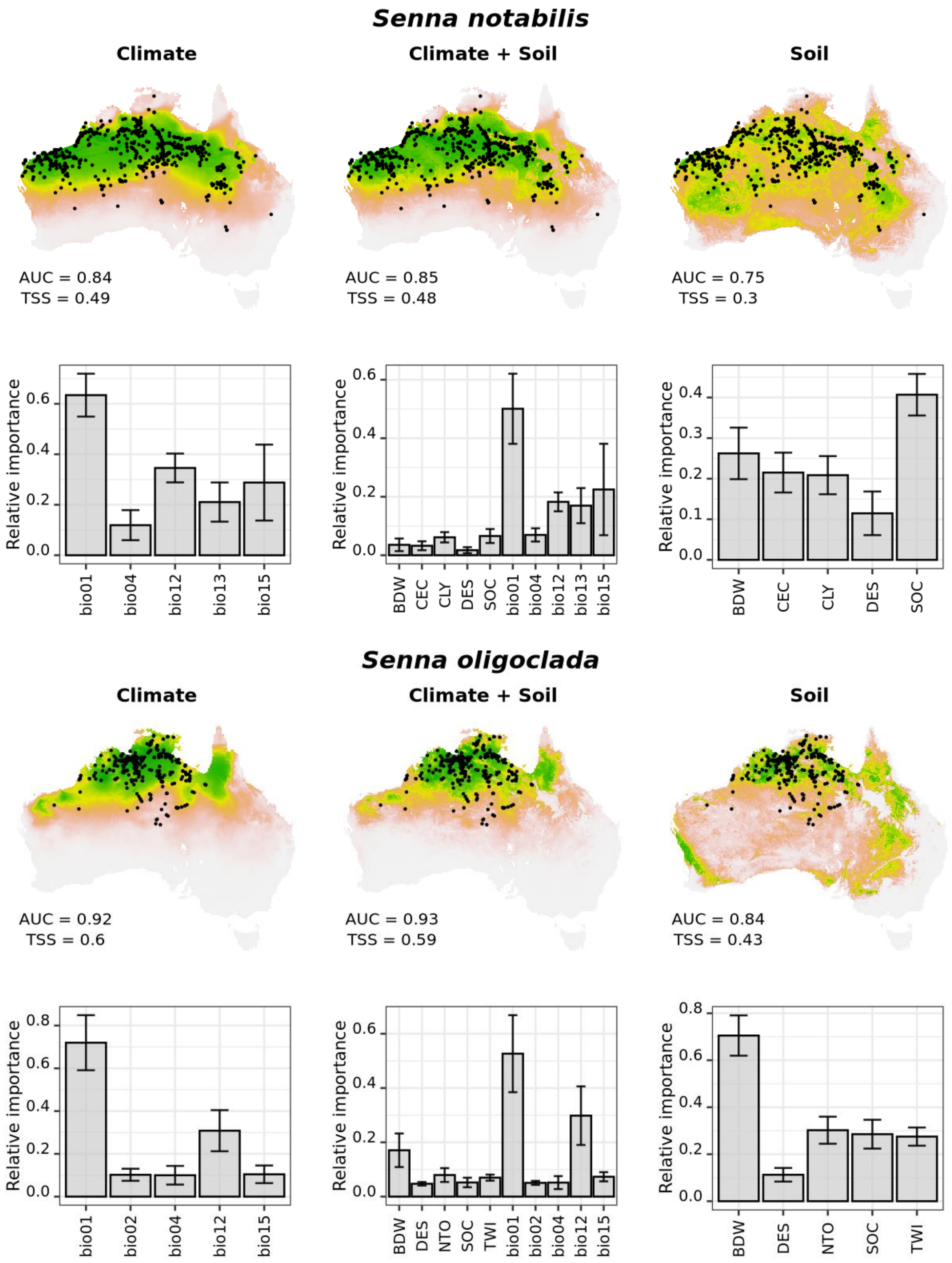
Senna notabilis
Climate
Climate + Soil
Soil
AUC = 0.84
TSS = 0.49
AUC = 0.85
TSS = 0.48
AUC = 0.75
TSS = 0.3
Relative importance
bio01 bio04 bio12 bio13 bio15
BDW CEC CLY DES SOC bio01 bio04 bio12 bio13 bio15
BDW CEC CLY DES SOC
Senna oligoclada
Climate
Climate + Soil
Soil
AUC = 0.92
TSS = 0.6
AUC = 0.93
TSS = 0.59
AUC = 0.84
TSS = 0.43
Relative importance
bio01 bio02 bio04 bio12 bio15
BDW DES NTO SOC TWI bio01 bio02 bio04 bio12 bio15
BDW DES NTO SOC TWI

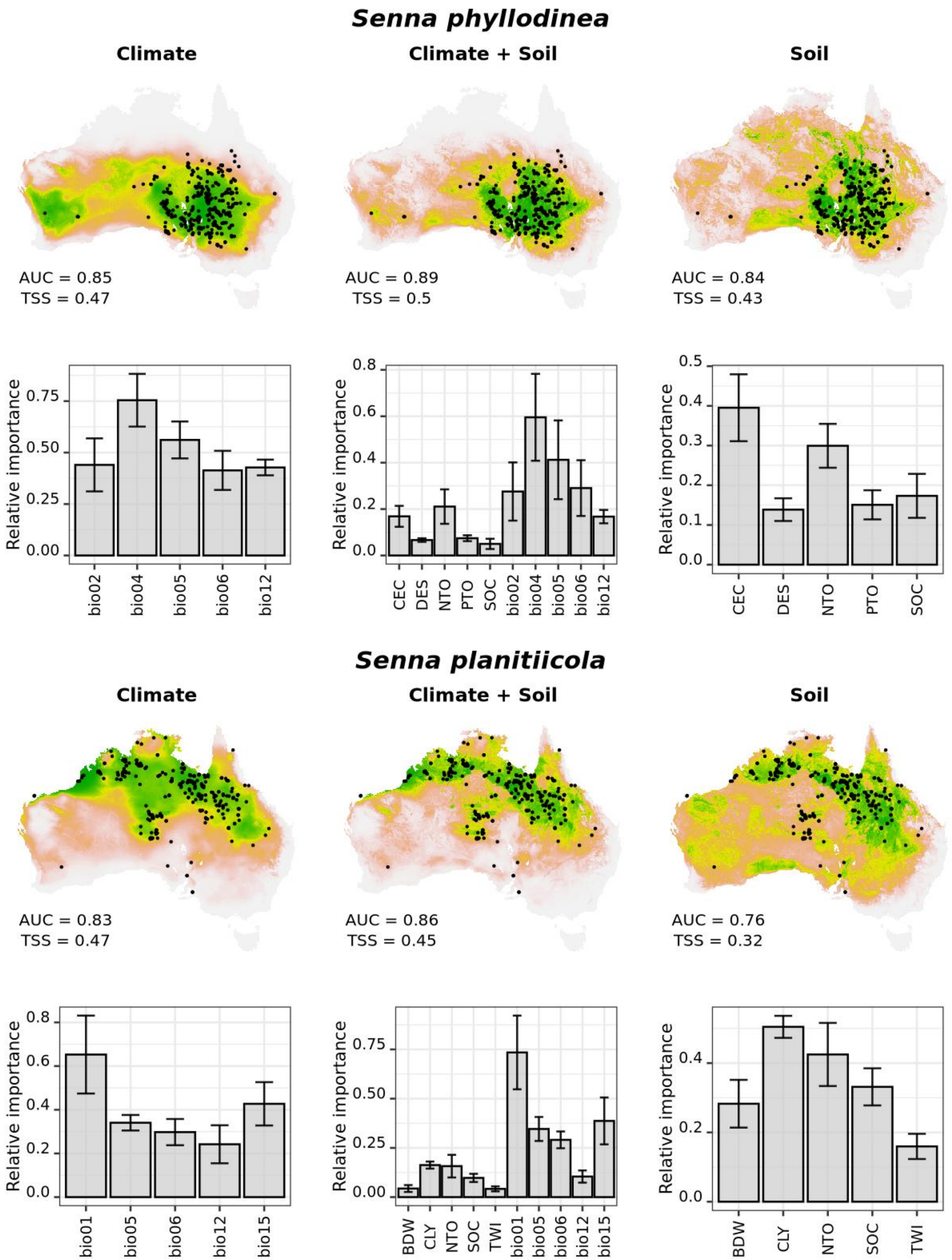
Senna phyllodinea
Climate
Climate + Soil
Soil
AUC = 0.85
TSS = 0.47
AUC = 0.89
TSS = 0.5
AUC = 0.84
TSS = 0.43
Relative importance
0.00
0.25
0.50
0.75
bio02
bio04
bio05
bio06
bio12
0.0
0.2
0.4
0.6
0.8
CEC
DES
NTO
PTO
SOC
0.1
0.3
0.5
Senna planitiicola
AUC = 0.83
TSS = 0.47
AUC = 0.86
TSS = 0.45
AUC = 0.76
TSS = 0.32
bio01
bio15
BDW
CLY
TWI

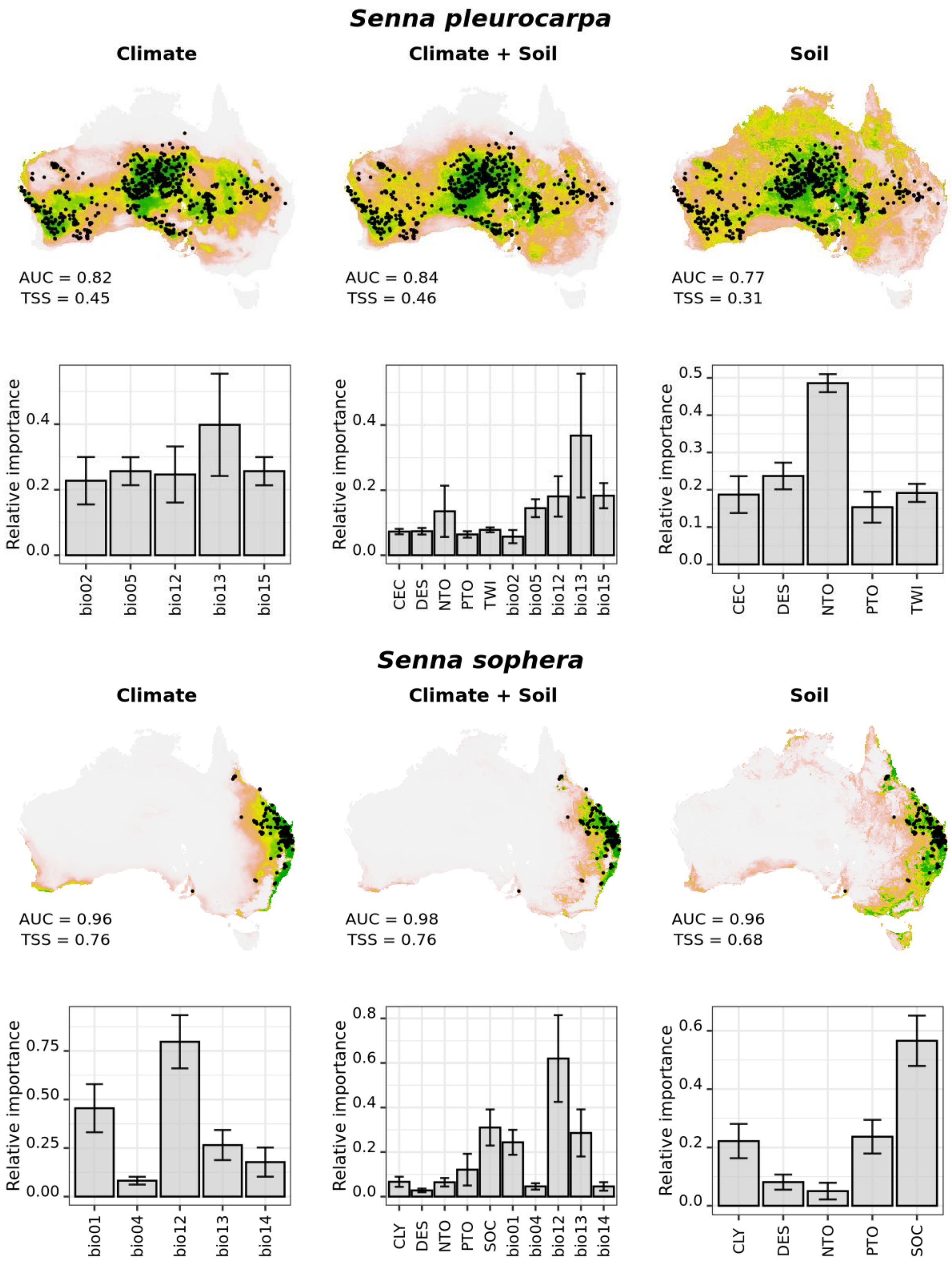

Senna pleurocarpa
Climate
Climate + Soil
Soil
AUC = 0.82
TSS = 0.45
AUC = 0.84
TSS = 0.46
AUC = 0.77
TSS = 0.31
Relative importance
0.0
0.2
0.4
bio02
bio05
bio12
bio13
bio15
Relative importance
0.0
0.2
0.4
CEC
DES
NTO
PTO
TWI
bio02
bio05
bio12
bio13
bio15
Relative importance
0.0
0.1
0.2
0.3
0.4
0.5
CEC
DES
NTO
PTO
TWI
Senna sophera
Climate
Climate + Soil
Soil
AUC = 0.96
TSS = 0.76
AUC = 0.98
TSS = 0.76
AUC = 0.96
TSS = 0.68
Relative importance
0.00
0.25
0.50
0.75
bio01
bio04
bio12
bio13
bio14
Relative importance
0.0
0.2
0.4
0.6
0.8
CLY
DES
NTO
PTO
SOC
bio01
bio04
bio12
bio13
bio14
Relative importance
0.0
0.2
0.4
0.6
CLY
DES
NTO
PTO
SOC

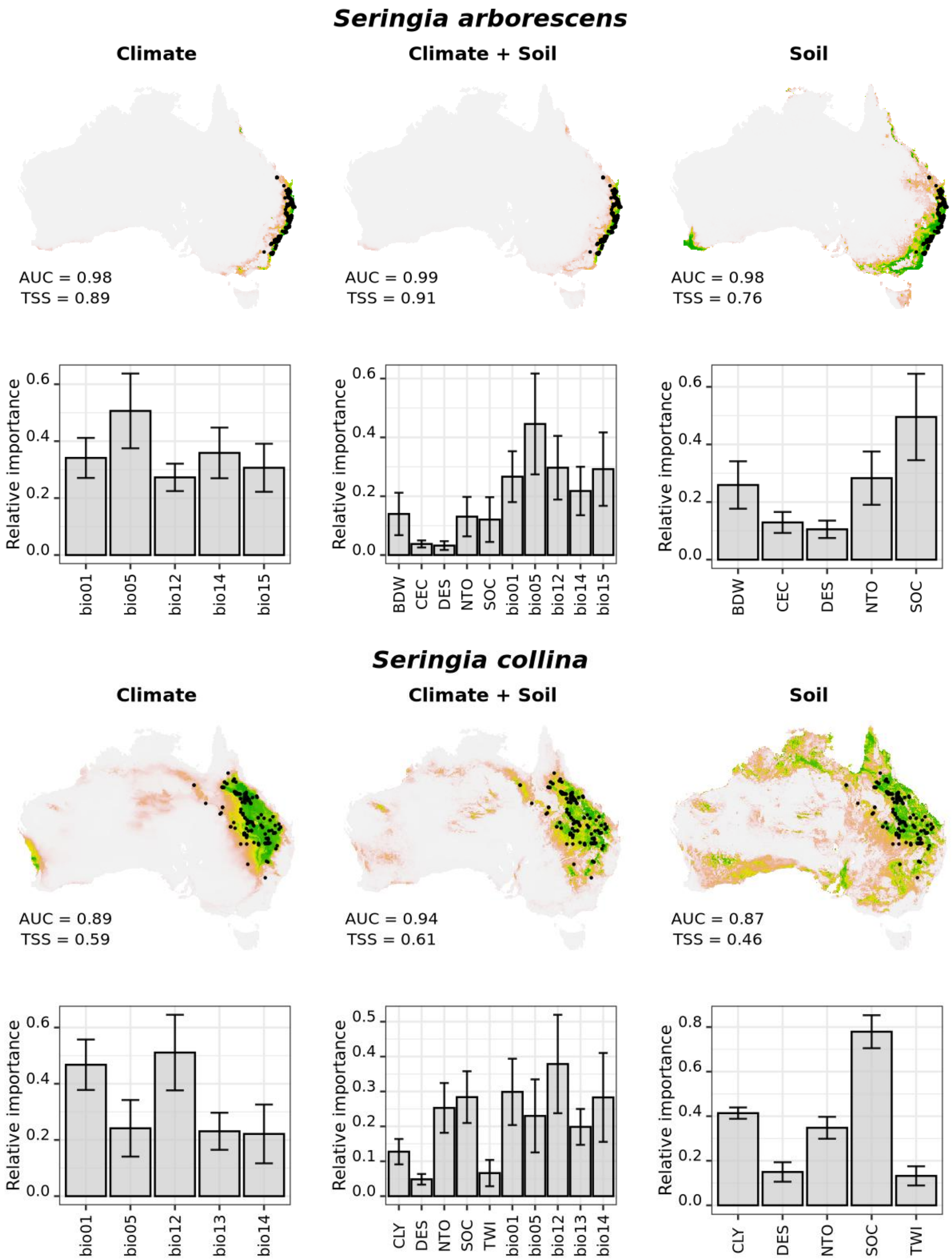
Seringia arborescens
Climate
Climate + Soil
Soil
AUC = 0.98
TSS = 0.89
AUC = 0.99
TSS = 0.91
AUC = 0.98
TSS = 0.76
Relative importance
0.0
0.2
0.4
0.6
bio01
bio05
bio12
bio14
bio15
BDW
CEC
DES
NTO
SOC
Seringia collina
AUC = 0.89
TSS = 0.59
AUC = 0.94
TSS = 0.61
AUC = 0.87
TSS = 0.46
0.1
0.3
0.5
0.8
bio13
CLY
TWI

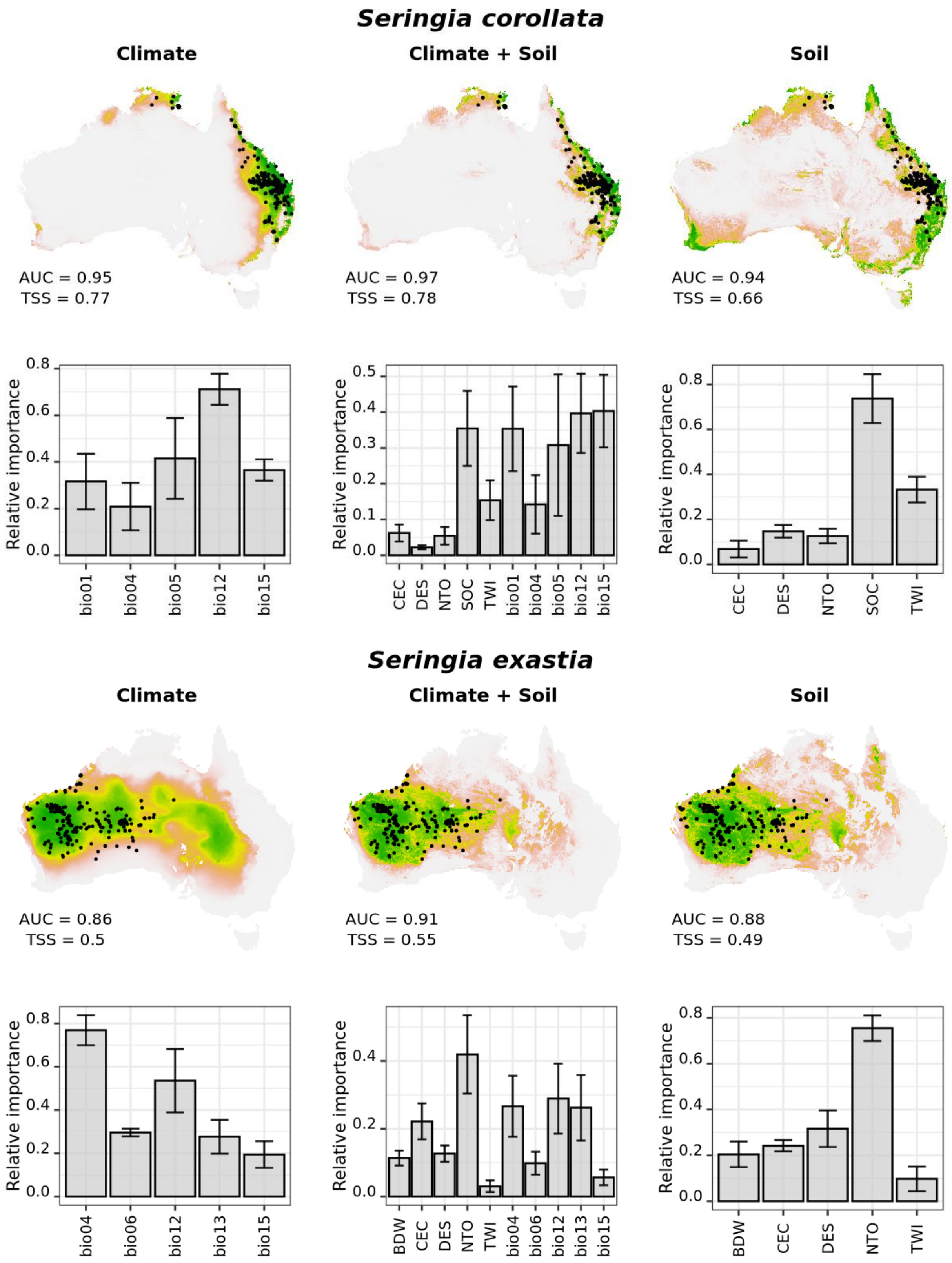

Seringia corollata
Climate
Climate + Soil
Soil
AUC = 0.95
TSS = 0.77
AUC = 0.97
TSS = 0.78
AUC = 0.94
TSS = 0.66
Relative importance
bio01 bio04 bio05 bio12 bio15
CEC DES NTO SOC TWI bio01 bio04 bio05 bio12 bio15
CEC DES NTO SOC TWI
Seringia exastia
Climate
Climate + Soil
Soil
AUC = 0.86
TSS = 0.5
AUC = 0.91
TSS = 0.55
AUC = 0.88
TSS = 0.49
Relative importance
bio04 bio06 bio12 bio13 bio15
BDW CEC DES NTO TWI bio04 bio06 bio12 bio13 bio15
BDW CEC DES NTO TWI

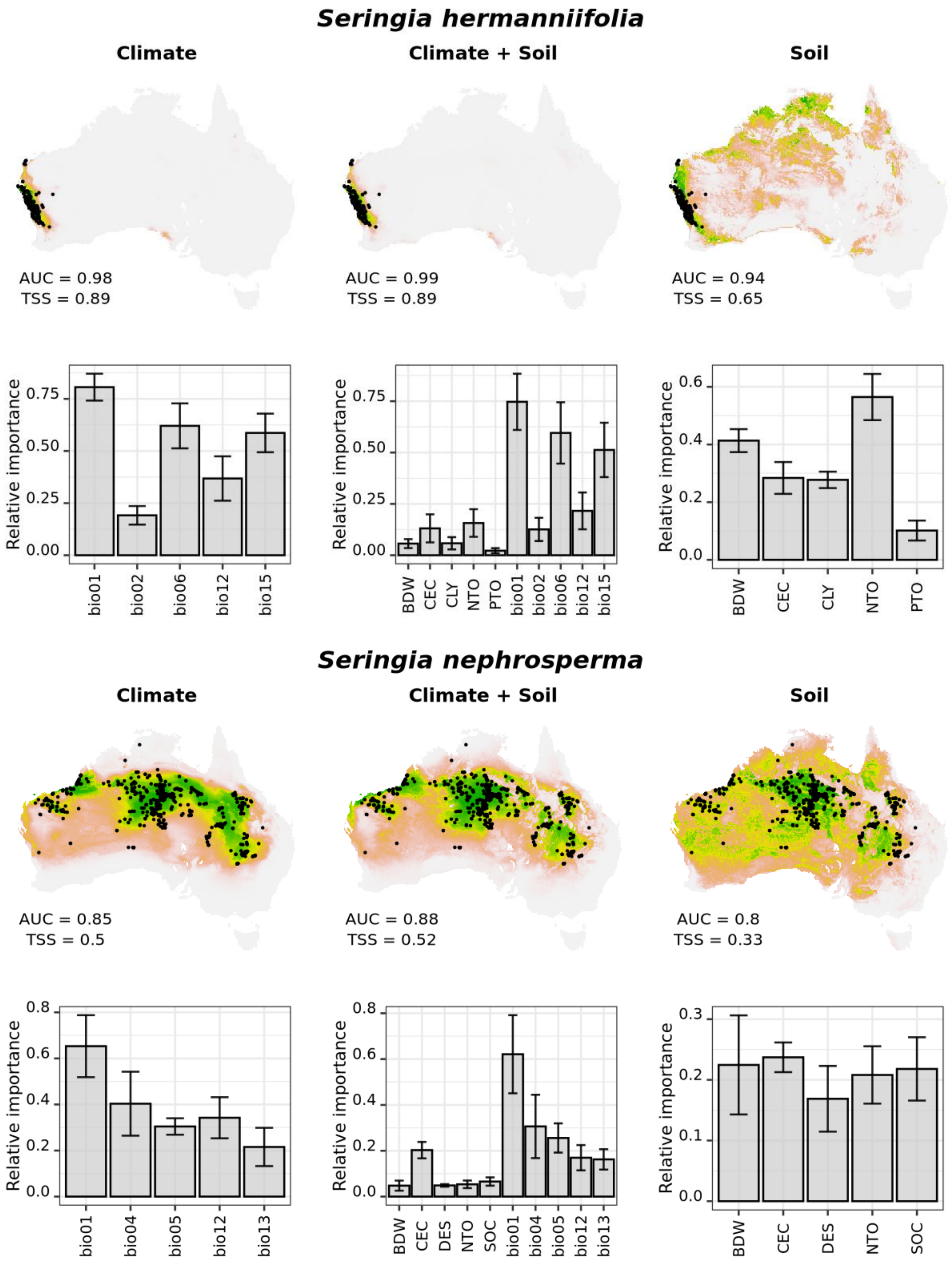
Seringia hermanniifolia
Climate
Climate + Soil
Soil
AUC = 0.98
TSS = 0.89
AUC = 0.99
TSS = 0.89
AUC = 0.94
TSS = 0.65
Relative importance
bio01
bio02
bio06
bio12
bio15
BDW
CEC
CLY
NTO
PTO
Seringia nephrosperma
Climate
Climate + Soil
Soil
AUC = 0.85
TSS = 0.5
AUC = 0.88
TSS = 0.52
AUC = 0.8
TSS = 0.33
Relative importance
bio01
bio04
bio05
bio12
bio13
BDW
CEC
DES
NTO
SOC

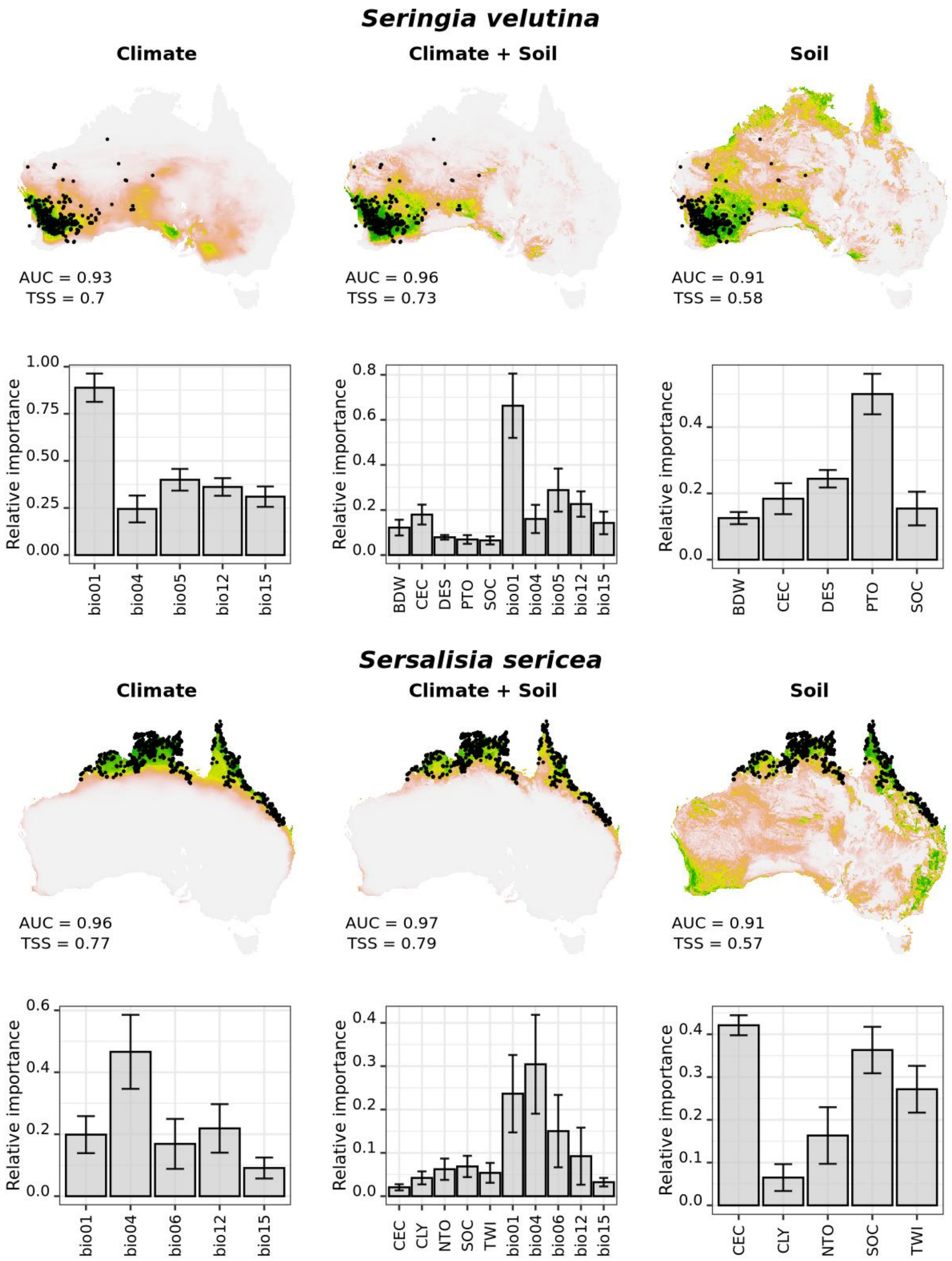
Seringia velutina
Climate
Climate + Soil
Soil
AUC = 0.93
TSS = 0.7
AUC = 0.96
TSS = 0.73
AUC = 0.91
TSS = 0.58
Relative importance
bio01
bio04
bio05
bio12
bio15
BDW
CEC
DES
PTO
SOC
Sersalisia sericea
Climate
Climate + Soil
Soil
AUC = 0.96
TSS = 0.77
AUC = 0.97
TSS = 0.79
AUC = 0.91
TSS = 0.57
Relative importance
bio01
bio04
bio06
bio12
bio15
CEC
CLY
NTO
SOC
TWI

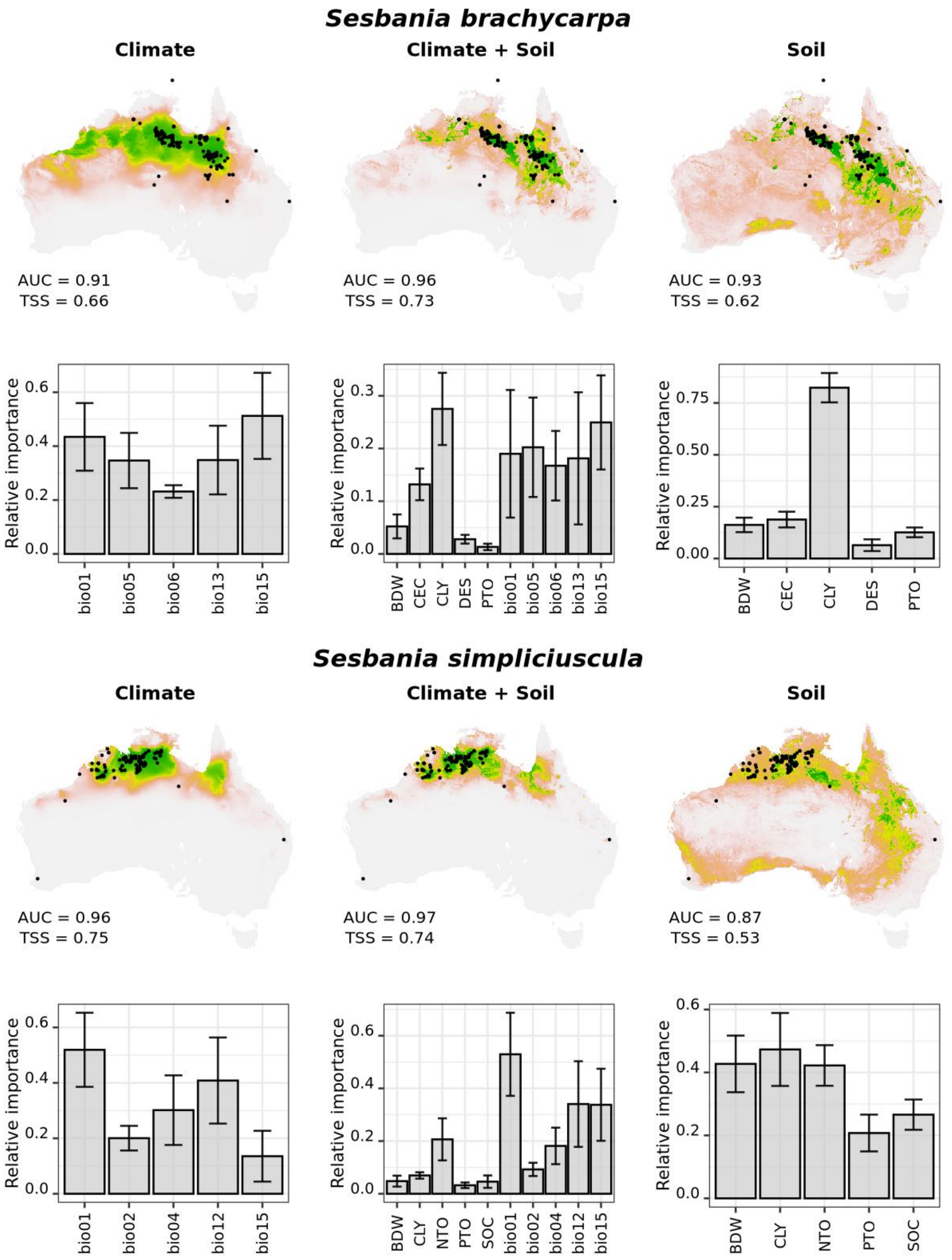
Sesbania brachycarpa
Climate
Climate + Soil
Soil
AUC = 0.91
TSS = 0.66
AUC = 0.96
TSS = 0.73
AUC = 0.93
TSS = 0.62
Relative importance
0.0
0.2
0.4
0.6
bio01
bio05
bio06
bio13
bio15
0.0
0.1
0.2
0.3
BDW
CEC
CLY
DES
PTO
bio01
bio05
bio06
bio13
bio15
0.00
0.25
0.50
0.75
BDW
CEC
CLY
DES
PTO
Sesbania simpliciuscula
Climate
Climate + Soil
Soil
AUC = 0.96
TSS = 0.75
AUC = 0.97
TSS = 0.74
AUC = 0.87
TSS = 0.53
Relative importance
0.0
0.2
0.4
0.6
bio01
bio02
bio04
bio12
bio15
BDW
CLY
NTO
PTO
SOC
bio01
bio02
bio04
bio12
bio15
BDW
CLY
NTO
PTO
SOC

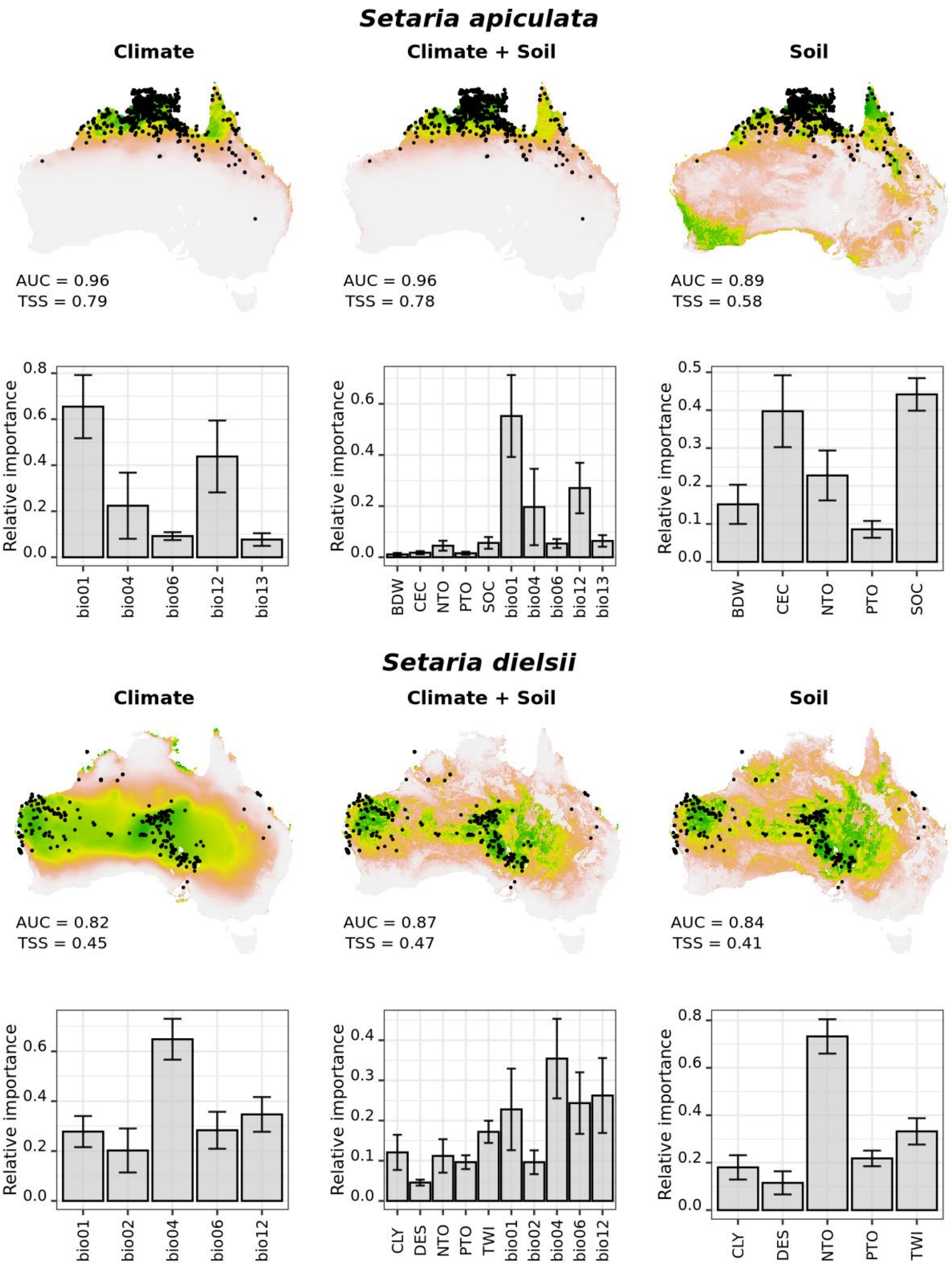
Setaria apiculata
Climate
Climate + Soil
Soil
AUC = 0.96
TSS = 0.79
AUC = 0.96
TSS = 0.78
AUC = 0.89
TSS = 0.58
Relative importance
bio01 bio04 bio06 bio12 bio13
BDW CEC NTO PTO SOC bio01 bio04 bio06 bio12 bio13
BDW CEC NTO PTO SOC
Setaria dielsii
Climate
Climate + Soil
Soil
AUC = 0.82
TSS = 0.45
AUC = 0.87
TSS = 0.47
AUC = 0.84
TSS = 0.41
Relative importance
bio01 bio02 bio04 bio06 bio12
CLY DES NTO PTO TWI bio01 bio02 bio04 bio06 bio12
CLY DES NTO PTO TWI

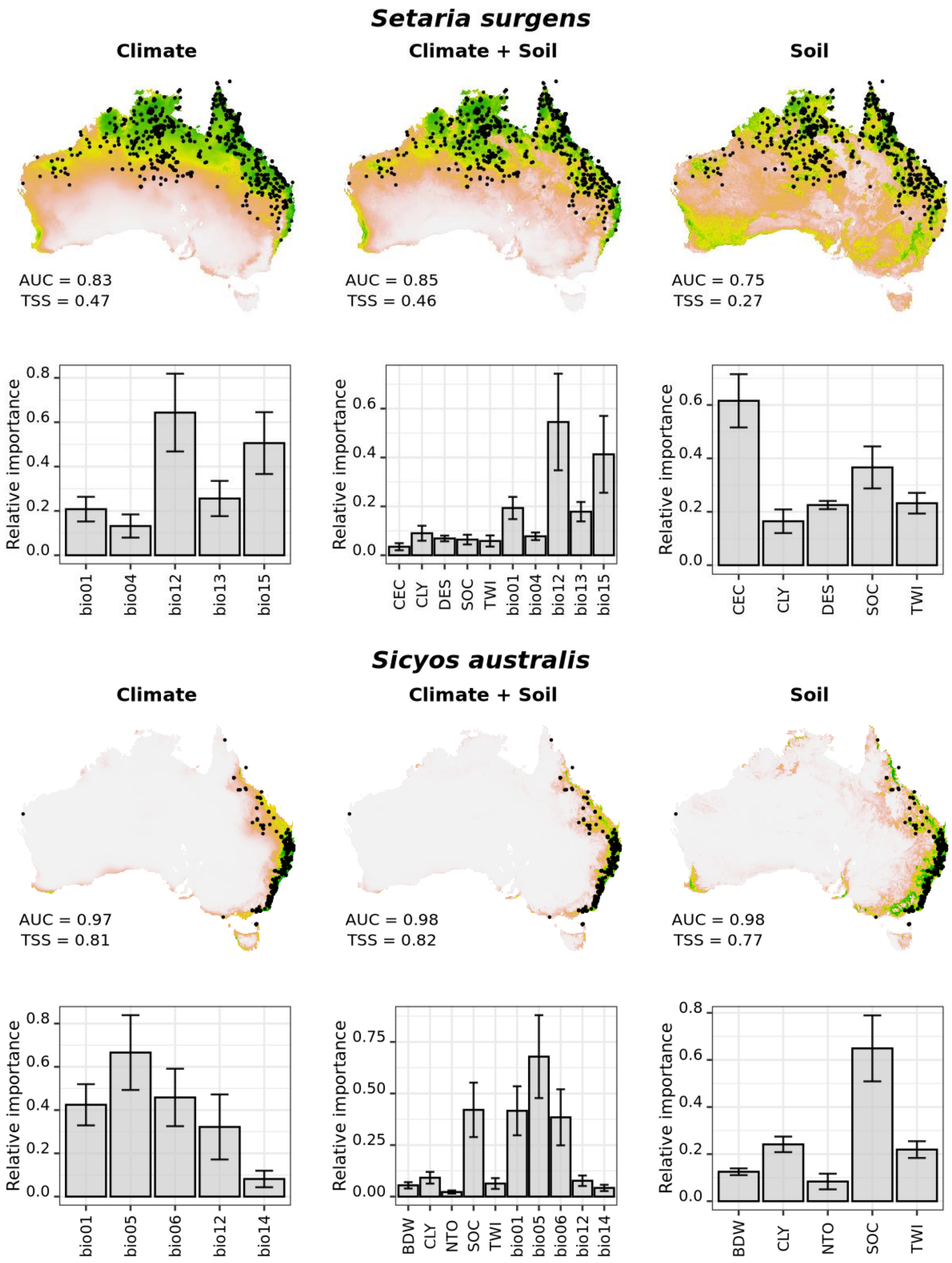
Setaria surgens
Climate
Climate + Soil
Soil
AUC = 0.83
TSS = 0.47
AUC = 0.85
TSS = 0.46
AUC = 0.75
TSS = 0.27
Relative importance
0.0
0.2
0.4
0.6
0.8
bio01
bio04
bio12
bio13
bio15
CEC
CLY
DES
SOC
TWI
Sicyos australis
Climate
Climate + Soil
Soil
AUC = 0.97
TSS = 0.81
AUC = 0.98
TSS = 0.82
AUC = 0.98
TSS = 0.77
0.00
0.25
0.50
0.75
bio05
bio06
bio14
BDW
NTO

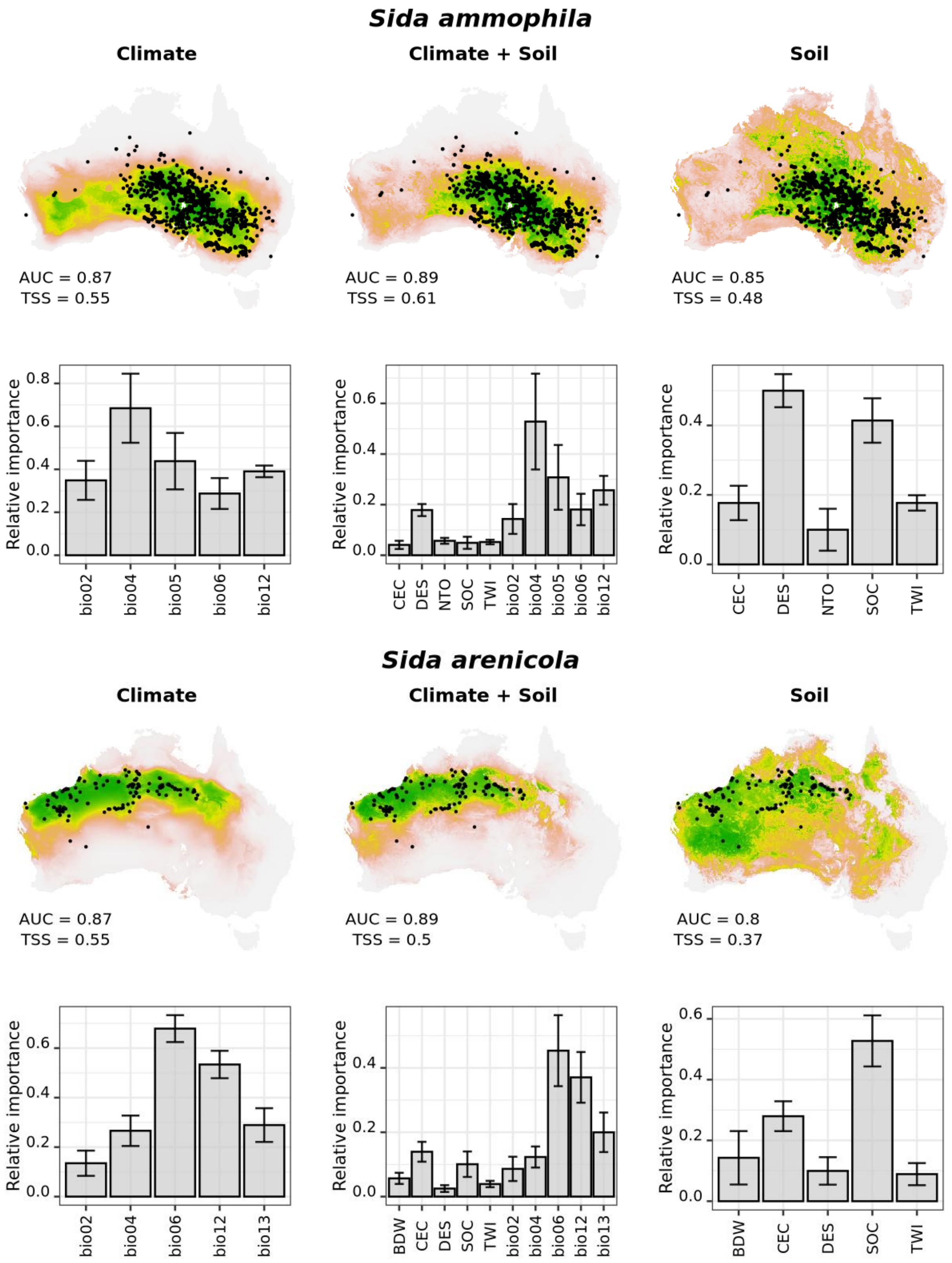
Sida ammophila
Climate
Climate + Soil
Soil
AUC = 0.87
TSS = 0.55
AUC = 0.89
TSS = 0.61
AUC = 0.85
TSS = 0.48
Relative importance
bio02
bio04
bio05
bio06
bio12
CEC
DES
NTO
SOC
TWI
Sida arenicola
Climate
Climate + Soil
Soil
AUC = 0.87
TSS = 0.55
AUC = 0.89
TSS = 0.5
AUC = 0.8
TSS = 0.37
Relative importance
bio02
bio04
bio06
bio12
bio13
BDW
CEC
DES
SOC
TWI

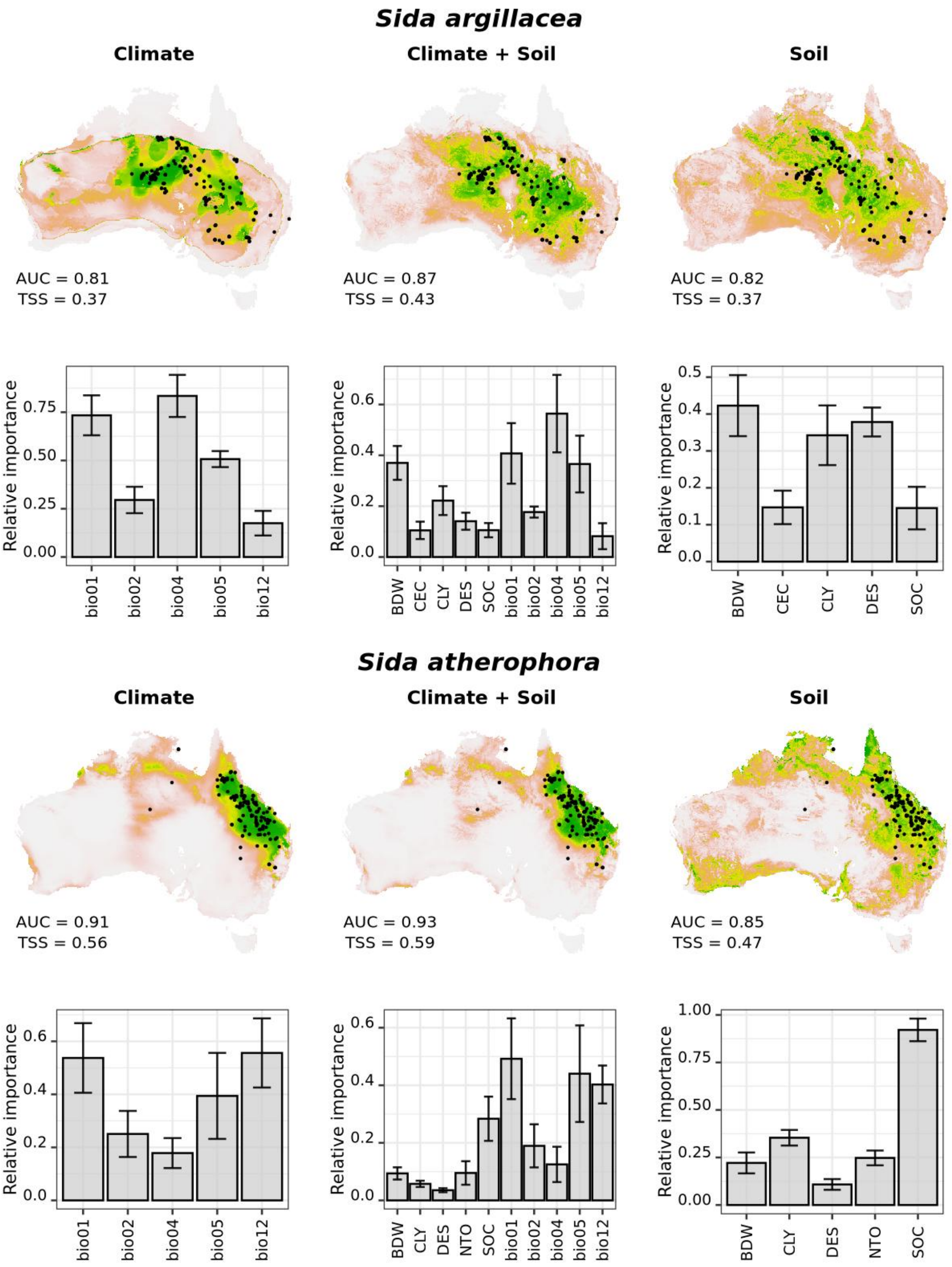
Sida argillacea
Climate
Climate + Soil
Soil
AUC = 0.81
TSS = 0.37
AUC = 0.87
TSS = 0.43
AUC = 0.82
TSS = 0.37
Relative importance
bio01
bio02
bio04
bio05
bio12
BDW
CEC
CLY
DES
SOC
Sida atherophora
Climate
Climate + Soil
Soil
AUC = 0.91
TSS = 0.56
AUC = 0.93
TSS = 0.59
AUC = 0.85
TSS = 0.47
Relative importance
bio01
bio02
bio04
bio05
bio12
BDW
CLY
DES
NTO
SOC

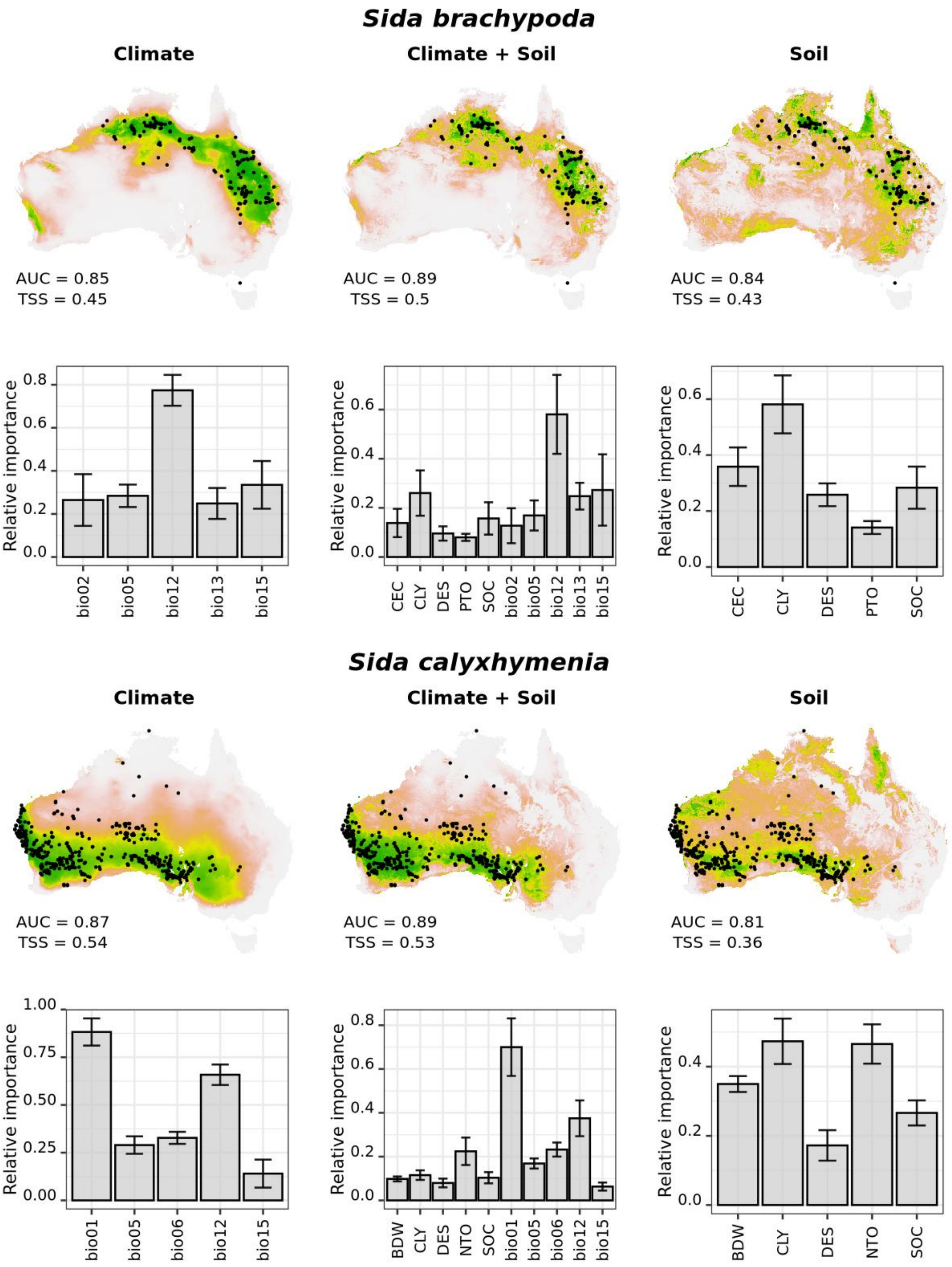
Sida brachypoda
Climate
Climate + Soil
Soil
AUC = 0.85
TSS = 0.45
AUC = 0.89
TSS = 0.5
AUC = 0.84
TSS = 0.43
Relative importance
0.0
0.2
0.4
0.6
0.8
bio02
bio05
bio12
bio13
bio15
CEC
CLY
DES
PTO
SOC
Sida calyxhymenia
Climate
Climate + Soil
Soil
AUC = 0.87
TSS = 0.54
AUC = 0.89
TSS = 0.53
AUC = 0.81
TSS = 0.36
Relative importance
0.00
0.25
0.50
0.75
1.00
bio01
bio05
bio06
bio12
bio15
BDW
CLY
DES
NTO
SOC

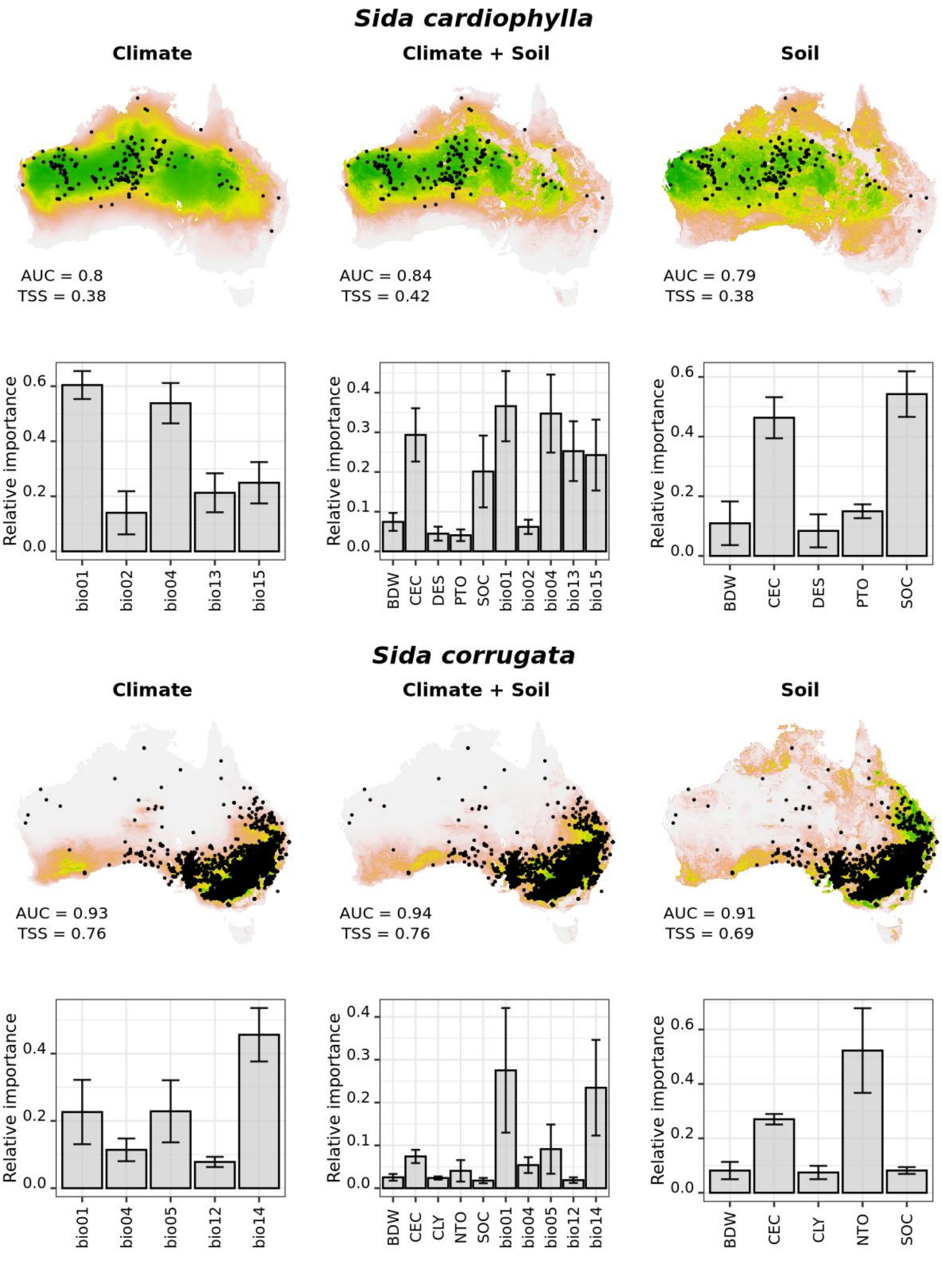
Sida cardiophylla
Climate
Climate + Soil
Soil
AUC = 0.8
TSS = 0.38
AUC = 0.84
TSS = 0.42
AUC = 0.79
TSS = 0.38
Relative importance
bio01
bio02
bio04
bio13
bio15
BDW
CEC
DES
PTO
SOC
Sida corrugata
Climate
Climate + Soil
Soil
AUC = 0.93
TSS = 0.76
AUC = 0.94
TSS = 0.76
AUC = 0.91
TSS = 0.69
Relative importance
bio01
bio04
bio05
bio12
bio14
BDW
CEC
CLY
NTO
SOC

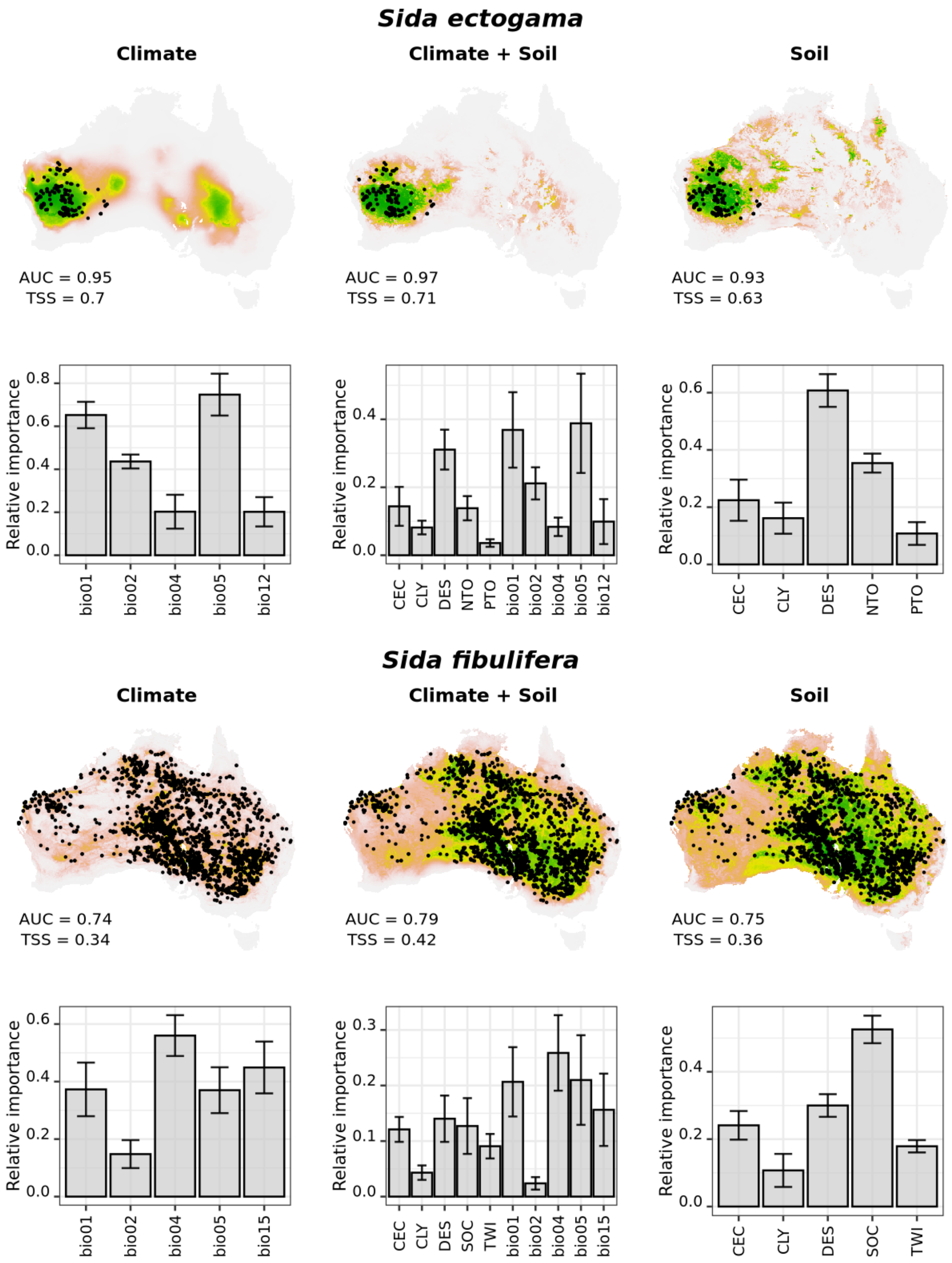
Sida ectogama
Climate
Climate + Soil
Soil
AUC = 0.95
TSS = 0.7
AUC = 0.97
TSS = 0.71
AUC = 0.93
TSS = 0.63
Relative importance
bio01
bio02
bio04
bio05
bio12
CEC
CLY
DES
NTO
PTO
Sida fibulifera
AUC = 0.74
TSS = 0.34
AUC = 0.79
TSS = 0.42
AUC = 0.75
TSS = 0.36
bio15
SOC
TWI

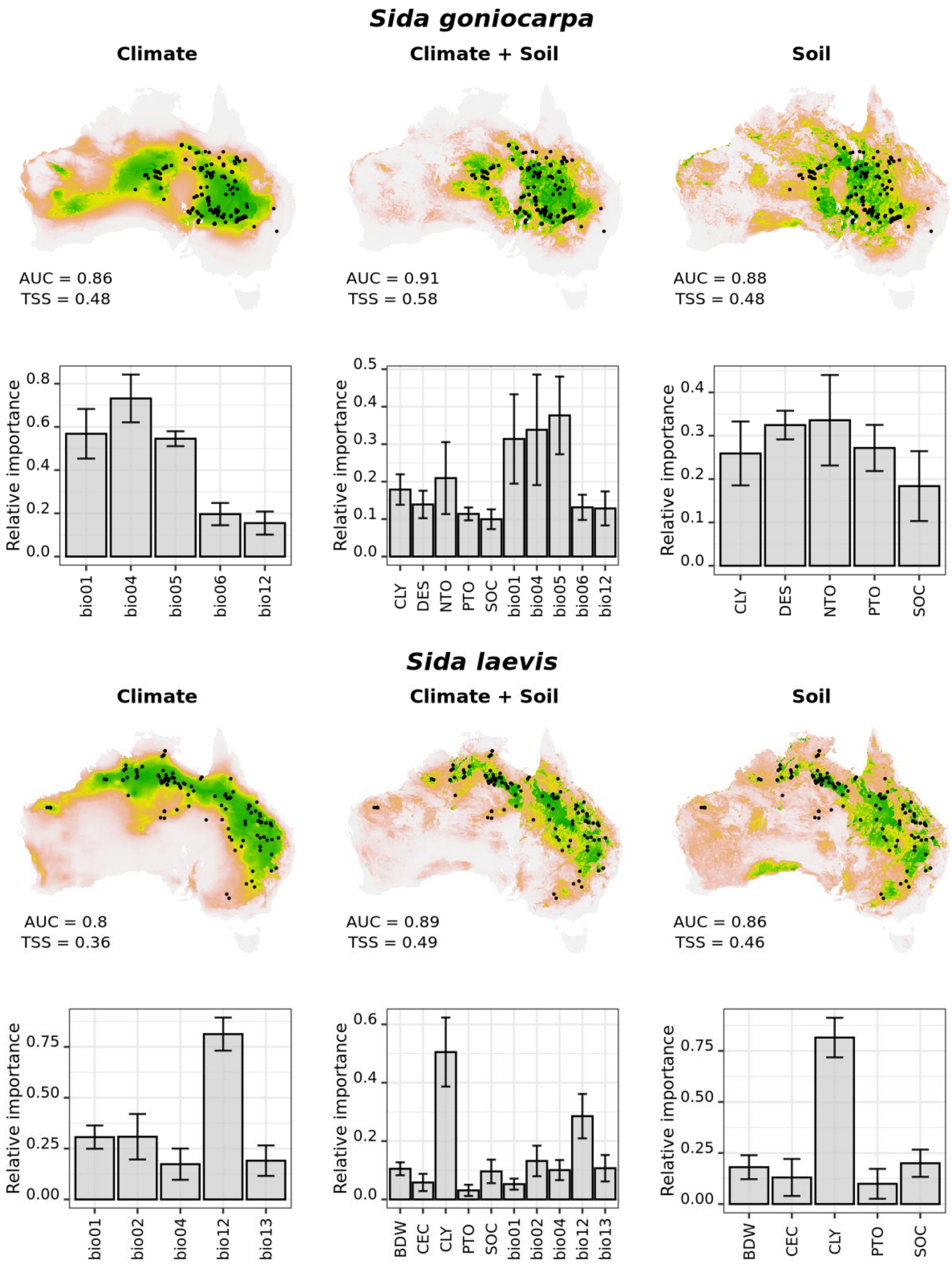
Sida goniocarpa
Climate
Climate + Soil
Soil
AUC = 0.86
TSS = 0.48
AUC = 0.91
TSS = 0.58
AUC = 0.88
TSS = 0.48
Relative importance
bio01 bio04 bio05 bio06 bio12
CLY DES NTO PTO SOC bio01 bio04 bio05 bio06 bio12
CLY DES NTO PTO SOC
Sida laevis
Climate
Climate + Soil
Soil
AUC = 0.8
TSS = 0.36
AUC = 0.89
TSS = 0.49
AUC = 0.86
TSS = 0.46
Relative importance
bio01 bio02 bio04 bio12 bio13
BDW CEC CLY PTO SOC bio01 bio02 bio04 bio12 bio13
BDW CEC CLY PTO SOC

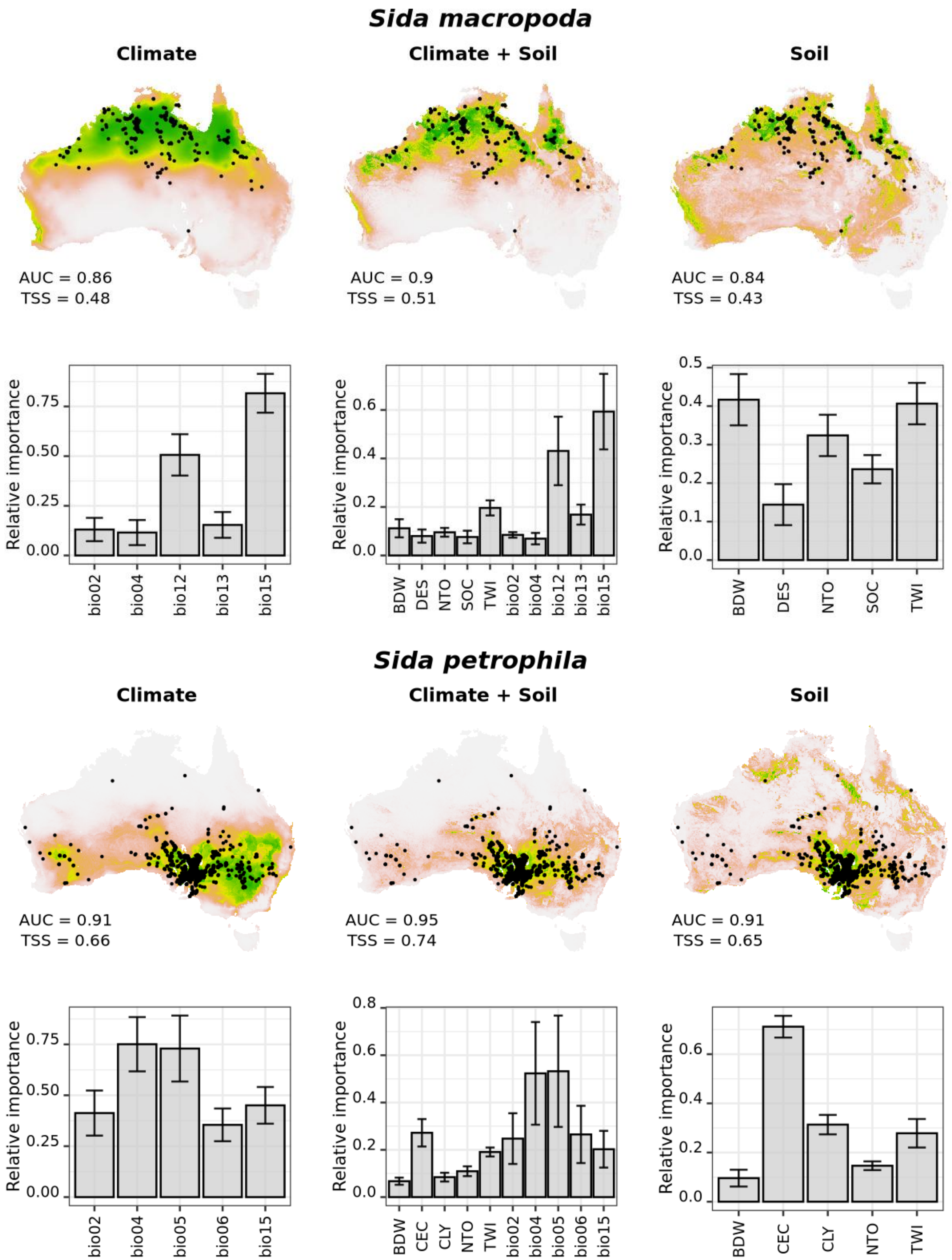

Sida macropoda
Climate
Climate + Soil
Soil
AUC = 0.86
TSS = 0.48
AUC = 0.9
TSS = 0.51
AUC = 0.84
TSS = 0.43
Relative importance
0.00
0.25
0.50
0.75
bio02
bio04
bio12
bio13
bio15
0.0
0.2
0.4
0.6
BDW
DES
NTO
SOC
TWI
bio02
bio04
bio12
bio13
bio15
0.0
0.1
0.2
0.3
0.4
0.5
BDW
DES
NTO
SOC
TWI
Sida petrophila
Climate
Climate + Soil
Soil
AUC = 0.91
TSS = 0.66
AUC = 0.95
TSS = 0.74
AUC = 0.91
TSS = 0.65
Relative importance
0.00
0.25
0.50
0.75
bio02
bio04
bio05
bio06
bio15
0.0
0.2
0.4
0.6
0.8
BDW
CEC
CLY
NTO
TWI
bio02
bio04
bio05
bio06
bio15
0.0
0.2
0.4
0.6
BDW
CEC
CLY
NTO
TWI

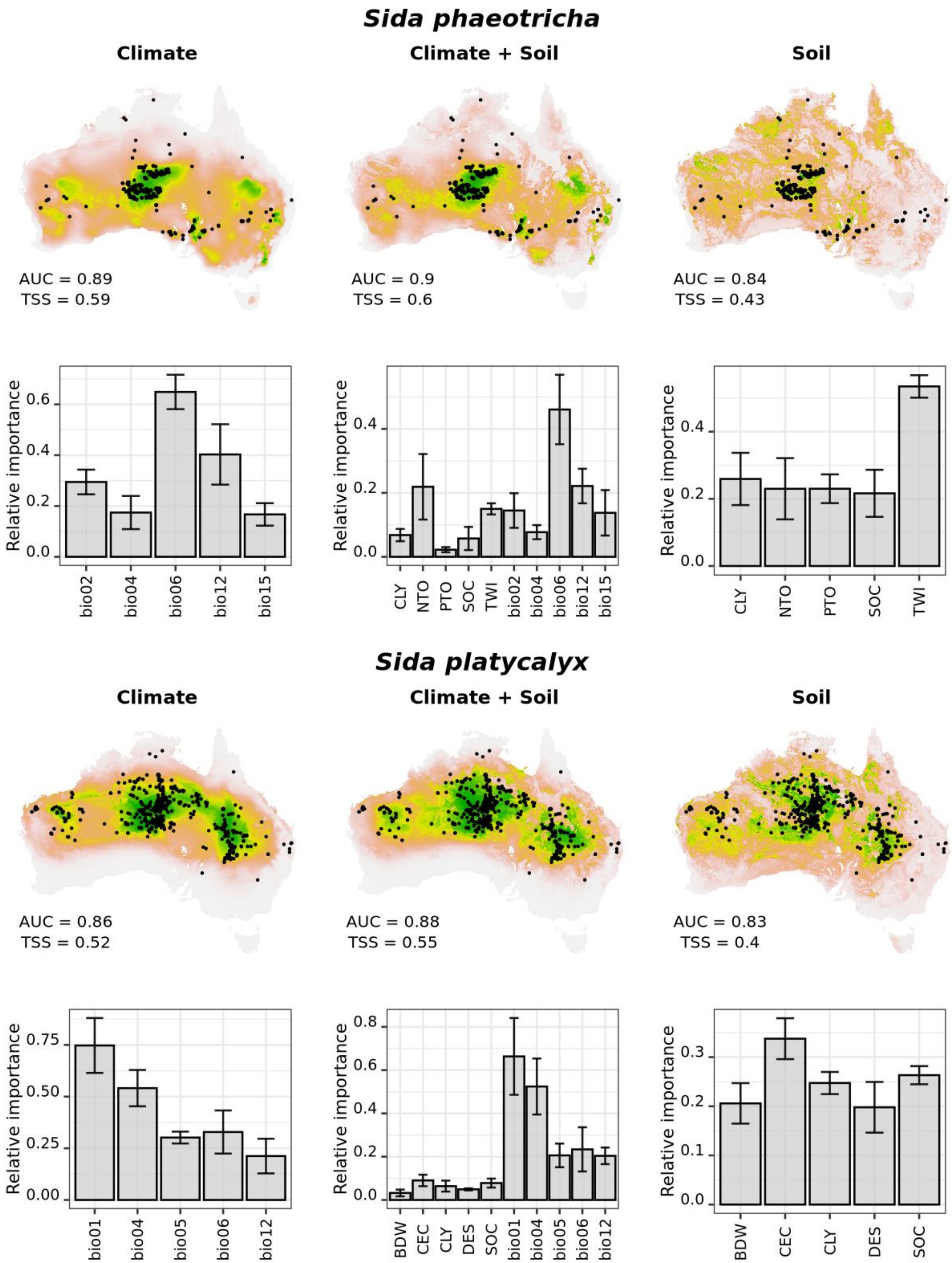
Sida phaeotricha
Climate
Climate + Soil
Soil
AUC = 0.89
TSS = 0.59
AUC = 0.9
TSS = 0.6
AUC = 0.84
TSS = 0.43
Relative importance
0.0
0.2
0.4
0.6
bio02
bio04
bio06
bio12
bio15
CLY
NTO
PTO
SOC
TWI
Sida platycalyx
AUC = 0.86
TSS = 0.52
AUC = 0.88
TSS = 0.55
AUC = 0.83
TSS = 0.4
0.00
0.25
0.50
0.75
0.8
0.1
0.3
bio01
bio05
BDW
CEC
DES

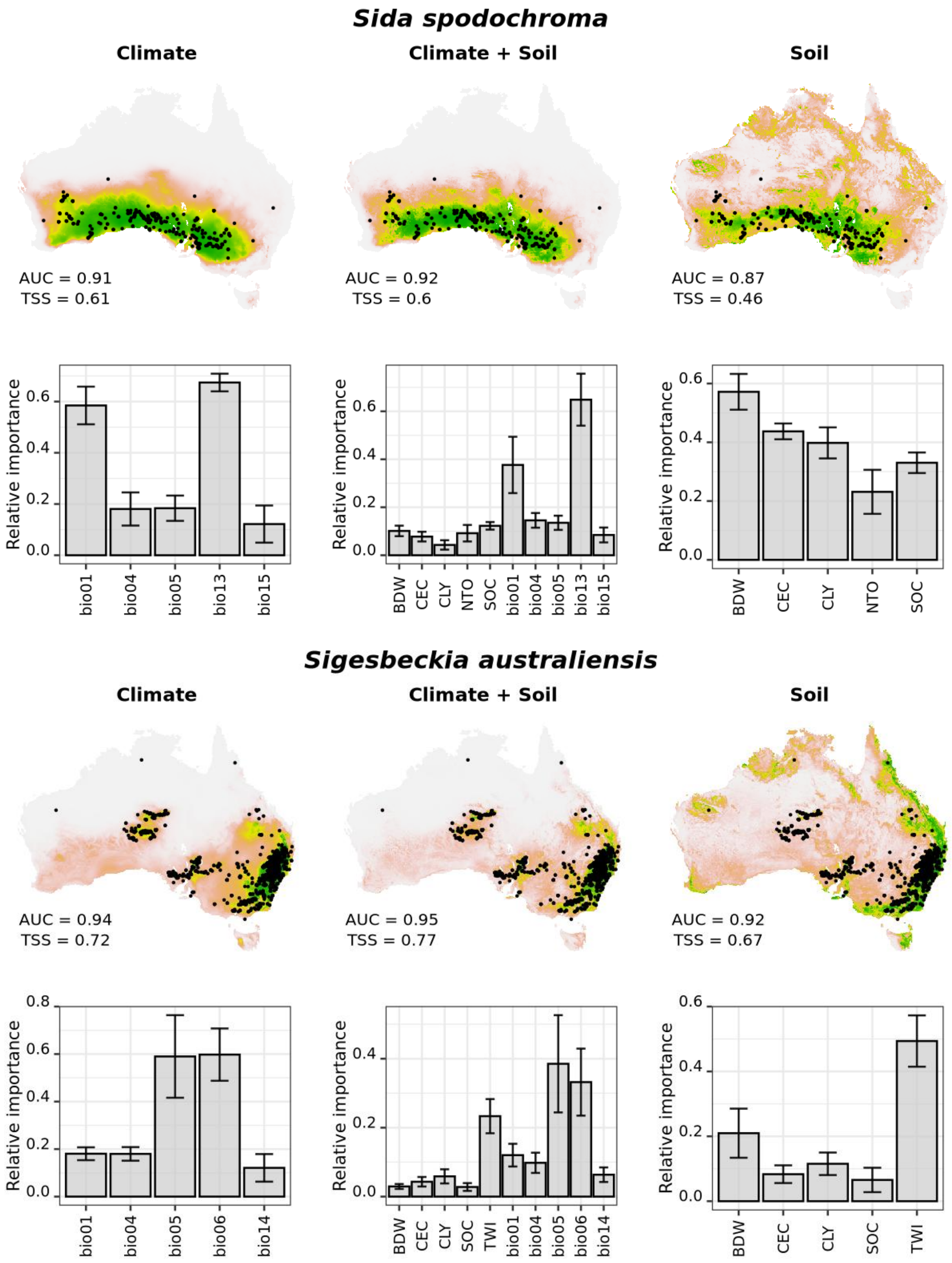

Sida spodochroma
Climate
Climate + Soil
Soil
AUC = 0.91
TSS = 0.61
AUC = 0.92
TSS = 0.6
AUC = 0.87
TSS = 0.46
Relative importance
bio01
bio04
bio05
bio13
bio15
BDW
CEC
CLY
NTO
SOC
Sigesbeckia australiensis
Climate
Climate + Soil
Soil
AUC = 0.94
TSS = 0.72
AUC = 0.95
TSS = 0.77
AUC = 0.92
TSS = 0.67
bio06
bio14
TWI

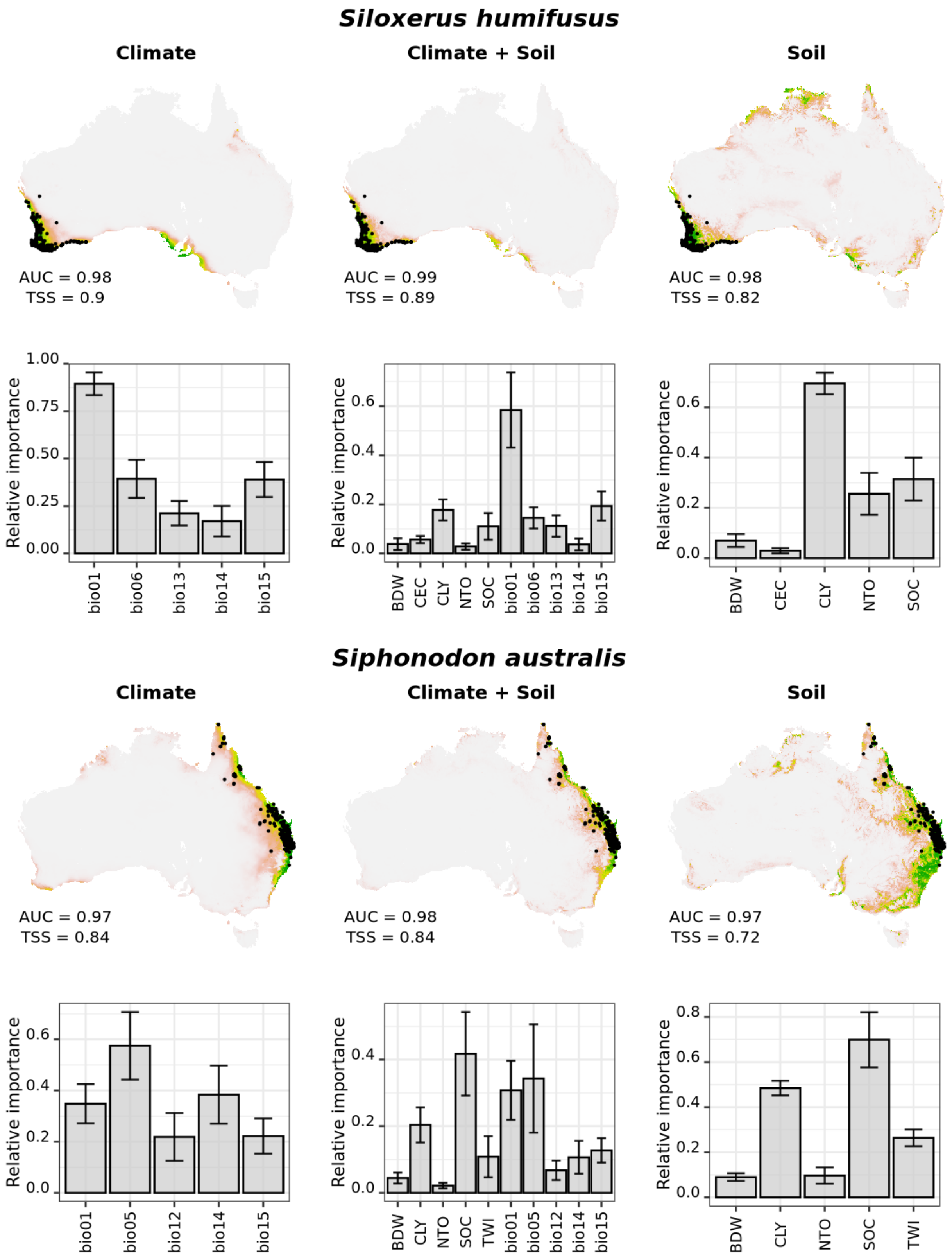
Siloxerus humifusus
Climate
Climate + Soil
Soil
AUC = 0.98
TSS = 0.9
AUC = 0.99
TSS = 0.89
AUC = 0.98
TSS = 0.82
Relative importance
1.00
0.75
0.50
0.25
0.00
bio01
bio06
bio13
bio14
bio15
0.6
0.4
0.2
0.0
BDW
CEC
CLY
NTO
SOC
bio01
bio06
bio13
bio14
bio15
BDW
CEC
CLY
NTO
SOC
Siphonodon australis
Climate
Climate + Soil
Soil
AUC = 0.97
TSS = 0.84
AUC = 0.98
TSS = 0.84
AUC = 0.97
TSS = 0.72
Relative importance
0.6
0.4
0.2
0.0
bio01
bio05
bio12
bio14
bio15
BDW
CLY
NTO
SOC
TWI
bio01
bio05
bio12
bio14
bio15
0.8
BDW
CLY
NTO
SOC
TWI

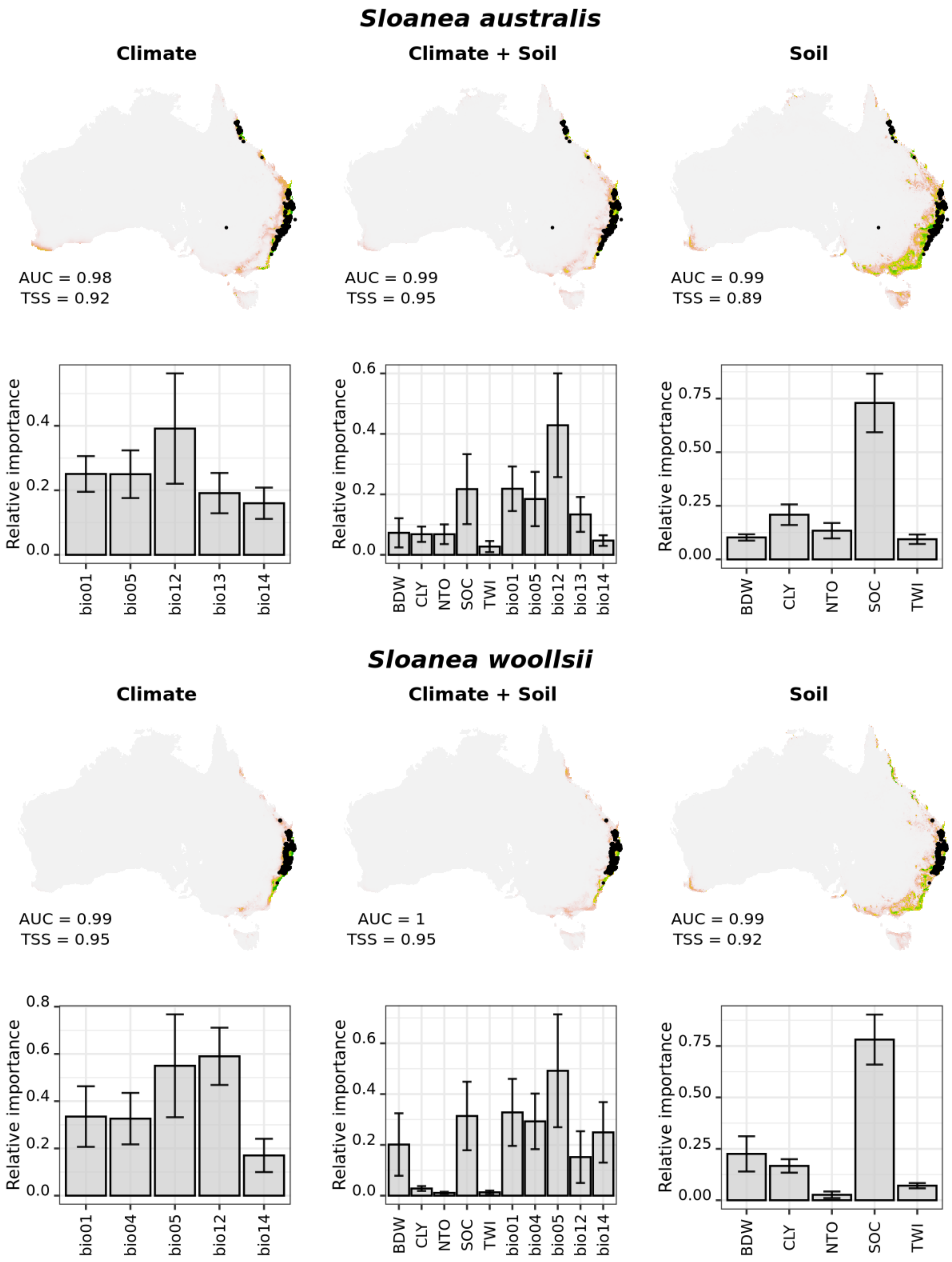

Sloanea australis
Climate
Climate + Soil
Soil
AUC = 0.98
TSS = 0.92
AUC = 0.99
TSS = 0.95
AUC = 0.99
TSS = 0.89
Relative importance
bio01
bio05
bio12
bio13
bio14
BDW
CLY
NTO
SOC
TWI
Sloanea woollsii
Climate
Climate + Soil
Soil
AUC = 0.99
TSS = 0.95
AUC = 1
TSS = 0.95
AUC = 0.99
TSS = 0.92
Relative importance
bio01
bio04
bio05
bio12
bio14
BDW
CLY
NTO
SOC
TWI

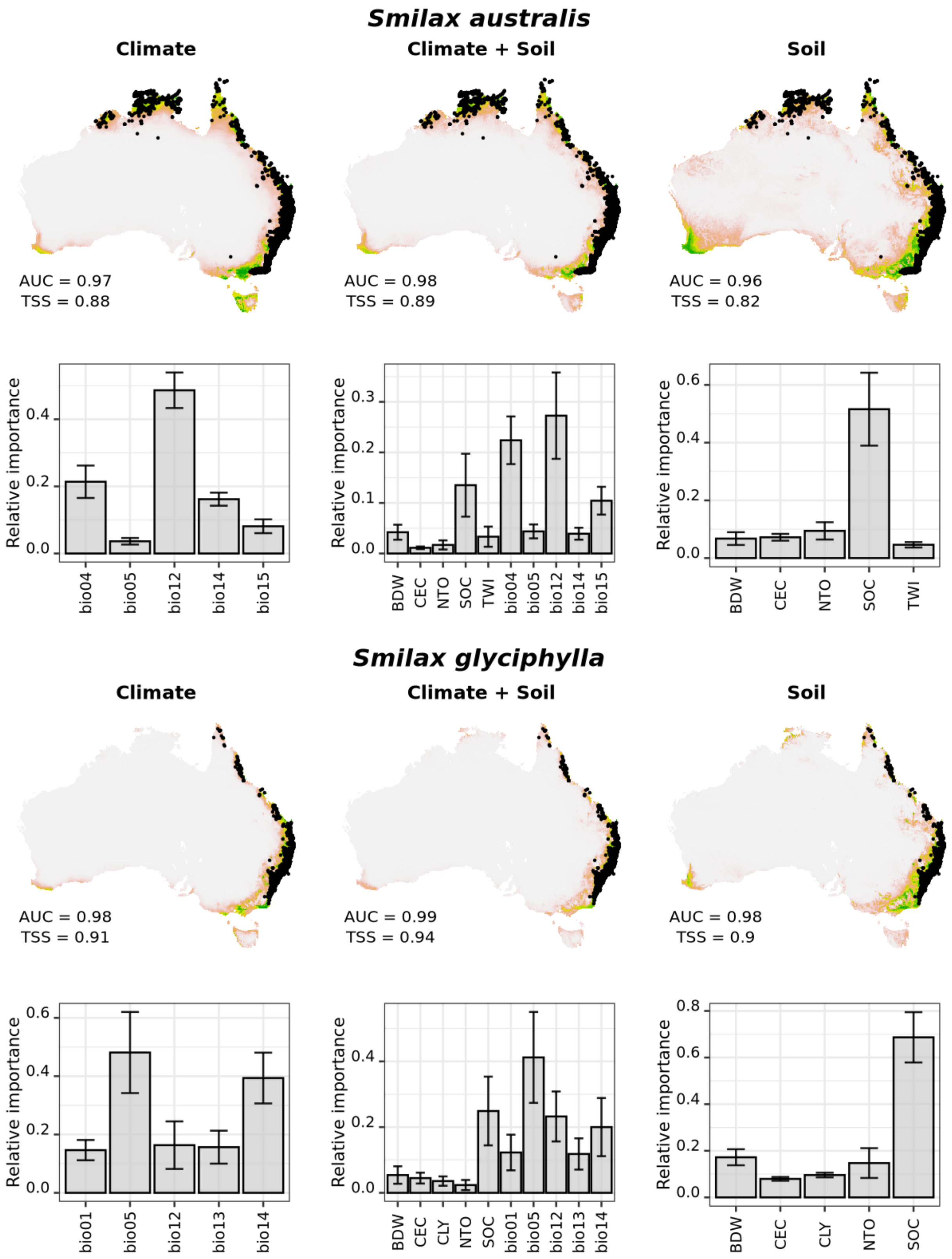

Smilax australis
Climate
Climate + Soil
Soil
AUC = 0.97
TSS = 0.88
AUC = 0.98
TSS = 0.89
AUC = 0.96
TSS = 0.82
Relative importance
bio04
bio05
bio12
bio14
bio15
BDW
CEC
NTO
SOC
TWI
Smilax glyciphylla
Climate
Climate + Soil
Soil
AUC = 0.98
TSS = 0.91
AUC = 0.99
TSS = 0.94
AUC = 0.98
TSS = 0.9
bio01
bio05
bio12
bio13
bio14
CLY

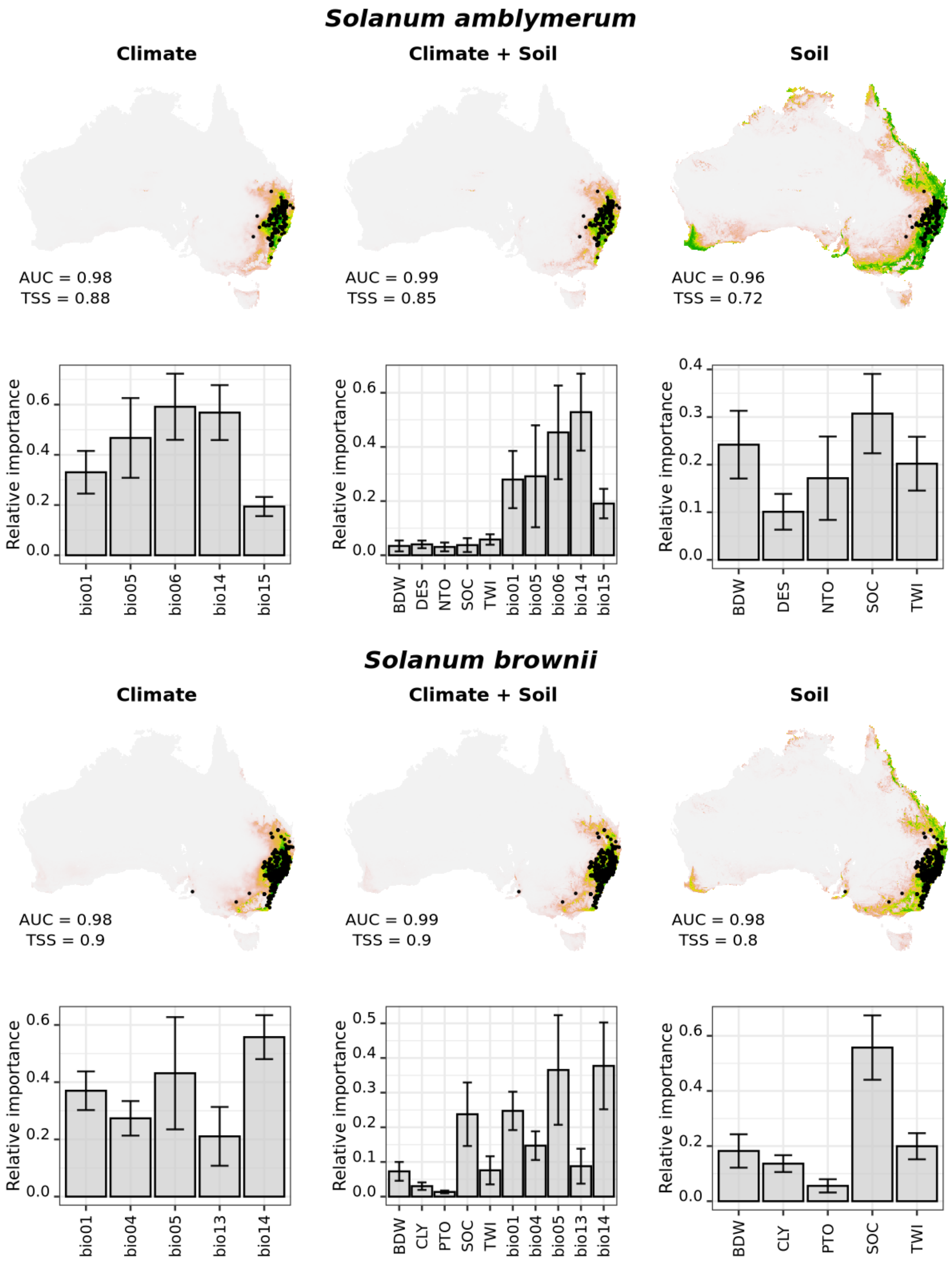

Solanum amblymerum
Climate
Climate + Soil
Soil
AUC = 0.98
TSS = 0.88
AUC = 0.99
TSS = 0.85
AUC = 0.96
TSS = 0.72
Relative importance
bio01
bio05
bio06
bio14
bio15
BDW
DES
NTO
SOC
TWI
Solanum brownii
Climate
Climate + Soil
Soil
AUC = 0.98
TSS = 0.9
AUC = 0.99
TSS = 0.9
AUC = 0.98
TSS = 0.8
Relative importance
bio01
bio04
bio05
bio13
bio14
BDW
CLY
PTO
SOC
TWI

Solanum callosum

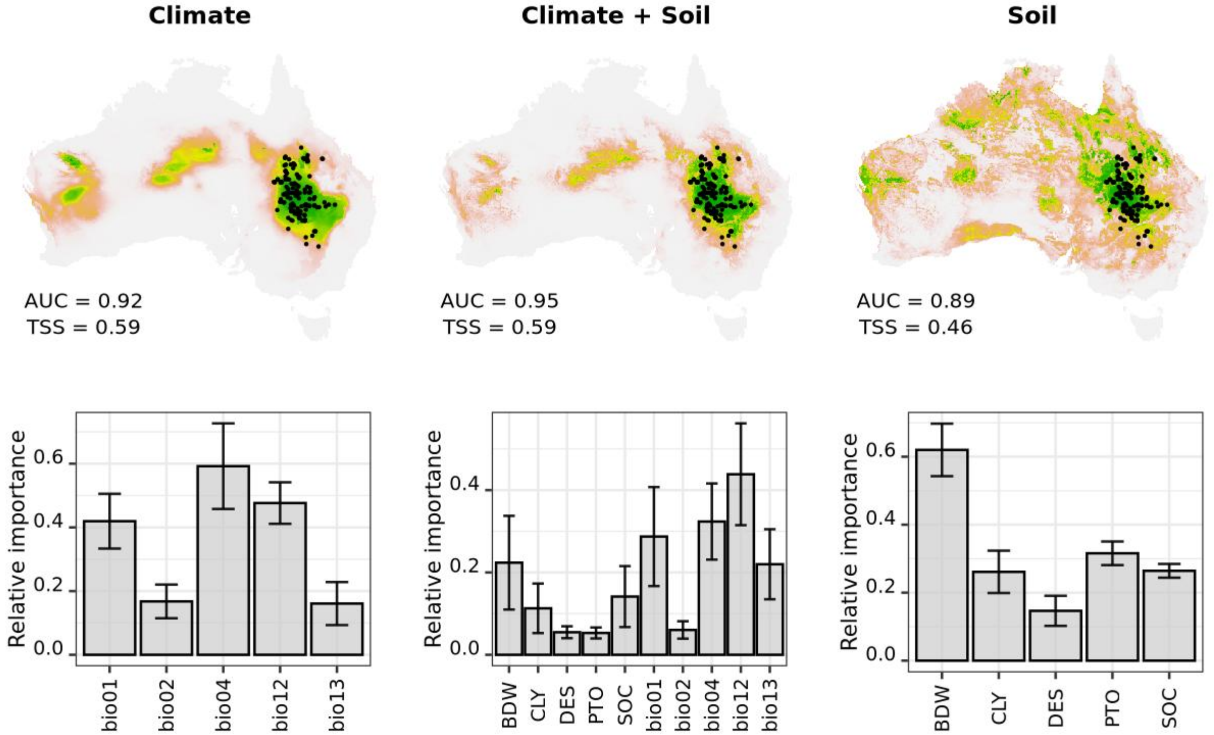

Solanum campanulatum

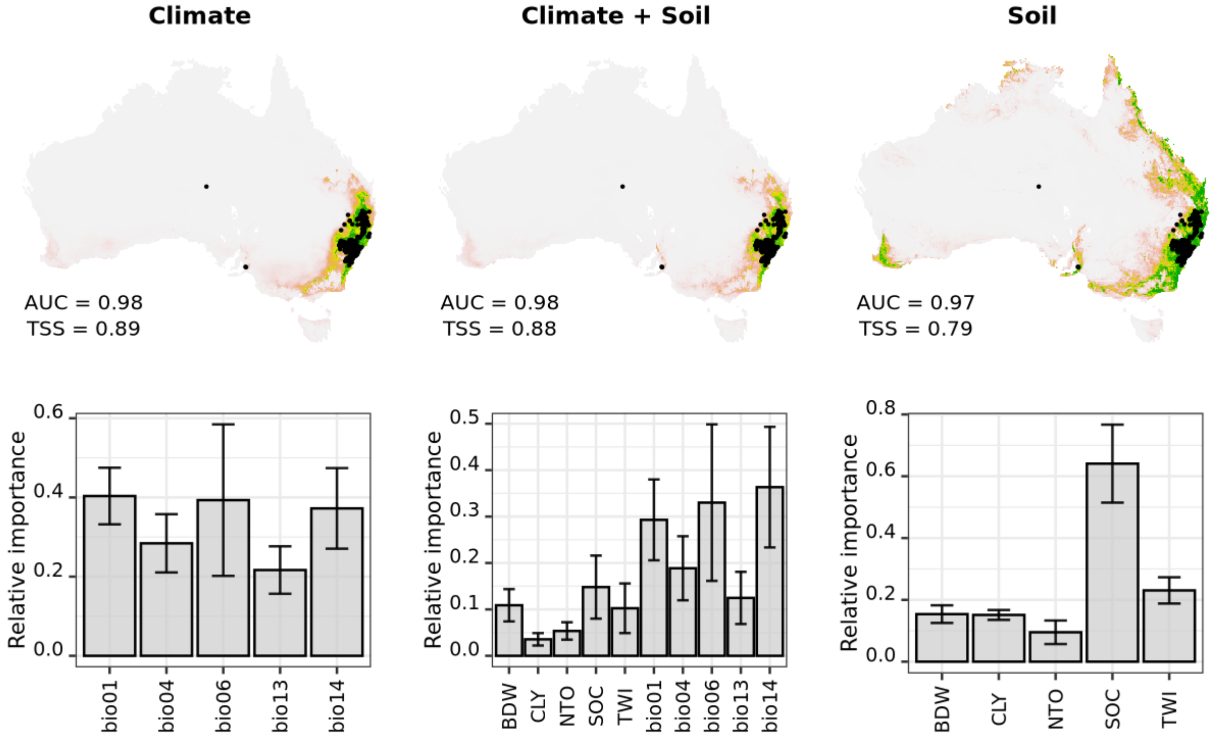

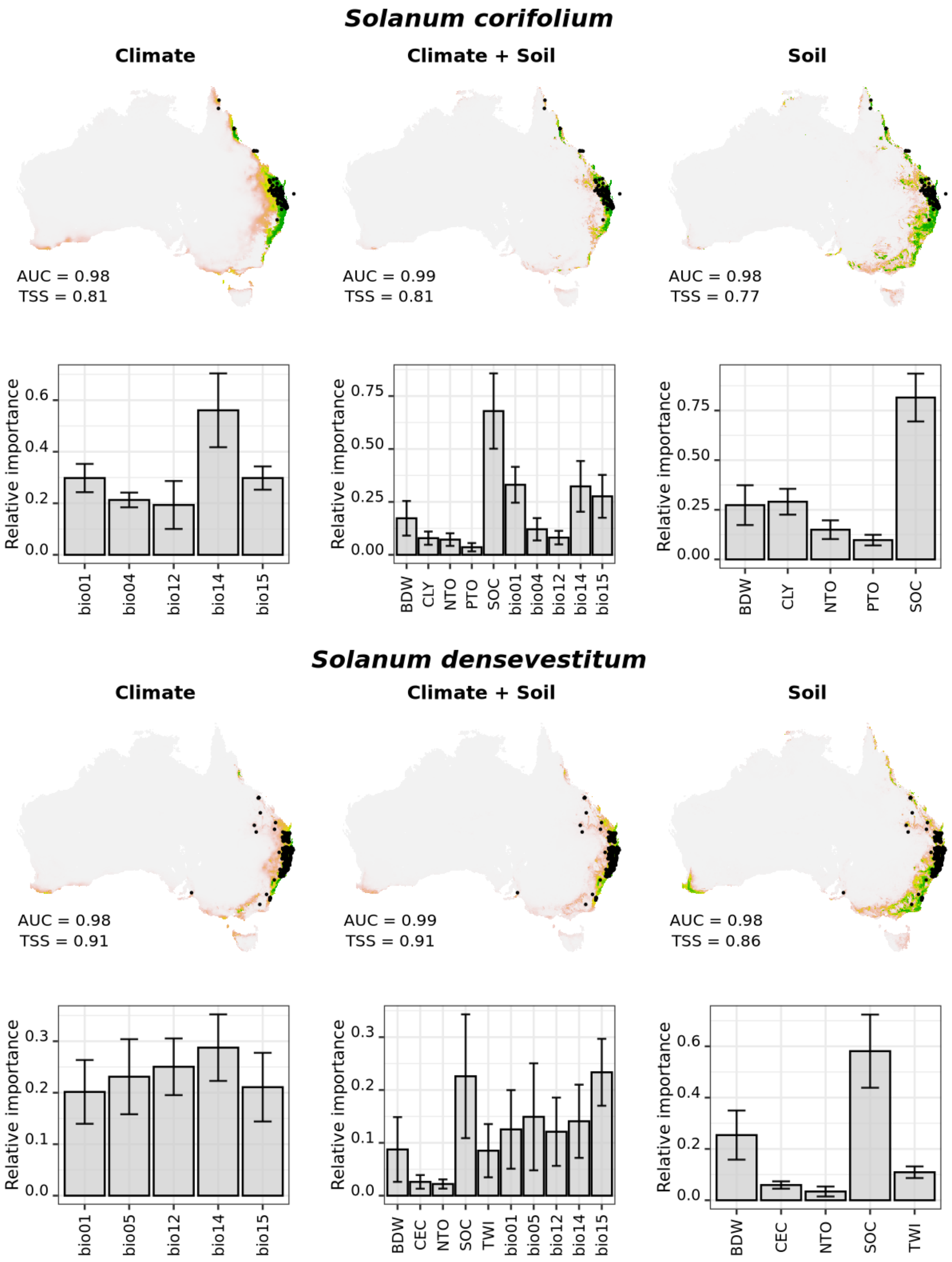

Solanum corifolium
Climate
Climate + Soil
Soil
AUC = 0.98
TSS = 0.81
AUC = 0.99
TSS = 0.81
AUC = 0.98
TSS = 0.77
Relative importance
0.0
0.2
0.4
0.6
bio01
bio04
bio12
bio14
bio15
0.00
0.25
0.50
0.75
BDW
CLY
NTO
PTO
SOC
bio01
bio04
bio12
bio14
bio15
BDW
CLY
NTO
PTO
SOC
Solanum densevestitum
Climate
Climate + Soil
Soil
AUC = 0.98
TSS = 0.91
AUC = 0.99
TSS = 0.91
AUC = 0.98
TSS = 0.86
Relative importance
0.0
0.1
0.2
0.3
bio01
bio05
bio12
bio14
bio15
BDW
CEC
NTO
SOC
TWI
bio01
bio05
bio12
bio14
bio15
0.4
0.6
BDW
CEC
NTO
SOC
TWI

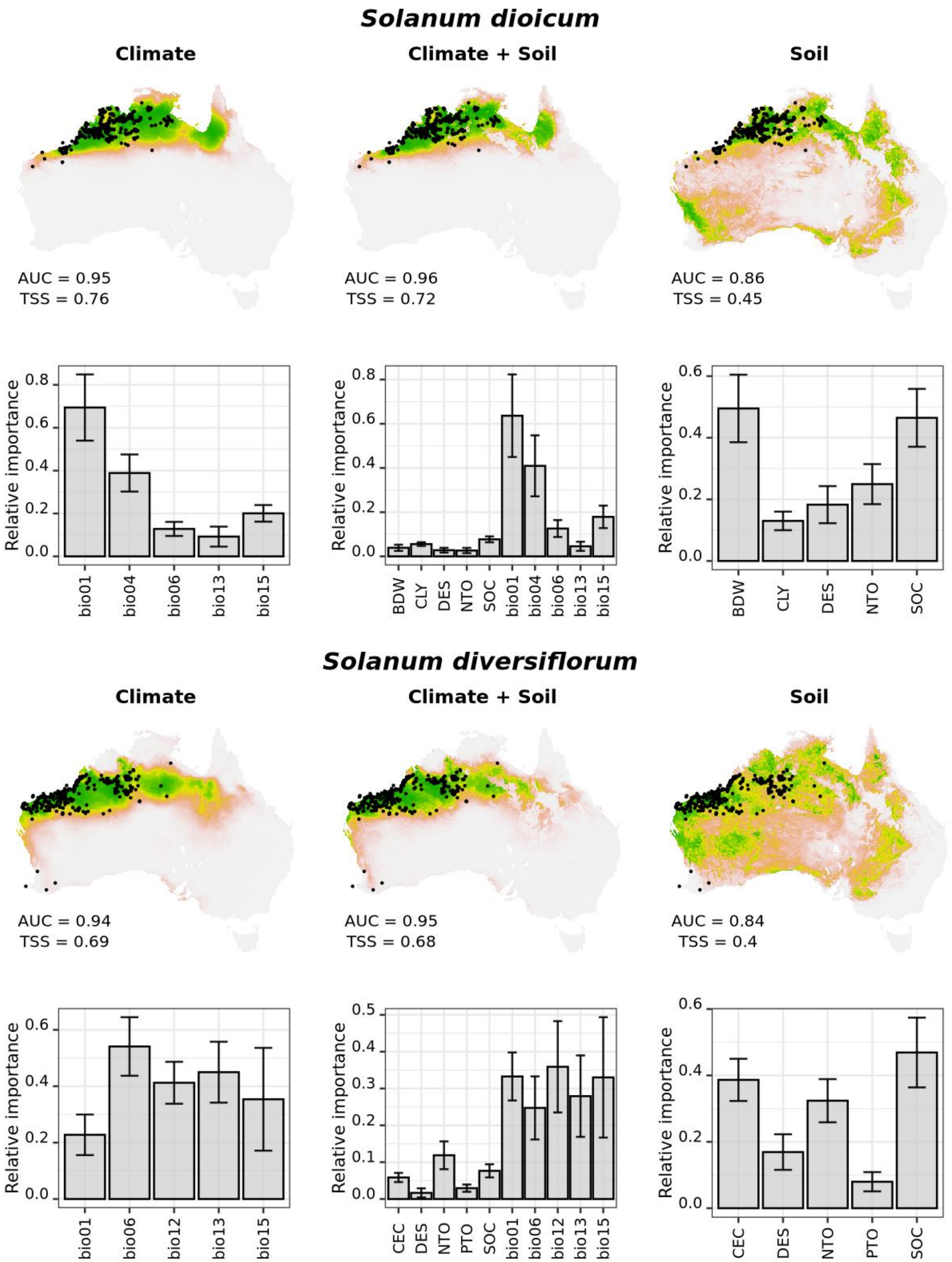
Solanum dioicum
Climate
Climate + Soil
Soil
AUC = 0.95
TSS = 0.76
AUC = 0.96
TSS = 0.72
AUC = 0.86
TSS = 0.45
Relative importance
bio01
bio04
bio06
bio13
bio15
BDW
CLY
DES
NTO
SOC
Solanum diversiflorum
Climate
Climate + Soil
Soil
AUC = 0.94
TSS = 0.69
AUC = 0.95
TSS = 0.68
AUC = 0.84
TSS = 0.4
Relative importance
bio01
bio06
bio12
bio13
bio15
CEC
DES
NTO
PTO
SOC

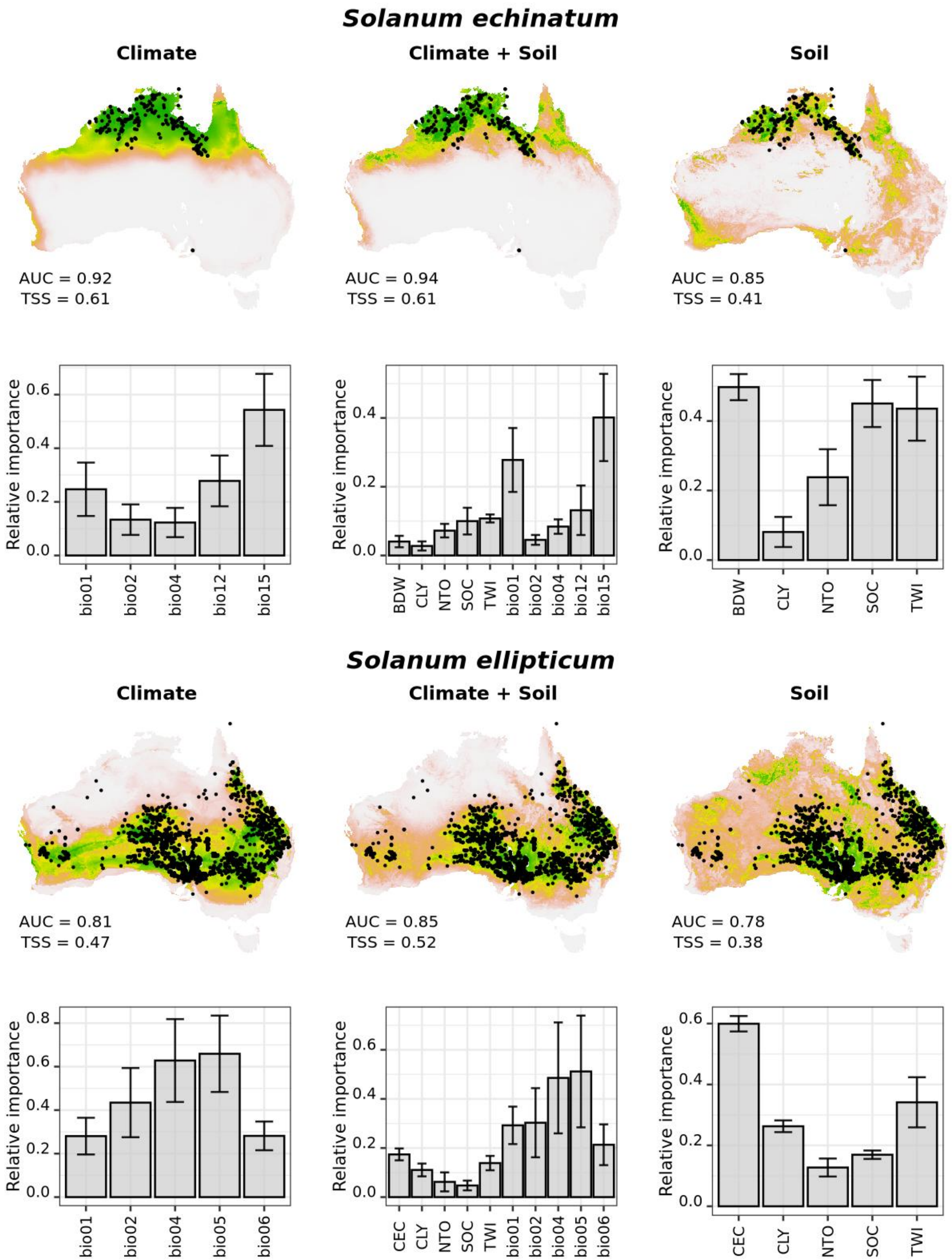
Solanum echinatum
Climate
Climate + Soil
Soil
AUC = 0.92
TSS = 0.61
AUC = 0.94
TSS = 0.61
AUC = 0.85
TSS = 0.41
Relative importance
bio01
bio02
bio04
bio12
bio15
BDW
CLY
NTO
SOC
TWI
Solanum ellipticum
Climate
Climate + Soil
Soil
AUC = 0.81
TSS = 0.47
AUC = 0.85
TSS = 0.52
AUC = 0.78
TSS = 0.38
Relative importance
bio01
bio02
bio04
bio05
bio06
CEC
CLY
NTO
SOC
TWI

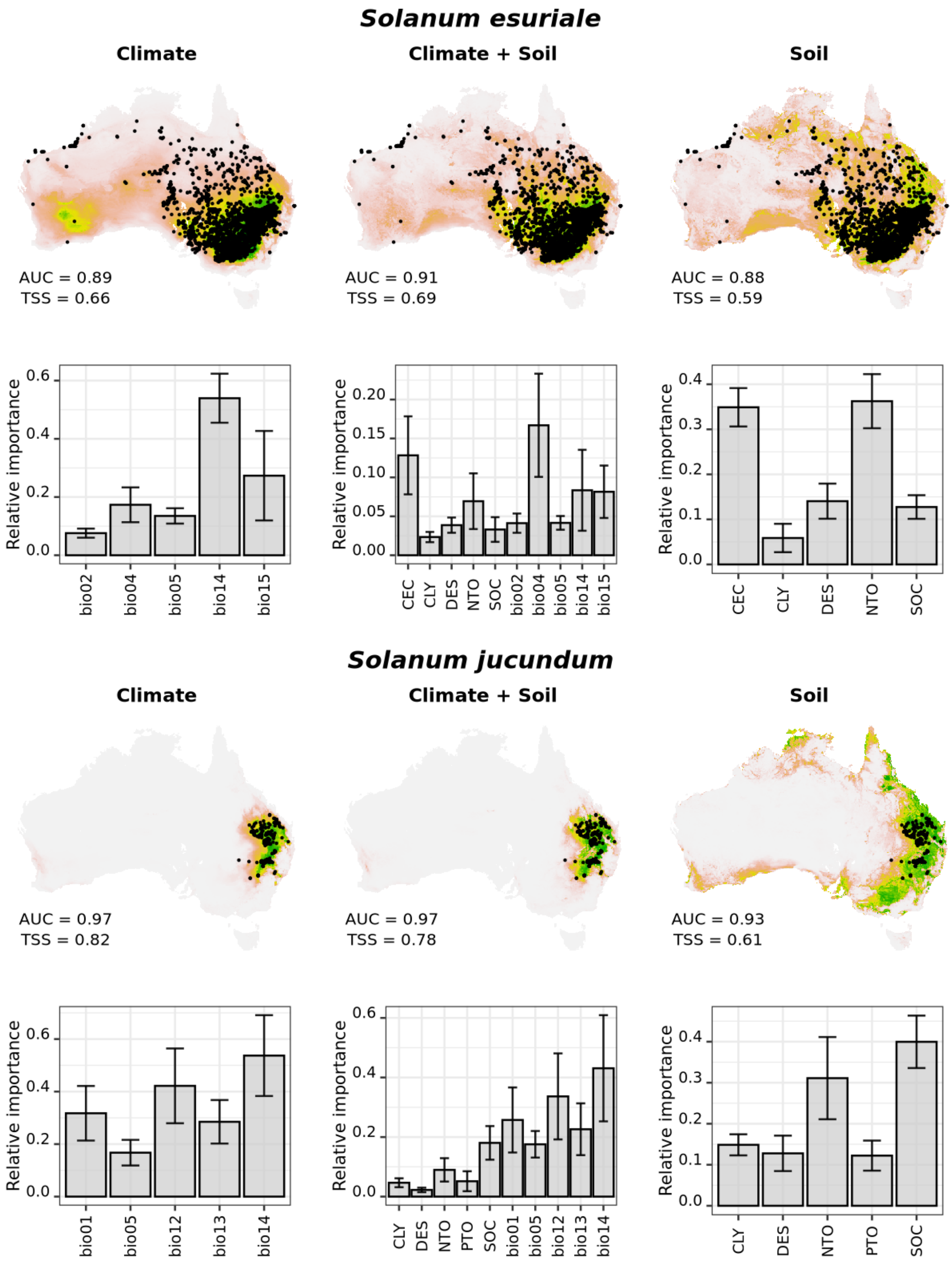

Solanum esuriale
Climate
Climate + Soil
Soil
AUC = 0.89
TSS = 0.66
AUC = 0.91
TSS = 0.69
AUC = 0.88
TSS = 0.59
Relative importance
bio02
bio04
bio05
bio14
bio15
CEC
CLY
DES
NTO
SOC
Solanum jucundum
AUC = 0.97
TSS = 0.82
AUC = 0.97
TSS = 0.78
AUC = 0.93
TSS = 0.61
bio01
bio12
bio13
PTO

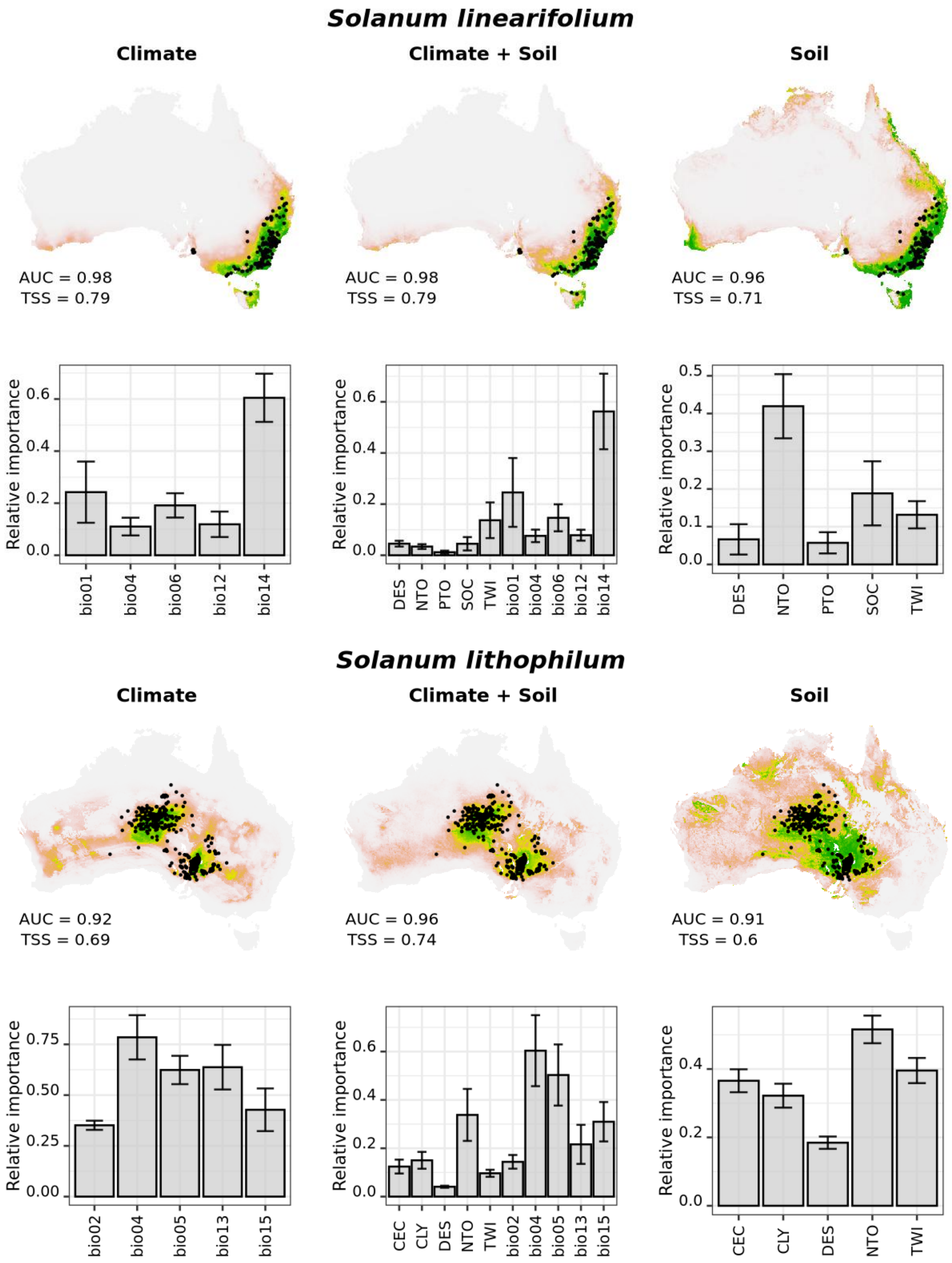

Solanum linearifolium
Climate
Climate + Soil
Soil
AUC = 0.98
TSS = 0.79
AUC = 0.98
TSS = 0.79
AUC = 0.96
TSS = 0.71
Relative importance
bio01
bio04
bio06
bio12
bio14
DES
NTO
PTO
SOC
TWI
Solanum lithophilum
Climate
Climate + Soil
Soil
AUC = 0.92
TSS = 0.69
AUC = 0.96
TSS = 0.74
AUC = 0.91
TSS = 0.6
Relative importance
bio02
bio04
bio05
bio13
bio15
CEC
CLY
DES
NTO
TWI

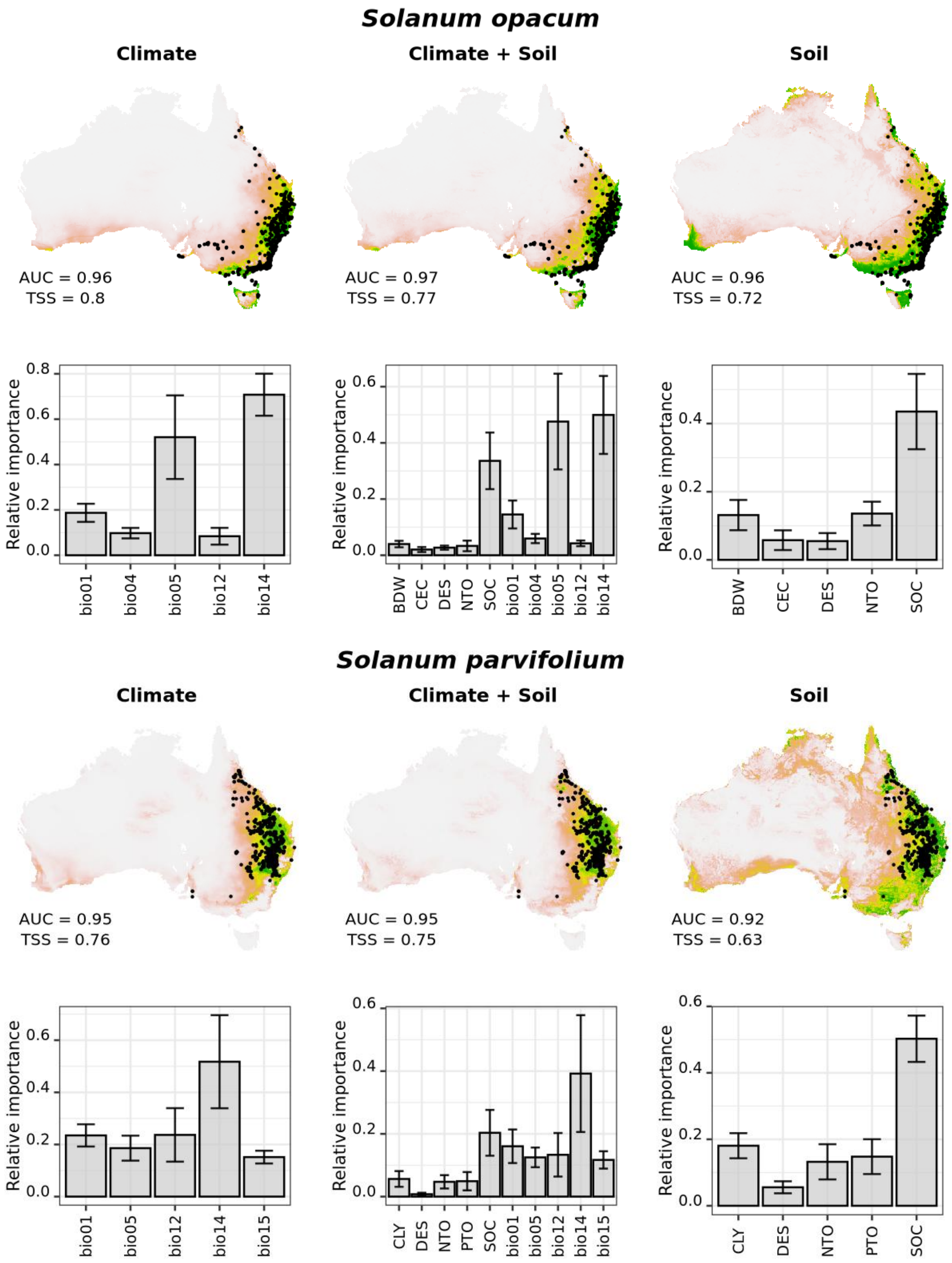

Solanum opacum
Climate
Climate + Soil
Soil
AUC = 0.96
TSS = 0.8
AUC = 0.97
TSS = 0.77
AUC = 0.96
TSS = 0.72
Relative importance
bio01
bio04
bio05
bio12
bio14
BDW
CEC
DES
NTO
SOC
Solanum parvifolium
Climate
Climate + Soil
Soil
AUC = 0.95
TSS = 0.76
AUC = 0.95
TSS = 0.75
AUC = 0.92
TSS = 0.63
Relative importance
bio01
bio05
bio12
bio14
bio15
CLY
DES
NTO
PTO
SOC

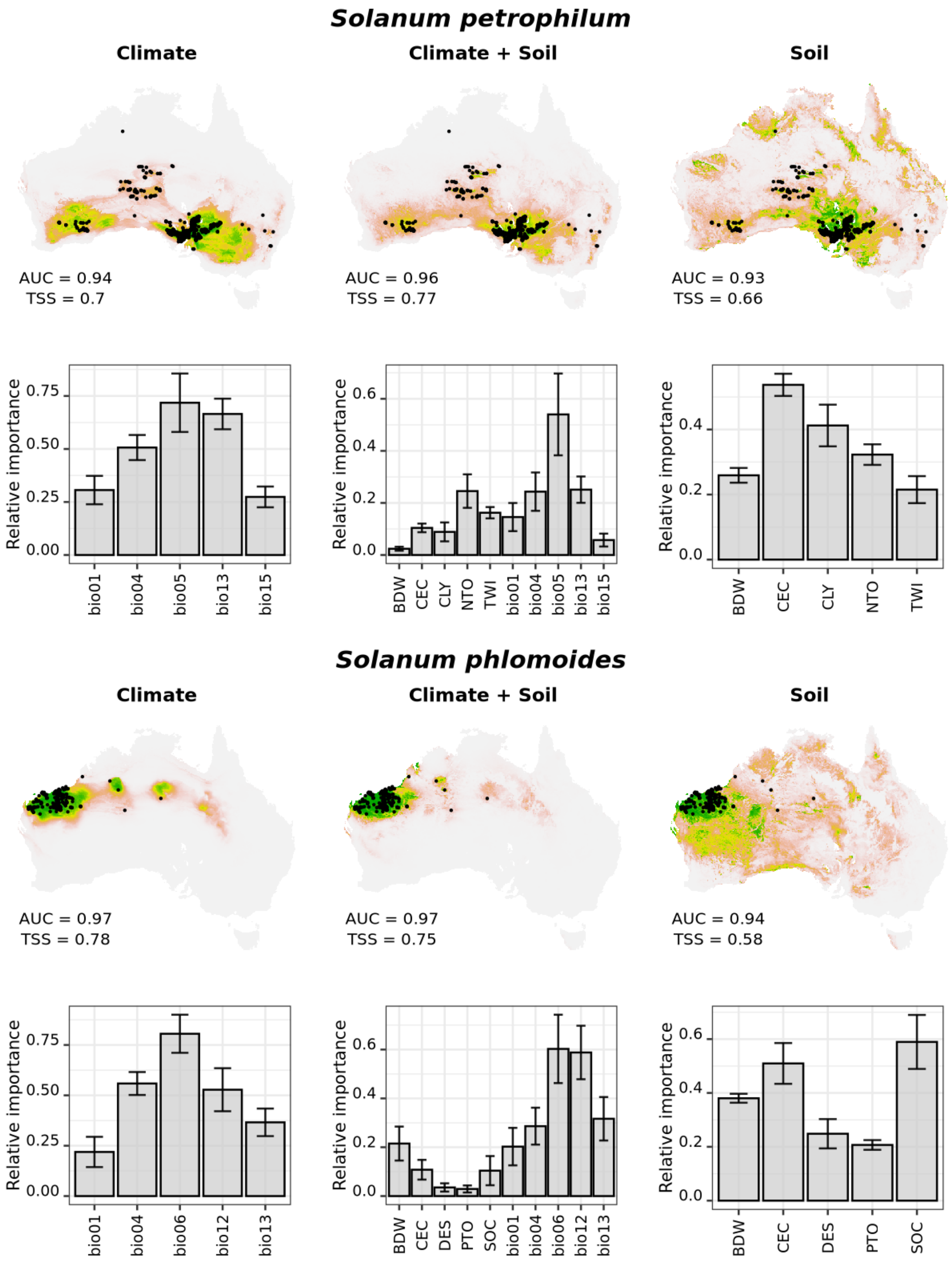

Solanum petrophilum
Climate
Climate + Soil
Soil
AUC = 0.94
TSS = 0.7
AUC = 0.96
TSS = 0.77
AUC = 0.93
TSS = 0.66
Relative importance
0.00 0.25 0.50 0.75
bio01 bio04 bio05 bio13 bio15
0.0 0.2 0.4 0.6
BDW CEC CLY NTO TWI bio01 bio04 bio05 bio13 bio15
0.0 0.2 0.4
BDW CEC CLY NTO TWI
Solanum phlomoides
Climate
Climate + Soil
Soil
AUC = 0.97
TSS = 0.78
AUC = 0.97
TSS = 0.75
AUC = 0.94
TSS = 0.58
Relative importance
0.00 0.25 0.50 0.75
bio01 bio04 bio06 bio12 bio13
0.0 0.2 0.4 0.6
BDW CEC DES PTO SOC bio01 bio04 bio06 bio12 bio13
0.0 0.2 0.4 0.6
BDW CEC DES PTO SOC

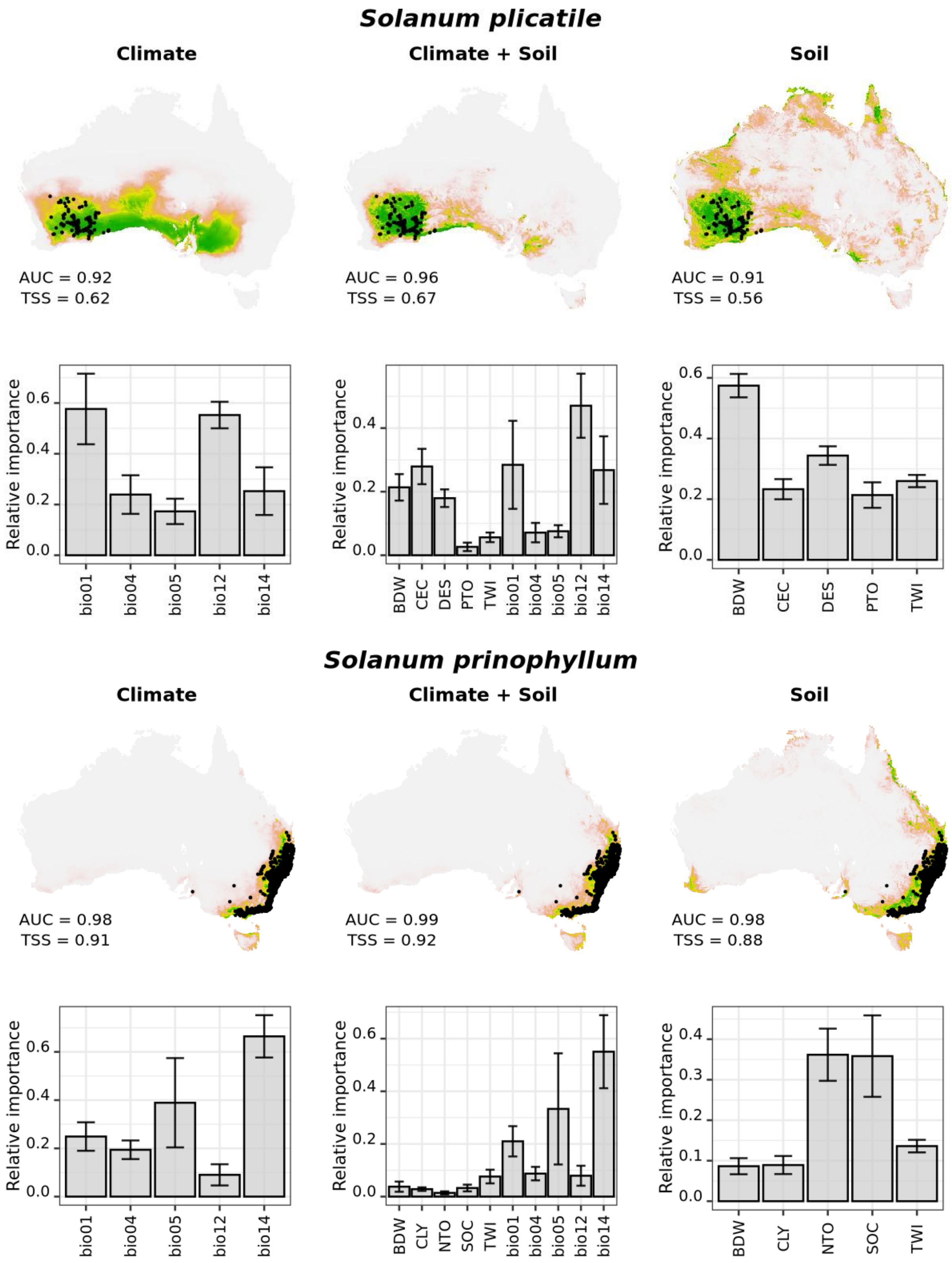

Solanum plicatile
Climate
Climate + Soil
Soil
AUC = 0.92
TSS = 0.62
AUC = 0.96
TSS = 0.67
AUC = 0.91
TSS = 0.56
Relative importance
0.0
0.2
0.4
0.6
bio01
bio04
bio05
bio12
bio14
BDW
CEC
DES
PTO
TWI
Solanum prinophyllum
Climate
Climate + Soil
Soil
AUC = 0.98
TSS = 0.91
AUC = 0.99
TSS = 0.92
AUC = 0.98
TSS = 0.88
Relative importance
0.0
0.1
0.2
0.3
0.4
0.6
BDW
CLY
NTO
SOC
TWI
bio01
bio04
bio05
bio12
bio14

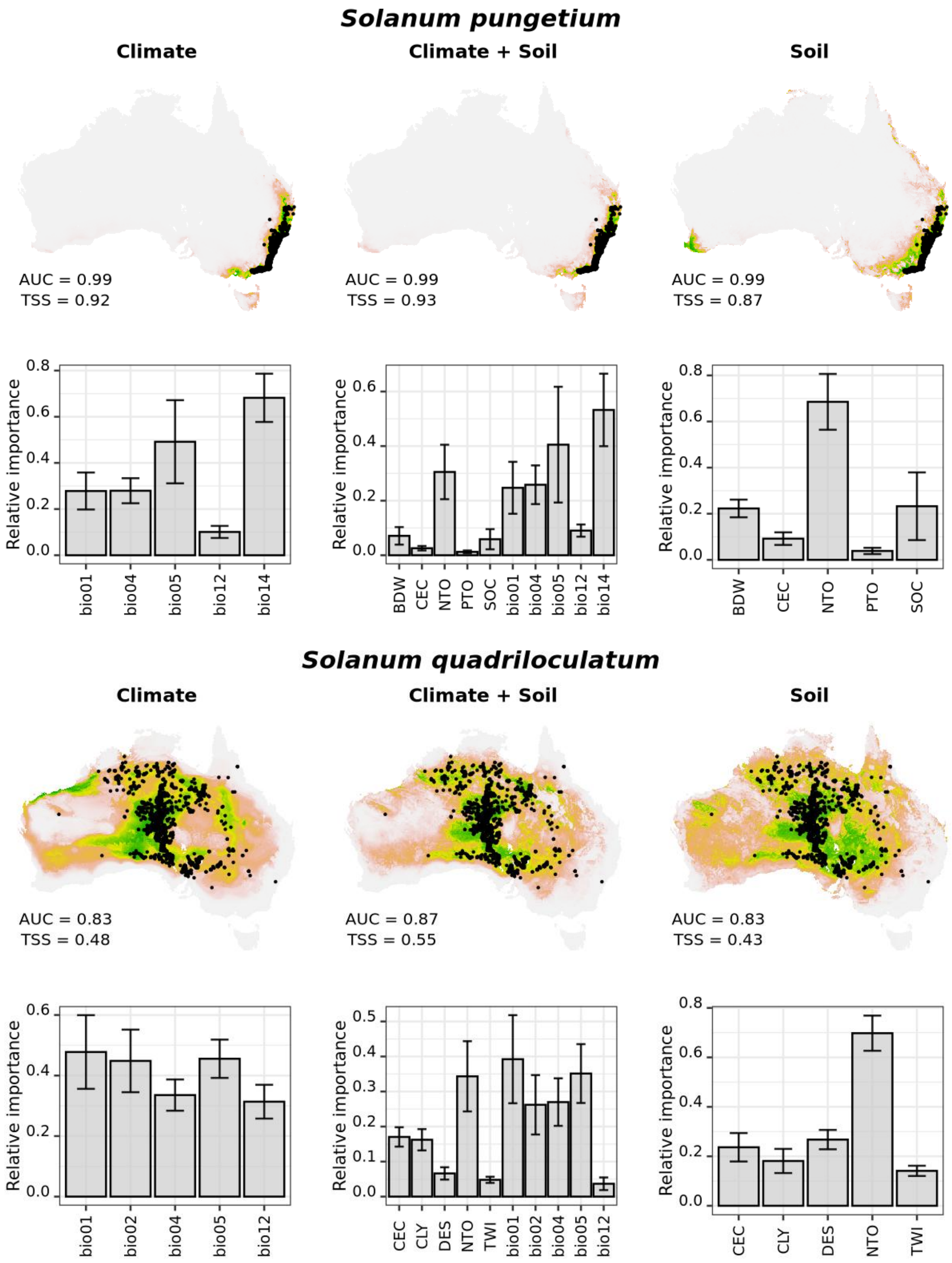
Solanum pungetium
Climate
Climate + Soil
Soil
AUC = 0.99
TSS = 0.92
AUC = 0.99
TSS = 0.93
AUC = 0.99
TSS = 0.87
Relative importance
bio01
bio04
bio05
bio12
bio14
BDW
CEC
NTO
PTO
SOC
Solanum quadriloculatum
AUC = 0.83
TSS = 0.48
AUC = 0.87
TSS = 0.55
AUC = 0.83
TSS = 0.43
bio02
CLY
DES
TWI

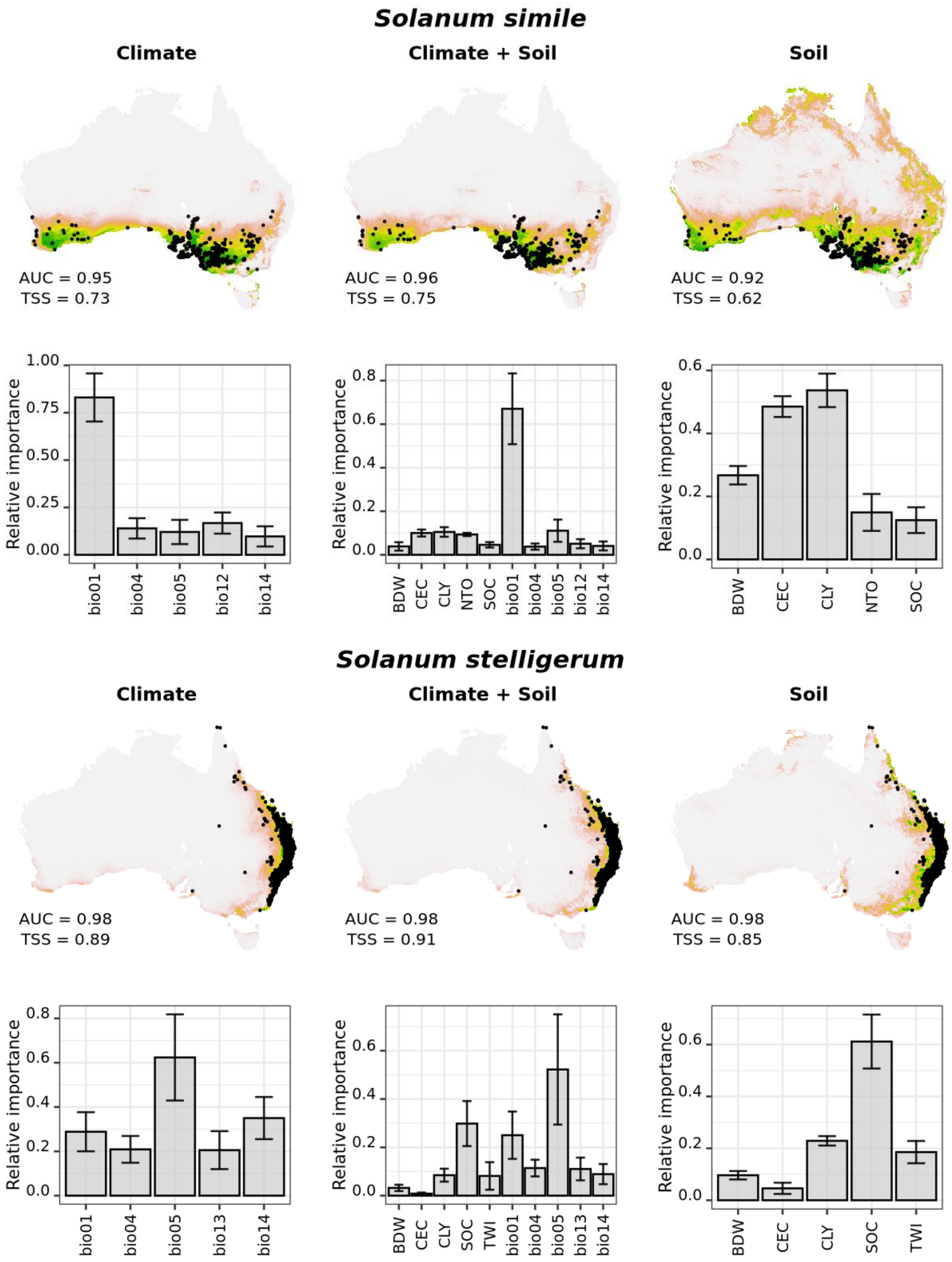
Solanum simile
Climate
Climate + Soil
Soil
AUC = 0.95
TSS = 0.73
AUC = 0.96
TSS = 0.75
AUC = 0.92
TSS = 0.62
Relative importance
1.00
0.75
0.50
0.25
0.00
bio01
bio04
bio05
bio12
bio14
0.8
0.6
0.4
0.2
0.0
BDW
CEC
CLY
NTO
SOC
bio01
bio04
bio05
bio12
bio14
0.6
0.4
0.2
0.0
BDW
CEC
CLY
NTO
SOC
Solanum stelligerum
Climate
Climate + Soil
Soil
AUC = 0.98
TSS = 0.89
AUC = 0.98
TSS = 0.91
AUC = 0.98
TSS = 0.85
Relative importance
0.8
0.6
0.4
0.2
0.0
bio01
bio04
bio05
bio13
bio14
0.6
0.4
0.2
0.0
BDW
CEC
CLY
SOC
TWI
bio01
bio04
bio05
bio13
bio14
0.6
0.4
0.2
0.0
BDW
CEC
CLY
SOC
TWI

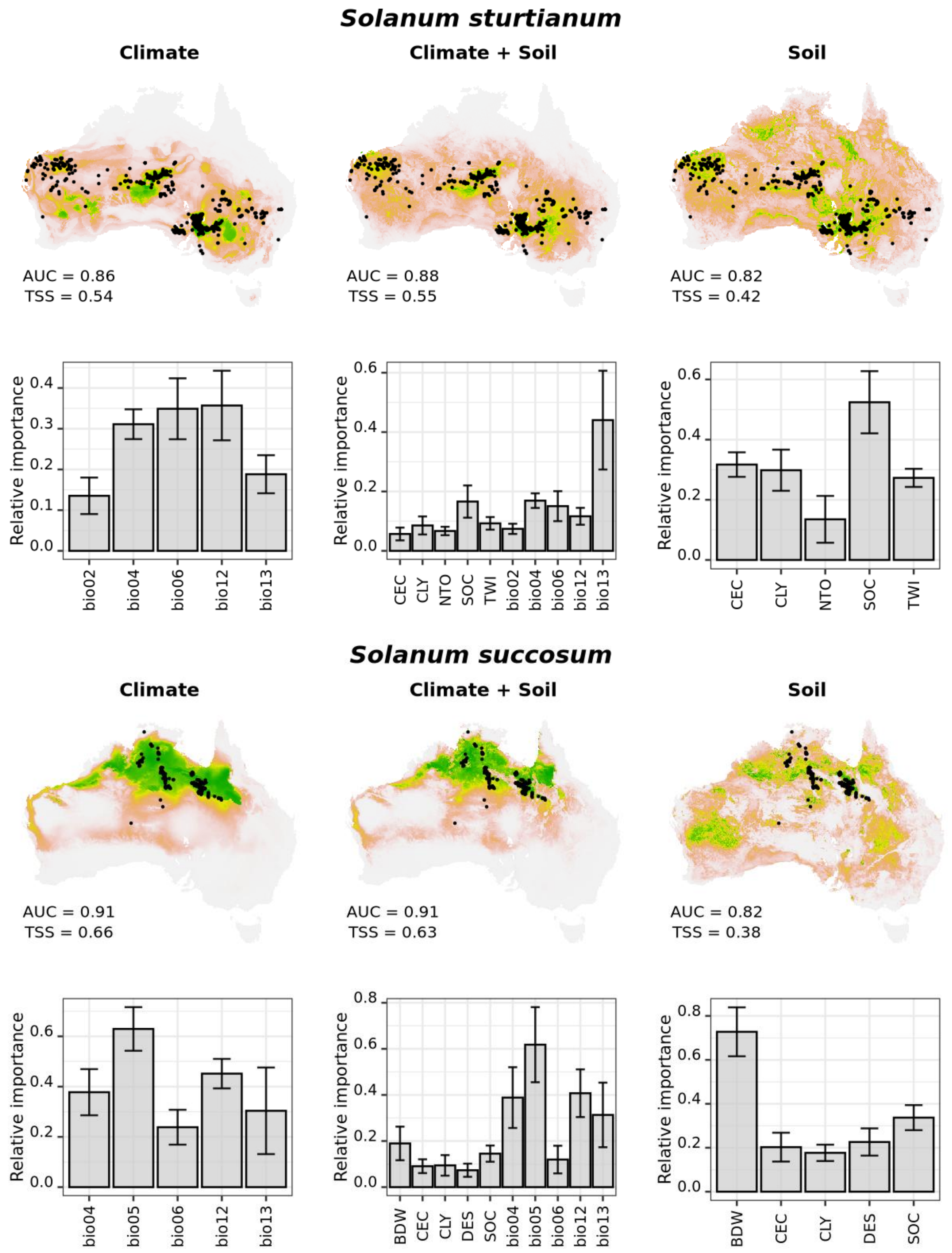
Solanum sturtianum
Climate
Climate + Soil
Soil
AUC = 0.86
TSS = 0.54
AUC = 0.88
TSS = 0.55
AUC = 0.82
TSS = 0.42
Relative importance
bio02
bio04
bio06
bio12
bio13
CEC
CLY
NTO
SOC
TWI
Solanum succosum
Climate
Climate + Soil
Soil
AUC = 0.91
TSS = 0.66
AUC = 0.91
TSS = 0.63
AUC = 0.82
TSS = 0.38
Relative importance
bio04
bio05
bio06
bio12
bio13
BDW
CEC
CLY
DES
SOC

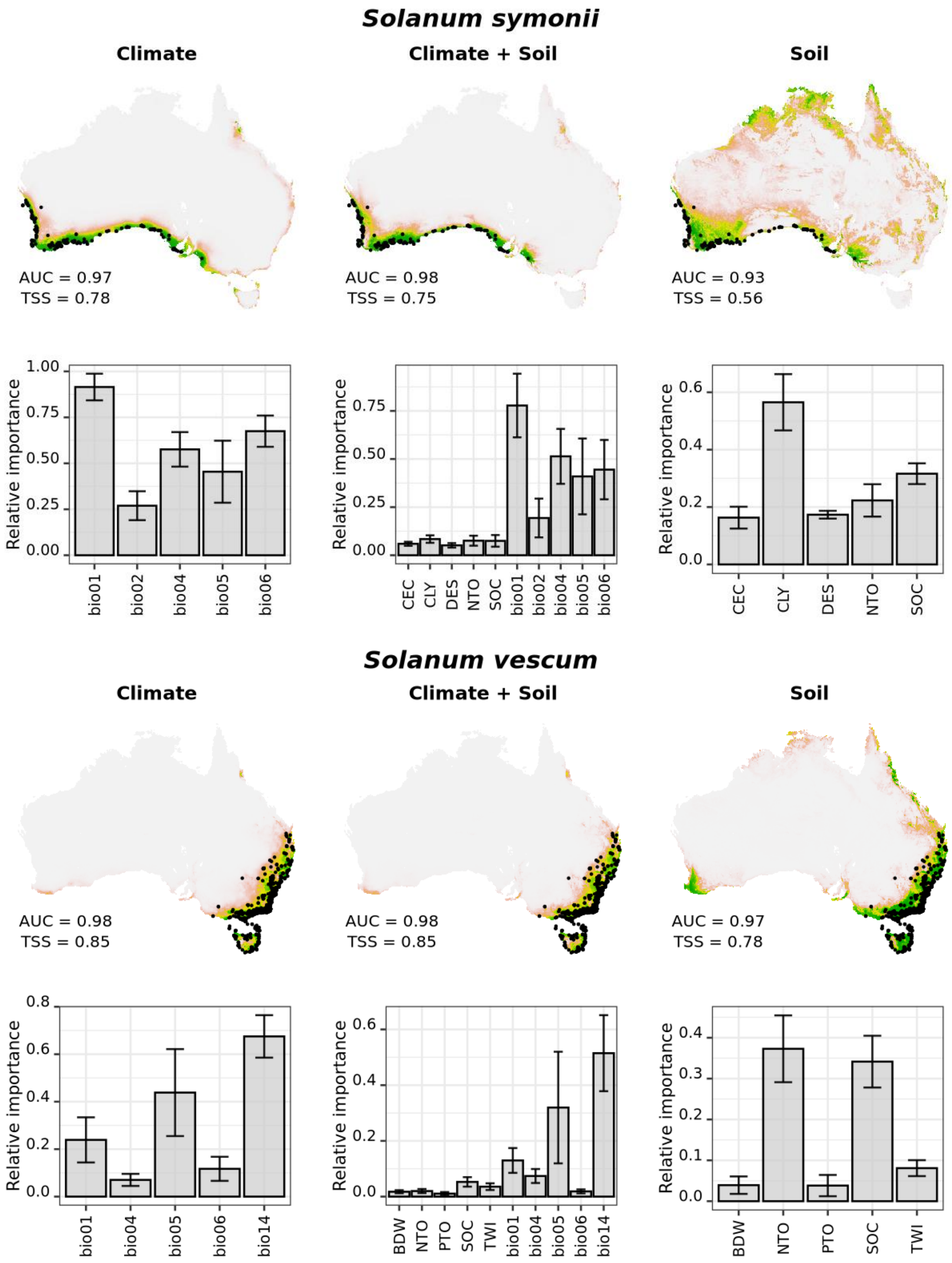
Solanum symonii
Climate
Climate + Soil
Soil
AUC = 0.97
TSS = 0.78
AUC = 0.98
TSS = 0.75
AUC = 0.93
TSS = 0.56
Relative importance
1.00
0.75
0.50
0.25
0.00
bio01
bio02
bio04
bio05
bio06
CEC
CLY
DES
NTO
SOC
0.6
0.4
0.2
0.0
Solanum vescum
Climate
Climate + Soil
Soil
AUC = 0.98
TSS = 0.85
AUC = 0.98
TSS = 0.85
AUC = 0.97
TSS = 0.78
0.8
0.3
0.1
bio14
BDW
PTO
TWI

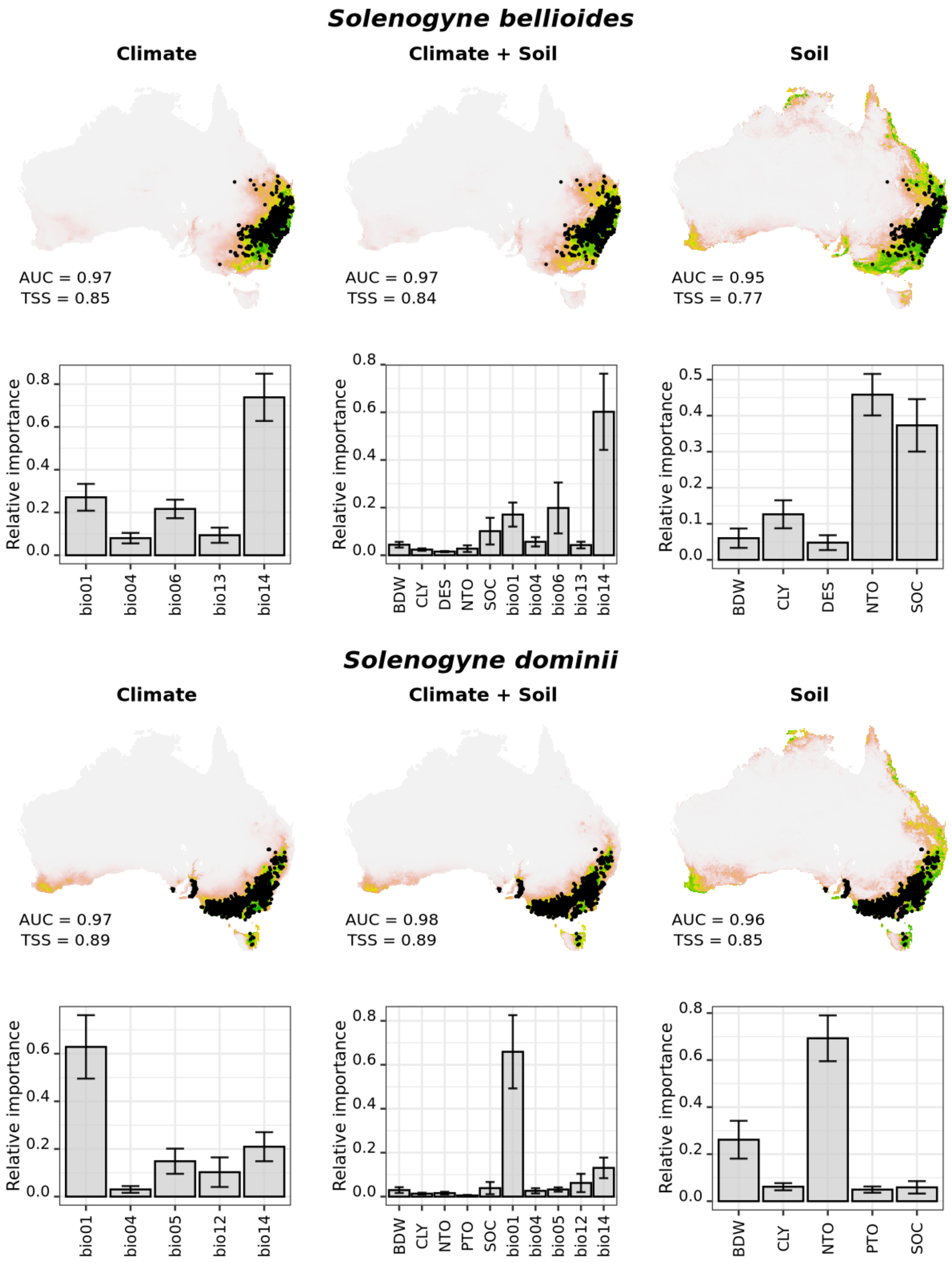

Solenogyne bellioides
Climate
Climate + Soil
Soil
AUC = 0.97
TSS = 0.85
AUC = 0.97
TSS = 0.84
AUC = 0.95
TSS = 0.77
Relative importance
bio01
bio04
bio06
bio13
bio14
BDW
CLY
DES
NTO
SOC
Solenogyne dominii
Climate
Climate + Soil
Soil
AUC = 0.97
TSS = 0.89
AUC = 0.98
TSS = 0.89
AUC = 0.96
TSS = 0.85
Relative importance
bio01
bio04
bio05
bio12
bio14
BDW
CLY
NTO
PTO
SOC

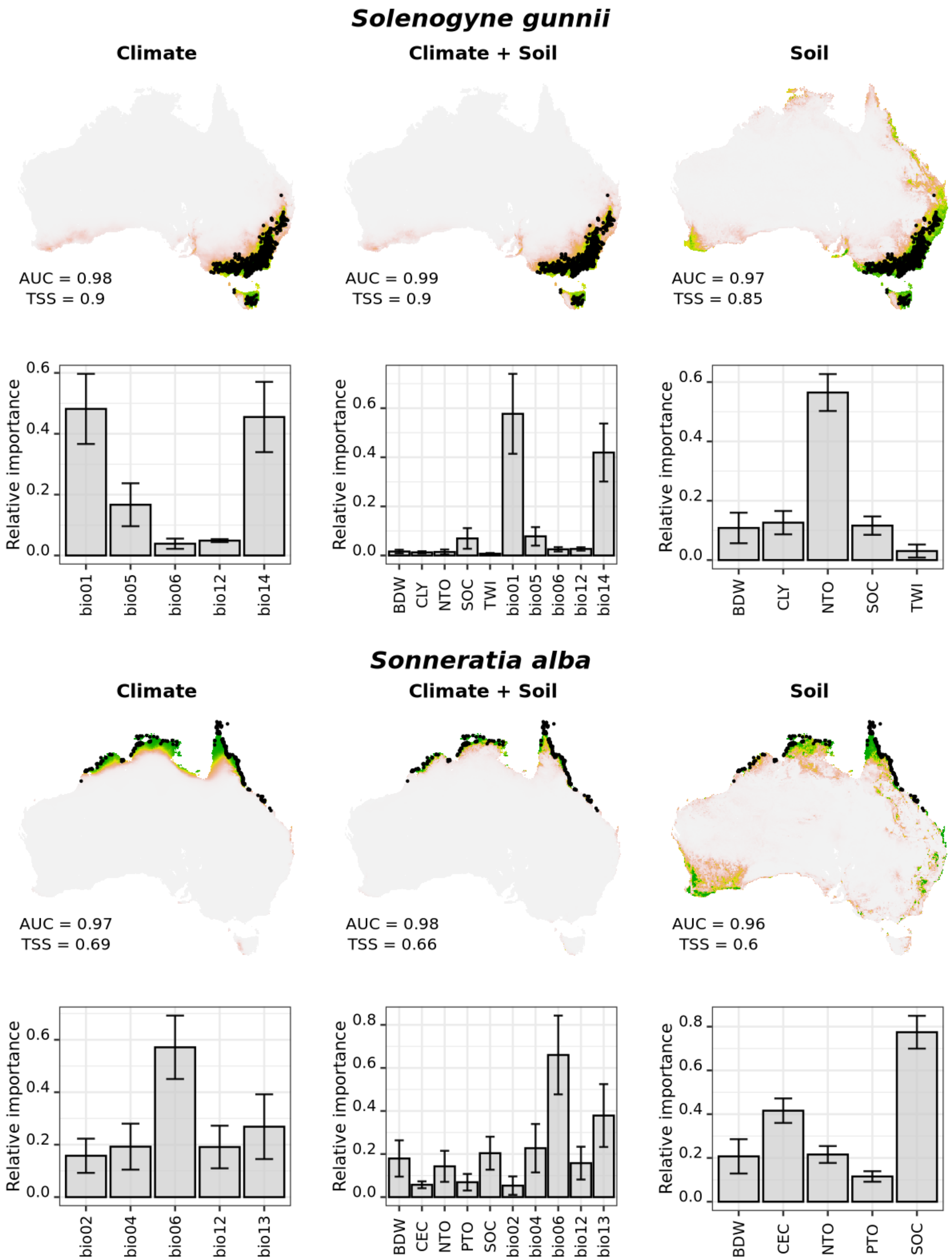
Solenogyne gunnii
Climate
Climate + Soil
Soil
AUC = 0.98
TSS = 0.9
AUC = 0.99
TSS = 0.9
AUC = 0.97
TSS = 0.85
Relative importance
0.0
0.2
0.4
0.6
bio01
bio05
bio06
bio12
bio14
BDW
CLY
NTO
SOC
TWI
Sonneratia alba
Climate
Climate + Soil
Soil
AUC = 0.97
TSS = 0.69
AUC = 0.98
TSS = 0.66
AUC = 0.96
TSS = 0.6
Relative importance
0.8
bio02
bio04
bio06
bio12
bio13
BDW
CEC
NTO
PTO
SOC

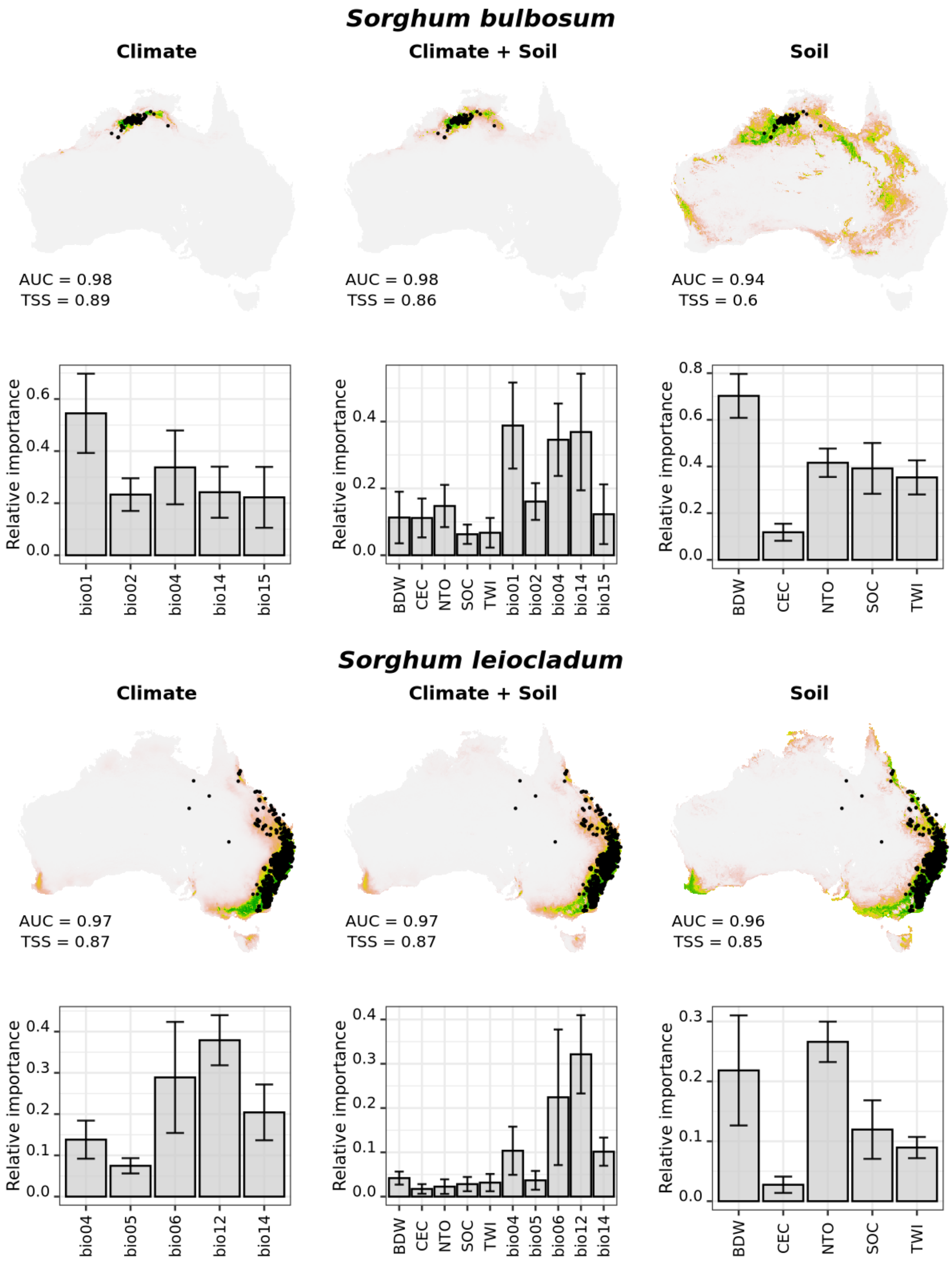

Sorghum bulbosum
Climate
Climate + Soil
Soil
AUC = 0.98
TSS = 0.89
AUC = 0.98
TSS = 0.86
AUC = 0.94
TSS = 0.6
Relative importance
bio01
bio02
bio04
bio14
bio15
BDW
CEC
NTO
SOC
TWI
Sorghum leiocladum
Climate
Climate + Soil
Soil
AUC = 0.97
TSS = 0.87
AUC = 0.97
TSS = 0.87
AUC = 0.96
TSS = 0.85
Relative importance
bio04
bio05
bio06
bio12
bio14
BDW
CEC
NTO
SOC
TWI

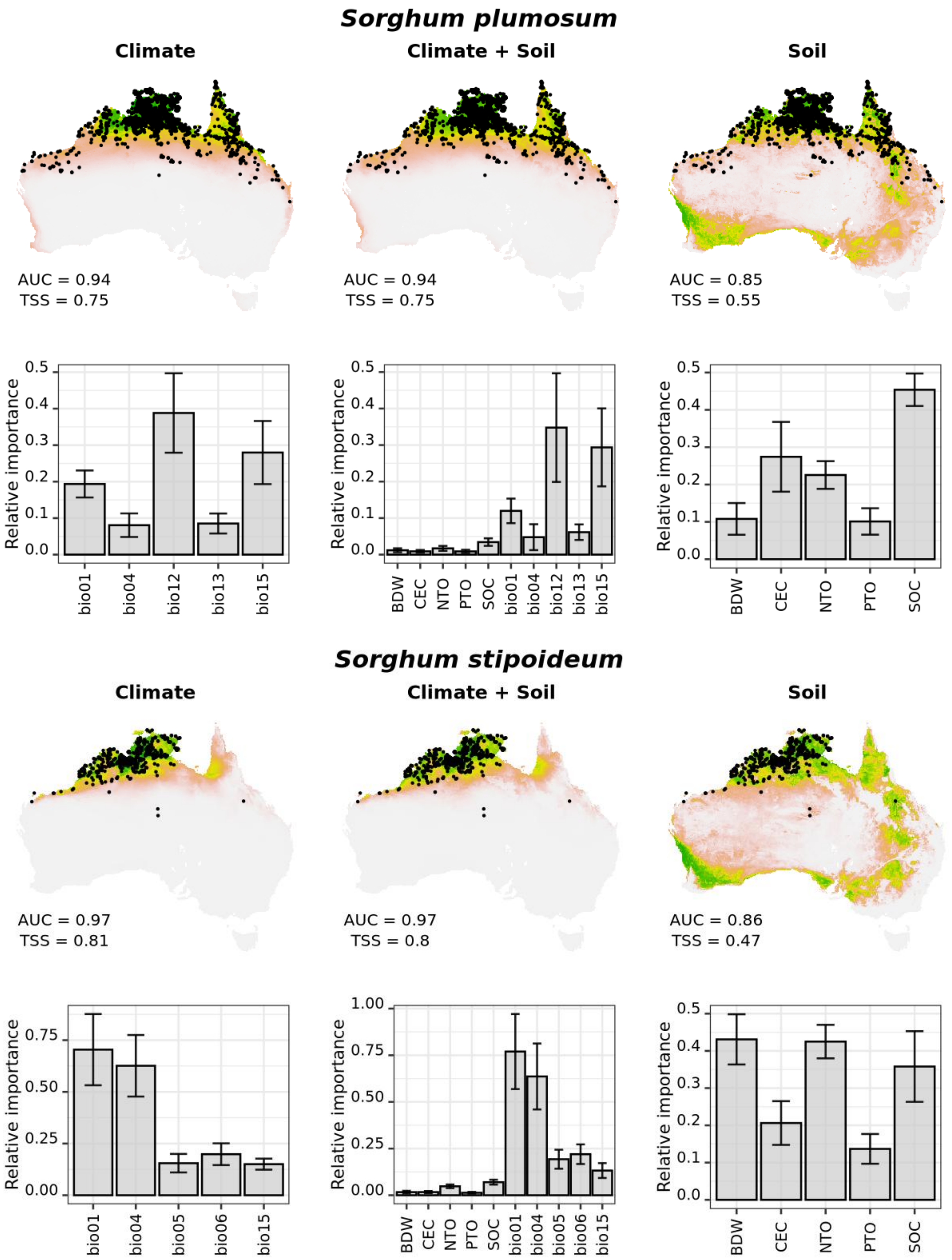
Sorghum plumosum
Climate
Climate + Soil
Soil
AUC = 0.94
TSS = 0.75
AUC = 0.94
TSS = 0.75
AUC = 0.85
TSS = 0.55
Relative importance
0.0
0.1
0.2
0.3
0.4
0.5
bio01
bio04
bio12
bio13
bio15
BDW
CEC
NTO
PTO
SOC
bio01
bio04
bio12
bio13
bio15
BDW
CEC
NTO
PTO
SOC
Sorghum stipoideum
Climate
Climate + Soil
Soil
AUC = 0.97
TSS = 0.81
AUC = 0.97
TSS = 0.8
AUC = 0.86
TSS = 0.47
Relative importance
0.00
0.25
0.50
0.75
1.00
bio01
bio04
bio05
bio06
bio15
BDW
CEC
NTO
PTO
SOC
bio01
bio04
bio05
bio06
bio15
0.0
0.1
0.2
0.3
0.4
0.5
BDW
CEC
NTO
PTO
SOC

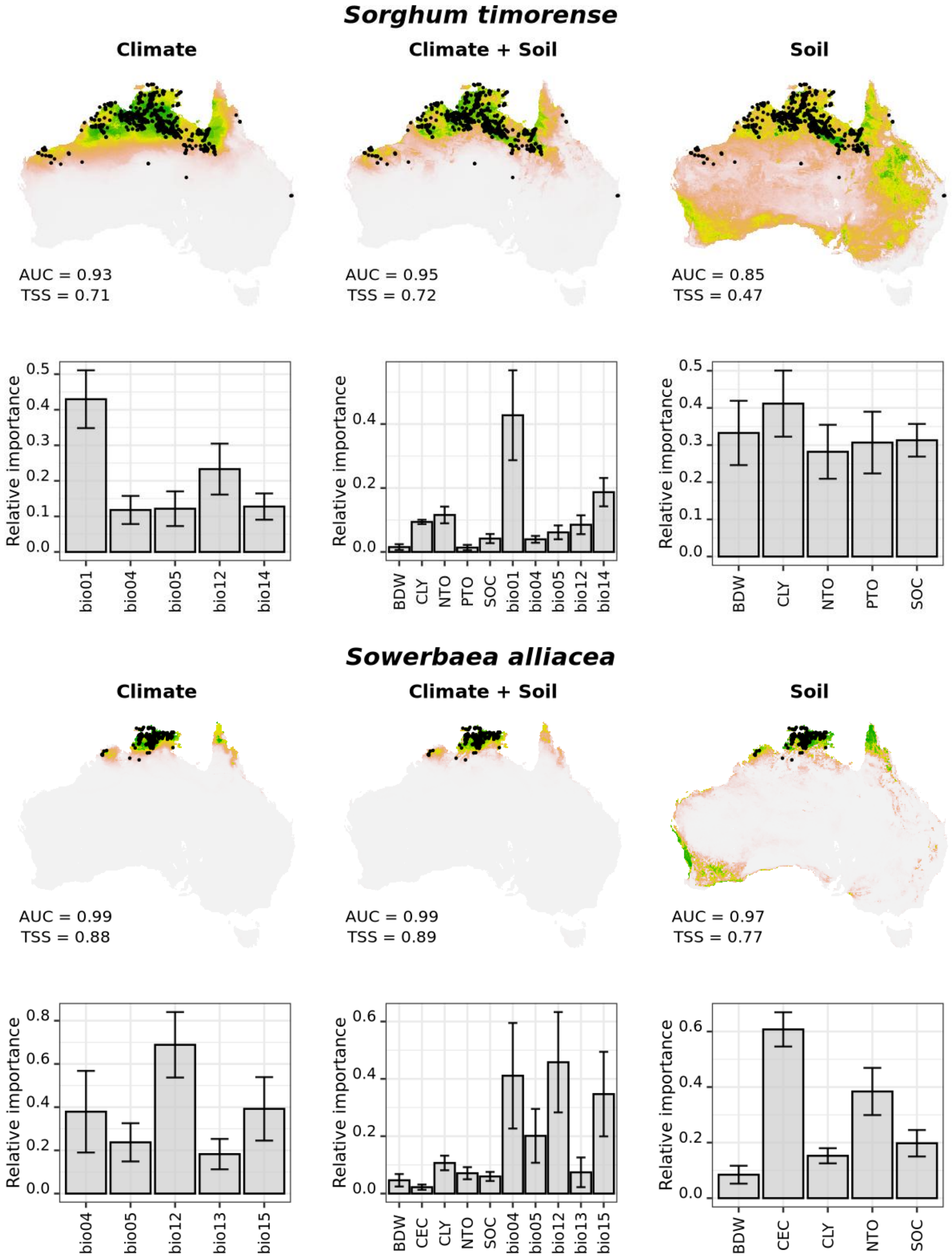
Sorghum timorense
Climate
Climate + Soil
Soil
AUC = 0.93
TSS = 0.71
AUC = 0.95
TSS = 0.72
AUC = 0.85
TSS = 0.47
Relative importance
bio01
bio04
bio05
bio12
bio14
BDW
CLY
NTO
PTO
SOC
Sowerbaea alliacea
Climate
Climate + Soil
Soil
AUC = 0.99
TSS = 0.88
AUC = 0.99
TSS = 0.89
AUC = 0.97
TSS = 0.77
Relative importance
bio04
bio05
bio12
bio13
bio15
BDW
CEC
CLY
NTO
SOC

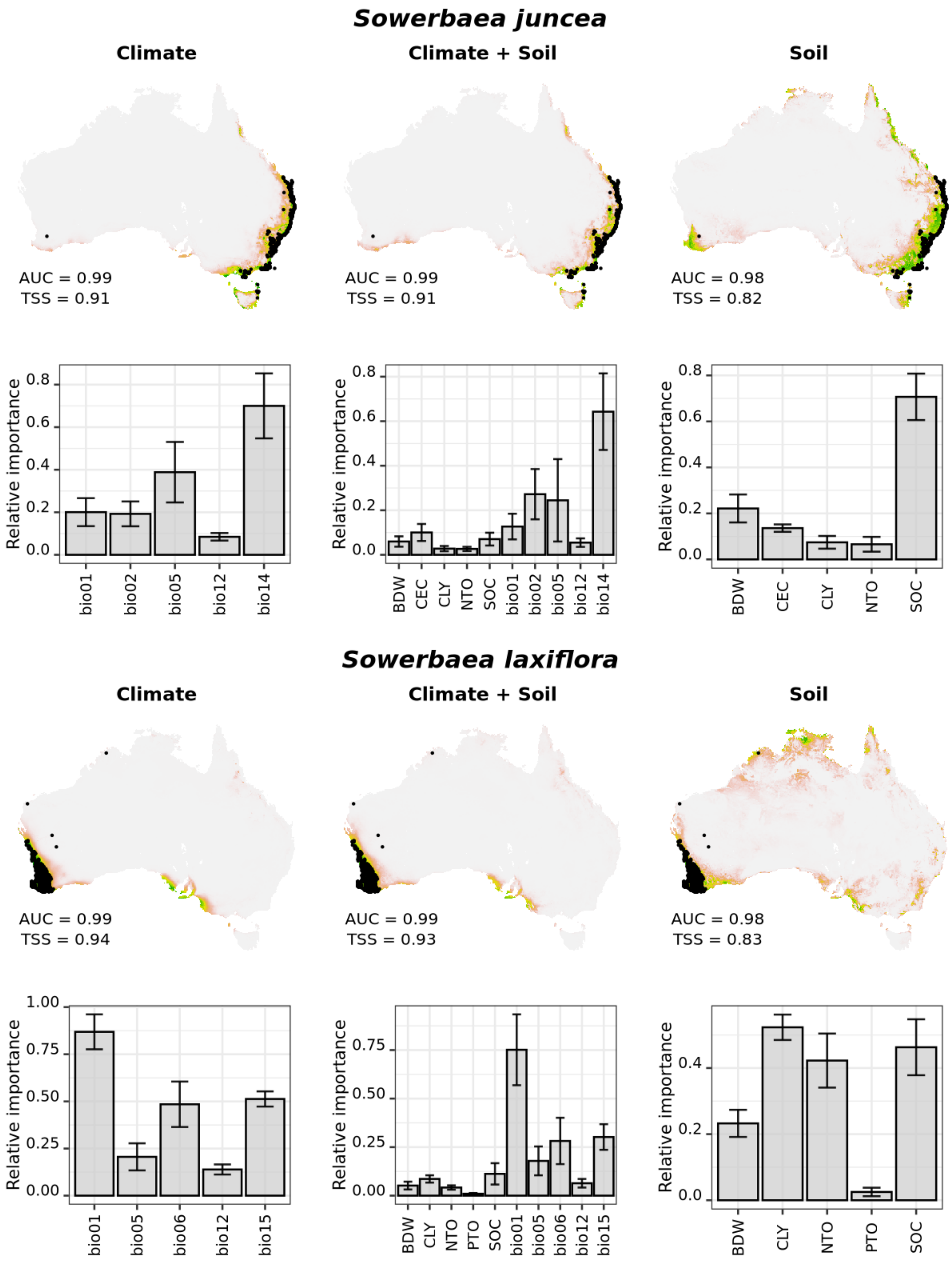

Sowerbaea juncea
Climate
Climate + Soil
Soil
AUC = 0.99
TSS = 0.91
AUC = 0.99
TSS = 0.91
AUC = 0.98
TSS = 0.82
Relative importance
bio01
bio02
bio05
bio12
bio14
BDW
CEC
CLY
NTO
SOC
Sowerbaea laxiflora
Climate
Climate + Soil
Soil
AUC = 0.99
TSS = 0.94
AUC = 0.99
TSS = 0.93
AUC = 0.98
TSS = 0.83
Relative importance
bio01
bio05
bio06
bio12
bio15
BDW
CLY
NTO
PTO
SOC

Spartochloa scirpoidea

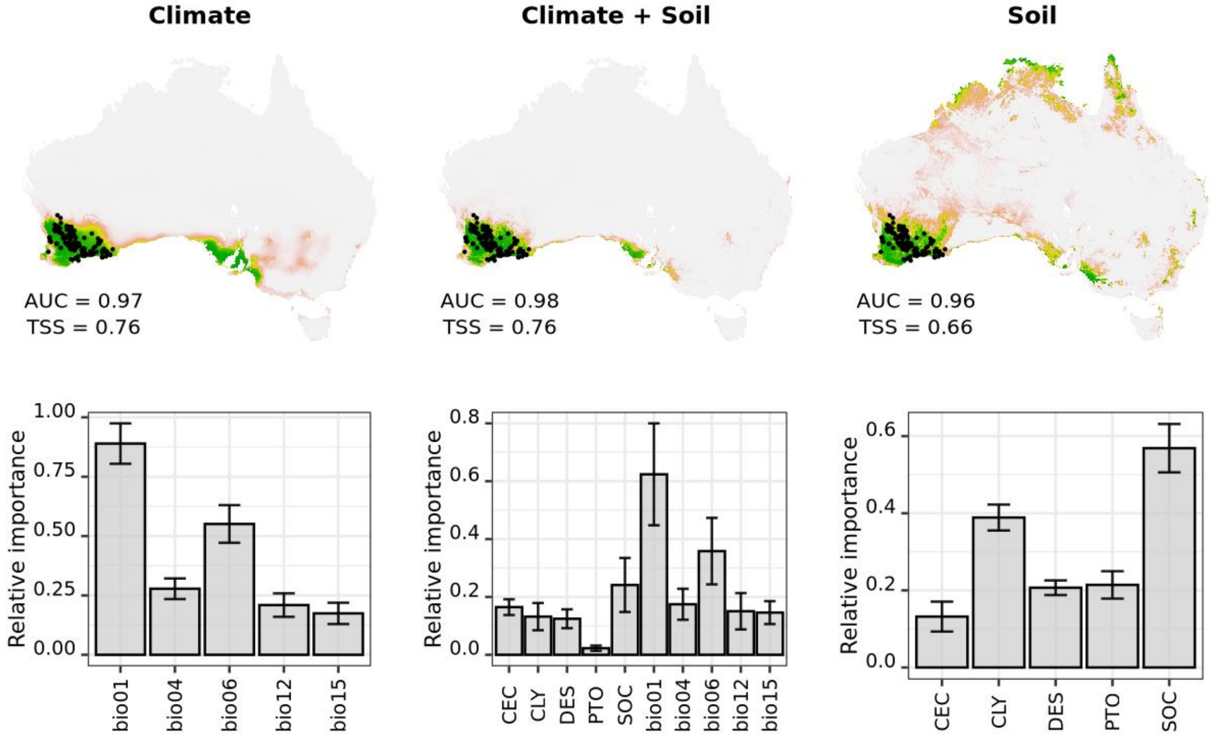

Spergularia brevifolia

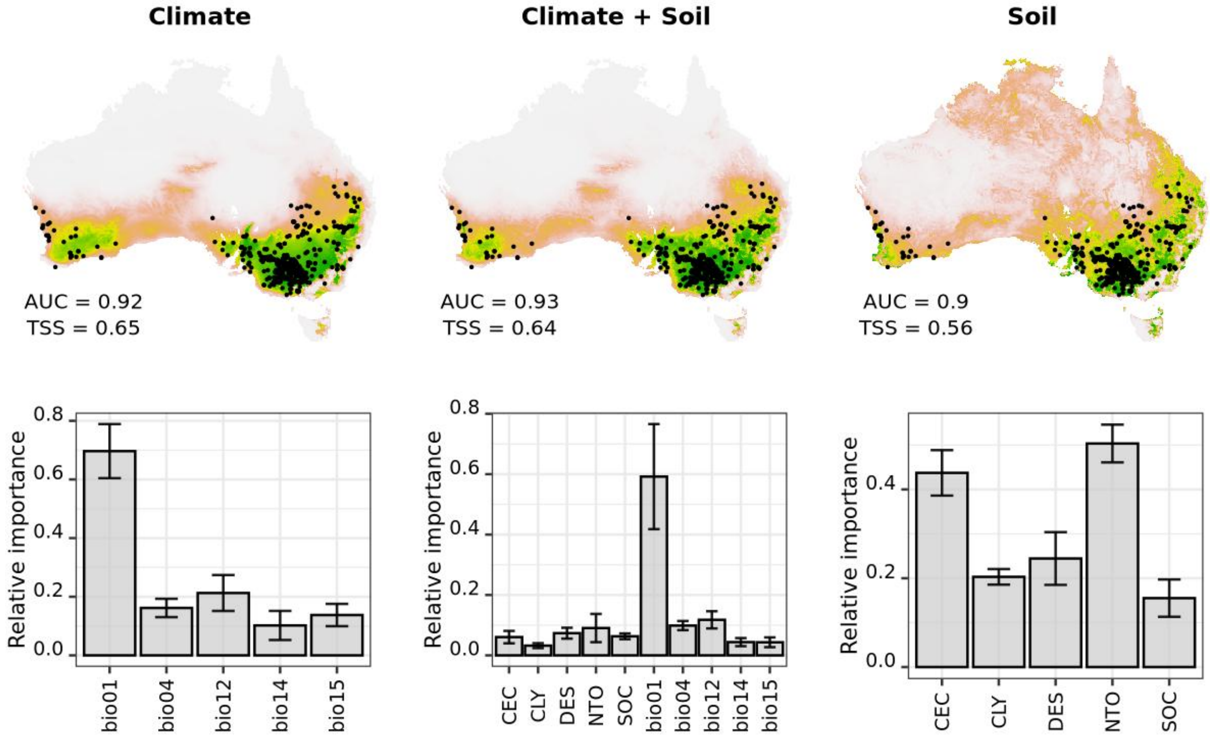

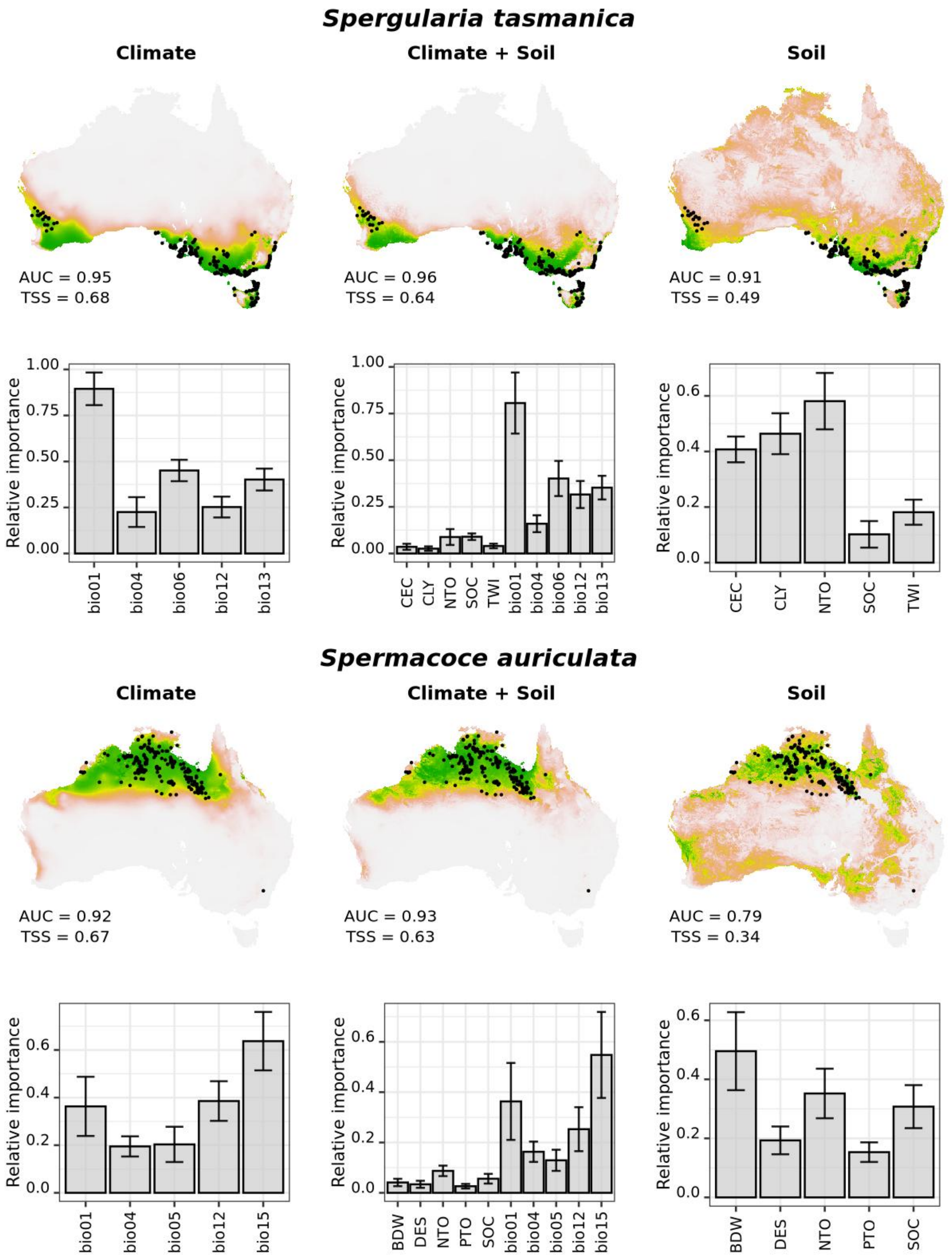
Spergularia tasmanica
Climate
Climate + Soil
Soil
AUC = 0.95
TSS = 0.68
AUC = 0.96
TSS = 0.64
AUC = 0.91
TSS = 0.49
Relative importance
1.00
0.75
0.50
0.25
0.00
bio01
bio04
bio06
bio12
bio13
CEC
CLY
NTO
SOC
TWI
0.6
0.4
0.2
0.0
Spermacoce auriculata
Climate
Climate + Soil
Soil
AUC = 0.92
TSS = 0.67
AUC = 0.93
TSS = 0.63
AUC = 0.79
TSS = 0.34
Relative importance
bio01
bio04
bio05
bio12
bio15
BDW
DES
NTO
PTO
SOC

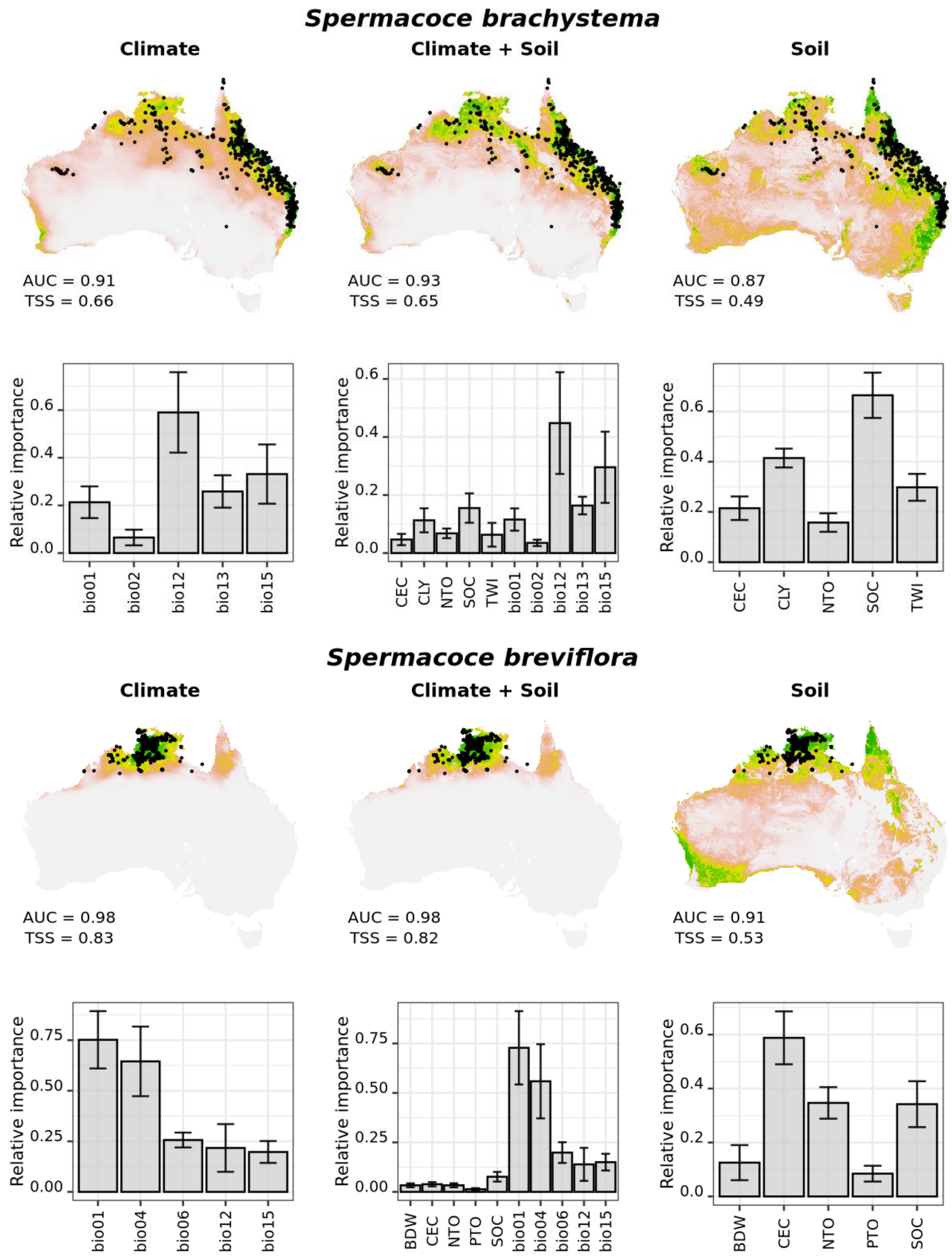
Spermacoce brachystema
Climate
Climate + Soil
Soil
AUC = 0.91
TSS = 0.66
AUC = 0.93
TSS = 0.65
AUC = 0.87
TSS = 0.49
Relative importance
0.0
0.2
0.4
0.6
bio01
bio02
bio12
bio13
bio15
CEC
CLY
NTO
SOC
TWI
bio01
bio02
bio12
bio13
bio15
CEC
CLY
NTO
SOC
TWI
Spermacoce breviflora
Climate
Climate + Soil
Soil
AUC = 0.98
TSS = 0.83
AUC = 0.98
TSS = 0.82
AUC = 0.91
TSS = 0.53
Relative importance
0.00
0.25
0.50
0.75
bio01
bio04
bio06
bio12
bio15
BDW
CEC
NTO
PTO
SOC
bio01
bio04
bio06
bio12
bio15
BDW
CEC
NTO
PTO
SOC

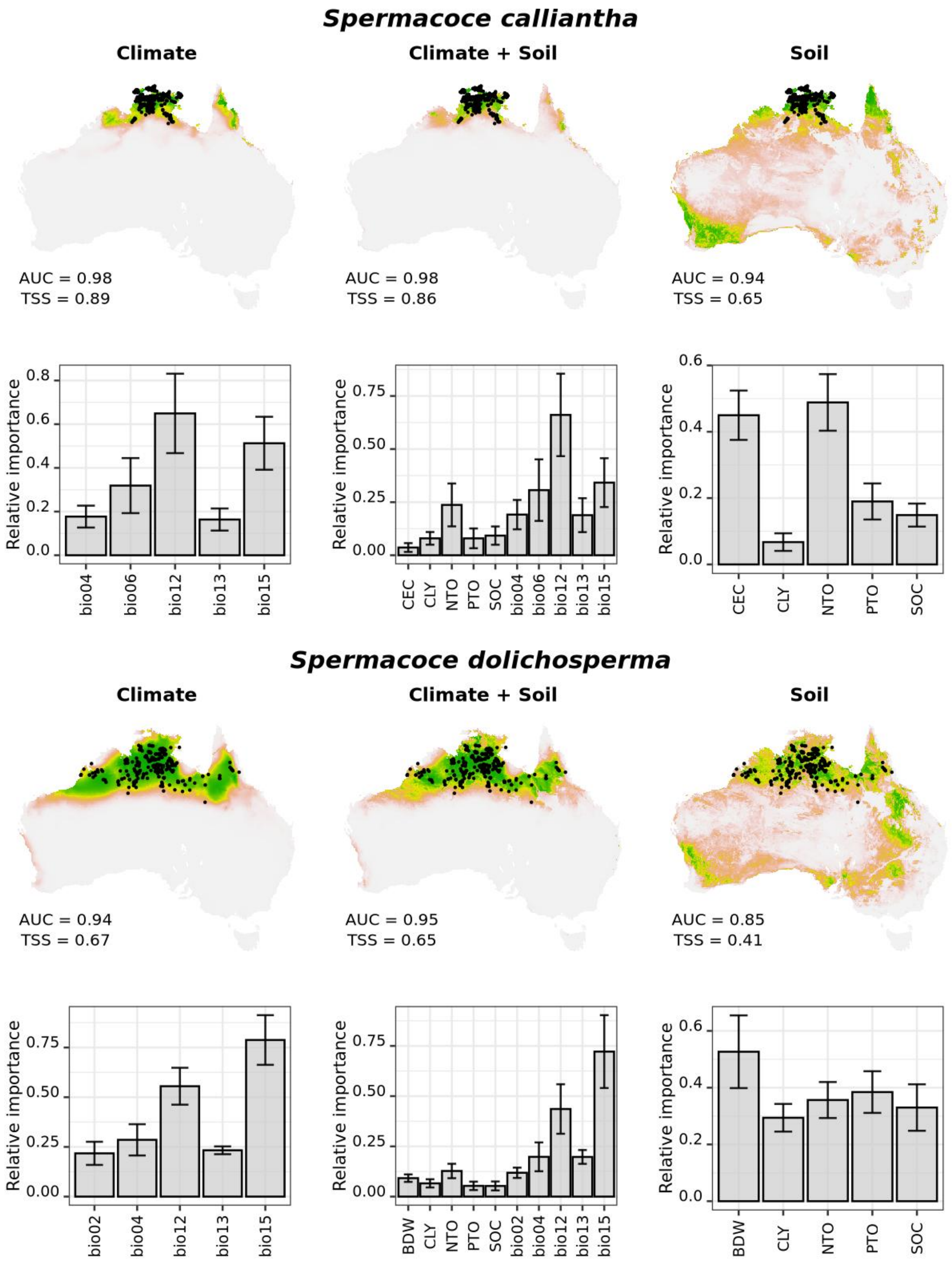

Spermacoce calliantha
Climate
Climate + Soil
Soil
AUC = 0.98
TSS = 0.89
AUC = 0.98
TSS = 0.86
AUC = 0.94
TSS = 0.65
Relative importance
bio04
bio06
bio12
bio13
bio15
CEC
CLY
NTO
PTO
SOC
Spermacoce dolichosperma
Climate
Climate + Soil
Soil
AUC = 0.94
TSS = 0.67
AUC = 0.95
TSS = 0.65
AUC = 0.85
TSS = 0.41
Relative importance
bio02
bio04
bio12
bio13
bio15
BDW
CLY
NTO
PTO
SOC

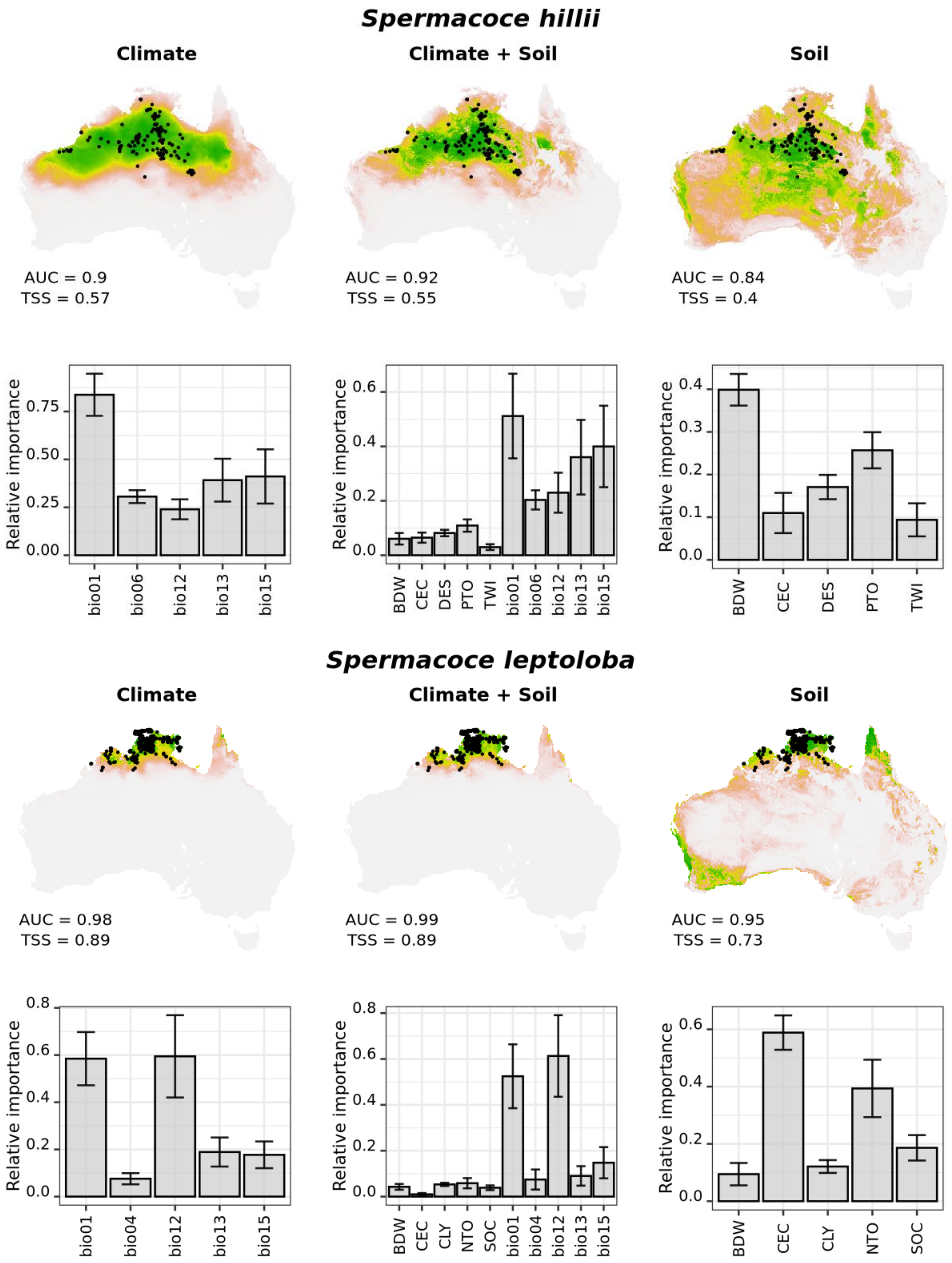

Spermacoce hillii
Climate
Climate + Soil
Soil
AUC = 0.9
TSS = 0.57
AUC = 0.92
TSS = 0.55
AUC = 0.84
TSS = 0.4
Relative importance
0.00
0.25
0.50
0.75
bio01
bio06
bio12
bio13
bio15
0.0
0.2
0.4
0.6
BDW
CEC
DES
PTO
TWI
bio01
bio06
bio12
bio13
bio15
0.0
0.1
0.2
0.3
0.4
BDW
CEC
DES
PTO
TWI
Spermacoce leptoloba
Climate
Climate + Soil
Soil
AUC = 0.98
TSS = 0.89
AUC = 0.99
TSS = 0.89
AUC = 0.95
TSS = 0.73
Relative importance
0.0
0.2
0.4
0.6
0.8
bio01
bio04
bio12
bio13
bio15
BDW
CEC
CLY
NTO
SOC
bio01
bio04
bio12
bio13
bio15
BDW
CEC
CLY
NTO
SOC

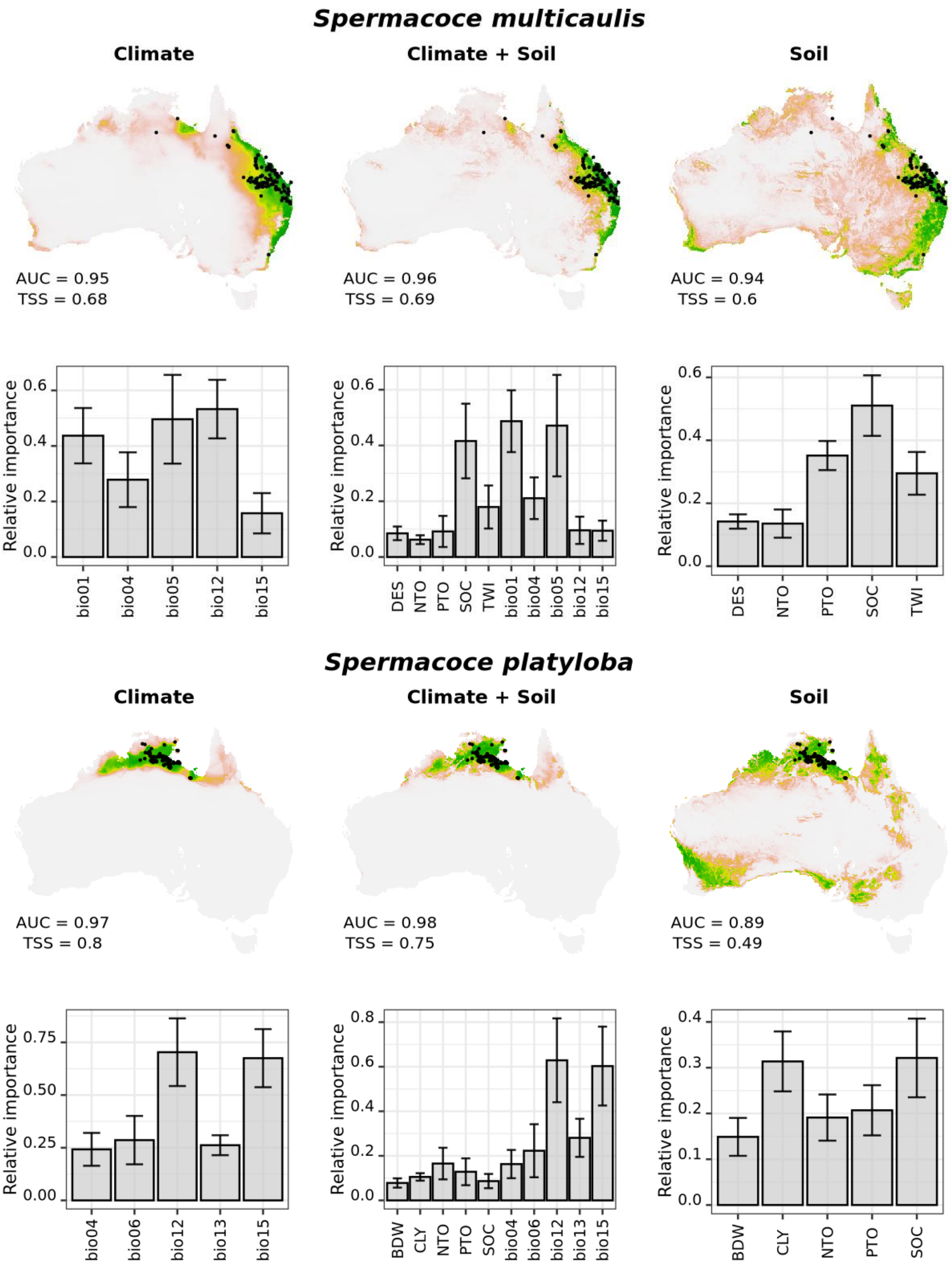
Spermacoce multicaulis
Climate
Climate + Soil
Soil
AUC = 0.95
TSS = 0.68
AUC = 0.96
TSS = 0.69
AUC = 0.94
TSS = 0.6
Relative importance
0.0
0.2
0.4
0.6
bio01
bio04
bio05
bio12
bio15
DES
NTO
PTO
SOC
TWI
Spermacoce platyloba
Climate
Climate + Soil
Soil
AUC = 0.97
TSS = 0.8
AUC = 0.98
TSS = 0.75
AUC = 0.89
TSS = 0.49
Relative importance
0.00
0.25
0.50
0.75
0.8
0.1
0.3
bio04
bio06
bio12
bio13
bio15
BDW
CLY
NTO
PTO
SOC

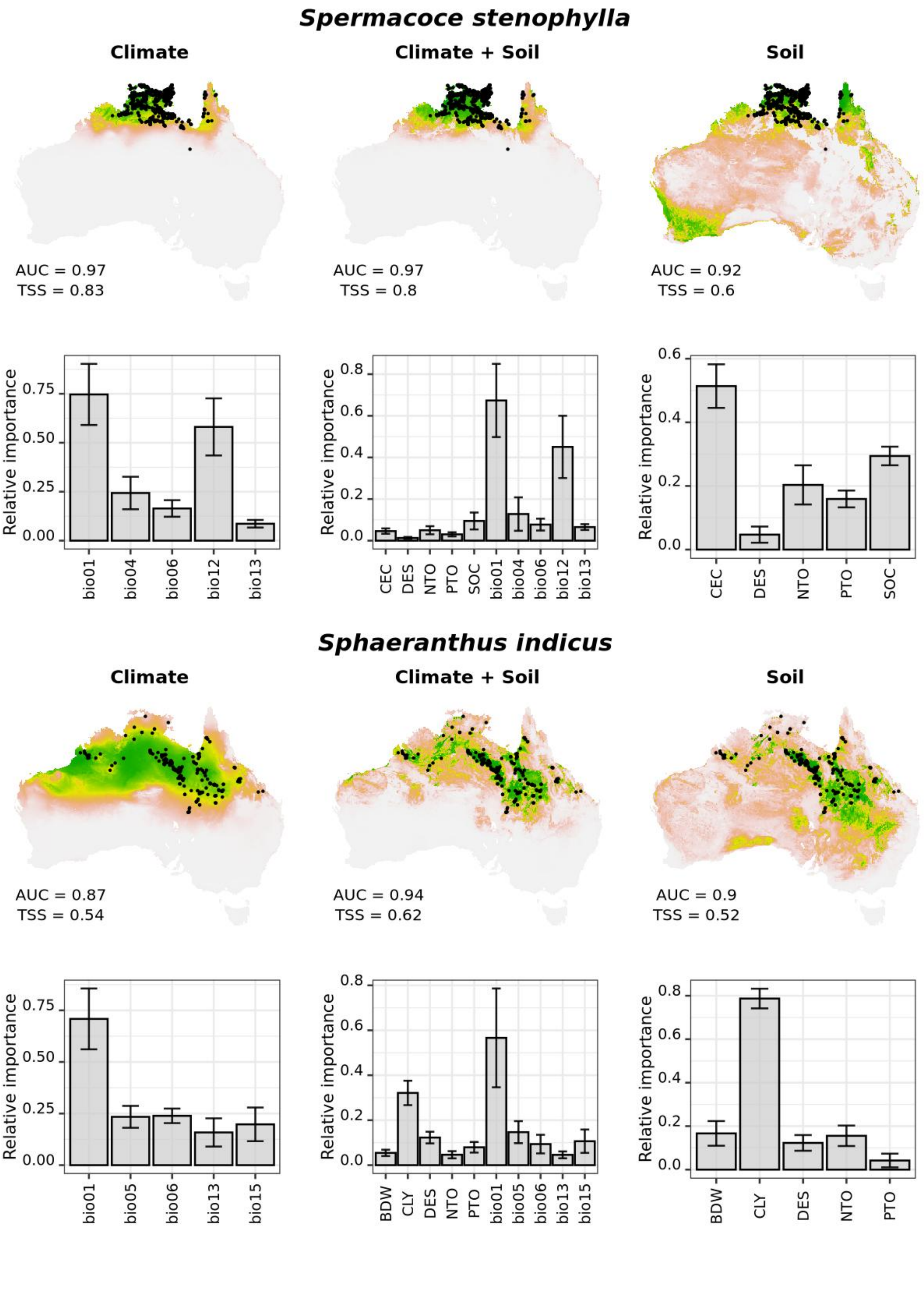
Spermacoce stenophylla
Climate
Climate + Soil
Soil
AUC = 0.97
TSS = 0.83
AUC = 0.97
TSS = 0.8
AUC = 0.92
TSS = 0.6
Relative importance
bio01 bio04 bio06 bio12 bio13
CEC DES NTO PTO SOC bio01 bio04 bio06 bio12 bio13
CEC DES NTO PTO SOC
Sphaeranthus indicus
Climate
Climate + Soil
Soil
AUC = 0.87
TSS = 0.54
AUC = 0.94
TSS = 0.62
AUC = 0.9
TSS = 0.52
Relative importance
bio01 bio05 bio06 bio13 bio15
BDW CLY DES NTO PTO bio01 bio05 bio06 bio13 bio15
BDW CLY DES NTO PTO

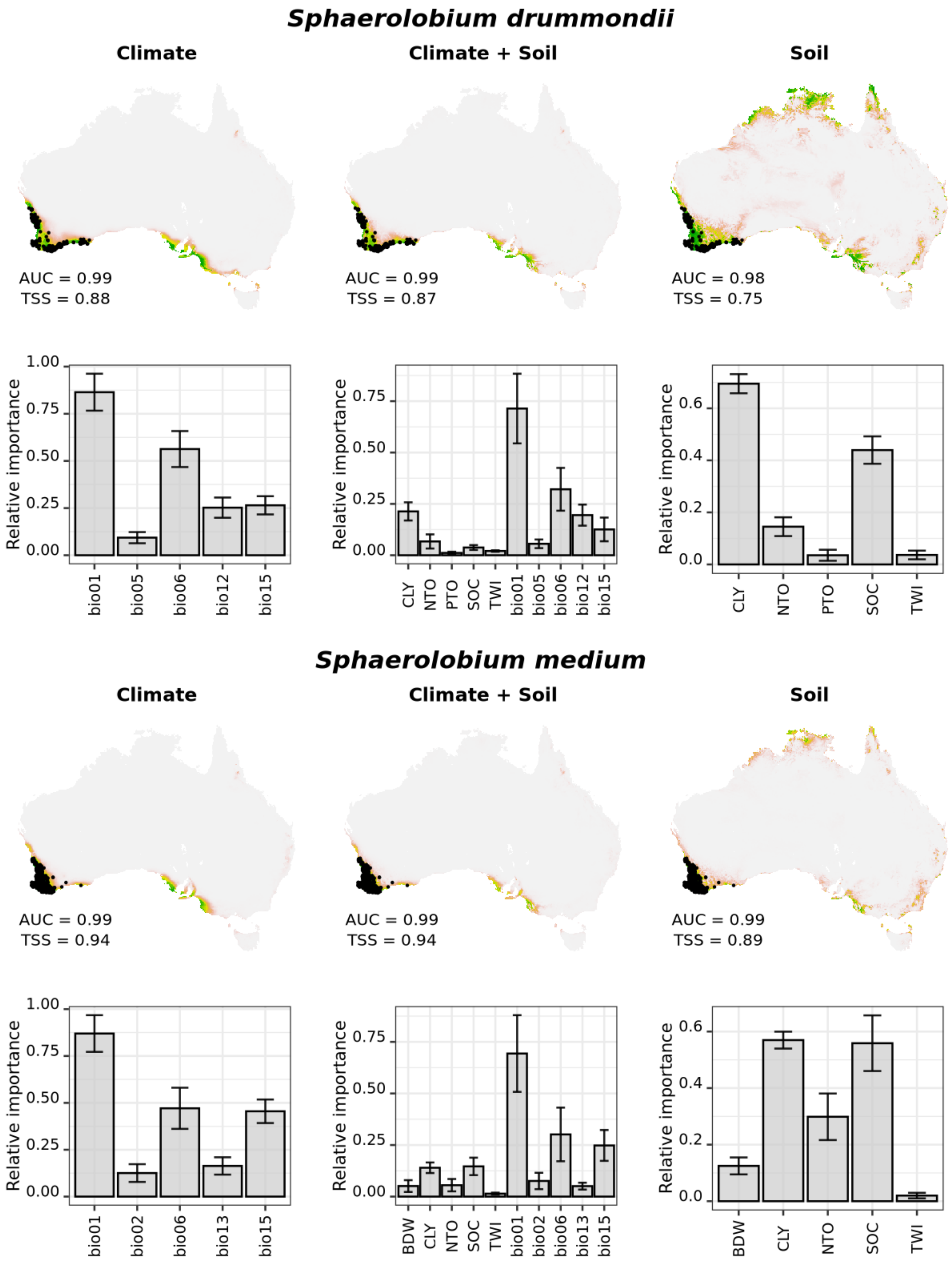
Sphaerolobium drummondii
Climate
Climate + Soil
Soil
AUC = 0.99
TSS = 0.88
AUC = 0.99
TSS = 0.87
AUC = 0.98
TSS = 0.75
Relative importance
1.00
0.75
0.50
0.25
0.00
bio01
bio05
bio06
bio12
bio15
CLY
NTO
PTO
SOC
TWI
0.6
0.4
0.2
0.0
Sphaerolobium medium
AUC = 0.99
TSS = 0.94
AUC = 0.99
TSS = 0.94
AUC = 0.99
TSS = 0.89
bio02
bio13
BDW

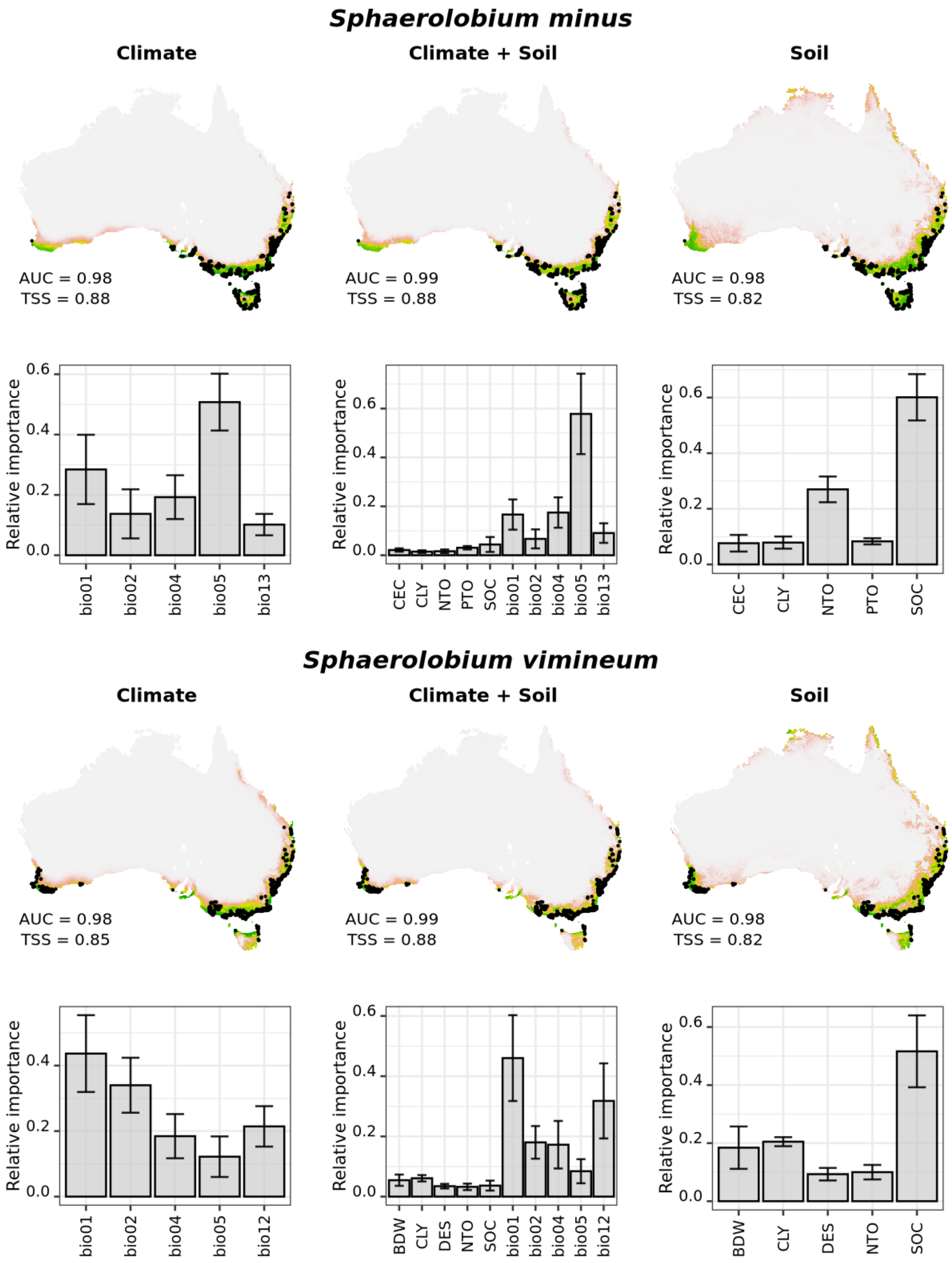
Sphaerolobium minus
Climate
Climate + Soil
Soil
AUC = 0.98
TSS = 0.88
AUC = 0.99
TSS = 0.88
AUC = 0.98
TSS = 0.82
Relative importance
0.0
0.2
0.4
0.6
bio01
bio02
bio04
bio05
bio13
CEC
CLY
NTO
PTO
SOC
bio01
bio02
bio04
bio05
bio13
CEC
CLY
NTO
PTO
SOC
Sphaerolobium vimineum
Climate
Climate + Soil
Soil
AUC = 0.98
TSS = 0.85
AUC = 0.99
TSS = 0.88
AUC = 0.98
TSS = 0.82
Relative importance
0.0
0.2
0.4
0.6
bio01
bio02
bio04
bio05
bio12
BDW
CLY
DES
NTO
SOC
bio01
bio02
bio04
bio05
bio12
BDW
CLY
DES
NTO
SOC

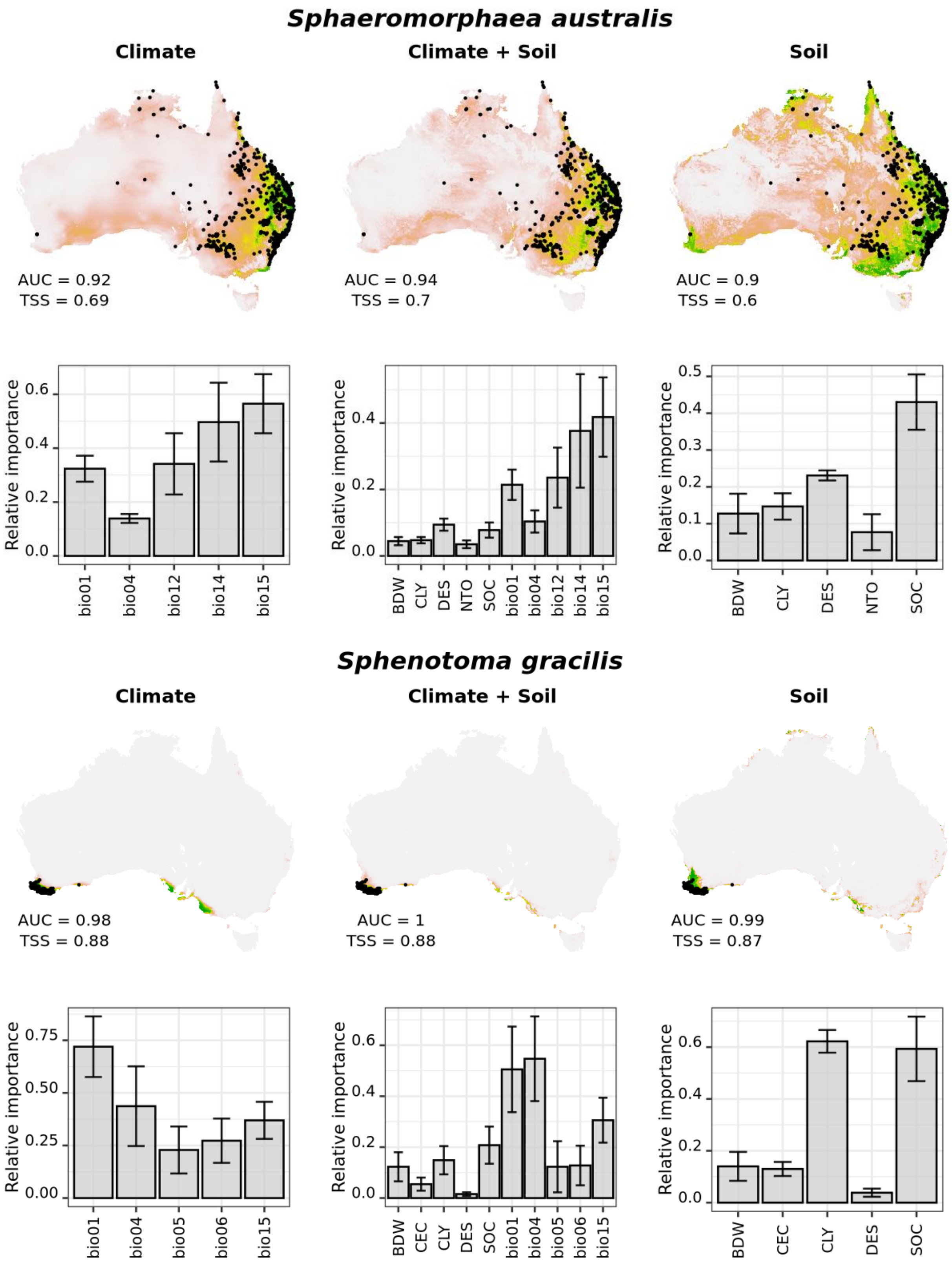
Sphaeromorphaea australis
Climate
Climate + Soil
Soil
AUC = 0.92
TSS = 0.69
AUC = 0.94
TSS = 0.7
AUC = 0.9
TSS = 0.6
Relative importance
bio01
bio04
bio12
bio14
bio15
BDW
CLY
DES
NTO
SOC
Sphenotoma gracilis
Climate
Climate + Soil
Soil
AUC = 0.98
TSS = 0.88
AUC = 1
TSS = 0.88
AUC = 0.99
TSS = 0.87
Relative importance
bio01
bio04
bio05
bio06
bio15
BDW
CEC
CLY
DES
SOC

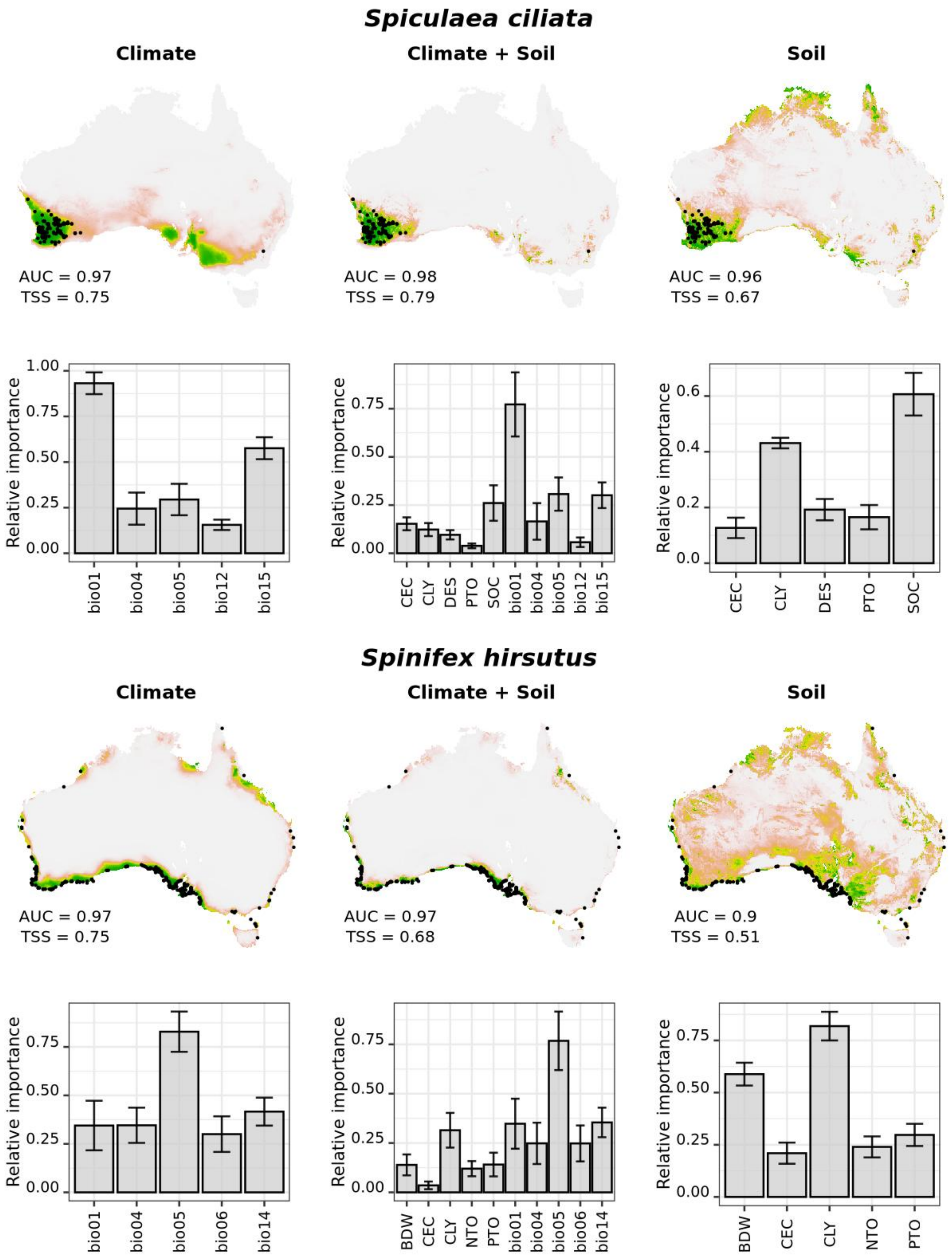
Spiculaea ciliata
Climate
Climate + Soil
Soil
AUC = 0.97
TSS = 0.75
AUC = 0.98
TSS = 0.79
AUC = 0.96
TSS = 0.67
Relative importance
1.00
0.75
0.50
0.25
0.00
bio01
bio04
bio05
bio12
bio15
CEC
CLY
DES
PTO
SOC
0.6
0.4
0.2
0.0
Spinifex hirsutus
Climate
Climate + Soil
Soil
AUC = 0.97
TSS = 0.75
AUC = 0.97
TSS = 0.68
AUC = 0.9
TSS = 0.51
bio06
bio14
BDW
NTO

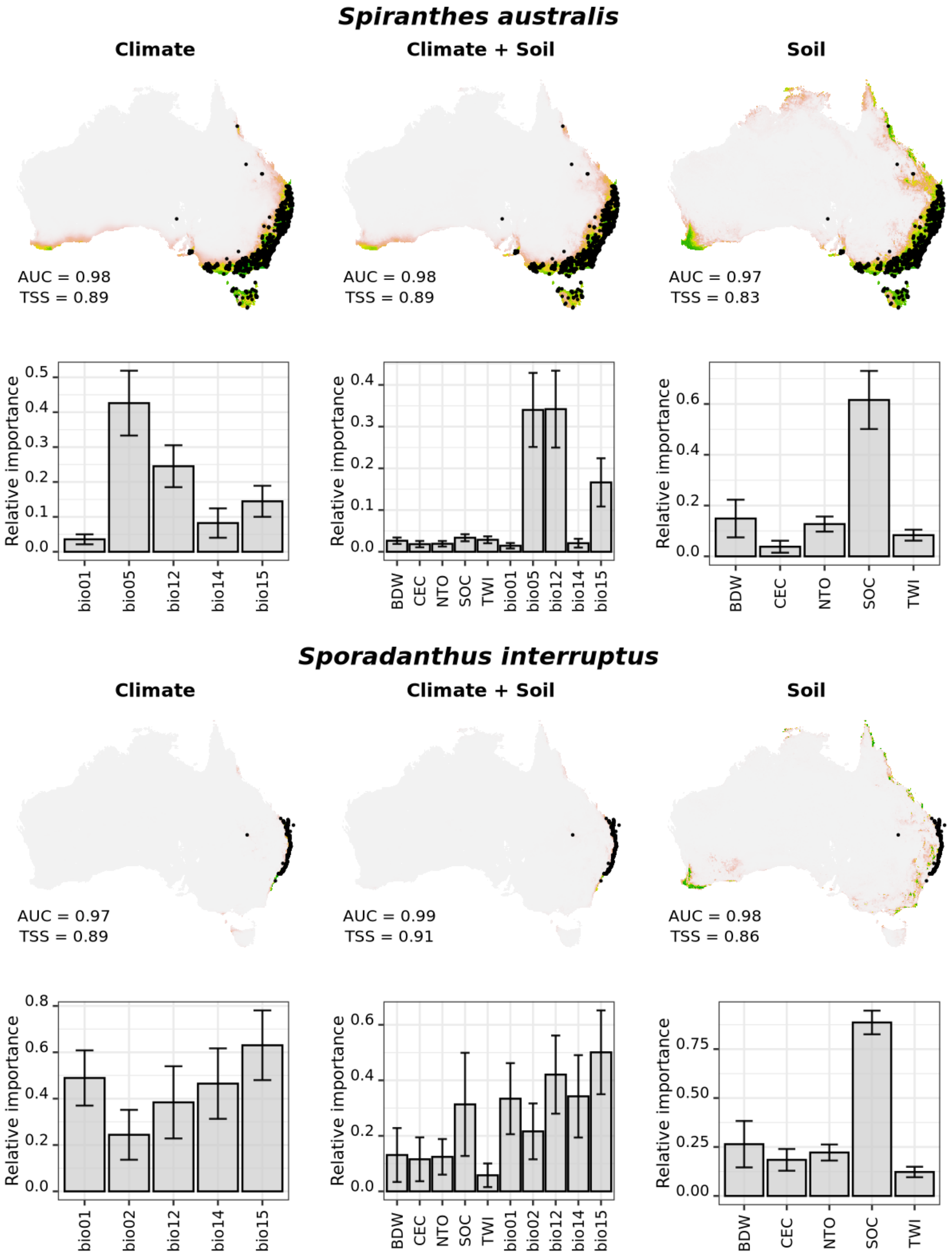
Spiranthes australis
Climate
Climate + Soil
Soil
AUC = 0.98
TSS = 0.89
AUC = 0.98
TSS = 0.89
AUC = 0.97
TSS = 0.83
Relative importance
bio01
bio05
bio12
bio14
bio15
BDW
CEC
NTO
SOC
TWI
Sporadanthus interruptus
Climate
Climate + Soil
Soil
AUC = 0.97
TSS = 0.89
AUC = 0.99
TSS = 0.91
AUC = 0.98
TSS = 0.86
Relative importance
bio01
bio02
bio12
bio14
bio15
BDW
CEC
NTO
SOC
TWI

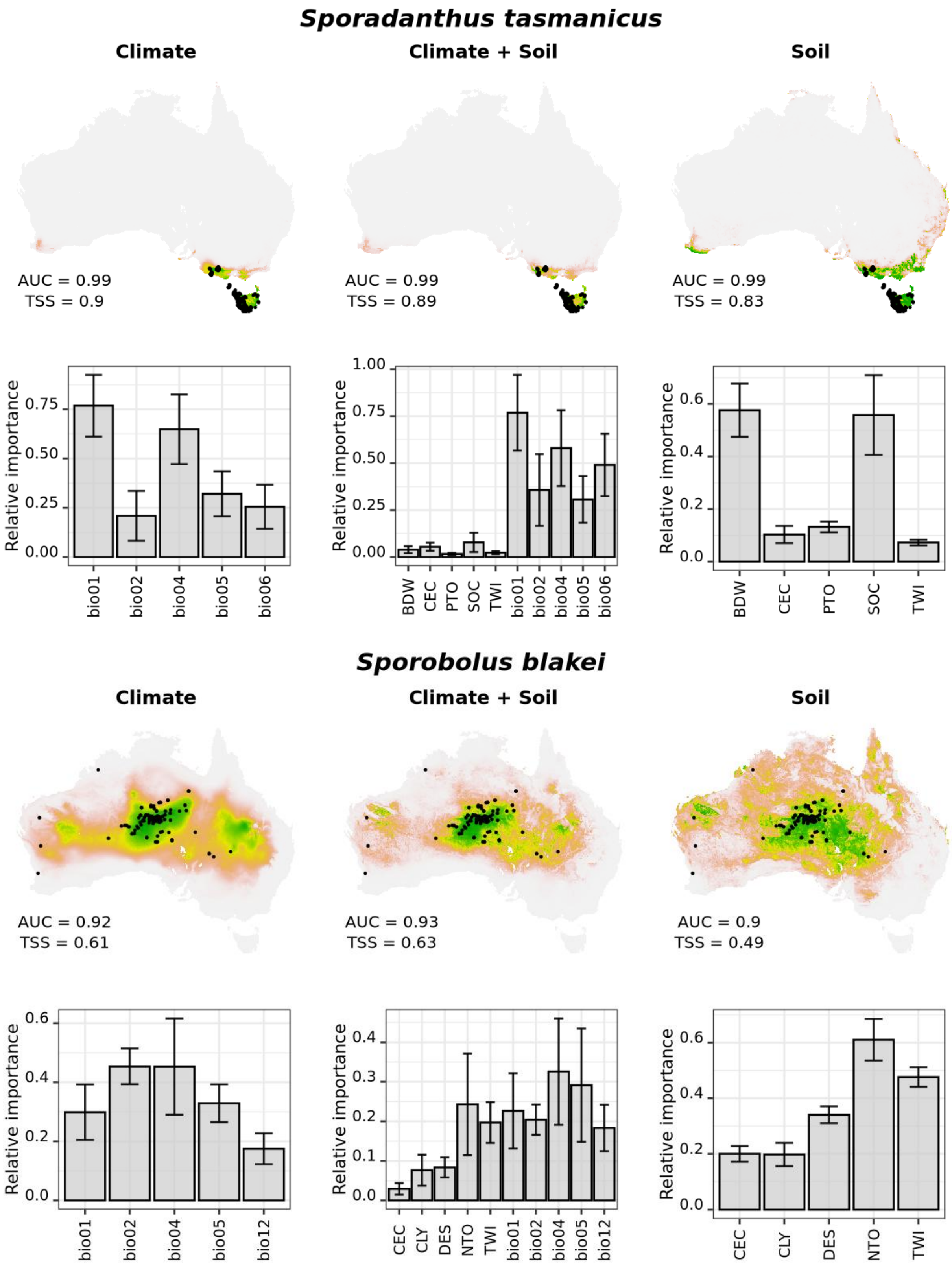
Sporadanthus tasmanicus
Climate
Climate + Soil
Soil
AUC = 0.99
TSS = 0.9
AUC = 0.99
TSS = 0.89
AUC = 0.99
TSS = 0.83
Relative importance
0.00
0.25
0.50
0.75
1.00
0.0
0.2
0.4
0.6
bio01
bio02
bio04
bio05
bio06
BDW
CEC
PTO
SOC
TWI
Sporobolus blakei
Climate
Climate + Soil
Soil
AUC = 0.92
TSS = 0.61
AUC = 0.93
TSS = 0.63
AUC = 0.9
TSS = 0.49
Relative importance
0.0
0.1
0.2
0.3
0.4
0.6
bio01
bio02
bio04
bio05
bio12
CEC
CLY
DES
NTO
TWI

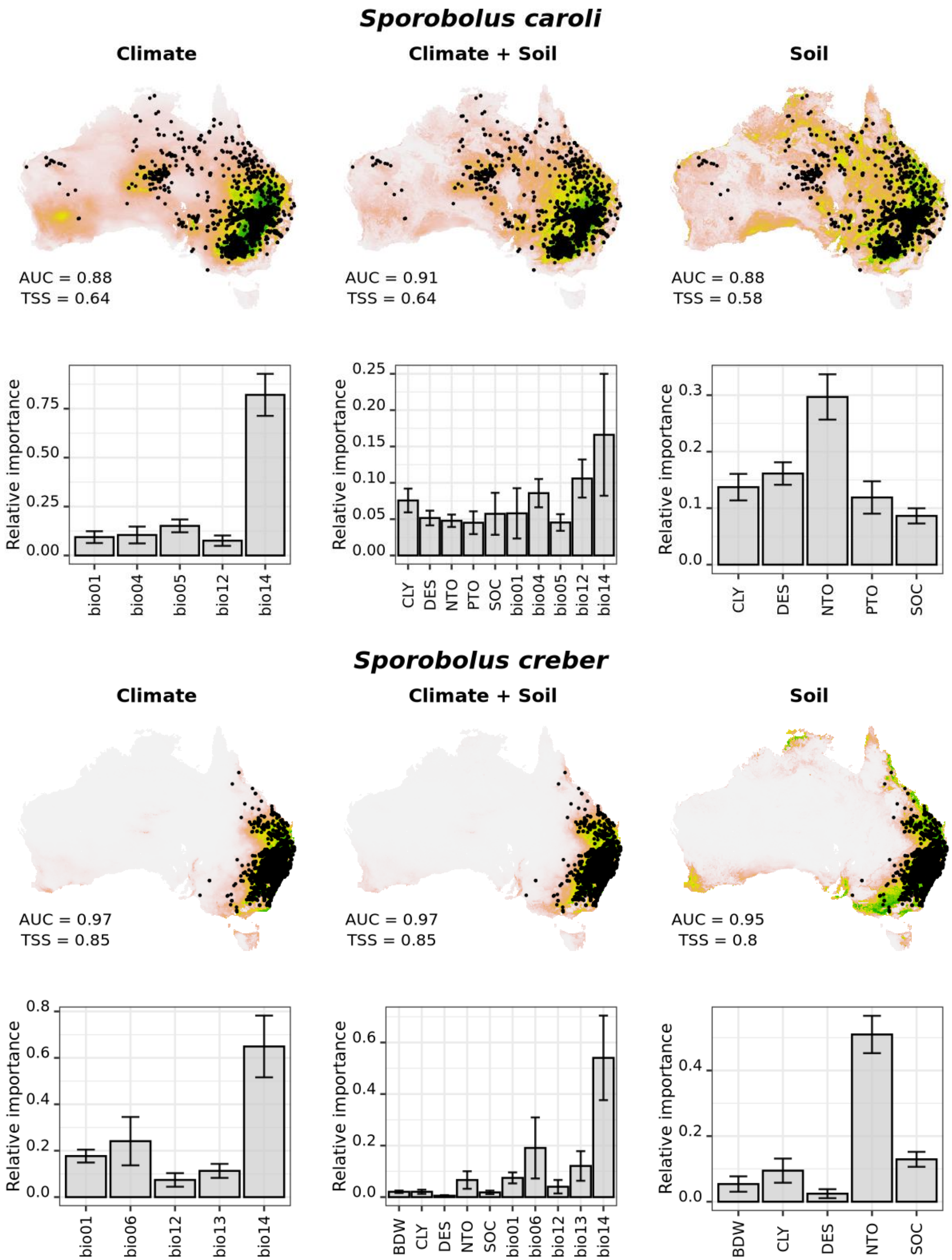
Sporobolus caroli
Climate
Climate + Soil
Soil
AUC = 0.88
TSS = 0.64
AUC = 0.91
TSS = 0.64
AUC = 0.88
TSS = 0.58
Relative importance
0.00
0.25
0.50
0.75
bio01
bio04
bio05
bio12
bio14
0.00
0.05
0.10
0.15
0.20
0.25
CLY
DES
NTO
PTO
SOC
bio01
bio04
bio05
bio12
bio14
0.0
0.1
0.2
0.3
CLY
DES
NTO
PTO
SOC
Sporobolus creber
Climate
Climate + Soil
Soil
AUC = 0.97
TSS = 0.85
AUC = 0.97
TSS = 0.85
AUC = 0.95
TSS = 0.8
Relative importance
0.0
0.2
0.4
0.6
0.8
bio01
bio06
bio12
bio13
bio14
0.0
0.2
0.4
0.6
BDW
CLY
DES
NTO
SOC
bio01
bio06
bio12
bio13
bio14
0.0
0.2
0.4
BDW
CLY
DES
NTO
SOC

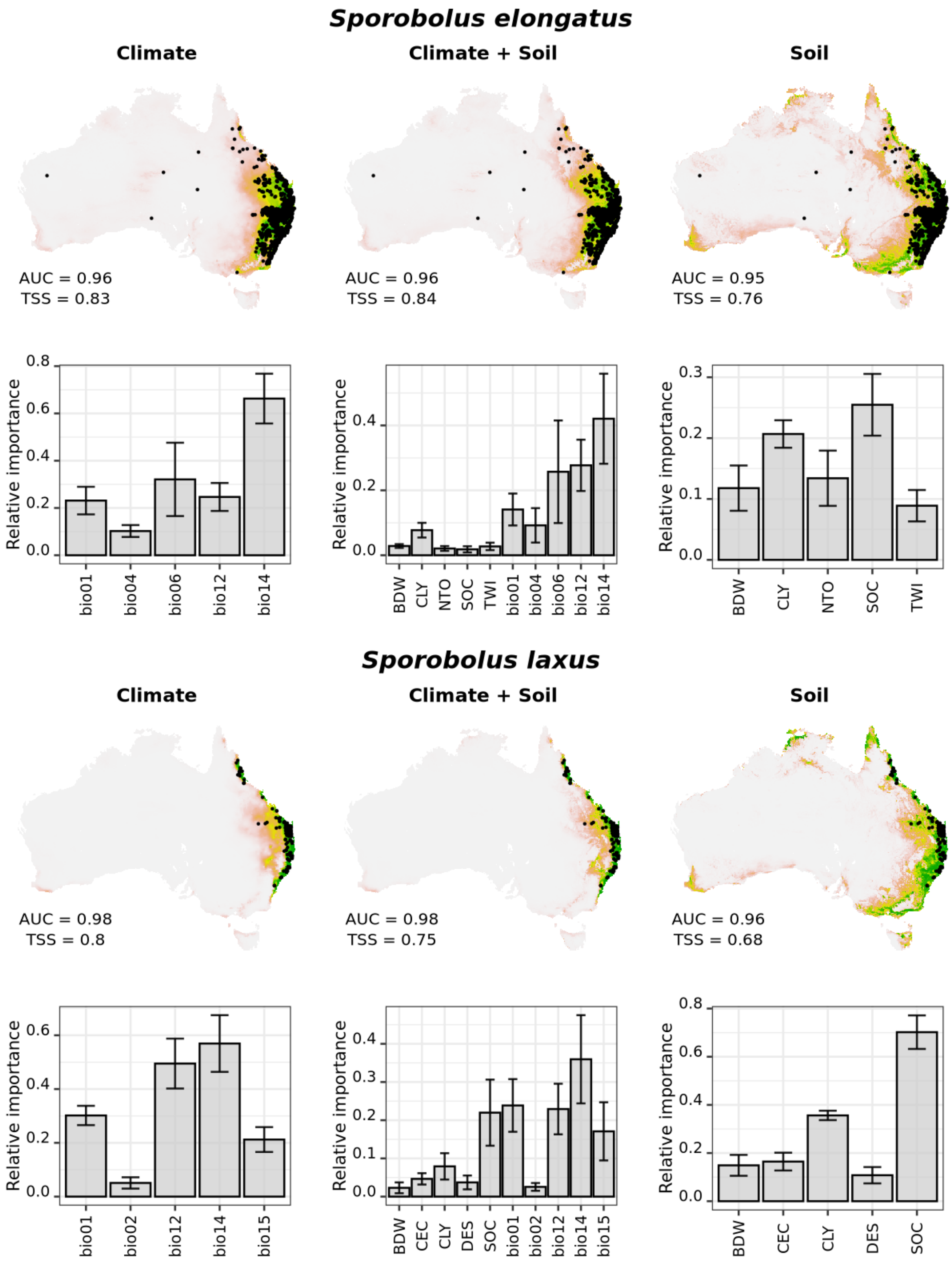

Sporobolus elongatus
Climate
Climate + Soil
Soil
AUC = 0.96
TSS = 0.83
AUC = 0.96
TSS = 0.84
AUC = 0.95
TSS = 0.76
Relative importance
bio01
bio04
bio06
bio12
bio14
BDW
CLY
NTO
SOC
TWI
Sporobolus laxus
AUC = 0.98
TSS = 0.8
AUC = 0.98
TSS = 0.75
AUC = 0.96
TSS = 0.68
bio02
bio15
CEC
DES

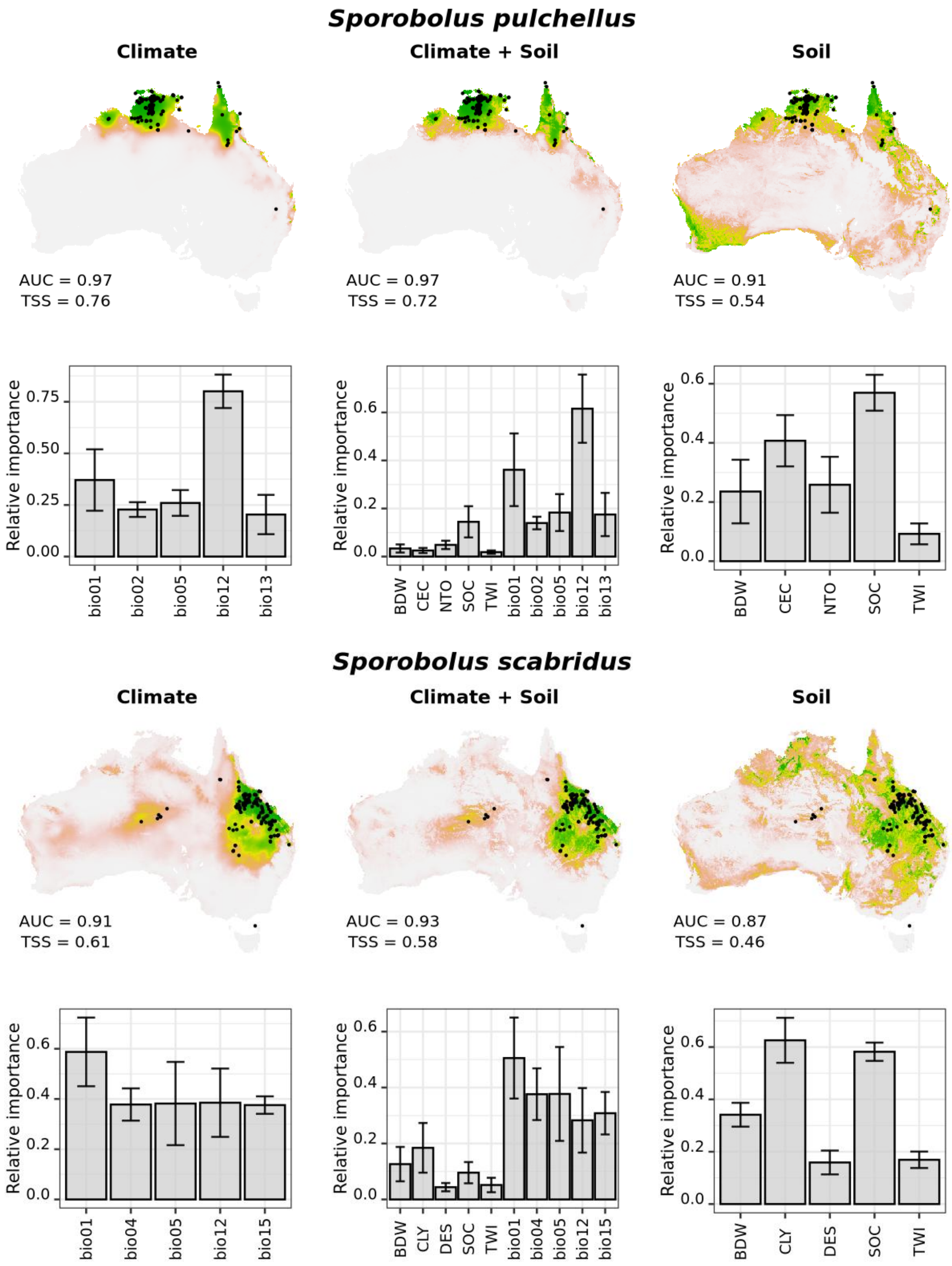
Sporobolus pulchellus
Climate
Climate + Soil
Soil
AUC = 0.97
TSS = 0.76
AUC = 0.97
TSS = 0.72
AUC = 0.91
TSS = 0.54
Relative importance
0.00
0.25
0.50
0.75
bio01
bio02
bio05
bio12
bio13
0.0
0.2
0.4
0.6
BDW
CEC
NTO
SOC
TWI
bio01
bio02
bio05
bio12
bio13
BDW
CEC
NTO
SOC
TWI
Sporobolus scabridus
Climate
Climate + Soil
Soil
AUC = 0.91
TSS = 0.61
AUC = 0.93
TSS = 0.58
AUC = 0.87
TSS = 0.46
Relative importance
0.0
0.2
0.4
0.6
bio01
bio04
bio05
bio12
bio15
BDW
CLY
DES
SOC
TWI
bio01
bio04
bio05
bio12
bio15
BDW
CLY
DES
SOC
TWI

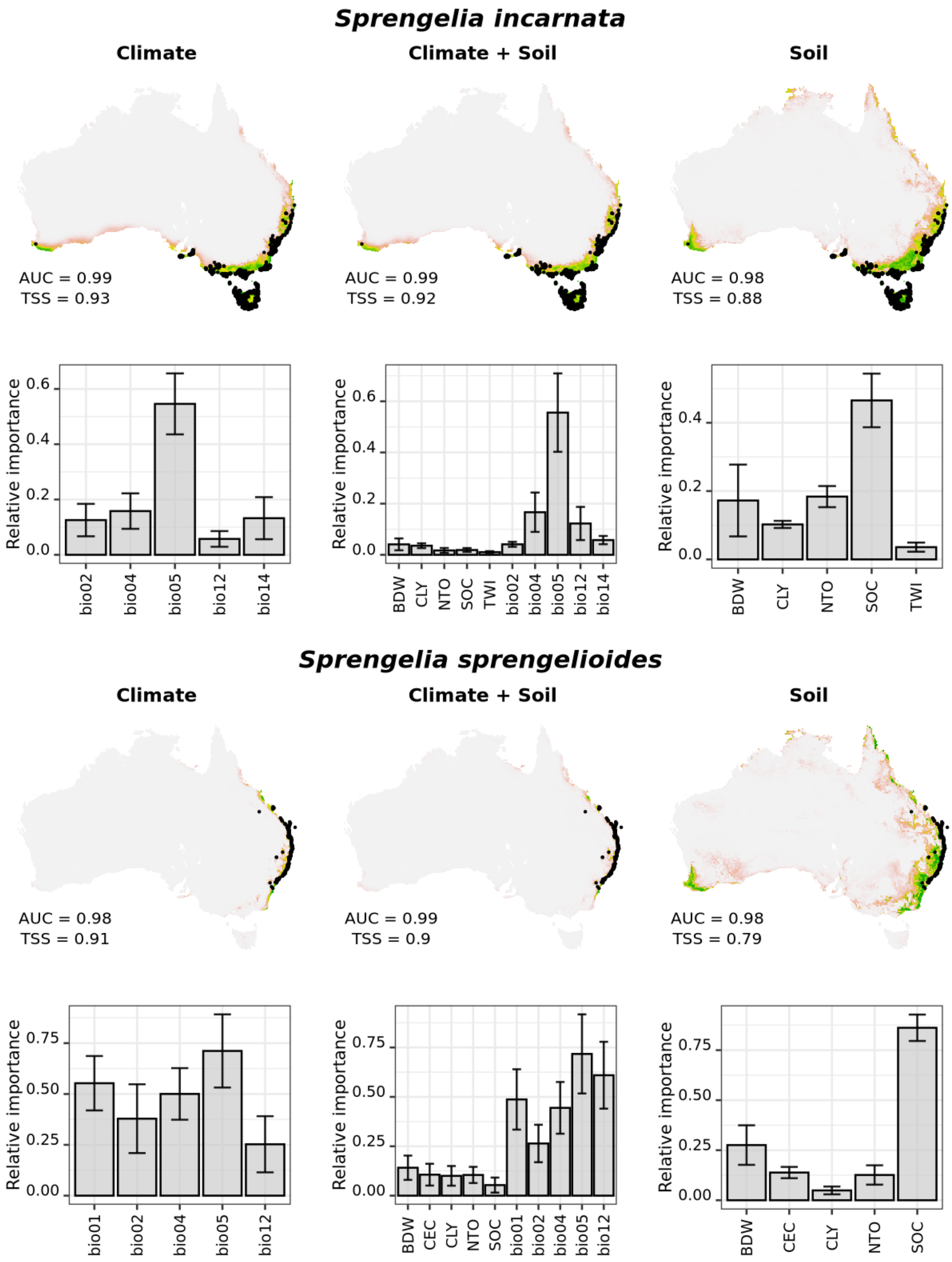
Sprengelia incarnata
Climate
Climate + Soil
Soil
AUC = 0.99
TSS = 0.93
AUC = 0.99
TSS = 0.92
AUC = 0.98
TSS = 0.88
Relative importance
0.0
0.2
0.4
0.6
bio02
bio04
bio05
bio12
bio14
BDW
CLY
NTO
SOC
TWI
Sprengelia sprengelioides
AUC = 0.98
TSS = 0.91
AUC = 0.99
TSS = 0.9
AUC = 0.98
TSS = 0.79
0.00
0.25
0.50
0.75
bio01
CEC

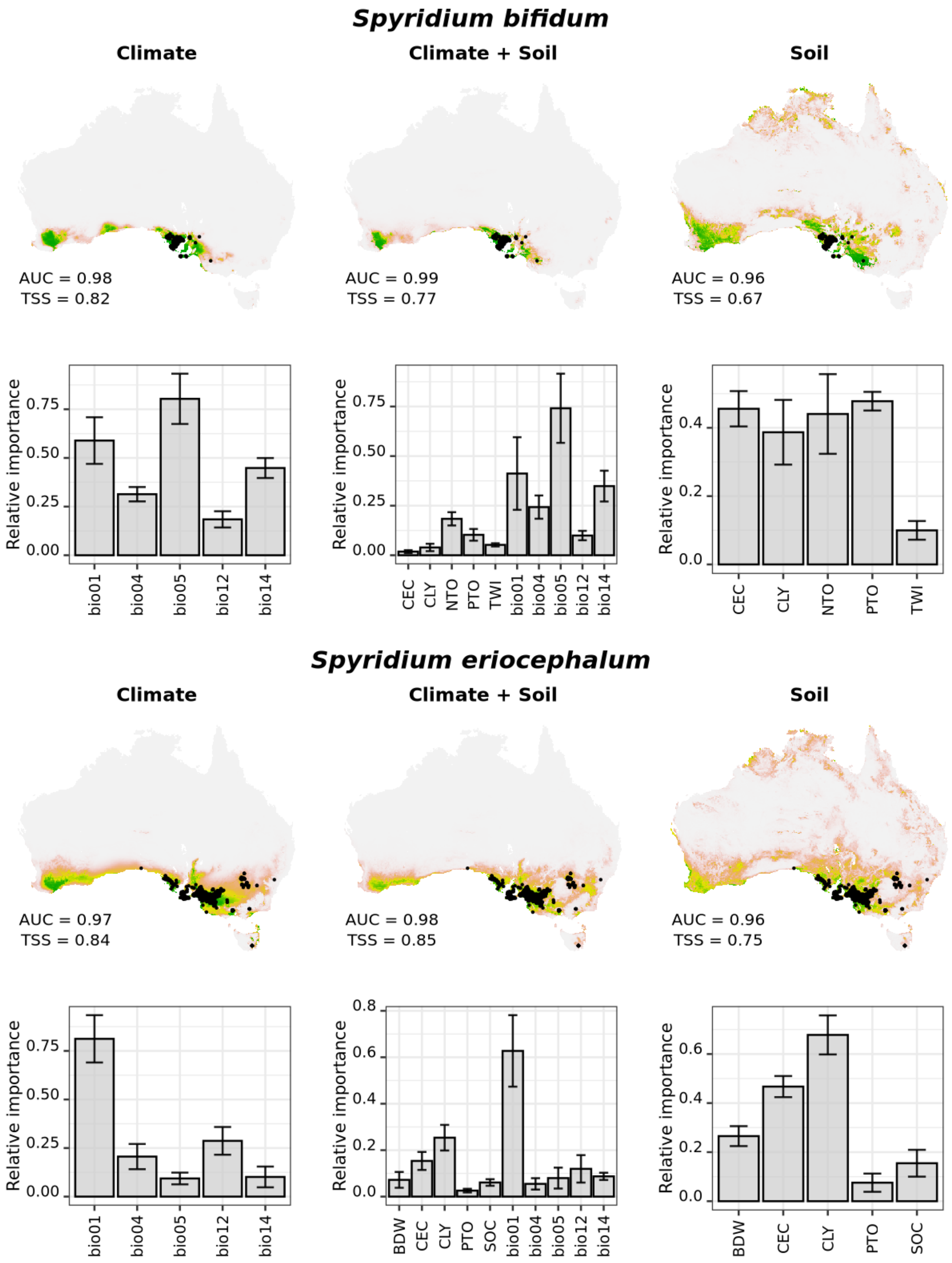

Spyridium bifidum
Climate
Climate + Soil
Soil
AUC = 0.98
TSS = 0.82
AUC = 0.99
TSS = 0.77
AUC = 0.96
TSS = 0.67
Relative importance
bio01 bio04 bio05 bio12 bio14
CEC CLY NTO PTO TWI bio01 bio04 bio05 bio12 bio14
CEC CLY NTO PTO TWI
Spyridium eriocephalum
Climate
Climate + Soil
Soil
AUC = 0.97
TSS = 0.84
AUC = 0.98
TSS = 0.85
AUC = 0.96
TSS = 0.75
Relative importance
bio01 bio04 bio05 bio12 bio14
BDW CEC CLY PTO SOC bio01 bio04 bio05 bio12 bio14
BDW CEC CLY PTO SOC

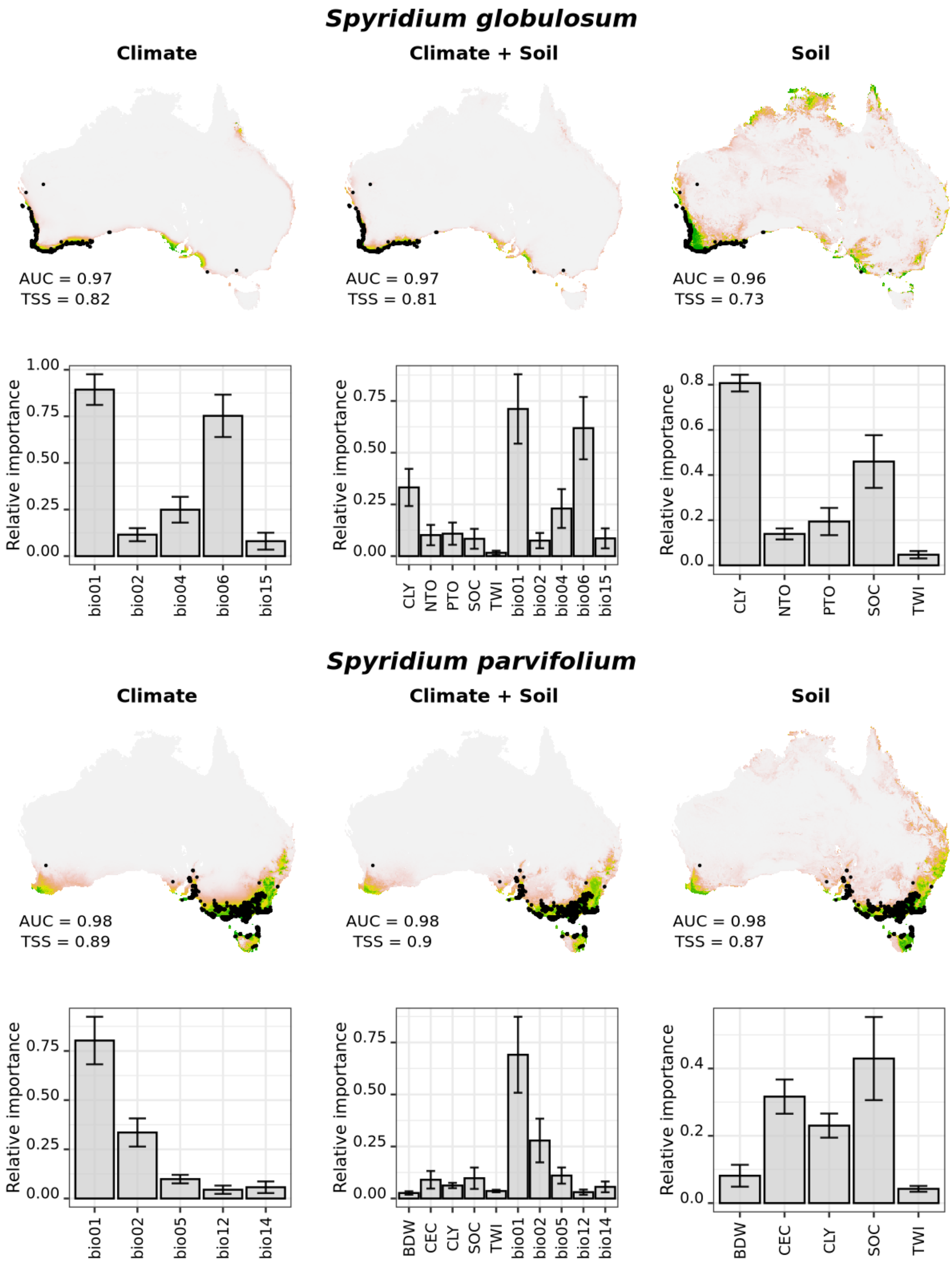
Spyridium globulosum
Climate
Climate + Soil
Soil
AUC = 0.97
TSS = 0.82
AUC = 0.97
TSS = 0.81
AUC = 0.96
TSS = 0.73
Relative importance
1.00
0.75
0.50
0.25
0.00
bio01
bio02
bio04
bio06
bio15
CLY
NTO
PTO
SOC
TWI
0.8
0.6
0.4
0.2
0.0
Spyridium parvifolium
Climate
Climate + Soil
Soil
AUC = 0.98
TSS = 0.89
AUC = 0.98
TSS = 0.9
AUC = 0.98
TSS = 0.87
bio05
bio12
bio14
BDW
CEC

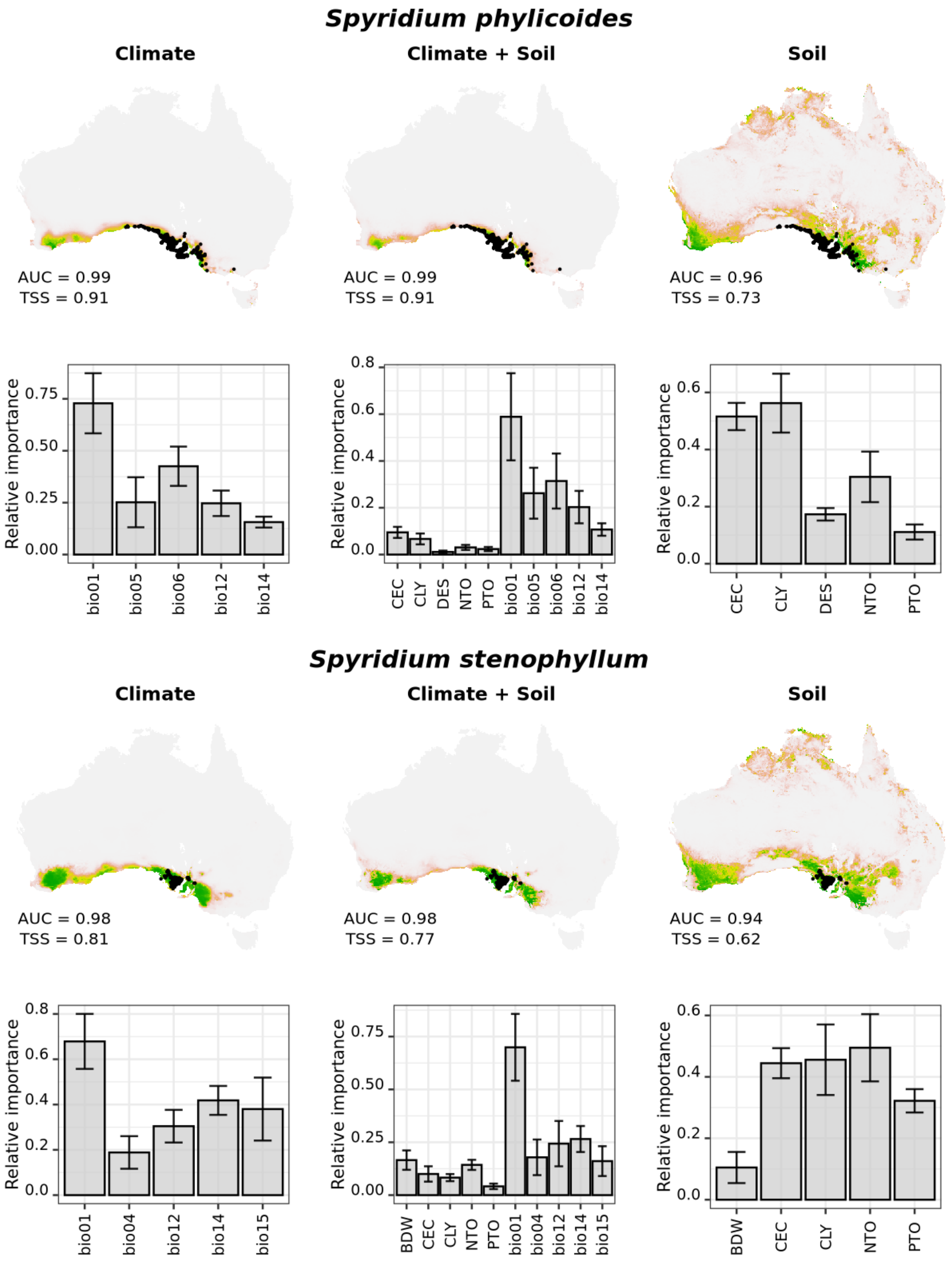

Spyridium phylicoides
Climate
Climate + Soil
Soil
AUC = 0.99
TSS = 0.91
AUC = 0.99
TSS = 0.91
AUC = 0.96
TSS = 0.73
Relative importance
0.00 0.25 0.50 0.75
bio01 bio05 bio06 bio12 bio14
0.0 0.2 0.4 0.6 0.8
CEC CLY DES NTO PTO bio01 bio05 bio06 bio12 bio14
0.0 0.2 0.4 0.6
CEC CLY DES NTO PTO
Spyridium stenophyllum
Climate
Climate + Soil
Soil
AUC = 0.98
TSS = 0.81
AUC = 0.98
TSS = 0.77
AUC = 0.94
TSS = 0.62
Relative importance
0.0 0.2 0.4 0.6 0.8
bio01 bio04 bio12 bio14 bio15
0.00 0.25 0.50 0.75
BDW CEC CLY NTO PTO bio01 bio04 bio12 bio14 bio15
0.0 0.2 0.4 0.6
BDW CEC CLY NTO PTO

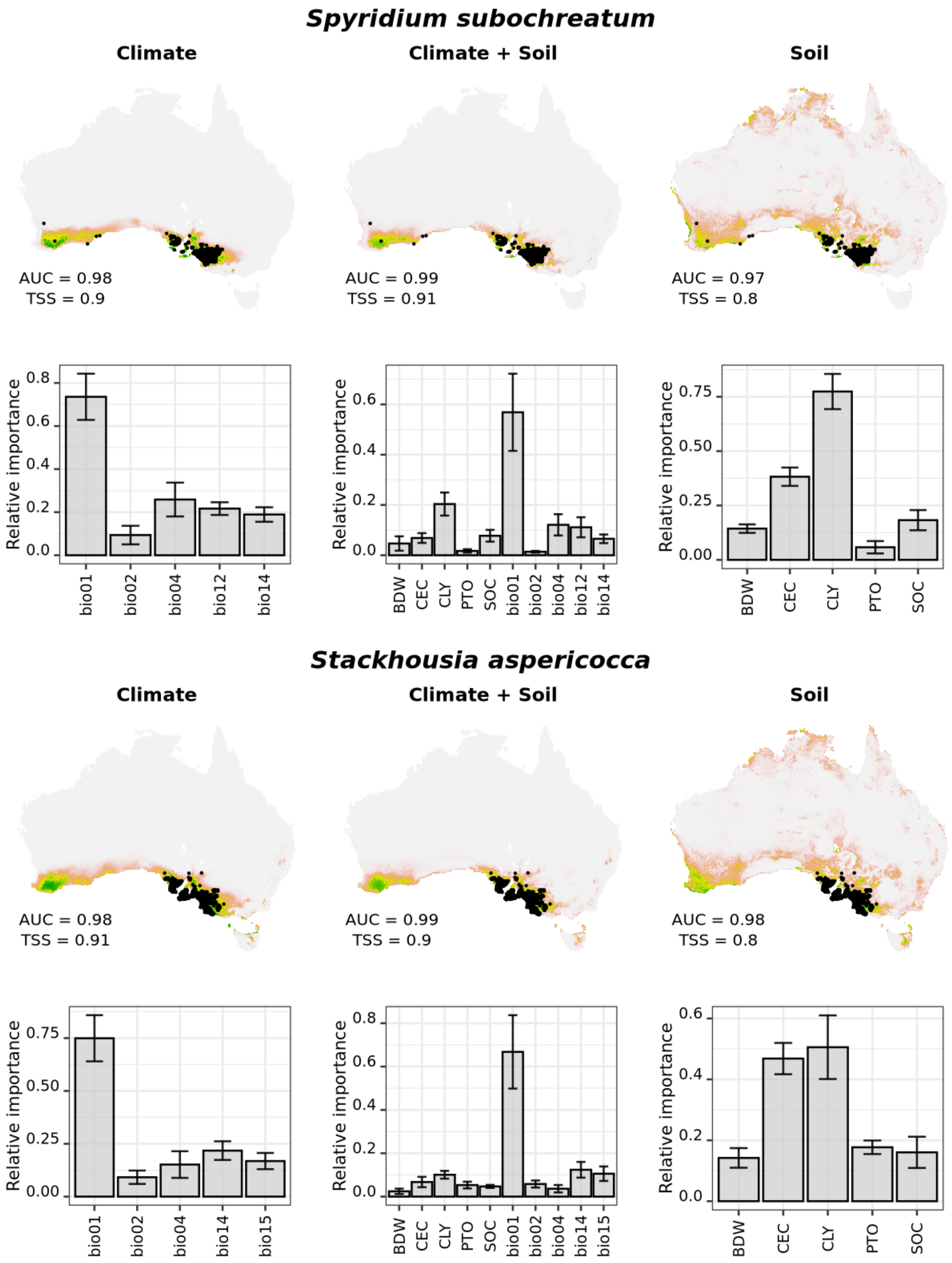

Spyridium subochreatum
Climate
Climate + Soil
Soil
AUC = 0.98
TSS = 0.9
AUC = 0.99
TSS = 0.91
AUC = 0.97
TSS = 0.8
Relative importance
bio01
bio02
bio04
bio12
bio14
BDW
CEC
CLY
PTO
SOC
Stackhousia aspericocca
Climate
Climate + Soil
Soil
AUC = 0.98
TSS = 0.91
AUC = 0.99
TSS = 0.9
AUC = 0.98
TSS = 0.8
Relative importance
bio01
bio02
bio04
bio14
bio15
BDW
CEC
CLY
PTO
SOC

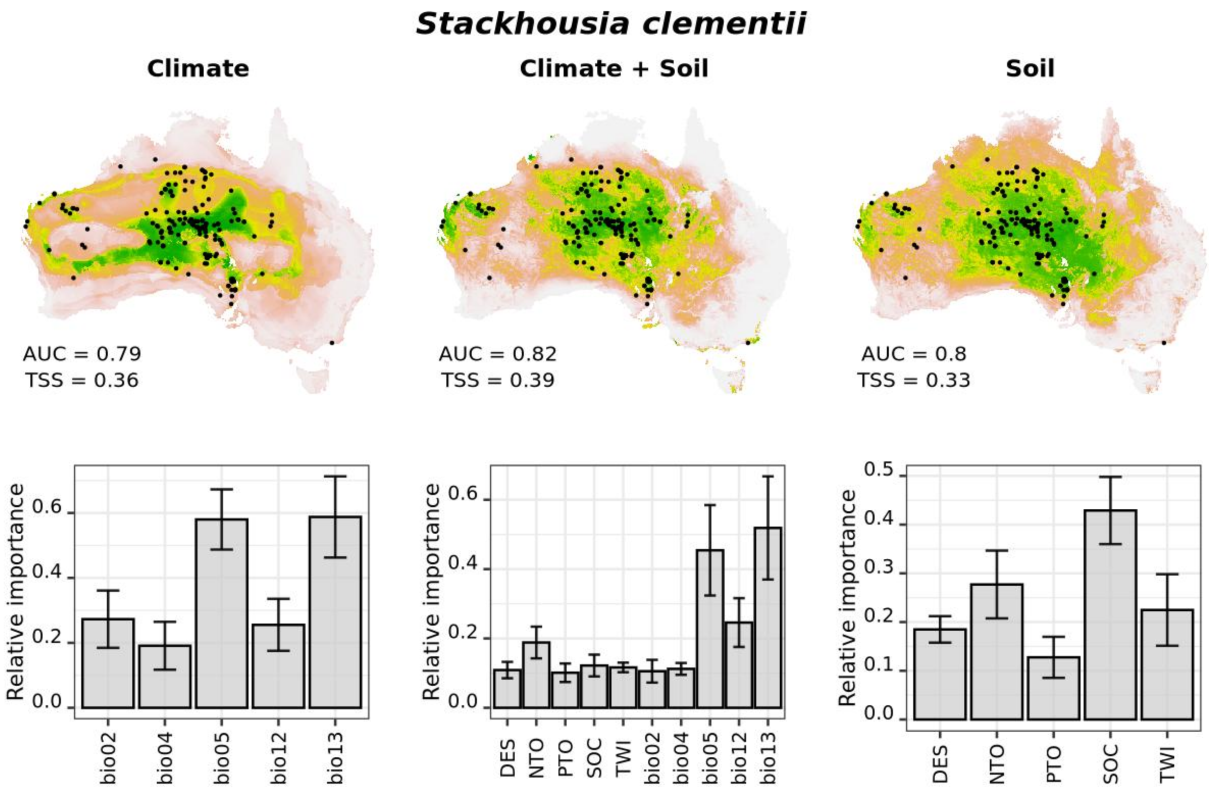
Stackhousia clementii
Climate
Climate + Soil
Soil
AUC = 0.79
TSS = 0.36
AUC = 0.82
TSS = 0.39
AUC = 0.8
TSS = 0.33
Relative importance
bio02
bio04
bio05
bio12
bio13
DES
NTO
PTO
SOC
TWI

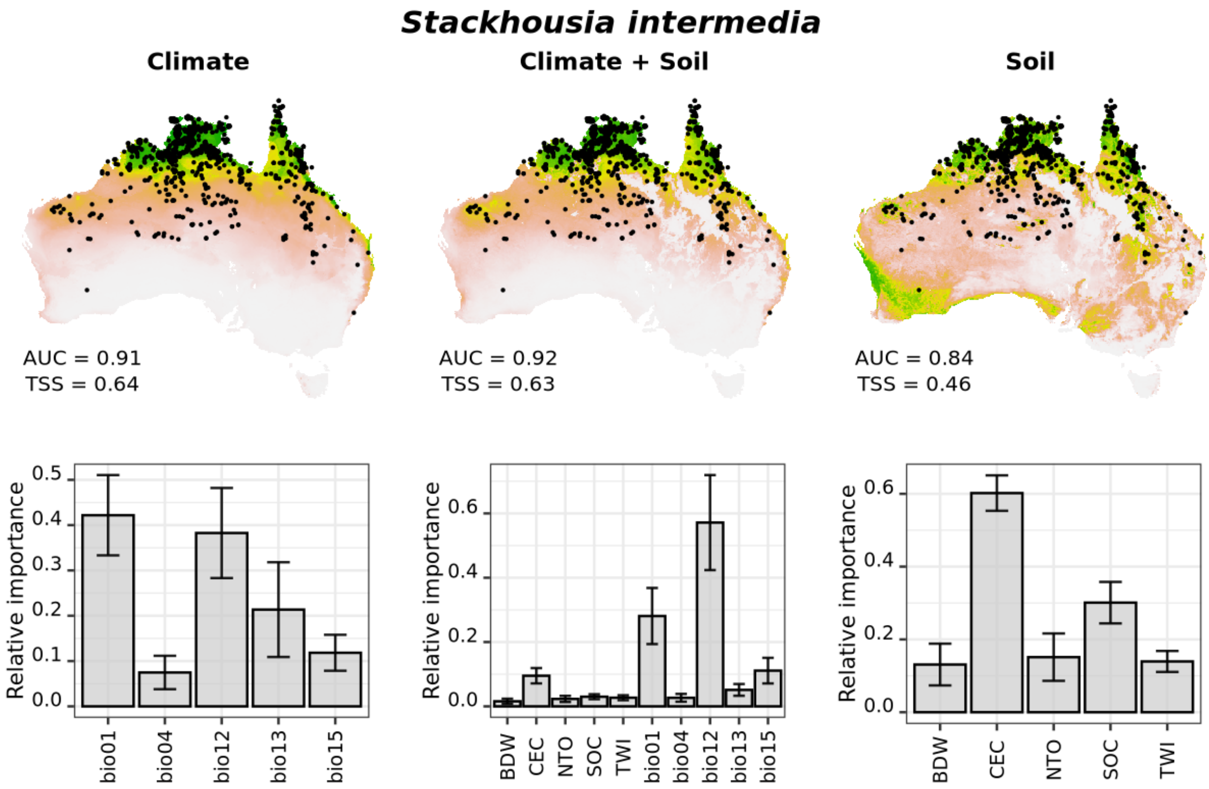
Stackhousia intermedia
Climate
Climate + Soil
Soil
AUC = 0.91
TSS = 0.64
AUC = 0.92
TSS = 0.63
AUC = 0.84
TSS = 0.46
Relative importance
bio01
bio04
bio12
bio13
bio15
BDW
CEC
NTO
SOC
TWI

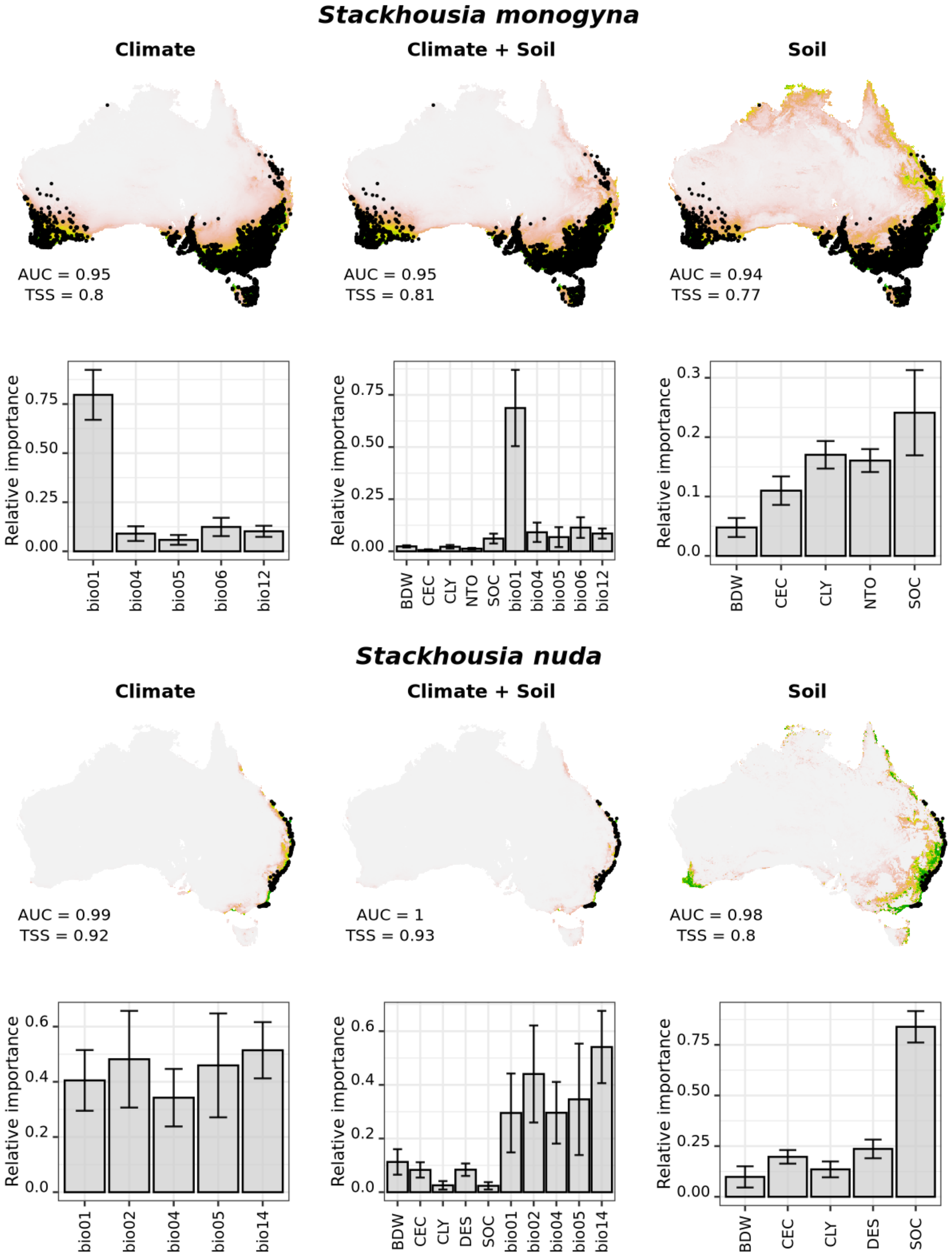

Stackhousia monogyna
Climate
Climate + Soil
Soil
AUC = 0.95
TSS = 0.8
AUC = 0.95
TSS = 0.81
AUC = 0.94
TSS = 0.77
Relative importance
bio01
bio04
bio05
bio06
bio12
BDW
CEC
CLY
NTO
SOC
Stackhousia nuda
Climate
Climate + Soil
Soil
AUC = 0.99
TSS = 0.92
AUC = 1
TSS = 0.93
AUC = 0.98
TSS = 0.8
Relative importance
bio01
bio02
bio04
bio05
bio14
BDW
CEC
CLY
DES
SOC

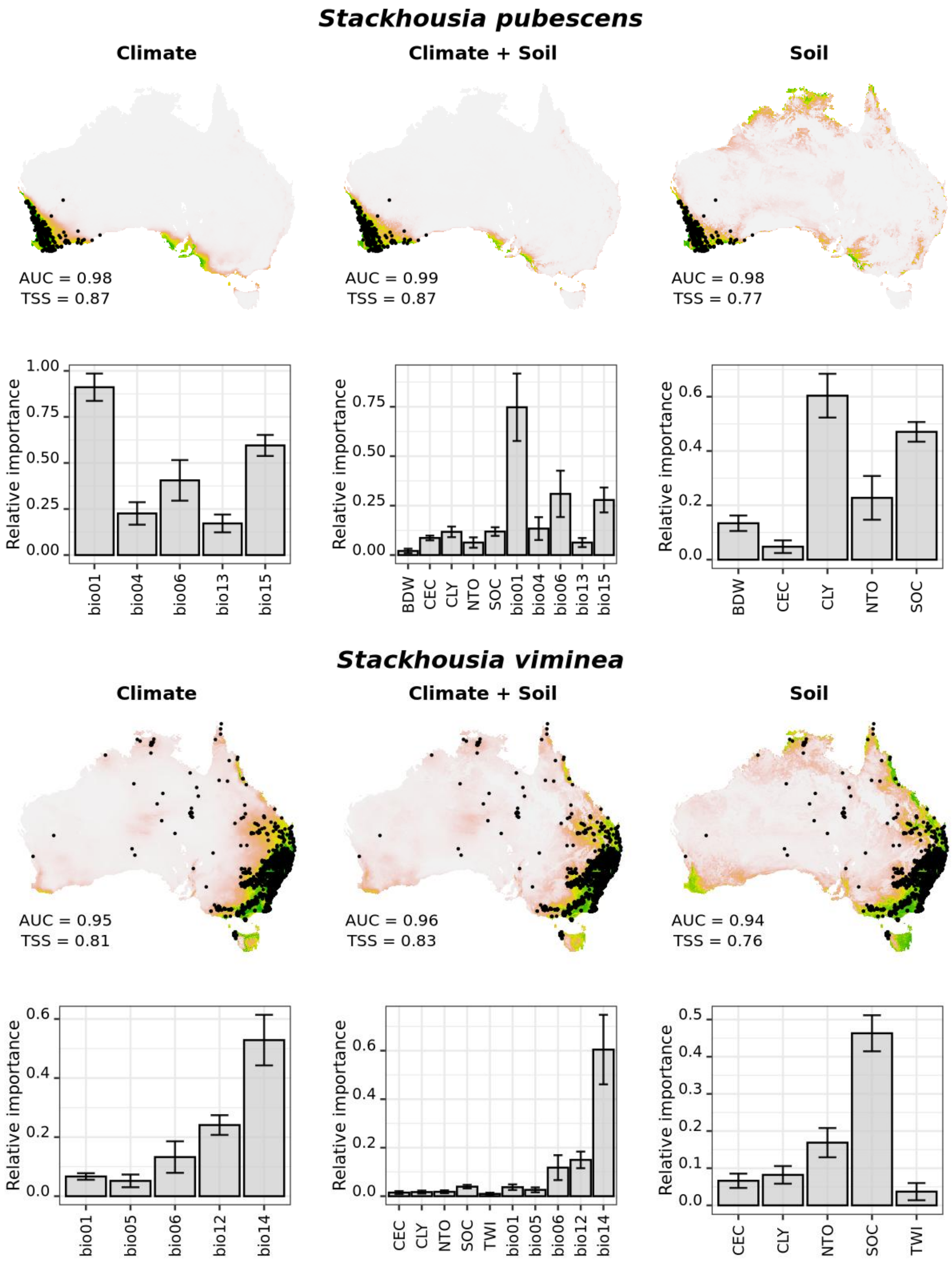

Stackhousia pubescens
Climate
Climate + Soil
Soil
AUC = 0.98
TSS = 0.87
AUC = 0.99
TSS = 0.87
AUC = 0.98
TSS = 0.77
Relative importance
bio01
bio04
bio06
bio13
bio15
BDW
CEC
CLY
NTO
SOC
Stackhousia viminea
Climate
Climate + Soil
Soil
AUC = 0.95
TSS = 0.81
AUC = 0.96
TSS = 0.83
AUC = 0.94
TSS = 0.76
Relative importance
bio01
bio05
bio06
bio12
bio14
CEC
CLY
NTO
SOC
TWI

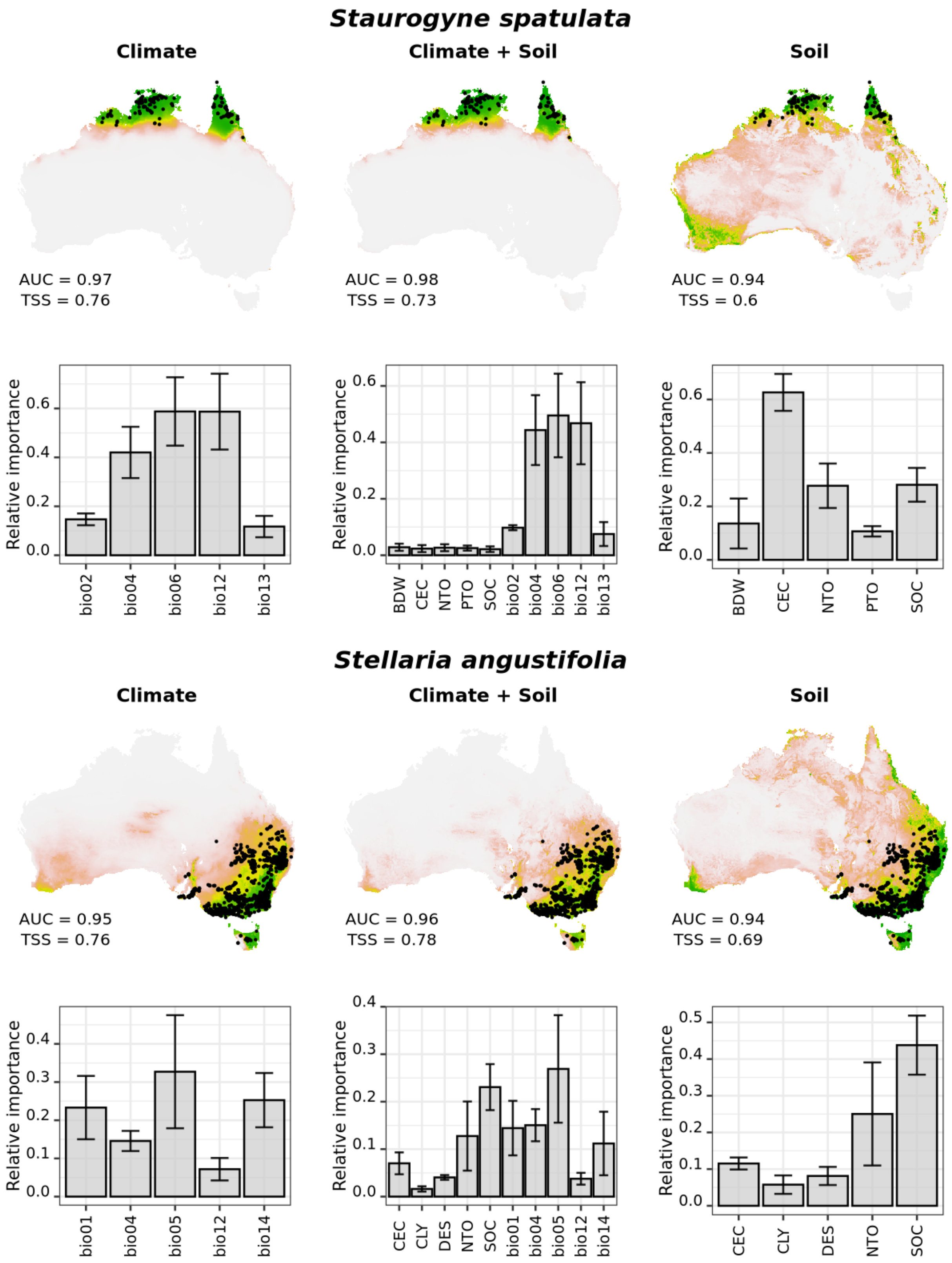
Staurogyne spatulata
Climate
Climate + Soil
Soil
AUC = 0.97
TSS = 0.76
AUC = 0.98
TSS = 0.73
AUC = 0.94
TSS = 0.6
Relative importance
0.0
0.2
0.4
0.6
bio02
bio04
bio06
bio12
bio13
BDW
CEC
NTO
PTO
SOC
Stellaria angustifolia
Climate
Climate + Soil
Soil
AUC = 0.95
TSS = 0.76
AUC = 0.96
TSS = 0.78
AUC = 0.94
TSS = 0.69
Relative importance
0.0
0.1
0.2
0.3
0.4
0.5
bio01
bio04
bio05
bio12
bio14
CEC
CLY
DES
NTO
SOC

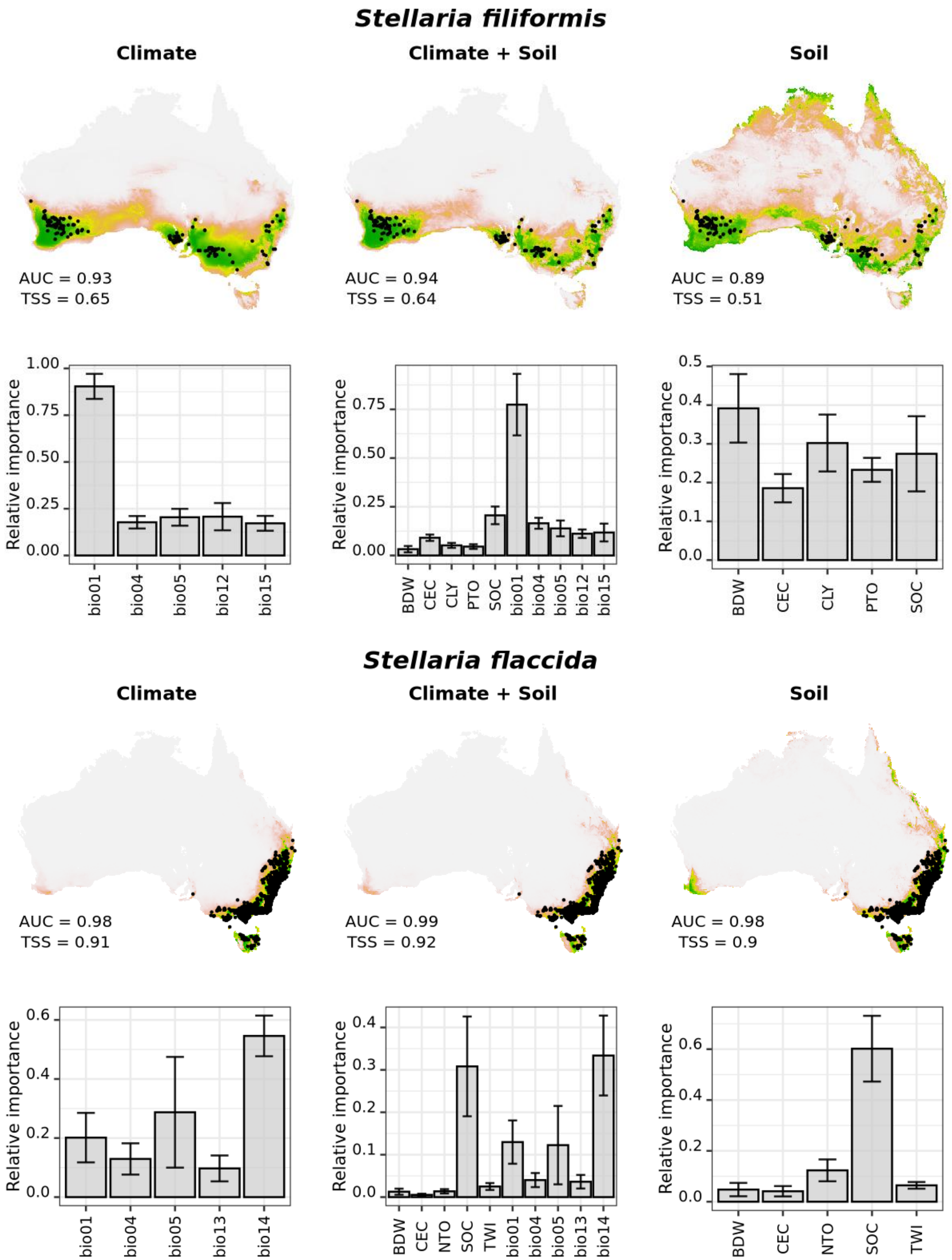

Stellaria filiformis
Climate
Climate + Soil
Soil
AUC = 0.93
TSS = 0.65
AUC = 0.94
TSS = 0.64
AUC = 0.89
TSS = 0.51
Relative importance
bio01
bio04
bio05
bio12
bio15
BDW
CEC
CLY
PTO
SOC
Stellaria flaccida
Climate
Climate + Soil
Soil
AUC = 0.98
TSS = 0.91
AUC = 0.99
TSS = 0.92
AUC = 0.98
TSS = 0.9
Relative importance
bio01
bio04
bio05
bio13
bio14
BDW
CEC
NTO
SOC
TWI

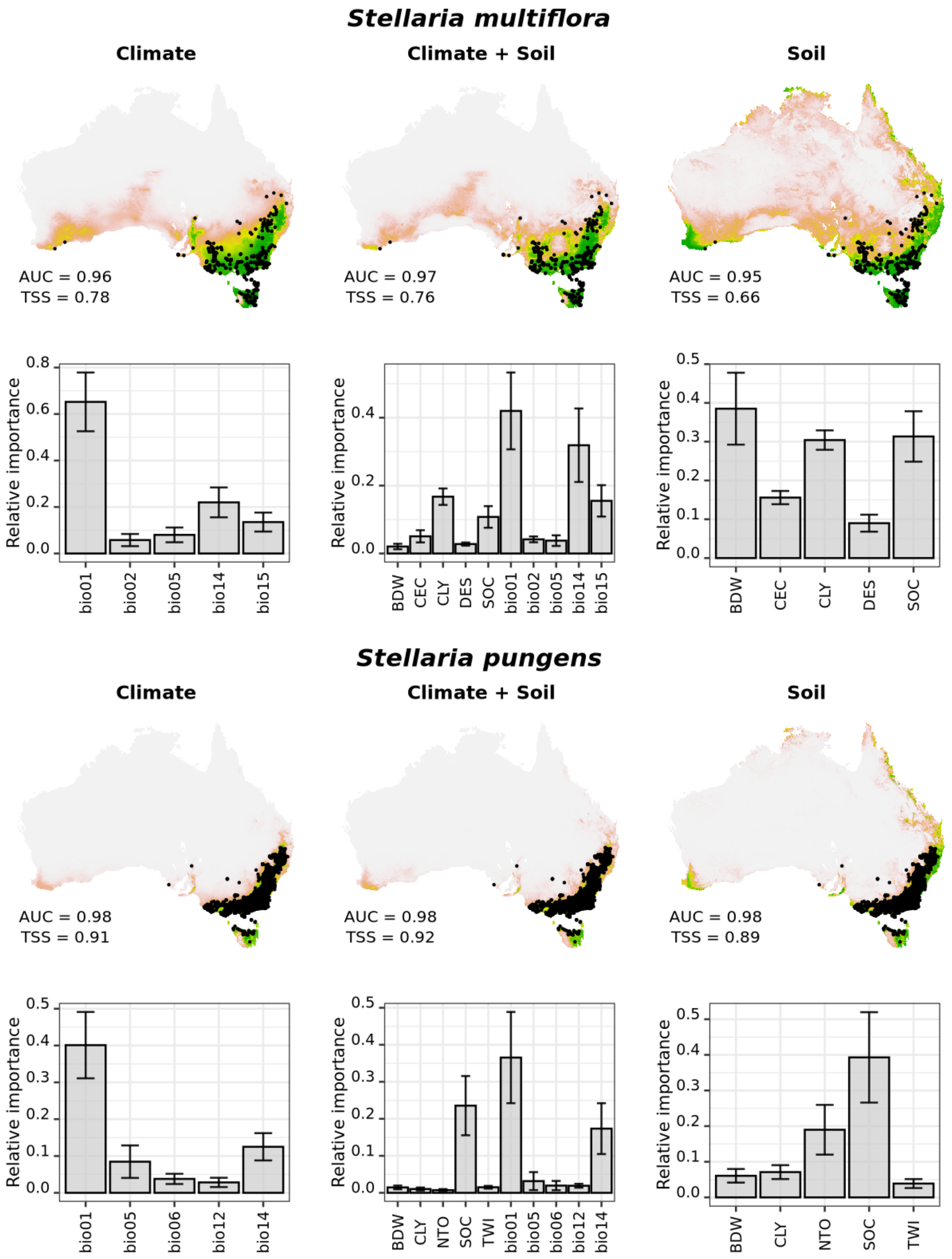
Stellaria multiflora
Climate
Climate + Soil
Soil
AUC = 0.96
TSS = 0.78
AUC = 0.97
TSS = 0.76
AUC = 0.95
TSS = 0.66
Relative importance
bio01
bio02
bio05
bio14
bio15
BDW
CEC
CLY
DES
SOC
Stellaria pungens
AUC = 0.98
TSS = 0.91
AUC = 0.98
TSS = 0.92
AUC = 0.98
TSS = 0.89
bio06
bio12
NTO
TWI

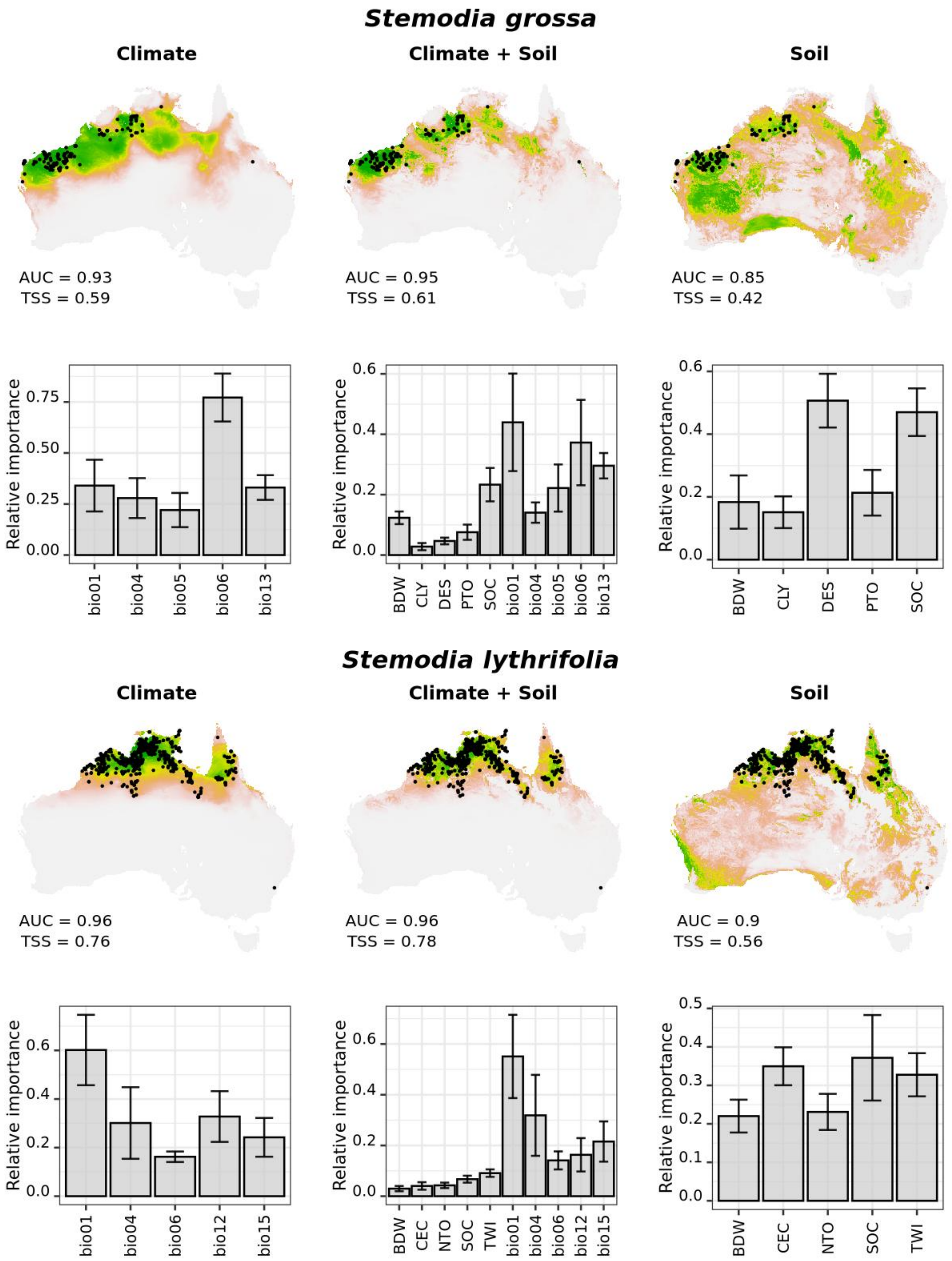
Stemodia grossa
Climate
Climate + Soil
Soil
AUC = 0.93
TSS = 0.59
AUC = 0.95
TSS = 0.61
AUC = 0.85
TSS = 0.42
Relative importance
bio01 bio04 bio05 bio06 bio13
BDW CLY DES PTO SOC bio01 bio04 bio05 bio06 bio13
BDW CLY DES PTO SOC
Stemodia lythrifolia
Climate
Climate + Soil
Soil
AUC = 0.96
TSS = 0.76
AUC = 0.96
TSS = 0.78
AUC = 0.9
TSS = 0.56
Relative importance
bio01 bio04 bio06 bio12 bio15
BDW CEC NTO SOC TWI bio01 bio04 bio06 bio12 bio15
BDW CEC NTO SOC TWI

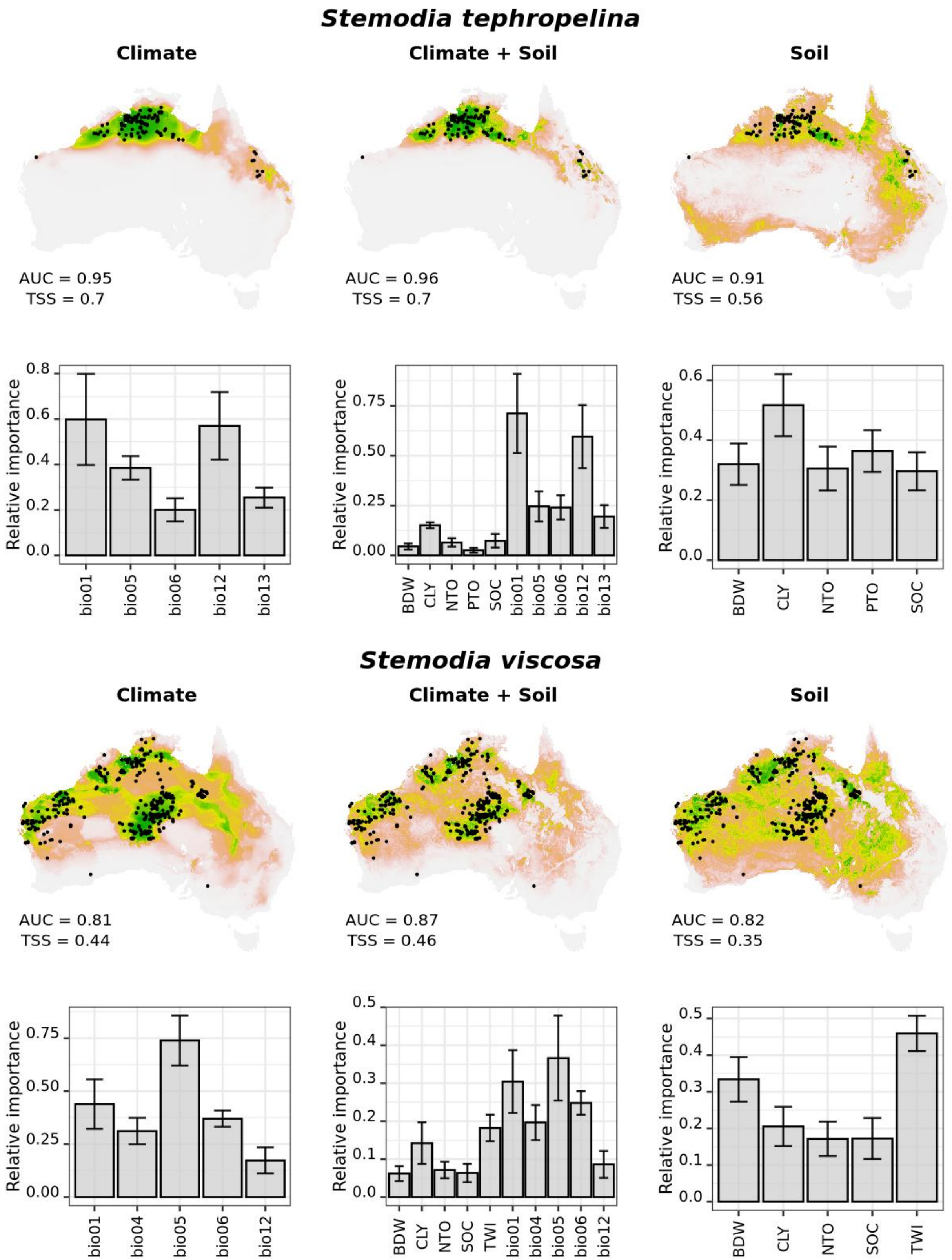
Stemodia tephropelina
Climate
Climate + Soil
Soil
AUC = 0.95
TSS = 0.7
AUC = 0.96
TSS = 0.7
AUC = 0.91
TSS = 0.56
Relative importance
0.0
0.2
0.4
0.6
0.8
bio01
bio05
bio06
bio12
bio13
0.00
0.25
0.50
0.75
BDW
CLY
NTO
PTO
SOC
bio01
bio05
bio06
bio12
bio13
0.0
0.2
0.4
0.6
BDW
CLY
NTO
PTO
SOC
Stemodia viscosa
Climate
Climate + Soil
Soil
AUC = 0.81
TSS = 0.44
AUC = 0.87
TSS = 0.46
AUC = 0.82
TSS = 0.35
Relative importance
0.00
0.25
0.50
0.75
bio01
bio04
bio05
bio06
bio12
0.0
0.1
0.2
0.3
0.4
0.5
BDW
CLY
NTO
SOC
TWI
bio01
bio04
bio05
bio06
bio12
0.0
0.1
0.2
0.3
0.4
0.5
BDW
CLY
NTO
SOC
TWI

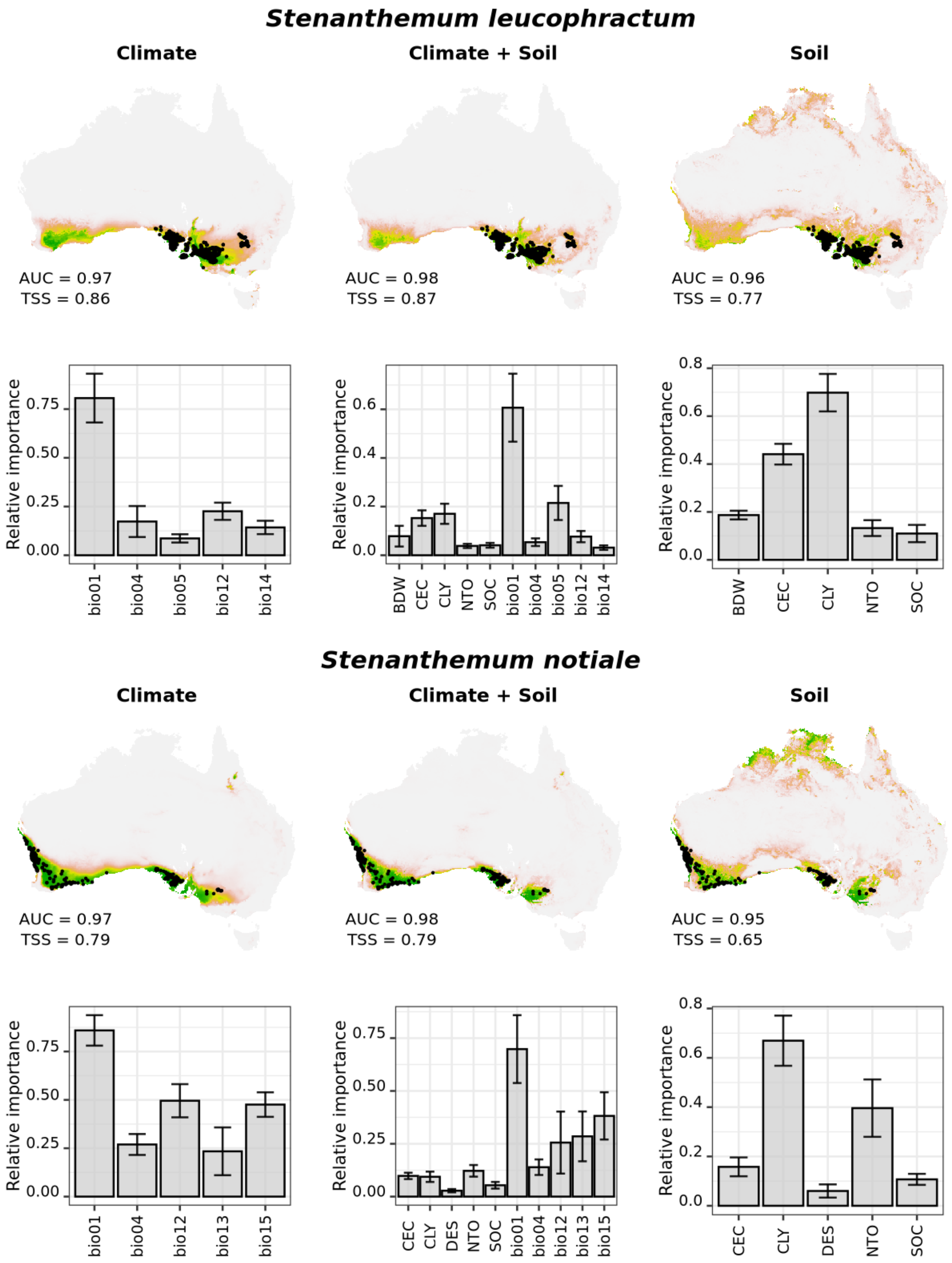

Stenanthemum leucophractum
Climate
Climate + Soil
Soil
AUC = 0.97
TSS = 0.86
AUC = 0.98
TSS = 0.87
AUC = 0.96
TSS = 0.77
Relative importance
0.00
0.25
0.50
0.75
bio01
bio04
bio05
bio12
bio14
0.0
0.2
0.4
0.6
BDW
CEC
CLY
NTO
SOC
bio01
bio04
bio05
bio12
bio14
0.8
BDW
CEC
CLY
NTO
SOC
Stenanthemum notiale
Climate
Climate + Soil
Soil
AUC = 0.97
TSS = 0.79
AUC = 0.98
TSS = 0.79
AUC = 0.95
TSS = 0.65
bio01
bio04
bio12
bio13
bio15
CEC
CLY
DES
NTO
SOC
bio01
bio04
bio12
bio13
bio15
CEC
CLY
DES
NTO
SOC

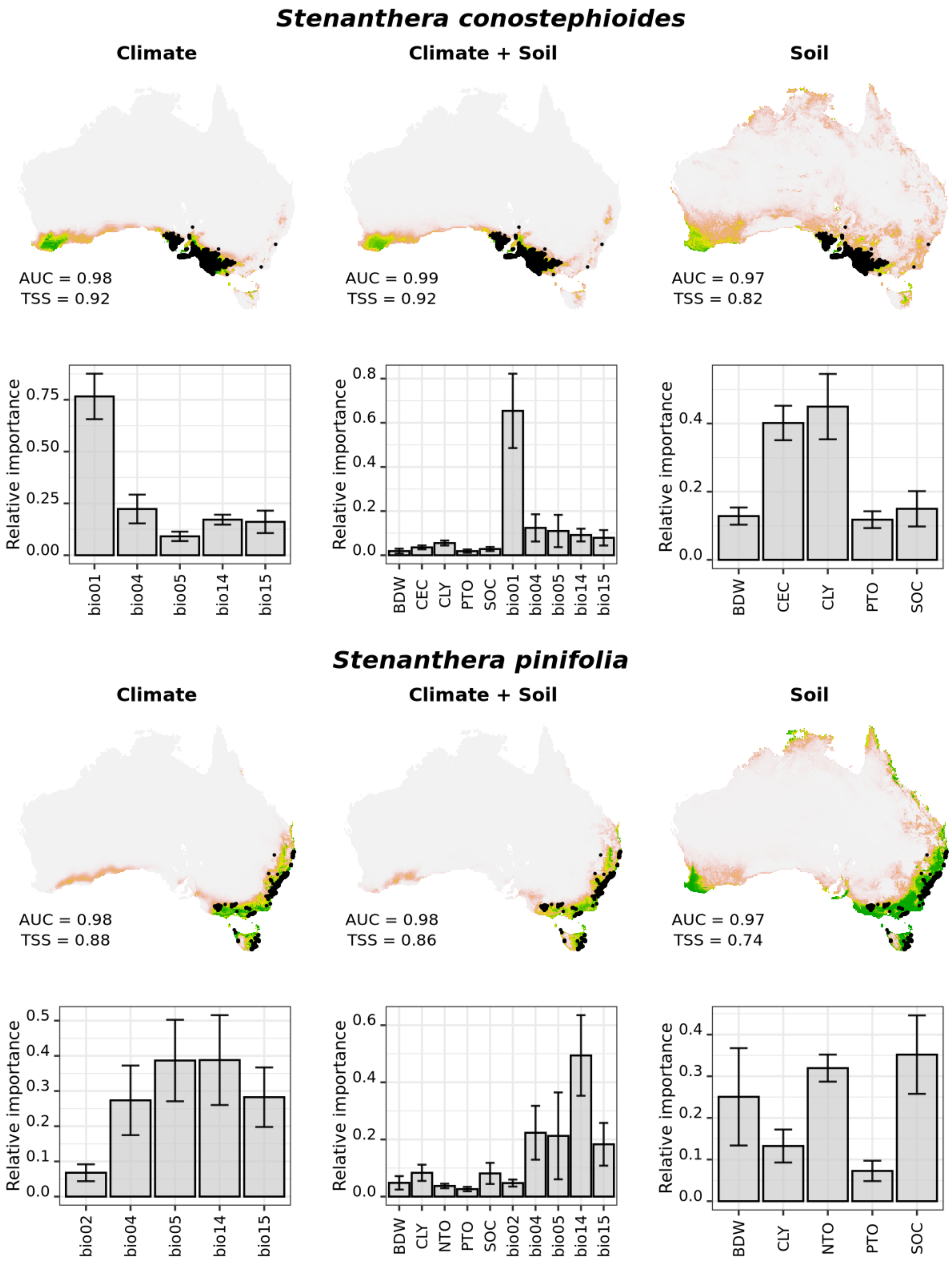
Stenanthera conostephioides
Climate
Climate + Soil
Soil
AUC = 0.98
TSS = 0.92
AUC = 0.99
TSS = 0.92
AUC = 0.97
TSS = 0.82
Relative importance
0.00
0.25
0.50
0.75
bio01
bio04
bio05
bio14
bio15
0.0
0.2
0.4
0.6
0.8
BDW
CEC
CLY
PTO
SOC
bio01
bio04
bio05
bio14
bio15
0.0
0.2
0.4
BDW
CEC
CLY
PTO
SOC
Stenanthera pinifolia
Climate
Climate + Soil
Soil
AUC = 0.98
TSS = 0.88
AUC = 0.98
TSS = 0.86
AUC = 0.97
TSS = 0.74
Relative importance
0.0
0.1
0.2
0.3
0.4
0.5
bio02
bio04
bio05
bio14
bio15
0.0
0.2
0.4
0.6
BDW
CLY
NTO
PTO
SOC
bio02
bio04
bio05
bio14
bio15
0.0
0.1
0.2
0.3
0.4
BDW
CLY
NTO
PTO
SOC

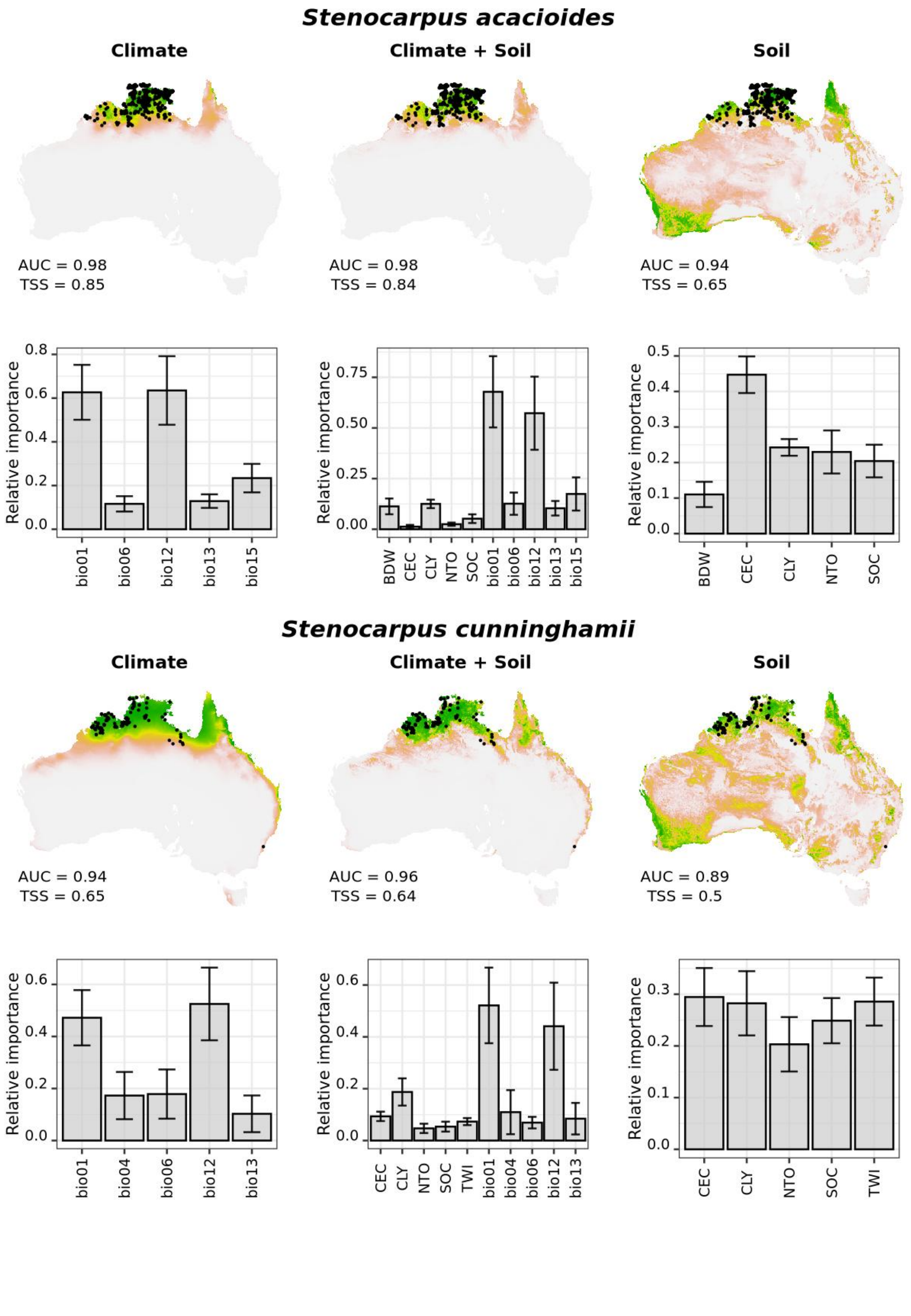
Stenocarpus acacioides
Climate
Climate + Soil
Soil
AUC = 0.98
TSS = 0.85
AUC = 0.98
TSS = 0.84
AUC = 0.94
TSS = 0.65
Relative importance
bio01 bio06 bio12 bio13 bio15
BDW CEC CLY NTO SOC bio01 bio06 bio12 bio13 bio15
BDW CEC CLY NTO SOC
Stenocarpus cunninghamii
Climate
Climate + Soil
Soil
AUC = 0.94
TSS = 0.65
AUC = 0.96
TSS = 0.64
AUC = 0.89
TSS = 0.5
Relative importance
bio01 bio04 bio06 bio12 bio13
CEC CLY NTO SOC TWI bio01 bio04 bio06 bio12 bio13
CEC CLY NTO SOC TWI

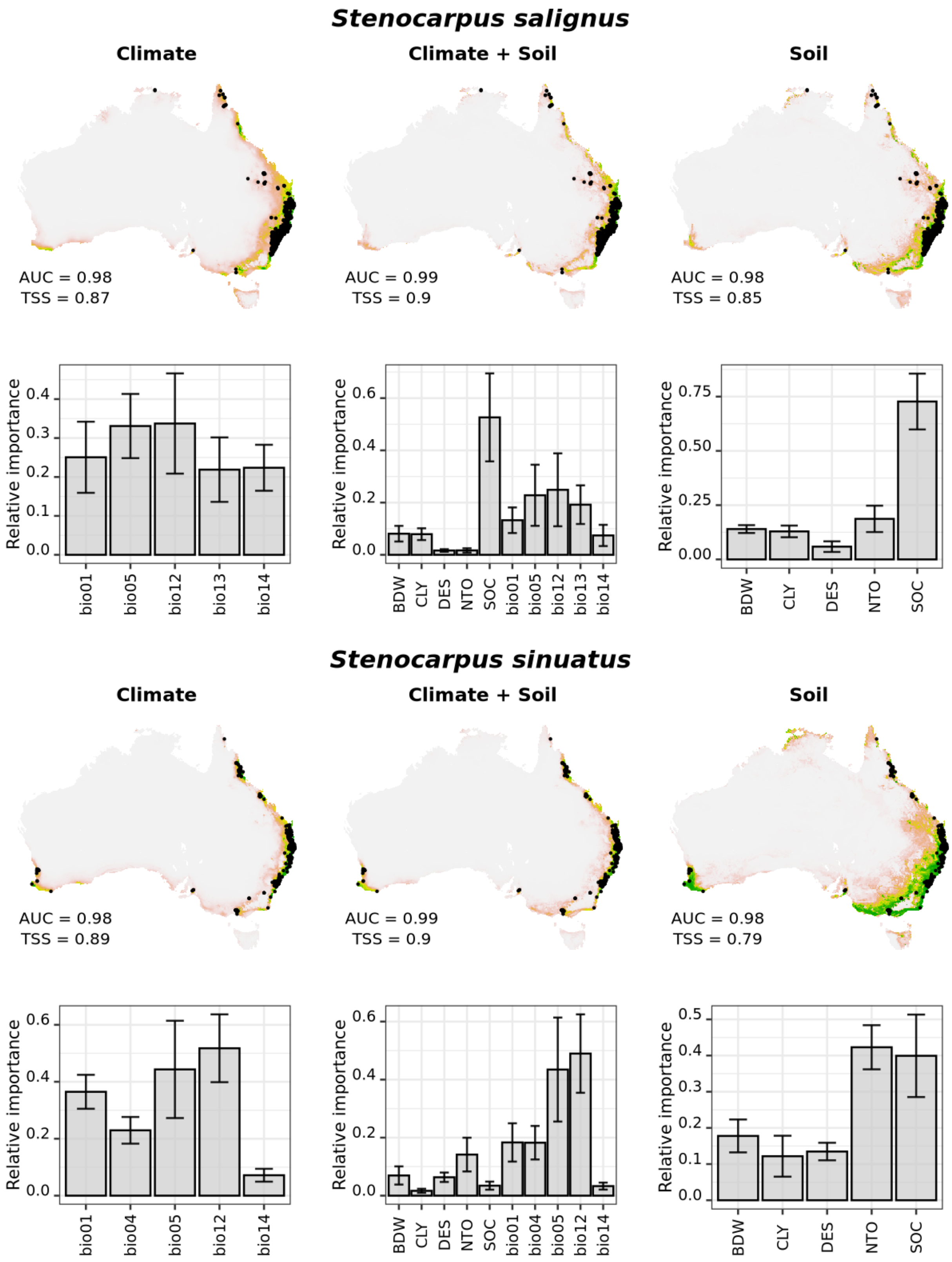
Stenocarpus salignus
Climate
Climate + Soil
Soil
AUC = 0.98
TSS = 0.87
AUC = 0.99
TSS = 0.9
AUC = 0.98
TSS = 0.85
Relative importance
bio01
bio05
bio12
bio13
bio14
BDW
CLY
DES
NTO
SOC
Stenocarpus sinuatus
AUC = 0.98
TSS = 0.89
AUC = 0.99
TSS = 0.9
AUC = 0.98
TSS = 0.79
bio04

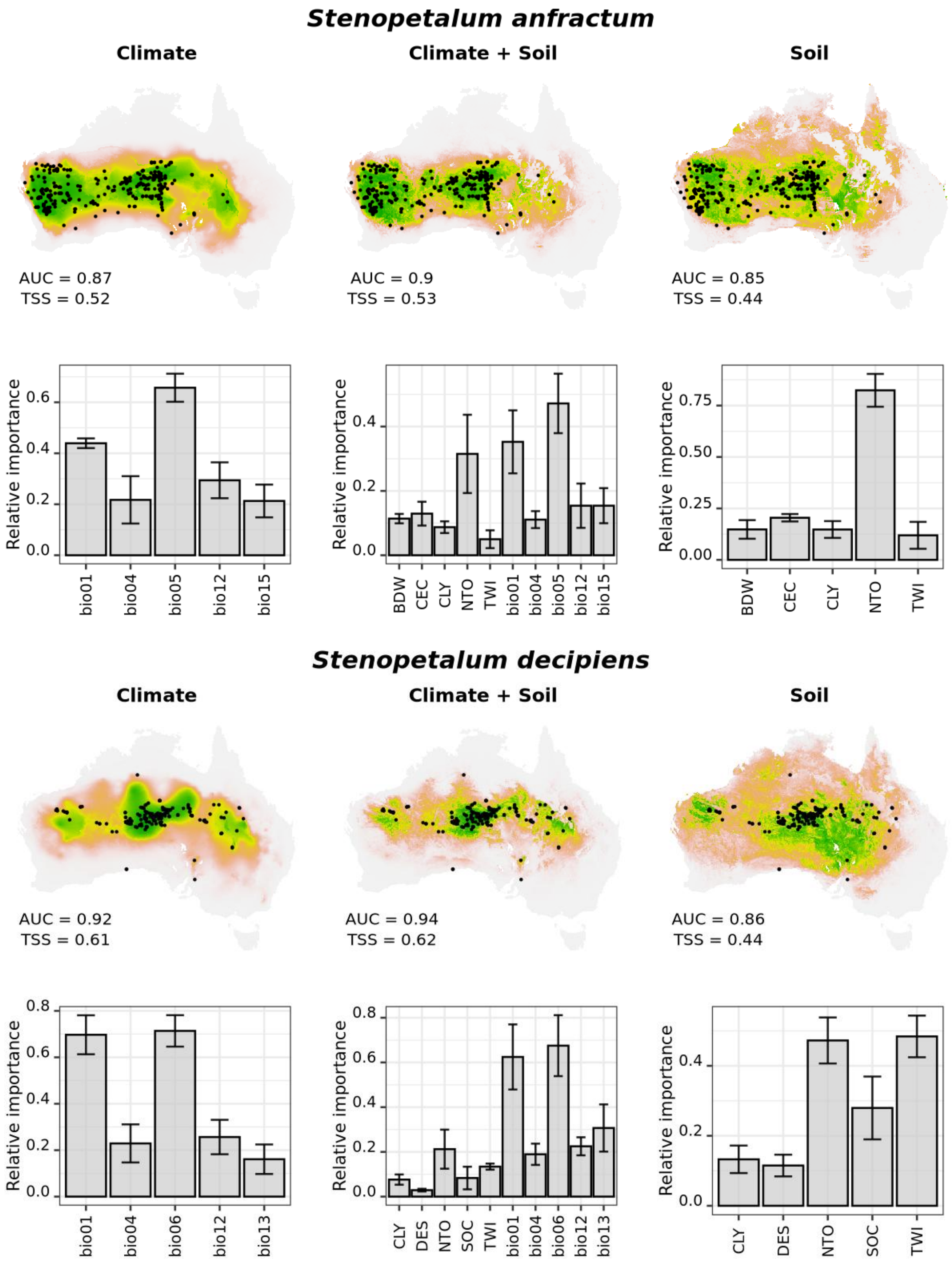
Stenopetalum anfractum
Climate
Climate + Soil
Soil
AUC = 0.87
TSS = 0.52
AUC = 0.9
TSS = 0.53
AUC = 0.85
TSS = 0.44
Relative importance
bio01 bio04 bio05 bio12 bio15
BDW CEC CLY NTO TWI bio01 bio04 bio05 bio12 bio15
BDW CEC CLY NTO TWI
Stenopetalum decipiens
Climate
Climate + Soil
Soil
AUC = 0.92
TSS = 0.61
AUC = 0.94
TSS = 0.62
AUC = 0.86
TSS = 0.44
Relative importance
bio01 bio04 bio06 bio12 bio13
CLY DES NTO SOC TWI bio01 bio04 bio06 bio12 bio13
CLY DES NTO SOC TWI

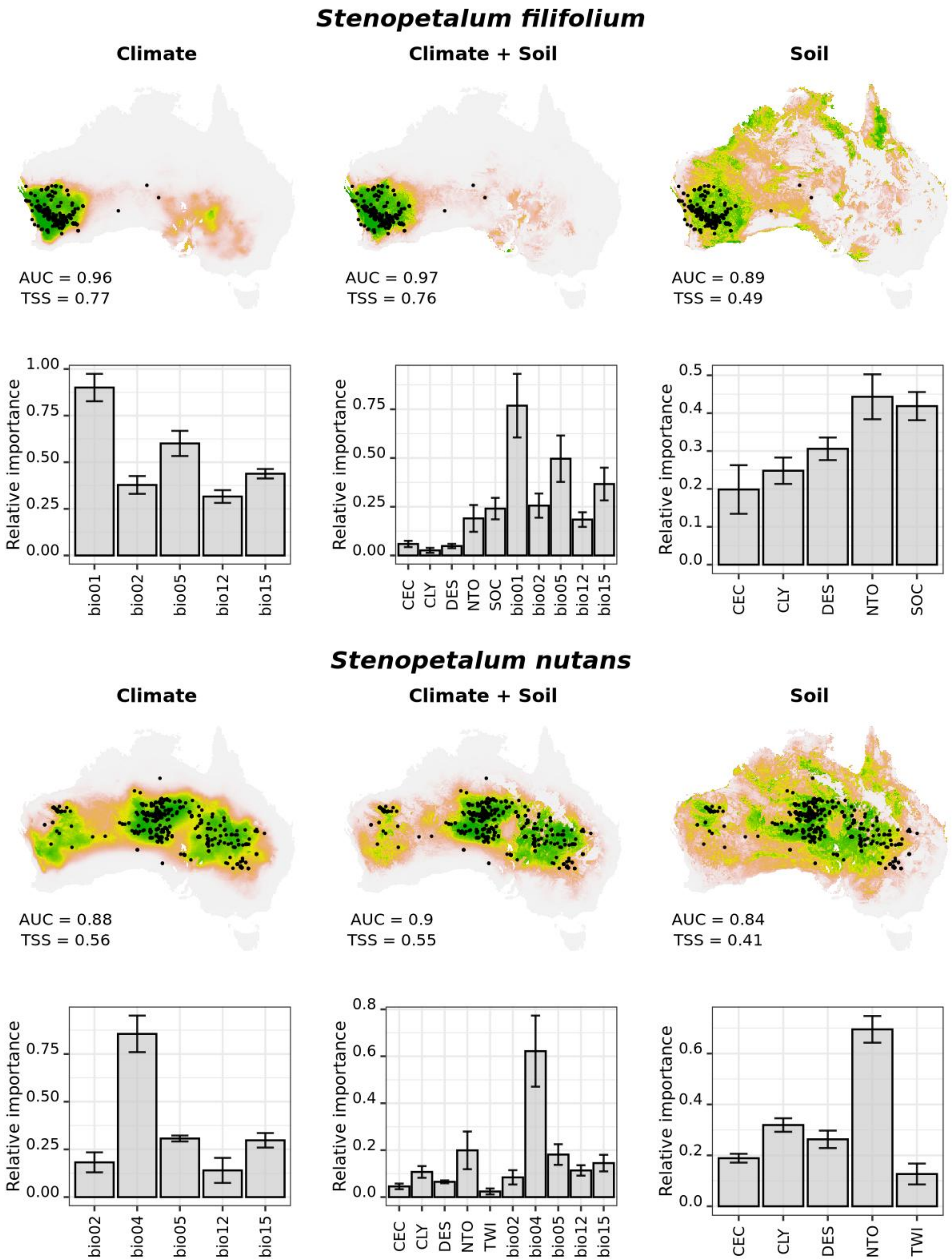
Stenopetalum filifolium
Climate
Climate + Soil
Soil
AUC = 0.96
TSS = 0.77
AUC = 0.97
TSS = 0.76
AUC = 0.89
TSS = 0.49
Relative importance
1.00
0.75
0.50
0.25
0.00
bio01
bio02
bio05
bio12
bio15
CEC
CLY
DES
NTO
SOC
0.5
0.4
0.3
0.2
0.1
0.0
Stenopetalum nutans
Climate
Climate + Soil
Soil
AUC = 0.88
TSS = 0.56
AUC = 0.9
TSS = 0.55
AUC = 0.84
TSS = 0.41
Relative importance
bio02
bio04
bio05
bio12
bio15
CEC
CLY
DES
NTO
TWI
0.8
0.6
0.4
0.2
0.0

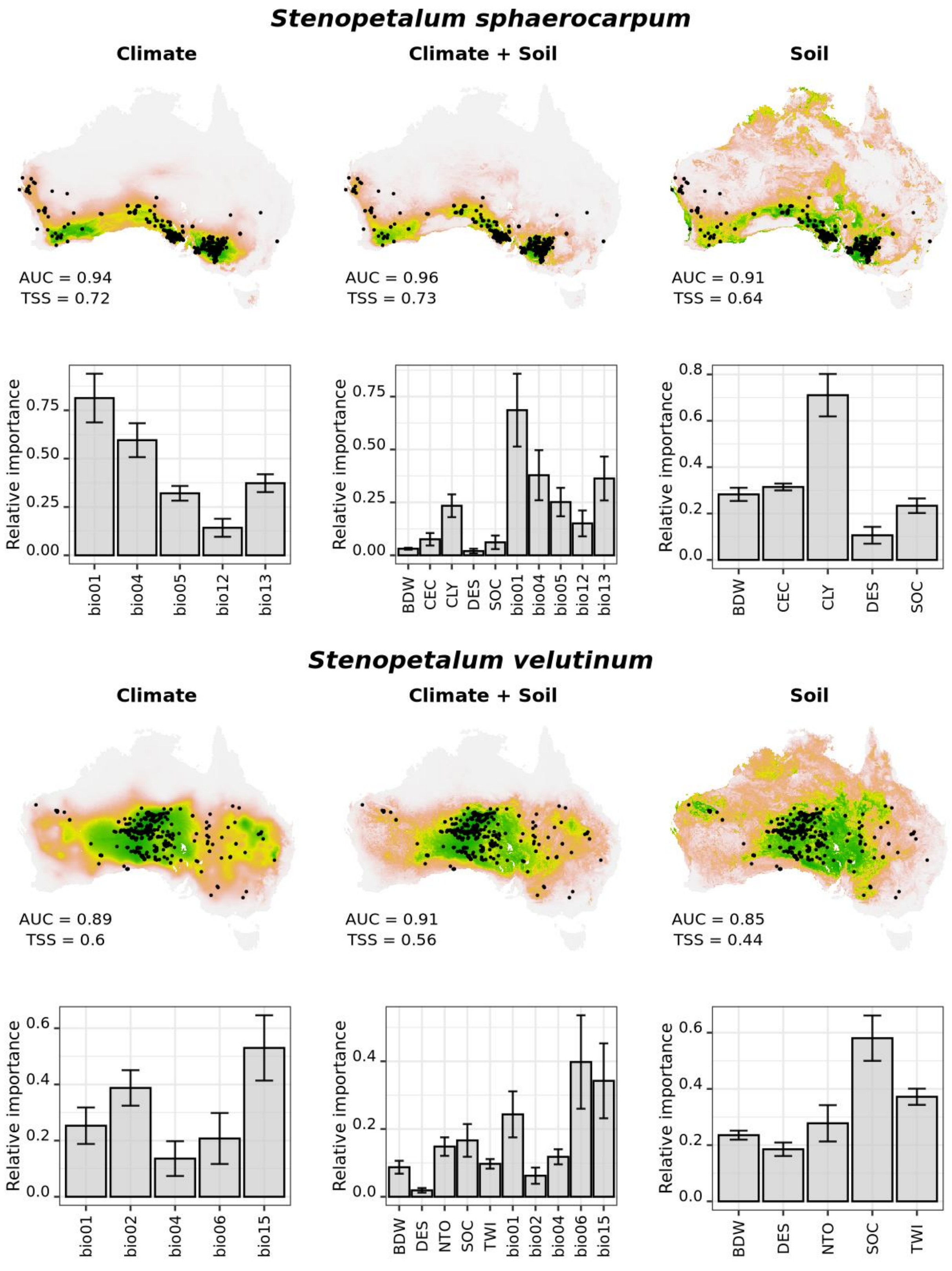
Stenopetalum sphaerocarpum
Climate
Climate + Soil
Soil
AUC = 0.94
TSS = 0.72
AUC = 0.96
TSS = 0.73
AUC = 0.91
TSS = 0.64
Relative importance
bio01
bio04
bio05
bio12
bio13
BDW
CEC
CLY
DES
SOC
Stenopetalum velutinum
Climate
Climate + Soil
Soil
AUC = 0.89
TSS = 0.6
AUC = 0.91
TSS = 0.56
AUC = 0.85
TSS = 0.44
Relative importance
bio01
bio02
bio04
bio06
bio15
BDW
DES
NTO
SOC
TWI

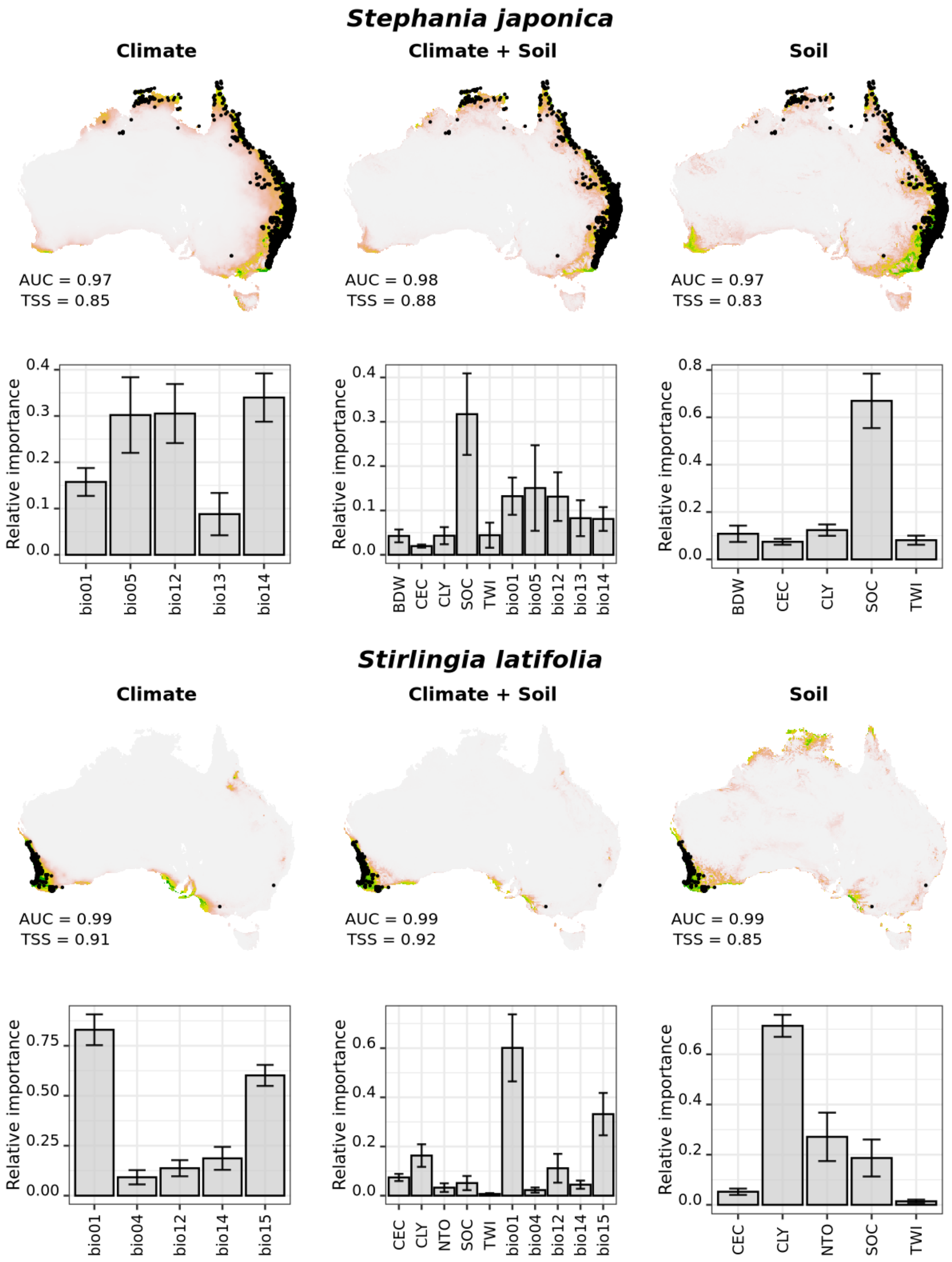

Stephania japonica
Climate
Climate + Soil
Soil
AUC = 0.97
TSS = 0.85
AUC = 0.98
TSS = 0.88
AUC = 0.97
TSS = 0.83
Relative importance
bio01 bio05 bio12 bio13 bio14
BDW CEC CLY SOC TWI bio01 bio05 bio12 bio13 bio14
BDW CEC CLY SOC TWI
Stirlingia latifolia
Climate
Climate + Soil
Soil
AUC = 0.99
TSS = 0.91
AUC = 0.99
TSS = 0.92
AUC = 0.99
TSS = 0.85
bio01 bio04 bio12 bio14 bio15
CEC CLY NTO SOC TWI bio01 bio04 bio12 bio14 bio15
CEC CLY NTO SOC TWI

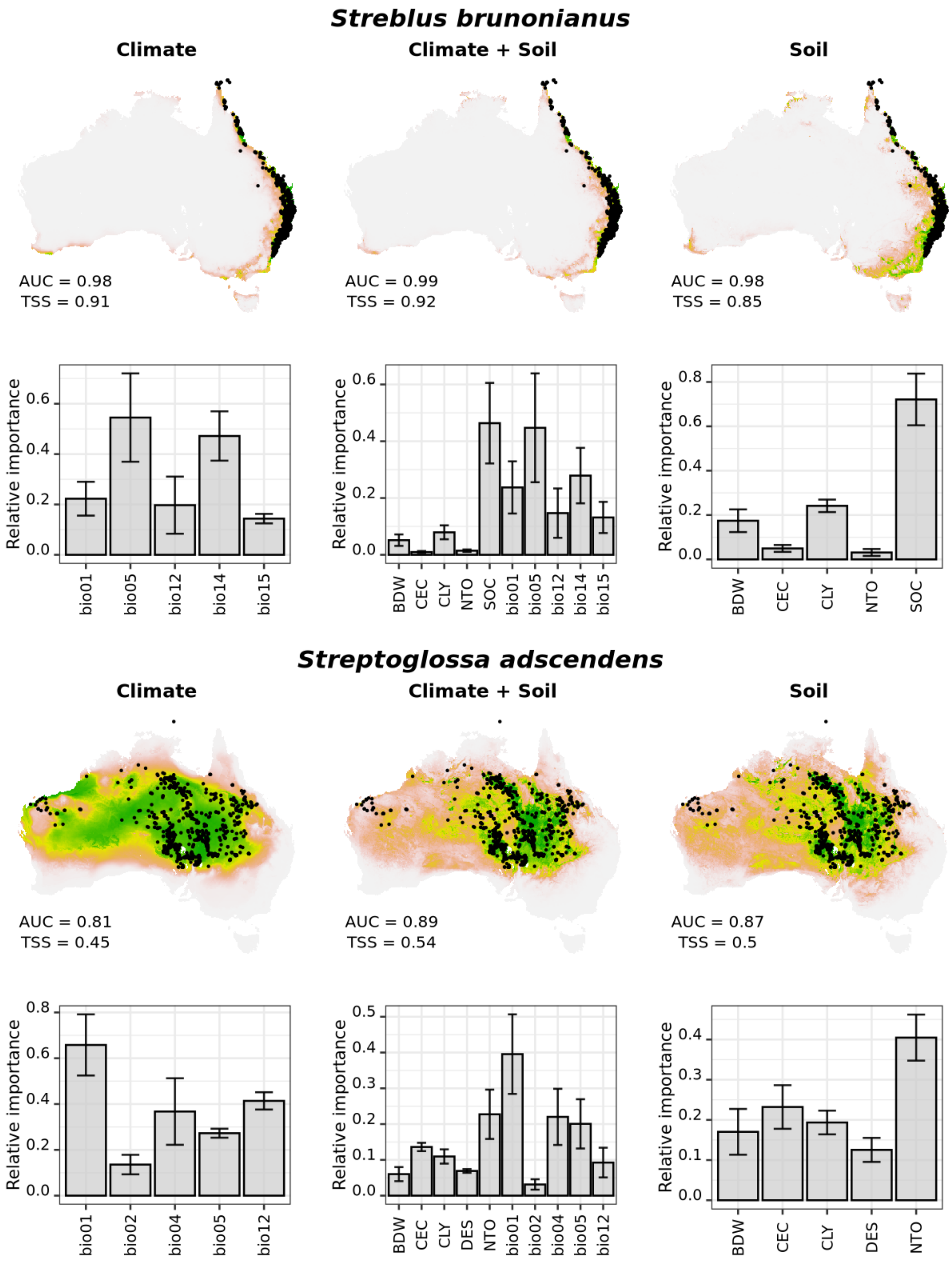
Streblus brunonianus
Climate
Climate + Soil
Soil
AUC = 0.98
TSS = 0.91
AUC = 0.99
TSS = 0.92
AUC = 0.98
TSS = 0.85
Relative importance
bio01
bio05
bio12
bio14
bio15
BDW
CEC
CLY
NTO
SOC
Streptoglossa adscendens
Climate
Climate + Soil
Soil
AUC = 0.81
TSS = 0.45
AUC = 0.89
TSS = 0.54
AUC = 0.87
TSS = 0.5
bio02
bio04
DES

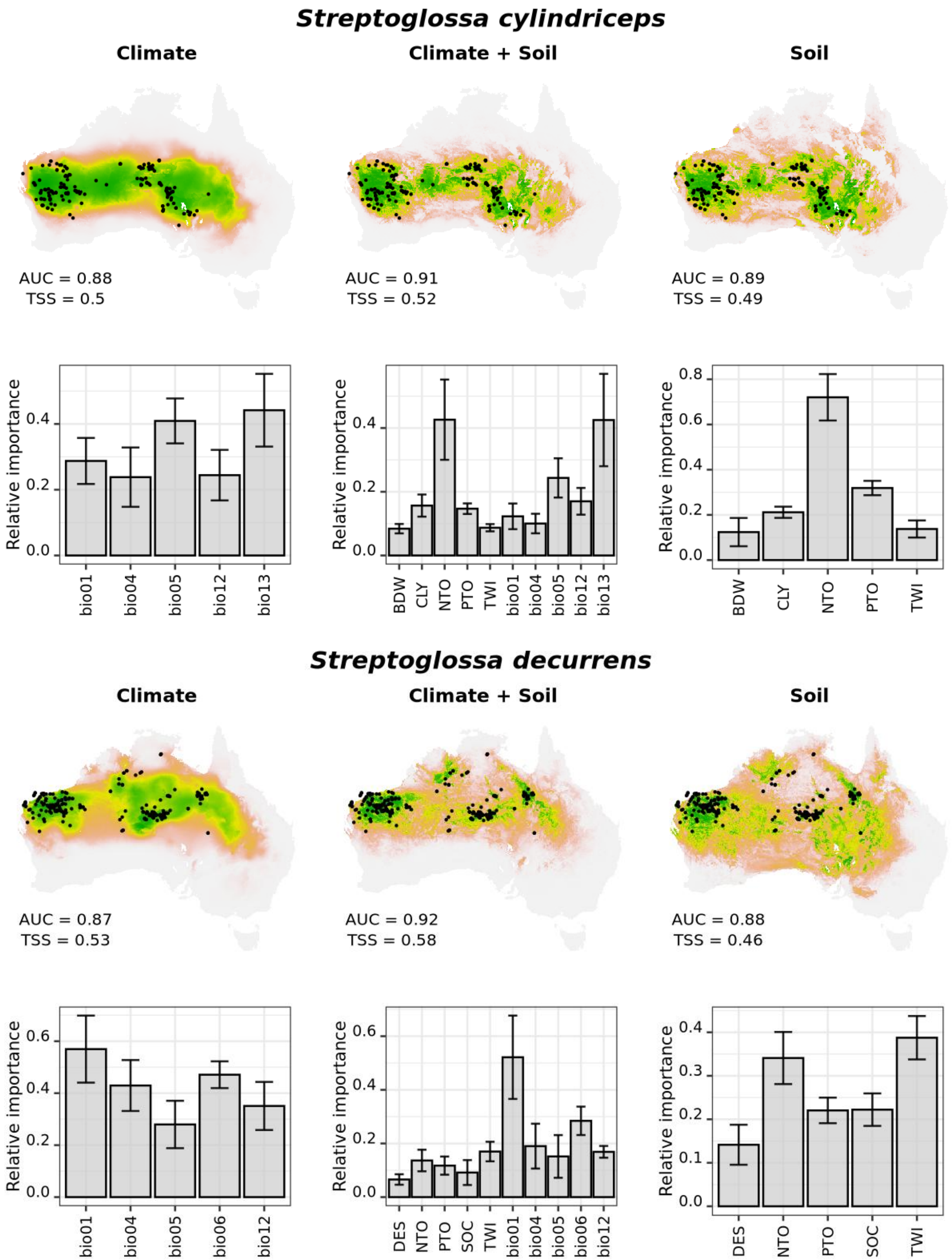

Streptoglossa cylindriceps
Climate
Climate + Soil
Soil
AUC = 0.88
TSS = 0.5
AUC = 0.91
TSS = 0.52
AUC = 0.89
TSS = 0.49
Relative importance
bio01
bio04
bio05
bio12
bio13
BDW
CLY
NTO
PTO
TWI
Streptoglossa decurrens
AUC = 0.87
TSS = 0.53
AUC = 0.92
TSS = 0.58
AUC = 0.88
TSS = 0.46
bio06
DES
SOC

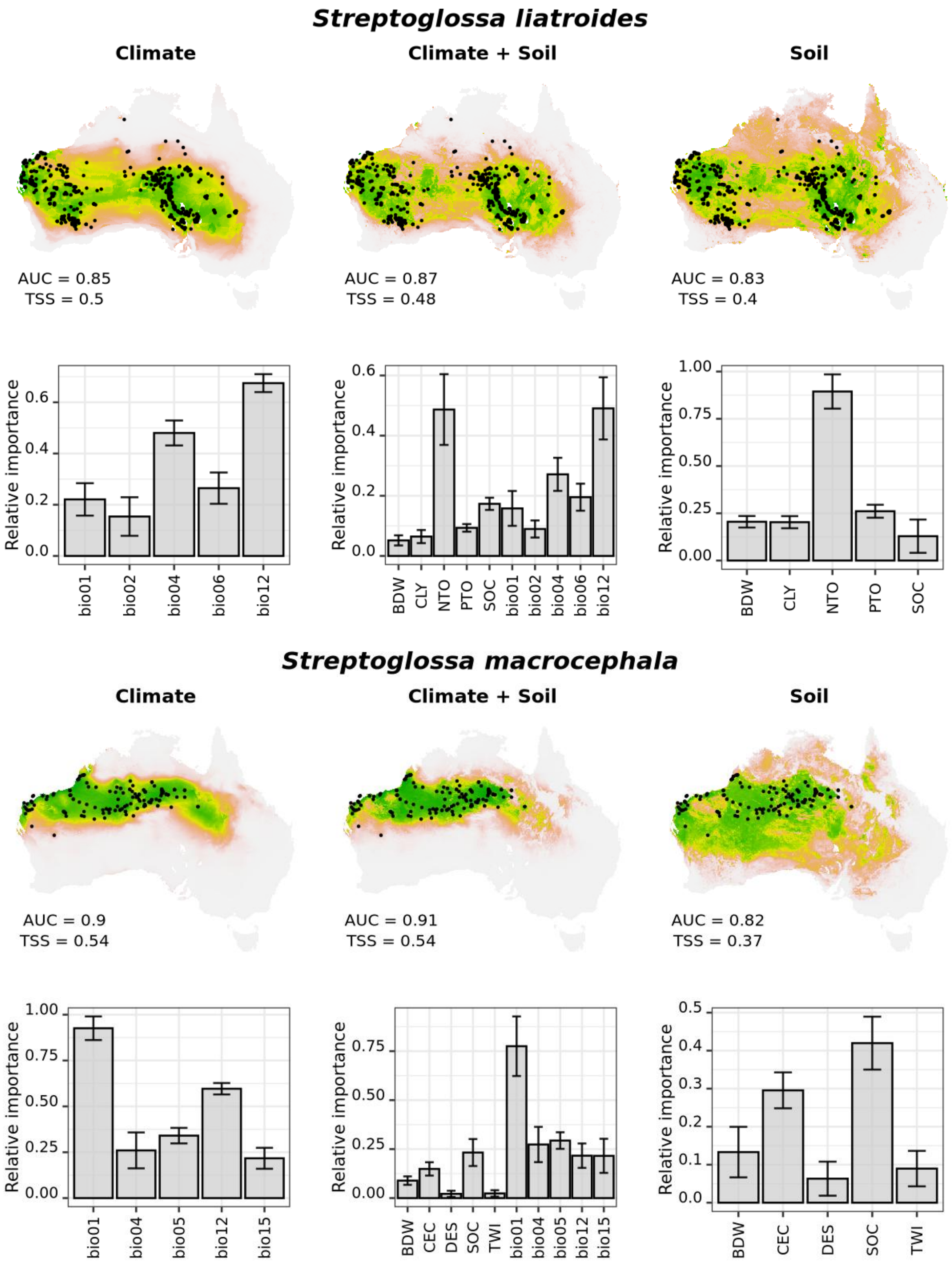

Streptoglossa liatroides
Climate
Climate + Soil
Soil
AUC = 0.85
TSS = 0.5
AUC = 0.87
TSS = 0.48
AUC = 0.83
TSS = 0.4
Relative importance
bio01
bio02
bio04
bio06
bio12
BDW
CLY
NTO
PTO
SOC
Streptoglossa macrocephala
Climate
Climate + Soil
Soil
AUC = 0.9
TSS = 0.54
AUC = 0.91
TSS = 0.54
AUC = 0.82
TSS = 0.37
Relative importance
bio01
bio04
bio05
bio12
bio15
BDW
CEC
DES
SOC
TWI

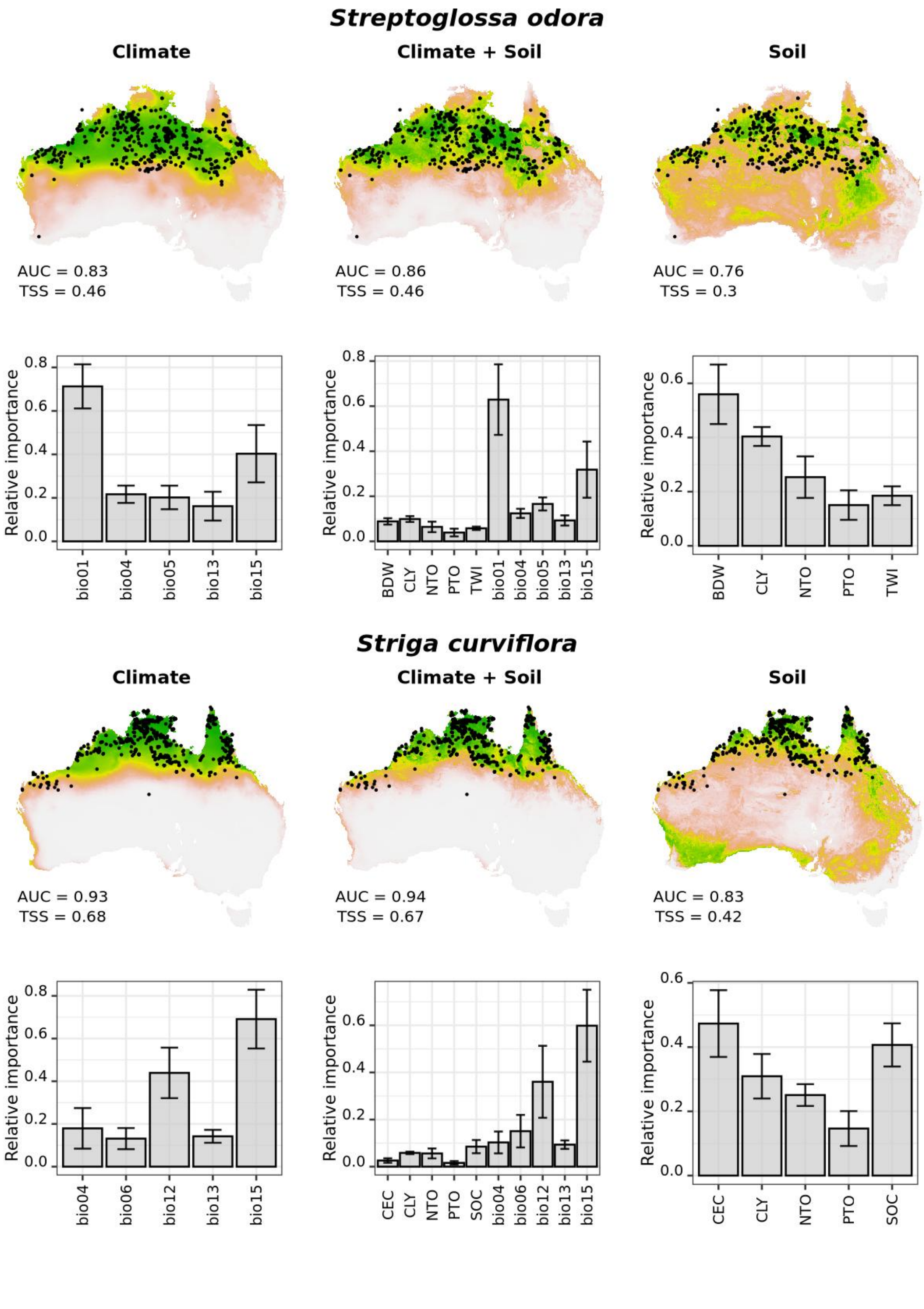
Streptoglossa odora
Climate
Climate + Soil
Soil
AUC = 0.83
TSS = 0.46
AUC = 0.86
TSS = 0.46
AUC = 0.76
TSS = 0.3
Relative importance
bio01
bio04
bio05
bio13
bio15
BDW
CLY
NTO
PTO
TWI
Striga curviflora
AUC = 0.93
TSS = 0.68
AUC = 0.94
TSS = 0.67
AUC = 0.83
TSS = 0.42
bio06
bio12
CEC
SOC

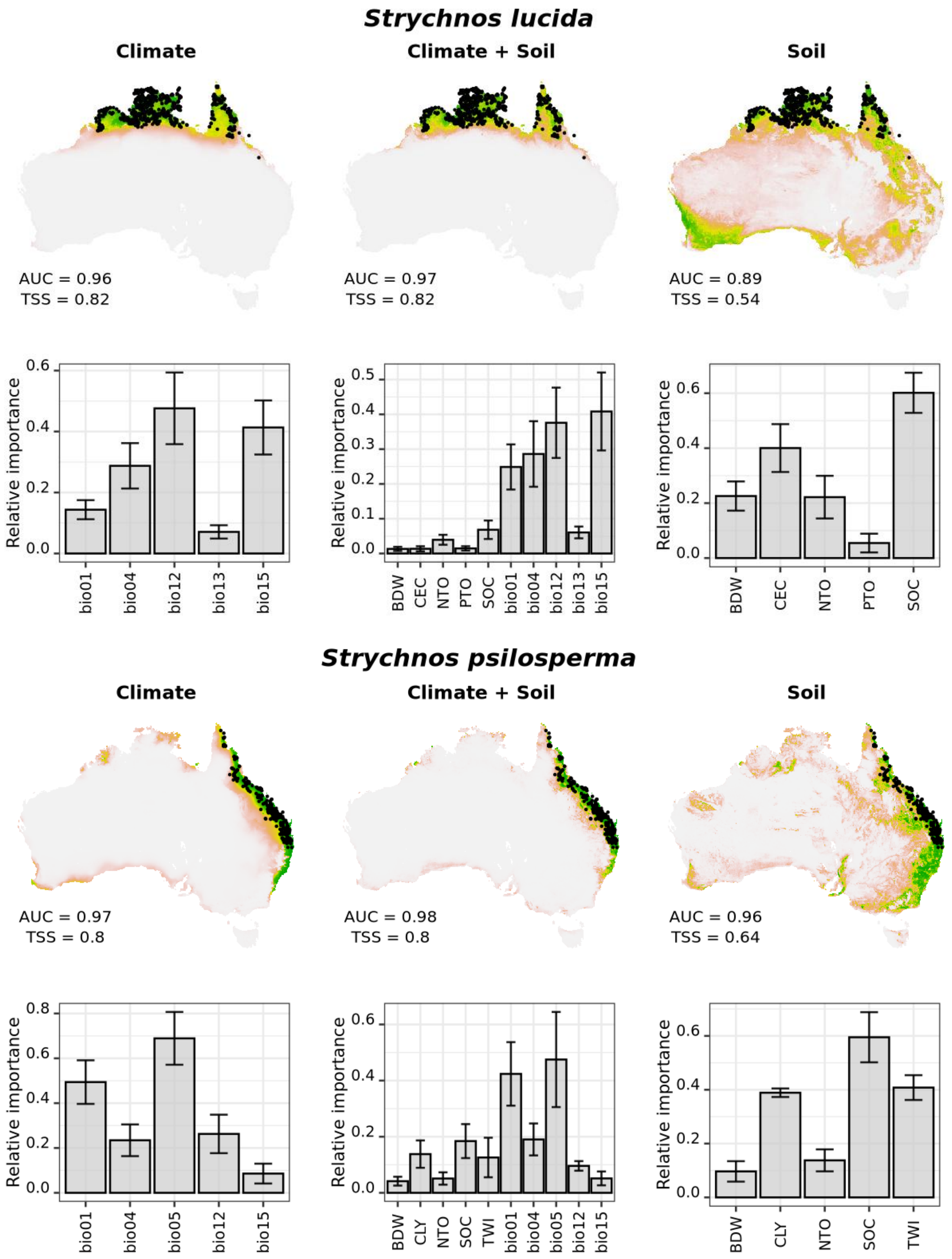

Strychnos lucida
Climate
Climate + Soil
Soil
AUC = 0.96
TSS = 0.82
AUC = 0.97
TSS = 0.82
AUC = 0.89
TSS = 0.54
Relative importance
bio01 bio04 bio12 bio13 bio15
BDW CEC NTO PTO SOC bio01 bio04 bio12 bio13 bio15
BDW CEC NTO PTO SOC
Strychnos psilosperma
Climate
Climate + Soil
Soil
AUC = 0.97
TSS = 0.8
AUC = 0.98
TSS = 0.8
AUC = 0.96
TSS = 0.64
Relative importance
bio01 bio04 bio05 bio12 bio15
BDW CLY NTO SOC TWI bio01 bio04 bio05 bio12 bio15
BDW CLY NTO SOC TWI

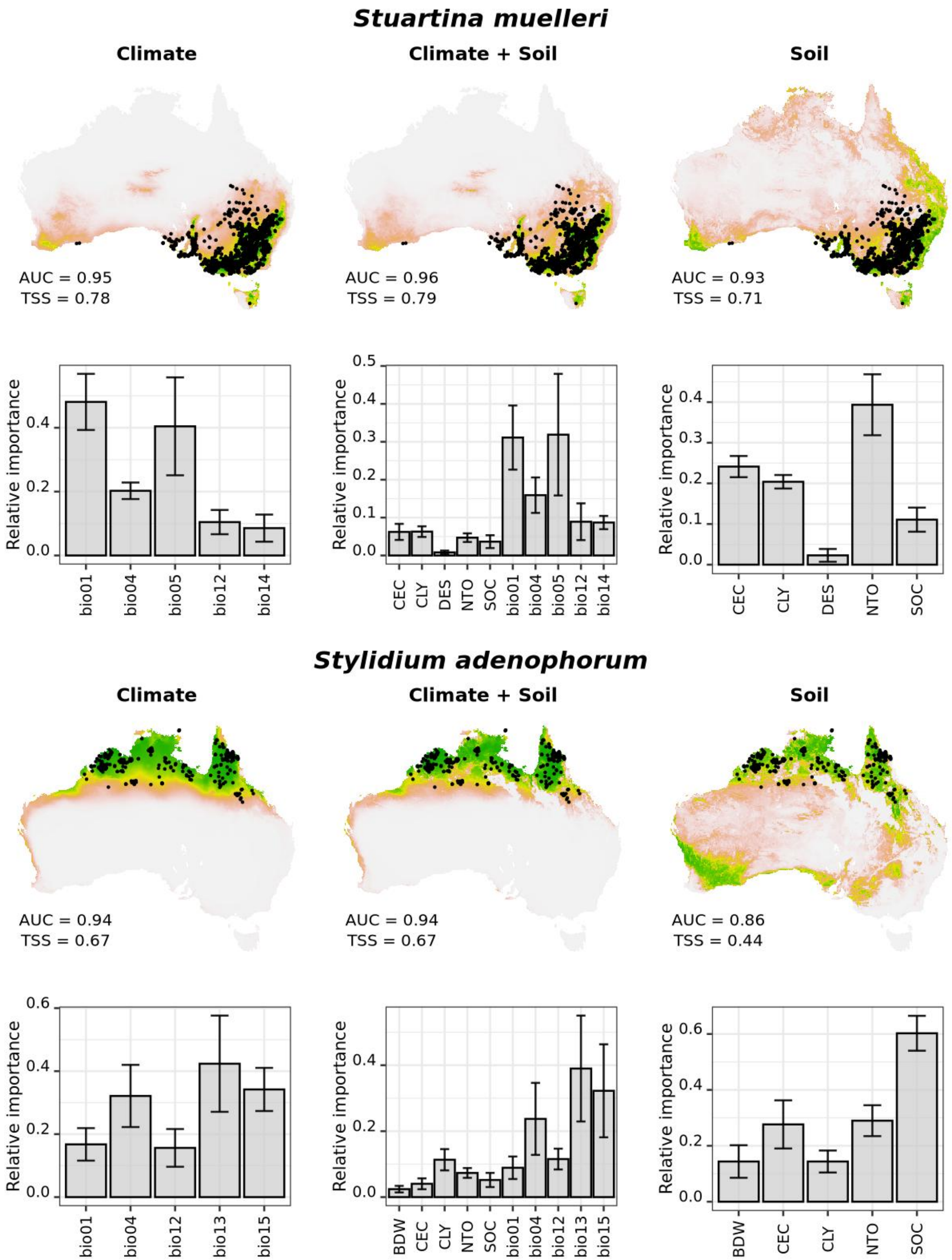
Stuartina muelleri
Climate
Climate + Soil
Soil
AUC = 0.95
TSS = 0.78
AUC = 0.96
TSS = 0.79
AUC = 0.93
TSS = 0.71
Relative importance
bio01 bio04 bio05 bio12 bio14
CEC CLY DES NTO SOC bio01 bio04 bio05 bio12 bio14
CEC CLY DES NTO SOC
Stylidium adenophorum
Climate
Climate + Soil
Soil
AUC = 0.94
TSS = 0.67
AUC = 0.94
TSS = 0.67
AUC = 0.86
TSS = 0.44
Relative importance
bio01 bio04 bio12 bio13 bio15
BDW CEC CLY NTO SOC bio01 bio04 bio12 bio13 bio15
BDW CEC CLY NTO SOC

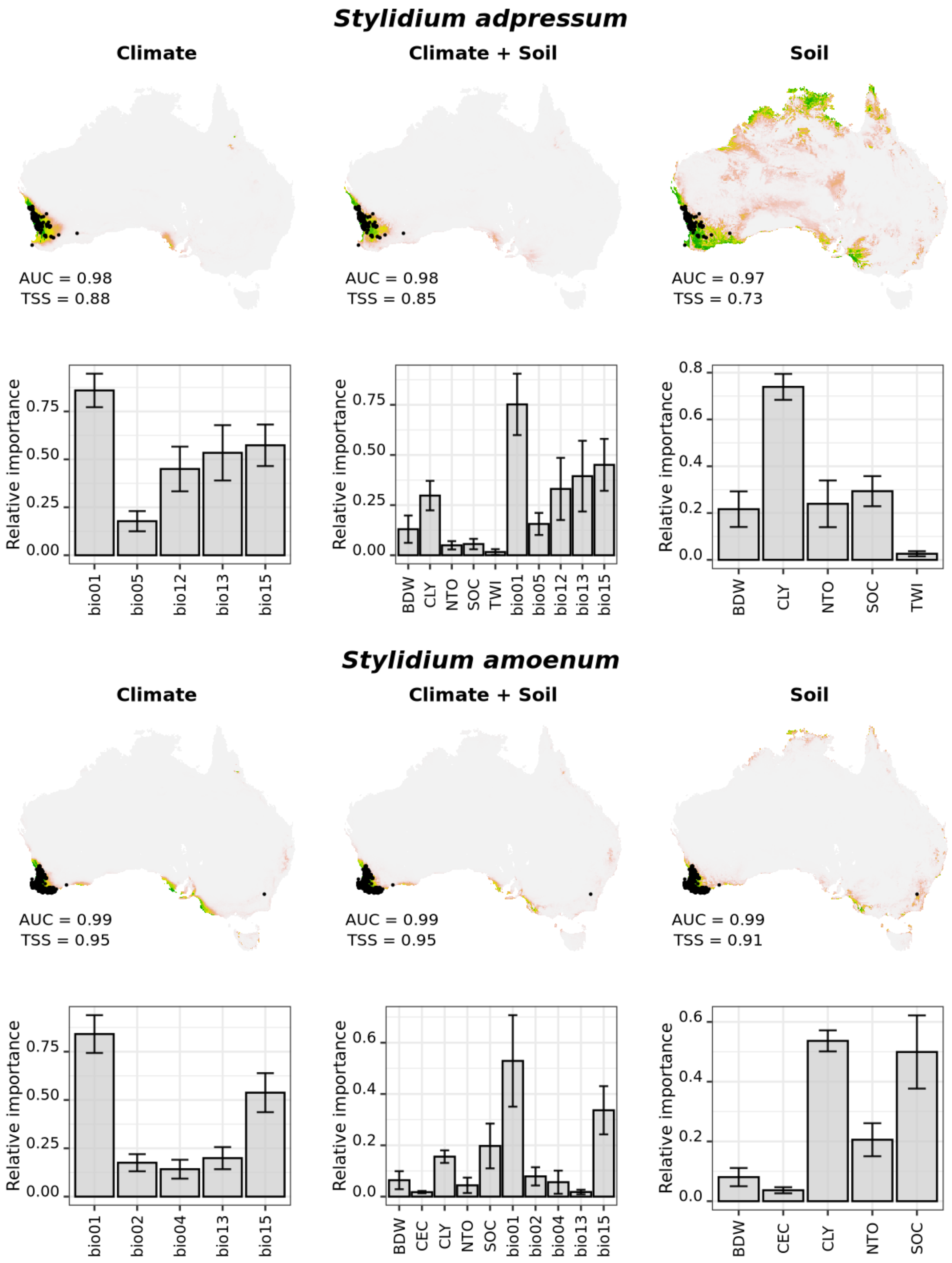
Stylidium adpressum
Climate
Climate + Soil
Soil
AUC = 0.98
TSS = 0.88
AUC = 0.98
TSS = 0.85
AUC = 0.97
TSS = 0.73
Relative importance
bio01
bio05
bio12
bio13
bio15
BDW
CLY
NTO
SOC
TWI
Stylidium amoenum
Climate
Climate + Soil
Soil
AUC = 0.99
TSS = 0.95
AUC = 0.99
TSS = 0.95
AUC = 0.99
TSS = 0.91
Relative importance
bio01
bio02
bio04
bio13
bio15
BDW
CEC
CLY
NTO
SOC

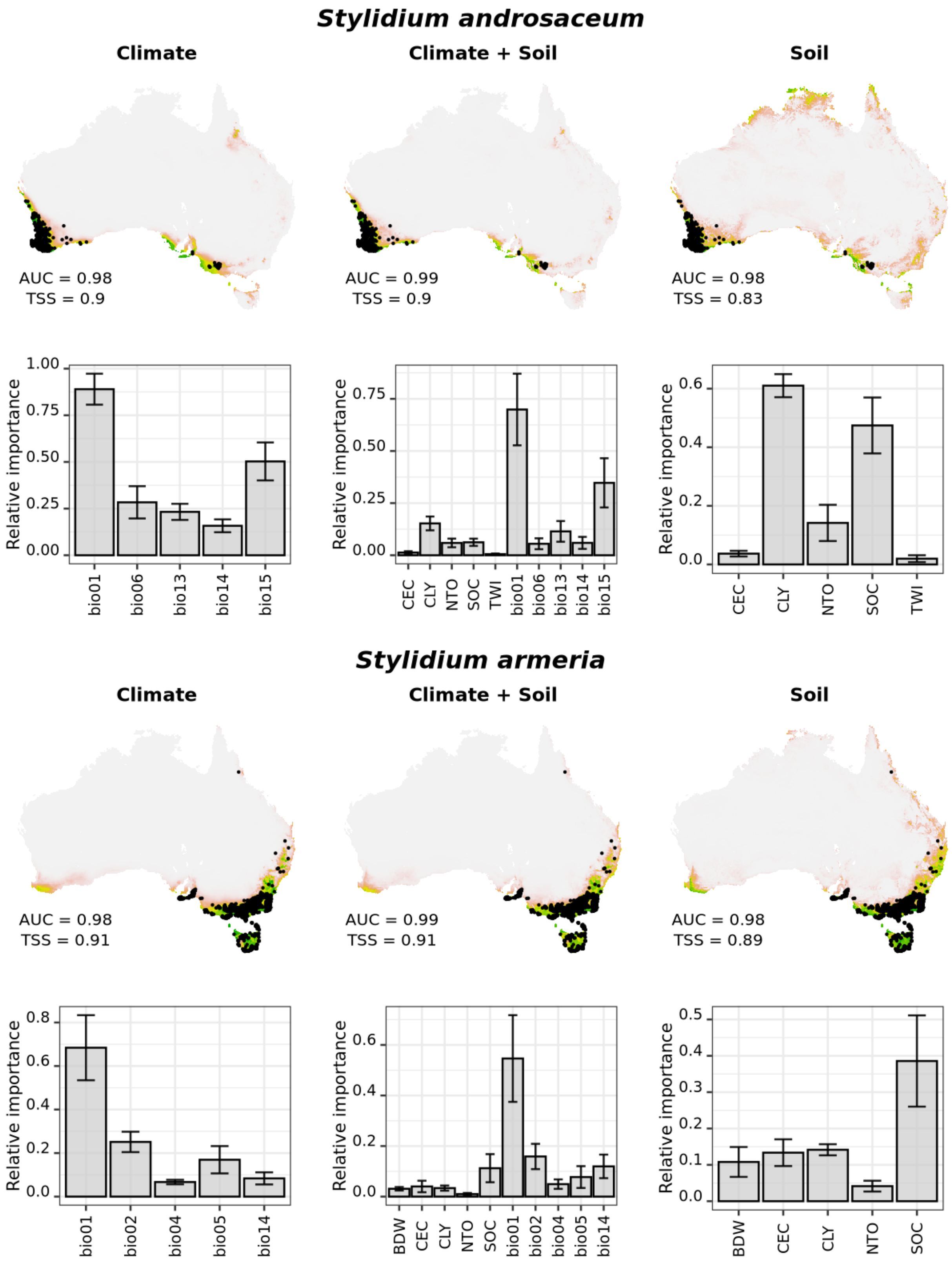

Stylidium androsaceum
Climate
Climate + Soil
Soil
AUC = 0.98
TSS = 0.9
AUC = 0.99
TSS = 0.9
AUC = 0.98
TSS = 0.83
Relative importance
1.00
0.75
0.50
0.25
0.00
bio01
bio06
bio13
bio14
bio15
CEC
CLY
NTO
SOC
TWI
0.6
0.4
0.2
0.0
Stylidium armeria
Climate
Climate + Soil
Soil
AUC = 0.98
TSS = 0.91
AUC = 0.99
TSS = 0.91
AUC = 0.98
TSS = 0.89
0.8
0.5
0.3
0.1
bio02
bio04
bio05
BDW

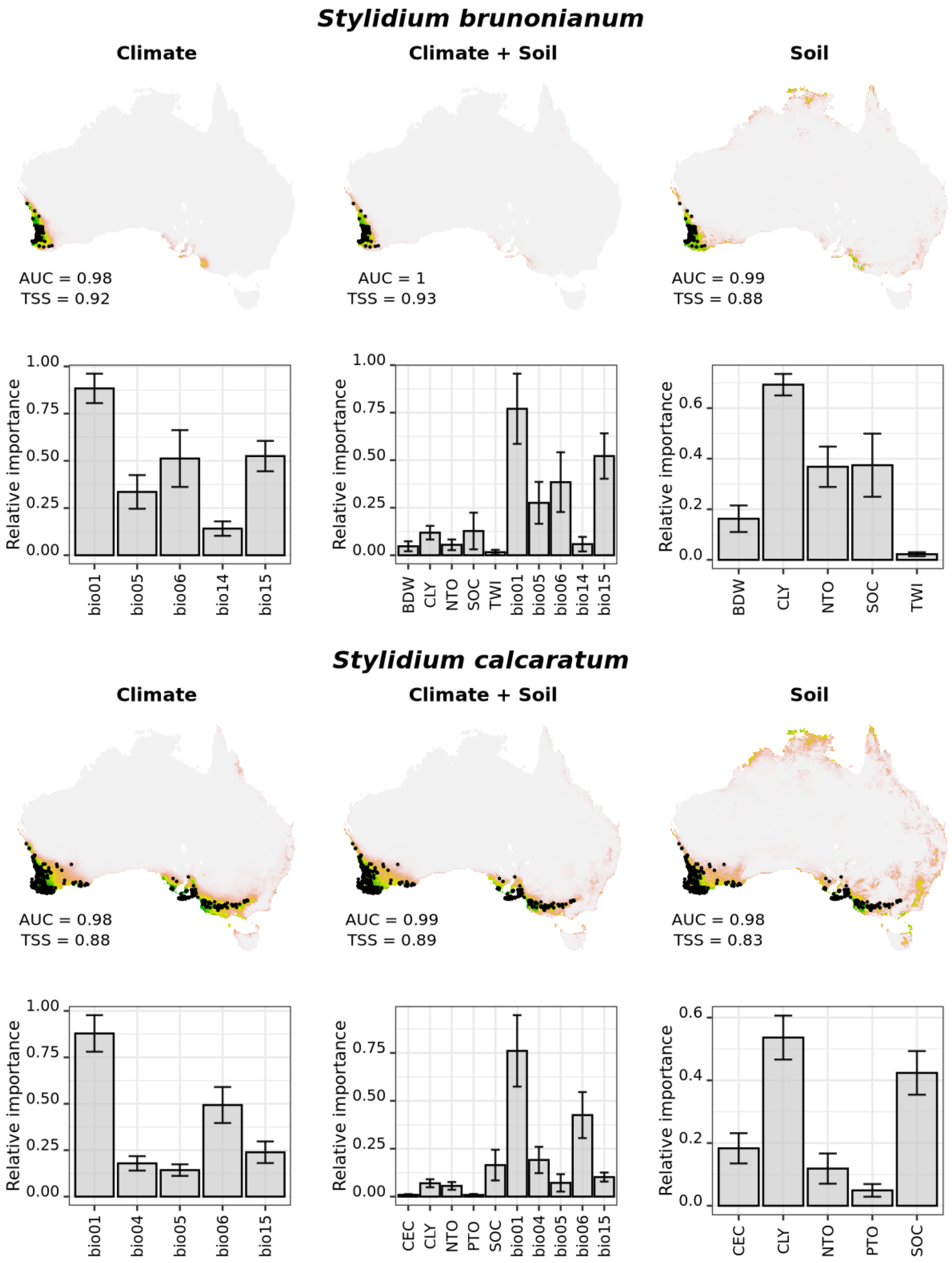
Stylidium brunonianum
Climate
Climate + Soil
Soil
AUC = 0.98
TSS = 0.92
AUC = 1
TSS = 0.93
AUC = 0.99
TSS = 0.88
Relative importance
bio01
bio05
bio06
bio14
bio15
BDW
CLY
NTO
SOC
TWI
Stylidium calcaratum
Climate
Climate + Soil
Soil
AUC = 0.98
TSS = 0.88
AUC = 0.99
TSS = 0.89
AUC = 0.98
TSS = 0.83
Relative importance
bio01
bio04
bio05
bio06
bio15
CEC
CLY
NTO
PTO
SOC

Stylidium caricifolium

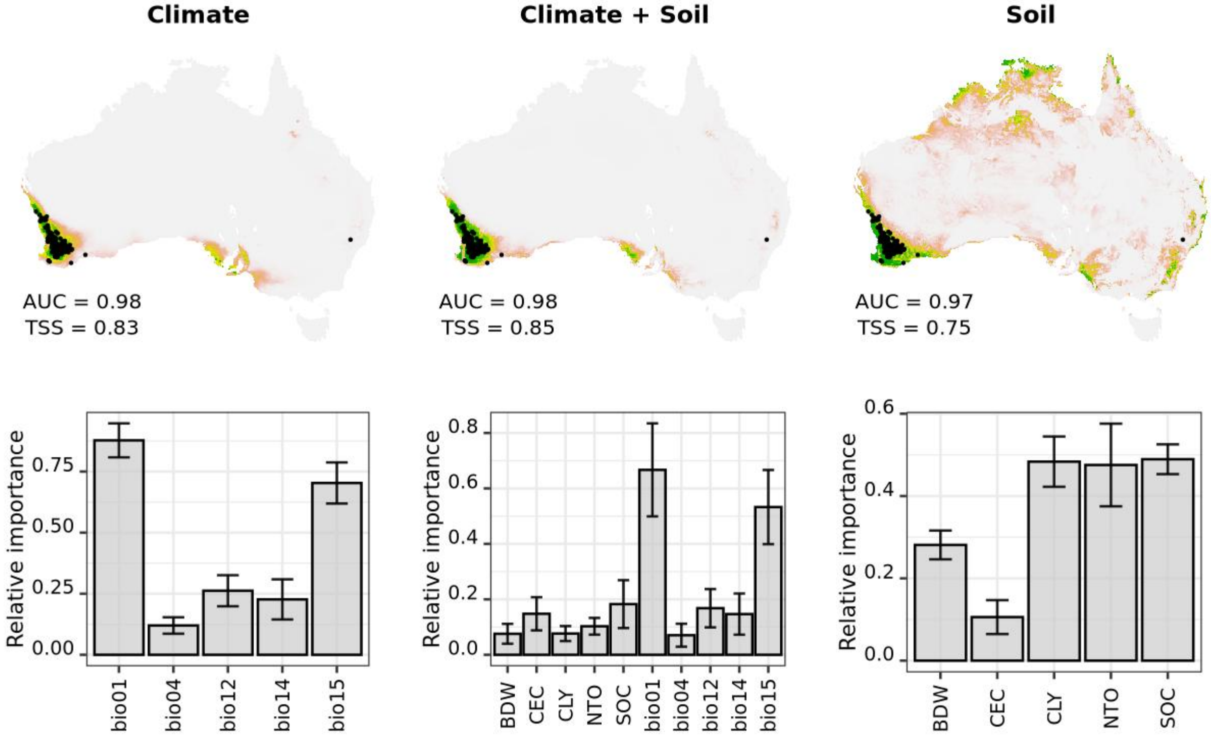

Stylidium ciliatum

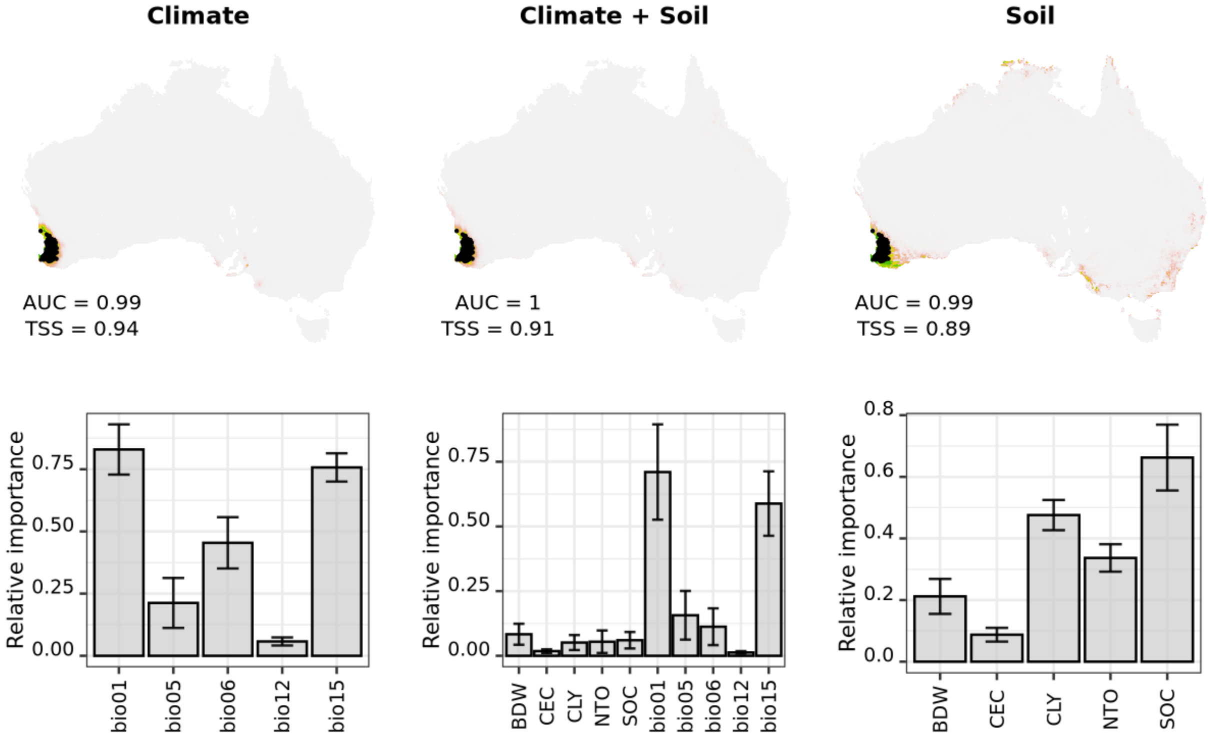

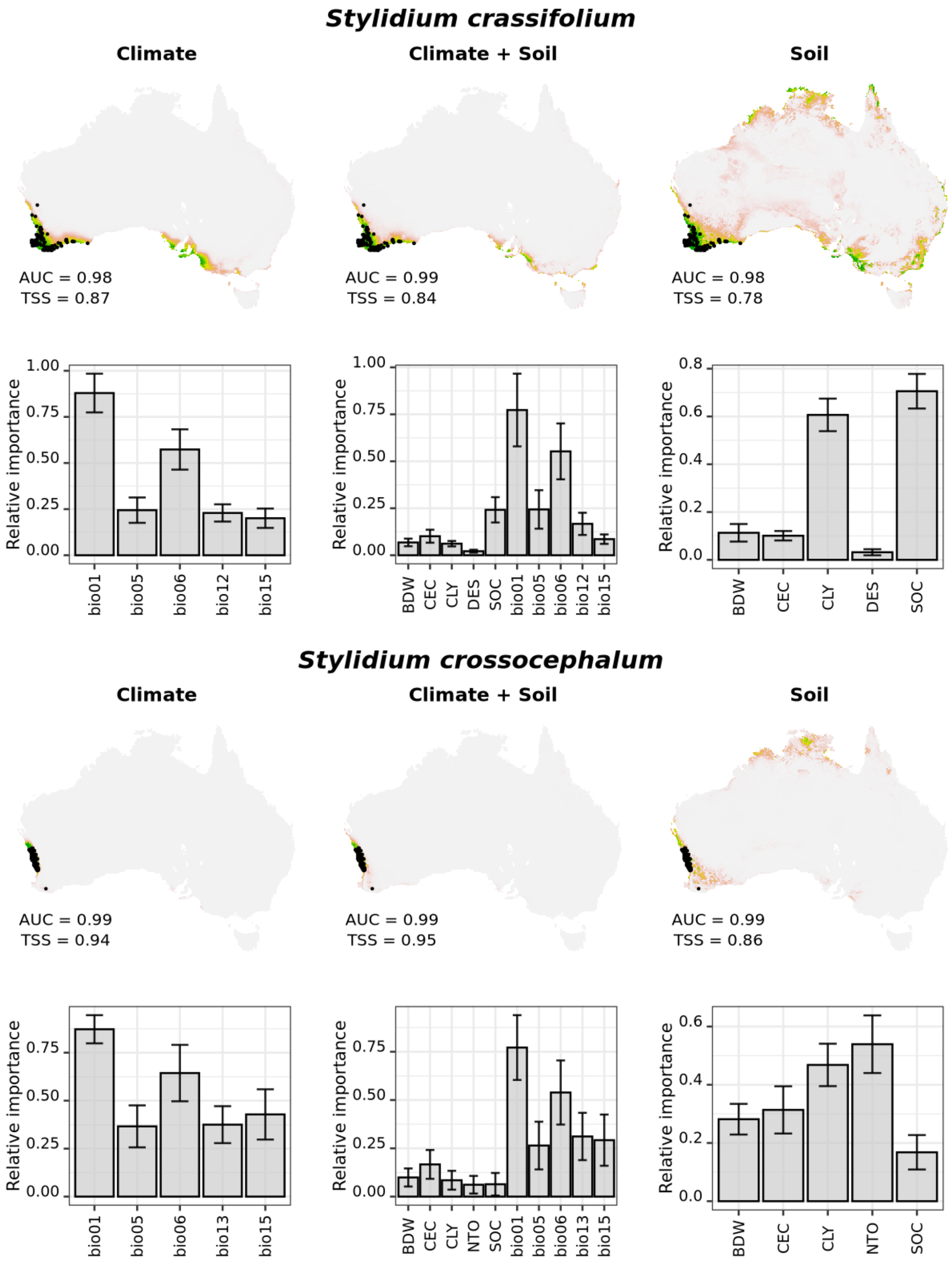
Stylidium crassifolium
Climate
Climate + Soil
Soil
AUC = 0.98
TSS = 0.87
AUC = 0.99
TSS = 0.84
AUC = 0.98
TSS = 0.78
Relative importance
1.00
0.75
0.50
0.25
0.00
bio01
bio05
bio06
bio12
bio15
BDW
CEC
CLY
DES
SOC
0.8
0.6
0.4
0.2
0.0
Stylidium crossocephalum
Climate
Climate + Soil
Soil
AUC = 0.99
TSS = 0.94
AUC = 0.99
TSS = 0.95
AUC = 0.99
TSS = 0.86
bio13
NTO

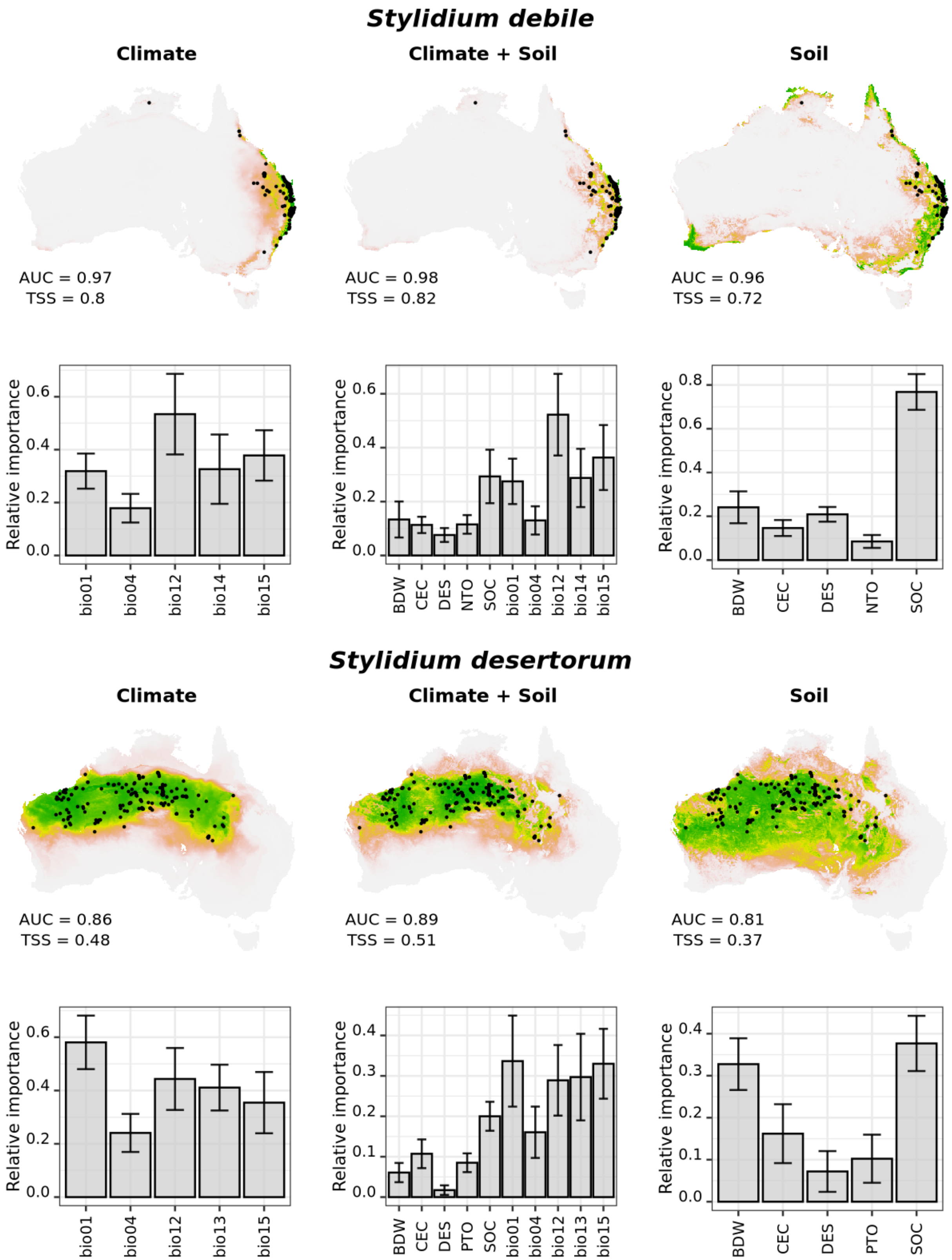
Stylidium debile
Climate
Climate + Soil
Soil
AUC = 0.97
TSS = 0.8
AUC = 0.98
TSS = 0.82
AUC = 0.96
TSS = 0.72
Relative importance
bio01 bio04 bio12 bio14 bio15
BDW CEC DES NTO SOC bio01 bio04 bio12 bio14 bio15
BDW CEC DES NTO SOC
Stylidium desertorum
Climate
Climate + Soil
Soil
AUC = 0.86
TSS = 0.48
AUC = 0.89
TSS = 0.51
AUC = 0.81
TSS = 0.37
Relative importance
bio01 bio04 bio12 bio13 bio15
BDW CEC DES PTO SOC bio01 bio04 bio12 bio13 bio15
BDW CEC DES PTO SOC

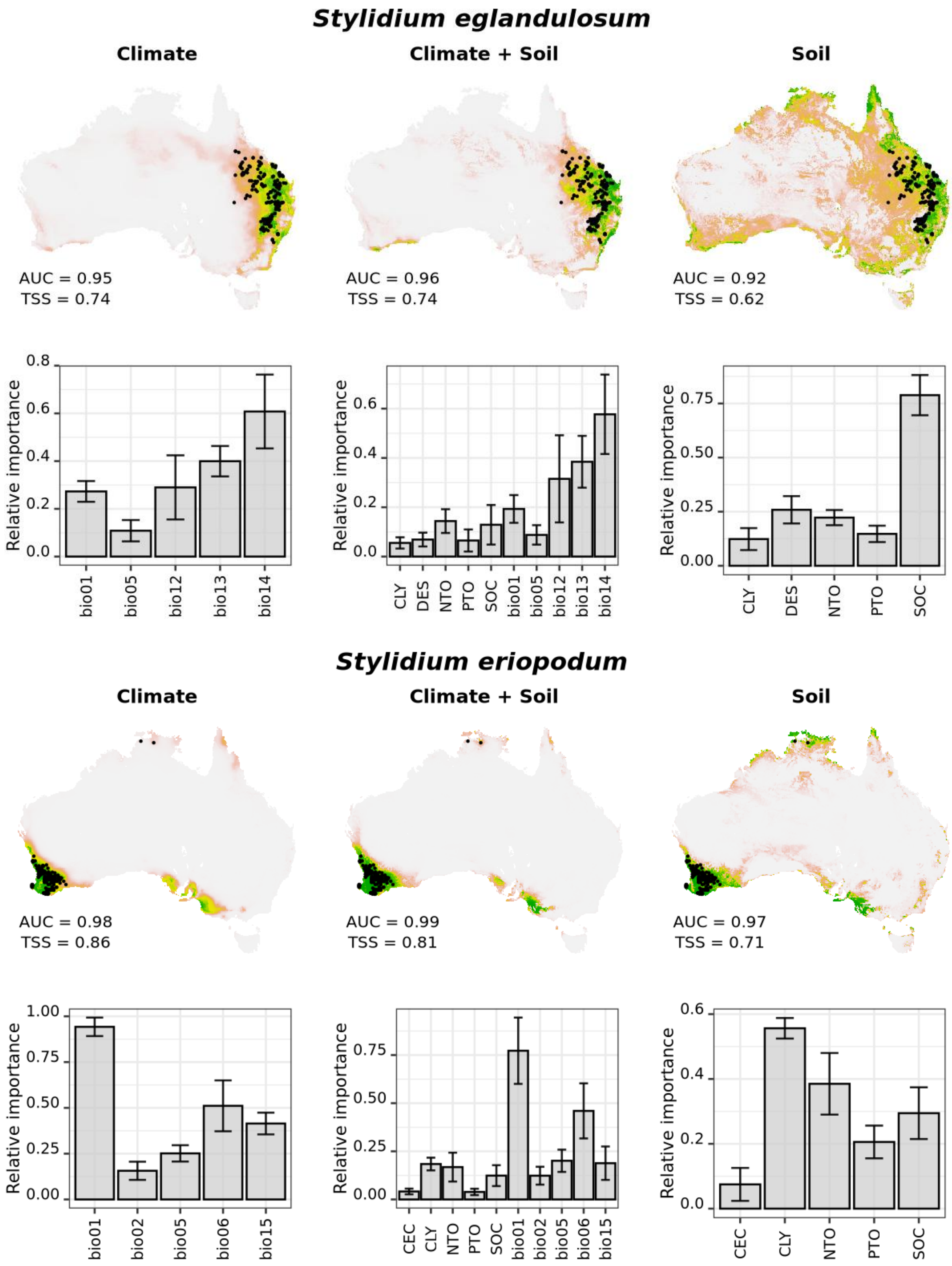
Stylidium eglandulosum
Climate
Climate + Soil
Soil
AUC = 0.95
TSS = 0.74
AUC = 0.96
TSS = 0.74
AUC = 0.92
TSS = 0.62
Relative importance
bio01
bio05
bio12
bio13
bio14
CLY
DES
NTO
PTO
SOC
Stylidium eriopodum
Climate
Climate + Soil
Soil
AUC = 0.98
TSS = 0.86
AUC = 0.99
TSS = 0.81
AUC = 0.97
TSS = 0.71
Relative importance
bio01
bio02
bio05
bio06
bio15
CEC
CLY
NTO
PTO
SOC

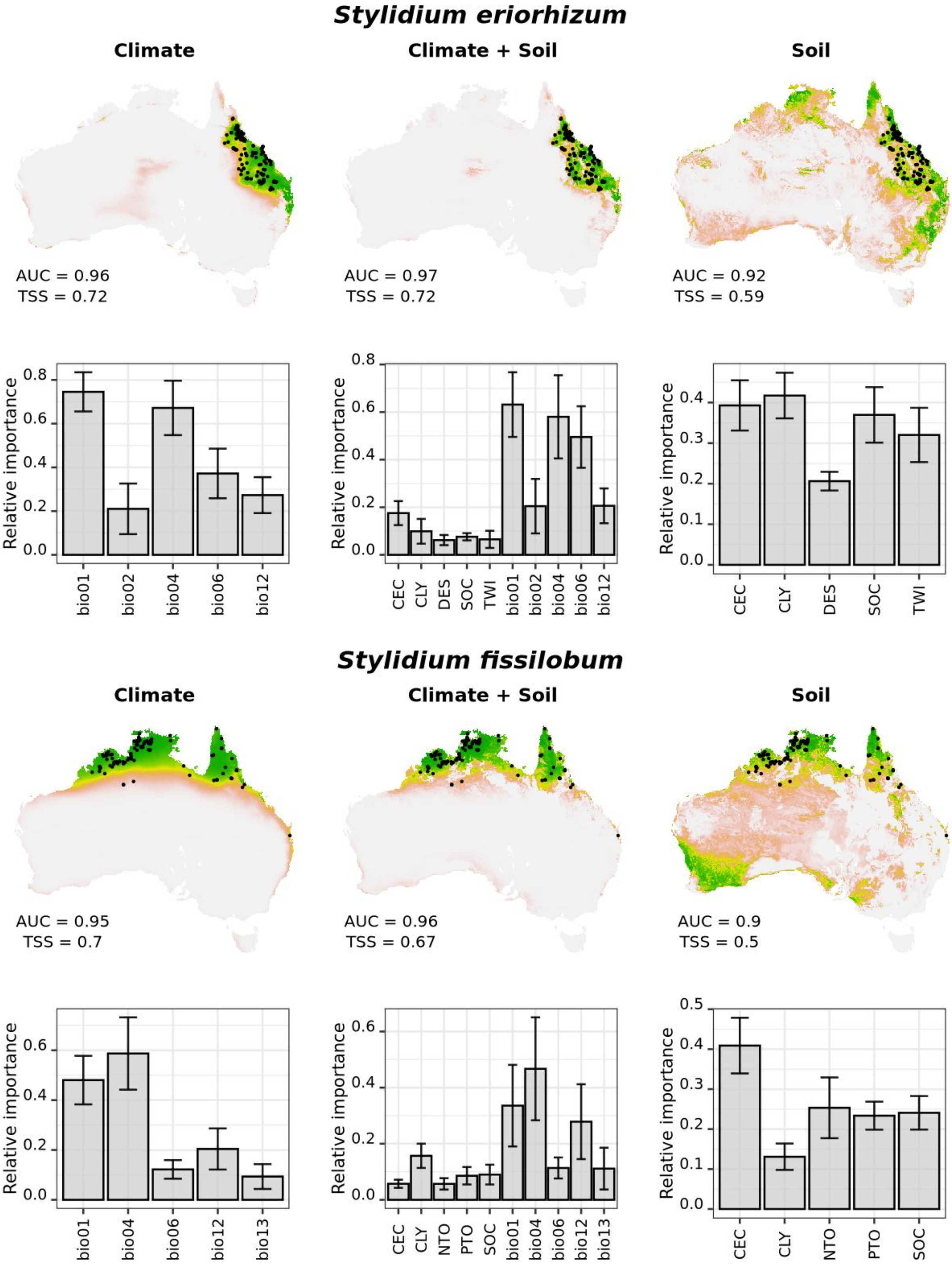

Stylidium eriorhizum
Climate
Climate + Soil
Soil
AUC = 0.96
TSS = 0.72
AUC = 0.97
TSS = 0.72
AUC = 0.92
TSS = 0.59
Relative importance
bio01 bio02 bio04 bio06 bio12
CEC CLY DES SOC TWI bio01 bio02 bio04 bio06 bio12
CEC CLY DES SOC TWI
Stylidium fissilobum
Climate
Climate + Soil
Soil
AUC = 0.95
TSS = 0.7
AUC = 0.96
TSS = 0.67
AUC = 0.9
TSS = 0.5
Relative importance
bio01 bio04 bio06 bio12 bio13
CEC CLY NTO PTO SOC bio01 bio04 bio06 bio12 bio13
CEC CLY NTO PTO SOC

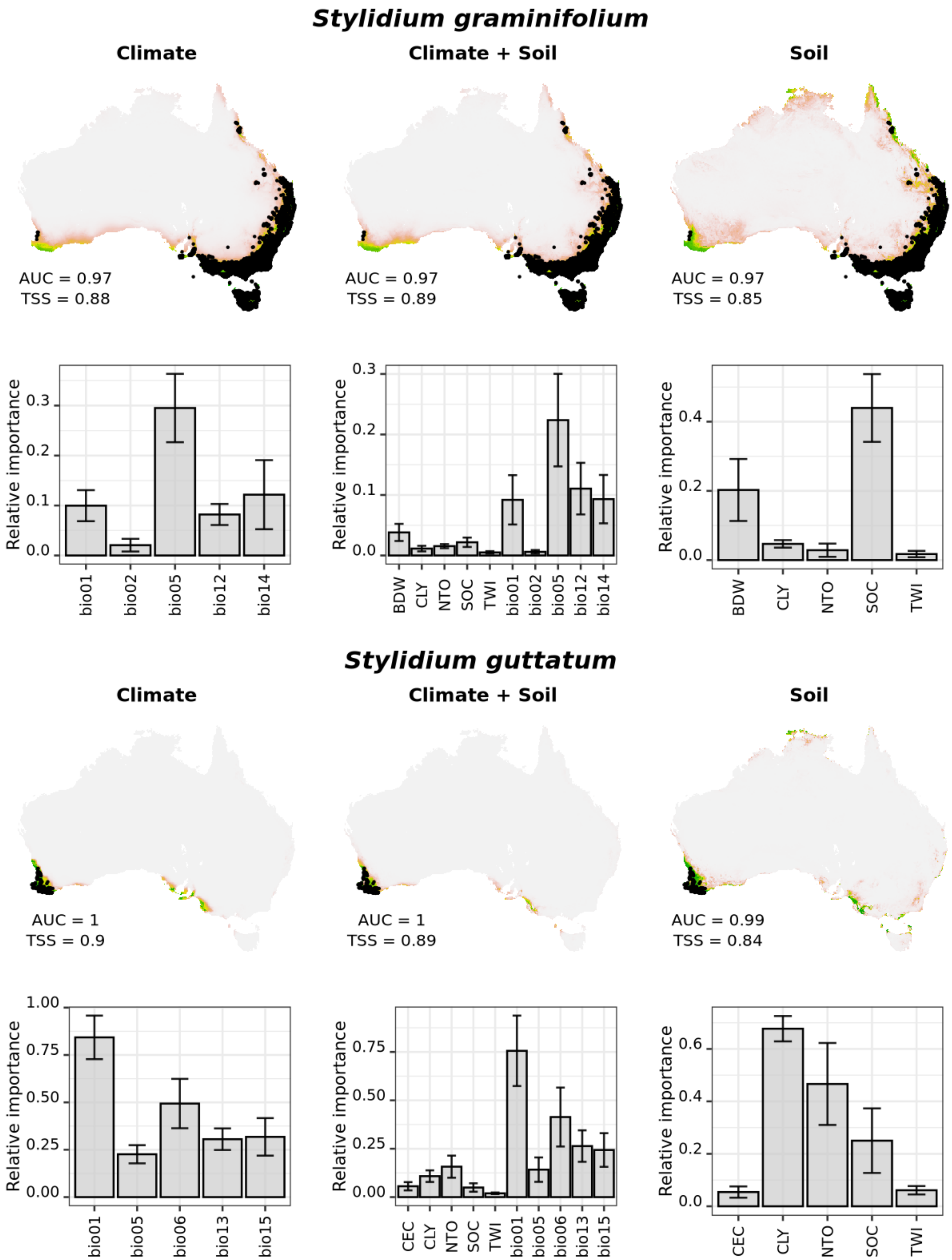
Stylidium graminifolium
Climate
Climate + Soil
Soil
AUC = 0.97
TSS = 0.88
AUC = 0.97
TSS = 0.89
AUC = 0.97
TSS = 0.85
Relative importance
bio01 bio02 bio05 bio12 bio14
BDW CLY NTO SOC TWI bio01 bio02 bio05 bio12 bio14
BDW CLY NTO SOC TWI
Stylidium guttatum
Climate
Climate + Soil
Soil
AUC = 1
TSS = 0.9
AUC = 1
TSS = 0.89
AUC = 0.99
TSS = 0.84
Relative importance
bio01 bio05 bio06 bio13 bio15
CEC CLY NTO SOC TWI bio01 bio05 bio06 bio13 bio15
CEC CLY NTO SOC TWI

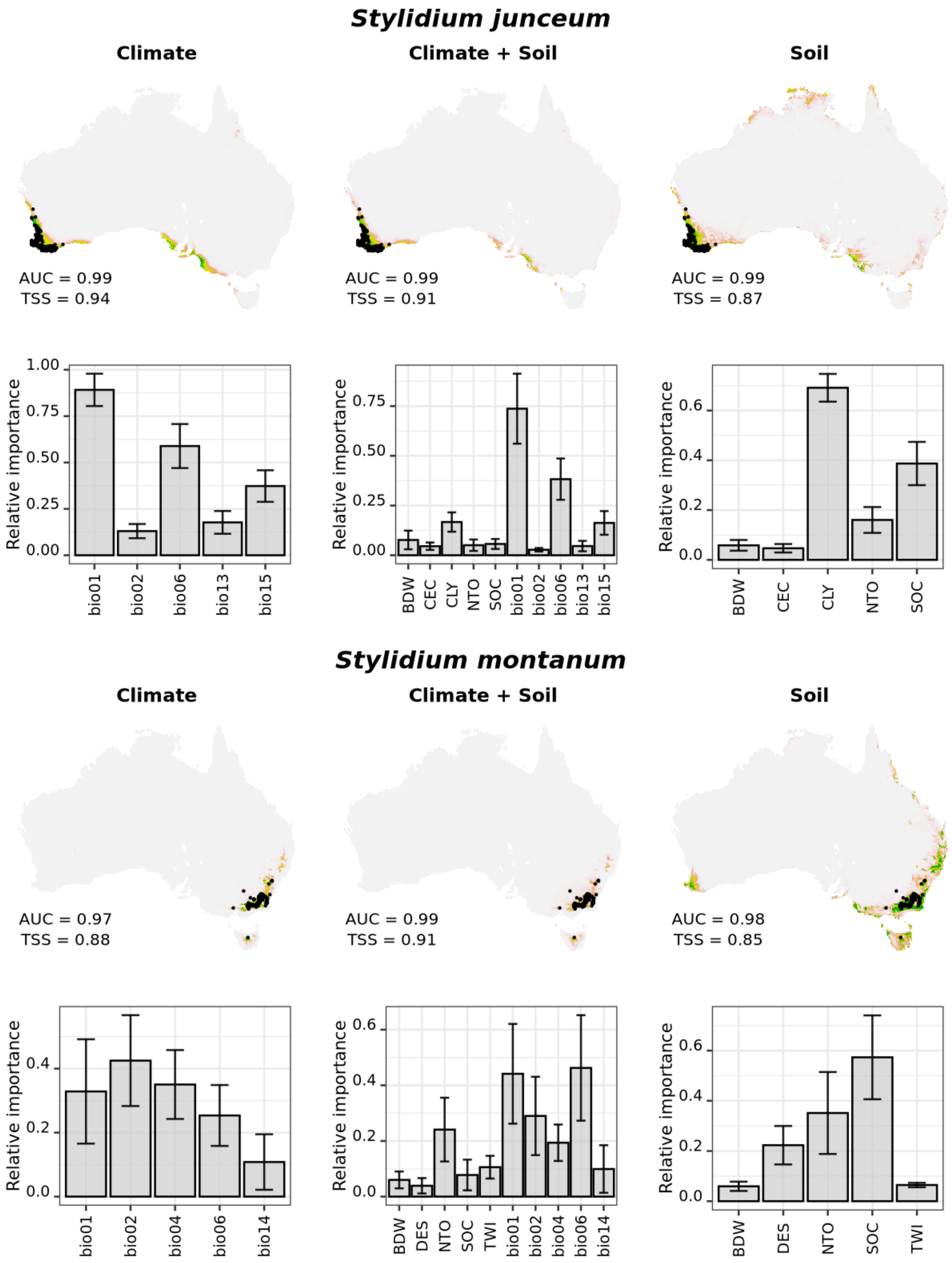

Stylidium junceum
Climate
Climate + Soil
Soil
AUC = 0.99
TSS = 0.94
AUC = 0.99
TSS = 0.91
AUC = 0.99
TSS = 0.87
Relative importance
1.00
0.75
0.50
0.25
0.00
bio01
bio02
bio06
bio13
bio15
BDW
CEC
CLY
NTO
SOC
0.6
0.4
0.2
0.0
Stylidium montanum
AUC = 0.97
TSS = 0.88
AUC = 0.99
TSS = 0.91
AUC = 0.98
TSS = 0.85
bio04
bio14
DES
TWI

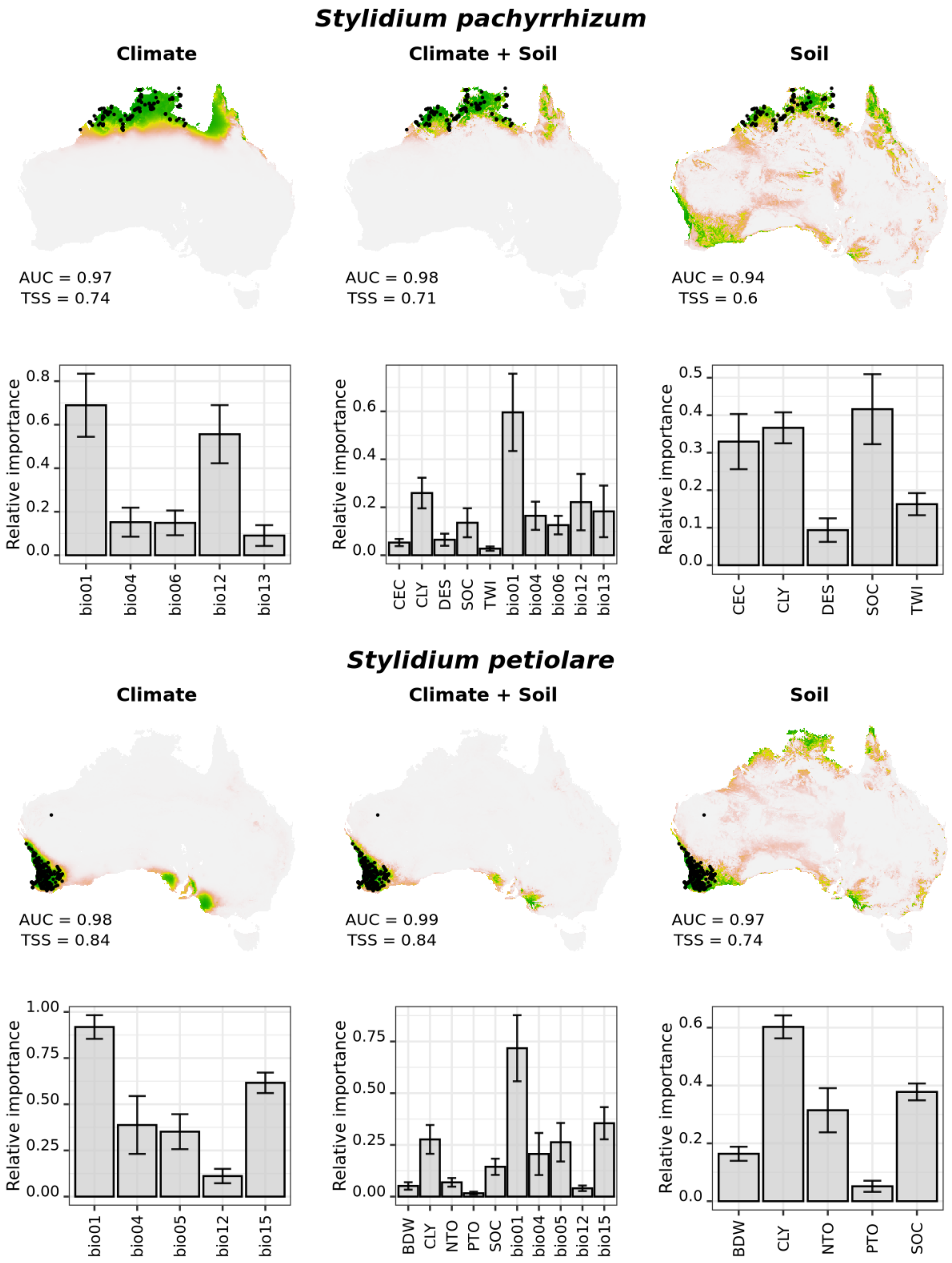

Stylidium pachyrrhizum
Climate
Climate + Soil
Soil
AUC = 0.97
TSS = 0.74
AUC = 0.98
TSS = 0.71
AUC = 0.94
TSS = 0.6
Relative importance
bio01
bio04
bio06
bio12
bio13
CEC
CLY
DES
SOC
TWI
Stylidium petiolare
Climate
Climate + Soil
Soil
AUC = 0.98
TSS = 0.84
AUC = 0.99
TSS = 0.84
AUC = 0.97
TSS = 0.74
Relative importance
bio01
bio04
bio05
bio12
bio15
BDW
CLY
NTO
PTO
SOC

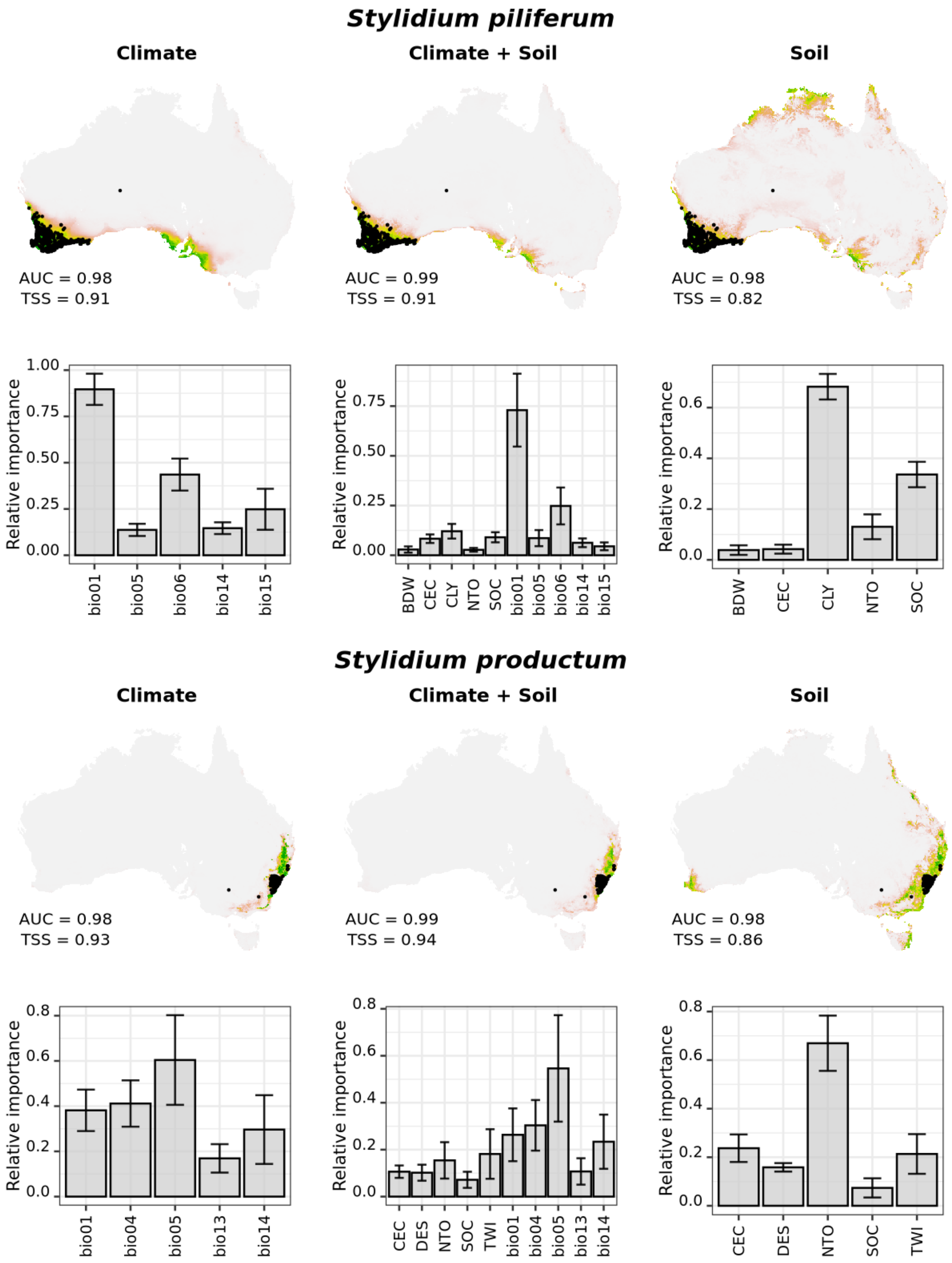

Stylidium piliferum
Climate
Climate + Soil
Soil
AUC = 0.98
TSS = 0.91
AUC = 0.99
TSS = 0.91
AUC = 0.98
TSS = 0.82
Relative importance
1.00
0.75
0.50
0.25
0.00
bio01
bio05
bio06
bio14
bio15
BDW
CEC
CLY
NTO
SOC
0.6
0.4
0.2
0.0
Stylidium productum
Climate
Climate + Soil
Soil
AUC = 0.98
TSS = 0.93
AUC = 0.99
TSS = 0.94
AUC = 0.98
TSS = 0.86
0.8
bio04
bio13
DES
TWI

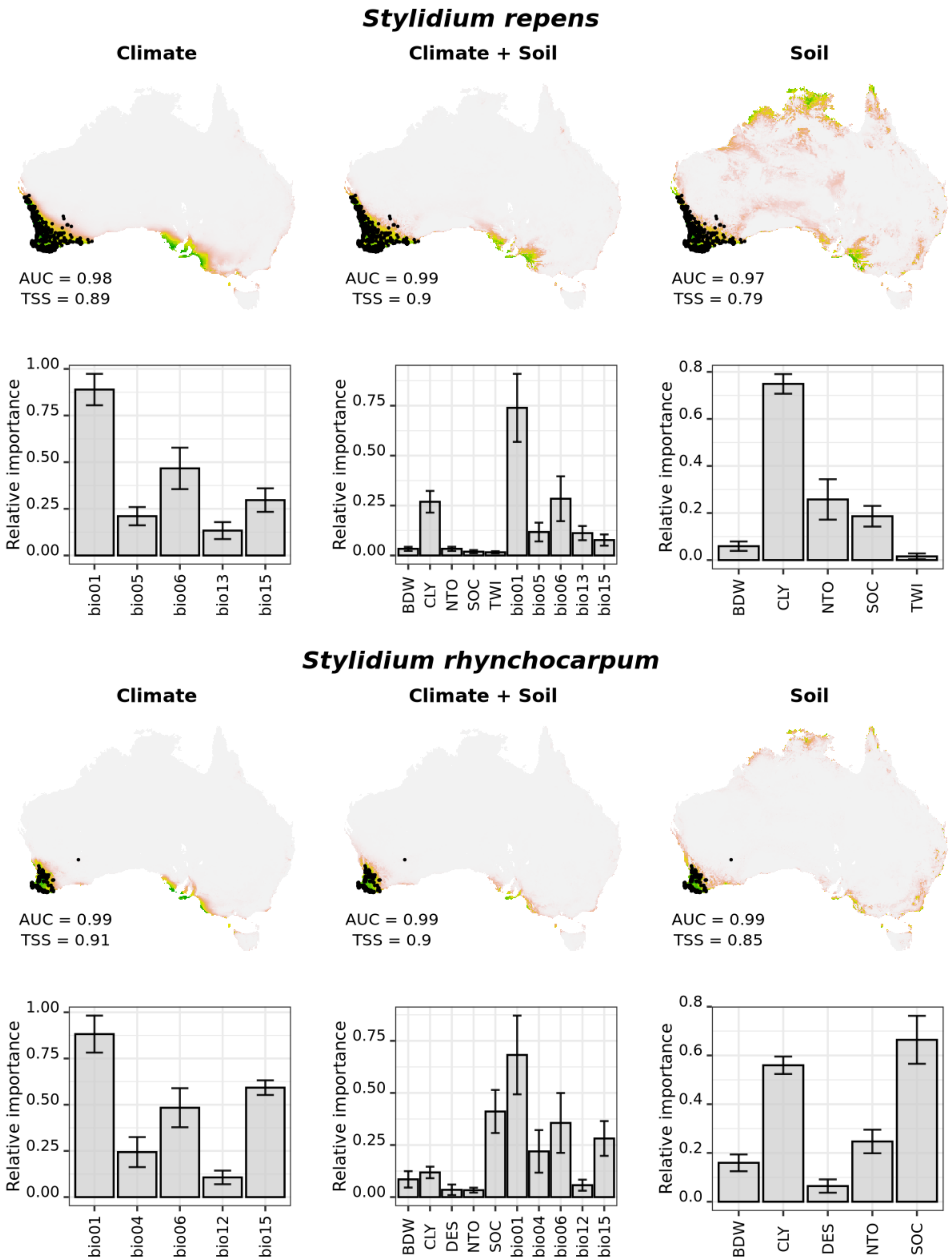
Stylidium repens
Climate
Climate + Soil
Soil
AUC = 0.98
TSS = 0.89
AUC = 0.99
TSS = 0.9
AUC = 0.97
TSS = 0.79
Relative importance
bio01
bio05
bio06
bio13
bio15
BDW
CLY
NTO
SOC
TWI
Stylidium rhynchocarpum
Climate
Climate + Soil
Soil
AUC = 0.99
TSS = 0.91
AUC = 0.99
TSS = 0.9
AUC = 0.99
TSS = 0.85
Relative importance
bio01
bio04
bio06
bio12
bio15
BDW
CLY
DES
NTO
SOC

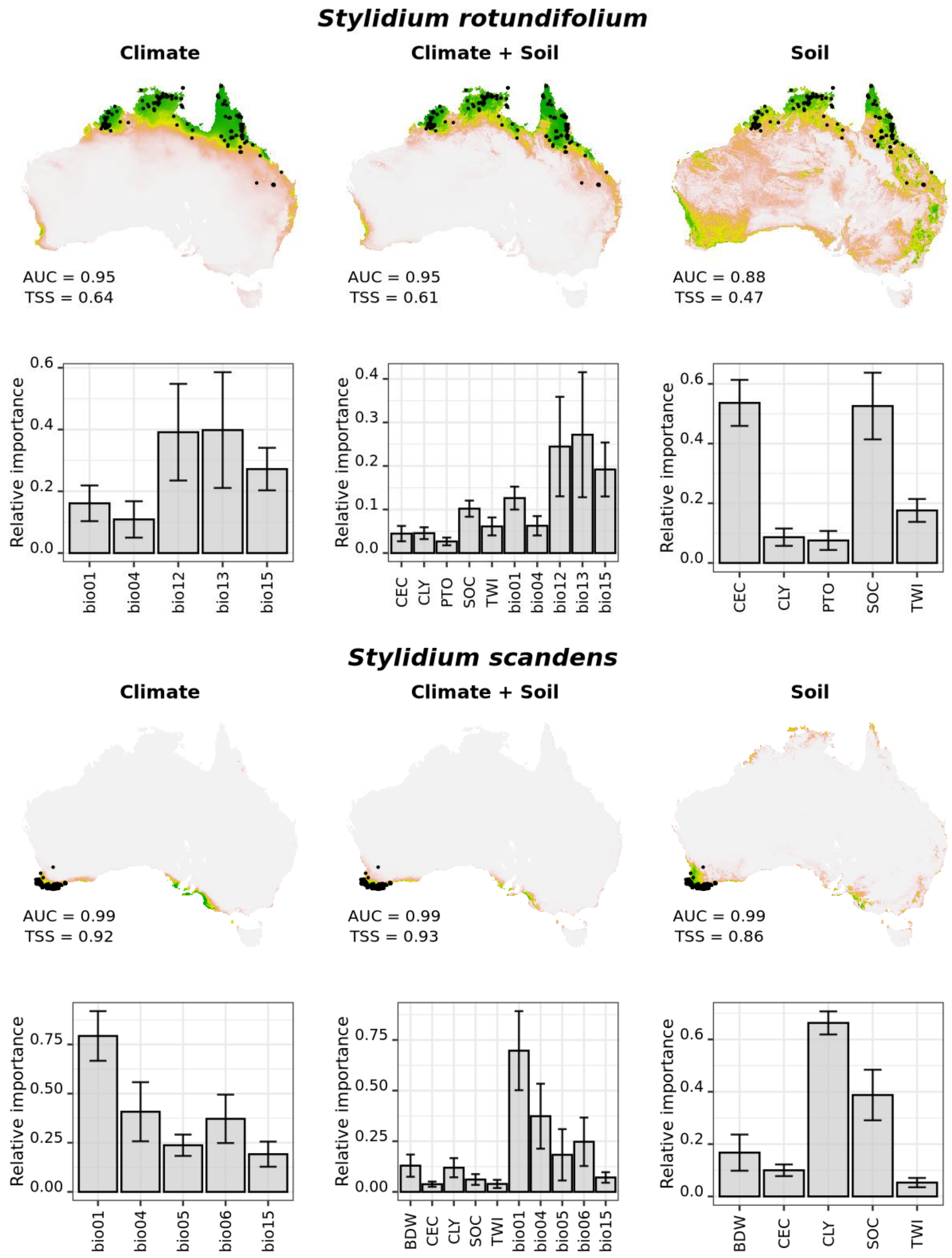

Stylidium rotundifolium
Climate
Climate + Soil
Soil
AUC = 0.95
TSS = 0.64
AUC = 0.95
TSS = 0.61
AUC = 0.88
TSS = 0.47
Relative importance
bio01
bio04
bio12
bio13
bio15
CEC
CLY
PTO
SOC
TWI
Stylidium scandens
Climate
Climate + Soil
Soil
AUC = 0.99
TSS = 0.92
AUC = 0.99
TSS = 0.93
AUC = 0.99
TSS = 0.86
Relative importance
bio01
bio04
bio05
bio06
bio15
BDW
CEC
CLY
SOC
TWI

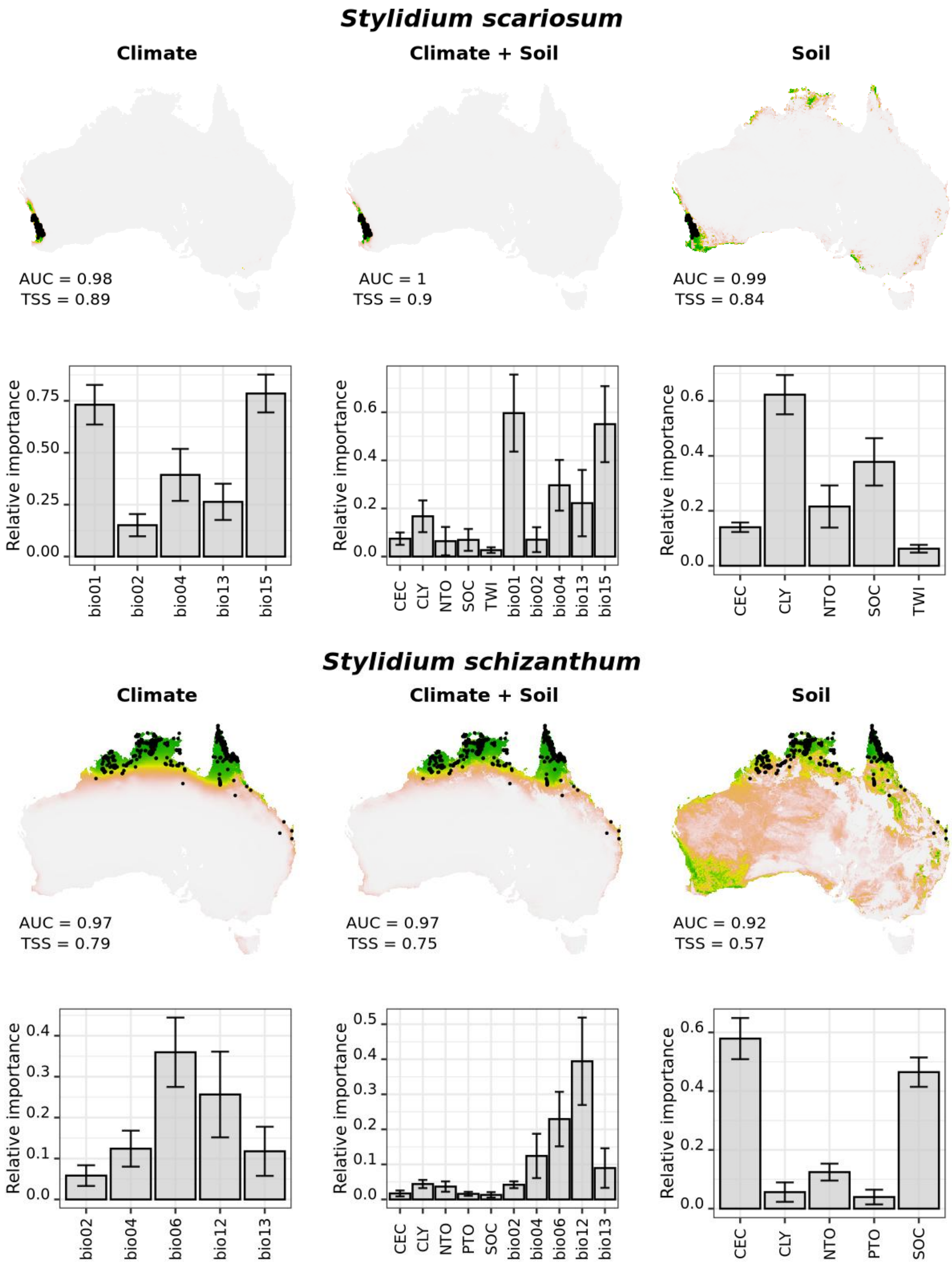

Stylidium scariosum
Climate
Climate + Soil
Soil
AUC = 0.98
TSS = 0.89
AUC = 1
TSS = 0.9
AUC = 0.99
TSS = 0.84
Relative importance
bio01
bio02
bio04
bio13
bio15
CEC
CLY
NTO
SOC
TWI
Stylidium schizanthum
Climate
Climate + Soil
Soil
AUC = 0.97
TSS = 0.79
AUC = 0.97
TSS = 0.75
AUC = 0.92
TSS = 0.57
Relative importance
bio02
bio04
bio06
bio12
bio13
CEC
CLY
NTO
PTO
SOC

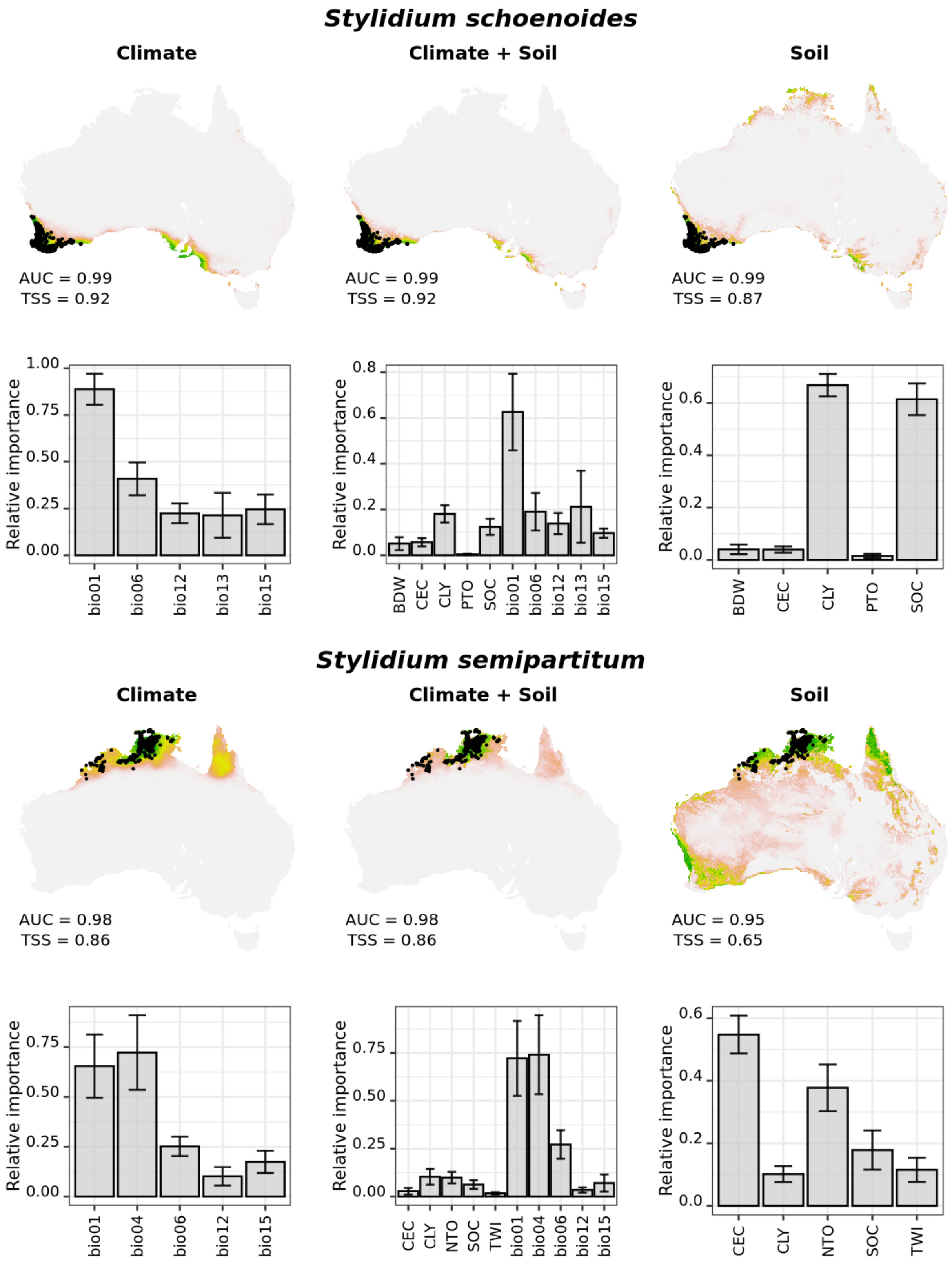
Stylidium schoenoides
Climate
Climate + Soil
Soil
AUC = 0.99
TSS = 0.92
AUC = 0.99
TSS = 0.92
AUC = 0.99
TSS = 0.87
Relative importance
1.00
0.75
0.50
0.25
0.00
bio01
bio06
bio12
bio13
bio15
0.8
0.6
0.4
0.2
0.0
BDW
CEC
CLY
PTO
SOC
bio01
bio06
bio12
bio13
bio15
0.6
0.4
0.2
0.0
BDW
CEC
CLY
PTO
SOC
Stylidium semipartitum
Climate
Climate + Soil
Soil
AUC = 0.98
TSS = 0.86
AUC = 0.98
TSS = 0.86
AUC = 0.95
TSS = 0.65
Relative importance
0.75
0.50
0.25
0.00
bio01
bio04
bio06
bio12
bio15
CEC
CLY
NTO
SOC
TWI
bio01
bio04
bio06
bio12
bio15
0.6
0.4
0.2
0.0
CEC
CLY
NTO
SOC
TWI

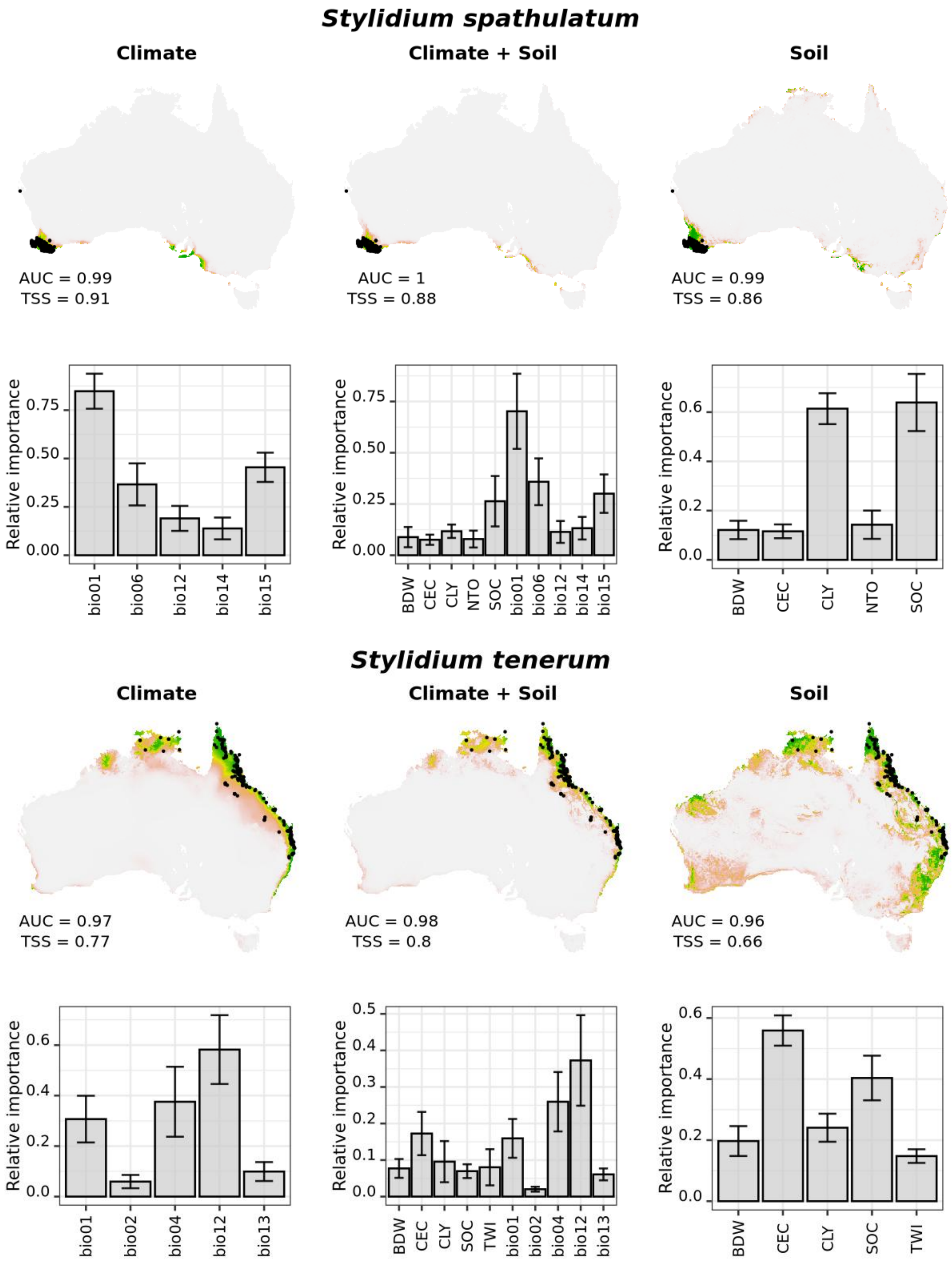

Stylidium spathulatum
Climate
Climate + Soil
Soil
AUC = 0.99
TSS = 0.91
AUC = 1
TSS = 0.88
AUC = 0.99
TSS = 0.86
Relative importance
0.00
0.25
0.50
0.75
bio01
bio06
bio12
bio14
bio15
BDW
CEC
CLY
NTO
SOC
0.0
0.2
0.4
0.6
Stylidium tenerum
AUC = 0.97
TSS = 0.77
AUC = 0.98
TSS = 0.8
AUC = 0.96
TSS = 0.66
0.1
0.3
0.5
bio02
bio04
bio13
TWI

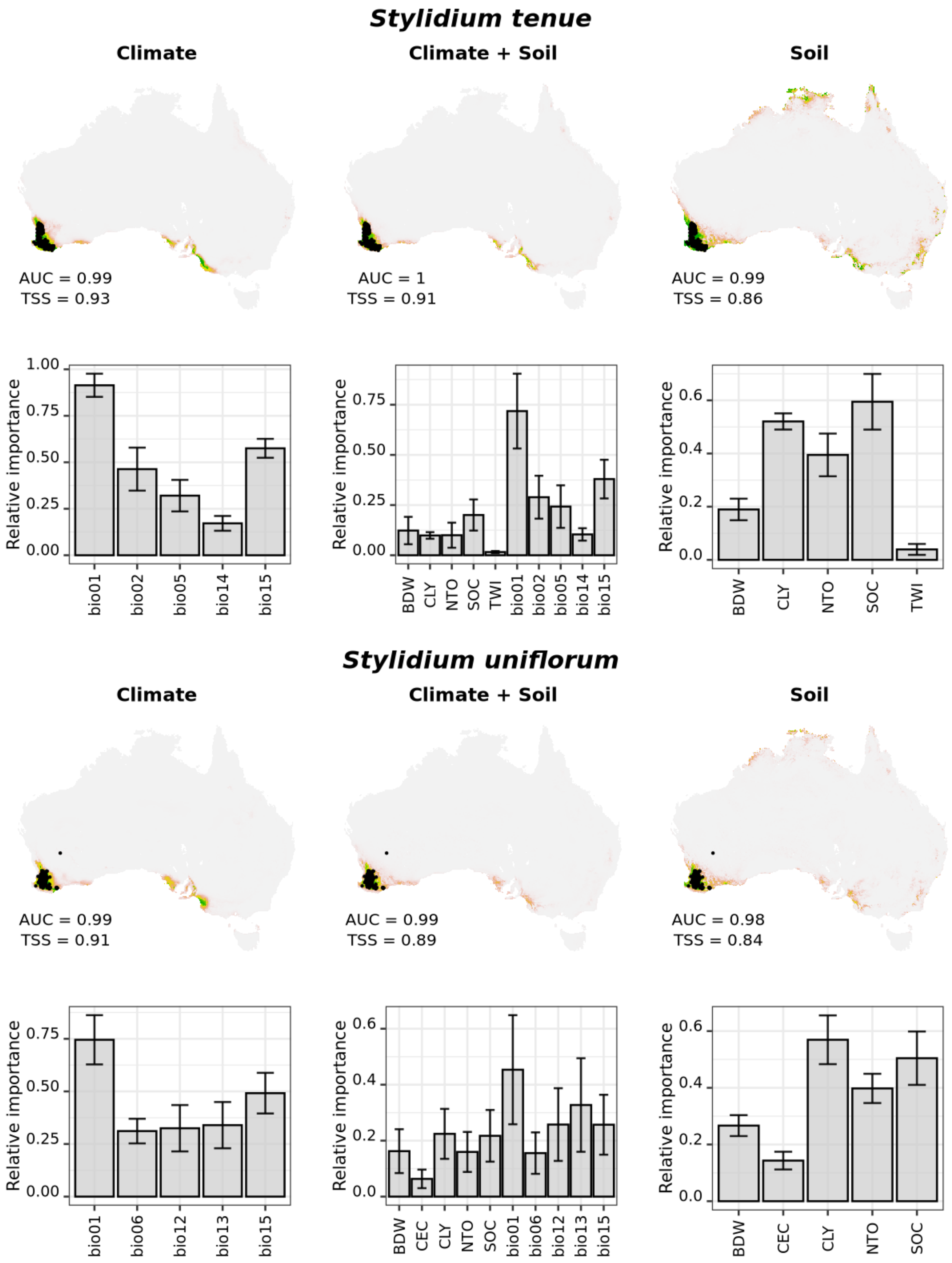

Stylidium tenue
Climate
Climate + Soil
Soil
AUC = 0.99
TSS = 0.93
AUC = 1
TSS = 0.91
AUC = 0.99
TSS = 0.86
Relative importance
1.00
0.75
0.50
0.25
0.00
bio01
bio02
bio05
bio14
bio15
BDW
CLY
NTO
SOC
TWI
0.6
0.4
0.2
0.0
Stylidium uniflorum
Climate
Climate + Soil
Soil
AUC = 0.99
TSS = 0.91
AUC = 0.99
TSS = 0.89
AUC = 0.98
TSS = 0.84
bio06
bio12
bio13
CEC

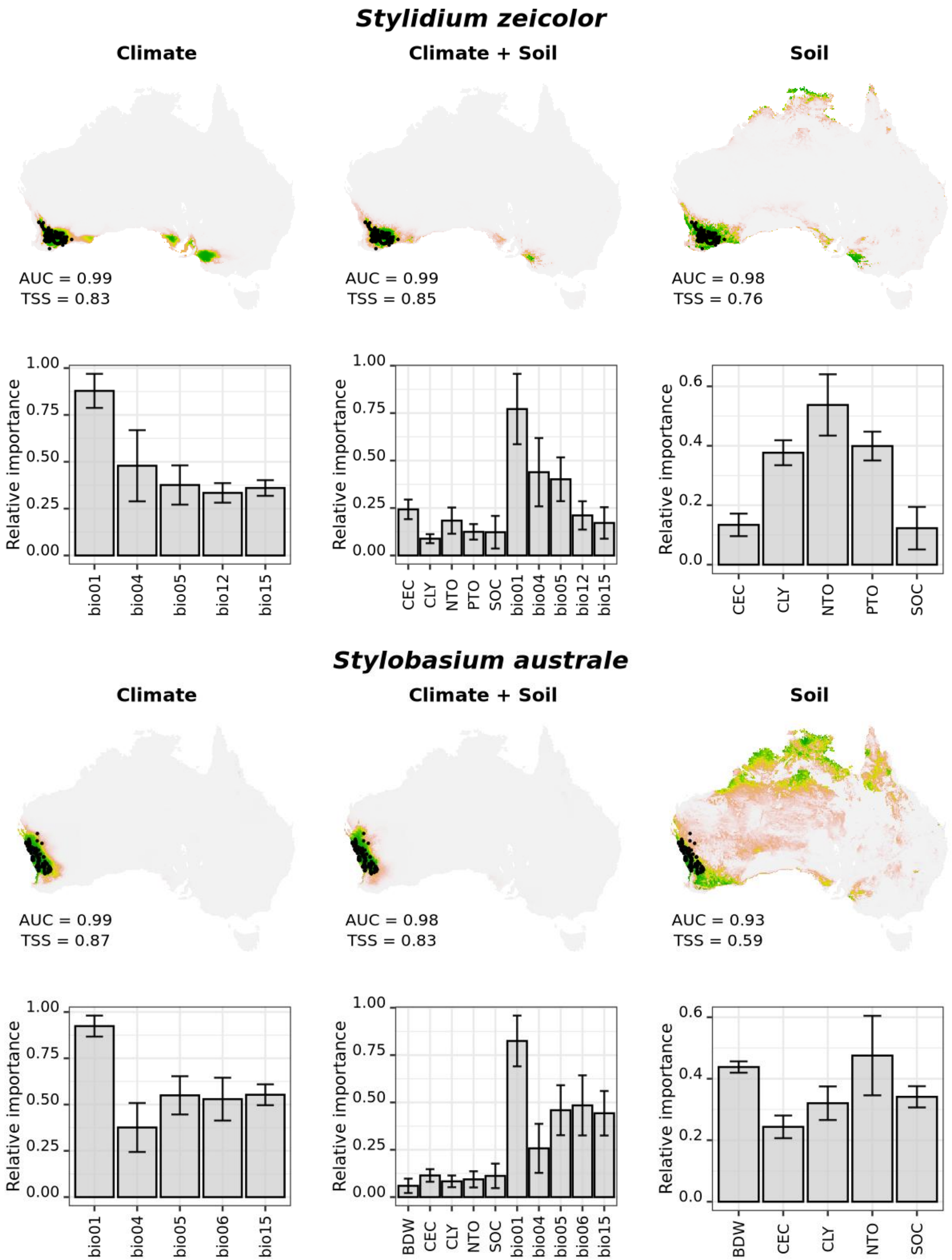
Stylidium zeicolor
Climate
Climate + Soil
Soil
AUC = 0.99
TSS = 0.83
AUC = 0.99
TSS = 0.85
AUC = 0.98
TSS = 0.76
Relative importance
0.00
0.25
0.50
0.75
1.00
0.0
0.2
0.4
0.6
bio01
bio04
bio05
bio12
bio15
CEC
CLY
NTO
PTO
SOC
Stylobasium australe
Climate
Climate + Soil
Soil
AUC = 0.99
TSS = 0.87
AUC = 0.98
TSS = 0.83
AUC = 0.93
TSS = 0.59
Relative importance
bio01
bio04
bio05
bio06
bio15
BDW
CEC
CLY
NTO
SOC

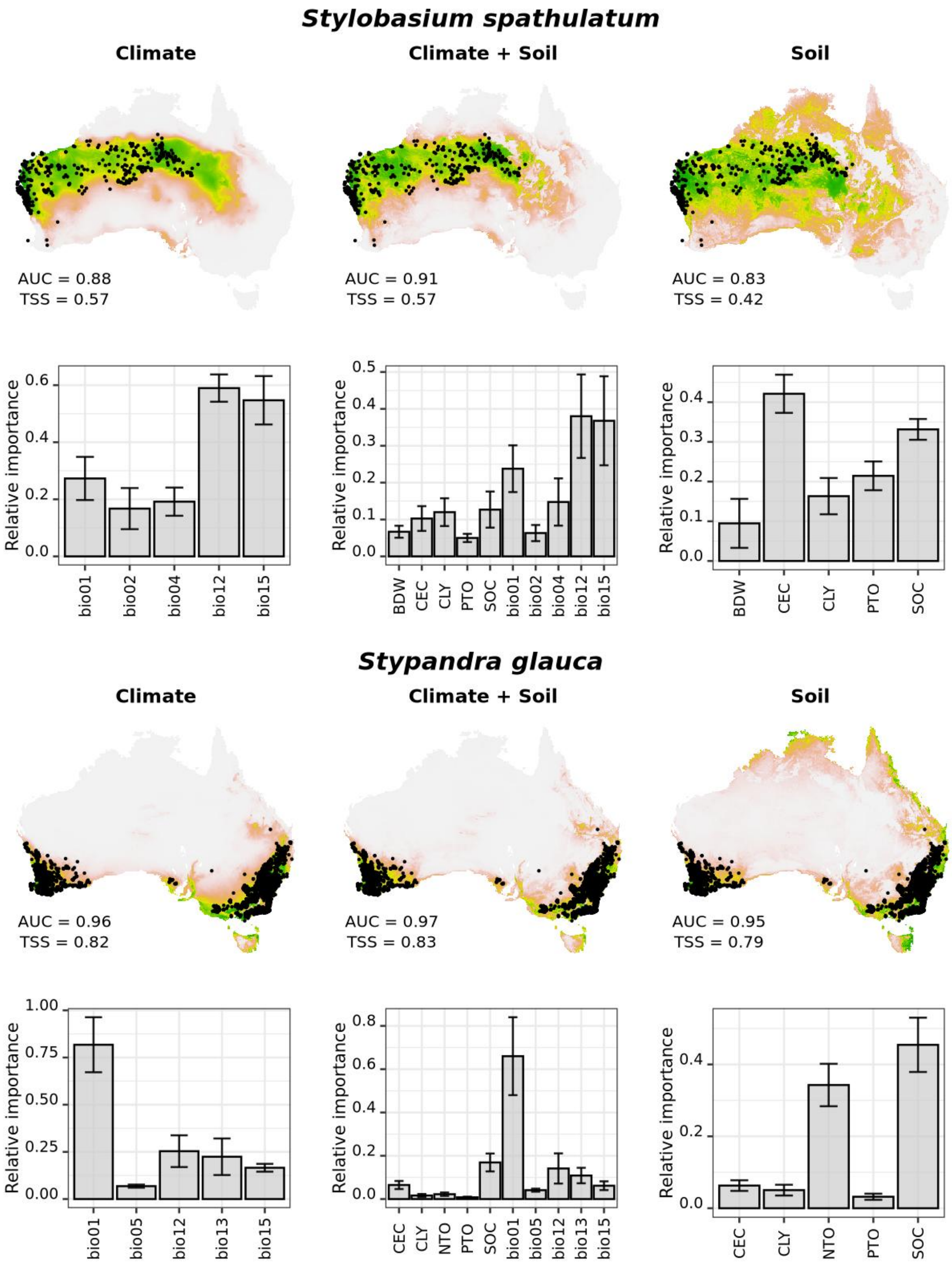
Stylobasium spathulatum
Climate
Climate + Soil
Soil
AUC = 0.88
TSS = 0.57
AUC = 0.91
TSS = 0.57
AUC = 0.83
TSS = 0.42
Relative importance
bio01 bio02 bio04 bio12 bio15
BDW CEC CLY PTO SOC bio01 bio02 bio04 bio12 bio15
BDW CEC CLY PTO SOC
Stypandra glauca
Climate
Climate + Soil
Soil
AUC = 0.96
TSS = 0.82
AUC = 0.97
TSS = 0.83
AUC = 0.95
TSS = 0.79
Relative importance
bio01 bio05 bio12 bio13 bio15
CEC CLY NTO PTO SOC bio01 bio05 bio12 bio13 bio15
CEC CLY NTO PTO SOC

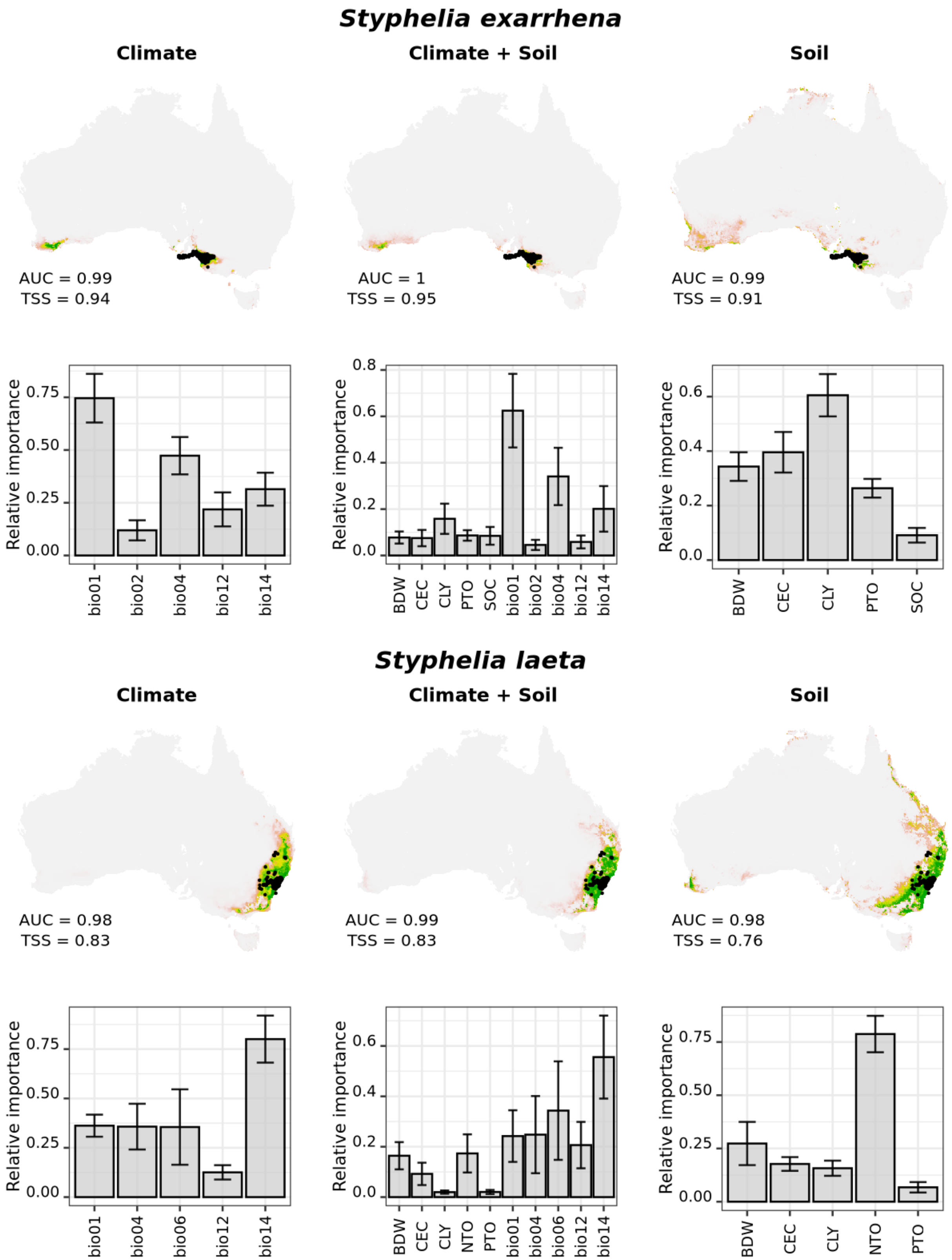
Styphelia exarrhena
Climate
Climate + Soil
Soil
AUC = 0.99
TSS = 0.94
AUC = 1
TSS = 0.95
AUC = 0.99
TSS = 0.91
Relative importance
0.00
0.25
0.50
0.75
bio01
bio02
bio04
bio12
bio14
0.0
0.2
0.4
0.6
0.8
BDW
CEC
CLY
PTO
SOC
bio01
bio02
bio04
bio12
bio14
0.0
0.2
0.4
0.6
BDW
CEC
CLY
PTO
SOC
Styphelia laeta
Climate
Climate + Soil
Soil
AUC = 0.98
TSS = 0.83
AUC = 0.99
TSS = 0.83
AUC = 0.98
TSS = 0.76
Relative importance
0.00
0.25
0.50
0.75
bio01
bio04
bio06
bio12
bio14
0.0
0.2
0.4
0.6
BDW
CEC
CLY
NTO
PTO
bio01
bio04
bio06
bio12
bio14
0.00
0.25
0.50
0.75
BDW
CEC
CLY
NTO
PTO

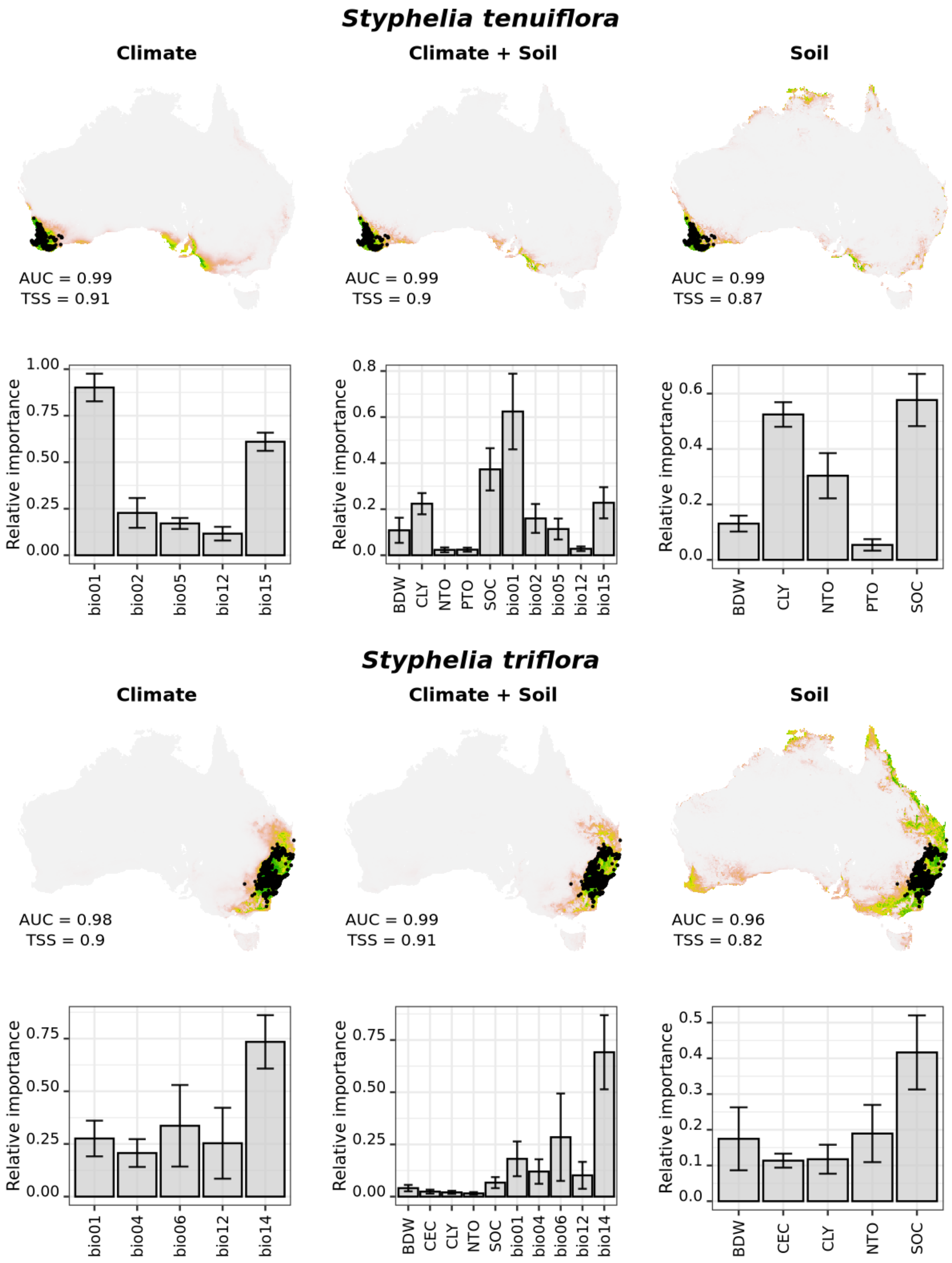
Styphelia tenuiflora
Climate
Climate + Soil
Soil
AUC = 0.99
TSS = 0.91
AUC = 0.99
TSS = 0.9
AUC = 0.99
TSS = 0.87
Relative importance
0.00
0.25
0.50
0.75
1.00
bio01
bio02
bio05
bio12
bio15
0.0
0.2
0.4
0.6
0.8
BDW
CLY
NTO
PTO
SOC
bio01
bio02
bio05
bio12
bio15
0.0
0.2
0.4
0.6
BDW
CLY
NTO
PTO
SOC
Styphelia triflora
Climate
Climate + Soil
Soil
AUC = 0.98
TSS = 0.9
AUC = 0.99
TSS = 0.91
AUC = 0.96
TSS = 0.82
Relative importance
0.00
0.25
0.50
0.75
bio01
bio04
bio06
bio12
bio14
0.00
0.25
0.50
0.75
BDW
CEC
CLY
NTO
SOC
bio01
bio04
bio06
bio12
bio14
0.0
0.1
0.2
0.3
0.4
0.5
BDW
CEC
CLY
NTO
SOC

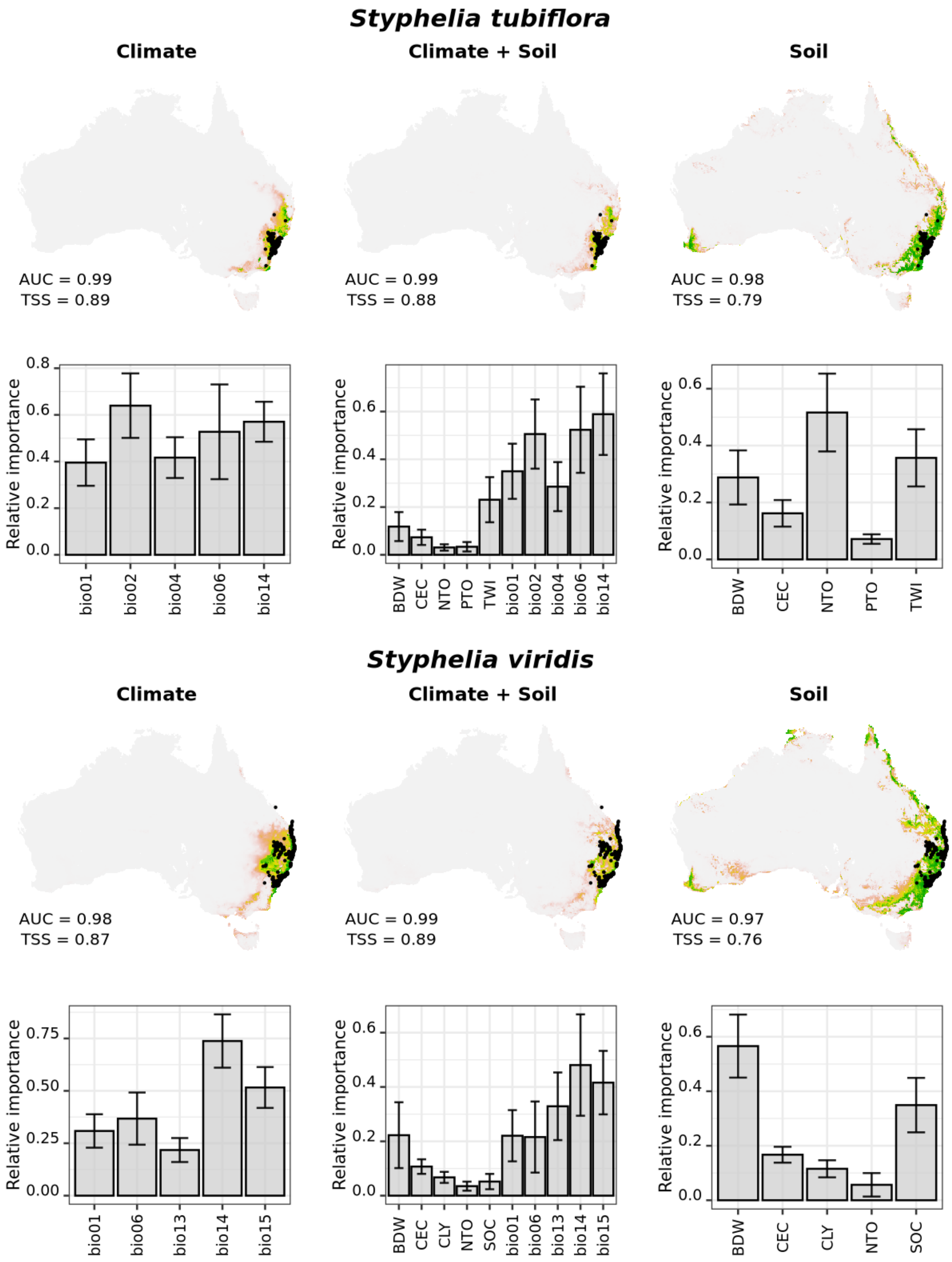
Styphelia tubiflora
Climate
Climate + Soil
Soil
AUC = 0.99
TSS = 0.89
AUC = 0.99
TSS = 0.88
AUC = 0.98
TSS = 0.79
Relative importance
bio01
bio02
bio04
bio06
bio14
BDW
CEC
NTO
PTO
TWI
bio01
bio02
bio04
bio06
bio14
BDW
CEC
NTO
PTO
TWI
Styphelia viridis
Climate
Climate + Soil
Soil
AUC = 0.98
TSS = 0.87
AUC = 0.99
TSS = 0.89
AUC = 0.97
TSS = 0.76
Relative importance
bio01
bio06
bio13
bio14
bio15
BDW
CEC
CLY
NTO
SOC
bio01
bio06
bio13
bio14
bio15
BDW
CEC
CLY
NTO
SOC

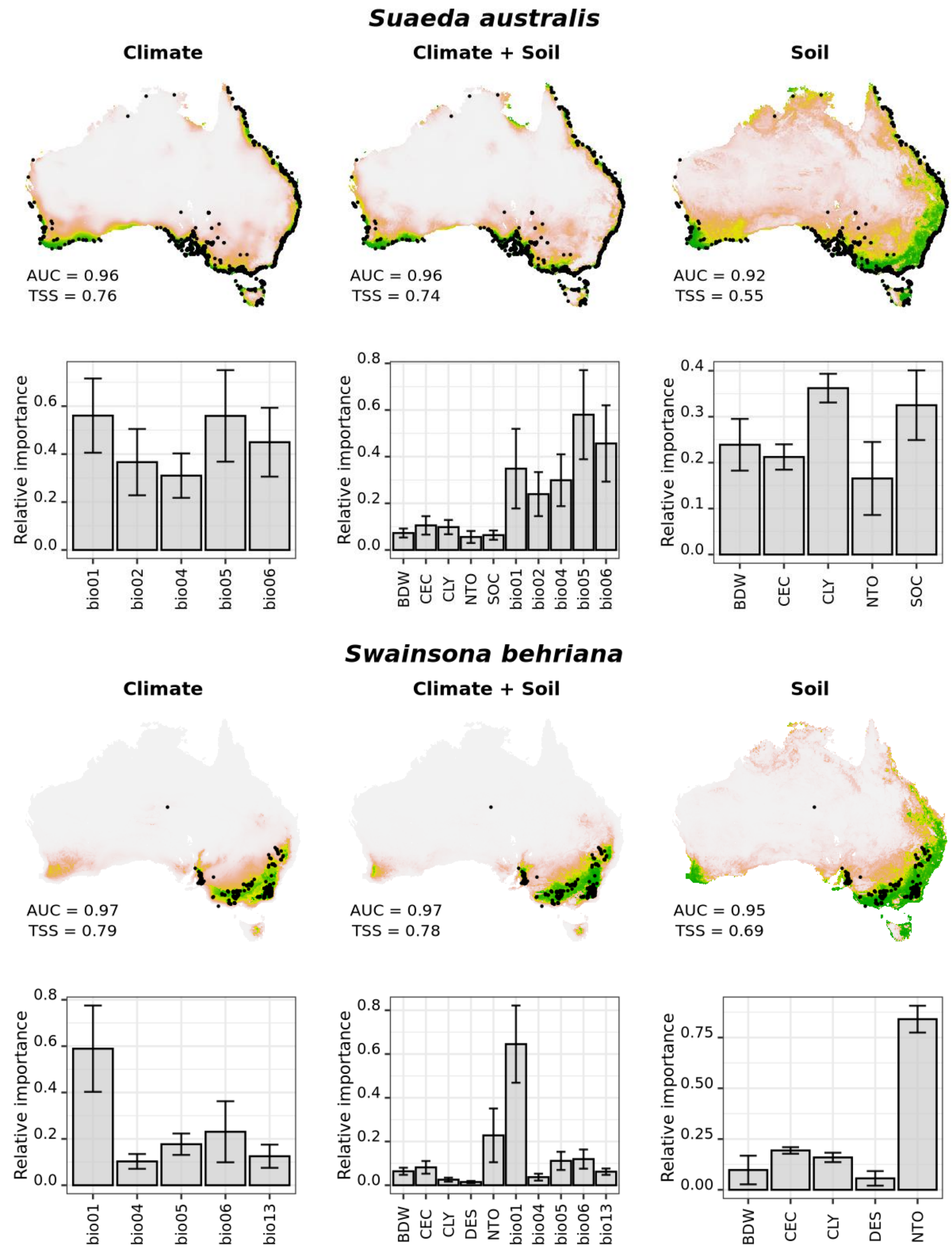
Suaeda australis
Climate
Climate + Soil
Soil
AUC = 0.96
TSS = 0.76
AUC = 0.96
TSS = 0.74
AUC = 0.92
TSS = 0.55
Relative importance
bio01 bio02 bio04 bio05 bio06
BDW CEC CLY NTO SOC bio01 bio02 bio04 bio05 bio06
BDW CEC CLY NTO SOC
Swainsona behriana
Climate
Climate + Soil
Soil
AUC = 0.97
TSS = 0.79
AUC = 0.97
TSS = 0.78
AUC = 0.95
TSS = 0.69
Relative importance
bio01 bio04 bio05 bio06 bio13
BDW CEC CLY DES NTO bio01 bio04 bio05 bio06 bio13
BDW CEC CLY DES NTO

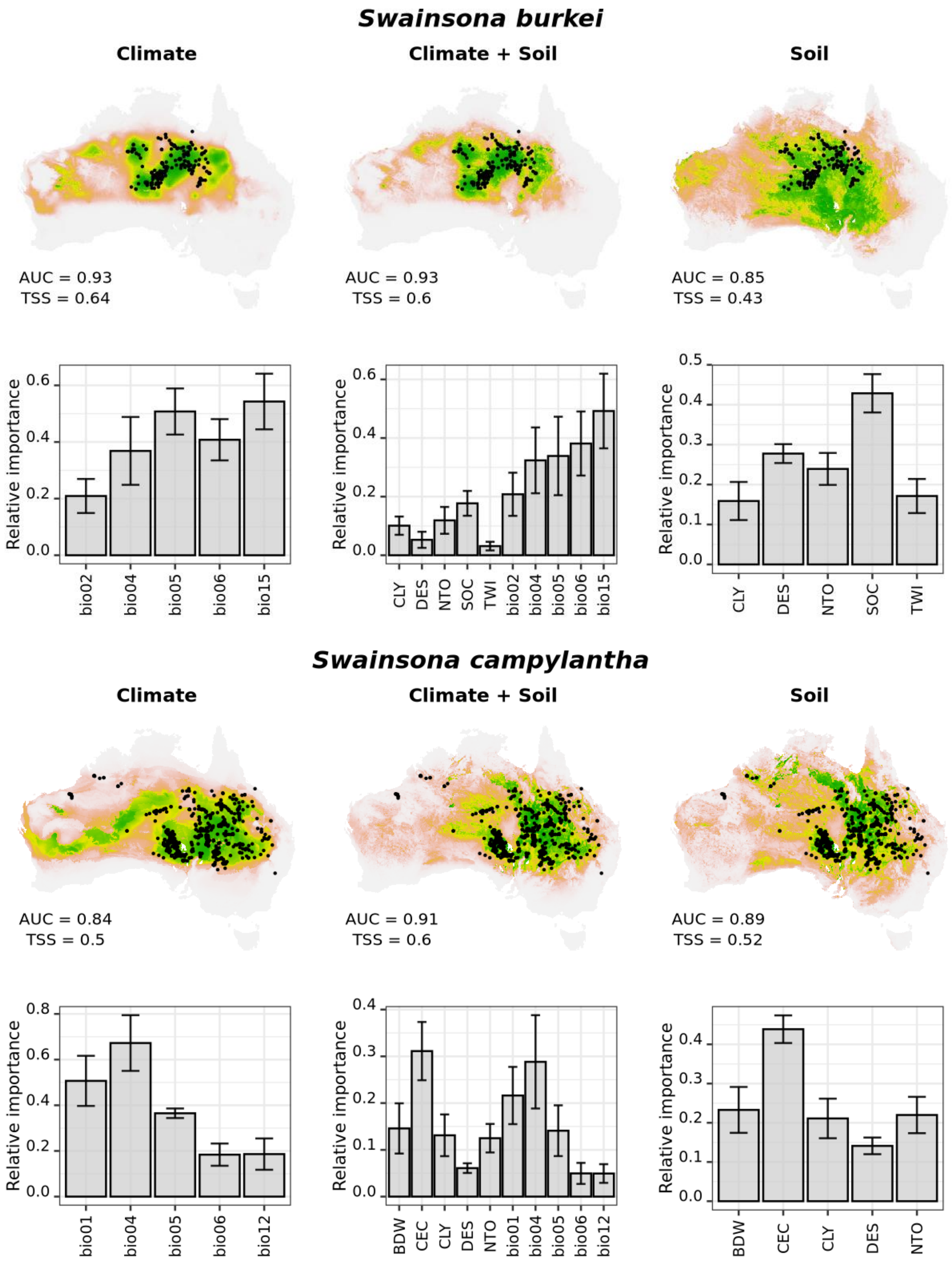
Swainsona burkei
Climate
Climate + Soil
Soil
AUC = 0.93
TSS = 0.64
AUC = 0.93
TSS = 0.6
AUC = 0.85
TSS = 0.43
Relative importance
0.0 0.2 0.4 0.6
bio02 bio04 bio05 bio06 bio15
CLY DES NTO SOC TWI bio02 bio04 bio05 bio06 bio15
0.0 0.1 0.2 0.3 0.4 0.5
CLY DES NTO SOC TWI
Swainsona campylantha
Climate
Climate + Soil
Soil
AUC = 0.84
TSS = 0.5
AUC = 0.91
TSS = 0.6
AUC = 0.89
TSS = 0.52
0.0 0.2 0.4 0.6 0.8
bio01 bio04 bio05 bio06 bio12
0.0 0.1 0.2 0.3 0.4
BDW CEC CLY DES NTO bio01 bio04 bio05 bio06 bio12
BDW CEC CLY DES NTO

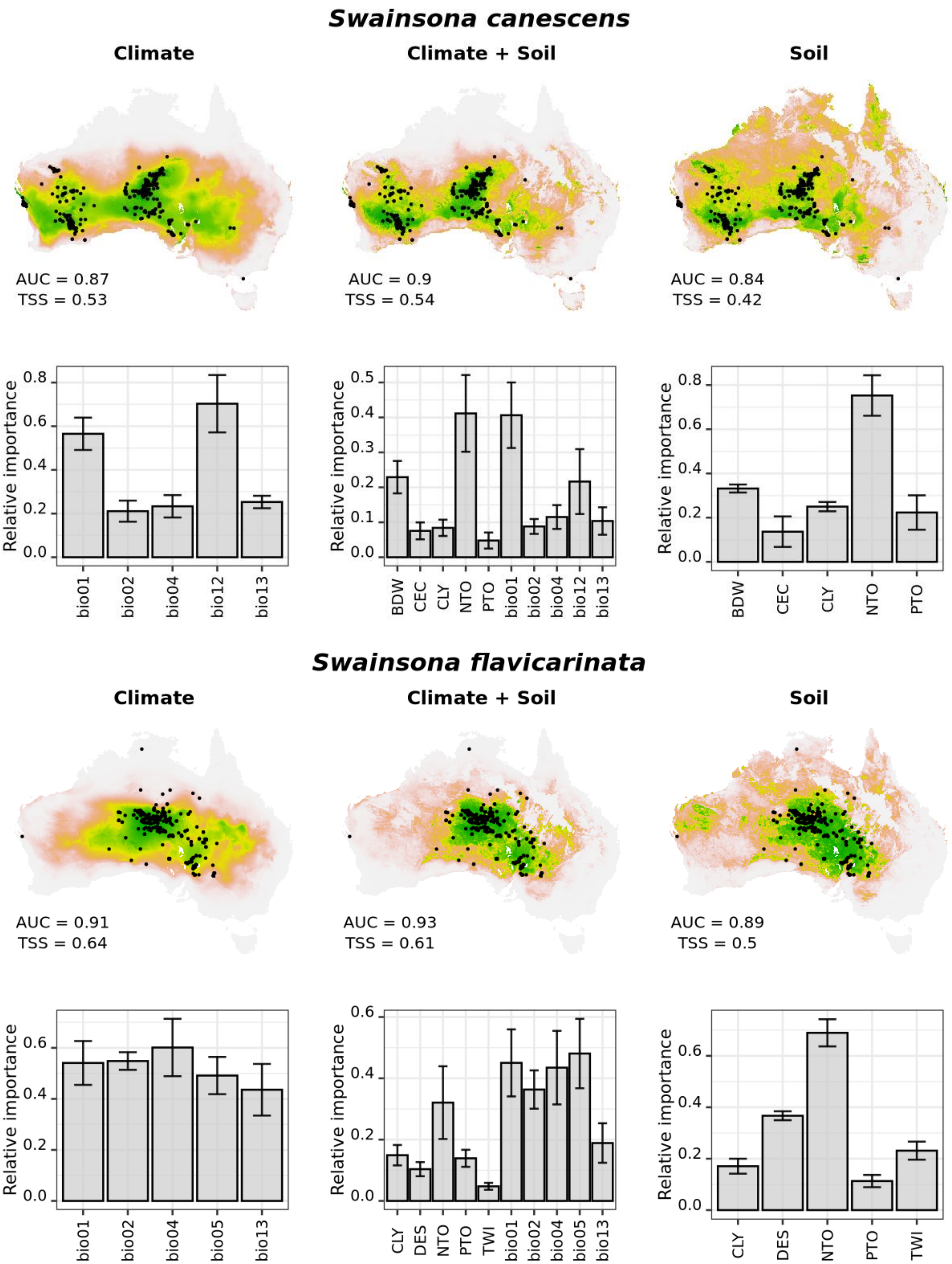

Swainsona canescens
Climate
Climate + Soil
Soil
AUC = 0.87
TSS = 0.53
AUC = 0.9
TSS = 0.54
AUC = 0.84
TSS = 0.42
Relative importance
bio01 bio02 bio04 bio12 bio13
BDW CEC CLY NTO PTO bio01 bio02 bio04 bio12 bio13
BDW CEC CLY NTO PTO
Swainsona flavicarinata
Climate
Climate + Soil
Soil
AUC = 0.91
TSS = 0.64
AUC = 0.93
TSS = 0.61
AUC = 0.89
TSS = 0.5
Relative importance
bio01 bio02 bio04 bio05 bio13
CLY DES NTO PTO TWI bio01 bio02 bio04 bio05 bio13
CLY DES NTO PTO TWI

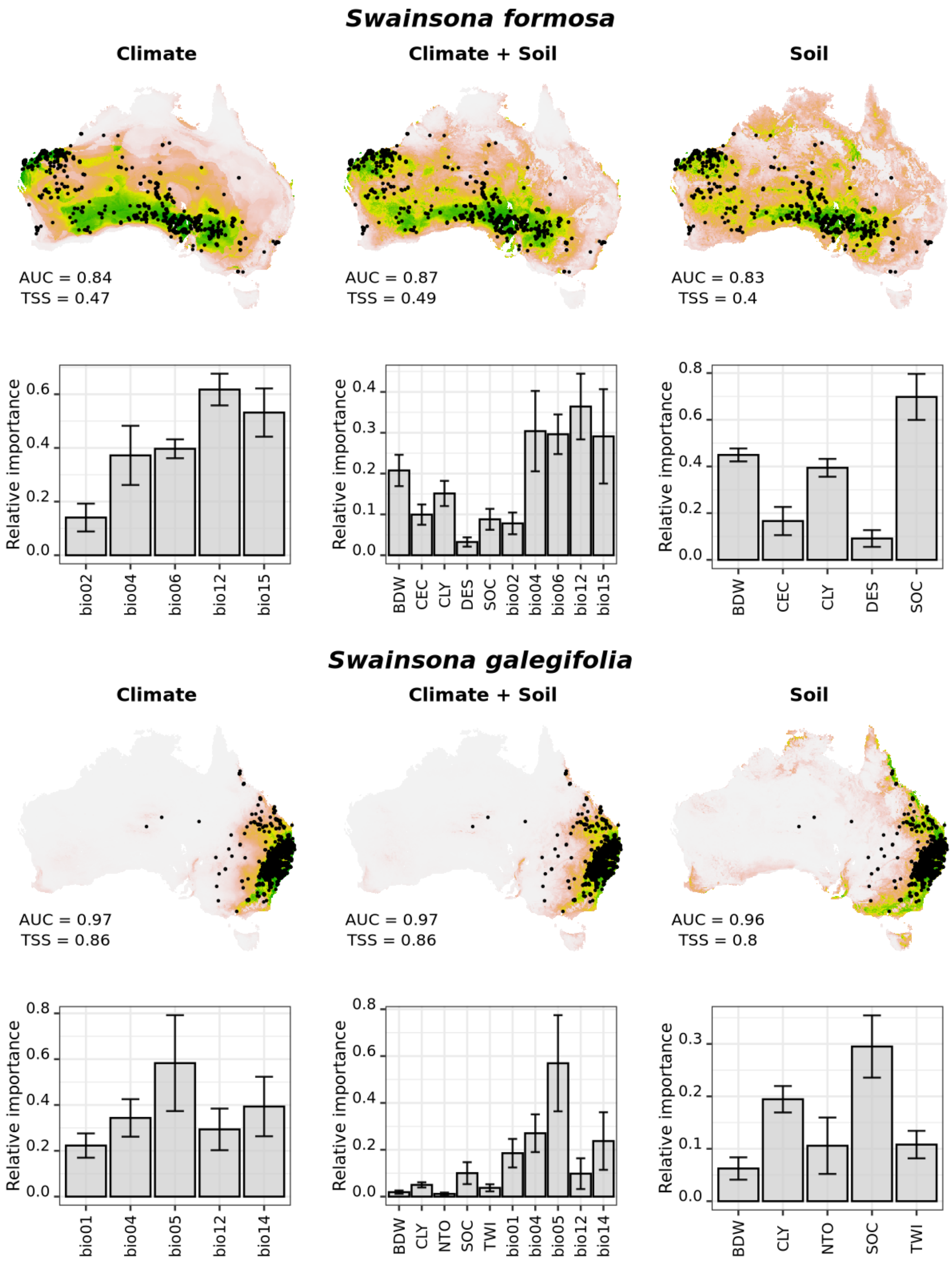

Swainsona formosa
Climate
Climate + Soil
Soil
AUC = 0.84
TSS = 0.47
AUC = 0.87
TSS = 0.49
AUC = 0.83
TSS = 0.4
Relative importance
0.0
0.2
0.4
0.6
bio02
bio04
bio06
bio12
bio15
Relative importance
0.0
0.1
0.2
0.3
0.4
BDW
CEC
CLY
DES
SOC
bio02
bio04
bio06
bio12
bio15
Relative importance
0.0
0.2
0.4
0.6
0.8
BDW
CEC
CLY
DES
SOC
Swainsona galegifolia
Climate
Climate + Soil
Soil
AUC = 0.97
TSS = 0.86
AUC = 0.97
TSS = 0.86
AUC = 0.96
TSS = 0.8
Relative importance
0.0
0.2
0.4
0.6
0.8
bio01
bio04
bio05
bio12
bio14
Relative importance
0.0
0.2
0.4
0.6
0.8
BDW
CLY
NTO
SOC
TWI
bio01
bio04
bio05
bio12
bio14
Relative importance
0.0
0.1
0.2
0.3
BDW
CLY
NTO
SOC
TWI

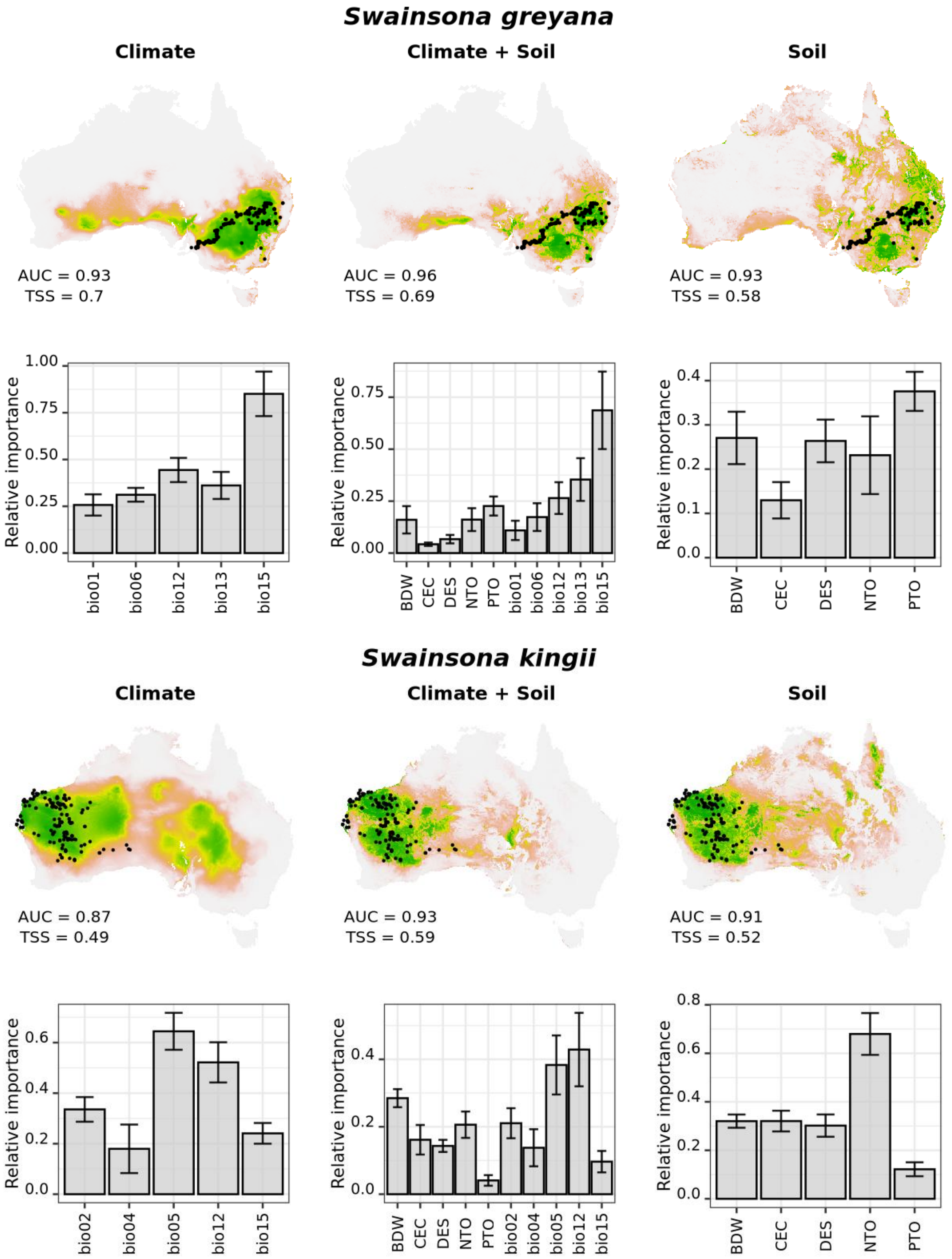

Swainsona greyana
Climate
Climate + Soil
Soil
AUC = 0.93
TSS = 0.7
AUC = 0.96
TSS = 0.69
AUC = 0.93
TSS = 0.58
Relative importance
1.00
0.75
0.50
0.25
0.00
bio01
bio06
bio12
bio13
bio15
BDW
CEC
DES
NTO
PTO
0.4
0.3
0.2
0.1
0.0
Swainsona kingii
Climate
Climate + Soil
Soil
AUC = 0.87
TSS = 0.49
AUC = 0.93
TSS = 0.59
AUC = 0.91
TSS = 0.52
Relative importance
0.8
0.6
0.4
0.2
0.0
bio02
bio04
bio05
bio12
bio15
BDW
CEC
DES
NTO
PTO

Swainsona laxa

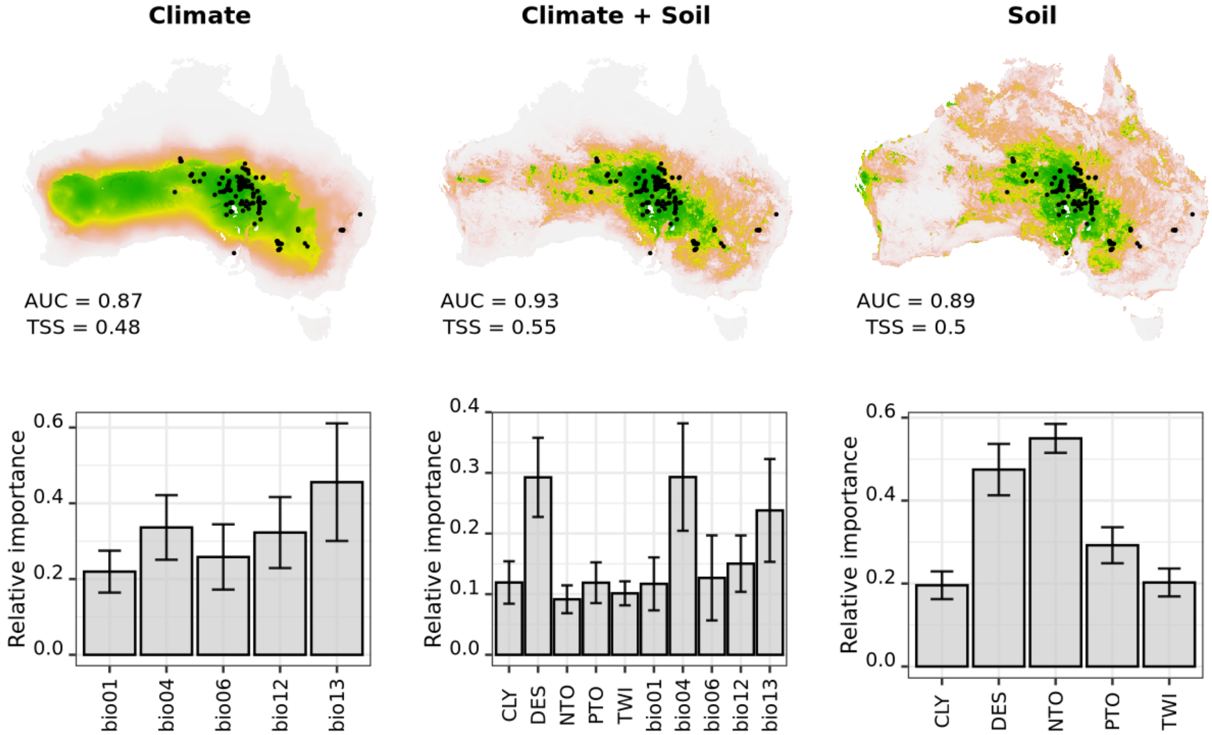

Swainsona monticola

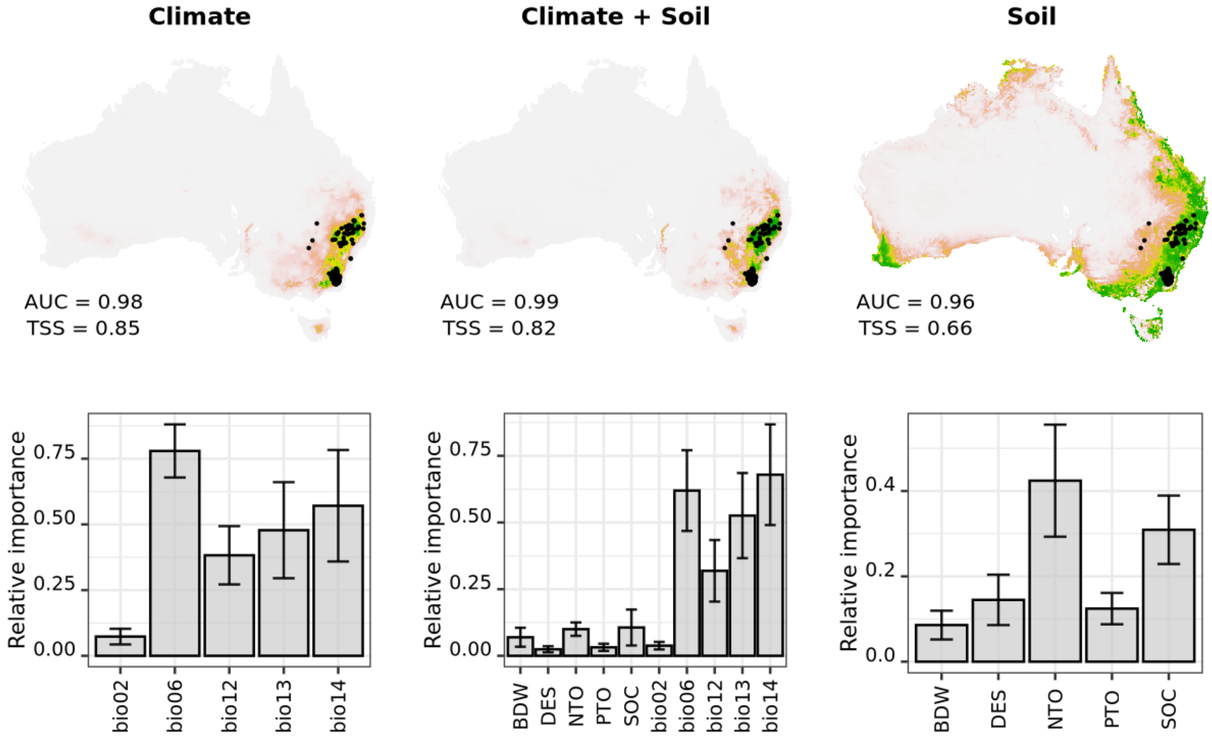

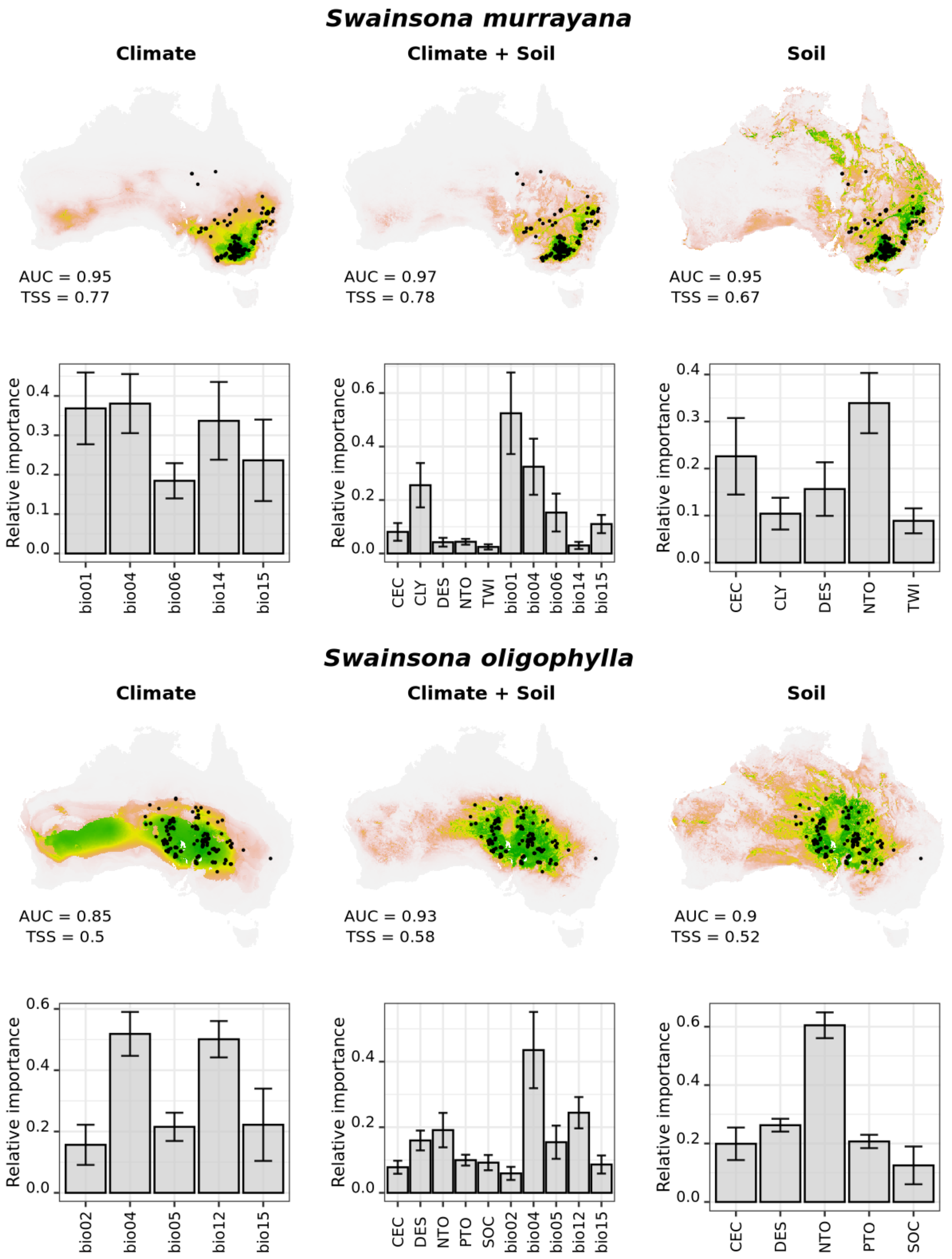
Swainsona murrayana
Climate
Climate + Soil
Soil
AUC = 0.95
TSS = 0.77
AUC = 0.97
TSS = 0.78
AUC = 0.95
TSS = 0.67
Relative importance
bio01 bio04 bio06 bio14 bio15
CEC CLY DES NTO TWI bio01 bio04 bio06 bio14 bio15
CEC CLY DES NTO TWI
Swainsona oligophylla
Climate
Climate + Soil
Soil
AUC = 0.85
TSS = 0.5
AUC = 0.93
TSS = 0.58
AUC = 0.9
TSS = 0.52
Relative importance
bio02 bio04 bio05 bio12 bio15
CEC DES NTO PTO SOC bio02 bio04 bio05 bio12 bio15
CEC DES NTO PTO SOC

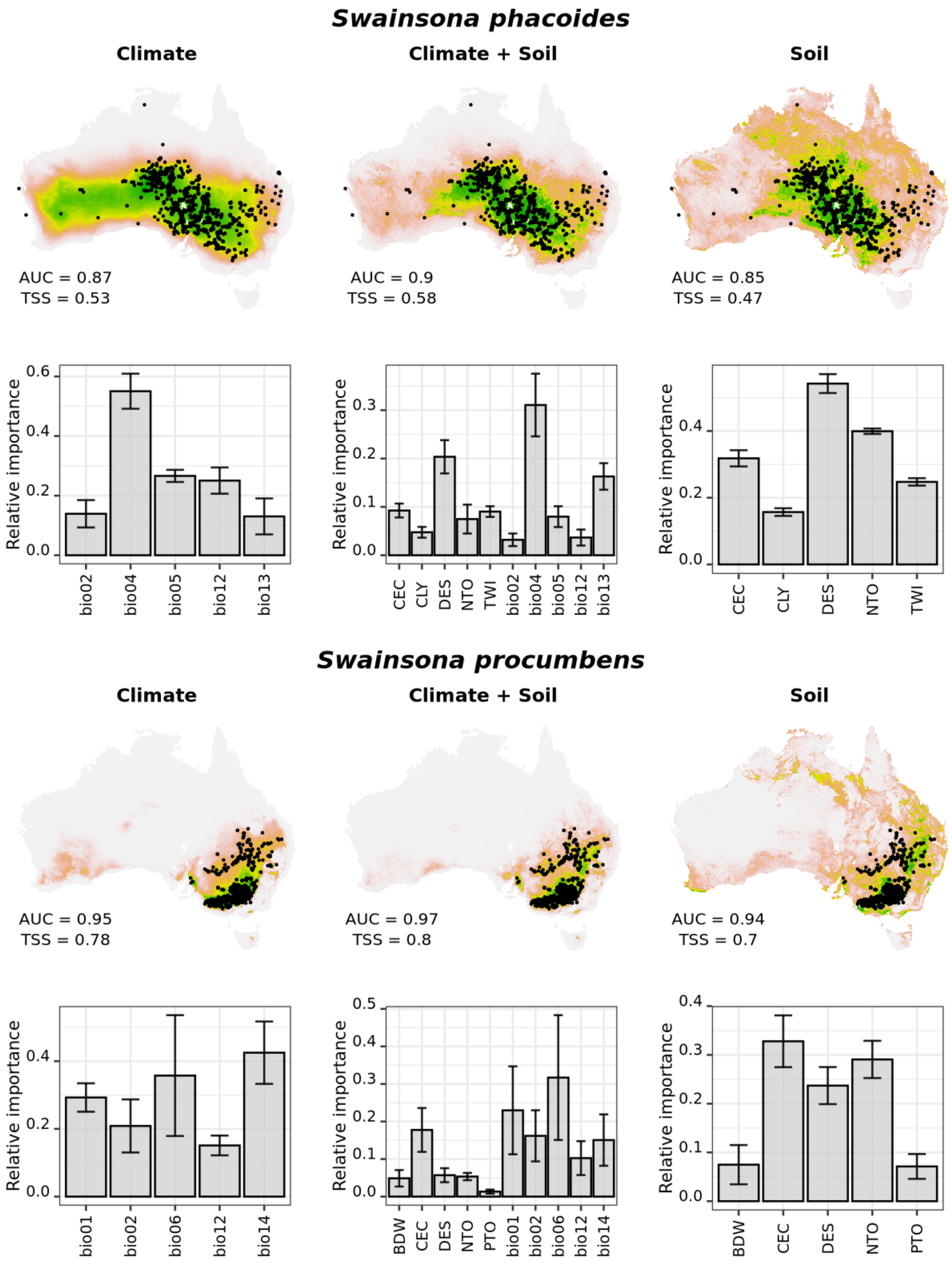
Swainsona phacoides
Climate
Climate + Soil
Soil
AUC = 0.87
TSS = 0.53
AUC = 0.9
TSS = 0.58
AUC = 0.85
TSS = 0.47
Relative importance
0.0
0.2
0.4
0.6
bio02
bio04
bio05
bio12
bio13
0.1
0.3
CEC
CLY
DES
NTO
TWI
Swainsona procumbens
AUC = 0.95
TSS = 0.78
AUC = 0.97
TSS = 0.8
AUC = 0.94
TSS = 0.7
bio01
bio06
bio14
0.5
BDW
PTO

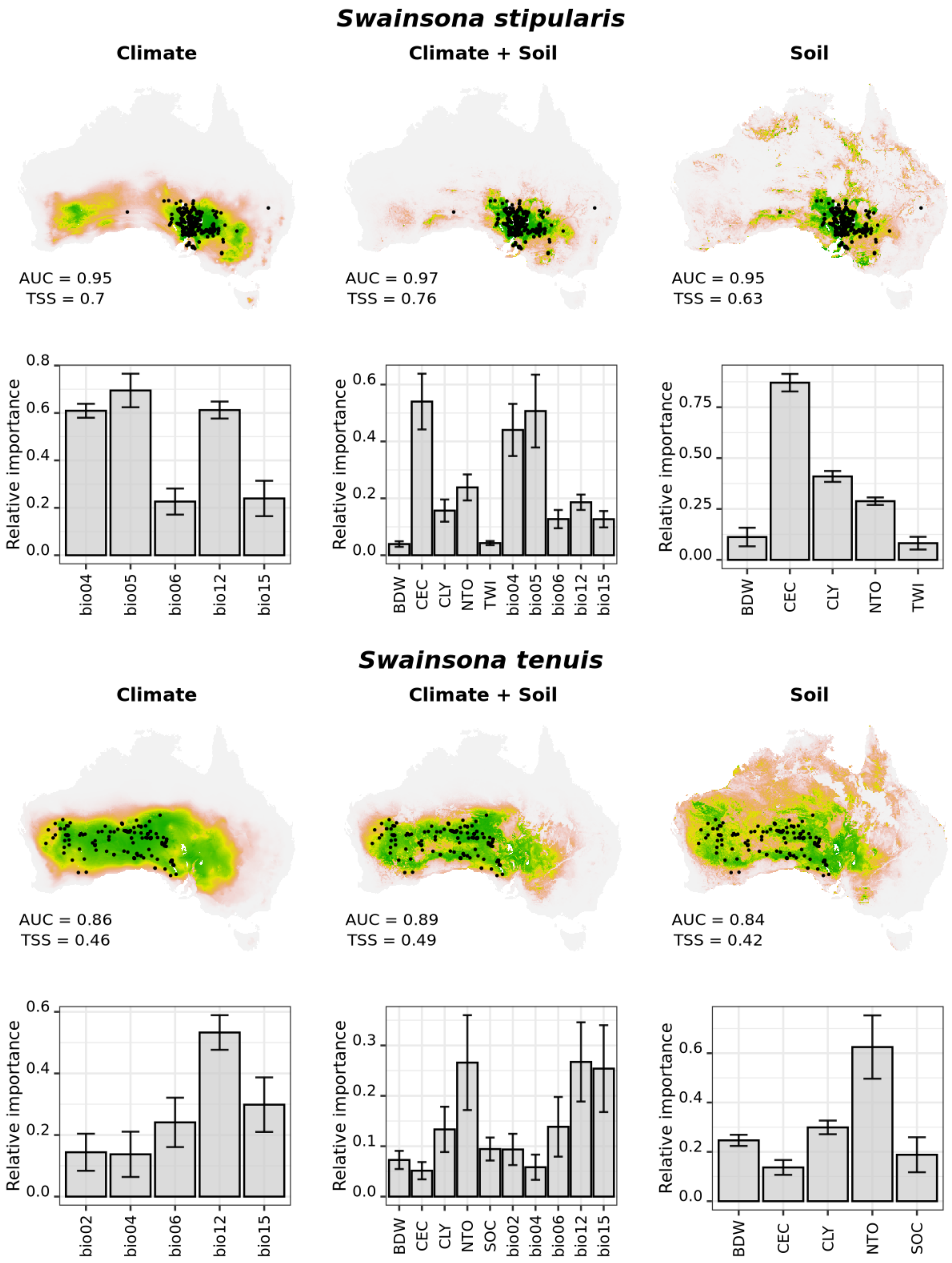
Swainsona stipularis
Climate
Climate + Soil
Soil
AUC = 0.95
TSS = 0.7
AUC = 0.97
TSS = 0.76
AUC = 0.95
TSS = 0.63
Relative importance
bio04
bio05
bio06
bio12
bio15
BDW
CEC
CLY
NTO
TWI
Swainsona tenuis
Climate
Climate + Soil
Soil
AUC = 0.86
TSS = 0.46
AUC = 0.89
TSS = 0.49
AUC = 0.84
TSS = 0.42
Relative importance
bio02
bio04
bio06
bio12
bio15
BDW
CEC
CLY
NTO
SOC

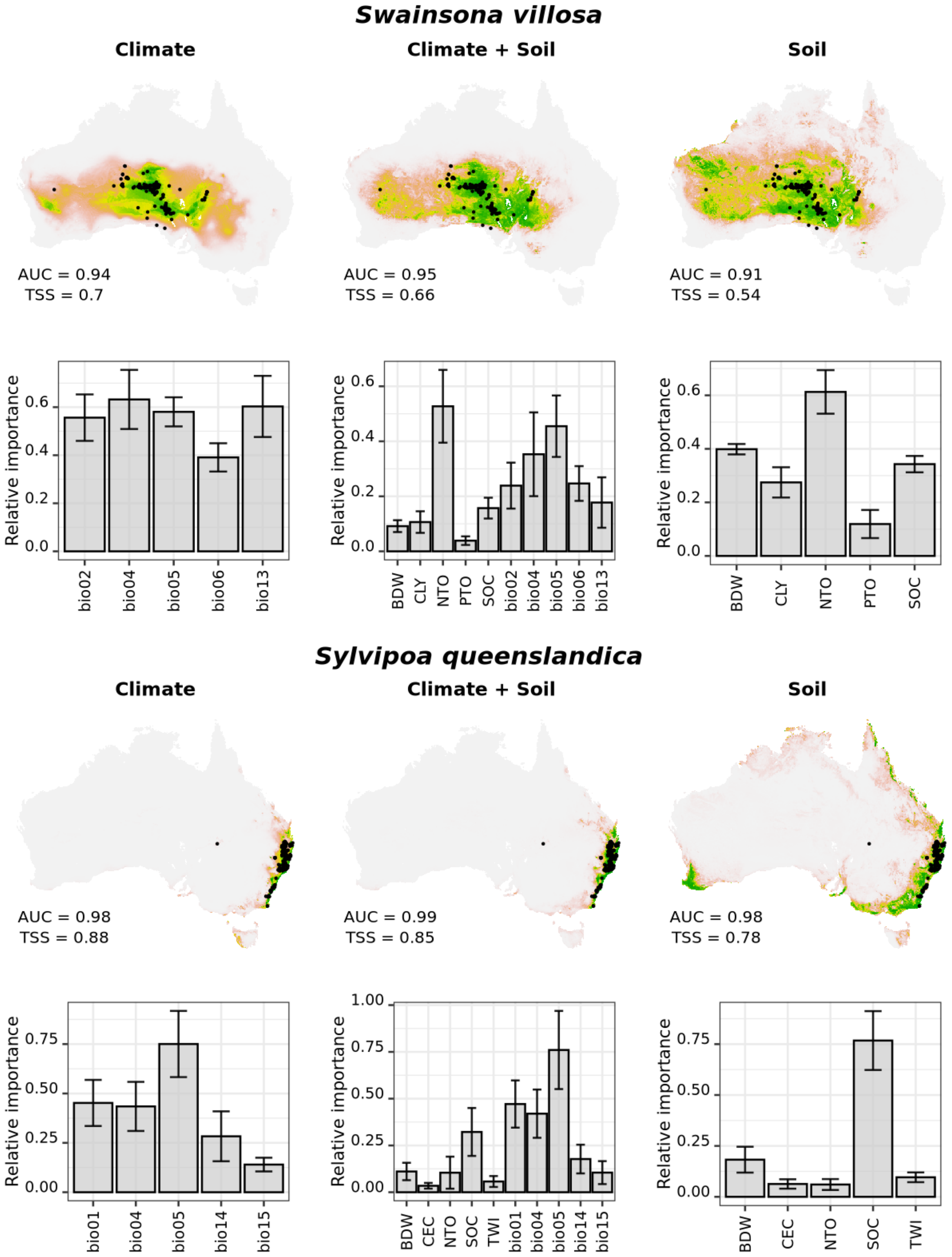

Swainsona villosa
Climate
Climate + Soil
Soil
AUC = 0.94
TSS = 0.7
AUC = 0.95
TSS = 0.66
AUC = 0.91
TSS = 0.54
Relative importance
bio02
bio04
bio05
bio06
bio13
BDW
CLY
NTO
PTO
SOC
Sylvipoa queenslandica
AUC = 0.98
TSS = 0.88
AUC = 0.99
TSS = 0.85
AUC = 0.98
TSS = 0.78
bio01
bio14
bio15
CEC
TWI

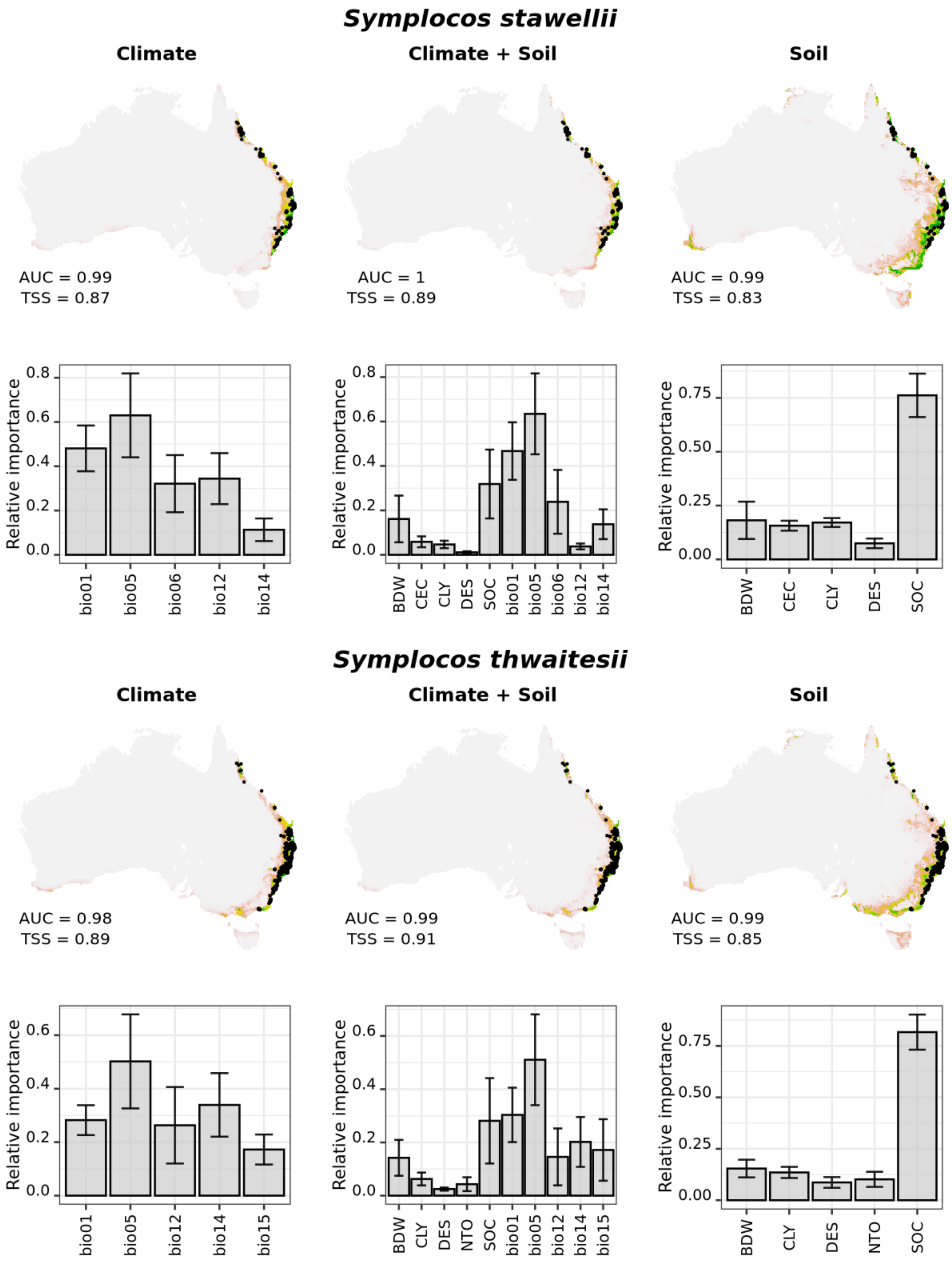
Symplocos stawellii
Climate
Climate + Soil
Soil
AUC = 0.99
TSS = 0.87
AUC = 1
TSS = 0.89
AUC = 0.99
TSS = 0.83
Relative importance
0.0
0.2
0.4
0.6
0.8
bio01
bio05
bio06
bio12
bio14
BDW
CEC
CLY
DES
SOC
bio01
bio05
bio06
bio12
bio14
0.00
0.25
0.50
0.75
BDW
CEC
CLY
DES
SOC
Symplocos thwaitesii
Climate
Climate + Soil
Soil
AUC = 0.98
TSS = 0.89
AUC = 0.99
TSS = 0.91
AUC = 0.99
TSS = 0.85
Relative importance
0.0
0.2
0.4
0.6
bio01
bio05
bio12
bio14
bio15
BDW
CLY
DES
NTO
SOC
bio01
bio05
bio12
bio14
bio15
0.00
0.25
0.50
0.75
BDW
CLY
DES
NTO
SOC

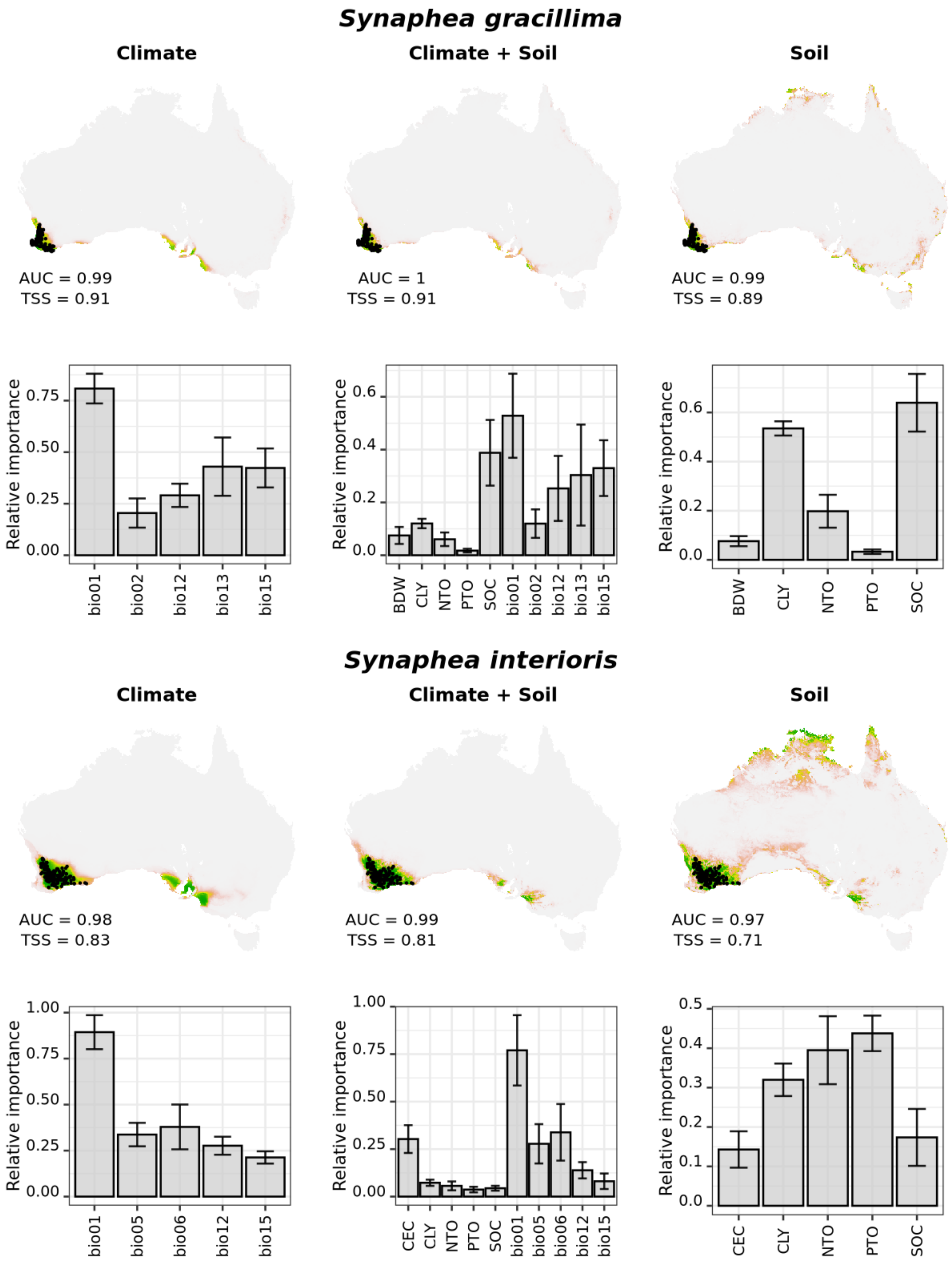

Synaphea gracillima
Climate
Climate + Soil
Soil
AUC = 0.99
TSS = 0.91
AUC = 1
TSS = 0.91
AUC = 0.99
TSS = 0.89
Relative importance
0.00
0.25
0.50
0.75
bio01
bio02
bio12
bio13
bio15
0.0
0.2
0.4
0.6
BDW
CLY
NTO
PTO
SOC
bio01
bio02
bio12
bio13
bio15
BDW
CLY
NTO
PTO
SOC
Synaphea interioris
Climate
Climate + Soil
Soil
AUC = 0.98
TSS = 0.83
AUC = 0.99
TSS = 0.81
AUC = 0.97
TSS = 0.71
Relative importance
1.00
bio01
bio05
bio06
bio12
bio15
CEC
CLY
NTO
PTO
SOC
bio01
bio05
bio06
bio12
bio15
0.0
0.1
0.2
0.3
0.4
0.5
CEC
CLY
NTO
PTO
SOC

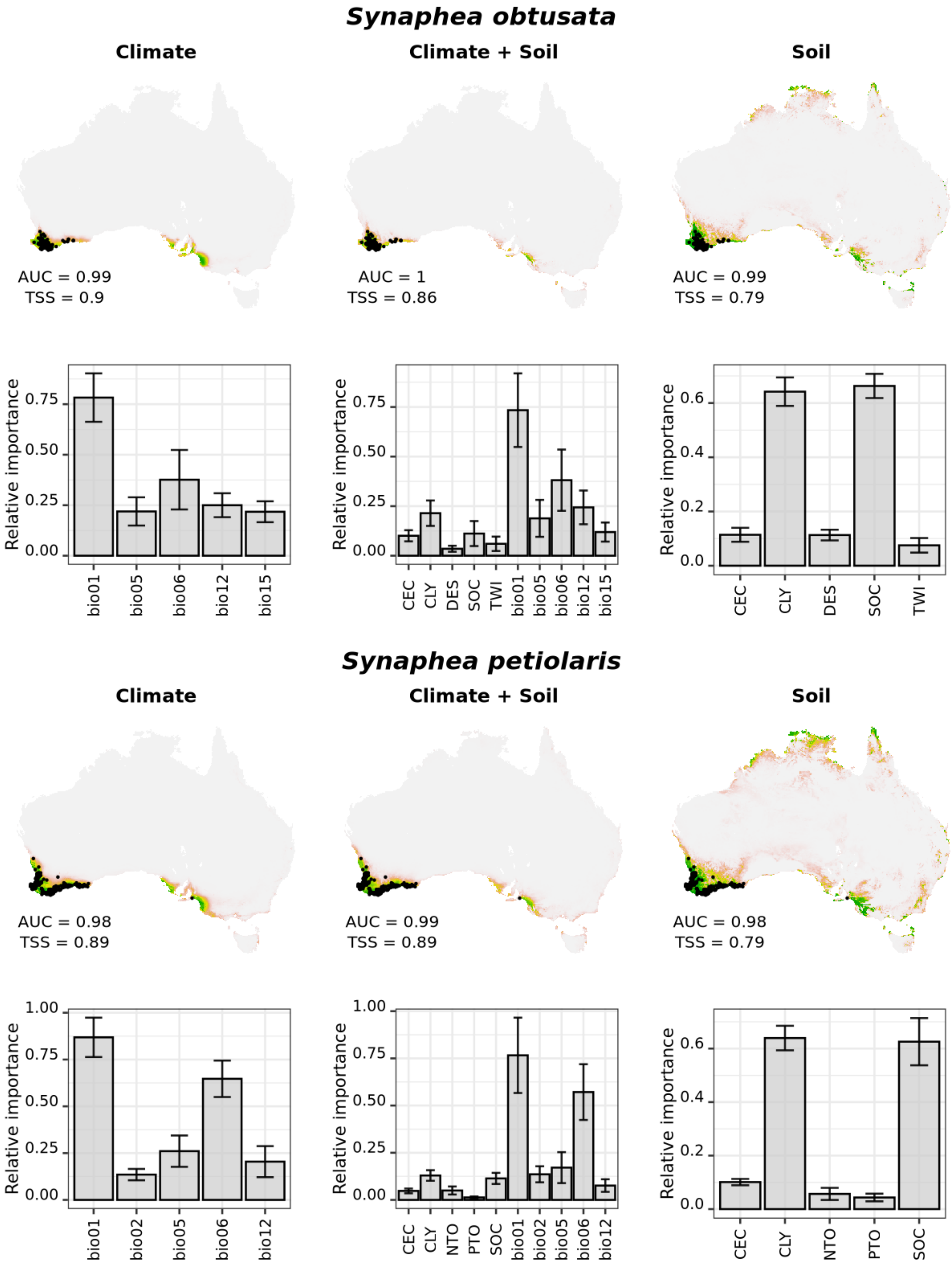

Synaphea obtusata
Climate
Climate + Soil
Soil
AUC = 0.99
TSS = 0.9
AUC = 1
TSS = 0.86
AUC = 0.99
TSS = 0.79
Relative importance
0.00
0.25
0.50
0.75
bio01
bio05
bio06
bio12
bio15
CEC
CLY
DES
SOC
TWI
0.0
0.2
0.4
0.6
Synaphea petiolaris
Climate
Climate + Soil
Soil
AUC = 0.98
TSS = 0.89
AUC = 0.99
TSS = 0.89
AUC = 0.98
TSS = 0.79
1.00
bio02
NTO
PTO

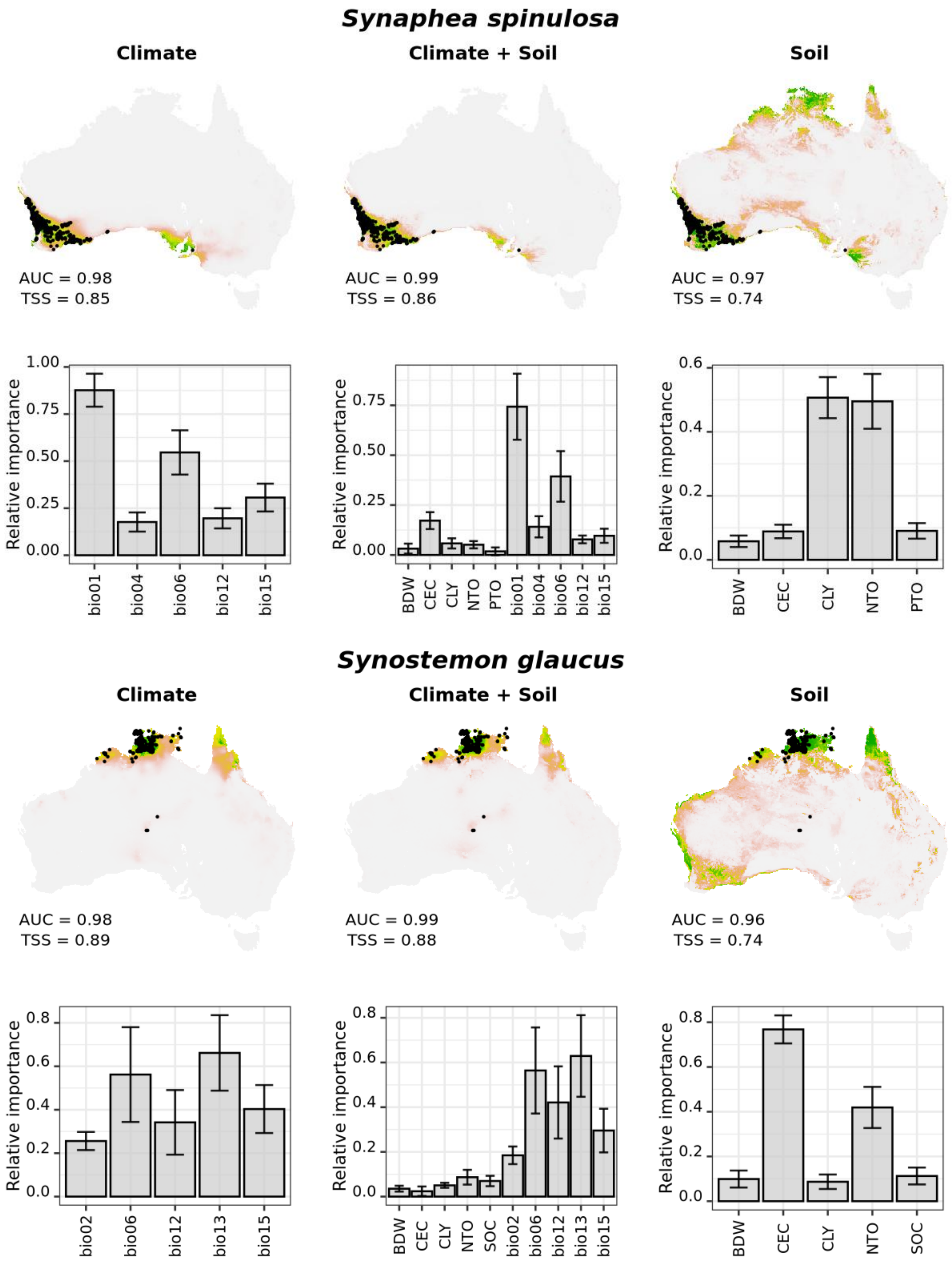
Synaphea spinulosa
Climate
Climate + Soil
Soil
AUC = 0.98
TSS = 0.85
AUC = 0.99
TSS = 0.86
AUC = 0.97
TSS = 0.74
Relative importance
1.00
0.75
0.50
0.25
0.00
bio01
bio04
bio06
bio12
bio15
BDW
CEC
CLY
NTO
PTO
0.6
0.4
0.2
0.0
Synostemon glaucus
Climate
Climate + Soil
Soil
AUC = 0.98
TSS = 0.89
AUC = 0.99
TSS = 0.88
AUC = 0.96
TSS = 0.74
0.8
0.6
0.4
0.2
0.0
bio02
bio06
bio12
bio13
bio15
BDW
CEC
CLY
NTO
SOC

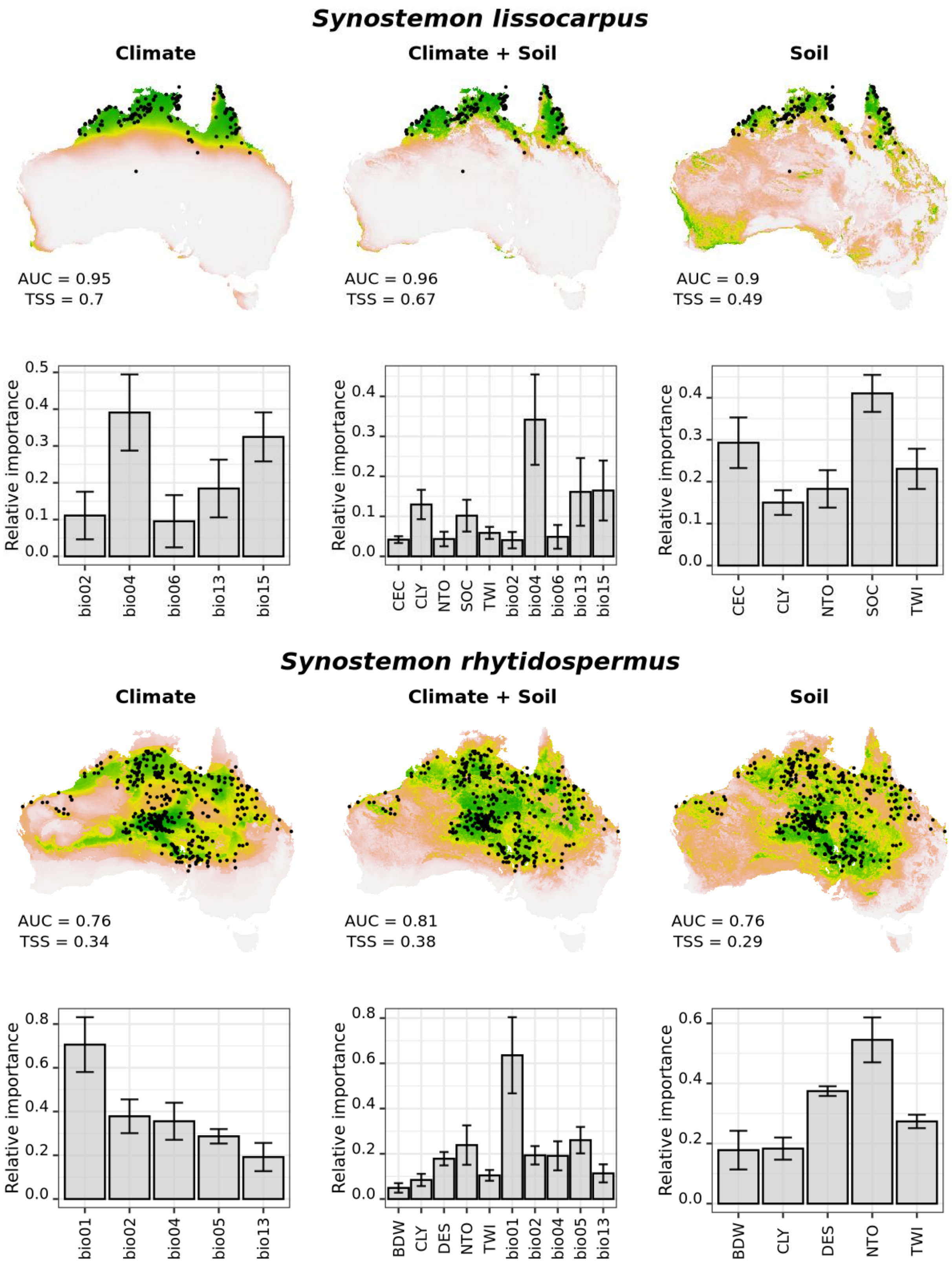

Synostemon lissocarpus
Climate
Climate + Soil
Soil
AUC = 0.95
TSS = 0.7
AUC = 0.96
TSS = 0.67
AUC = 0.9
TSS = 0.49
Relative importance
bio02
bio04
bio06
bio13
bio15
CEC
CLY
NTO
SOC
TWI
bio02
bio04
bio06
bio13
bio15
CEC
CLY
NTO
SOC
TWI
Synostemon rhytidospermus
Climate
Climate + Soil
Soil
AUC = 0.76
TSS = 0.34
AUC = 0.81
TSS = 0.38
AUC = 0.76
TSS = 0.29
Relative importance
bio01
bio02
bio04
bio05
bio13
BDW
CLY
DES
NTO
TWI
bio01
bio02
bio04
bio05
bio13
BDW
CLY
DES
NTO
TWI

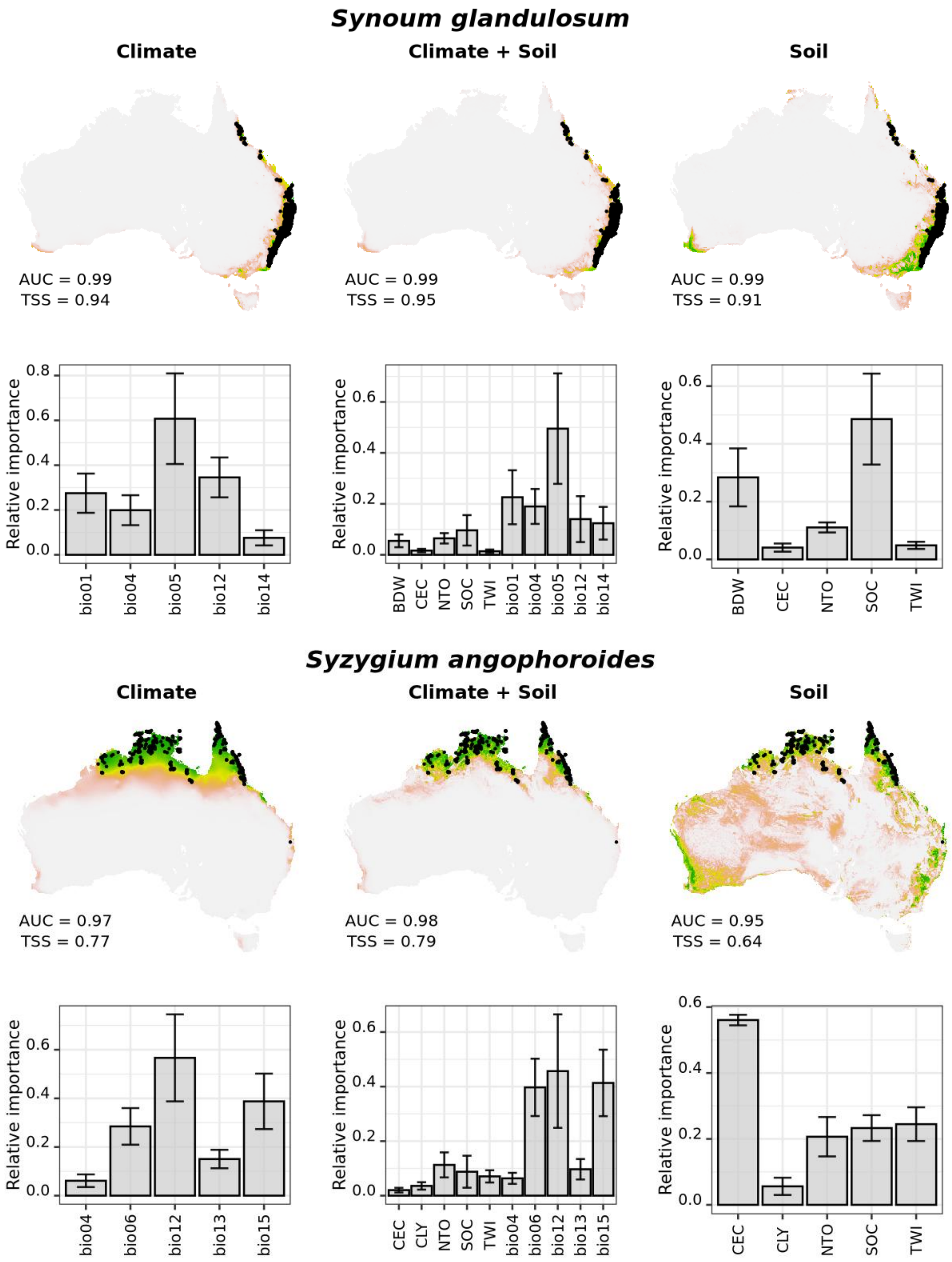

Synoum glandulosum
Climate
Climate + Soil
Soil
AUC = 0.99
TSS = 0.94
AUC = 0.99
TSS = 0.95
AUC = 0.99
TSS = 0.91
Relative importance
bio01
bio04
bio05
bio12
bio14
BDW
CEC
NTO
SOC
TWI
Syzygium angophoroides
Climate
Climate + Soil
Soil
AUC = 0.97
TSS = 0.77
AUC = 0.98
TSS = 0.79
AUC = 0.95
TSS = 0.64
bio04
bio06
bio12
bio13
bio15
CEC
CLY
NTO
SOC
TWI

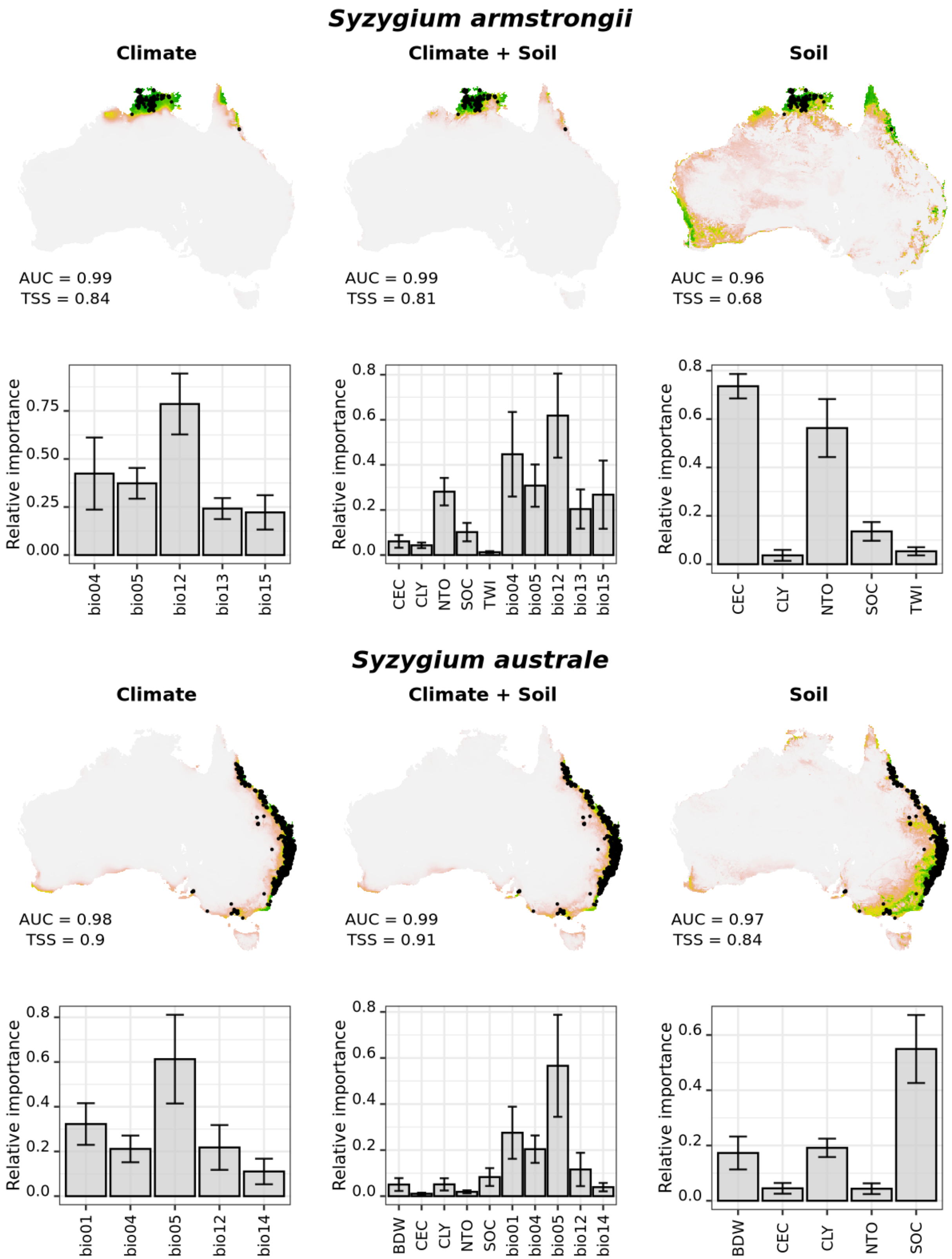

Syzygium armstrongii
Climate
Climate + Soil
Soil
AUC = 0.99
TSS = 0.84
AUC = 0.99
TSS = 0.81
AUC = 0.96
TSS = 0.68
Relative importance
bio04
bio05
bio12
bio13
bio15
CEC
CLY
NTO
SOC
TWI
Syzygium australe
AUC = 0.98
TSS = 0.9
AUC = 0.99
TSS = 0.91
AUC = 0.97
TSS = 0.84
bio01
bio14
BDW

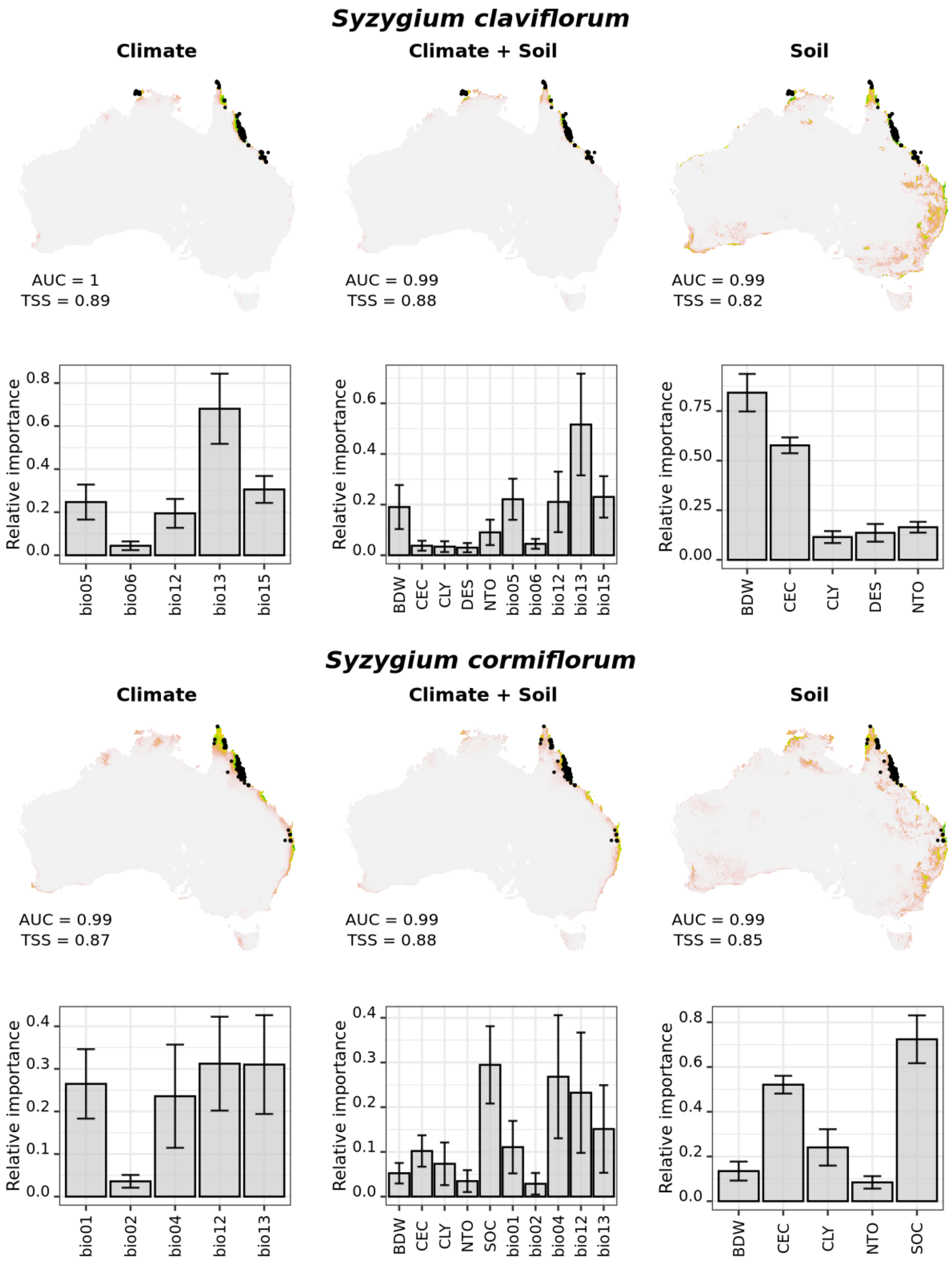
Syzygium claviflorum
Climate
Climate + Soil
Soil
AUC = 1
TSS = 0.89
AUC = 0.99
TSS = 0.88
AUC = 0.99
TSS = 0.82
Relative importance
bio05
bio06
bio12
bio13
bio15
BDW
CEC
CLY
DES
NTO
Syzygium cormiflorum
Climate
Climate + Soil
Soil
AUC = 0.99
TSS = 0.87
AUC = 0.99
TSS = 0.88
AUC = 0.99
TSS = 0.85
bio01
bio02
bio04
SOC

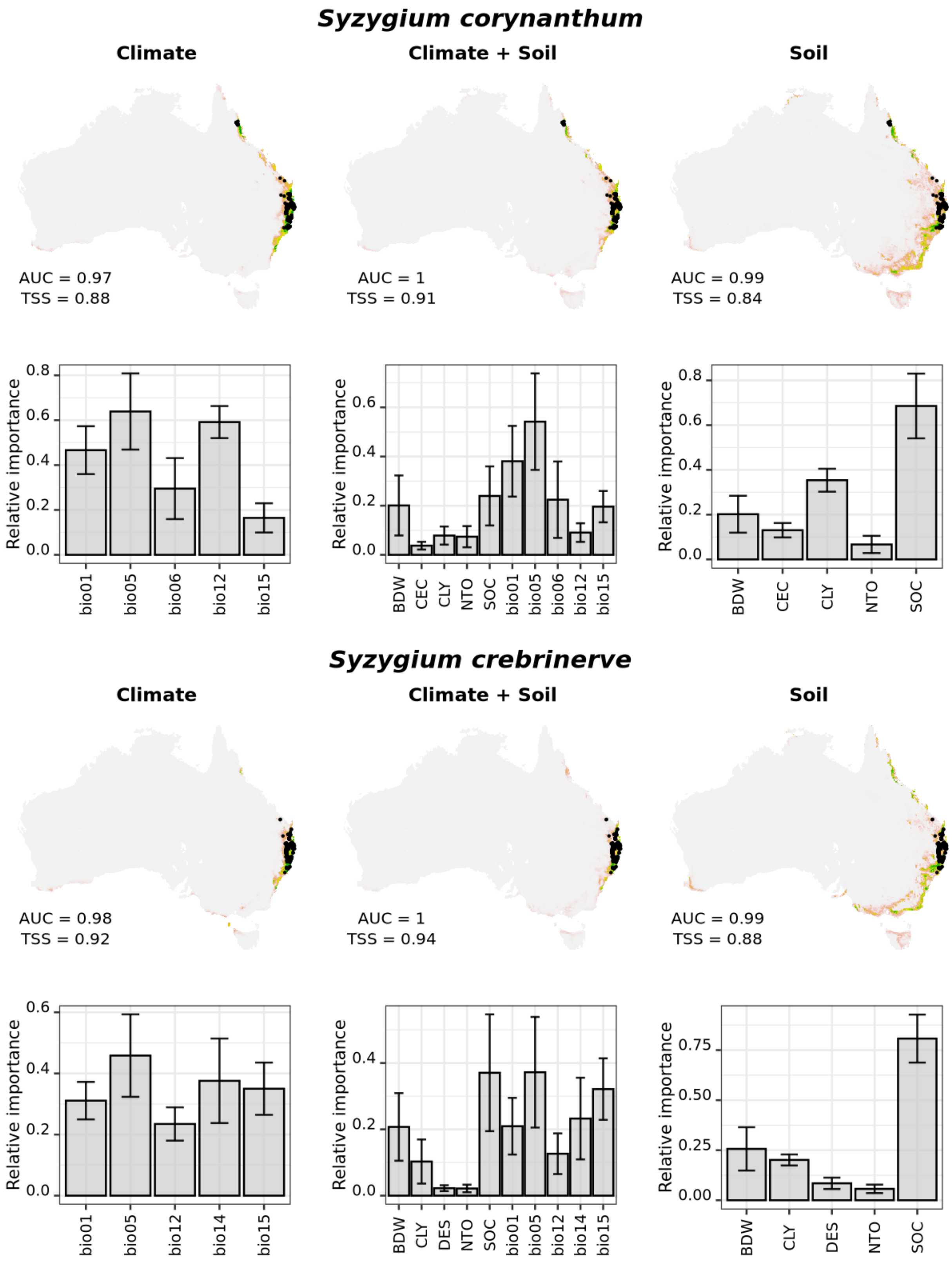
Syzygium corynanthum
Climate
Climate + Soil
Soil
AUC = 0.97
TSS = 0.88
AUC = 1
TSS = 0.91
AUC = 0.99
TSS = 0.84
Relative importance
bio01
bio05
bio06
bio12
bio15
BDW
CEC
CLY
NTO
SOC
Syzygium crebrinerve
Climate
Climate + Soil
Soil
AUC = 0.98
TSS = 0.92
AUC = 1
TSS = 0.94
AUC = 0.99
TSS = 0.88
Relative importance
bio01
bio05
bio12
bio14
bio15
BDW
CLY
DES
NTO
SOC

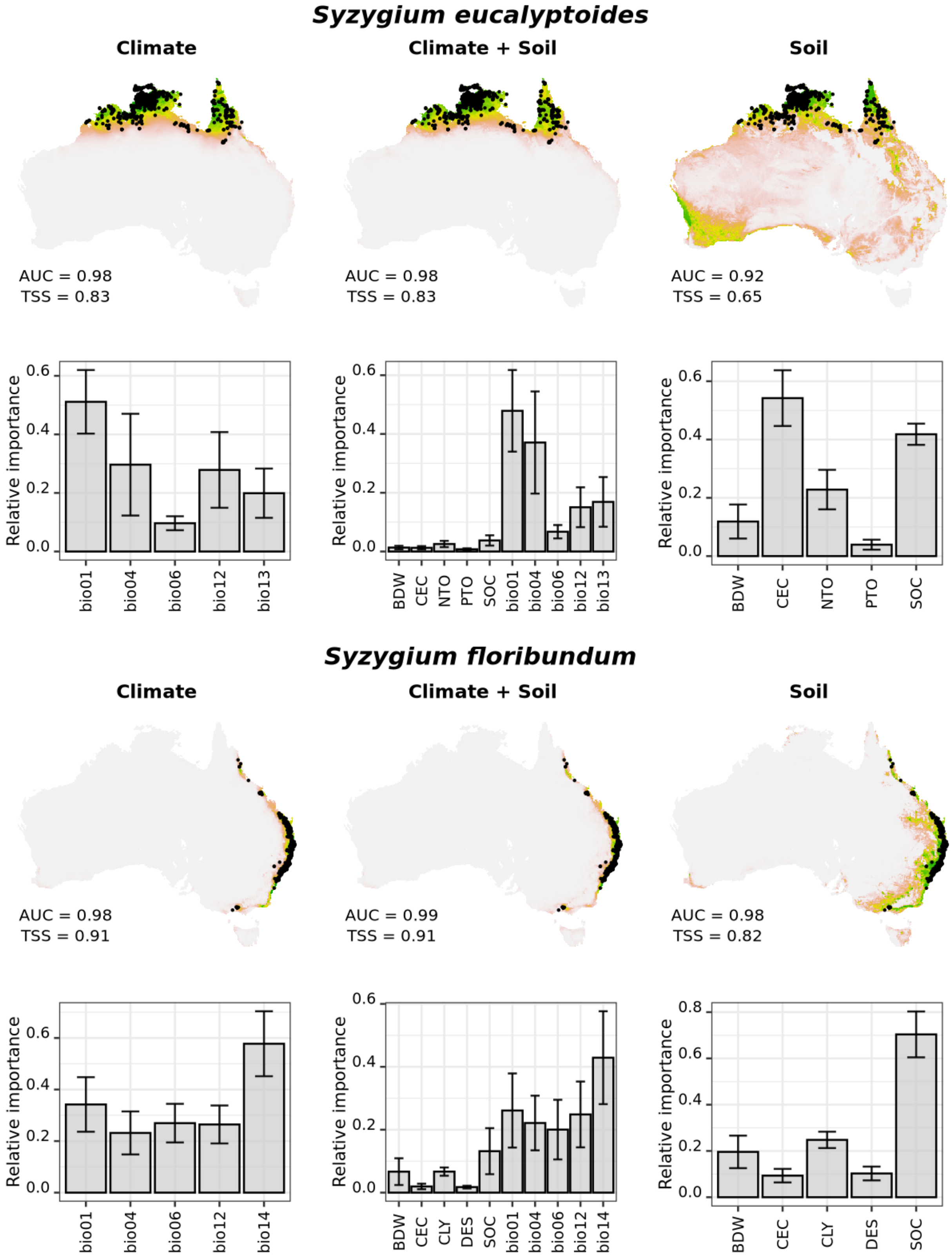
Syzygium eucalyptoides
Climate
Climate + Soil
Soil
AUC = 0.98
TSS = 0.83
AUC = 0.98
TSS = 0.83
AUC = 0.92
TSS = 0.65
Relative importance
0.0
0.2
0.4
0.6
bio01
bio04
bio06
bio12
bio13
BDW
CEC
NTO
PTO
SOC
Syzygium floribundum
Climate
Climate + Soil
Soil
AUC = 0.98
TSS = 0.91
AUC = 0.99
TSS = 0.91
AUC = 0.98
TSS = 0.82
Relative importance
0.0
0.2
0.4
0.6
0.8
bio01
bio04
bio06
bio12
bio14
BDW
CEC
CLY
DES
SOC

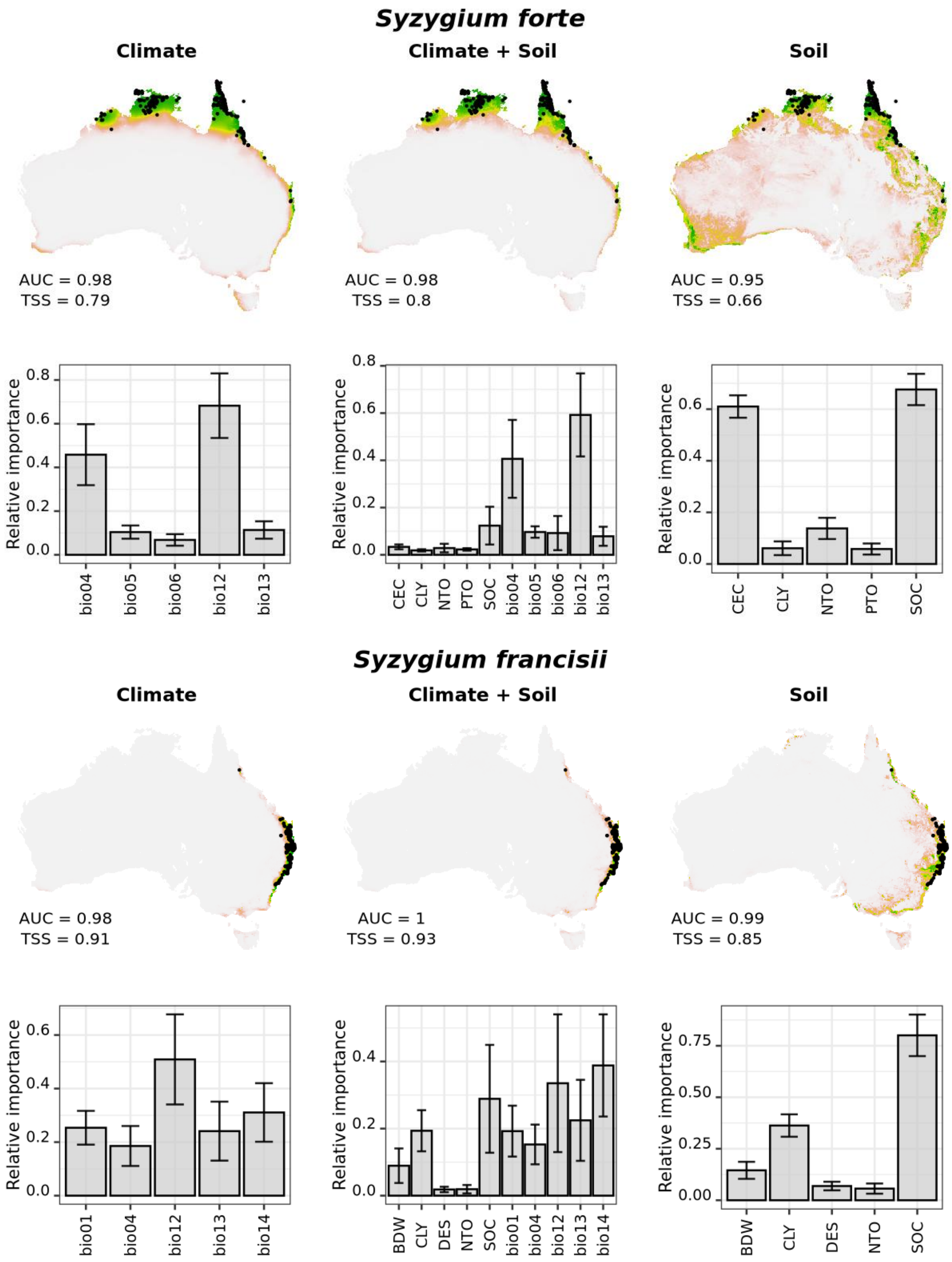
Syzygium forte
Climate
Climate + Soil
Soil
AUC = 0.98
TSS = 0.79
AUC = 0.98
TSS = 0.8
AUC = 0.95
TSS = 0.66
Relative importance
bio04 bio05 bio06 bio12 bio13
CEC CLY NTO PTO SOC bio04 bio05 bio06 bio12 bio13
CEC CLY NTO PTO SOC
Syzygium francisii
Climate
Climate + Soil
Soil
AUC = 0.98
TSS = 0.91
AUC = 1
TSS = 0.93
AUC = 0.99
TSS = 0.85
Relative importance
bio01 bio04 bio12 bio13 bio14
BDW CLY DES NTO SOC bio01 bio04 bio12 bio13 bio14
BDW CLY DES NTO SOC

Syzygium hemilamprum

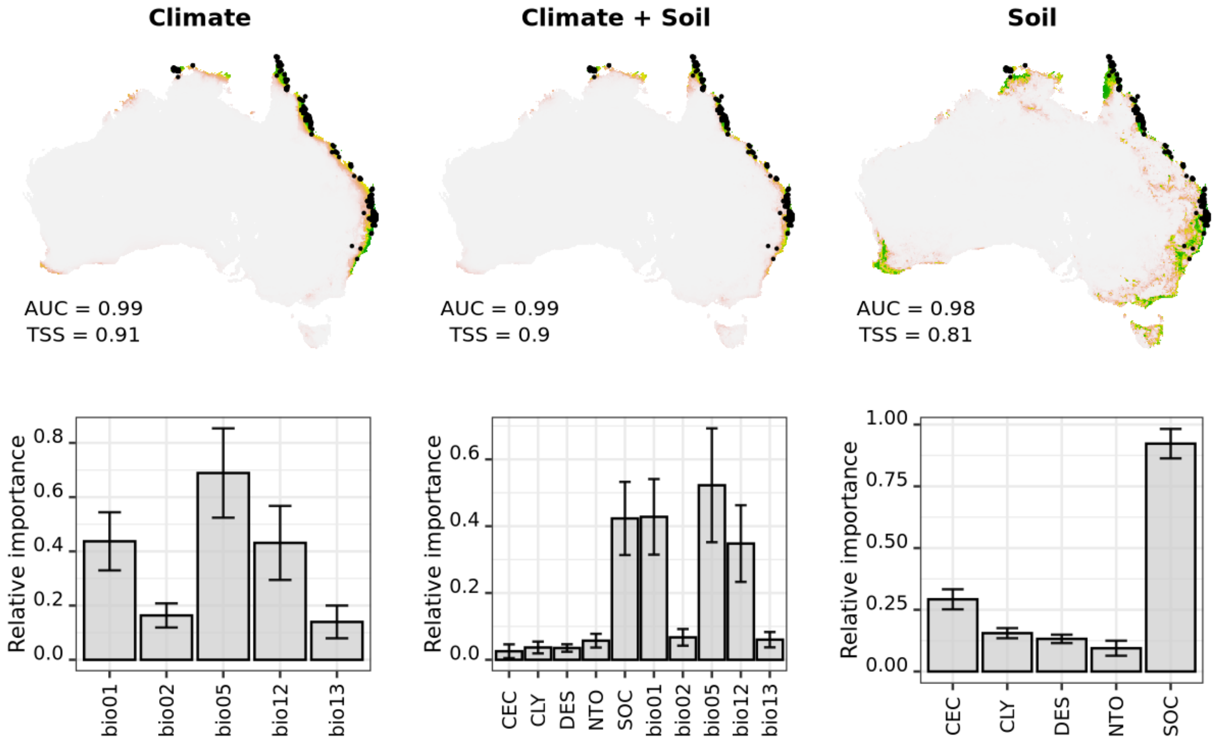

Syzygium ingens

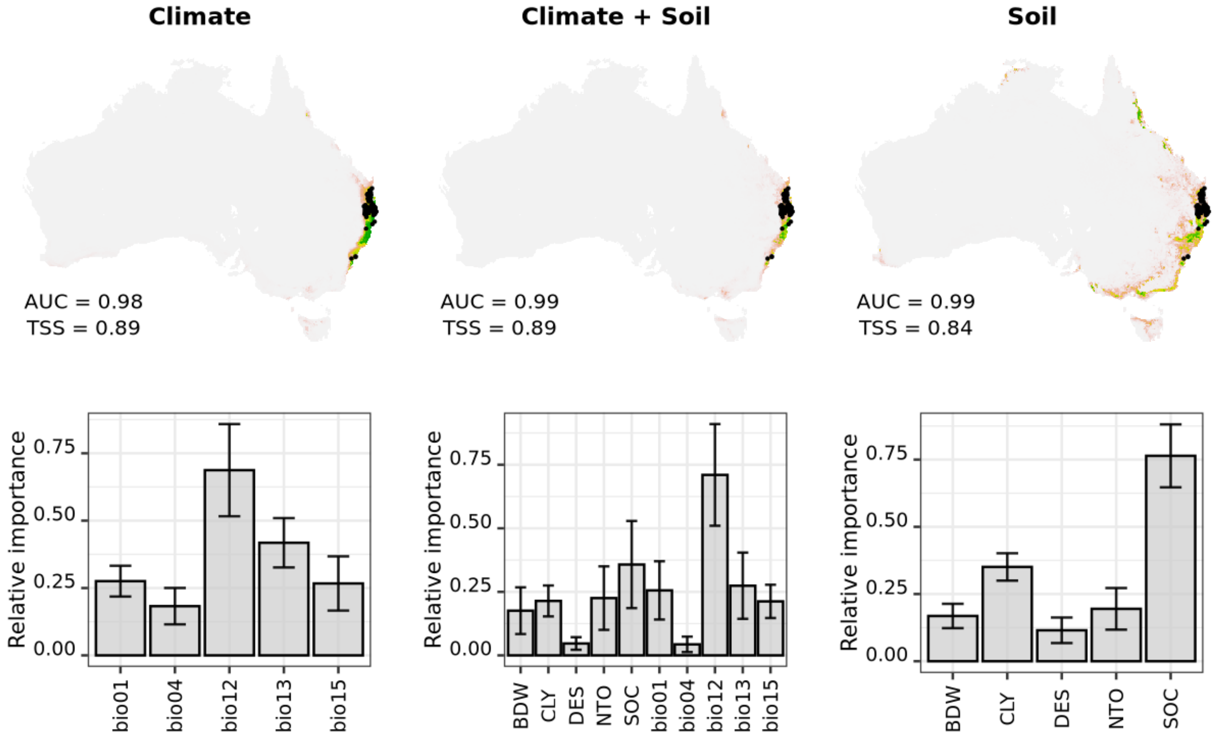

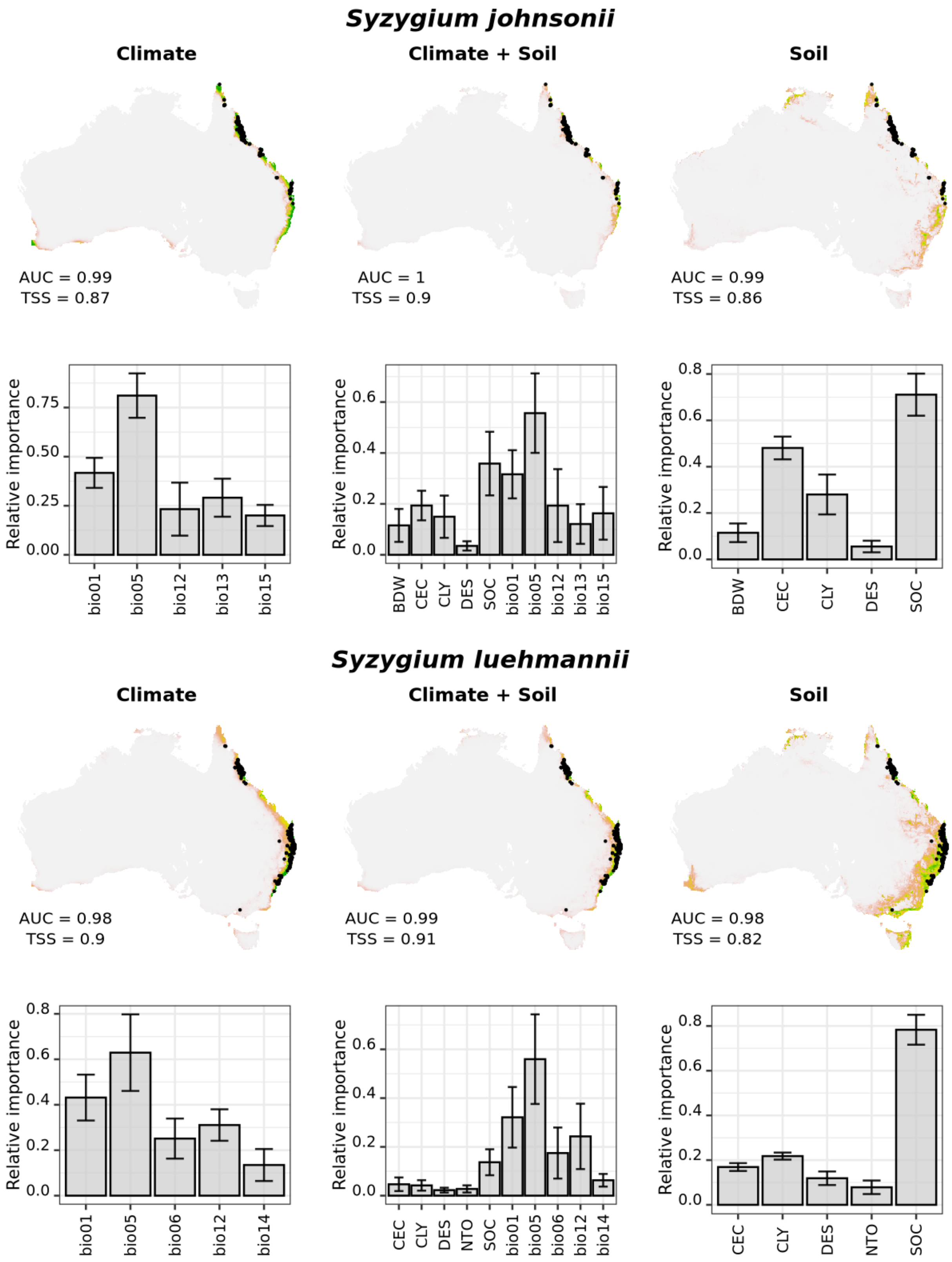
Syzygium johnsonii
Climate
Climate + Soil
Soil
AUC = 0.99
TSS = 0.87
AUC = 1
TSS = 0.9
AUC = 0.99
TSS = 0.86
Relative importance
bio01
bio05
bio12
bio13
bio15
BDW
CEC
CLY
DES
SOC
Syzygium luehmannii
Climate
Climate + Soil
Soil
AUC = 0.98
TSS = 0.9
AUC = 0.99
TSS = 0.91
AUC = 0.98
TSS = 0.82
Relative importance
bio01
bio05
bio06
bio12
bio14
CEC
CLY
DES
NTO
SOC

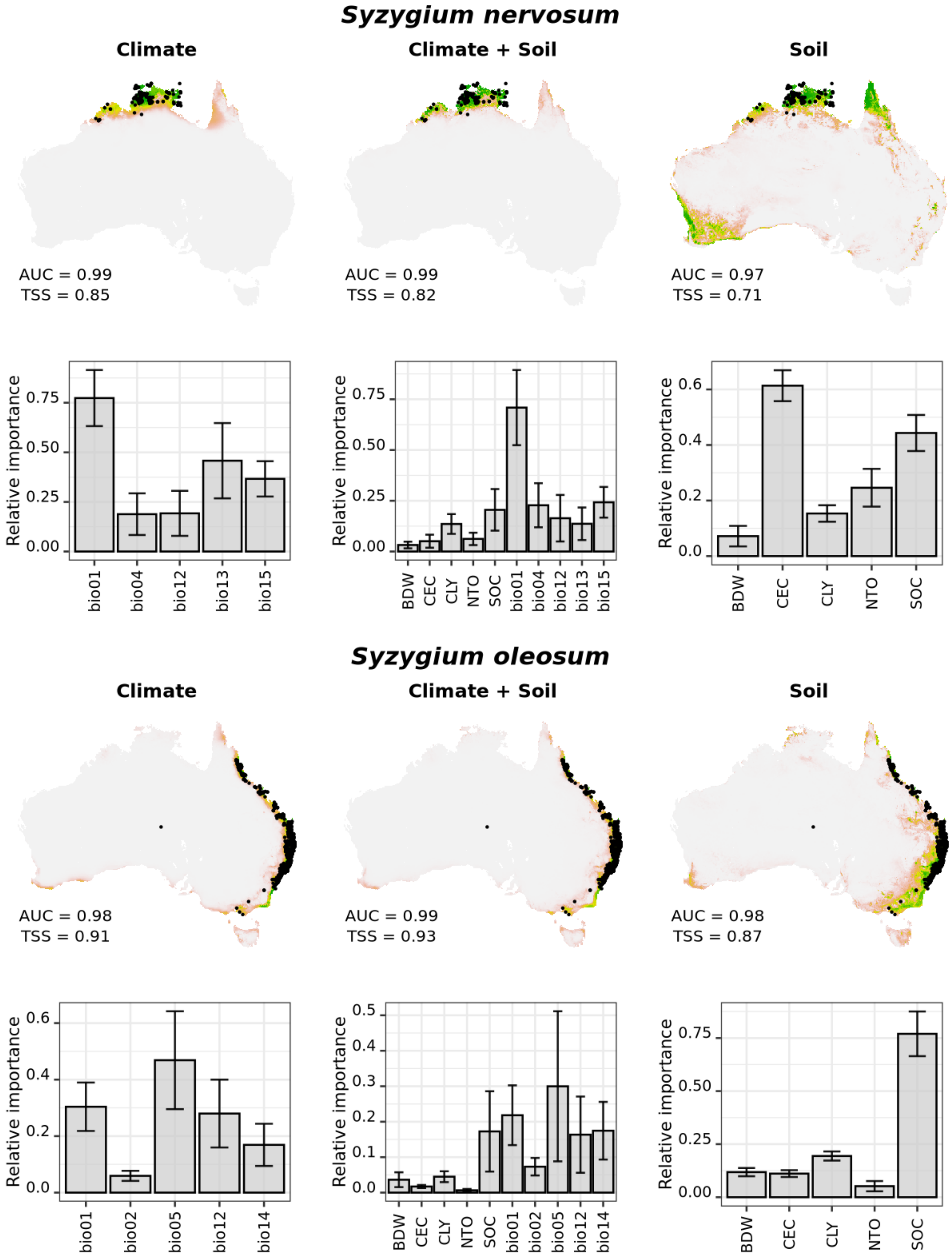
Syzygium nervosum
Climate
Climate + Soil
Soil
AUC = 0.99
TSS = 0.85
AUC = 0.99
TSS = 0.82
AUC = 0.97
TSS = 0.71
Relative importance
0.00
0.25
0.50
0.75
bio01
bio04
bio12
bio13
bio15
Relative importance
0.00
0.25
0.50
0.75
BDW
CEC
CLY
NTO
SOC
bio01
bio04
bio12
bio13
bio15
Relative importance
0.0
0.2
0.4
0.6
BDW
CEC
CLY
NTO
SOC
Syzygium oleosum
Climate
Climate + Soil
Soil
AUC = 0.98
TSS = 0.91
AUC = 0.99
TSS = 0.93
AUC = 0.98
TSS = 0.87
Relative importance
0.0
0.2
0.4
0.6
bio01
bio02
bio05
bio12
bio14
Relative importance
0.0
0.1
0.2
0.3
0.4
0.5
BDW
CEC
CLY
NTO
SOC
bio01
bio02
bio05
bio12
bio14
Relative importance
0.00
0.25
0.50
0.75
BDW
CEC
CLY
NTO
SOC

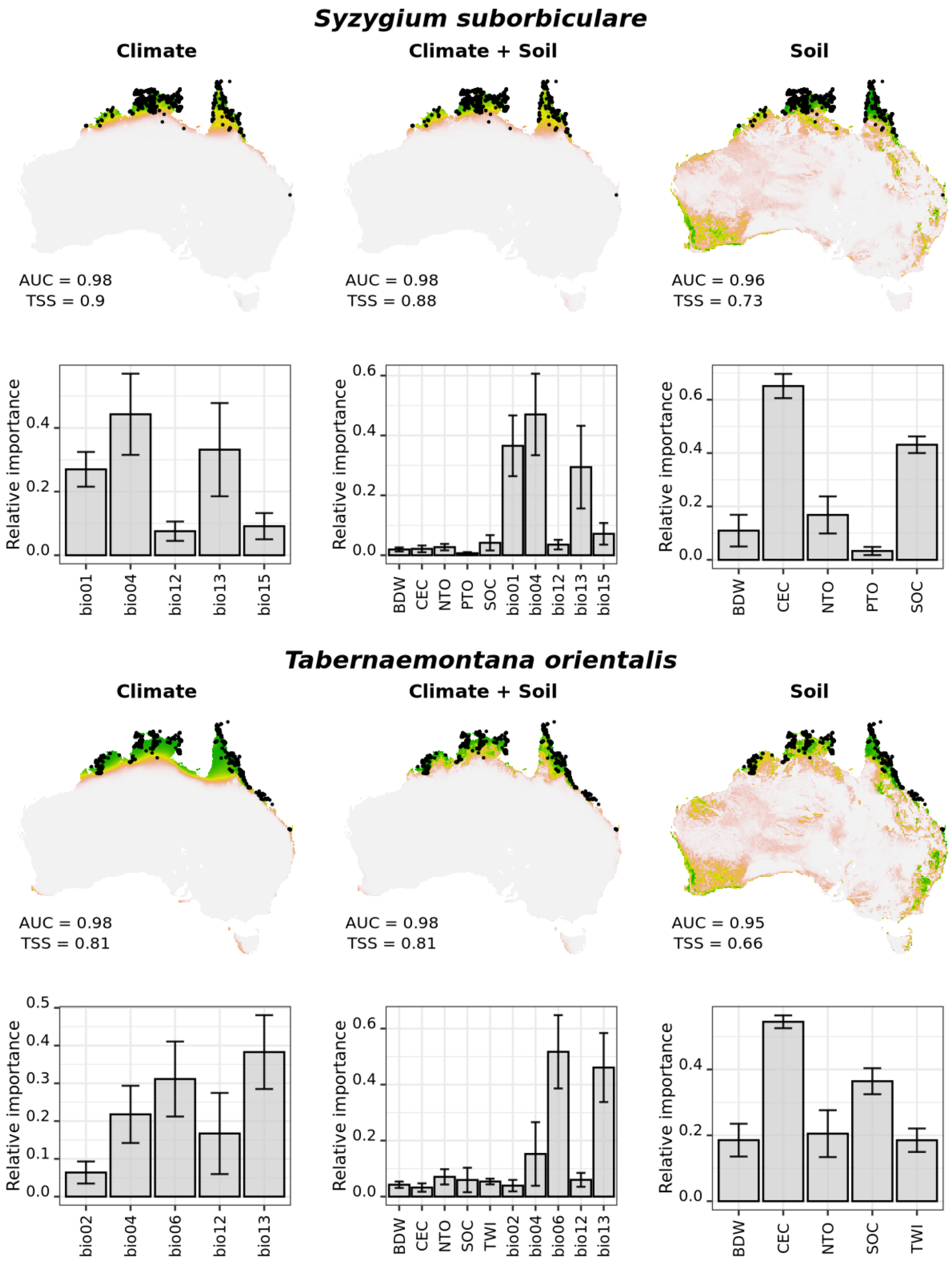

Syzygium suborbiculare
Climate
Climate + Soil
Soil
AUC = 0.98
TSS = 0.9
AUC = 0.98
TSS = 0.88
AUC = 0.96
TSS = 0.73
Relative importance
bio01 bio04 bio12 bio13 bio15
BDW CEC NTO PTO SOC bio01 bio04 bio12 bio13 bio15
BDW CEC NTO PTO SOC
Tabernaemontana orientalis
Climate
Climate + Soil
Soil
AUC = 0.98
TSS = 0.81
AUC = 0.98
TSS = 0.81
AUC = 0.95
TSS = 0.66
Relative importance
bio02 bio04 bio06 bio12 bio13
BDW CEC NTO SOC TWI bio02 bio04 bio06 bio12 bio13
BDW CEC NTO SOC TWI

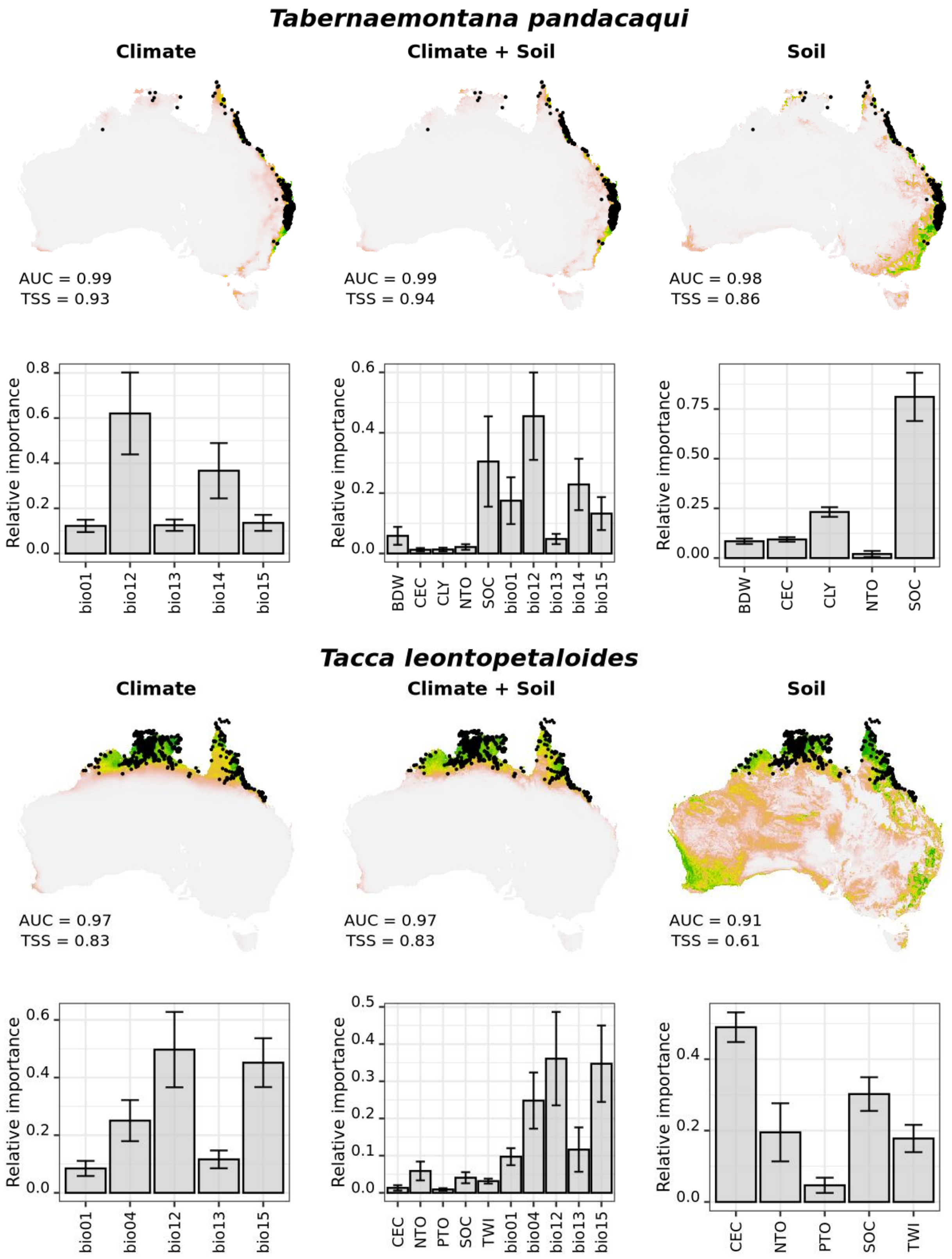

Tabernaemontana pandacaqui
Climate
Climate + Soil
Soil
AUC = 0.99
TSS = 0.93
AUC = 0.99
TSS = 0.94
AUC = 0.98
TSS = 0.86
Relative importance
0.0 0.2 0.4 0.6 0.8
bio01 bio12 bio13 bio14 bio15
0.0 0.2 0.4 0.6
BDW CEC CLY NTO SOC bio01 bio12 bio13 bio14 bio15
0.00 0.25 0.50 0.75
BDW CEC CLY NTO SOC
Tacca leontopetaloides
Climate
Climate + Soil
Soil
AUC = 0.97
TSS = 0.83
AUC = 0.97
TSS = 0.83
AUC = 0.91
TSS = 0.61
Relative importance
0.0 0.2 0.4 0.6
bio01 bio04 bio12 bio13 bio15
0.0 0.1 0.2 0.3 0.4 0.5
CEC NTO PTO SOC TWI bio01 bio04 bio12 bio13 bio15
0.0 0.2 0.4
CEC NTO PTO SOC TWI

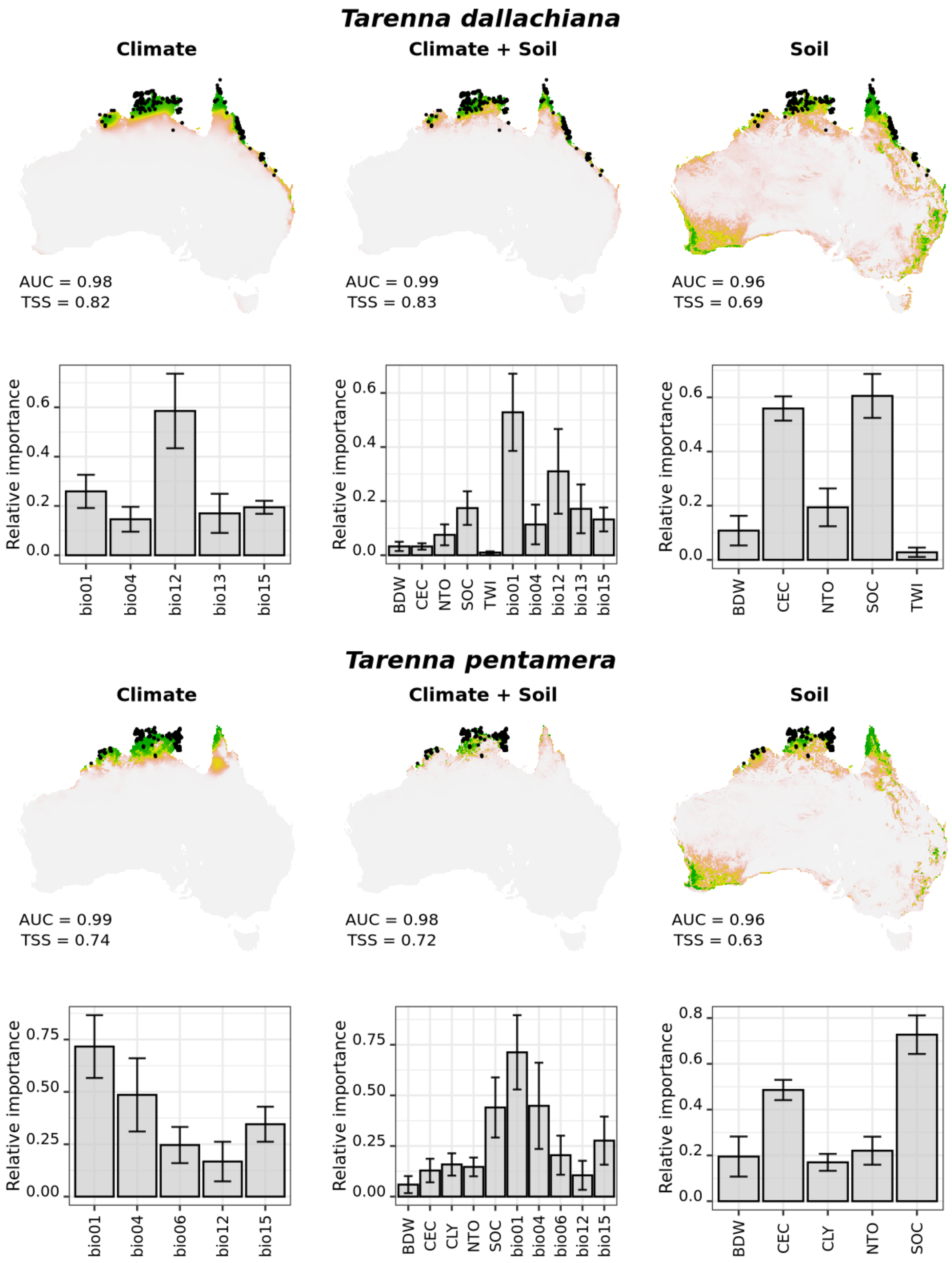

Tarenna dallachiana
Climate
Climate + Soil
Soil
AUC = 0.98
TSS = 0.82
AUC = 0.99
TSS = 0.83
AUC = 0.96
TSS = 0.69
Relative importance
bio01
bio04
bio12
bio13
bio15
BDW
CEC
NTO
SOC
TWI
Tarenna pentamera
AUC = 0.99
TSS = 0.74
AUC = 0.98
TSS = 0.72
AUC = 0.96
TSS = 0.63
bio06
CLY

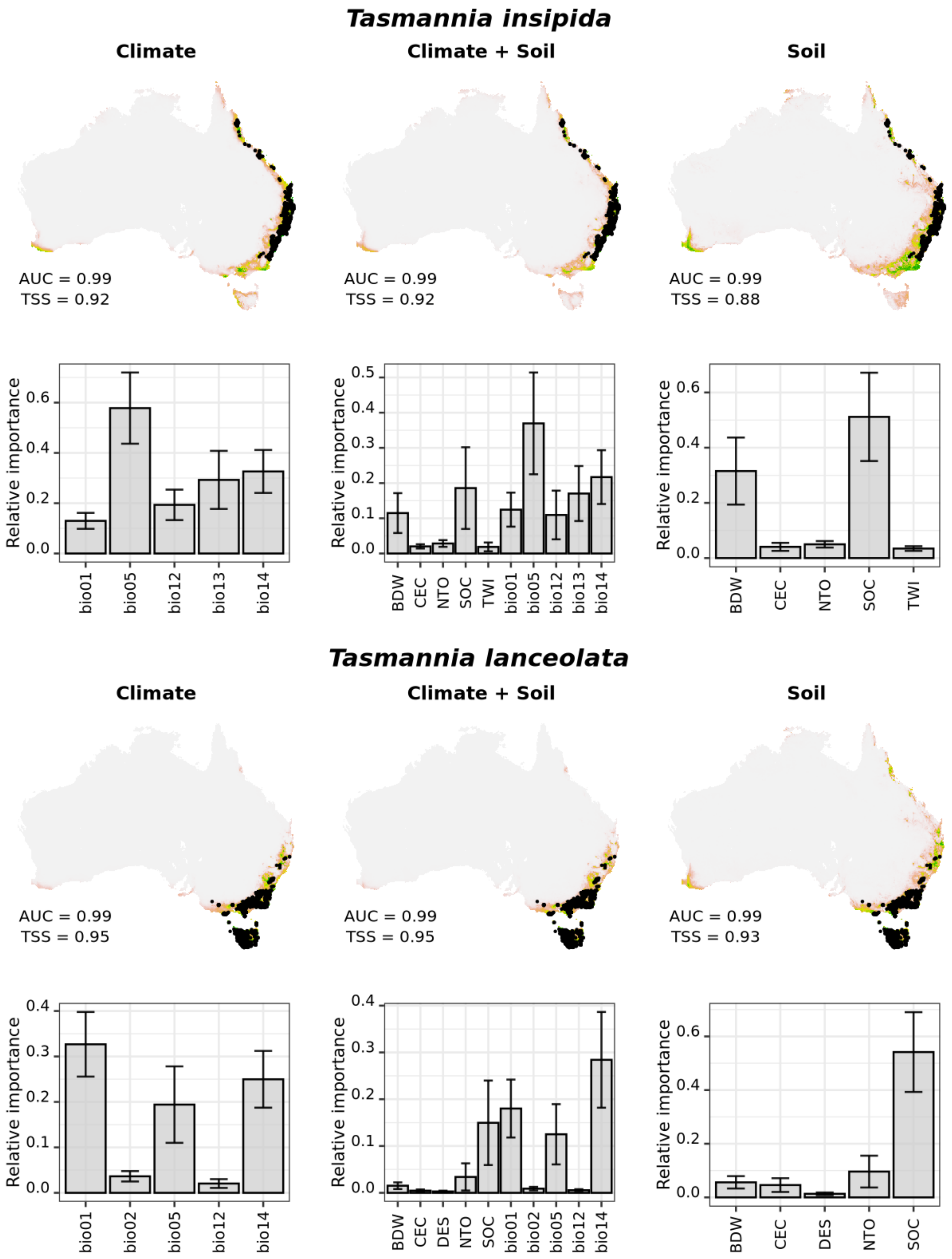
Tasmannia insipida
Climate
Climate + Soil
Soil
AUC = 0.99
TSS = 0.92
AUC = 0.99
TSS = 0.92
AUC = 0.99
TSS = 0.88
Relative importance
bio01
bio05
bio12
bio13
bio14
BDW
CEC
NTO
SOC
TWI
Tasmannia lanceolata
Climate
Climate + Soil
Soil
AUC = 0.99
TSS = 0.95
AUC = 0.99
TSS = 0.95
AUC = 0.99
TSS = 0.93
Relative importance
bio01
bio02
bio05
bio12
bio14
BDW
CEC
DES
NTO
SOC

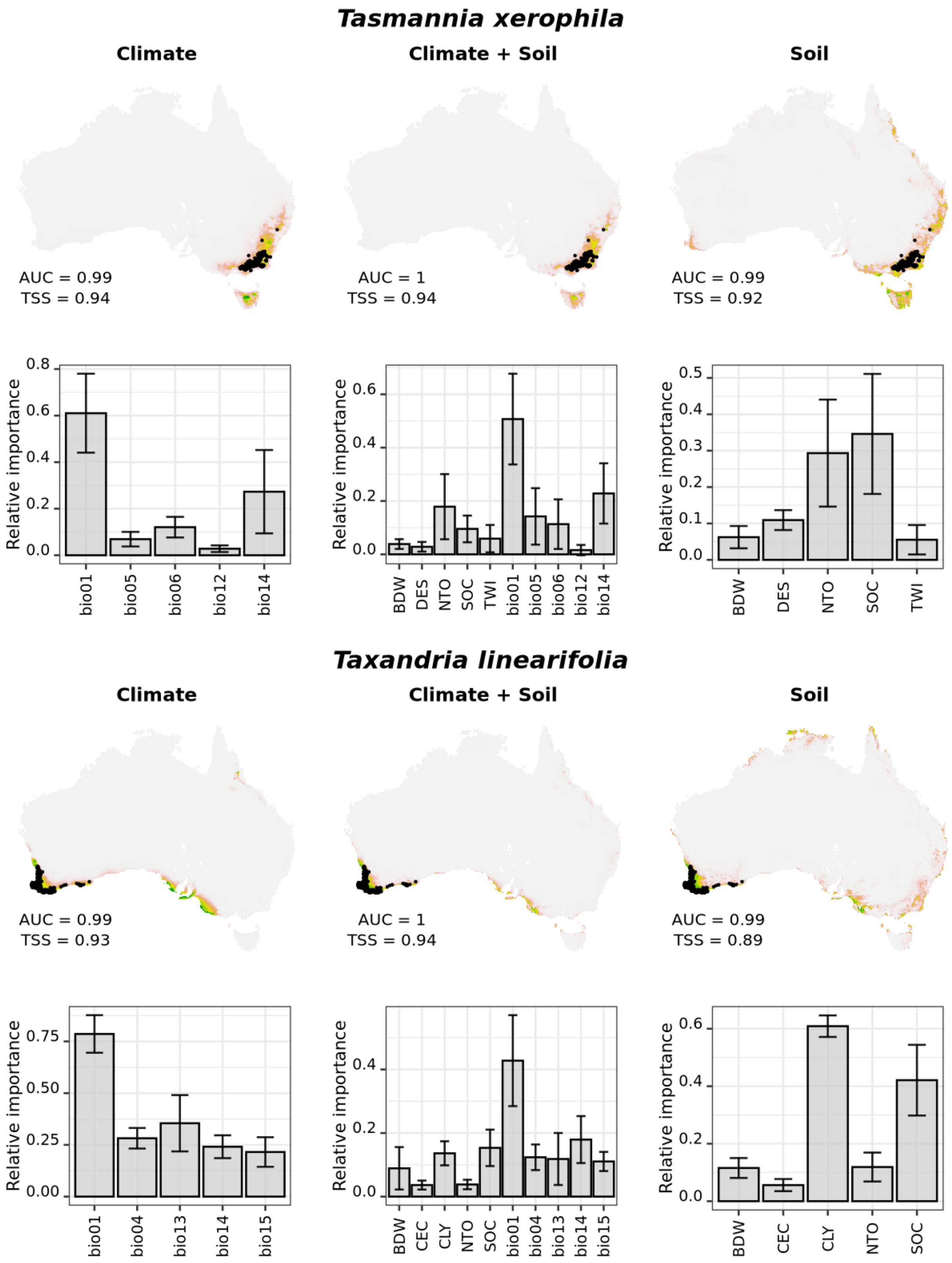

Tasmannia xerophila
Climate
Climate + Soil
Soil
AUC = 0.99
TSS = 0.94
AUC = 1
TSS = 0.94
AUC = 0.99
TSS = 0.92
Relative importance
bio01
bio05
bio06
bio12
bio14
BDW
DES
NTO
SOC
TWI
Taxandria linearifolia
Climate
Climate + Soil
Soil
AUC = 0.99
TSS = 0.93
AUC = 1
TSS = 0.94
AUC = 0.99
TSS = 0.89
Relative importance
bio01
bio04
bio13
bio14
bio15
BDW
CEC
CLY
NTO
SOC

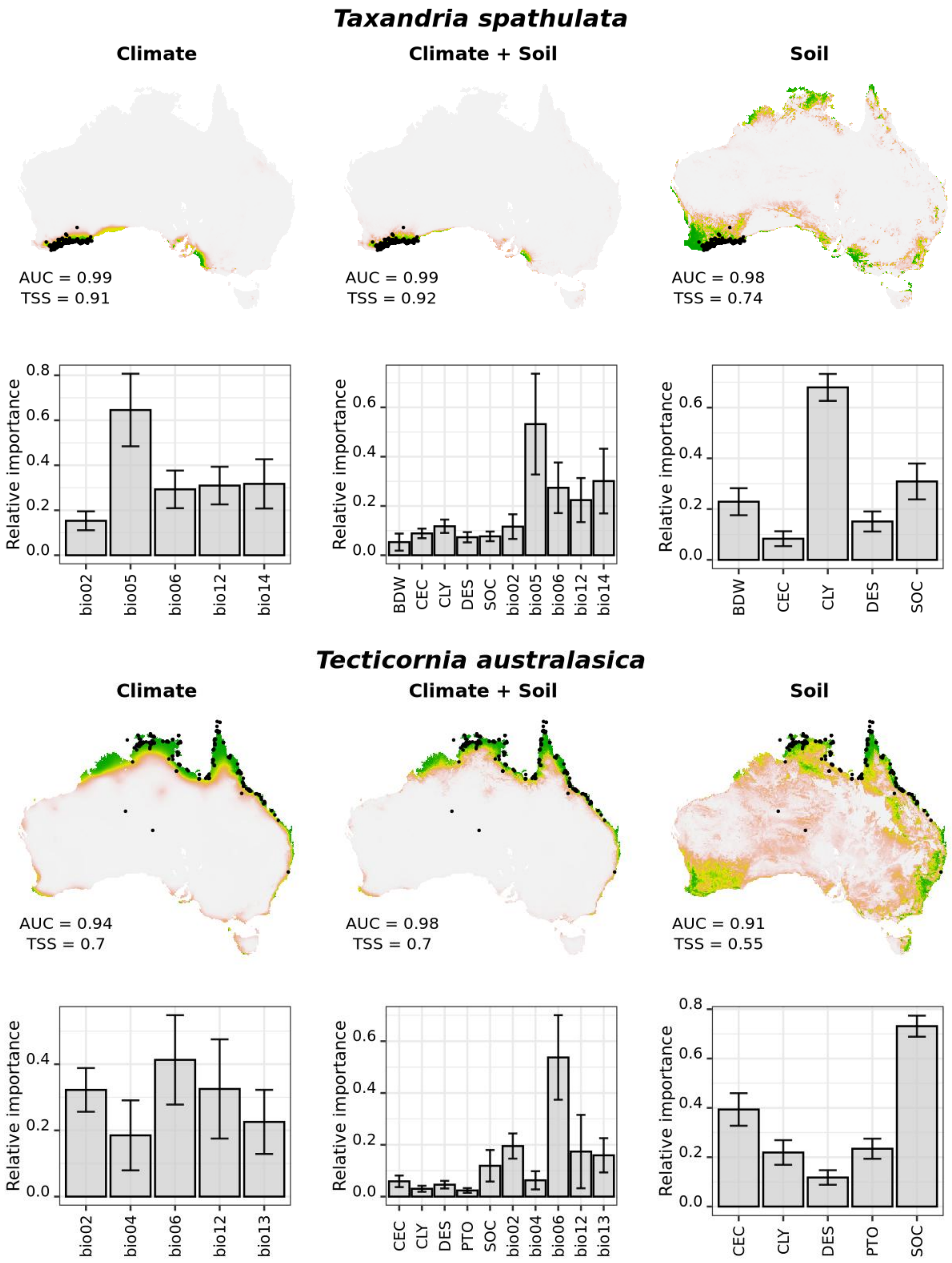
Taxandria spathulata
Climate
Climate + Soil
Soil
AUC = 0.99
TSS = 0.91
AUC = 0.99
TSS = 0.92
AUC = 0.98
TSS = 0.74
Relative importance
bio02 bio05 bio06 bio12 bio14
BDW CEC CLY DES SOC bio02 bio05 bio06 bio12 bio14
BDW CEC CLY DES SOC
Tecticornia australasica
Climate
Climate + Soil
Soil
AUC = 0.94
TSS = 0.7
AUC = 0.98
TSS = 0.7
AUC = 0.91
TSS = 0.55
Relative importance
bio02 bio04 bio06 bio12 bio13
CEC CLY DES PTO SOC bio02 bio04 bio06 bio12 bio13
CEC CLY DES PTO SOC

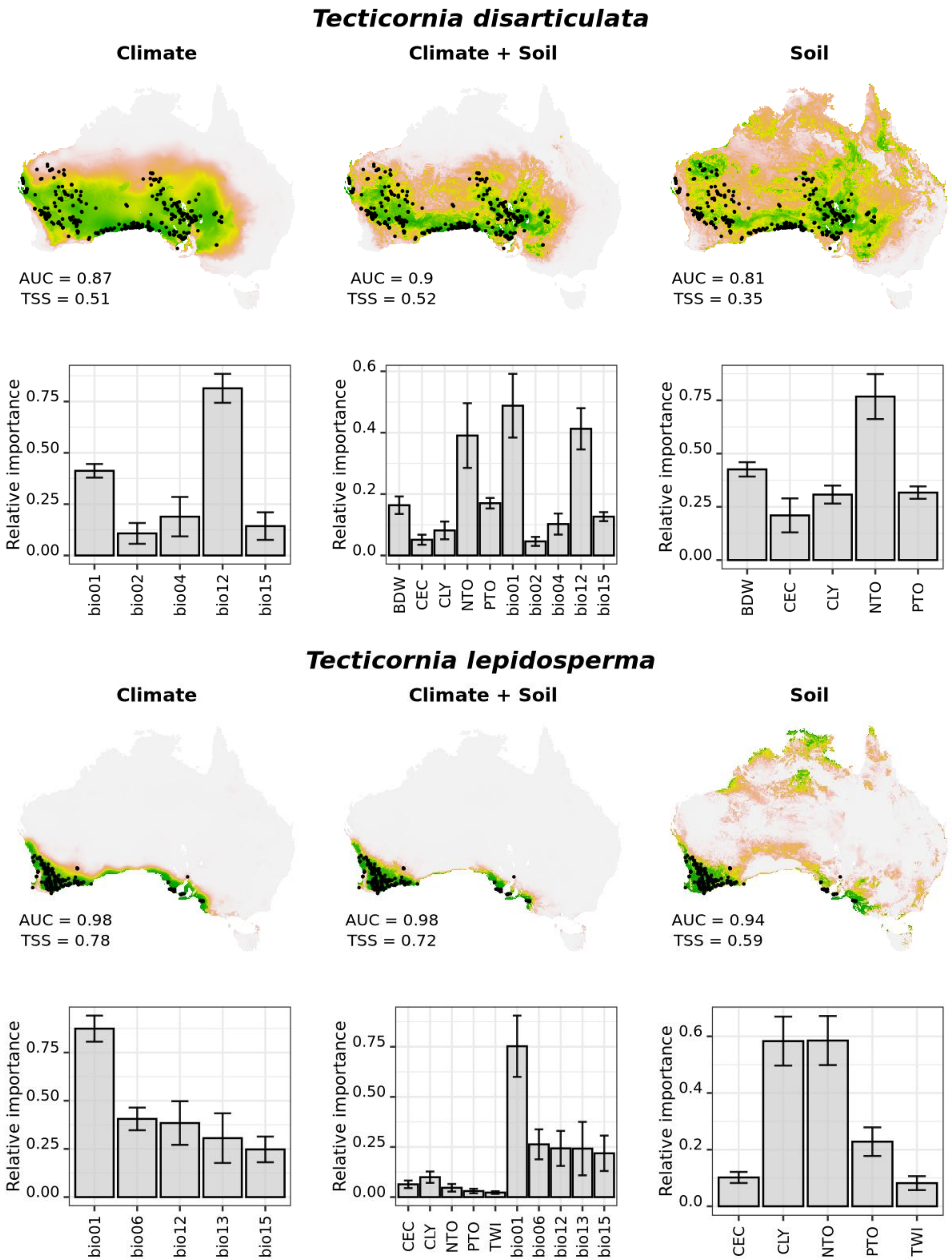

Tecticornia disarticulata
Climate
Climate + Soil
Soil
AUC = 0.87
TSS = 0.51
AUC = 0.9
TSS = 0.52
AUC = 0.81
TSS = 0.35
Relative importance
bio01
bio02
bio04
bio12
bio15
BDW
CEC
CLY
NTO
PTO
Tecticornia lepidosperma
Climate
Climate + Soil
Soil
AUC = 0.98
TSS = 0.78
AUC = 0.98
TSS = 0.72
AUC = 0.94
TSS = 0.59
Relative importance
bio01
bio06
bio12
bio13
bio15
CEC
CLY
NTO
PTO
TWI

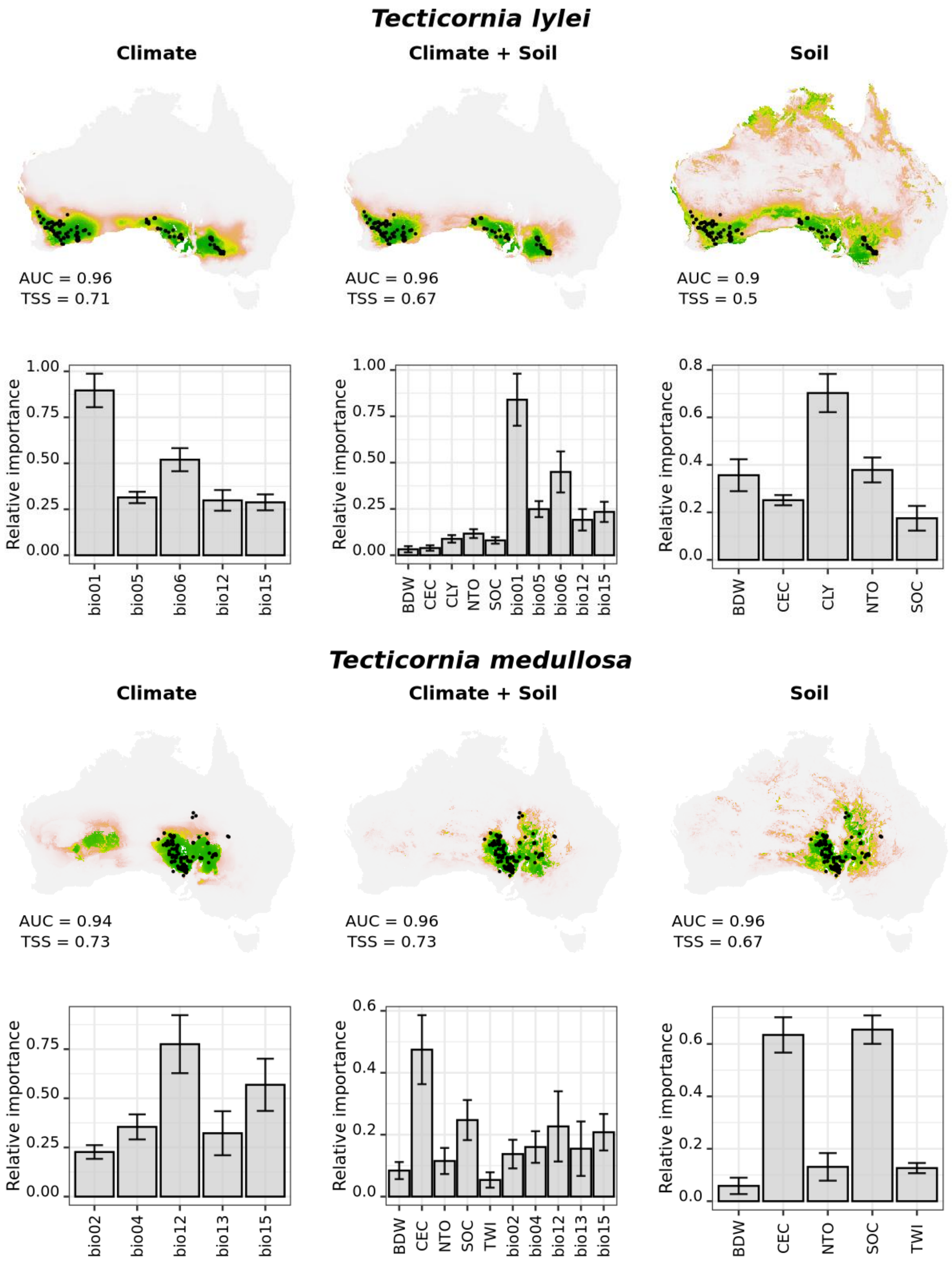

Tecticornia lylei
Climate
Climate + Soil
Soil
AUC = 0.96
TSS = 0.71
AUC = 0.96
TSS = 0.67
AUC = 0.9
TSS = 0.5
Relative importance
0.00
0.25
0.50
0.75
1.00
bio01
bio05
bio06
bio12
bio15
BDW
CEC
CLY
NTO
SOC
0.0
0.2
0.4
0.6
0.8
Tecticornia medullosa
Climate
Climate + Soil
Soil
AUC = 0.94
TSS = 0.73
AUC = 0.96
TSS = 0.73
AUC = 0.96
TSS = 0.67
bio02
bio04
bio12
bio13
bio15
TWI

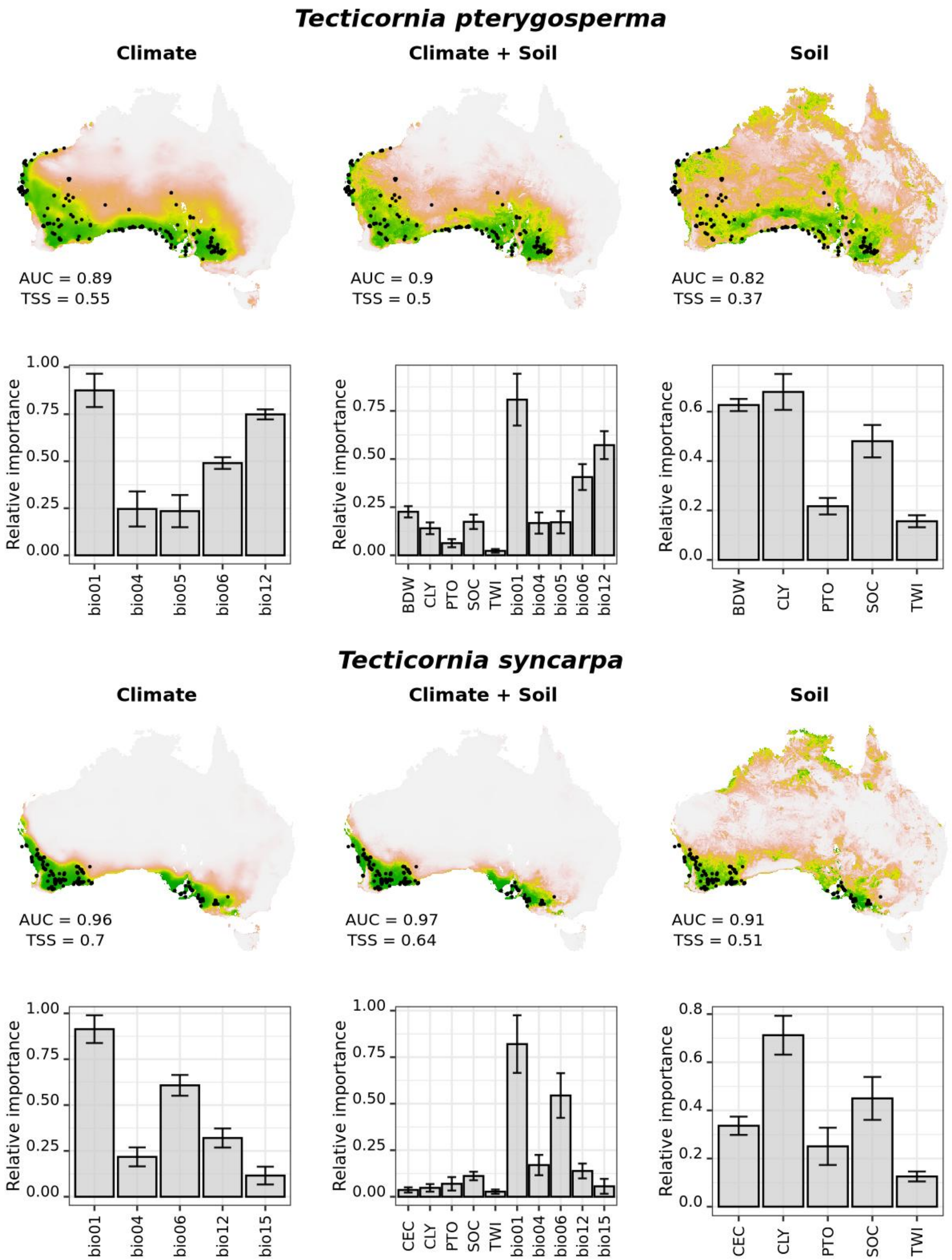
Tecticornia pterygosperma
Climate
Climate + Soil
Soil
AUC = 0.89
TSS = 0.55
AUC = 0.9
TSS = 0.5
AUC = 0.82
TSS = 0.37
Relative importance
bio01 bio04 bio05 bio06 bio12
BDW CLY PTO SOC TWI bio01 bio04 bio05 bio06 bio12
BDW CLY PTO SOC TWI
Tecticornia syncarpa
Climate
Climate + Soil
Soil
AUC = 0.96
TSS = 0.7
AUC = 0.97
TSS = 0.64
AUC = 0.91
TSS = 0.51
Relative importance
bio01 bio04 bio06 bio12 bio15
CEC CLY PTO SOC TWI bio01 bio04 bio06 bio12 bio15
CEC CLY PTO SOC TWI

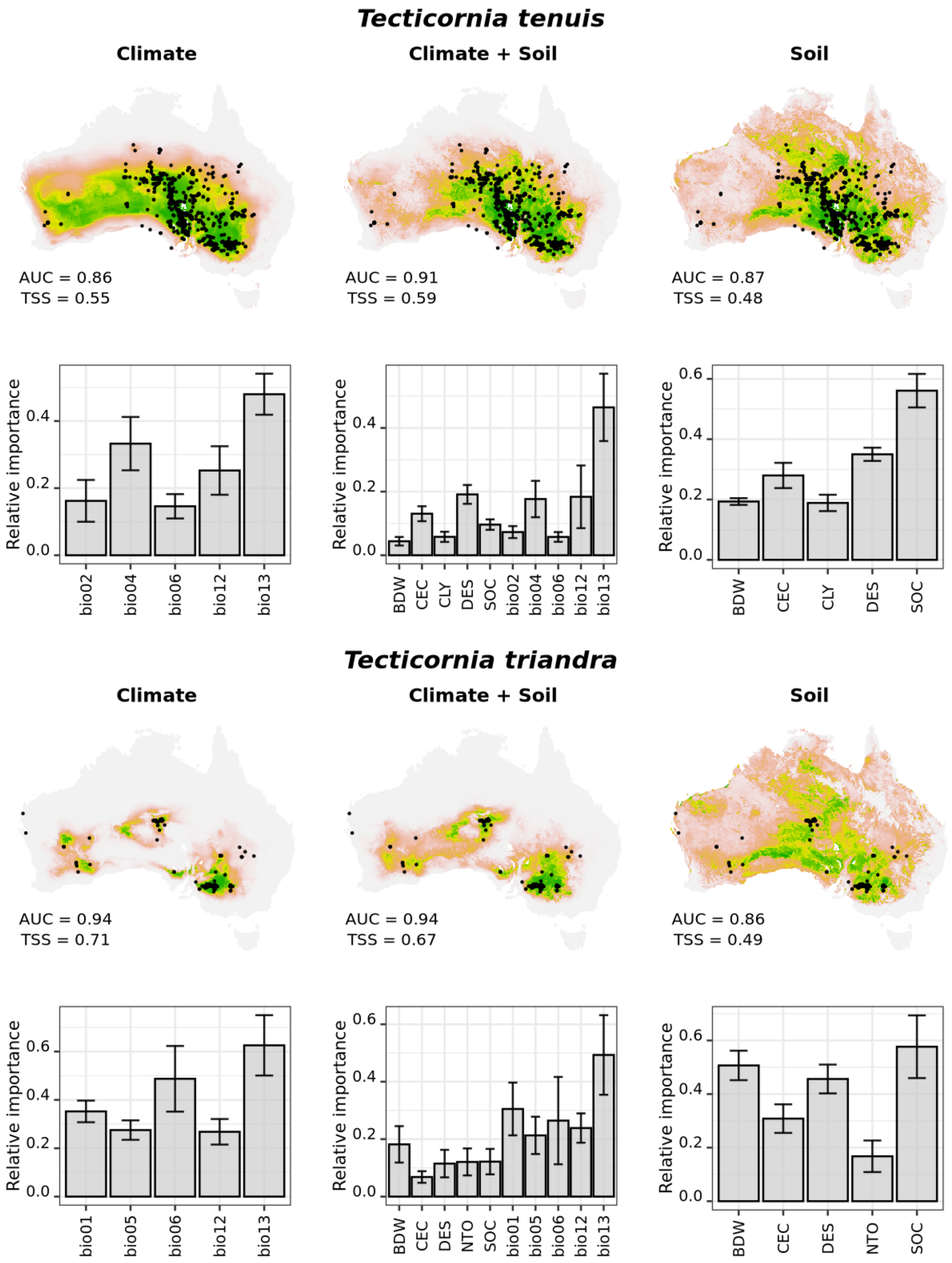
Tecticornia tenuis
Climate
Climate + Soil
Soil
AUC = 0.86
TSS = 0.55
AUC = 0.91
TSS = 0.59
AUC = 0.87
TSS = 0.48
Relative importance
0.0
0.2
0.4
0.6
bio02
bio04
bio06
bio12
bio13
BDW
CEC
CLY
DES
SOC
Tecticornia triandra
AUC = 0.94
TSS = 0.71
AUC = 0.94
TSS = 0.67
AUC = 0.86
TSS = 0.49
bio01
bio05
NTO

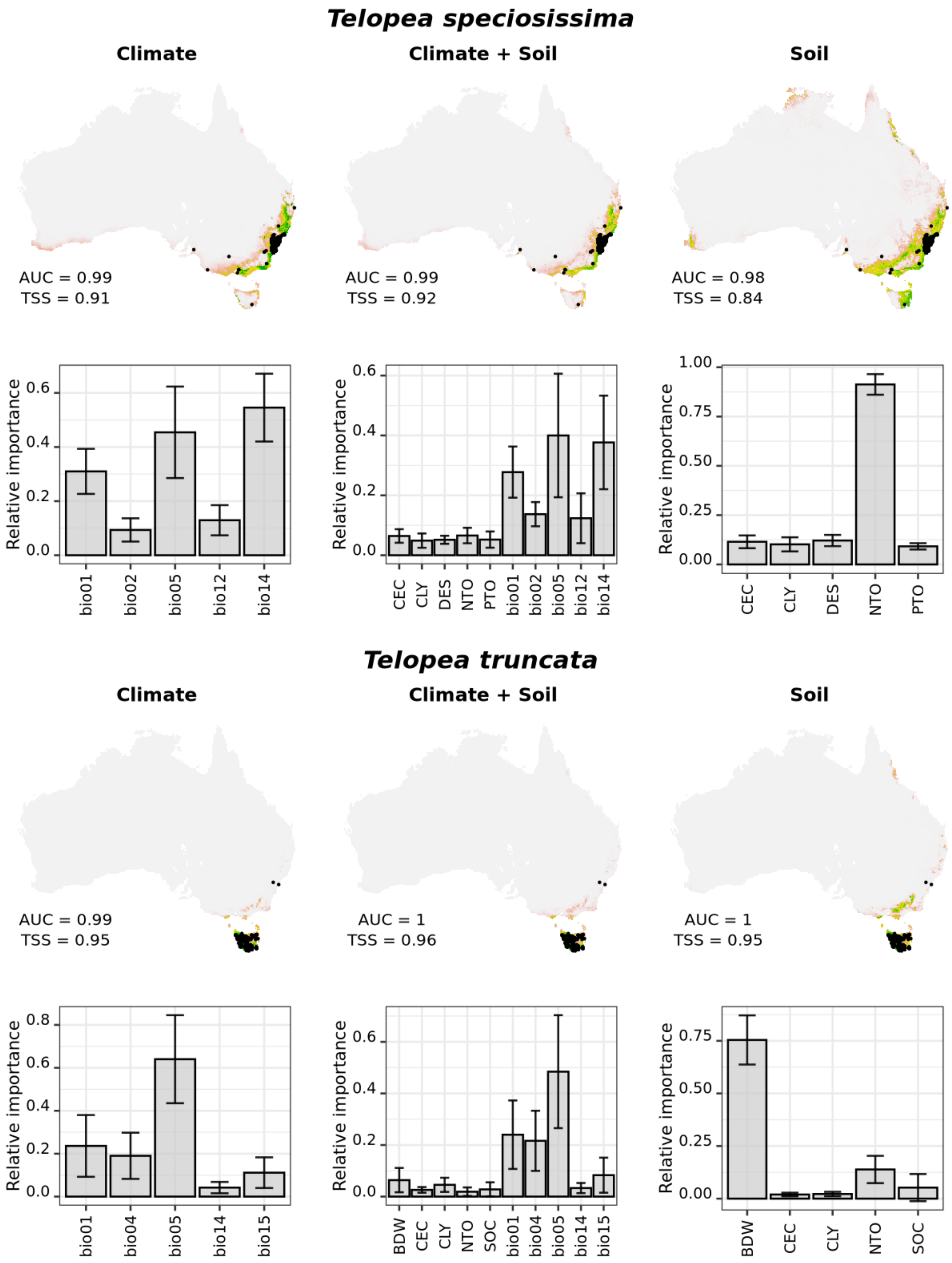

Telopea speciosissima
Climate
Climate + Soil
Soil
AUC = 0.99
TSS = 0.91
AUC = 0.99
TSS = 0.92
AUC = 0.98
TSS = 0.84
Relative importance
bio01
bio02
bio05
bio12
bio14
CEC
CLY
DES
NTO
PTO
Telopea truncata
AUC = 0.99
TSS = 0.95
AUC = 1
TSS = 0.96
AUC = 1
TSS = 0.95
bio04
bio15
BDW
SOC

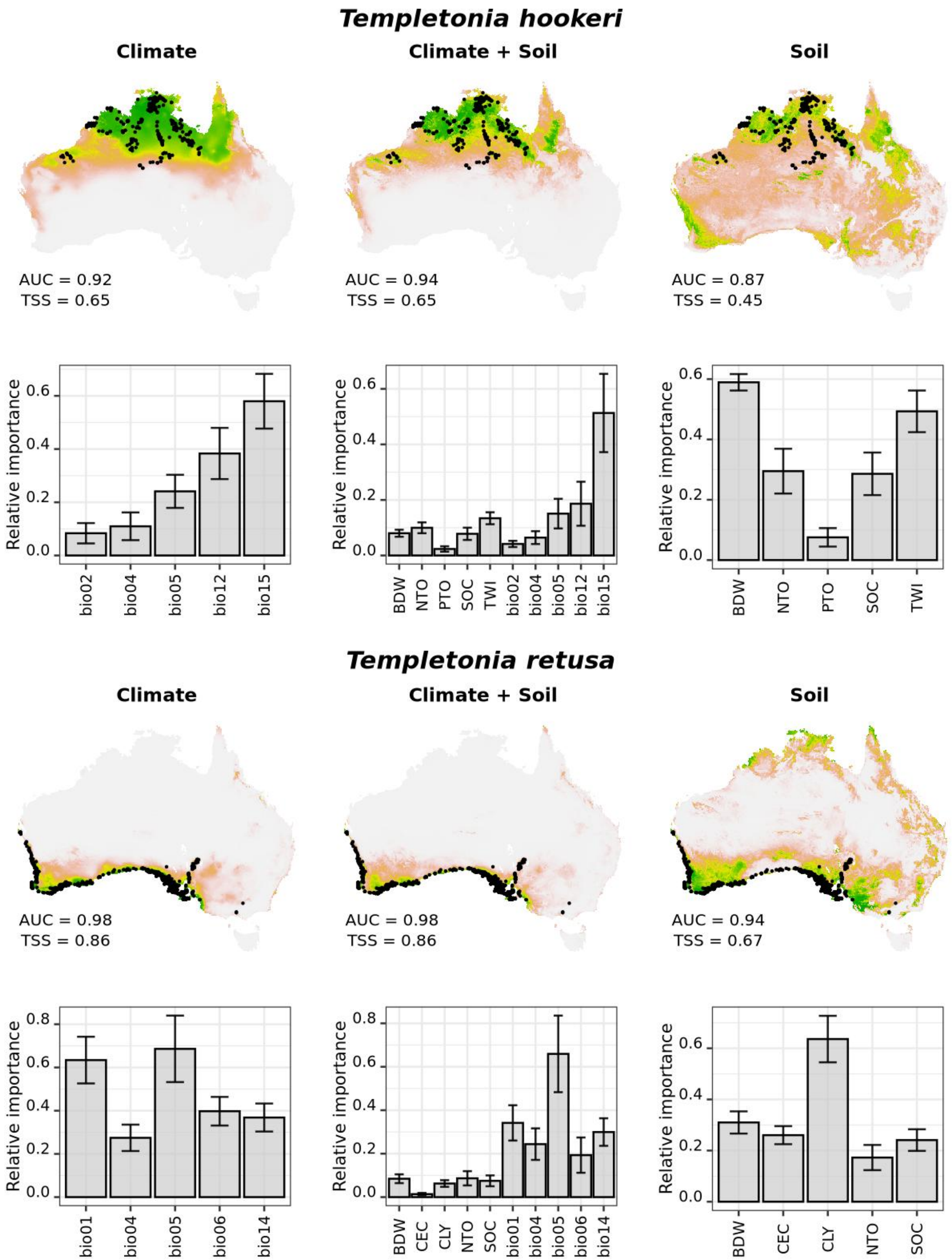

Templetonia hookeri
Climate
Climate + Soil
Soil
AUC = 0.92
TSS = 0.65
AUC = 0.94
TSS = 0.65
AUC = 0.87
TSS = 0.45
Relative importance
bio02
bio04
bio05
bio12
bio15
BDW
NTO
PTO
SOC
TWI
Templetonia retusa
Climate
Climate + Soil
Soil
AUC = 0.98
TSS = 0.86
AUC = 0.98
TSS = 0.86
AUC = 0.94
TSS = 0.67
Relative importance
bio01
bio04
bio05
bio06
bio14
BDW
CEC
CLY
NTO
SOC

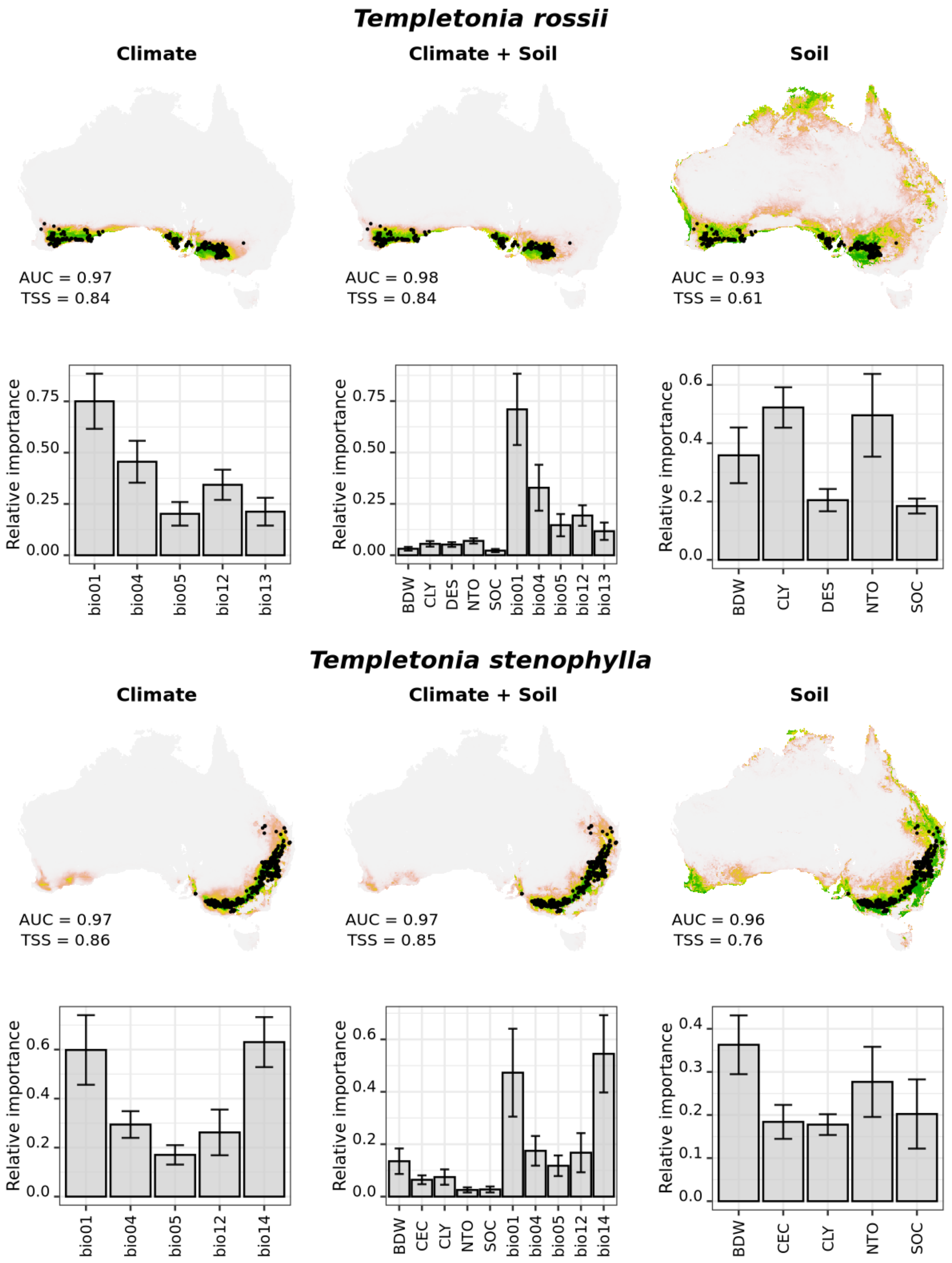
Templetonia rossii
Climate
Climate + Soil
Soil
AUC = 0.97
TSS = 0.84
AUC = 0.98
TSS = 0.84
AUC = 0.93
TSS = 0.61
Relative importance
bio01 bio04 bio05 bio12 bio13
BDW CLY DES NTO SOC bio01 bio04 bio05 bio12 bio13
BDW CLY DES NTO SOC
Templetonia stenophylla
Climate
Climate + Soil
Soil
AUC = 0.97
TSS = 0.86
AUC = 0.97
TSS = 0.85
AUC = 0.96
TSS = 0.76
Relative importance
bio01 bio04 bio05 bio12 bio14
BDW CEC CLY NTO SOC bio01 bio04 bio05 bio12 bio14
BDW CEC CLY NTO SOC

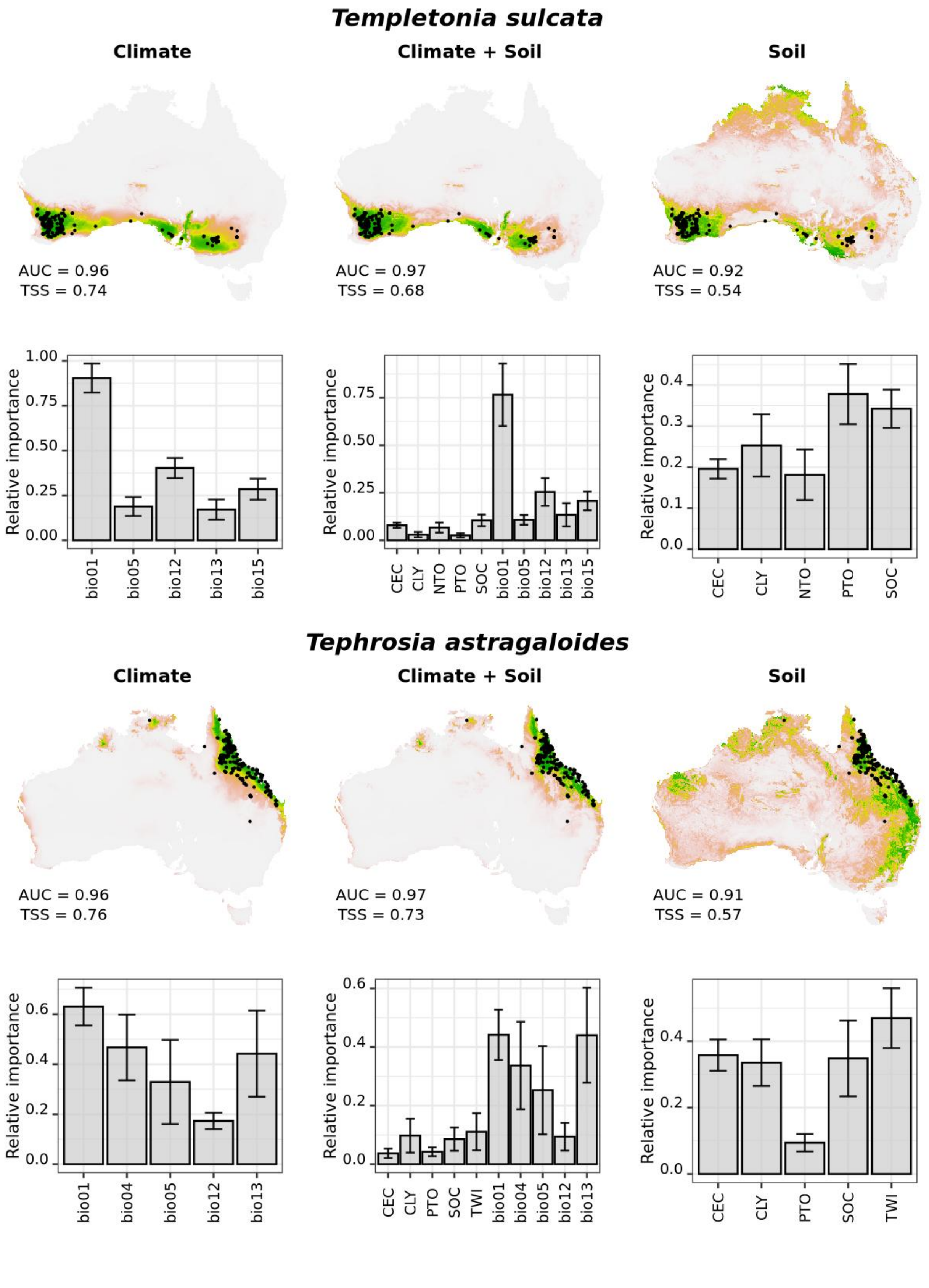

Templetonia sulcata
Climate
Climate + Soil
Soil
AUC = 0.96
TSS = 0.74
AUC = 0.97
TSS = 0.68
AUC = 0.92
TSS = 0.54
Relative importance
1.00
0.75
0.50
0.25
0.00
bio01
bio05
bio12
bio13
bio15
Relative importance
0.75
0.50
0.25
0.00
CEC
CLY
NTO
PTO
SOC
bio01
bio05
bio12
bio13
bio15
Relative importance
0.4
0.3
0.2
0.1
0.0
CEC
CLY
NTO
PTO
SOC
Tephrosia astragaloides
Climate
Climate + Soil
Soil
AUC = 0.96
TSS = 0.76
AUC = 0.97
TSS = 0.73
AUC = 0.91
TSS = 0.57
Relative importance
0.6
0.4
0.2
0.0
bio01
bio04
bio05
bio12
bio13
Relative importance
0.6
0.4
0.2
0.0
CEC
CLY
PTO
SOC
TWI
bio01
bio04
bio05
bio12
bio13
Relative importance
0.4
0.2
0.0
CEC
CLY
PTO
SOC
TWI

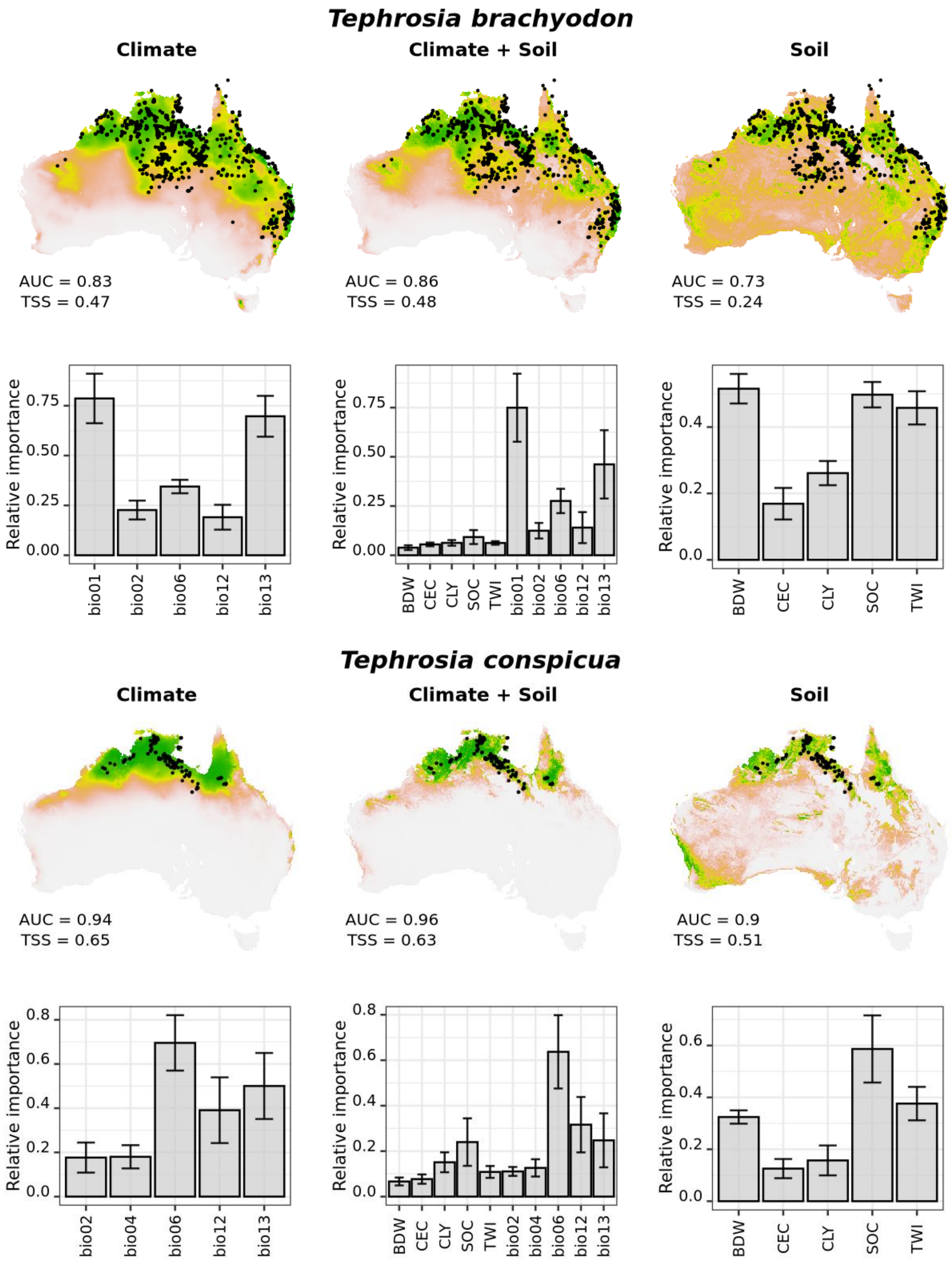

Tephrosia brachyodon
Climate
Climate + Soil
Soil
AUC = 0.83
TSS = 0.47
AUC = 0.86
TSS = 0.48
AUC = 0.73
TSS = 0.24
Relative importance
0.00
0.25
0.50
0.75
bio01
bio02
bio06
bio12
bio13
BDW
CEC
CLY
SOC
TWI
0.0
0.2
0.4
Tephrosia conspicua
Climate
Climate + Soil
Soil
AUC = 0.94
TSS = 0.65
AUC = 0.96
TSS = 0.63
AUC = 0.9
TSS = 0.51
Relative importance
0.0
0.2
0.4
0.6
0.8
bio02
bio04
bio06
bio12
bio13
BDW
CEC
CLY
SOC
TWI

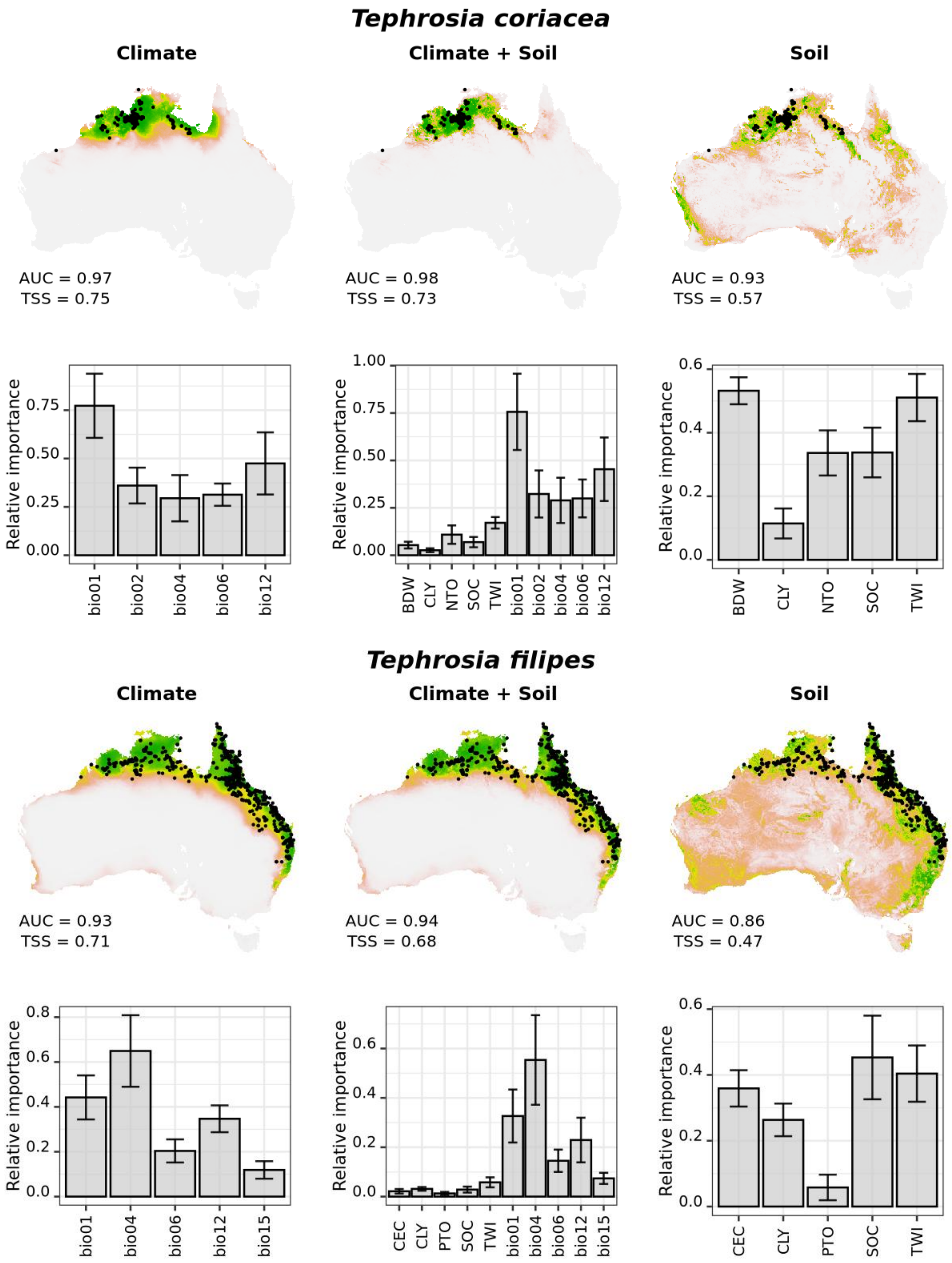
Tephrosia coriacea
Climate
Climate + Soil
Soil
AUC = 0.97
TSS = 0.75
AUC = 0.98
TSS = 0.73
AUC = 0.93
TSS = 0.57
Relative importance
0.00
0.25
0.50
0.75
1.00
bio01
bio02
bio04
bio06
bio12
BDW
CLY
NTO
SOC
TWI
0.0
0.2
0.4
0.6
Tephrosia filipes
Climate
Climate + Soil
Soil
AUC = 0.93
TSS = 0.71
AUC = 0.94
TSS = 0.68
AUC = 0.86
TSS = 0.47
Relative importance
0.0
0.2
0.4
0.6
0.8
bio01
bio04
bio06
bio12
bio15
CEC
CLY
PTO
SOC
TWI

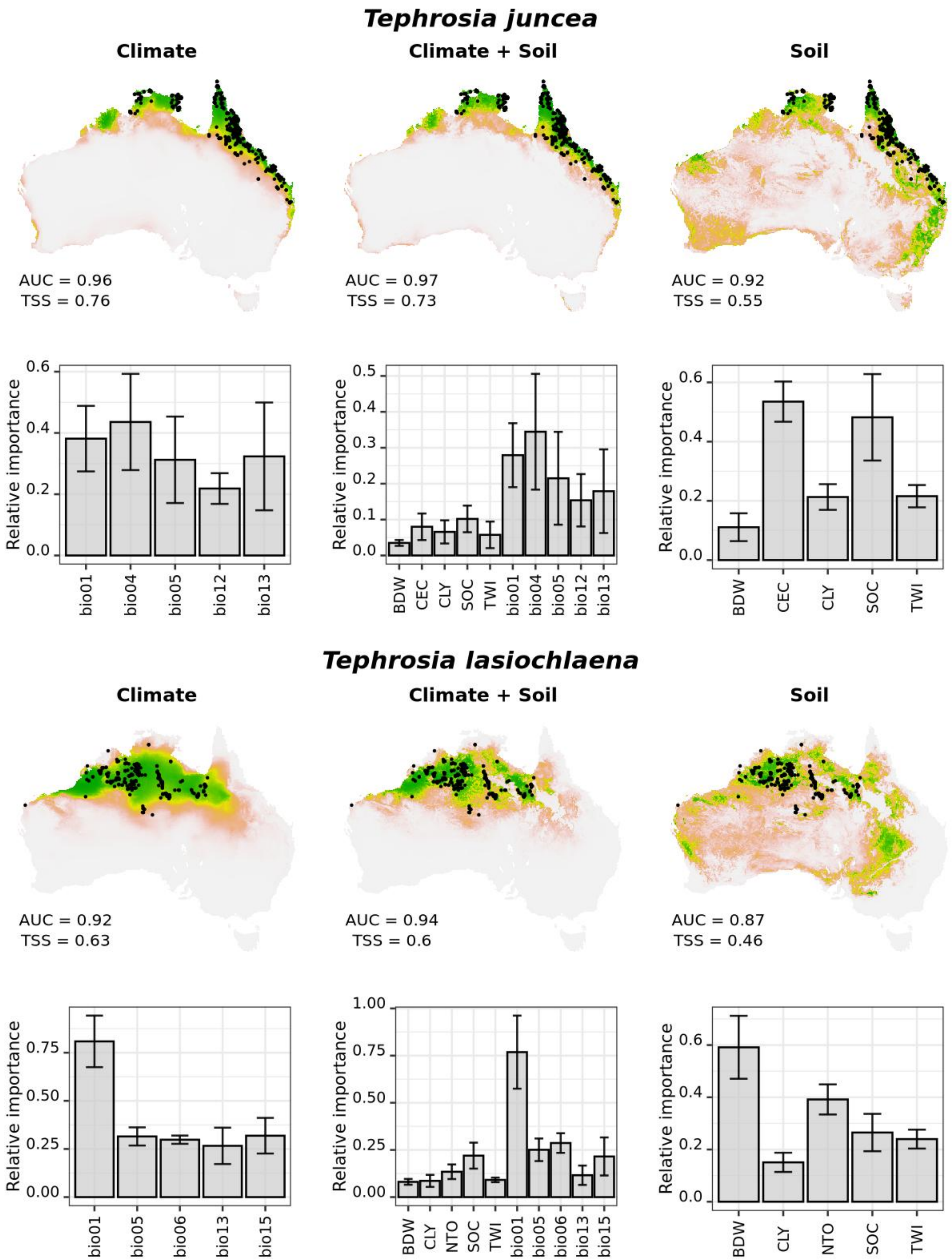
Tephrosia juncea
Climate
Climate + Soil
Soil
AUC = 0.96
TSS = 0.76
AUC = 0.97
TSS = 0.73
AUC = 0.92
TSS = 0.55
Relative importance
bio01 bio04 bio05 bio12 bio13
BDW CEC CLY SOC TWI bio01 bio04 bio05 bio12 bio13
BDW CEC CLY SOC TWI
Tephrosia lasiochlaena
Climate
Climate + Soil
Soil
AUC = 0.92
TSS = 0.63
AUC = 0.94
TSS = 0.6
AUC = 0.87
TSS = 0.46
Relative importance
bio01 bio05 bio06 bio13 bio15
BDW CLY NTO SOC TWI bio01 bio05 bio06 bio13 bio15
BDW CLY NTO SOC TWI

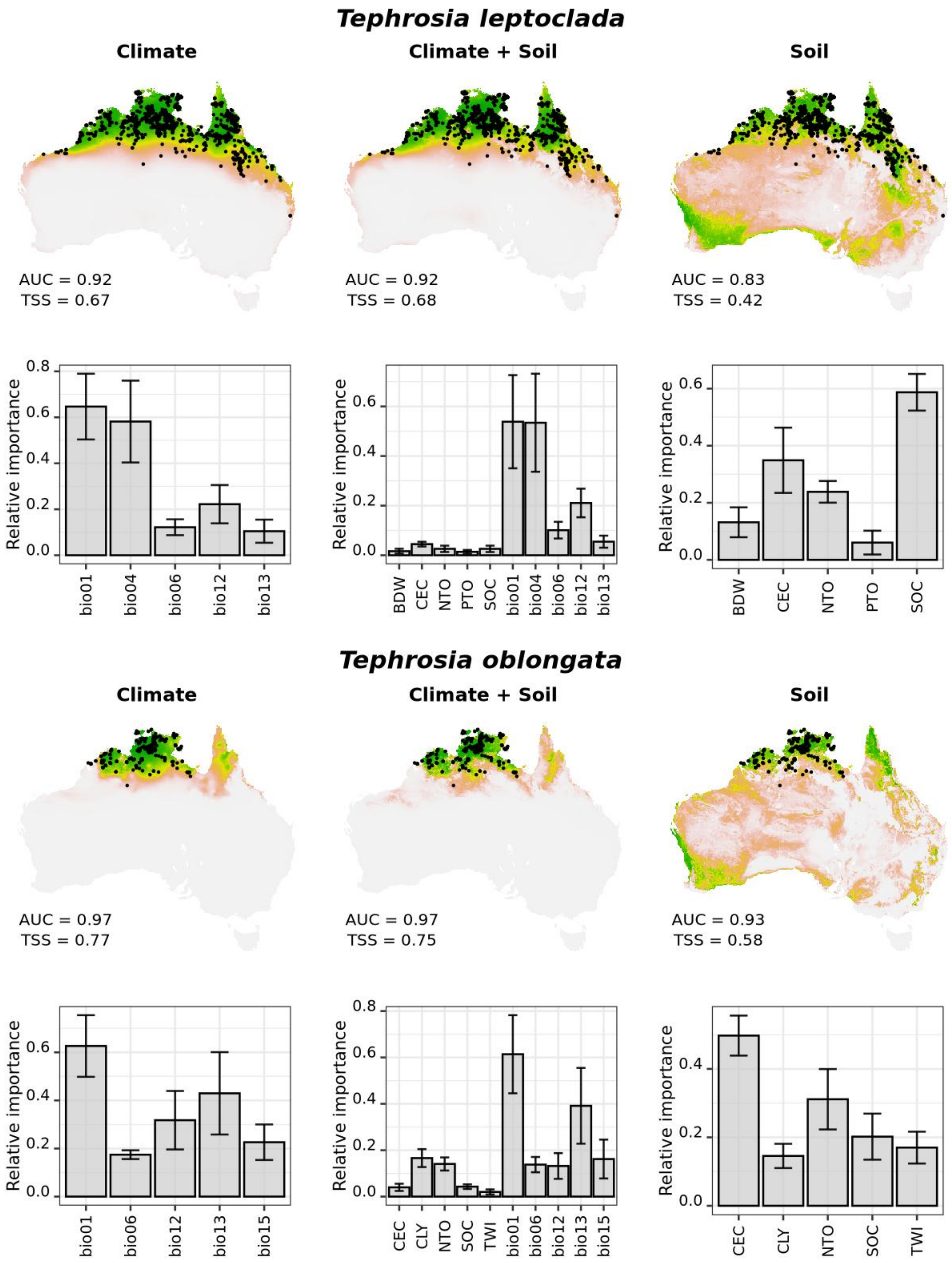

Tephrosia leptoclada
Climate
Climate + Soil
Soil
AUC = 0.92
TSS = 0.67
AUC = 0.92
TSS = 0.68
AUC = 0.83
TSS = 0.42
Relative importance
bio01 bio04 bio06 bio12 bio13
BDW CEC NTO PTO SOC bio01 bio04 bio06 bio12 bio13
BDW CEC NTO PTO SOC
Tephrosia oblongata
Climate
Climate + Soil
Soil
AUC = 0.97
TSS = 0.77
AUC = 0.97
TSS = 0.75
AUC = 0.93
TSS = 0.58
Relative importance
bio01 bio06 bio12 bio13 bio15
CEC CLY NTO SOC TWI bio01 bio06 bio12 bio13 bio15
CEC CLY NTO SOC TWI

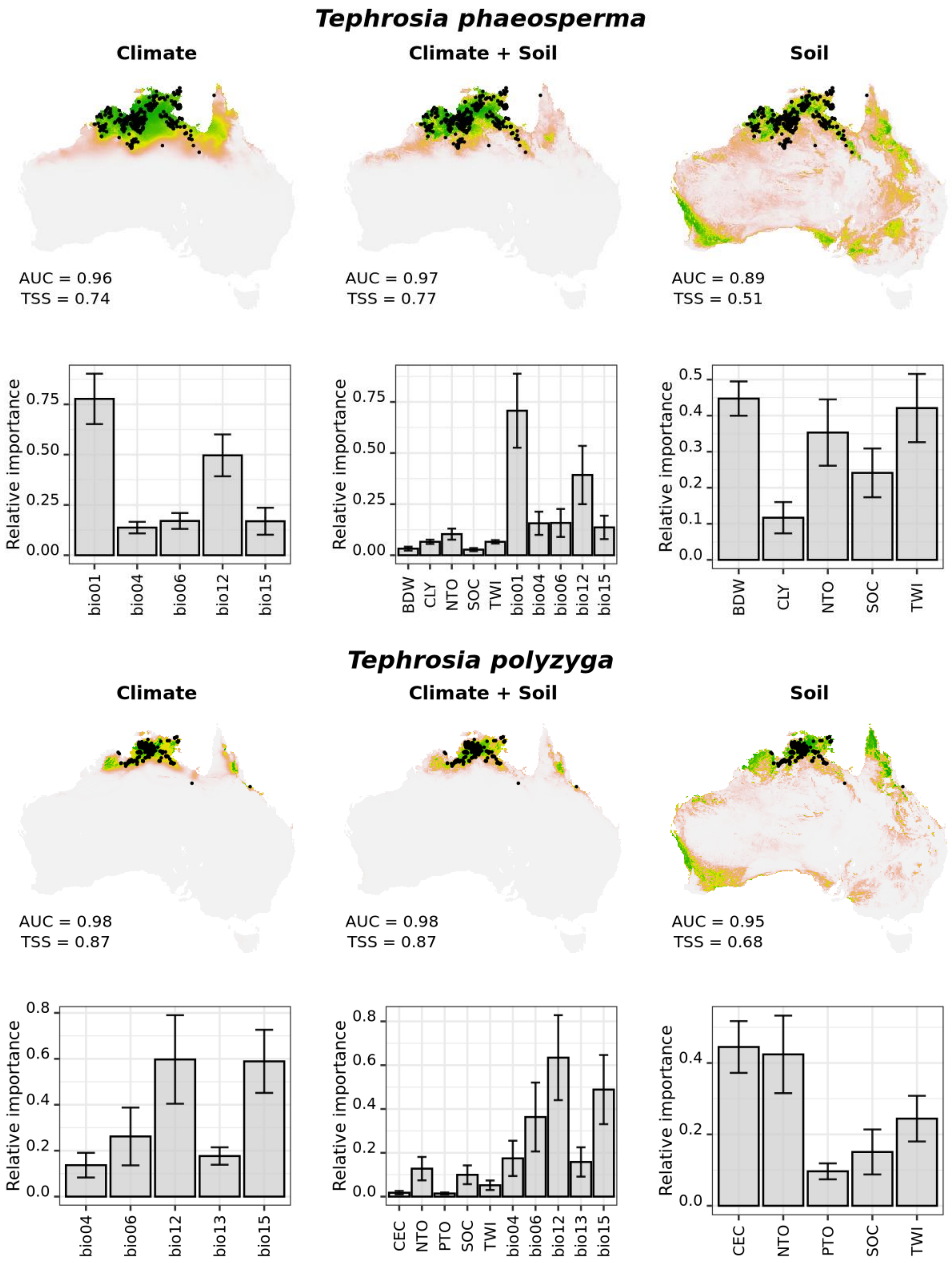

Tephrosia phaeosperma
Climate
Climate + Soil
Soil
AUC = 0.96
TSS = 0.74
AUC = 0.97
TSS = 0.77
AUC = 0.89
TSS = 0.51
Relative importance
0.00
0.25
0.50
0.75
bio01
bio04
bio06
bio12
bio15
BDW
CLY
NTO
SOC
TWI
0.0
0.1
0.2
0.3
0.4
0.5
Tephrosia polyzyga
Climate
Climate + Soil
Soil
AUC = 0.98
TSS = 0.87
AUC = 0.98
TSS = 0.87
AUC = 0.95
TSS = 0.68
Relative importance
0.0
0.2
0.4
0.6
0.8
bio04
bio06
bio12
bio13
bio15
CEC
NTO
PTO
SOC
TWI

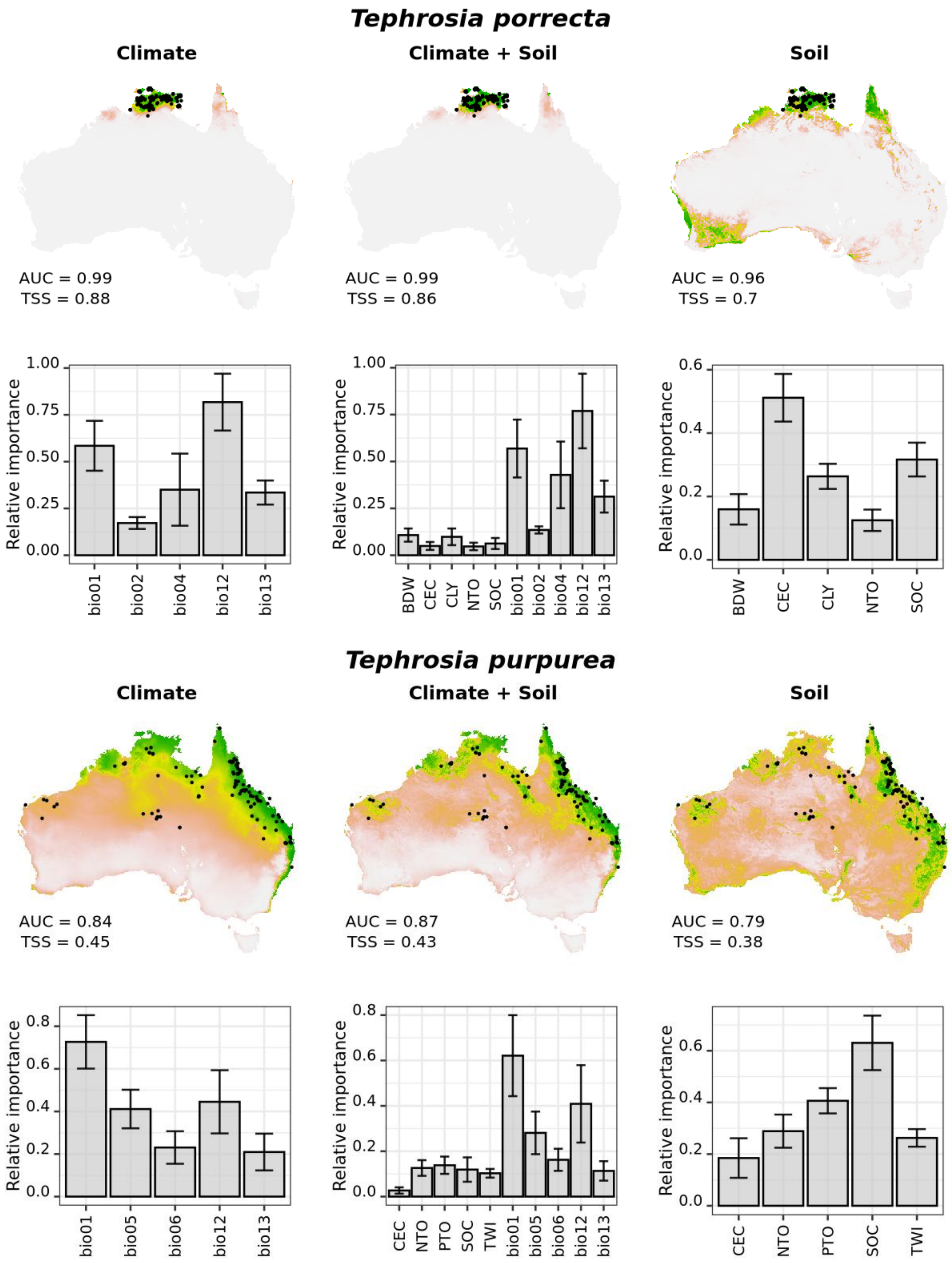

Tephrosia porrecta
Climate
Climate + Soil
Soil
AUC = 0.99
TSS = 0.88
AUC = 0.99
TSS = 0.86
AUC = 0.96
TSS = 0.7
Relative importance
1.00
0.75
0.50
0.25
0.00
bio01
bio02
bio04
bio12
bio13
BDW
CEC
CLY
NTO
SOC
0.6
0.4
0.2
0.0
Tephrosia purpurea
Climate
Climate + Soil
Soil
AUC = 0.84
TSS = 0.45
AUC = 0.87
TSS = 0.43
AUC = 0.79
TSS = 0.38
0.8
0.6
0.4
0.2
0.0
bio01
bio05
bio06
bio12
bio13
CEC
NTO
PTO
SOC
TWI

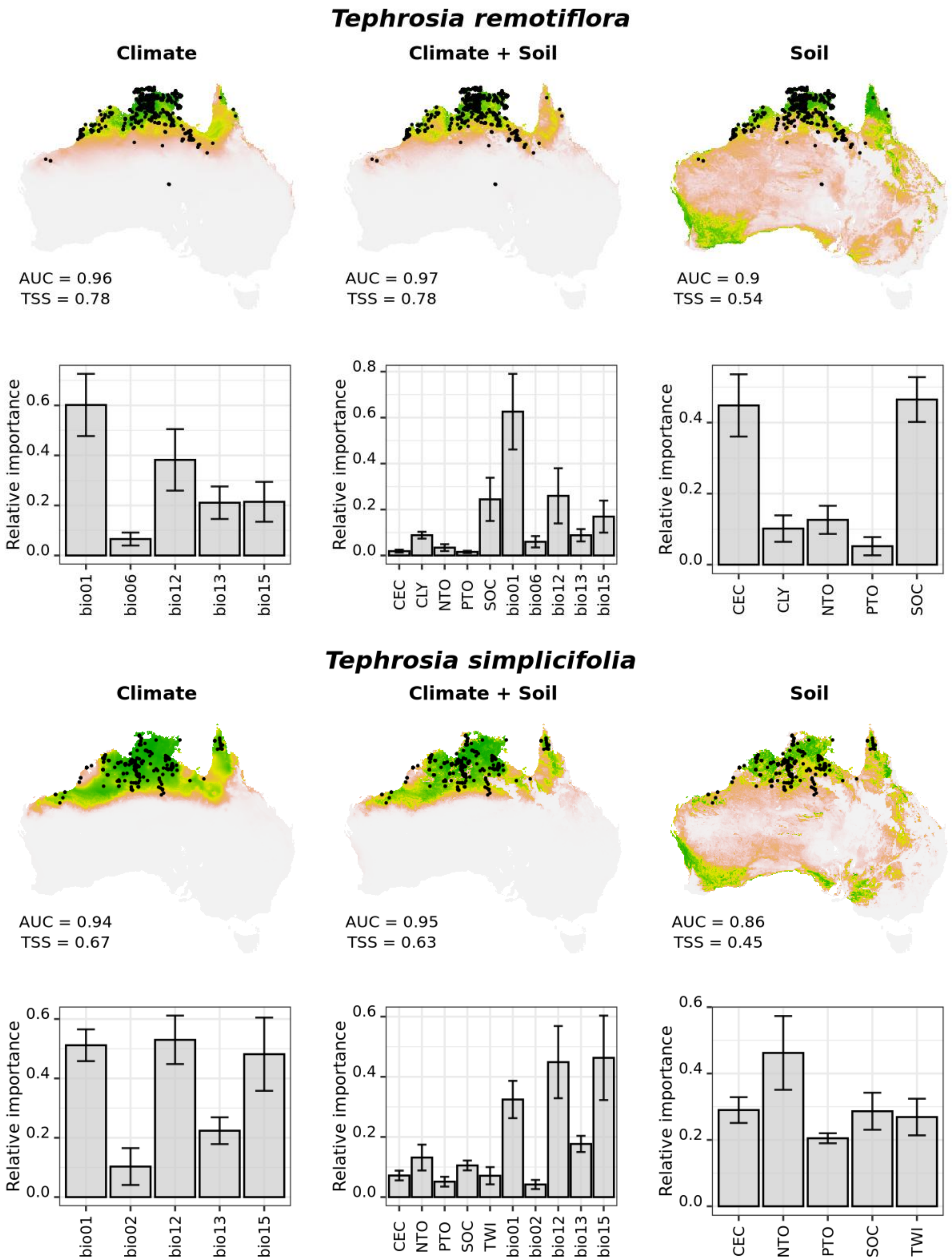

Tephrosia remotiflora
Climate
Climate + Soil
Soil
AUC = 0.96
TSS = 0.78
AUC = 0.97
TSS = 0.78
AUC = 0.9
TSS = 0.54
Relative importance
bio01
bio06
bio12
bio13
bio15
CEC
CLY
NTO
PTO
SOC
bio01
bio06
bio12
bio13
bio15
CEC
CLY
NTO
PTO
SOC
Tephrosia simplicifolia
Climate
Climate + Soil
Soil
AUC = 0.94
TSS = 0.67
AUC = 0.95
TSS = 0.63
AUC = 0.86
TSS = 0.45
Relative importance
bio01
bio02
bio12
bio13
bio15
CEC
NTO
PTO
SOC
TWI
bio01
bio02
bio12
bio13
bio15
CEC
NTO
PTO
SOC
TWI

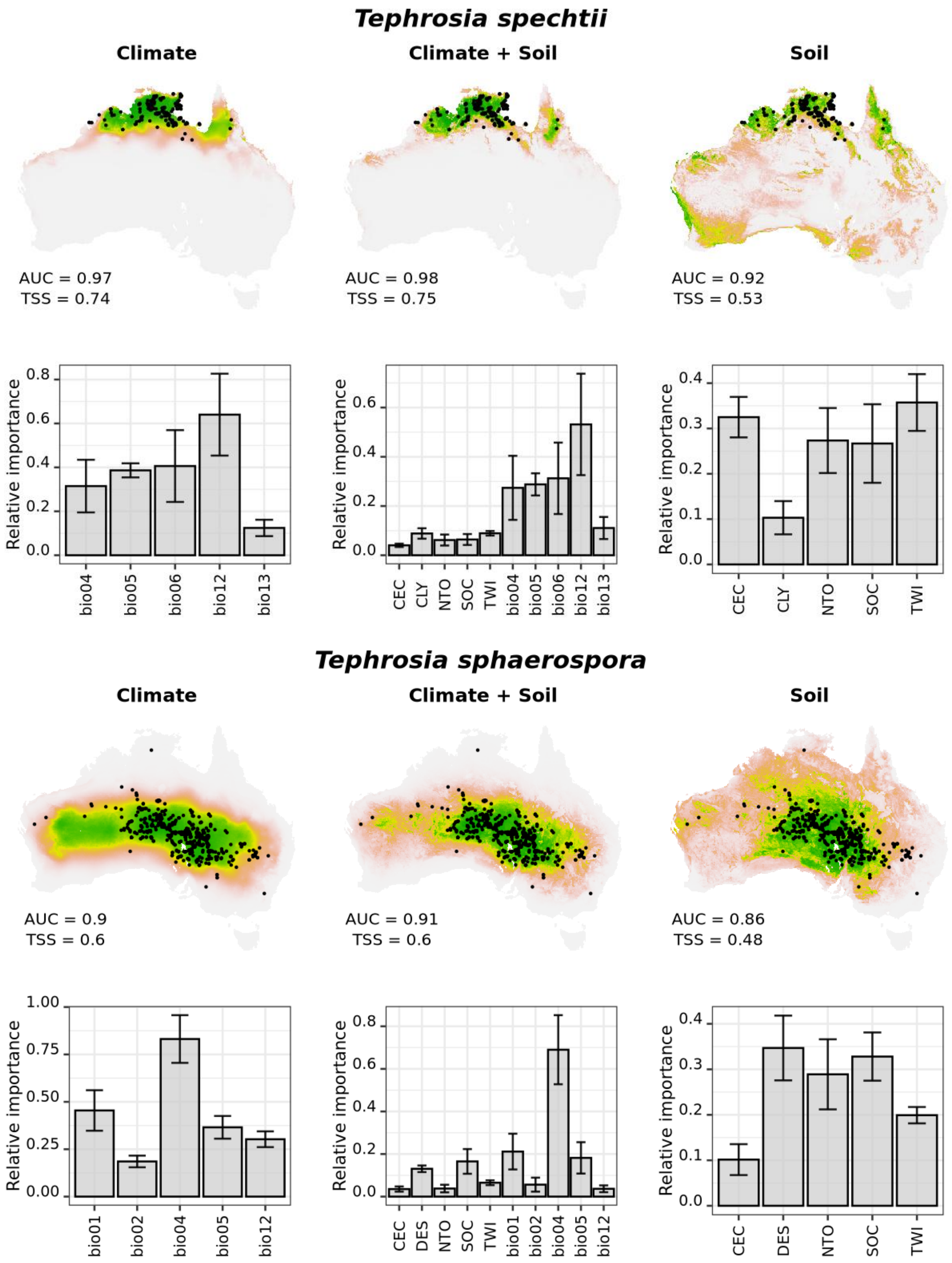
Tephrosia spechtii
Climate
Climate + Soil
Soil
AUC = 0.97
TSS = 0.74
AUC = 0.98
TSS = 0.75
AUC = 0.92
TSS = 0.53
Relative importance
bio04
bio05
bio06
bio12
bio13
CEC
CLY
NTO
SOC
TWI
bio04
bio05
bio06
bio12
bio13
CEC
CLY
NTO
SOC
TWI
Tephrosia sphaerospora
Climate
Climate + Soil
Soil
AUC = 0.9
TSS = 0.6
AUC = 0.91
TSS = 0.6
AUC = 0.86
TSS = 0.48
Relative importance
bio01
bio02
bio04
bio05
bio12
CEC
DES
NTO
SOC
TWI
bio01
bio02
bio04
bio05
bio12
CEC
DES
NTO
SOC
TWI

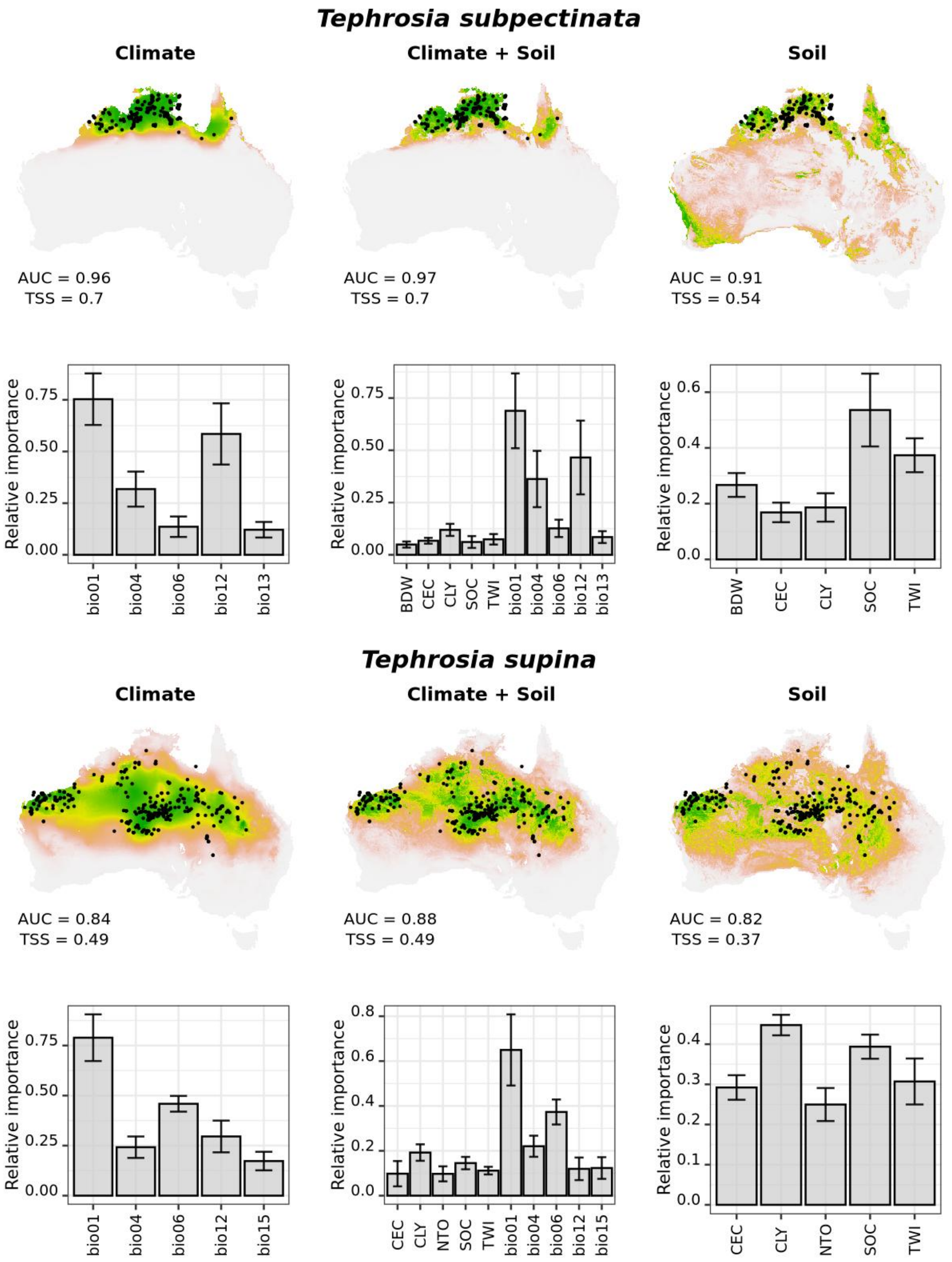

Tephrosia subpectinata
Climate
Climate + Soil
Soil
AUC = 0.96
TSS = 0.7
AUC = 0.97
TSS = 0.7
AUC = 0.91
TSS = 0.54
Relative importance
0.00
0.25
0.50
0.75
bio01
bio04
bio06
bio12
bio13
BDW
CEC
CLY
SOC
TWI
0.0
0.2
0.4
0.6
Tephrosia supina
Climate
Climate + Soil
Soil
AUC = 0.84
TSS = 0.49
AUC = 0.88
TSS = 0.49
AUC = 0.82
TSS = 0.37
Relative importance
bio15
NTO
0.8
0.1
0.3

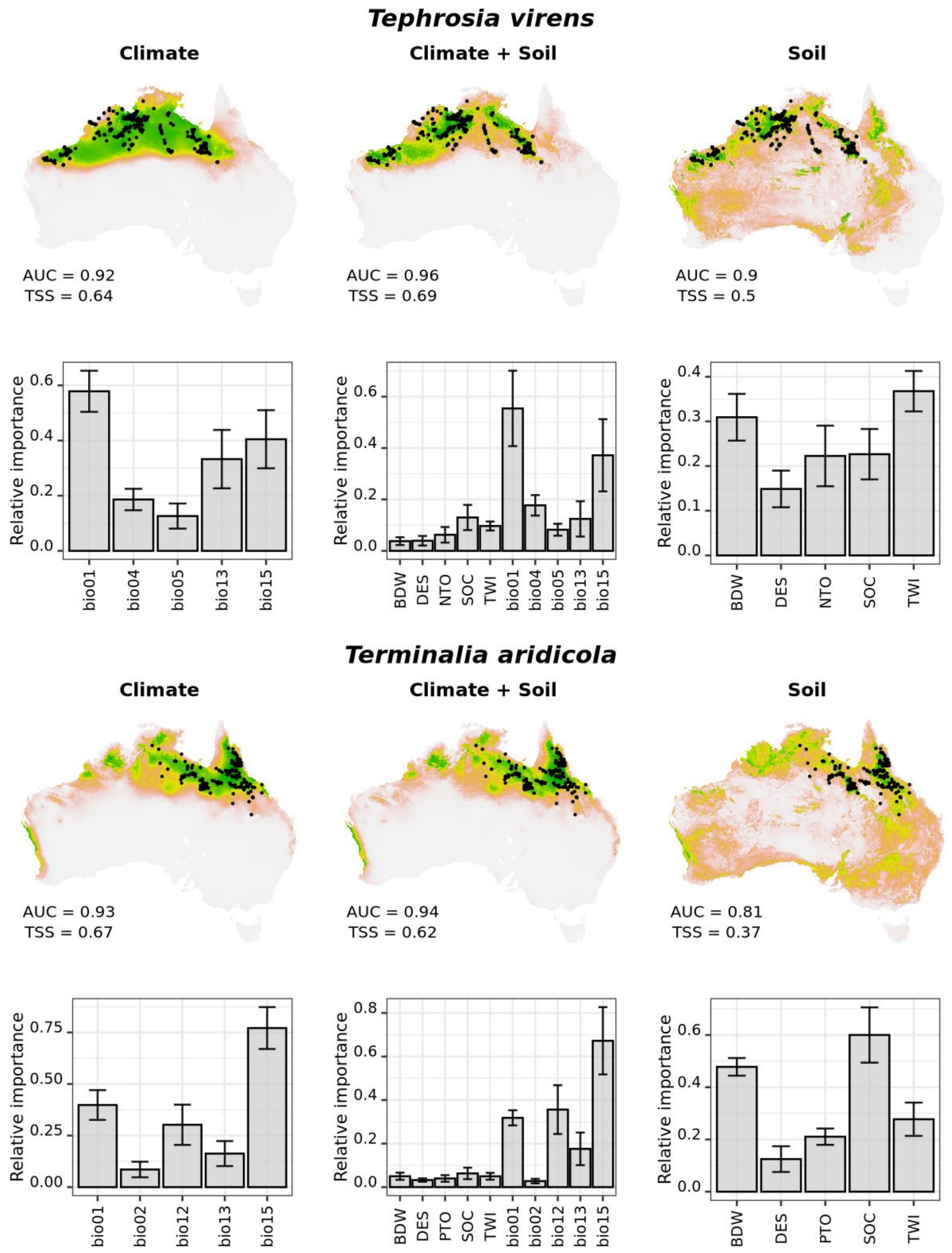
Tephrosia virens
Climate
Climate + Soil
Soil
AUC = 0.92
TSS = 0.64
AUC = 0.96
TSS = 0.69
AUC = 0.9
TSS = 0.5
Relative importance
bio01
bio04
bio05
bio13
bio15
BDW
DES
NTO
SOC
TWI
Terminalia aridicola
Climate
Climate + Soil
Soil
AUC = 0.93
TSS = 0.67
AUC = 0.94
TSS = 0.62
AUC = 0.81
TSS = 0.37
Relative importance
bio01
bio02
bio12
bio13
bio15
BDW
DES
PTO
SOC
TWI

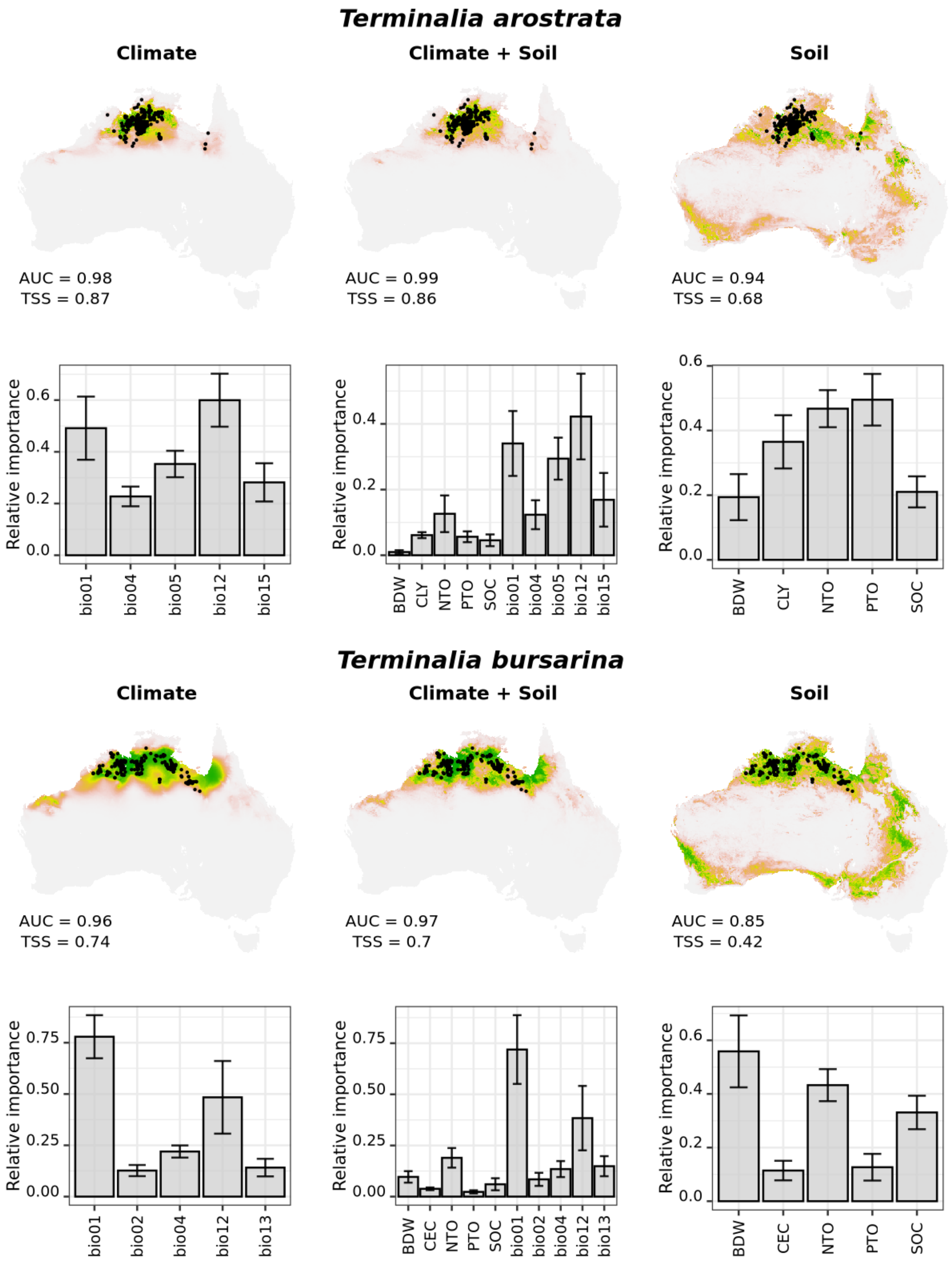

Terminalia arostrata
Climate
Climate + Soil
Soil
AUC = 0.98
TSS = 0.87
AUC = 0.99
TSS = 0.86
AUC = 0.94
TSS = 0.68
Relative importance
0.0
0.2
0.4
0.6
bio01
bio04
bio05
bio12
bio15
BDW
CLY
NTO
PTO
SOC
Terminalia bursarina
Climate
Climate + Soil
Soil
AUC = 0.96
TSS = 0.74
AUC = 0.97
TSS = 0.7
AUC = 0.85
TSS = 0.42
Relative importance
0.00
0.25
0.50
0.75
bio01
bio02
bio04
bio12
bio13
BDW
CEC
NTO
PTO
SOC

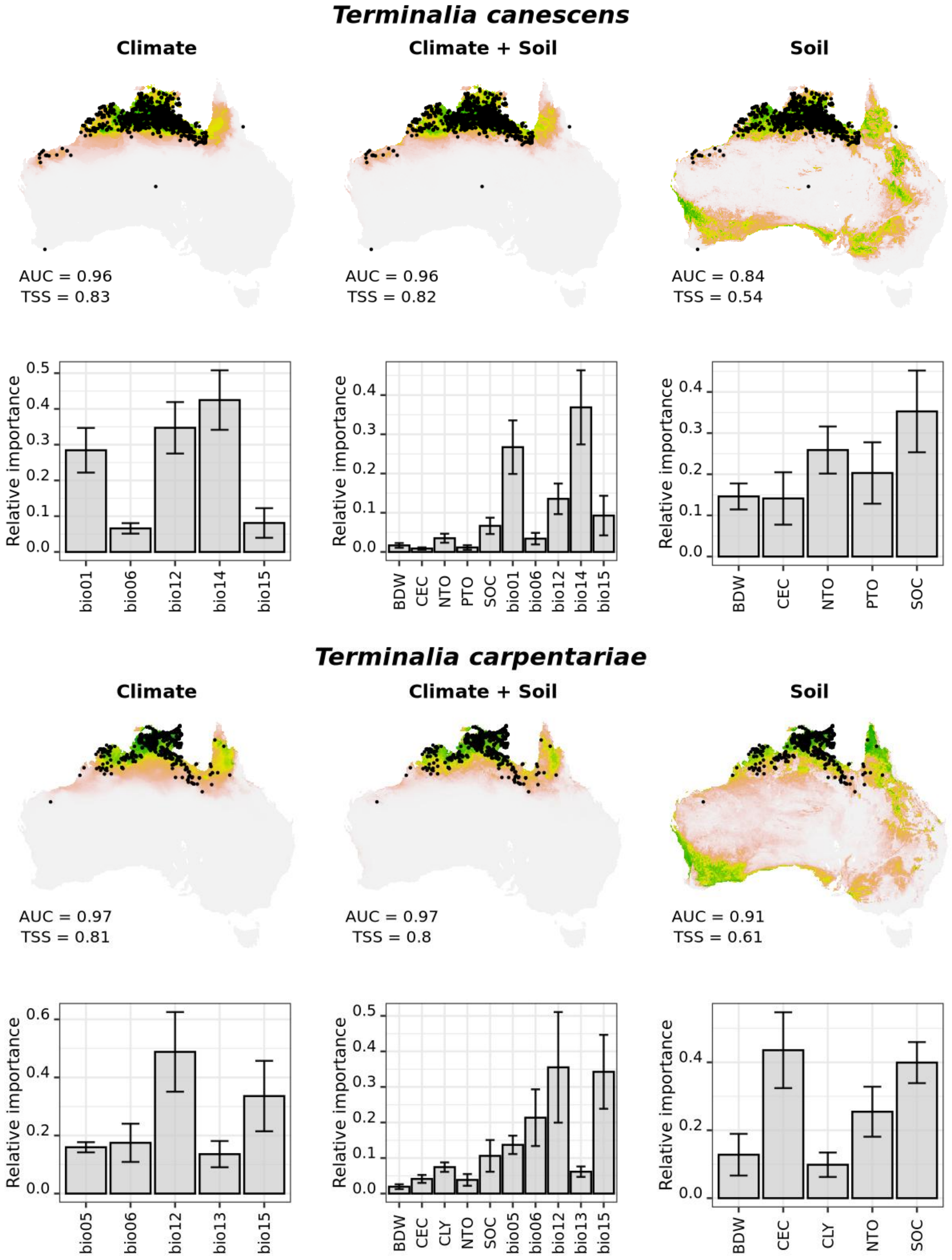
Terminalia canescens
Climate
Climate + Soil
Soil
AUC = 0.96
TSS = 0.83
AUC = 0.96
TSS = 0.82
AUC = 0.84
TSS = 0.54
Relative importance
bio01 bio06 bio12 bio14 bio15
BDW CEC NTO PTO SOC bio01 bio06 bio12 bio14 bio15
BDW CEC NTO PTO SOC
Terminalia carpentariae
Climate
Climate + Soil
Soil
AUC = 0.97
TSS = 0.81
AUC = 0.97
TSS = 0.8
AUC = 0.91
TSS = 0.61
Relative importance
bio05 bio06 bio12 bio13 bio15
BDW CEC CLY NTO SOC bio05 bio06 bio12 bio13 bio15
BDW CEC CLY NTO SOC

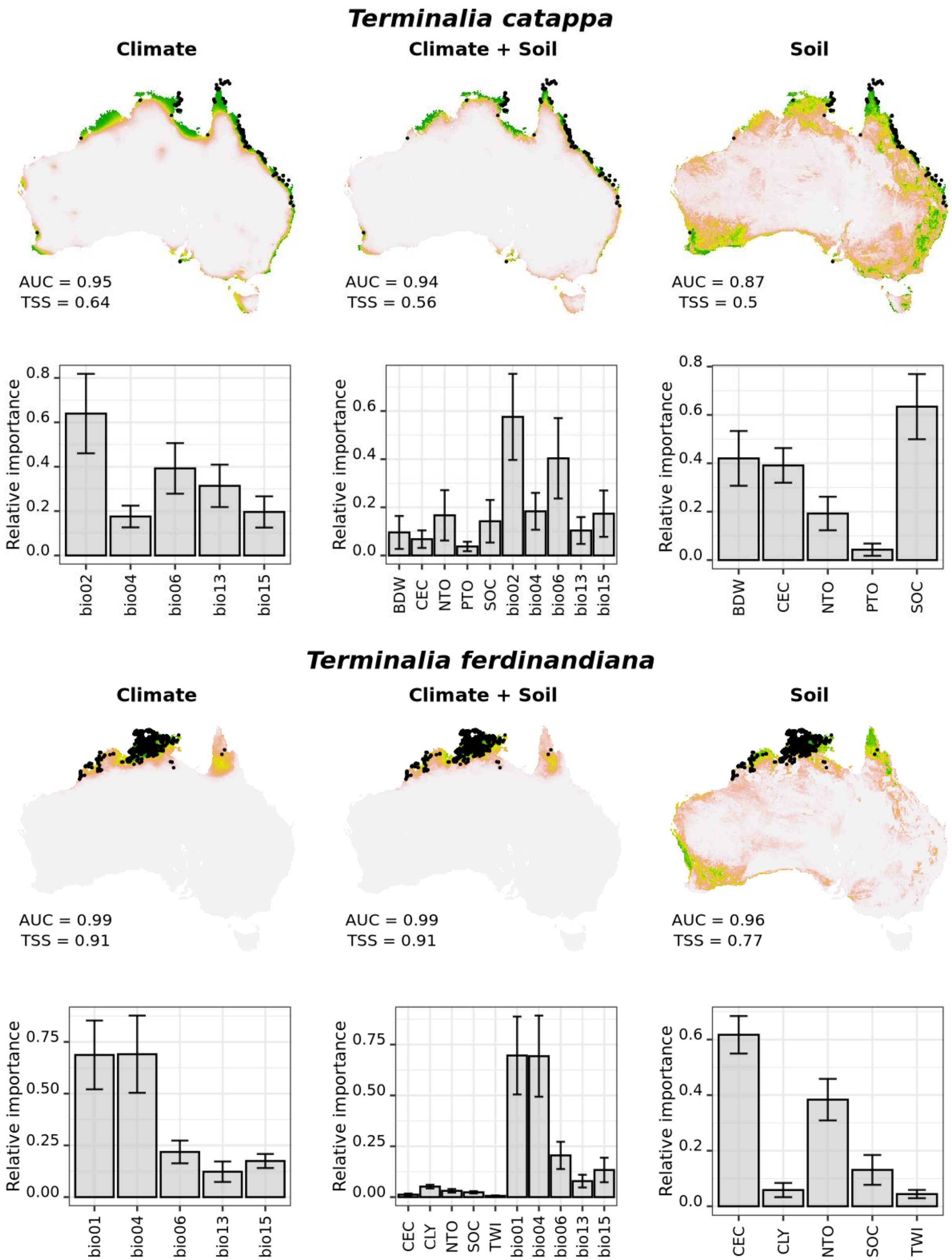

Terminalia catappa
Climate
Climate + Soil
Soil
AUC = 0.95
TSS = 0.64
AUC = 0.94
TSS = 0.56
AUC = 0.87
TSS = 0.5
Relative importance
bio02
bio04
bio06
bio13
bio15
BDW
CEC
NTO
PTO
SOC
Terminalia ferdinandiana
Climate
Climate + Soil
Soil
AUC = 0.99
TSS = 0.91
AUC = 0.99
TSS = 0.91
AUC = 0.96
TSS = 0.77
bio01
CLY
TWI

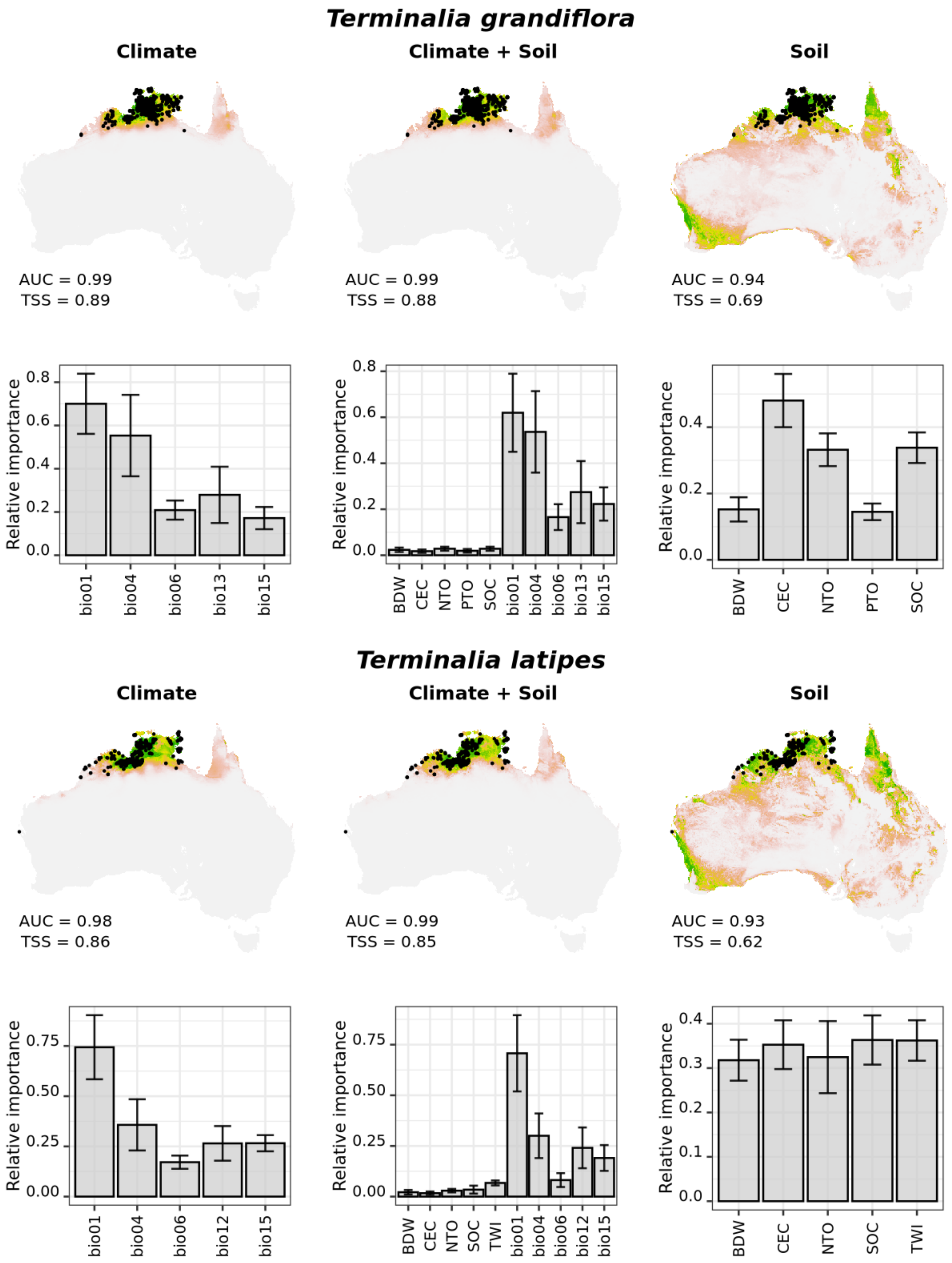

Terminalia grandiflora
Climate
Climate + Soil
Soil
AUC = 0.99
TSS = 0.89
AUC = 0.99
TSS = 0.88
AUC = 0.94
TSS = 0.69
Relative importance
bio01
bio04
bio06
bio13
bio15
BDW
CEC
NTO
PTO
SOC
Terminalia latipes
Climate
Climate + Soil
Soil
AUC = 0.98
TSS = 0.86
AUC = 0.99
TSS = 0.85
AUC = 0.93
TSS = 0.62
Relative importance
bio01
bio04
bio06
bio12
bio15
BDW
CEC
NTO
SOC
TWI

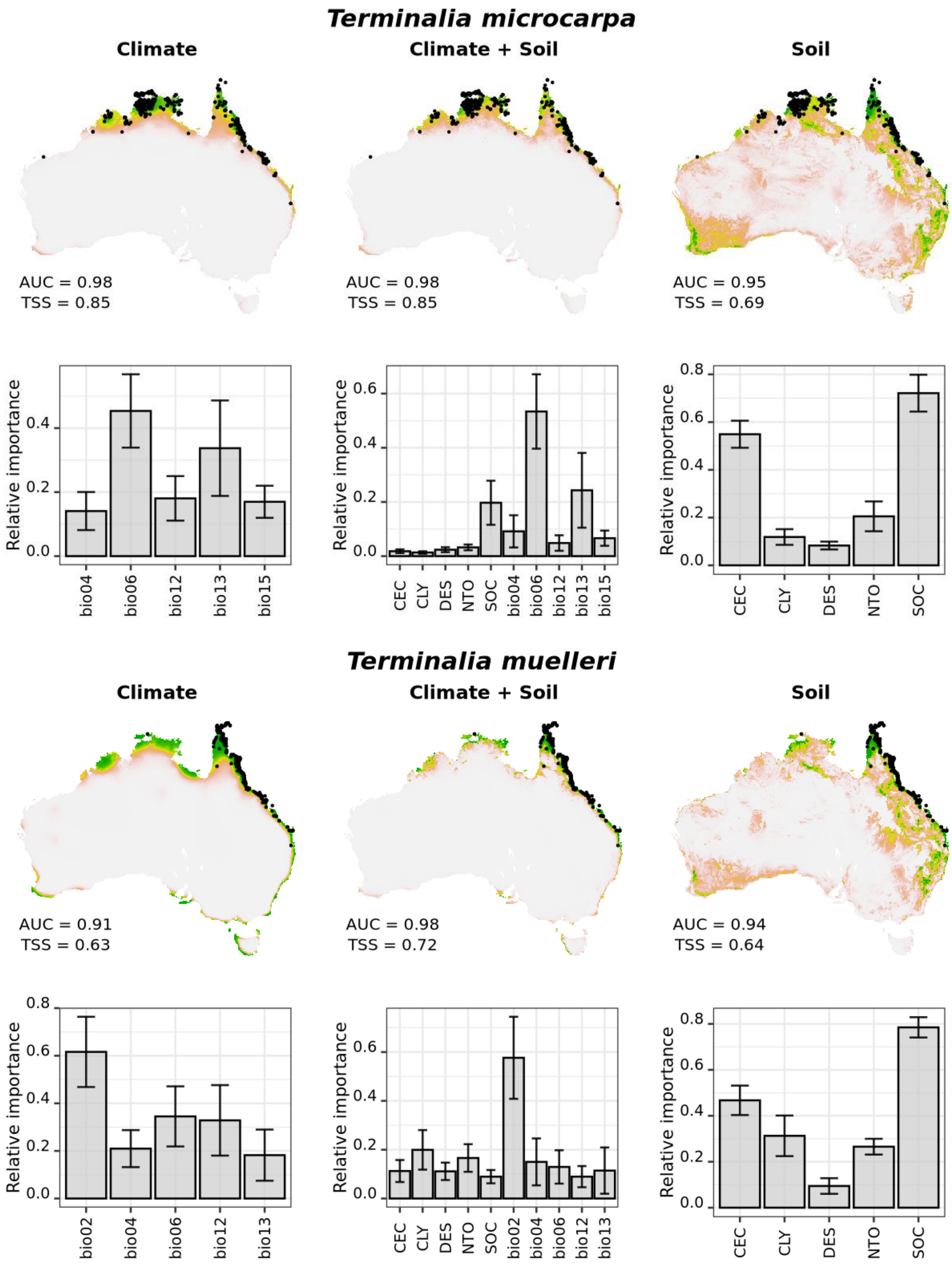

Terminalia microcarpa
Climate
Climate + Soil
Soil
AUC = 0.98
TSS = 0.85
AUC = 0.98
TSS = 0.85
AUC = 0.95
TSS = 0.69
Relative importance
bio04
bio06
bio12
bio13
bio15
CEC
CLY
DES
NTO
SOC
Terminalia muelleri
Climate
Climate + Soil
Soil
AUC = 0.91
TSS = 0.63
AUC = 0.98
TSS = 0.72
AUC = 0.94
TSS = 0.64
Relative importance
bio02
bio04
bio06
bio12
bio13
CEC
CLY
DES
NTO
SOC

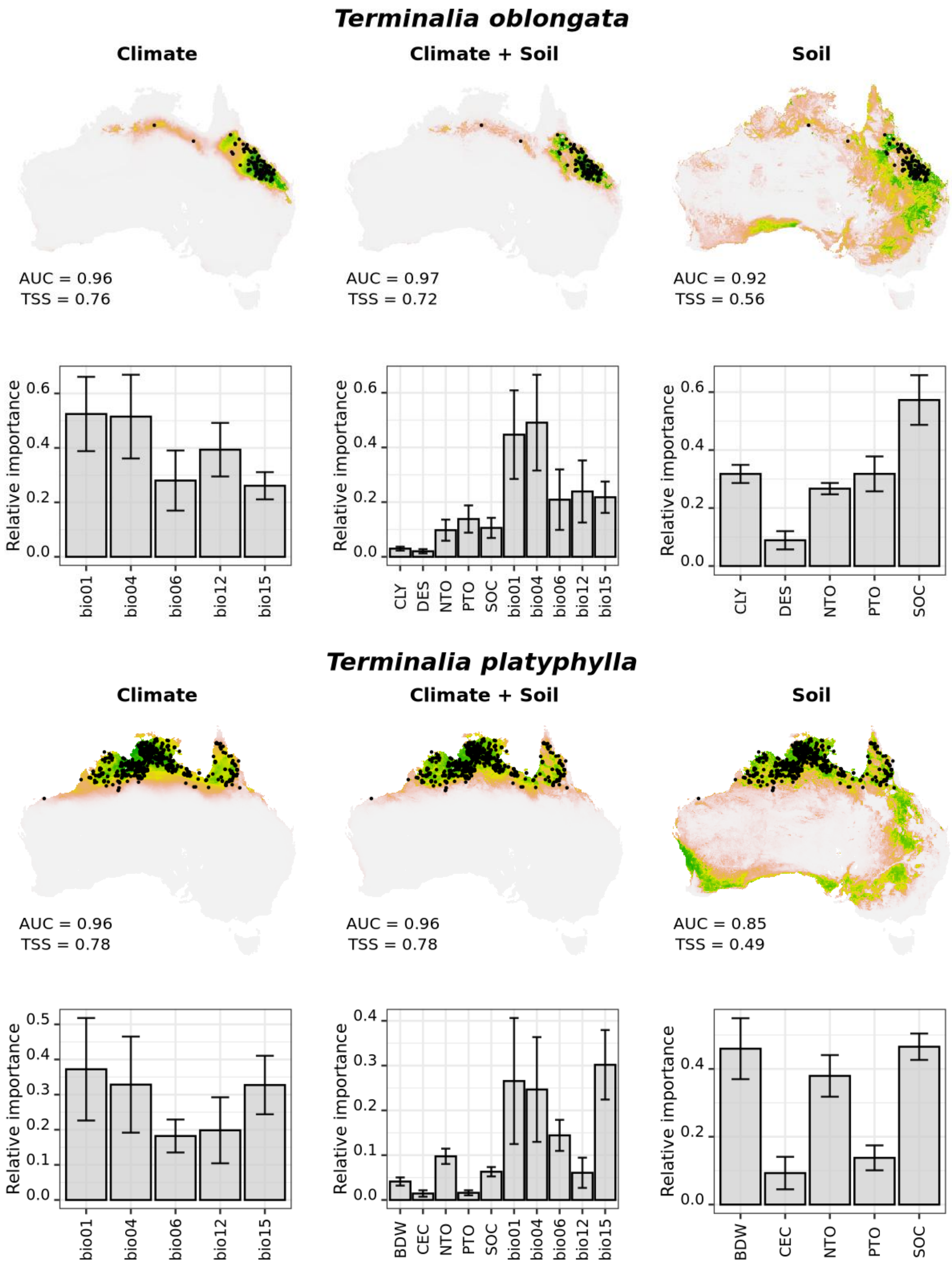

Terminalia oblongata
Climate
Climate + Soil
Soil
AUC = 0.96
TSS = 0.76
AUC = 0.97
TSS = 0.72
AUC = 0.92
TSS = 0.56
Relative importance
0.0
0.2
0.4
0.6
bio01
bio04
bio06
bio12
bio15
CLY
DES
NTO
PTO
SOC
Terminalia platyphylla
Climate
Climate + Soil
Soil
AUC = 0.96
TSS = 0.78
AUC = 0.96
TSS = 0.78
AUC = 0.85
TSS = 0.49
Relative importance
0.0
0.1
0.2
0.3
0.4
0.5
bio01
bio04
bio06
bio12
bio15
BDW
CEC
NTO
PTO
SOC

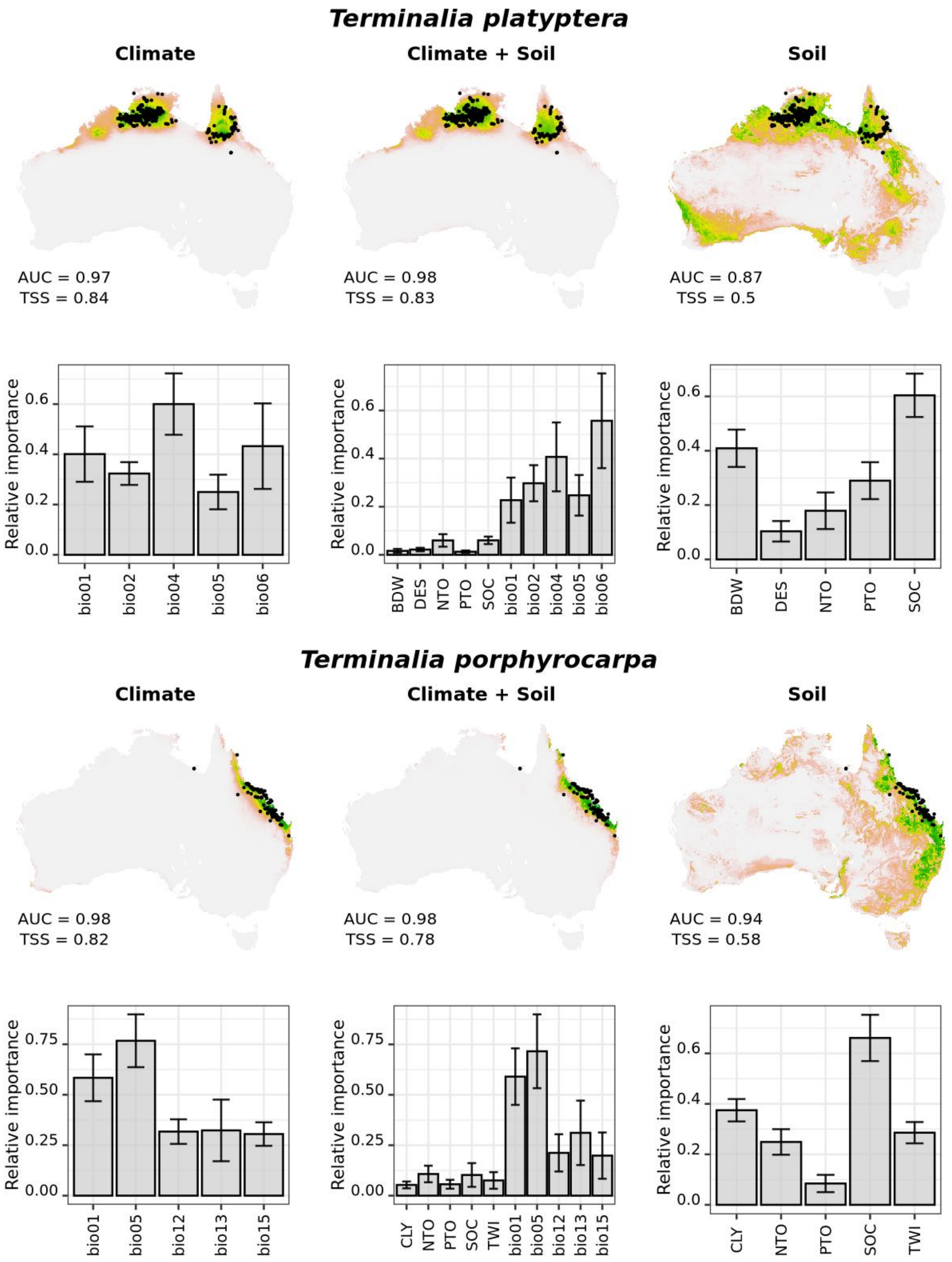
Terminalia platyptera
Climate
Climate + Soil
Soil
AUC = 0.97
TSS = 0.84
AUC = 0.98
TSS = 0.83
AUC = 0.87
TSS = 0.5
Relative importance
bio01
bio02
bio04
bio05
bio06
BDW
DES
NTO
PTO
SOC
Terminalia porphyrocarpa
Climate
Climate + Soil
Soil
AUC = 0.98
TSS = 0.82
AUC = 0.98
TSS = 0.78
AUC = 0.94
TSS = 0.58
Relative importance
bio01
bio05
bio12
bio13
bio15
CLY
NTO
PTO
SOC
TWI

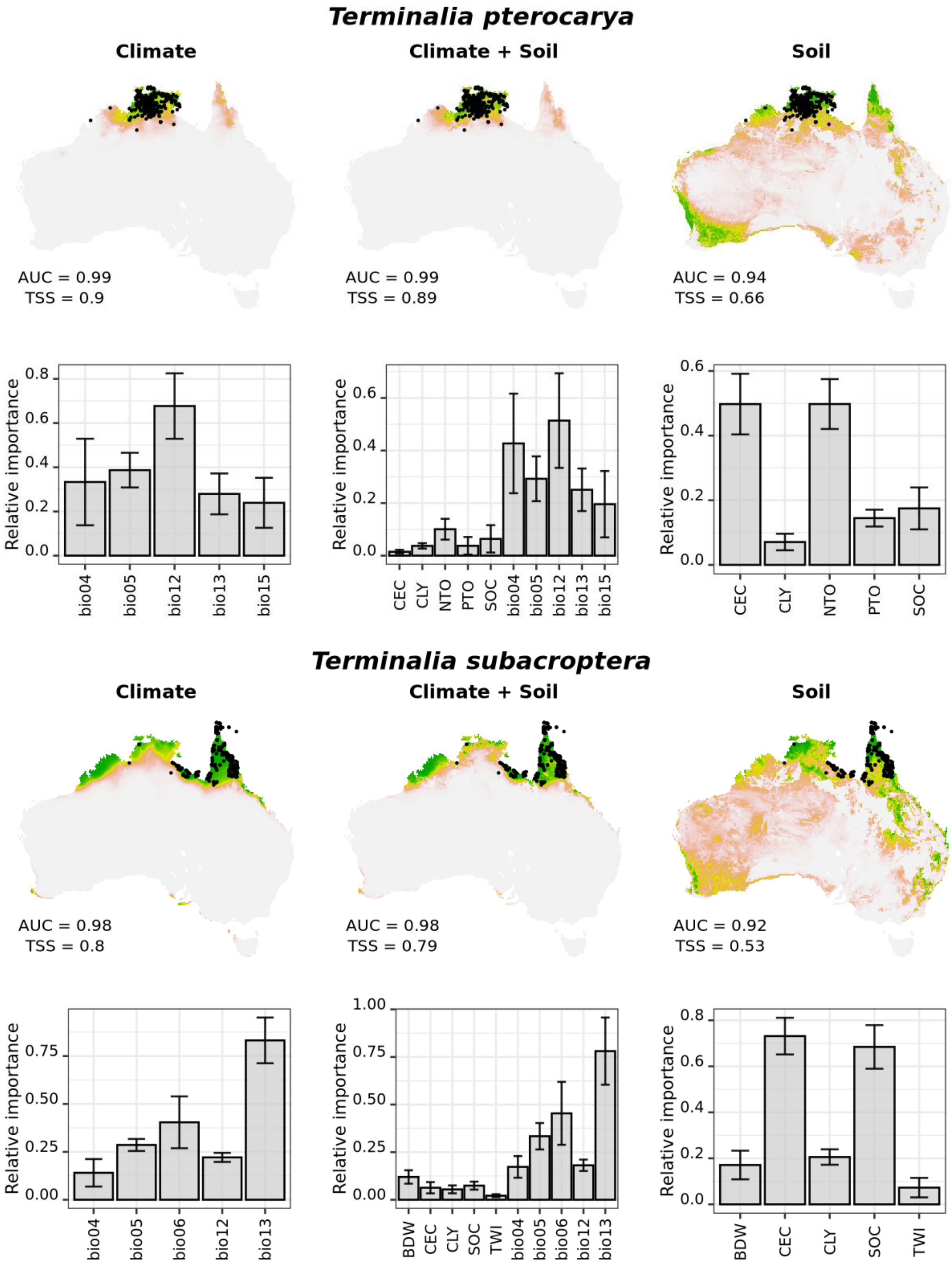

Terminalia pterocarya
Climate
Climate + Soil
Soil
AUC = 0.99
TSS = 0.9
AUC = 0.99
TSS = 0.89
AUC = 0.94
TSS = 0.66
Relative importance
bio04
bio05
bio12
bio13
bio15
CEC
CLY
NTO
PTO
SOC
Terminalia subacroptera
Climate
Climate + Soil
Soil
AUC = 0.98
TSS = 0.8
AUC = 0.98
TSS = 0.79
AUC = 0.92
TSS = 0.53
Relative importance
bio04
bio05
bio06
bio12
bio13
BDW
CEC
CLY
SOC
TWI

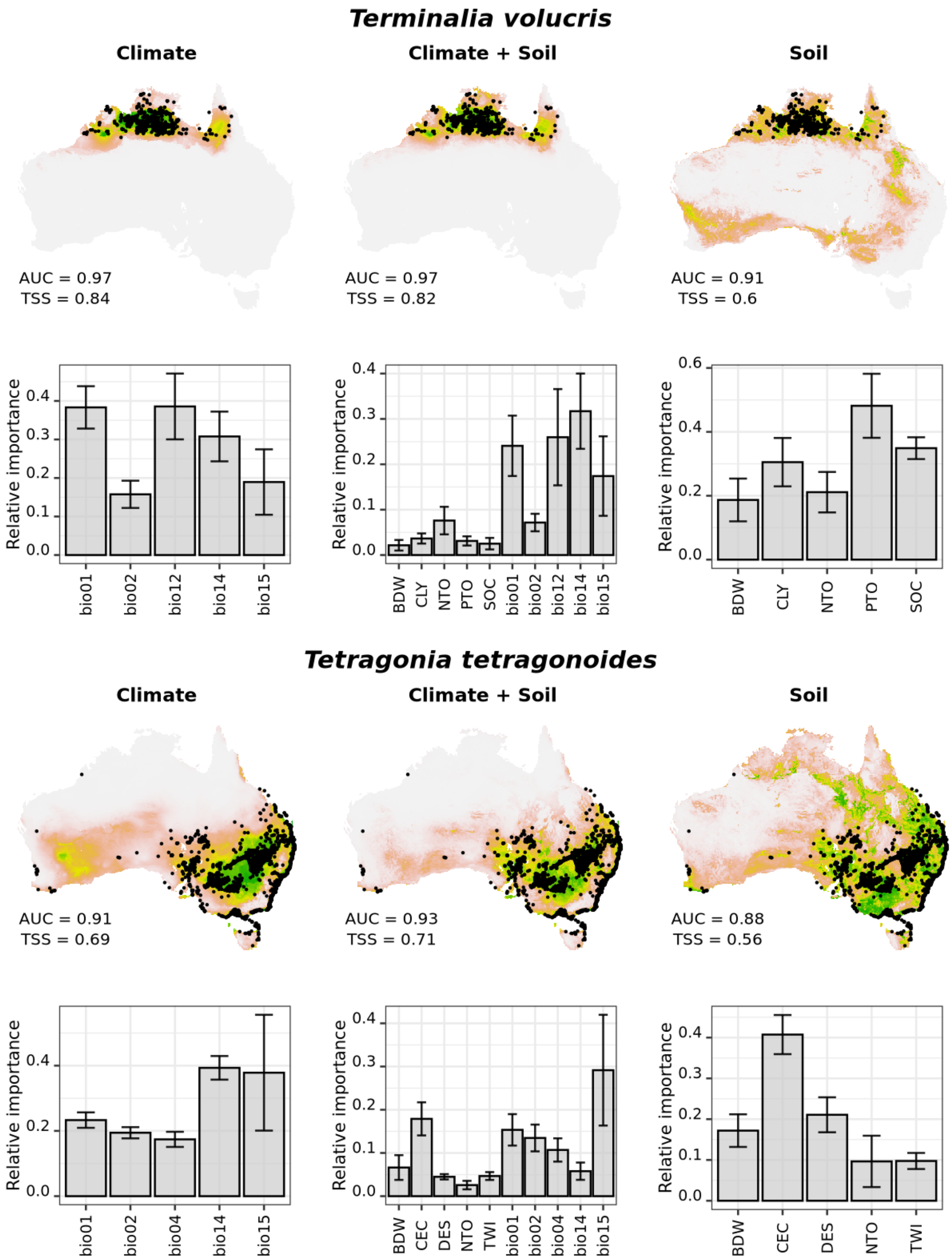
Terminalia volucris
Climate
Climate + Soil
Soil
AUC = 0.97
TSS = 0.84
AUC = 0.97
TSS = 0.82
AUC = 0.91
TSS = 0.6
Relative importance
bio01
bio02
bio12
bio14
bio15
BDW
CLY
NTO
PTO
SOC
Tetragonia tetragonoides
Climate
Climate + Soil
Soil
AUC = 0.91
TSS = 0.69
AUC = 0.93
TSS = 0.71
AUC = 0.88
TSS = 0.56
Relative importance
bio01
bio02
bio04
bio14
bio15
BDW
CEC
DES
NTO
TWI

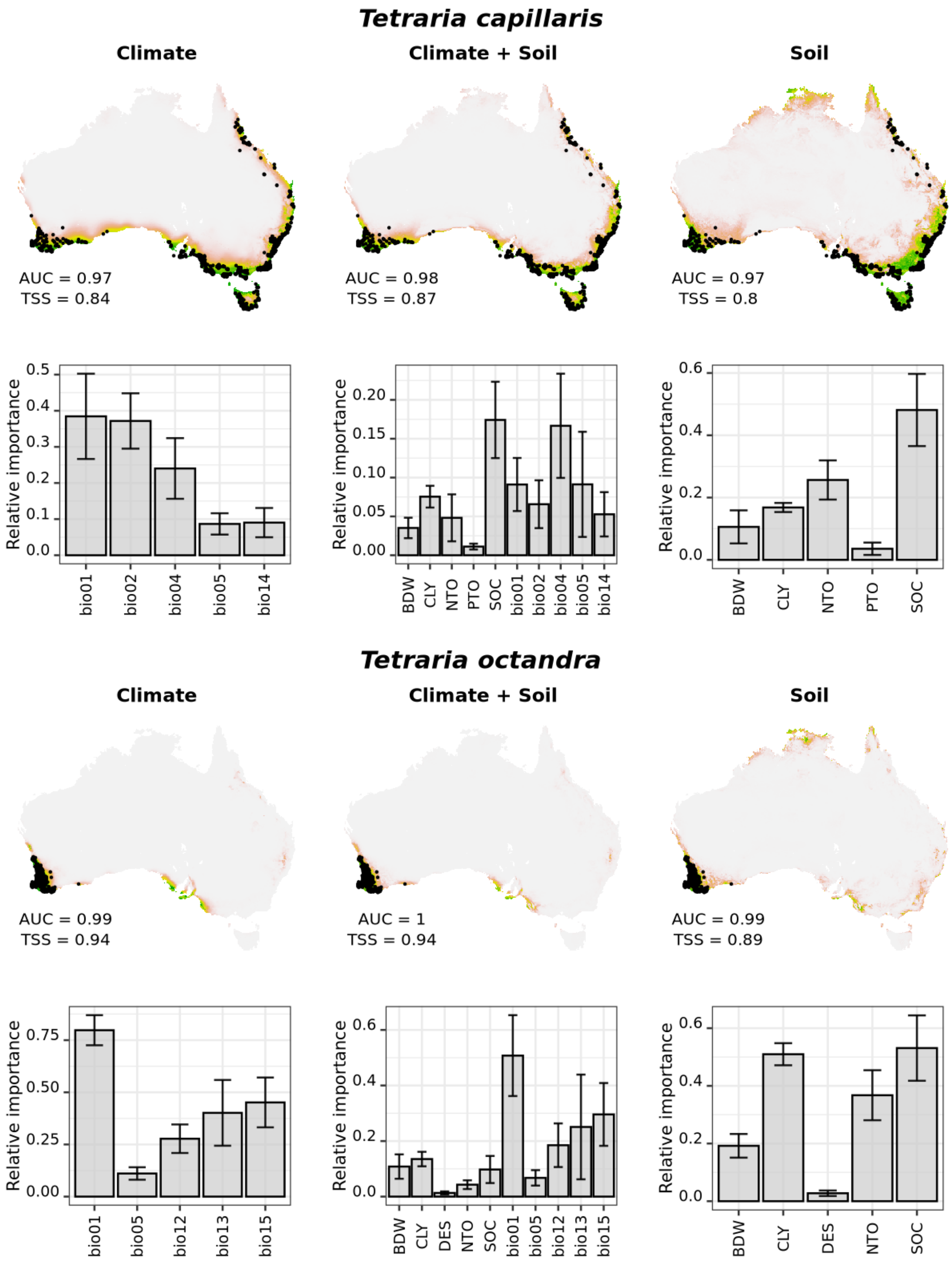
Tetraria capillaris
Climate
Climate + Soil
Soil
AUC = 0.97
TSS = 0.84
AUC = 0.98
TSS = 0.87
AUC = 0.97
TSS = 0.8
Relative importance
0.5
0.4
0.3
0.2
0.1
0.0
bio01
bio02
bio04
bio05
bio14
Relative importance
0.20
0.15
0.10
0.05
0.00
BDW
CLY
NTO
PTO
SOC
bio01
bio02
bio04
bio05
bio14
Relative importance
0.6
0.4
0.2
0.0
BDW
CLY
NTO
PTO
SOC
Tetraria octandra
Climate
Climate + Soil
Soil
AUC = 0.99
TSS = 0.94
AUC = 1
TSS = 0.94
AUC = 0.99
TSS = 0.89
Relative importance
0.75
0.50
0.25
0.00
bio01
bio05
bio12
bio13
bio15
Relative importance
0.6
0.4
0.2
0.0
BDW
CLY
DES
NTO
SOC
bio01
bio05
bio12
bio13
bio15
Relative importance
0.6
0.4
0.2
0.0
BDW
CLY
DES
NTO
SOC

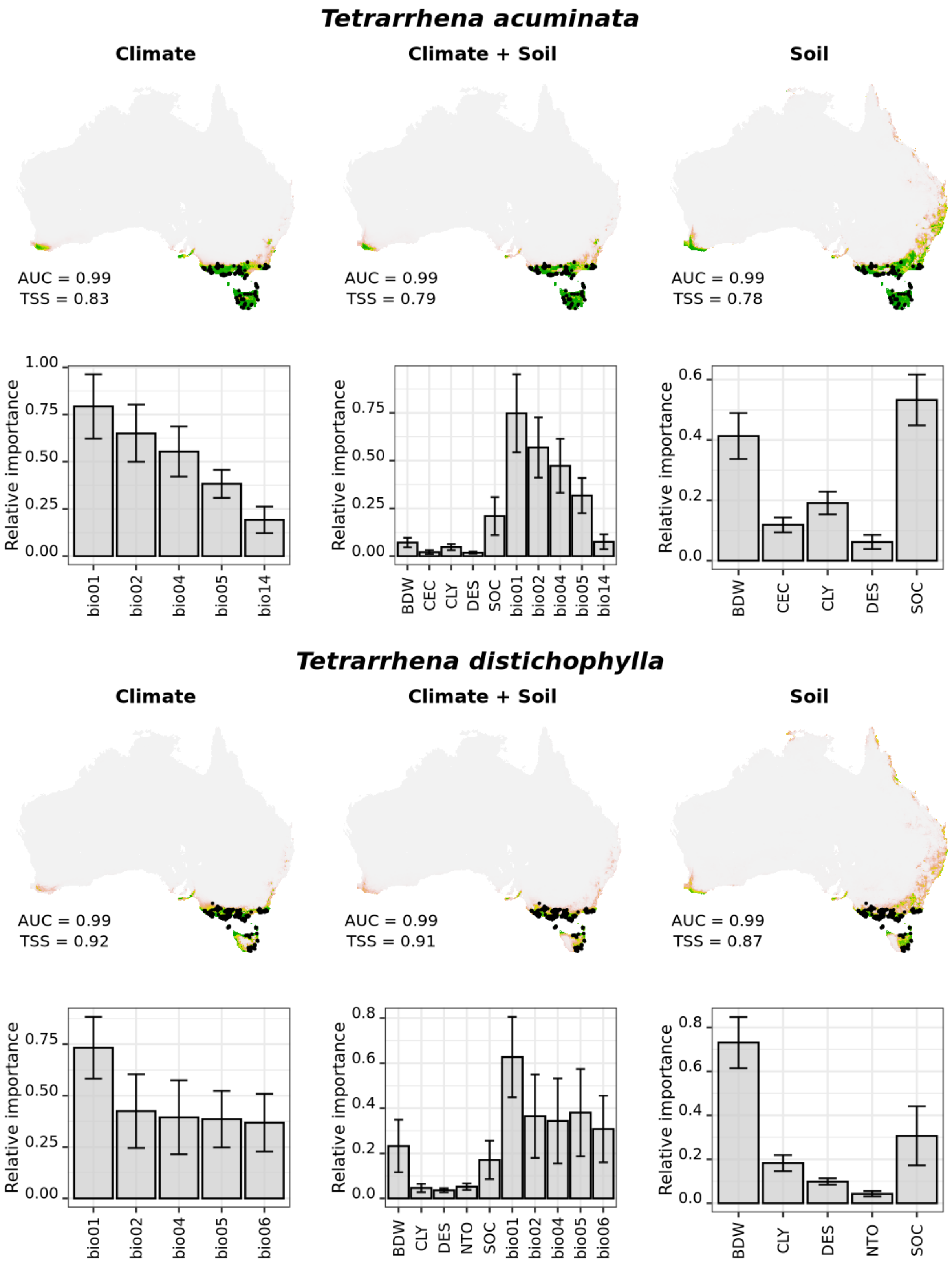
Tetrarrhena acuminata
Climate
Climate + Soil
Soil
AUC = 0.99
TSS = 0.83
AUC = 0.99
TSS = 0.79
AUC = 0.99
TSS = 0.78
Relative importance
1.00
0.75
0.50
0.25
0.00
bio01
bio02
bio04
bio05
bio14
BDW
CEC
CLY
DES
SOC
0.6
0.4
0.2
0.0
Tetrarrhena distichophylla
Climate
Climate + Soil
Soil
AUC = 0.99
TSS = 0.92
AUC = 0.99
TSS = 0.91
AUC = 0.99
TSS = 0.87
0.8
NTO
bio06

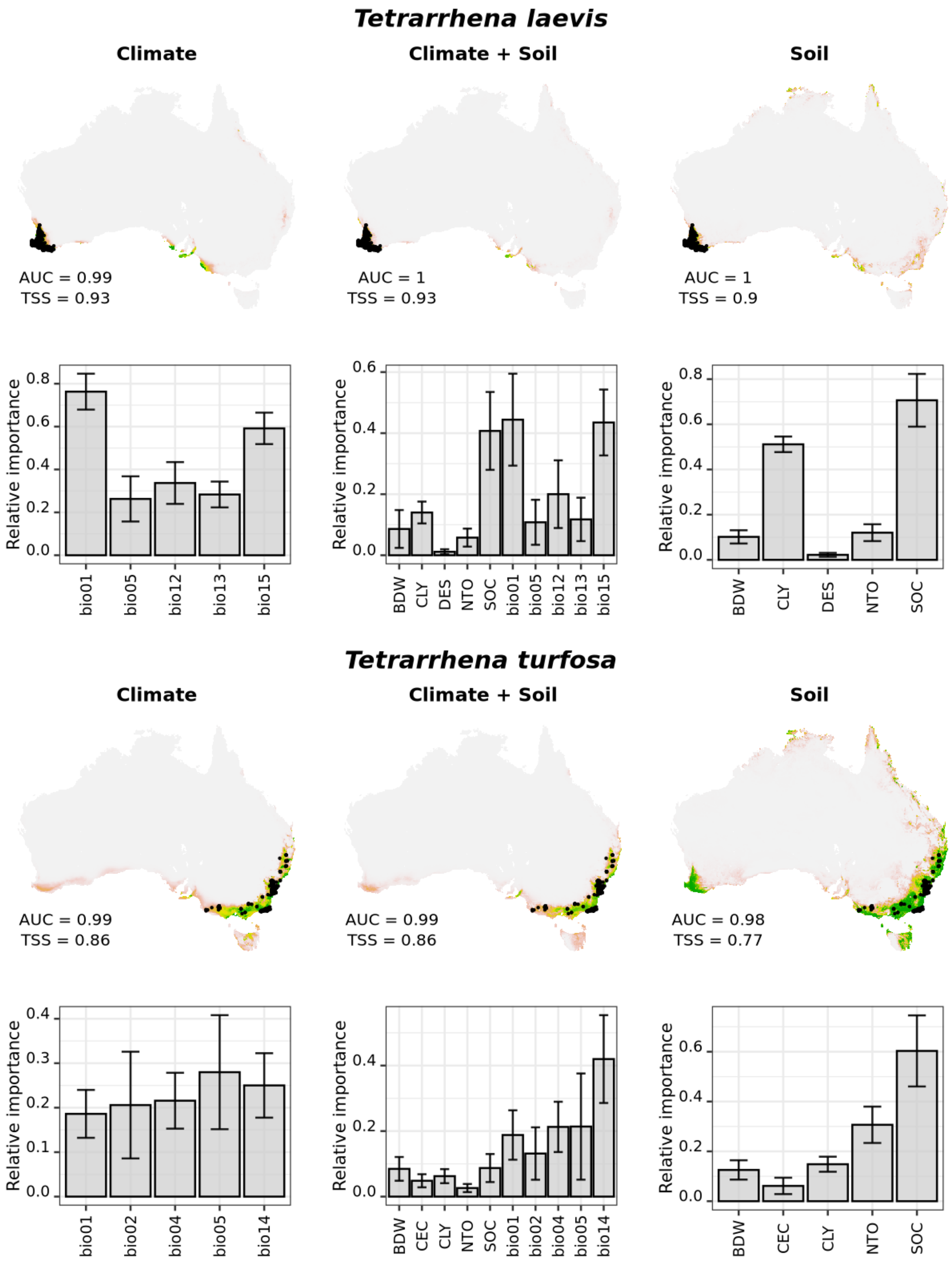
Tetrarrhena laevis
Climate
Climate + Soil
Soil
AUC = 0.99
TSS = 0.93
AUC = 1
TSS = 0.93
AUC = 1
TSS = 0.9
Relative importance
bio01
bio05
bio12
bio13
bio15
BDW
CLY
DES
NTO
SOC
Tetrarrhena turfosa
Climate
Climate + Soil
Soil
AUC = 0.99
TSS = 0.86
AUC = 0.99
TSS = 0.86
AUC = 0.98
TSS = 0.77
Relative importance
bio01
bio02
bio04
bio05
bio14
BDW
CEC
CLY
NTO
SOC

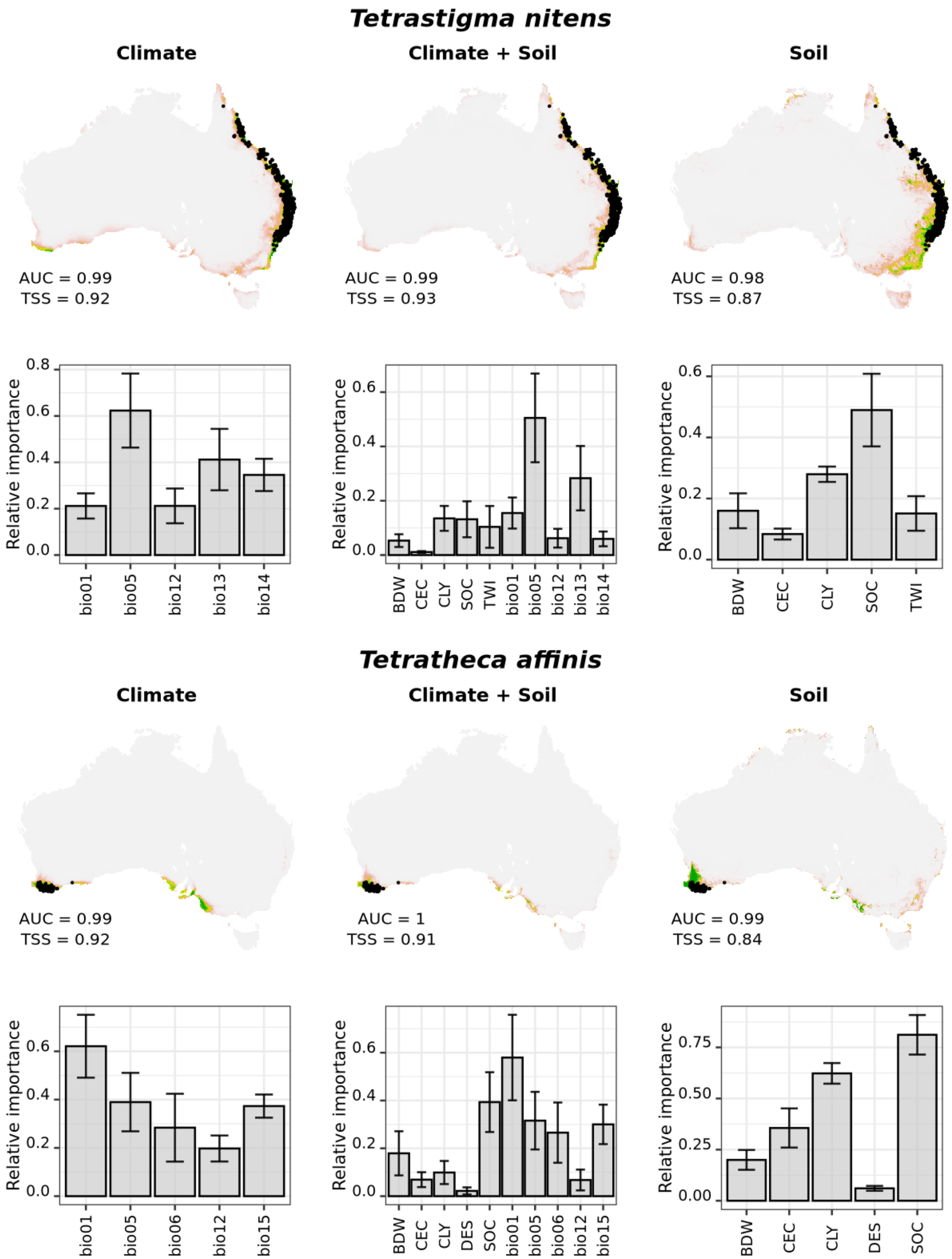
Tetrastigma nitens
Climate
Climate + Soil
Soil
AUC = 0.99
TSS = 0.92
AUC = 0.99
TSS = 0.93
AUC = 0.98
TSS = 0.87
Relative importance
0.0
0.2
0.4
0.6
0.8
bio01
bio05
bio12
bio13
bio14
BDW
CEC
CLY
SOC
TWI
bio01
bio05
bio12
bio13
bio14
BDW
CEC
CLY
SOC
TWI
Tetratheca affinis
Climate
Climate + Soil
Soil
AUC = 0.99
TSS = 0.92
AUC = 1
TSS = 0.91
AUC = 0.99
TSS = 0.84
Relative importance
0.00
0.25
0.50
0.75
bio01
bio05
bio06
bio12
bio15
BDW
CEC
CLY
DES
SOC
bio01
bio05
bio06
bio12
bio15
BDW
CEC
CLY
DES
SOC

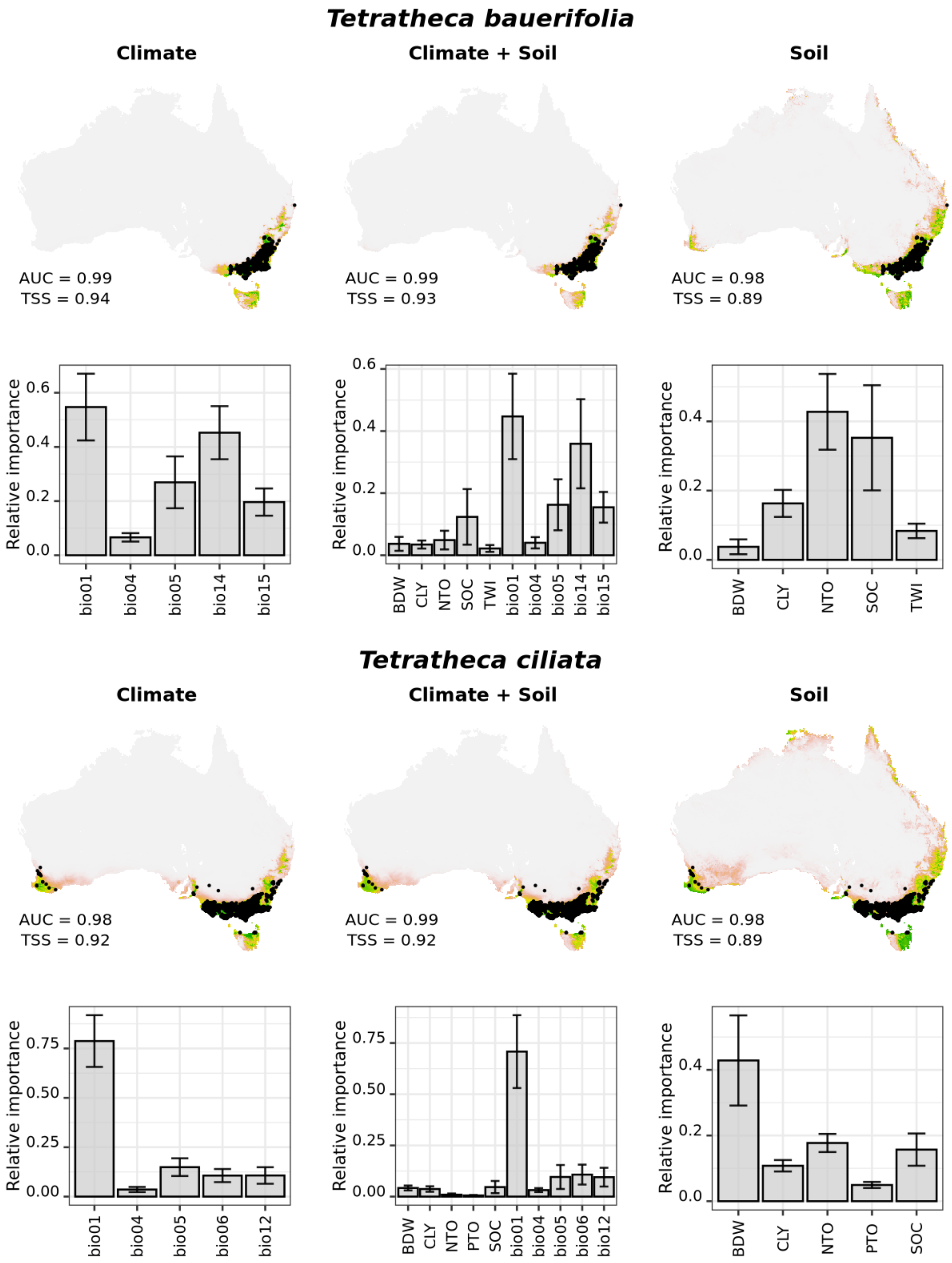
Tetratheca bauerifolia
Climate
Climate + Soil
Soil
AUC = 0.99
TSS = 0.94
AUC = 0.99
TSS = 0.93
AUC = 0.98
TSS = 0.89
Relative importance
0.0
0.2
0.4
0.6
bio01
bio04
bio05
bio14
bio15
BDW
CLY
NTO
SOC
TWI
Tetratheca ciliata
Climate
Climate + Soil
Soil
AUC = 0.98
TSS = 0.92
AUC = 0.99
TSS = 0.92
AUC = 0.98
TSS = 0.89
Relative importance
0.00
0.25
0.50
0.75
bio01
bio04
bio05
bio06
bio12
BDW
CLY
NTO
PTO
SOC

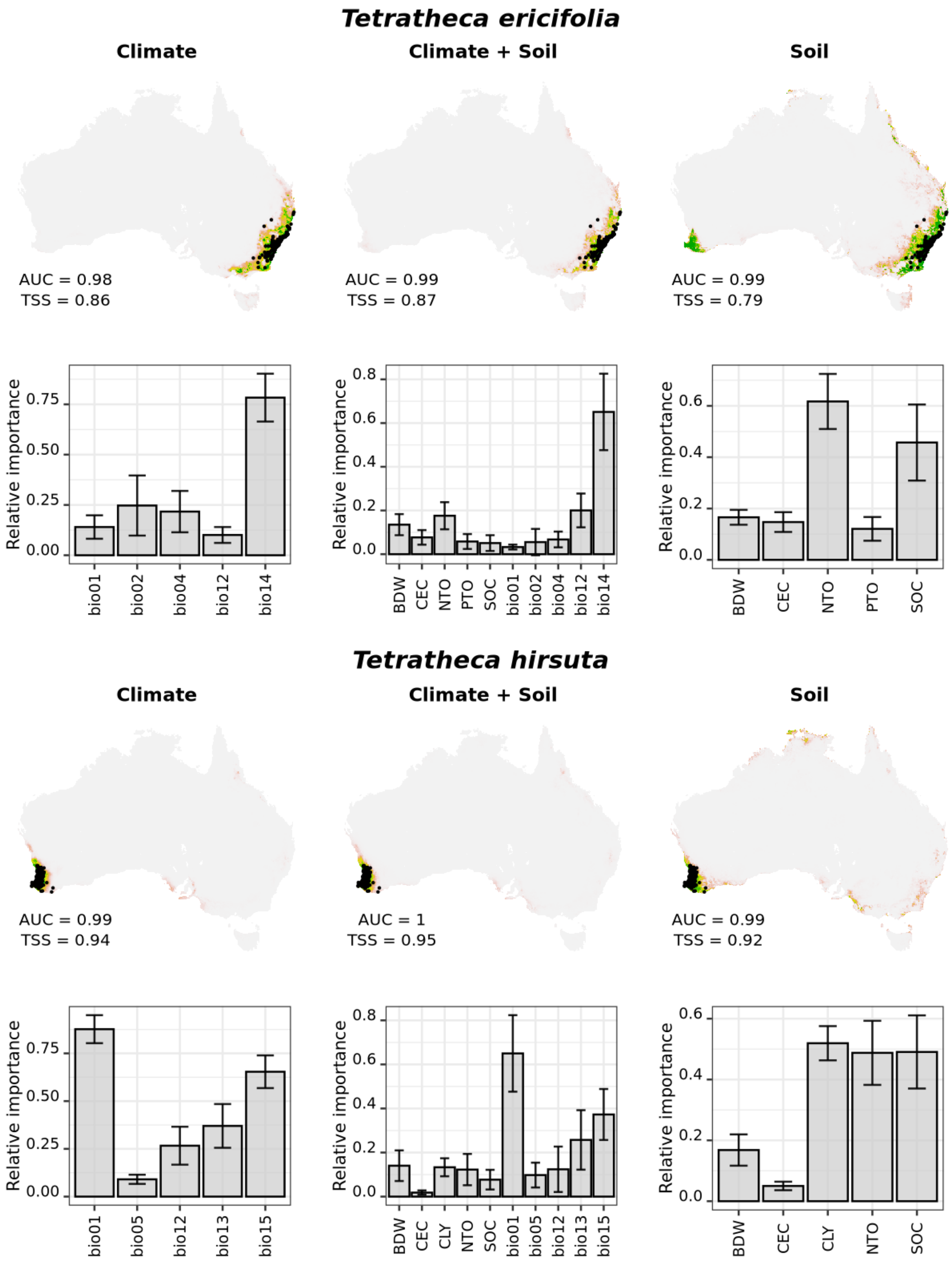

Tetratheca ericifolia
Climate
Climate + Soil
Soil
AUC = 0.98
TSS = 0.86
AUC = 0.99
TSS = 0.87
AUC = 0.99
TSS = 0.79
Relative importance
0.00
0.25
0.50
0.75
bio01
bio02
bio04
bio12
bio14
0.0
0.2
0.4
0.6
0.8
BDW
CEC
NTO
PTO
SOC
bio01
bio02
bio04
bio12
bio14
0.0
0.2
0.4
0.6
BDW
CEC
NTO
PTO
SOC
Tetratheca hirsuta
Climate
Climate + Soil
Soil
AUC = 0.99
TSS = 0.94
AUC = 1
TSS = 0.95
AUC = 0.99
TSS = 0.92
Relative importance
0.00
0.25
0.50
0.75
bio01
bio05
bio12
bio13
bio15
0.0
0.2
0.4
0.6
0.8
BDW
CEC
CLY
NTO
SOC
bio01
bio05
bio12
bio13
bio15
0.0
0.2
0.4
0.6
BDW
CEC
CLY
NTO
SOC

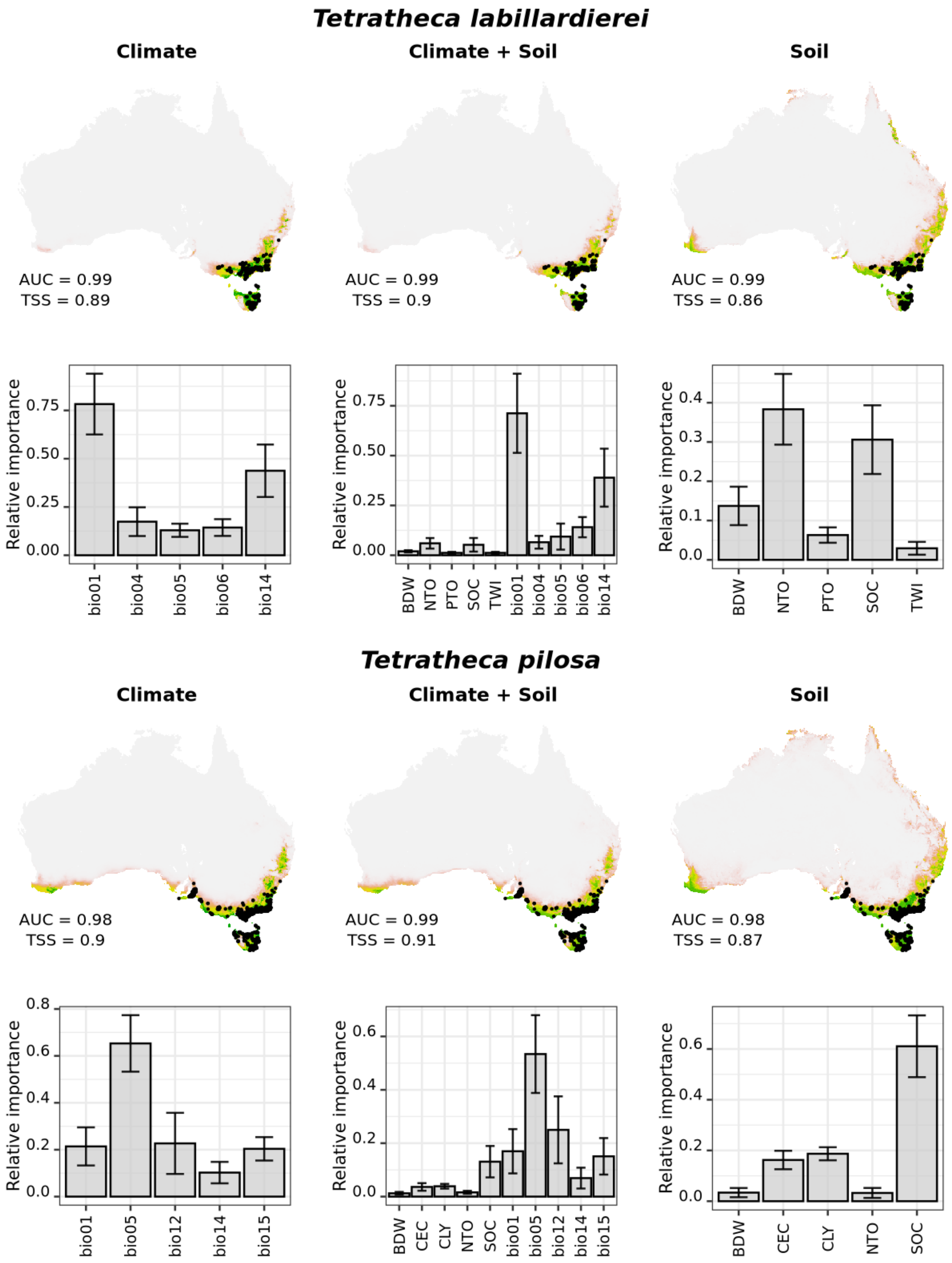

Tetratheca labillardierei
Climate
Climate + Soil
Soil
AUC = 0.99
TSS = 0.89
AUC = 0.99
TSS = 0.9
AUC = 0.99
TSS = 0.86
Relative importance
bio01 bio04 bio05 bio06 bio14
BDW NTO PTO SOC TWI bio01 bio04 bio05 bio06 bio14
BDW NTO PTO SOC TWI
Tetratheca pilosa
Climate
Climate + Soil
Soil
AUC = 0.98
TSS = 0.9
AUC = 0.99
TSS = 0.91
AUC = 0.98
TSS = 0.87
Relative importance
bio01 bio05 bio12 bio14 bio15
BDW CEC CLY NTO SOC bio01 bio05 bio12 bio14 bio15
BDW CEC CLY NTO SOC

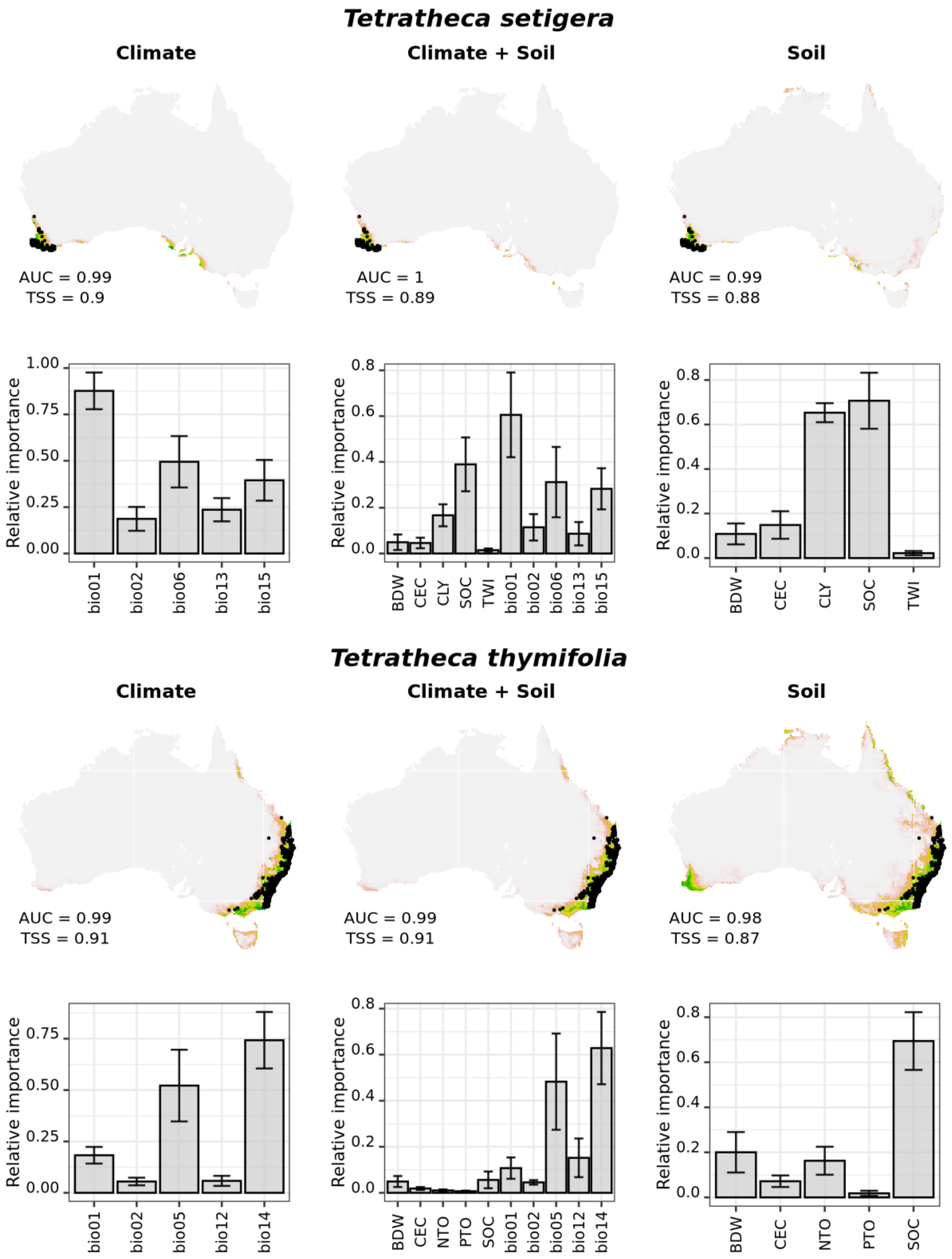

Tetratheca setigera
Climate
Climate + Soil
Soil
AUC = 0.99
TSS = 0.9
AUC = 1
TSS = 0.89
AUC = 0.99
TSS = 0.88
Relative importance
bio01
bio02
bio06
bio13
bio15
BDW
CEC
CLY
SOC
TWI
Tetratheca thymifolia
AUC = 0.99
TSS = 0.91
AUC = 0.99
TSS = 0.91
AUC = 0.98
TSS = 0.87
bio05
bio12
bio14
NTO
PTO

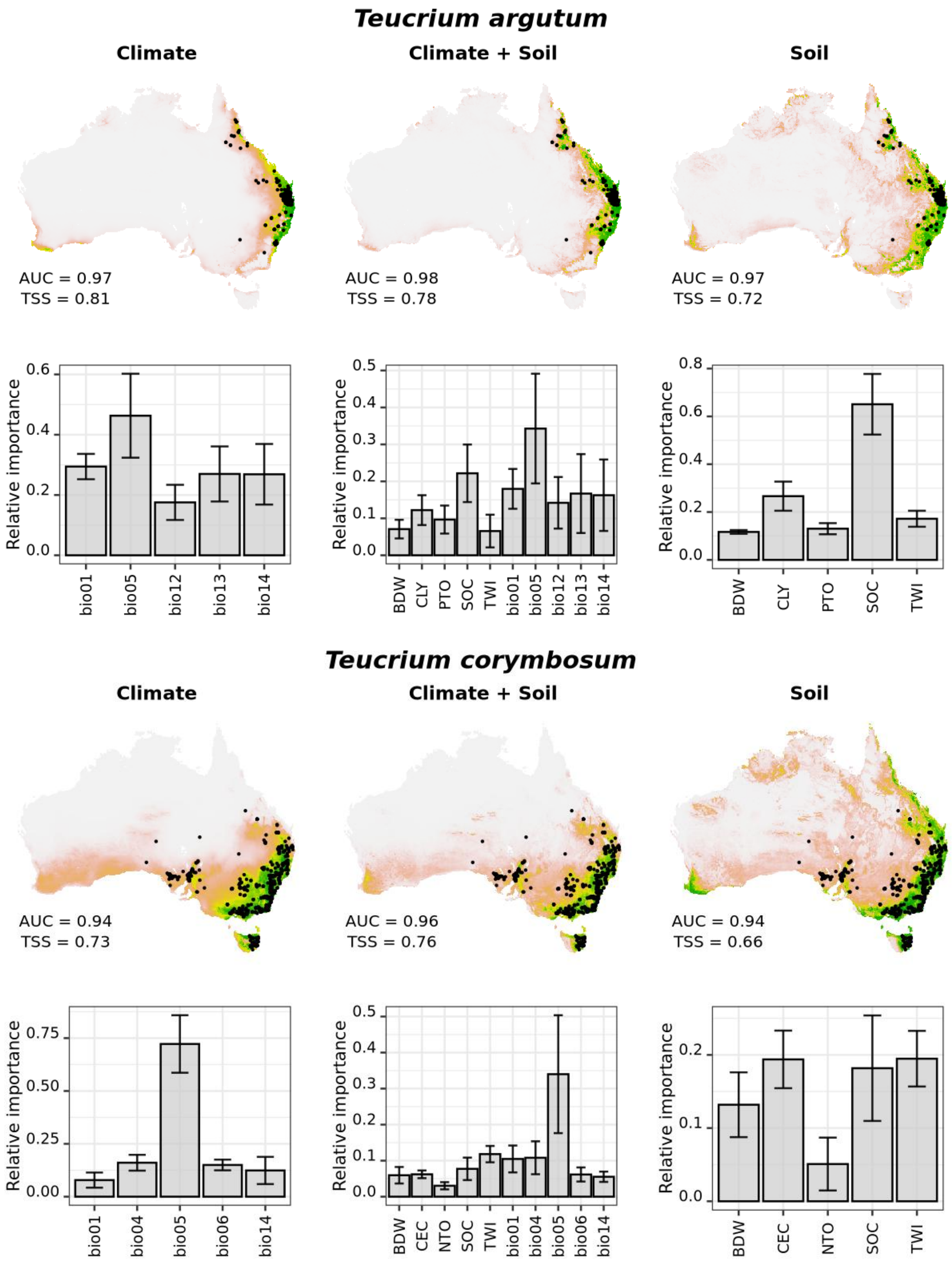
Teucrium argutum
Climate
Climate + Soil
Soil
AUC = 0.97
TSS = 0.81
AUC = 0.98
TSS = 0.78
AUC = 0.97
TSS = 0.72
Relative importance
bio01
bio05
bio12
bio13
bio14
BDW
CLY
PTO
SOC
TWI
Teucrium corymbosum
Climate
Climate + Soil
Soil
AUC = 0.94
TSS = 0.73
AUC = 0.96
TSS = 0.76
AUC = 0.94
TSS = 0.66
Relative importance
bio01
bio04
bio05
bio06
bio14
BDW
CEC
NTO
SOC
TWI

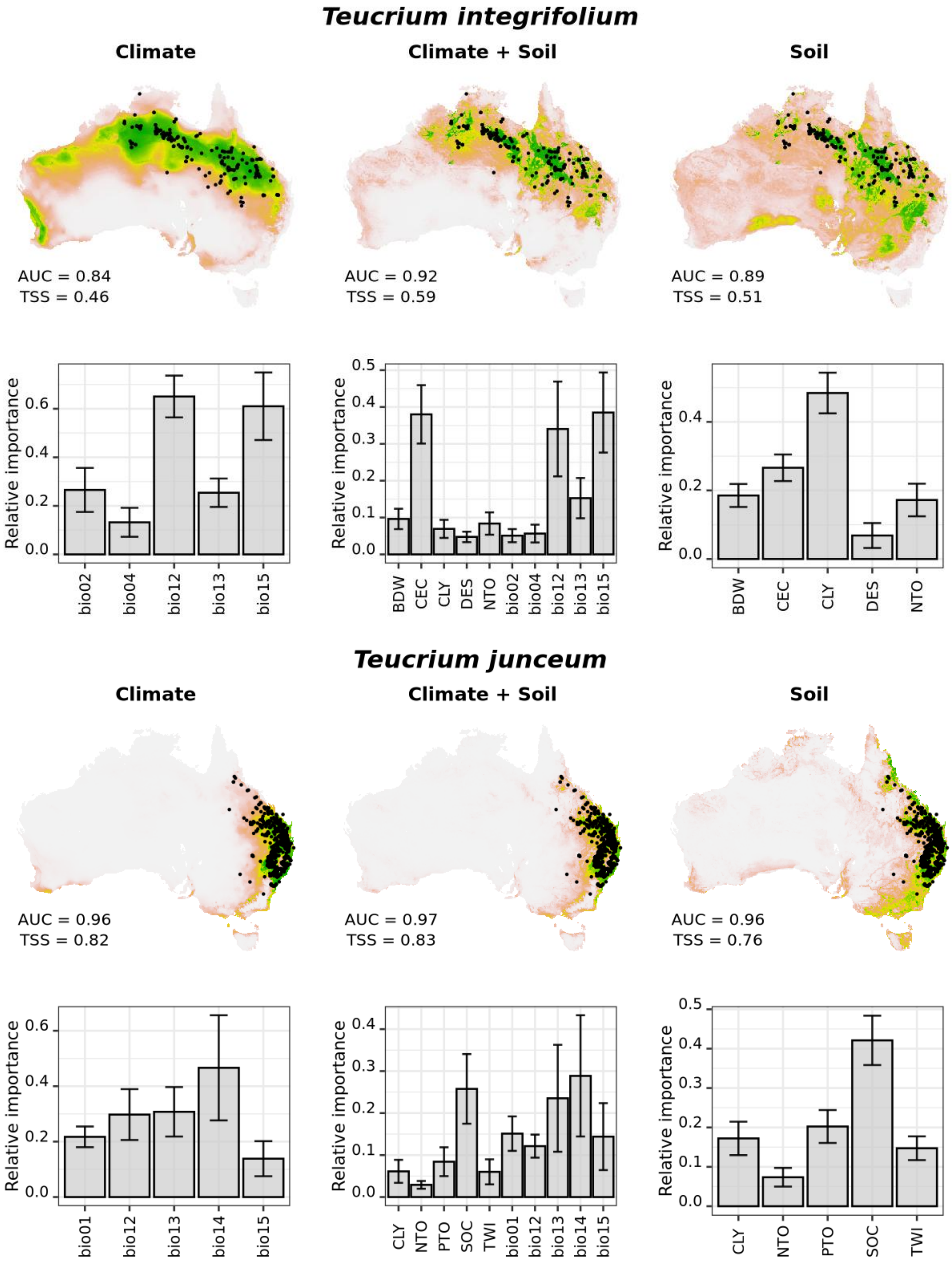

Teucrium integrifolium
Climate
Climate + Soil
Soil
AUC = 0.84
TSS = 0.46
AUC = 0.92
TSS = 0.59
AUC = 0.89
TSS = 0.51
Relative importance
bio02
bio04
bio12
bio13
bio15
BDW
CEC
CLY
DES
NTO
Teucrium junceum
Climate
Climate + Soil
Soil
AUC = 0.96
TSS = 0.82
AUC = 0.97
TSS = 0.83
AUC = 0.96
TSS = 0.76
Relative importance
bio01
bio12
bio13
bio14
bio15
CLY
NTO
PTO
SOC
TWI

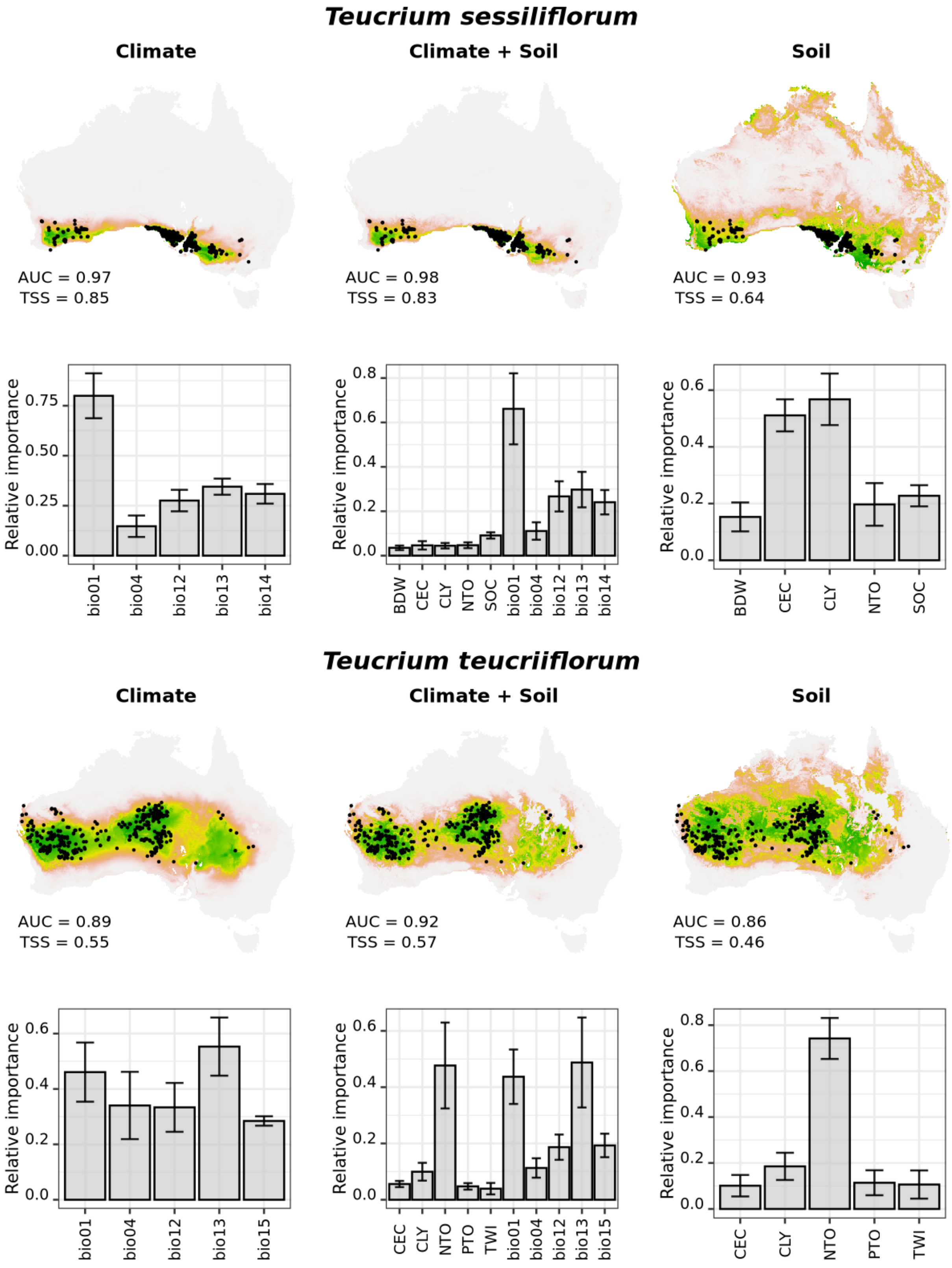

Teucrium sessiliflorum
Climate
Climate + Soil
Soil
AUC = 0.97
TSS = 0.85
AUC = 0.98
TSS = 0.83
AUC = 0.93
TSS = 0.64
Relative importance
0.00
0.25
0.50
0.75
bio01
bio04
bio12
bio13
bio14
Relative importance
0.0
0.2
0.4
0.6
0.8
BDW
CEC
CLY
NTO
SOC
bio01
bio04
bio12
bio13
bio14
Relative importance
0.0
0.2
0.4
0.6
BDW
CEC
CLY
NTO
SOC
Teucrium teucriiflorum
Climate
Climate + Soil
Soil
AUC = 0.89
TSS = 0.55
AUC = 0.92
TSS = 0.57
AUC = 0.86
TSS = 0.46
Relative importance
0.0
0.2
0.4
0.6
bio01
bio04
bio12
bio13
bio15
Relative importance
0.0
0.2
0.4
0.6
CEC
CLY
NTO
PTO
TWI
bio01
bio04
bio12
bio13
bio15
Relative importance
0.0
0.2
0.4
0.6
0.8
CEC
CLY
NTO
PTO
TWI

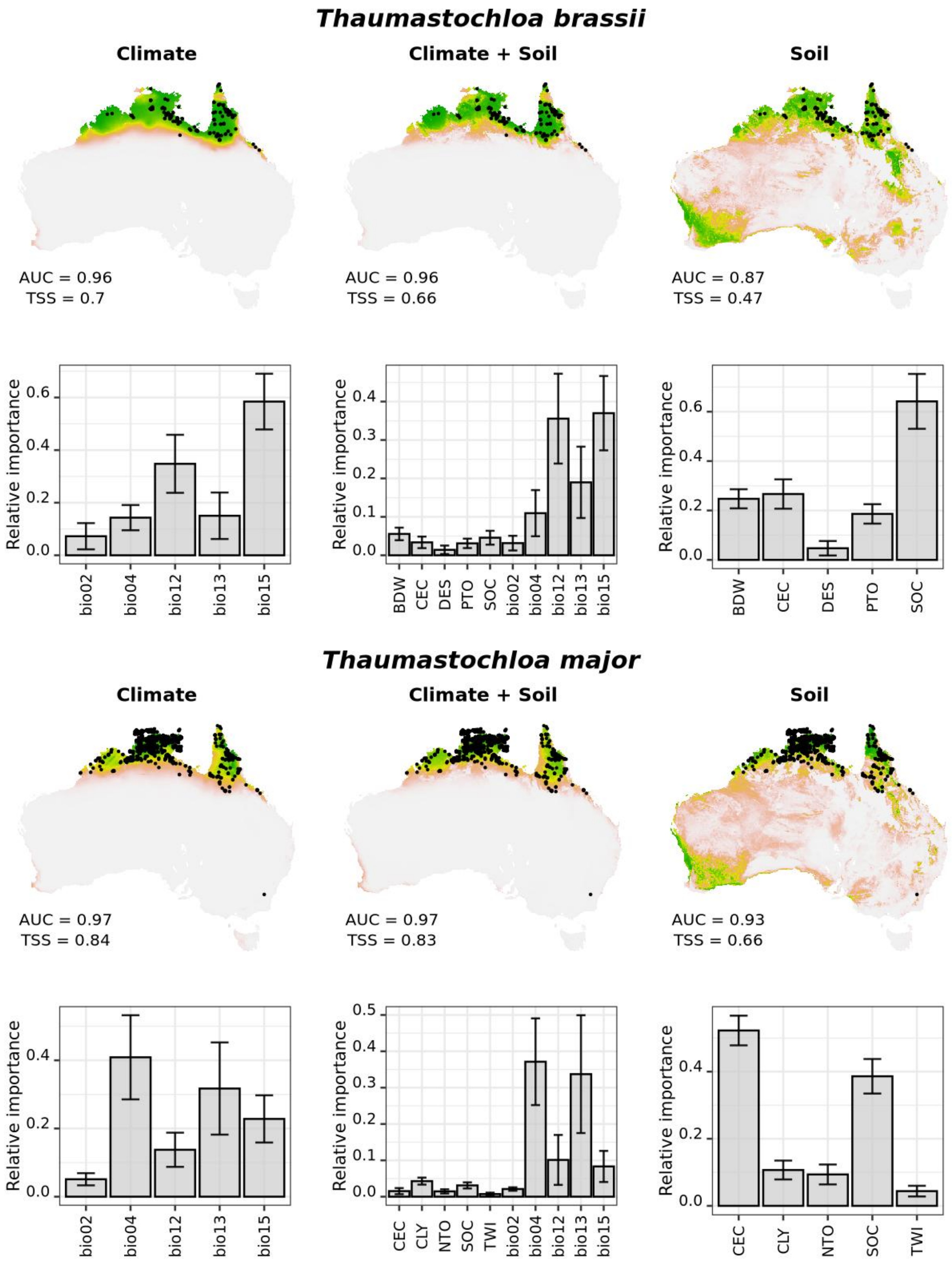
Thaumastochloa brassii
Climate
Climate + Soil
Soil
AUC = 0.96
TSS = 0.7
AUC = 0.96
TSS = 0.66
AUC = 0.87
TSS = 0.47
Relative importance
0.0
0.2
0.4
0.6
bio02
bio04
bio12
bio13
bio15
Relative importance
0.0
0.1
0.2
0.3
0.4
BDW
CEC
DES
PTO
SOC
bio02
bio04
bio12
bio13
bio15
Relative importance
0.0
0.2
0.4
0.6
BDW
CEC
DES
PTO
SOC
Thaumastochloa major
Climate
Climate + Soil
Soil
AUC = 0.97
TSS = 0.84
AUC = 0.97
TSS = 0.83
AUC = 0.93
TSS = 0.66
Relative importance
0.0
0.2
0.4
bio02
bio04
bio12
bio13
bio15
Relative importance
0.0
0.1
0.2
0.3
0.4
0.5
CEC
CLY
NTO
SOC
TWI
bio02
bio04
bio12
bio13
bio15
Relative importance
0.0
0.2
0.4
CEC
CLY
NTO
SOC
TWI

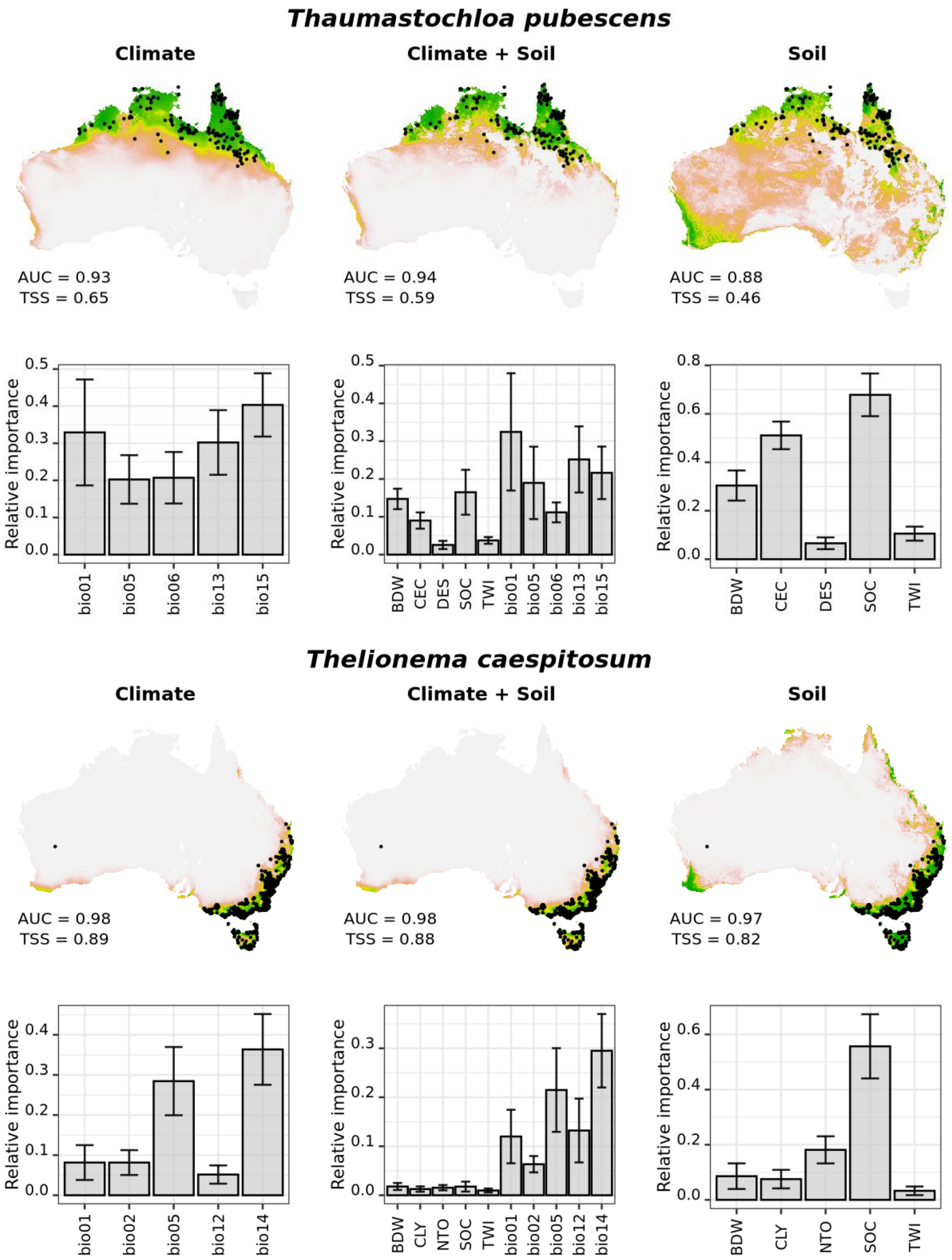
Thaumastochloa pubescens
Climate
Climate + Soil
Soil
AUC = 0.93
TSS = 0.65
AUC = 0.94
TSS = 0.59
AUC = 0.88
TSS = 0.46
Relative importance
bio01
bio05
bio06
bio13
bio15
BDW
CEC
DES
SOC
TWI
Thelionema caespitosum
Climate
Climate + Soil
Soil
AUC = 0.98
TSS = 0.89
AUC = 0.98
TSS = 0.88
AUC = 0.97
TSS = 0.82
Relative importance
bio01
bio02
bio05
bio12
bio14
BDW
CLY
NTO
SOC
TWI

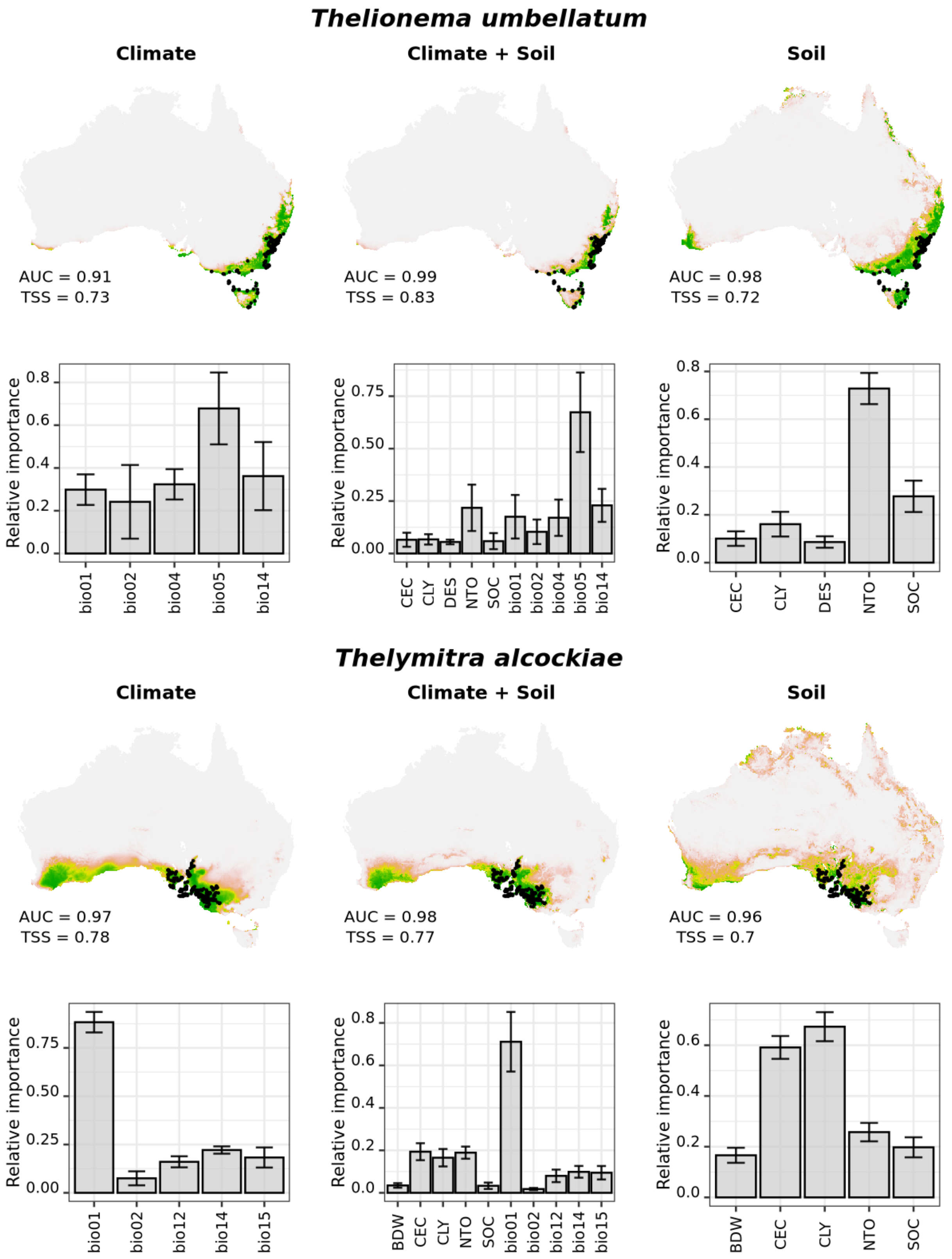

Thelionema umbellatum
Climate
Climate + Soil
Soil
AUC = 0.91
TSS = 0.73
AUC = 0.99
TSS = 0.83
AUC = 0.98
TSS = 0.72
Relative importance
0.0
0.2
0.4
0.6
0.8
bio01
bio02
bio04
bio05
bio14
0.00
0.25
0.50
0.75
CEC
CLY
DES
NTO
SOC
bio01
bio02
bio04
bio05
bio14
CEC
CLY
DES
NTO
SOC
Thelymitra alcockiae
Climate
Climate + Soil
Soil
AUC = 0.97
TSS = 0.78
AUC = 0.98
TSS = 0.77
AUC = 0.96
TSS = 0.7
Relative importance
0.00
0.25
0.50
0.75
bio01
bio02
bio12
bio14
bio15
0.0
0.2
0.4
0.6
0.8
BDW
CEC
CLY
NTO
SOC
bio01
bio02
bio12
bio14
bio15
BDW
CEC
CLY
NTO
SOC

Thelymitra antennifera

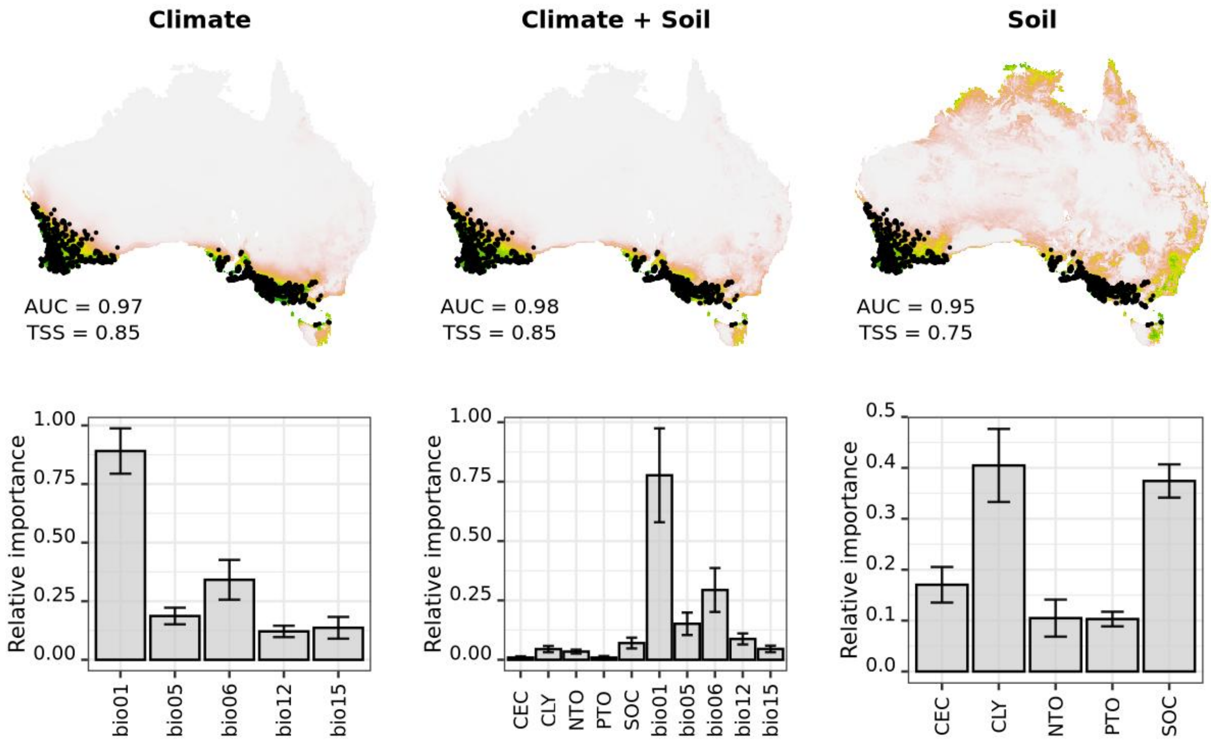

Thelymitra arenaria

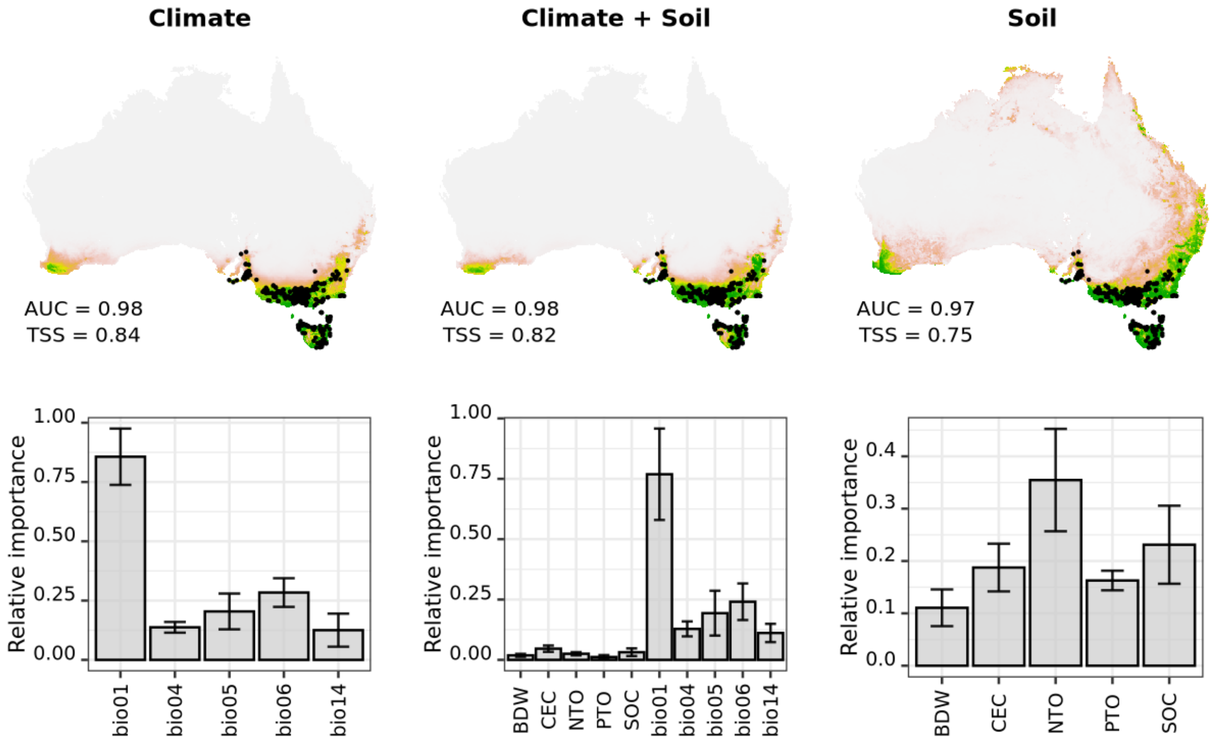

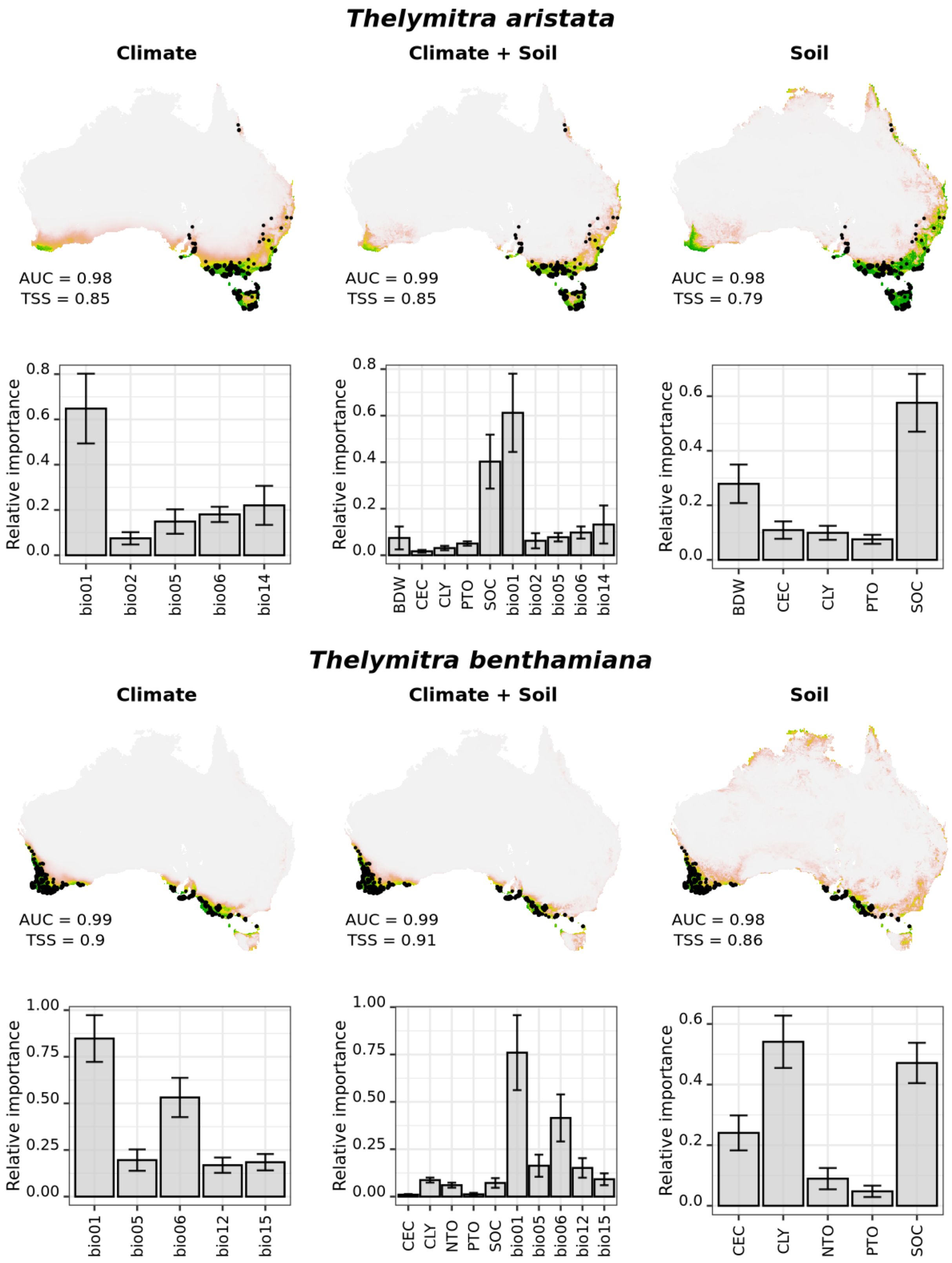

Thelymitra aristata
Climate
Climate + Soil
Soil
AUC = 0.98
TSS = 0.85
AUC = 0.99
TSS = 0.85
AUC = 0.98
TSS = 0.79
Relative importance
0.0
0.2
0.4
0.6
0.8
bio01
bio02
bio05
bio06
bio14
BDW
CEC
CLY
PTO
SOC
bio01
bio02
bio05
bio06
bio14
BDW
CEC
CLY
PTO
SOC
Thelymitra benthamiana
Climate
Climate + Soil
Soil
AUC = 0.99
TSS = 0.9
AUC = 0.99
TSS = 0.91
AUC = 0.98
TSS = 0.86
Relative importance
0.00
0.25
0.50
0.75
1.00
bio01
bio05
bio06
bio12
bio15
CEC
CLY
NTO
PTO
SOC
bio01
bio05
bio06
bio12
bio15
0.0
0.2
0.4
0.6
CEC
CLY
NTO
PTO
SOC

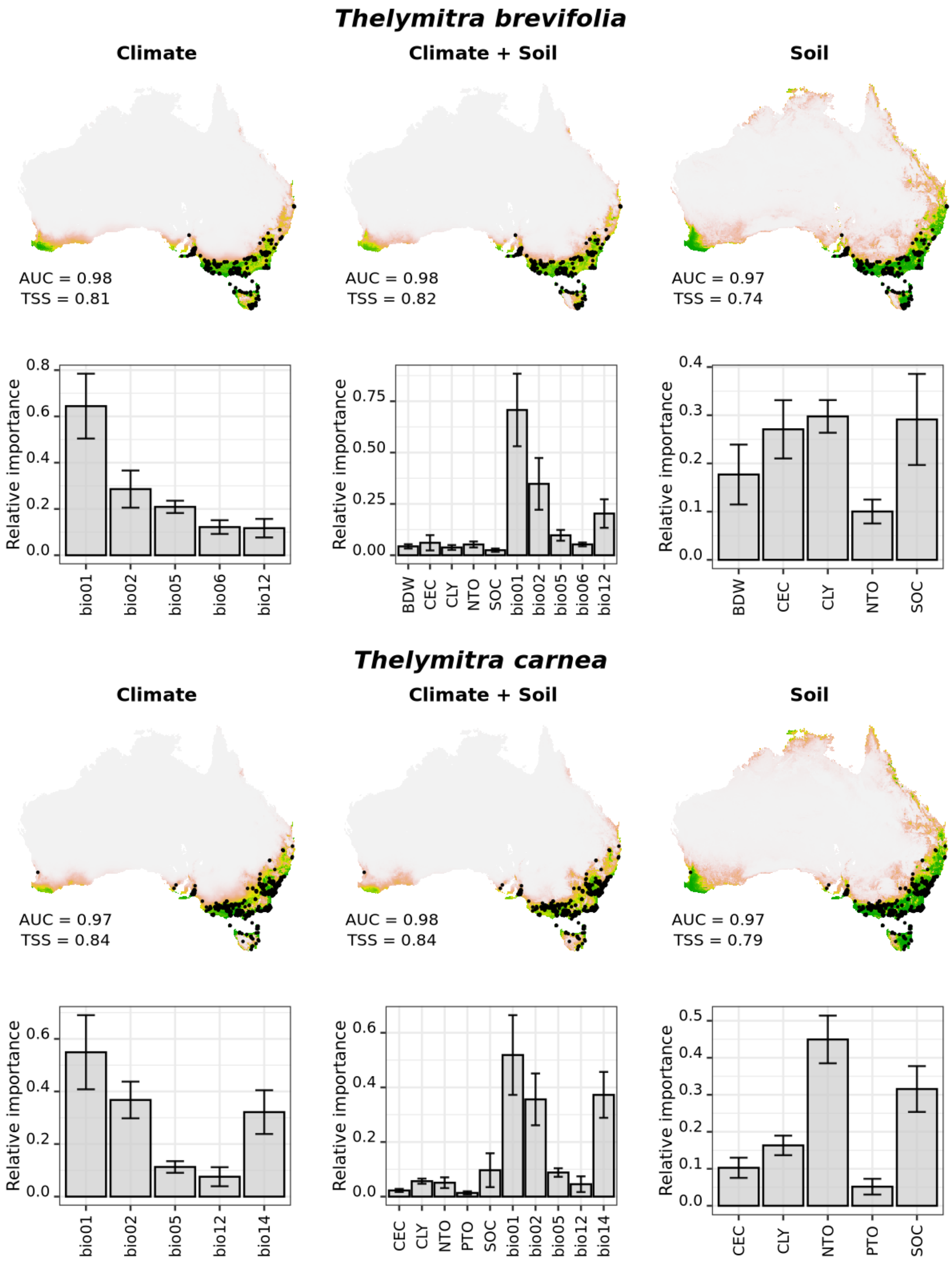
Thelymitra brevifolia
Climate
Climate + Soil
Soil
AUC = 0.98
TSS = 0.81
AUC = 0.98
TSS = 0.82
AUC = 0.97
TSS = 0.74
Relative importance
0.0 0.2 0.4 0.6 0.8
bio01 bio02 bio05 bio06 bio12
0.00 0.25 0.50 0.75
BDW CEC CLY NTO SOC bio01 bio02 bio05 bio06 bio12
0.0 0.1 0.2 0.3 0.4
BDW CEC CLY NTO SOC
Thelymitra carnea
Climate
Climate + Soil
Soil
AUC = 0.97
TSS = 0.84
AUC = 0.98
TSS = 0.84
AUC = 0.97
TSS = 0.79
Relative importance
0.0 0.2 0.4 0.6
bio01 bio02 bio05 bio12 bio14
0.0 0.2 0.4 0.6
CEC CLY NTO PTO SOC bio01 bio02 bio05 bio12 bio14
0.0 0.1 0.2 0.3 0.4 0.5
CEC CLY NTO PTO SOC

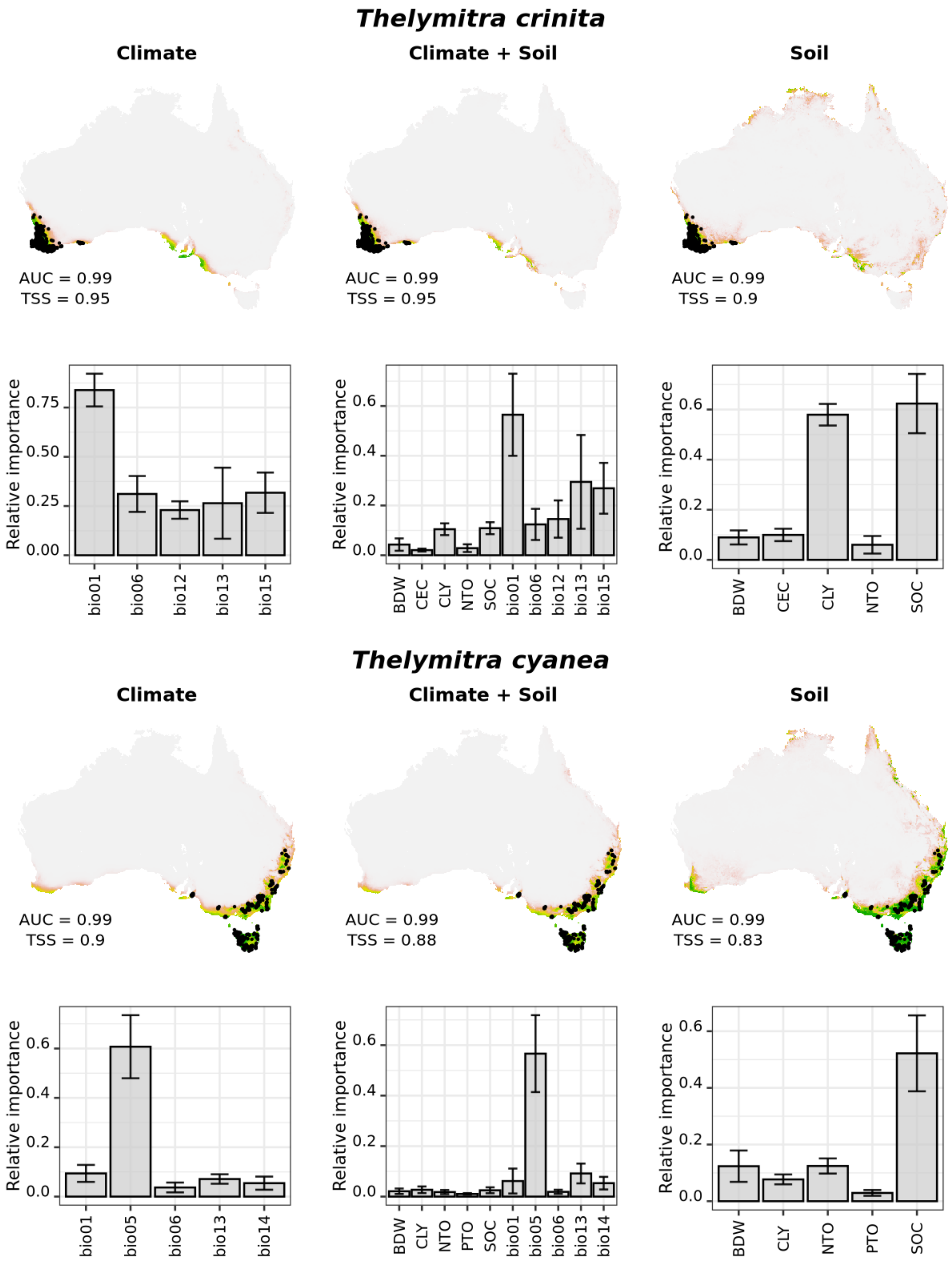

Thelymitra crinita
Climate
Climate + Soil
Soil
AUC = 0.99
TSS = 0.95
AUC = 0.99
TSS = 0.95
AUC = 0.99
TSS = 0.9
Relative importance
0.00
0.25
0.50
0.75
bio01
bio06
bio12
bio13
bio15
0.0
0.2
0.4
0.6
BDW
CEC
CLY
NTO
SOC
bio01
bio06
bio12
bio13
bio15
BDW
CEC
CLY
NTO
SOC
Thelymitra cyanea
Climate
Climate + Soil
Soil
AUC = 0.99
TSS = 0.9
AUC = 0.99
TSS = 0.88
AUC = 0.99
TSS = 0.83
bio01
bio05
bio06
bio13
bio14
BDW
CLY
NTO
PTO
SOC
bio01
bio05
bio06
bio13
bio14
BDW
CLY
NTO
PTO
SOC

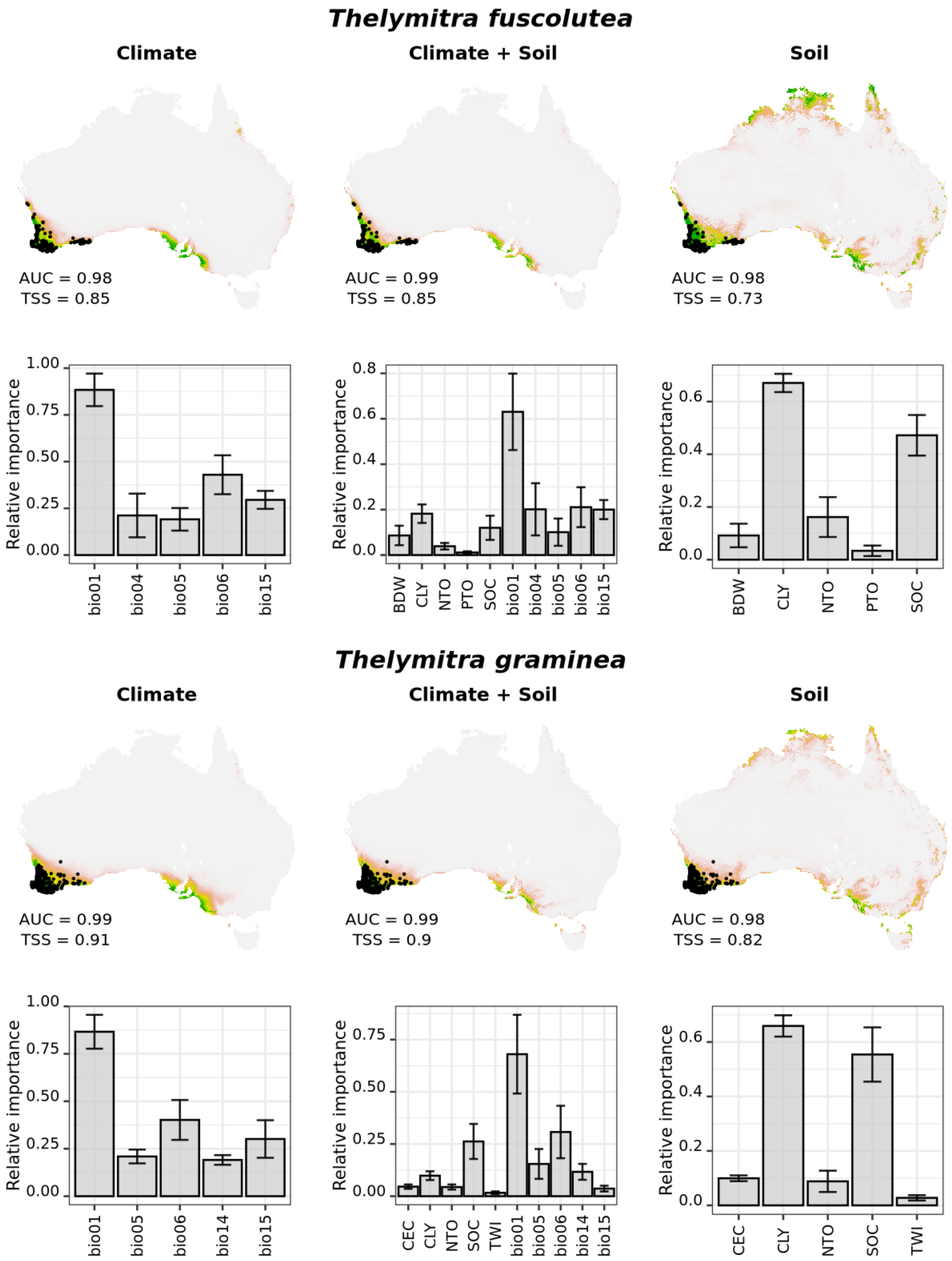
Thelymitra fuscolutea
Climate
Climate + Soil
Soil
AUC = 0.98
TSS = 0.85
AUC = 0.99
TSS = 0.85
AUC = 0.98
TSS = 0.73
Relative importance
1.00
0.75
0.50
0.25
0.00
bio01
bio04
bio05
bio06
bio15
0.8
0.6
0.4
0.2
0.0
BDW
CLY
NTO
PTO
SOC
bio01
bio04
bio05
bio06
bio15
0.6
0.4
0.2
0.0
BDW
CLY
NTO
PTO
SOC
Thelymitra graminea
Climate
Climate + Soil
Soil
AUC = 0.99
TSS = 0.91
AUC = 0.99
TSS = 0.9
AUC = 0.98
TSS = 0.82
Relative importance
1.00
0.75
0.50
0.25
0.00
bio01
bio05
bio06
bio14
bio15
0.75
0.50
0.25
0.00
CEC
CLY
NTO
SOC
TWI
bio01
bio05
bio06
bio14
bio15
0.6
0.4
0.2
0.0
CEC
CLY
NTO
SOC
TWI

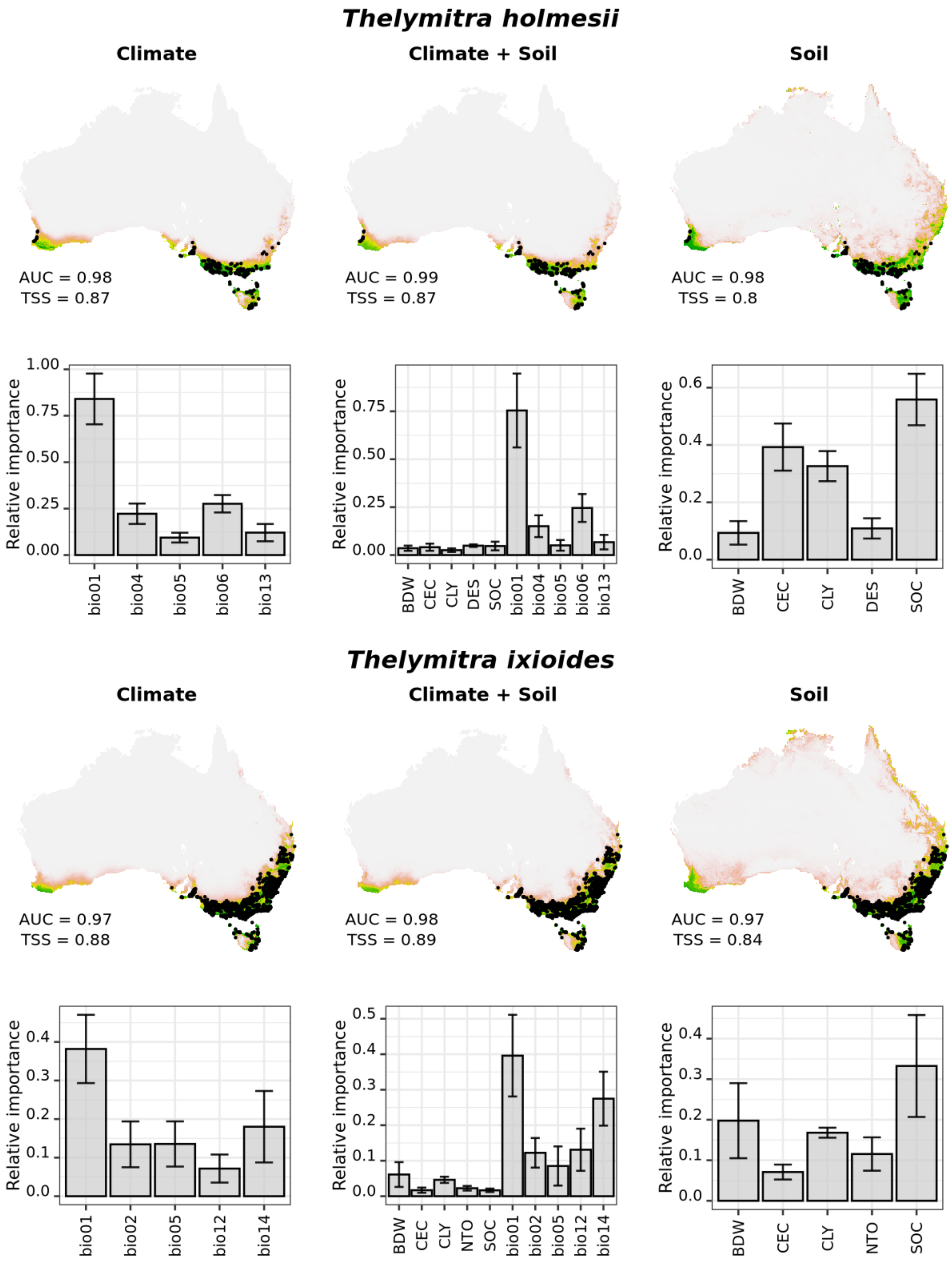

Thelymitra holmesii
Climate
Climate + Soil
Soil
AUC = 0.98
TSS = 0.87
AUC = 0.99
TSS = 0.87
AUC = 0.98
TSS = 0.8
Relative importance
1.00
0.75
0.50
0.25
0.00
bio01
bio04
bio05
bio06
bio13
Relative importance
0.75
0.50
0.25
0.00
BDW
CEC
CLY
DES
SOC
bio01
bio04
bio05
bio06
bio13
Relative importance
0.6
0.4
0.2
0.0
BDW
CEC
CLY
DES
SOC
Thelymitra ixioides
Climate
Climate + Soil
Soil
AUC = 0.97
TSS = 0.88
AUC = 0.98
TSS = 0.89
AUC = 0.97
TSS = 0.84
Relative importance
0.4
0.3
0.2
0.1
0.0
bio01
bio02
bio05
bio12
bio14
Relative importance
0.5
0.4
0.3
0.2
0.1
0.0
BDW
CEC
CLY
NTO
SOC
bio01
bio02
bio05
bio12
bio14
Relative importance
0.4
0.3
0.2
0.1
0.0
BDW
CEC
CLY
NTO
SOC

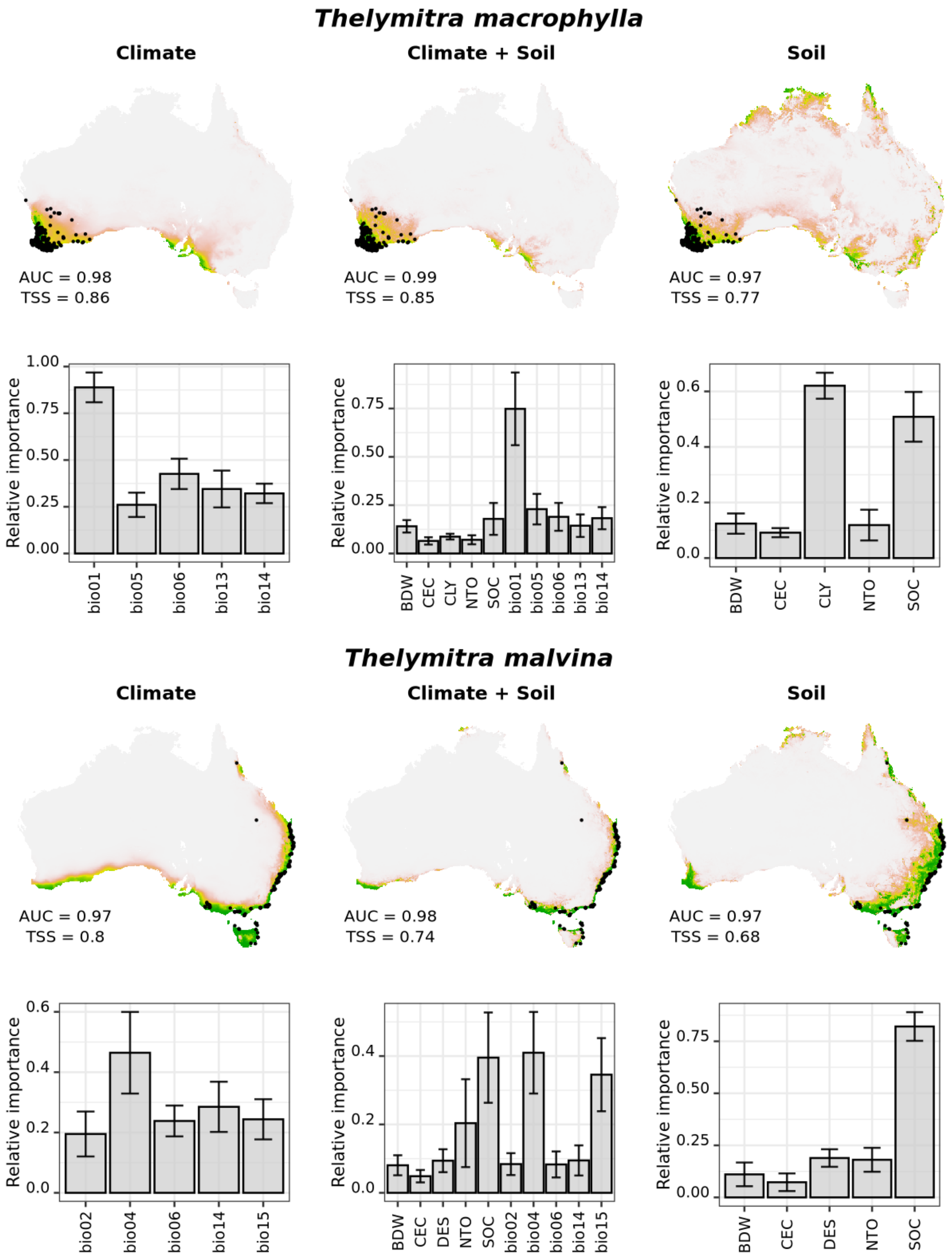
Thelymitra macrophylla
Climate
Climate + Soil
Soil
AUC = 0.98
TSS = 0.86
AUC = 0.99
TSS = 0.85
AUC = 0.97
TSS = 0.77
Relative importance
1.00
0.75
0.50
0.25
0.00
bio01
bio05
bio06
bio13
bio14
BDW
CEC
CLY
NTO
SOC
0.6
0.4
0.2
0.0
Thelymitra malvina
AUC = 0.97
TSS = 0.8
AUC = 0.98
TSS = 0.74
AUC = 0.97
TSS = 0.68
bio02
bio04
bio15
DES

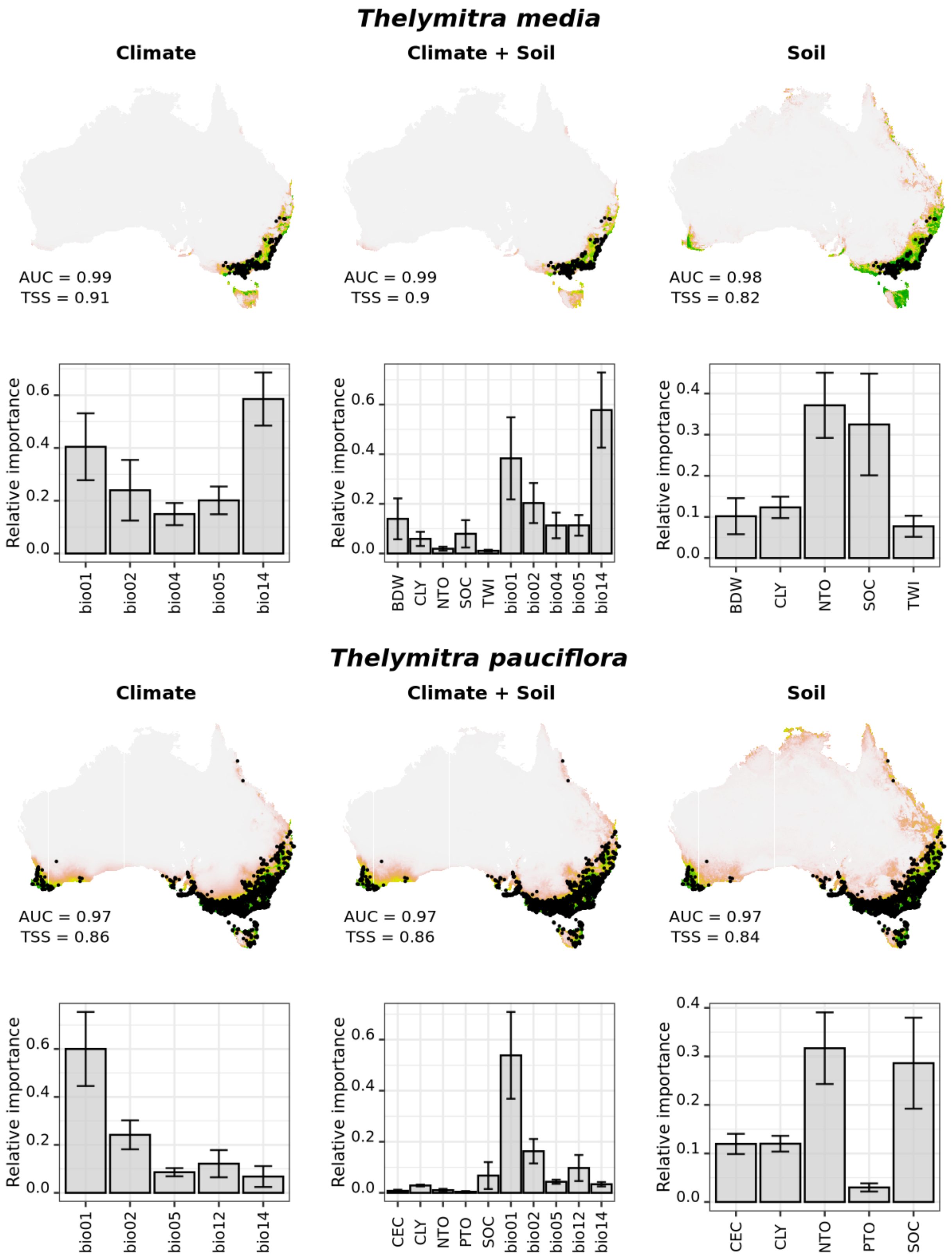
Thelymitra media
Climate
Climate + Soil
Soil
AUC = 0.99
TSS = 0.91
AUC = 0.99
TSS = 0.9
AUC = 0.98
TSS = 0.82
Relative importance
bio01
bio02
bio04
bio05
bio14
BDW
CLY
NTO
SOC
TWI
Thelymitra pauciflora
Climate
Climate + Soil
Soil
AUC = 0.97
TSS = 0.86
AUC = 0.97
TSS = 0.86
AUC = 0.97
TSS = 0.84
Relative importance
bio01
bio02
bio05
bio12
bio14
CEC
CLY
NTO
PTO
SOC

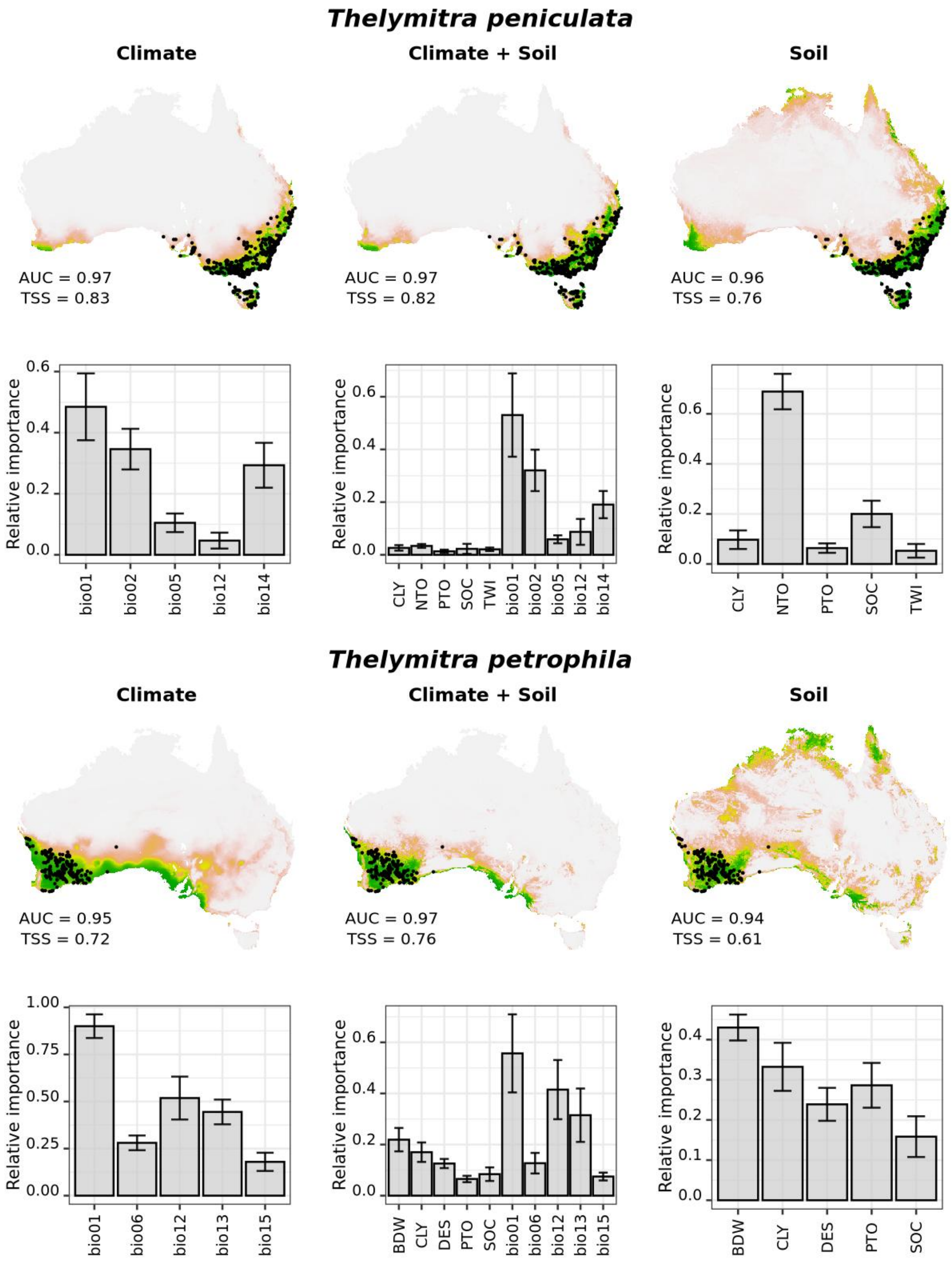

Thelymitra peniculata
Climate
Climate + Soil
Soil
AUC = 0.97
TSS = 0.83
AUC = 0.97
TSS = 0.82
AUC = 0.96
TSS = 0.76
Relative importance
0.0
0.2
0.4
0.6
bio01
bio02
bio05
bio12
bio14
CLY
NTO
PTO
SOC
TWI
Thelymitra petrophila
Climate
Climate + Soil
Soil
AUC = 0.95
TSS = 0.72
AUC = 0.97
TSS = 0.76
AUC = 0.94
TSS = 0.61
Relative importance
0.00
0.25
0.50
0.75
1.00
0.1
0.3
bio01
bio06
bio12
bio13
bio15
BDW
CLY
DES
PTO
SOC

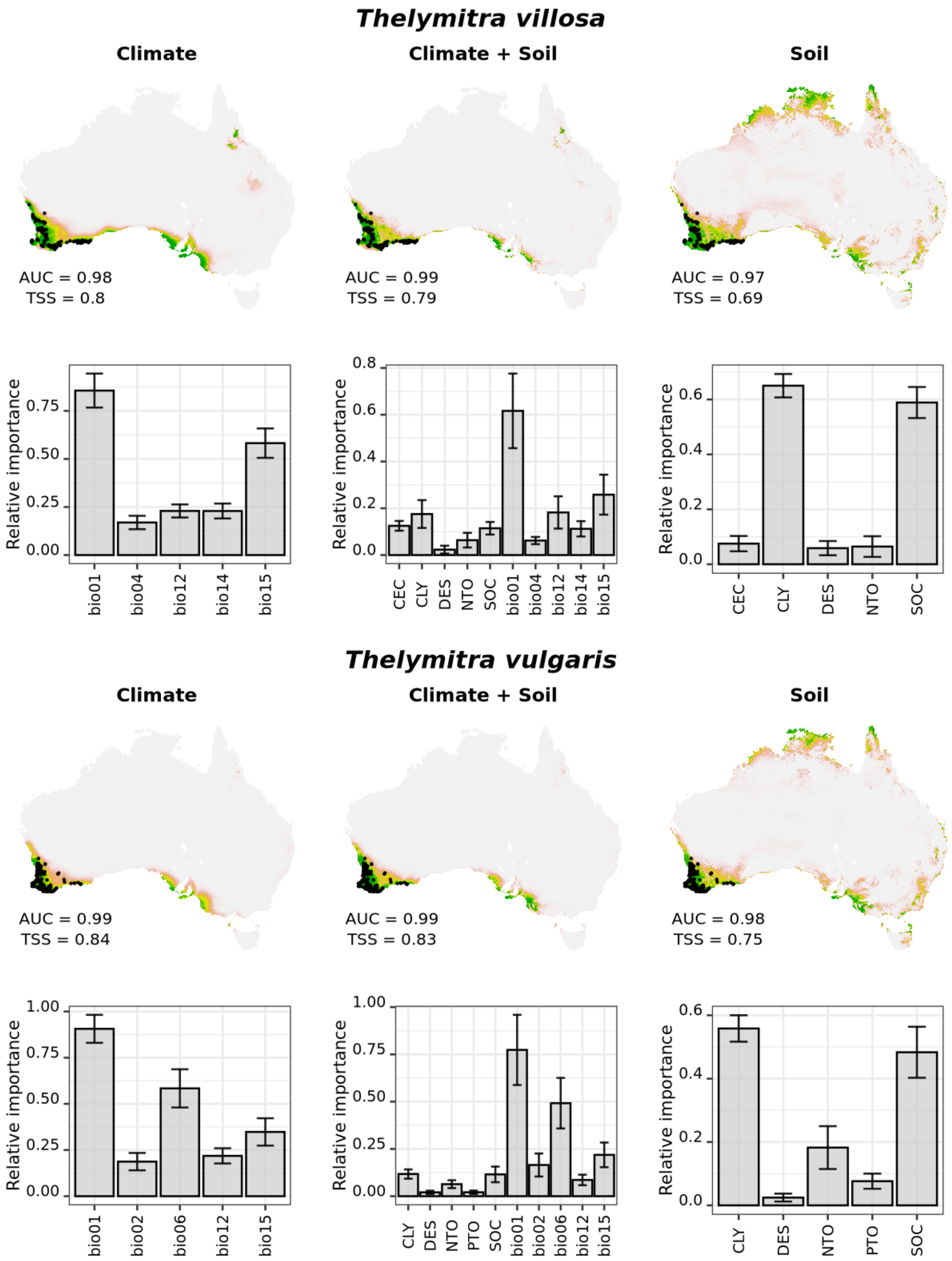
Thelymitra villosa
Climate
Climate + Soil
Soil
AUC = 0.98
TSS = 0.8
AUC = 0.99
TSS = 0.79
AUC = 0.97
TSS = 0.69
Relative importance
bio01 bio04 bio12 bio14 bio15
CEC CLY DES NTO SOC bio01 bio04 bio12 bio14 bio15
CEC CLY DES NTO SOC
Thelymitra vulgaris
Climate
Climate + Soil
Soil
AUC = 0.99
TSS = 0.84
AUC = 0.99
TSS = 0.83
AUC = 0.98
TSS = 0.75
Relative importance
bio01 bio02 bio06 bio12 bio15
CLY DES NTO PTO SOC bio01 bio02 bio06 bio12 bio15
CLY DES NTO PTO SOC

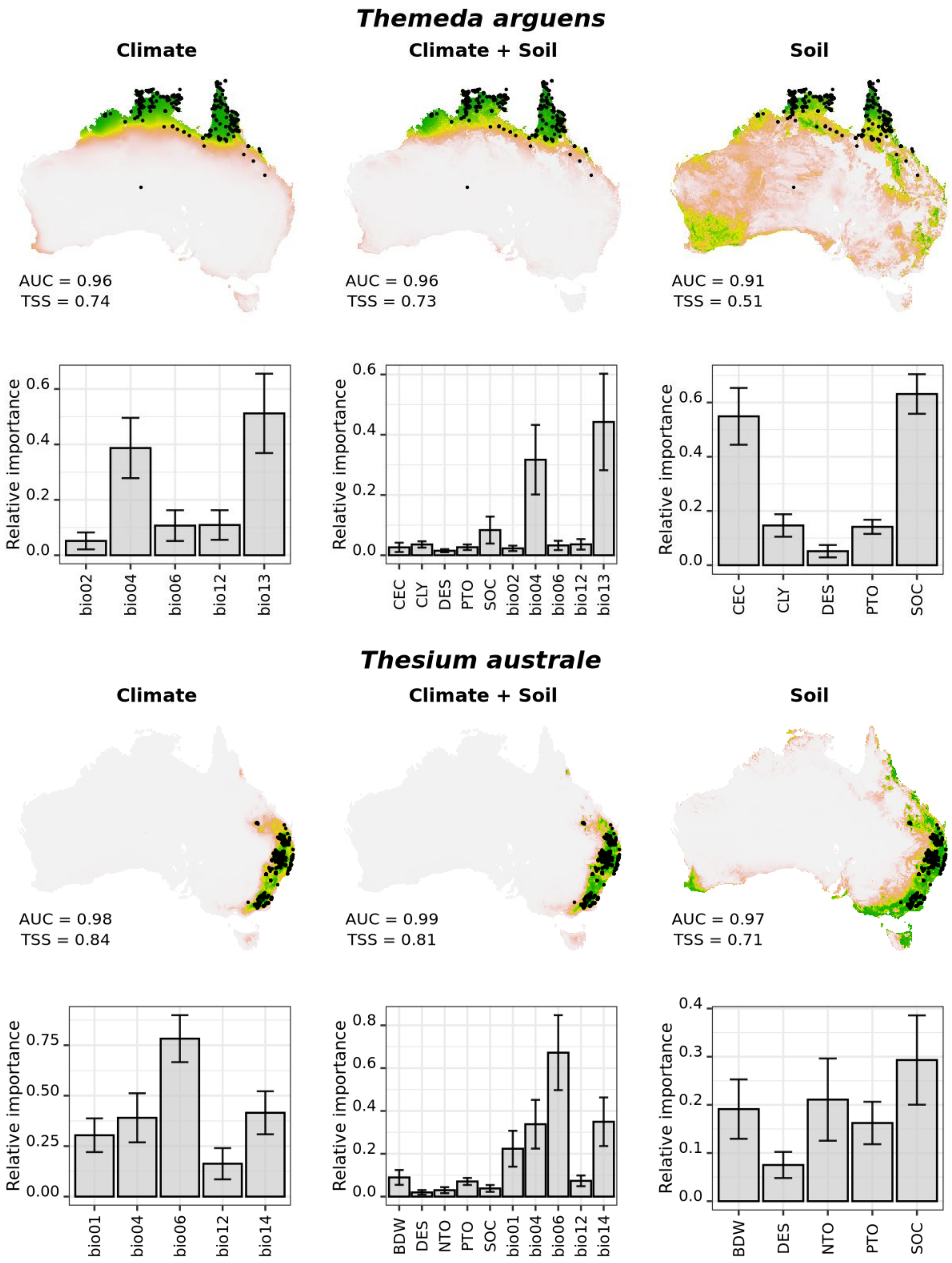
Themeda arguens
Climate
Climate + Soil
Soil
AUC = 0.96
TSS = 0.74
AUC = 0.96
TSS = 0.73
AUC = 0.91
TSS = 0.51
Relative importance
bio02
bio04
bio06
bio12
bio13
CEC
CLY
DES
PTO
SOC
Thesium australe
Climate
Climate + Soil
Soil
AUC = 0.98
TSS = 0.84
AUC = 0.99
TSS = 0.81
AUC = 0.97
TSS = 0.71
Relative importance
bio01
bio04
bio06
bio12
bio14
BDW
DES
NTO
PTO
SOC

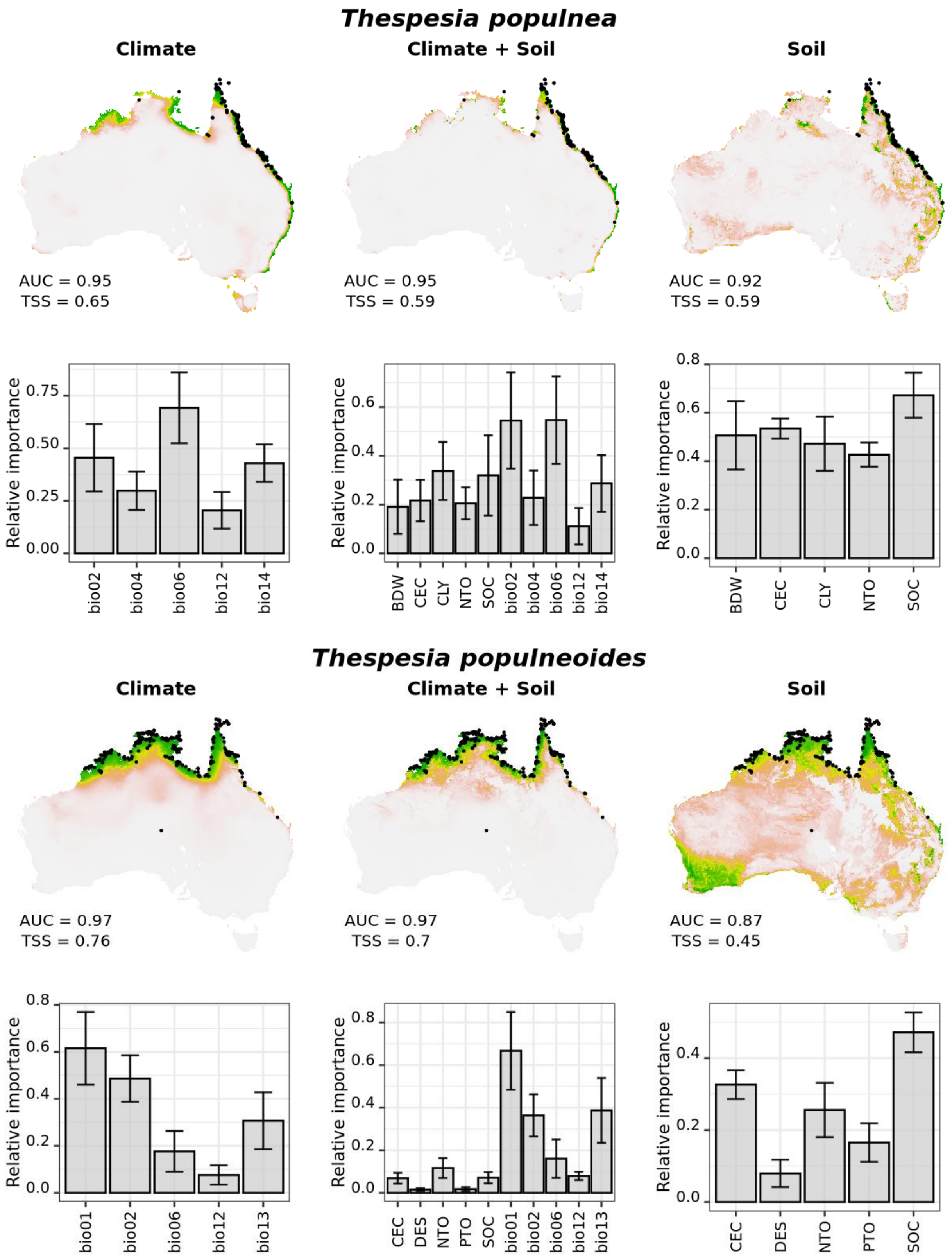
Thespesia populnea
Climate
Climate + Soil
Soil
AUC = 0.95
TSS = 0.65
AUC = 0.95
TSS = 0.59
AUC = 0.92
TSS = 0.59
Relative importance
bio02
bio04
bio06
bio12
bio14
BDW
CEC
CLY
NTO
SOC
Thespesia populneoides
AUC = 0.97
TSS = 0.76
AUC = 0.97
TSS = 0.7
AUC = 0.87
TSS = 0.45
bio01
bio13
DES
PTO

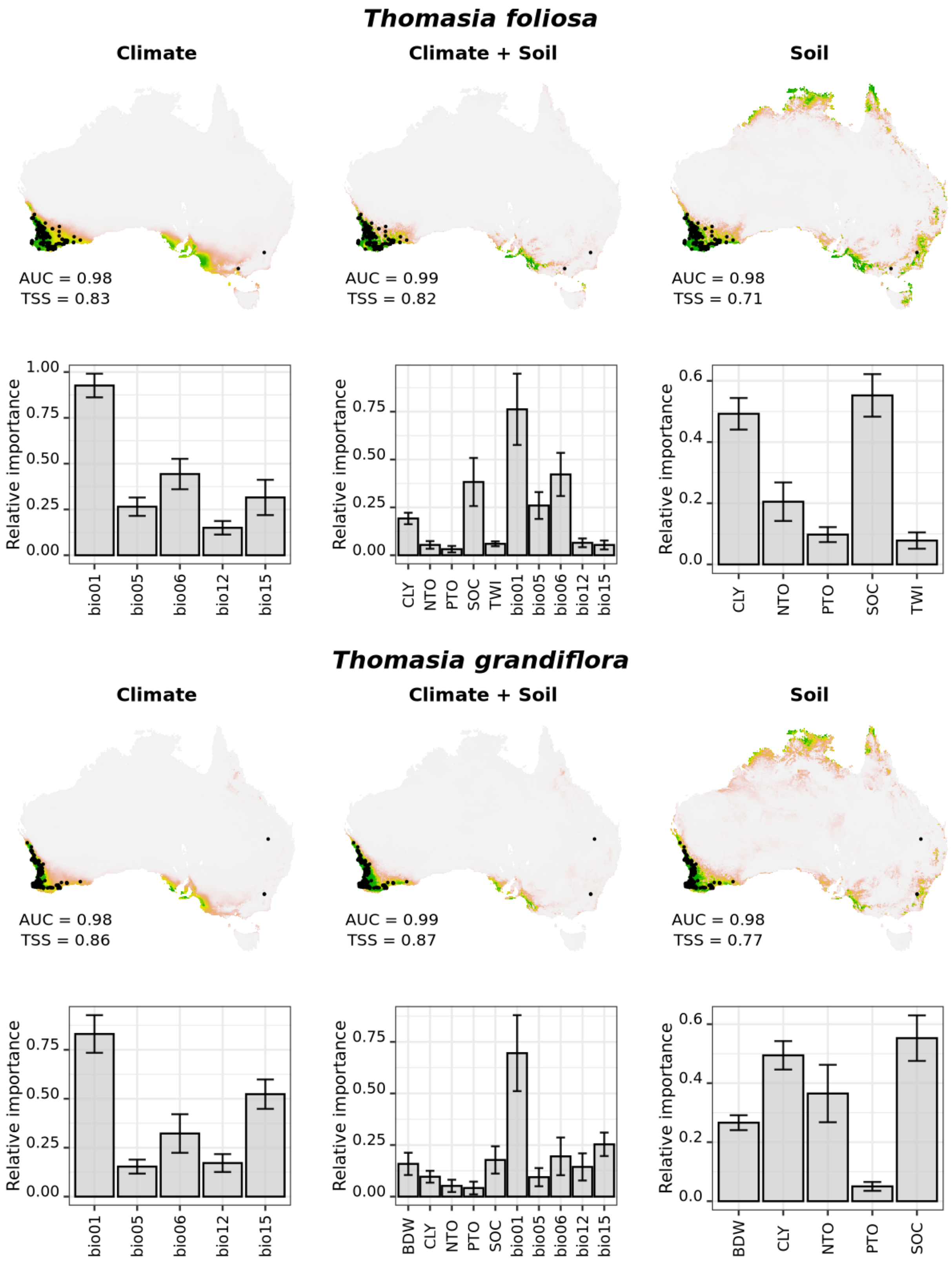
Thomasia foliosa
Climate
Climate + Soil
Soil
AUC = 0.98
TSS = 0.83
AUC = 0.99
TSS = 0.82
AUC = 0.98
TSS = 0.71
Relative importance
bio01
bio05
bio06
bio12
bio15
CLY
NTO
PTO
SOC
TWI
Thomasia grandiflora
Climate
Climate + Soil
Soil
AUC = 0.98
TSS = 0.86
AUC = 0.99
TSS = 0.87
AUC = 0.98
TSS = 0.77
Relative importance
BDW
CLY
NTO
PTO
SOC
bio01
bio05
bio06
bio12
bio15

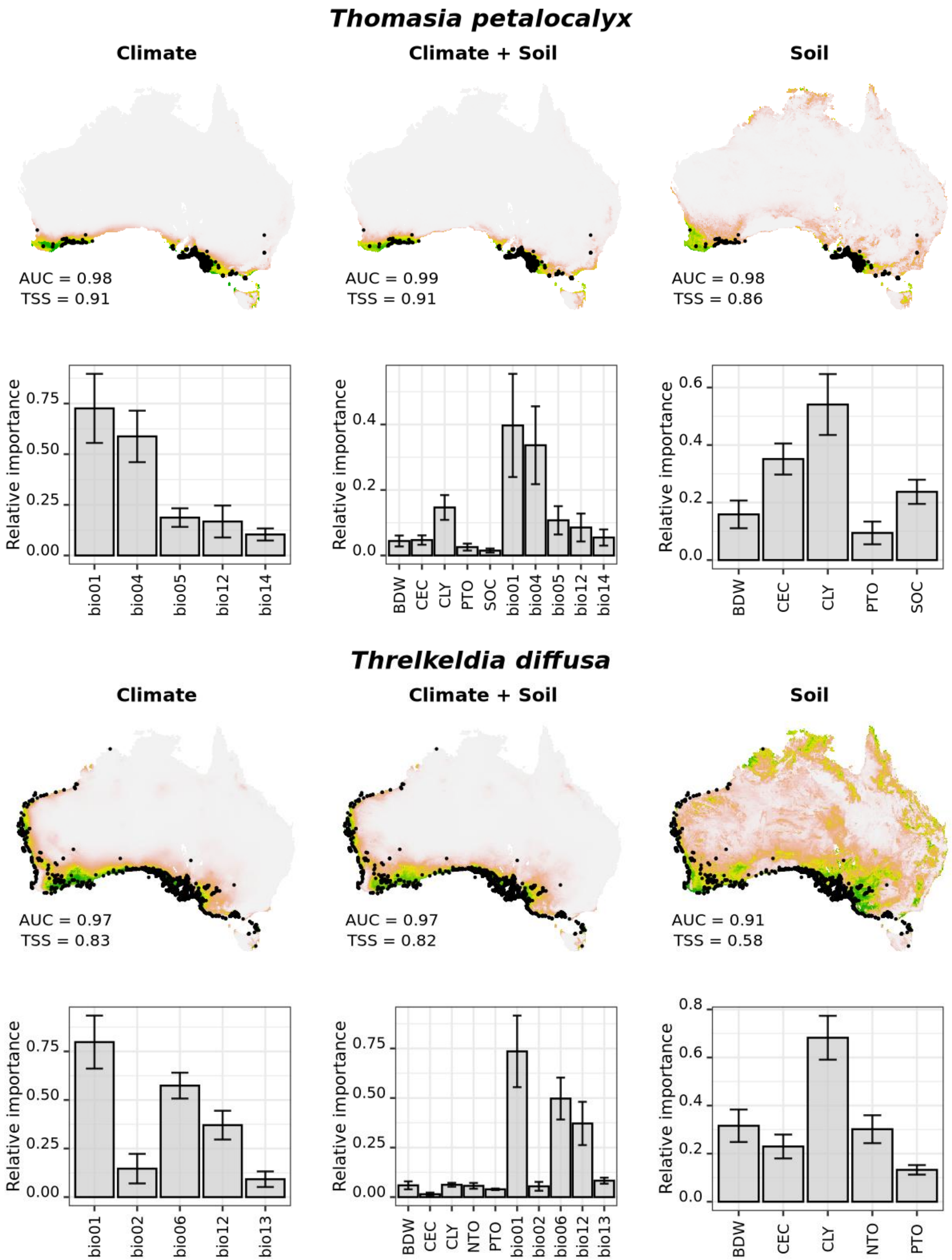
Thomasia petalocalyx
Climate
Climate + Soil
Soil
AUC = 0.98
TSS = 0.91
AUC = 0.99
TSS = 0.91
AUC = 0.98
TSS = 0.86
Relative importance
0.00
0.25
0.50
0.75
bio01
bio04
bio05
bio12
bio14
0.0
0.2
0.4
BDW
CEC
CLY
PTO
SOC
bio01
bio04
bio05
bio12
bio14
0.0
0.2
0.4
0.6
BDW
CEC
CLY
PTO
SOC
Threlkeldia diffusa
Climate
Climate + Soil
Soil
AUC = 0.97
TSS = 0.83
AUC = 0.97
TSS = 0.82
AUC = 0.91
TSS = 0.58
Relative importance
0.00
0.25
0.50
0.75
bio01
bio02
bio06
bio12
bio13
BDW
CEC
CLY
NTO
PTO
bio01
bio02
bio06
bio12
bio13
0.0
0.2
0.4
0.6
0.8
BDW
CEC
CLY
NTO
PTO

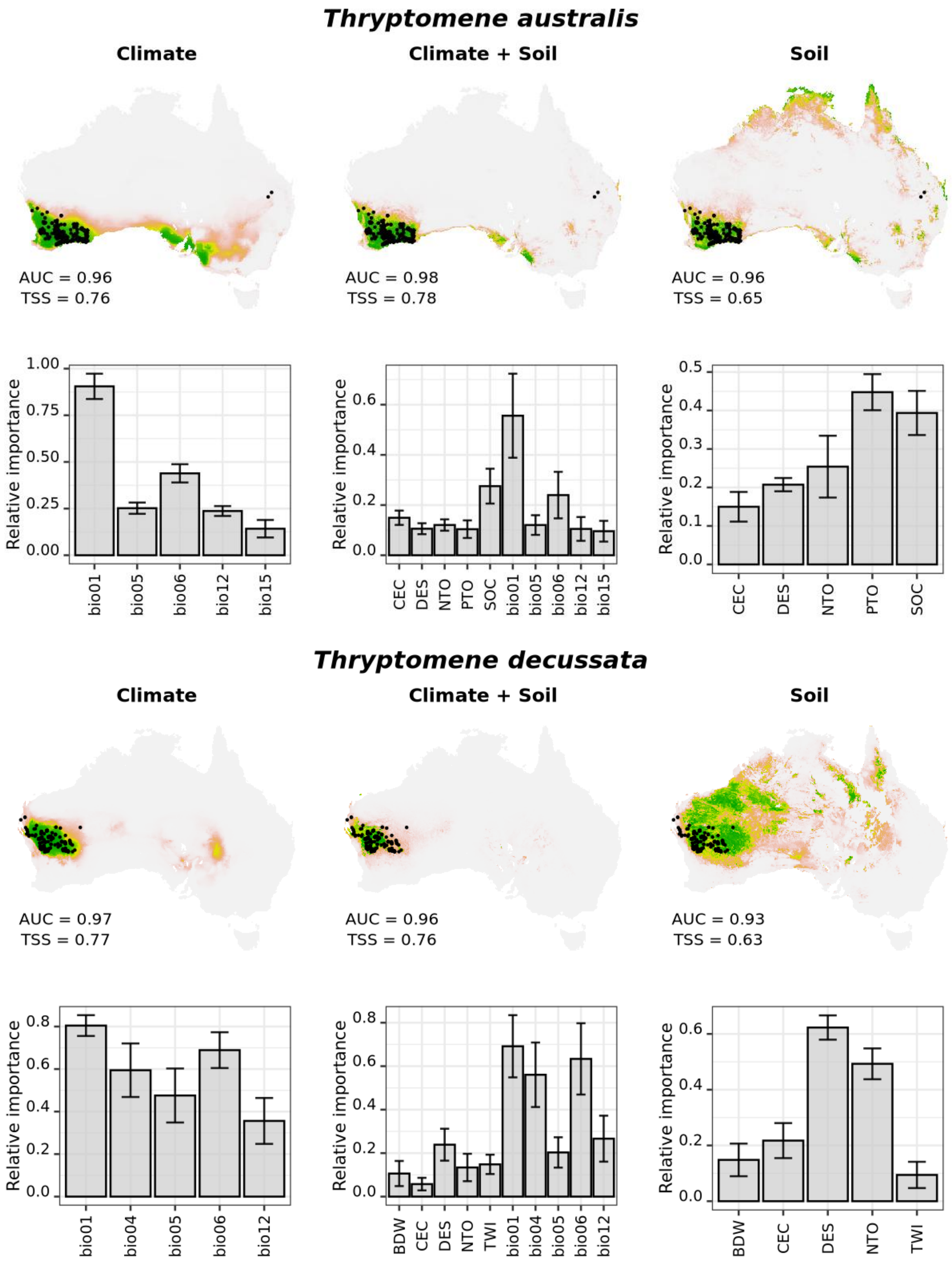

Thryptomene australis
Climate
Climate + Soil
Soil
AUC = 0.96
TSS = 0.76
AUC = 0.98
TSS = 0.78
AUC = 0.96
TSS = 0.65
Relative importance
bio01
bio05
bio06
bio12
bio15
CEC
DES
NTO
PTO
SOC
Thryptomene decussata
Climate
Climate + Soil
Soil
AUC = 0.97
TSS = 0.77
AUC = 0.96
TSS = 0.76
AUC = 0.93
TSS = 0.63
Relative importance
bio01
bio04
bio05
bio06
bio12
BDW
CEC
DES
NTO
TWI

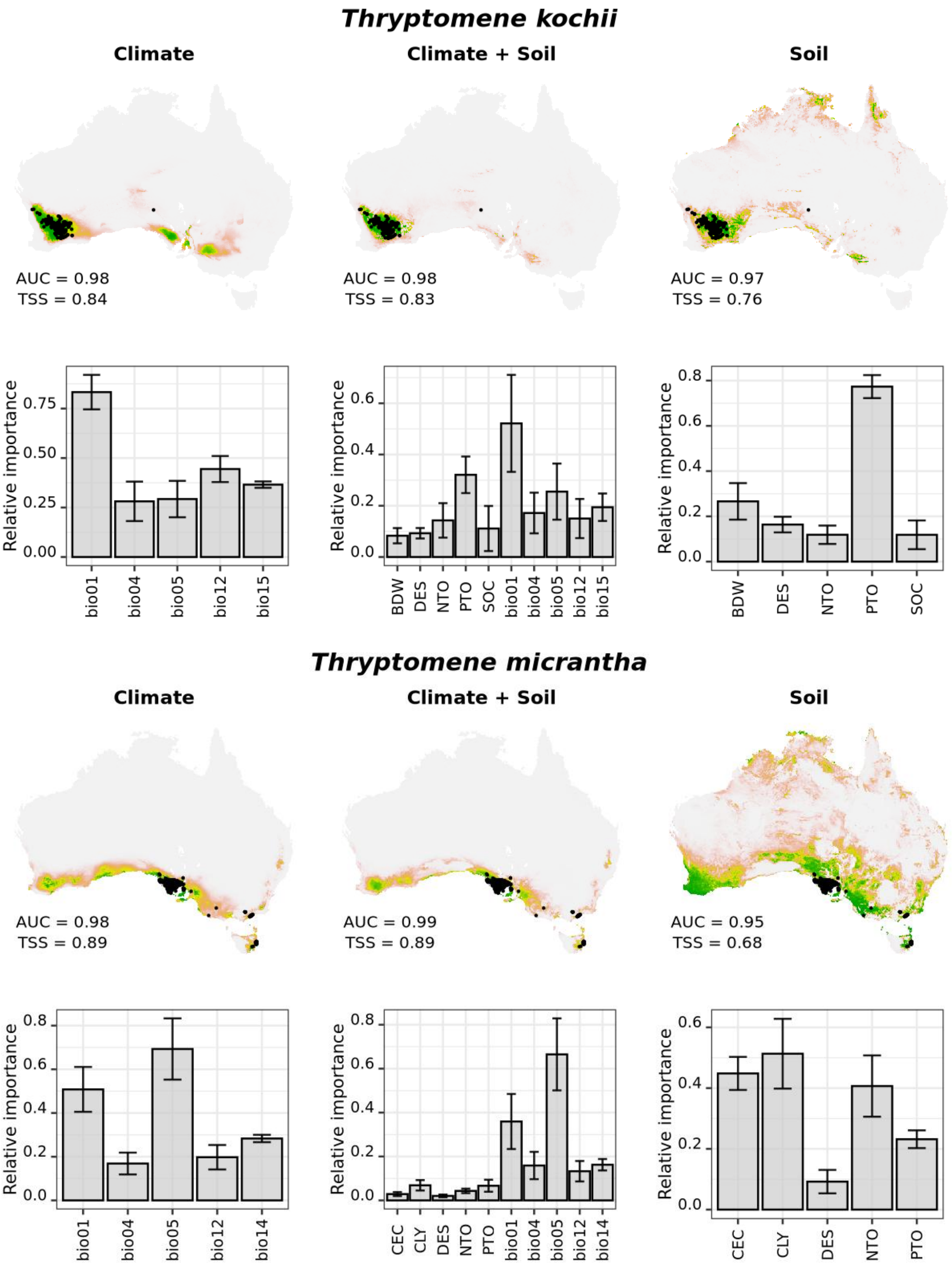
Thryptomene kochii
Climate
Climate + Soil
Soil
AUC = 0.98
TSS = 0.84
AUC = 0.98
TSS = 0.83
AUC = 0.97
TSS = 0.76
Relative importance
0.00
0.25
0.50
0.75
bio01
bio04
bio05
bio12
bio15
0.0
0.2
0.4
0.6
BDW
DES
NTO
PTO
SOC
0.8
Thryptomene micrantha
AUC = 0.98
TSS = 0.89
AUC = 0.99
TSS = 0.89
AUC = 0.95
TSS = 0.68
bio14
CEC
CLY

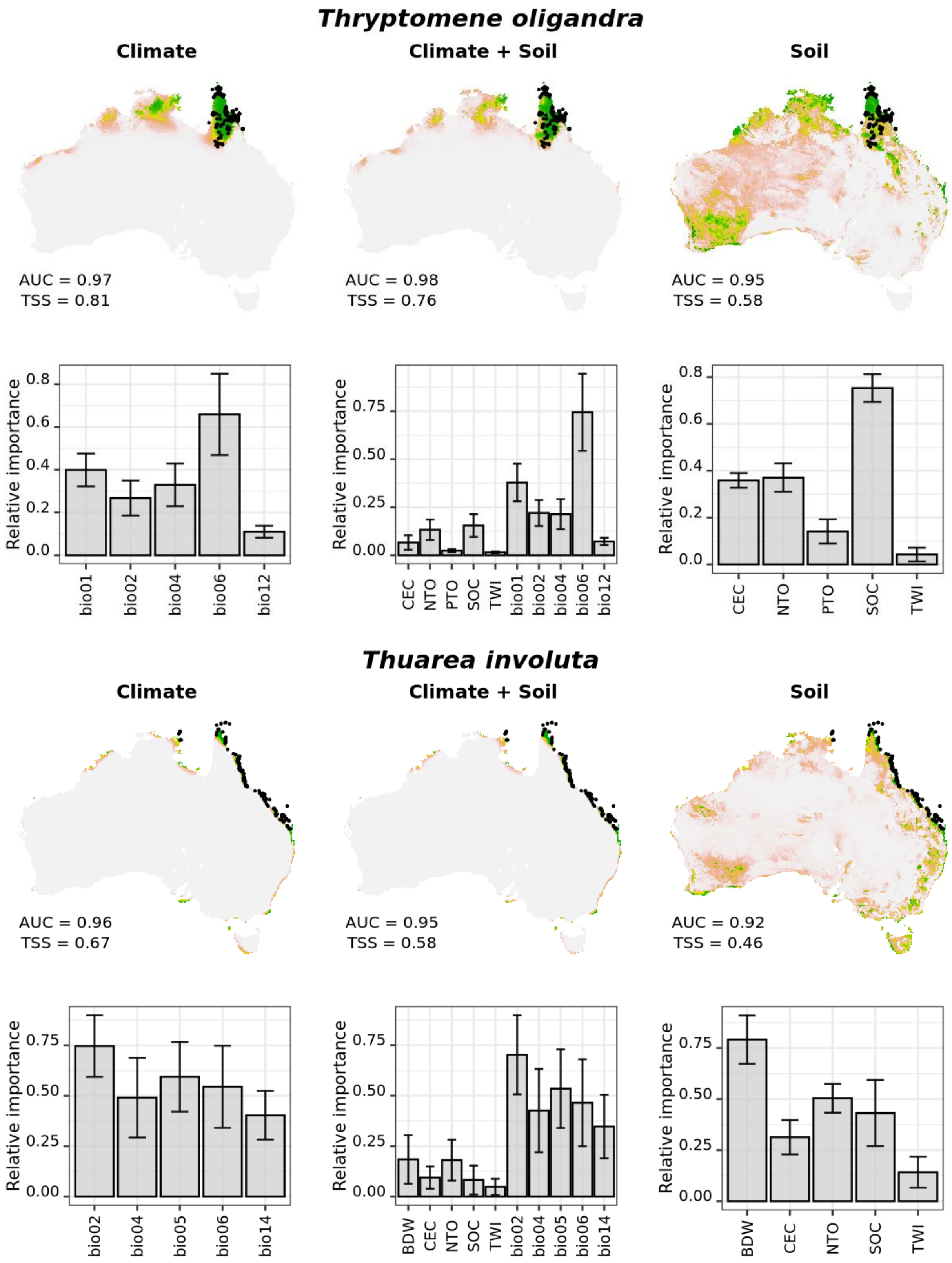
Thryptomene oligandra
Climate
Climate + Soil
Soil
AUC = 0.97
TSS = 0.81
AUC = 0.98
TSS = 0.76
AUC = 0.95
TSS = 0.58
Relative importance
0.0
0.2
0.4
0.6
0.8
bio01
bio02
bio04
bio06
bio12
0.00
0.25
0.50
0.75
CEC
NTO
PTO
SOC
TWI
bio01
bio02
bio04
bio06
bio12
CEC
NTO
PTO
SOC
TWI
Thuarea involuta
Climate
Climate + Soil
Soil
AUC = 0.96
TSS = 0.67
AUC = 0.95
TSS = 0.58
AUC = 0.92
TSS = 0.46
Relative importance
0.00
0.25
0.50
0.75
bio02
bio04
bio05
bio06
bio14
BDW
CEC
NTO
SOC
TWI
bio02
bio04
bio05
bio06
bio14
BDW
CEC
NTO
SOC
TWI

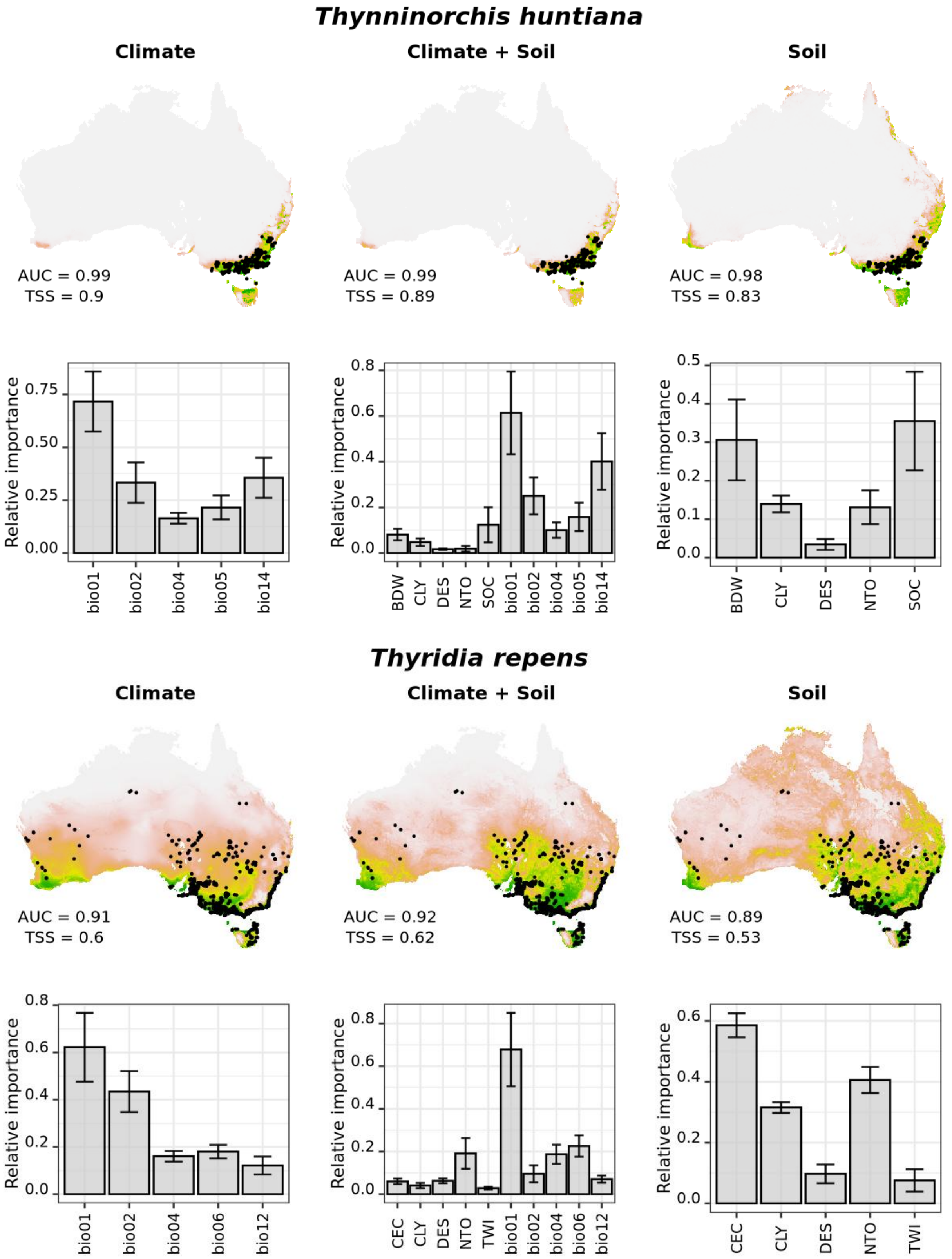
Thynninorchis huntiana
Climate
Climate + Soil
Soil
AUC = 0.99
TSS = 0.9
AUC = 0.99
TSS = 0.89
AUC = 0.98
TSS = 0.83
Relative importance
bio01 bio02 bio04 bio05 bio14
BDW CLY DES NTO SOC bio01 bio02 bio04 bio05 bio14
BDW CLY DES NTO SOC
Thyridia repens
Climate
Climate + Soil
Soil
AUC = 0.91
TSS = 0.6
AUC = 0.92
TSS = 0.62
AUC = 0.89
TSS = 0.53
Relative importance
bio01 bio02 bio04 bio06 bio12
CEC CLY DES NTO TWI bio01 bio02 bio04 bio06 bio12
CEC CLY DES NTO TWI

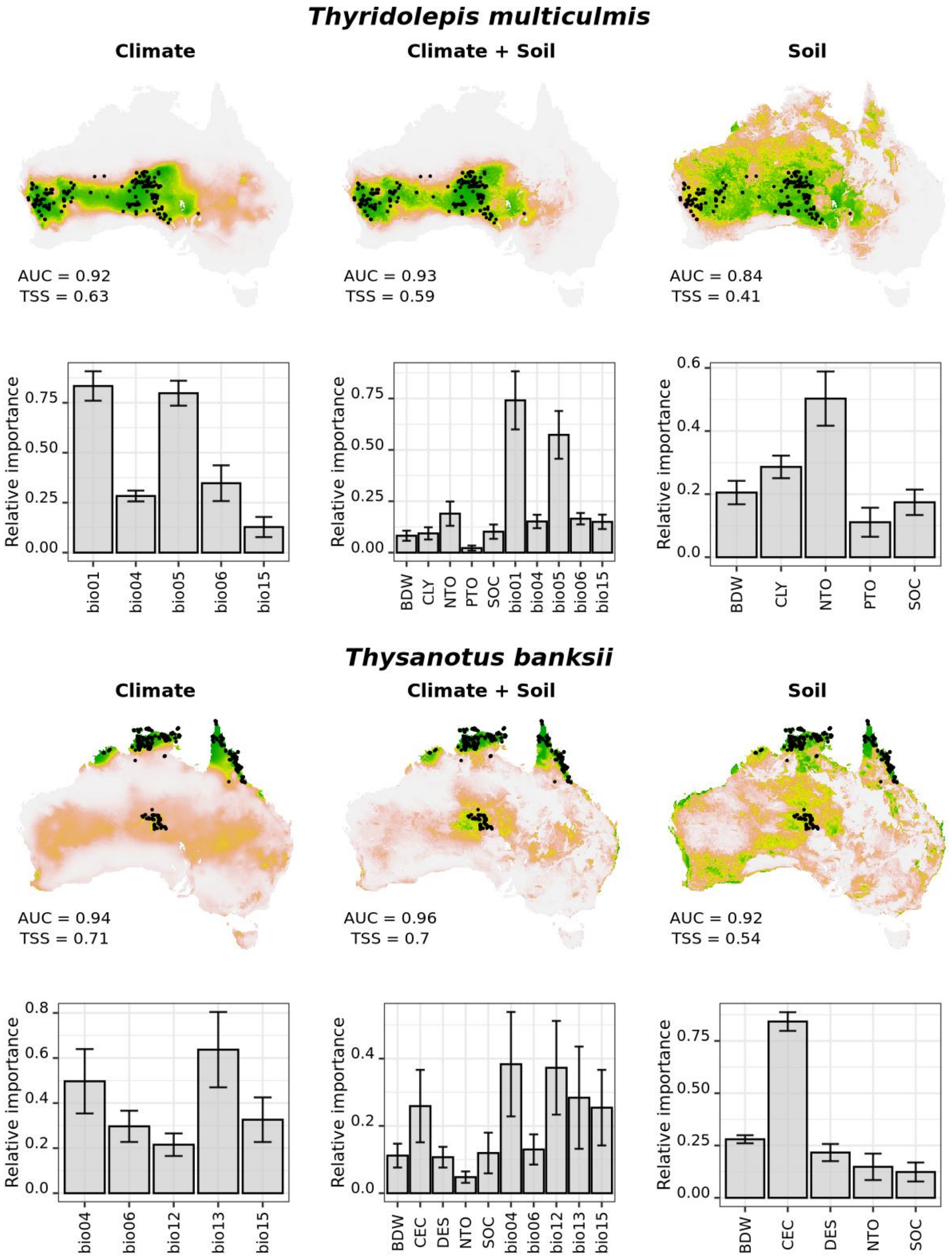
Thyridolepis multiculmis
Climate
Climate + Soil
Soil
AUC = 0.92
TSS = 0.63
AUC = 0.93
TSS = 0.59
AUC = 0.84
TSS = 0.41
Relative importance
bio01
bio04
bio05
bio06
bio15
BDW
CLY
NTO
PTO
SOC
Thysanotus banksii
Climate
Climate + Soil
Soil
AUC = 0.94
TSS = 0.71
AUC = 0.96
TSS = 0.7
AUC = 0.92
TSS = 0.54
Relative importance
bio04
bio06
bio12
bio13
bio15
BDW
CEC
DES
NTO
SOC

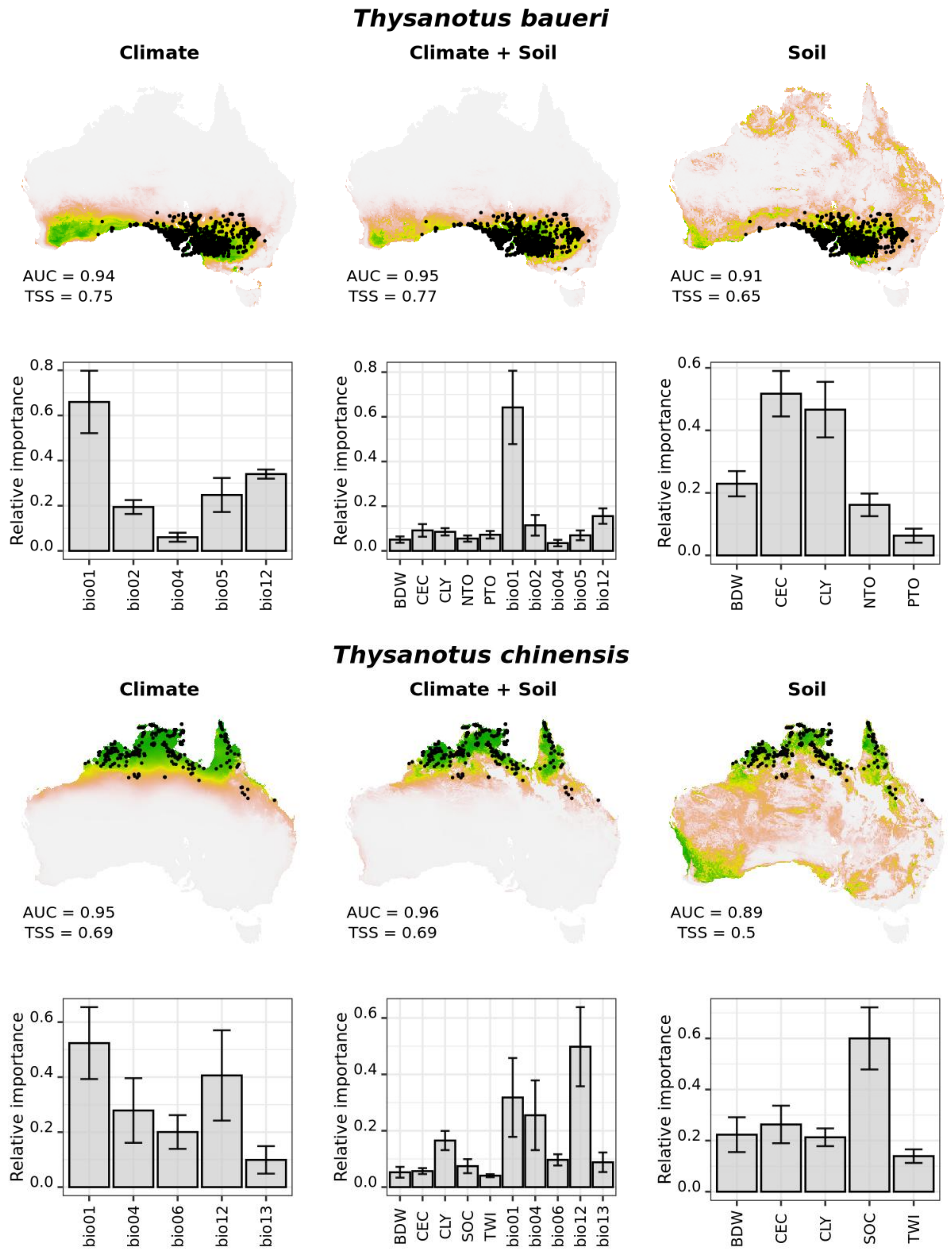
Thysanotus baueri
Climate
Climate + Soil
Soil
AUC = 0.94
TSS = 0.75
AUC = 0.95
TSS = 0.77
AUC = 0.91
TSS = 0.65
Relative importance
bio01 bio02 bio04 bio05 bio12
BDW CEC CLY NTO PTO bio01 bio02 bio04 bio05 bio12
BDW CEC CLY NTO PTO
Thysanotus chinensis
Climate
Climate + Soil
Soil
AUC = 0.95
TSS = 0.69
AUC = 0.96
TSS = 0.69
AUC = 0.89
TSS = 0.5
Relative importance
bio01 bio04 bio06 bio12 bio13
BDW CEC CLY SOC TWI bio01 bio04 bio06 bio12 bio13
BDW CEC CLY SOC TWI

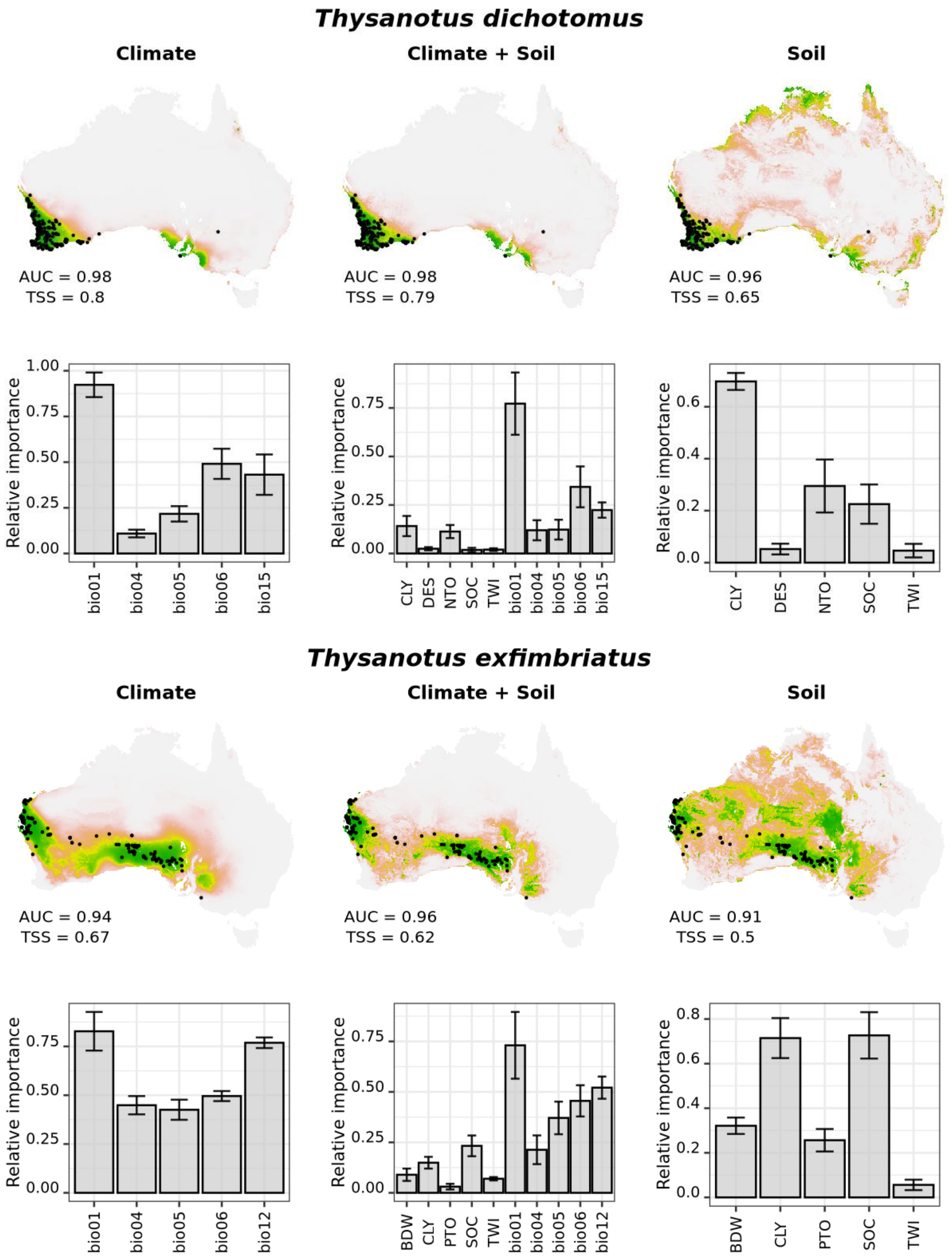
Thysanotus dichotomus
Climate
Climate + Soil
Soil
AUC = 0.98
TSS = 0.8
AUC = 0.98
TSS = 0.79
AUC = 0.96
TSS = 0.65
Relative importance
1.00
0.75
0.50
0.25
0.00
bio01
bio04
bio05
bio06
bio15
CLY
DES
NTO
SOC
TWI
0.6
0.4
0.2
0.0
Thysanotus exfimbriatus
Climate
Climate + Soil
Soil
AUC = 0.94
TSS = 0.67
AUC = 0.96
TSS = 0.62
AUC = 0.91
TSS = 0.5
bio12
BDW
PTO
0.8

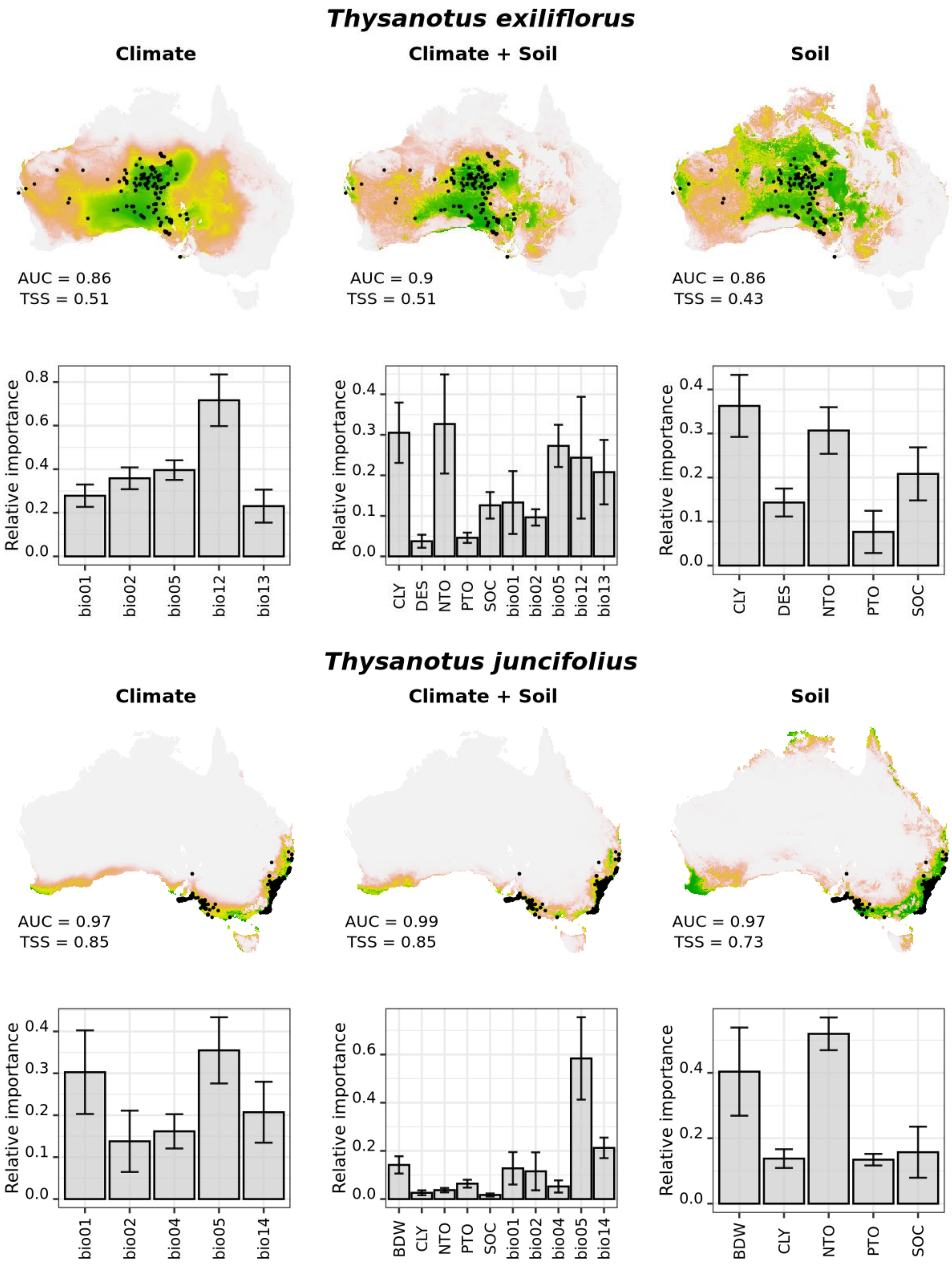

Thysanotus exiliflorus
Climate
Climate + Soil
Soil
AUC = 0.86
TSS = 0.51
AUC = 0.9
TSS = 0.51
AUC = 0.86
TSS = 0.43
Relative importance
bio01
bio02
bio05
bio12
bio13
CLY
DES
NTO
PTO
SOC
Thysanotus juncifolius
Climate
Climate + Soil
Soil
AUC = 0.97
TSS = 0.85
AUC = 0.99
TSS = 0.85
AUC = 0.97
TSS = 0.73
Relative importance
bio01
bio02
bio04
bio05
bio14
BDW
CLY
NTO
PTO
SOC

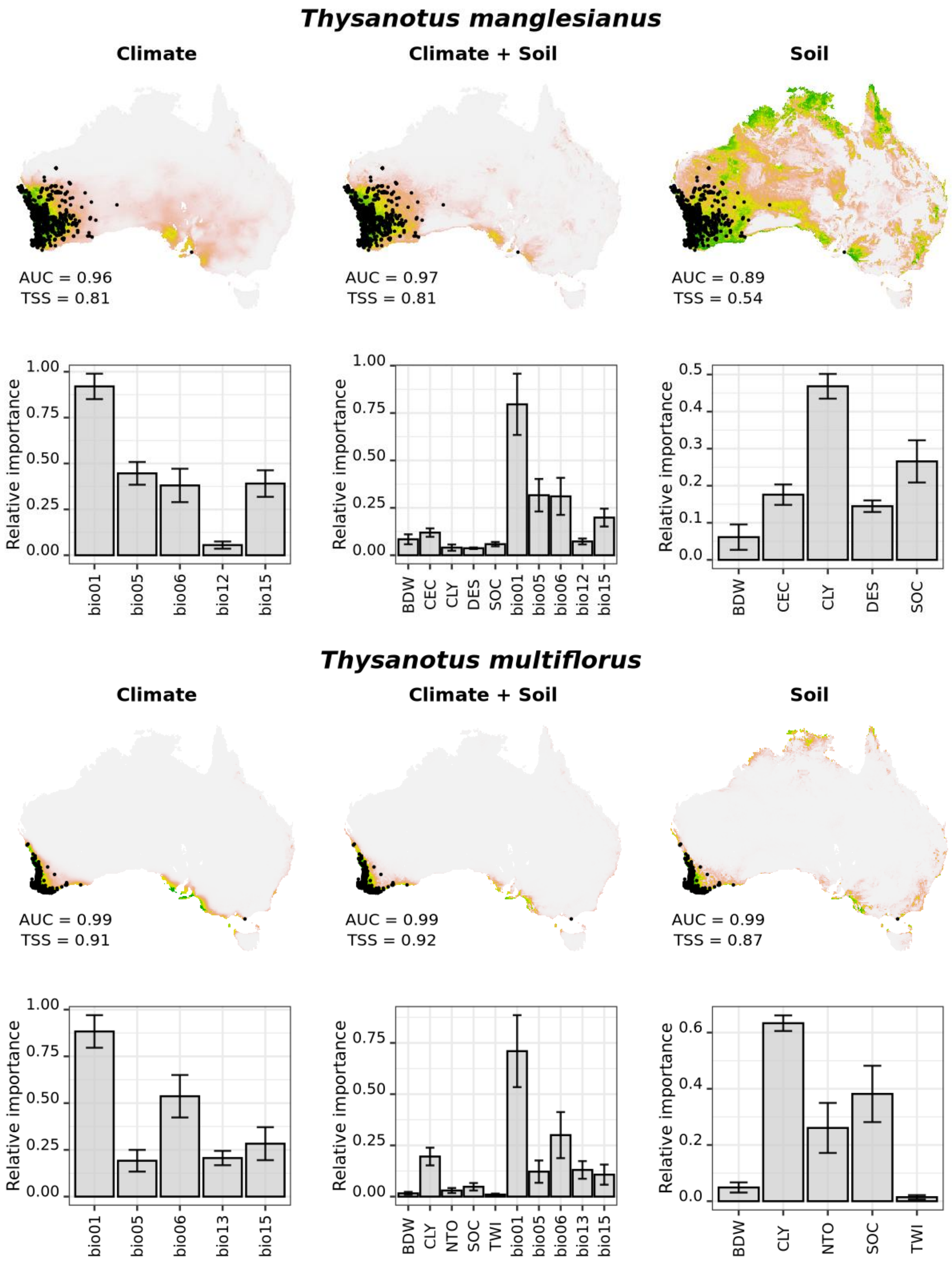

Thysanotus manglesianus
Climate
Climate + Soil
Soil
AUC = 0.96
TSS = 0.81
AUC = 0.97
TSS = 0.81
AUC = 0.89
TSS = 0.54
Relative importance
1.00
0.75
0.50
0.25
0.00
bio01
bio05
bio06
bio12
bio15
BDW
CEC
CLY
DES
SOC
0.5
0.4
0.3
0.2
0.1
0.0
Thysanotus multiflorus
Climate
Climate + Soil
Soil
AUC = 0.99
TSS = 0.91
AUC = 0.99
TSS = 0.92
AUC = 0.99
TSS = 0.87
bio13
NTO
TWI
0.6

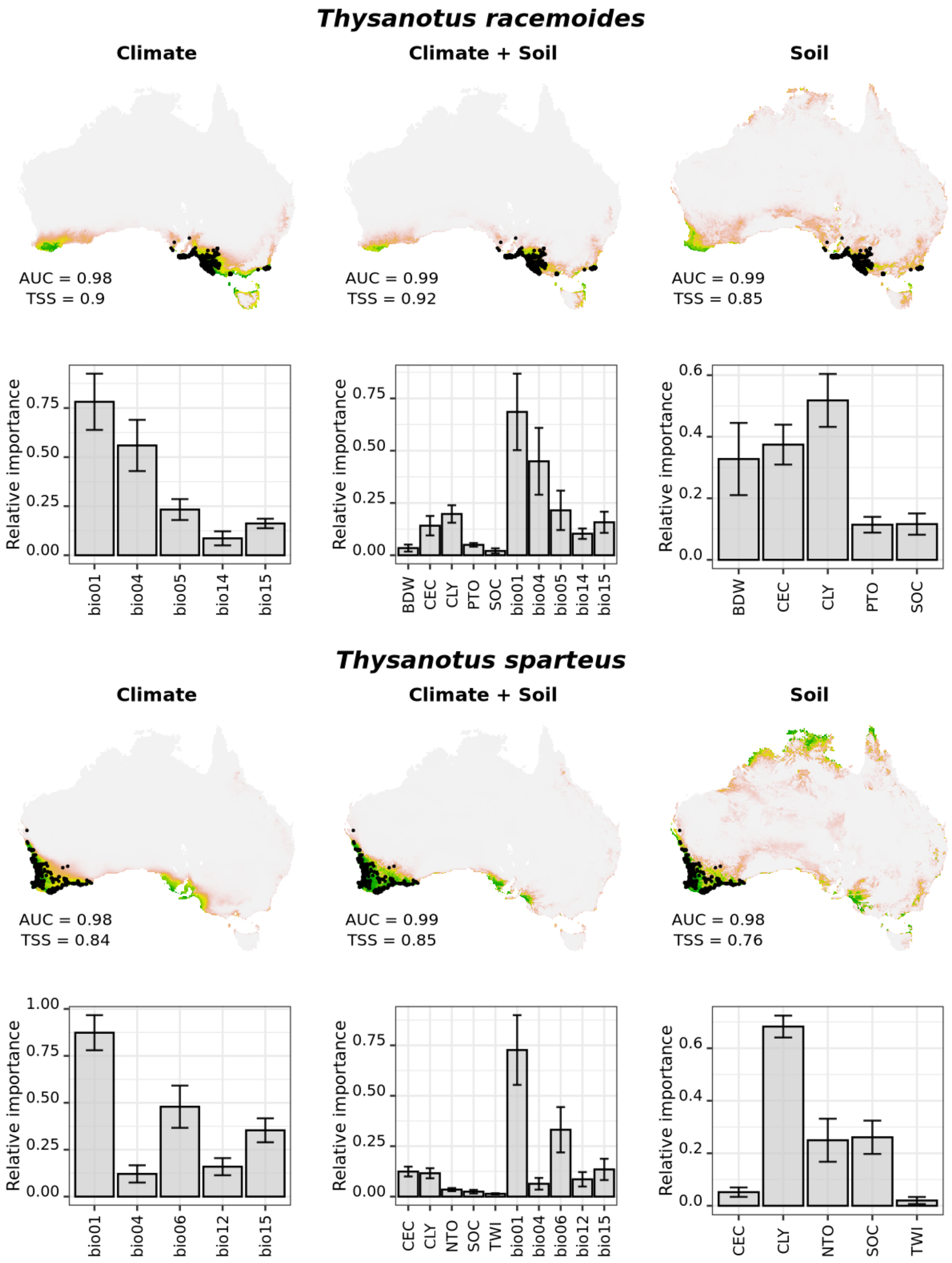
Thysanotus racemoides
Climate
Climate + Soil
Soil
AUC = 0.98
TSS = 0.9
AUC = 0.99
TSS = 0.92
AUC = 0.99
TSS = 0.85
Relative importance
0.00
0.25
0.50
0.75
bio01
bio04
bio05
bio14
bio15
BDW
CEC
CLY
PTO
SOC
0.0
0.2
0.4
0.6
Thysanotus sparteus
AUC = 0.98
TSS = 0.84
AUC = 0.99
TSS = 0.85
AUC = 0.98
TSS = 0.76
1.00
bio06
bio12
NTO
TWI

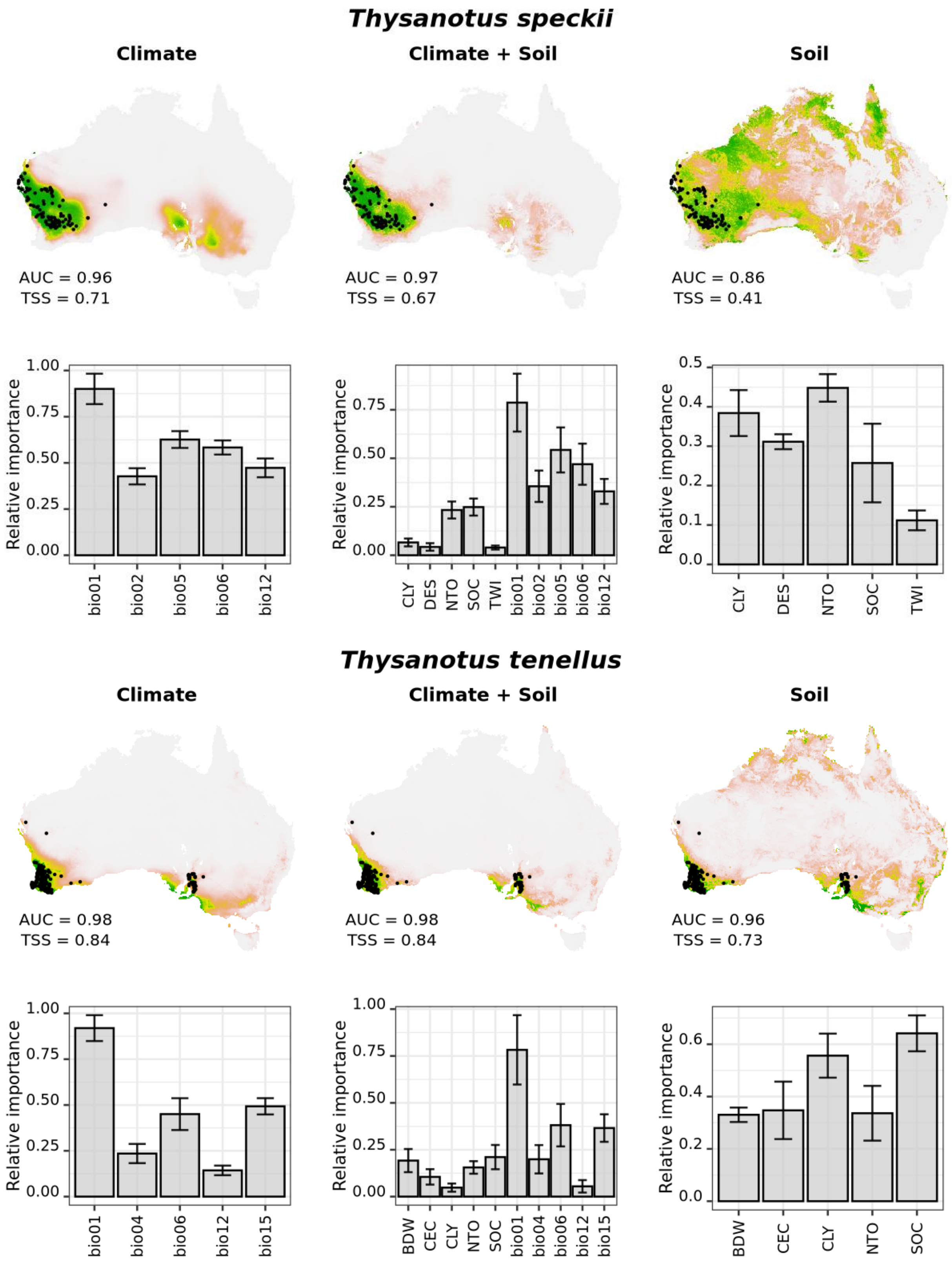

Thysanotus speckii
Climate
Climate + Soil
Soil
AUC = 0.96
TSS = 0.71
AUC = 0.97
TSS = 0.67
AUC = 0.86
TSS = 0.41
Relative importance
bio01 bio02 bio05 bio06 bio12
CLY DES NTO SOC TWI bio01 bio02 bio05 bio06 bio12
CLY DES NTO SOC TWI
Thysanotus tenellus
Climate
Climate + Soil
Soil
AUC = 0.98
TSS = 0.84
AUC = 0.98
TSS = 0.84
AUC = 0.96
TSS = 0.73
Relative importance
bio01 bio04 bio06 bio12 bio15
BDW CEC CLY NTO SOC bio01 bio04 bio06 bio12 bio15
BDW CEC CLY NTO SOC

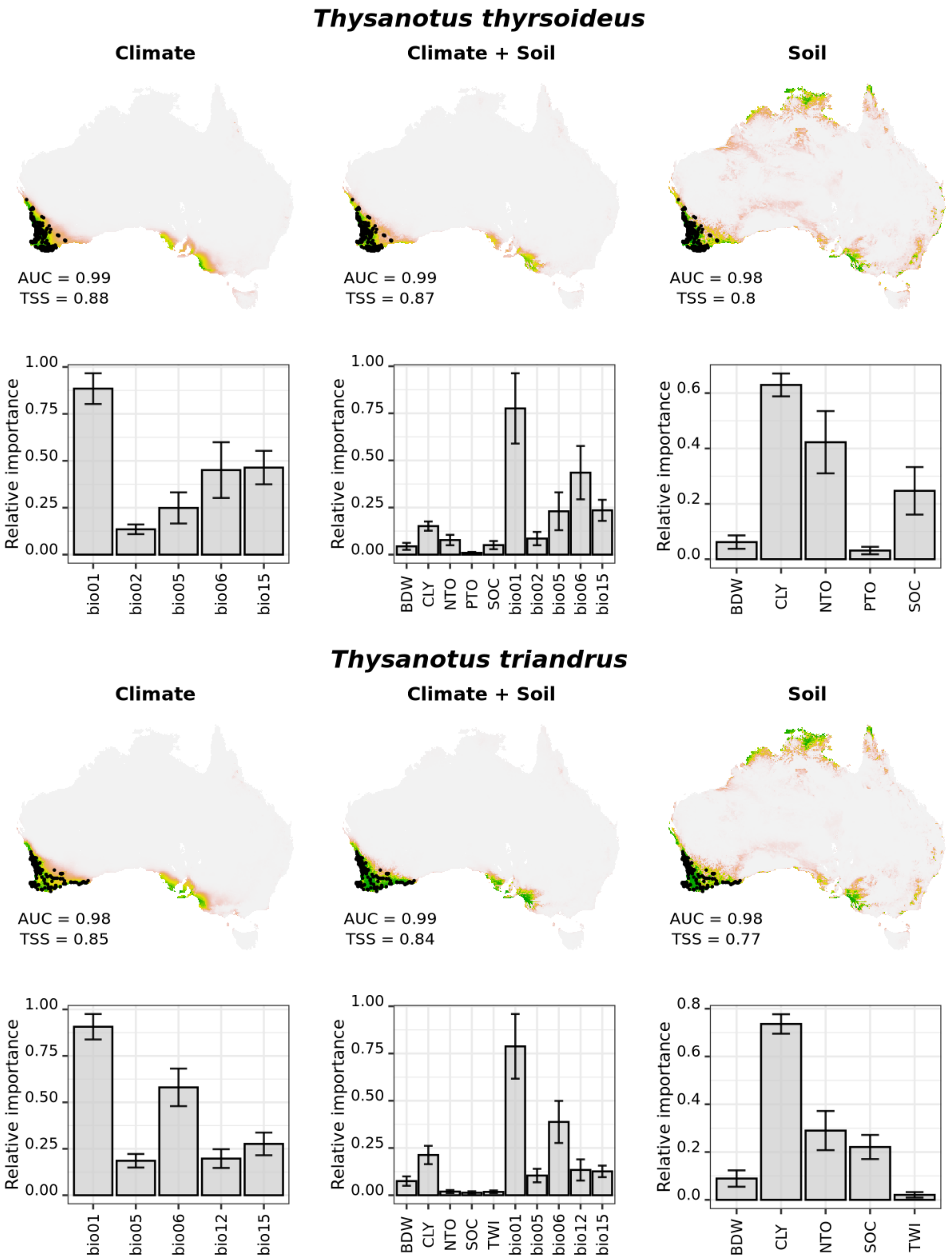

Thysanotus thyrsoideus
Climate
Climate + Soil
Soil
AUC = 0.99
TSS = 0.88
AUC = 0.99
TSS = 0.87
AUC = 0.98
TSS = 0.8
Relative importance
bio01
bio02
bio05
bio06
bio15
BDW
CLY
NTO
PTO
SOC
Thysanotus triandrus
Climate
Climate + Soil
Soil
AUC = 0.98
TSS = 0.85
AUC = 0.99
TSS = 0.84
AUC = 0.98
TSS = 0.77
Relative importance
bio01
bio05
bio06
bio12
bio15
BDW
CLY
NTO
SOC
TWI

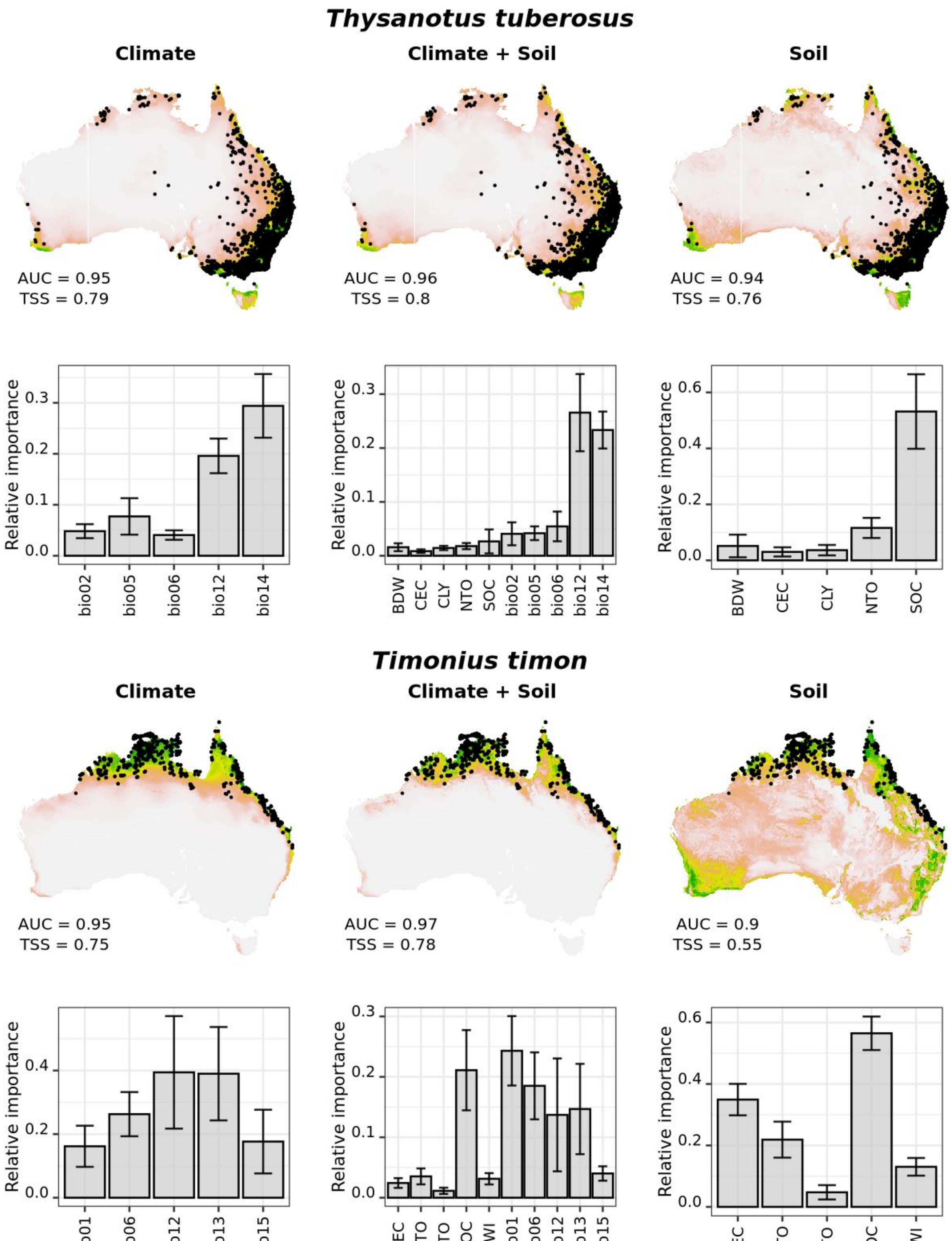
Thysanotus tuberosus
Climate
Climate + Soil
Soil
AUC = 0.95
TSS = 0.79
AUC = 0.96
TSS = 0.8
AUC = 0.94
TSS = 0.76
Relative importance
bio02
bio05
bio06
bio12
bio14
BDW
CEC
CLY
NTO
SOC
Timonius timon
AUC = 0.95
TSS = 0.75
AUC = 0.97
TSS = 0.78
AUC = 0.9
TSS = 0.55
bio01
bio13
bio15
PTO
TWI

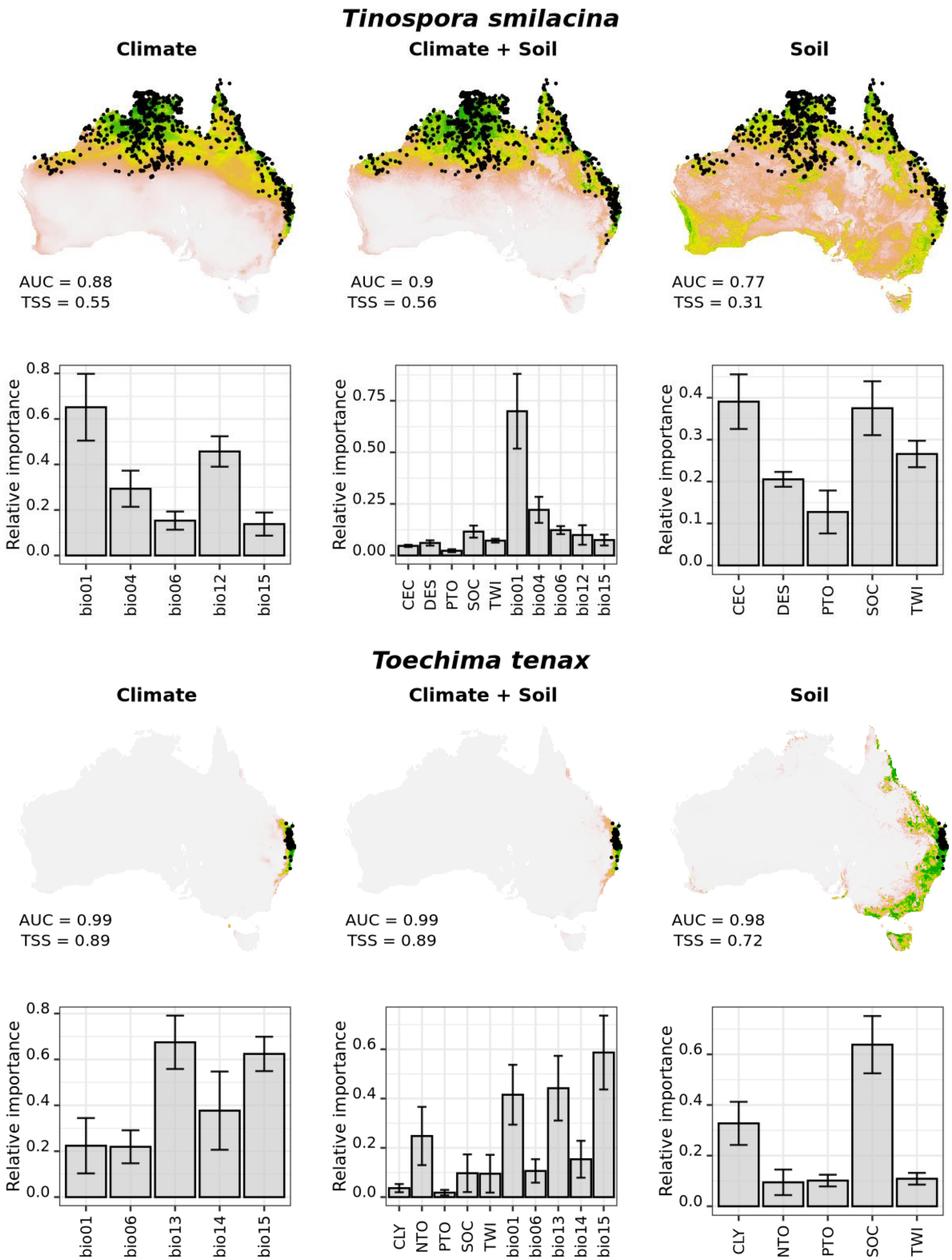

Tinospora smilacina
Climate
Climate + Soil
Soil
AUC = 0.88
TSS = 0.55
AUC = 0.9
TSS = 0.56
AUC = 0.77
TSS = 0.31
Relative importance
bio01
bio04
bio06
bio12
bio15
CEC
DES
PTO
SOC
TWI
bio01
bio04
bio06
bio12
bio15
CEC
DES
PTO
SOC
TWI
Toechima tenax
Climate
Climate + Soil
Soil
AUC = 0.99
TSS = 0.89
AUC = 0.99
TSS = 0.89
AUC = 0.98
TSS = 0.72
Relative importance
bio01
bio06
bio13
bio14
bio15
CLY
NTO
PTO
SOC
TWI
bio01
bio06
bio13
bio14
bio15
CLY
NTO
PTO
SOC
TWI

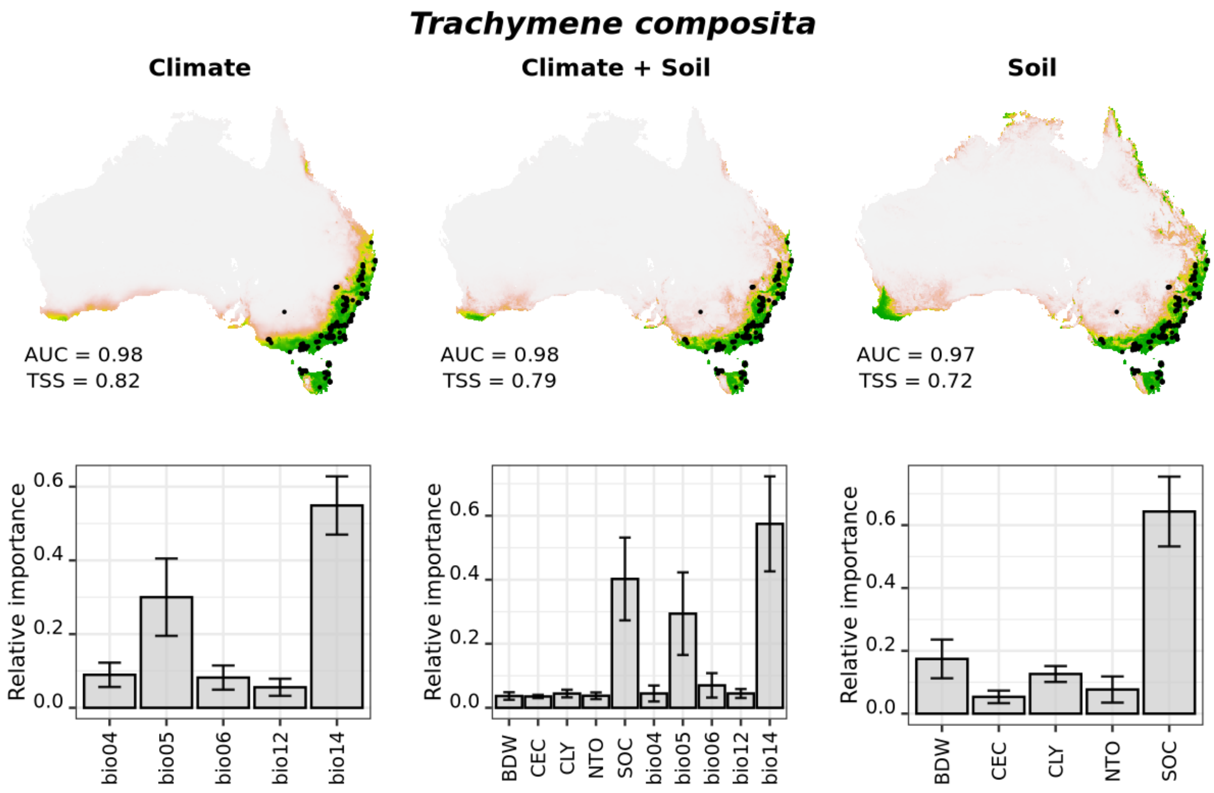

Trachymene composita
Climate
Climate + Soil
Soil
AUC = 0.98
TSS = 0.82
AUC = 0.98
TSS = 0.79
AUC = 0.97
TSS = 0.72
Relative importance
0.0
0.2
0.4
0.6
bio04
bio05
bio06
bio12
bio14
BDW
CEC
CLY
NTO
SOC

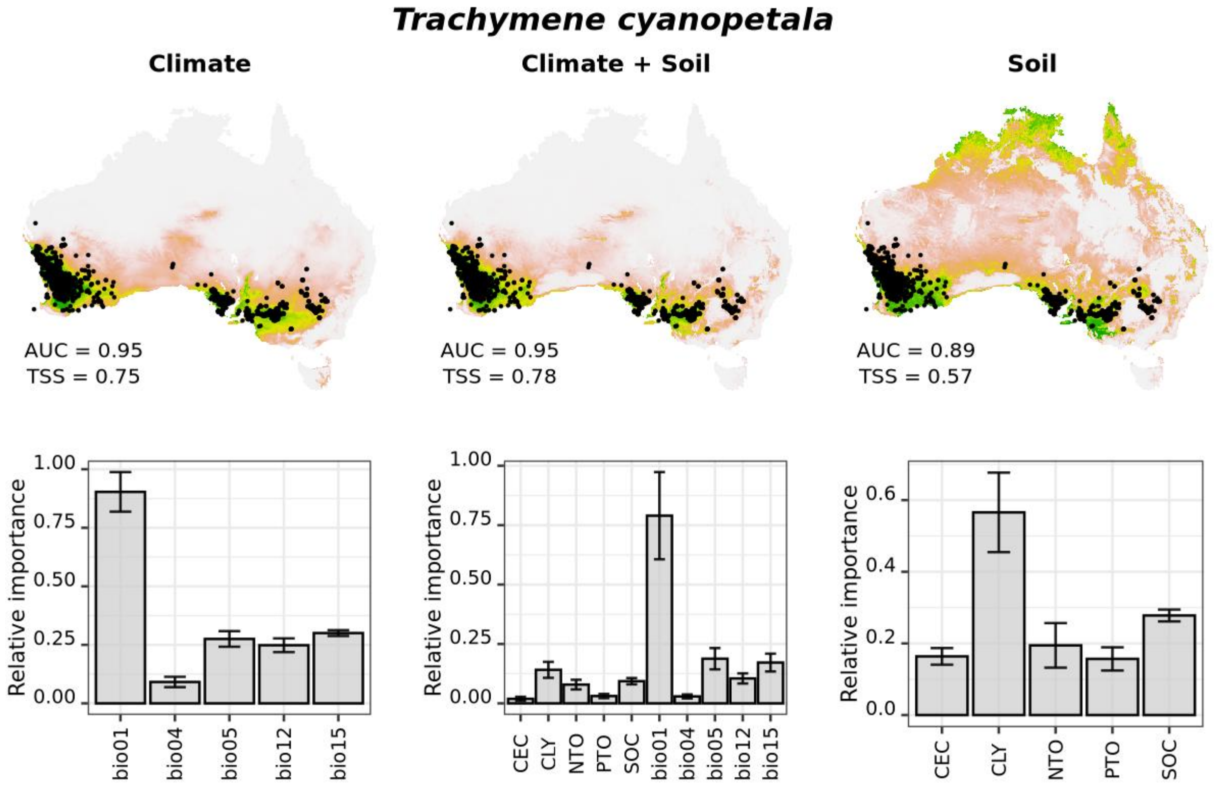

Trachymene cyanopetala
Climate
Climate + Soil
Soil
AUC = 0.95
TSS = 0.75
AUC = 0.95
TSS = 0.78
AUC = 0.89
TSS = 0.57
Relative importance
0.00
0.25
0.50
0.75
1.00
0.0
0.2
0.4
0.6
bio01
bio04
bio05
bio12
bio15
CEC
CLY
NTO
PTO
SOC

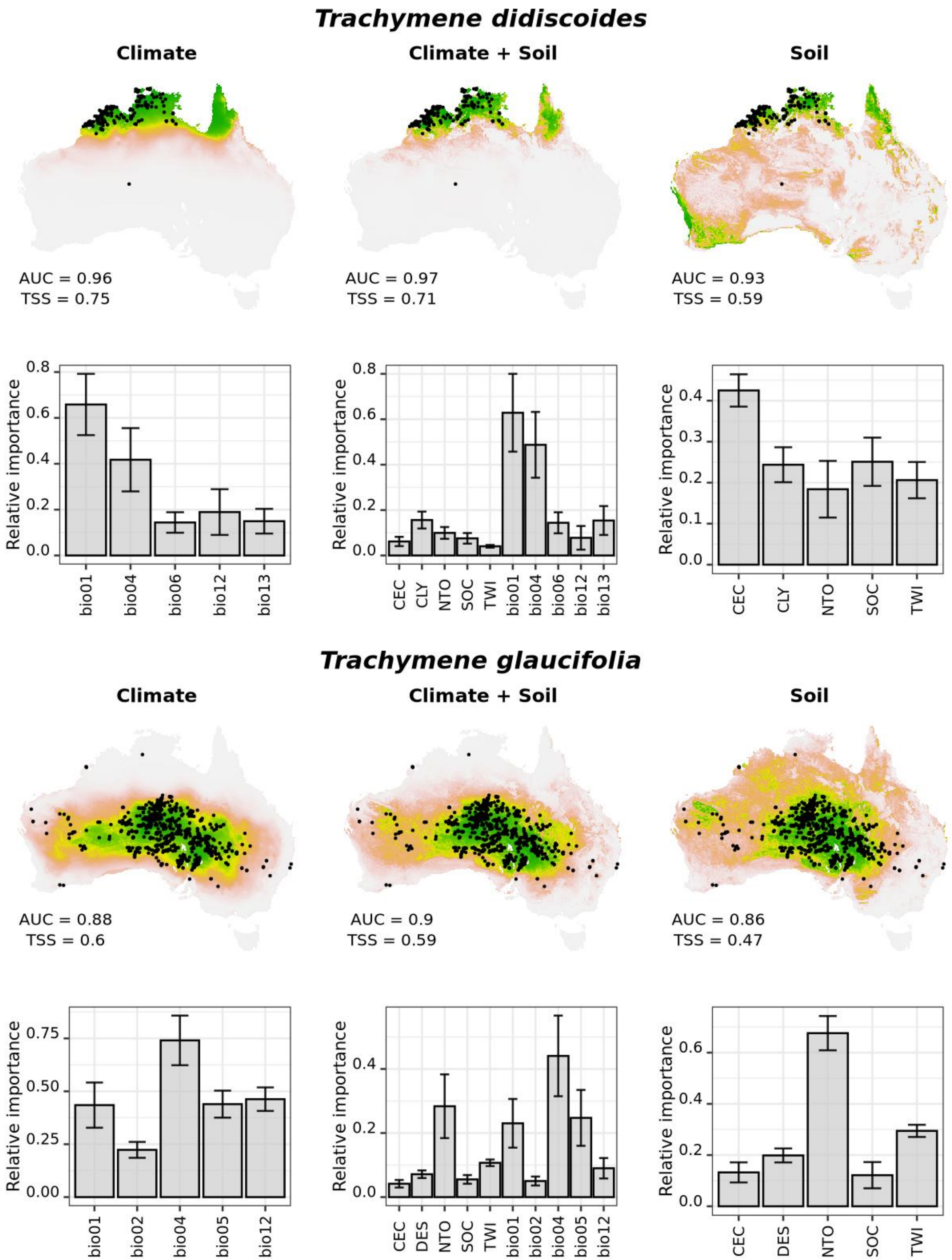
Trachymene didiscoides
Climate
Climate + Soil
Soil
AUC = 0.96
TSS = 0.75
AUC = 0.97
TSS = 0.71
AUC = 0.93
TSS = 0.59
Relative importance
bio01
bio04
bio06
bio12
bio13
CEC
CLY
NTO
SOC
TWI
Trachymene glaucifolia
Climate
Climate + Soil
Soil
AUC = 0.88
TSS = 0.6
AUC = 0.9
TSS = 0.59
AUC = 0.86
TSS = 0.47
Relative importance
bio01
bio02
bio04
bio05
bio12
CEC
DES
NTO
SOC
TWI

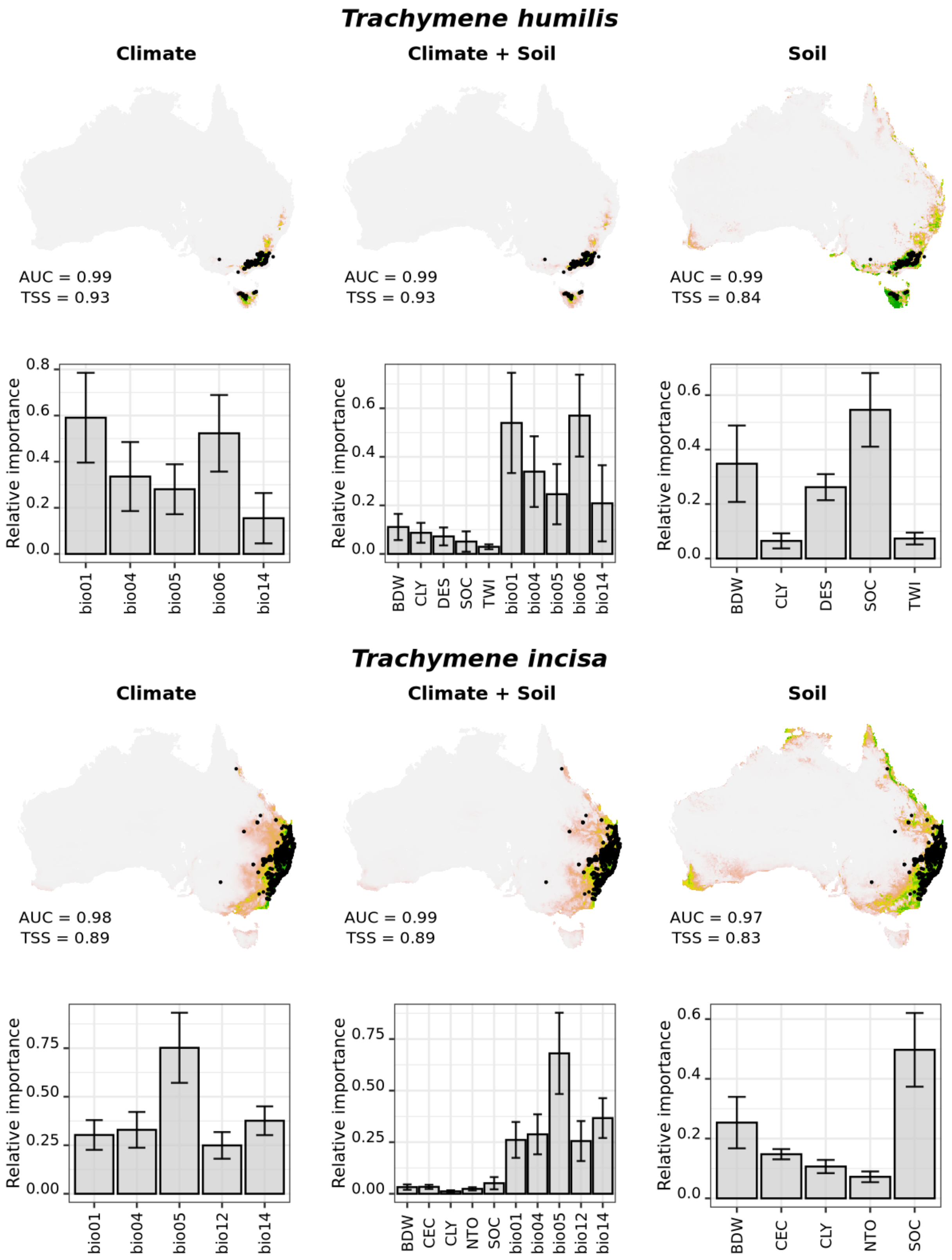
Trachymene humilis
Climate
Climate + Soil
Soil
AUC = 0.99
TSS = 0.93
AUC = 0.99
TSS = 0.93
AUC = 0.99
TSS = 0.84
Relative importance
bio01
bio04
bio05
bio06
bio14
BDW
CLY
DES
SOC
TWI
Trachymene incisa
Climate
Climate + Soil
Soil
AUC = 0.98
TSS = 0.89
AUC = 0.99
TSS = 0.89
AUC = 0.97
TSS = 0.83
Relative importance
bio01
bio04
bio05
bio12
bio14
BDW
CEC
CLY
NTO
SOC

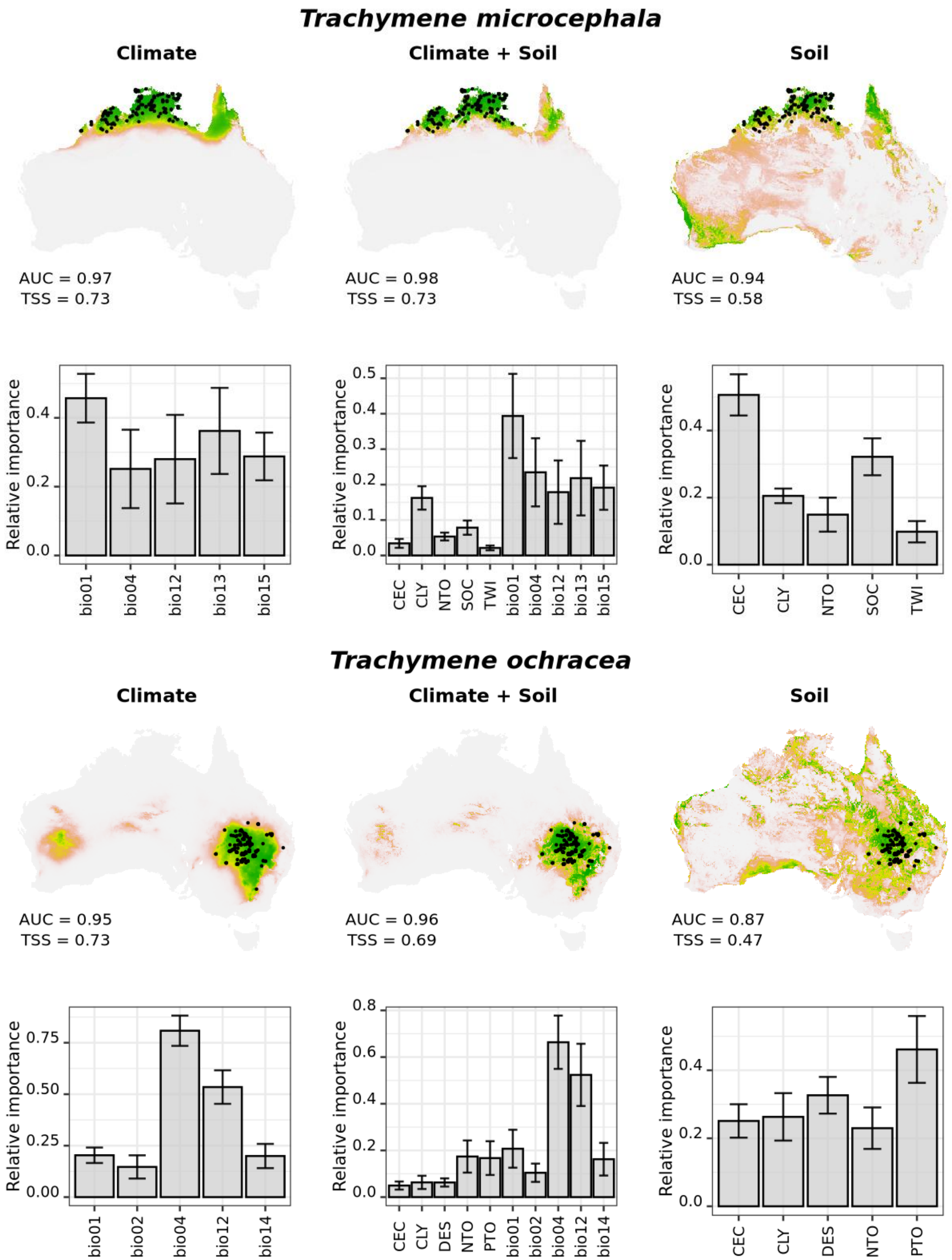

Trachymene microcephala
Climate
Climate + Soil
Soil
AUC = 0.97
TSS = 0.73
AUC = 0.98
TSS = 0.73
AUC = 0.94
TSS = 0.58
Relative importance
0.0
0.2
0.4
bio01
bio04
bio12
bio13
bio15
Relative importance
0.0
0.1
0.2
0.3
0.4
0.5
CEC
CLY
NTO
SOC
TWI
bio01
bio04
bio12
bio13
bio15
Relative importance
0.0
0.2
0.4
CEC
CLY
NTO
SOC
TWI
Trachymene ochracea
Climate
Climate + Soil
Soil
AUC = 0.95
TSS = 0.73
AUC = 0.96
TSS = 0.69
AUC = 0.87
TSS = 0.47
Relative importance
0.00
0.25
0.50
0.75
bio01
bio02
bio04
bio12
bio14
Relative importance
0.0
0.2
0.4
0.6
0.8
CEC
CLY
DES
NTO
PTO
bio01
bio02
bio04
bio12
bio14
Relative importance
0.0
0.2
0.4
CEC
CLY
DES
NTO
PTO

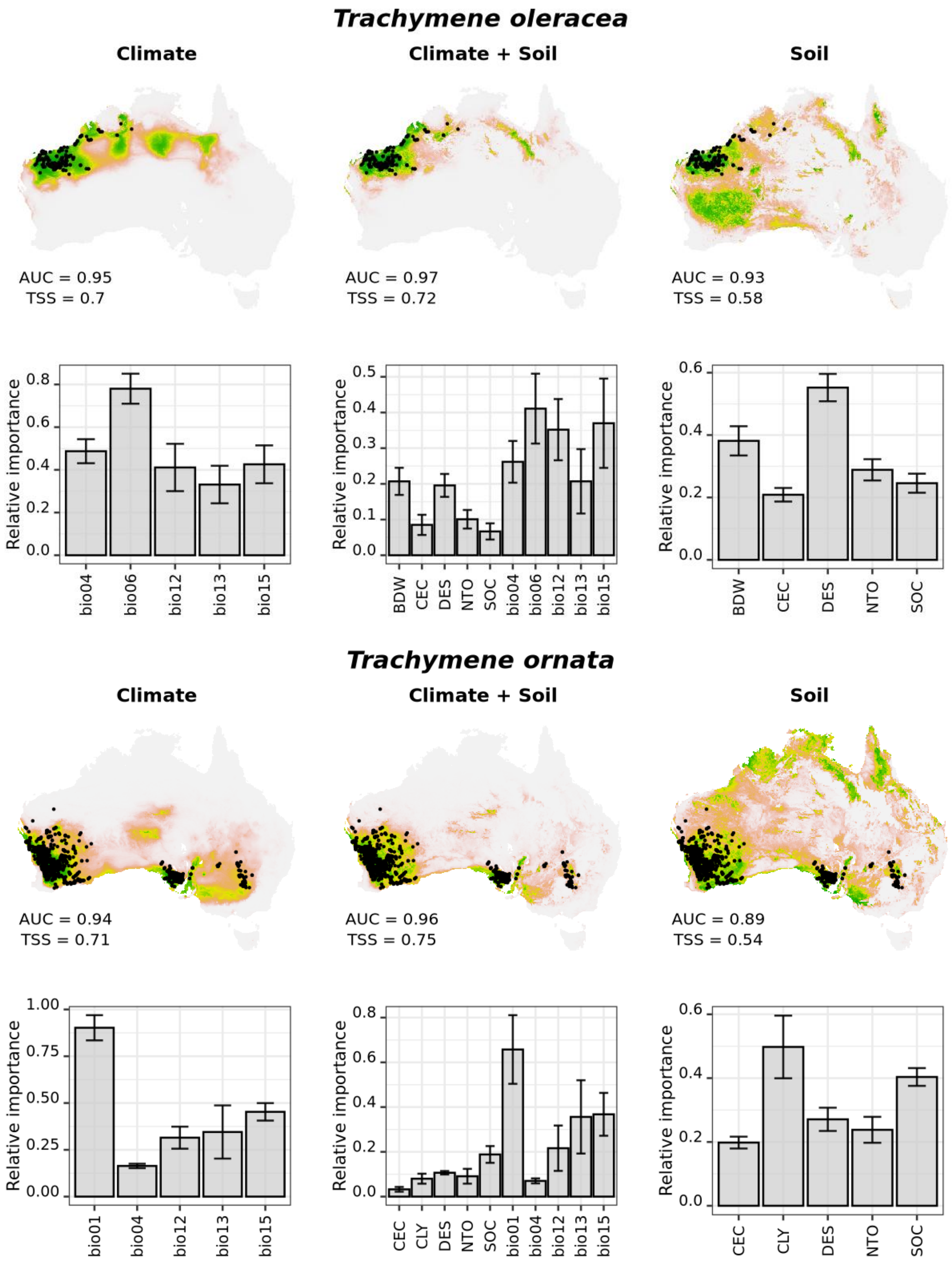
Trachymene oleracea
Climate
Climate + Soil
Soil
AUC = 0.95
TSS = 0.7
AUC = 0.97
TSS = 0.72
AUC = 0.93
TSS = 0.58
Relative importance
bio04
bio06
bio12
bio13
bio15
BDW
CEC
DES
NTO
SOC
Trachymene ornata
Climate
Climate + Soil
Soil
AUC = 0.94
TSS = 0.71
AUC = 0.96
TSS = 0.75
AUC = 0.89
TSS = 0.54
Relative importance
bio01
CLY

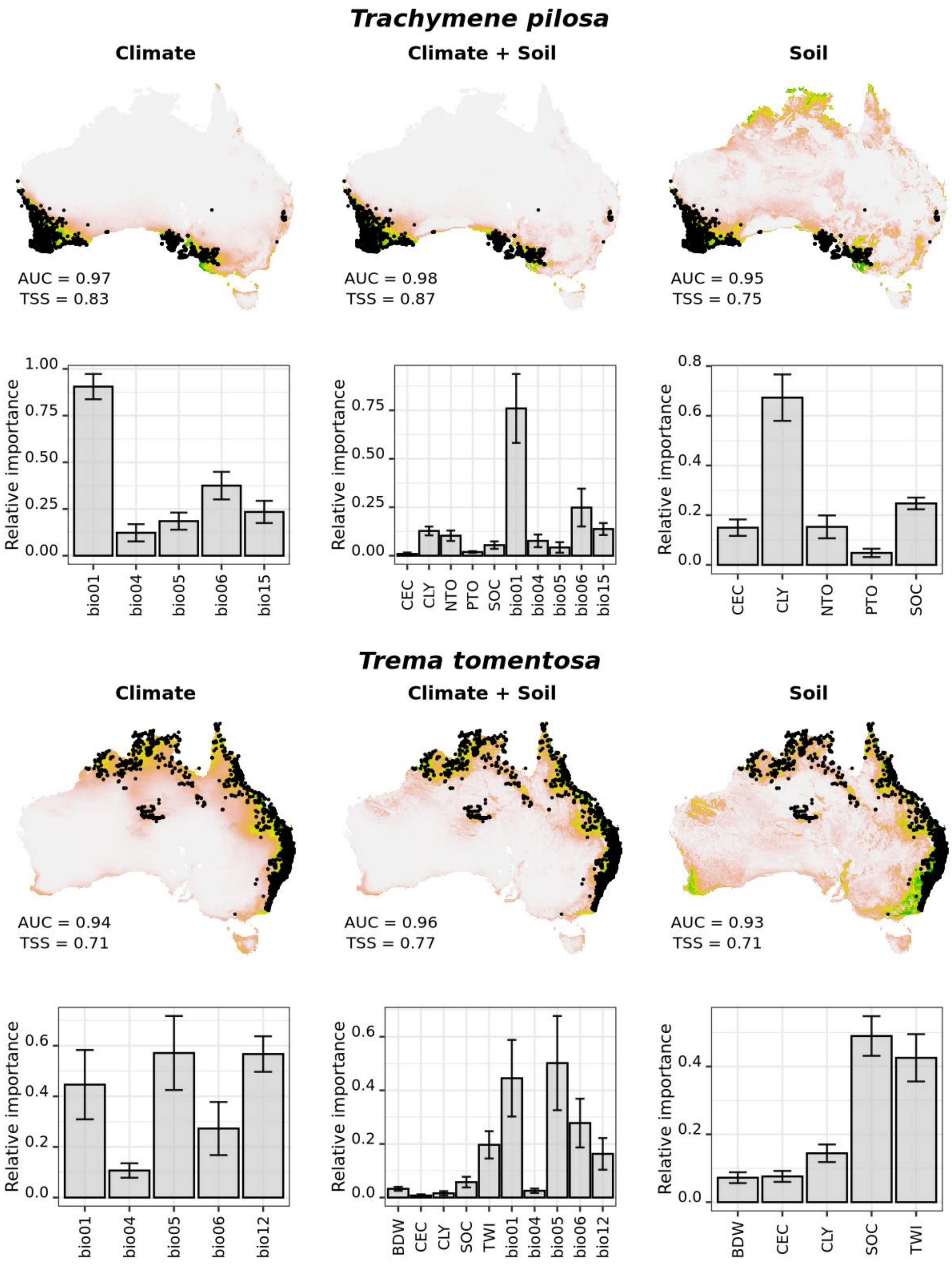
Trachymene pilosa
Climate
Climate + Soil
Soil
AUC = 0.97
TSS = 0.83
AUC = 0.98
TSS = 0.87
AUC = 0.95
TSS = 0.75
Relative importance
bio01 bio04 bio05 bio06 bio15
CEC CLY NTO PTO SOC bio01 bio04 bio05 bio06 bio15
CEC CLY NTO PTO SOC
Trema tomentosa
Climate
Climate + Soil
Soil
AUC = 0.94
TSS = 0.71
AUC = 0.96
TSS = 0.77
AUC = 0.93
TSS = 0.71
Relative importance
bio01 bio04 bio05 bio06 bio12
BDW CEC CLY SOC TWI bio01 bio04 bio05 bio06 bio12
BDW CEC CLY SOC TWI

Tremandra stelligera

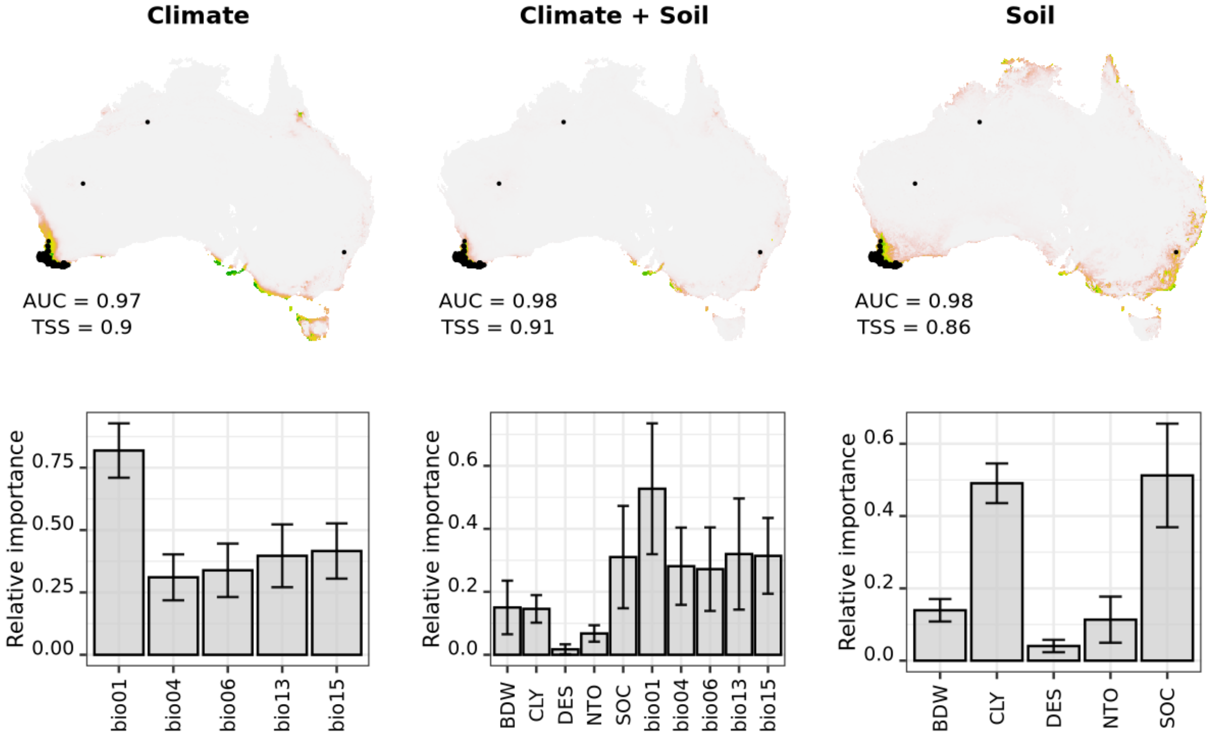

Trianthema pilosum

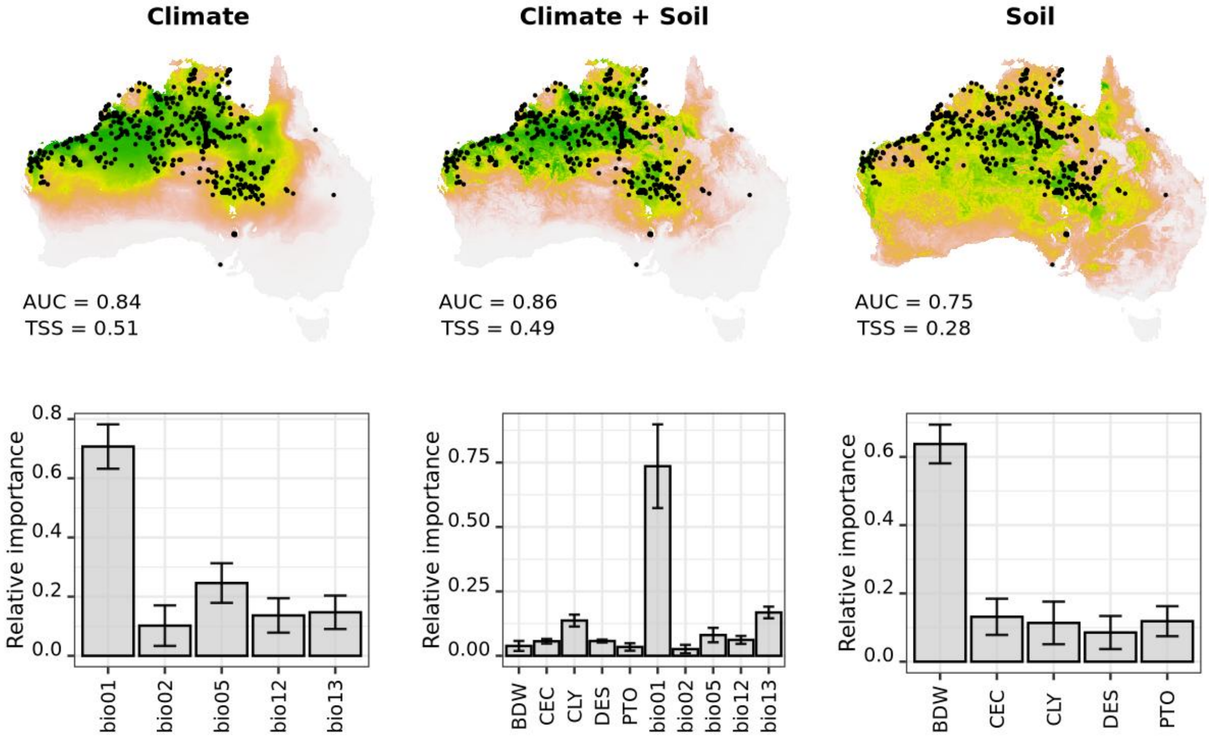

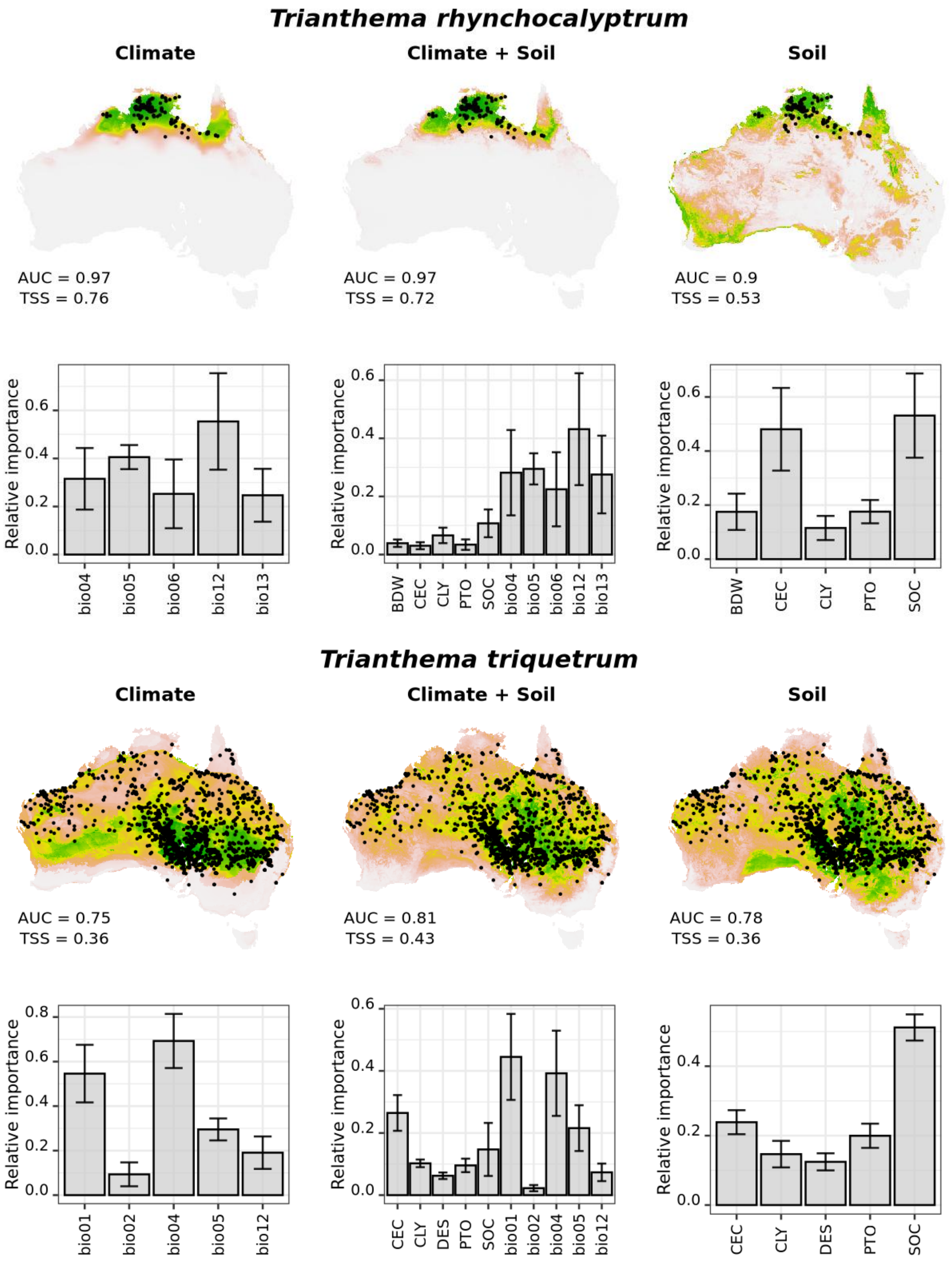

Trianthema rhynchocalyptrum
Climate
Climate + Soil
Soil
AUC = 0.97
TSS = 0.76
AUC = 0.97
TSS = 0.72
AUC = 0.9
TSS = 0.53
Relative importance
bio04
bio05
bio06
bio12
bio13
BDW
CEC
CLY
PTO
SOC
Trianthema triquetrum
Climate
Climate + Soil
Soil
AUC = 0.75
TSS = 0.36
AUC = 0.81
TSS = 0.43
AUC = 0.78
TSS = 0.36
Relative importance
bio01
bio02
bio04
bio05
bio12
CEC
CLY
DES
PTO
SOC

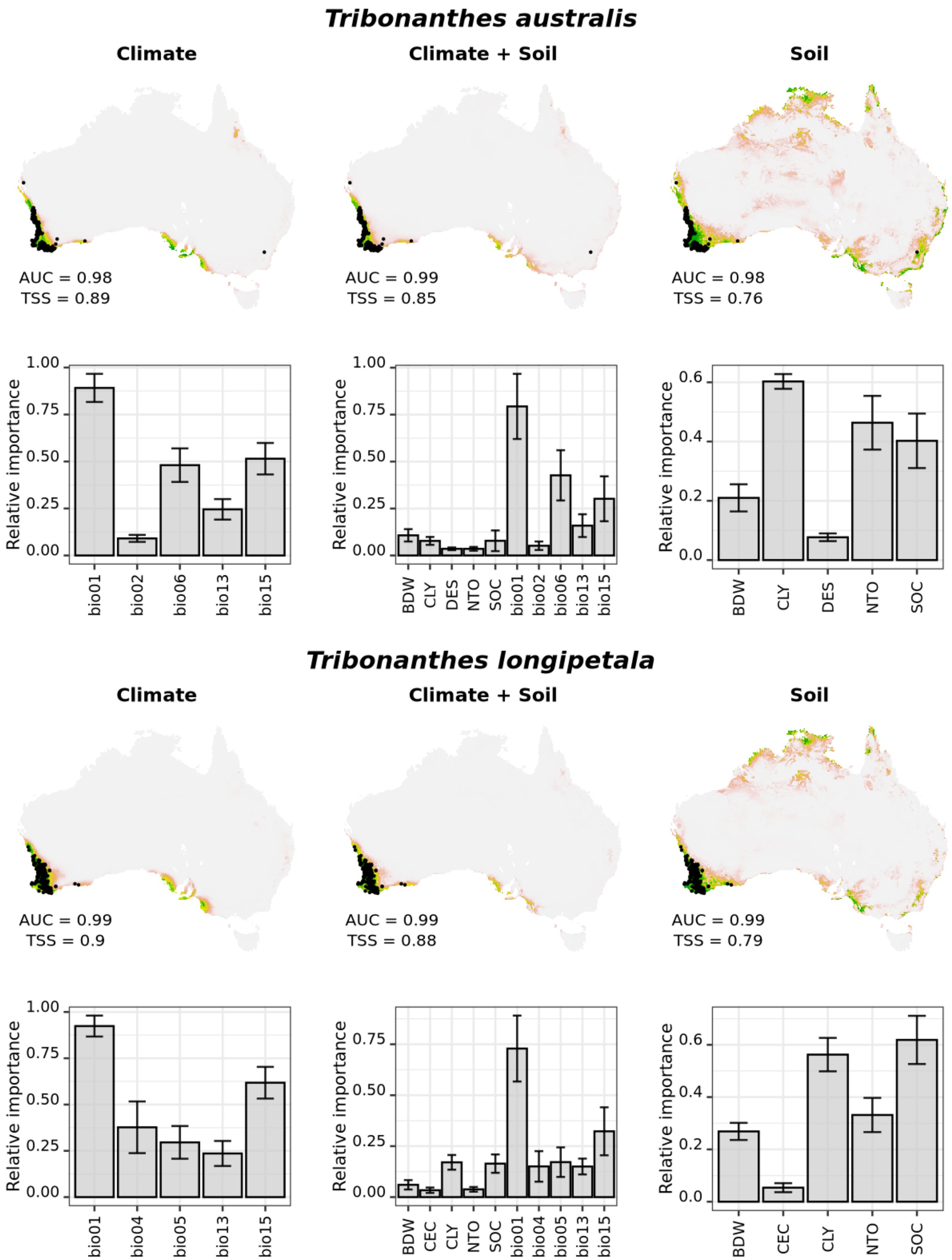

Tribonanthes australis
Climate
Climate + Soil
Soil
AUC = 0.98
TSS = 0.89
AUC = 0.99
TSS = 0.85
AUC = 0.98
TSS = 0.76
Relative importance
bio01 bio02 bio06 bio13 bio15
BDW CLY DES NTO SOC bio01 bio02 bio06 bio13 bio15
BDW CLY DES NTO SOC
Tribonanthes longipetala
Climate
Climate + Soil
Soil
AUC = 0.99
TSS = 0.9
AUC = 0.99
TSS = 0.88
AUC = 0.99
TSS = 0.79
Relative importance
bio01 bio04 bio05 bio13 bio15
BDW CEC CLY NTO SOC bio01 bio04 bio05 bio13 bio15
BDW CEC CLY NTO SOC

Tribulopis angustifolia

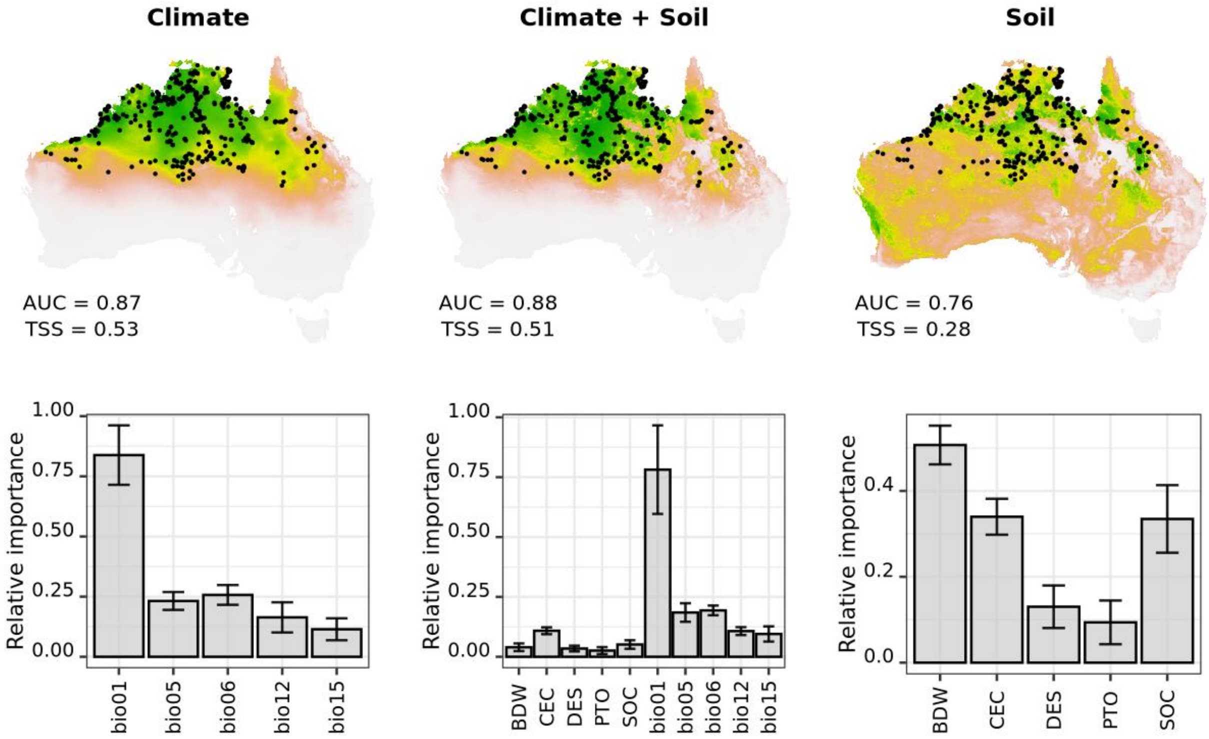

Tribulopis pentandra

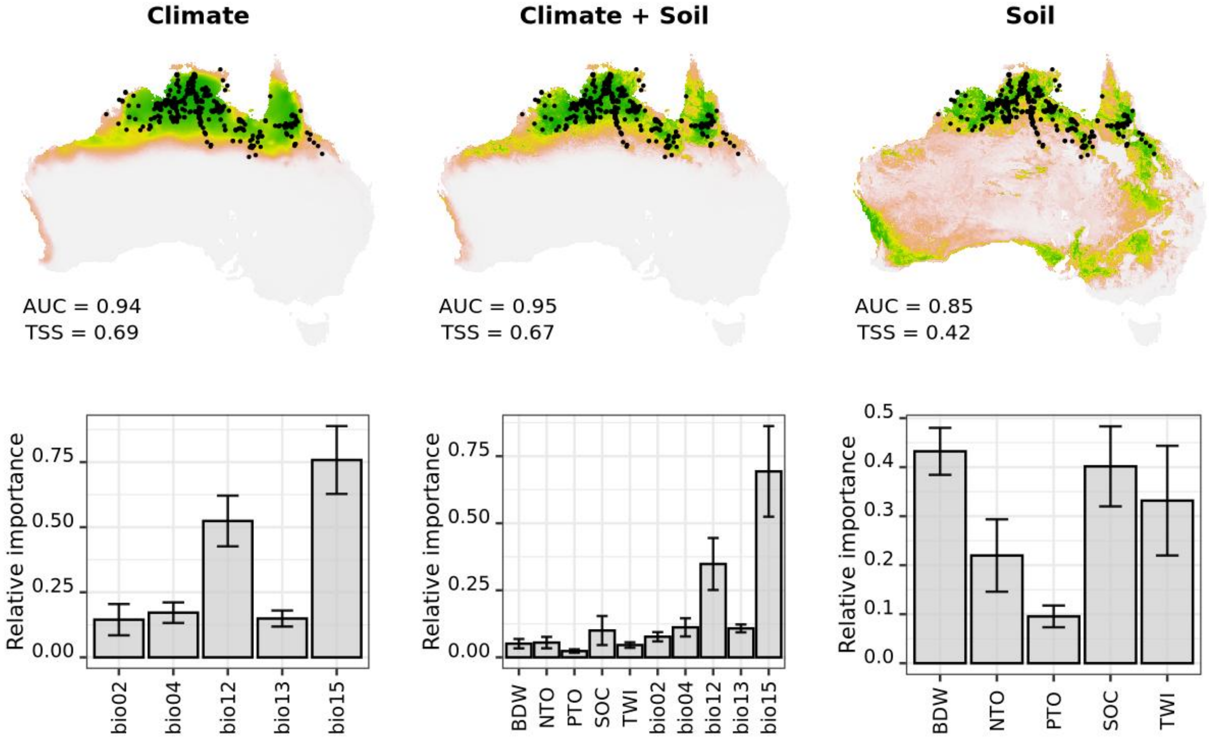

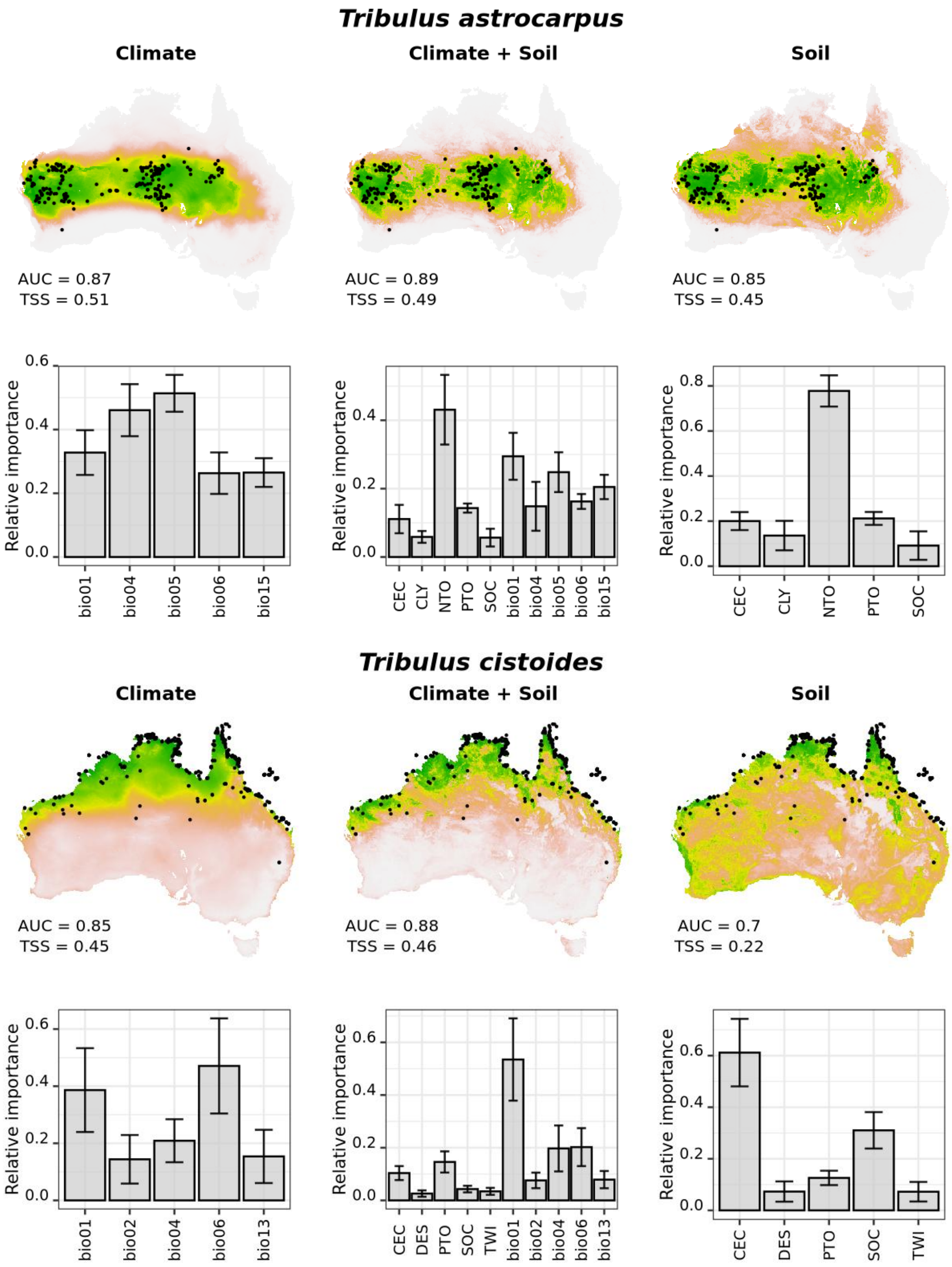
Tribulus astrocarpus
Climate
Climate + Soil
Soil
AUC = 0.87
TSS = 0.51
AUC = 0.89
TSS = 0.49
AUC = 0.85
TSS = 0.45
Relative importance
bio01
bio04
bio05
bio06
bio15
CEC
CLY
NTO
PTO
SOC
Tribulus cistoides
AUC = 0.85
TSS = 0.45
AUC = 0.88
TSS = 0.46
AUC = 0.7
TSS = 0.22
bio02
bio13
DES
TWI

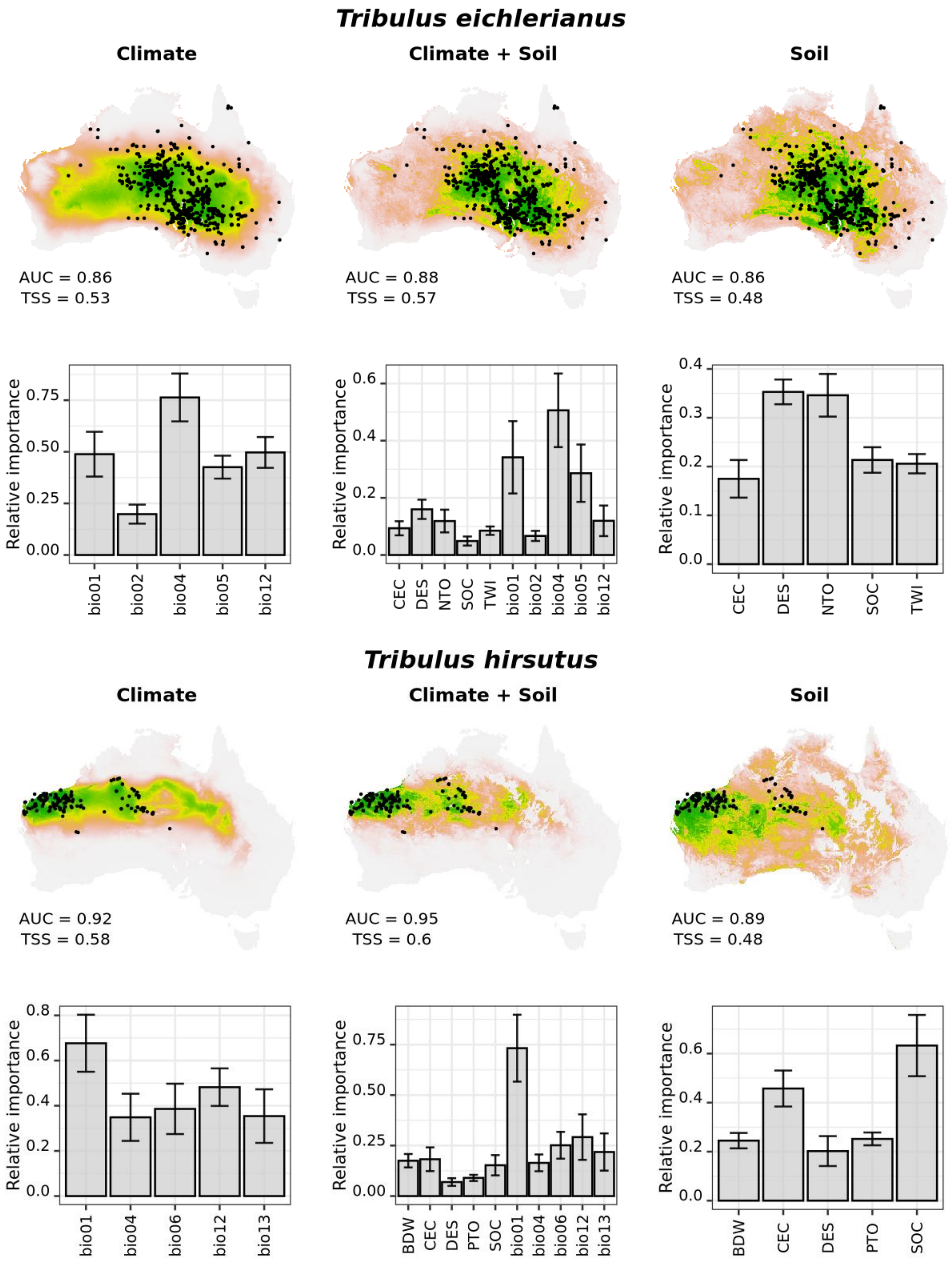
Tribulus eichlerianus
Climate
Climate + Soil
Soil
AUC = 0.86
TSS = 0.53
AUC = 0.88
TSS = 0.57
AUC = 0.86
TSS = 0.48
Relative importance
0.00
0.25
0.50
0.75
bio01
bio02
bio04
bio05
bio12
0.0
0.2
0.4
0.6
CEC
DES
NTO
SOC
TWI
bio01
bio02
bio04
bio05
bio12
0.0
0.1
0.2
0.3
0.4
CEC
DES
NTO
SOC
TWI
Tribulus hirsutus
Climate
Climate + Soil
Soil
AUC = 0.92
TSS = 0.58
AUC = 0.95
TSS = 0.6
AUC = 0.89
TSS = 0.48
Relative importance
0.0
0.2
0.4
0.6
0.8
bio01
bio04
bio06
bio12
bio13
0.00
0.25
0.50
0.75
BDW
CEC
DES
PTO
SOC
bio01
bio04
bio06
bio12
bio13
0.0
0.2
0.4
0.6
BDW
CEC
DES
PTO
SOC

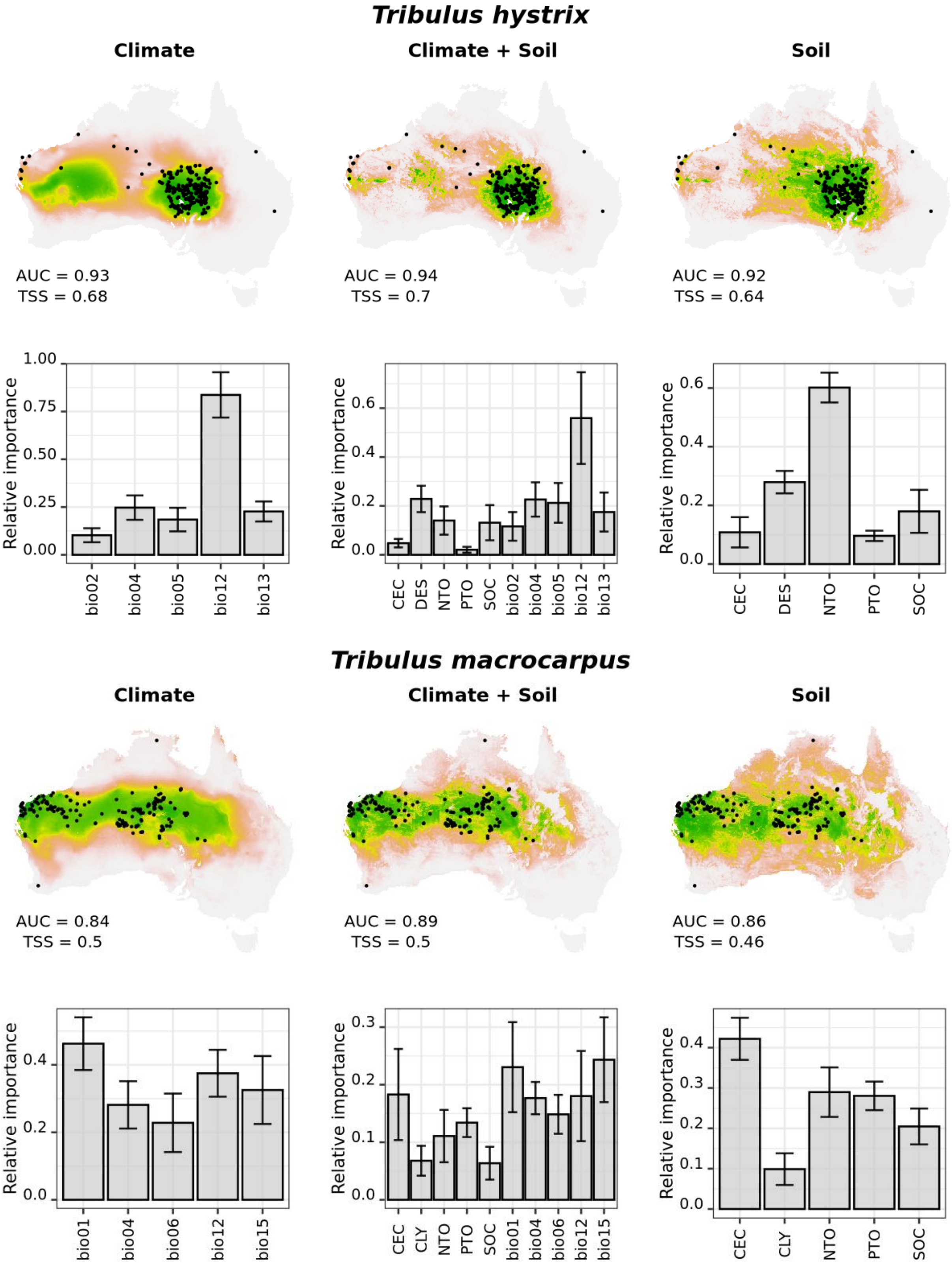
Tribulus hystrix
Climate
Climate + Soil
Soil
AUC = 0.93
TSS = 0.68
AUC = 0.94
TSS = 0.7
AUC = 0.92
TSS = 0.64
Relative importance
1.00
0.75
0.50
0.25
0.00
bio02
bio04
bio05
bio12
bio13
0.6
0.4
0.2
0.0
CEC
DES
NTO
PTO
SOC
bio02
bio04
bio05
bio12
bio13
CEC
DES
NTO
PTO
SOC
Tribulus macrocarpus
Climate
Climate + Soil
Soil
AUC = 0.84
TSS = 0.5
AUC = 0.89
TSS = 0.5
AUC = 0.86
TSS = 0.46
Relative importance
0.4
0.2
0.0
bio01
bio04
bio06
bio12
bio15
0.3
0.2
0.1
0.0
CEC
CLY
NTO
PTO
SOC
bio01
bio04
bio06
bio12
bio15
0.4
0.3
0.2
0.1
0.0
CEC
CLY
NTO
PTO
SOC

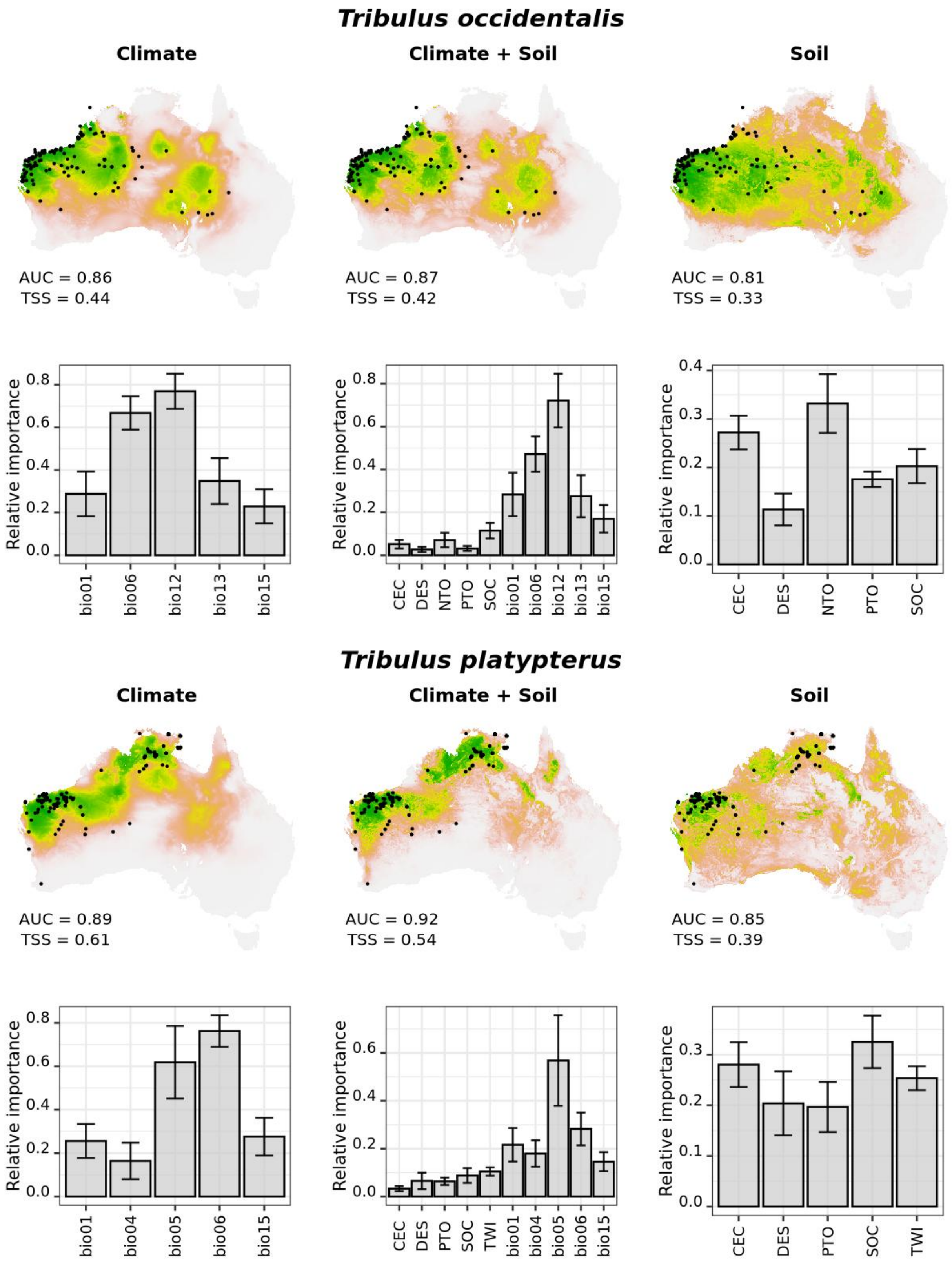

Tribulus occidentalis
Climate
Climate + Soil
Soil
AUC = 0.86
TSS = 0.44
AUC = 0.87
TSS = 0.42
AUC = 0.81
TSS = 0.33
Relative importance
bio01
bio06
bio12
bio13
bio15
CEC
DES
NTO
PTO
SOC
Tribulus platypterus
Climate
Climate + Soil
Soil
AUC = 0.89
TSS = 0.61
AUC = 0.92
TSS = 0.54
AUC = 0.85
TSS = 0.39
bio04
bio05
TWI

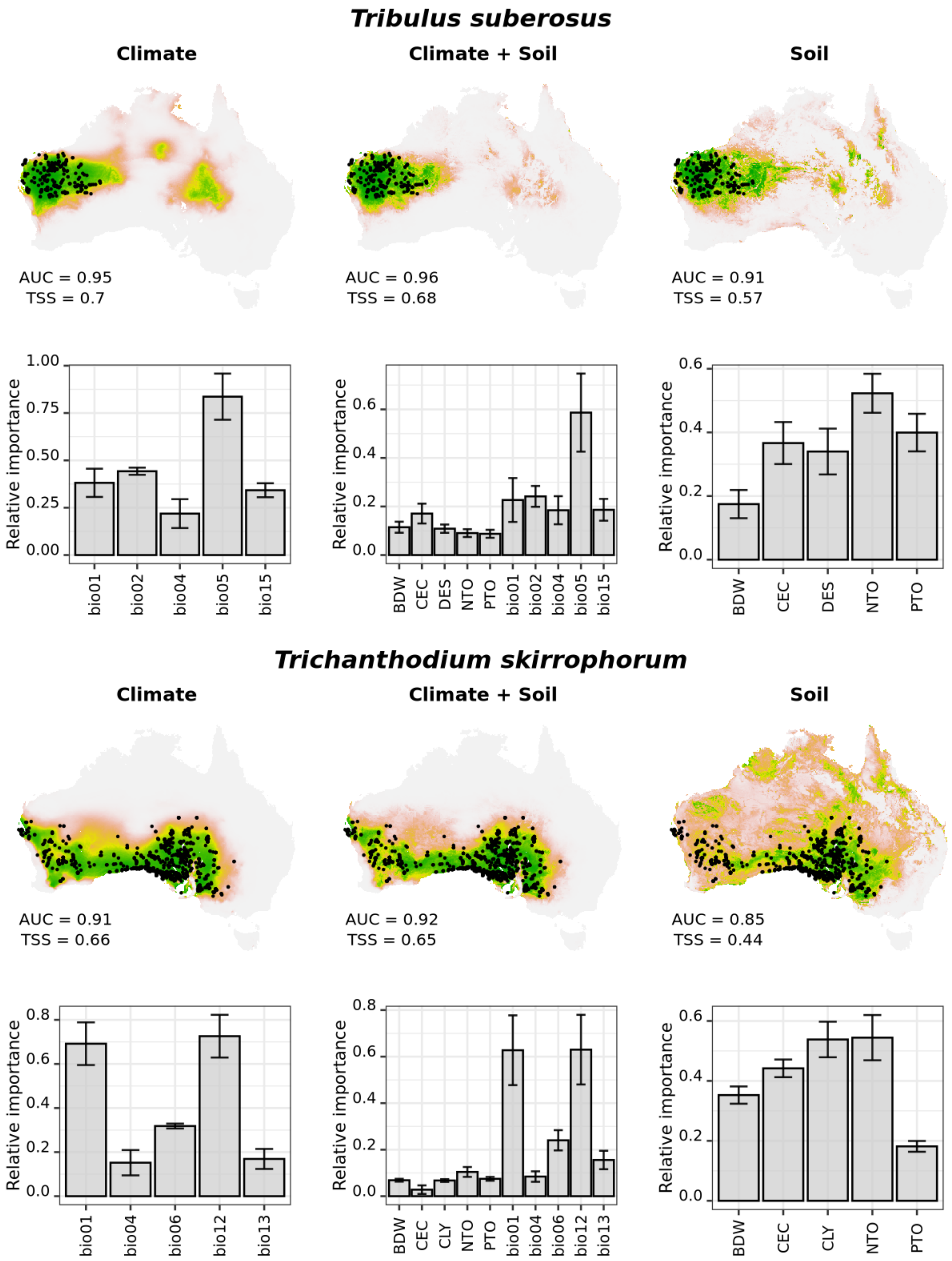

Tribulus suberosus
Climate
Climate + Soil
Soil
AUC = 0.95
TSS = 0.7
AUC = 0.96
TSS = 0.68
AUC = 0.91
TSS = 0.57
Relative importance
1.00
0.75
0.50
0.25
0.00
bio01
bio02
bio04
bio05
bio15
0.6
0.4
0.2
0.0
BDW
CEC
DES
NTO
PTO
bio01
bio02
bio04
bio05
bio15
BDW
CEC
DES
NTO
PTO
Trichanthodium skirrophorum
Climate
Climate + Soil
Soil
AUC = 0.91
TSS = 0.66
AUC = 0.92
TSS = 0.65
AUC = 0.85
TSS = 0.44
0.8
0.6
0.4
0.2
0.0
bio01
bio04
bio06
bio12
bio13
BDW
CEC
CLY
NTO
PTO
bio01
bio04
bio06
bio12
bio13
BDW
CEC
CLY
NTO
PTO

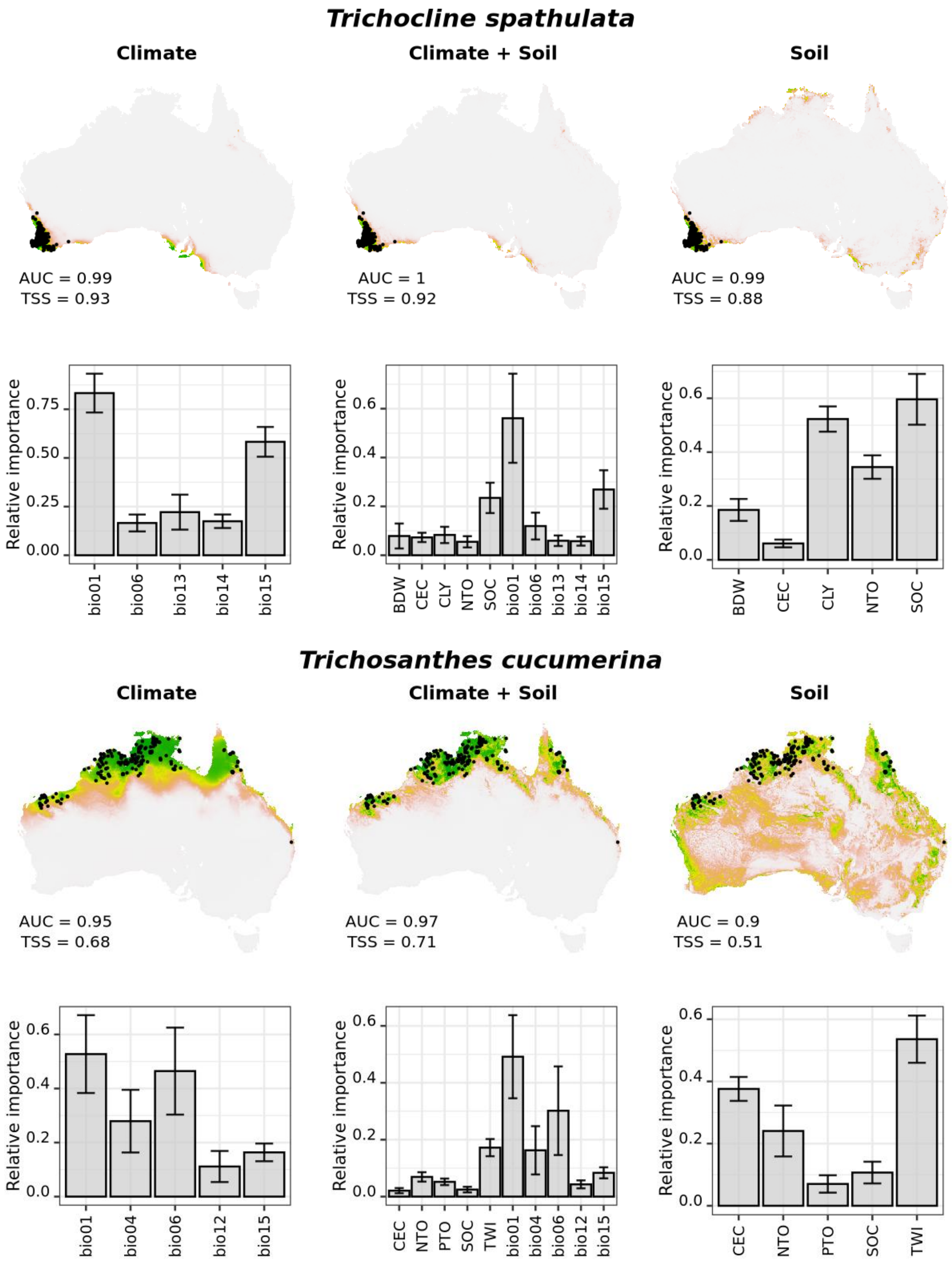
Trichocline spathulata
Climate
Climate + Soil
Soil
AUC = 0.99
TSS = 0.93
AUC = 1
TSS = 0.92
AUC = 0.99
TSS = 0.88
Relative importance
bio01
bio06
bio13
bio14
bio15
BDW
CEC
CLY
NTO
SOC
Trichosanthes cucumerina
Climate
Climate + Soil
Soil
AUC = 0.95
TSS = 0.68
AUC = 0.97
TSS = 0.71
AUC = 0.9
TSS = 0.51
Relative importance
bio01
bio04
bio06
bio12
bio15
CEC
NTO
PTO
SOC
TWI

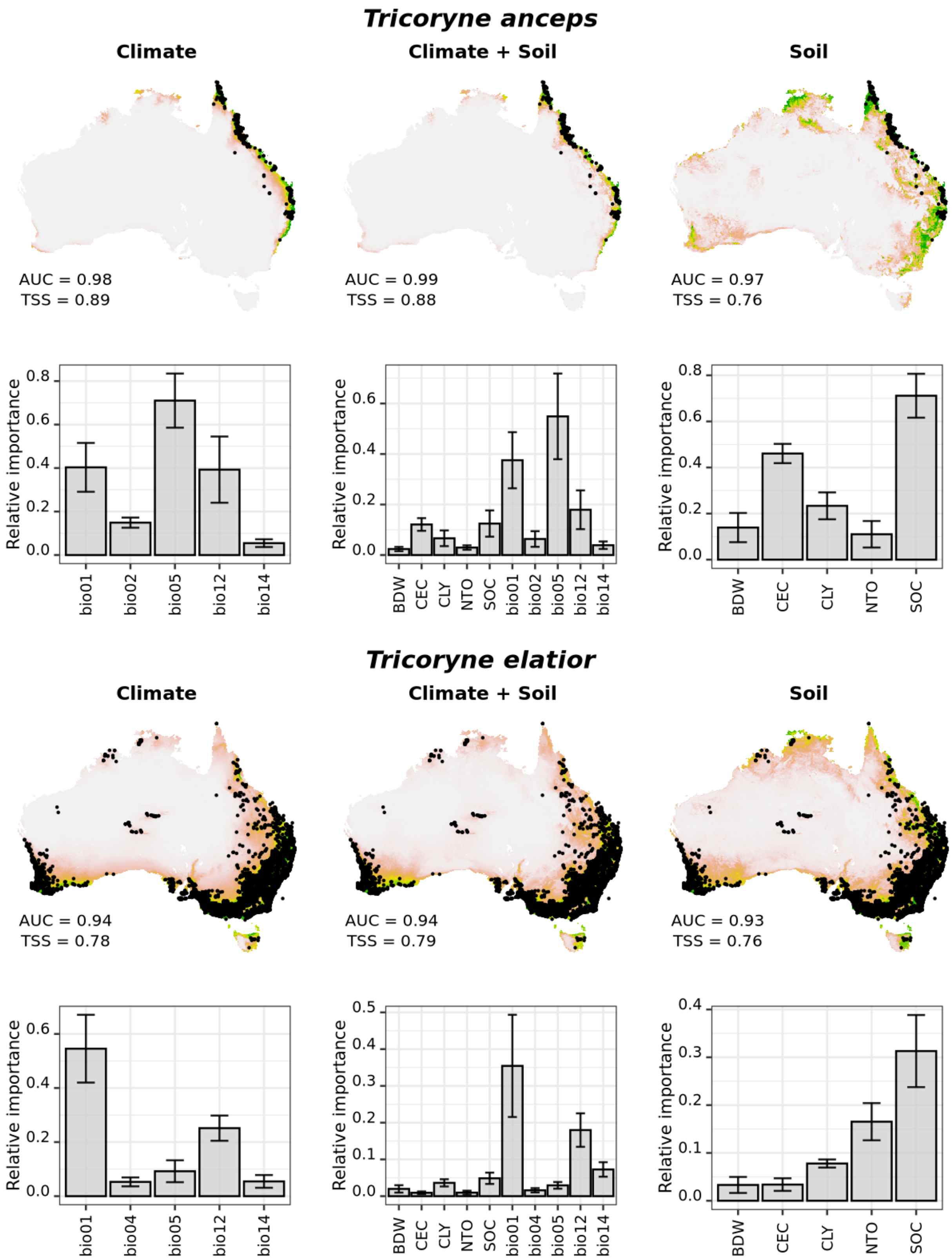
Tricoryne anceps
Climate
Climate + Soil
Soil
AUC = 0.98
TSS = 0.89
AUC = 0.99
TSS = 0.88
AUC = 0.97
TSS = 0.76
Relative importance
bio01
bio02
bio05
bio12
bio14
BDW
CEC
CLY
NTO
SOC
Tricoryne elatior
Climate
Climate + Soil
Soil
AUC = 0.94
TSS = 0.78
AUC = 0.94
TSS = 0.79
AUC = 0.93
TSS = 0.76
Relative importance
bio01
bio04
bio05
bio12
bio14
BDW
CEC
CLY
NTO
SOC

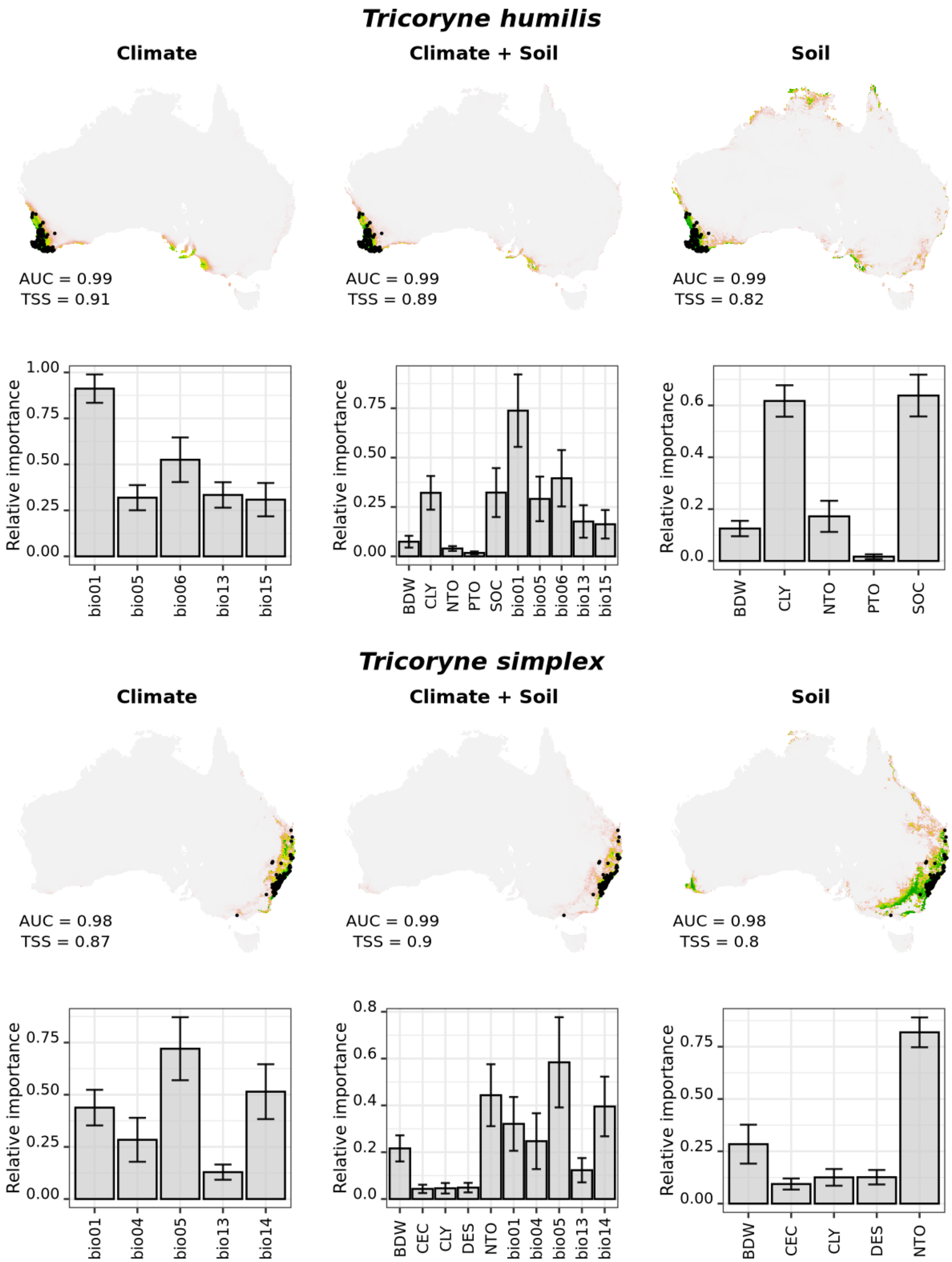
Tricoryne humilis
Climate
Climate + Soil
Soil
AUC = 0.99
TSS = 0.91
AUC = 0.99
TSS = 0.89
AUC = 0.99
TSS = 0.82
Relative importance
1.00
0.75
0.50
0.25
0.00
bio01
bio05
bio06
bio13
bio15
BDW
CLY
NTO
PTO
SOC
0.6
0.4
0.2
0.0
Tricoryne simplex
AUC = 0.98
TSS = 0.87
AUC = 0.99
TSS = 0.9
AUC = 0.98
TSS = 0.8
bio04
bio14
0.8
CEC
DES

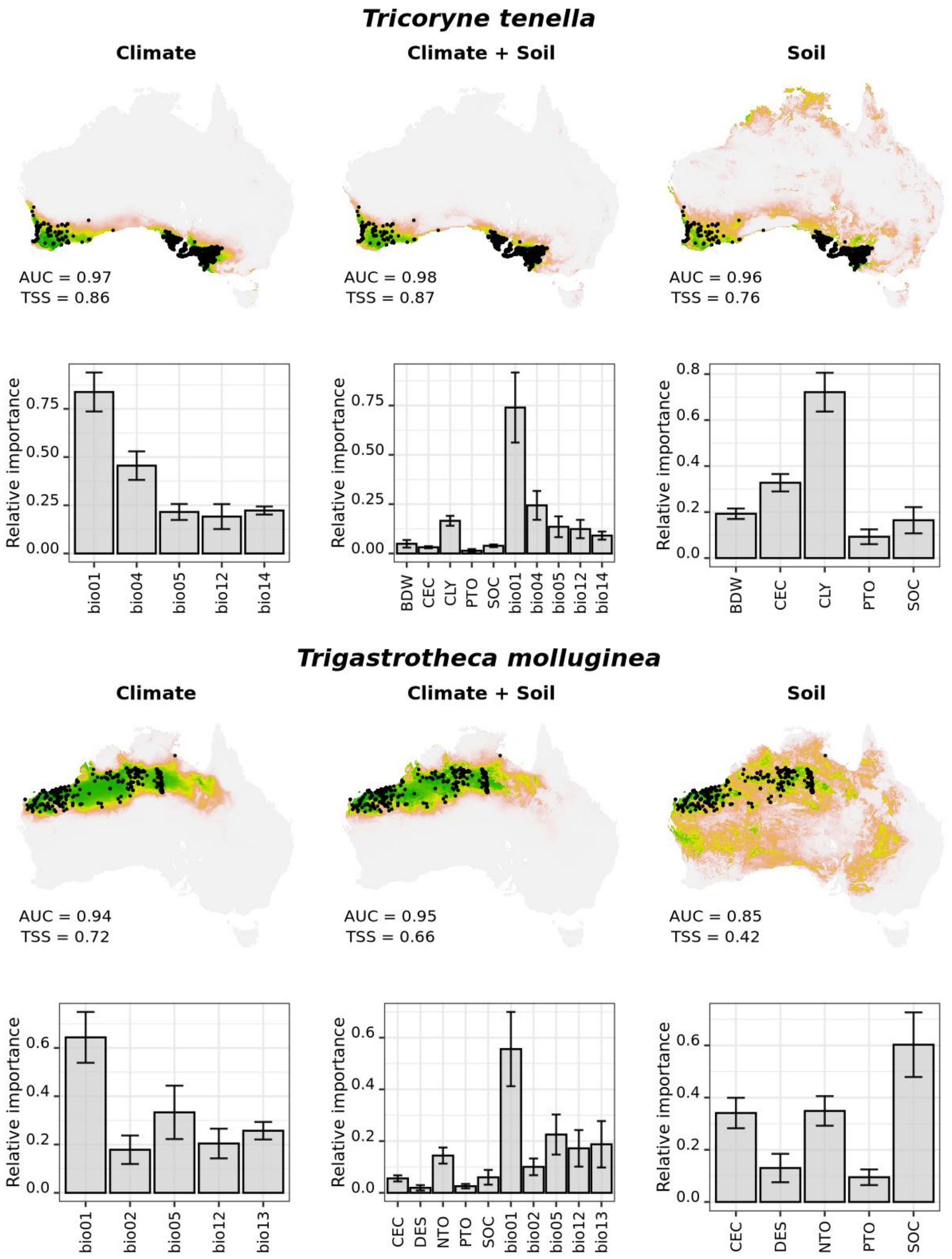
Tricoryne tenella
Climate
Climate + Soil
Soil
AUC = 0.97
TSS = 0.86
AUC = 0.98
TSS = 0.87
AUC = 0.96
TSS = 0.76
Relative importance
bio01
bio04
bio05
bio12
bio14
BDW
CEC
CLY
PTO
SOC
Trigastrotheca molluginea
Climate
Climate + Soil
Soil
AUC = 0.94
TSS = 0.72
AUC = 0.95
TSS = 0.66
AUC = 0.85
TSS = 0.42
Relative importance
bio01
bio02
bio05
bio12
bio13
CEC
DES
NTO
PTO
SOC

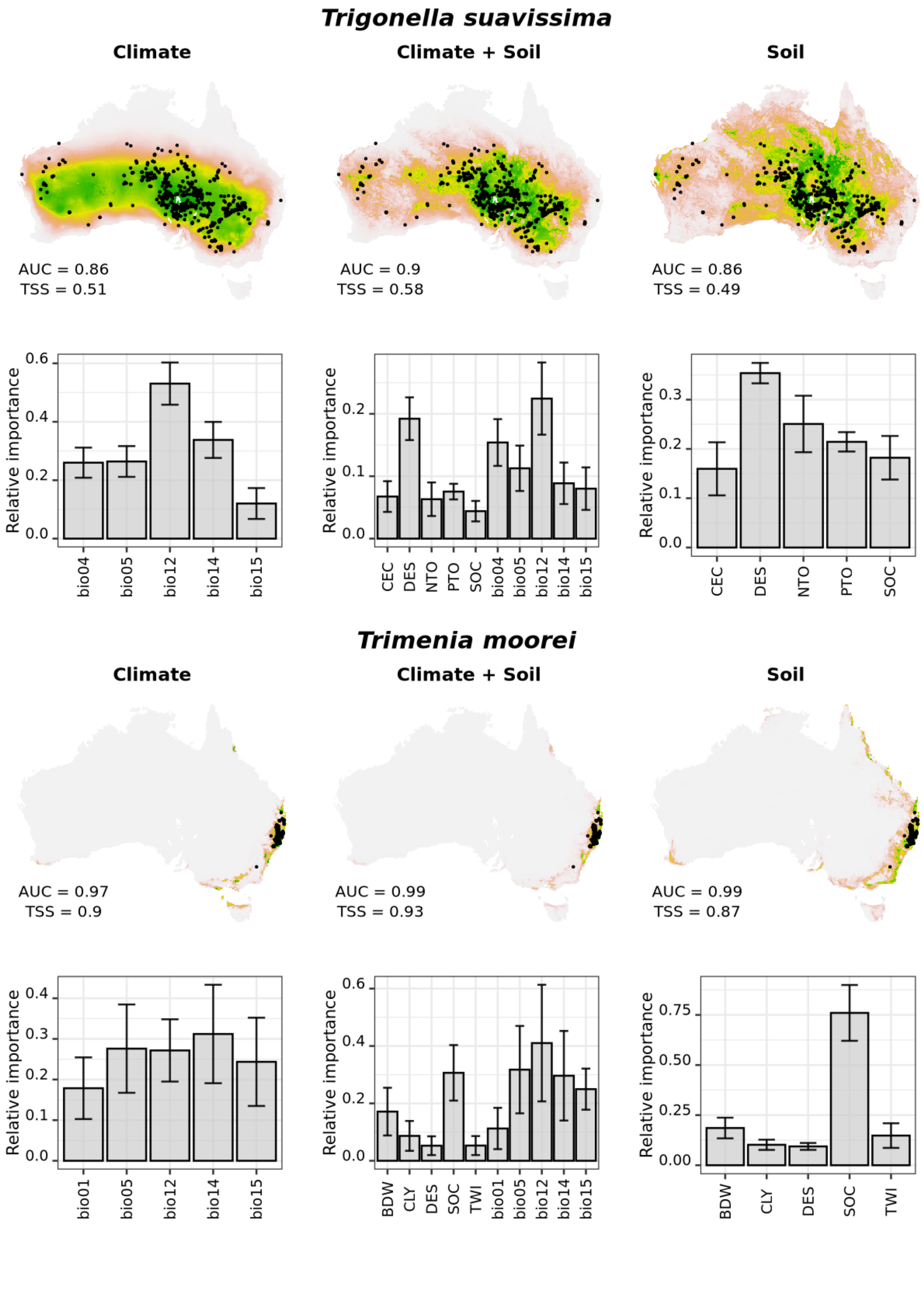
Trigonella suavissima
Climate
Climate + Soil
Soil
AUC = 0.86
TSS = 0.51
AUC = 0.9
TSS = 0.58
AUC = 0.86
TSS = 0.49
Relative importance
bio04 bio05 bio12 bio14 bio15
CEC DES NTO PTO SOC bio04 bio05 bio12 bio14 bio15
CEC DES NTO PTO SOC
Trimenia moorei
Climate
Climate + Soil
Soil
AUC = 0.97
TSS = 0.9
AUC = 0.99
TSS = 0.93
AUC = 0.99
TSS = 0.87
Relative importance
bio01 bio05 bio12 bio14 bio15
BDW CLY DES SOC TWI bio01 bio05 bio12 bio14 bio15
BDW CLY DES SOC TWI

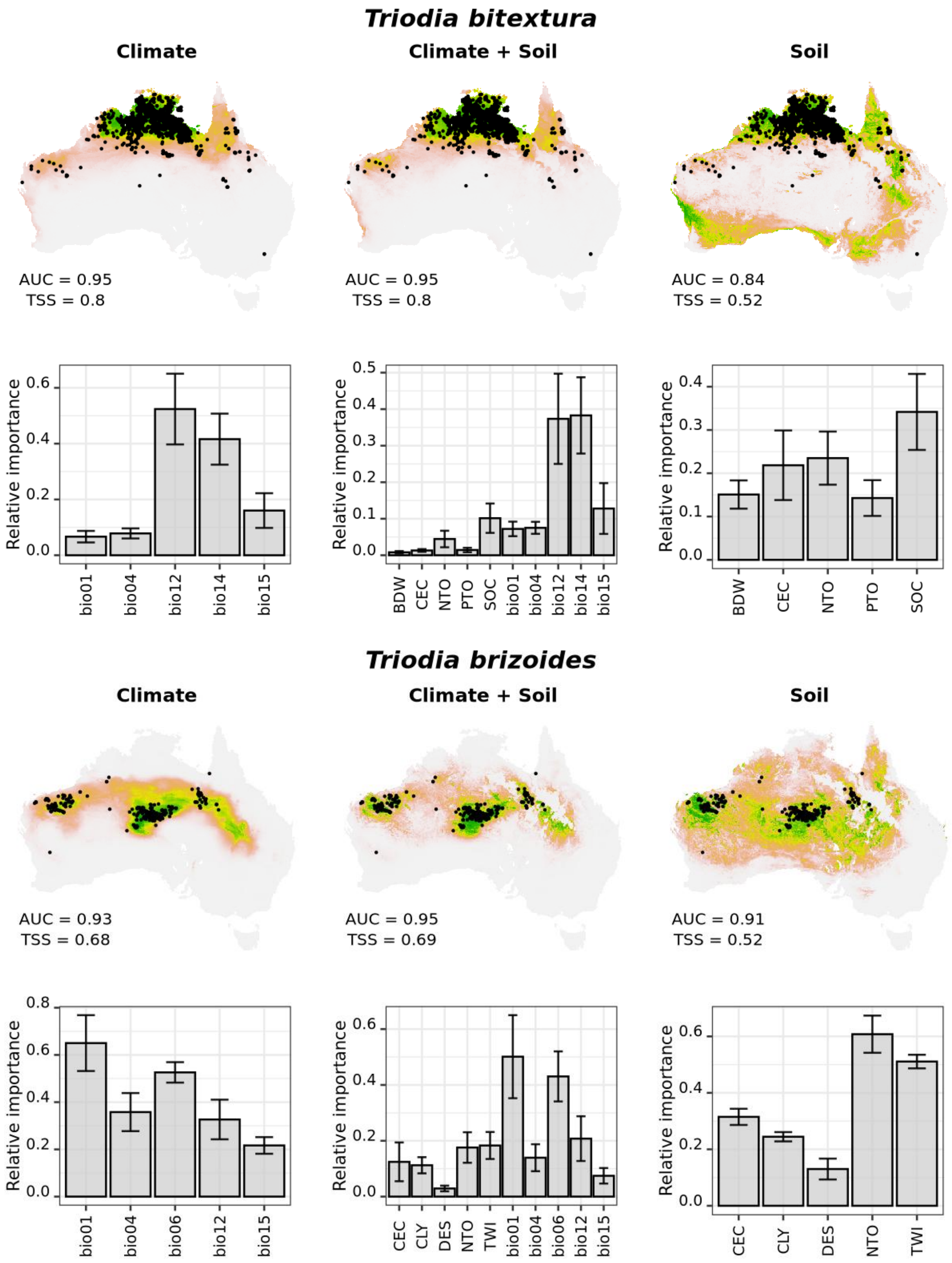

Triodia bitextura
Climate
Climate + Soil
Soil
AUC = 0.95
TSS = 0.8
AUC = 0.95
TSS = 0.8
AUC = 0.84
TSS = 0.52
Relative importance
0.0
0.2
0.4
0.6
bio01
bio04
bio12
bio14
bio15
0.0
0.1
0.2
0.3
0.4
0.5
BDW
CEC
NTO
PTO
SOC
bio01
bio04
bio12
bio14
bio15
0.0
0.1
0.2
0.3
0.4
BDW
CEC
NTO
PTO
SOC
Triodia brizoides
Climate
Climate + Soil
Soil
AUC = 0.93
TSS = 0.68
AUC = 0.95
TSS = 0.69
AUC = 0.91
TSS = 0.52
Relative importance
0.0
0.2
0.4
0.6
0.8
bio01
bio04
bio06
bio12
bio15
0.0
0.2
0.4
0.6
CEC
CLY
DES
NTO
TWI
bio01
bio04
bio06
bio12
bio15
0.0
0.2
0.4
0.6
CEC
CLY
DES
NTO
TWI

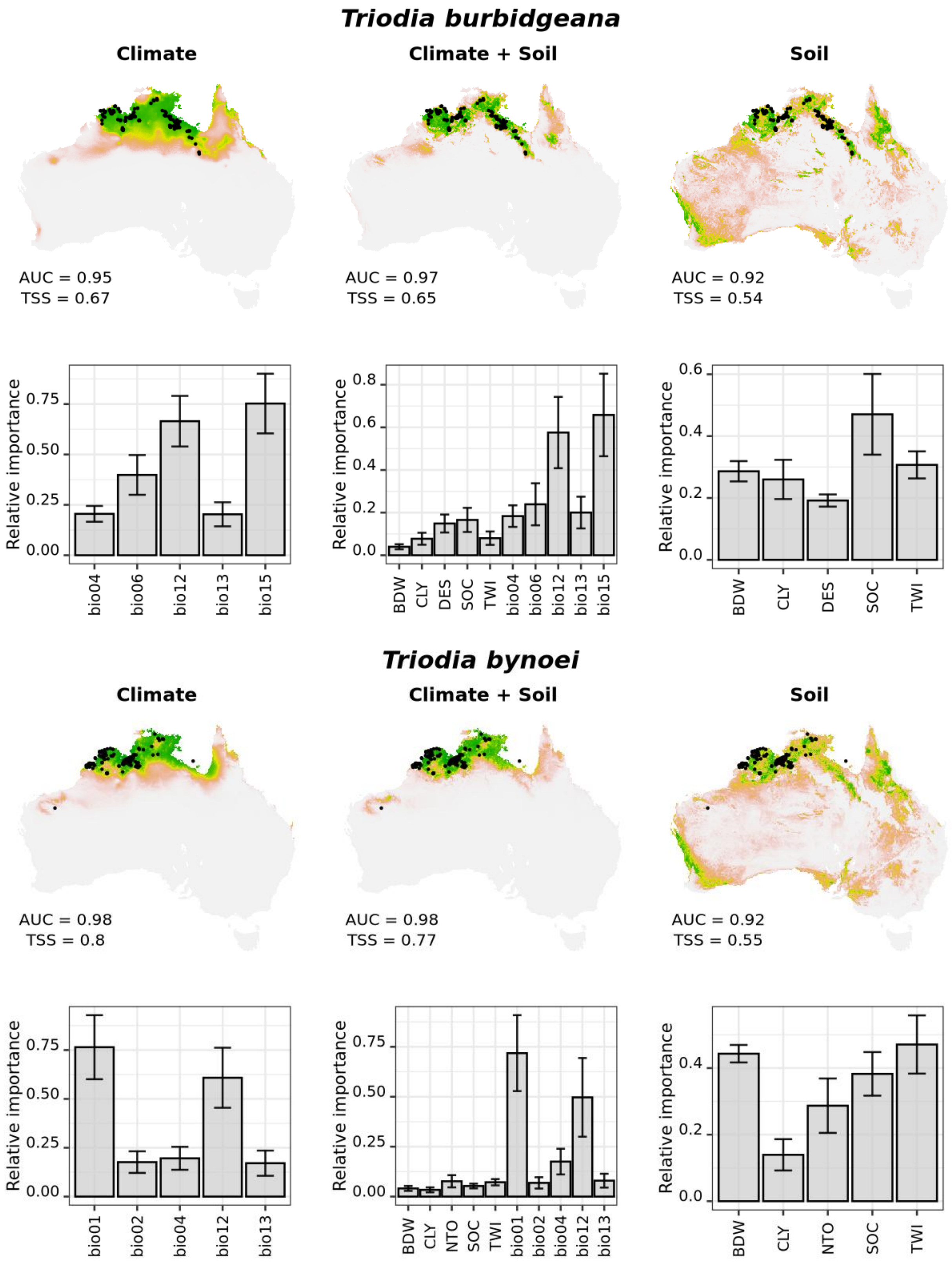

Triodia burbidgeana
Climate
Climate + Soil
Soil
AUC = 0.95
TSS = 0.67
AUC = 0.97
TSS = 0.65
AUC = 0.92
TSS = 0.54
Relative importance
0.00
0.25
0.50
0.75
bio04
bio06
bio12
bio13
bio15
Relative importance
0.0
0.2
0.4
0.6
0.8
BDW
CLY
DES
SOC
TWI
bio04
bio06
bio12
bio13
bio15
Relative importance
0.0
0.2
0.4
0.6
BDW
CLY
DES
SOC
TWI
Triodia bynoei
Climate
Climate + Soil
Soil
AUC = 0.98
TSS = 0.8
AUC = 0.98
TSS = 0.77
AUC = 0.92
TSS = 0.55
Relative importance
0.00
0.25
0.50
0.75
bio01
bio02
bio04
bio12
bio13
Relative importance
0.00
0.25
0.50
0.75
BDW
CLY
NTO
SOC
TWI
bio01
bio02
bio04
bio12
bio13
Relative importance
0.0
0.2
0.4
BDW
CLY
NTO
SOC
TWI

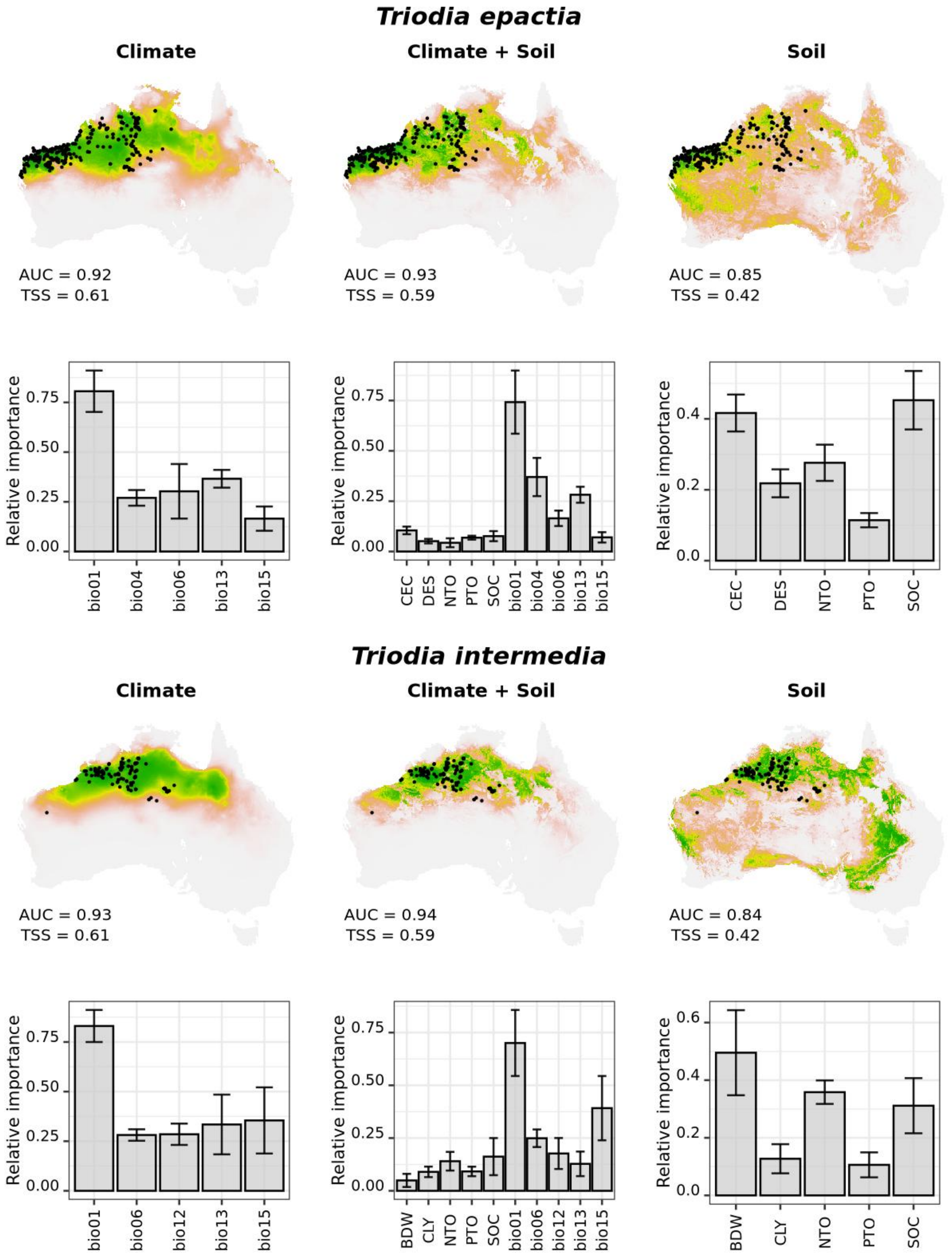
Triodia epactia
Climate
Climate + Soil
Soil
AUC = 0.92
TSS = 0.61
AUC = 0.93
TSS = 0.59
AUC = 0.85
TSS = 0.42
Relative importance
0.00
0.25
0.50
0.75
bio01
bio04
bio06
bio13
bio15
CEC
DES
NTO
PTO
SOC
0.0
0.2
0.4
Triodia intermedia
Climate
Climate + Soil
Soil
AUC = 0.93
TSS = 0.61
AUC = 0.94
TSS = 0.59
AUC = 0.84
TSS = 0.42
Relative importance
bio01
bio06
bio12
bio13
bio15
BDW
CLY
NTO
PTO
SOC
0.6

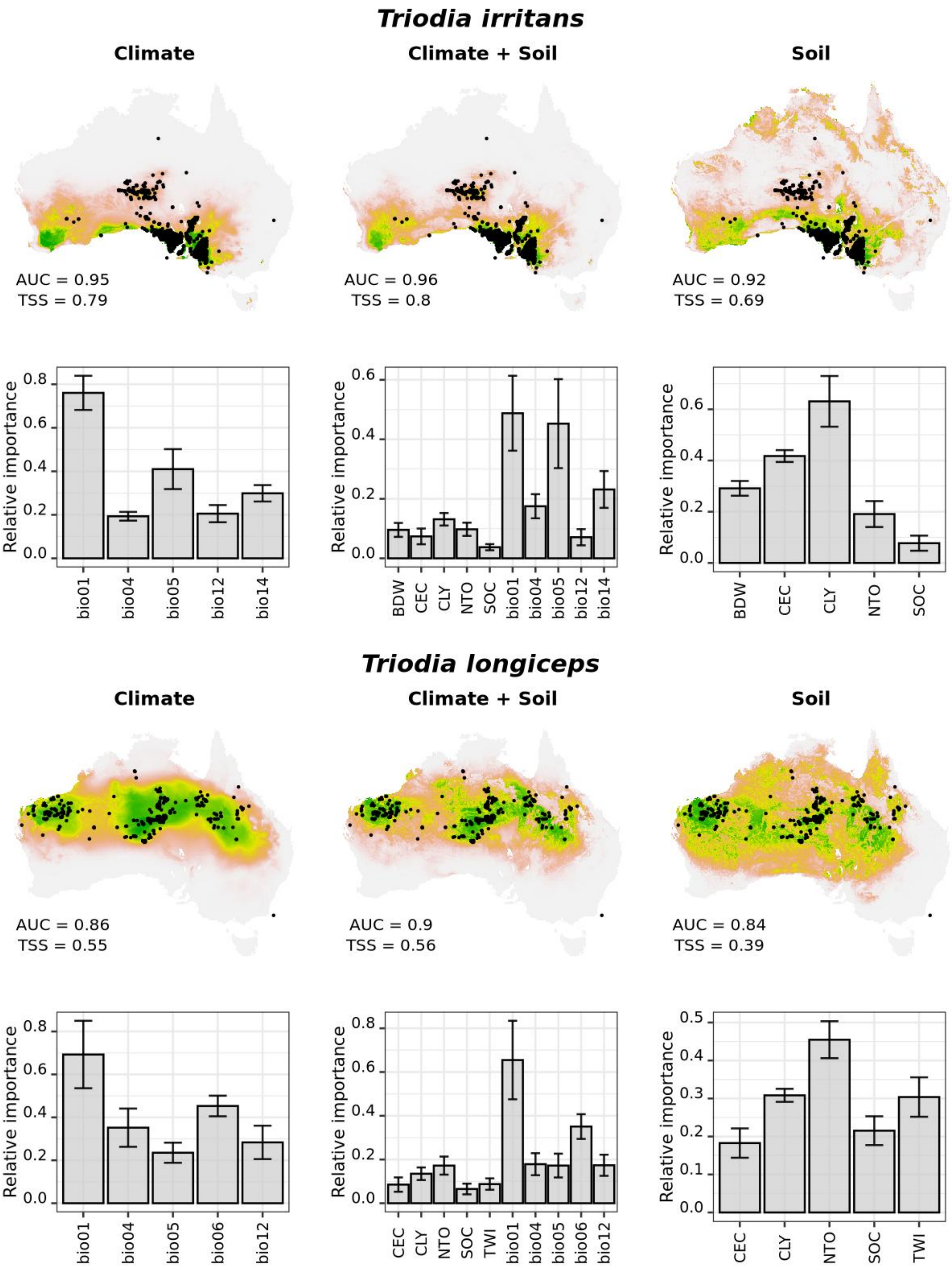

Triodia irritans
Climate
Climate + Soil
Soil
AUC = 0.95
TSS = 0.79
AUC = 0.96
TSS = 0.8
AUC = 0.92
TSS = 0.69
Relative importance
0.0
0.2
0.4
0.6
0.8
bio01
bio04
bio05
bio12
bio14
BDW
CEC
CLY
NTO
SOC
Triodia longiceps
AUC = 0.86
TSS = 0.55
AUC = 0.9
TSS = 0.56
AUC = 0.84
TSS = 0.39
bio06
TWI
0.1
0.3
0.5

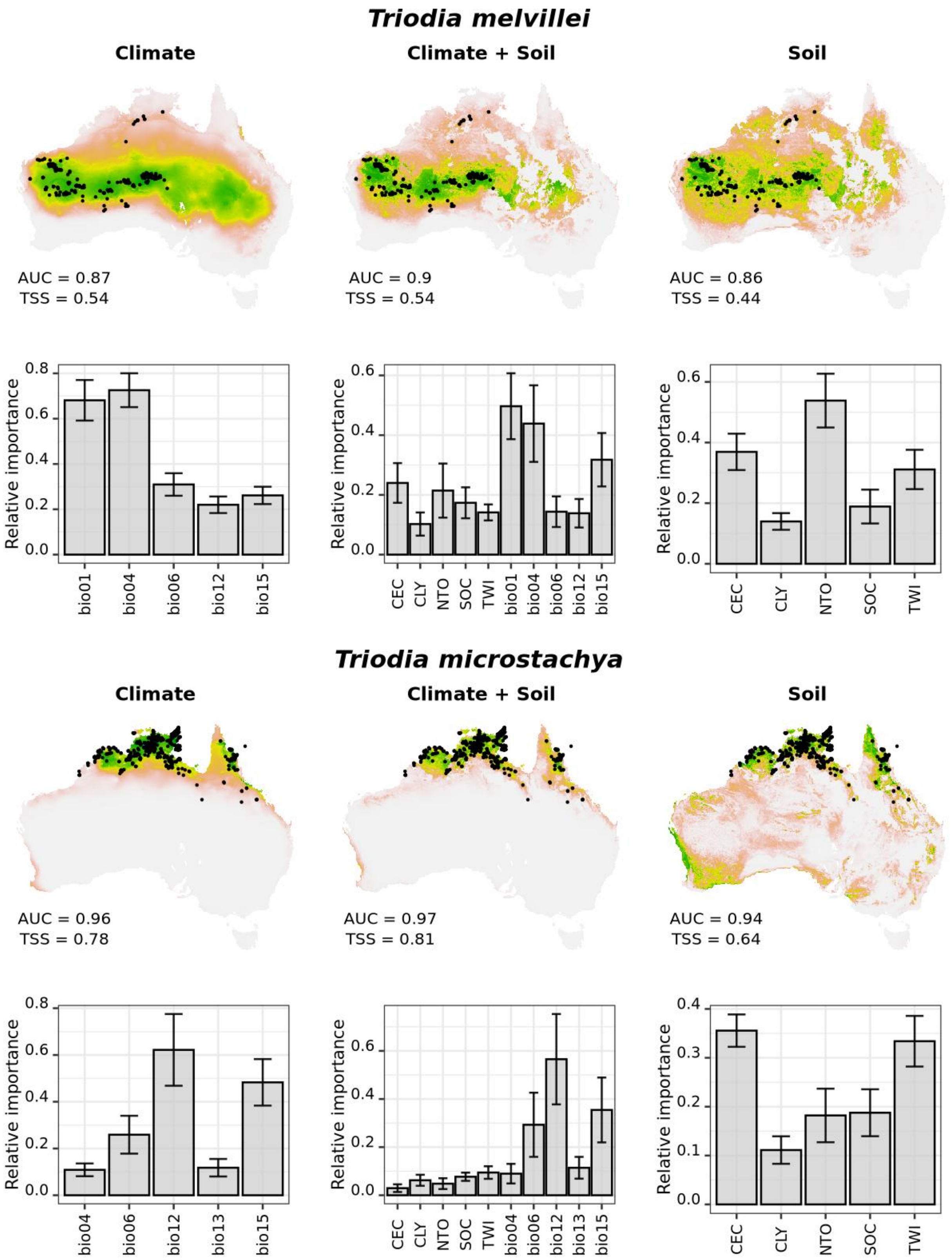
Triodia melvillei
Climate
Climate + Soil
Soil
AUC = 0.87
TSS = 0.54
AUC = 0.9
TSS = 0.54
AUC = 0.86
TSS = 0.44
Relative importance
bio01
bio04
bio06
bio12
bio15
CEC
CLY
NTO
SOC
TWI
Triodia microstachya
Climate
Climate + Soil
Soil
AUC = 0.96
TSS = 0.78
AUC = 0.97
TSS = 0.81
AUC = 0.94
TSS = 0.64
Relative importance
bio04
bio06
bio12
bio13
bio15
CEC
CLY
NTO
SOC
TWI

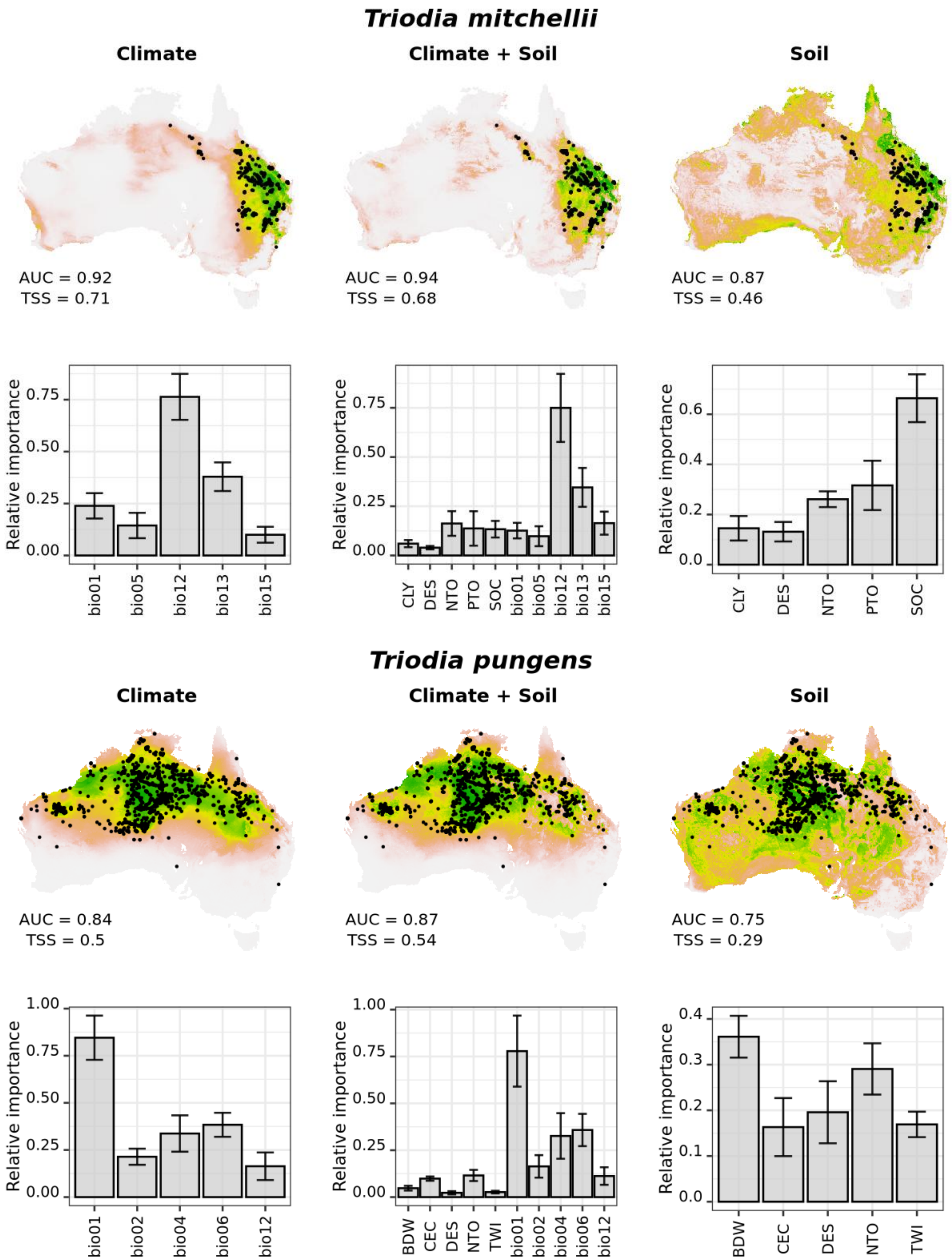
Triodia mitchellii
Climate
Climate + Soil
Soil
AUC = 0.92
TSS = 0.71
AUC = 0.94
TSS = 0.68
AUC = 0.87
TSS = 0.46
Relative importance
bio01
bio05
bio12
bio13
bio15
CLY
DES
NTO
PTO
SOC
Triodia pungens
AUC = 0.84
TSS = 0.5
AUC = 0.87
TSS = 0.54
AUC = 0.75
TSS = 0.29
bio02
bio04
bio06
BDW
CEC
TWI

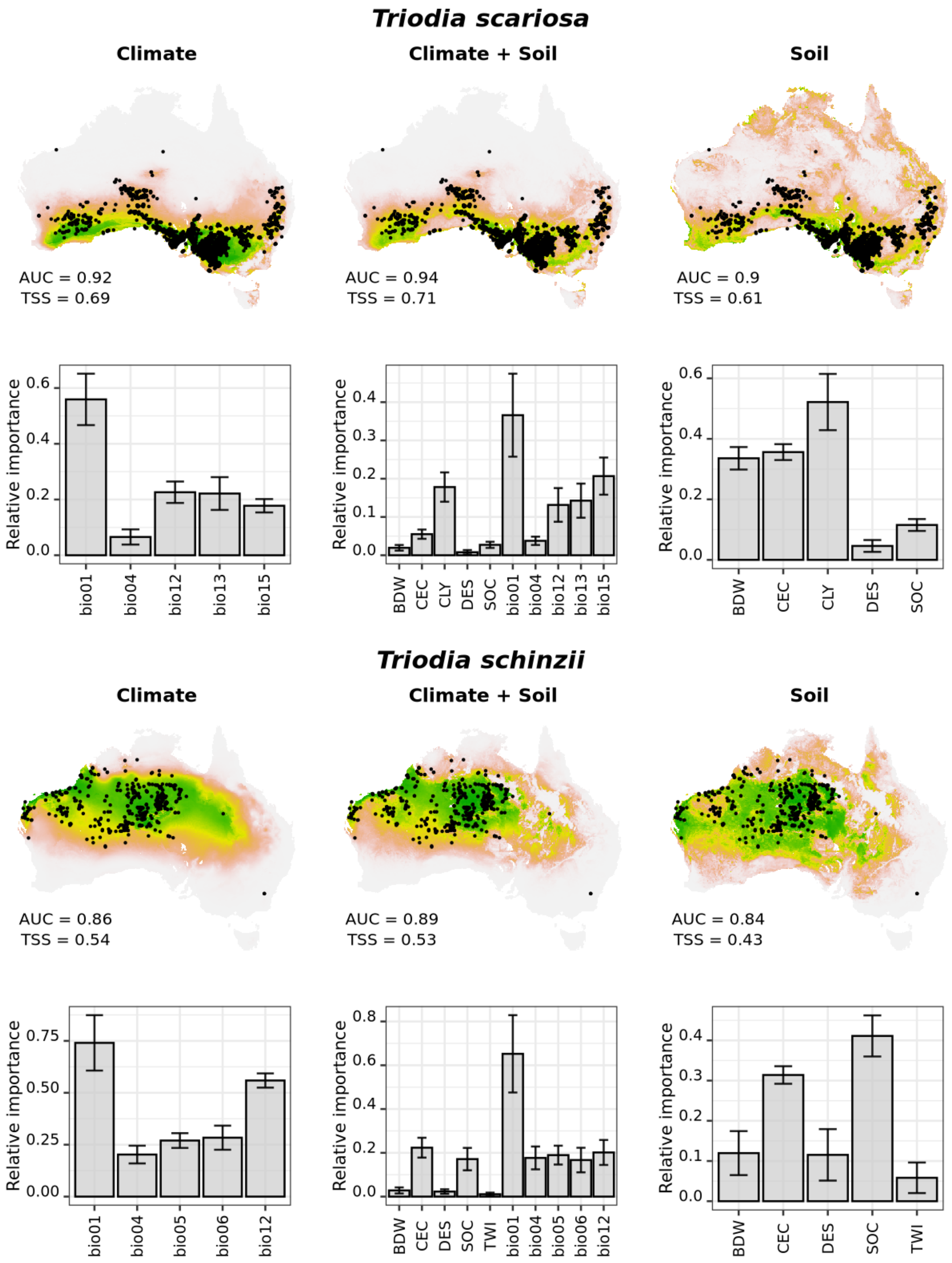
Triodia scariosa
Climate
Climate + Soil
Soil
AUC = 0.92
TSS = 0.69
AUC = 0.94
TSS = 0.71
AUC = 0.9
TSS = 0.61
Relative importance
0.0
0.1
0.2
0.3
0.4
0.6
bio01
bio04
bio12
bio13
bio15
BDW
CEC
CLY
DES
SOC
Triodia schinzii
AUC = 0.86
TSS = 0.54
AUC = 0.89
TSS = 0.53
AUC = 0.84
TSS = 0.43
0.00
0.25
0.50
0.75
0.8
bio05
bio06
TWI

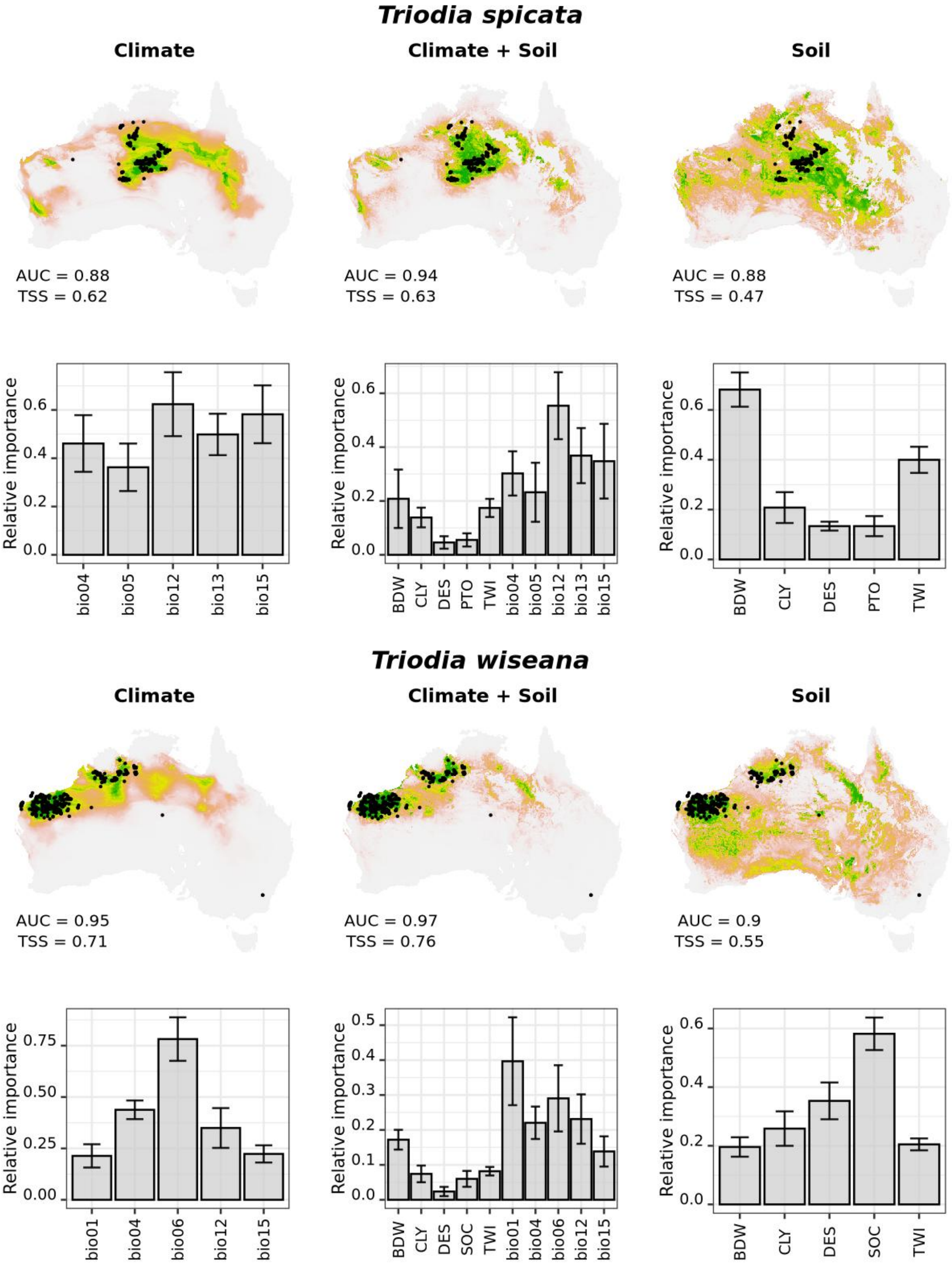

Triodia spicata
Climate
Climate + Soil
Soil
AUC = 0.88
TSS = 0.62
AUC = 0.94
TSS = 0.63
AUC = 0.88
TSS = 0.47
Relative importance
bio04
bio05
bio12
bio13
bio15
BDW
CLY
DES
PTO
TWI
Triodia wiseana
AUC = 0.95
TSS = 0.71
AUC = 0.97
TSS = 0.76
AUC = 0.9
TSS = 0.55
bio01
bio06
SOC

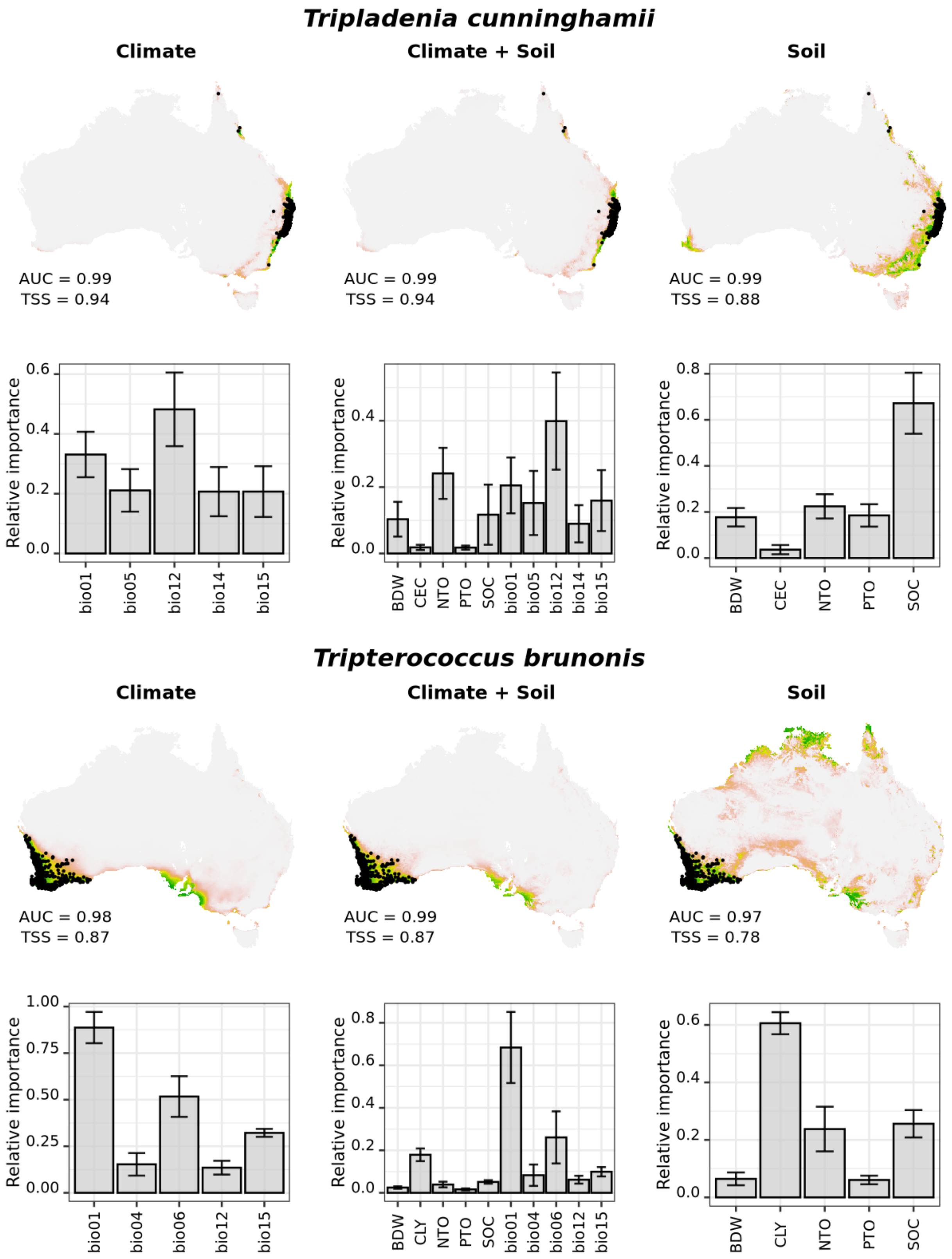
Tripladenia cunninghamii
Climate
Climate + Soil
Soil
AUC = 0.99
TSS = 0.94
AUC = 0.99
TSS = 0.94
AUC = 0.99
TSS = 0.88
Relative importance
bio01
bio05
bio12
bio14
bio15
BDW
CEC
NTO
PTO
SOC
Tripterococcus brunonis
AUC = 0.98
TSS = 0.87
AUC = 0.99
TSS = 0.87
AUC = 0.97
TSS = 0.78
bio04
bio06
CLY

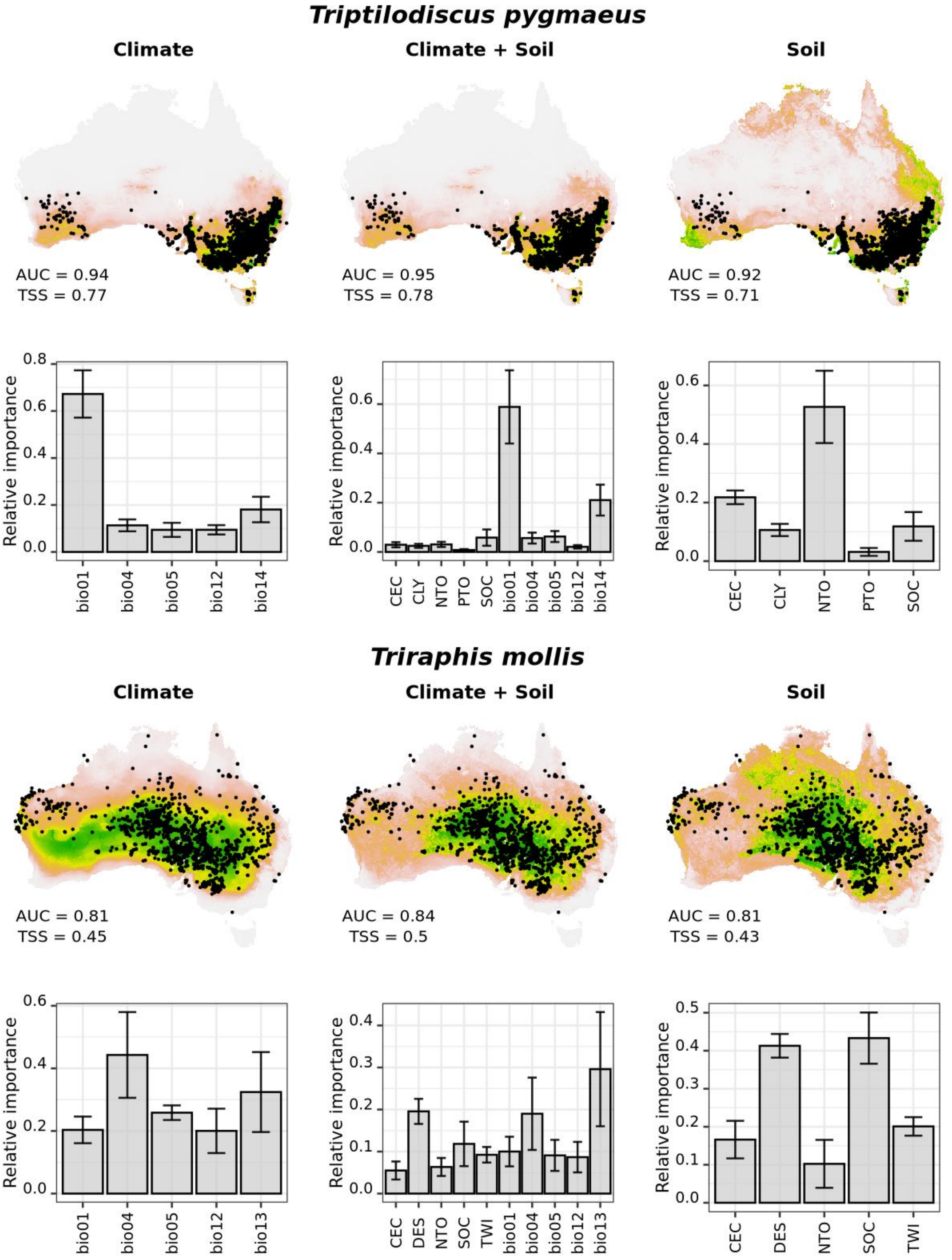
Triptilodiscus pygmaeus
Climate
Climate + Soil
Soil
AUC = 0.94
TSS = 0.77
AUC = 0.95
TSS = 0.78
AUC = 0.92
TSS = 0.71
Relative importance
bio01
bio04
bio05
bio12
bio14
CEC
CLY
NTO
PTO
SOC
Triraphis mollis
AUC = 0.81
TSS = 0.45
AUC = 0.84
TSS = 0.5
AUC = 0.81
TSS = 0.43
bio13
DES
TWI

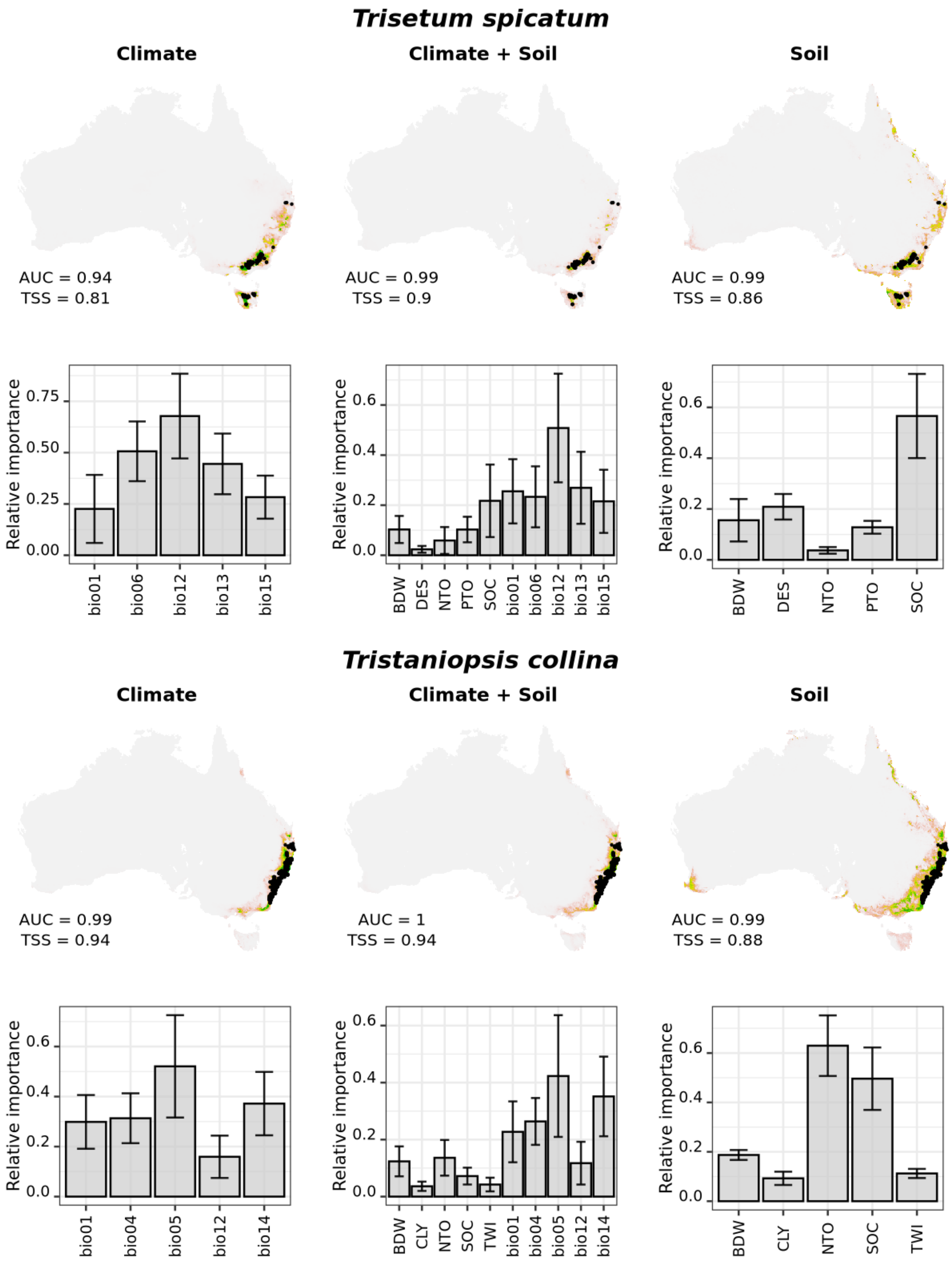
Trisetum spicatum
Climate
Climate + Soil
Soil
AUC = 0.94
TSS = 0.81
AUC = 0.99
TSS = 0.9
AUC = 0.99
TSS = 0.86
Relative importance
0.00
0.25
0.50
0.75
bio01
bio06
bio12
bio13
bio15
0.0
0.2
0.4
0.6
BDW
DES
NTO
PTO
SOC
bio01
bio06
bio12
bio13
bio15
BDW
DES
NTO
PTO
SOC
Tristaniopsis collina
Climate
Climate + Soil
Soil
AUC = 0.99
TSS = 0.94
AUC = 1
TSS = 0.94
AUC = 0.99
TSS = 0.88
Relative importance
0.0
0.2
0.4
0.6
bio01
bio04
bio05
bio12
bio14
BDW
CLY
NTO
SOC
TWI
bio01
bio04
bio05
bio12
bio14
BDW
CLY
NTO
SOC
TWI

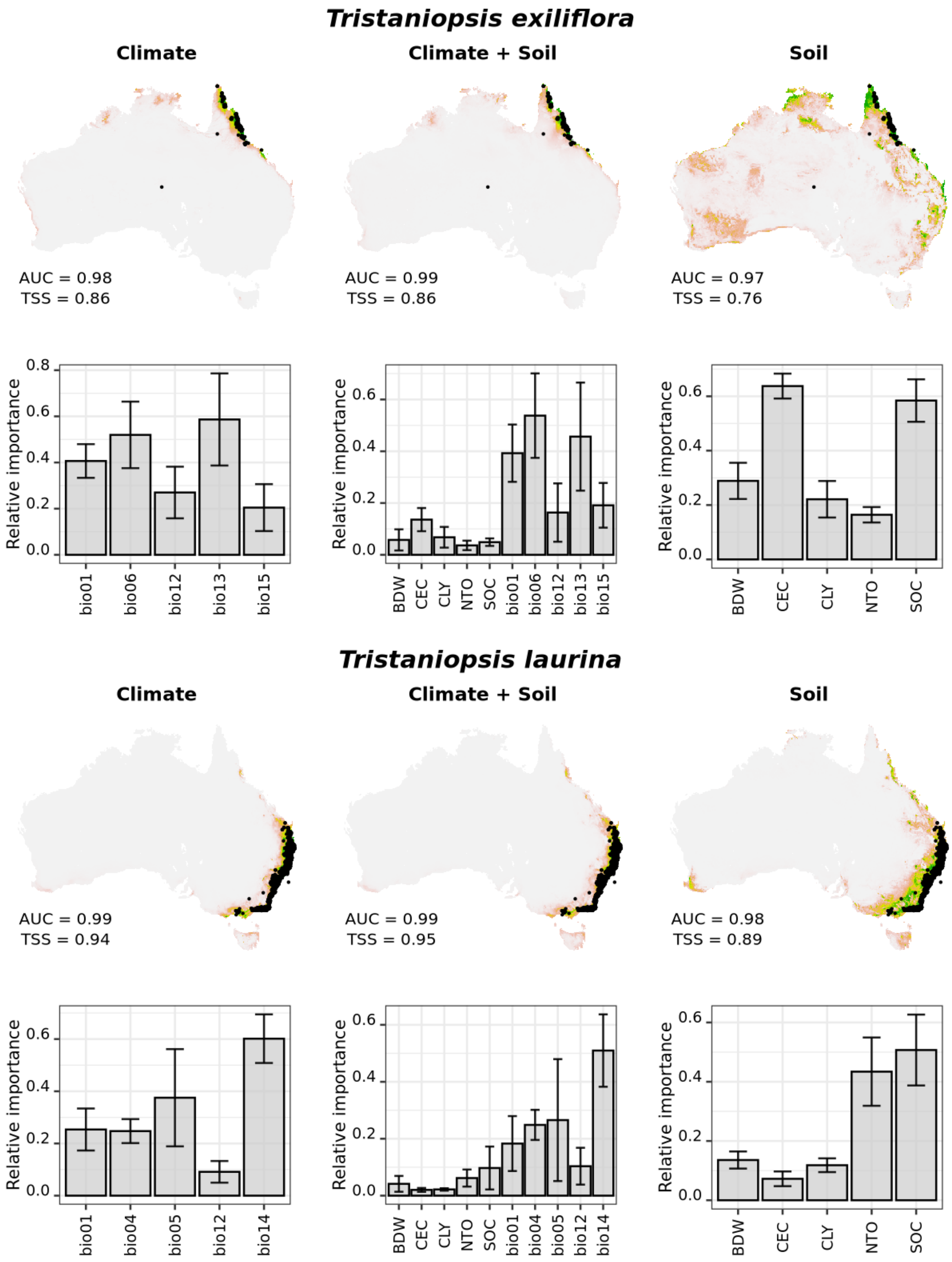

Tristaniopsis exiliflora
Climate
Climate + Soil
Soil
AUC = 0.98
TSS = 0.86
AUC = 0.99
TSS = 0.86
AUC = 0.97
TSS = 0.76
Relative importance
bio01
bio06
bio12
bio13
bio15
BDW
CEC
CLY
NTO
SOC
Tristaniopsis laurina
Climate
Climate + Soil
Soil
AUC = 0.99
TSS = 0.94
AUC = 0.99
TSS = 0.95
AUC = 0.98
TSS = 0.89
Relative importance
bio01
bio04
bio05
bio12
bio14
BDW
CEC
CLY
NTO
SOC

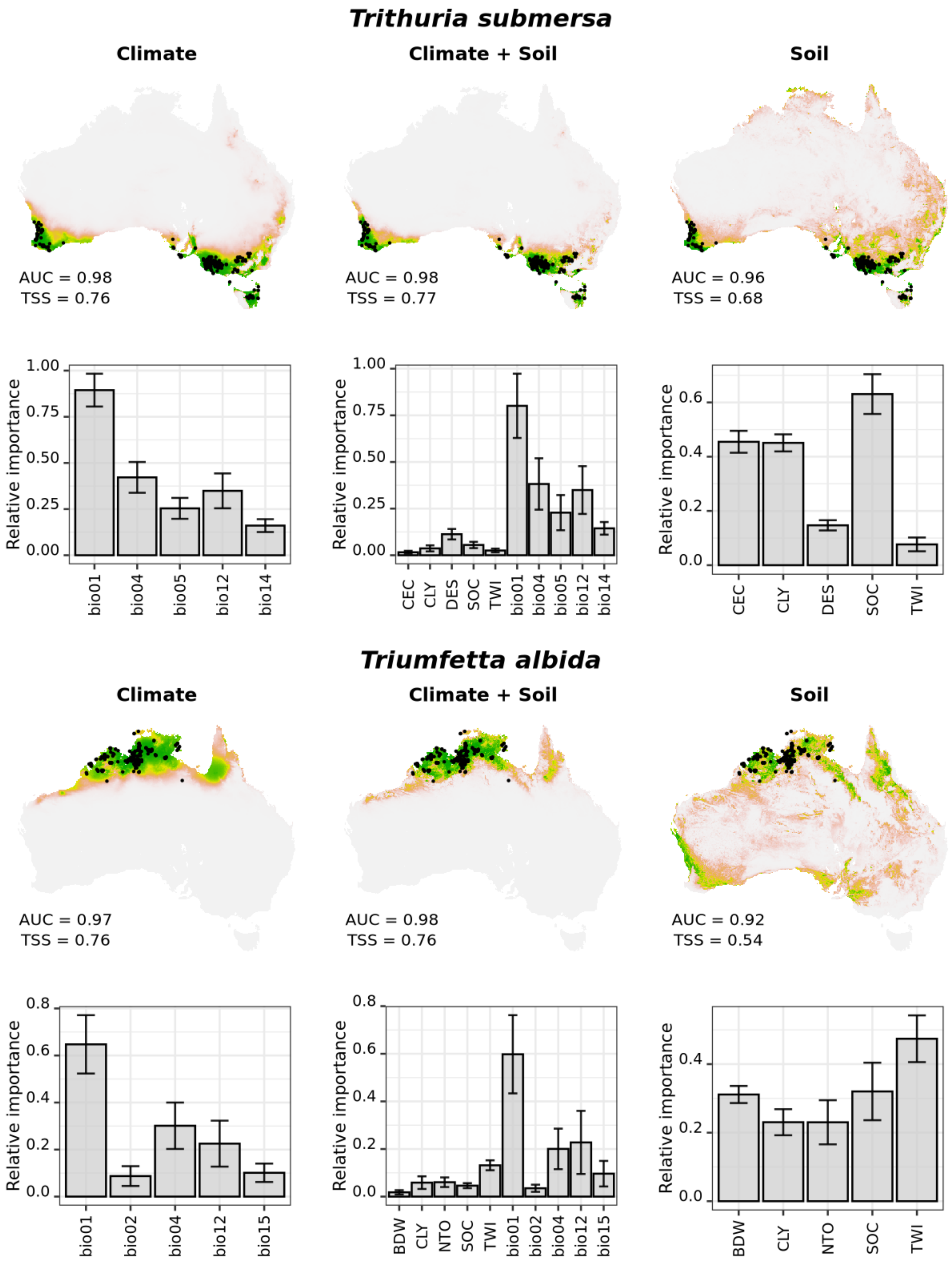

Trithuria submersa
Climate
Climate + Soil
Soil
AUC = 0.98
TSS = 0.76
AUC = 0.98
TSS = 0.77
AUC = 0.96
TSS = 0.68
Relative importance
bio01
bio04
bio05
bio12
bio14
CEC
CLY
DES
SOC
TWI
Triumfetta albida
Climate
Climate + Soil
Soil
AUC = 0.97
TSS = 0.76
AUC = 0.98
TSS = 0.76
AUC = 0.92
TSS = 0.54
Relative importance
bio01
bio02
bio04
bio12
bio15
BDW
CLY
NTO
SOC
TWI

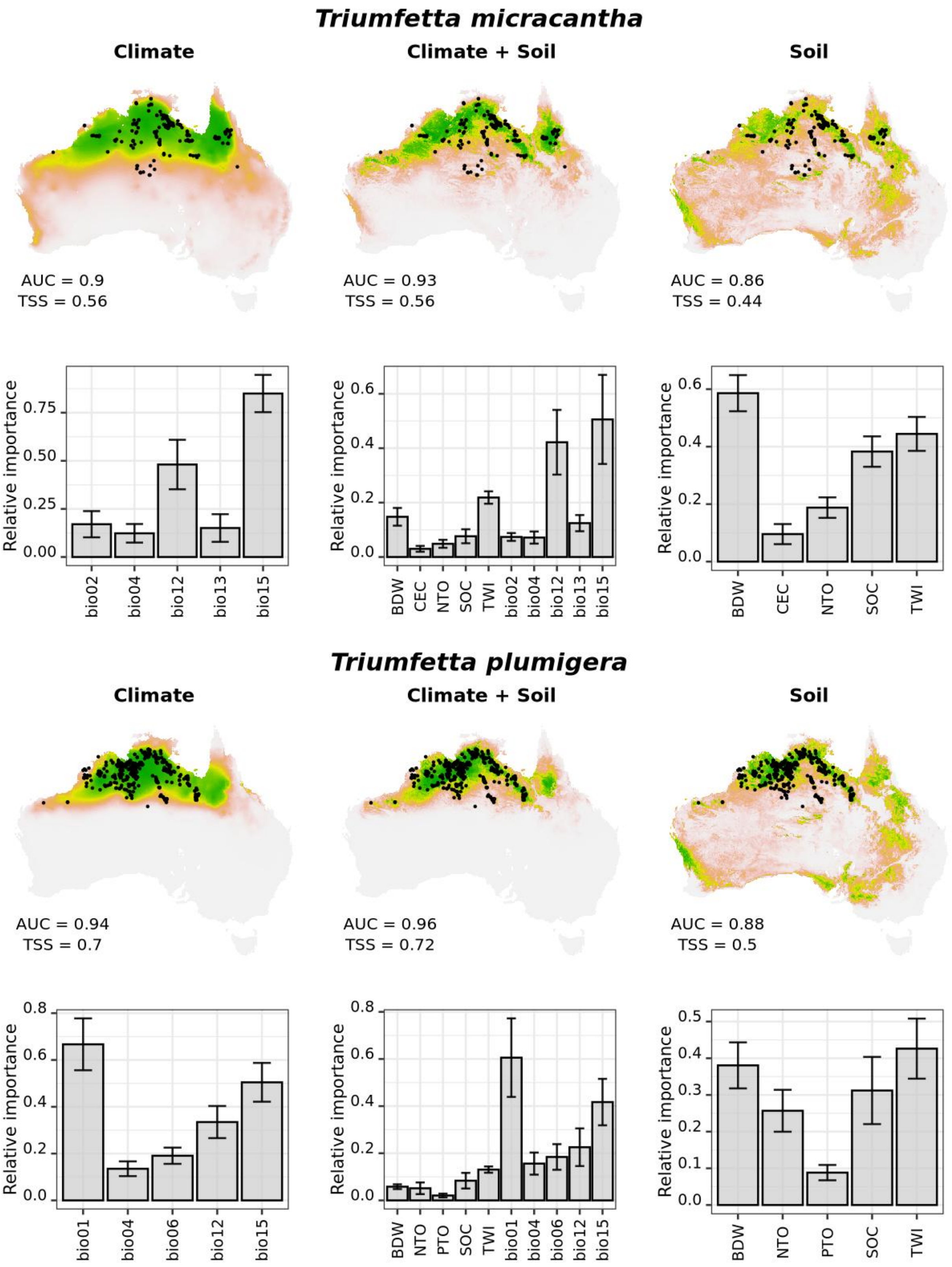
Triumfetta micracantha
Climate
Climate + Soil
Soil
AUC = 0.9
TSS = 0.56
AUC = 0.93
TSS = 0.56
AUC = 0.86
TSS = 0.44
Relative importance
0.00
0.25
0.50
0.75
bio02
bio04
bio12
bio13
bio15
0.0
0.2
0.4
0.6
BDW
CEC
NTO
SOC
TWI
bio02
bio04
bio12
bio13
bio15
BDW
CEC
NTO
SOC
TWI
Triumfetta plumigera
Climate
Climate + Soil
Soil
AUC = 0.94
TSS = 0.7
AUC = 0.96
TSS = 0.72
AUC = 0.88
TSS = 0.5
0.0
0.2
0.4
0.6
0.8
bio01
bio04
bio06
bio12
bio15
BDW
NTO
PTO
SOC
TWI
bio01
bio04
bio06
bio12
bio15
0.0
0.1
0.2
0.3
0.4
0.5
BDW
NTO
PTO
SOC
TWI

Trochocarpa laurina

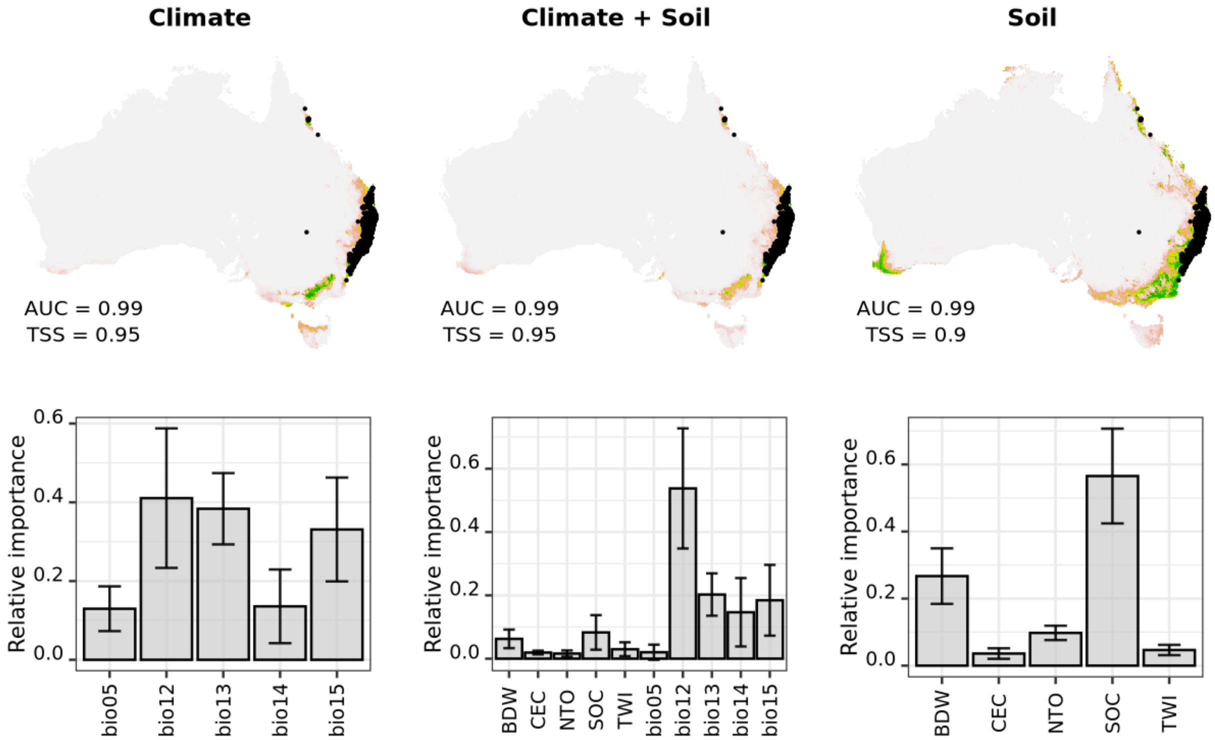

Trymalium ledifolium

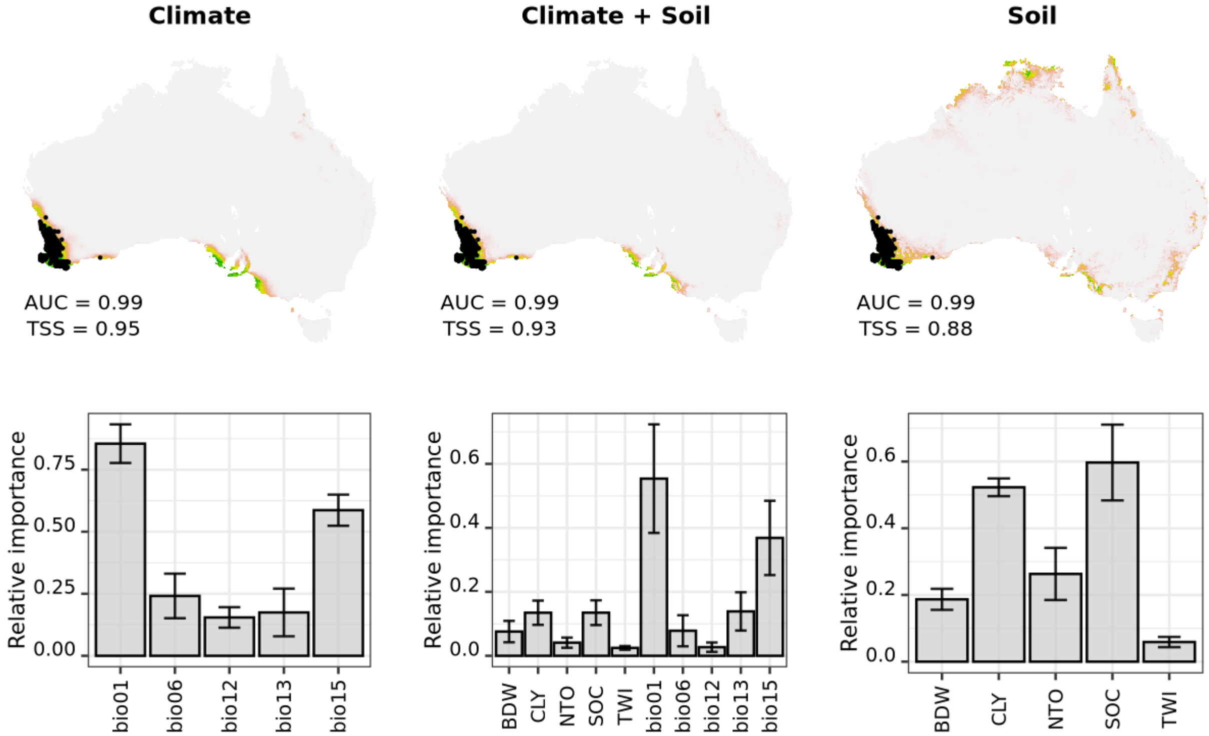

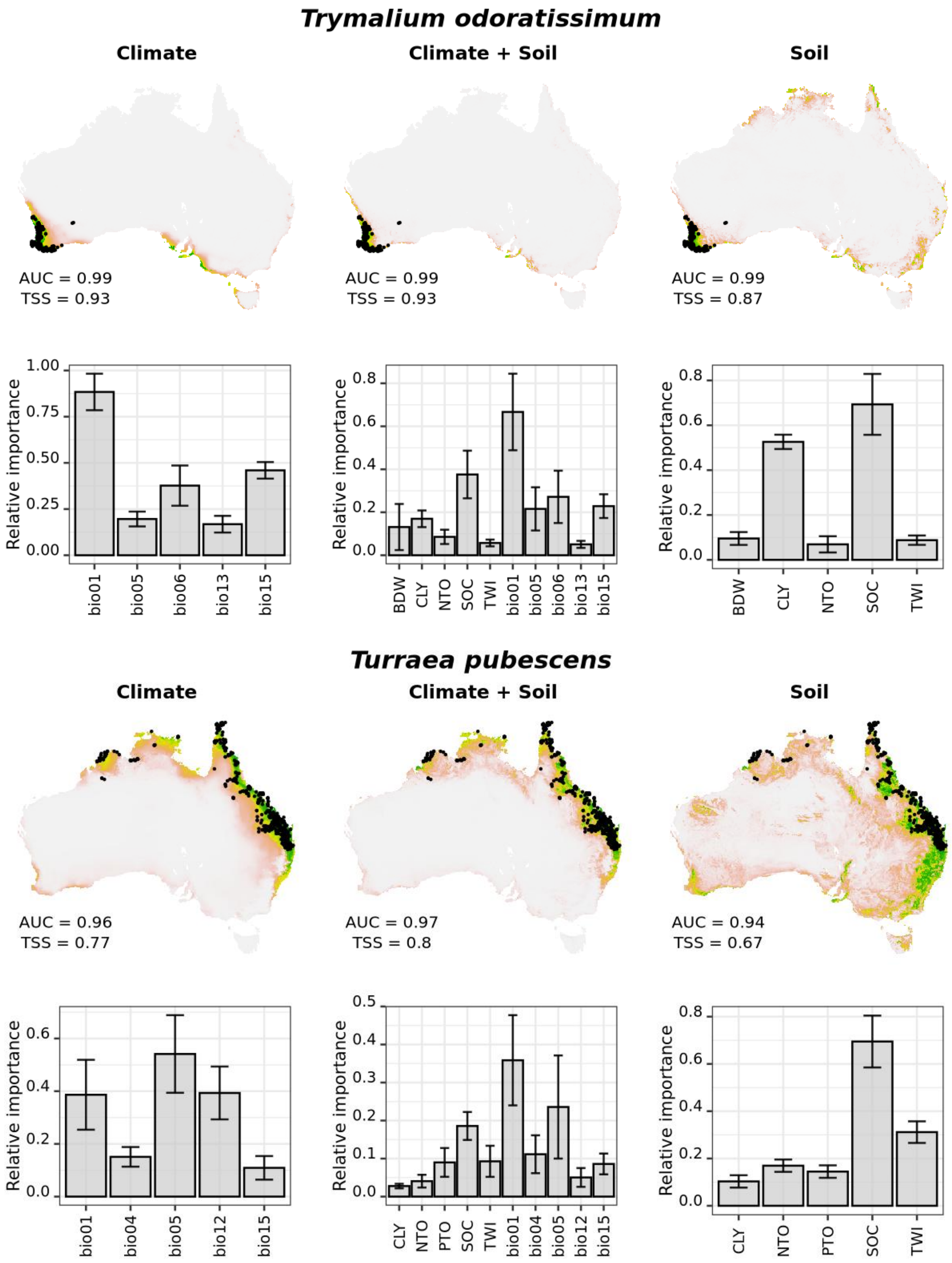
Trymalium odoratissimum
Climate
Climate + Soil
Soil
AUC = 0.99
TSS = 0.93
AUC = 0.99
TSS = 0.93
AUC = 0.99
TSS = 0.87
Relative importance
bio01 bio05 bio06 bio13 bio15
BDW CLY NTO SOC TWI bio01 bio05 bio06 bio13 bio15
BDW CLY NTO SOC TWI
Turraea pubescens
Climate
Climate + Soil
Soil
AUC = 0.96
TSS = 0.77
AUC = 0.97
TSS = 0.8
AUC = 0.94
TSS = 0.67
Relative importance
bio01 bio04 bio05 bio12 bio15
CLY NTO PTO SOC TWI bio01 bio04 bio05 bio12 bio15
CLY NTO PTO SOC TWI

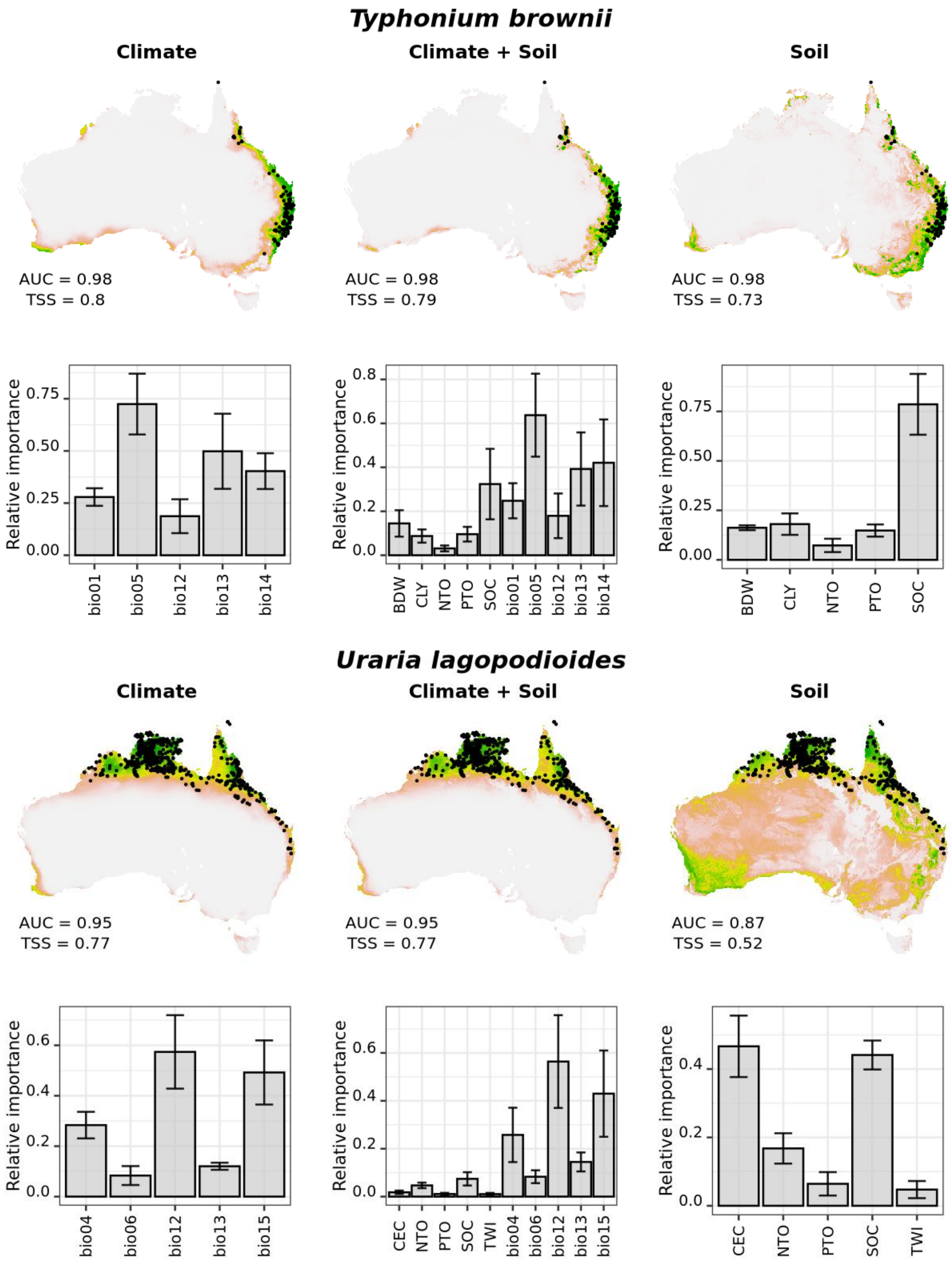

Typhonium brownii
Climate
Climate + Soil
Soil
AUC = 0.98
TSS = 0.8
AUC = 0.98
TSS = 0.79
AUC = 0.98
TSS = 0.73
Relative importance
bio01 bio05 bio12 bio13 bio14
BDW CLY NTO PTO SOC bio01 bio05 bio12 bio13 bio14
BDW CLY NTO PTO SOC
Uraria lagopodioides
Climate
Climate + Soil
Soil
AUC = 0.95
TSS = 0.77
AUC = 0.95
TSS = 0.77
AUC = 0.87
TSS = 0.52
Relative importance
bio04 bio06 bio12 bio13 bio15
CEC NTO PTO SOC TWI bio04 bio06 bio12 bio13 bio15
CEC NTO PTO SOC TWI

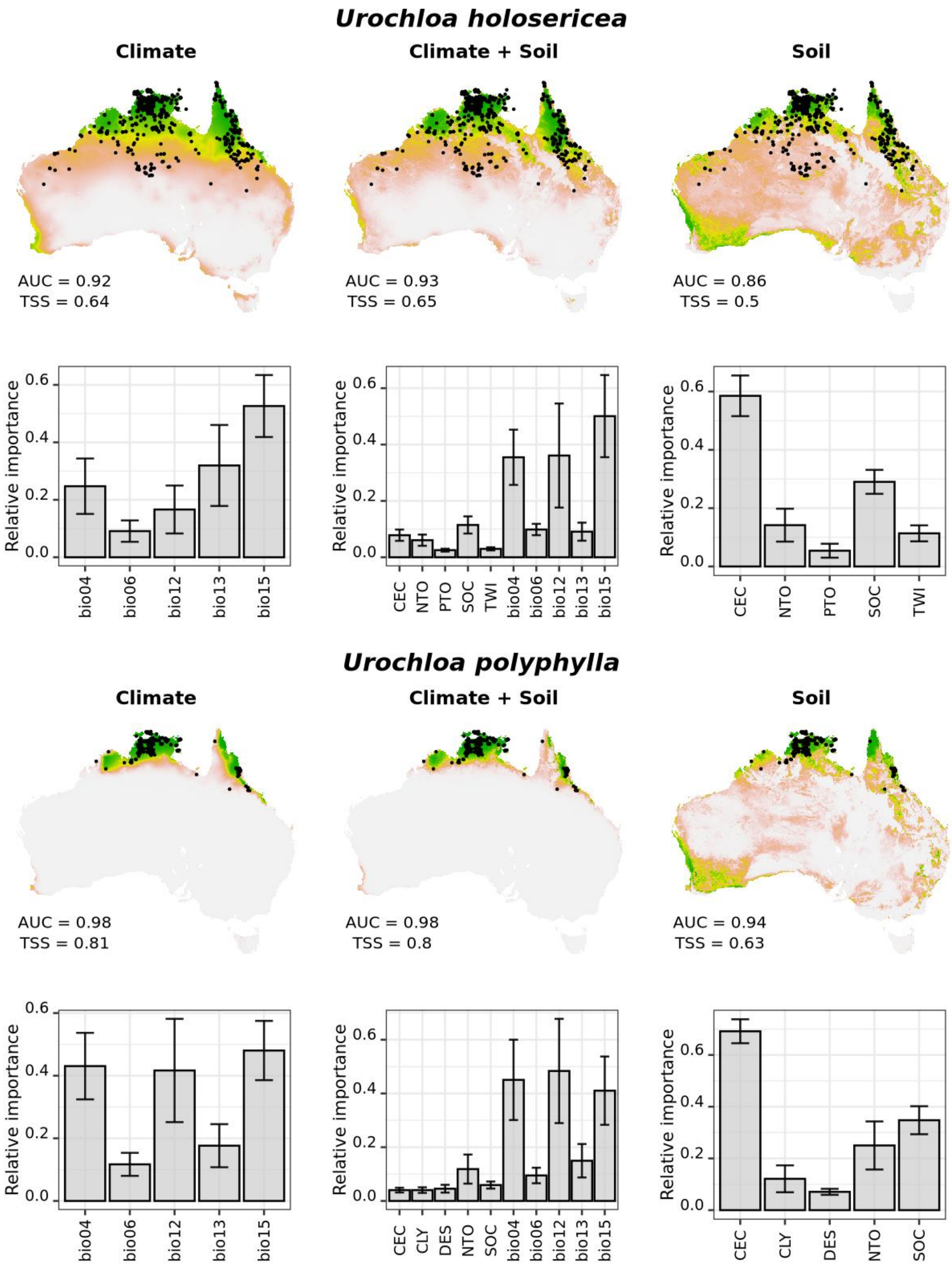
Urochloa holosericea
Climate
Climate + Soil
Soil
AUC = 0.92
TSS = 0.64
AUC = 0.93
TSS = 0.65
AUC = 0.86
TSS = 0.5
Relative importance
0.0
0.2
0.4
0.6
bio04
bio06
bio12
bio13
bio15
CEC
NTO
PTO
SOC
TWI
Urochloa polyphylla
Climate
Climate + Soil
Soil
AUC = 0.98
TSS = 0.81
AUC = 0.98
TSS = 0.8
AUC = 0.94
TSS = 0.63
Relative importance
CLY
DES

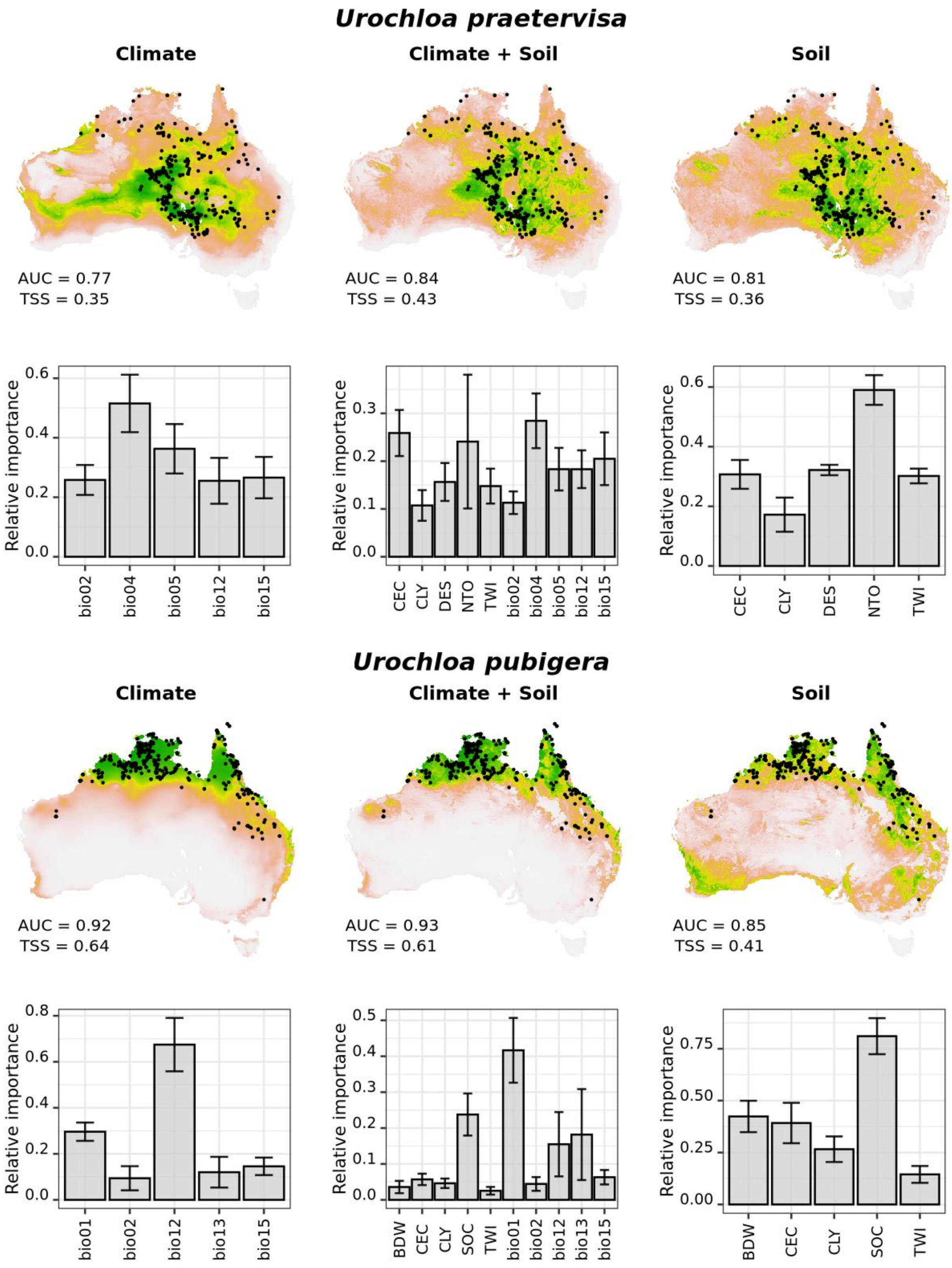
Urochloa praetervisa
Climate
Climate + Soil
Soil
AUC = 0.77
TSS = 0.35
AUC = 0.84
TSS = 0.43
AUC = 0.81
TSS = 0.36
Relative importance
bio02
bio04
bio05
bio12
bio15
CEC
CLY
DES
NTO
TWI
bio02
bio04
bio05
bio12
bio15
CEC
CLY
DES
NTO
TWI
Urochloa pubigera
Climate
Climate + Soil
Soil
AUC = 0.92
TSS = 0.64
AUC = 0.93
TSS = 0.61
AUC = 0.85
TSS = 0.41
Relative importance
bio01
bio02
bio12
bio13
bio15
BDW
CEC
CLY
SOC
TWI
bio01
bio02
bio12
bio13
bio15
BDW
CEC
CLY
SOC
TWI

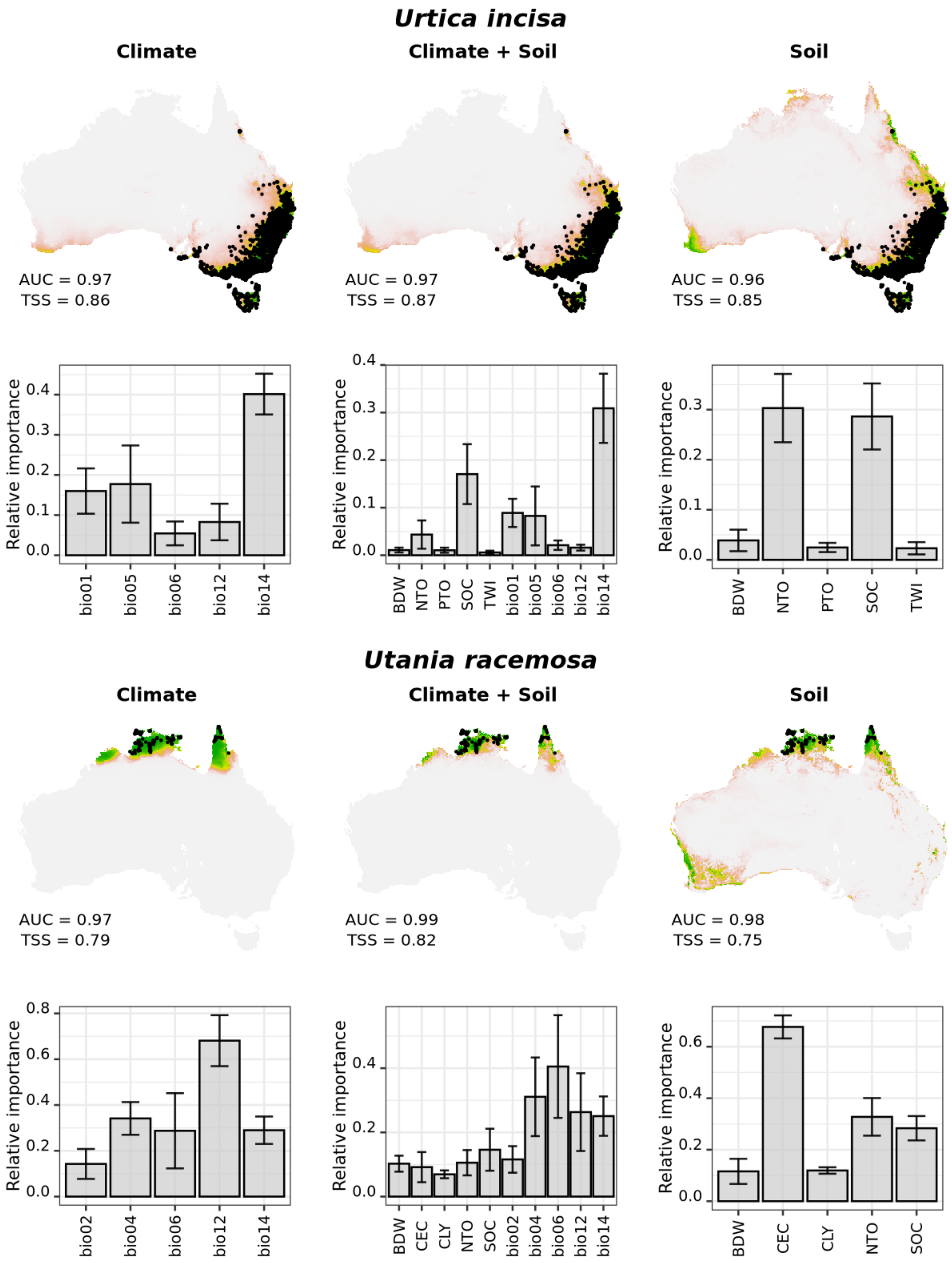
Urtica incisa
Climate
Climate + Soil
Soil
AUC = 0.97
TSS = 0.86
AUC = 0.97
TSS = 0.87
AUC = 0.96
TSS = 0.85
Relative importance
0.0
0.1
0.2
0.3
0.4
bio01
bio05
bio06
bio12
bio14
BDW
NTO
PTO
SOC
TWI
bio01
bio05
bio06
bio12
bio14
BDW
NTO
PTO
SOC
TWI
Utania racemosa
Climate
Climate + Soil
Soil
AUC = 0.97
TSS = 0.79
AUC = 0.99
TSS = 0.82
AUC = 0.98
TSS = 0.75
Relative importance
0.0
0.2
0.4
0.6
0.8
bio02
bio04
bio06
bio12
bio14
BDW
CEC
CLY
NTO
SOC
bio02
bio04
bio06
bio12
bio14
BDW
CEC
CLY
NTO
SOC

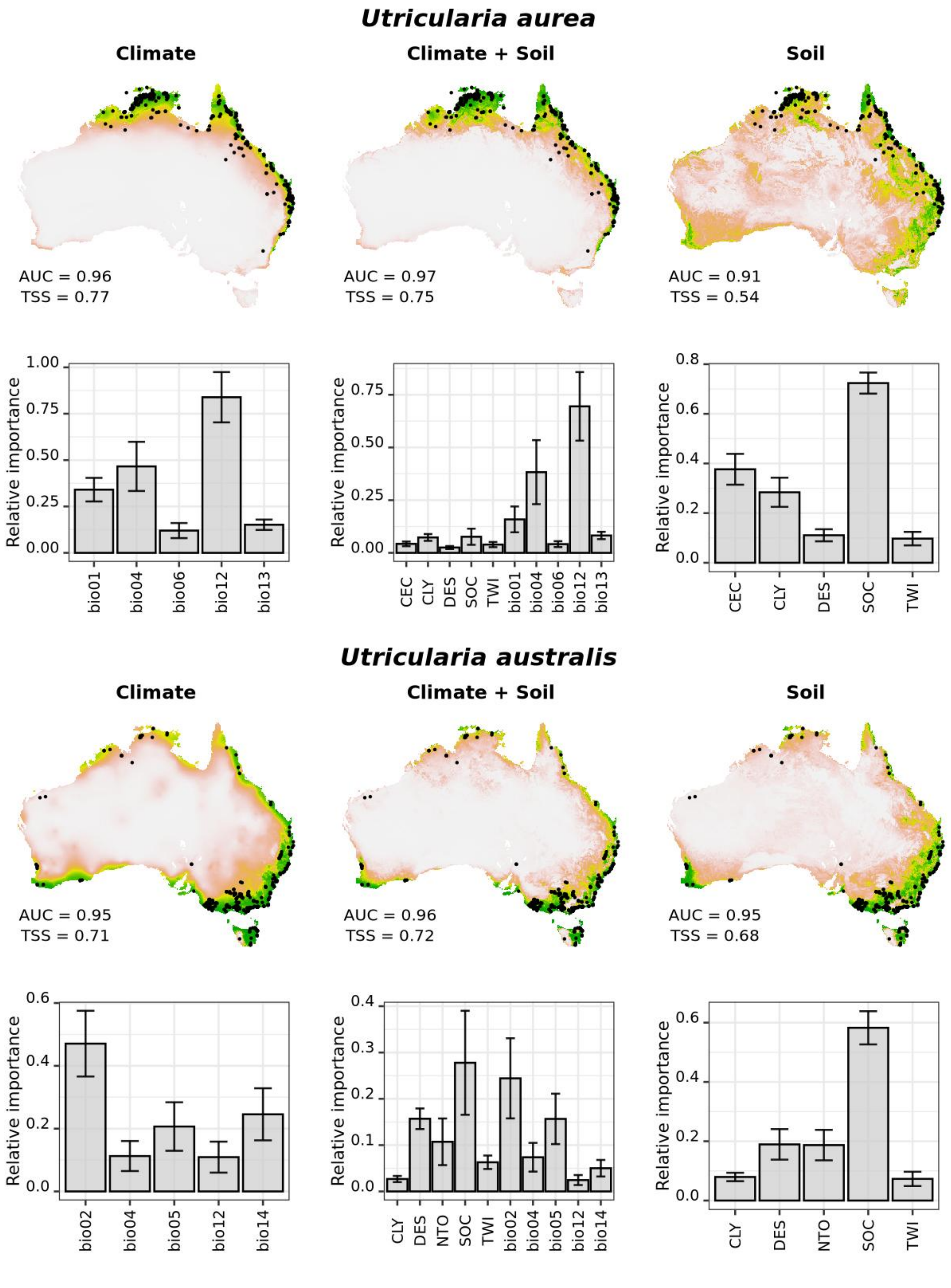
Utricularia aurea
Climate
Climate + Soil
Soil
AUC = 0.96
TSS = 0.77
AUC = 0.97
TSS = 0.75
AUC = 0.91
TSS = 0.54
Relative importance
bio01 bio04 bio06 bio12 bio13
CEC CLY DES SOC TWI bio01 bio04 bio06 bio12 bio13
CEC CLY DES SOC TWI
Utricularia australis
Climate
Climate + Soil
Soil
AUC = 0.95
TSS = 0.71
AUC = 0.96
TSS = 0.72
AUC = 0.95
TSS = 0.68
Relative importance
bio02 bio04 bio05 bio12 bio14
CLY DES NTO SOC TWI bio02 bio04 bio05 bio12 bio14
CLY DES NTO SOC TWI

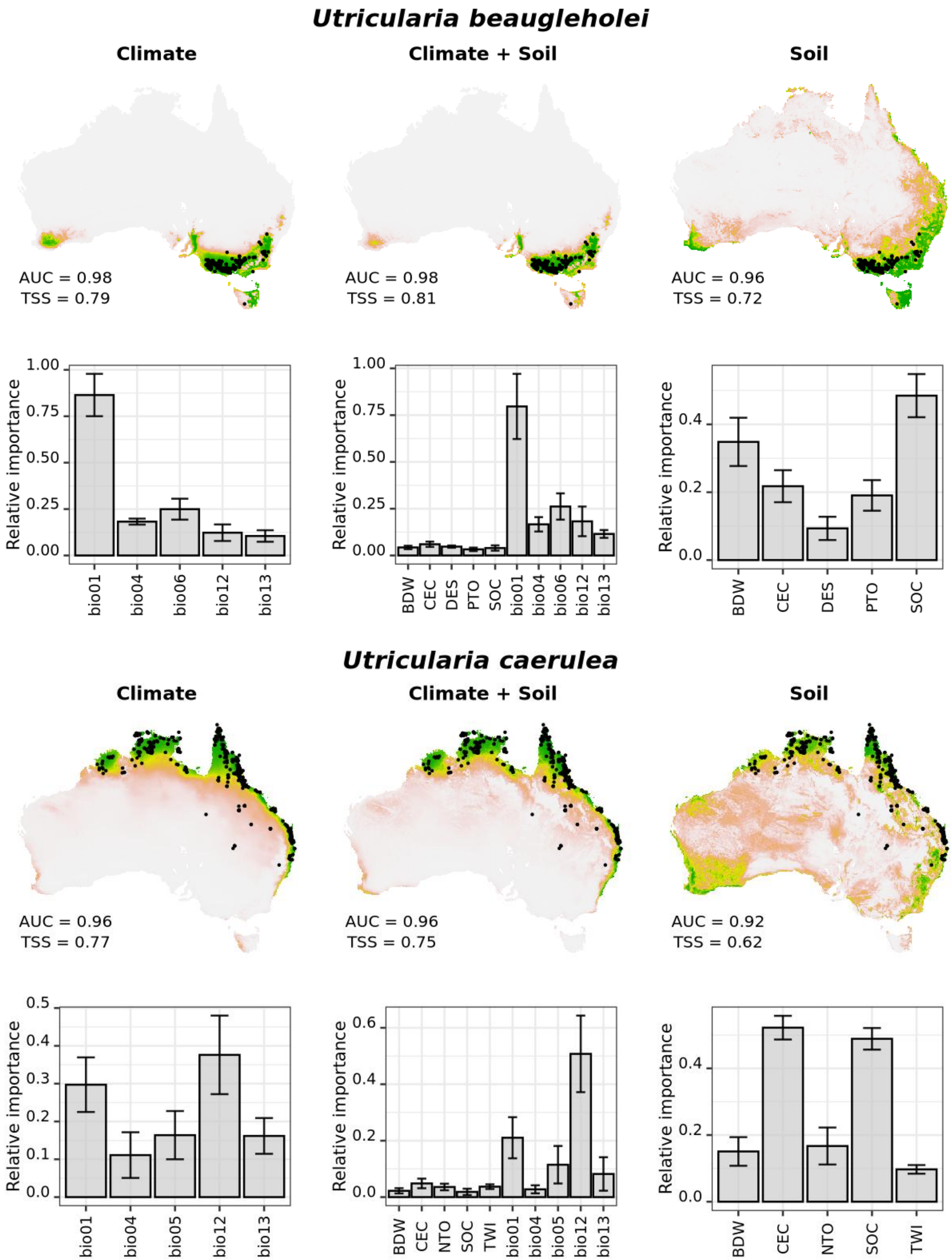
Utricularia beaugleholei
Climate
Climate + Soil
Soil
AUC = 0.98
TSS = 0.79
AUC = 0.98
TSS = 0.81
AUC = 0.96
TSS = 0.72
Relative importance
bio01
bio04
bio06
bio12
bio13
BDW
CEC
DES
PTO
SOC
Utricularia caerulea
Climate
Climate + Soil
Soil
AUC = 0.96
TSS = 0.77
AUC = 0.96
TSS = 0.75
AUC = 0.92
TSS = 0.62
Relative importance
bio01
bio04
bio05
bio12
bio13
BDW
CEC
NTO
SOC
TWI

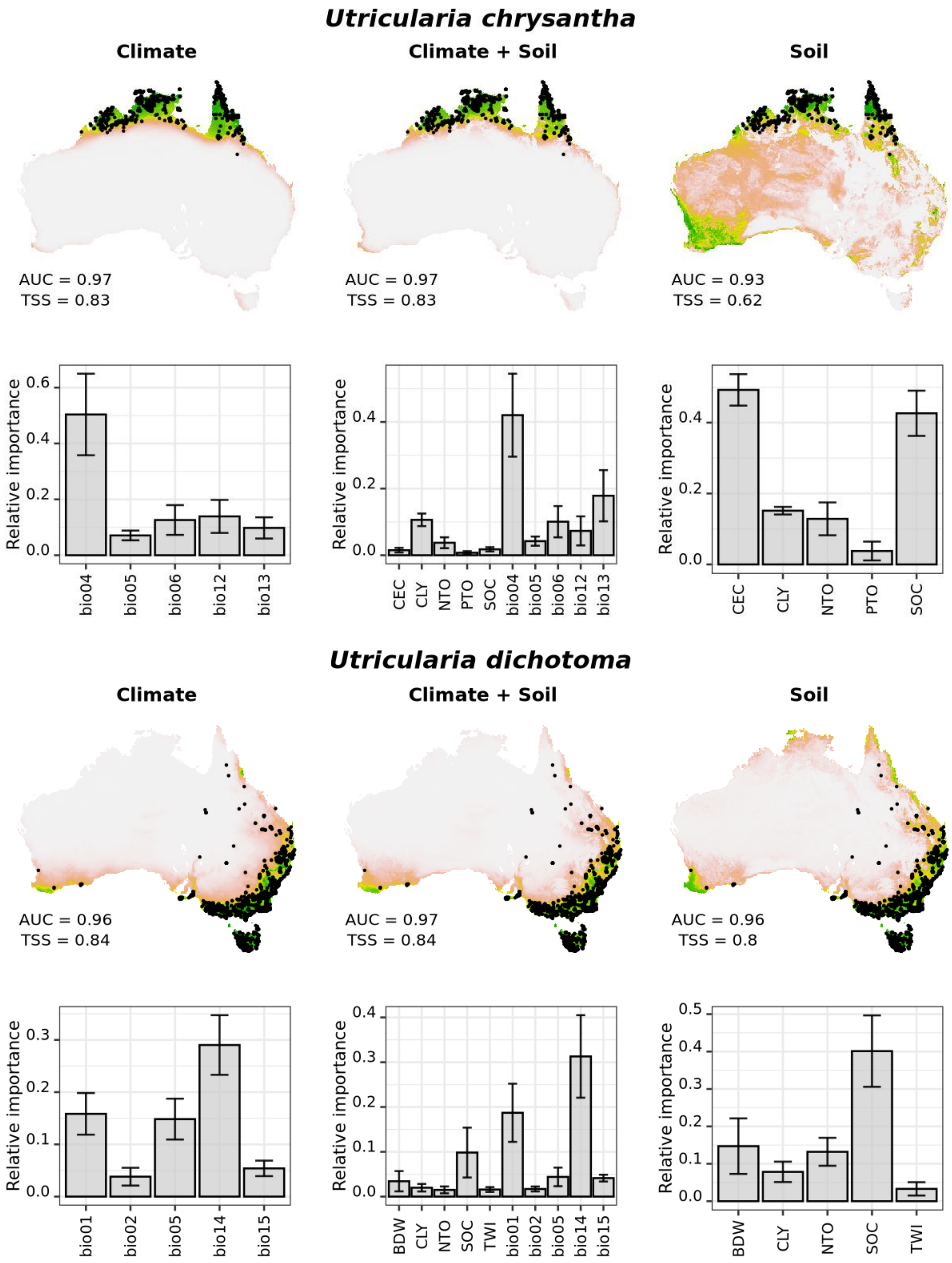

Utricularia chrysantha
Climate
Climate + Soil
Soil
AUC = 0.97
TSS = 0.83
AUC = 0.97
TSS = 0.83
AUC = 0.93
TSS = 0.62
Relative importance
bio04
bio05
bio06
bio12
bio13
CEC
CLY
NTO
PTO
SOC
Utricularia dichotoma
Climate
Climate + Soil
Soil
AUC = 0.96
TSS = 0.84
AUC = 0.97
TSS = 0.84
AUC = 0.96
TSS = 0.8
Relative importance
bio01
bio02
bio05
bio14
bio15
BDW
CLY
NTO
SOC
TWI

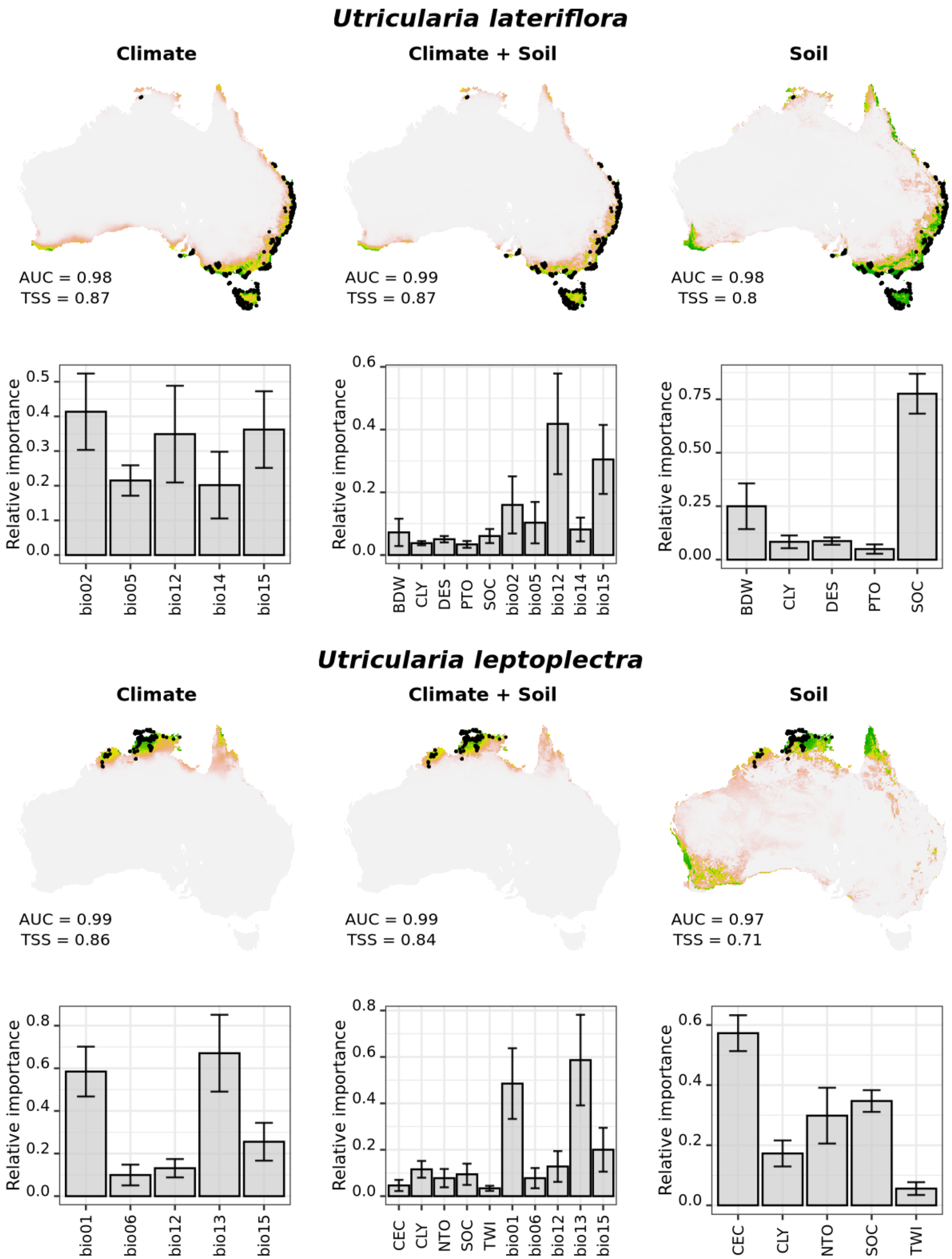
Utricularia lateriflora
Climate
Climate + Soil
Soil
AUC = 0.98
TSS = 0.87
AUC = 0.99
TSS = 0.87
AUC = 0.98
TSS = 0.8
Relative importance
0.0
0.1
0.2
0.3
0.4
0.5
bio02
bio05
bio12
bio14
bio15
0.0
0.2
0.4
0.6
BDW
CLY
DES
PTO
SOC
bio02
bio05
bio12
bio14
bio15
0.00
0.25
0.50
0.75
BDW
CLY
DES
PTO
SOC
Utricularia leptoplectra
Climate
Climate + Soil
Soil
AUC = 0.99
TSS = 0.86
AUC = 0.99
TSS = 0.84
AUC = 0.97
TSS = 0.71
0.0
0.2
0.4
0.6
0.8
bio01
bio06
bio12
bio13
bio15
CEC
CLY
NTO
SOC
TWI
bio01
bio06
bio12
bio13
bio15
CEC
CLY
NTO
SOC
TWI

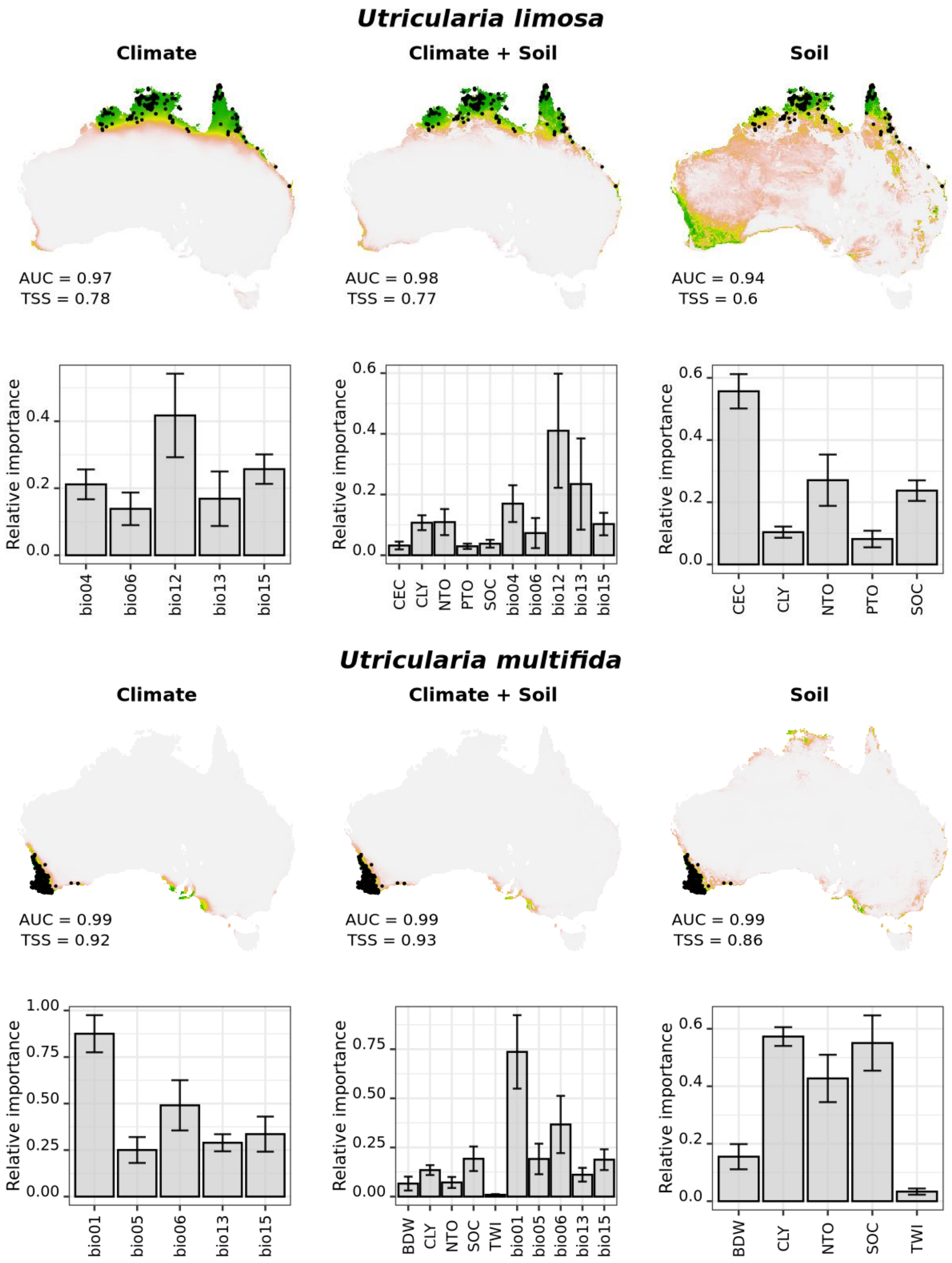

Utricularia limosa
Climate
Climate + Soil
Soil
AUC = 0.97
TSS = 0.78
AUC = 0.98
TSS = 0.77
AUC = 0.94
TSS = 0.6
Relative importance
bio04
bio06
bio12
bio13
bio15
CEC
CLY
NTO
PTO
SOC
Utricularia multifida
AUC = 0.99
TSS = 0.92
AUC = 0.99
TSS = 0.93
AUC = 0.99
TSS = 0.86
bio01
bio05
BDW
TWI

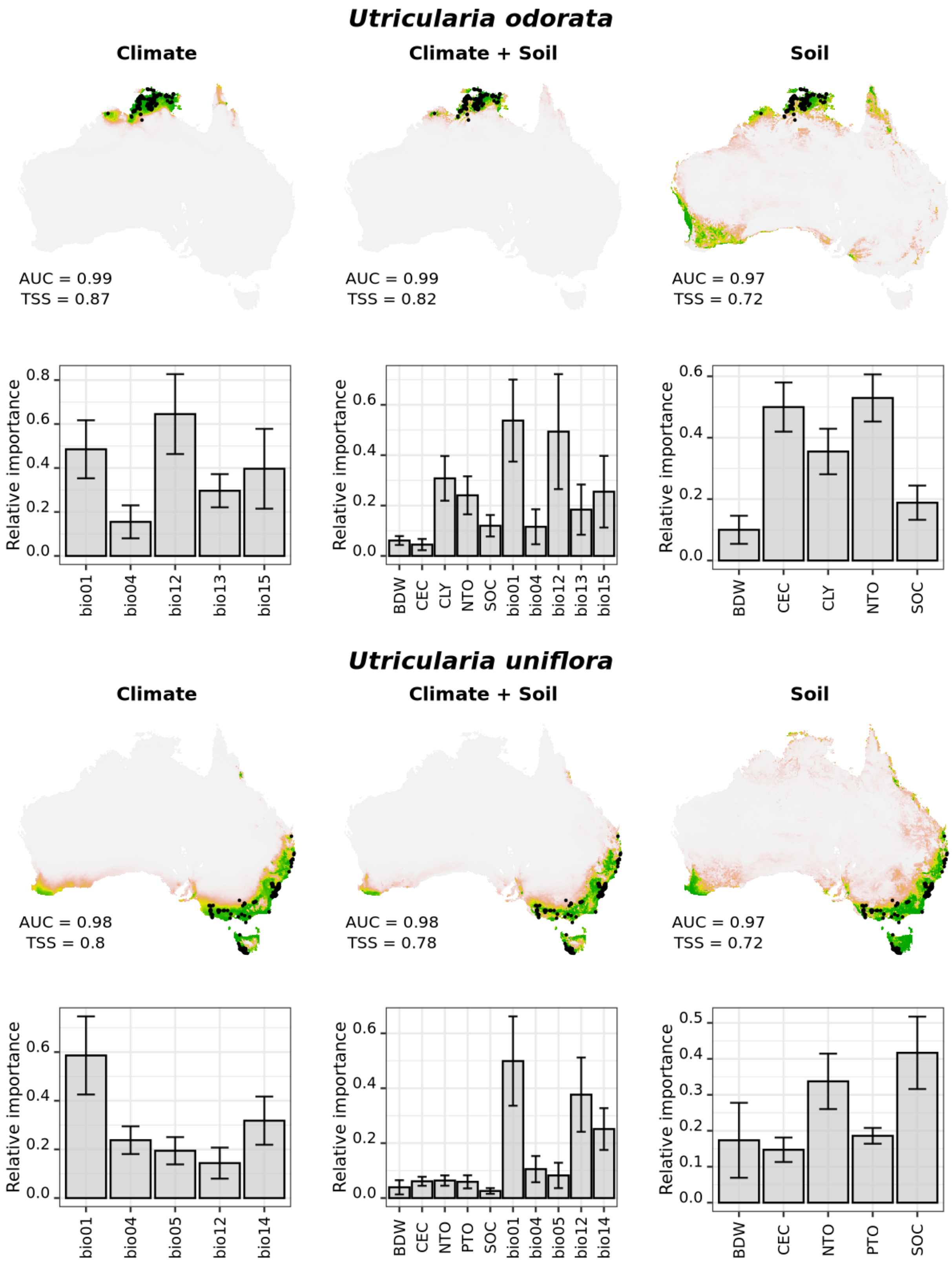
Utricularia odorata
Climate
Climate + Soil
Soil
AUC = 0.99
TSS = 0.87
AUC = 0.99
TSS = 0.82
AUC = 0.97
TSS = 0.72
Relative importance
bio01
bio04
bio12
bio13
bio15
BDW
CEC
CLY
NTO
SOC
Utricularia uniflora
Climate
Climate + Soil
Soil
AUC = 0.98
TSS = 0.8
AUC = 0.98
TSS = 0.78
AUC = 0.97
TSS = 0.72
Relative importance
bio01
bio04
bio05
bio12
bio14
BDW
CEC
NTO
PTO
SOC

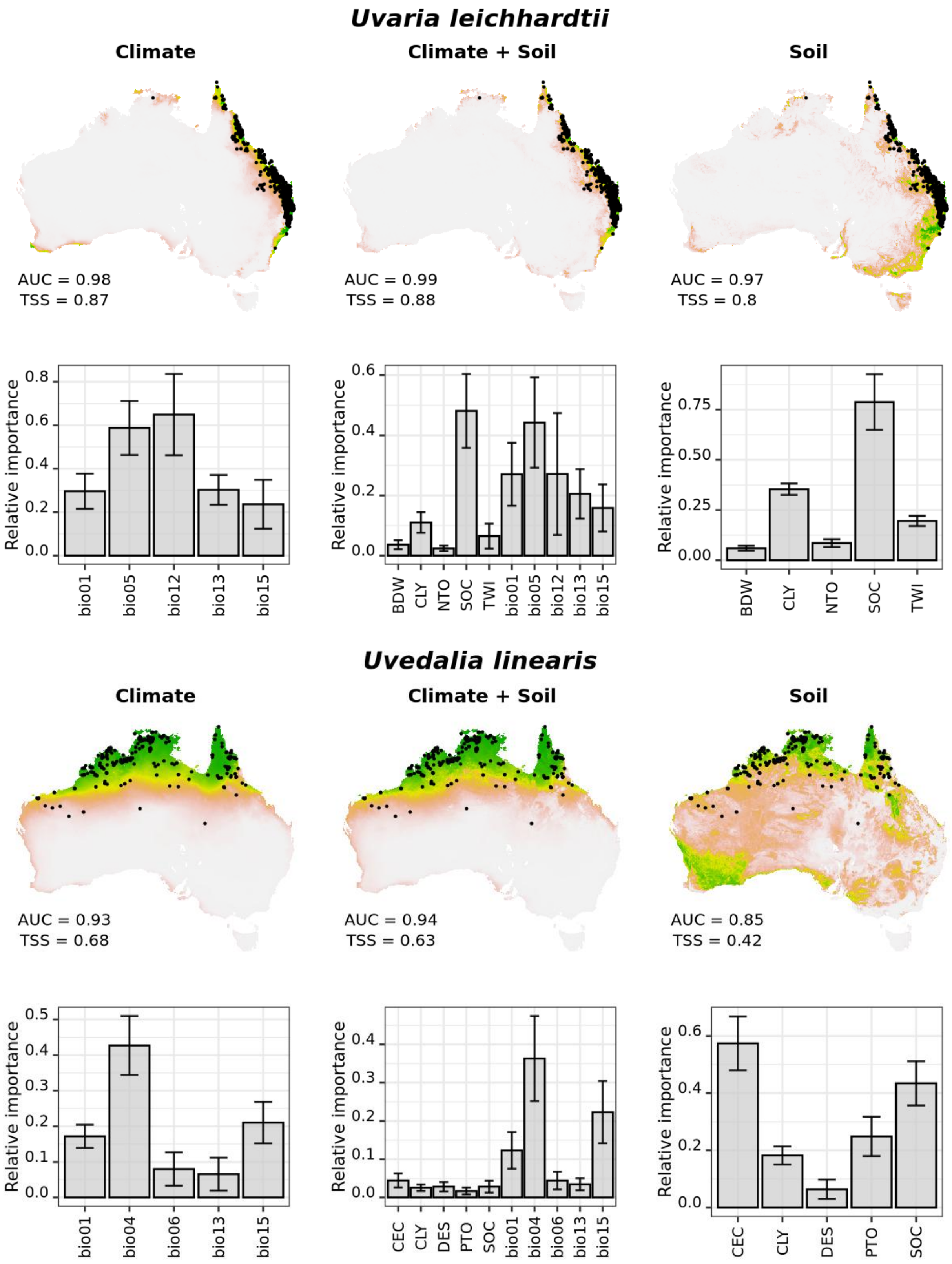

Uvaria leichhardtii
Climate
Climate + Soil
Soil
AUC = 0.98
TSS = 0.87
AUC = 0.99
TSS = 0.88
AUC = 0.97
TSS = 0.8
Relative importance
bio01 bio05 bio12 bio13 bio15
BDW CLY NTO SOC TWI bio01 bio05 bio12 bio13 bio15
BDW CLY NTO SOC TWI
Uvedalia linearis
Climate
Climate + Soil
Soil
AUC = 0.93
TSS = 0.68
AUC = 0.94
TSS = 0.63
AUC = 0.85
TSS = 0.42
Relative importance
bio01 bio04 bio06 bio13 bio15
CEC CLY DES PTO SOC bio01 bio04 bio06 bio13 bio15
CEC CLY DES PTO SOC

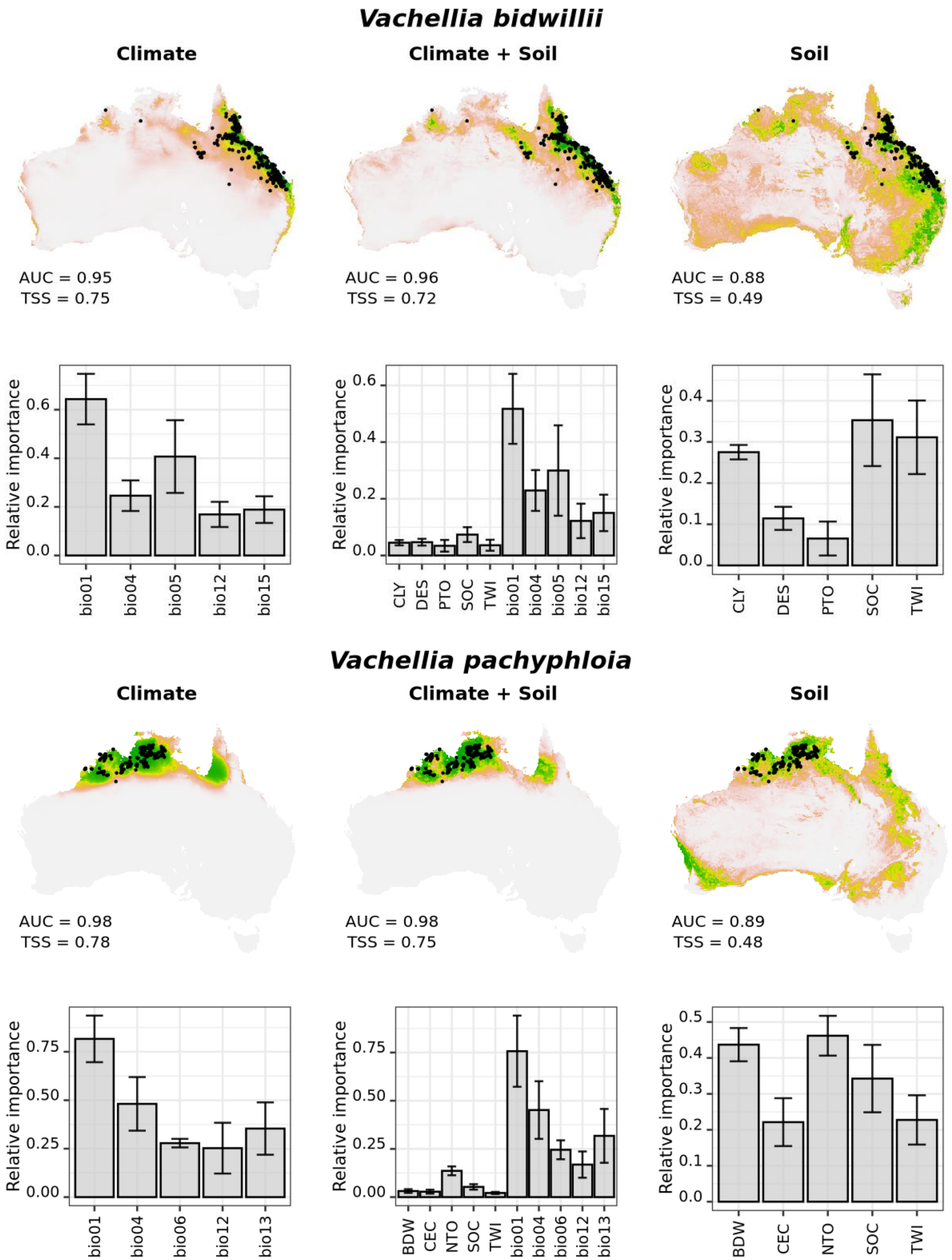
Vachellia bidwillii
Climate
Climate + Soil
Soil
AUC = 0.95
TSS = 0.75
AUC = 0.96
TSS = 0.72
AUC = 0.88
TSS = 0.49
Relative importance
bio01
bio04
bio05
bio12
bio15
CLY
DES
PTO
SOC
TWI
Vachellia pachyphloia
Climate
Climate + Soil
Soil
AUC = 0.98
TSS = 0.78
AUC = 0.98
TSS = 0.75
AUC = 0.89
TSS = 0.48
Relative importance
bio01
bio04
bio06
bio12
bio13
BDW
CEC
NTO
SOC
TWI

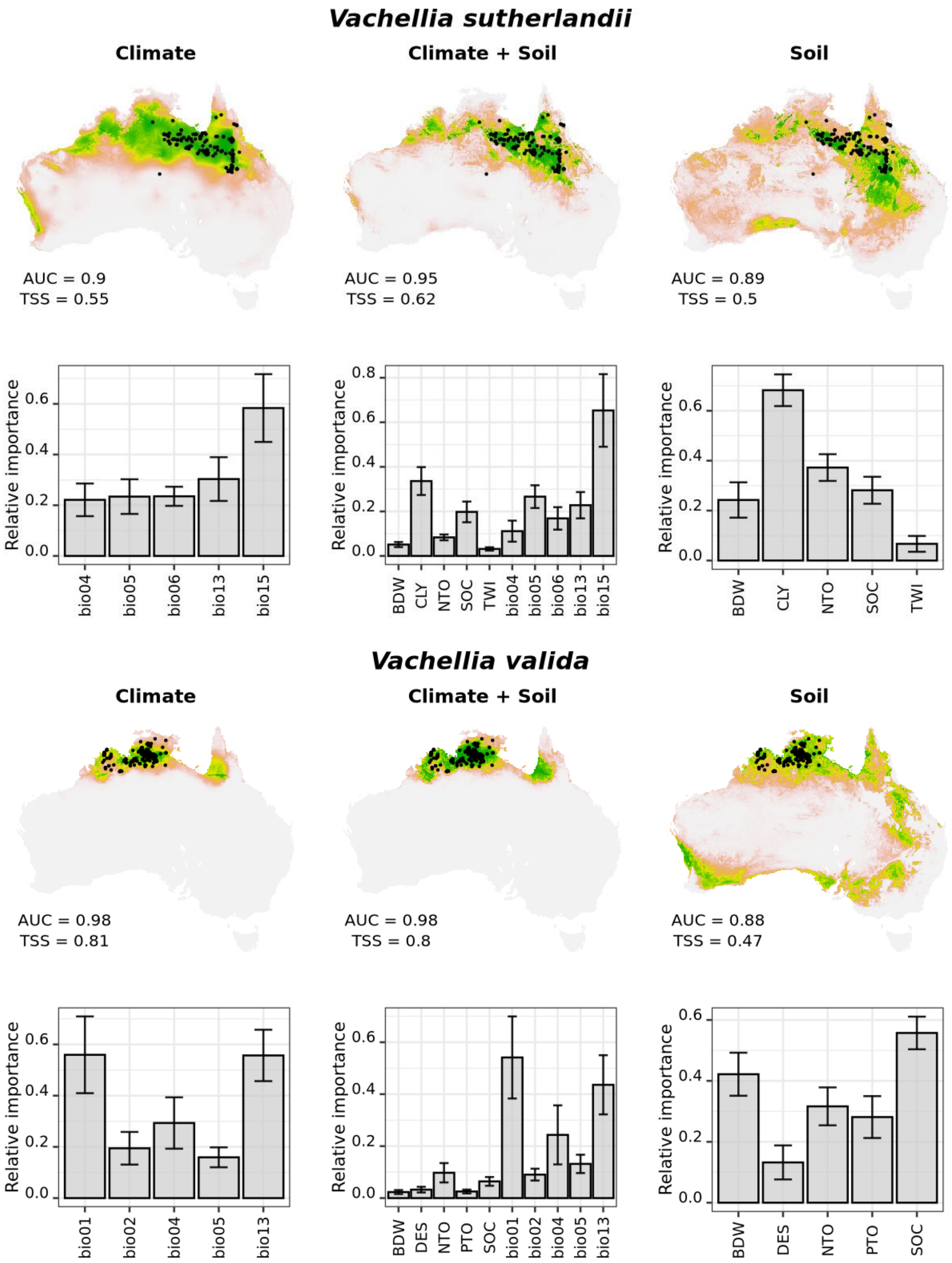
Vachellia sutherlandii
Climate
Climate + Soil
Soil
AUC = 0.9
TSS = 0.55
AUC = 0.95
TSS = 0.62
AUC = 0.89
TSS = 0.5
Relative importance
bio04
bio05
bio06
bio13
bio15
BDW
CLY
NTO
SOC
TWI
Vachellia valida
Climate
Climate + Soil
Soil
AUC = 0.98
TSS = 0.81
AUC = 0.98
TSS = 0.8
AUC = 0.88
TSS = 0.47
Relative importance
bio01
bio02
bio04
bio05
bio13
BDW
DES
NTO
PTO
SOC

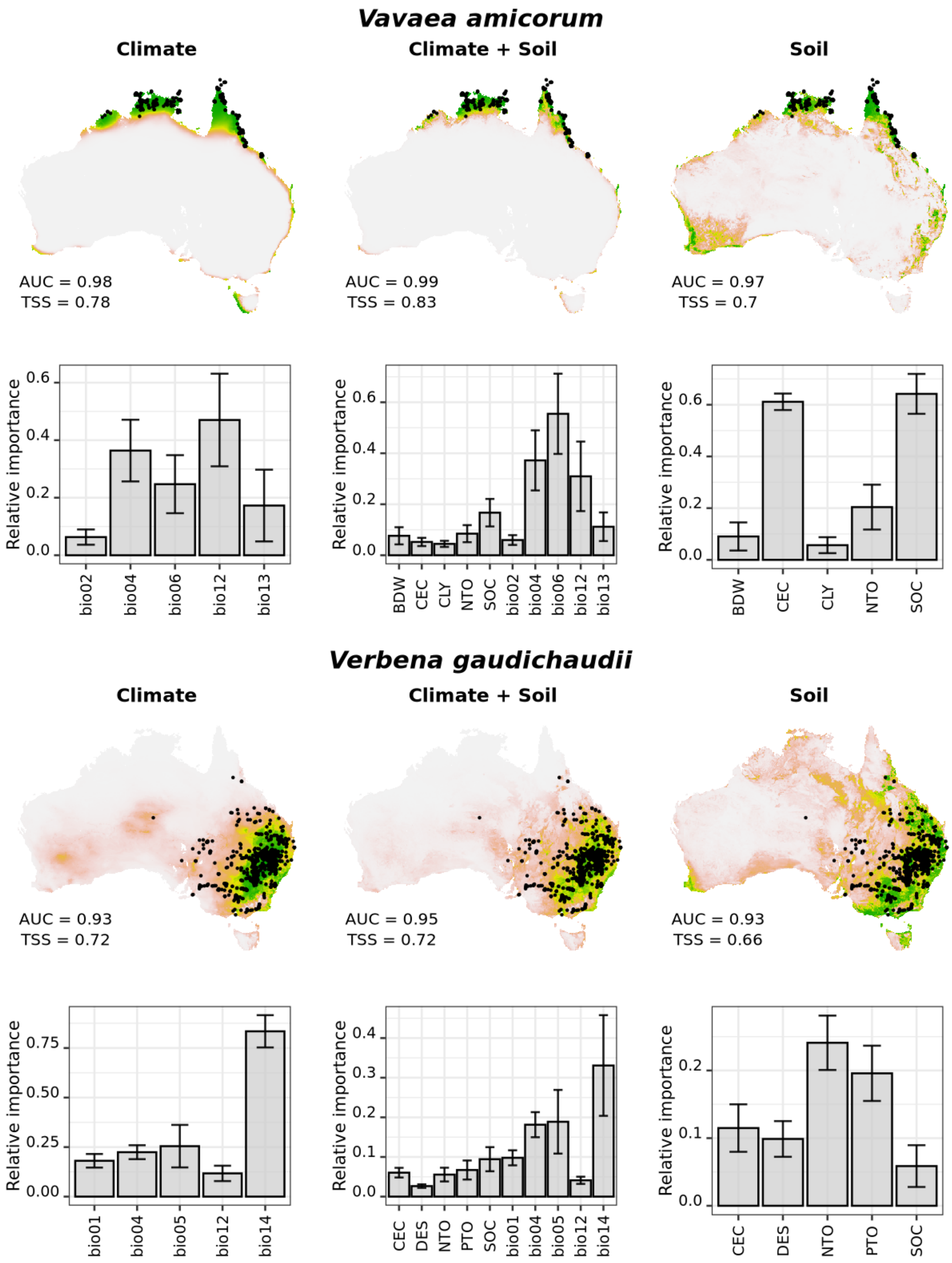

Vavaea amicorum
Climate
Climate + Soil
Soil
AUC = 0.98
TSS = 0.78
AUC = 0.99
TSS = 0.83
AUC = 0.97
TSS = 0.7
Relative importance
0.0
0.2
0.4
0.6
bio02
bio04
bio06
bio12
bio13
BDW
CEC
CLY
NTO
SOC
bio02
bio04
bio06
bio12
bio13
BDW
CEC
CLY
NTO
SOC
Verbena gaudichaudii
Climate
Climate + Soil
Soil
AUC = 0.93
TSS = 0.72
AUC = 0.95
TSS = 0.72
AUC = 0.93
TSS = 0.66
0.00
0.25
0.50
0.75
0.1
0.3
bio01
bio04
bio05
bio12
bio14
CEC
DES
NTO
PTO
SOC
bio01
bio04
bio05
bio12
bio14
CEC
DES
NTO
PTO
SOC

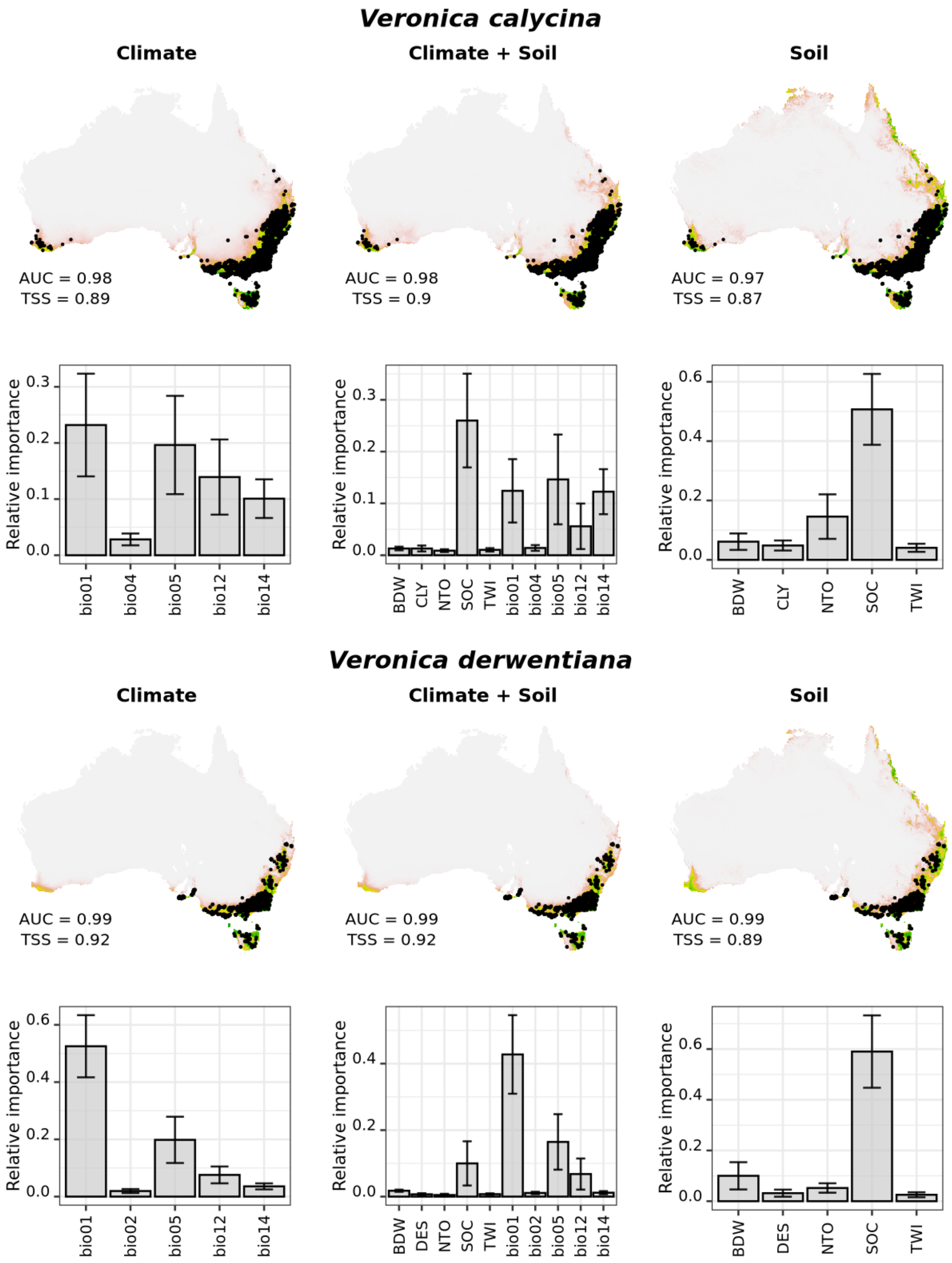

Veronica calycina
Climate
Climate + Soil
Soil
AUC = 0.98
TSS = 0.89
AUC = 0.98
TSS = 0.9
AUC = 0.97
TSS = 0.87
Relative importance
bio01
bio04
bio05
bio12
bio14
BDW
CLY
NTO
SOC
TWI
Veronica derwentiana
Climate
Climate + Soil
Soil
AUC = 0.99
TSS = 0.92
AUC = 0.99
TSS = 0.92
AUC = 0.99
TSS = 0.89
Relative importance
bio01
bio02
bio05
bio12
bio14
BDW
DES
NTO
SOC
TWI

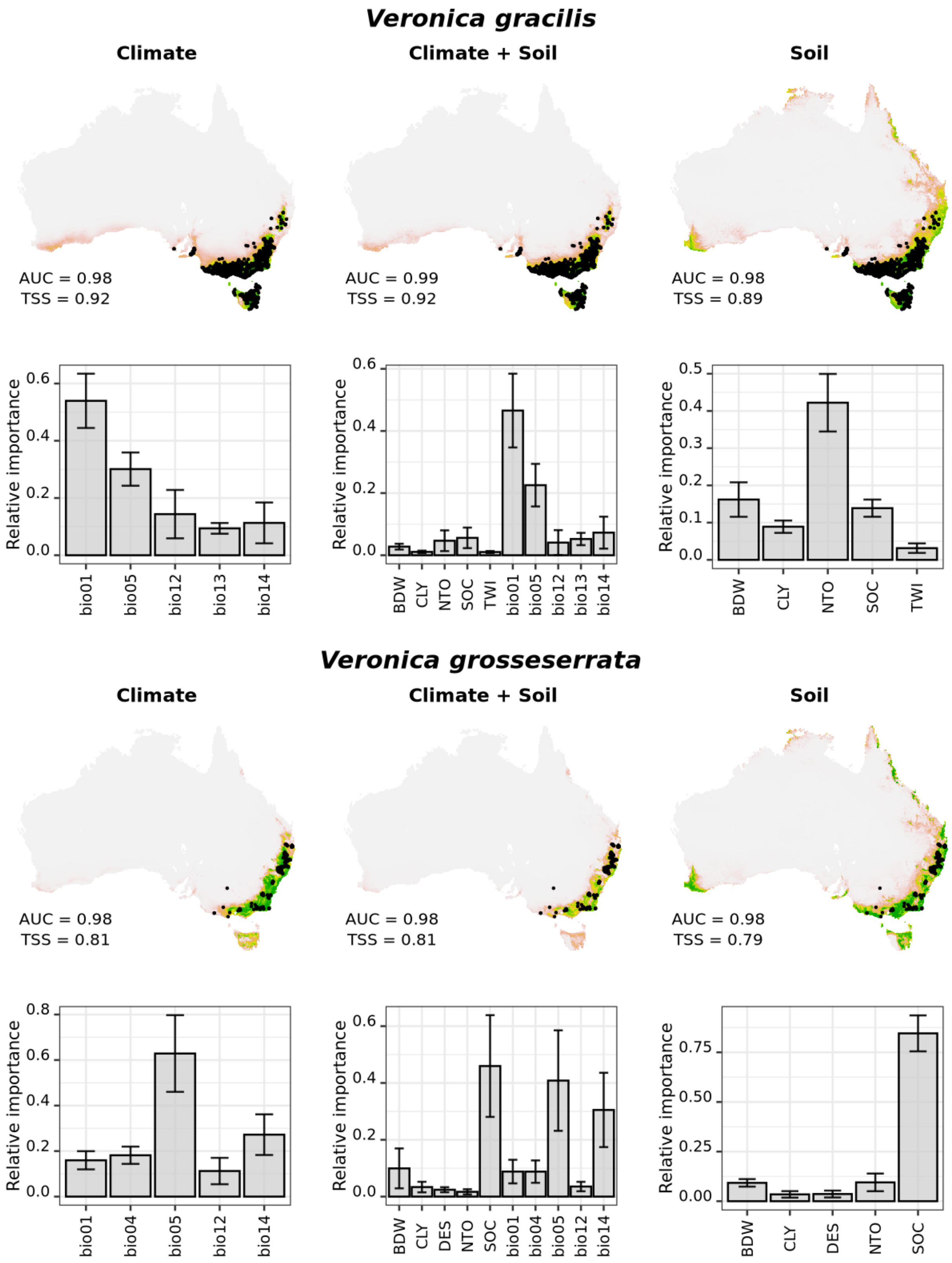

Veronica gracilis
Climate
Climate + Soil
Soil
AUC = 0.98
TSS = 0.92
AUC = 0.99
TSS = 0.92
AUC = 0.98
TSS = 0.89
Relative importance
bio01
bio05
bio12
bio13
bio14
BDW
CLY
NTO
SOC
TWI
Veronica grosseserrata
Climate
Climate + Soil
Soil
AUC = 0.98
TSS = 0.81
AUC = 0.98
TSS = 0.81
AUC = 0.98
TSS = 0.79
Relative importance
bio01
bio04
bio05
bio12
bio14
BDW
CLY
DES
NTO
SOC

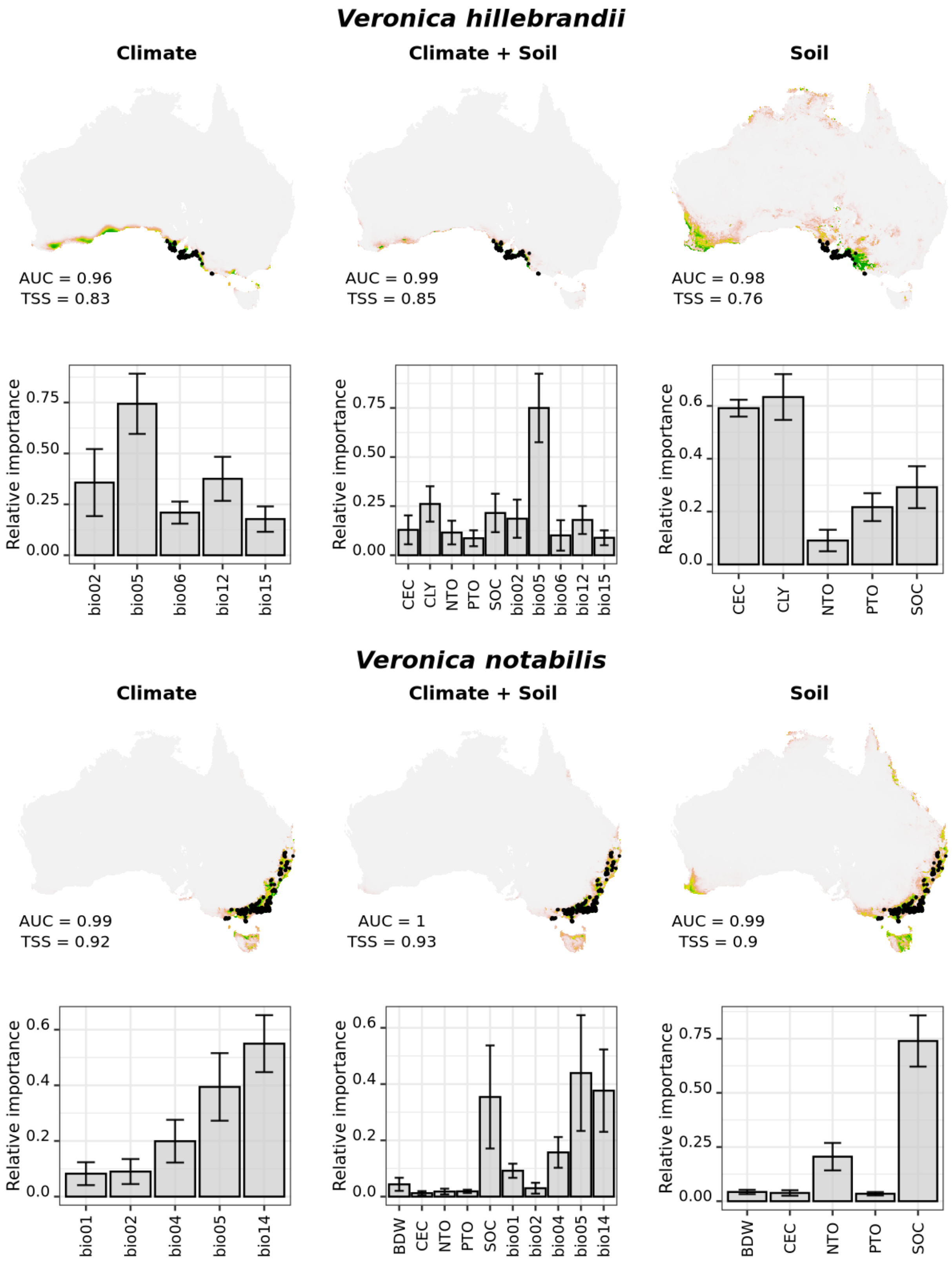
Veronica hillebrandii
Climate
Climate + Soil
Soil
AUC = 0.96
TSS = 0.83
AUC = 0.99
TSS = 0.85
AUC = 0.98
TSS = 0.76
Relative importance
0.00
0.25
0.50
0.75
bio02
bio05
bio06
bio12
bio15
CEC
CLY
NTO
PTO
SOC
bio02
bio05
bio06
bio12
bio15
0.0
0.2
0.4
0.6
CEC
CLY
NTO
PTO
SOC
Veronica notabilis
Climate
Climate + Soil
Soil
AUC = 0.99
TSS = 0.92
AUC = 1
TSS = 0.93
AUC = 0.99
TSS = 0.9
Relative importance
0.0
0.2
0.4
0.6
bio01
bio02
bio04
bio05
bio14
BDW
CEC
NTO
PTO
SOC
bio01
bio02
bio04
bio05
bio14
0.00
0.25
0.50
0.75
BDW
CEC
NTO
PTO
SOC

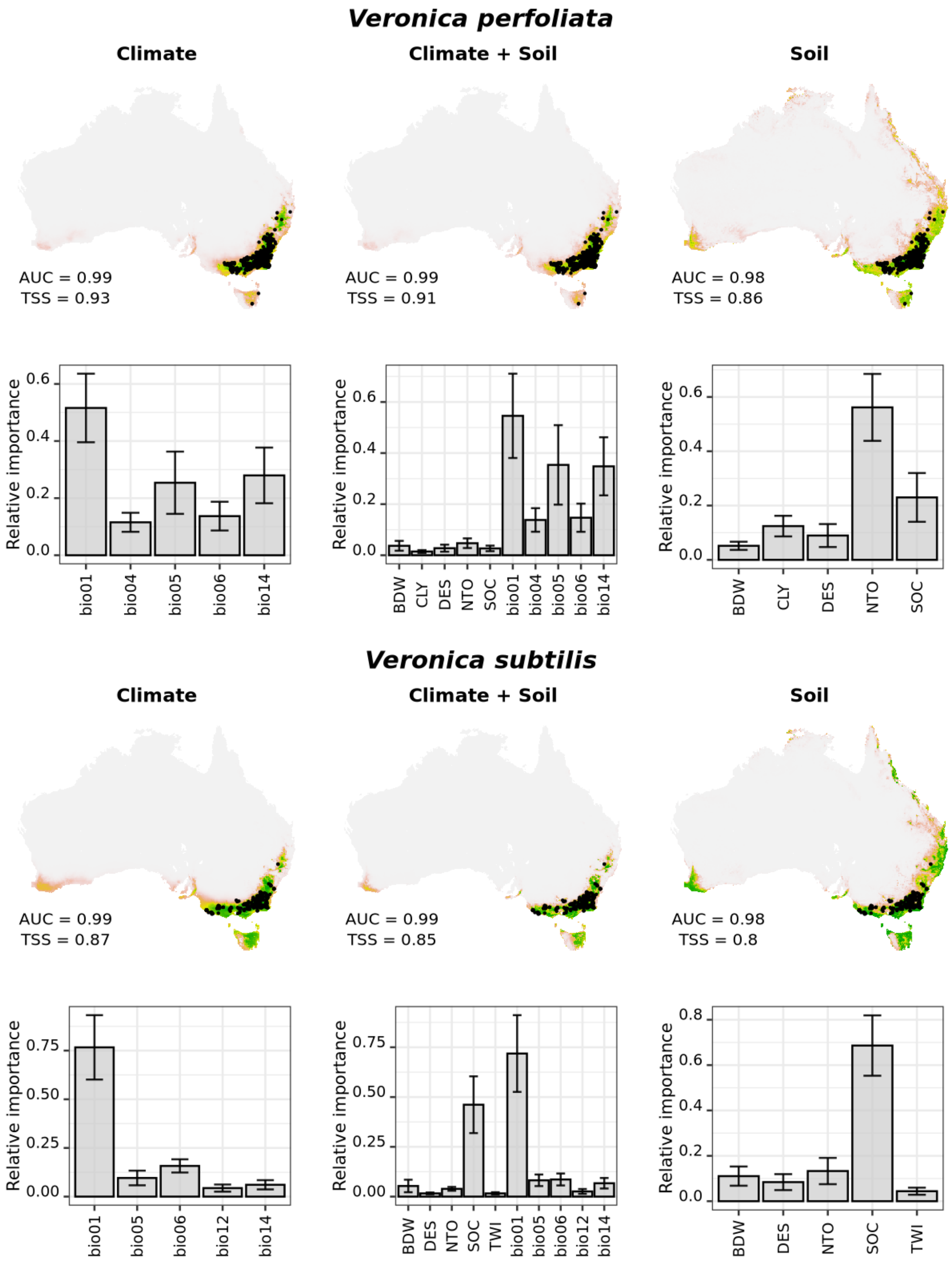

Veronica perfoliata
Climate
Climate + Soil
Soil
AUC = 0.99
TSS = 0.93
AUC = 0.99
TSS = 0.91
AUC = 0.98
TSS = 0.86
Relative importance
0.0
0.2
0.4
0.6
bio01
bio04
bio05
bio06
bio14
BDW
CLY
DES
NTO
SOC
Veronica subtilis
Climate
Climate + Soil
Soil
AUC = 0.99
TSS = 0.87
AUC = 0.99
TSS = 0.85
AUC = 0.98
TSS = 0.8
Relative importance
0.00
0.25
0.50
0.75
0.8
bio12
TWI

Verticordia acerosa

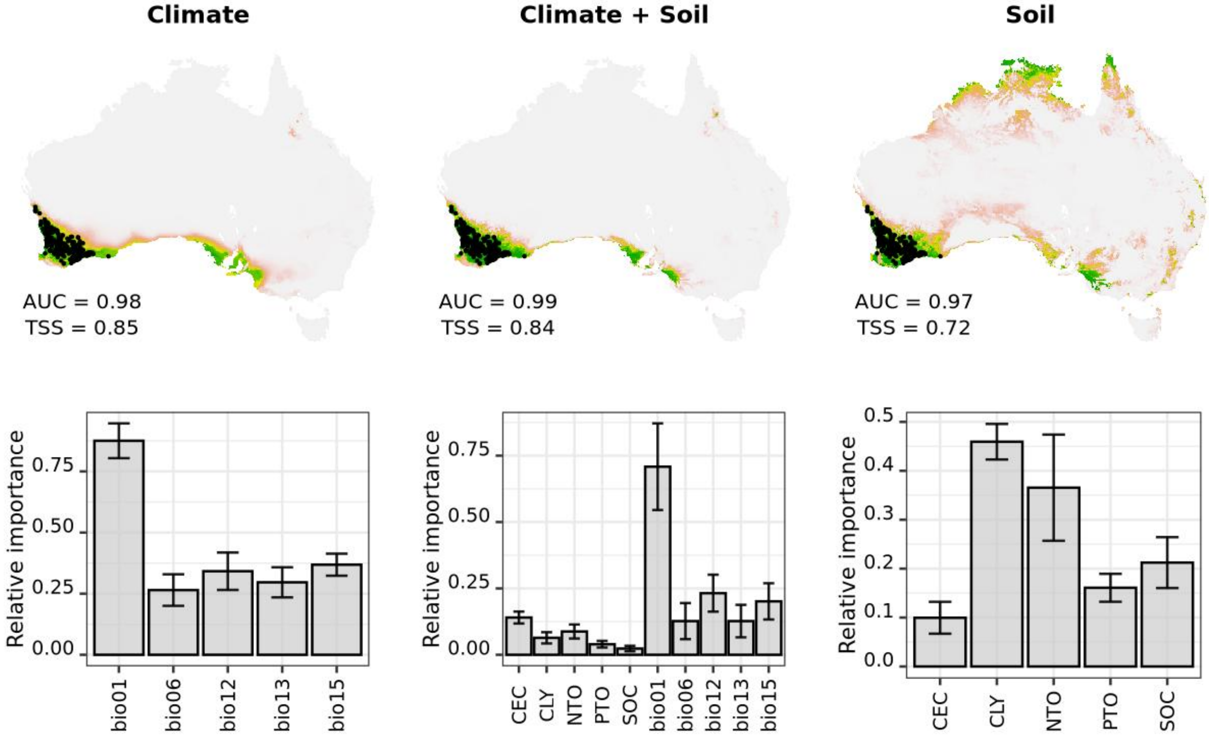

Verticordia chrysantha

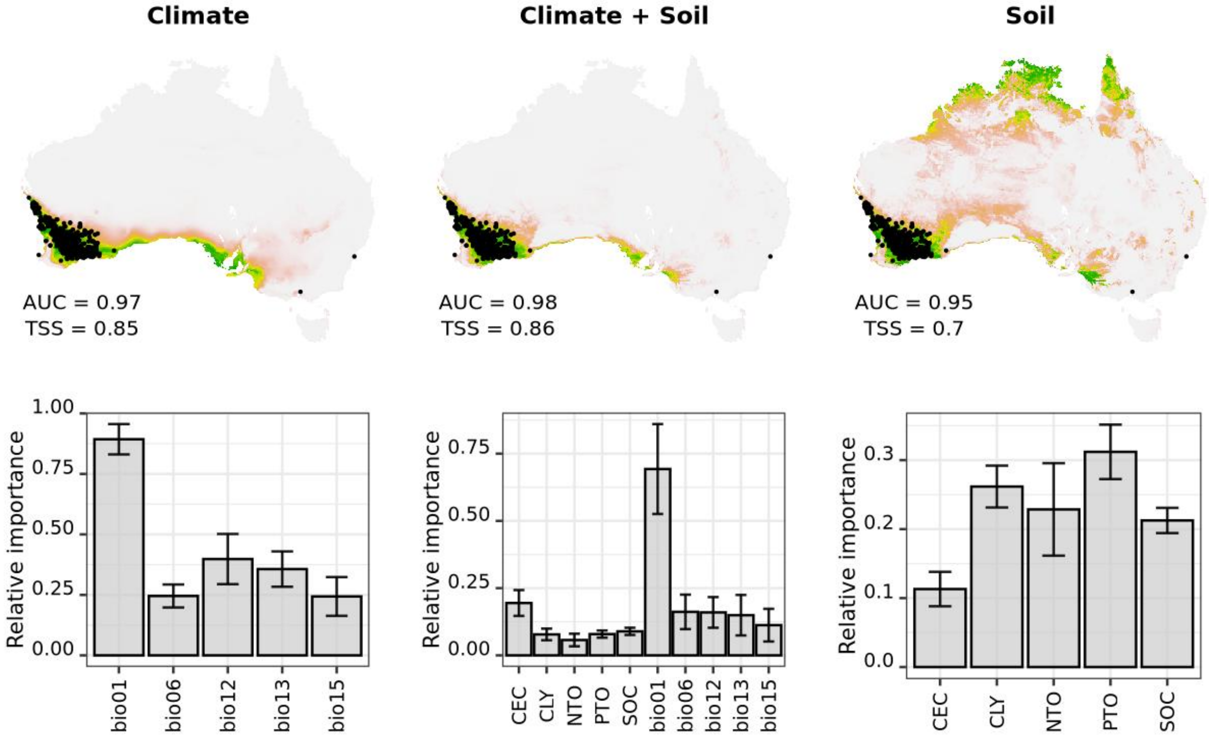

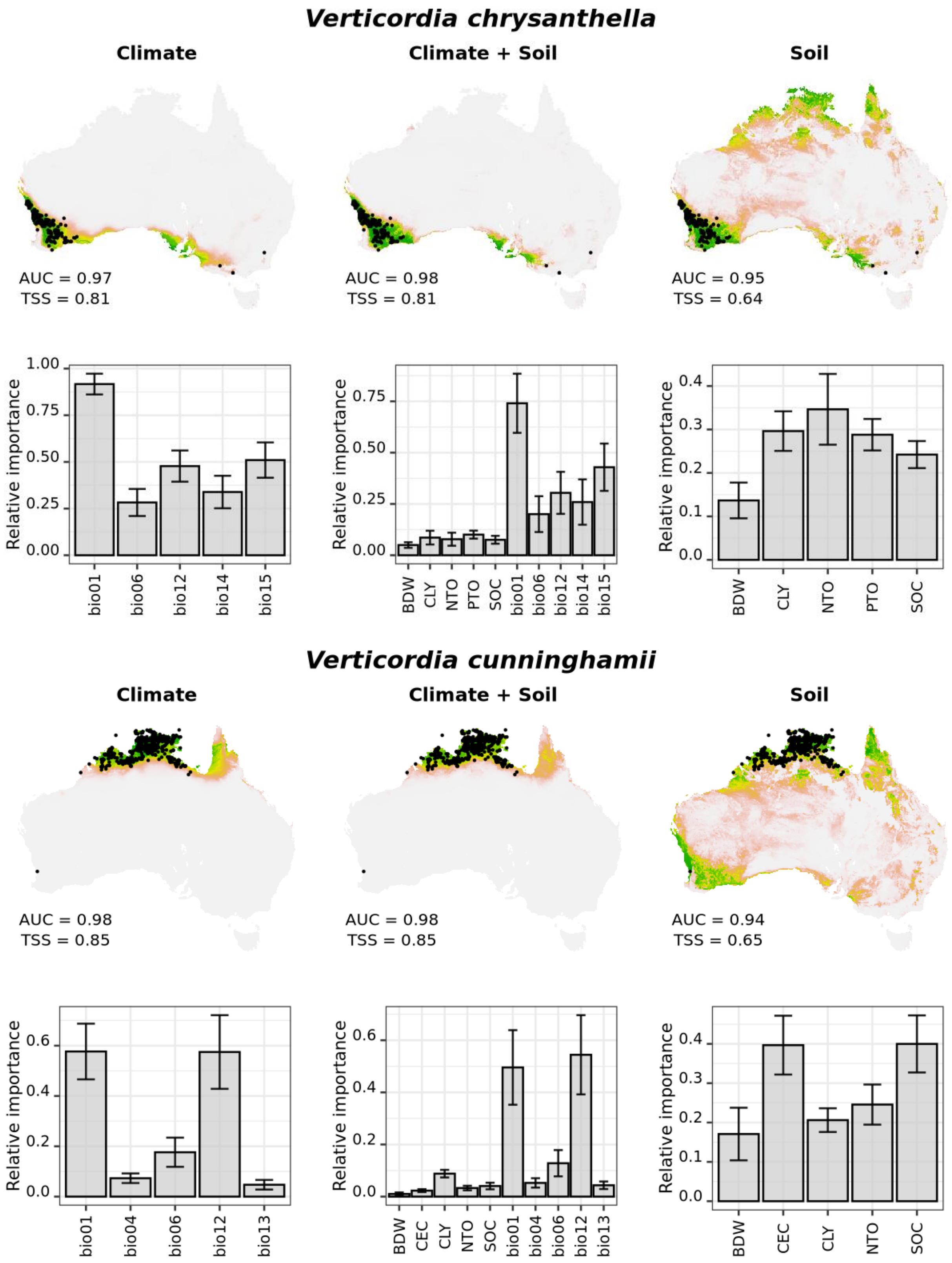
Verticordia chrysanthella
Climate
Climate + Soil
Soil
AUC = 0.97
TSS = 0.81
AUC = 0.98
TSS = 0.81
AUC = 0.95
TSS = 0.64
Relative importance
bio01
bio06
bio12
bio14
bio15
BDW
CLY
NTO
PTO
SOC
Verticordia cunninghamii
Climate
Climate + Soil
Soil
AUC = 0.98
TSS = 0.85
AUC = 0.98
TSS = 0.85
AUC = 0.94
TSS = 0.65
Relative importance
bio01
bio04
bio06
bio12
bio13
BDW
CEC
CLY
NTO
SOC

Verticordia densiflora

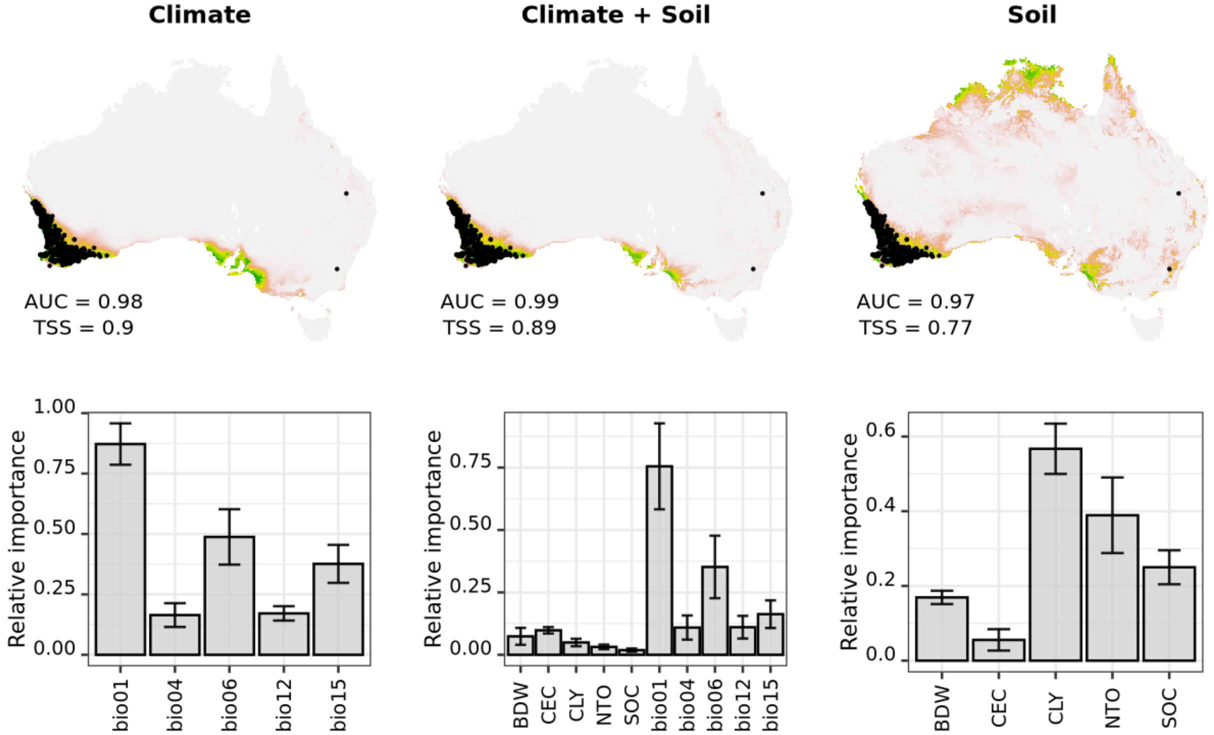

Verticordia endlicheriana

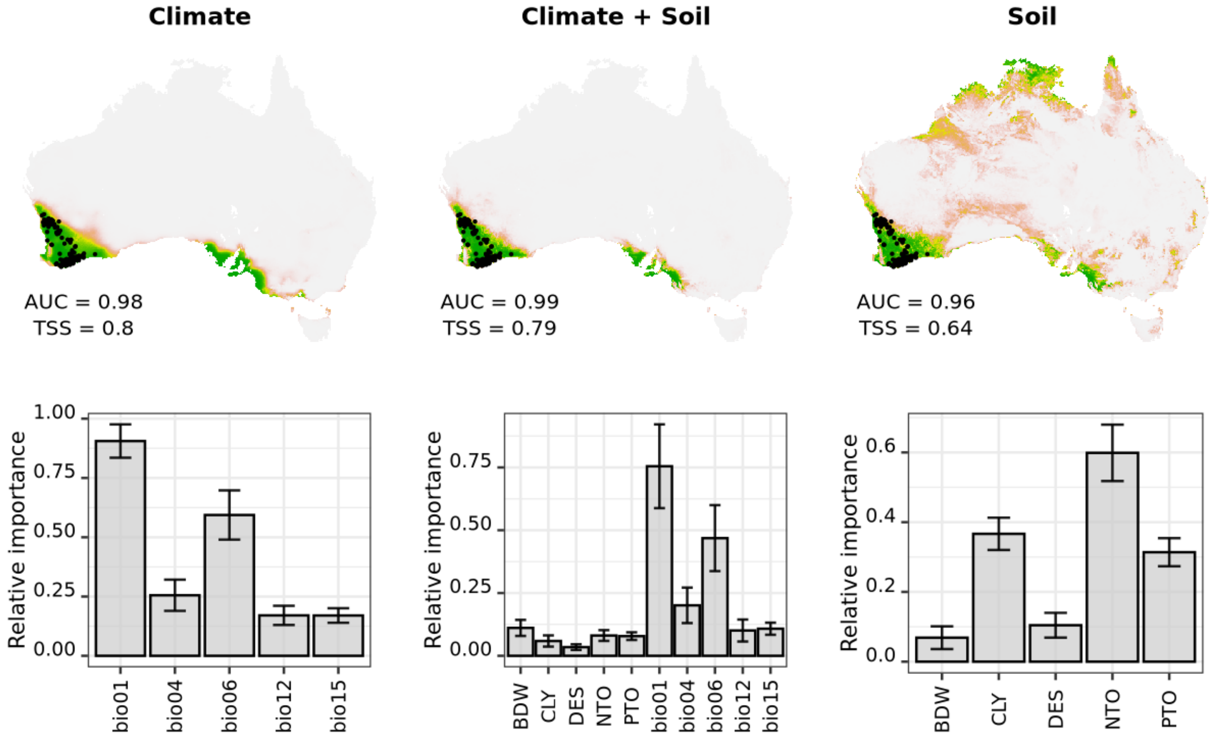

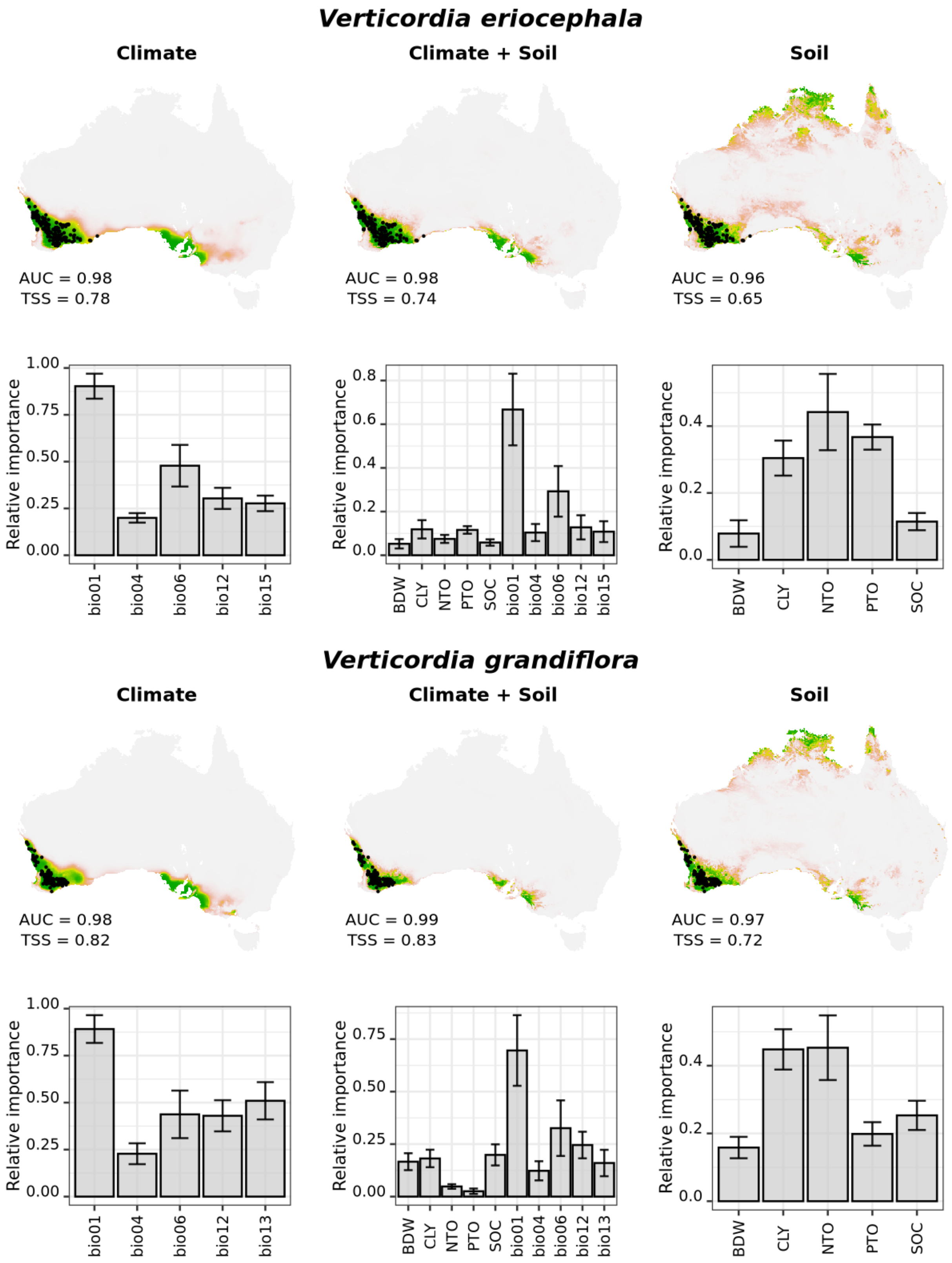

Verticordia eriocephala
Climate
Climate + Soil
Soil
AUC = 0.98
TSS = 0.78
AUC = 0.98
TSS = 0.74
AUC = 0.96
TSS = 0.65
Relative importance
1.00
0.75
0.50
0.25
0.00
bio01
bio04
bio06
bio12
bio15
Relative importance
0.8
0.6
0.4
0.2
0.0
BDW
CLY
NTO
PTO
SOC
bio01
bio04
bio06
bio12
bio15
Relative importance
0.4
0.2
0.0
BDW
CLY
NTO
PTO
SOC
Verticordia grandiflora
Climate
Climate + Soil
Soil
AUC = 0.98
TSS = 0.82
AUC = 0.99
TSS = 0.83
AUC = 0.97
TSS = 0.72
Relative importance
1.00
0.75
0.50
0.25
0.00
bio01
bio04
bio06
bio12
bio13
Relative importance
0.75
0.50
0.25
0.00
BDW
CLY
NTO
PTO
SOC
bio01
bio04
bio06
bio12
bio13
Relative importance
0.4
0.2
0.0
BDW
CLY
NTO
PTO
SOC

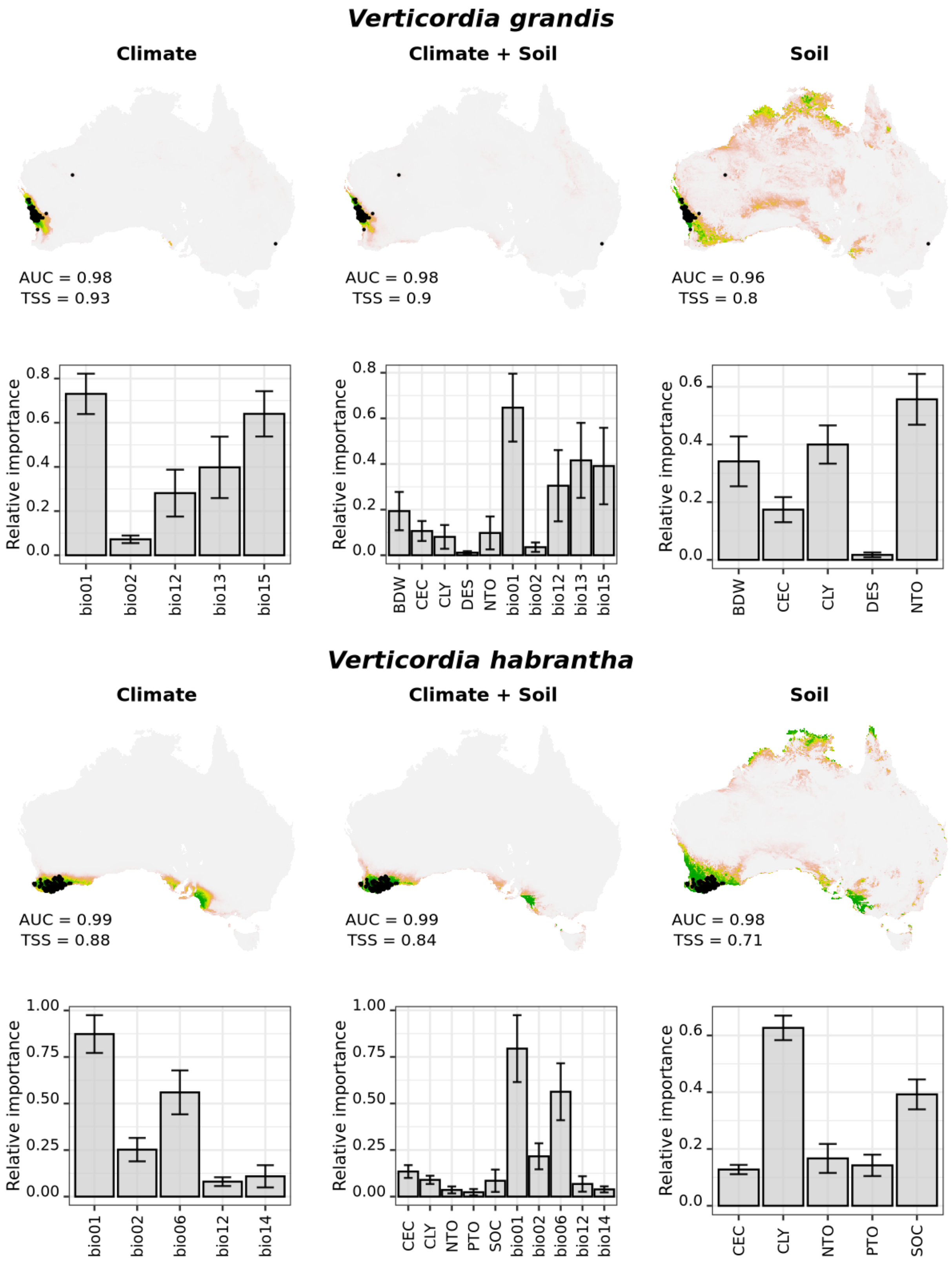

Verticordia grandis
Climate
Climate + Soil
Soil
AUC = 0.98
TSS = 0.93
AUC = 0.98
TSS = 0.9
AUC = 0.96
TSS = 0.8
Relative importance
bio01
bio02
bio12
bio13
bio15
BDW
CEC
CLY
DES
NTO
Verticordia habrantha
Climate
Climate + Soil
Soil
AUC = 0.99
TSS = 0.88
AUC = 0.99
TSS = 0.84
AUC = 0.98
TSS = 0.71
Relative importance
bio01
bio02
bio06
bio12
bio14
CEC
CLY
NTO
PTO
SOC

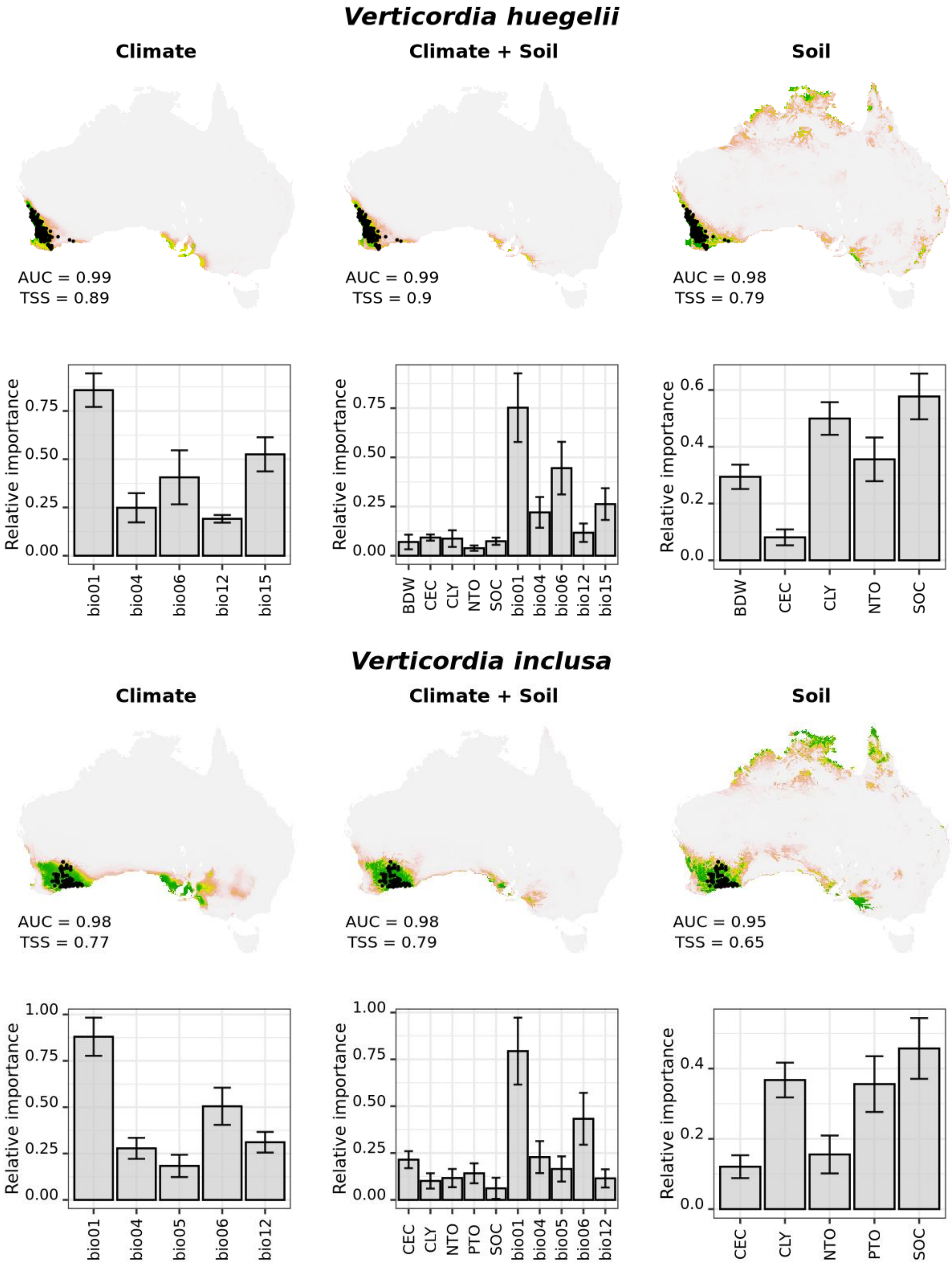
Verticordia huegelii
Climate
Climate + Soil
Soil
AUC = 0.99
TSS = 0.89
AUC = 0.99
TSS = 0.9
AUC = 0.98
TSS = 0.79
Relative importance
0.00
0.25
0.50
0.75
bio01
bio04
bio06
bio12
bio15
BDW
CEC
CLY
NTO
SOC
0.0
0.2
0.4
0.6
Verticordia inclusa
Climate
Climate + Soil
Soil
AUC = 0.98
TSS = 0.77
AUC = 0.98
TSS = 0.79
AUC = 0.95
TSS = 0.65
Relative importance
0.00
0.25
0.50
0.75
1.00
bio01
bio04
bio05
bio06
bio12
CEC
CLY
NTO
PTO
SOC
0.0
0.2
0.4

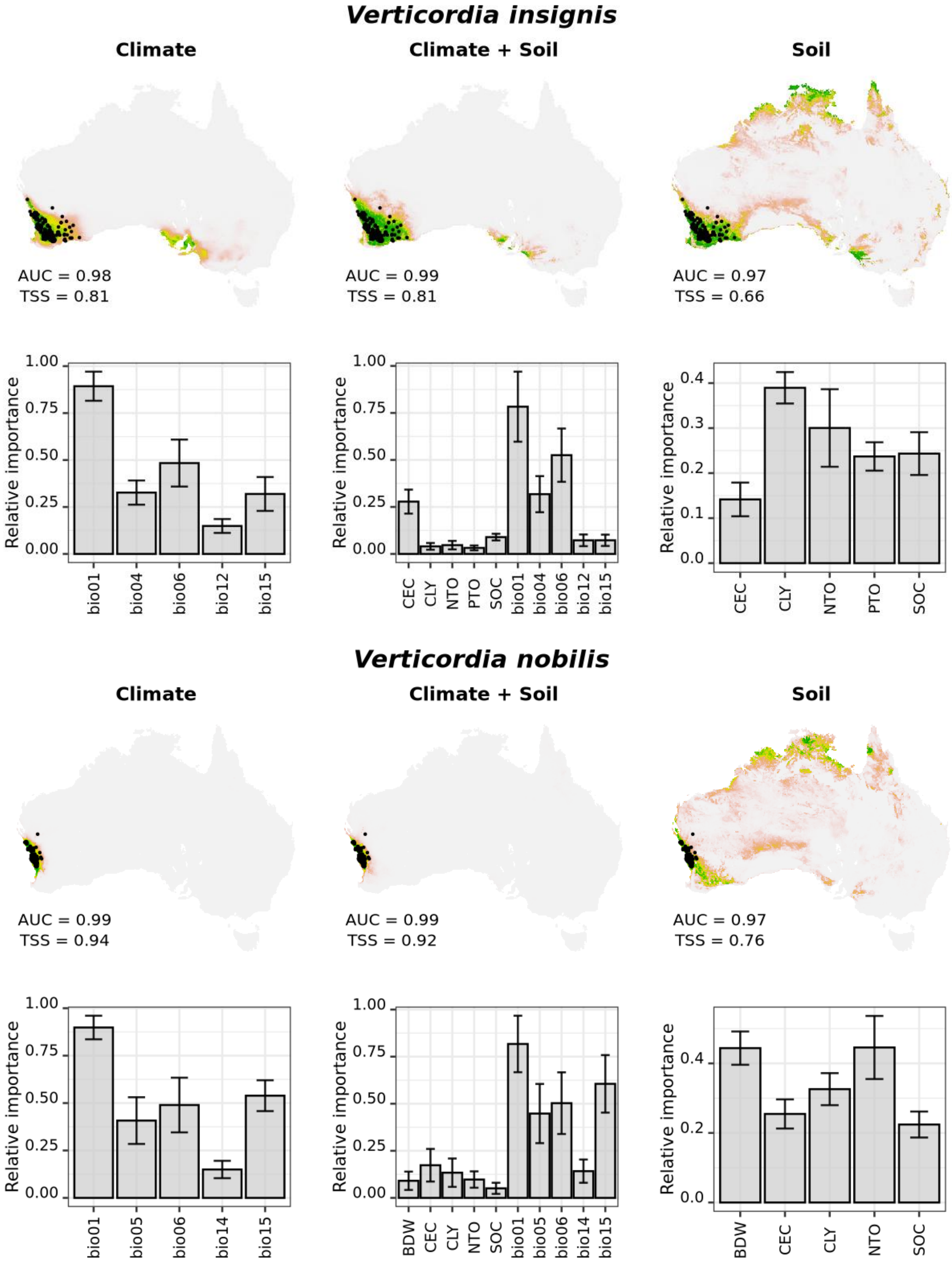
Verticordia insignis
Climate
Climate + Soil
Soil
AUC = 0.98
TSS = 0.81
AUC = 0.99
TSS = 0.81
AUC = 0.97
TSS = 0.66
Relative importance
0.00
0.25
0.50
0.75
1.00
bio01
bio04
bio06
bio12
bio15
CEC
CLY
NTO
PTO
SOC
0.0
0.1
0.2
0.3
0.4
Verticordia nobilis
Climate
Climate + Soil
Soil
AUC = 0.99
TSS = 0.94
AUC = 0.99
TSS = 0.92
AUC = 0.97
TSS = 0.76
bio01
bio05
bio06
bio14
bio15
BDW
CEC
CLY
NTO
SOC
0.0
0.2
0.4

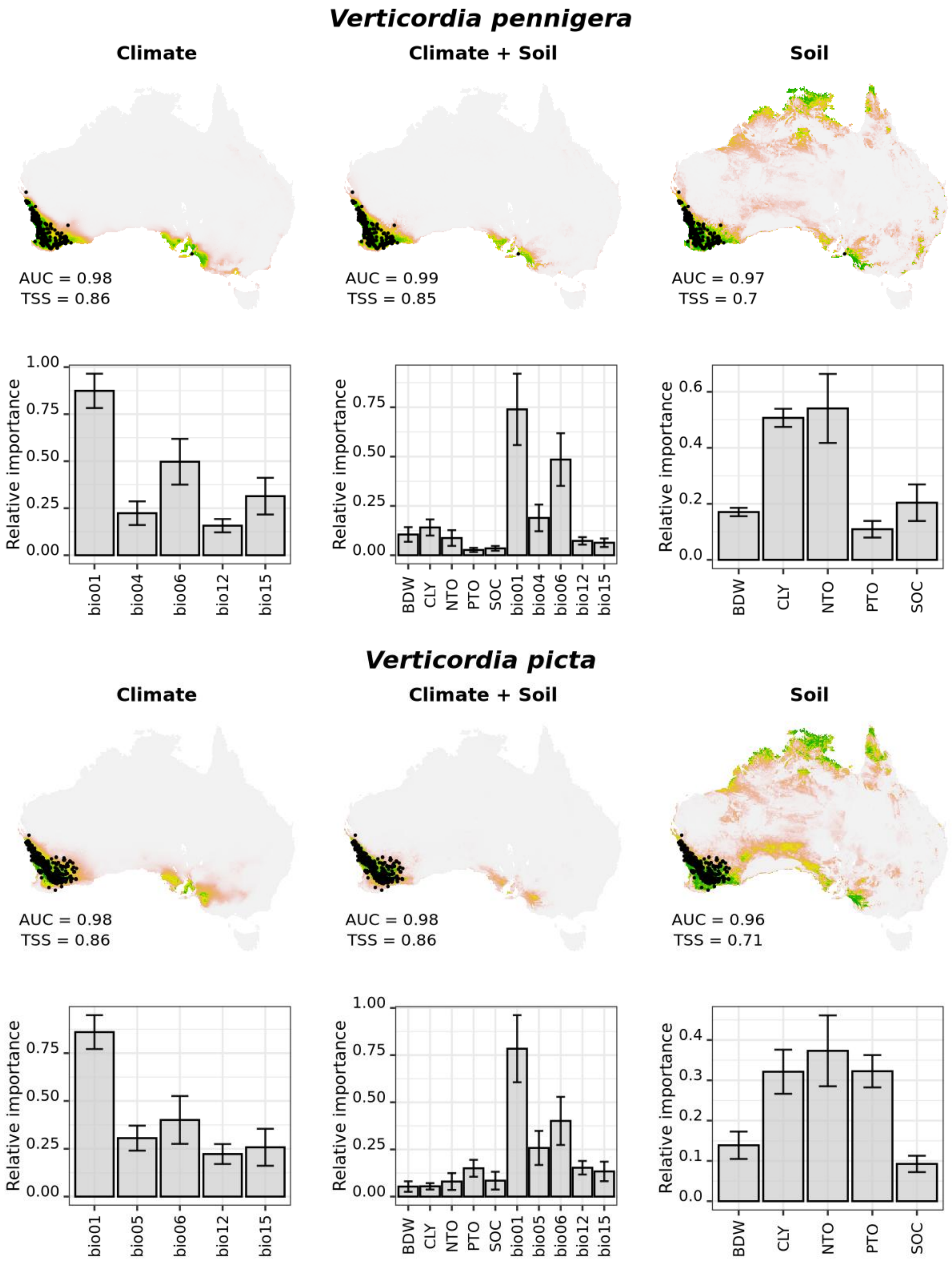

Verticordia pennigera
Climate
Climate + Soil
Soil
AUC = 0.98
TSS = 0.86
AUC = 0.99
TSS = 0.85
AUC = 0.97
TSS = 0.7
Relative importance
1.00
0.75
0.50
0.25
0.00
bio01
bio04
bio06
bio12
bio15
BDW
CLY
NTO
PTO
SOC
0.6
0.4
0.2
0.0
Verticordia picta
Climate
Climate + Soil
Soil
AUC = 0.98
TSS = 0.86
AUC = 0.98
TSS = 0.86
AUC = 0.96
TSS = 0.71
Relative importance
bio01
bio05
bio06
bio12
bio15
BDW
CLY
NTO
PTO
SOC
0.4
0.3
0.2
0.1
0.0

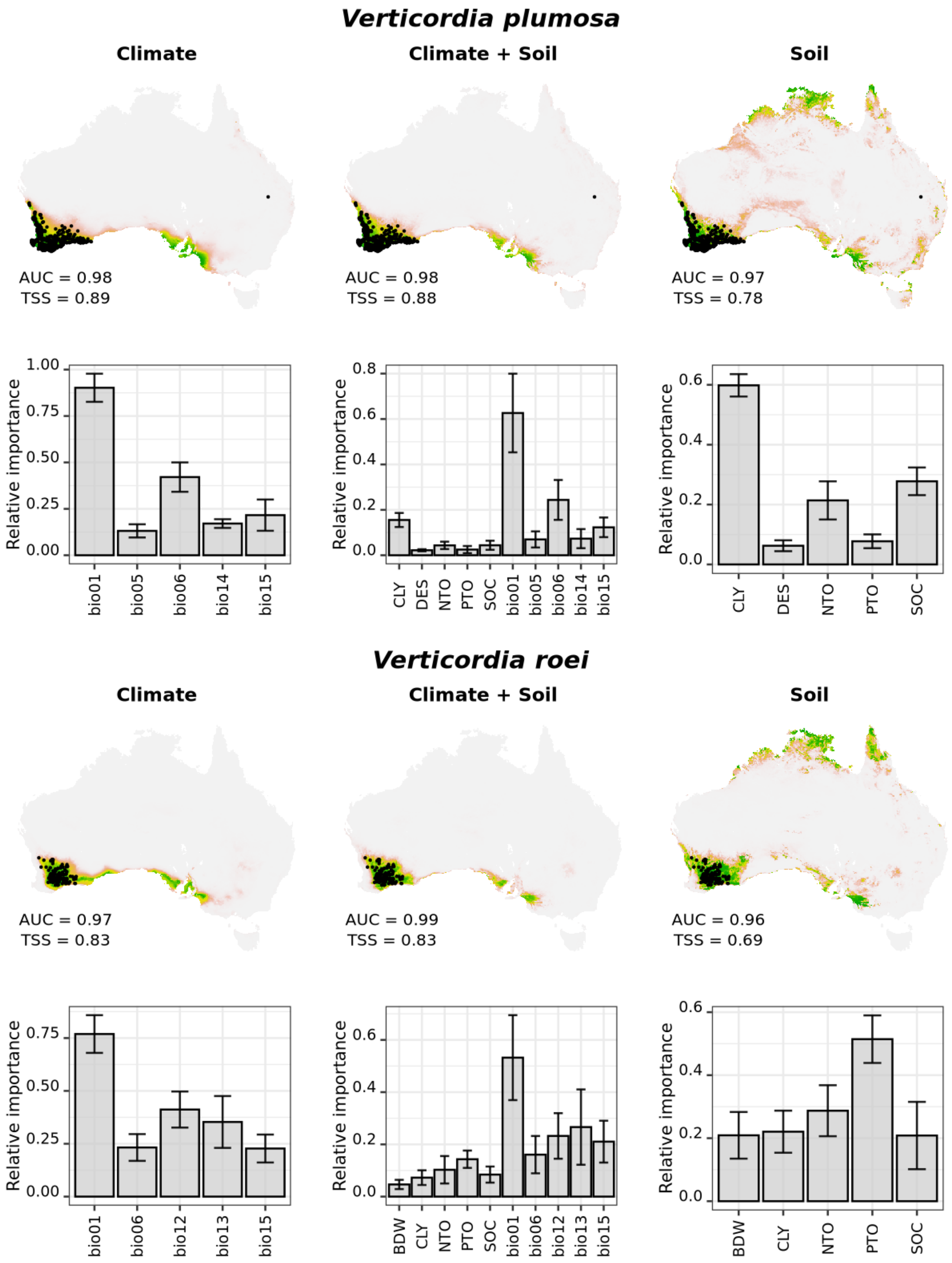
Verticordia plumosa
Climate
Climate + Soil
Soil
AUC = 0.98
TSS = 0.89
AUC = 0.98
TSS = 0.88
AUC = 0.97
TSS = 0.78
Relative importance
bio01 bio05 bio06 bio14 bio15
CLY DES NTO PTO SOC bio01 bio05 bio06 bio14 bio15
CLY DES NTO PTO SOC
Verticordia roei
Climate
Climate + Soil
Soil
AUC = 0.97
TSS = 0.83
AUC = 0.99
TSS = 0.83
AUC = 0.96
TSS = 0.69
Relative importance
bio01 bio06 bio12 bio13 bio15
BDW CLY NTO PTO SOC bio01 bio06 bio12 bio13 bio15
BDW CLY NTO PTO SOC

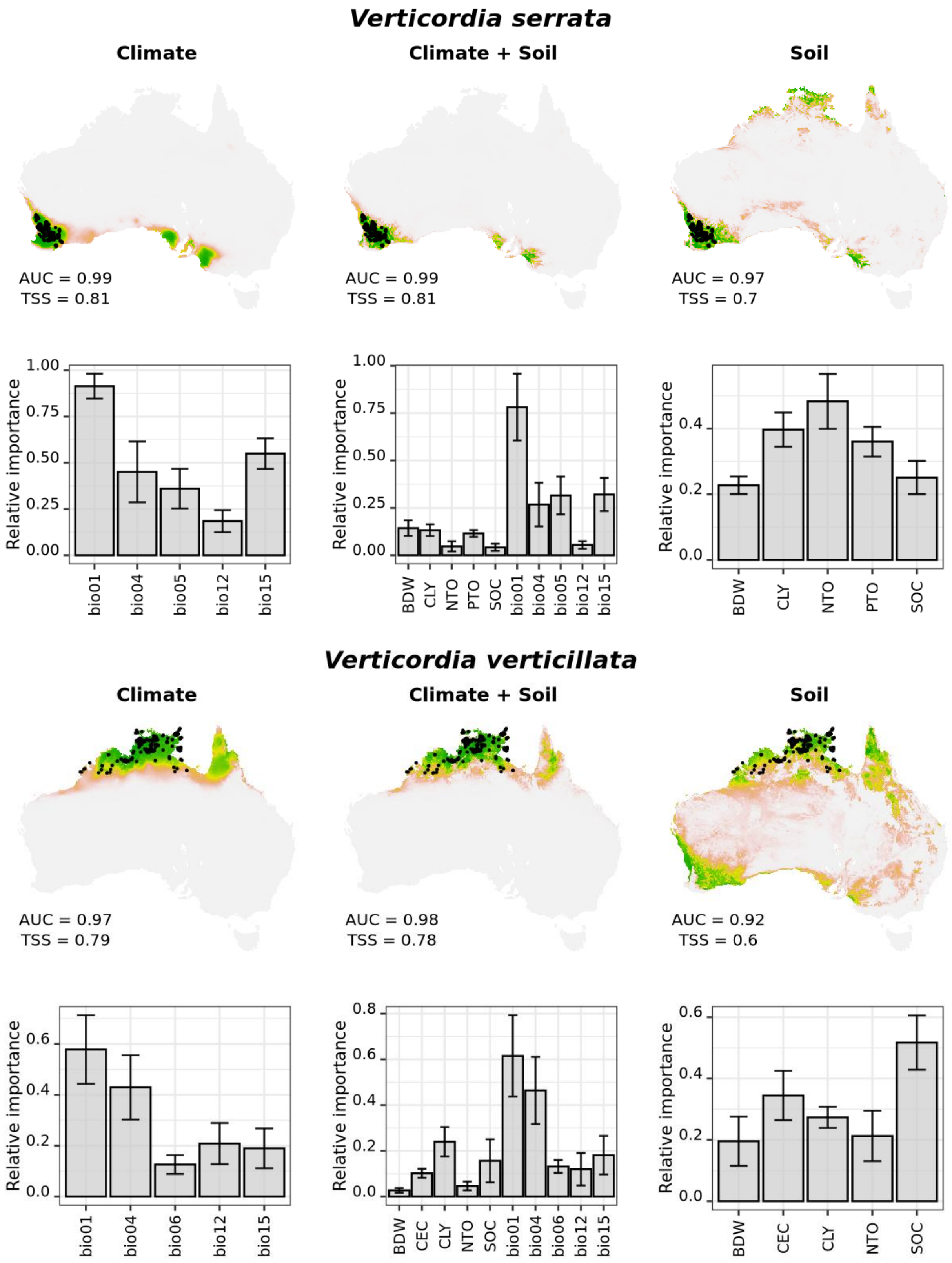
Verticordia serrata
Climate
Climate + Soil
Soil
AUC = 0.99
TSS = 0.81
AUC = 0.99
TSS = 0.81
AUC = 0.97
TSS = 0.7
Relative importance
bio01
bio04
bio05
bio12
bio15
BDW
CLY
NTO
PTO
SOC
Verticordia verticillata
Climate
Climate + Soil
Soil
AUC = 0.97
TSS = 0.79
AUC = 0.98
TSS = 0.78
AUC = 0.92
TSS = 0.6
Relative importance
bio01
bio04
bio06
bio12
bio15
BDW
CEC
CLY
NTO
SOC

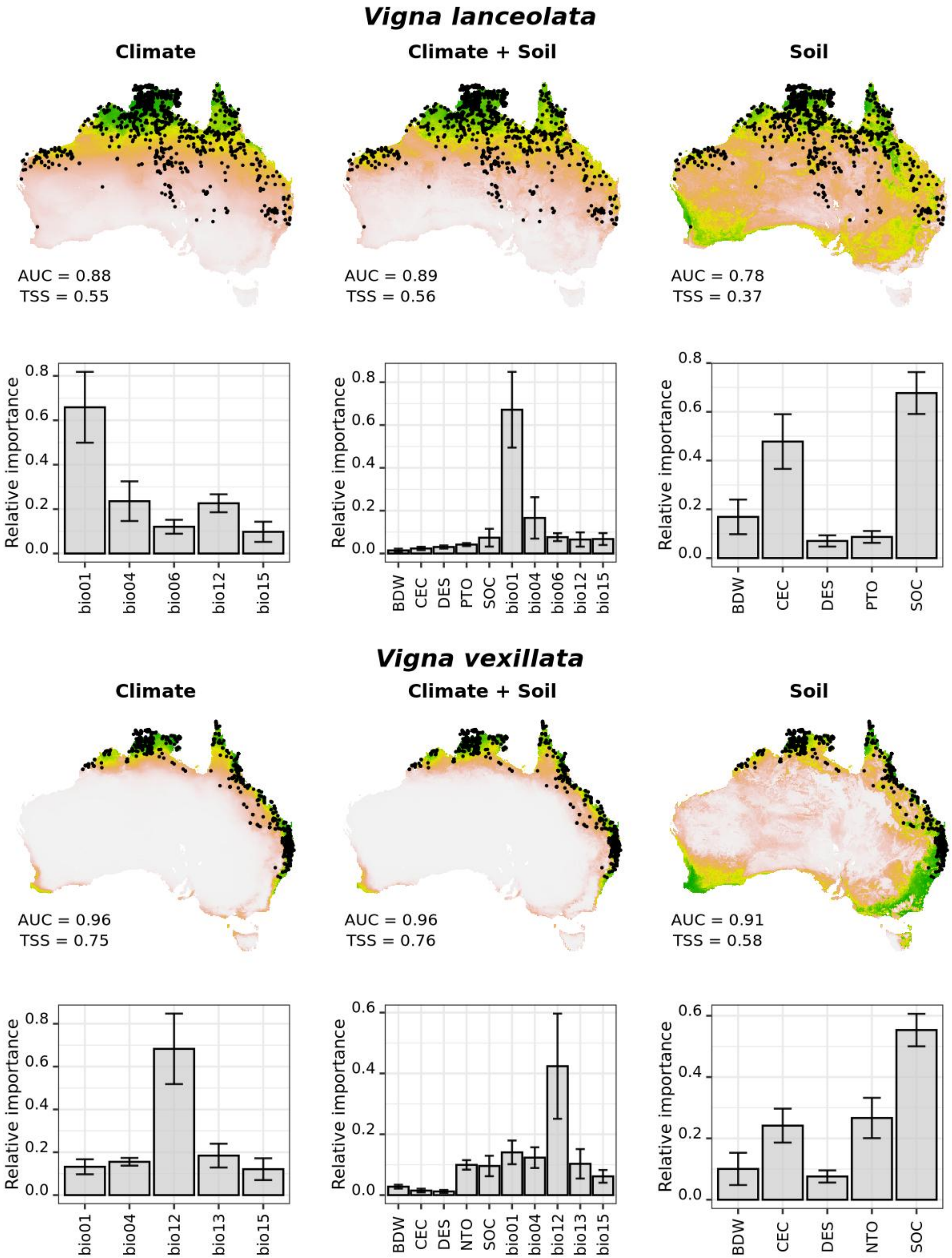
Vigna lanceolata
Climate
Climate + Soil
Soil
AUC = 0.88
TSS = 0.55
AUC = 0.89
TSS = 0.56
AUC = 0.78
TSS = 0.37
Relative importance
0.0
0.2
0.4
0.6
0.8
bio01
bio04
bio06
bio12
bio15
BDW
CEC
DES
PTO
SOC
Vigna vexillata
Climate
Climate + Soil
Soil
AUC = 0.96
TSS = 0.75
AUC = 0.96
TSS = 0.76
AUC = 0.91
TSS = 0.58
Relative importance
bio01
bio04
bio12
bio13
bio15
BDW
CEC
DES
NTO
SOC

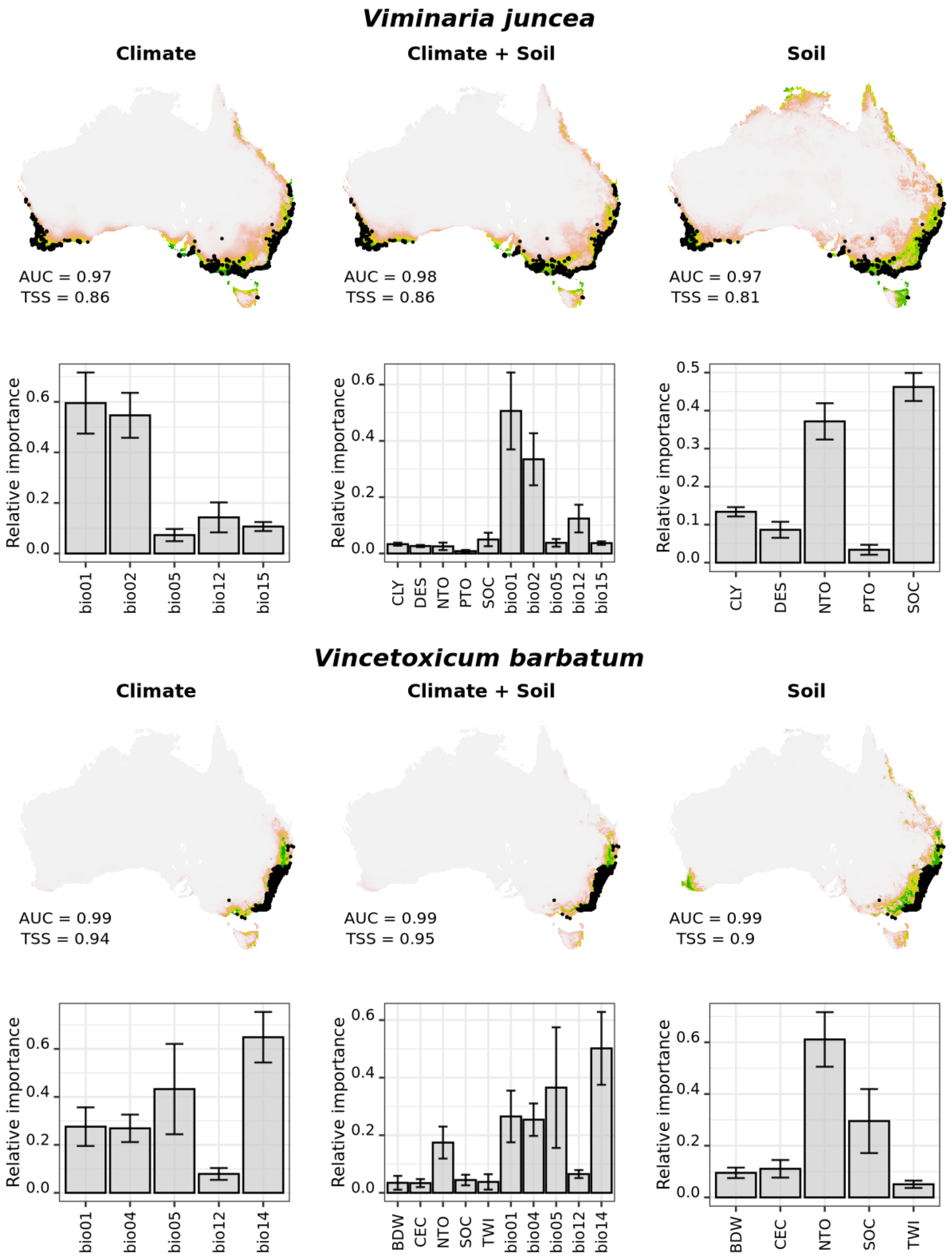

Viminaria juncea
Climate
Climate + Soil
Soil
AUC = 0.97
TSS = 0.86
AUC = 0.98
TSS = 0.86
AUC = 0.97
TSS = 0.81
Relative importance
bio01
bio02
bio05
bio12
bio15
CLY
DES
NTO
PTO
SOC
Vincetoxicum barbatum
Climate
Climate + Soil
Soil
AUC = 0.99
TSS = 0.94
AUC = 0.99
TSS = 0.95
AUC = 0.99
TSS = 0.9
Relative importance
bio01
bio04
bio05
bio12
bio14
BDW
CEC
NTO
SOC
TWI

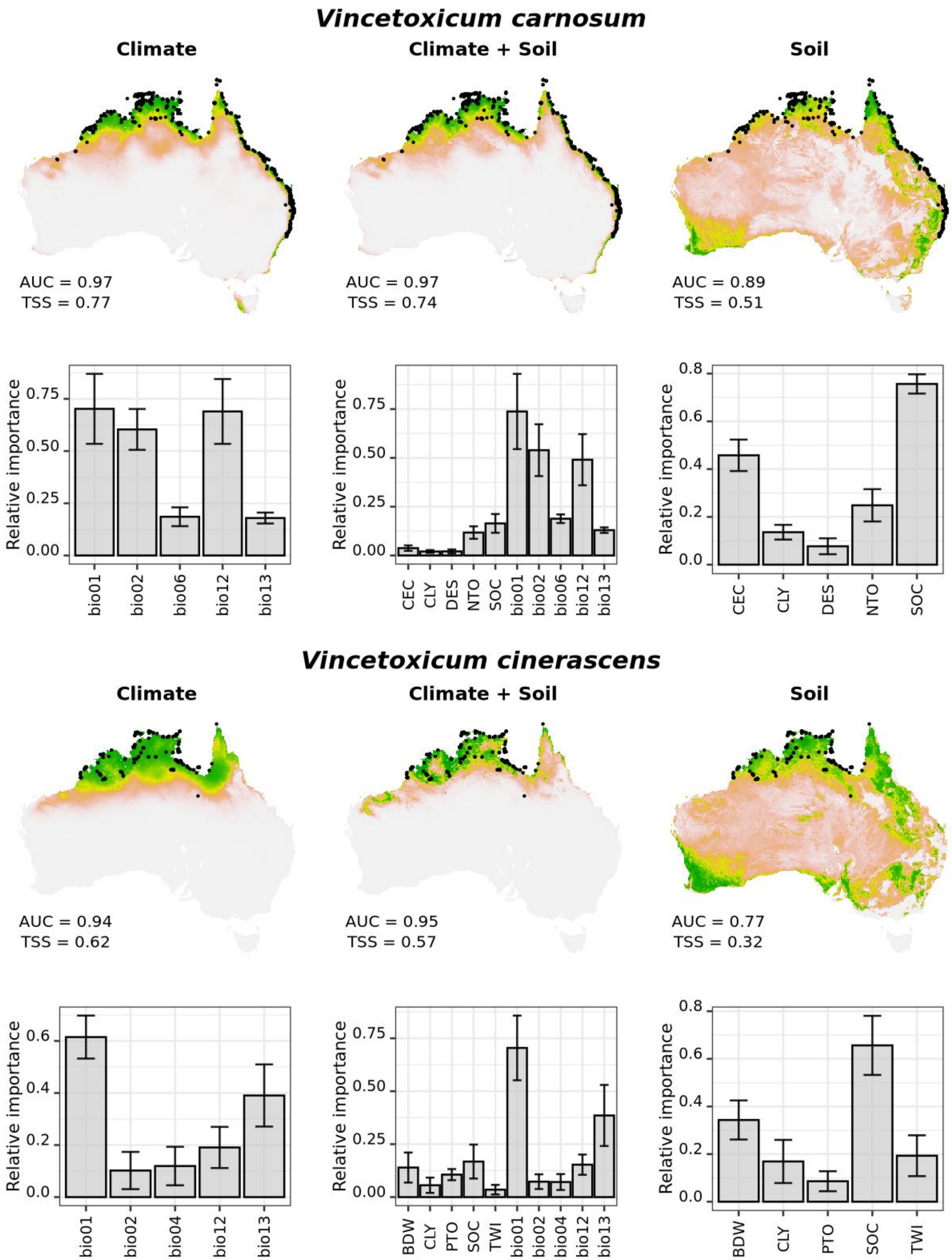
Vincetoxicum carnosum
Climate
Climate + Soil
Soil
AUC = 0.97
TSS = 0.77
AUC = 0.97
TSS = 0.74
AUC = 0.89
TSS = 0.51
Relative importance
bio01
bio02
bio06
bio12
bio13
CEC
CLY
DES
NTO
SOC
bio01
bio02
bio06
bio12
bio13
CEC
CLY
DES
NTO
SOC
Vincetoxicum cinerascens
Climate
Climate + Soil
Soil
AUC = 0.94
TSS = 0.62
AUC = 0.95
TSS = 0.57
AUC = 0.77
TSS = 0.32
Relative importance
bio01
bio02
bio04
bio12
bio13
BDW
CLY
PTO
SOC
TWI
bio01
bio02
bio04
bio12
bio13
BDW
CLY
PTO
SOC
TWI

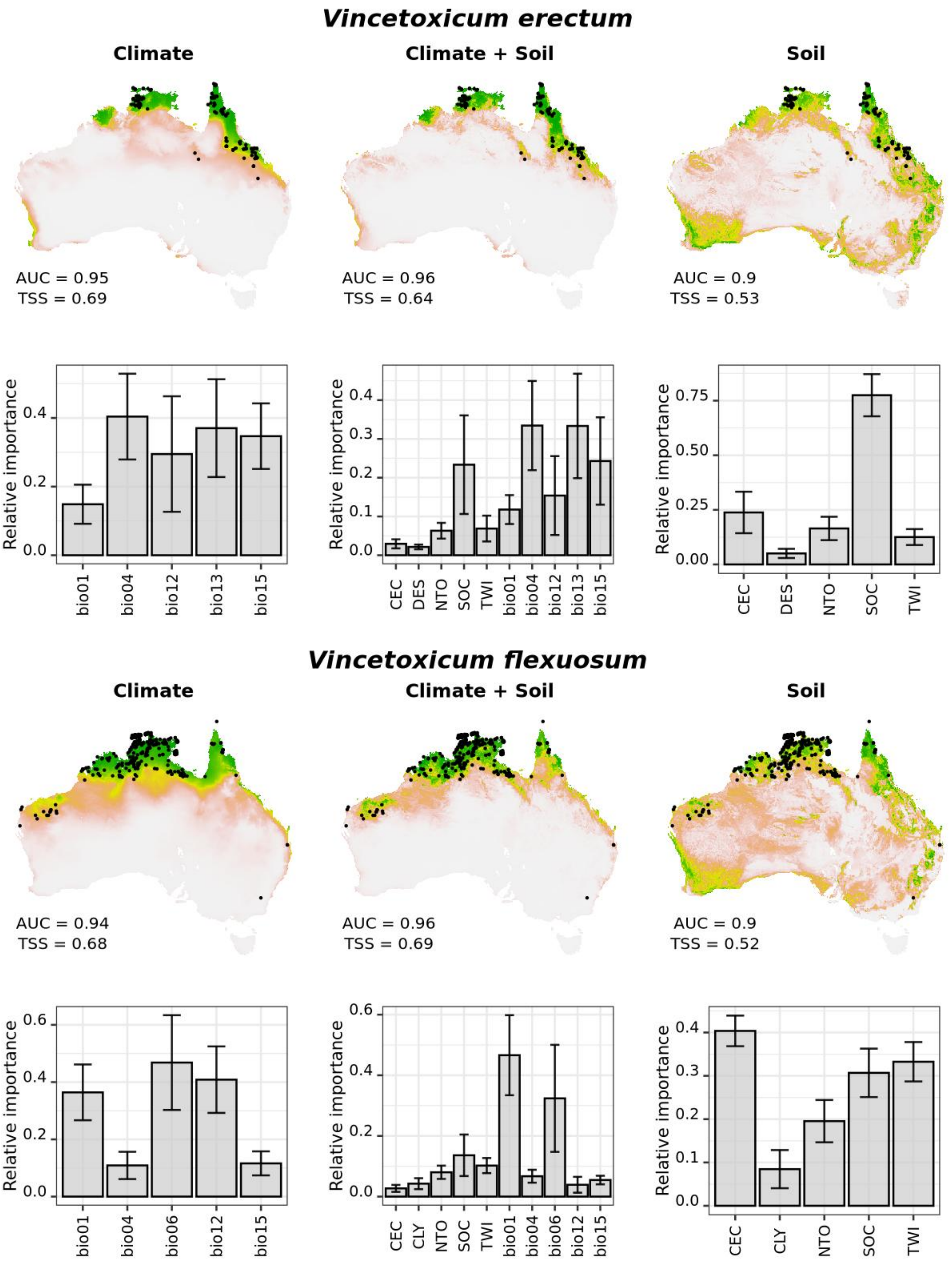
Vincetoxicum erectum
Climate
Climate + Soil
Soil
AUC = 0.95
TSS = 0.69
AUC = 0.96
TSS = 0.64
AUC = 0.9
TSS = 0.53
Relative importance
bio01
bio04
bio12
bio13
bio15
CEC
DES
NTO
SOC
TWI
Vincetoxicum flexuosum
Climate
Climate + Soil
Soil
AUC = 0.94
TSS = 0.68
AUC = 0.96
TSS = 0.69
AUC = 0.9
TSS = 0.52
Relative importance
bio01
bio04
bio06
bio12
bio15
CEC
CLY
NTO
SOC
TWI

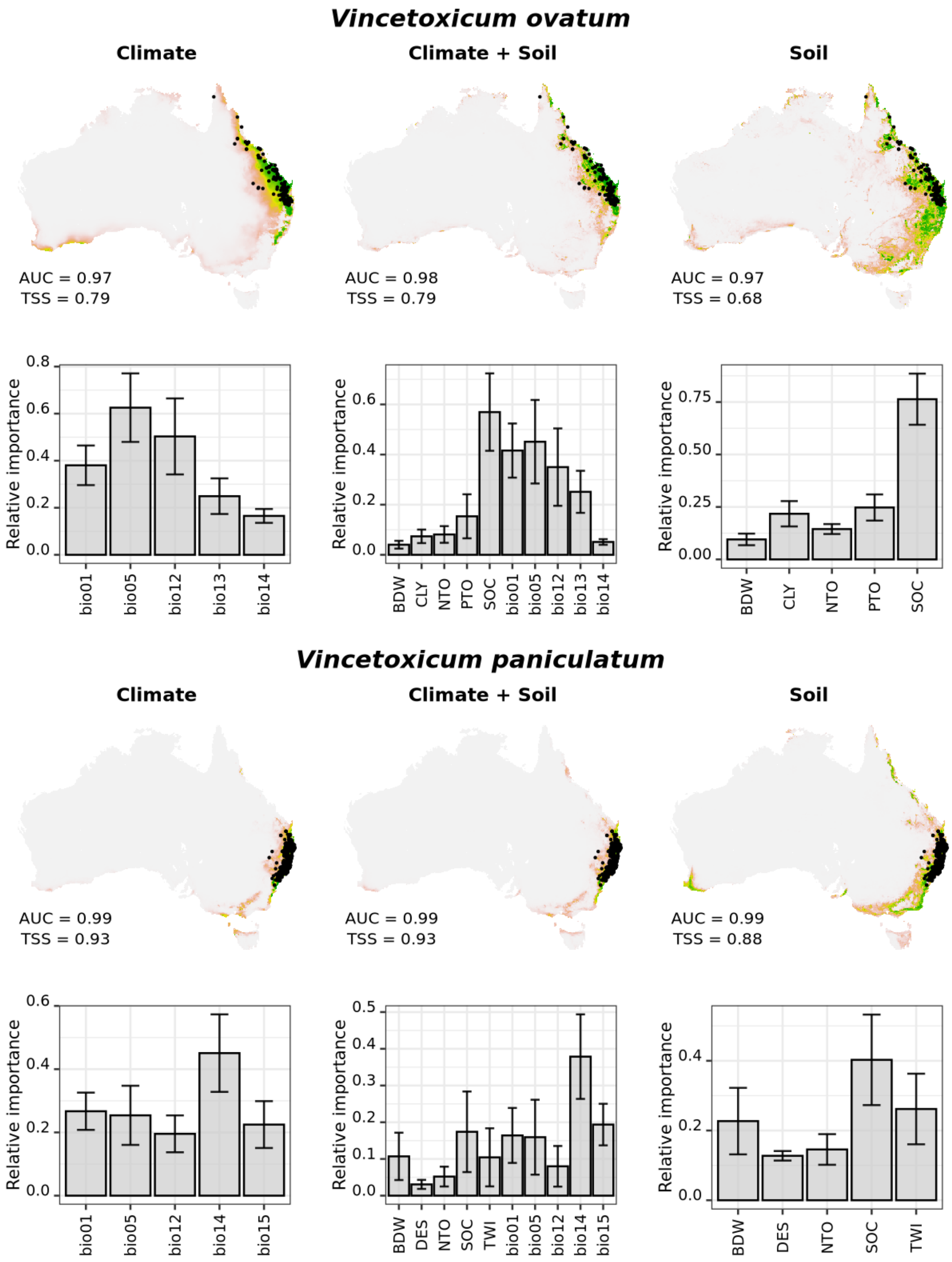

Vincetoxicum ovatum
Climate
Climate + Soil
Soil
AUC = 0.97
TSS = 0.79
AUC = 0.98
TSS = 0.79
AUC = 0.97
TSS = 0.68
Relative importance
0.0
0.2
0.4
0.6
0.8
bio01
bio05
bio12
bio13
bio14
BDW
CLY
NTO
PTO
SOC
0.00
0.25
0.50
0.75
Vincetoxicum paniculatum
AUC = 0.99
TSS = 0.93
AUC = 0.99
TSS = 0.93
AUC = 0.99
TSS = 0.88
0.1
0.3
0.5
bio15
DES
TWI

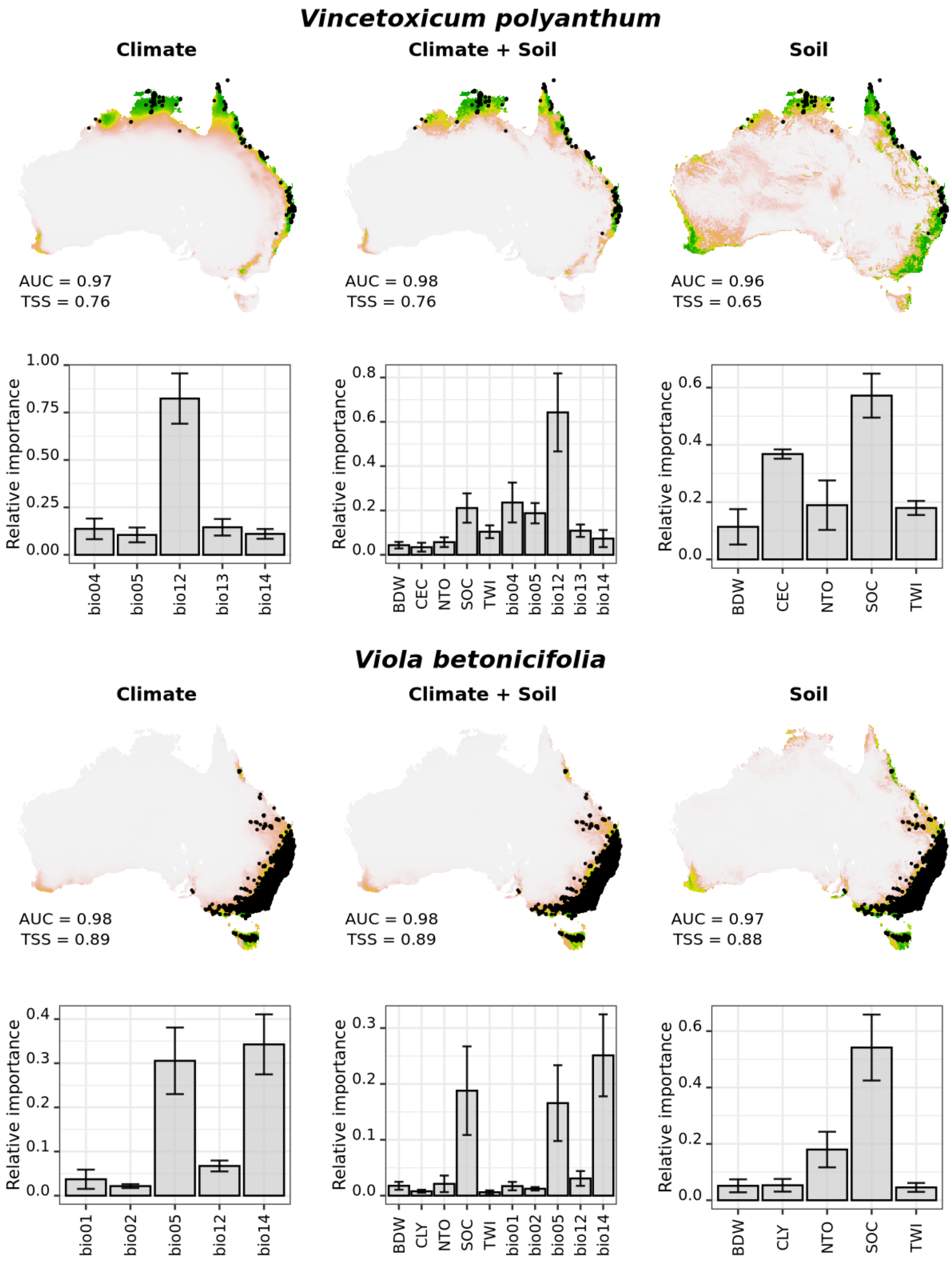

Vincetoxicum polyanthum
Climate
Climate + Soil
Soil
AUC = 0.97
TSS = 0.76
AUC = 0.98
TSS = 0.76
AUC = 0.96
TSS = 0.65
Relative importance
bio04
bio05
bio12
bio13
bio14
BDW
CEC
NTO
SOC
TWI
Viola betonicifolia
AUC = 0.98
TSS = 0.89
AUC = 0.98
TSS = 0.89
AUC = 0.97
TSS = 0.88
bio01
bio02
CLY

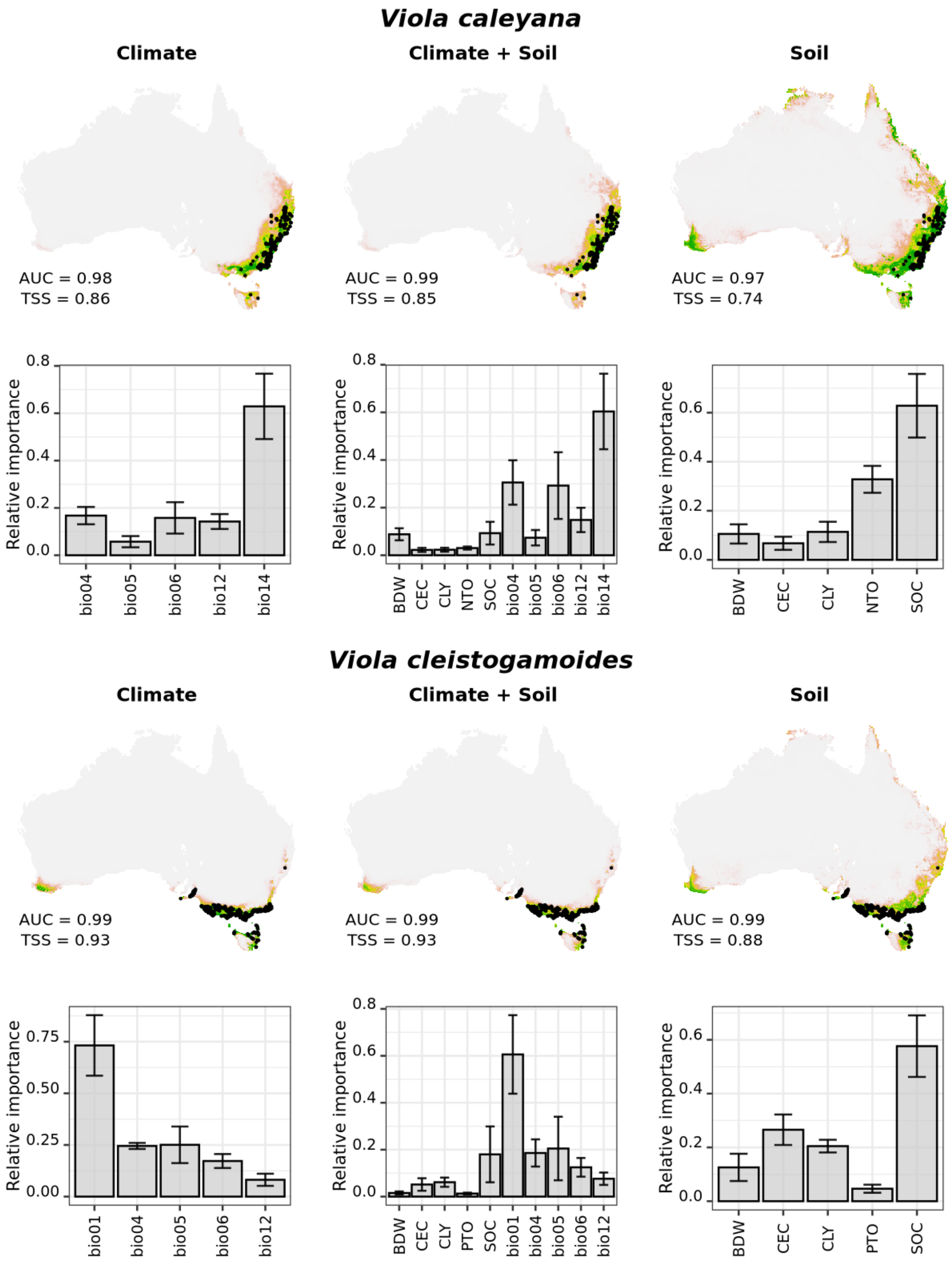

Viola caleyana
Climate
Climate + Soil
Soil
AUC = 0.98
TSS = 0.86
AUC = 0.99
TSS = 0.85
AUC = 0.97
TSS = 0.74
Relative importance
0.0
0.2
0.4
0.6
0.8
bio04
bio05
bio06
bio12
bio14
BDW
CEC
CLY
NTO
SOC
Viola cleistogamoides
Climate
Climate + Soil
Soil
AUC = 0.99
TSS = 0.93
AUC = 0.99
TSS = 0.93
AUC = 0.99
TSS = 0.88
0.00
0.25
0.50
0.75
bio01
PTO

Viola eminens

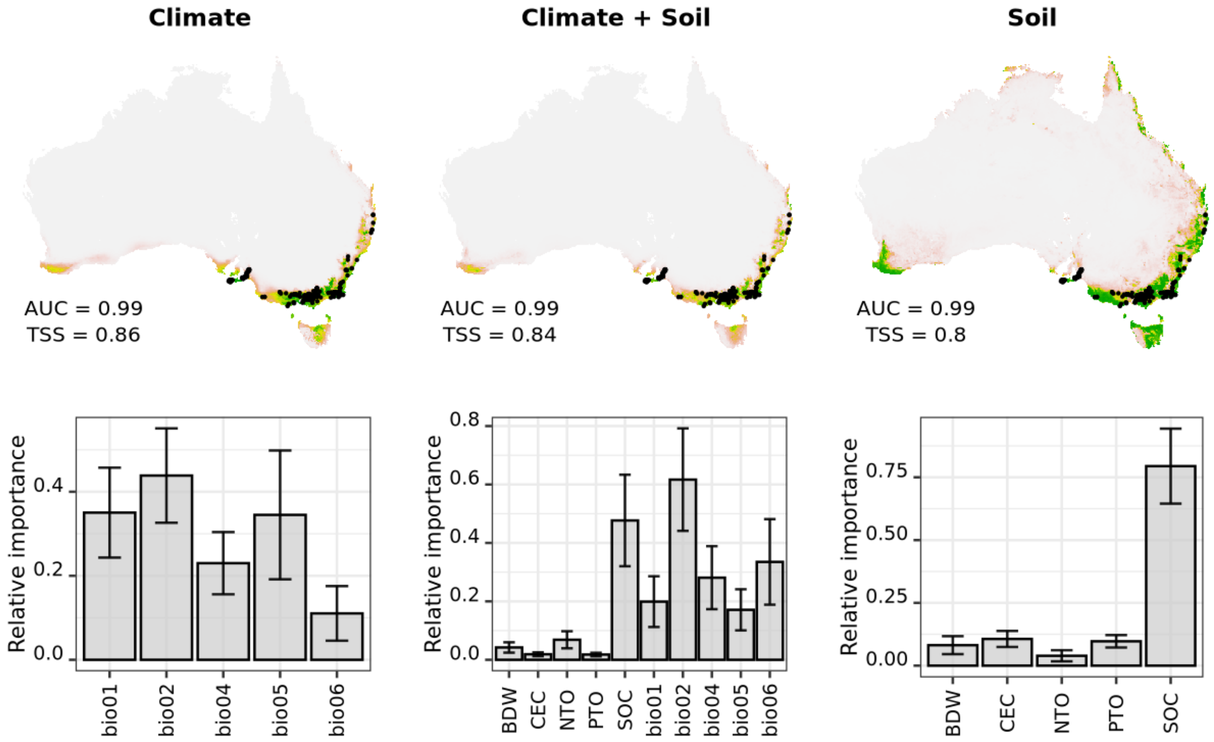

Viola fuscoviolacea

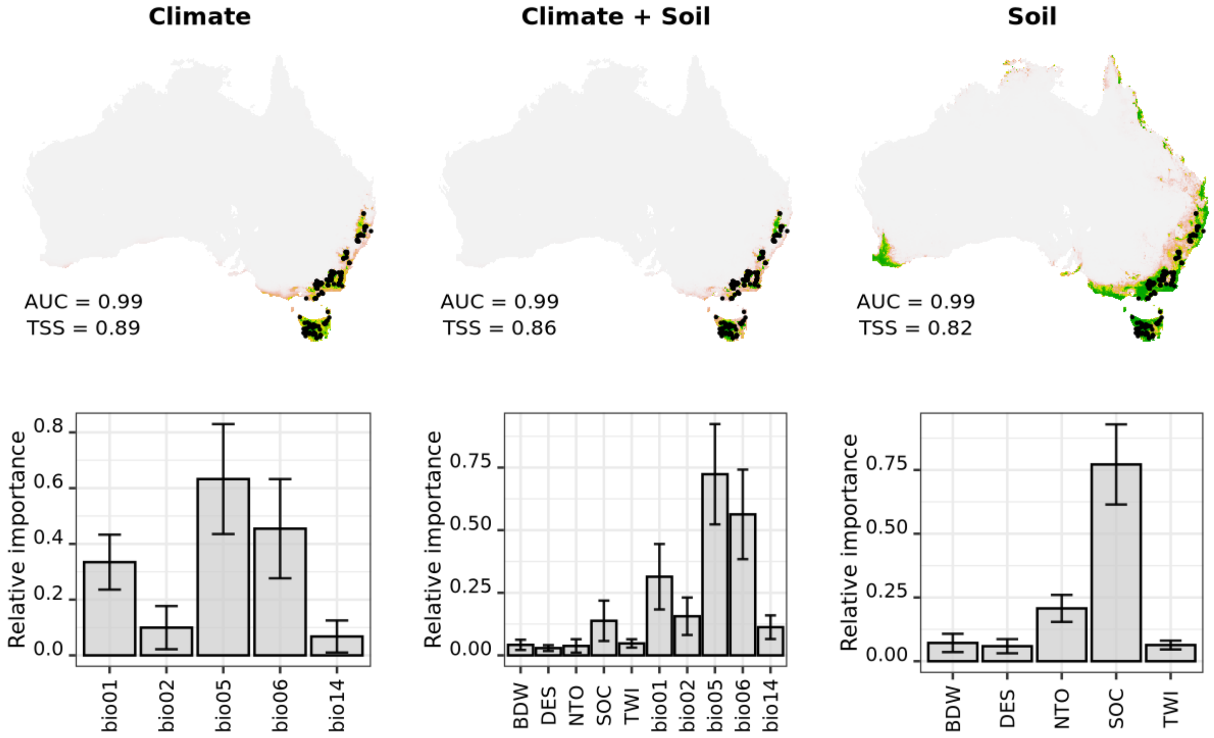

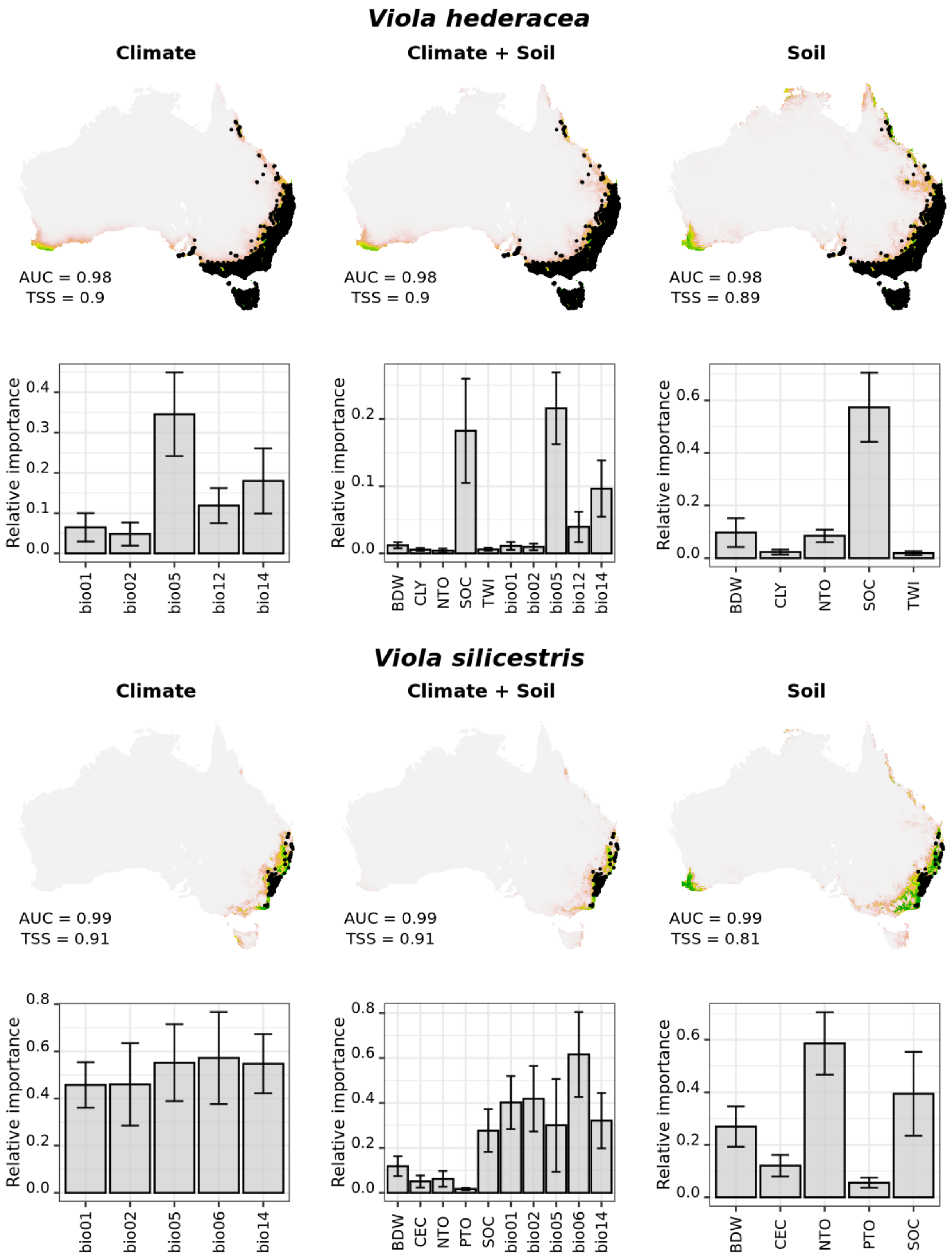
Viola hederacea
Climate
Climate + Soil
Soil
AUC = 0.98
TSS = 0.9
AUC = 0.98
TSS = 0.9
AUC = 0.98
TSS = 0.89
Relative importance
0.0
0.1
0.2
0.3
0.4
bio01
bio02
bio05
bio12
bio14
Relative importance
0.0
0.1
0.2
BDW
CLY
NTO
SOC
TWI
bio01
bio02
bio05
bio12
bio14
Relative importance
0.0
0.2
0.4
0.6
BDW
CLY
NTO
SOC
TWI
Viola silicestris
Climate
Climate + Soil
Soil
AUC = 0.99
TSS = 0.91
AUC = 0.99
TSS = 0.91
AUC = 0.99
TSS = 0.81
Relative importance
0.0
0.2
0.4
0.6
0.8
bio01
bio02
bio05
bio06
bio14
Relative importance
0.0
0.2
0.4
0.6
0.8
BDW
CEC
NTO
PTO
SOC
bio01
bio02
bio05
bio06
bio14
Relative importance
0.0
0.2
0.4
0.6
BDW
CEC
NTO
PTO
SOC

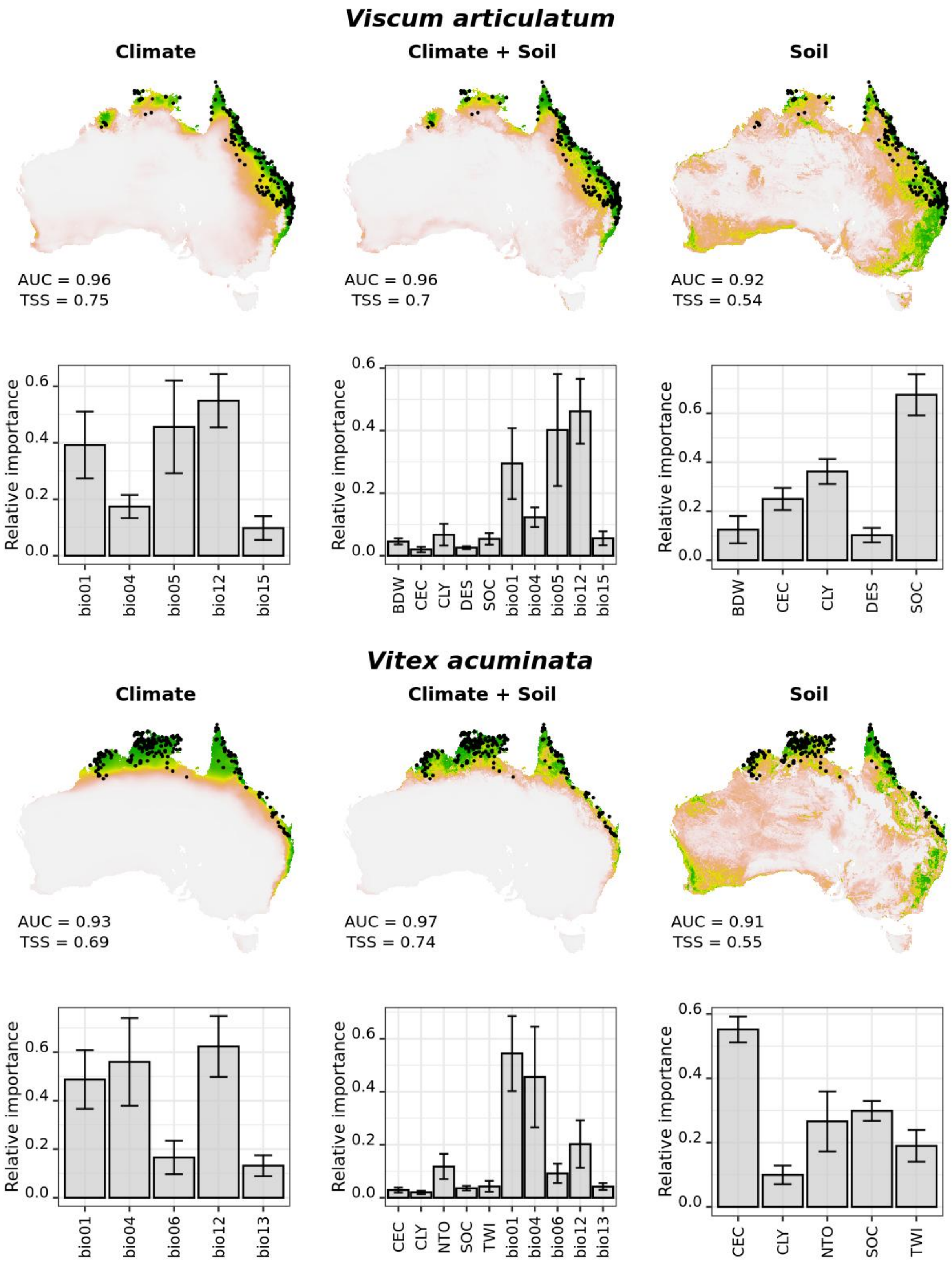
Viscum articulatum
Climate
Climate + Soil
Soil
AUC = 0.96
TSS = 0.75
AUC = 0.96
TSS = 0.7
AUC = 0.92
TSS = 0.54
Relative importance
bio01
bio04
bio05
bio12
bio15
BDW
CEC
CLY
DES
SOC
Vitex acuminata
Climate
Climate + Soil
Soil
AUC = 0.93
TSS = 0.69
AUC = 0.97
TSS = 0.74
AUC = 0.91
TSS = 0.55
Relative importance
bio01
bio04
bio06
bio12
bio13
CEC
CLY
NTO
SOC
TWI

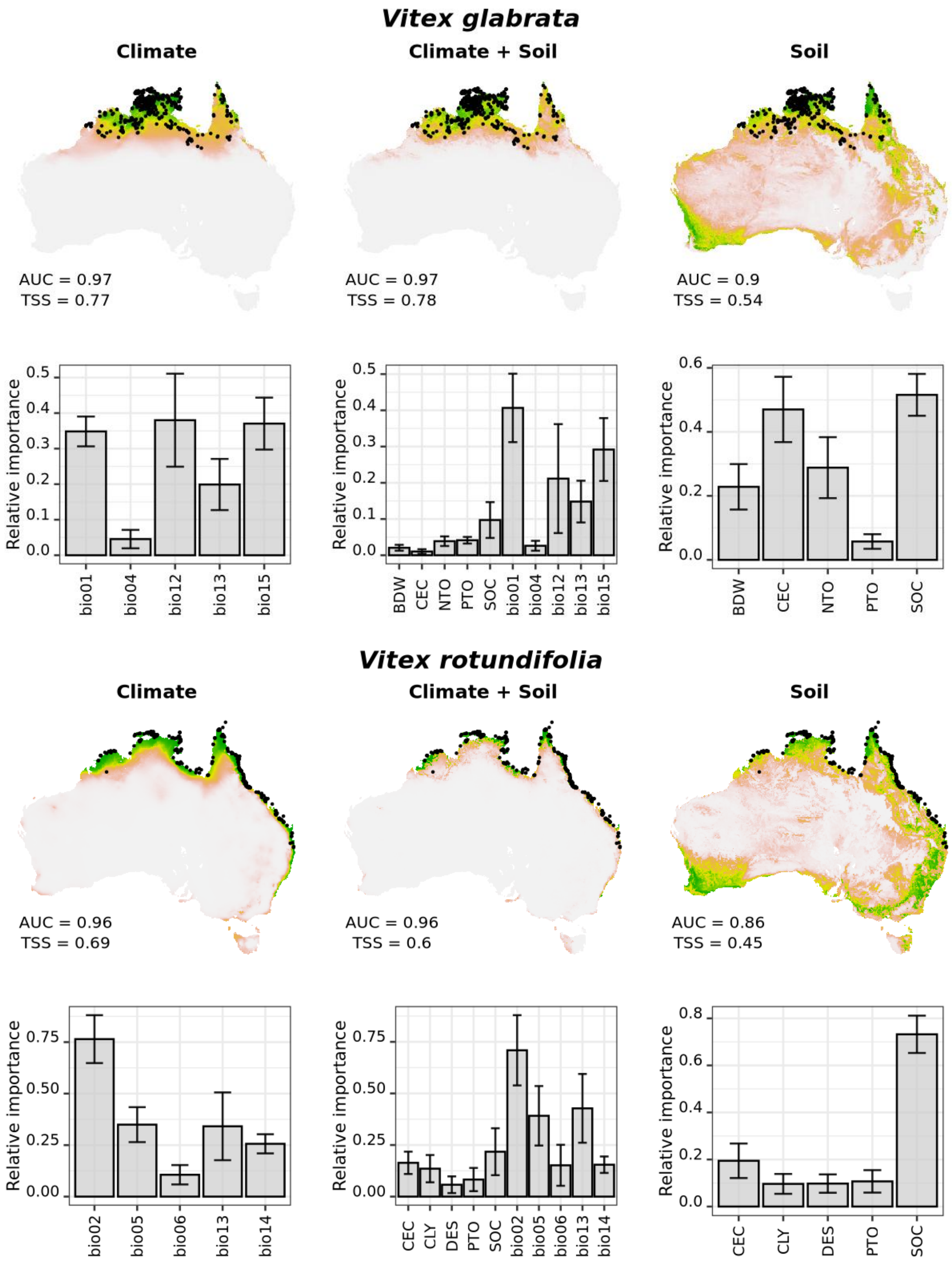
Vitex glabrata
Climate
Climate + Soil
Soil
AUC = 0.97
TSS = 0.77
AUC = 0.97
TSS = 0.78
AUC = 0.9
TSS = 0.54
Relative importance
bio01 bio04 bio12 bio13 bio15
BDW CEC NTO PTO SOC bio01 bio04 bio12 bio13 bio15
BDW CEC NTO PTO SOC
Vitex rotundifolia
Climate
Climate + Soil
Soil
AUC = 0.96
TSS = 0.69
AUC = 0.96
TSS = 0.6
AUC = 0.86
TSS = 0.45
Relative importance
bio02 bio05 bio06 bio13 bio14
CEC CLY DES PTO SOC bio02 bio05 bio06 bio13 bio14
CEC CLY DES PTO SOC

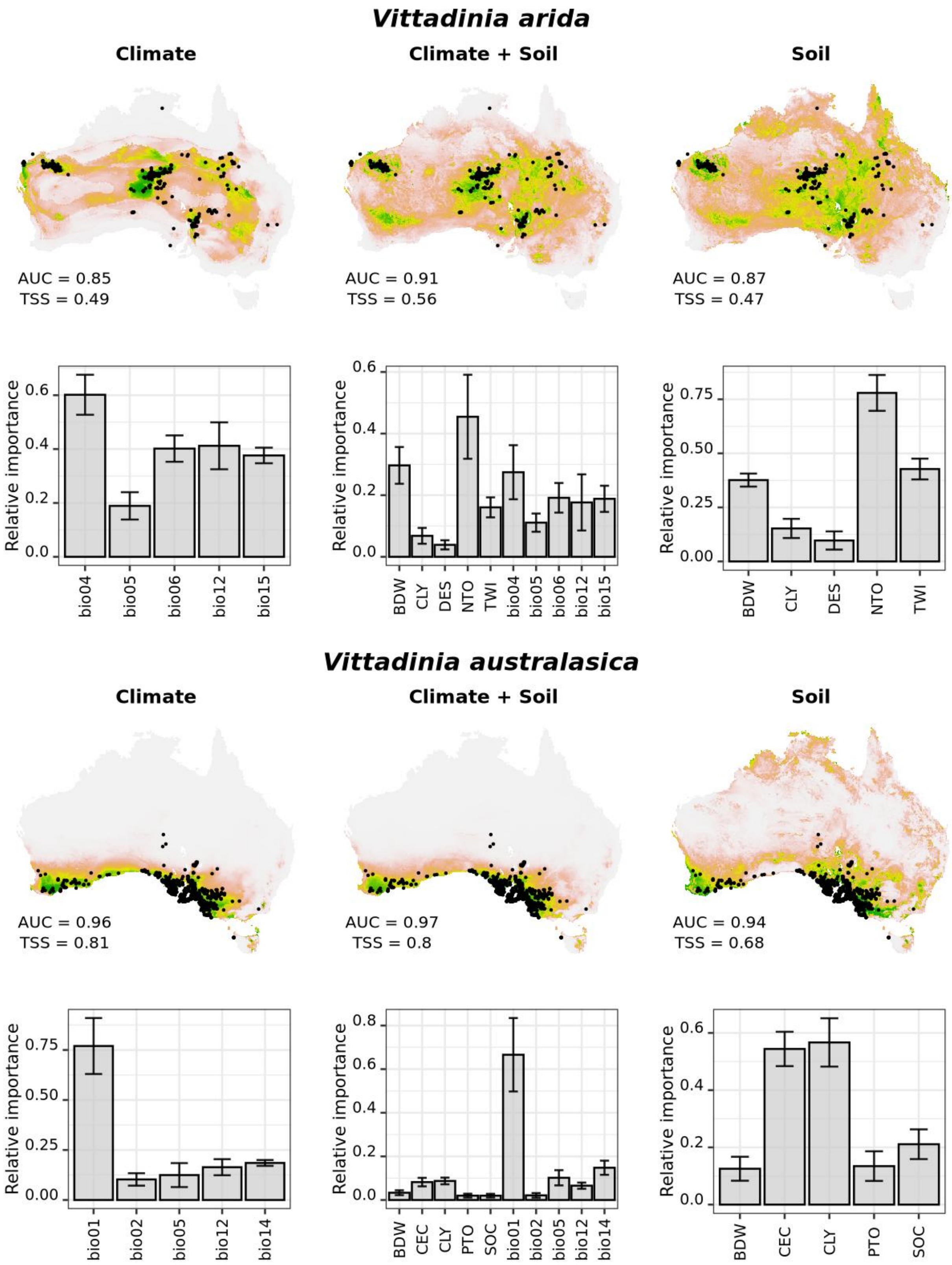

Vittadinia arida
Climate
Climate + Soil
Soil
AUC = 0.85
TSS = 0.49
AUC = 0.91
TSS = 0.56
AUC = 0.87
TSS = 0.47
Relative importance
bio04 bio05 bio06 bio12 bio15
BDW CLY DES NTO TWI bio04 bio05 bio06 bio12 bio15
BDW CLY DES NTO TWI
Vittadinia australasica
Climate
Climate + Soil
Soil
AUC = 0.96
TSS = 0.81
AUC = 0.97
TSS = 0.8
AUC = 0.94
TSS = 0.68
Relative importance
bio01 bio02 bio05 bio12 bio14
BDW CEC CLY PTO SOC bio01 bio02 bio05 bio12 bio14
BDW CEC CLY PTO SOC

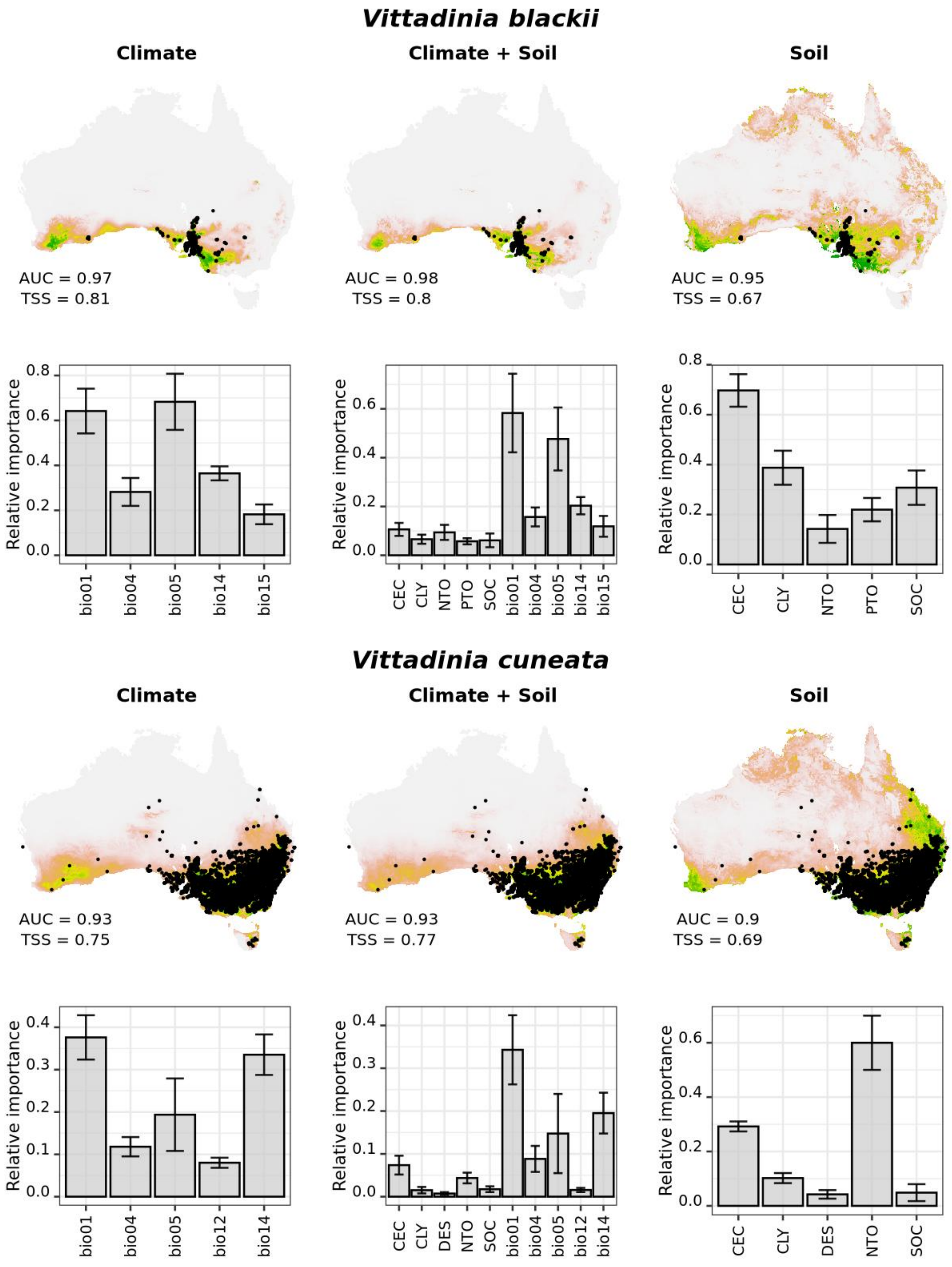

Vittadinia blackii
Climate
Climate + Soil
Soil
AUC = 0.97
TSS = 0.81
AUC = 0.98
TSS = 0.8
AUC = 0.95
TSS = 0.67
Relative importance
bio01
bio04
bio05
bio14
bio15
CEC
CLY
NTO
PTO
SOC
Vittadinia cuneata
Climate
Climate + Soil
Soil
AUC = 0.93
TSS = 0.75
AUC = 0.93
TSS = 0.77
AUC = 0.9
TSS = 0.69
Relative importance
bio01
bio04
bio05
bio12
bio14
CEC
CLY
DES
NTO
SOC

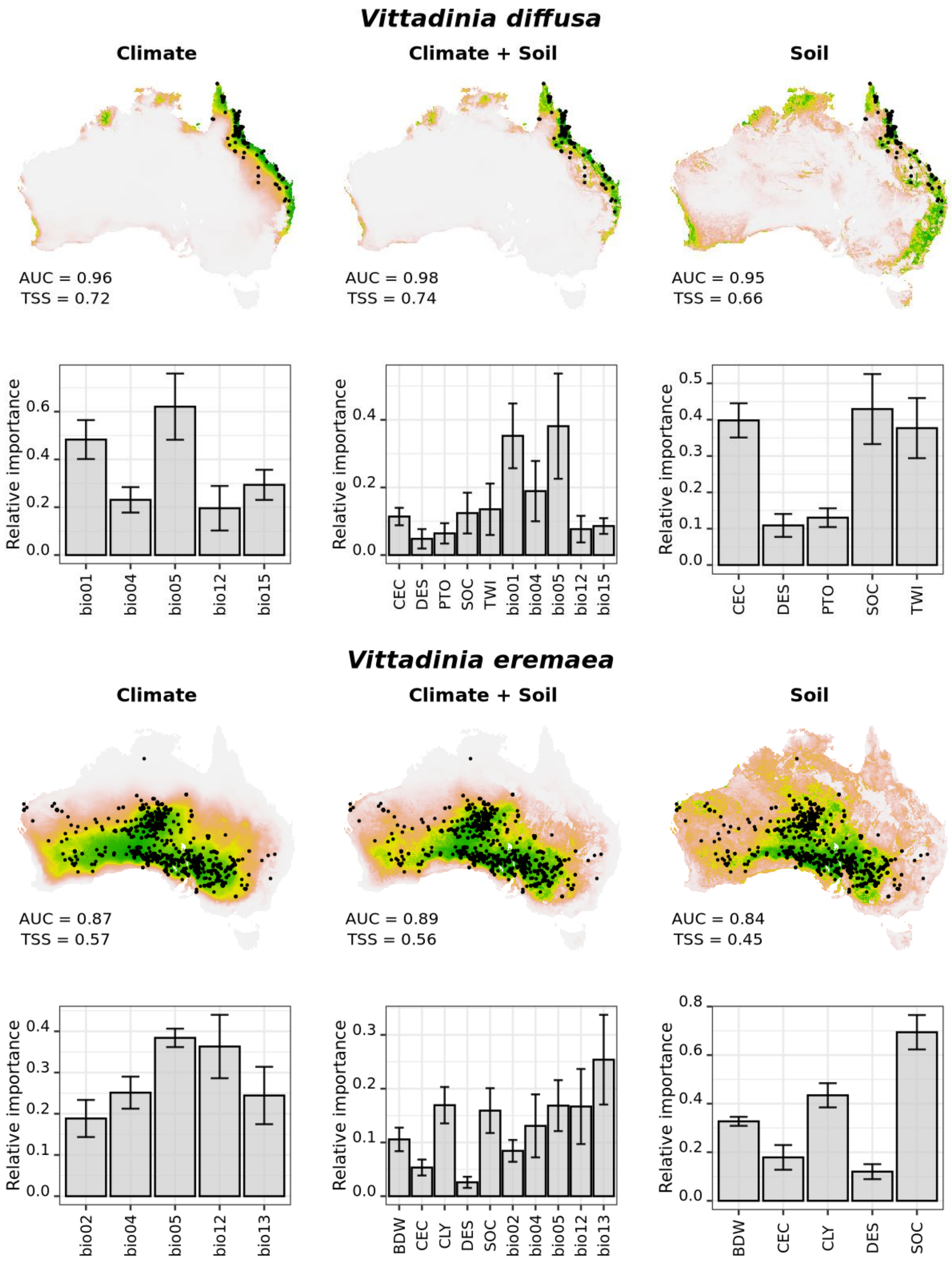
Vittadinia diffusa
Climate
Climate + Soil
Soil
AUC = 0.96
TSS = 0.72
AUC = 0.98
TSS = 0.74
AUC = 0.95
TSS = 0.66
Relative importance
bio01
bio04
bio05
bio12
bio15
CEC
DES
PTO
SOC
TWI
Vittadinia eremaea
Climate
Climate + Soil
Soil
AUC = 0.87
TSS = 0.57
AUC = 0.89
TSS = 0.56
AUC = 0.84
TSS = 0.45
Relative importance
bio02
bio04
bio05
bio12
bio13
BDW
CEC
CLY
DES
SOC

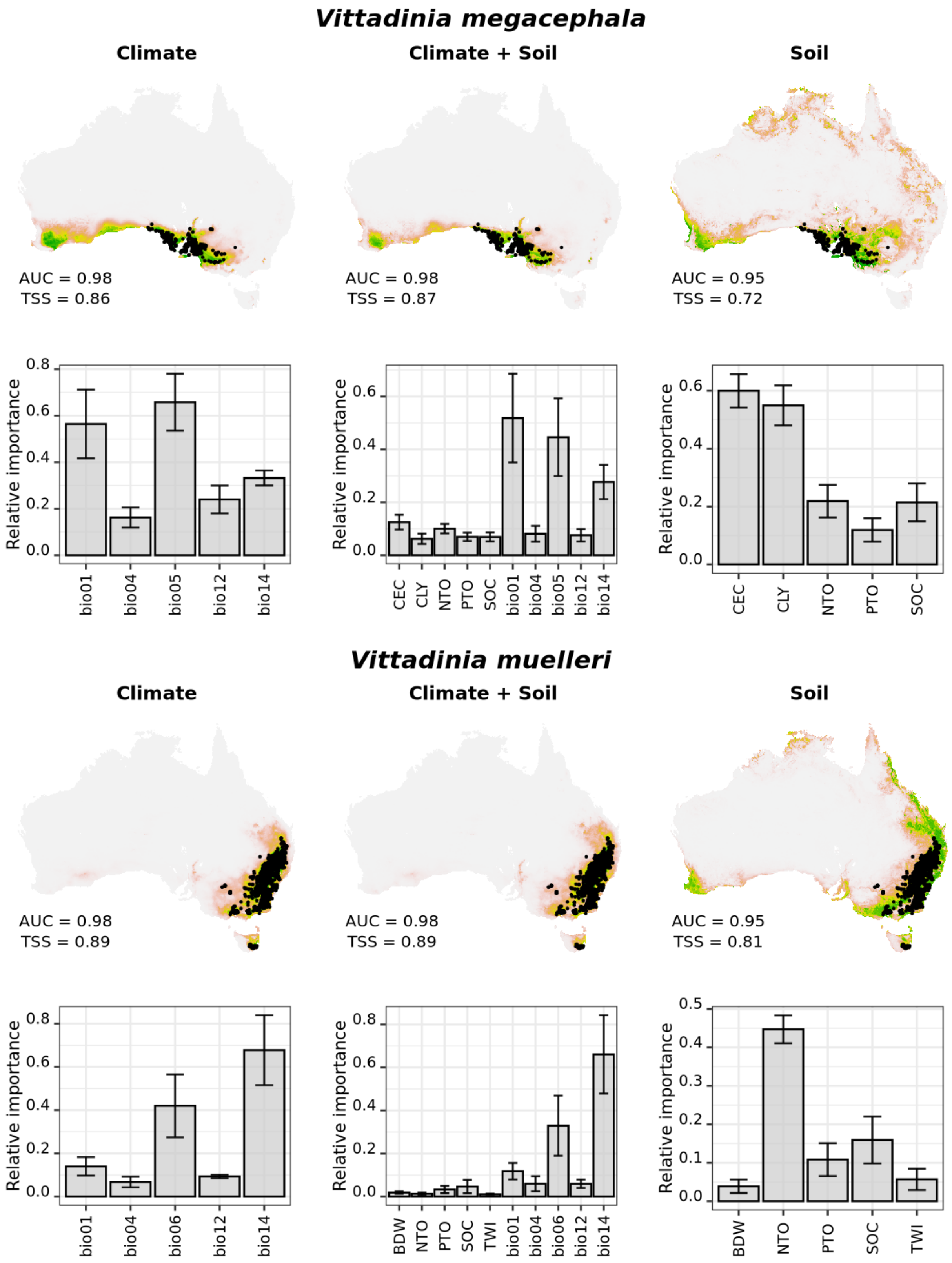

Vittadinia megacephala
Climate
Climate + Soil
Soil
AUC = 0.98
TSS = 0.86
AUC = 0.98
TSS = 0.87
AUC = 0.95
TSS = 0.72
Relative importance
bio01 bio04 bio05 bio12 bio14
CEC CLY NTO PTO SOC bio01 bio04 bio05 bio12 bio14
CEC CLY NTO PTO SOC
Vittadinia muelleri
Climate
Climate + Soil
Soil
AUC = 0.98
TSS = 0.89
AUC = 0.98
TSS = 0.89
AUC = 0.95
TSS = 0.81
Relative importance
bio01 bio04 bio06 bio12 bio14
BDW NTO PTO SOC TWI bio01 bio04 bio06 bio12 bio14
BDW NTO PTO SOC TWI

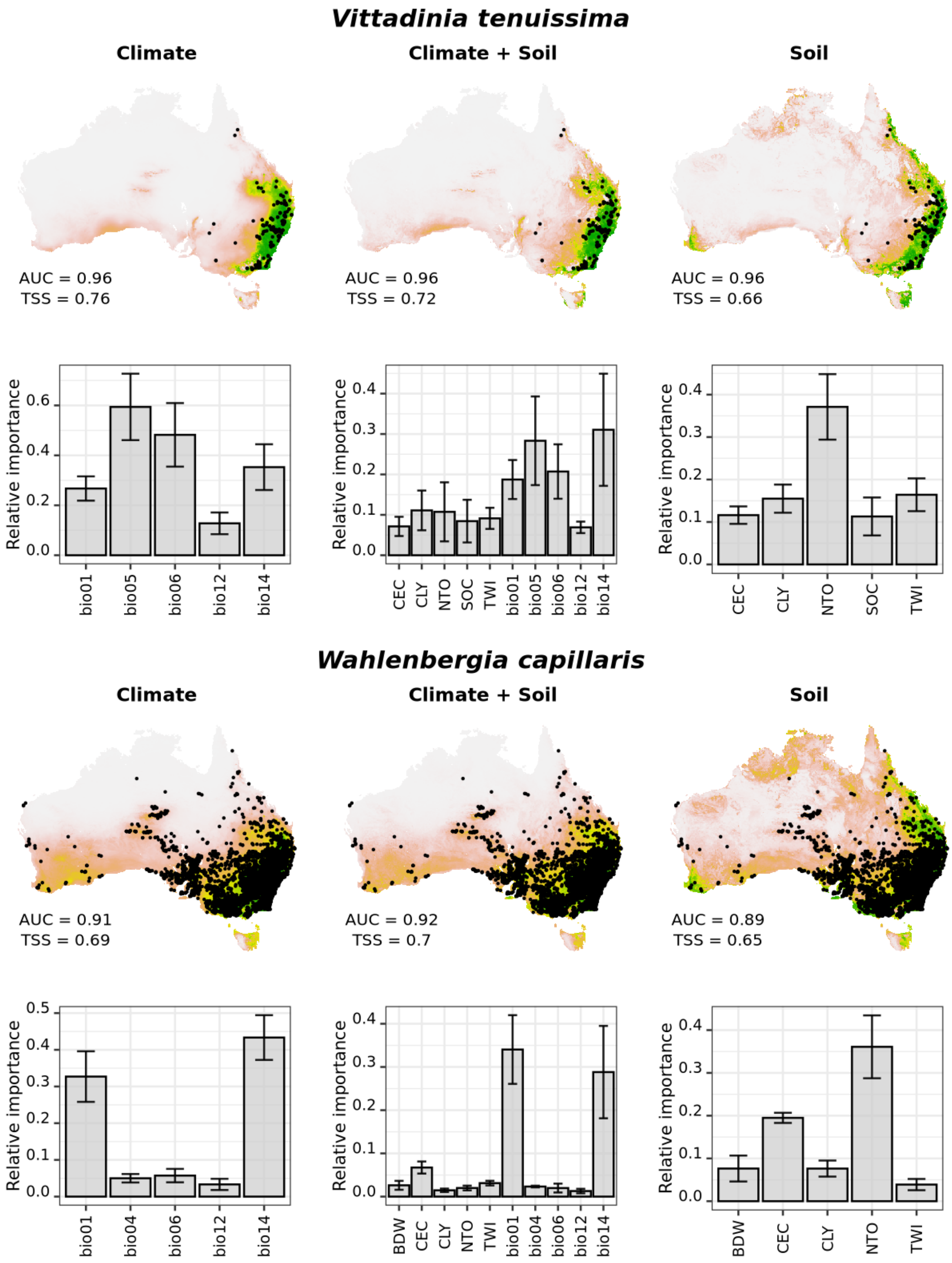

Vittadinia tenuissima
Climate
Climate + Soil
Soil
AUC = 0.96
TSS = 0.76
AUC = 0.96
TSS = 0.72
AUC = 0.96
TSS = 0.66
Relative importance
0.0
0.2
0.4
0.6
bio01
bio05
bio06
bio12
bio14
Relative importance
0.0
0.1
0.2
0.3
0.4
CEC
CLY
NTO
SOC
TWI
bio01
bio05
bio06
bio12
bio14
Relative importance
0.0
0.1
0.2
0.3
0.4
CEC
CLY
NTO
SOC
TWI
Wahlenbergia capillaris
Climate
Climate + Soil
Soil
AUC = 0.91
TSS = 0.69
AUC = 0.92
TSS = 0.7
AUC = 0.89
TSS = 0.65
Relative importance
0.0
0.1
0.2
0.3
0.4
0.5
bio01
bio04
bio06
bio12
bio14
Relative importance
0.0
0.1
0.2
0.3
0.4
BDW
CEC
CLY
NTO
TWI
bio01
bio04
bio06
bio12
bio14
Relative importance
0.0
0.1
0.2
0.3
0.4
BDW
CEC
CLY
NTO
TWI

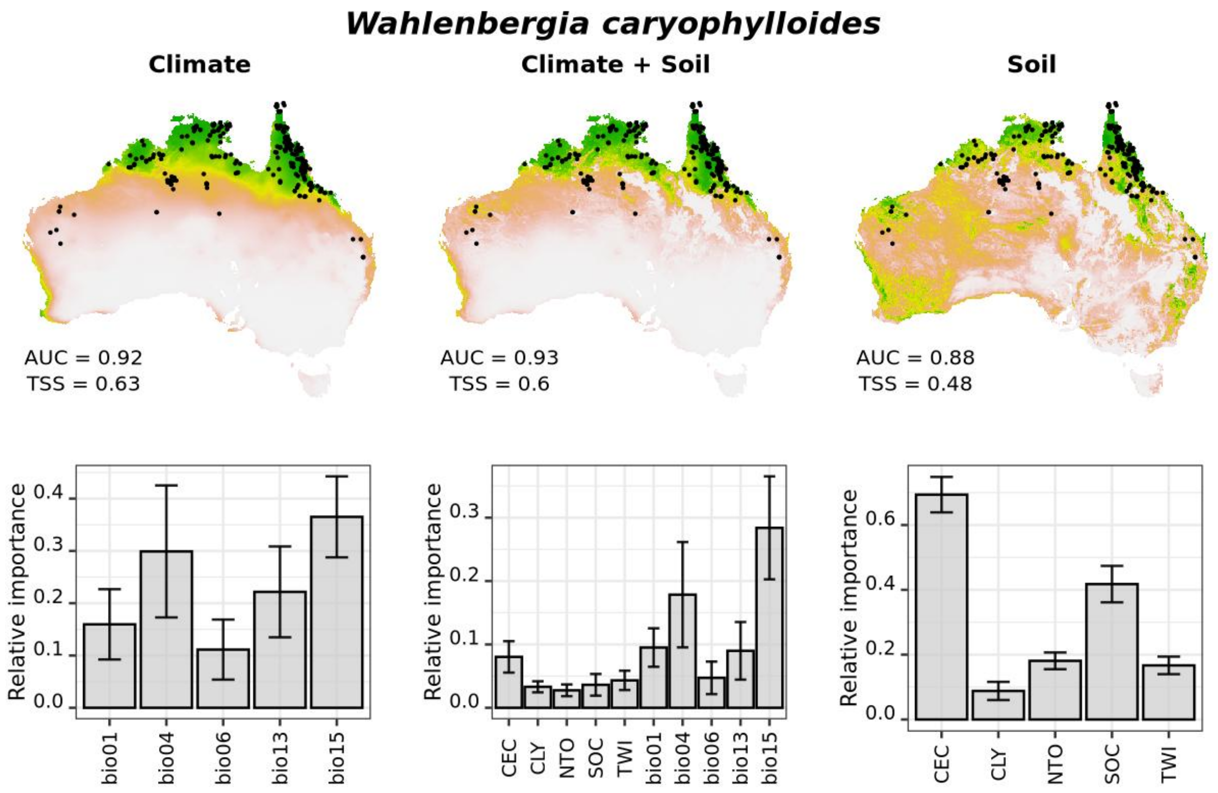
Wahlenbergia caryophylloides
Climate
Climate + Soil
Soil
AUC = 0.92
TSS = 0.63
AUC = 0.93
TSS = 0.6
AUC = 0.88
TSS = 0.48
Relative importance
0.0
0.1
0.2
0.3
0.4
0.6
bio01
bio04
bio06
bio13
bio15
CEC
CLY
NTO
SOC
TWI

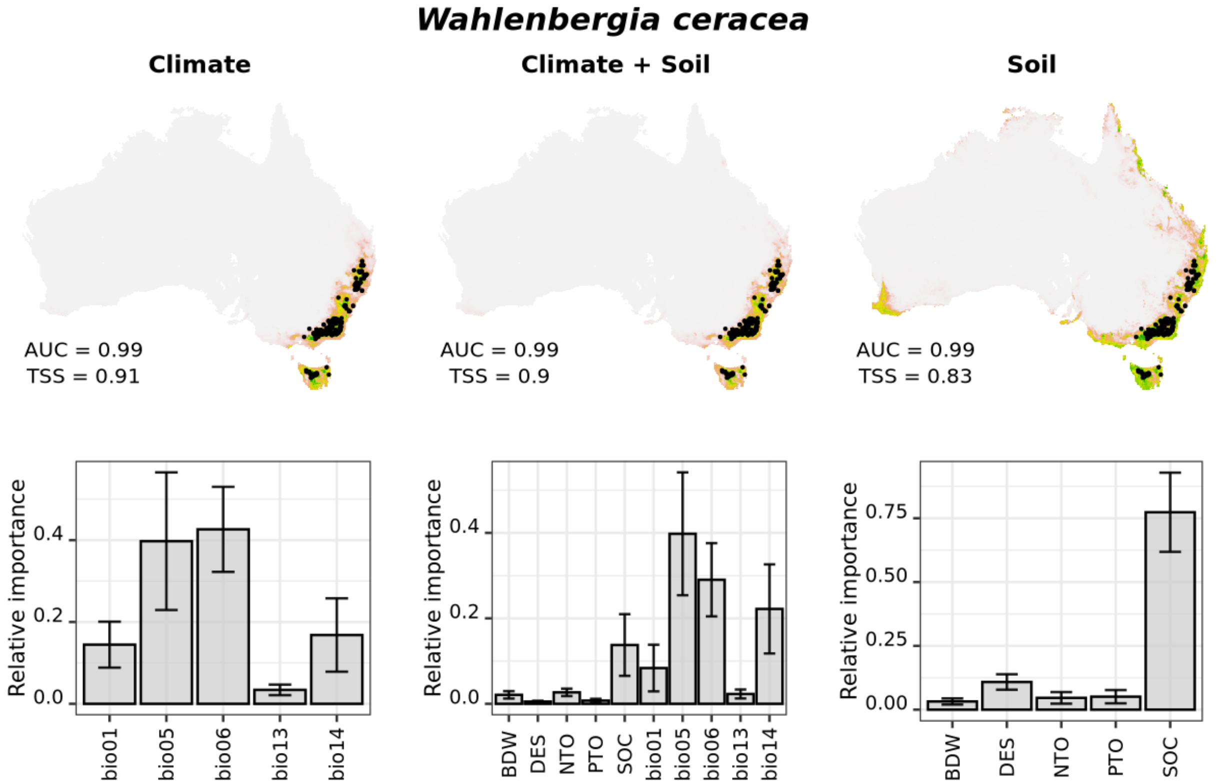
Wahlenbergia ceracea
Climate
Climate + Soil
Soil
AUC = 0.99
TSS = 0.91
AUC = 0.99
TSS = 0.9
AUC = 0.99
TSS = 0.83
Relative importance
0.0
0.2
0.4
0.00
0.25
0.50
0.75
bio01
bio05
bio06
bio13
bio14
BDW
DES
NTO
PTO
SOC

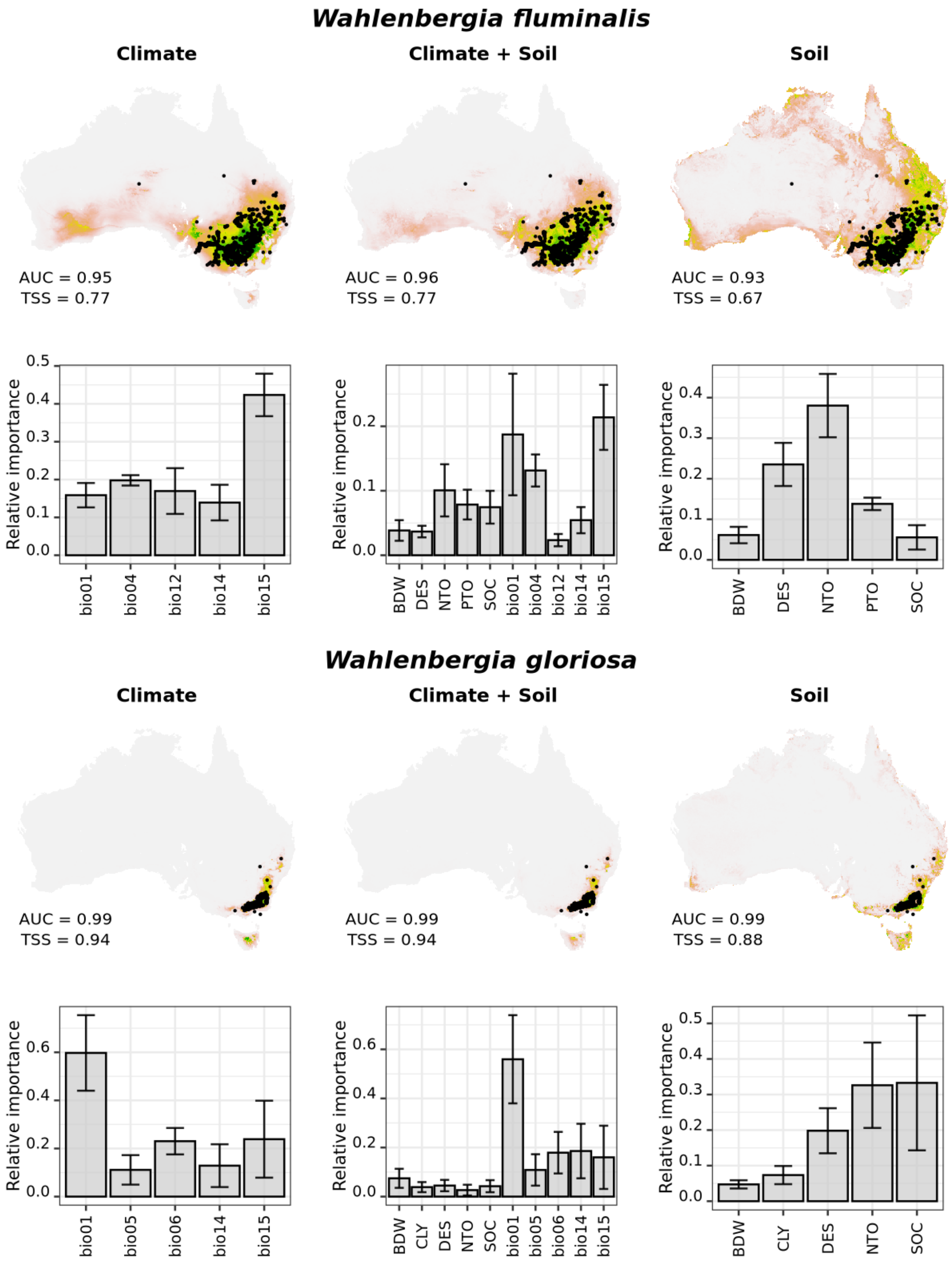
Wahlenbergia fluminalis
Climate
Climate + Soil
Soil
AUC = 0.95
TSS = 0.77
AUC = 0.96
TSS = 0.77
AUC = 0.93
TSS = 0.67
Relative importance
0.0
0.1
0.2
0.3
0.4
0.5
bio01
bio04
bio12
bio14
bio15
BDW
DES
NTO
PTO
SOC
bio01
bio04
bio12
bio14
bio15
BDW
DES
NTO
PTO
SOC
Wahlenbergia gloriosa
Climate
Climate + Soil
Soil
AUC = 0.99
TSS = 0.94
AUC = 0.99
TSS = 0.94
AUC = 0.99
TSS = 0.88
Relative importance
0.0
0.2
0.4
0.6
bio01
bio05
bio06
bio14
bio15
BDW
CLY
DES
NTO
SOC
bio01
bio05
bio06
bio14
bio15
BDW
CLY
DES
NTO
SOC

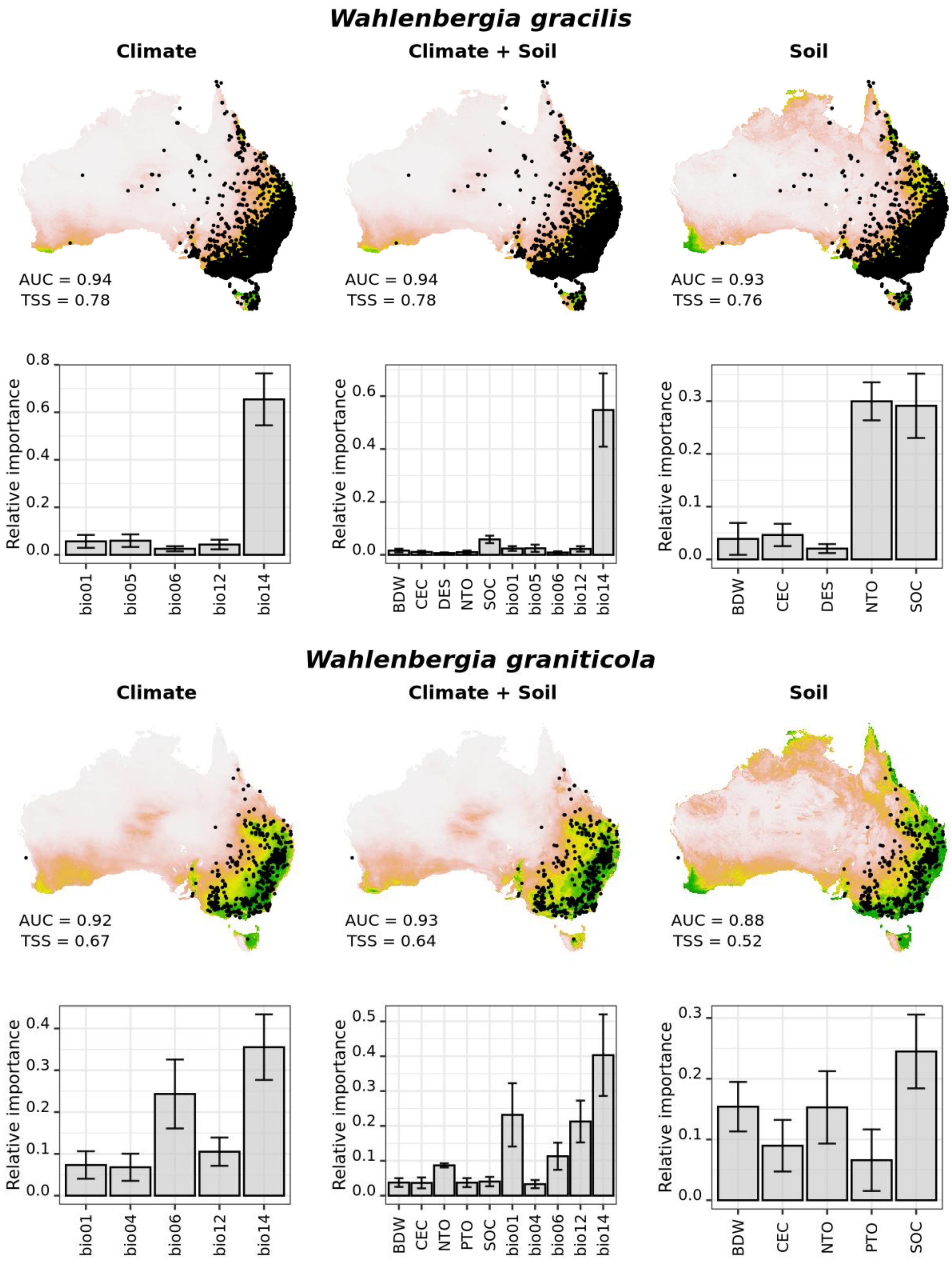

Wahlenbergia gracilis
Climate
Climate + Soil
Soil
AUC = 0.94
TSS = 0.78
AUC = 0.94
TSS = 0.78
AUC = 0.93
TSS = 0.76
Relative importance
bio01 bio05 bio06 bio12 bio14
BDW CEC DES NTO SOC bio01 bio05 bio06 bio12 bio14
BDW CEC DES NTO SOC
Wahlenbergia graniticola
Climate
Climate + Soil
Soil
AUC = 0.92
TSS = 0.67
AUC = 0.93
TSS = 0.64
AUC = 0.88
TSS = 0.52
Relative importance
bio01 bio04 bio06 bio12 bio14
BDW CEC NTO PTO SOC bio01 bio04 bio06 bio12 bio14
BDW CEC NTO PTO SOC

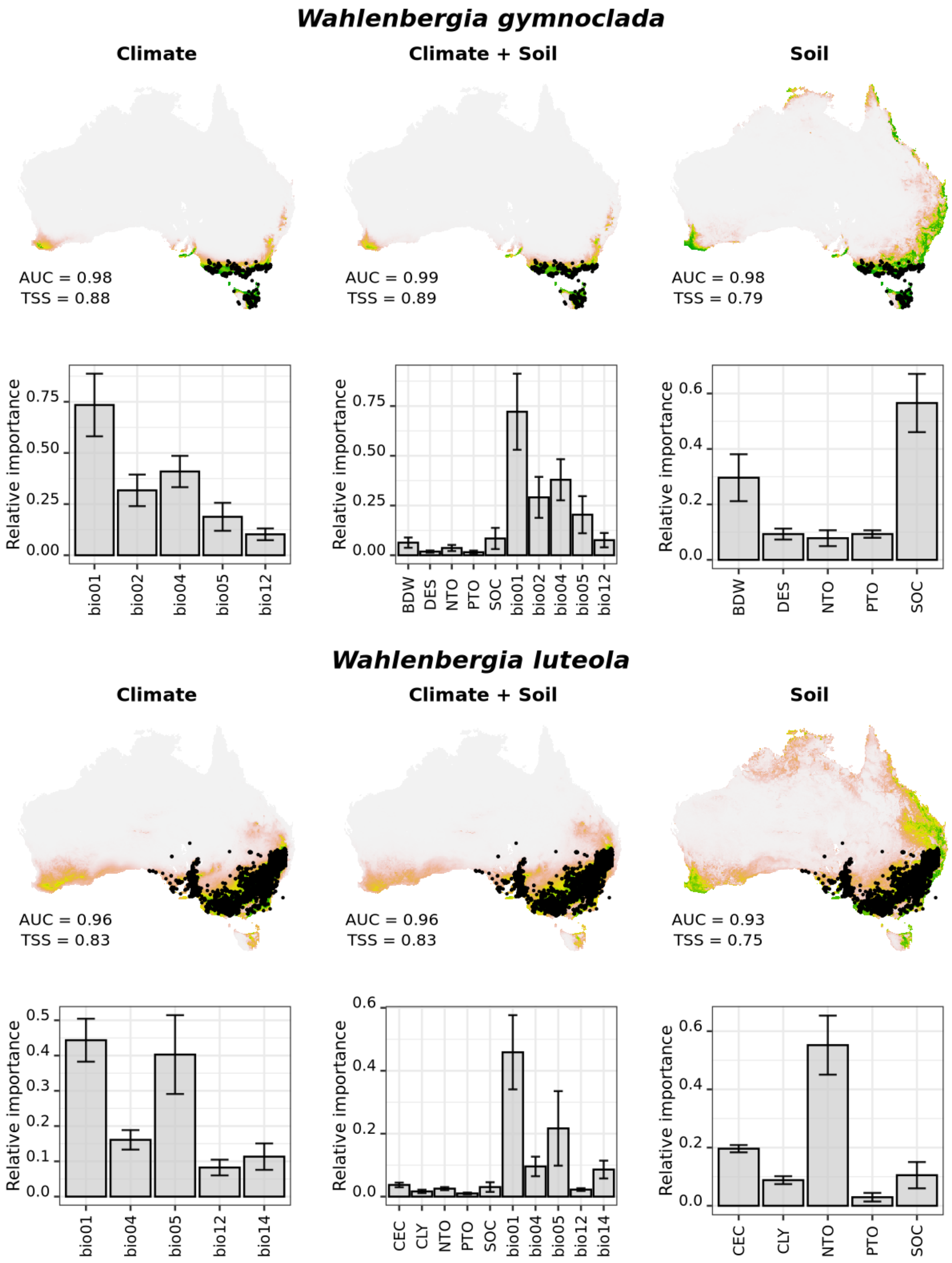

Wahlenbergia gymnoclada
Climate
Climate + Soil
Soil
AUC = 0.98
TSS = 0.88
AUC = 0.99
TSS = 0.89
AUC = 0.98
TSS = 0.79
Relative importance
0.00
0.25
0.50
0.75
bio01
bio02
bio04
bio05
bio12
BDW
DES
NTO
PTO
SOC
0.0
0.2
0.4
0.6
Wahlenbergia luteola
Climate
Climate + Soil
Soil
AUC = 0.96
TSS = 0.83
AUC = 0.96
TSS = 0.83
AUC = 0.93
TSS = 0.75
Relative importance
0.0
0.1
0.2
0.3
0.4
0.5
0.6
bio01
bio04
bio05
bio12
bio14
CEC
CLY
NTO
PTO
SOC

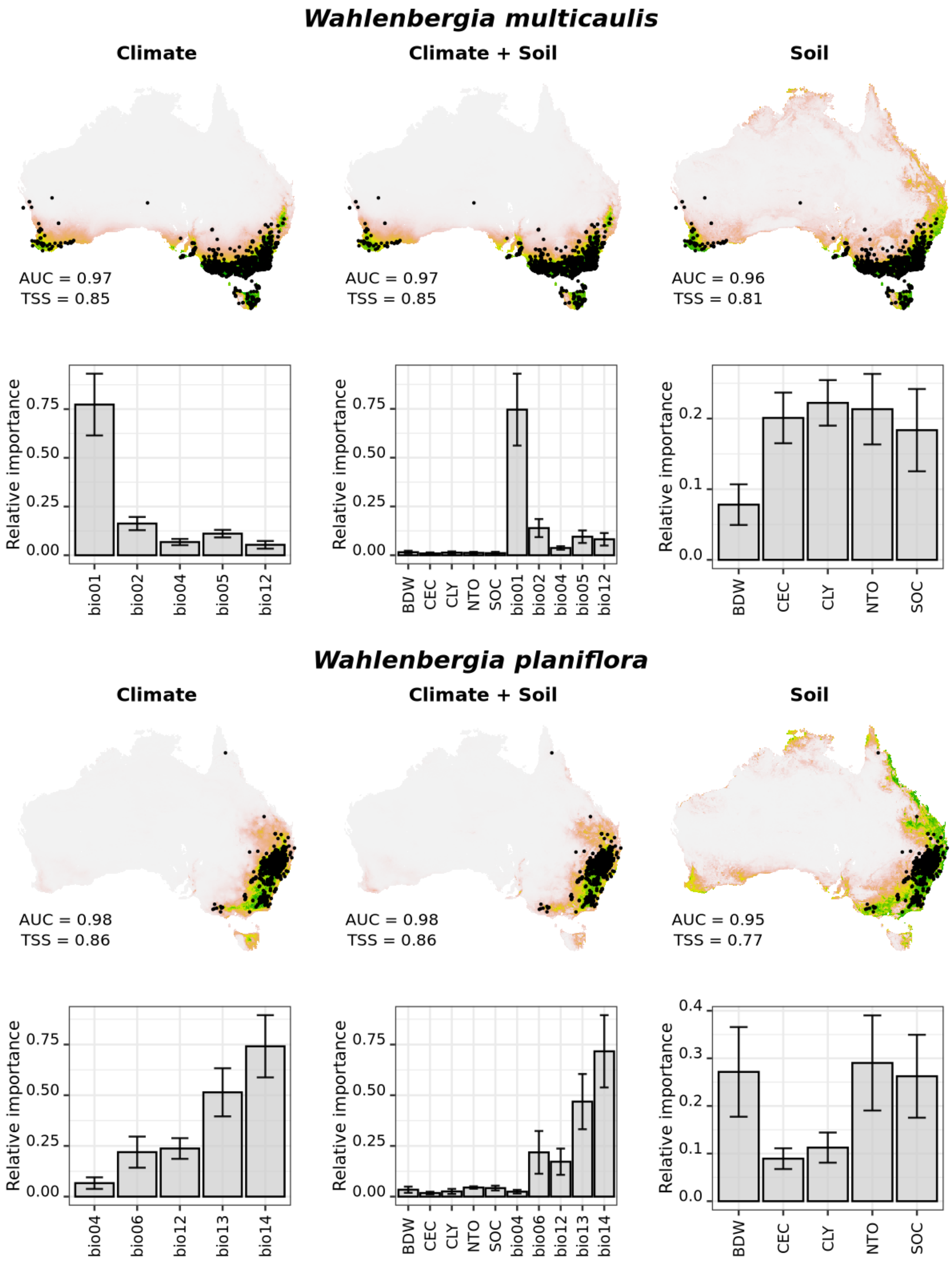
Wahlenbergia multicaulis
Climate
Climate + Soil
Soil
AUC = 0.97
TSS = 0.85
AUC = 0.97
TSS = 0.85
AUC = 0.96
TSS = 0.81
Relative importance
0.00
0.25
0.50
0.75
bio01
bio02
bio04
bio05
bio12
BDW
CEC
CLY
NTO
SOC
0.0
0.1
0.2
Wahlenbergia planiflora
Climate
Climate + Soil
Soil
AUC = 0.98
TSS = 0.86
AUC = 0.98
TSS = 0.86
AUC = 0.95
TSS = 0.77
Relative importance
0.00
0.25
0.50
0.75
bio04
bio06
bio12
bio13
bio14
BDW
CEC
CLY
NTO
SOC
0.0
0.1
0.2
0.3
0.4

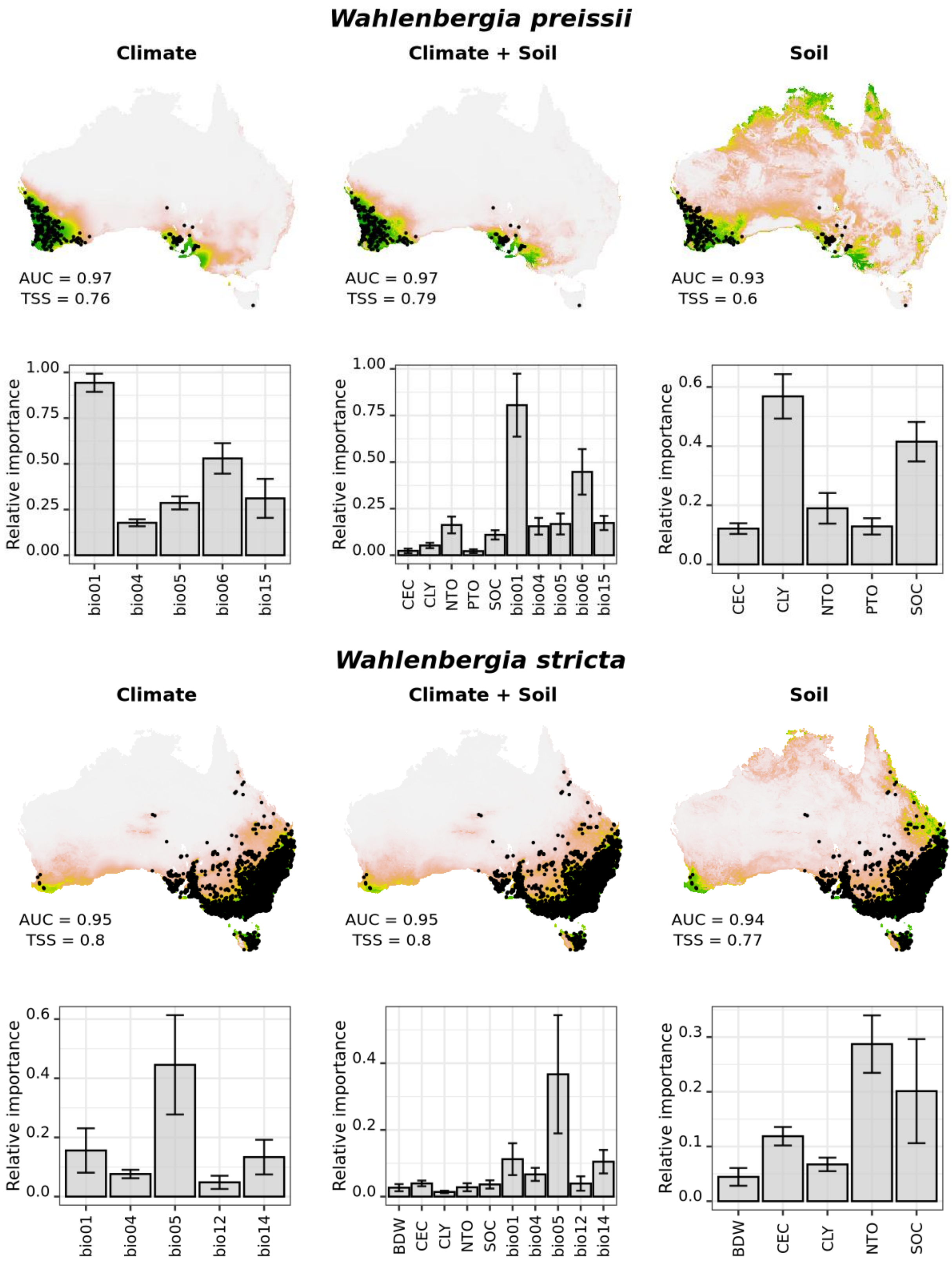

Wahlenbergia preissii
Climate
Climate + Soil
Soil
AUC = 0.97
TSS = 0.76
AUC = 0.97
TSS = 0.79
AUC = 0.93
TSS = 0.6
Relative importance
bio01
bio04
bio05
bio06
bio15
CEC
CLY
NTO
PTO
SOC
Wahlenbergia stricta
Climate
Climate + Soil
Soil
AUC = 0.95
TSS = 0.8
AUC = 0.95
TSS = 0.8
AUC = 0.94
TSS = 0.77
Relative importance
bio01
bio04
bio05
bio12
bio14
BDW
CEC
CLY
NTO
SOC

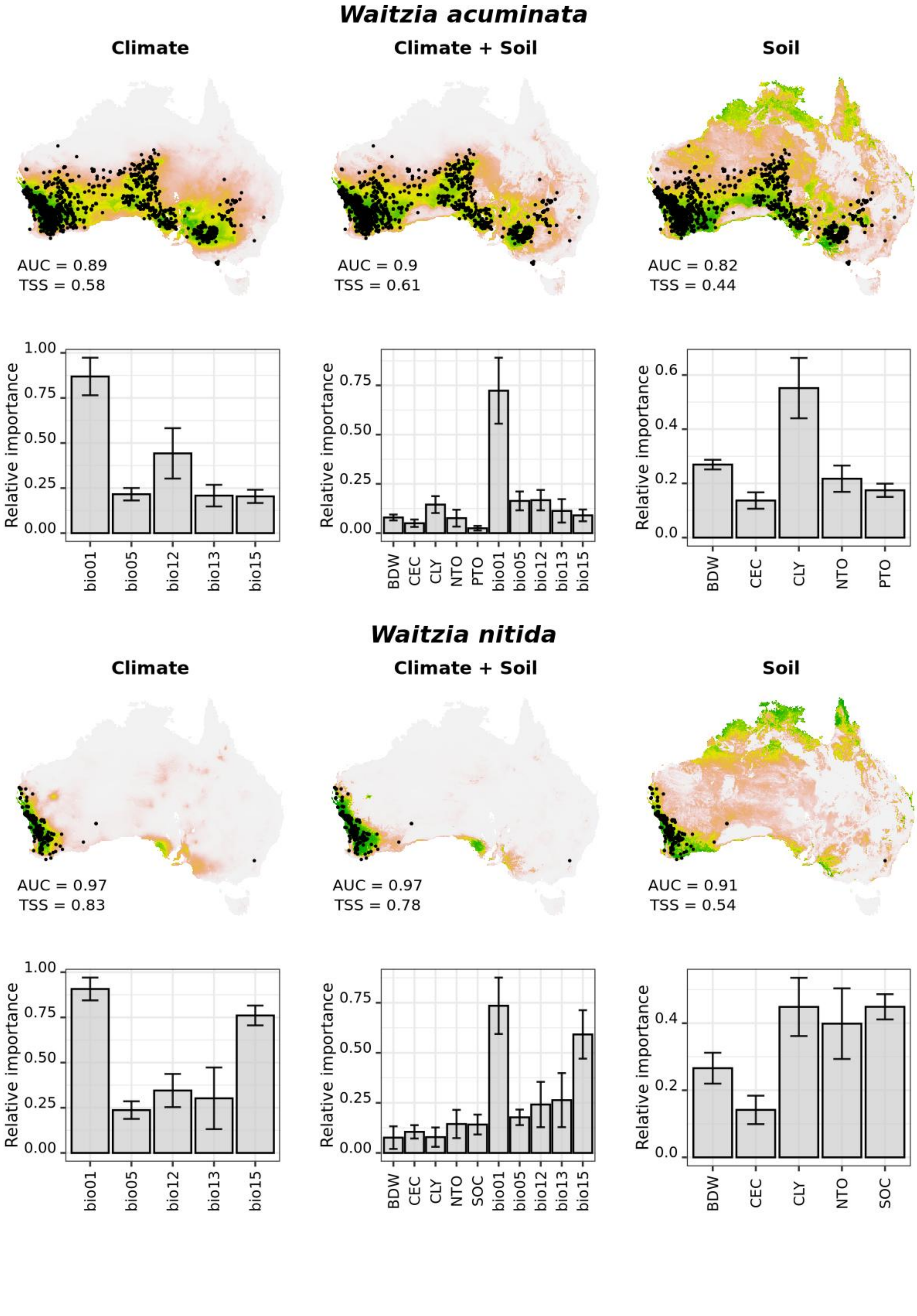
Waitzia acuminata
Climate
Climate + Soil
Soil
AUC = 0.89
TSS = 0.58
AUC = 0.9
TSS = 0.61
AUC = 0.82
TSS = 0.44
Relative importance
1.00
0.75
0.50
0.25
0.00
bio01
bio05
bio12
bio13
bio15
0.6
0.4
0.2
0.0
BDW
CEC
CLY
NTO
PTO
Waitzia nitida
AUC = 0.97
TSS = 0.83
AUC = 0.97
TSS = 0.78
AUC = 0.91
TSS = 0.54
SOC

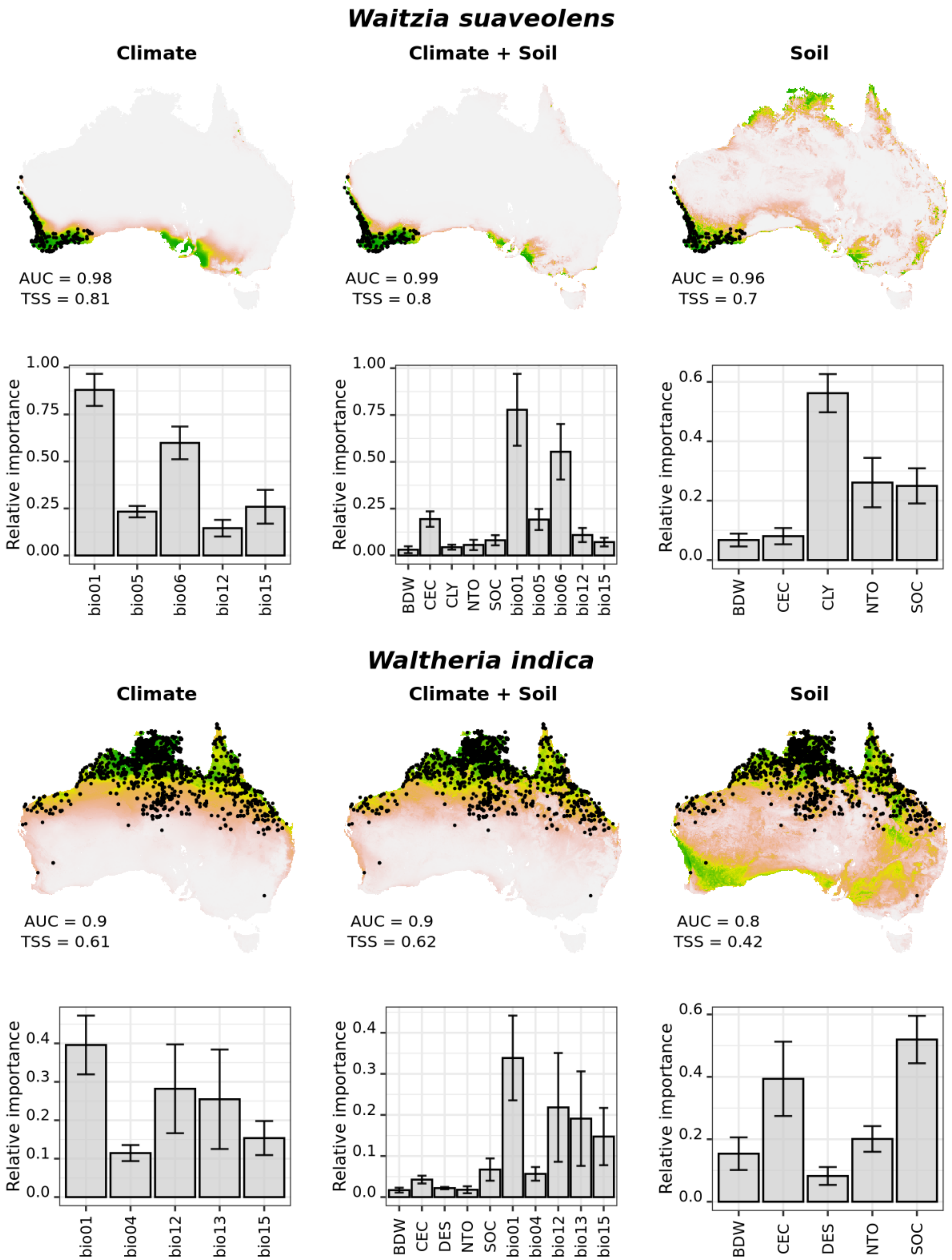
Waitzia suaveolens
Climate
Climate + Soil
Soil
AUC = 0.98
TSS = 0.81
AUC = 0.99
TSS = 0.8
AUC = 0.96
TSS = 0.7
Relative importance
bio01 bio05 bio06 bio12 bio15
BDW CEC CLY NTO SOC bio01 bio05 bio06 bio12 bio15
BDW CEC CLY NTO SOC
Waltheria indica
Climate
Climate + Soil
Soil
AUC = 0.9
TSS = 0.61
AUC = 0.9
TSS = 0.62
AUC = 0.8
TSS = 0.42
Relative importance
bio01 bio04 bio12 bio13 bio15
BDW CEC DES NTO SOC bio01 bio04 bio12 bio13 bio15
BDW CEC DES NTO SOC

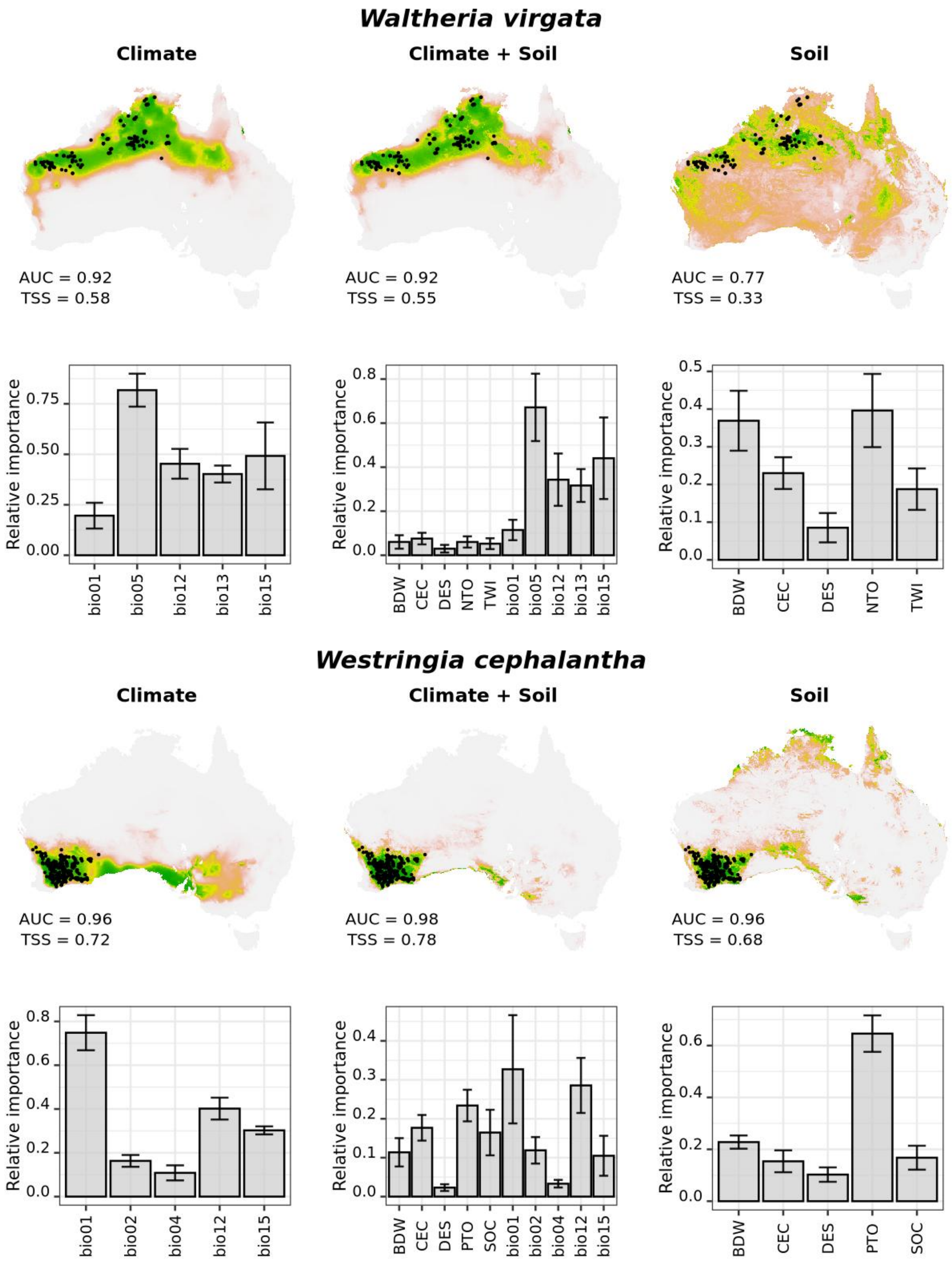
Waltheria virgata
Climate
Climate + Soil
Soil
AUC = 0.92
TSS = 0.58
AUC = 0.92
TSS = 0.55
AUC = 0.77
TSS = 0.33
Relative importance
bio01
bio05
bio12
bio13
bio15
BDW
CEC
DES
NTO
TWI
Westringia cephalantha
Climate
Climate + Soil
Soil
AUC = 0.96
TSS = 0.72
AUC = 0.98
TSS = 0.78
AUC = 0.96
TSS = 0.68
Relative importance
bio01
bio02
bio04
bio12
bio15
BDW
CEC
DES
PTO
SOC

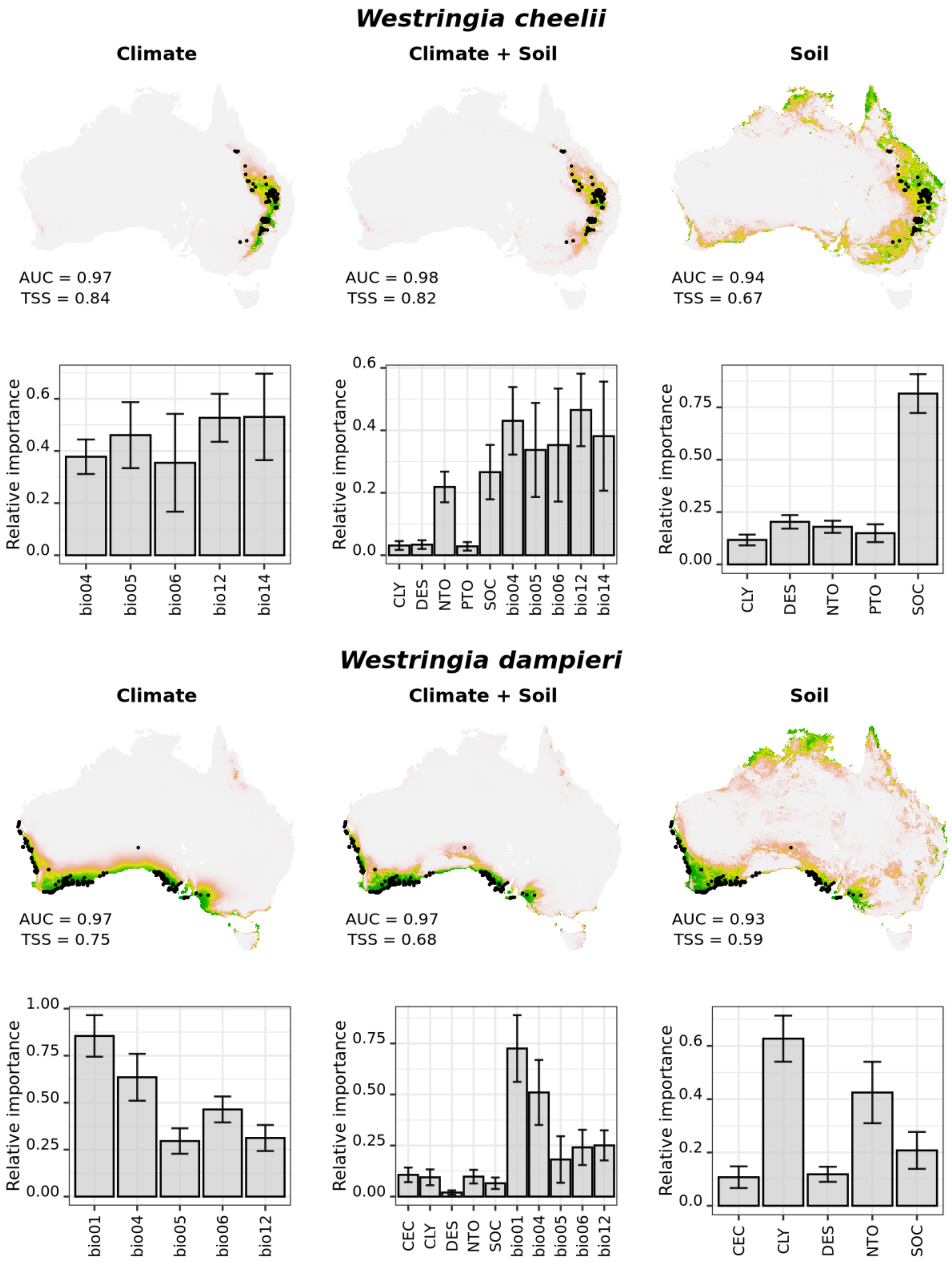
Westringia cheelii
Climate
Climate + Soil
Soil
AUC = 0.97
TSS = 0.84
AUC = 0.98
TSS = 0.82
AUC = 0.94
TSS = 0.67
Relative importance
bio04
bio05
bio06
bio12
bio14
CLY
DES
NTO
PTO
SOC
Westringia dampieri
AUC = 0.97
TSS = 0.75
AUC = 0.97
TSS = 0.68
AUC = 0.93
TSS = 0.59
bio01
CEC

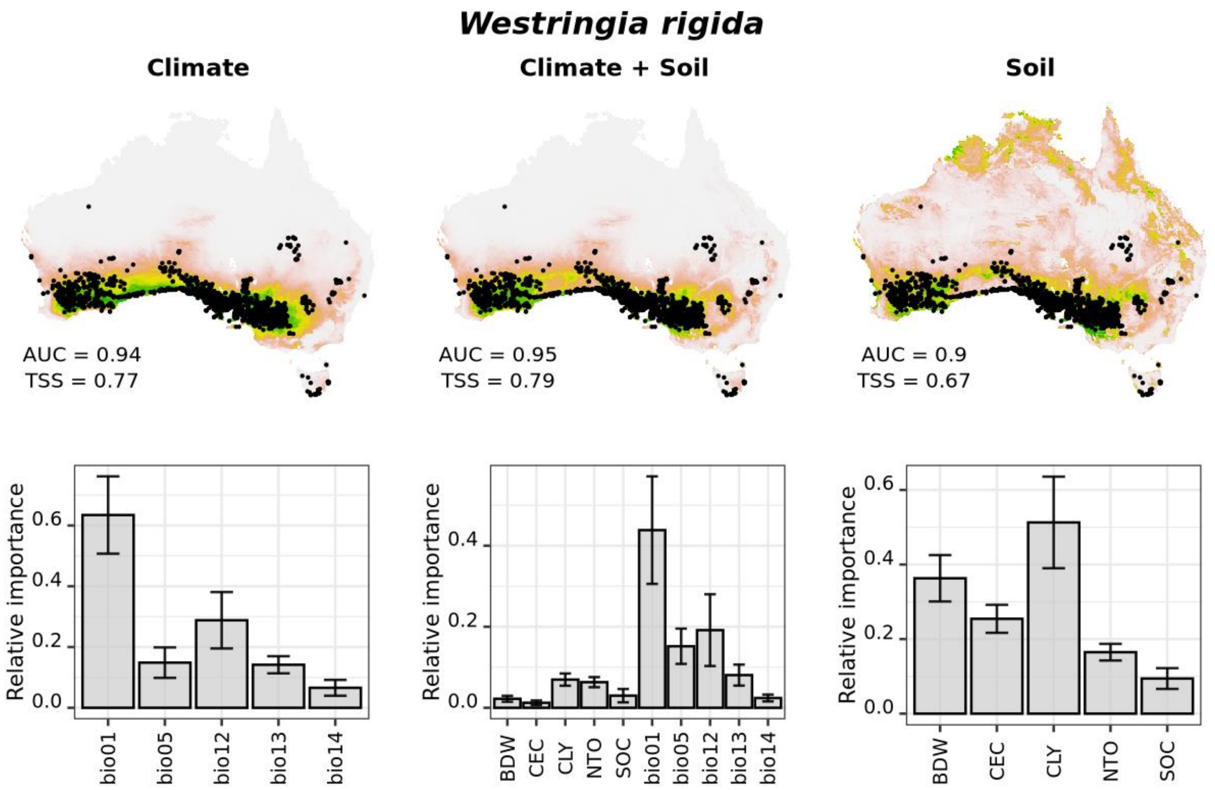
Westringia rigida
Climate
Climate + Soil
Soil
AUC = 0.94
TSS = 0.77
AUC = 0.95
TSS = 0.79
AUC = 0.9
TSS = 0.67
Relative importance
bio01 bio05 bio12 bio13 bio14
BDW CEC CLY NTO SOC bio01 bio05 bio12 bio13 bio14
BDW CEC CLY NTO SOC

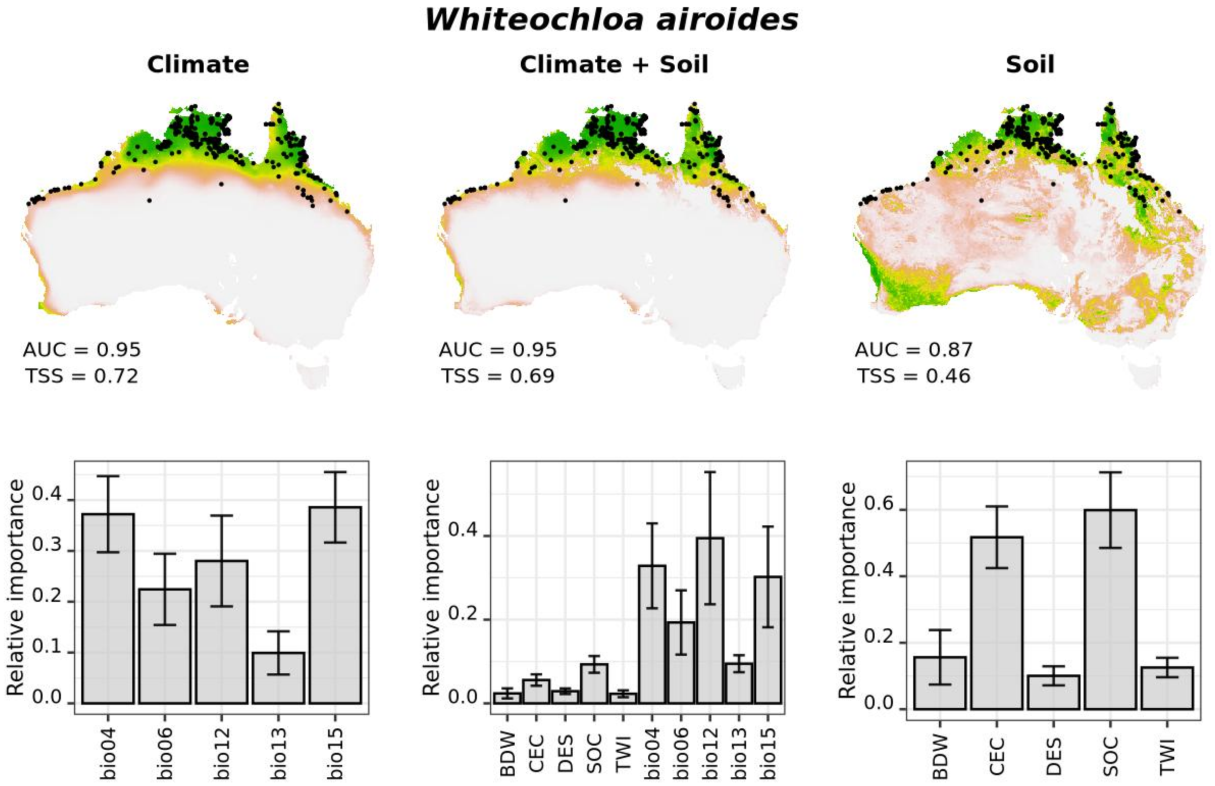
Whiteochloa airoides
Climate
Climate + Soil
Soil
AUC = 0.95
TSS = 0.72
AUC = 0.95
TSS = 0.69
AUC = 0.87
TSS = 0.46
Relative importance
bio04 bio06 bio12 bio13 bio15
BDW CEC DES SOC TWI bio04 bio06 bio12 bio13 bio15
BDW CEC DES SOC TWI

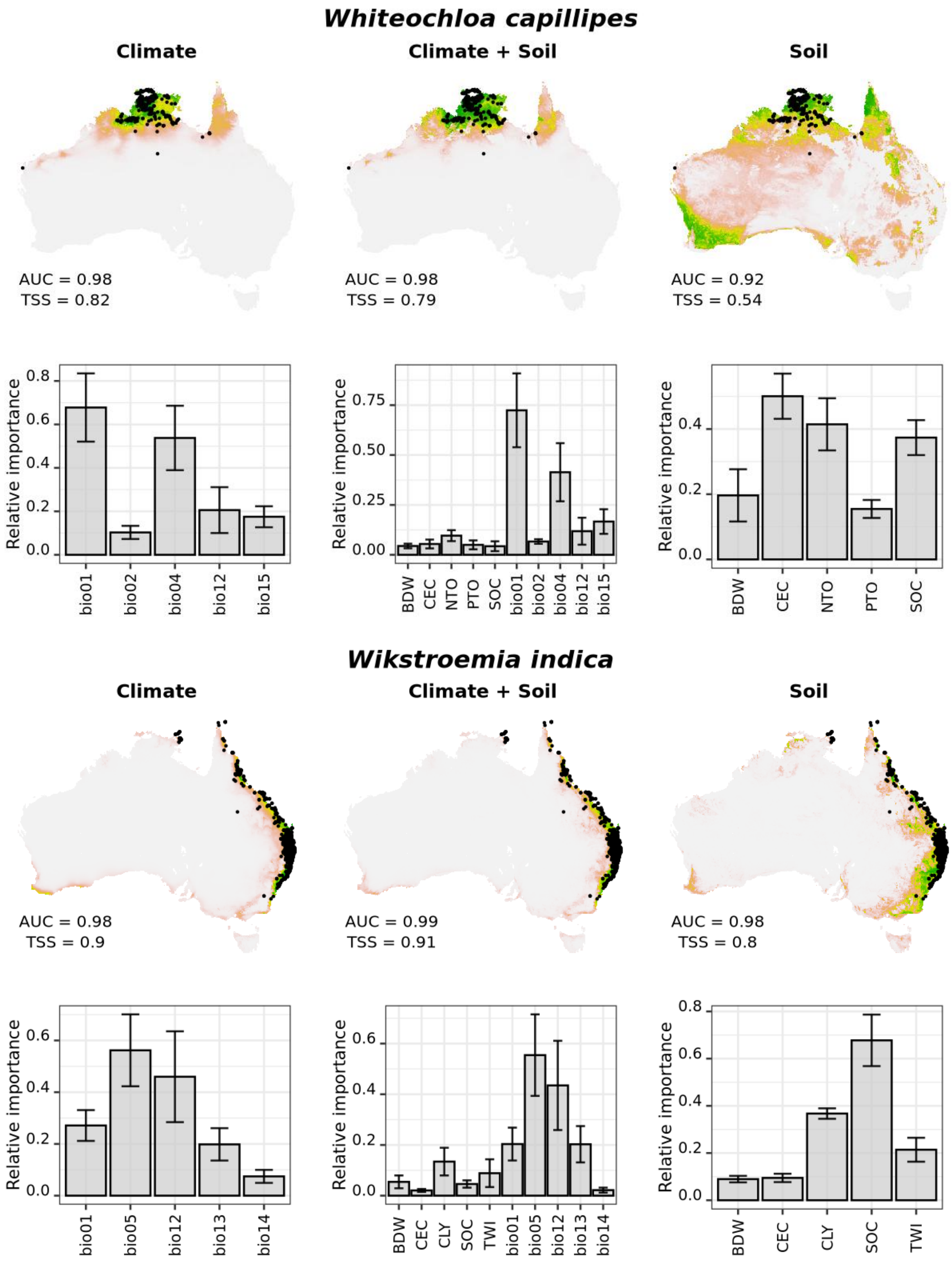
Whiteochloa capillipes
Climate
Climate + Soil
Soil
AUC = 0.98
TSS = 0.82
AUC = 0.98
TSS = 0.79
AUC = 0.92
TSS = 0.54
Relative importance
bio01
bio02
bio04
bio12
bio15
BDW
CEC
NTO
PTO
SOC
Wikstroemia indica
Climate
Climate + Soil
Soil
AUC = 0.98
TSS = 0.9
AUC = 0.99
TSS = 0.91
AUC = 0.98
TSS = 0.8
Relative importance
bio01
bio05
bio12
bio13
bio14
BDW
CEC
CLY
SOC
TWI

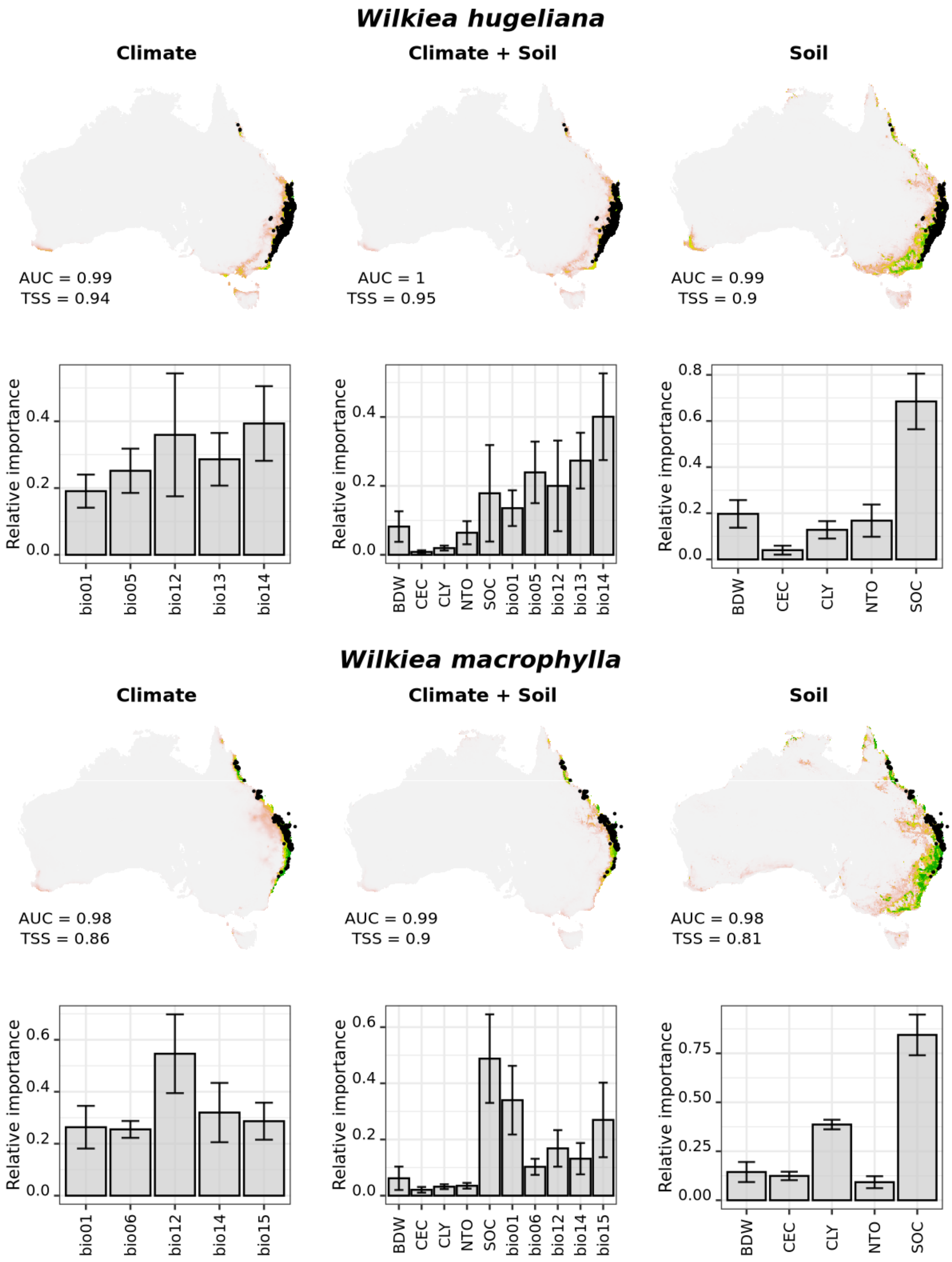

Wilkiea hugeliana
Climate
Climate + Soil
Soil
AUC = 0.99
TSS = 0.94
AUC = 1
TSS = 0.95
AUC = 0.99
TSS = 0.9
Relative importance
bio01
bio05
bio12
bio13
bio14
BDW
CEC
CLY
NTO
SOC
Wilkiea macrophylla
Climate
Climate + Soil
Soil
AUC = 0.98
TSS = 0.86
AUC = 0.99
TSS = 0.9
AUC = 0.98
TSS = 0.81
Relative importance
bio01
bio06
bio12
bio14
bio15
BDW
CEC
CLY
NTO
SOC

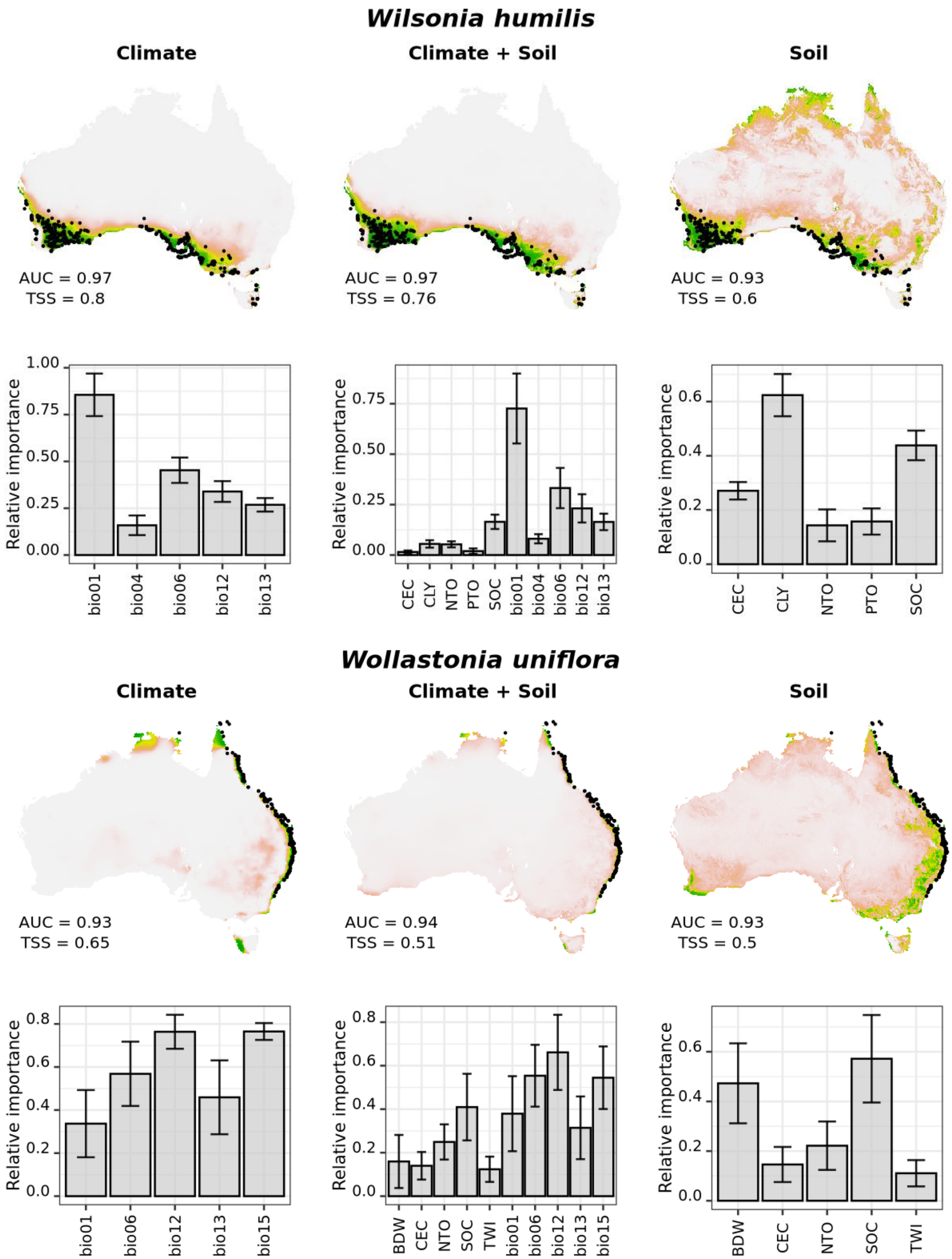

Wilsonia humilis
Climate
Climate + Soil
Soil
AUC = 0.97
TSS = 0.8
AUC = 0.97
TSS = 0.76
AUC = 0.93
TSS = 0.6
Relative importance
bio01
bio04
bio06
bio12
bio13
CEC
CLY
NTO
PTO
SOC
Wollastonia uniflora
Climate
Climate + Soil
Soil
AUC = 0.93
TSS = 0.65
AUC = 0.94
TSS = 0.51
AUC = 0.93
TSS = 0.5
Relative importance
bio01
bio06
bio12
bio13
bio15
BDW
CEC
NTO
SOC
TWI

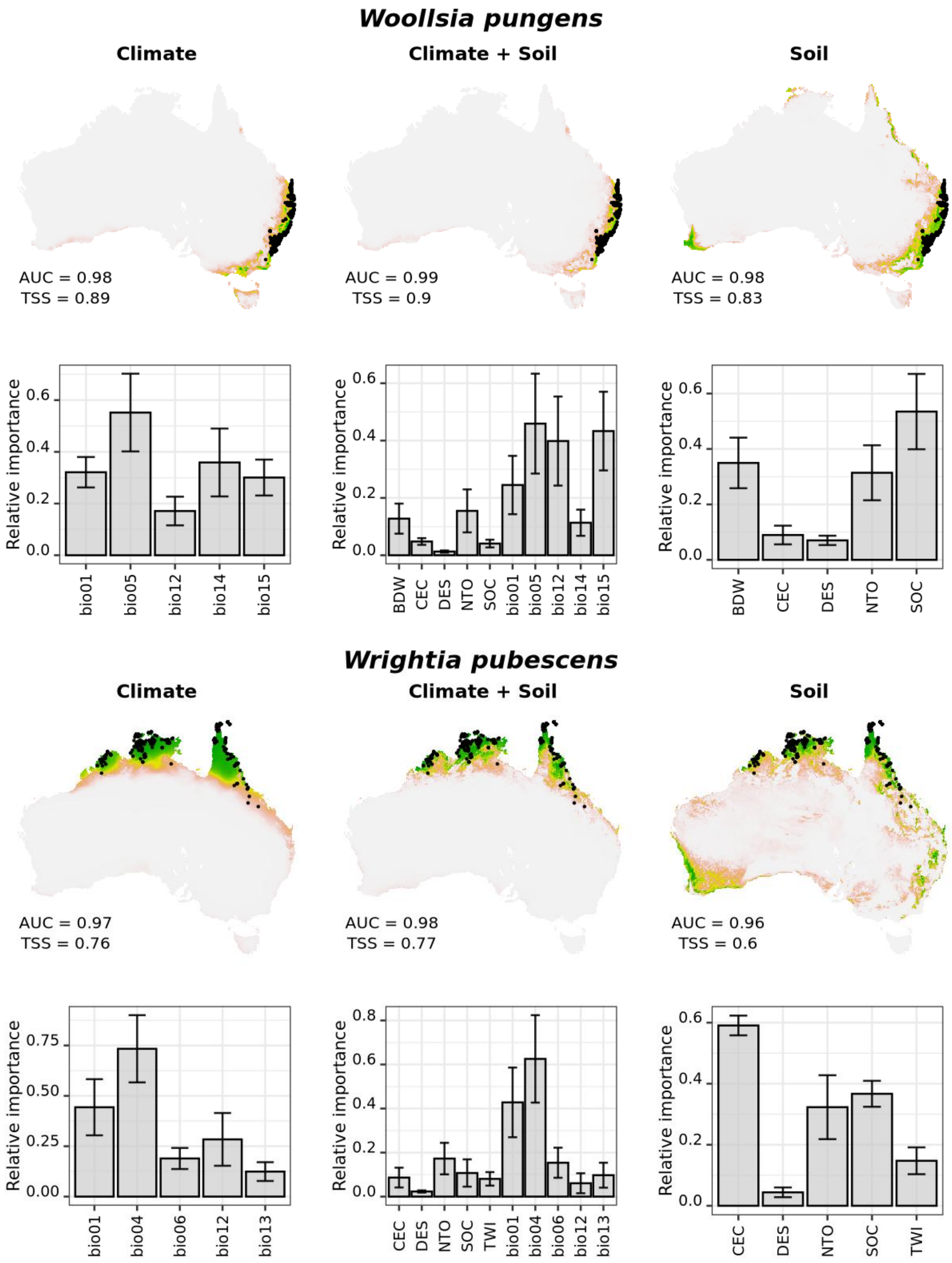
Woollsia pungens
Climate
Climate + Soil
Soil
AUC = 0.98
TSS = 0.89
AUC = 0.99
TSS = 0.9
AUC = 0.98
TSS = 0.83
Relative importance
bio01
bio05
bio12
bio14
bio15
BDW
CEC
DES
NTO
SOC
bio01
bio05
bio12
bio14
bio15
BDW
CEC
DES
NTO
SOC
Wrightia pubescens
Climate
Climate + Soil
Soil
AUC = 0.97
TSS = 0.76
AUC = 0.98
TSS = 0.77
AUC = 0.96
TSS = 0.6
Relative importance
bio01
bio04
bio06
bio12
bio13
CEC
DES
NTO
SOC
TWI
bio01
bio04
bio06
bio12
bio13
CEC
DES
NTO
SOC
TWI

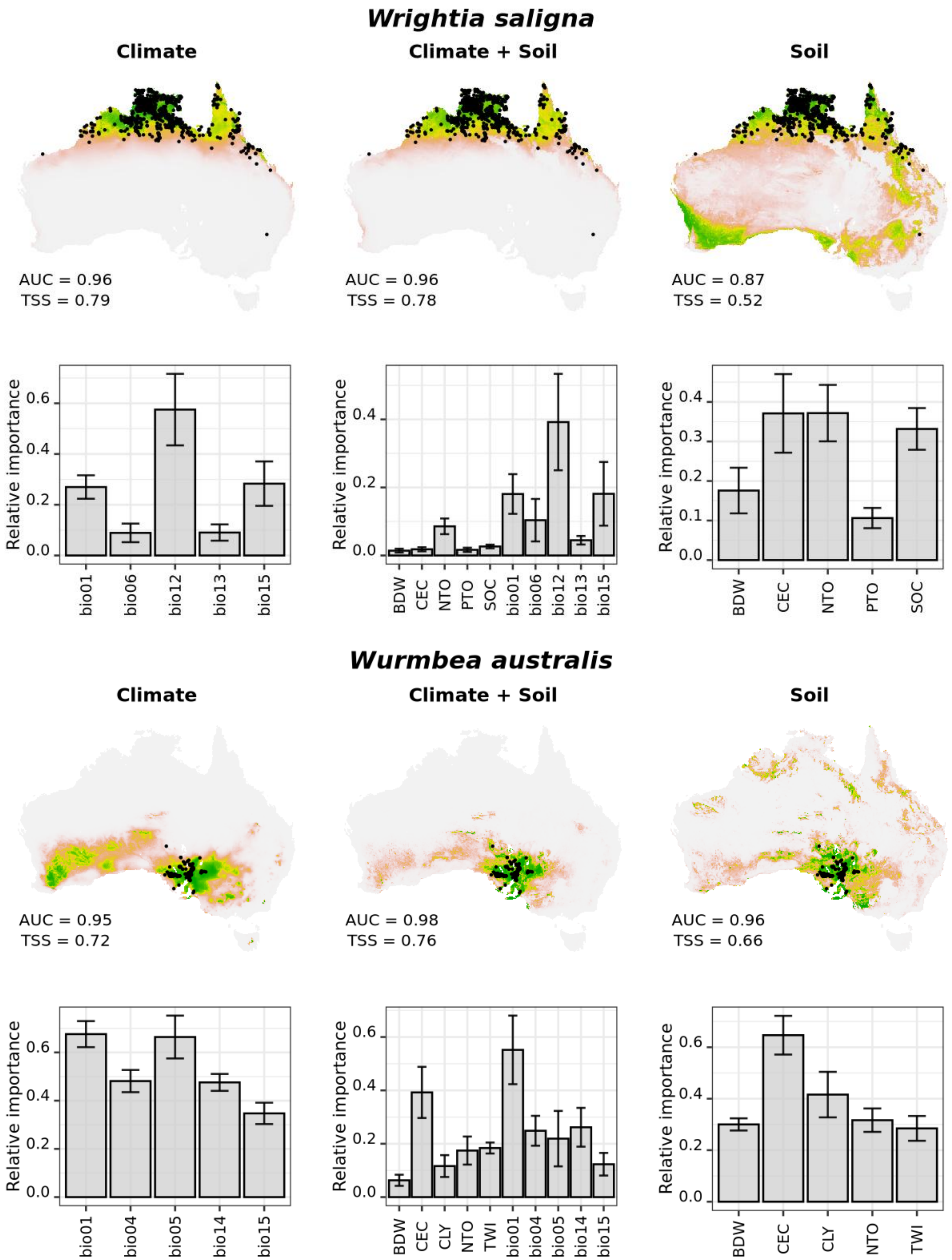

Wrightia saligna
Climate
Climate + Soil
Soil
AUC = 0.96
TSS = 0.79
AUC = 0.96
TSS = 0.78
AUC = 0.87
TSS = 0.52
Relative importance
bio01
bio06
bio12
bio13
bio15
BDW
CEC
NTO
PTO
SOC
Wurmbea australis
Climate
Climate + Soil
Soil
AUC = 0.95
TSS = 0.72
AUC = 0.98
TSS = 0.76
AUC = 0.96
TSS = 0.66
Relative importance
bio01
bio04
bio05
bio14
bio15
BDW
CEC
CLY
NTO
TWI

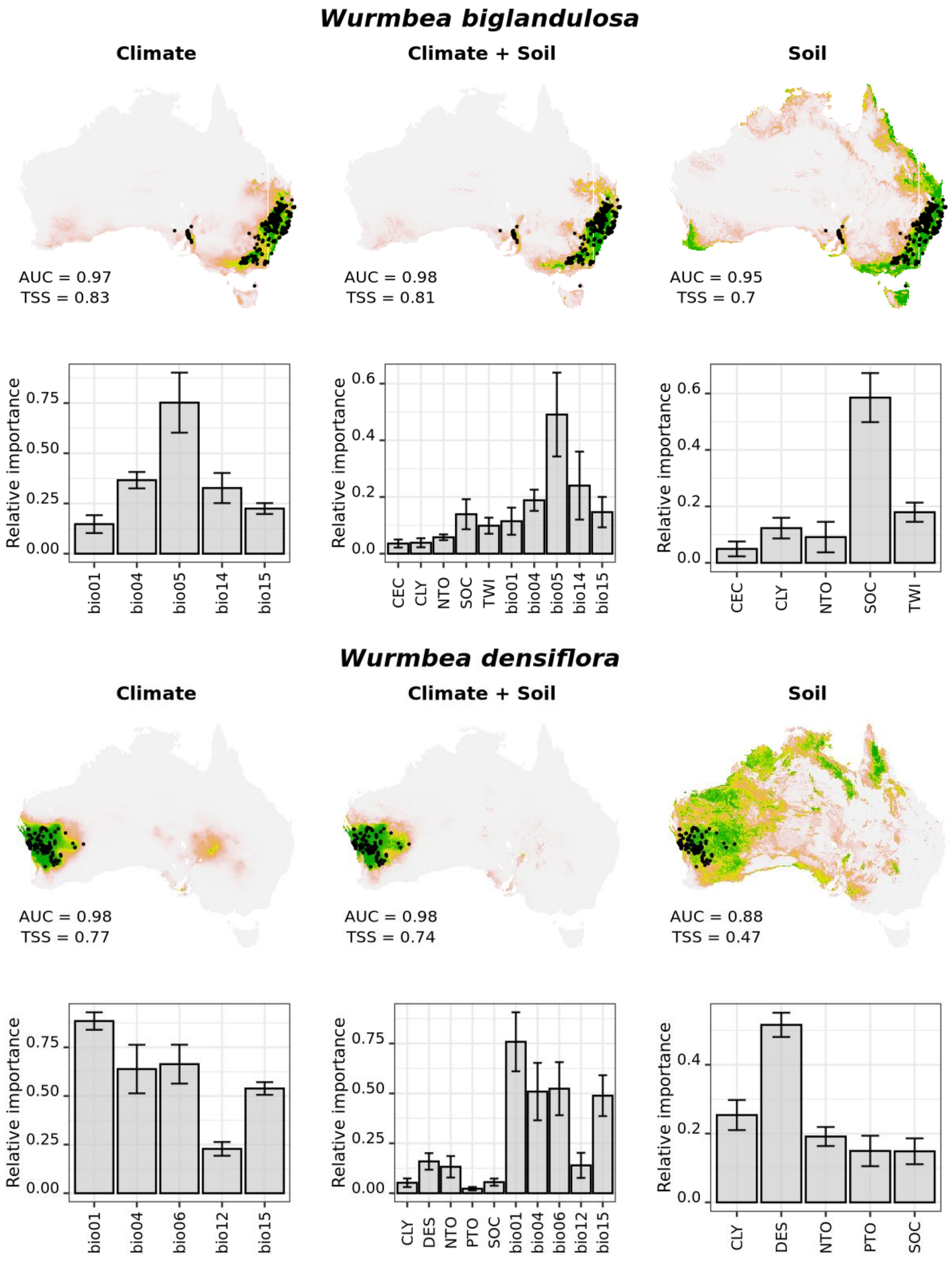
Wurmbea biglandulosa
Climate
Climate + Soil
Soil
AUC = 0.97
TSS = 0.83
AUC = 0.98
TSS = 0.81
AUC = 0.95
TSS = 0.7
Relative importance
0.00
0.25
0.50
0.75
bio01
bio04
bio05
bio14
bio15
0.0
0.2
0.4
0.6
CEC
CLY
NTO
SOC
TWI
bio01
bio04
bio05
bio14
bio15
CEC
CLY
NTO
SOC
TWI
Wurmbea densiflora
Climate
Climate + Soil
Soil
AUC = 0.98
TSS = 0.77
AUC = 0.98
TSS = 0.74
AUC = 0.88
TSS = 0.47
bio01
bio04
bio06
bio12
bio15
CLY
DES
NTO
PTO
SOC
bio01
bio04
bio06
bio12
bio15
CLY
DES
NTO
PTO
SOC

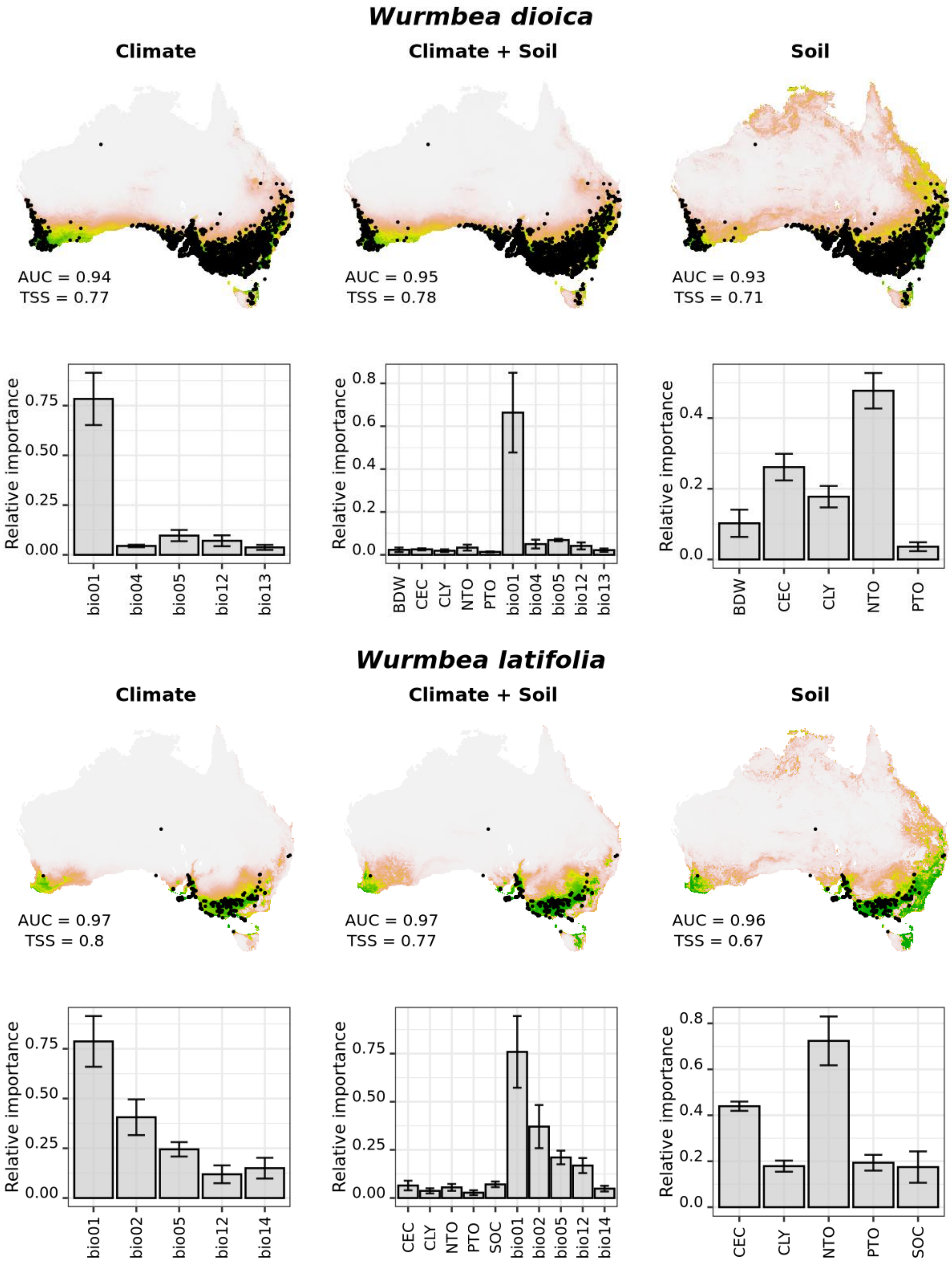
Wurmbea dioica
Climate
Climate + Soil
Soil
AUC = 0.94
TSS = 0.77
AUC = 0.95
TSS = 0.78
AUC = 0.93
TSS = 0.71
Relative importance
0.00
0.25
0.50
0.75
bio01
bio04
bio05
bio12
bio13
0.0
0.2
0.4
0.6
0.8
BDW
CEC
CLY
NTO
PTO
bio01
bio04
bio05
bio12
bio13
0.0
0.2
0.4
BDW
CEC
CLY
NTO
PTO
Wurmbea latifolia
Climate
Climate + Soil
Soil
AUC = 0.97
TSS = 0.8
AUC = 0.97
TSS = 0.77
AUC = 0.96
TSS = 0.67
Relative importance
0.00
0.25
0.50
0.75
bio01
bio02
bio05
bio12
bio14
CEC
CLY
NTO
PTO
SOC
bio01
bio02
bio05
bio12
bio14
0.0
0.2
0.4
0.6
0.8
CEC
CLY
NTO
PTO
SOC

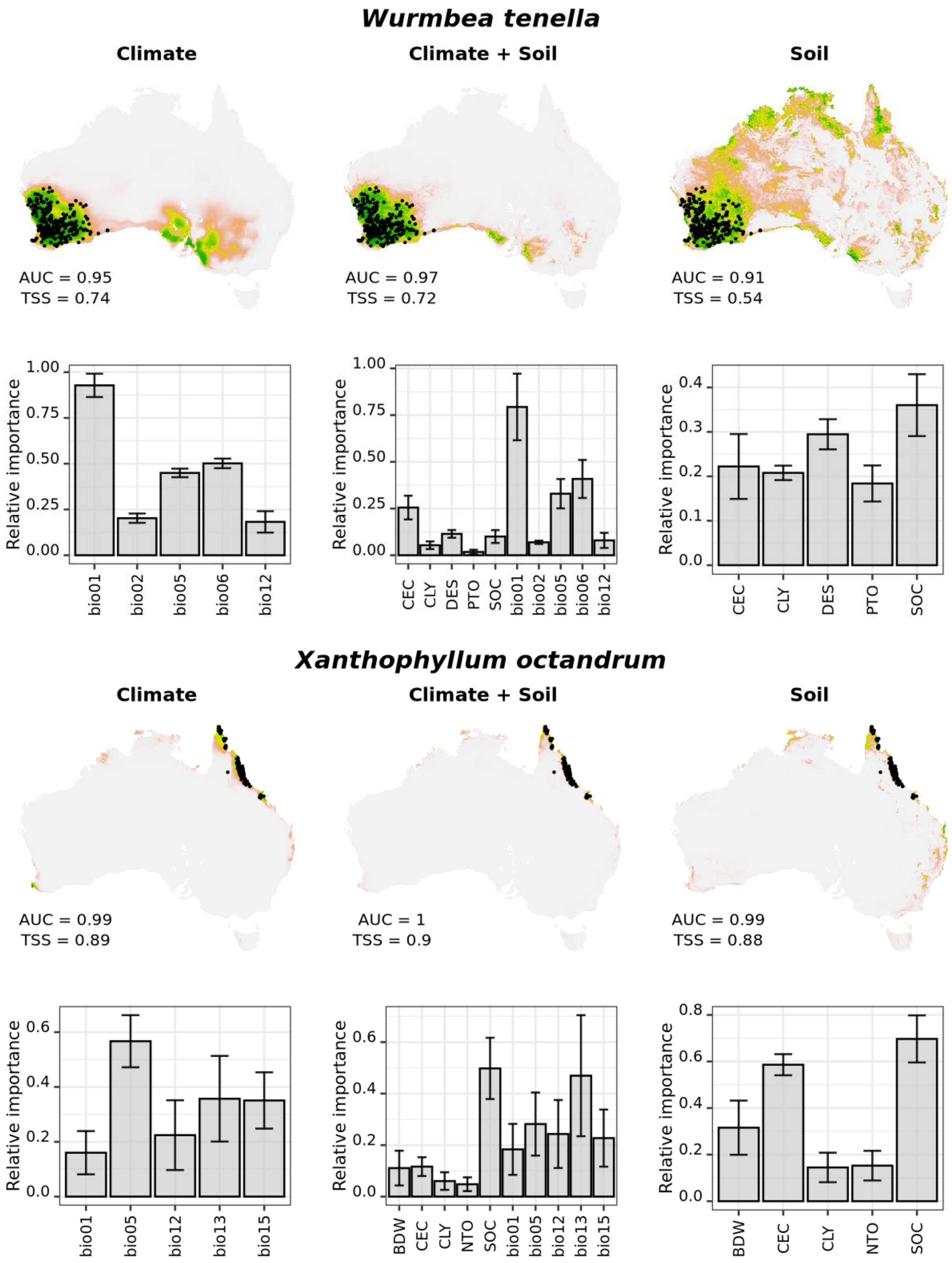

Wurmbea tenella
Climate
Climate + Soil
Soil
AUC = 0.95
TSS = 0.74
AUC = 0.97
TSS = 0.72
AUC = 0.91
TSS = 0.54
Relative importance
bio01 bio02 bio05 bio06 bio12
CEC CLY DES PTO SOC bio01 bio02 bio05 bio06 bio12
CEC CLY DES PTO SOC
Xanthophyllum octandrum
Climate
Climate + Soil
Soil
AUC = 0.99
TSS = 0.89
AUC = 1
TSS = 0.9
AUC = 0.99
TSS = 0.88
Relative importance
bio01 bio05 bio12 bio13 bio15
BDW CEC CLY NTO SOC bio01 bio05 bio12 bio13 bio15
BDW CEC CLY NTO SOC

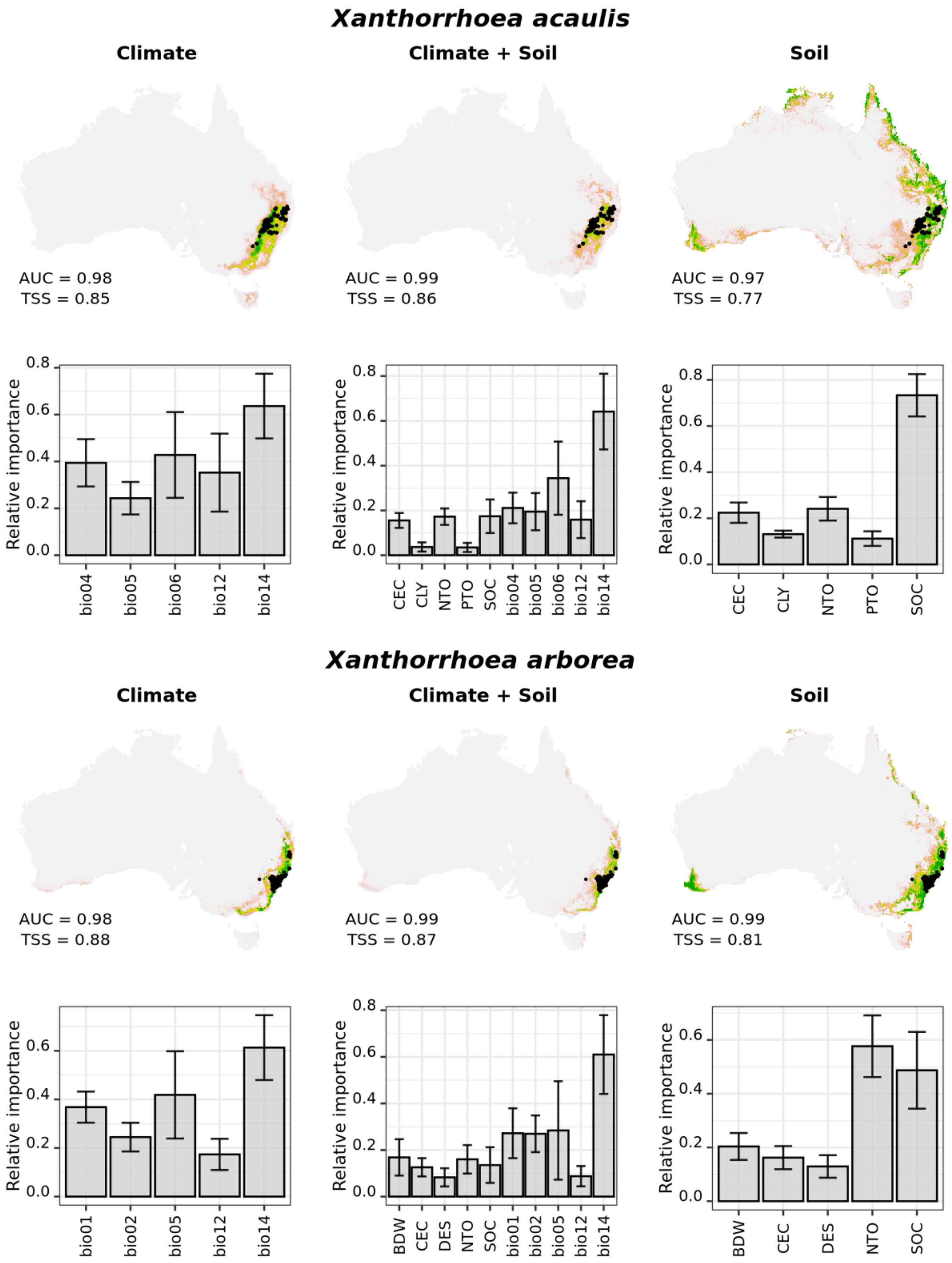
Xanthorrhoea acaulis
Climate
Climate + Soil
Soil
AUC = 0.98
TSS = 0.85
AUC = 0.99
TSS = 0.86
AUC = 0.97
TSS = 0.77
Relative importance
bio04
bio05
bio06
bio12
bio14
CEC
CLY
NTO
PTO
SOC
bio04
bio05
bio06
bio12
bio14
CEC
CLY
NTO
PTO
SOC
Xanthorrhoea arborea
Climate
Climate + Soil
Soil
AUC = 0.98
TSS = 0.88
AUC = 0.99
TSS = 0.87
AUC = 0.99
TSS = 0.81
Relative importance
bio01
bio02
bio05
bio12
bio14
BDW
CEC
DES
NTO
SOC
bio01
bio02
bio05
bio12
bio14
BDW
CEC
DES
NTO
SOC

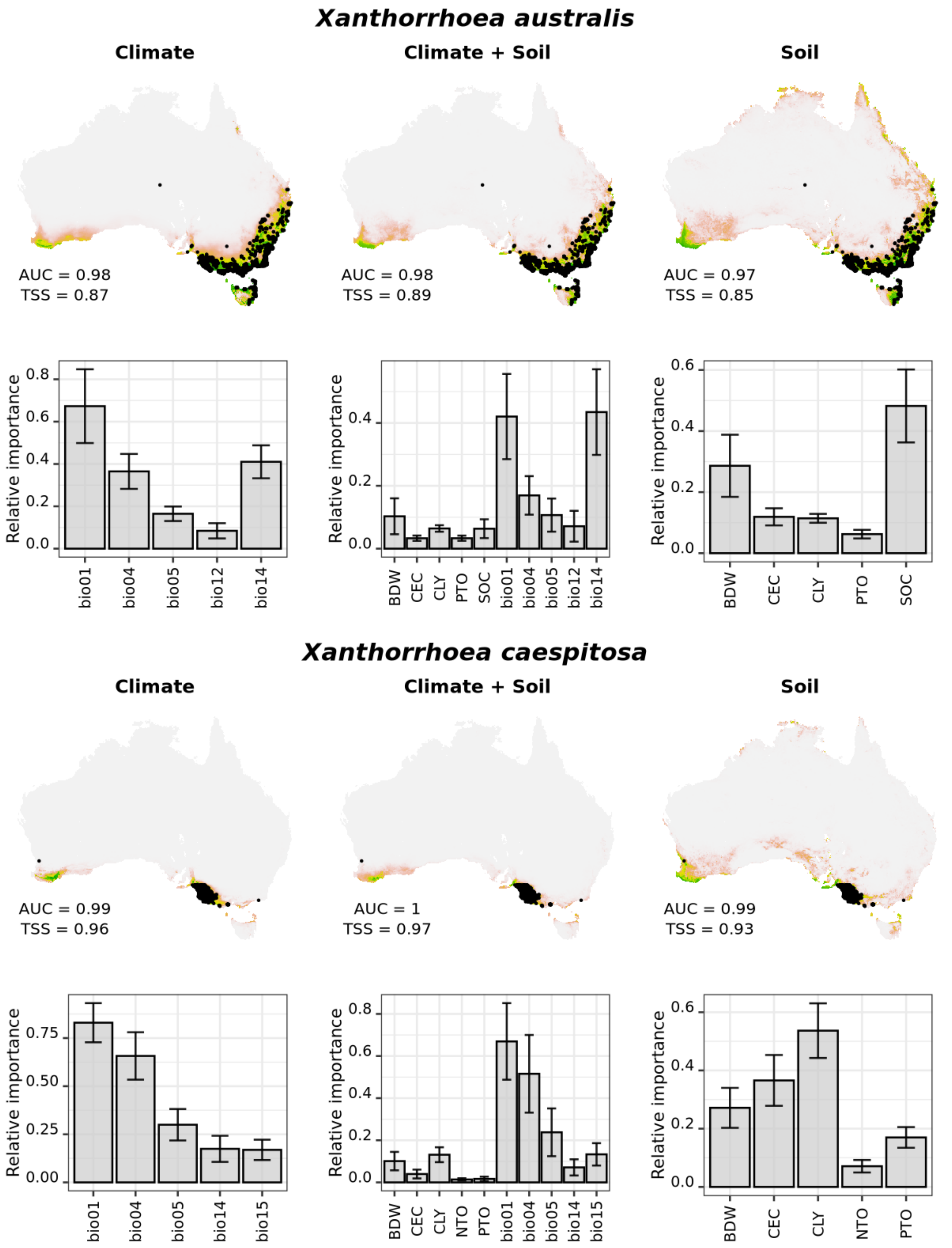
Xanthorrhoea australis
Climate
Climate + Soil
Soil
AUC = 0.98
TSS = 0.87
AUC = 0.98
TSS = 0.89
AUC = 0.97
TSS = 0.85
Relative importance
0.0 0.2 0.4 0.6 0.8
bio01 bio04 bio05 bio12 bio14
0.0 0.2 0.4
BDW CEC CLY PTO SOC bio01 bio04 bio05 bio12 bio14
0.0 0.2 0.4 0.6
BDW CEC CLY PTO SOC
Xanthorrhoea caespitosa
Climate
Climate + Soil
Soil
AUC = 0.99
TSS = 0.96
AUC = 1
TSS = 0.97
AUC = 0.99
TSS = 0.93
0.00 0.25 0.50 0.75
bio01 bio04 bio05 bio14 bio15
0.0 0.2 0.4 0.6 0.8
BDW CEC CLY NTO PTO bio01 bio04 bio05 bio14 bio15
0.0 0.2 0.4 0.6
BDW CEC CLY NTO PTO

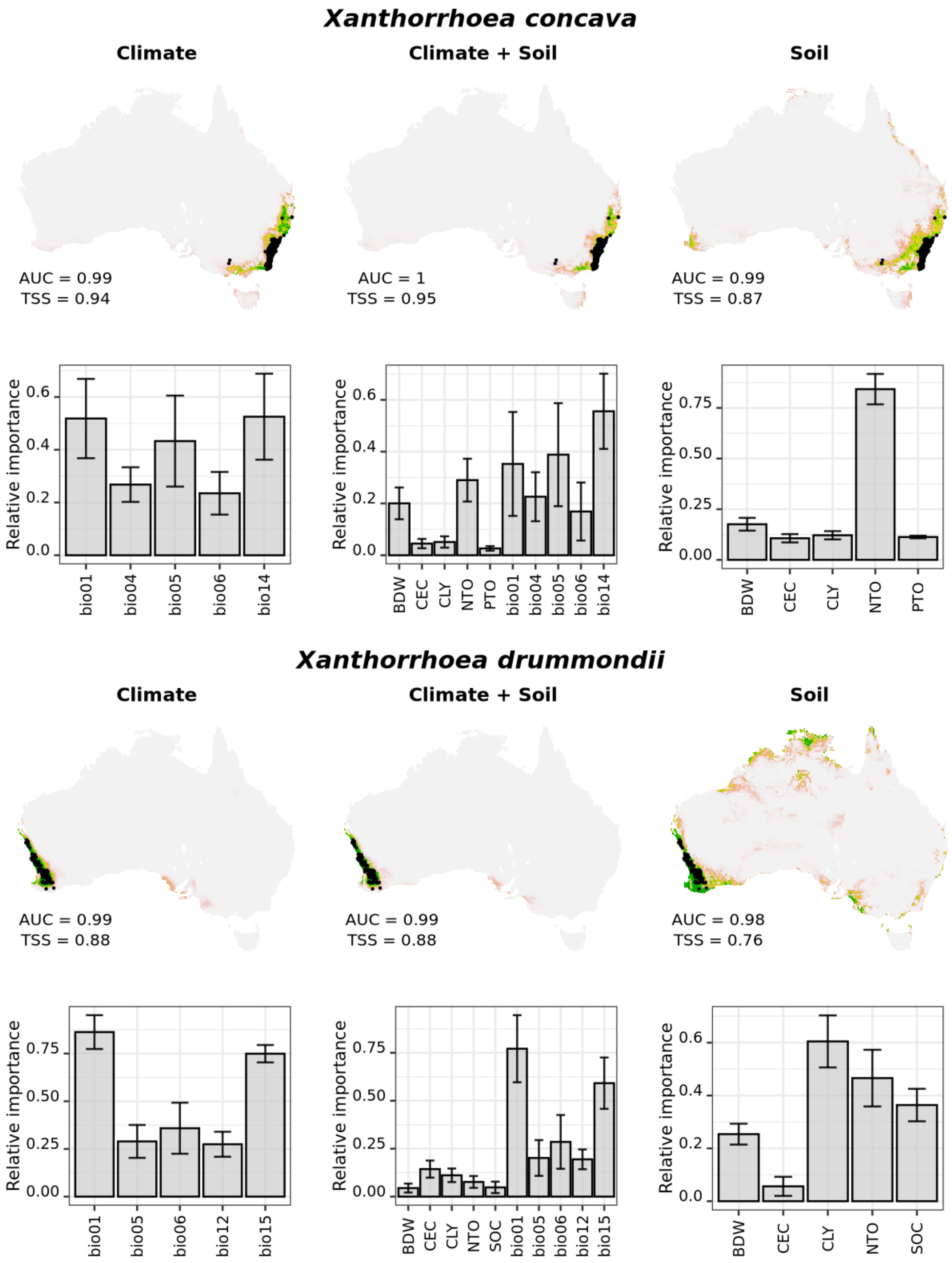

Xanthorrhoea concava
Climate
Climate + Soil
Soil
AUC = 0.99
TSS = 0.94
AUC = 1
TSS = 0.95
AUC = 0.99
TSS = 0.87
Relative importance
bio01
bio04
bio05
bio06
bio14
BDW
CEC
CLY
NTO
PTO
Xanthorrhoea drummondii
Climate
Climate + Soil
Soil
AUC = 0.99
TSS = 0.88
AUC = 0.99
TSS = 0.88
AUC = 0.98
TSS = 0.76
Relative importance
bio01
bio05
bio06
bio12
bio15
BDW
CEC
CLY
NTO
SOC

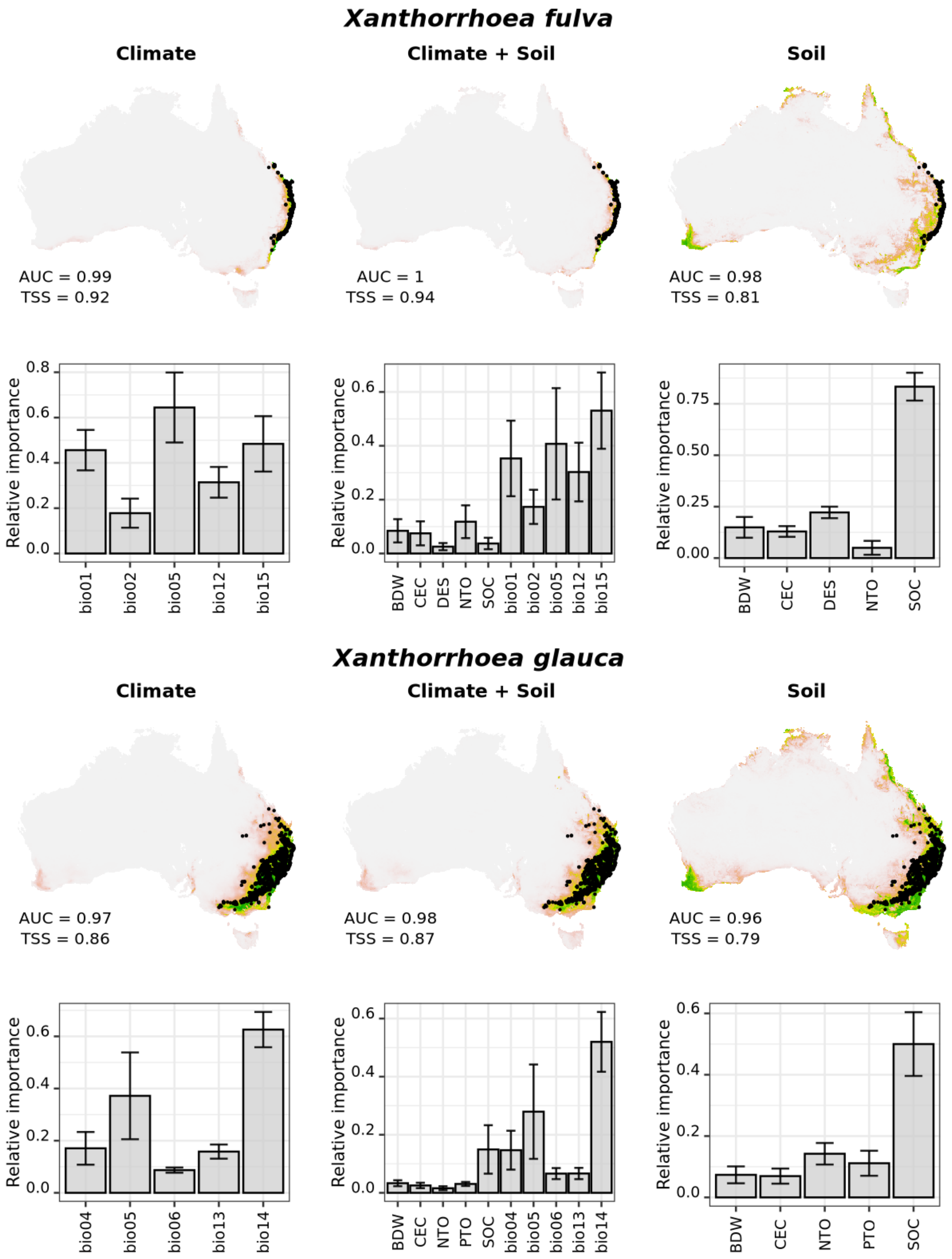
Xanthorrhoea fulva
Climate
Climate + Soil
Soil
AUC = 0.99
TSS = 0.92
AUC = 1
TSS = 0.94
AUC = 0.98
TSS = 0.81
Relative importance
bio01
bio02
bio05
bio12
bio15
BDW
CEC
DES
NTO
SOC
Xanthorrhoea glauca
Climate
Climate + Soil
Soil
AUC = 0.97
TSS = 0.86
AUC = 0.98
TSS = 0.87
AUC = 0.96
TSS = 0.79
bio04
bio05
bio06
bio13
bio14
PTO

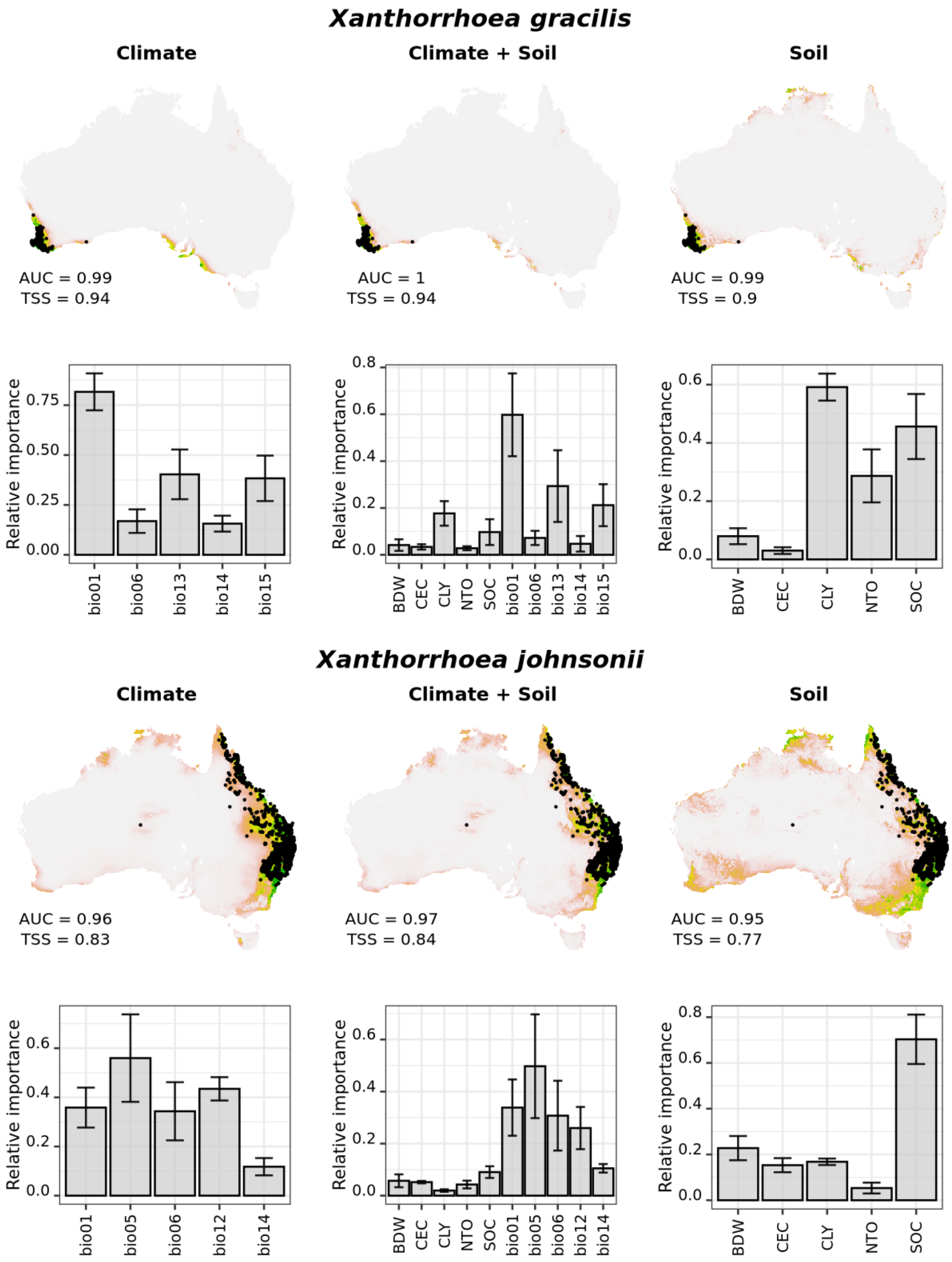
Xanthorrhoea gracilis
Climate
Climate + Soil
Soil
AUC = 0.99
TSS = 0.94
AUC = 1
TSS = 0.94
AUC = 0.99
TSS = 0.9
Relative importance
bio01
bio06
bio13
bio14
bio15
BDW
CEC
CLY
NTO
SOC
Xanthorrhoea johnsonii
Climate
Climate + Soil
Soil
AUC = 0.96
TSS = 0.83
AUC = 0.97
TSS = 0.84
AUC = 0.95
TSS = 0.77
Relative importance
bio01
bio05
bio06
bio12
bio14
BDW
CEC
CLY
NTO
SOC

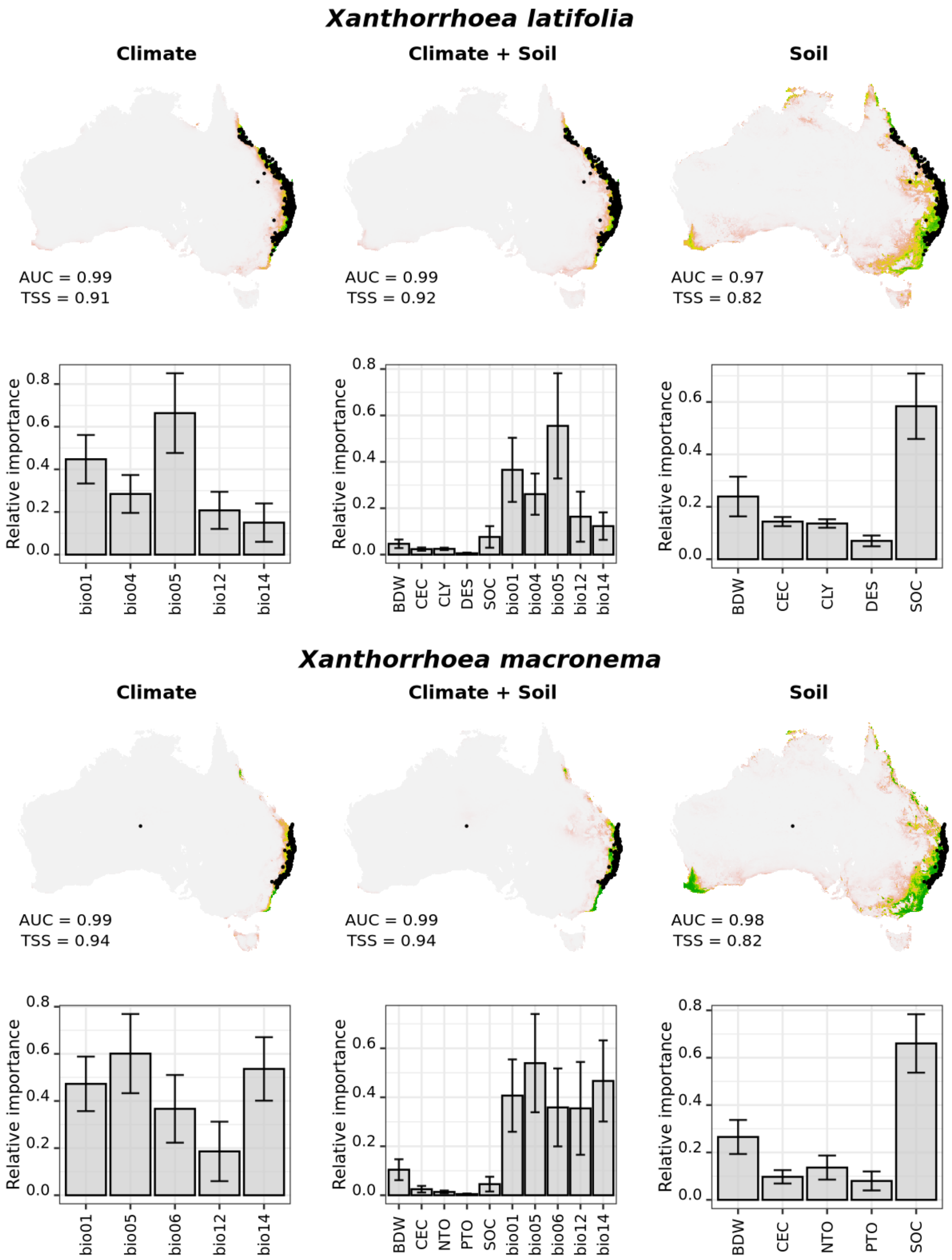

Xanthorrhoea latifolia
Climate
Climate + Soil
Soil
AUC = 0.99
TSS = 0.91
AUC = 0.99
TSS = 0.92
AUC = 0.97
TSS = 0.82
Relative importance
0.0
0.2
0.4
0.6
0.8
bio01
bio04
bio05
bio12
bio14
BDW
CEC
CLY
DES
SOC
Xanthorrhoea macronema
Climate
Climate + Soil
Soil
AUC = 0.99
TSS = 0.94
AUC = 0.99
TSS = 0.94
AUC = 0.98
TSS = 0.82
Relative importance
bio01
bio05
bio06
bio12
bio14
BDW
CEC
NTO
PTO
SOC

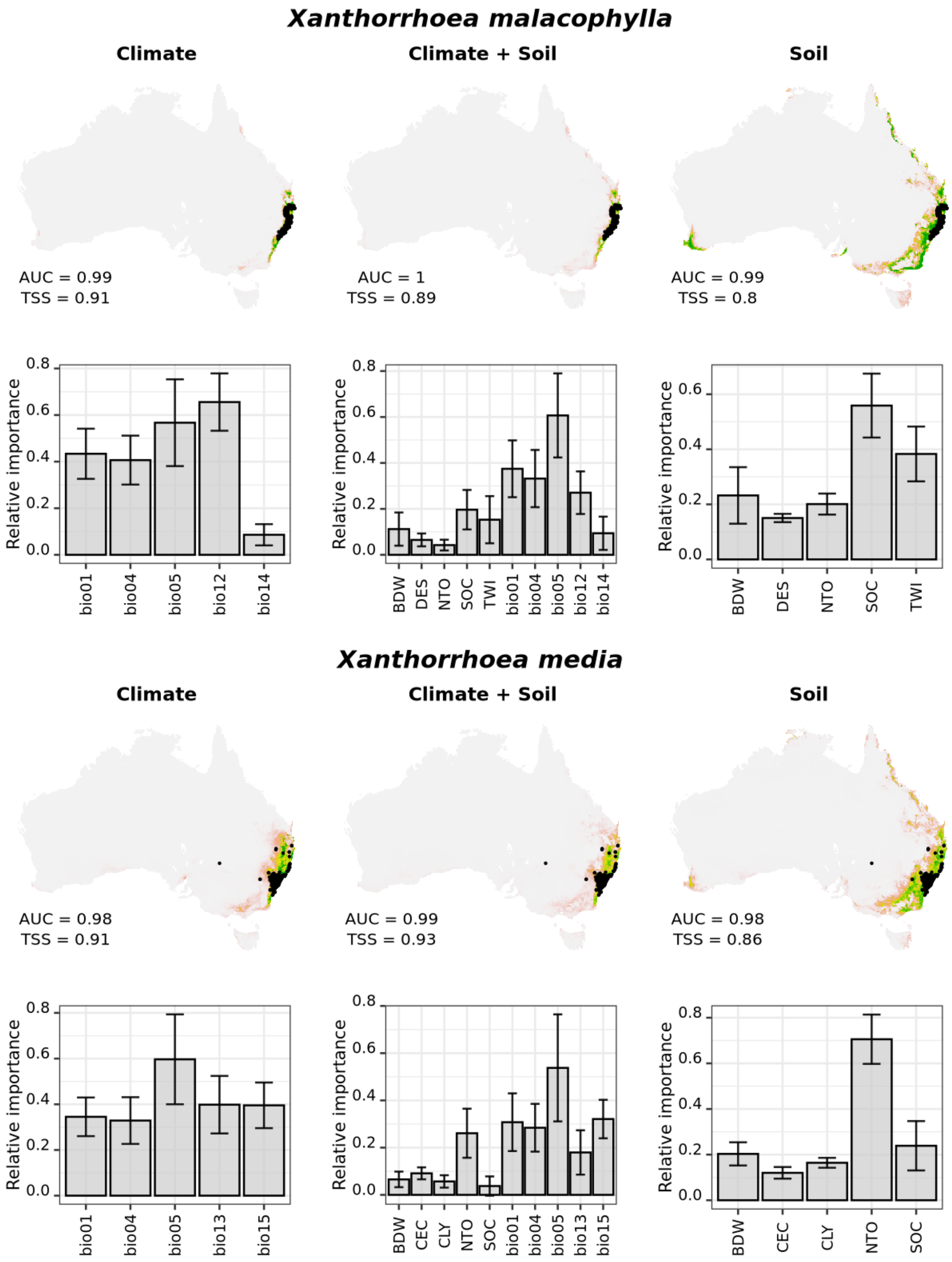
Xanthorrhoea malacophylla
Climate
Climate + Soil
Soil
AUC = 0.99
TSS = 0.91
AUC = 1
TSS = 0.89
AUC = 0.99
TSS = 0.8
Relative importance
0.0
0.2
0.4
0.6
0.8
bio01
bio04
bio05
bio12
bio14
BDW
DES
NTO
SOC
TWI
Xanthorrhoea media
AUC = 0.98
TSS = 0.91
AUC = 0.99
TSS = 0.93
AUC = 0.98
TSS = 0.86
bio13
bio15
CEC
CLY

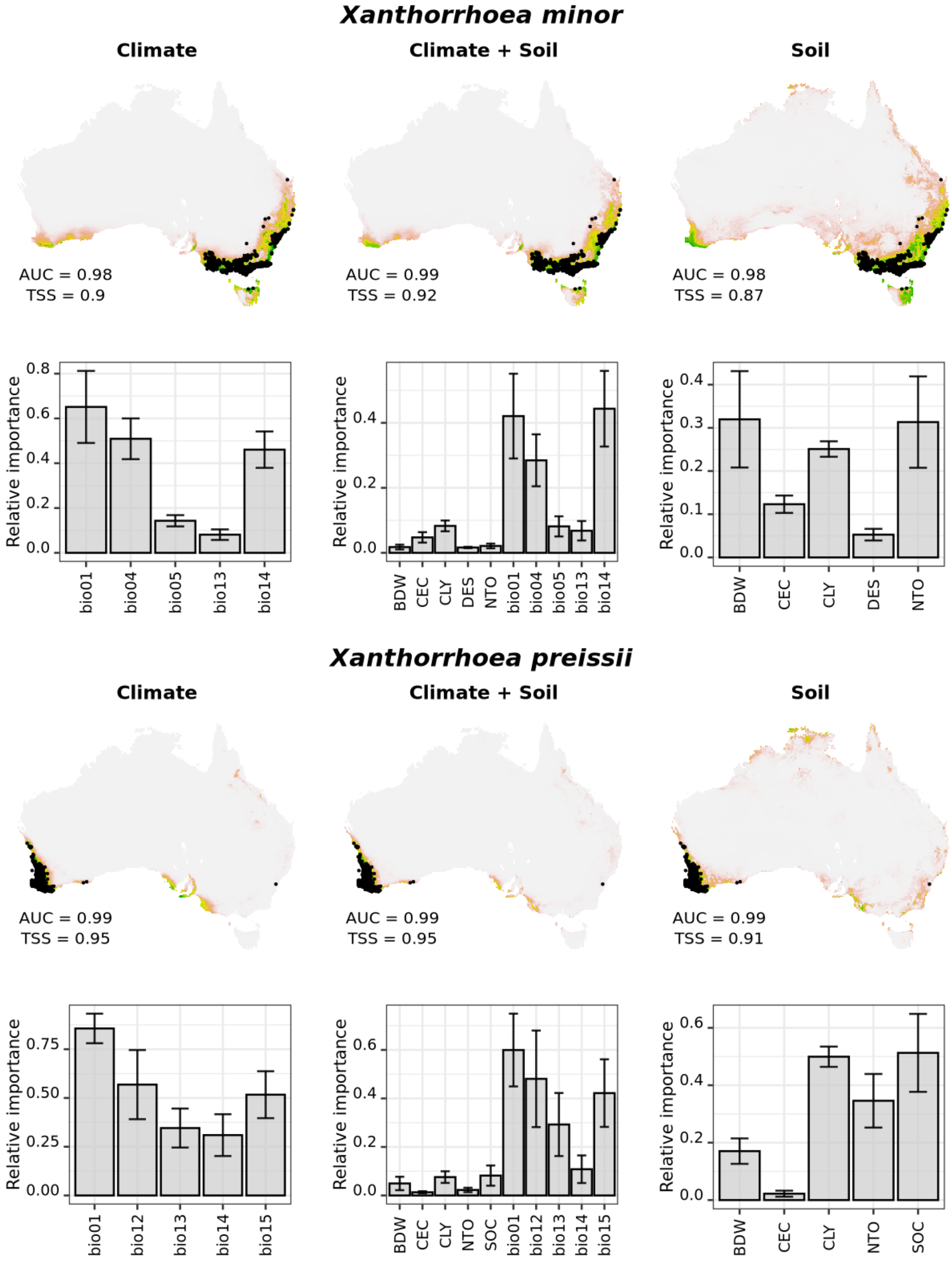

Xanthorrhoea minor
Climate
Climate + Soil
Soil
AUC = 0.98
TSS = 0.9
AUC = 0.99
TSS = 0.92
AUC = 0.98
TSS = 0.87
Relative importance
bio01 bio04 bio05 bio13 bio14
BDW CEC CLY DES NTO bio01 bio04 bio05 bio13 bio14
BDW CEC CLY DES NTO
Xanthorrhoea preissii
Climate
Climate + Soil
Soil
AUC = 0.99
TSS = 0.95
AUC = 0.99
TSS = 0.95
AUC = 0.99
TSS = 0.91
Relative importance
bio01 bio12 bio13 bio14 bio15
BDW CEC CLY NTO SOC bio01 bio12 bio13 bio14 bio15
BDW CEC CLY NTO SOC

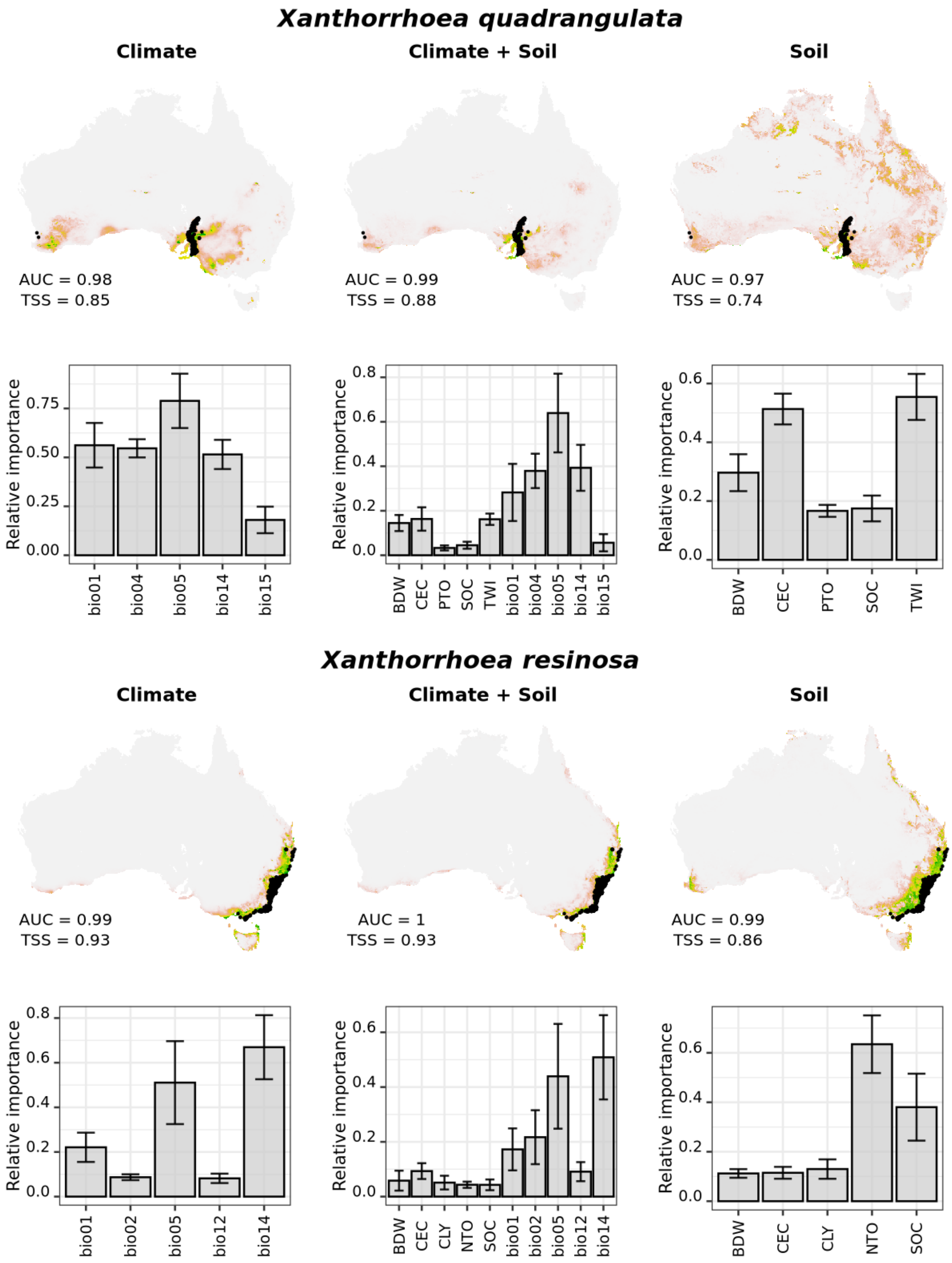

Xanthorrhoea quadrangulata
Climate
Climate + Soil
Soil
AUC = 0.98
TSS = 0.85
AUC = 0.99
TSS = 0.88
AUC = 0.97
TSS = 0.74
Relative importance
bio01
bio04
bio05
bio14
bio15
BDW
CEC
PTO
SOC
TWI
Xanthorrhoea resinosa
Climate
Climate + Soil
Soil
AUC = 0.99
TSS = 0.93
AUC = 1
TSS = 0.93
AUC = 0.99
TSS = 0.86
Relative importance
bio01
bio02
bio05
bio12
bio14
BDW
CEC
CLY
NTO
SOC

Xanthorrhoea semiplana

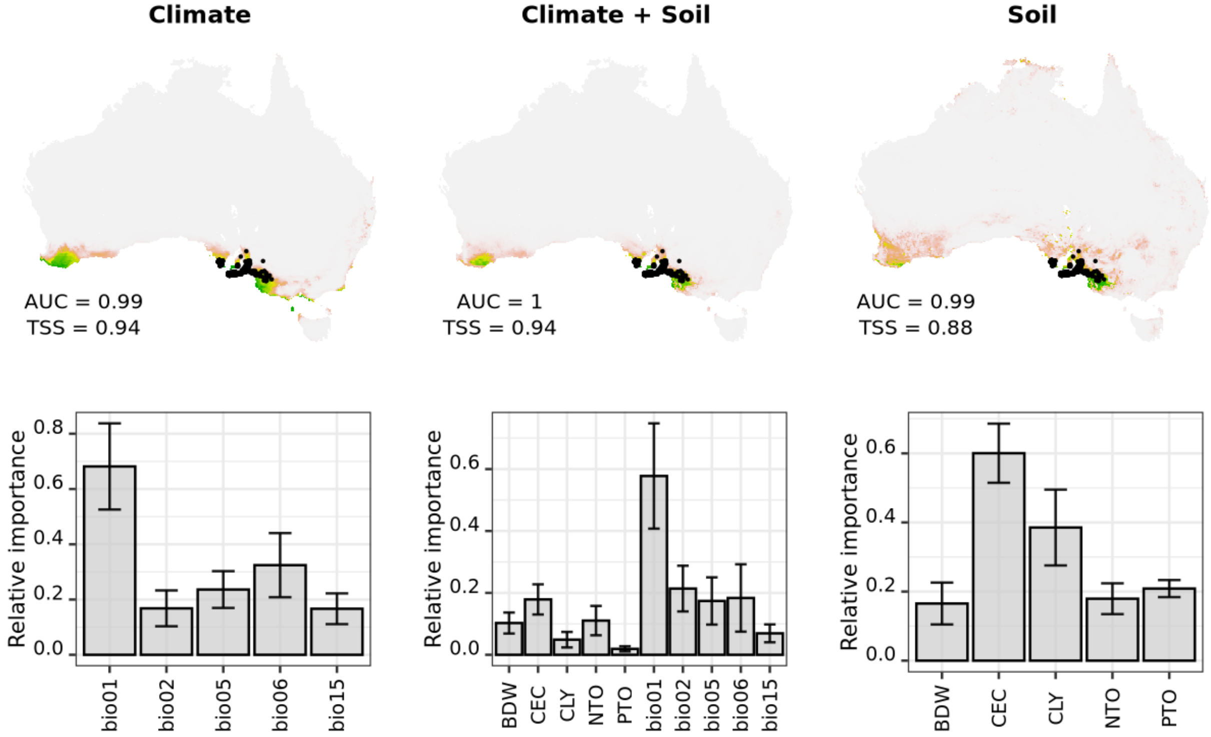

Xanthosia atkinsoniana

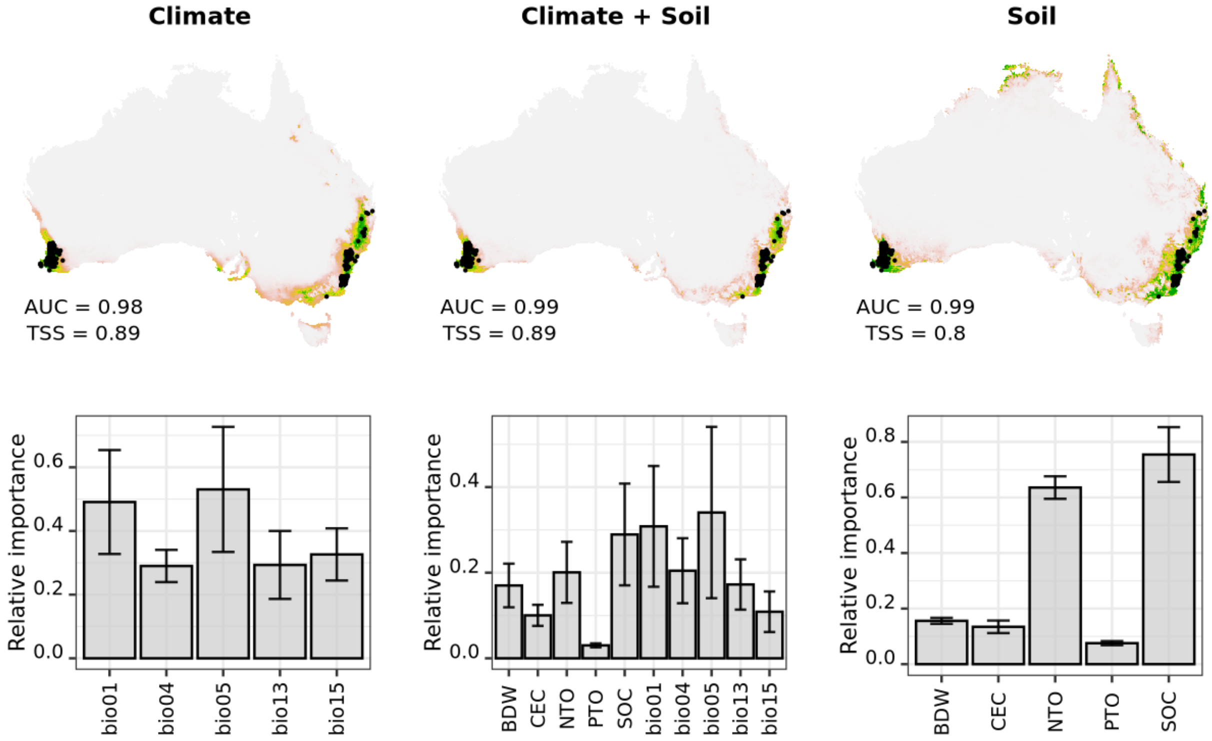

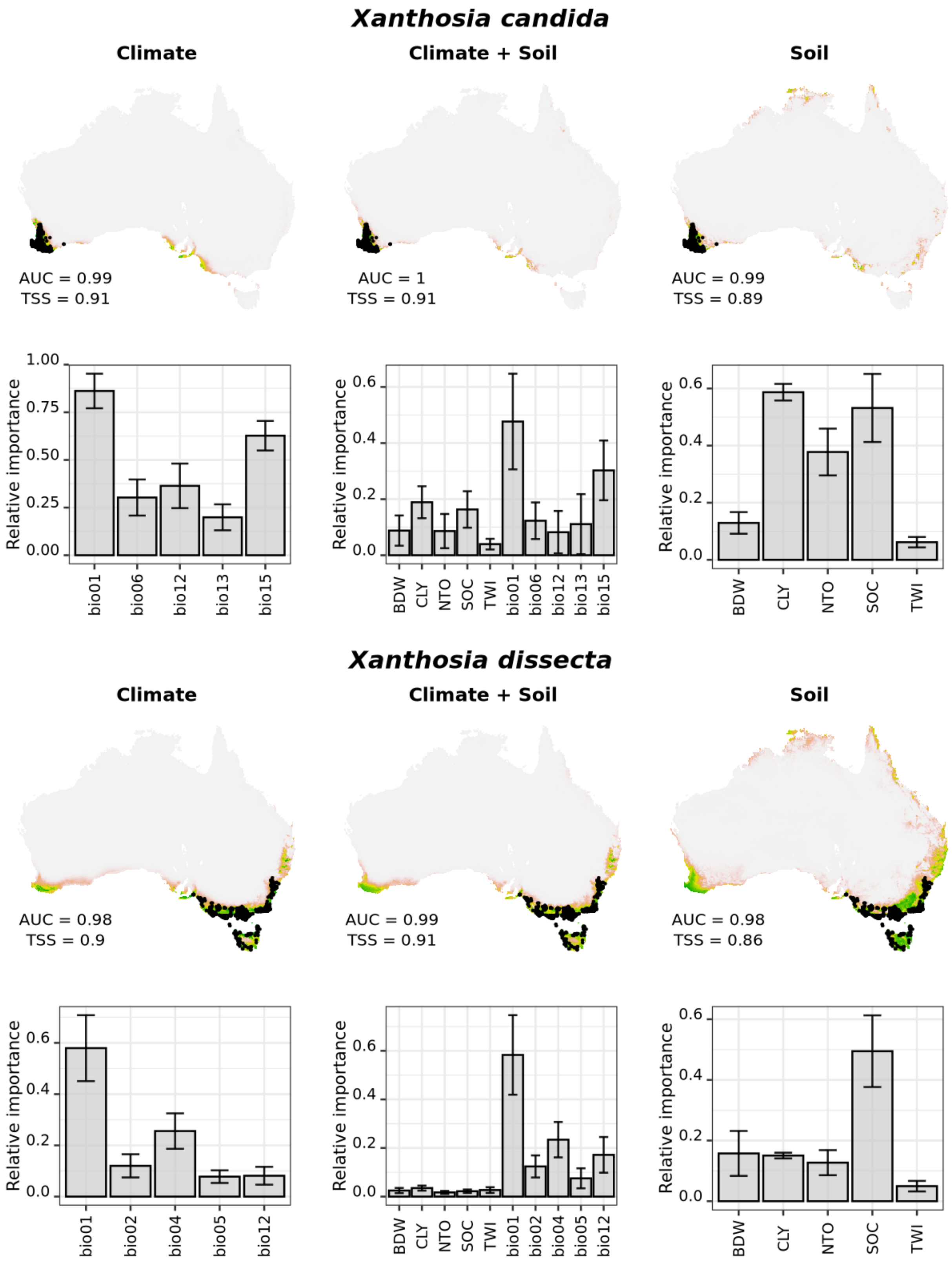

Xanthosia candida
Climate
Climate + Soil
Soil
AUC = 0.99
TSS = 0.91
AUC = 1
TSS = 0.91
AUC = 0.99
TSS = 0.89
Relative importance
1.00
0.75
0.50
0.25
0.00
bio01
bio06
bio12
bio13
bio15
0.6
0.4
0.2
0.0
BDW
CLY
NTO
SOC
TWI
Xanthosia dissecta
AUC = 0.98
TSS = 0.9
AUC = 0.99
TSS = 0.91
AUC = 0.98
TSS = 0.86
bio02
bio04
bio05

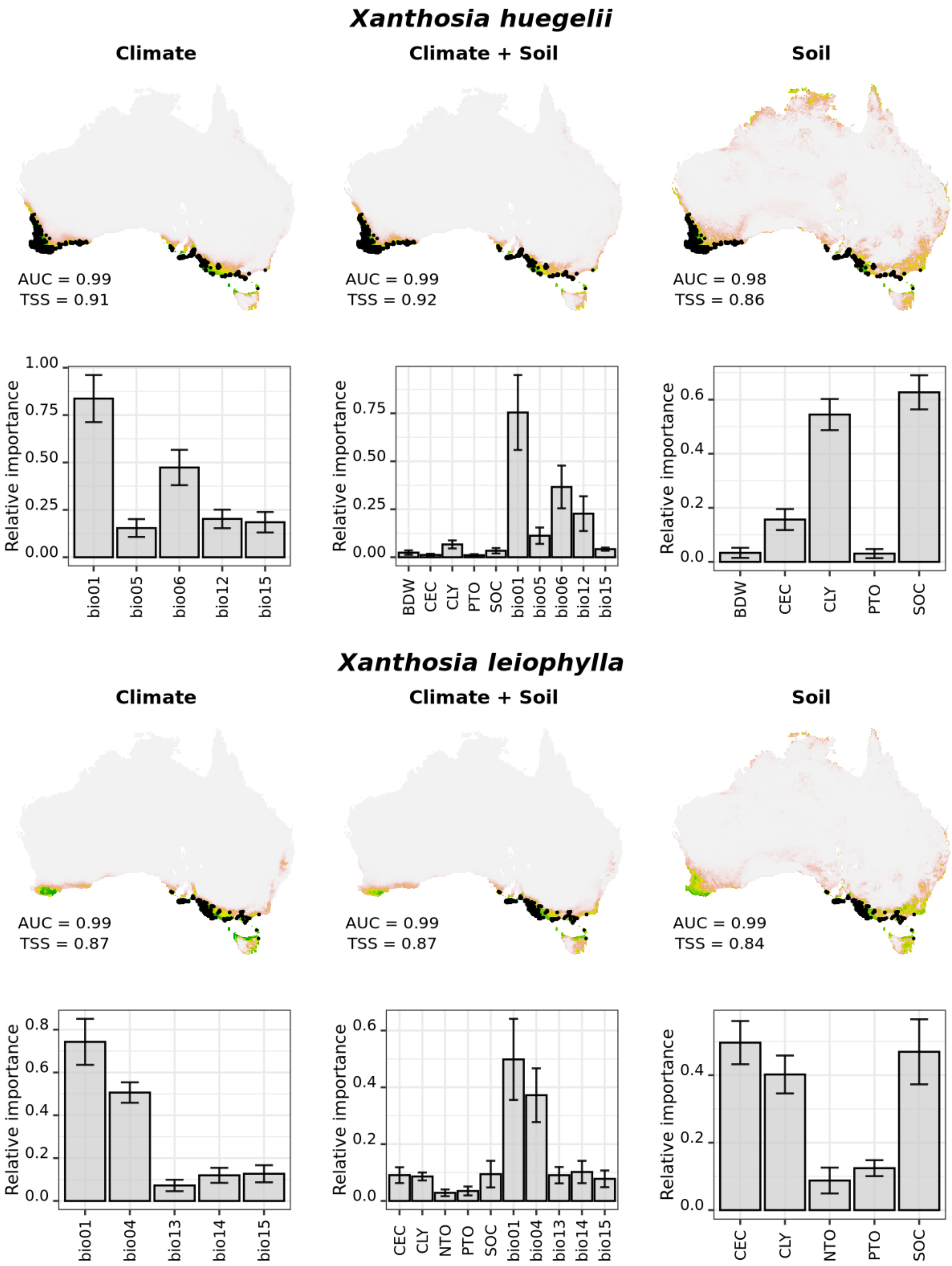
Xanthosia huegelii
Climate
Climate + Soil
Soil
AUC = 0.99
TSS = 0.91
AUC = 0.99
TSS = 0.92
AUC = 0.98
TSS = 0.86
Relative importance
1.00
0.75
0.50
0.25
0.00
bio01
bio05
bio06
bio12
bio15
BDW
CEC
CLY
PTO
SOC
0.6
0.4
0.2
0.0
Xanthosia leiophylla
Climate
Climate + Soil
Soil
AUC = 0.99
TSS = 0.87
AUC = 0.99
TSS = 0.87
AUC = 0.99
TSS = 0.84
0.8
bio04
bio13
bio14
NTO

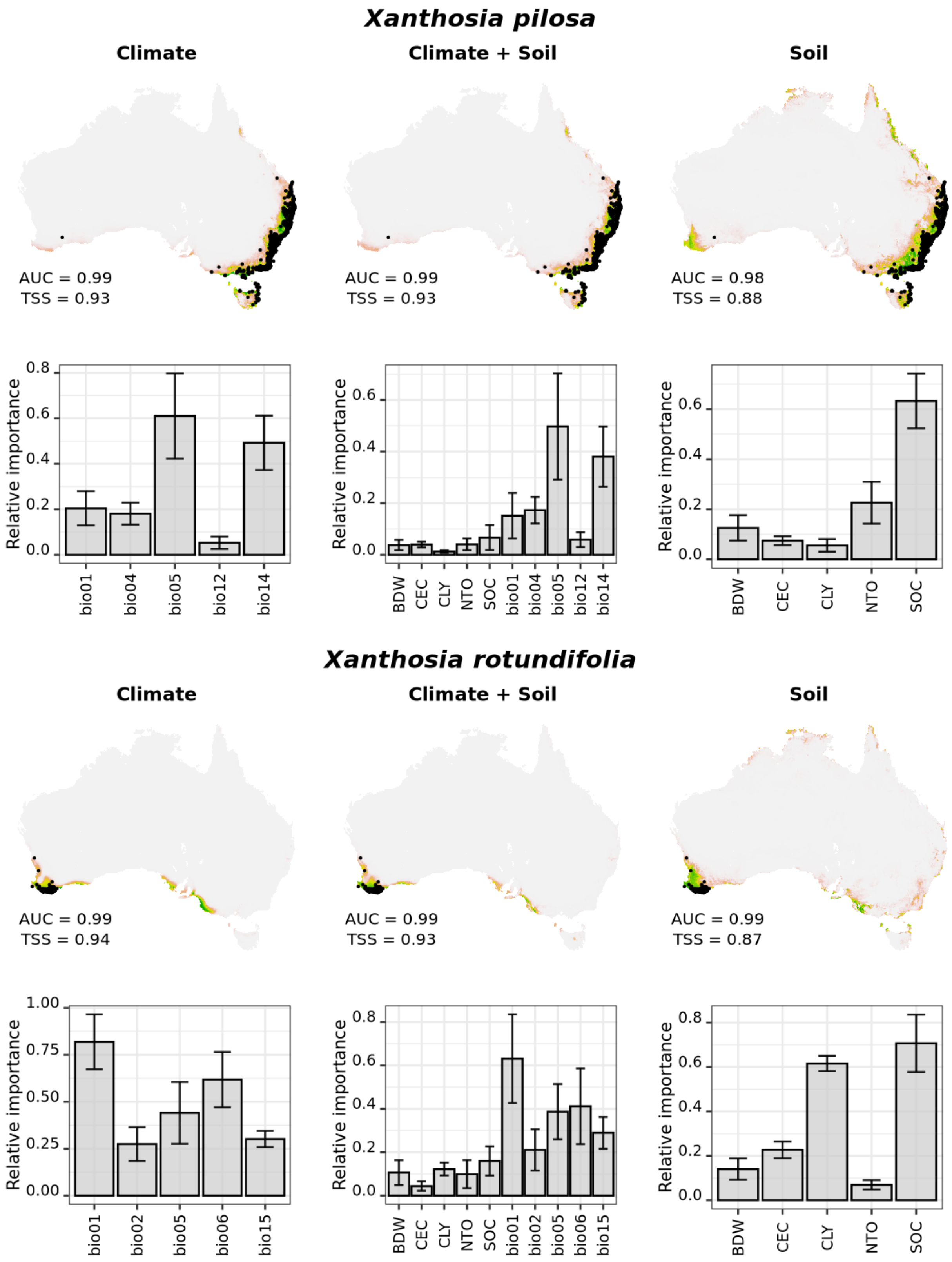
Xanthosia pilosa
Climate
Climate + Soil
Soil
AUC = 0.99
TSS = 0.93
AUC = 0.99
TSS = 0.93
AUC = 0.98
TSS = 0.88
Relative importance
0.0
0.2
0.4
0.6
0.8
bio01
bio04
bio05
bio12
bio14
BDW
CEC
CLY
NTO
SOC
Xanthosia rotundifolia
Climate
Climate + Soil
Soil
AUC = 0.99
TSS = 0.94
AUC = 0.99
TSS = 0.93
AUC = 0.99
TSS = 0.87
Relative importance
0.00
0.25
0.50
0.75
1.00
bio01
bio02
bio05
bio06
bio15
BDW
CEC
CLY
NTO
SOC

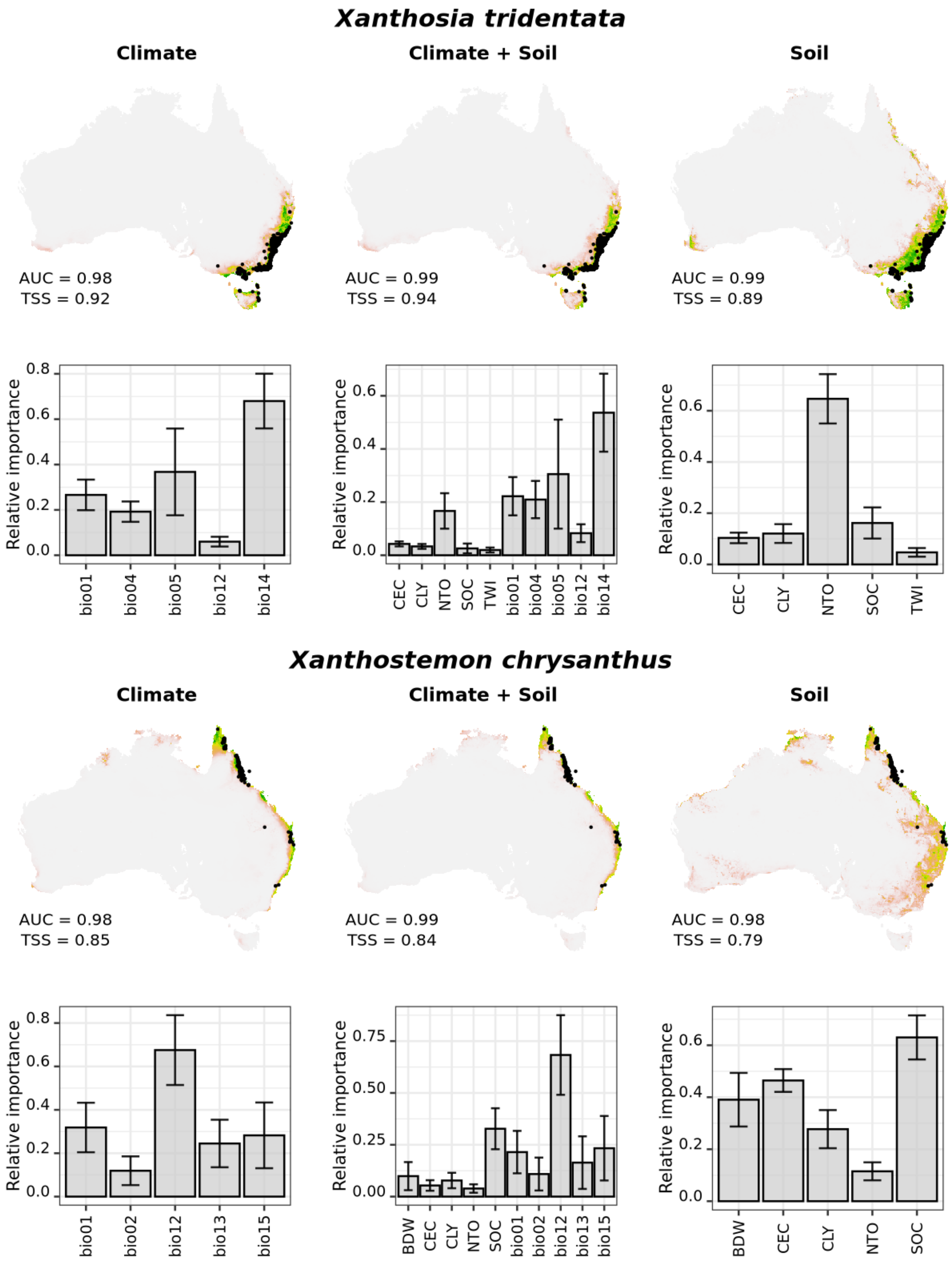

Xanthosia tridentata
Climate
Climate + Soil
Soil
AUC = 0.98
TSS = 0.92
AUC = 0.99
TSS = 0.94
AUC = 0.99
TSS = 0.89
Relative importance
0.0
0.2
0.4
0.6
0.8
bio01
bio04
bio05
bio12
bio14
Relative importance
0.0
0.2
0.4
0.6
CEC
CLY
NTO
SOC
TWI
bio01
bio04
bio05
bio12
bio14
Relative importance
0.0
0.2
0.4
0.6
CEC
CLY
NTO
SOC
TWI
Xanthostemon chrysanthus
Climate
Climate + Soil
Soil
AUC = 0.98
TSS = 0.85
AUC = 0.99
TSS = 0.84
AUC = 0.98
TSS = 0.79
Relative importance
0.0
0.2
0.4
0.6
0.8
bio01
bio02
bio12
bio13
bio15
Relative importance
0.00
0.25
0.50
0.75
BDW
CEC
CLY
NTO
SOC
bio01
bio02
bio12
bio13
bio15
Relative importance
0.0
0.2
0.4
0.6
BDW
CEC
CLY
NTO
SOC

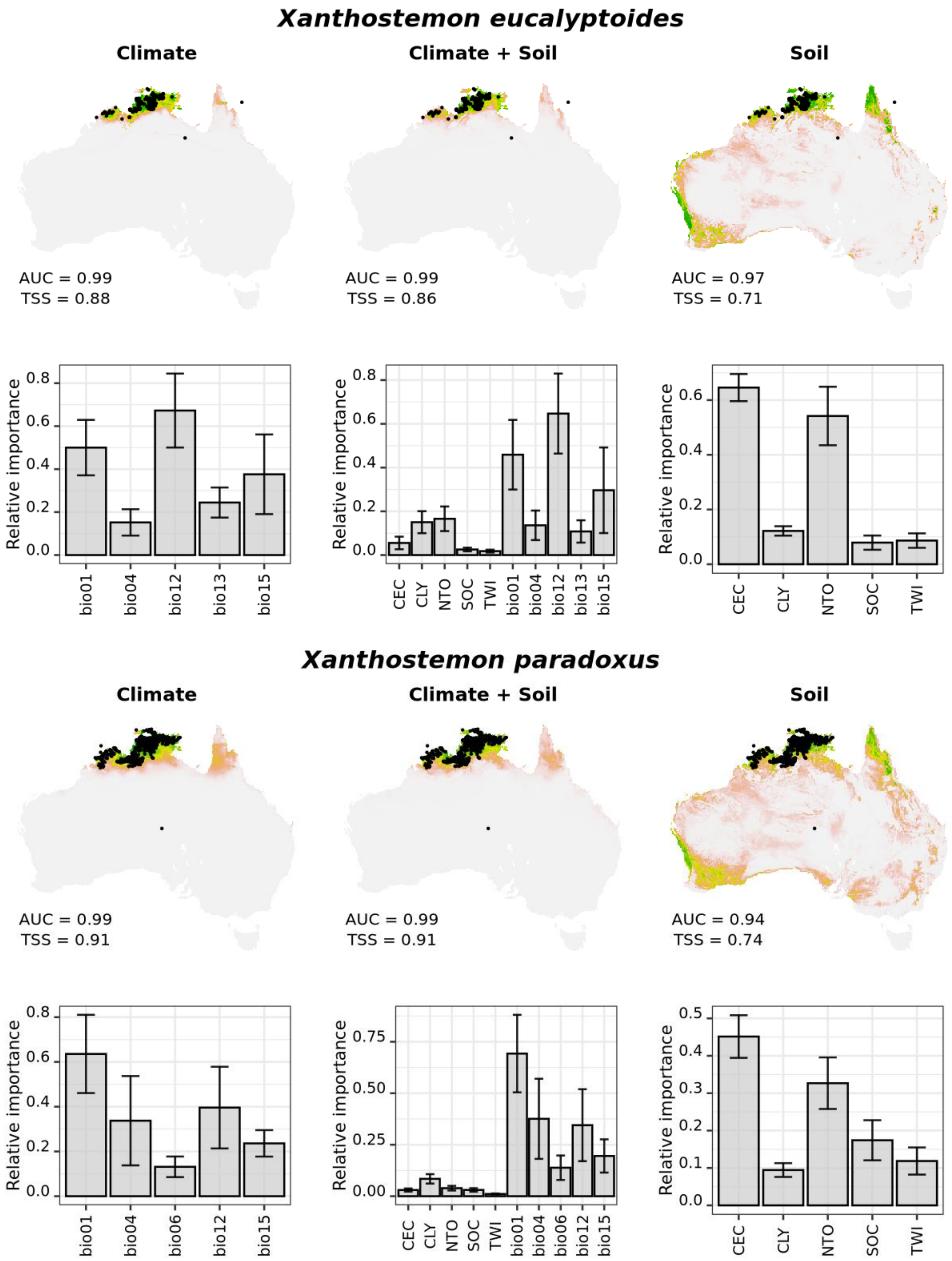
Xanthostemon eucalyptoides
Climate
Climate + Soil
Soil
AUC = 0.99
TSS = 0.88
AUC = 0.99
TSS = 0.86
AUC = 0.97
TSS = 0.71
Relative importance
0.0
0.2
0.4
0.6
0.8
bio01
bio04
bio12
bio13
bio15
CEC
CLY
NTO
SOC
TWI
Xanthostemon paradoxus
Climate
Climate + Soil
Soil
AUC = 0.99
TSS = 0.91
AUC = 0.99
TSS = 0.91
AUC = 0.94
TSS = 0.74
Relative importance
0.00
0.25
0.50
0.75
0.1
0.3
0.5
bio01
bio04
bio06
bio12
bio15
CEC
CLY
NTO
SOC
TWI

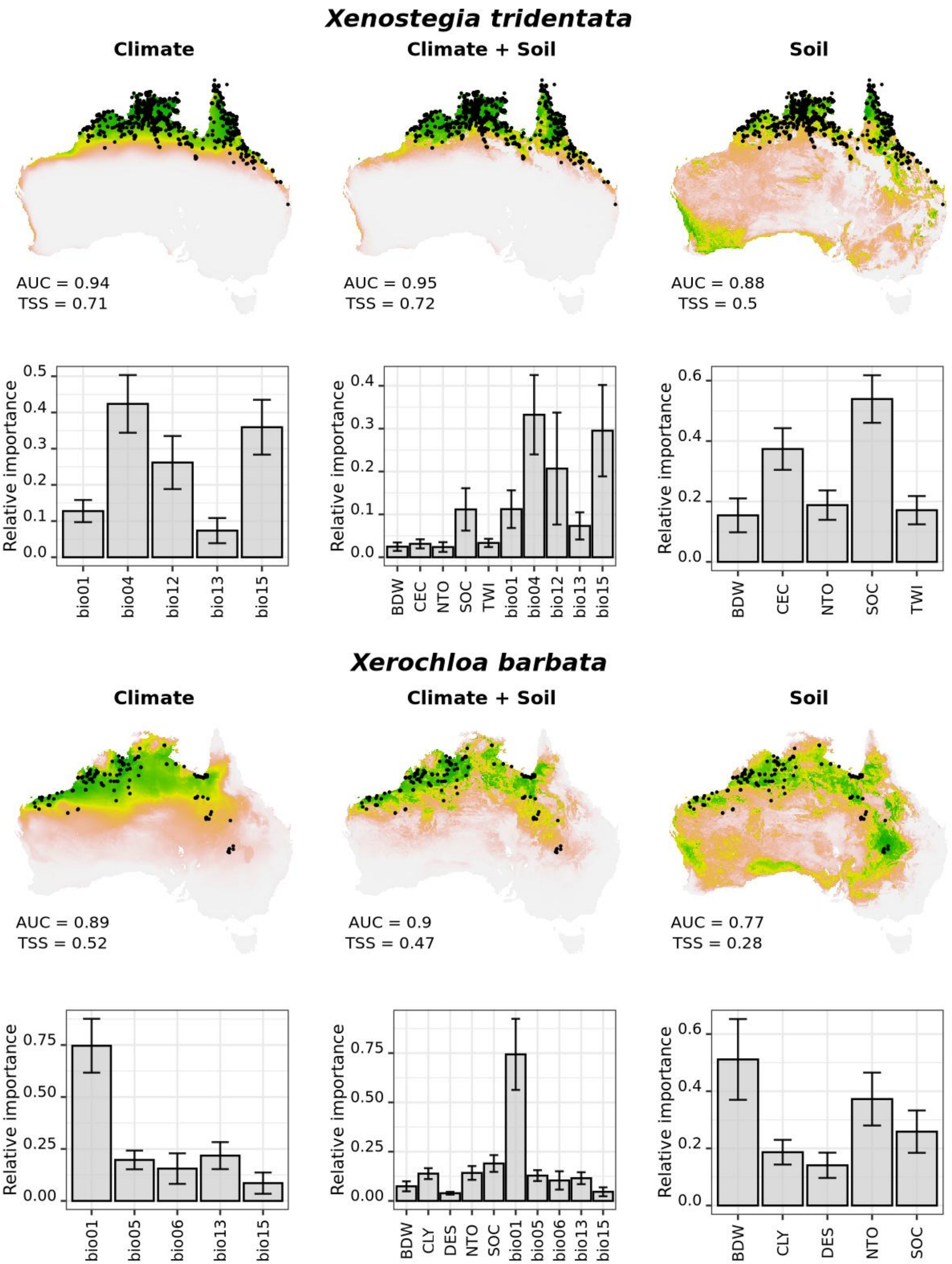

Xenostegia tridentata
Climate
Climate + Soil
Soil
AUC = 0.94
TSS = 0.71
AUC = 0.95
TSS = 0.72
AUC = 0.88
TSS = 0.5
Relative importance
bio01 bio04 bio12 bio13 bio15
BDW CEC NTO SOC TWI bio01 bio04 bio12 bio13 bio15
BDW CEC NTO SOC TWI
Xerochloa barbata
Climate
Climate + Soil
Soil
AUC = 0.89
TSS = 0.52
AUC = 0.9
TSS = 0.47
AUC = 0.77
TSS = 0.28
Relative importance
bio01 bio05 bio06 bio13 bio15
BDW CLY DES NTO SOC bio01 bio05 bio06 bio13 bio15
BDW CLY DES NTO SOC

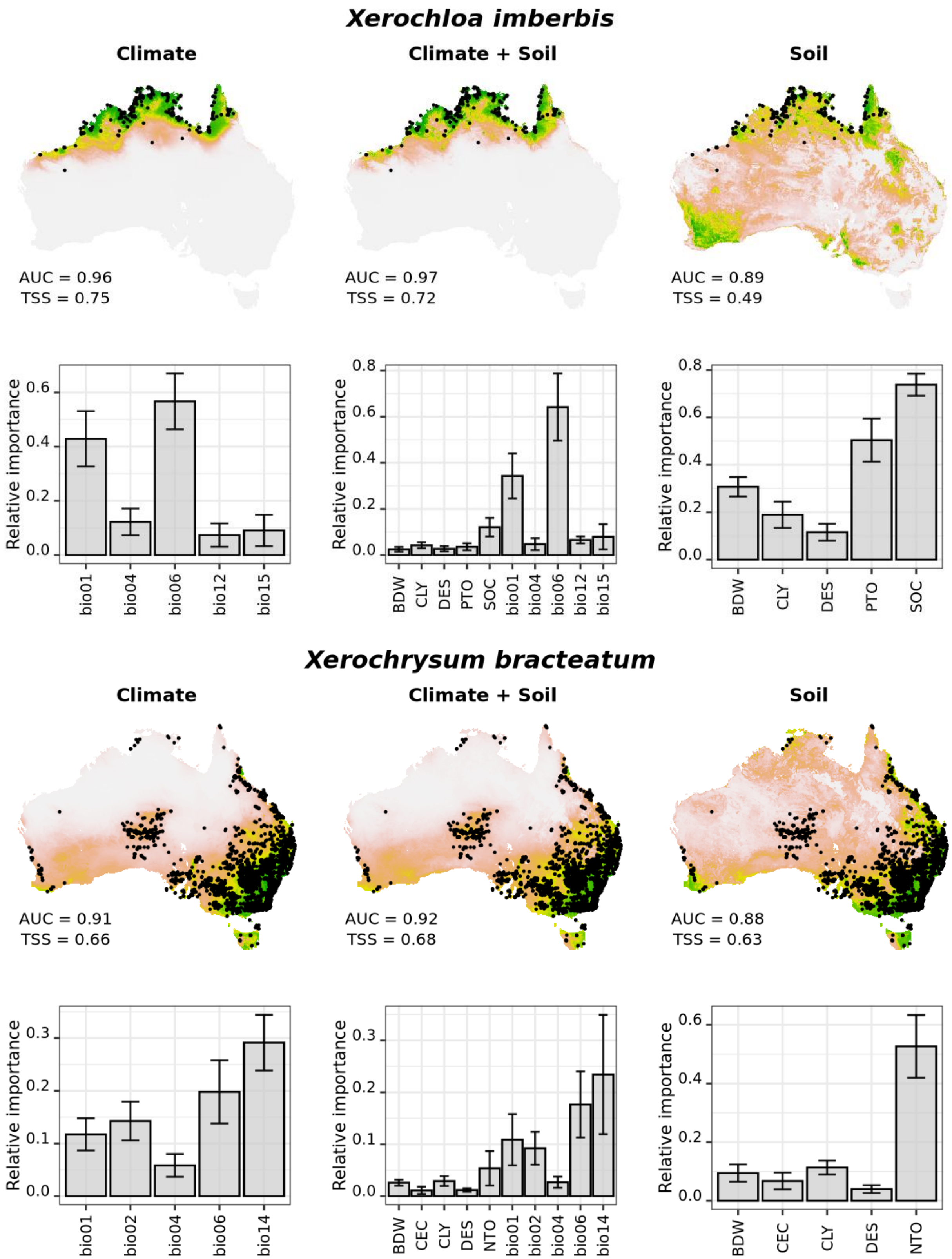

Xerochloa imberbis
Climate
Climate + Soil
Soil
AUC = 0.96
TSS = 0.75
AUC = 0.97
TSS = 0.72
AUC = 0.89
TSS = 0.49
Relative importance
bio01
bio04
bio06
bio12
bio15
BDW
CLY
DES
PTO
SOC
Xerochrysum bracteatum
Climate
Climate + Soil
Soil
AUC = 0.91
TSS = 0.66
AUC = 0.92
TSS = 0.68
AUC = 0.88
TSS = 0.63
Relative importance
bio01
bio02
bio04
bio06
bio14
BDW
CEC
CLY
DES
NTO

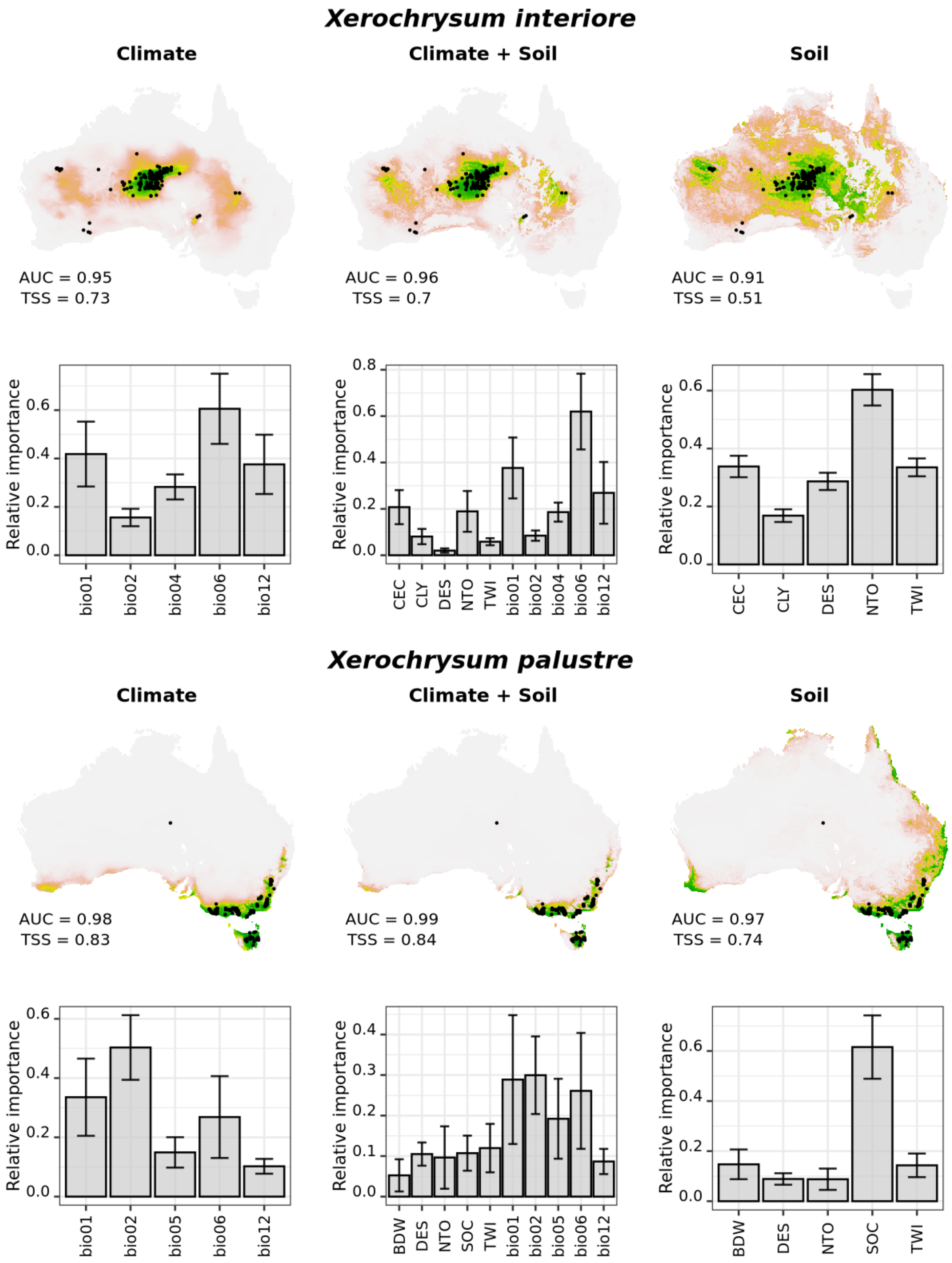

Xerochrysum interiore
Climate
Climate + Soil
Soil
AUC = 0.95
TSS = 0.73
AUC = 0.96
TSS = 0.7
AUC = 0.91
TSS = 0.51
Relative importance
0.0
0.2
0.4
0.6
0.8
bio01
bio02
bio04
bio06
bio12
CEC
CLY
DES
NTO
TWI
Xerochrysum palustre
Climate
Climate + Soil
Soil
AUC = 0.98
TSS = 0.83
AUC = 0.99
TSS = 0.84
AUC = 0.97
TSS = 0.74
Relative importance
0.0
0.1
0.2
0.3
0.4
0.6
bio01
bio02
bio05
bio06
bio12
BDW
DES
NTO
SOC
TWI

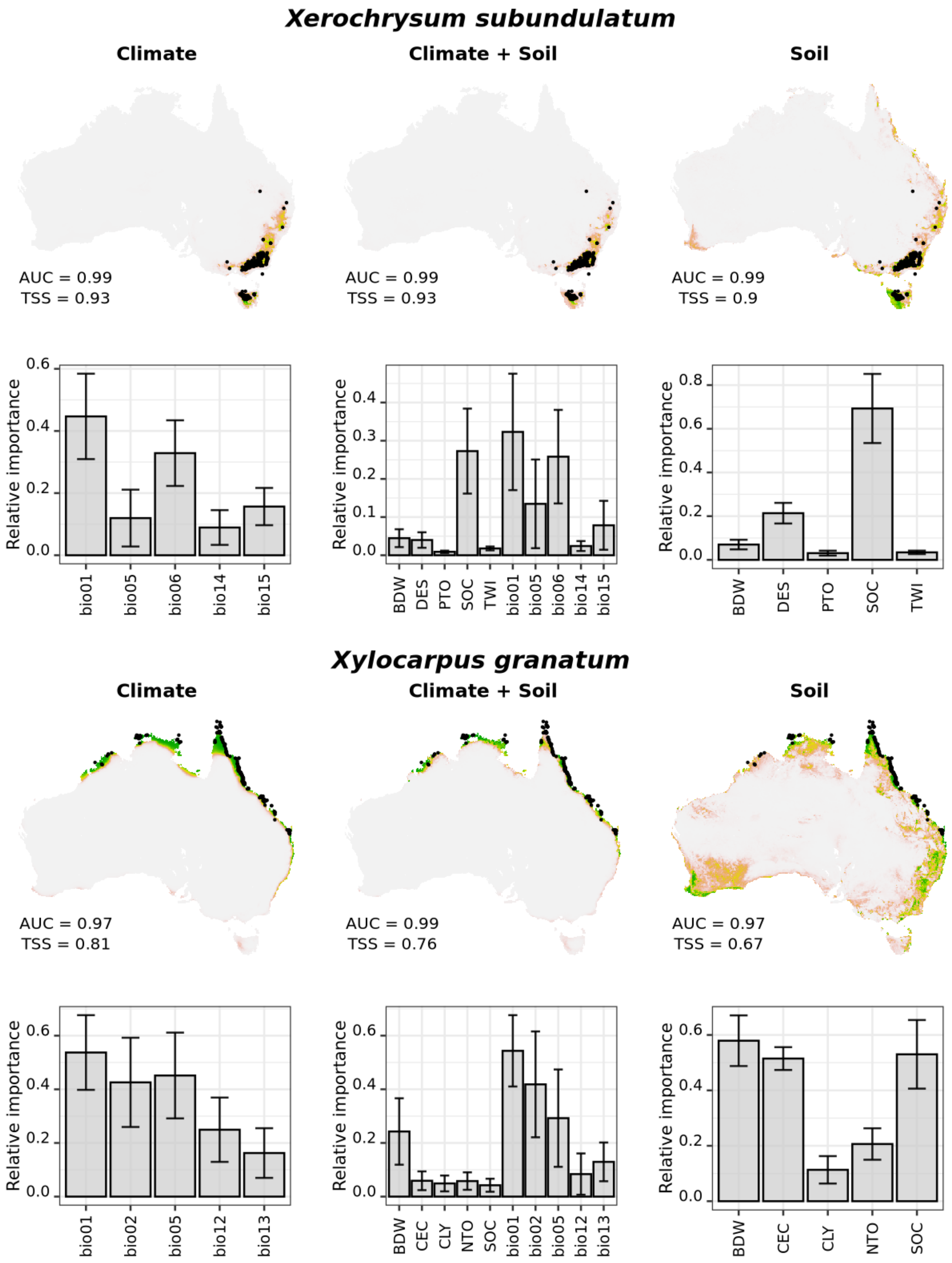
Xerochrysum subundulatum
Climate
Climate + Soil
Soil
AUC = 0.99
TSS = 0.93
AUC = 0.99
TSS = 0.93
AUC = 0.99
TSS = 0.9
Relative importance
bio01
bio05
bio06
bio14
bio15
BDW
DES
PTO
SOC
TWI
Xylocarpus granatum
Climate
Climate + Soil
Soil
AUC = 0.97
TSS = 0.81
AUC = 0.99
TSS = 0.76
AUC = 0.97
TSS = 0.67
bio01
bio02
bio05
bio12
bio13
BDW
CEC
CLY
NTO
SOC

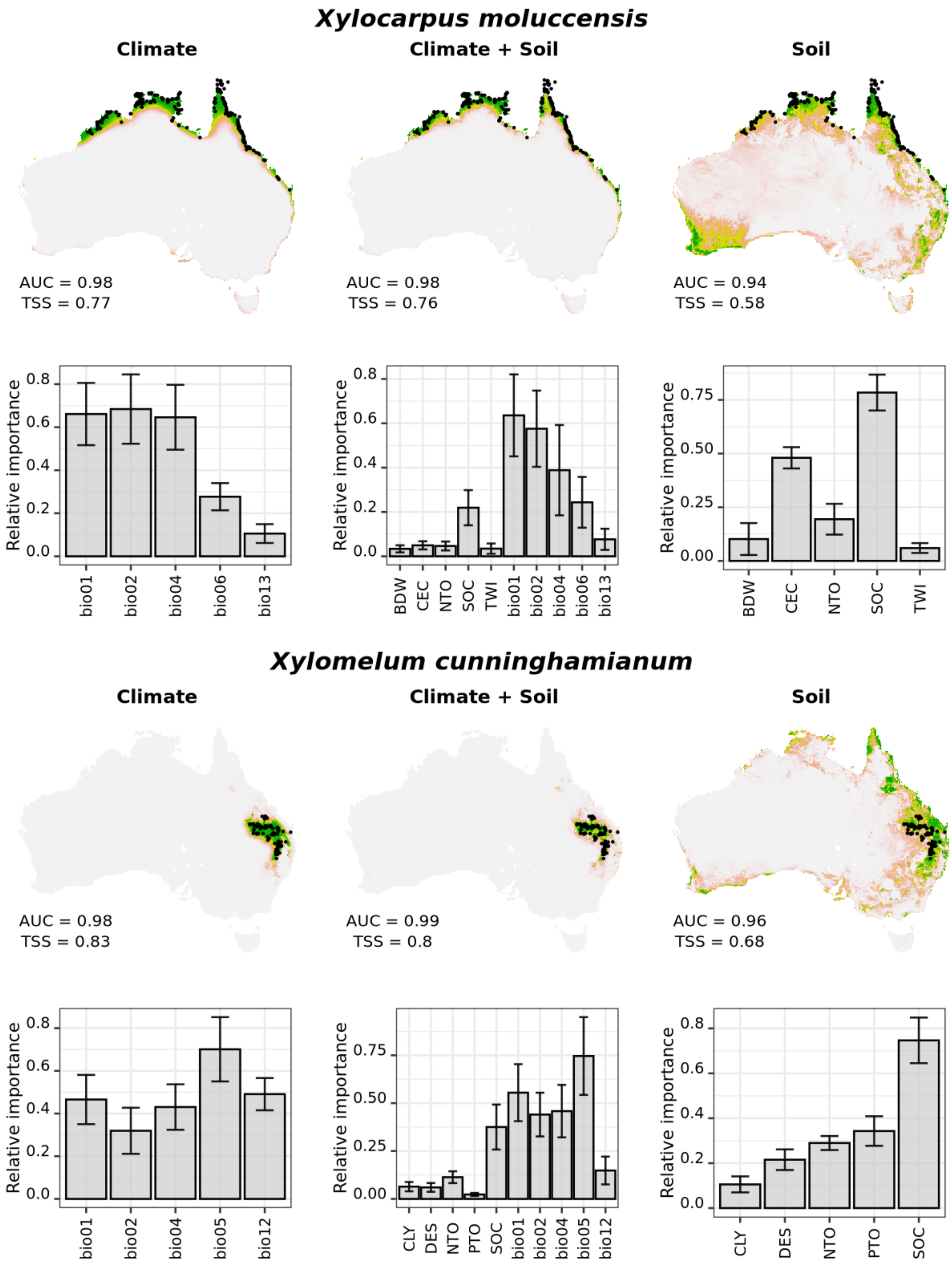

Xylocarpus moluccensis
Climate
Climate + Soil
Soil
AUC = 0.98
TSS = 0.77
AUC = 0.98
TSS = 0.76
AUC = 0.94
TSS = 0.58
Relative importance
0.0
0.2
0.4
0.6
0.8
bio01
bio02
bio04
bio06
bio13
BDW
CEC
NTO
SOC
TWI
0.00
0.25
0.50
0.75
Xylomelum cunninghamianum
AUC = 0.98
TSS = 0.83
AUC = 0.99
TSS = 0.8
AUC = 0.96
TSS = 0.68
bio05
bio12
CLY
DES
PTO

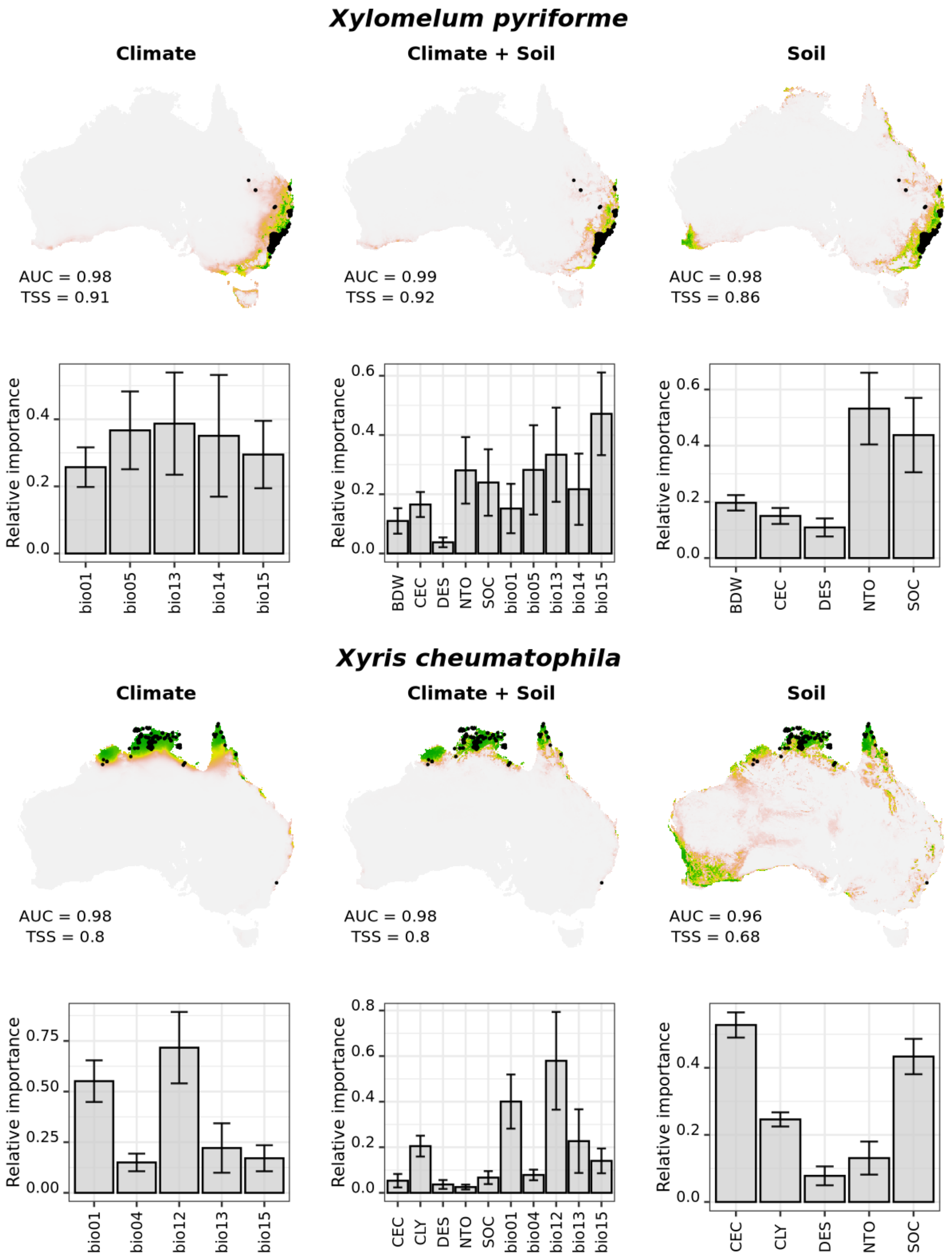
Xylomelum pyriforme
Climate
Climate + Soil
Soil
AUC = 0.98
TSS = 0.91
AUC = 0.99
TSS = 0.92
AUC = 0.98
TSS = 0.86
Relative importance
0.0
0.2
0.4
bio01
bio05
bio13
bio14
bio15
Relative importance
0.0
0.2
0.4
0.6
BDW
CEC
DES
NTO
SOC
bio01
bio05
bio13
bio14
bio15
Relative importance
0.0
0.2
0.4
0.6
BDW
CEC
DES
NTO
SOC
Xyris cheumatophila
Climate
Climate + Soil
Soil
AUC = 0.98
TSS = 0.8
AUC = 0.98
TSS = 0.8
AUC = 0.96
TSS = 0.68
Relative importance
0.00
0.25
0.50
0.75
bio01
bio04
bio12
bio13
bio15
Relative importance
0.0
0.2
0.4
0.6
0.8
CEC
CLY
DES
NTO
SOC
bio01
bio04
bio12
bio13
bio15
Relative importance
0.0
0.2
0.4
CEC
CLY
DES
NTO
SOC

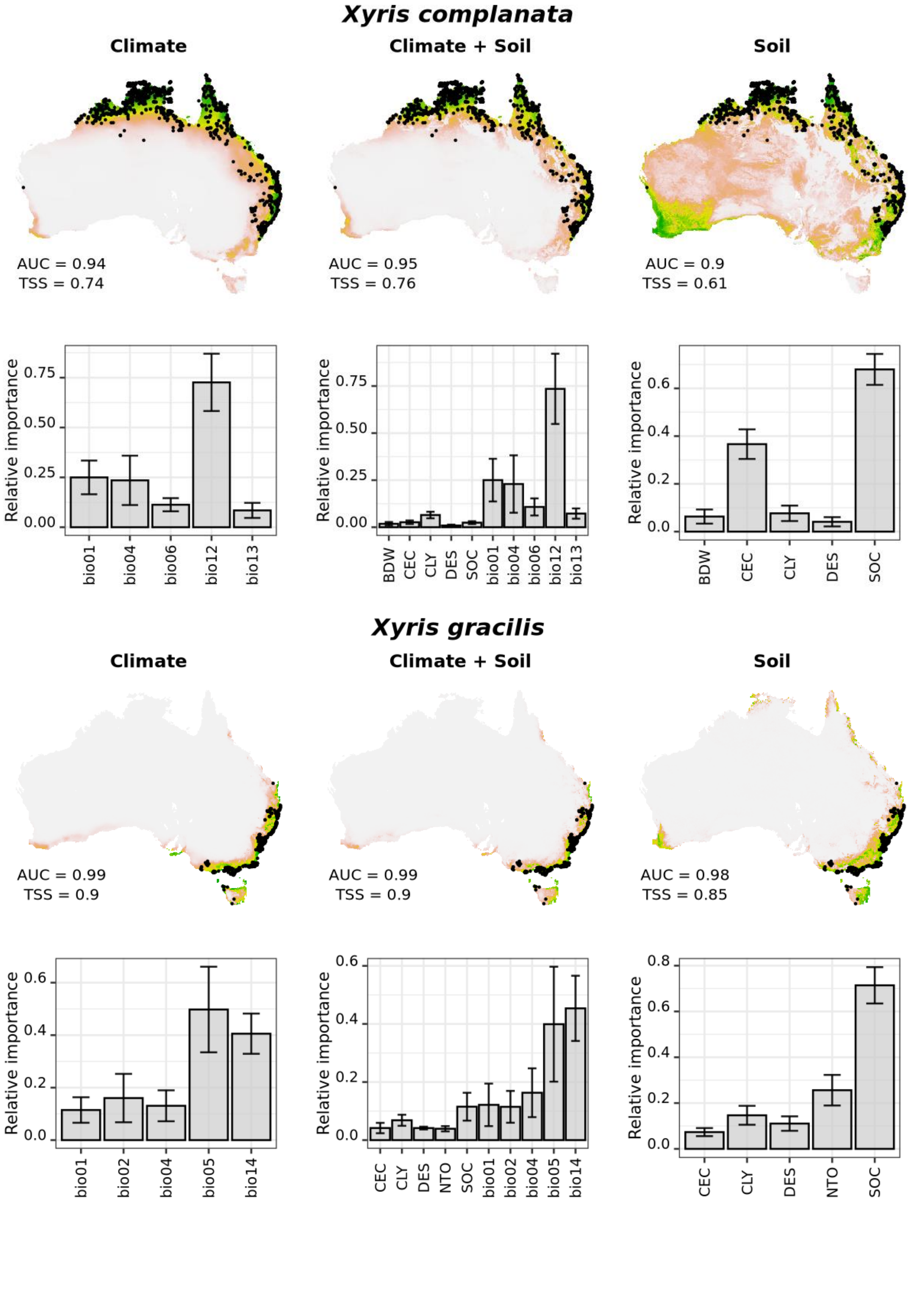

Xyris complanata
Climate
Climate + Soil
Soil
AUC = 0.94
TSS = 0.74
AUC = 0.95
TSS = 0.76
AUC = 0.9
TSS = 0.61
Relative importance
0.00
0.25
0.50
0.75
bio01
bio04
bio06
bio12
bio13
BDW
CEC
CLY
DES
SOC
0.0
0.2
0.4
0.6
Xyris gracilis
Climate
Climate + Soil
Soil
AUC = 0.99
TSS = 0.9
AUC = 0.99
TSS = 0.9
AUC = 0.98
TSS = 0.85
Relative importance
bio01
bio02
bio04
bio05
bio14
CEC
CLY
DES
NTO
SOC
0.8

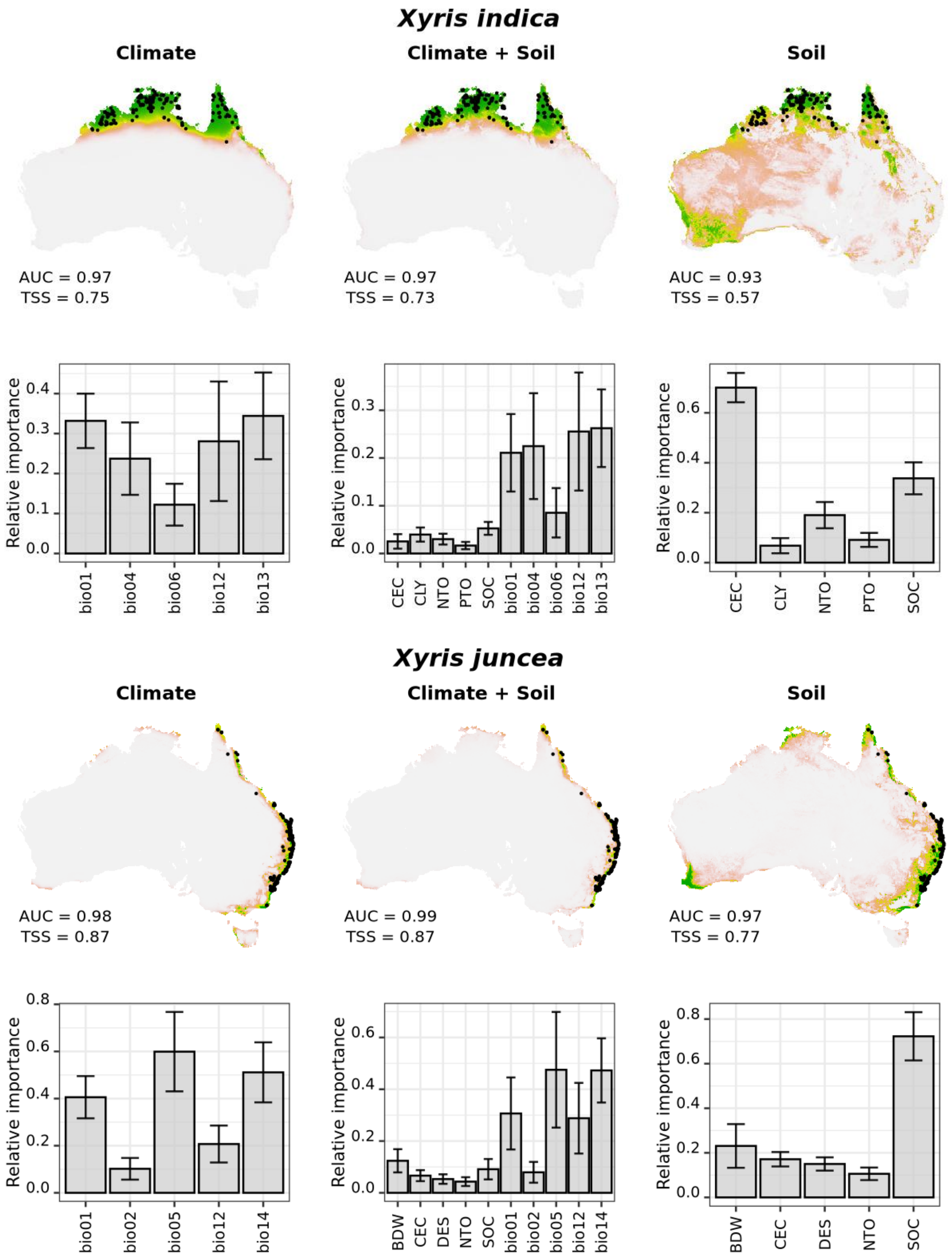
Xyris indica
Climate
Climate + Soil
Soil
AUC = 0.97
TSS = 0.75
AUC = 0.97
TSS = 0.73
AUC = 0.93
TSS = 0.57
Relative importance
bio01 bio04 bio06 bio12 bio13
CEC CLY NTO PTO SOC bio01 bio04 bio06 bio12 bio13
CEC CLY NTO PTO SOC
Xyris juncea
Climate
Climate + Soil
Soil
AUC = 0.98
TSS = 0.87
AUC = 0.99
TSS = 0.87
AUC = 0.97
TSS = 0.77
Relative importance
bio01 bio02 bio05 bio12 bio14
BDW CEC DES NTO SOC bio01 bio02 bio05 bio12 bio14
BDW CEC DES NTO SOC

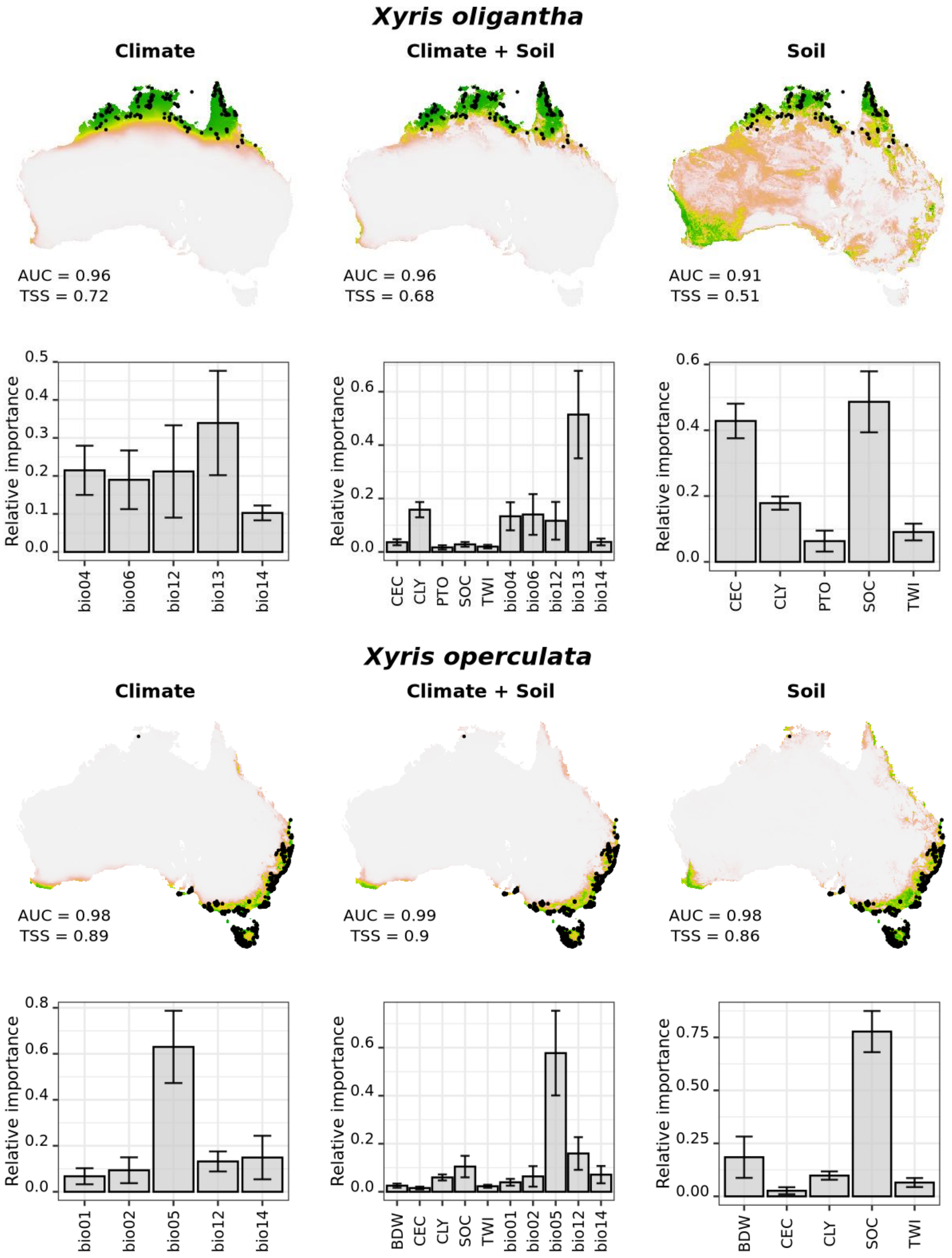

Xyris oligantha
Climate
Climate + Soil
Soil
AUC = 0.96
TSS = 0.72
AUC = 0.96
TSS = 0.68
AUC = 0.91
TSS = 0.51
Relative importance
bio04
bio06
bio12
bio13
bio14
CEC
CLY
PTO
SOC
TWI
Xyris operculata
Climate
Climate + Soil
Soil
AUC = 0.98
TSS = 0.89
AUC = 0.99
TSS = 0.9
AUC = 0.98
TSS = 0.86
Relative importance
bio01
bio02
bio05
bio12
bio14
BDW
CEC
CLY
SOC
TWI

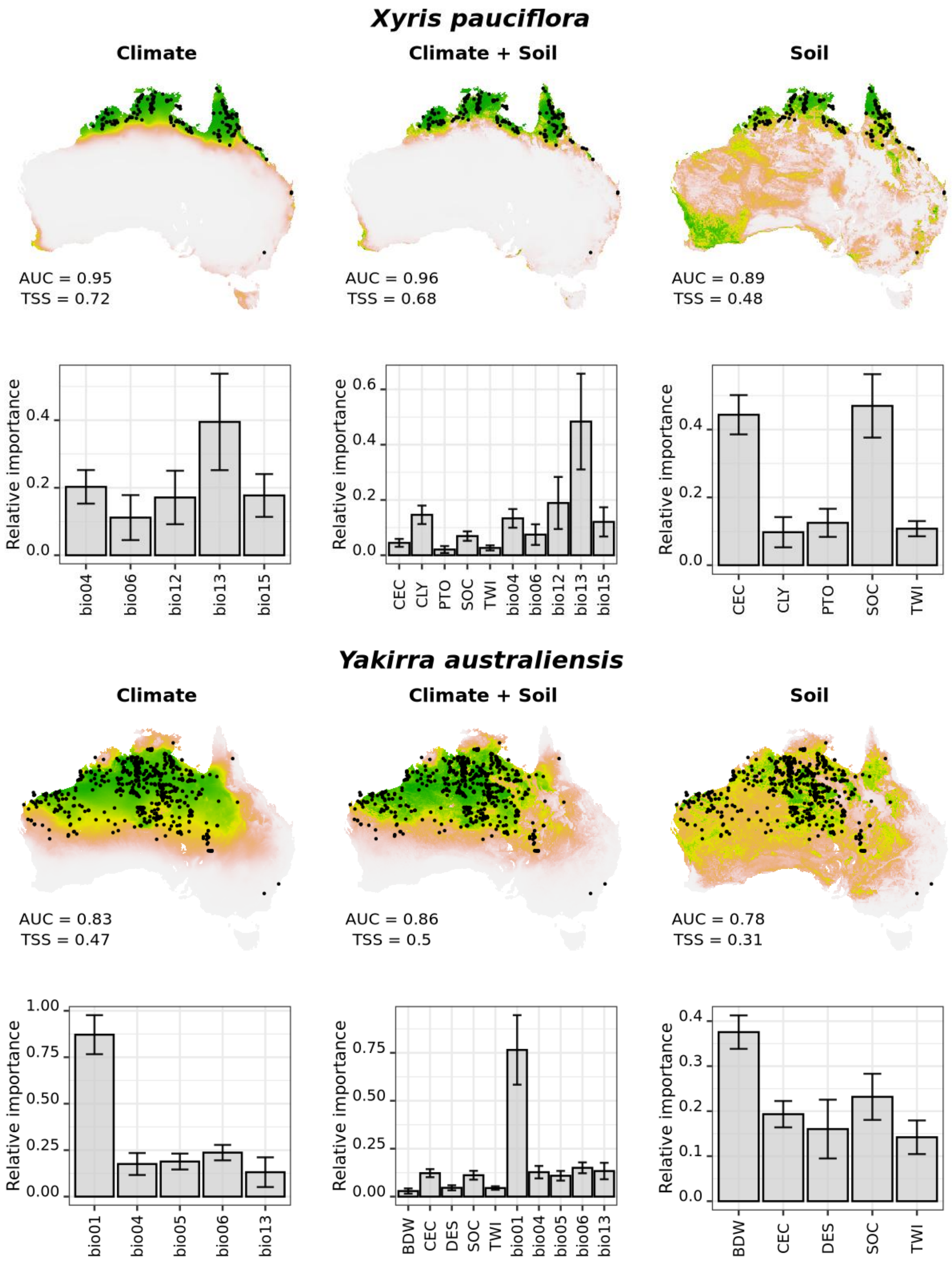
Xyris pauciflora
Climate
Climate + Soil
Soil
AUC = 0.95
TSS = 0.72
AUC = 0.96
TSS = 0.68
AUC = 0.89
TSS = 0.48
Relative importance
bio04
bio06
bio12
bio13
bio15
CEC
CLY
PTO
SOC
TWI
Yakirra australiensis
Climate
Climate + Soil
Soil
AUC = 0.83
TSS = 0.47
AUC = 0.86
TSS = 0.5
AUC = 0.78
TSS = 0.31
Relative importance
bio01
bio04
bio05
bio06
bio13
BDW
CEC
DES
SOC
TWI

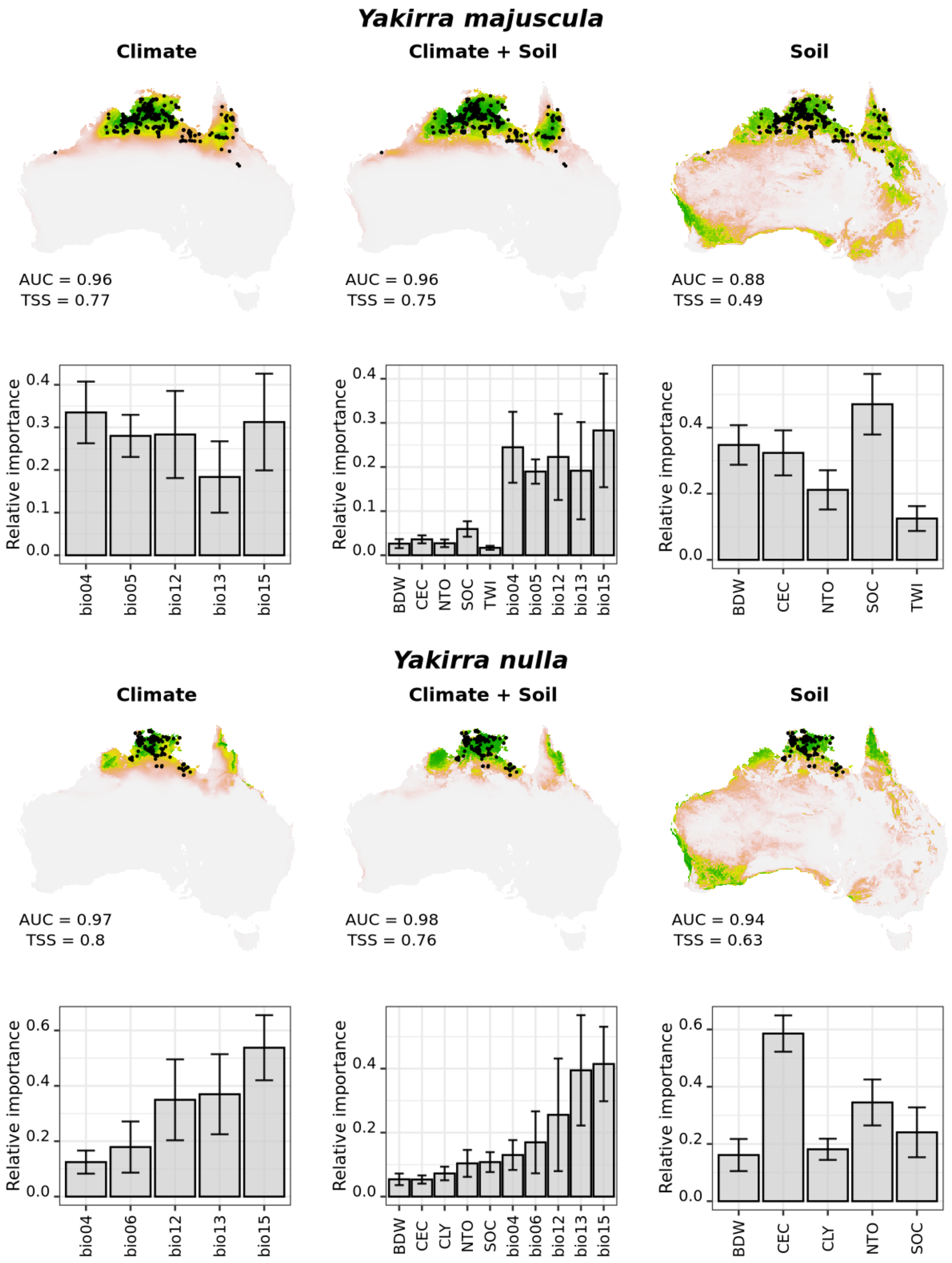

Yakirra majuscula
Climate
Climate + Soil
Soil
AUC = 0.96
TSS = 0.77
AUC = 0.96
TSS = 0.75
AUC = 0.88
TSS = 0.49
Relative importance
bio04
bio05
bio12
bio13
bio15
BDW
CEC
NTO
SOC
TWI
Yakirra nulla
Climate
Climate + Soil
Soil
AUC = 0.97
TSS = 0.8
AUC = 0.98
TSS = 0.76
AUC = 0.94
TSS = 0.63
Relative importance
bio04
bio06
bio12
bio13
bio15
BDW
CEC
CLY
NTO
SOC

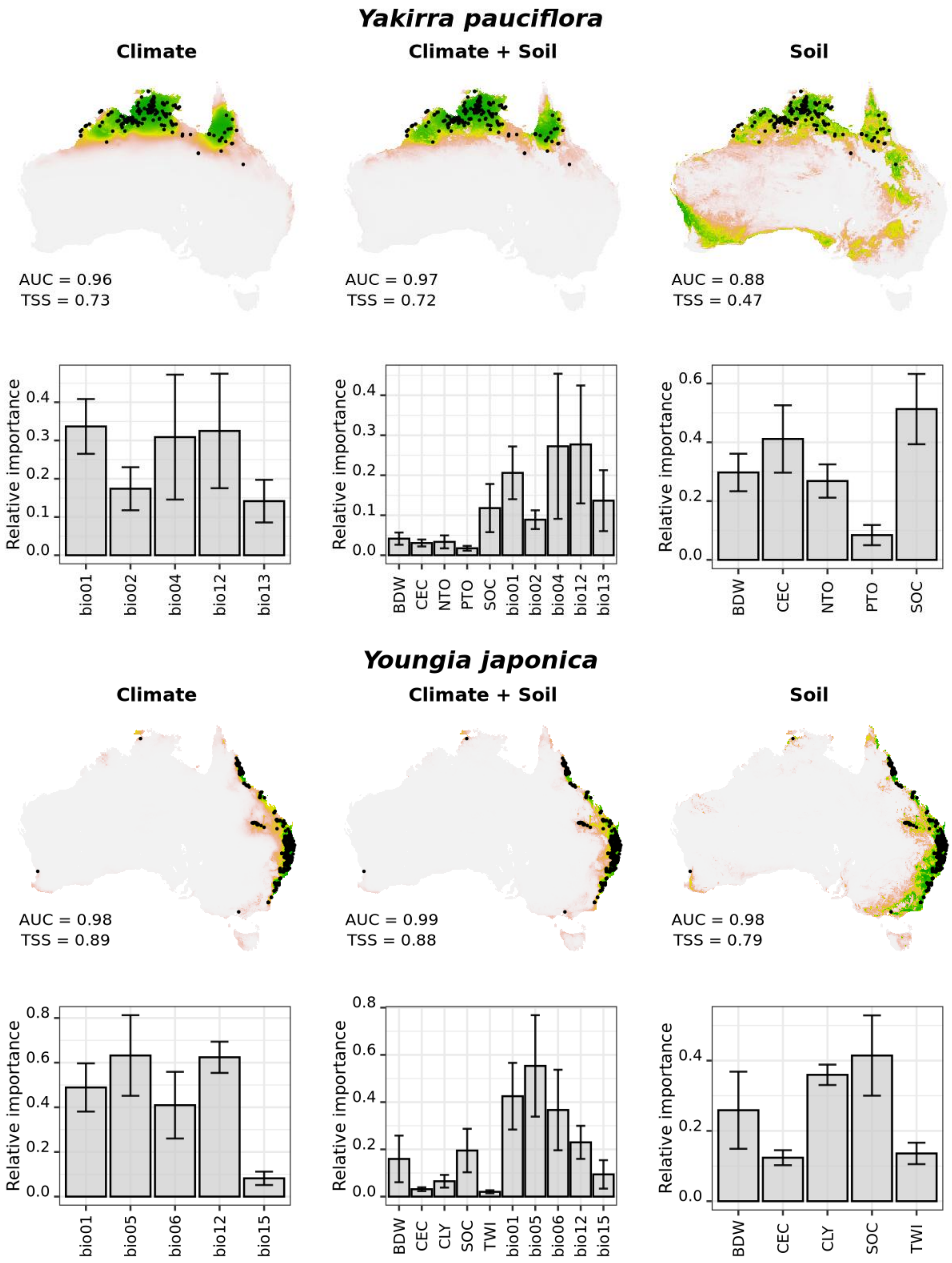
Yakirra pauciflora
Climate
Climate + Soil
Soil
AUC = 0.96
TSS = 0.73
AUC = 0.97
TSS = 0.72
AUC = 0.88
TSS = 0.47
Relative importance
bio01
bio02
bio04
bio12
bio13
BDW
CEC
NTO
PTO
SOC
Youngia japonica
Climate
Climate + Soil
Soil
AUC = 0.98
TSS = 0.89
AUC = 0.99
TSS = 0.88
AUC = 0.98
TSS = 0.79
Relative importance
bio01
bio05
bio06
bio12
bio15
BDW
CEC
CLY
SOC
TWI

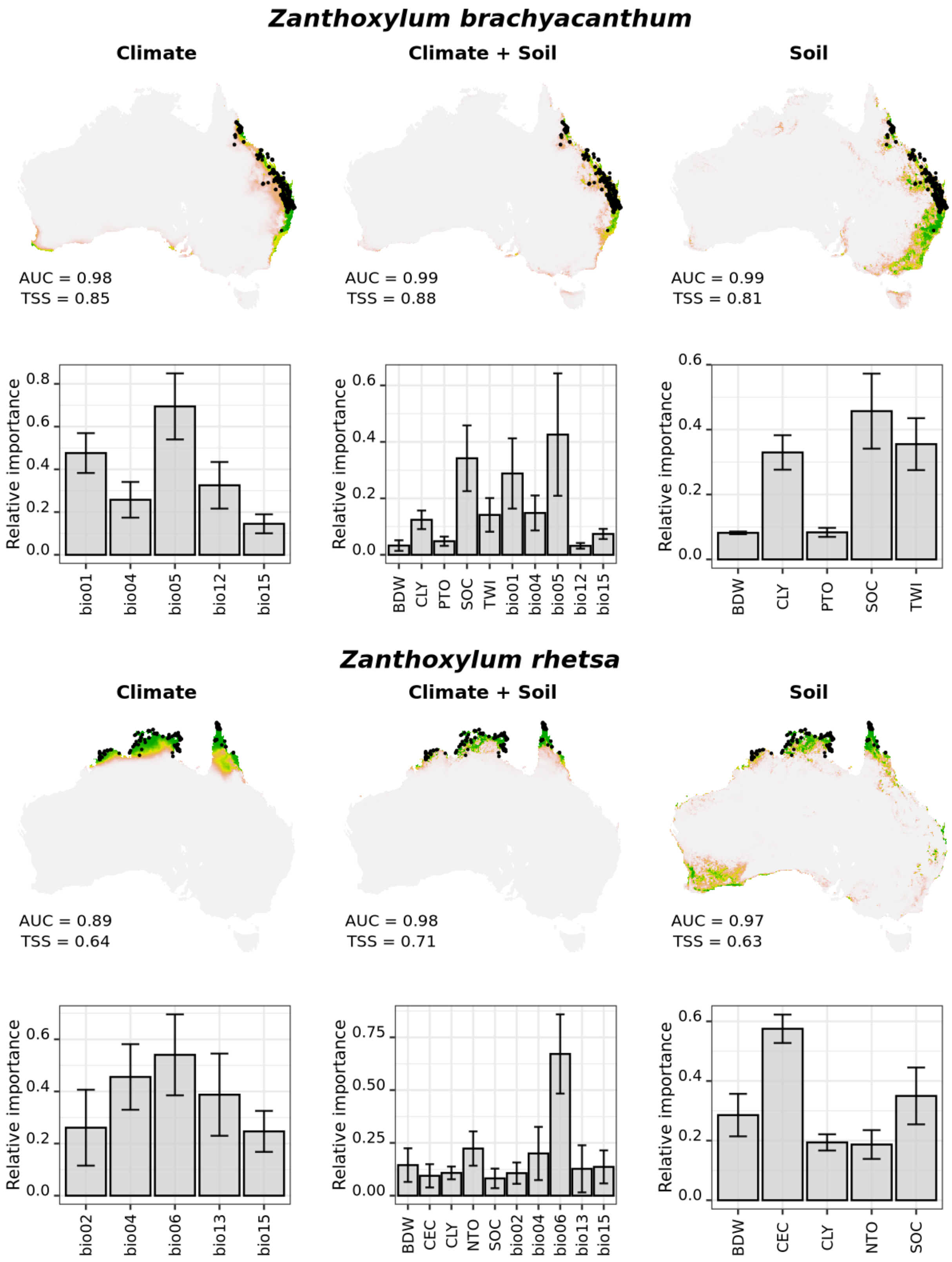

Zanthoxylum brachyacanthum
Climate
Climate + Soil
Soil
AUC = 0.98
TSS = 0.85
AUC = 0.99
TSS = 0.88
AUC = 0.99
TSS = 0.81
Relative importance
0.0
0.2
0.4
0.6
0.8
bio01
bio04
bio05
bio12
bio15
BDW
CLY
PTO
SOC
TWI
bio01
bio04
bio05
bio12
bio15
Zanthoxylum rhetsa
Climate
Climate + Soil
Soil
AUC = 0.89
TSS = 0.64
AUC = 0.98
TSS = 0.71
AUC = 0.97
TSS = 0.63
0.00
0.25
0.50
0.75
bio02
bio04
bio06
bio13
bio15
BDW
CEC
CLY
NTO
SOC

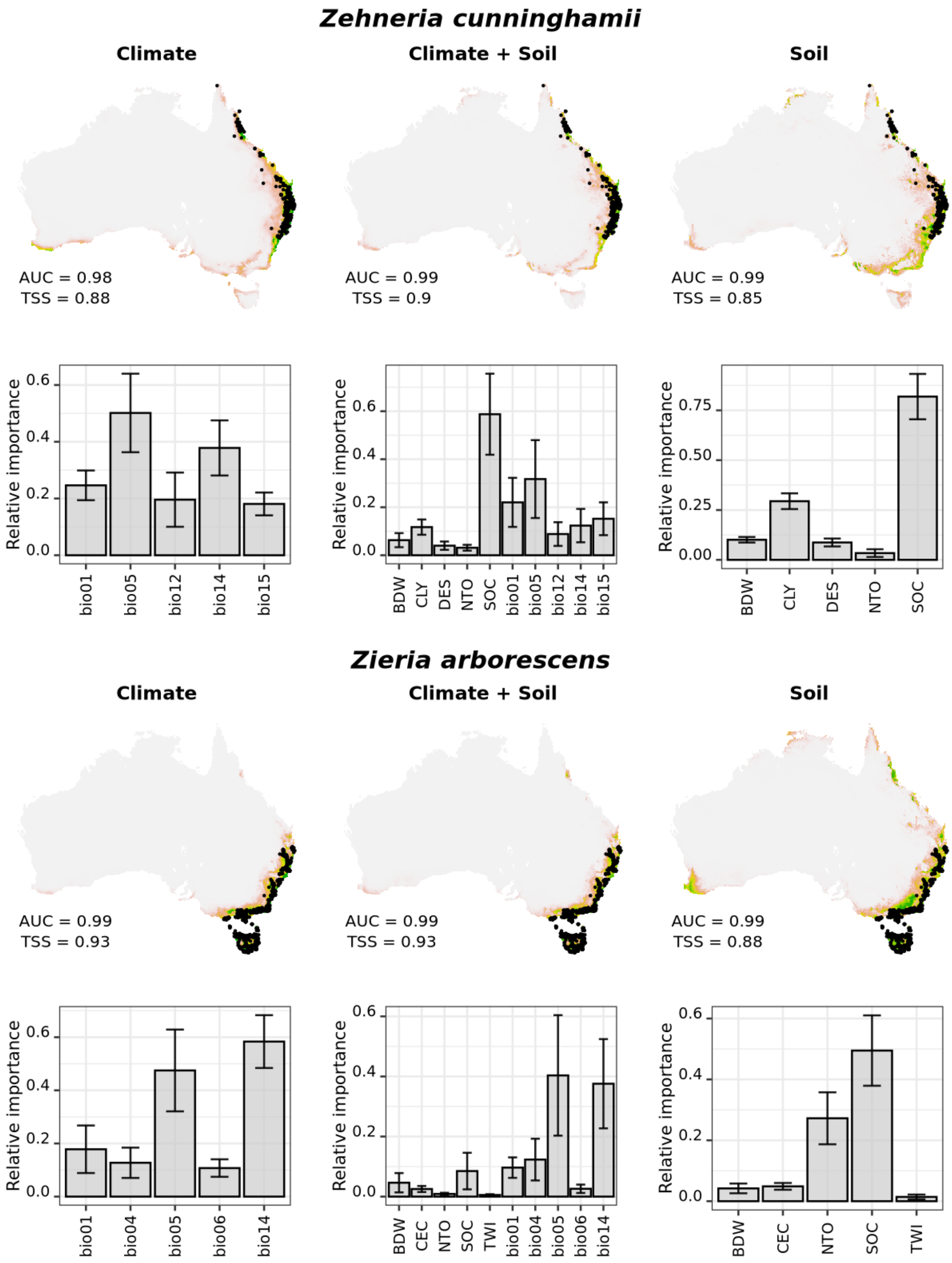
Zehneria cunninghamii
Climate
Climate + Soil
Soil
AUC = 0.98
TSS = 0.88
AUC = 0.99
TSS = 0.9
AUC = 0.99
TSS = 0.85
Relative importance
bio01
bio05
bio12
bio14
bio15
BDW
CLY
DES
NTO
SOC
Zieria arborescens
Climate
Climate + Soil
Soil
AUC = 0.99
TSS = 0.93
AUC = 0.99
TSS = 0.93
AUC = 0.99
TSS = 0.88
Relative importance
bio01
bio04
bio05
bio06
bio14
BDW
CEC
NTO
SOC
TWI

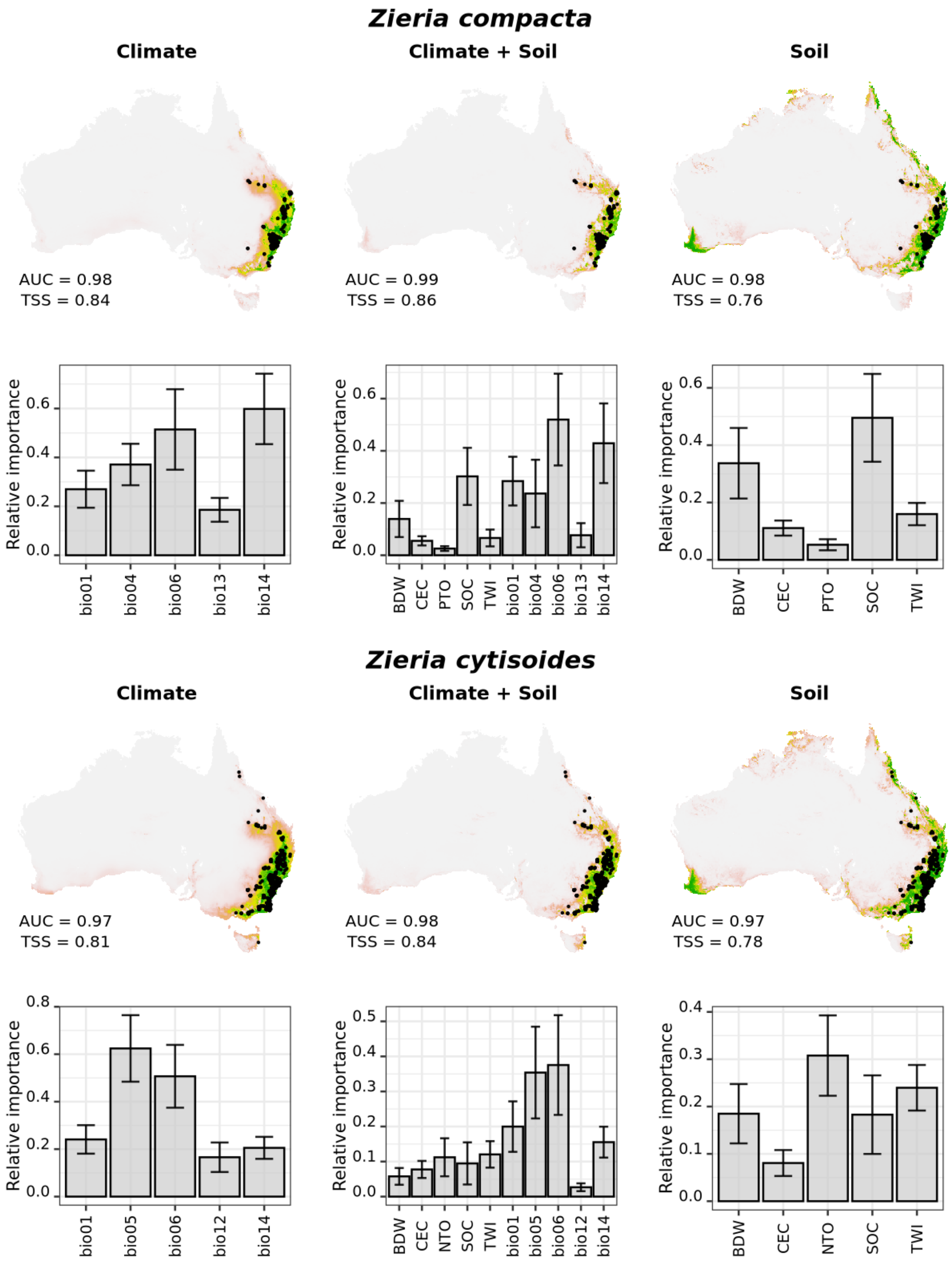

Zieria compacta
Climate
Climate + Soil
Soil
AUC = 0.98
TSS = 0.84
AUC = 0.99
TSS = 0.86
AUC = 0.98
TSS = 0.76
Relative importance
bio01
bio04
bio06
bio13
bio14
BDW
CEC
PTO
SOC
TWI
Zieria cytisoides
AUC = 0.97
TSS = 0.81
AUC = 0.98
TSS = 0.84
AUC = 0.97
TSS = 0.78
bio05
bio12
NTO

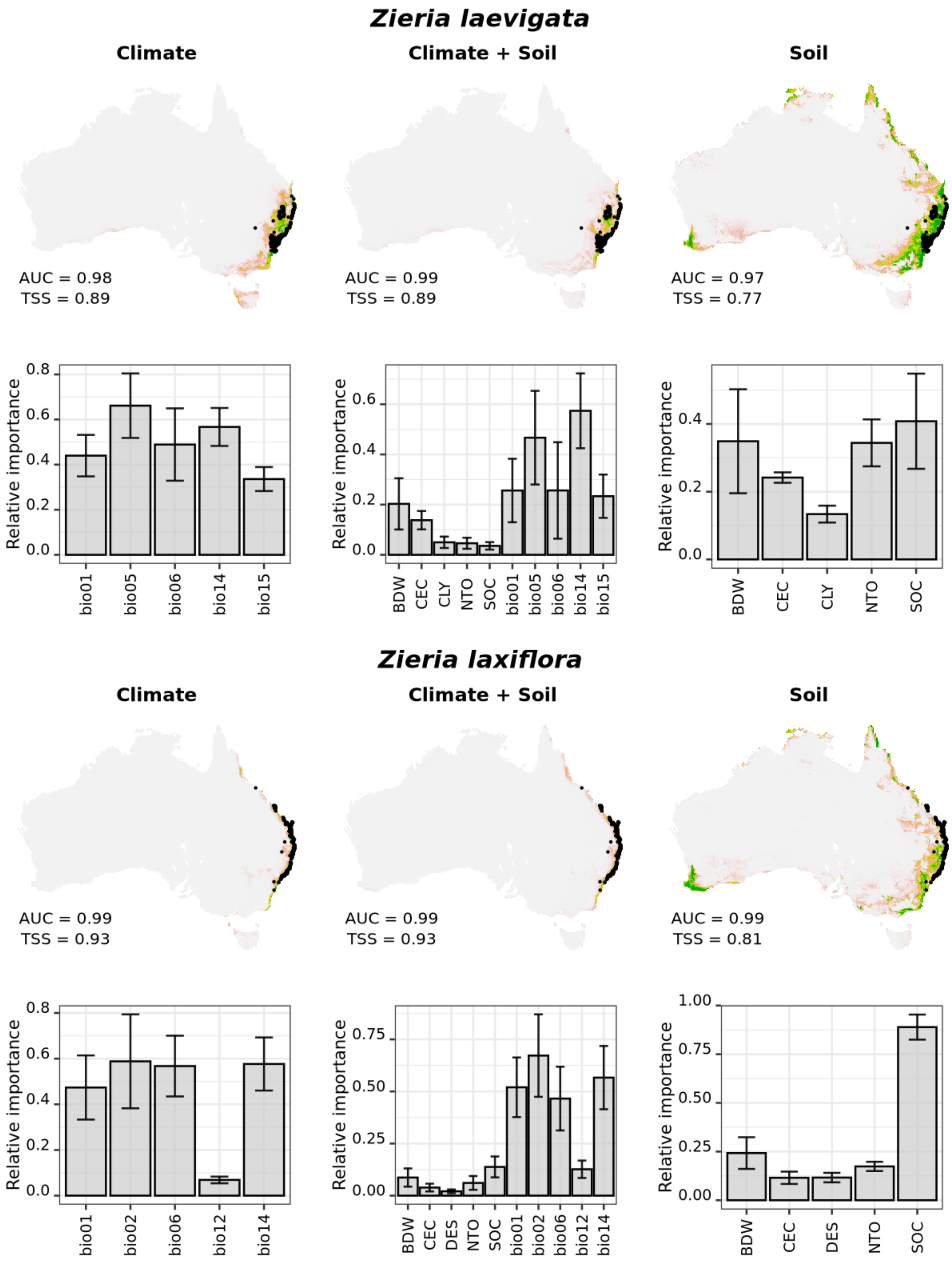

Zieria laevigata
Climate
Climate + Soil
Soil
AUC = 0.98
TSS = 0.89
AUC = 0.99
TSS = 0.89
AUC = 0.97
TSS = 0.77
Relative importance
bio01 bio05 bio06 bio14 bio15
BDW CEC CLY NTO SOC bio01 bio05 bio06 bio14 bio15
BDW CEC CLY NTO SOC
Zieria laxiflora
Climate
Climate + Soil
Soil
AUC = 0.99
TSS = 0.93
AUC = 0.99
TSS = 0.93
AUC = 0.99
TSS = 0.81
Relative importance
bio01 bio02 bio06 bio12 bio14
BDW CEC DES NTO SOC bio01 bio02 bio06 bio12 bio14
BDW CEC DES NTO SOC

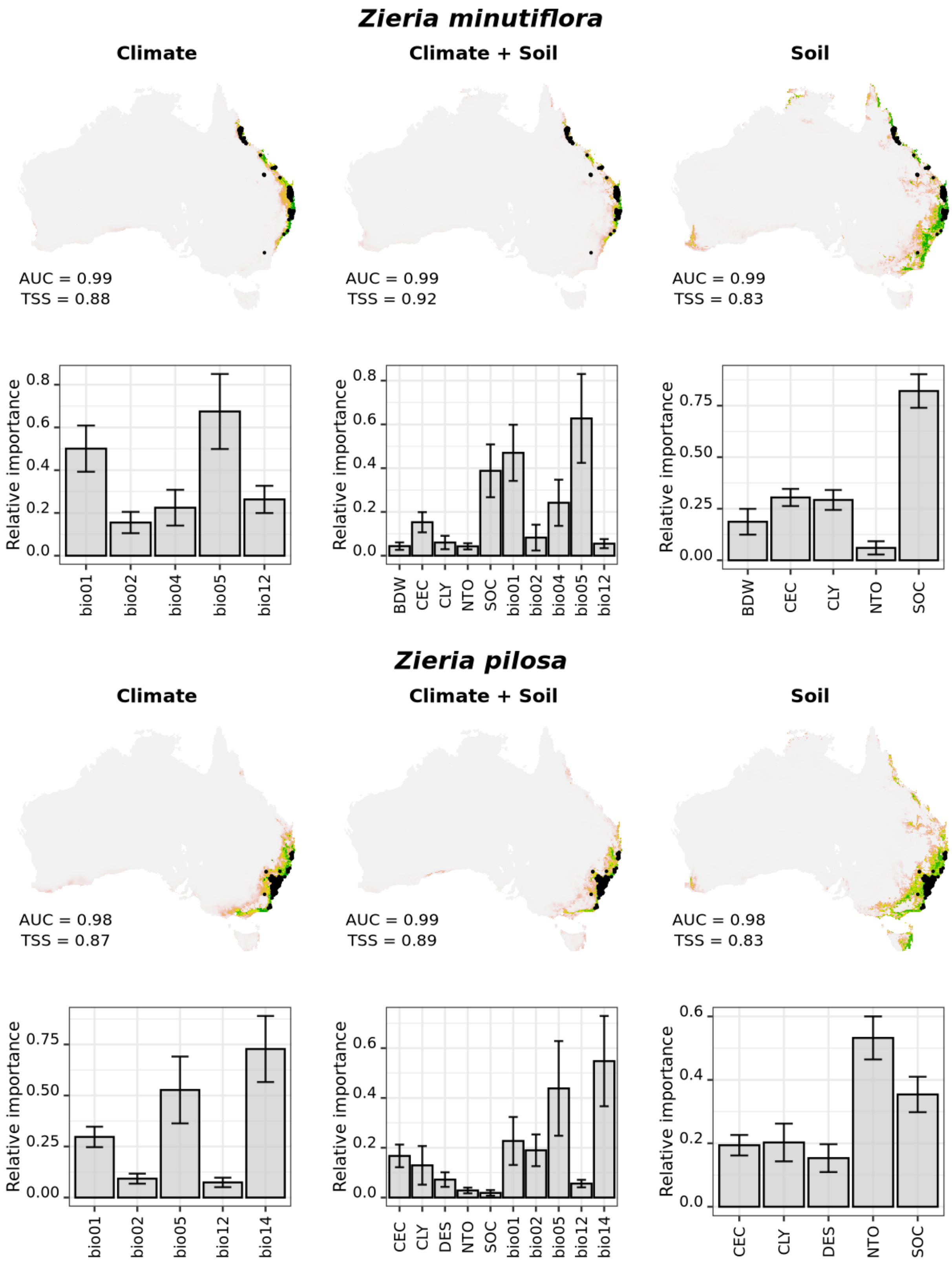

Zieria minutiflora
Climate
Climate + Soil
Soil
AUC = 0.99
TSS = 0.88
AUC = 0.99
TSS = 0.92
AUC = 0.99
TSS = 0.83
Relative importance
0.0
0.2
0.4
0.6
0.8
bio01
bio02
bio04
bio05
bio12
BDW
CEC
CLY
NTO
SOC
bio01
bio02
bio04
bio05
bio12
0.00
0.25
0.50
0.75
BDW
CEC
CLY
NTO
SOC
Zieria pilosa
Climate
Climate + Soil
Soil
AUC = 0.98
TSS = 0.87
AUC = 0.99
TSS = 0.89
AUC = 0.98
TSS = 0.83
Relative importance
0.00
0.25
0.50
0.75
bio01
bio02
bio05
bio12
bio14
0.0
0.2
0.4
0.6
CEC
CLY
DES
NTO
SOC
bio01
bio02
bio05
bio12
bio14
CEC
CLY
DES
NTO
SOC

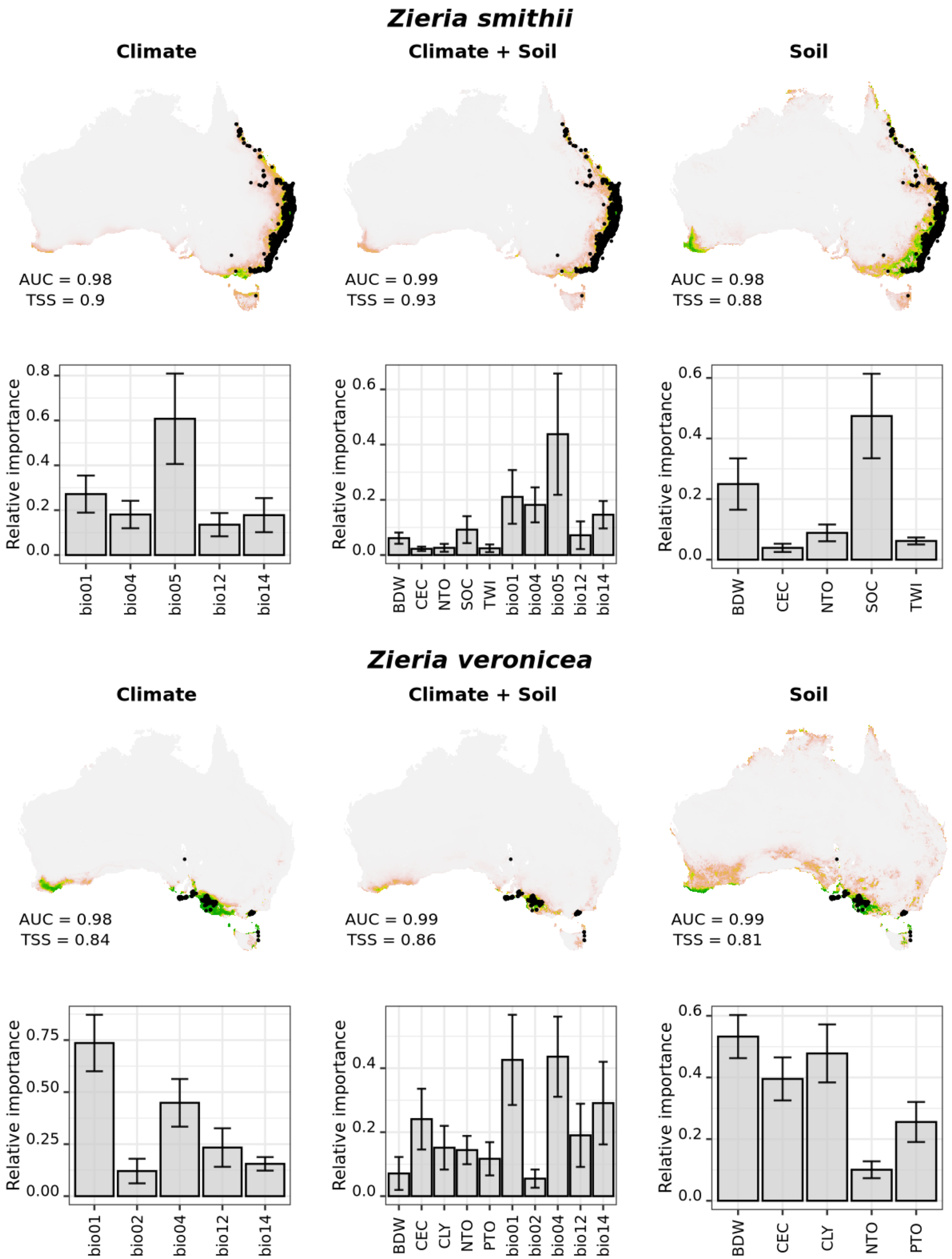
Zieria smithii
Climate
Climate + Soil
Soil
AUC = 0.98
TSS = 0.9
AUC = 0.99
TSS = 0.93
AUC = 0.98
TSS = 0.88
Relative importance
bio01
bio04
bio05
bio12
bio14
BDW
CEC
NTO
SOC
TWI
Zieria veronicea
Climate
Climate + Soil
Soil
AUC = 0.98
TSS = 0.84
AUC = 0.99
TSS = 0.86
AUC = 0.99
TSS = 0.81
Relative importance
bio01
bio02
bio04
bio12
bio14
BDW
CEC
CLY
NTO
PTO

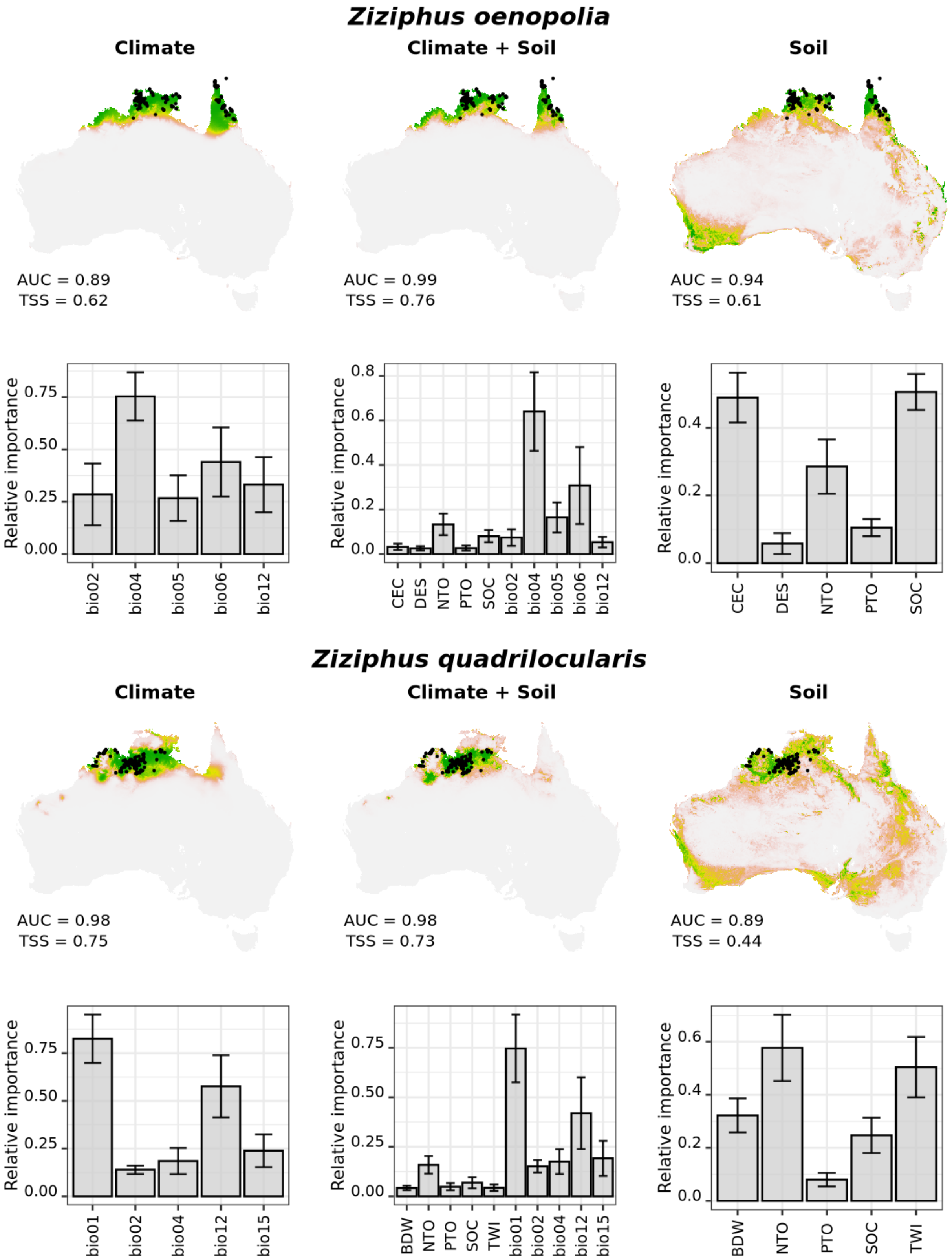
Ziziphus oenopolia
Climate
Climate + Soil
Soil
AUC = 0.89
TSS = 0.62
AUC = 0.99
TSS = 0.76
AUC = 0.94
TSS = 0.61
Relative importance
0.00
0.25
0.50
0.75
bio02
bio04
bio05
bio06
bio12
0.0
0.2
0.4
0.6
0.8
CEC
DES
NTO
PTO
SOC
bio02
bio04
bio05
bio06
bio12
0.0
0.2
0.4
CEC
DES
NTO
PTO
SOC
Ziziphus quadrilocularis
Climate
Climate + Soil
Soil
AUC = 0.98
TSS = 0.75
AUC = 0.98
TSS = 0.73
AUC = 0.89
TSS = 0.44
Relative importance
0.00
0.25
0.50
0.75
bio01
bio02
bio04
bio12
bio15
BDW
NTO
PTO
SOC
TWI
bio01
bio02
bio04
bio12
bio15
0.0
0.2
0.4
0.6
BDW
NTO
PTO
SOC
TWI

Zornia albiflora

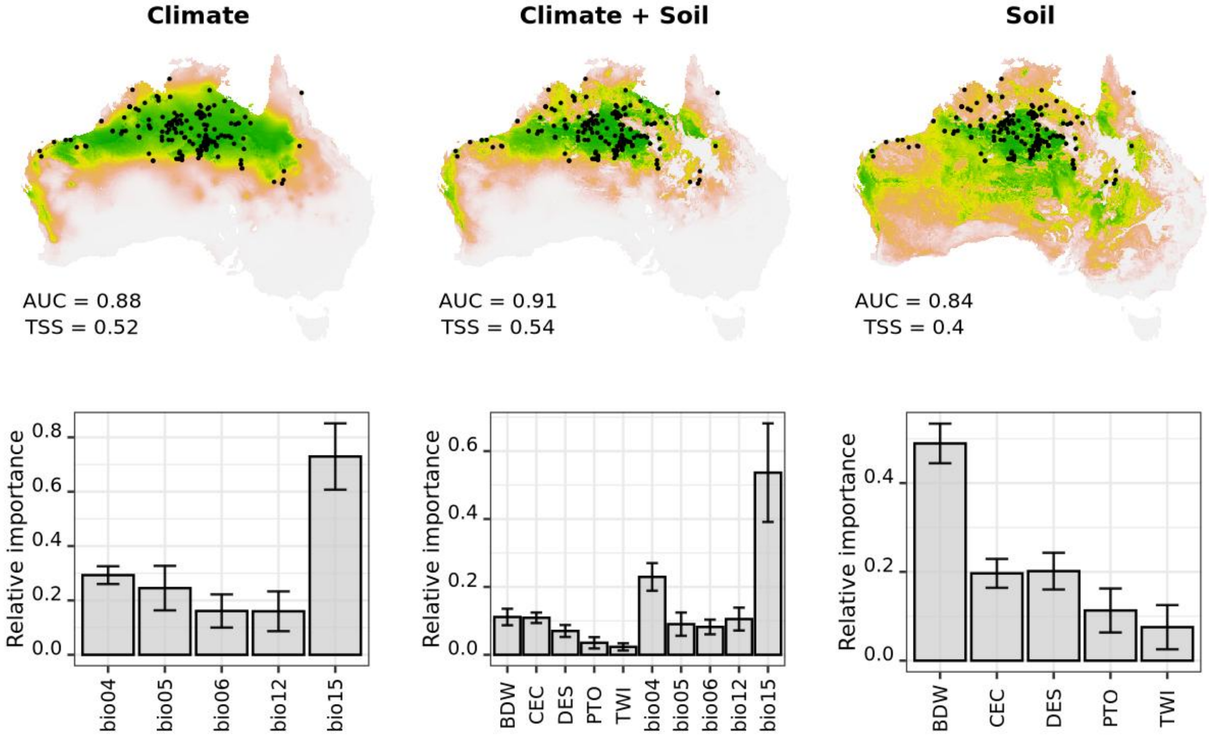

Zornia areolata

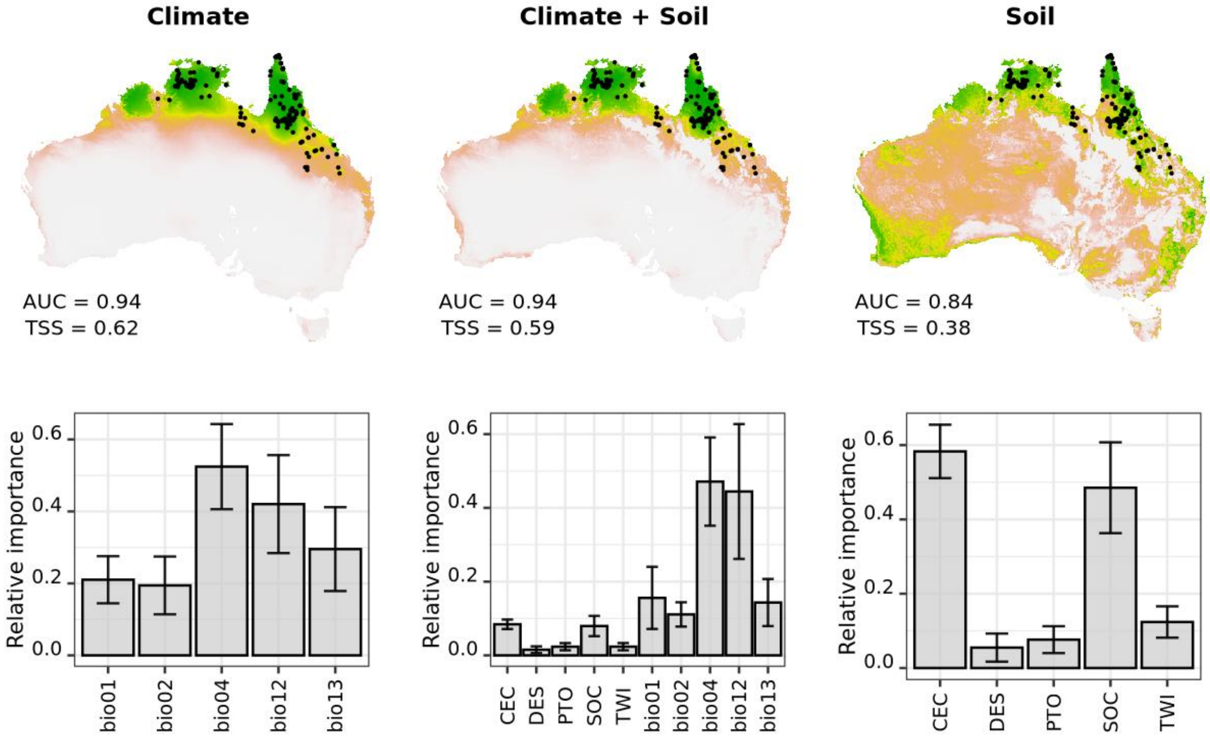

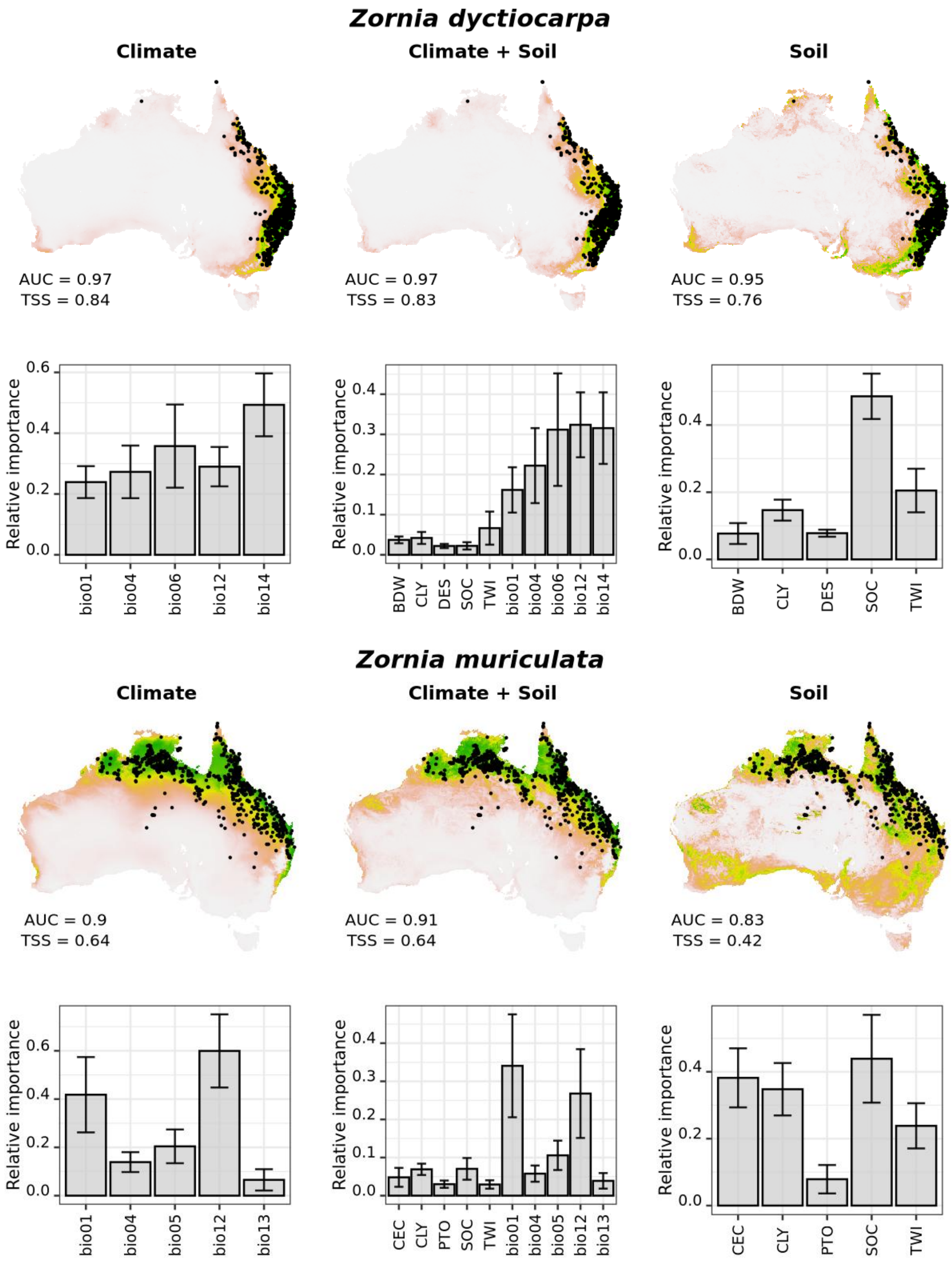
Zornia dyctiocarpa
Climate
Climate + Soil
Soil
AUC = 0.97
TSS = 0.84
AUC = 0.97
TSS = 0.83
AUC = 0.95
TSS = 0.76
Relative importance
bio01
bio04
bio06
bio12
bio14
BDW
CLY
DES
SOC
TWI
Zornia muriculata
Climate
Climate + Soil
Soil
AUC = 0.9
TSS = 0.64
AUC = 0.91
TSS = 0.64
AUC = 0.83
TSS = 0.42
Relative importance
bio01
bio04
bio05
bio12
bio13
CEC
CLY
PTO
SOC
TWI

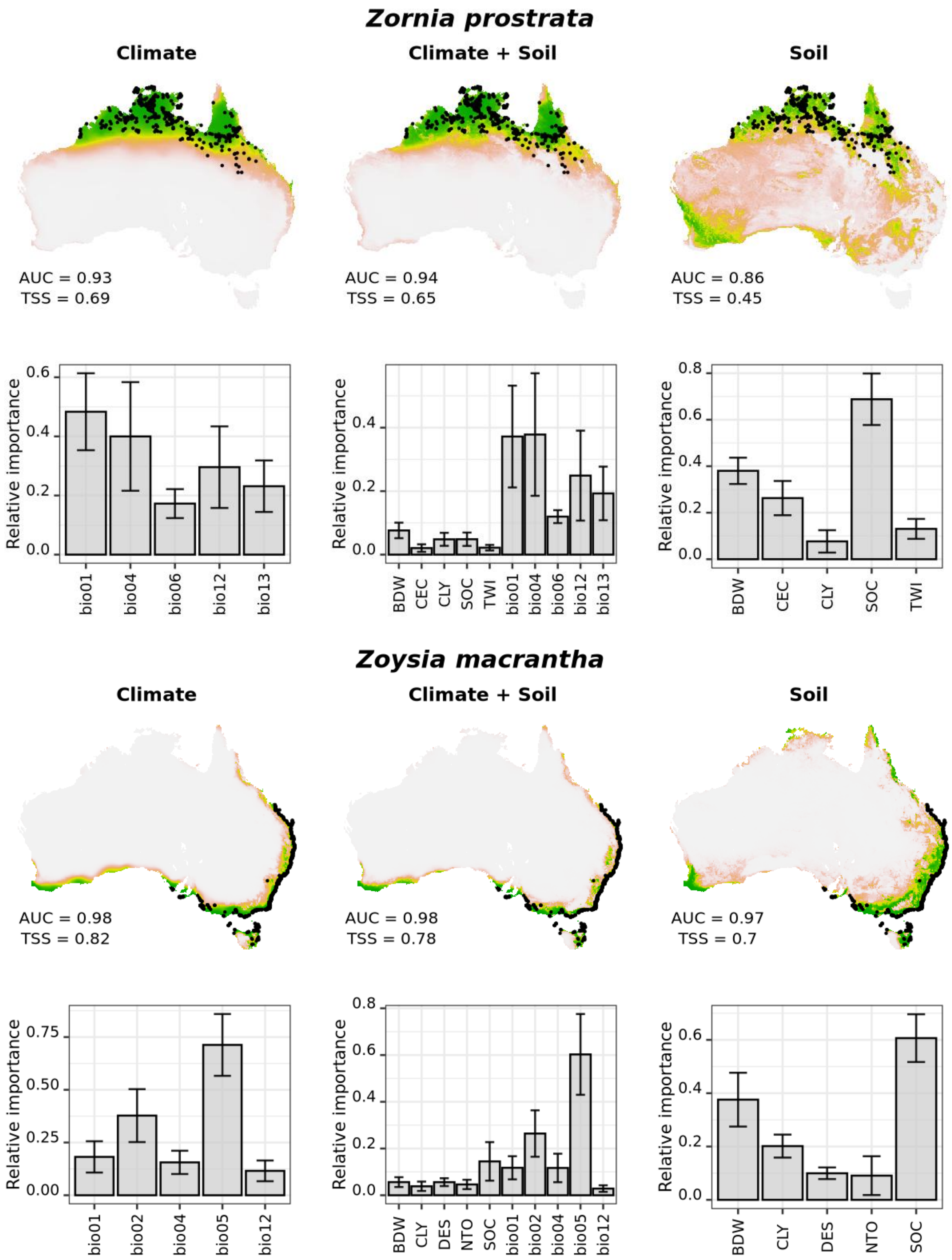

Zornia prostrata
Climate
Climate + Soil
Soil
AUC = 0.93
TSS = 0.69
AUC = 0.94
TSS = 0.65
AUC = 0.86
TSS = 0.45
Relative importance
bio01
bio04
bio06
bio12
bio13
BDW
CEC
CLY
SOC
TWI
Zoysia macrantha
Climate
Climate + Soil
Soil
AUC = 0.98
TSS = 0.82
AUC = 0.98
TSS = 0.78
AUC = 0.97
TSS = 0.7
Relative importance
bio01
bio02
bio04
bio05
bio12
BDW
CLY
DES
NTO
SOC

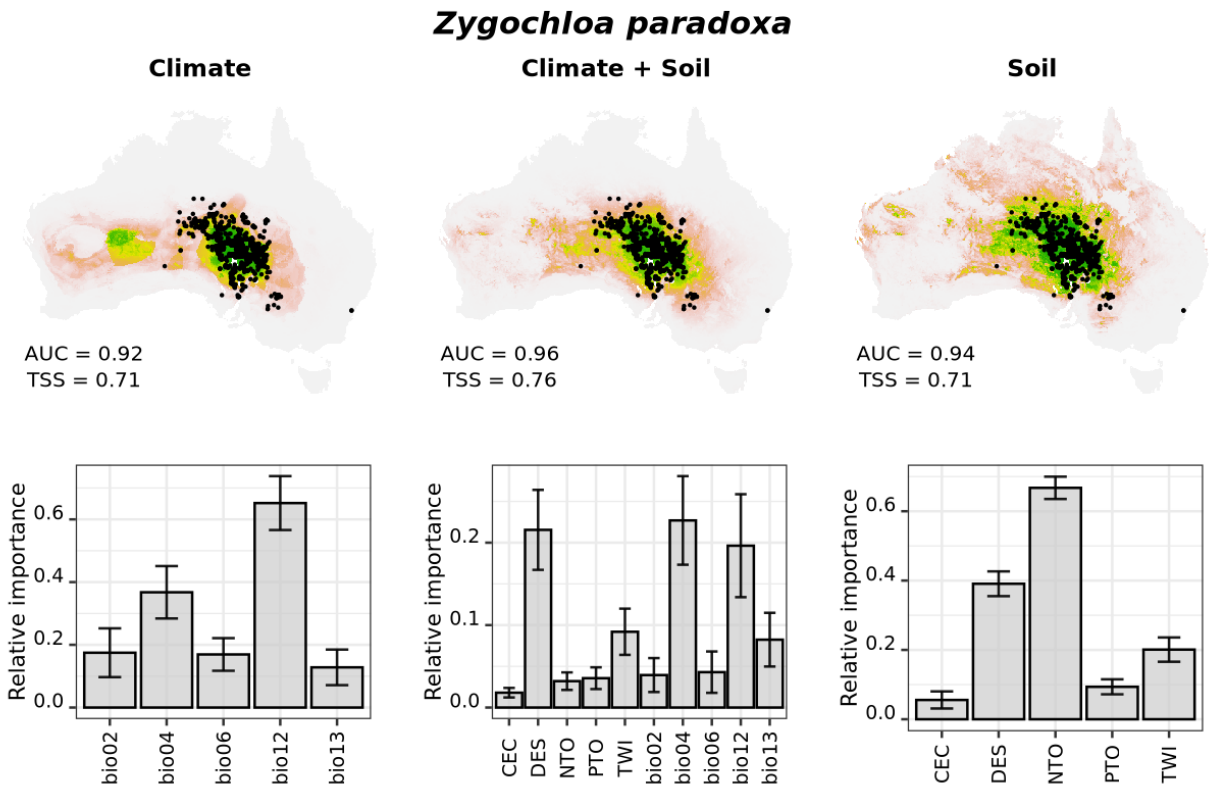

Figure S4. Ensemble species distribution modeling results, highlighting the relative importance of climate variables, soil variables, and their combined effects (climate + soil), along with the spatial distribution of 5,033 native Australian species.

Table S5. List of gbif occurrence downloads used in this study

1	GBIF.org (18 June 2024) GBIF Occurrence Download https://doi.org/10.15468/dl.zp4ffq	7	GBIF.org (16 June 2024) GBIF Occurrence Download https://doi.org/10.15468/dl.9ed2su
2	GBIF.org (13 June 2024) GBIF Occurrence Download https://doi.org/10.15468/dl.v2bhjc	8	GBIF.org (16 June 2024) GBIF Occurrence Download https://doi.org/10.15468/dl.u2xhc2
3	GBIF.org (13 June 2024) GBIF Occurrence Download https://doi.org/10.15468/dl.qrrrwv	9	GBIF.org (16 June 2024) GBIF Occurrence Download https://doi.org/10.15468/dl.ru9q2n
4	GBIF.org (13 June 2024) GBIF Occurrence Download https://doi.org/10.15468/dl.eekx54	10	GBIF.org (16 June 2024) GBIF Occurrence Download https://doi.org/10.15468/dl.nexqrc
5	GBIF.org (13 June 2024) GBIF Occurrence Download https://doi.org/10.15468/dl.7eab72	11	GBIF.org (18 June 2024) GBIF Occurrence Download https://doi.org/10.15468/dl.ktjv2e
6	GBIF.org (13 June 2024) GBIF Occurrence Download https://doi.org/10.15468/dl.88kb6f		

References

Anpsa. (2024) Australian Native Plants Society. Available from: https://anpsa.org.au/ [Accessed 10 July 2024]

Cai, L., Kreft, H., Taylor, A., Denelle, P., Schrader, J., Essl, F., Van Kleunen, M., Pergl, J., Pyšek, P., Stein, A., Winter, M., Barcelona, J. F., Fuentes, N., Inderjit, Karger, D. N., Kartesz, J., Kuprijanov, A., Nishino, M., Nickrent, D., Nowak, A., Patzelt, A., Pelser, P. B., Singh, P., Wieringa, J. J. & Weigelt, P. 2023. Global models and predictions of plant diversity based on advanced machine learning techniques. *New Phytologist,* 237**,** 1432-1445.

Conservation;, D. O. E. A. 2012. A guide to managing and restoring wetlands in Western Australia. Perth, Western, Australia.: Department of Environment and Conservation.

Eflorasa. (2024) Electronic Flora of South Australia Available from: https://ecobits.net.au/eflorasa-electronic-flora-of-south-australia/ [Accessed 12 July 2024]

Gbif. (2024) Occurrence Download. The Global Biodiversity Information Facility. Available from: https://doi.org/10.15468/dl.zp4ffq [Accessed 18 June 2024]

Jin, Y. & Qian, H. 2022. V.PhyloMaker2: An updated and enlarged R package that can generate very large phylogenies for vascular plants. *Plant Divers,* 44**,** 335-339.

Jin, Y. & Qian, H. 2023. U.PhyloMaker: An R package that can generate large phylogenetic trees for plants and animals. *Plant Diversity,* 45**,** 347-352.

Ling, J. E., Casanova, M. T., Shannon, I. & Powell, M. 2019. Development of a wetland plant indicator list to inform the delineation of wetlands in New South Wales. *Marine and Freshwater Research,* 70**,** 322-344.

Lyons, M., Gibson, N., Keighery, G. & Lyons, S. D. 2004. Wetland flora and vegetation of the Western Australian wheatbelt. *Records of the Western Australian Museum, Supplement,* 67**,** 39.

Mf, D. S. (2009) Flora of Tasmania Online. (Tasmanian Herbarium, Tasmanian Museum & Art Gallery: Hobart). Available from: https://flora.tmag.tas.gov.au/ [Accessed 13 July 2024]

Powo. (2017) Plants of the World Online. the Royal Botanic Gardens, Kew. Available from: https://powo.science.kew.org/ [Accessed 10 July 2024]

Qian, H. & Jin, Y. 2021. Are phylogenies resolved at the genus level appropriate for studies on phylogenetic structure of species assemblages? *Plant Diversity,* 43**,** 255-263.

Qian, H., Kessler, M., Zhang, J., Jin, Y., Soltis, D. E., Qian, S., Zhou, Y. & Soltis, P. S. 2023a. Angiosperm phylogenetic diversity is lower in Africa than South America. *Science Advances,* 9**,** eadj1022.

Qian, H., Zhang, J. & Jiang, M. 2023b. Global patterns of taxonomic and phylogenetic diversity of flowering plants: Biodiversity hotspots and coldspots. *Plant Diversity,* 45**,** 265-271.

Smith, S. A. & Brown, J. W. 2018. Constructing a broadly inclusive seed plant phylogeny. *Am J Bot,* 105**,** 302-314.

Vicflora. (2024) Flora of Victoria. Available from: https://vicflora.rbg.vic.gov.au/ [Accessed 12 July 2024]

Victoria, D. O. S. a. E. & Rylah, I. F. E. R. 2012. *A field guide to Victorian wetland ecological vegetation classes : for the Index of wetland condition*, Dept. of Sustainability and Environment, State Government of Victoria.

Wetlandinfo. (2017) Department of Environment, Science and Innovation, Queensland. Available from: https://wetlandinfo.des.qld.gov.au/wetlands/ [Accessed 15 July 2024]

Zhou, Y.-D., Qian, H., Jin, Y., Xiao, K.-Y., Yan, X. & Wang, Q.-F. 2023. Geographic patterns of taxonomic and phylogenetic β-diversity of aquatic angiosperms in China. *Plant Diversity*, 45**,** 177-184.
